# Serial Block-Face Scanning Electron Microscopy to Reconstruct Three-Dimensional Tissue Nanostructure

**DOI:** 10.1371/journal.pbio.0020329

**Published:** 2004-10-19

**Authors:** Winfried Denk, Heinz Horstmann

**Affiliations:** **1**Max Planck Institute for Medical ResearchHeidelbergGermany

## Abstract

Three-dimensional (3D) structural information on many length scales is of central importance in biological research. Excellent methods exist to obtain structures of molecules at atomic, organelles at electron microscopic, and tissue at light-microscopic resolution. A gap exists, however, when 3D tissue structure needs to be reconstructed over hundreds of micrometers with a resolution sufficient to follow the thinnest cellular processes and to identify small organelles such as synaptic vesicles. Such 3D data are, however, essential to understand cellular networks that, particularly in the nervous system, need to be completely reconstructed throughout a substantial spatial volume. Here we demonstrate that datasets meeting these requirements can be obtained by automated block-face imaging combined with serial sectioning inside the chamber of a scanning electron microscope. Backscattering contrast is used to visualize the heavy-metal staining of tissue prepared using techniques that are routine for transmission electron microscopy. Low-vacuum (20–60 Pa H_2_O) conditions prevent charging of the uncoated block face. The resolution is sufficient to trace even the thinnest axons and to identify synapses. Stacks of several hundred sections, 50–70 nm thick, have been obtained at a lateral position jitter of typically under 10 nm. This opens the possibility of automatically obtaining the electron-microscope-level 3D datasets needed to completely reconstruct the connectivity of neuronal circuits.

## Abstract 2

A new method combines automated imaging with serial sectioning inside the chamber of a scanning electron microscope

## Introduction

Many cellular structures are so small that they can only be resolved in the electron microscope. Furthermore, it is often crucial to visualize and reconstruct the three-dimensional (3D) structure of biological tissue. One prime example of where 3D information is indispensable is in exploring the connectivity of local networks of neurons. While axonal and dendritic processes have been traced using the light microscope from the very beginning of cellular neuroscience (Cajal 1911), light microscopic tracing is only possible if staining is restricted to a small subset of cells, as results, for example, from the Golgi method (Golgi 1873) or from the mosaic expression of fluorescent proteins (Feng et al. 2000). However, in many cases, in order to understand computational algorithms, the reconstruction of a complete neural circuit may be necessary. For this, the resolution of the light microscope is insufficient because dendritic and axonal processes can have diameters that are substantially below the wavelength of light. This lack of resolution (1) results in the inability to resolve densely packed neighboring processes, which is absolutely necessary to reconstruct network topology, and (2) does not allow a sufficiently precise estimation of the neuronal geometry, which may be necessary for biophysical modeling of cellular behavior. So far, only the electron microscope (EM) can provide the spatial resolution needed to track neural processes or to identify synapses unambiguously. Most commonly used to image biological tissue is the transmission electron microscope (TEM) (Ruska and Knoll 1932), in which a broad beam of electrons is directed at a sample that is thin enough to allow a substantial fraction of the electrons to pass through and then be focused onto film or another electron-sensitive spatially resolving detector. Specimens are typically thin slices that are cut from blocks of plastic-embedded tissue, with the resulting electron micrographs providing a two-dimensional cross section through the tissue. Scanning electron microscopy (SEM) (Ardenne 1938a, 1938b), in which a tightly focused beam of electrons is raster-scanned over the specimen while secondary or backscattered electrons are detected, is used in biological imaging mostly as a surface visualization tool, creating a 3D appearance but no actual 3D datasets.

Truly 3D information in the TEM can be obtained using either tilt-series-based tomography (Hoppe 1981; Frank 1992; Baumeister 2002) or serial ultrathin sections (Sjostrand 1958; Ware and Lopresti 1975; Stevens et al. 1980). Tomography is a very promising technique for obtaining high-resolution structural data of macromolecules, organelles, and small cells but may not be applicable when larger volumes need to be reconstructed, because section thickness is limited to around 1 μm. In the high-voltage EM, somewhat thicker sections can be viewed, which facilitates the correlation with light microcopy (Martone et al. 2000).

Serial-sectioning TEM, which can be used to obtain 3D data of much thicker volumes than is possible with tomography, is a mostly mature technology and has contributed enormously to our understanding of the local 3D ultrastructure, for example of dendritic spines (Harris 1999). A whole animal, the nematode Caenorhabditis elegans has been reconstructed in this way (White et al. 1986). This is still considered a seminal effort, partly because serial sectioning is such a labor-intensive and time-consuming method, which requires intensive operator involvement in cutting sections, gathering data, and reconstructing volumes. The number of sections that need to be handled can be reduced by combining tomography with serial sectioning (Soto et al. 1994), but the need to manually handle the sections remains. For these reasons the number of larger-scale 3D serial reconstructions has been rather limited, even though many biological problems, not only the tracing of neural circuits, require truly 3D information.

Volume information equivalent to that from reconstructing serial sections can, of course, be obtained if the sections are imaged before being cut, that is, by repeatedly imaging the block face. Block-face imaging is used even in light microscopy (Odgaard et al. 1990; Ewald et al. 2002), where optical-sectioning techniques, such as confocal (Minski 1961) or multi-photon microcopy (Denk et al. 1990), are readily available. It is, of course, impossible to image the block face in the TEM. The SEM, as a surface-imaging technique, is, on the other hand, well suited for this task. This was recognized several decades ago by Leighton, who also constructed a microtome for cutting sections inside the microscope chamber (Leighton 1981). Imaging the block face removes not only the need to deal manually with ribbons of fragile sections but also the difficulty of aligning the images of sections, which are often distorted. The prevalent contrast mode in the SEM is the detection of so-called secondary electrons, which are low-energy electrons that are emitted when the primary electron beam strikes the sample surface. The secondary-electron signal depends strongly on the orientation of the surface, leading to topographic images that characteristically resemble obliquely illuminated solid objects. Since the microtome-cut block surface is devoid of topographic features, very little contrast is generated unless an additional preparatory step, such a plasma etching (Kuzirian and Leighton 1983; Hukui 1996), is used.

At the time of Leighton's original work, no stacks of volumetric data were presented. One reason might have been that low-vacuum SEMs (also called atmospheric [Robinson 1975; Danilatos 1980] or environmental SEMs; for a recent review see Donald [2003]), which can be used to image nonconducting specimens (see below), were not widely available. In the original report (Leighton 1981), the sample had to be removed from the SEM chamber for coating with a conducting layer. Furthermore, digital image acquisition, storage, and processing were in their infancy and quite limited in capacity.

In this paper we show, first, that by using backscattering contrast and low-vacuum operation we can obtain high-contrast images from cut block faces and, second, that in combination with a custom-designed microtome we can obtain 3D ultrastructural data with a resolution and from volumes appropriate for the 3D reconstruction of local neural circuits.

## Results

As a first step, we explored whether sufficient contrast and stable images can be obtained from uncoated block faces of plastic-embedded tissue, prepared essentially as for TEM. There are a number of techniques known that allow imaging of nonconducting specimens in the SEM. One technique relies on choosing an accelerating voltage at which the introduction of charge by the electron beam is just balanced by the sum of backscattered and secondary electrons (for a review see Joy and Joy [1996]). We first tried the charge-balance method (electron energies around 600 eV) but found that the secondary-electron signal provides, not surprisingly, unsatisfactory contrast (data not shown). The secondary-electron signal is the only contrast available because low-energy backscattered electrons (BSEs) cannot be easily detected at such low voltages. Furthermore, residual charging, which may not be strong enough to preclude the formation of an image, can still lead to slight shifts between images by electrostatic beam deflection. This then destroys the alignment between successive images, which is one of the main reasons to establish serial block-face imaging.

Another set of techniques relies on providing ions in the specimen chamber to neutralize charge on the sample rather than avoiding net charge introduction by the electron beam. This can, for example, be achieved by maintaining a low concentration of gas in the chamber (Robinson 1975; Moncrieff et al. 1978) at a pressure (10–100 Pa) that is low enough for a substantial fraction of the beam's electrons to reach the sample unscattered and thus be able to form a tight focus. Ions needed for discharging the sample are generated by those electrons that do strike gas molecules or atoms.

**Figure 1:**
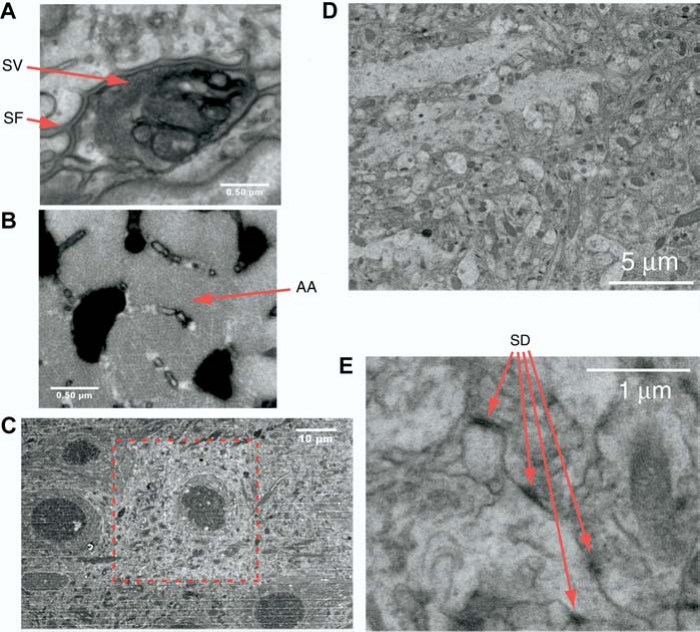
Resolution and Contrast Using the Backscattered Electron Signal (A and B) Presynaptic vesicles (SV) and postsynaptic folds (SF) are clearly visible (A) in a motor endplate preparation embedded in Spurr's resin. Similarly, the hexagonal array of actin filaments (AA) can be clearly resolved (B) in a different region from the same image (both images were smoothed using the ImageJ “smooth” command). Imaging conditions for (A) and (B): electron energy, 7.5 keV; spot, 3.5; chamber pressure, 30 Pa (H_2_O); pixel dwell time, 30 μs. The scanning resolution was 6.7 nm/pixel. (C) The effect of beam exposure on the block surface. Note the increased brightness and the lack of chatter in the central region (inside the dashed rectangle), from which a stack was acquired at higher resolution before taking the image shown. The tissue was rat neocortex embedded in Spurr's resin. Imaging conditions for (C): electron energy, 7.5 keV; spot, 3; digital resolution for stack acquisition, 26.7 nm/pixel; dwell time, 30 μs. (D and E) Cortical tissue embedded in Epon. Synapses (SD) are clearly discernable (E). Imaging conditions for (D) and (E): electron energy, 7.5 keV beam current; spot, 3; chamber pressure, 30Pa (H_2_O); pixel dwell time, 30 μs. The scanning resolution was 9.5 nm/pixel. Note that more backscattering corresponds to darker pixels in (A), (B), (D), and (E) but to brighter pixels in (C).

The residual-gas method (also called low-vacuum or variable-pressure mode) can be used at higher accelerating voltages and is, therefore, compatible with BSE contrast. Since scattering of electrons is strongly dependent on the charge of the atomic nucleus, the BSE signal provides a clear distinction between heavy-metal-stained and unstained structures (Figures 1 and S1) that, after contrast reversal, look very similar to traditional transmission electron micrographs. Images of uncoated block faces (Figure 1) resemble scanning electron micrographs obtained from block faces of similar specimens coated with a very thin conducting film and imaged under high-vacuum conditions (Richards and ap Gwynn 1996). Our BSE images also demonstrate that the resolution under these conditions is sufficient to resolve structures such as synaptic vesicles (Figure 1A) or the array of actin filaments characteristic of skeletal muscle (Figure 1B). Synaptic contacts in cortical tissue can be clearly identified as well (Figure 1D and 1E).

These results show that we can obtain sufficient contrast from uncoated block faces. On this basis we decided to construct an ultra-microtome appropriate for sequential sectioning inside the sample chamber of an SEM. There are a number of special requirements for such a microtome. (1) The position of the block has to remain stable or be returned to the same location after each image is taken in order to ensure alignment of subsequent images, which is one of the primary objectives of serial block-face imaging. (2) The distance between the block face and the SEM objective lens has to be sufficiently small to allow high-resolution imaging and the efficient collection of backscattered electrons under low-vacuum conditions. (3) The block face has to be perpendicular to the optical axis of the electron optical column to keep the whole block face in focus; for all commercial SEM instruments this means horizontal orientation. (4) The operation of the microtome must be remotely controllable to allow automation. (5) The microtome has to be compatible with low vacuum. Finally, (6) the range of the fine advance mechanism has to be at least several hundred microns to permit continuous sectioning of volumes large enough to be of interest in the study of local neuronal circuits. Conditions 1, 2, and 3 rule out the modification of a conventional ultra-microtome, in which the sample block, its face oriented vertically, is moved against a stationary knife. Condition 2, in addition, rules out the use of a conventionally mounted diamond knife, since the boat would protrude upwards, colliding with the BSE detector and even with the objective lens.

**Figure 2:**
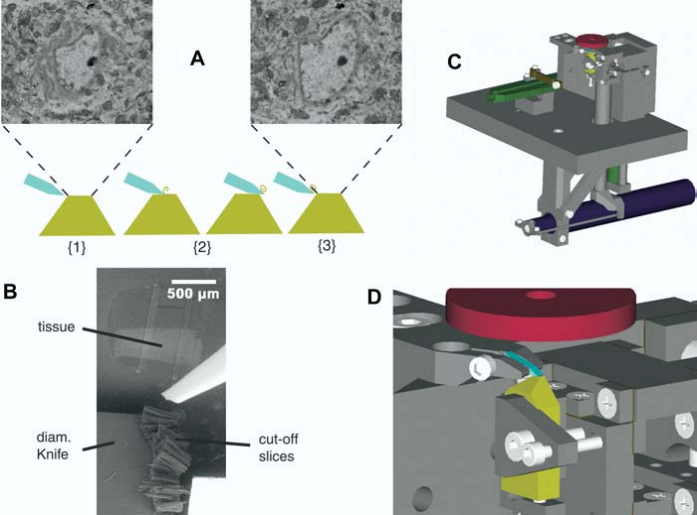
SEM Microtomy (A) Principle of SBFSEM operation: (1) a SEM image is taken of the surface of the plastic-embedded tissue preparation (amber trapezoid). (2) Then with a diamond knife (blue) an ultrathin slice is cut off the top of the block. (3) After retraction of the knife, the next picture is taken. The pictures shown are from an actual stack (cerebellar cortex) but are not successive slices; rather, they are spaced by five images (about 315 nm) to make the changes more apparent. (B) Usually cut-off slices pile up on the top of the knife. Protruding into the picture from the right is a puffer pipette, occasionally used to remove debris from the knife. (C and D) The mechanical design for the in-chamber microtome is shown in an overview (C) and a close-up of knife and sample (D) in renderings from the computer-aided design software. Most parts are nonmagnetic stainless steel (grey). A large-motion leveraged piezo actuator (green part on the left) drives the knife holder back and forth. The custom diamond knife (light blue) is clamped in a special holder. The sample (amber) advance is driven via a lever by a direct-current-motor-driven micrometer (dark blue). The retraction during the backwards knife motion is again piezo actuated (green cylinder in the lower right of [C]). Bearing springs are brown. The BSE detector (red) is depicted schematically above the sample. Not shown is the lateral positioning mechanism.

We, therefore, decided to construct a microtome de novo (Figure 2; for details see Materials and Methods), which does share some design features with the instrument built by Leighton (1981). A custom knife that could fit under the detector was designed in cooperation with the Diatome company and fabricated by Diatome. To meet condition 1 above, the knife is moved for cutting. In the z-direction, sample advance rather than knife advance is used in order to keep the cutting plane, and hence the location of the block surface, constant. This removes the need for refocusing the SEM, thereby contributing to image stability and registration. To avoid sliding friction, crossed leave flexures were used for the knife arm and for the sample advance mechanism. To provide advance motion with sufficiently fine resolution, we used a lever mechanism that scaled the motion of a motorized micrometer down by a factor of roughly 1/9. To minimize heat production, which can lead to thermal drift and is hard to dissipate in vacuum, we used a direct-current motor drive.

Preparation of samples for the serial block-face SEM (SBFSEM) was done using procedures common for preparing samples for viewing in the TEM (see, for example, Hayat 2000). Since SBFSEM does not allow contrast enhancement after cutting, which is often used in the TEM, we tested several methods of enhancing the contrast of the whole block and found that exposing the tissue to uranyl acetate (see Materials and Methods and also Hayat [2000], p. 342ff) leads to excellent contrast in BSE mode (see Figure 1).

We could reliably cut serial sections thicker than about 50 nm, the exact lower thickness limit depending on the embedding material and the beam exposure (an upper thickness limit was not established). When trying to cut thinner sections the actual thickness became uneven or the knife would skip every other cut, as recognized by the lack of change in structural detail. Another indication of uneven cutting is alternating brightness of the whole images or image regions. The reason for this is that the backscattering efficiency generally increases after beam exposure (see Figure 1C), which may be because a selective ablation of the embedding matrix leads to an effective increase in the heavy-metal concentration.

**Figure 3:**
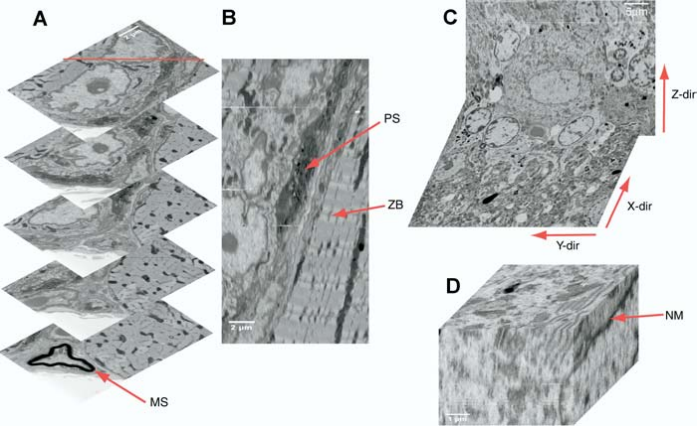
3D Datasets: Five Slices (A) Five slices from a stack containing a total of 365 slices at 63-nm section thickness; same tissue as in Figure 1A and 1B. Note beginning of myelin sheath (MS) in the lowest slice. Imaging conditions as in Figures 1A and 1B except resolution is 13.4 nm/pixel. (B) Reslice of the same dataset along the red line show in the top image of (A). Arrows point to the presynaptic ending (PS) and the z-band (ZB). Image corners in (A) touch the reslice (B) at the depths at which they were taken. (C and D) Cerebellar tissue displayed at low (C) and high (D) resolution; note nuclear envelope (NM). Note differences in lateral and vertical resolution. Imaging conditions for (C) and (D): electron energy, 7.5 keV; spot size, 3.5; digital resolution, 12.7 nm/pixel.

Usually the cut-off slices pile up on the top surface of the knife (see Figure 2B). Only rarely does a sliver get deposited on the block surface. When this happens it results in the loss of only one image since the sliver is reliably removed by the next passage of the knife. During the cutting of the stack used for Figure 3A and 3B, three instances of deposited debris occurred (white horizontal streaks in Figure 3B). Currently efforts are underway to devise methods (such as using a brief puff of gas from a glass pipette, such as can be seen in Figure 2B) to reliably remove the debris from the knife after each cut.

We found, somewhat surprisingly, that cutting quality was often better in the area scanned by the electron beam than outside (see Figure 1C), presumably because of a modification of the mechanical properties of the embedding material. We have successfully cut a number of different embedding materials (Araldite, Epon, and Spurr) and tissue types (mammalian muscle, cortex, cerebellum, retina, zebrafish brain, and fly brain) using our SBFSEM setup. Figure 3 shows examples of 3D datasets.

**Figure 4:**
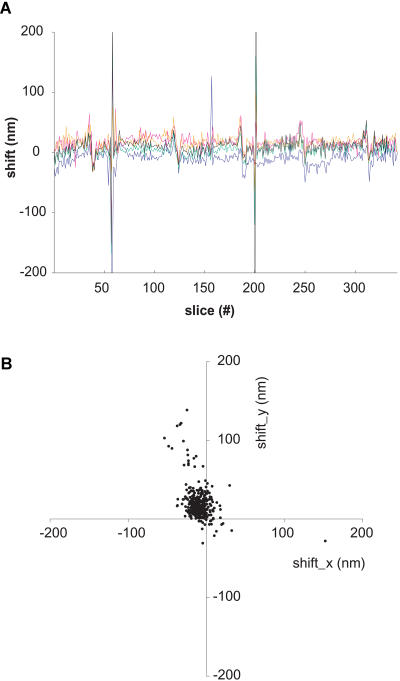
The Alignment of Successive Images in a Stack Shifts between images were quantified using the positions of the peaks of the cross correlation (see Materials and Methods); same dataset as in Figure 3A and 3B. (A) The peak shifts in x-direction are shown for five different subregions distributed over the field of view. Four of the regions have a size of 256 × 256 pixels, one has a size of 512 × 512 (black trace). The peaks around slices 59 and 202 are caused by slice debris on the block face (see also streaks in Figure 3B). (B) The x/y displacement for the 512 × 512 region is shown in a scatter plot. For the central cluster the standard deviations are 10.9 nm and 11.8 nm for x and y, respectively.

Since lateral registration between successive slices is crucial for the applicability of the SBFSEM technique for automated 3D reconstruction, we quantified the registration using cross correlation. Figure 4 shows the registration precision throughout a stack of images from muscle tissue. The fluctuations are mostly around 10 nm in size (standard deviation). Large fluctuations (greater than 60 nm) are seen occasionally but can be traced to temperature drifts or are spurious values caused by debris on the block face. Some of the apparent position shifts and fluctuations do not, furthermore, represent registration errors but are caused by systematic or random pattern shifts. An example is the shift of the central cloud of points in Figure 4B, which is caused by the tilt of the actin fibers from the block-face normal.

The depth resolution depends, of course, on the section thickness but also on the depth below the sample surface from which the BSE signal originates, which, in turn, scales with the electron penetration (see chapter 3 of Goldstein et al. [2003]). For our early experiments we had used the electron energy of 7.5 keV because lower voltages gave unsatisfactory signals. At 7.5 keV the resolution along the z-axis is not very good, as is apparent from Figure 3D. To improve the z-resolution one could conceivably use deconvolution techniques because the point spread function contains a sharp edge and hence high spatial frequencies along the z-axis, even if the electron penetration into the sample is large. The reason is that while a structural detail can contribute to the signal if buried in the sample, the contribution from that detail drops immediately to zero as soon as the slice containing the detail is removed.

**Figure 5:**
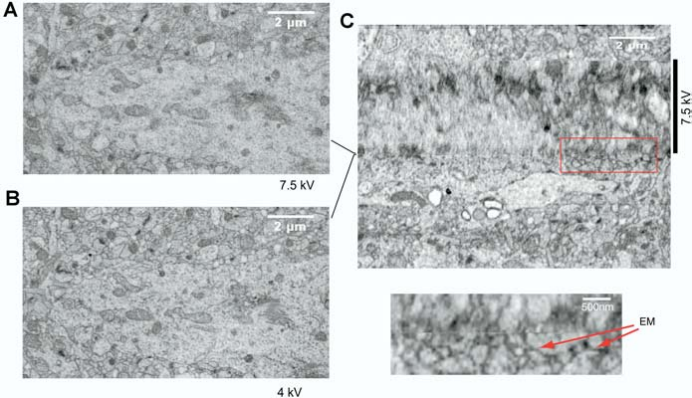
Energy Dependence of the Depth Resolution The lateral resolution does not change very much as the electron energy is reduced from 7.5 keV (A) to 4 keV (B), but the resolution along the z-direction is dramatically different (C). The lines between (A), (B), and (C) indicate the z-positions in the stack from which (A) and (B) were taken. In the high-resolution view (lower panel in [C]) membranes that were en-face (EM) in the original slices can be clearly recognized. The nominal slice thickness was 55 nm. Tissue is rat cortex. Imaging conditions: dwell time, 25 μs; spot sizes, 3.5 and 2.8 for 4 and 7.5 keV, respectively.

Electron penetration depends strongly (Kanaya and Okayama 1972) on the energy (scaling as *E* ^1.67^). This means, for example, that at 4 keV the depth is reduced to about one-third of that at 7.5 keV. The actual value for the depth penetration is difficult to estimate for a composite material such as plastic-embedded heavy-metal-stained tissue. For a detailed discussion of the issues surrounding depth penetration see, for example, chapter 3 of Goldstein et al. (2003). We, therefore, decided to operate the SBFESM at lower voltages. In order to obtain sufficient signal at the lower electron energy, where the efficiency of semiconductor-diode BSE detectors declines steeply (Funsten et al. 1997), we replaced the detector amplifier with an instrument originally designed to detect single ion-channel currents (commonly called a patch-clamp amplifier [Hamill et al. 1981]) and found that we could now get satisfactory images for electron energies of 4 keV. In Figure 5 the depth resolutions at 7.5 and 4 keV are directly compared for the same sample. It is particularly encouraging that at 4 keV it is possible to recognize clearly membranes that are parallel to the block surface (en-face membranes), which is crucial for the successful reconstruction of cellular topology.

## Discussion

We have shown that fully automated acquisition of truly 3D datasets at nanoscopic resolution is possible with serial block-face imaging in the SEM. The lateral resolution is sufficient to recognize most cellular organelles and synaptic specializations and does not appear to be limited by the beam spot diameter but by the spread of the beam in the sample (Kanaya and Okayama 1972; see also Joy and Joy 1996). This spread could be further reduced by using lower beam energies, provided that more sensitive detectors for such low electron energies can be obtained (Funsten et al. 1997). Lower beam energies also would further improve the z-resolution, even though at some point elemental contrast in the BSE signal begins to decline (Joy and Joy 1996). The resolution is, in practice, also limited by the radiation dose that a specimen can sustain. Since at lower electron energies the beam's energy is deposited into a rapidly shrinking volume, heating effects could become an issue. Nonetheless, it appears conceivable that nearly isotropic resolution can be achieved if the sectioning thickness can be reduced to around 20 nm (see below), with imaging then done at primary electron energies of around 2 keV. Because the registration between successive slices (see Figure 4) is mostly better than the resolution, the 3D data stacks can be used without further alignment and distortion correction (for the complete set of raw data see Datasets S1–S20; slices through this dataset are shown in Figures S2–S4) . This is crucial if automated reconstruction is to be performed, since alignment of serial sections often requires manually identified landmarks.

It would be highly desirable to reliably cut sections thinner than 50 nm, which appears to be the current limit in our hands. The exact reason for this thickness limit is not clear as yet. Much thinner sections have been cut from plastic blocks (Frosch et al. 1985), albeit under atmospheric pressure with the sections floated onto water. An important difference between standard ultra-microtomy and SBFSEM is that, because of the inevitable exposure of the block face to the electron beam, the mechanical properties of the block surface are changed, affecting cutting of subsequent sections. While at very high doses these effects are deleterious, they can be helpful at intermediate levels for suppressing chatter (see Figure 1C). To suppress chatter and to achieve thinner sectioning, we have begun to use the oscillating-knife technique (Studer and Gnaegi 2000) (see Figures 5 and S2–S4). It might also help to reduce the sample temperature, which, in addition to affecting cutting properties directly by changing the polymer stiffness, might substantially improve the resistance to radiation damage (Lamvik 1991). SBFSEM might ultimately allow a smaller section thickness than is routinely possible with serial sectioning for the TEM, since there the mechanical integrity of the cut sections has to be ensured. Alternatively, one might use nonmechanical surface-ablation techniques such as plasma etching, reactive or focused ion beam etching, or ion milling to achieve smaller depth increments. Nonmechanical techniques do, however, suffer from a dependence of the ablation rate on the composition, which is, by the very nature of embedded tissue, rather inhomogeneous.

In spite of the similarity in appearance, it is important to keep in mind a number of differences between transmission imaging of sections and backscattered SBFSEM. First, in the SEM, a large fraction of the entire beam energy is deposited inside the sample, leading to much increased radiation damage per primary electron. This is partly counteracted by the fact that BSE imaging is a “dark field” technique and, therefore, each detected electron carries more information. Furthermore, because the primary-electron energies can be lower by a large factor in the SEM (4 keV) than in the TEM (typically about 80 keV but routinely as high as 300 keV and up to 3 MeV in special cases), scattering cross sections are much larger in the SEM. This may, however, not always translate into improved contrast, since in the SEM only electrons scattered by angles larger than 90° can contribute to the image, while rather small deflections are enough to remove an electron from the beam in the TEM. A major practical advantage of SEM block-face imaging is that there is no need to manually handle sections, and no occlusion occurs by EM-grid bars. On the other hand, it is, unlike in TEM, impossible to reimage a section at higher resolution. Finally, it is unlikely that SEM surface imaging will ever reach the lateral resolution that can be achieved by imaging thin sections in the TEM.

The total volume that can be reconstructed by SBFSEM is currently limited in the lateral dimension by the digital resolution available on commercial SEM instruments. It should, however, be straightforward to increase the digital resolution by modifying the scan and data-collection cir-cuitry. Eventually, off-axis electron-optical aberrations will become significant, but the total field of view can then be increased by mechanical translation of the microtome and tiling of multiple images. In the z-direction the stack height that can be cut continuously is, first, limited by the travel of the fine advance. Second, and more fundamentally, the cutting pyramid becomes unstable and too compliant if it is too high. However, a single step of image realignment after retrimming and repositioning will allow the continuation in the z-direction for almost unlimited distances.

A major practical limitation is, of course, the acquisition speed of the SEM, which is ultimately dependent on the required signal-to-noise ratio *(R_SN_).* We can estimate the minimally achievable voxel dwell time *(τ_d_)* using the backscattering coefficient (η) as follows:



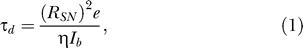



where *I_B_* is the beam current and *e* is the elementary charge. The available beam current depends on the electron-gun type, the desired resolution, and the electron energy, but a value of *I_B_* = 1 nA is realistic for our purposes. We set η to 0.1, which is the value for carbon and thus a lower limit since backscattering is, of course, higher for the heavy-metal-stained areas (Drescher et al. 1970; see also chapter 3 in Goldstein et al. [2003]). To get *R_SN_* =100 we then need a dwell time of 16 μs, allowing data rates slightly above 50,000 voxels/s. A cube 200 μm on a side imaged at a resolution of 10 nm × 10 nm × 50 nm, which corresponds to 1.6 teravoxels, would then require a total scan time of 25,600,000 s, which is on the order of one year. However, the tracing of axons probably can, with the appropriate staining, be achieved at 20-nm lateral resolution and with an *R_SN_* of ten or less. This reduces the estimated time by a factor of 400, to less than one day. While in some cases the resolution provided by conventional (tungsten filament) electron guns may be sufficient, we feel that the long-term stability of field-emission emitters is essential for imaging large volumes.

The approach described here will speed up the collection of medium-to-high resolution 3D electron microscopic datasets by several orders of magnitude. The acquired data will, in addition, not require time-consuming and error-prone alignment and distortion correction. This should have wide-ranging applications not only in biomedical research but also in materials characterization (Alkemper and Voorhees 2001). We are particularly interested in the complete reconstruction of local neural circuits such as those that underlie the detection of motion in the retina (Barlow et al. 1964; Euler et al. 2002). This requires the imaging of volumes containing at least one complete dendritic tree and is therefore virtually impossible using conventional electron microscopic methods. The data quality using current staining techniques is good enough to manually trace neuronal processes in many cases (see Figure S5). One of the major challenges will be the automation of data analysis, which could well require the development of novel or the further optimization of existing staining techniques that highlight the plasma membranes or the extracellular space (Brightman and Reese 1969).

It is quite likely that scanning-probe methods such as atomic-force or near-field microscopy could be used instead of SEM to image the block face. It would be particularly interesting if multiple fluorescent dyes could be detected and discriminated in this way since they still provide unmatched specificity in labeling biological samples.

## Materials and Methods

### 

#### Imaging

All data shown were taken on a environmental SEM with a field-emission electron gun (QuantaFEG 200, FEI, Eindhoven, the Netherlands) mostly at an electron energy of 7.5 keV (exceptions as noted, see Figures 5 and S2–S4, Datasets S1–S20, and Video 2). For data in Figures 5 and S2–S4, Datasets S1–S20, and Video 2, a highly sensitive current amplifier was used (Axopatch200B, Axon Instruments, Union City, California , United States) to amplify the current from the solid-state backscattered electron detector (type L2, K. E. Developments, Cambridge, United Kingdom). The beam current values for the parameters used were roughly interpolated from manufacturer's data, yielding estimated beam currents of 190, 430, and 880 pA for spot sizes of 2.5, 3, and 3.5, respectively, at a beam energy of 7.5 keV, and 150, 330, and 645 pA for spot sizes of 2.5, 3, and 3.5, respectively, at a beam energy of 4 keV.

**Video 2:** Cortical Tissue A movie sequence through a stack of block-face images from cortical tissue (same dataset as in Figure 5). The beginning and the second half of the stack were taken at 4 keV electron energy, the middle part at 7.5 keV (see also Figure 5C). The dimensions of this volume are 11.64 μm laterally and 9 μm vertically (see also Figure S5).

**Video 1:** Neuromuscular Junction A movie sequence through a stack of block-face images from muscle tissue (same dataset as in Figures 1A, 1B, 3A, and 3B).

#### Specimen preparation

Muscle tissue was prepared as described in Schwarz et al. (2000). For the preparation of rodent brain tissue the animals were perfused transcardially first with 30 ml of phosphate-buffered saline and then with 40 ml of fixative solution (4% paraformaldehyde in 0.1M PBS [pH 7.4]). The brain tissue was then removed and kept in fixative over night at 4 °C. After being washed twice in PBS, tissue slices (0.2 to 1.5 mm thick) were cut on a vibratome (752 M Vibroslice, Campden Instruments, Leichester, United Kingdom) and kept for 24 h in PBS at 4 °C. Pieces about 1.5 mm in size were then excised and washed three times for 30 min each in cacodylate buffer at pH 7.4.The tissue was postfixed for 2 h in 2% osmium tetroxide/1.5% potassium ferric cyanide in aqueous solution at room temperature. Then the tissue was subjected to a contrast enhancement step by soaking it over night in a solution of 4% uranyl acetate in a 25% methanol/75% water mixture (Stempak and Ward 1964) at room temperature. After that the tissue was dehydrated in a methanol sequence (25%, 70%, 90%, and 100% for 30 min each) followed by infiltration of the epoxy (Spurr, Epon 812, or Araldite, all from Serva, Heidelberg, Germany) monomer (epoxy/methanol 1:1, for 3 h rotation at room temperature; epoxy/methanol 3:1, overnight at 4°C; pure epoxy, 3 h rotating at room temperature). Polymerization was 48 h at 60 °C for Epon and at 70 °C for Spurr and Araldite. The block face was trimmed to a width of several hundred microns and a length of about 500 μm using either a conventional microtome or a sharp knife. SEM images of the untrimmed block face can be used to select the desired field of view before the final trimming step producing the desired small cutting pyramid.

#### Data analysis

Reslicing of image stacks (see Figures 2B–2D and 5C, as well as S2–S4) was done using the ImageJ reslicing command, which interpolates the z-axis data so that the digital resolution matches that of the lateral direction. For calibration of the z-axis, see below. Image shifts (see Figure 4) were measured by cross-correlating subregions (256 × 256 or 512 × 512) of subsequent slices. The peak positions were determined by first normalizing the cross correlations, then raising the values to the 64th power, and finally calculating the barycenter. All calculations were performed using ImageJ (version 1.32g). The neurite reconstruction shown in Figure S5 was done using Amira 3.1 (Mercury Computer Systems, TGS Unit, Düsseldorf, Germany).

#### Microtome

The microtome was constructed using mostly custom-machined parts made from nonmagnetic stainless steel. The leave springs were made out of bronze. The specimen advance contained a lever mechanism that reduced the motion of the motorized micrometer (M227.10 with controller C862, Physik Instrumente, Karlsruhe, Germany) by a factor of 0.11. This scaled the position uncertainty of the motorized micrometer (50 nm) down to a value 5.5 nm. Retraction of the sample during reverse motion of the diamond knife was driven by a closed-loop piezo element (P841.10, with controller E-610.S0, Physik Instrumente). The cutting motion was driven by a large-displacement piezo element (PX-1500, Piezojena, Jena, Germany). Suspension of the microtome on steel balls sliding on sapphire plates allowed lateral positioning driven by piezo-actuated slip-stick motors (Picomotor 8321-V, New Focus, San Jose, California, United States). The microtome was controlled using a computer interface designed originally for electrophysiology applications (1401 power, CED, Cambridge, United Kingdom) using scripts written in the Spike2 programming environment (CED). Analog voltages that were generated by the computer interface drove the cutting and the retraction piezos. The specimen-advance motor was controlled via serial interface. During automated stack acquisition the QuantaFEG 200 microscope was controlled using a modified keyboard that allowed simulated key pressings via a serial interface. Both serial interface connections were driven by commands in Spike2 software scripts.

The diamond knife used was custom made by Diatome, derived from their ultra 35° type. The cutting edge was 1.5 mm long. Unlike in the standard knife, in which the diamond is soldered to a piece of hard metal—increasing the clearance height necessary above the cutting edge and thereby increasing the working distance—our diamond was directly clamped in a custom-made stainless-steel holder. The clearance angle was fixed at 6°. The section thickness values quoted were calculated using the nominal micrometer position change and the mechanical reduction ratio of the advance mechanism. For some of the data (Figures 5 and S2–S5; Datasets S1–S20) the knife was oscillated (about 300 nm pp at 12 kHz) along the line of the cutting edge using a modified, piezo-driven knife holder.

## Supporting Information

Dataset S1 Cortical Tissue Slices 1–99 (248.1 MB ZIP).

Dataset S2 Cortical Tissue Slices 100–199 (252.6 MB ZIP).

Dataset S3 Cortical Tissue Slices 200–299 (252.7 MB ZIP).

Dataset S4 Cortical Tissue Slices 300–399 (252.6 MB ZIP).

Dataset S5 Cortical Tissue Slices 400–499 (251.9 MB ZIP).

Dataset S6 Cortical Tissue Slices 500–599 (252.2 MB ZIP).

Dataset S7 Cortical Tissue Slices 600–699 (253.7 MB ZIP).

Dataset S8 Cortical Tissue Slices 700–799 (255.9 MB ZIP).

Dataset S9 Cortical Tissue Slices 800–899 (256.1 MB ZIP).

Dataset S10 Cortical Tissue Slices 900–999 (253.8 MB ZIP).

Dataset S11 Cortical Tissue Slices 1,000–1,099 (252.6 MB ZIP).

Dataset S12 Cortical Tissue Slices 1,100–1,199 (252.6 MB ZIP).

Dataset S13 Cortical Tissue Slices 1,200–1,299 (251.9 MB ZIP).

Dataset S14 Cortical Tissue Slices 1,300–1,399 (251.8 MB ZIP).

Dataset S15 Cortical Tissue Slices 1,400–1,499 (250.7 MB ZIP).

Dataset S16 Cortical Tissue Slices 1,500–1,599 (251.4 MB ZIP).

Dataset S17 Cortical Tissue Slices 1,600–1,699 (252.7 MB ZIP).

Dataset S18 Cortical Tissue Slices 1,700–1,799 (250.5 MB ZIP).

Dataset S19 Cortical Tissue Slices 1,800–1,899 (253.4 MB ZIP).

Dataset S20 Cortical Tissue Slices 1,900–2,000 (254.9 MB ZIP).

Figure S1 Muscle Tissue Complete field of view for dataset underlying Figure 1A and 1B. Grayscale is inverted from data taken; no smoothing or contrast enhancement was applied.
(10.8 MB TIF).

Figure S2 Large Volume of Cortical Tissue Bottom slice of a stack of 2,000 images taken at 4 keV. Slice thickness was 55 nm. Spotsize was 3.4. Pixel size is 26.7 nm. The pixels correspond to the original data. The area shown corresponds to 54.8 × 47.3 μm. The raw data can be found as numbered TIF images in Datasets S1–S20.
(10.6 MB TIF).

Figure S3 Large Volume of Cortical Tissue: X-Resliced Stack Same volume data as used for Figure S2. The stack was resliced along the horizontal dotted line shown in Figure S2. In the vertical direction the data were interpolated so that each slice now corresponds to slightly more that two pixels. Horizontal white lines are slices with deposited debris. The total stack height was 110 μm.
(8.2 MB TIF).

Figure S4 Large Volume of Cortical Tissue: Y-Resliced Stack Same as Figure S3 but now resliced along the vertical dotted line in Figure S2.
(7.1 MB TIF).

Figure S5 Neurite Reconstruction Manual reconstruction of selected processes in cortical tissue (data from Video 2 and Figure 5). Blue, portion of proximal apical dendrite; green, secondary dendrite with three synaptically connected axons (yellow, ocher, and mauve). Insets show the synaptic contacts. Also shown is a passing axon that is not synaptically connected within the volume analyzed. Only the lower part of the stack, which was taken at 4 keV electron energy, was used.
(17.5 MB TIF).

## Abbreviations

3D: three-dimensional
BSE: backscattered electron
EM: electron microscope
SBFSEM: serial block-face scanning electron microscope
SEM: scanning electron microscopy
TEM: transmission electron microscope
