# Supplementary figures and images for: Serial Block-Face Scanning Electron Microscopy to Reconstruct Three-Dimensional Tissue Nanostructure (part 1 of 21)
Source: PLoS Biol. 2004 Oct 19;2(11):e329. doi: 10.1371/journal.pbio.0020329 (PMC524270; doi:10.1371/journal.pbio.0020329)

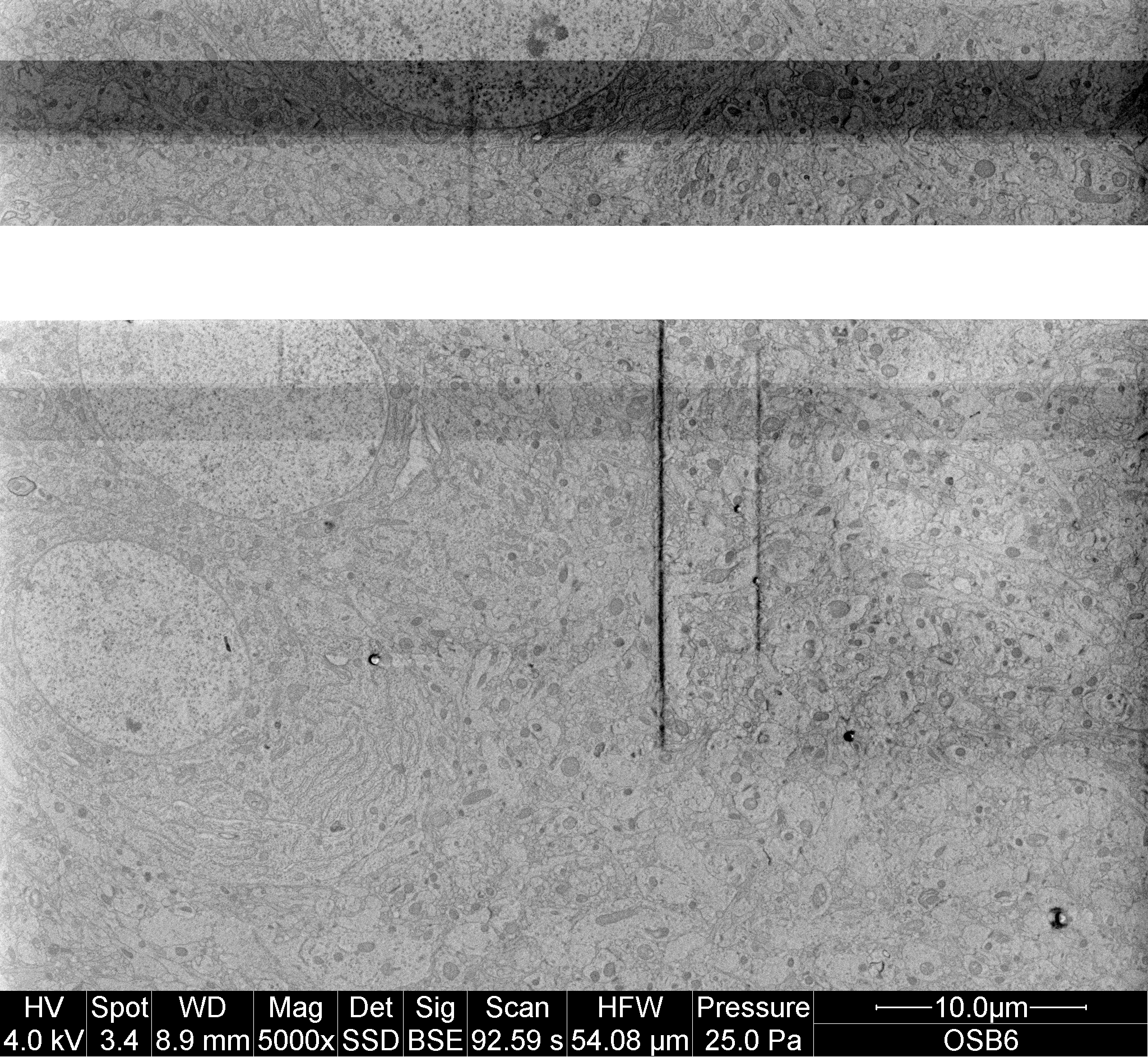

Supplement: Dataset S1 — (248.1 MB ZIP). [file pbio.0020329.sd001.zip › 040604_OS5_st1_001.tif]

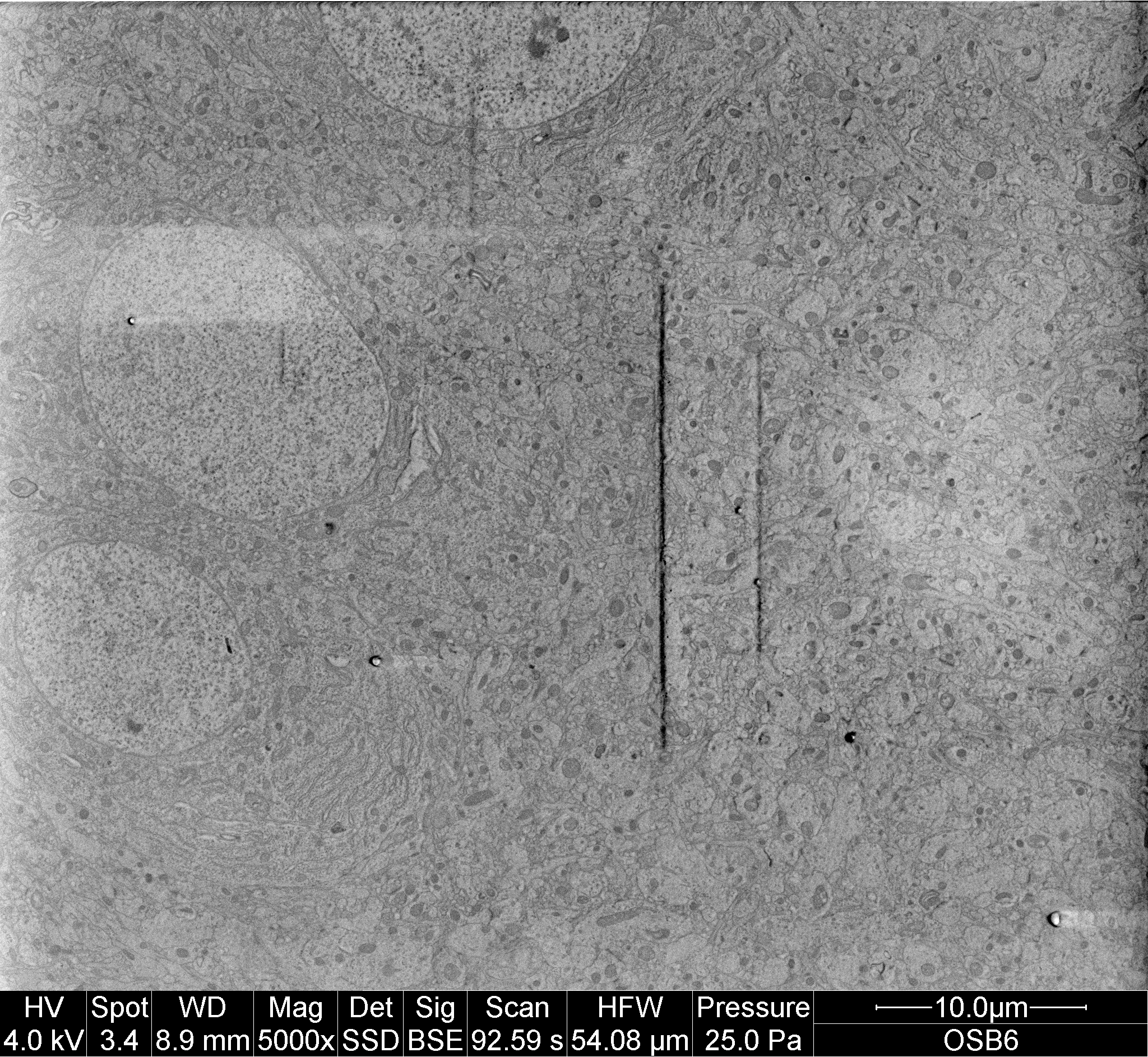

Supplement: Dataset S1 — (248.1 MB ZIP). [file pbio.0020329.sd001.zip › 040604_OS5_st1_002.tif]

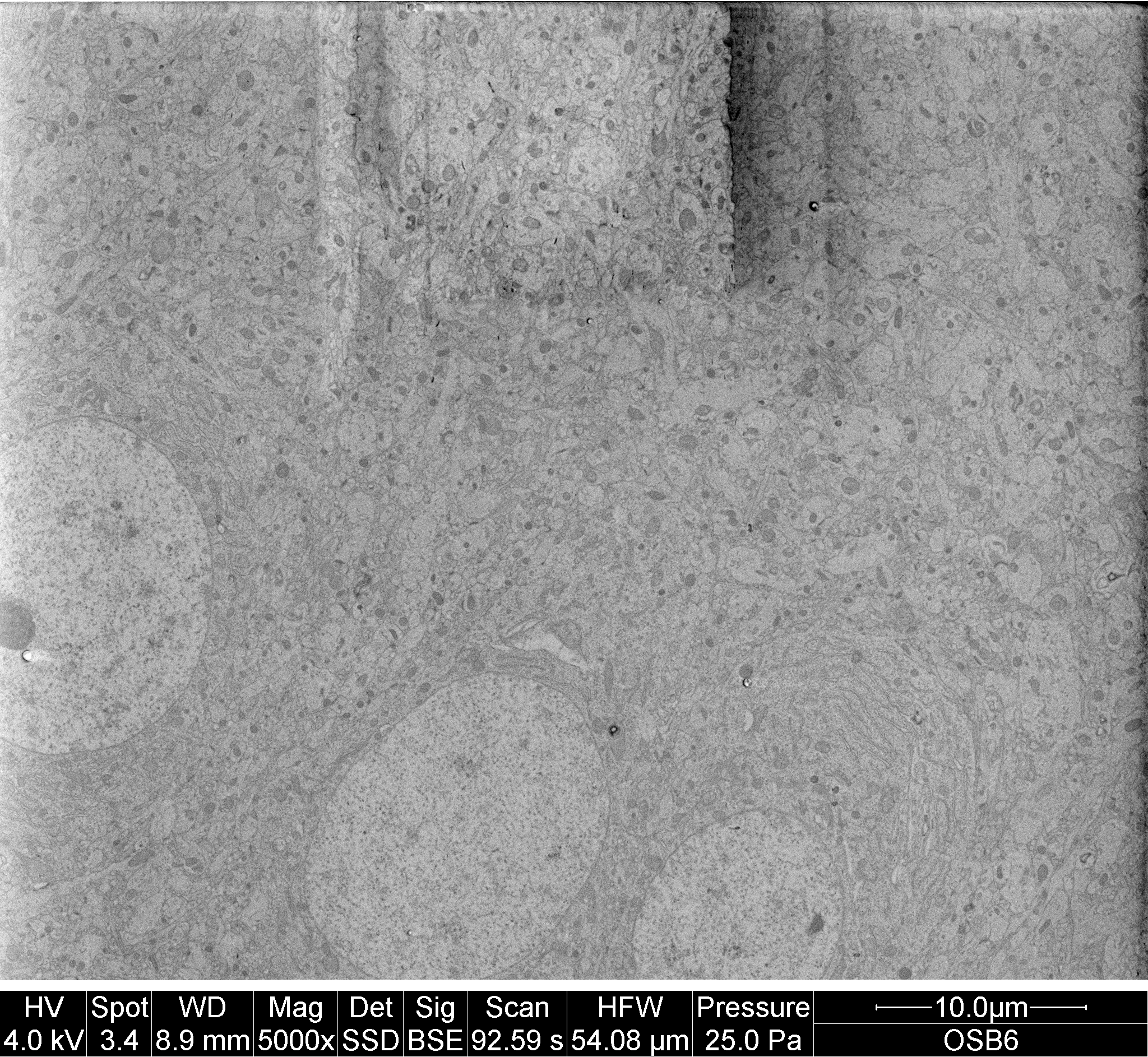

Supplement: Dataset S1 — (248.1 MB ZIP). [file pbio.0020329.sd001.zip › 040604_OS5_st1_003.tif]

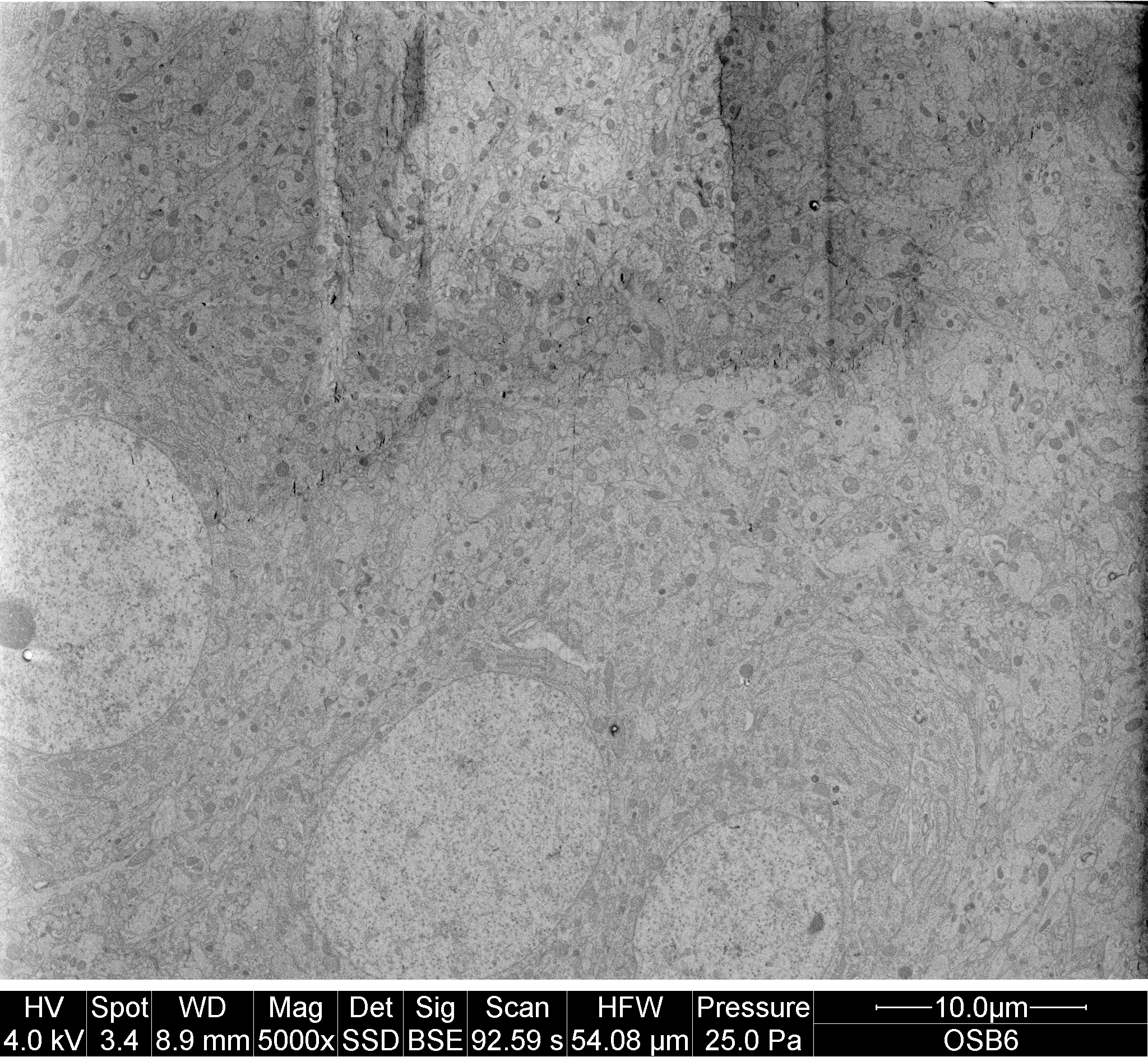

Supplement: Dataset S1 — (248.1 MB ZIP). [file pbio.0020329.sd001.zip › 040604_OS5_st1_004.tif]

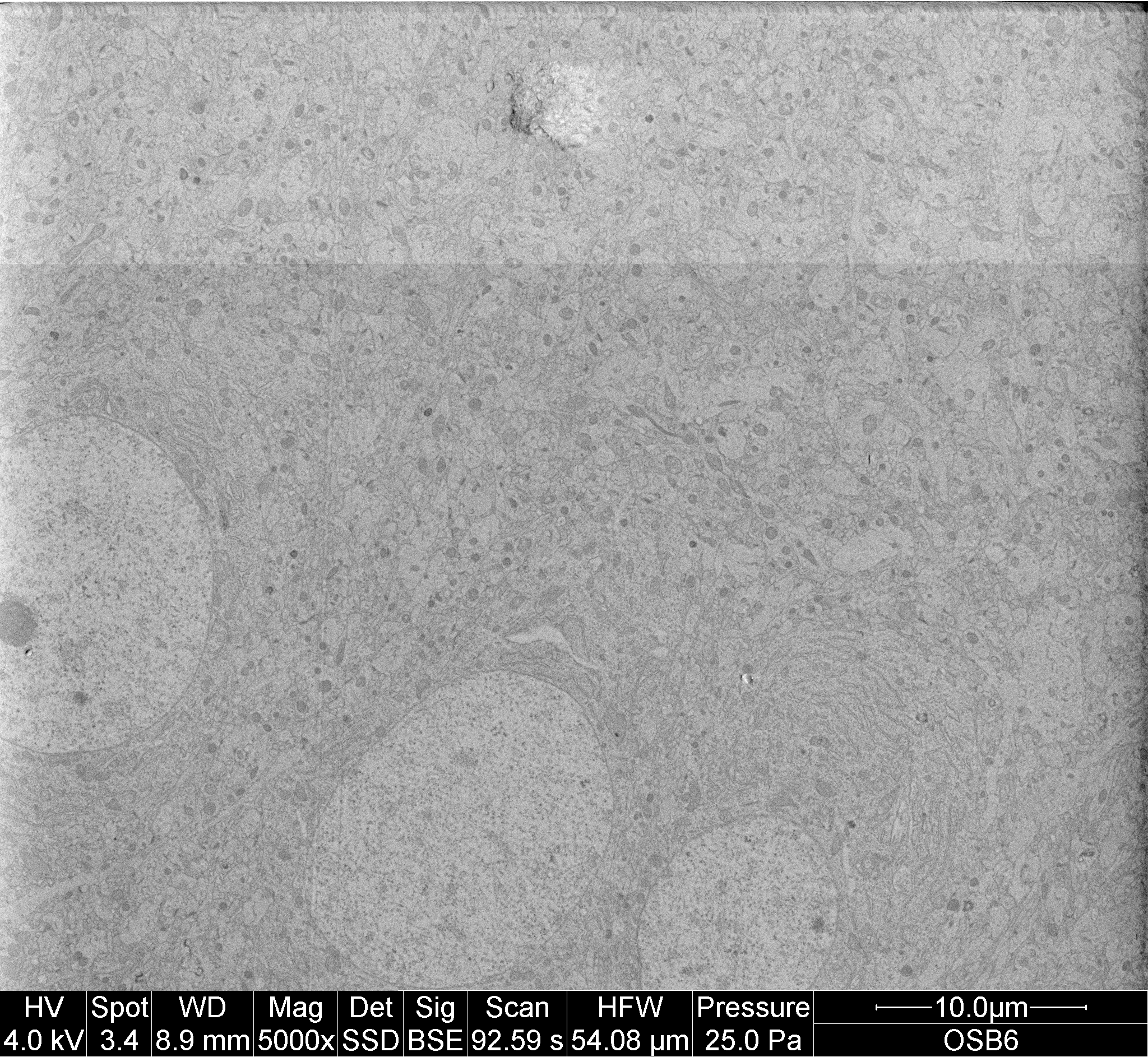

Supplement: Dataset S1 — (248.1 MB ZIP). [file pbio.0020329.sd001.zip › 040604_OS5_st1_005.tif]

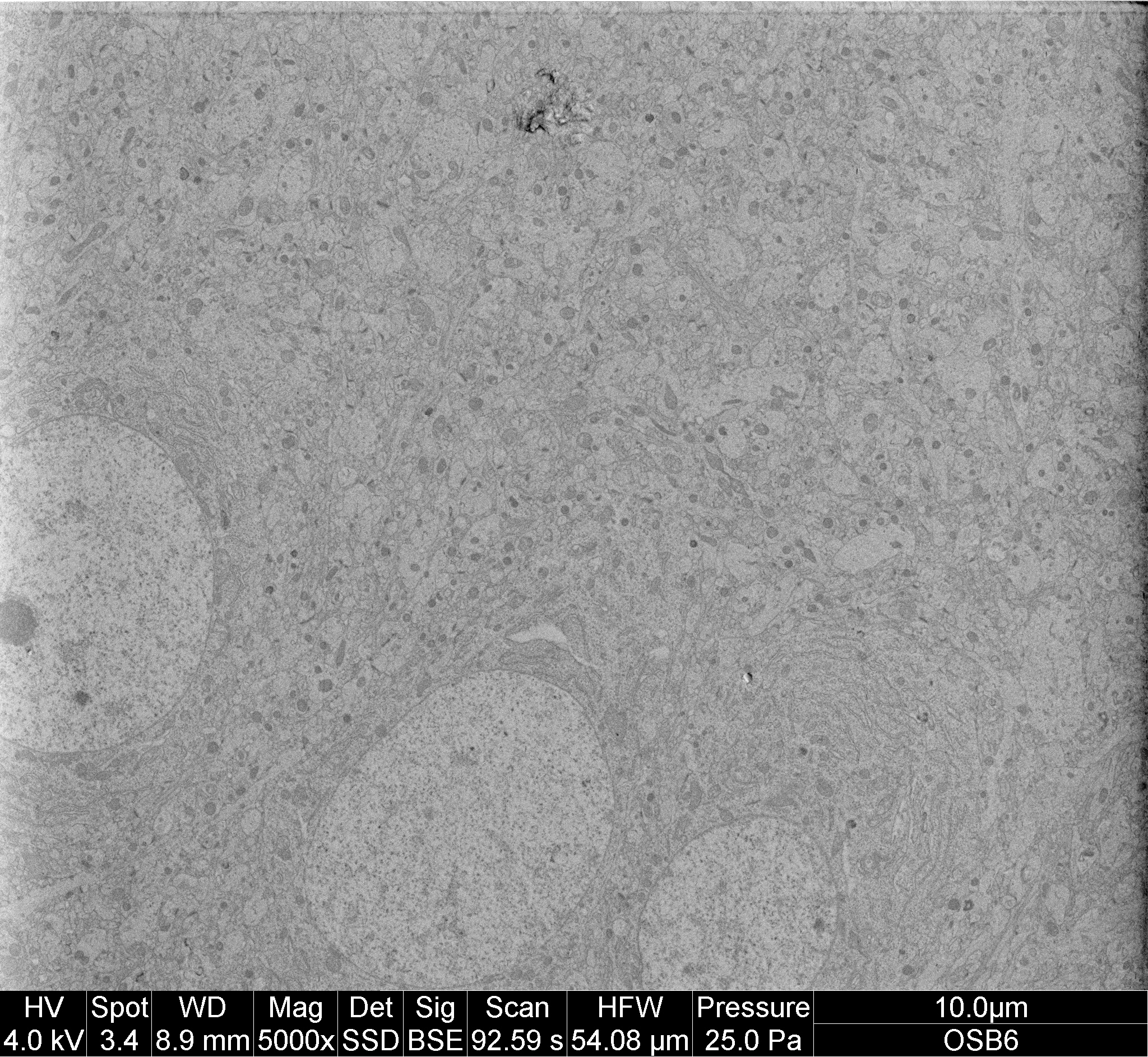

Supplement: Dataset S1 — (248.1 MB ZIP). [file pbio.0020329.sd001.zip › 040604_OS5_st1_006.tif]

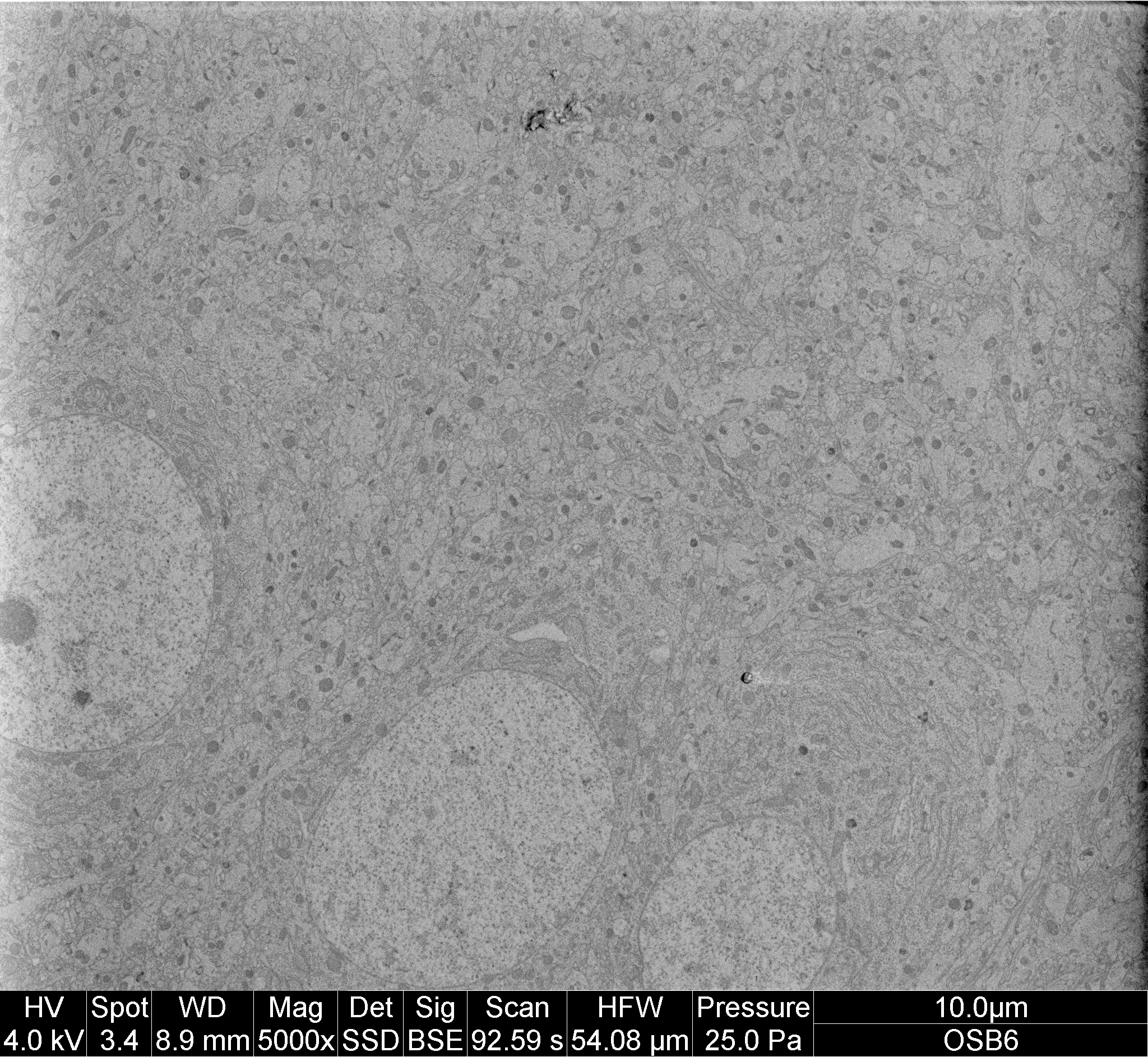

Supplement: Dataset S1 — (248.1 MB ZIP). [file pbio.0020329.sd001.zip › 040604_OS5_st1_007.tif]

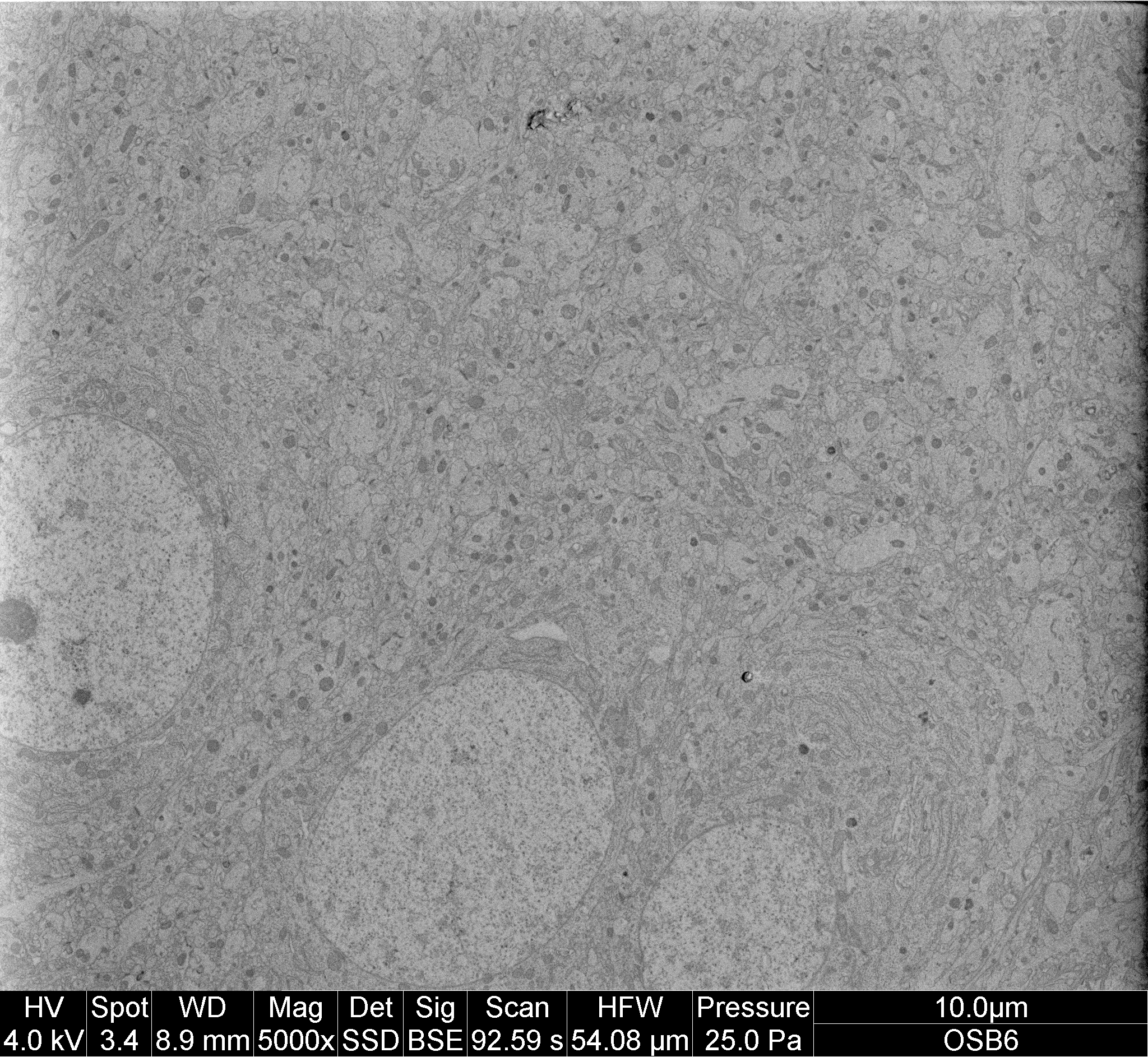

Supplement: Dataset S1 — (248.1 MB ZIP). [file pbio.0020329.sd001.zip › 040604_OS5_st1_008.tif]

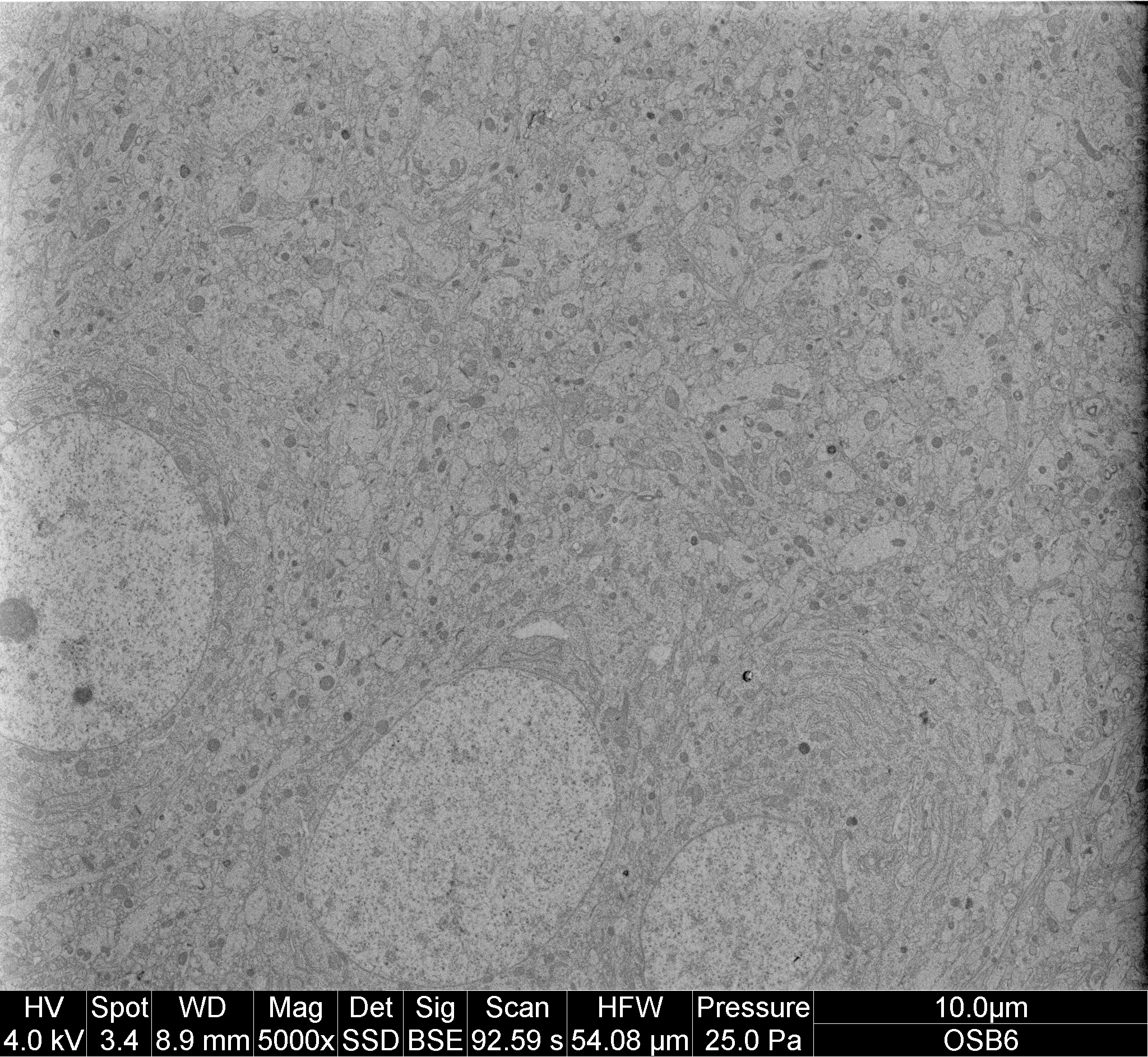

Supplement: Dataset S1 — (248.1 MB ZIP). [file pbio.0020329.sd001.zip › 040604_OS5_st1_009.tif]

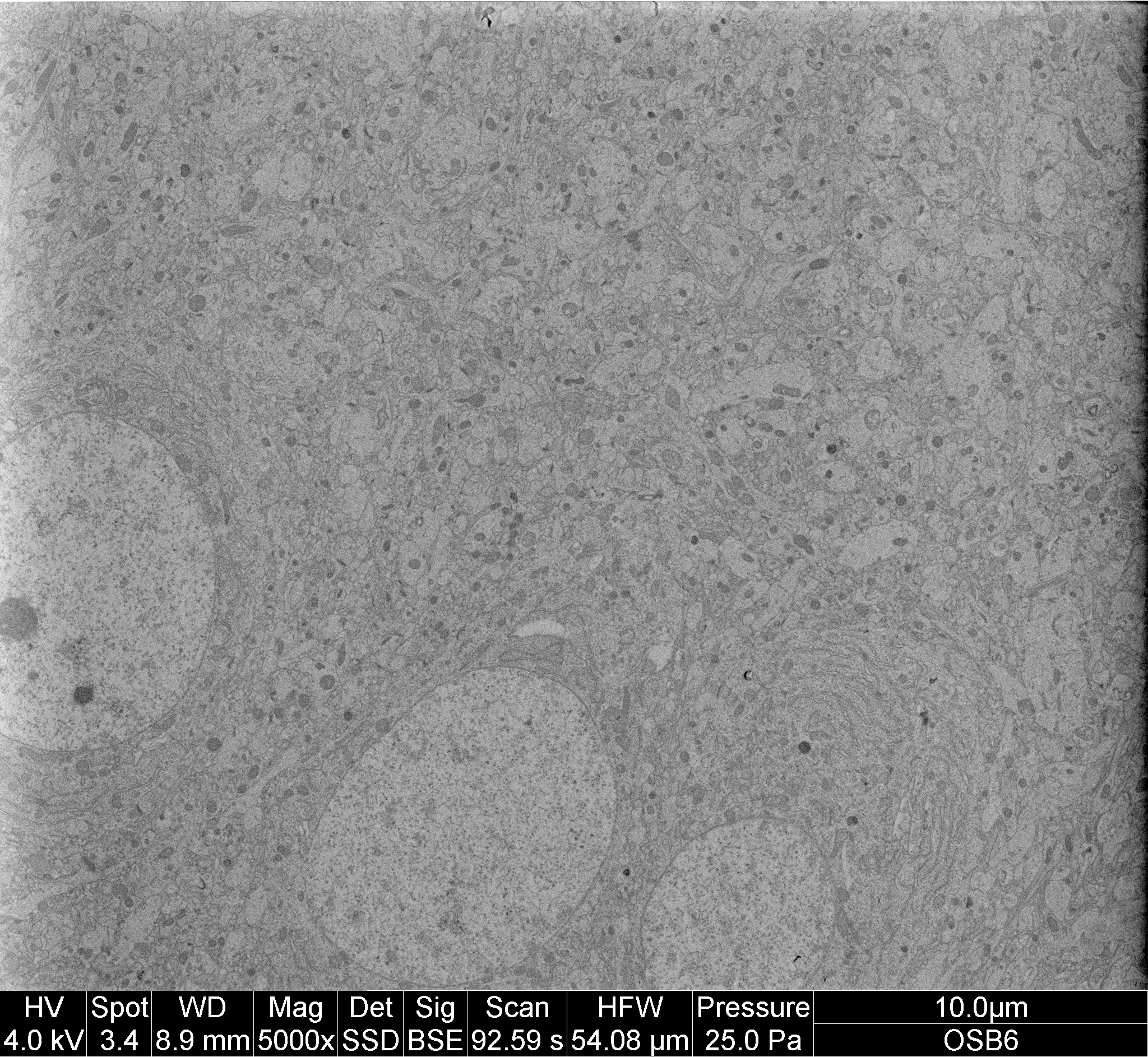

Supplement: Dataset S1 — (248.1 MB ZIP). [file pbio.0020329.sd001.zip › 040604_OS5_st1_010.tif]

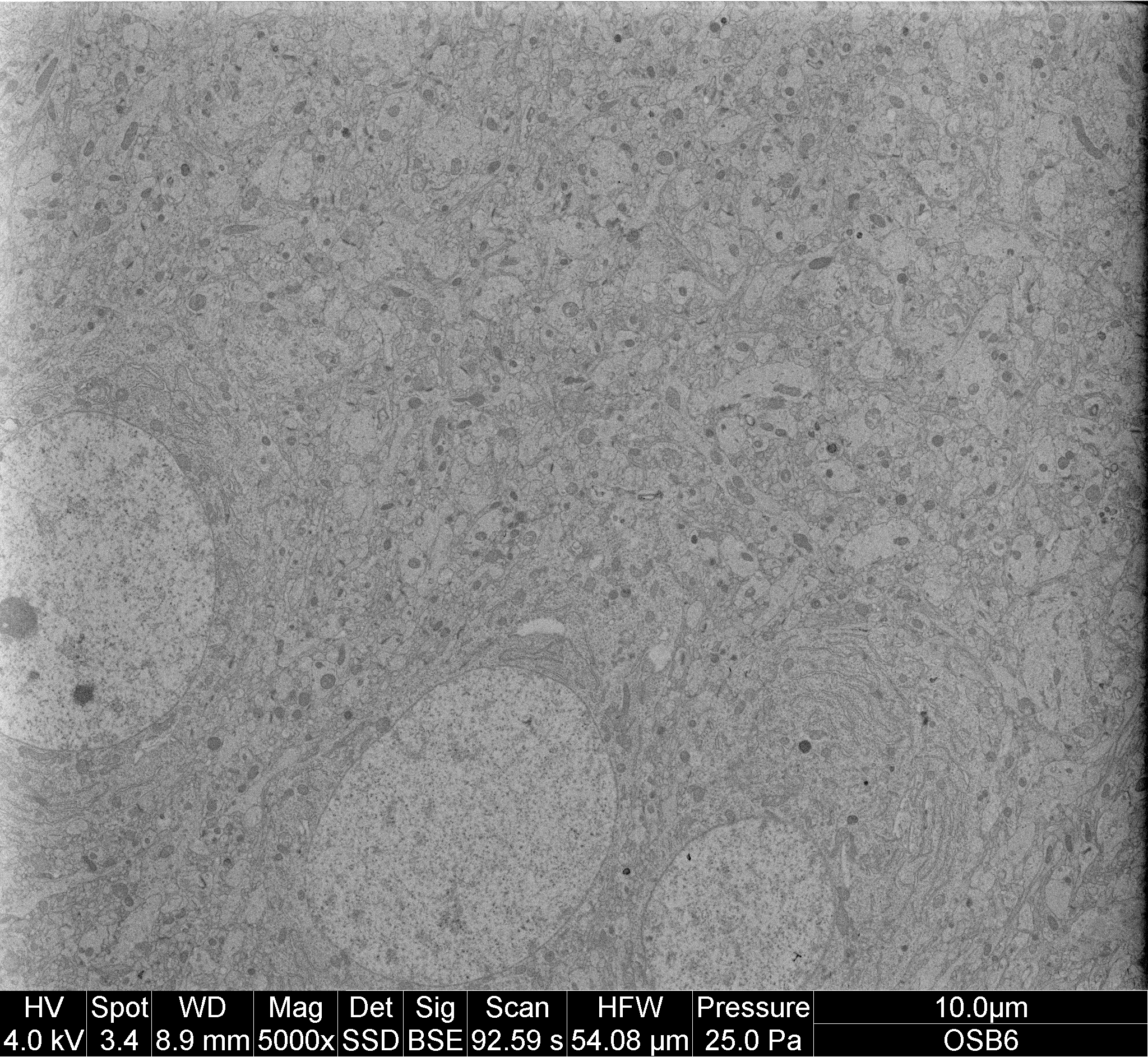

Supplement: Dataset S1 — (248.1 MB ZIP). [file pbio.0020329.sd001.zip › 040604_OS5_st1_011.tif]

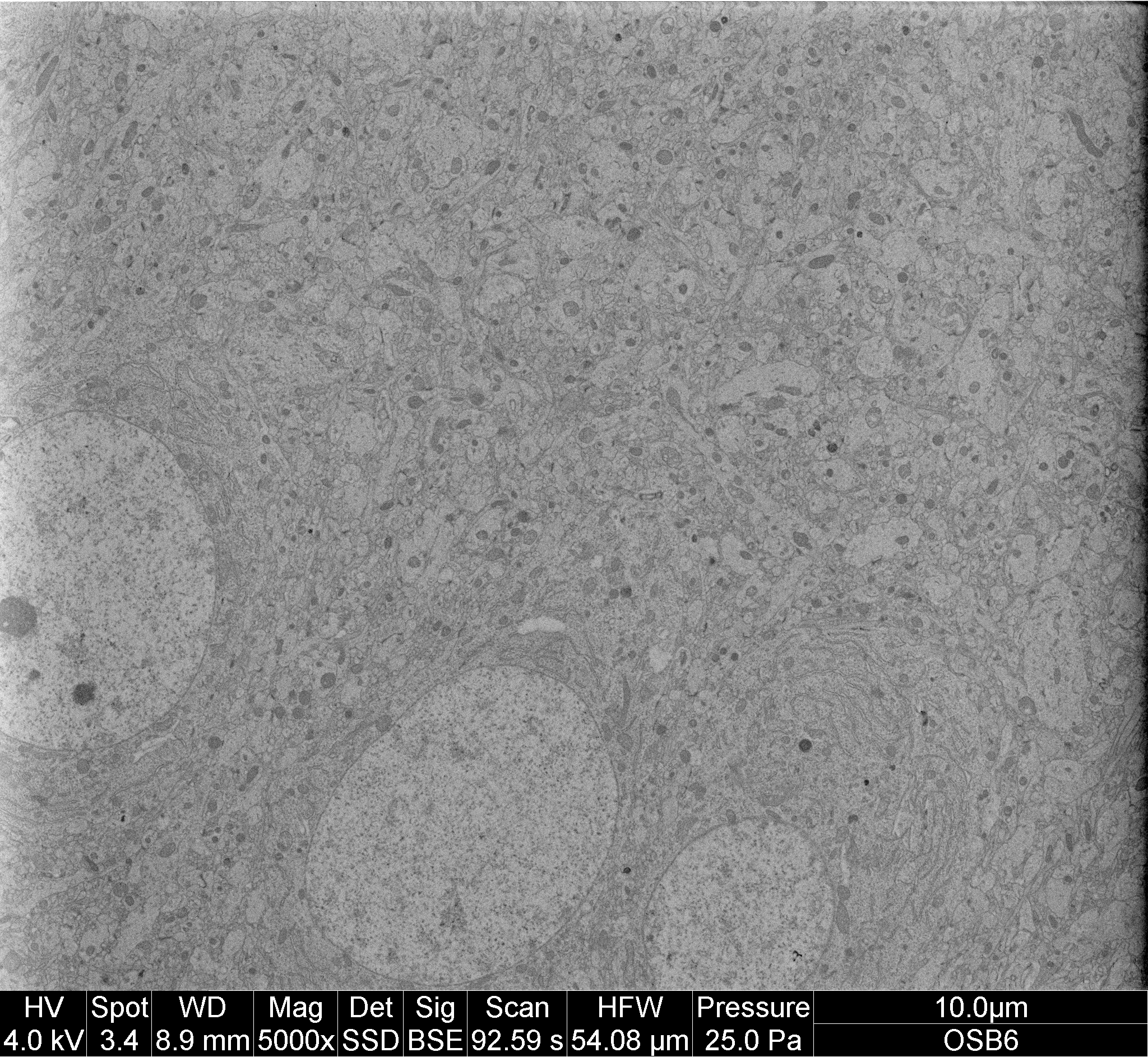

Supplement: Dataset S1 — (248.1 MB ZIP). [file pbio.0020329.sd001.zip › 040604_OS5_st1_012.tif]

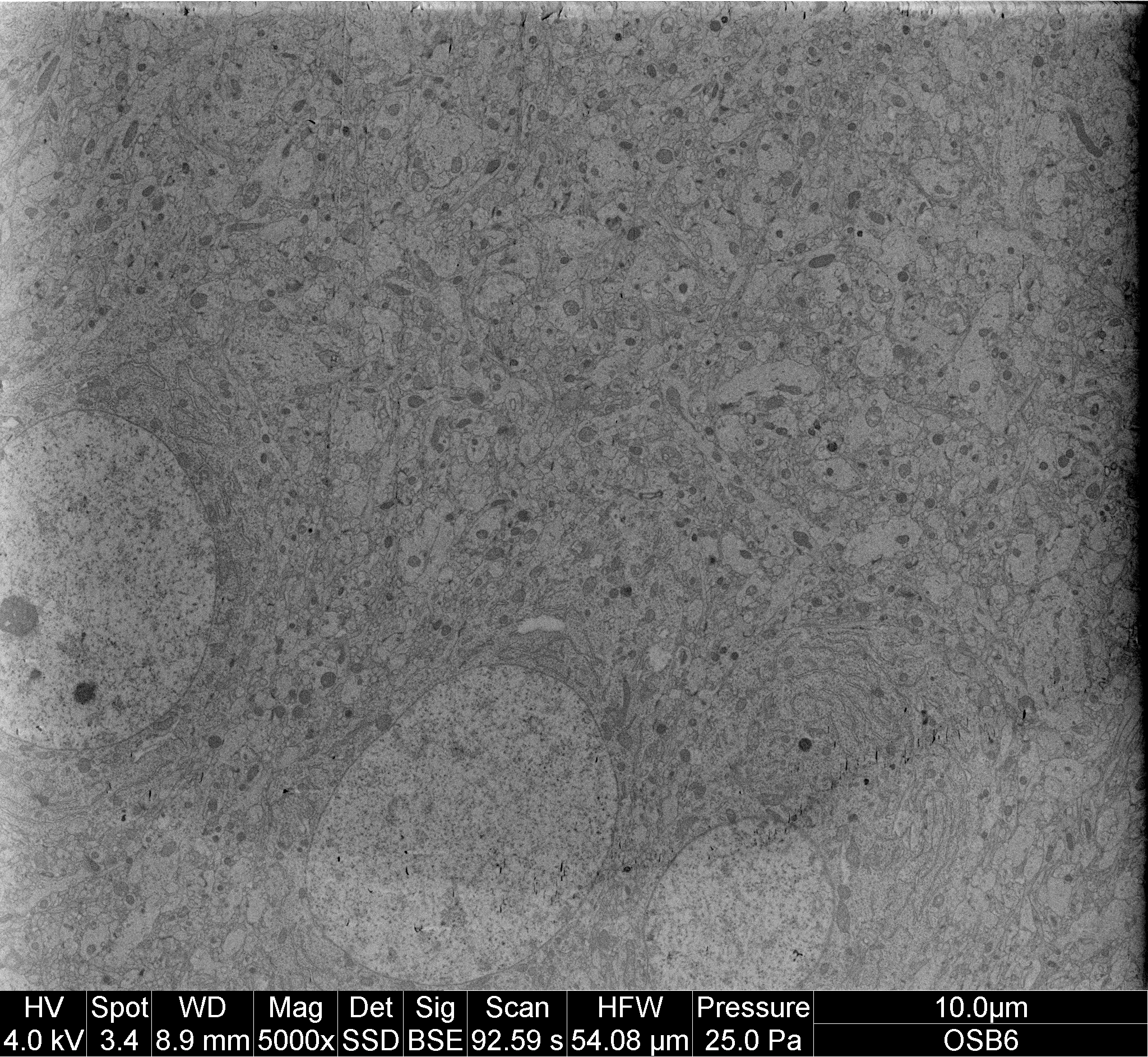

Supplement: Dataset S1 — (248.1 MB ZIP). [file pbio.0020329.sd001.zip › 040604_OS5_st1_013.tif]

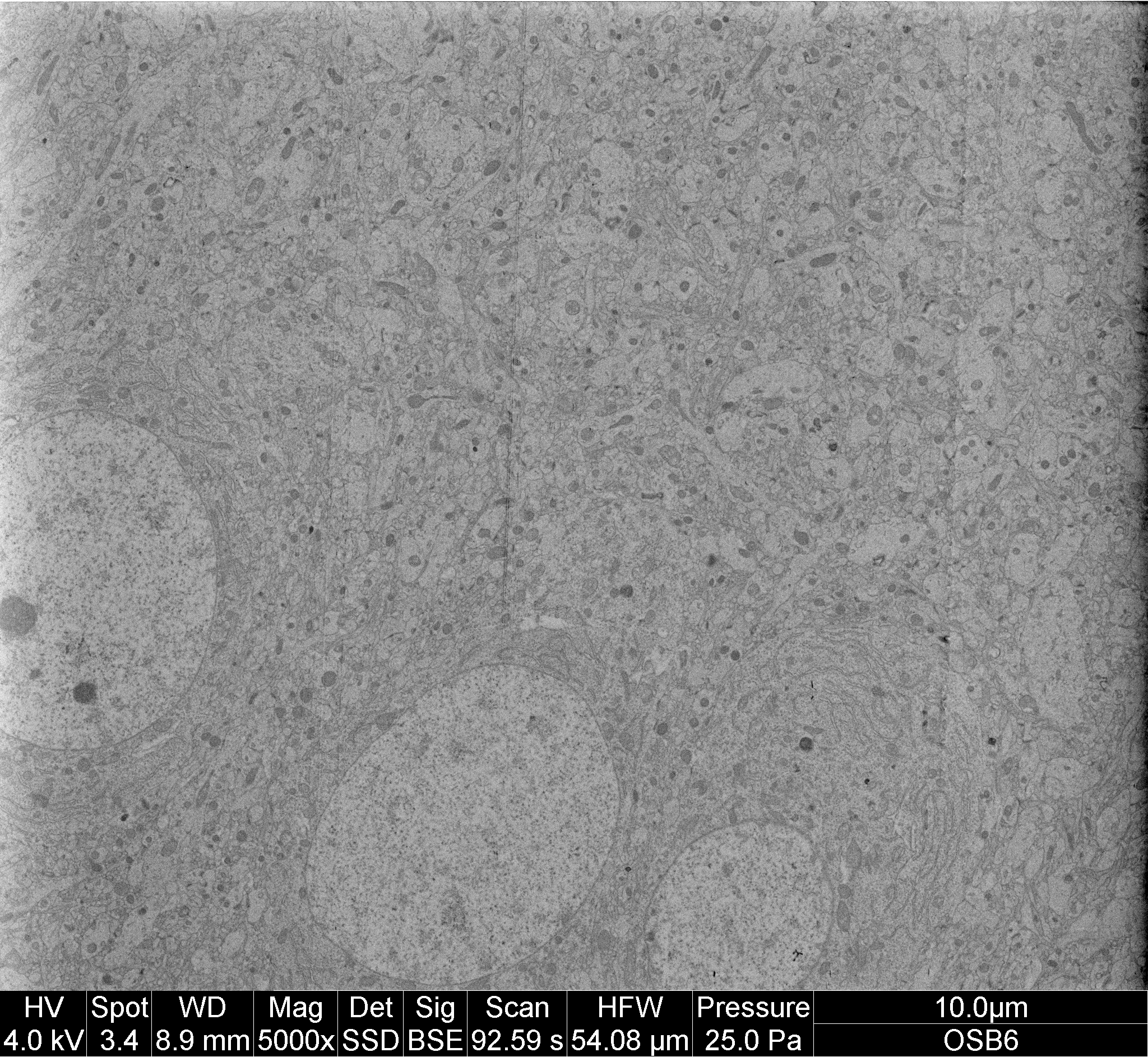

Supplement: Dataset S1 — (248.1 MB ZIP). [file pbio.0020329.sd001.zip › 040604_OS5_st1_014.tif]

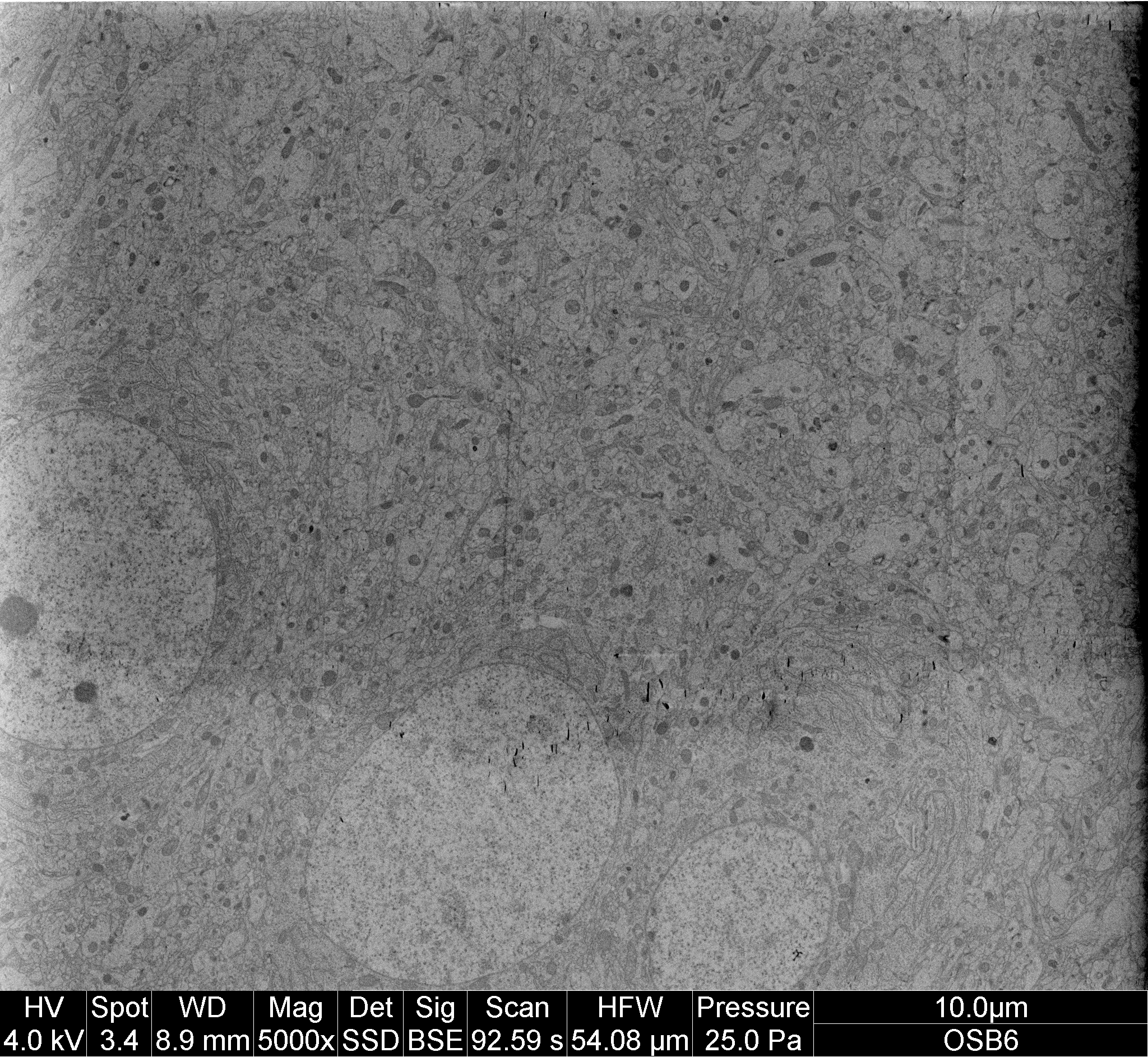

Supplement: Dataset S1 — (248.1 MB ZIP). [file pbio.0020329.sd001.zip › 040604_OS5_st1_015.tif]

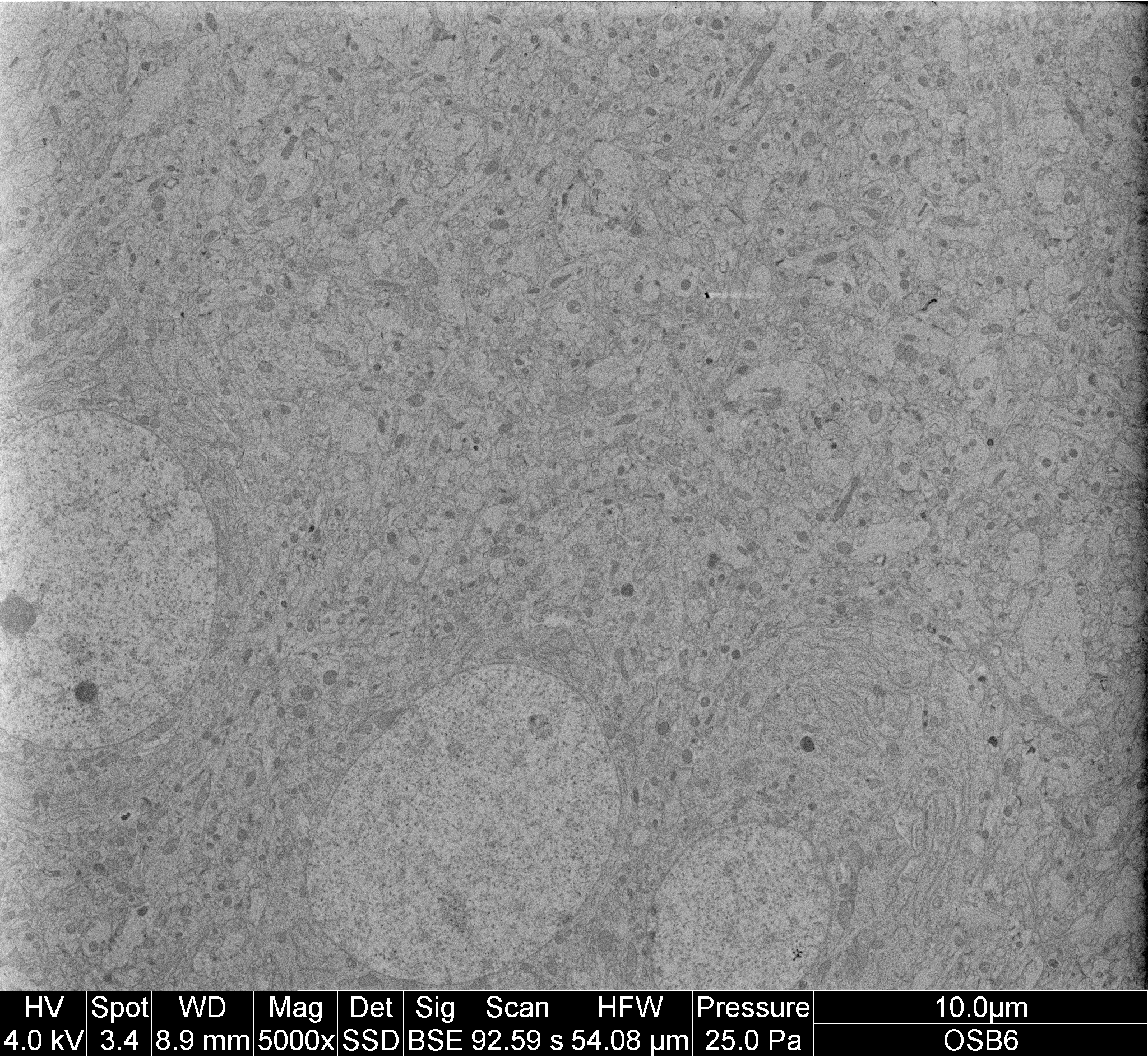

Supplement: Dataset S1 — (248.1 MB ZIP). [file pbio.0020329.sd001.zip › 040604_OS5_st1_016.tif]

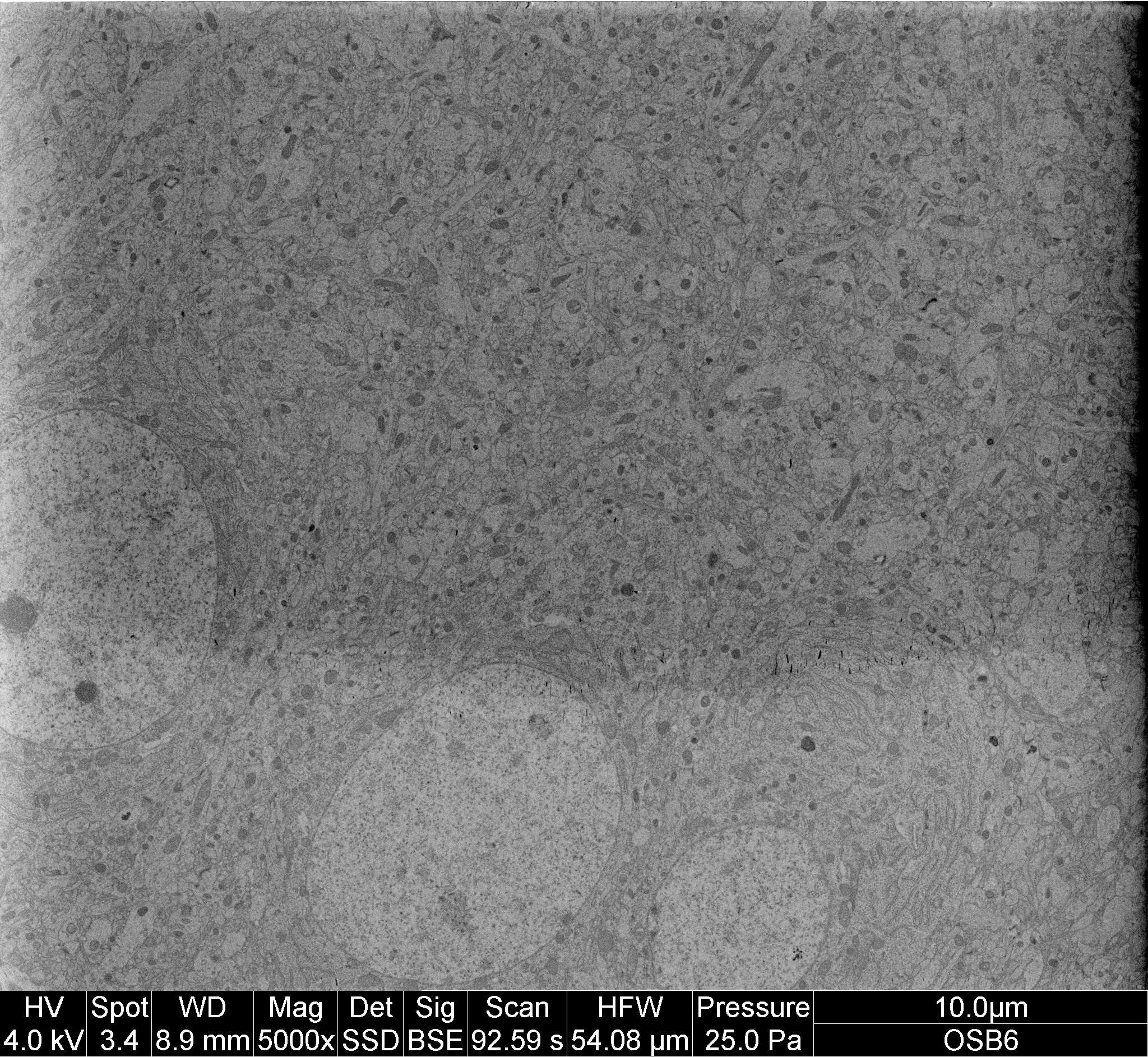

Supplement: Dataset S1 — (248.1 MB ZIP). [file pbio.0020329.sd001.zip › 040604_OS5_st1_017.tif]

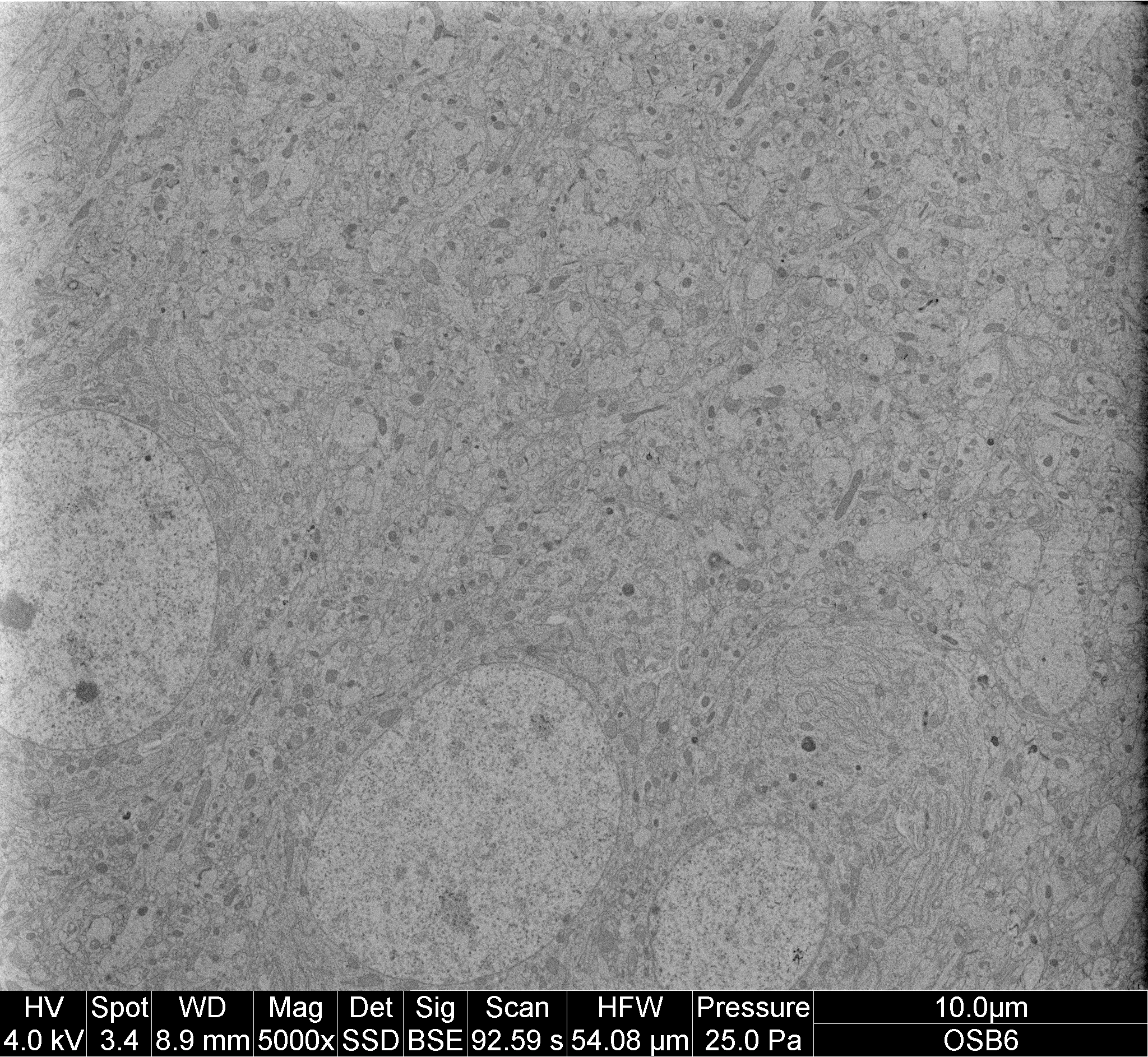

Supplement: Dataset S1 — (248.1 MB ZIP). [file pbio.0020329.sd001.zip › 040604_OS5_st1_018.tif]

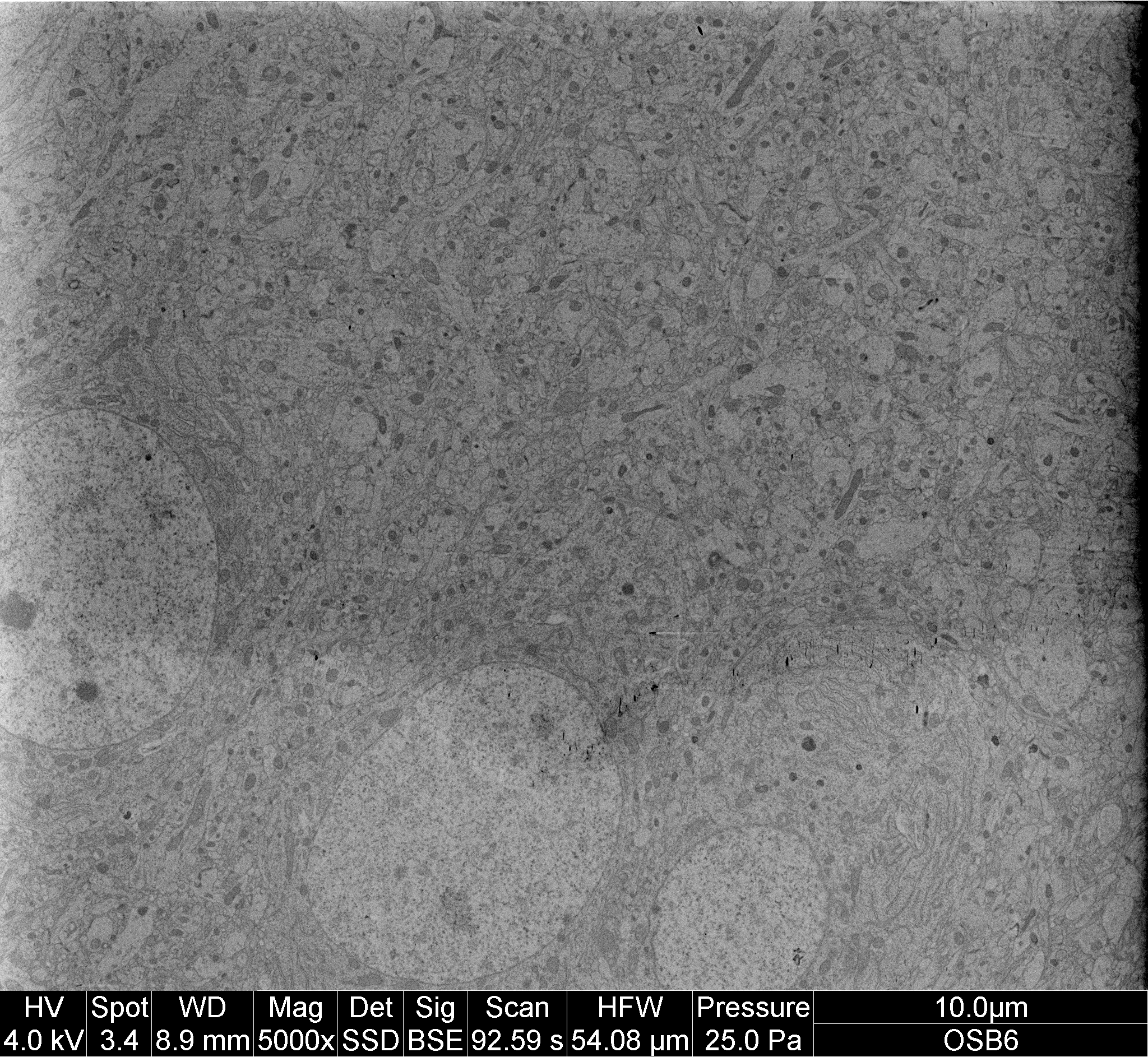

Supplement: Dataset S1 — (248.1 MB ZIP). [file pbio.0020329.sd001.zip › 040604_OS5_st1_019.tif]

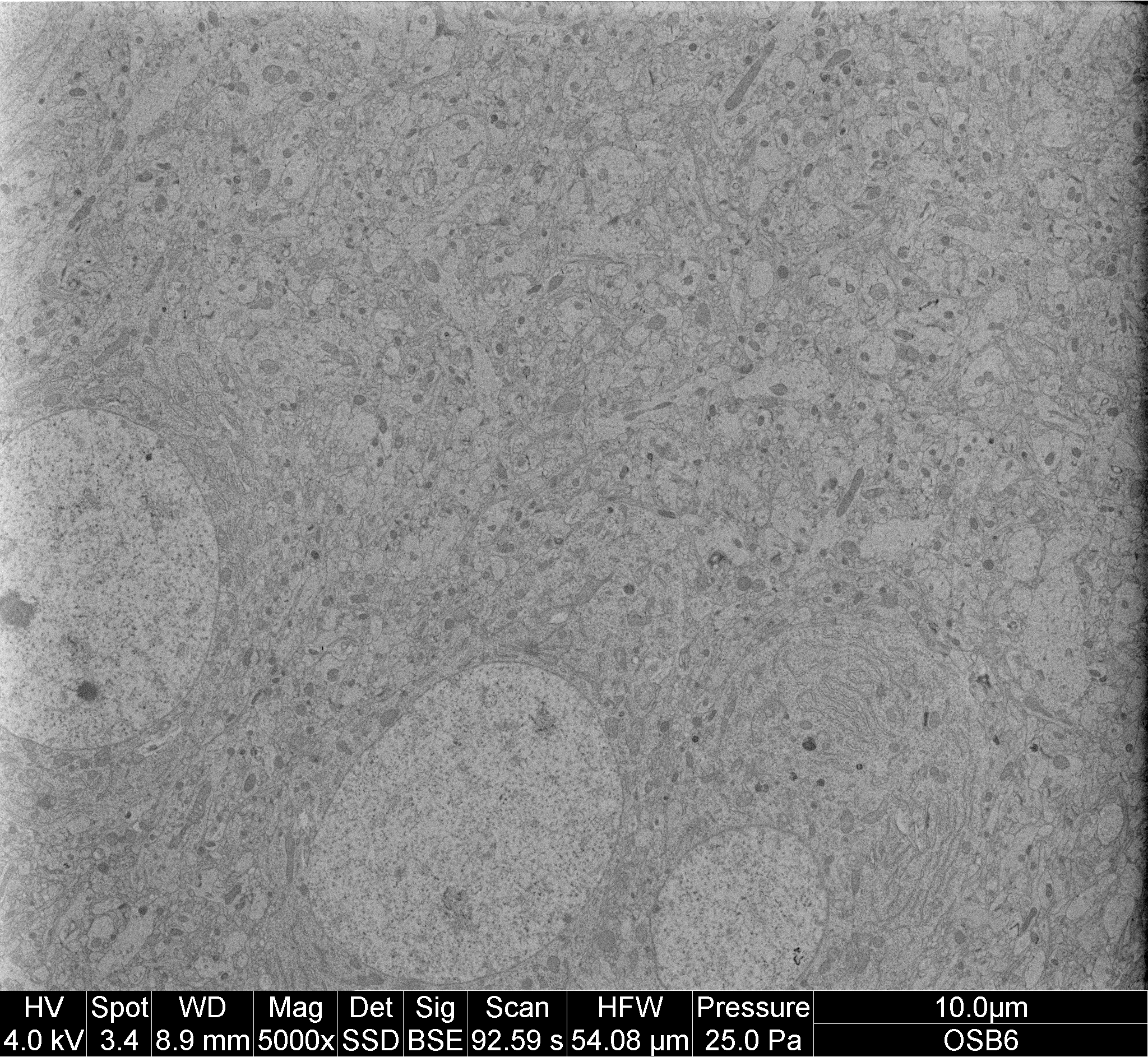

Supplement: Dataset S1 — (248.1 MB ZIP). [file pbio.0020329.sd001.zip › 040604_OS5_st1_020.tif]

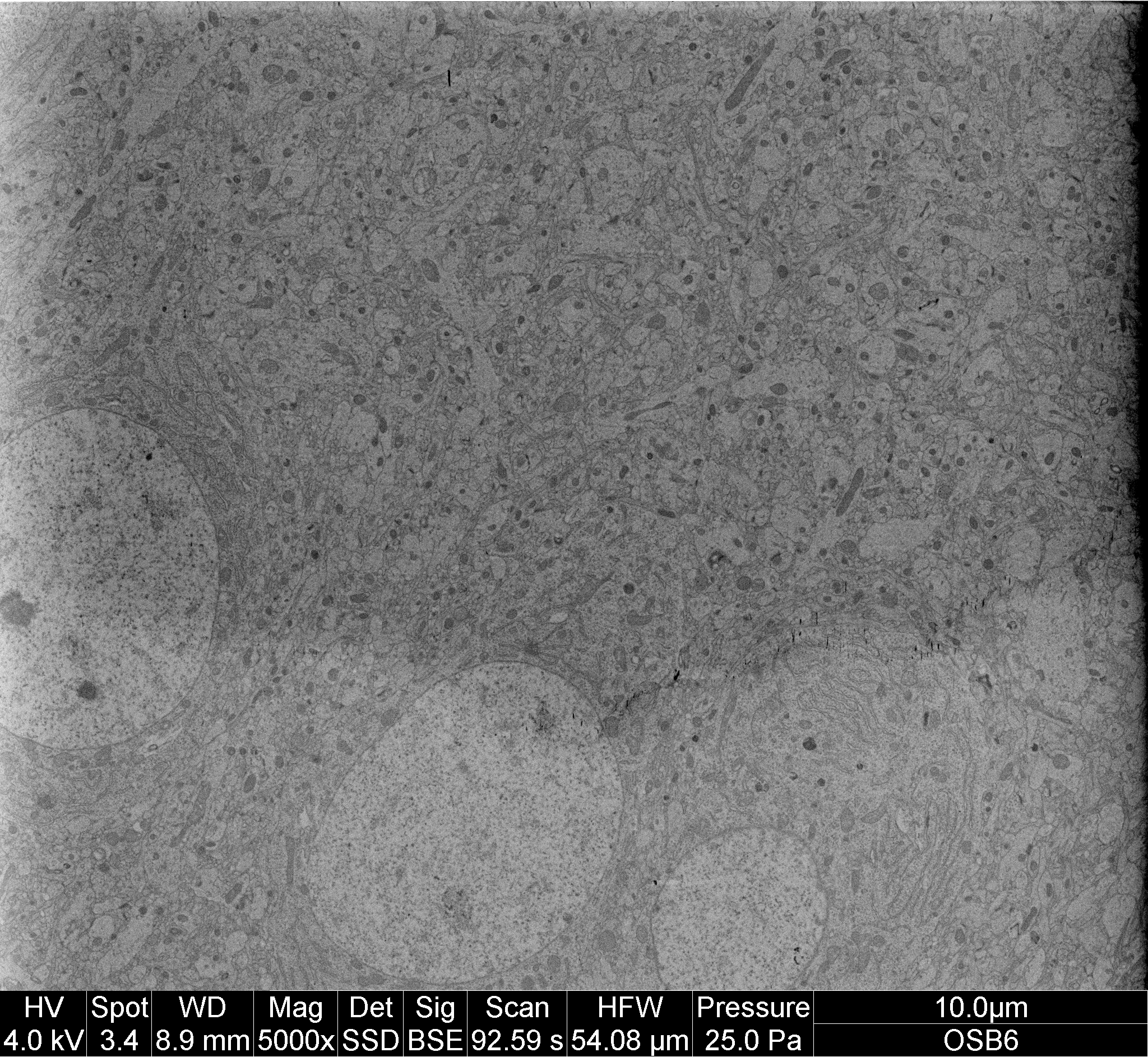

Supplement: Dataset S1 — (248.1 MB ZIP). [file pbio.0020329.sd001.zip › 040604_OS5_st1_021.tif]

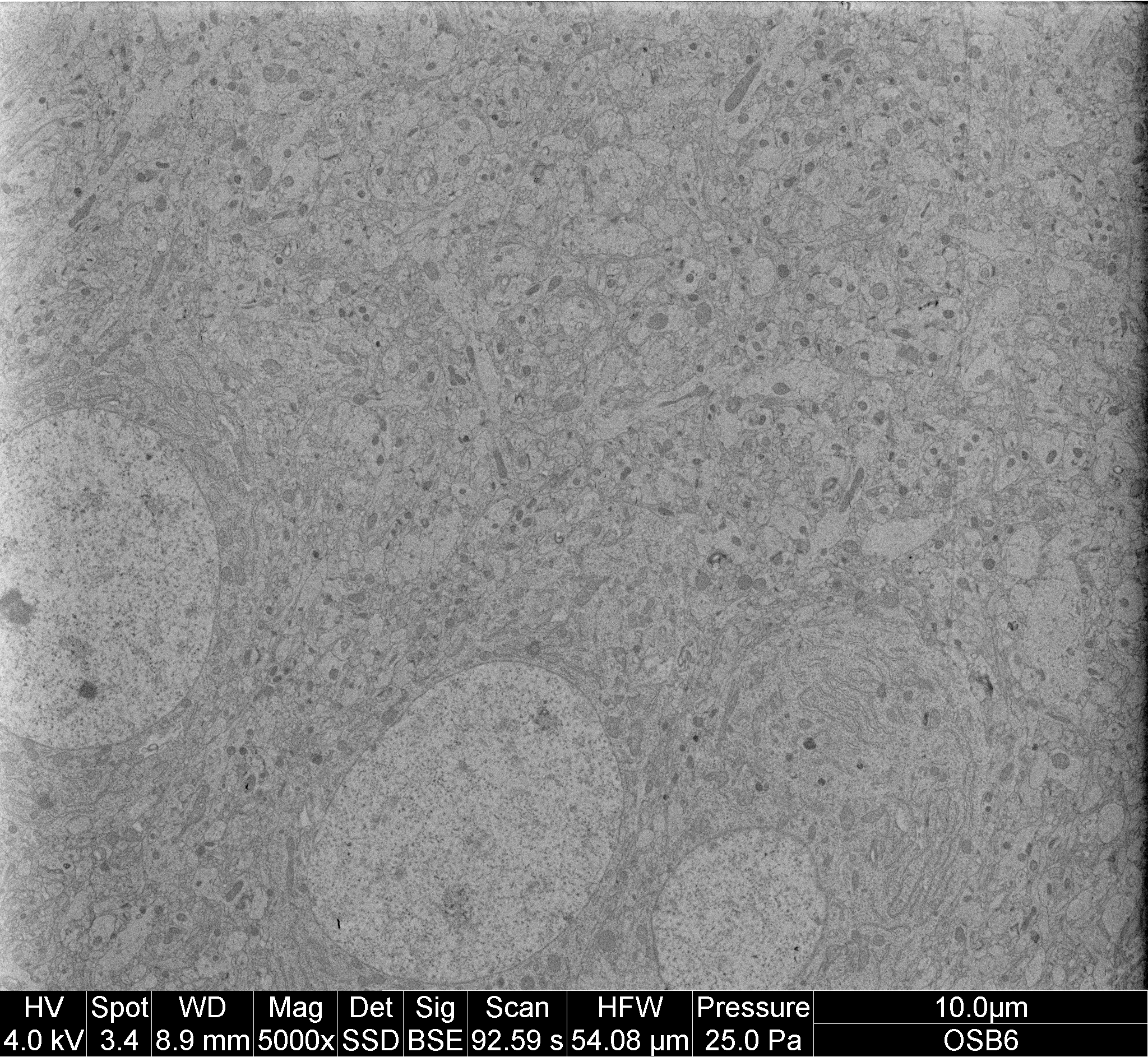

Supplement: Dataset S1 — (248.1 MB ZIP). [file pbio.0020329.sd001.zip › 040604_OS5_st1_022.tif]

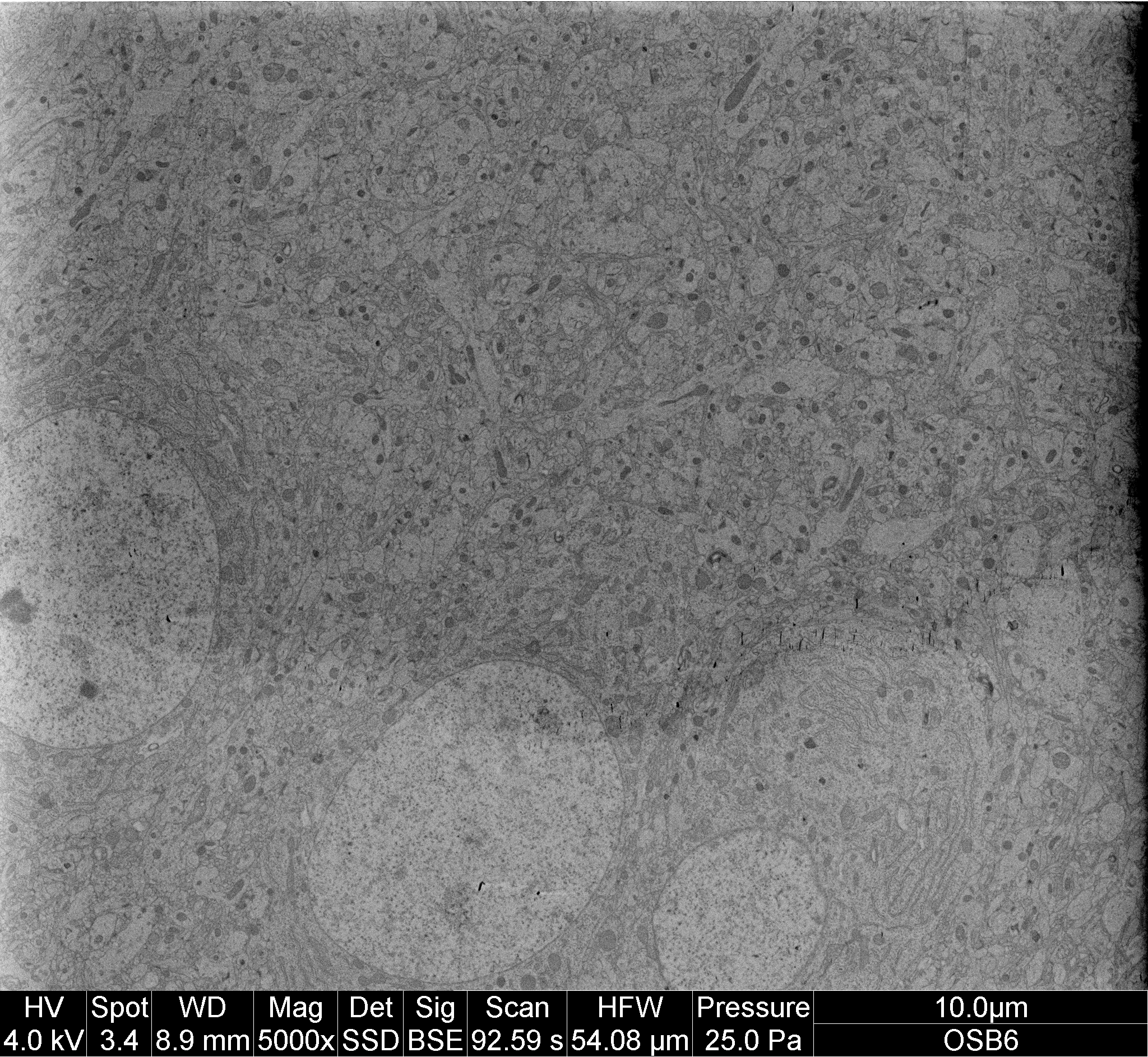

Supplement: Dataset S1 — (248.1 MB ZIP). [file pbio.0020329.sd001.zip › 040604_OS5_st1_023.tif]

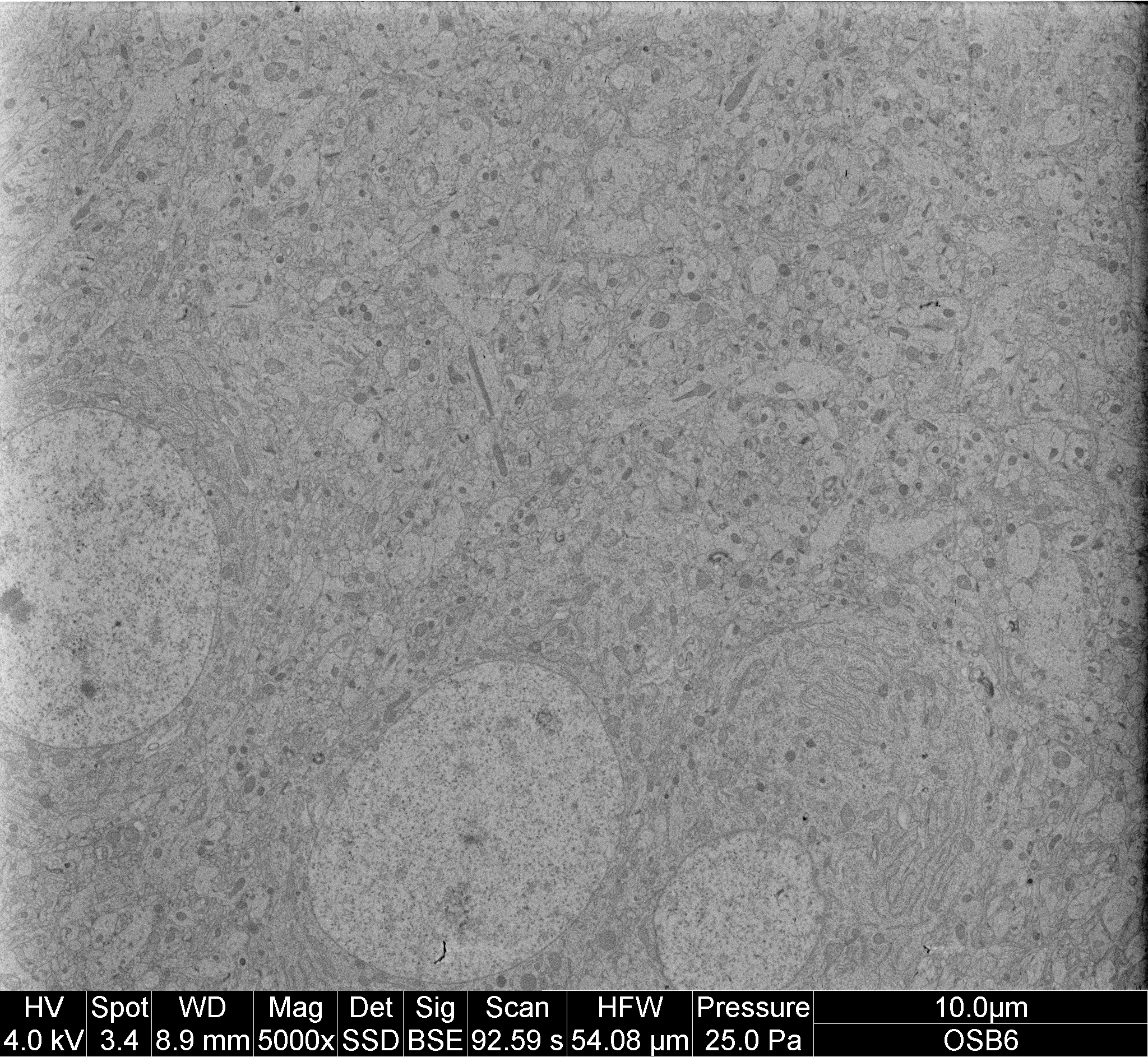

Supplement: Dataset S1 — (248.1 MB ZIP). [file pbio.0020329.sd001.zip › 040604_OS5_st1_024.tif]

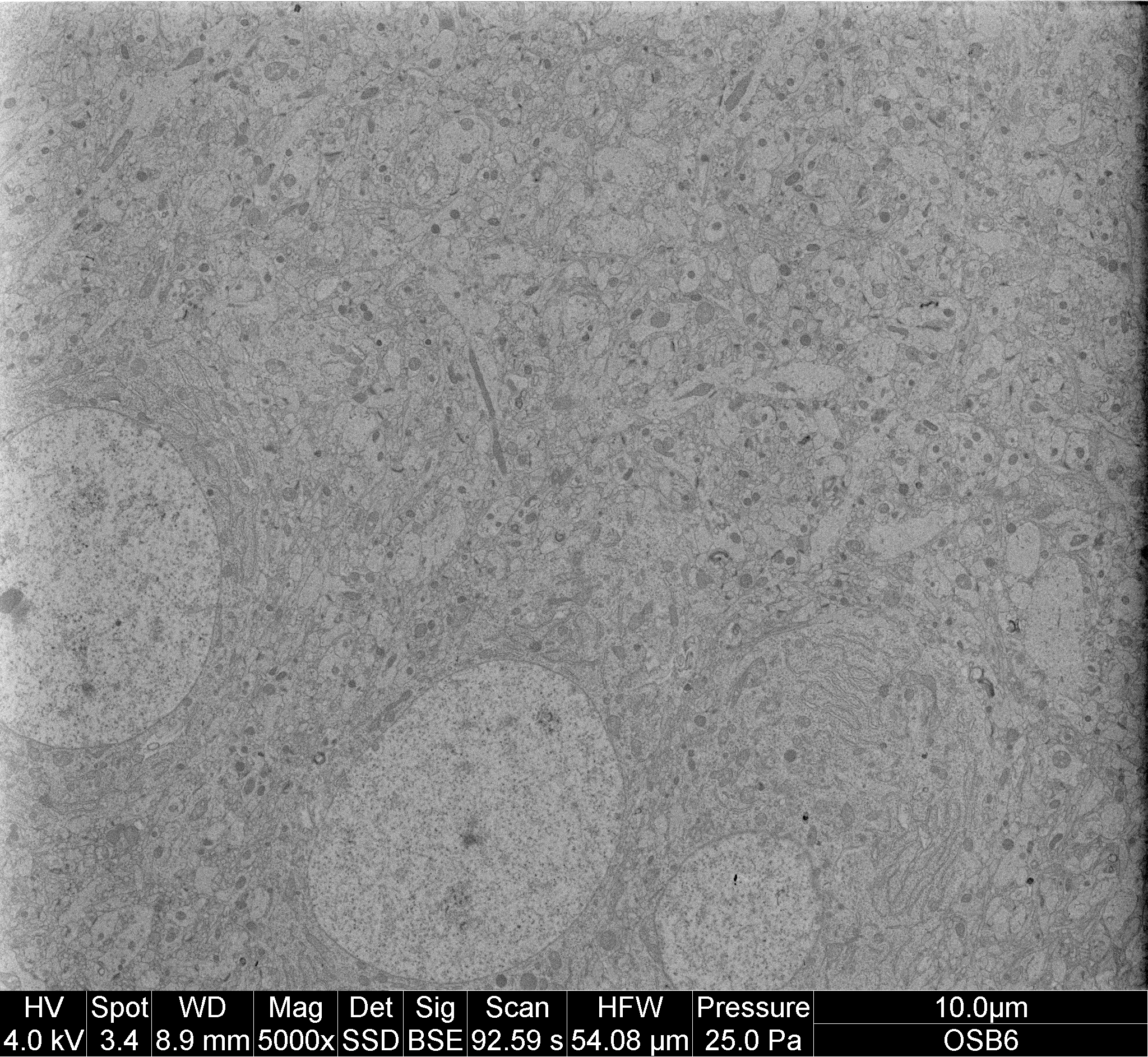

Supplement: Dataset S1 — (248.1 MB ZIP). [file pbio.0020329.sd001.zip › 040604_OS5_st1_025.tif]

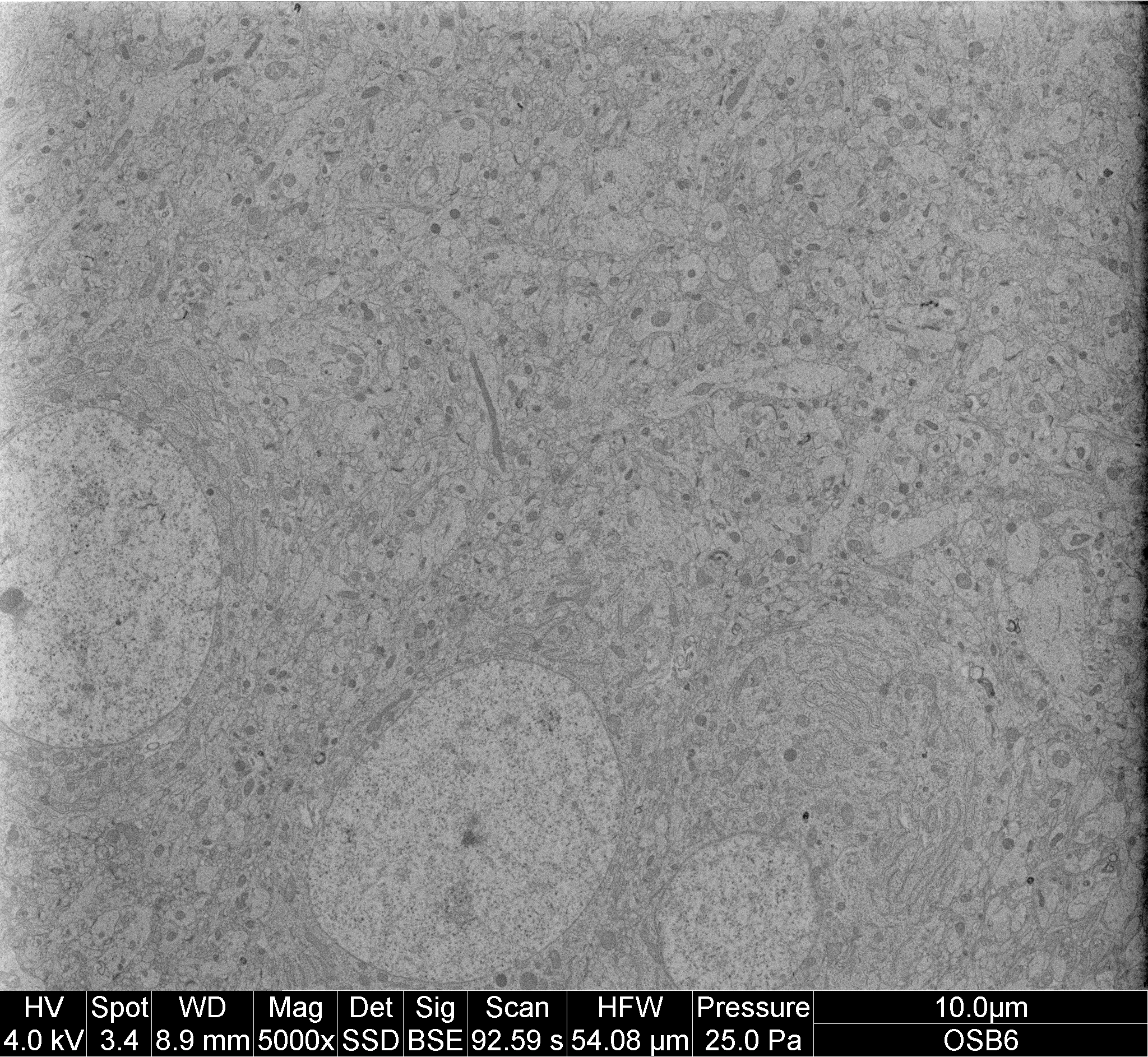

Supplement: Dataset S1 — (248.1 MB ZIP). [file pbio.0020329.sd001.zip › 040604_OS5_st1_026.tif]

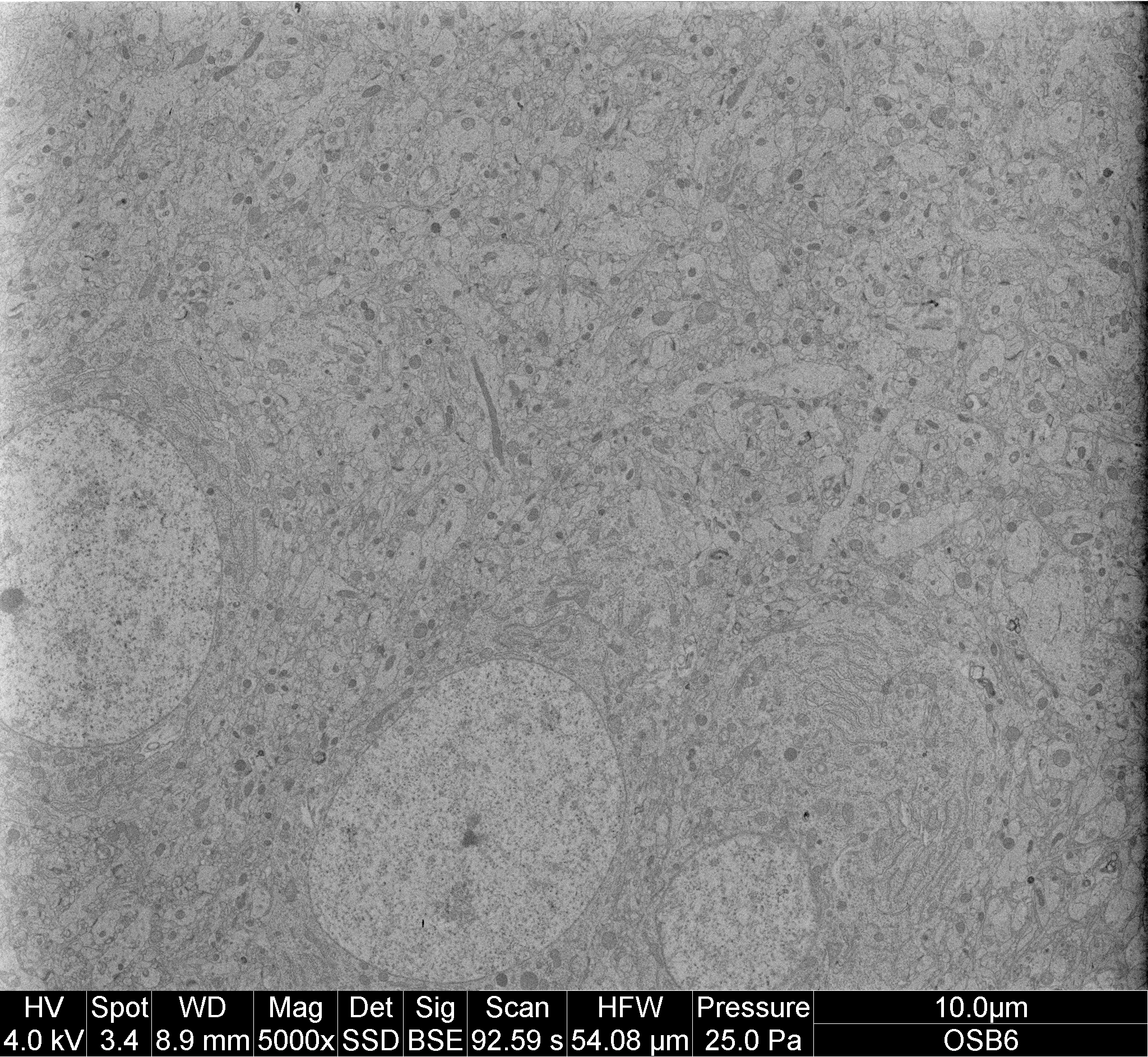

Supplement: Dataset S1 — (248.1 MB ZIP). [file pbio.0020329.sd001.zip › 040604_OS5_st1_027.tif]

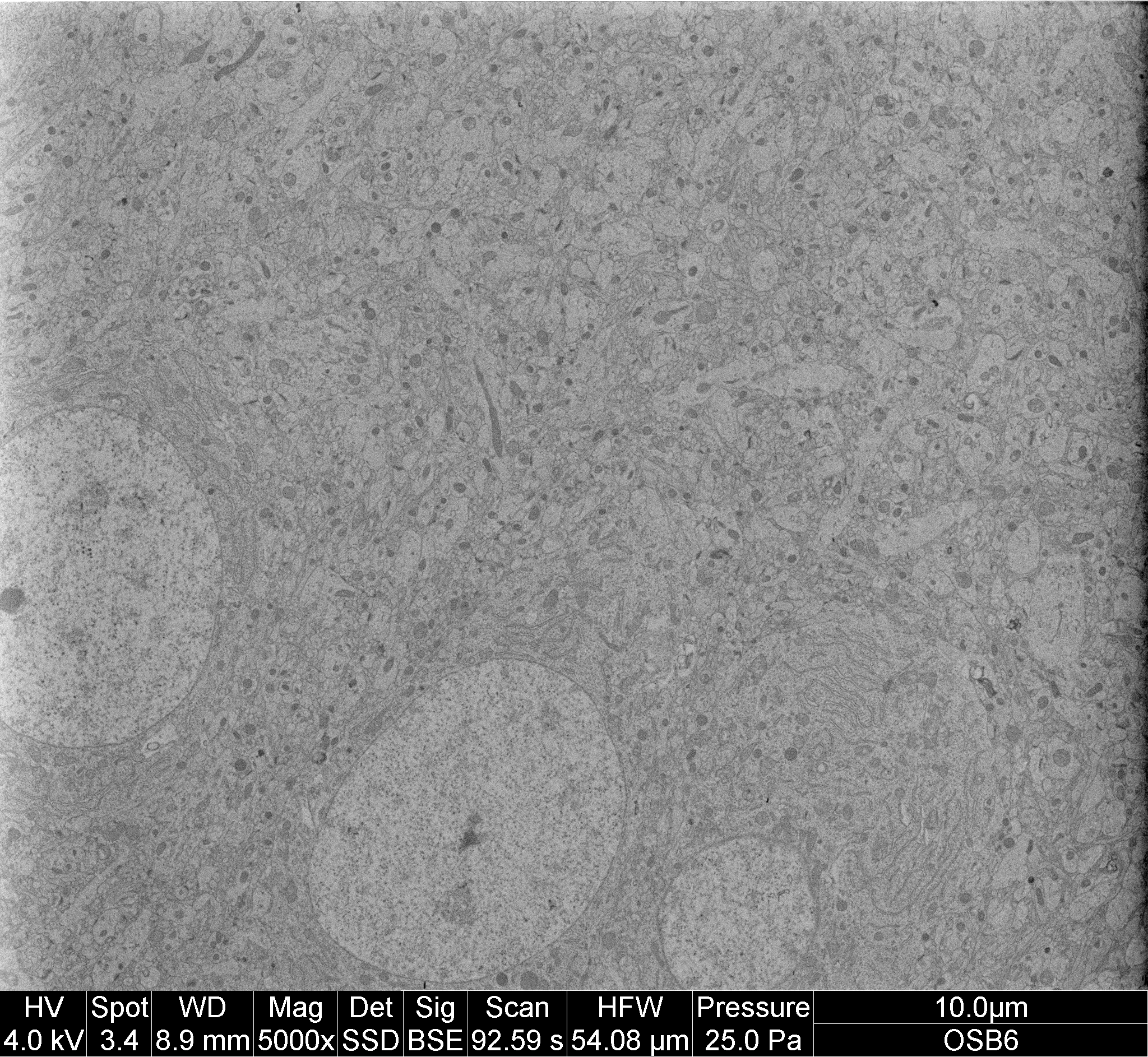

Supplement: Dataset S1 — (248.1 MB ZIP). [file pbio.0020329.sd001.zip › 040604_OS5_st1_028.tif]

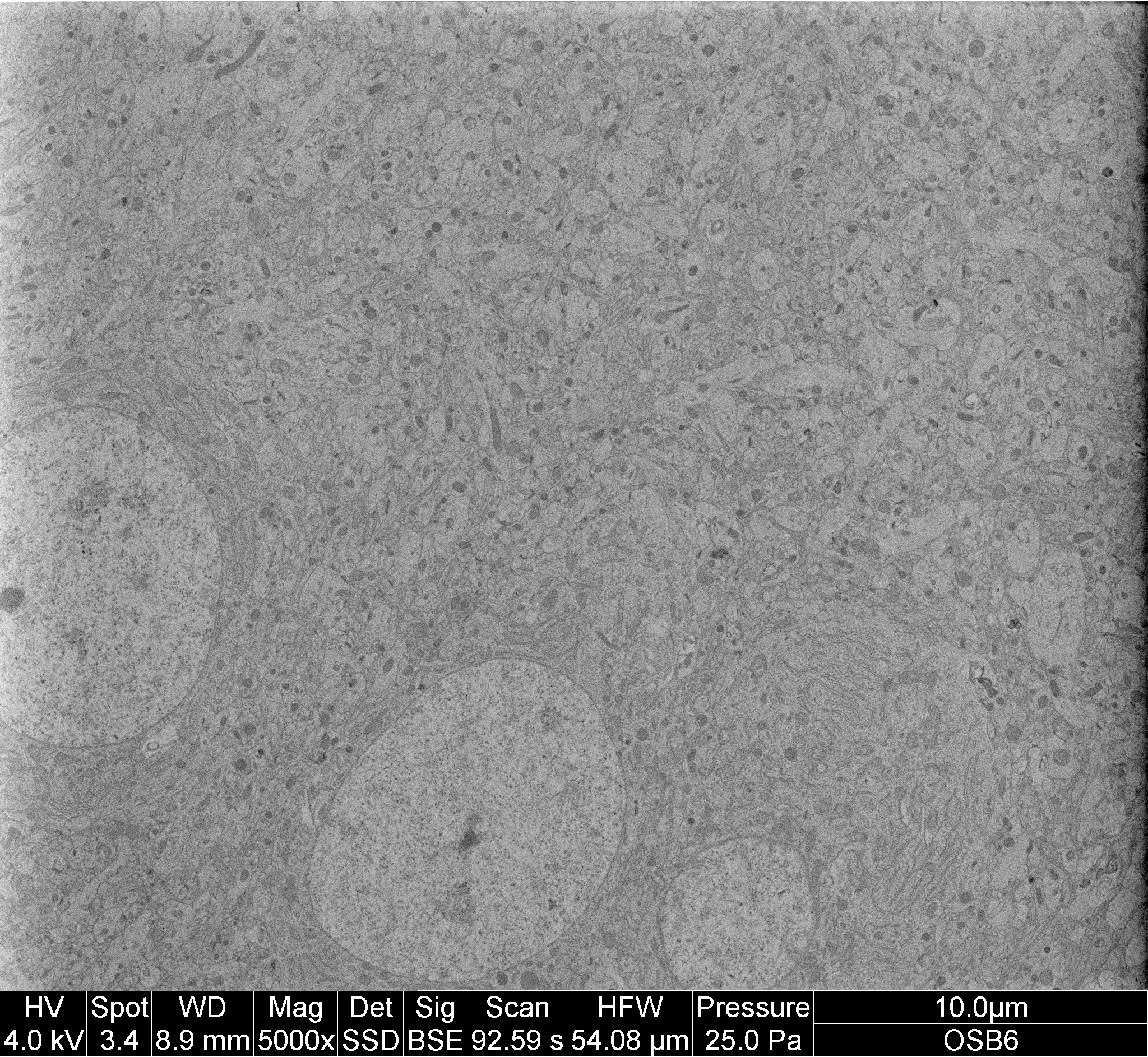

Supplement: Dataset S1 — (248.1 MB ZIP). [file pbio.0020329.sd001.zip › 040604_OS5_st1_029.tif]

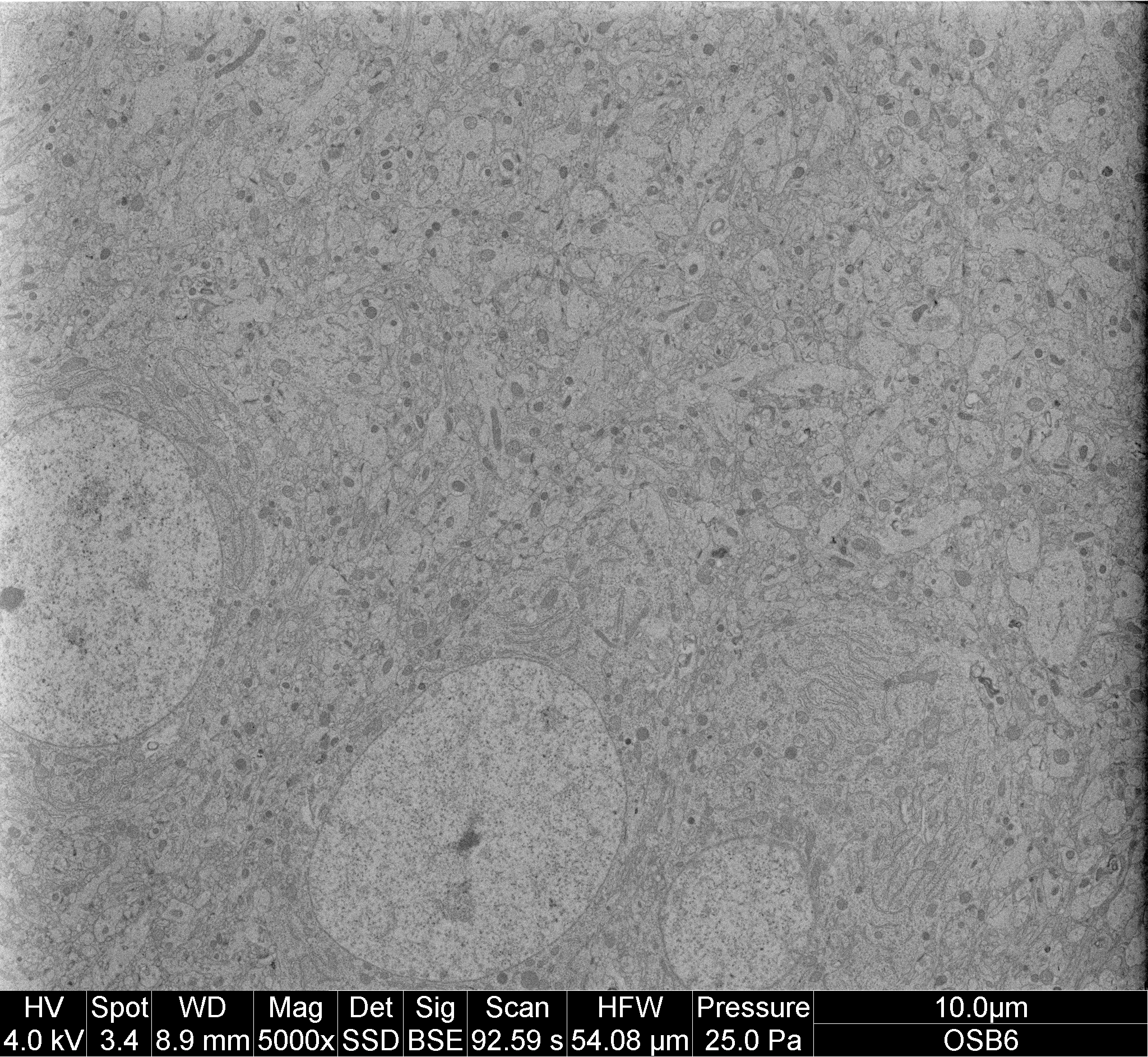

Supplement: Dataset S1 — (248.1 MB ZIP). [file pbio.0020329.sd001.zip › 040604_OS5_st1_030.tif]

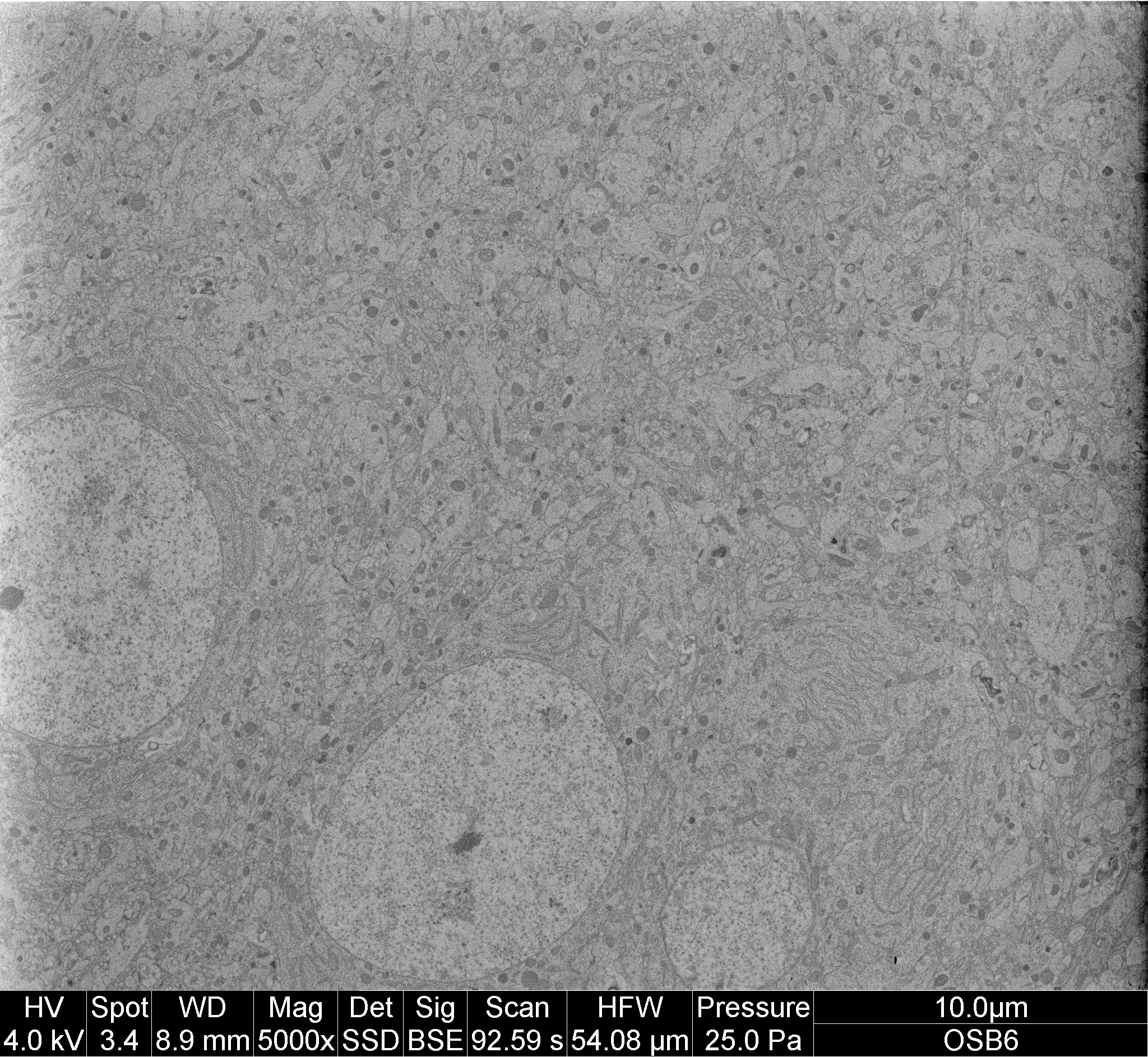

Supplement: Dataset S1 — (248.1 MB ZIP). [file pbio.0020329.sd001.zip › 040604_OS5_st1_031.tif]

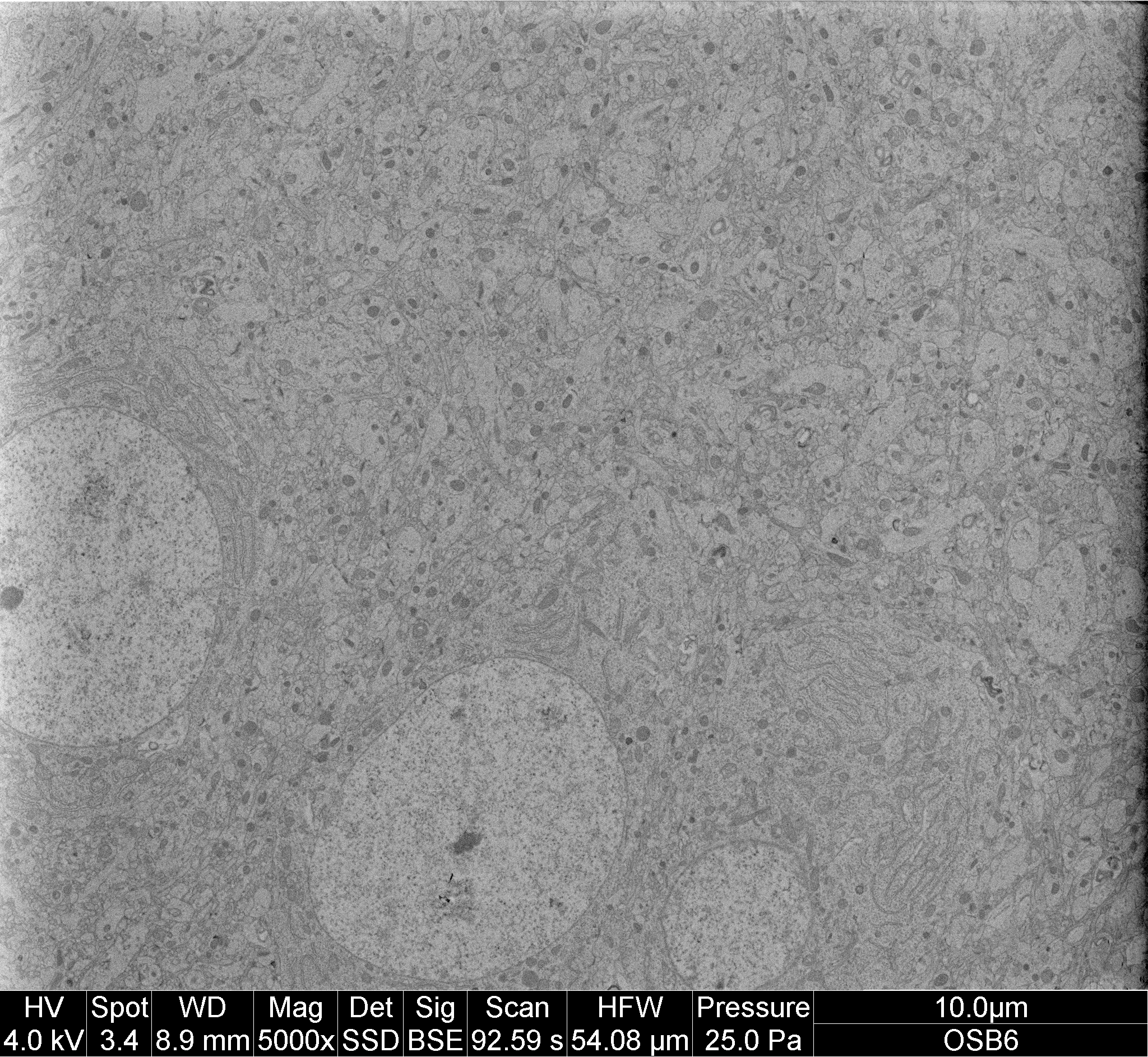

Supplement: Dataset S1 — (248.1 MB ZIP). [file pbio.0020329.sd001.zip › 040604_OS5_st1_032.tif]

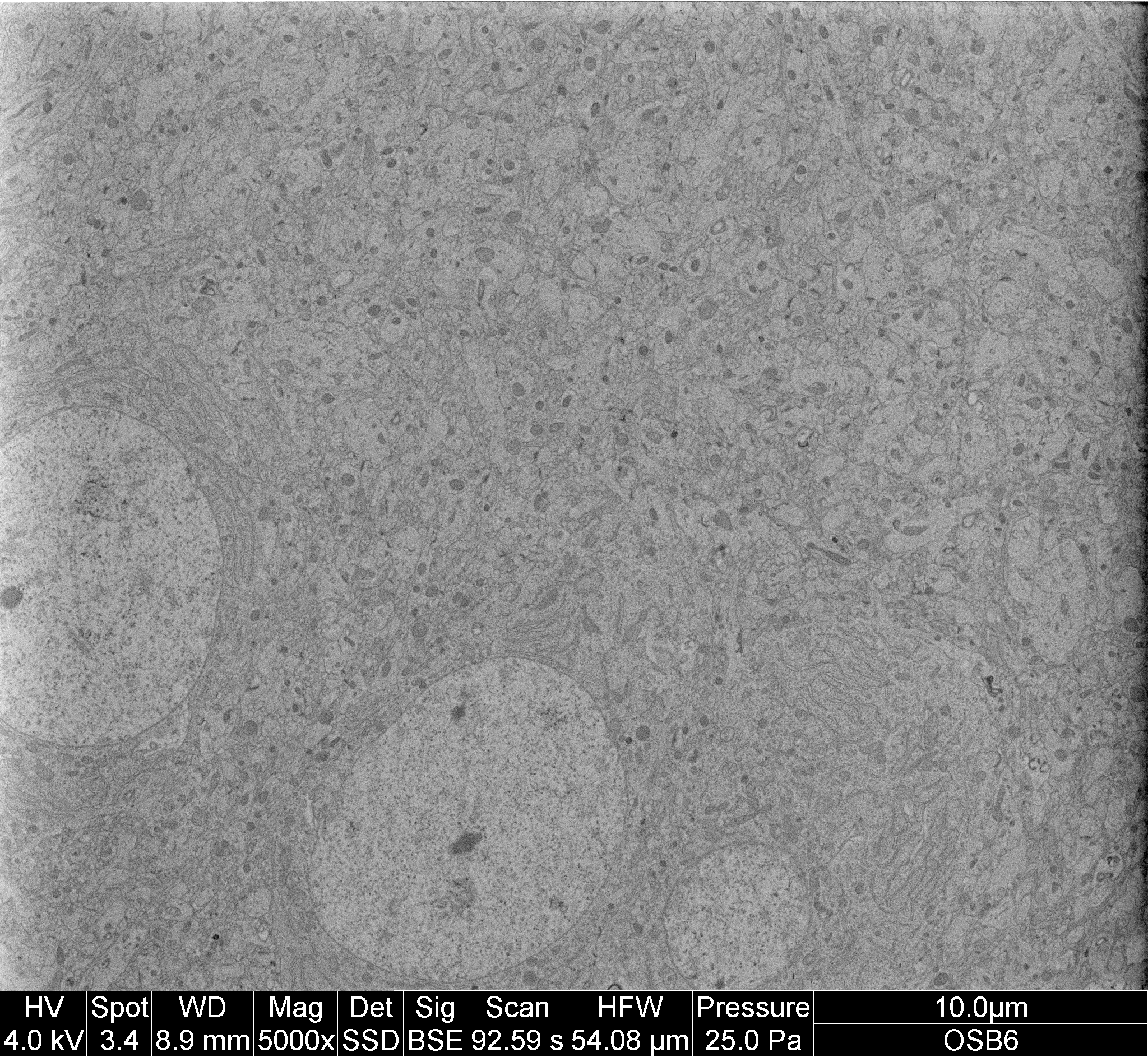

Supplement: Dataset S1 — (248.1 MB ZIP). [file pbio.0020329.sd001.zip › 040604_OS5_st1_033.tif]

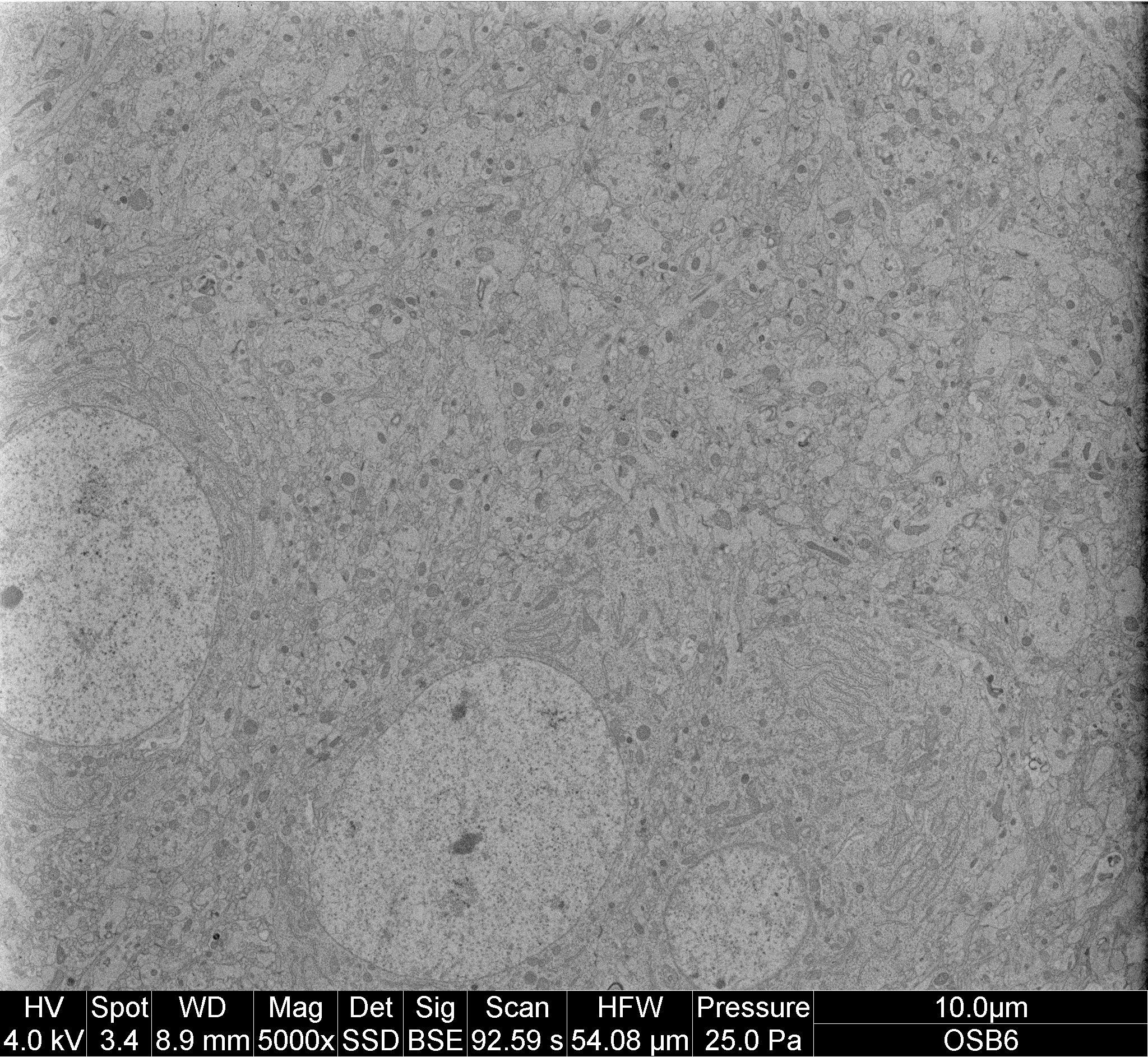

Supplement: Dataset S1 — (248.1 MB ZIP). [file pbio.0020329.sd001.zip › 040604_OS5_st1_034.tif]

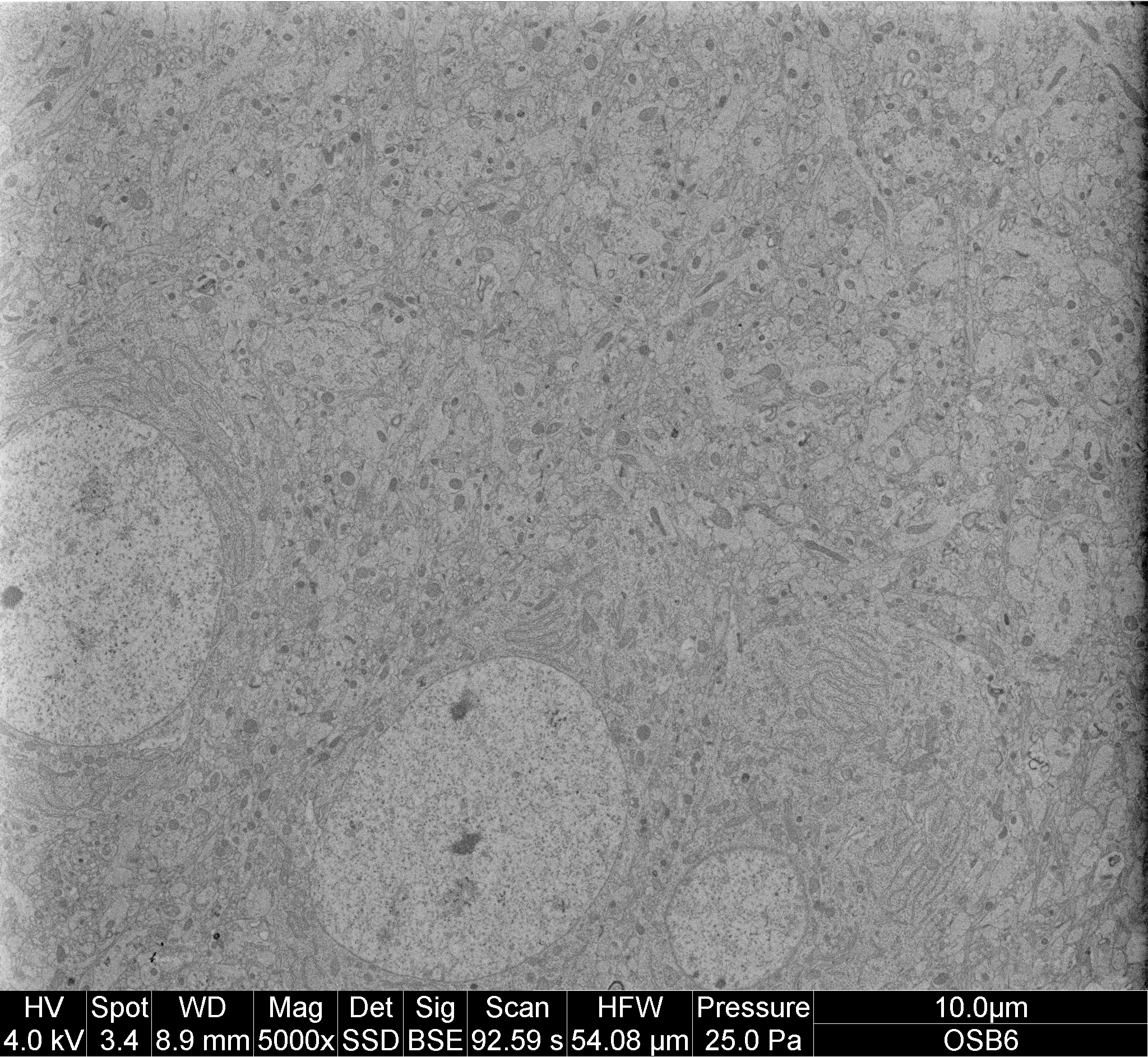

Supplement: Dataset S1 — (248.1 MB ZIP). [file pbio.0020329.sd001.zip › 040604_OS5_st1_035.tif]

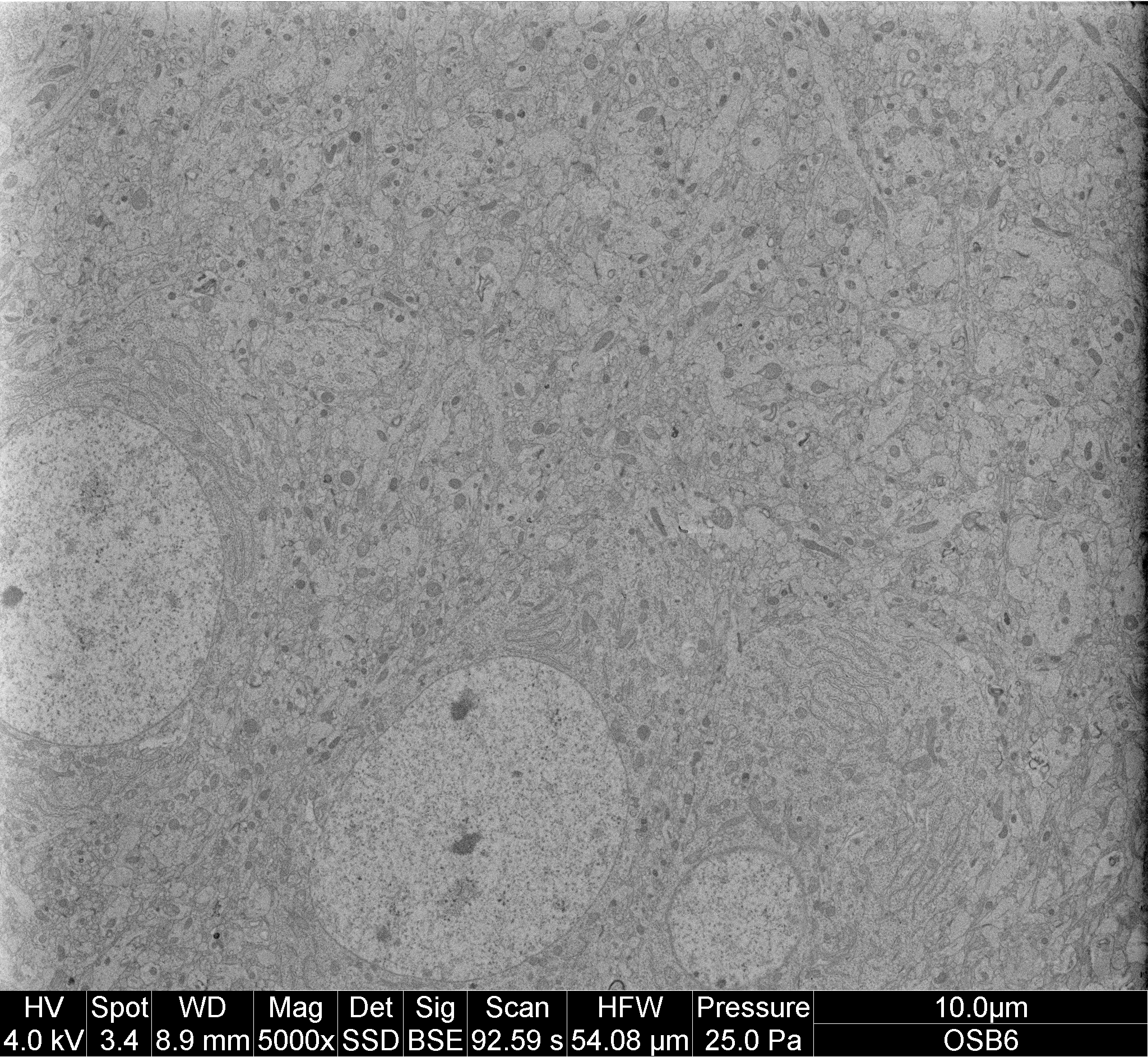

Supplement: Dataset S1 — (248.1 MB ZIP). [file pbio.0020329.sd001.zip › 040604_OS5_st1_036.tif]

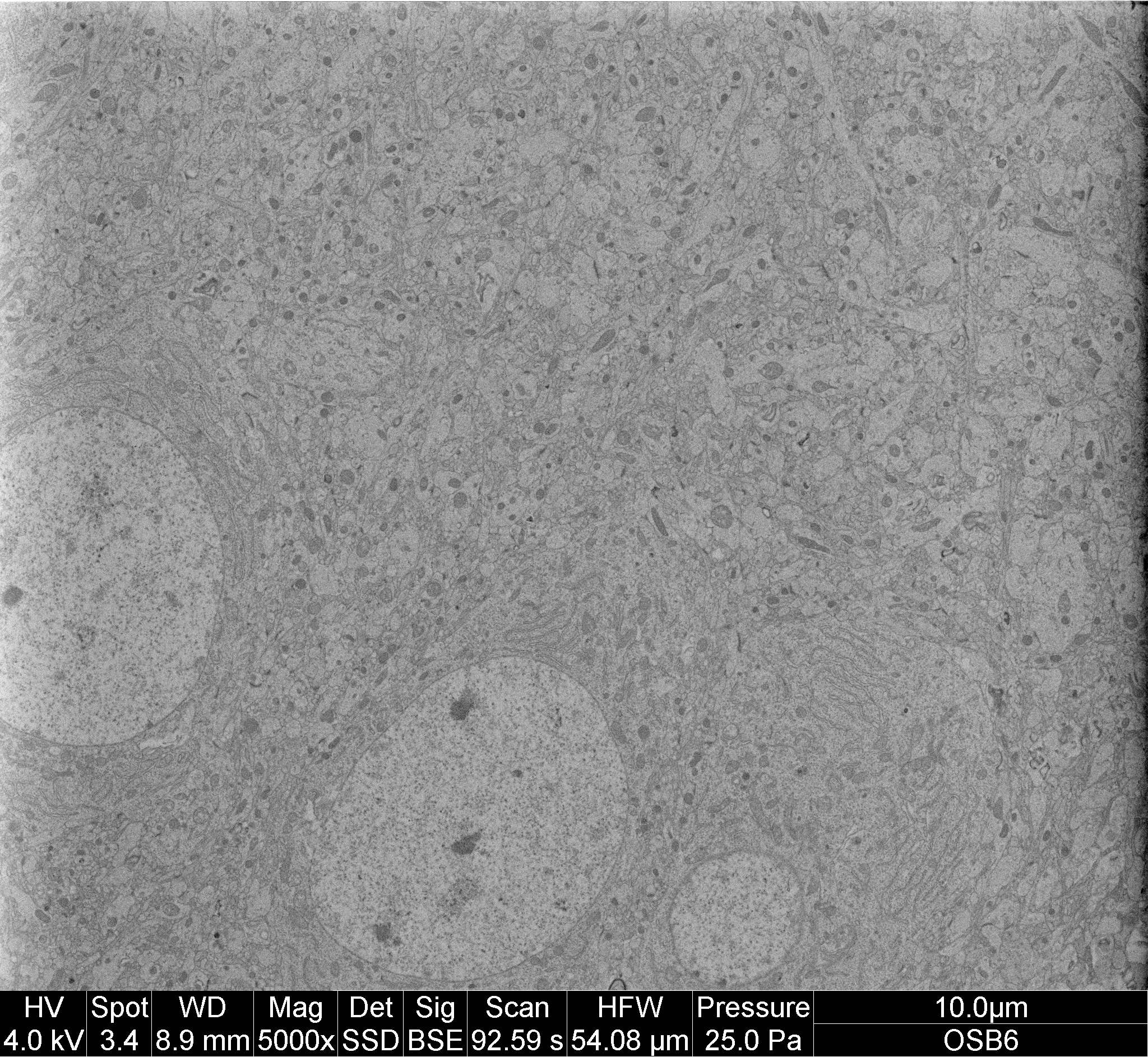

Supplement: Dataset S1 — (248.1 MB ZIP). [file pbio.0020329.sd001.zip › 040604_OS5_st1_037.tif]

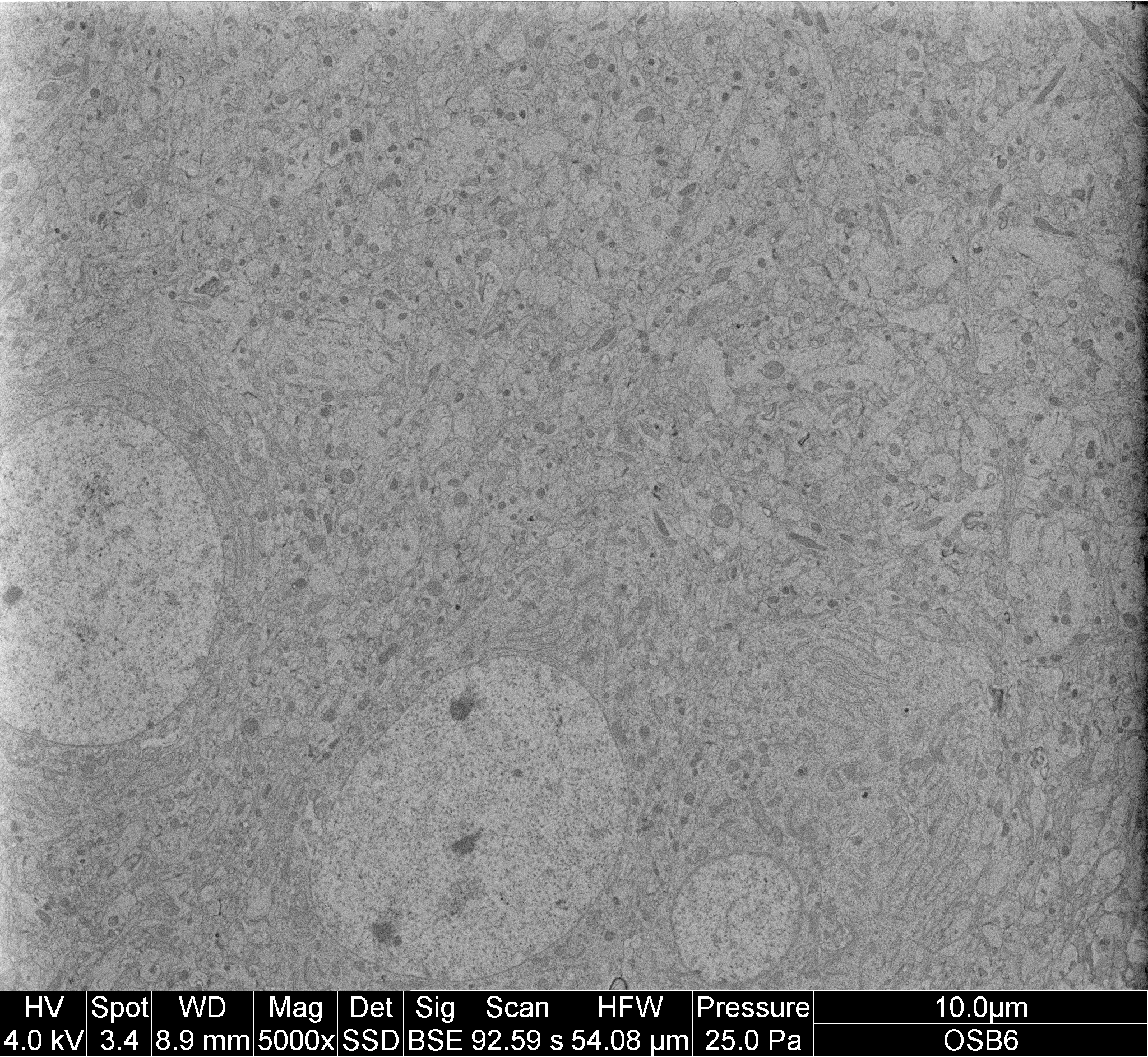

Supplement: Dataset S1 — (248.1 MB ZIP). [file pbio.0020329.sd001.zip › 040604_OS5_st1_038.tif]

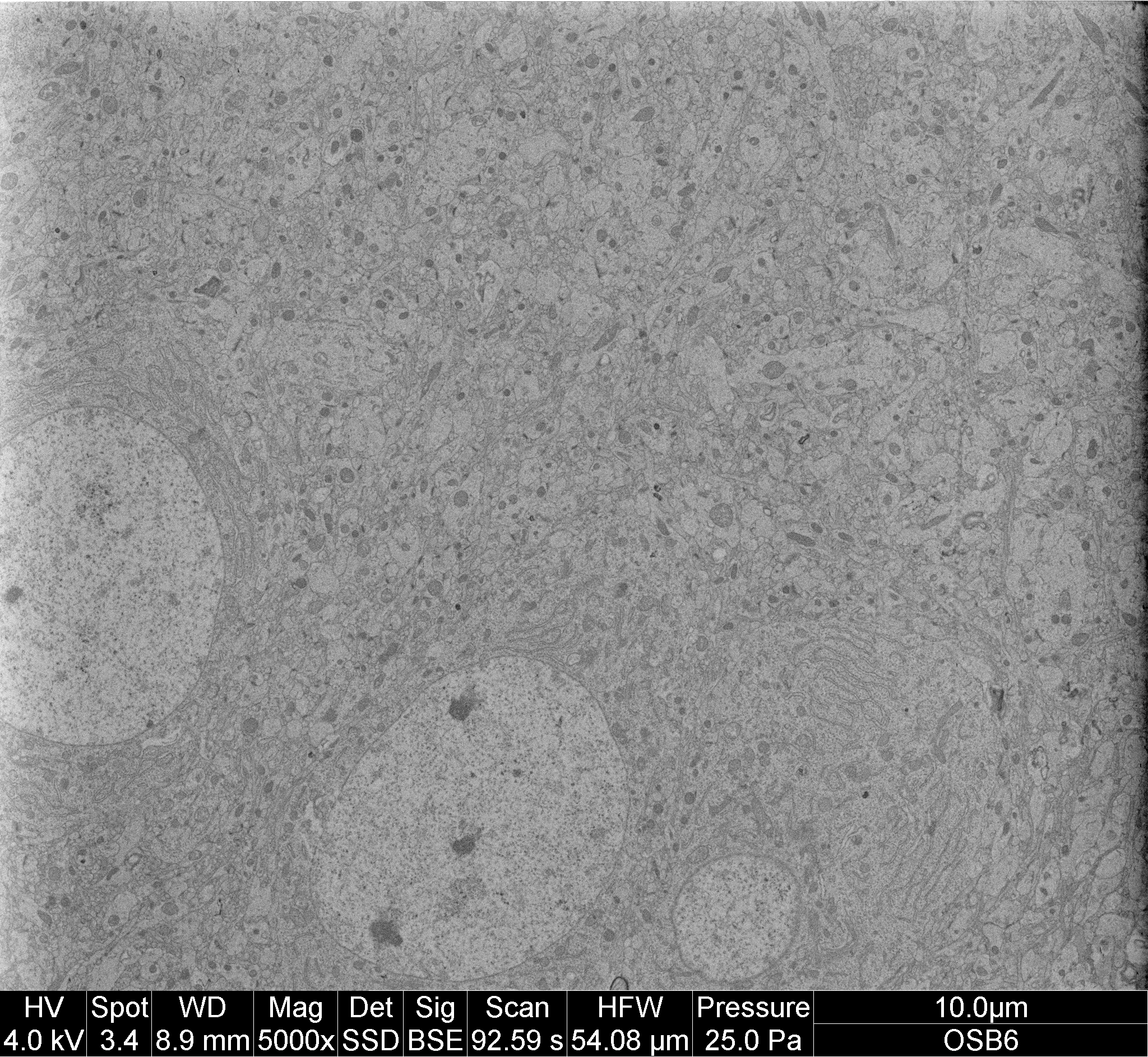

Supplement: Dataset S1 — (248.1 MB ZIP). [file pbio.0020329.sd001.zip › 040604_OS5_st1_039.tif]

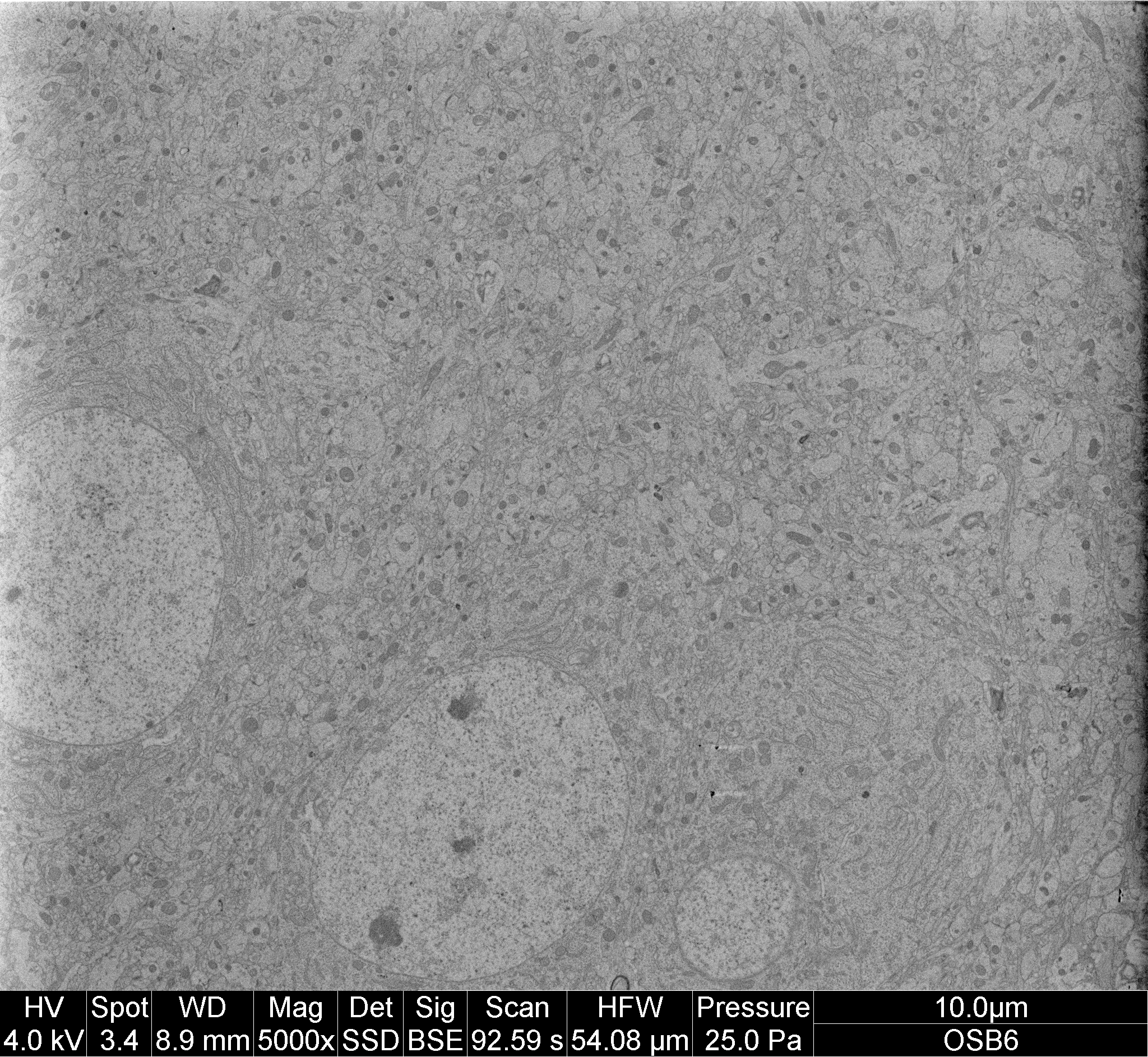

Supplement: Dataset S1 — (248.1 MB ZIP). [file pbio.0020329.sd001.zip › 040604_OS5_st1_040.tif]

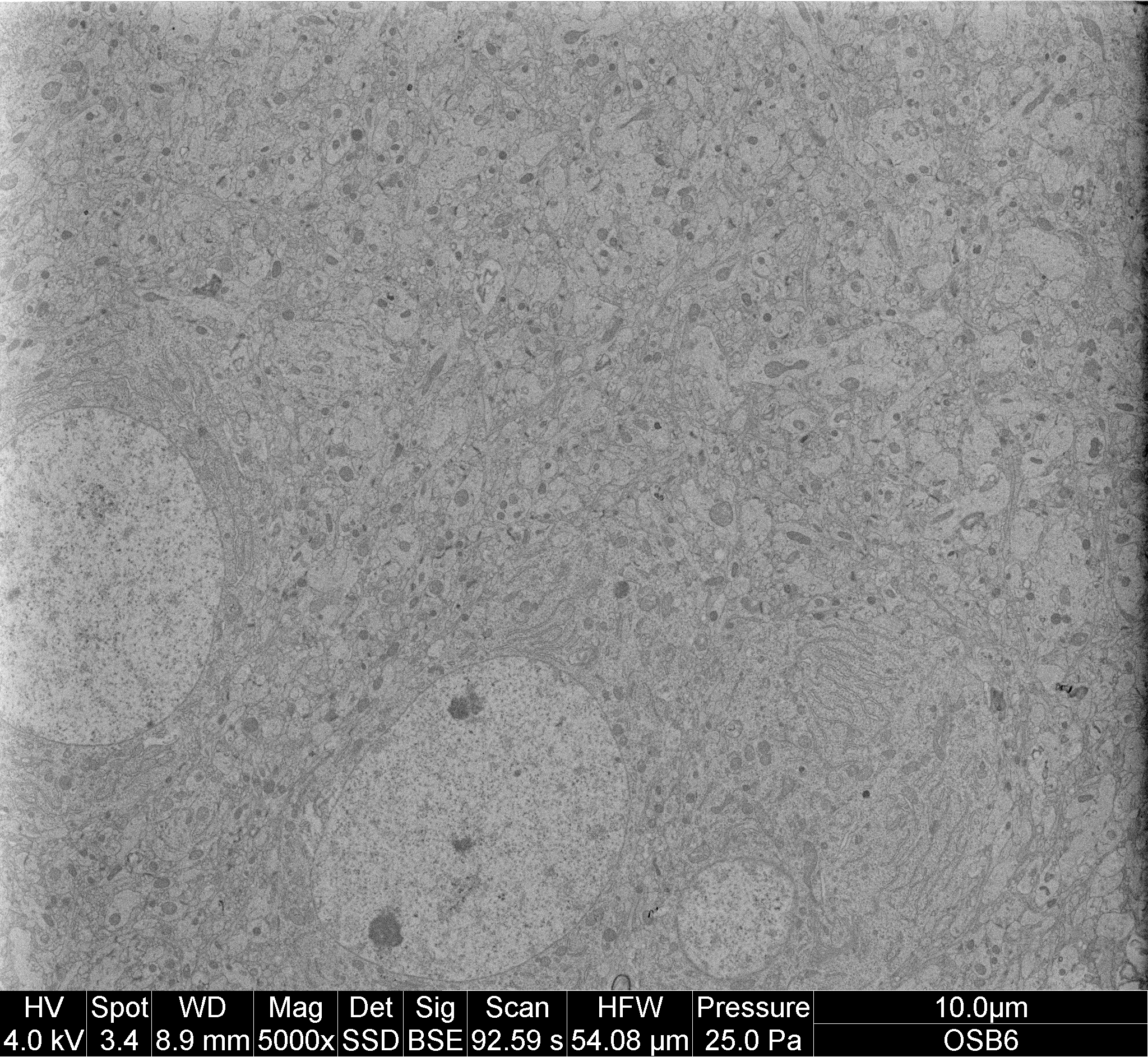

Supplement: Dataset S1 — (248.1 MB ZIP). [file pbio.0020329.sd001.zip › 040604_OS5_st1_041.tif]

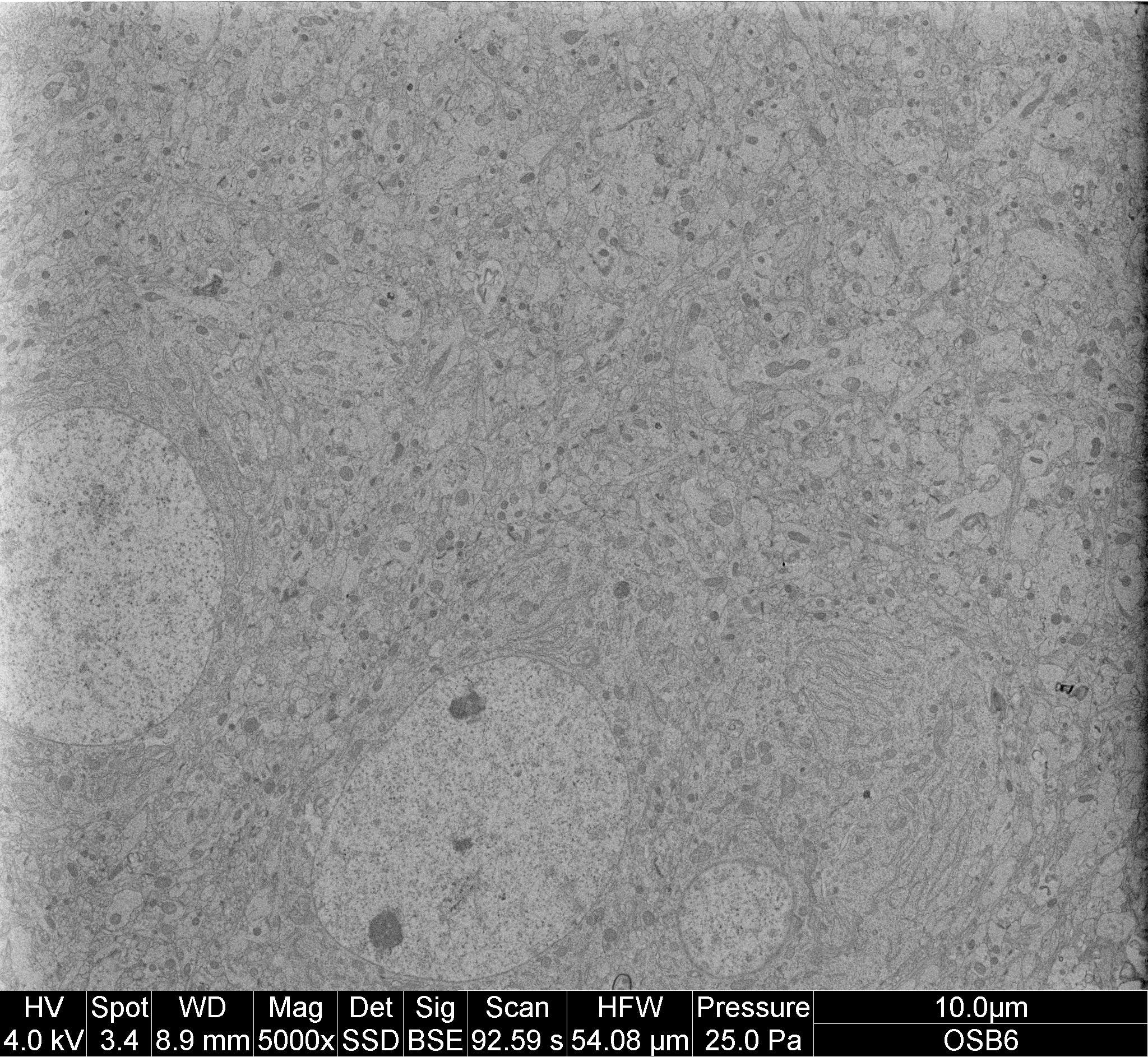

Supplement: Dataset S1 — (248.1 MB ZIP). [file pbio.0020329.sd001.zip › 040604_OS5_st1_042.tif]

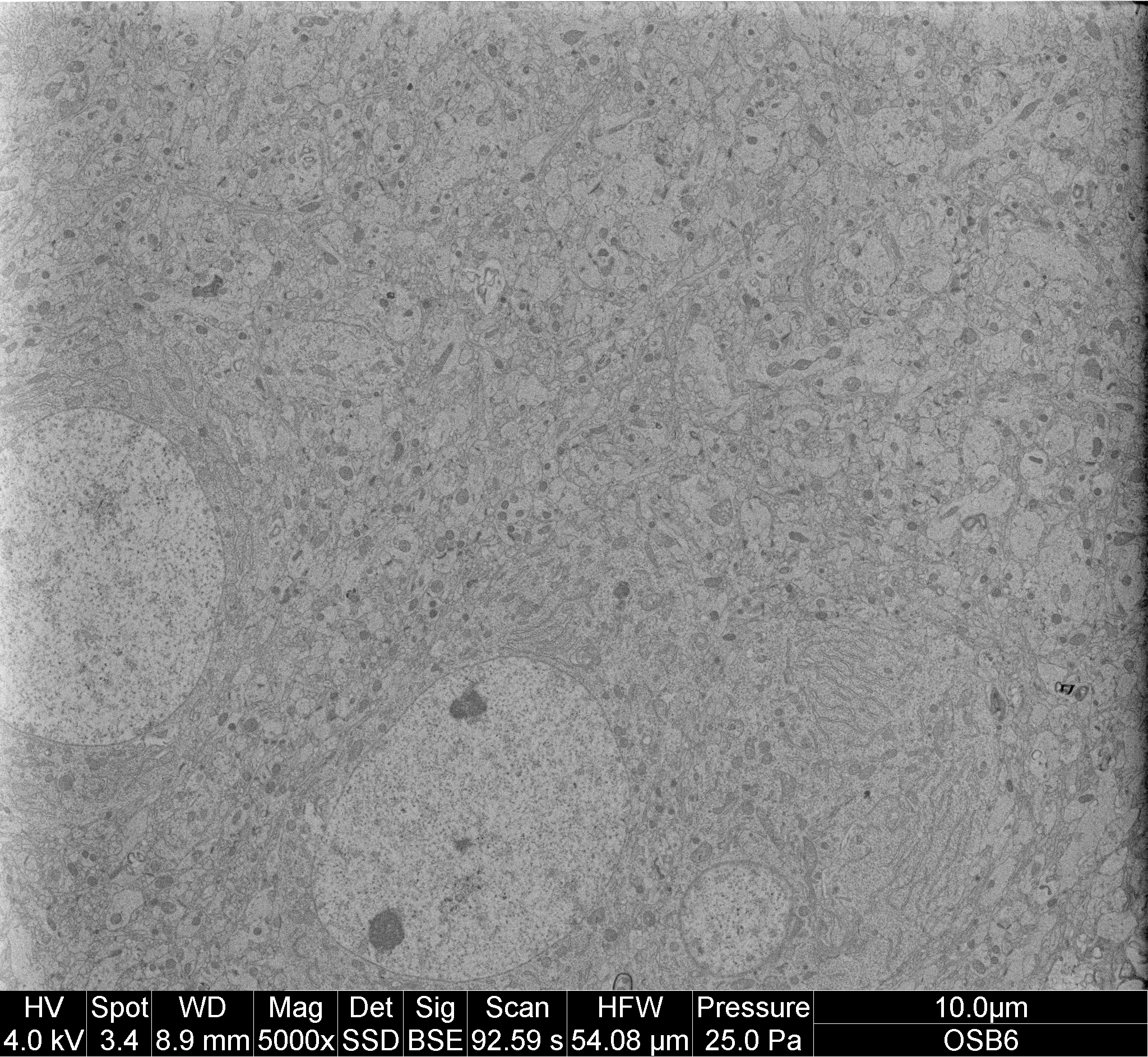

Supplement: Dataset S1 — (248.1 MB ZIP). [file pbio.0020329.sd001.zip › 040604_OS5_st1_043.tif]

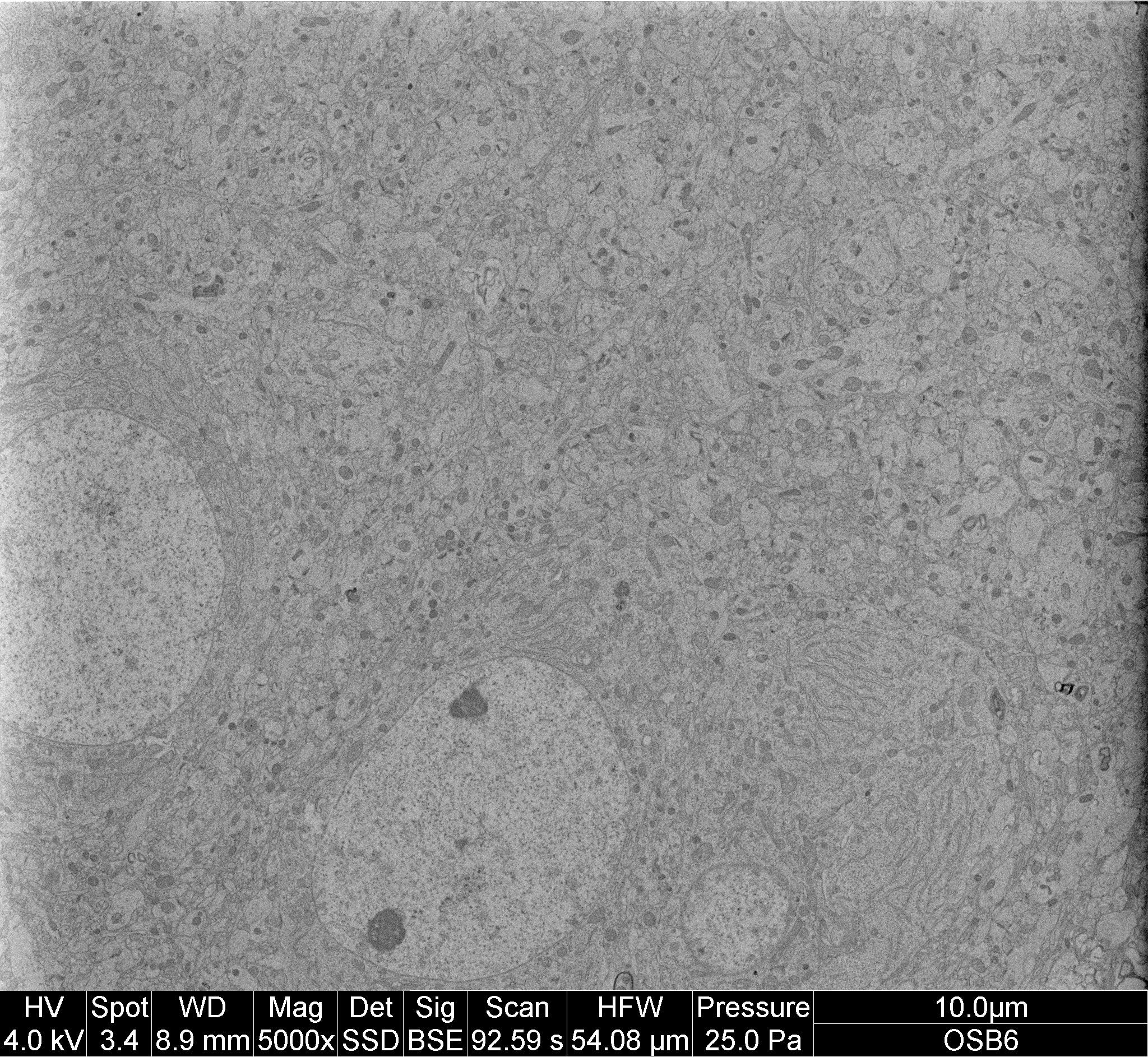

Supplement: Dataset S1 — (248.1 MB ZIP). [file pbio.0020329.sd001.zip › 040604_OS5_st1_044.tif]

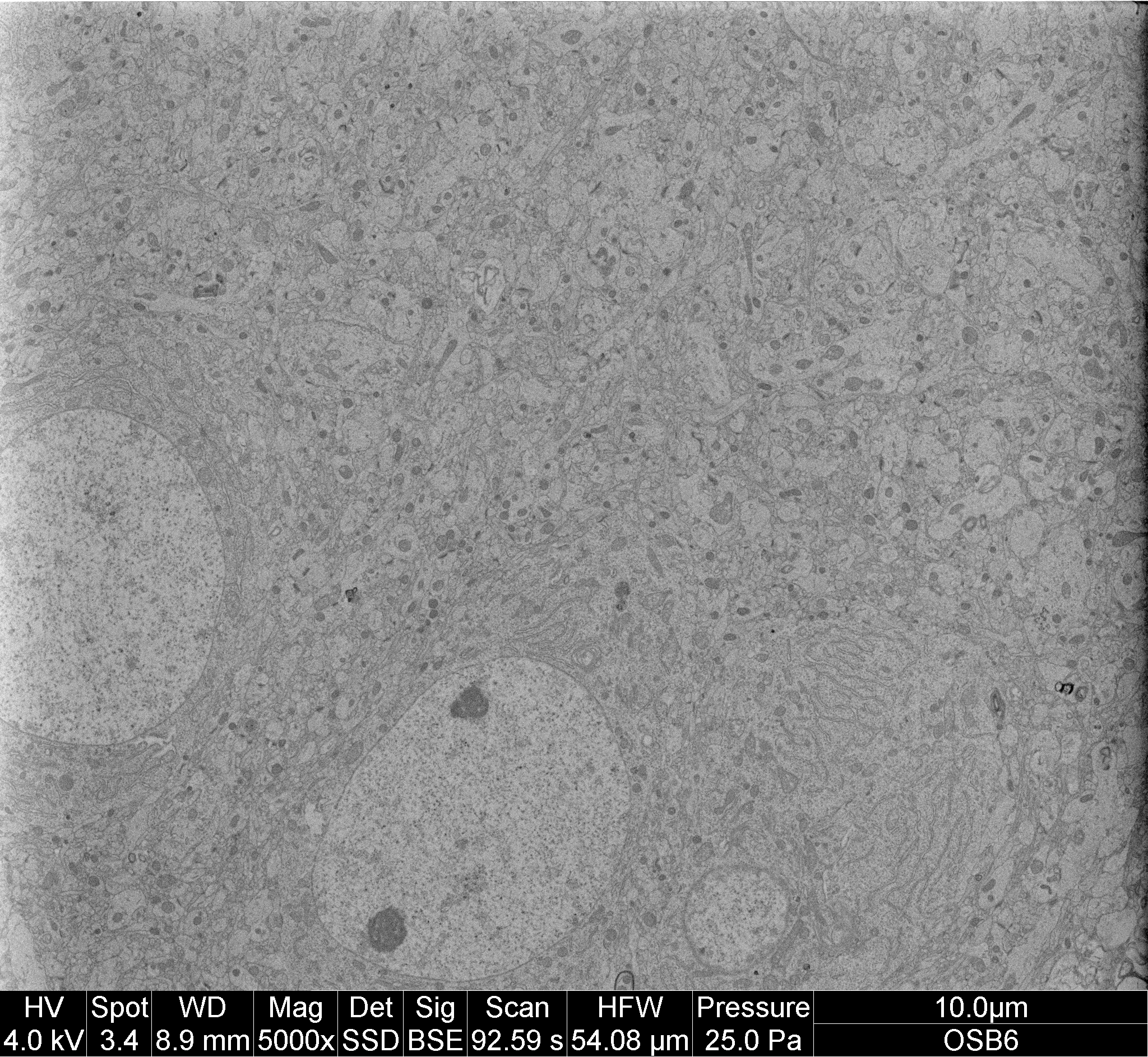

Supplement: Dataset S1 — (248.1 MB ZIP). [file pbio.0020329.sd001.zip › 040604_OS5_st1_045.tif]

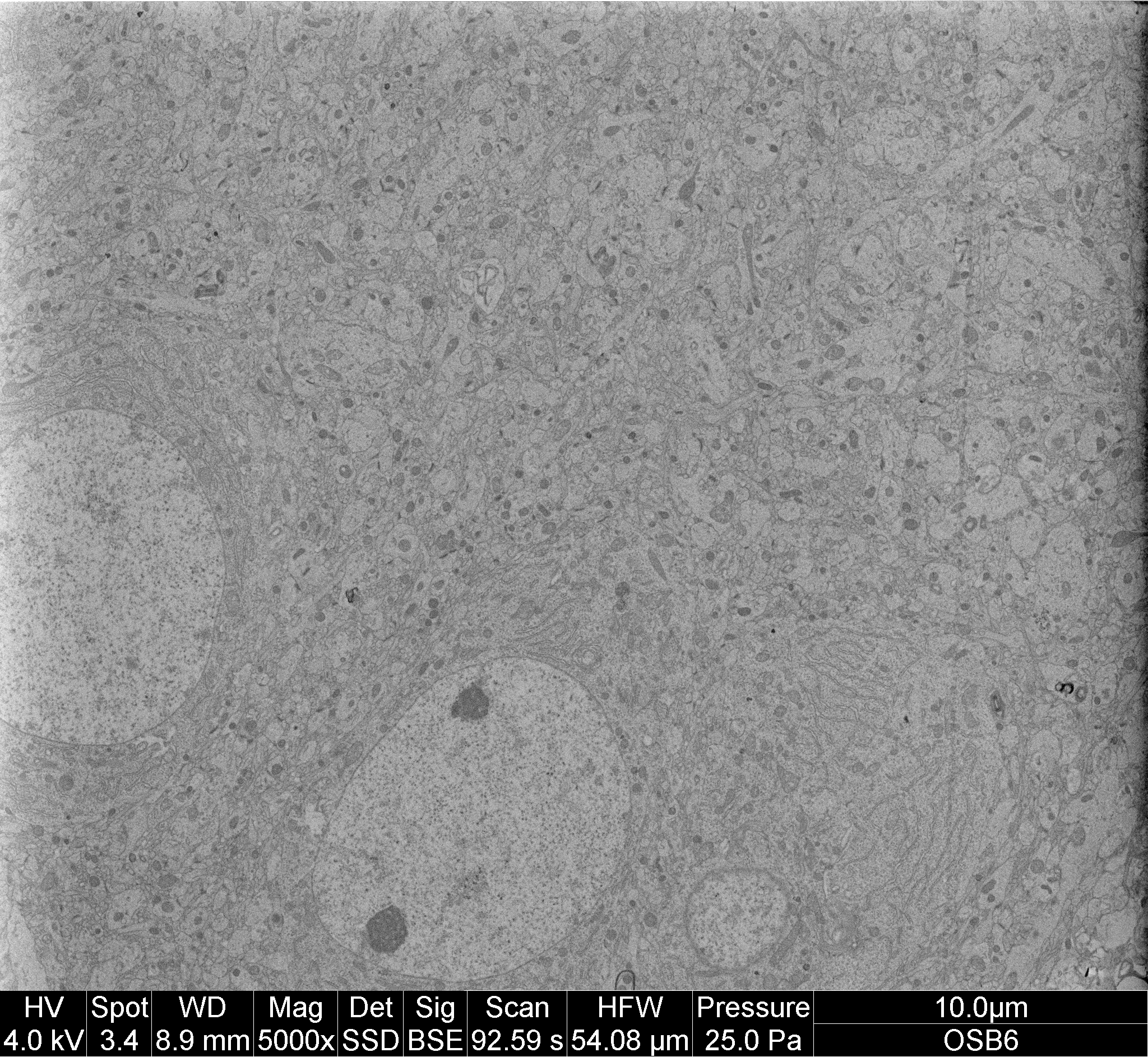

Supplement: Dataset S1 — (248.1 MB ZIP). [file pbio.0020329.sd001.zip › 040604_OS5_st1_046.tif]

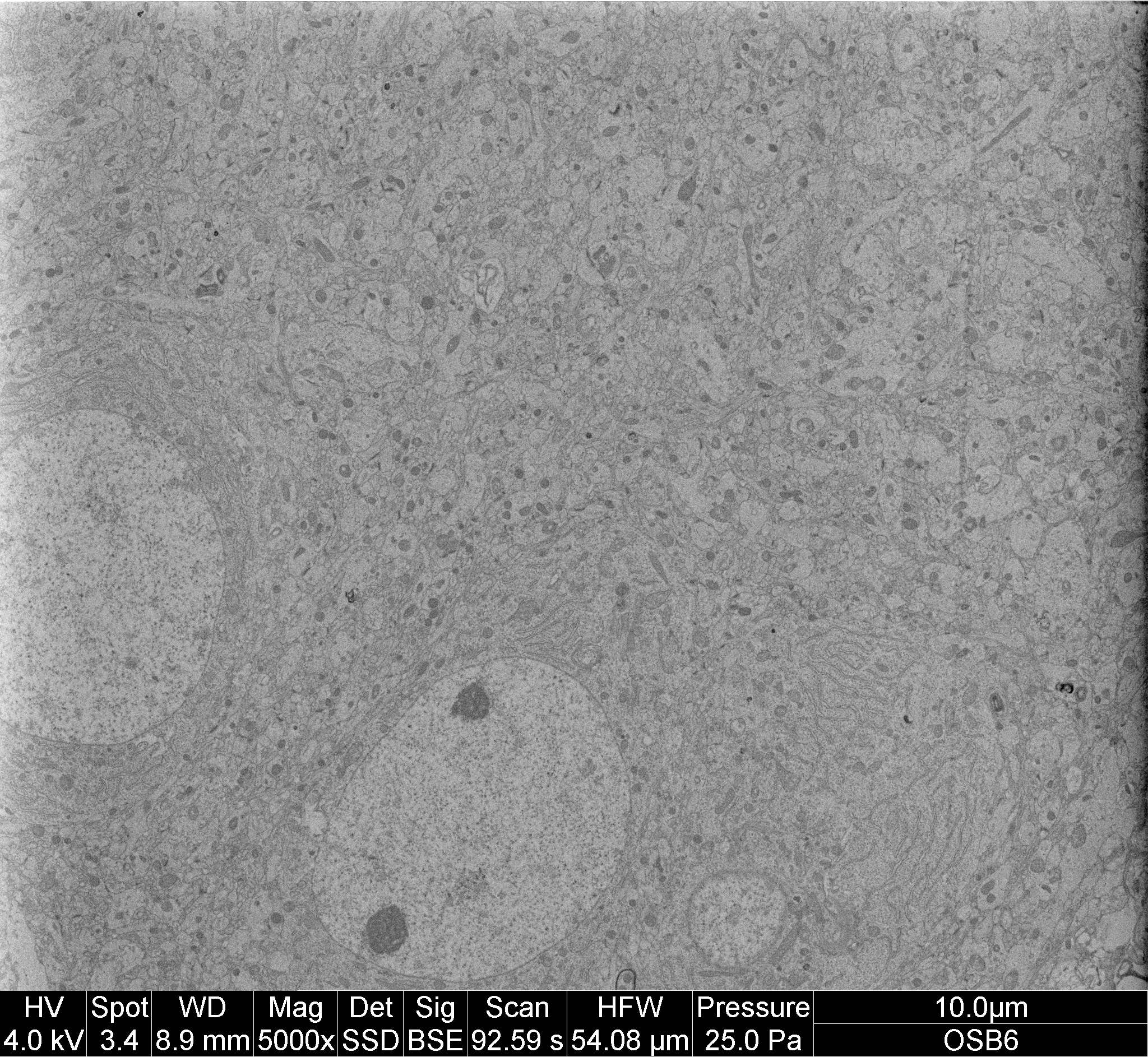

Supplement: Dataset S1 — (248.1 MB ZIP). [file pbio.0020329.sd001.zip › 040604_OS5_st1_047.tif]

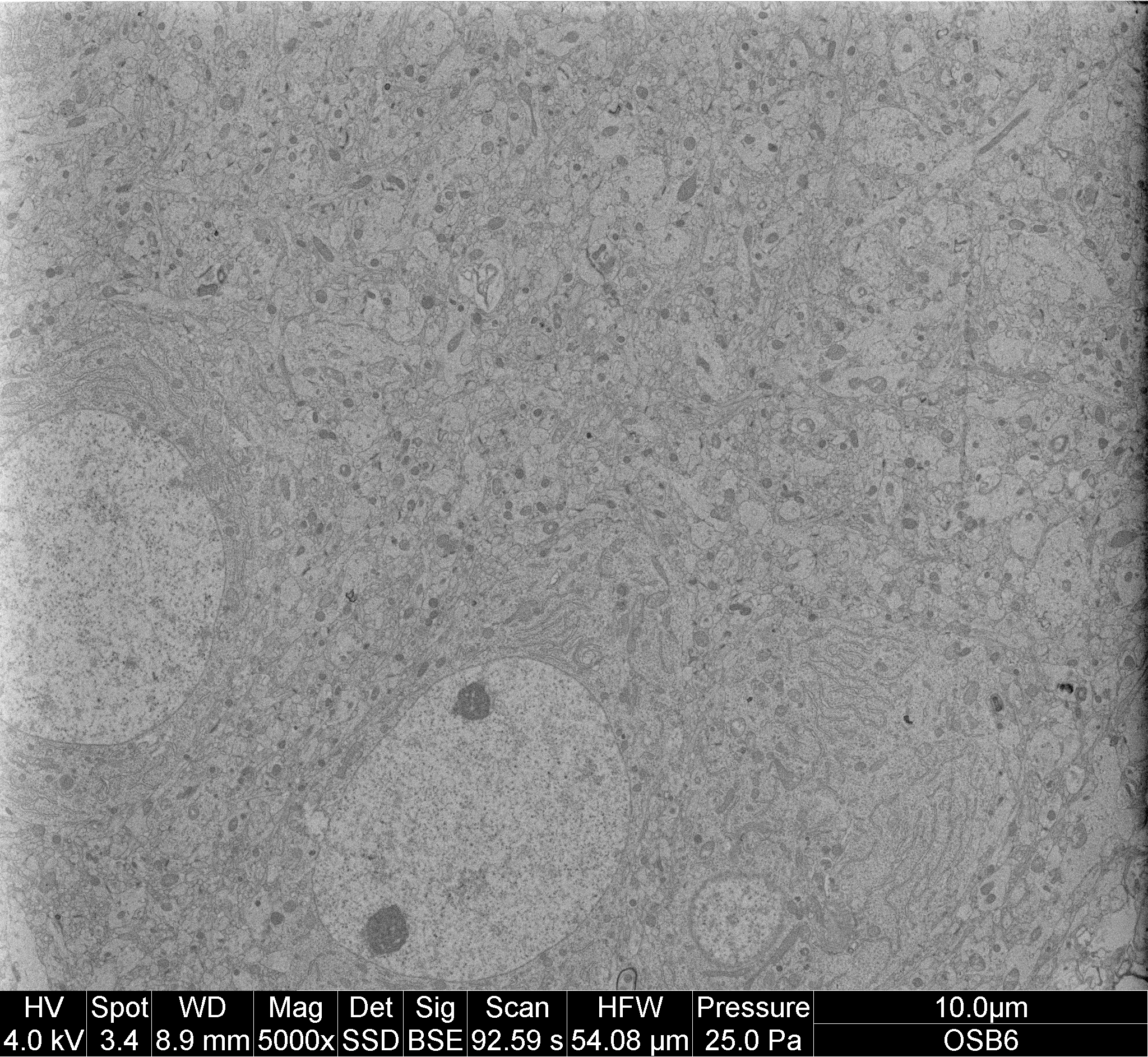

Supplement: Dataset S1 — (248.1 MB ZIP). [file pbio.0020329.sd001.zip › 040604_OS5_st1_048.tif]

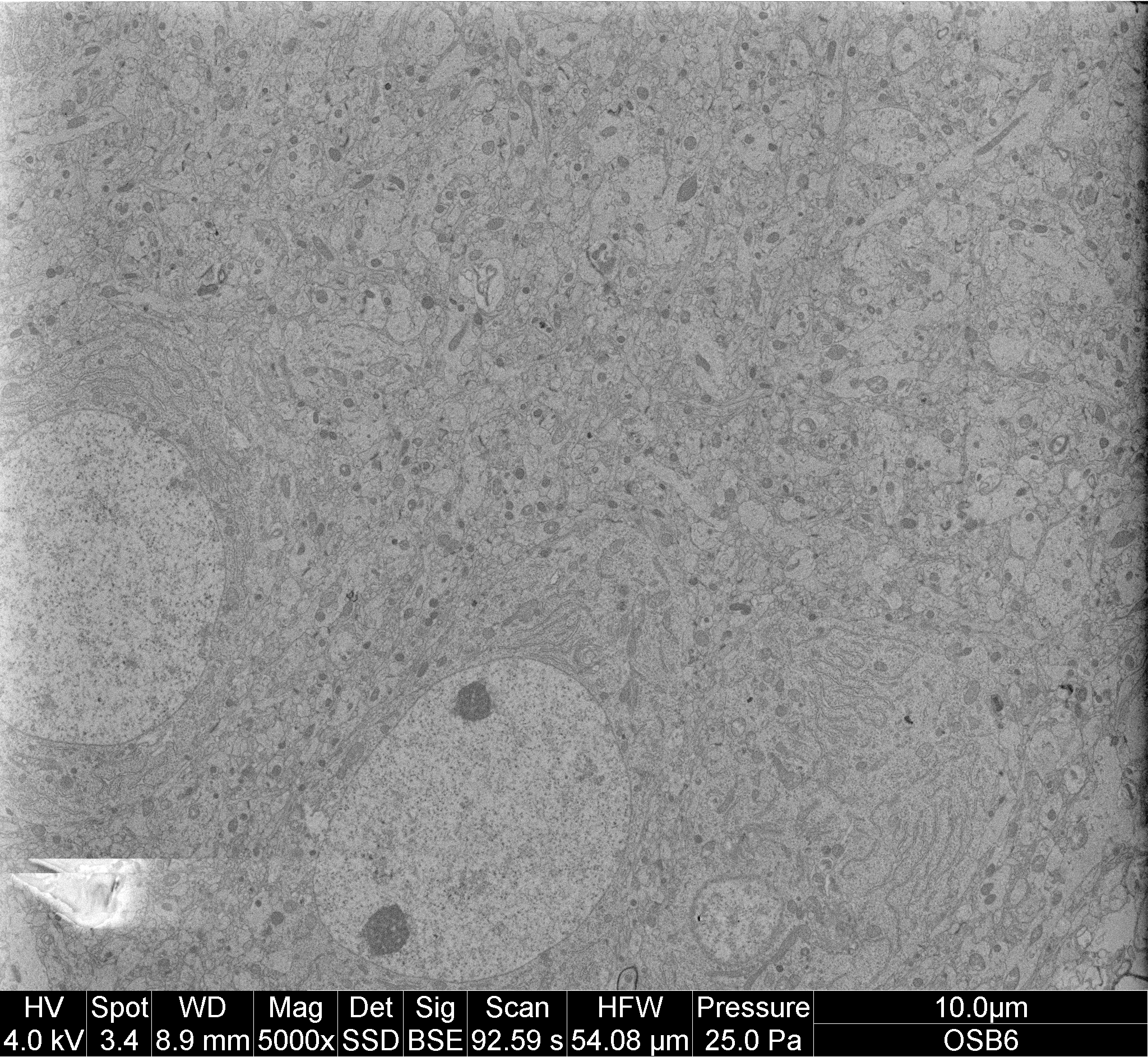

Supplement: Dataset S1 — (248.1 MB ZIP). [file pbio.0020329.sd001.zip › 040604_OS5_st1_049.tif]

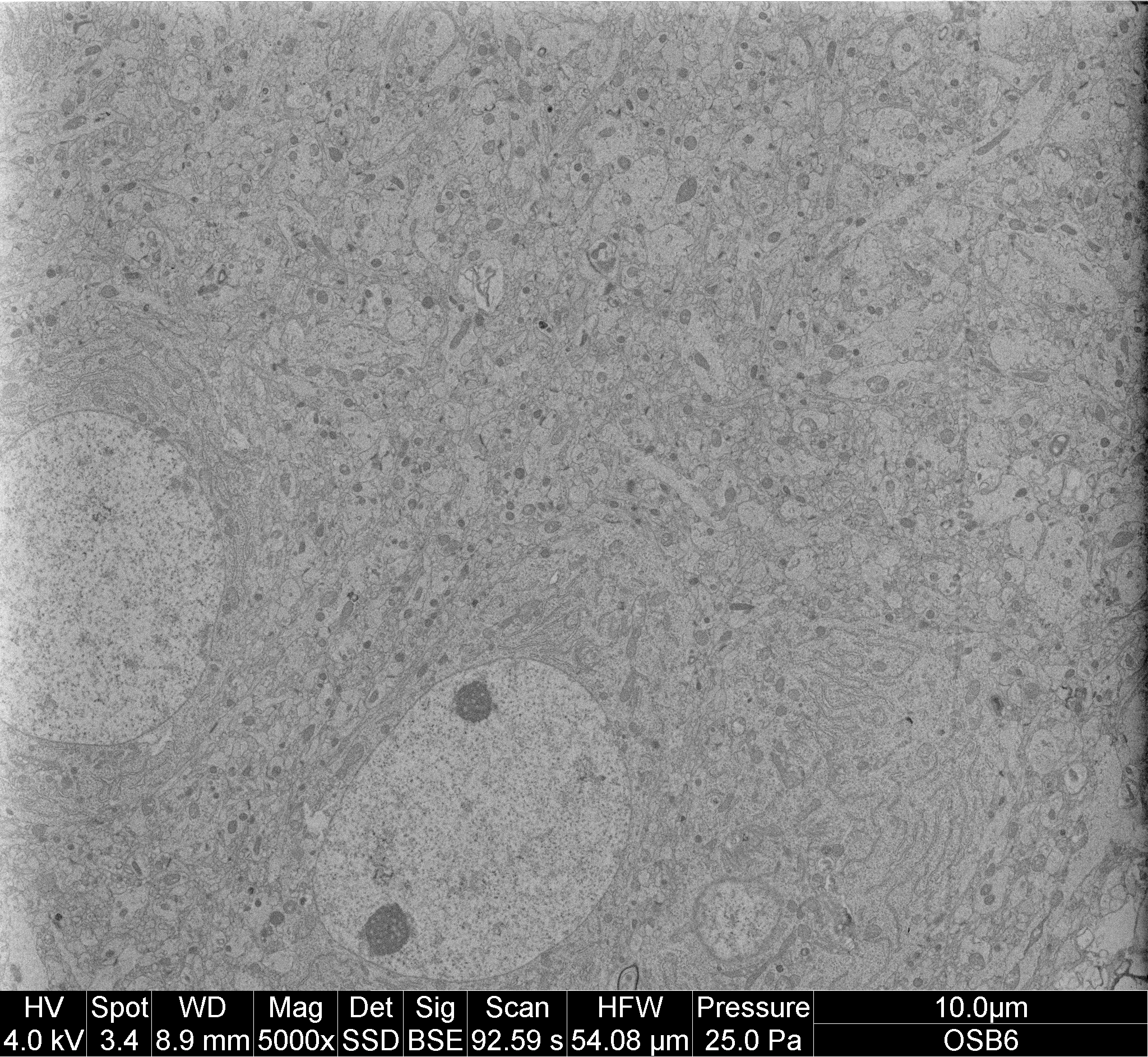

Supplement: Dataset S1 — (248.1 MB ZIP). [file pbio.0020329.sd001.zip › 040604_OS5_st1_050.tif]

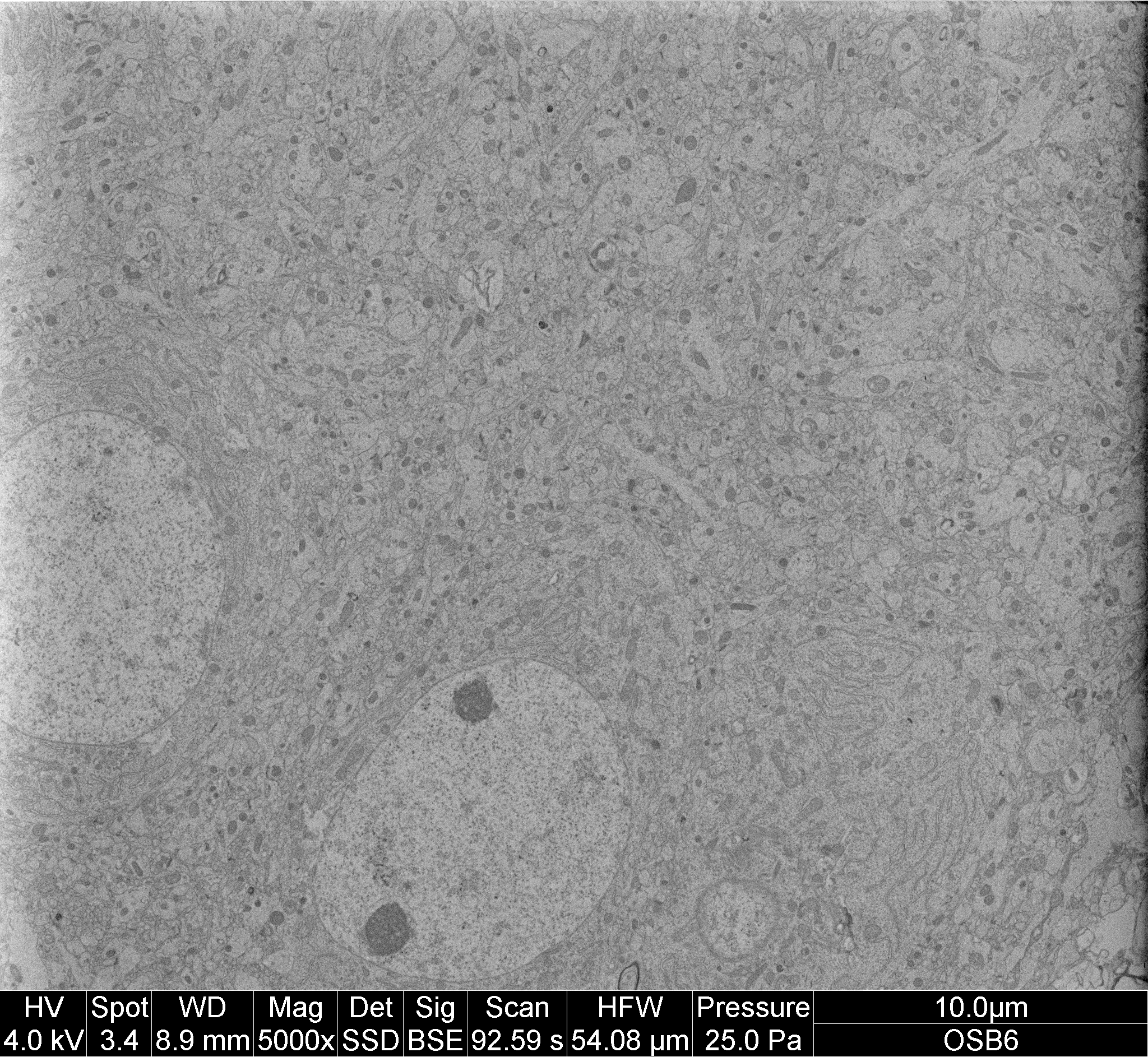

Supplement: Dataset S1 — (248.1 MB ZIP). [file pbio.0020329.sd001.zip › 040604_OS5_st1_051.tif]

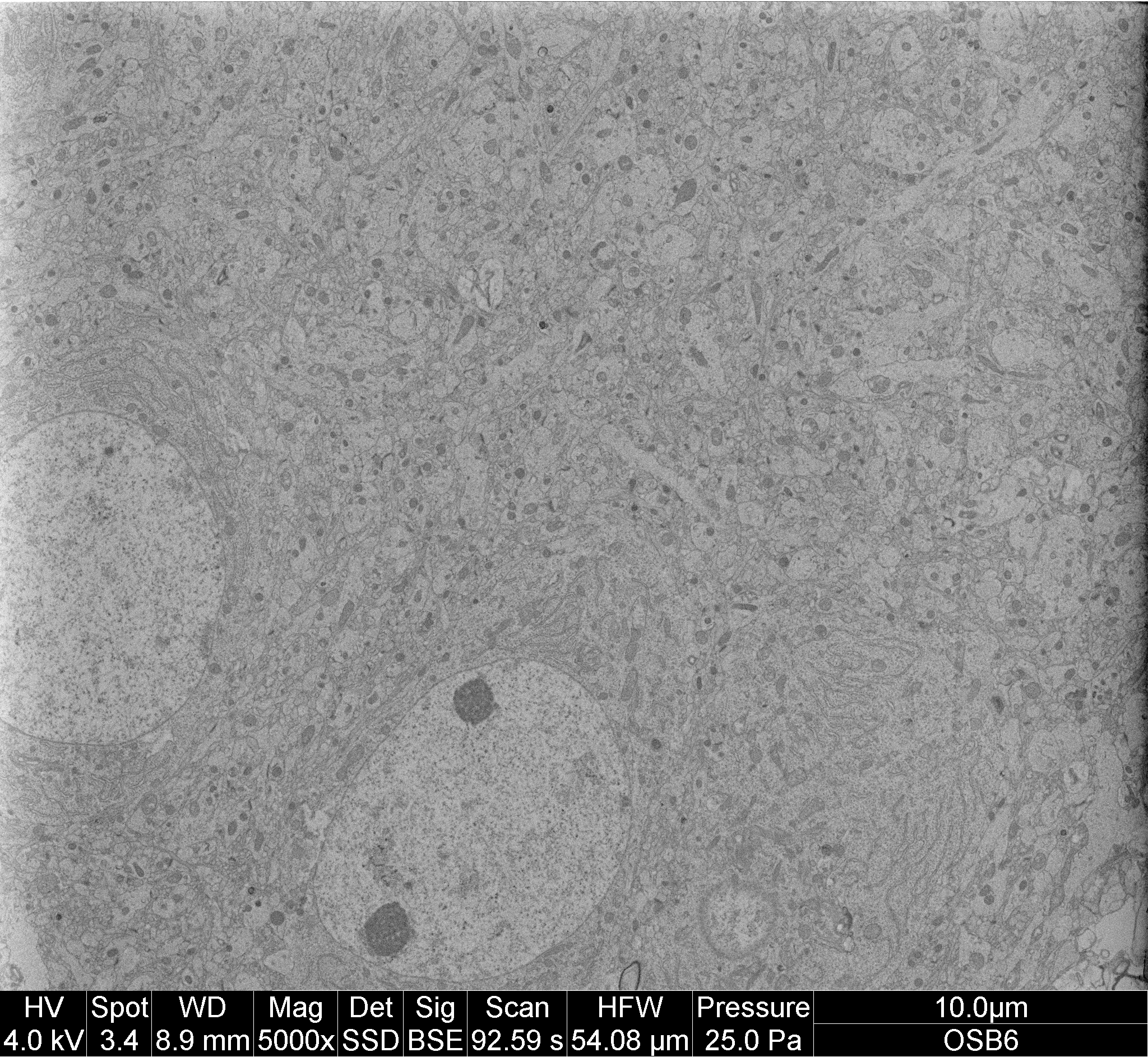

Supplement: Dataset S1 — (248.1 MB ZIP). [file pbio.0020329.sd001.zip › 040604_OS5_st1_052.tif]

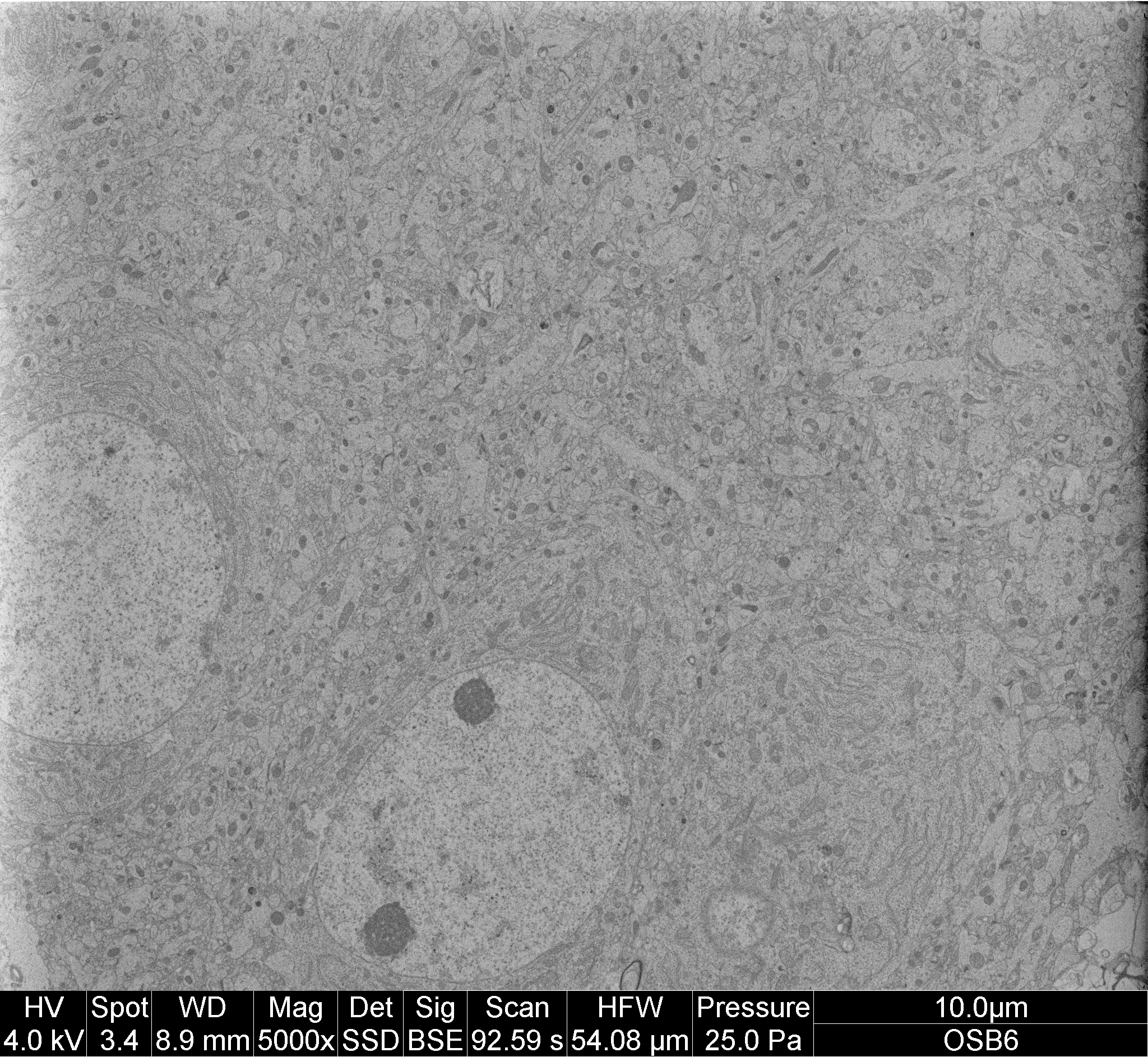

Supplement: Dataset S1 — (248.1 MB ZIP). [file pbio.0020329.sd001.zip › 040604_OS5_st1_053.tif]

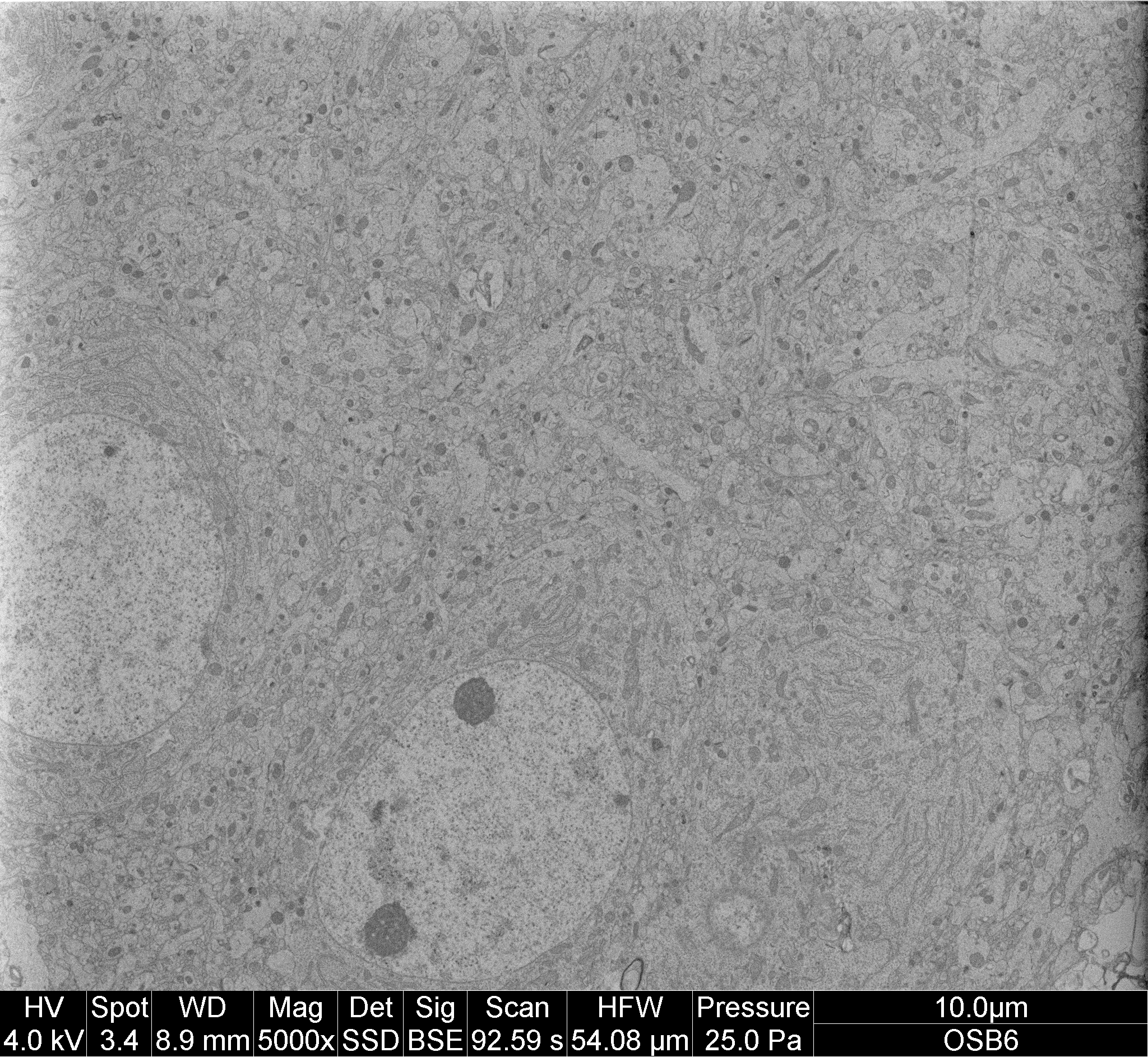

Supplement: Dataset S1 — (248.1 MB ZIP). [file pbio.0020329.sd001.zip › 040604_OS5_st1_054.tif]

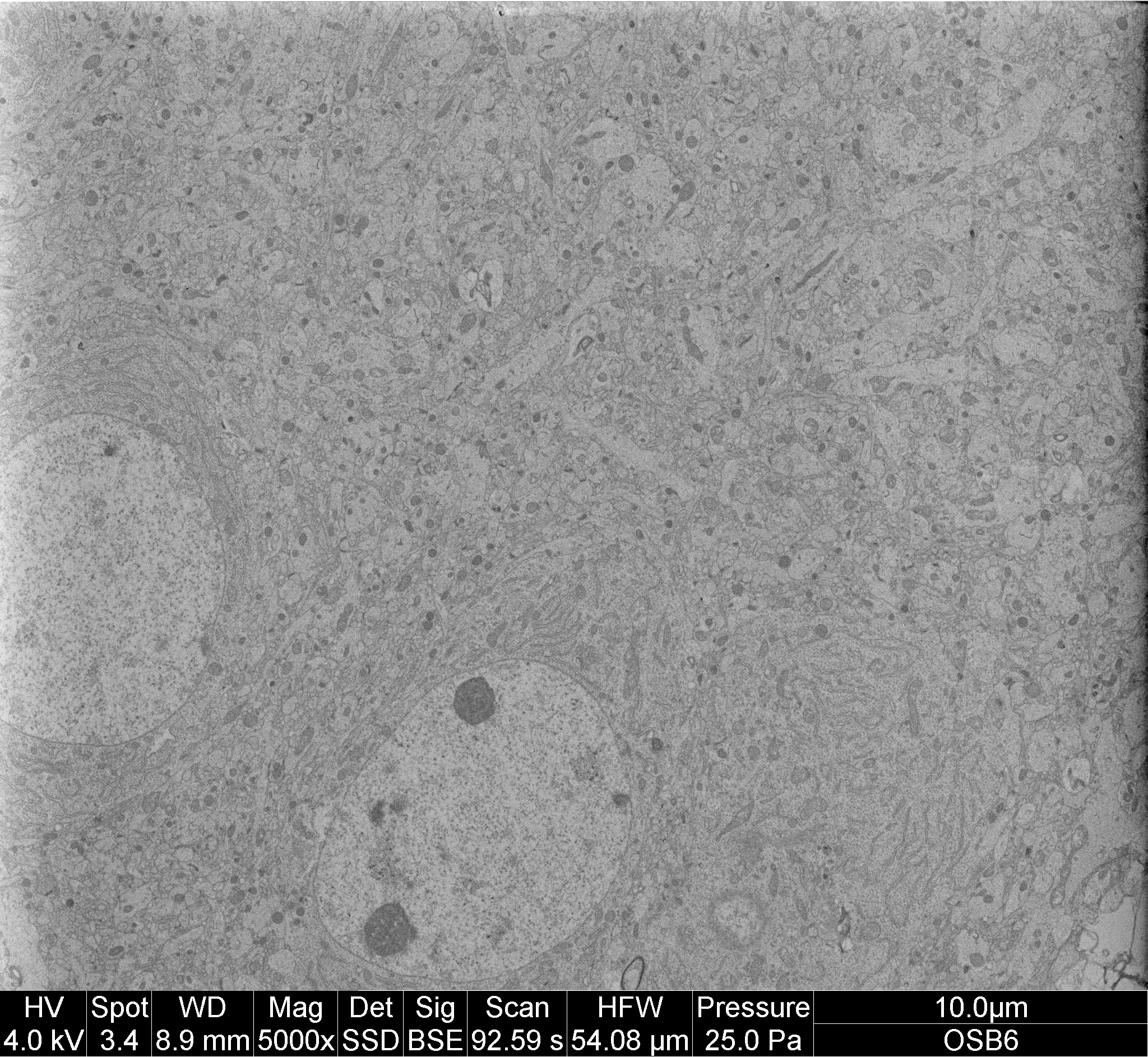

Supplement: Dataset S1 — (248.1 MB ZIP). [file pbio.0020329.sd001.zip › 040604_OS5_st1_055.tif]

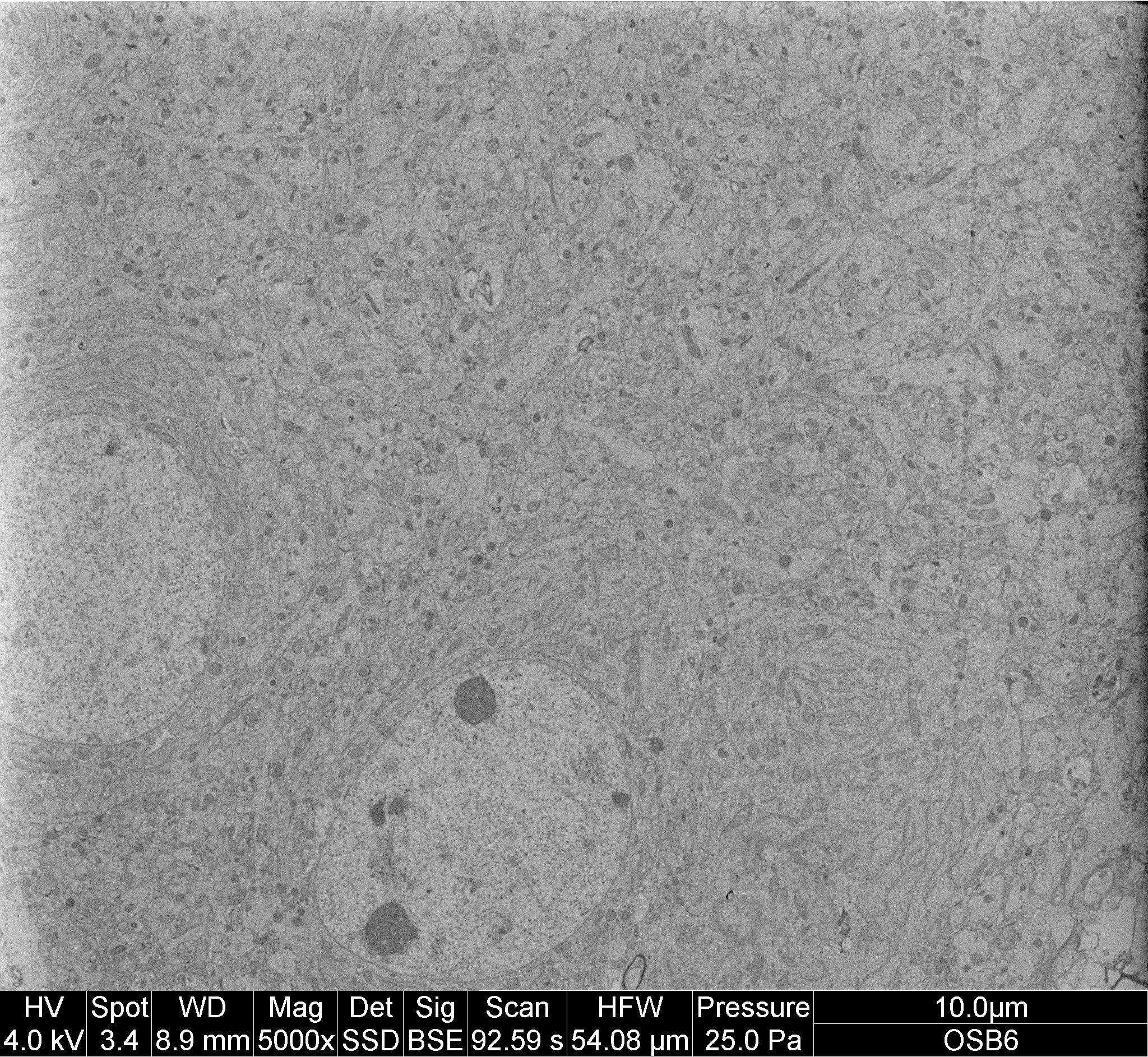

Supplement: Dataset S1 — (248.1 MB ZIP). [file pbio.0020329.sd001.zip › 040604_OS5_st1_056.tif]

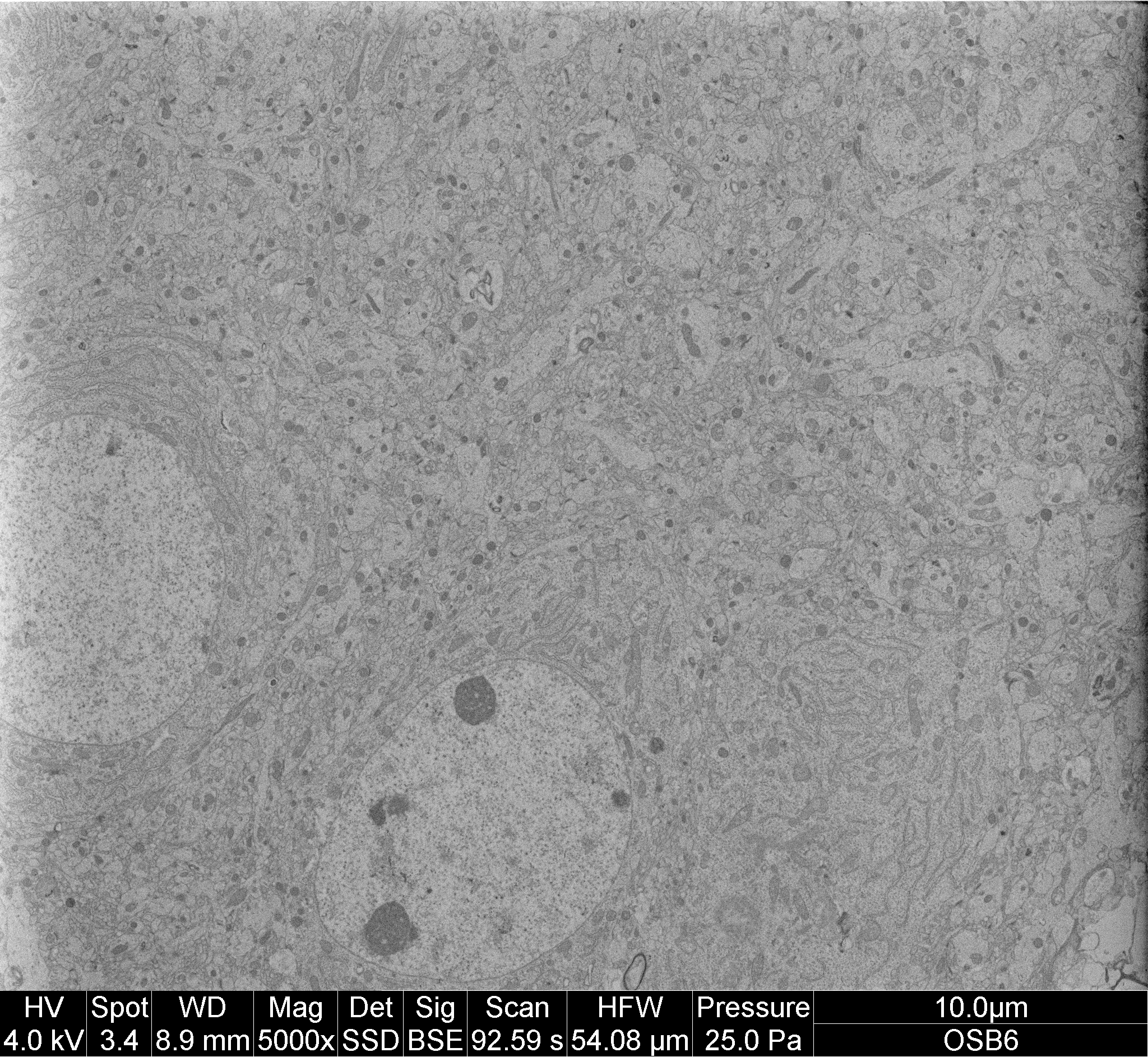

Supplement: Dataset S1 — (248.1 MB ZIP). [file pbio.0020329.sd001.zip › 040604_OS5_st1_057.tif]

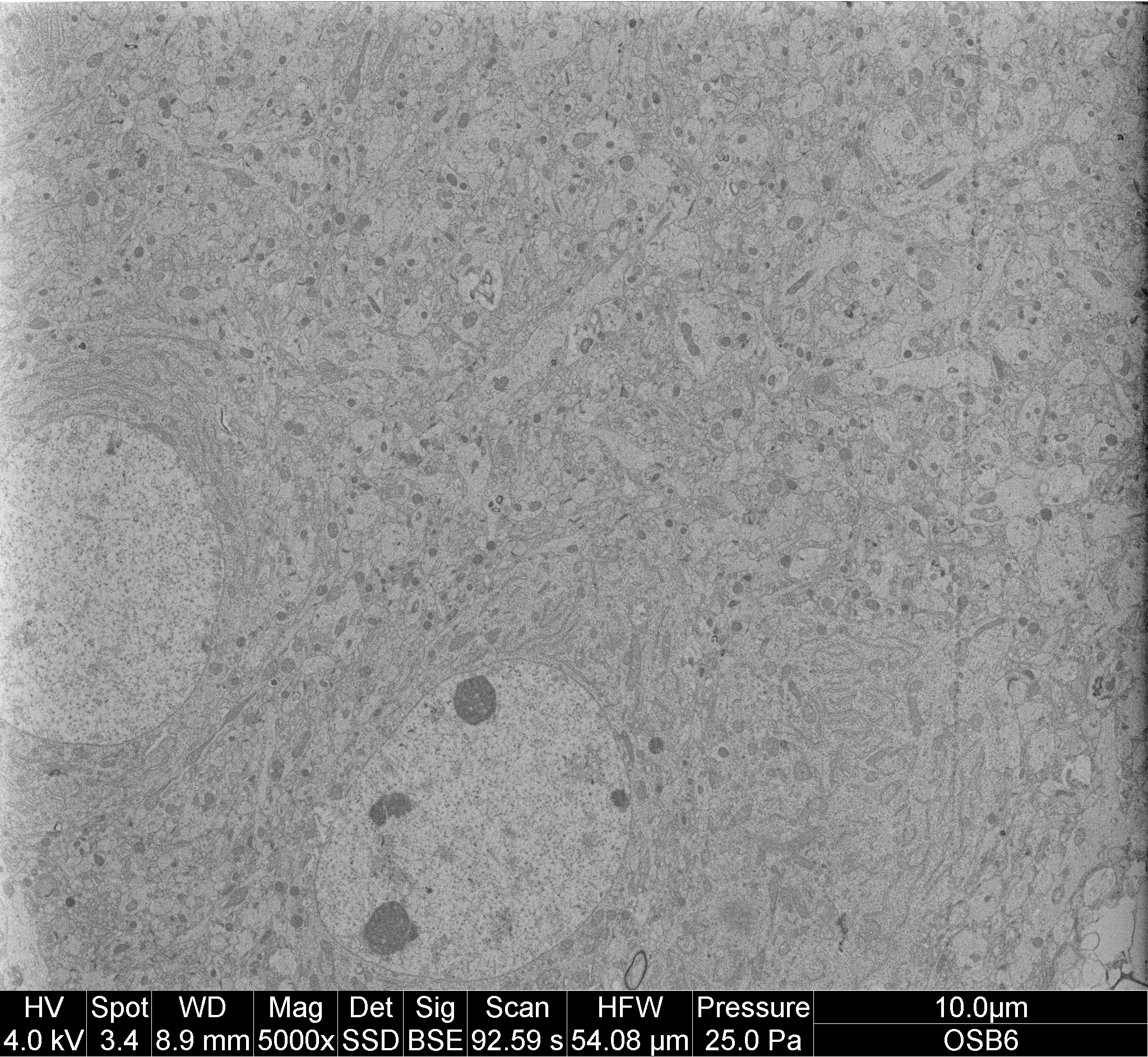

Supplement: Dataset S1 — (248.1 MB ZIP). [file pbio.0020329.sd001.zip › 040604_OS5_st1_058.tif]

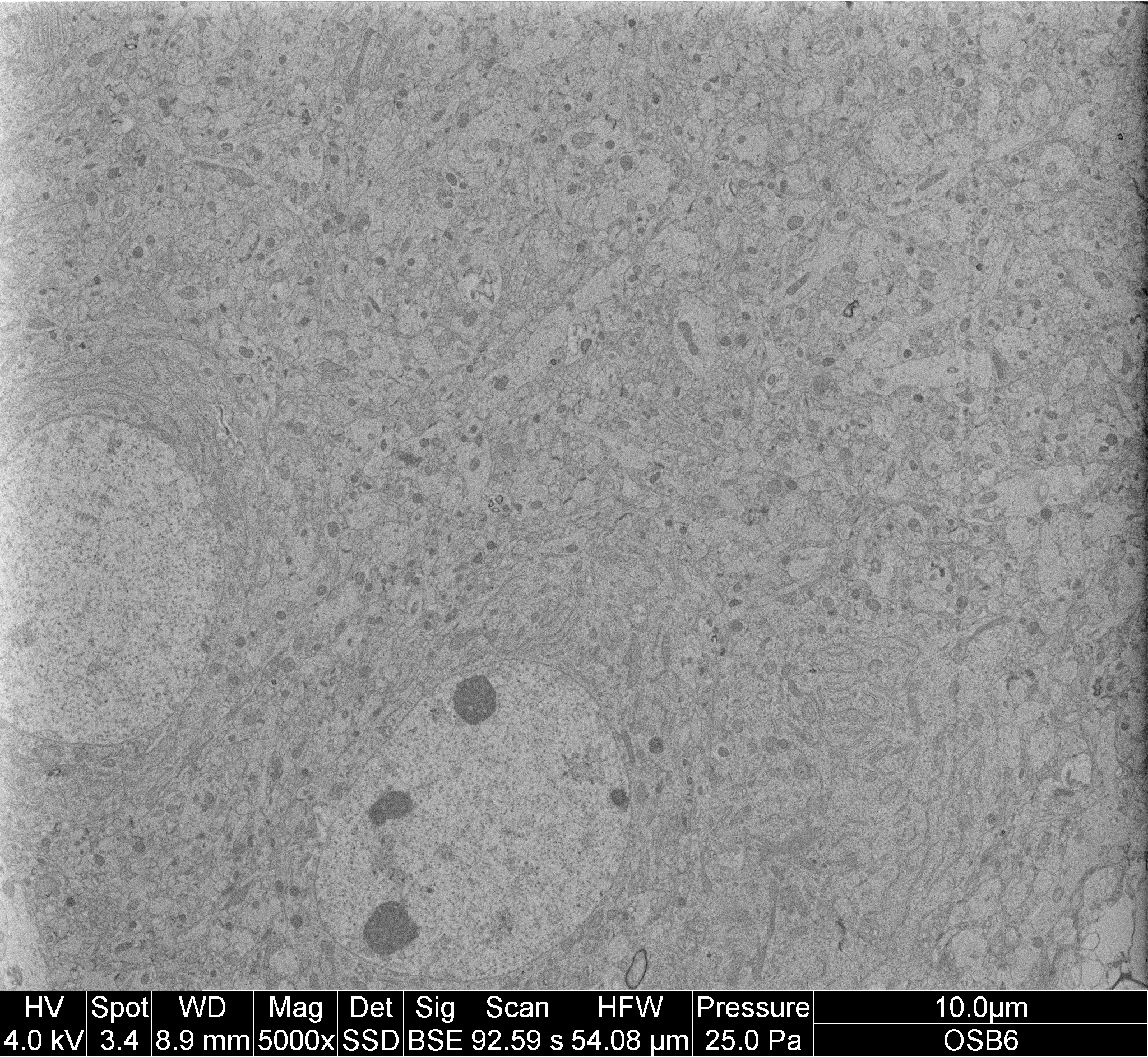

Supplement: Dataset S1 — (248.1 MB ZIP). [file pbio.0020329.sd001.zip › 040604_OS5_st1_059.tif]

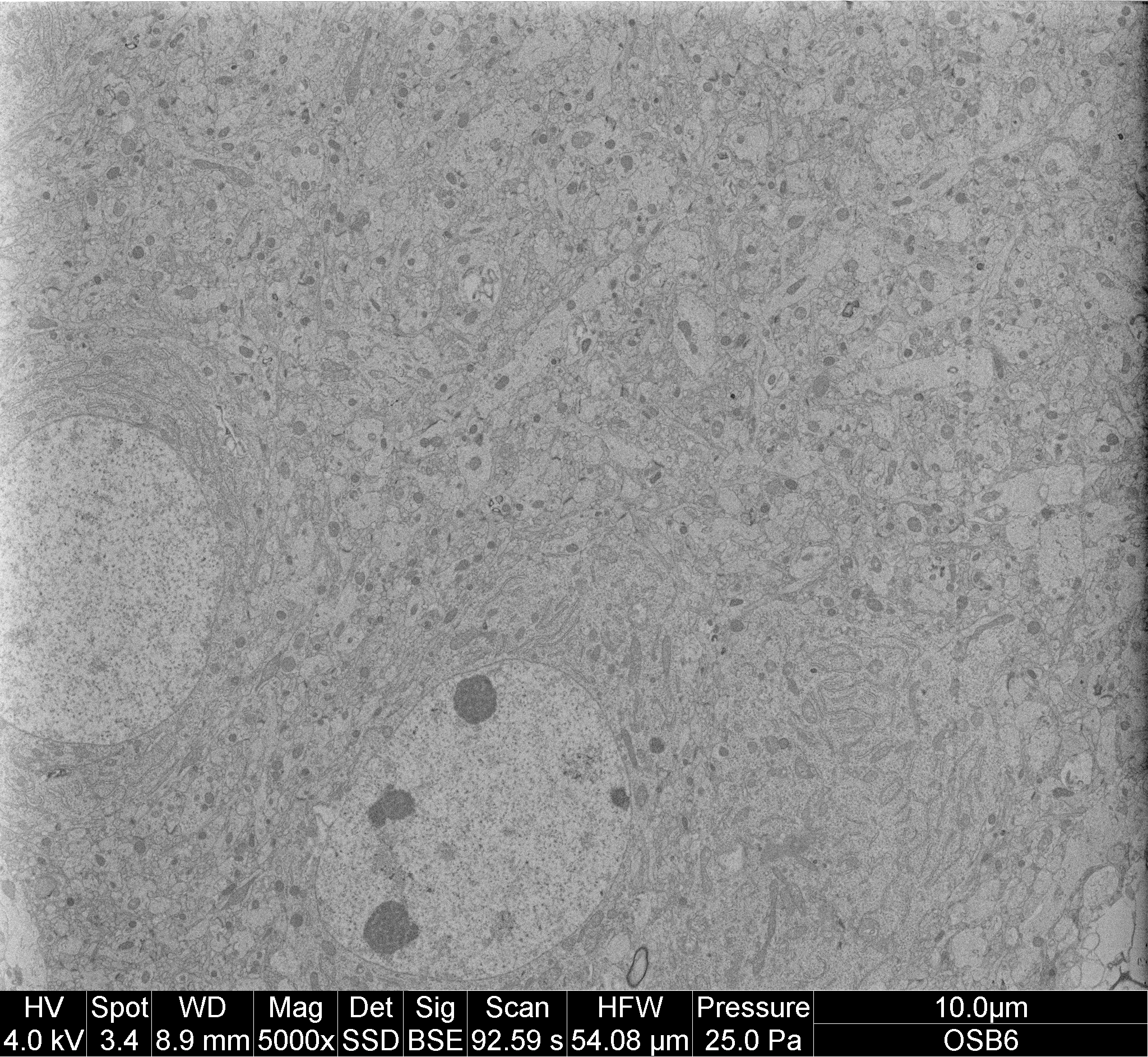

Supplement: Dataset S1 — (248.1 MB ZIP). [file pbio.0020329.sd001.zip › 040604_OS5_st1_060.tif]

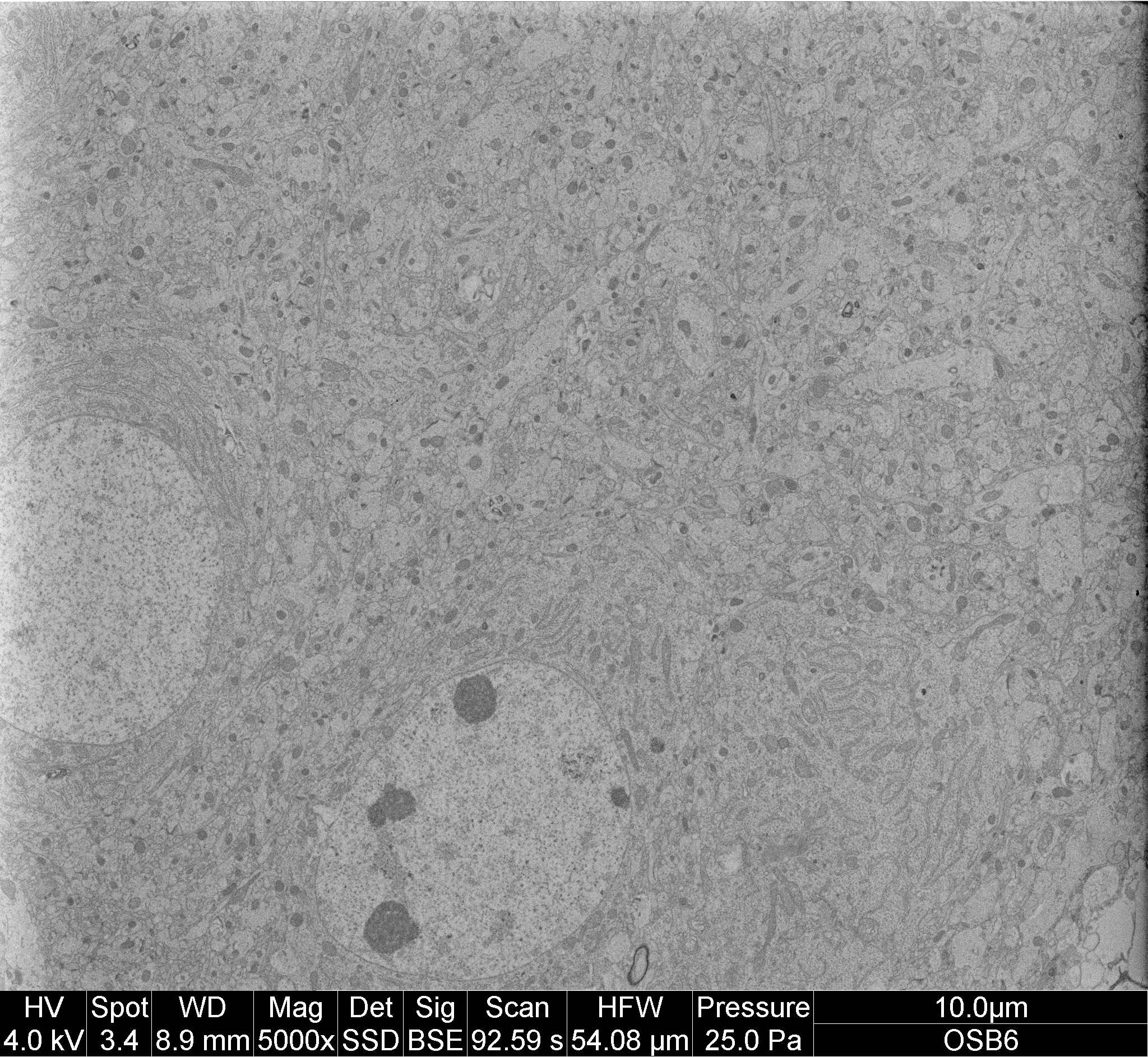

Supplement: Dataset S1 — (248.1 MB ZIP). [file pbio.0020329.sd001.zip › 040604_OS5_st1_061.tif]

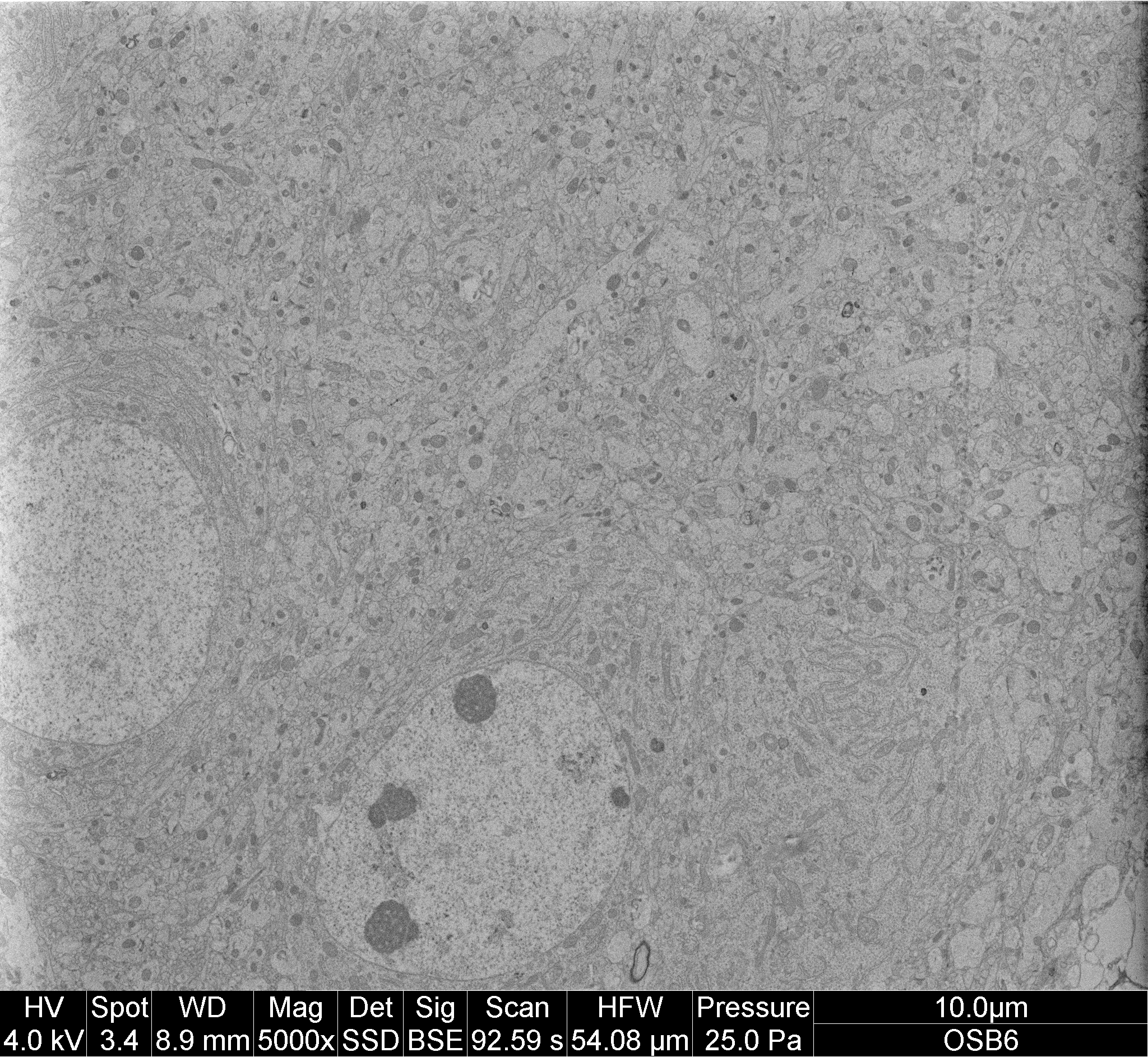

Supplement: Dataset S1 — (248.1 MB ZIP). [file pbio.0020329.sd001.zip › 040604_OS5_st1_062.tif]

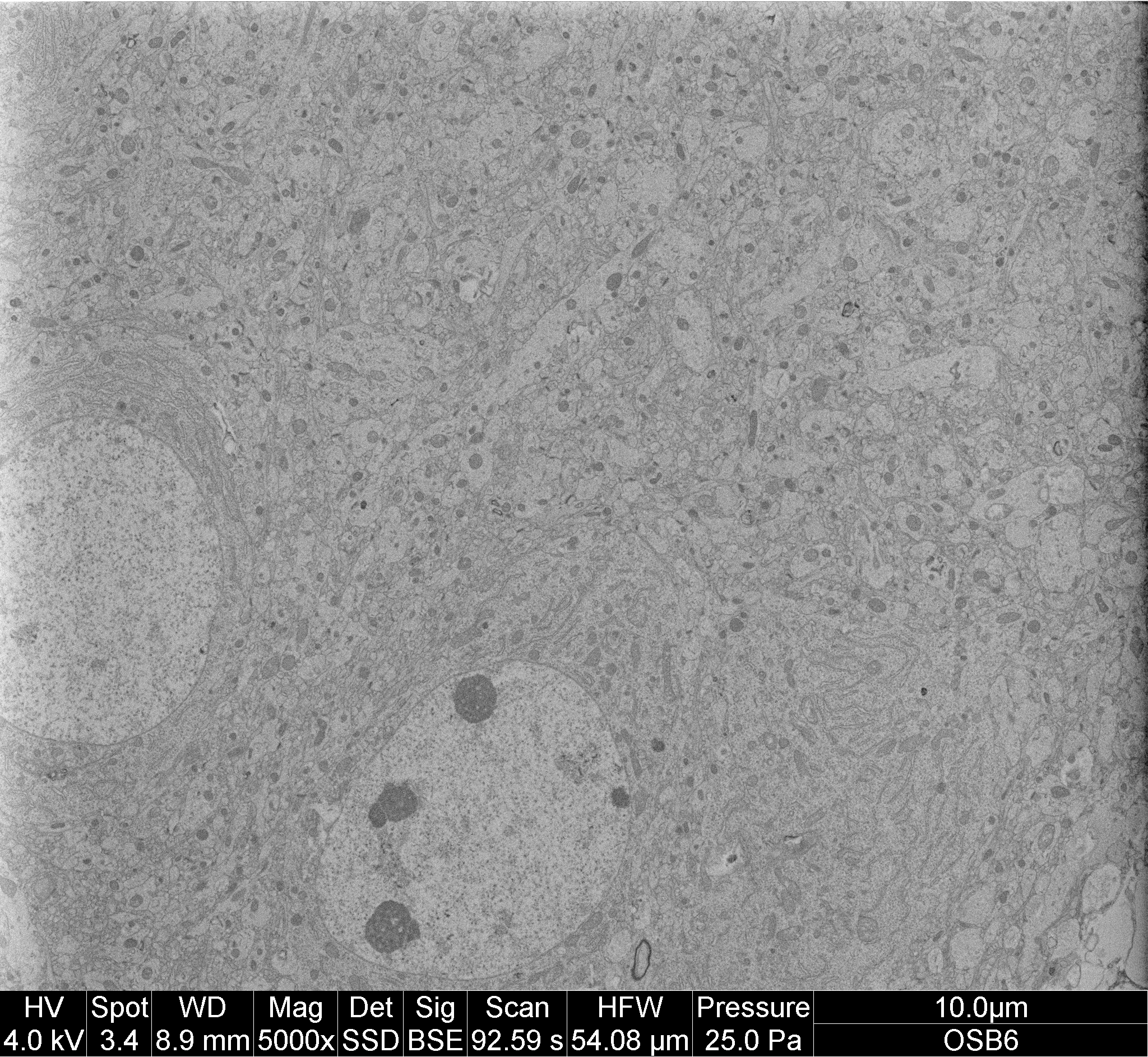

Supplement: Dataset S1 — (248.1 MB ZIP). [file pbio.0020329.sd001.zip › 040604_OS5_st1_063.tif]

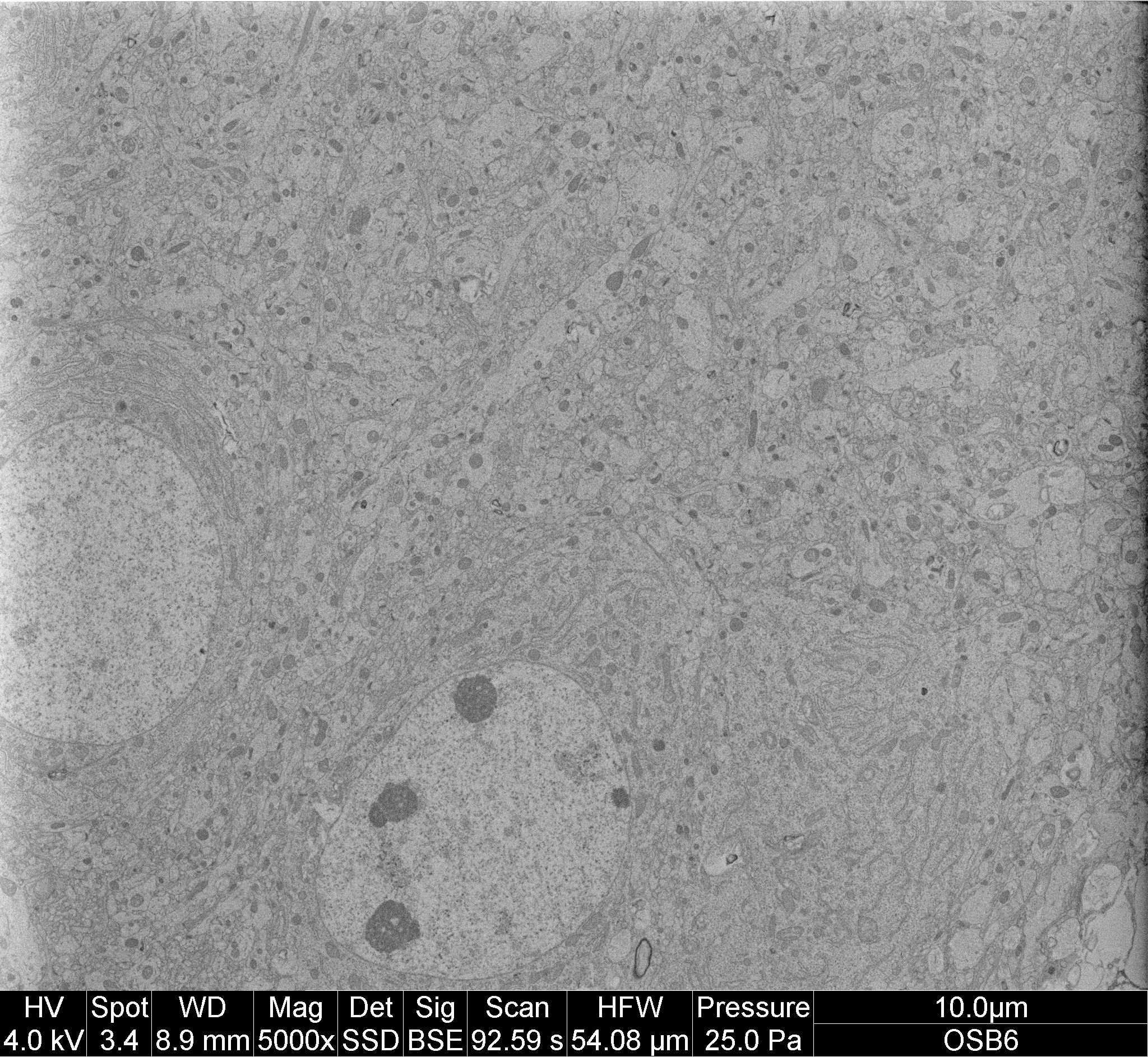

Supplement: Dataset S1 — (248.1 MB ZIP). [file pbio.0020329.sd001.zip › 040604_OS5_st1_064.tif]

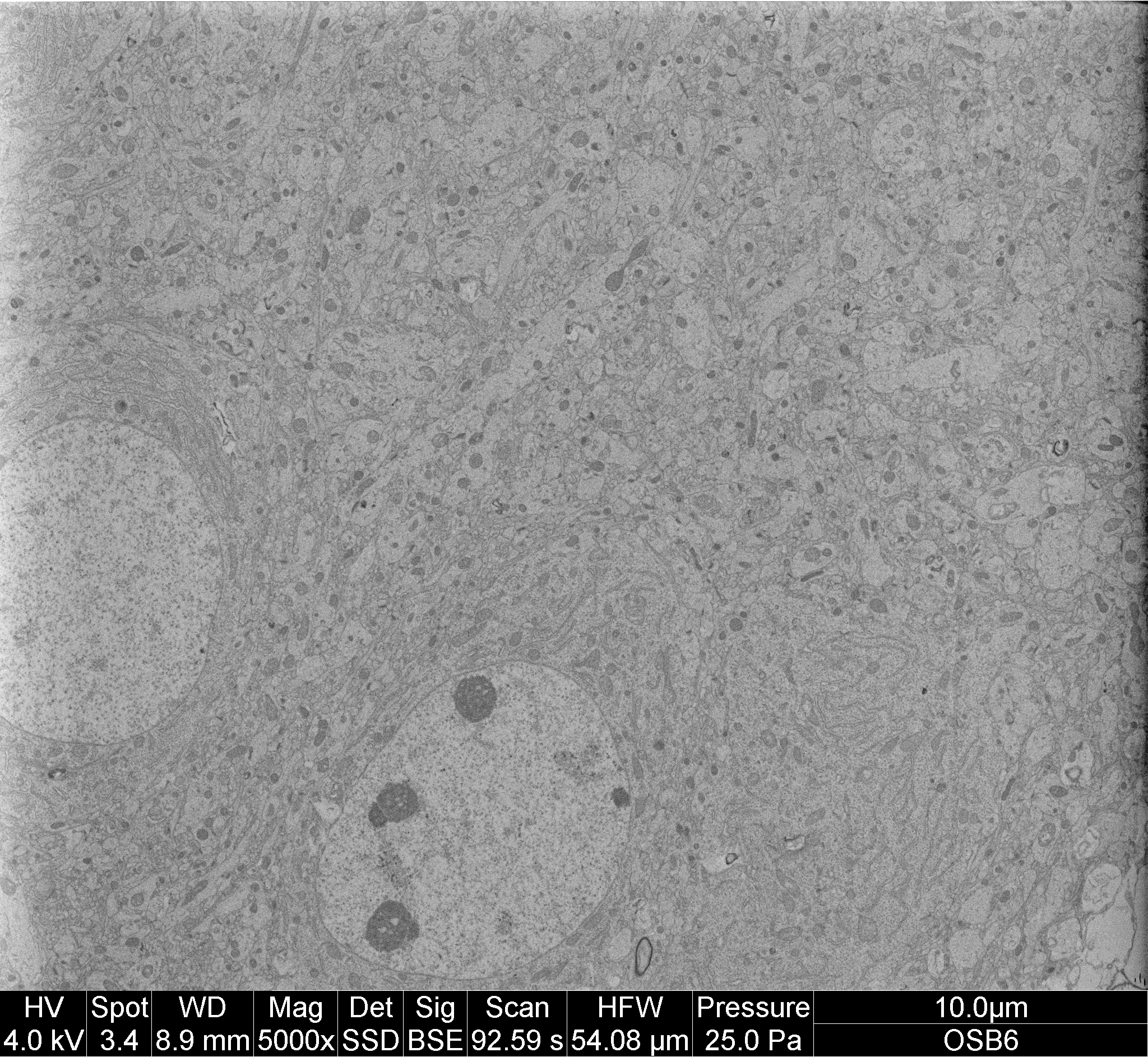

Supplement: Dataset S1 — (248.1 MB ZIP). [file pbio.0020329.sd001.zip › 040604_OS5_st1_065.tif]

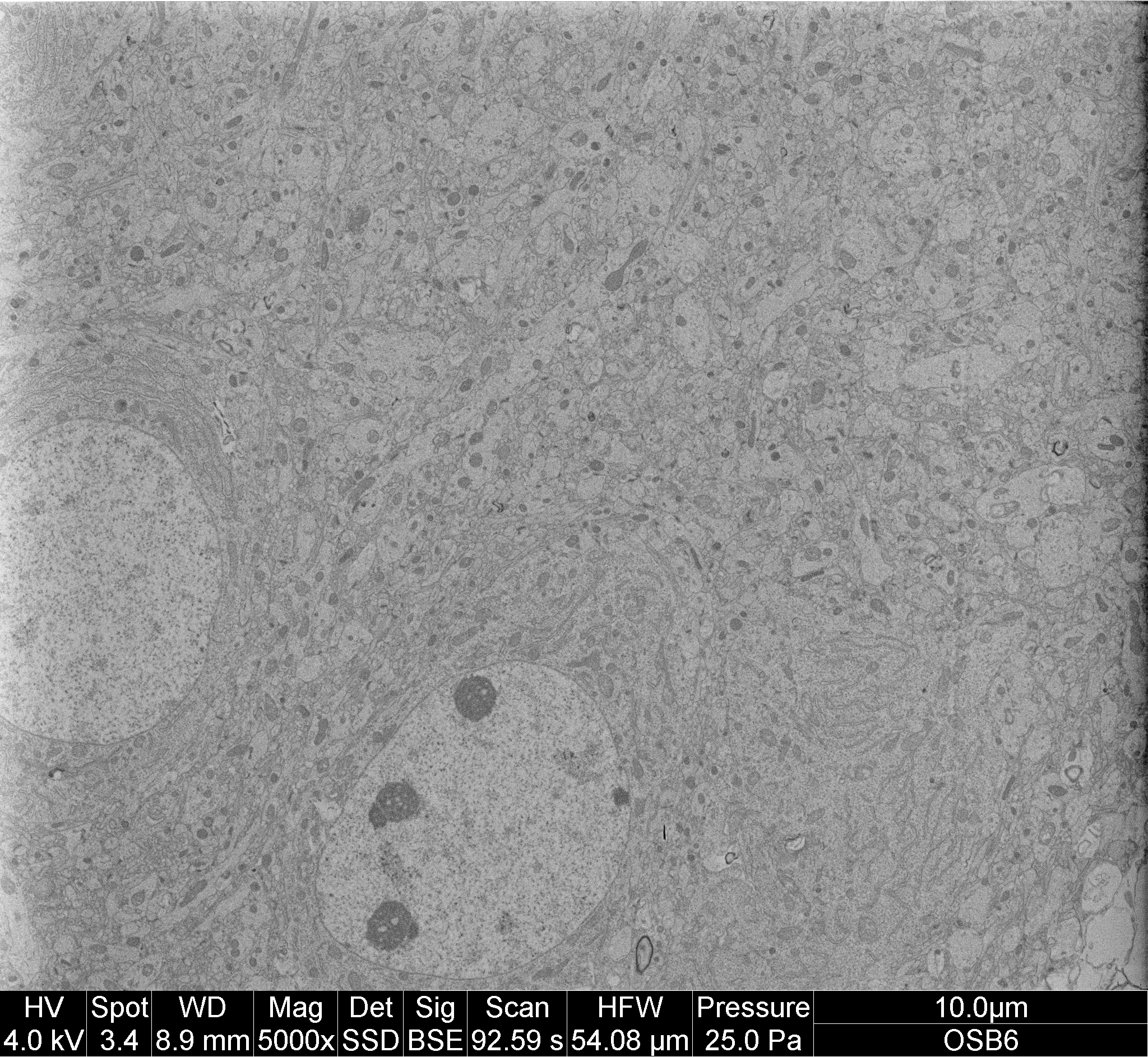

Supplement: Dataset S1 — (248.1 MB ZIP). [file pbio.0020329.sd001.zip › 040604_OS5_st1_066.tif]

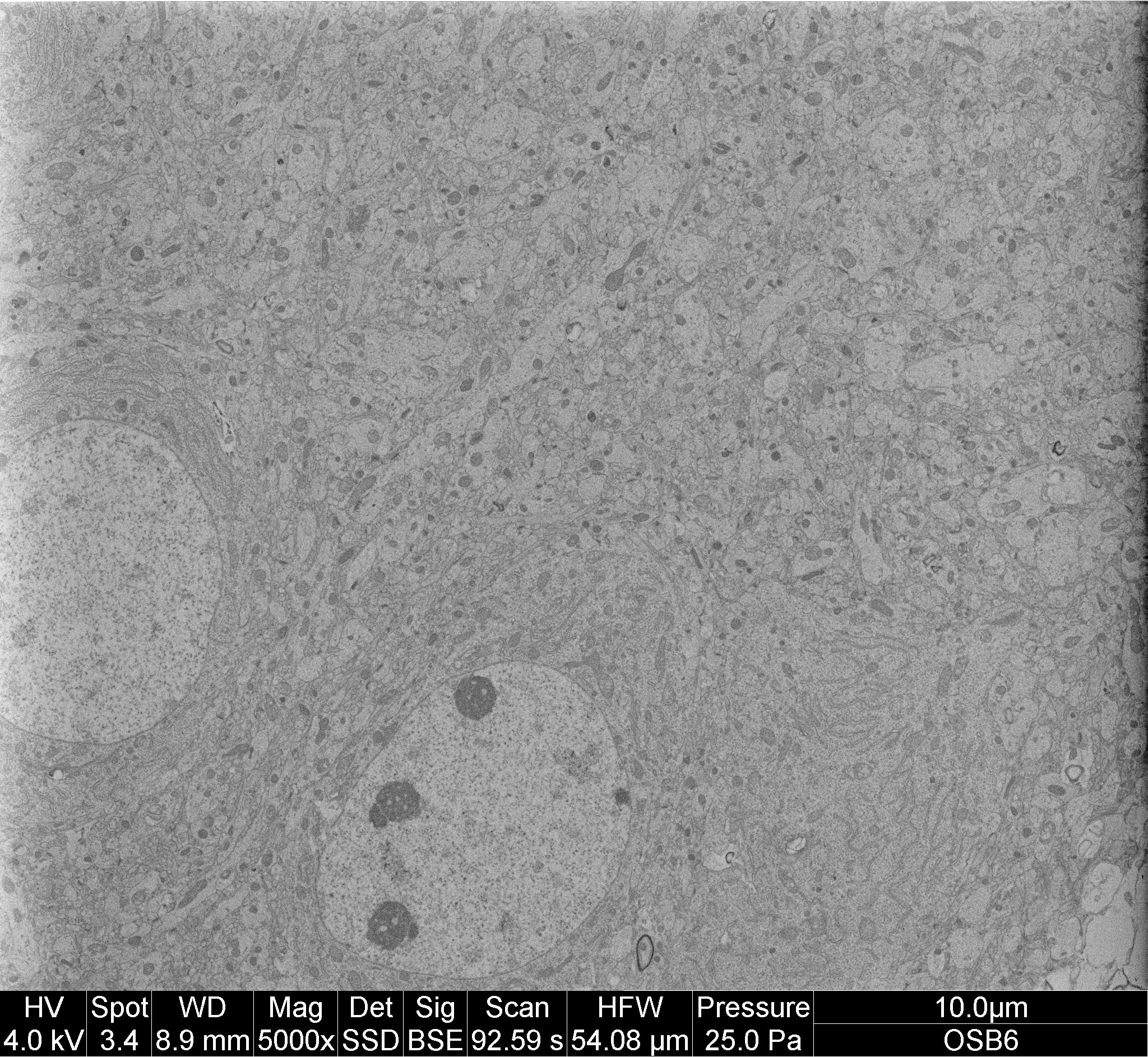

Supplement: Dataset S1 — (248.1 MB ZIP). [file pbio.0020329.sd001.zip › 040604_OS5_st1_067.tif]

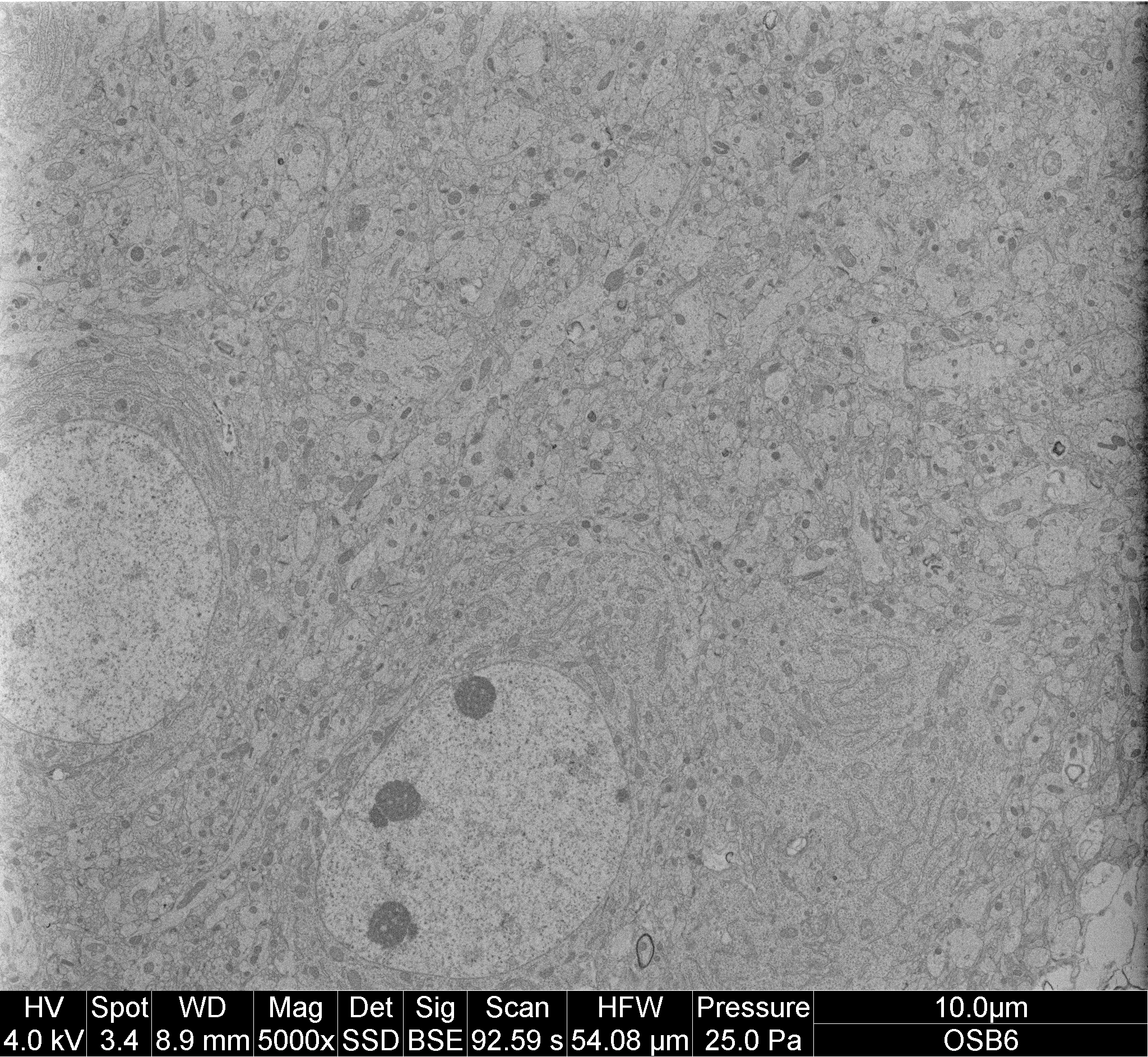

Supplement: Dataset S1 — (248.1 MB ZIP). [file pbio.0020329.sd001.zip › 040604_OS5_st1_068.tif]

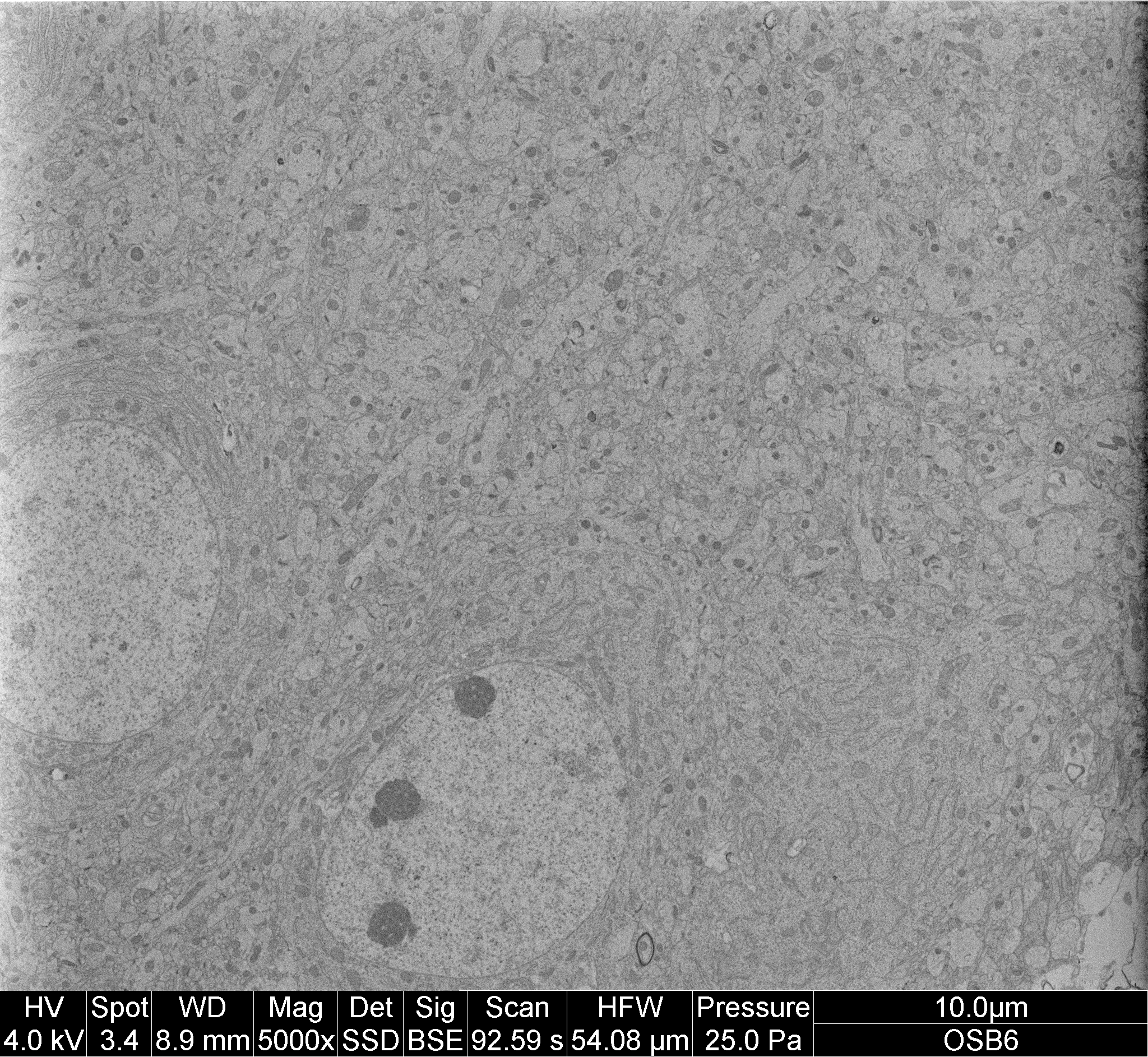

Supplement: Dataset S1 — (248.1 MB ZIP). [file pbio.0020329.sd001.zip › 040604_OS5_st1_069.tif]

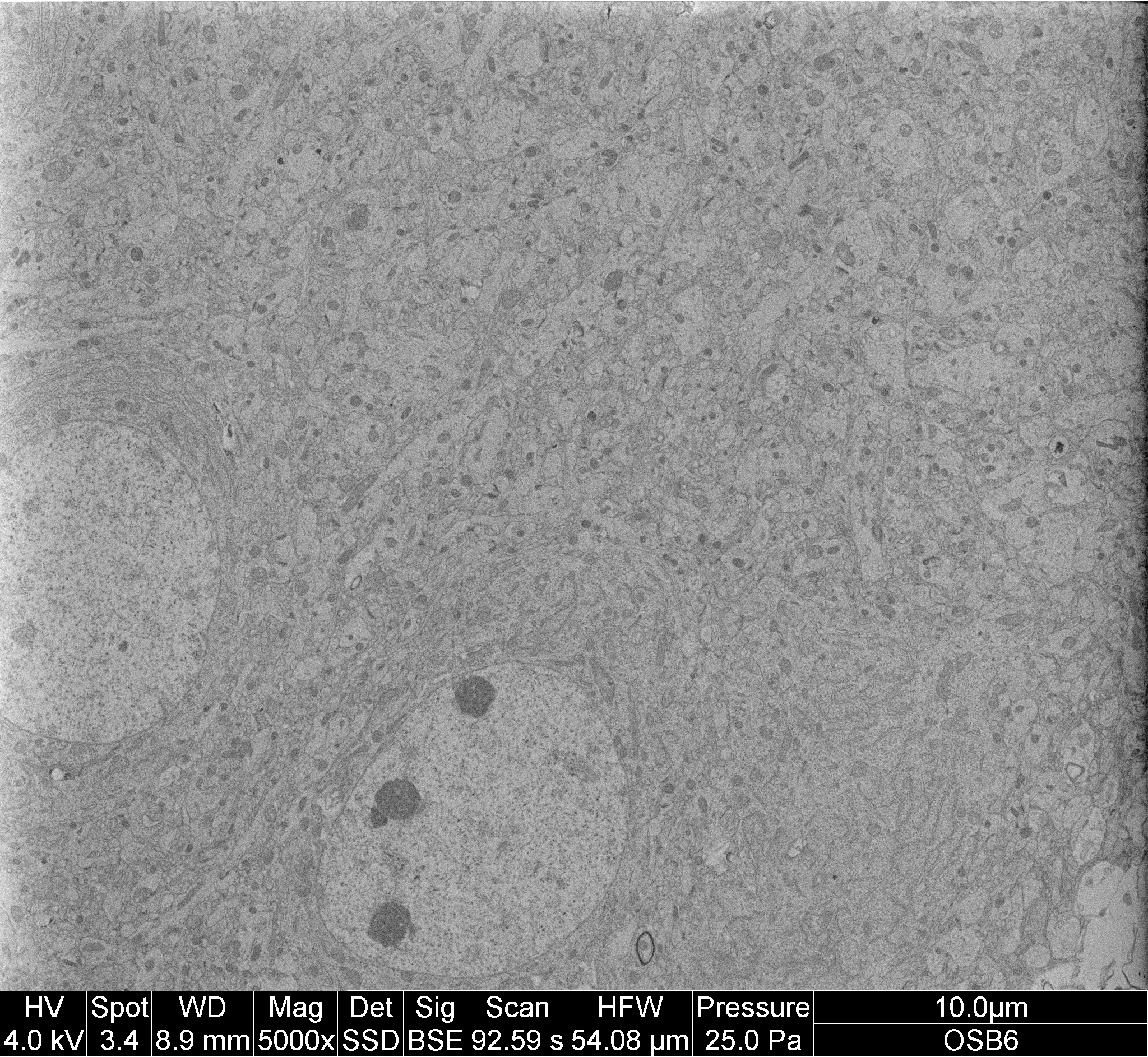

Supplement: Dataset S1 — (248.1 MB ZIP). [file pbio.0020329.sd001.zip › 040604_OS5_st1_070.tif]

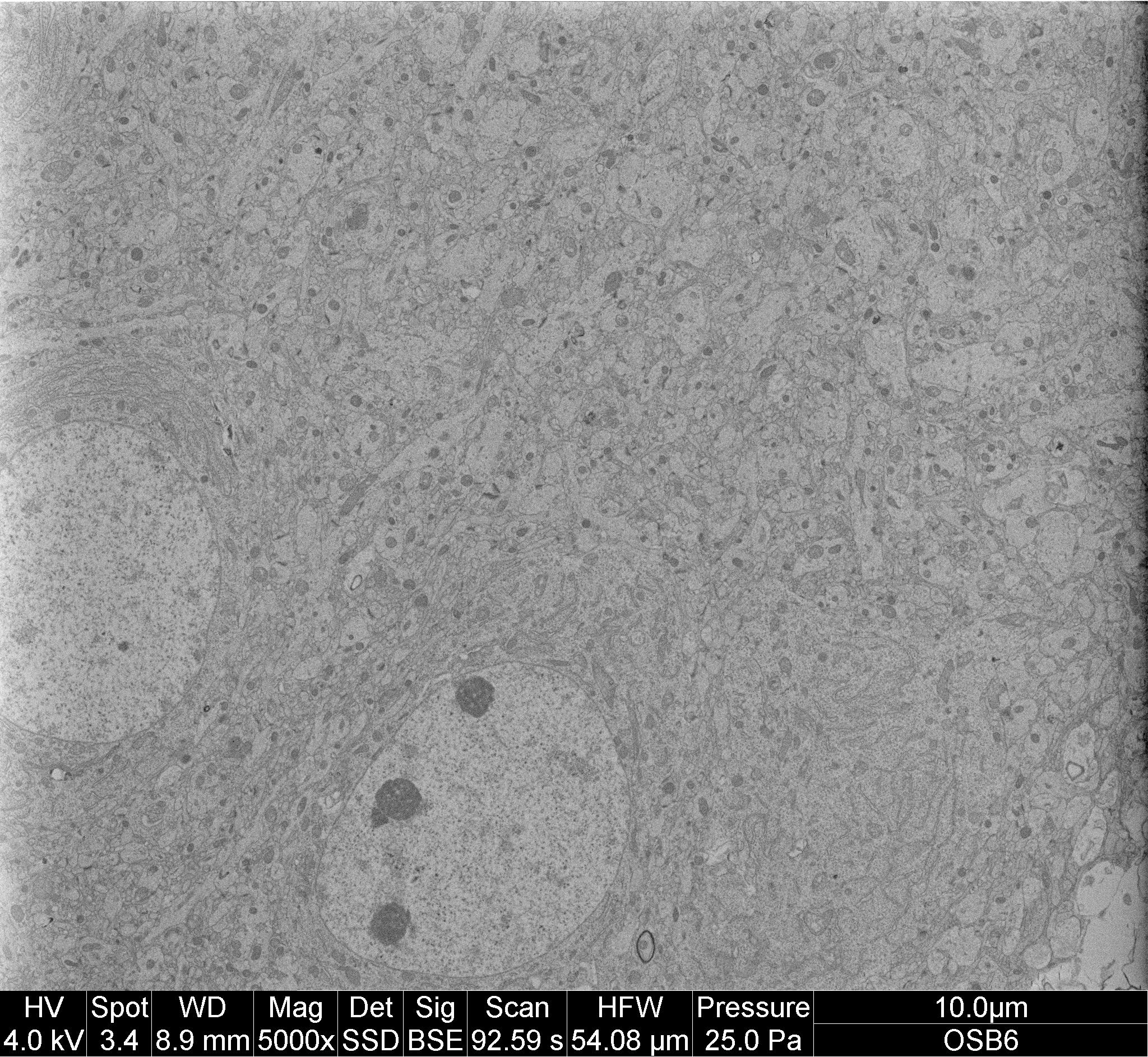

Supplement: Dataset S1 — (248.1 MB ZIP). [file pbio.0020329.sd001.zip › 040604_OS5_st1_071.tif]

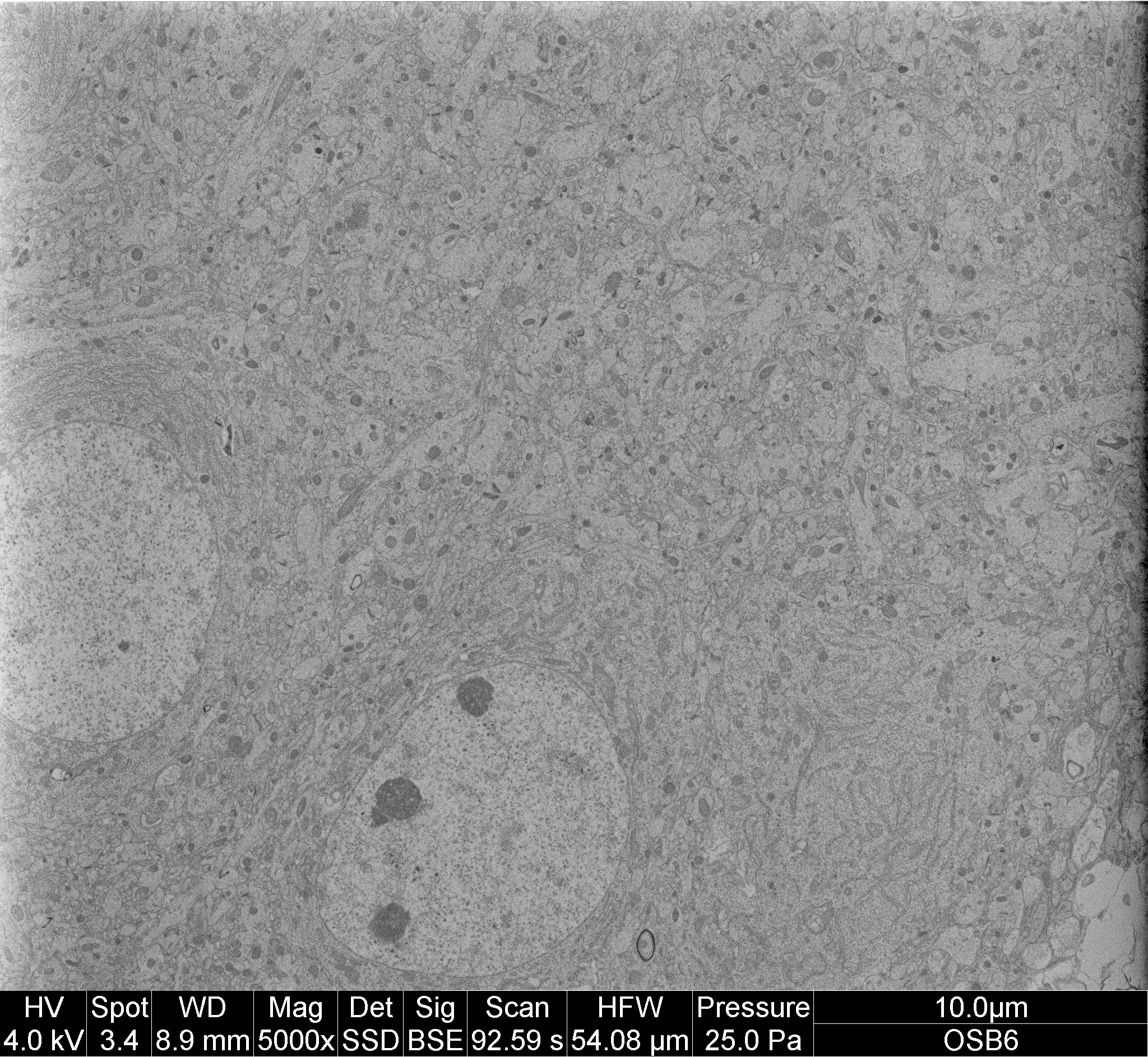

Supplement: Dataset S1 — (248.1 MB ZIP). [file pbio.0020329.sd001.zip › 040604_OS5_st1_072.tif]

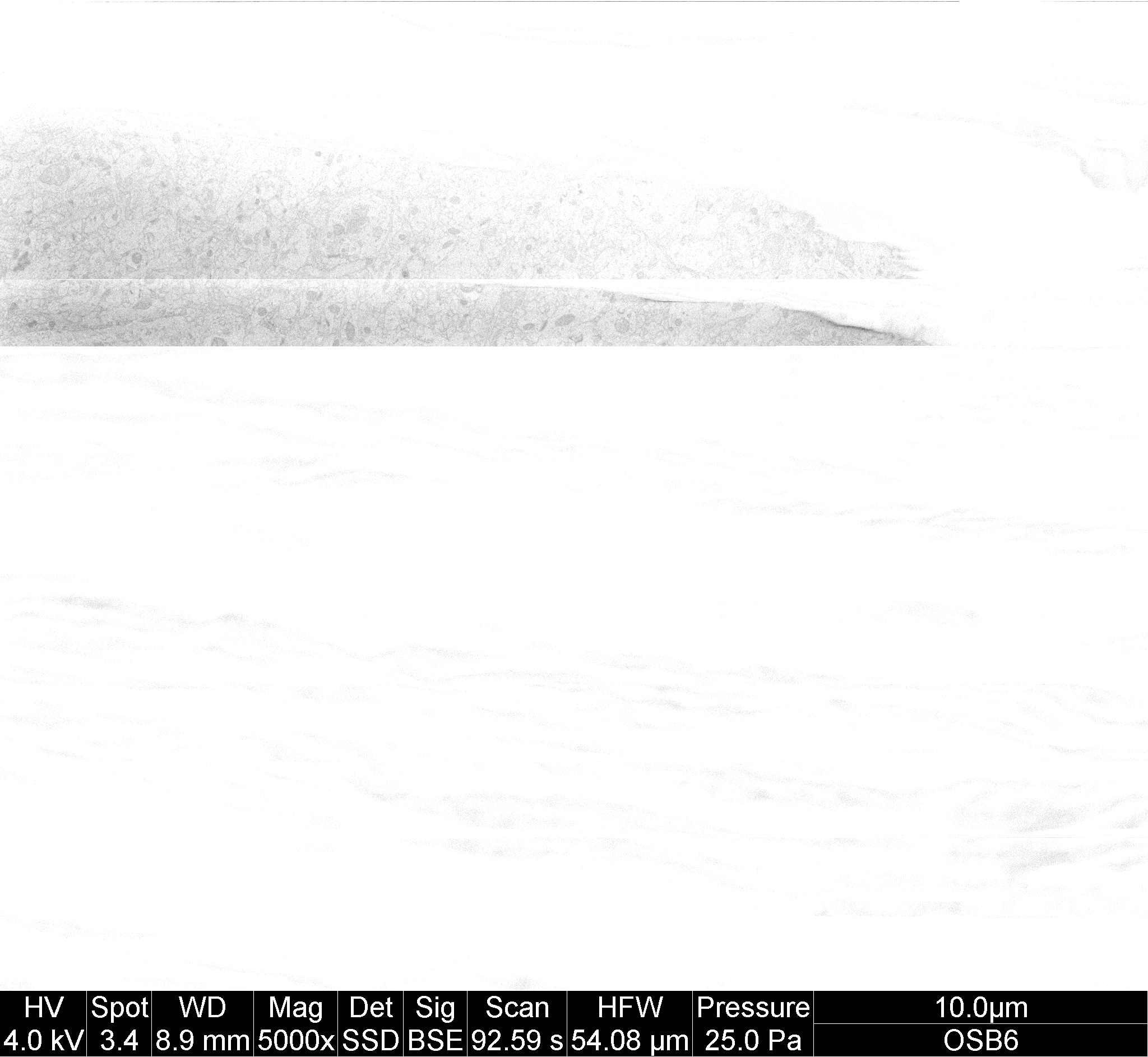

Supplement: Dataset S1 — (248.1 MB ZIP). [file pbio.0020329.sd001.zip › 040604_OS5_st1_073.tif]

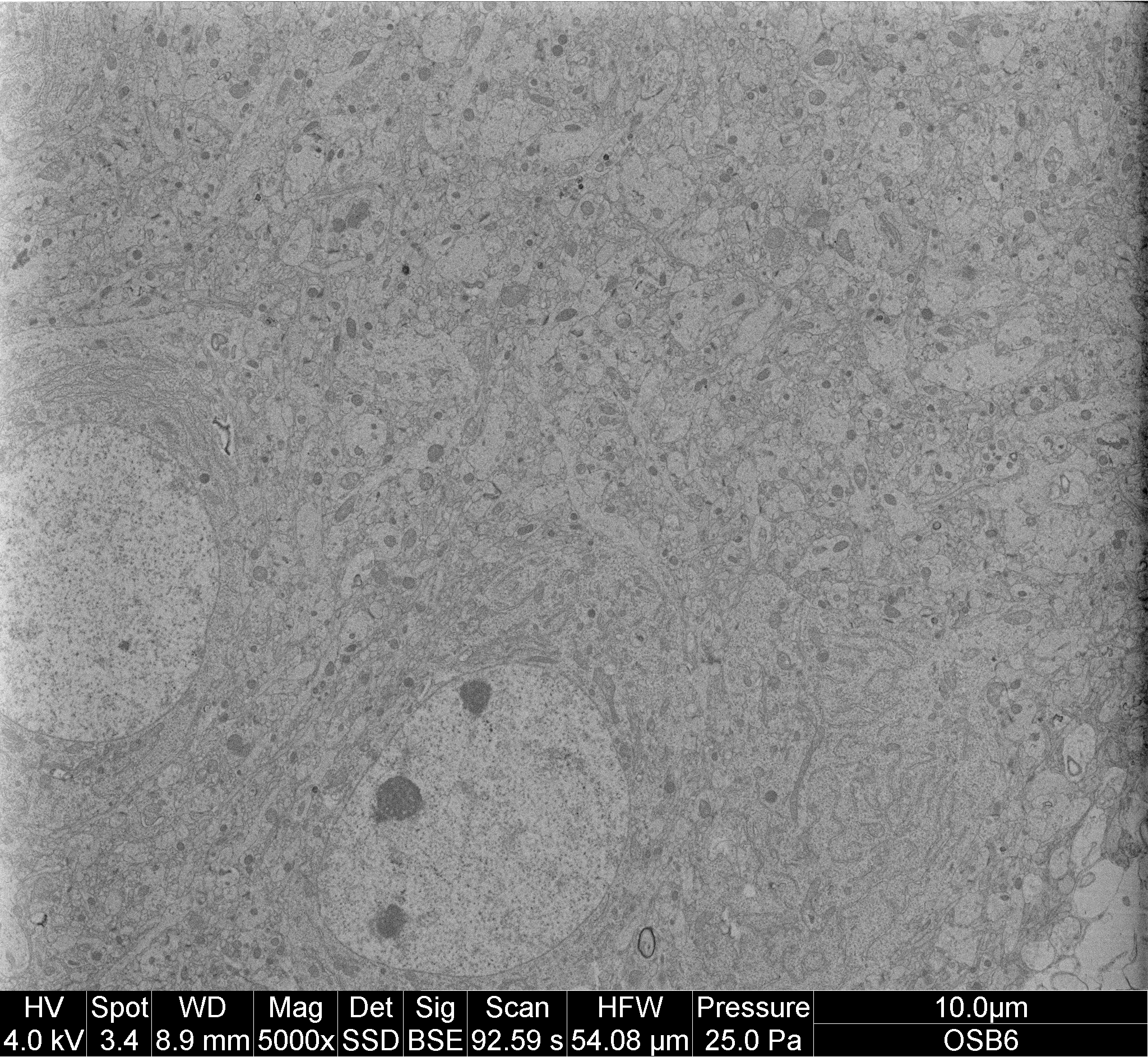

Supplement: Dataset S1 — (248.1 MB ZIP). [file pbio.0020329.sd001.zip › 040604_OS5_st1_074.tif]

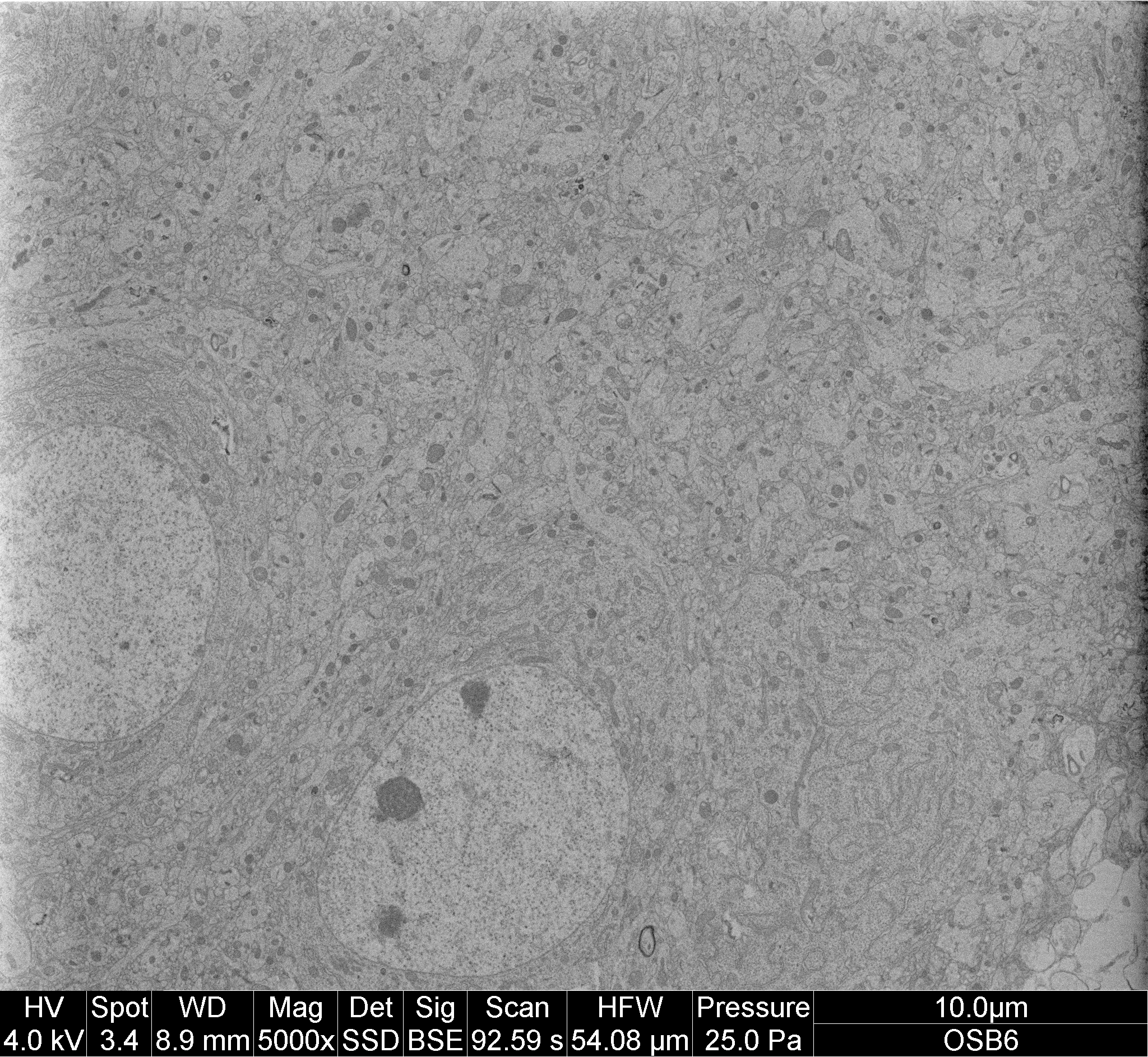

Supplement: Dataset S1 — (248.1 MB ZIP). [file pbio.0020329.sd001.zip › 040604_OS5_st1_075.tif]

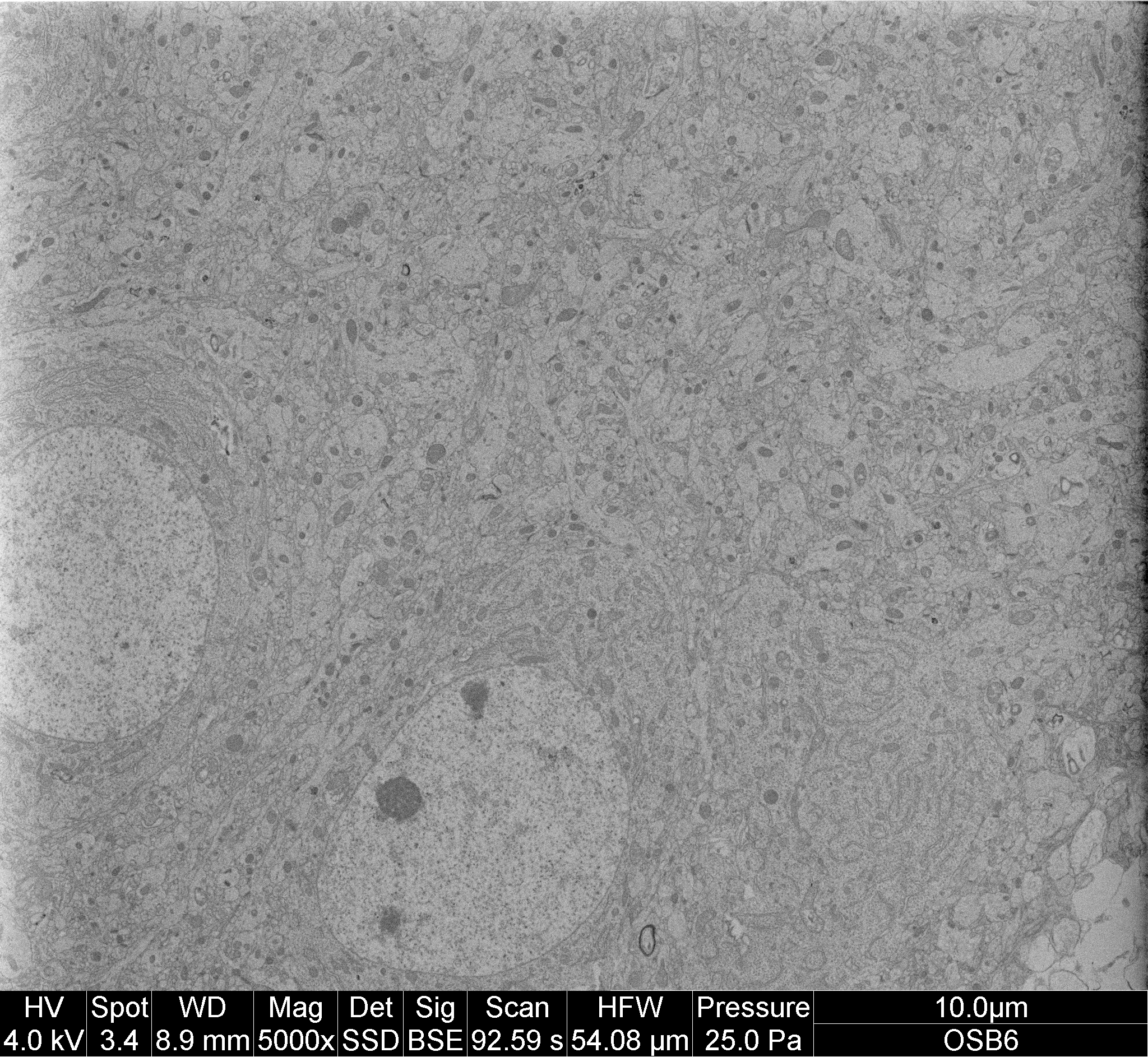

Supplement: Dataset S1 — (248.1 MB ZIP). [file pbio.0020329.sd001.zip › 040604_OS5_st1_076.tif]

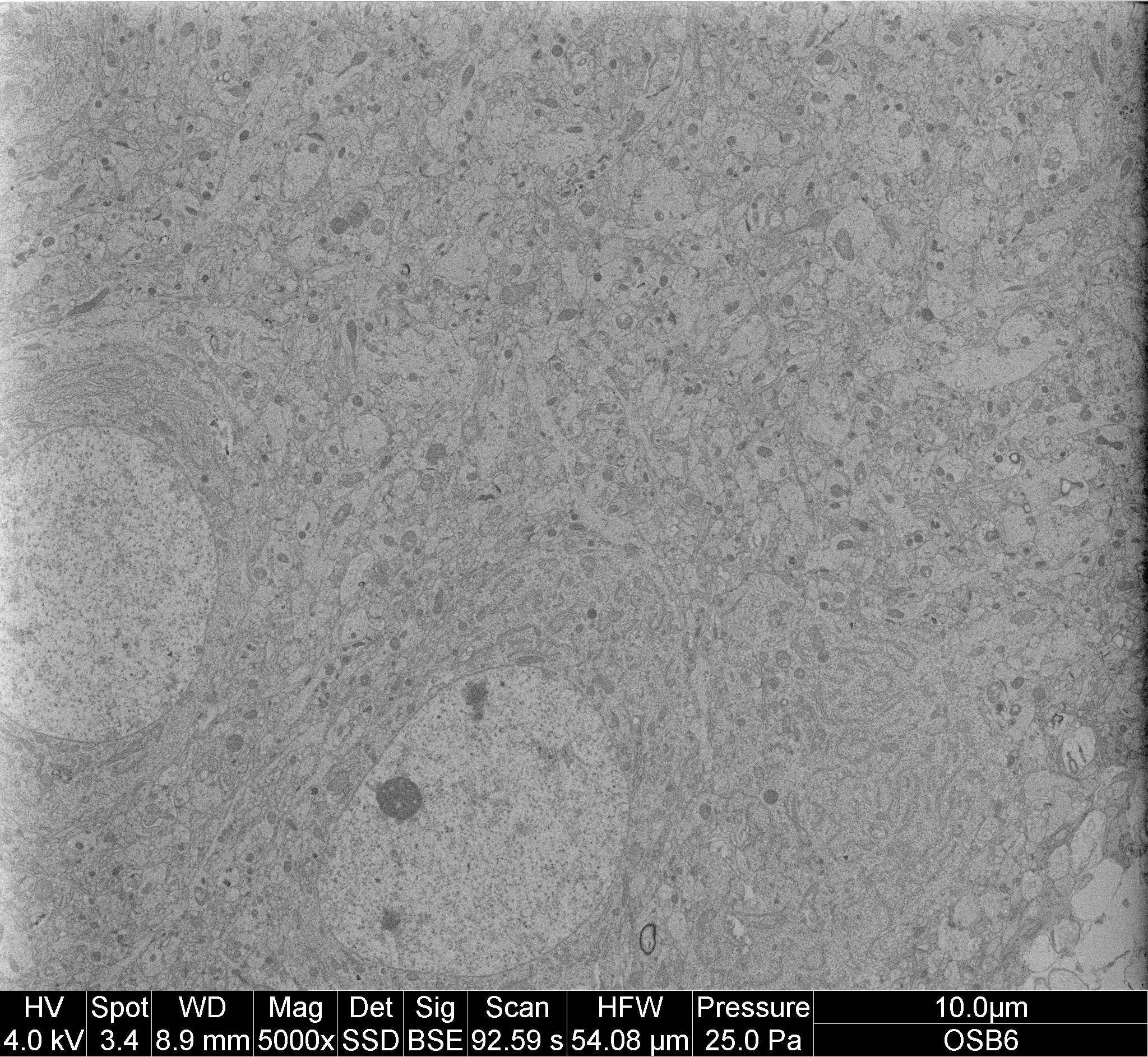

Supplement: Dataset S1 — (248.1 MB ZIP). [file pbio.0020329.sd001.zip › 040604_OS5_st1_077.tif]

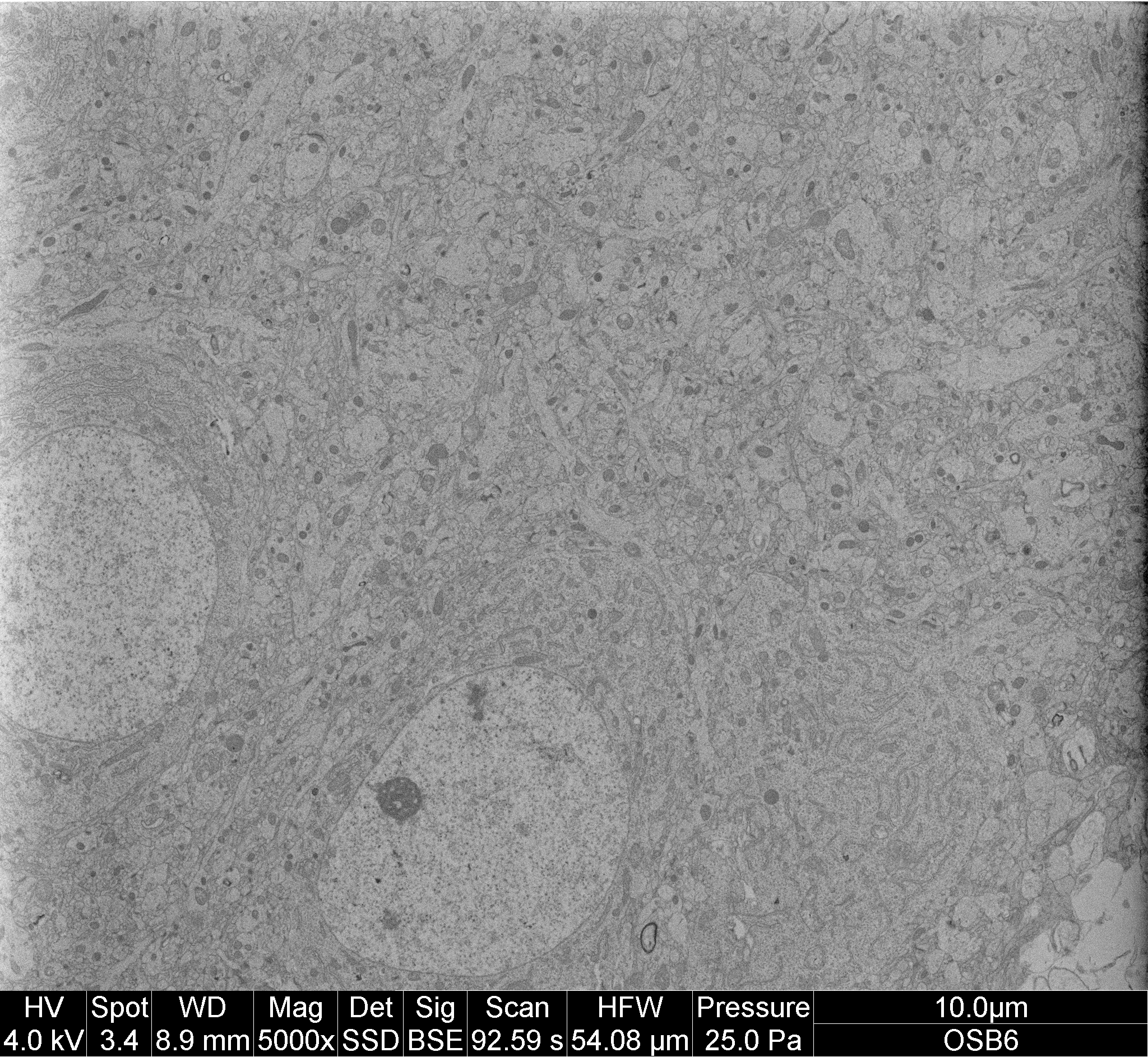

Supplement: Dataset S1 — (248.1 MB ZIP). [file pbio.0020329.sd001.zip › 040604_OS5_st1_078.tif]

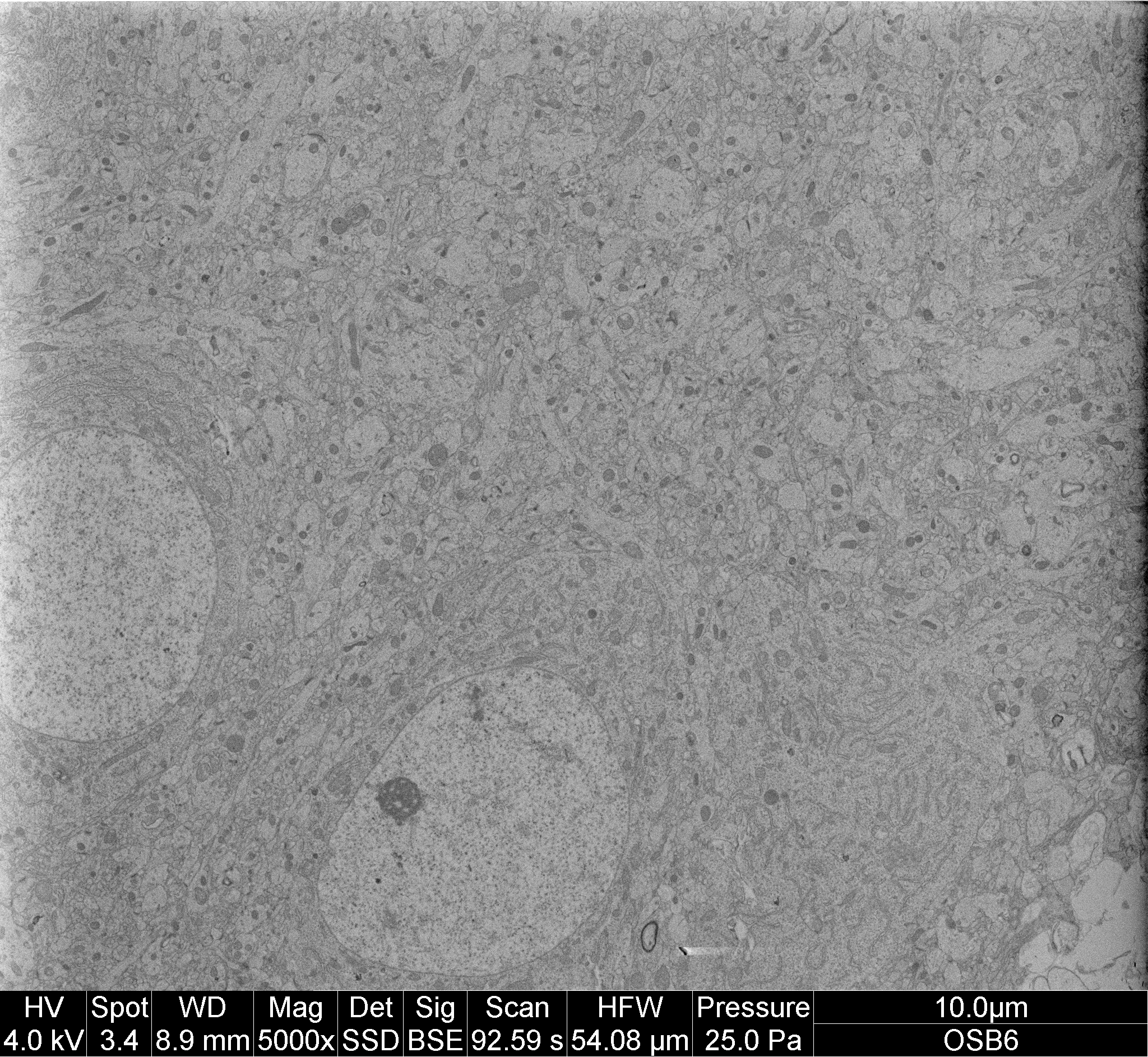

Supplement: Dataset S1 — (248.1 MB ZIP). [file pbio.0020329.sd001.zip › 040604_OS5_st1_079.tif]

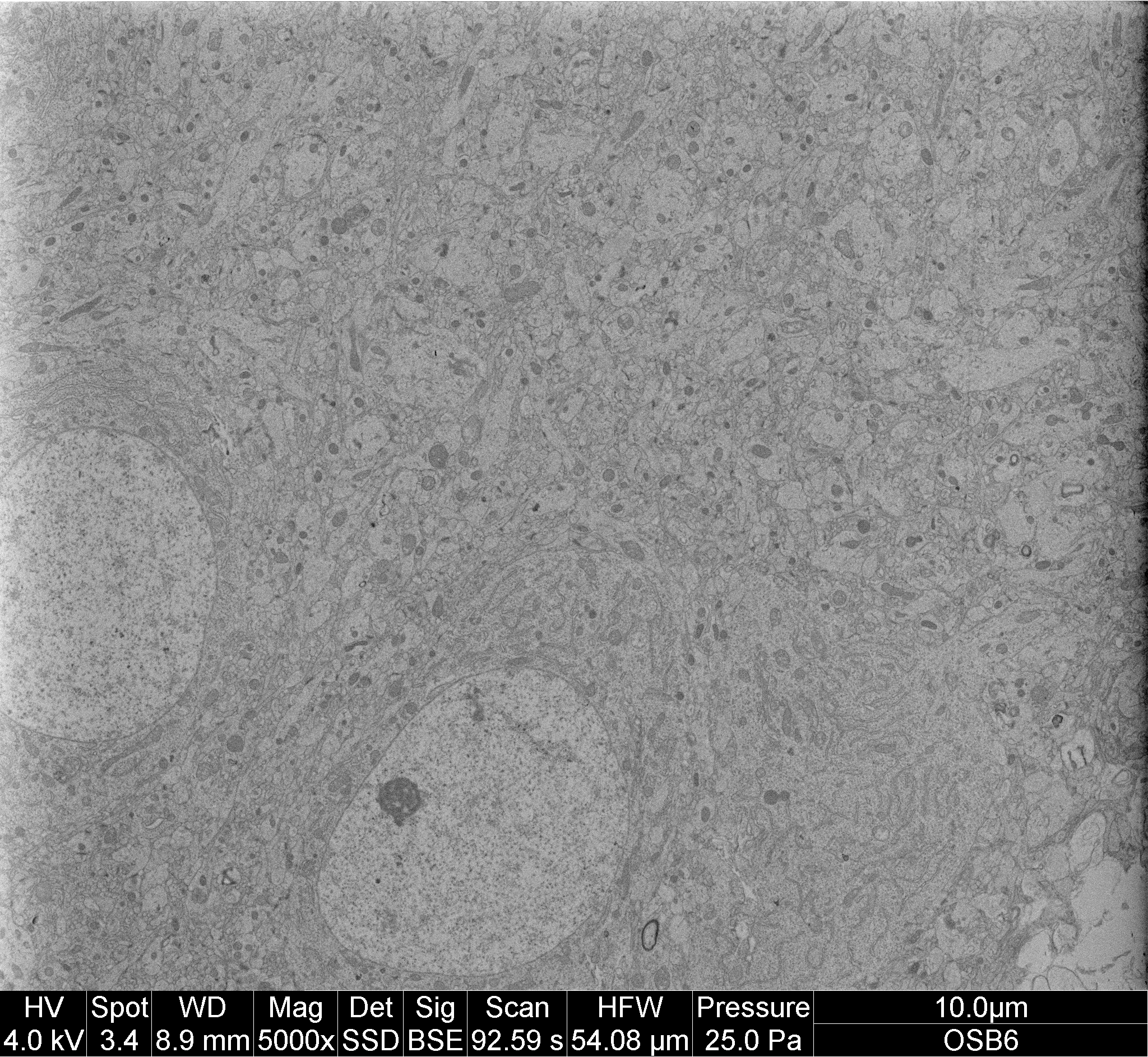

Supplement: Dataset S1 — (248.1 MB ZIP). [file pbio.0020329.sd001.zip › 040604_OS5_st1_080.tif]

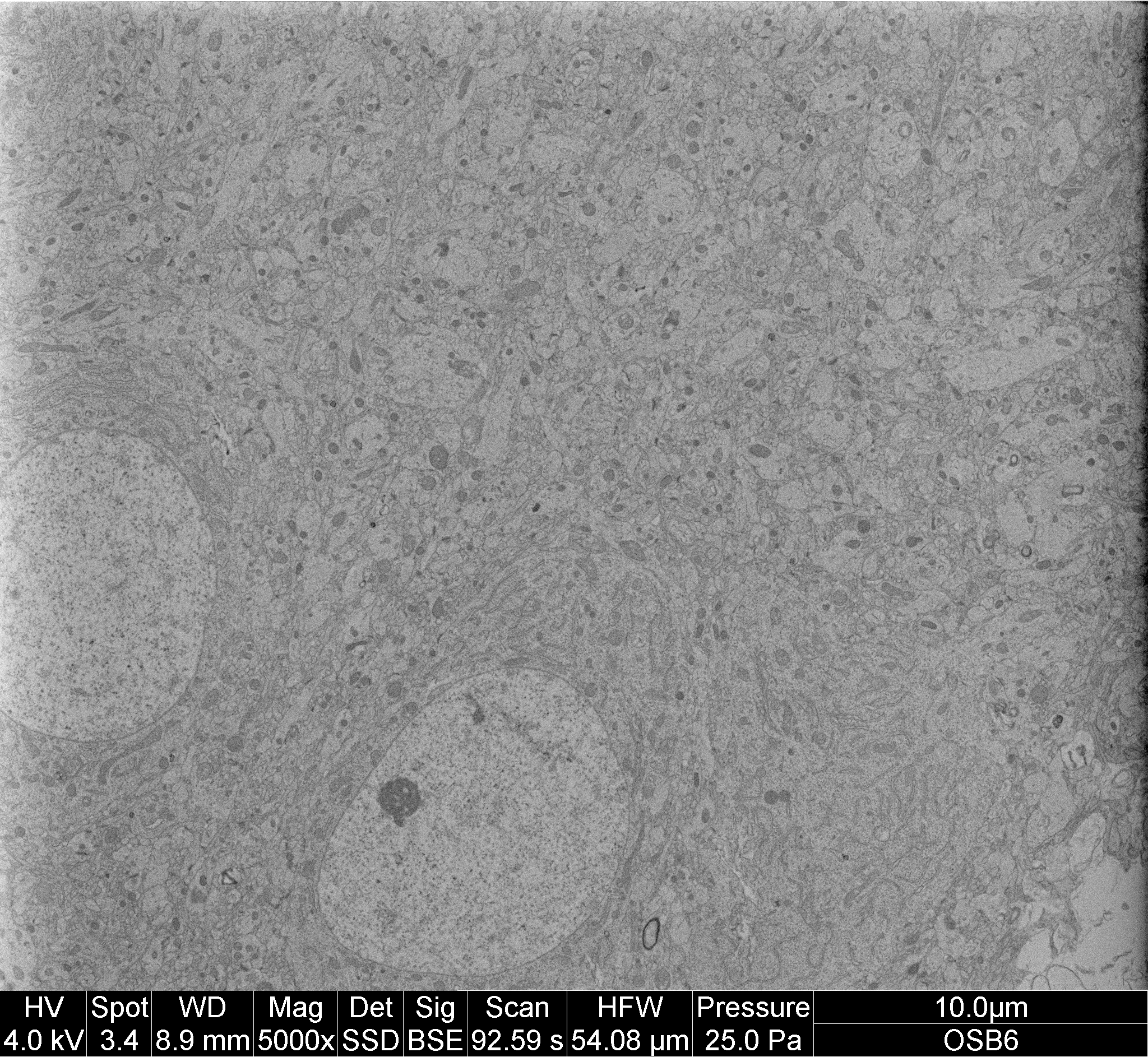

Supplement: Dataset S1 — (248.1 MB ZIP). [file pbio.0020329.sd001.zip › 040604_OS5_st1_081.tif]

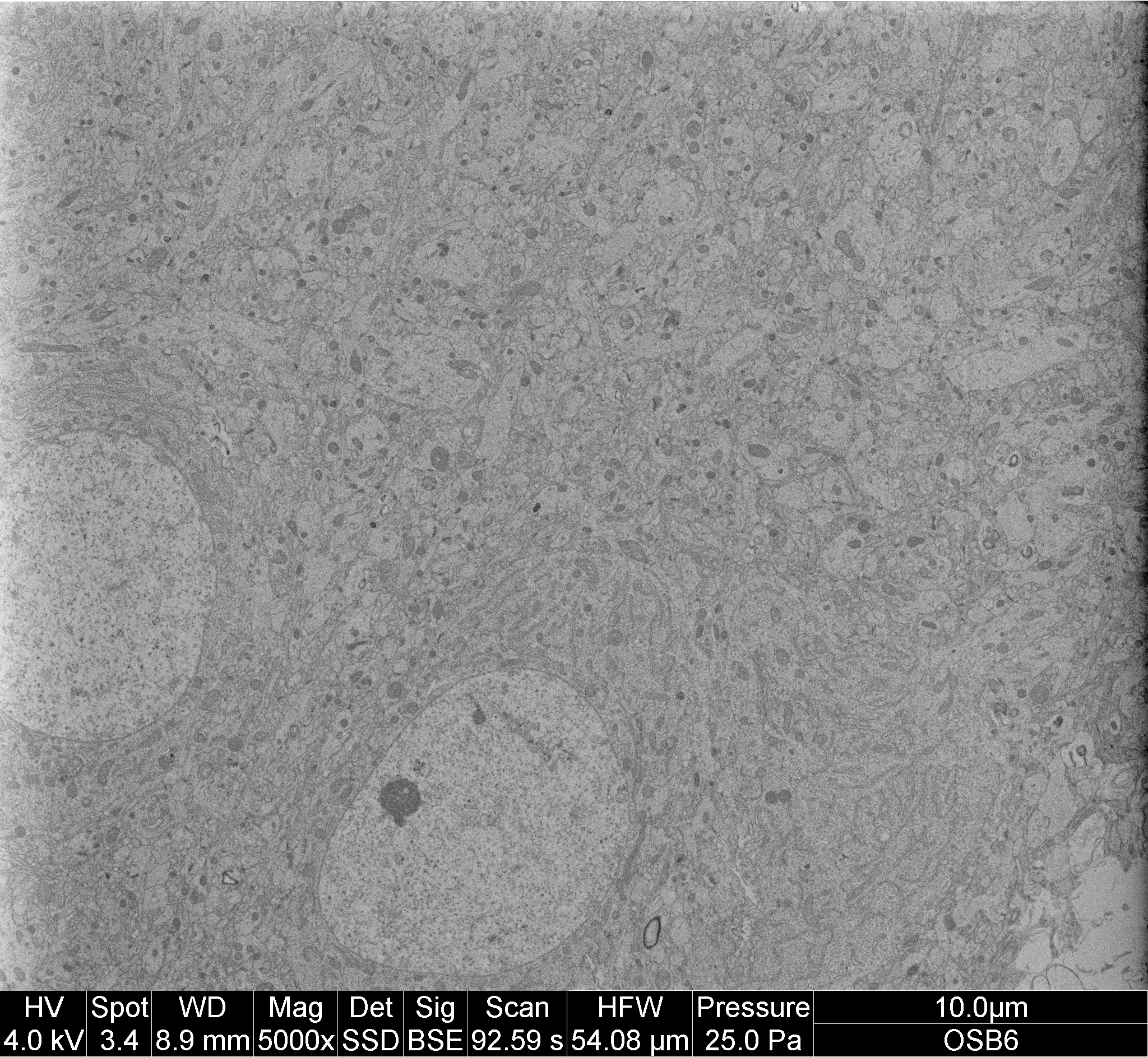

Supplement: Dataset S1 — (248.1 MB ZIP). [file pbio.0020329.sd001.zip › 040604_OS5_st1_082.tif]

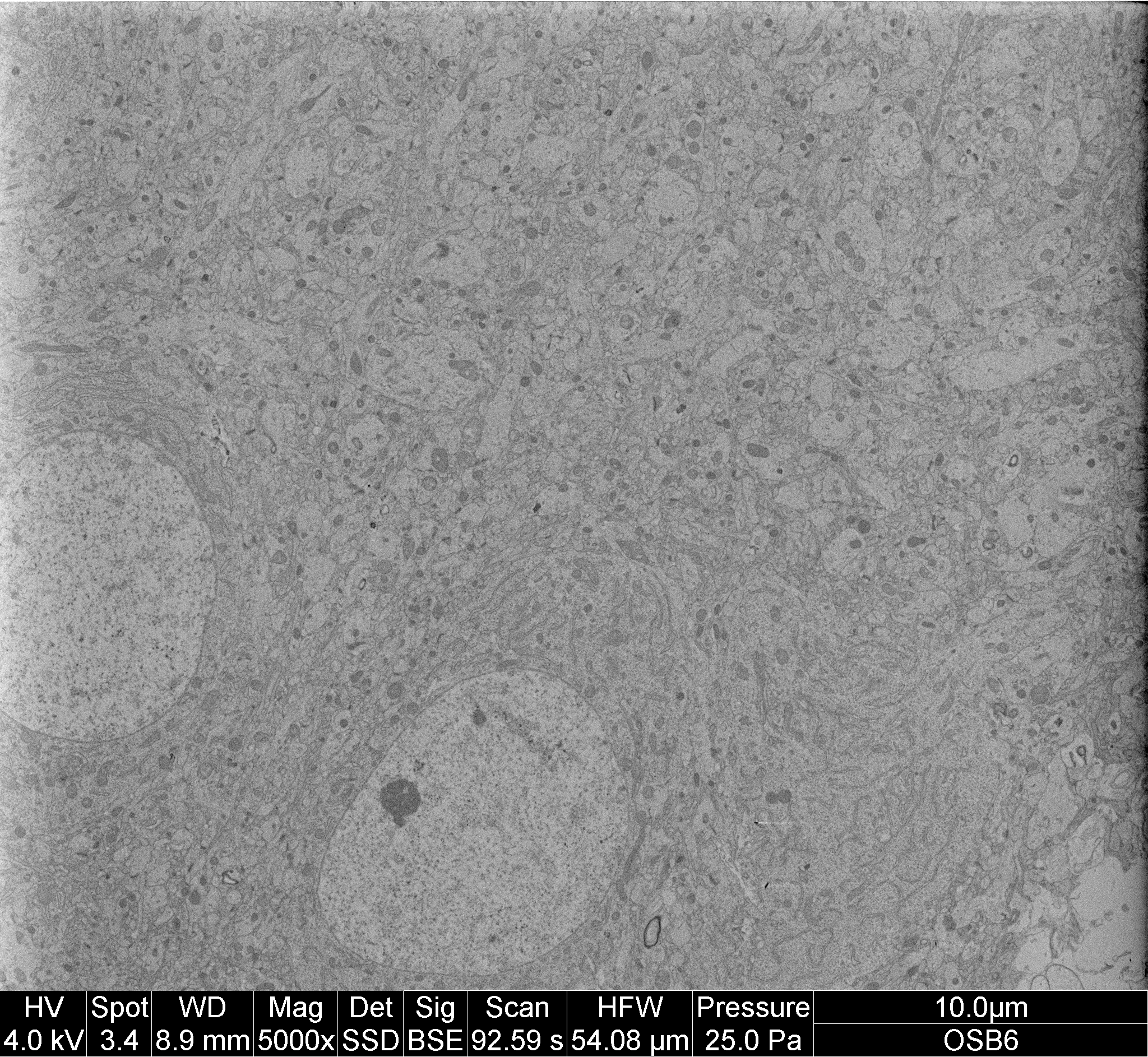

Supplement: Dataset S1 — (248.1 MB ZIP). [file pbio.0020329.sd001.zip › 040604_OS5_st1_083.tif]

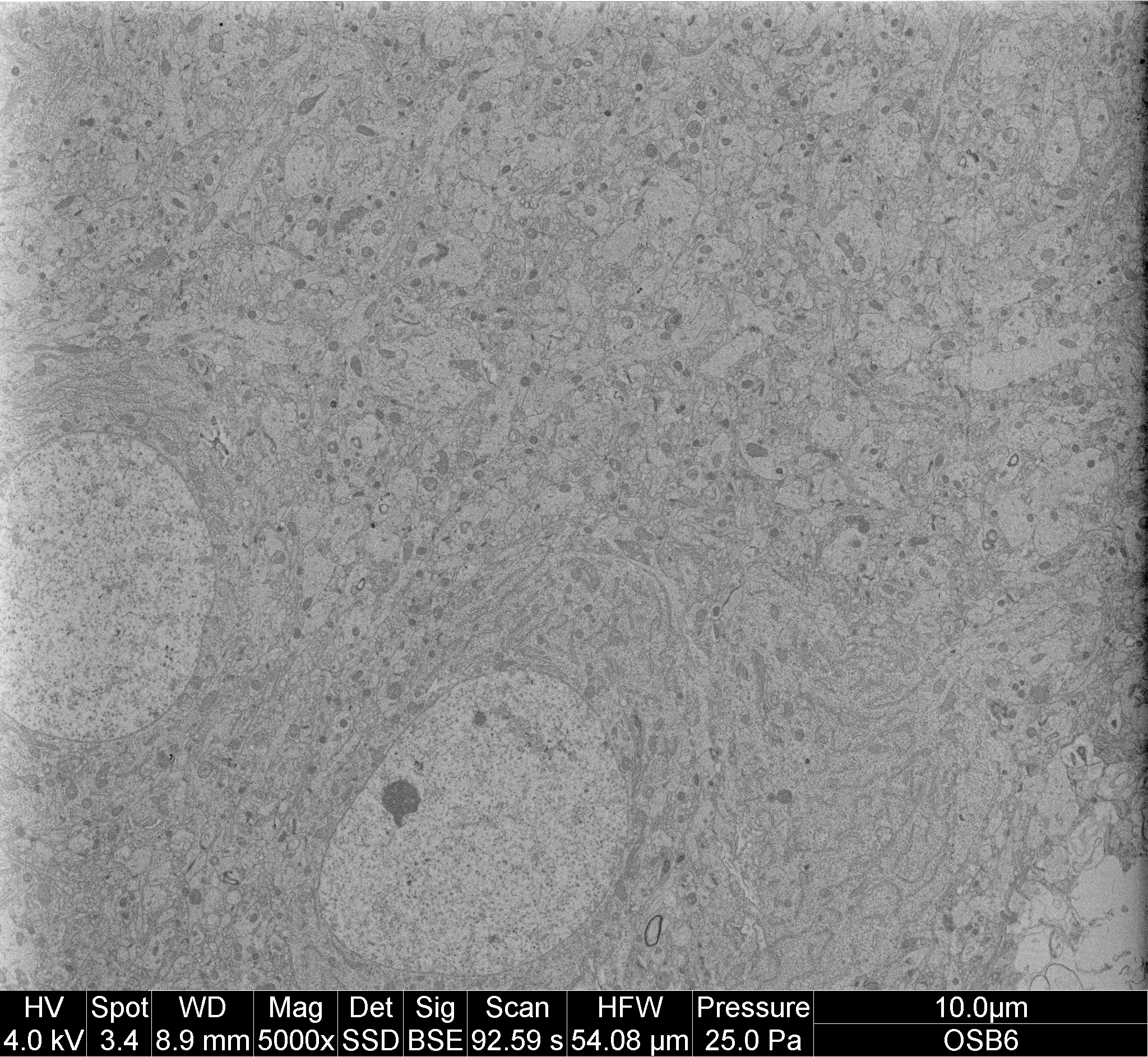

Supplement: Dataset S1 — (248.1 MB ZIP). [file pbio.0020329.sd001.zip › 040604_OS5_st1_084.tif]

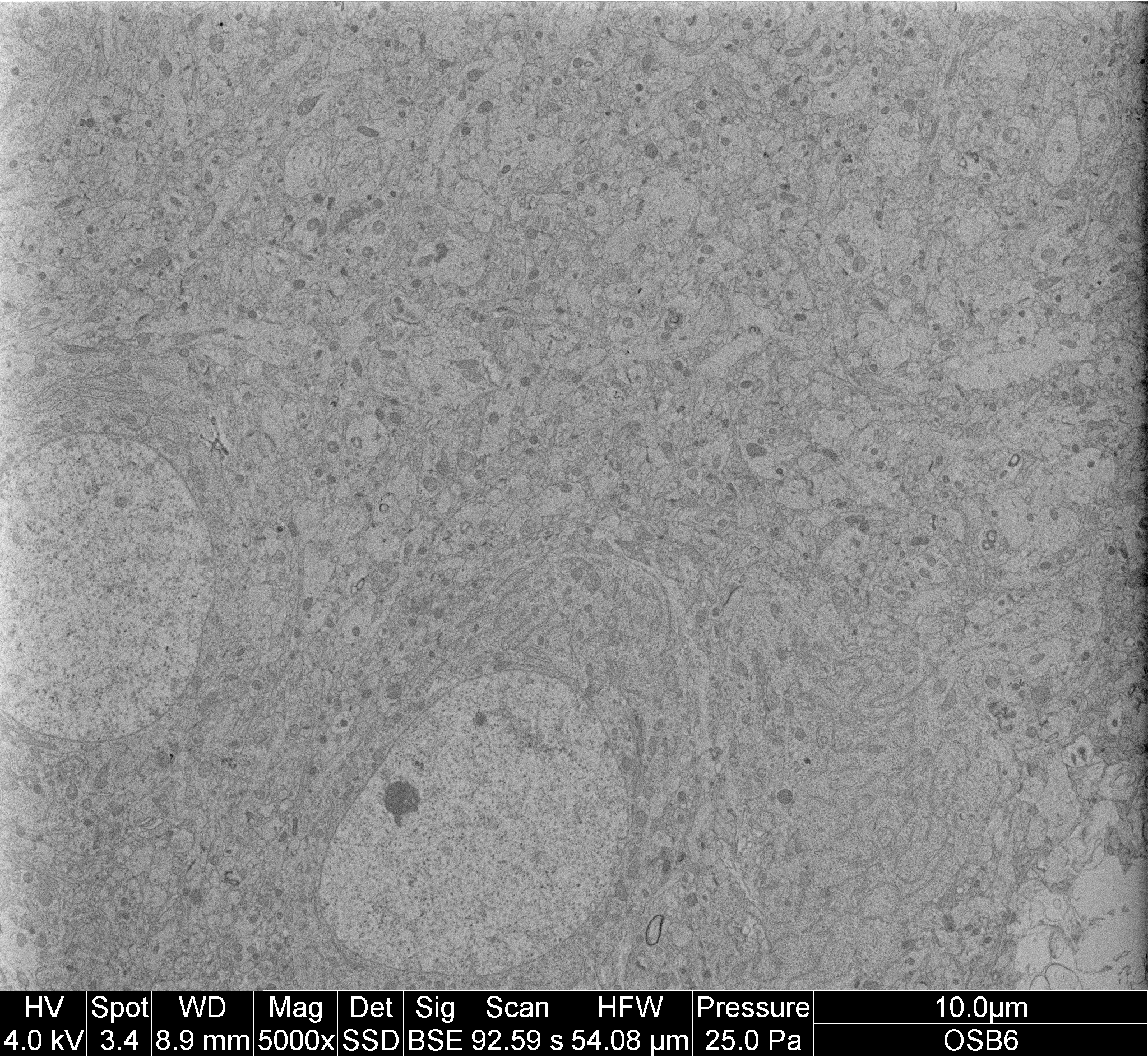

Supplement: Dataset S1 — (248.1 MB ZIP). [file pbio.0020329.sd001.zip › 040604_OS5_st1_085.tif]

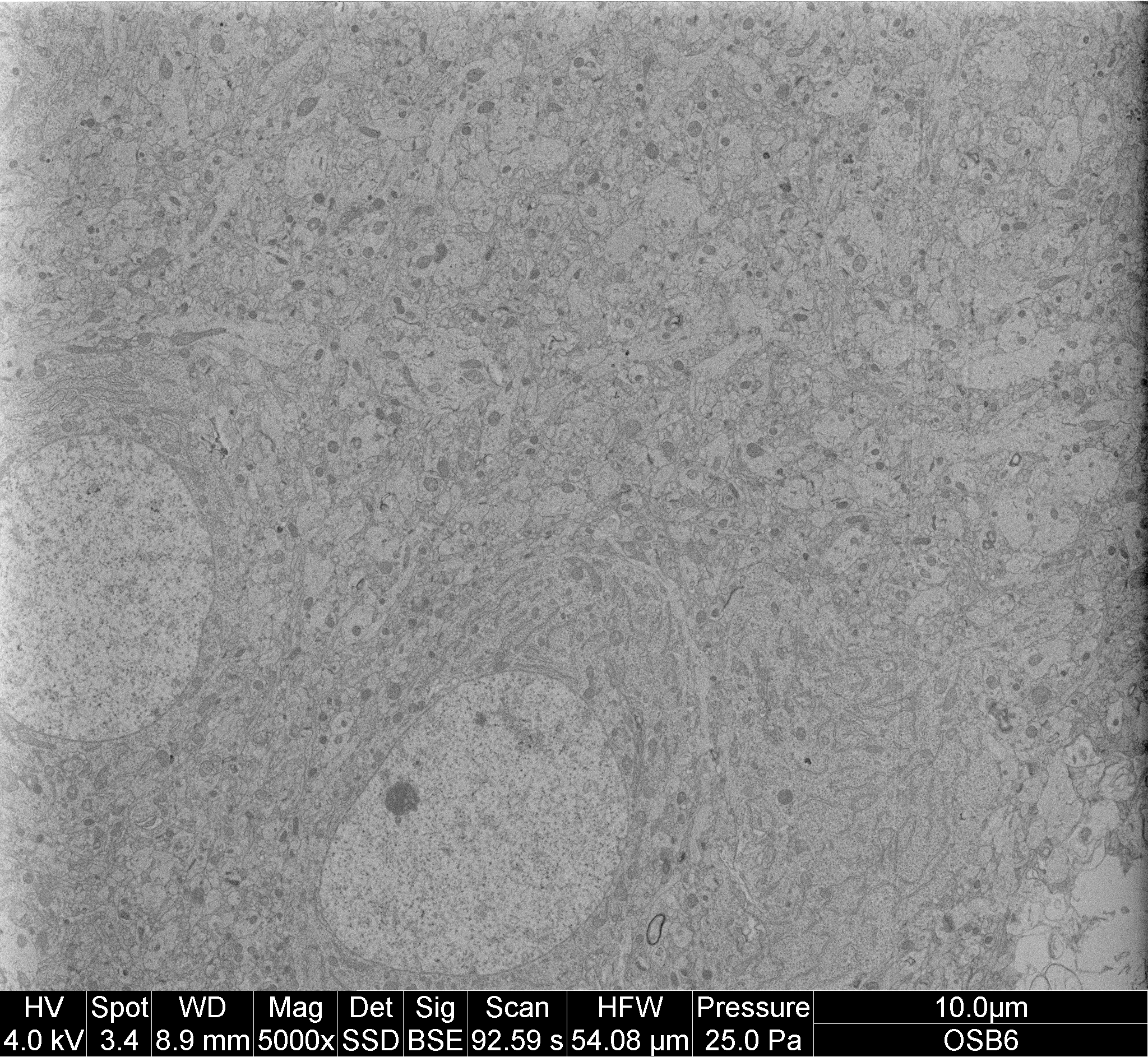

Supplement: Dataset S1 — (248.1 MB ZIP). [file pbio.0020329.sd001.zip › 040604_OS5_st1_086.tif]

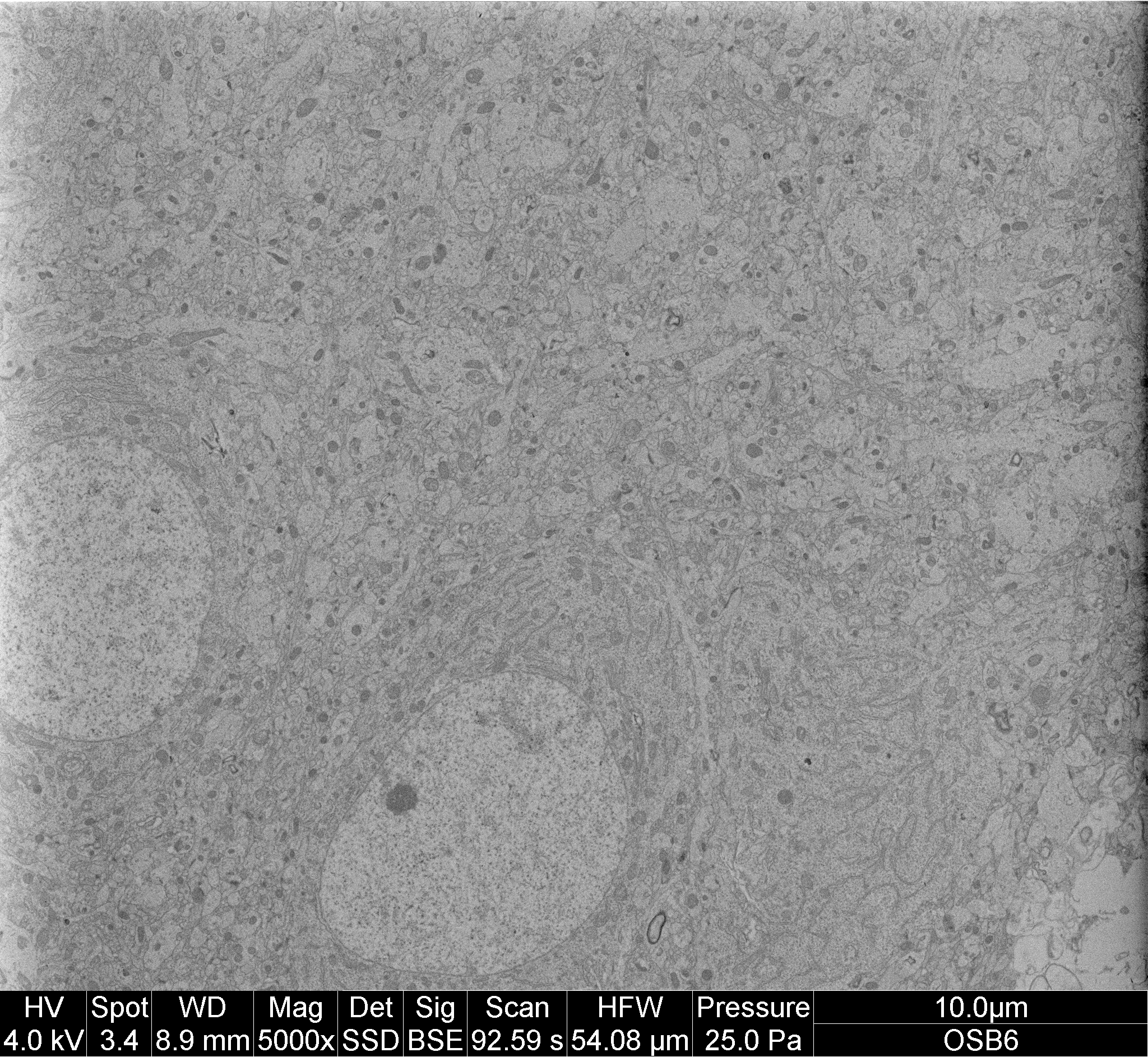

Supplement: Dataset S1 — (248.1 MB ZIP). [file pbio.0020329.sd001.zip › 040604_OS5_st1_087.tif]

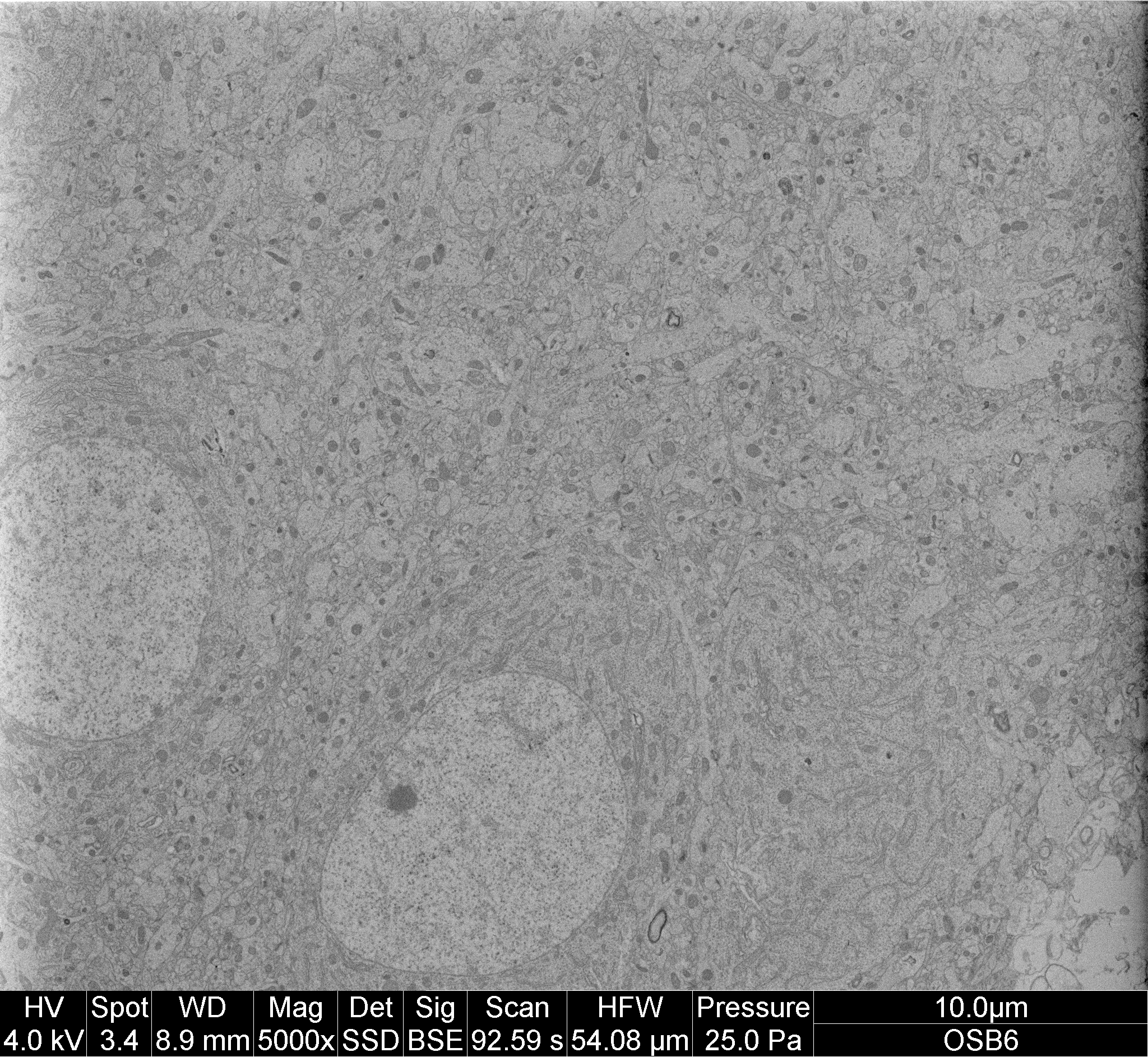

Supplement: Dataset S1 — (248.1 MB ZIP). [file pbio.0020329.sd001.zip › 040604_OS5_st1_088.tif]

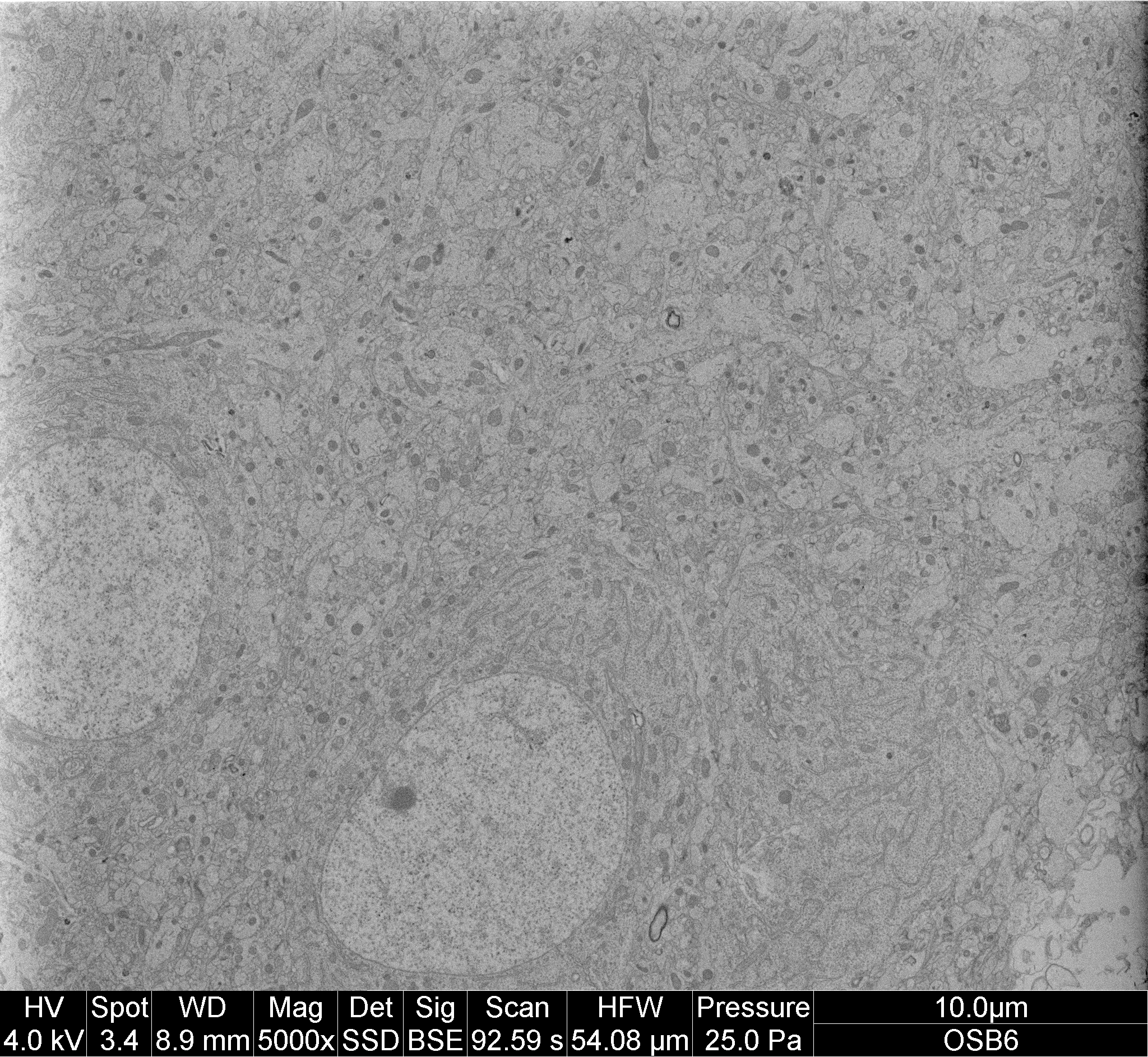

Supplement: Dataset S1 — (248.1 MB ZIP). [file pbio.0020329.sd001.zip › 040604_OS5_st1_089.tif]

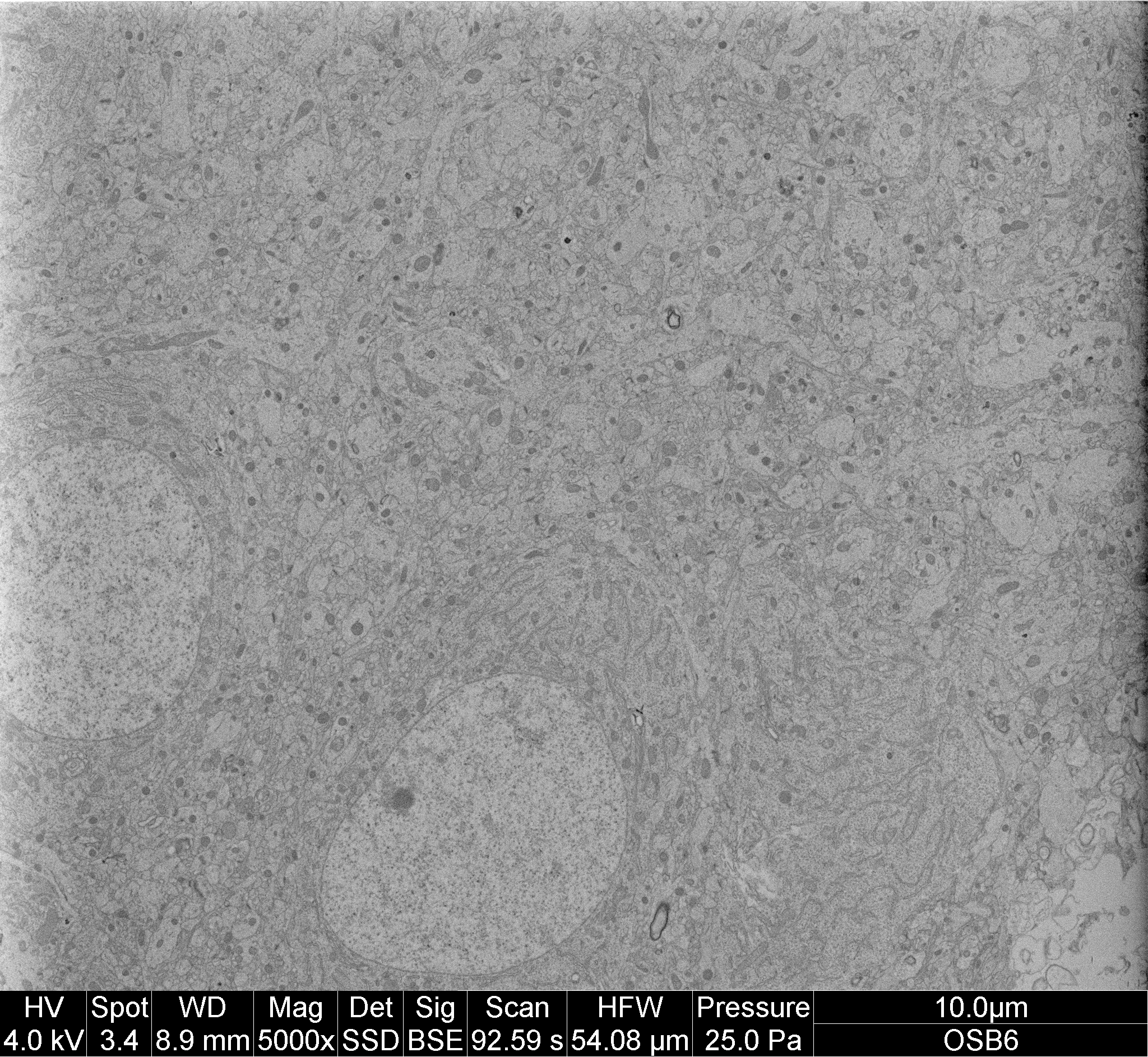

Supplement: Dataset S1 — (248.1 MB ZIP). [file pbio.0020329.sd001.zip › 040604_OS5_st1_090.tif]

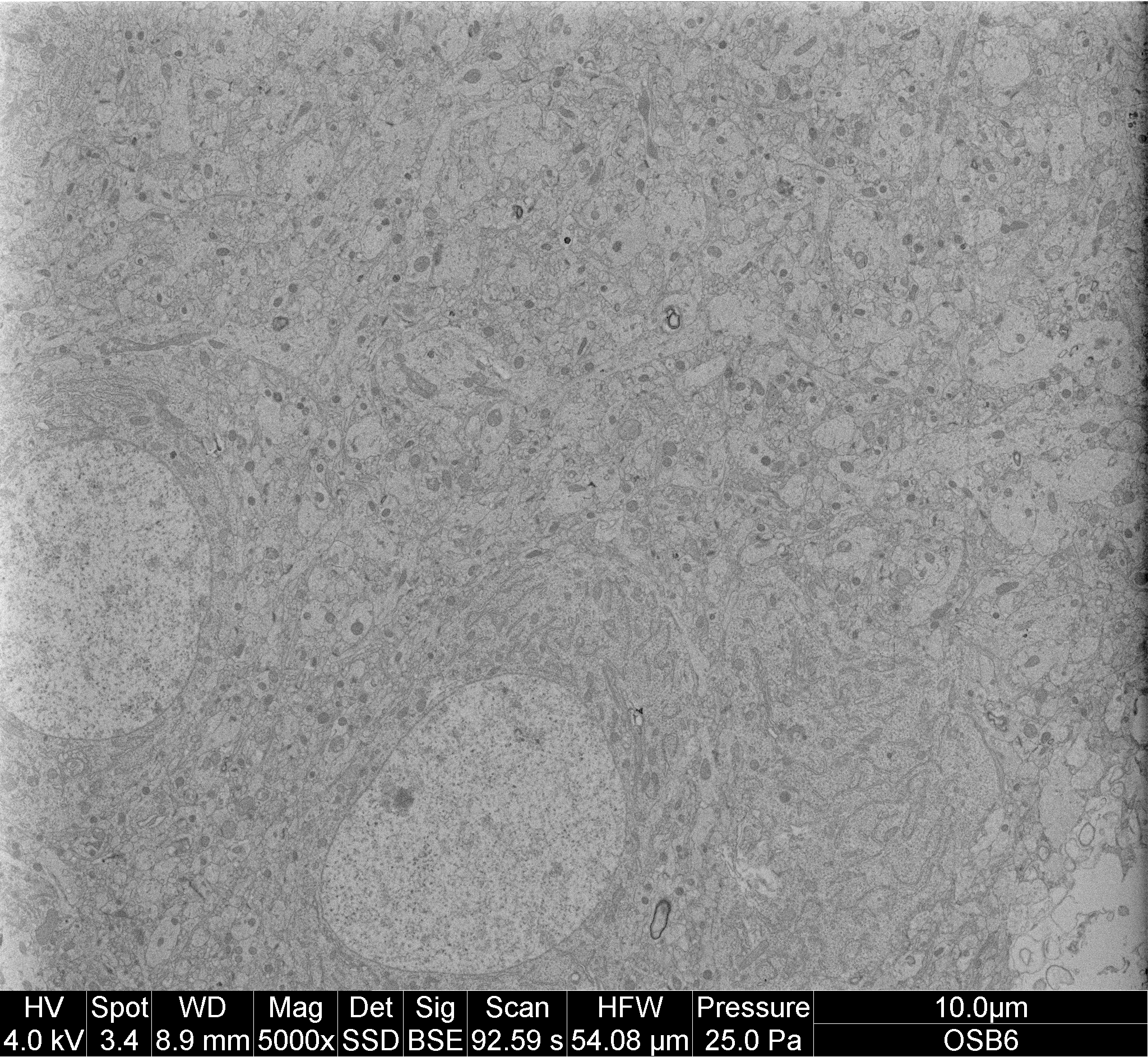

Supplement: Dataset S1 — (248.1 MB ZIP). [file pbio.0020329.sd001.zip › 040604_OS5_st1_091.tif]

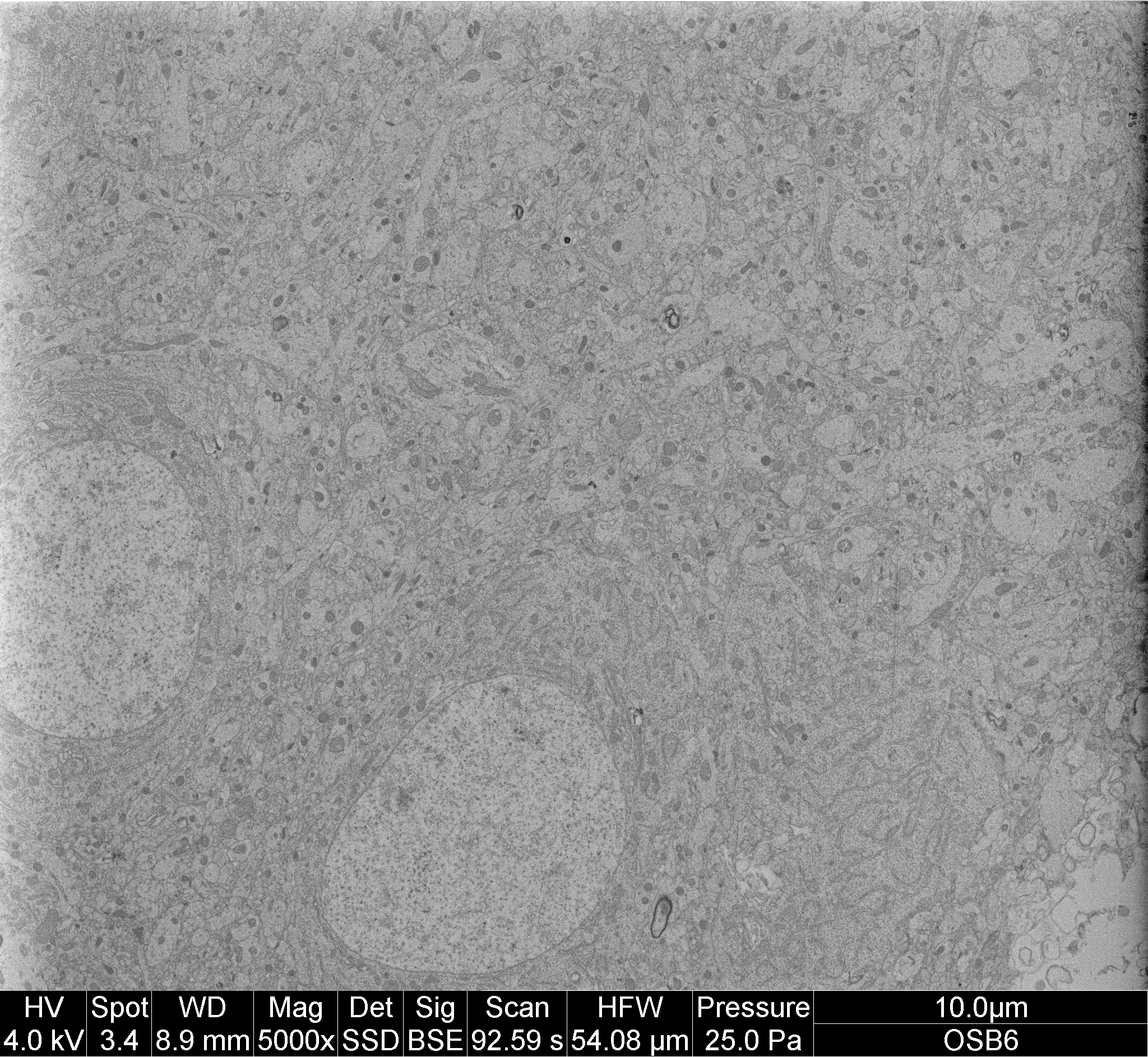

Supplement: Dataset S1 — (248.1 MB ZIP). [file pbio.0020329.sd001.zip › 040604_OS5_st1_092.tif]

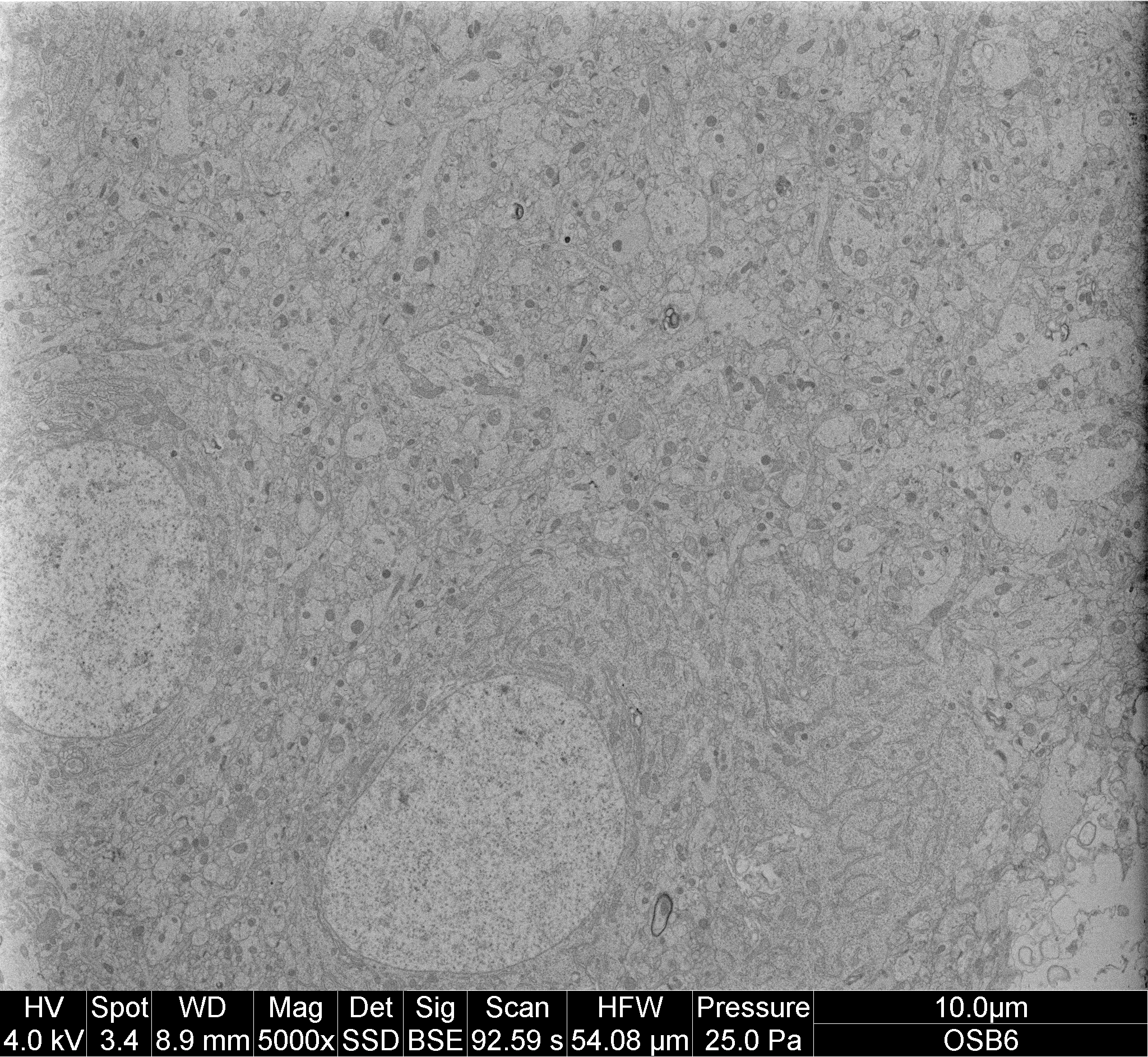

Supplement: Dataset S1 — (248.1 MB ZIP). [file pbio.0020329.sd001.zip › 040604_OS5_st1_093.tif]

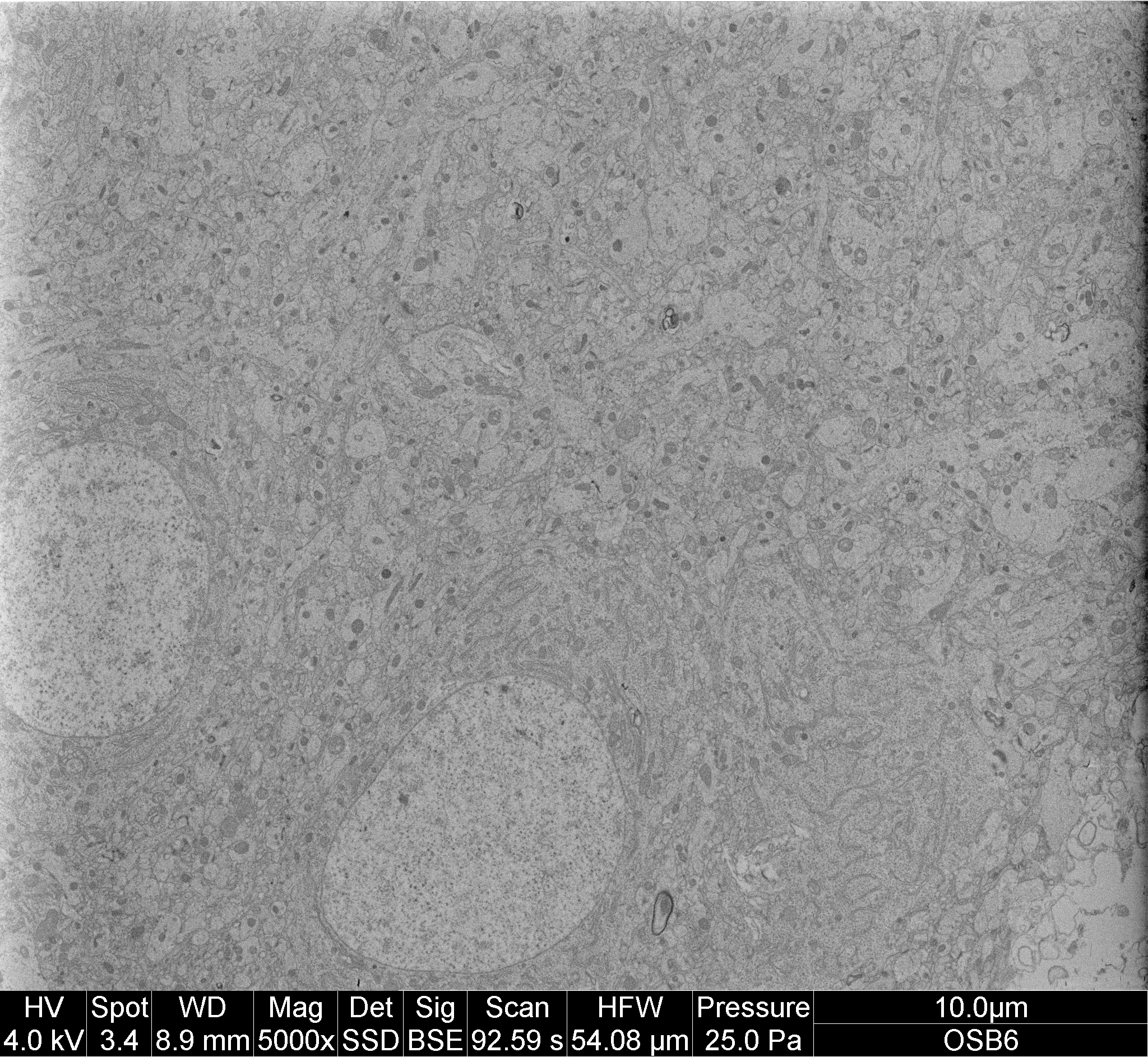

Supplement: Dataset S1 — (248.1 MB ZIP). [file pbio.0020329.sd001.zip › 040604_OS5_st1_094.tif]

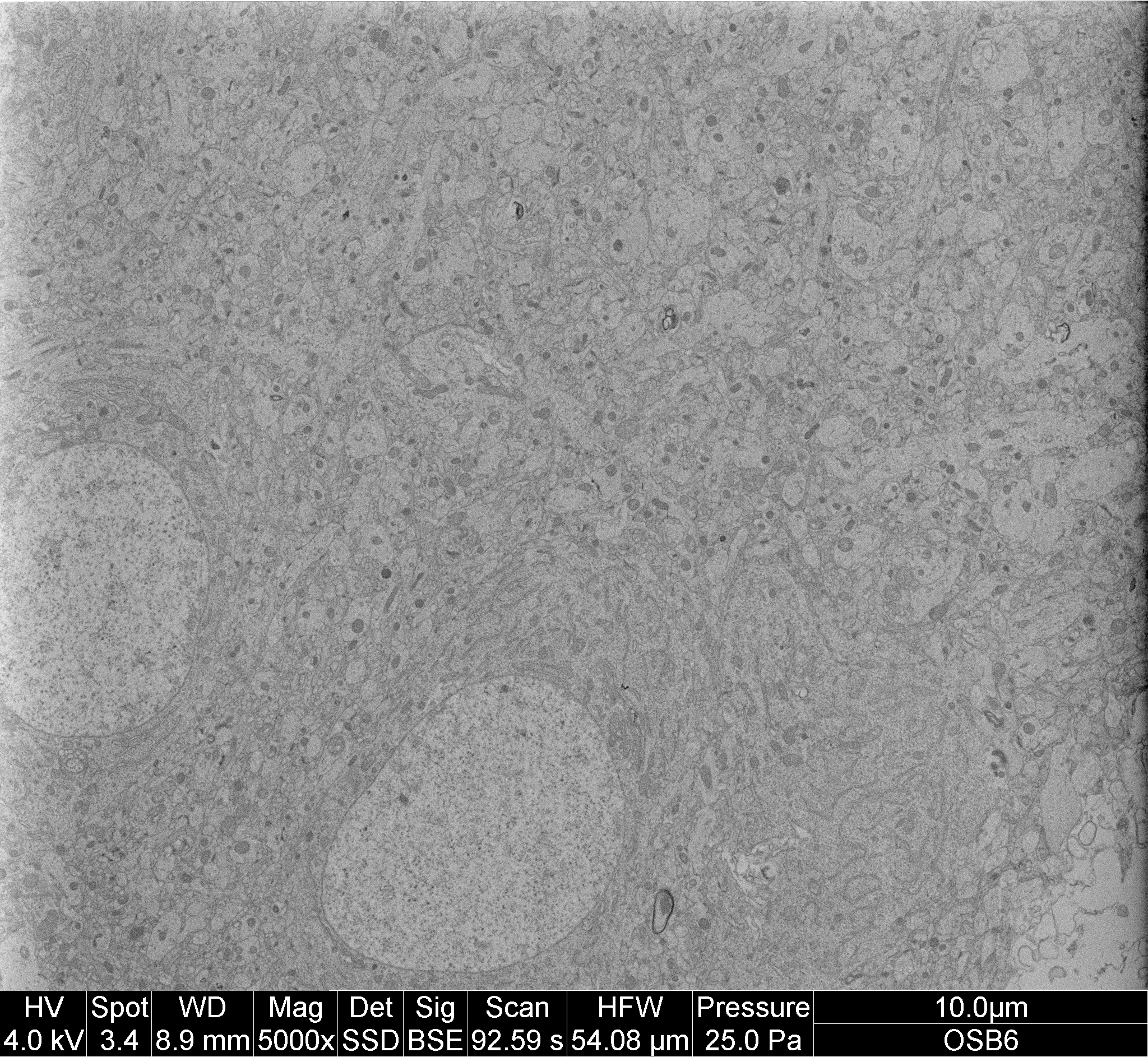

Supplement: Dataset S1 — (248.1 MB ZIP). [file pbio.0020329.sd001.zip › 040604_OS5_st1_095.tif]

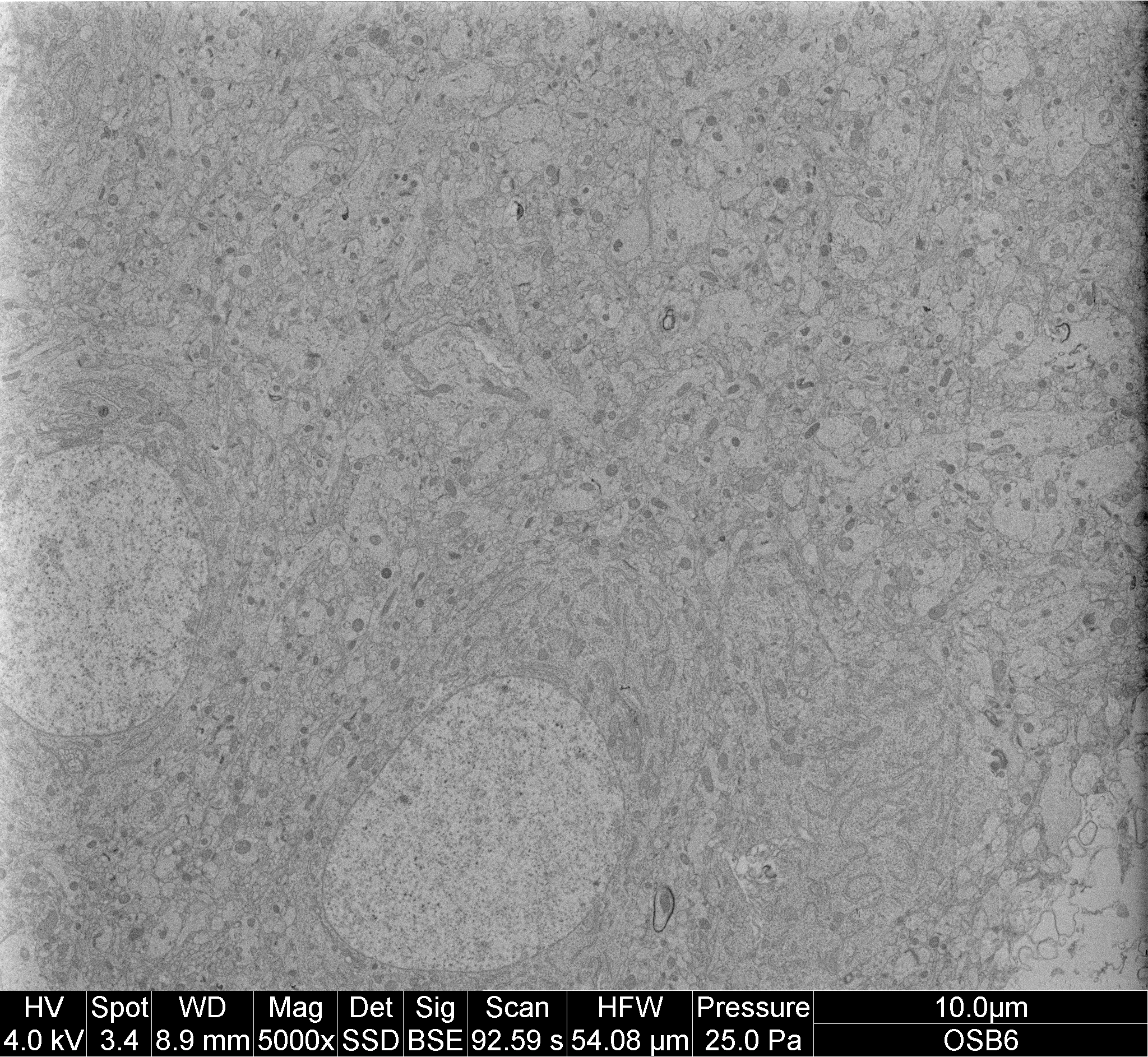

Supplement: Dataset S1 — (248.1 MB ZIP). [file pbio.0020329.sd001.zip › 040604_OS5_st1_096.tif]

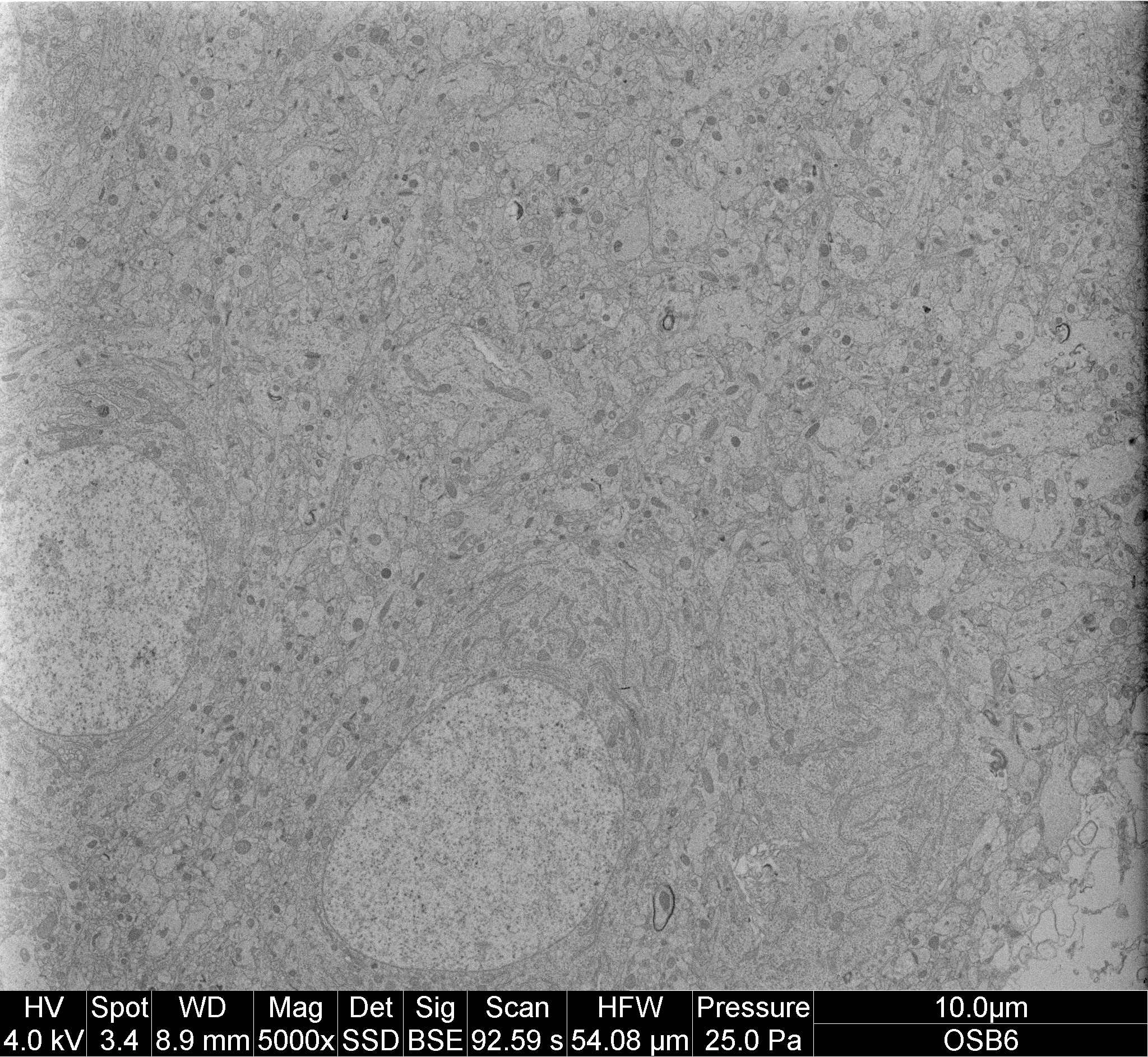

Supplement: Dataset S1 — (248.1 MB ZIP). [file pbio.0020329.sd001.zip › 040604_OS5_st1_097.tif]

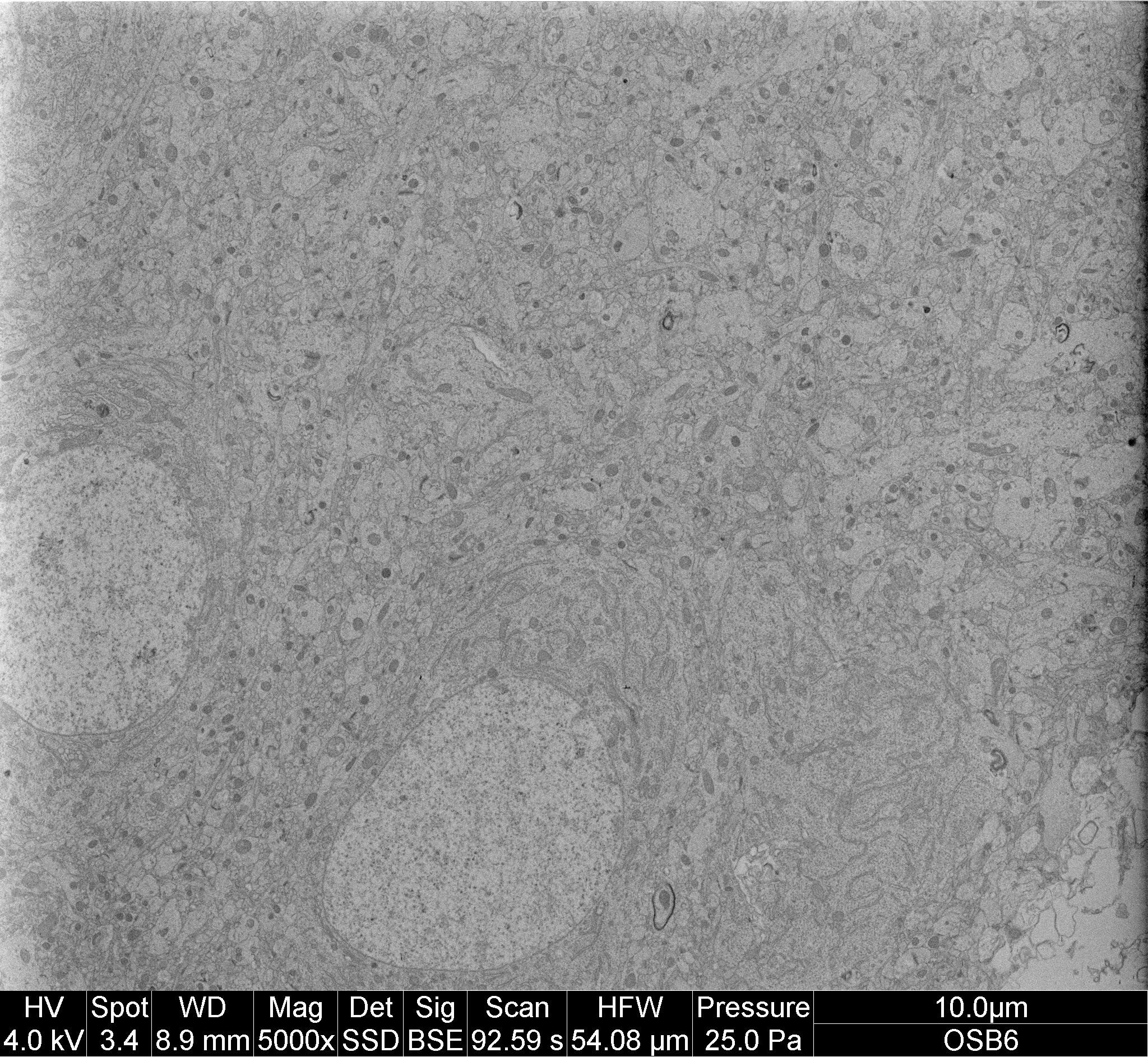

Supplement: Dataset S1 — (248.1 MB ZIP). [file pbio.0020329.sd001.zip › 040604_OS5_st1_098.tif]

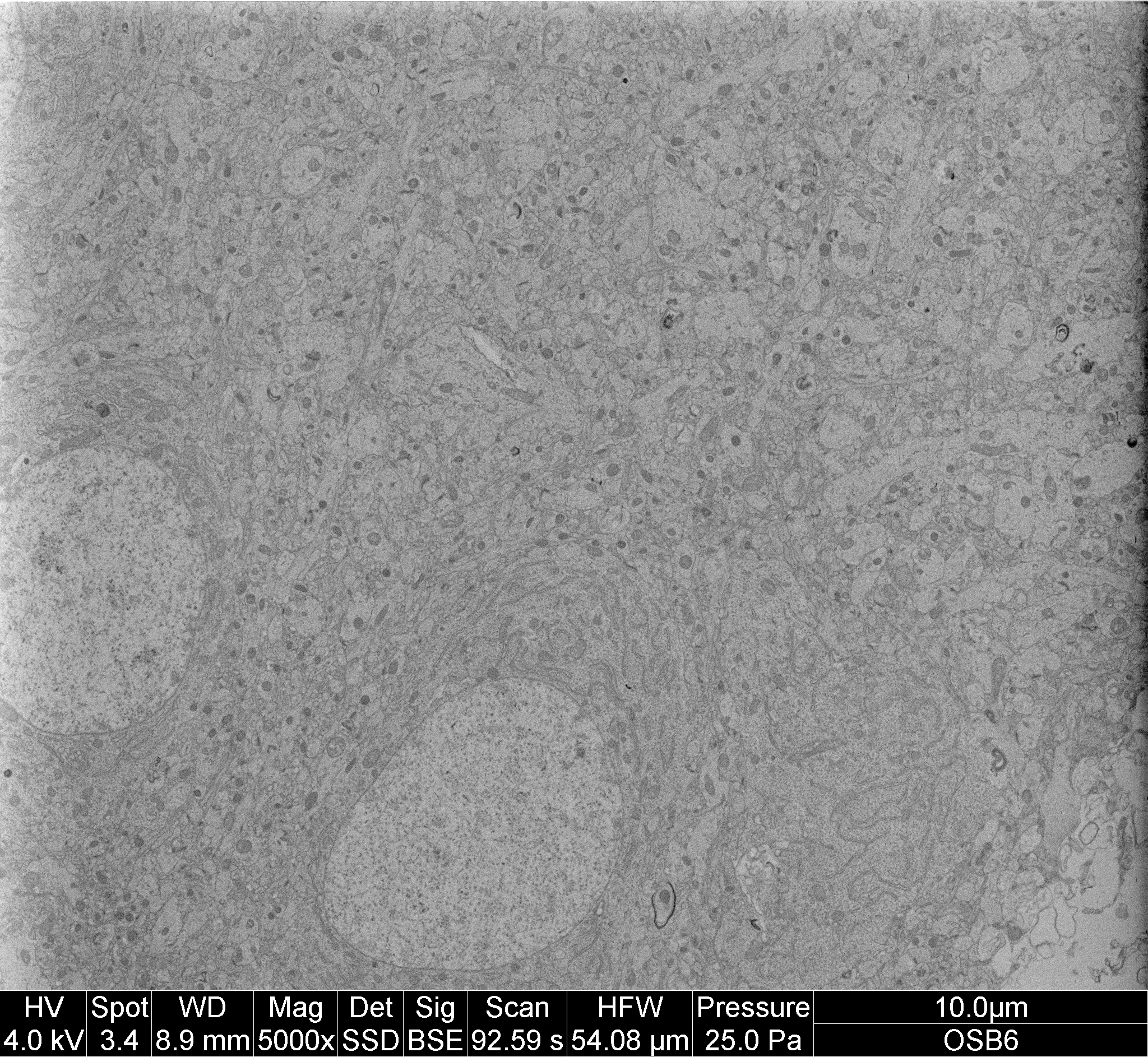

Supplement: Dataset S1 — (248.1 MB ZIP). [file pbio.0020329.sd001.zip › 040604_OS5_st1_099.tif]

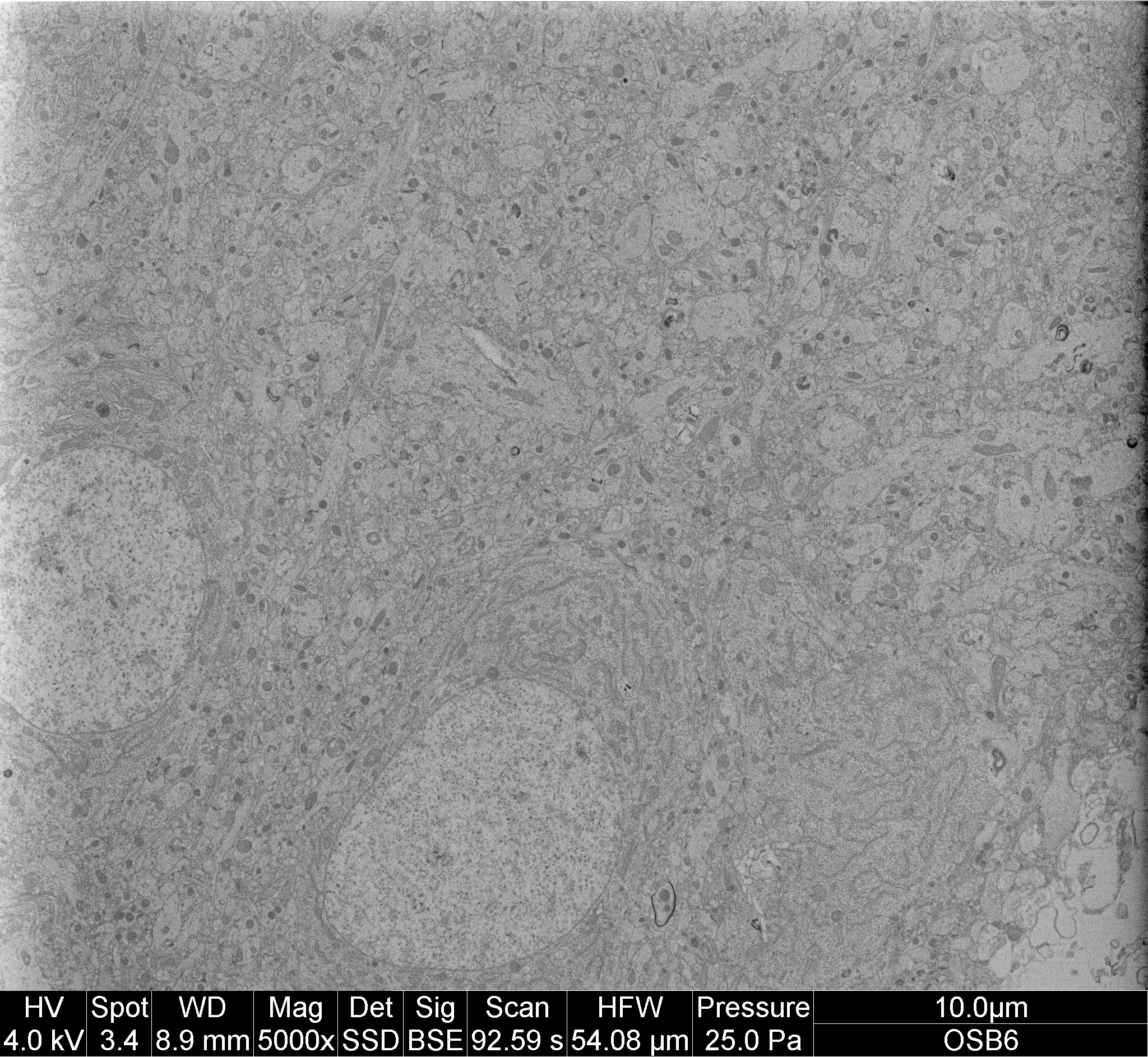

Supplement: Dataset S2 — (252.6 MB ZIP). [file pbio.0020329.sd002.zip › 040604_OS5_st1_100.tif]
