# Supplementary figures and images for: Serial Block-Face Scanning Electron Microscopy to Reconstruct Three-Dimensional Tissue Nanostructure (part 2 of 21)
Source: PLoS Biol. 2004 Oct 19;2(11):e329. doi: 10.1371/journal.pbio.0020329 (PMC524270; doi:10.1371/journal.pbio.0020329)

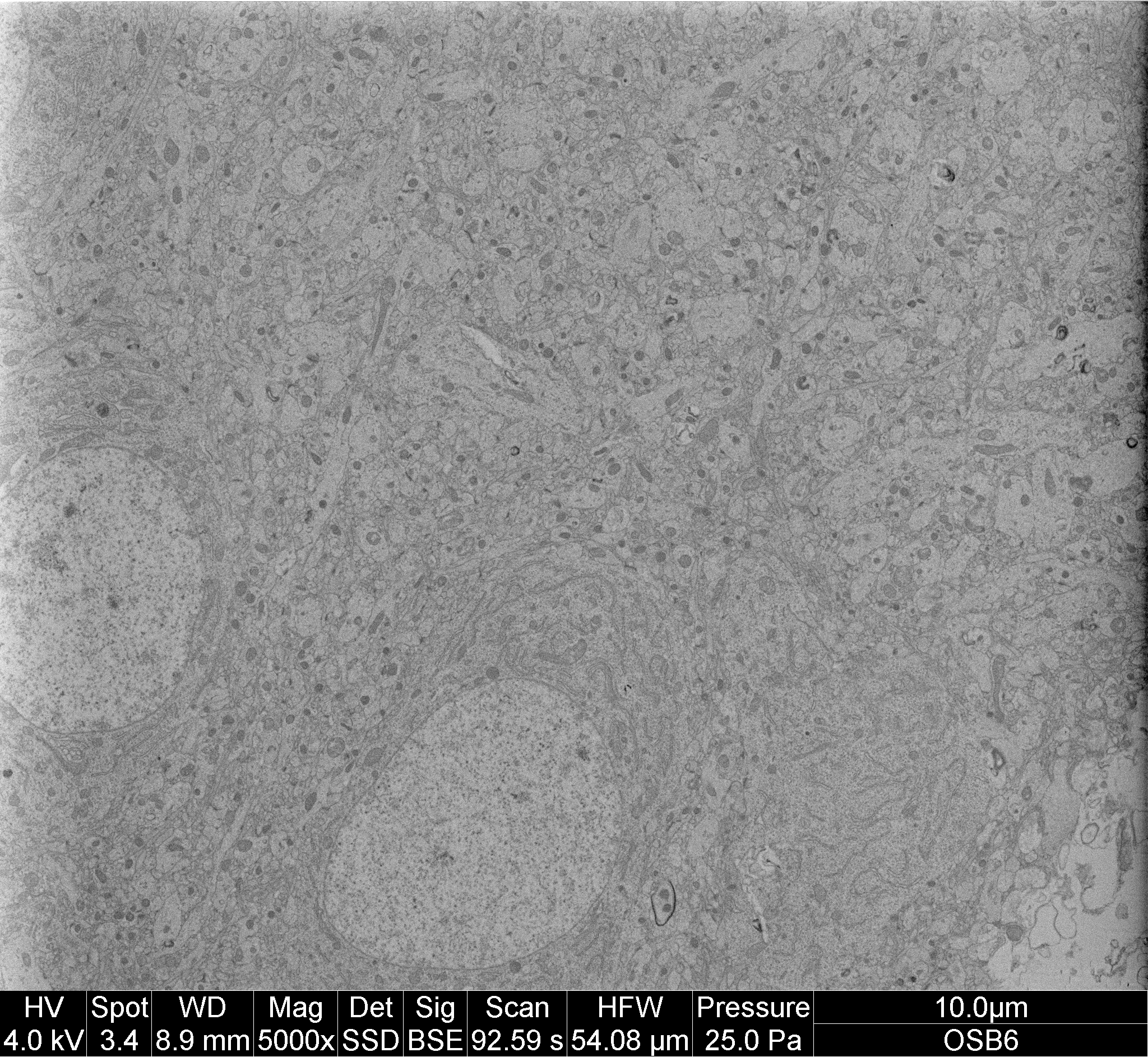

Supplement: Dataset S2 — (252.6 MB ZIP). [file pbio.0020329.sd002.zip › 040604_OS5_st1_101.tif]

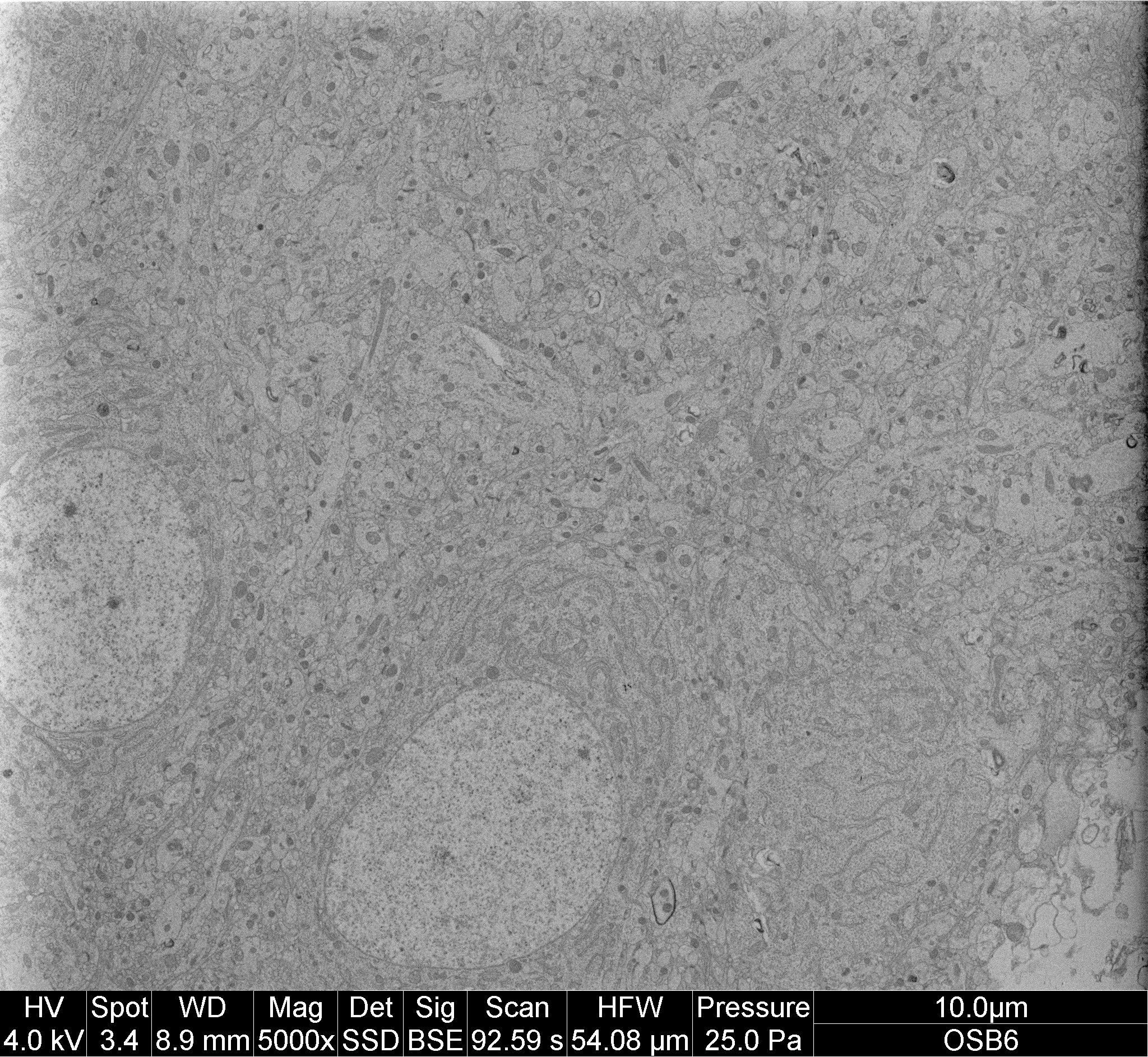

Supplement: Dataset S2 — (252.6 MB ZIP). [file pbio.0020329.sd002.zip › 040604_OS5_st1_102.tif]

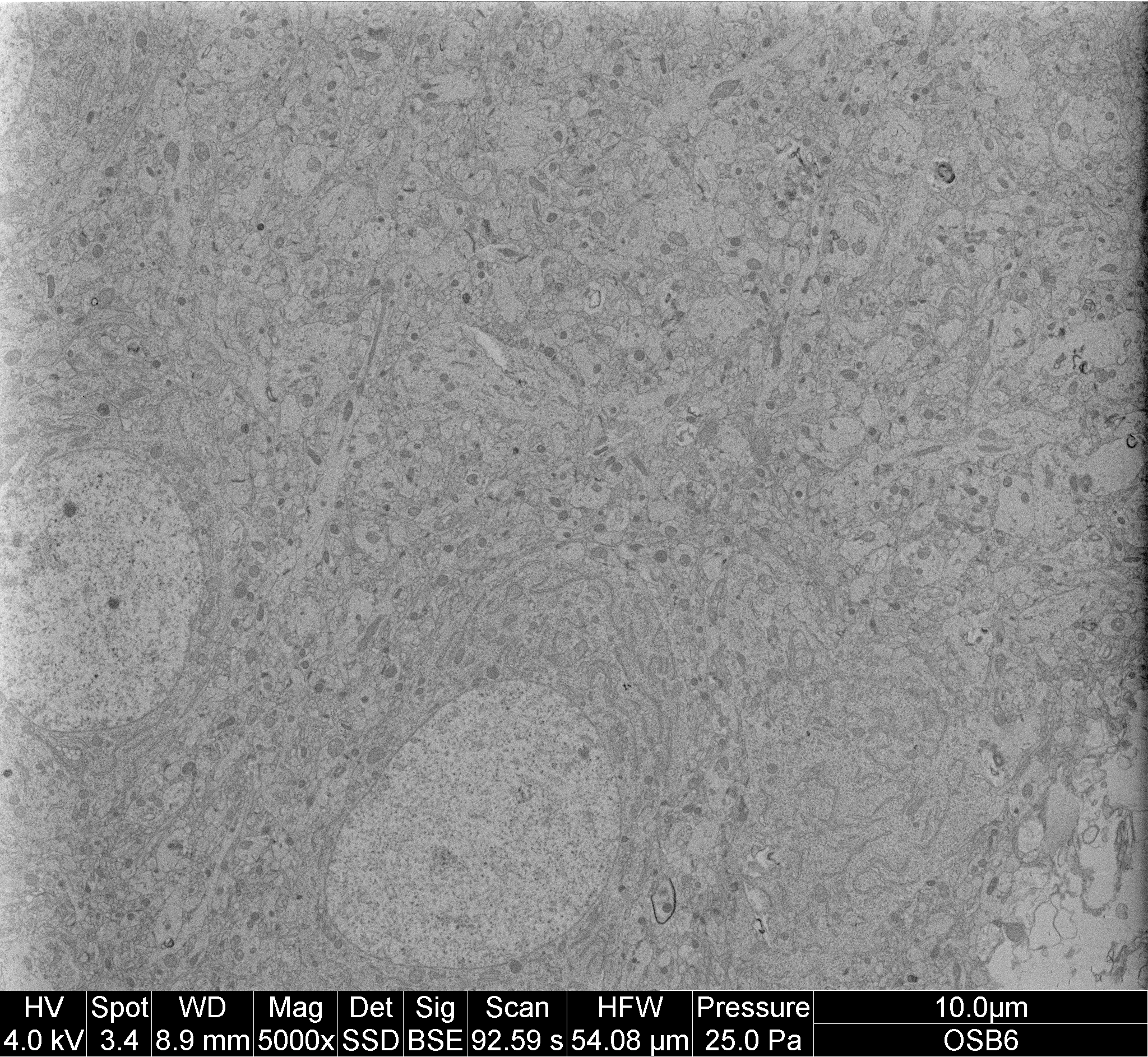

Supplement: Dataset S2 — (252.6 MB ZIP). [file pbio.0020329.sd002.zip › 040604_OS5_st1_103.tif]

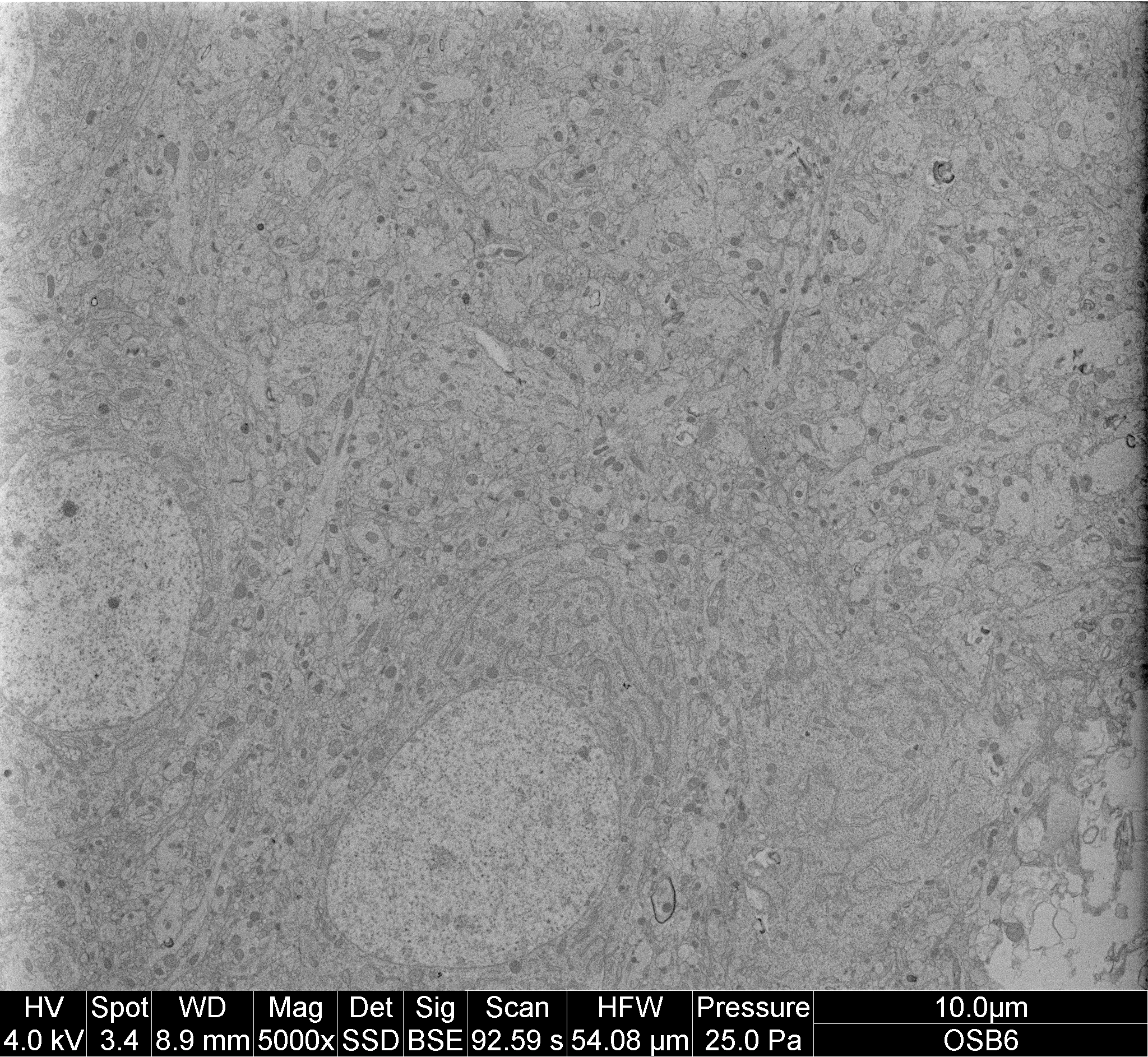

Supplement: Dataset S2 — (252.6 MB ZIP). [file pbio.0020329.sd002.zip › 040604_OS5_st1_104.tif]

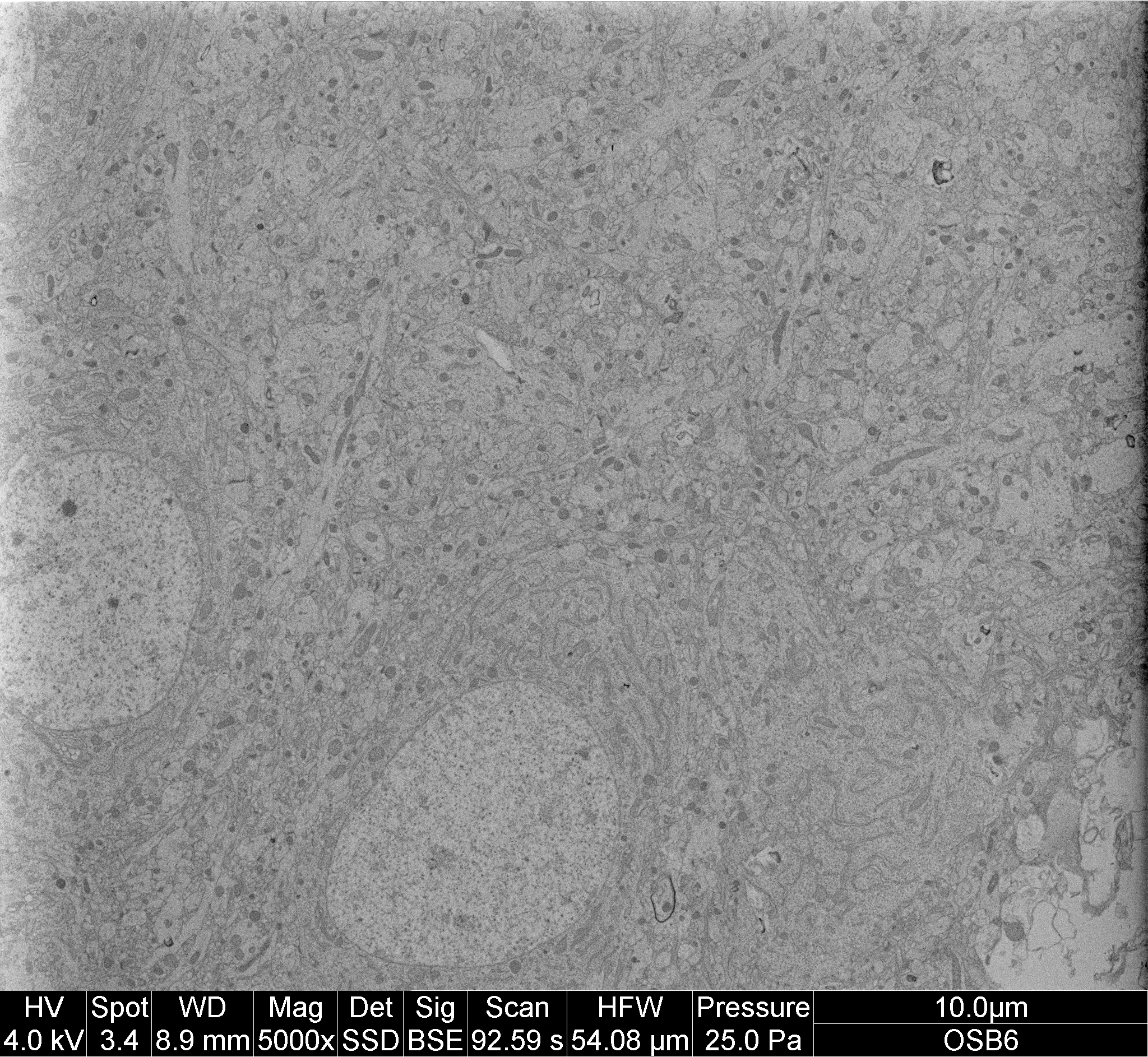

Supplement: Dataset S2 — (252.6 MB ZIP). [file pbio.0020329.sd002.zip › 040604_OS5_st1_105.tif]

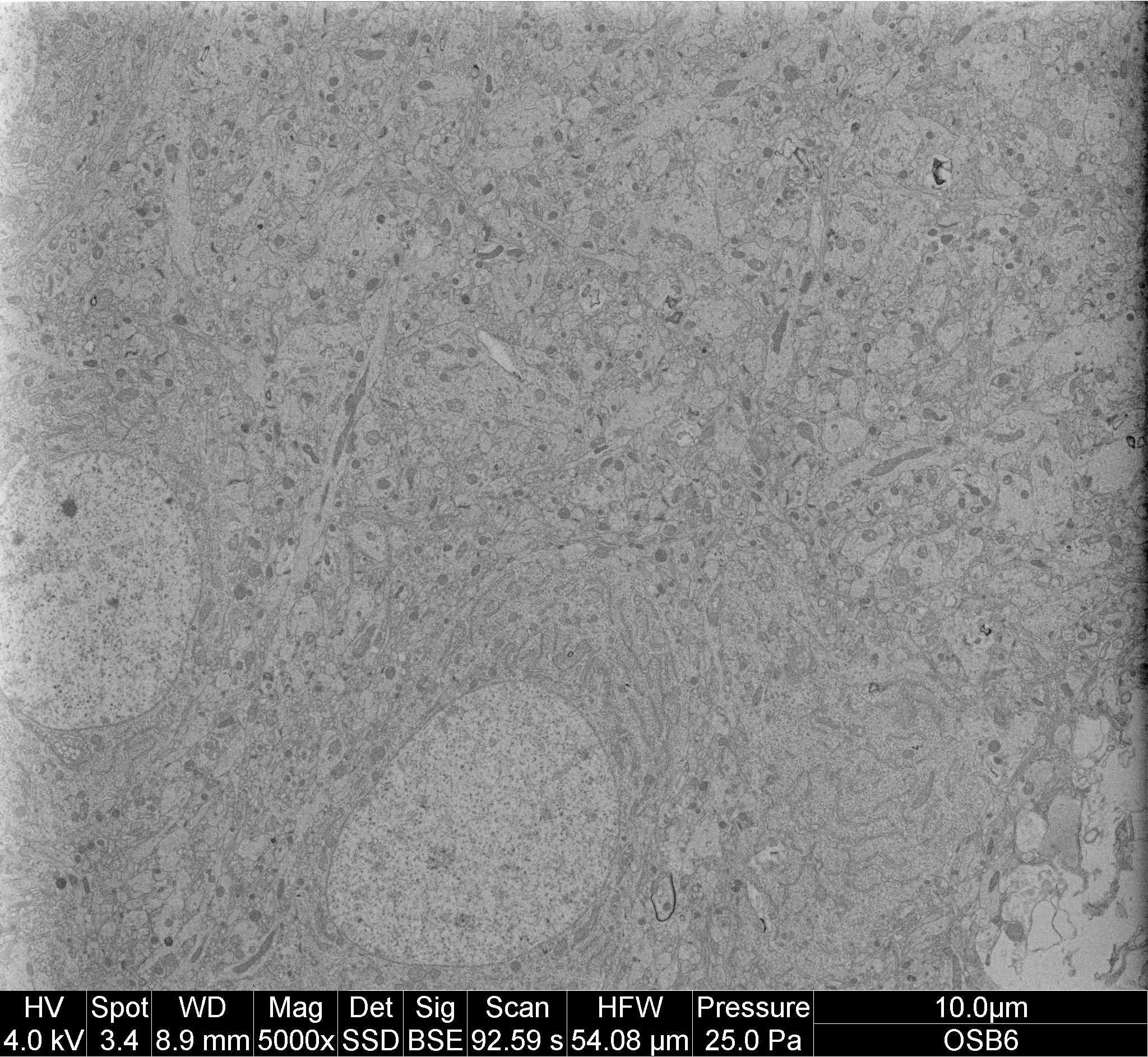

Supplement: Dataset S2 — (252.6 MB ZIP). [file pbio.0020329.sd002.zip › 040604_OS5_st1_106.tif]

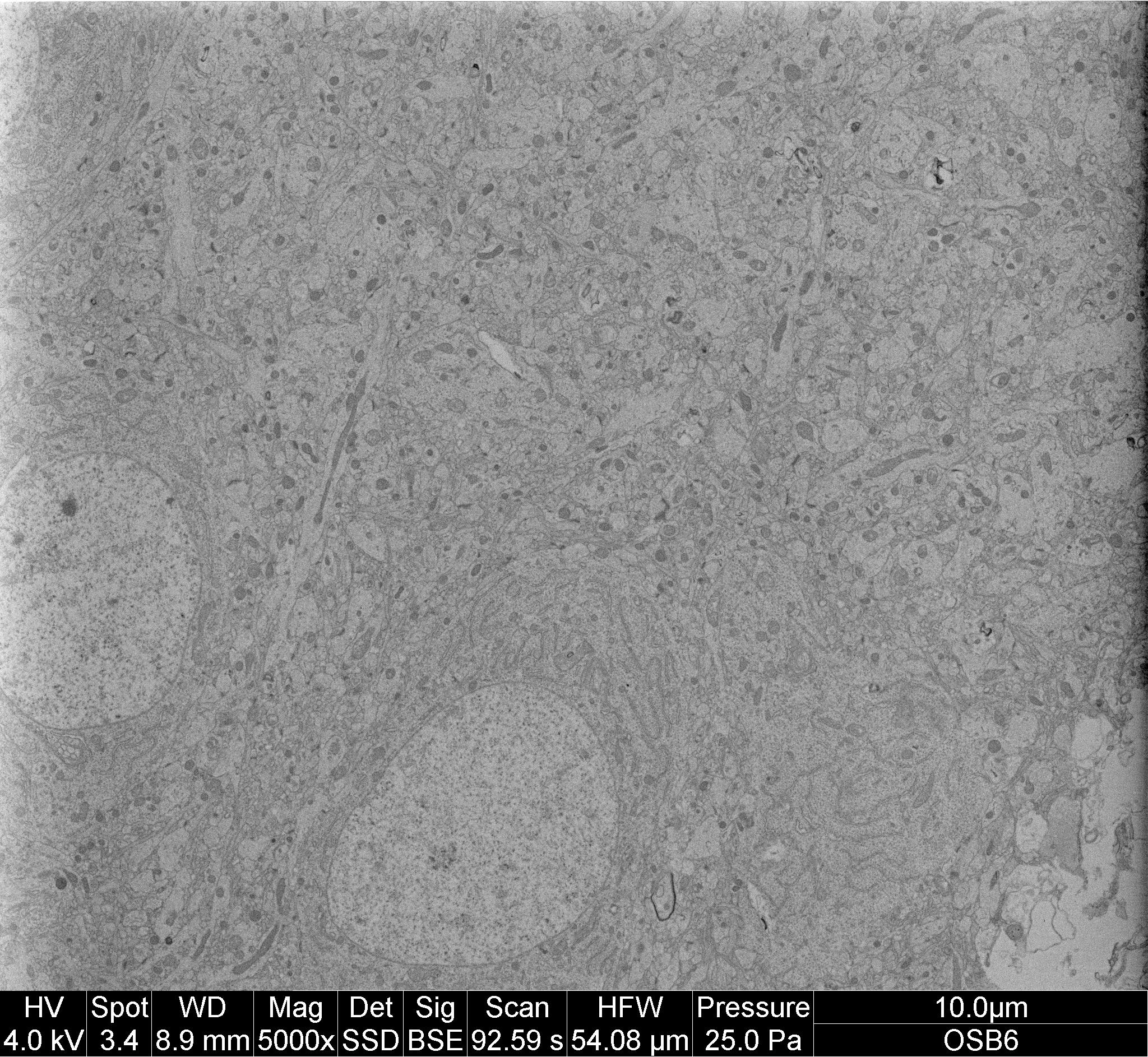

Supplement: Dataset S2 — (252.6 MB ZIP). [file pbio.0020329.sd002.zip › 040604_OS5_st1_107.tif]

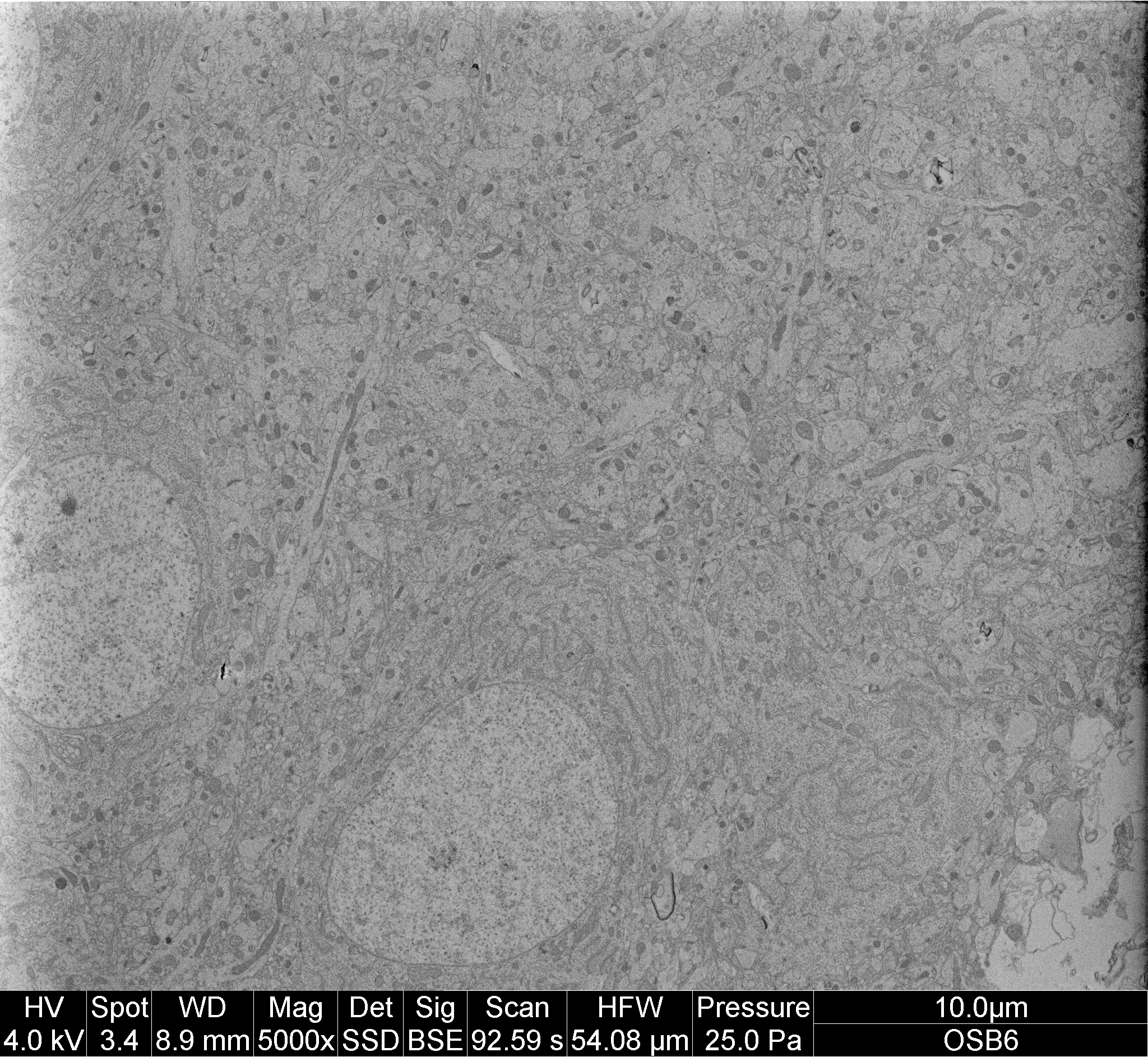

Supplement: Dataset S2 — (252.6 MB ZIP). [file pbio.0020329.sd002.zip › 040604_OS5_st1_108.tif]

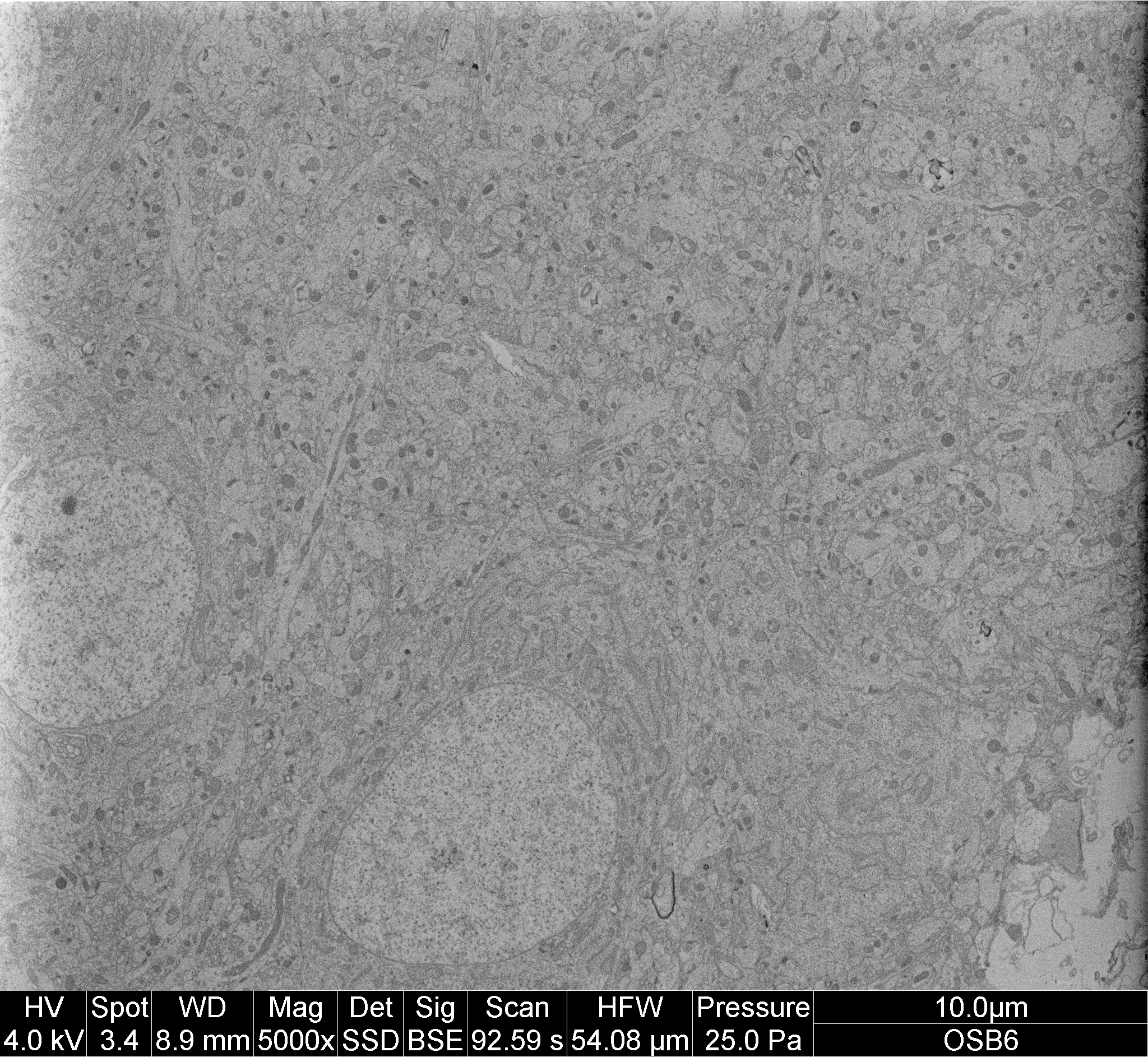

Supplement: Dataset S2 — (252.6 MB ZIP). [file pbio.0020329.sd002.zip › 040604_OS5_st1_109.tif]

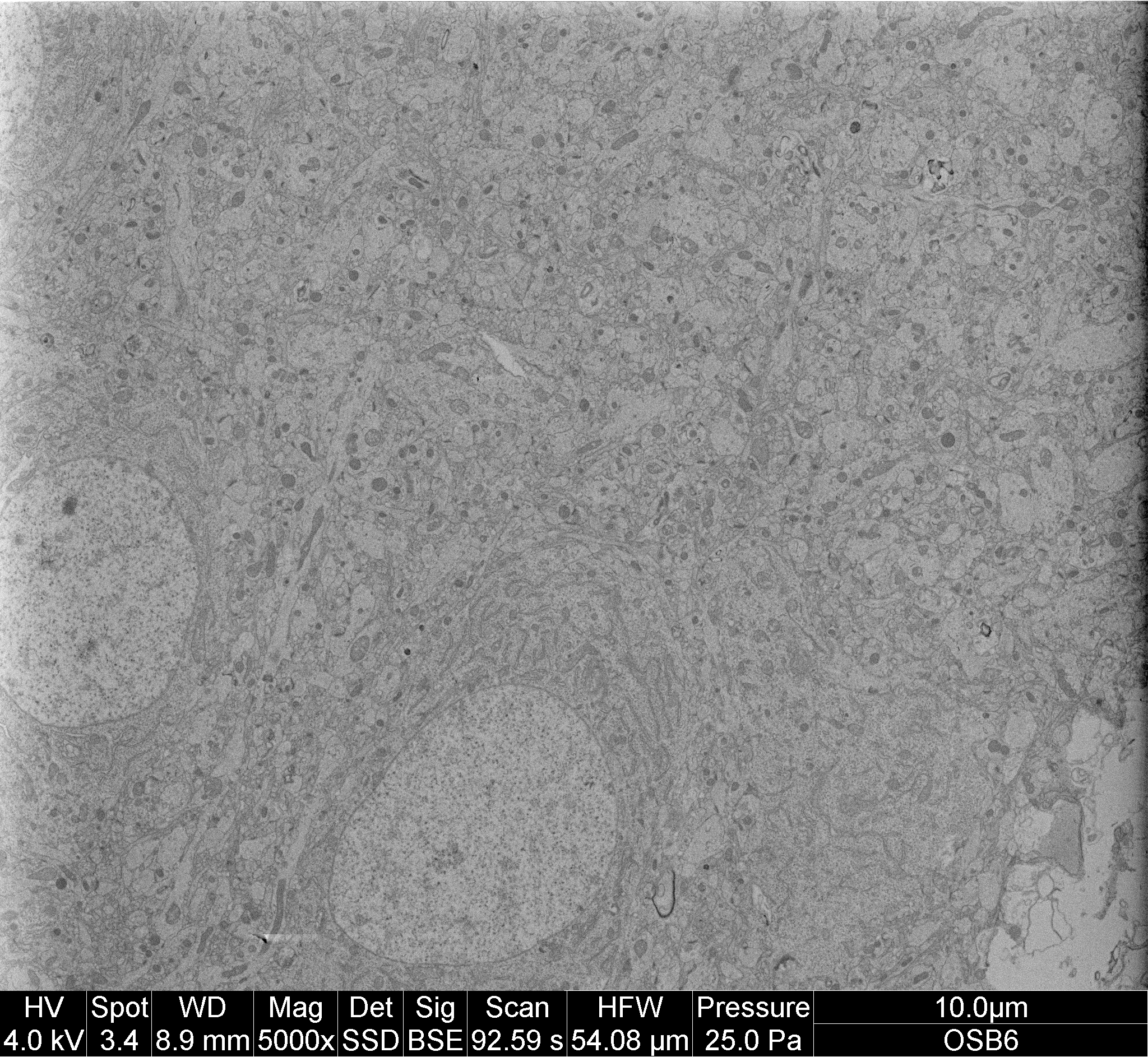

Supplement: Dataset S2 — (252.6 MB ZIP). [file pbio.0020329.sd002.zip › 040604_OS5_st1_110.tif]

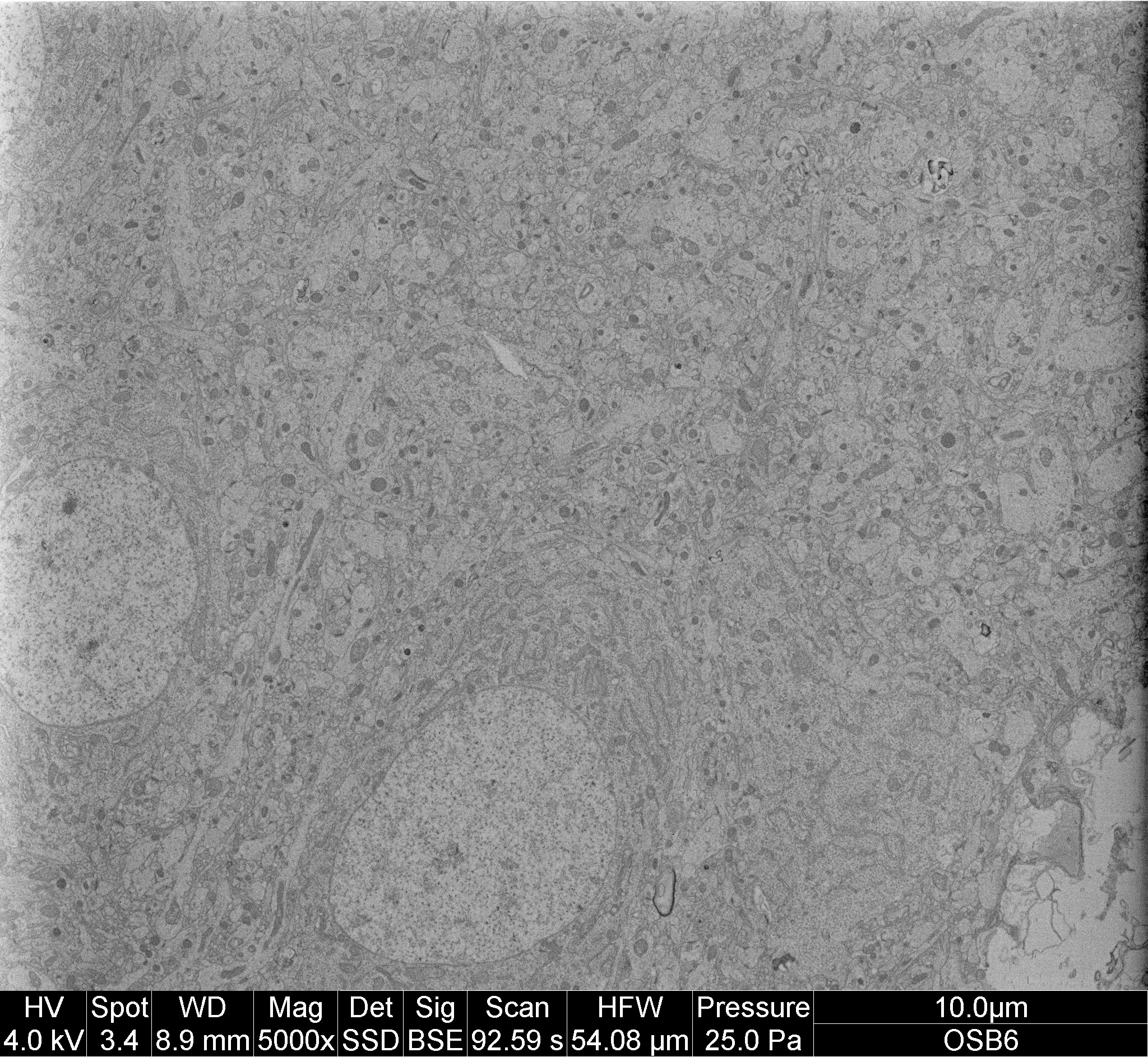

Supplement: Dataset S2 — (252.6 MB ZIP). [file pbio.0020329.sd002.zip › 040604_OS5_st1_111.tif]

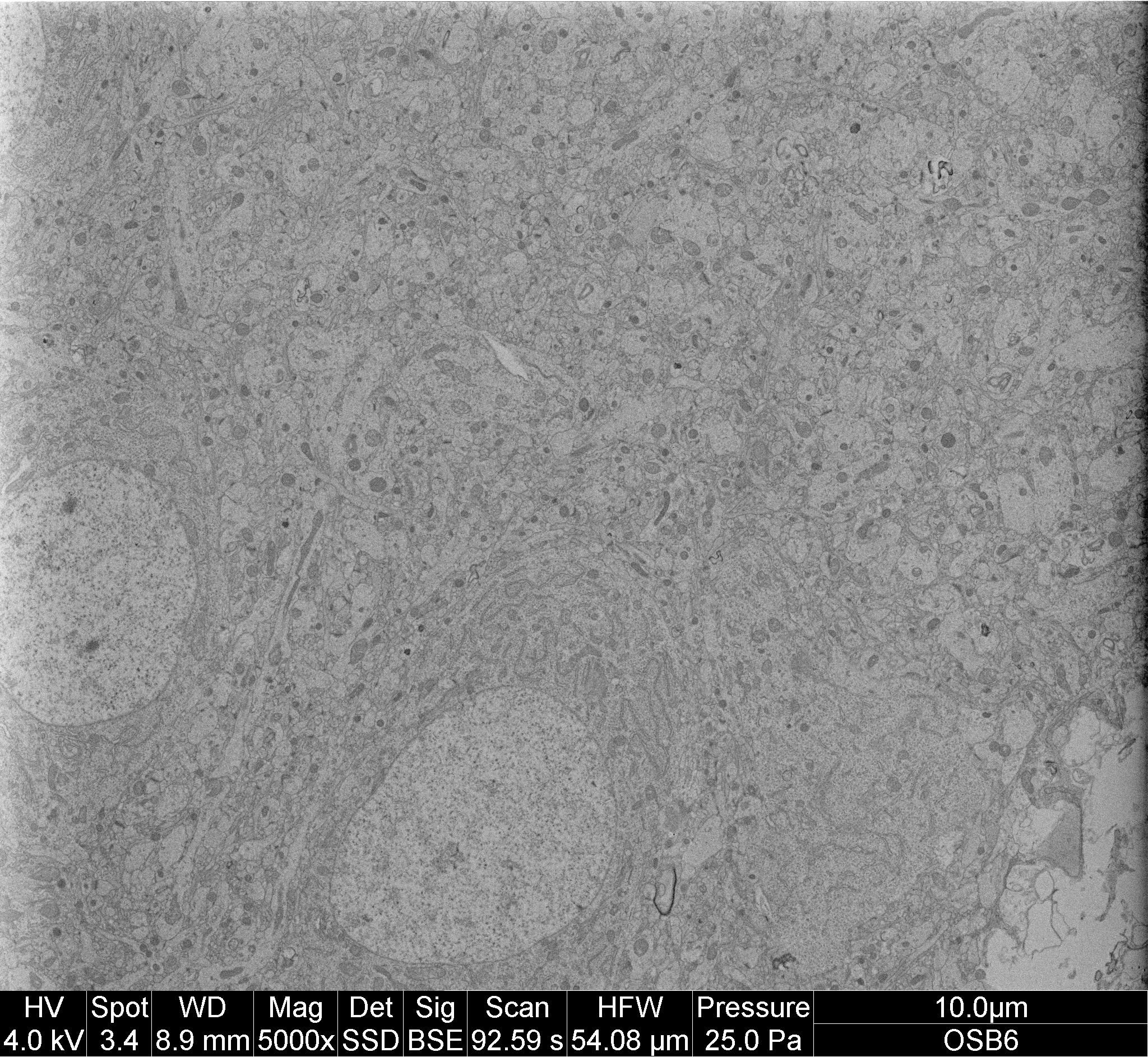

Supplement: Dataset S2 — (252.6 MB ZIP). [file pbio.0020329.sd002.zip › 040604_OS5_st1_112.tif]

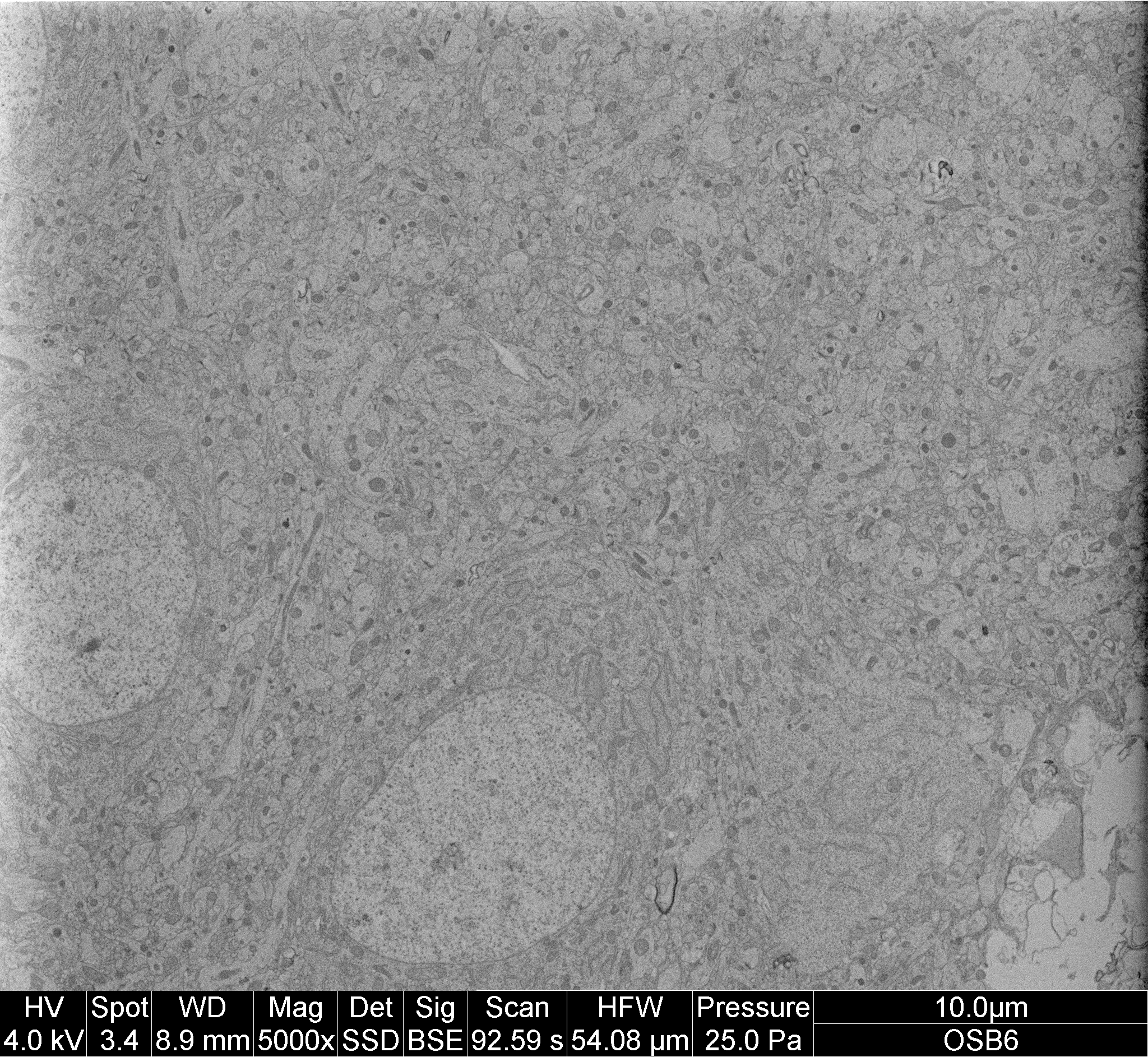

Supplement: Dataset S2 — (252.6 MB ZIP). [file pbio.0020329.sd002.zip › 040604_OS5_st1_113.tif]

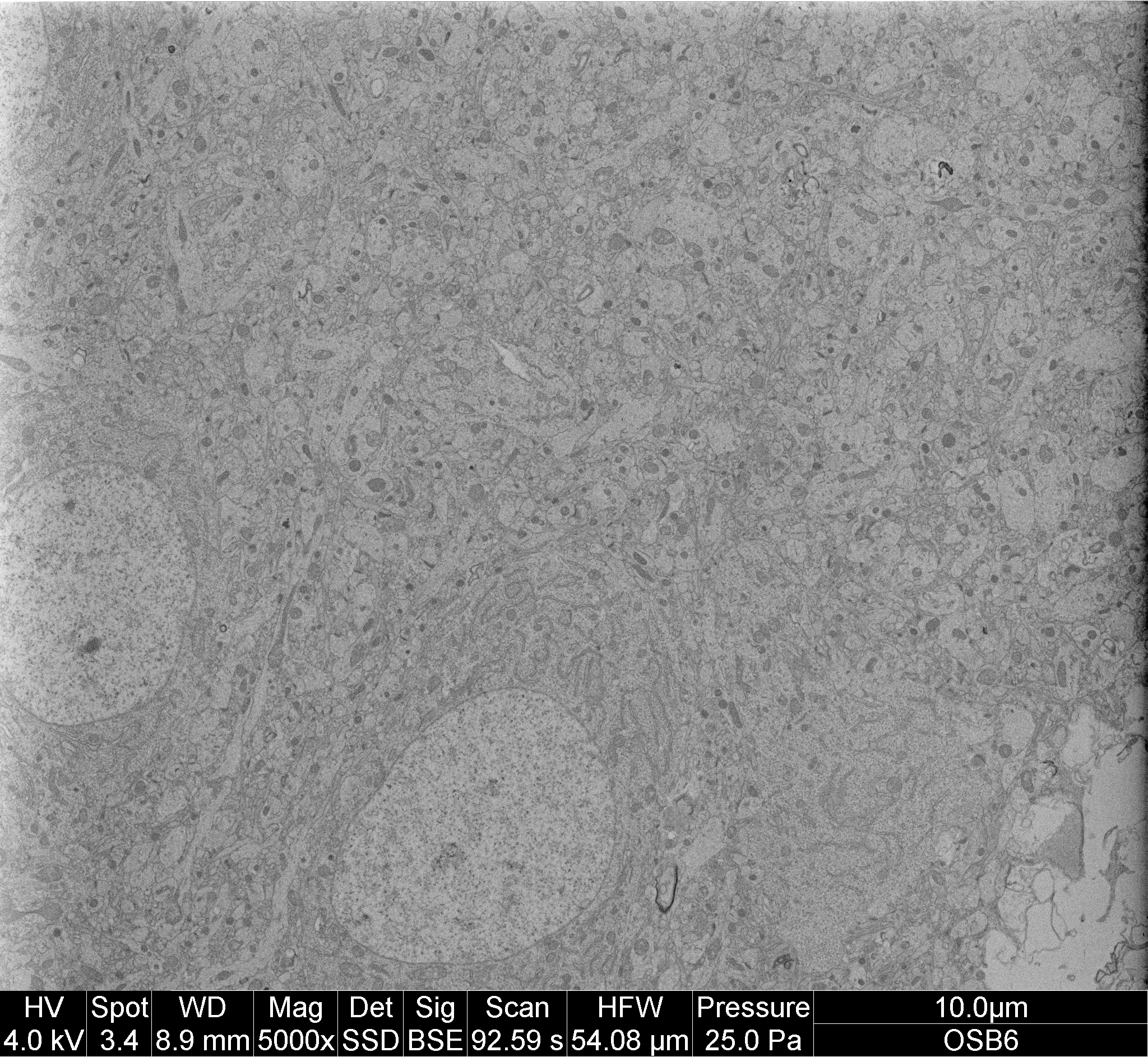

Supplement: Dataset S2 — (252.6 MB ZIP). [file pbio.0020329.sd002.zip › 040604_OS5_st1_114.tif]

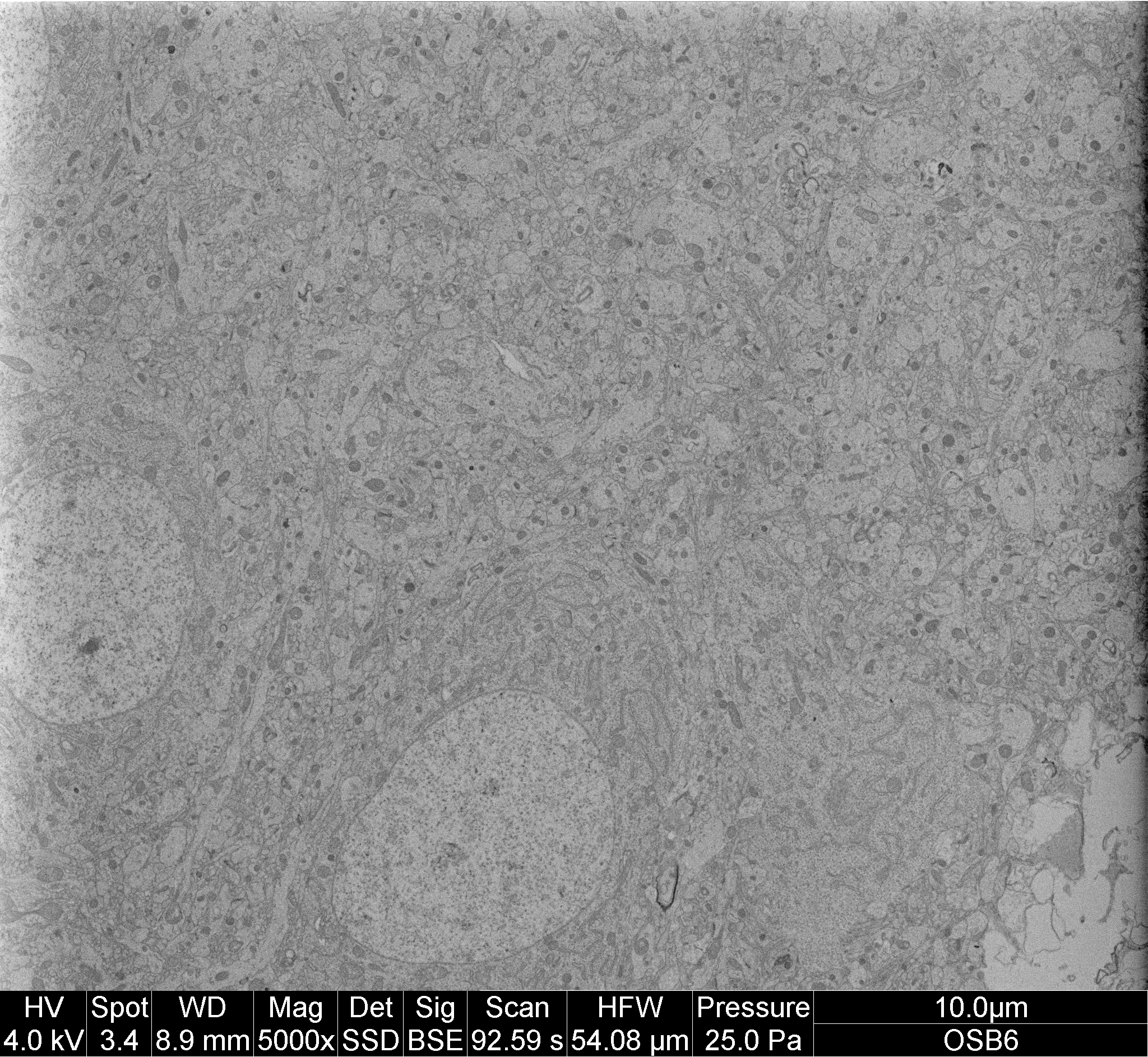

Supplement: Dataset S2 — (252.6 MB ZIP). [file pbio.0020329.sd002.zip › 040604_OS5_st1_115.tif]

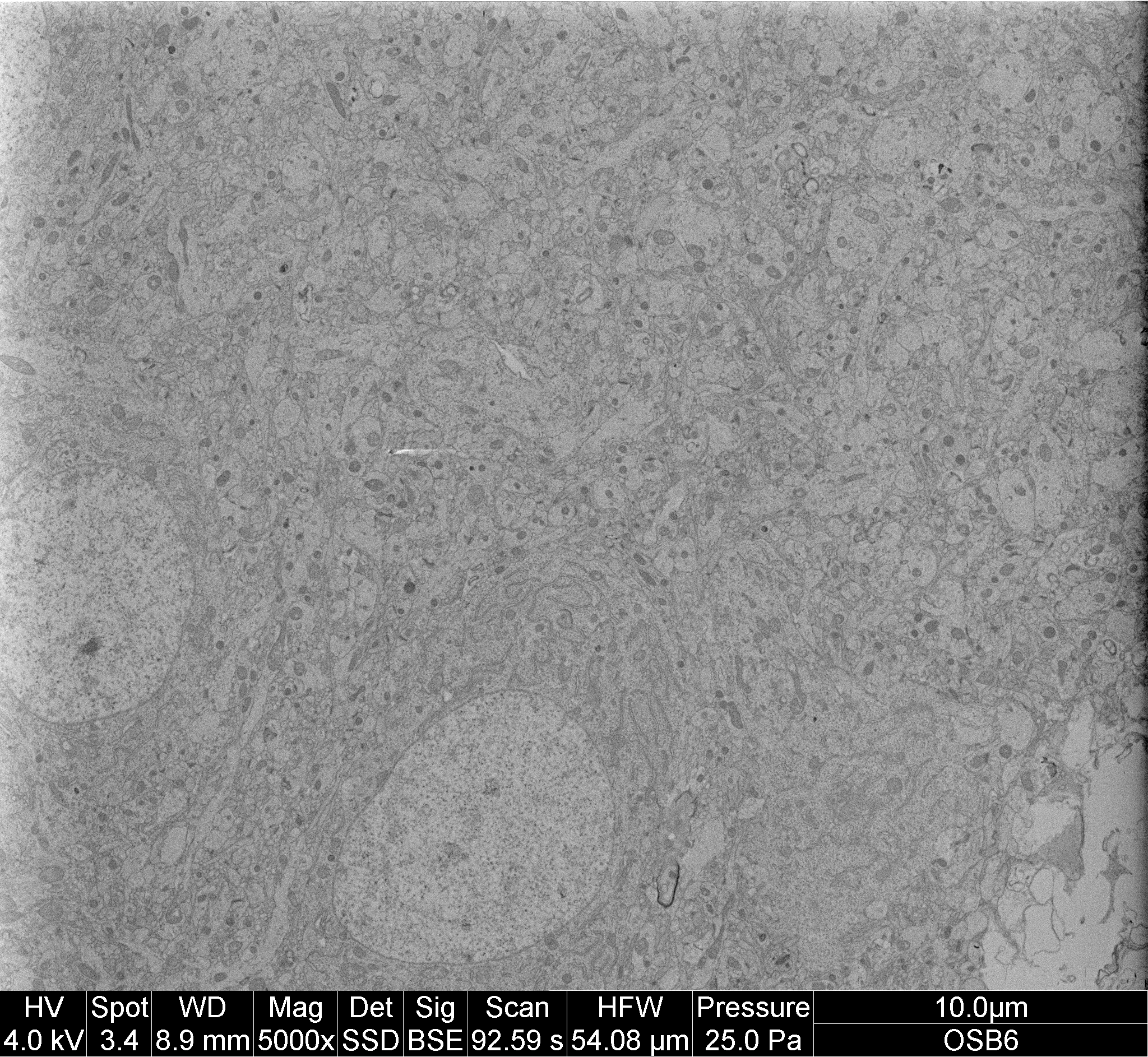

Supplement: Dataset S2 — (252.6 MB ZIP). [file pbio.0020329.sd002.zip › 040604_OS5_st1_116.tif]

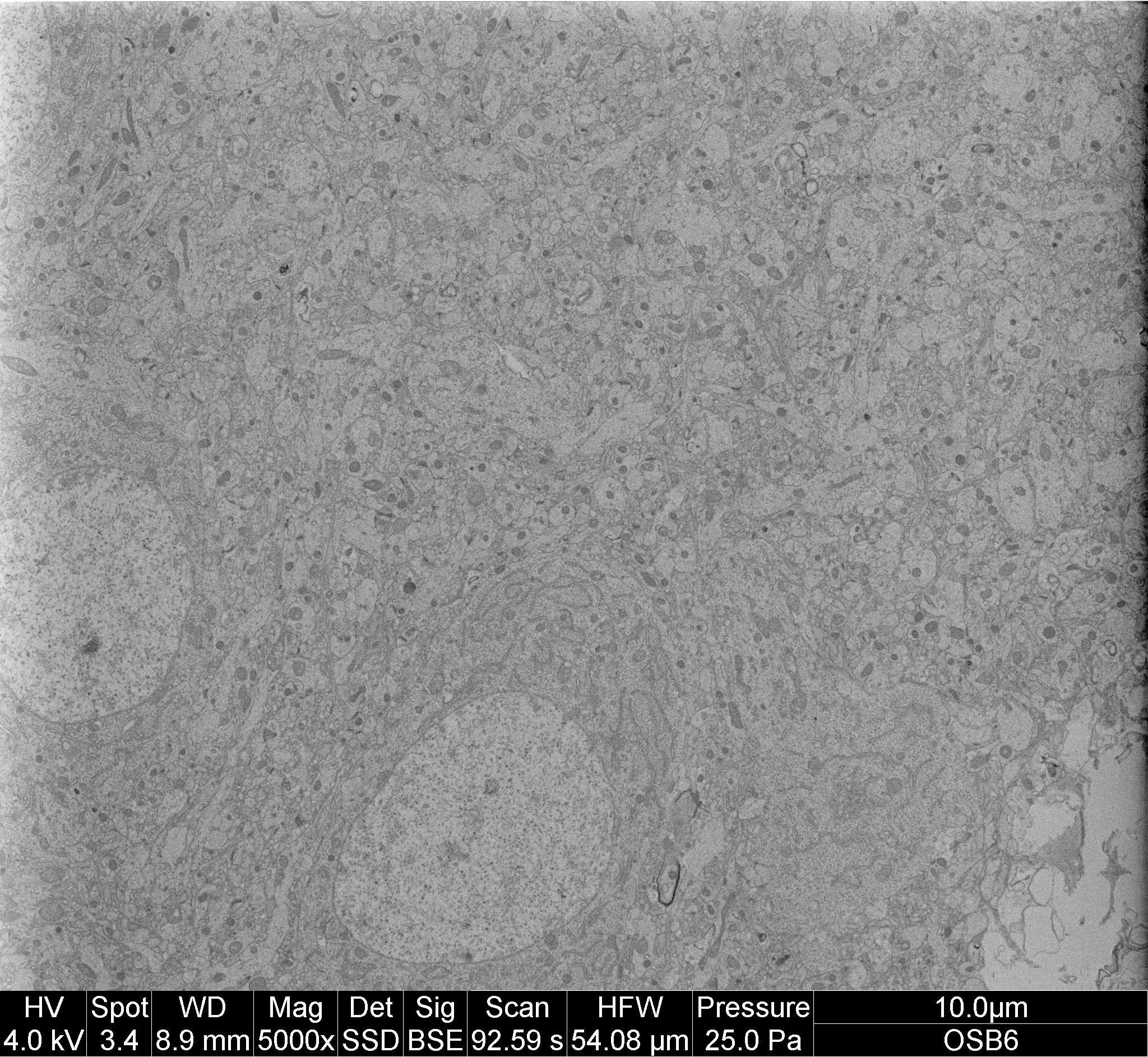

Supplement: Dataset S2 — (252.6 MB ZIP). [file pbio.0020329.sd002.zip › 040604_OS5_st1_117.tif]

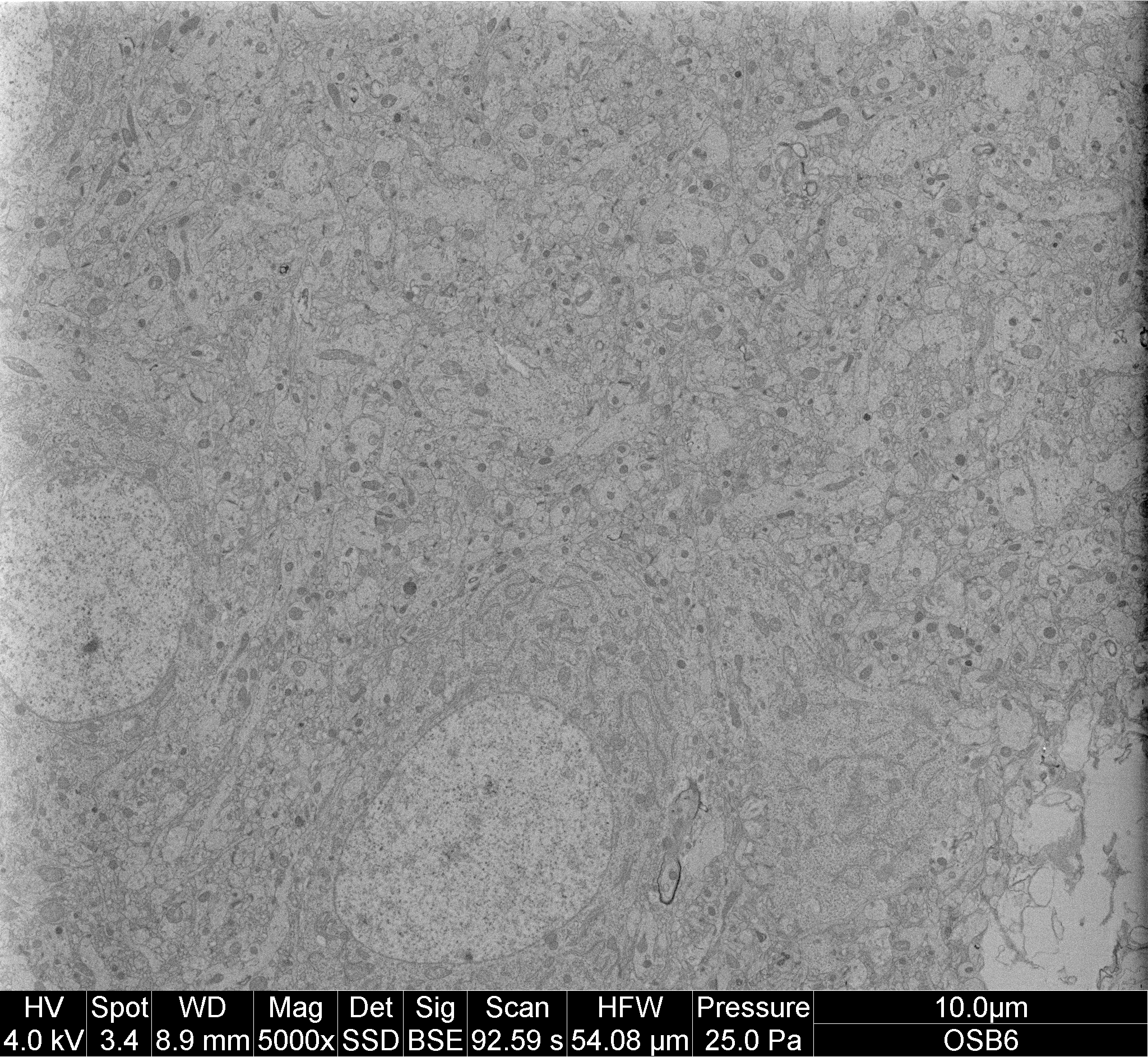

Supplement: Dataset S2 — (252.6 MB ZIP). [file pbio.0020329.sd002.zip › 040604_OS5_st1_118.tif]

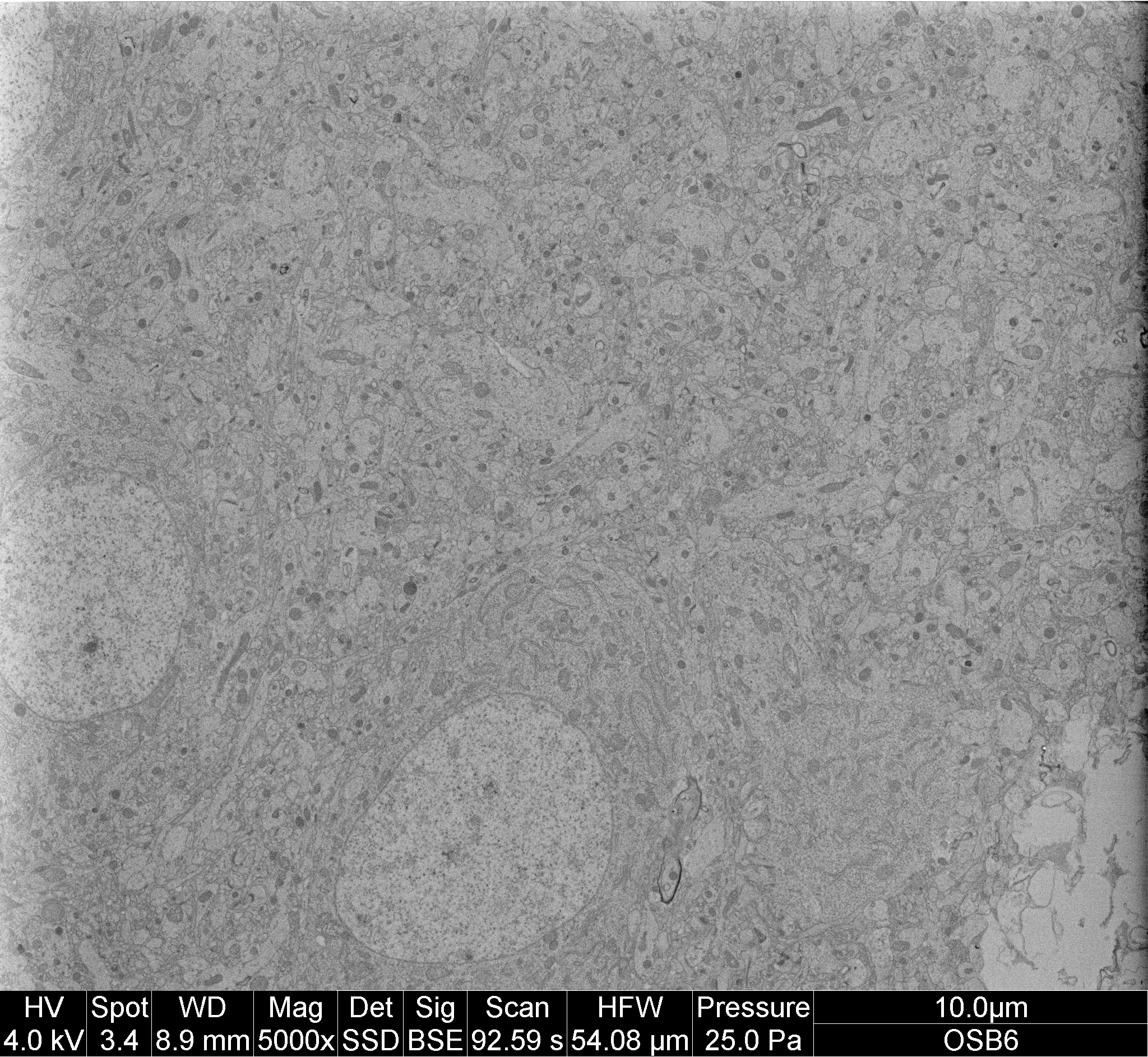

Supplement: Dataset S2 — (252.6 MB ZIP). [file pbio.0020329.sd002.zip › 040604_OS5_st1_119.tif]

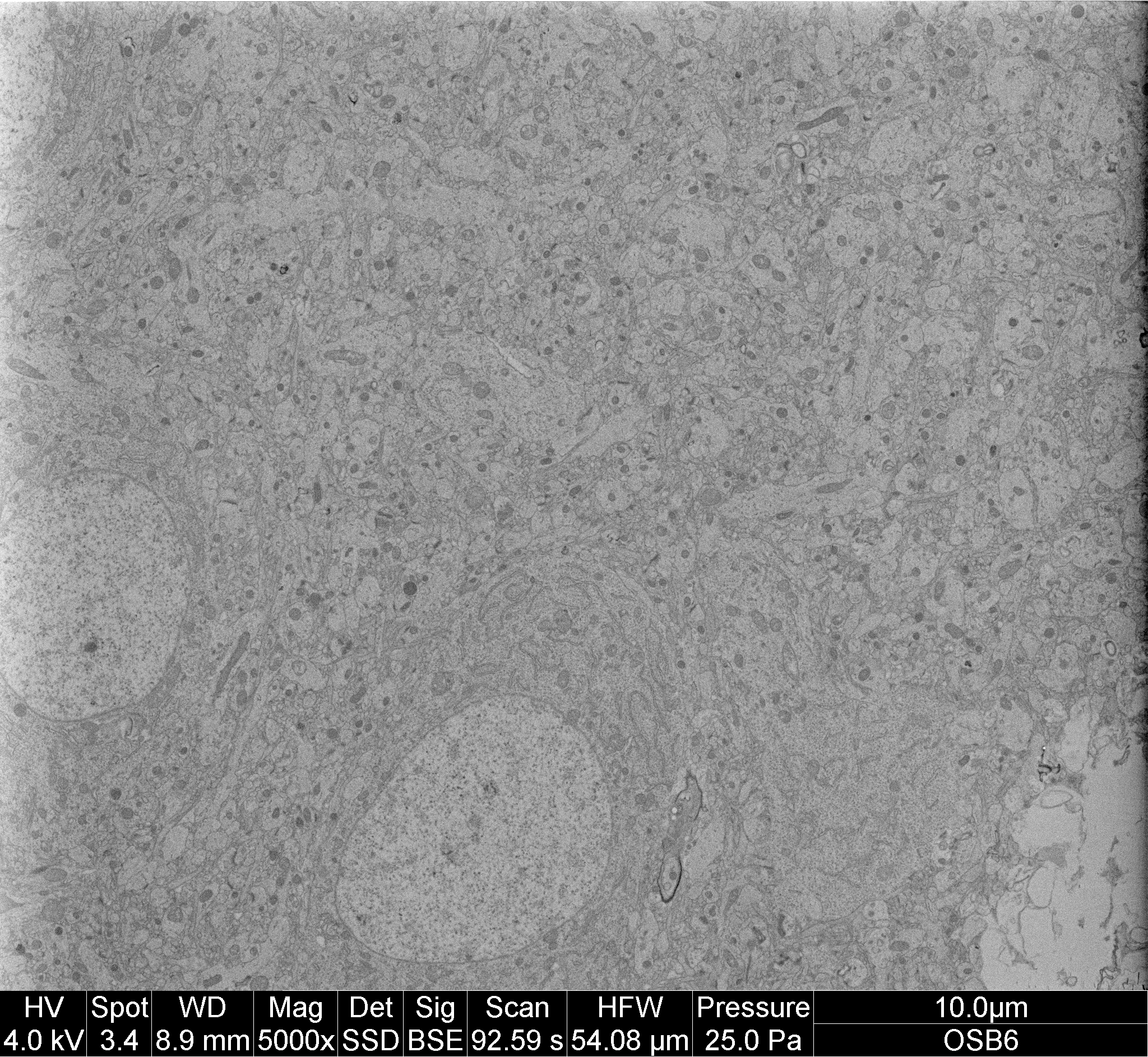

Supplement: Dataset S2 — (252.6 MB ZIP). [file pbio.0020329.sd002.zip › 040604_OS5_st1_120.tif]

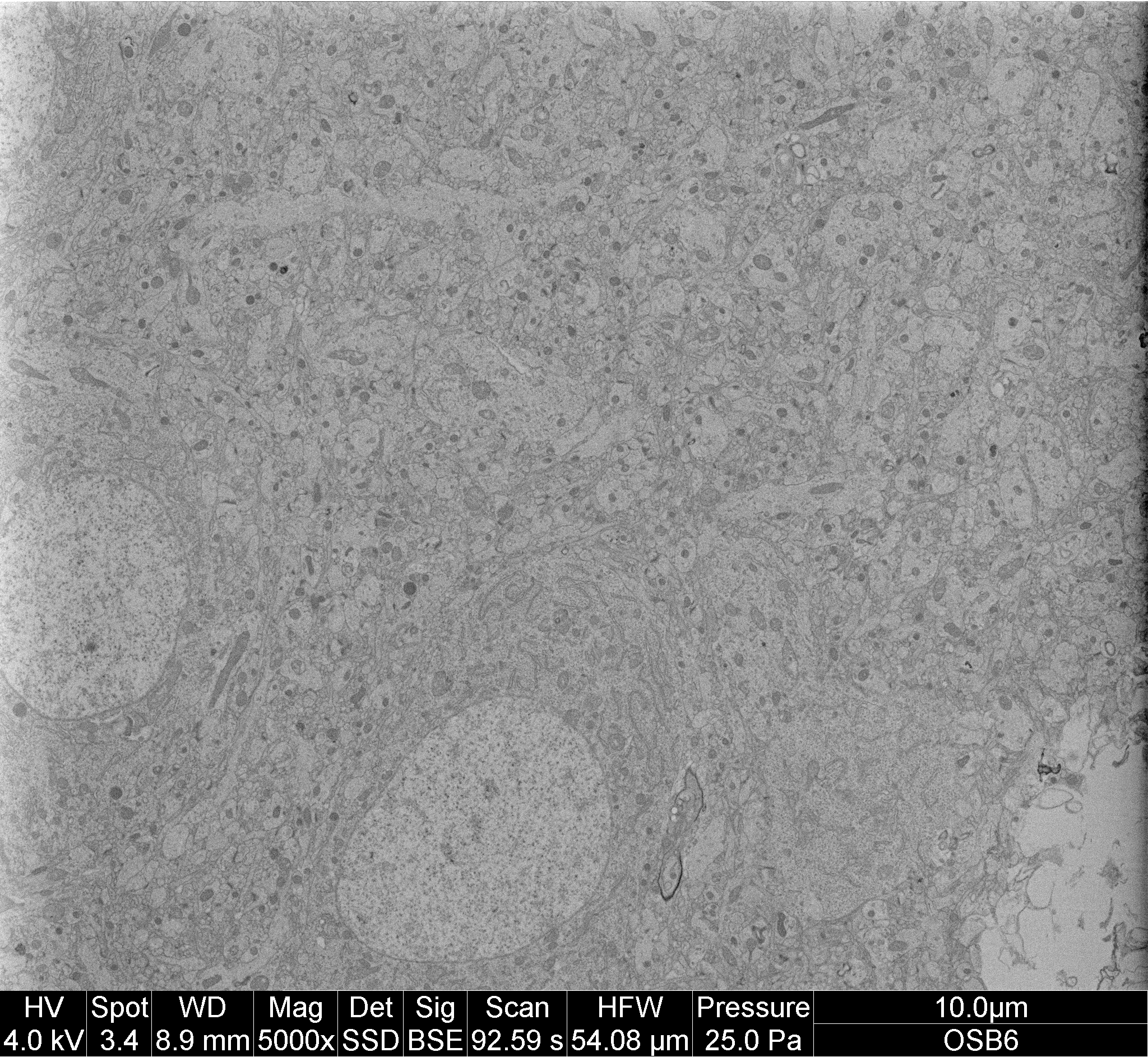

Supplement: Dataset S2 — (252.6 MB ZIP). [file pbio.0020329.sd002.zip › 040604_OS5_st1_121.tif]

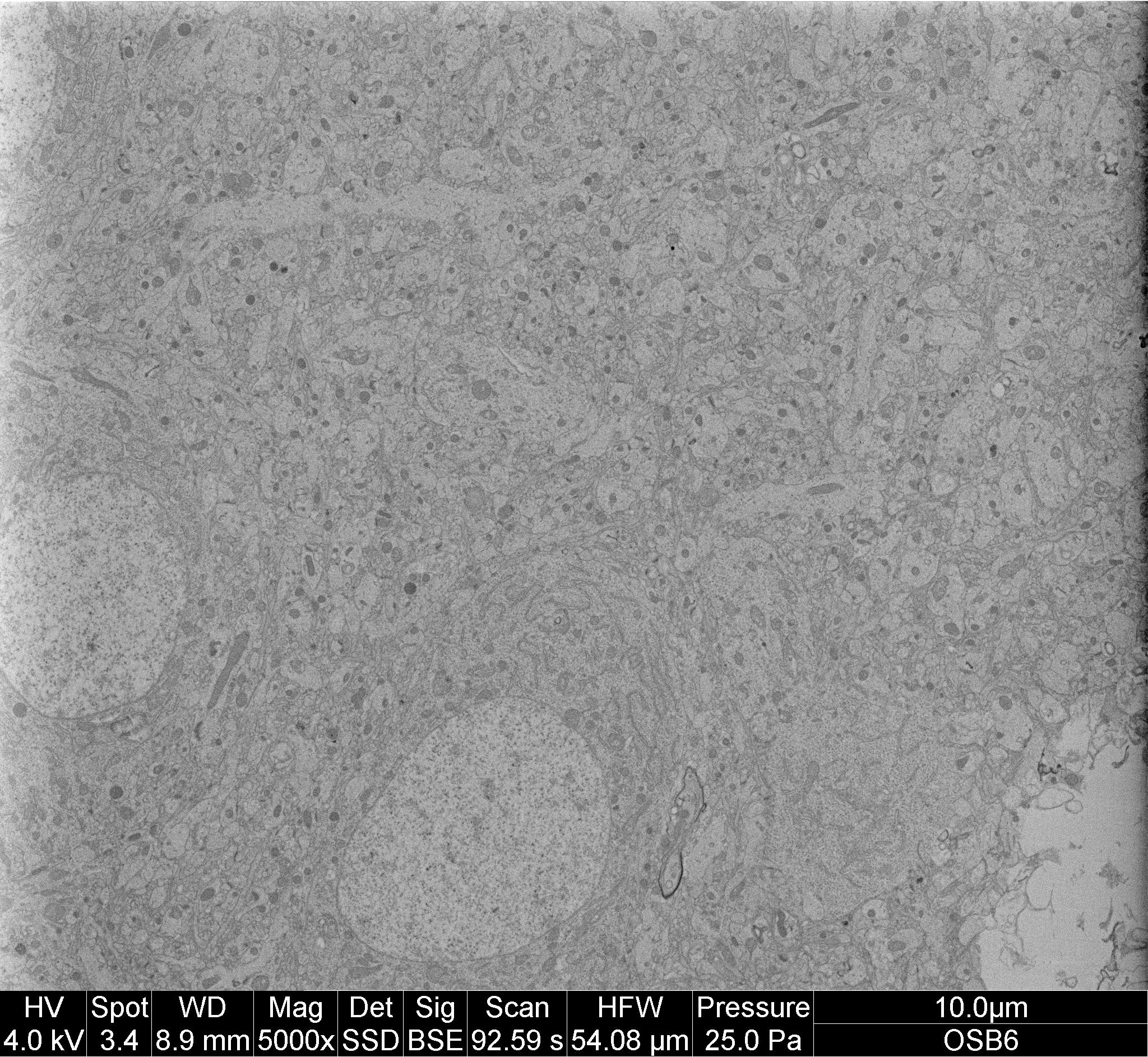

Supplement: Dataset S2 — (252.6 MB ZIP). [file pbio.0020329.sd002.zip › 040604_OS5_st1_122.tif]

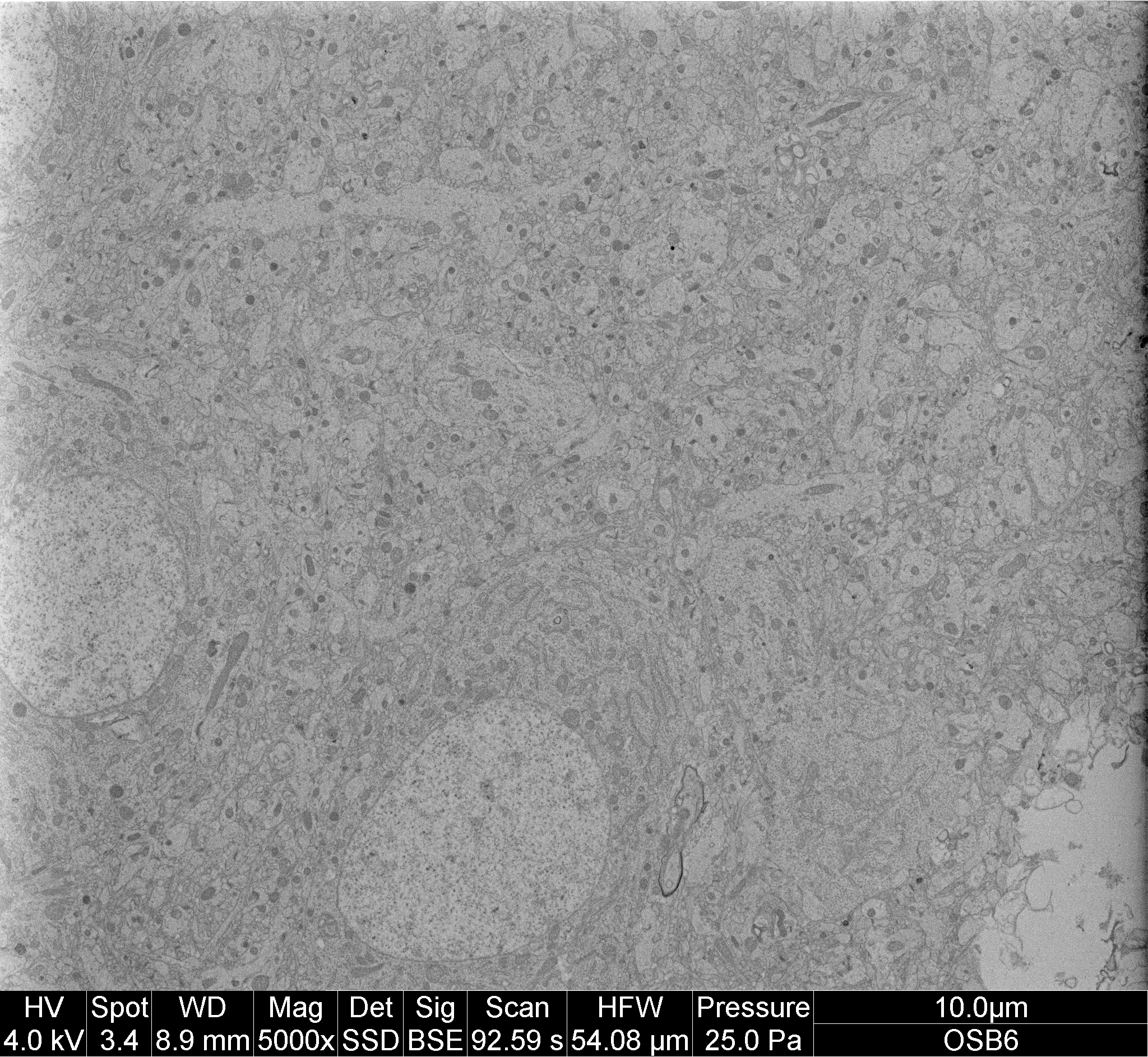

Supplement: Dataset S2 — (252.6 MB ZIP). [file pbio.0020329.sd002.zip › 040604_OS5_st1_123.tif]

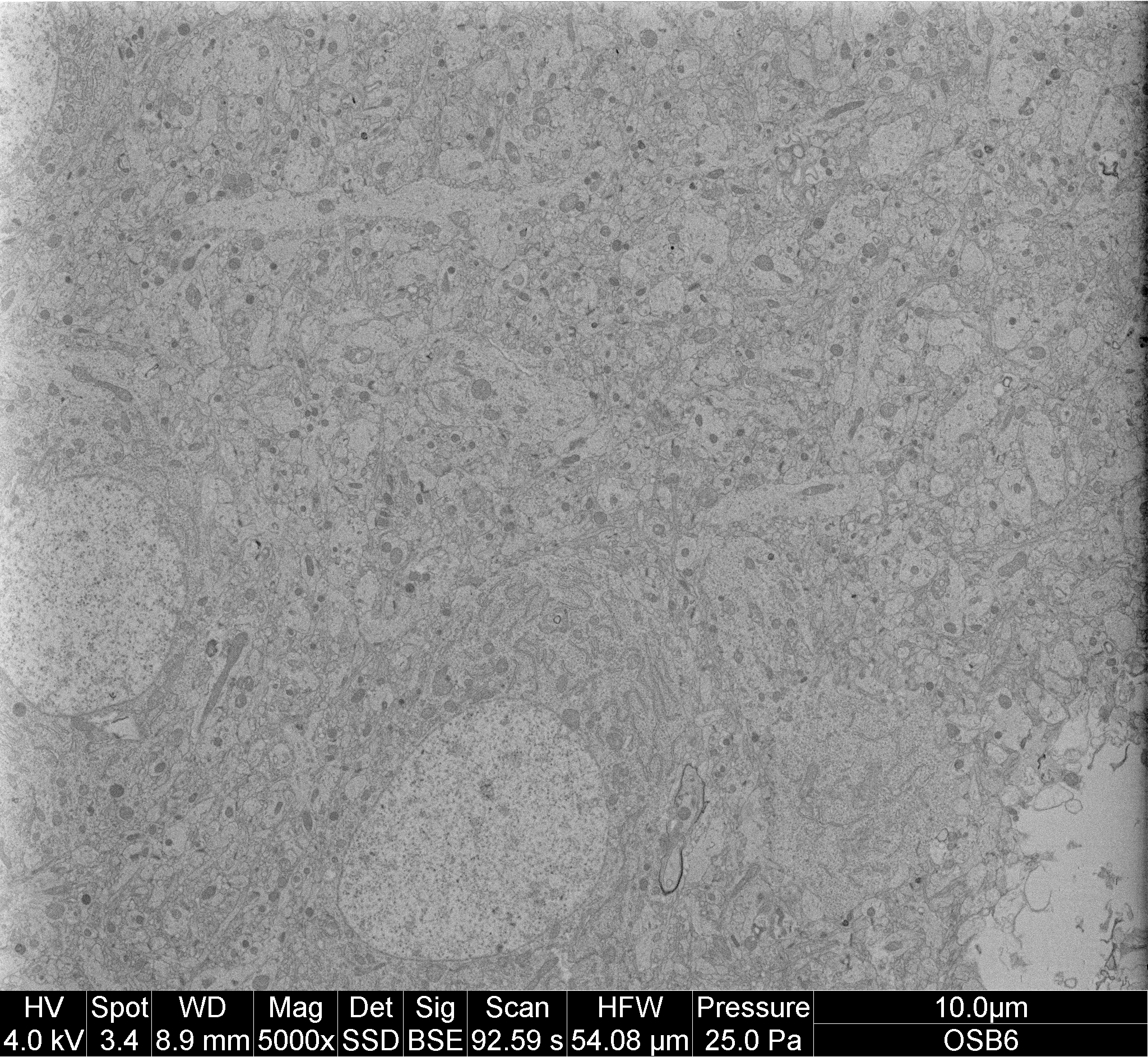

Supplement: Dataset S2 — (252.6 MB ZIP). [file pbio.0020329.sd002.zip › 040604_OS5_st1_124.tif]

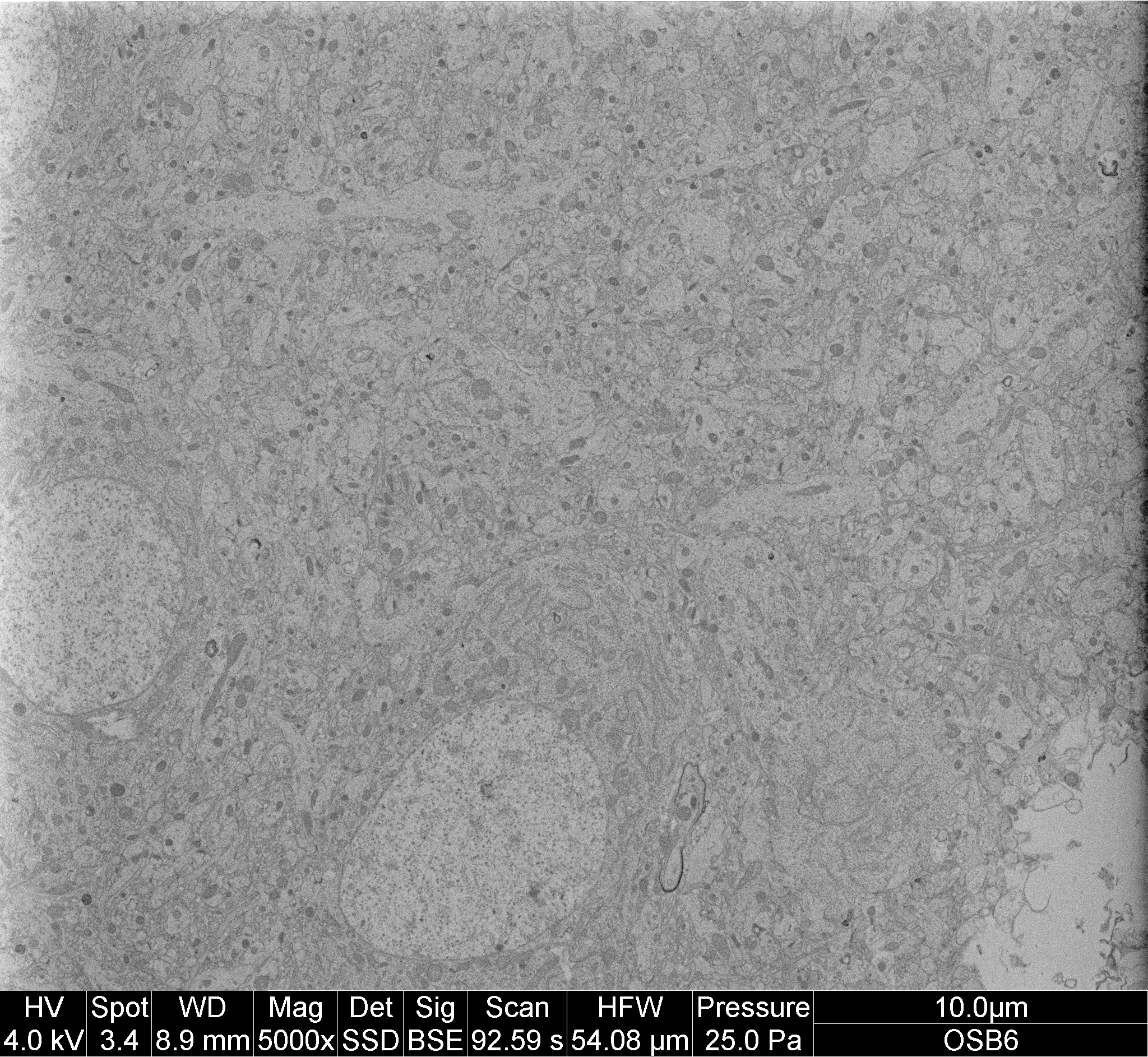

Supplement: Dataset S2 — (252.6 MB ZIP). [file pbio.0020329.sd002.zip › 040604_OS5_st1_125.tif]

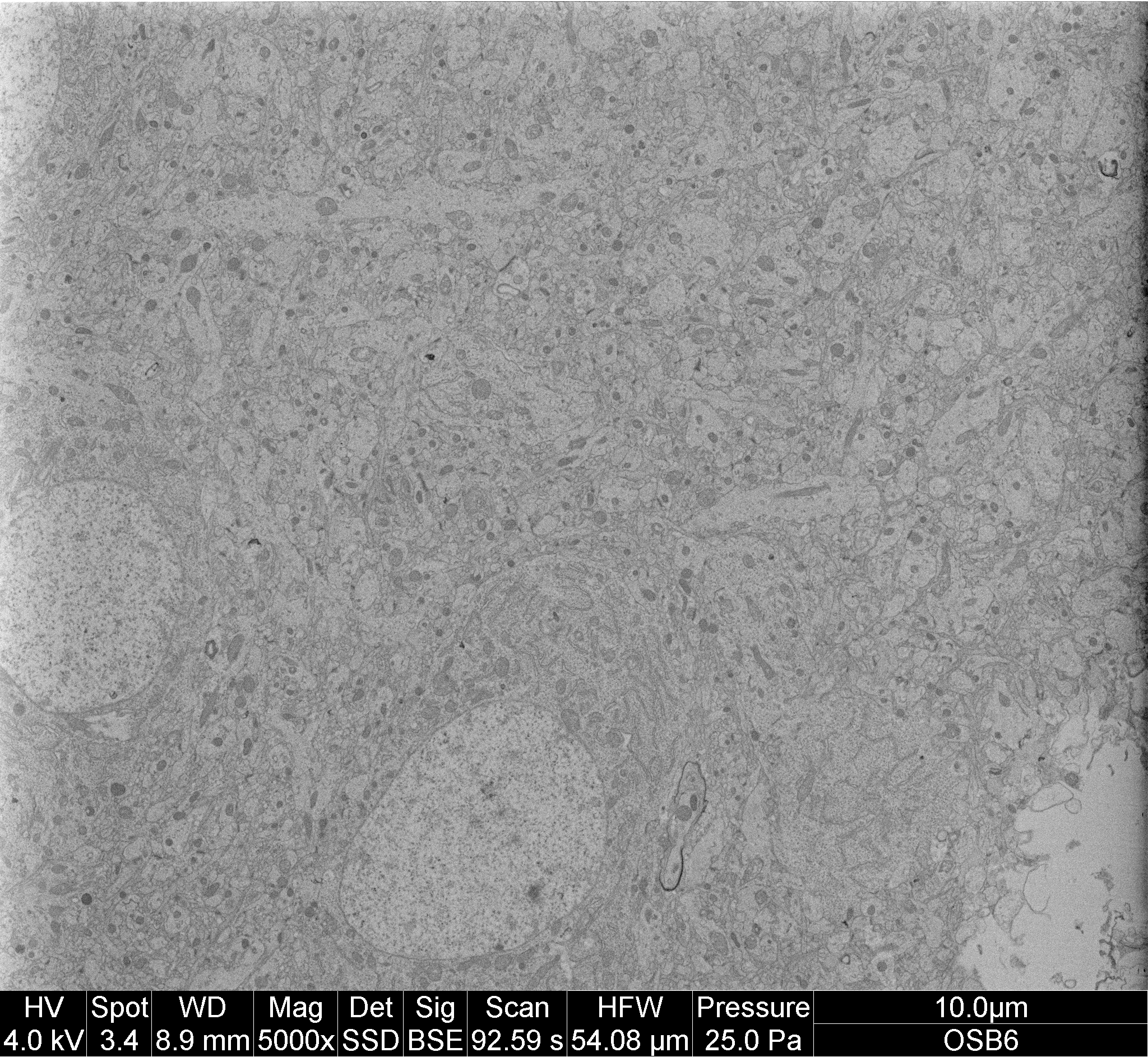

Supplement: Dataset S2 — (252.6 MB ZIP). [file pbio.0020329.sd002.zip › 040604_OS5_st1_126.tif]

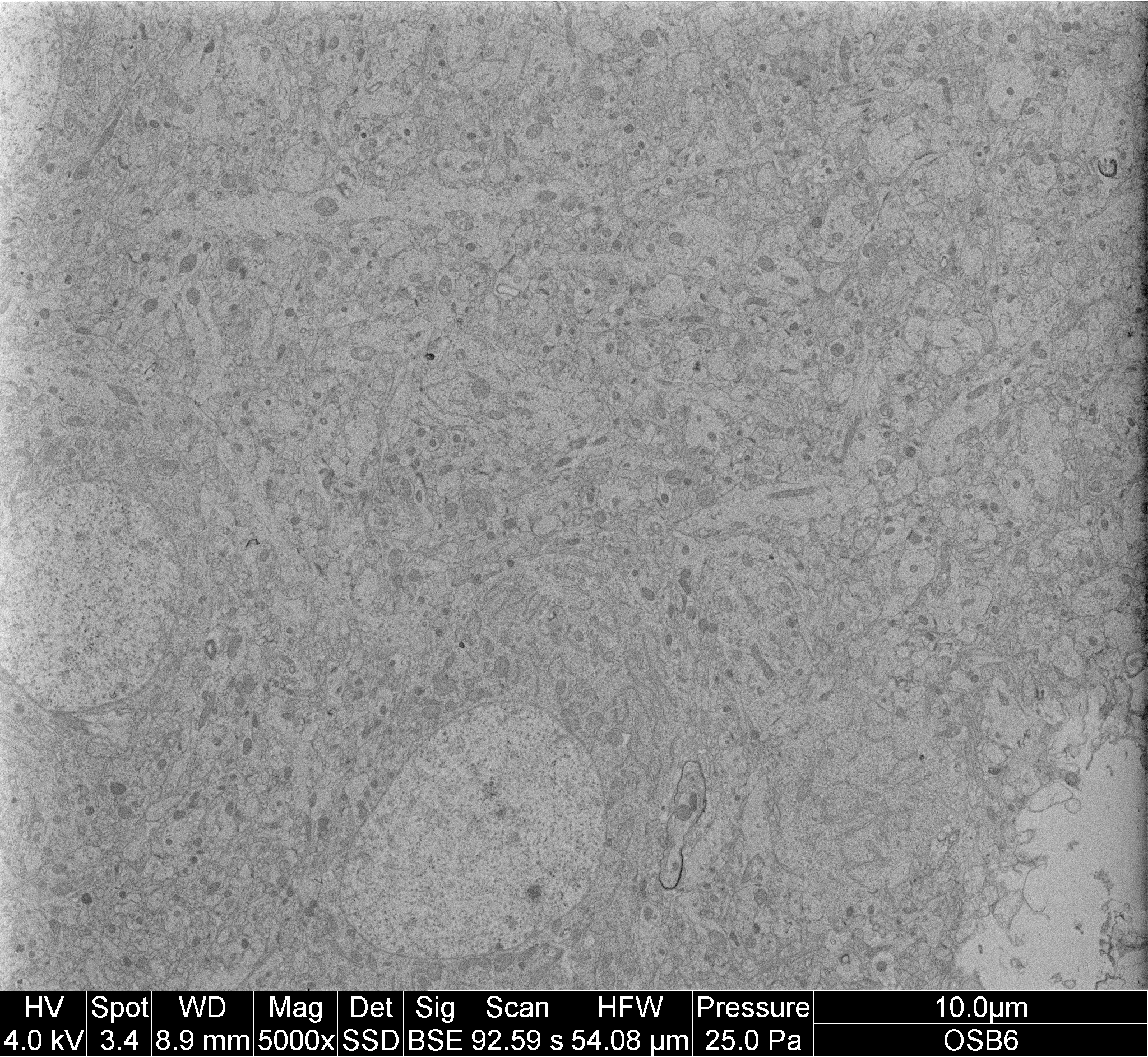

Supplement: Dataset S2 — (252.6 MB ZIP). [file pbio.0020329.sd002.zip › 040604_OS5_st1_127.tif]

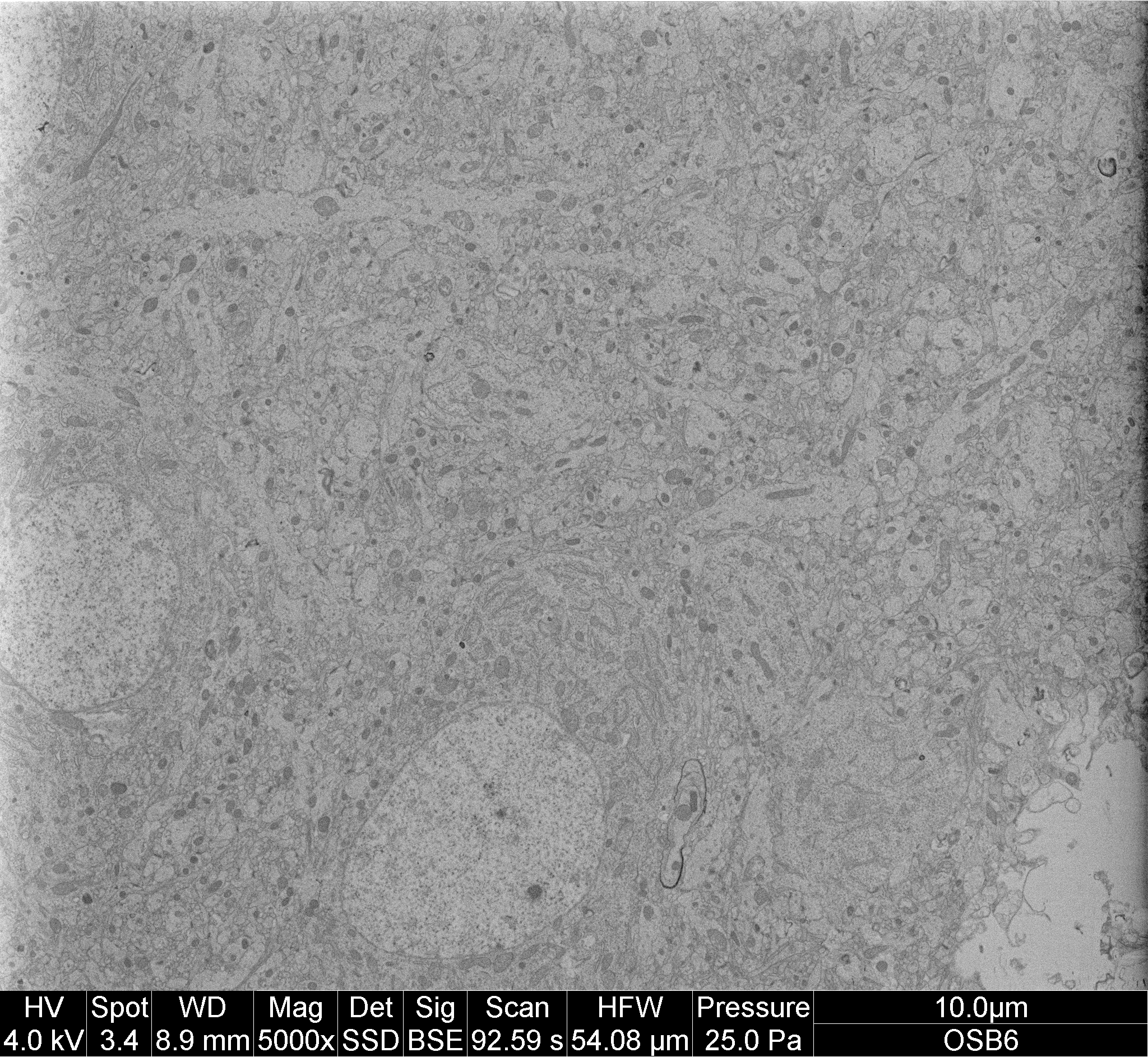

Supplement: Dataset S2 — (252.6 MB ZIP). [file pbio.0020329.sd002.zip › 040604_OS5_st1_128.tif]

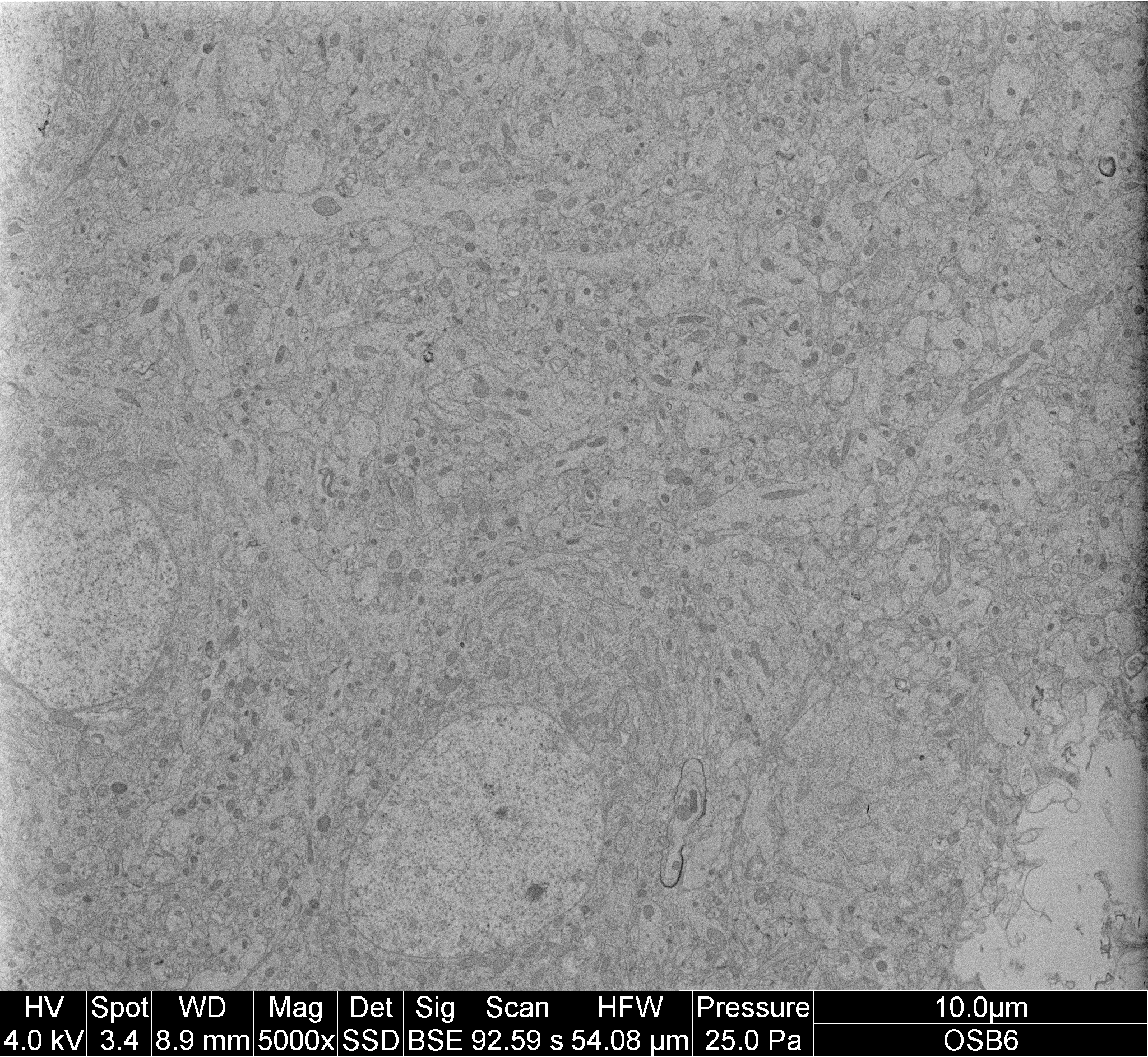

Supplement: Dataset S2 — (252.6 MB ZIP). [file pbio.0020329.sd002.zip › 040604_OS5_st1_129.tif]

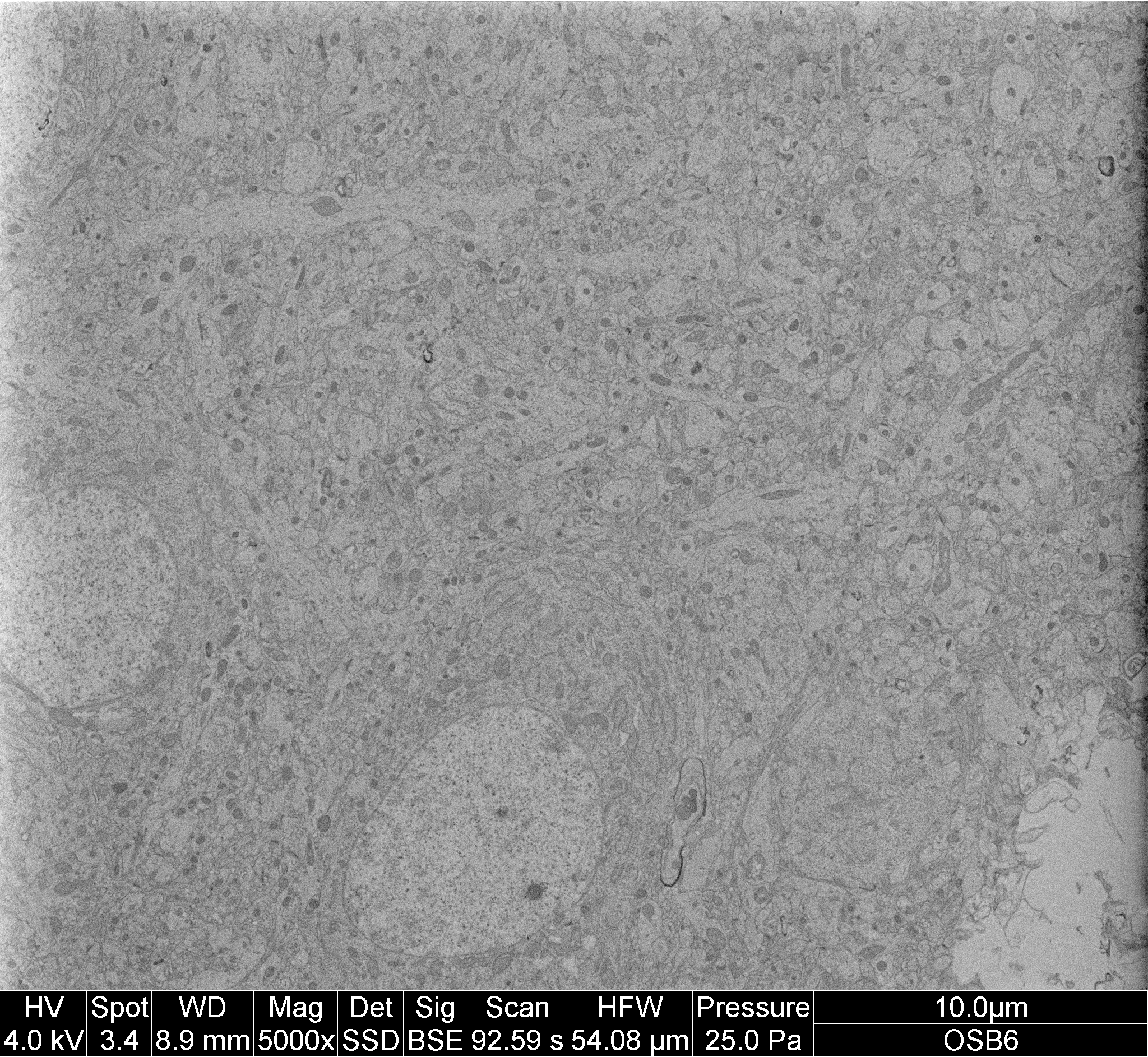

Supplement: Dataset S2 — (252.6 MB ZIP). [file pbio.0020329.sd002.zip › 040604_OS5_st1_130.tif]

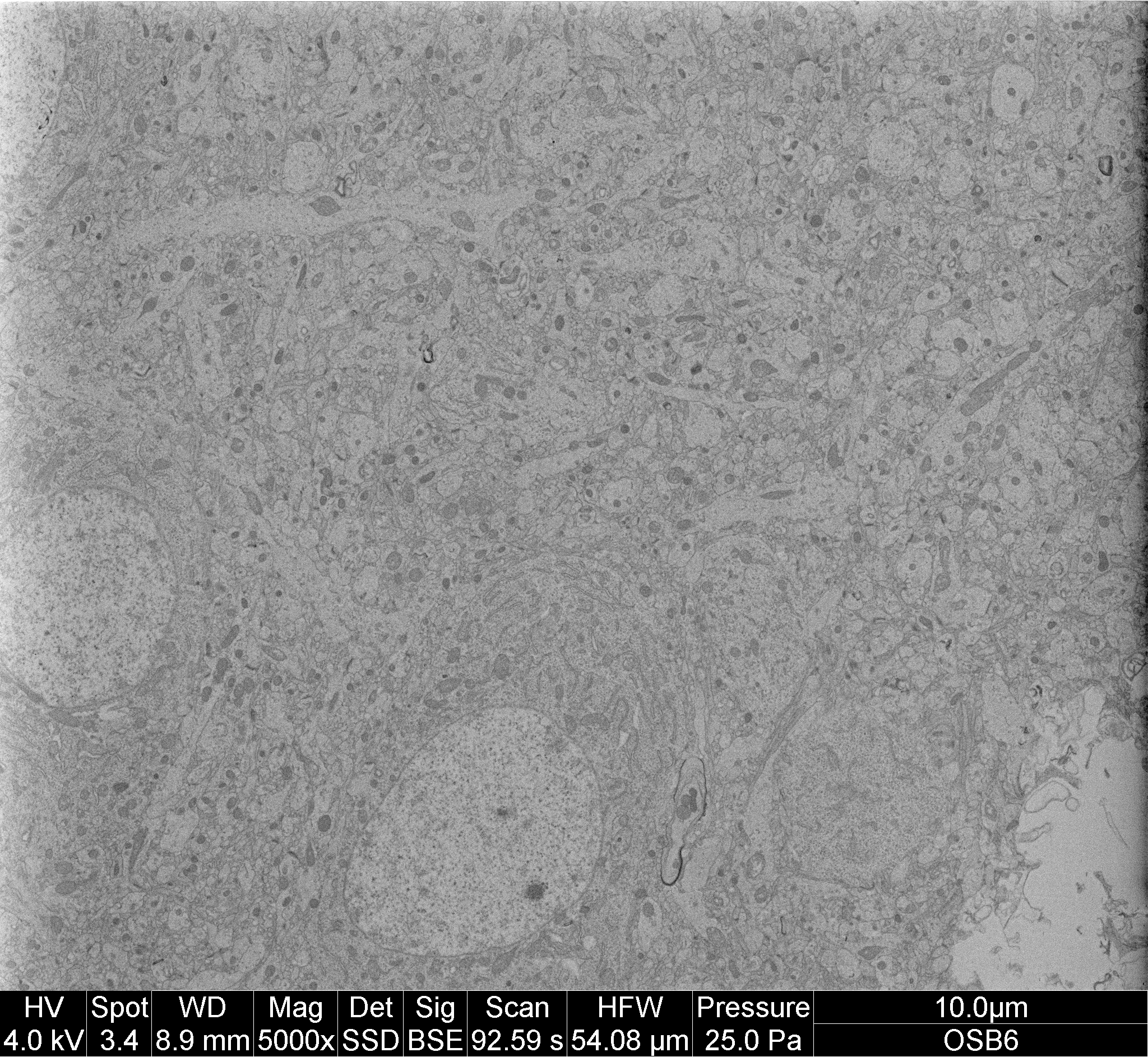

Supplement: Dataset S2 — (252.6 MB ZIP). [file pbio.0020329.sd002.zip › 040604_OS5_st1_131.tif]

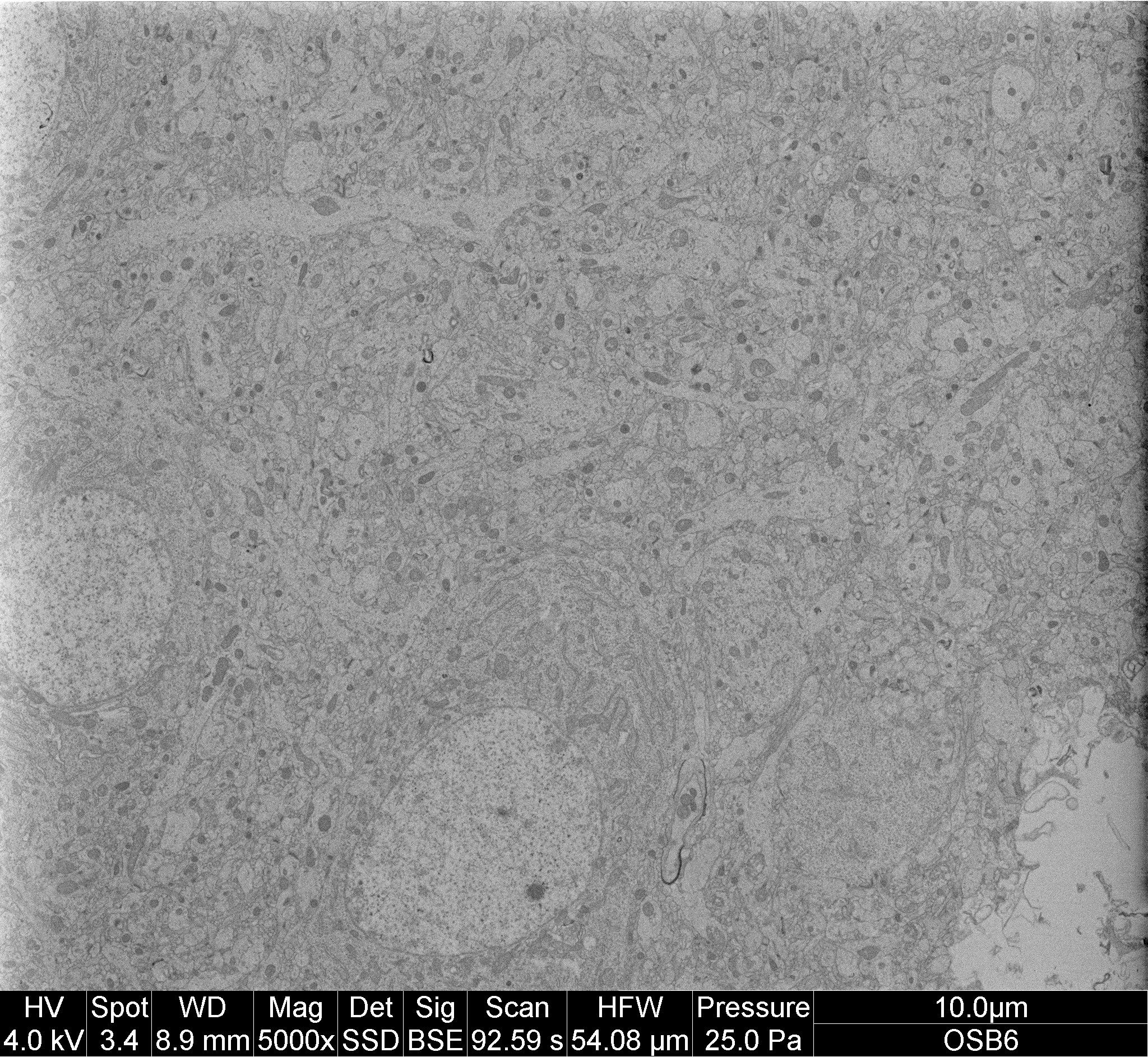

Supplement: Dataset S2 — (252.6 MB ZIP). [file pbio.0020329.sd002.zip › 040604_OS5_st1_132.tif]

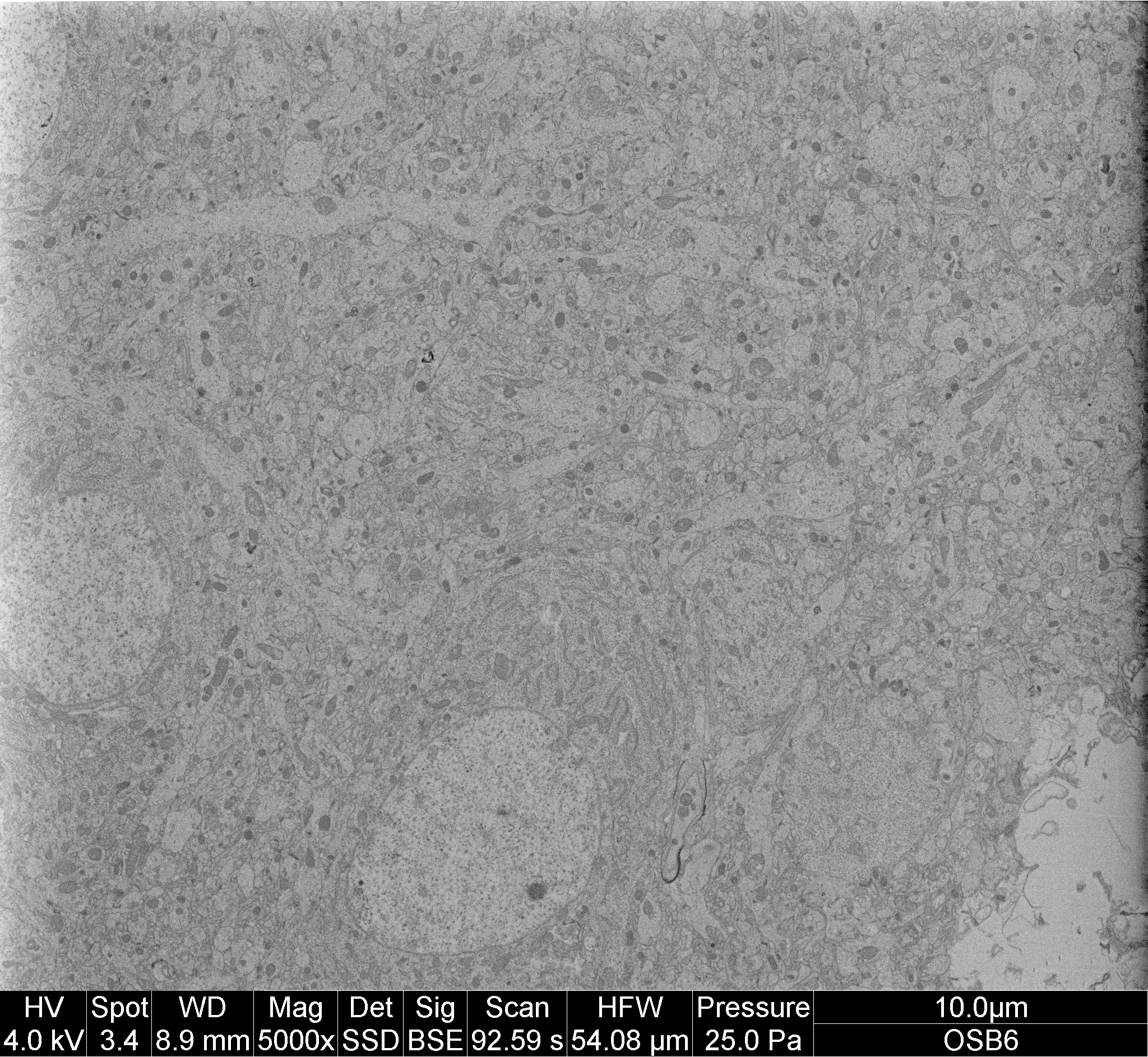

Supplement: Dataset S2 — (252.6 MB ZIP). [file pbio.0020329.sd002.zip › 040604_OS5_st1_133.tif]

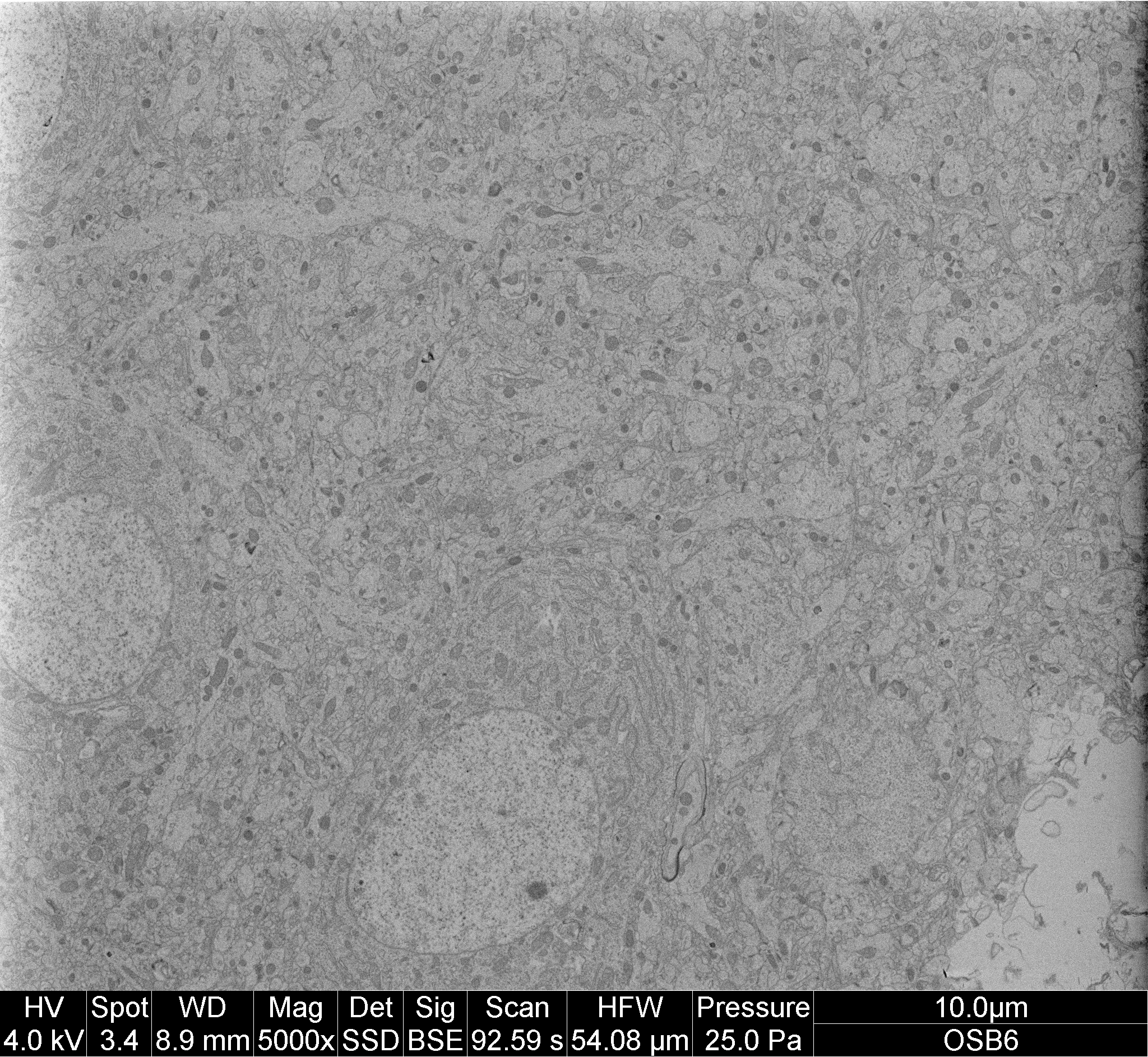

Supplement: Dataset S2 — (252.6 MB ZIP). [file pbio.0020329.sd002.zip › 040604_OS5_st1_134.tif]

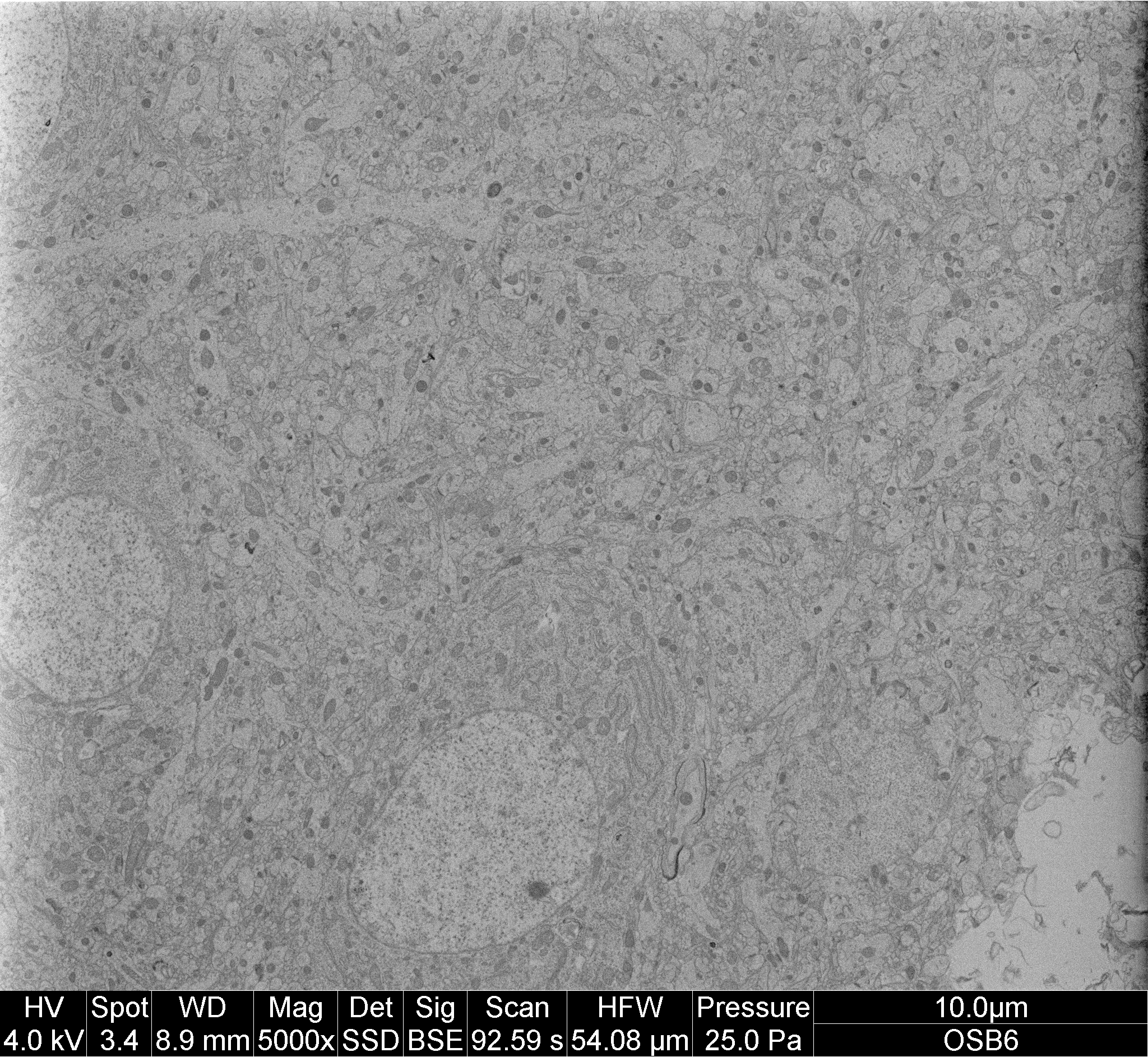

Supplement: Dataset S2 — (252.6 MB ZIP). [file pbio.0020329.sd002.zip › 040604_OS5_st1_135.tif]

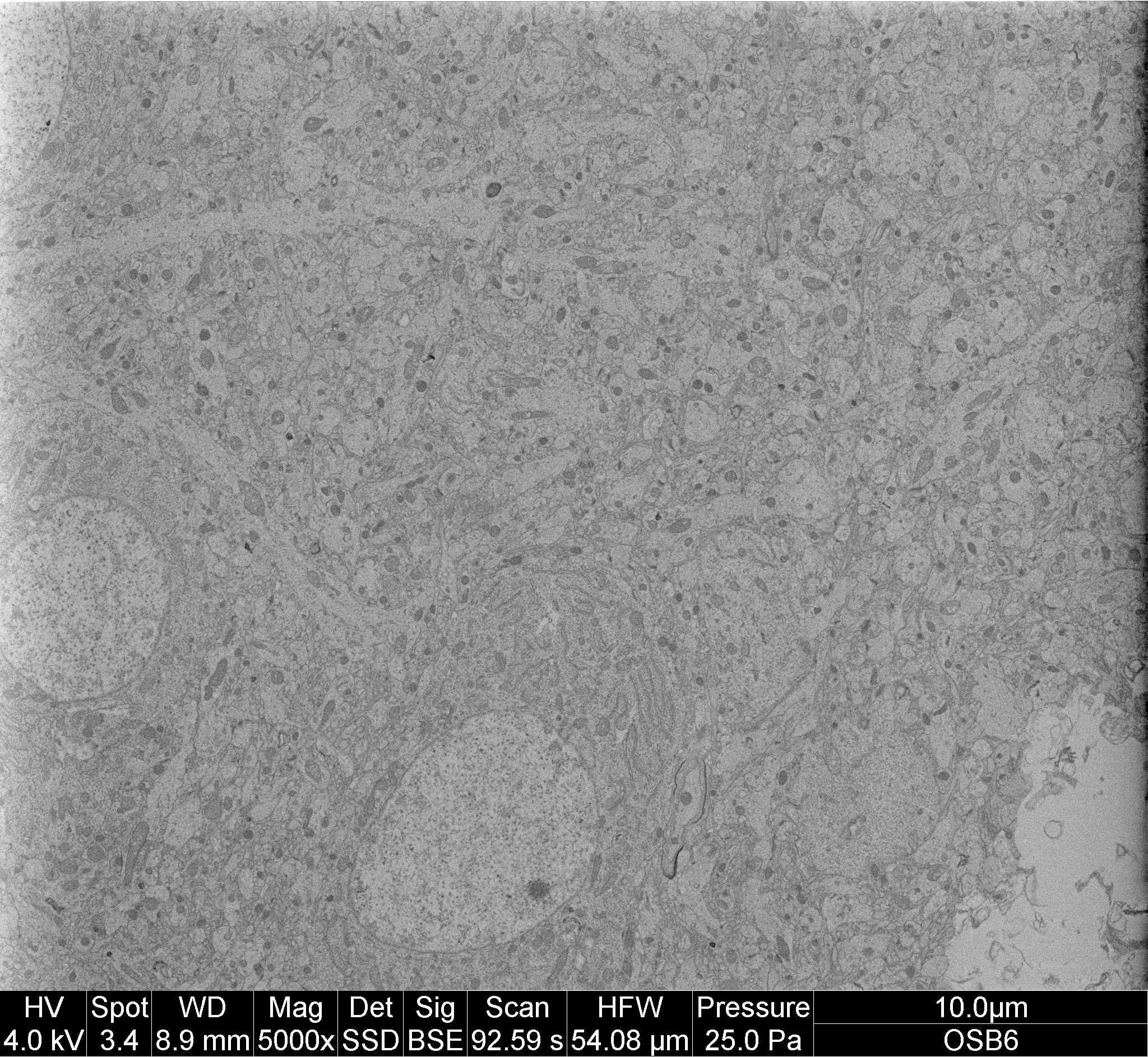

Supplement: Dataset S2 — (252.6 MB ZIP). [file pbio.0020329.sd002.zip › 040604_OS5_st1_136.tif]

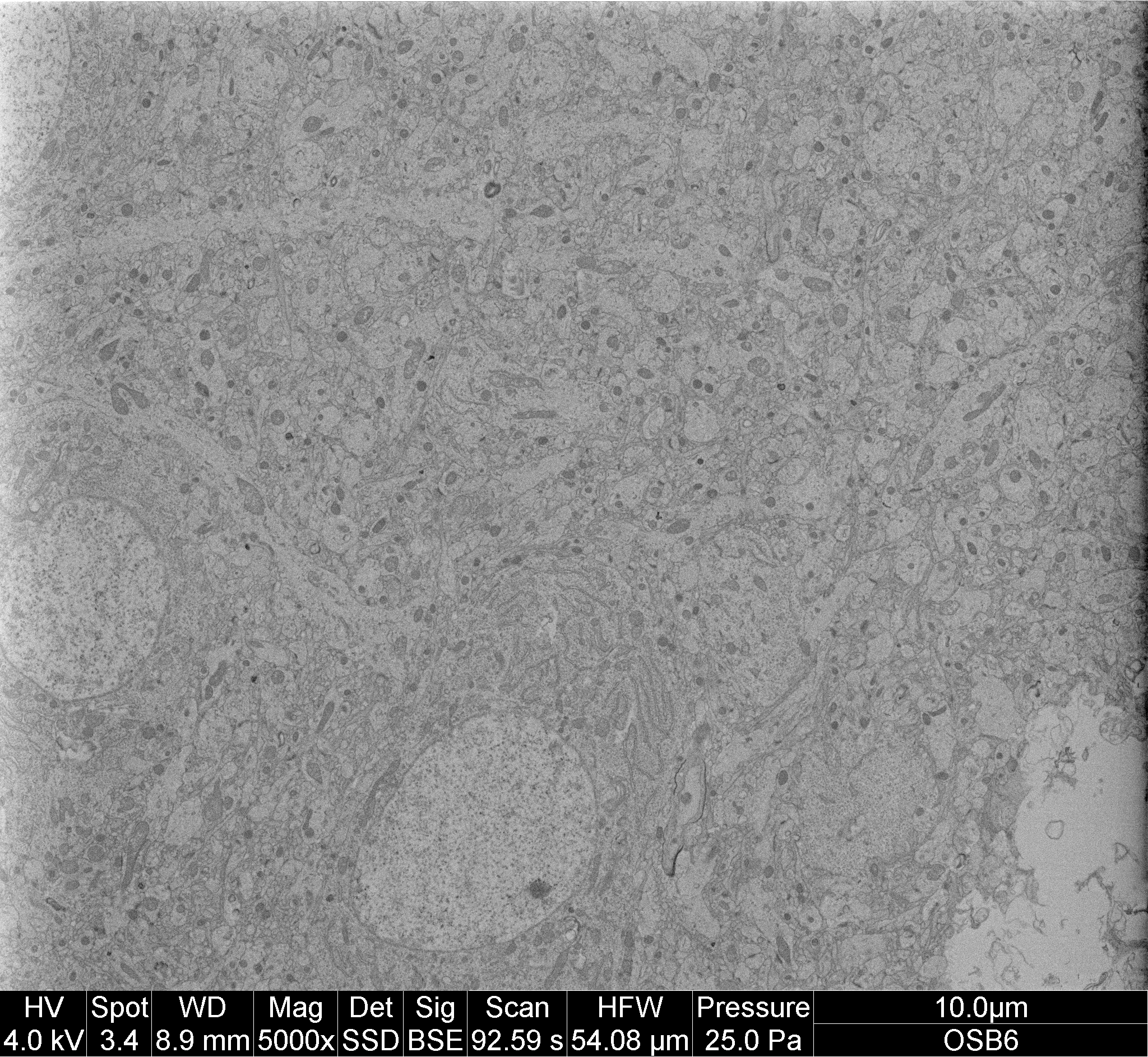

Supplement: Dataset S2 — (252.6 MB ZIP). [file pbio.0020329.sd002.zip › 040604_OS5_st1_137.tif]

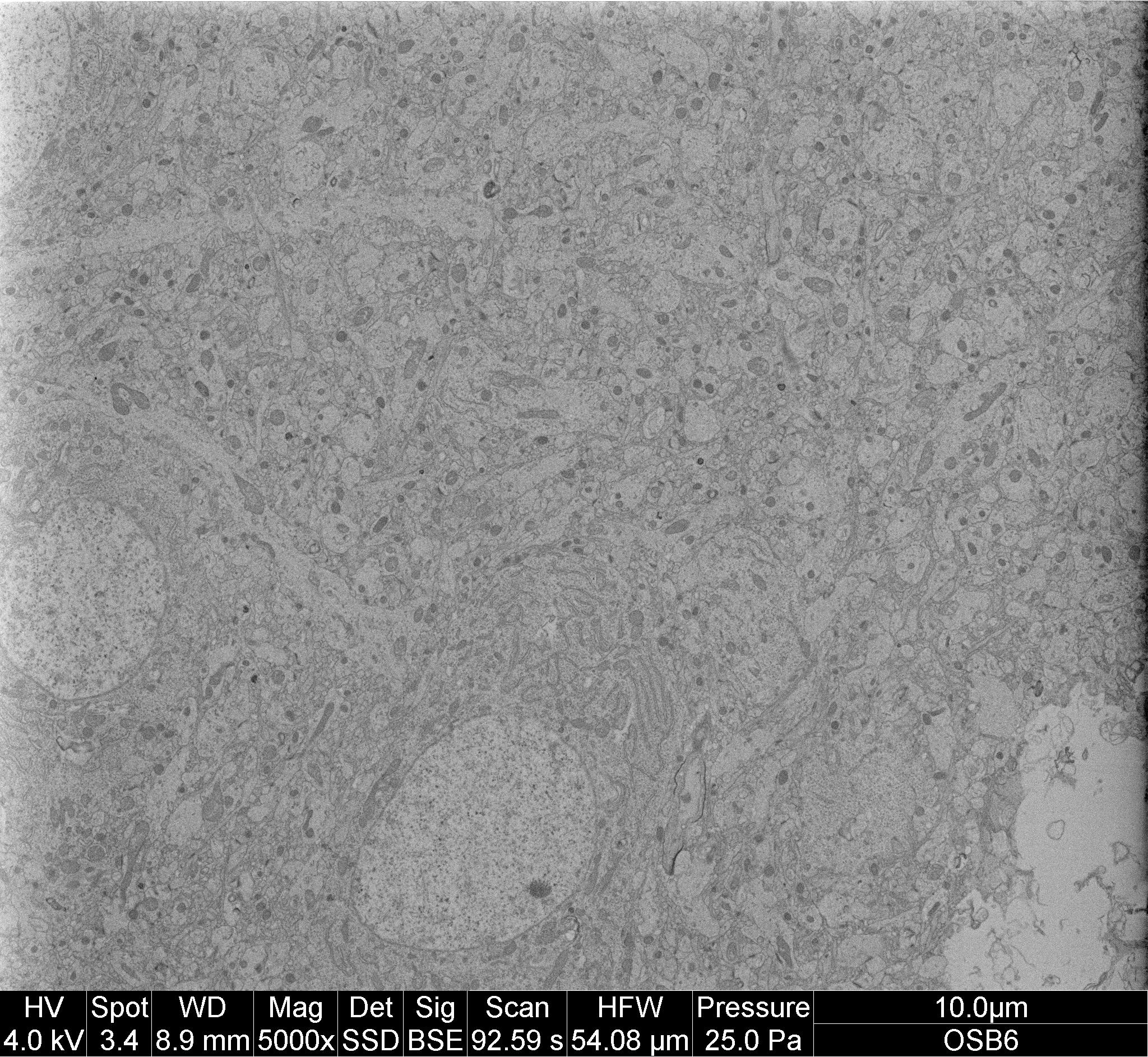

Supplement: Dataset S2 — (252.6 MB ZIP). [file pbio.0020329.sd002.zip › 040604_OS5_st1_138.tif]

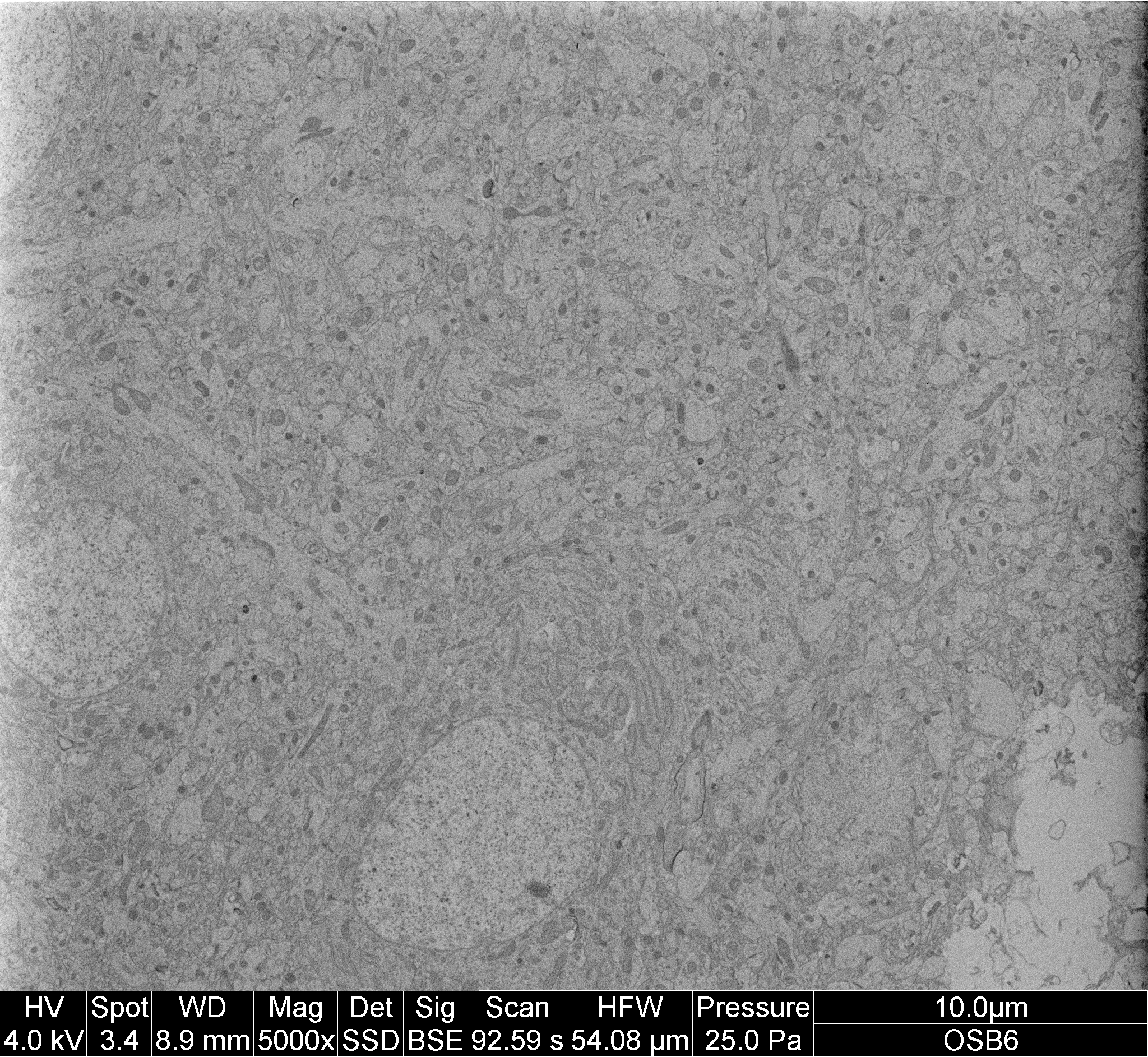

Supplement: Dataset S2 — (252.6 MB ZIP). [file pbio.0020329.sd002.zip › 040604_OS5_st1_139.tif]

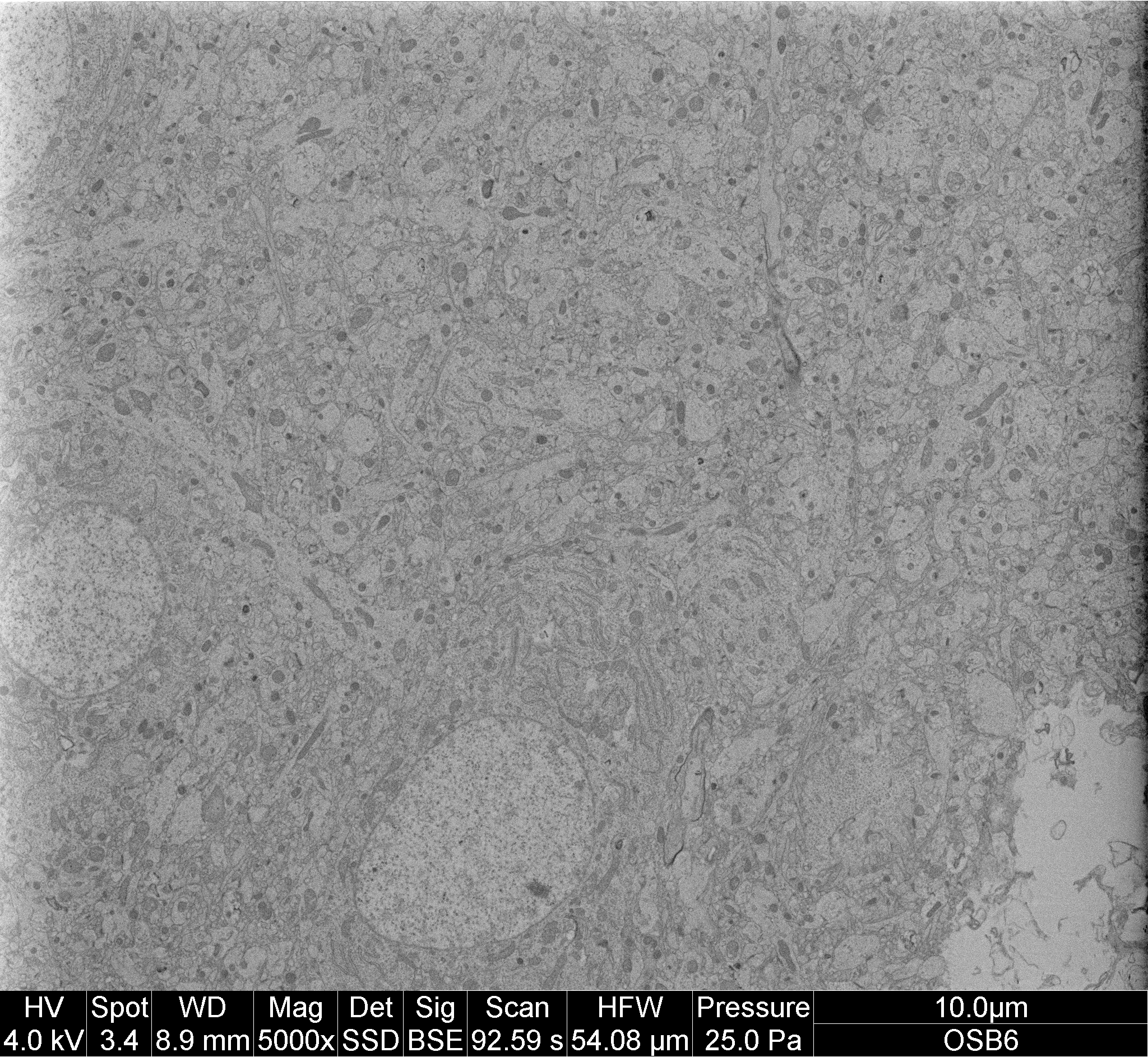

Supplement: Dataset S2 — (252.6 MB ZIP). [file pbio.0020329.sd002.zip › 040604_OS5_st1_140.tif]

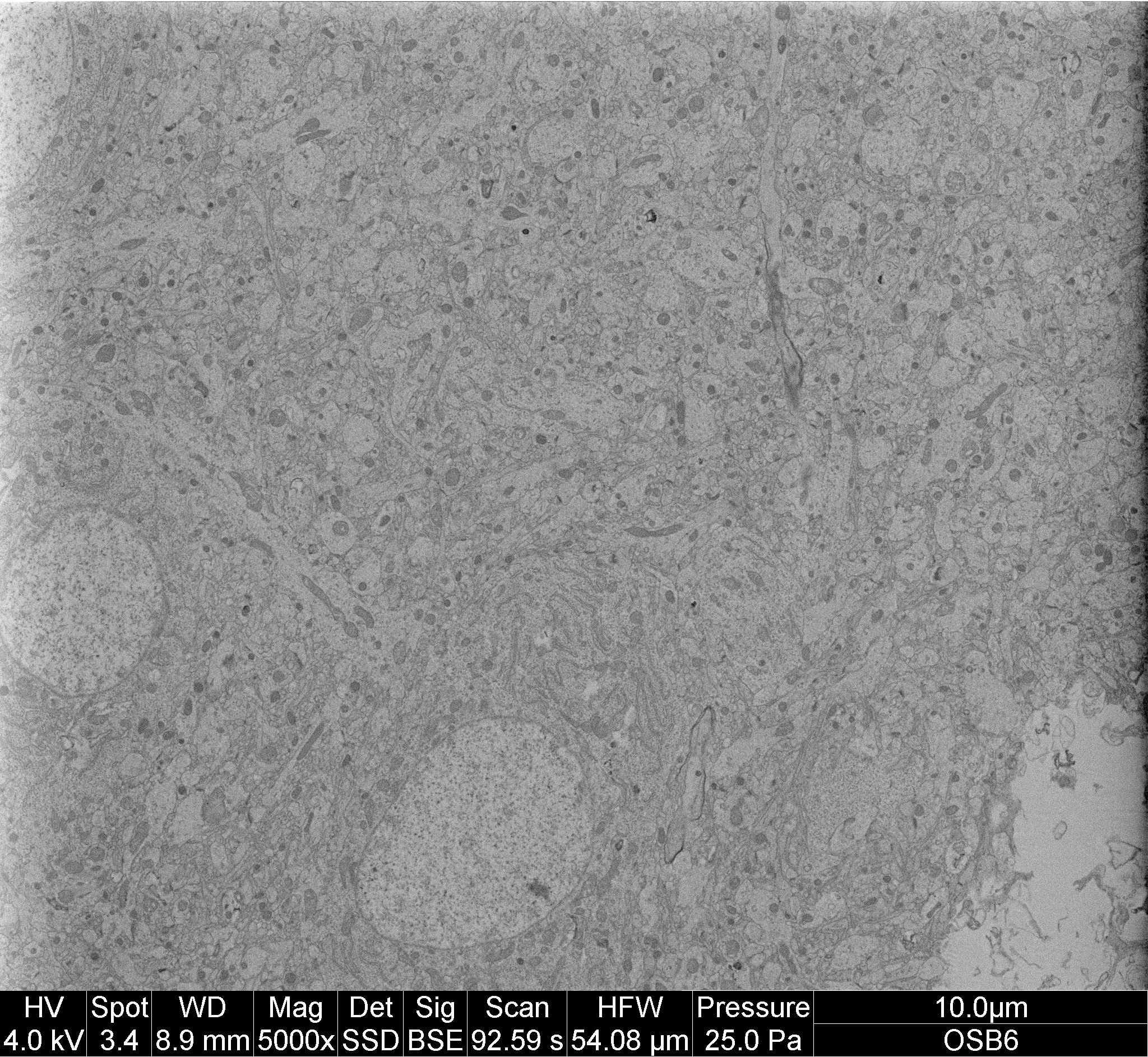

Supplement: Dataset S2 — (252.6 MB ZIP). [file pbio.0020329.sd002.zip › 040604_OS5_st1_141.tif]

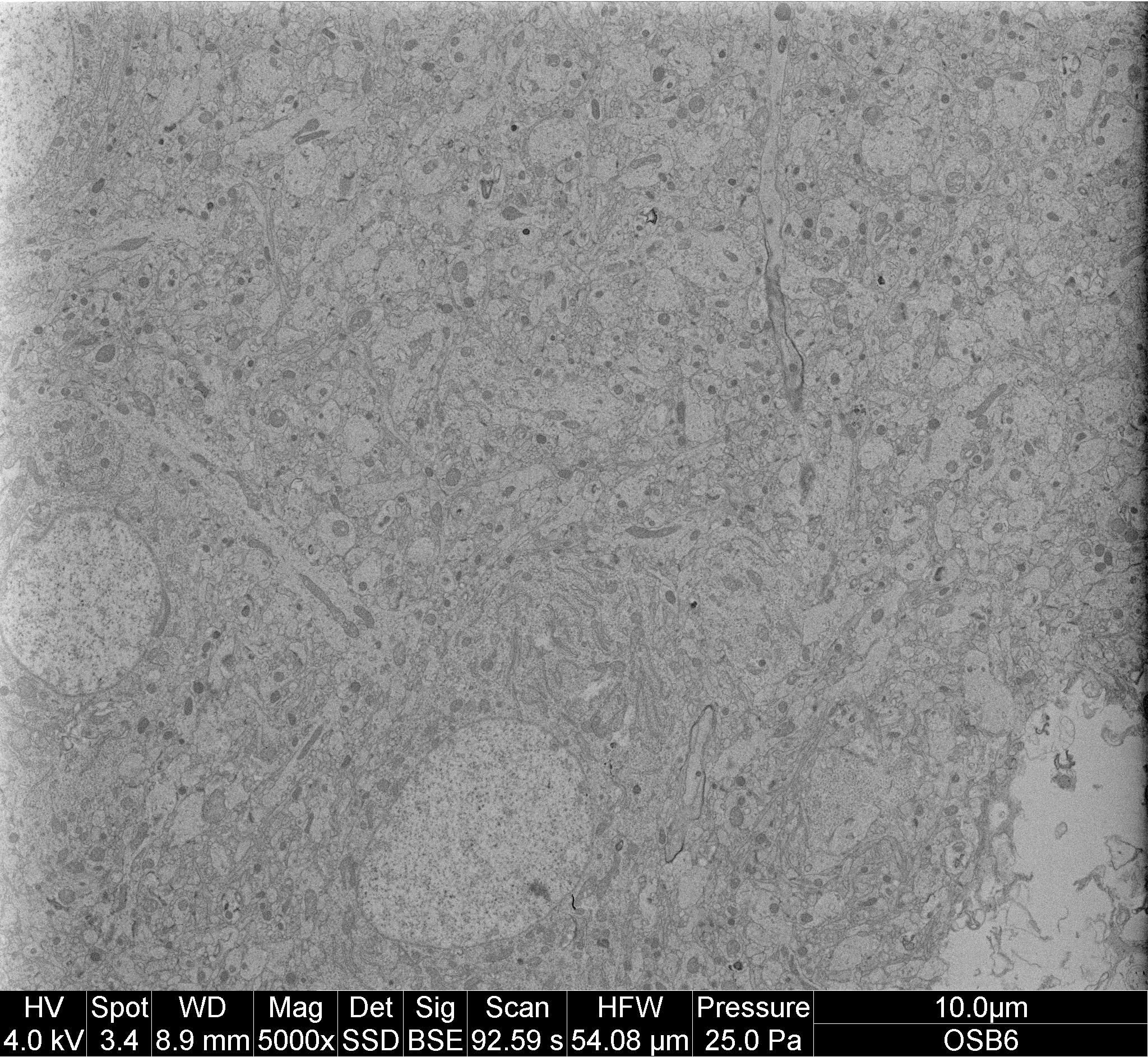

Supplement: Dataset S2 — (252.6 MB ZIP). [file pbio.0020329.sd002.zip › 040604_OS5_st1_142.tif]

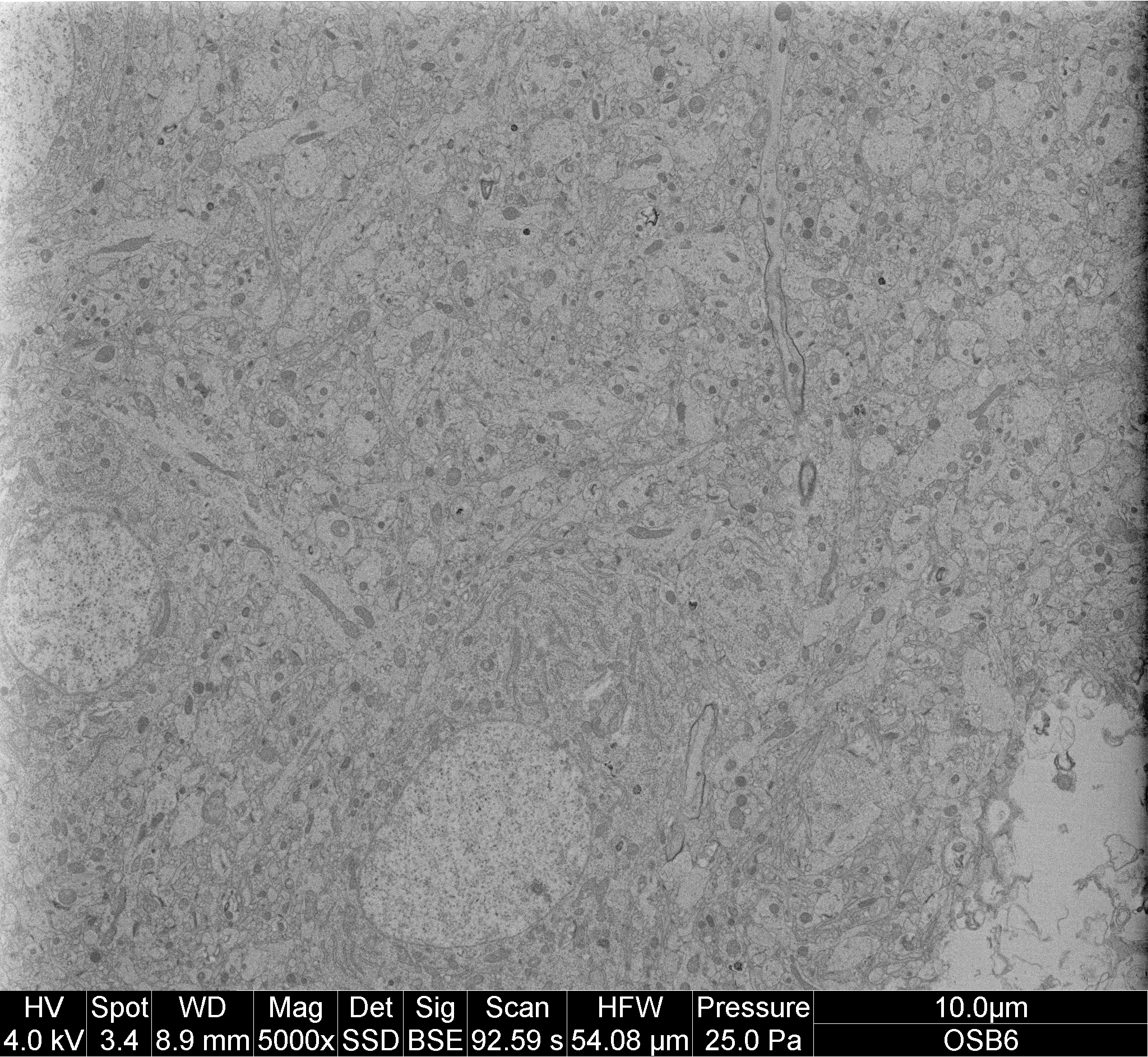

Supplement: Dataset S2 — (252.6 MB ZIP). [file pbio.0020329.sd002.zip › 040604_OS5_st1_143.tif]

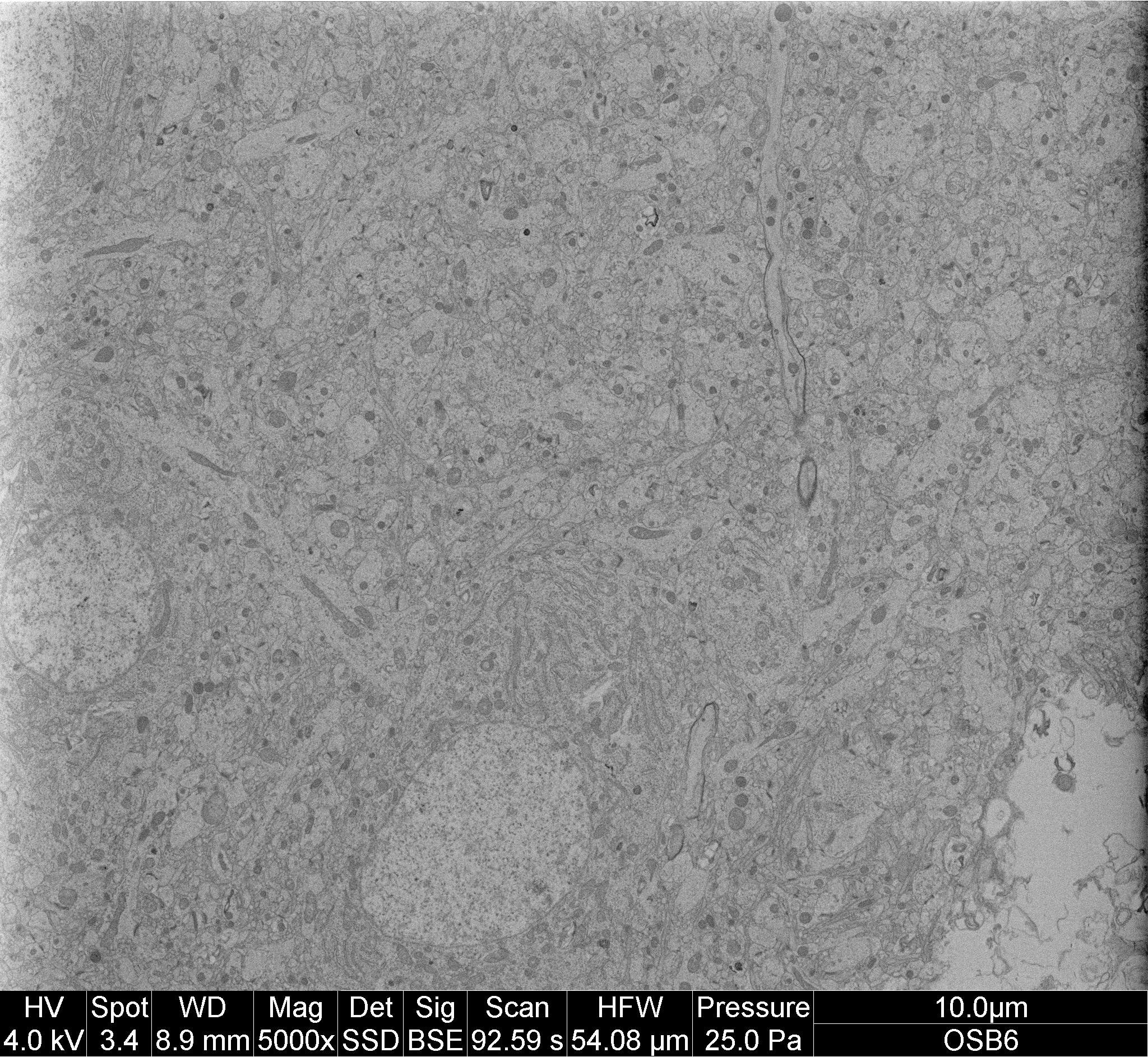

Supplement: Dataset S2 — (252.6 MB ZIP). [file pbio.0020329.sd002.zip › 040604_OS5_st1_144.tif]

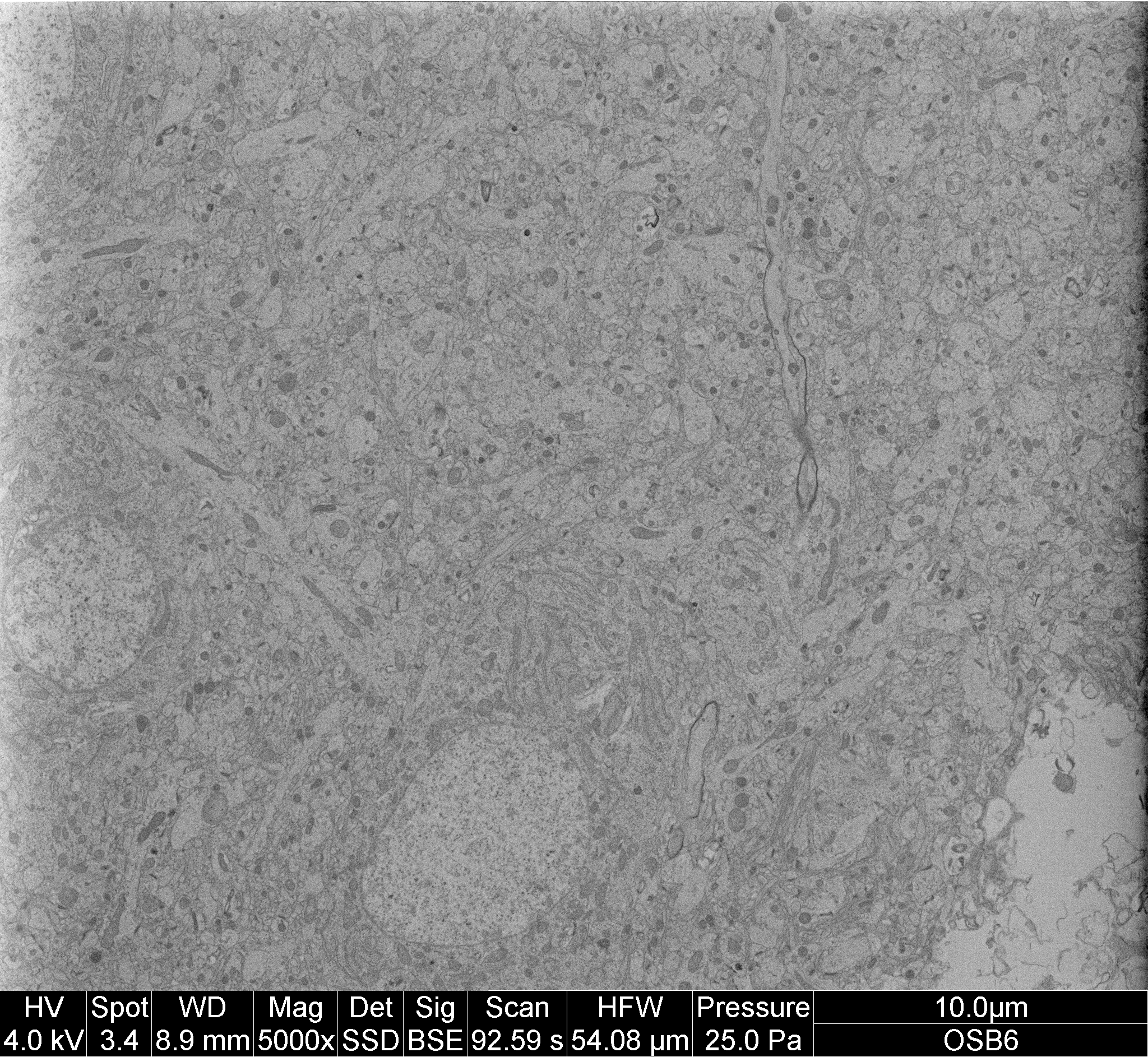

Supplement: Dataset S2 — (252.6 MB ZIP). [file pbio.0020329.sd002.zip › 040604_OS5_st1_145.tif]

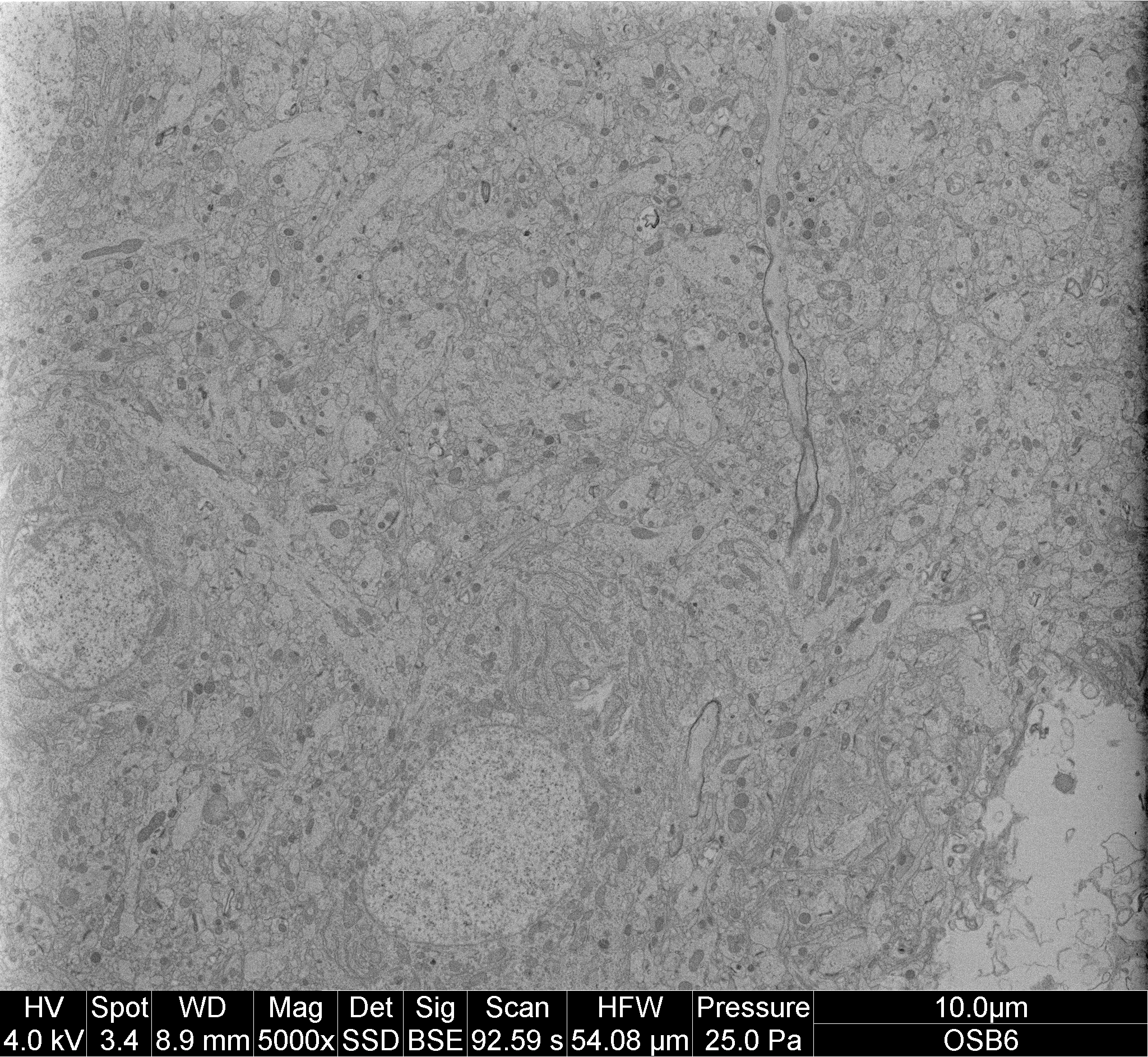

Supplement: Dataset S2 — (252.6 MB ZIP). [file pbio.0020329.sd002.zip › 040604_OS5_st1_146.tif]

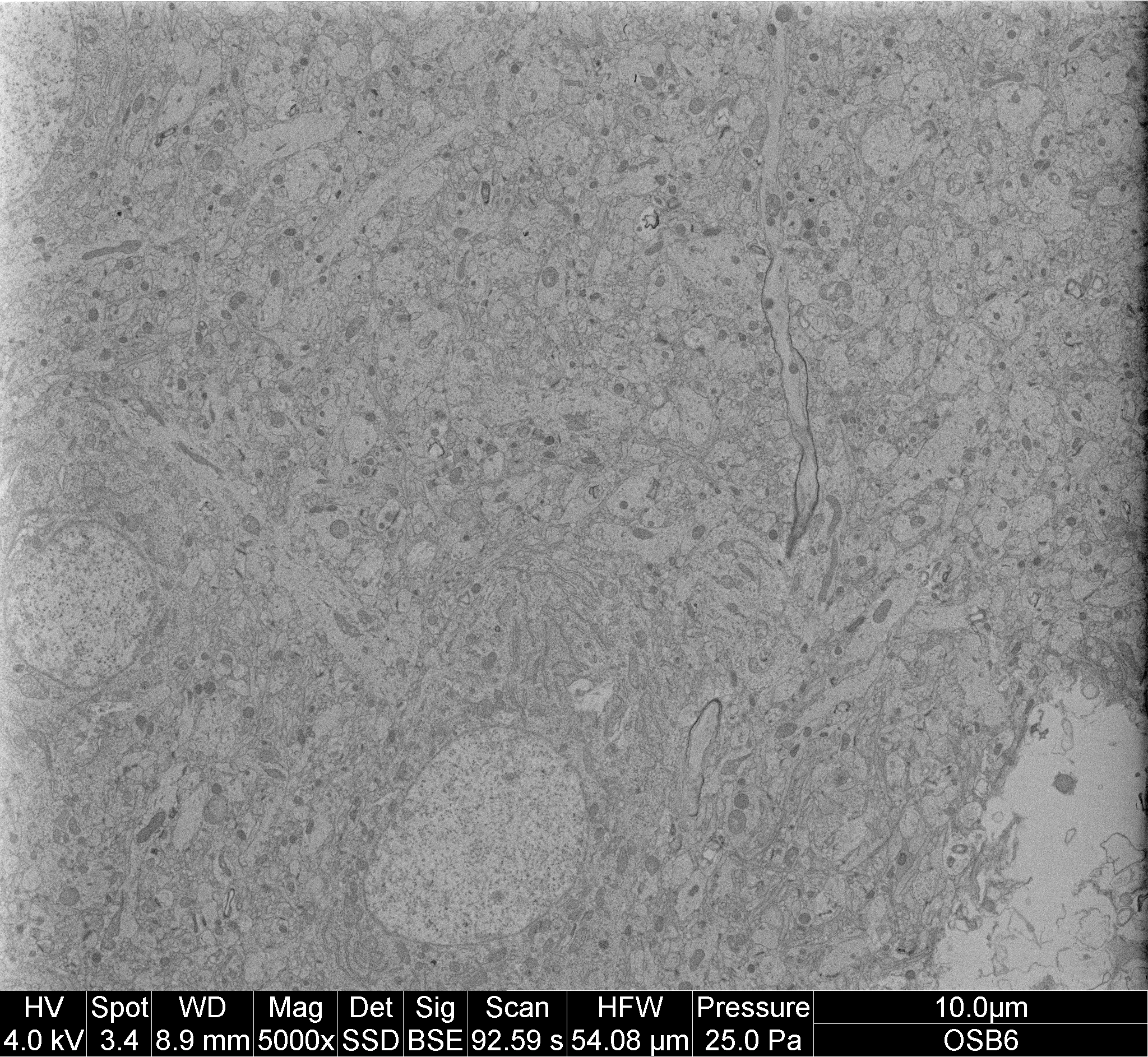

Supplement: Dataset S2 — (252.6 MB ZIP). [file pbio.0020329.sd002.zip › 040604_OS5_st1_147.tif]

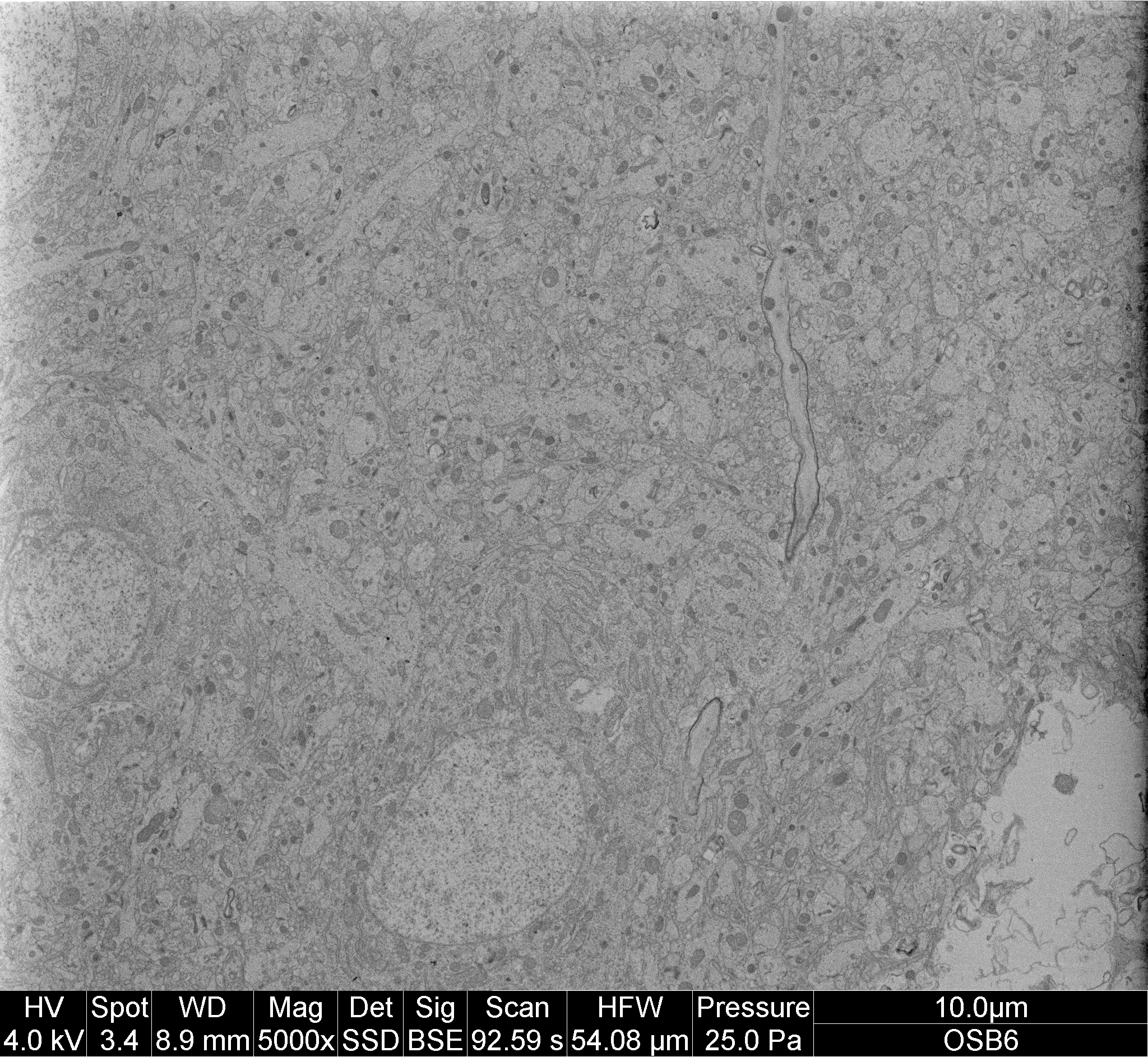

Supplement: Dataset S2 — (252.6 MB ZIP). [file pbio.0020329.sd002.zip › 040604_OS5_st1_148.tif]

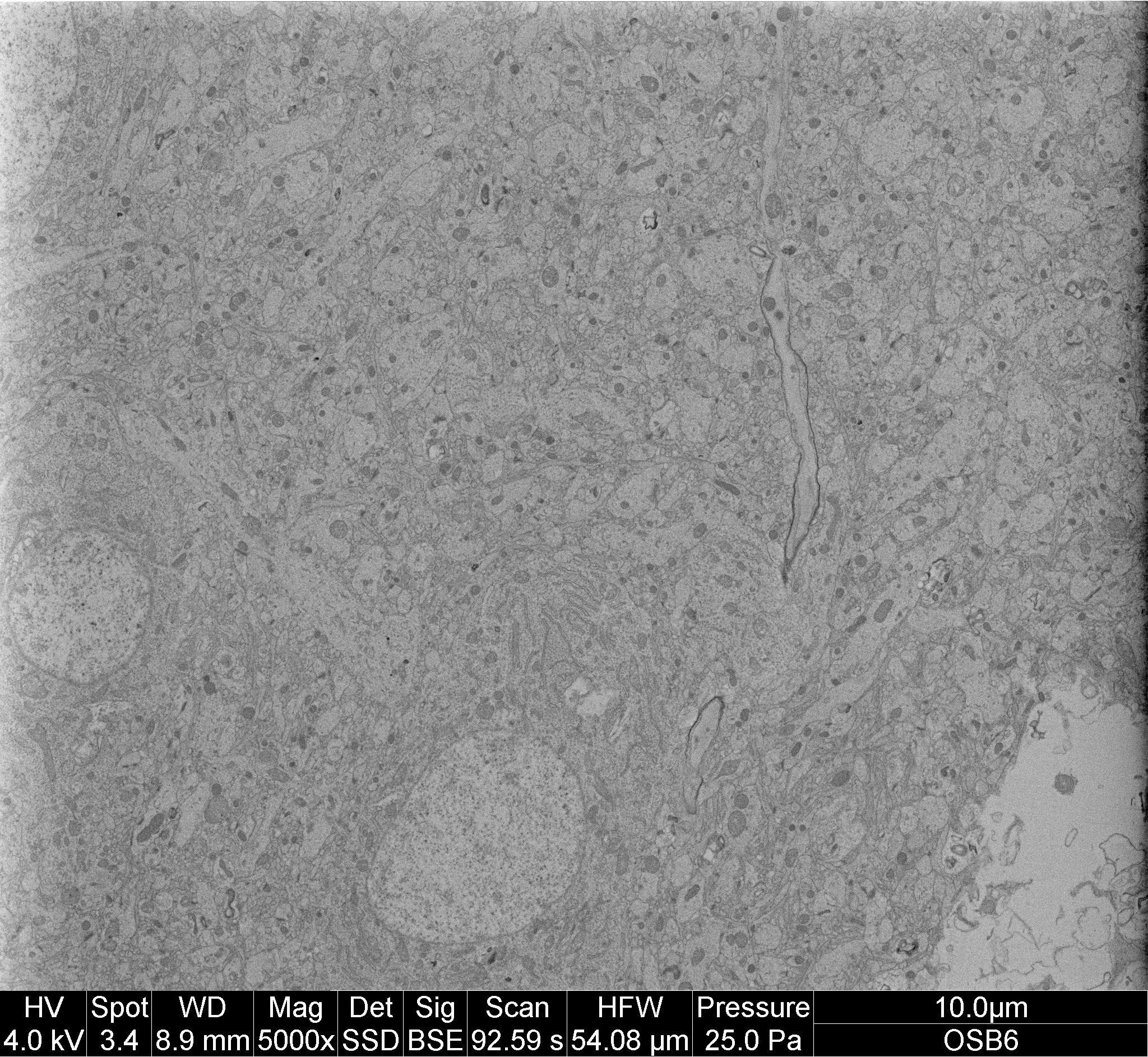

Supplement: Dataset S2 — (252.6 MB ZIP). [file pbio.0020329.sd002.zip › 040604_OS5_st1_149.tif]

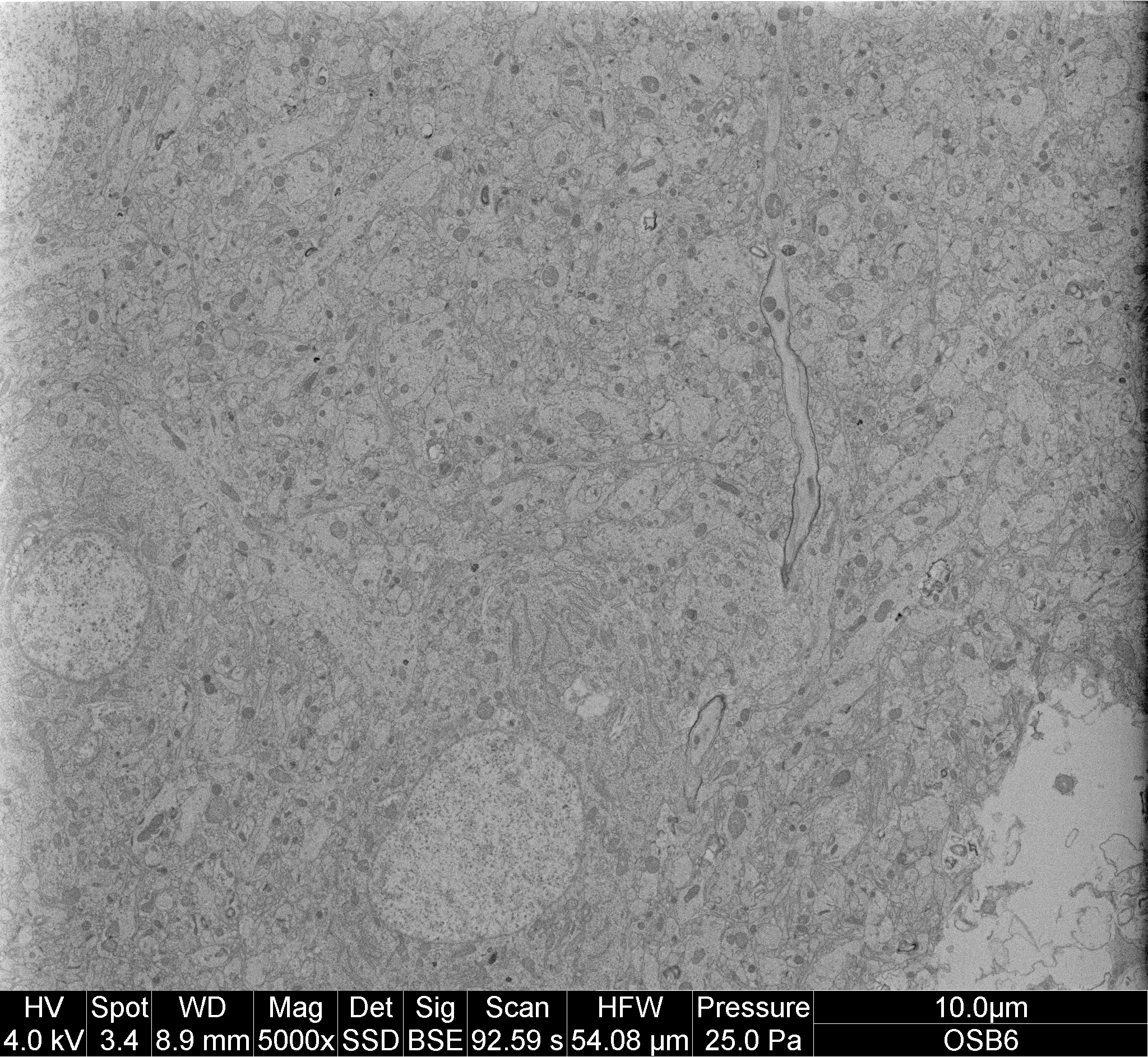

Supplement: Dataset S2 — (252.6 MB ZIP). [file pbio.0020329.sd002.zip › 040604_OS5_st1_150.tif]

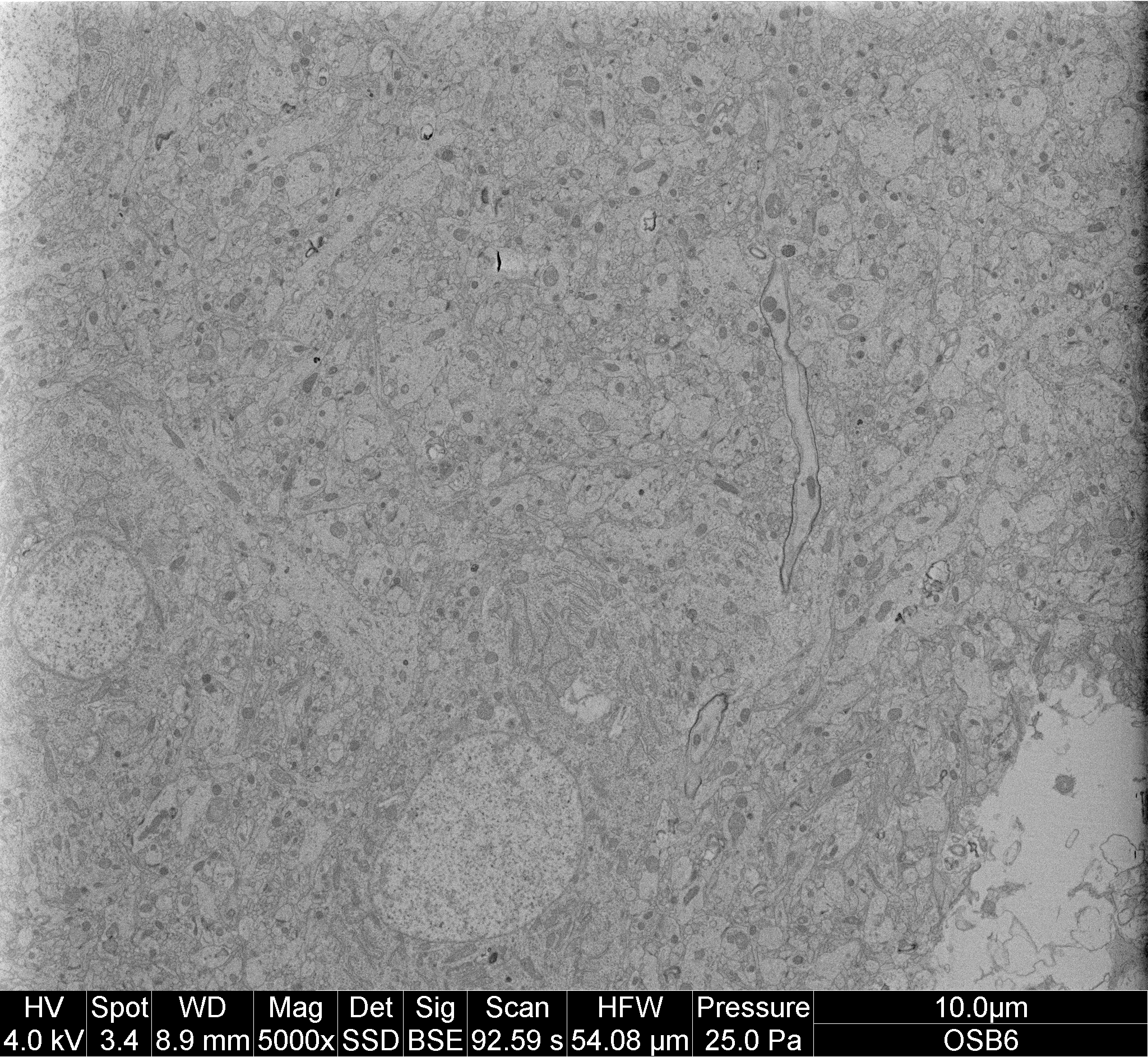

Supplement: Dataset S2 — (252.6 MB ZIP). [file pbio.0020329.sd002.zip › 040604_OS5_st1_151.tif]

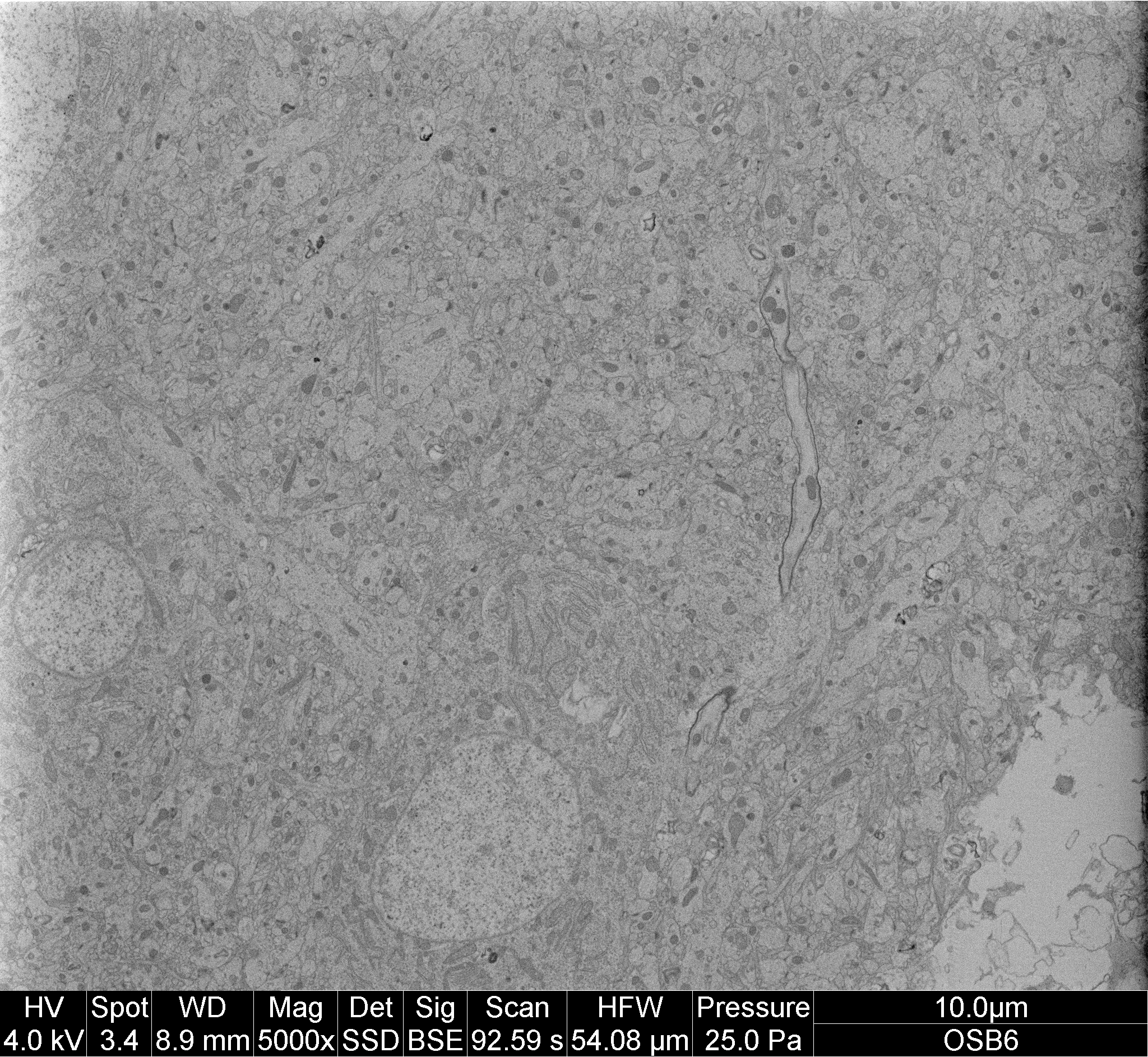

Supplement: Dataset S2 — (252.6 MB ZIP). [file pbio.0020329.sd002.zip › 040604_OS5_st1_152.tif]

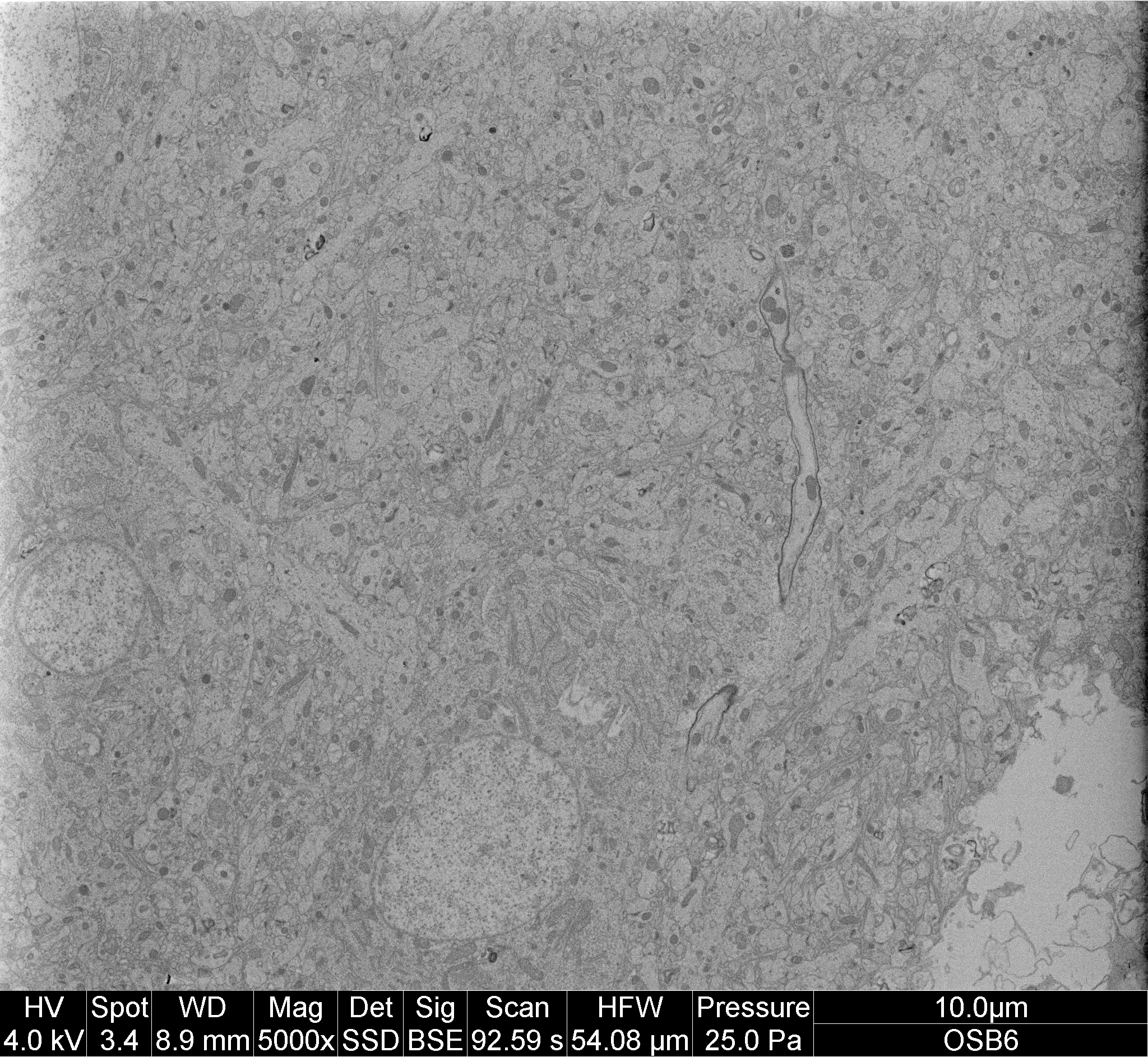

Supplement: Dataset S2 — (252.6 MB ZIP). [file pbio.0020329.sd002.zip › 040604_OS5_st1_153.tif]

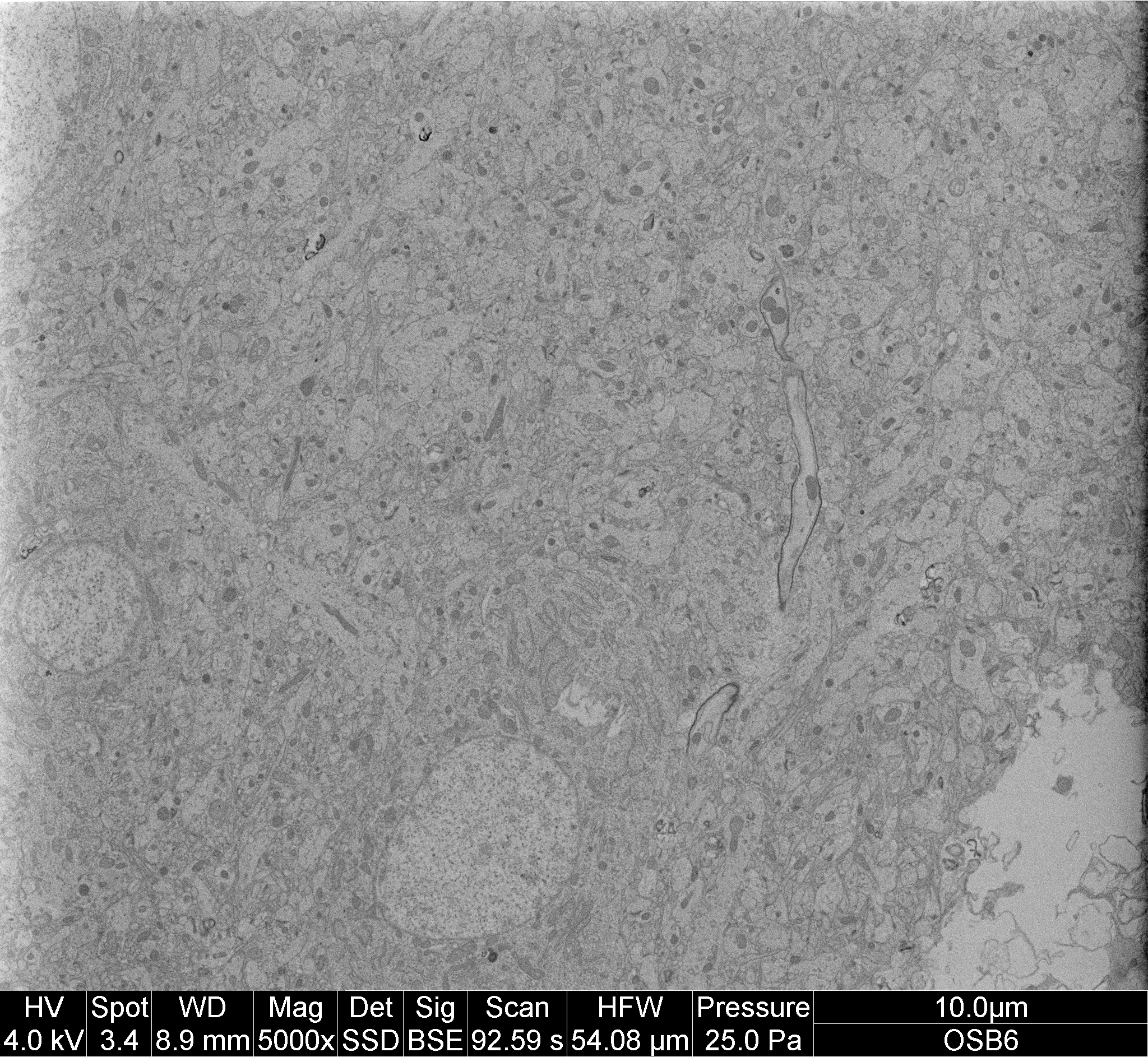

Supplement: Dataset S2 — (252.6 MB ZIP). [file pbio.0020329.sd002.zip › 040604_OS5_st1_154.tif]

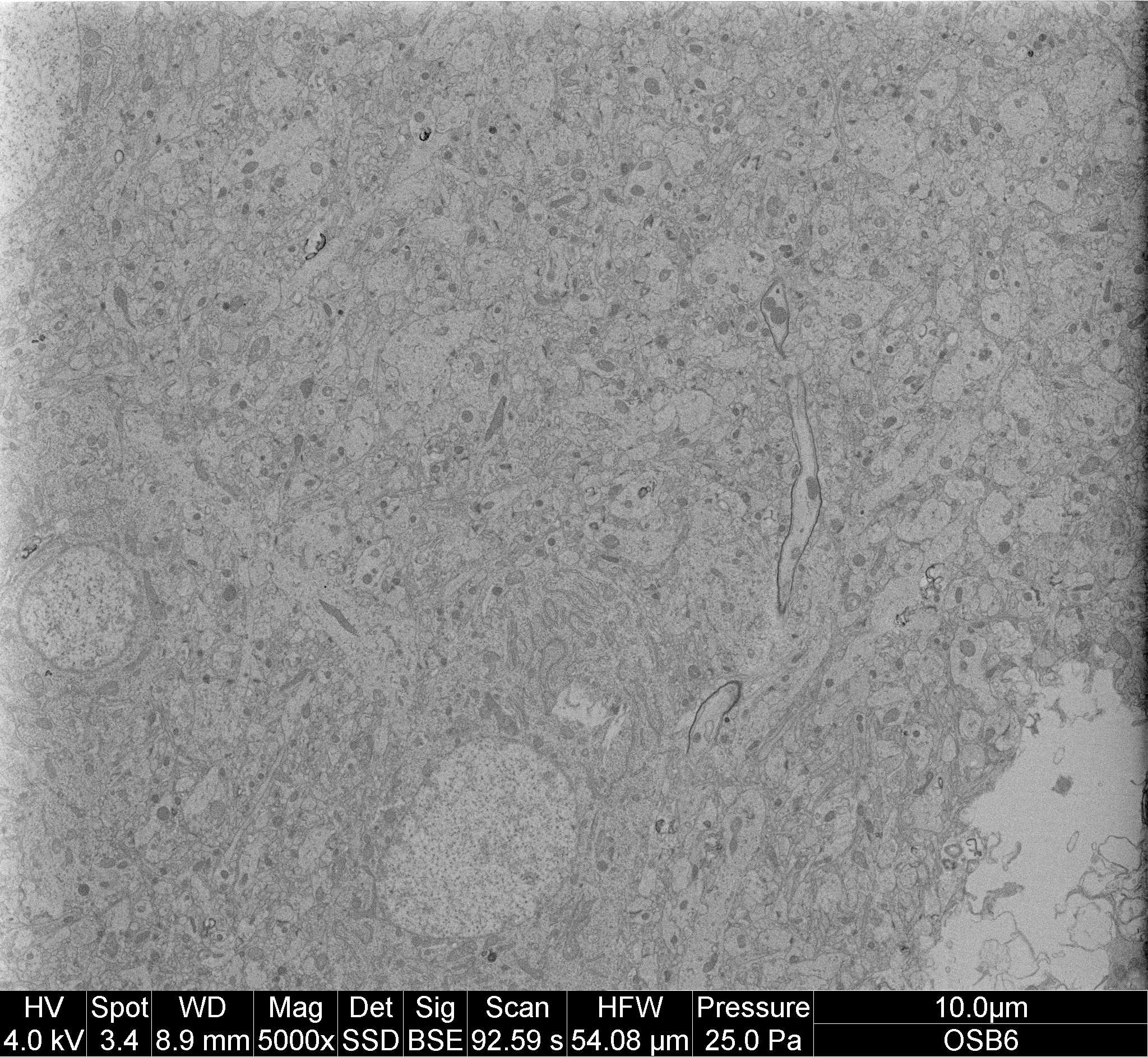

Supplement: Dataset S2 — (252.6 MB ZIP). [file pbio.0020329.sd002.zip › 040604_OS5_st1_155.tif]

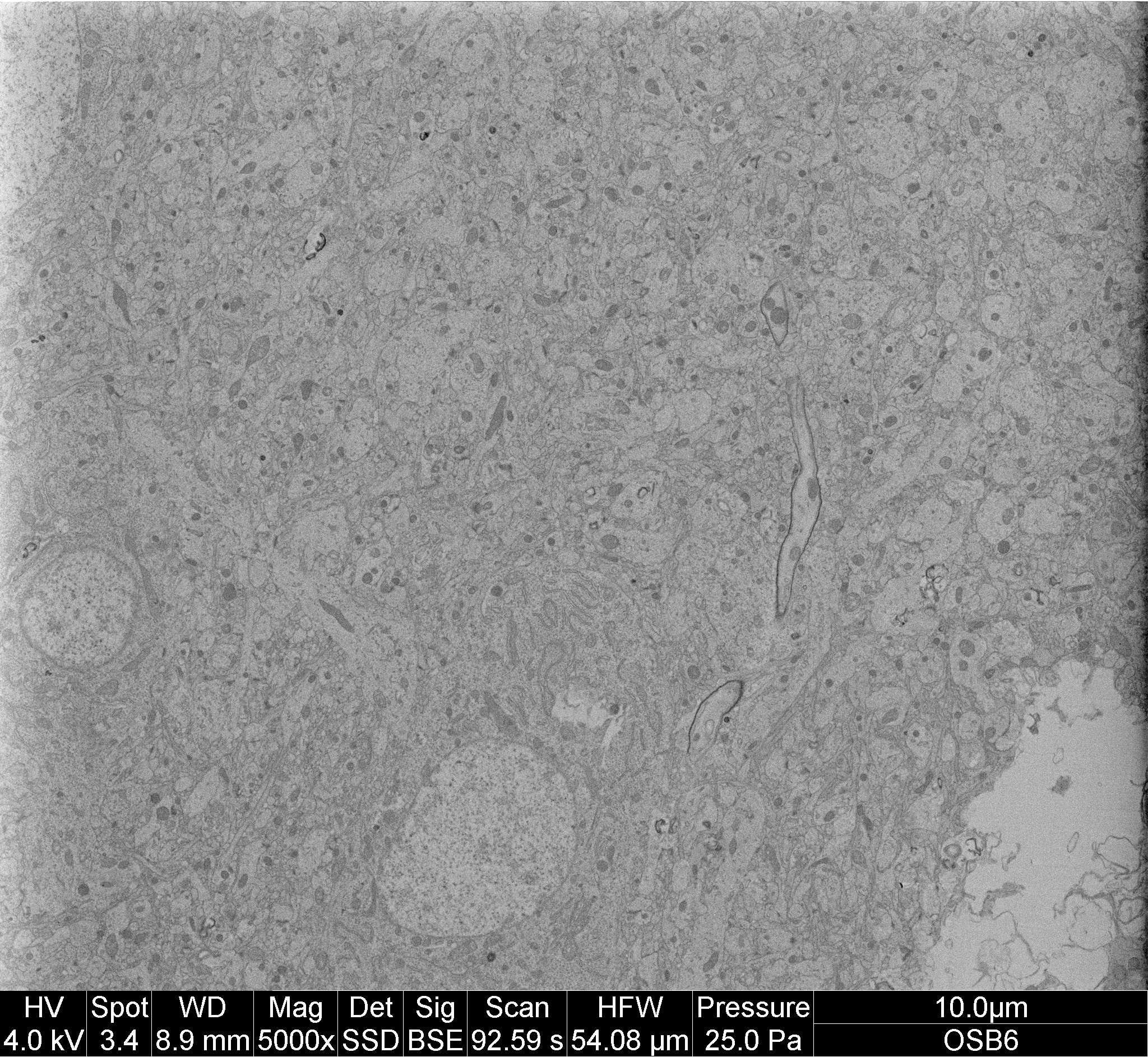

Supplement: Dataset S2 — (252.6 MB ZIP). [file pbio.0020329.sd002.zip › 040604_OS5_st1_156.tif]

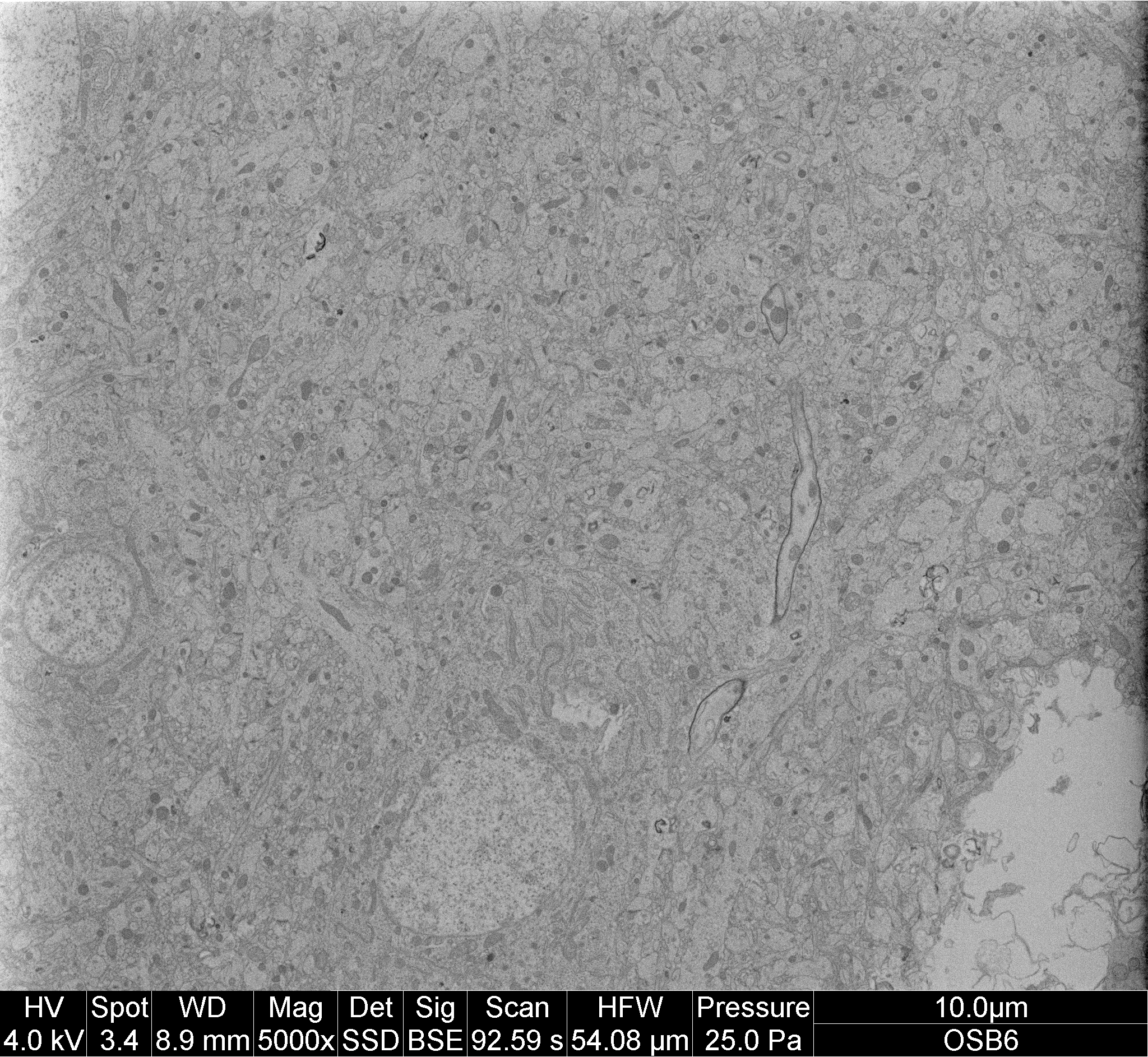

Supplement: Dataset S2 — (252.6 MB ZIP). [file pbio.0020329.sd002.zip › 040604_OS5_st1_157.tif]

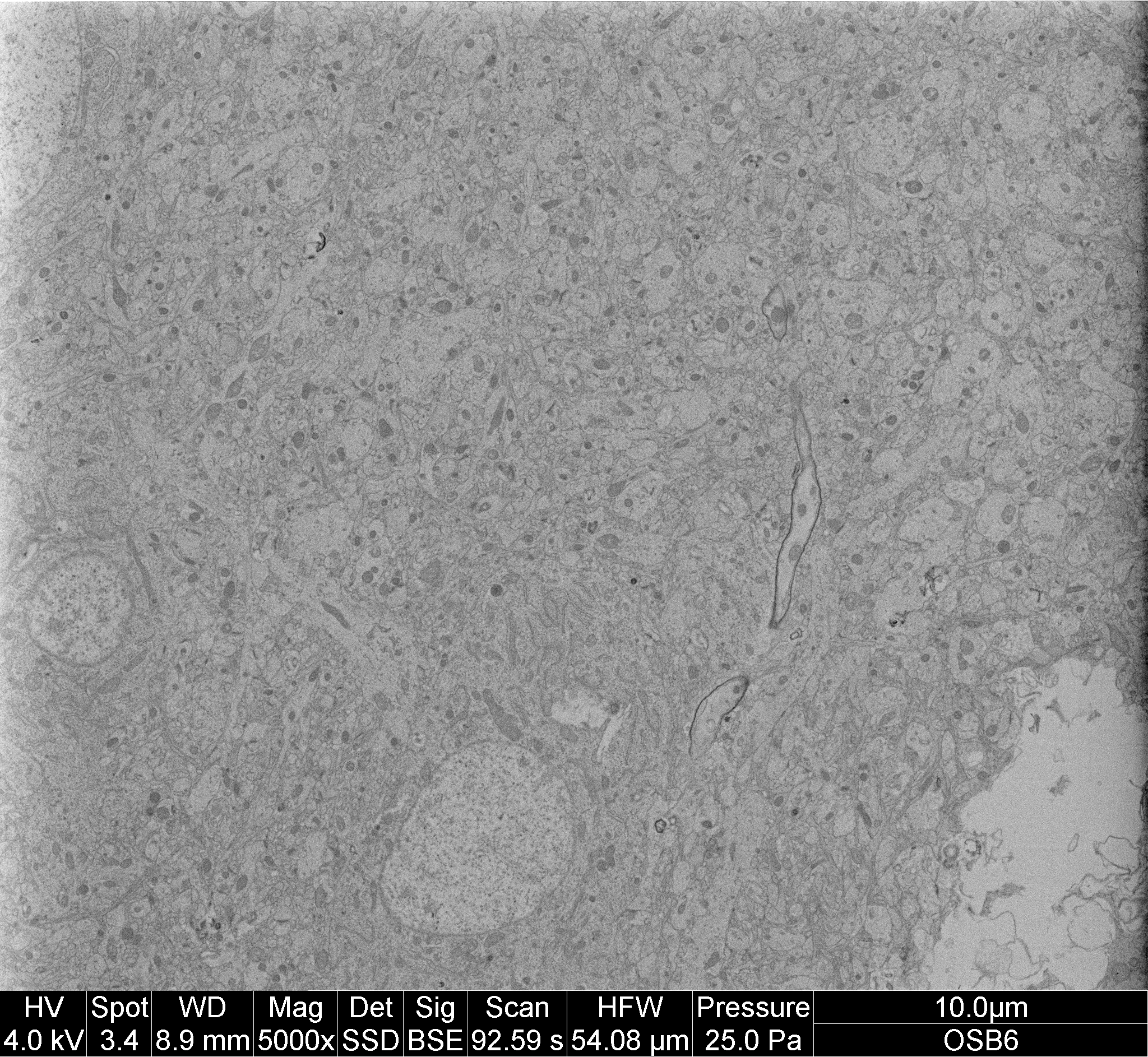

Supplement: Dataset S2 — (252.6 MB ZIP). [file pbio.0020329.sd002.zip › 040604_OS5_st1_158.tif]

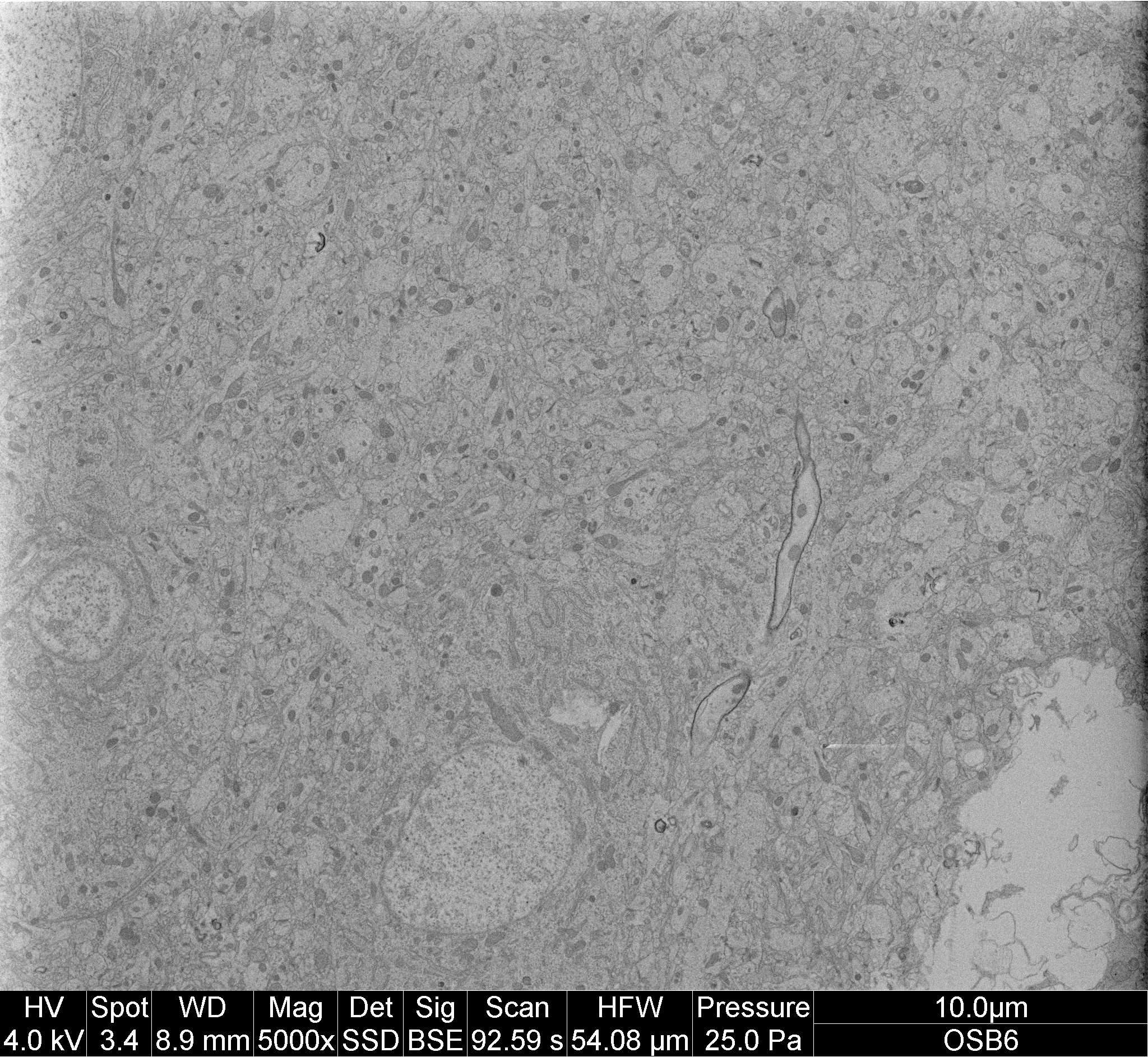

Supplement: Dataset S2 — (252.6 MB ZIP). [file pbio.0020329.sd002.zip › 040604_OS5_st1_159.tif]

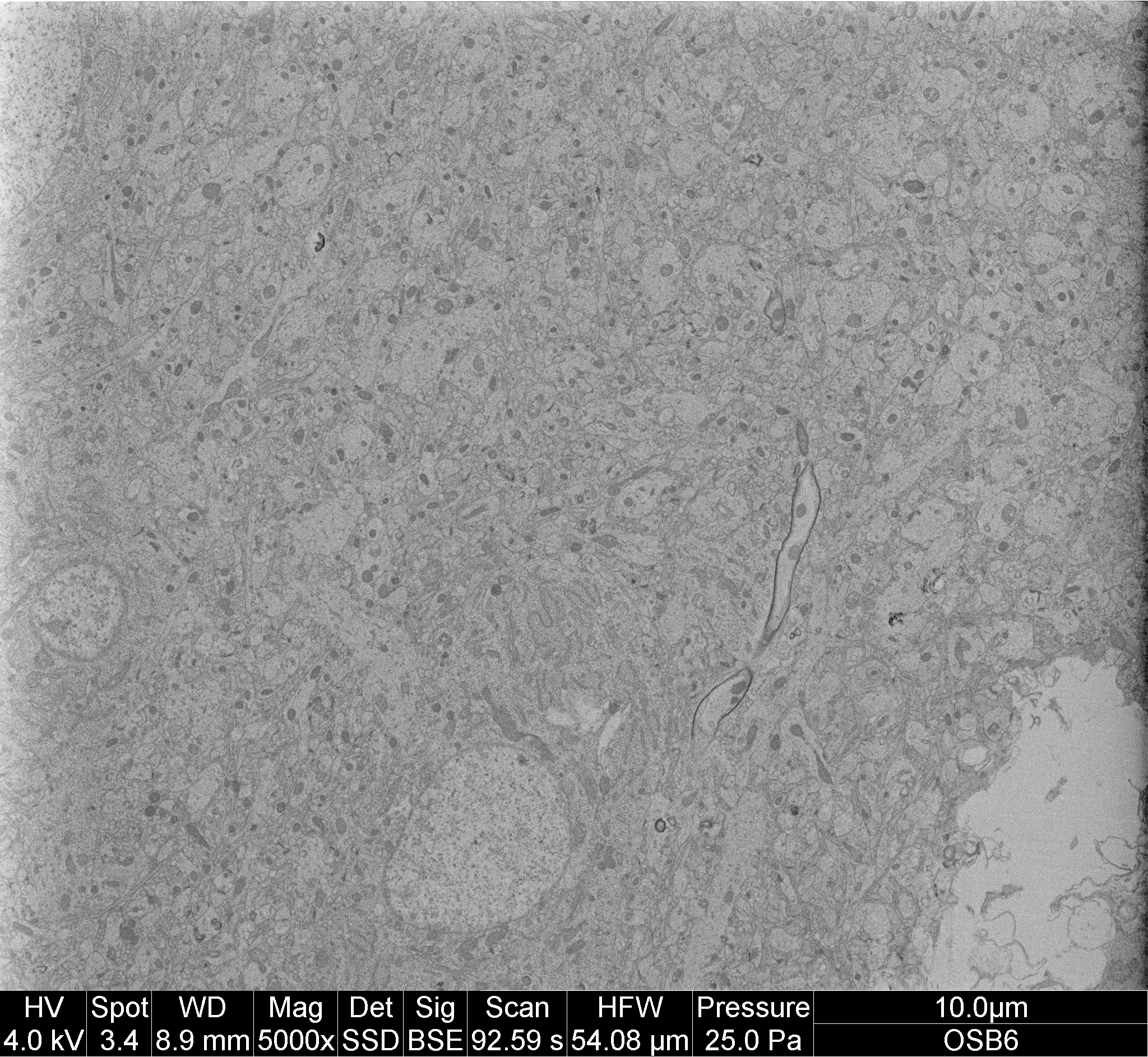

Supplement: Dataset S2 — (252.6 MB ZIP). [file pbio.0020329.sd002.zip › 040604_OS5_st1_160.tif]

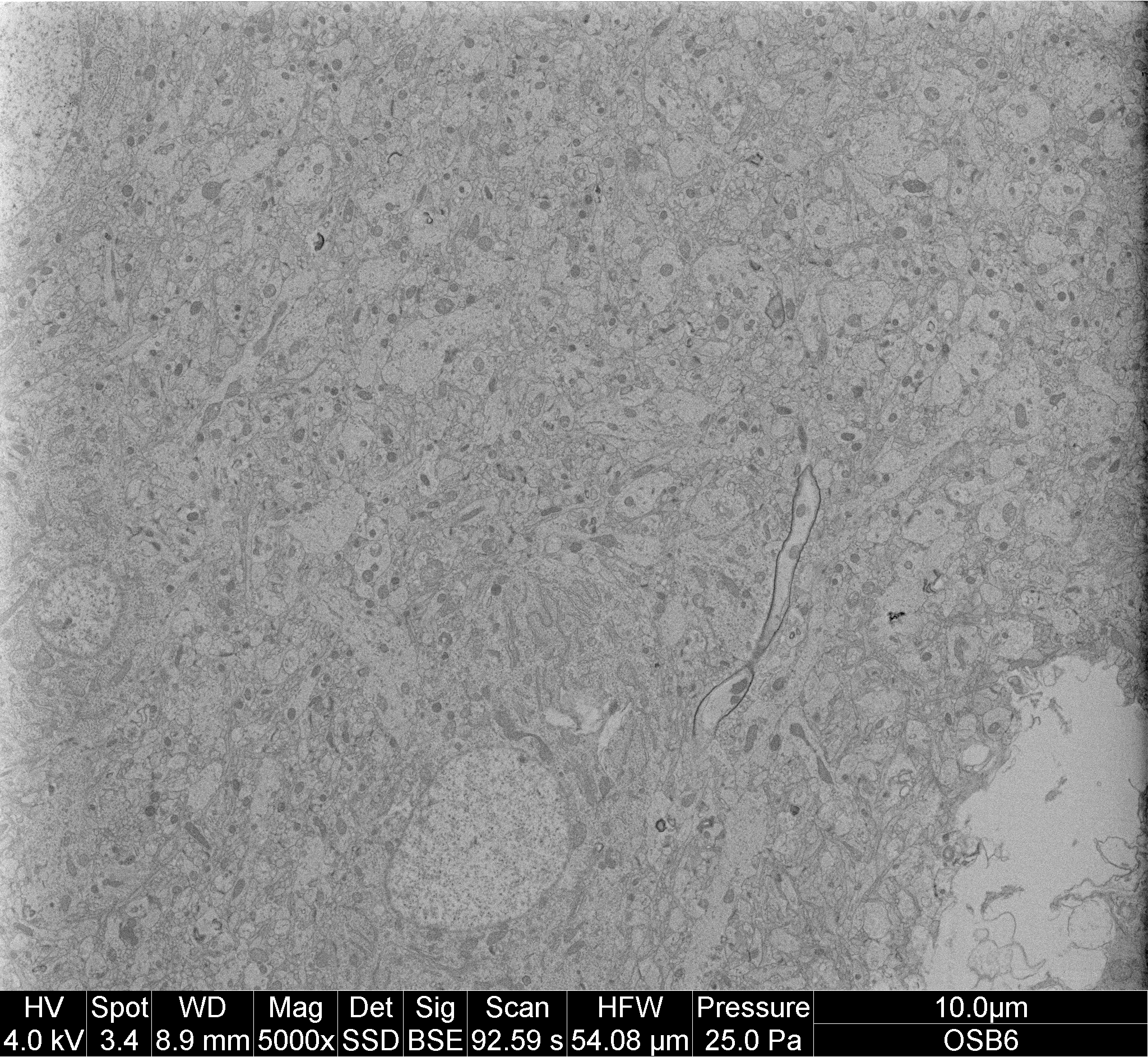

Supplement: Dataset S2 — (252.6 MB ZIP). [file pbio.0020329.sd002.zip › 040604_OS5_st1_161.tif]

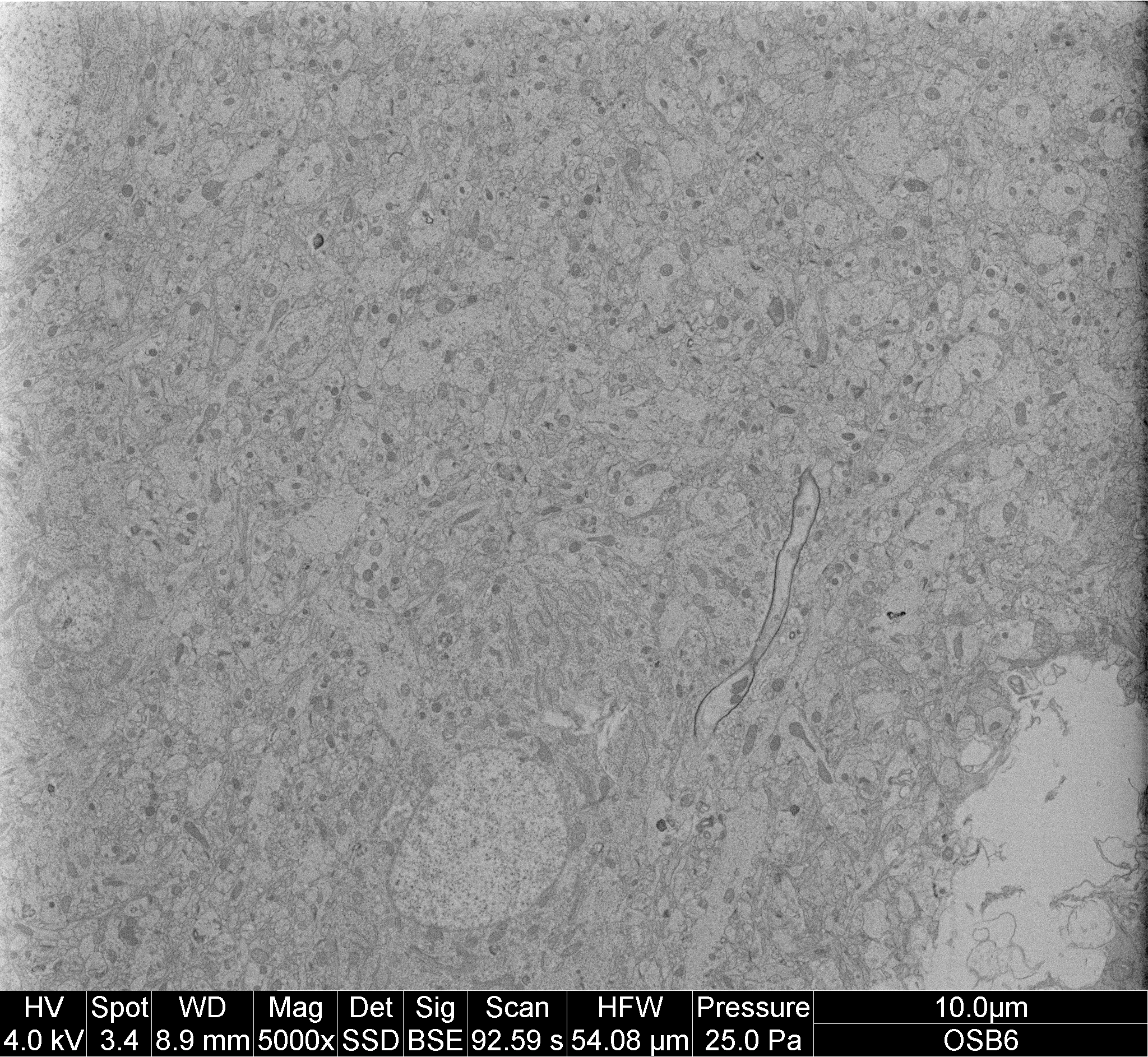

Supplement: Dataset S2 — (252.6 MB ZIP). [file pbio.0020329.sd002.zip › 040604_OS5_st1_162.tif]

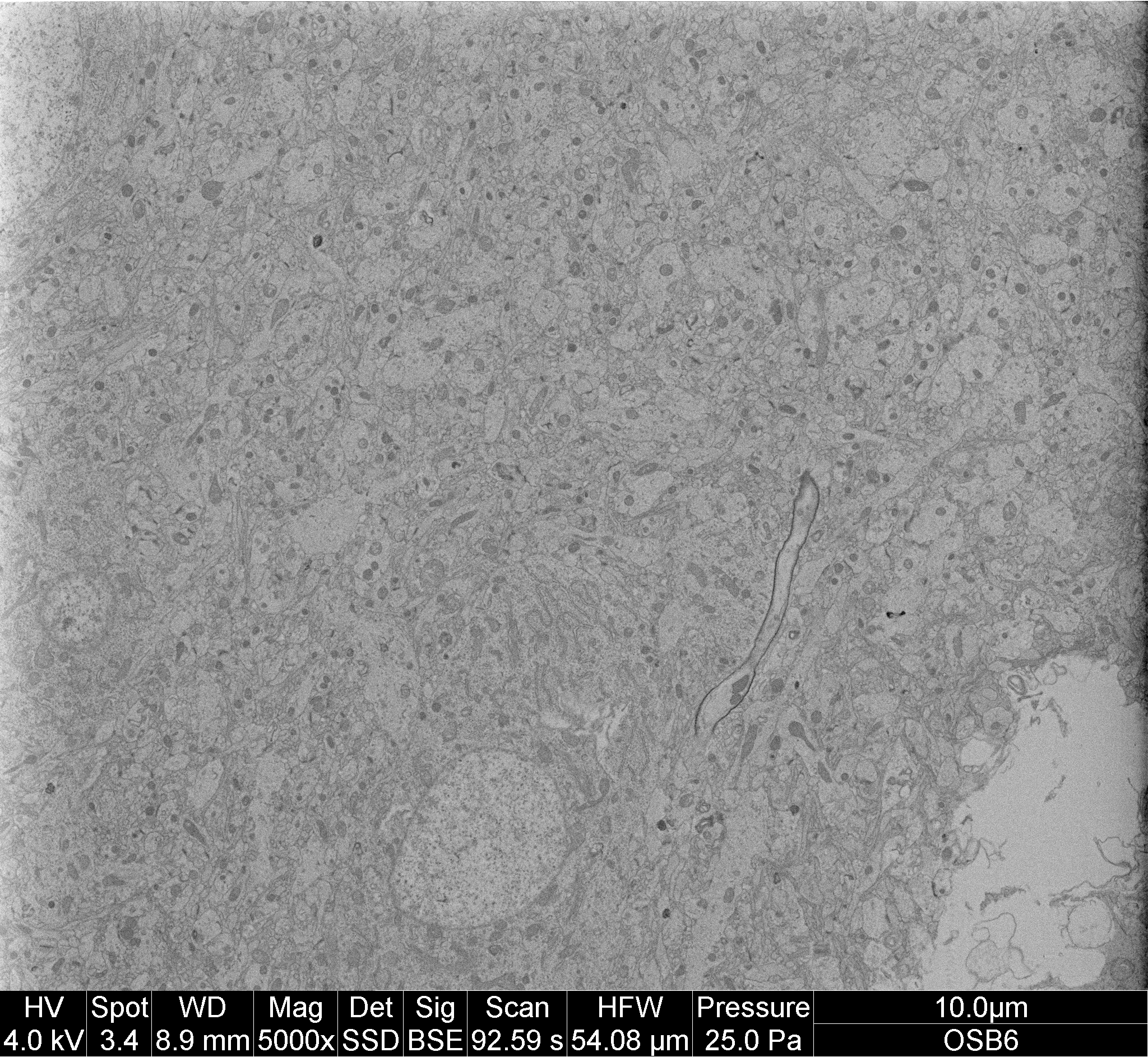

Supplement: Dataset S2 — (252.6 MB ZIP). [file pbio.0020329.sd002.zip › 040604_OS5_st1_163.tif]

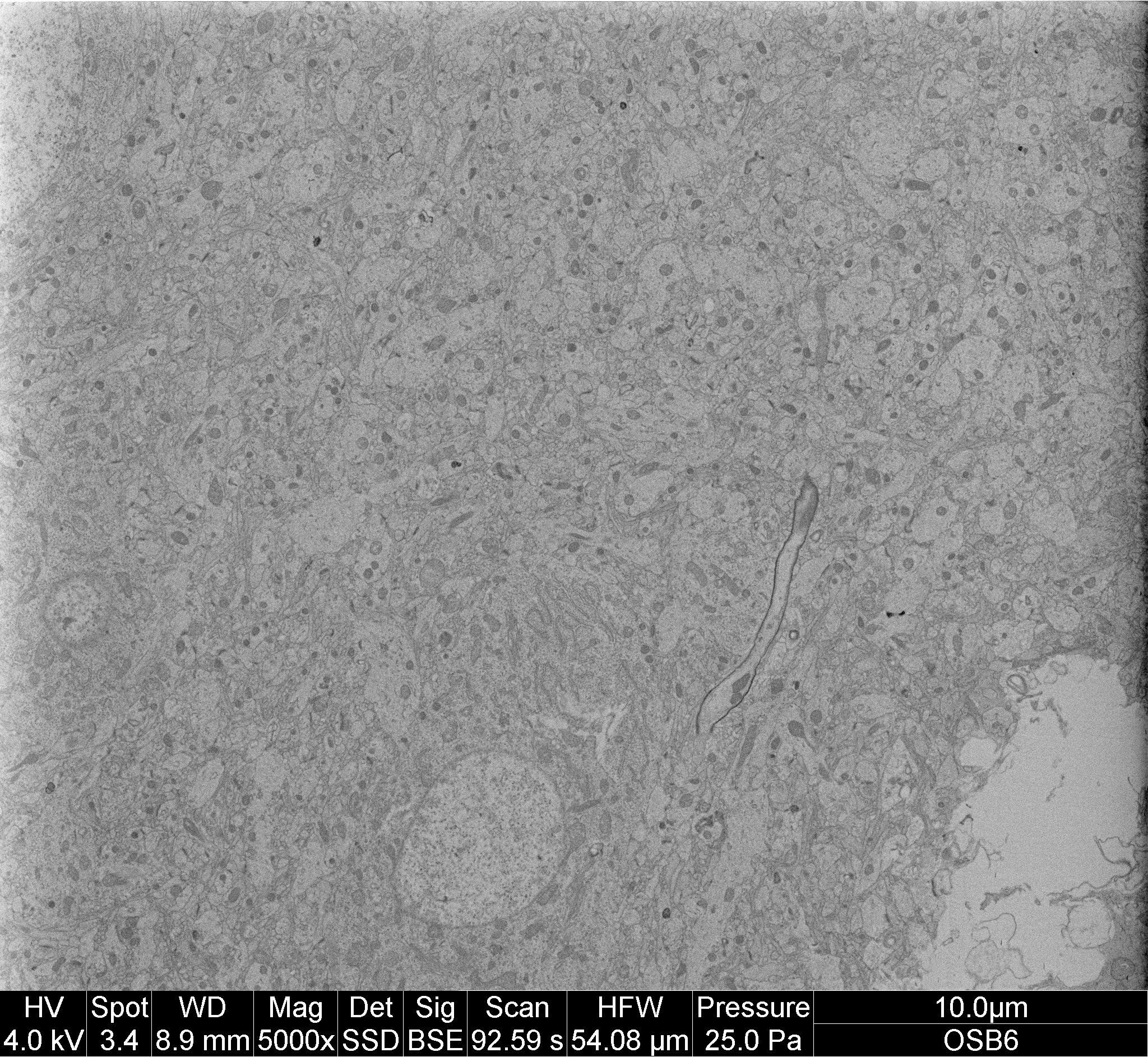

Supplement: Dataset S2 — (252.6 MB ZIP). [file pbio.0020329.sd002.zip › 040604_OS5_st1_164.tif]

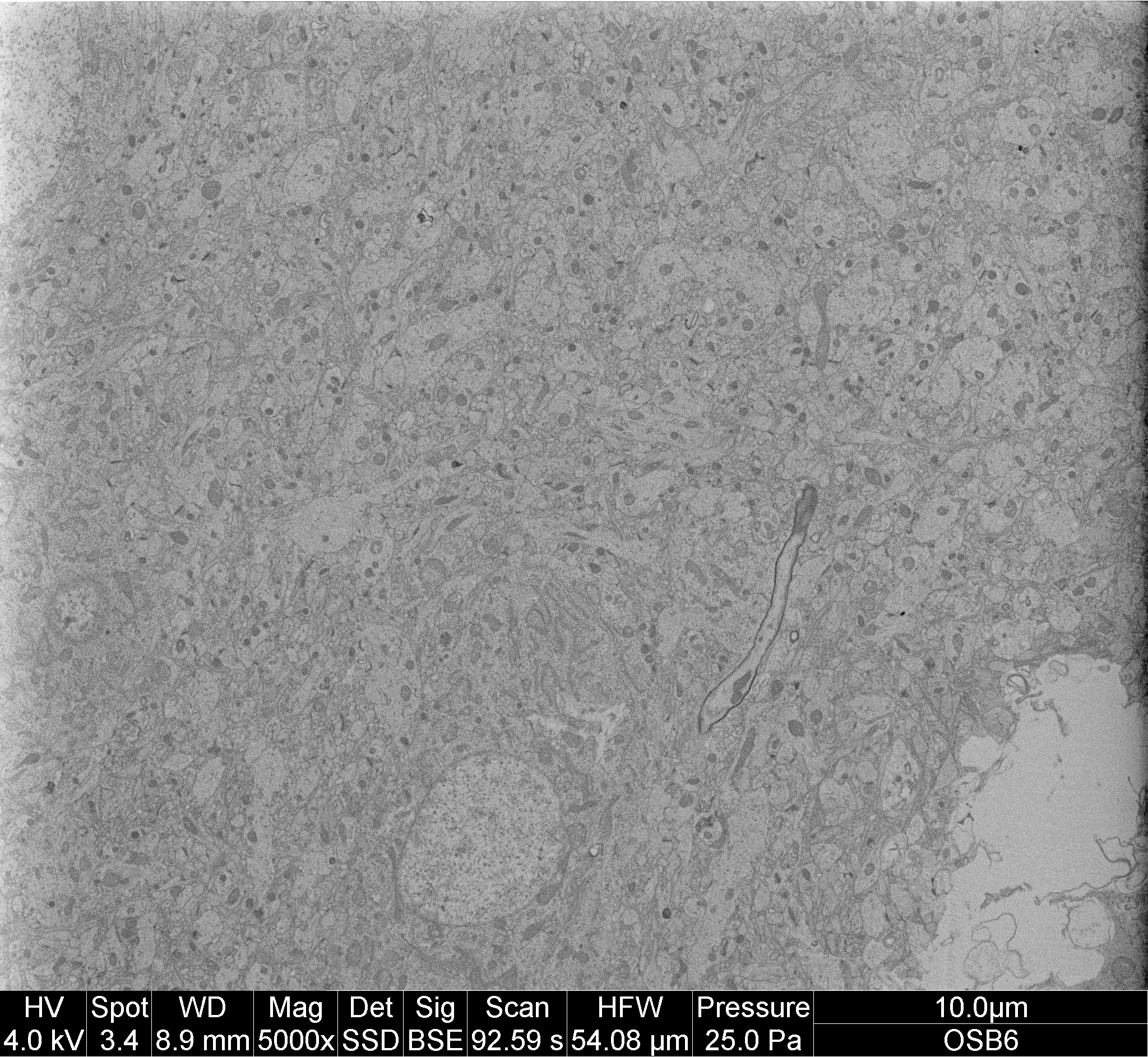

Supplement: Dataset S2 — (252.6 MB ZIP). [file pbio.0020329.sd002.zip › 040604_OS5_st1_165.tif]

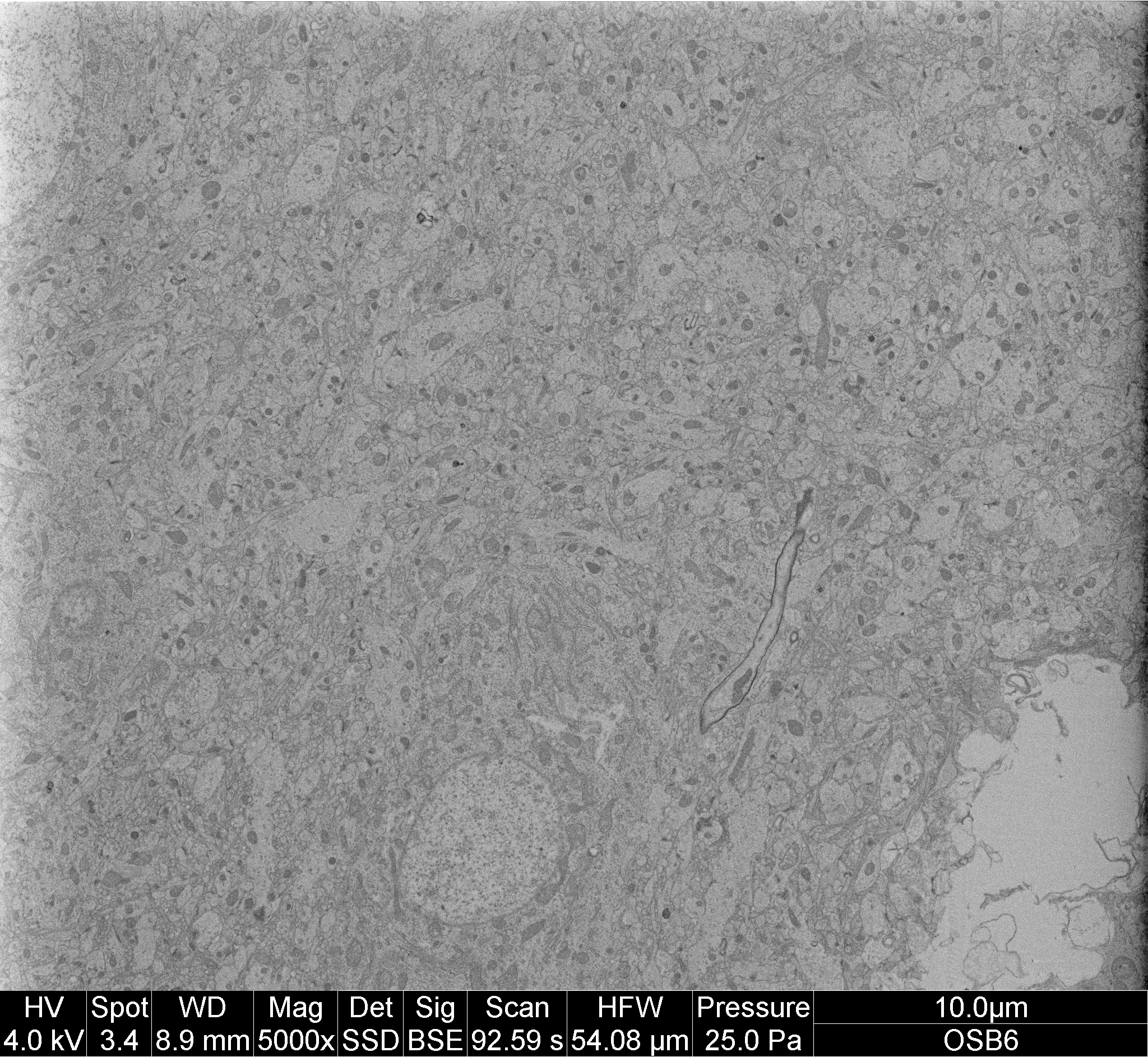

Supplement: Dataset S2 — (252.6 MB ZIP). [file pbio.0020329.sd002.zip › 040604_OS5_st1_166.tif]

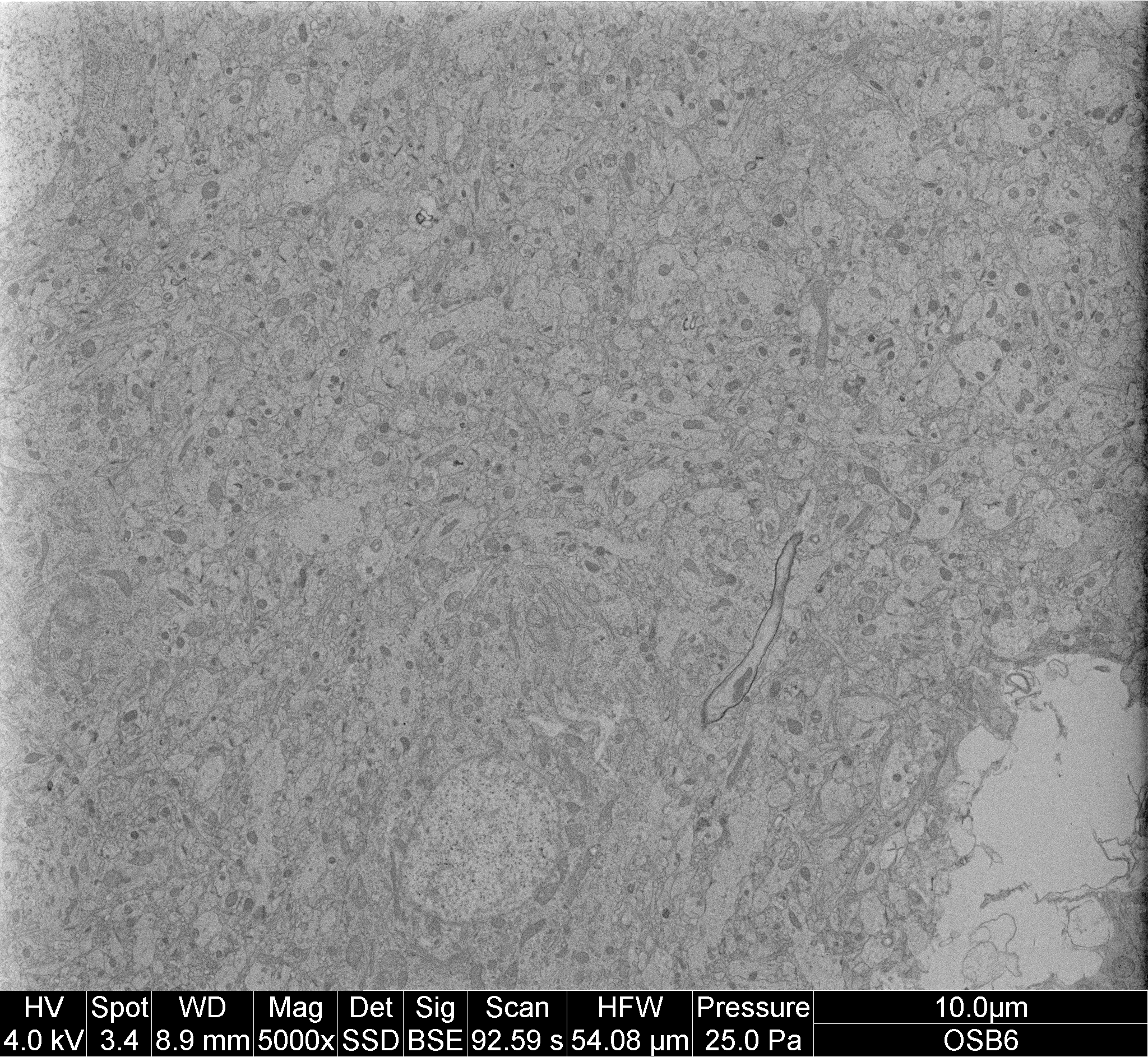

Supplement: Dataset S2 — (252.6 MB ZIP). [file pbio.0020329.sd002.zip › 040604_OS5_st1_167.tif]

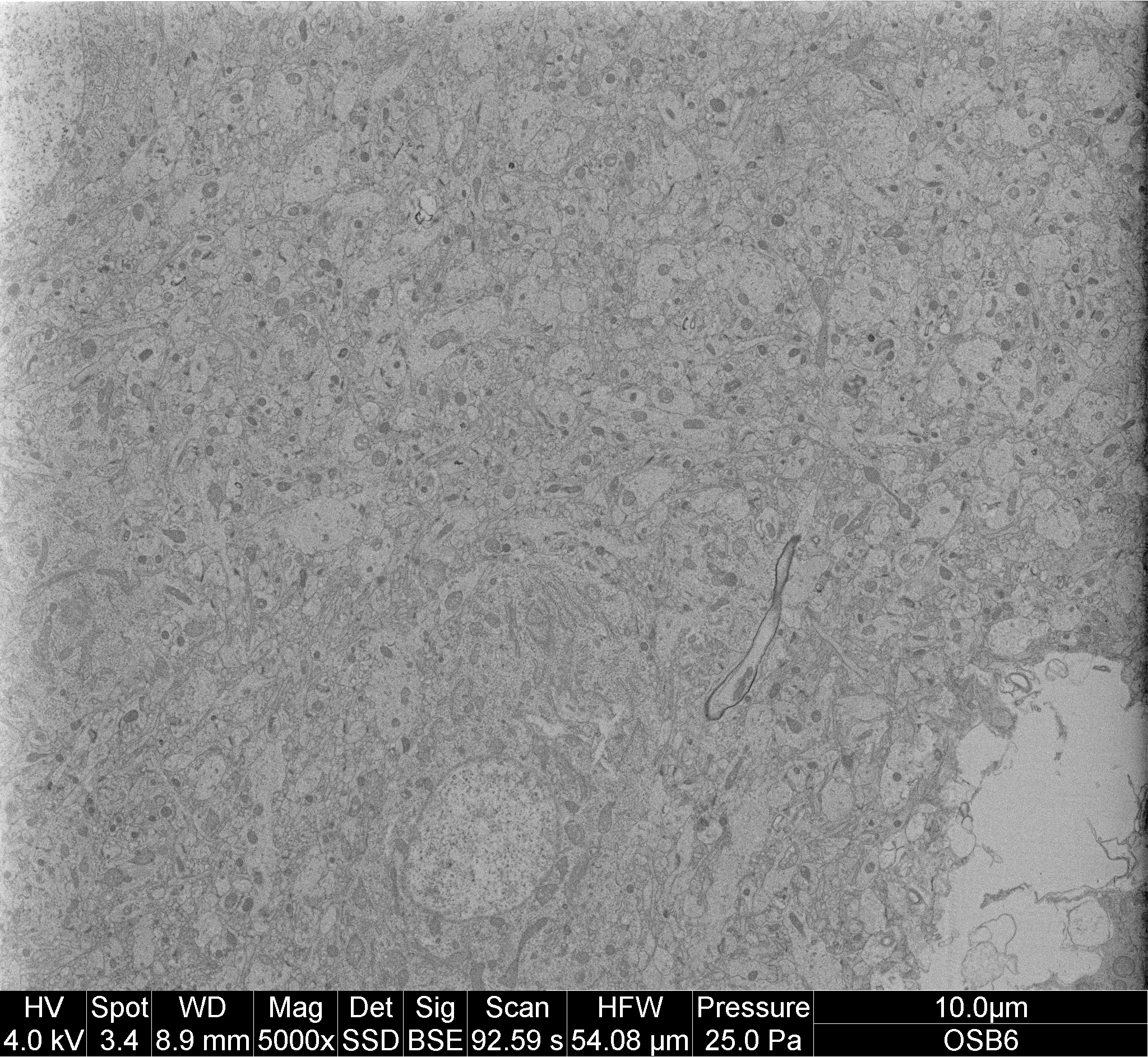

Supplement: Dataset S2 — (252.6 MB ZIP). [file pbio.0020329.sd002.zip › 040604_OS5_st1_168.tif]

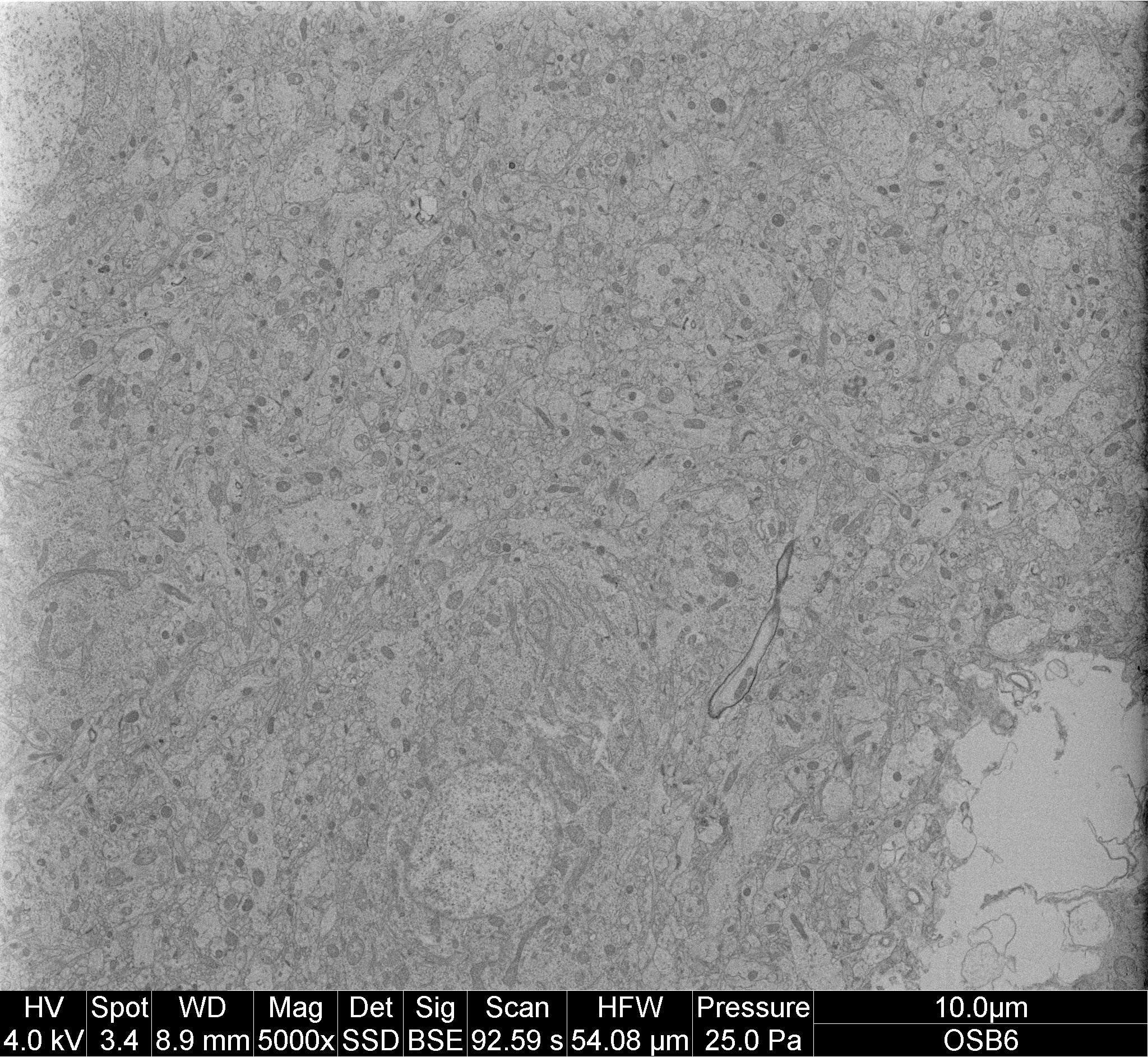

Supplement: Dataset S2 — (252.6 MB ZIP). [file pbio.0020329.sd002.zip › 040604_OS5_st1_169.tif]

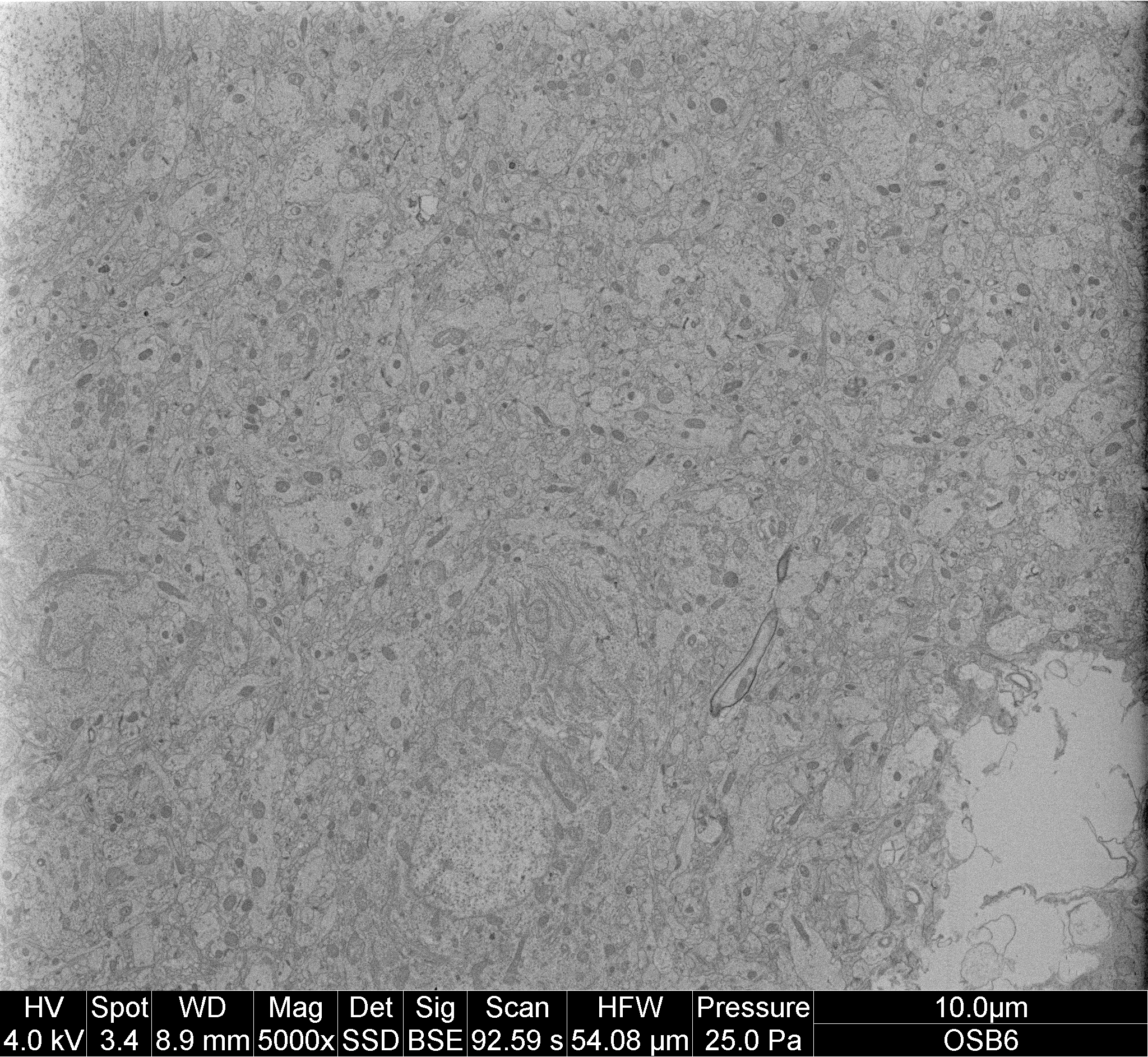

Supplement: Dataset S2 — (252.6 MB ZIP). [file pbio.0020329.sd002.zip › 040604_OS5_st1_170.tif]

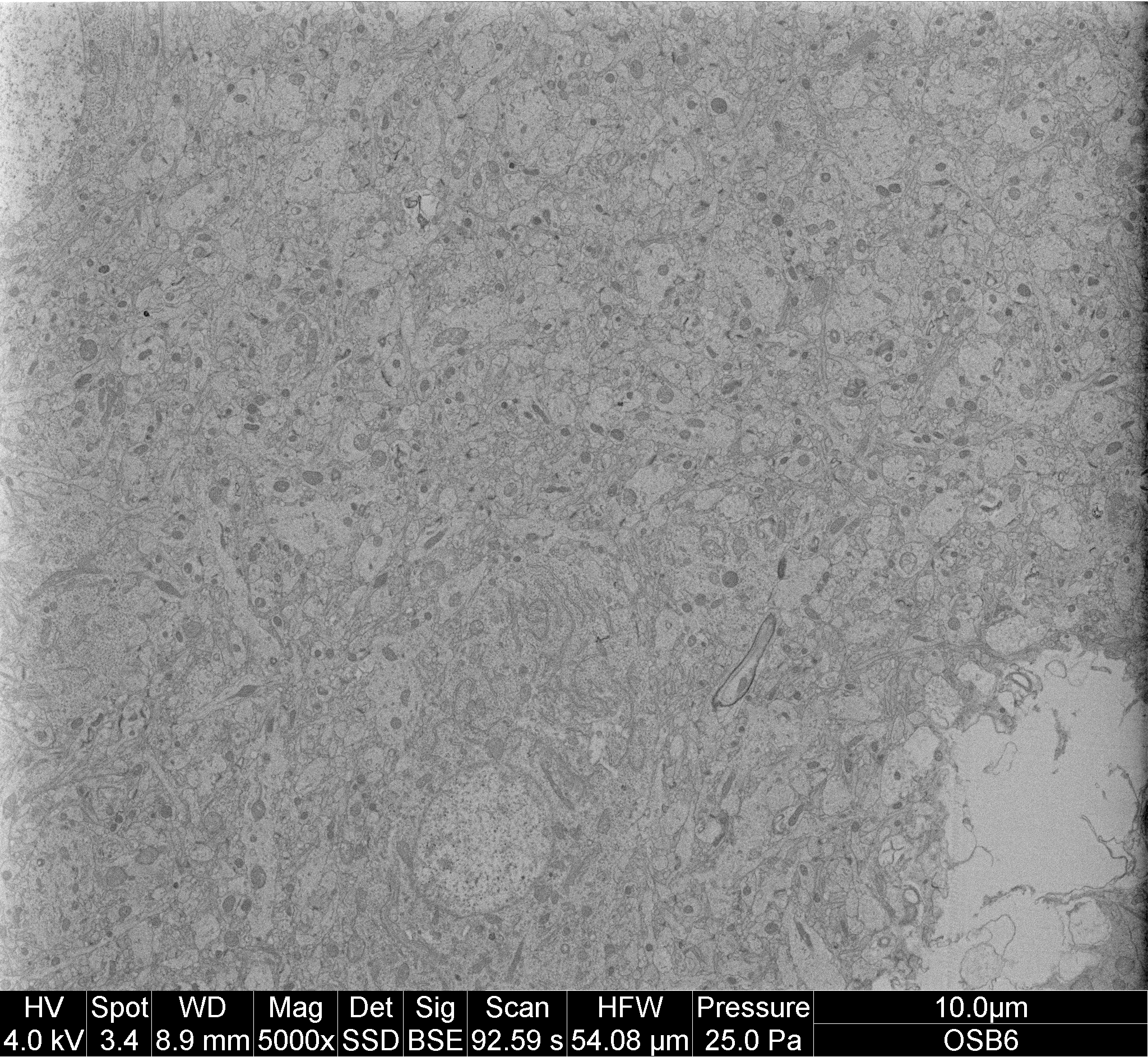

Supplement: Dataset S2 — (252.6 MB ZIP). [file pbio.0020329.sd002.zip › 040604_OS5_st1_171.tif]

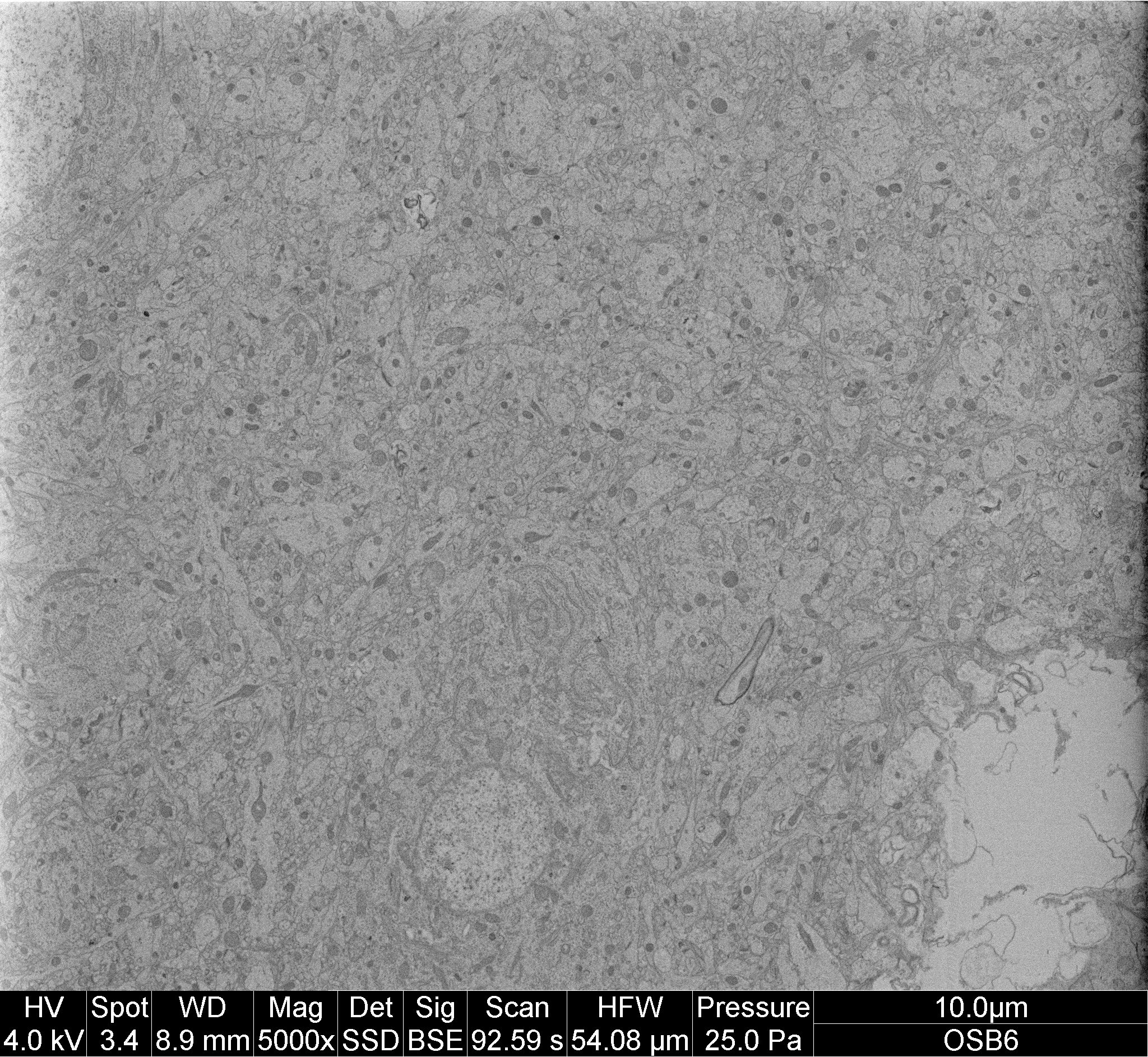

Supplement: Dataset S2 — (252.6 MB ZIP). [file pbio.0020329.sd002.zip › 040604_OS5_st1_172.tif]

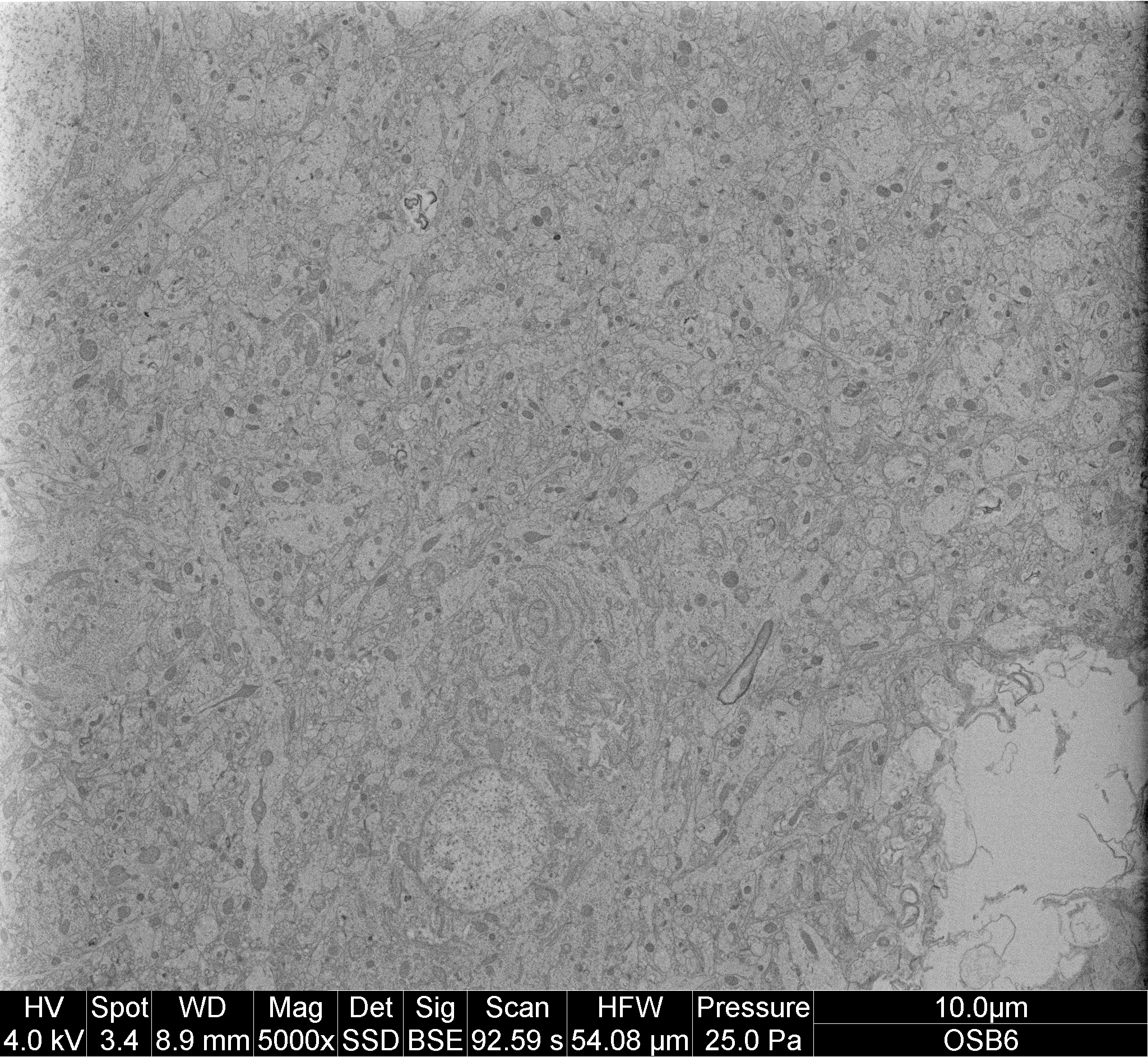

Supplement: Dataset S2 — (252.6 MB ZIP). [file pbio.0020329.sd002.zip › 040604_OS5_st1_173.tif]

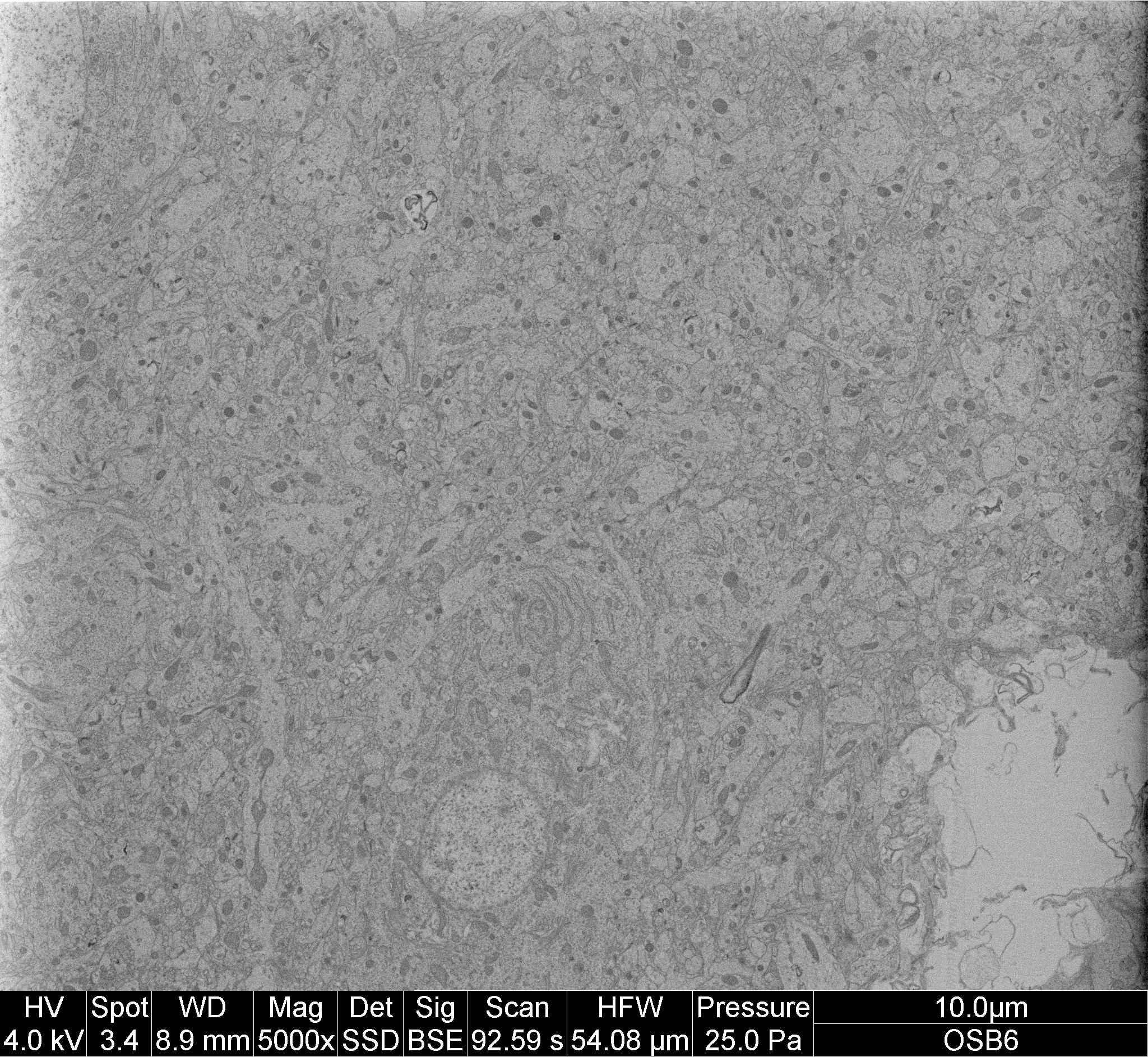

Supplement: Dataset S2 — (252.6 MB ZIP). [file pbio.0020329.sd002.zip › 040604_OS5_st1_174.tif]

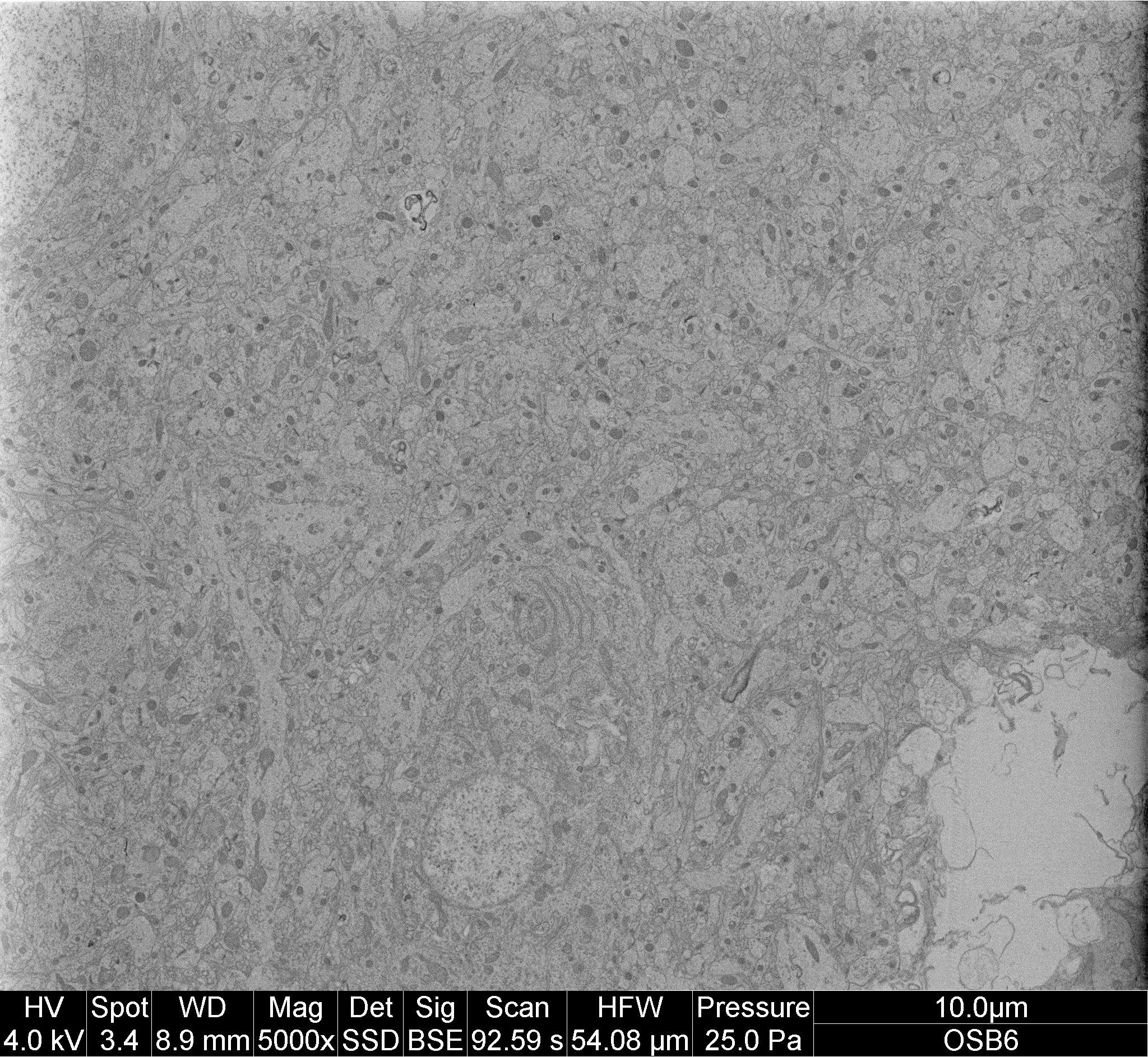

Supplement: Dataset S2 — (252.6 MB ZIP). [file pbio.0020329.sd002.zip › 040604_OS5_st1_175.tif]

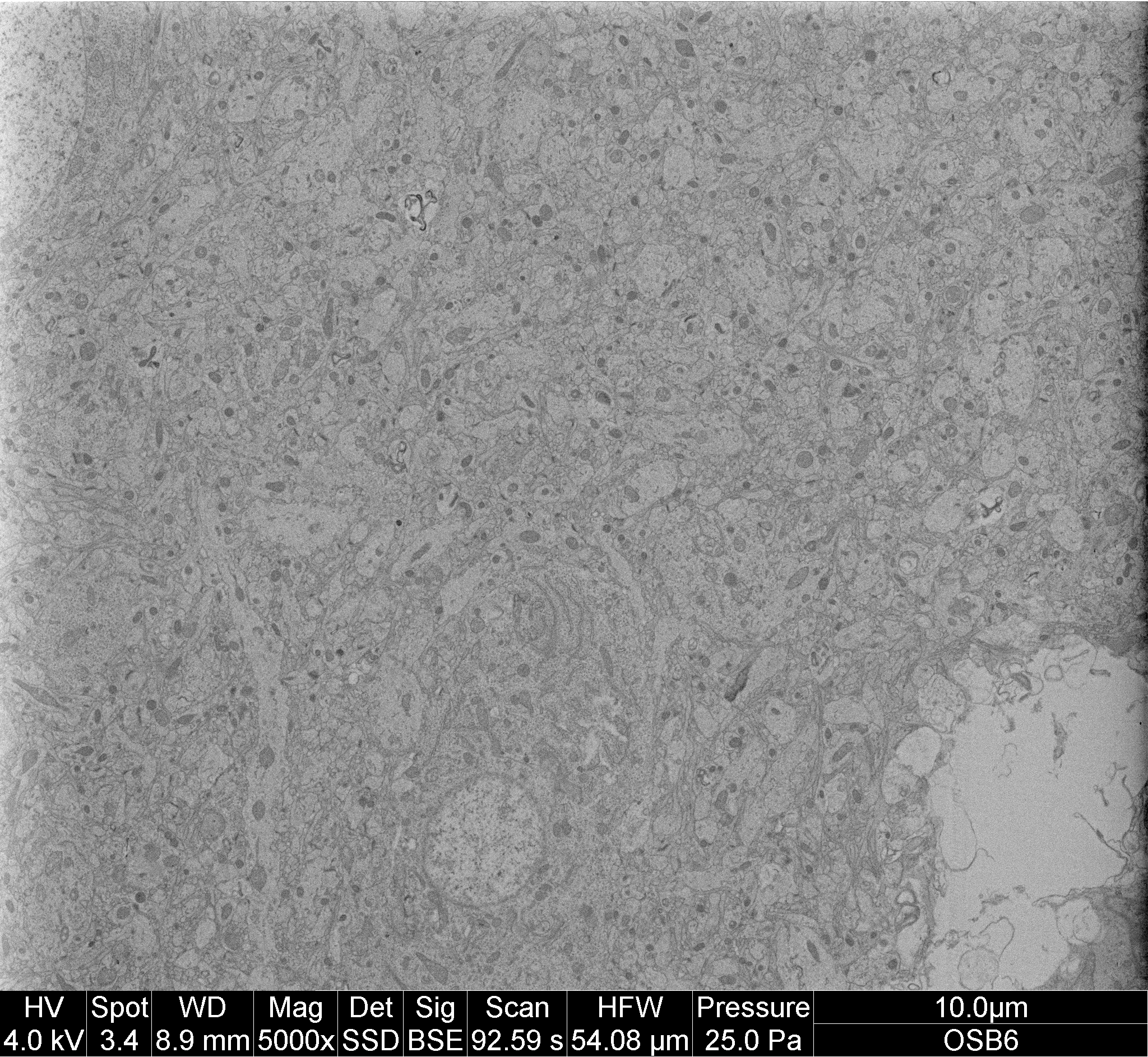

Supplement: Dataset S2 — (252.6 MB ZIP). [file pbio.0020329.sd002.zip › 040604_OS5_st1_176.tif]

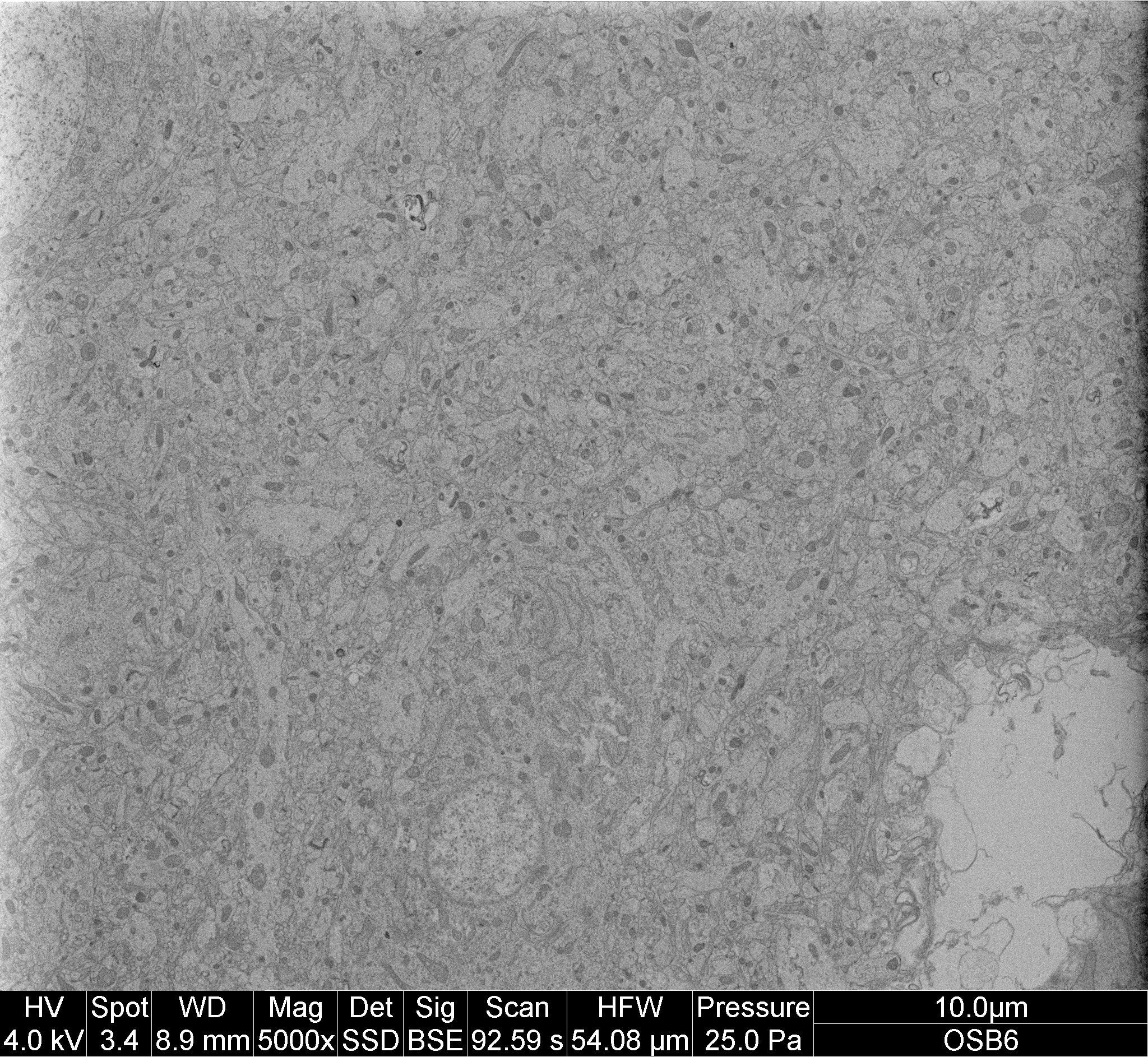

Supplement: Dataset S2 — (252.6 MB ZIP). [file pbio.0020329.sd002.zip › 040604_OS5_st1_177.tif]

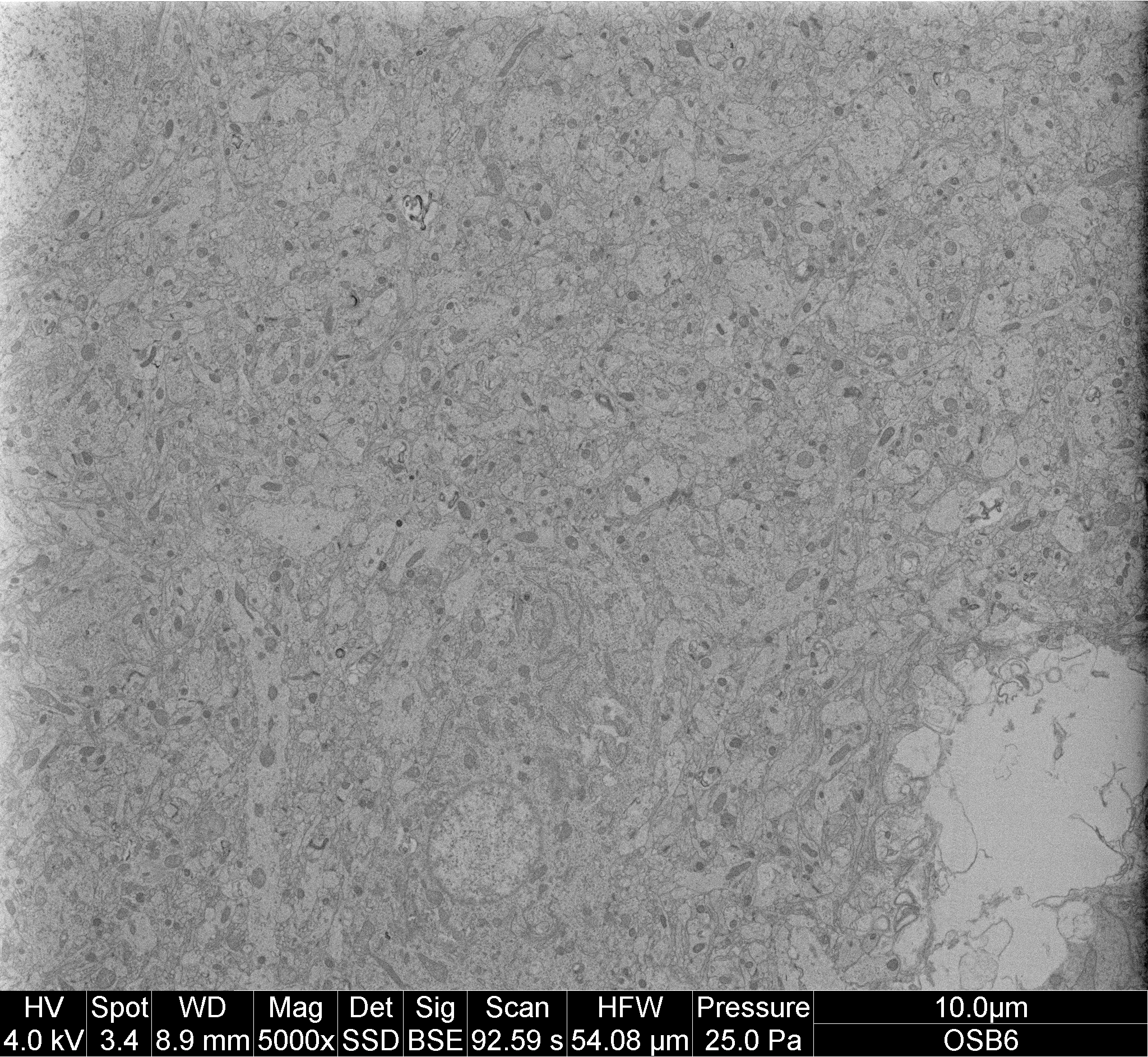

Supplement: Dataset S2 — (252.6 MB ZIP). [file pbio.0020329.sd002.zip › 040604_OS5_st1_178.tif]

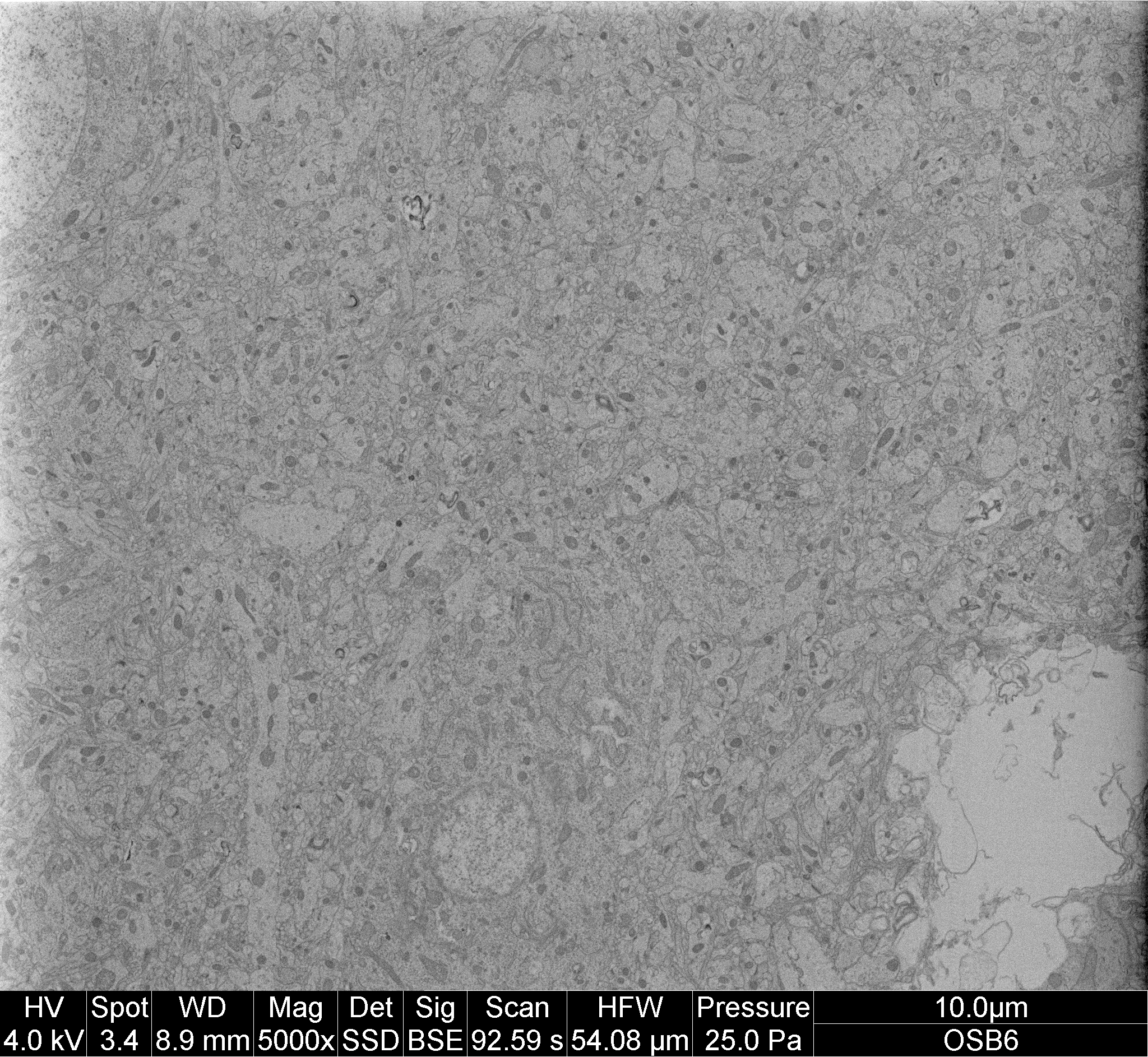

Supplement: Dataset S2 — (252.6 MB ZIP). [file pbio.0020329.sd002.zip › 040604_OS5_st1_179.tif]

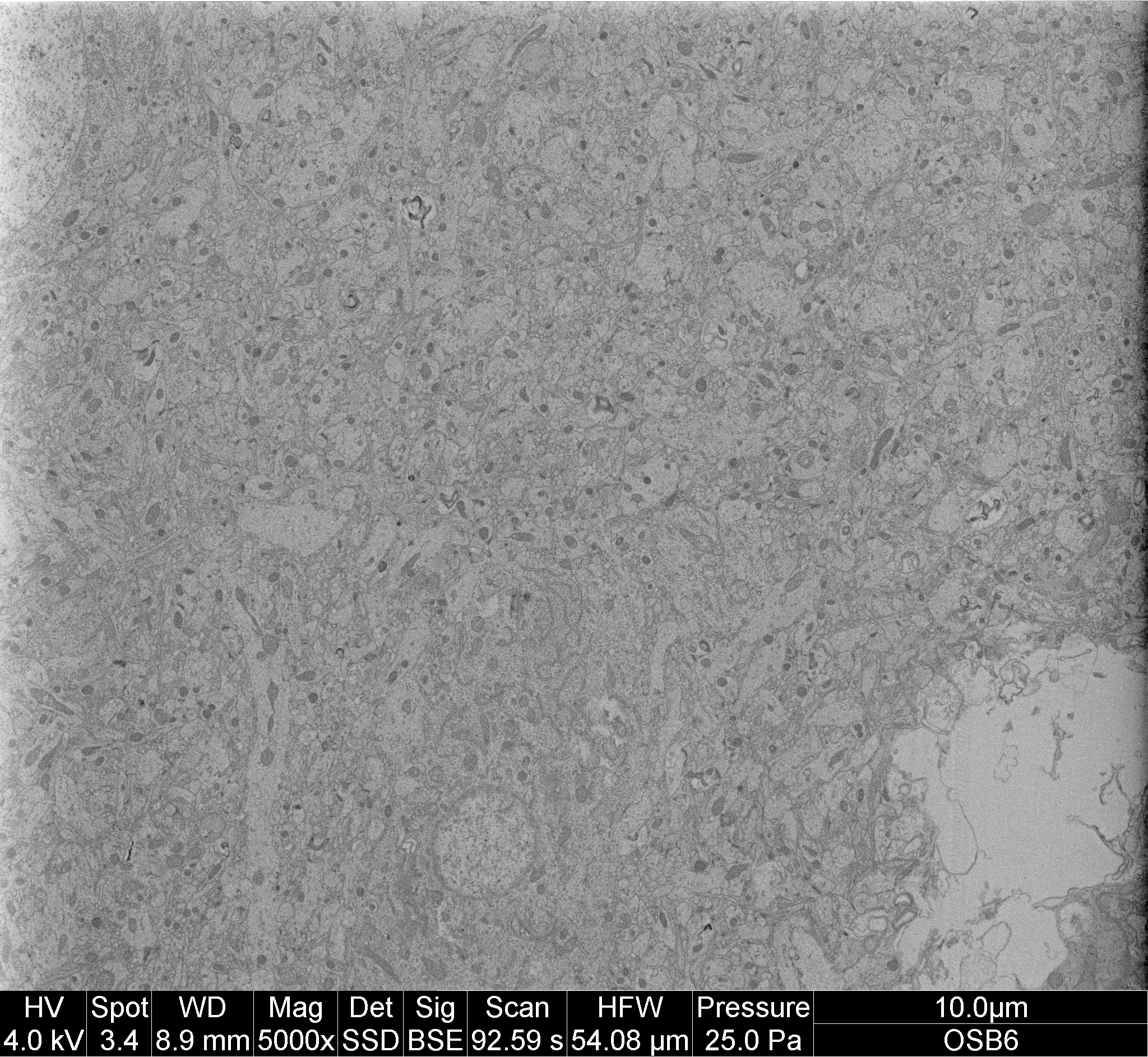

Supplement: Dataset S2 — (252.6 MB ZIP). [file pbio.0020329.sd002.zip › 040604_OS5_st1_180.tif]

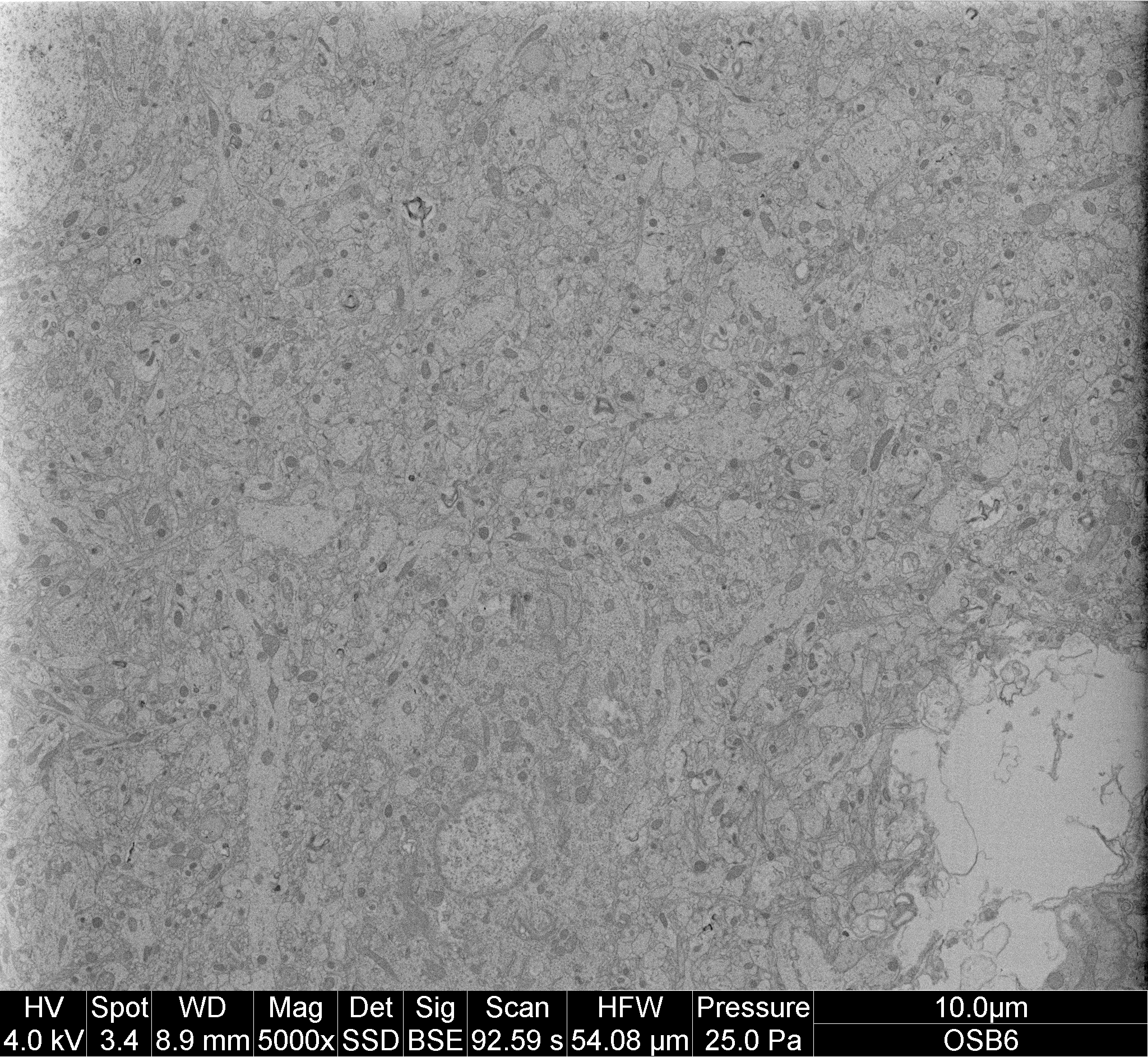

Supplement: Dataset S2 — (252.6 MB ZIP). [file pbio.0020329.sd002.zip › 040604_OS5_st1_181.tif]

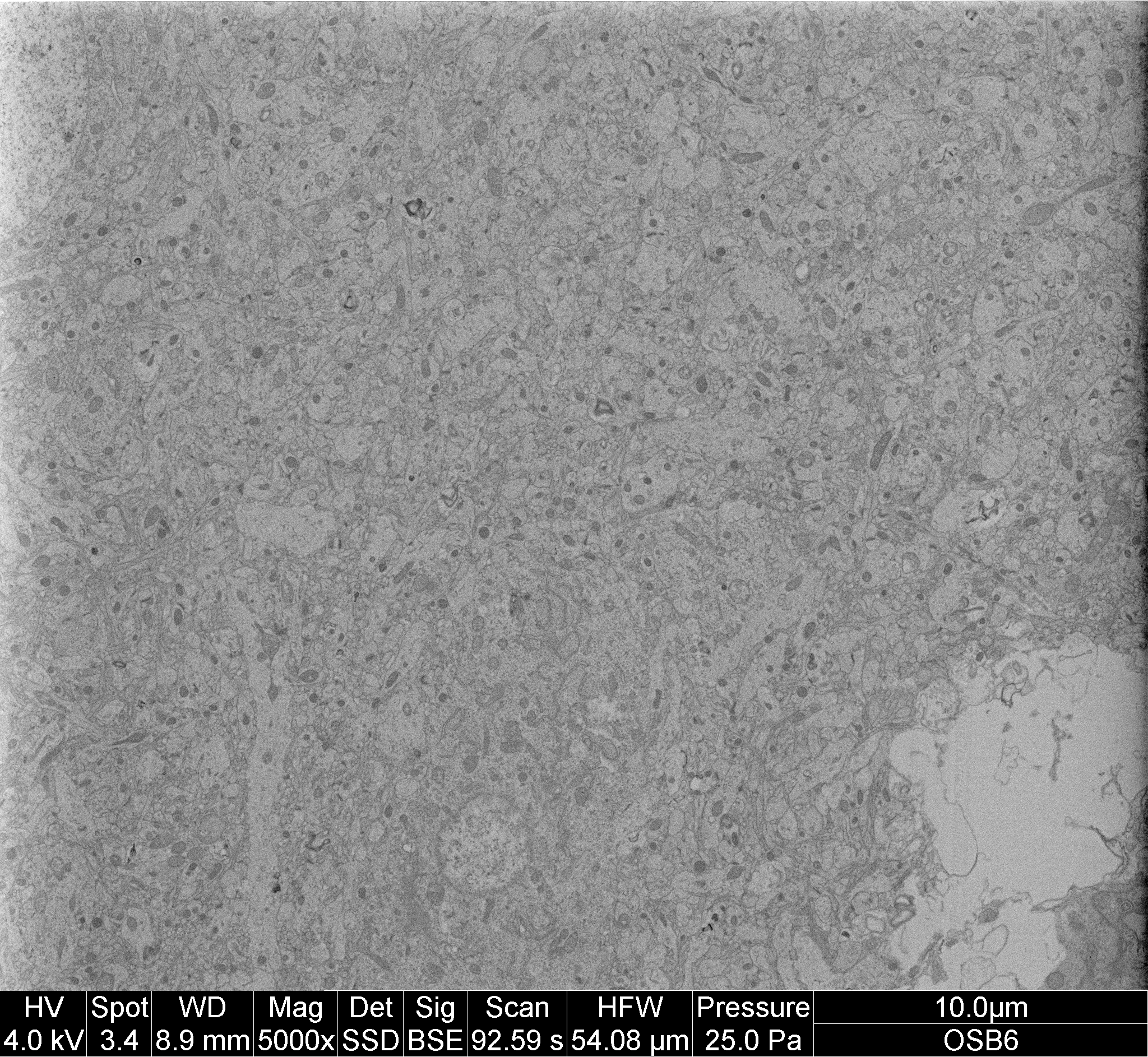

Supplement: Dataset S2 — (252.6 MB ZIP). [file pbio.0020329.sd002.zip › 040604_OS5_st1_182.tif]

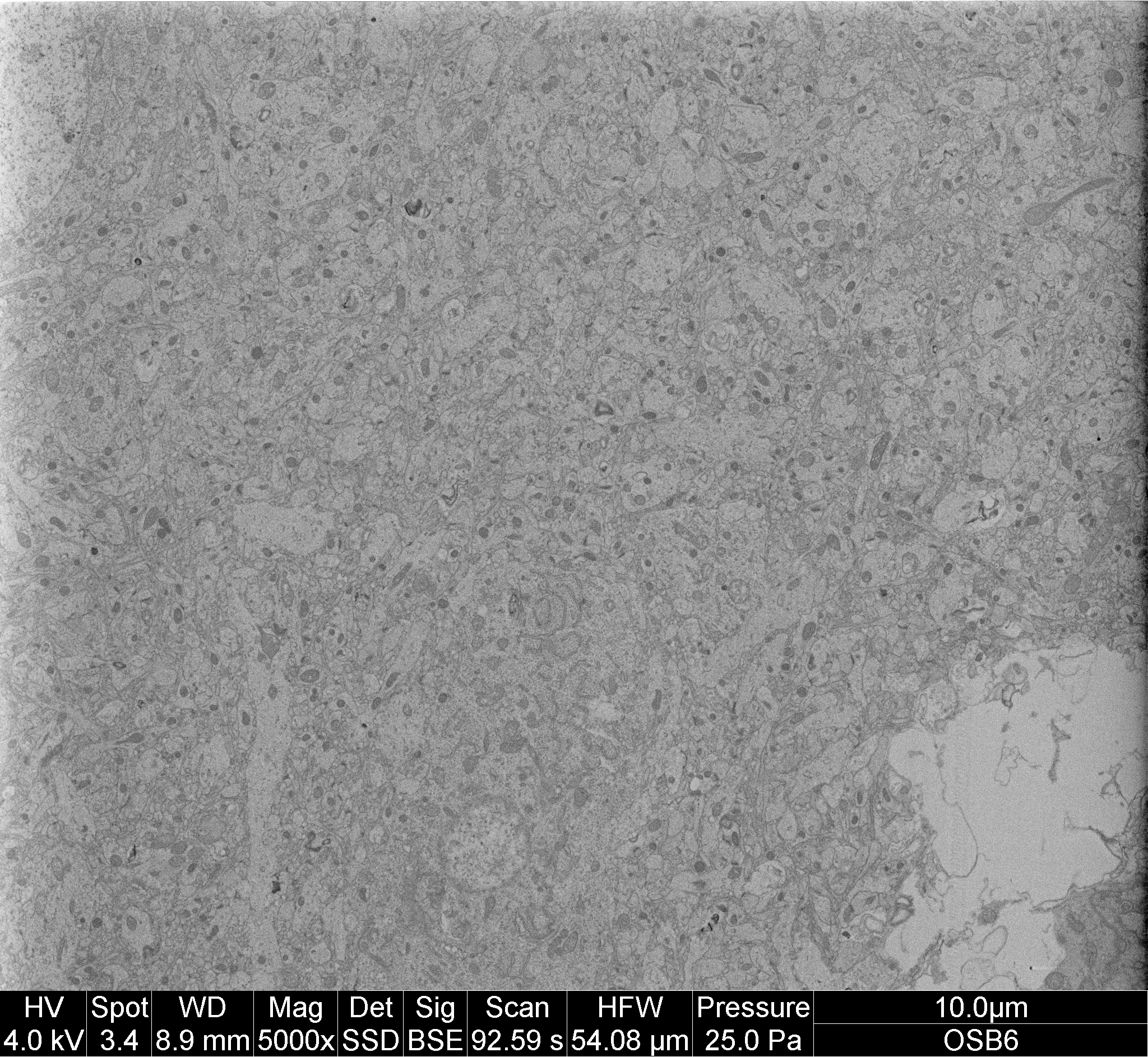

Supplement: Dataset S2 — (252.6 MB ZIP). [file pbio.0020329.sd002.zip › 040604_OS5_st1_183.tif]

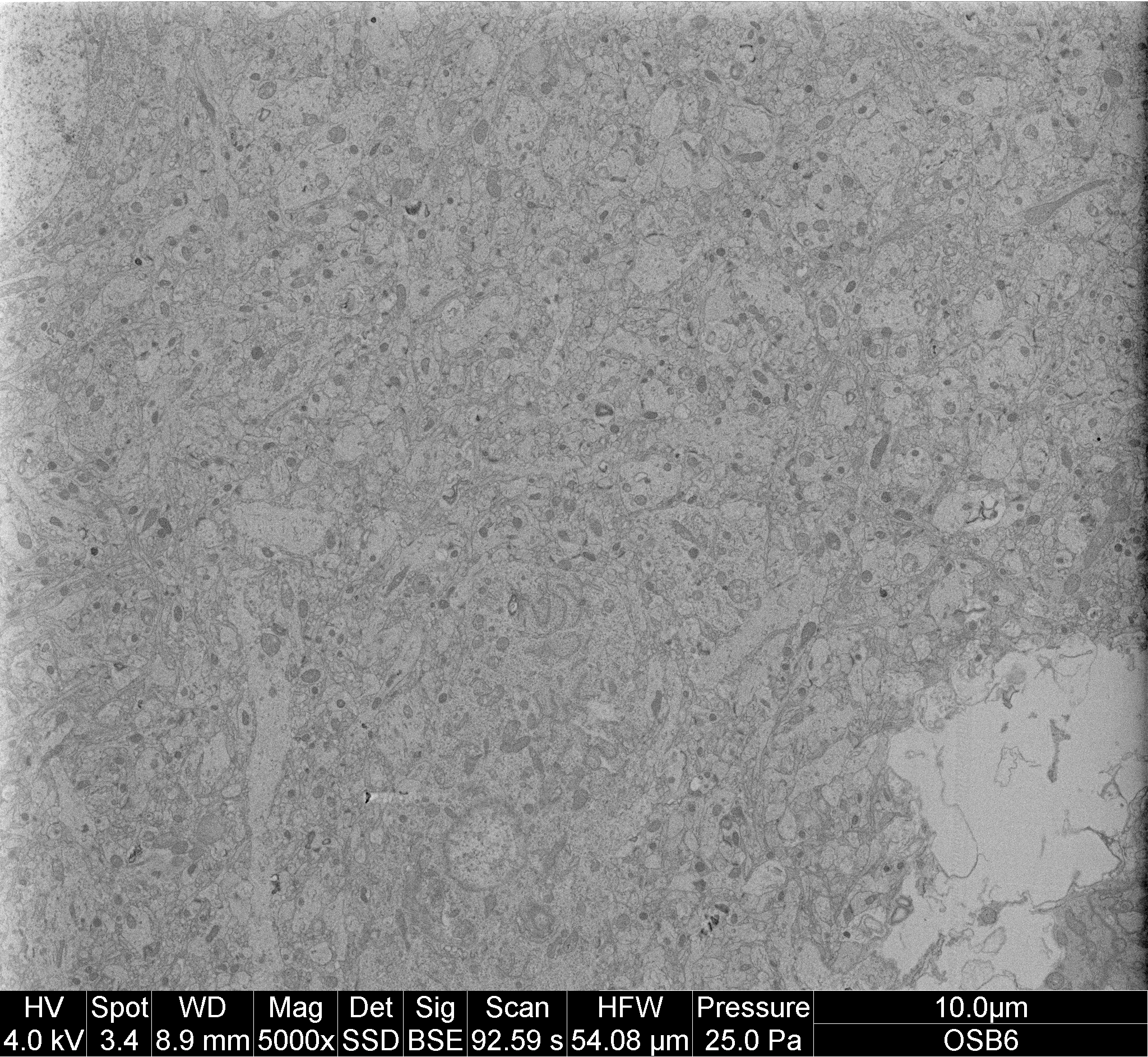

Supplement: Dataset S2 — (252.6 MB ZIP). [file pbio.0020329.sd002.zip › 040604_OS5_st1_184.tif]

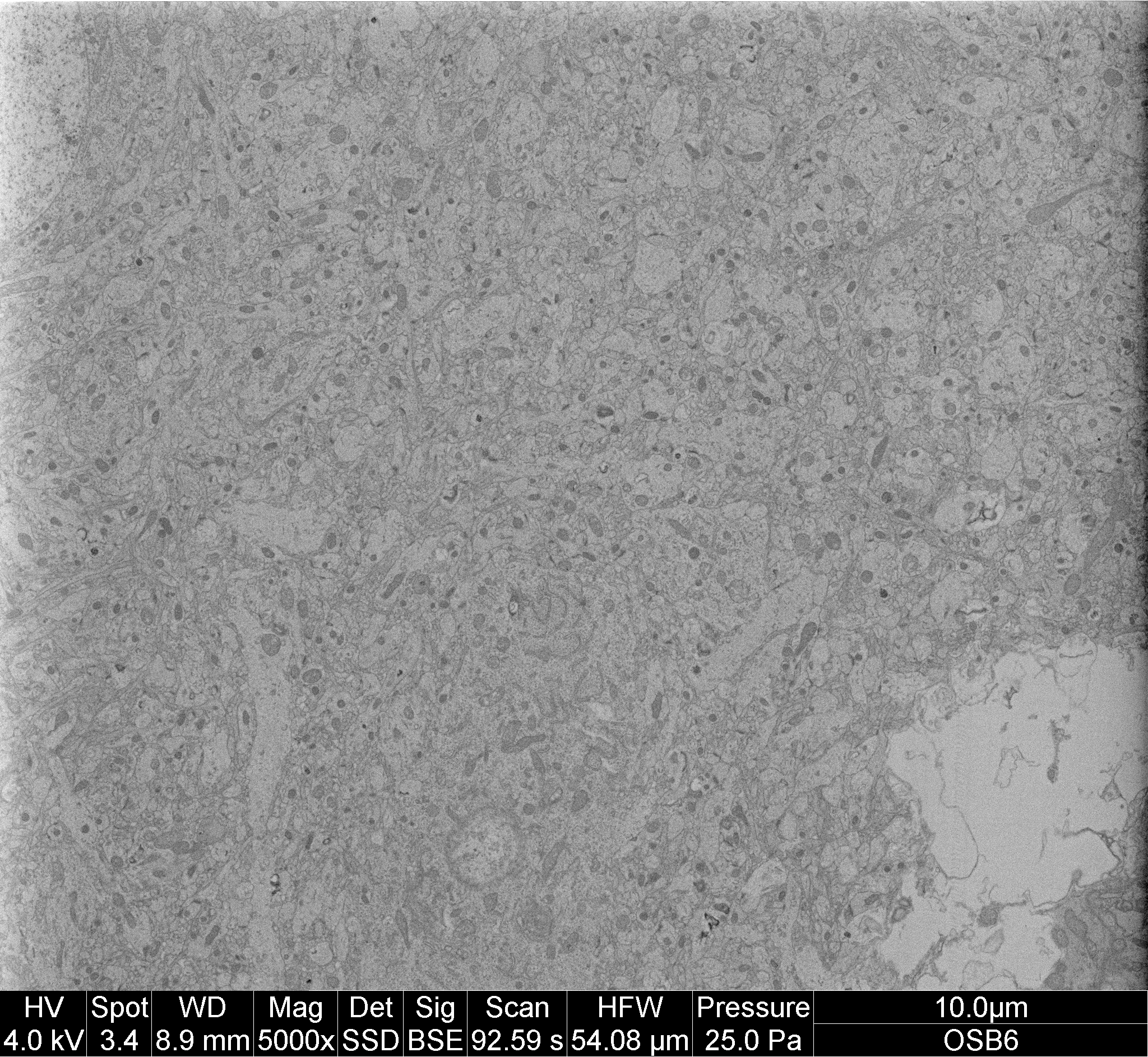

Supplement: Dataset S2 — (252.6 MB ZIP). [file pbio.0020329.sd002.zip › 040604_OS5_st1_185.tif]

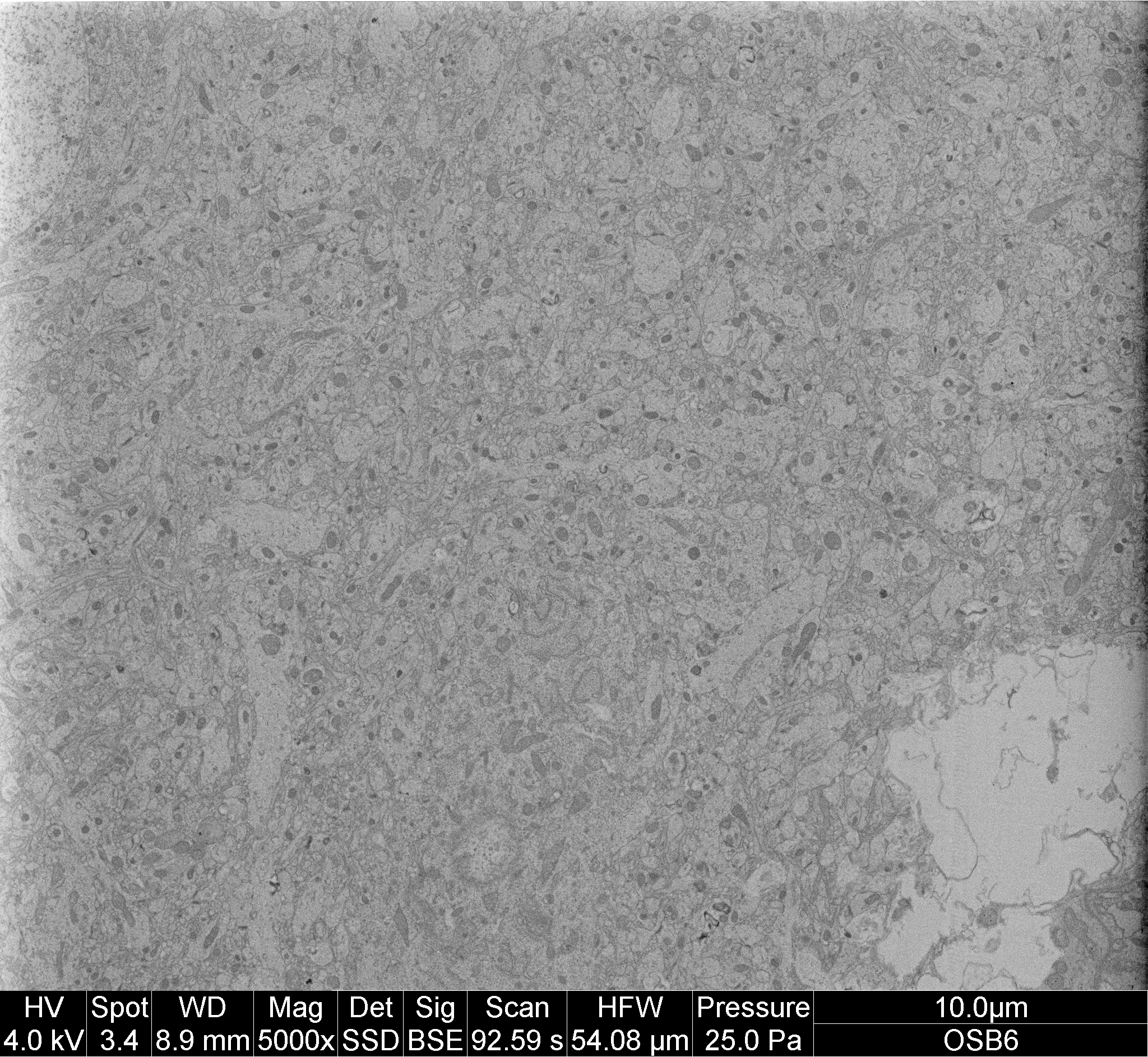

Supplement: Dataset S2 — (252.6 MB ZIP). [file pbio.0020329.sd002.zip › 040604_OS5_st1_186.tif]

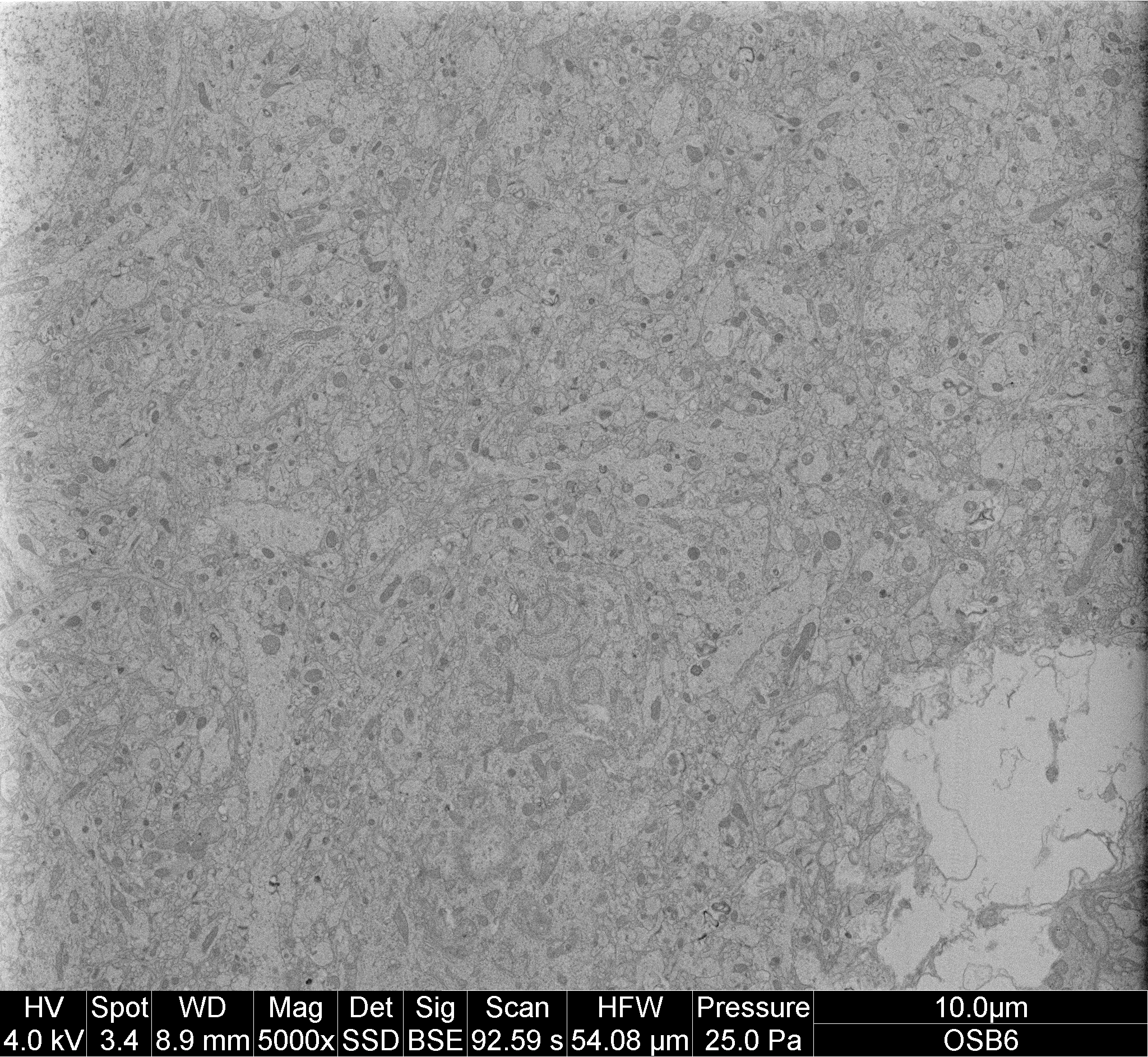

Supplement: Dataset S2 — (252.6 MB ZIP). [file pbio.0020329.sd002.zip › 040604_OS5_st1_187.tif]

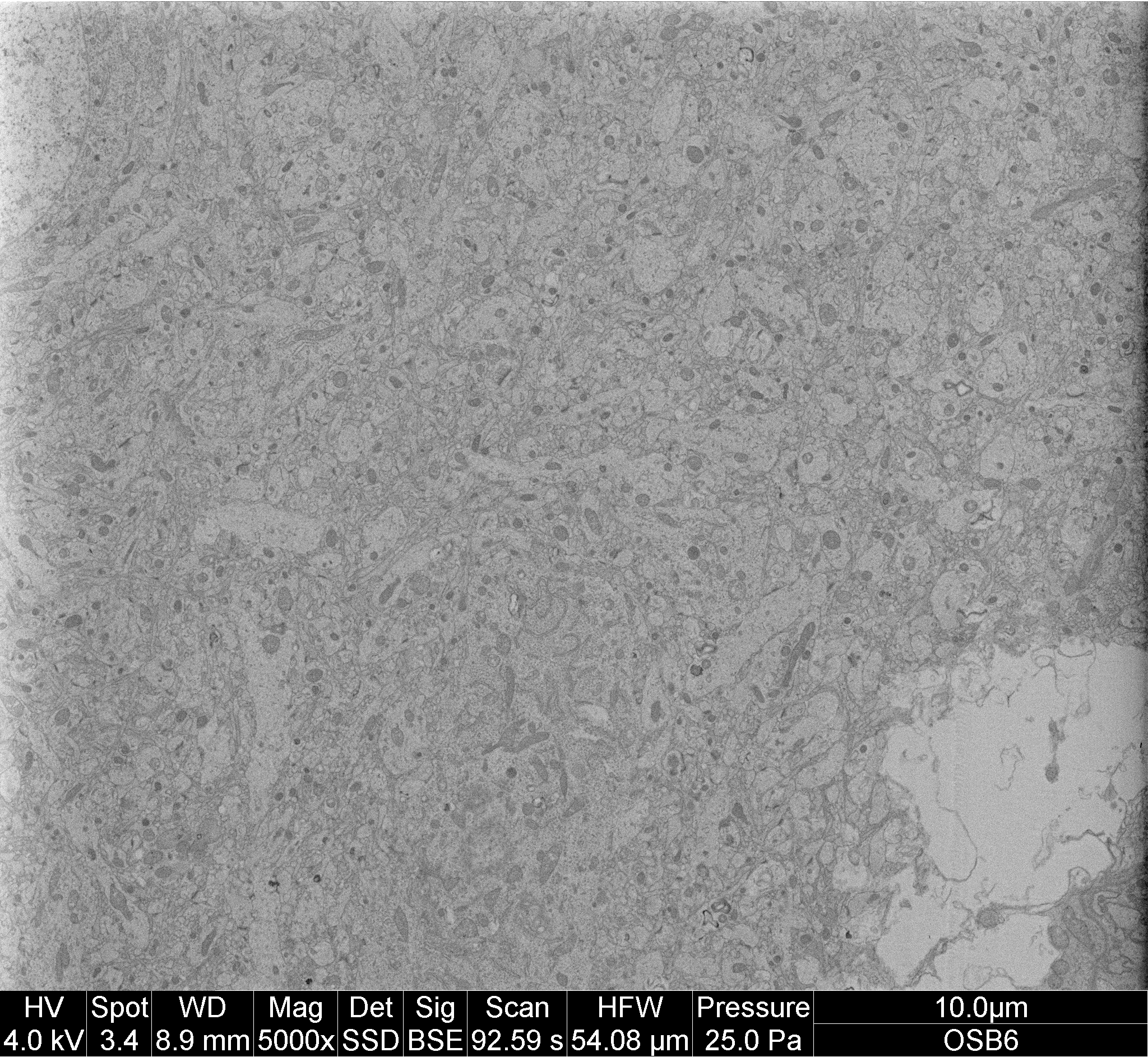

Supplement: Dataset S2 — (252.6 MB ZIP). [file pbio.0020329.sd002.zip › 040604_OS5_st1_188.tif]

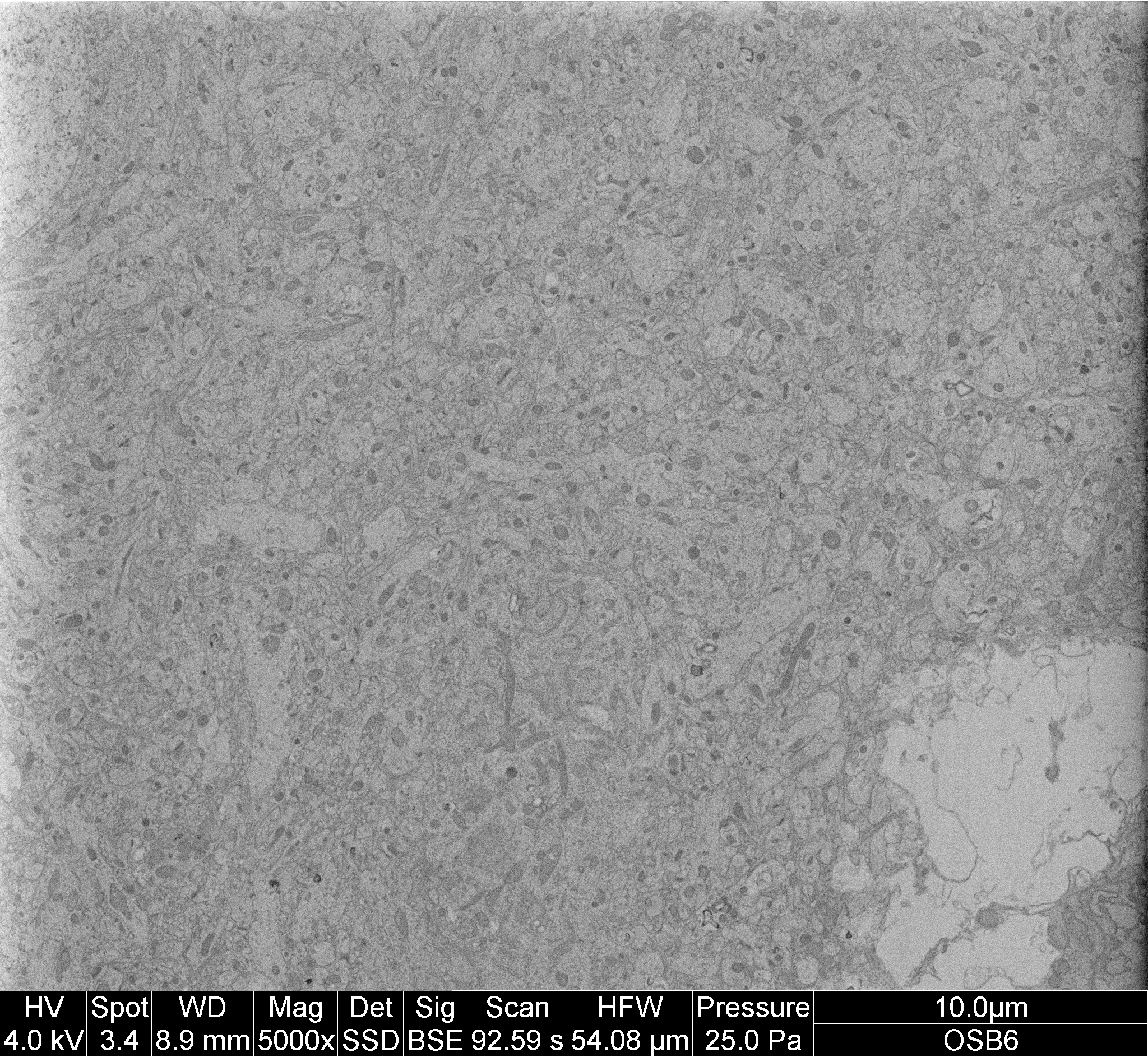

Supplement: Dataset S2 — (252.6 MB ZIP). [file pbio.0020329.sd002.zip › 040604_OS5_st1_189.tif]

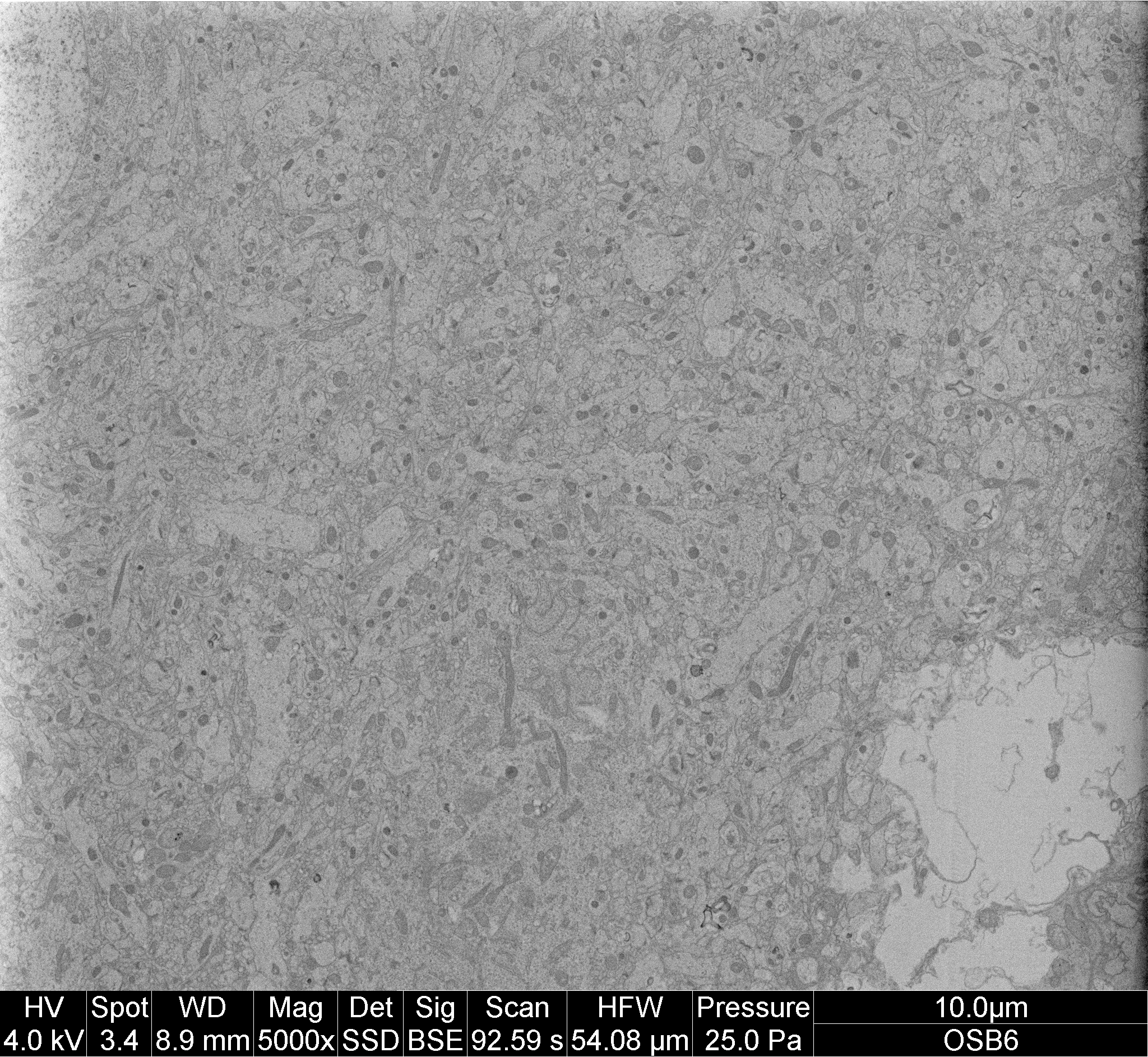

Supplement: Dataset S2 — (252.6 MB ZIP). [file pbio.0020329.sd002.zip › 040604_OS5_st1_190.tif]

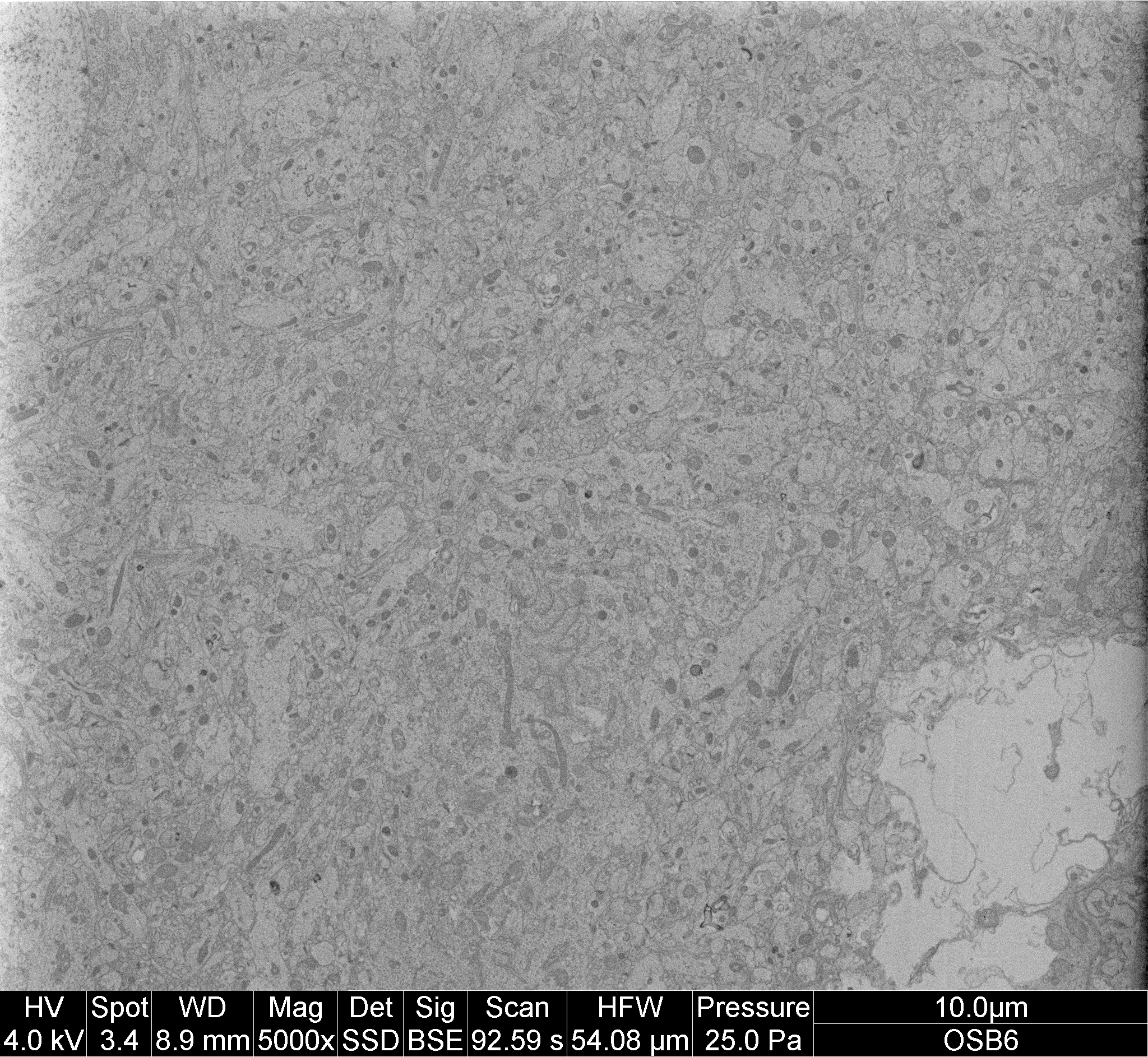

Supplement: Dataset S2 — (252.6 MB ZIP). [file pbio.0020329.sd002.zip › 040604_OS5_st1_191.tif]

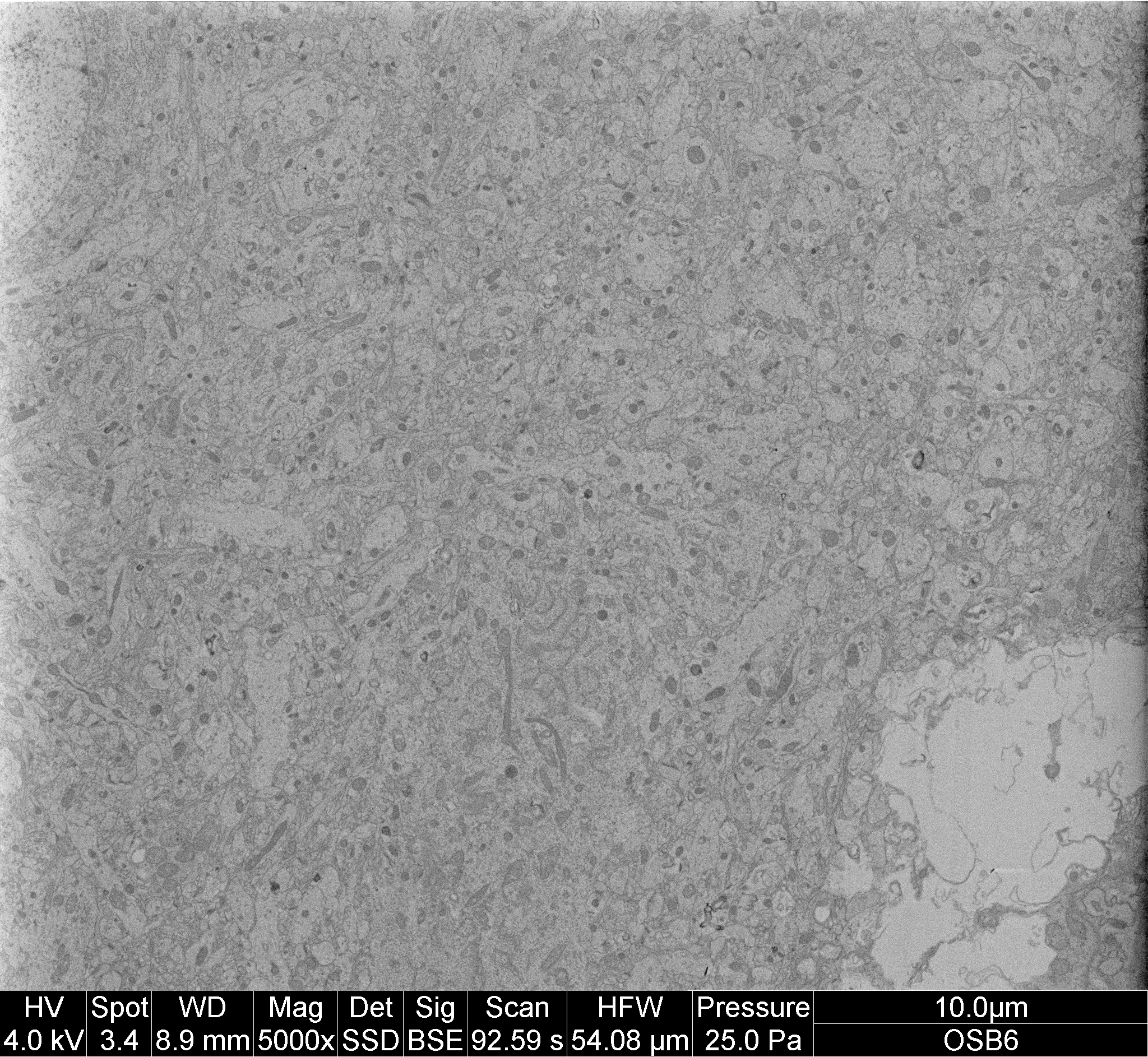

Supplement: Dataset S2 — (252.6 MB ZIP). [file pbio.0020329.sd002.zip › 040604_OS5_st1_192.tif]

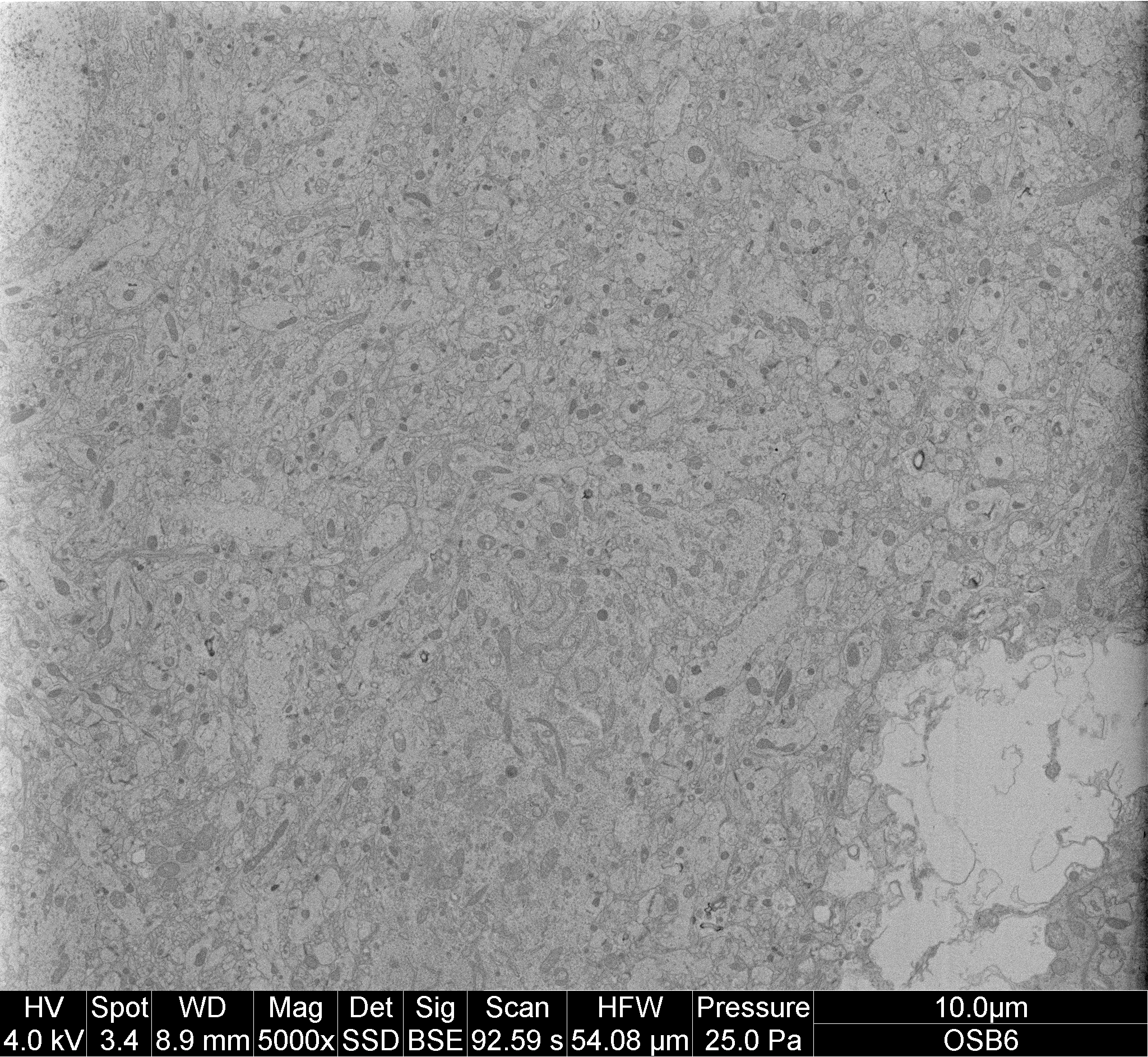

Supplement: Dataset S2 — (252.6 MB ZIP). [file pbio.0020329.sd002.zip › 040604_OS5_st1_193.tif]

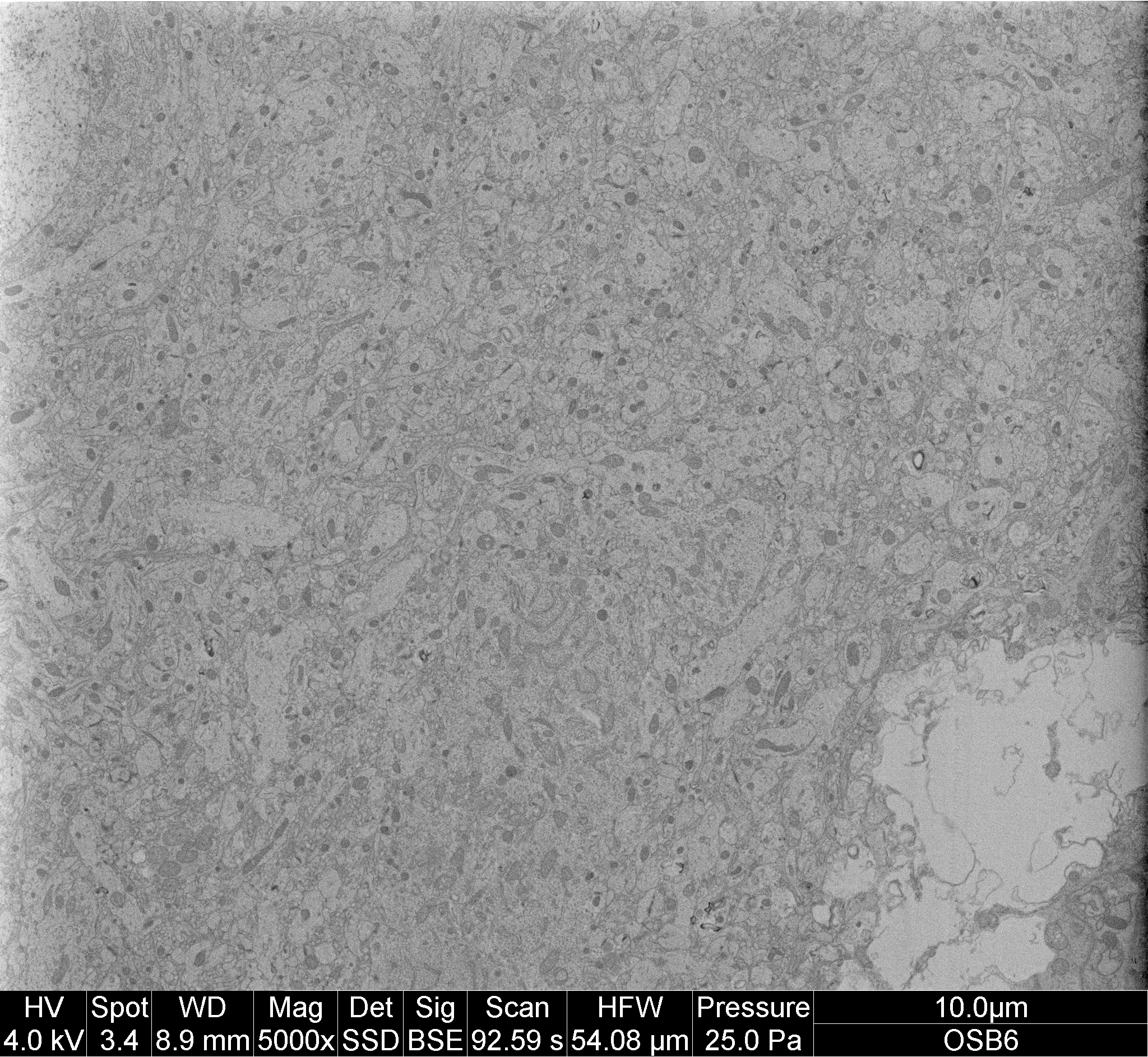

Supplement: Dataset S2 — (252.6 MB ZIP). [file pbio.0020329.sd002.zip › 040604_OS5_st1_194.tif]

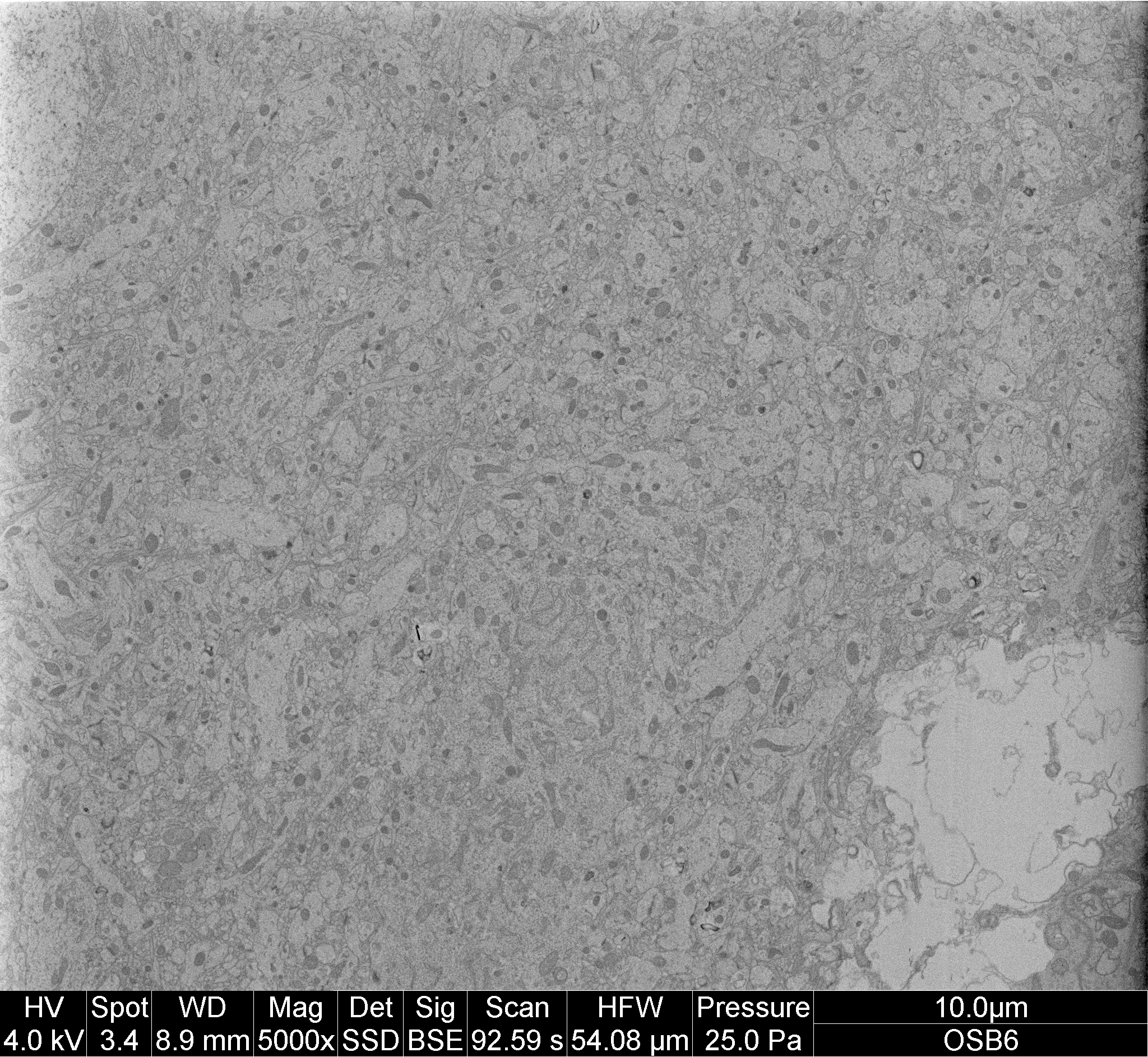

Supplement: Dataset S2 — (252.6 MB ZIP). [file pbio.0020329.sd002.zip › 040604_OS5_st1_195.tif]

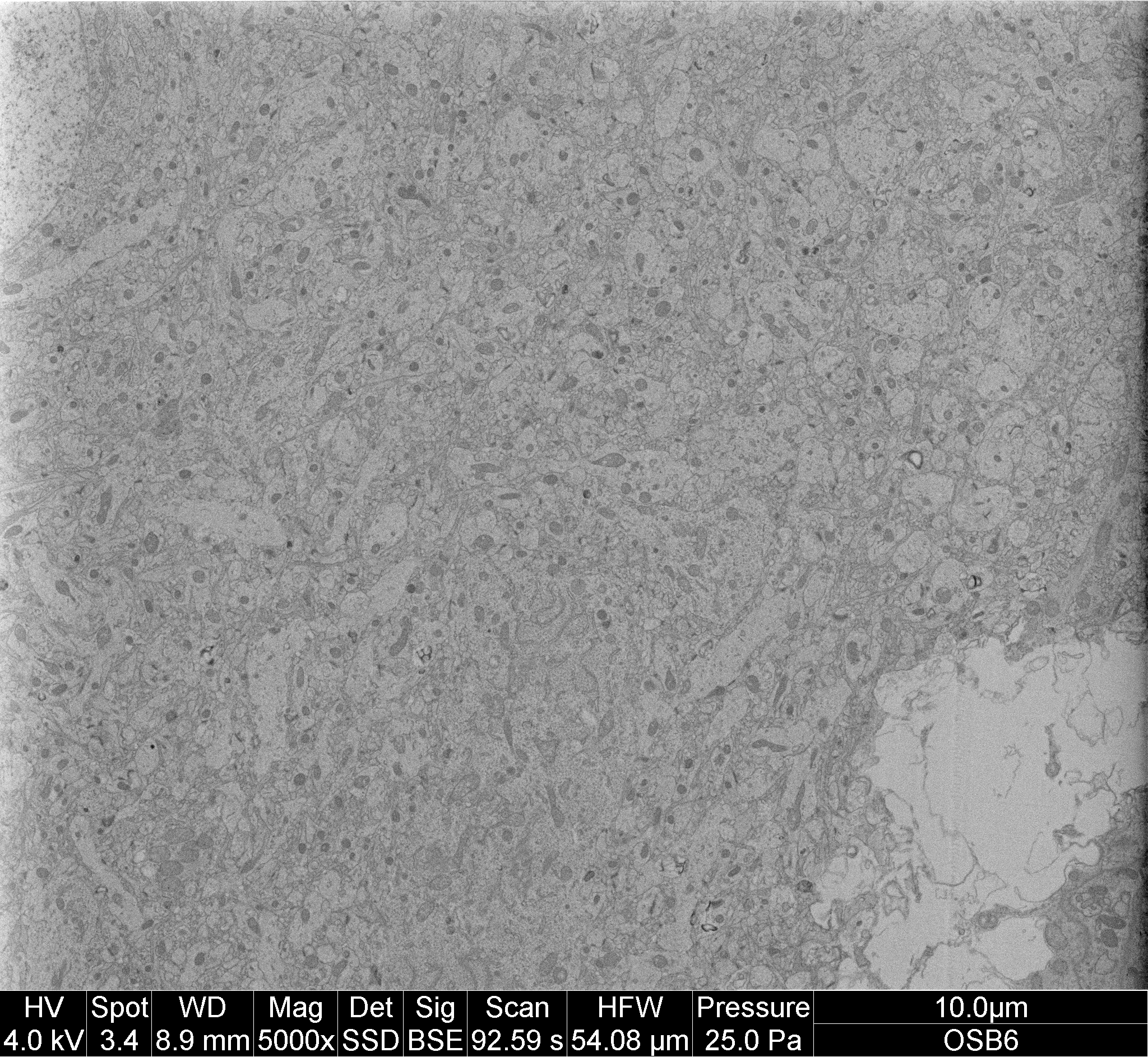

Supplement: Dataset S2 — (252.6 MB ZIP). [file pbio.0020329.sd002.zip › 040604_OS5_st1_196.tif]

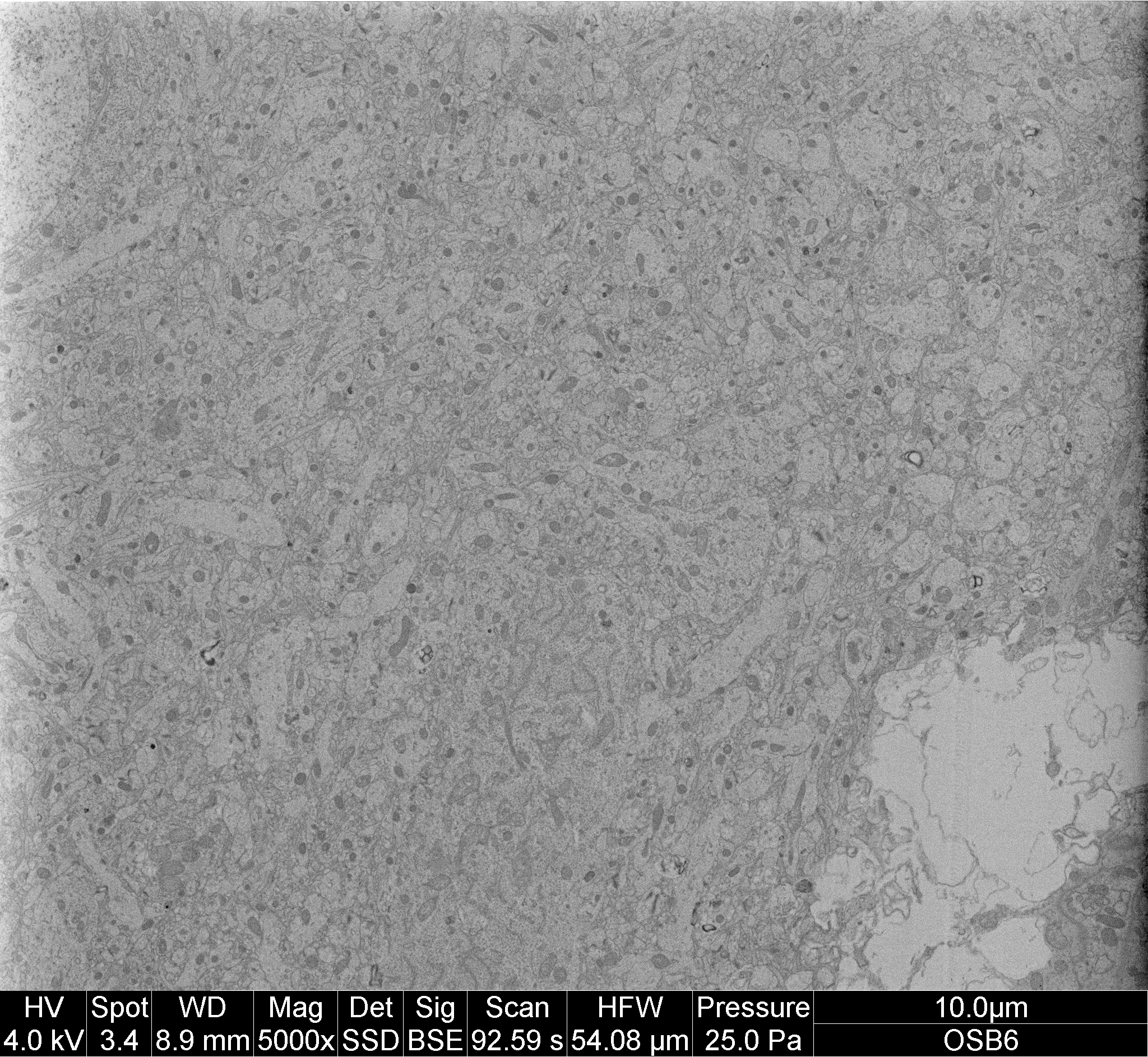

Supplement: Dataset S2 — (252.6 MB ZIP). [file pbio.0020329.sd002.zip › 040604_OS5_st1_197.tif]

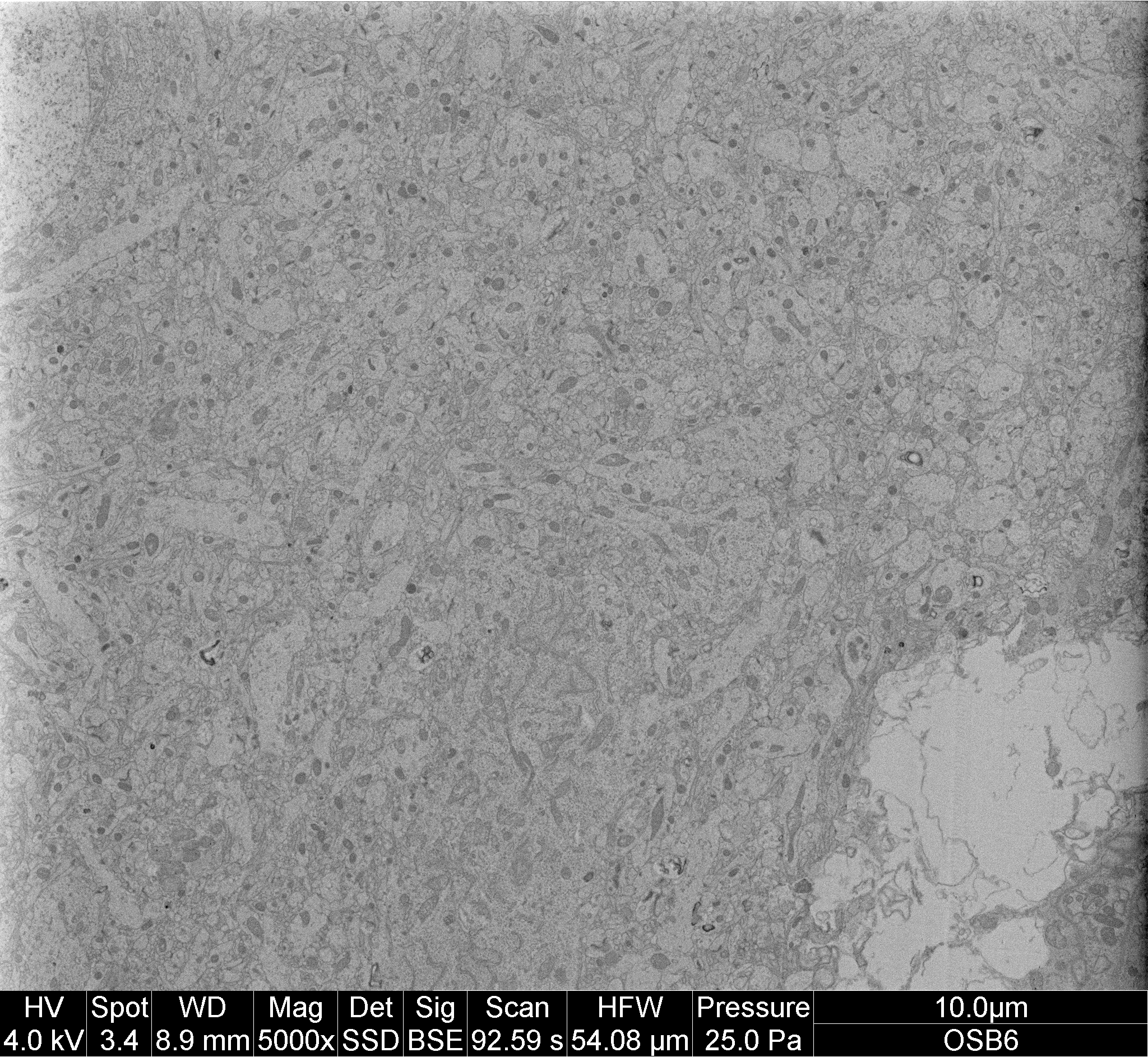

Supplement: Dataset S2 — (252.6 MB ZIP). [file pbio.0020329.sd002.zip › 040604_OS5_st1_198.tif]

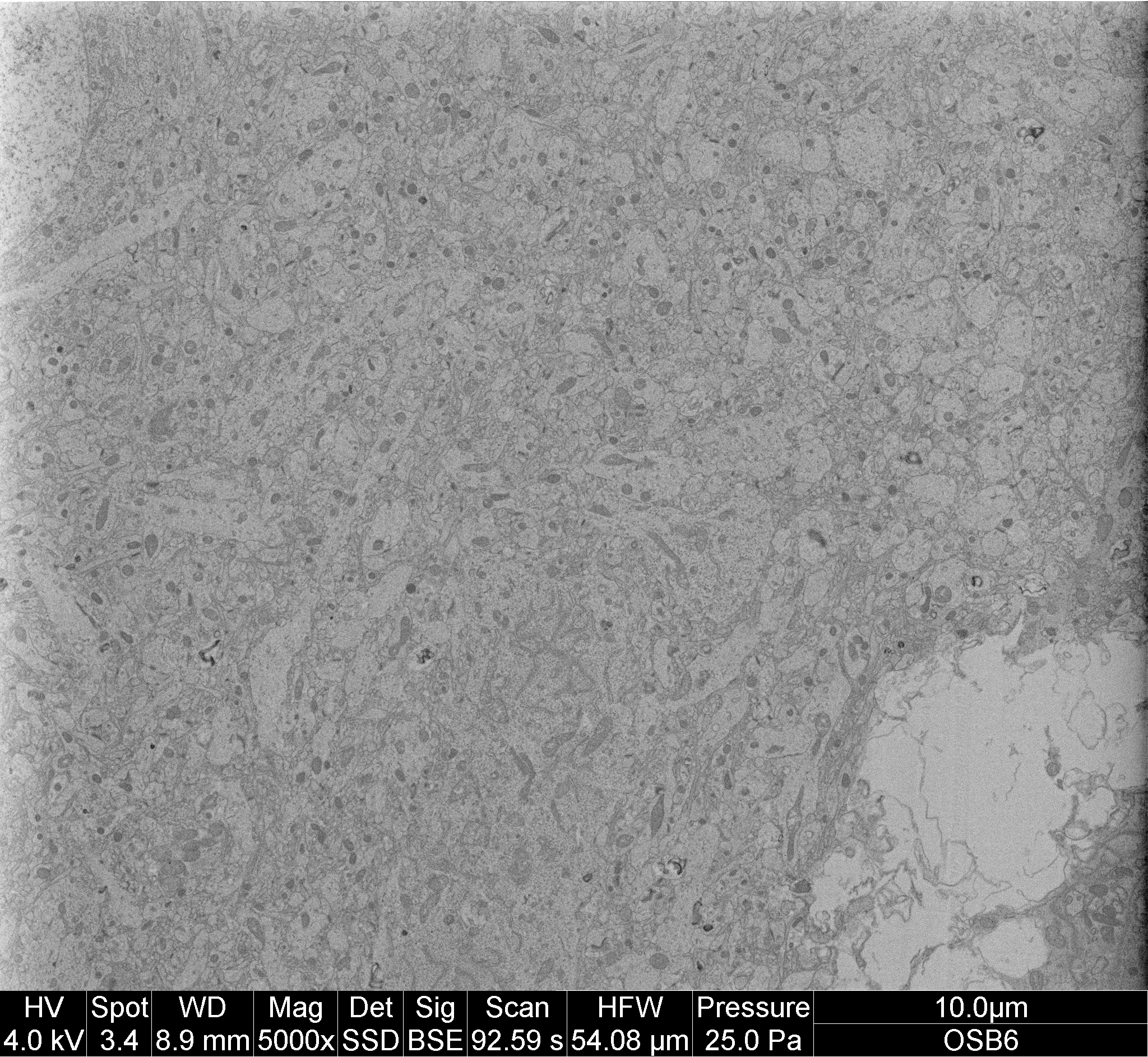

Supplement: Dataset S2 — (252.6 MB ZIP). [file pbio.0020329.sd002.zip › 040604_OS5_st1_199.tif]

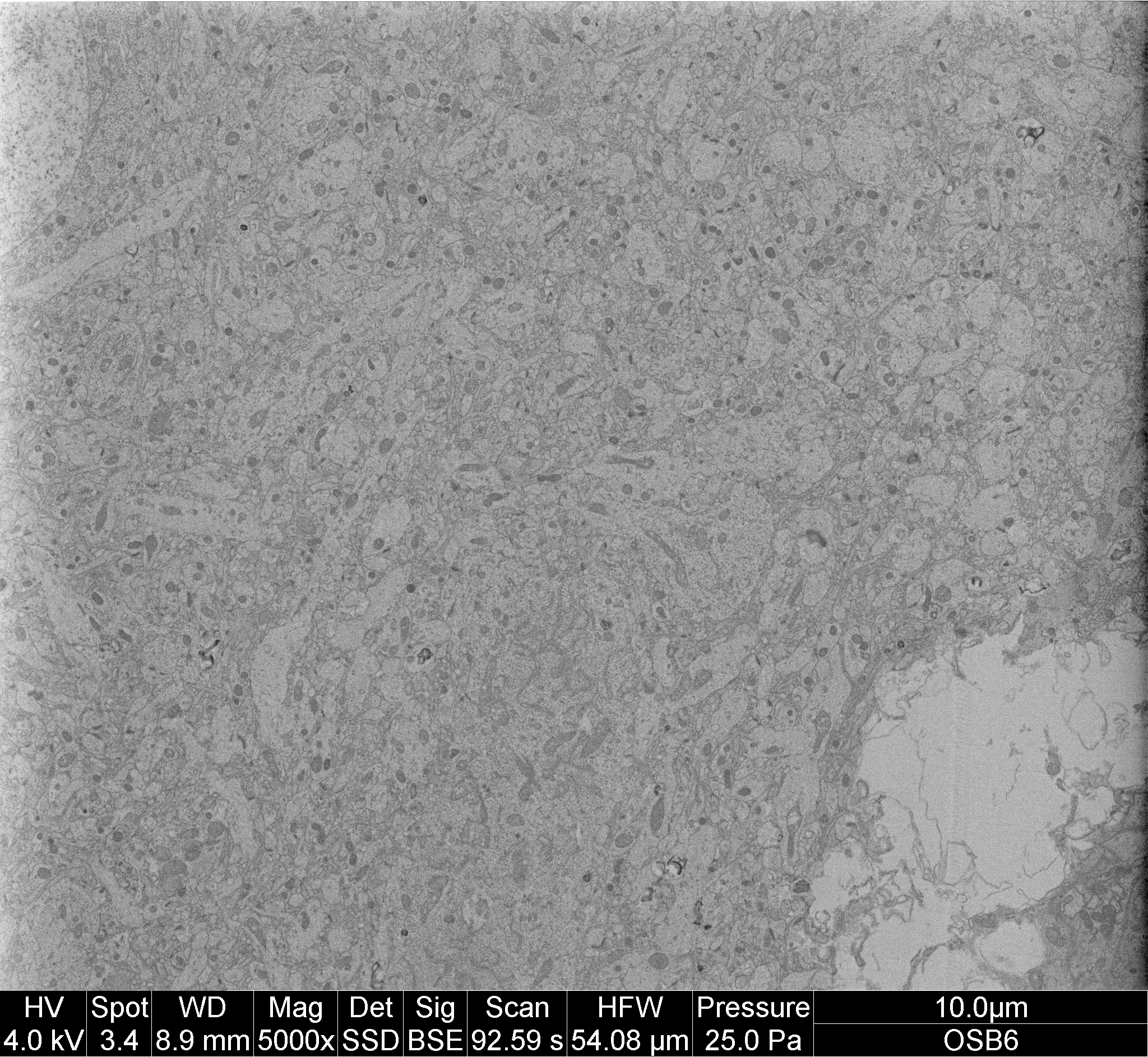

Supplement: Dataset S3 — (252.7 MB ZIP). [file pbio.0020329.sd003.zip › 040604_OS5_st1_200.tif]
