# Supplementary figures and images for: Serial Block-Face Scanning Electron Microscopy to Reconstruct Three-Dimensional Tissue Nanostructure (part 3 of 21)
Source: PLoS Biol. 2004 Oct 19;2(11):e329. doi: 10.1371/journal.pbio.0020329 (PMC524270; doi:10.1371/journal.pbio.0020329)

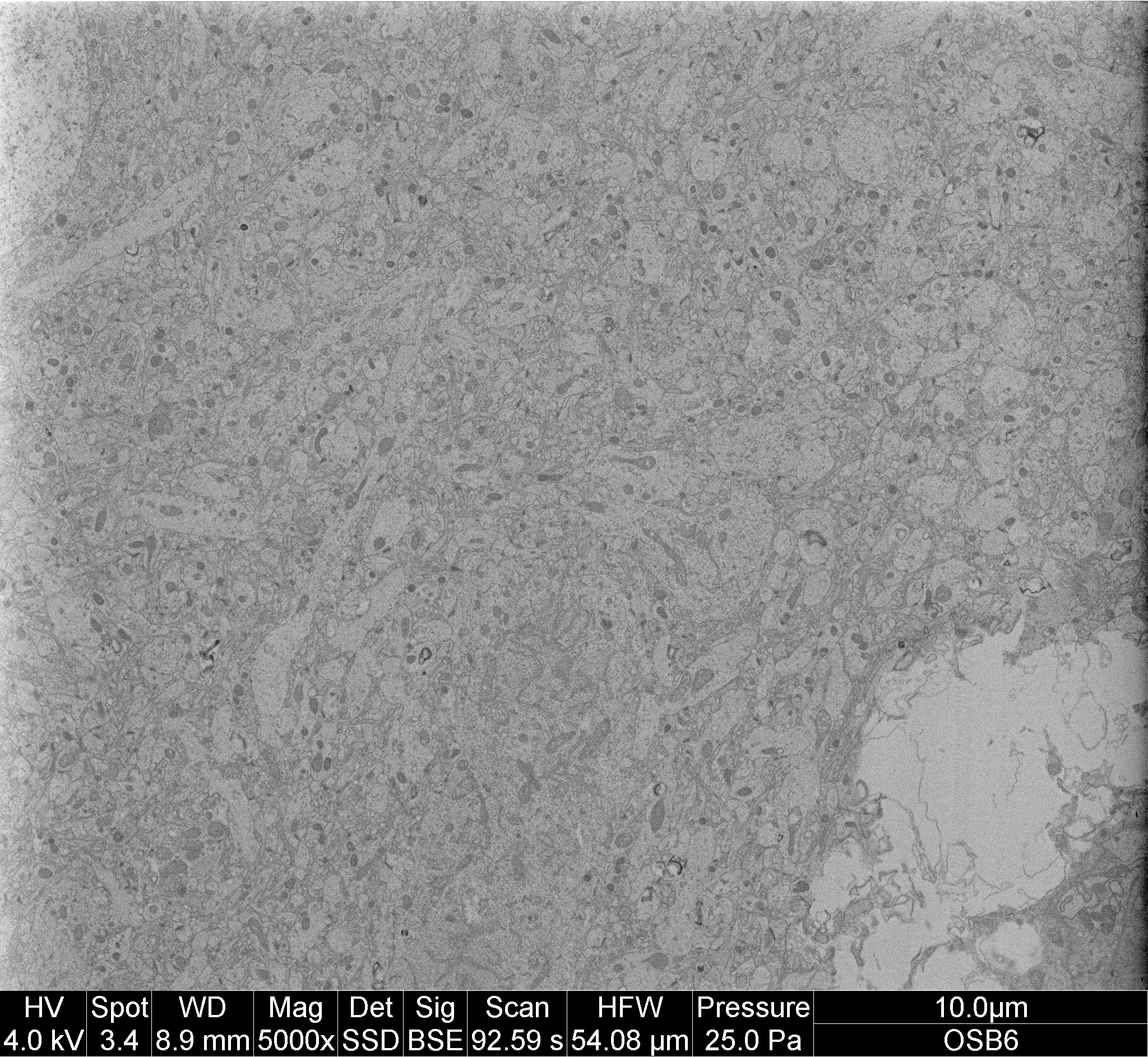

Supplement: Dataset S3 — (252.7 MB ZIP). [file pbio.0020329.sd003.zip › 040604_OS5_st1_201.tif]

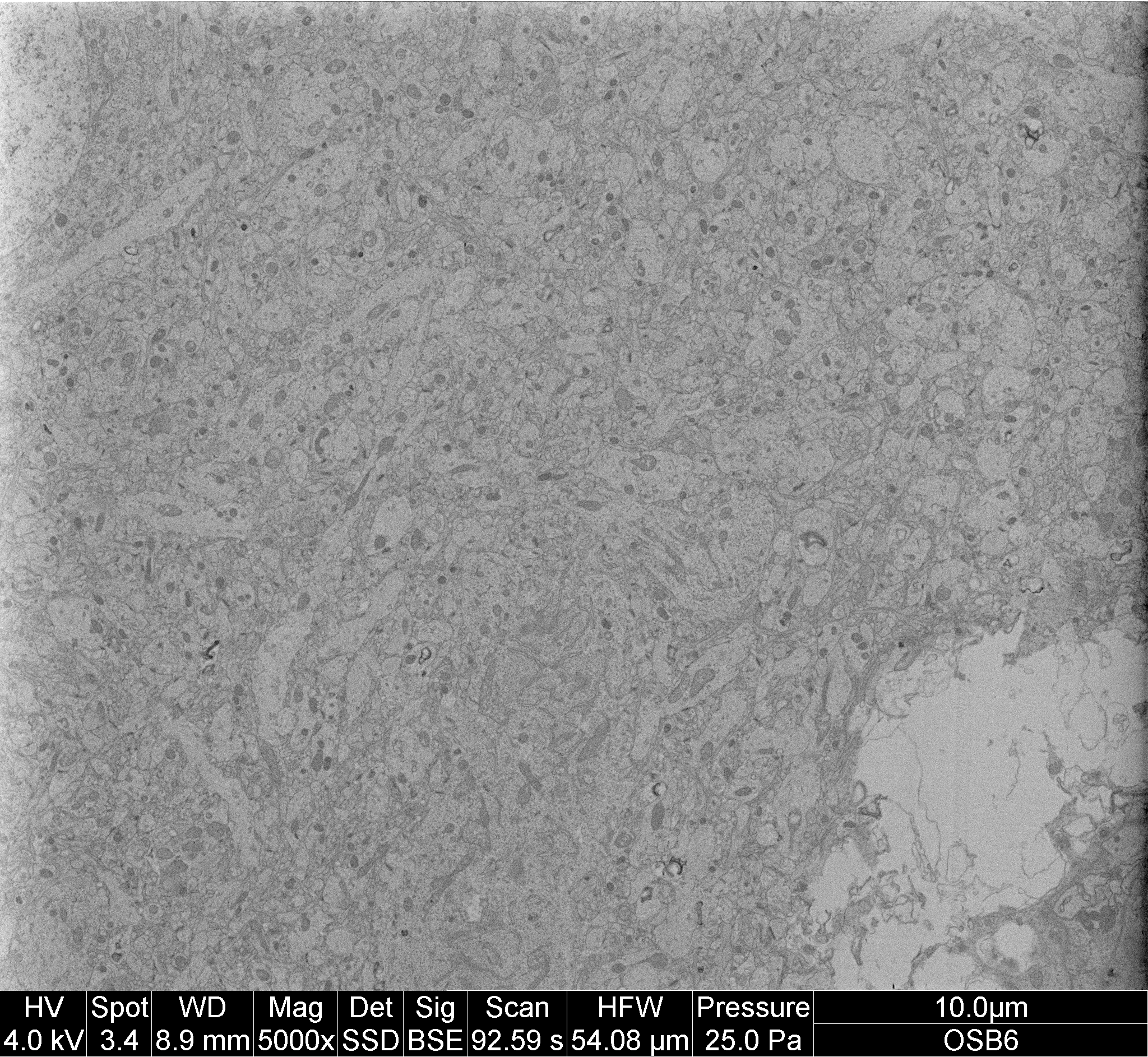

Supplement: Dataset S3 — (252.7 MB ZIP). [file pbio.0020329.sd003.zip › 040604_OS5_st1_202.tif]

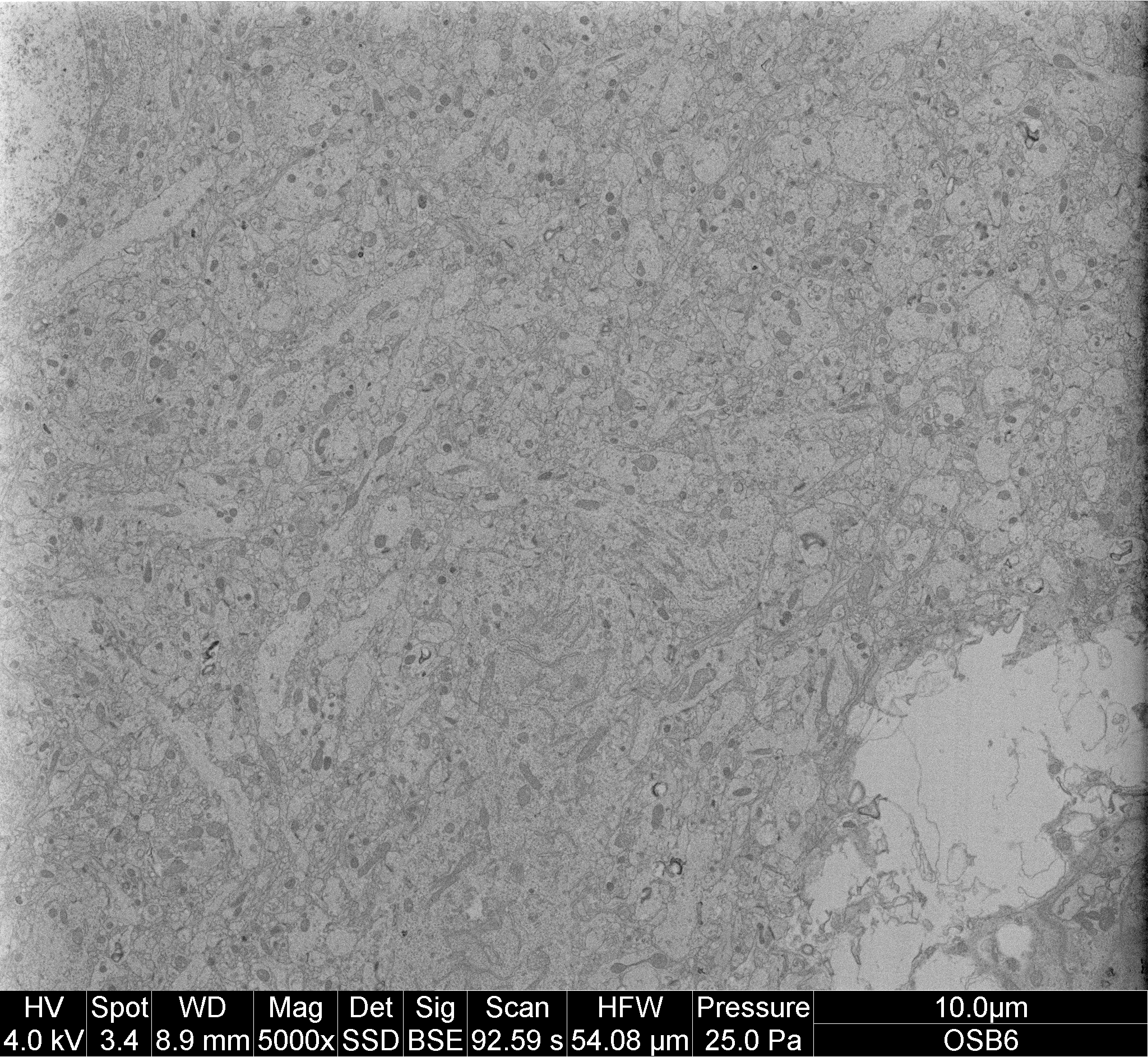

Supplement: Dataset S3 — (252.7 MB ZIP). [file pbio.0020329.sd003.zip › 040604_OS5_st1_203.tif]

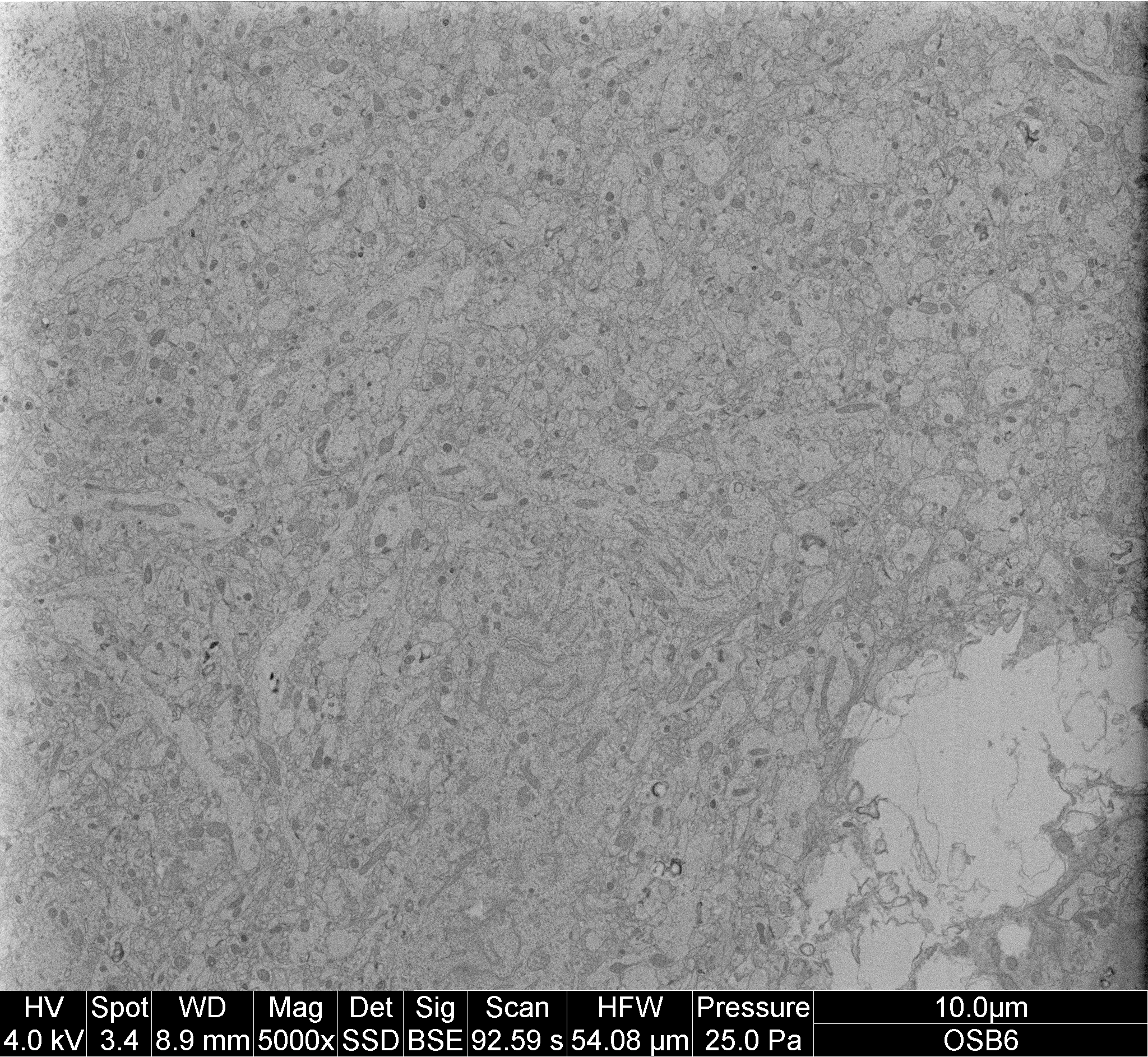

Supplement: Dataset S3 — (252.7 MB ZIP). [file pbio.0020329.sd003.zip › 040604_OS5_st1_204.tif]

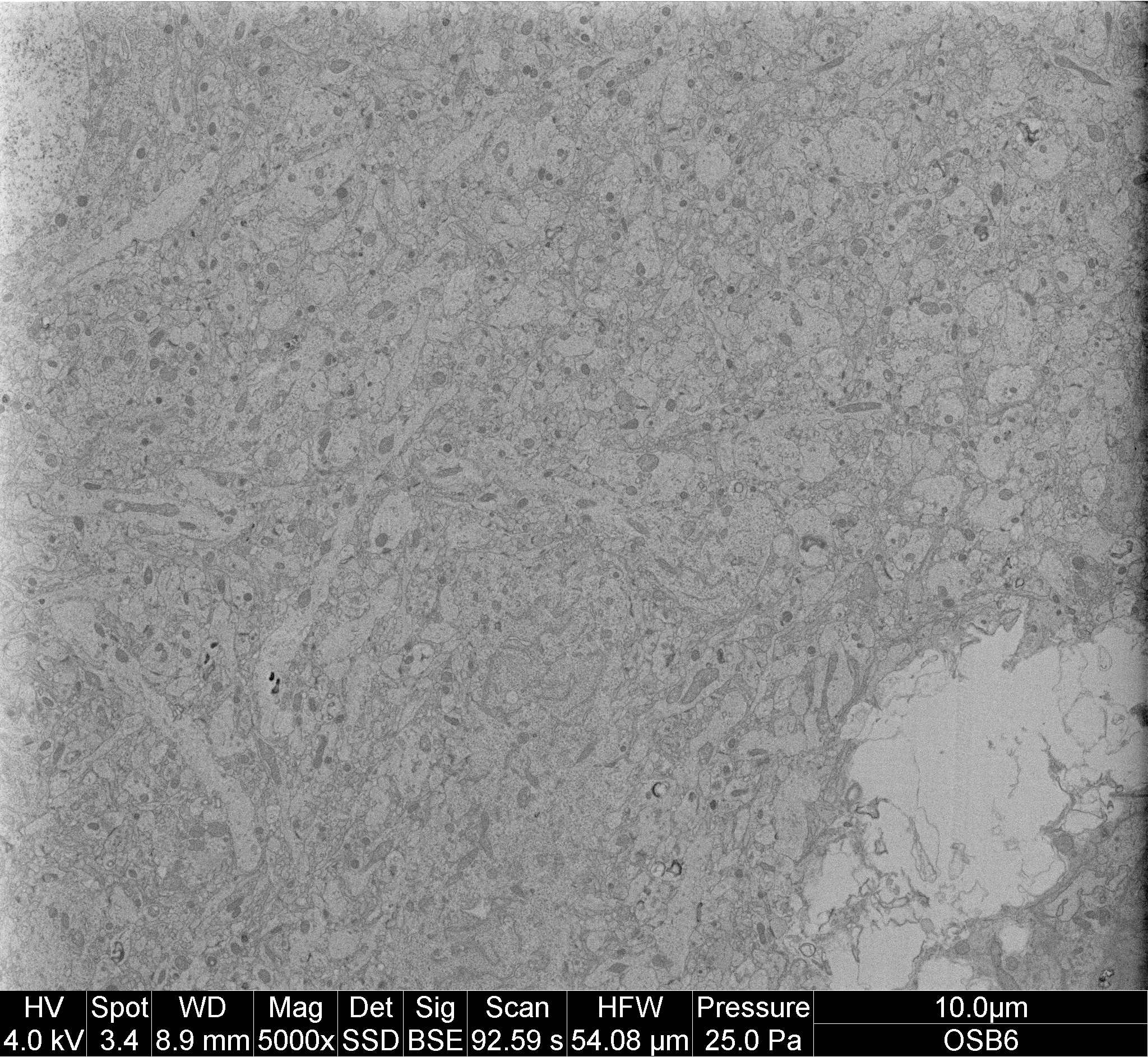

Supplement: Dataset S3 — (252.7 MB ZIP). [file pbio.0020329.sd003.zip › 040604_OS5_st1_205.tif]

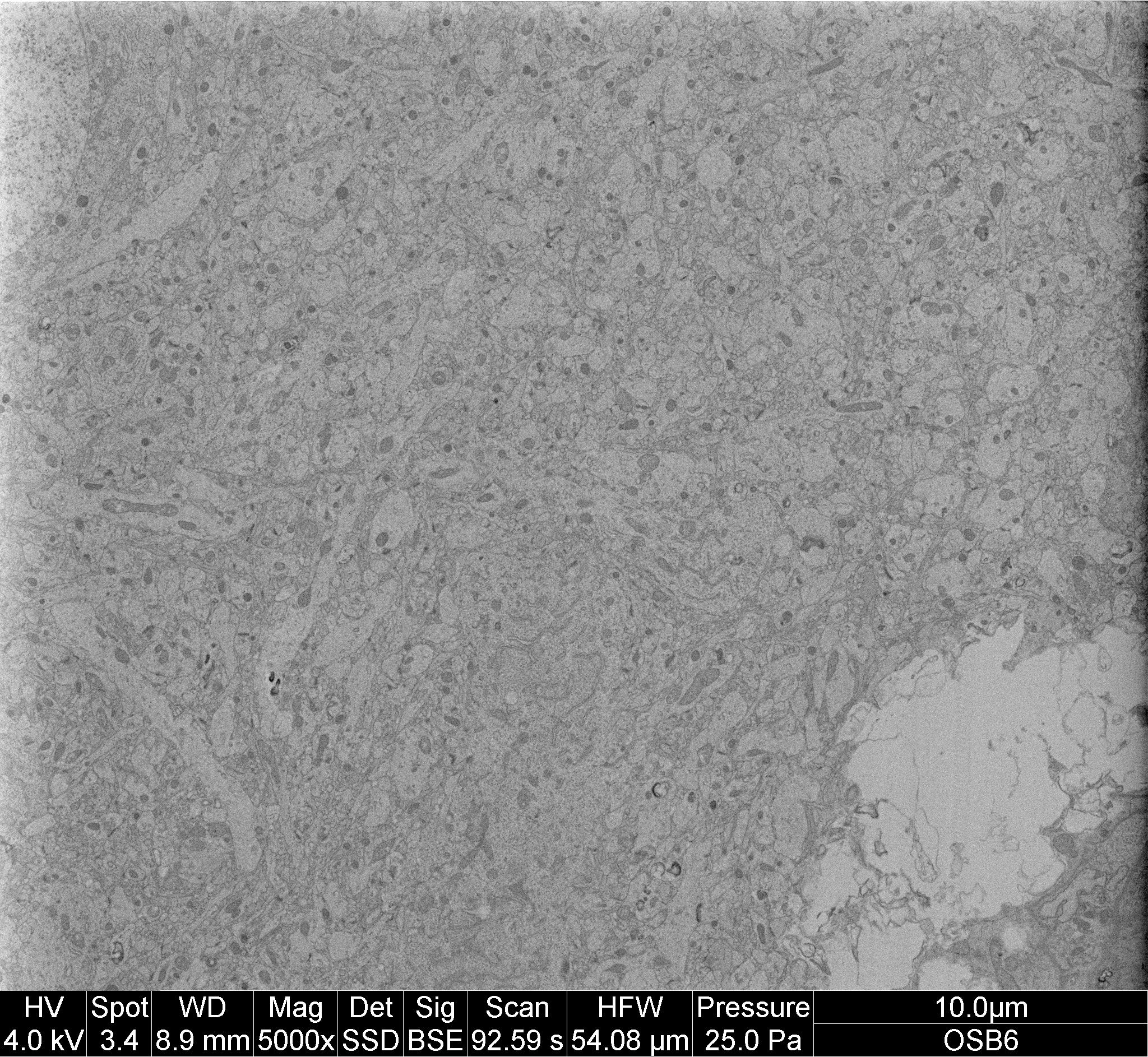

Supplement: Dataset S3 — (252.7 MB ZIP). [file pbio.0020329.sd003.zip › 040604_OS5_st1_206.tif]

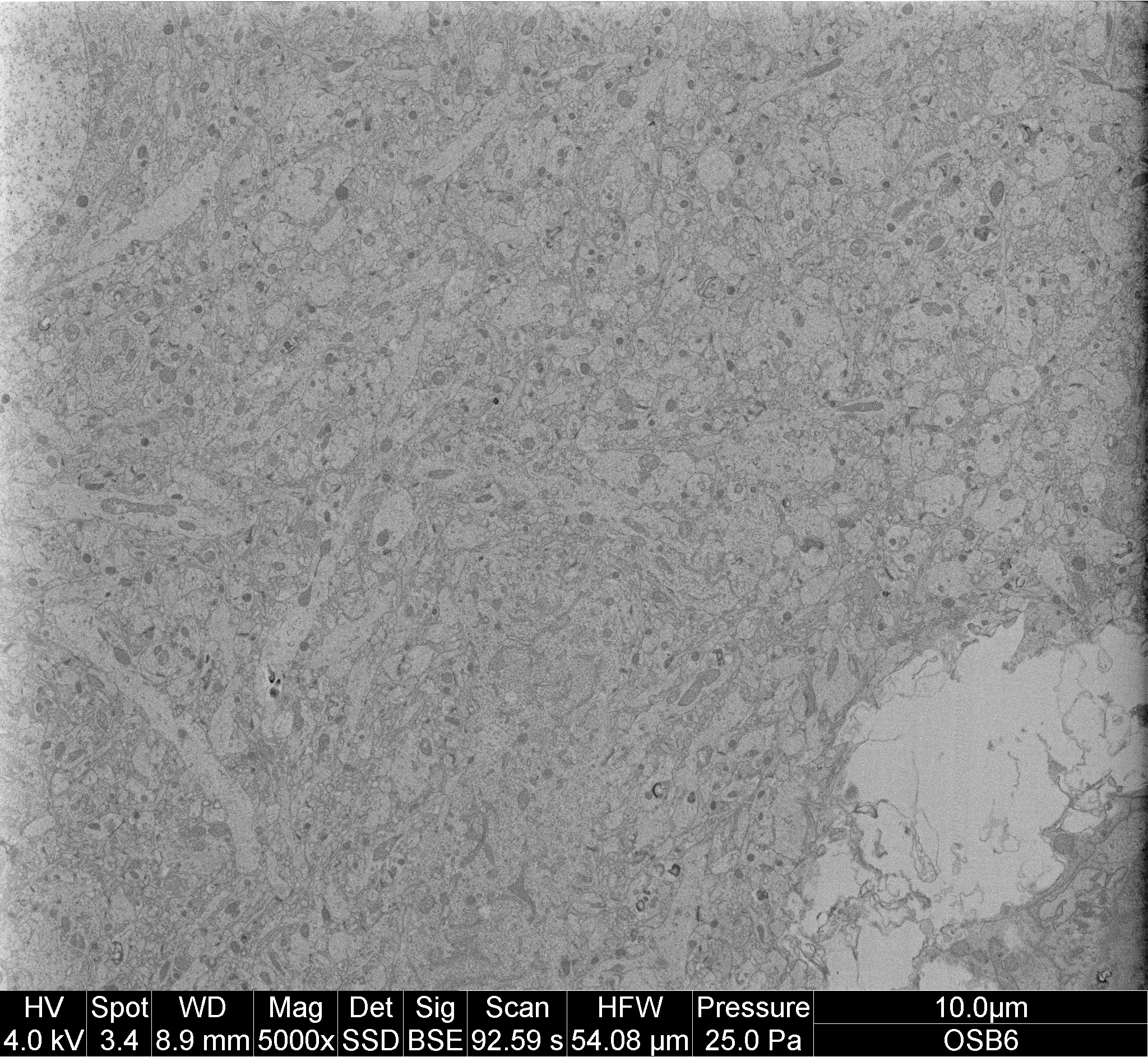

Supplement: Dataset S3 — (252.7 MB ZIP). [file pbio.0020329.sd003.zip › 040604_OS5_st1_207.tif]

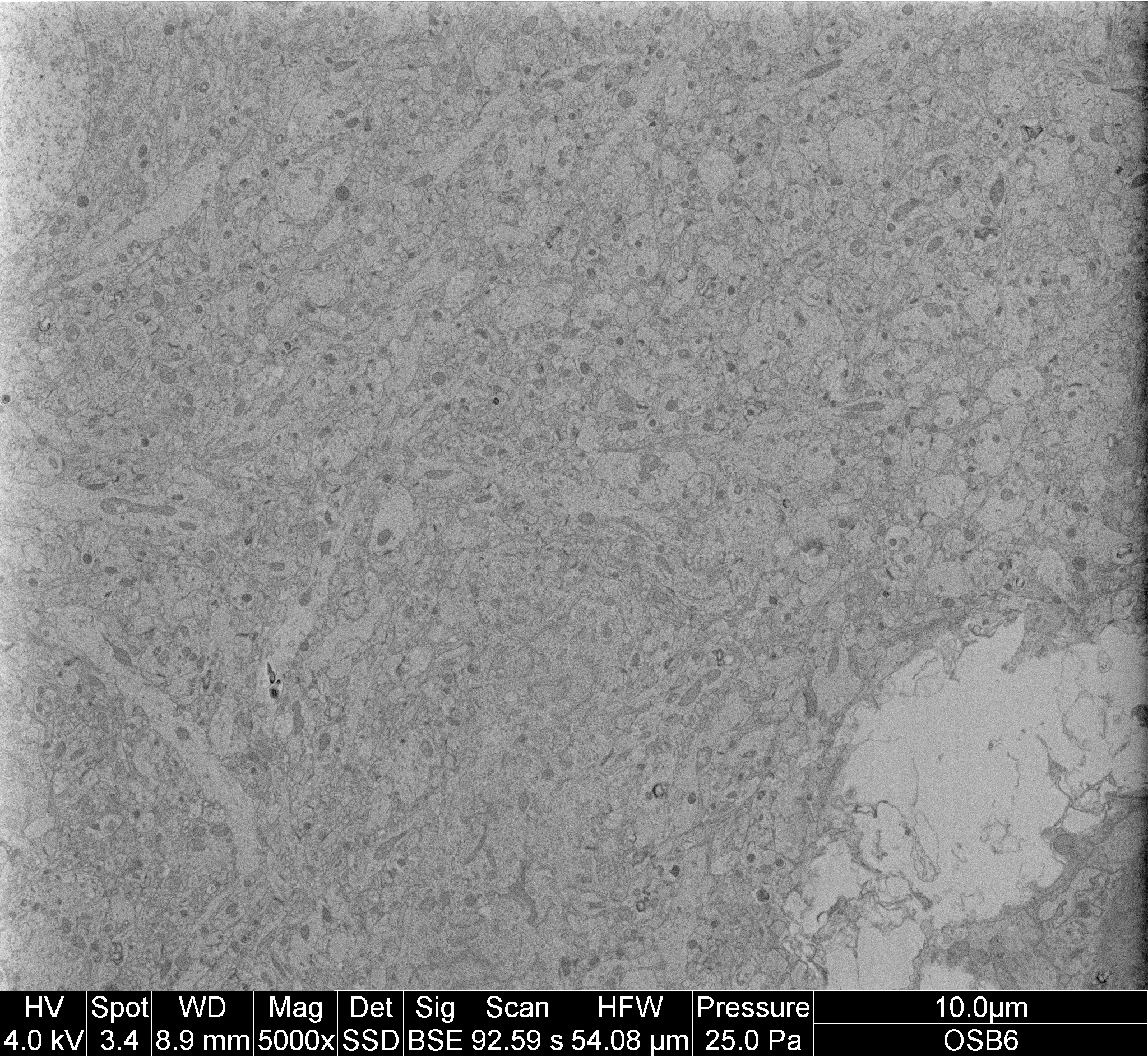

Supplement: Dataset S3 — (252.7 MB ZIP). [file pbio.0020329.sd003.zip › 040604_OS5_st1_208.tif]

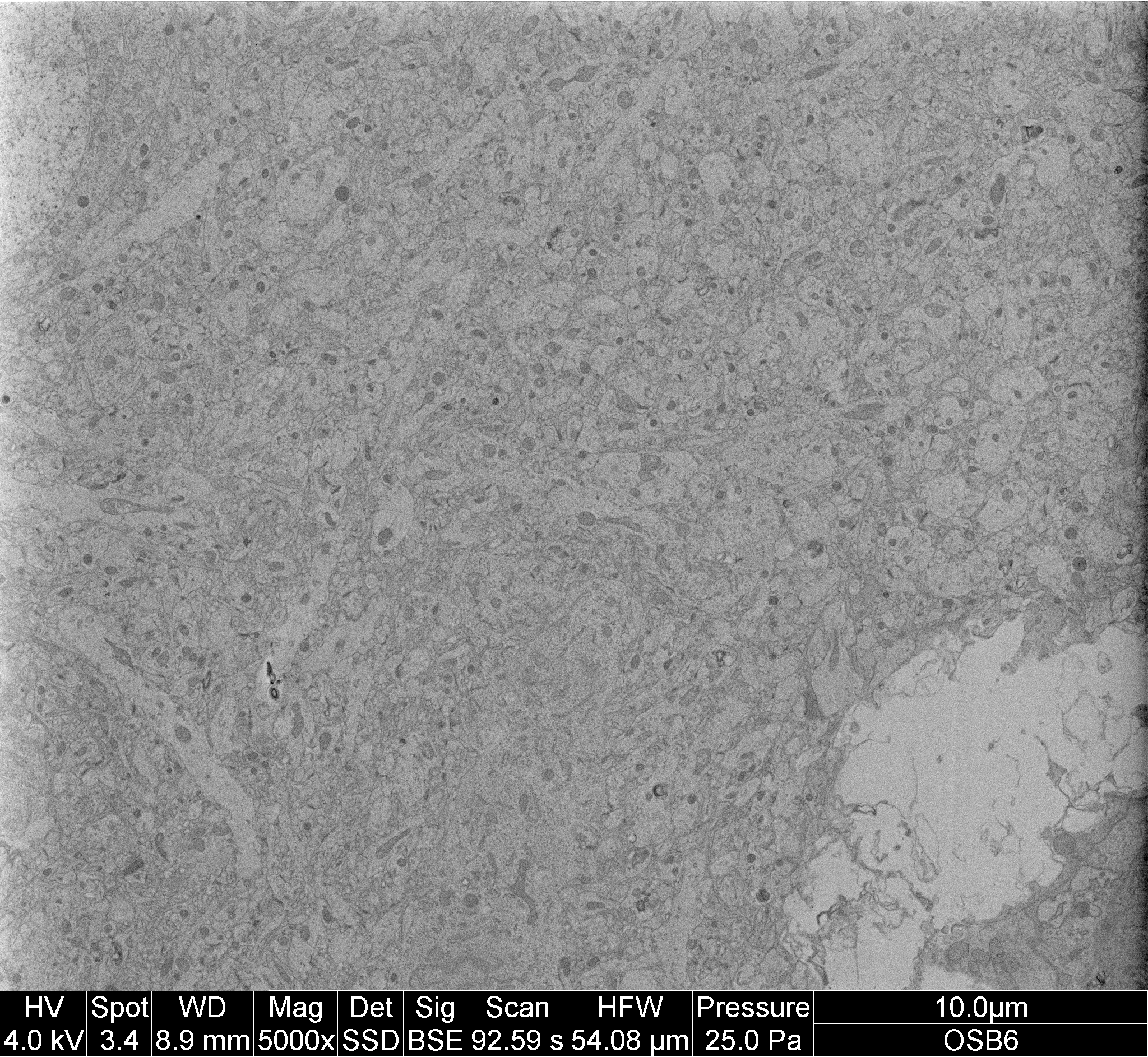

Supplement: Dataset S3 — (252.7 MB ZIP). [file pbio.0020329.sd003.zip › 040604_OS5_st1_209.tif]

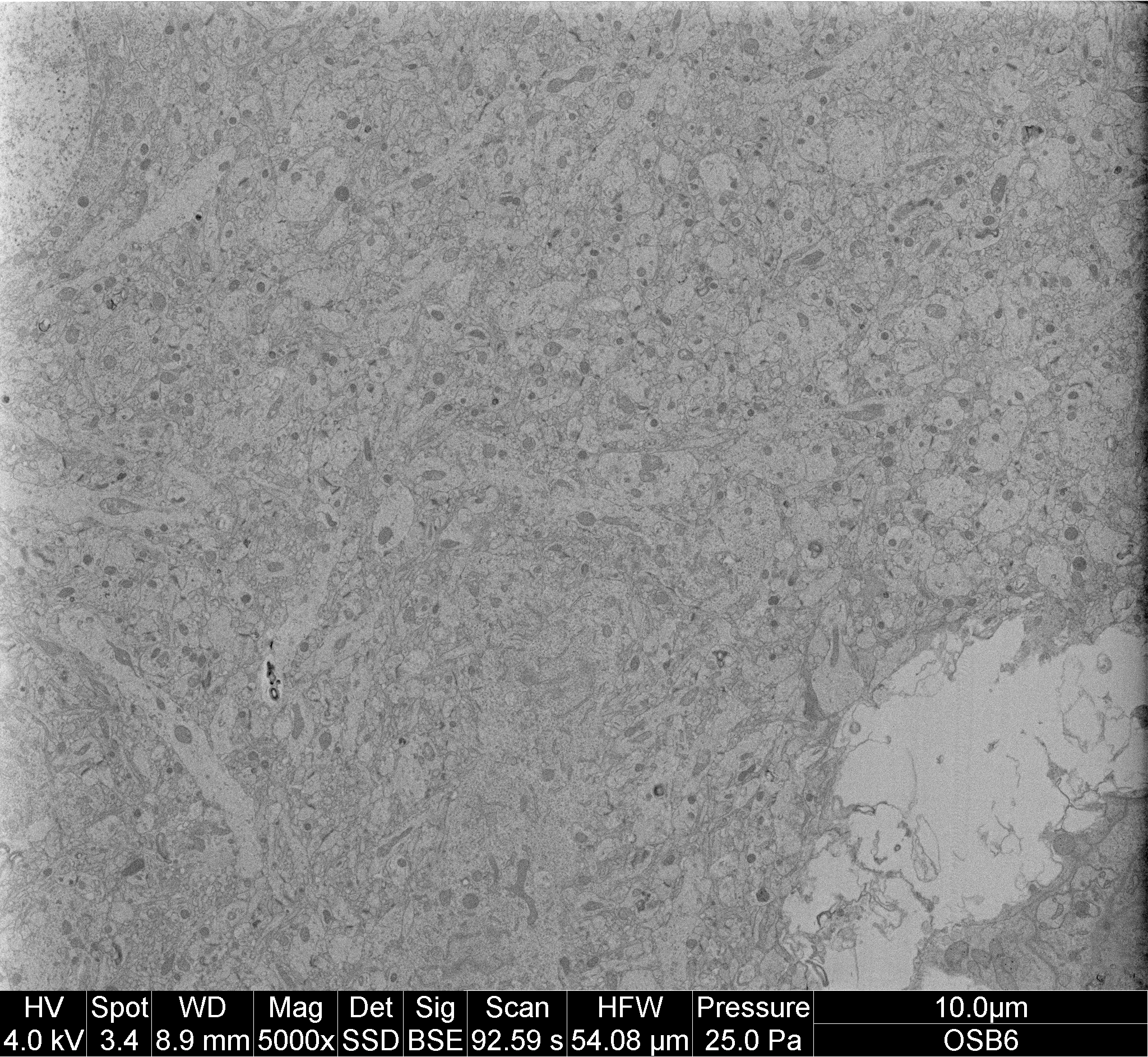

Supplement: Dataset S3 — (252.7 MB ZIP). [file pbio.0020329.sd003.zip › 040604_OS5_st1_210.tif]

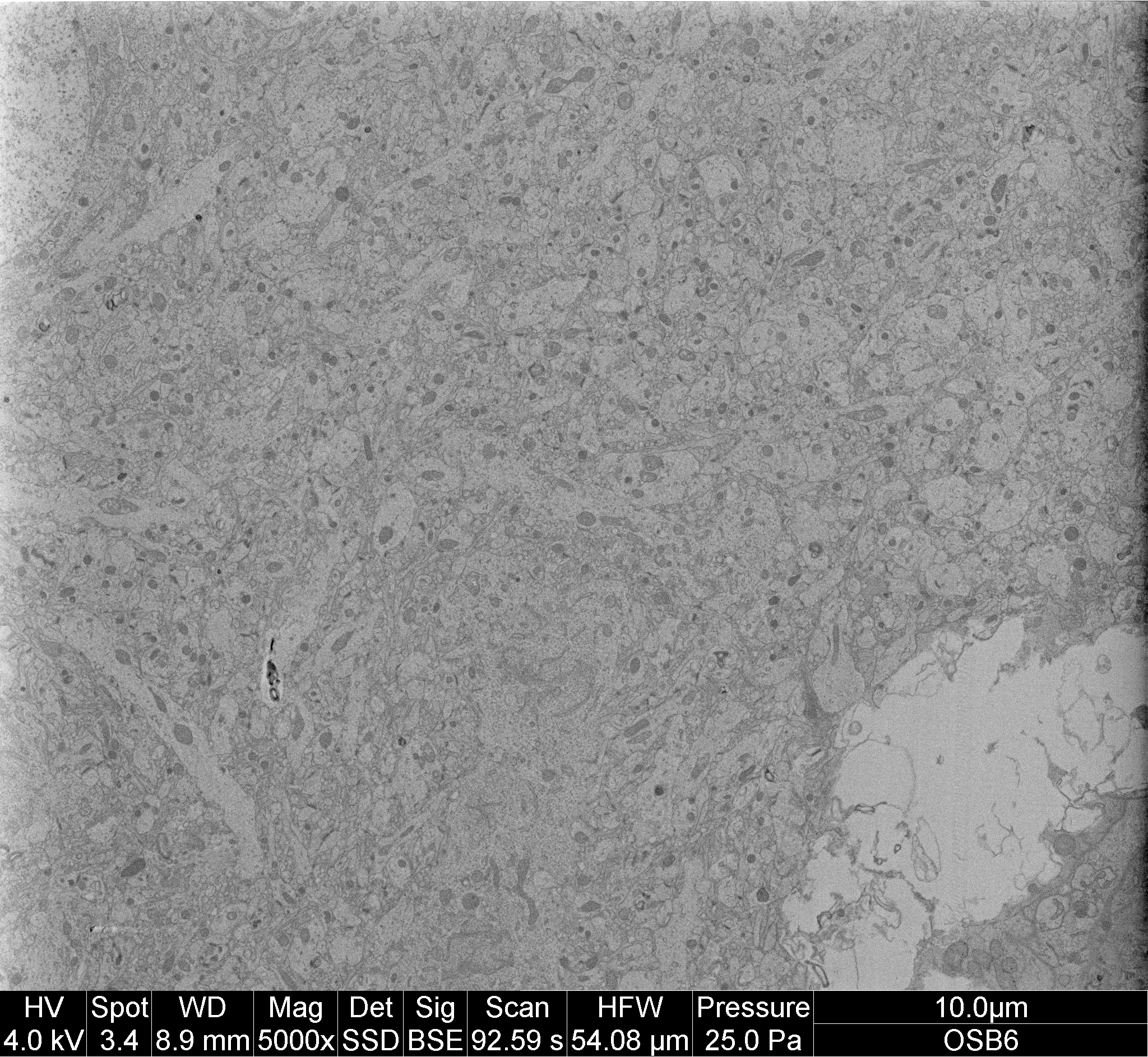

Supplement: Dataset S3 — (252.7 MB ZIP). [file pbio.0020329.sd003.zip › 040604_OS5_st1_211.tif]

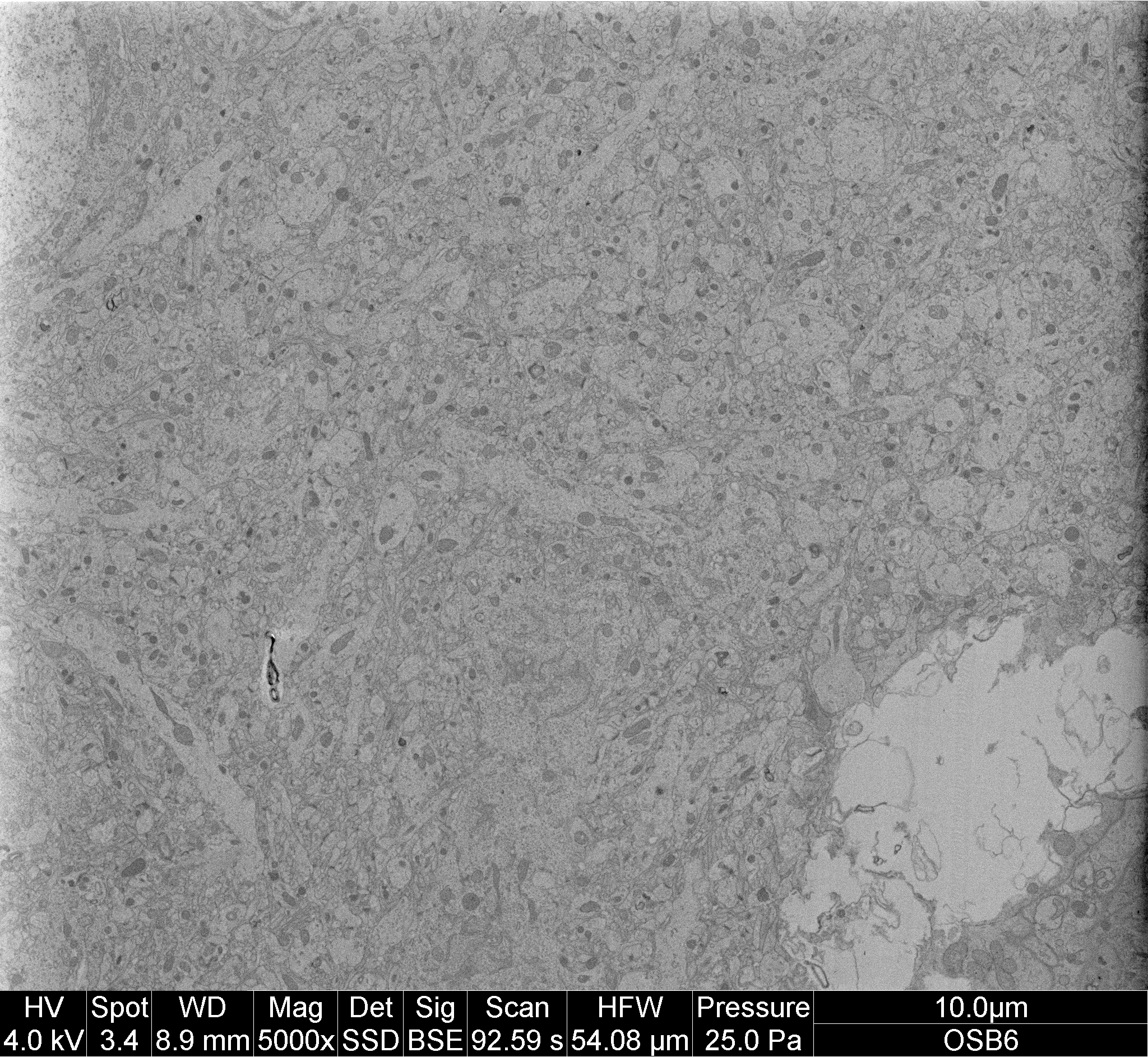

Supplement: Dataset S3 — (252.7 MB ZIP). [file pbio.0020329.sd003.zip › 040604_OS5_st1_212.tif]

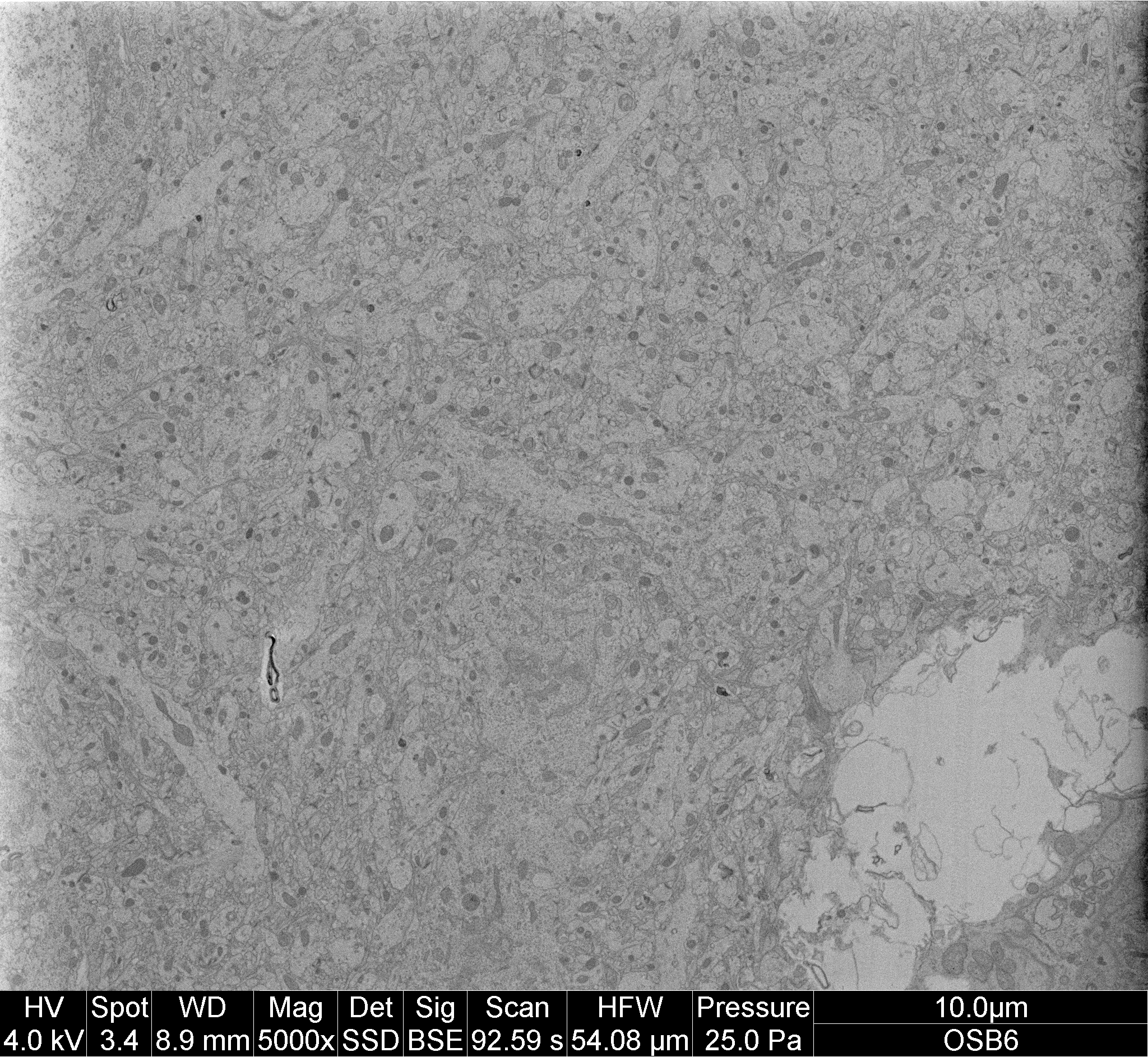

Supplement: Dataset S3 — (252.7 MB ZIP). [file pbio.0020329.sd003.zip › 040604_OS5_st1_213.tif]

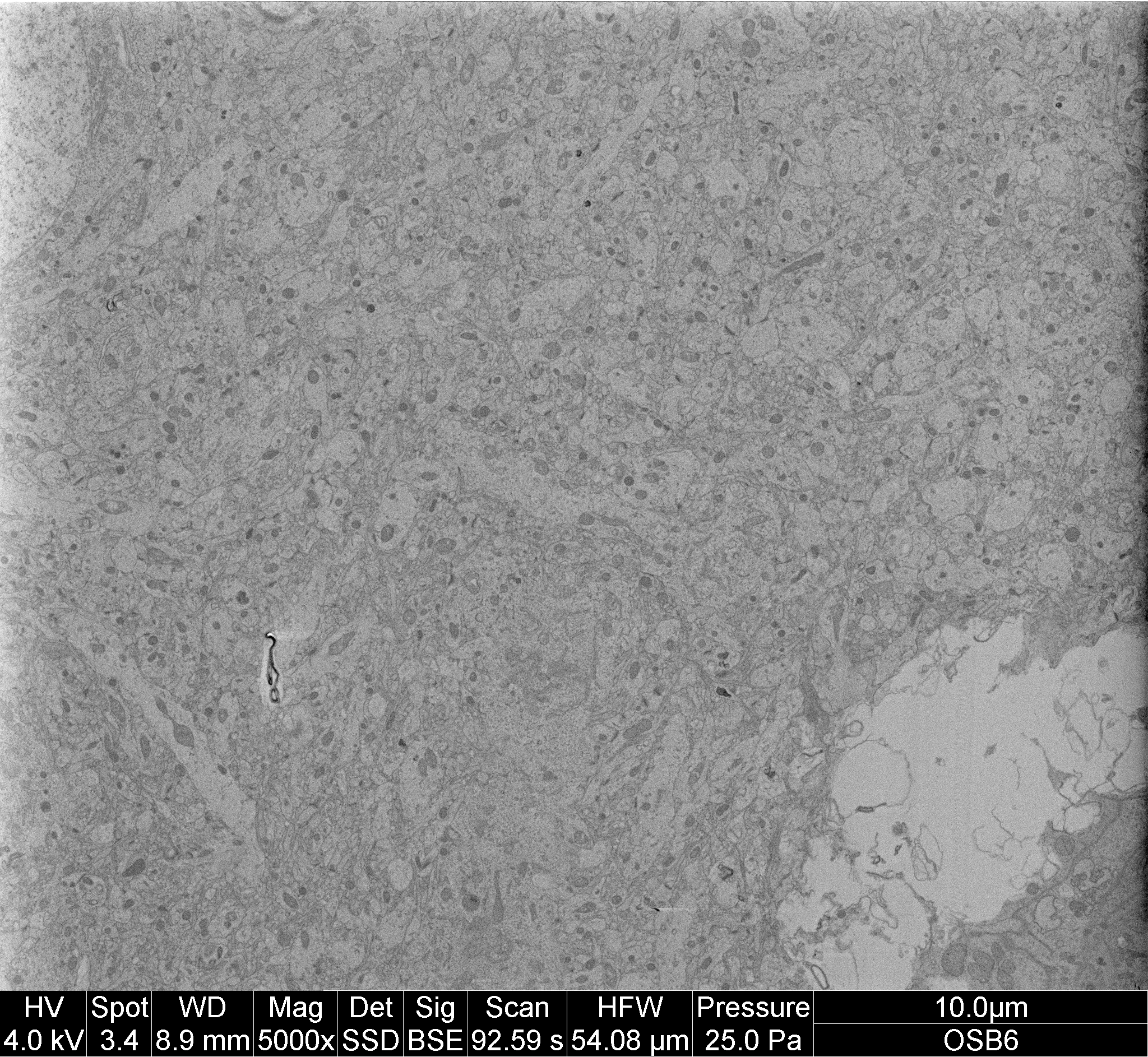

Supplement: Dataset S3 — (252.7 MB ZIP). [file pbio.0020329.sd003.zip › 040604_OS5_st1_214.tif]

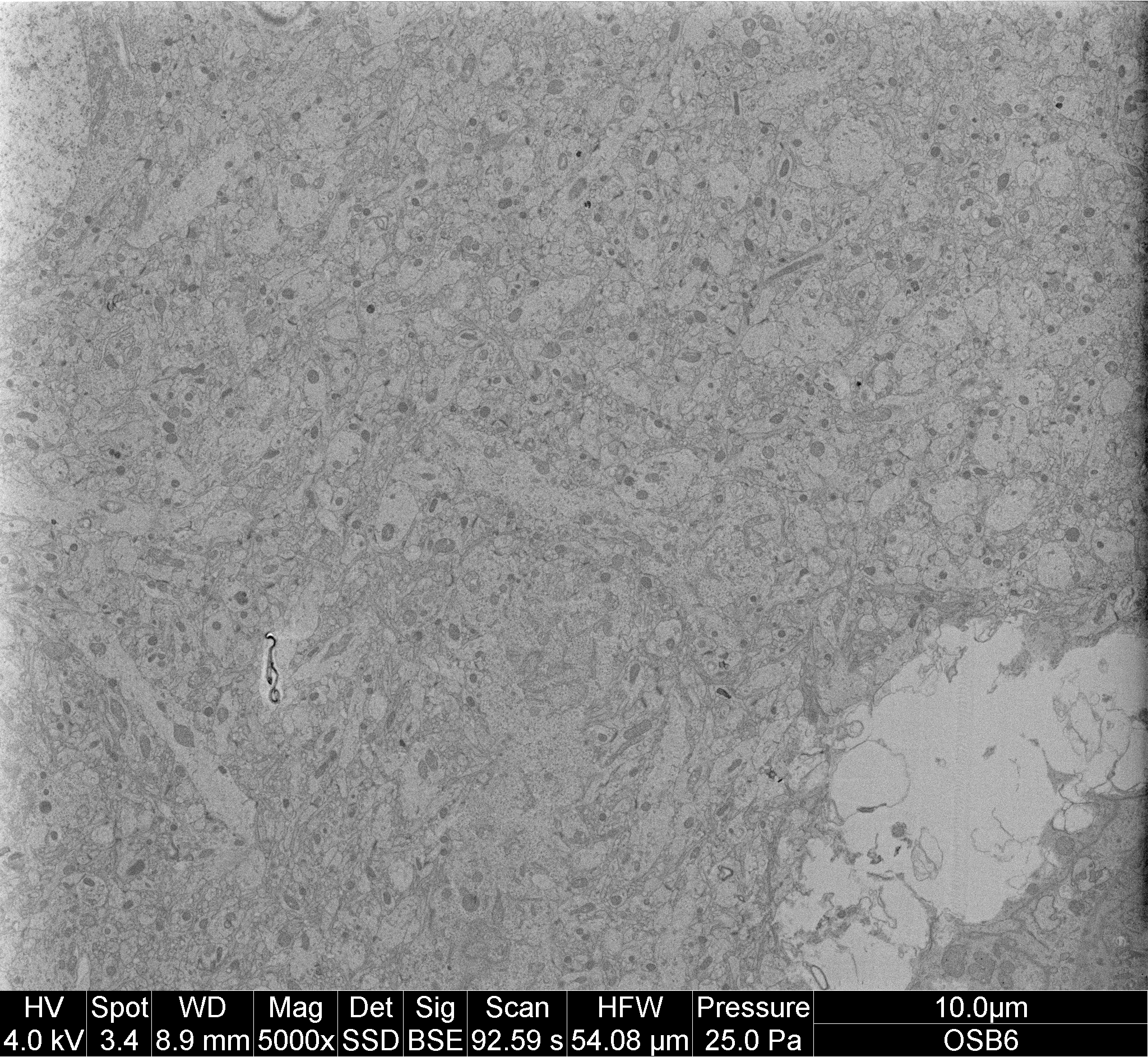

Supplement: Dataset S3 — (252.7 MB ZIP). [file pbio.0020329.sd003.zip › 040604_OS5_st1_215.tif]

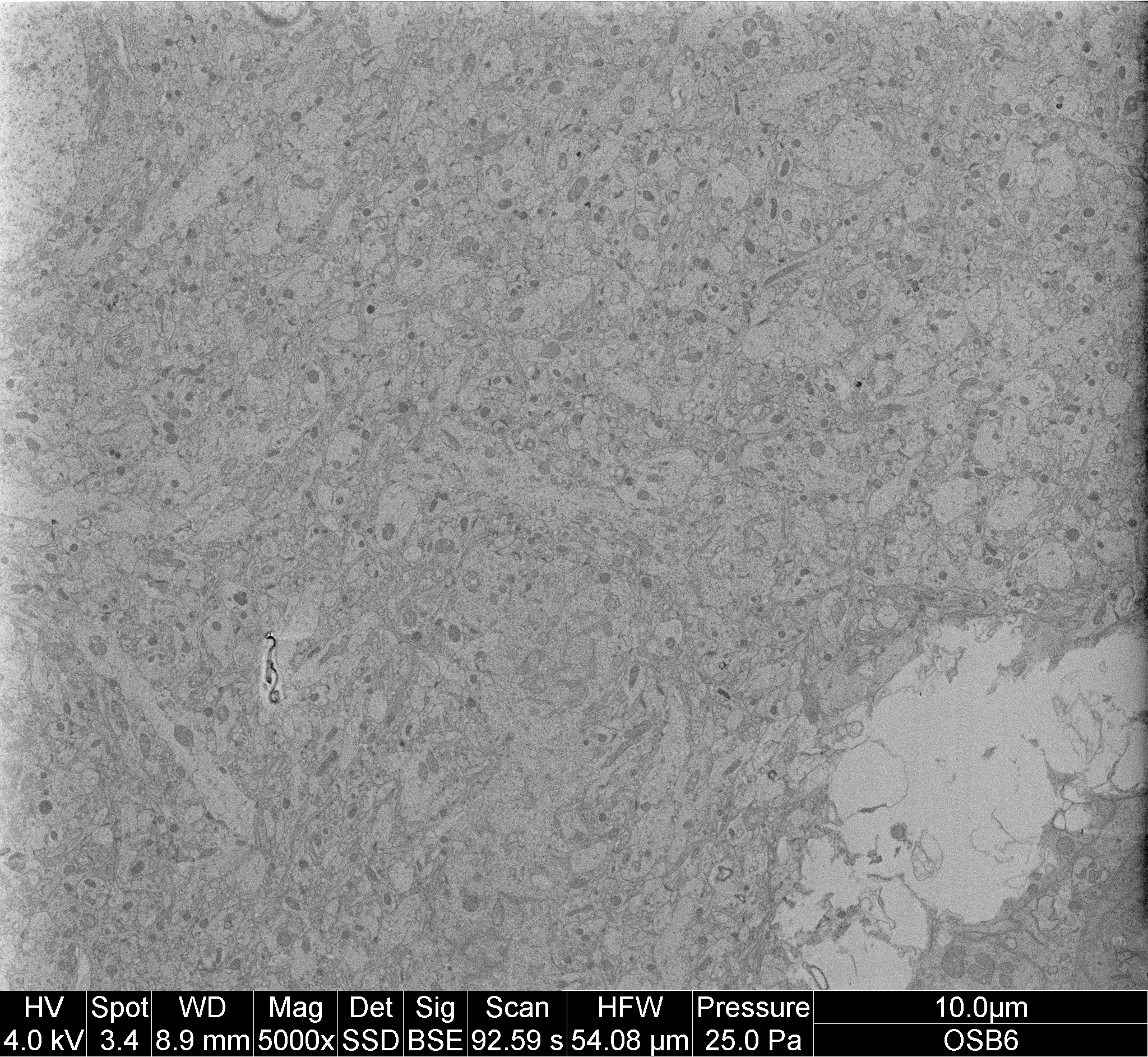

Supplement: Dataset S3 — (252.7 MB ZIP). [file pbio.0020329.sd003.zip › 040604_OS5_st1_216.tif]

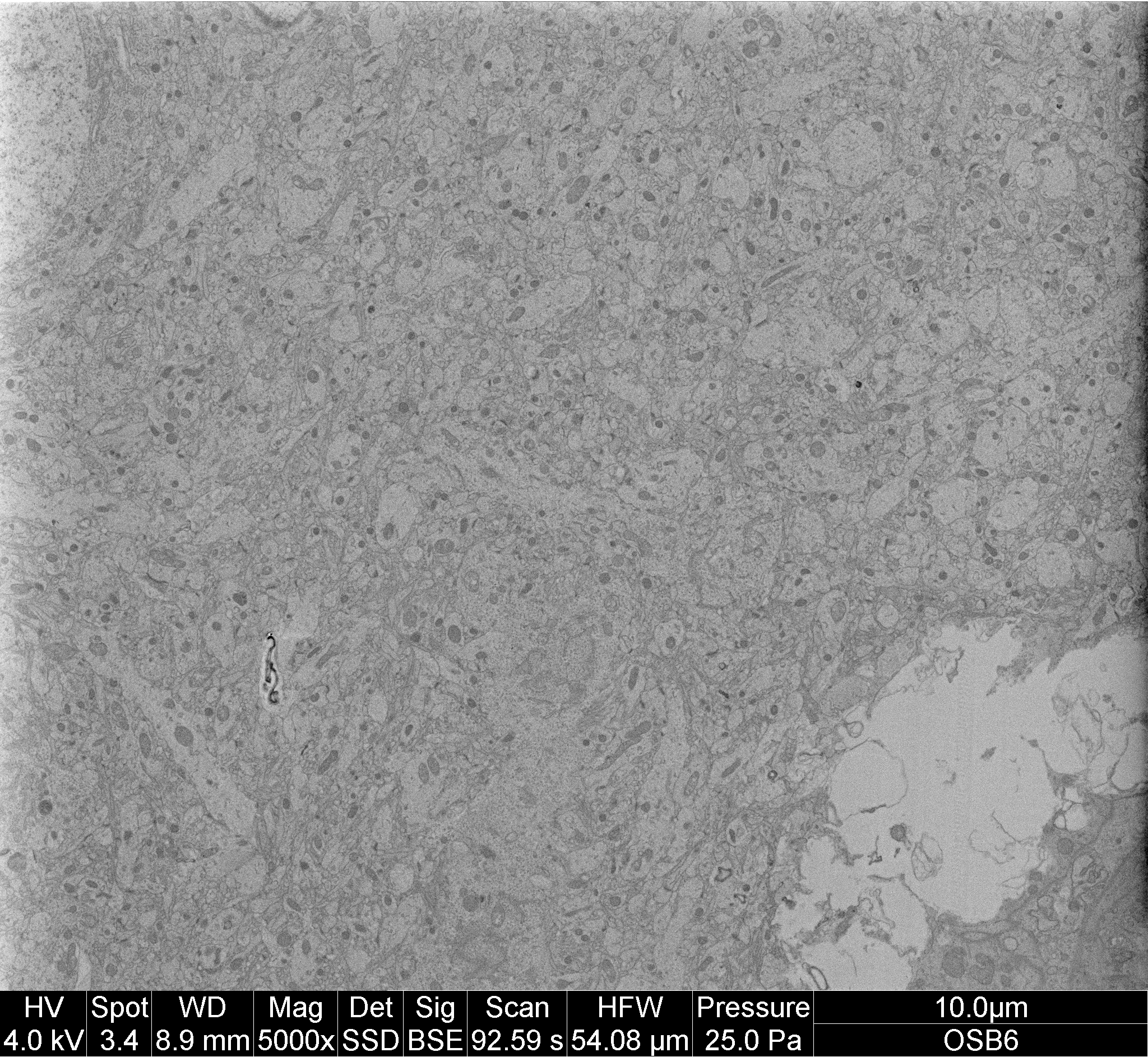

Supplement: Dataset S3 — (252.7 MB ZIP). [file pbio.0020329.sd003.zip › 040604_OS5_st1_217.tif]

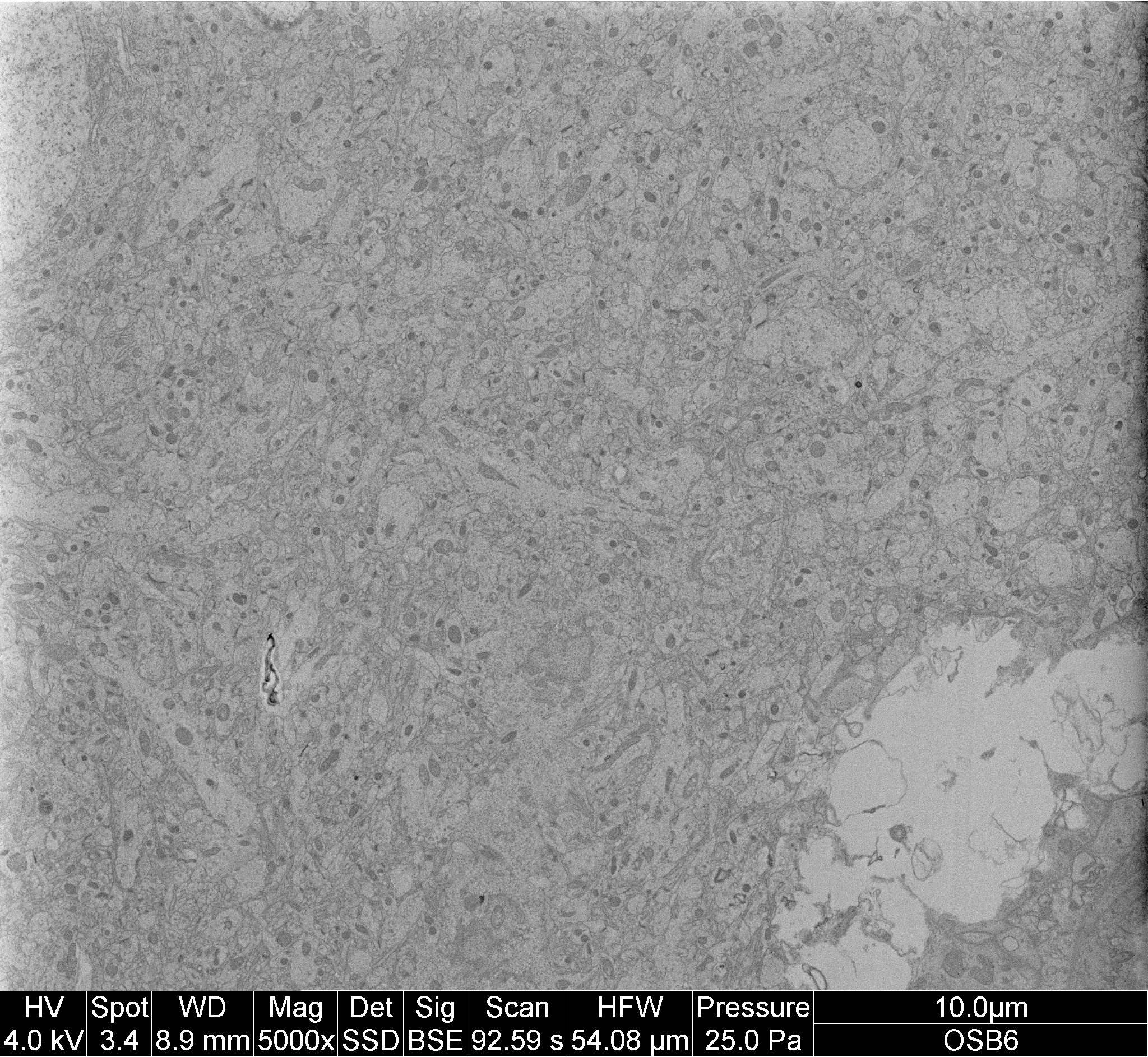

Supplement: Dataset S3 — (252.7 MB ZIP). [file pbio.0020329.sd003.zip › 040604_OS5_st1_218.tif]

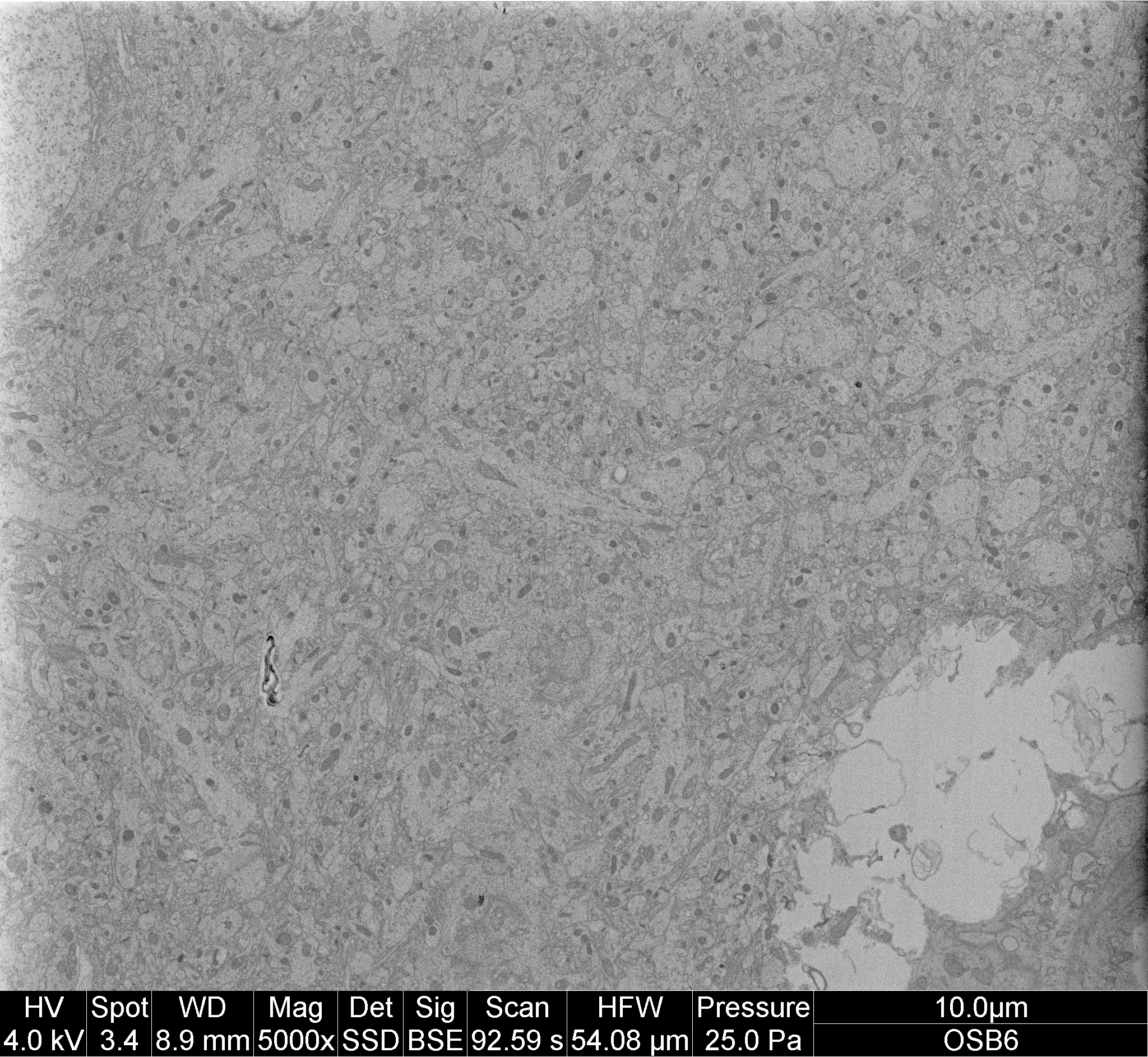

Supplement: Dataset S3 — (252.7 MB ZIP). [file pbio.0020329.sd003.zip › 040604_OS5_st1_219.tif]

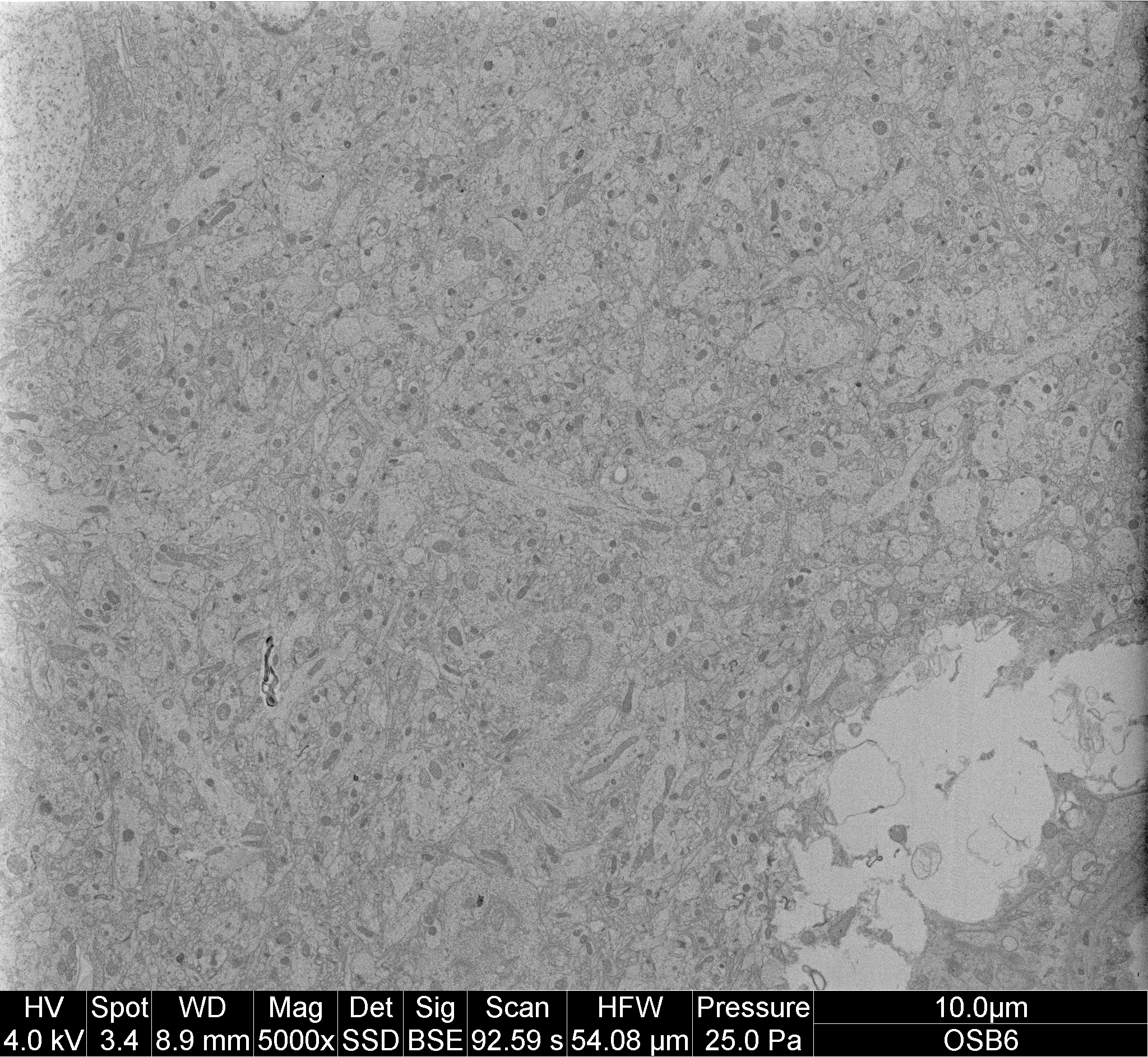

Supplement: Dataset S3 — (252.7 MB ZIP). [file pbio.0020329.sd003.zip › 040604_OS5_st1_220.tif]

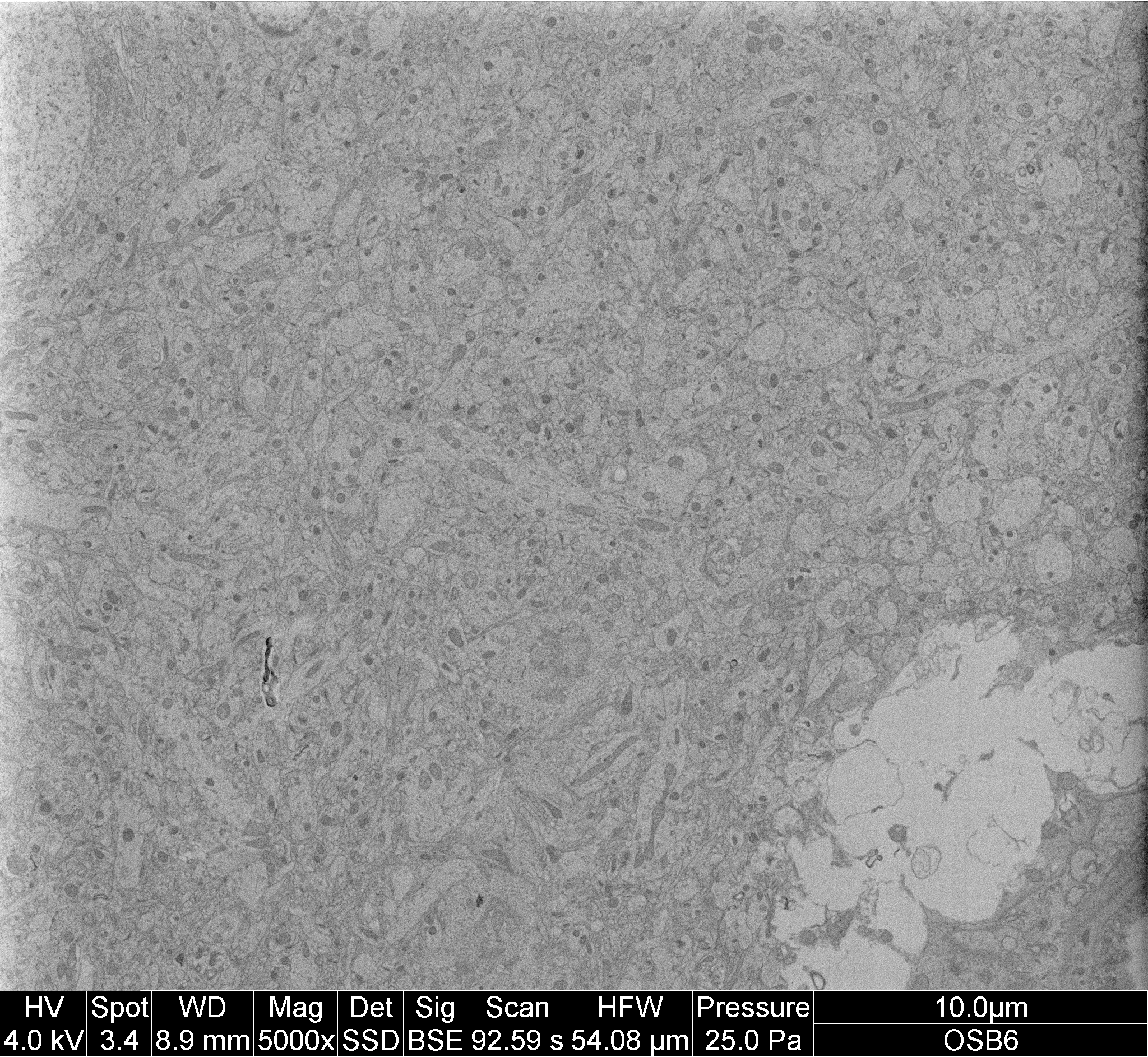

Supplement: Dataset S3 — (252.7 MB ZIP). [file pbio.0020329.sd003.zip › 040604_OS5_st1_221.tif]

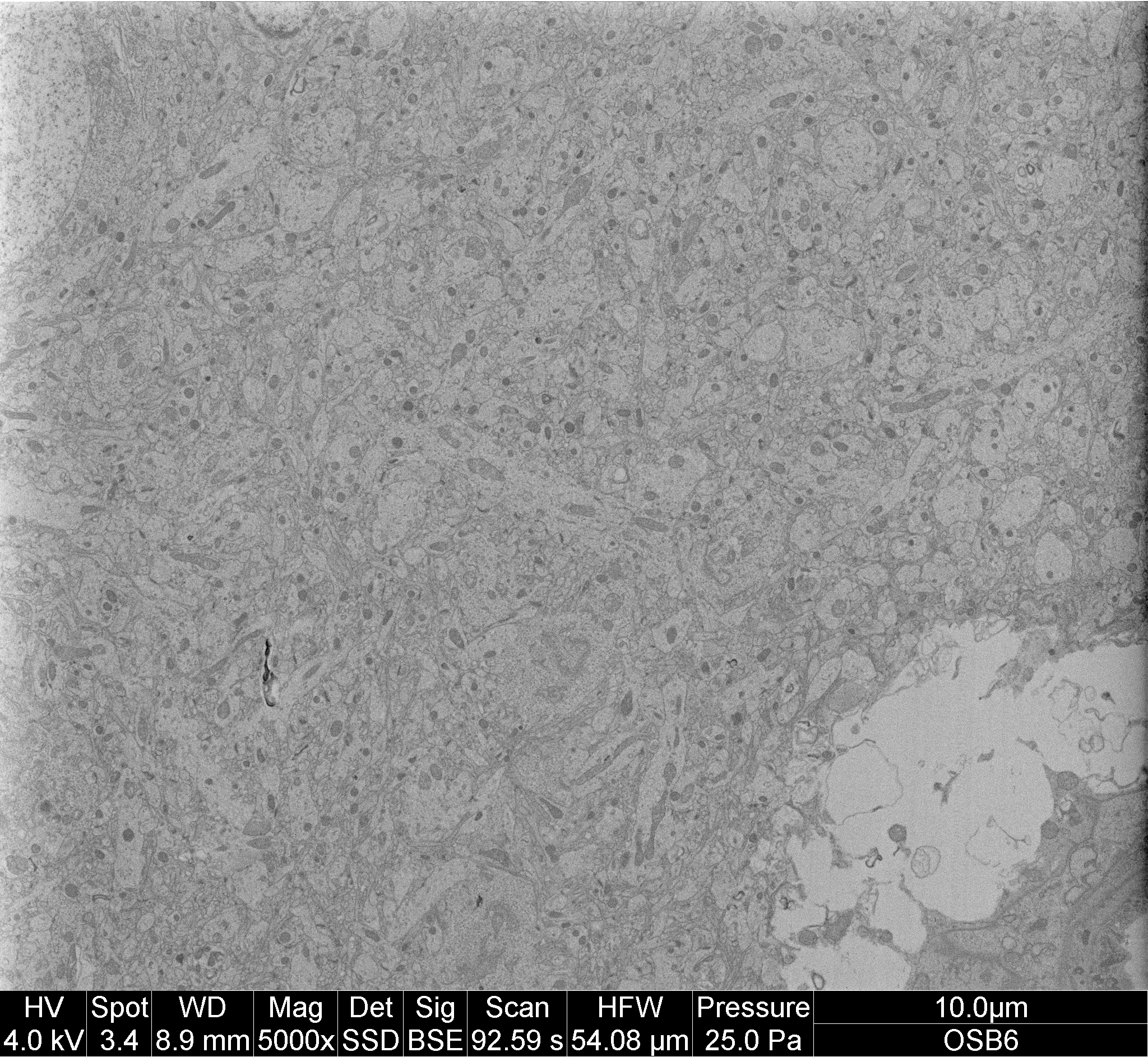

Supplement: Dataset S3 — (252.7 MB ZIP). [file pbio.0020329.sd003.zip › 040604_OS5_st1_222.tif]

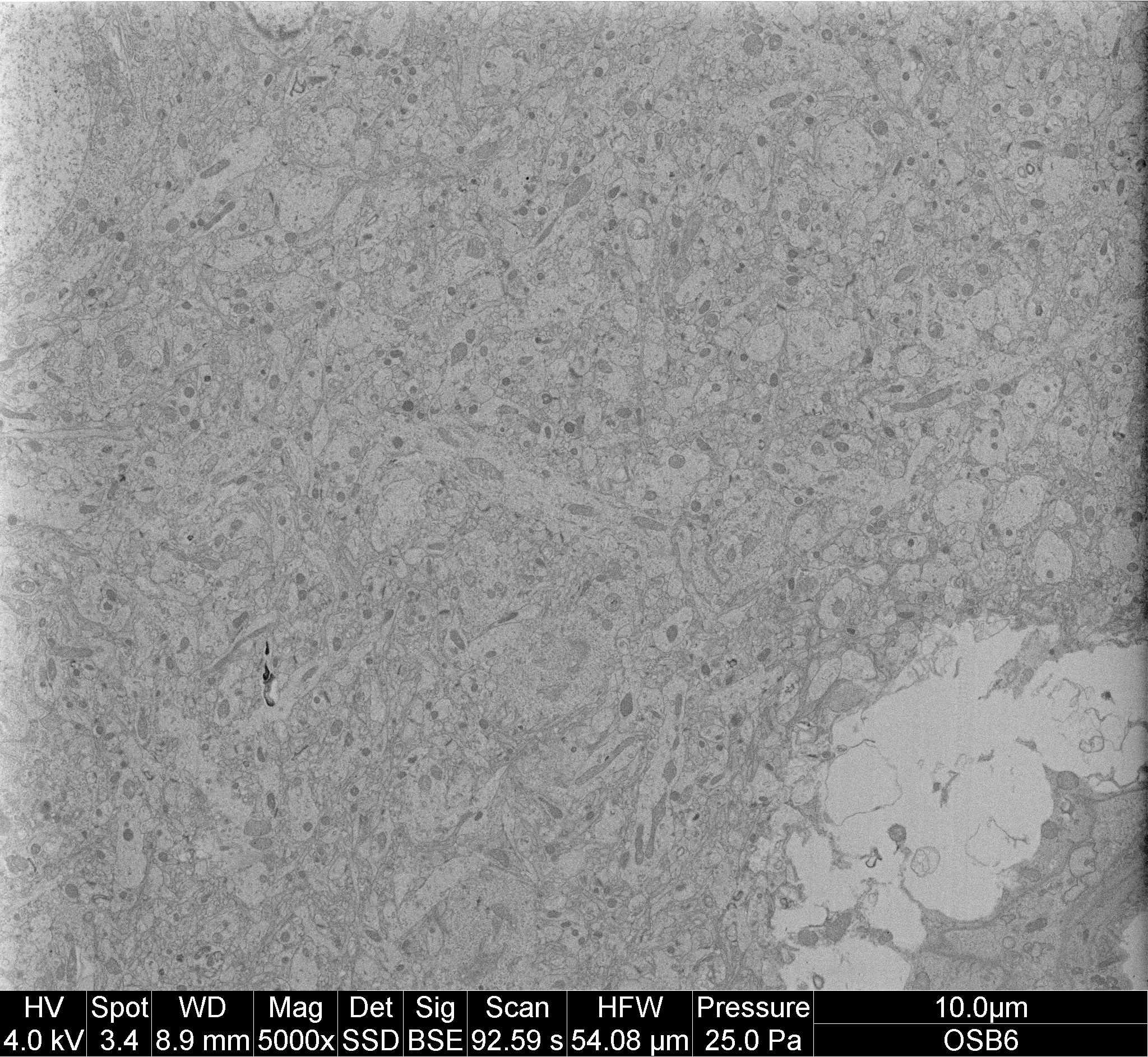

Supplement: Dataset S3 — (252.7 MB ZIP). [file pbio.0020329.sd003.zip › 040604_OS5_st1_223.tif]

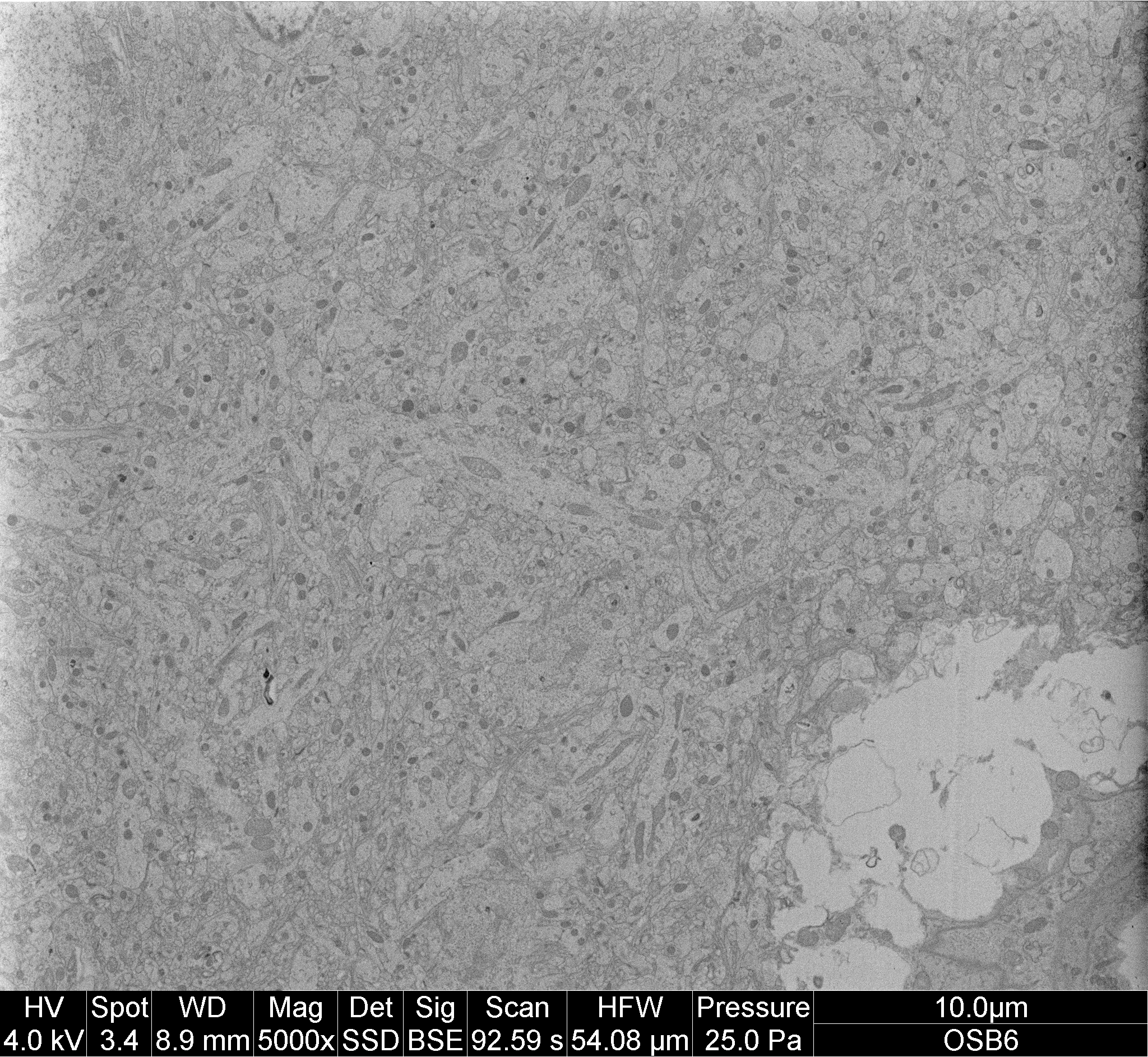

Supplement: Dataset S3 — (252.7 MB ZIP). [file pbio.0020329.sd003.zip › 040604_OS5_st1_224.tif]

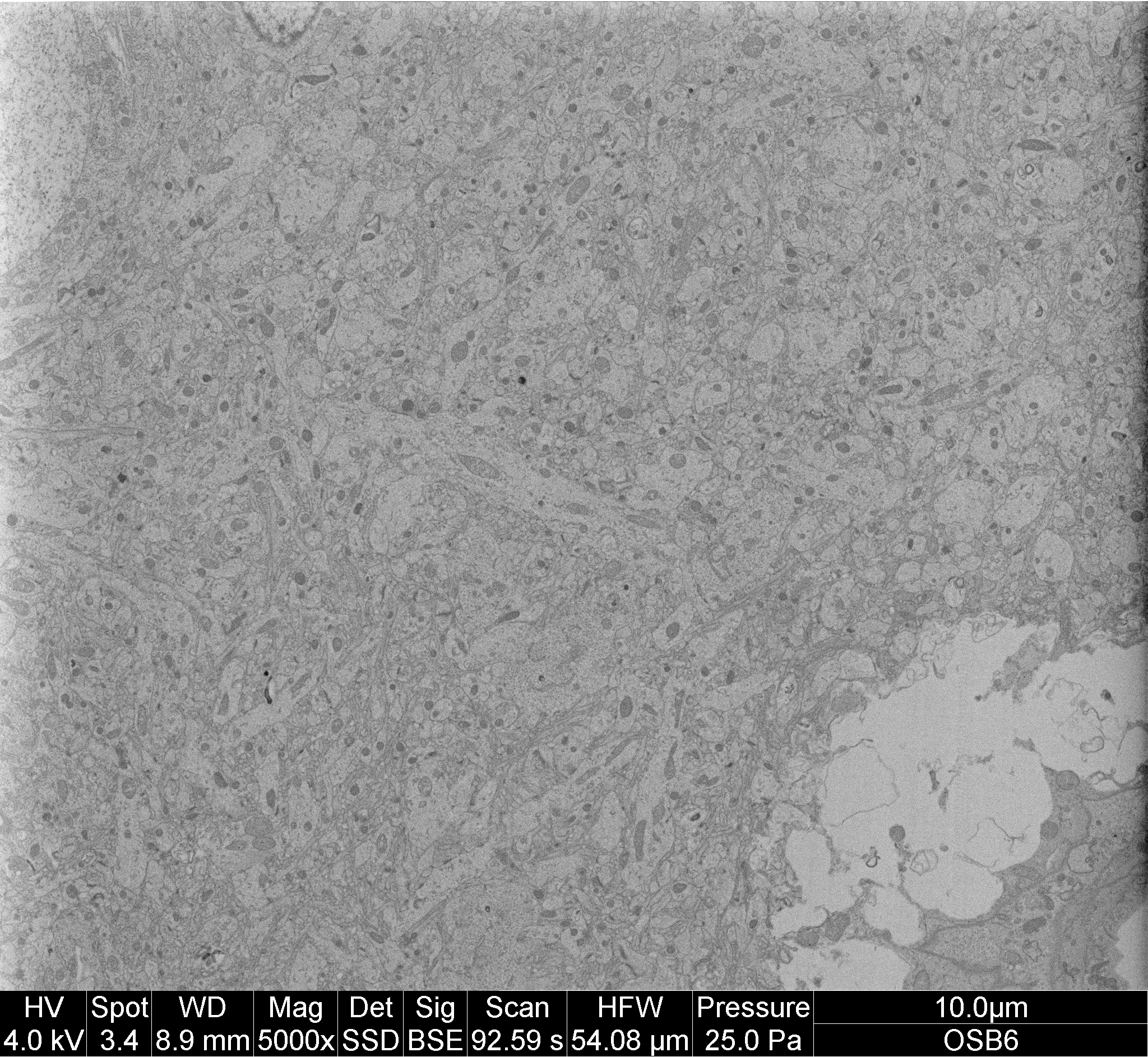

Supplement: Dataset S3 — (252.7 MB ZIP). [file pbio.0020329.sd003.zip › 040604_OS5_st1_225.tif]

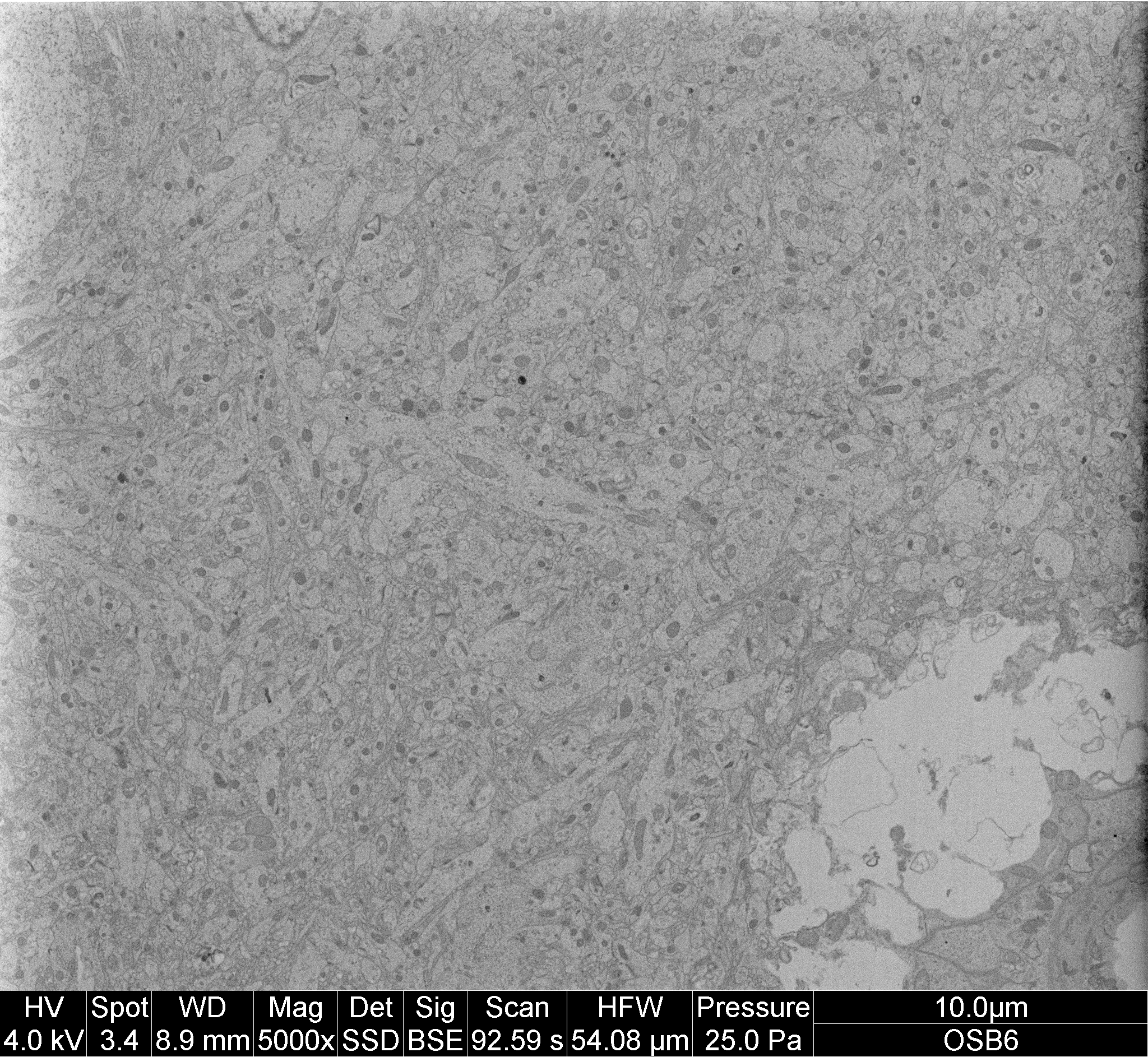

Supplement: Dataset S3 — (252.7 MB ZIP). [file pbio.0020329.sd003.zip › 040604_OS5_st1_226.tif]

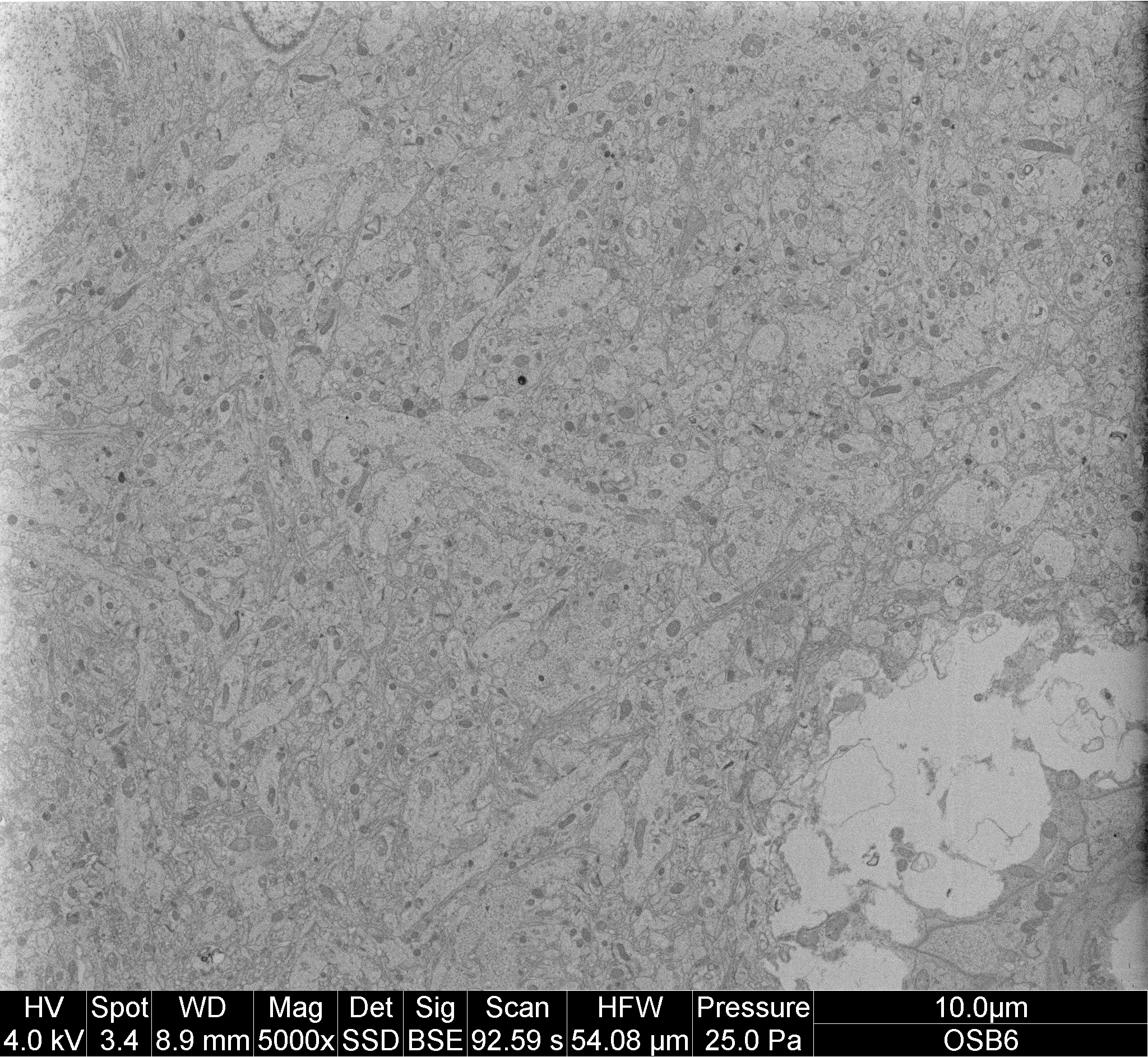

Supplement: Dataset S3 — (252.7 MB ZIP). [file pbio.0020329.sd003.zip › 040604_OS5_st1_227.tif]

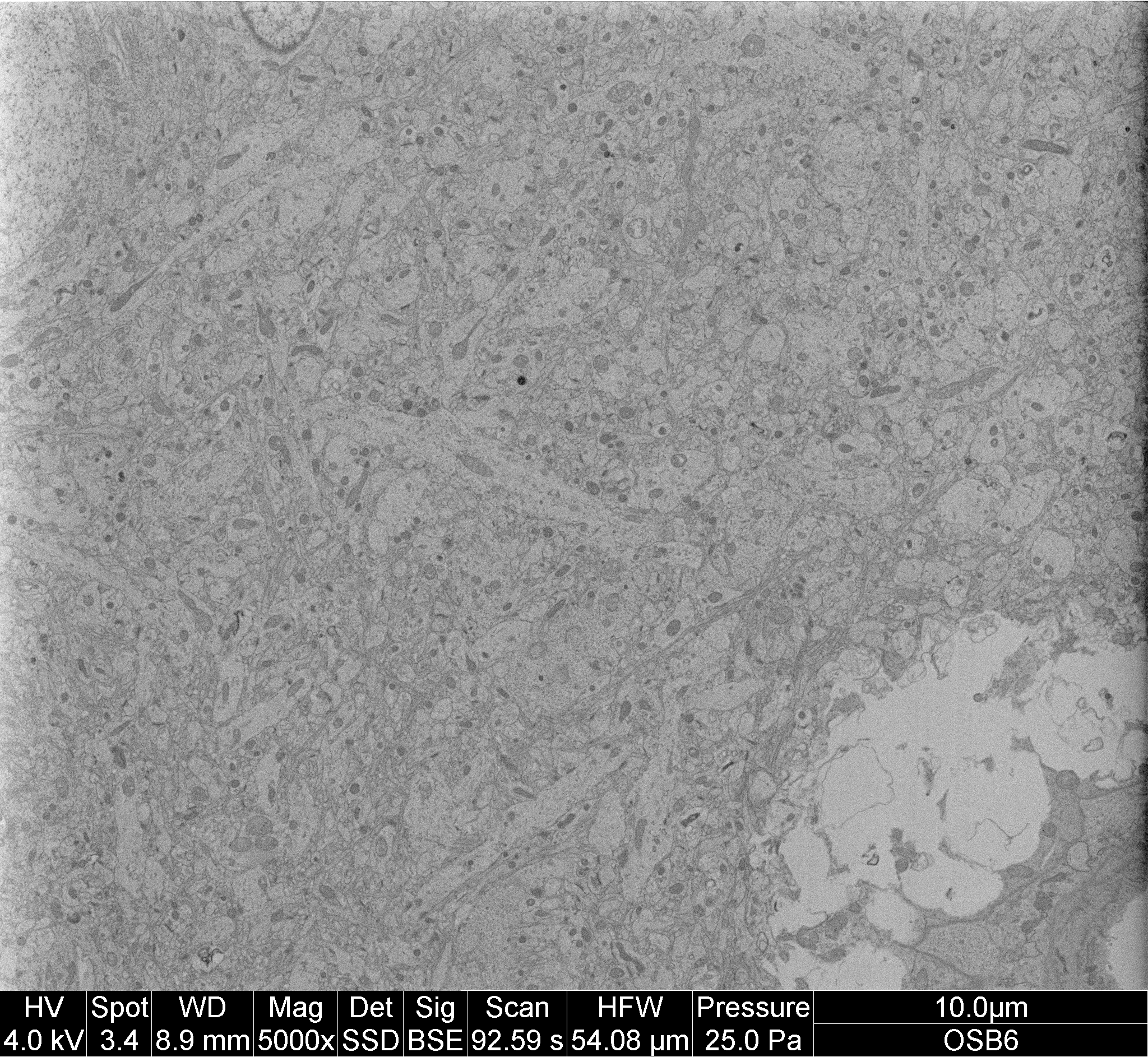

Supplement: Dataset S3 — (252.7 MB ZIP). [file pbio.0020329.sd003.zip › 040604_OS5_st1_228.tif]

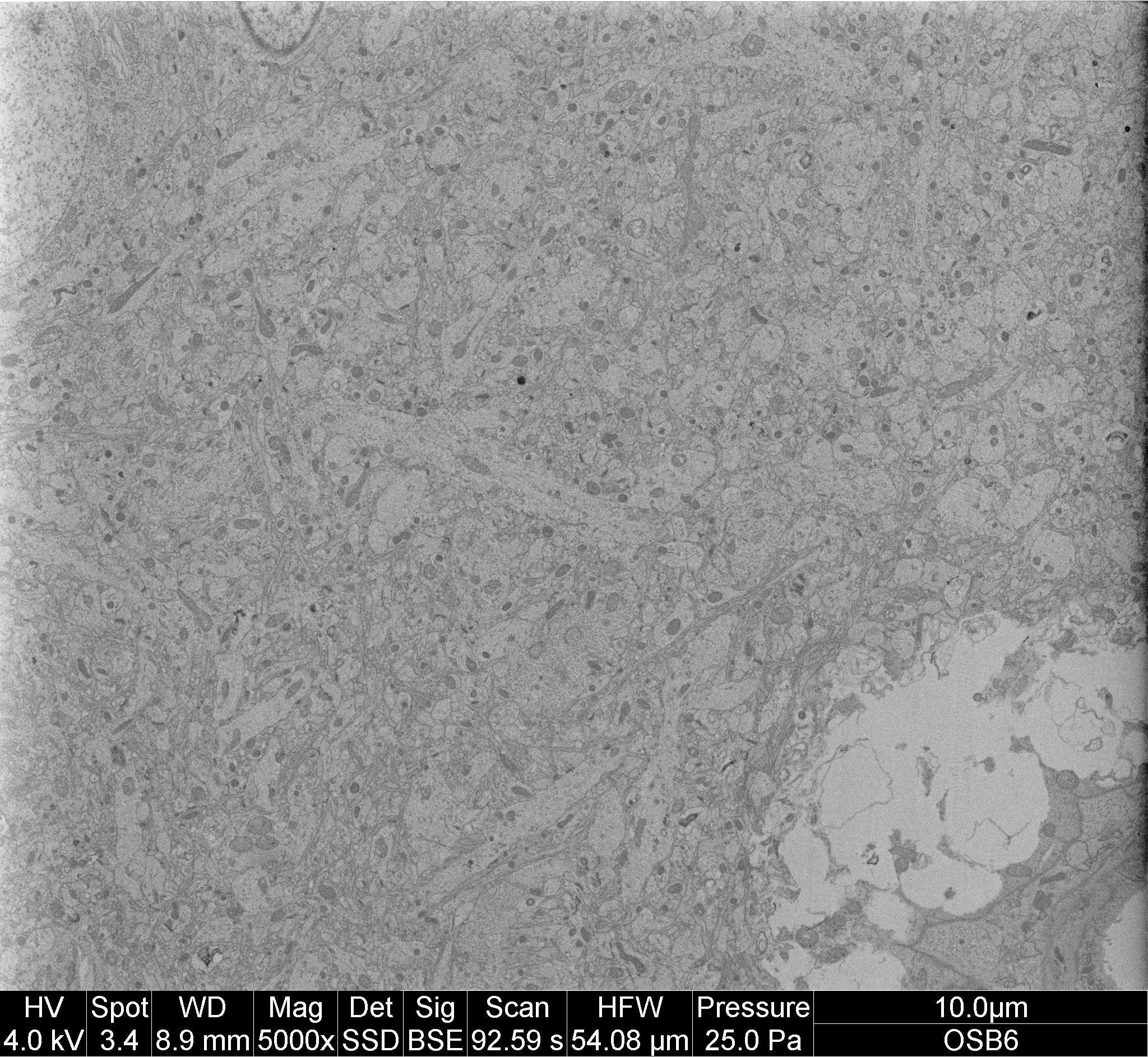

Supplement: Dataset S3 — (252.7 MB ZIP). [file pbio.0020329.sd003.zip › 040604_OS5_st1_229.tif]

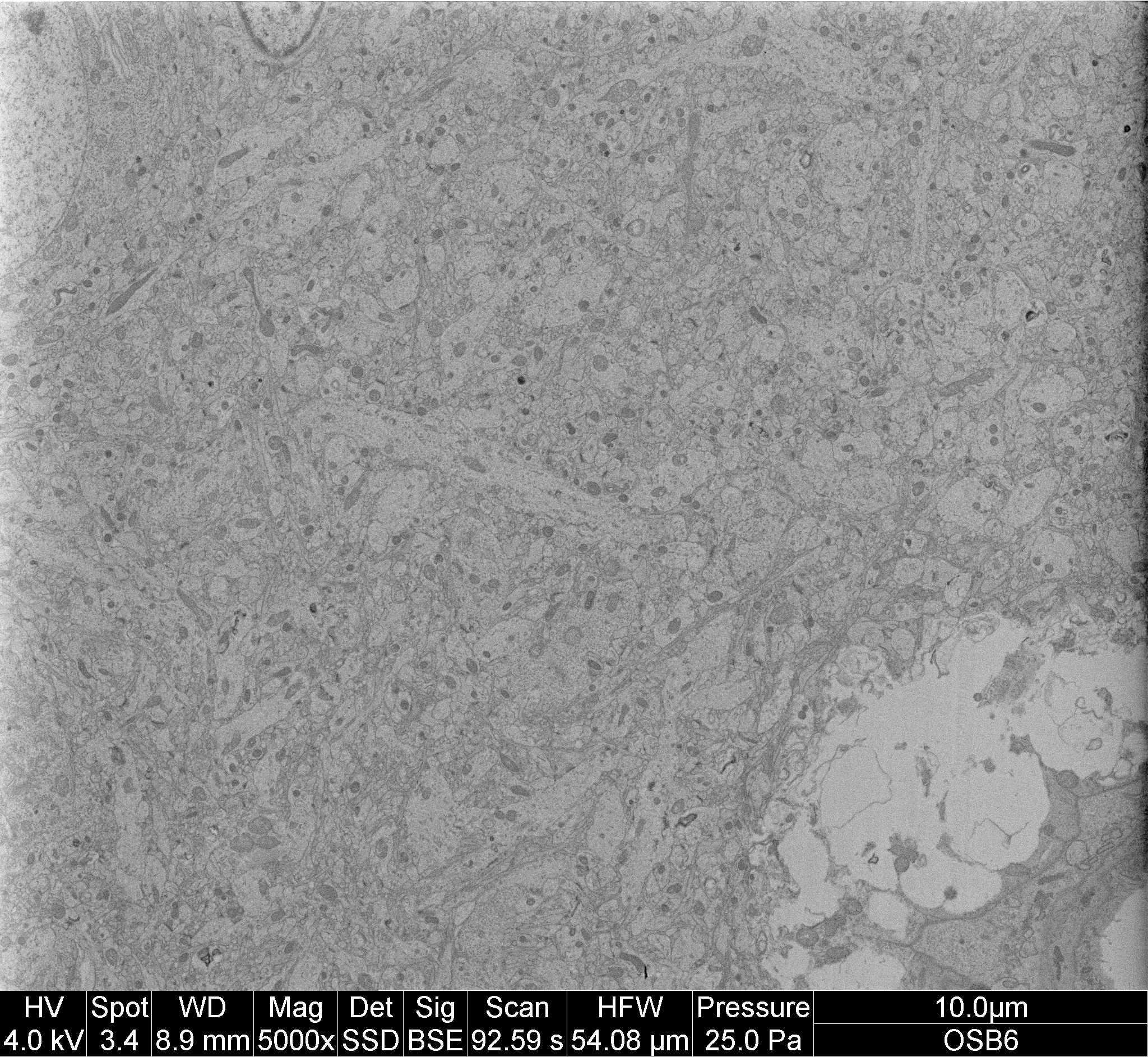

Supplement: Dataset S3 — (252.7 MB ZIP). [file pbio.0020329.sd003.zip › 040604_OS5_st1_230.tif]

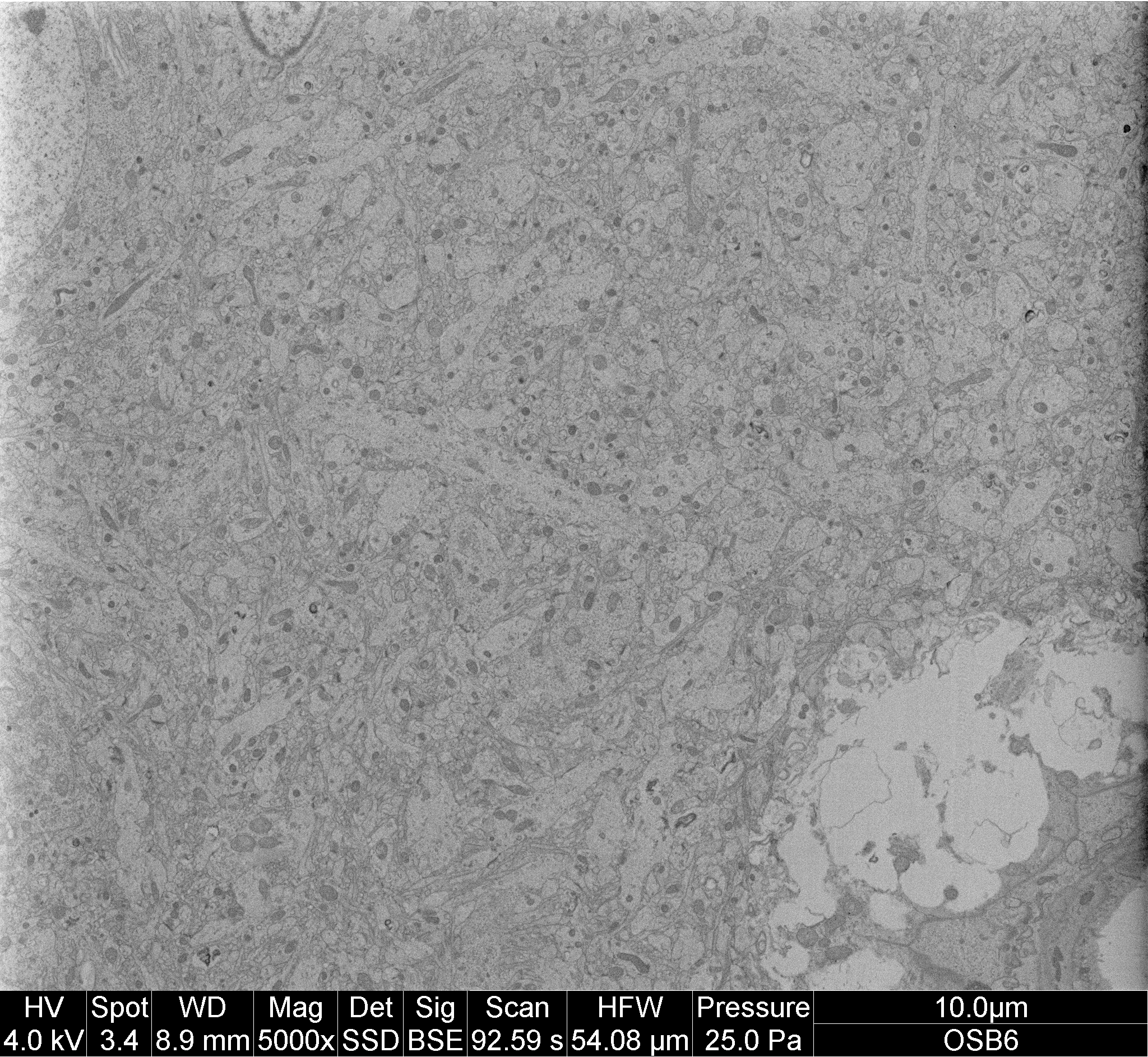

Supplement: Dataset S3 — (252.7 MB ZIP). [file pbio.0020329.sd003.zip › 040604_OS5_st1_231.tif]

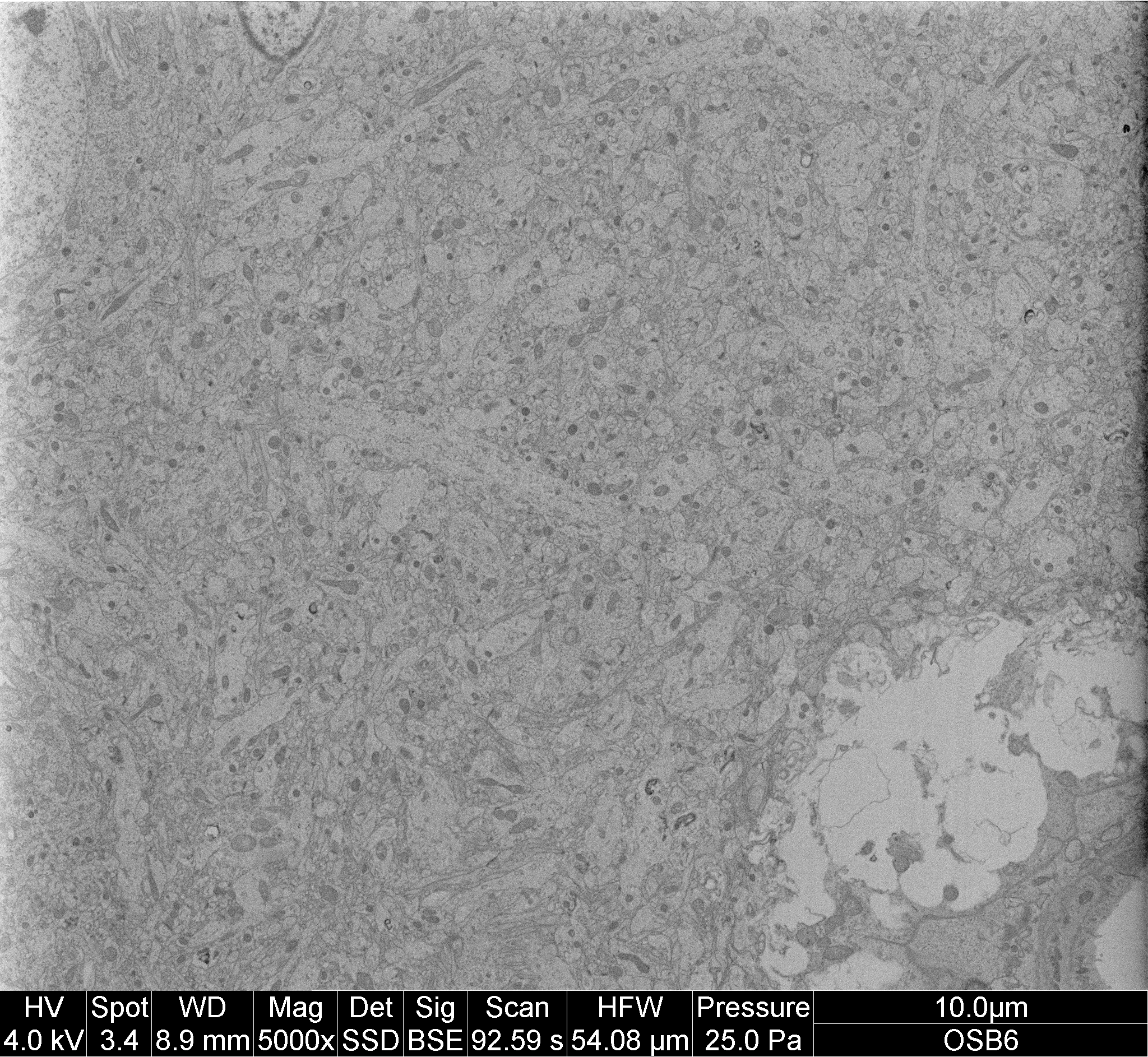

Supplement: Dataset S3 — (252.7 MB ZIP). [file pbio.0020329.sd003.zip › 040604_OS5_st1_232.tif]

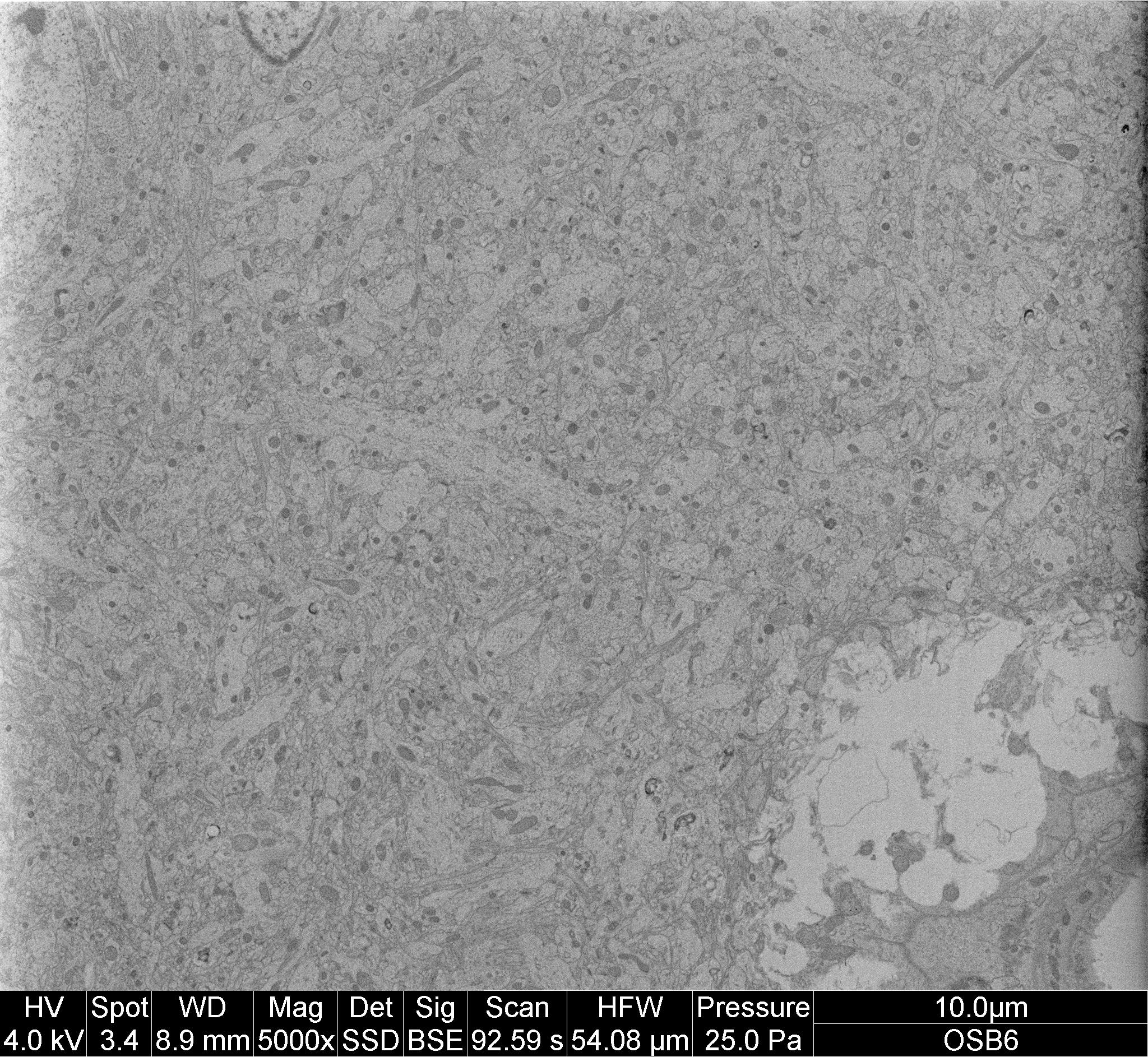

Supplement: Dataset S3 — (252.7 MB ZIP). [file pbio.0020329.sd003.zip › 040604_OS5_st1_233.tif]

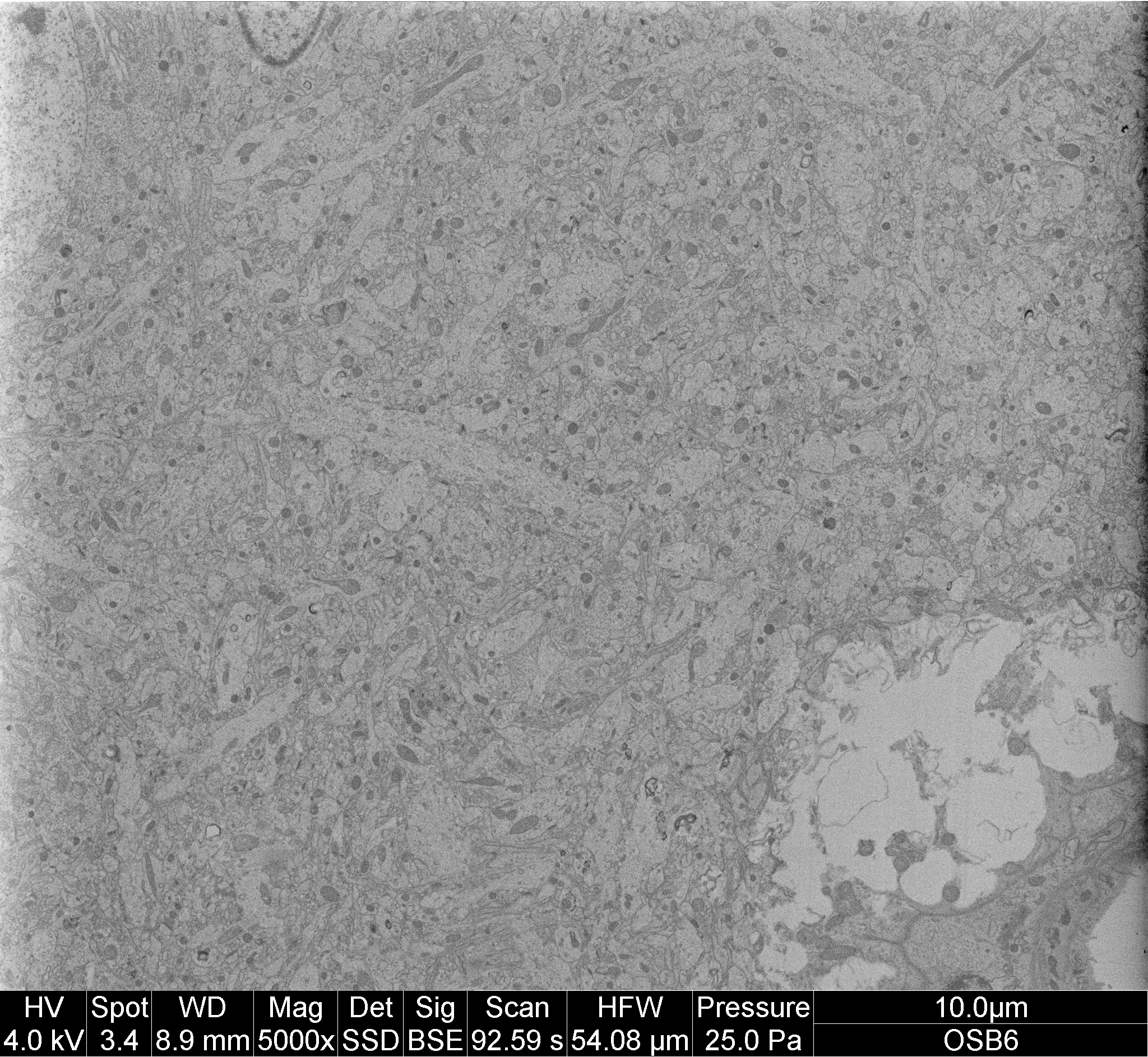

Supplement: Dataset S3 — (252.7 MB ZIP). [file pbio.0020329.sd003.zip › 040604_OS5_st1_234.tif]

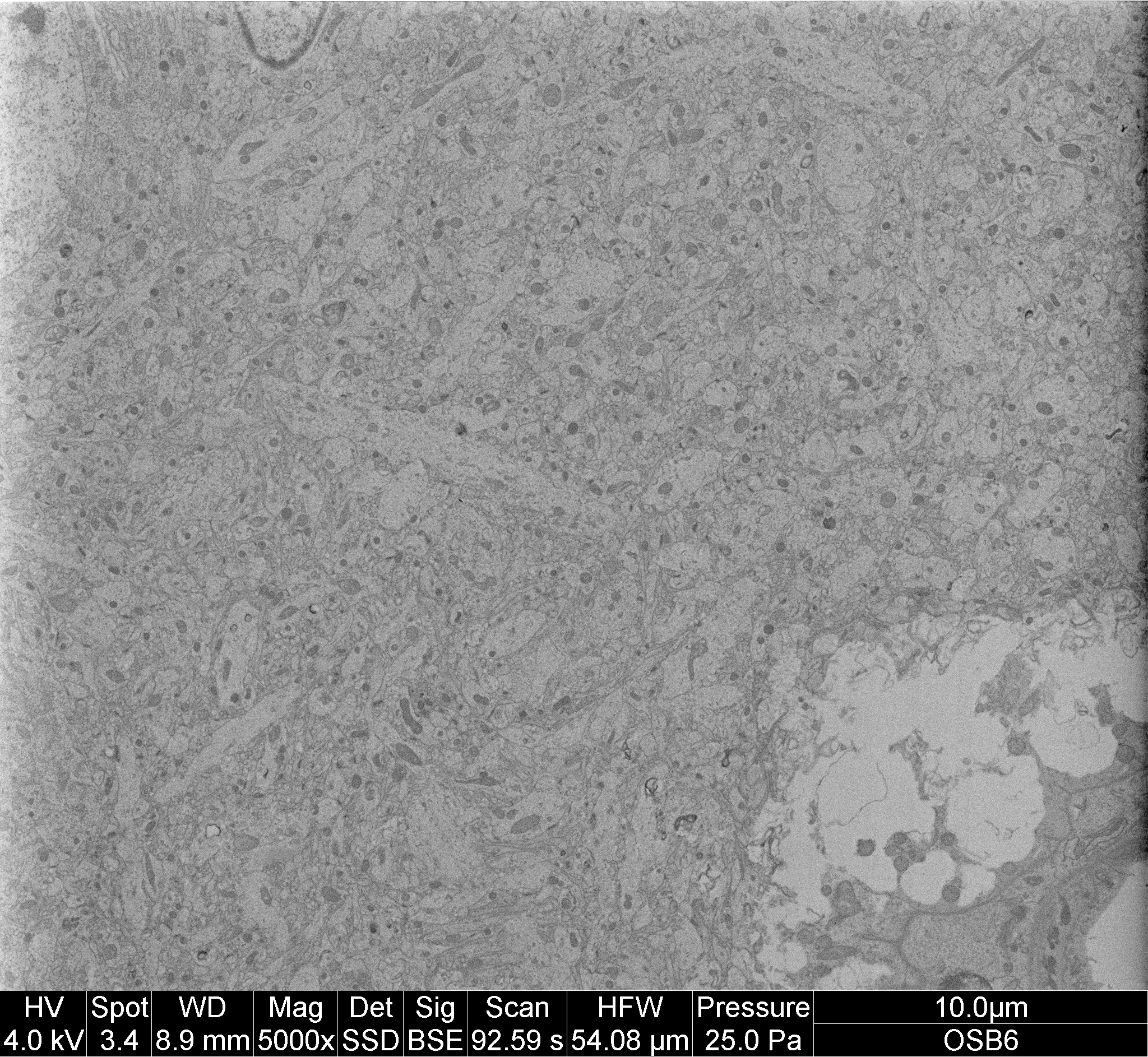

Supplement: Dataset S3 — (252.7 MB ZIP). [file pbio.0020329.sd003.zip › 040604_OS5_st1_235.tif]

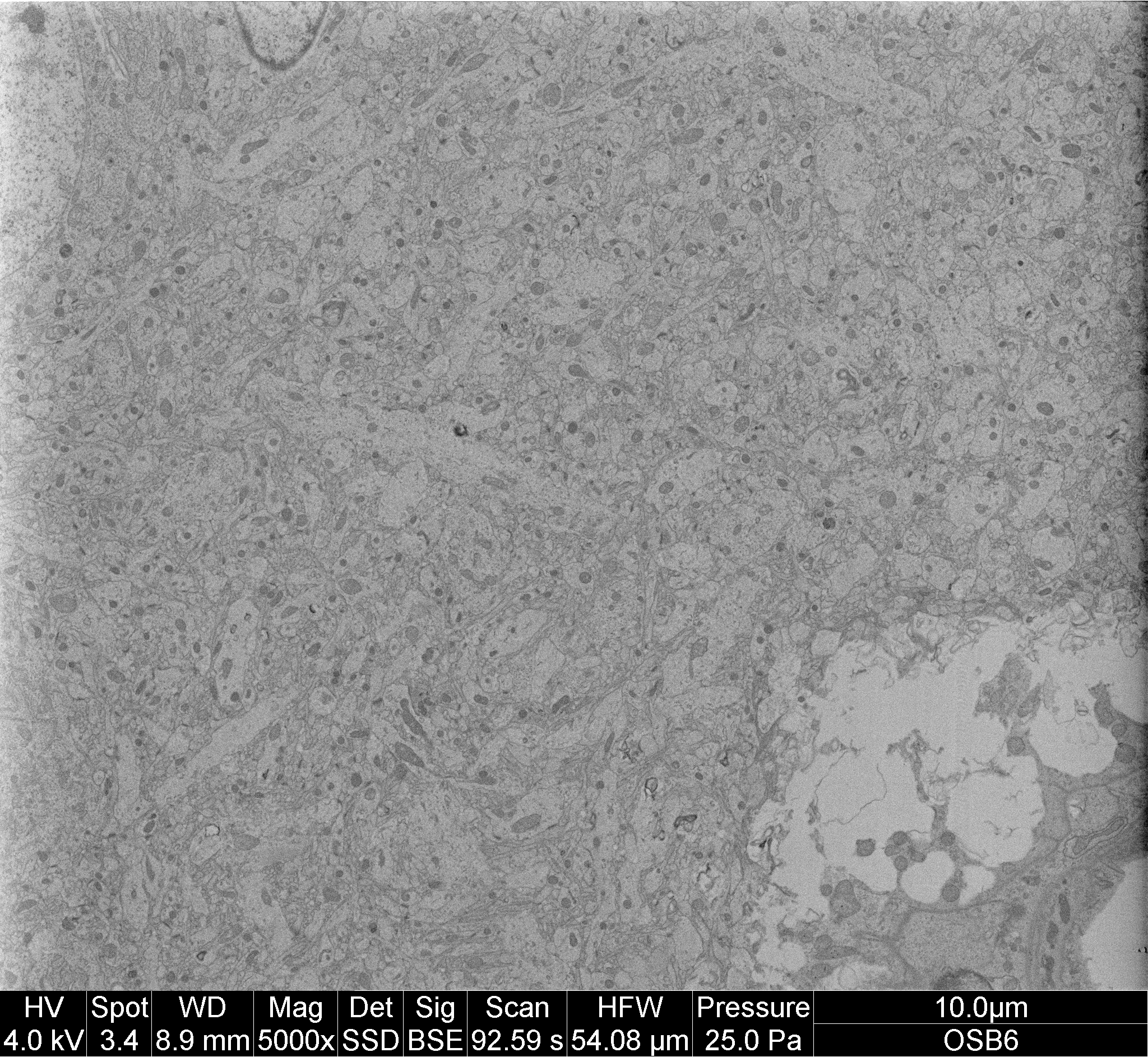

Supplement: Dataset S3 — (252.7 MB ZIP). [file pbio.0020329.sd003.zip › 040604_OS5_st1_236.tif]

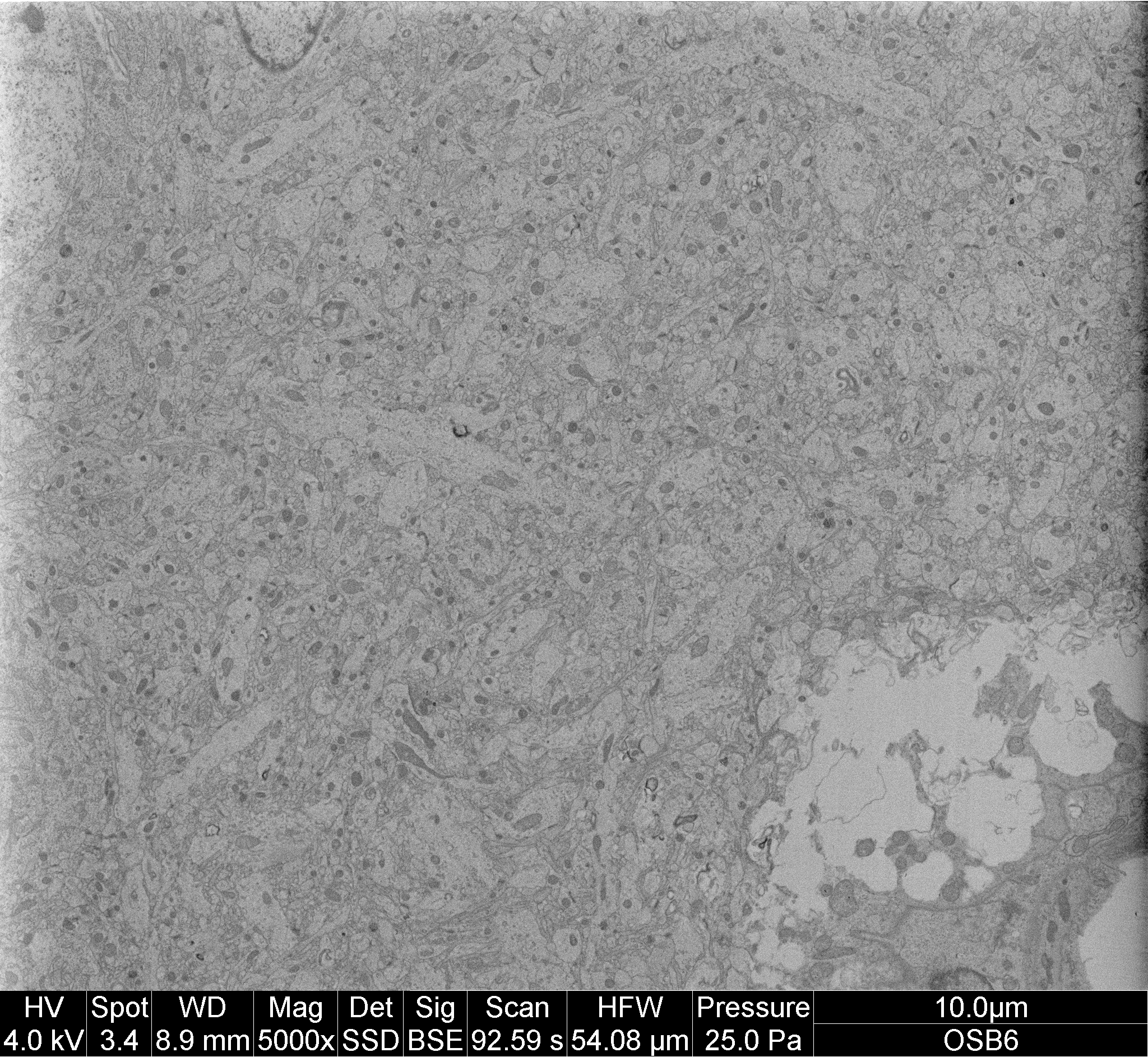

Supplement: Dataset S3 — (252.7 MB ZIP). [file pbio.0020329.sd003.zip › 040604_OS5_st1_237.tif]

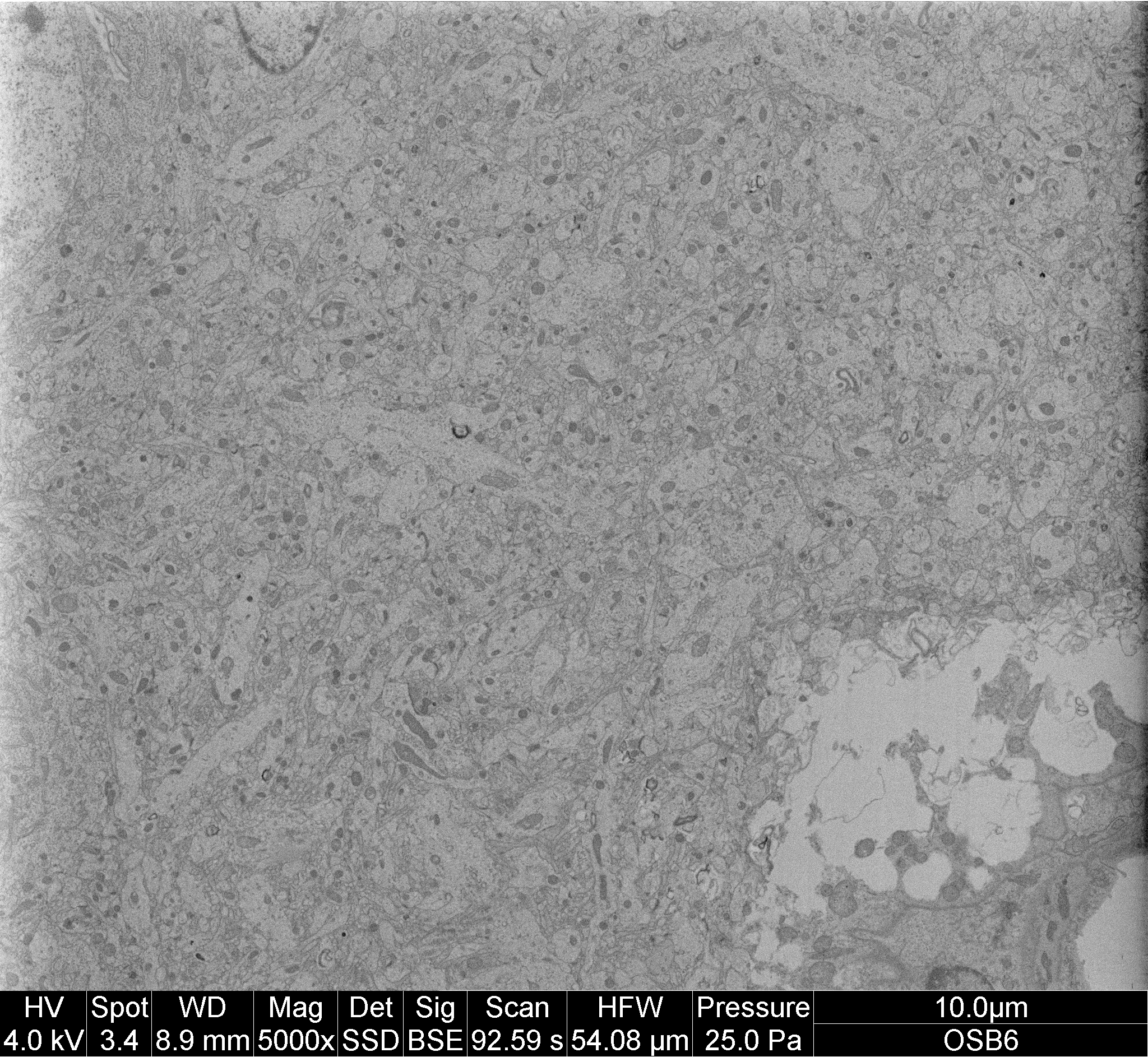

Supplement: Dataset S3 — (252.7 MB ZIP). [file pbio.0020329.sd003.zip › 040604_OS5_st1_238.tif]

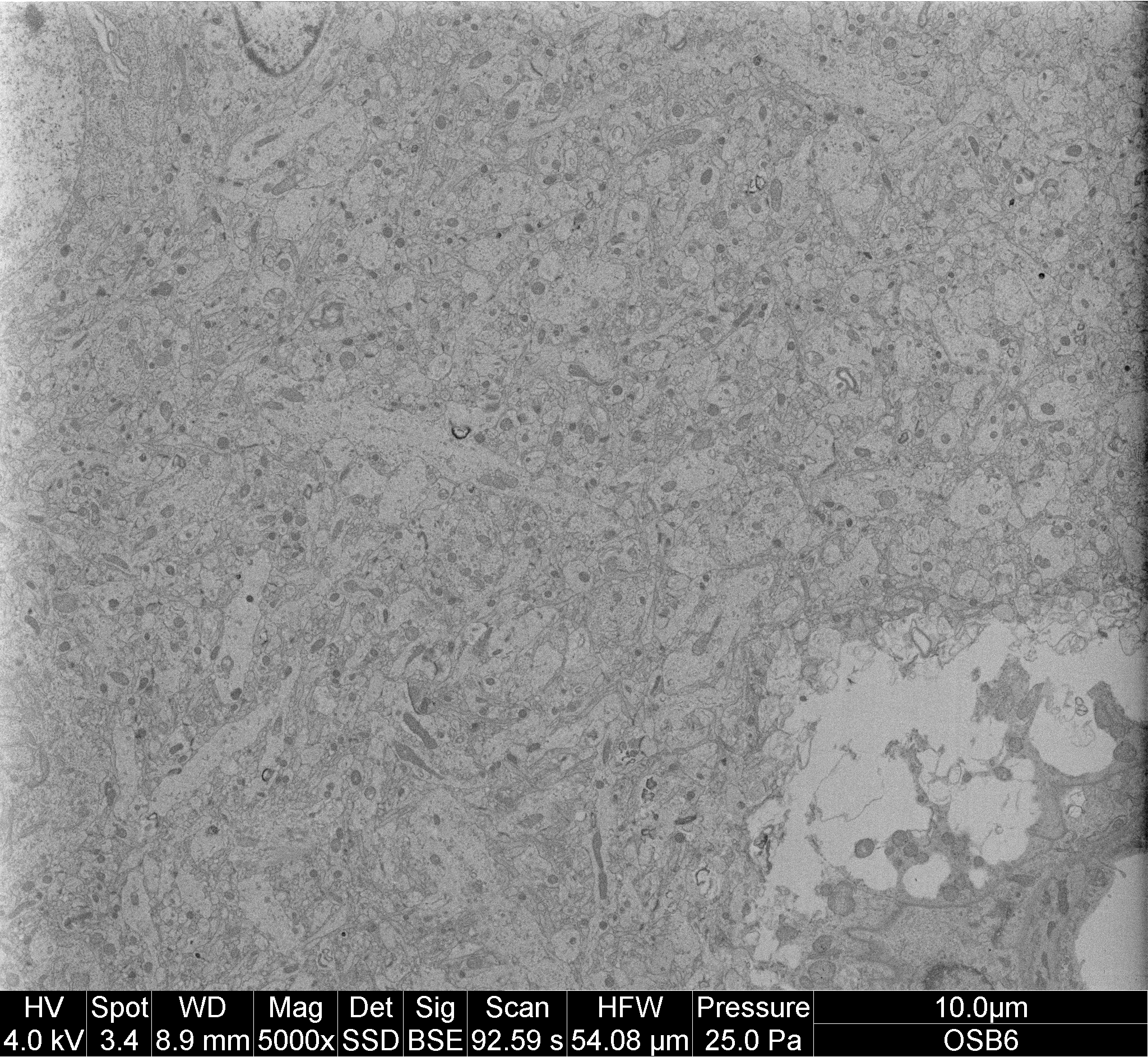

Supplement: Dataset S3 — (252.7 MB ZIP). [file pbio.0020329.sd003.zip › 040604_OS5_st1_239.tif]

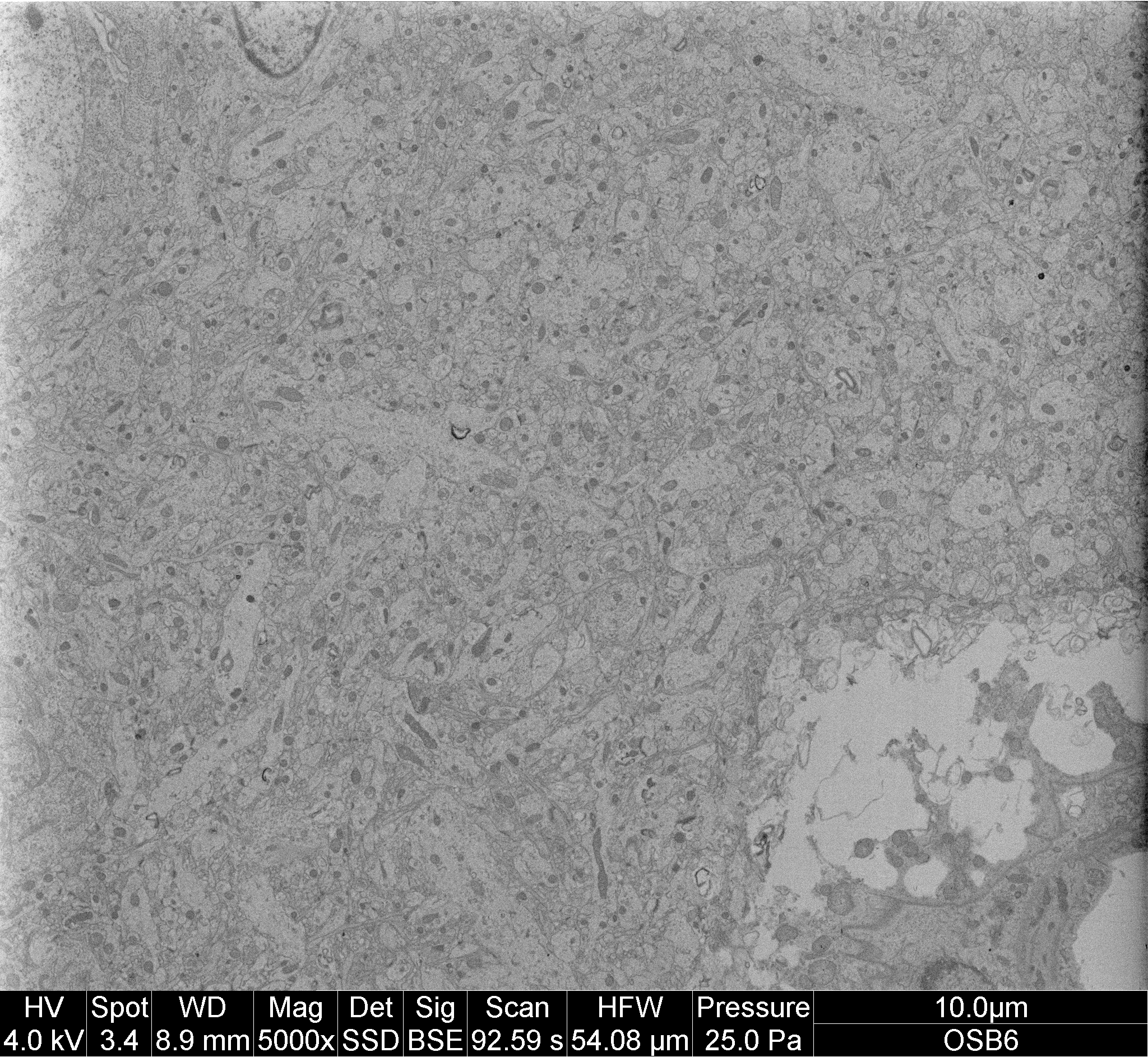

Supplement: Dataset S3 — (252.7 MB ZIP). [file pbio.0020329.sd003.zip › 040604_OS5_st1_240.tif]

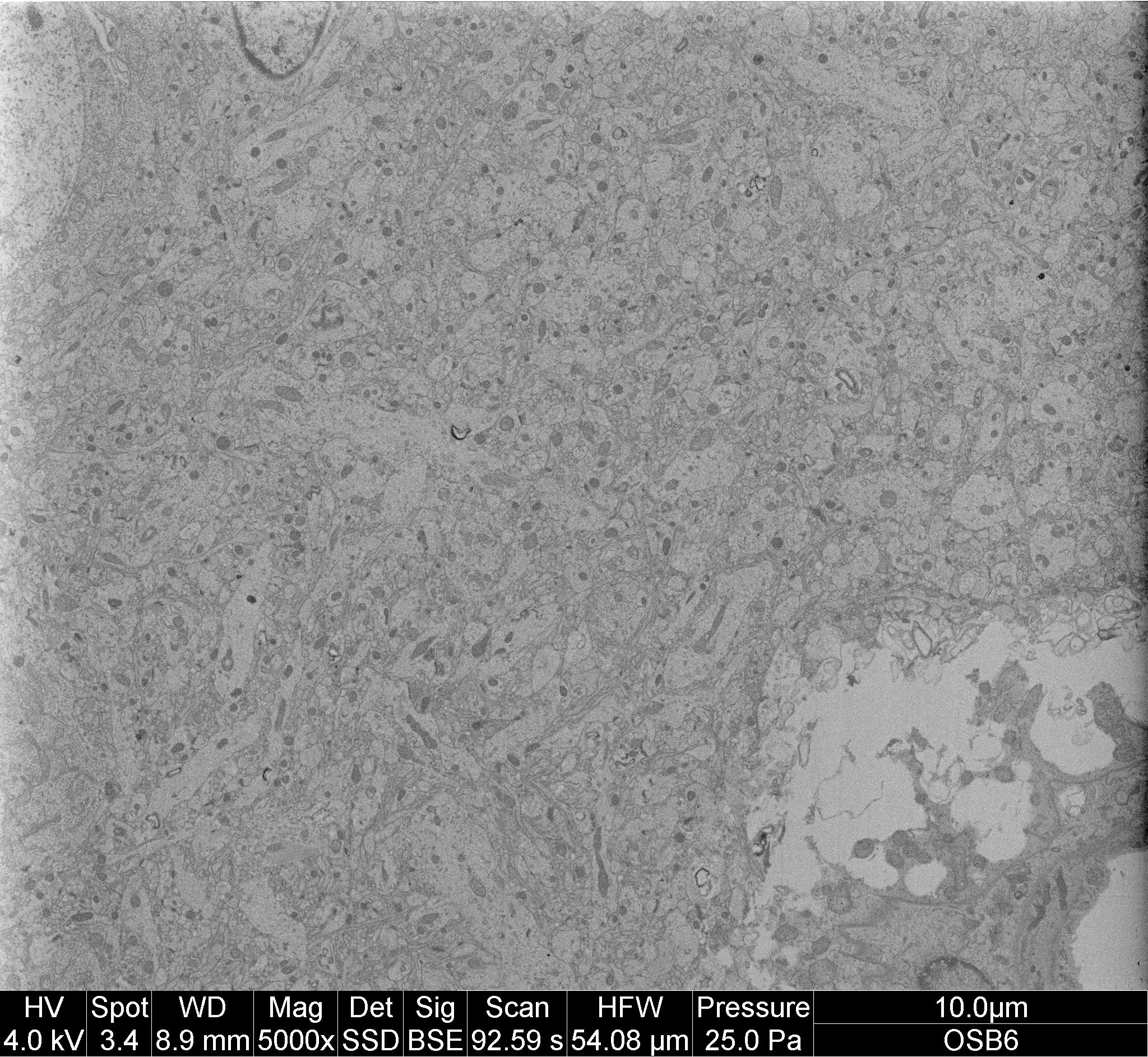

Supplement: Dataset S3 — (252.7 MB ZIP). [file pbio.0020329.sd003.zip › 040604_OS5_st1_241.tif]

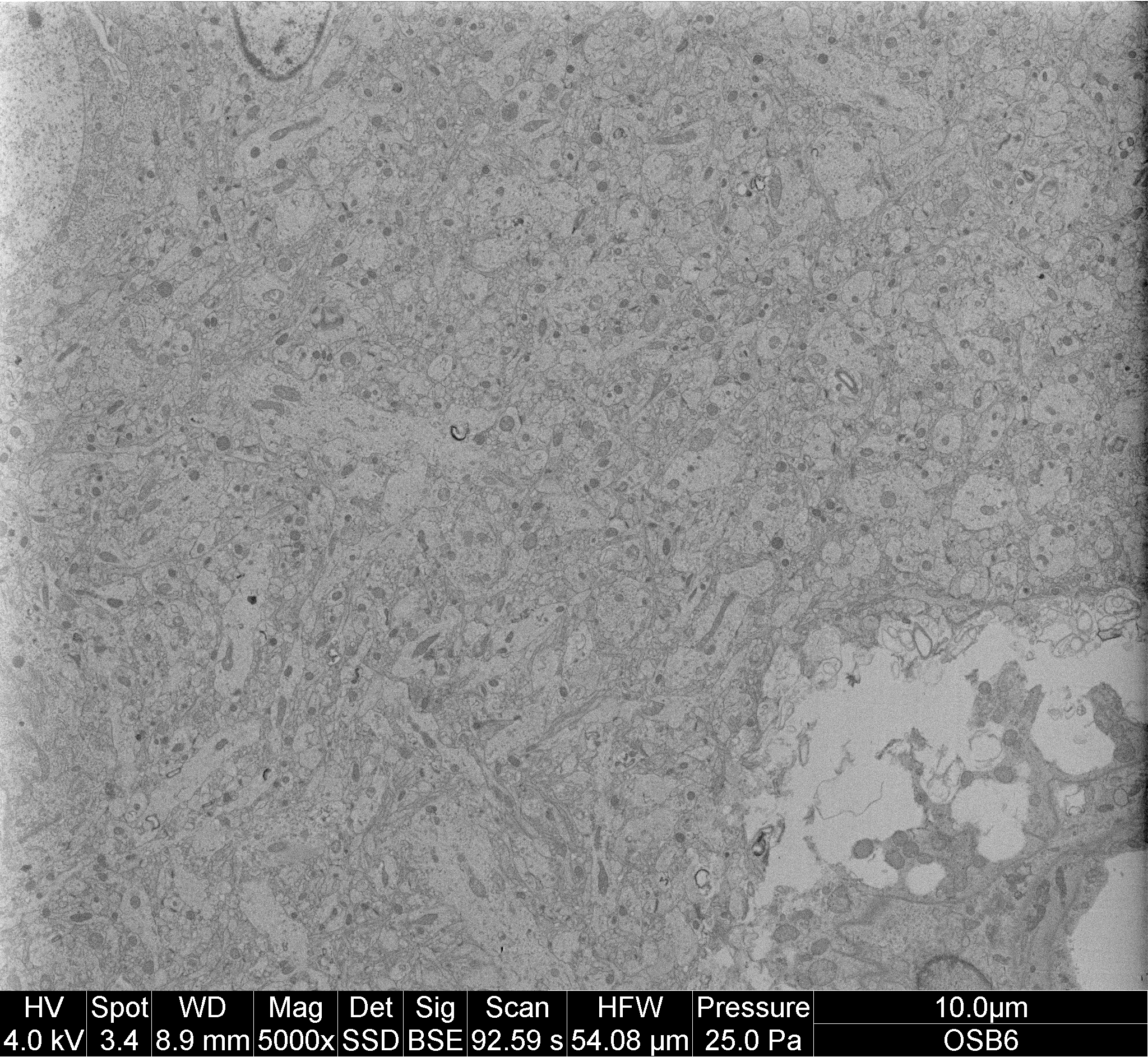

Supplement: Dataset S3 — (252.7 MB ZIP). [file pbio.0020329.sd003.zip › 040604_OS5_st1_242.tif]

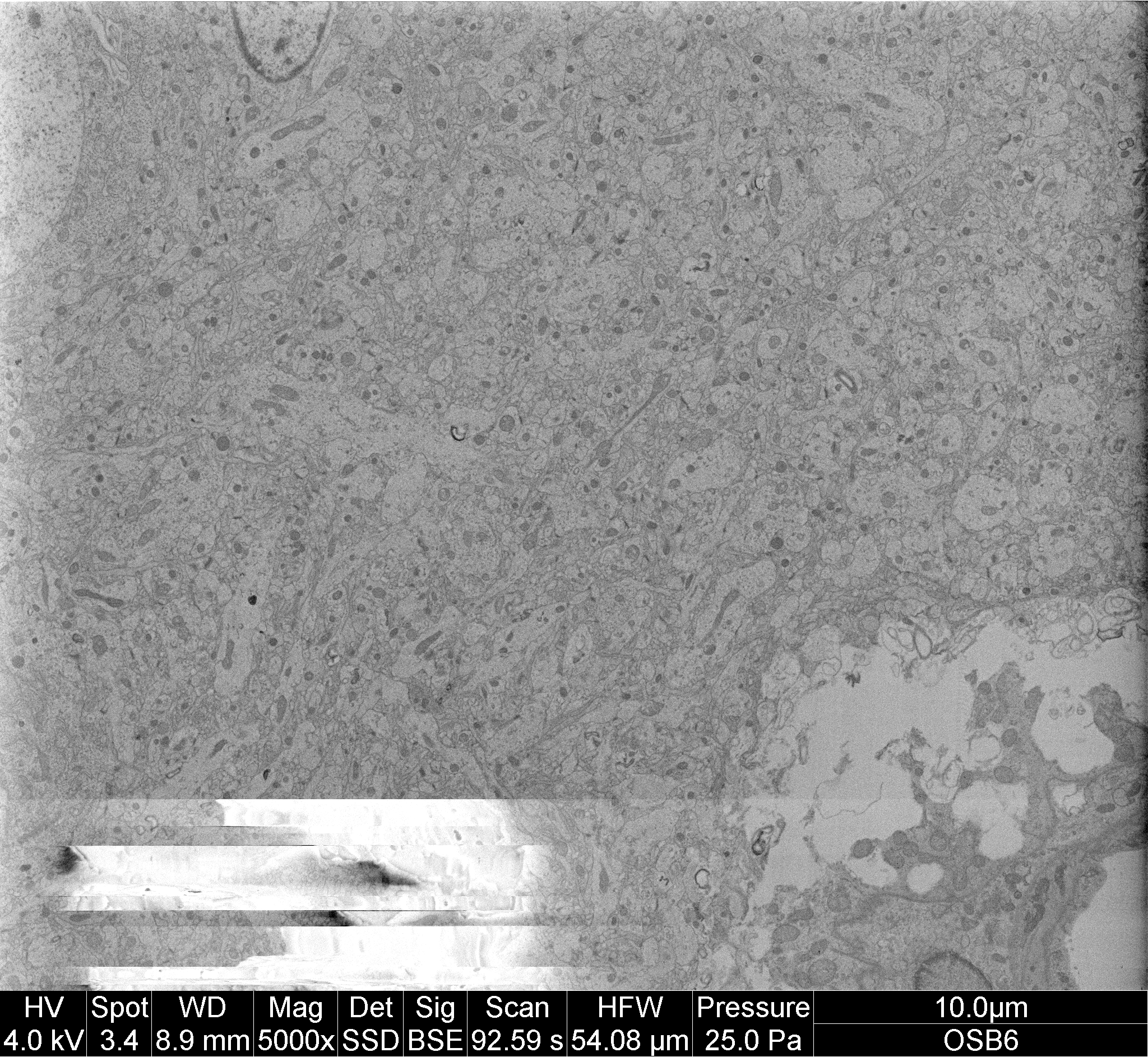

Supplement: Dataset S3 — (252.7 MB ZIP). [file pbio.0020329.sd003.zip › 040604_OS5_st1_243.tif]

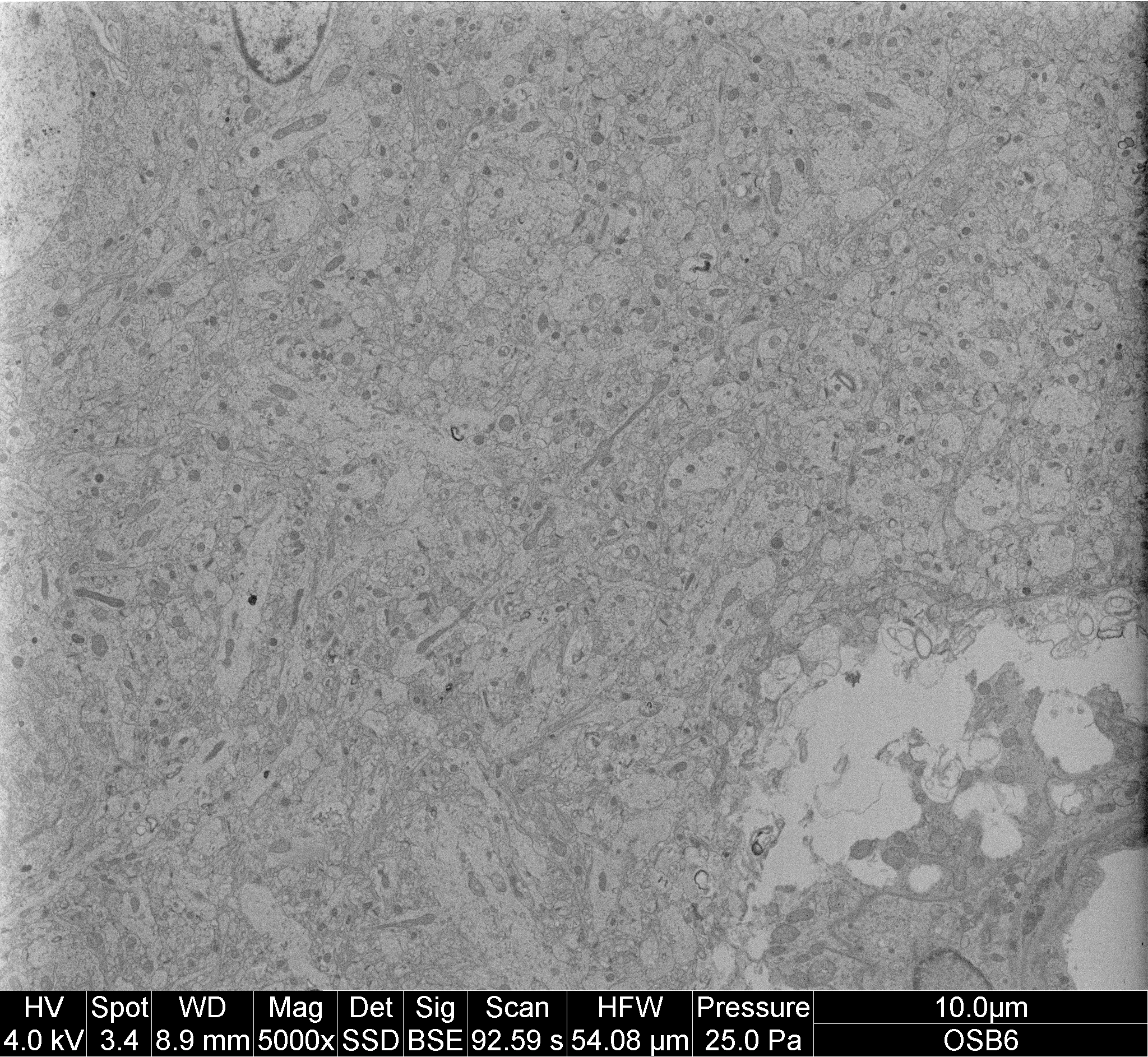

Supplement: Dataset S3 — (252.7 MB ZIP). [file pbio.0020329.sd003.zip › 040604_OS5_st1_244.tif]

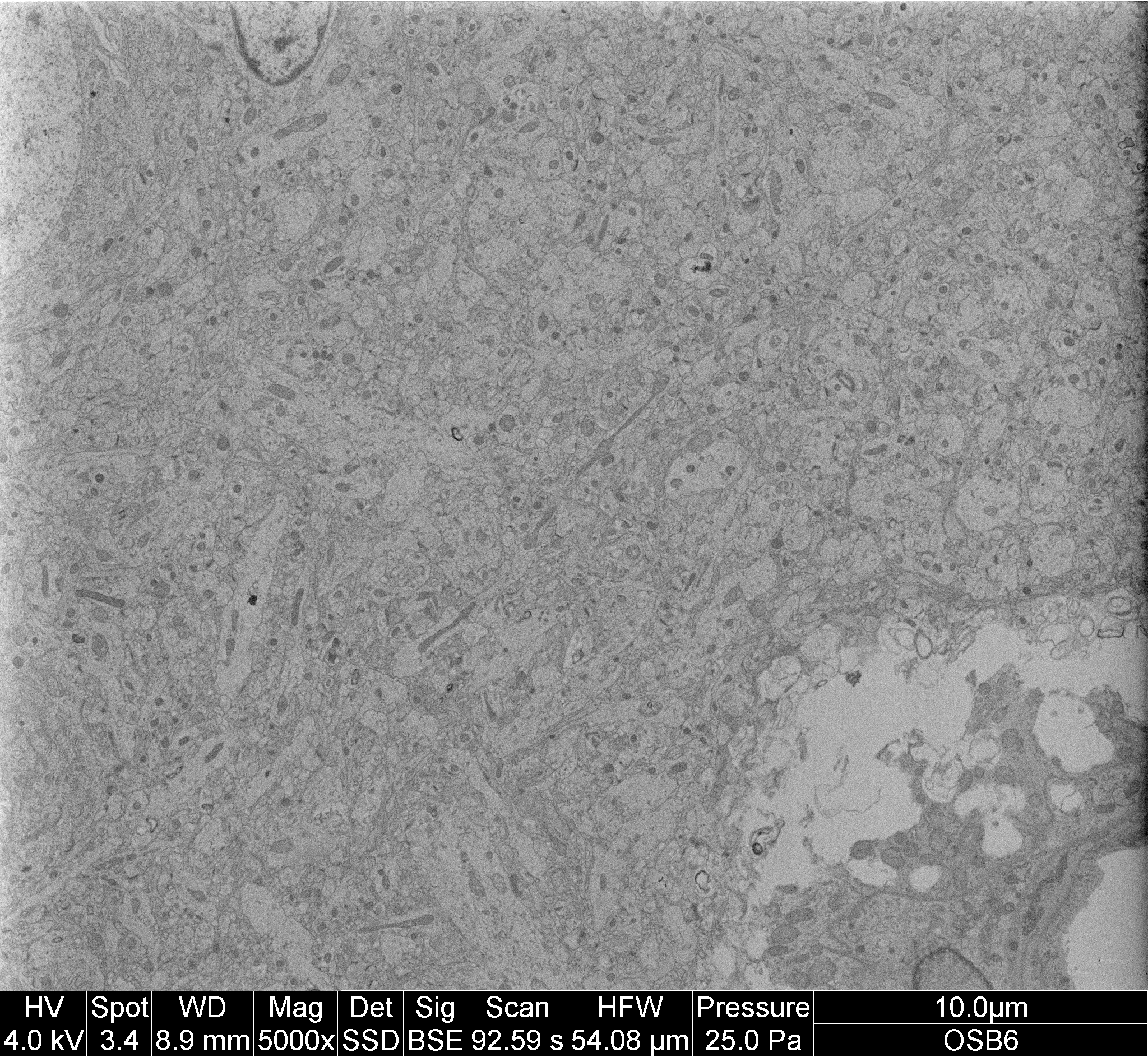

Supplement: Dataset S3 — (252.7 MB ZIP). [file pbio.0020329.sd003.zip › 040604_OS5_st1_245.tif]

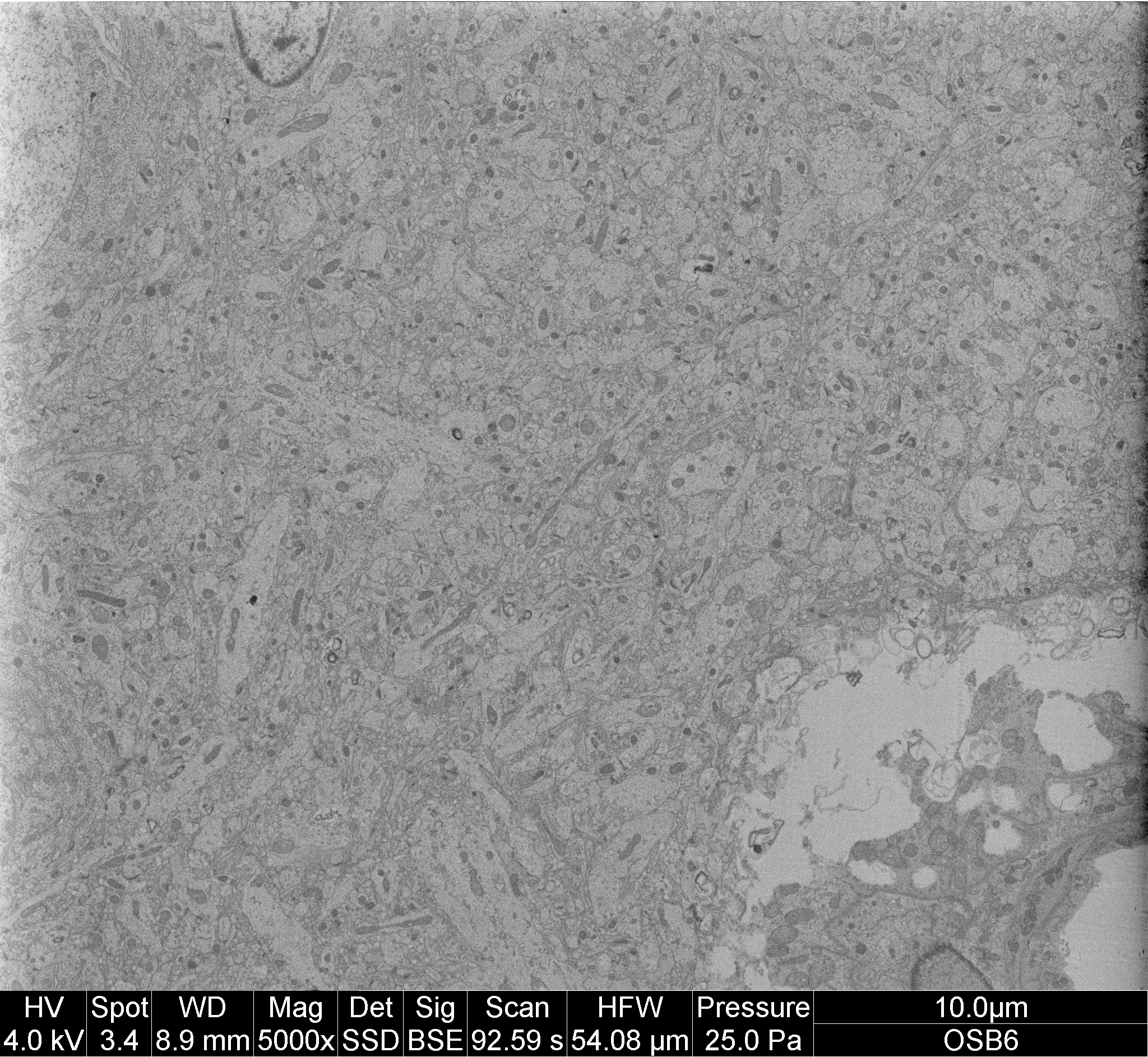

Supplement: Dataset S3 — (252.7 MB ZIP). [file pbio.0020329.sd003.zip › 040604_OS5_st1_246.tif]

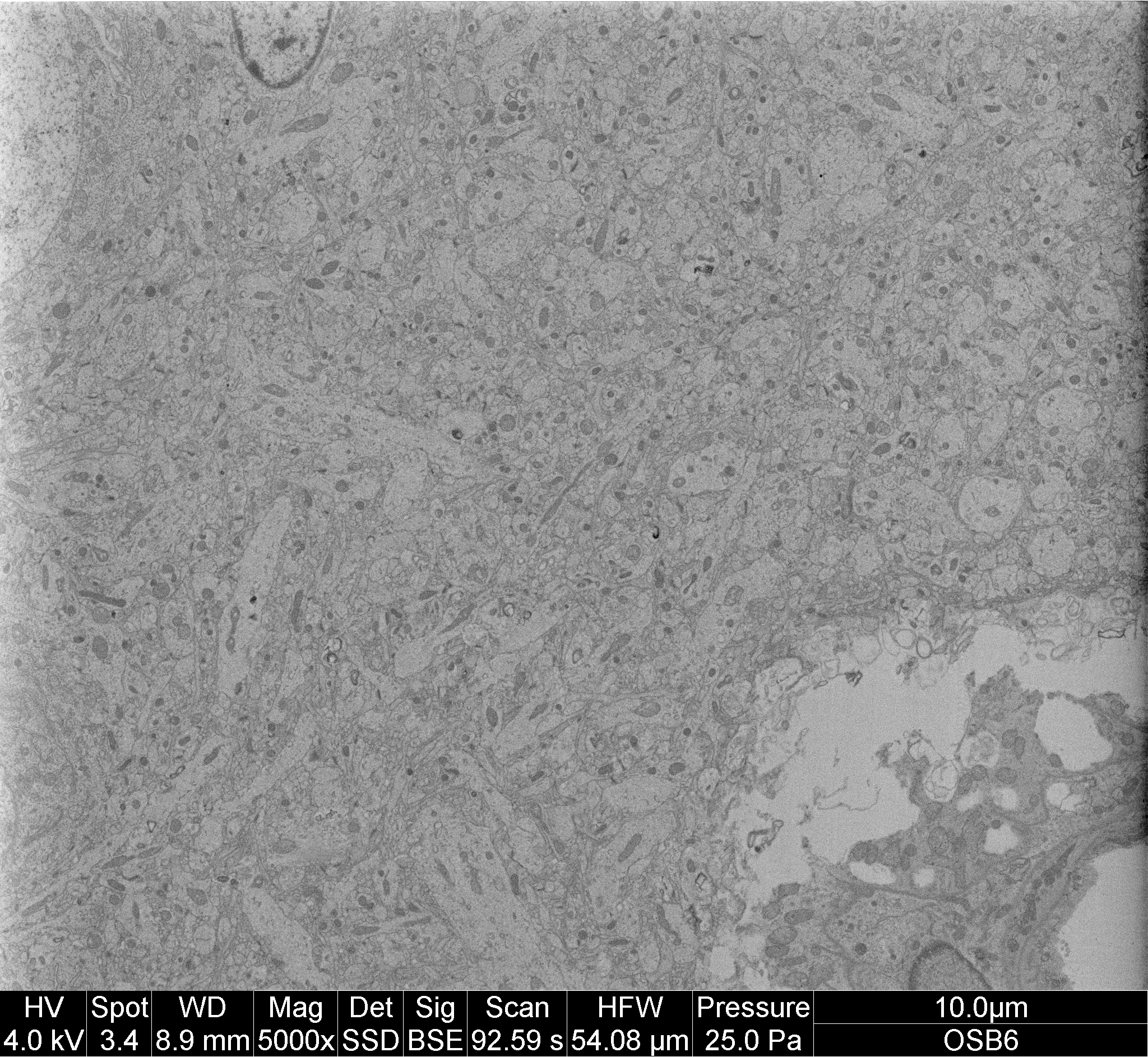

Supplement: Dataset S3 — (252.7 MB ZIP). [file pbio.0020329.sd003.zip › 040604_OS5_st1_247.tif]

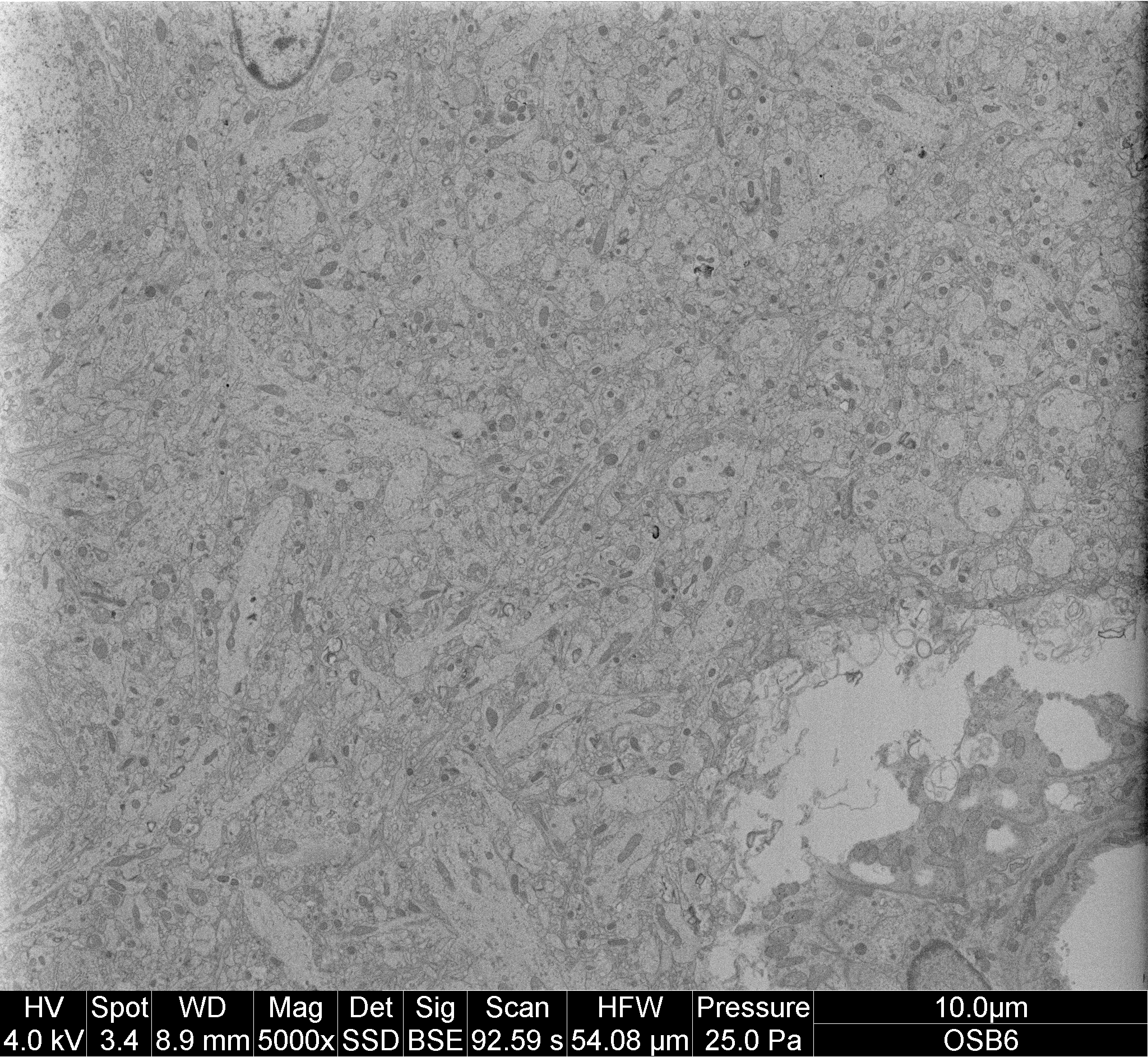

Supplement: Dataset S3 — (252.7 MB ZIP). [file pbio.0020329.sd003.zip › 040604_OS5_st1_248.tif]

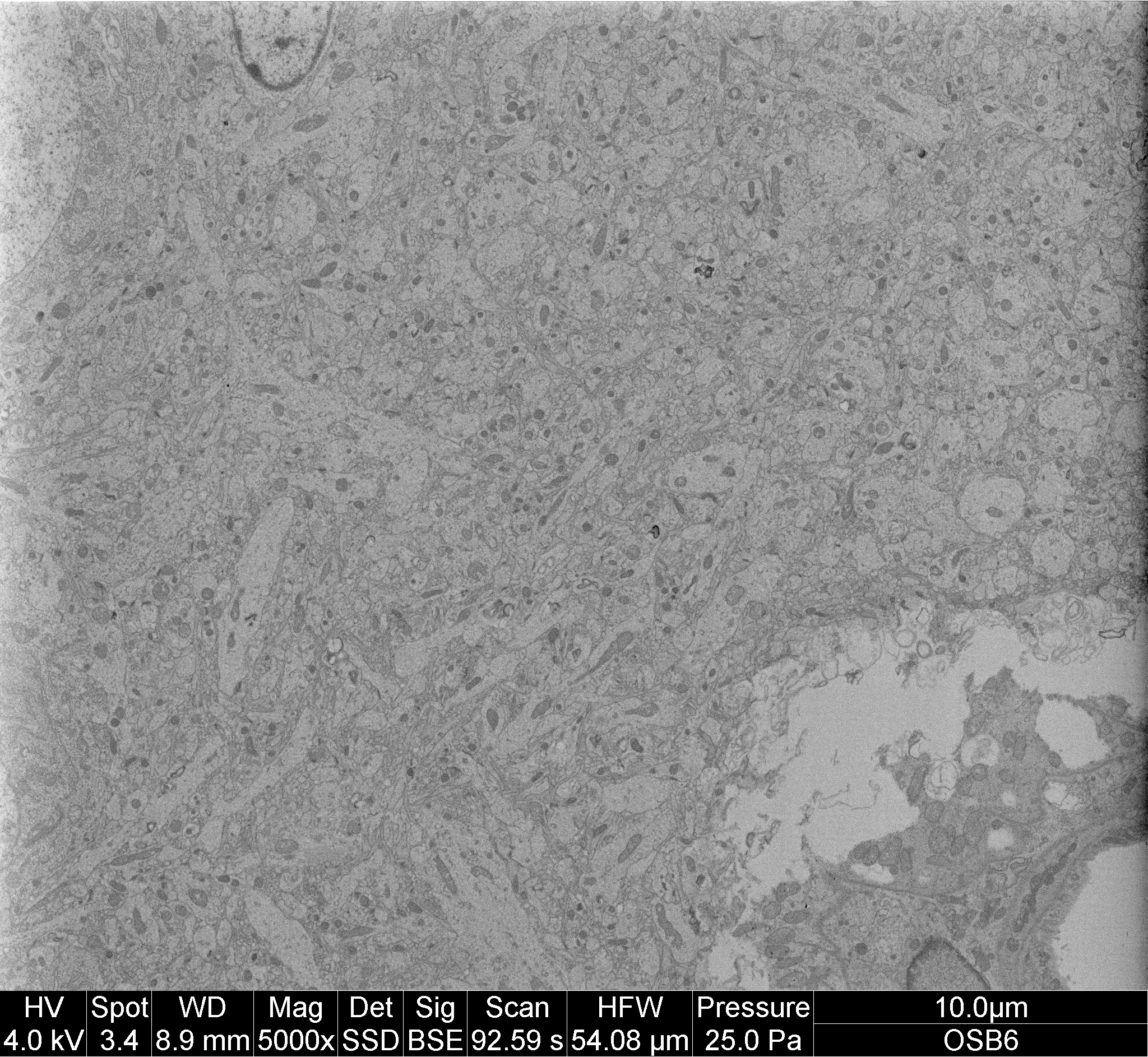

Supplement: Dataset S3 — (252.7 MB ZIP). [file pbio.0020329.sd003.zip › 040604_OS5_st1_249.tif]

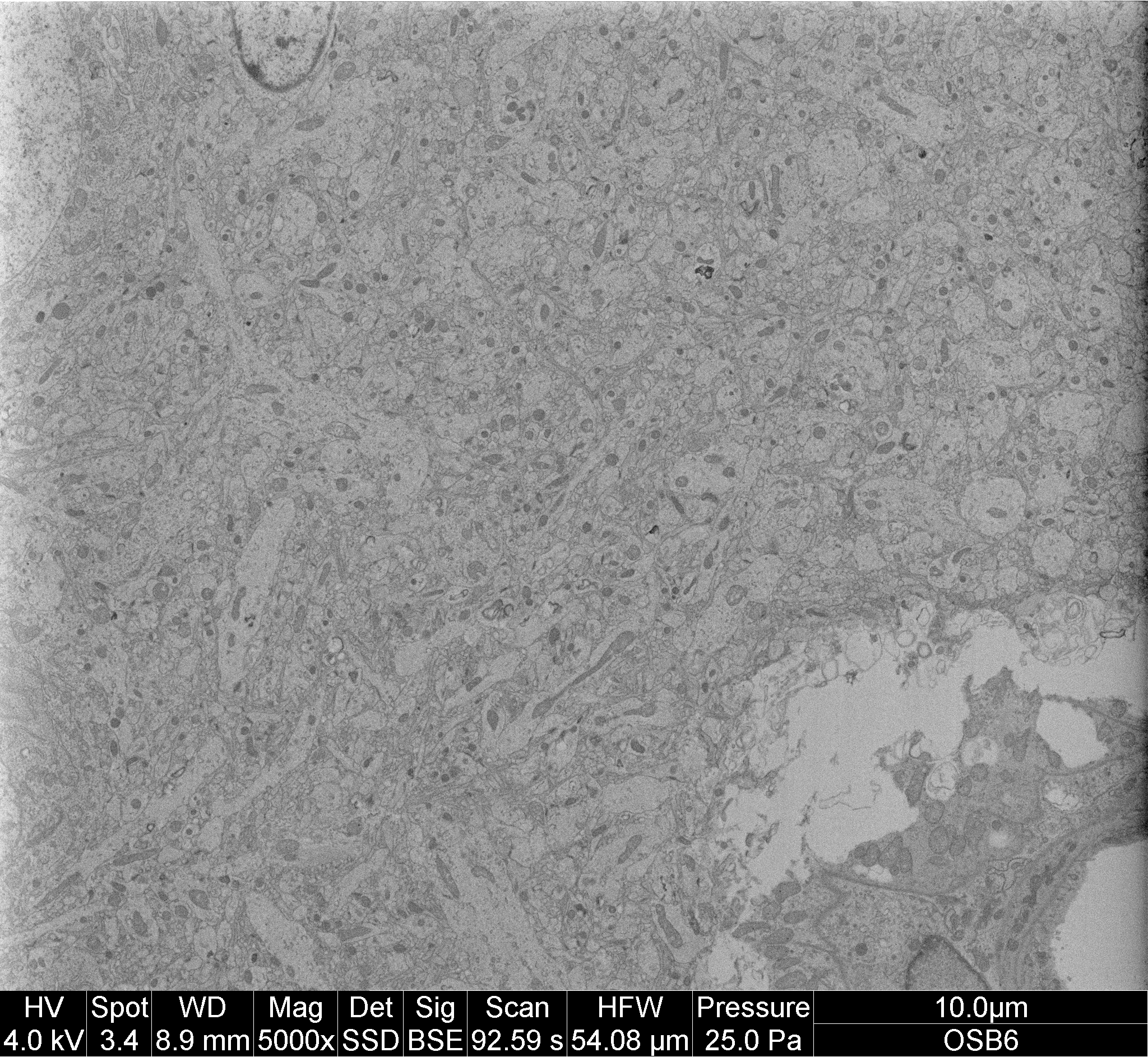

Supplement: Dataset S3 — (252.7 MB ZIP). [file pbio.0020329.sd003.zip › 040604_OS5_st1_250.tif]

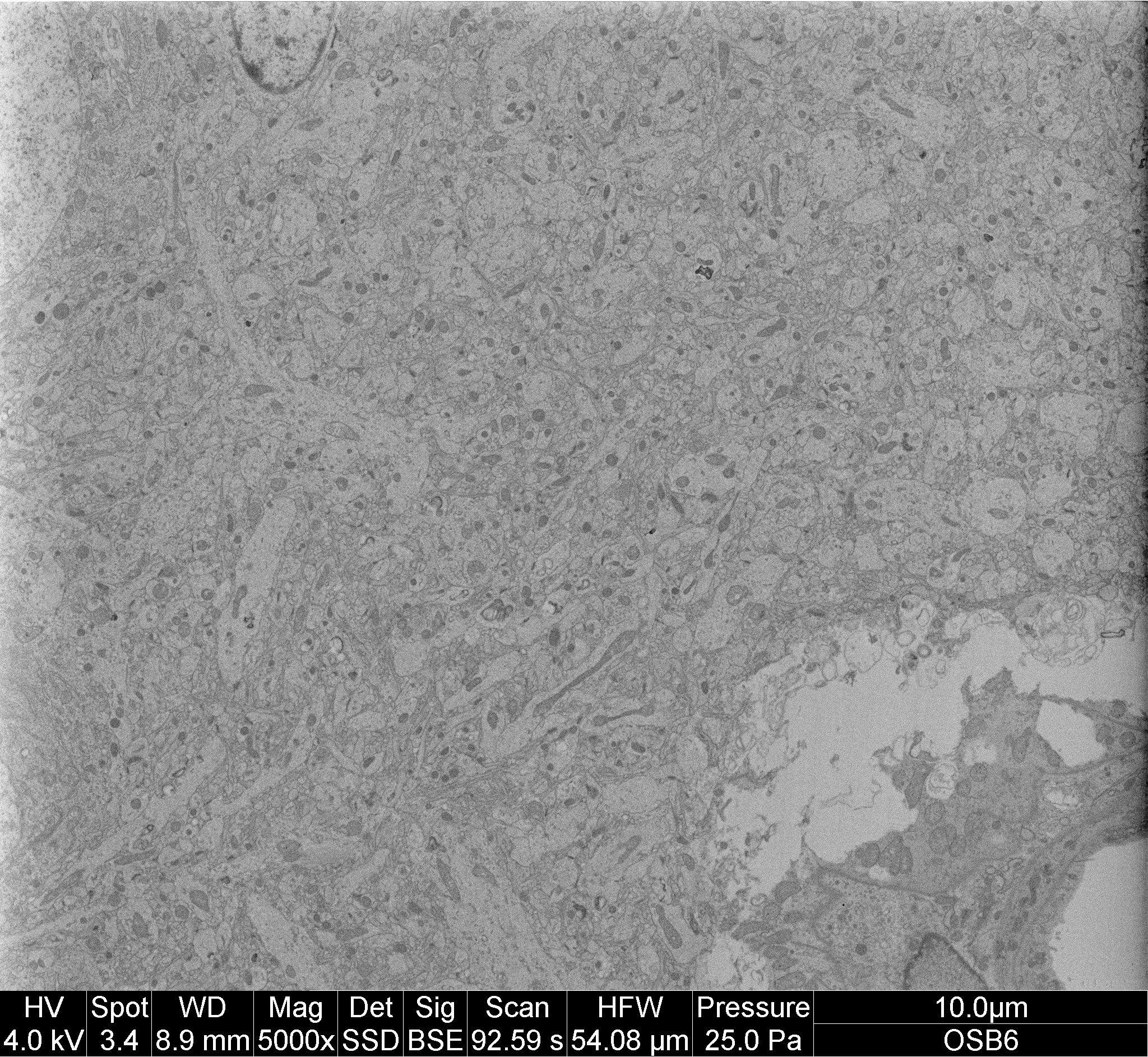

Supplement: Dataset S3 — (252.7 MB ZIP). [file pbio.0020329.sd003.zip › 040604_OS5_st1_251.tif]

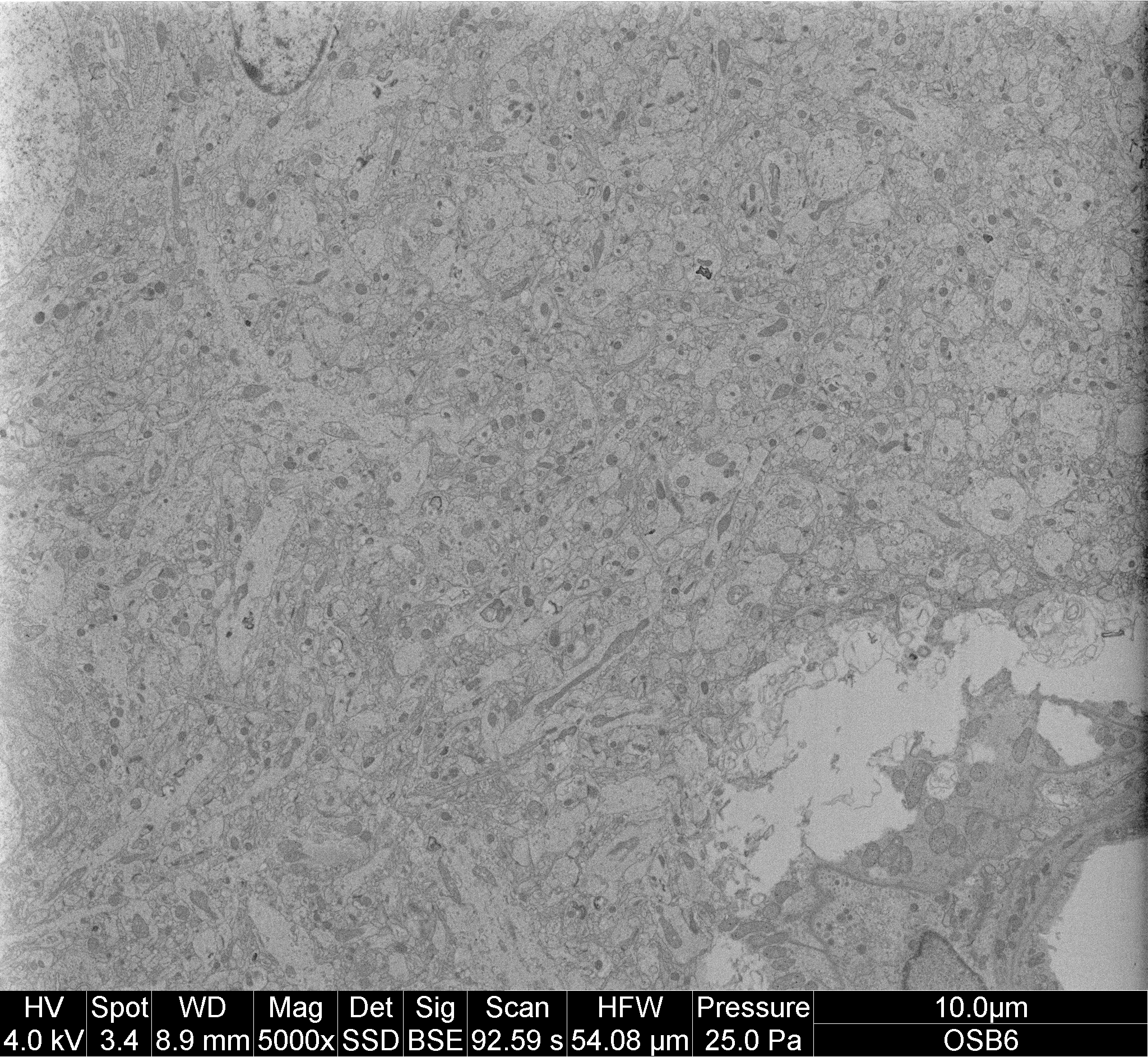

Supplement: Dataset S3 — (252.7 MB ZIP). [file pbio.0020329.sd003.zip › 040604_OS5_st1_252.tif]

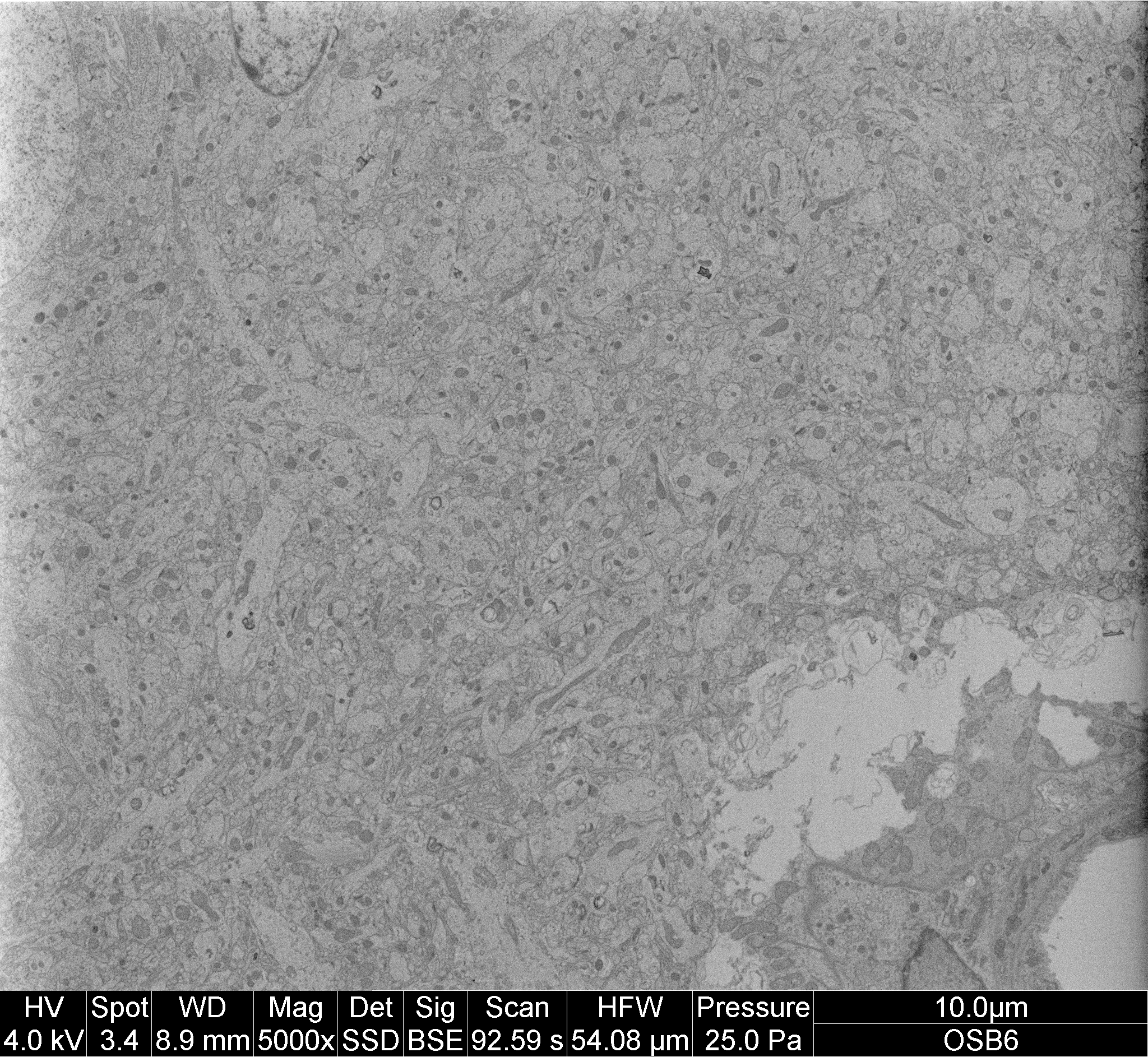

Supplement: Dataset S3 — (252.7 MB ZIP). [file pbio.0020329.sd003.zip › 040604_OS5_st1_253.tif]

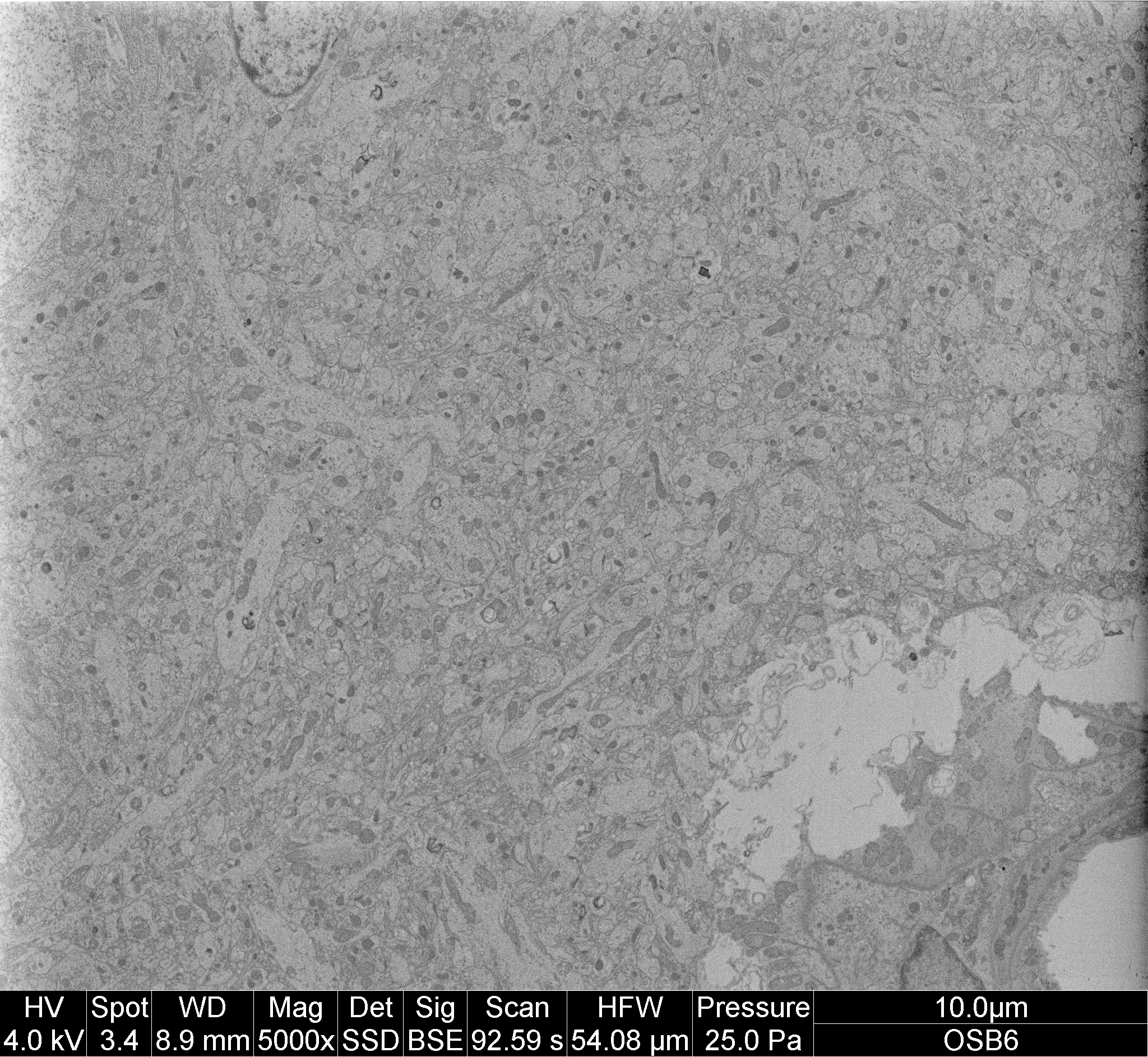

Supplement: Dataset S3 — (252.7 MB ZIP). [file pbio.0020329.sd003.zip › 040604_OS5_st1_254.tif]

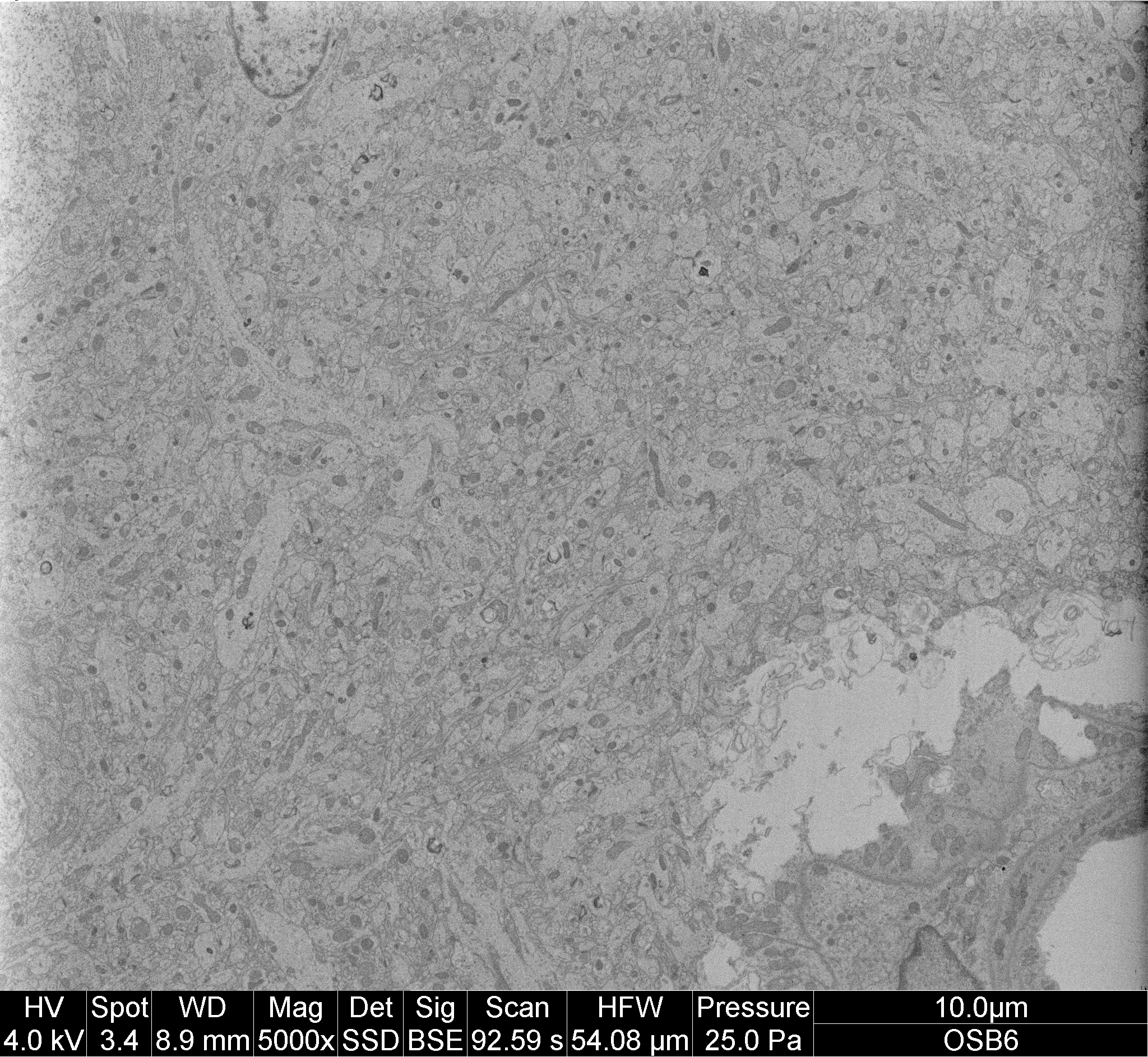

Supplement: Dataset S3 — (252.7 MB ZIP). [file pbio.0020329.sd003.zip › 040604_OS5_st1_255.tif]

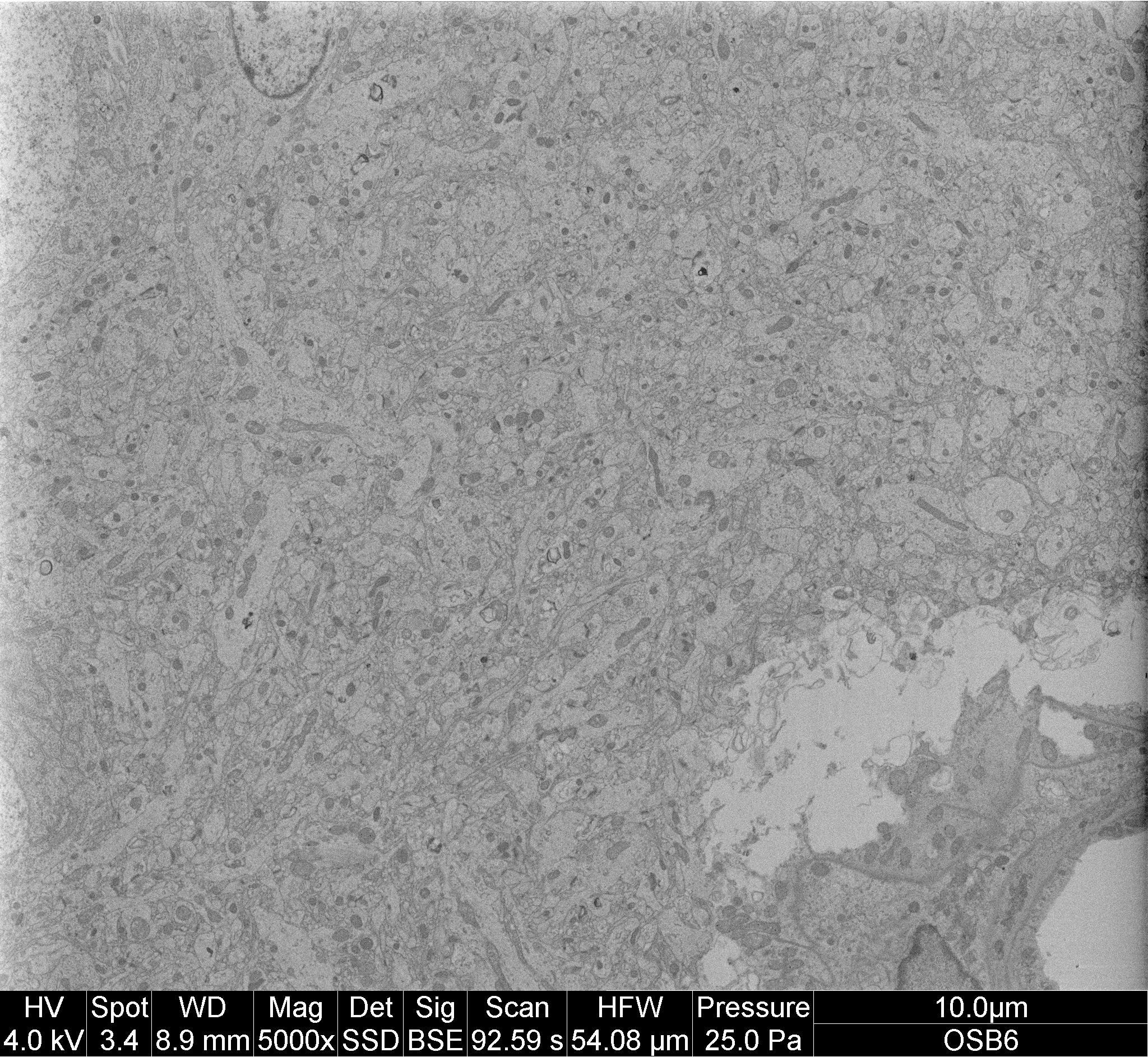

Supplement: Dataset S3 — (252.7 MB ZIP). [file pbio.0020329.sd003.zip › 040604_OS5_st1_256.tif]

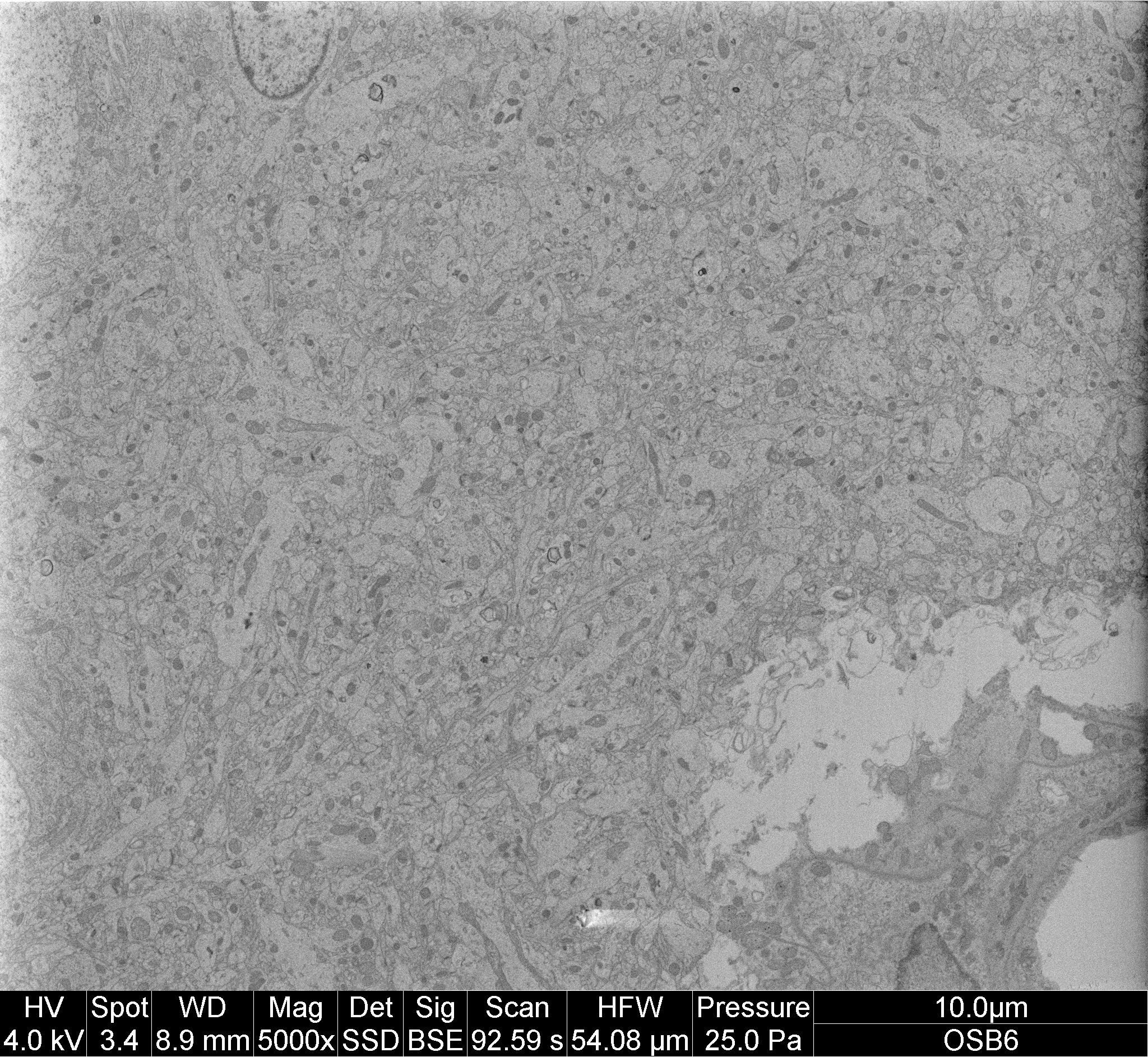

Supplement: Dataset S3 — (252.7 MB ZIP). [file pbio.0020329.sd003.zip › 040604_OS5_st1_257.tif]

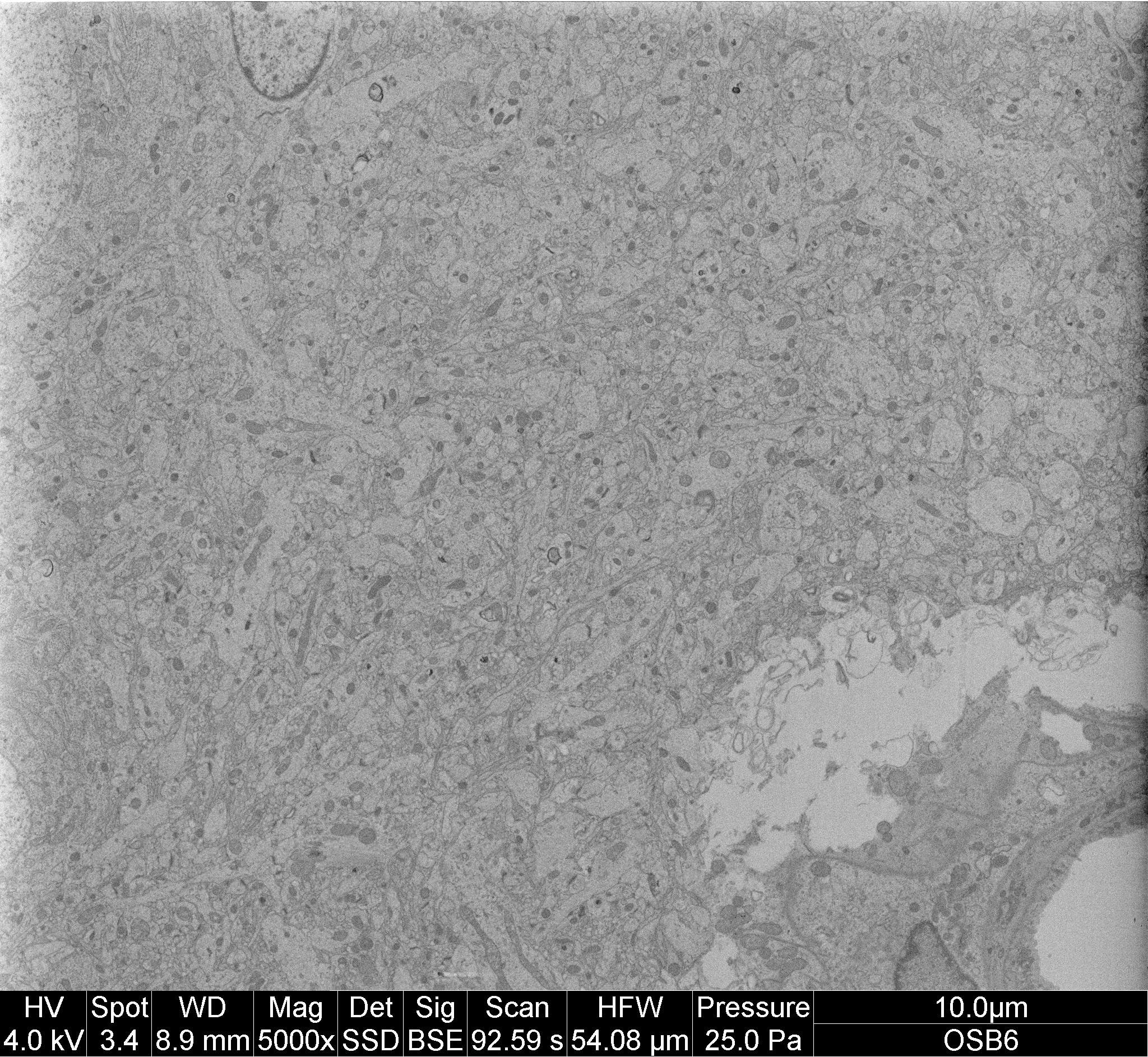

Supplement: Dataset S3 — (252.7 MB ZIP). [file pbio.0020329.sd003.zip › 040604_OS5_st1_258.tif]

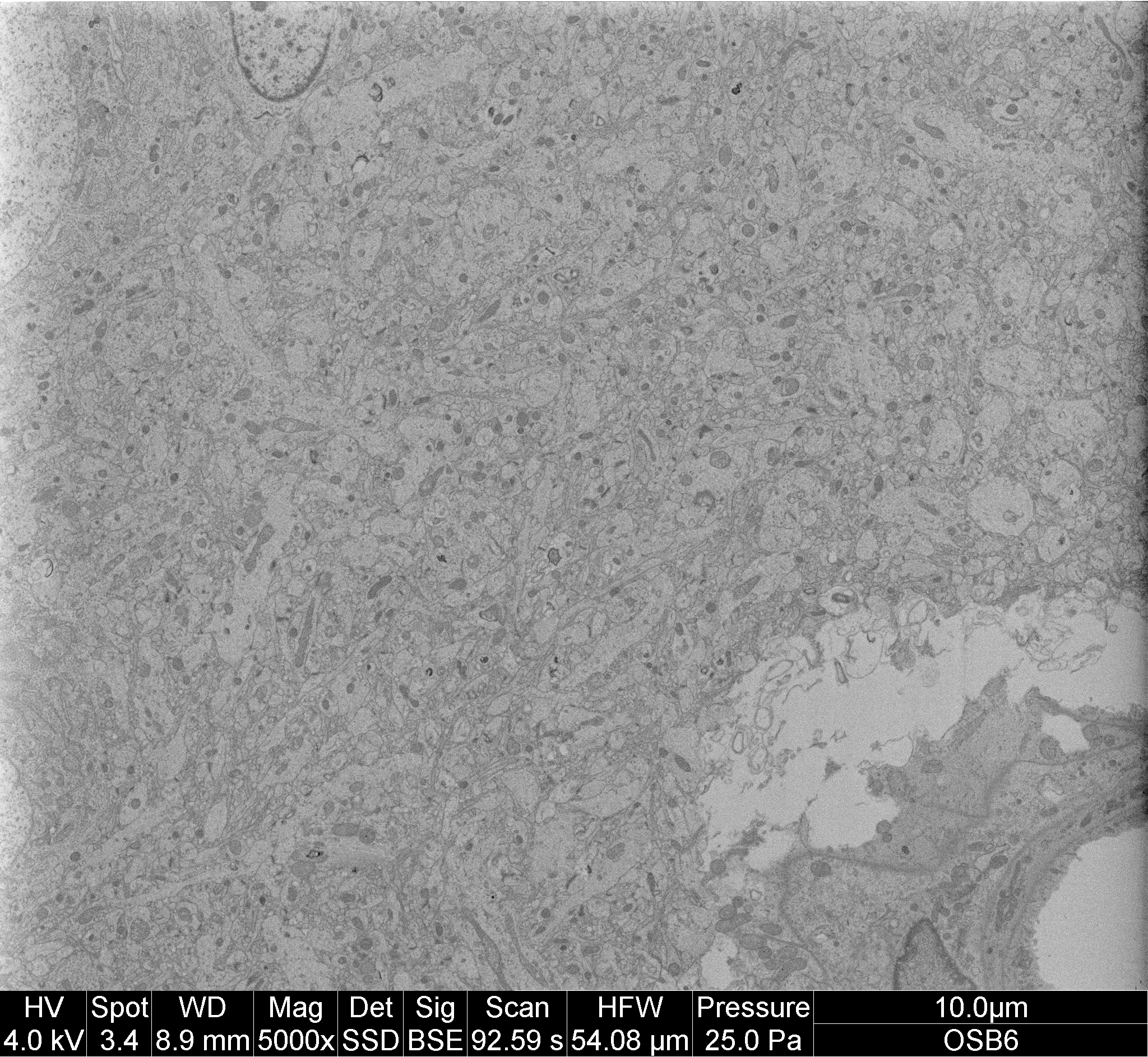

Supplement: Dataset S3 — (252.7 MB ZIP). [file pbio.0020329.sd003.zip › 040604_OS5_st1_259.tif]

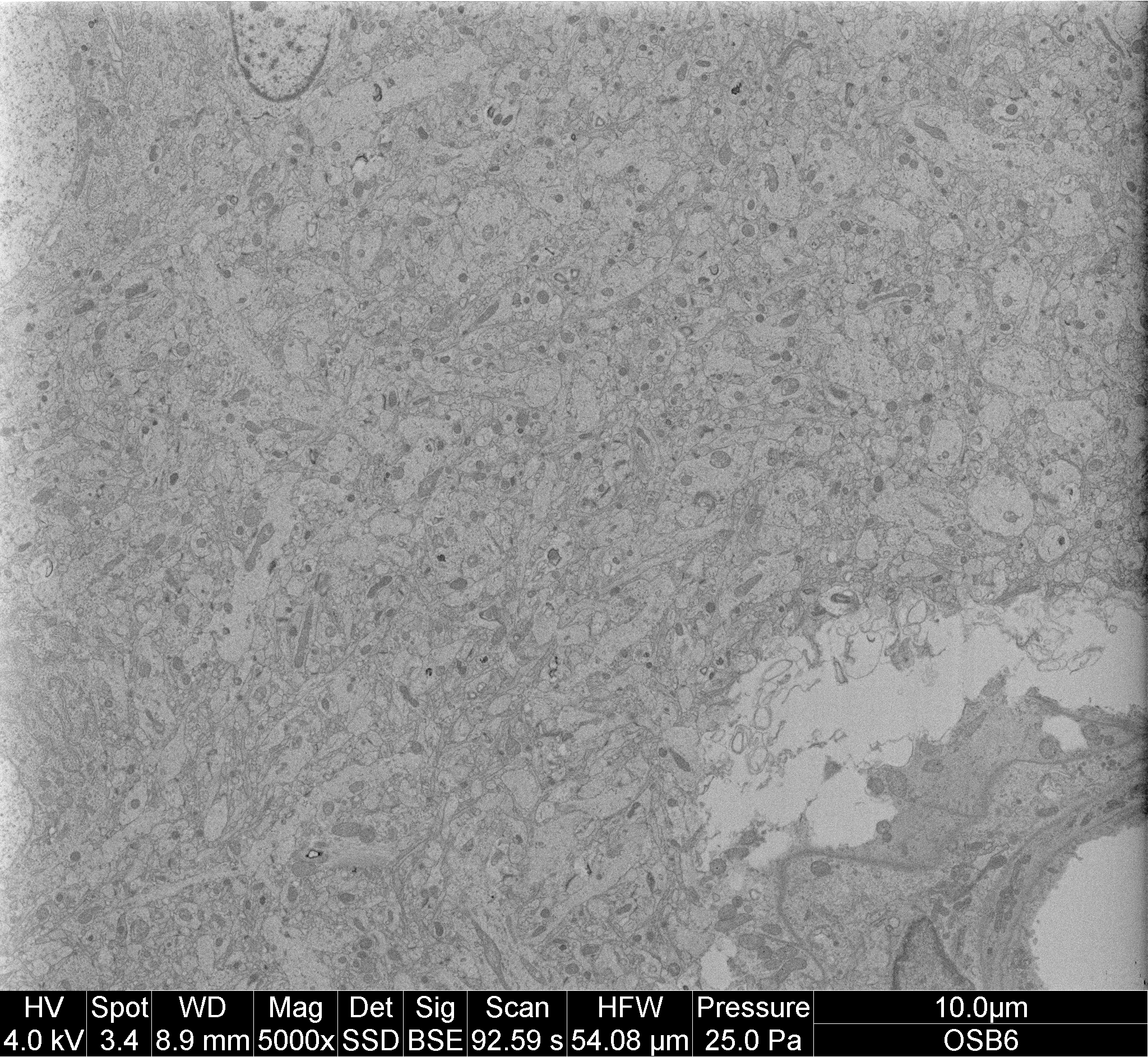

Supplement: Dataset S3 — (252.7 MB ZIP). [file pbio.0020329.sd003.zip › 040604_OS5_st1_260.tif]

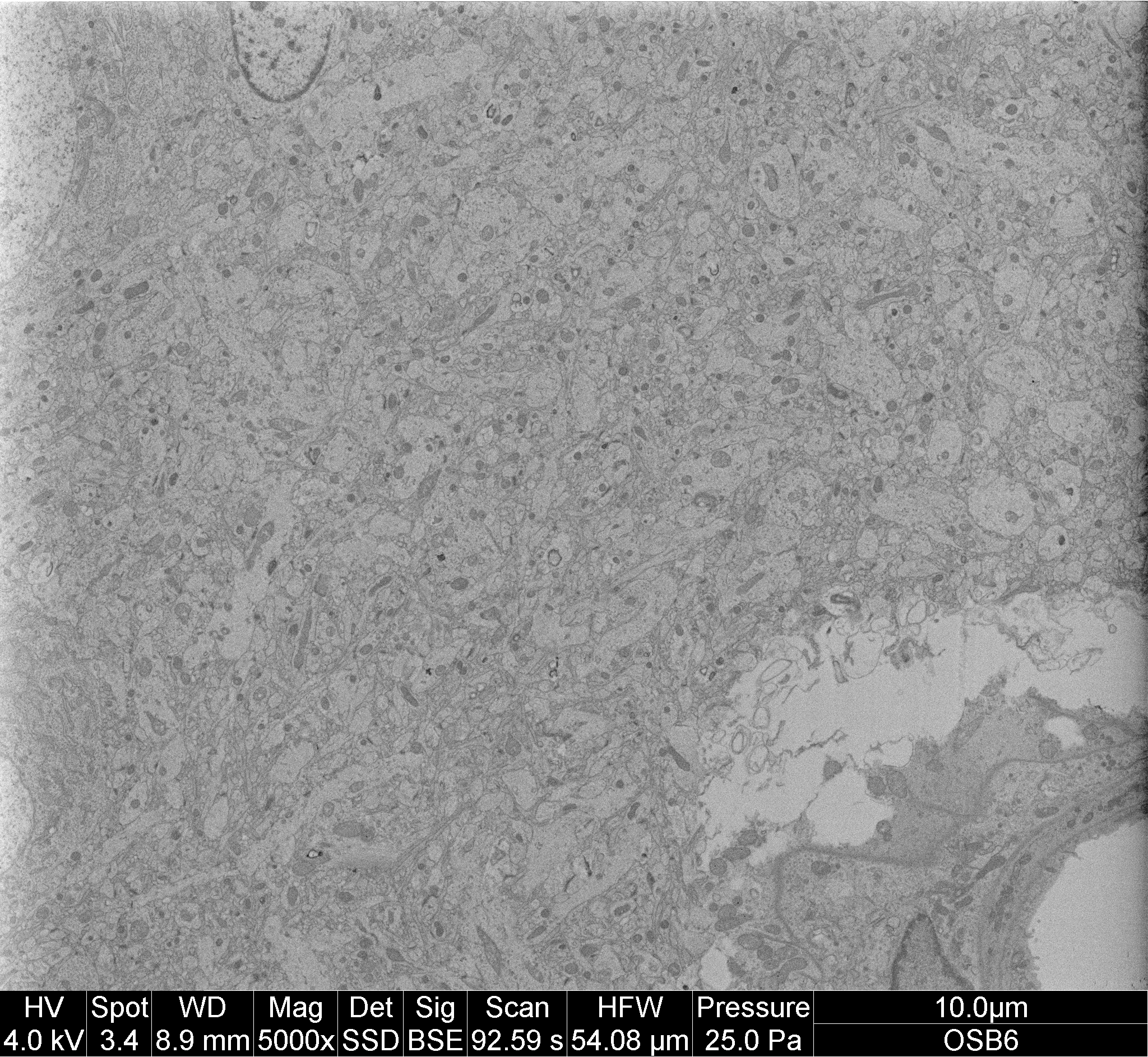

Supplement: Dataset S3 — (252.7 MB ZIP). [file pbio.0020329.sd003.zip › 040604_OS5_st1_261.tif]

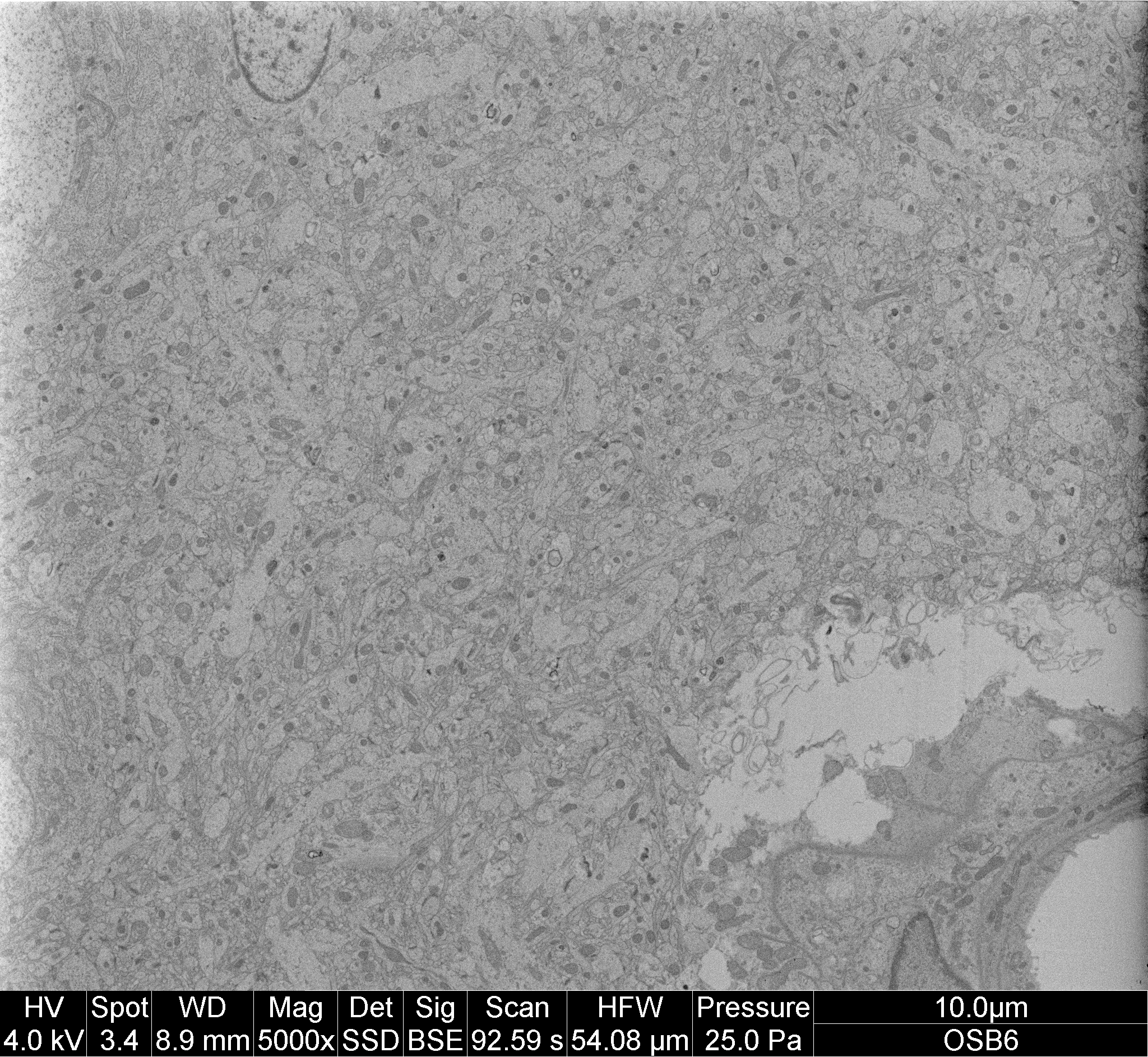

Supplement: Dataset S3 — (252.7 MB ZIP). [file pbio.0020329.sd003.zip › 040604_OS5_st1_262.tif]

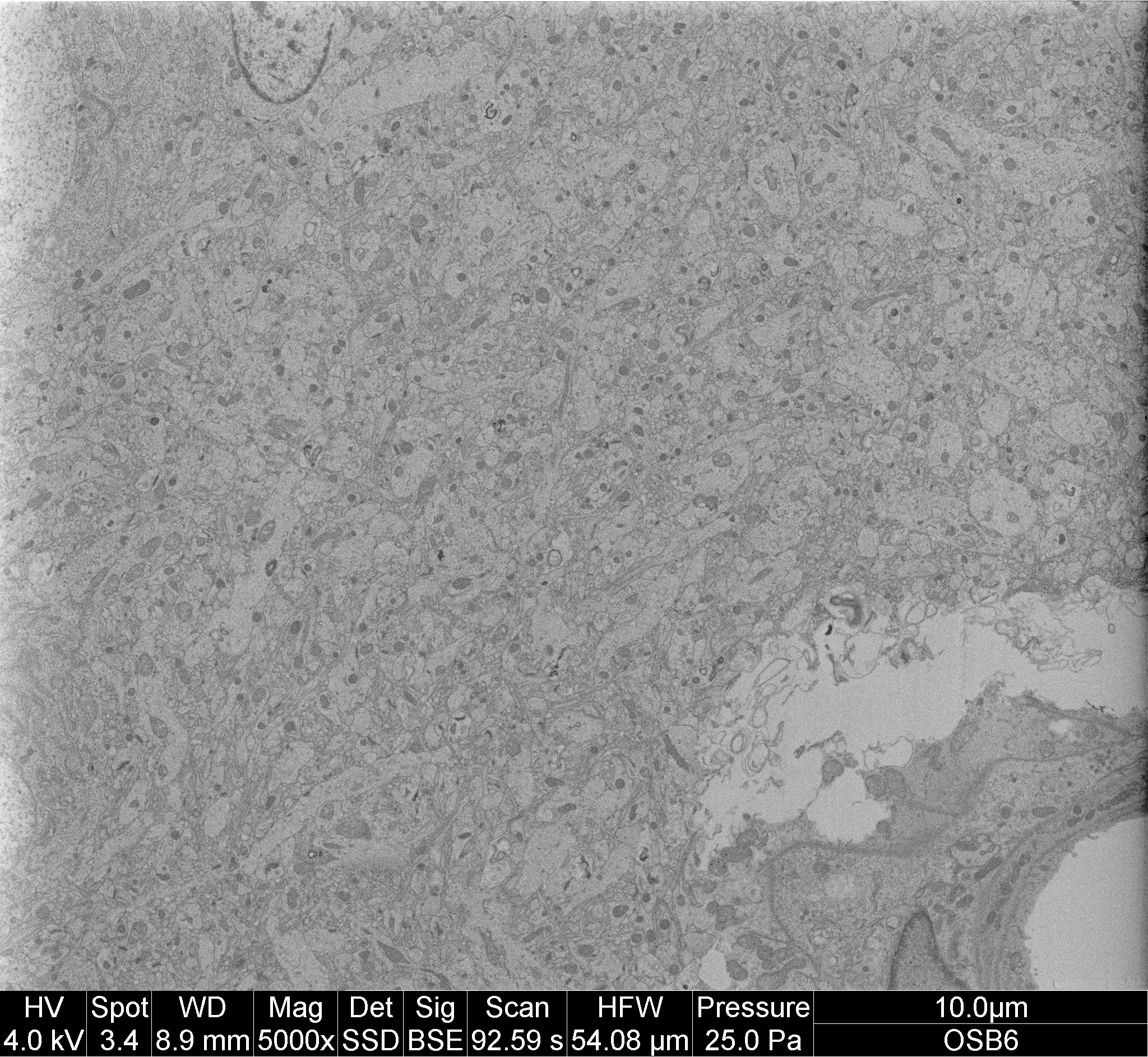

Supplement: Dataset S3 — (252.7 MB ZIP). [file pbio.0020329.sd003.zip › 040604_OS5_st1_263.tif]

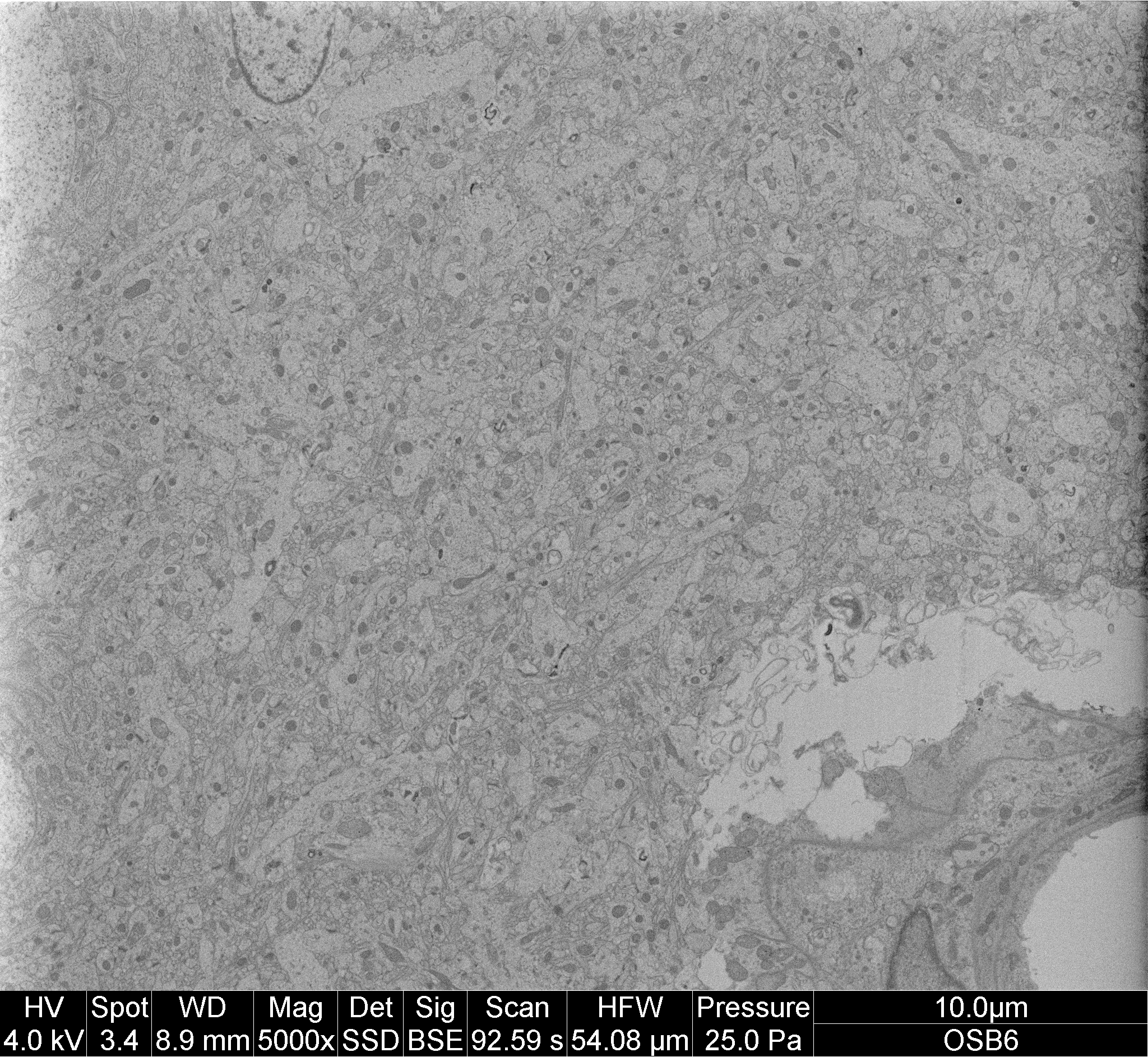

Supplement: Dataset S3 — (252.7 MB ZIP). [file pbio.0020329.sd003.zip › 040604_OS5_st1_264.tif]

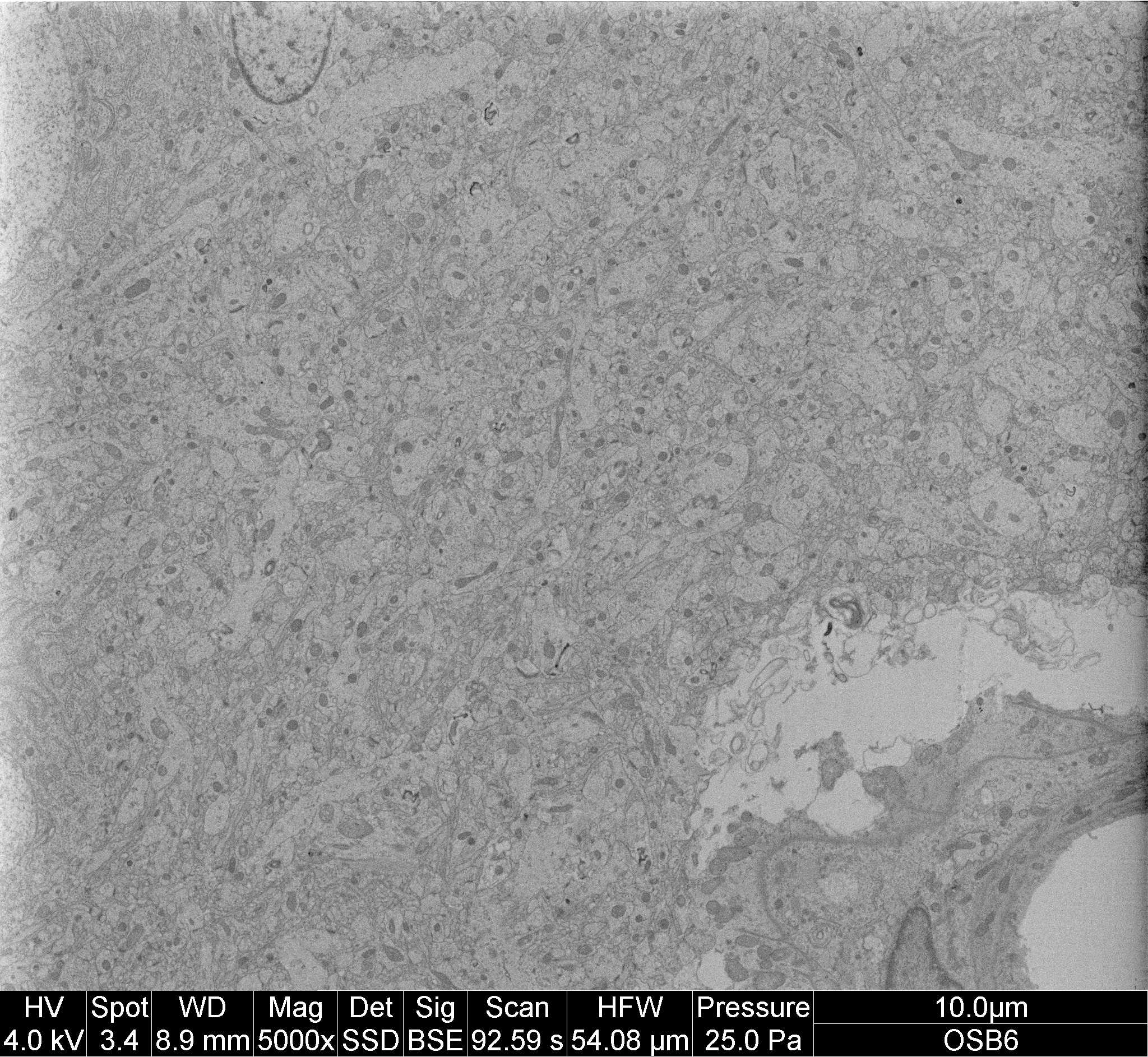

Supplement: Dataset S3 — (252.7 MB ZIP). [file pbio.0020329.sd003.zip › 040604_OS5_st1_265.tif]

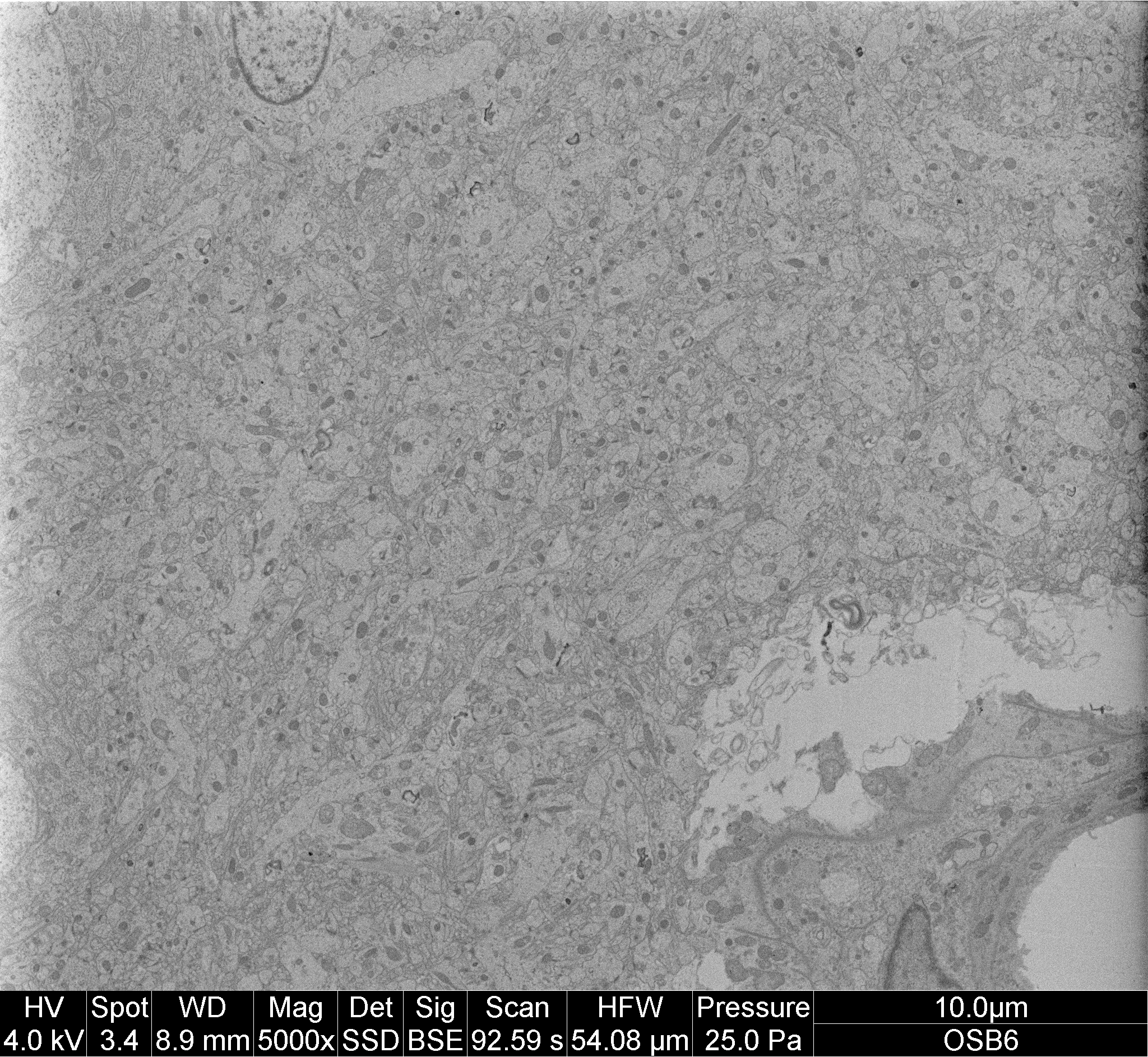

Supplement: Dataset S3 — (252.7 MB ZIP). [file pbio.0020329.sd003.zip › 040604_OS5_st1_266.tif]

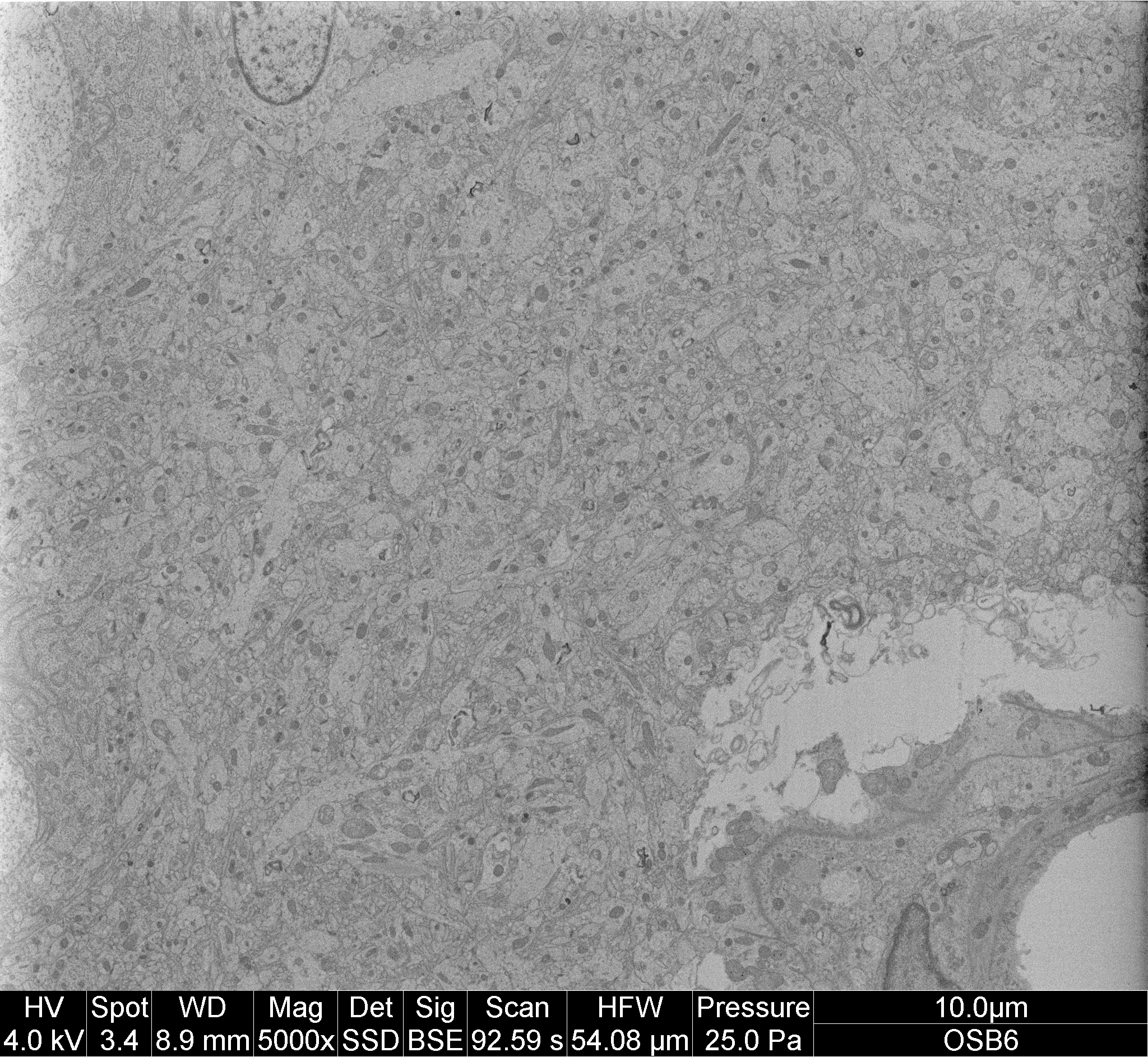

Supplement: Dataset S3 — (252.7 MB ZIP). [file pbio.0020329.sd003.zip › 040604_OS5_st1_267.tif]

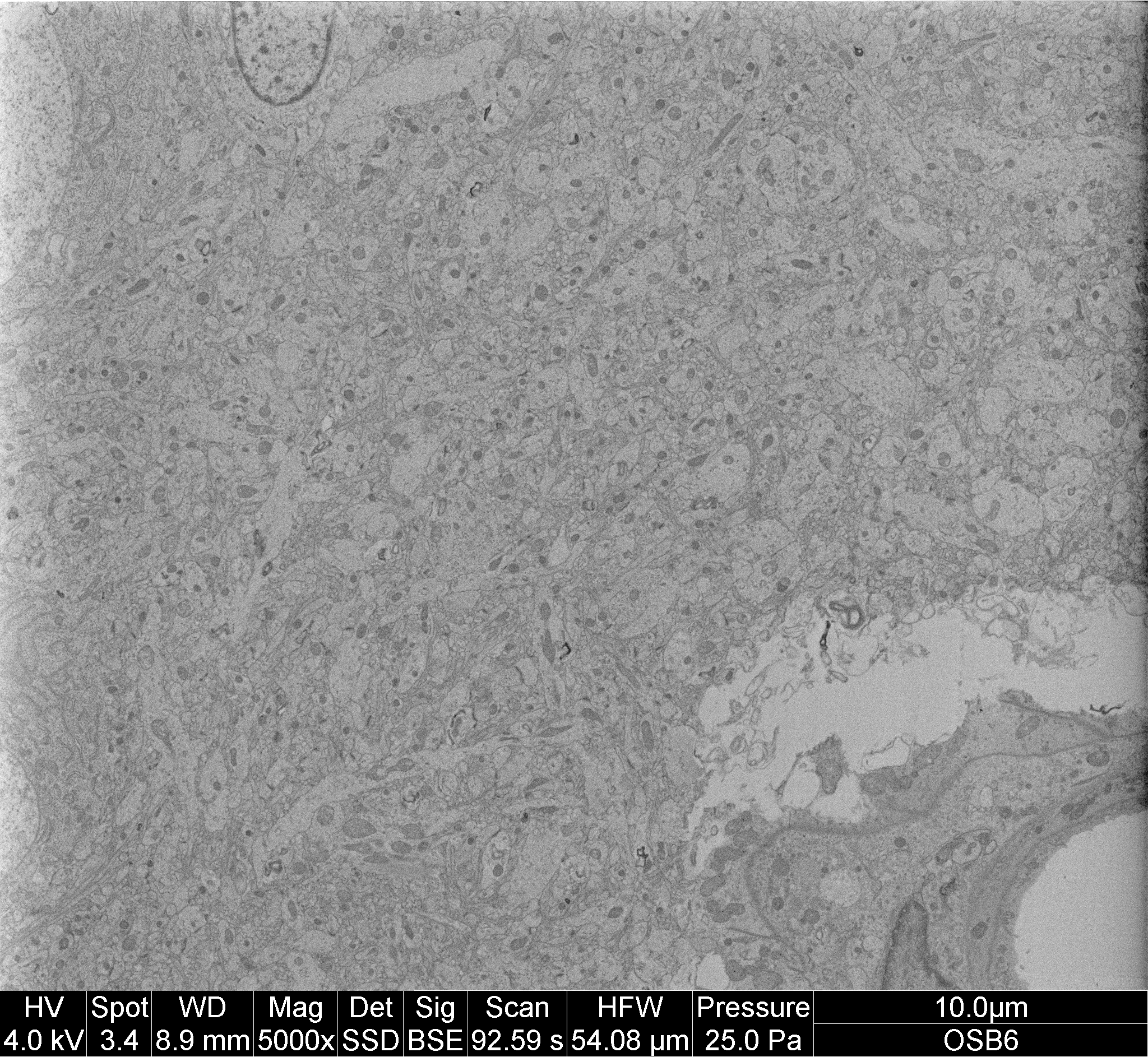

Supplement: Dataset S3 — (252.7 MB ZIP). [file pbio.0020329.sd003.zip › 040604_OS5_st1_268.tif]

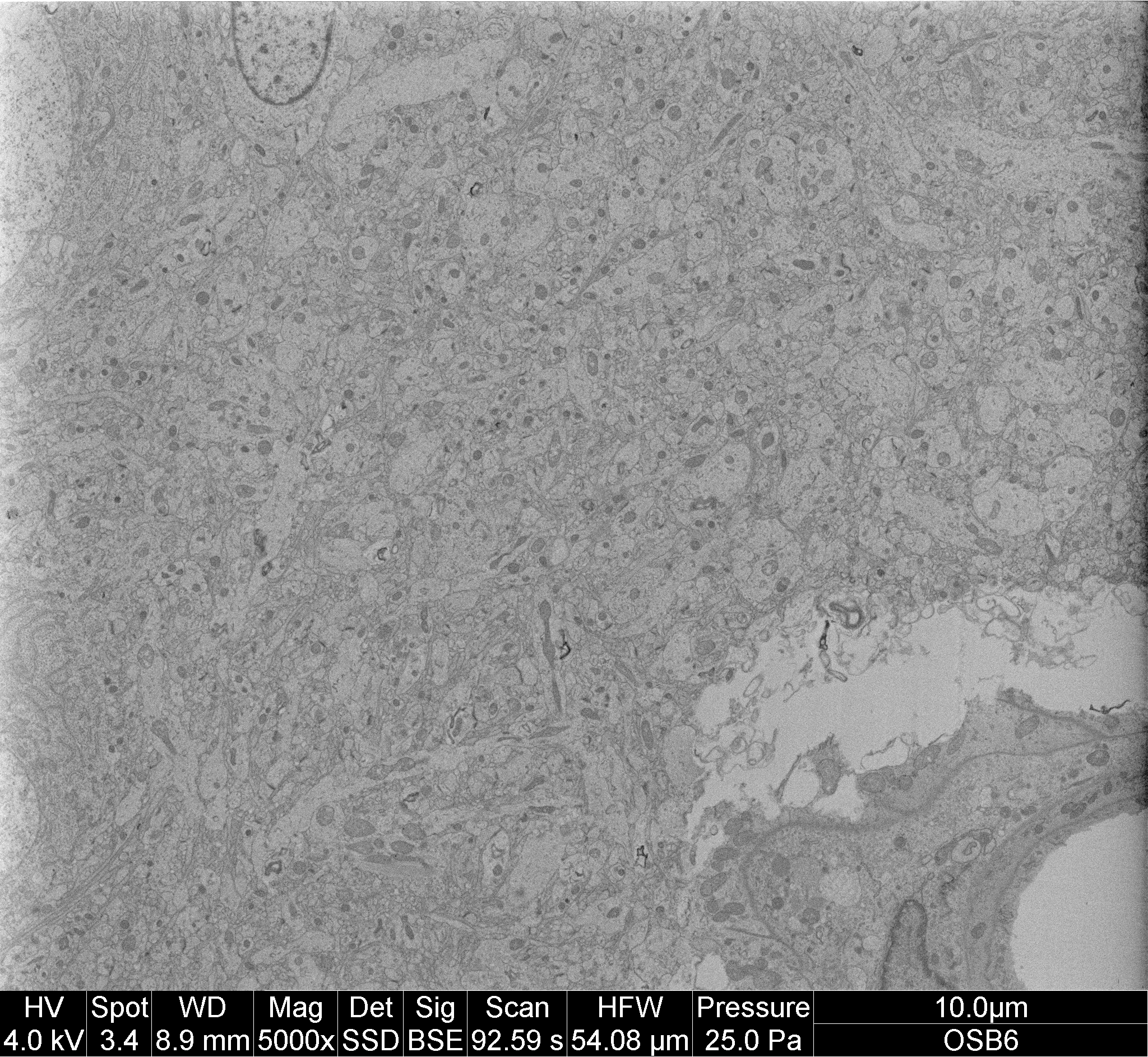

Supplement: Dataset S3 — (252.7 MB ZIP). [file pbio.0020329.sd003.zip › 040604_OS5_st1_269.tif]

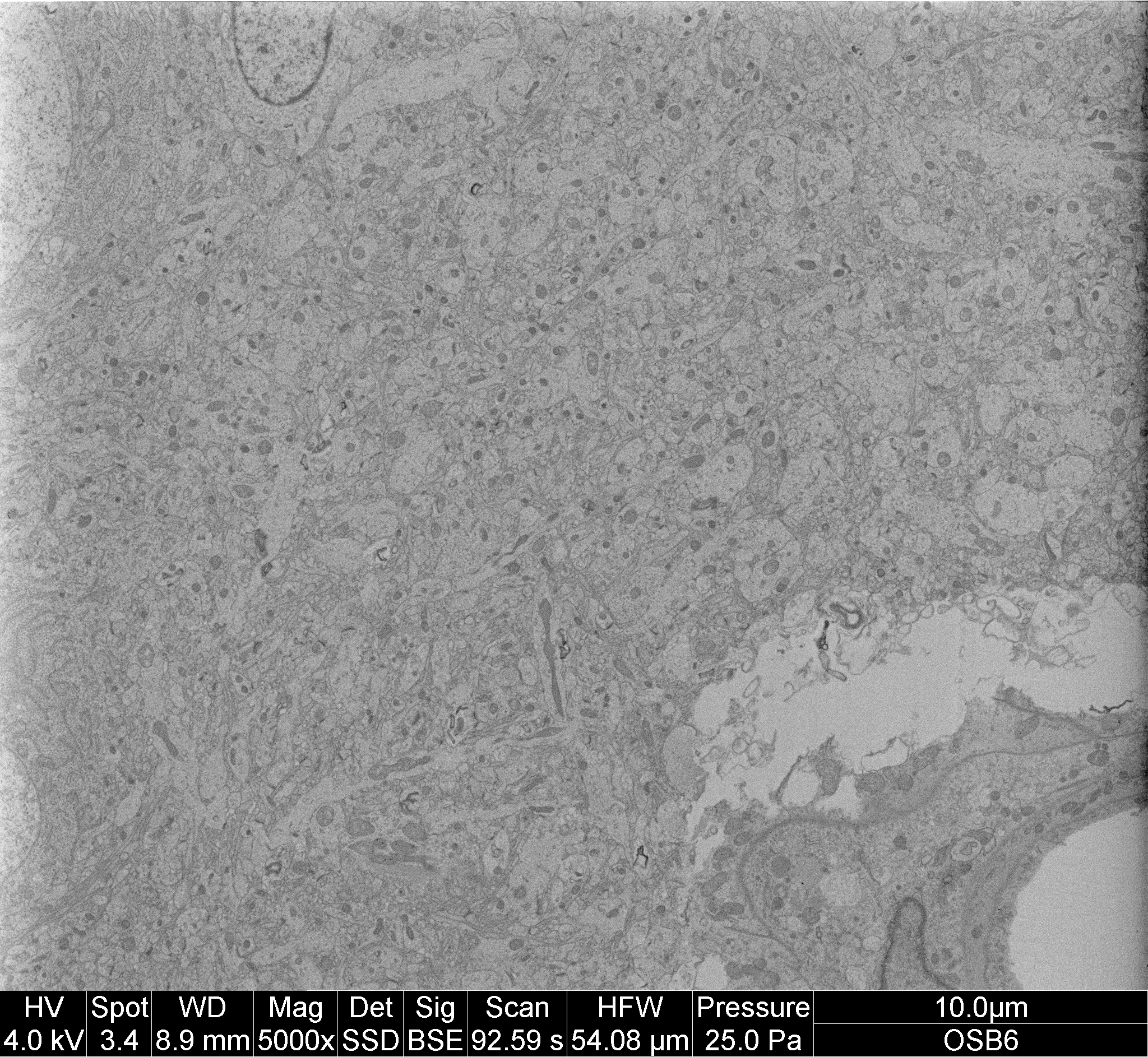

Supplement: Dataset S3 — (252.7 MB ZIP). [file pbio.0020329.sd003.zip › 040604_OS5_st1_270.tif]

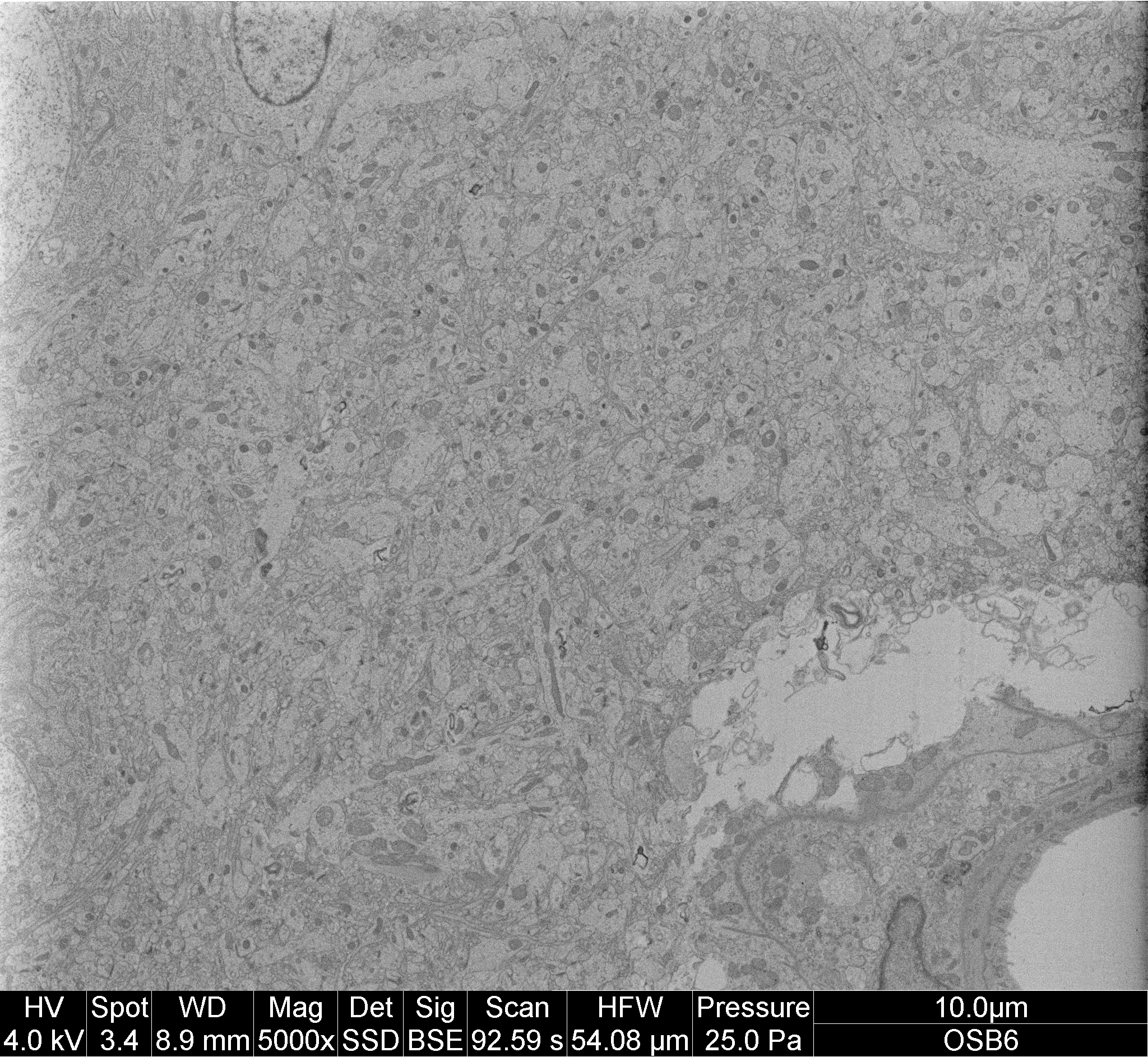

Supplement: Dataset S3 — (252.7 MB ZIP). [file pbio.0020329.sd003.zip › 040604_OS5_st1_271.tif]

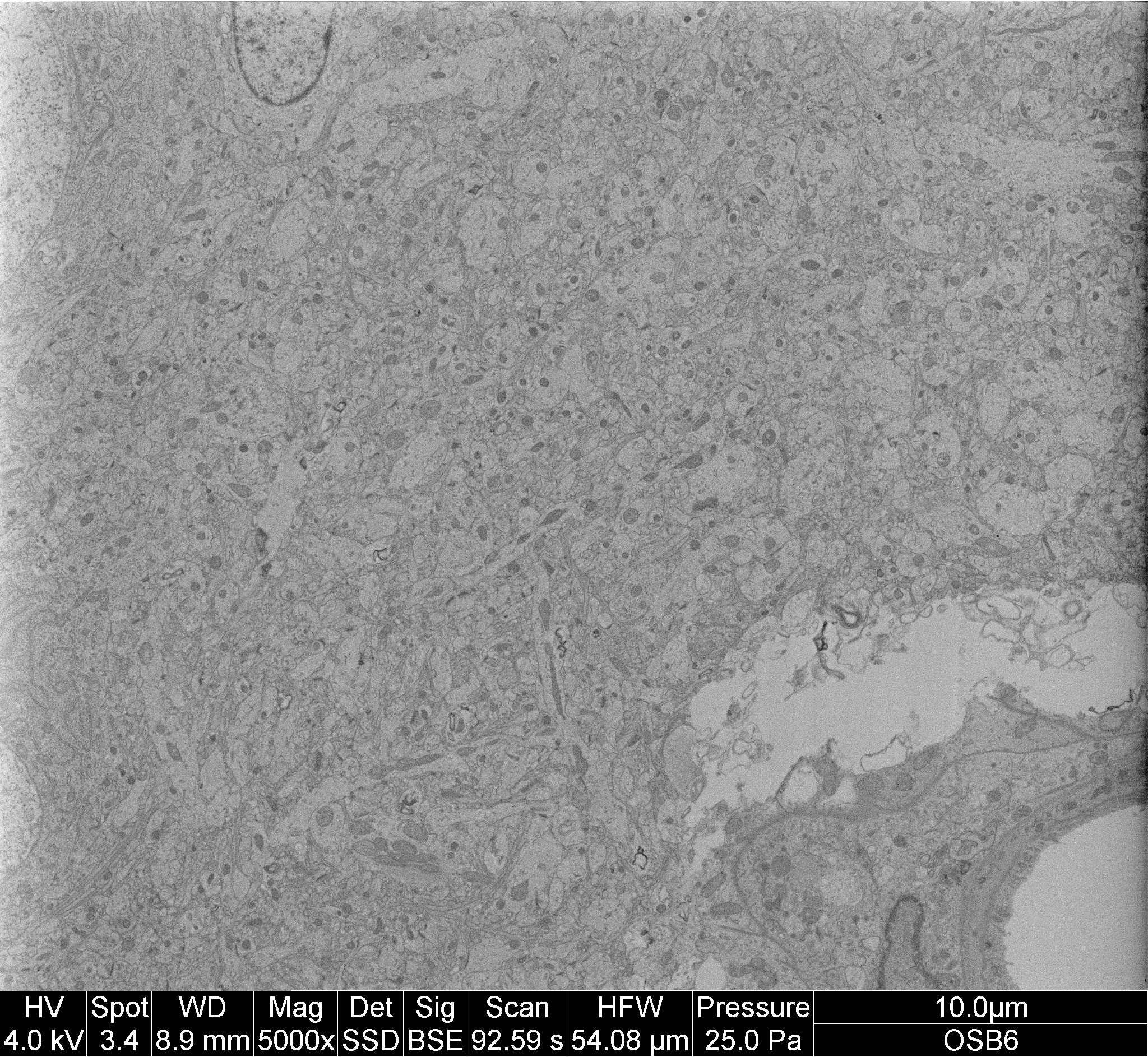

Supplement: Dataset S3 — (252.7 MB ZIP). [file pbio.0020329.sd003.zip › 040604_OS5_st1_272.tif]

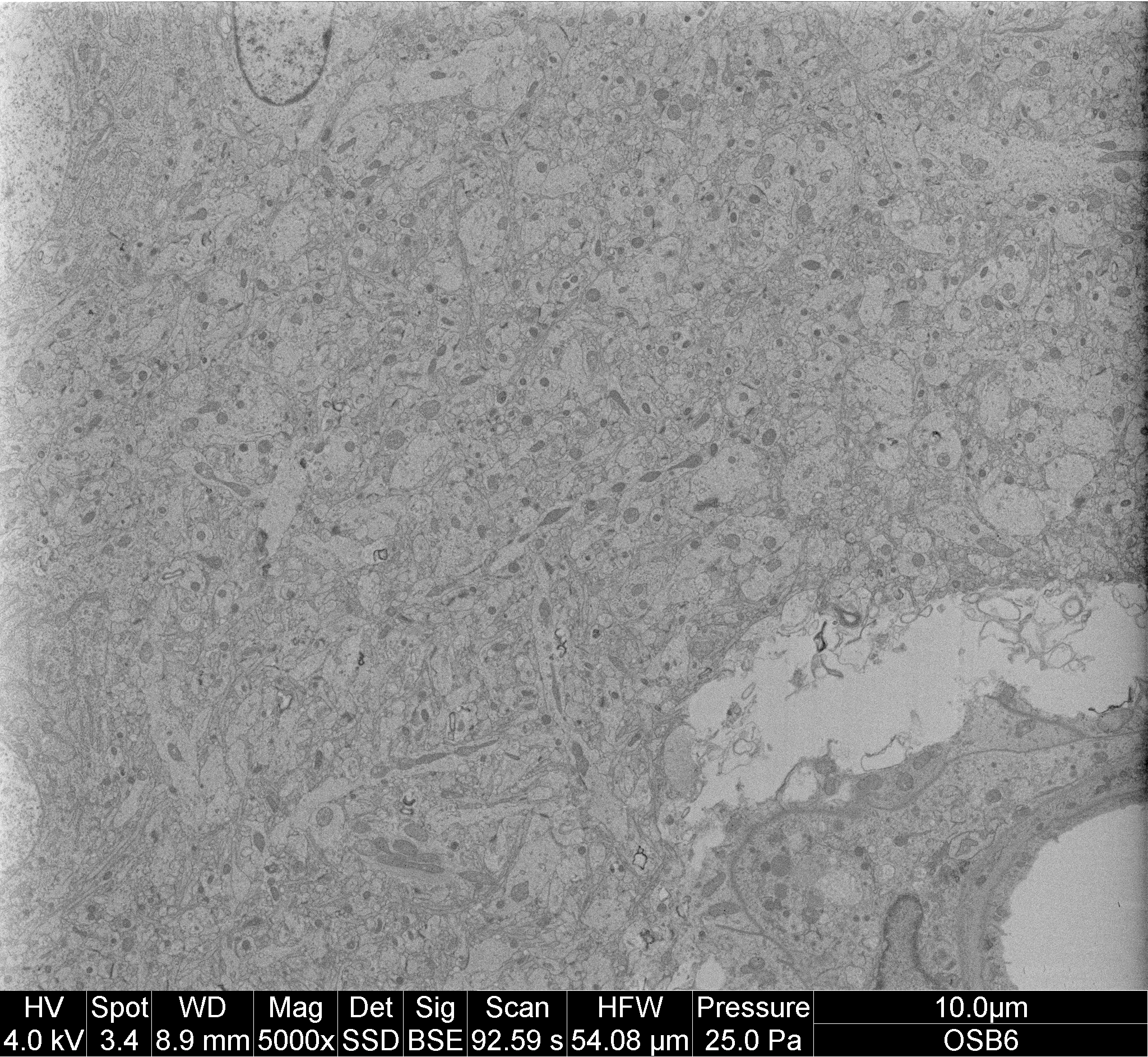

Supplement: Dataset S3 — (252.7 MB ZIP). [file pbio.0020329.sd003.zip › 040604_OS5_st1_273.tif]

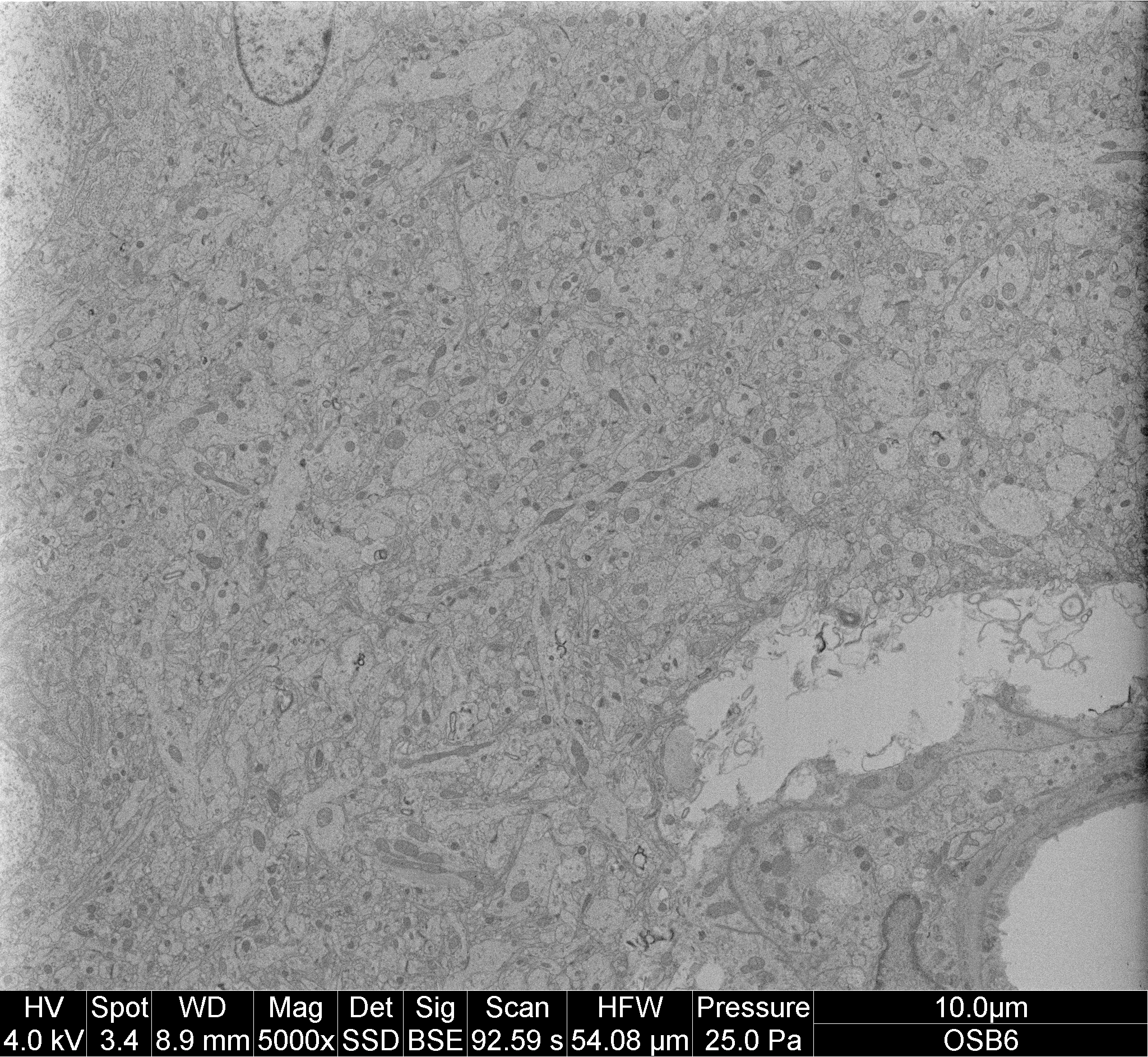

Supplement: Dataset S3 — (252.7 MB ZIP). [file pbio.0020329.sd003.zip › 040604_OS5_st1_274.tif]

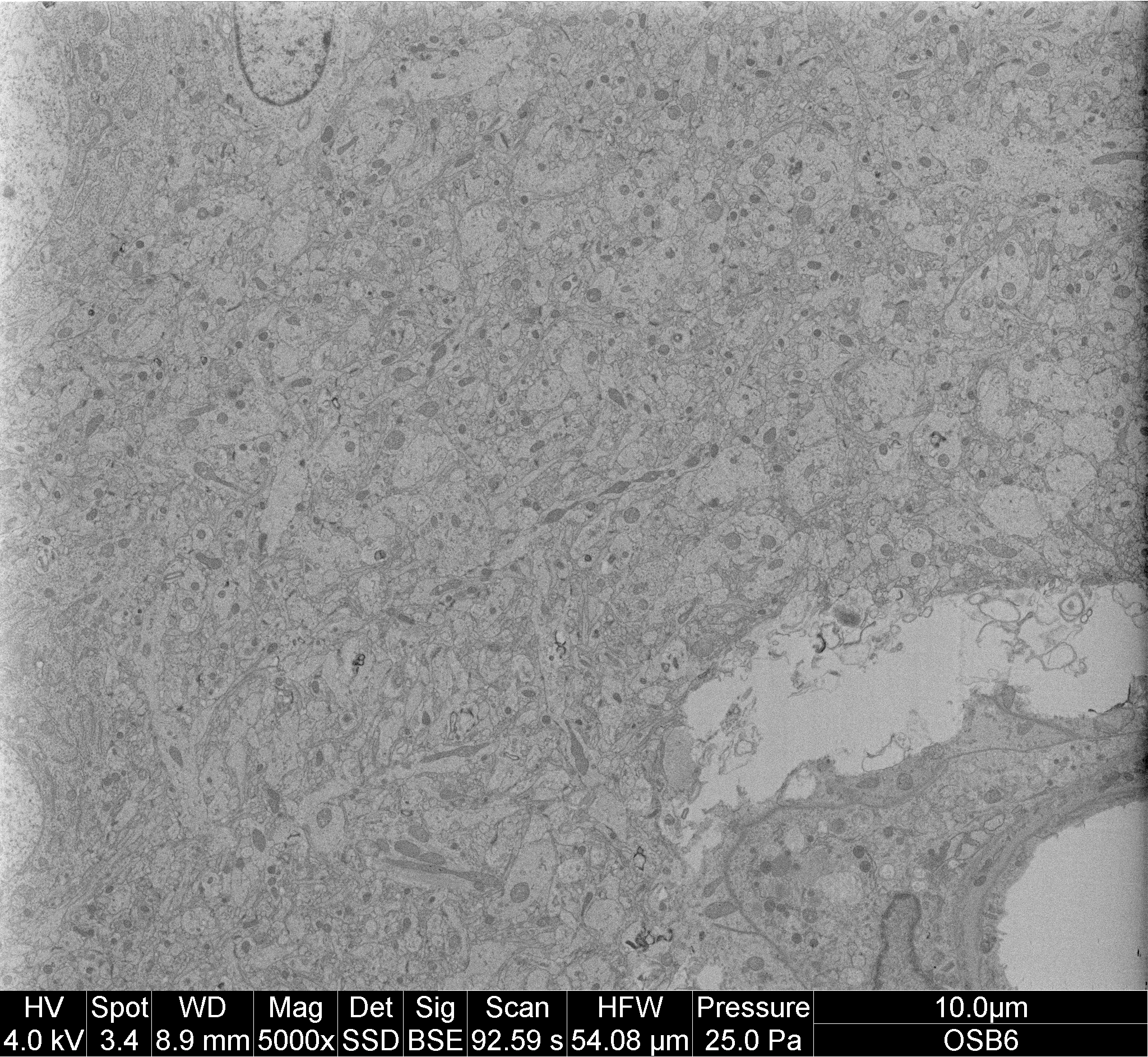

Supplement: Dataset S3 — (252.7 MB ZIP). [file pbio.0020329.sd003.zip › 040604_OS5_st1_275.tif]

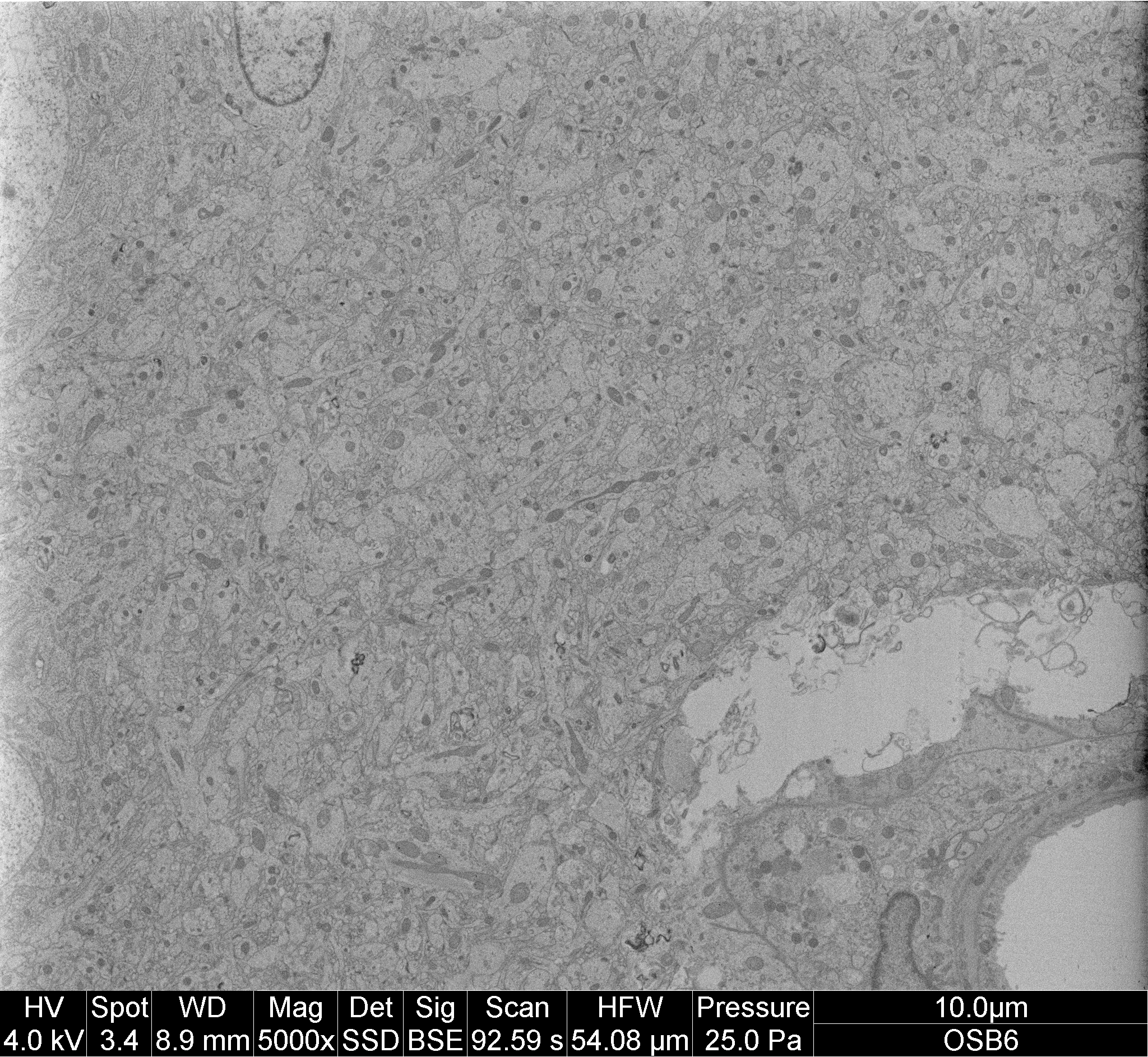

Supplement: Dataset S3 — (252.7 MB ZIP). [file pbio.0020329.sd003.zip › 040604_OS5_st1_276.tif]

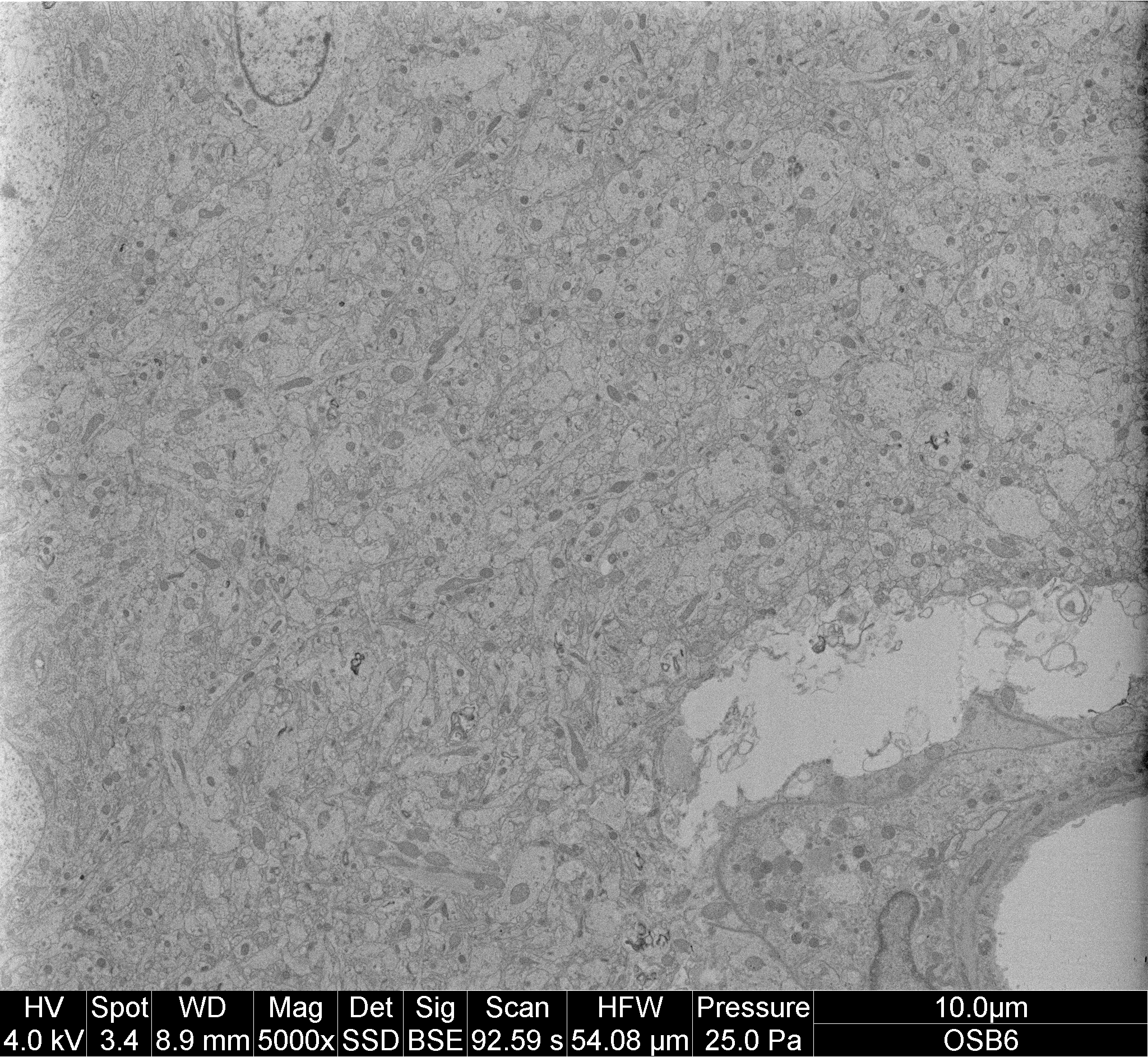

Supplement: Dataset S3 — (252.7 MB ZIP). [file pbio.0020329.sd003.zip › 040604_OS5_st1_277.tif]

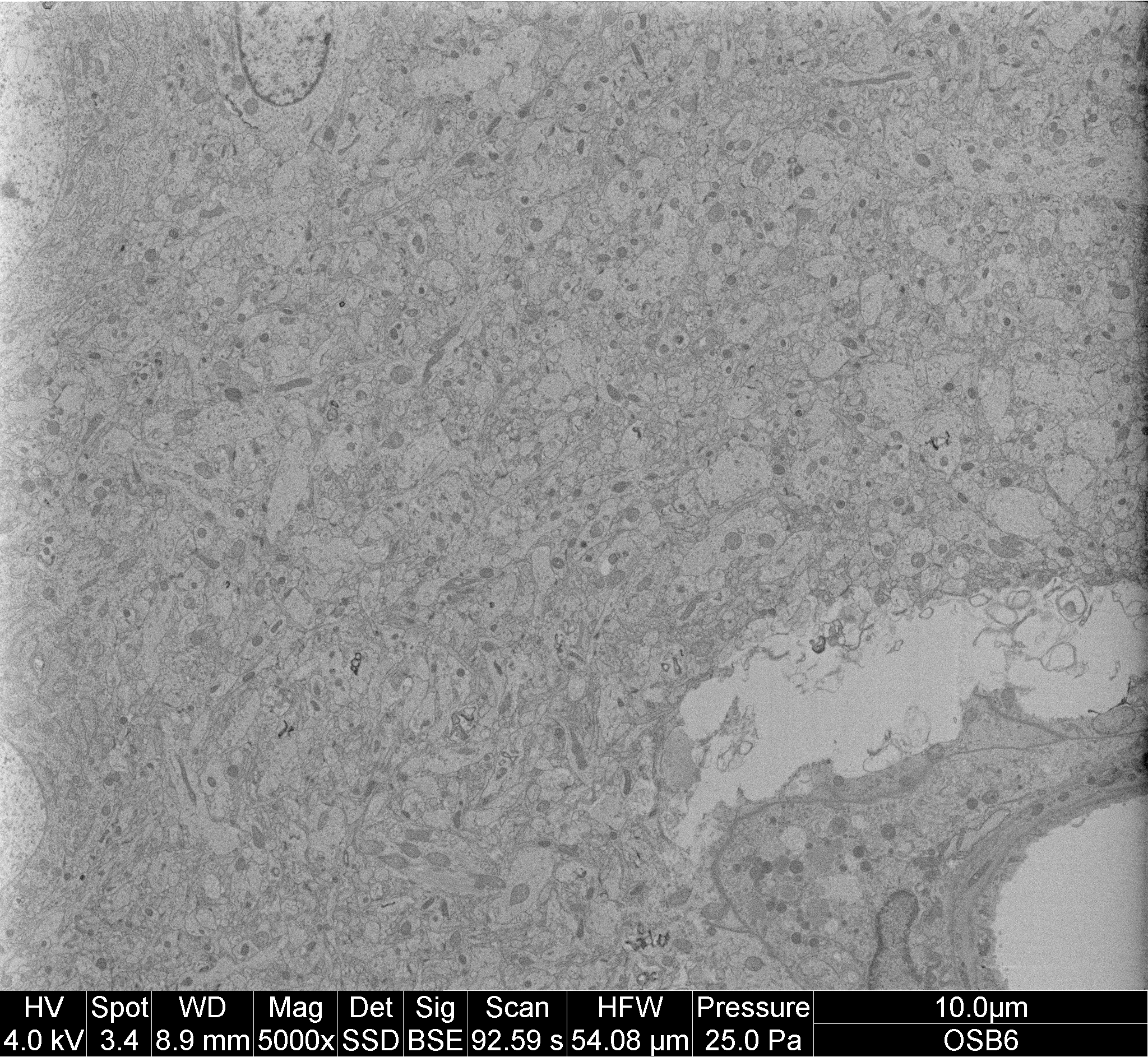

Supplement: Dataset S3 — (252.7 MB ZIP). [file pbio.0020329.sd003.zip › 040604_OS5_st1_278.tif]

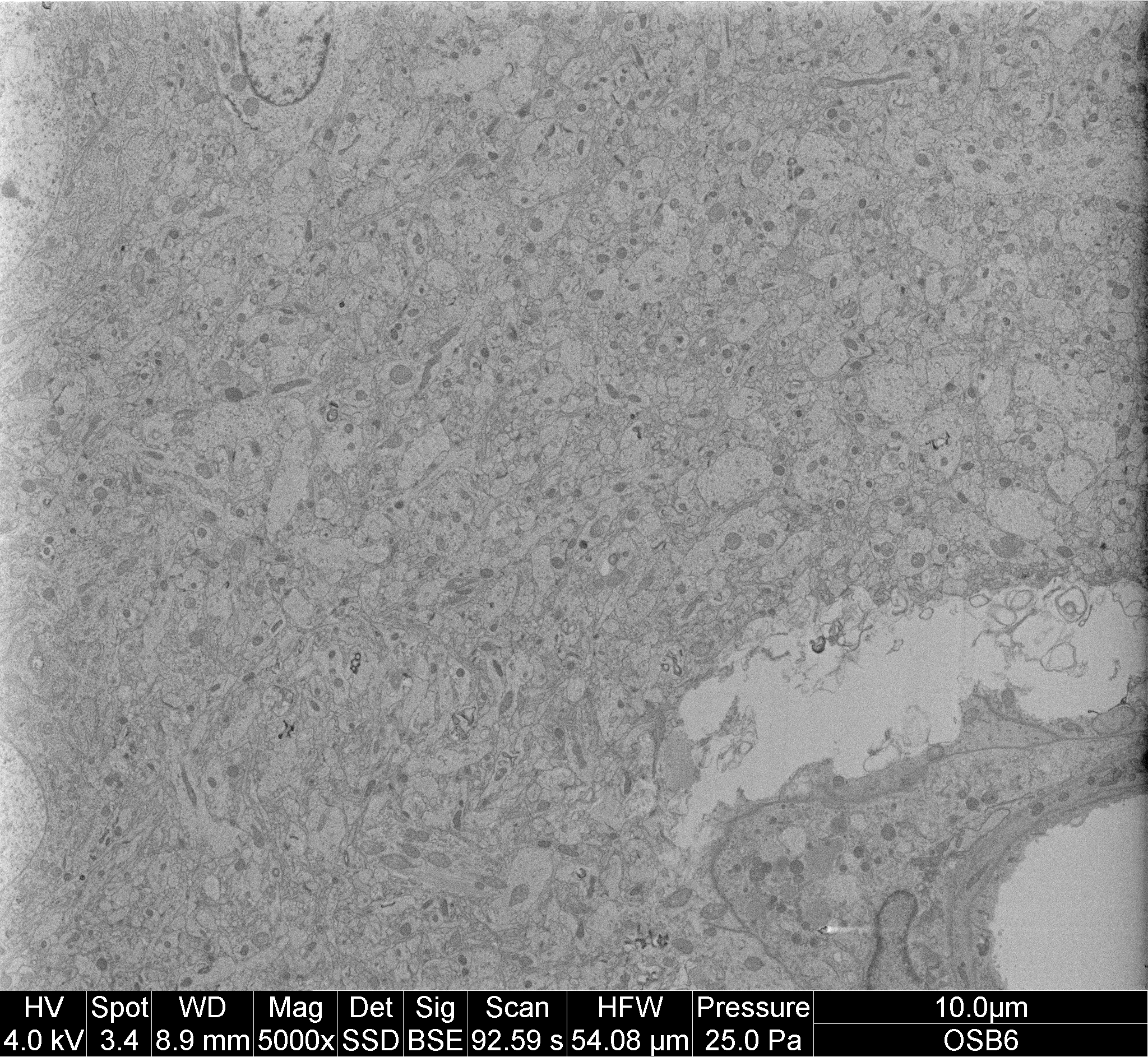

Supplement: Dataset S3 — (252.7 MB ZIP). [file pbio.0020329.sd003.zip › 040604_OS5_st1_279.tif]

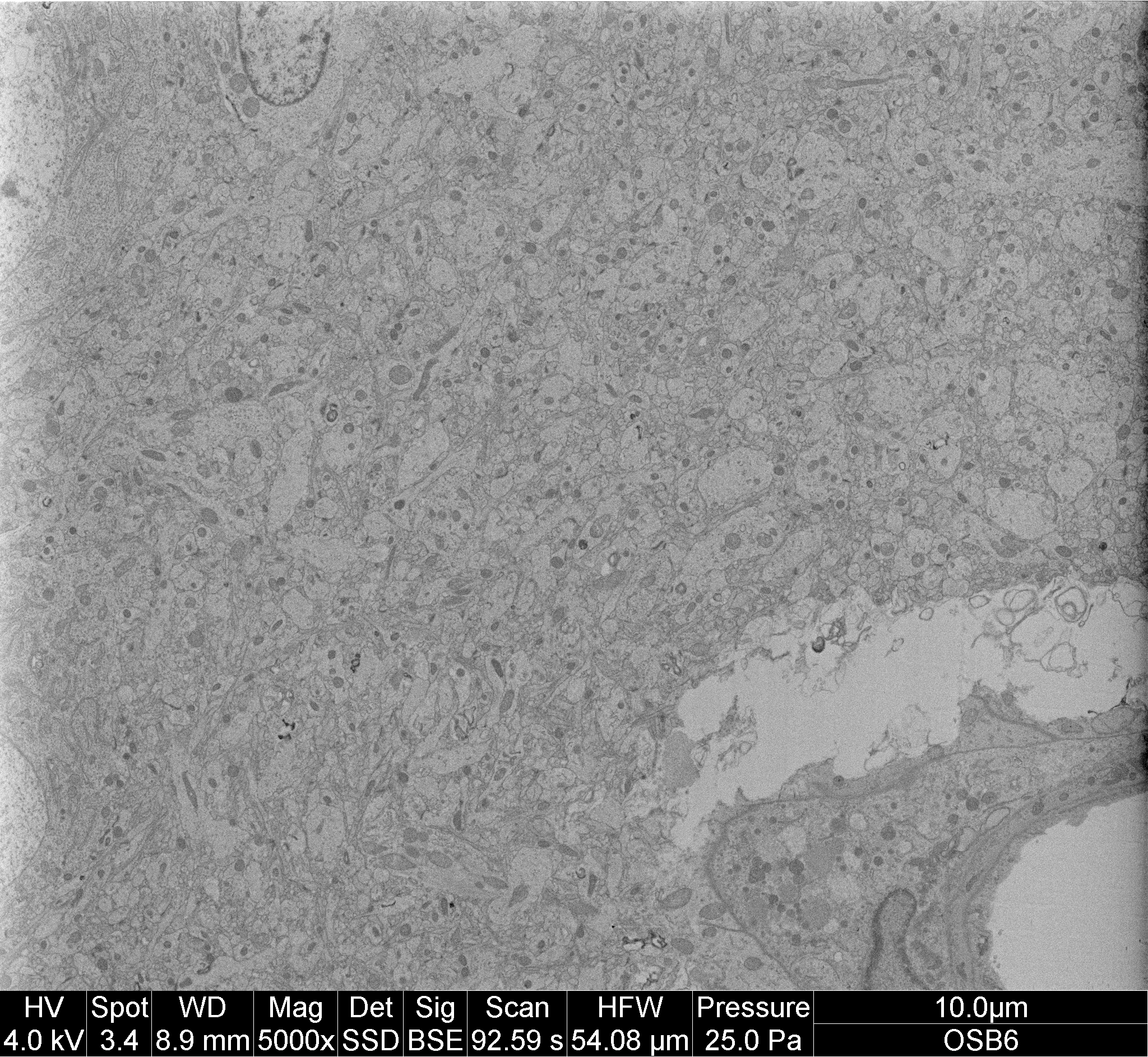

Supplement: Dataset S3 — (252.7 MB ZIP). [file pbio.0020329.sd003.zip › 040604_OS5_st1_280.tif]

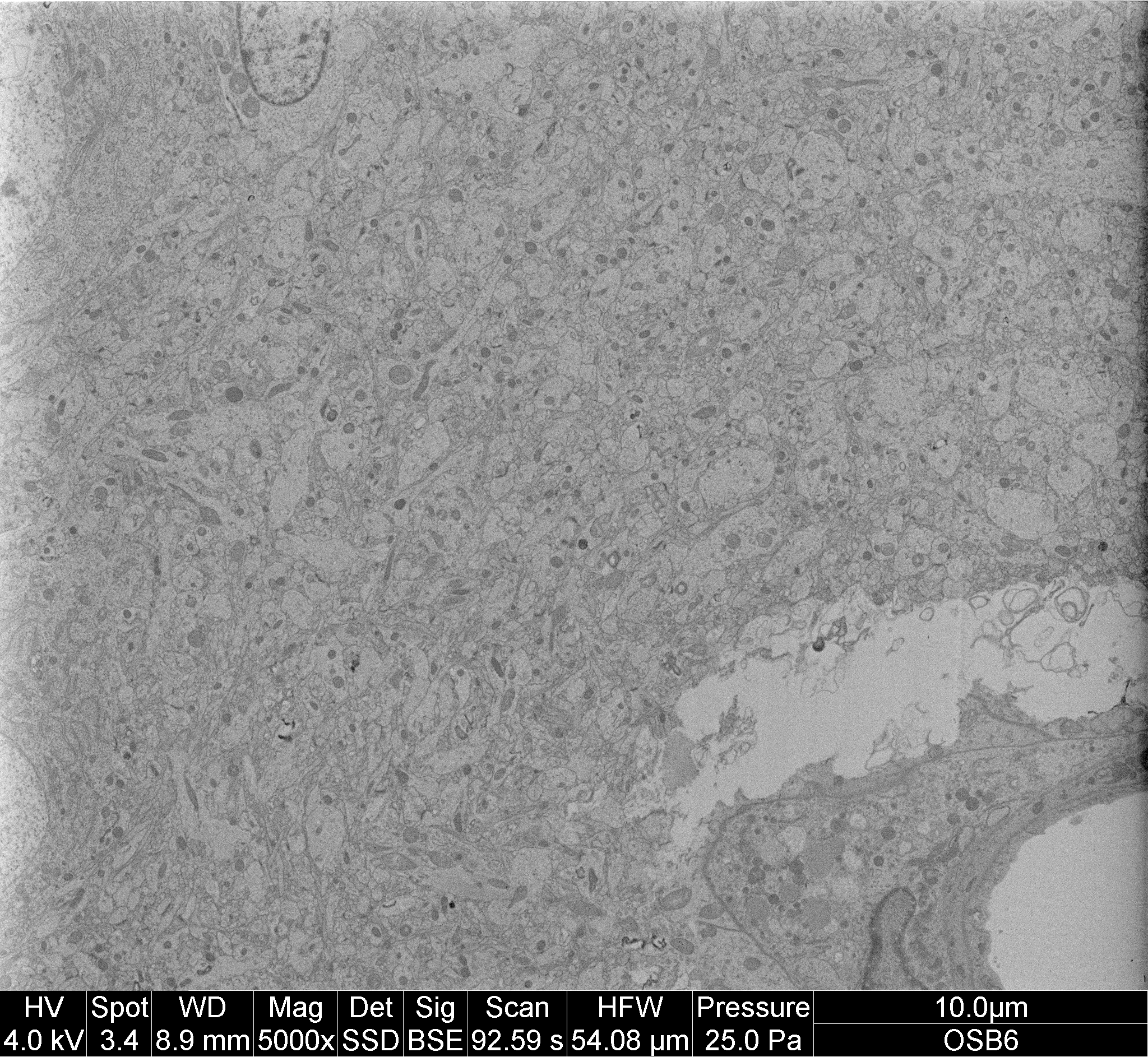

Supplement: Dataset S3 — (252.7 MB ZIP). [file pbio.0020329.sd003.zip › 040604_OS5_st1_281.tif]

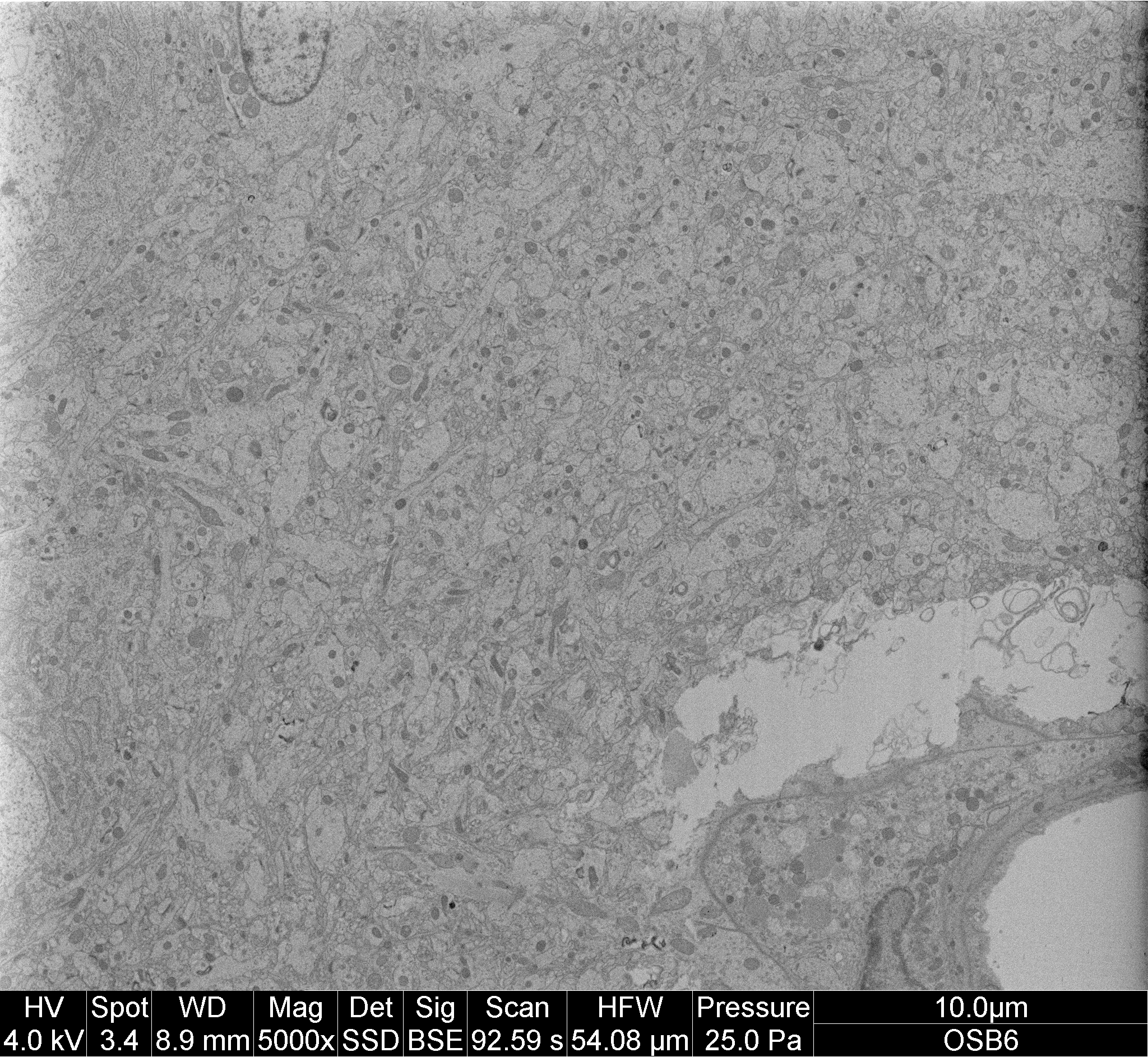

Supplement: Dataset S3 — (252.7 MB ZIP). [file pbio.0020329.sd003.zip › 040604_OS5_st1_282.tif]

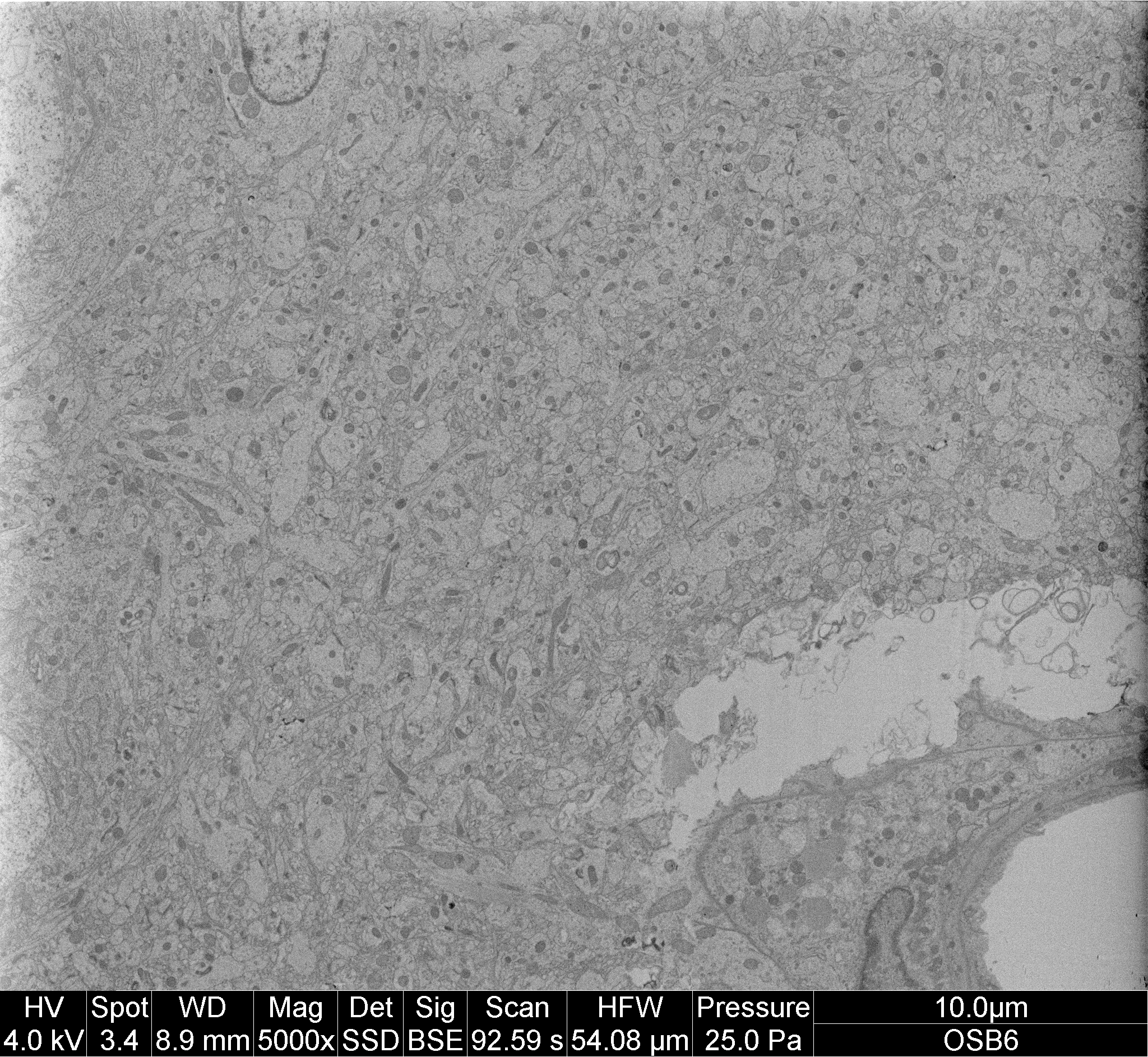

Supplement: Dataset S3 — (252.7 MB ZIP). [file pbio.0020329.sd003.zip › 040604_OS5_st1_283.tif]

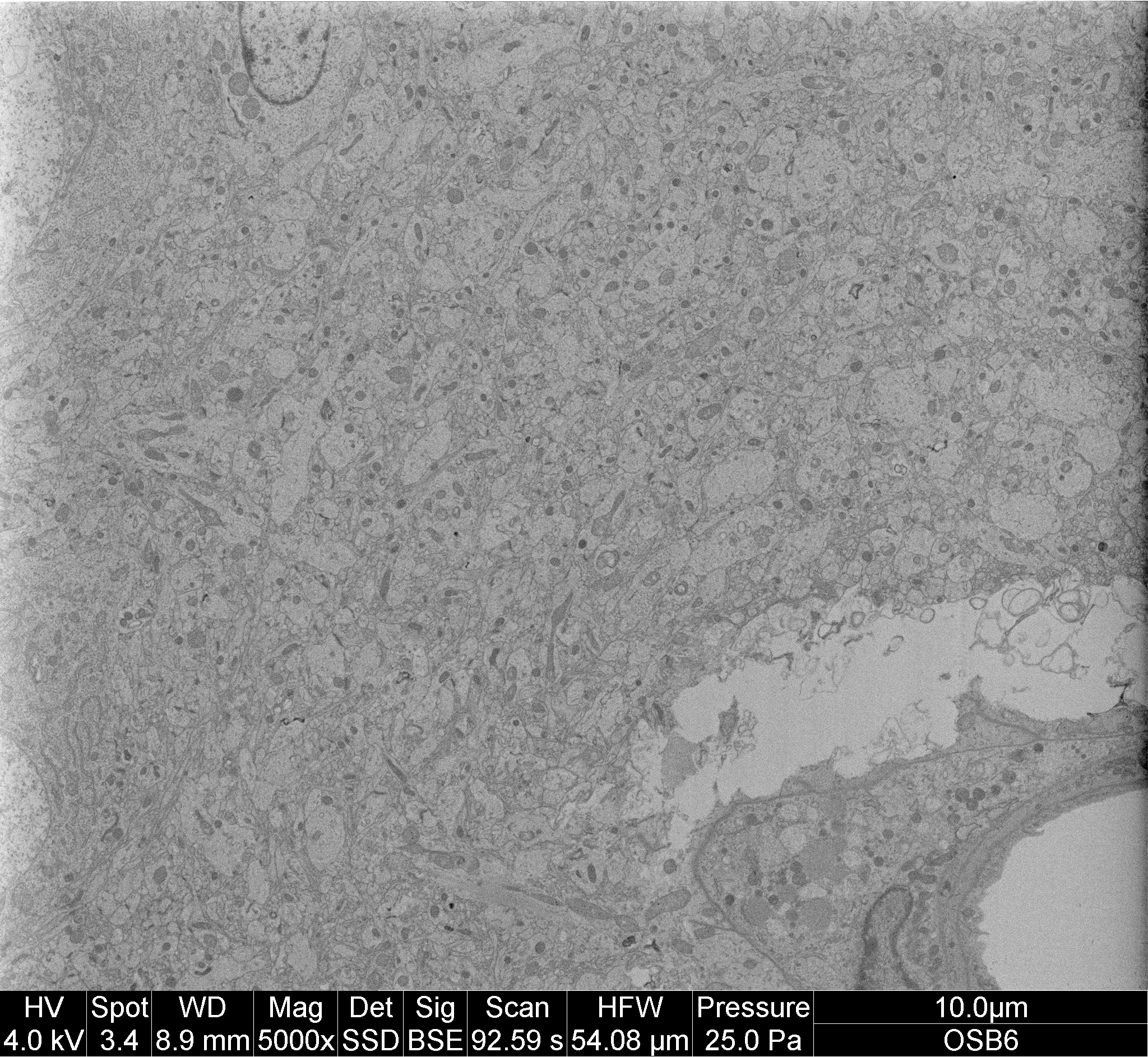

Supplement: Dataset S3 — (252.7 MB ZIP). [file pbio.0020329.sd003.zip › 040604_OS5_st1_284.tif]

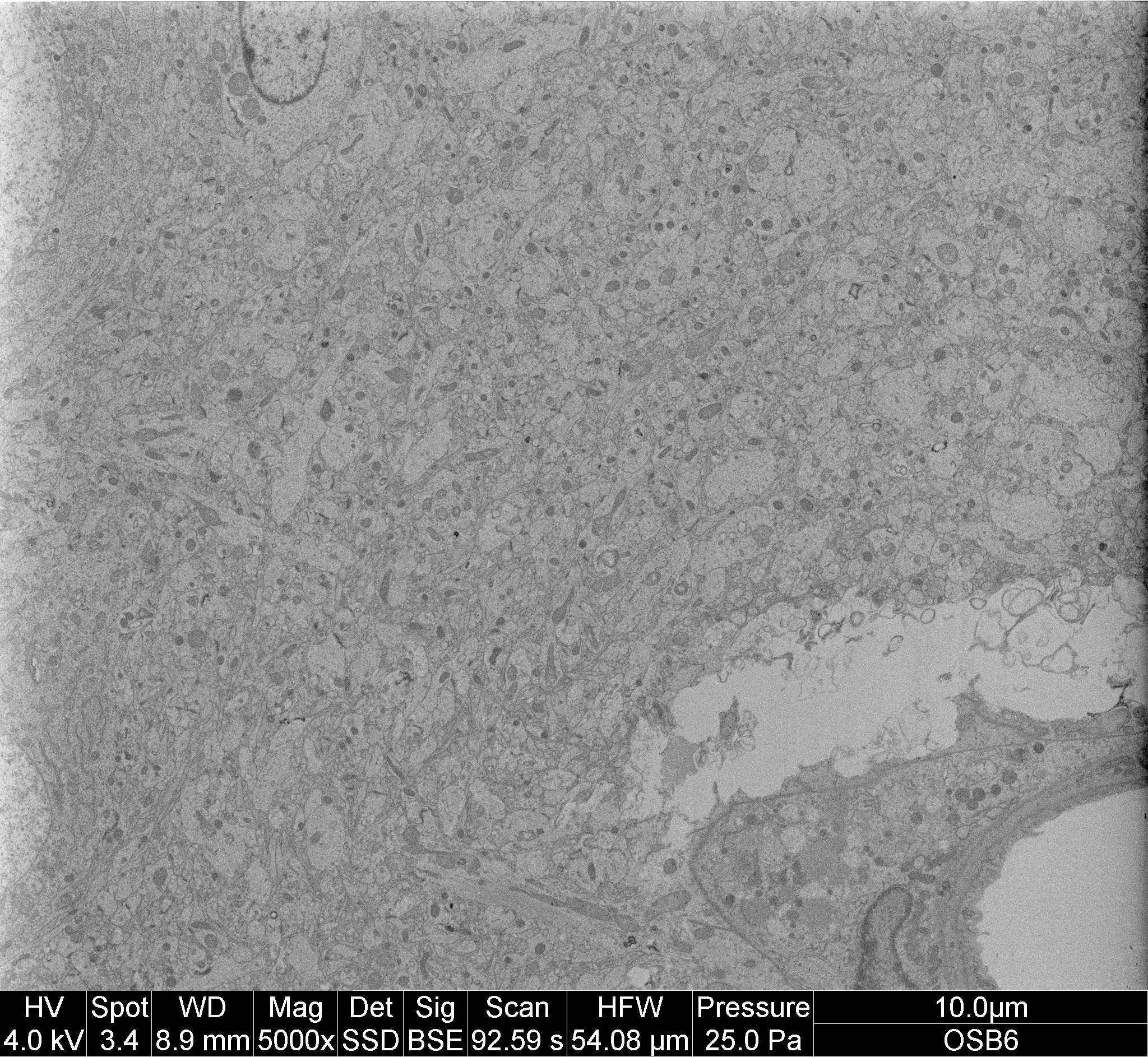

Supplement: Dataset S3 — (252.7 MB ZIP). [file pbio.0020329.sd003.zip › 040604_OS5_st1_285.tif]

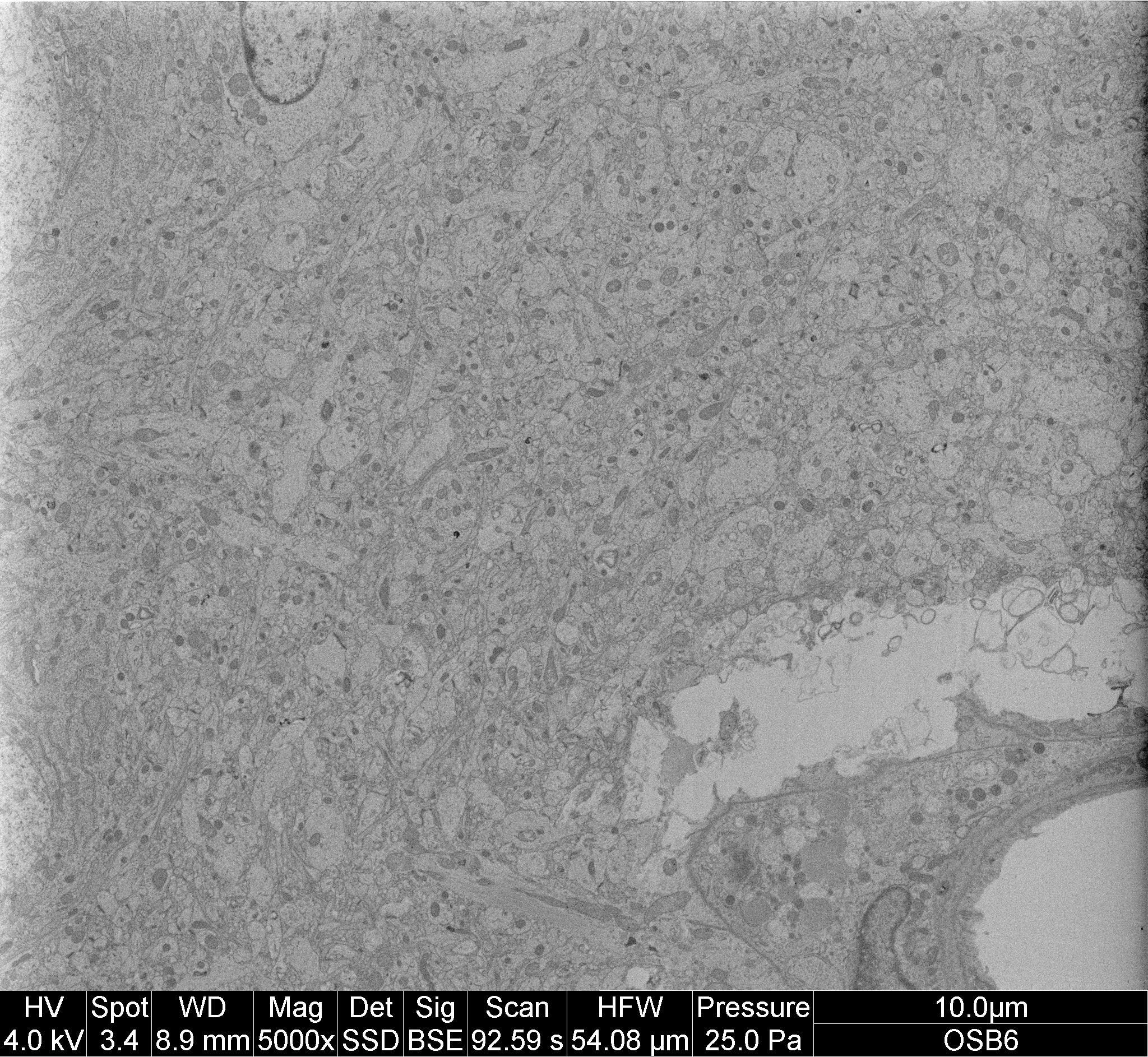

Supplement: Dataset S3 — (252.7 MB ZIP). [file pbio.0020329.sd003.zip › 040604_OS5_st1_286.tif]

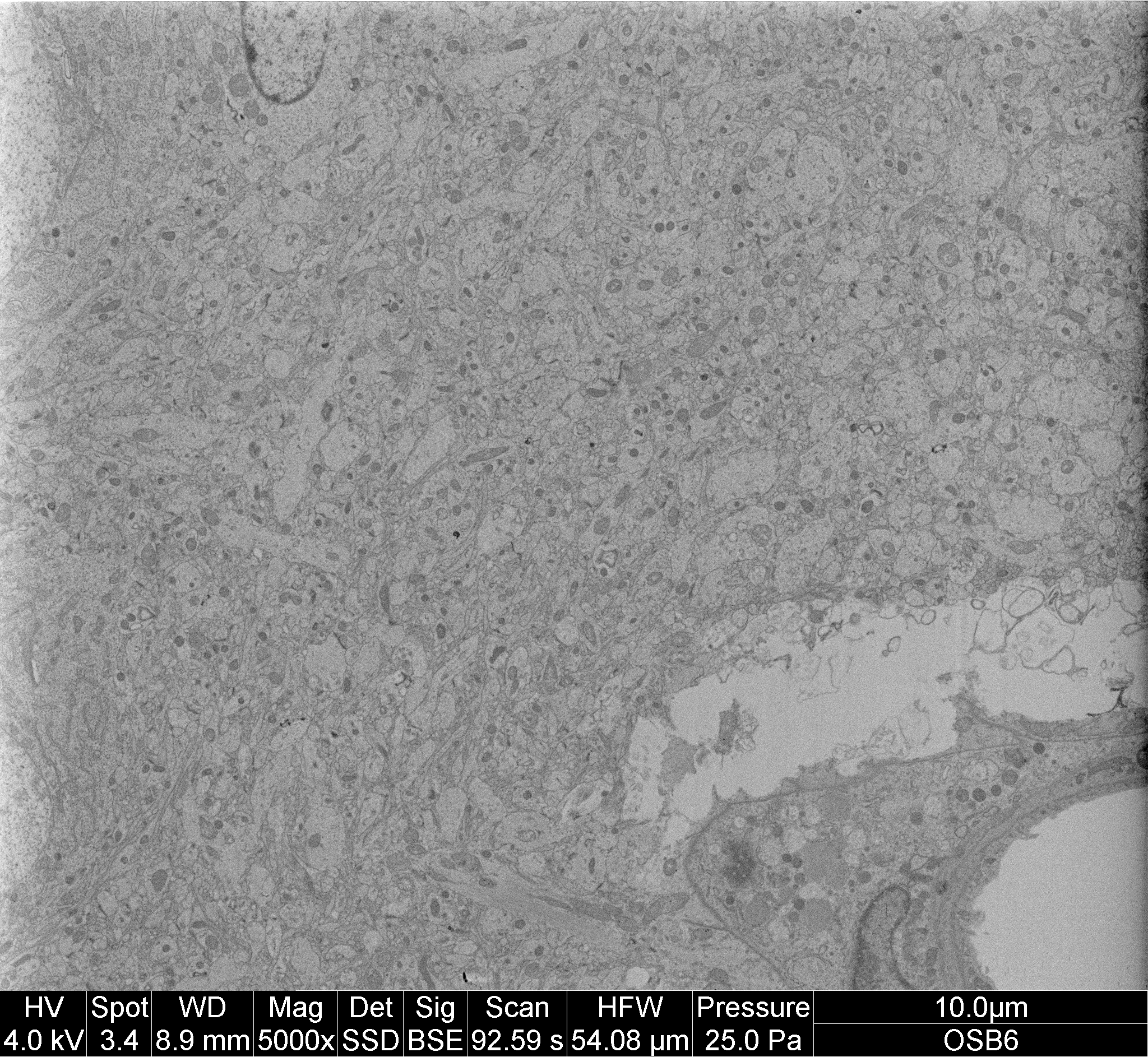

Supplement: Dataset S3 — (252.7 MB ZIP). [file pbio.0020329.sd003.zip › 040604_OS5_st1_287.tif]

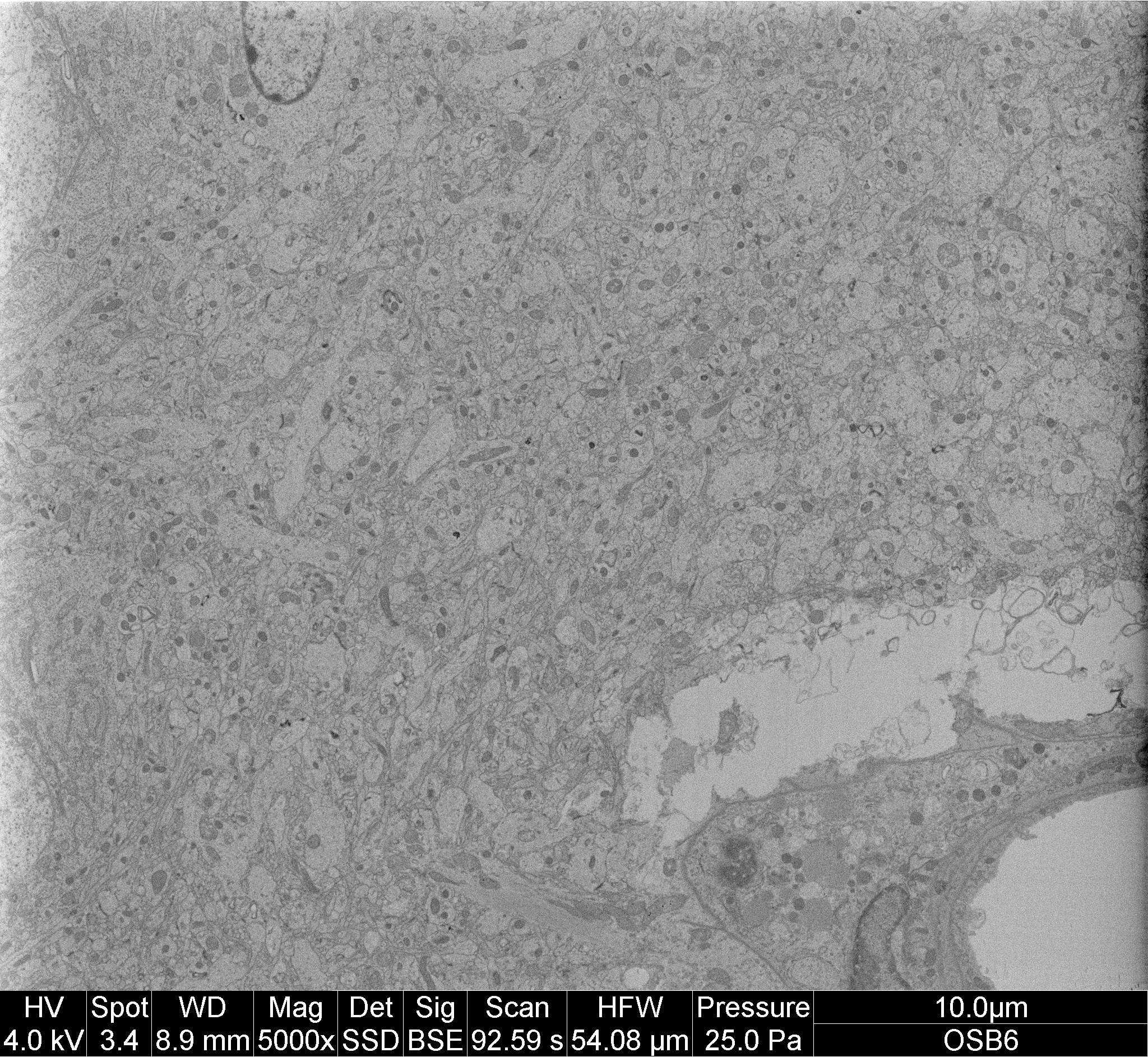

Supplement: Dataset S3 — (252.7 MB ZIP). [file pbio.0020329.sd003.zip › 040604_OS5_st1_288.tif]

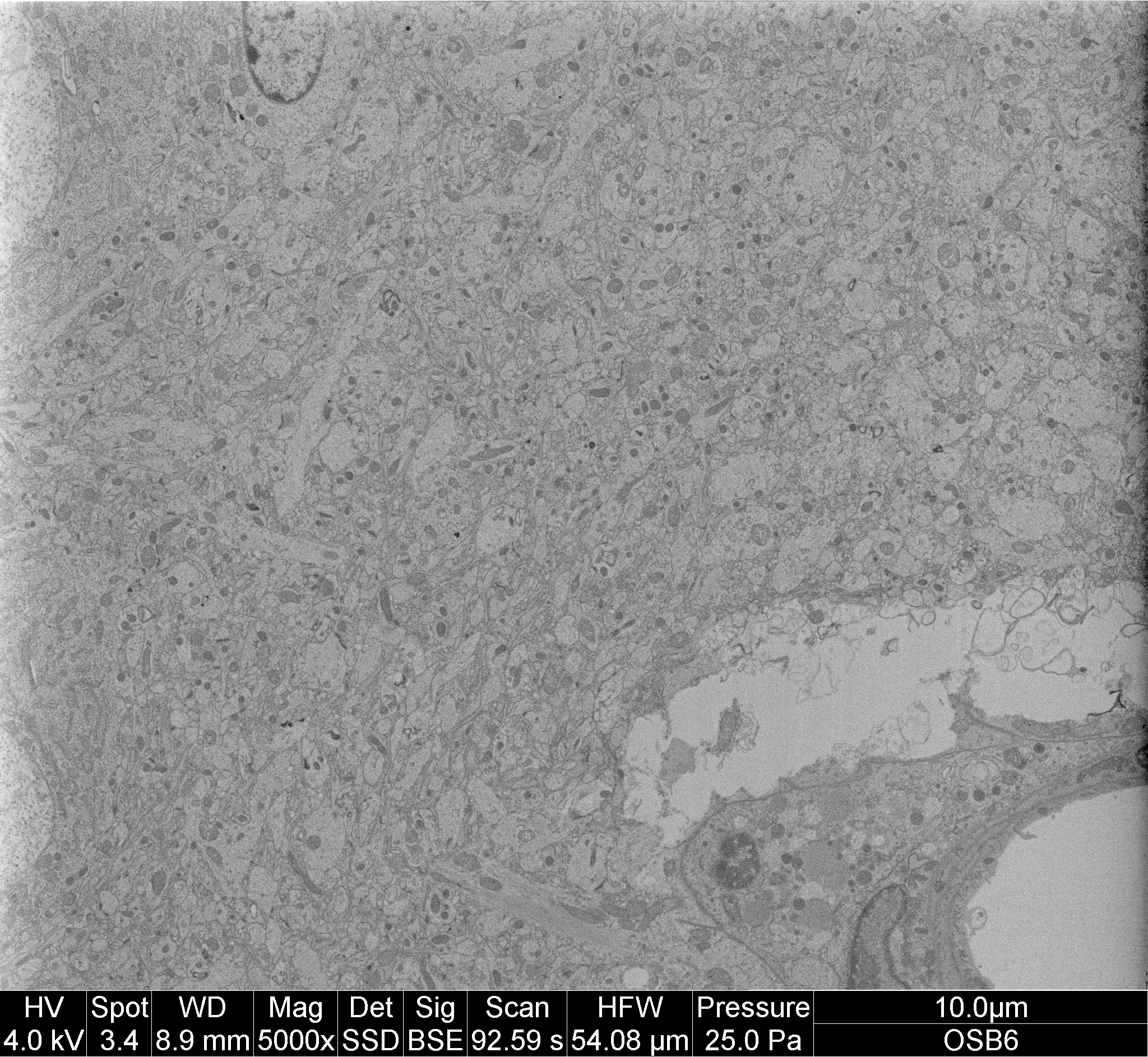

Supplement: Dataset S3 — (252.7 MB ZIP). [file pbio.0020329.sd003.zip › 040604_OS5_st1_289.tif]

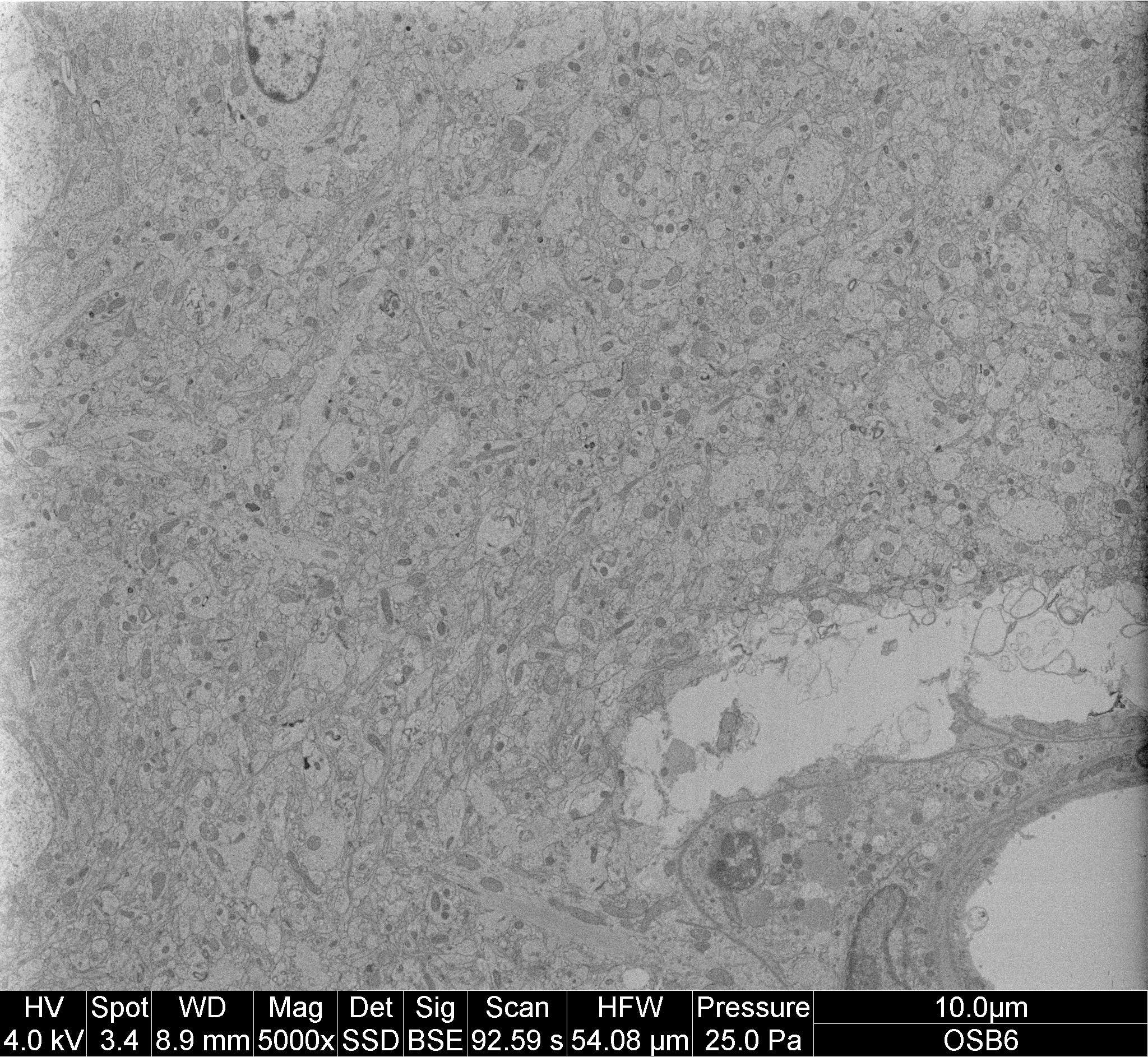

Supplement: Dataset S3 — (252.7 MB ZIP). [file pbio.0020329.sd003.zip › 040604_OS5_st1_290.tif]

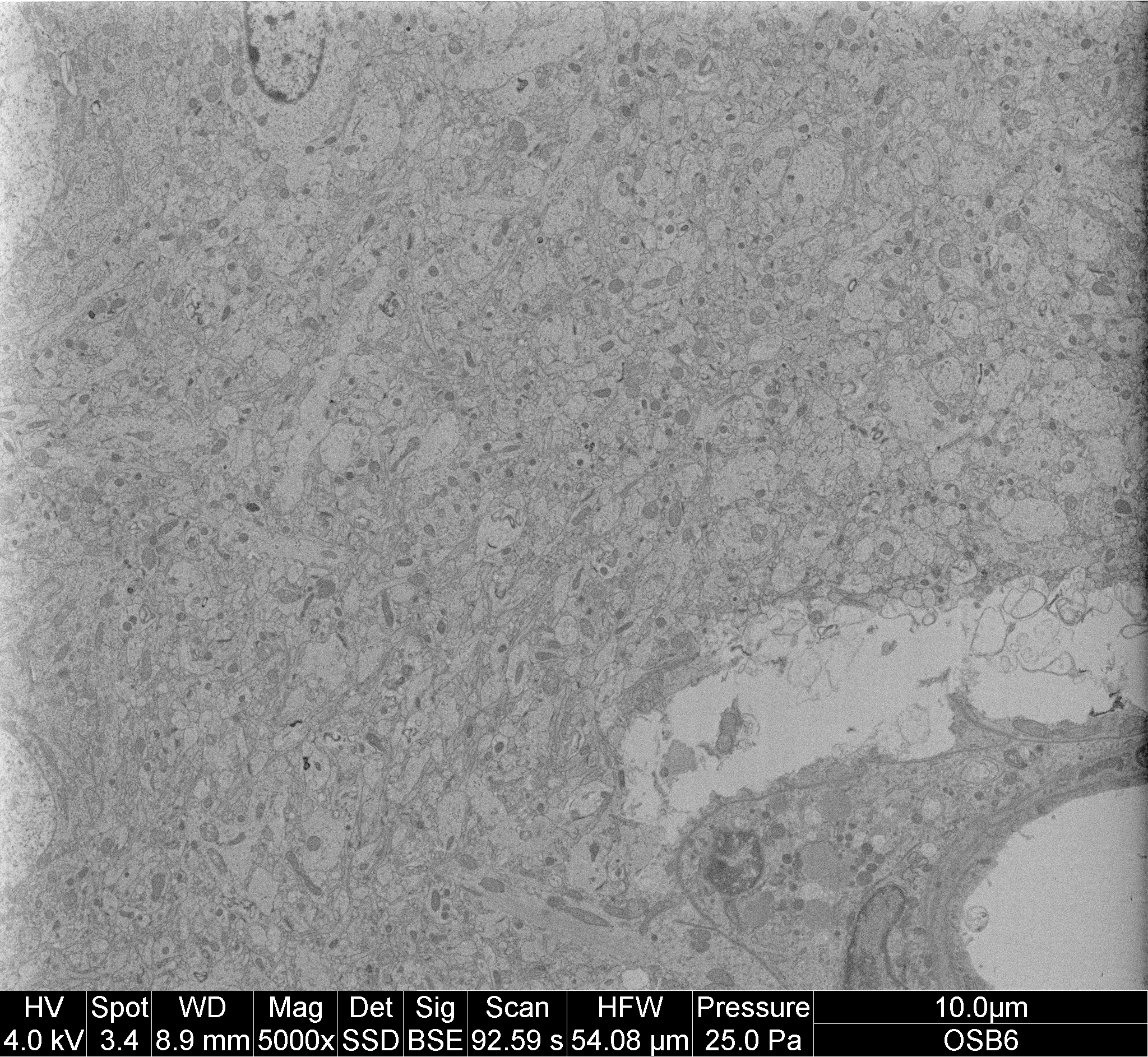

Supplement: Dataset S3 — (252.7 MB ZIP). [file pbio.0020329.sd003.zip › 040604_OS5_st1_291.tif]

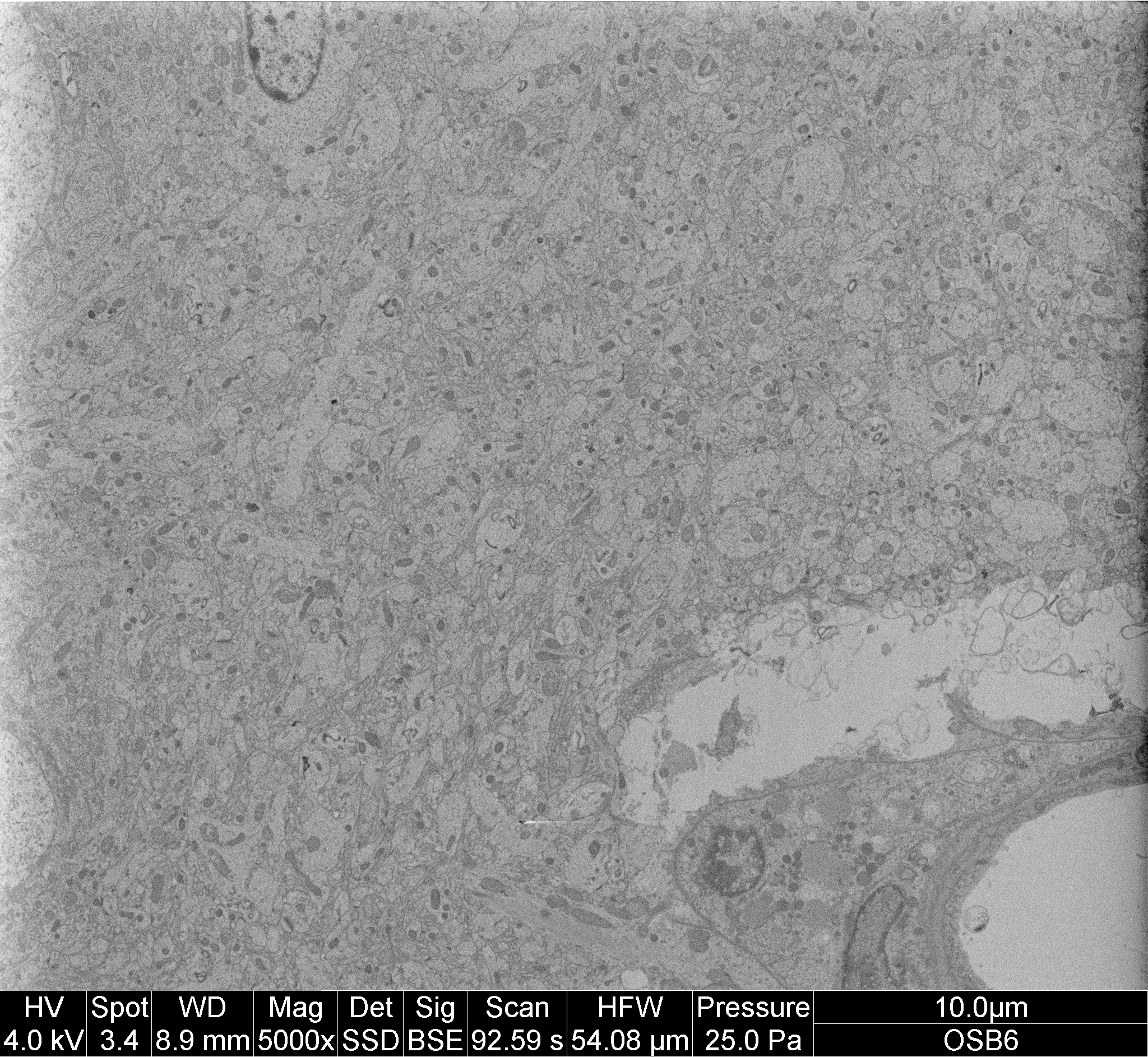

Supplement: Dataset S3 — (252.7 MB ZIP). [file pbio.0020329.sd003.zip › 040604_OS5_st1_292.tif]

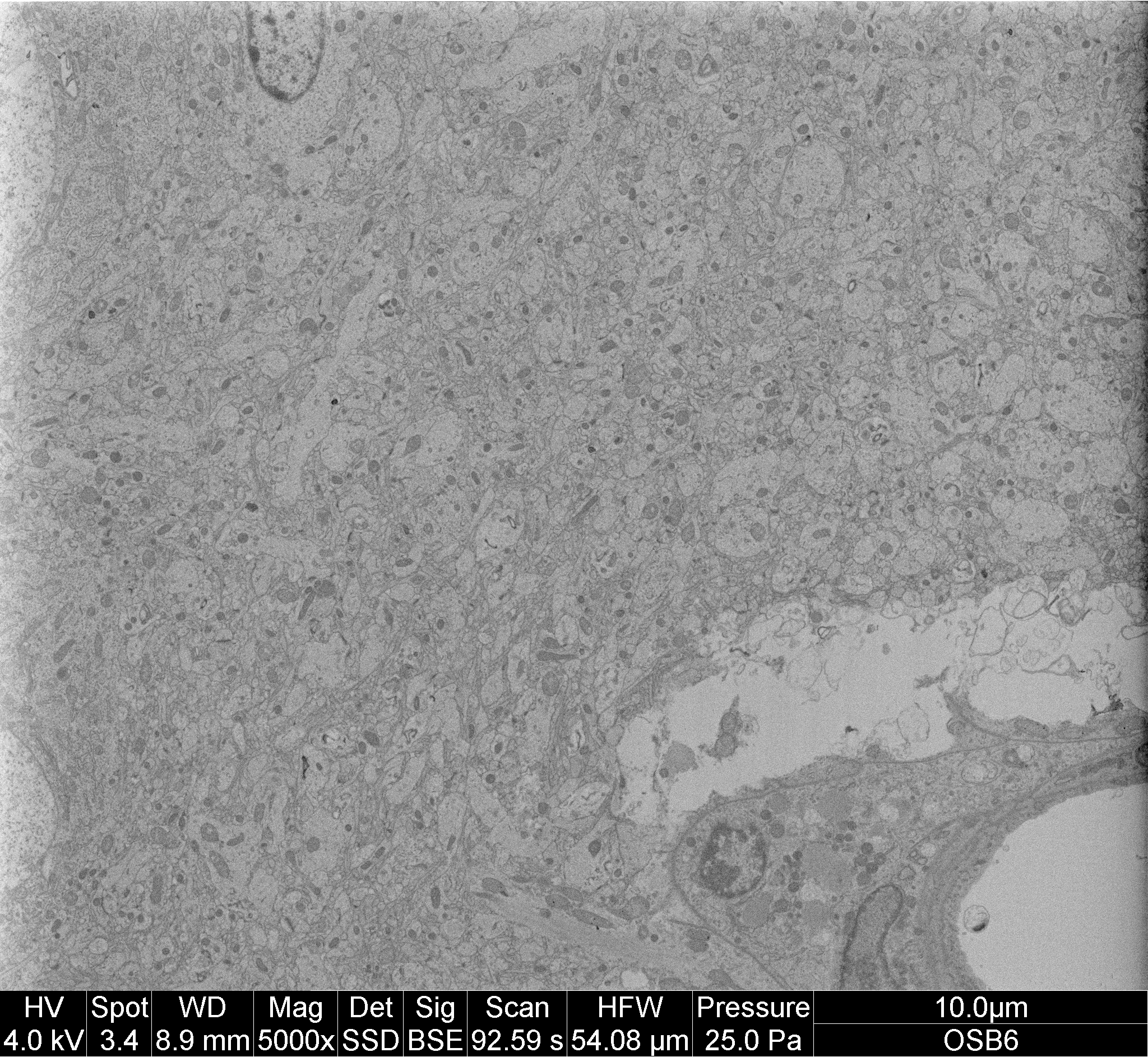

Supplement: Dataset S3 — (252.7 MB ZIP). [file pbio.0020329.sd003.zip › 040604_OS5_st1_293.tif]

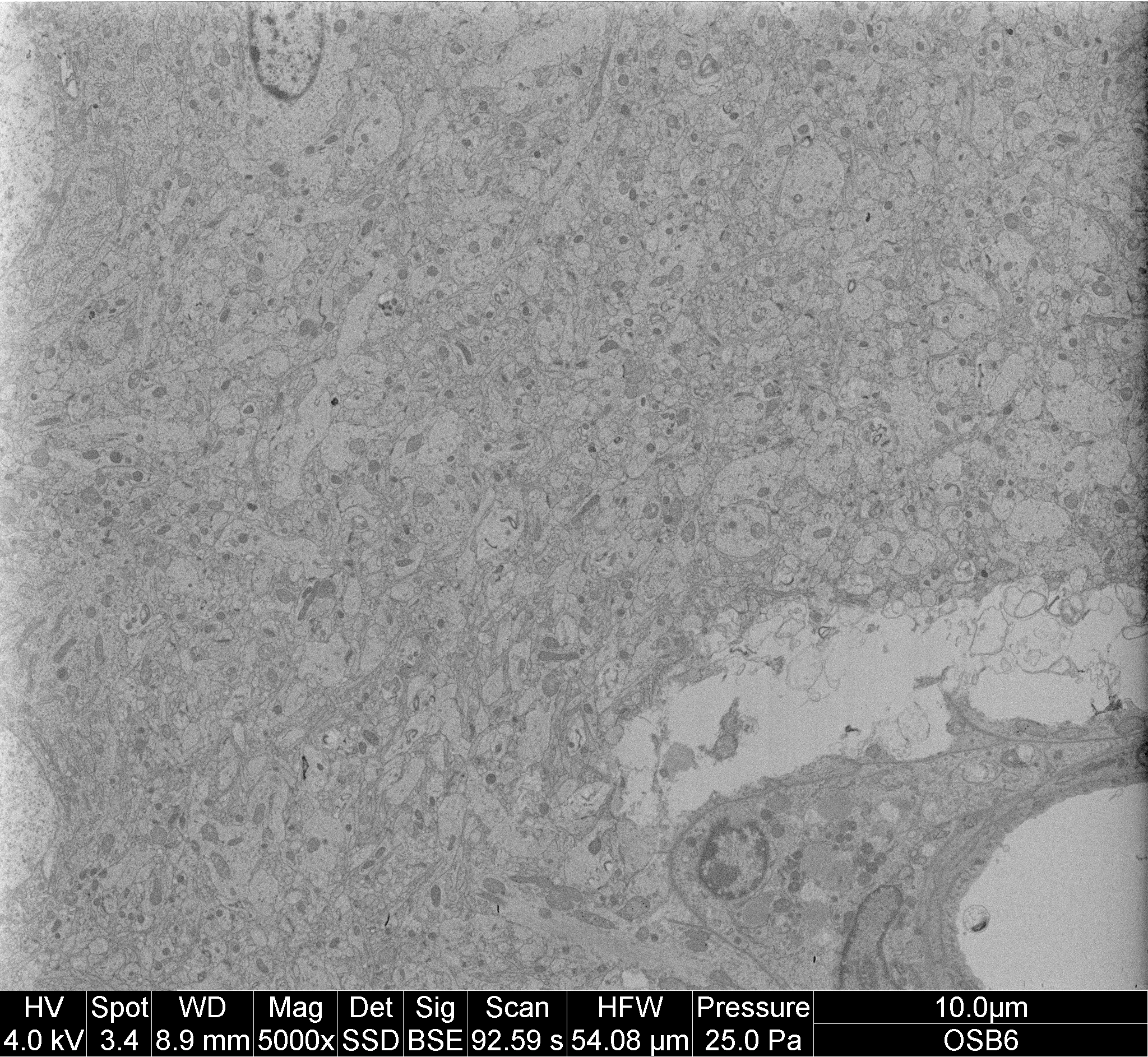

Supplement: Dataset S3 — (252.7 MB ZIP). [file pbio.0020329.sd003.zip › 040604_OS5_st1_294.tif]

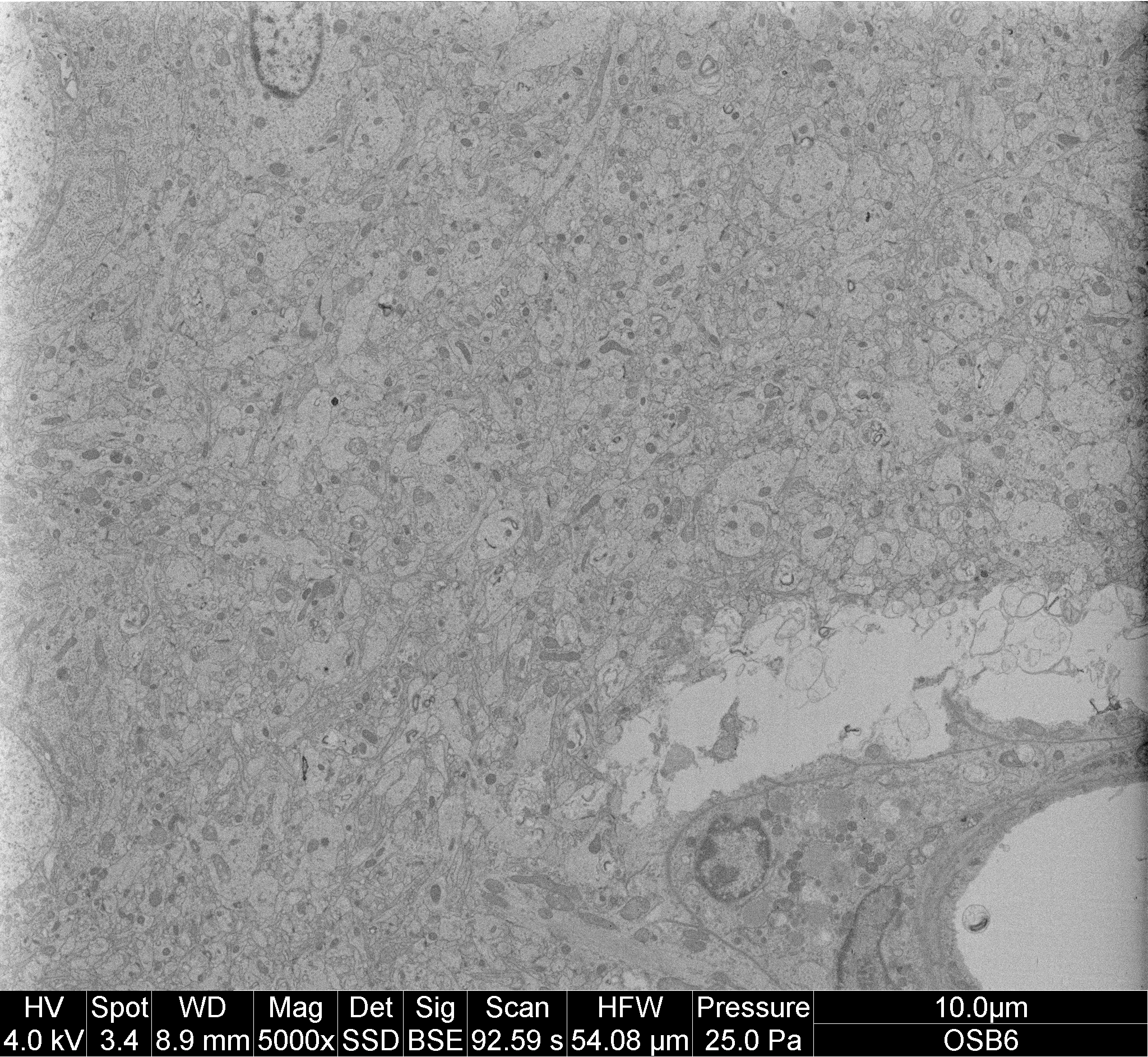

Supplement: Dataset S3 — (252.7 MB ZIP). [file pbio.0020329.sd003.zip › 040604_OS5_st1_295.tif]

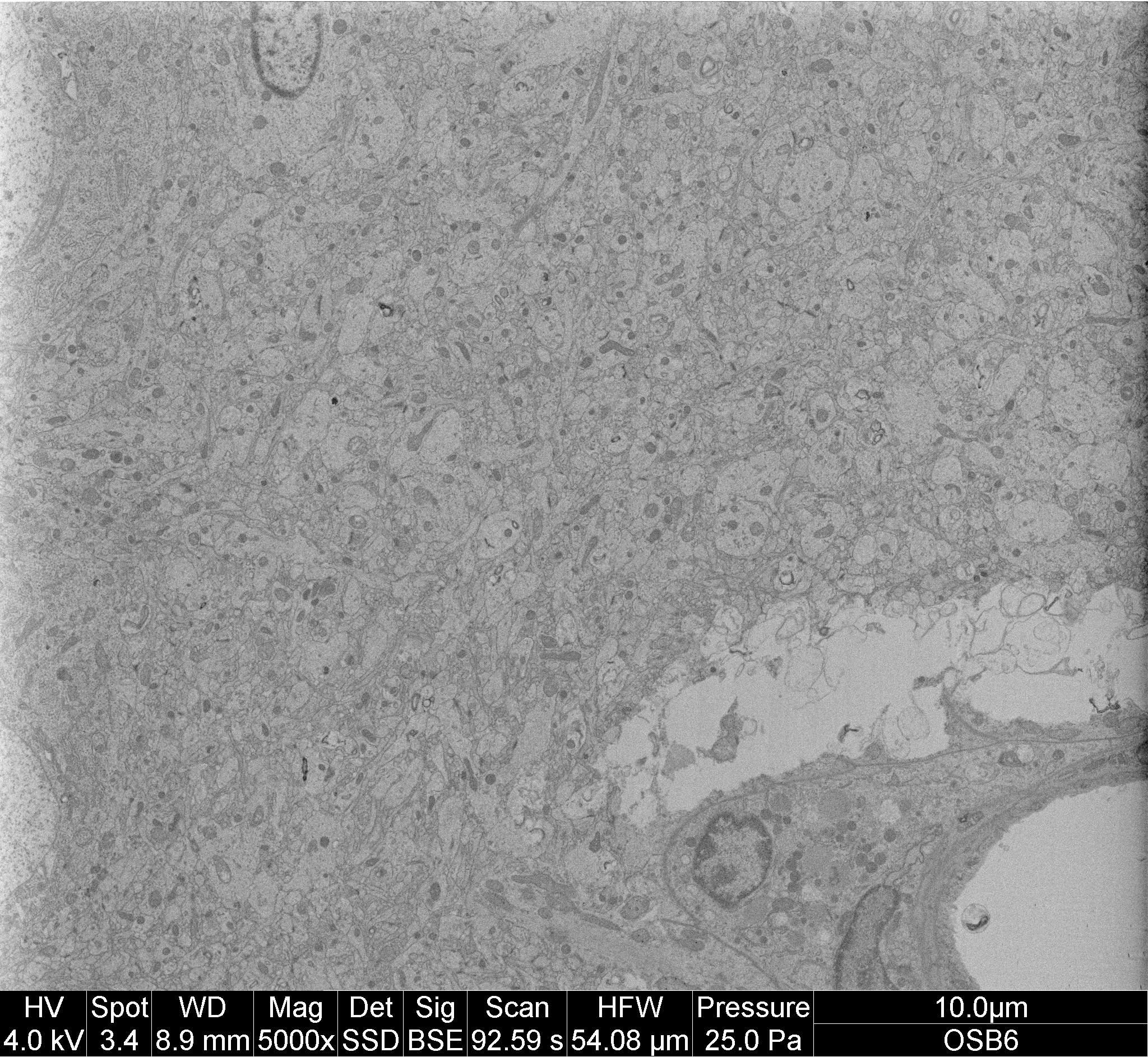

Supplement: Dataset S3 — (252.7 MB ZIP). [file pbio.0020329.sd003.zip › 040604_OS5_st1_296.tif]

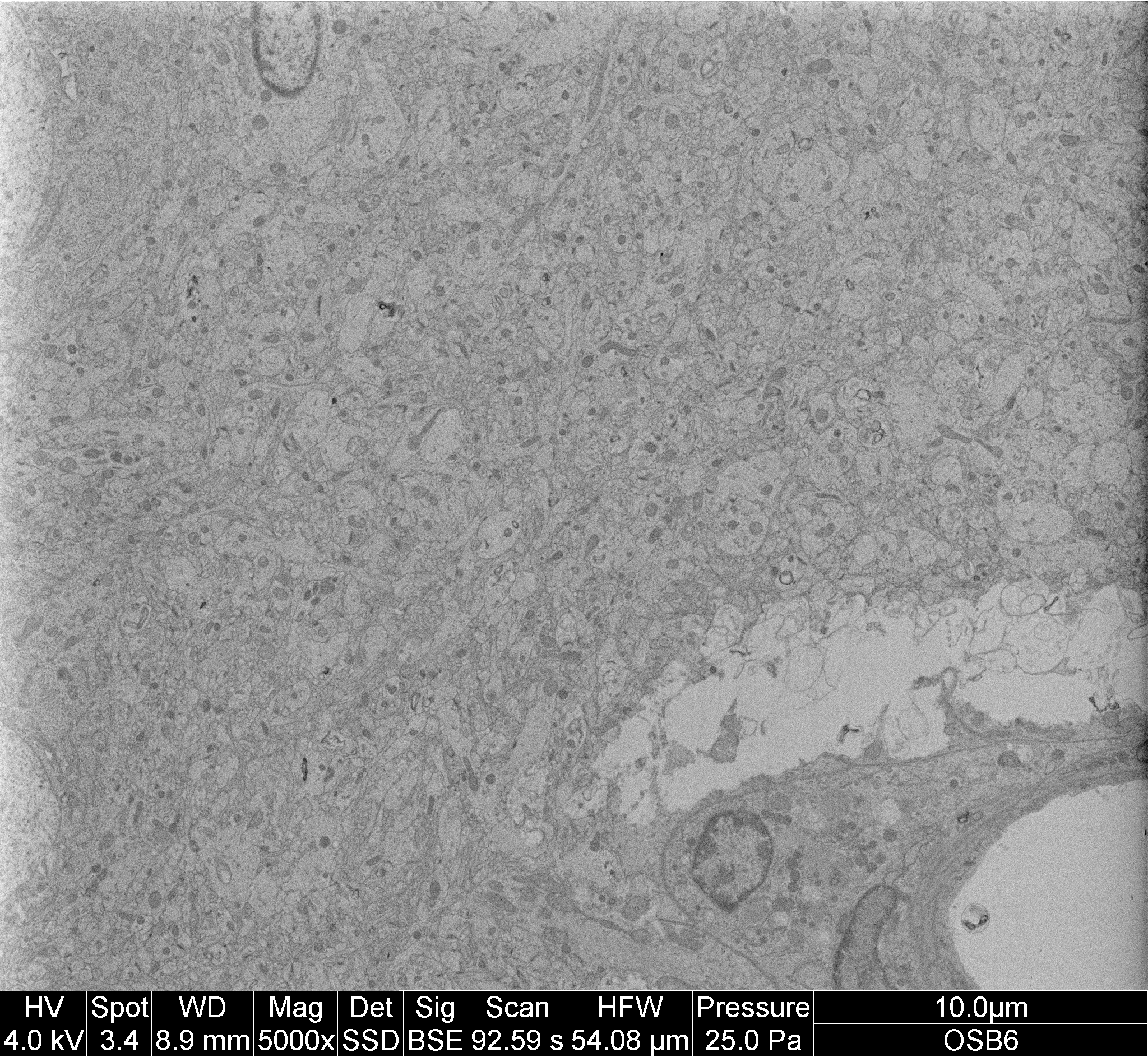

Supplement: Dataset S3 — (252.7 MB ZIP). [file pbio.0020329.sd003.zip › 040604_OS5_st1_297.tif]

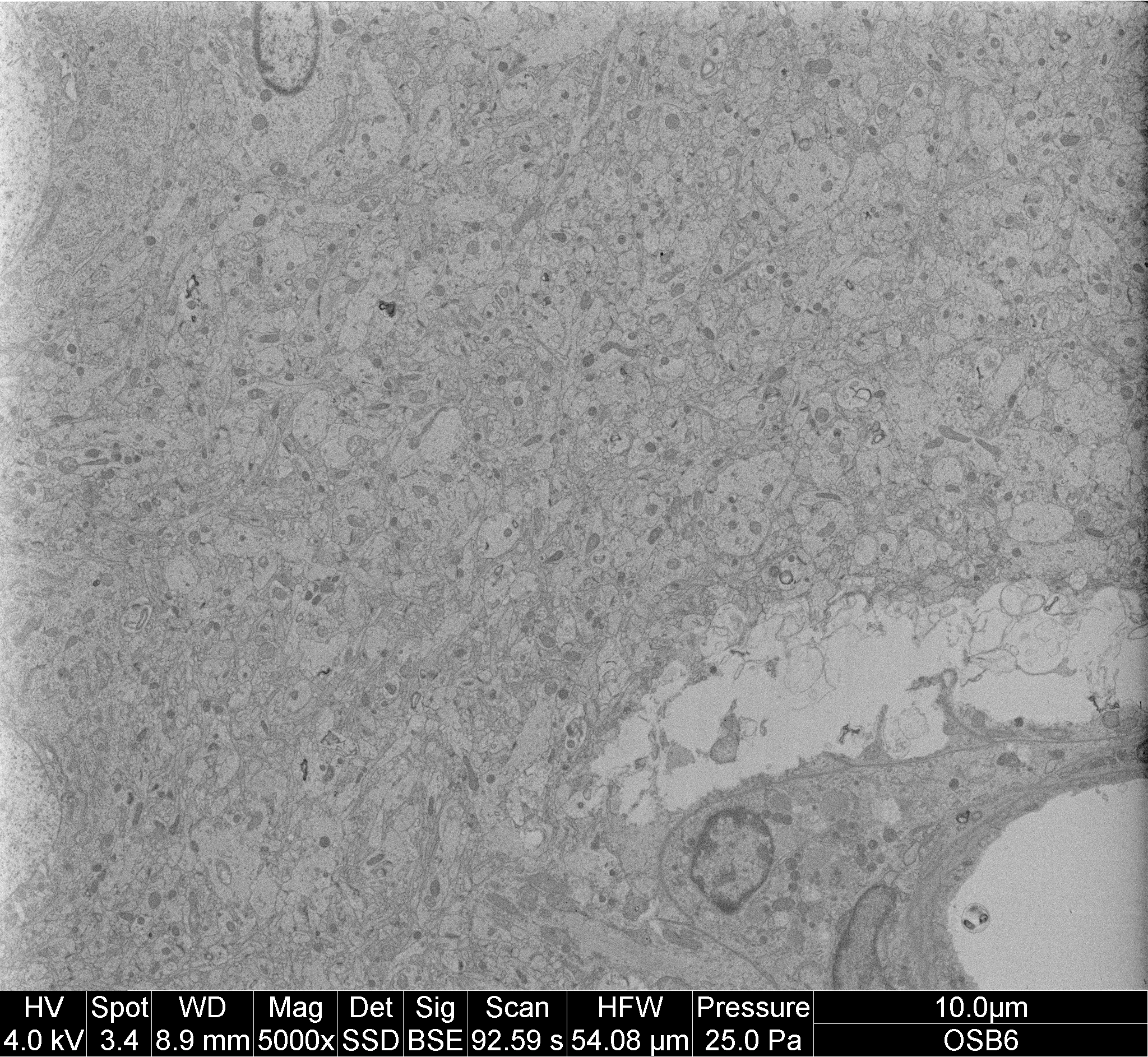

Supplement: Dataset S3 — (252.7 MB ZIP). [file pbio.0020329.sd003.zip › 040604_OS5_st1_298.tif]

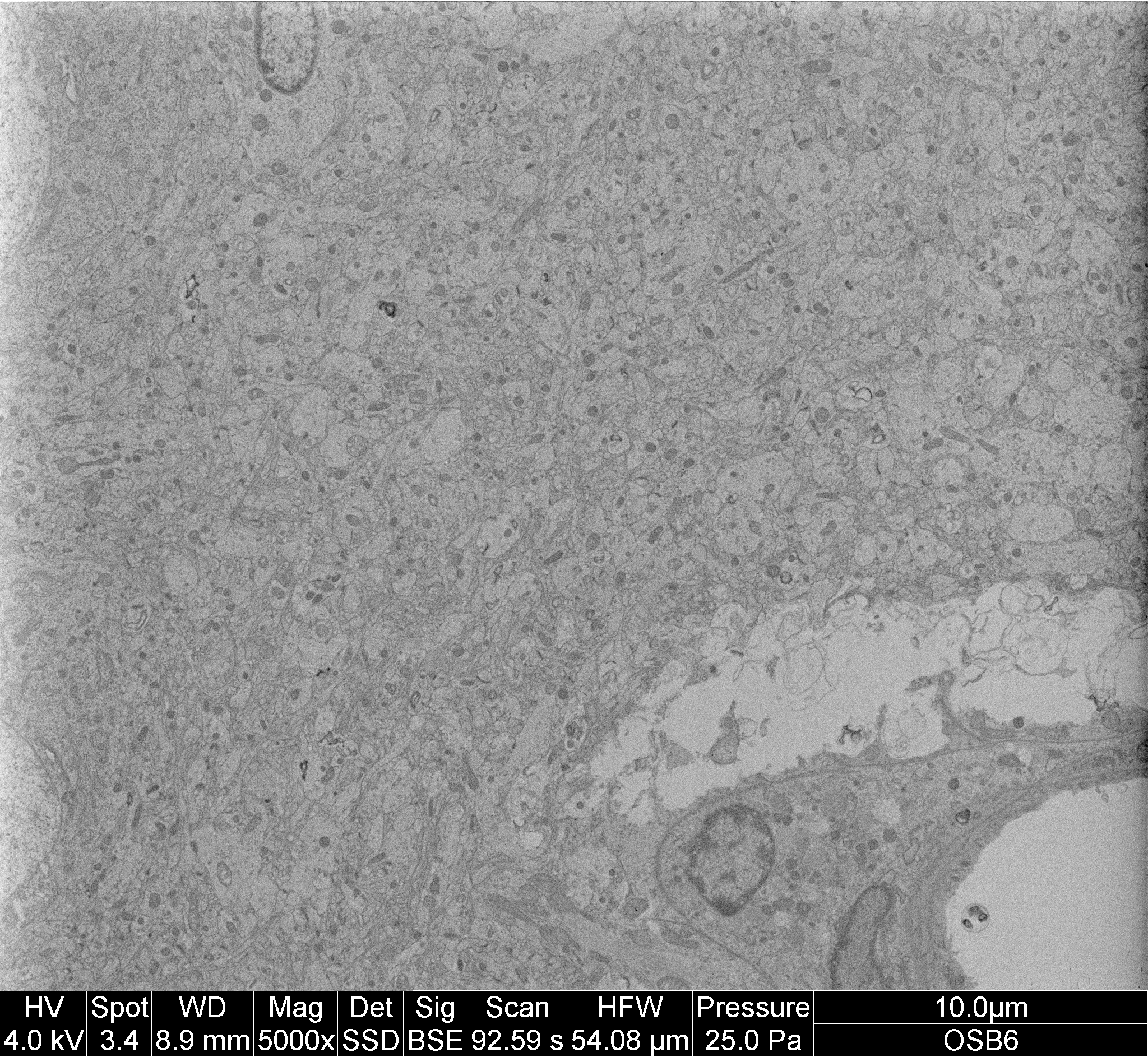

Supplement: Dataset S3 — (252.7 MB ZIP). [file pbio.0020329.sd003.zip › 040604_OS5_st1_299.tif]

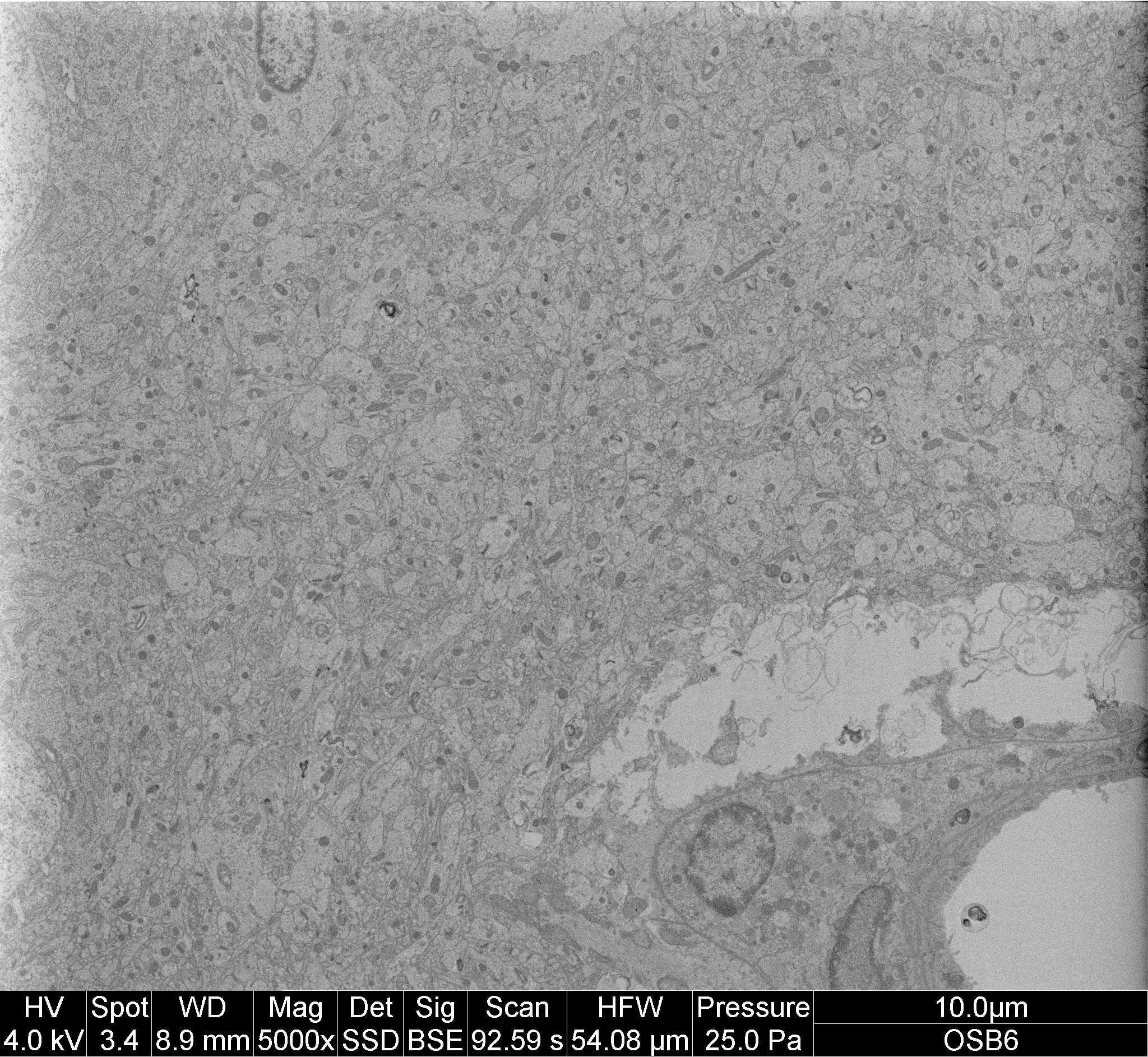

Supplement: Dataset S4 — (252.6 MB ZIP). [file pbio.0020329.sd004.zip › 040604_OS5_st1_300.tif]
