# Supplementary figures and images for: Serial Block-Face Scanning Electron Microscopy to Reconstruct Three-Dimensional Tissue Nanostructure (part 4 of 21)
Source: PLoS Biol. 2004 Oct 19;2(11):e329. doi: 10.1371/journal.pbio.0020329 (PMC524270; doi:10.1371/journal.pbio.0020329)

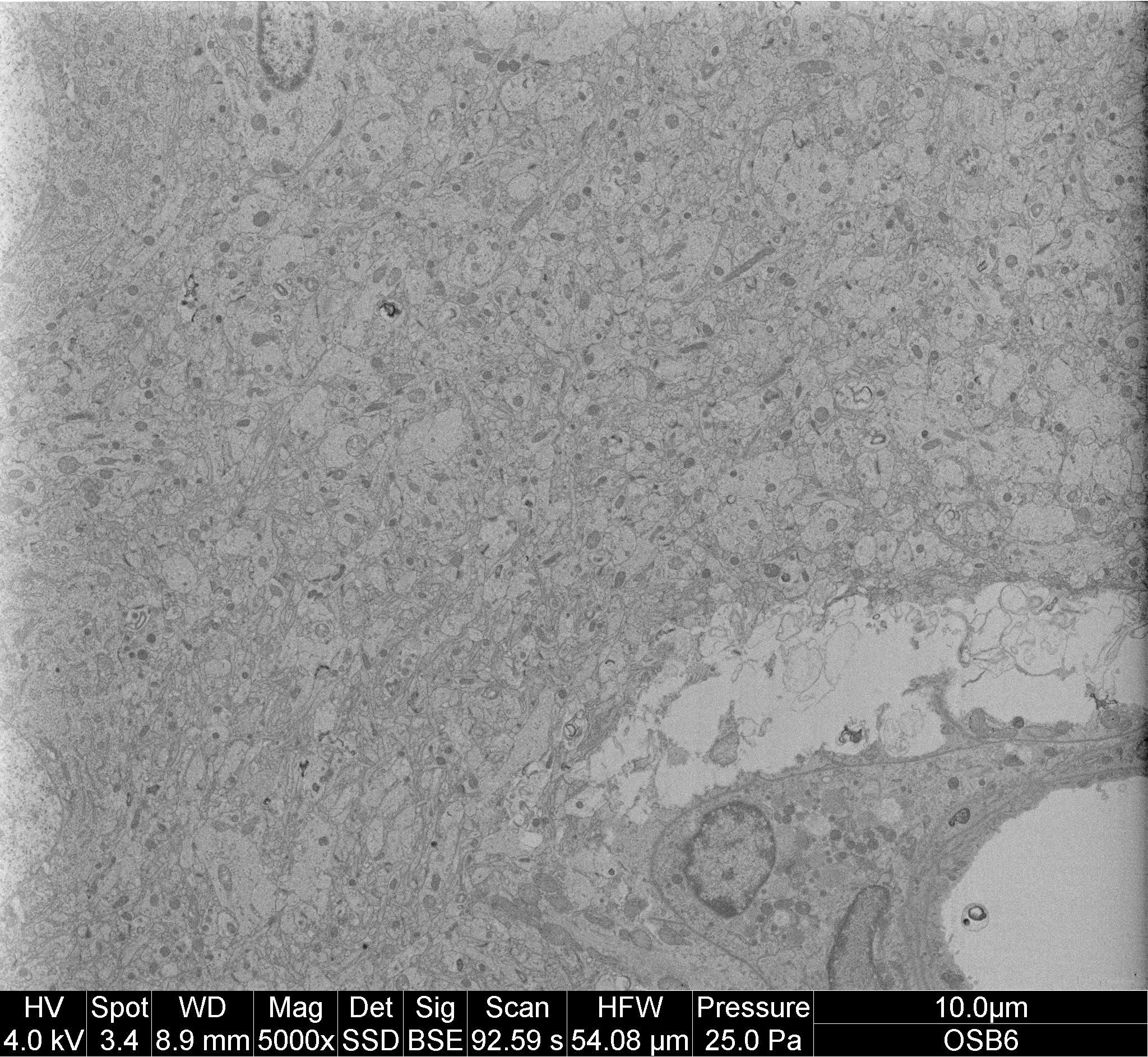

Supplement: Dataset S4 — (252.6 MB ZIP). [file pbio.0020329.sd004.zip › 040604_OS5_st1_301.tif]

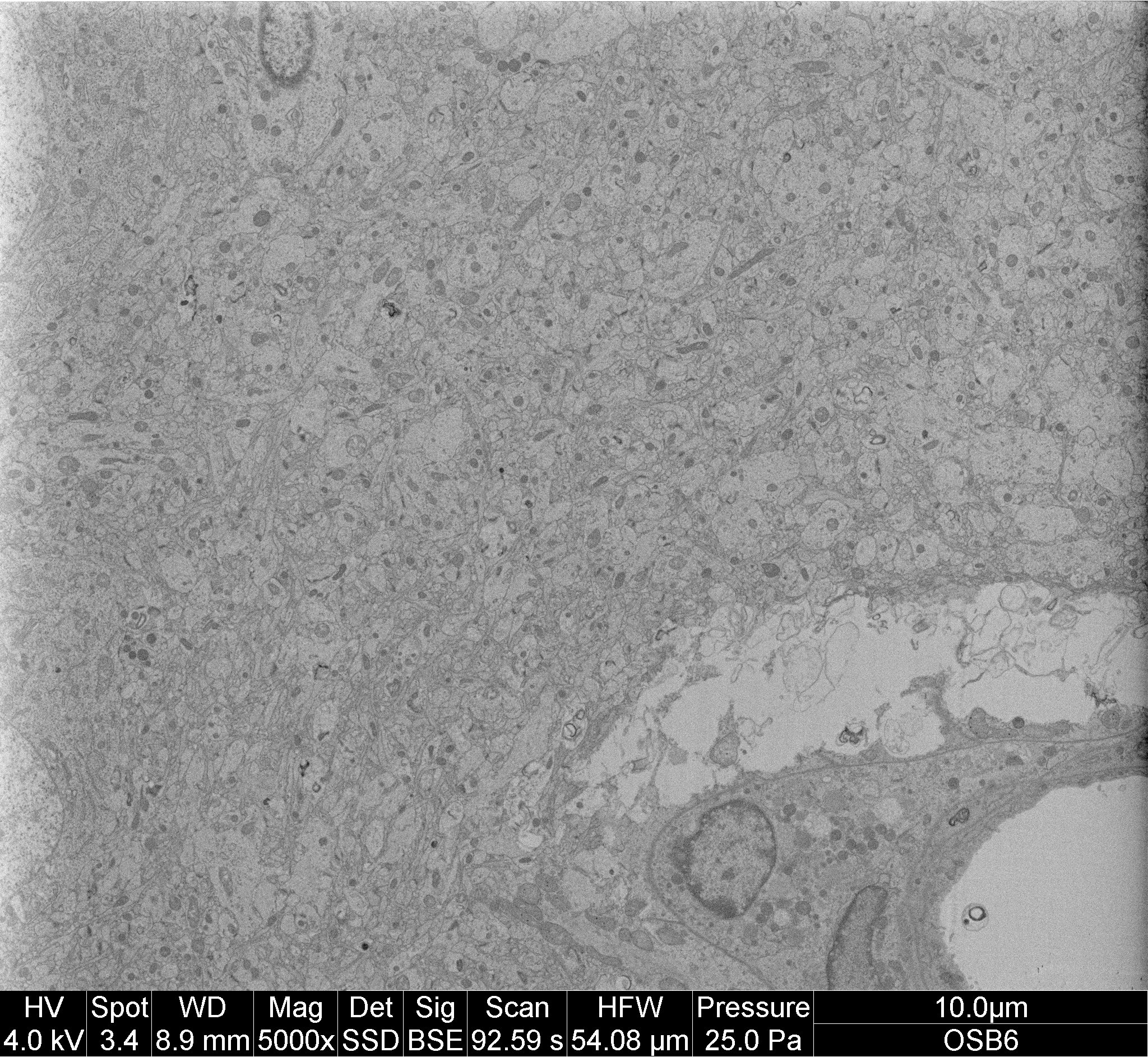

Supplement: Dataset S4 — (252.6 MB ZIP). [file pbio.0020329.sd004.zip › 040604_OS5_st1_302.tif]

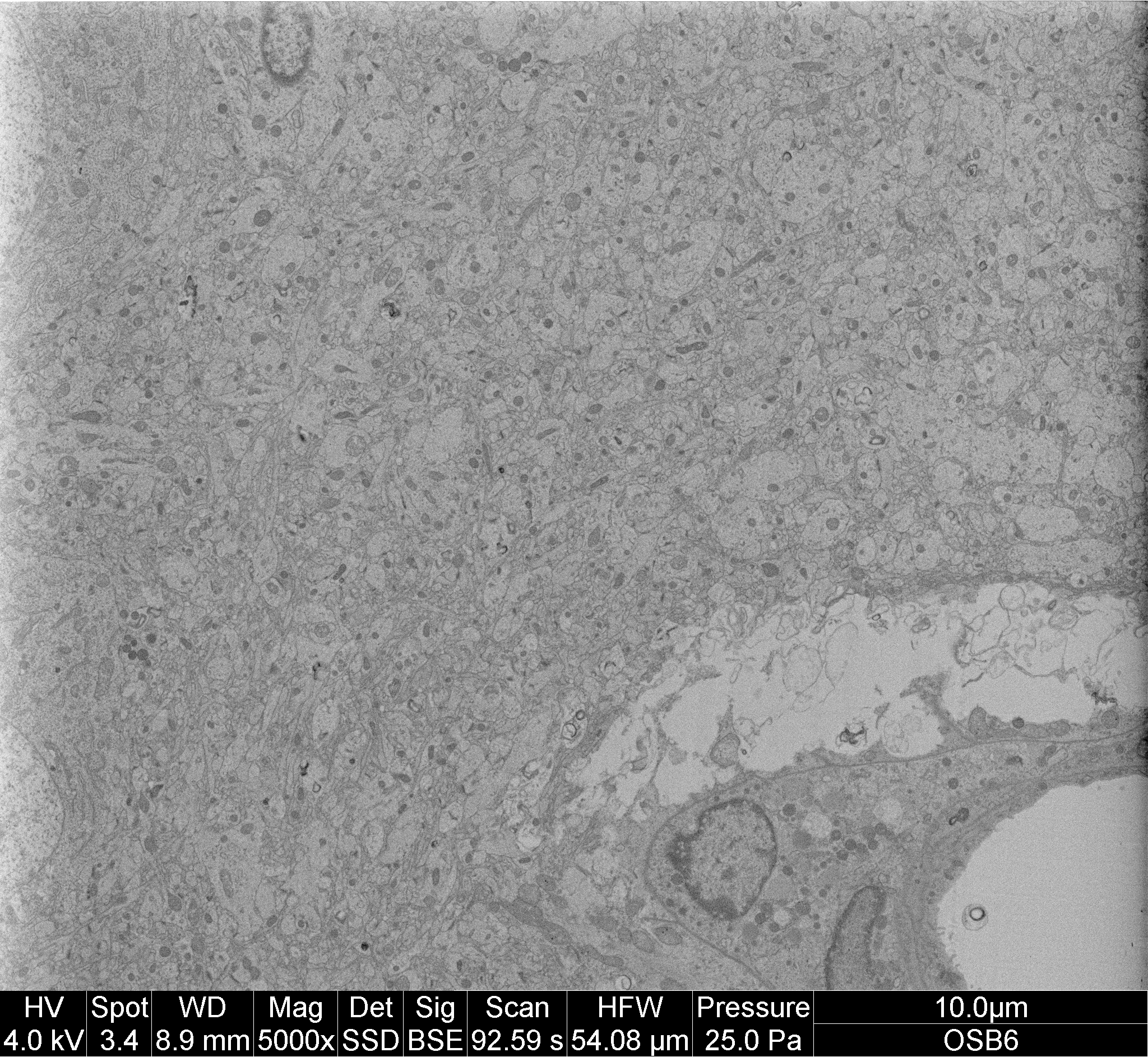

Supplement: Dataset S4 — (252.6 MB ZIP). [file pbio.0020329.sd004.zip › 040604_OS5_st1_303.tif]

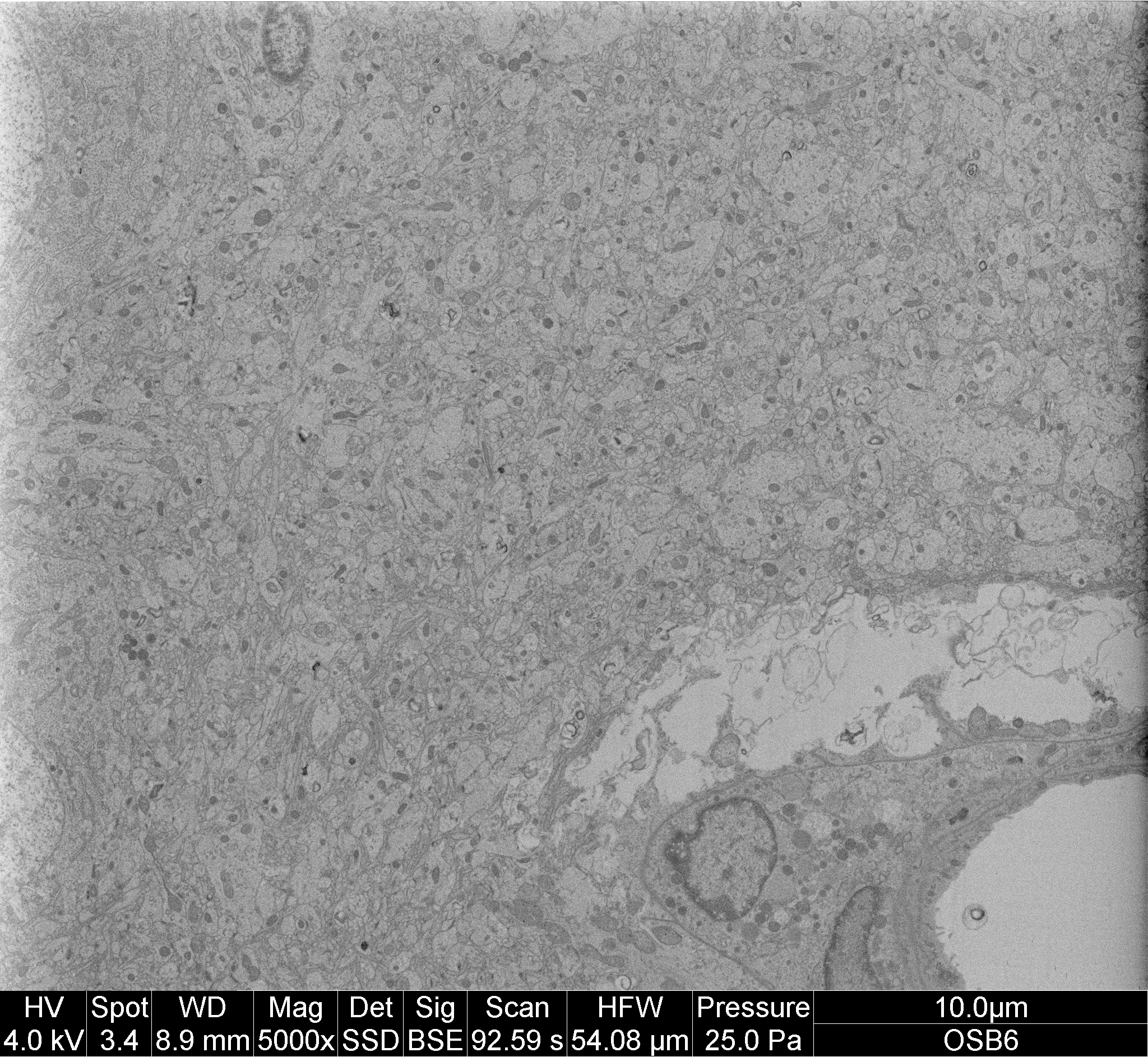

Supplement: Dataset S4 — (252.6 MB ZIP). [file pbio.0020329.sd004.zip › 040604_OS5_st1_304.tif]

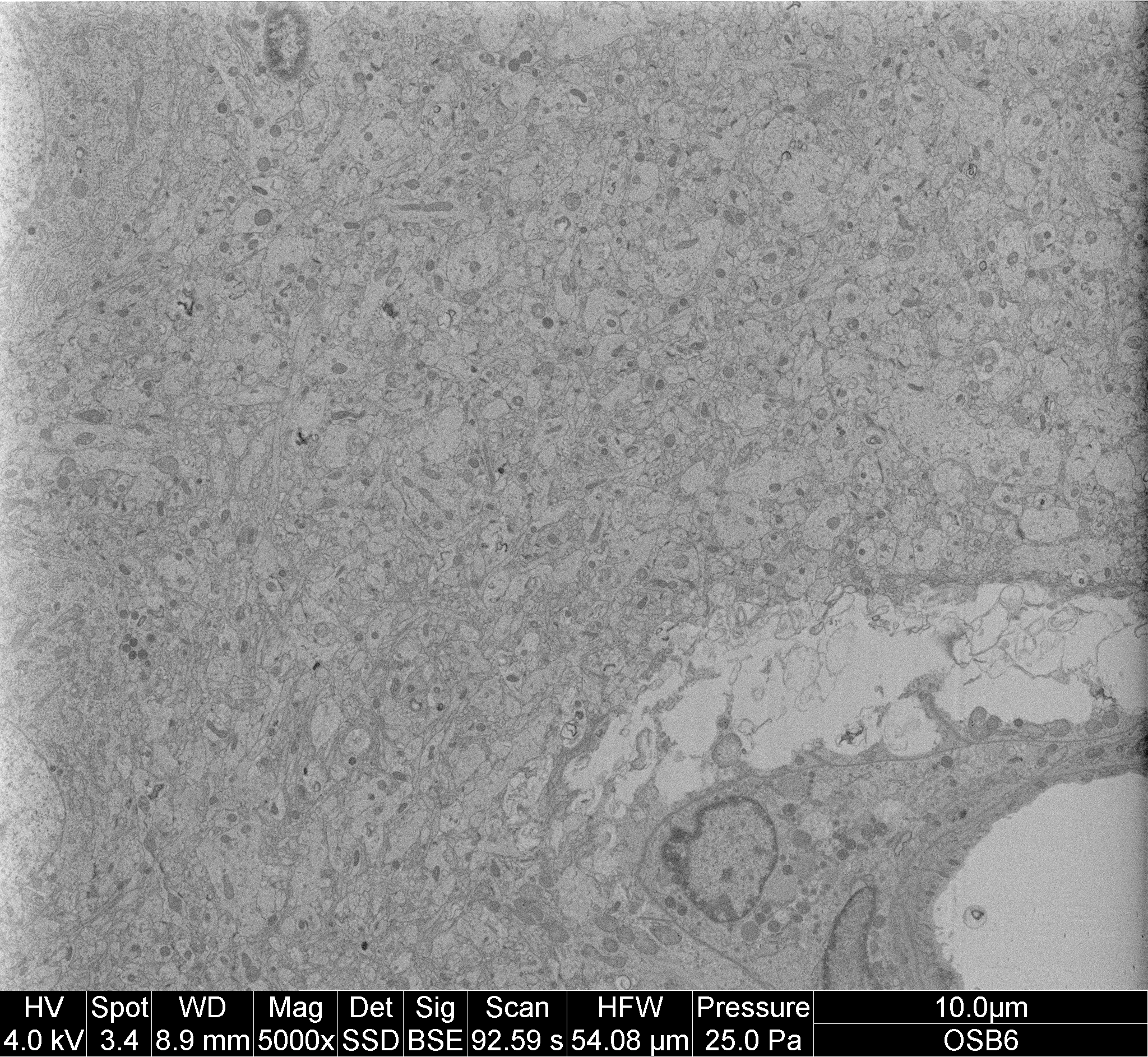

Supplement: Dataset S4 — (252.6 MB ZIP). [file pbio.0020329.sd004.zip › 040604_OS5_st1_305.tif]

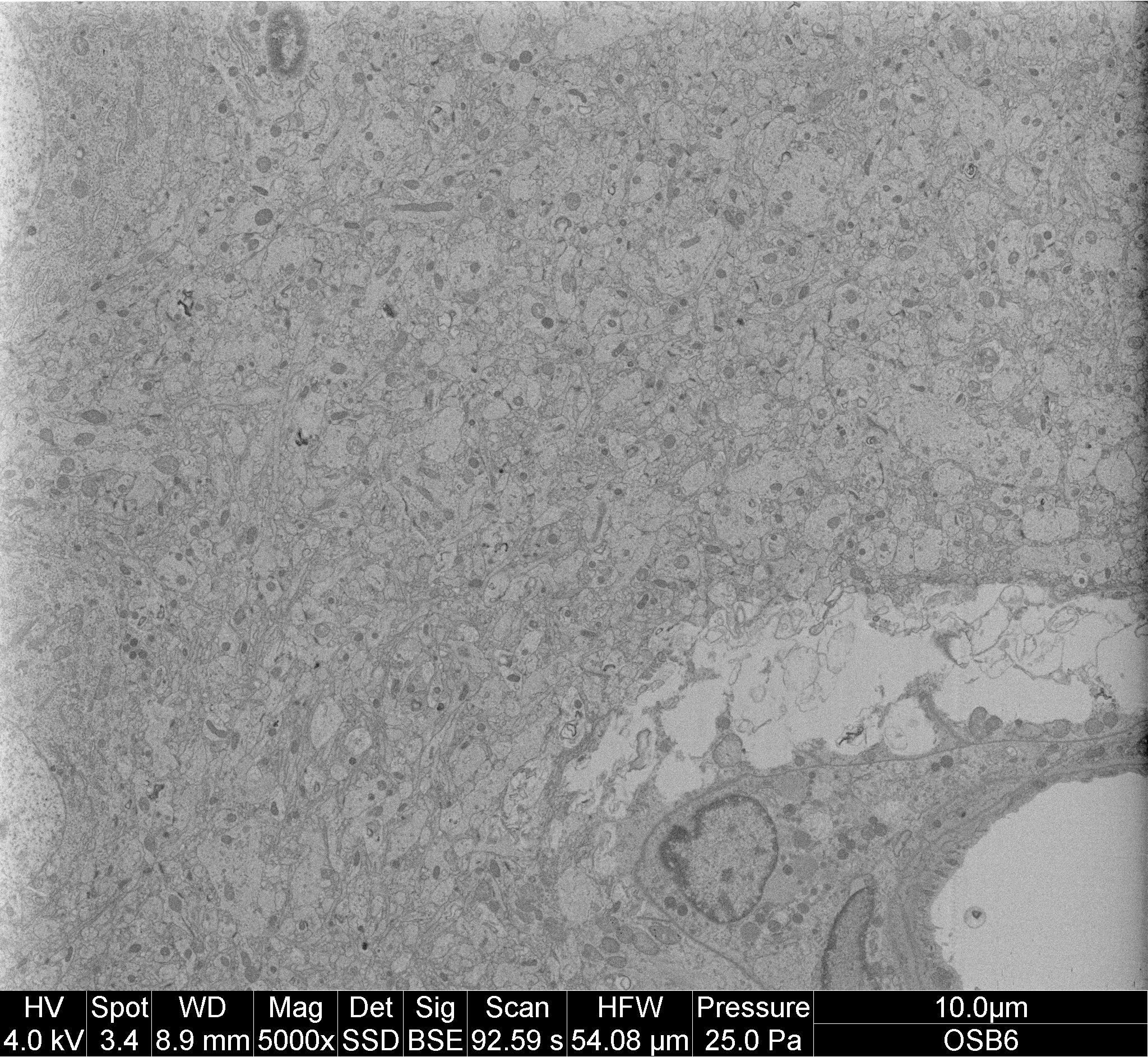

Supplement: Dataset S4 — (252.6 MB ZIP). [file pbio.0020329.sd004.zip › 040604_OS5_st1_306.tif]

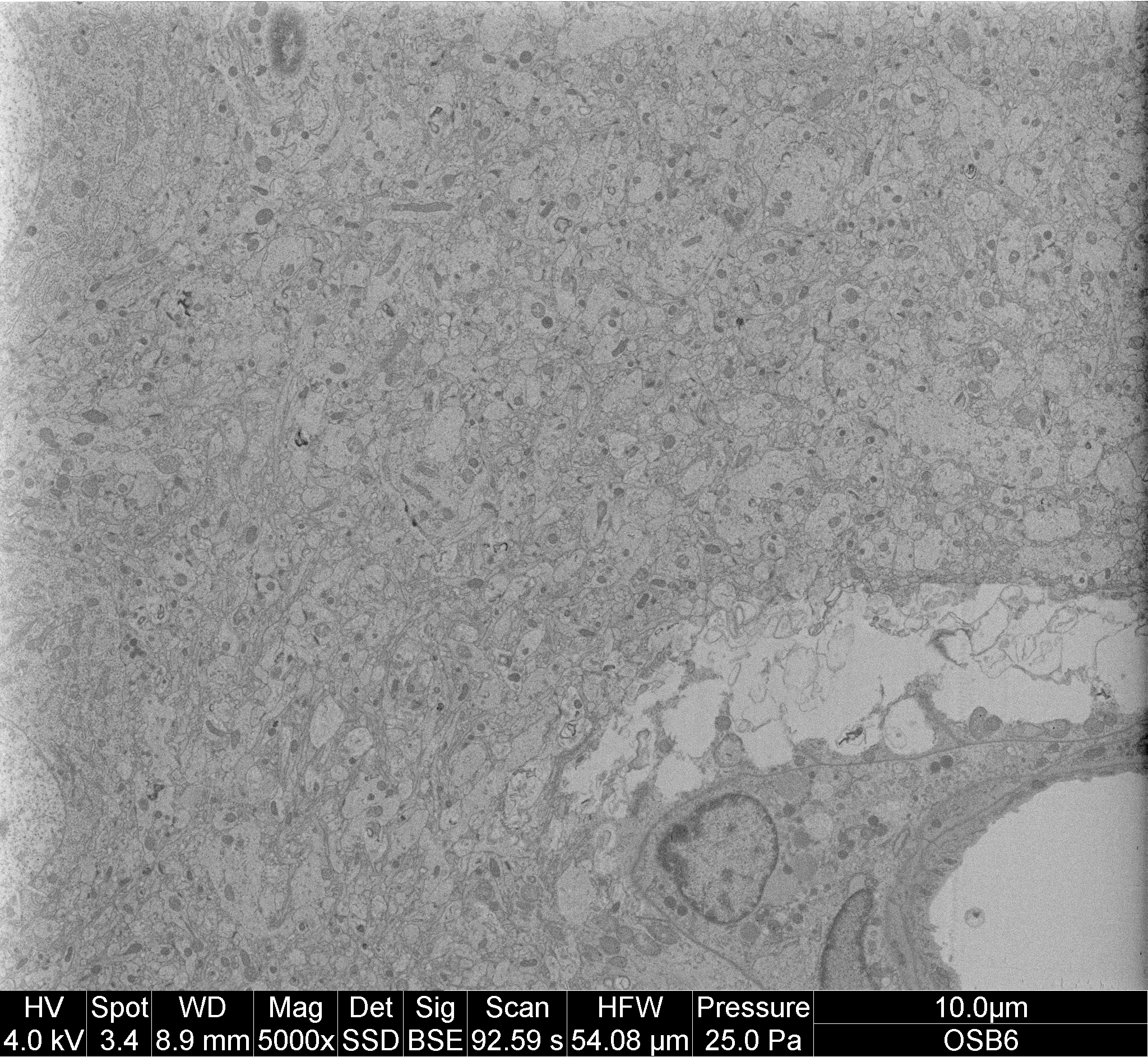

Supplement: Dataset S4 — (252.6 MB ZIP). [file pbio.0020329.sd004.zip › 040604_OS5_st1_307.tif]

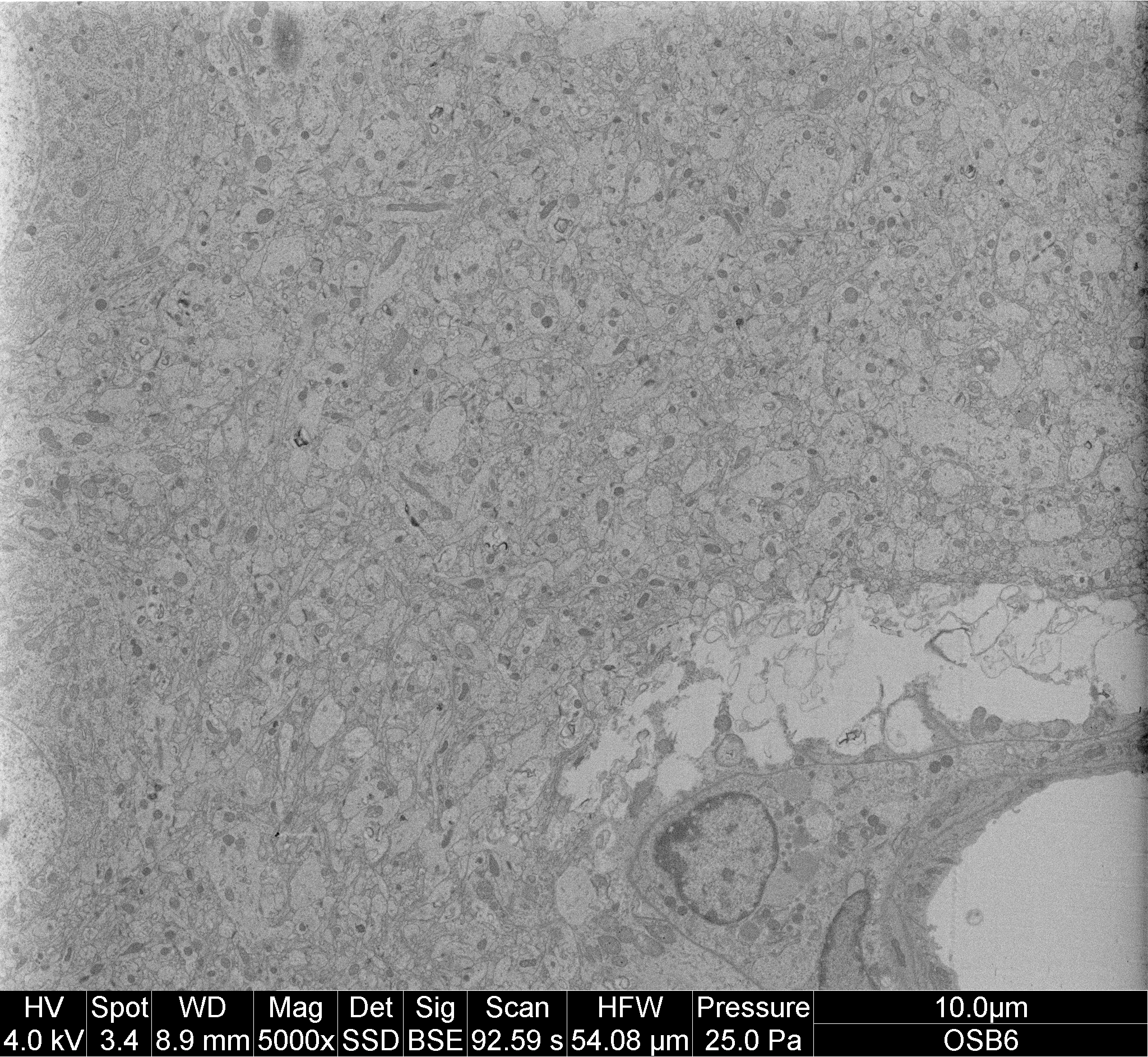

Supplement: Dataset S4 — (252.6 MB ZIP). [file pbio.0020329.sd004.zip › 040604_OS5_st1_308.tif]

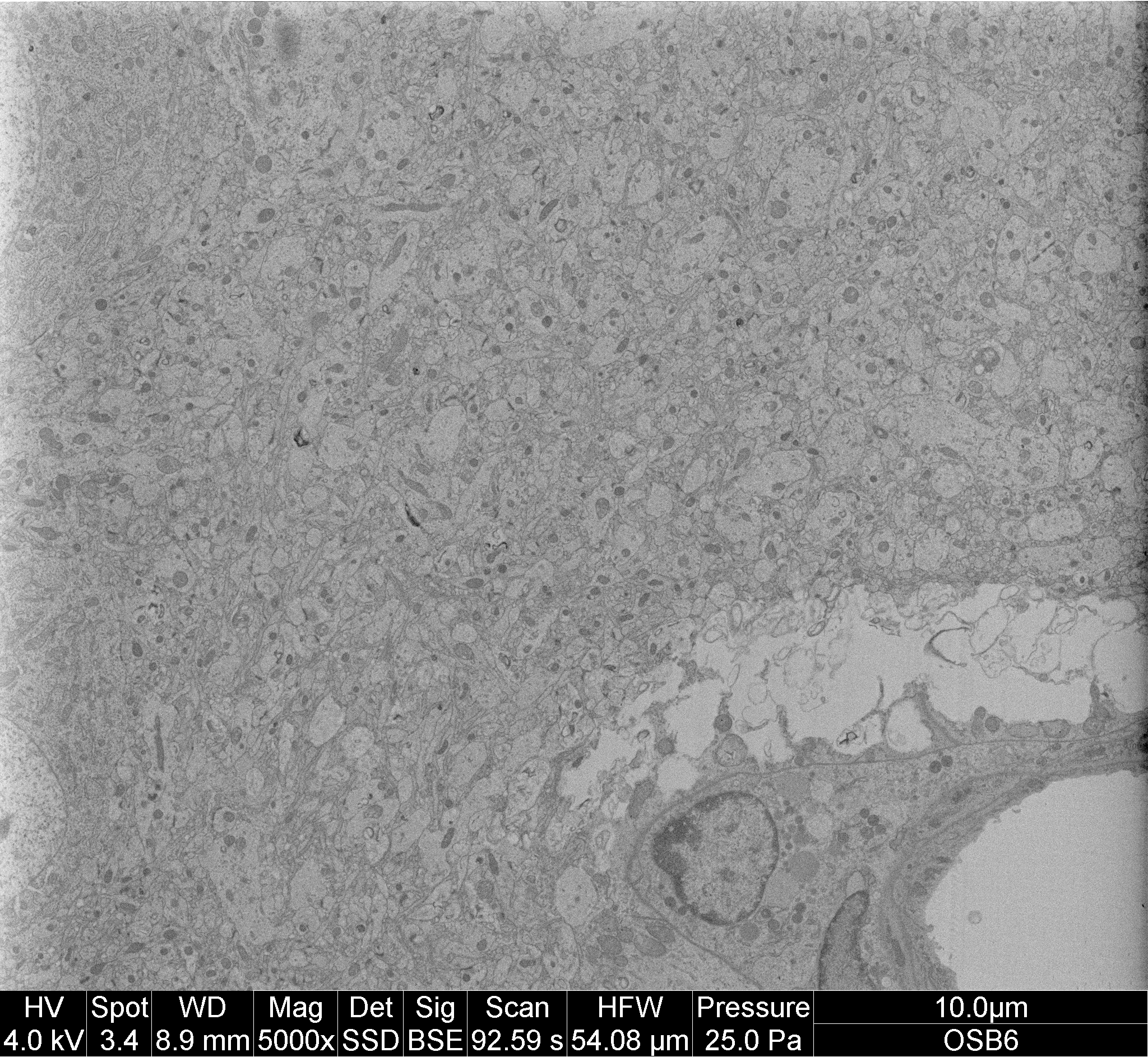

Supplement: Dataset S4 — (252.6 MB ZIP). [file pbio.0020329.sd004.zip › 040604_OS5_st1_309.tif]

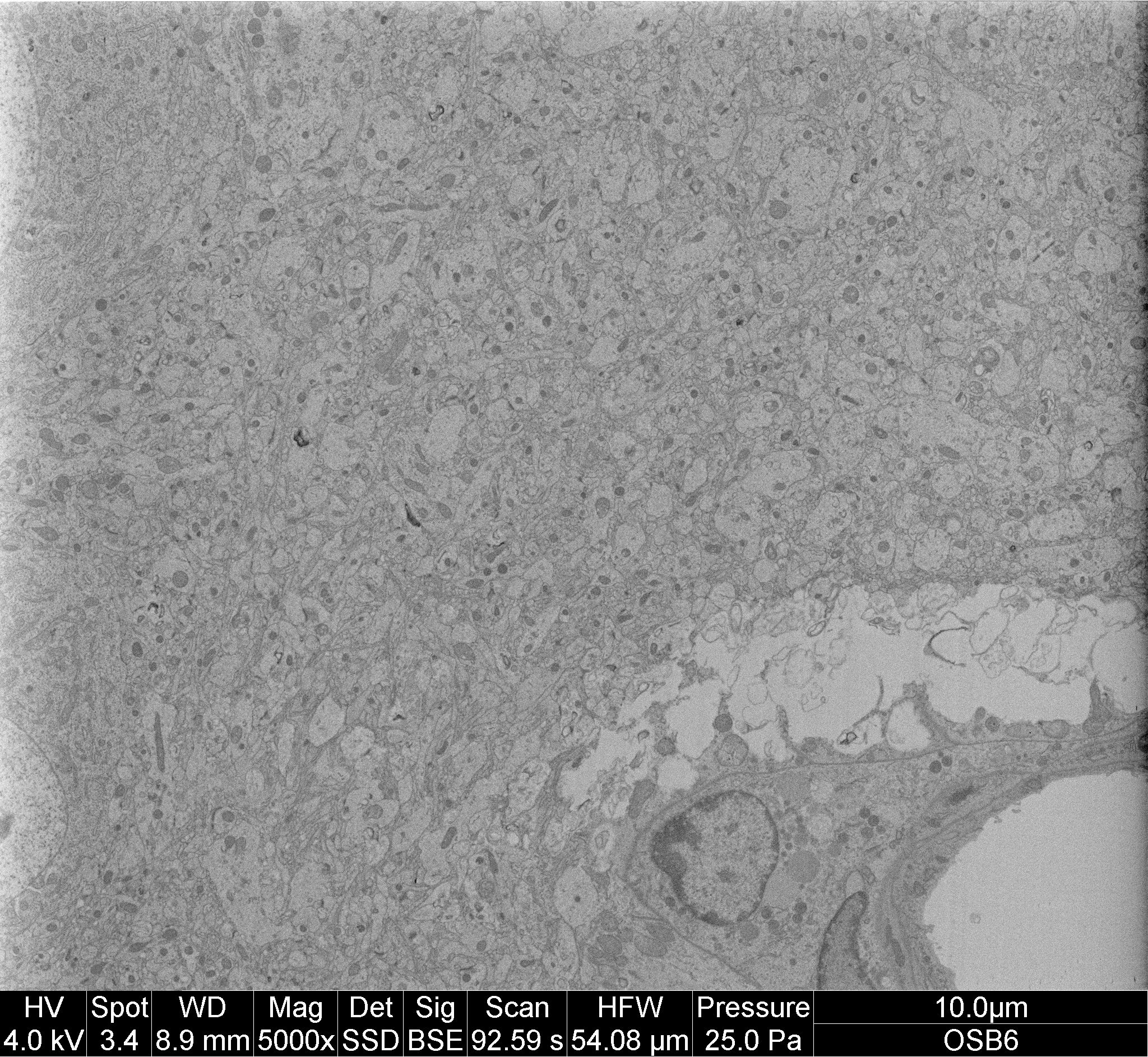

Supplement: Dataset S4 — (252.6 MB ZIP). [file pbio.0020329.sd004.zip › 040604_OS5_st1_310.tif]

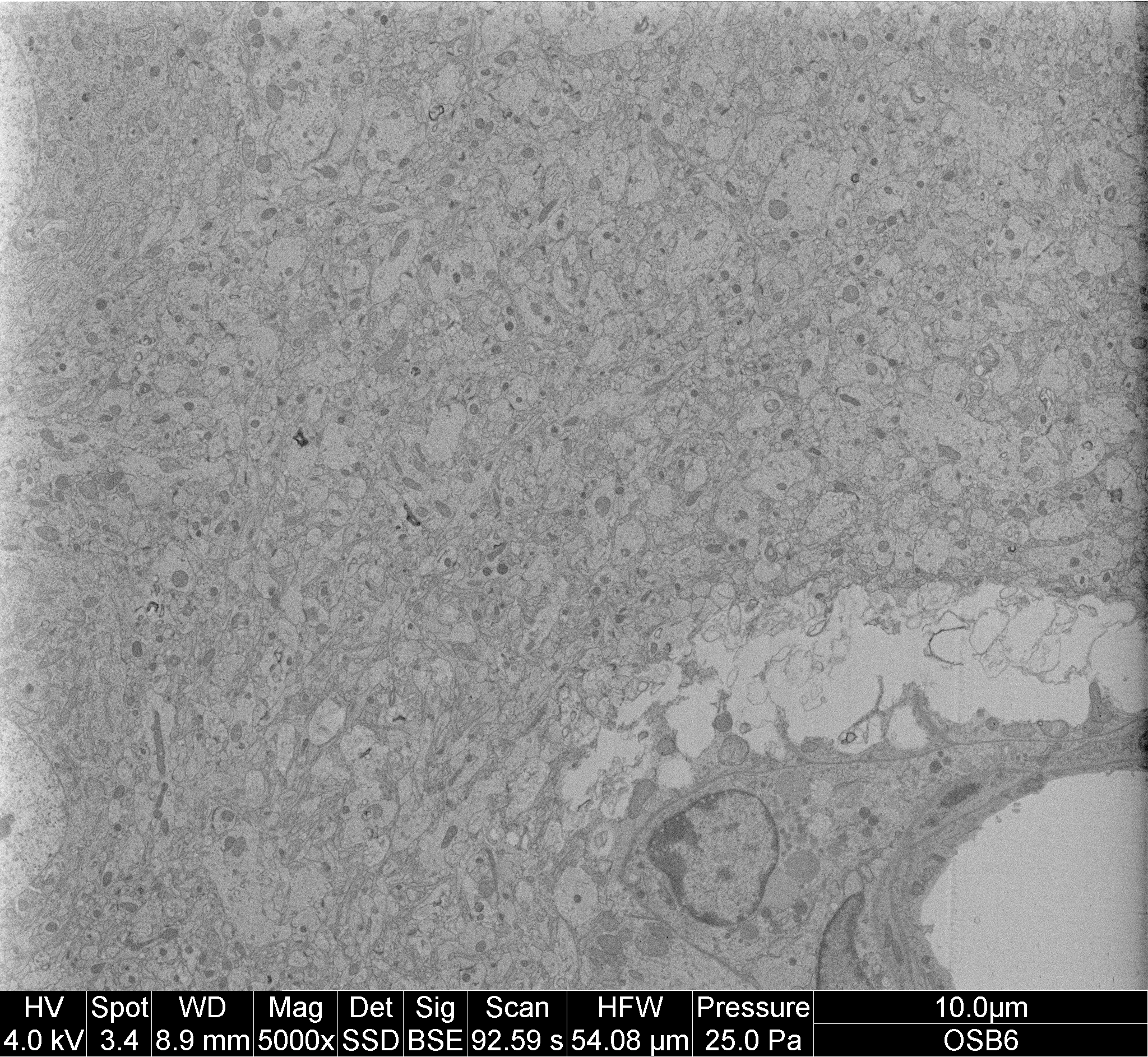

Supplement: Dataset S4 — (252.6 MB ZIP). [file pbio.0020329.sd004.zip › 040604_OS5_st1_311.tif]

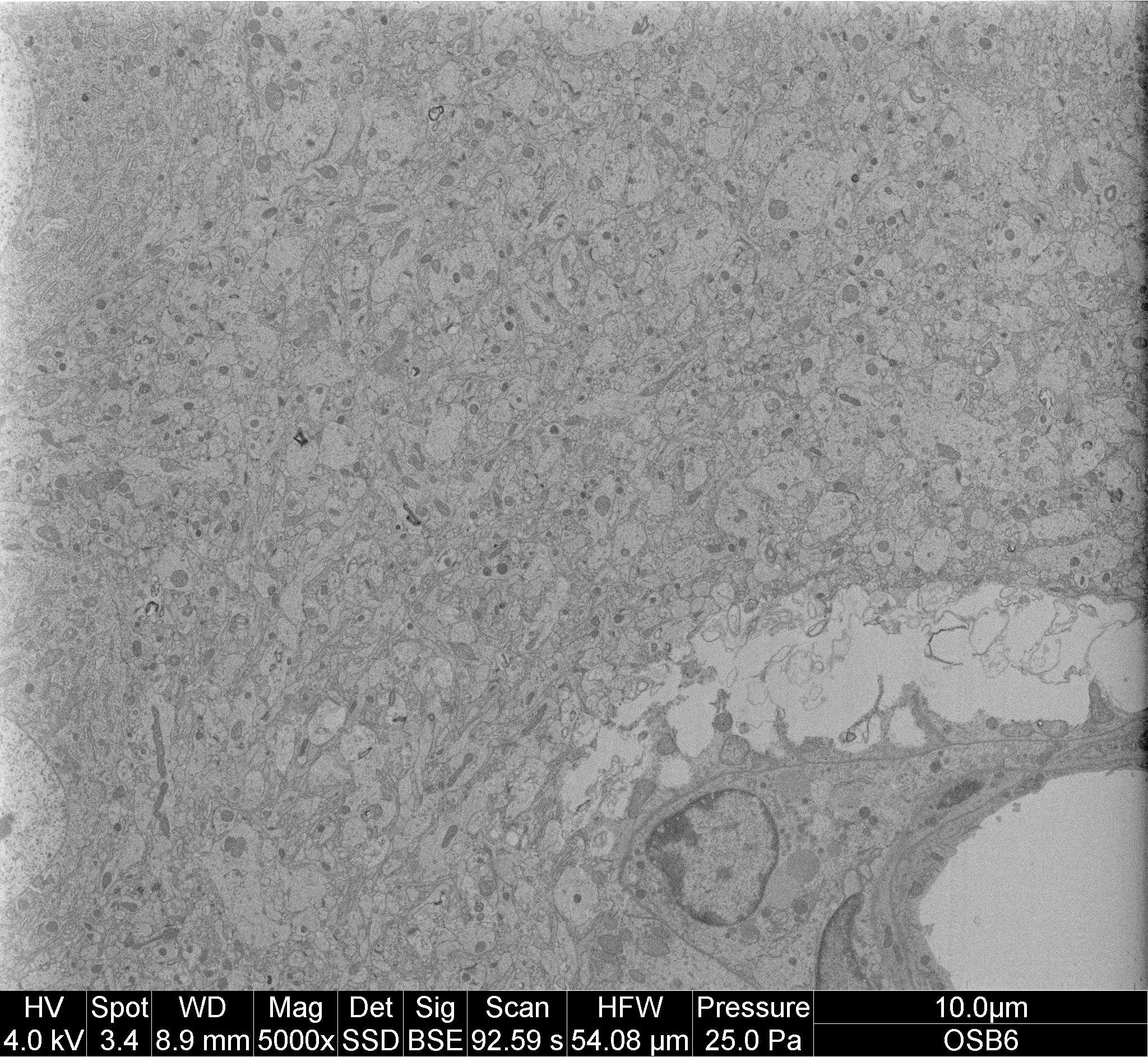

Supplement: Dataset S4 — (252.6 MB ZIP). [file pbio.0020329.sd004.zip › 040604_OS5_st1_312.tif]

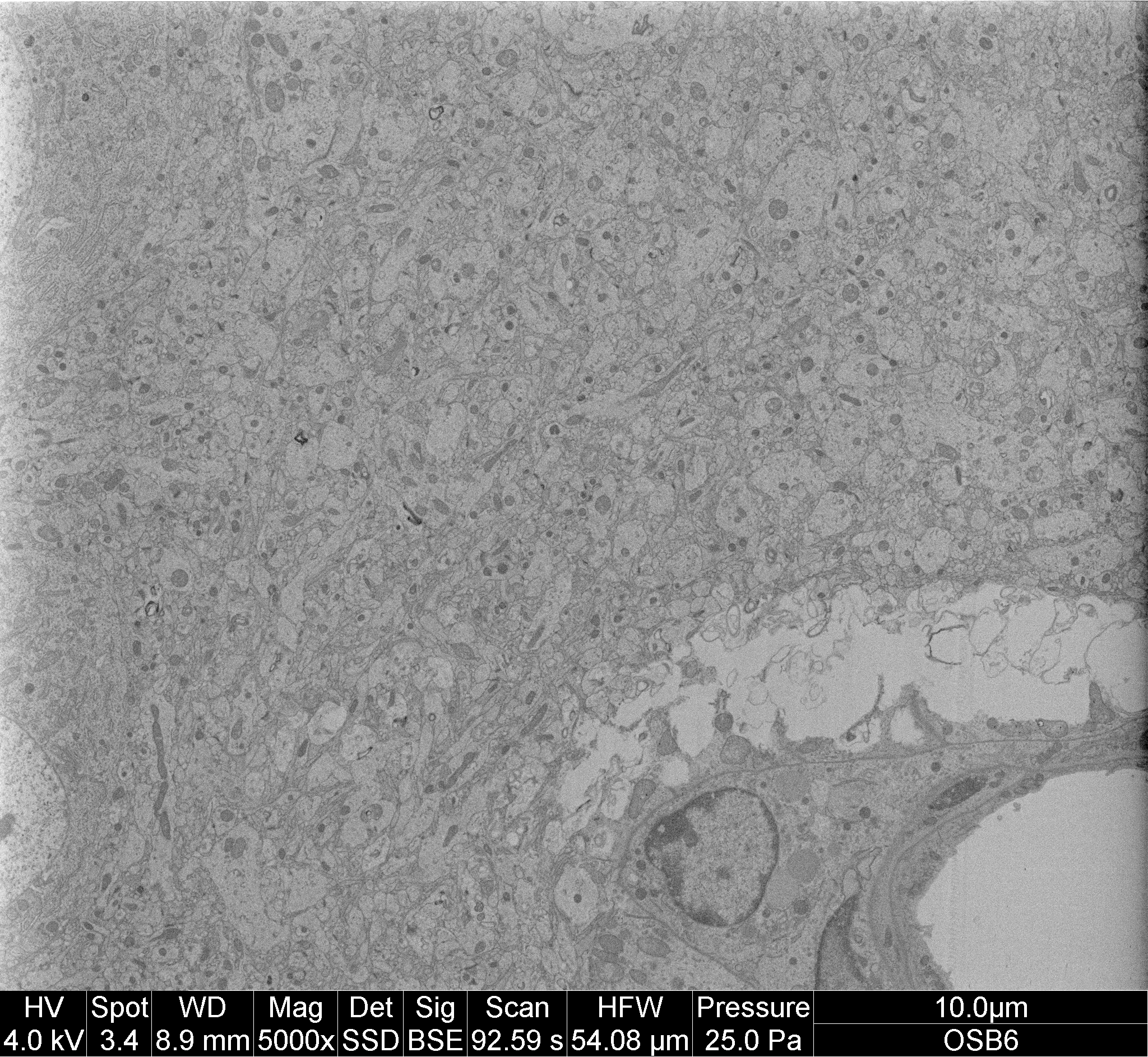

Supplement: Dataset S4 — (252.6 MB ZIP). [file pbio.0020329.sd004.zip › 040604_OS5_st1_313.tif]

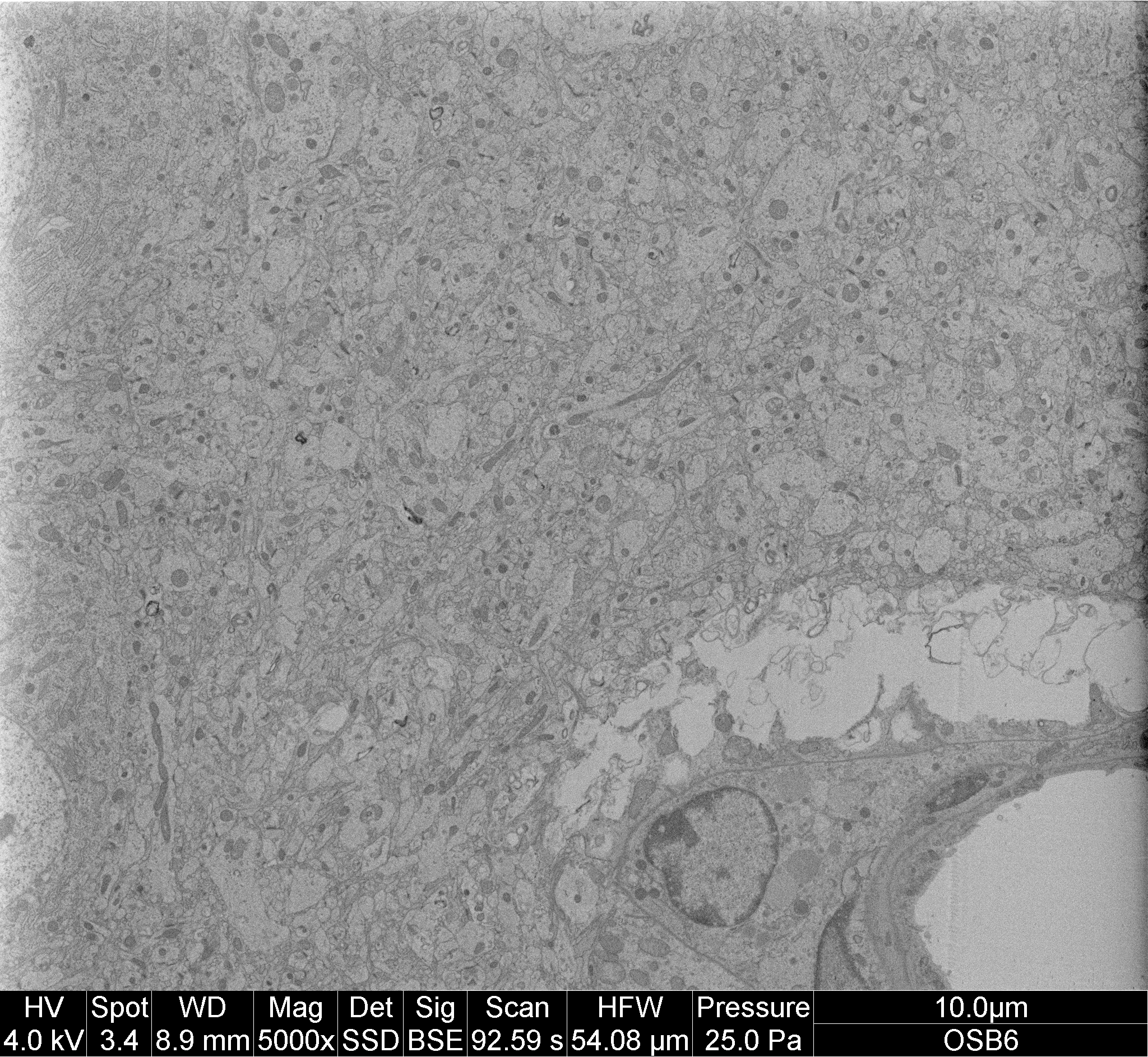

Supplement: Dataset S4 — (252.6 MB ZIP). [file pbio.0020329.sd004.zip › 040604_OS5_st1_314.tif]

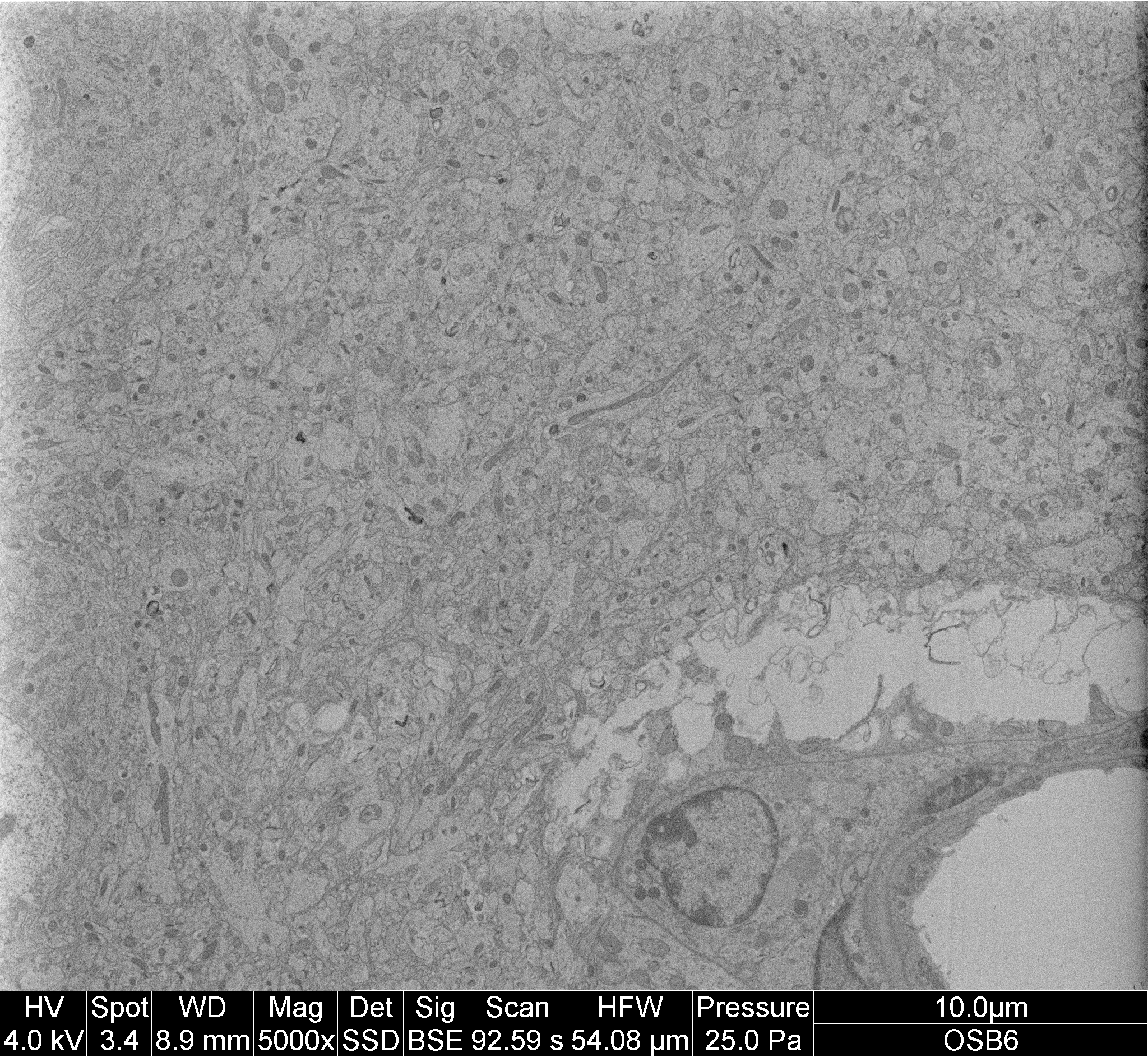

Supplement: Dataset S4 — (252.6 MB ZIP). [file pbio.0020329.sd004.zip › 040604_OS5_st1_315.tif]

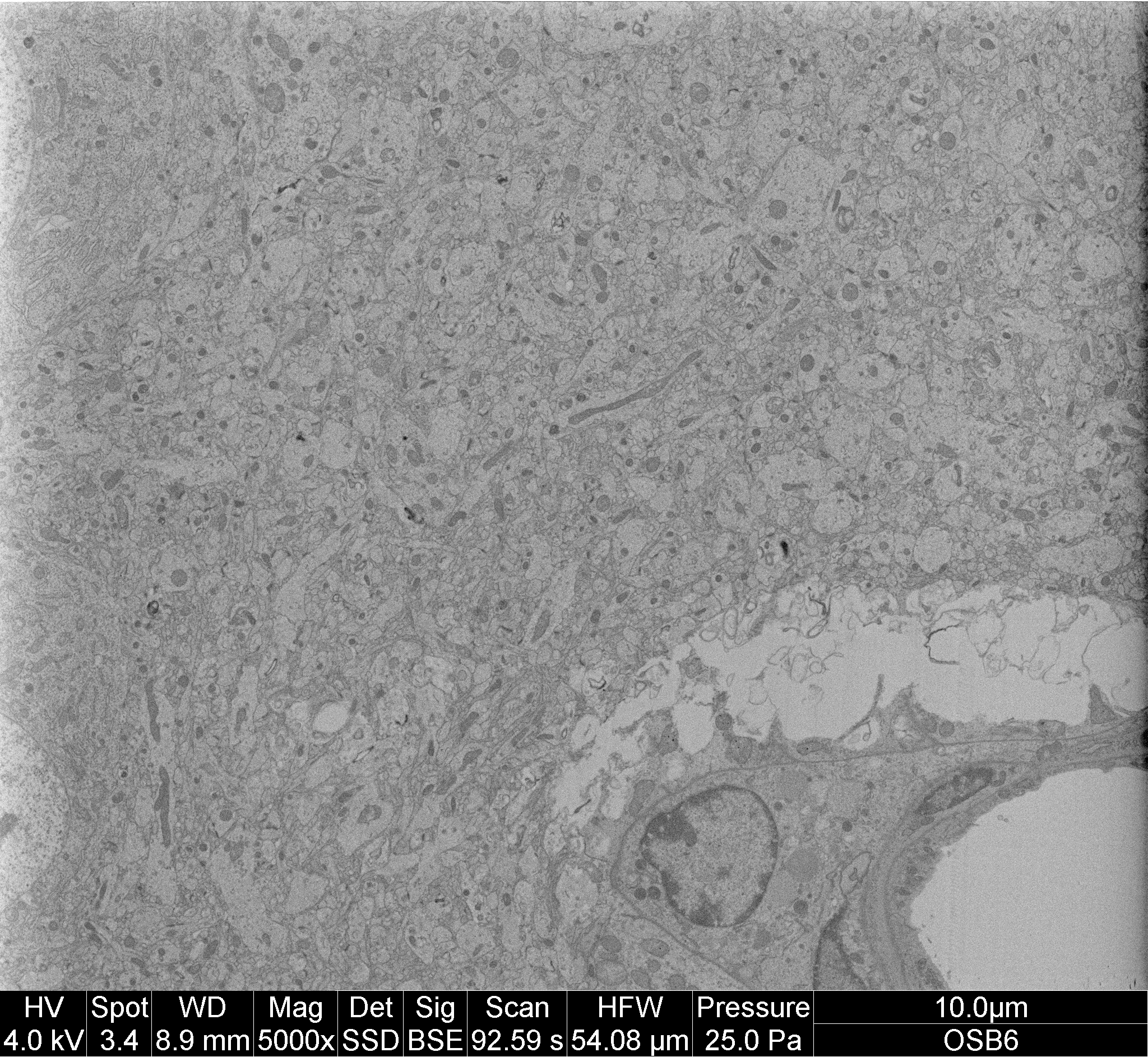

Supplement: Dataset S4 — (252.6 MB ZIP). [file pbio.0020329.sd004.zip › 040604_OS5_st1_316.tif]

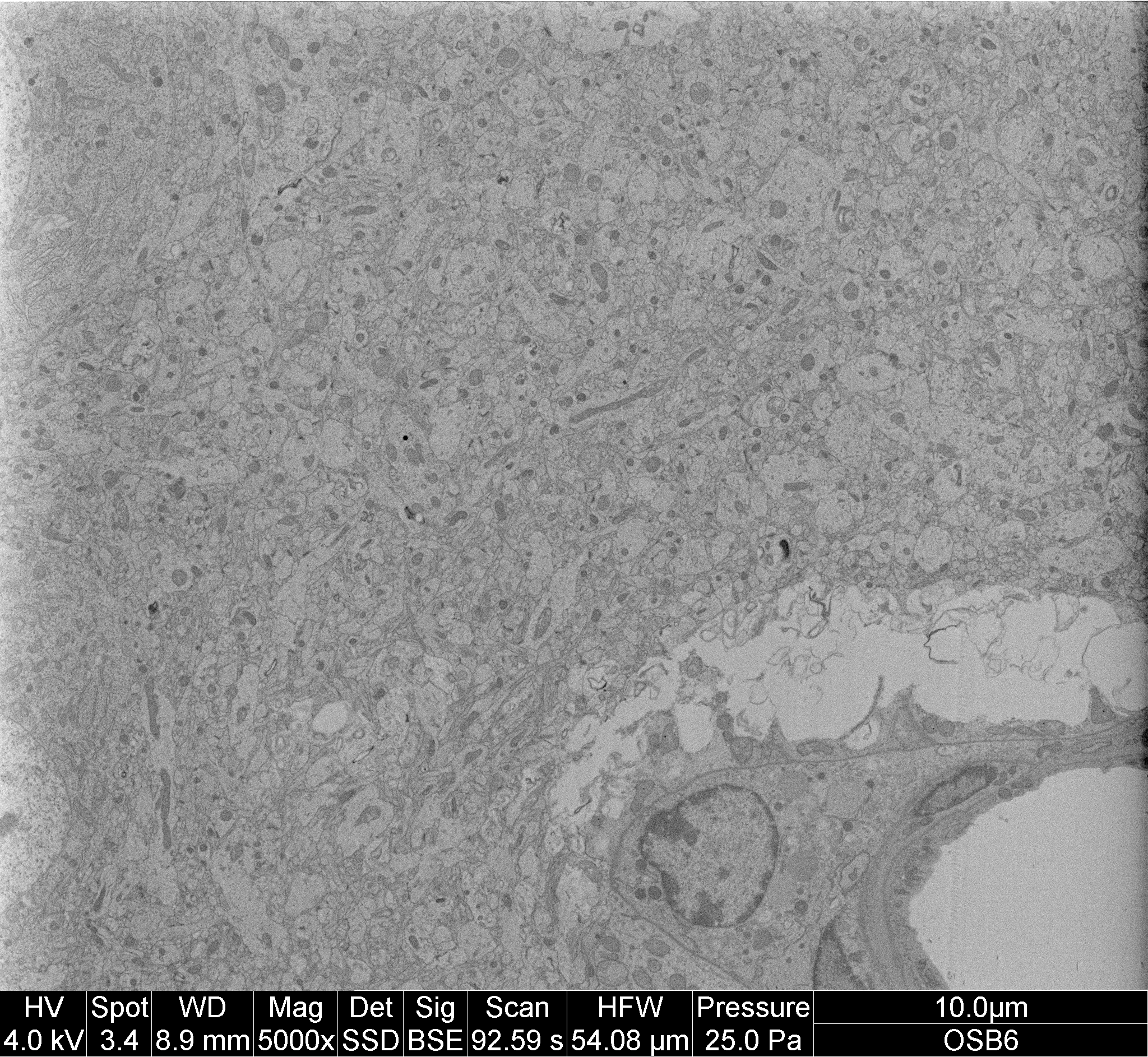

Supplement: Dataset S4 — (252.6 MB ZIP). [file pbio.0020329.sd004.zip › 040604_OS5_st1_317.tif]

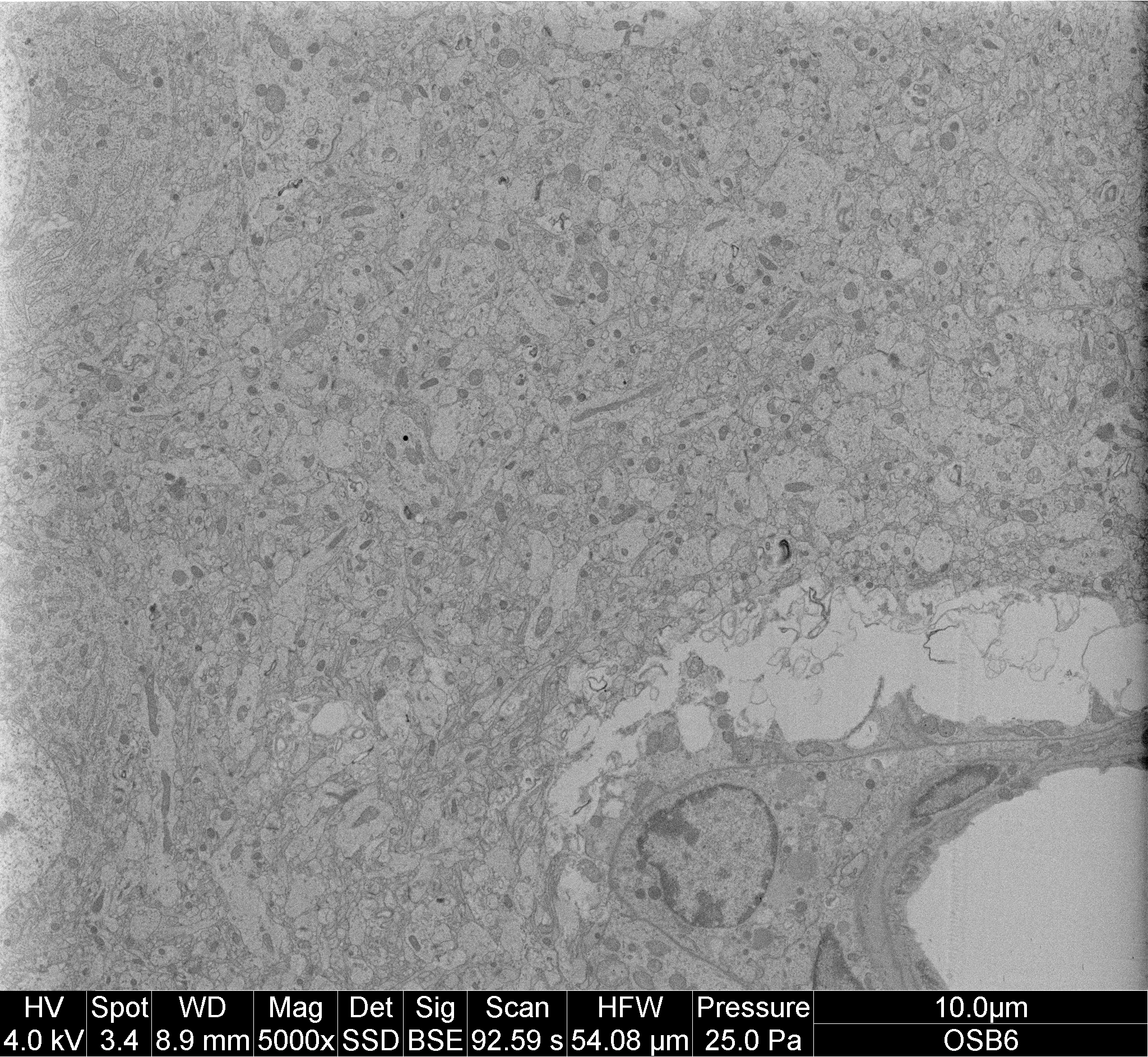

Supplement: Dataset S4 — (252.6 MB ZIP). [file pbio.0020329.sd004.zip › 040604_OS5_st1_318.tif]

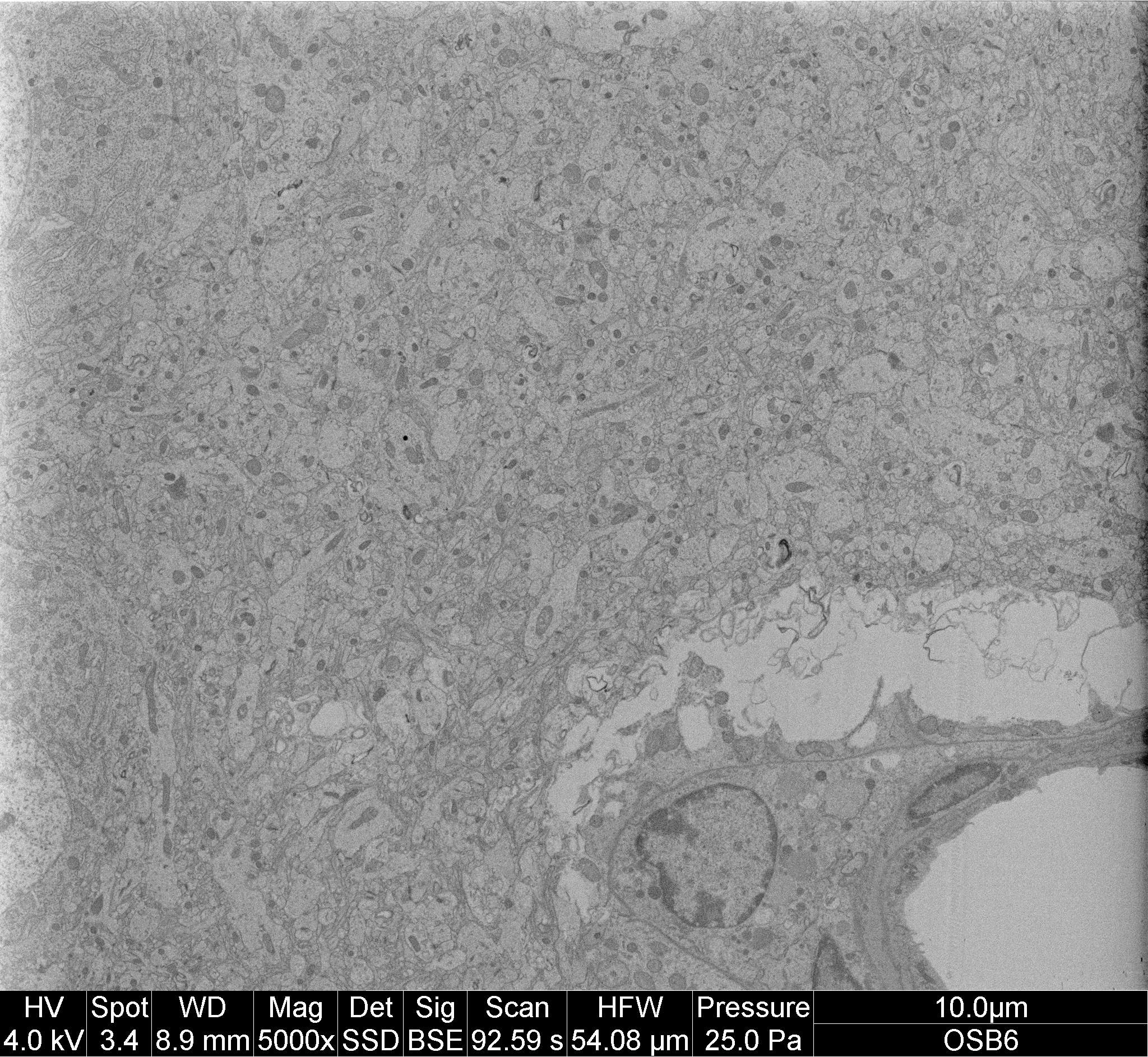

Supplement: Dataset S4 — (252.6 MB ZIP). [file pbio.0020329.sd004.zip › 040604_OS5_st1_319.tif]

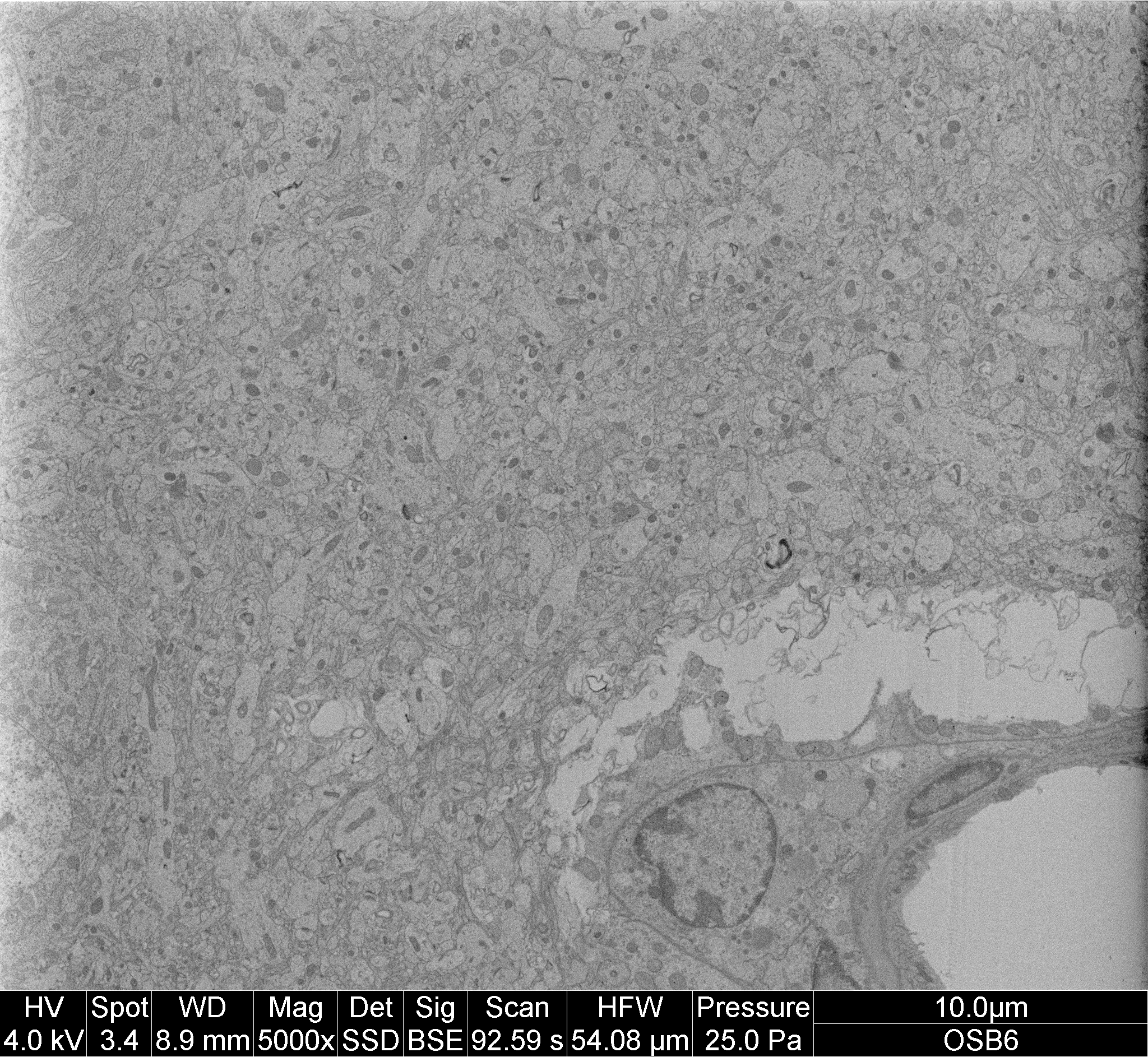

Supplement: Dataset S4 — (252.6 MB ZIP). [file pbio.0020329.sd004.zip › 040604_OS5_st1_320.tif]

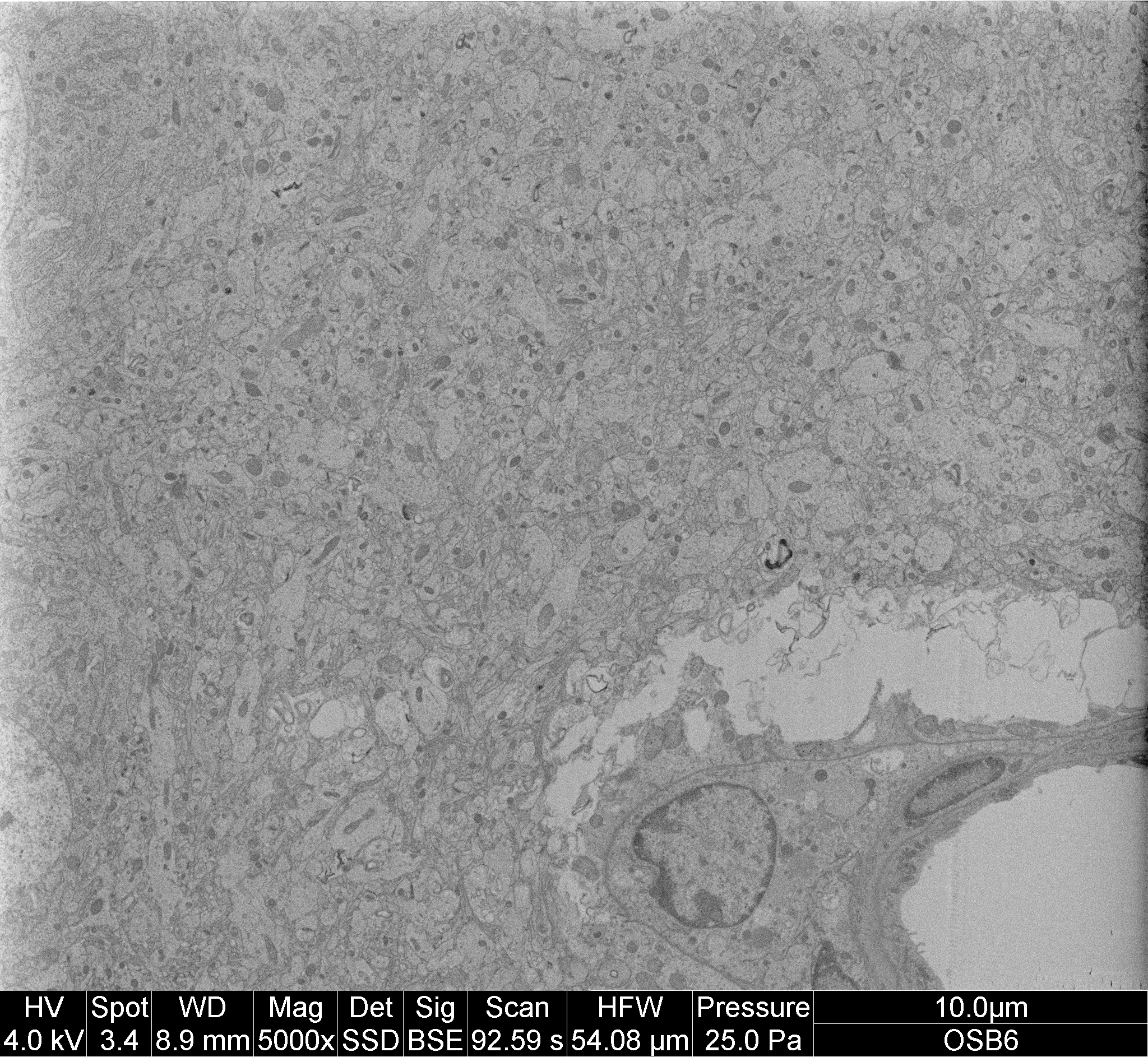

Supplement: Dataset S4 — (252.6 MB ZIP). [file pbio.0020329.sd004.zip › 040604_OS5_st1_321.tif]

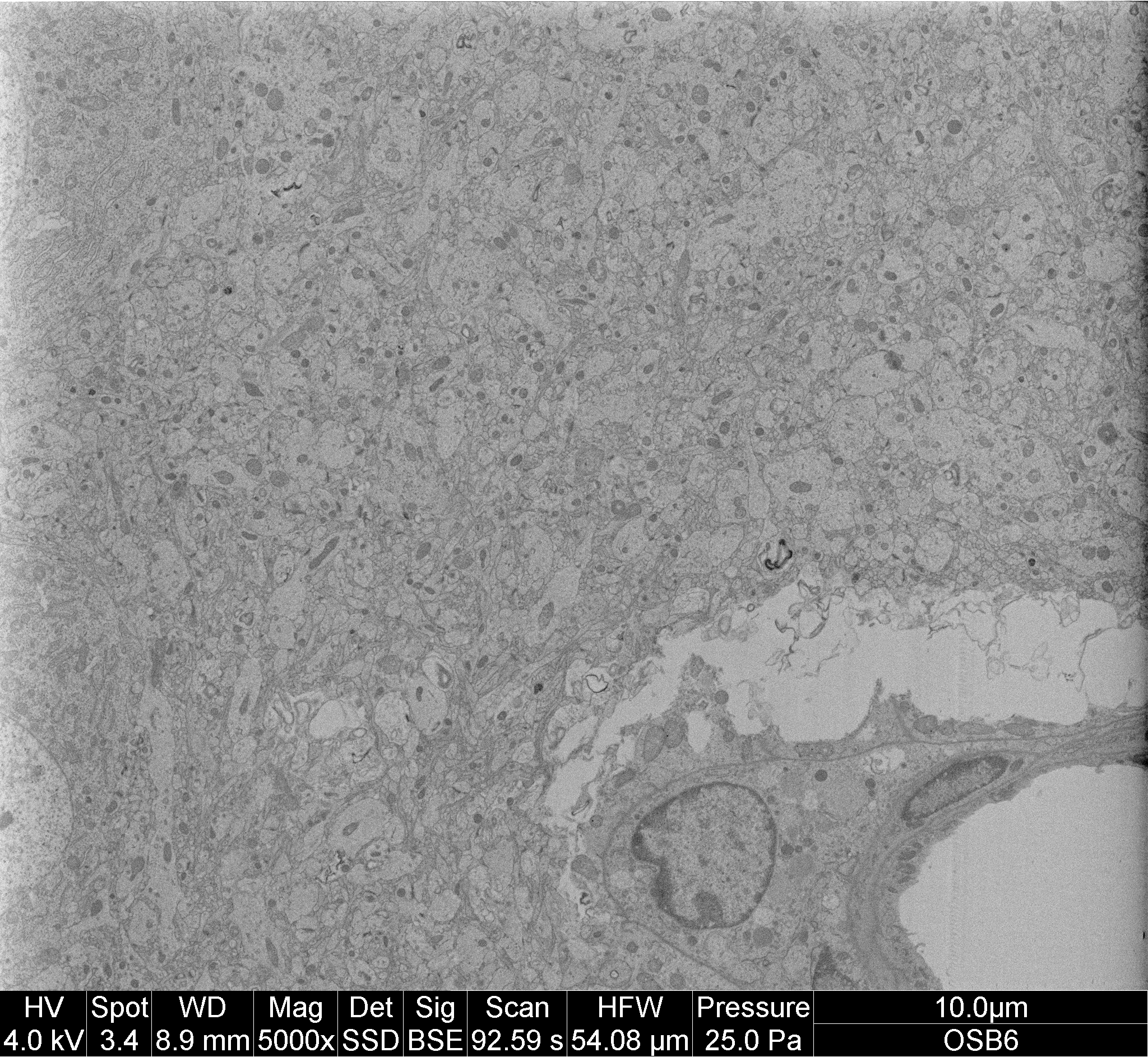

Supplement: Dataset S4 — (252.6 MB ZIP). [file pbio.0020329.sd004.zip › 040604_OS5_st1_322.tif]

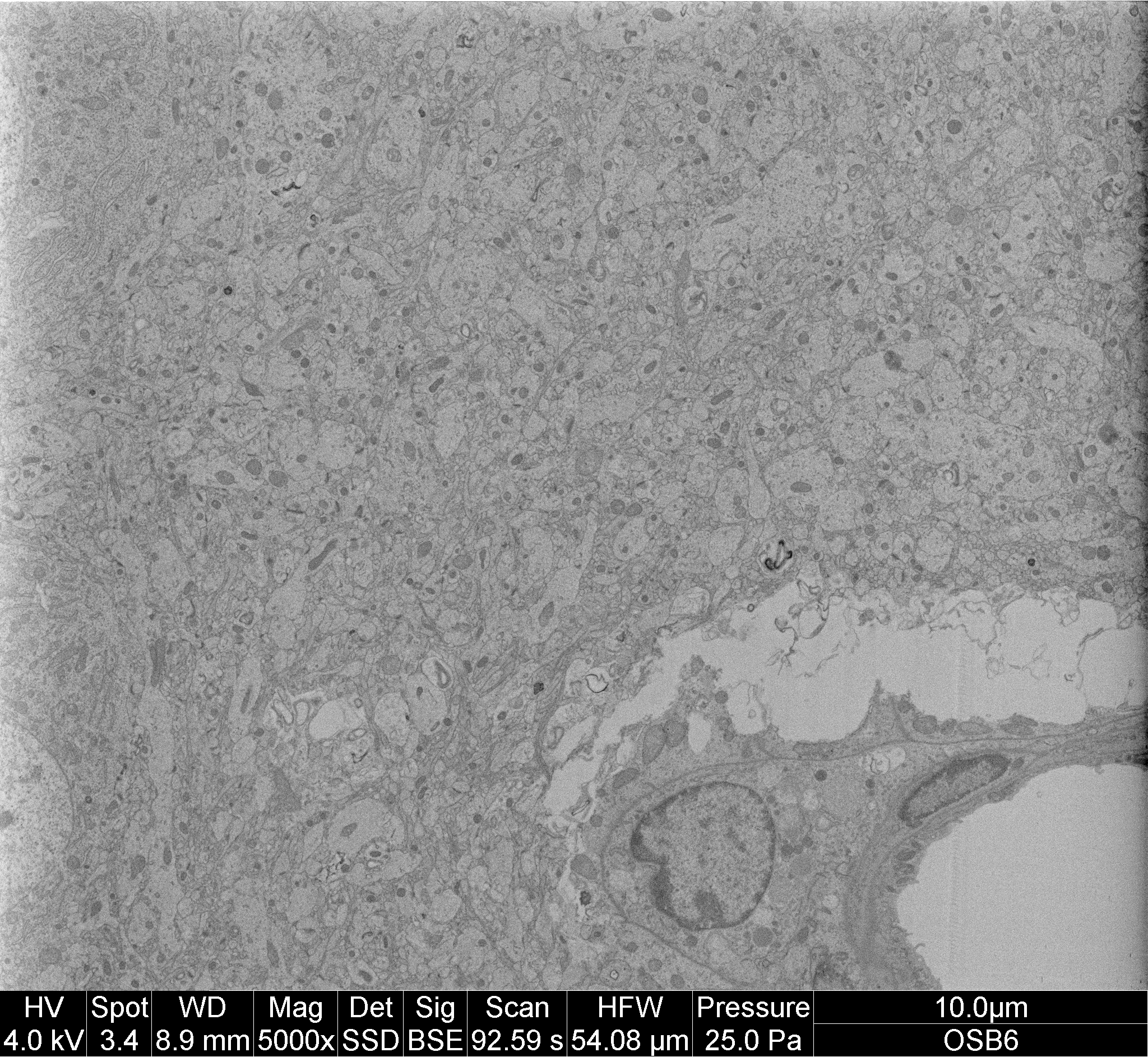

Supplement: Dataset S4 — (252.6 MB ZIP). [file pbio.0020329.sd004.zip › 040604_OS5_st1_323.tif]

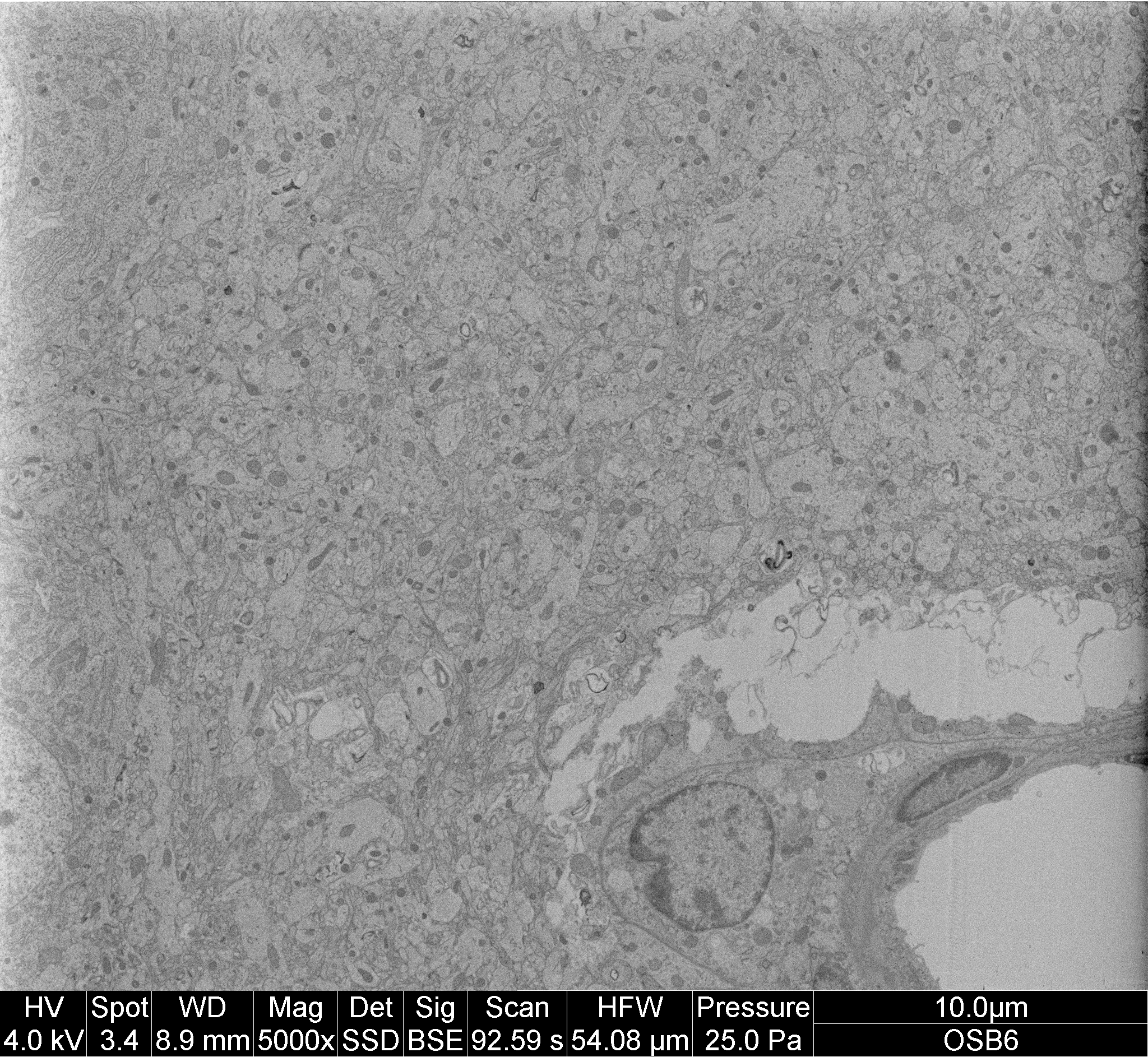

Supplement: Dataset S4 — (252.6 MB ZIP). [file pbio.0020329.sd004.zip › 040604_OS5_st1_324.tif]

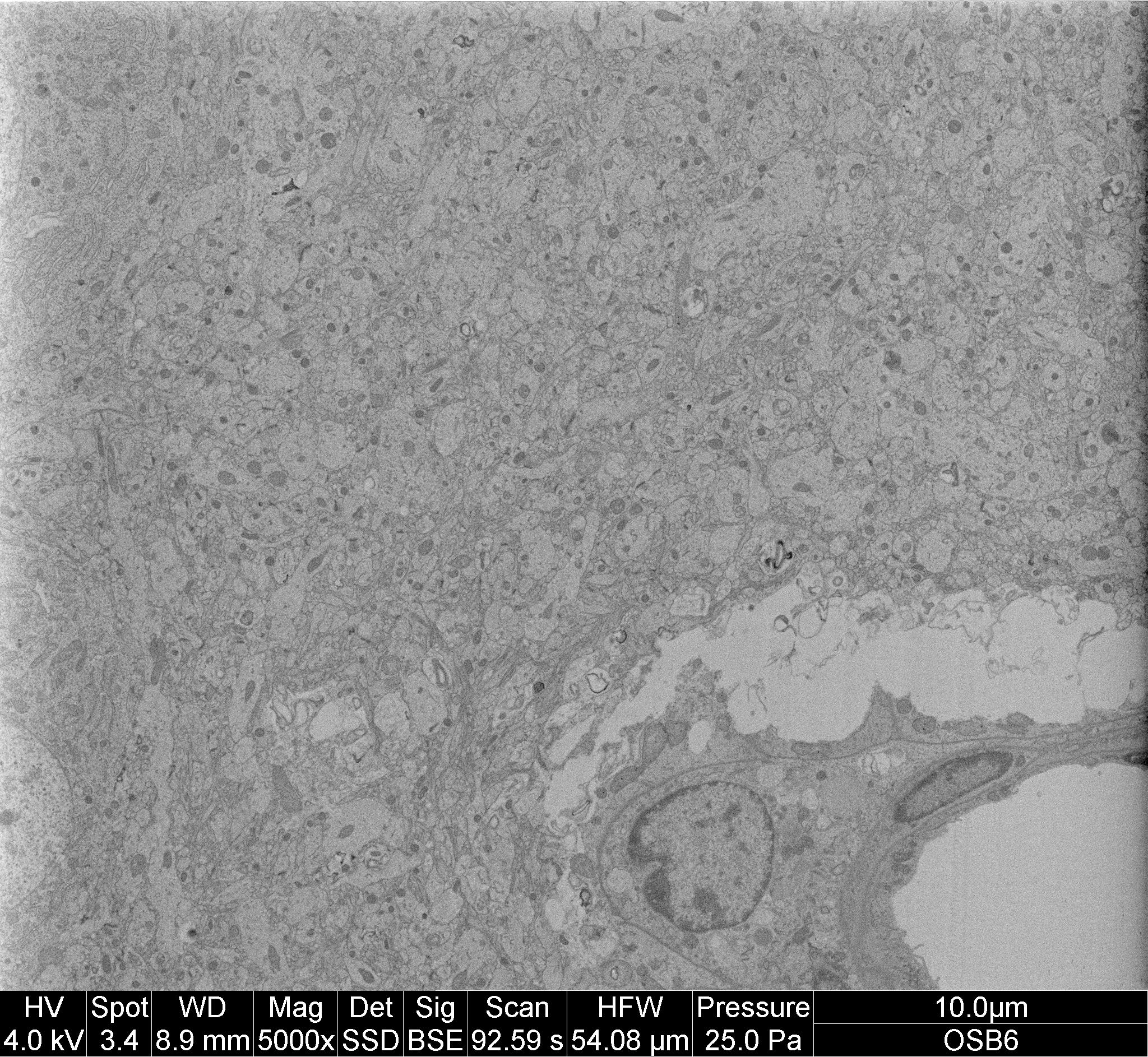

Supplement: Dataset S4 — (252.6 MB ZIP). [file pbio.0020329.sd004.zip › 040604_OS5_st1_325.tif]

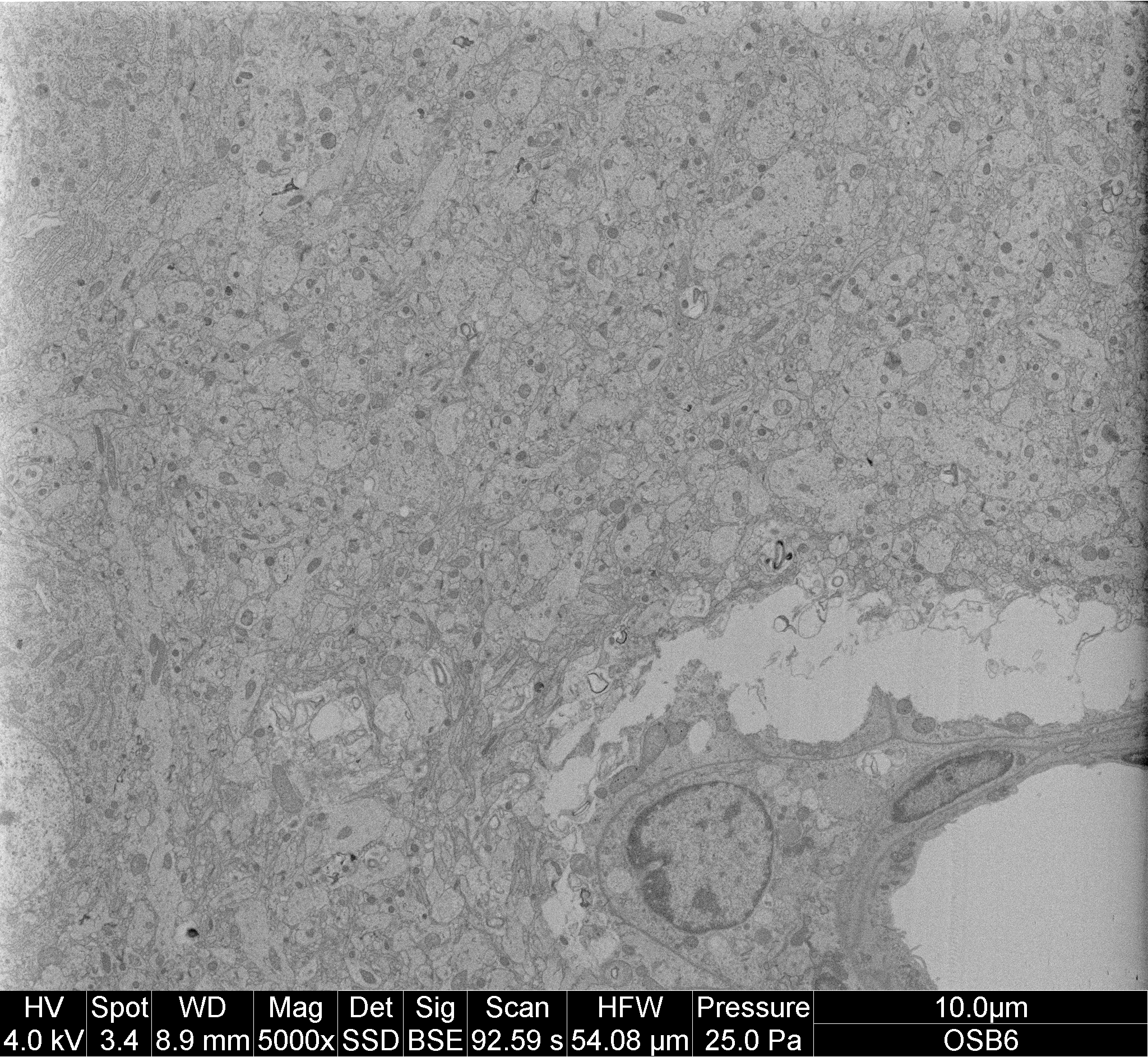

Supplement: Dataset S4 — (252.6 MB ZIP). [file pbio.0020329.sd004.zip › 040604_OS5_st1_326.tif]

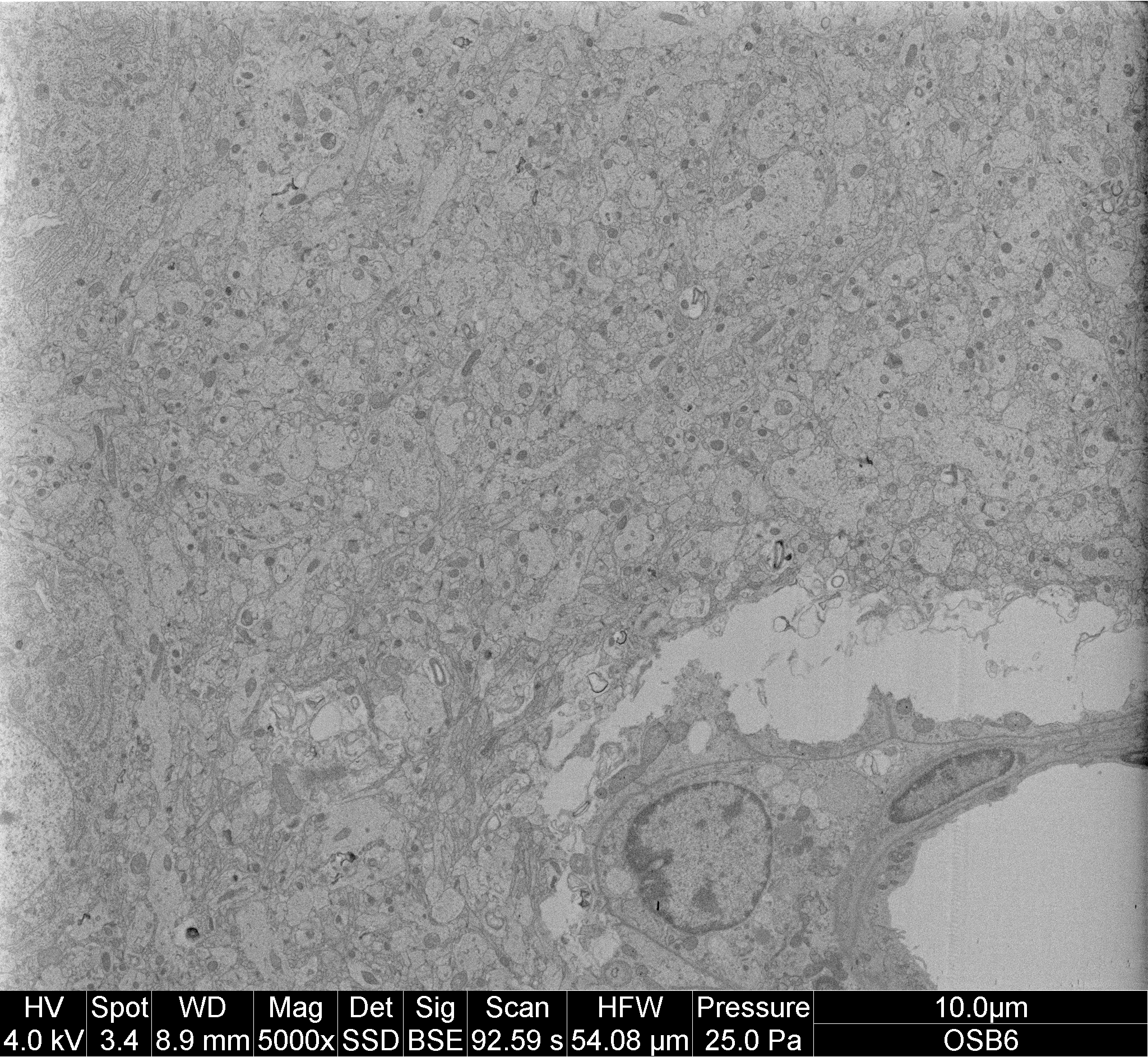

Supplement: Dataset S4 — (252.6 MB ZIP). [file pbio.0020329.sd004.zip › 040604_OS5_st1_327.tif]

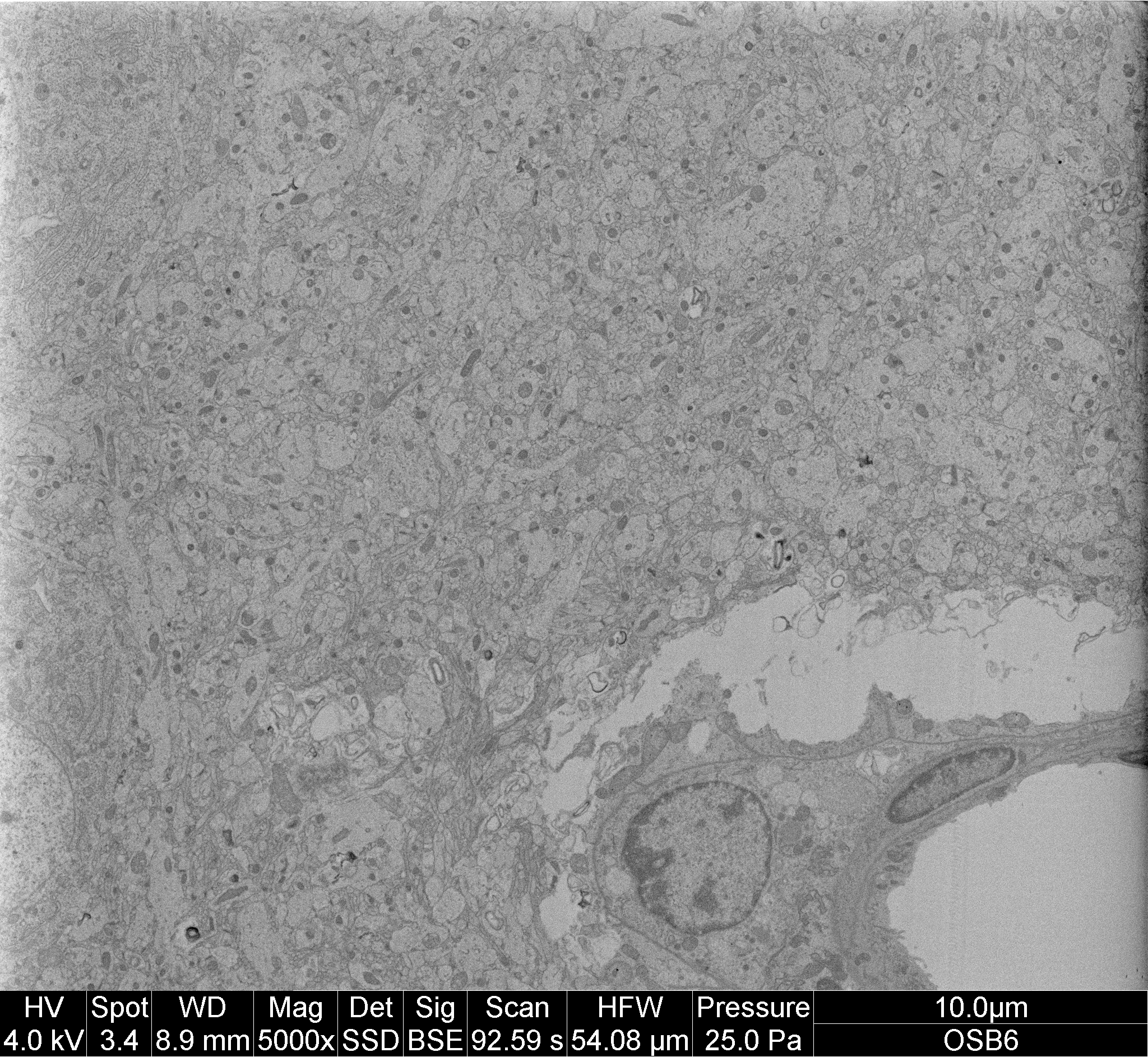

Supplement: Dataset S4 — (252.6 MB ZIP). [file pbio.0020329.sd004.zip › 040604_OS5_st1_328.tif]

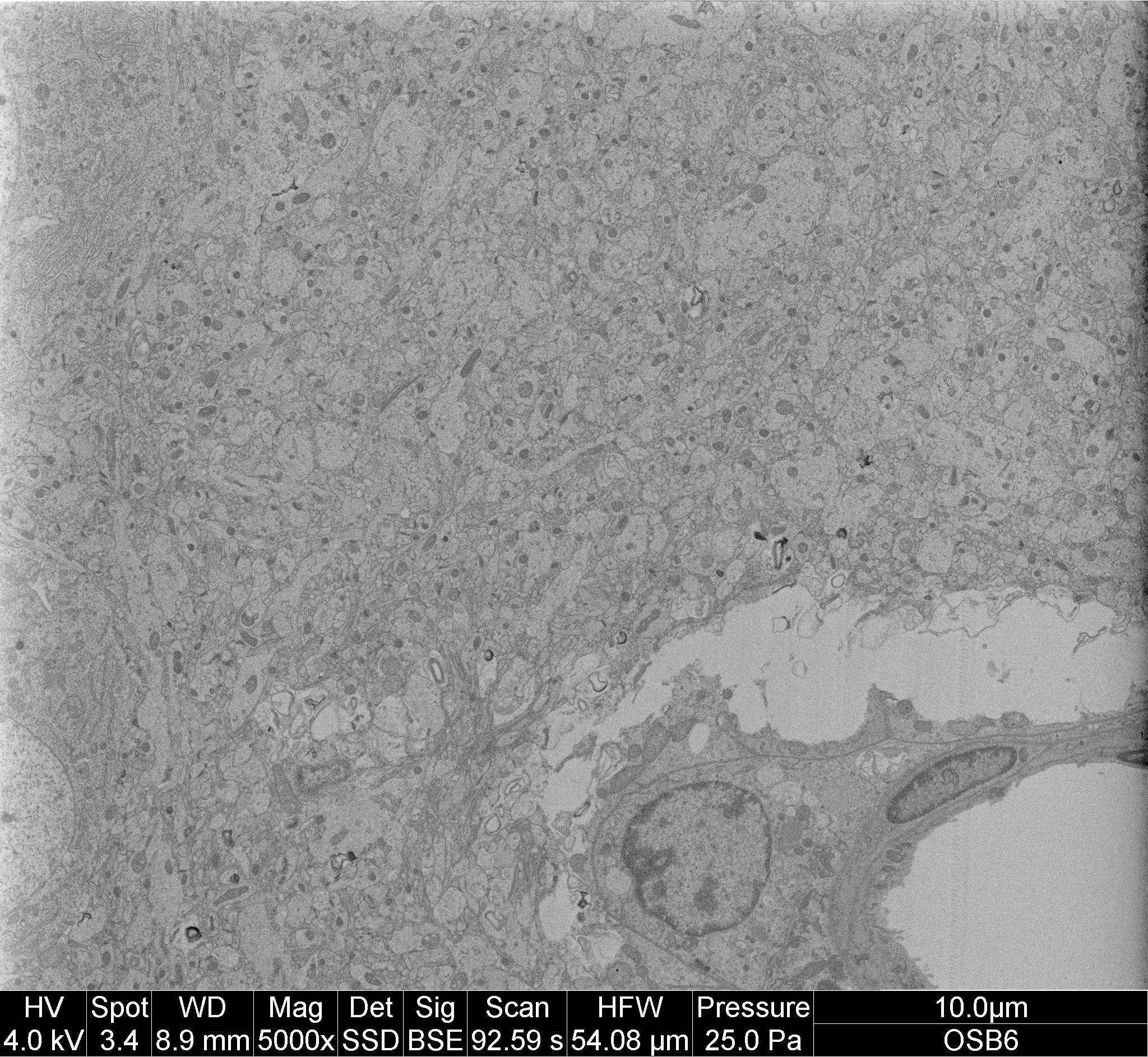

Supplement: Dataset S4 — (252.6 MB ZIP). [file pbio.0020329.sd004.zip › 040604_OS5_st1_329.tif]

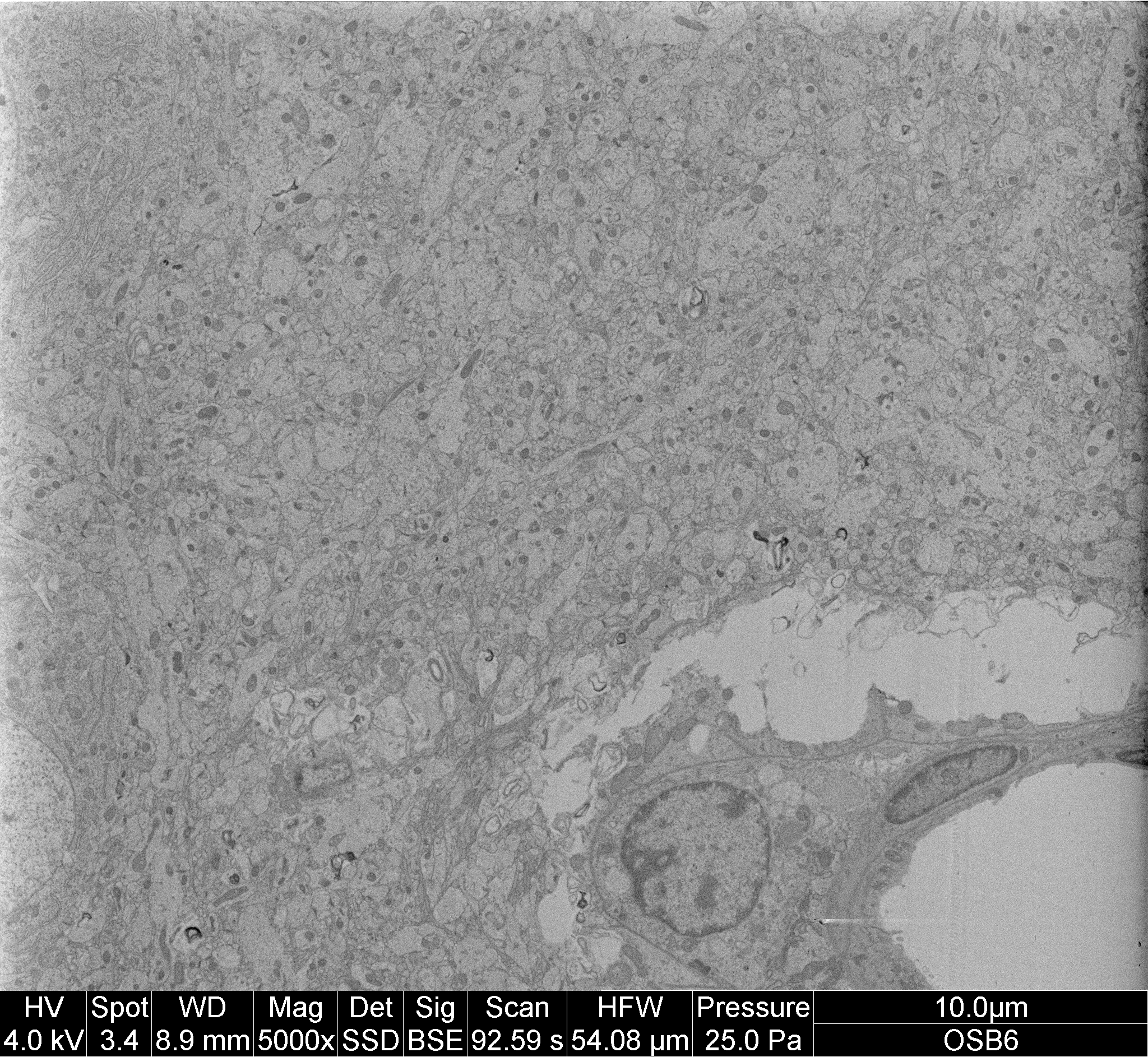

Supplement: Dataset S4 — (252.6 MB ZIP). [file pbio.0020329.sd004.zip › 040604_OS5_st1_330.tif]

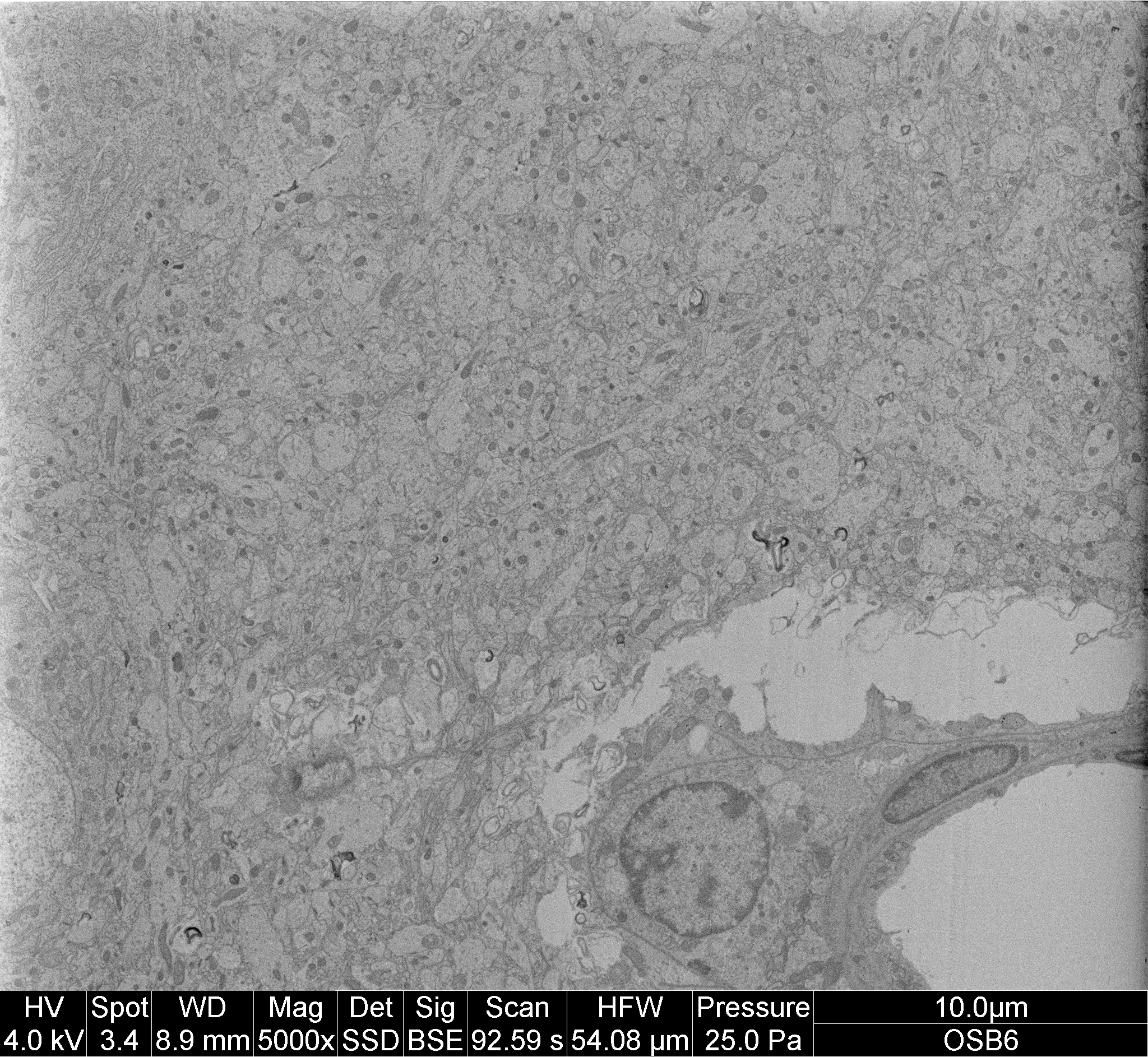

Supplement: Dataset S4 — (252.6 MB ZIP). [file pbio.0020329.sd004.zip › 040604_OS5_st1_331.tif]

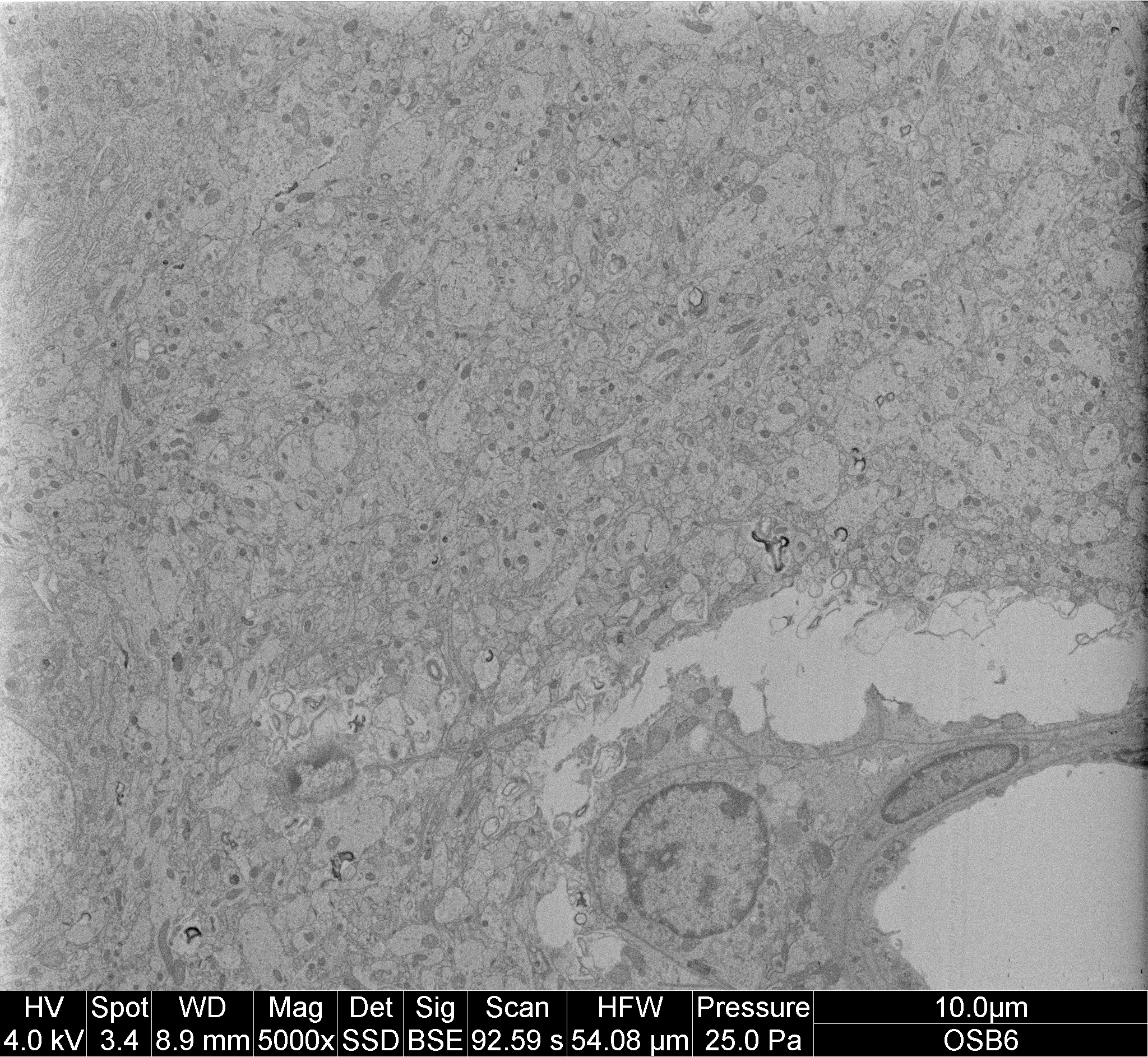

Supplement: Dataset S4 — (252.6 MB ZIP). [file pbio.0020329.sd004.zip › 040604_OS5_st1_332.tif]

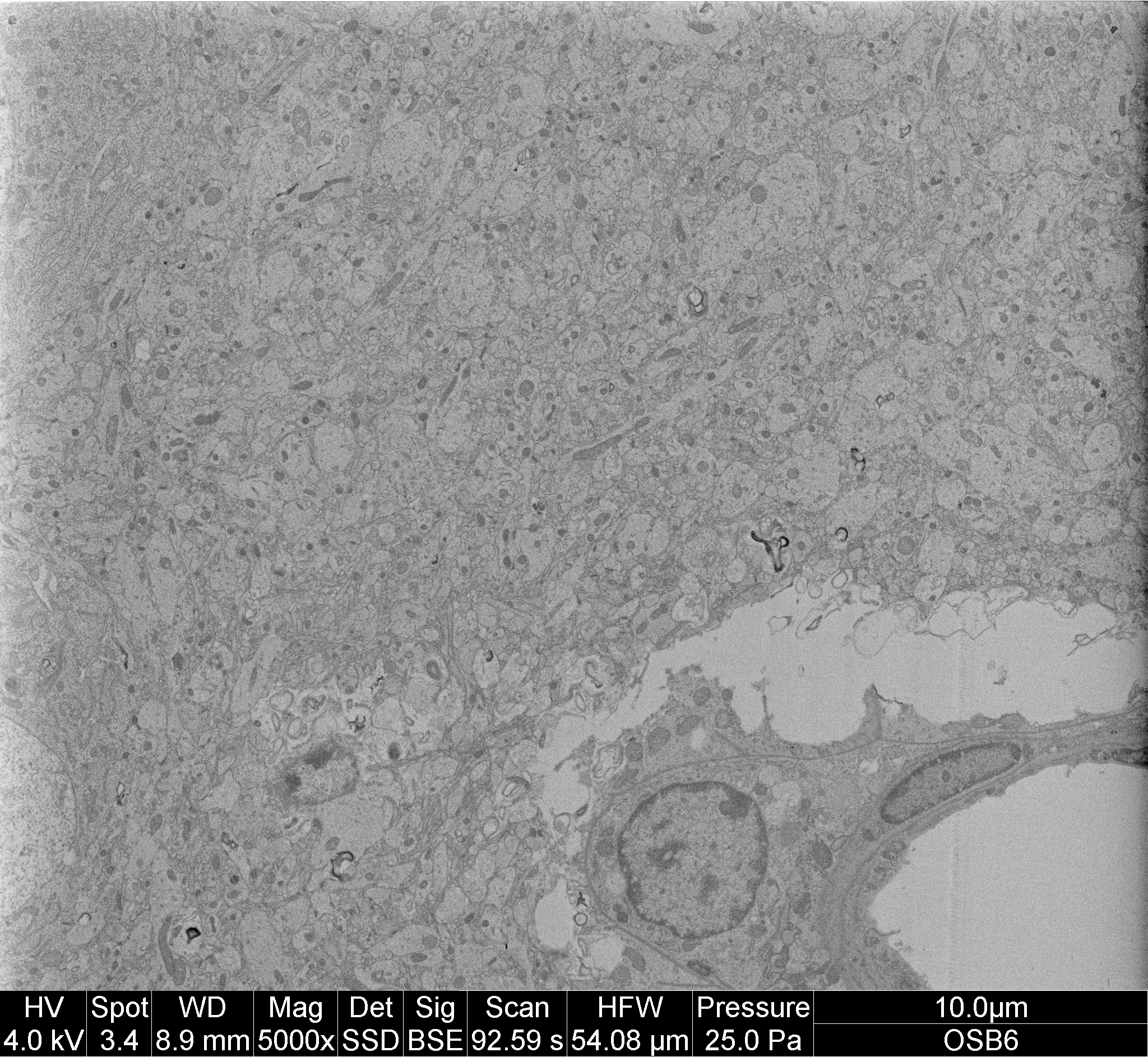

Supplement: Dataset S4 — (252.6 MB ZIP). [file pbio.0020329.sd004.zip › 040604_OS5_st1_333.tif]

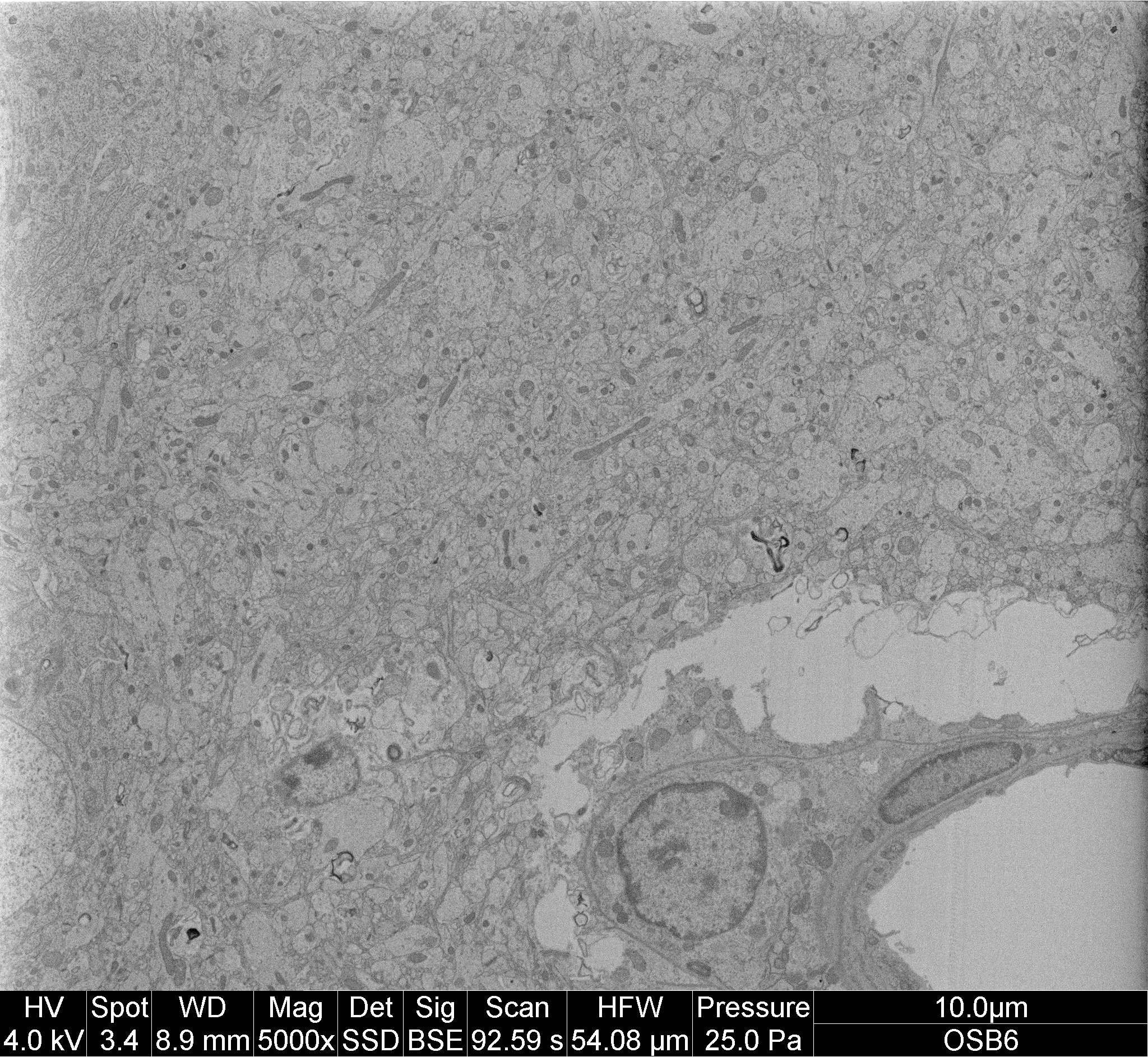

Supplement: Dataset S4 — (252.6 MB ZIP). [file pbio.0020329.sd004.zip › 040604_OS5_st1_334.tif]

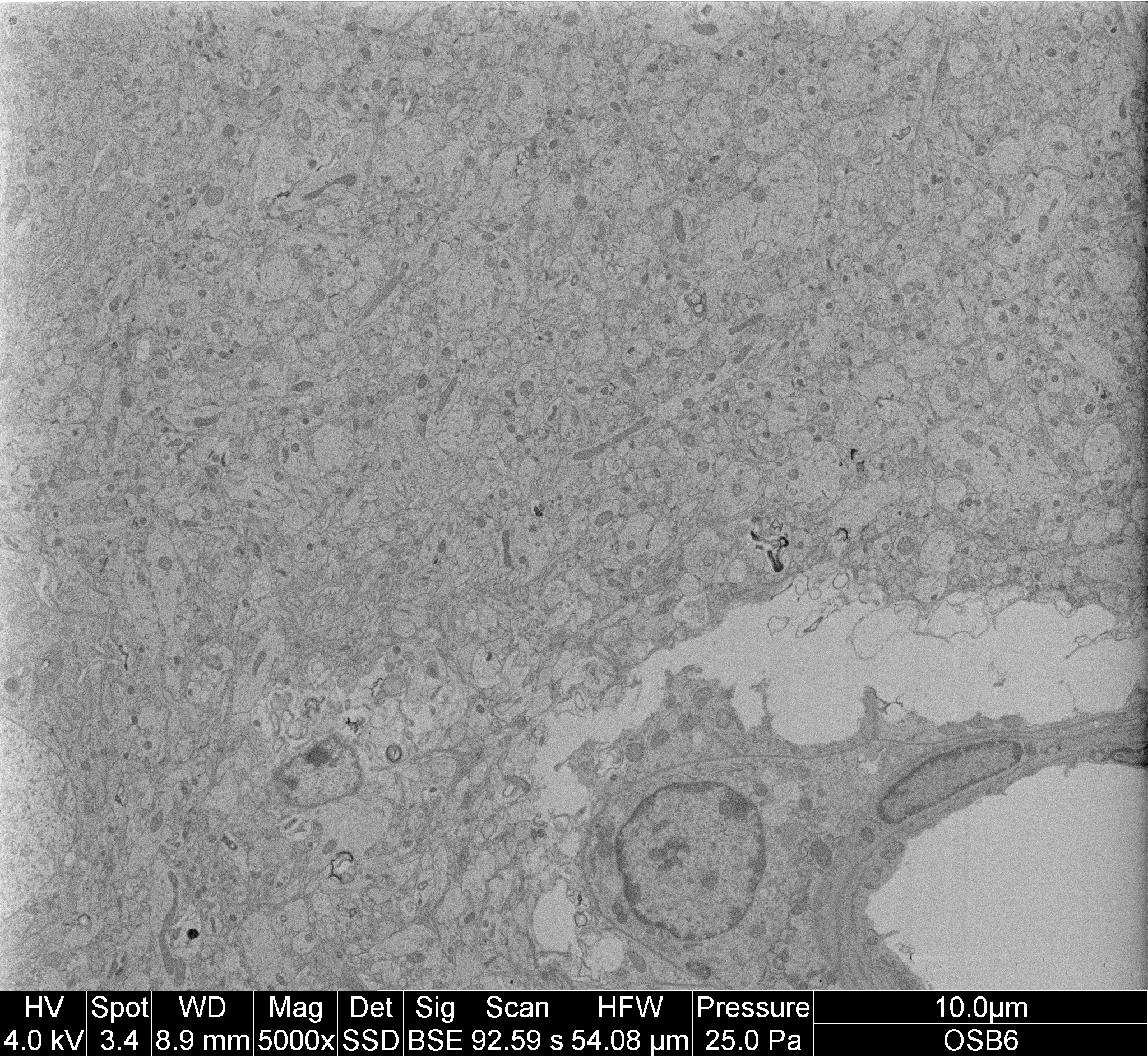

Supplement: Dataset S4 — (252.6 MB ZIP). [file pbio.0020329.sd004.zip › 040604_OS5_st1_335.tif]

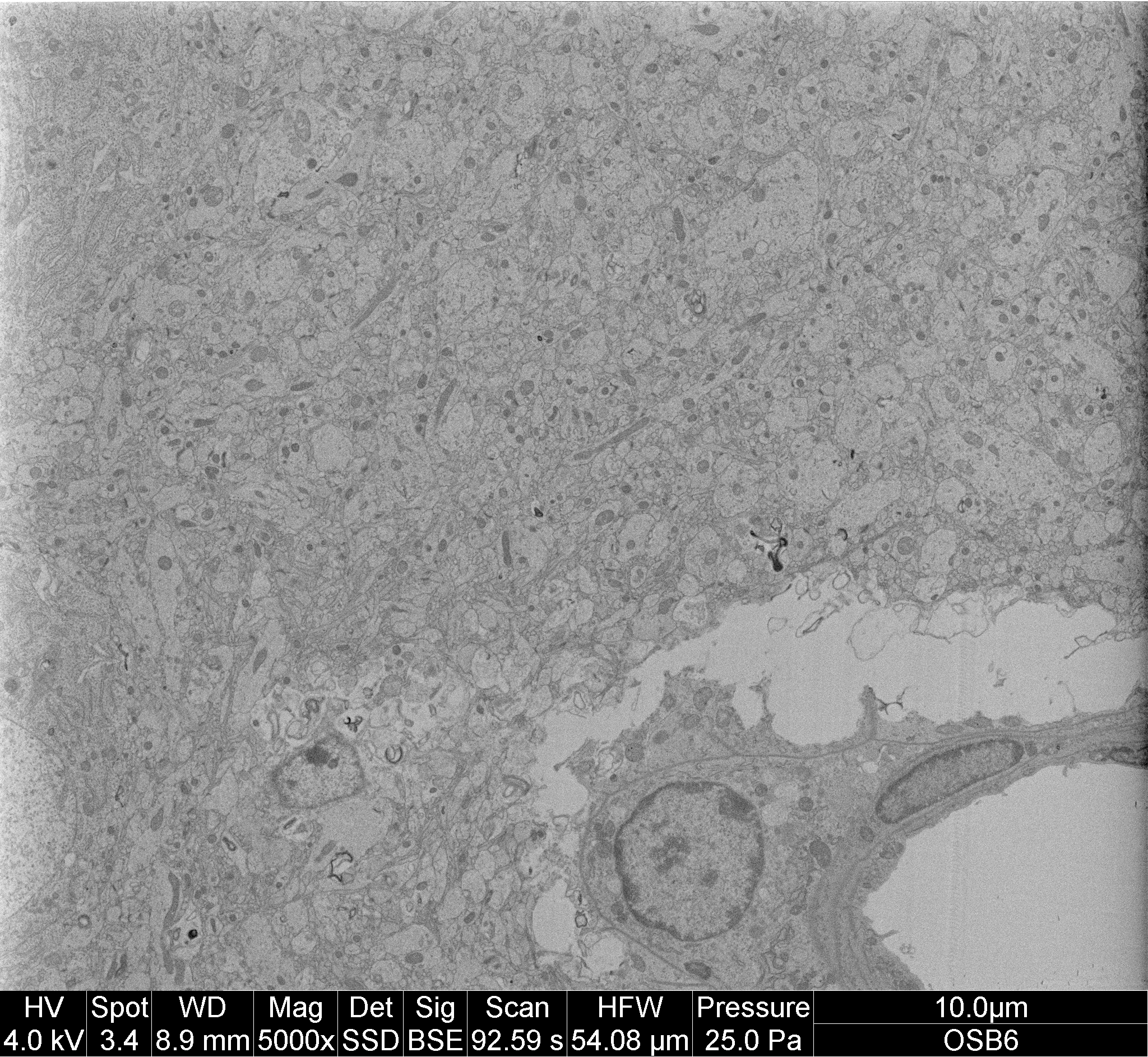

Supplement: Dataset S4 — (252.6 MB ZIP). [file pbio.0020329.sd004.zip › 040604_OS5_st1_336.tif]

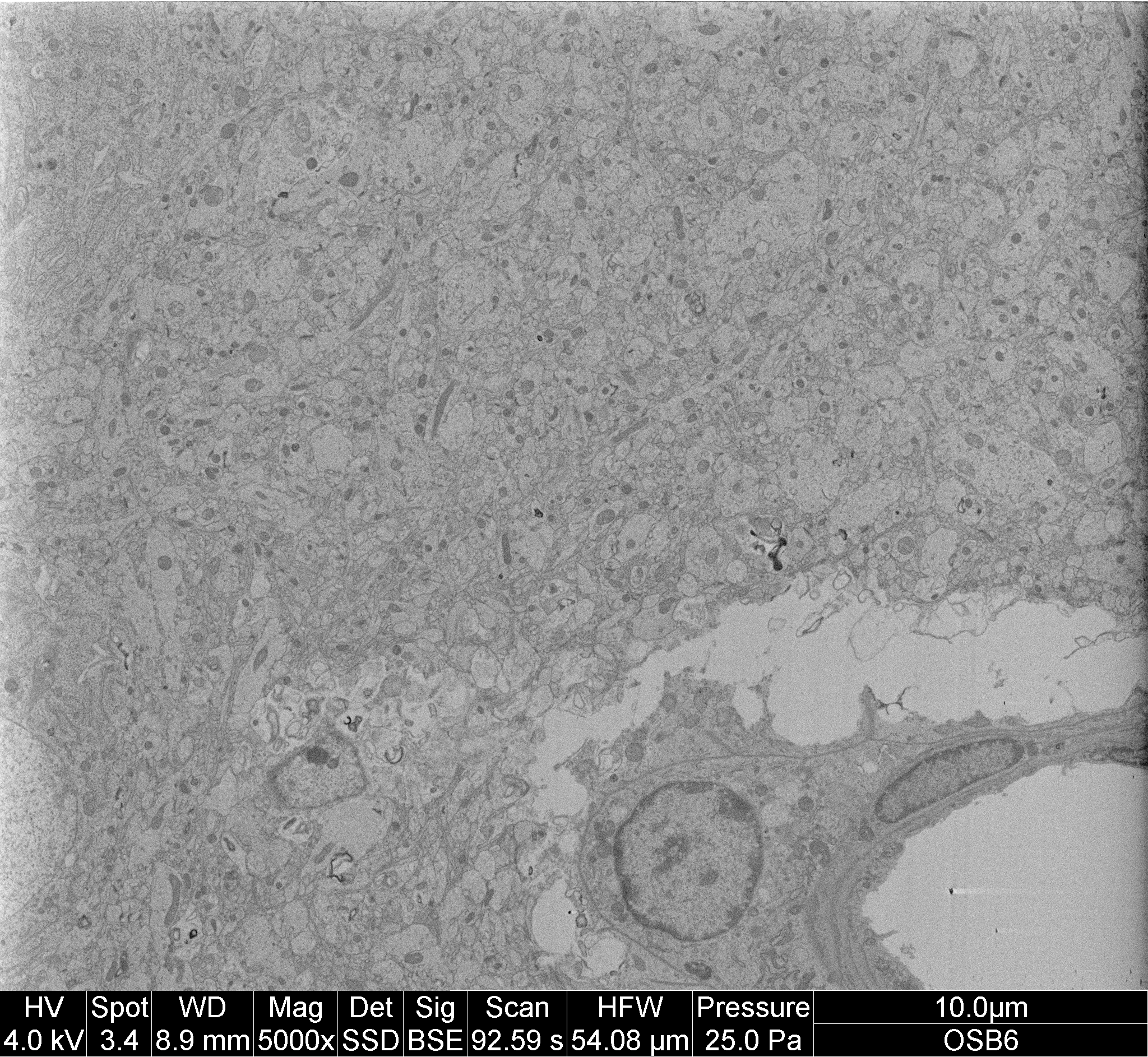

Supplement: Dataset S4 — (252.6 MB ZIP). [file pbio.0020329.sd004.zip › 040604_OS5_st1_337.tif]

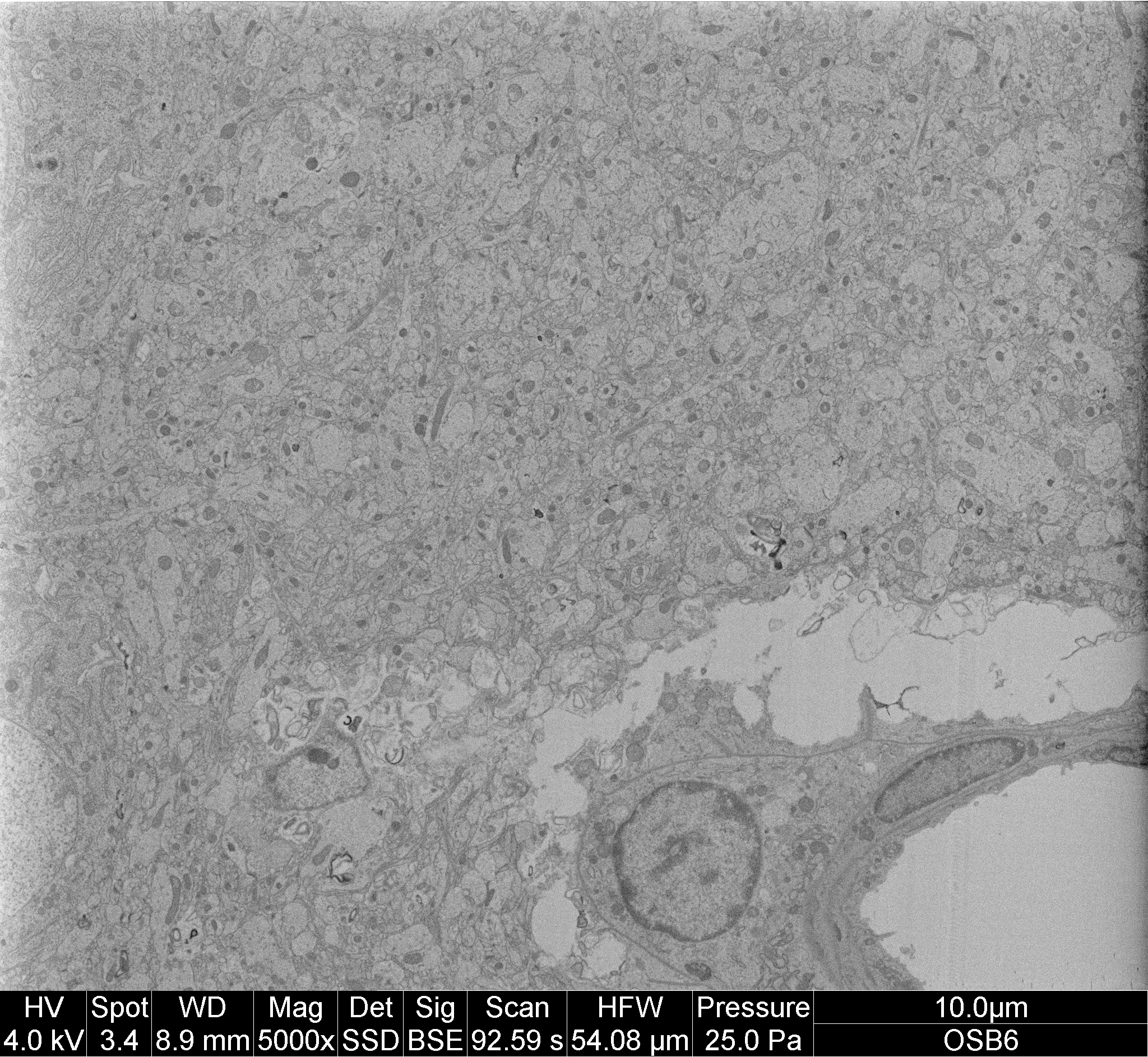

Supplement: Dataset S4 — (252.6 MB ZIP). [file pbio.0020329.sd004.zip › 040604_OS5_st1_338.tif]

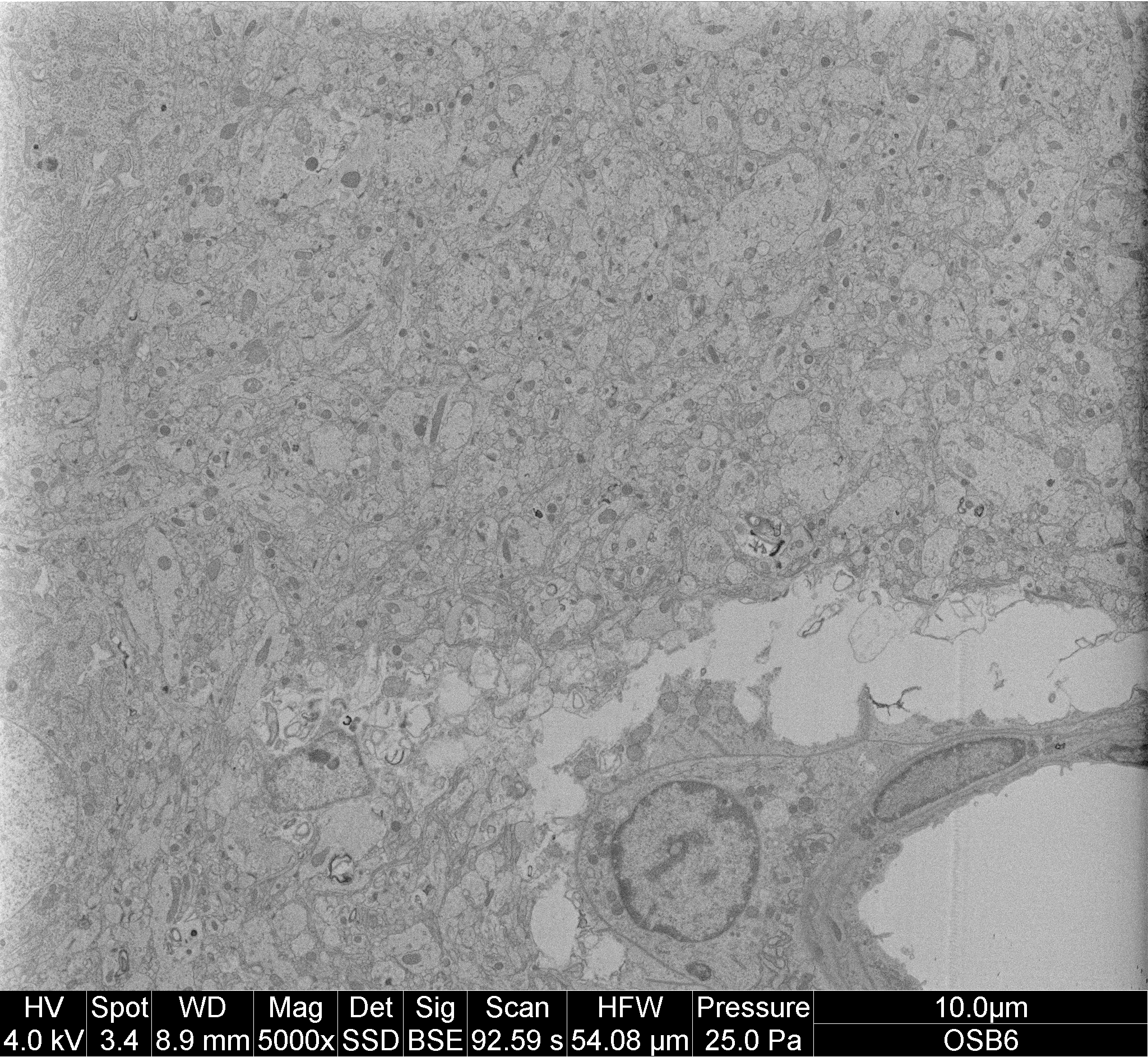

Supplement: Dataset S4 — (252.6 MB ZIP). [file pbio.0020329.sd004.zip › 040604_OS5_st1_339.tif]

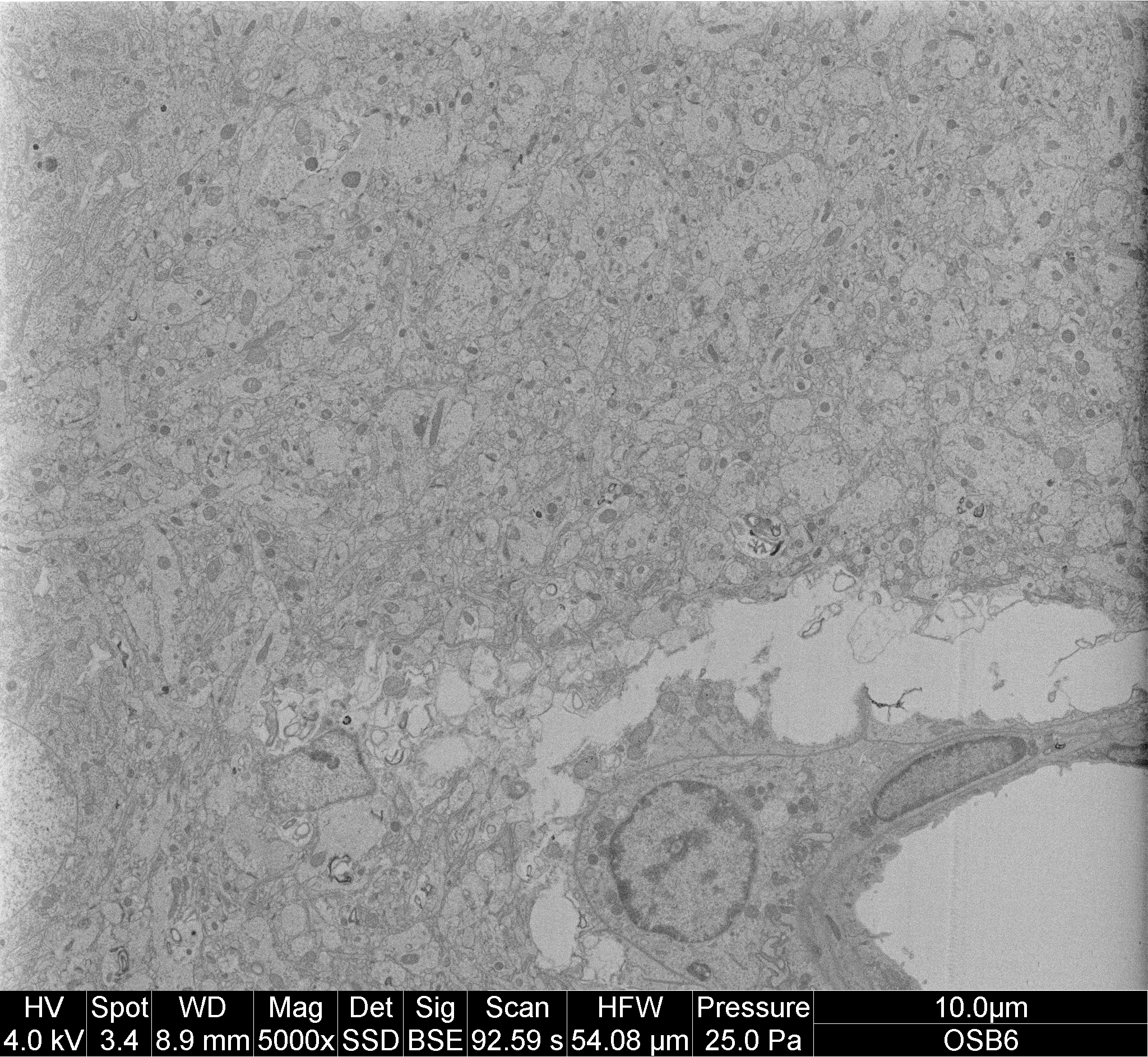

Supplement: Dataset S4 — (252.6 MB ZIP). [file pbio.0020329.sd004.zip › 040604_OS5_st1_340.tif]

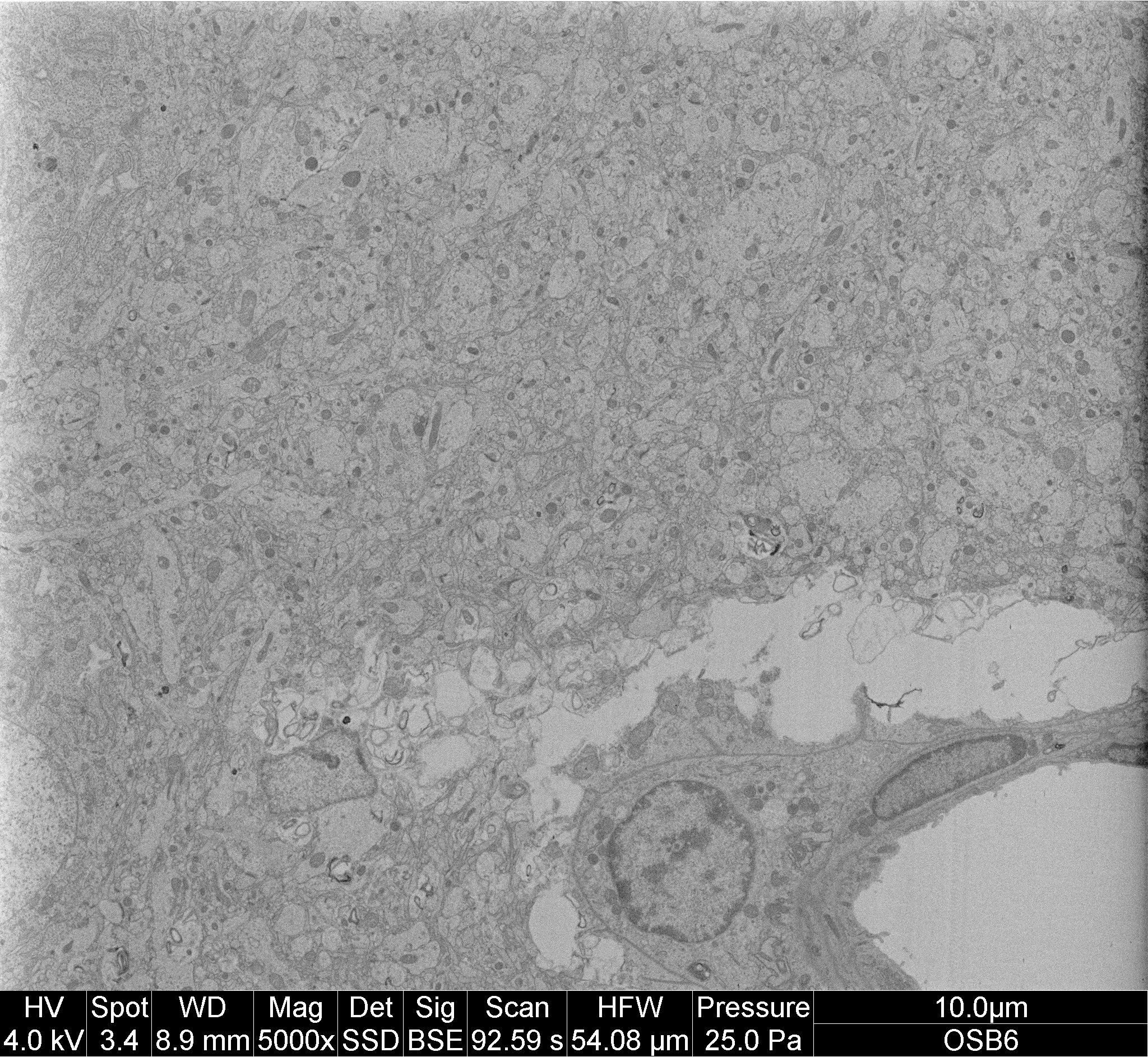

Supplement: Dataset S4 — (252.6 MB ZIP). [file pbio.0020329.sd004.zip › 040604_OS5_st1_341.tif]

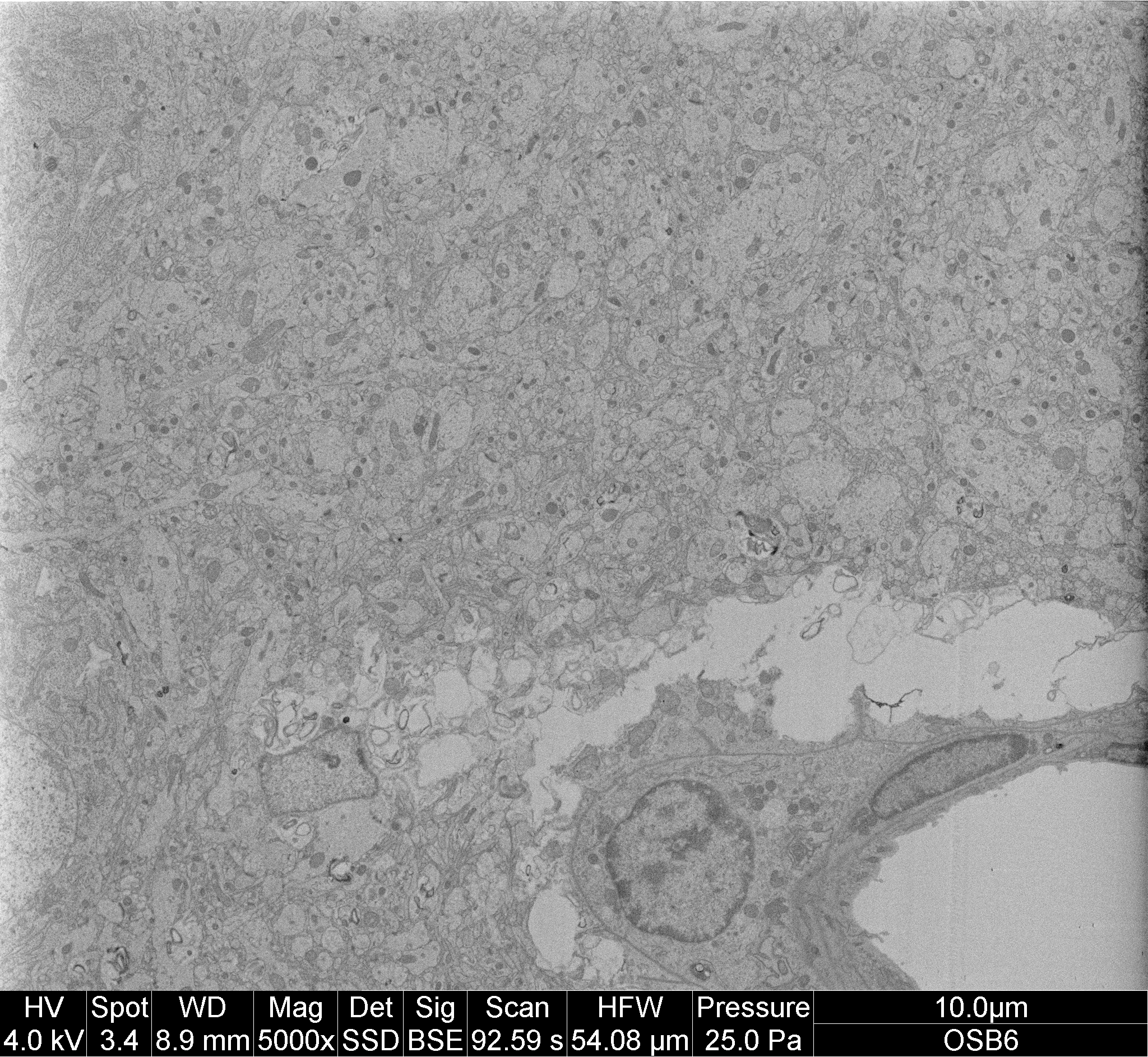

Supplement: Dataset S4 — (252.6 MB ZIP). [file pbio.0020329.sd004.zip › 040604_OS5_st1_342.tif]

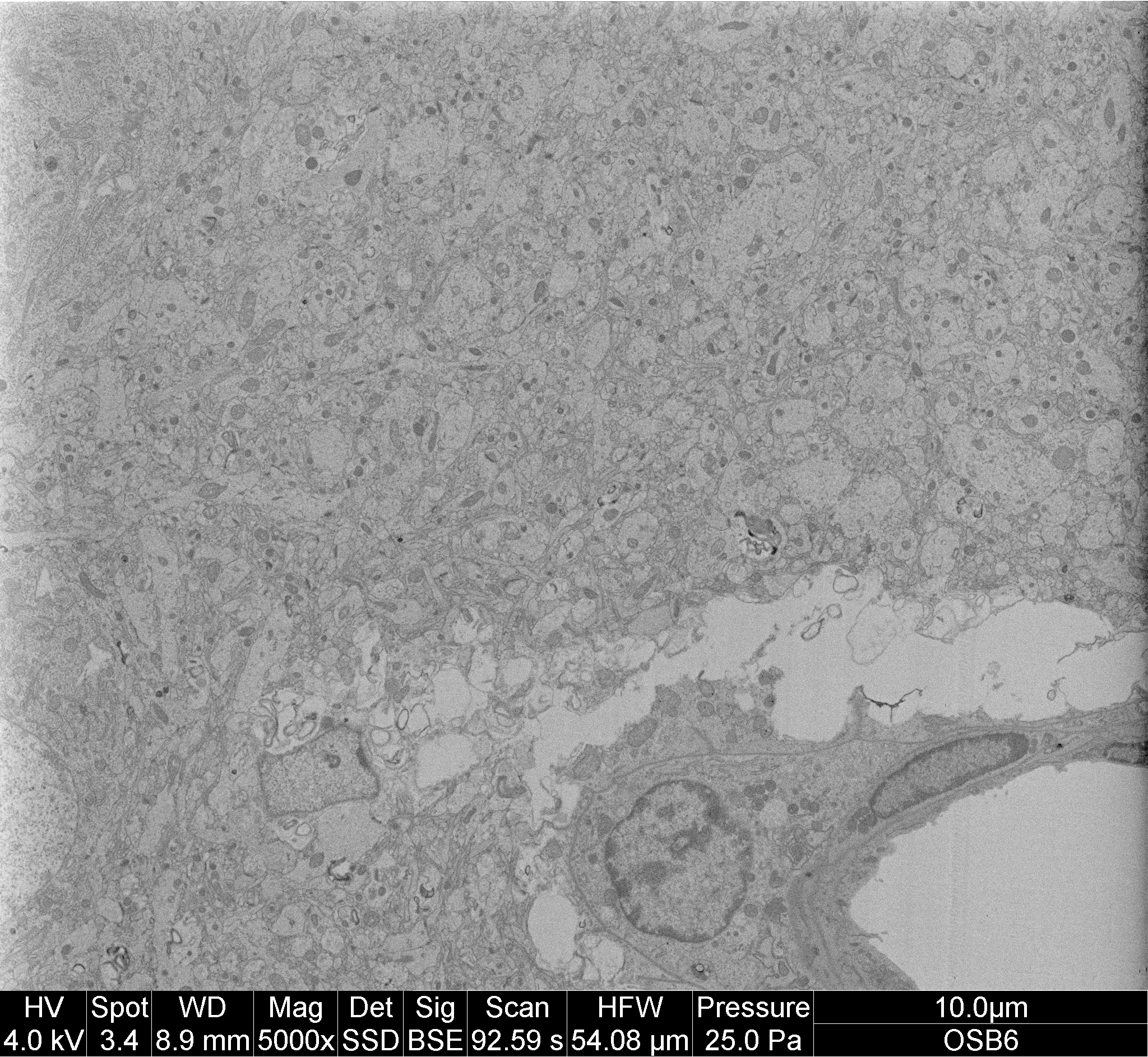

Supplement: Dataset S4 — (252.6 MB ZIP). [file pbio.0020329.sd004.zip › 040604_OS5_st1_343.tif]

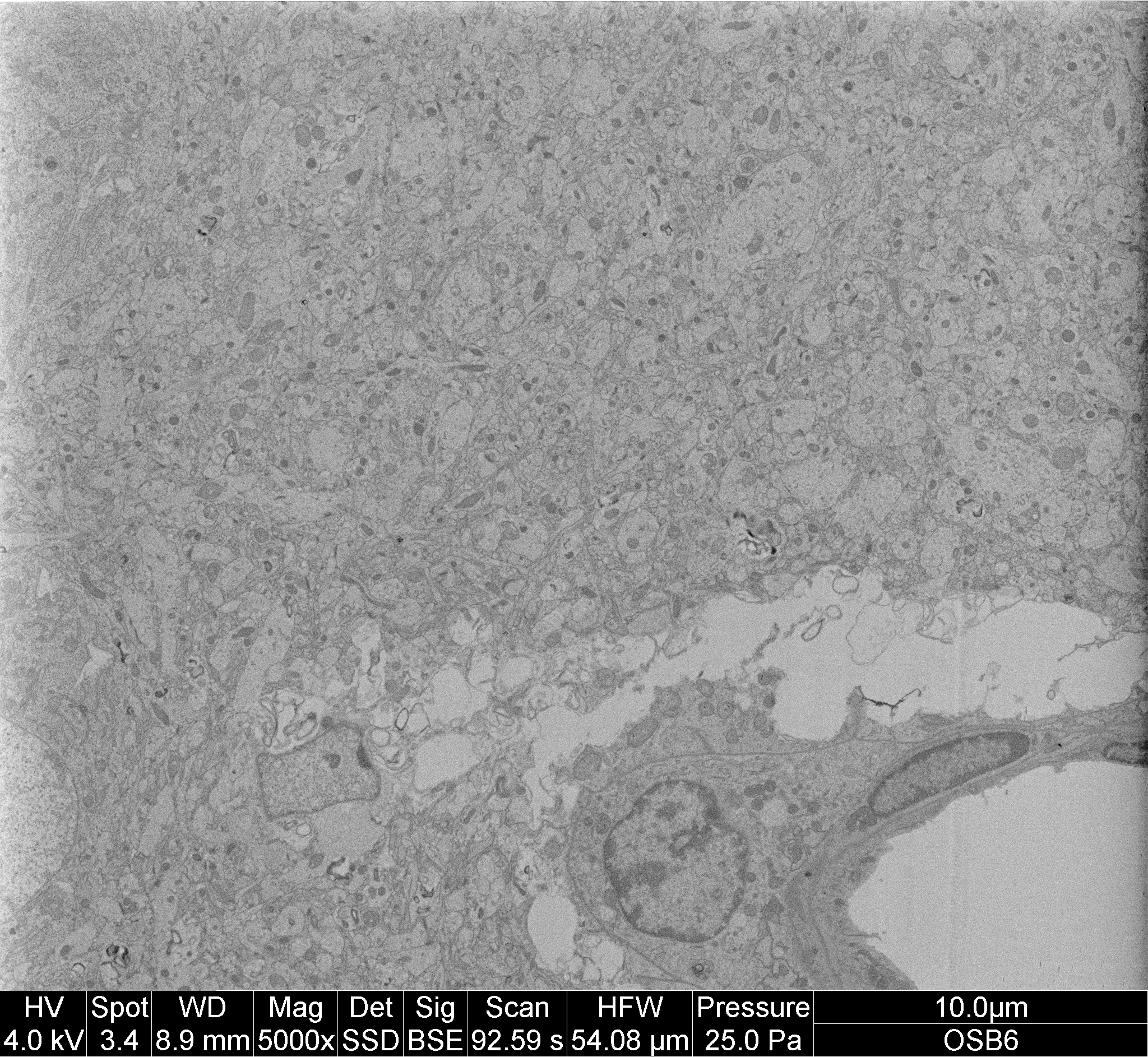

Supplement: Dataset S4 — (252.6 MB ZIP). [file pbio.0020329.sd004.zip › 040604_OS5_st1_344.tif]

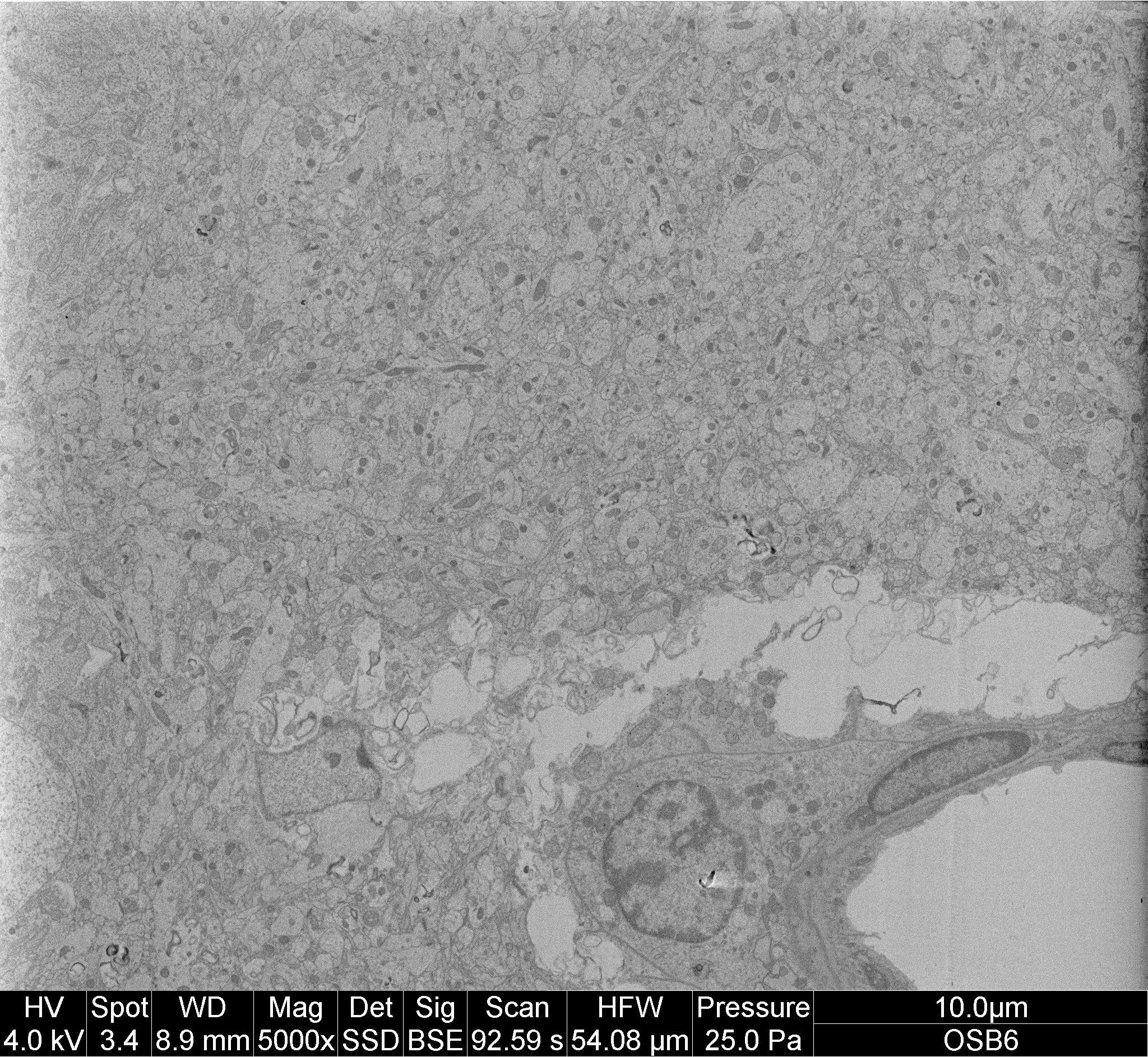

Supplement: Dataset S4 — (252.6 MB ZIP). [file pbio.0020329.sd004.zip › 040604_OS5_st1_345.tif]

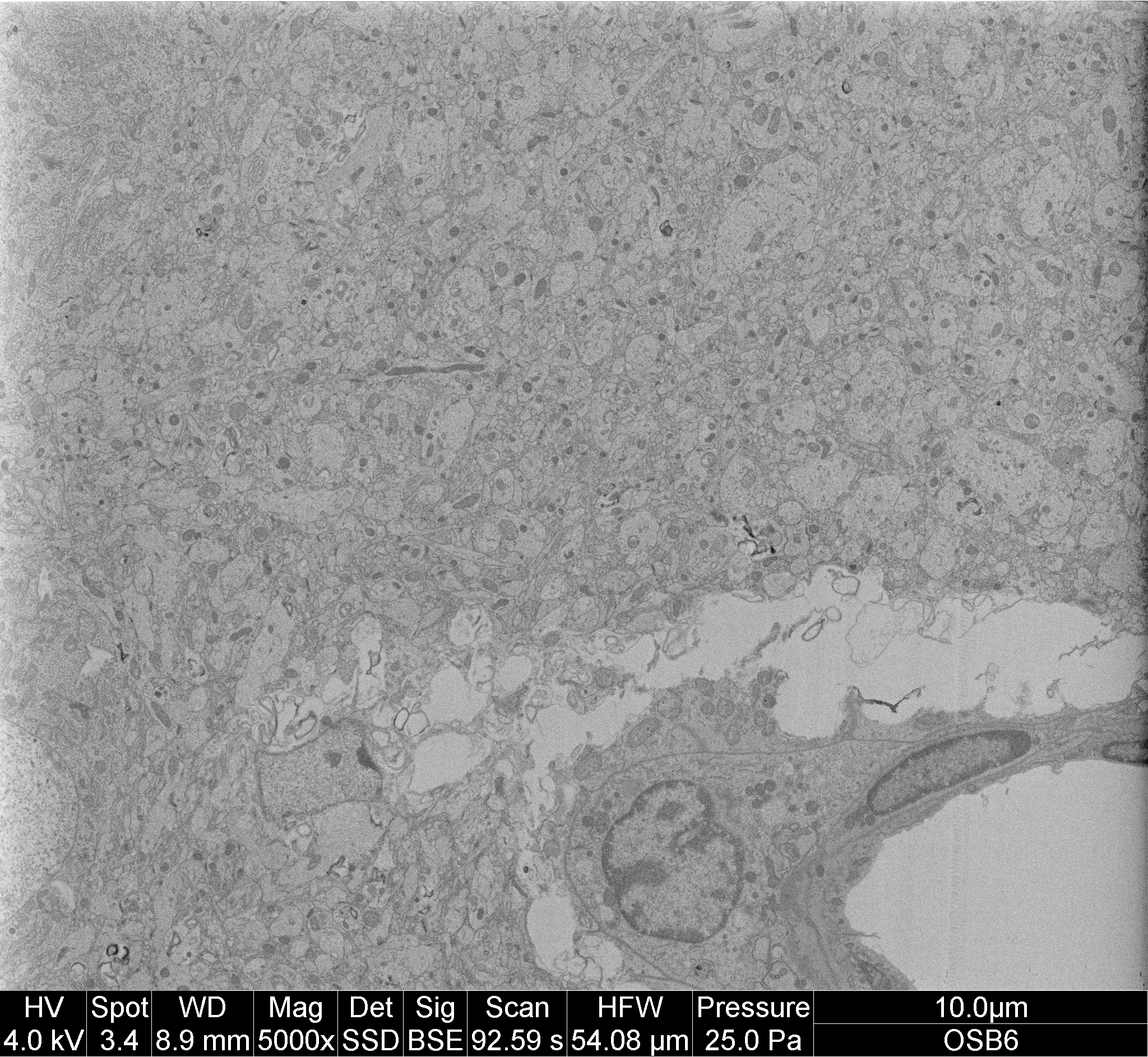

Supplement: Dataset S4 — (252.6 MB ZIP). [file pbio.0020329.sd004.zip › 040604_OS5_st1_346.tif]

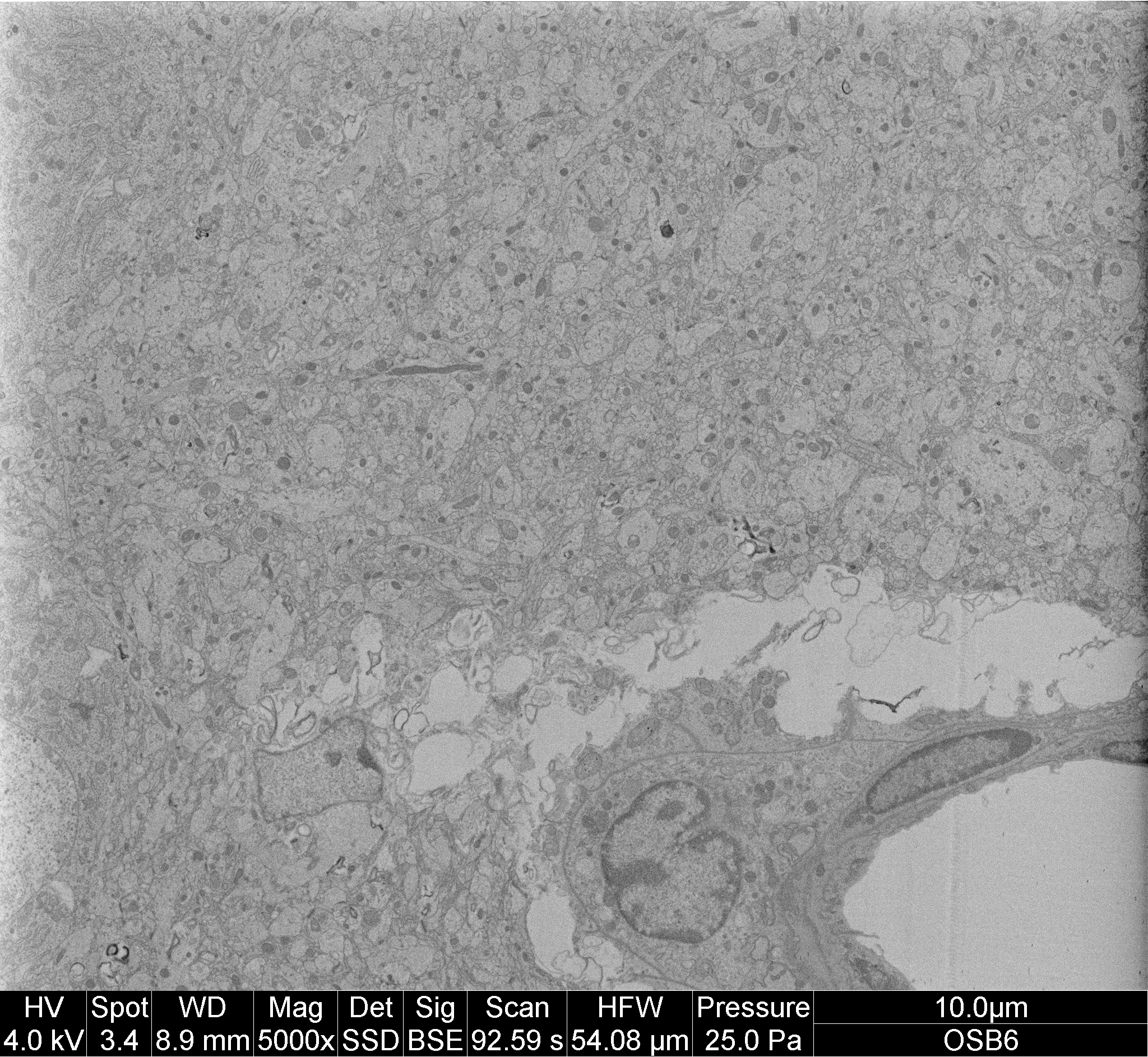

Supplement: Dataset S4 — (252.6 MB ZIP). [file pbio.0020329.sd004.zip › 040604_OS5_st1_347.tif]

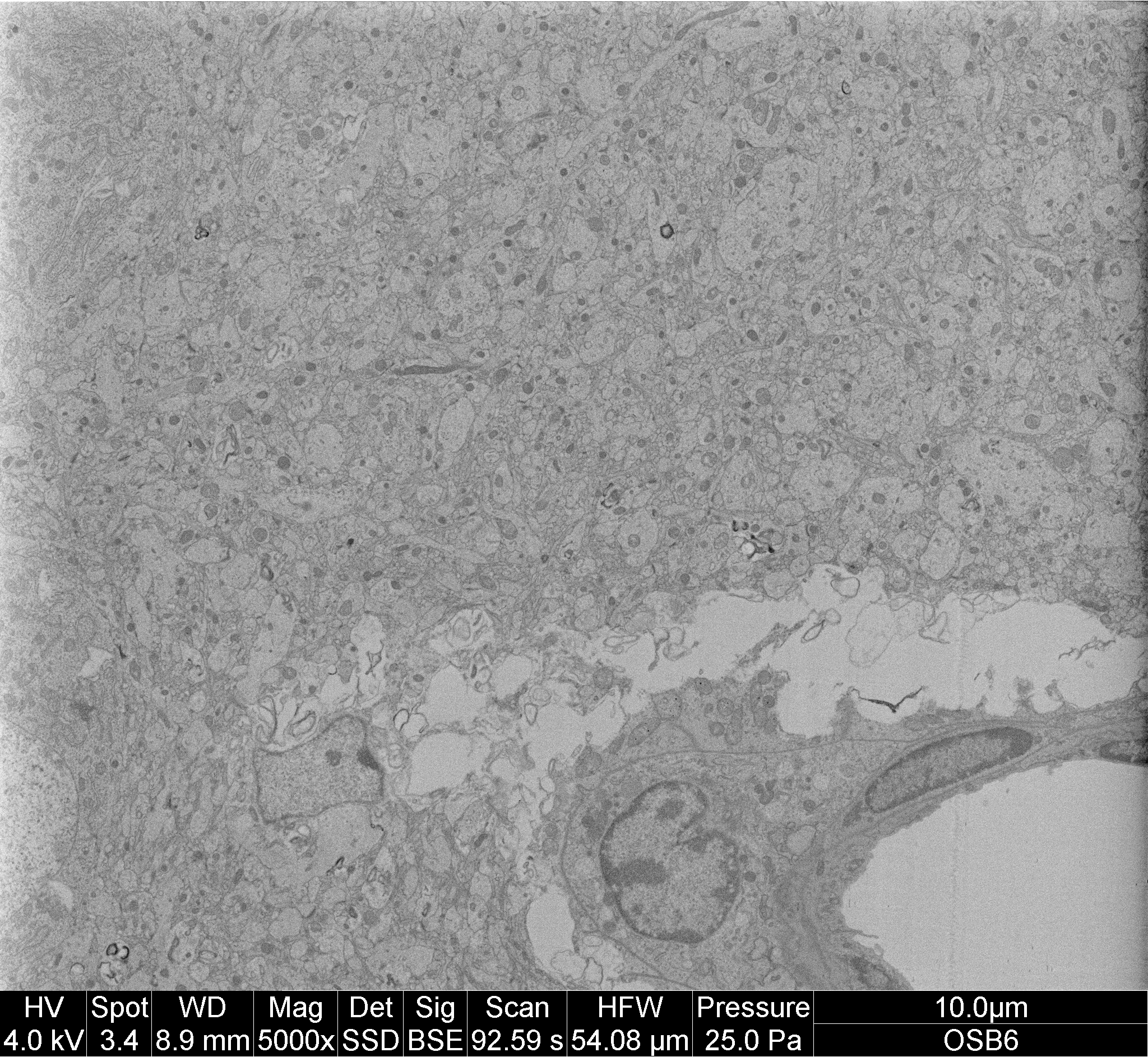

Supplement: Dataset S4 — (252.6 MB ZIP). [file pbio.0020329.sd004.zip › 040604_OS5_st1_348.tif]

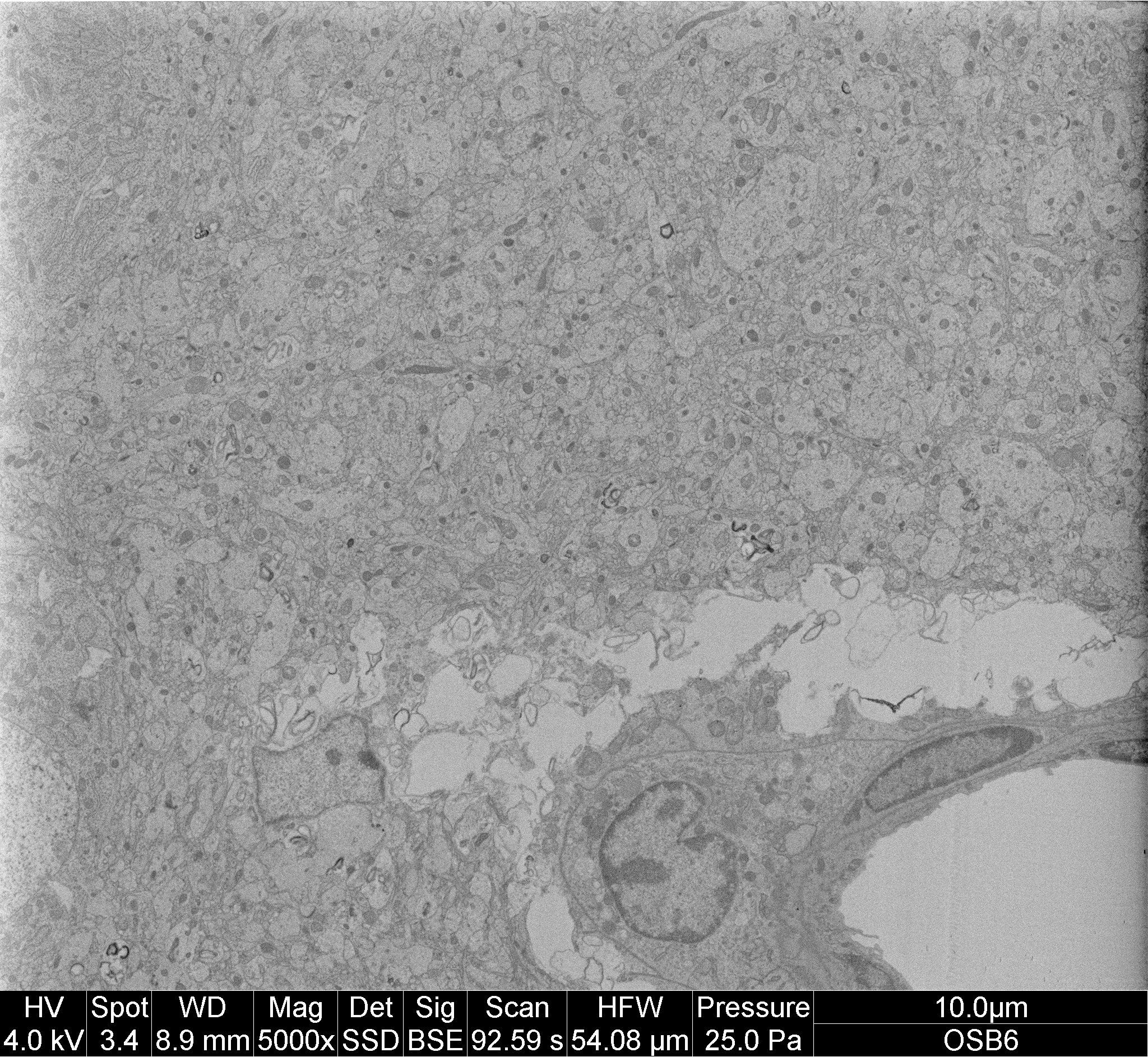

Supplement: Dataset S4 — (252.6 MB ZIP). [file pbio.0020329.sd004.zip › 040604_OS5_st1_349.tif]

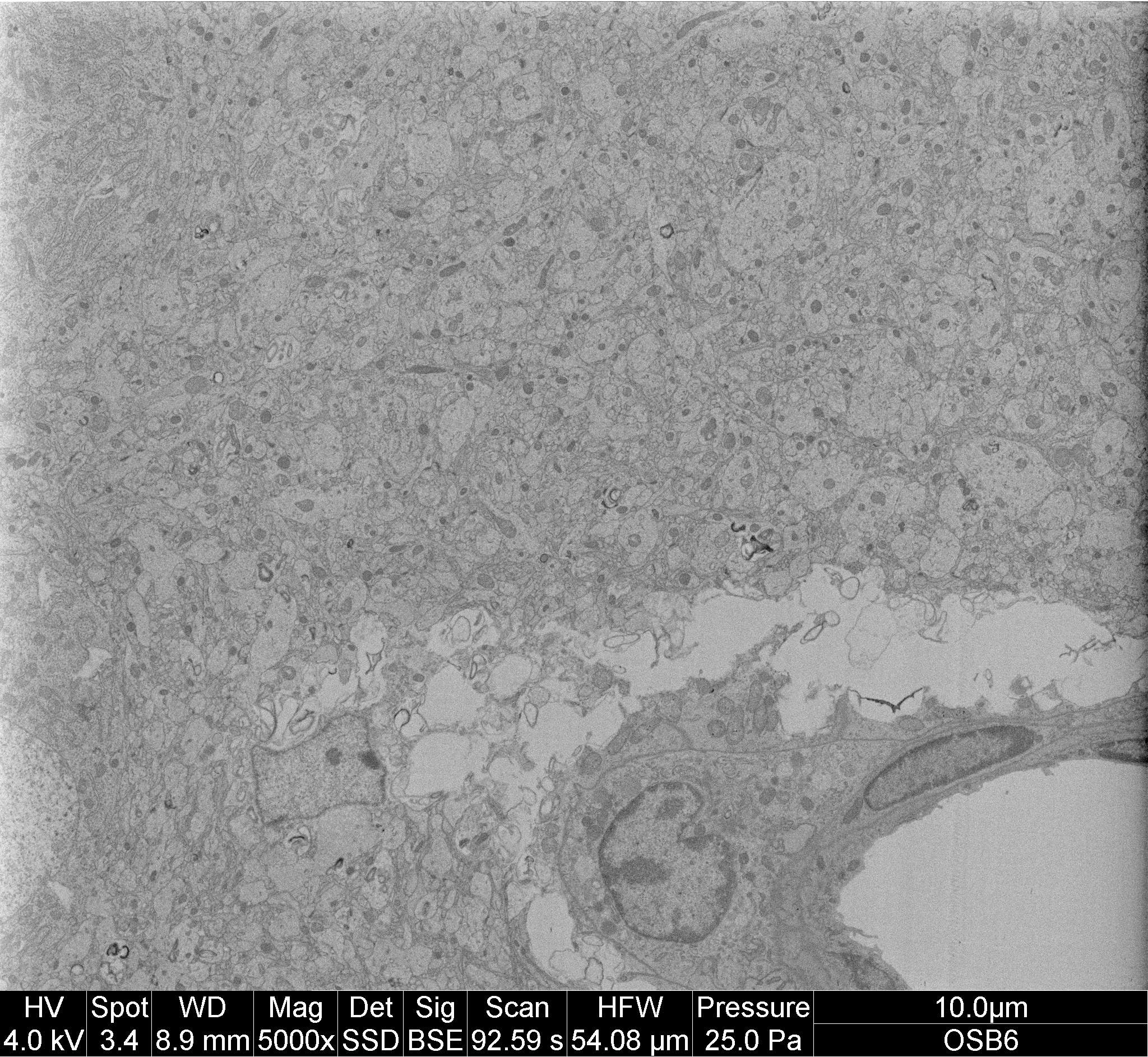

Supplement: Dataset S4 — (252.6 MB ZIP). [file pbio.0020329.sd004.zip › 040604_OS5_st1_350.tif]

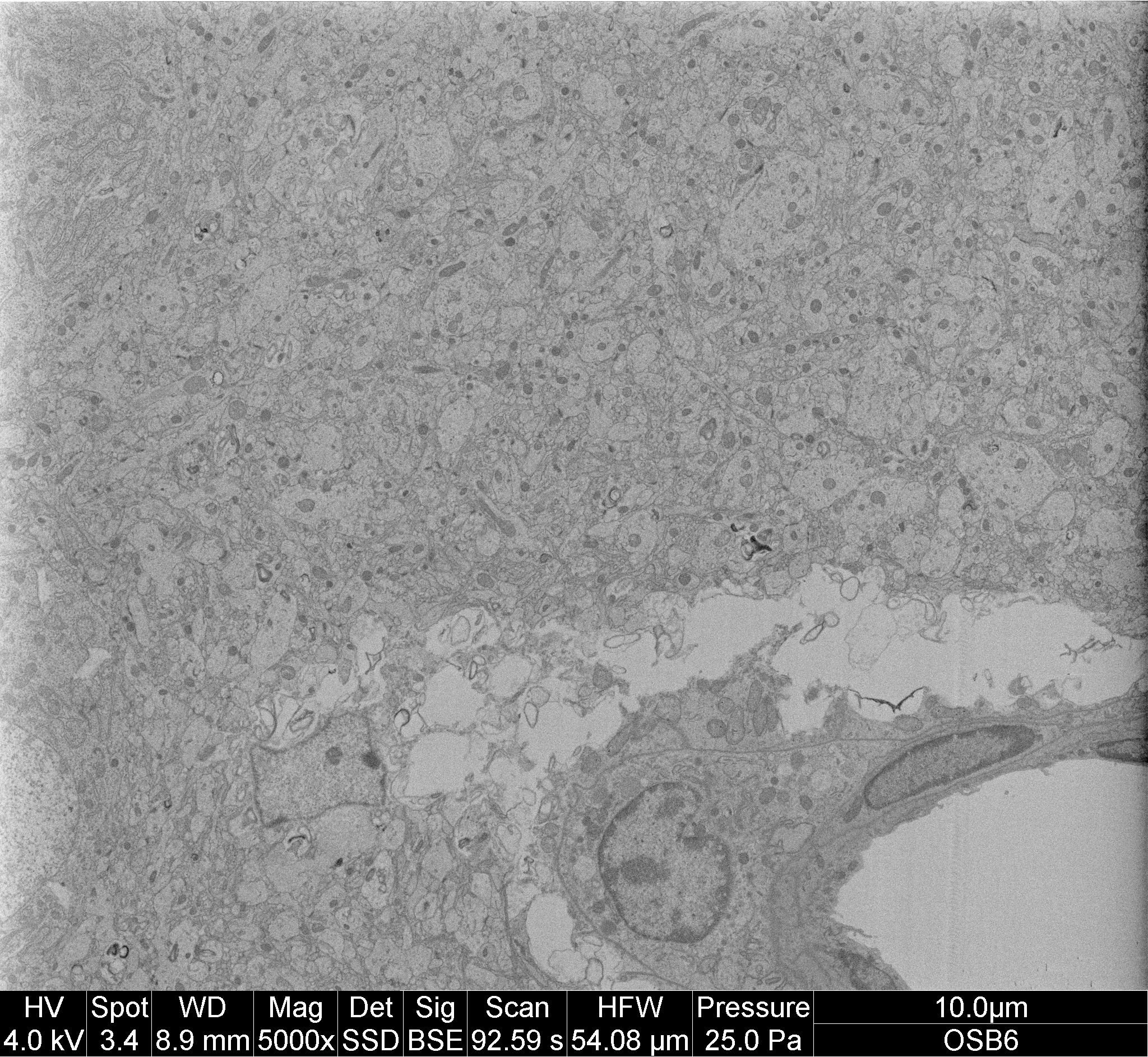

Supplement: Dataset S4 — (252.6 MB ZIP). [file pbio.0020329.sd004.zip › 040604_OS5_st1_351.tif]

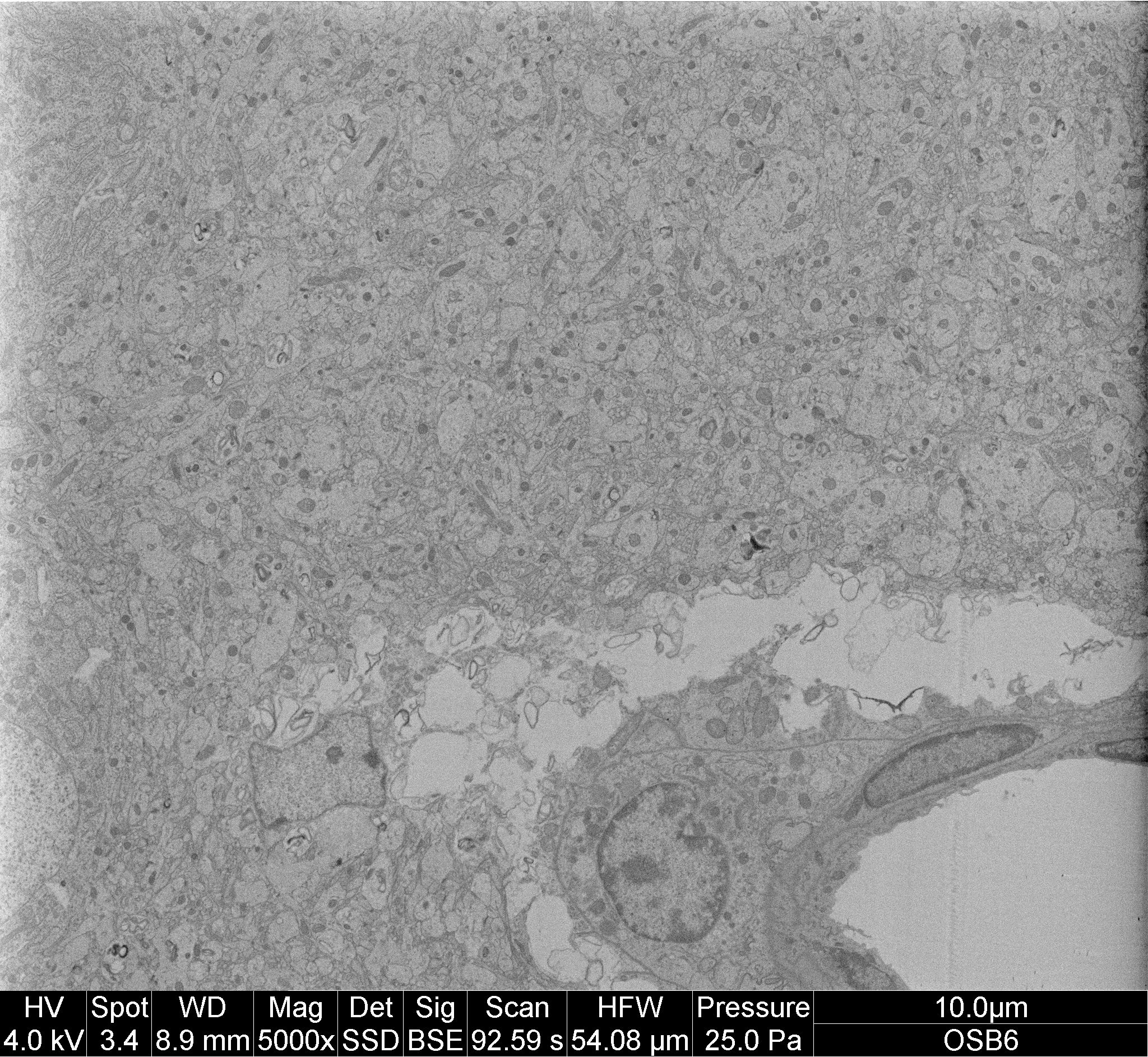

Supplement: Dataset S4 — (252.6 MB ZIP). [file pbio.0020329.sd004.zip › 040604_OS5_st1_352.tif]

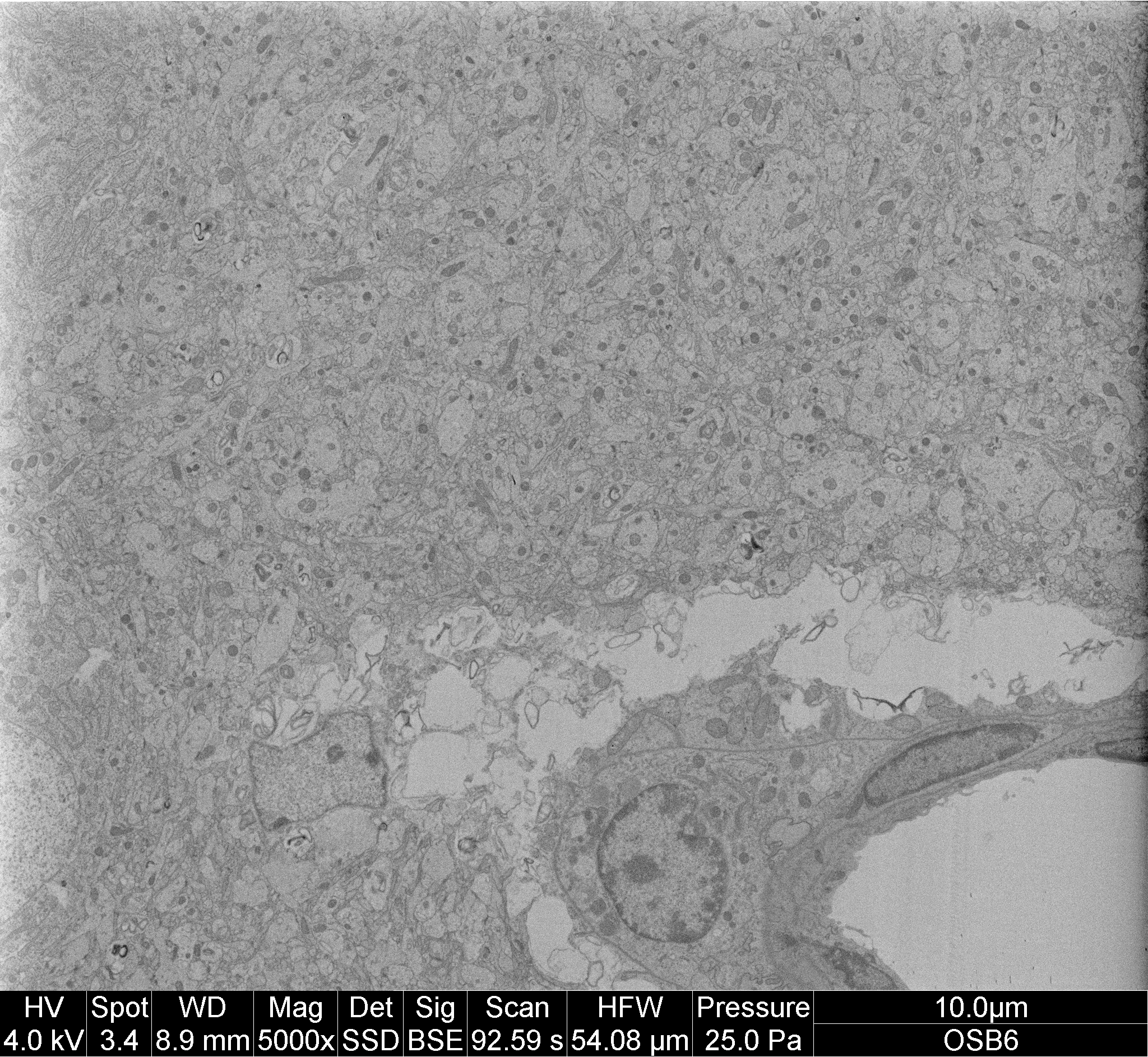

Supplement: Dataset S4 — (252.6 MB ZIP). [file pbio.0020329.sd004.zip › 040604_OS5_st1_353.tif]

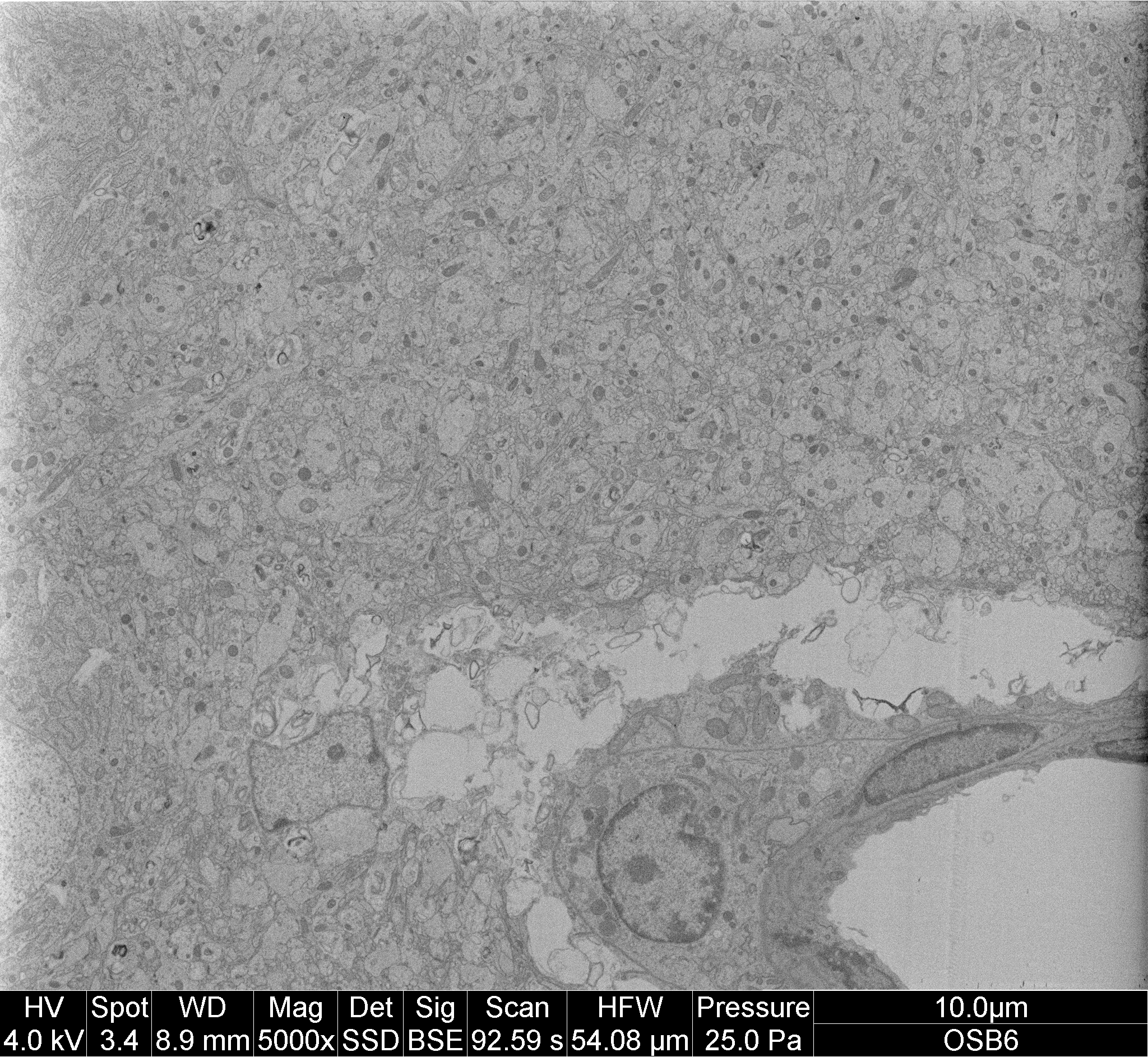

Supplement: Dataset S4 — (252.6 MB ZIP). [file pbio.0020329.sd004.zip › 040604_OS5_st1_354.tif]

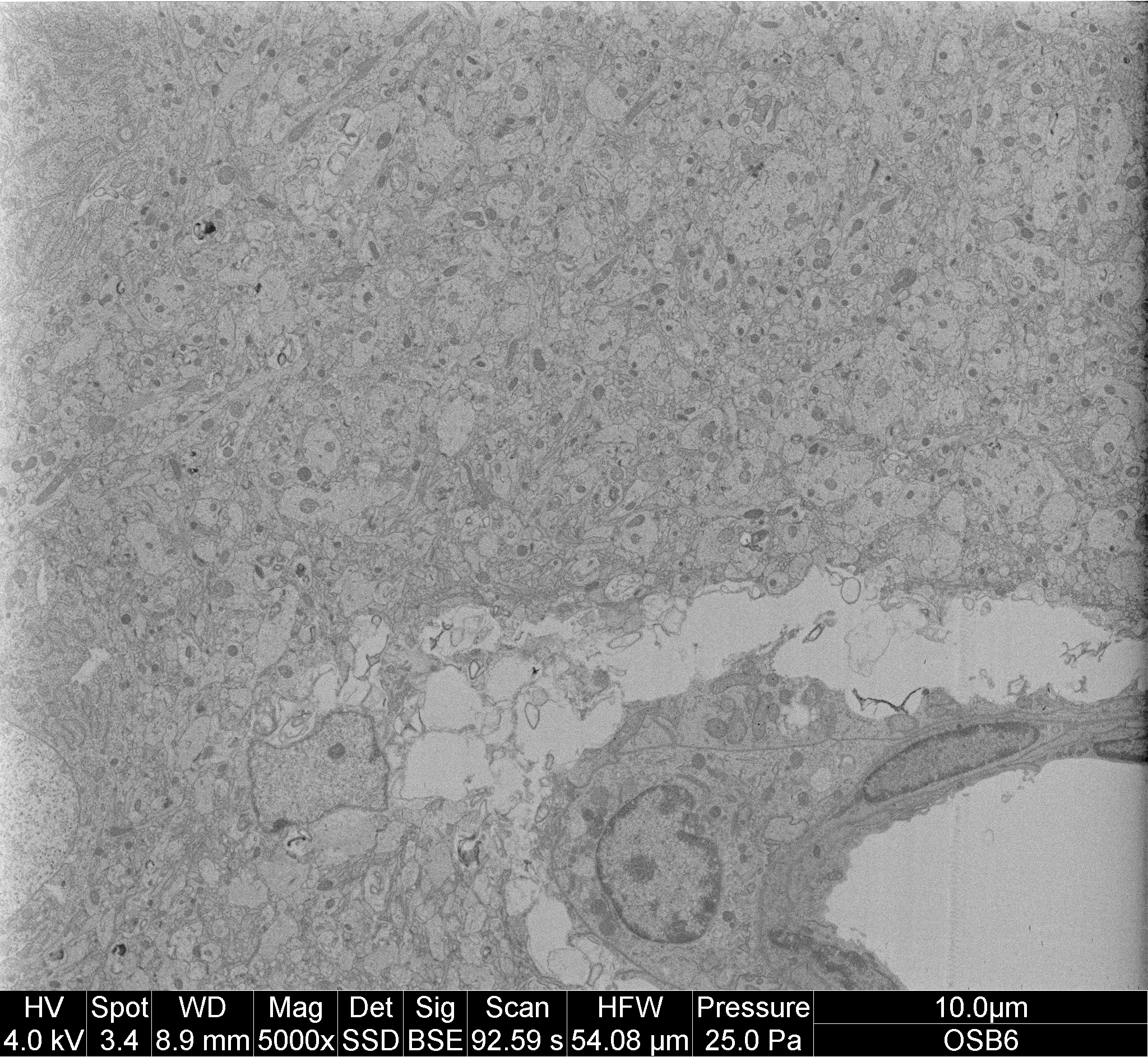

Supplement: Dataset S4 — (252.6 MB ZIP). [file pbio.0020329.sd004.zip › 040604_OS5_st1_355.tif]

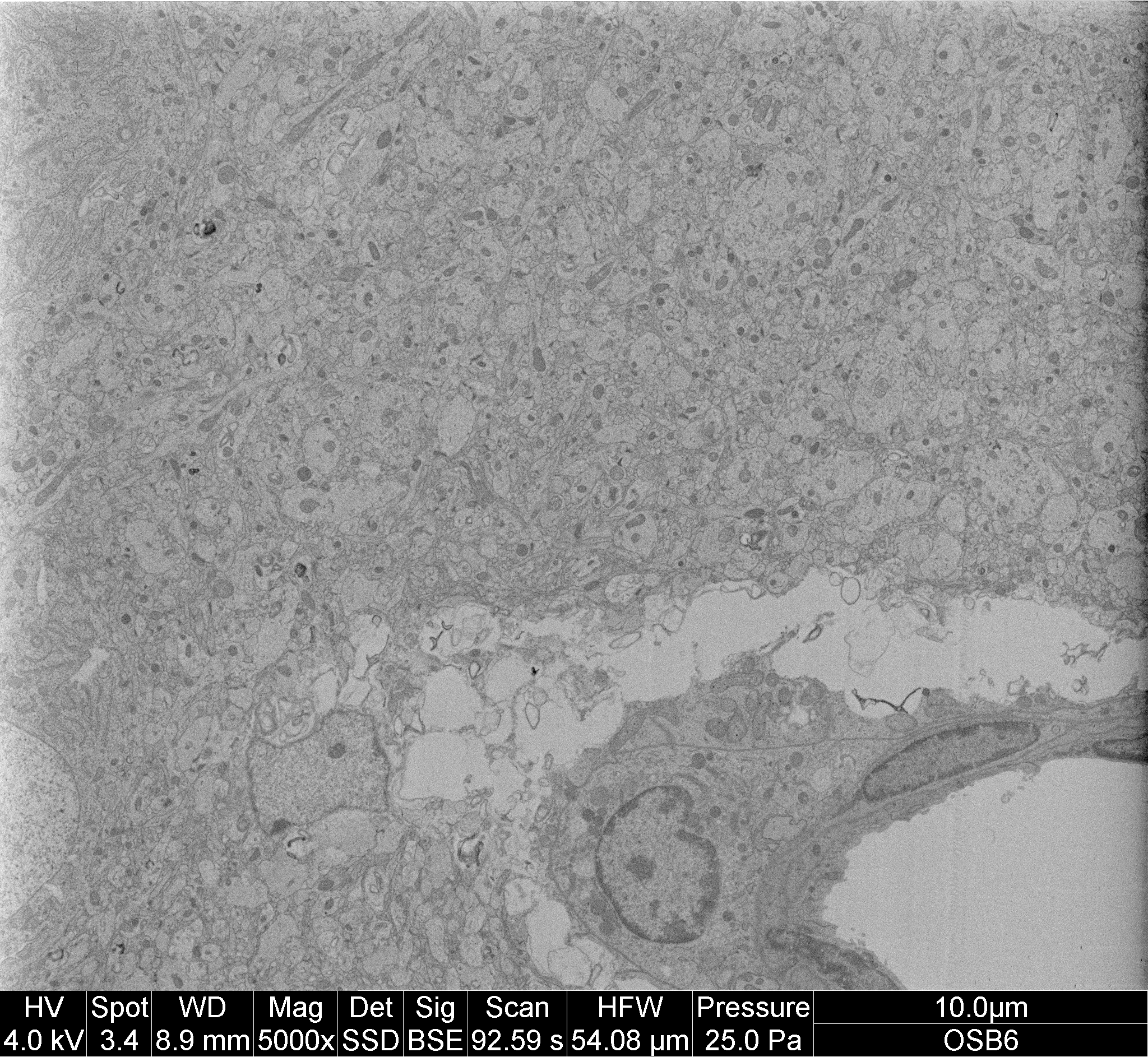

Supplement: Dataset S4 — (252.6 MB ZIP). [file pbio.0020329.sd004.zip › 040604_OS5_st1_356.tif]

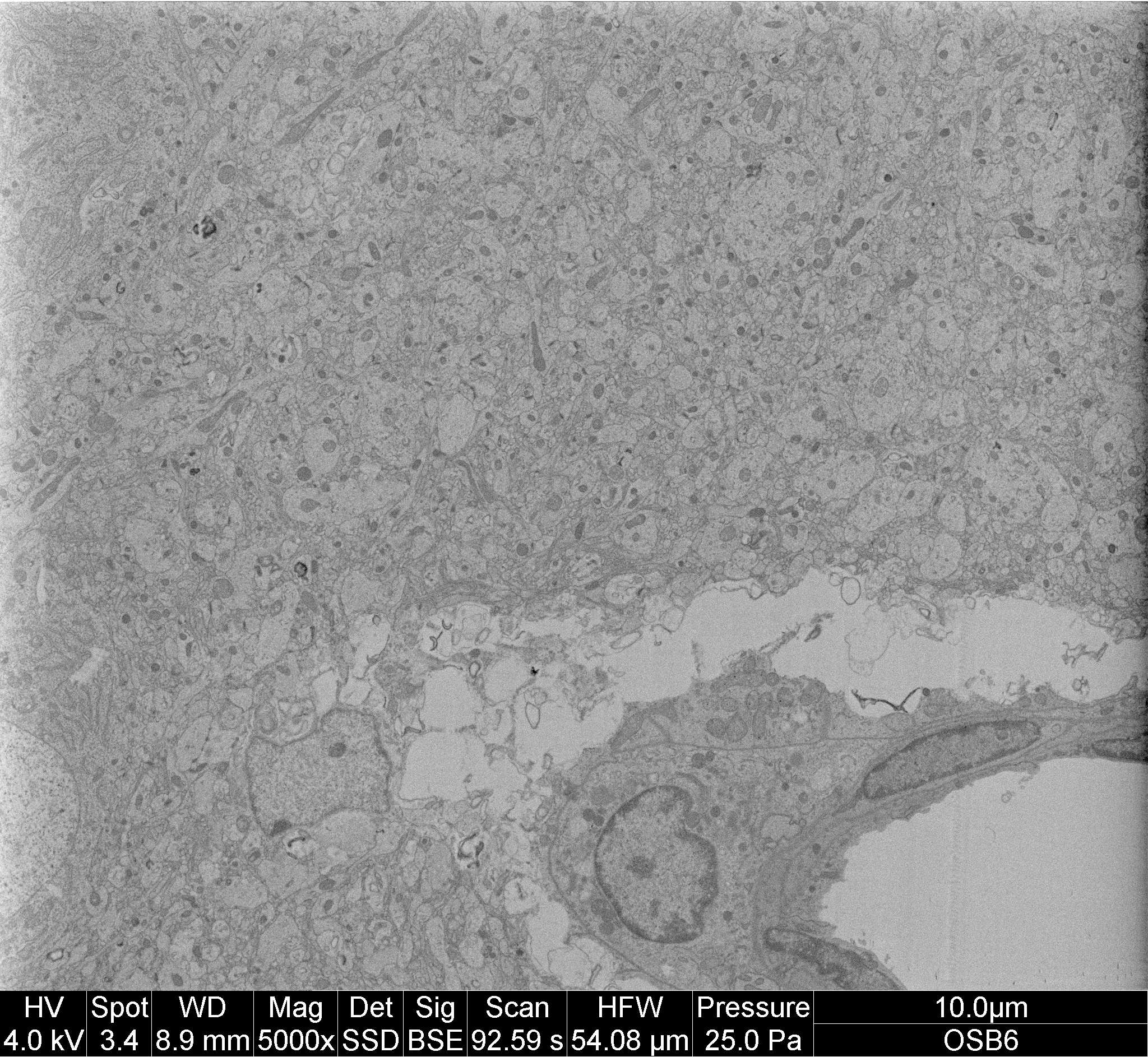

Supplement: Dataset S4 — (252.6 MB ZIP). [file pbio.0020329.sd004.zip › 040604_OS5_st1_357.tif]

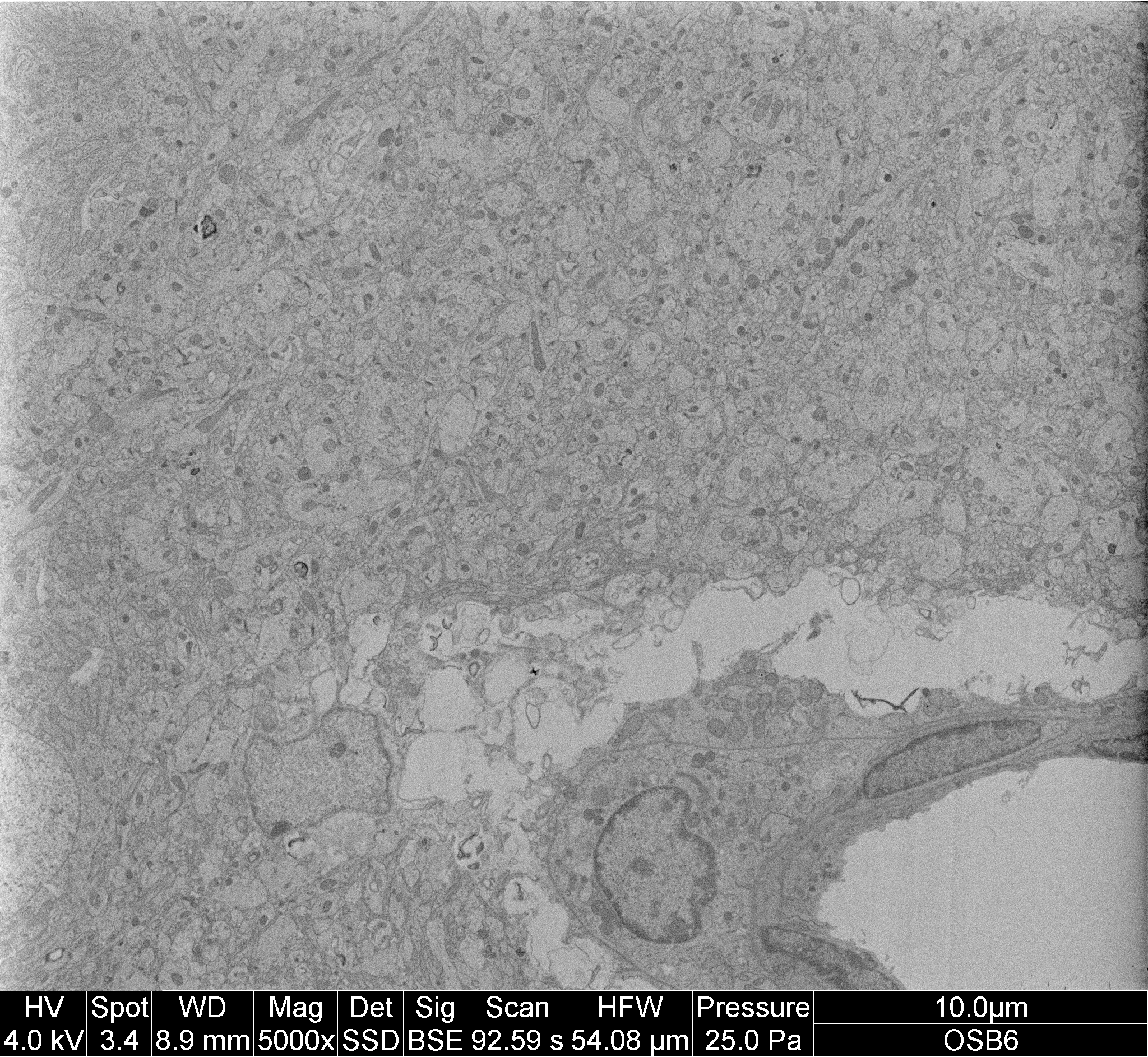

Supplement: Dataset S4 — (252.6 MB ZIP). [file pbio.0020329.sd004.zip › 040604_OS5_st1_358.tif]

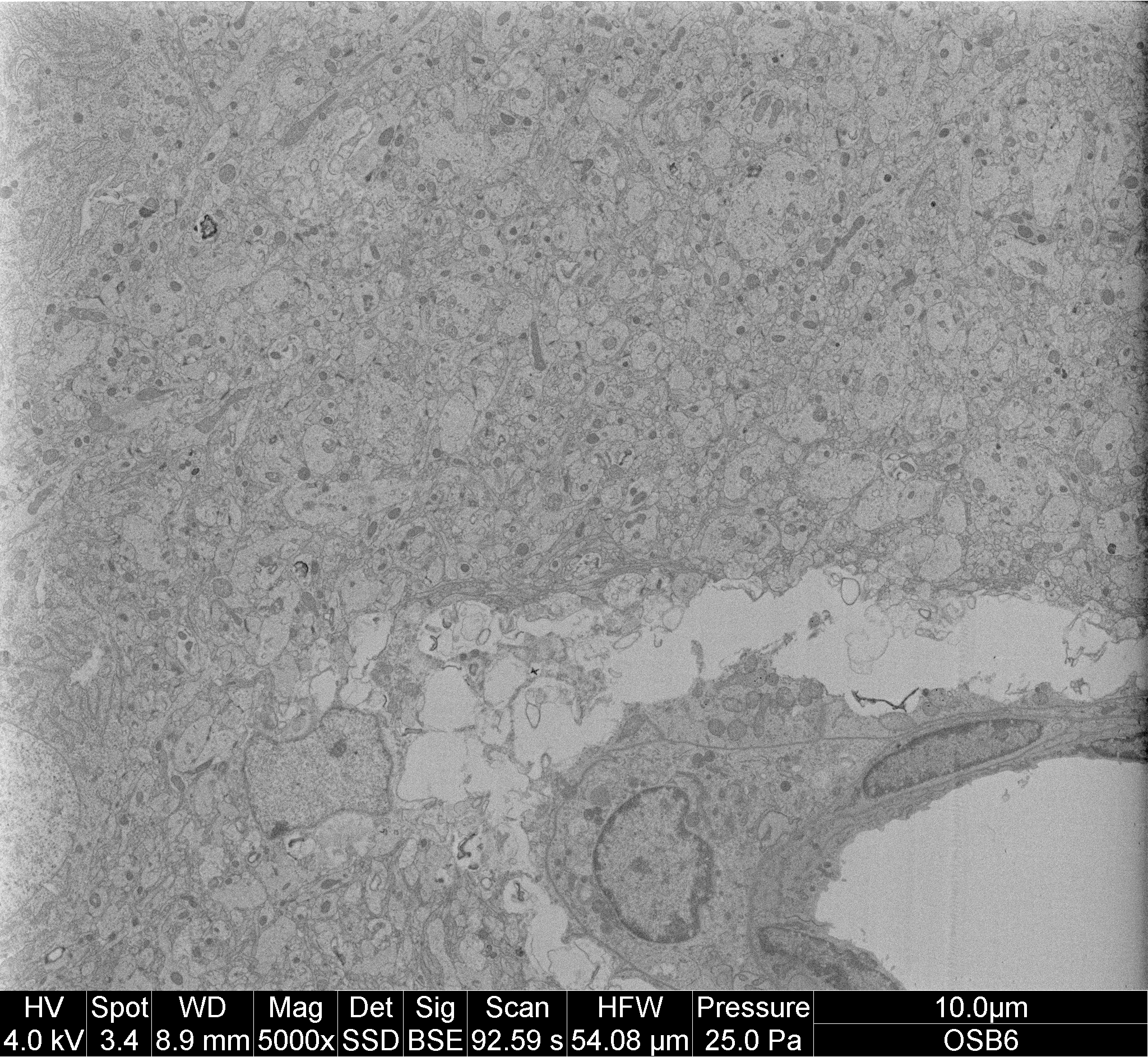

Supplement: Dataset S4 — (252.6 MB ZIP). [file pbio.0020329.sd004.zip › 040604_OS5_st1_359.tif]

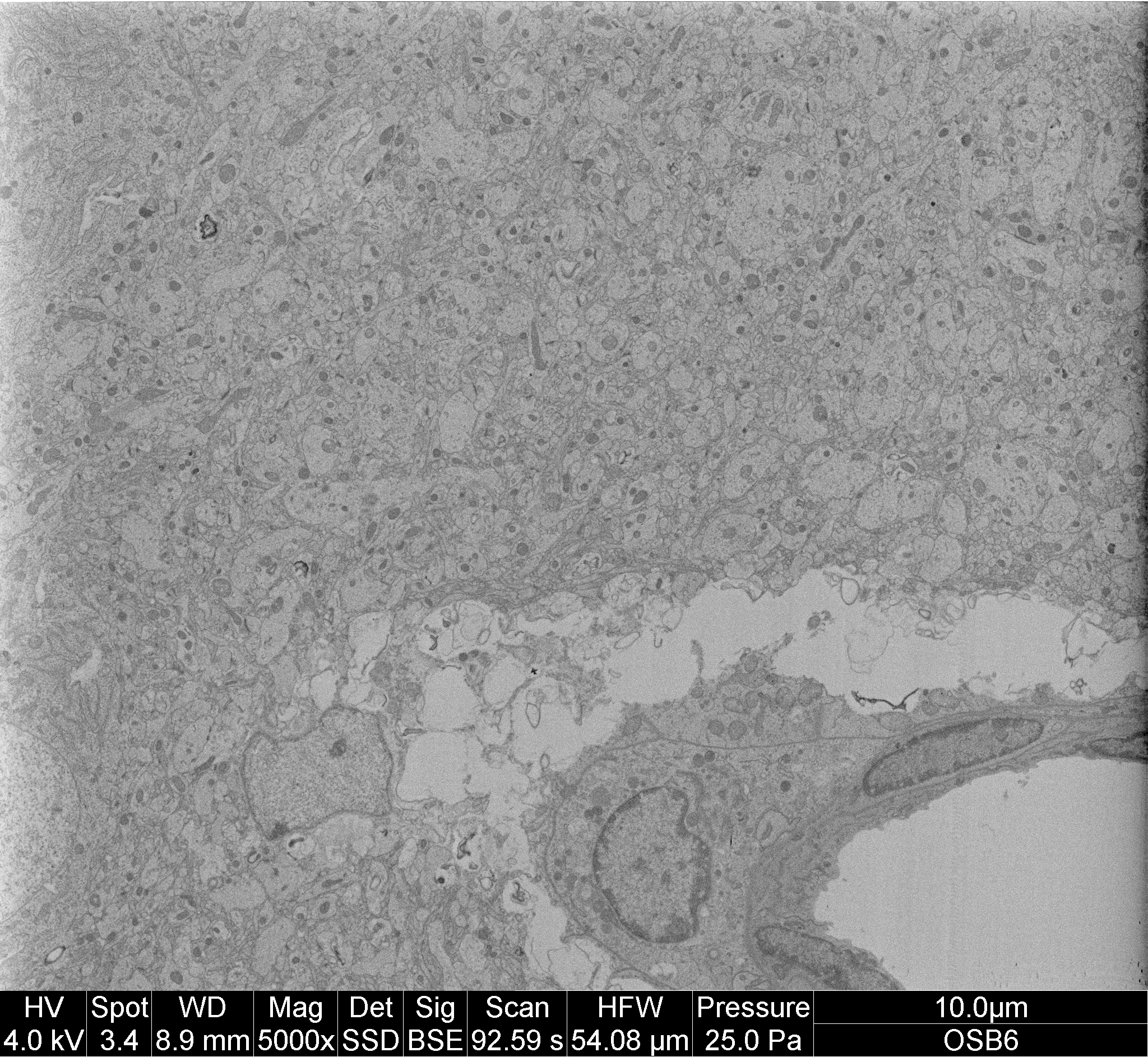

Supplement: Dataset S4 — (252.6 MB ZIP). [file pbio.0020329.sd004.zip › 040604_OS5_st1_360.tif]

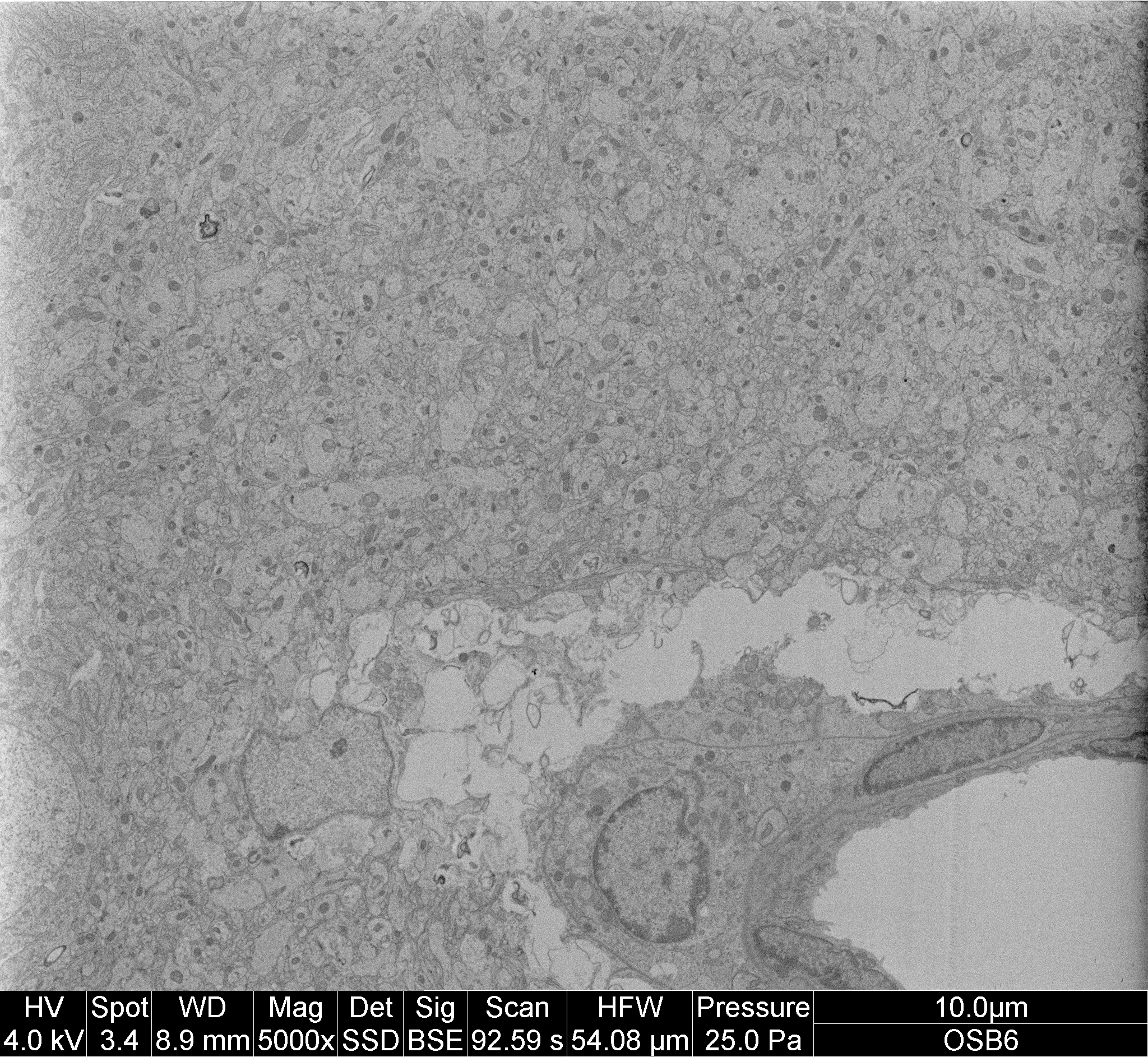

Supplement: Dataset S4 — (252.6 MB ZIP). [file pbio.0020329.sd004.zip › 040604_OS5_st1_361.tif]

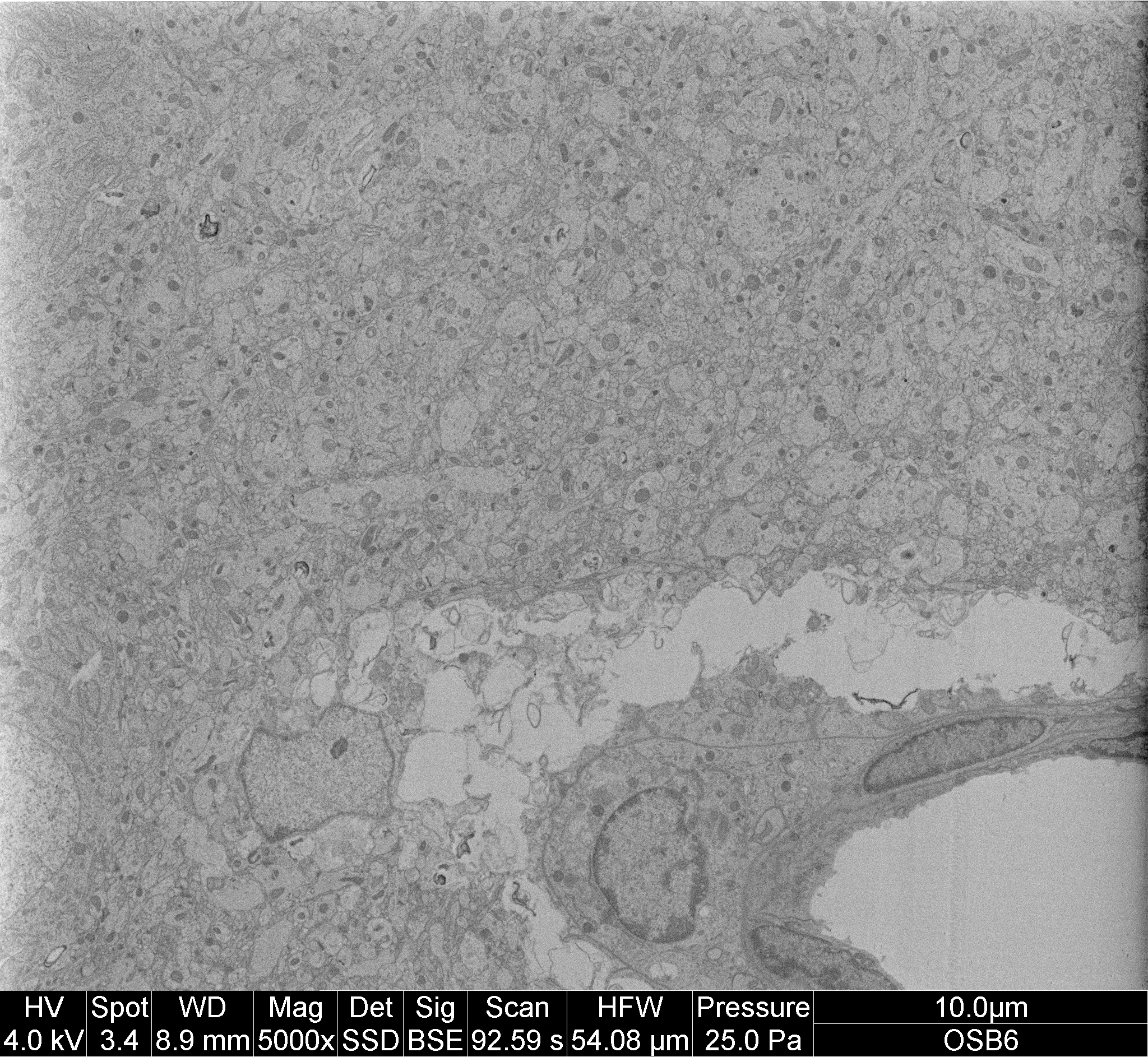

Supplement: Dataset S4 — (252.6 MB ZIP). [file pbio.0020329.sd004.zip › 040604_OS5_st1_362.tif]

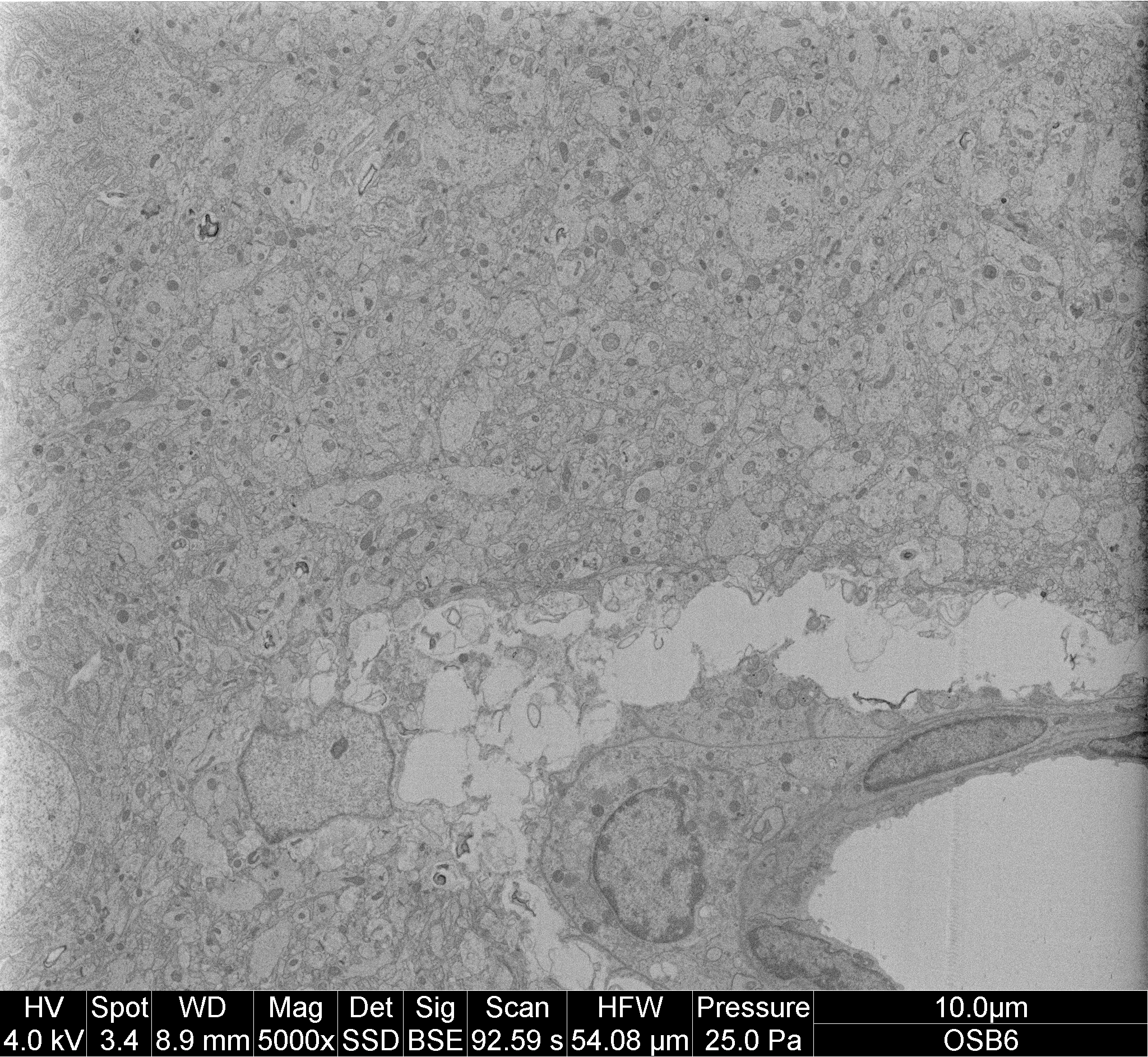

Supplement: Dataset S4 — (252.6 MB ZIP). [file pbio.0020329.sd004.zip › 040604_OS5_st1_363.tif]

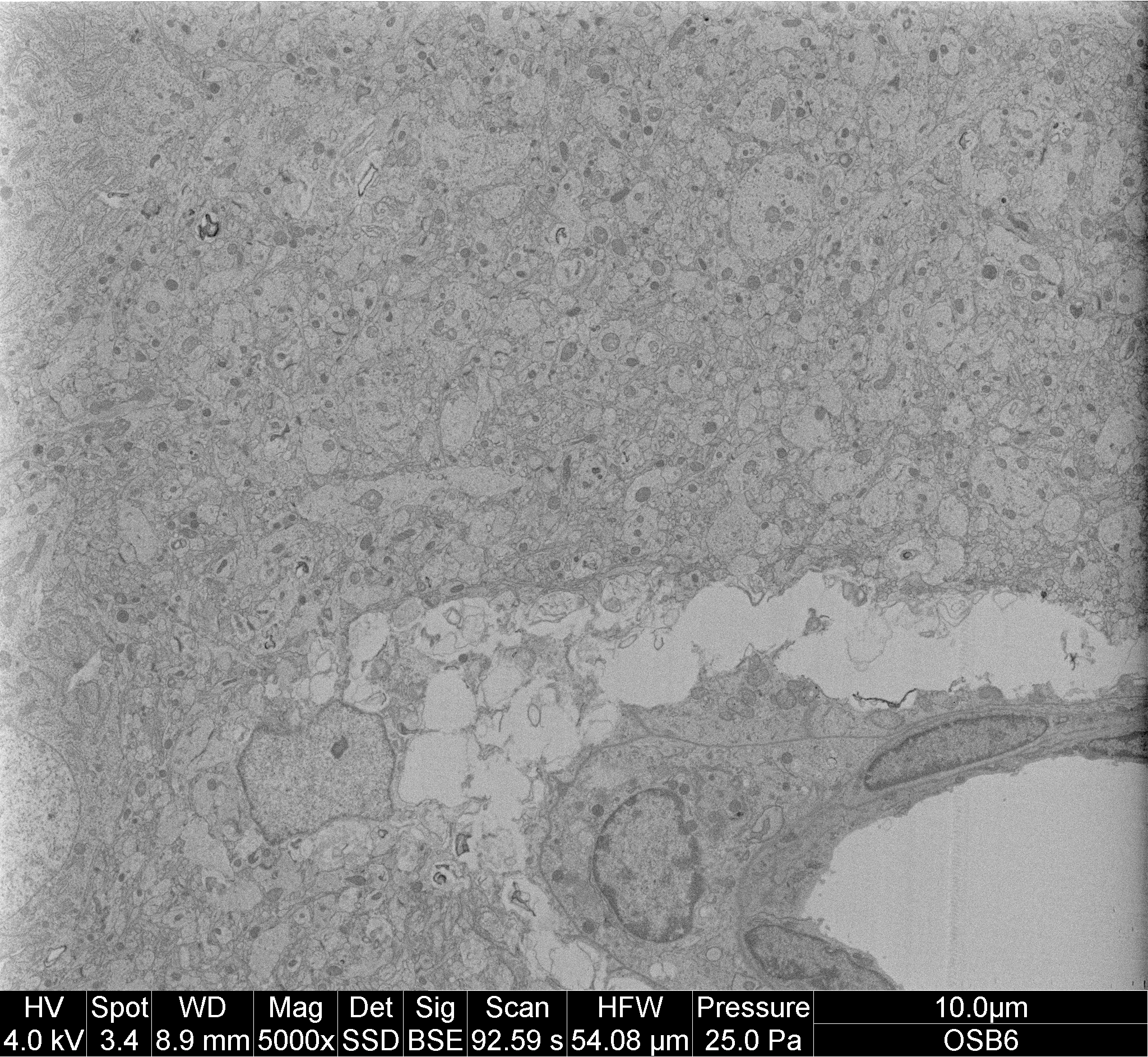

Supplement: Dataset S4 — (252.6 MB ZIP). [file pbio.0020329.sd004.zip › 040604_OS5_st1_364.tif]

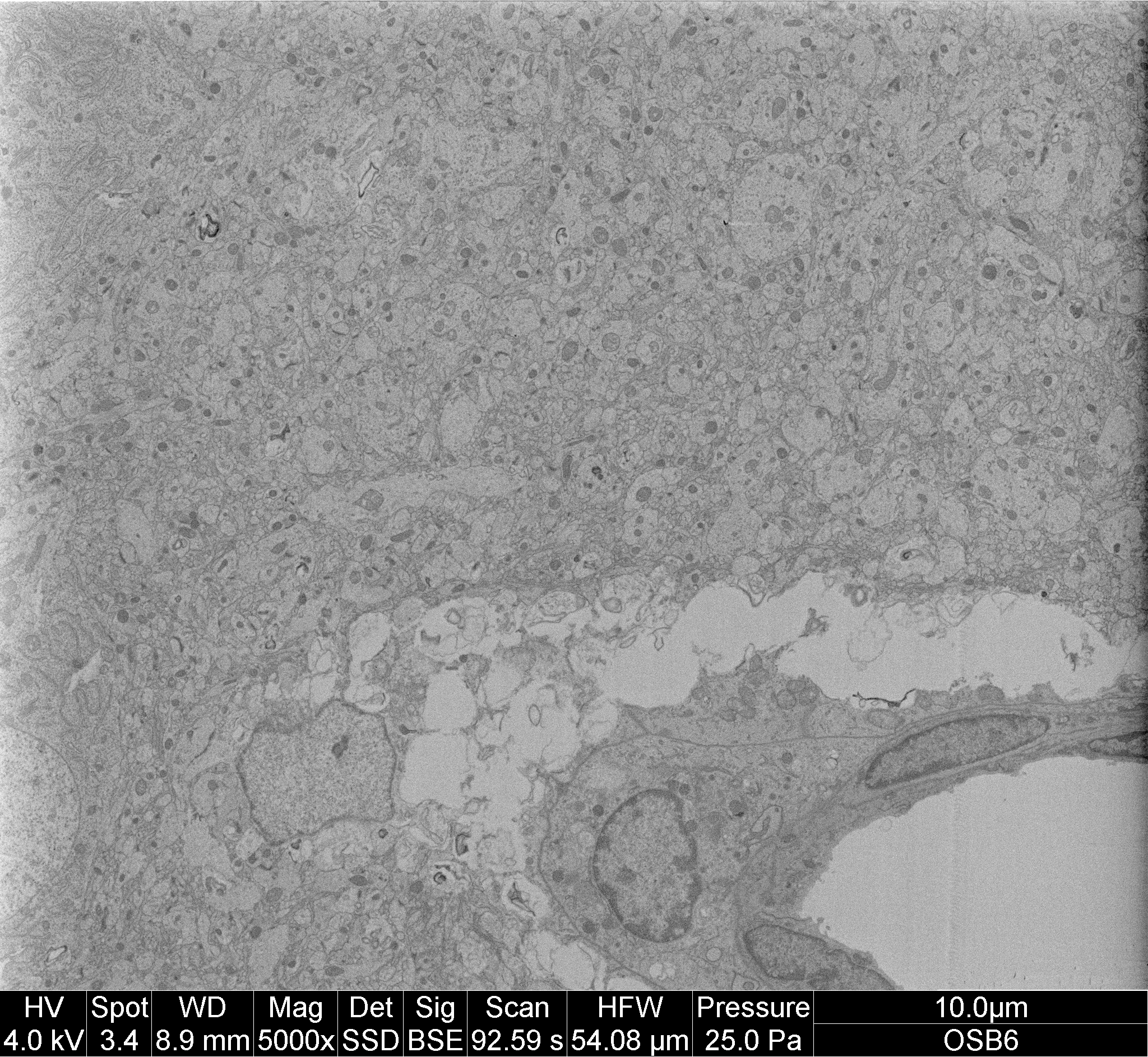

Supplement: Dataset S4 — (252.6 MB ZIP). [file pbio.0020329.sd004.zip › 040604_OS5_st1_365.tif]

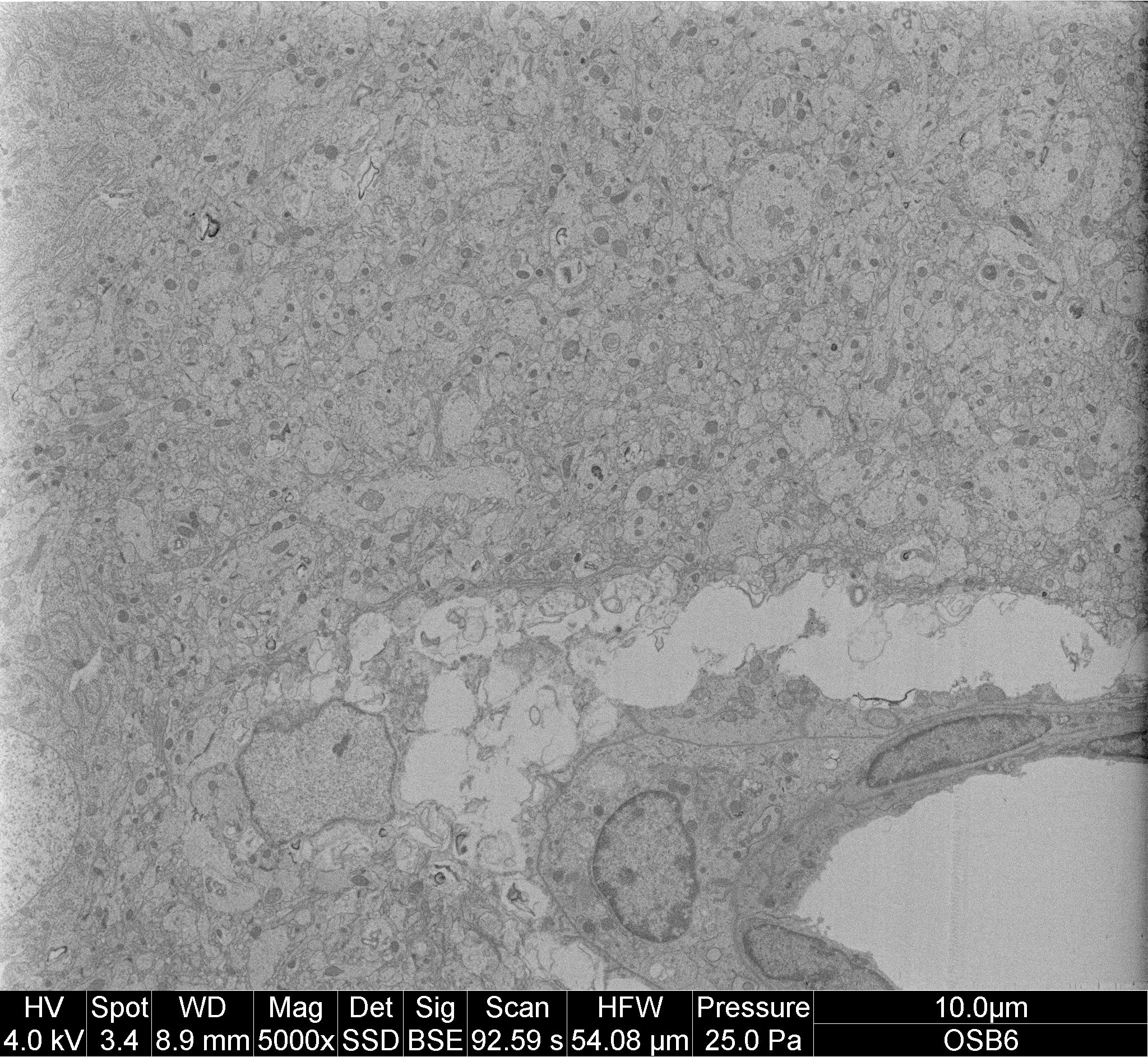

Supplement: Dataset S4 — (252.6 MB ZIP). [file pbio.0020329.sd004.zip › 040604_OS5_st1_366.tif]

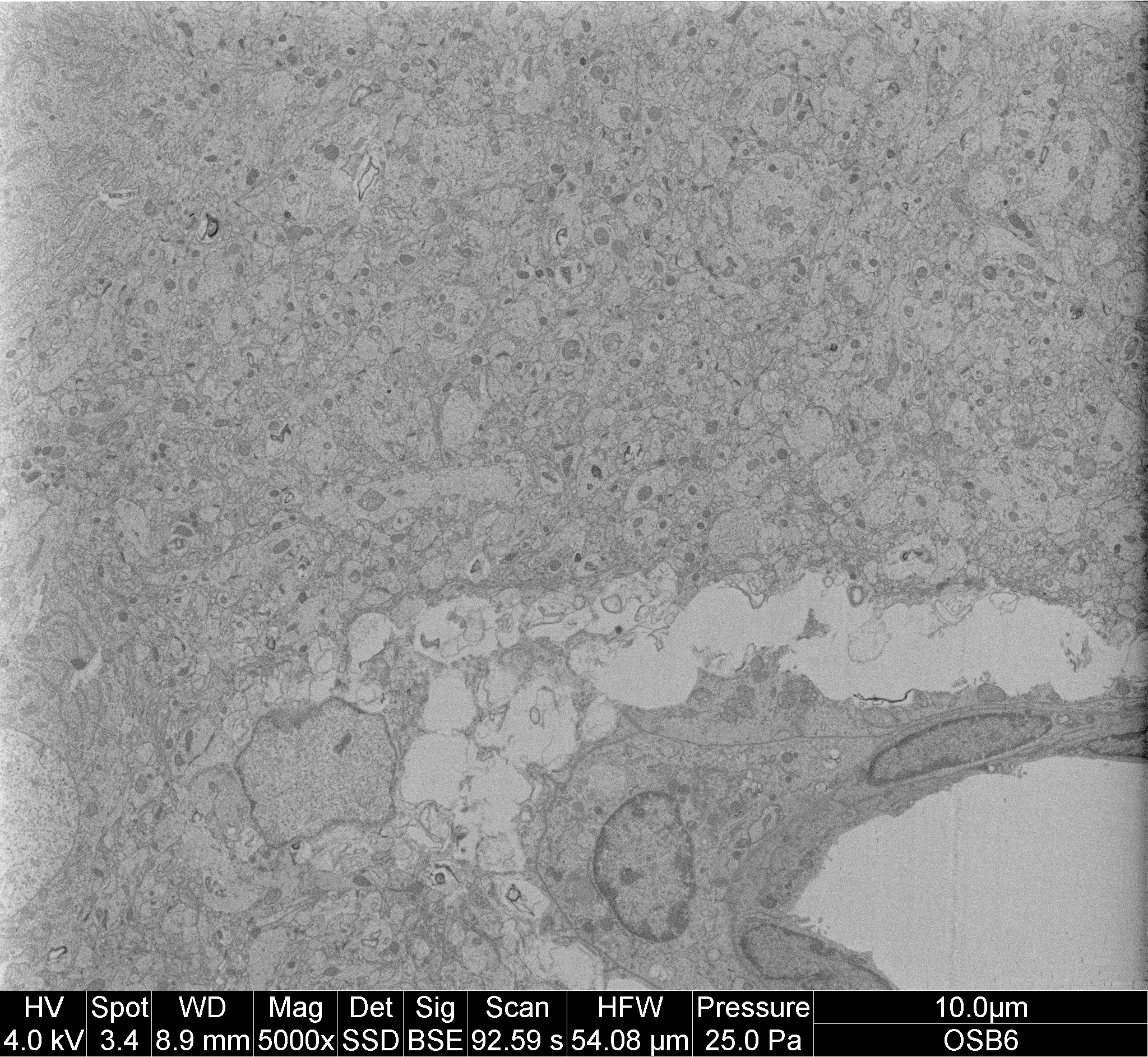

Supplement: Dataset S4 — (252.6 MB ZIP). [file pbio.0020329.sd004.zip › 040604_OS5_st1_367.tif]

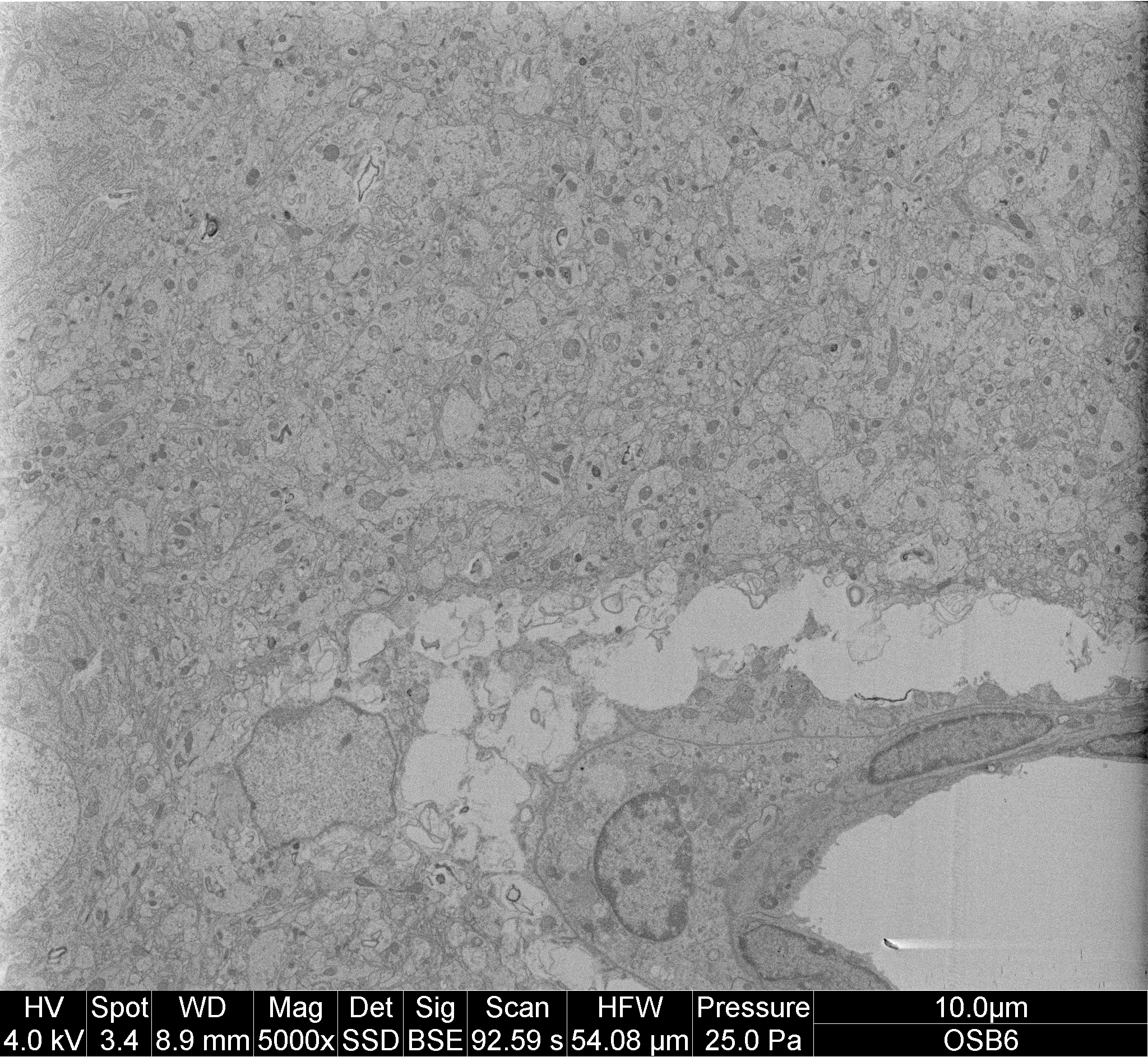

Supplement: Dataset S4 — (252.6 MB ZIP). [file pbio.0020329.sd004.zip › 040604_OS5_st1_368.tif]

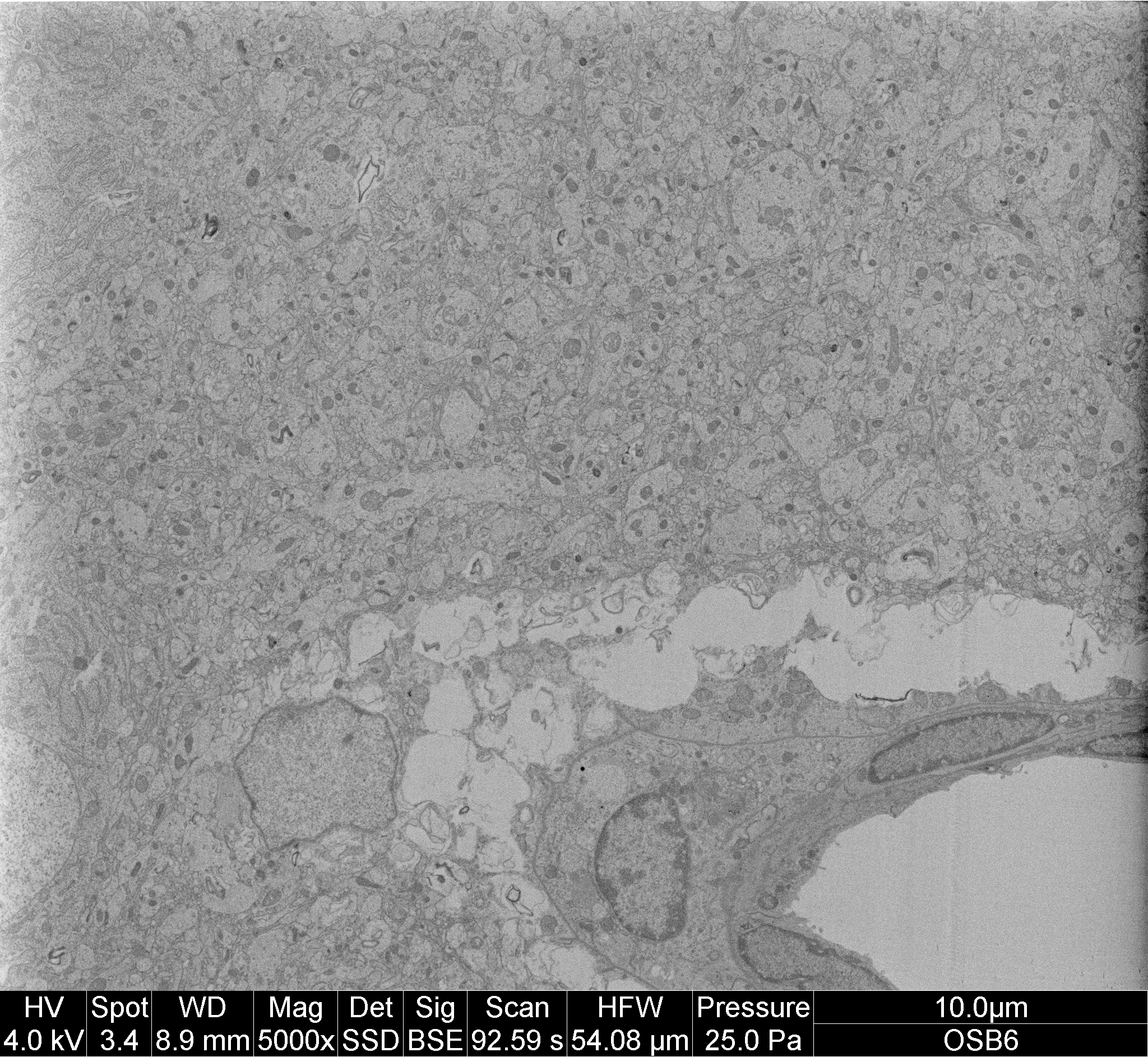

Supplement: Dataset S4 — (252.6 MB ZIP). [file pbio.0020329.sd004.zip › 040604_OS5_st1_369.tif]

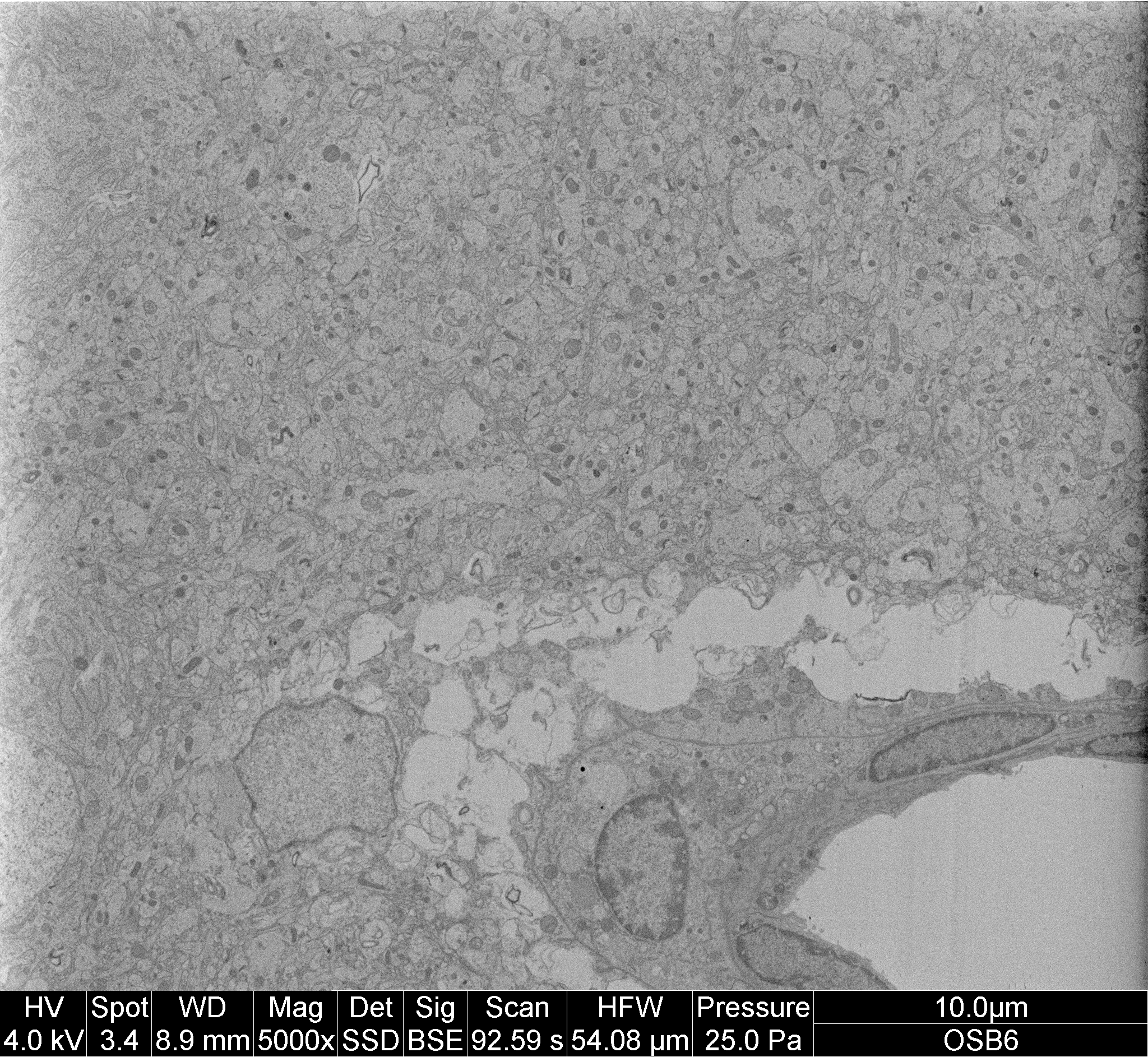

Supplement: Dataset S4 — (252.6 MB ZIP). [file pbio.0020329.sd004.zip › 040604_OS5_st1_370.tif]

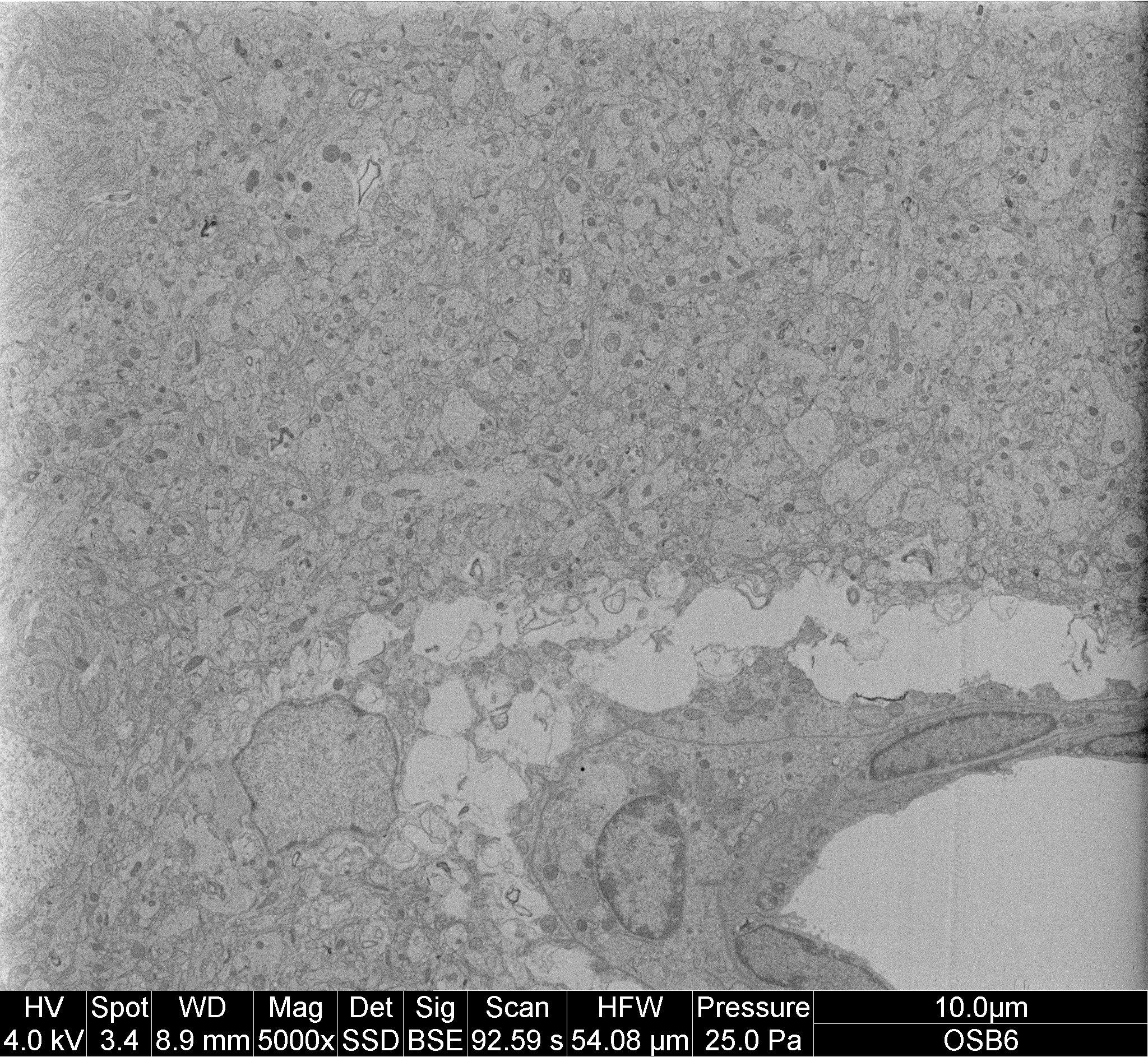

Supplement: Dataset S4 — (252.6 MB ZIP). [file pbio.0020329.sd004.zip › 040604_OS5_st1_371.tif]

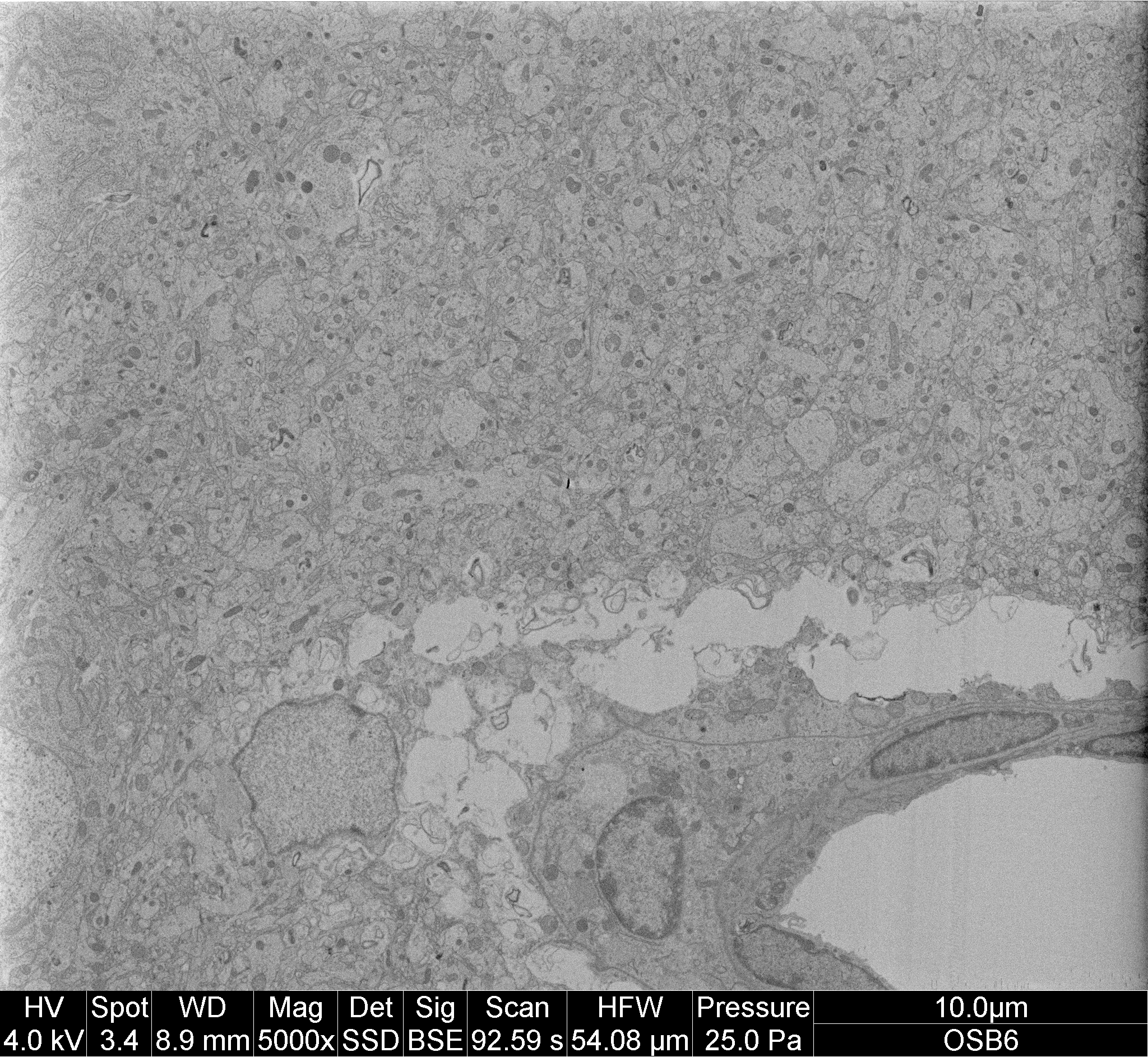

Supplement: Dataset S4 — (252.6 MB ZIP). [file pbio.0020329.sd004.zip › 040604_OS5_st1_372.tif]

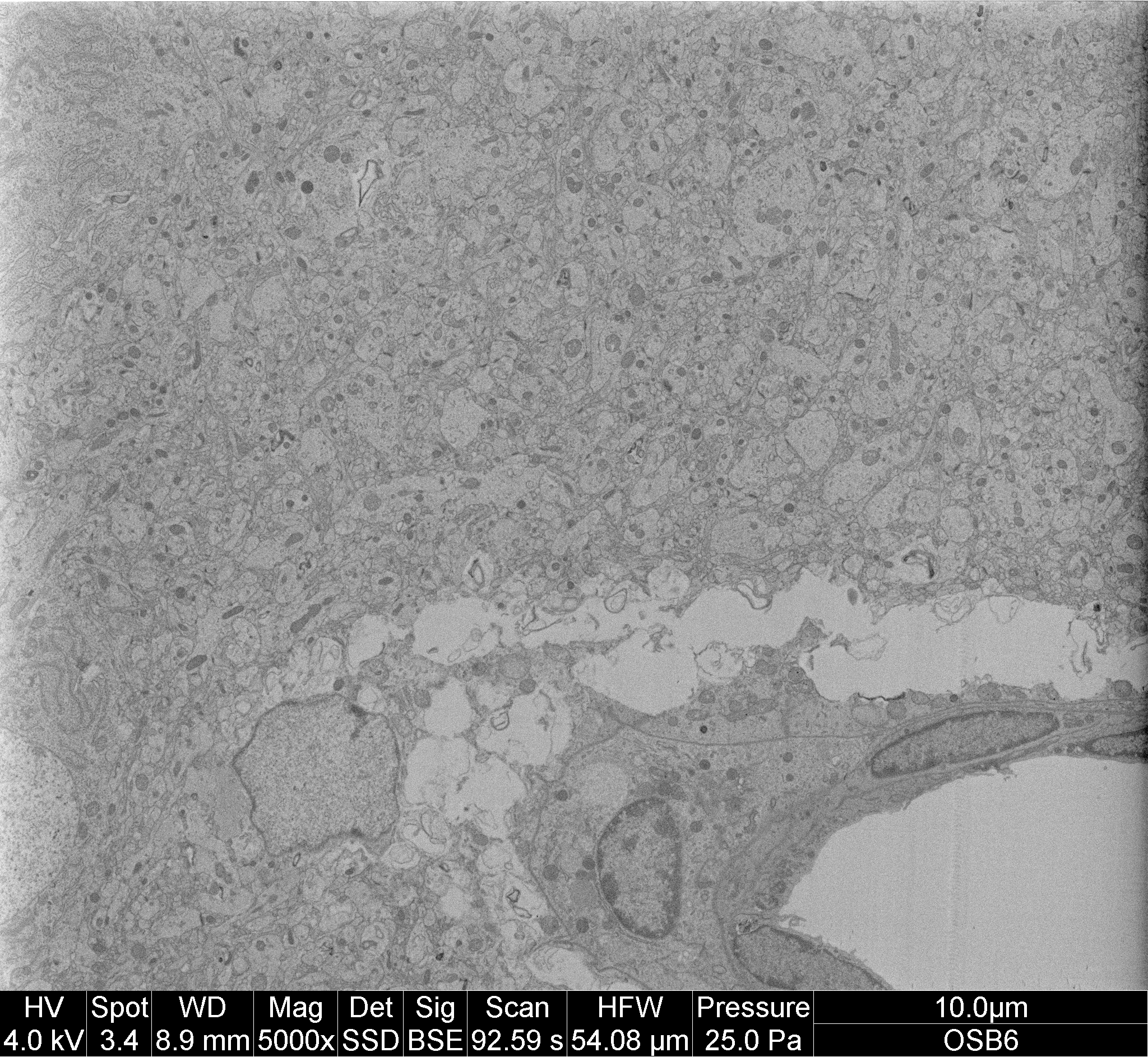

Supplement: Dataset S4 — (252.6 MB ZIP). [file pbio.0020329.sd004.zip › 040604_OS5_st1_373.tif]

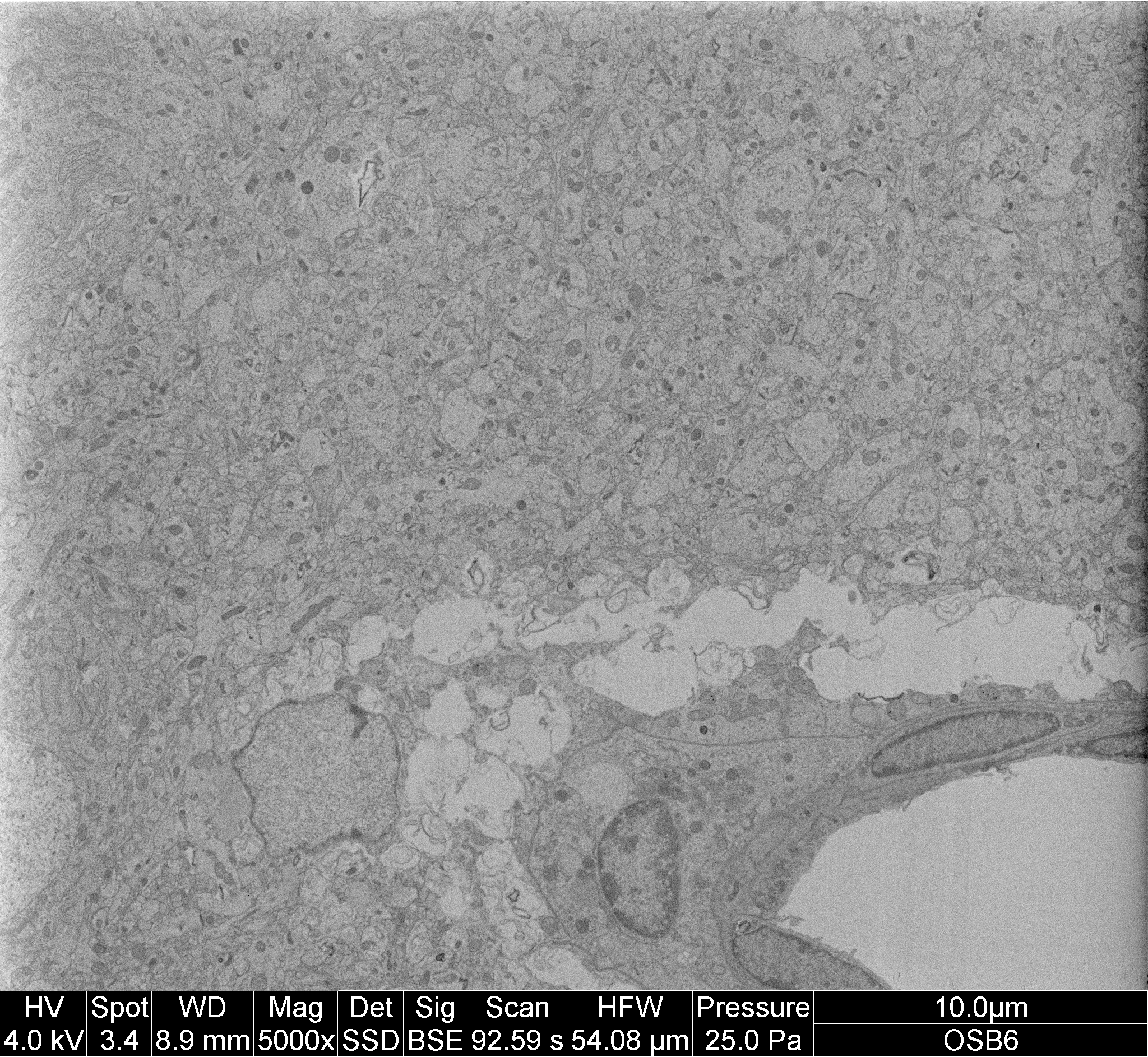

Supplement: Dataset S4 — (252.6 MB ZIP). [file pbio.0020329.sd004.zip › 040604_OS5_st1_374.tif]

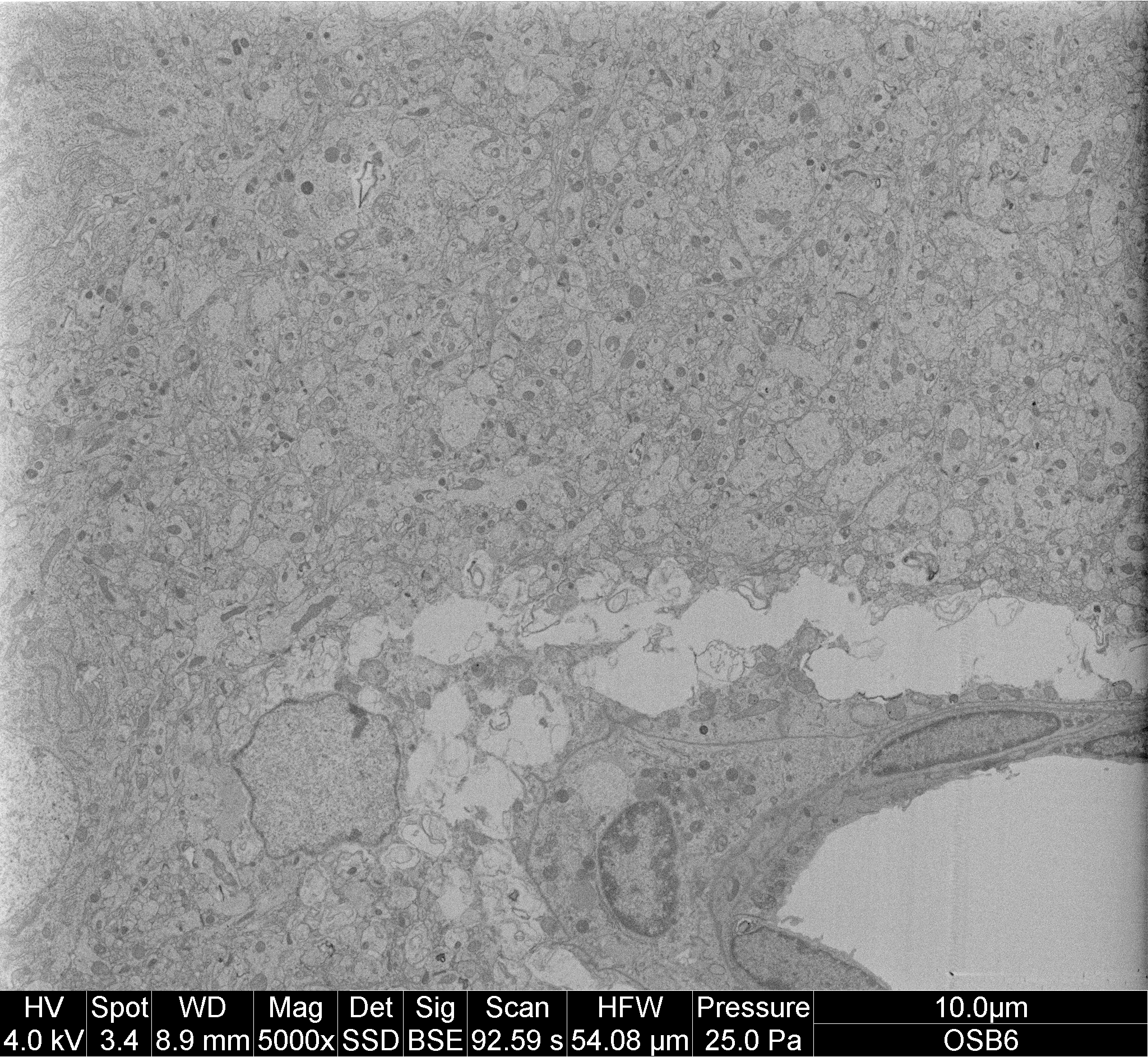

Supplement: Dataset S4 — (252.6 MB ZIP). [file pbio.0020329.sd004.zip › 040604_OS5_st1_375.tif]

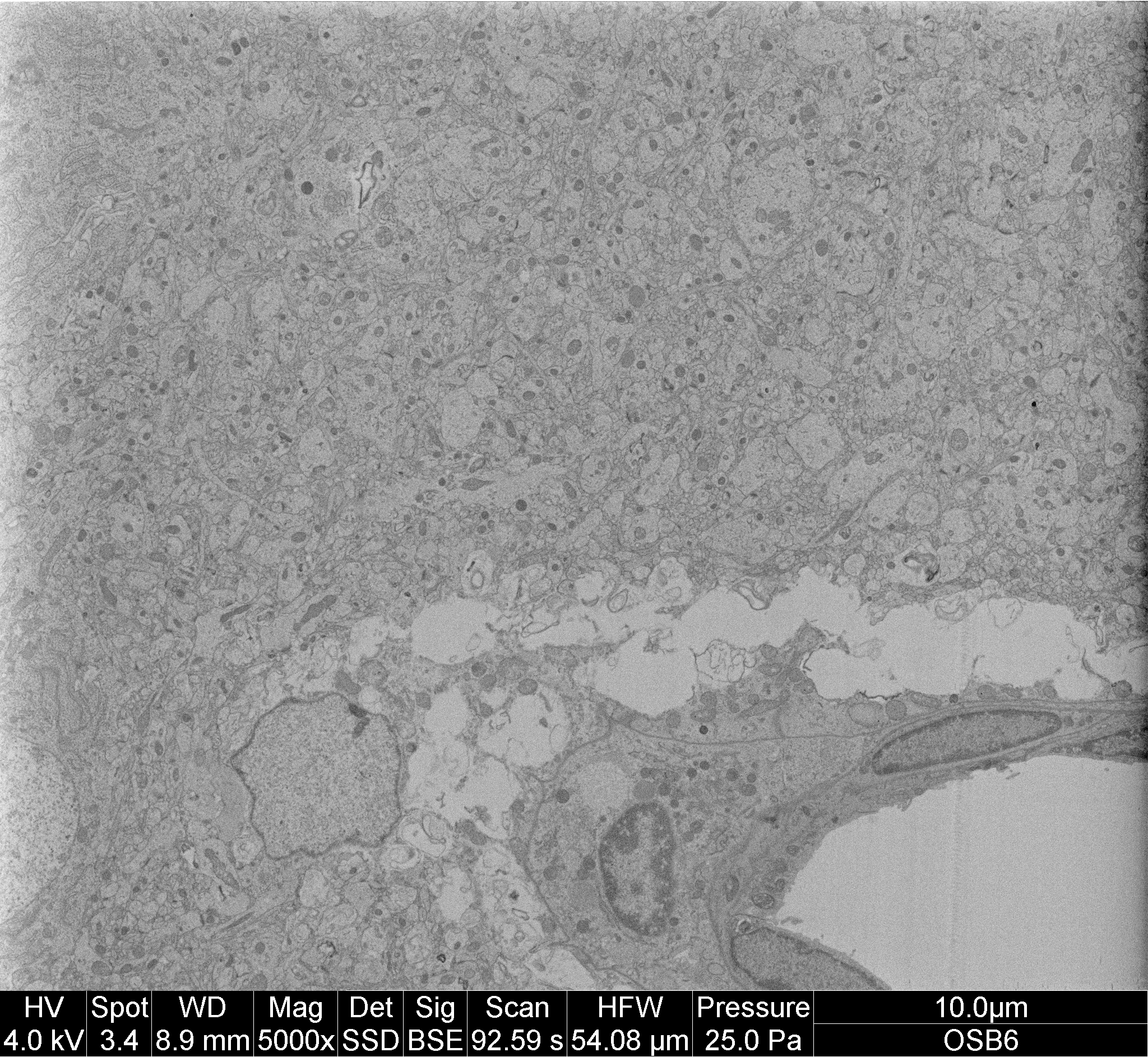

Supplement: Dataset S4 — (252.6 MB ZIP). [file pbio.0020329.sd004.zip › 040604_OS5_st1_376.tif]

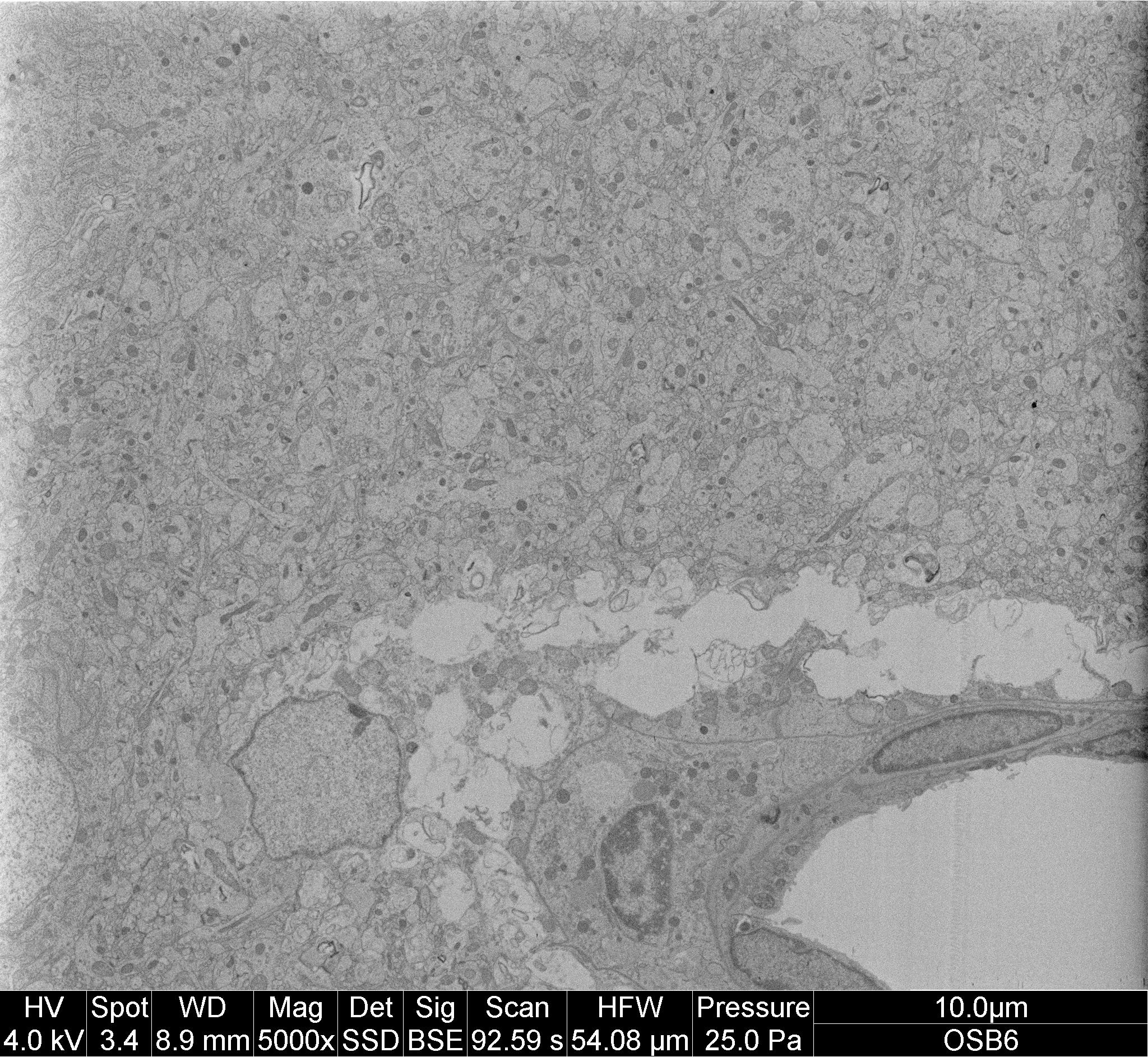

Supplement: Dataset S4 — (252.6 MB ZIP). [file pbio.0020329.sd004.zip › 040604_OS5_st1_377.tif]

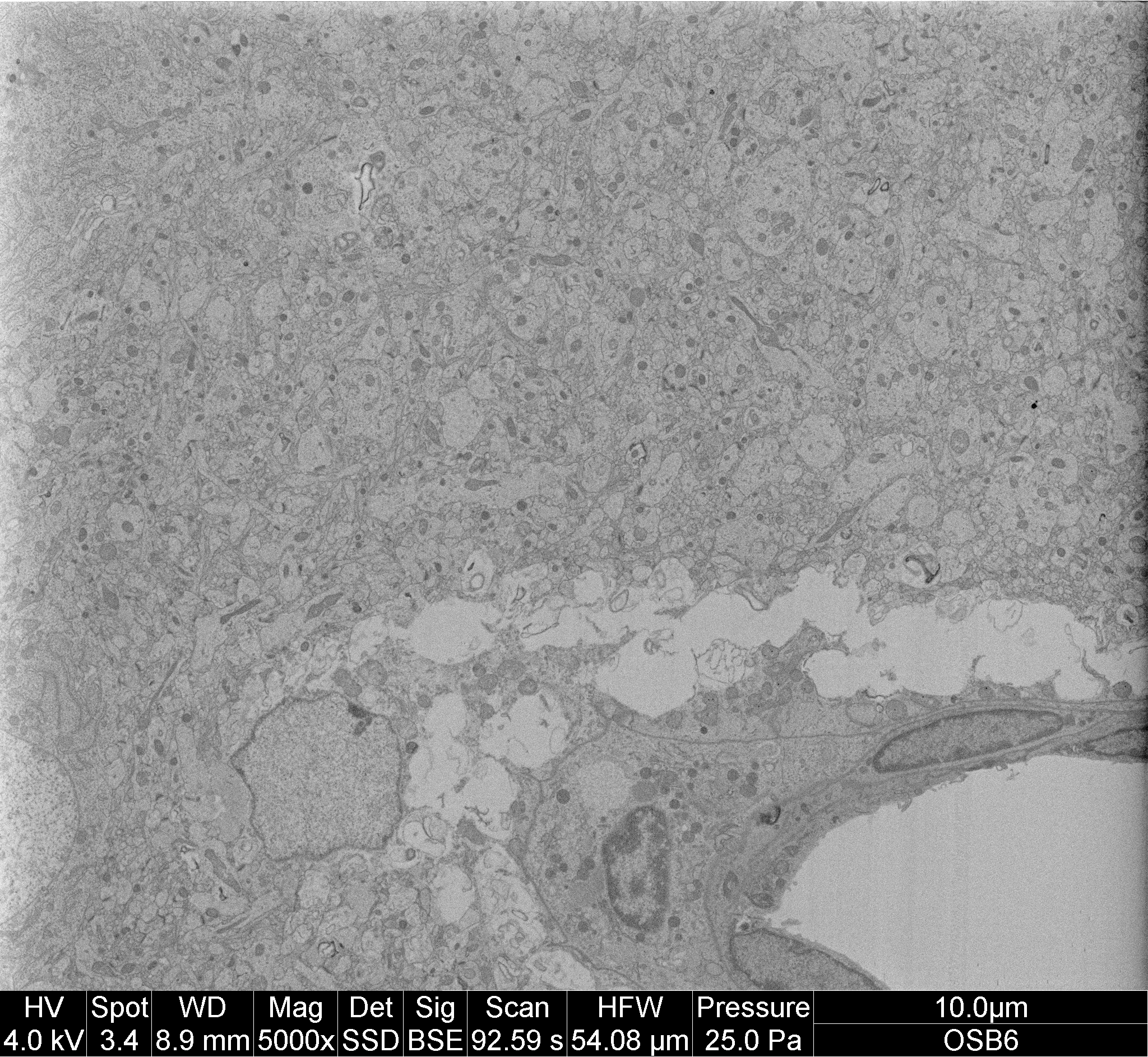

Supplement: Dataset S4 — (252.6 MB ZIP). [file pbio.0020329.sd004.zip › 040604_OS5_st1_378.tif]

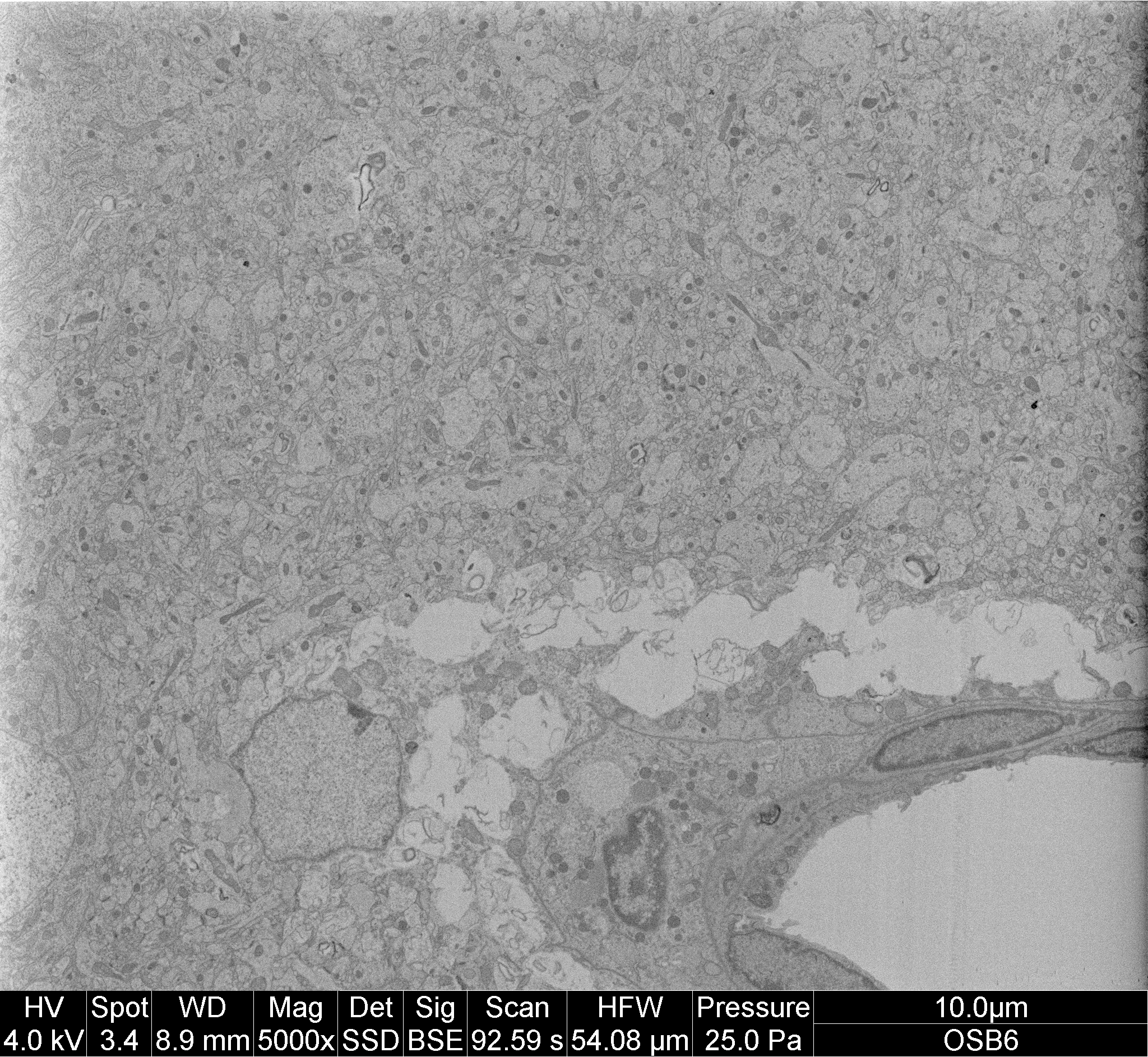

Supplement: Dataset S4 — (252.6 MB ZIP). [file pbio.0020329.sd004.zip › 040604_OS5_st1_379.tif]

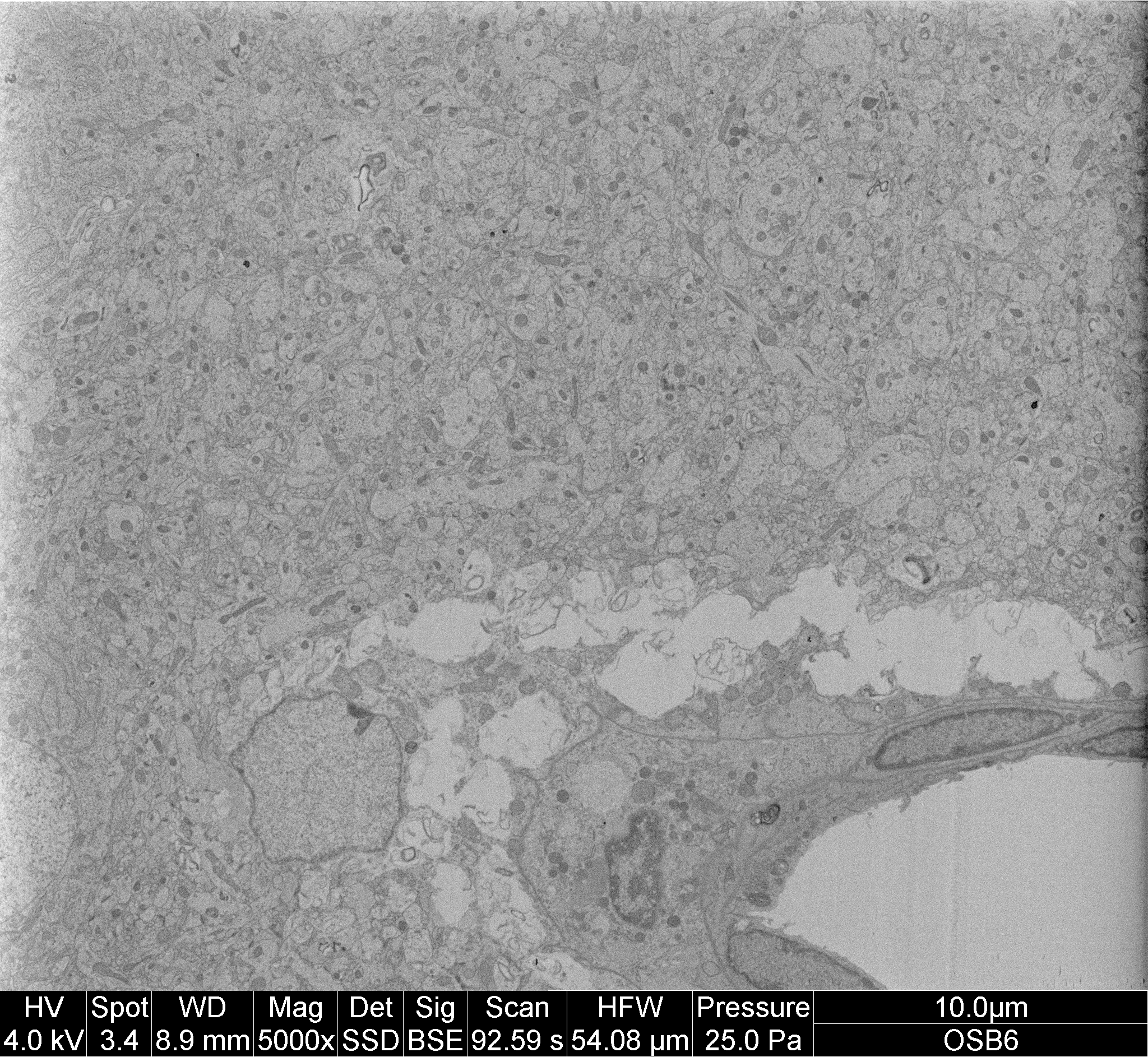

Supplement: Dataset S4 — (252.6 MB ZIP). [file pbio.0020329.sd004.zip › 040604_OS5_st1_380.tif]

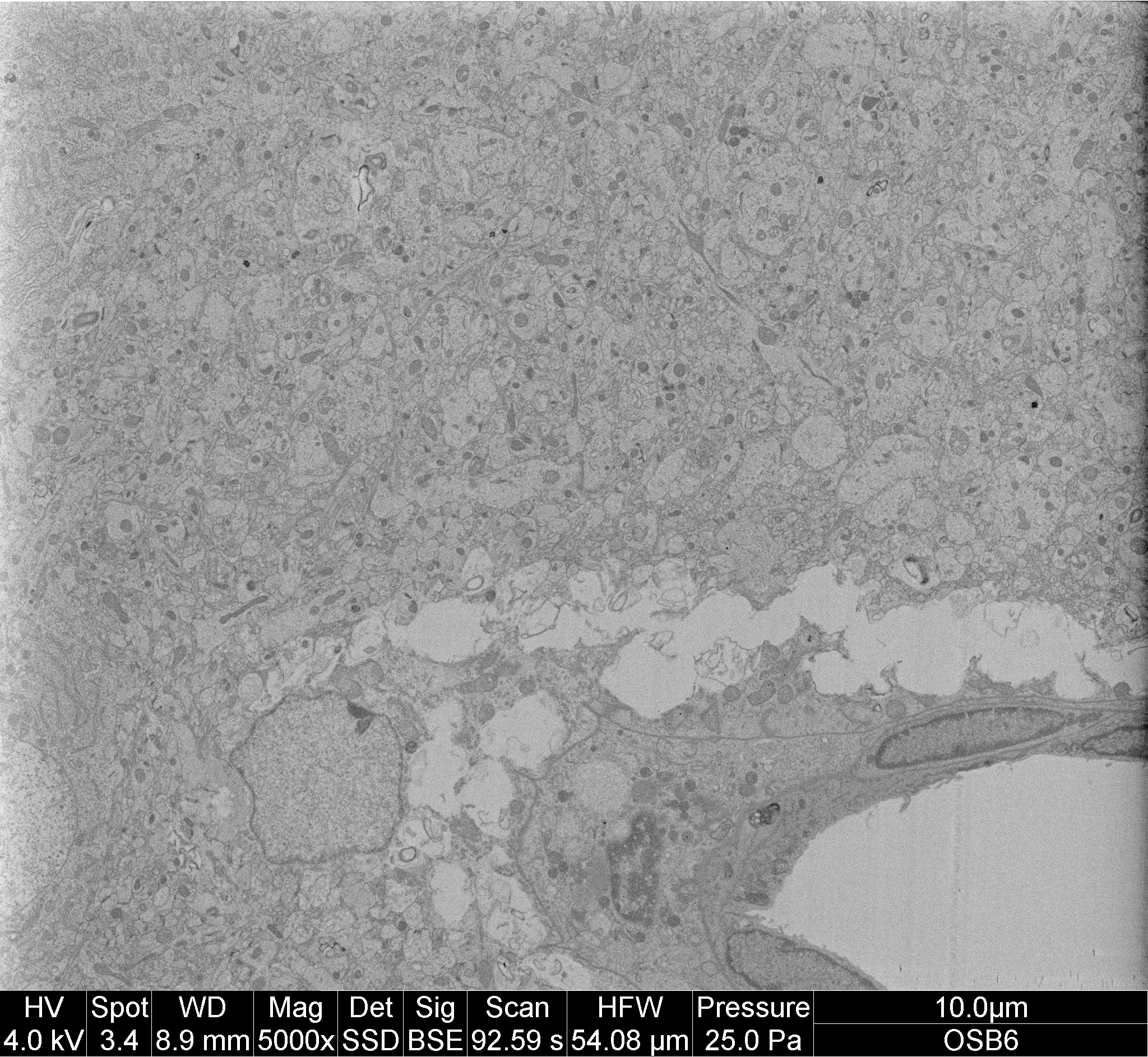

Supplement: Dataset S4 — (252.6 MB ZIP). [file pbio.0020329.sd004.zip › 040604_OS5_st1_381.tif]

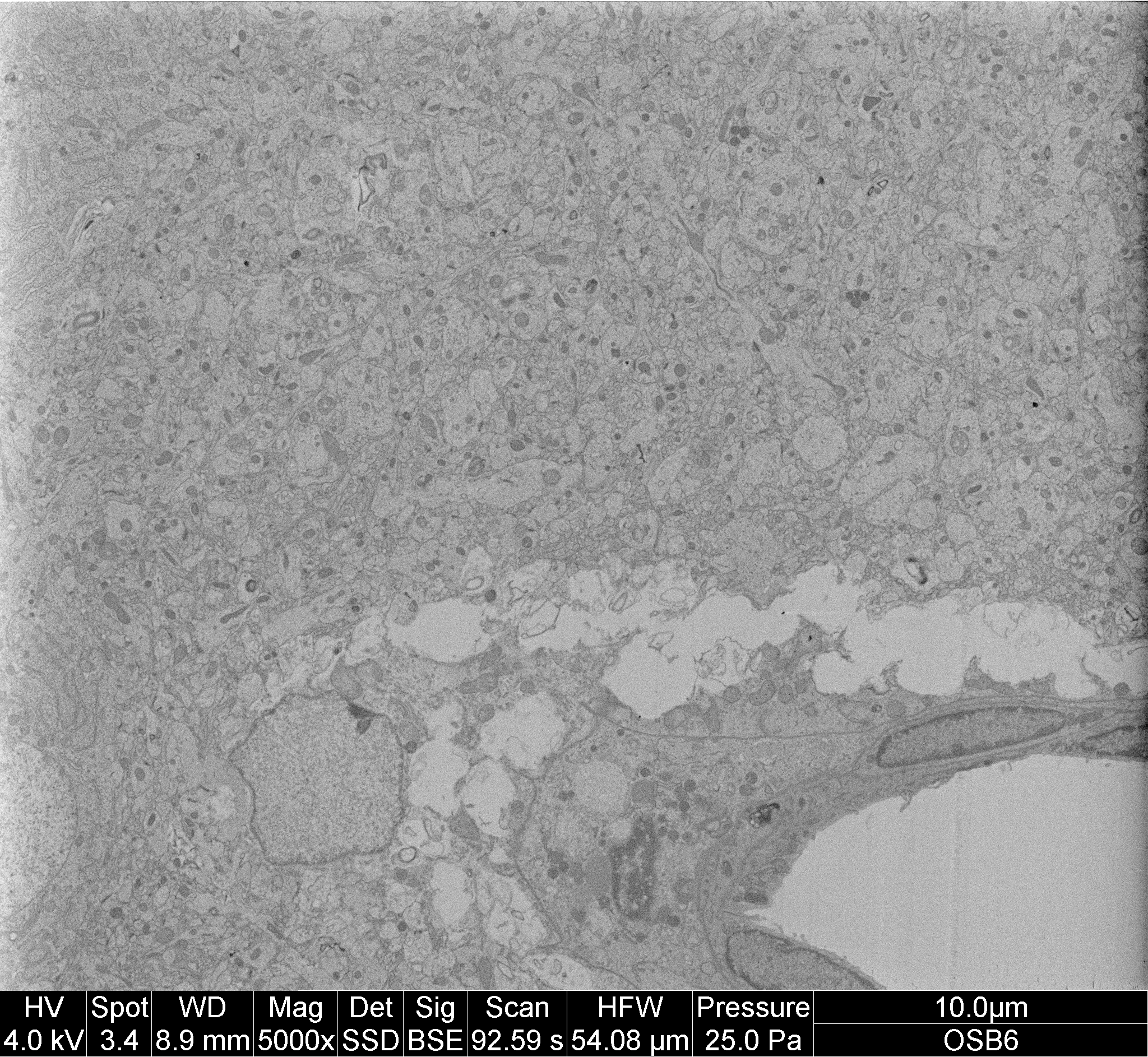

Supplement: Dataset S4 — (252.6 MB ZIP). [file pbio.0020329.sd004.zip › 040604_OS5_st1_382.tif]

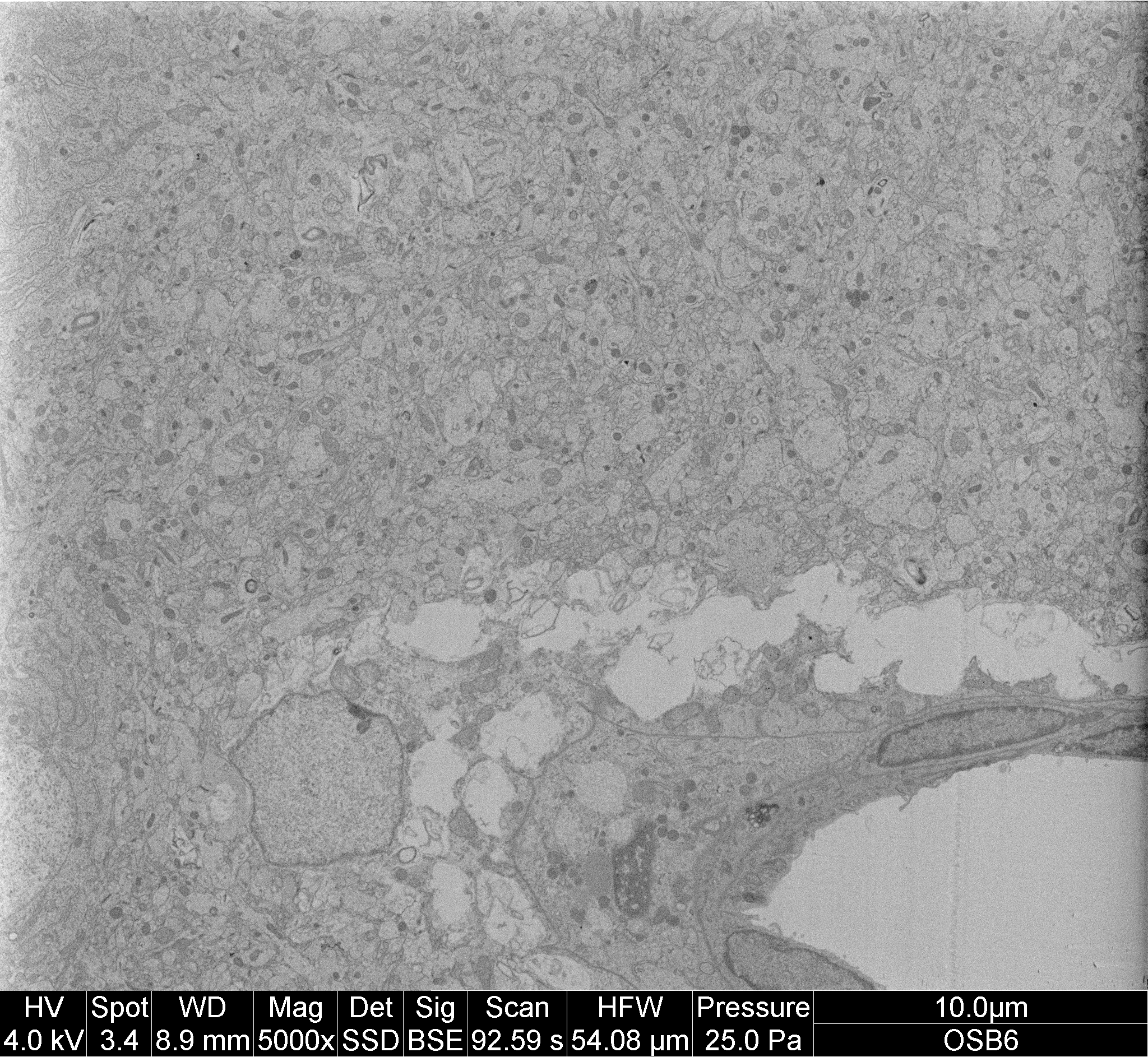

Supplement: Dataset S4 — (252.6 MB ZIP). [file pbio.0020329.sd004.zip › 040604_OS5_st1_383.tif]

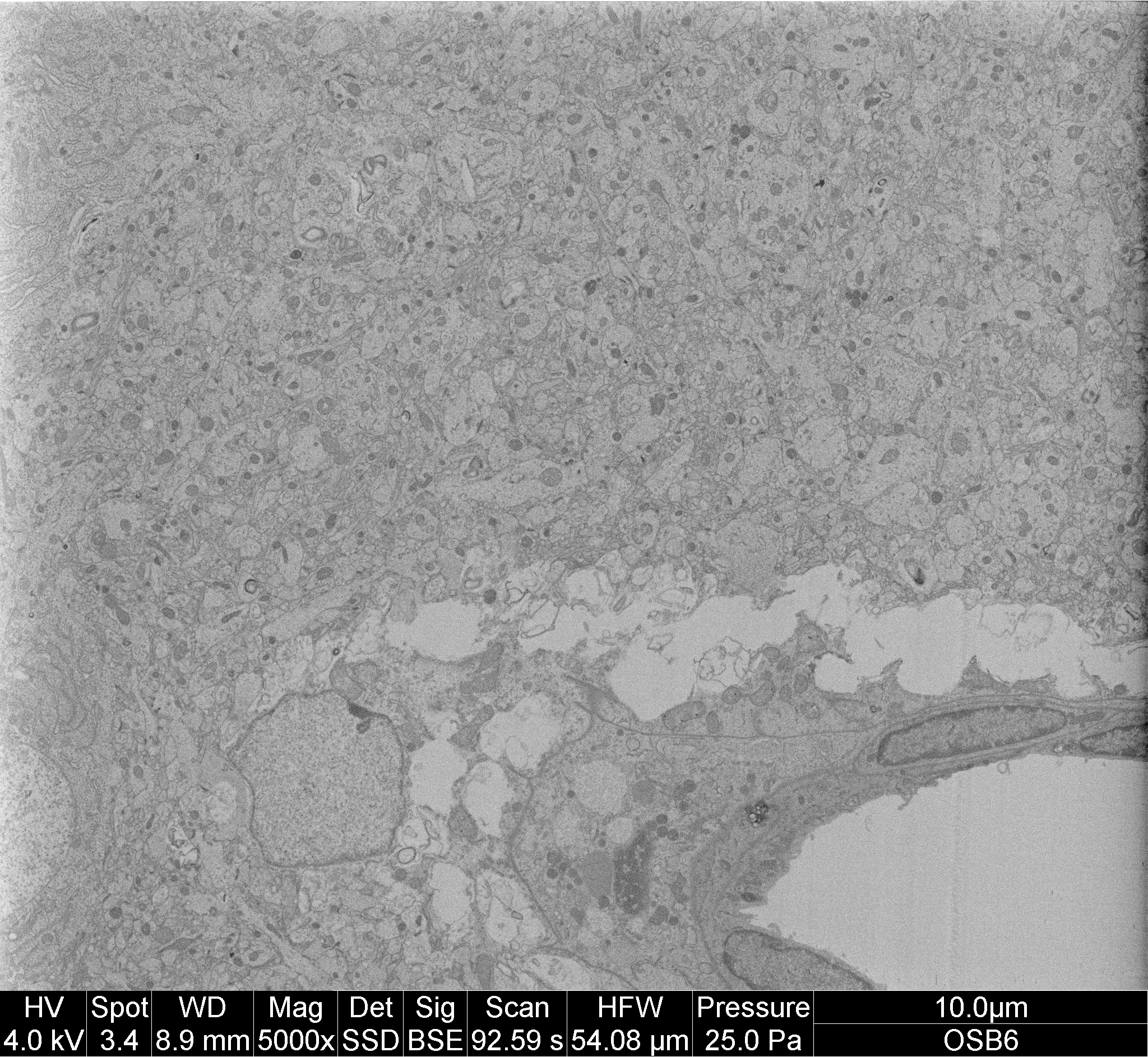

Supplement: Dataset S4 — (252.6 MB ZIP). [file pbio.0020329.sd004.zip › 040604_OS5_st1_384.tif]

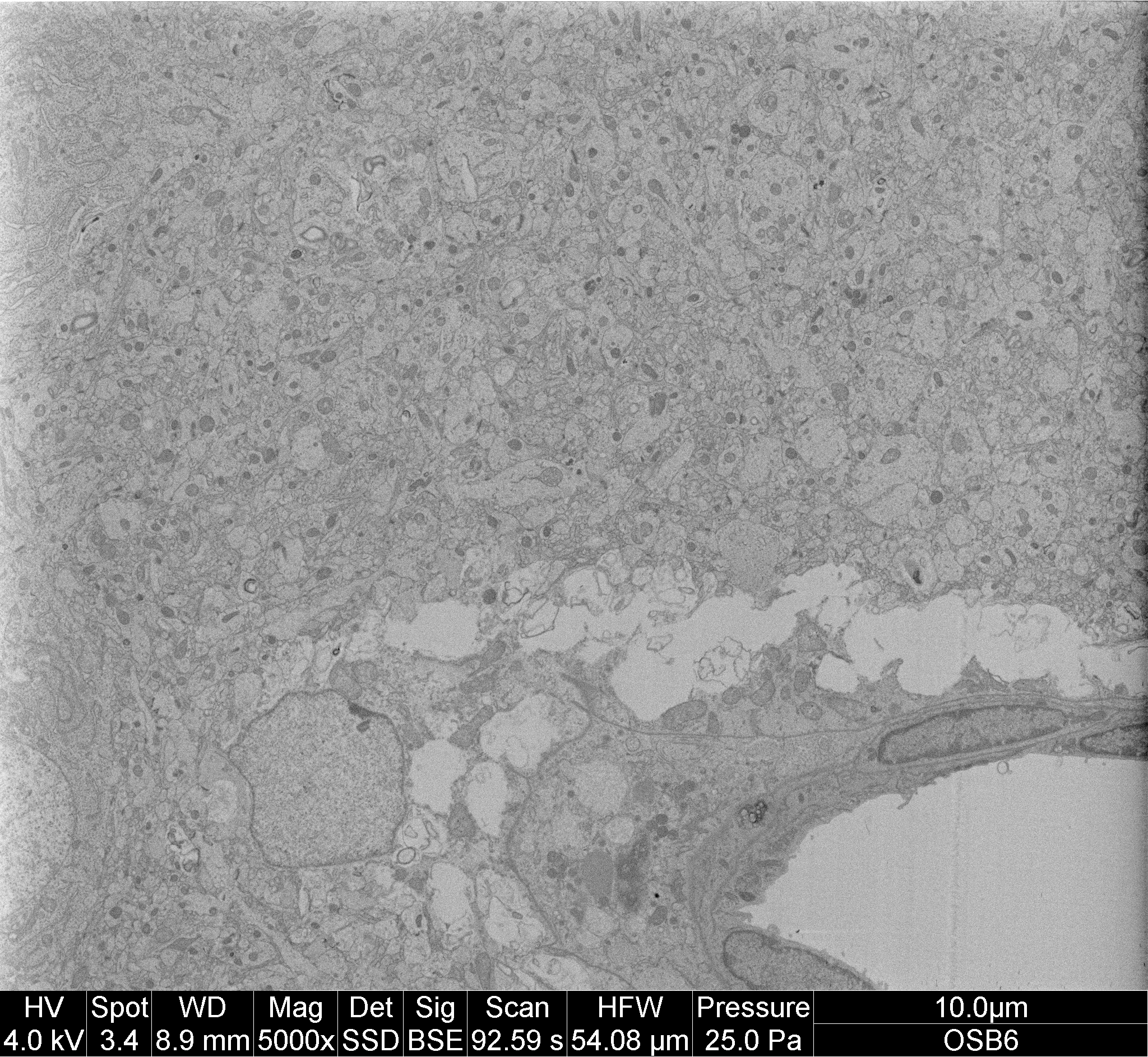

Supplement: Dataset S4 — (252.6 MB ZIP). [file pbio.0020329.sd004.zip › 040604_OS5_st1_385.tif]

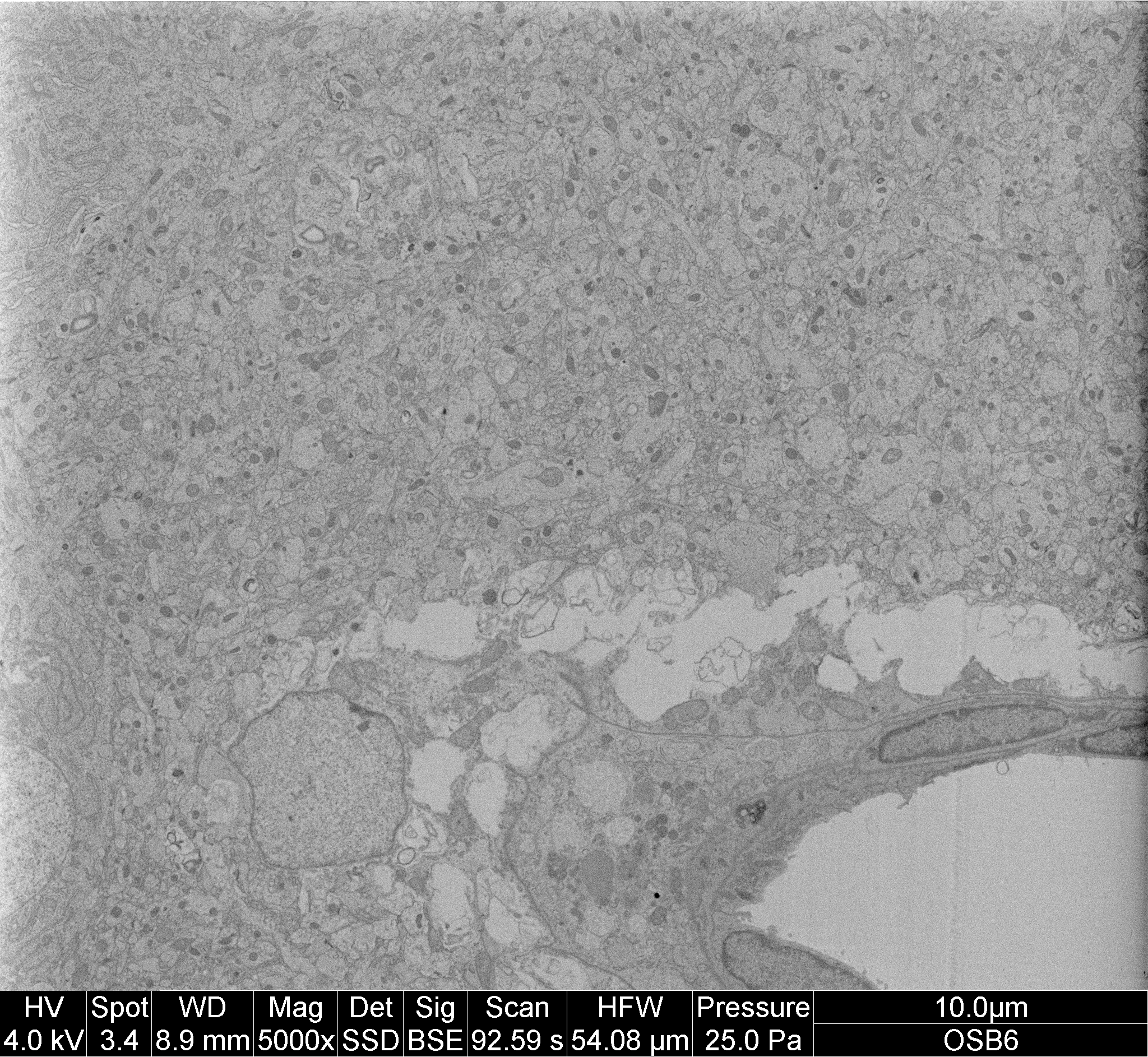

Supplement: Dataset S4 — (252.6 MB ZIP). [file pbio.0020329.sd004.zip › 040604_OS5_st1_386.tif]

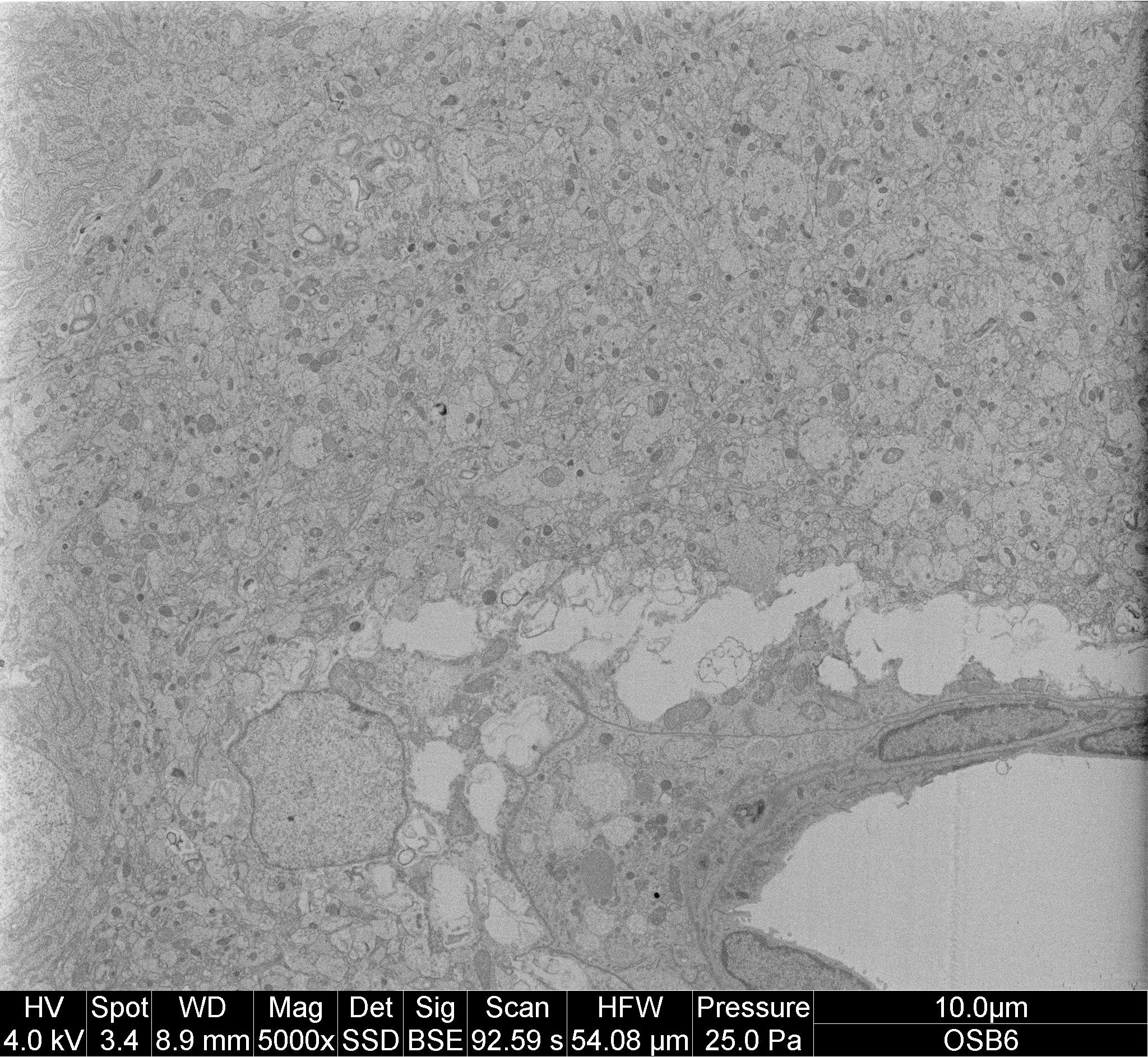

Supplement: Dataset S4 — (252.6 MB ZIP). [file pbio.0020329.sd004.zip › 040604_OS5_st1_387.tif]

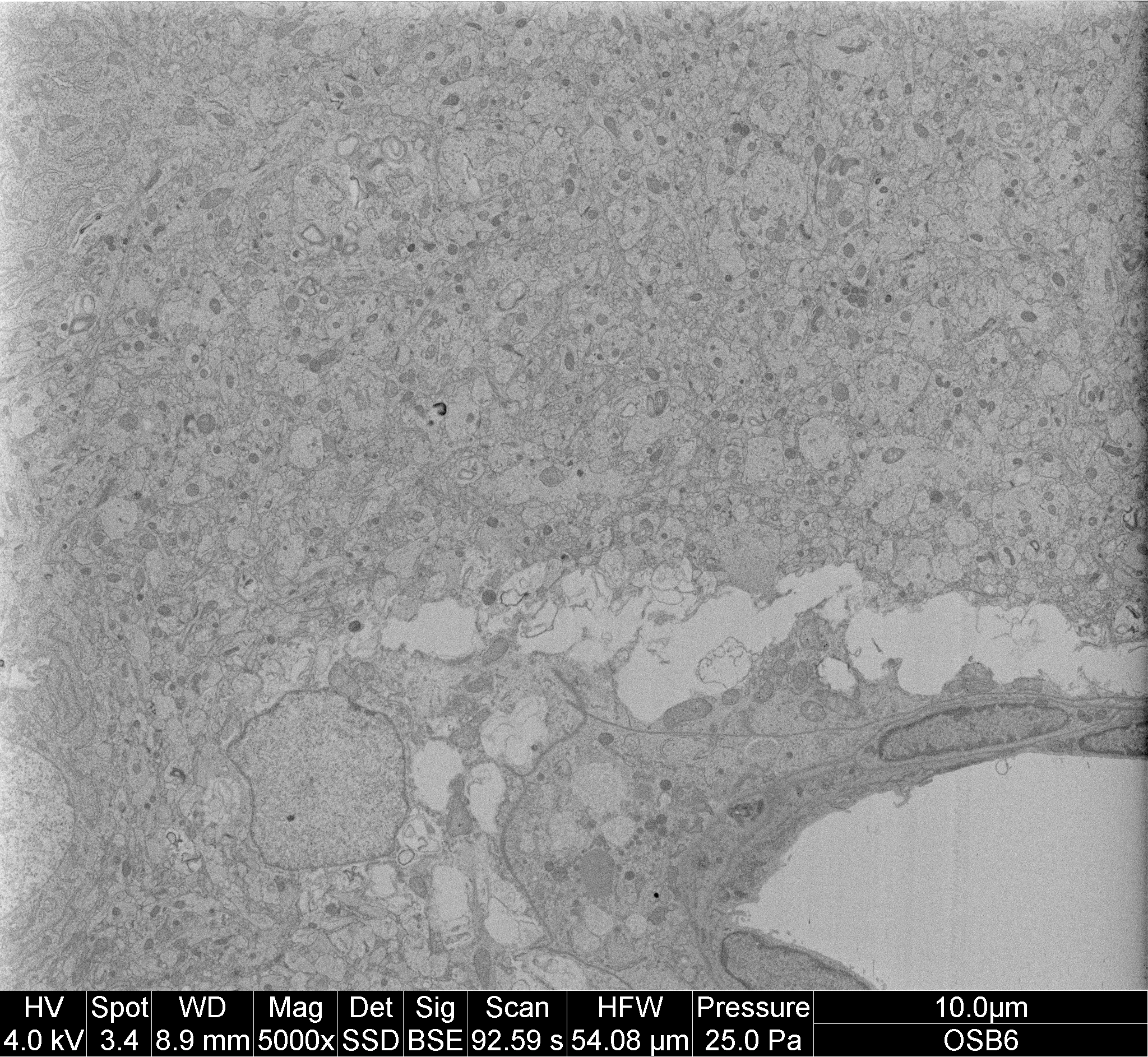

Supplement: Dataset S4 — (252.6 MB ZIP). [file pbio.0020329.sd004.zip › 040604_OS5_st1_388.tif]

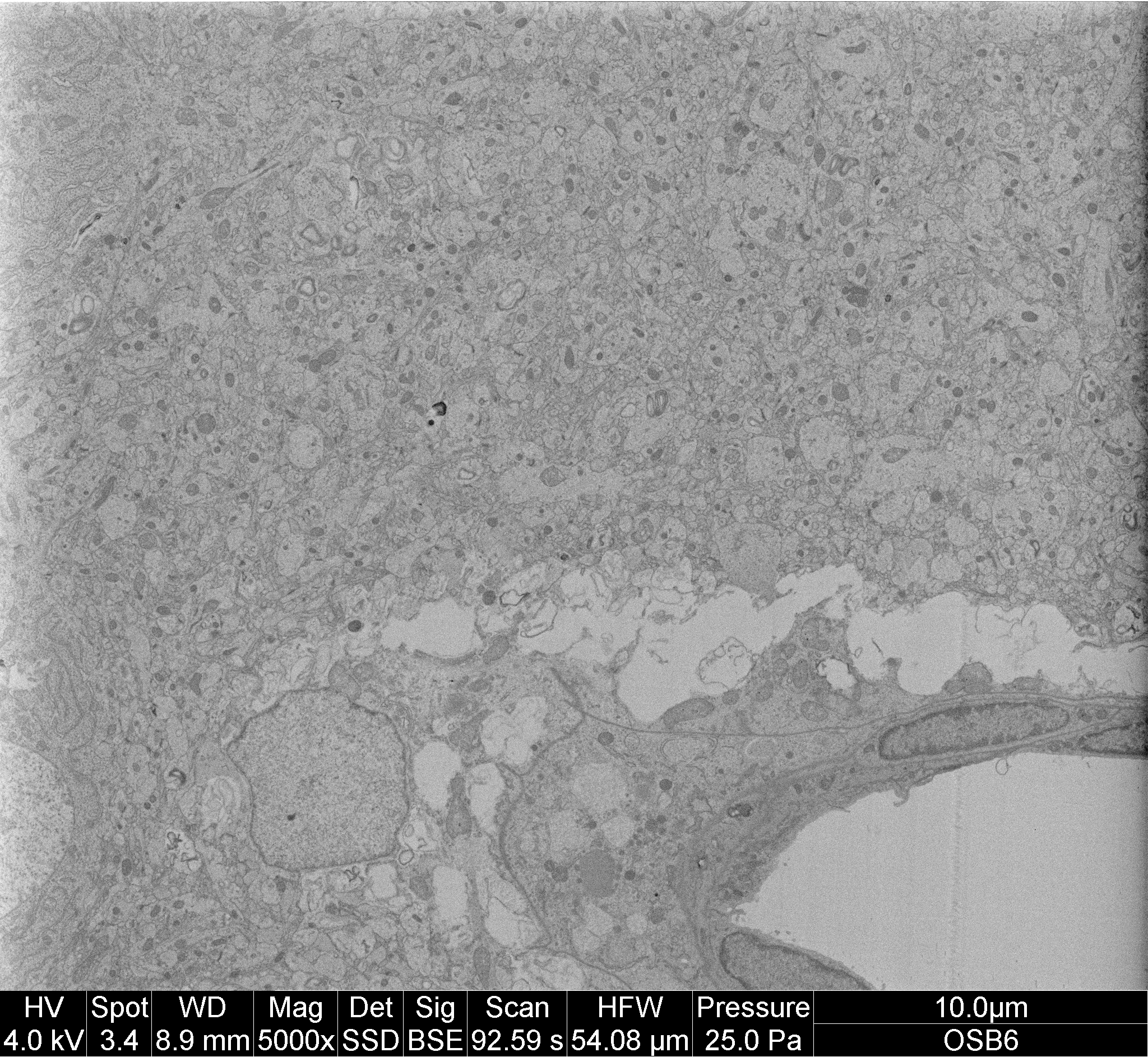

Supplement: Dataset S4 — (252.6 MB ZIP). [file pbio.0020329.sd004.zip › 040604_OS5_st1_389.tif]

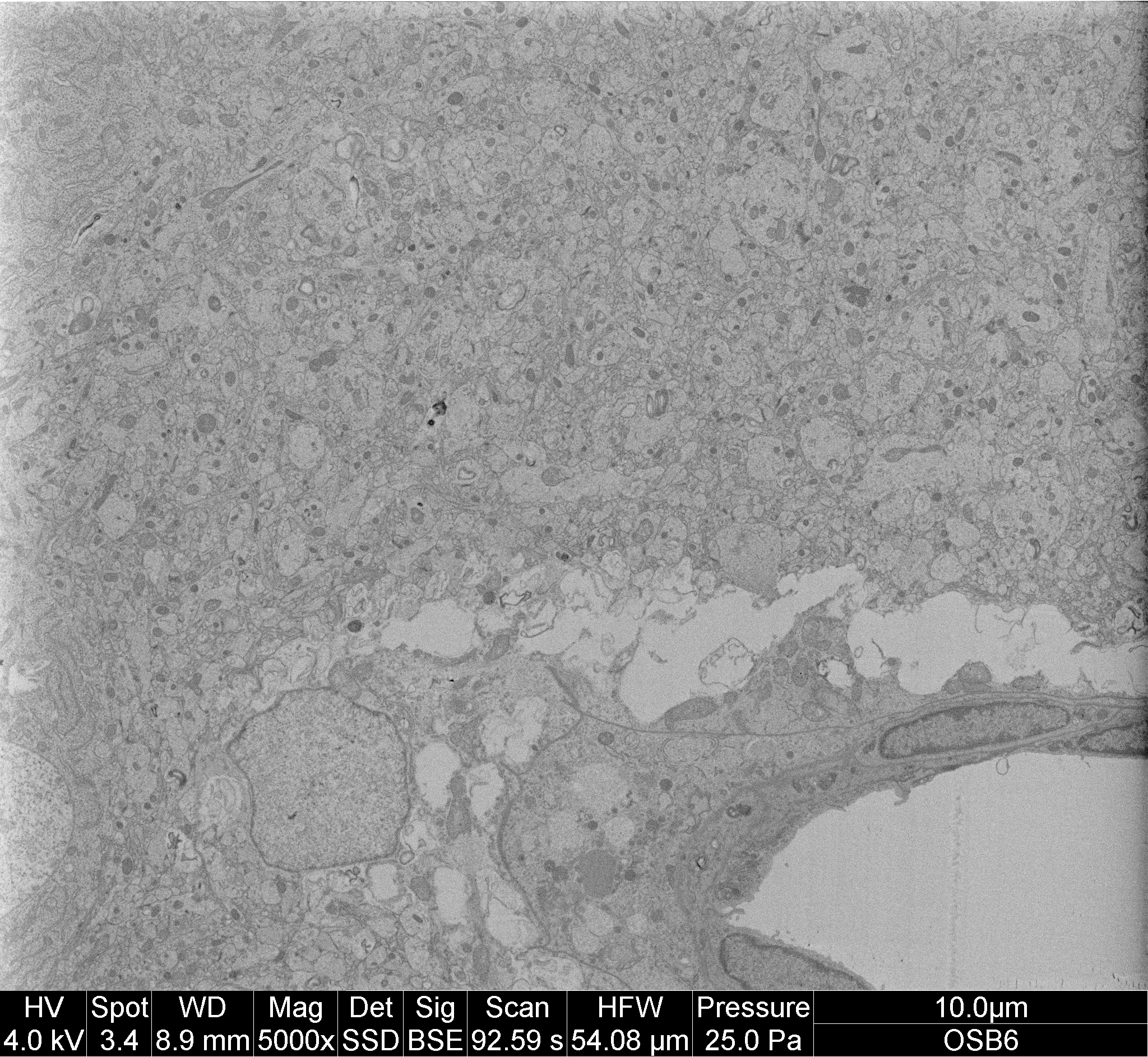

Supplement: Dataset S4 — (252.6 MB ZIP). [file pbio.0020329.sd004.zip › 040604_OS5_st1_390.tif]

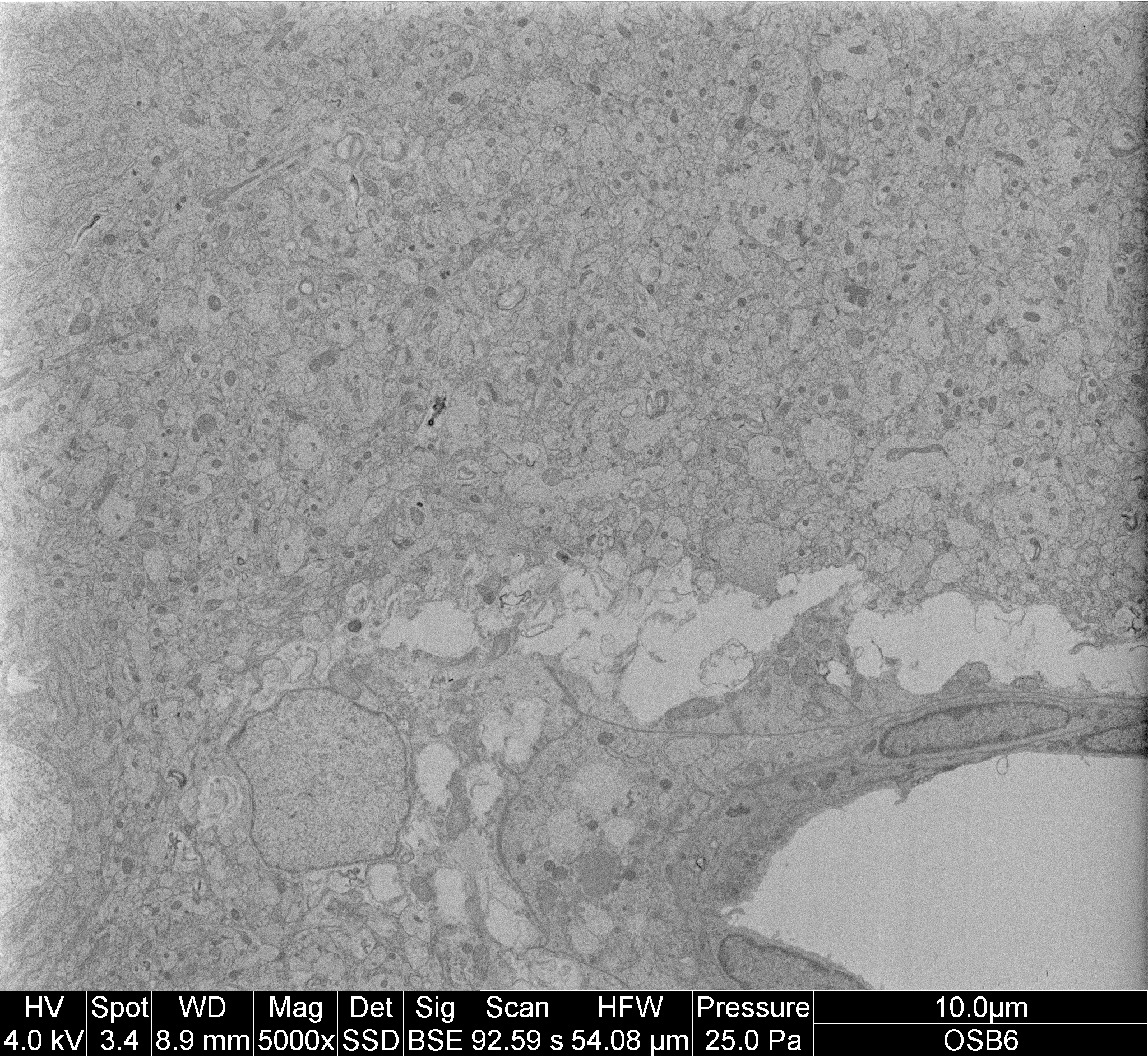

Supplement: Dataset S4 — (252.6 MB ZIP). [file pbio.0020329.sd004.zip › 040604_OS5_st1_391.tif]

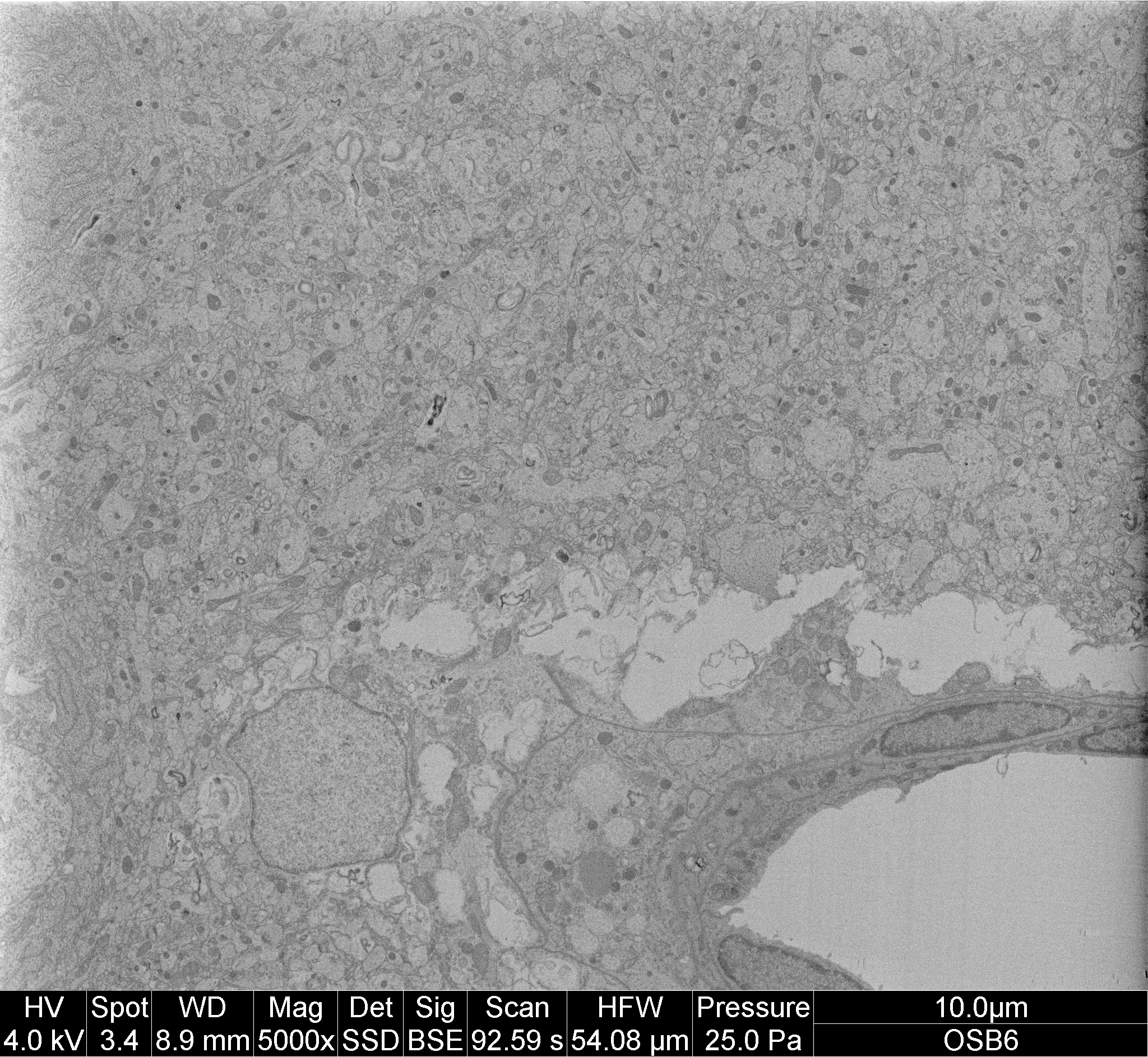

Supplement: Dataset S4 — (252.6 MB ZIP). [file pbio.0020329.sd004.zip › 040604_OS5_st1_392.tif]

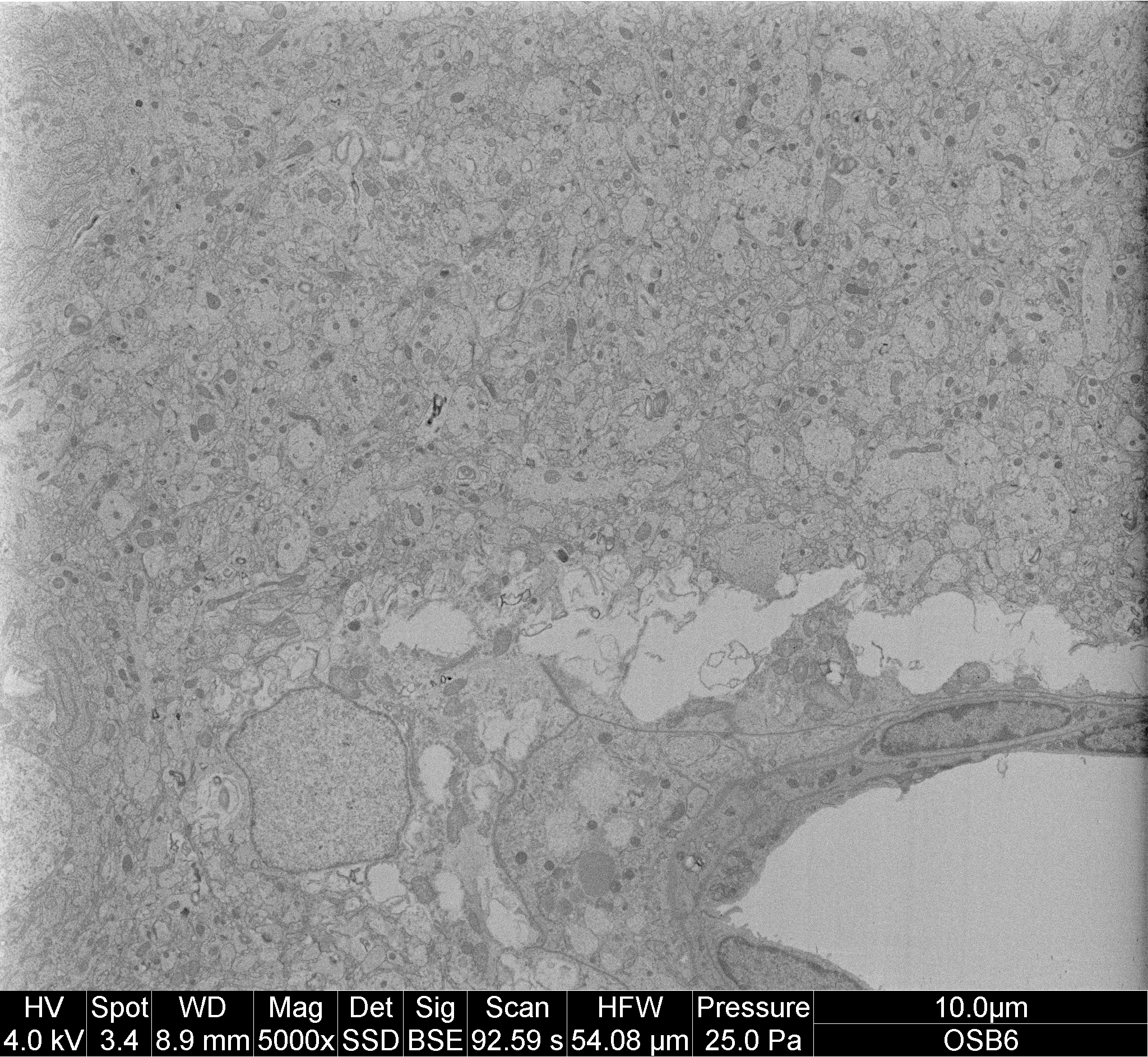

Supplement: Dataset S4 — (252.6 MB ZIP). [file pbio.0020329.sd004.zip › 040604_OS5_st1_393.tif]

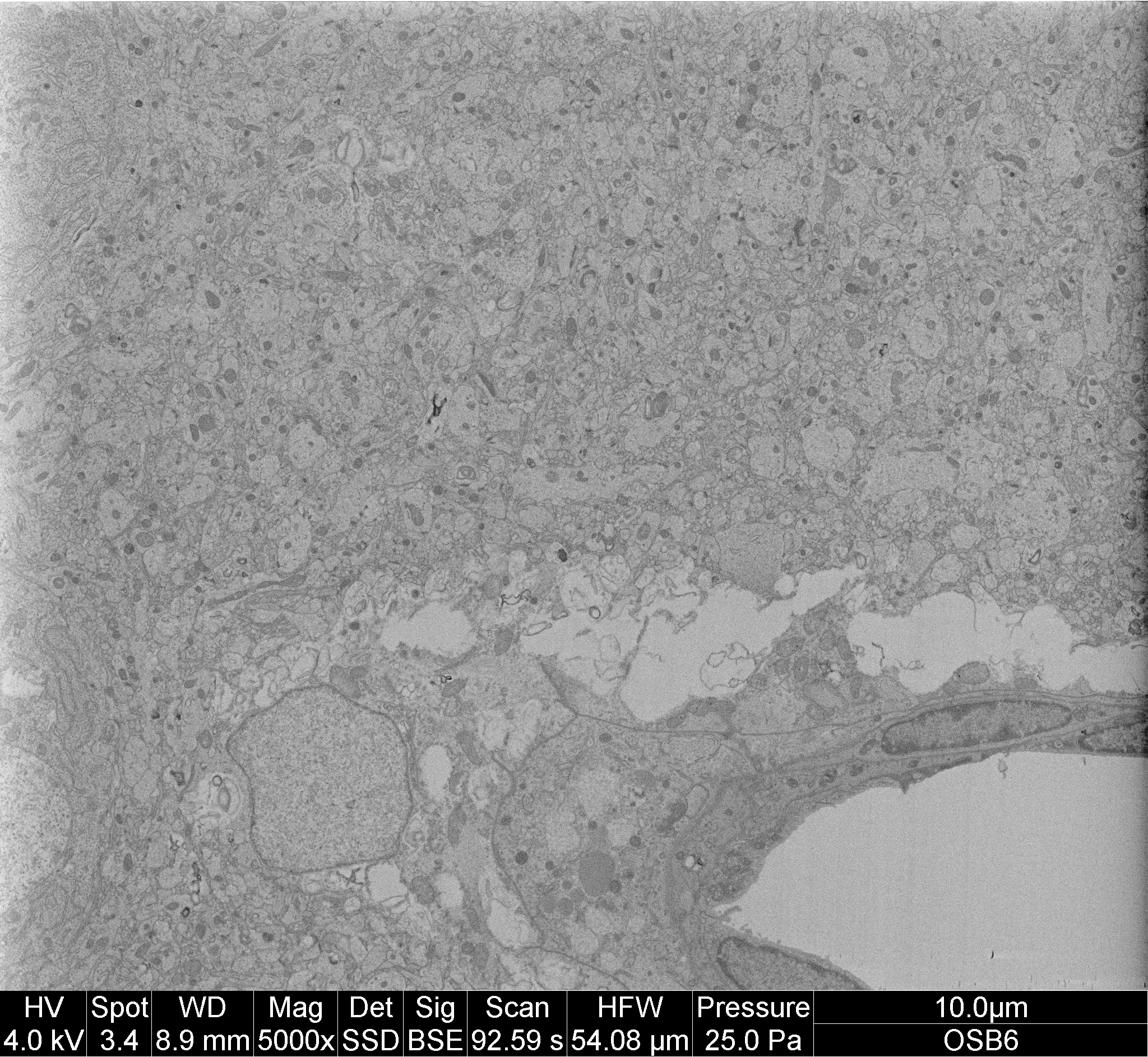

Supplement: Dataset S4 — (252.6 MB ZIP). [file pbio.0020329.sd004.zip › 040604_OS5_st1_394.tif]

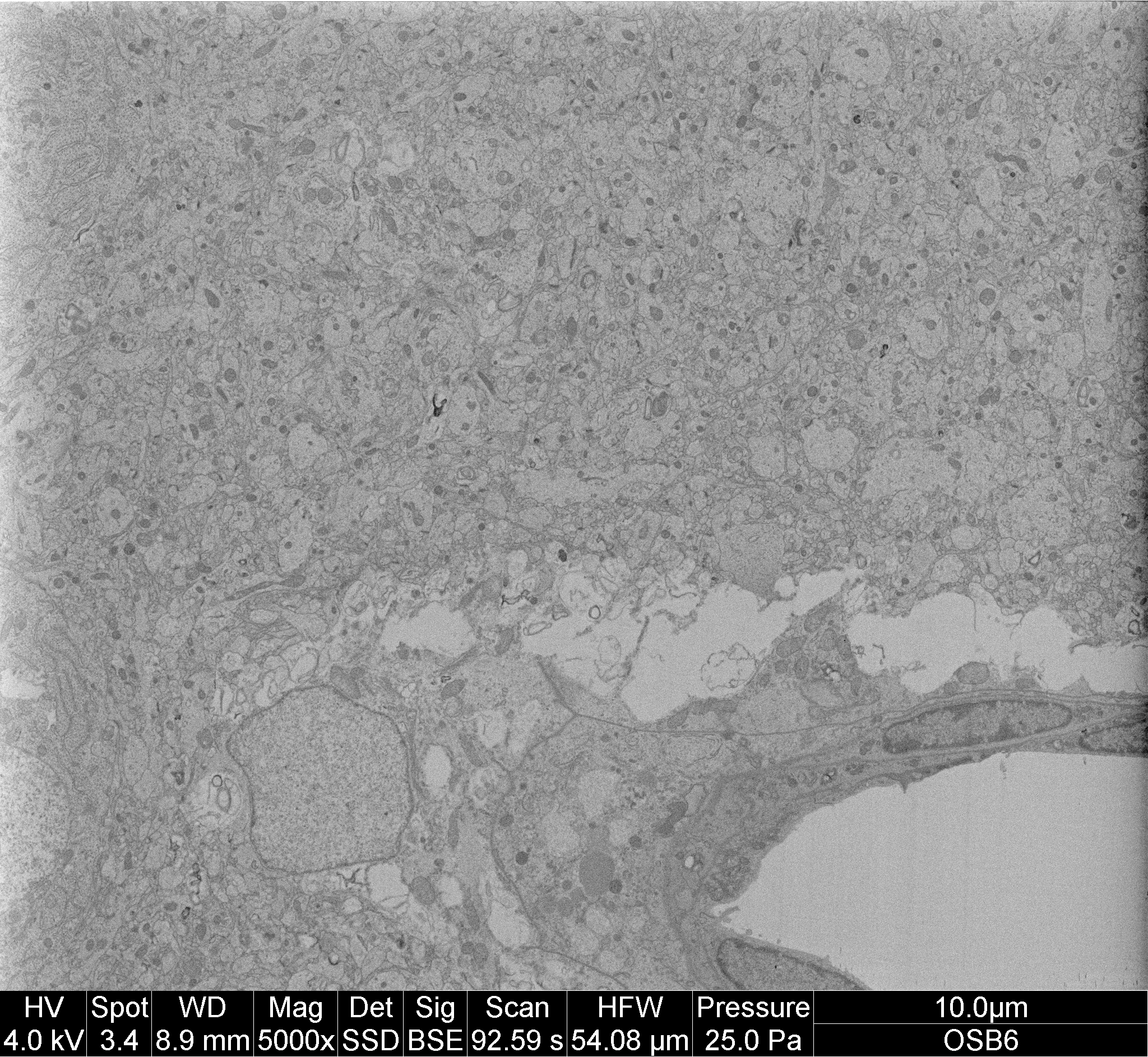

Supplement: Dataset S4 — (252.6 MB ZIP). [file pbio.0020329.sd004.zip › 040604_OS5_st1_395.tif]

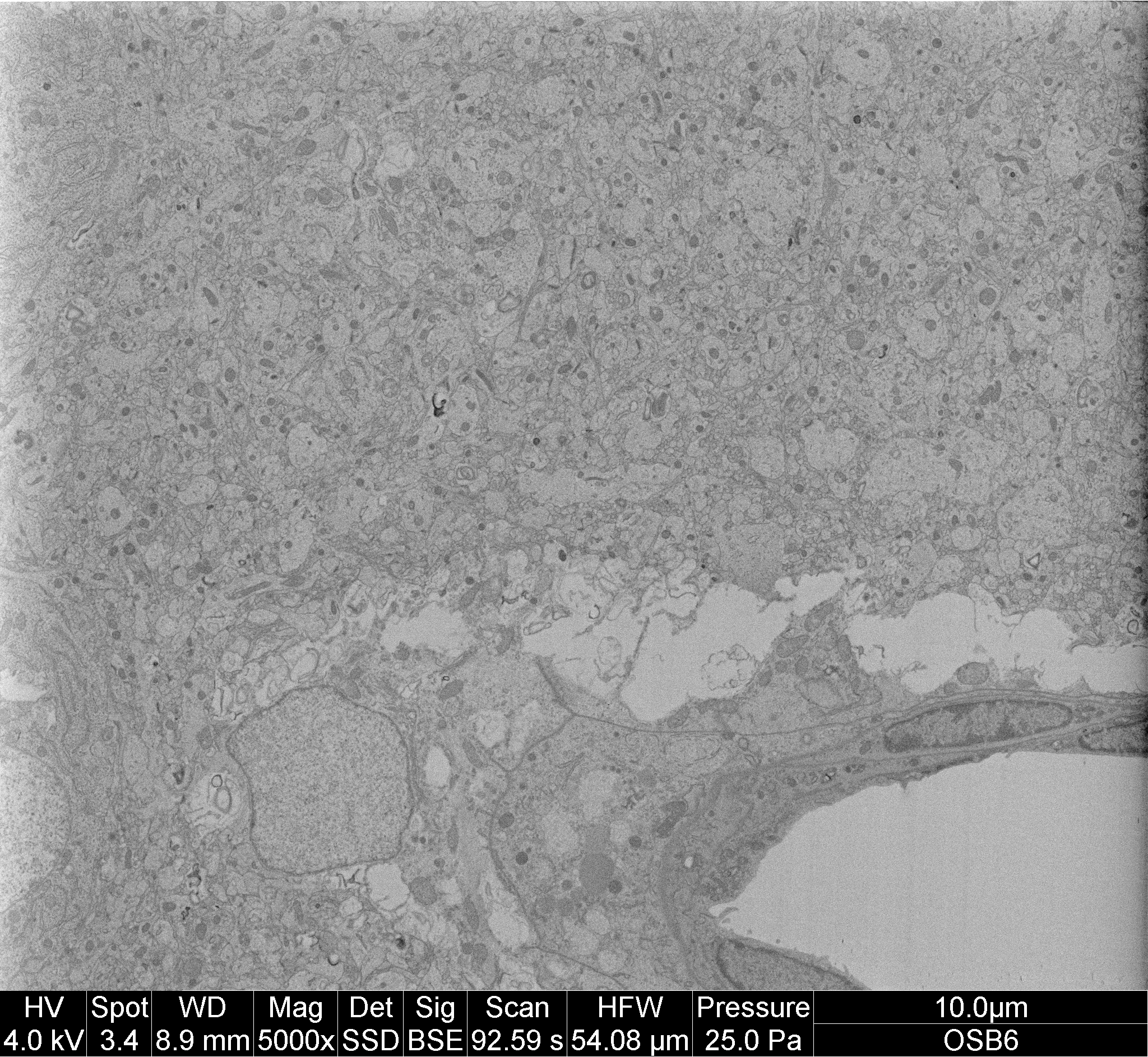

Supplement: Dataset S4 — (252.6 MB ZIP). [file pbio.0020329.sd004.zip › 040604_OS5_st1_396.tif]

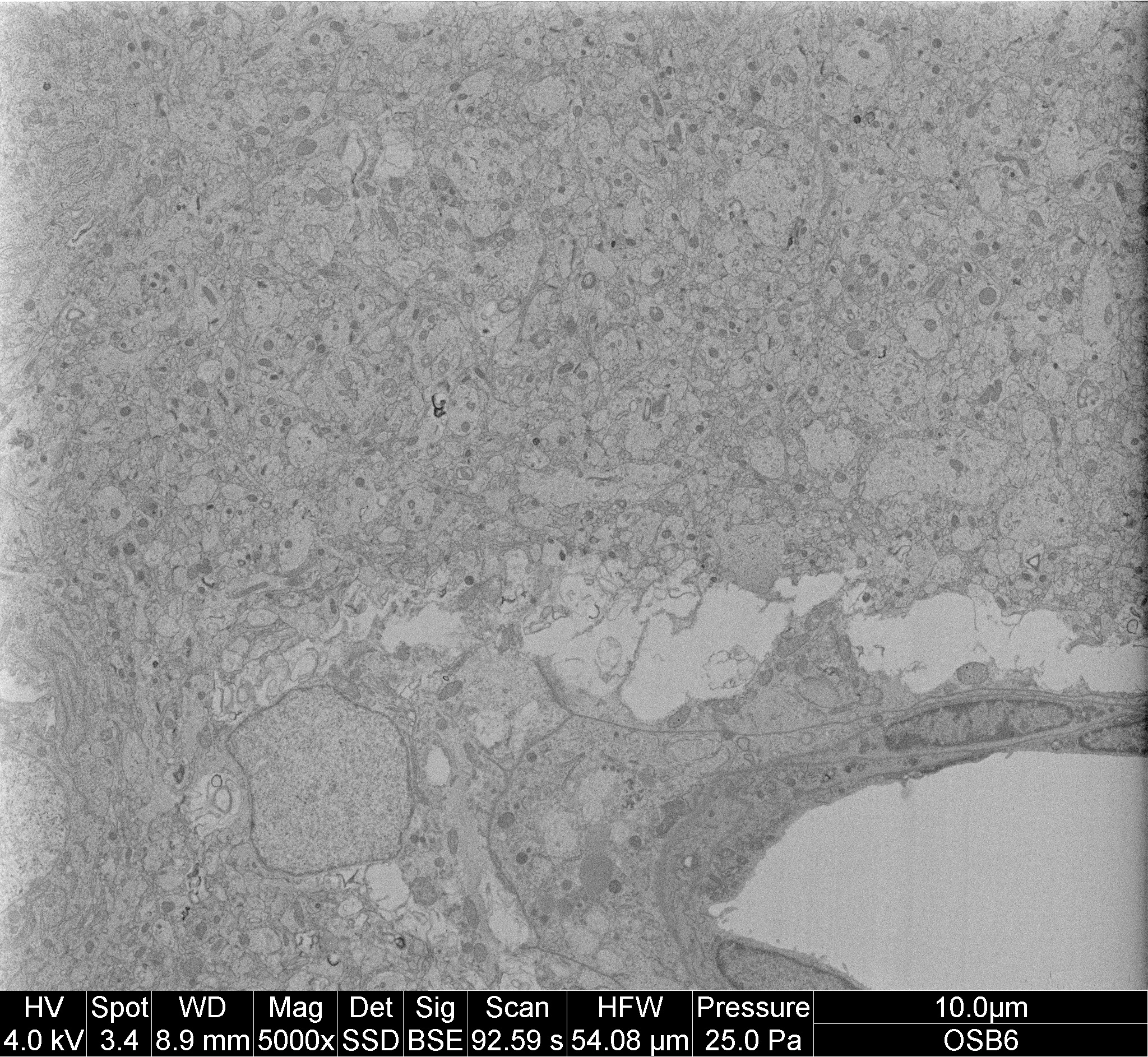

Supplement: Dataset S4 — (252.6 MB ZIP). [file pbio.0020329.sd004.zip › 040604_OS5_st1_397.tif]

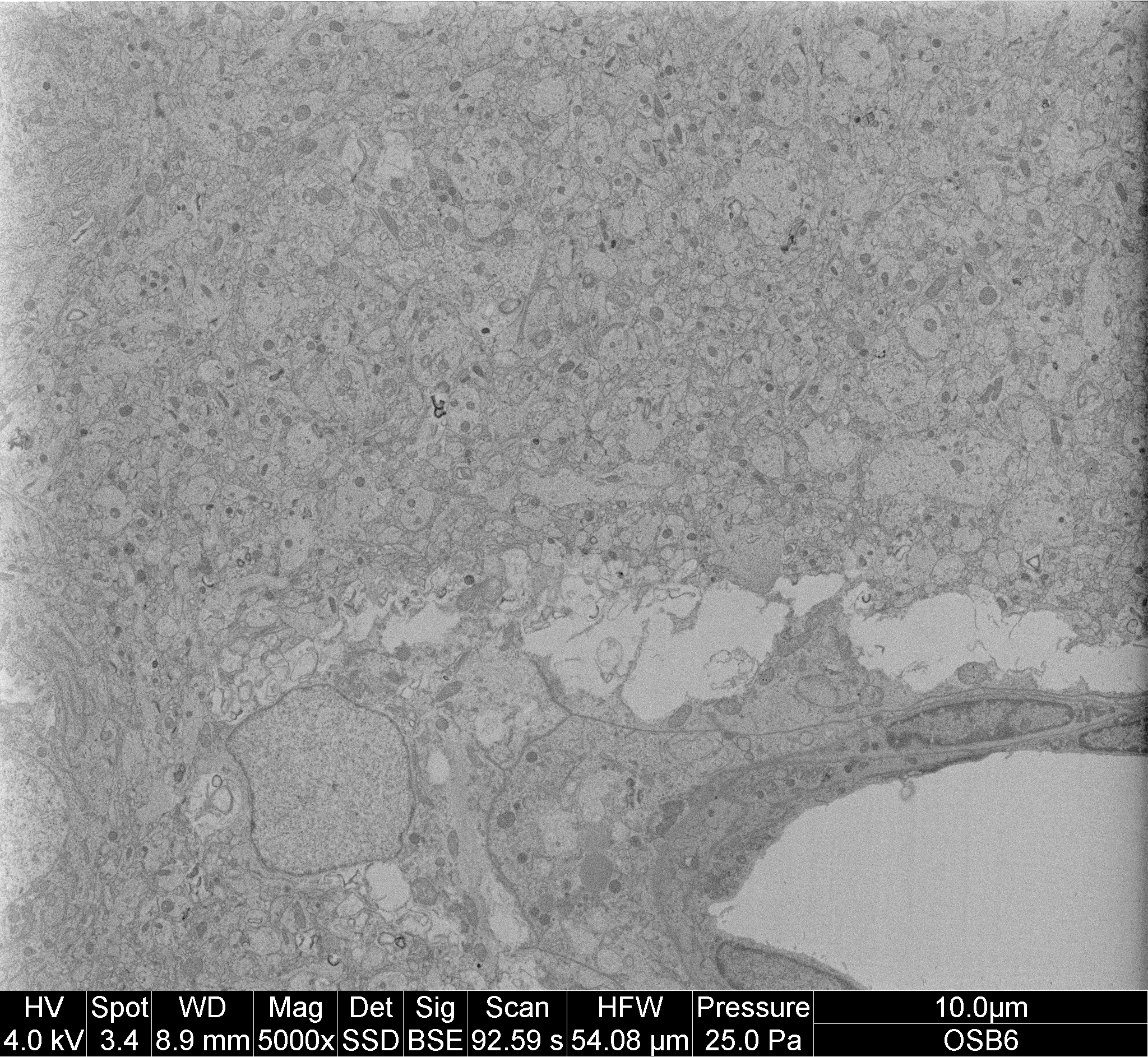

Supplement: Dataset S4 — (252.6 MB ZIP). [file pbio.0020329.sd004.zip › 040604_OS5_st1_398.tif]

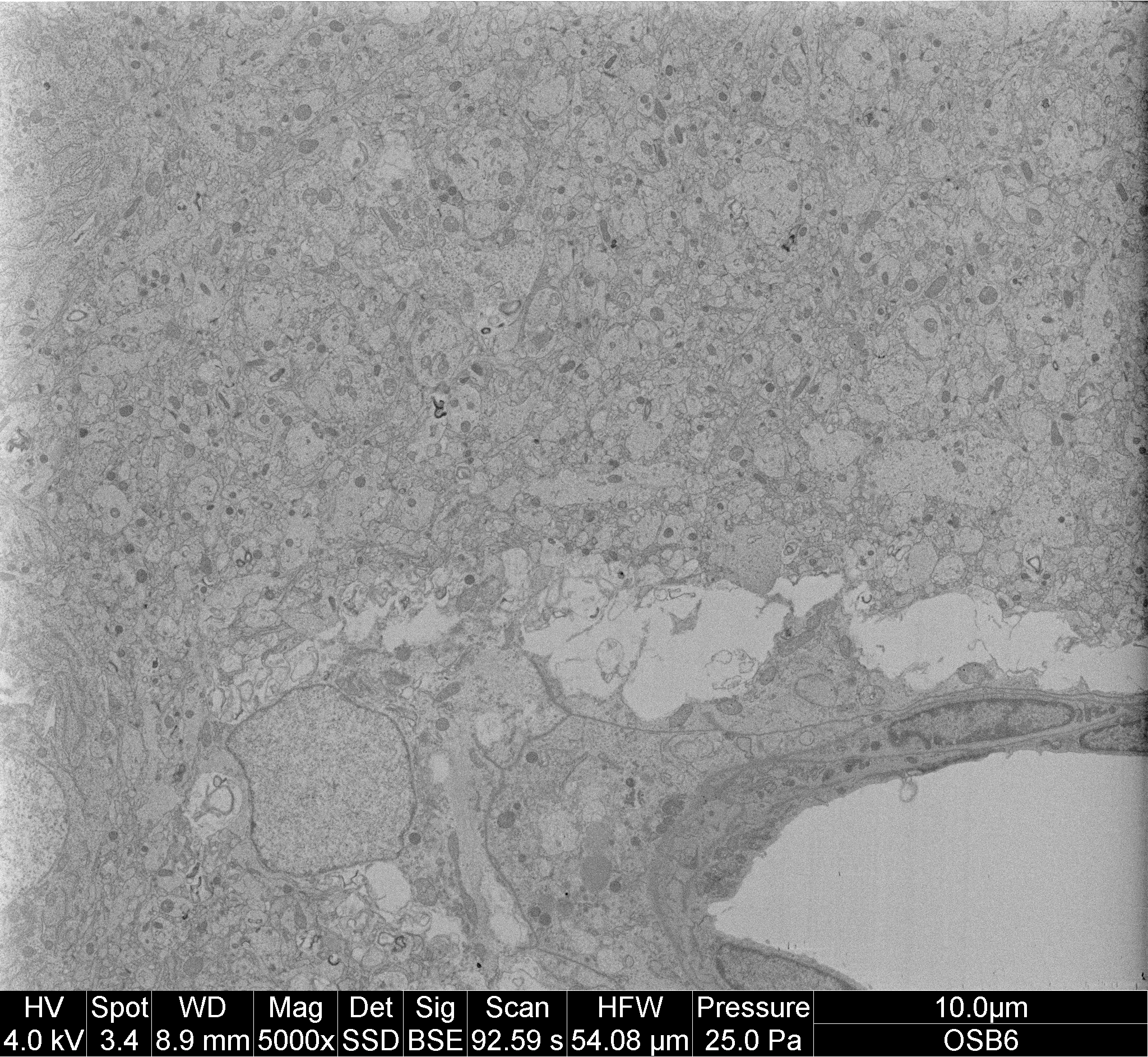

Supplement: Dataset S4 — (252.6 MB ZIP). [file pbio.0020329.sd004.zip › 040604_OS5_st1_399.tif]

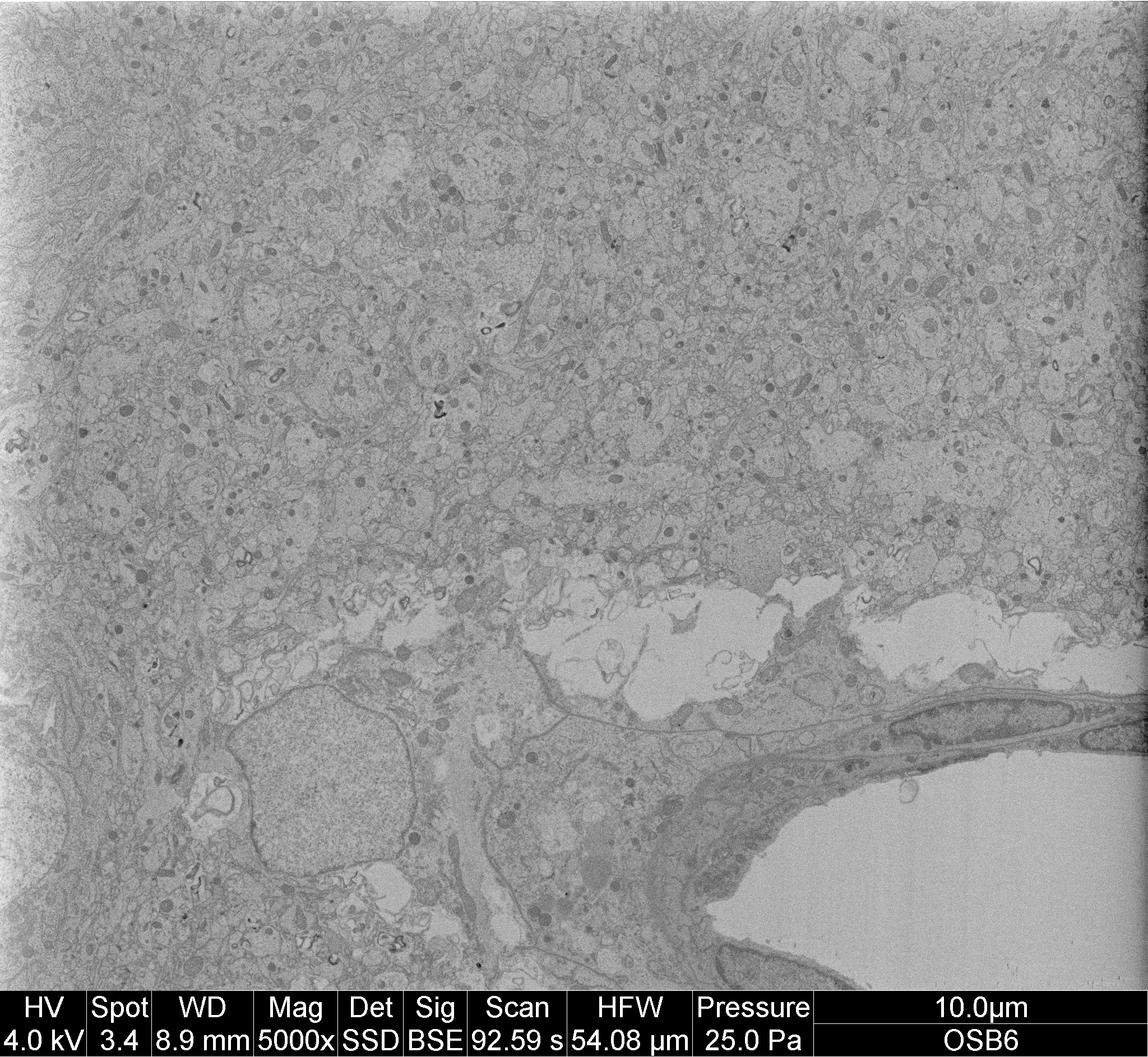

Supplement: Dataset S5 — (251.9 MB ZIP). [file pbio.0020329.sd005.zip › 040604_OS5_st1_400.tif]
