# Supplementary figures and images for: Serial Block-Face Scanning Electron Microscopy to Reconstruct Three-Dimensional Tissue Nanostructure (part 5 of 21)
Source: PLoS Biol. 2004 Oct 19;2(11):e329. doi: 10.1371/journal.pbio.0020329 (PMC524270; doi:10.1371/journal.pbio.0020329)

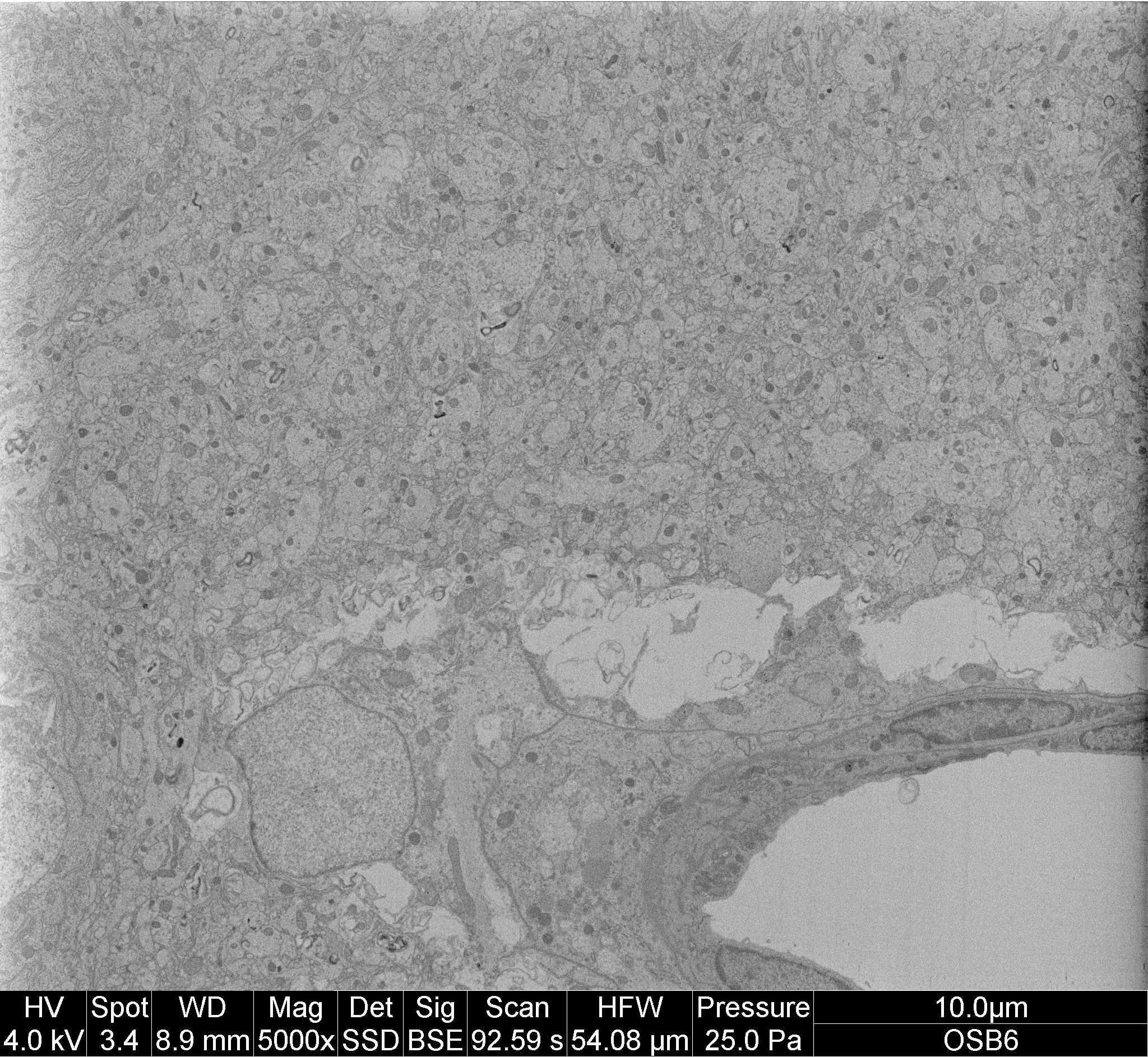

Supplement: Dataset S5 — (251.9 MB ZIP). [file pbio.0020329.sd005.zip › 040604_OS5_st1_401.tif]

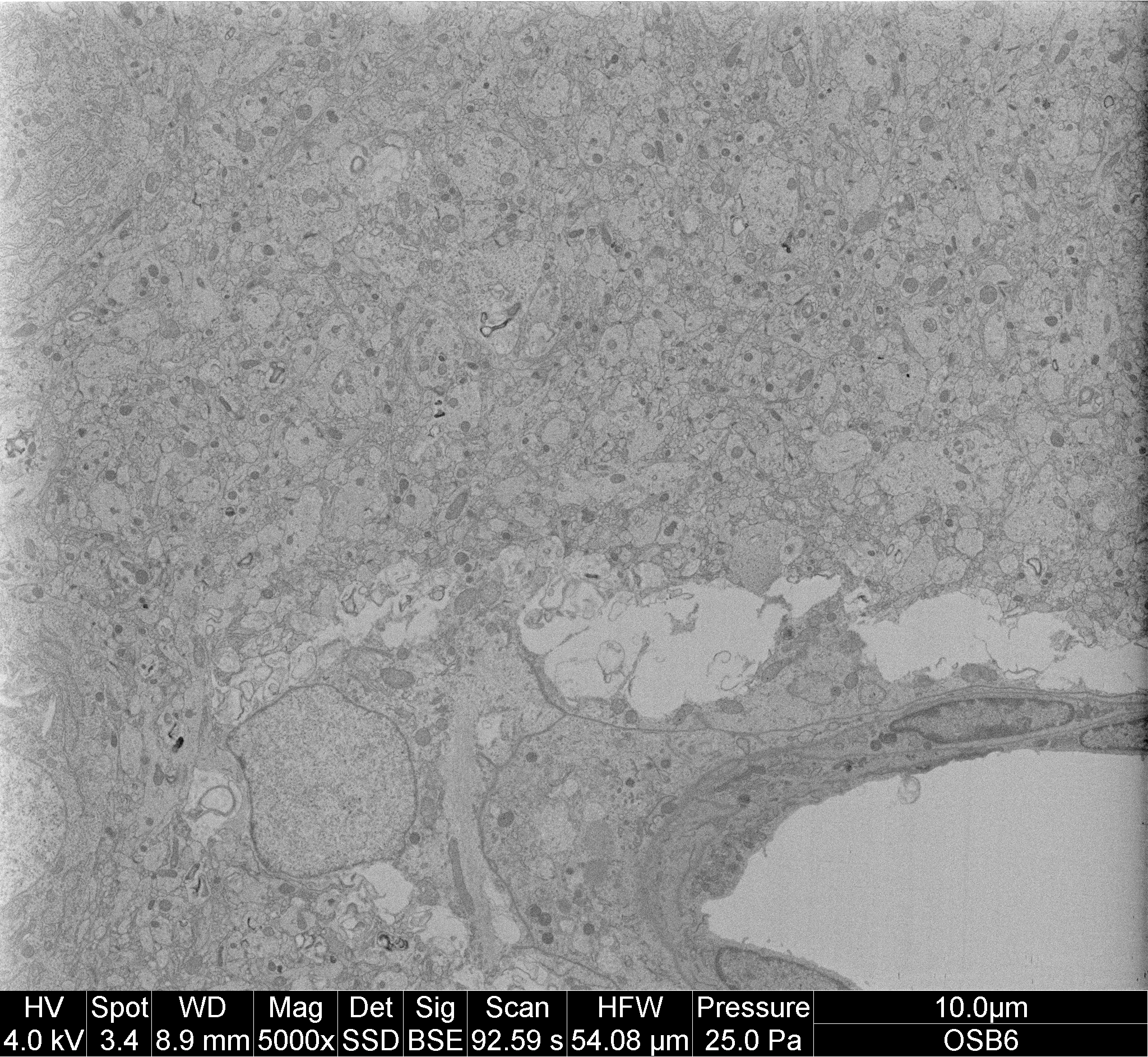

Supplement: Dataset S5 — (251.9 MB ZIP). [file pbio.0020329.sd005.zip › 040604_OS5_st1_402.tif]

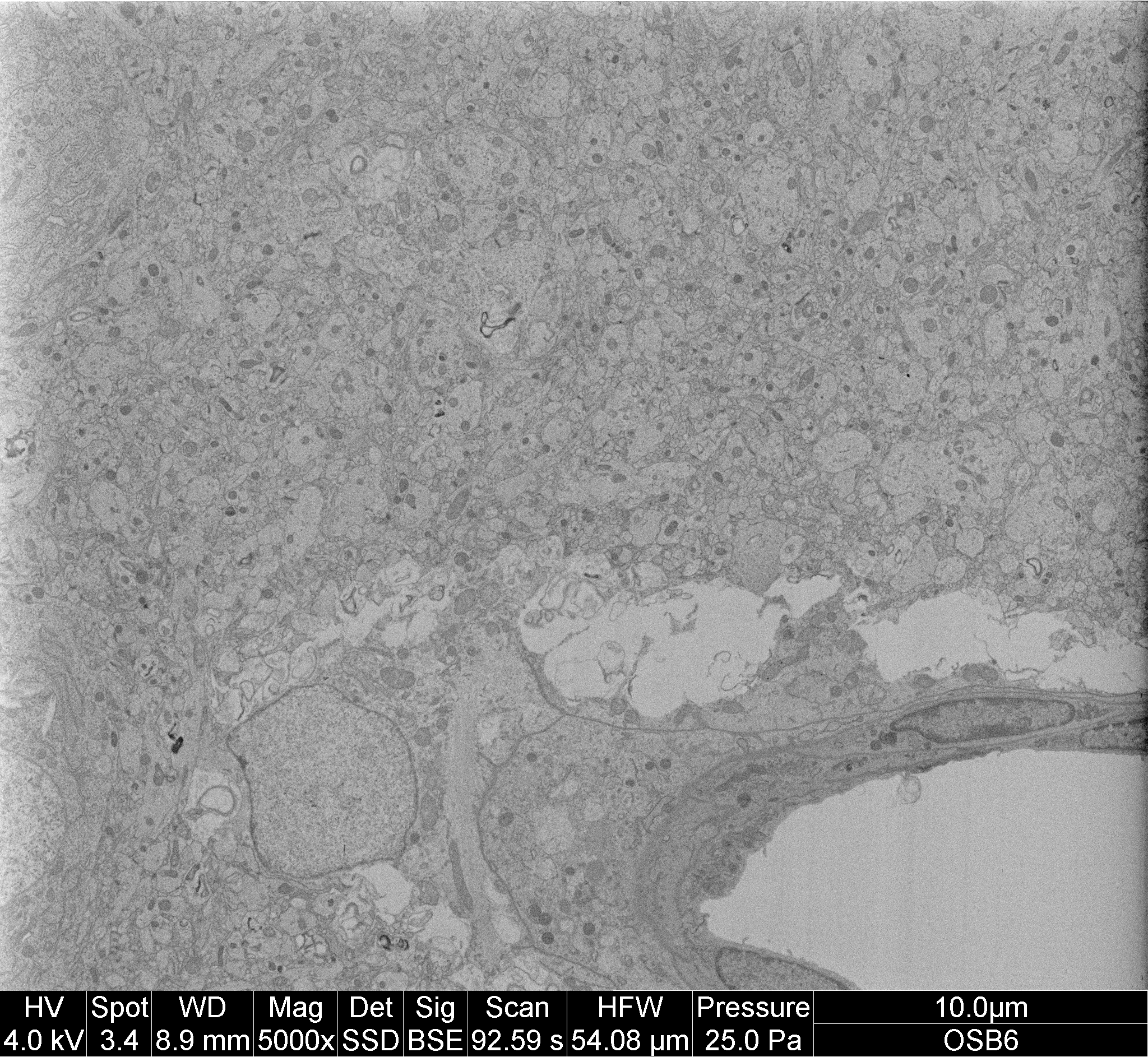

Supplement: Dataset S5 — (251.9 MB ZIP). [file pbio.0020329.sd005.zip › 040604_OS5_st1_403.tif]

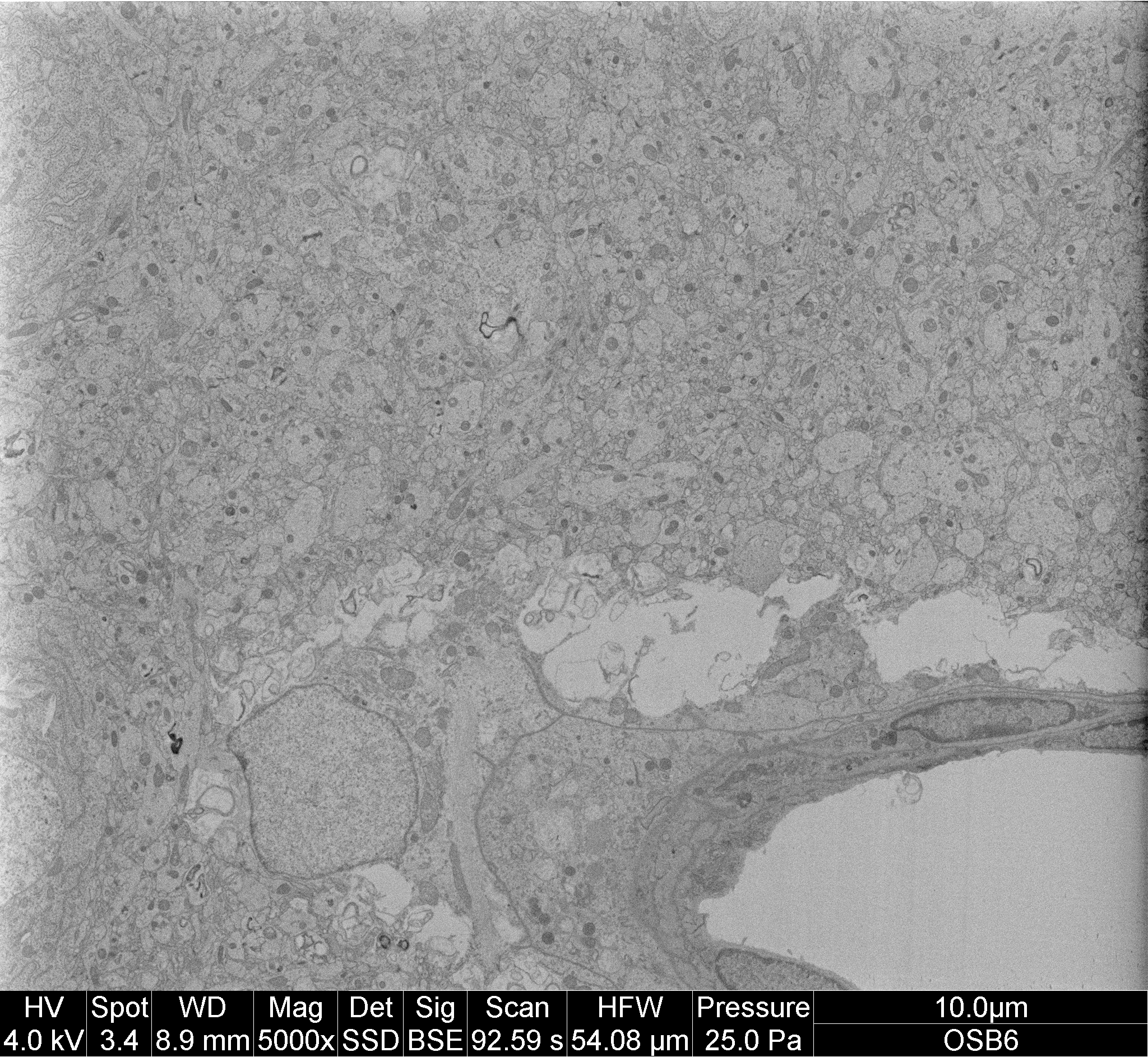

Supplement: Dataset S5 — (251.9 MB ZIP). [file pbio.0020329.sd005.zip › 040604_OS5_st1_404.tif]

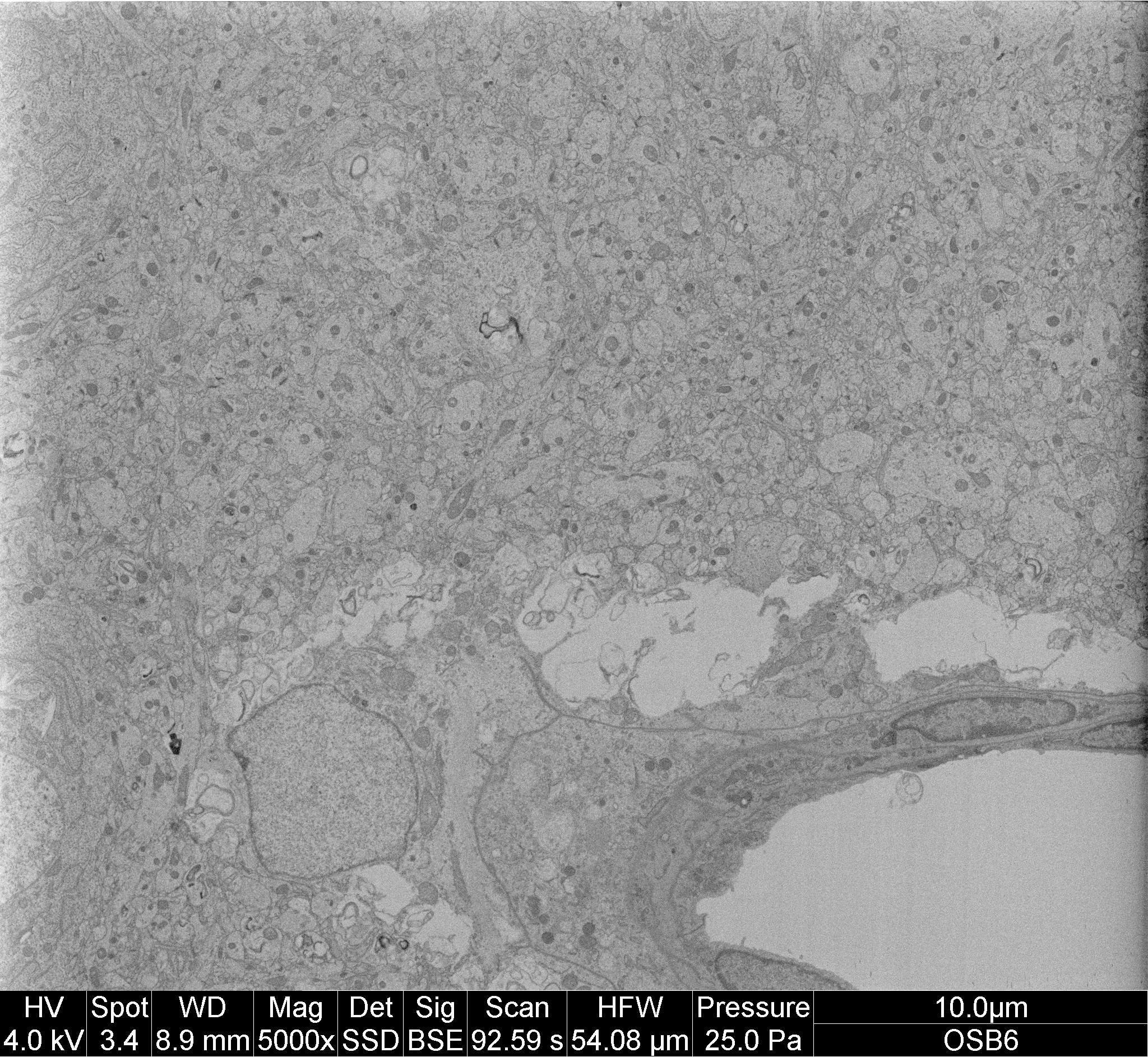

Supplement: Dataset S5 — (251.9 MB ZIP). [file pbio.0020329.sd005.zip › 040604_OS5_st1_405.tif]

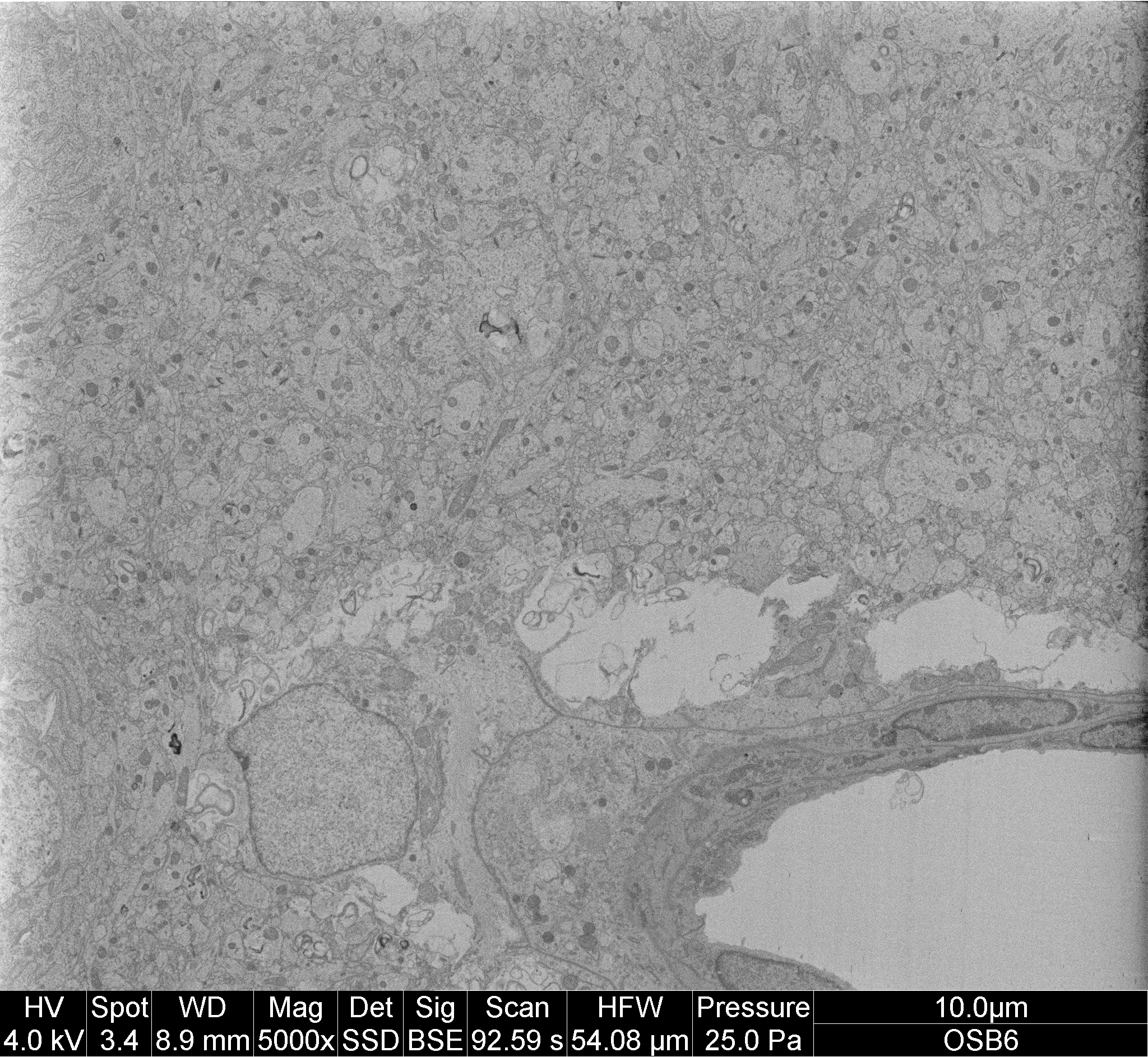

Supplement: Dataset S5 — (251.9 MB ZIP). [file pbio.0020329.sd005.zip › 040604_OS5_st1_406.tif]

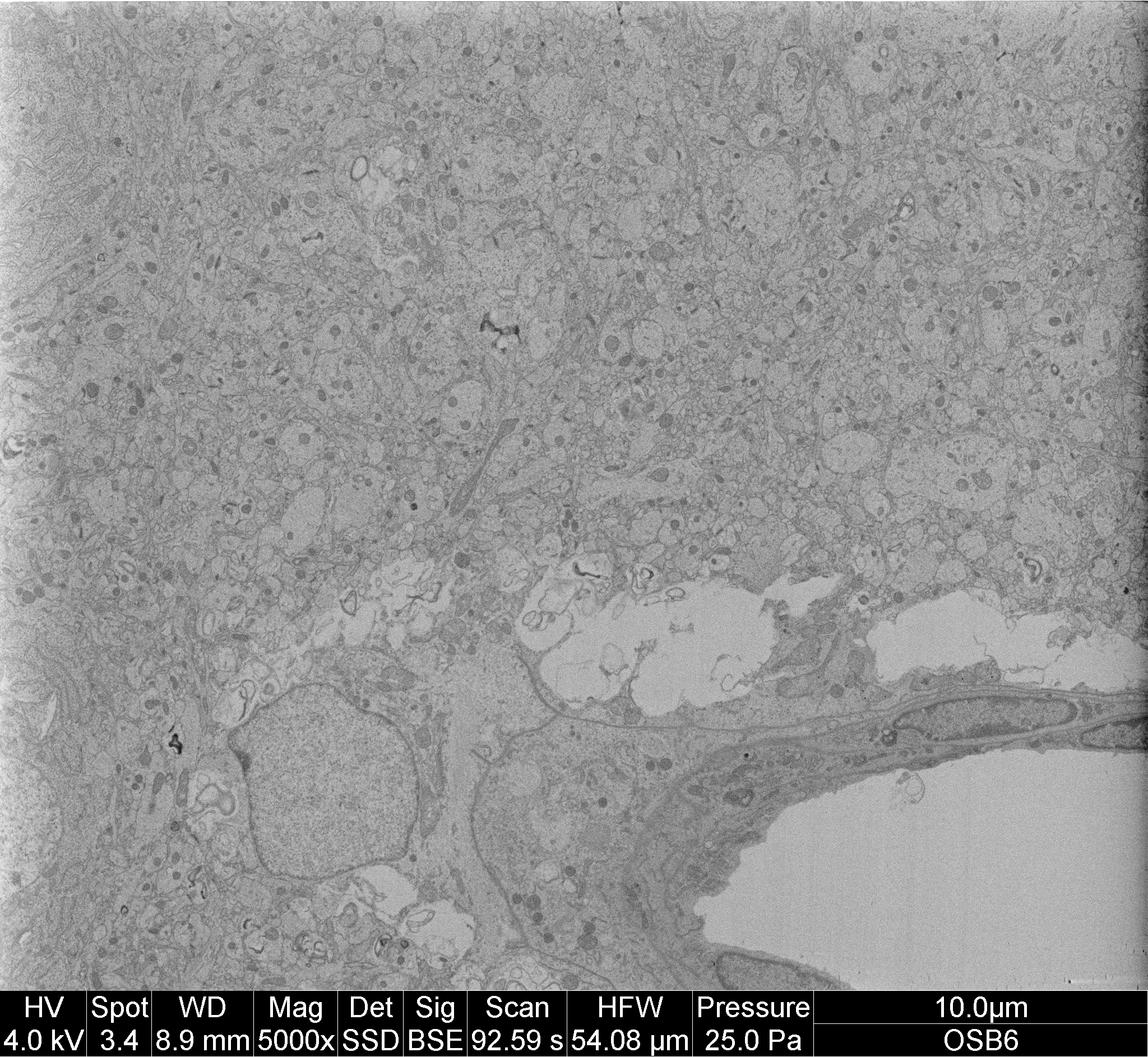

Supplement: Dataset S5 — (251.9 MB ZIP). [file pbio.0020329.sd005.zip › 040604_OS5_st1_407.tif]

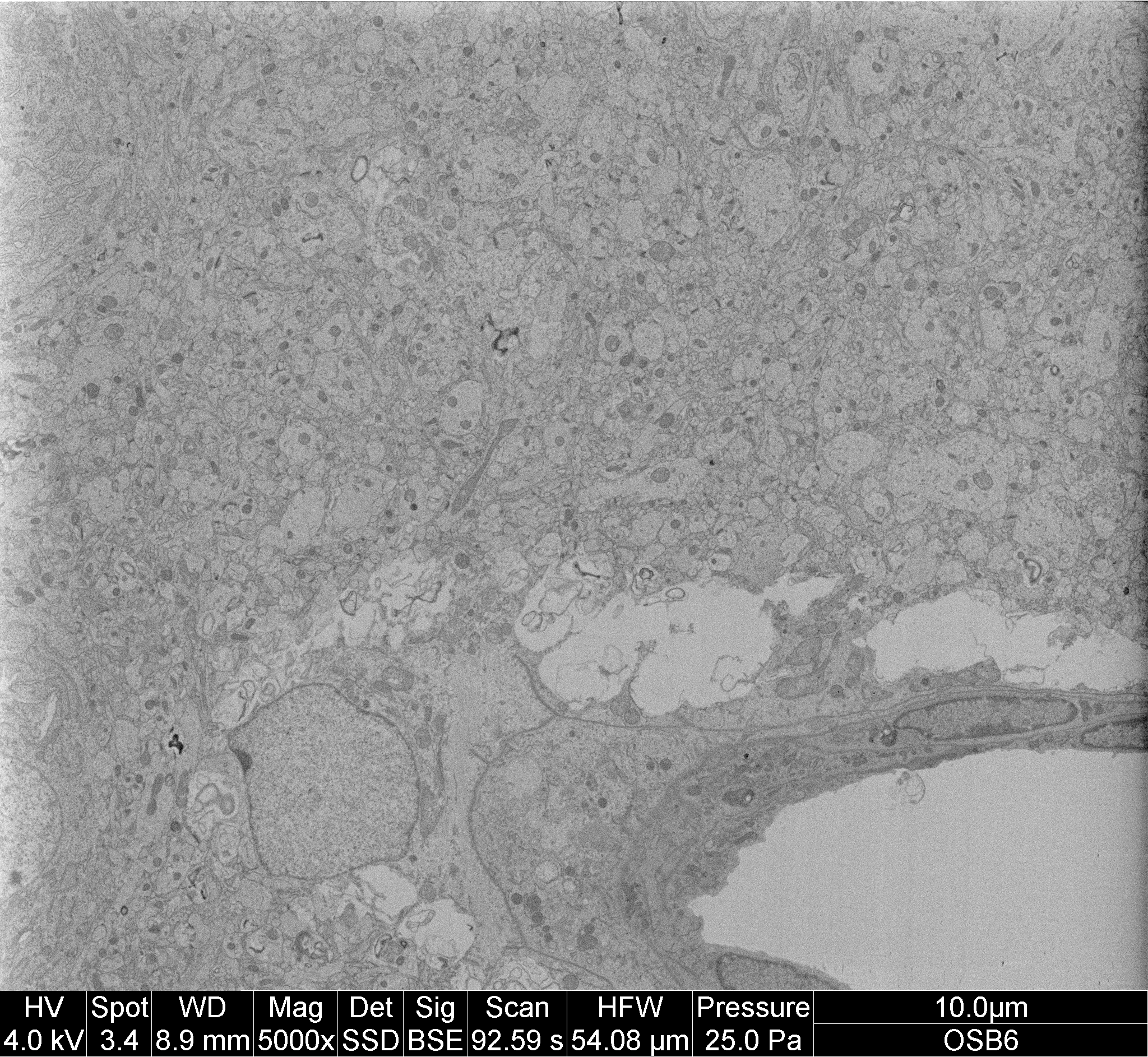

Supplement: Dataset S5 — (251.9 MB ZIP). [file pbio.0020329.sd005.zip › 040604_OS5_st1_408.tif]

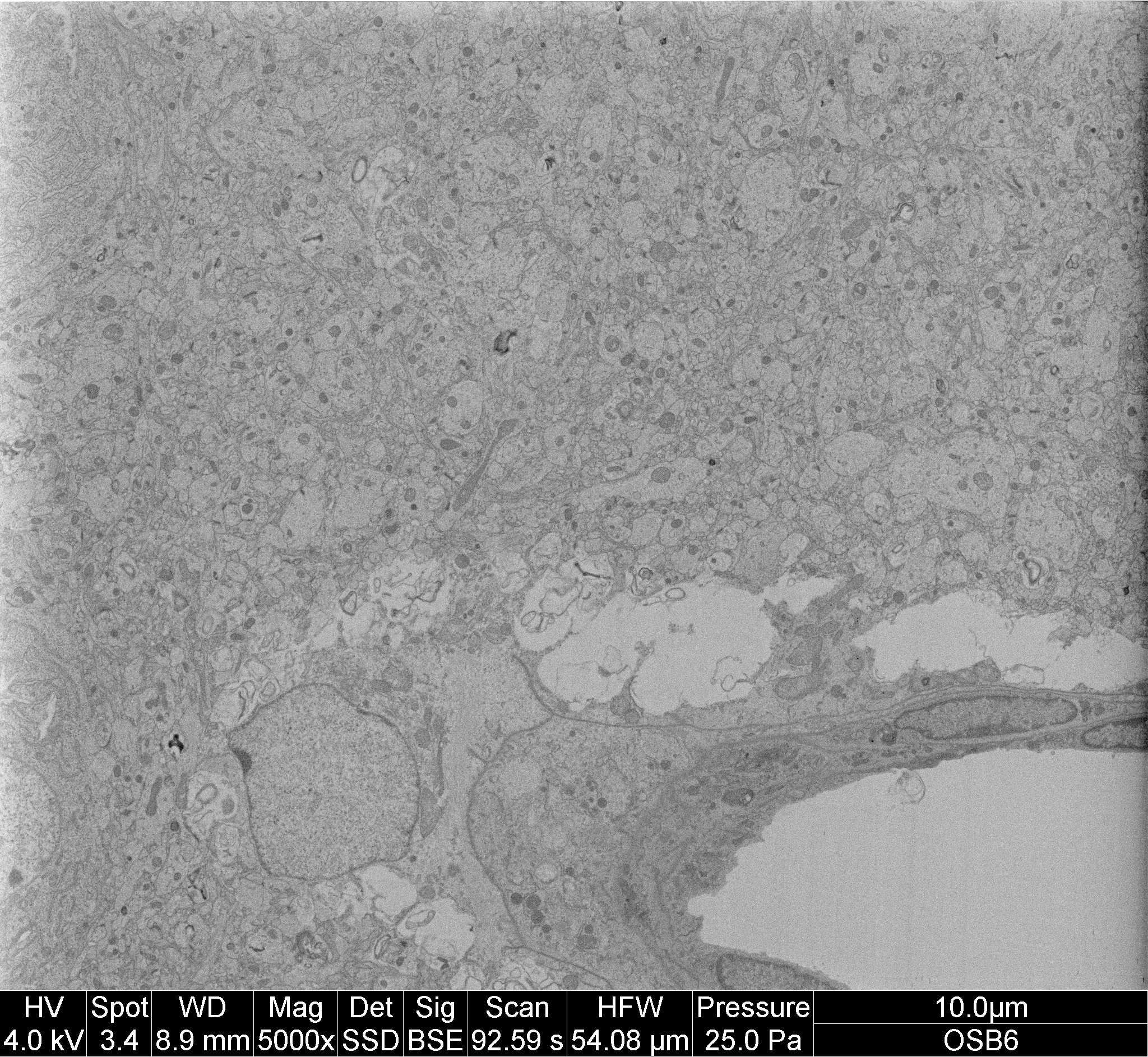

Supplement: Dataset S5 — (251.9 MB ZIP). [file pbio.0020329.sd005.zip › 040604_OS5_st1_409.tif]

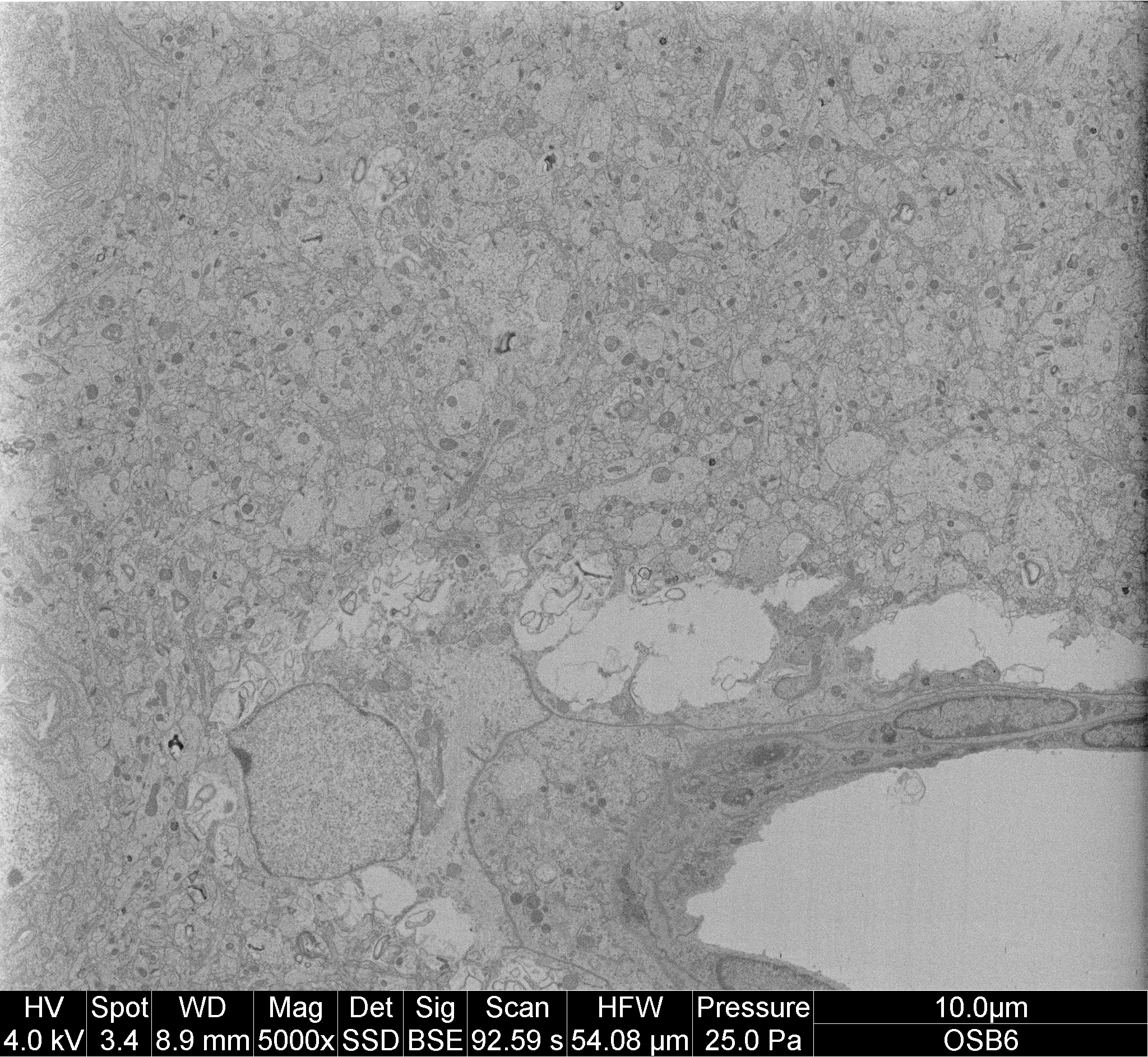

Supplement: Dataset S5 — (251.9 MB ZIP). [file pbio.0020329.sd005.zip › 040604_OS5_st1_410.tif]

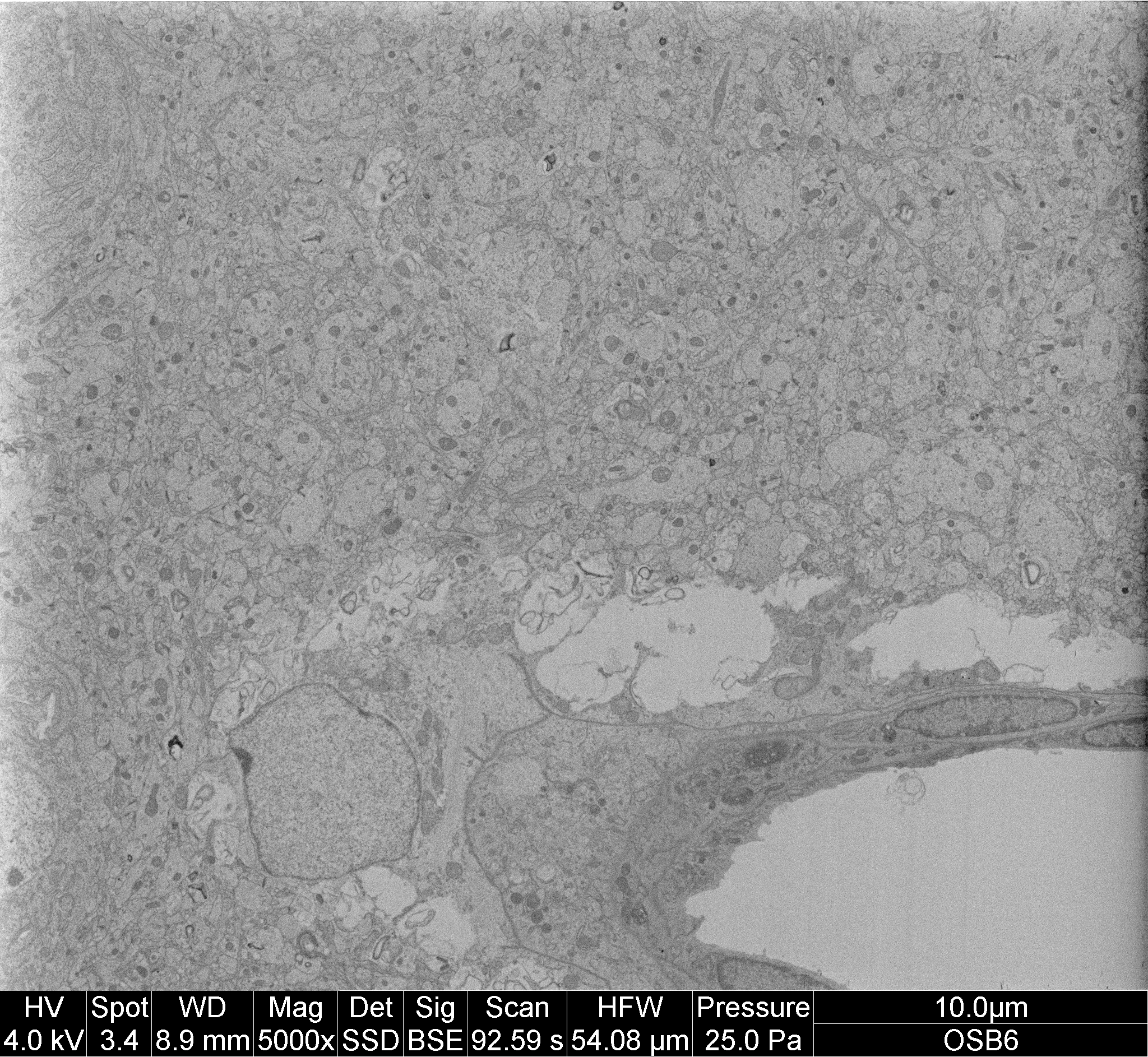

Supplement: Dataset S5 — (251.9 MB ZIP). [file pbio.0020329.sd005.zip › 040604_OS5_st1_411.tif]

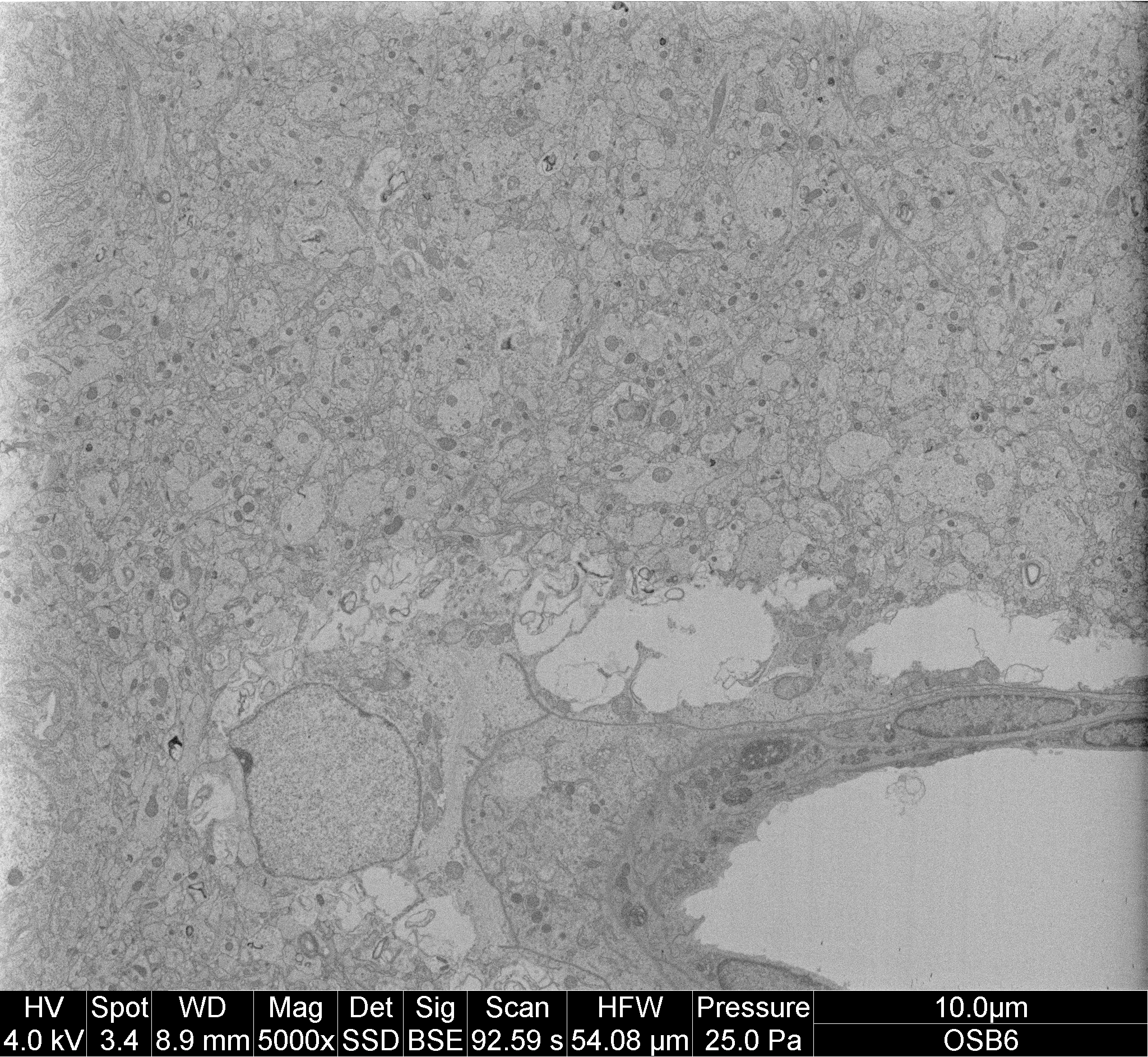

Supplement: Dataset S5 — (251.9 MB ZIP). [file pbio.0020329.sd005.zip › 040604_OS5_st1_412.tif]

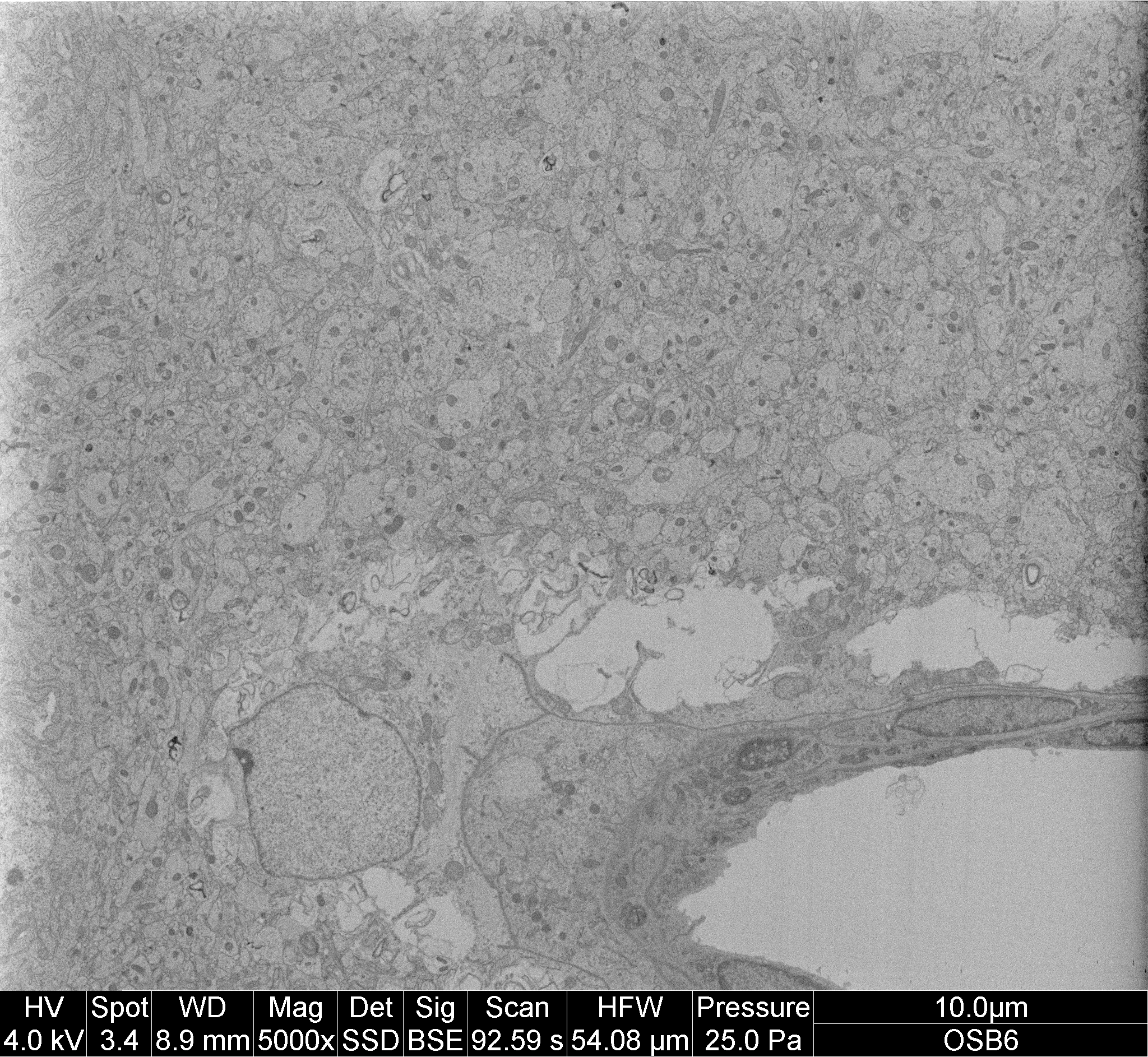

Supplement: Dataset S5 — (251.9 MB ZIP). [file pbio.0020329.sd005.zip › 040604_OS5_st1_413.tif]

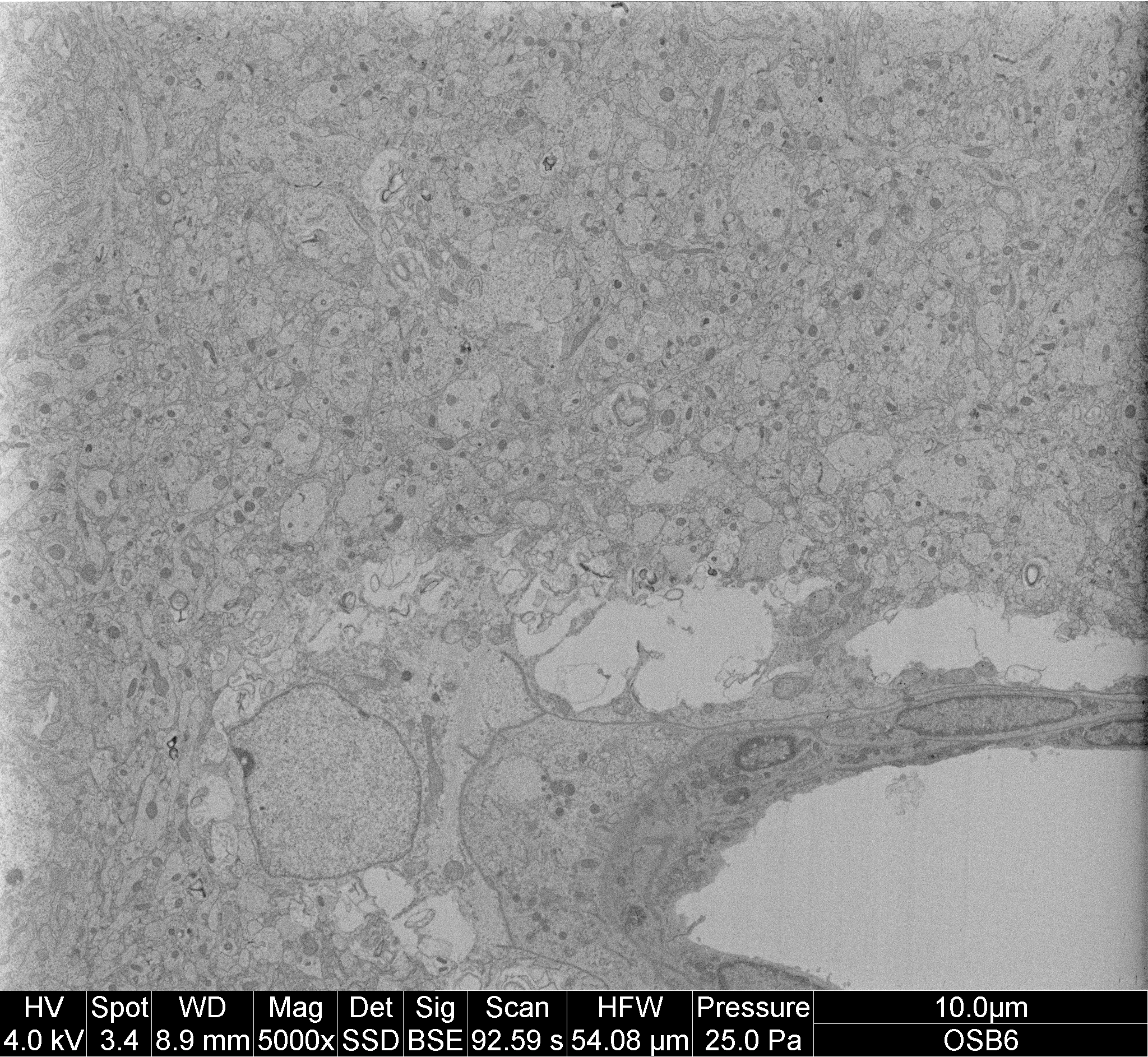

Supplement: Dataset S5 — (251.9 MB ZIP). [file pbio.0020329.sd005.zip › 040604_OS5_st1_414.tif]

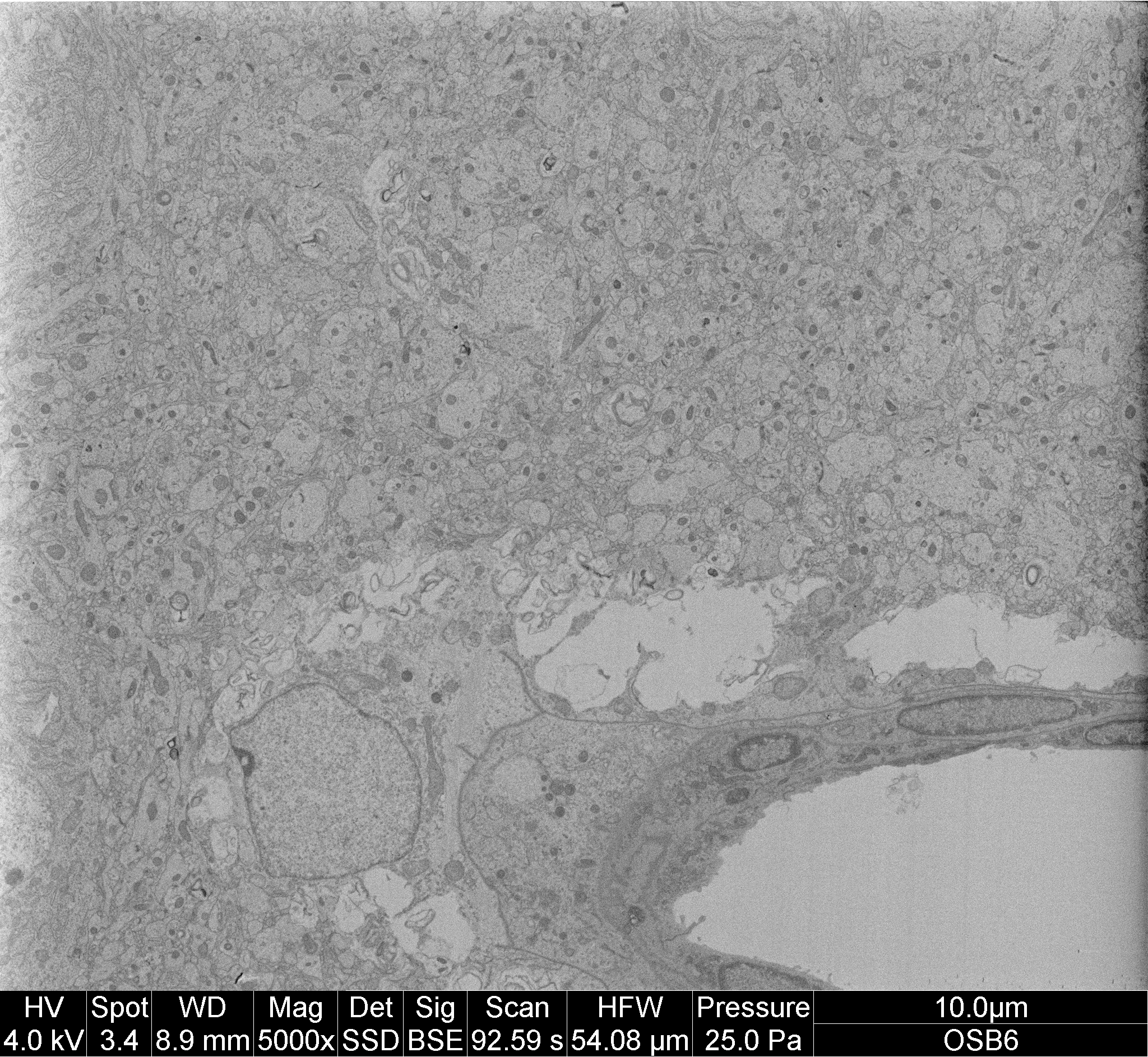

Supplement: Dataset S5 — (251.9 MB ZIP). [file pbio.0020329.sd005.zip › 040604_OS5_st1_415.tif]

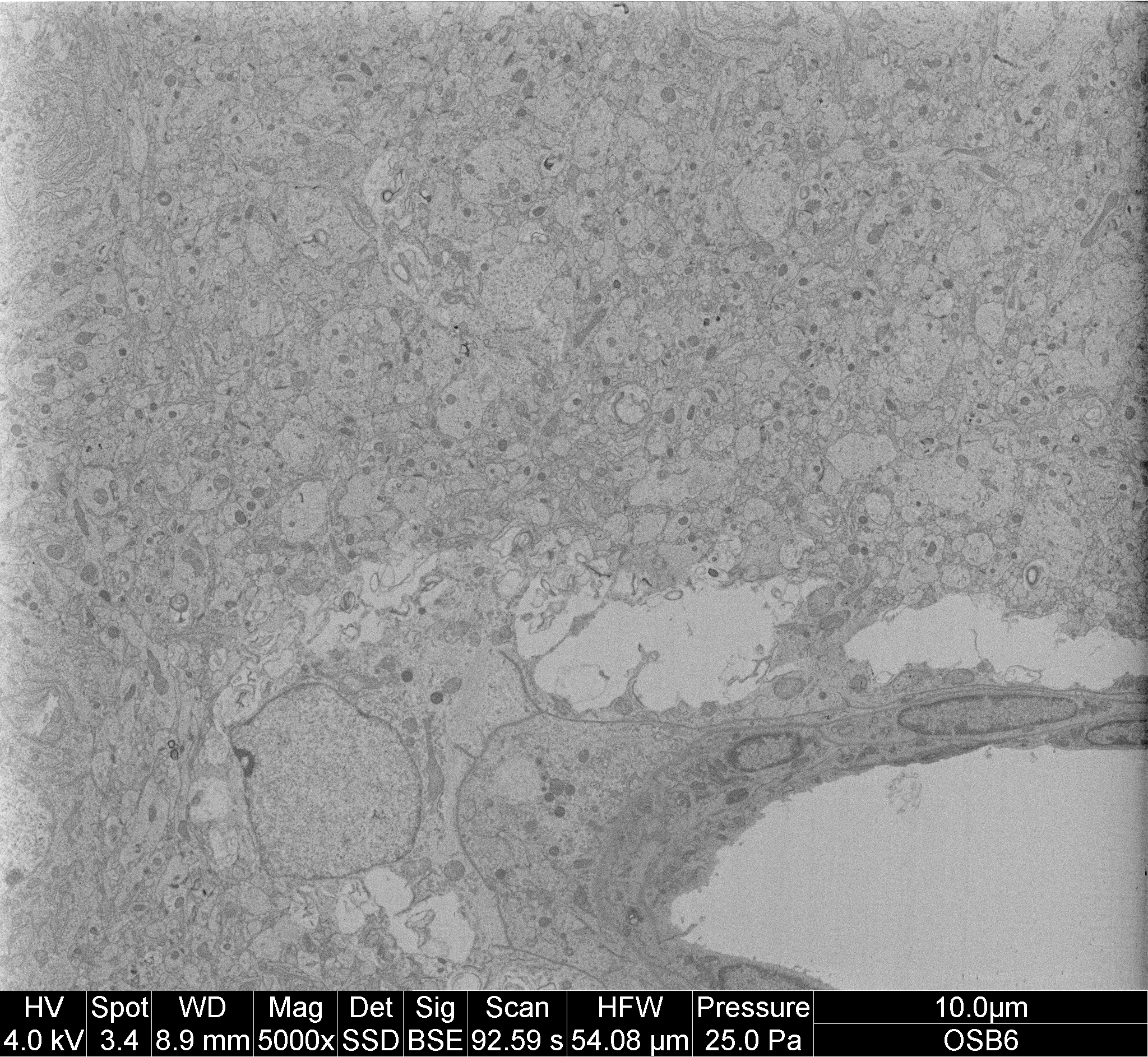

Supplement: Dataset S5 — (251.9 MB ZIP). [file pbio.0020329.sd005.zip › 040604_OS5_st1_416.tif]

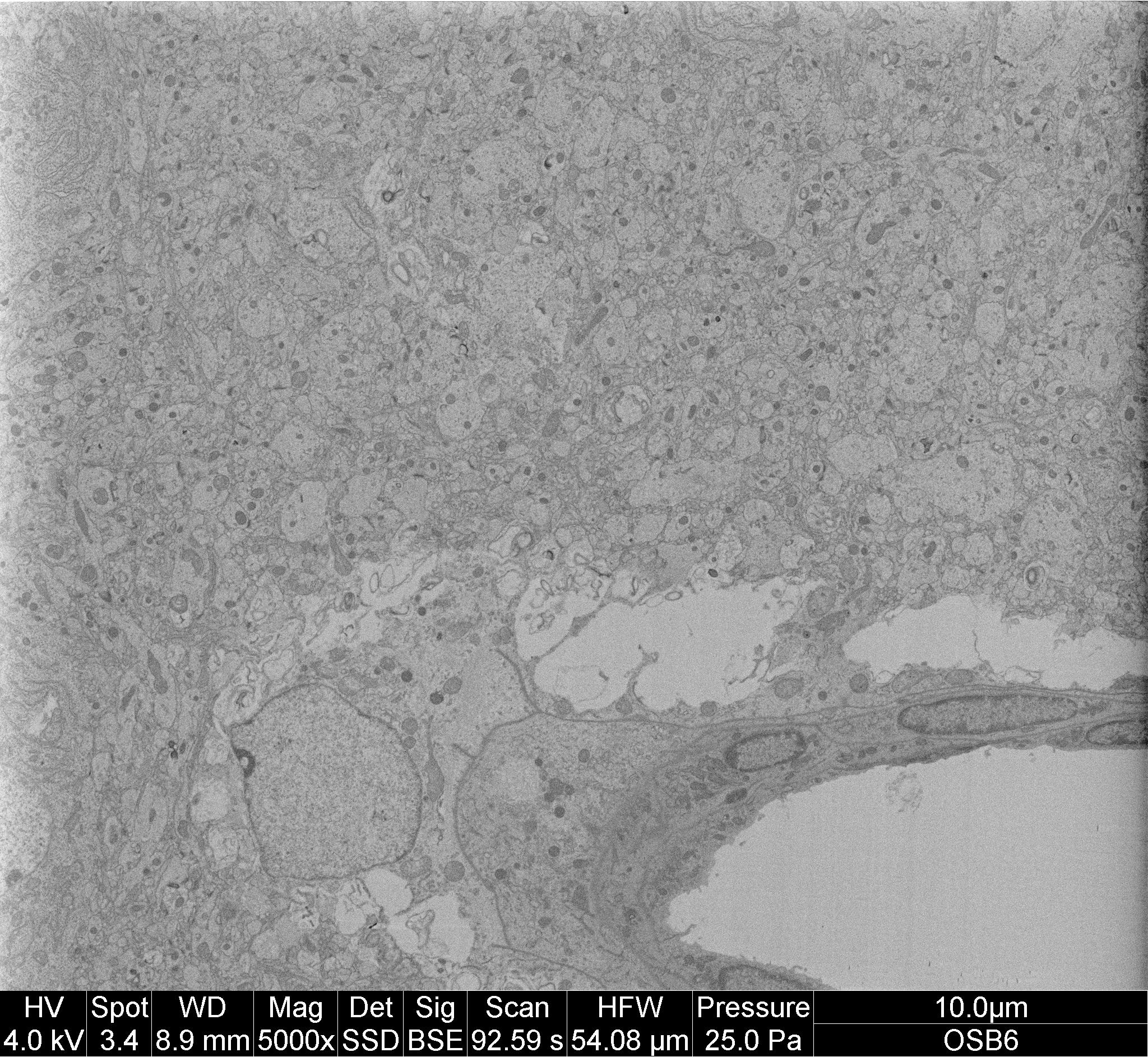

Supplement: Dataset S5 — (251.9 MB ZIP). [file pbio.0020329.sd005.zip › 040604_OS5_st1_417.tif]

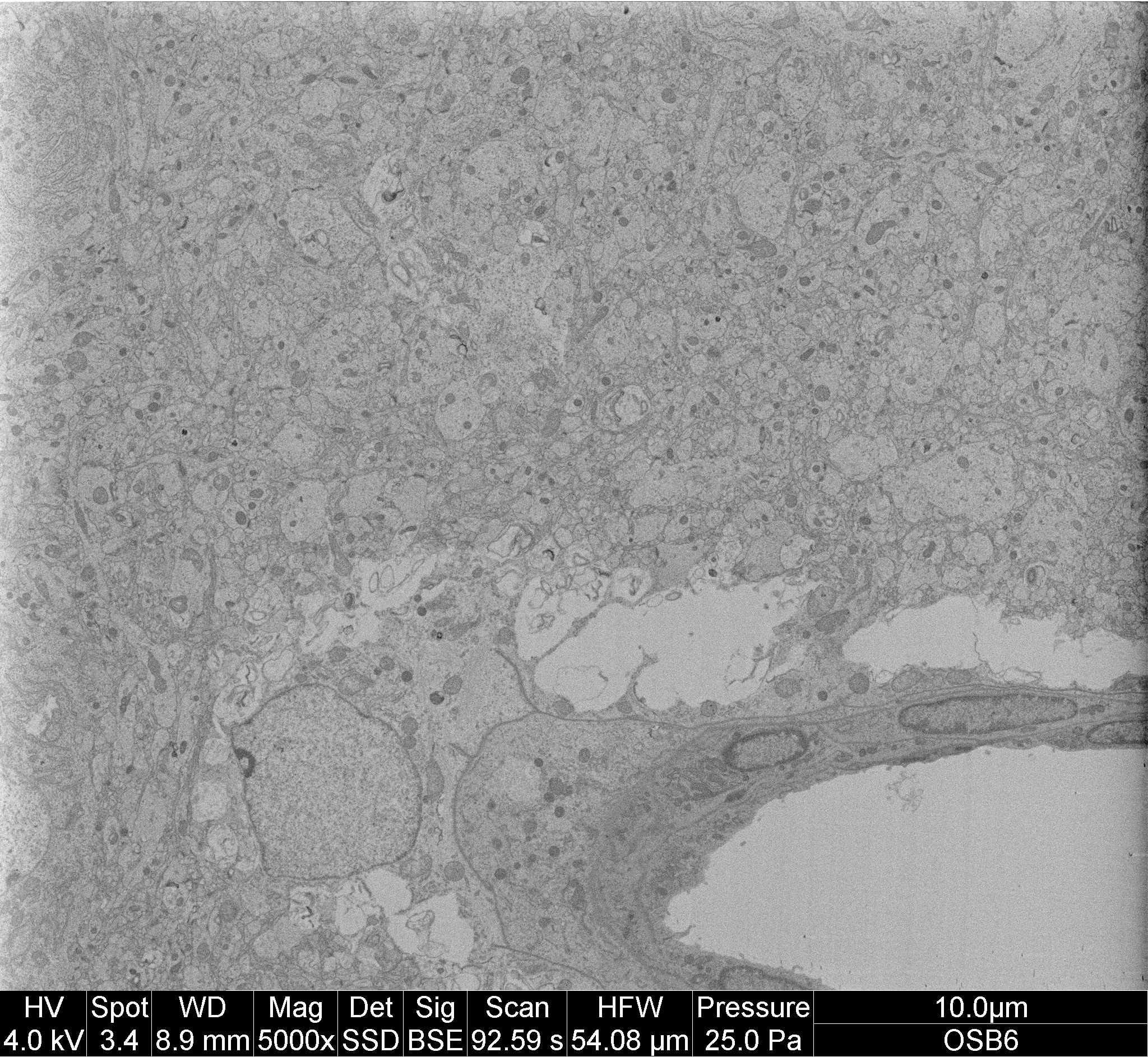

Supplement: Dataset S5 — (251.9 MB ZIP). [file pbio.0020329.sd005.zip › 040604_OS5_st1_418.tif]

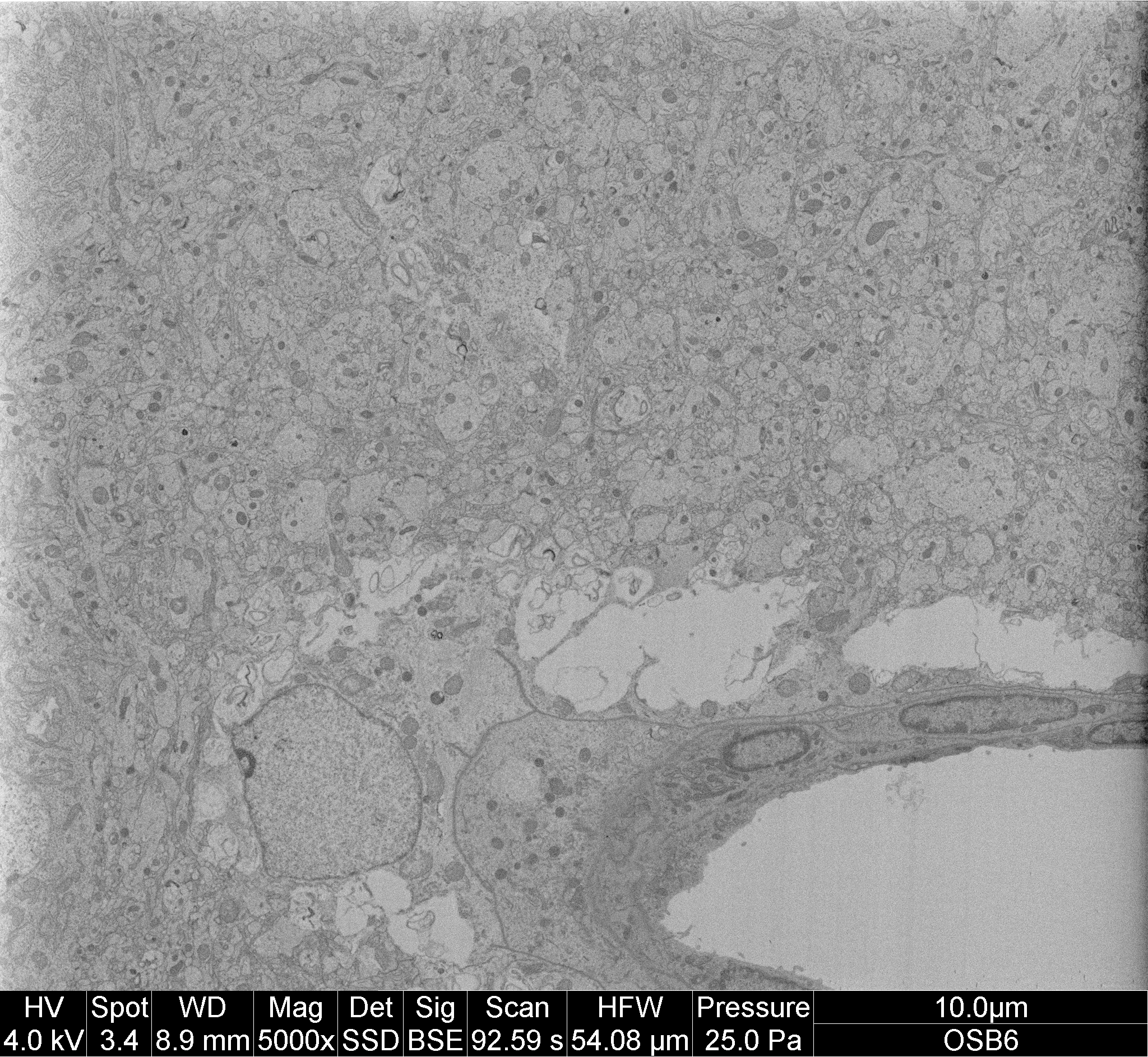

Supplement: Dataset S5 — (251.9 MB ZIP). [file pbio.0020329.sd005.zip › 040604_OS5_st1_419.tif]

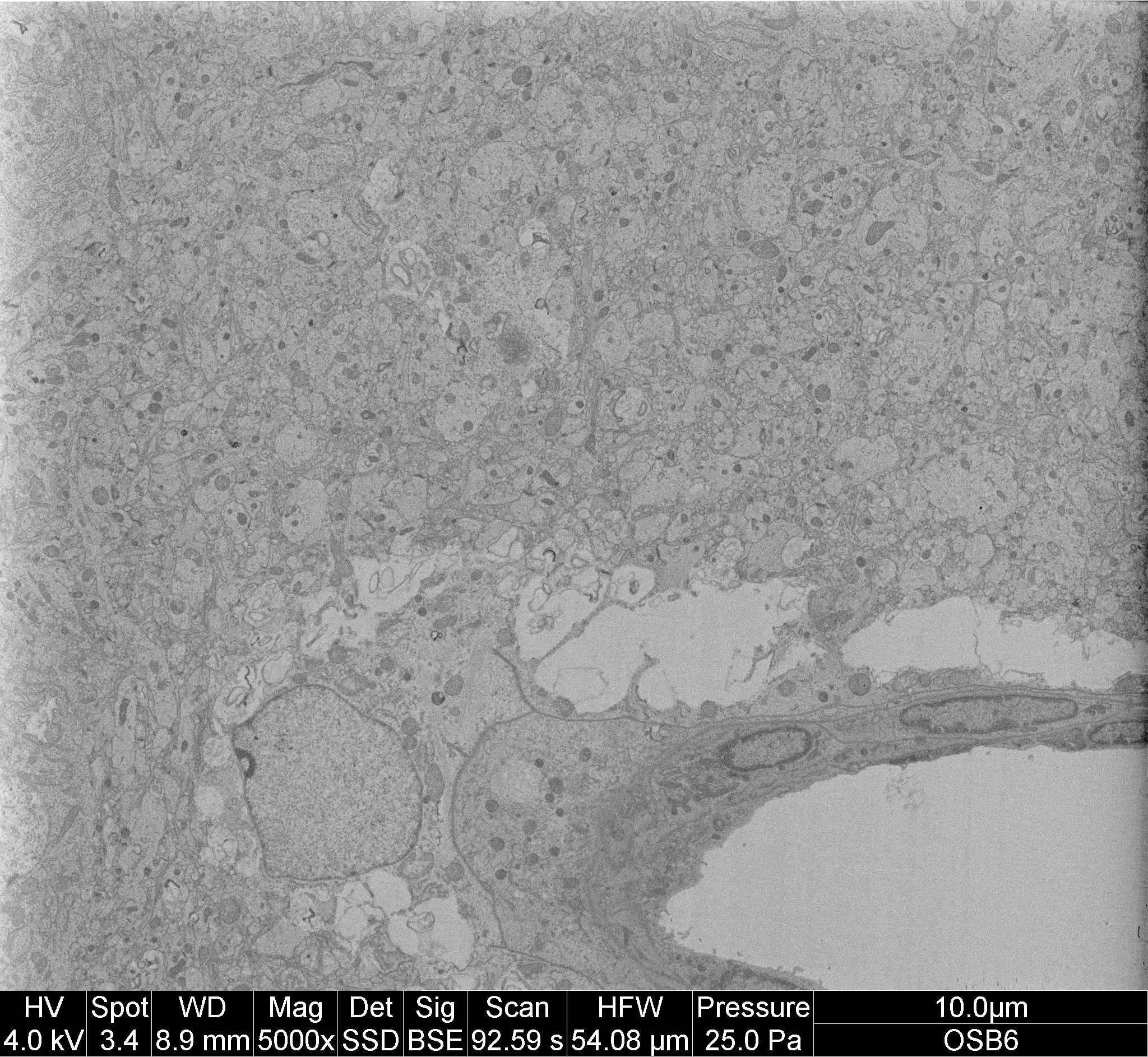

Supplement: Dataset S5 — (251.9 MB ZIP). [file pbio.0020329.sd005.zip › 040604_OS5_st1_420.tif]

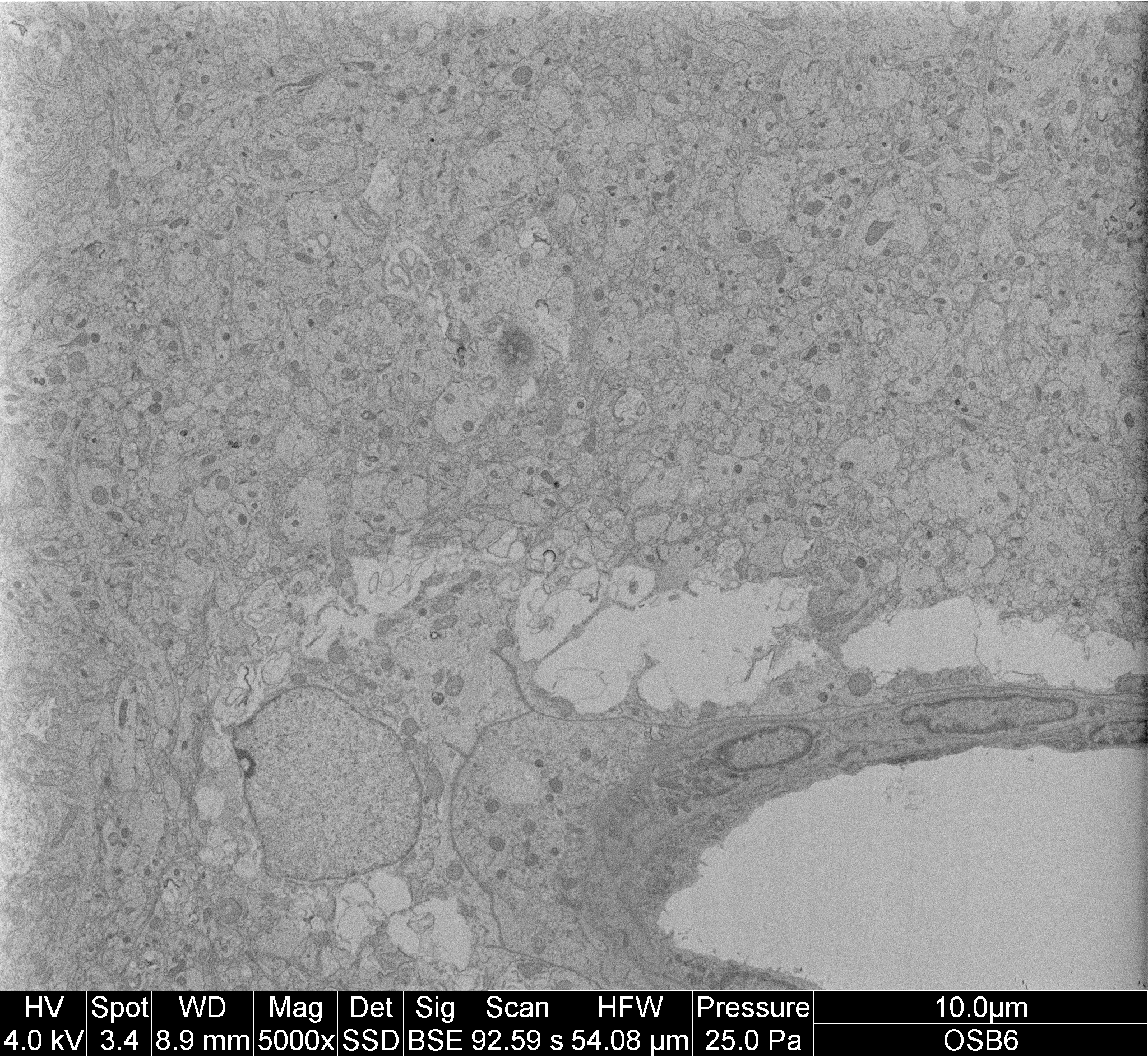

Supplement: Dataset S5 — (251.9 MB ZIP). [file pbio.0020329.sd005.zip › 040604_OS5_st1_421.tif]

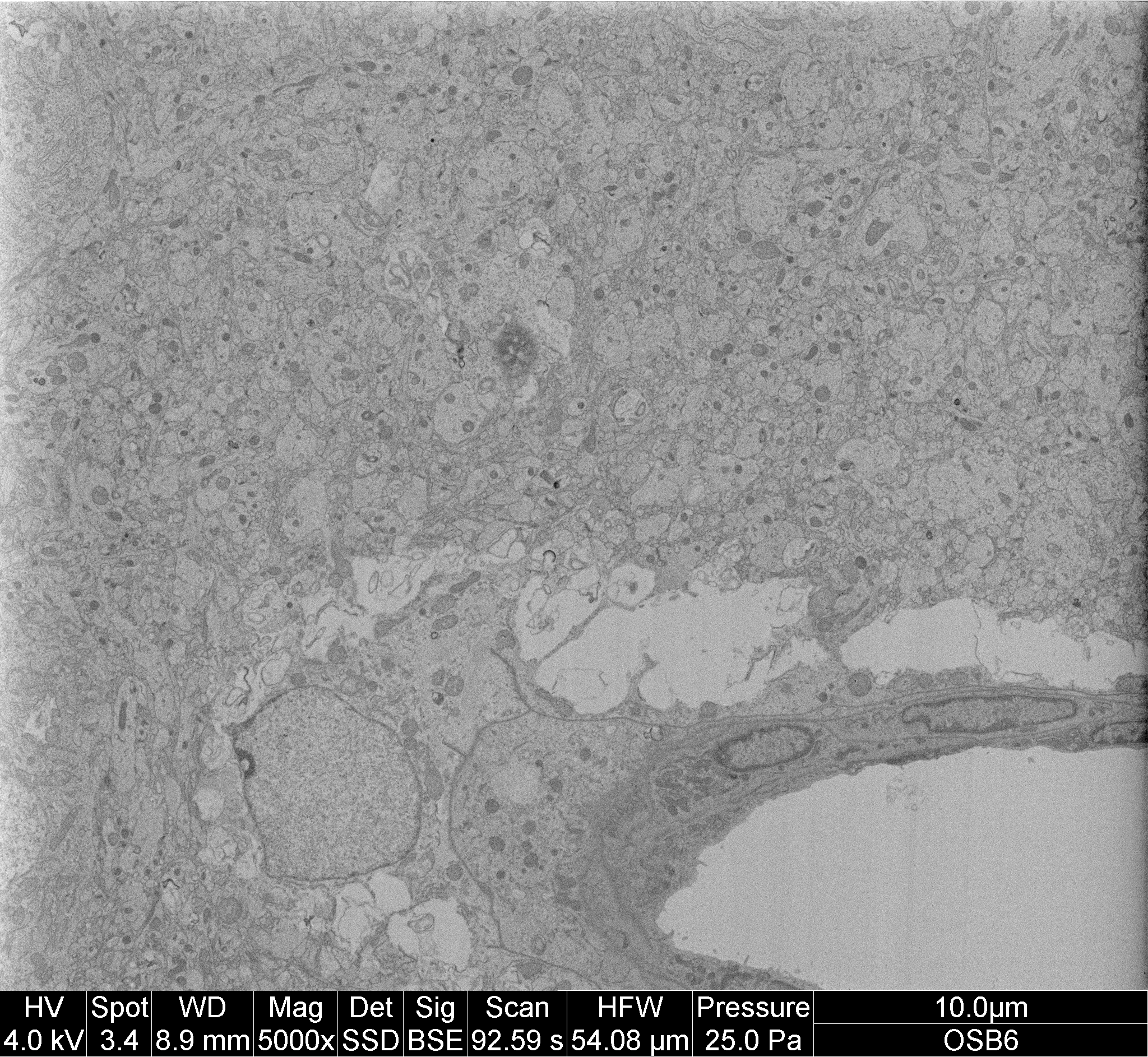

Supplement: Dataset S5 — (251.9 MB ZIP). [file pbio.0020329.sd005.zip › 040604_OS5_st1_422.tif]

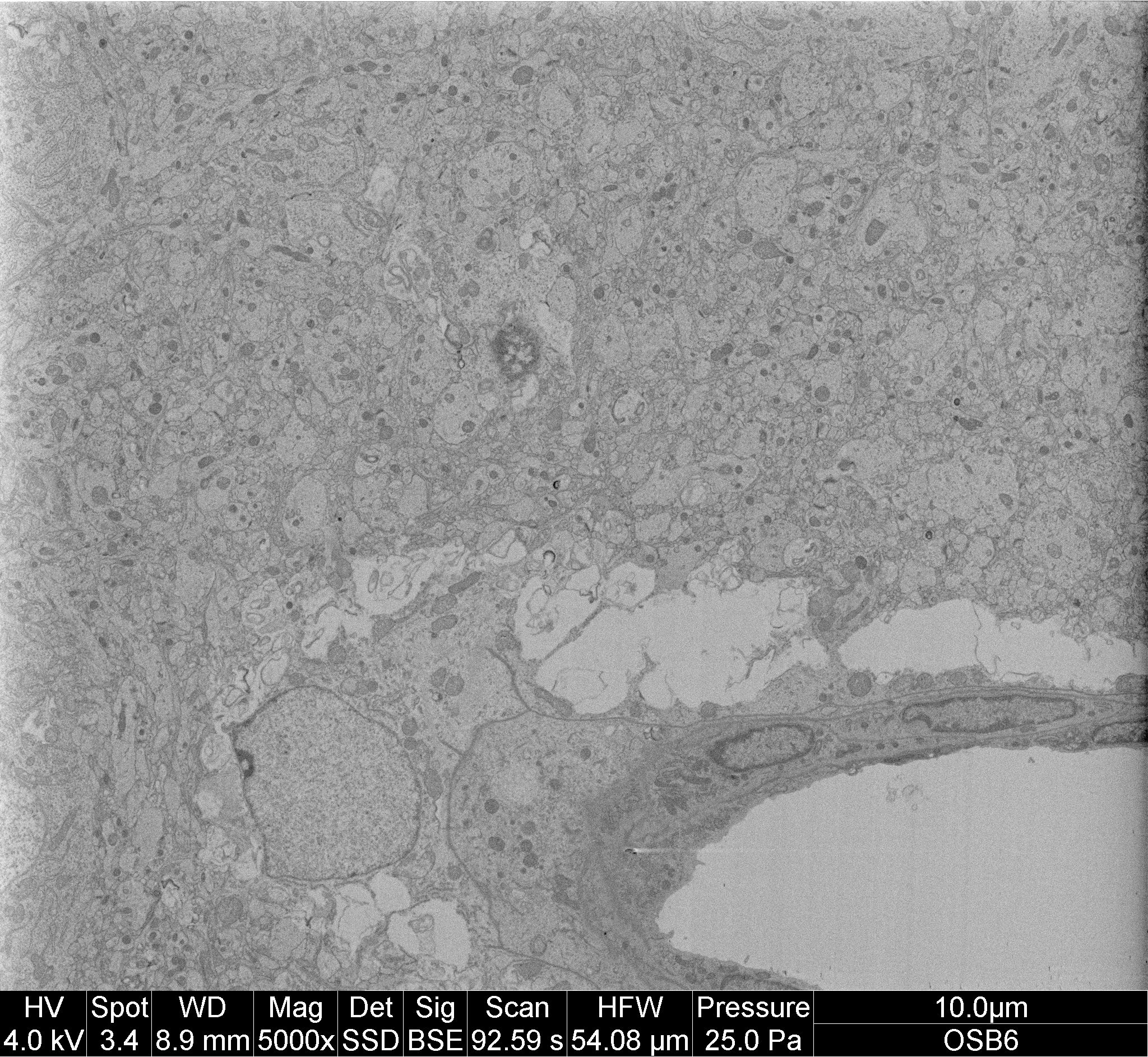

Supplement: Dataset S5 — (251.9 MB ZIP). [file pbio.0020329.sd005.zip › 040604_OS5_st1_423.tif]

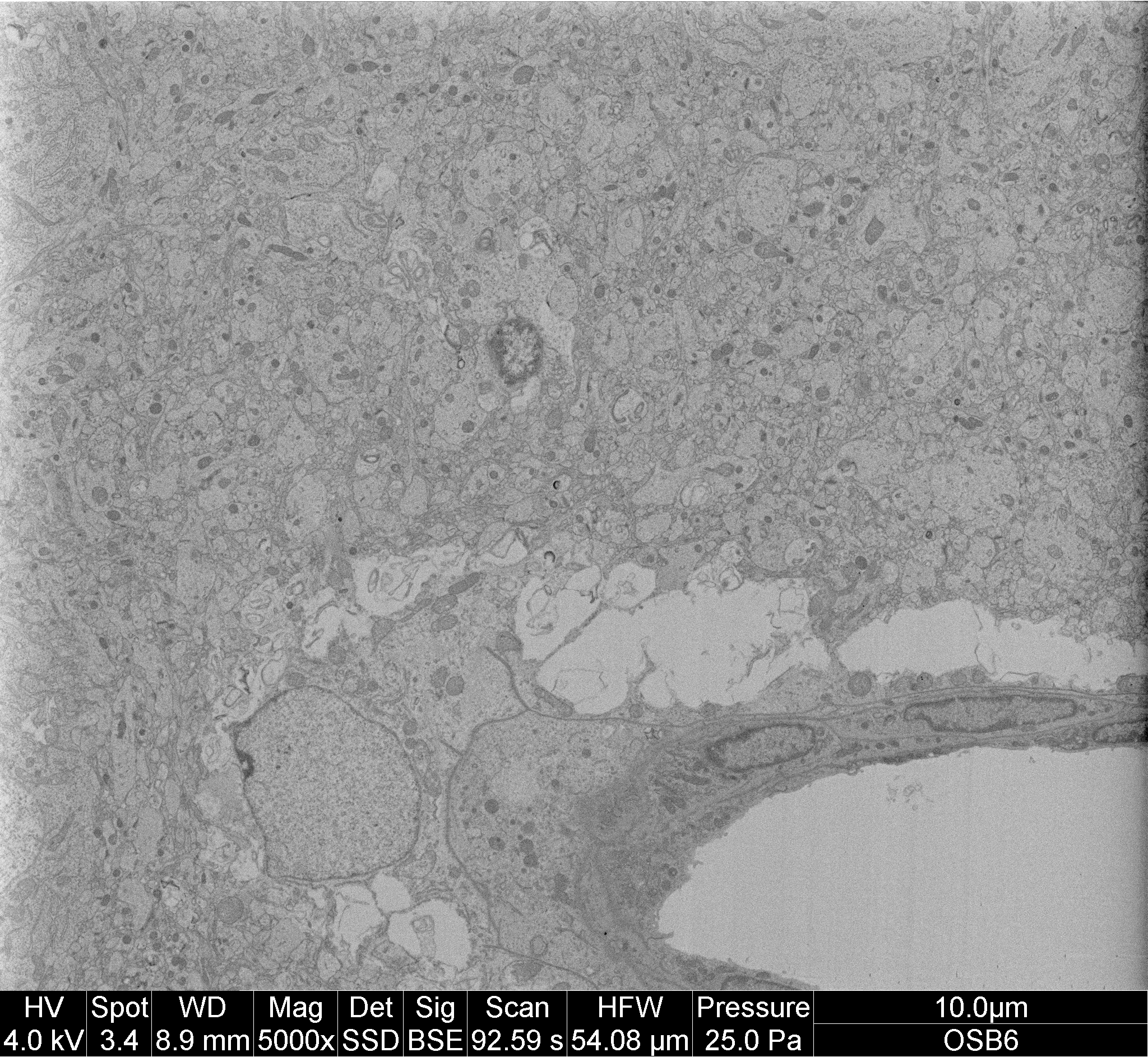

Supplement: Dataset S5 — (251.9 MB ZIP). [file pbio.0020329.sd005.zip › 040604_OS5_st1_424.tif]

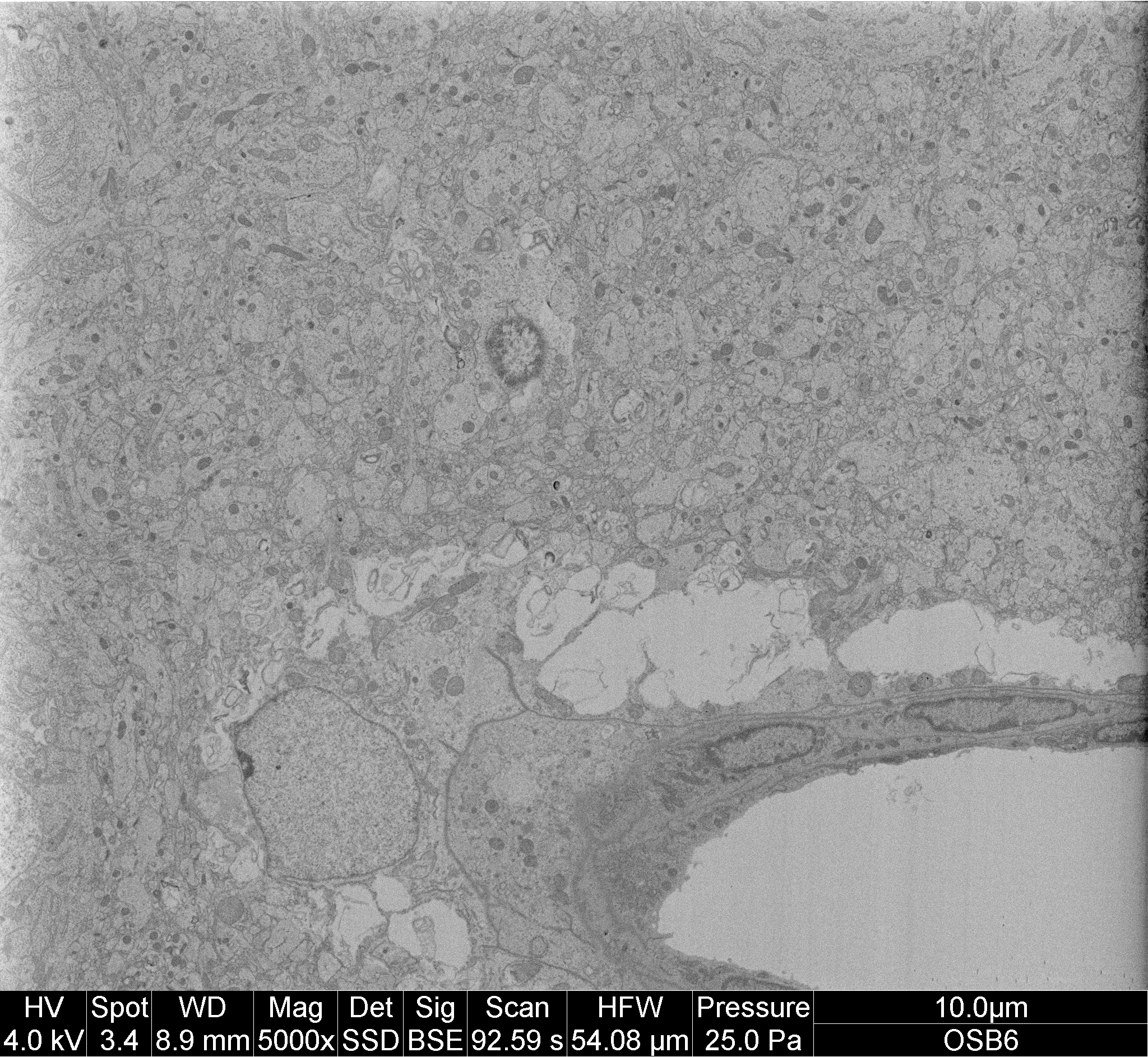

Supplement: Dataset S5 — (251.9 MB ZIP). [file pbio.0020329.sd005.zip › 040604_OS5_st1_425.tif]

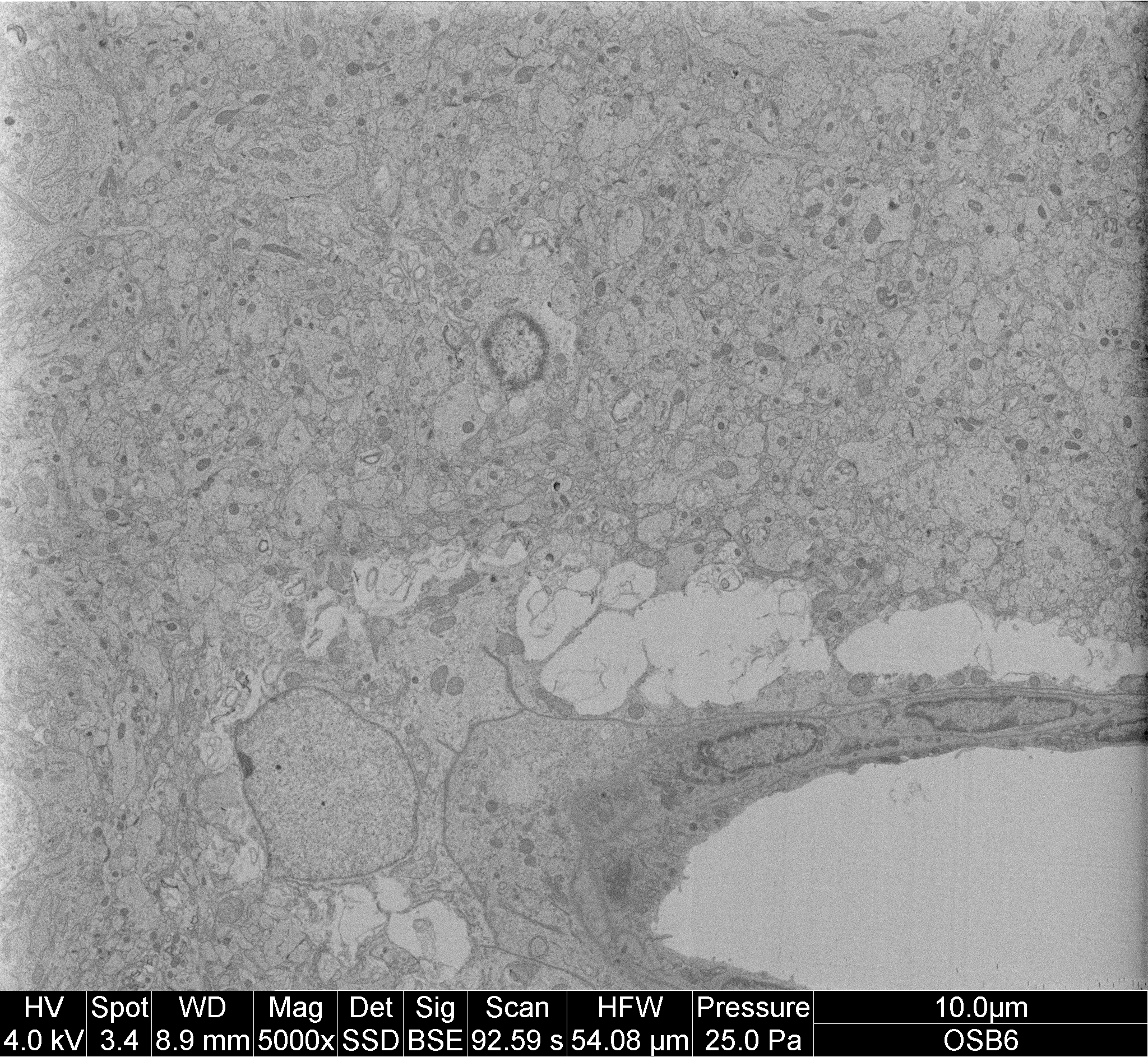

Supplement: Dataset S5 — (251.9 MB ZIP). [file pbio.0020329.sd005.zip › 040604_OS5_st1_426.tif]

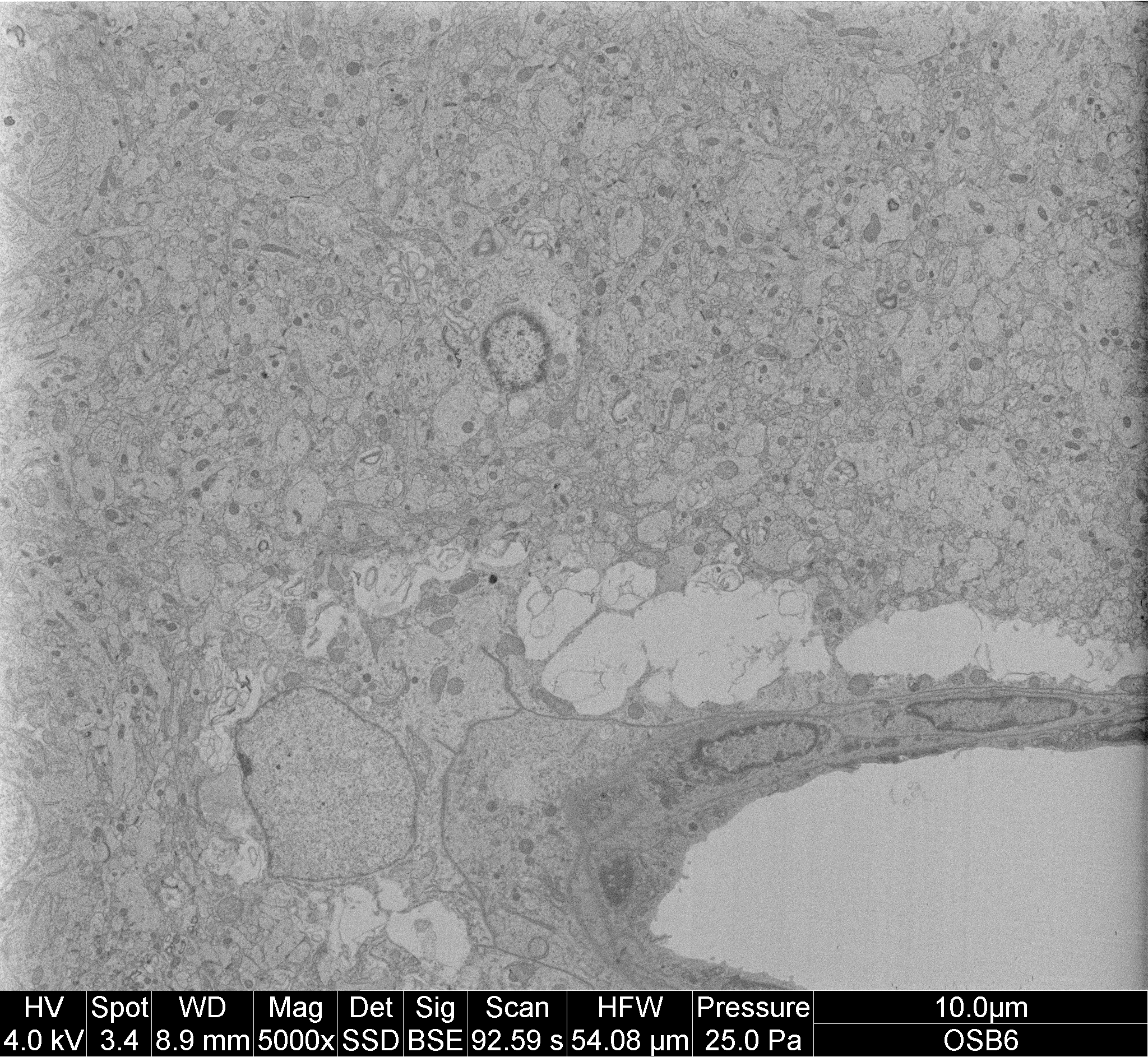

Supplement: Dataset S5 — (251.9 MB ZIP). [file pbio.0020329.sd005.zip › 040604_OS5_st1_427.tif]

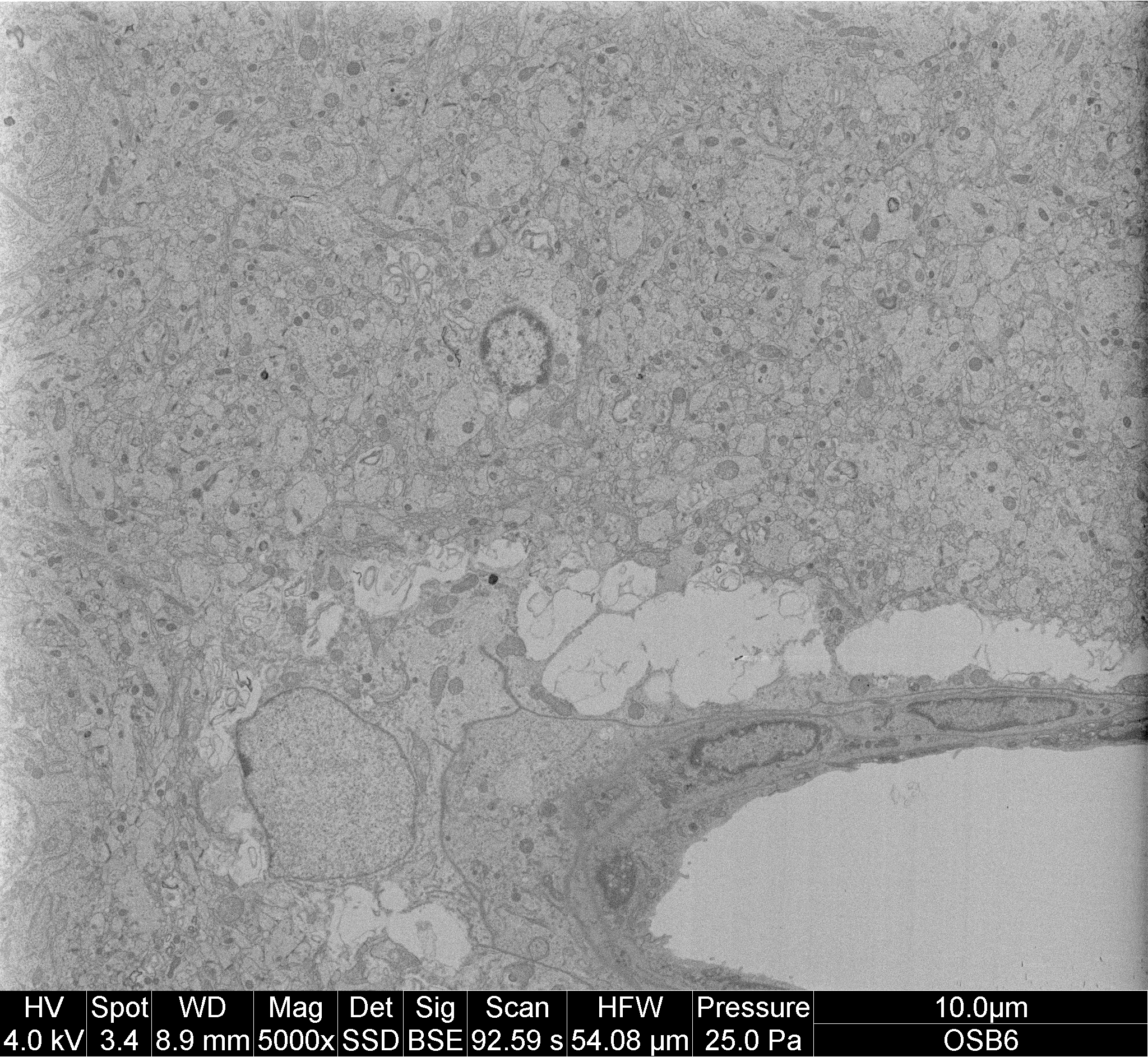

Supplement: Dataset S5 — (251.9 MB ZIP). [file pbio.0020329.sd005.zip › 040604_OS5_st1_428.tif]

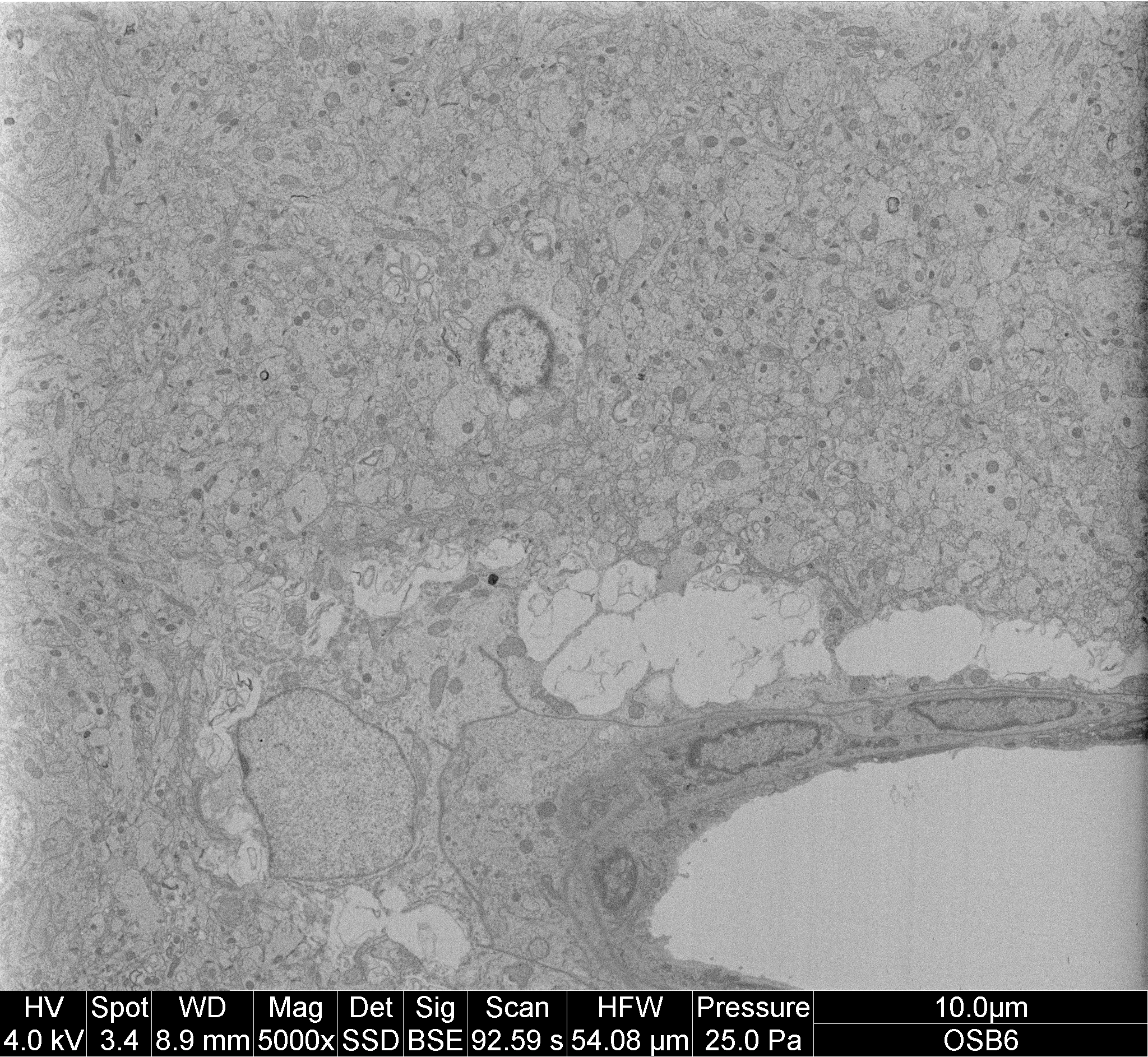

Supplement: Dataset S5 — (251.9 MB ZIP). [file pbio.0020329.sd005.zip › 040604_OS5_st1_429.tif]

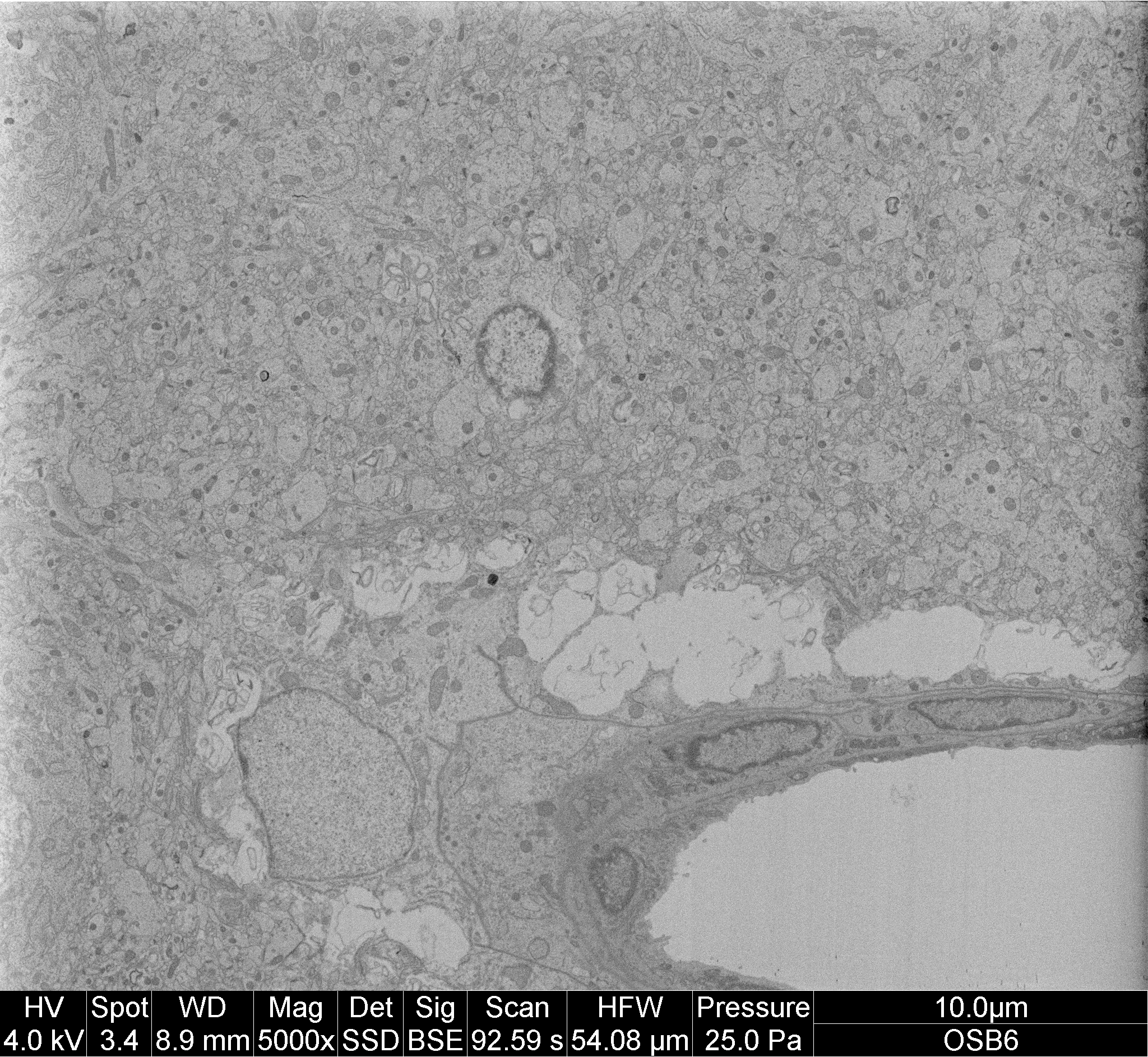

Supplement: Dataset S5 — (251.9 MB ZIP). [file pbio.0020329.sd005.zip › 040604_OS5_st1_430.tif]

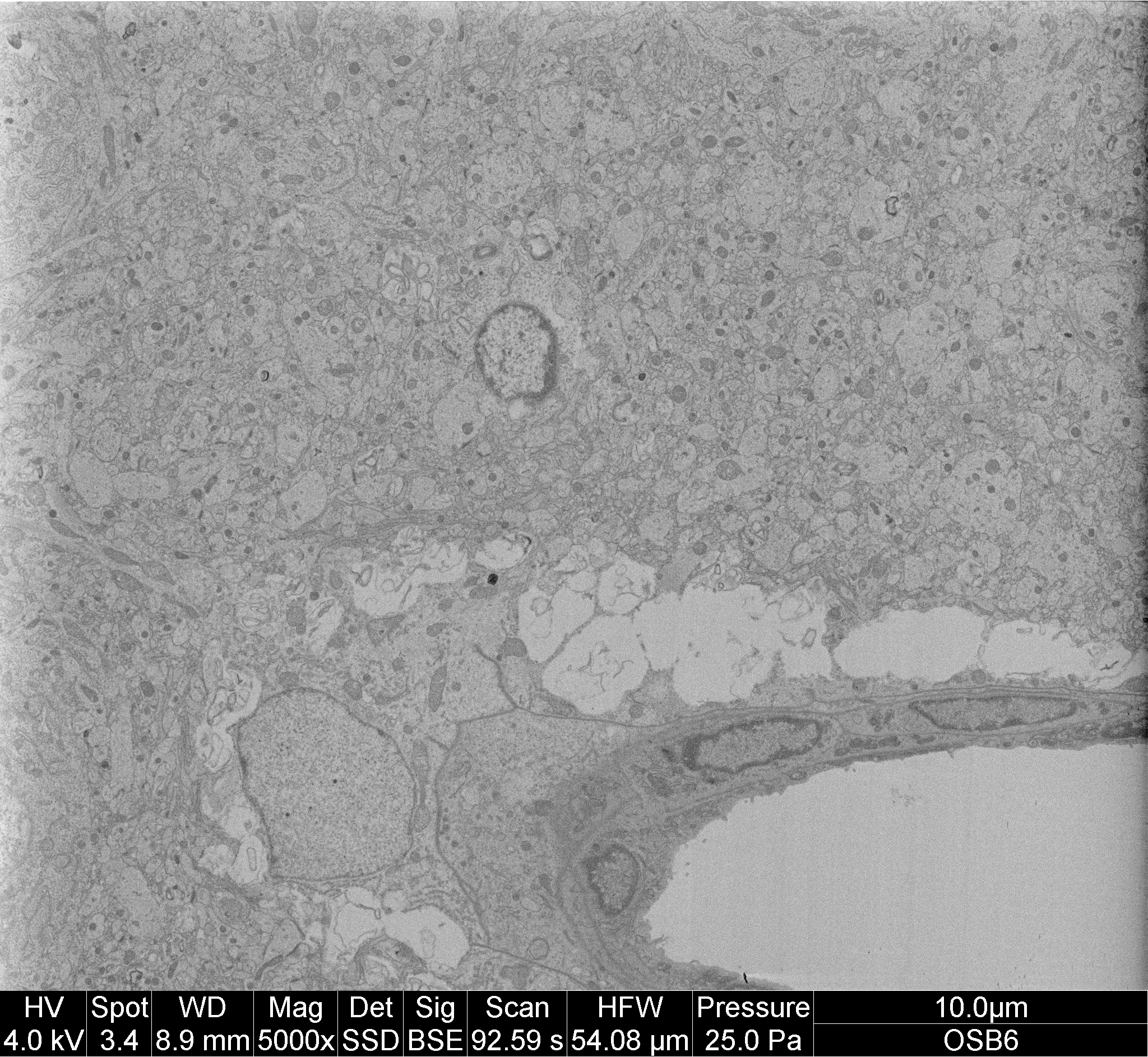

Supplement: Dataset S5 — (251.9 MB ZIP). [file pbio.0020329.sd005.zip › 040604_OS5_st1_431.tif]

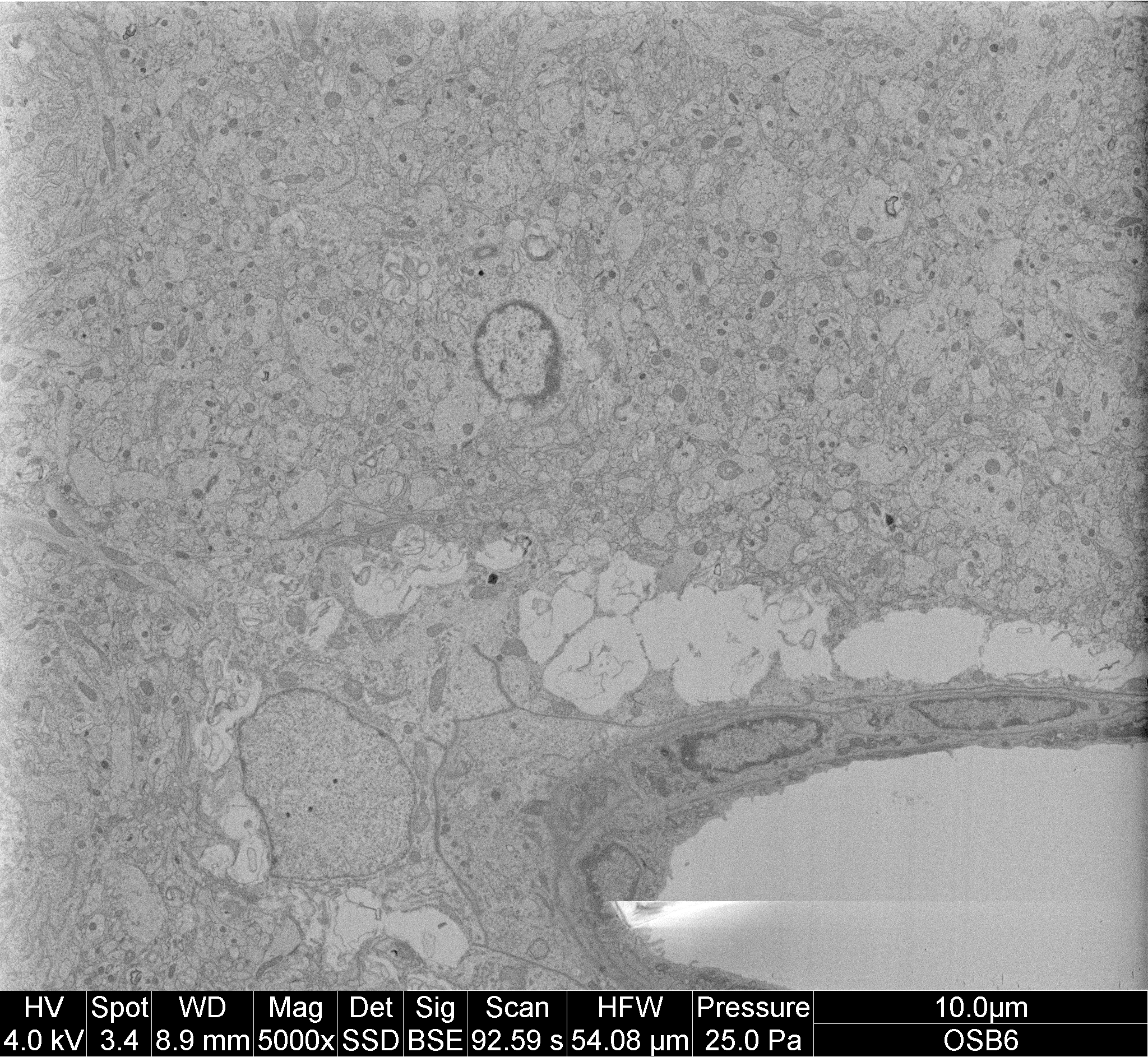

Supplement: Dataset S5 — (251.9 MB ZIP). [file pbio.0020329.sd005.zip › 040604_OS5_st1_432.tif]

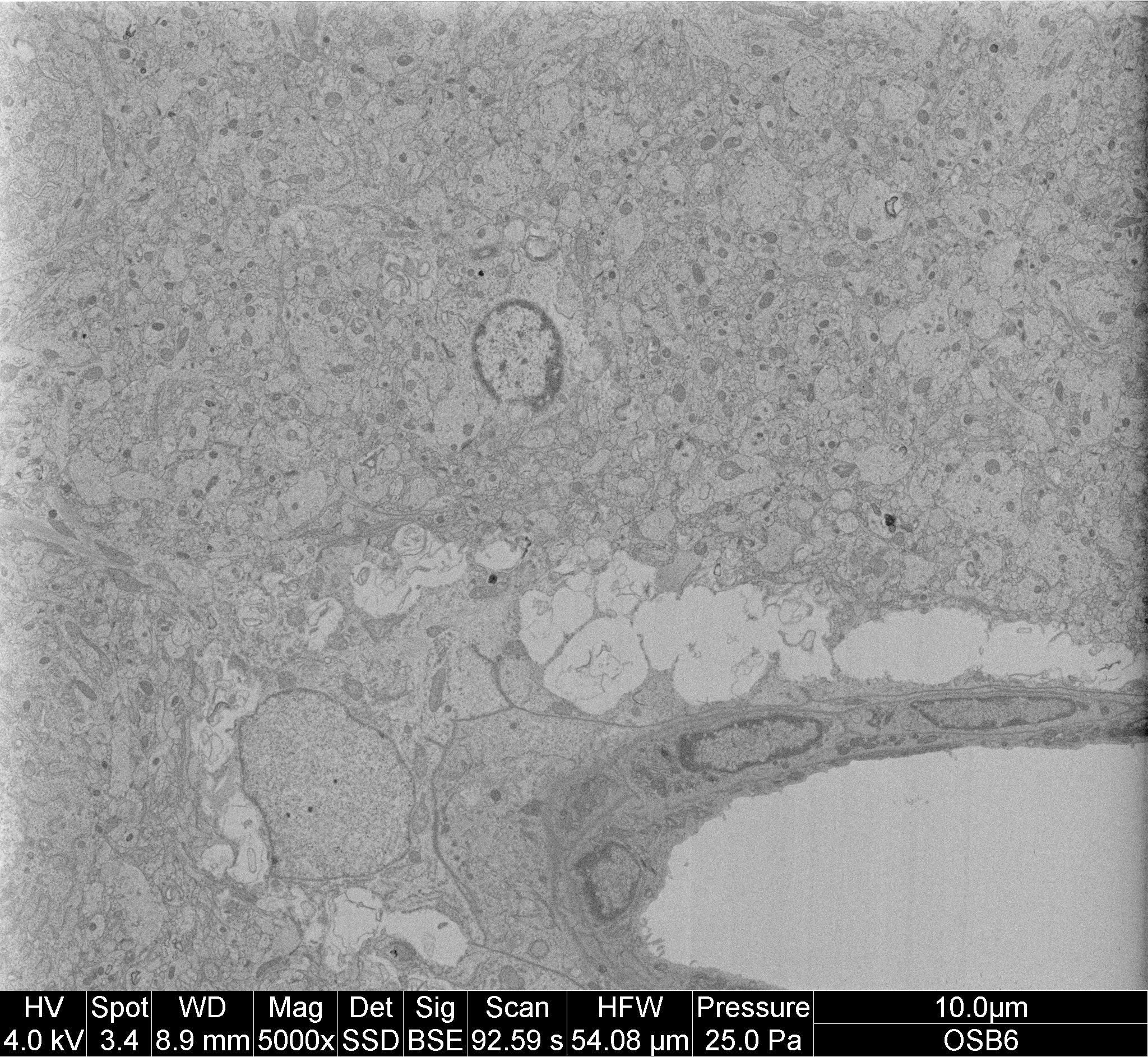

Supplement: Dataset S5 — (251.9 MB ZIP). [file pbio.0020329.sd005.zip › 040604_OS5_st1_433.tif]

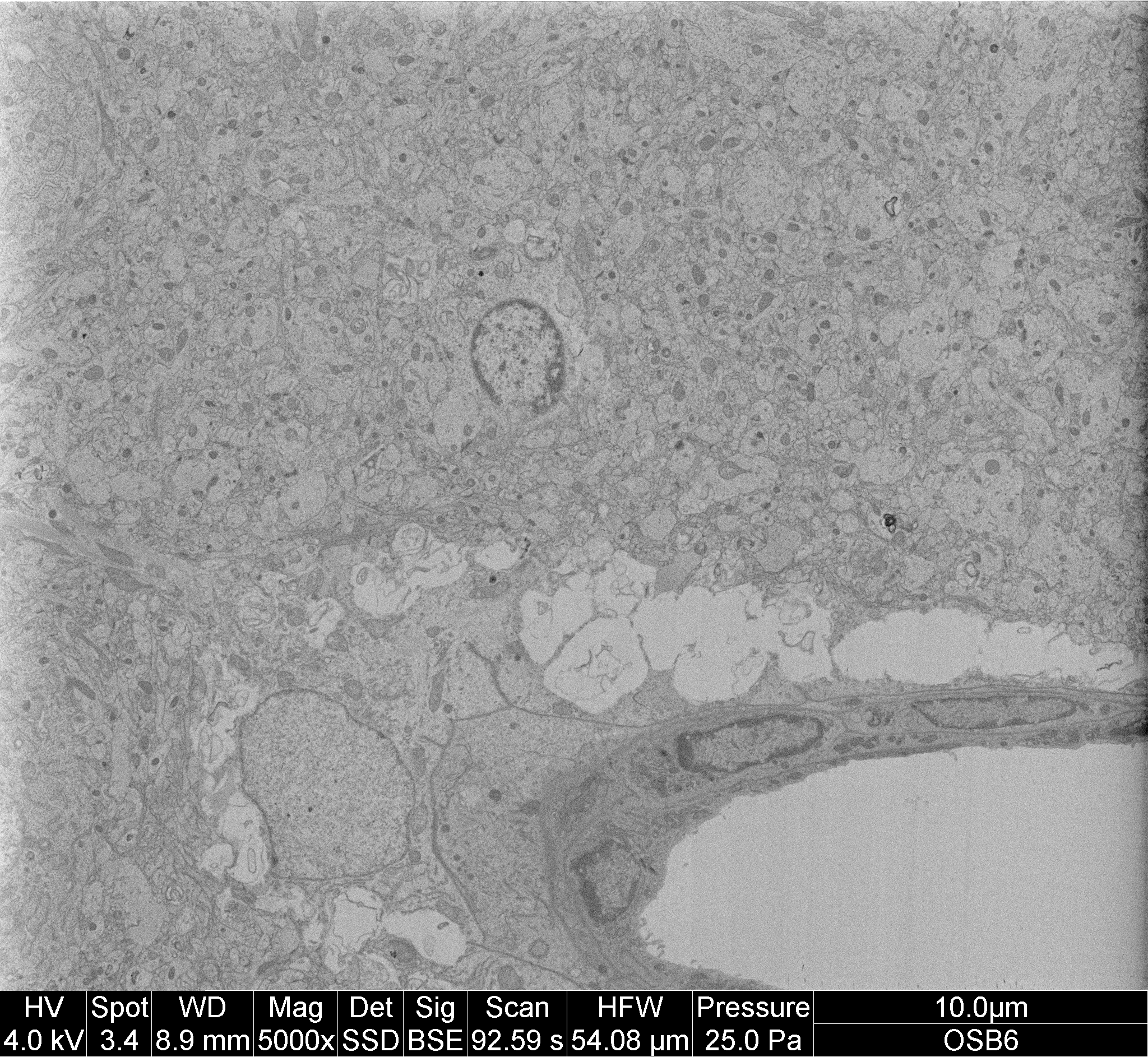

Supplement: Dataset S5 — (251.9 MB ZIP). [file pbio.0020329.sd005.zip › 040604_OS5_st1_434.tif]

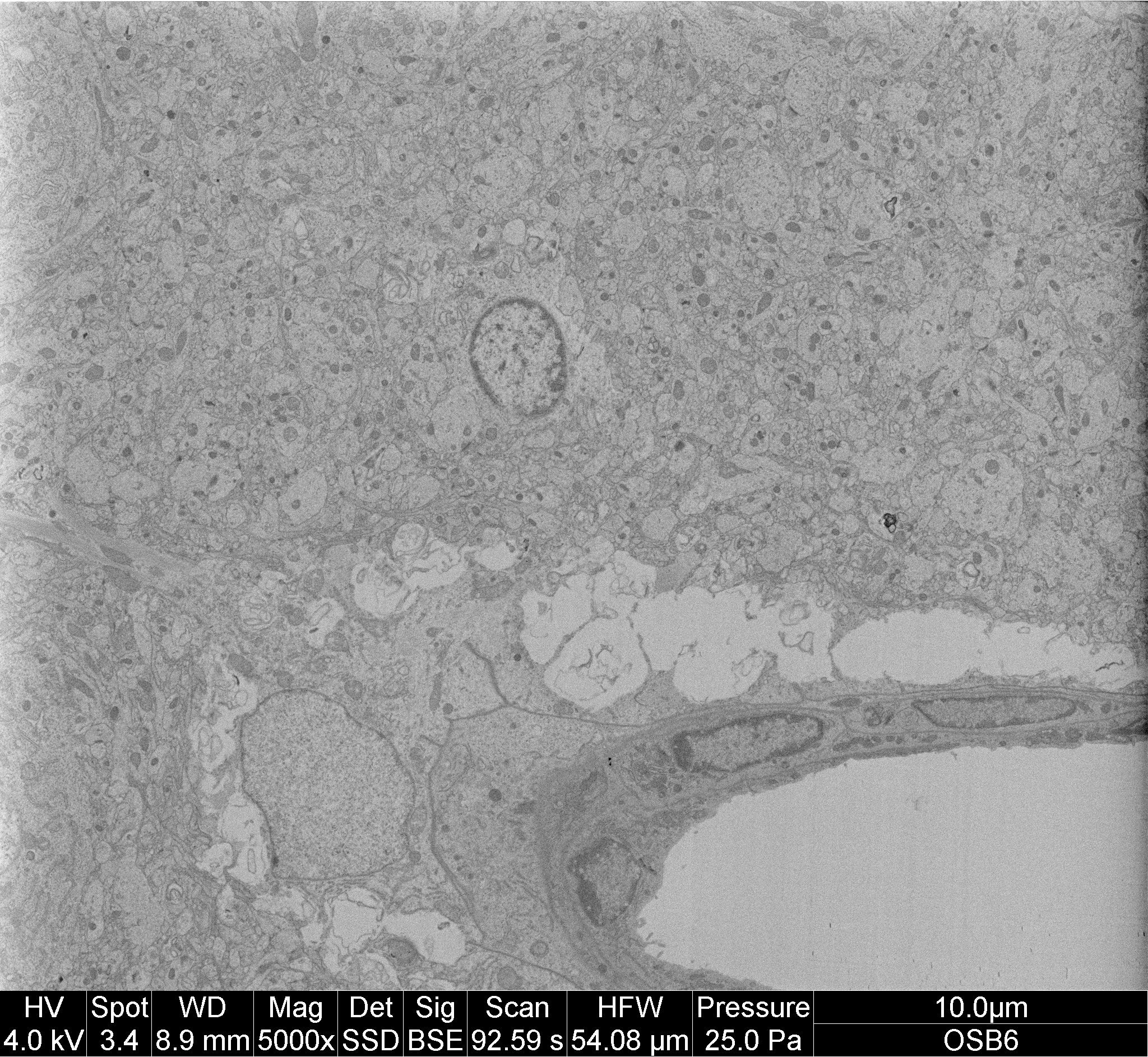

Supplement: Dataset S5 — (251.9 MB ZIP). [file pbio.0020329.sd005.zip › 040604_OS5_st1_435.tif]

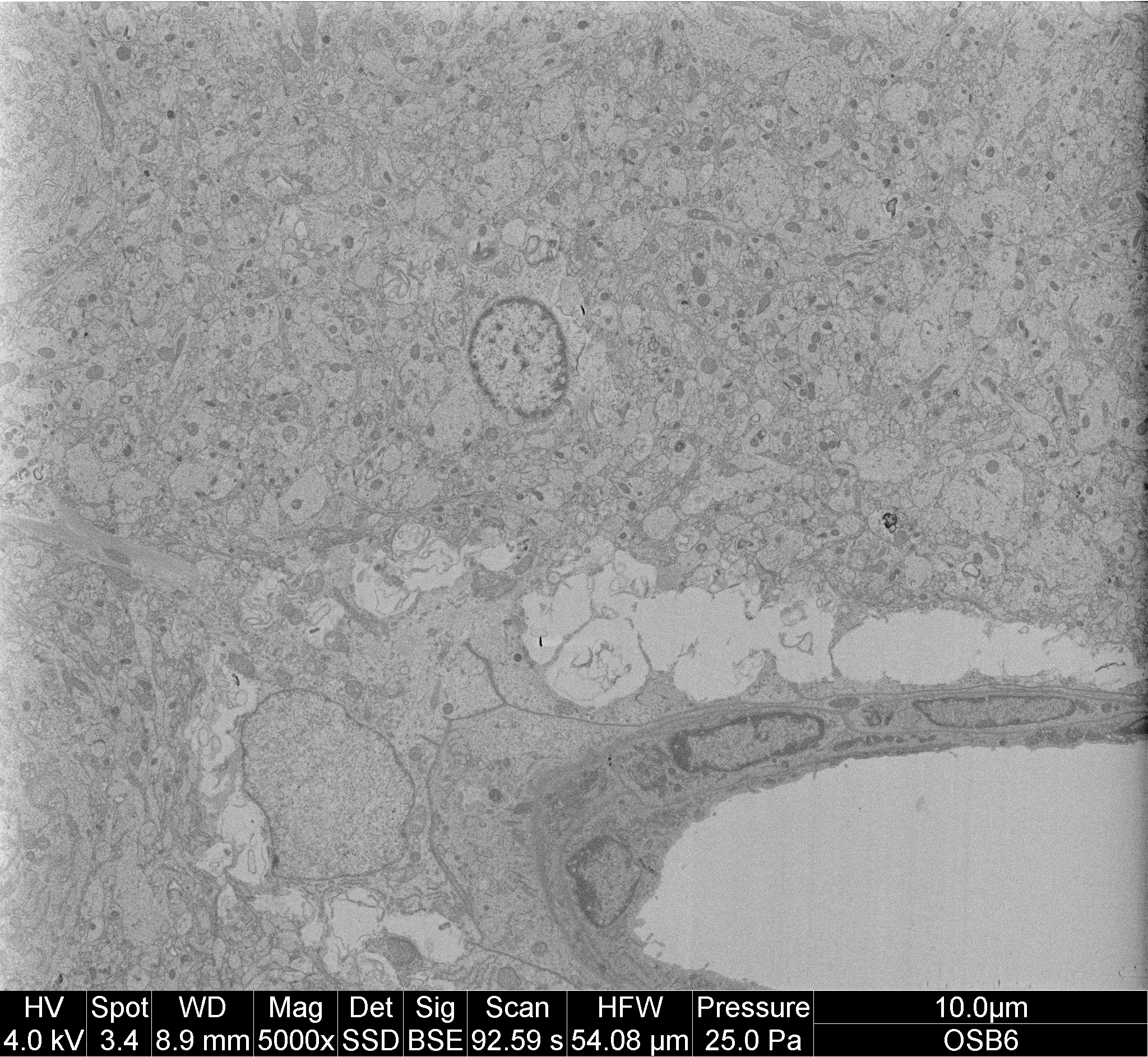

Supplement: Dataset S5 — (251.9 MB ZIP). [file pbio.0020329.sd005.zip › 040604_OS5_st1_436.tif]

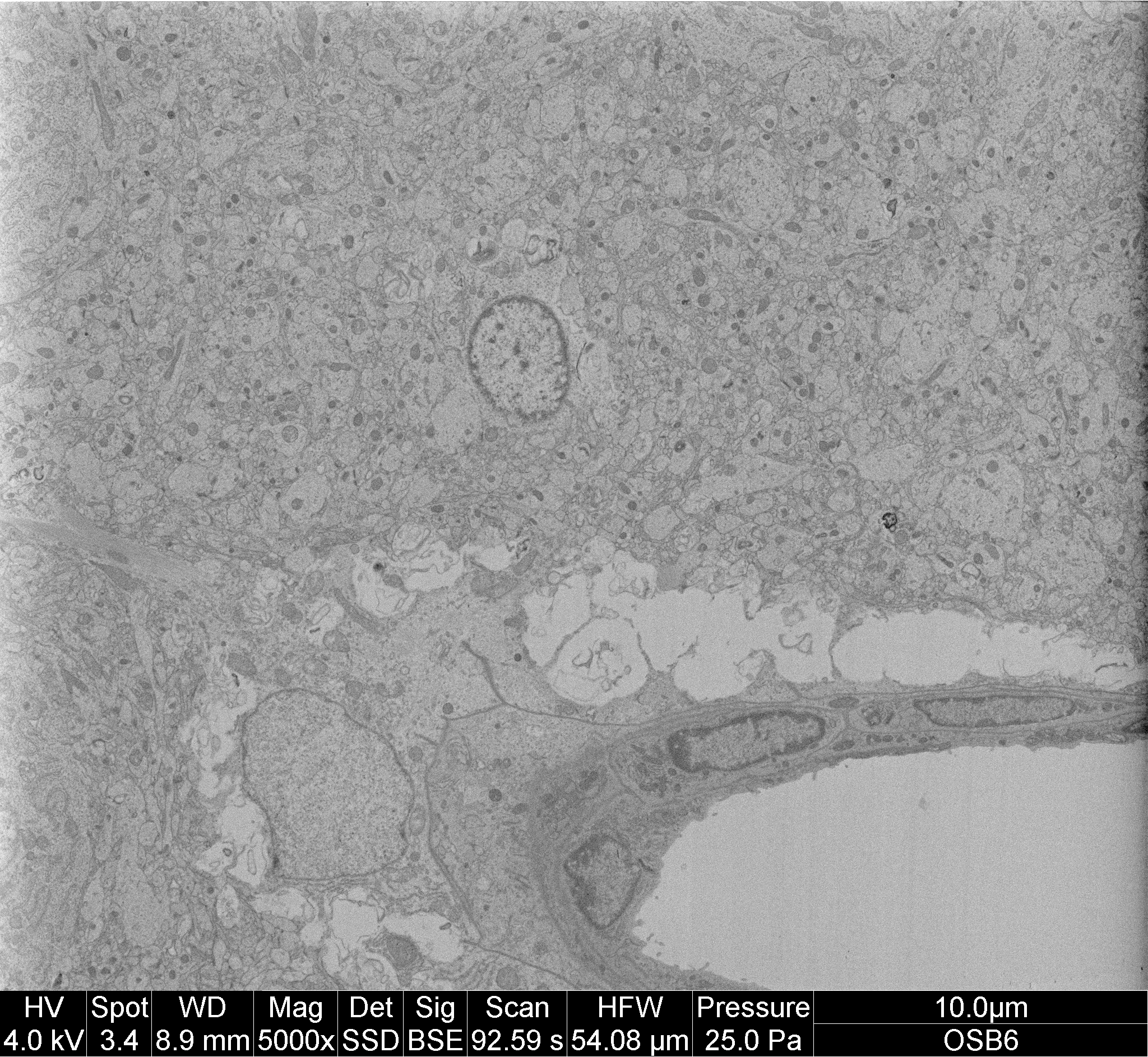

Supplement: Dataset S5 — (251.9 MB ZIP). [file pbio.0020329.sd005.zip › 040604_OS5_st1_437.tif]

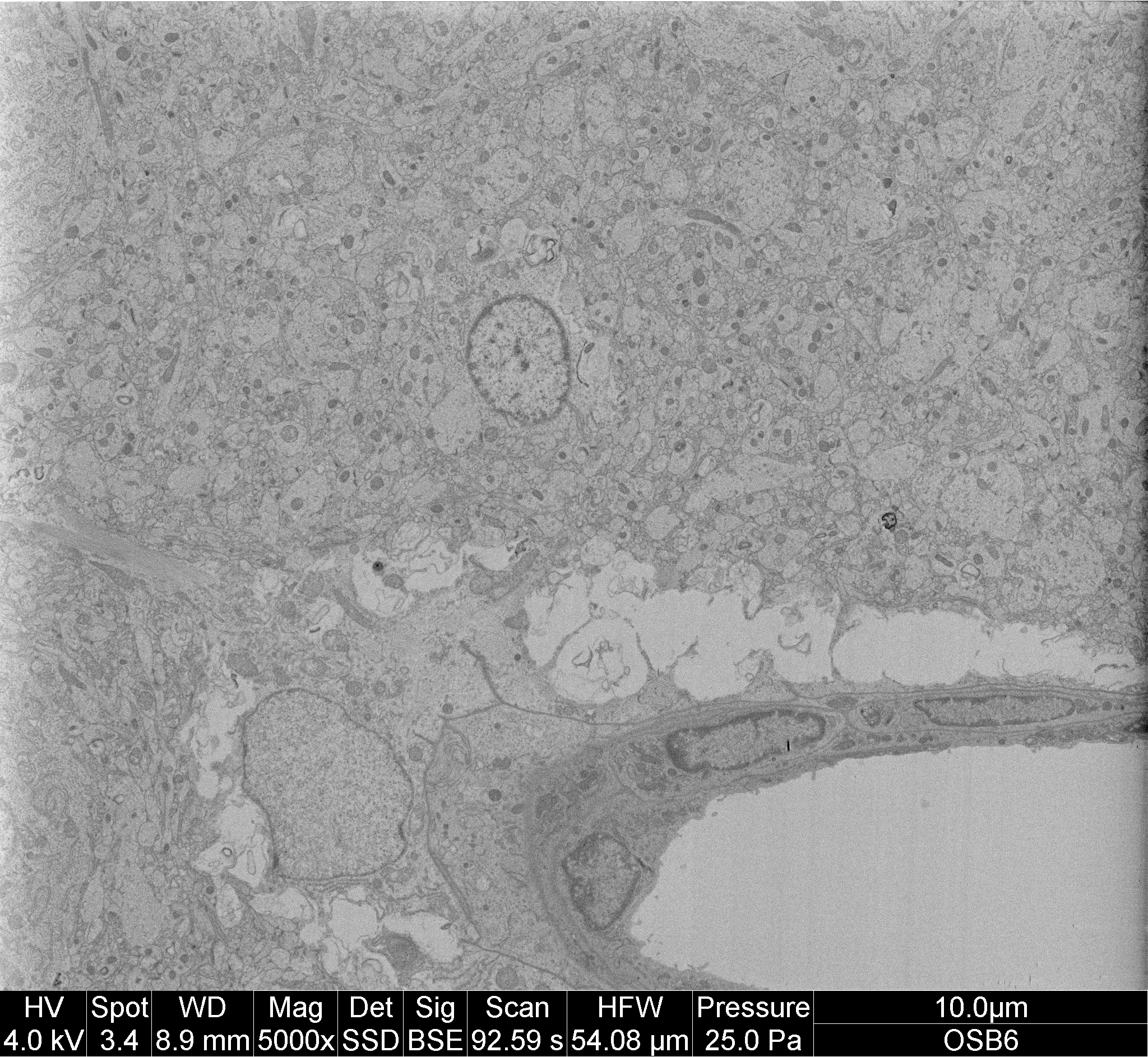

Supplement: Dataset S5 — (251.9 MB ZIP). [file pbio.0020329.sd005.zip › 040604_OS5_st1_438.tif]

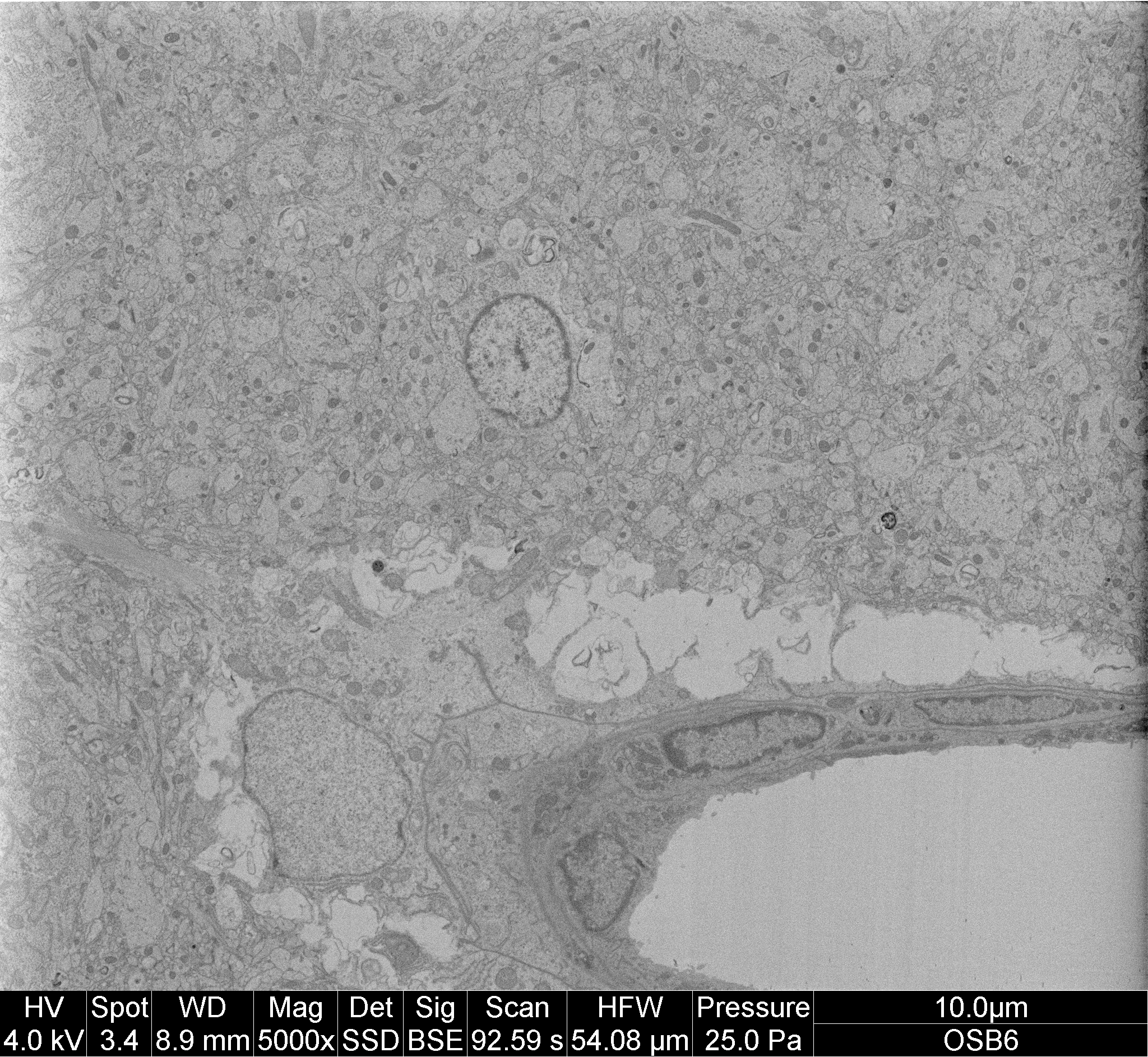

Supplement: Dataset S5 — (251.9 MB ZIP). [file pbio.0020329.sd005.zip › 040604_OS5_st1_439.tif]

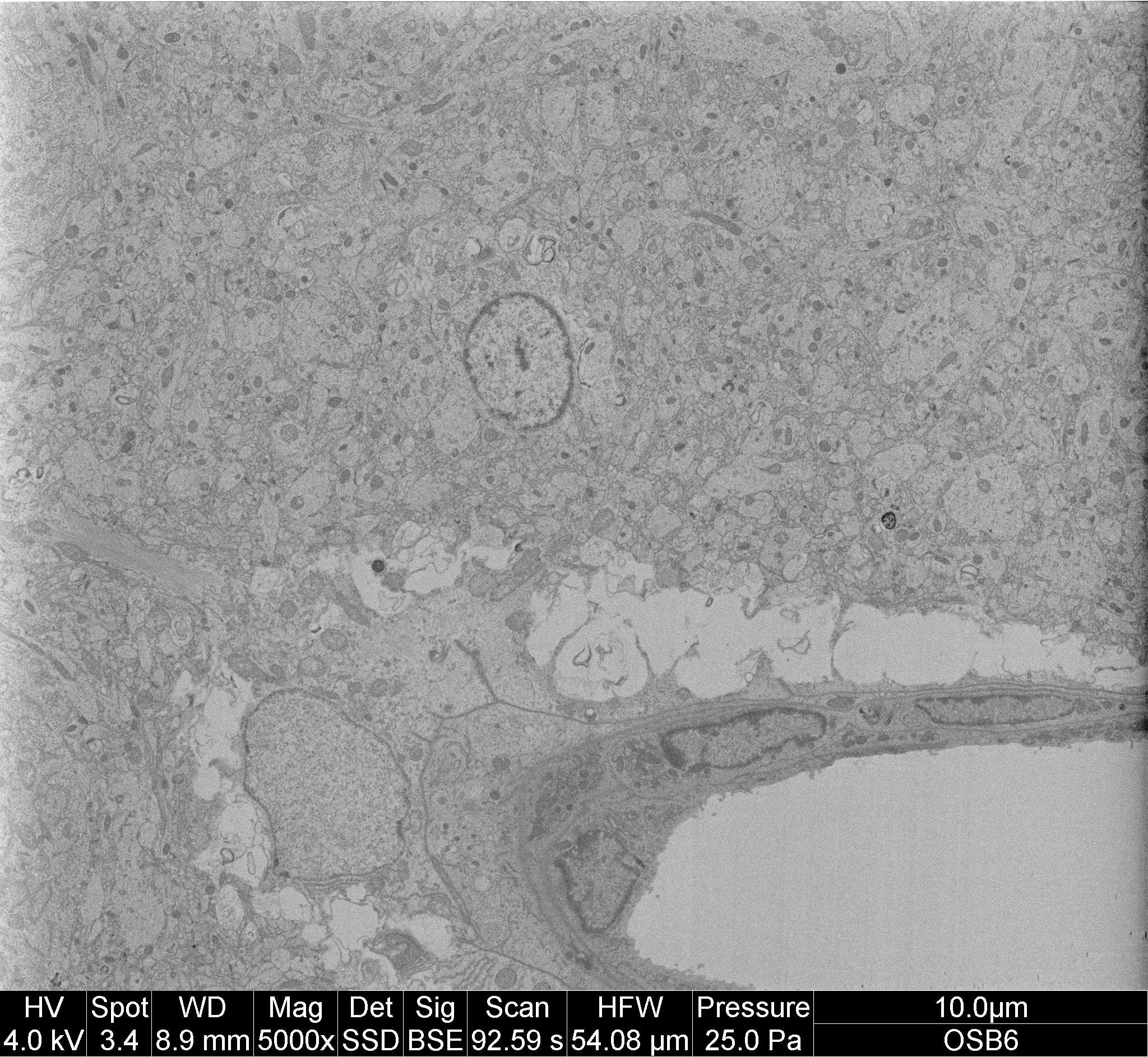

Supplement: Dataset S5 — (251.9 MB ZIP). [file pbio.0020329.sd005.zip › 040604_OS5_st1_440.tif]

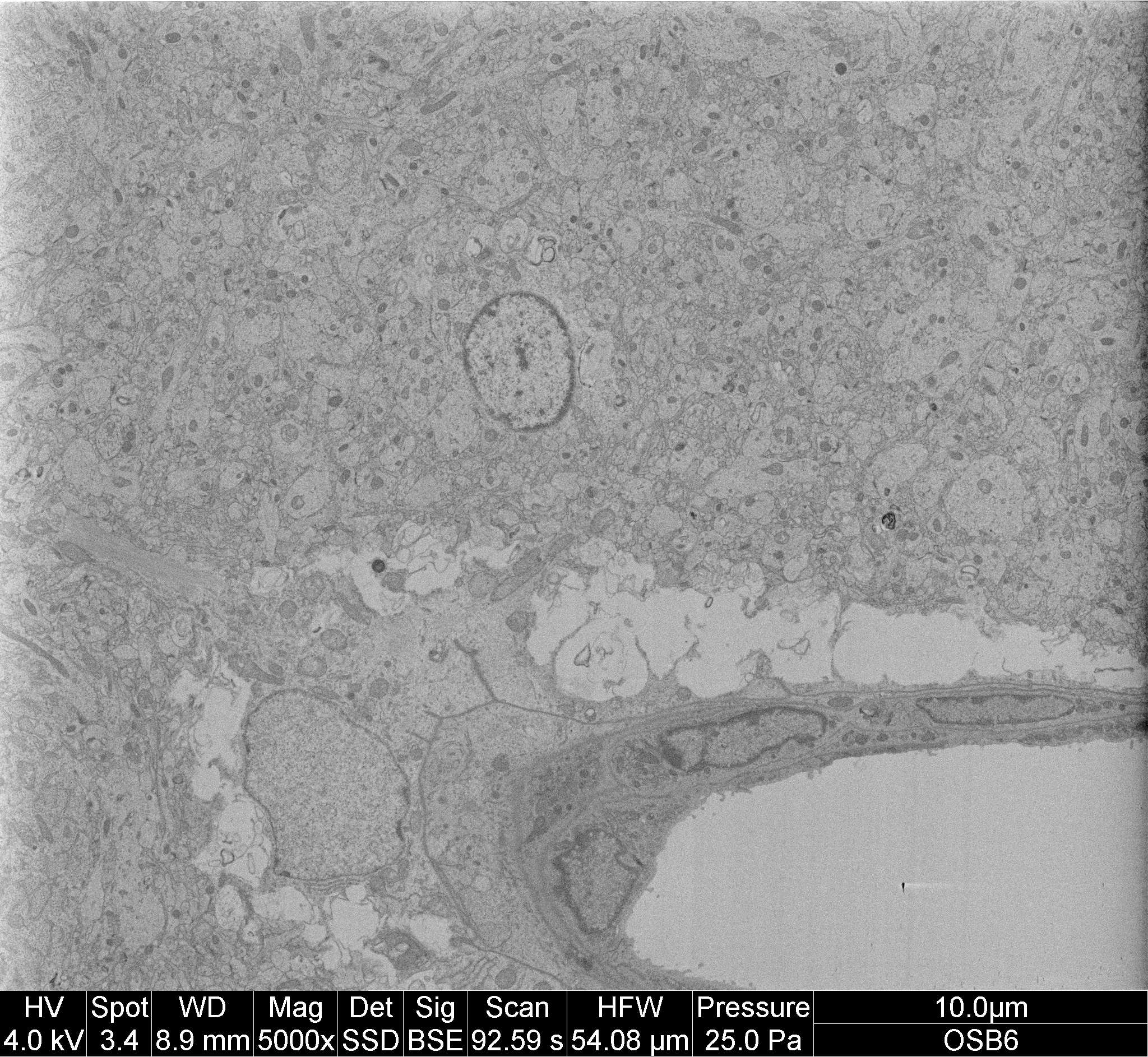

Supplement: Dataset S5 — (251.9 MB ZIP). [file pbio.0020329.sd005.zip › 040604_OS5_st1_441.tif]

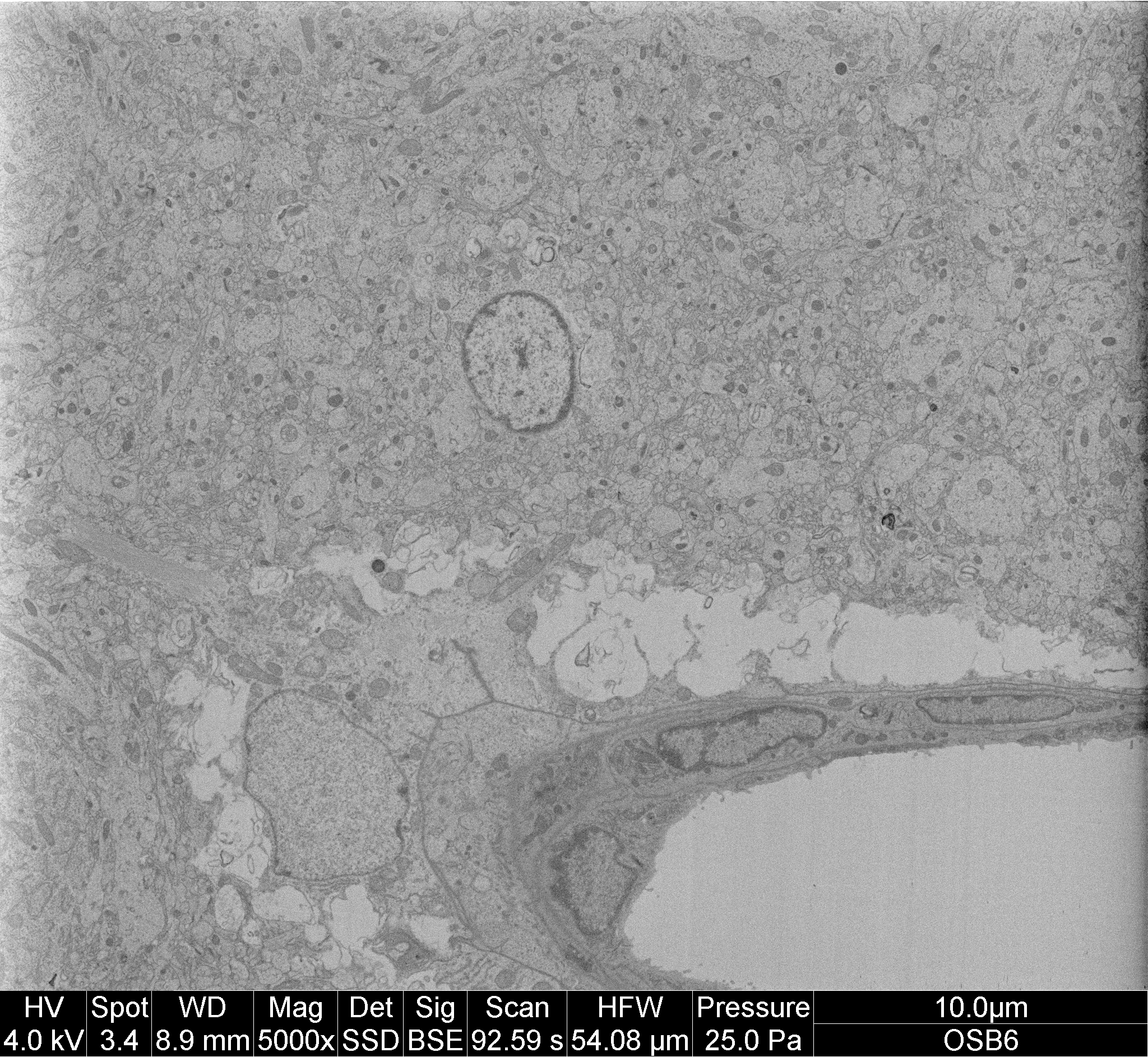

Supplement: Dataset S5 — (251.9 MB ZIP). [file pbio.0020329.sd005.zip › 040604_OS5_st1_442.tif]

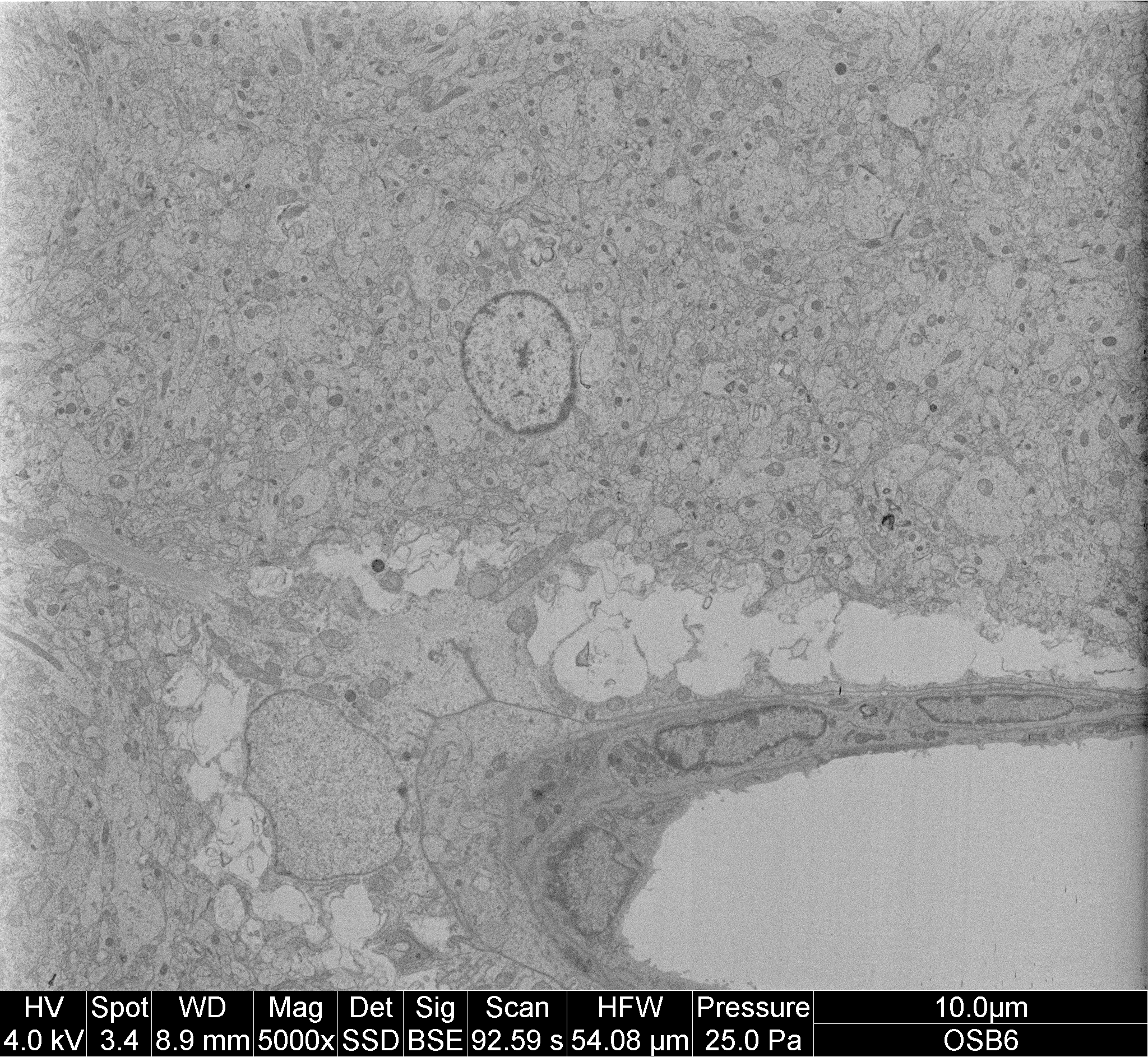

Supplement: Dataset S5 — (251.9 MB ZIP). [file pbio.0020329.sd005.zip › 040604_OS5_st1_443.tif]

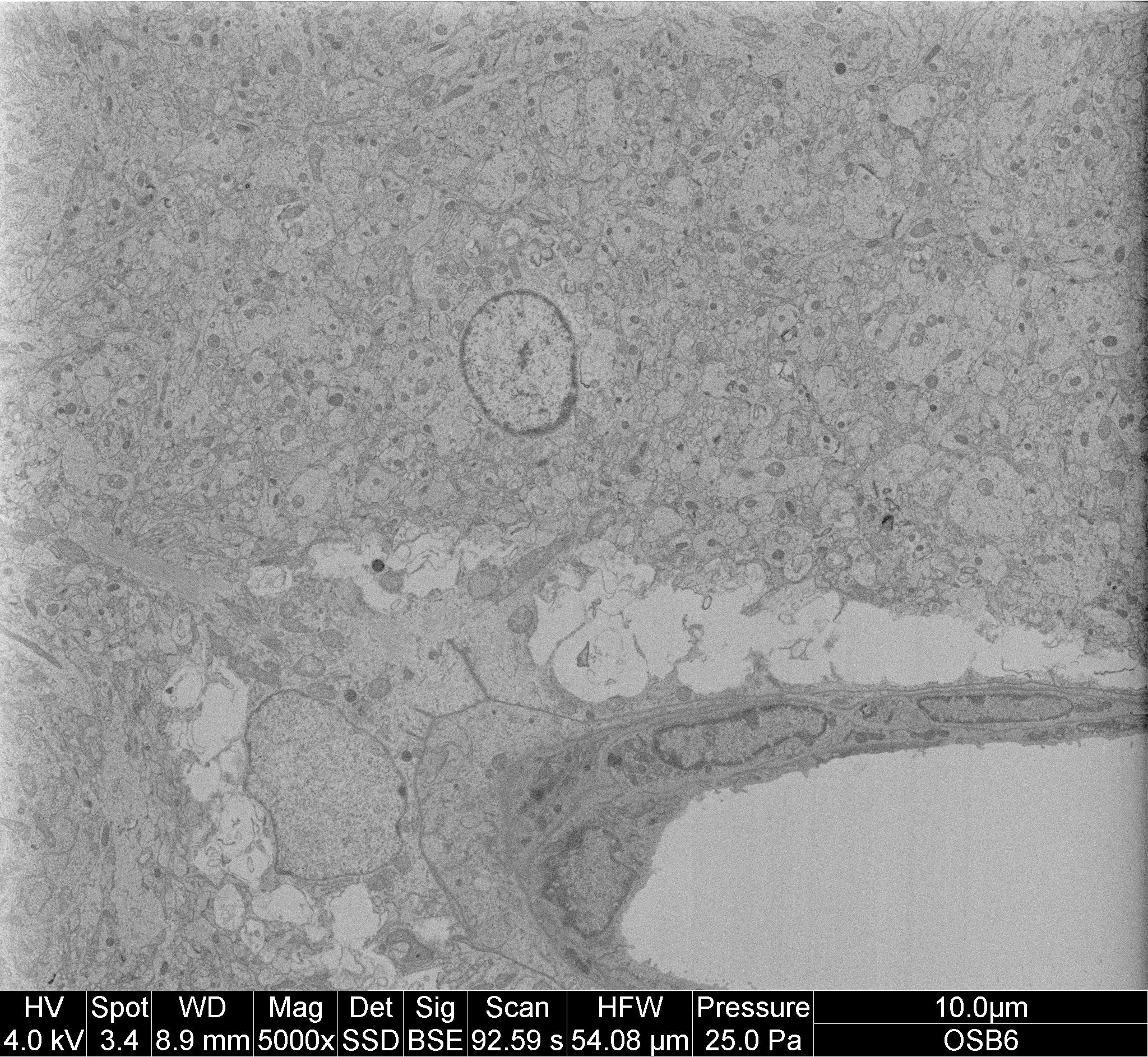

Supplement: Dataset S5 — (251.9 MB ZIP). [file pbio.0020329.sd005.zip › 040604_OS5_st1_444.tif]

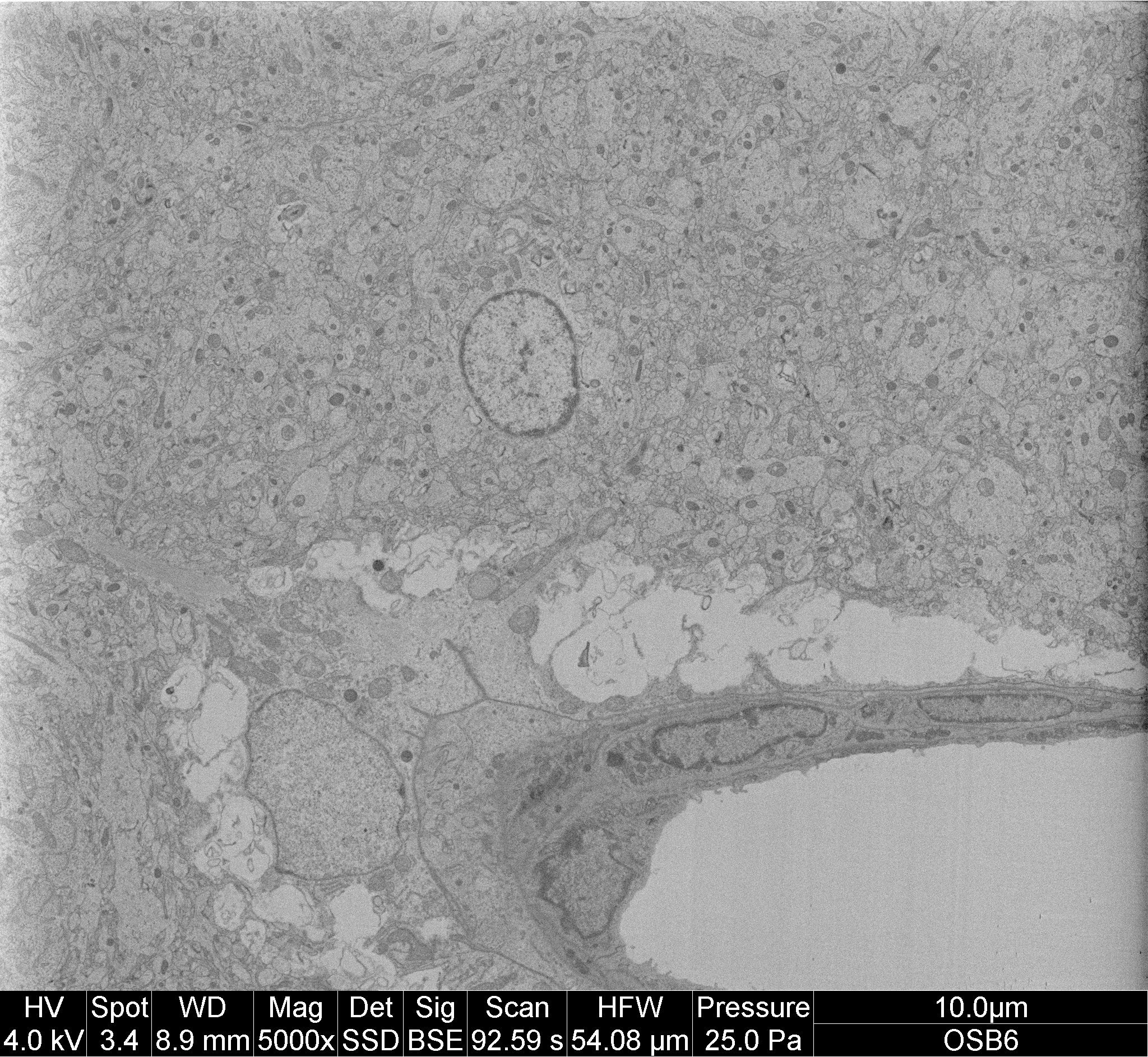

Supplement: Dataset S5 — (251.9 MB ZIP). [file pbio.0020329.sd005.zip › 040604_OS5_st1_445.tif]

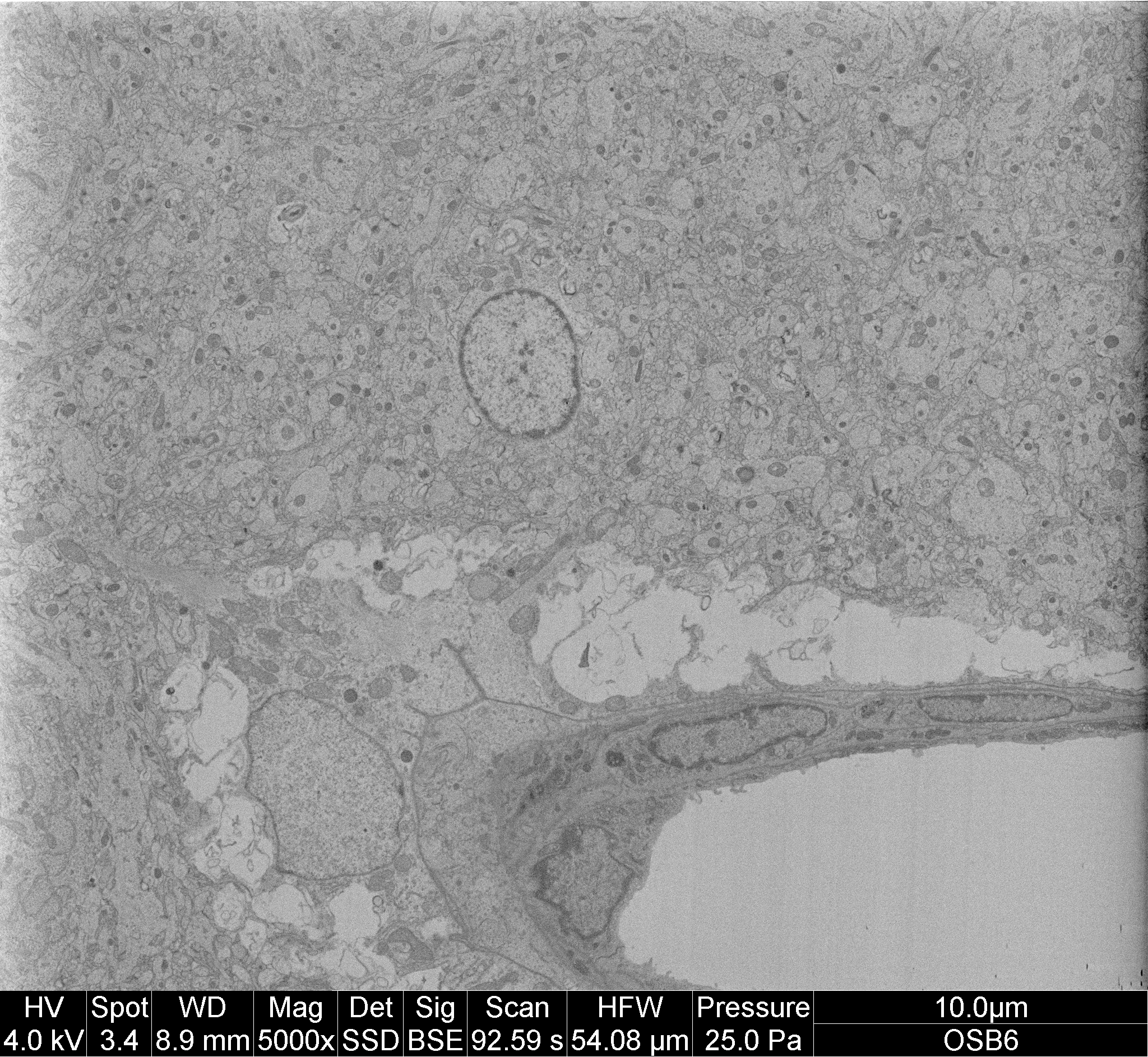

Supplement: Dataset S5 — (251.9 MB ZIP). [file pbio.0020329.sd005.zip › 040604_OS5_st1_446.tif]

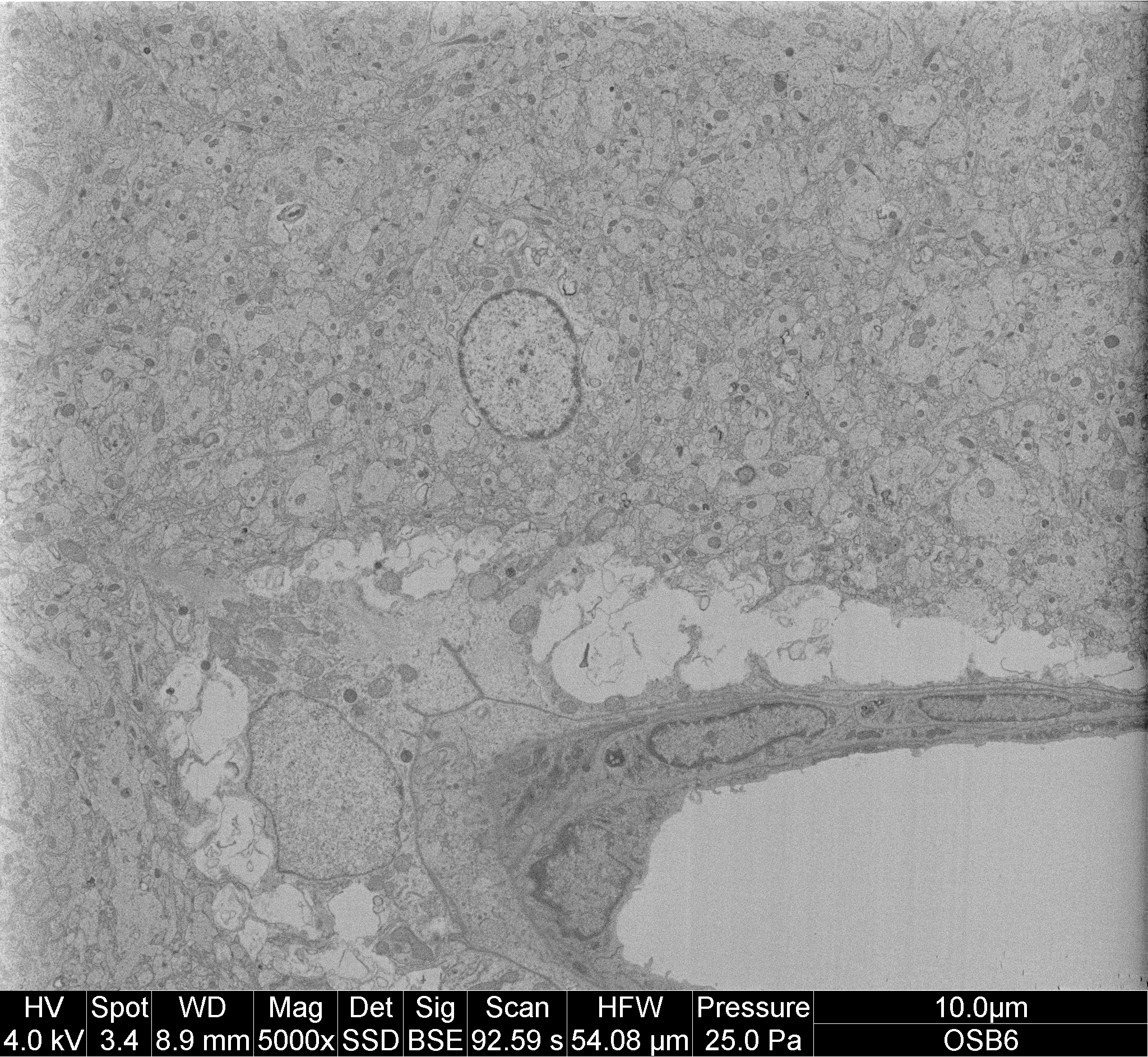

Supplement: Dataset S5 — (251.9 MB ZIP). [file pbio.0020329.sd005.zip › 040604_OS5_st1_447.tif]

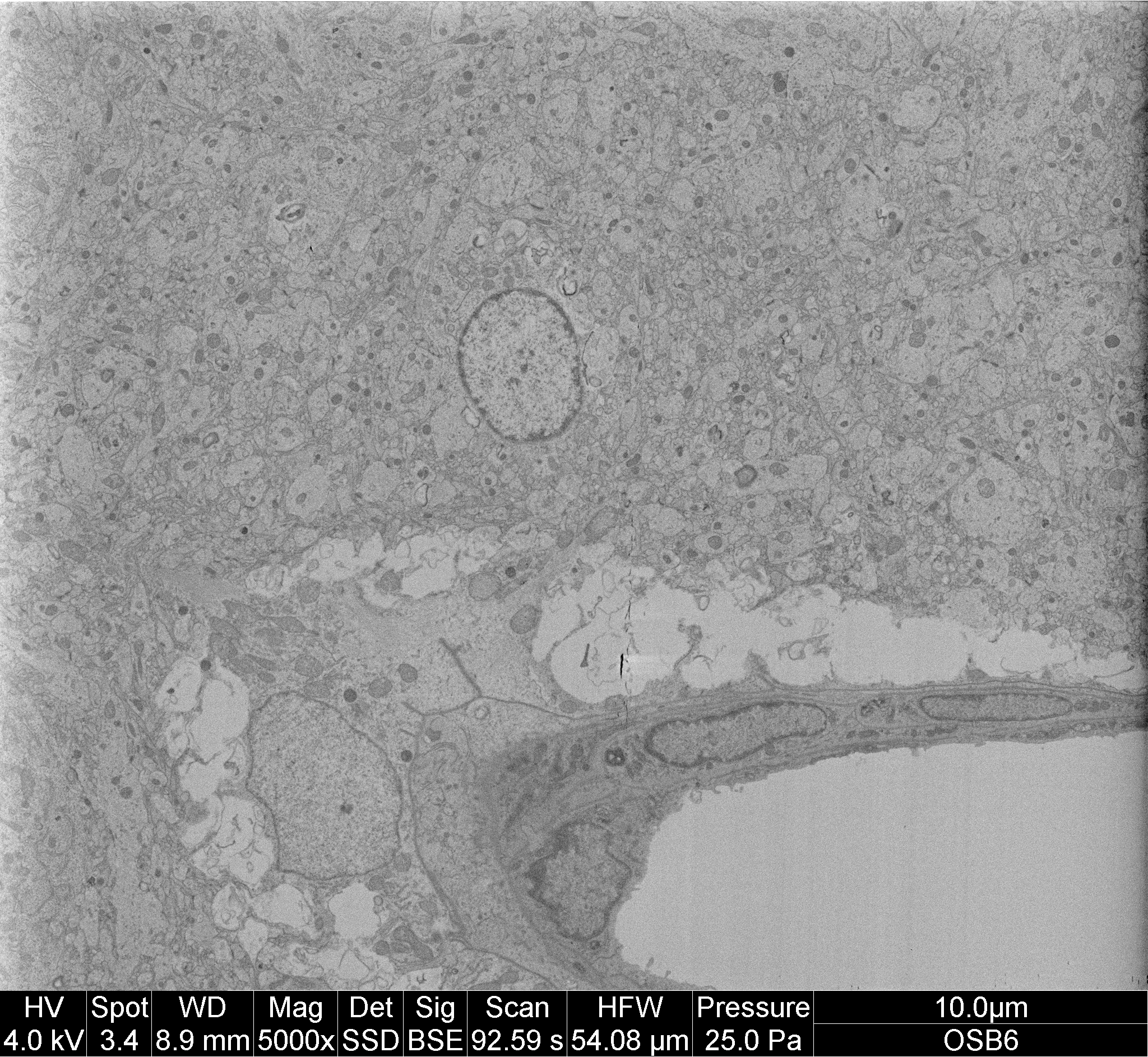

Supplement: Dataset S5 — (251.9 MB ZIP). [file pbio.0020329.sd005.zip › 040604_OS5_st1_448.tif]

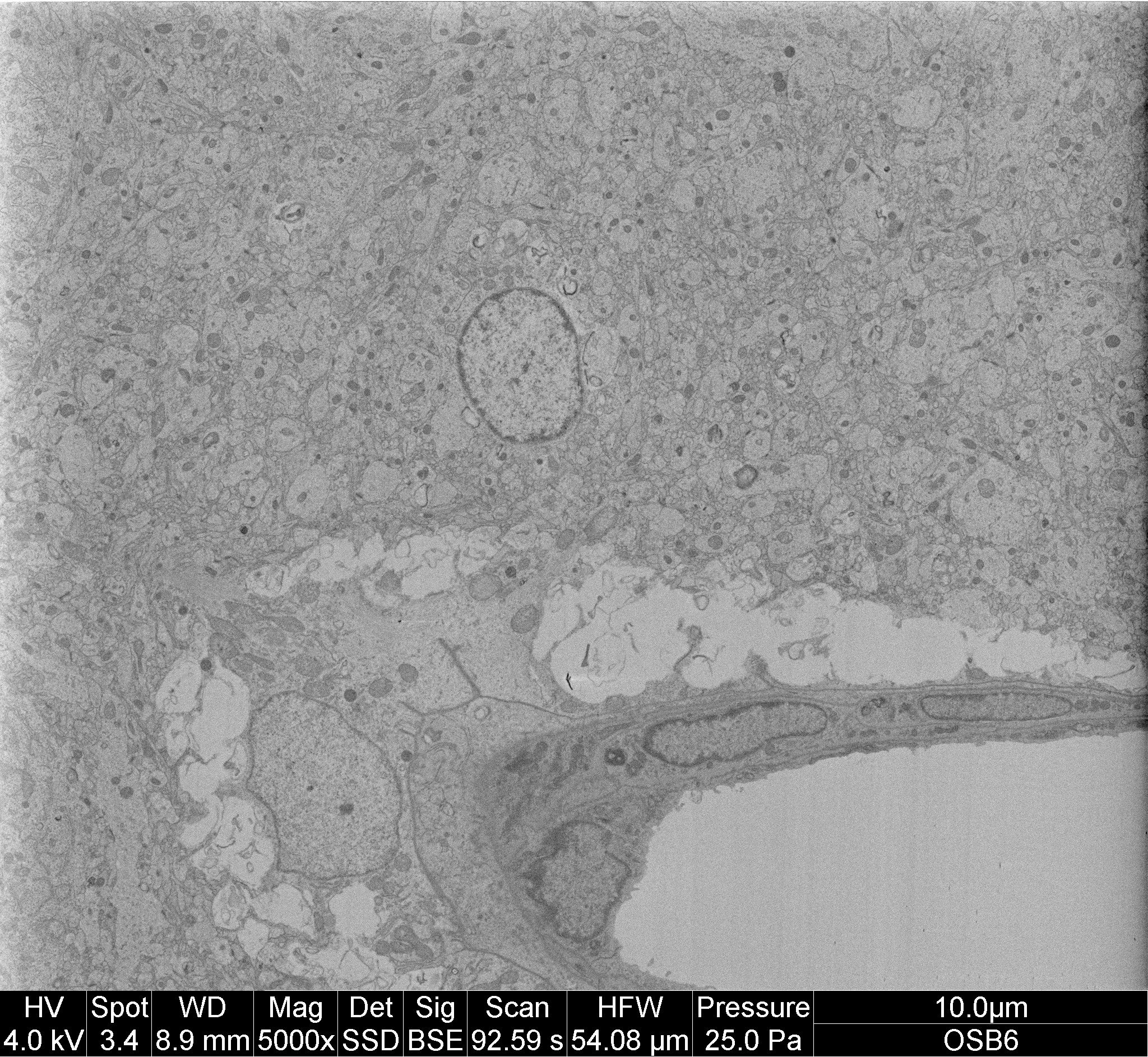

Supplement: Dataset S5 — (251.9 MB ZIP). [file pbio.0020329.sd005.zip › 040604_OS5_st1_449.tif]

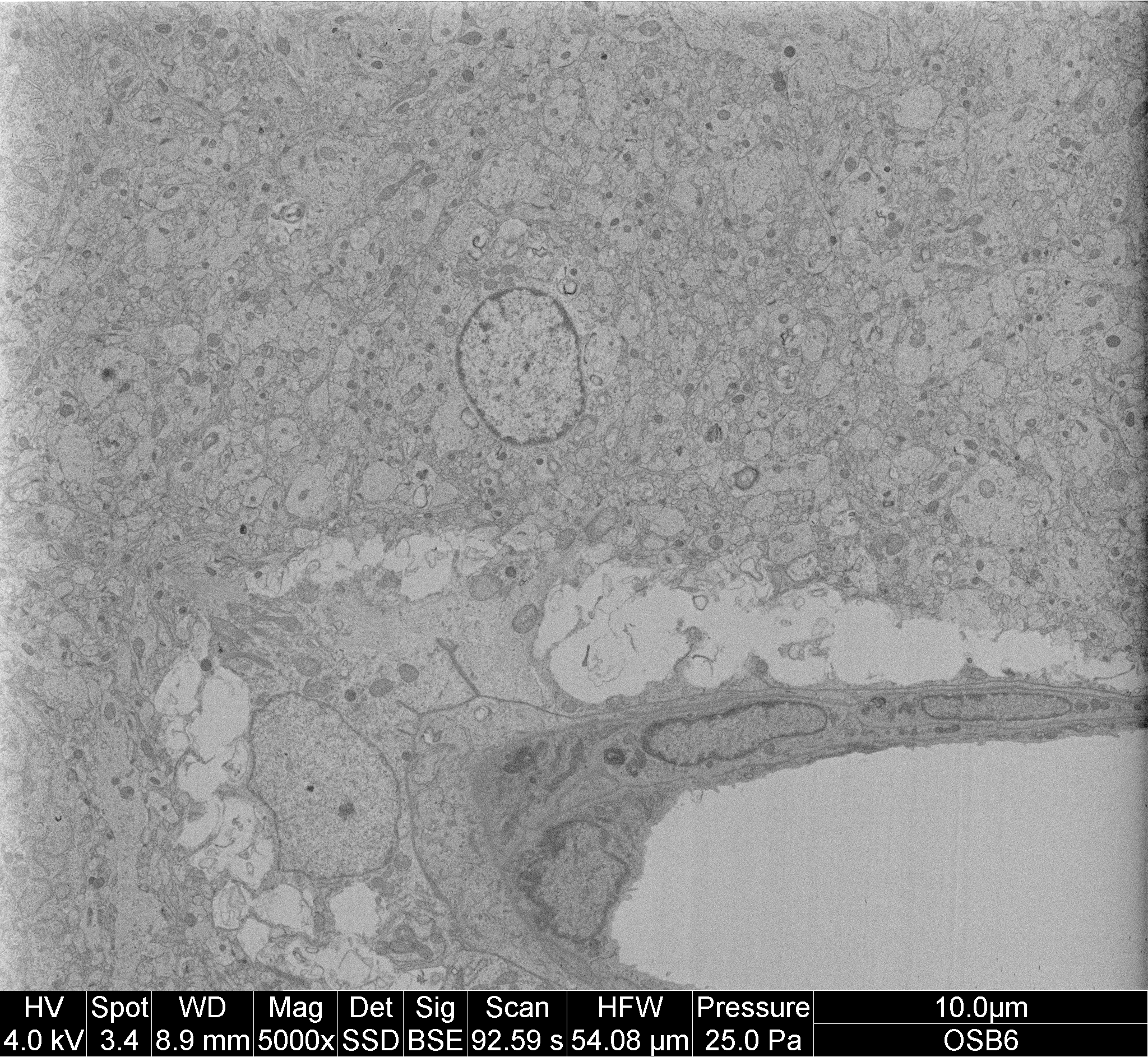

Supplement: Dataset S5 — (251.9 MB ZIP). [file pbio.0020329.sd005.zip › 040604_OS5_st1_450.tif]

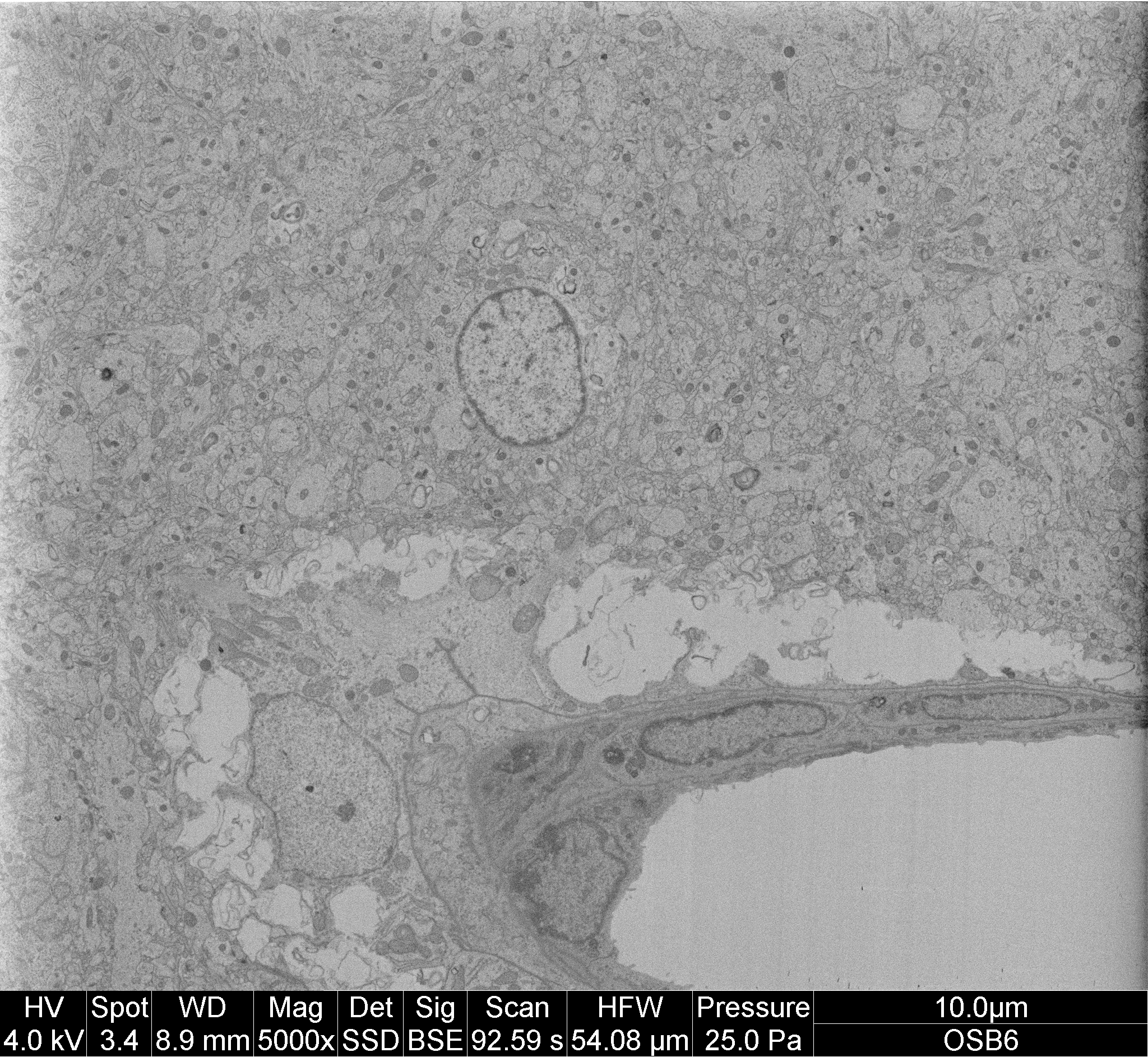

Supplement: Dataset S5 — (251.9 MB ZIP). [file pbio.0020329.sd005.zip › 040604_OS5_st1_451.tif]

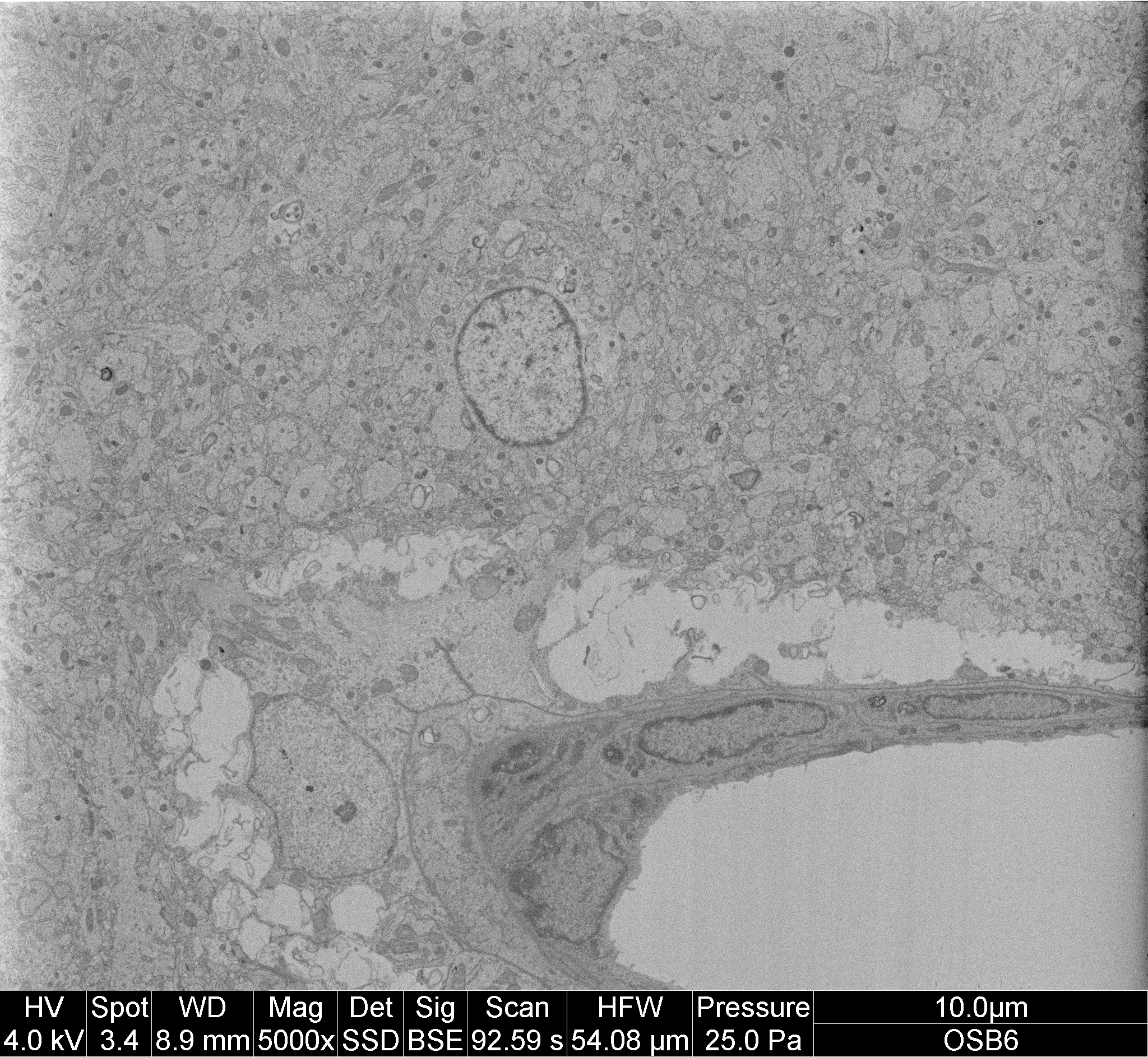

Supplement: Dataset S5 — (251.9 MB ZIP). [file pbio.0020329.sd005.zip › 040604_OS5_st1_452.tif]

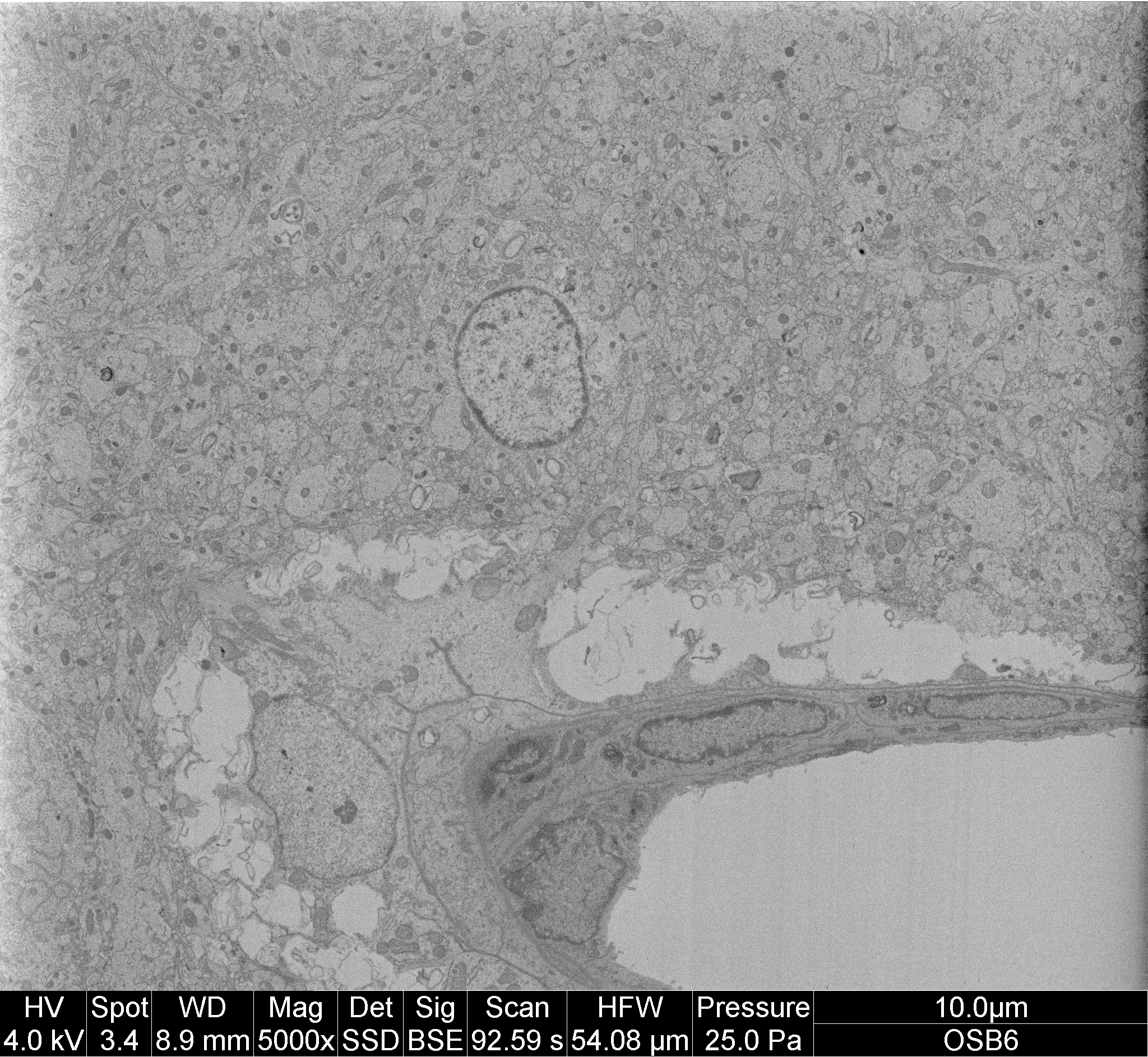

Supplement: Dataset S5 — (251.9 MB ZIP). [file pbio.0020329.sd005.zip › 040604_OS5_st1_453.tif]

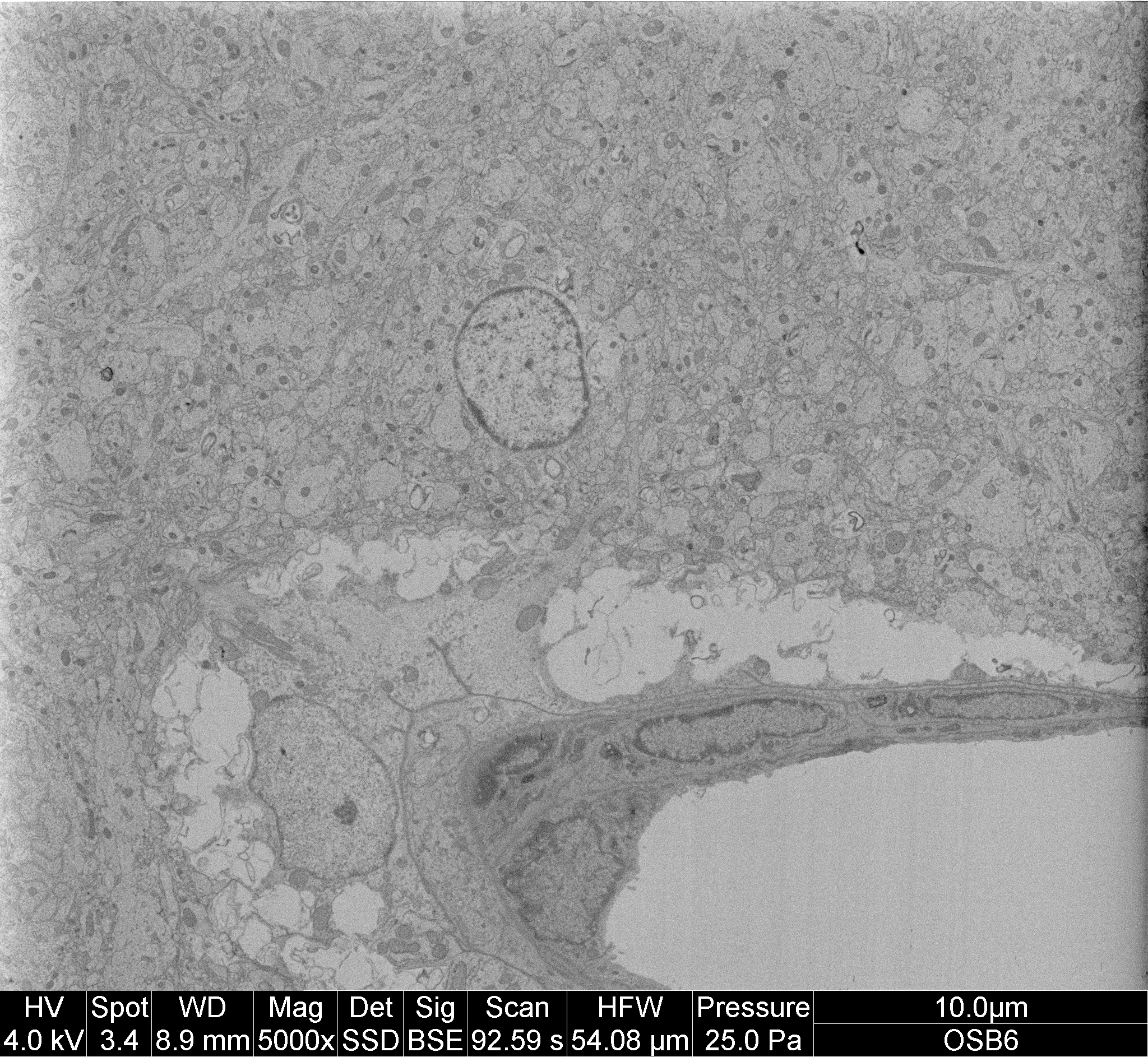

Supplement: Dataset S5 — (251.9 MB ZIP). [file pbio.0020329.sd005.zip › 040604_OS5_st1_454.tif]

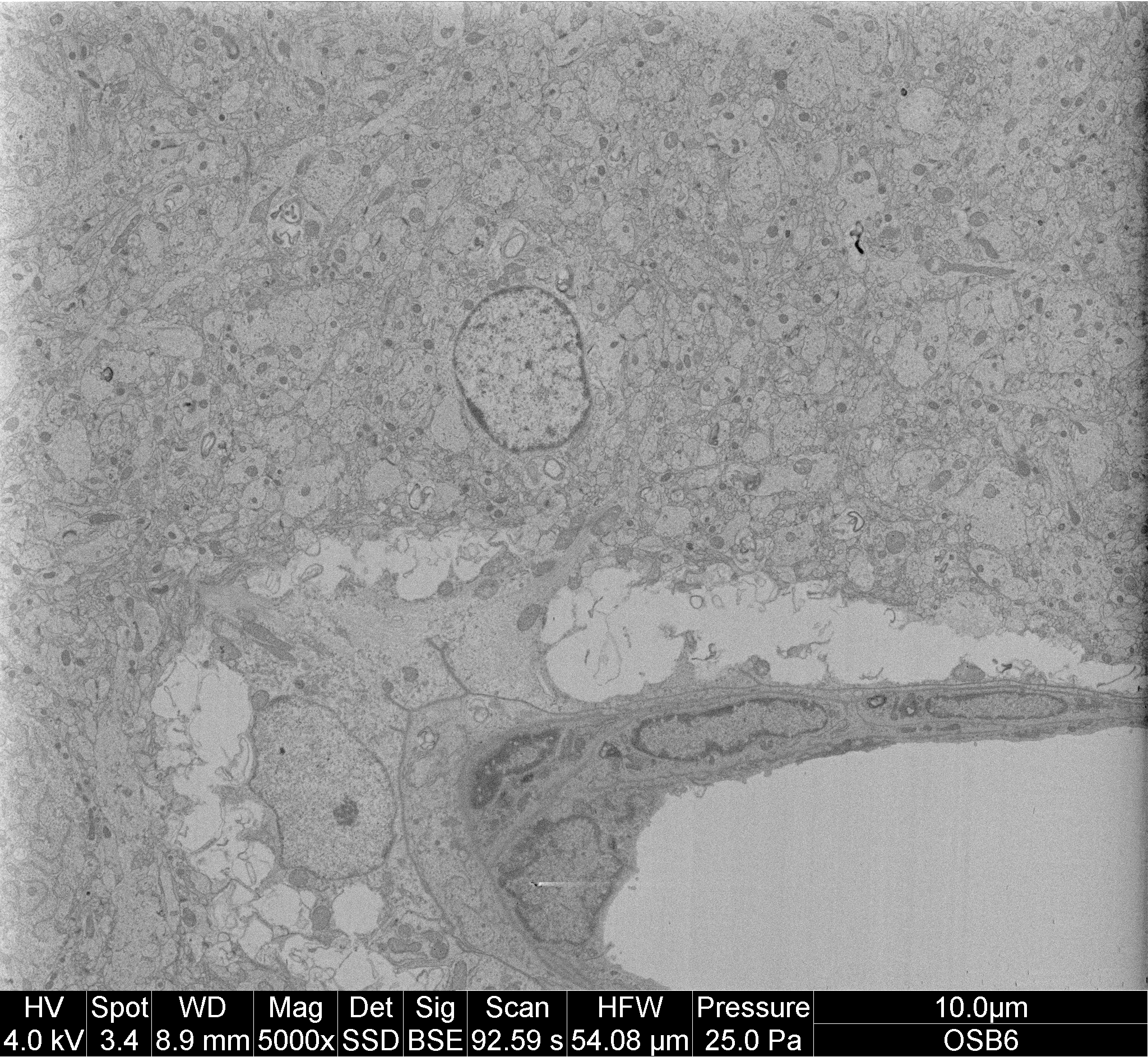

Supplement: Dataset S5 — (251.9 MB ZIP). [file pbio.0020329.sd005.zip › 040604_OS5_st1_455.tif]

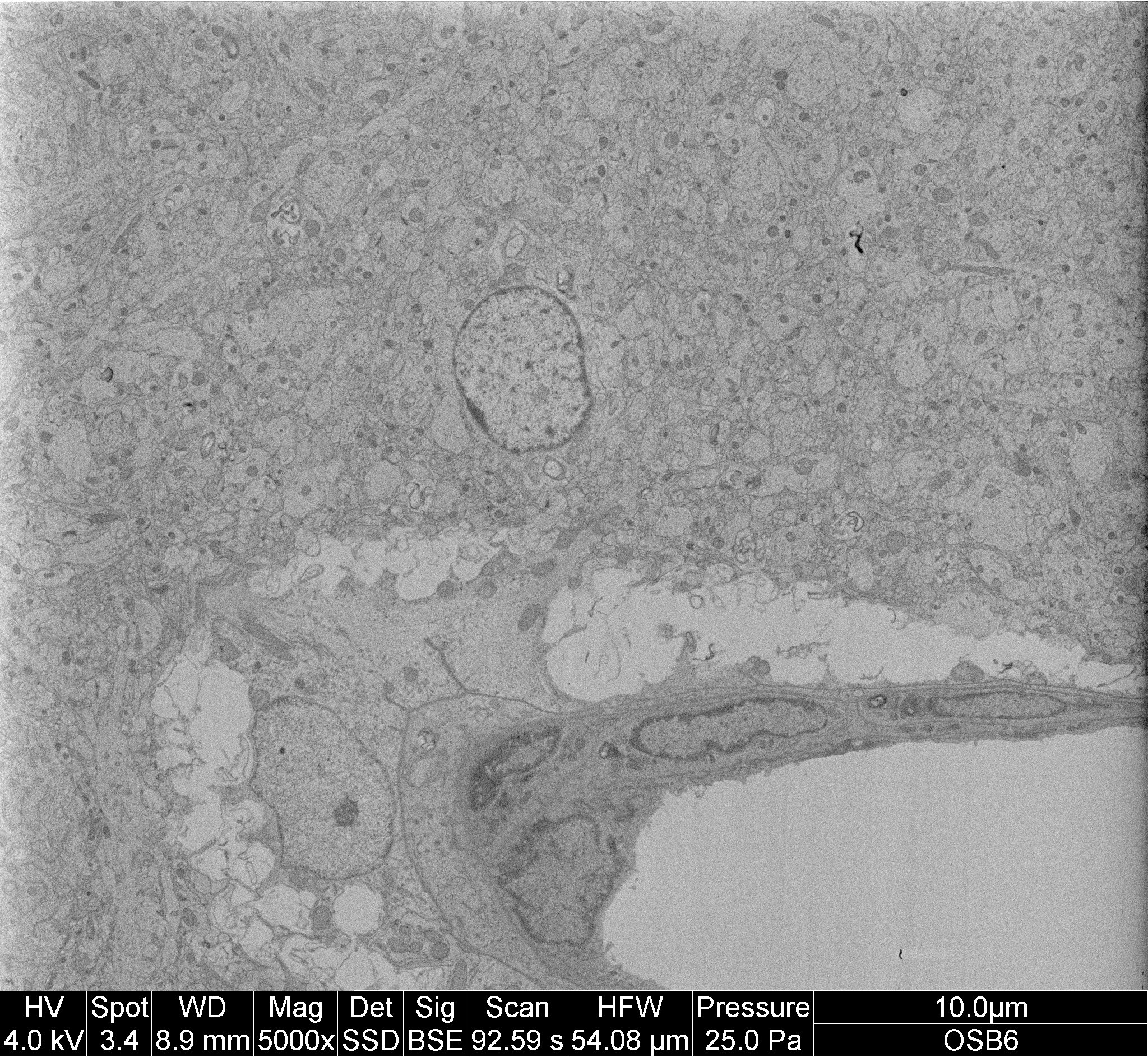

Supplement: Dataset S5 — (251.9 MB ZIP). [file pbio.0020329.sd005.zip › 040604_OS5_st1_456.tif]

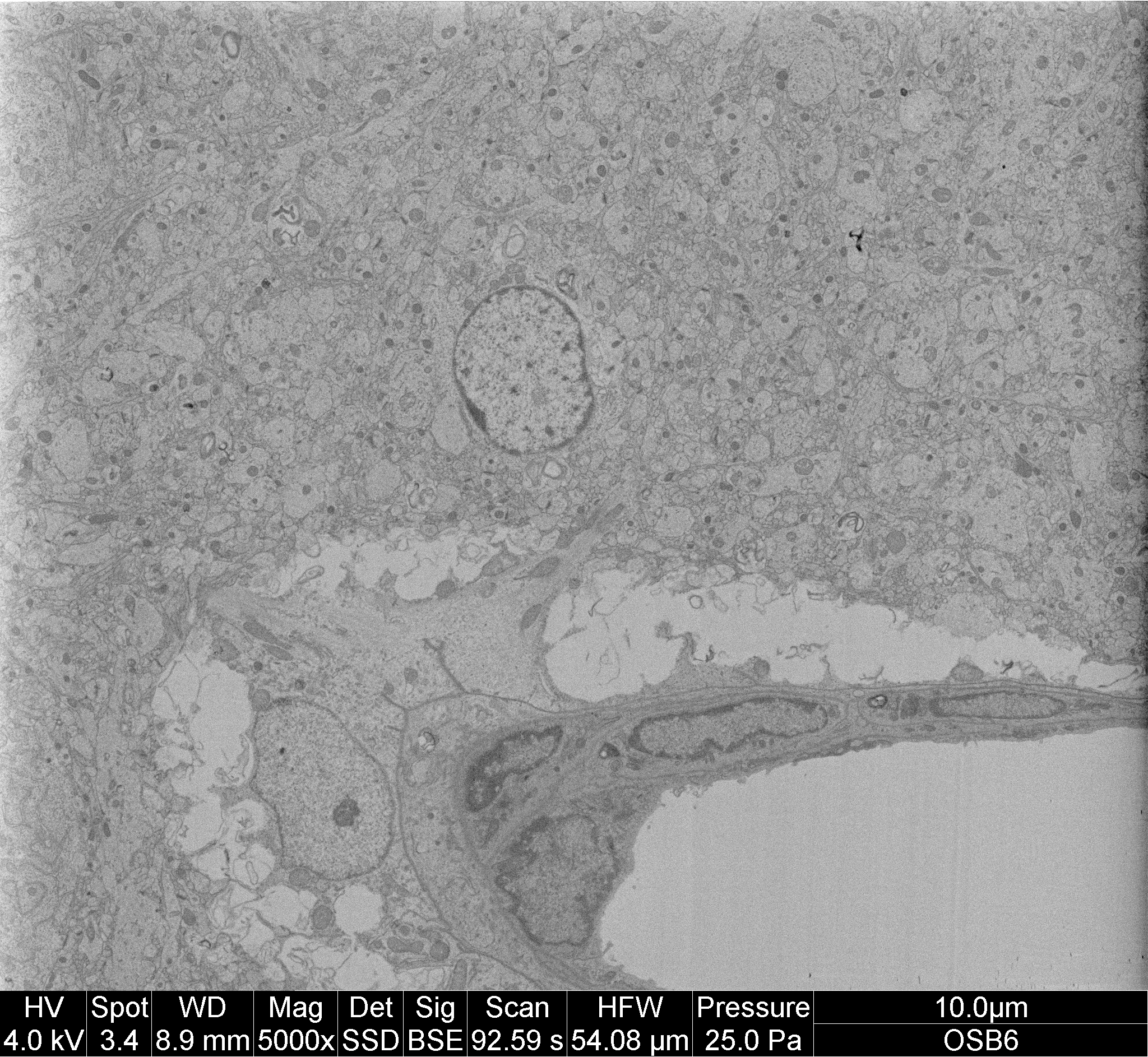

Supplement: Dataset S5 — (251.9 MB ZIP). [file pbio.0020329.sd005.zip › 040604_OS5_st1_457.tif]

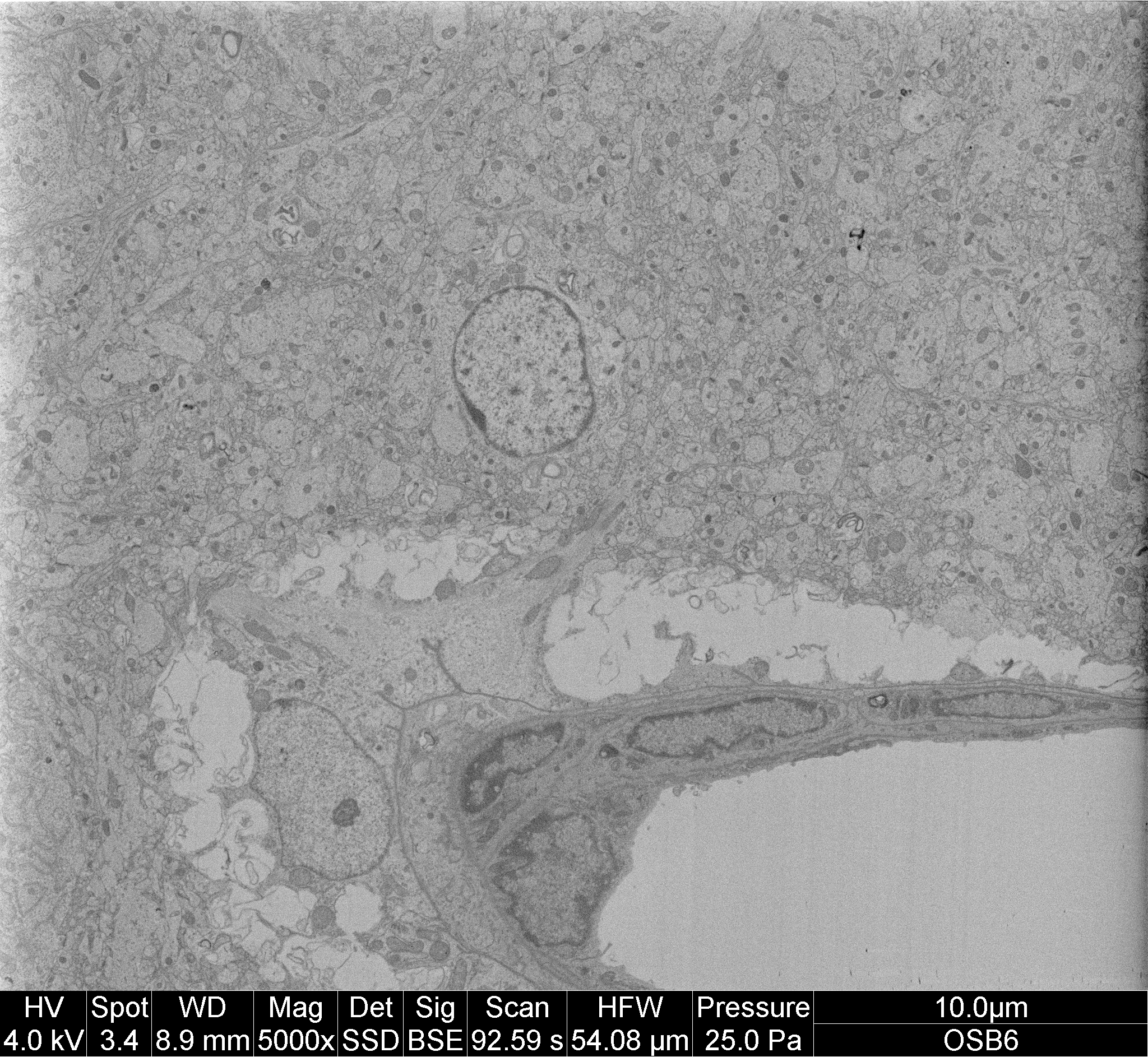

Supplement: Dataset S5 — (251.9 MB ZIP). [file pbio.0020329.sd005.zip › 040604_OS5_st1_458.tif]

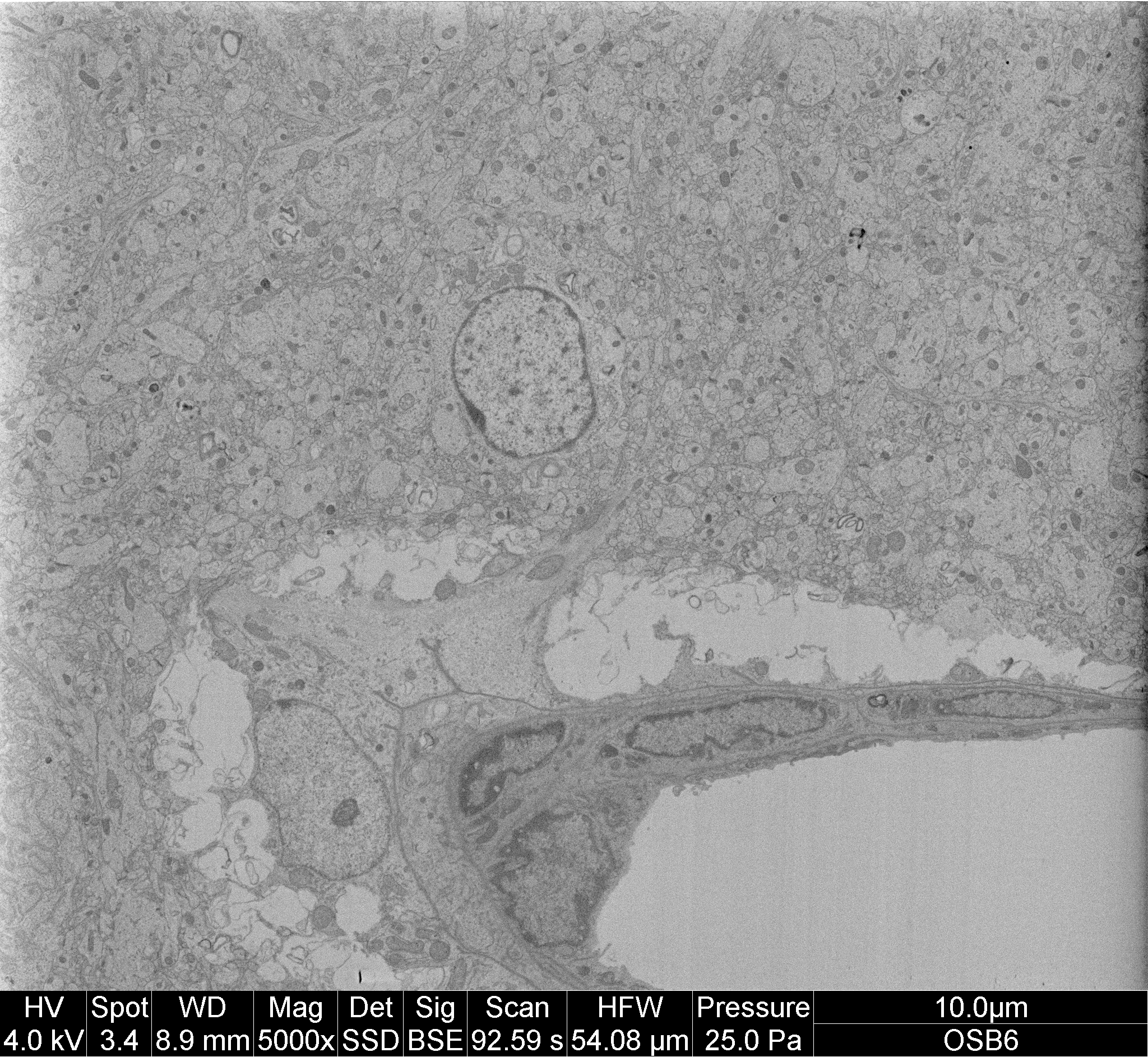

Supplement: Dataset S5 — (251.9 MB ZIP). [file pbio.0020329.sd005.zip › 040604_OS5_st1_459.tif]

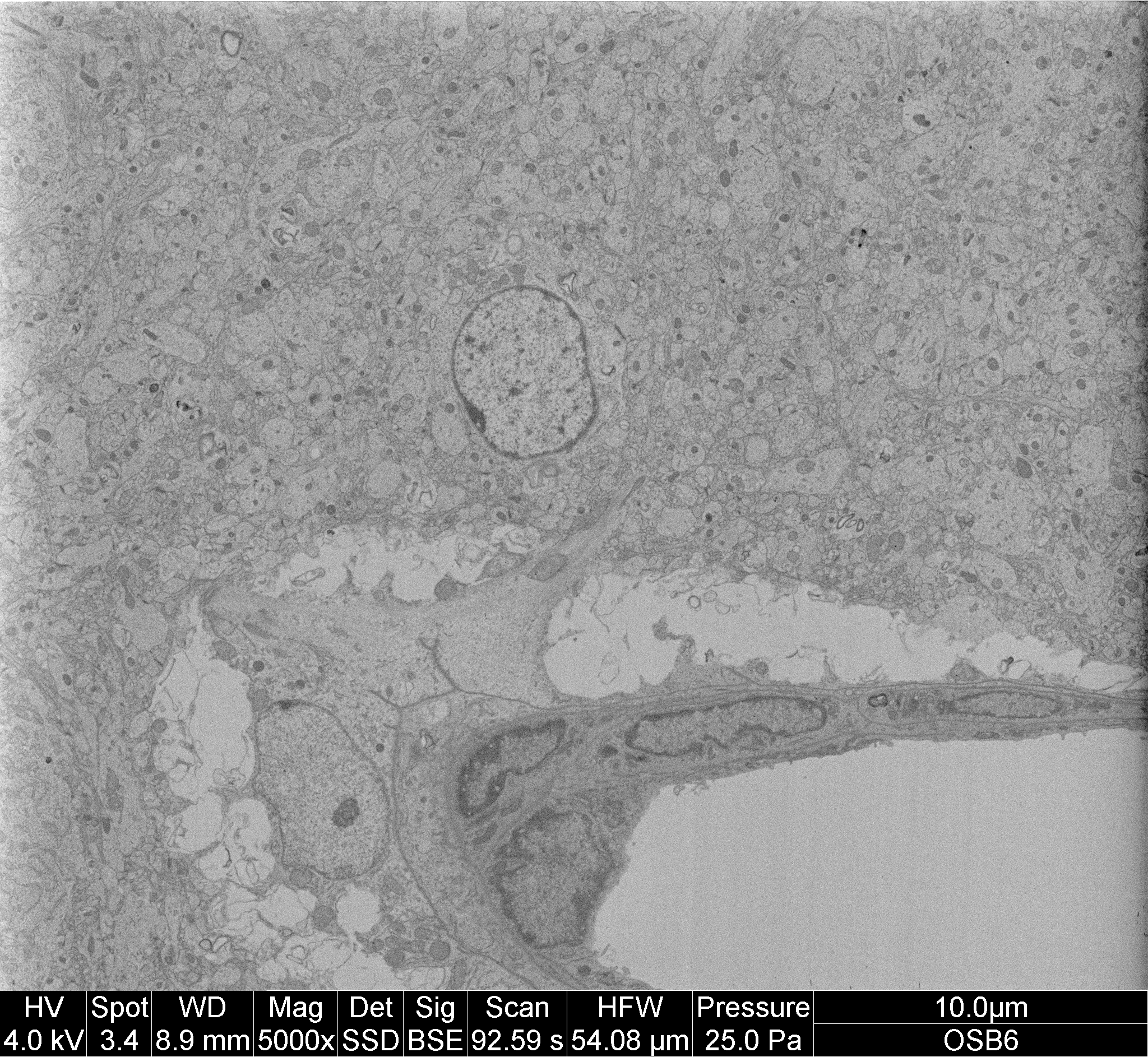

Supplement: Dataset S5 — (251.9 MB ZIP). [file pbio.0020329.sd005.zip › 040604_OS5_st1_460.tif]

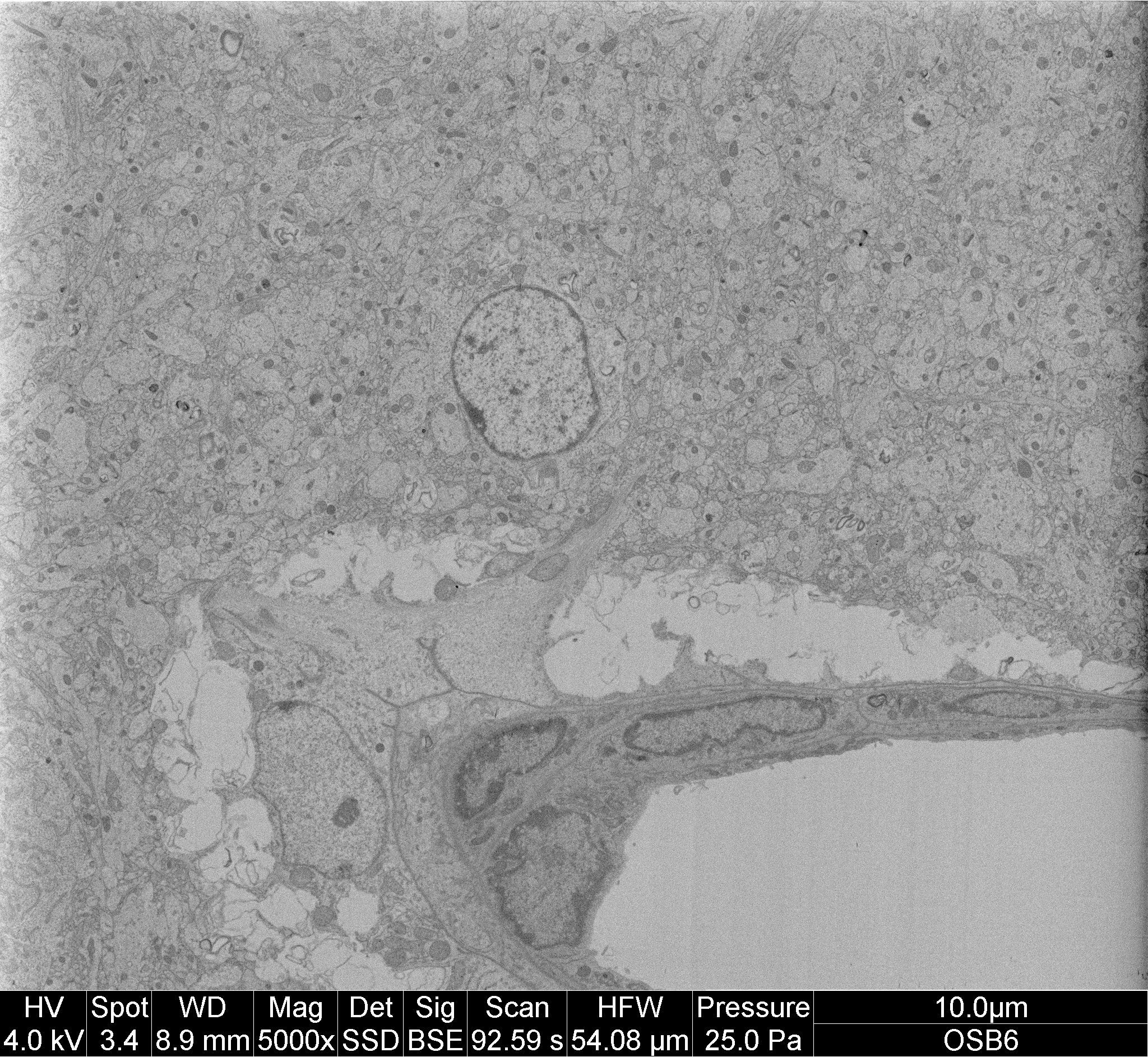

Supplement: Dataset S5 — (251.9 MB ZIP). [file pbio.0020329.sd005.zip › 040604_OS5_st1_461.tif]

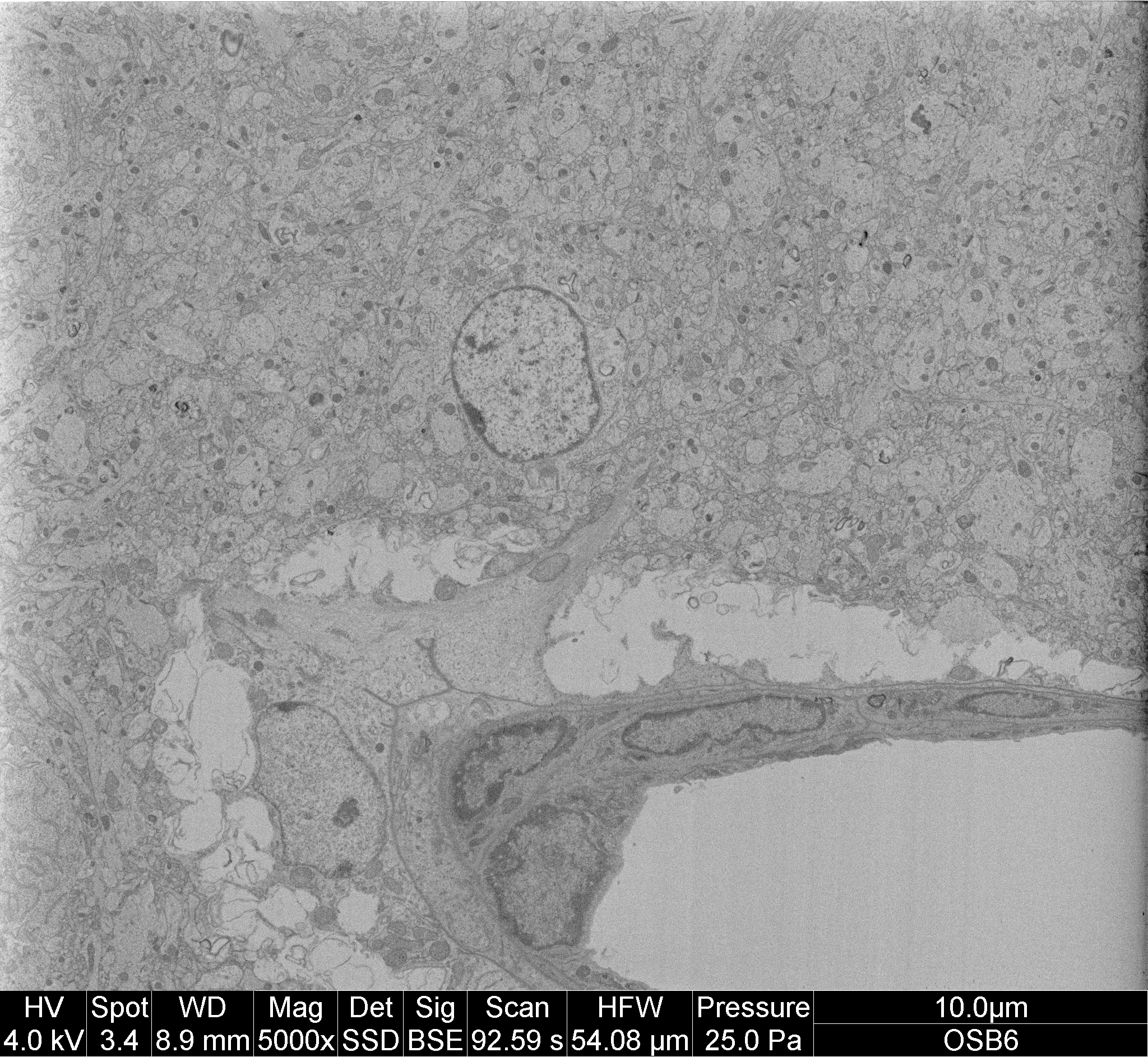

Supplement: Dataset S5 — (251.9 MB ZIP). [file pbio.0020329.sd005.zip › 040604_OS5_st1_462.tif]

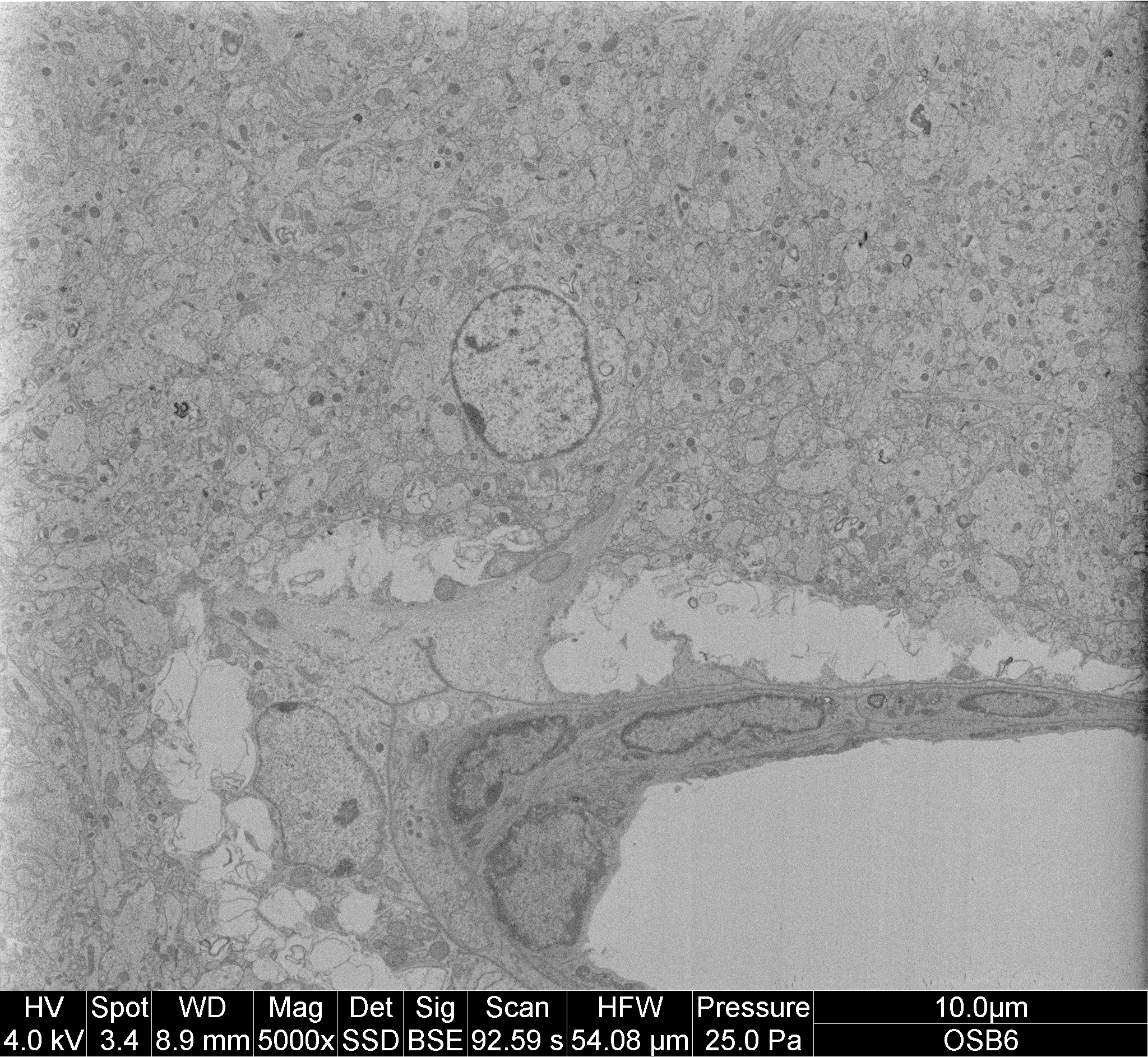

Supplement: Dataset S5 — (251.9 MB ZIP). [file pbio.0020329.sd005.zip › 040604_OS5_st1_463.tif]

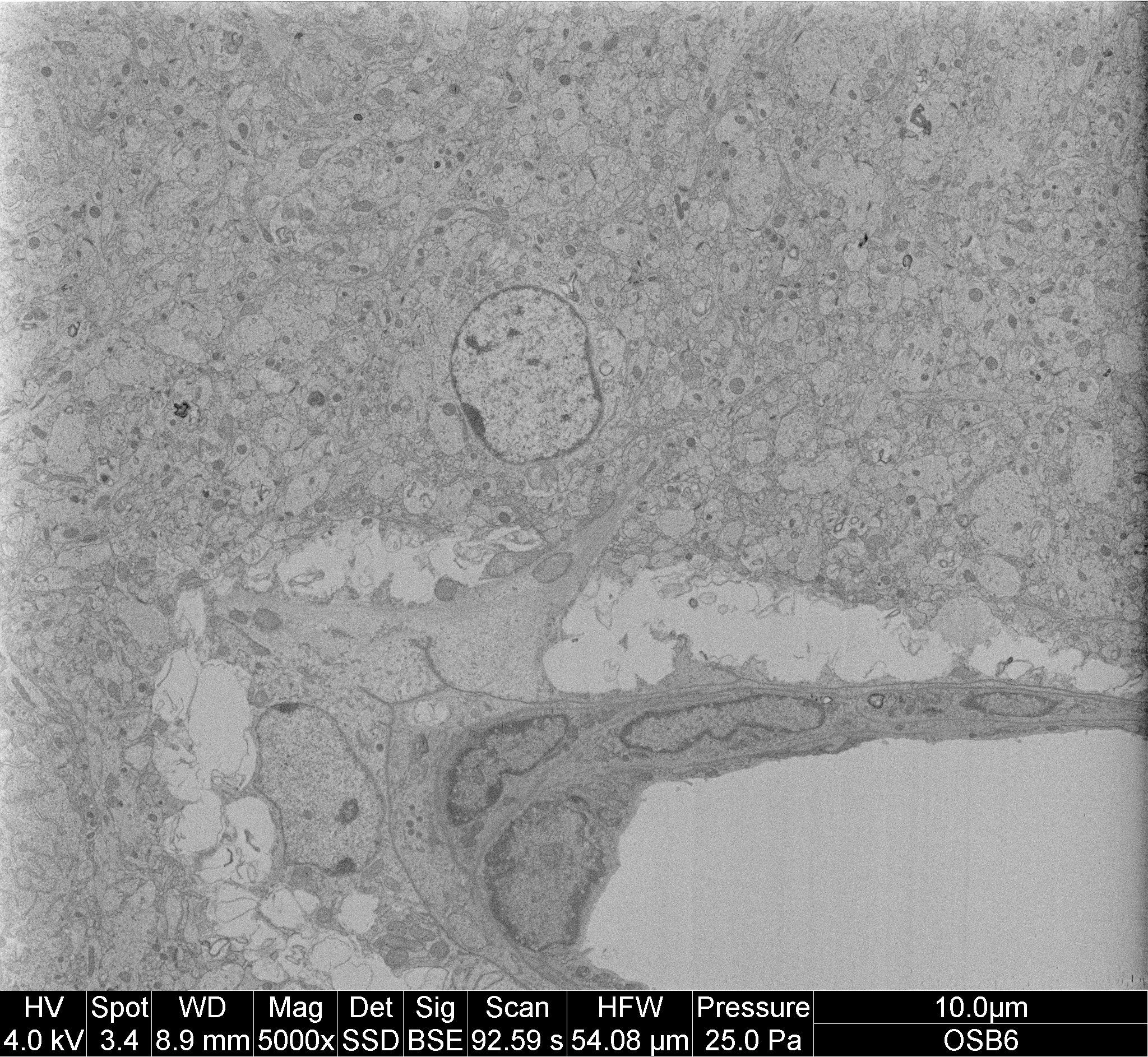

Supplement: Dataset S5 — (251.9 MB ZIP). [file pbio.0020329.sd005.zip › 040604_OS5_st1_464.tif]

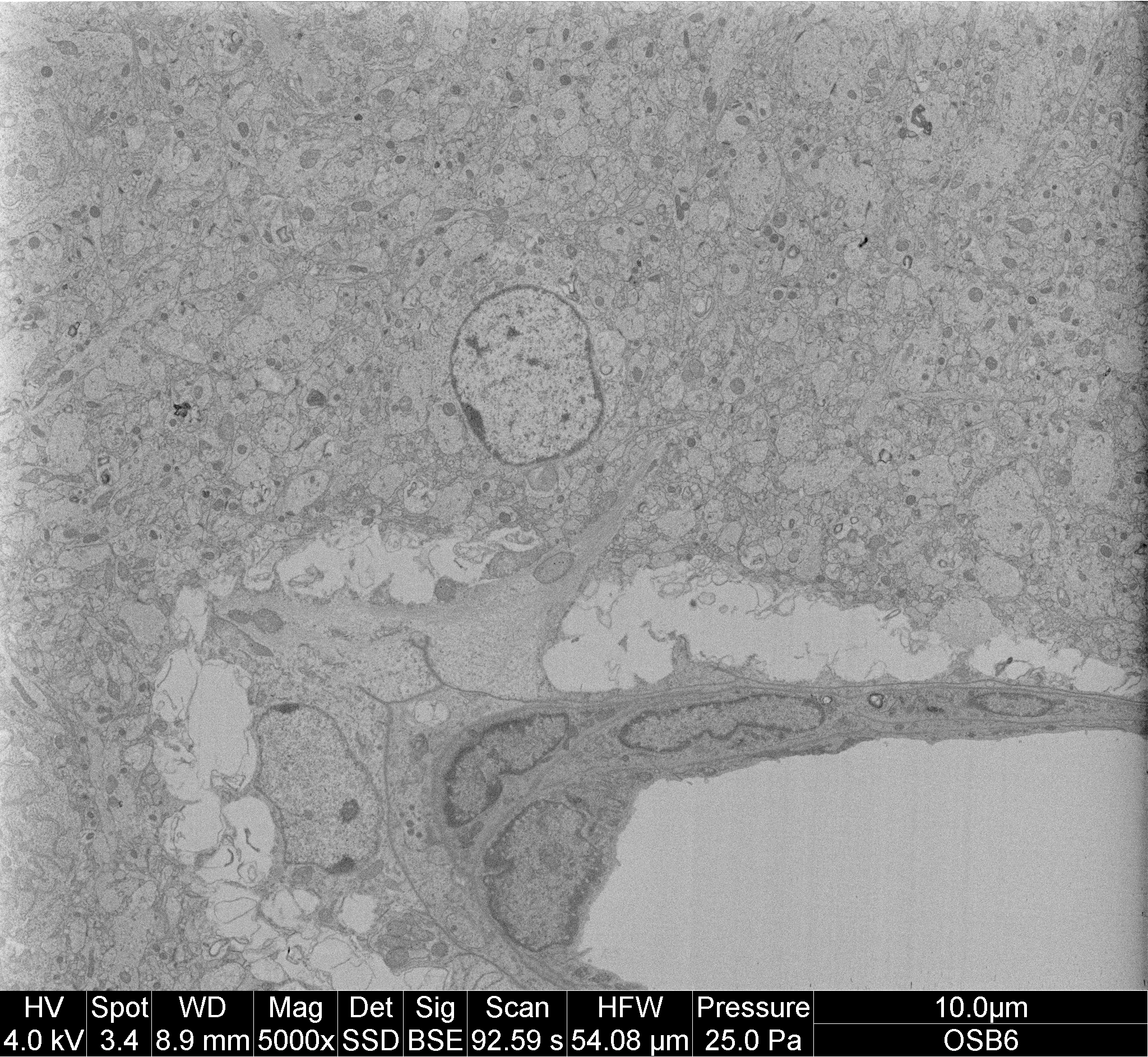

Supplement: Dataset S5 — (251.9 MB ZIP). [file pbio.0020329.sd005.zip › 040604_OS5_st1_465.tif]

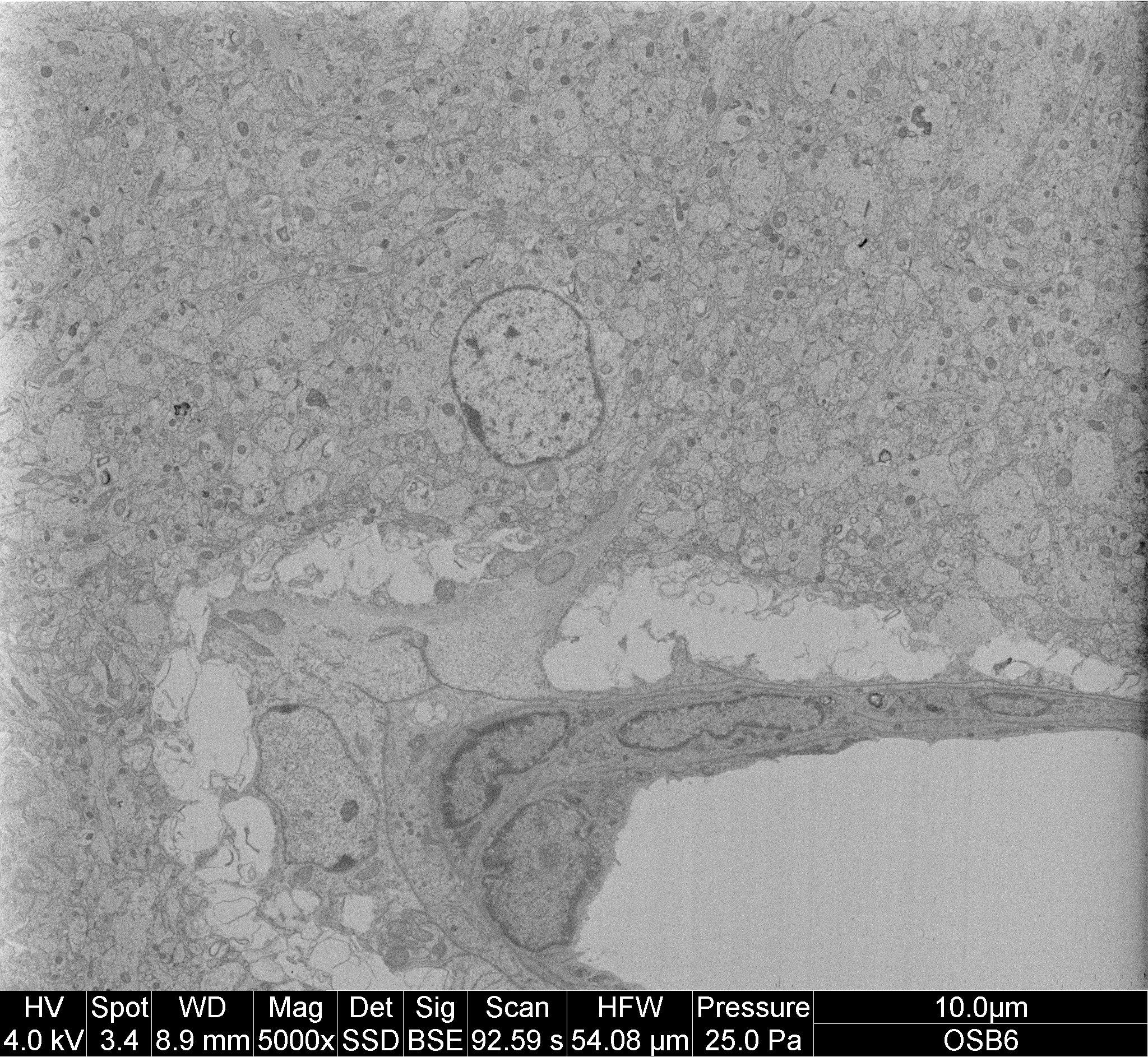

Supplement: Dataset S5 — (251.9 MB ZIP). [file pbio.0020329.sd005.zip › 040604_OS5_st1_466.tif]

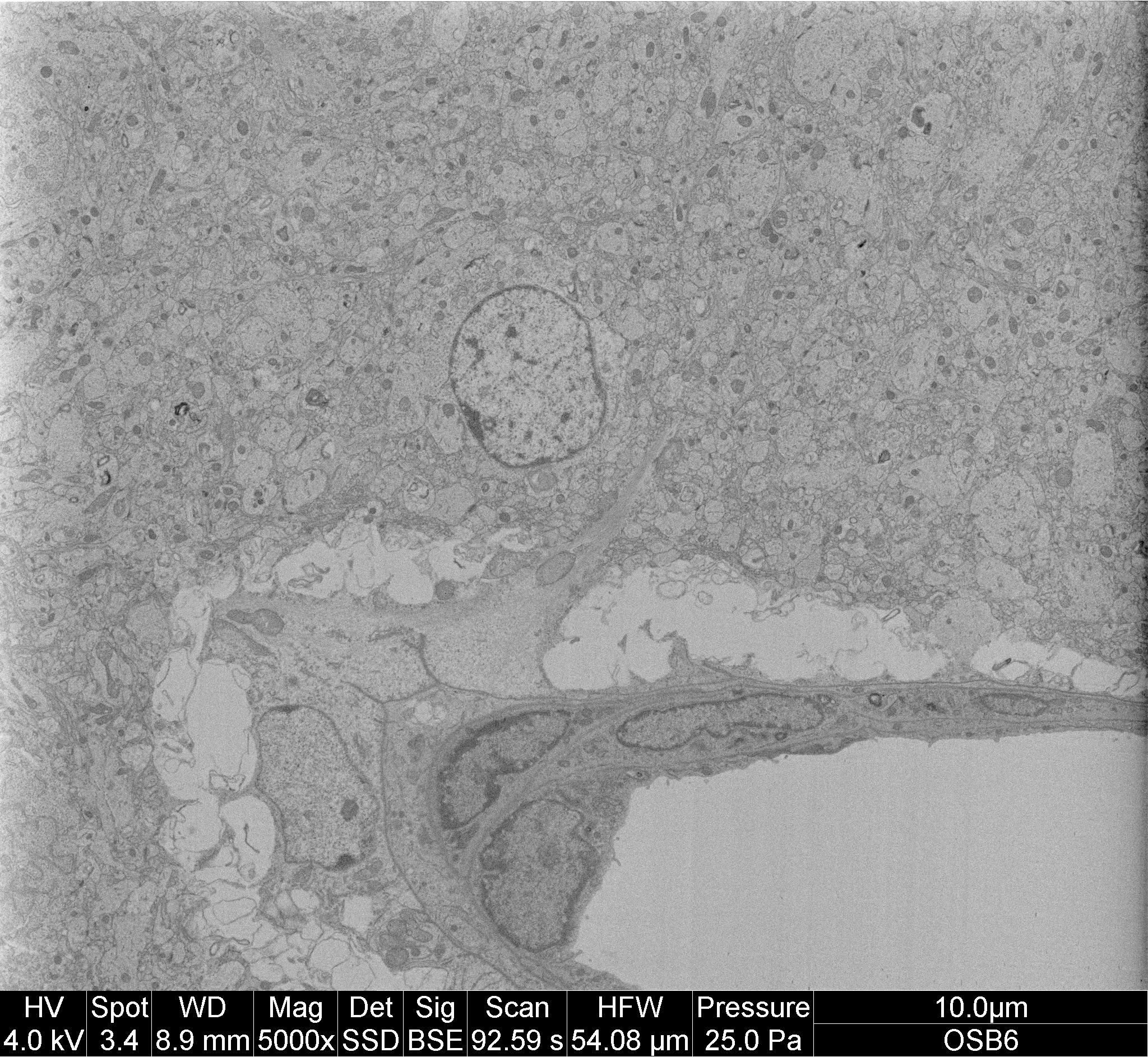

Supplement: Dataset S5 — (251.9 MB ZIP). [file pbio.0020329.sd005.zip › 040604_OS5_st1_467.tif]

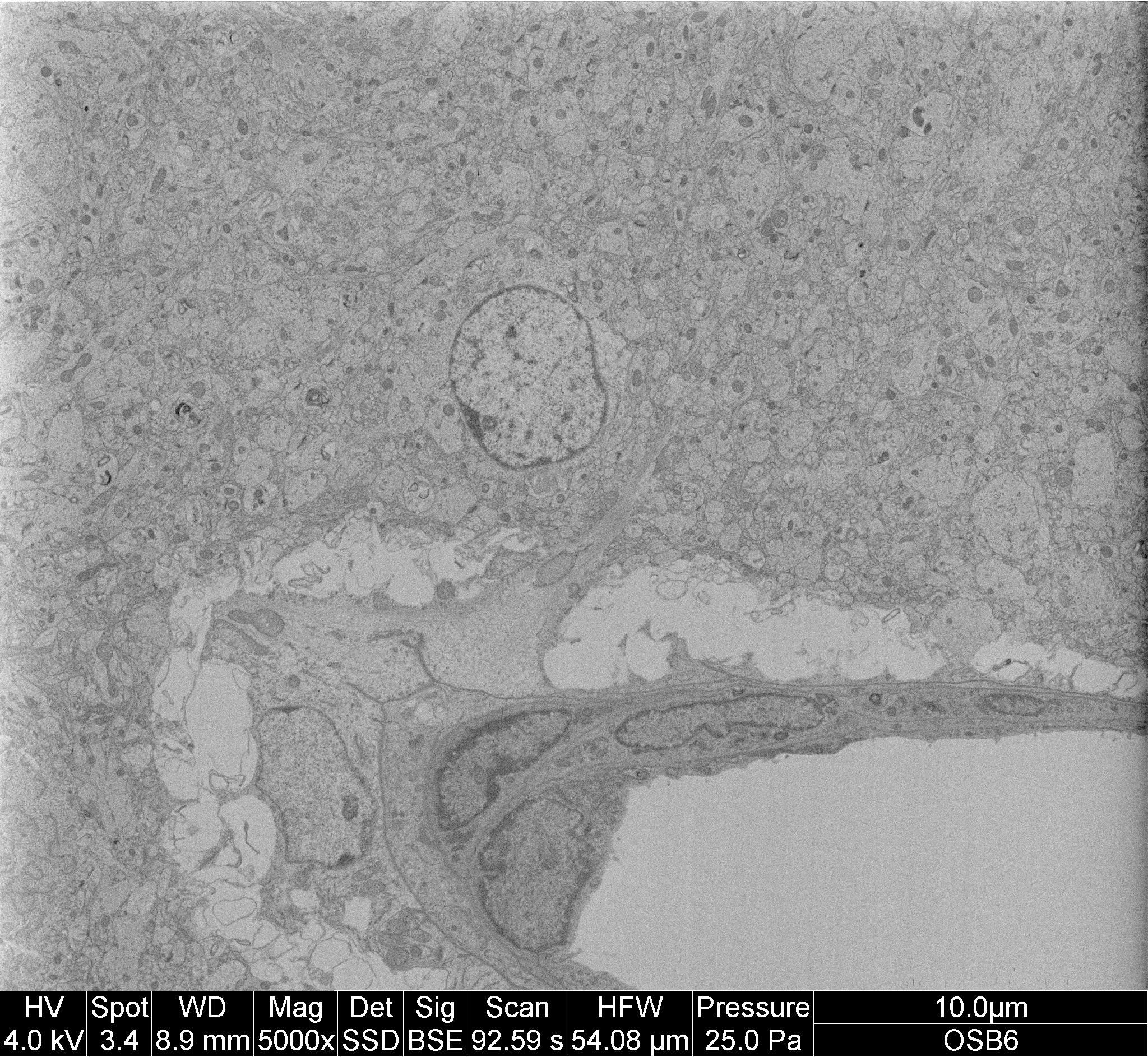

Supplement: Dataset S5 — (251.9 MB ZIP). [file pbio.0020329.sd005.zip › 040604_OS5_st1_468.tif]

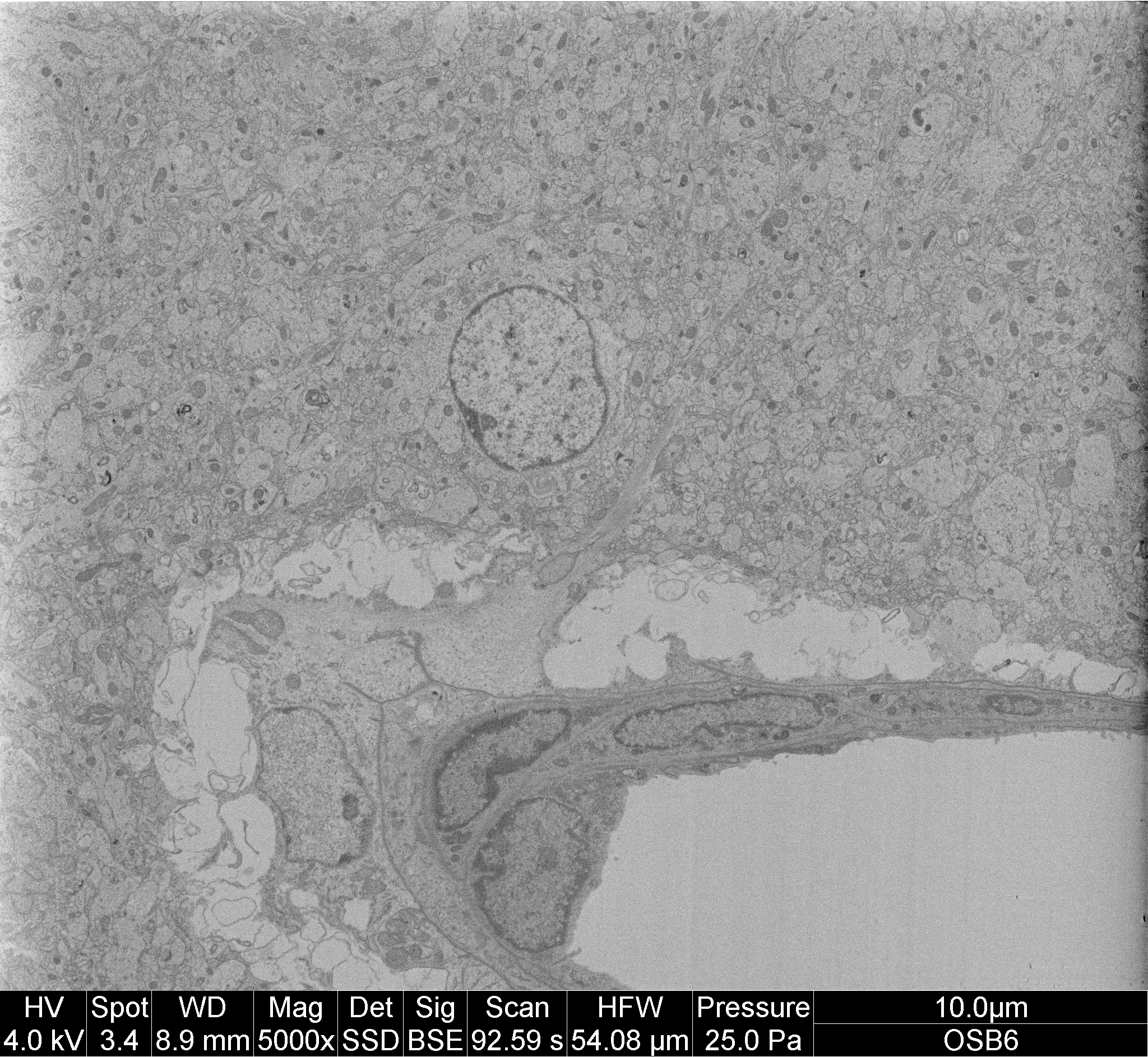

Supplement: Dataset S5 — (251.9 MB ZIP). [file pbio.0020329.sd005.zip › 040604_OS5_st1_469.tif]

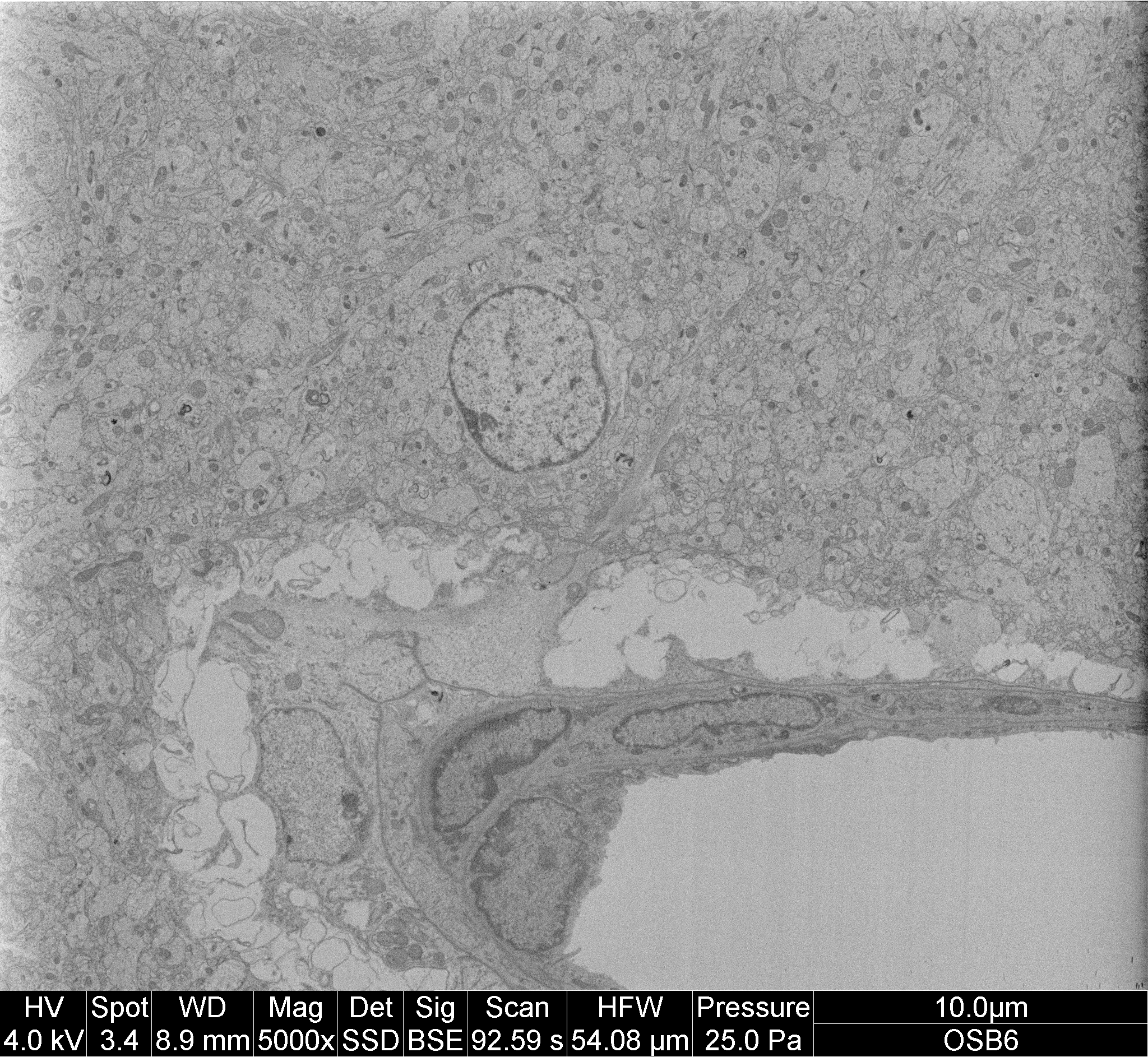

Supplement: Dataset S5 — (251.9 MB ZIP). [file pbio.0020329.sd005.zip › 040604_OS5_st1_470.tif]

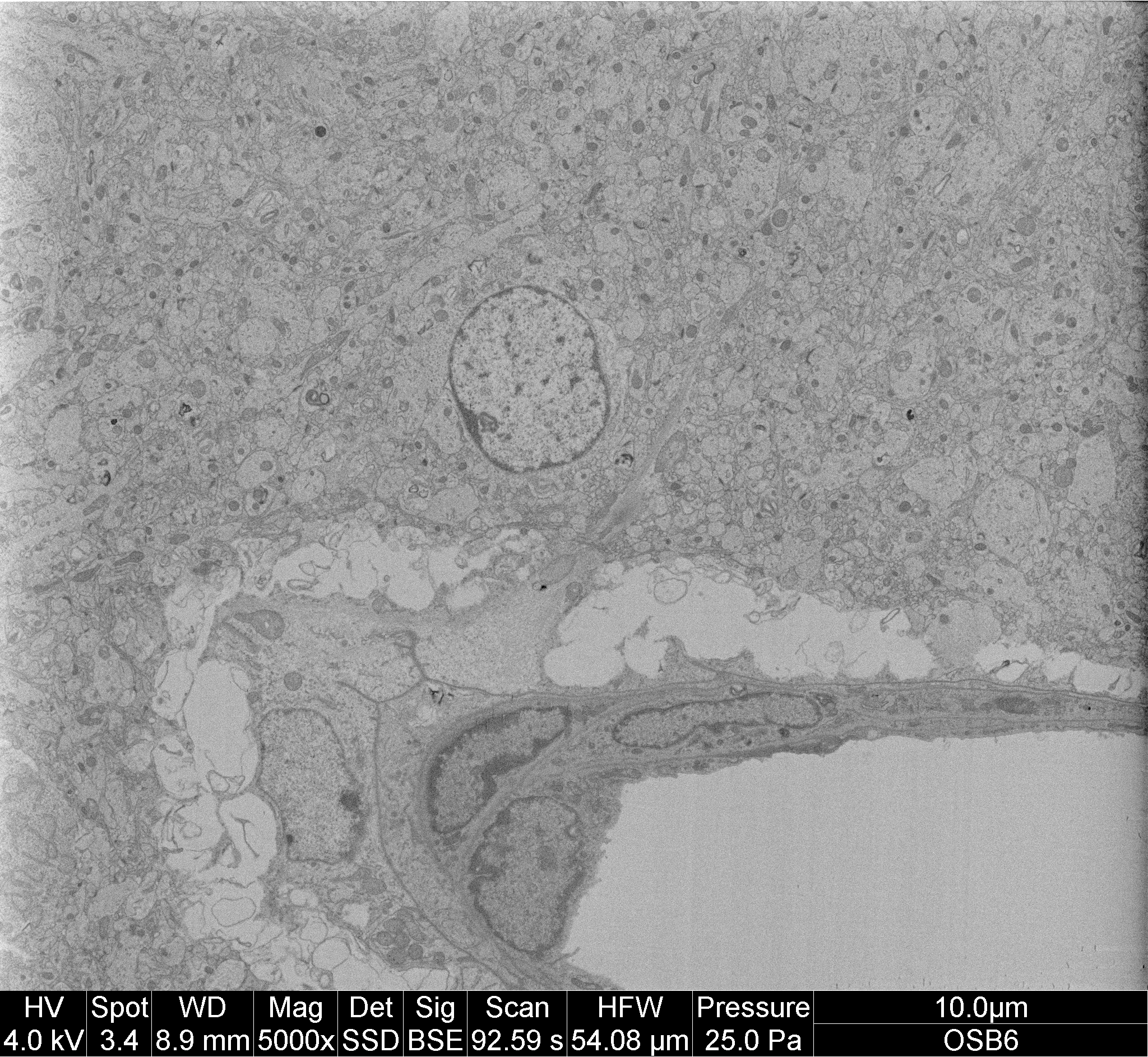

Supplement: Dataset S5 — (251.9 MB ZIP). [file pbio.0020329.sd005.zip › 040604_OS5_st1_471.tif]

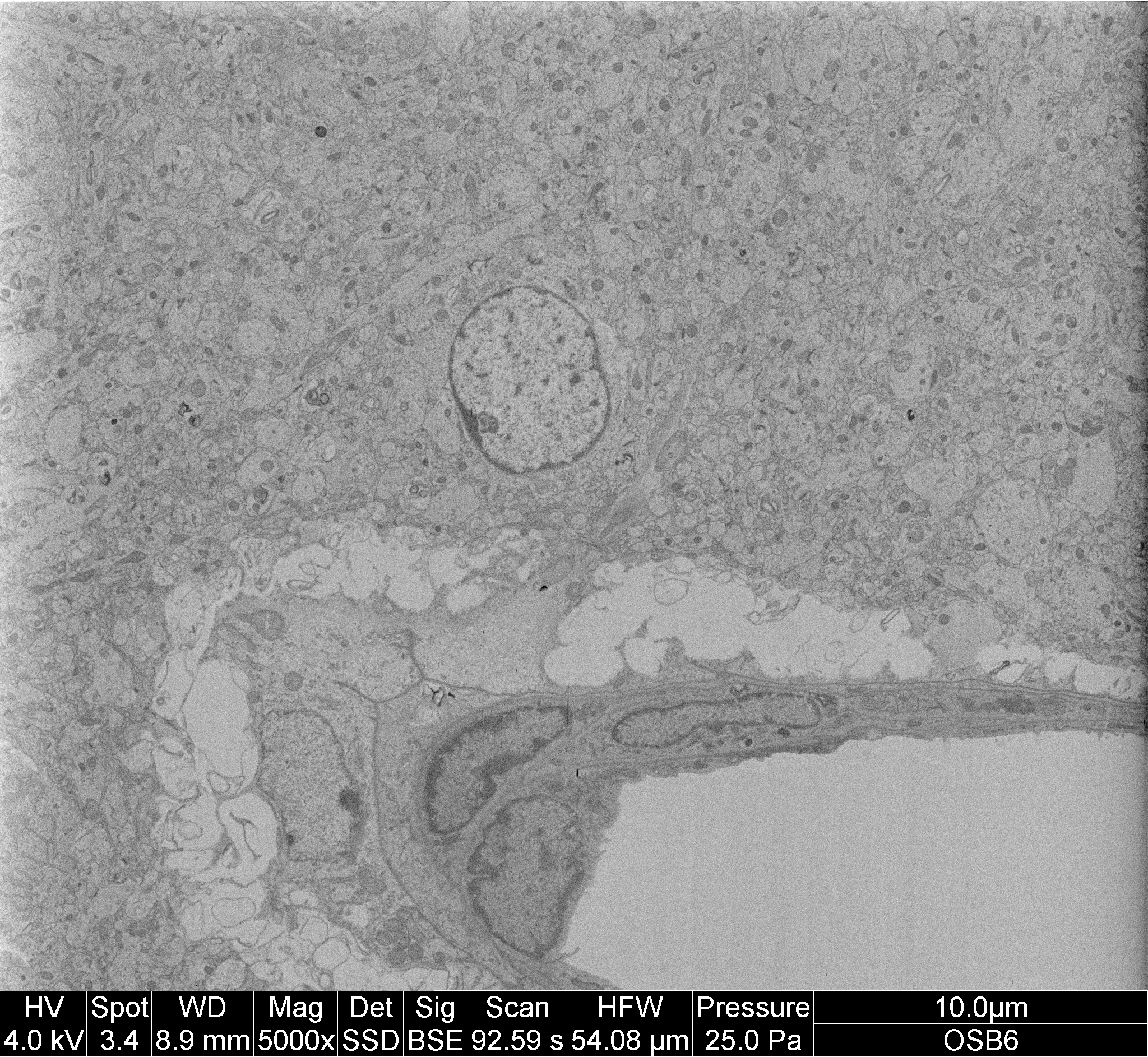

Supplement: Dataset S5 — (251.9 MB ZIP). [file pbio.0020329.sd005.zip › 040604_OS5_st1_472.tif]

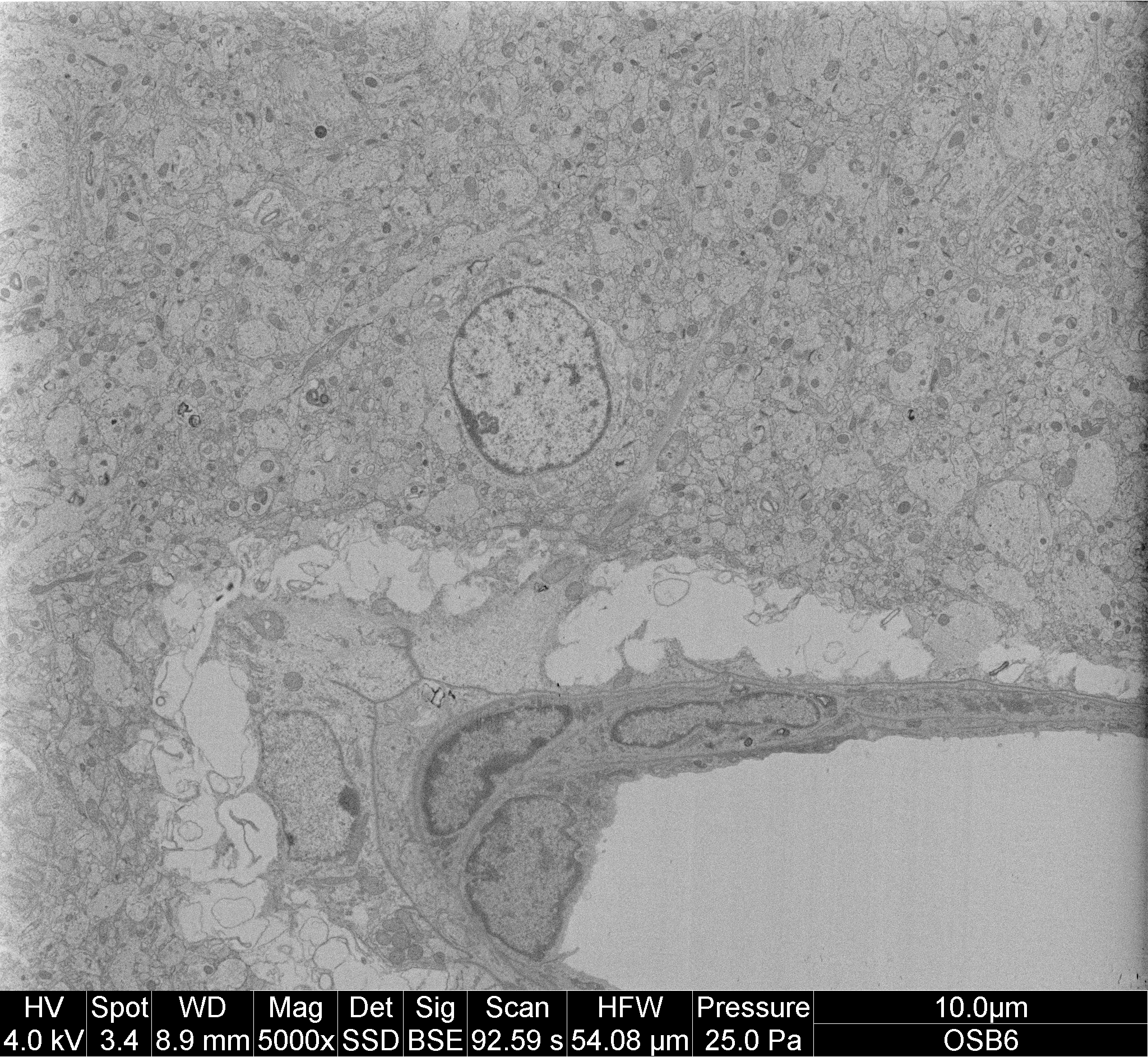

Supplement: Dataset S5 — (251.9 MB ZIP). [file pbio.0020329.sd005.zip › 040604_OS5_st1_473.tif]

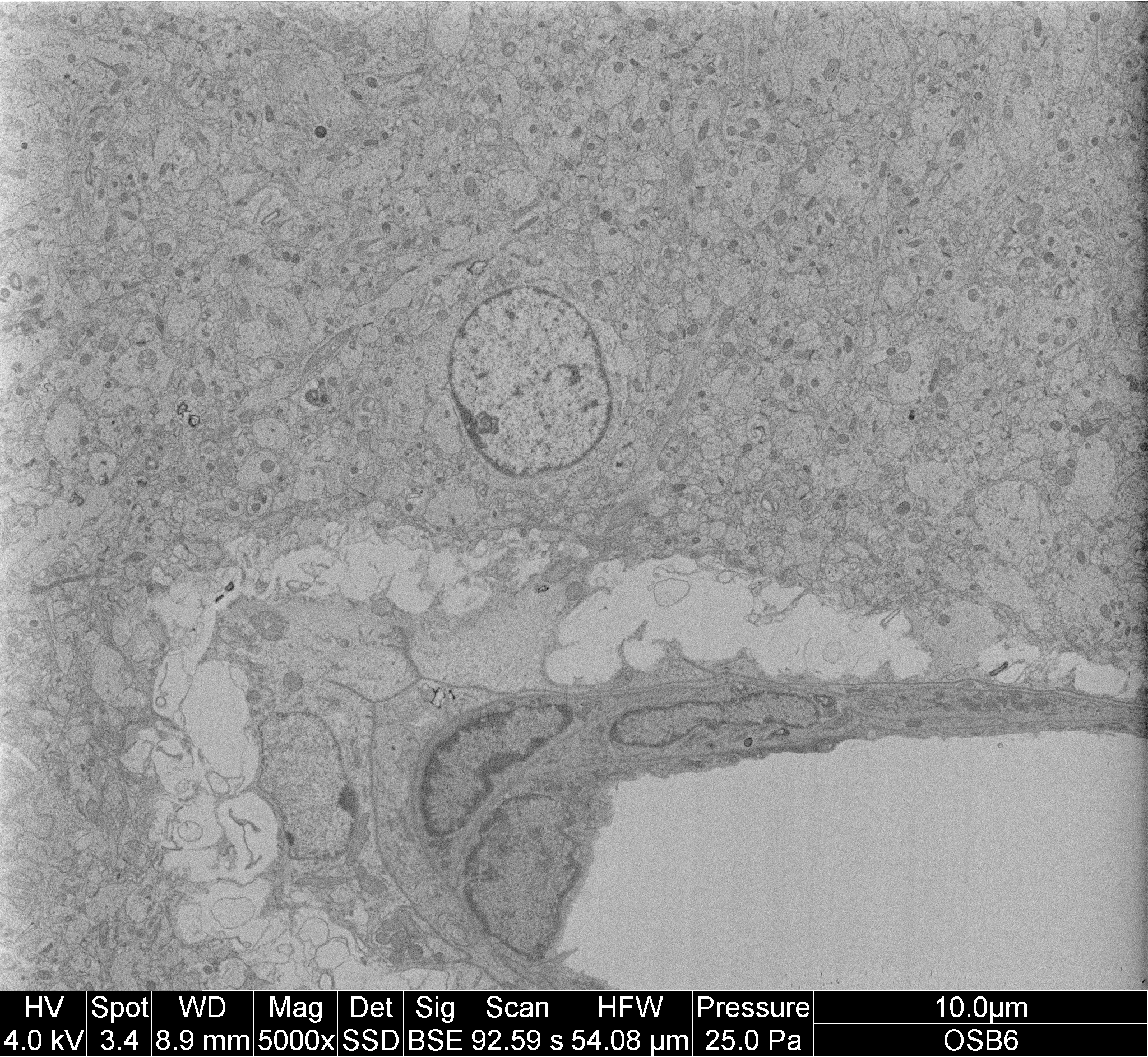

Supplement: Dataset S5 — (251.9 MB ZIP). [file pbio.0020329.sd005.zip › 040604_OS5_st1_474.tif]

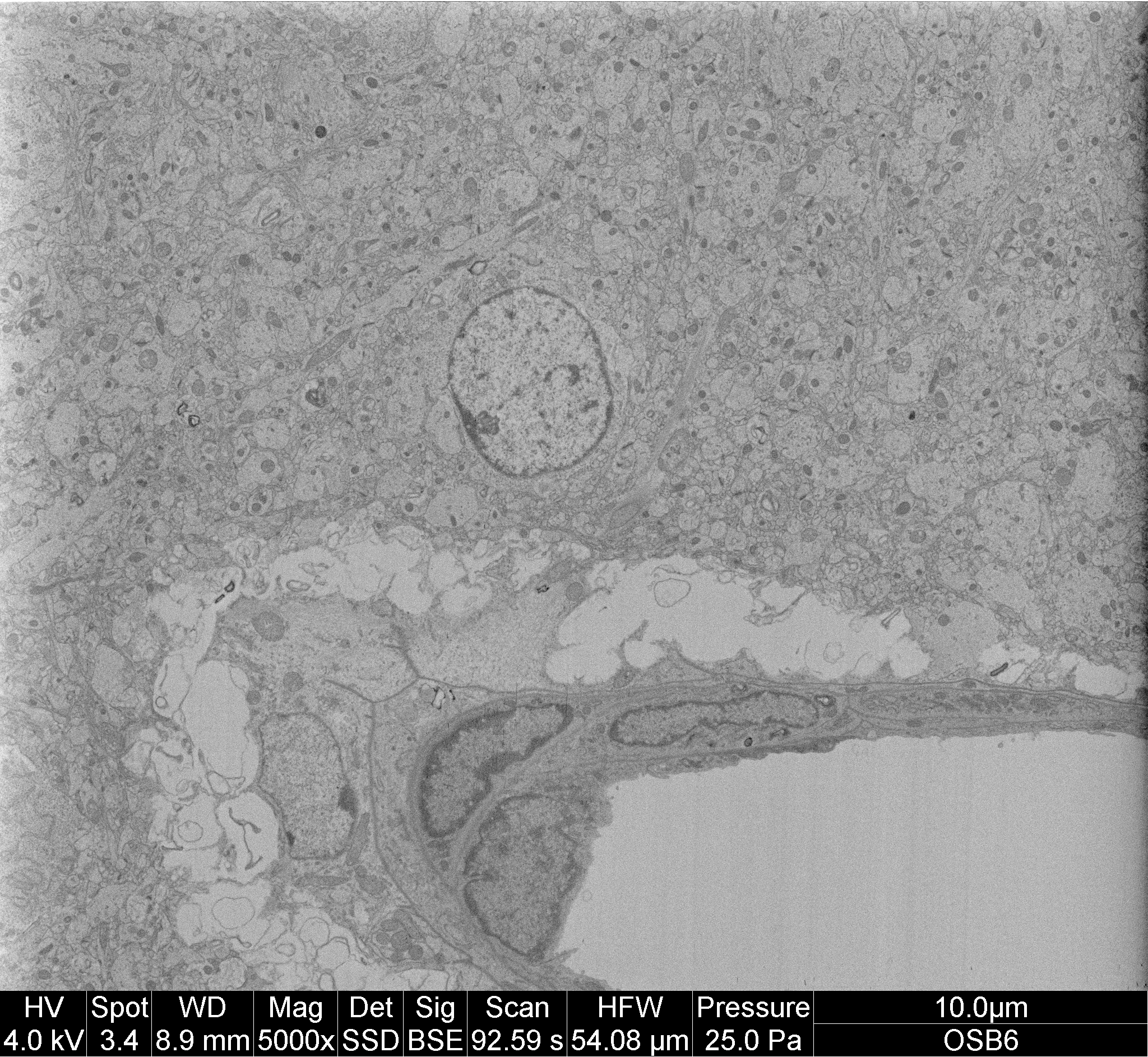

Supplement: Dataset S5 — (251.9 MB ZIP). [file pbio.0020329.sd005.zip › 040604_OS5_st1_475.tif]

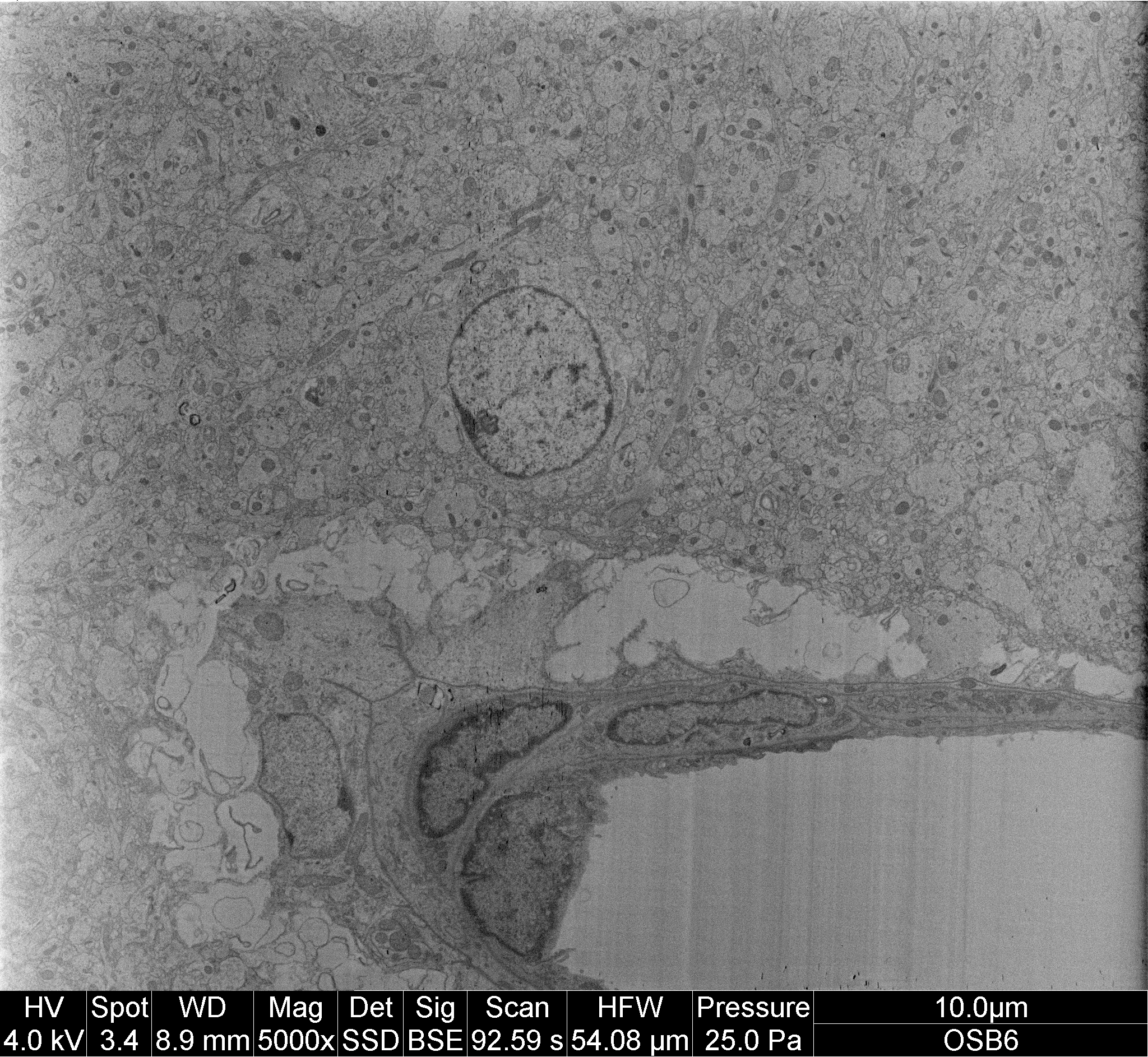

Supplement: Dataset S5 — (251.9 MB ZIP). [file pbio.0020329.sd005.zip › 040604_OS5_st1_476.tif]

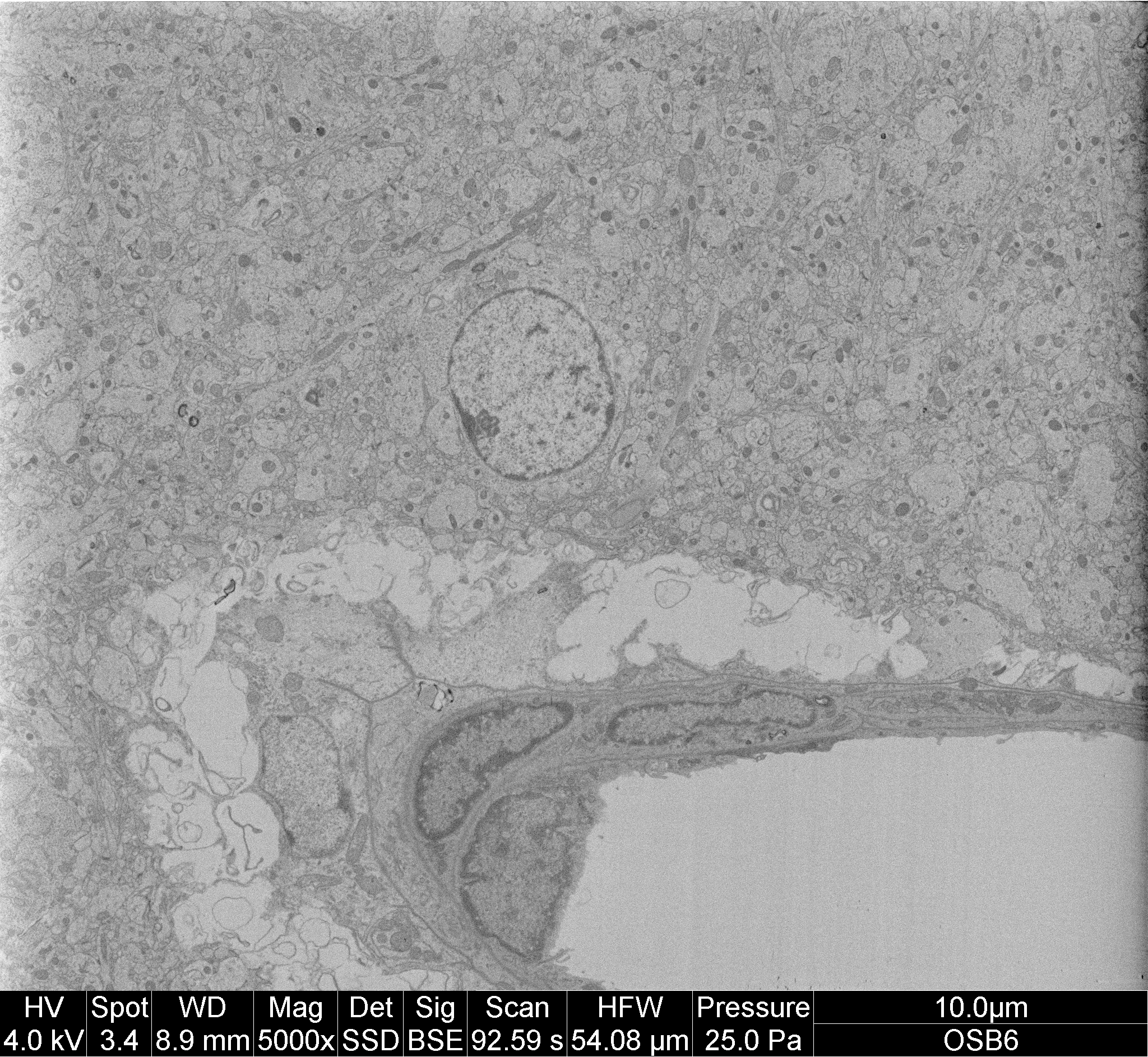

Supplement: Dataset S5 — (251.9 MB ZIP). [file pbio.0020329.sd005.zip › 040604_OS5_st1_477.tif]

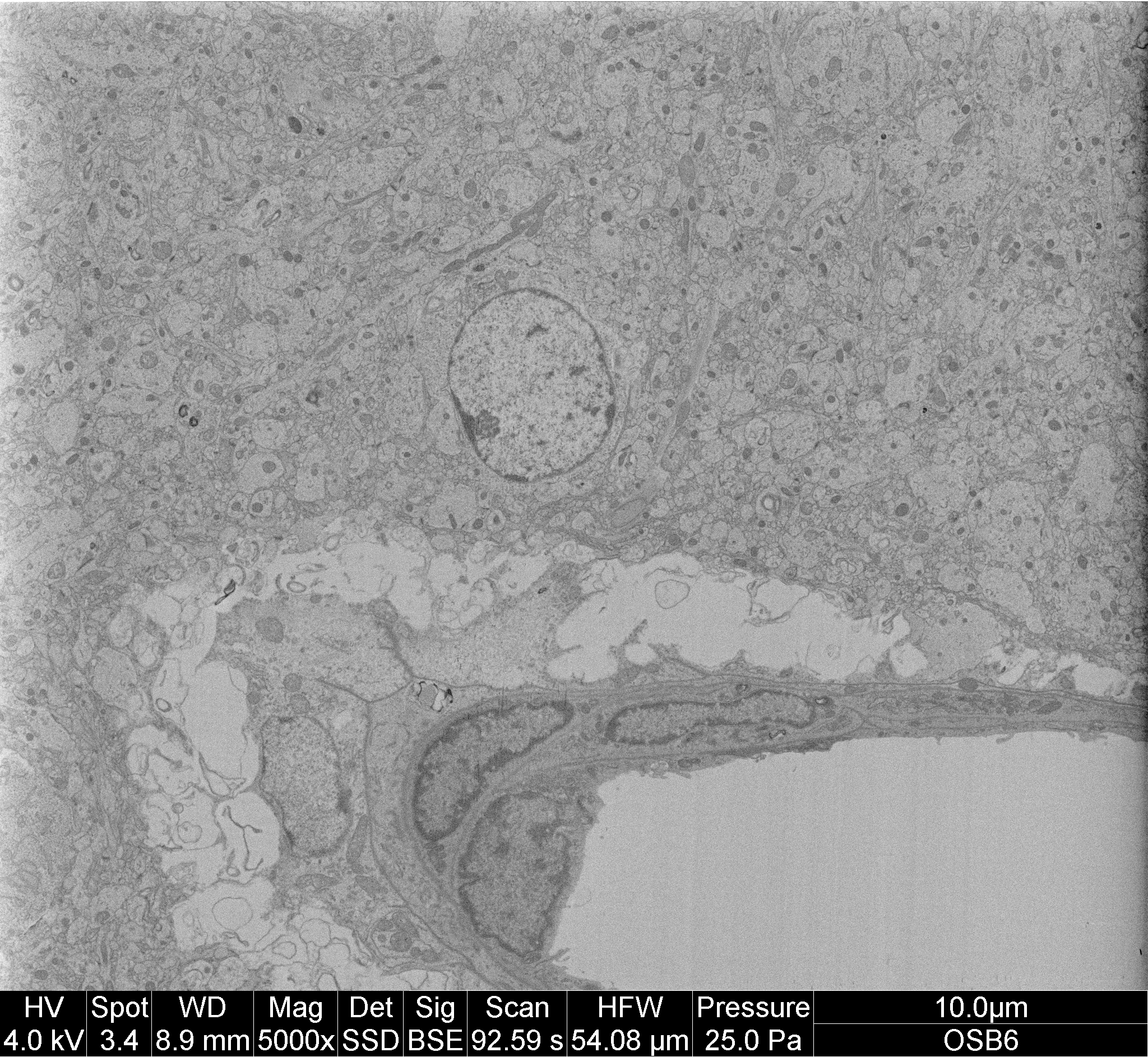

Supplement: Dataset S5 — (251.9 MB ZIP). [file pbio.0020329.sd005.zip › 040604_OS5_st1_478.tif]

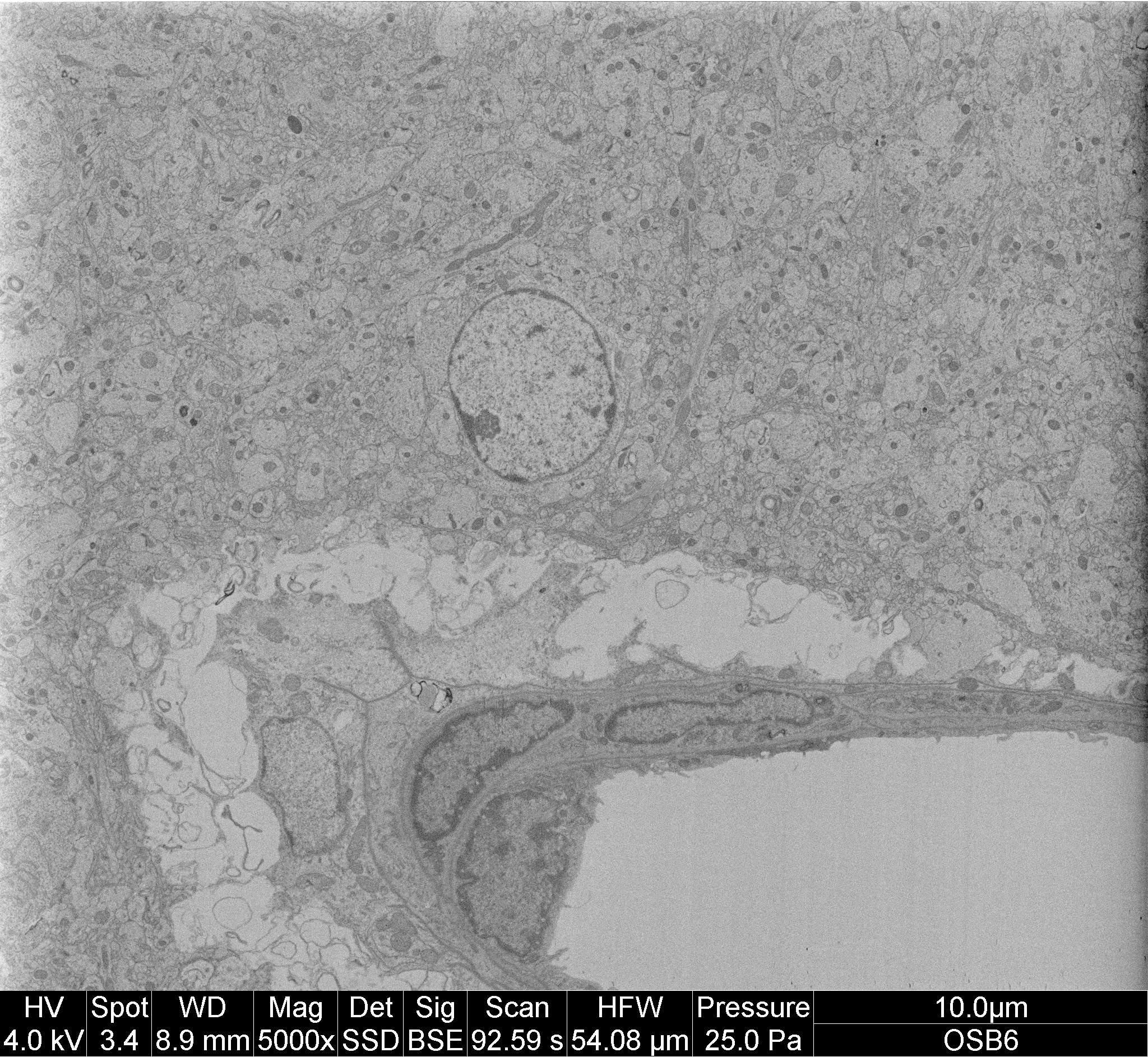

Supplement: Dataset S5 — (251.9 MB ZIP). [file pbio.0020329.sd005.zip › 040604_OS5_st1_479.tif]

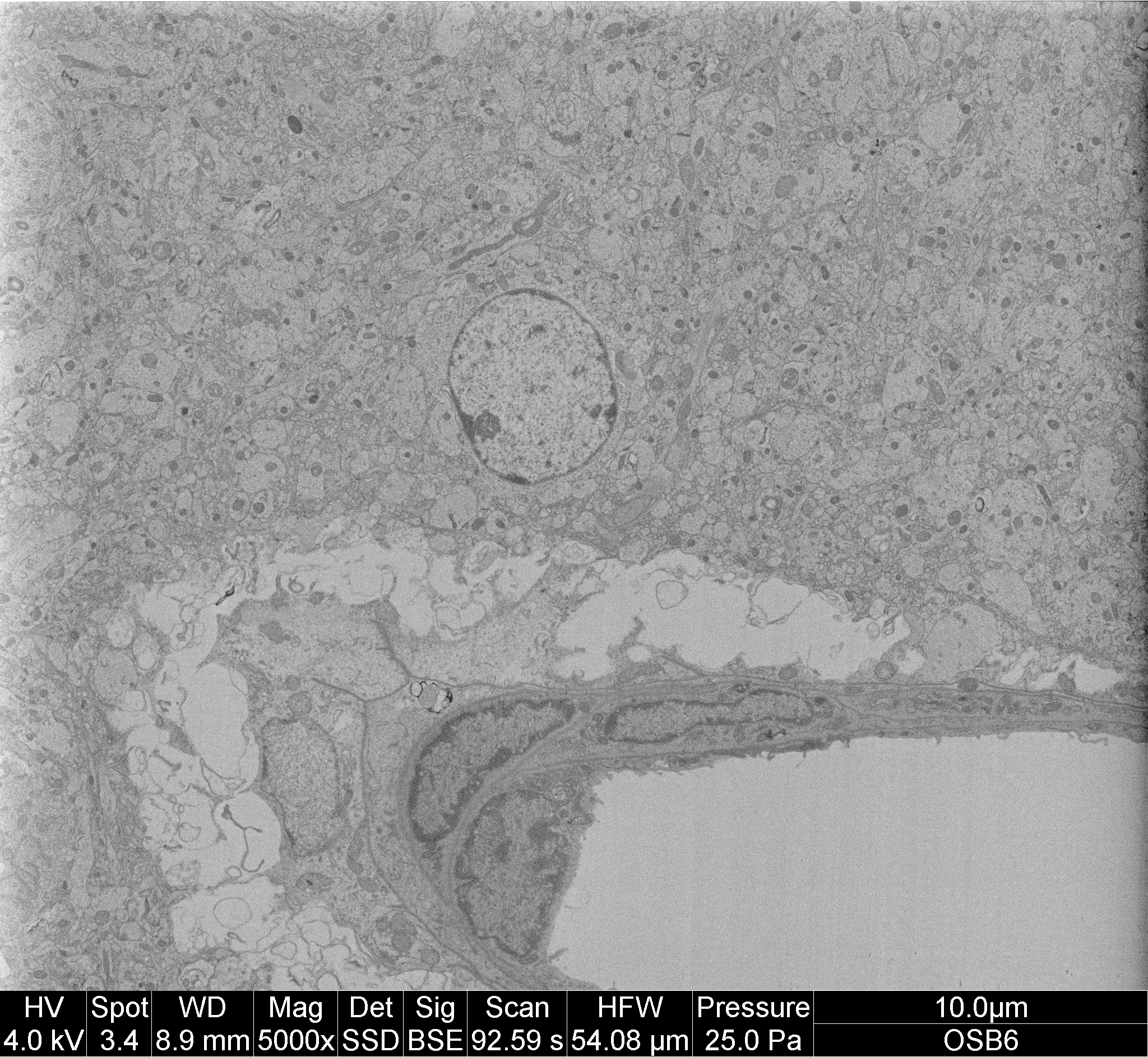

Supplement: Dataset S5 — (251.9 MB ZIP). [file pbio.0020329.sd005.zip › 040604_OS5_st1_480.tif]

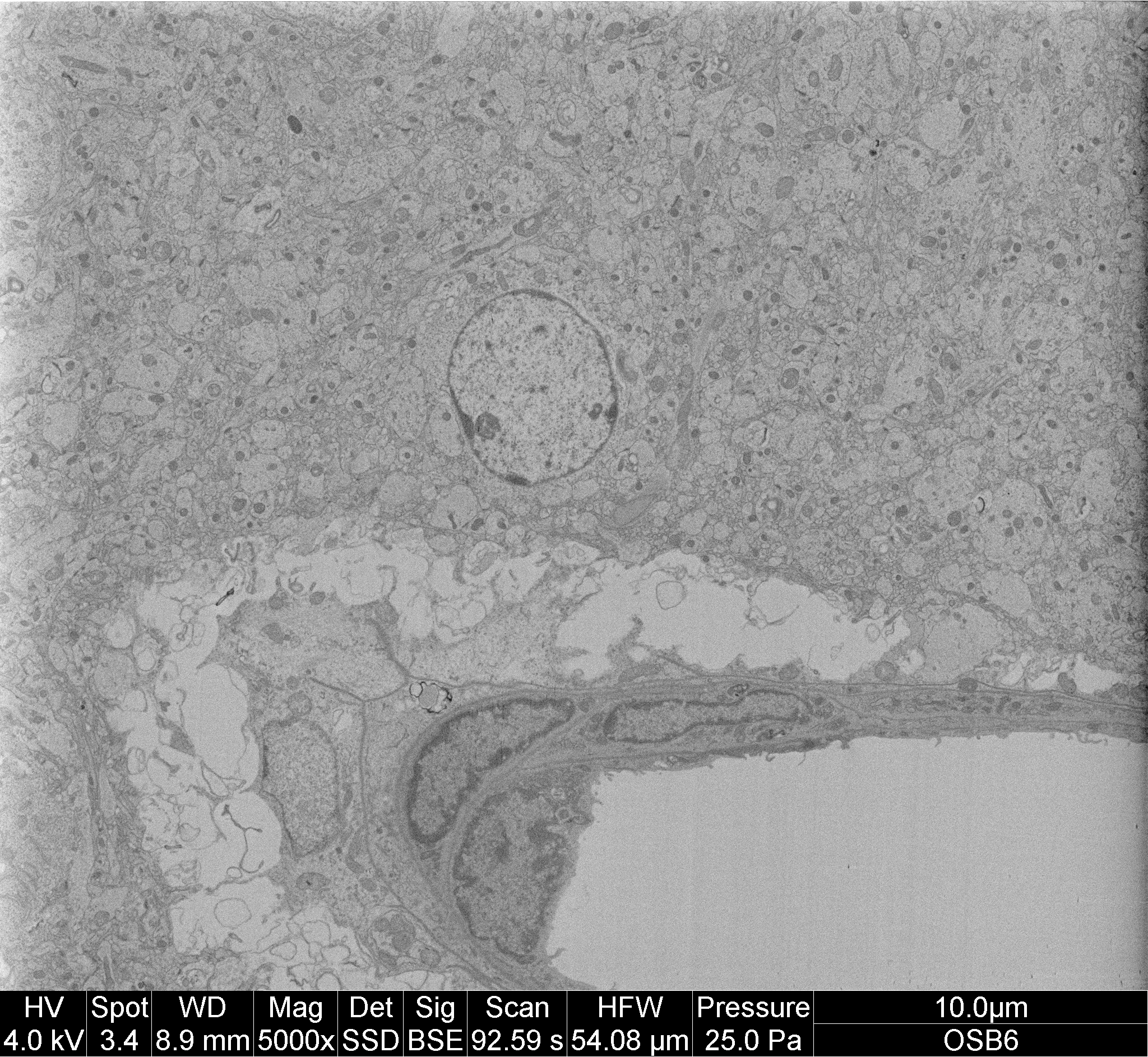

Supplement: Dataset S5 — (251.9 MB ZIP). [file pbio.0020329.sd005.zip › 040604_OS5_st1_481.tif]

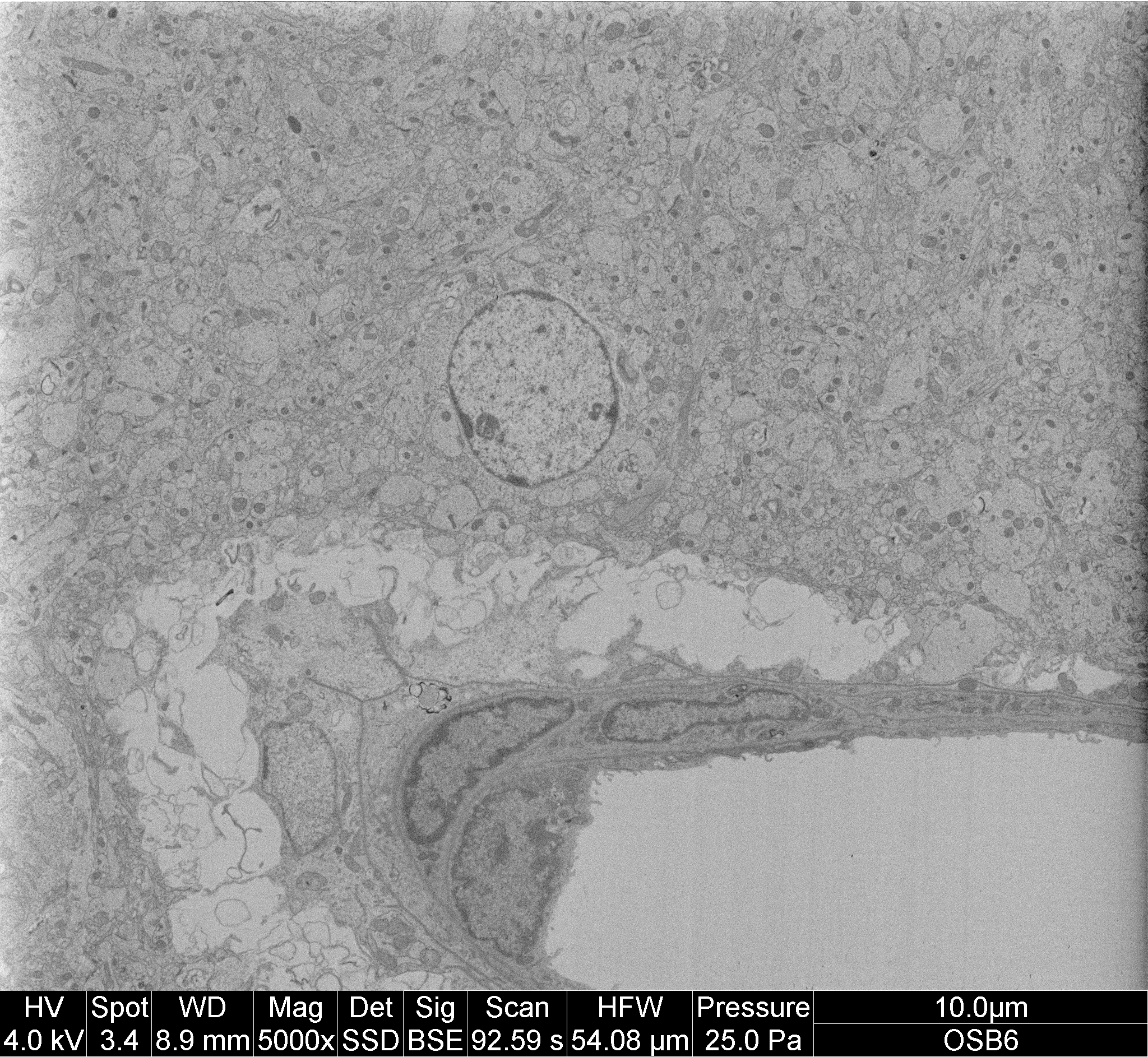

Supplement: Dataset S5 — (251.9 MB ZIP). [file pbio.0020329.sd005.zip › 040604_OS5_st1_482.tif]

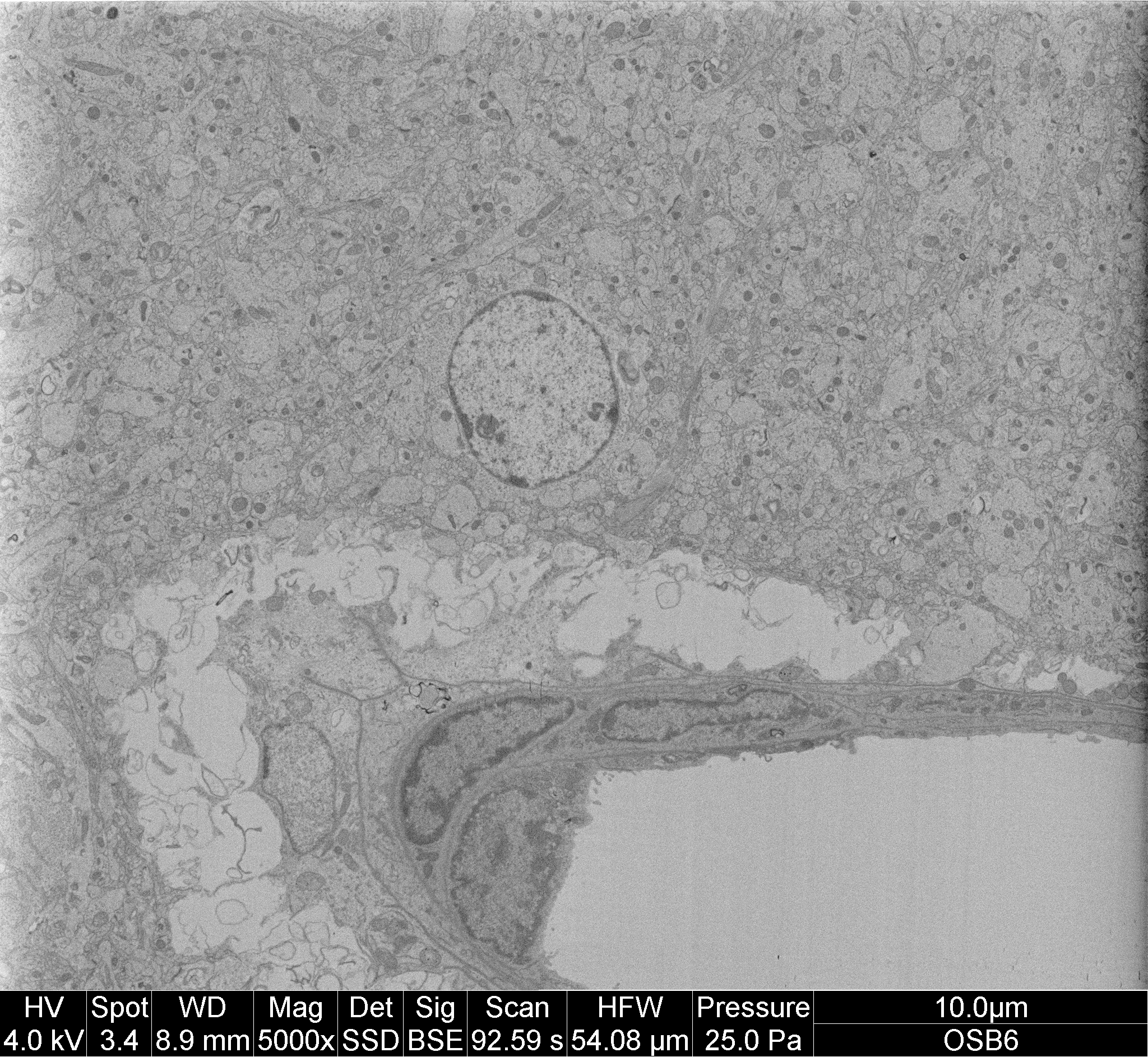

Supplement: Dataset S5 — (251.9 MB ZIP). [file pbio.0020329.sd005.zip › 040604_OS5_st1_483.tif]

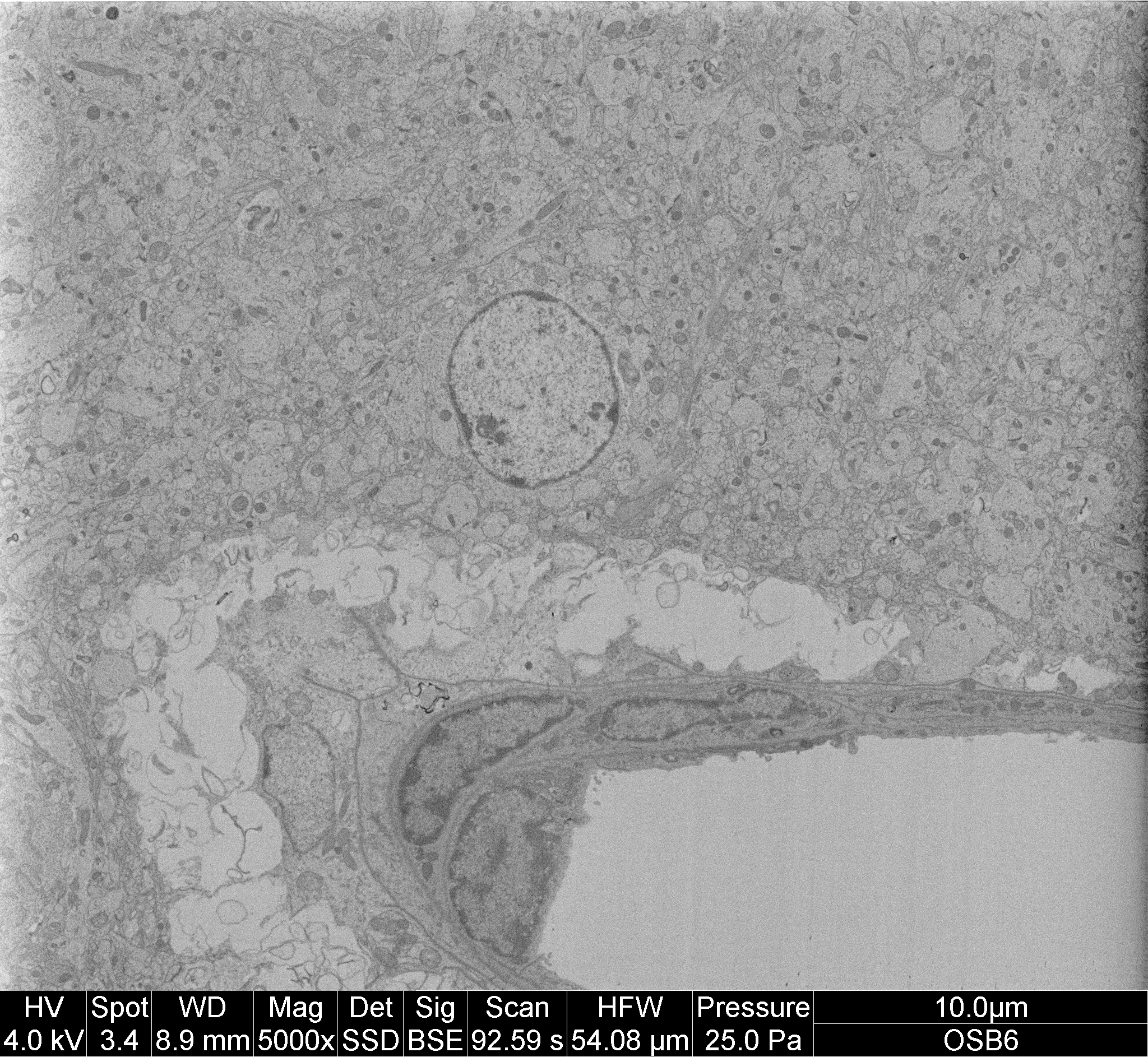

Supplement: Dataset S5 — (251.9 MB ZIP). [file pbio.0020329.sd005.zip › 040604_OS5_st1_484.tif]

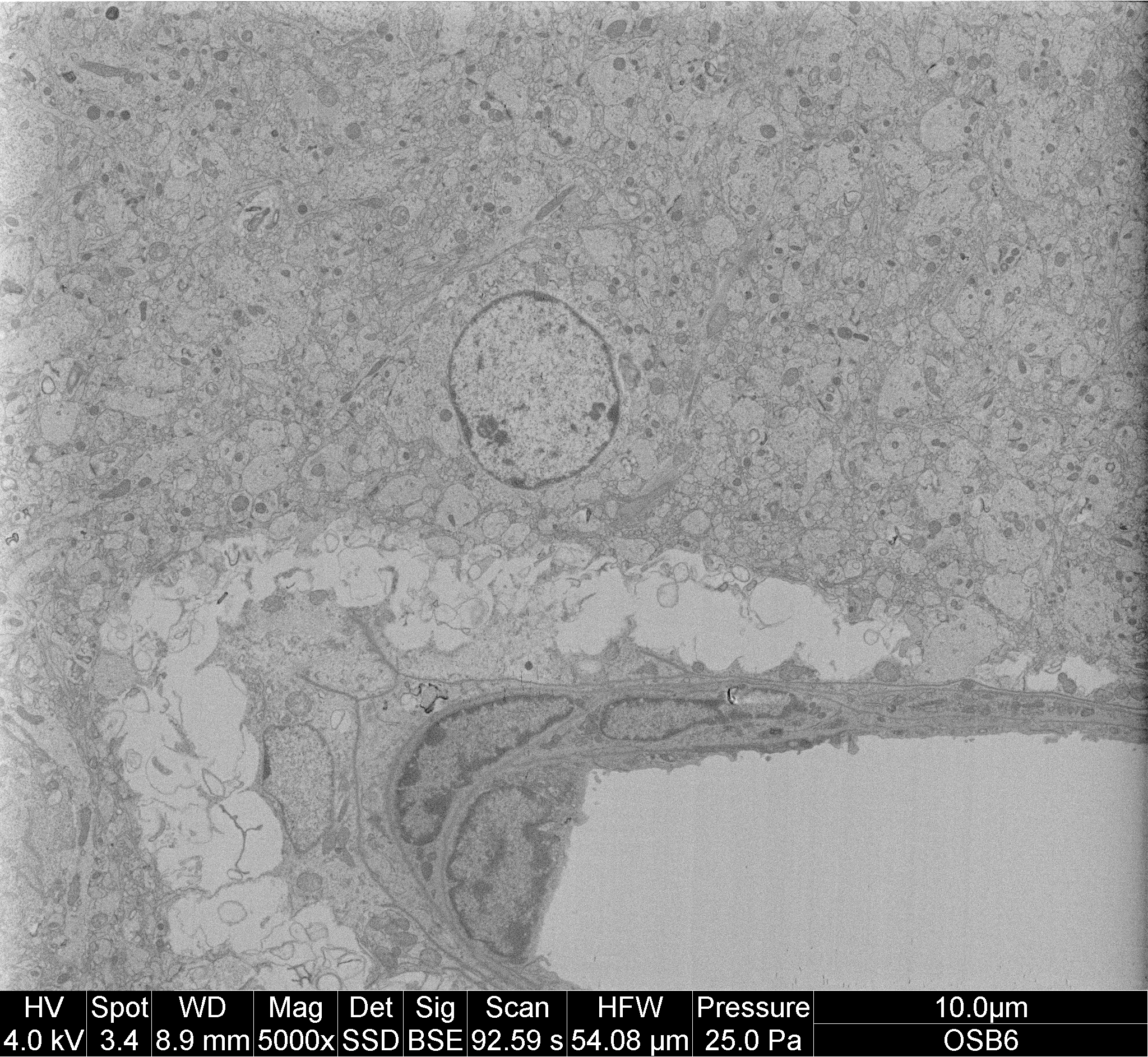

Supplement: Dataset S5 — (251.9 MB ZIP). [file pbio.0020329.sd005.zip › 040604_OS5_st1_485.tif]

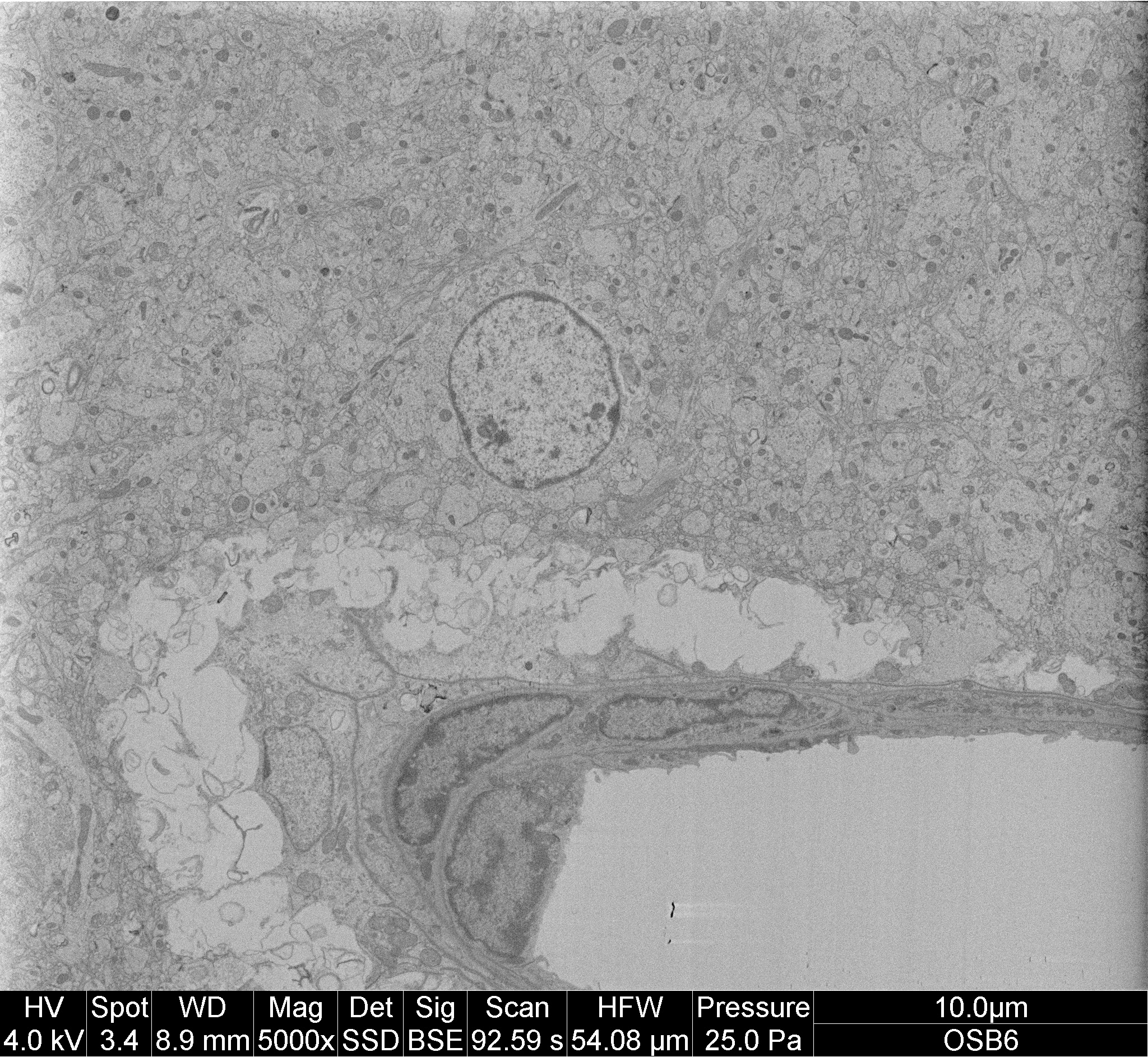

Supplement: Dataset S5 — (251.9 MB ZIP). [file pbio.0020329.sd005.zip › 040604_OS5_st1_486.tif]

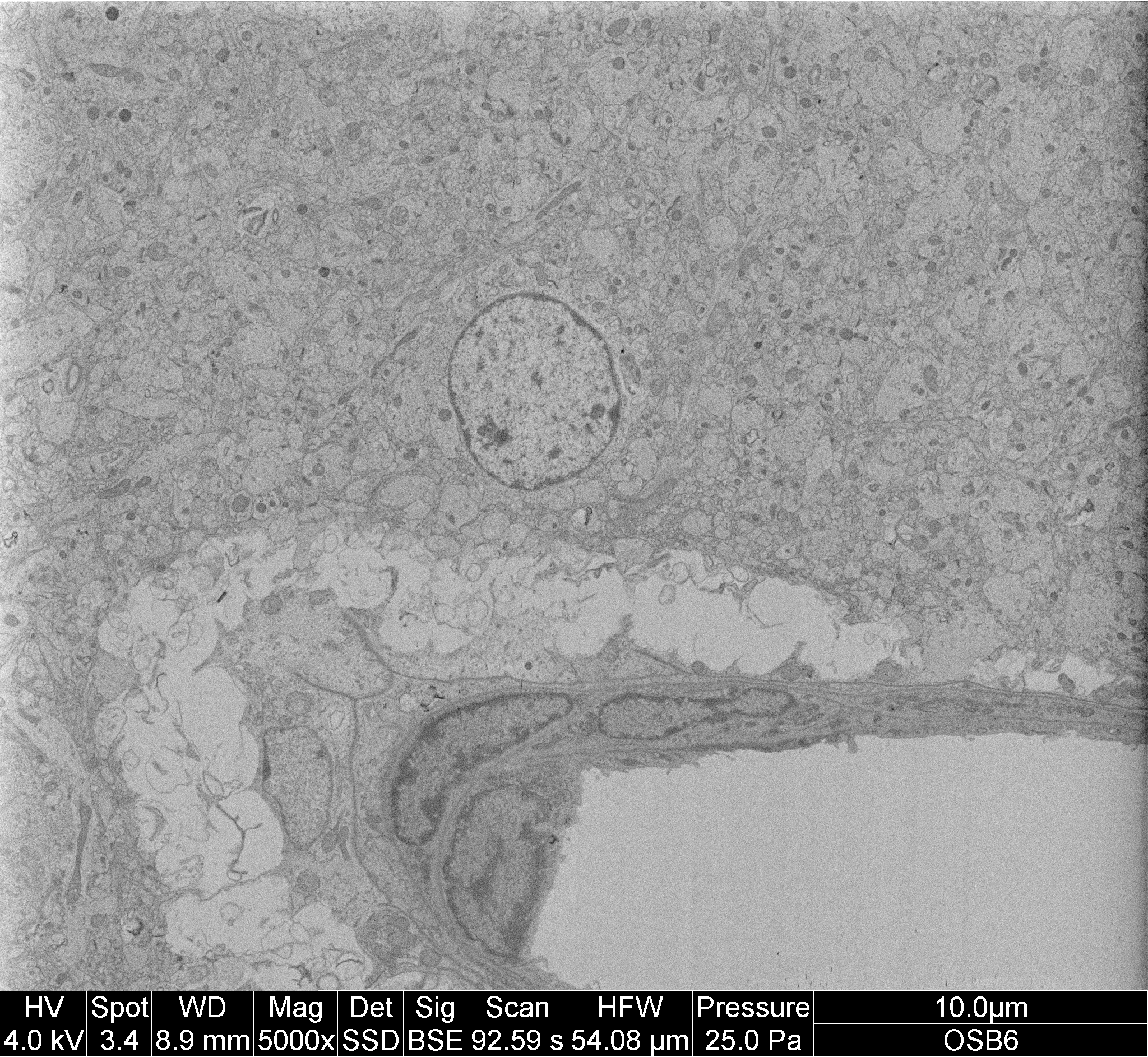

Supplement: Dataset S5 — (251.9 MB ZIP). [file pbio.0020329.sd005.zip › 040604_OS5_st1_487.tif]

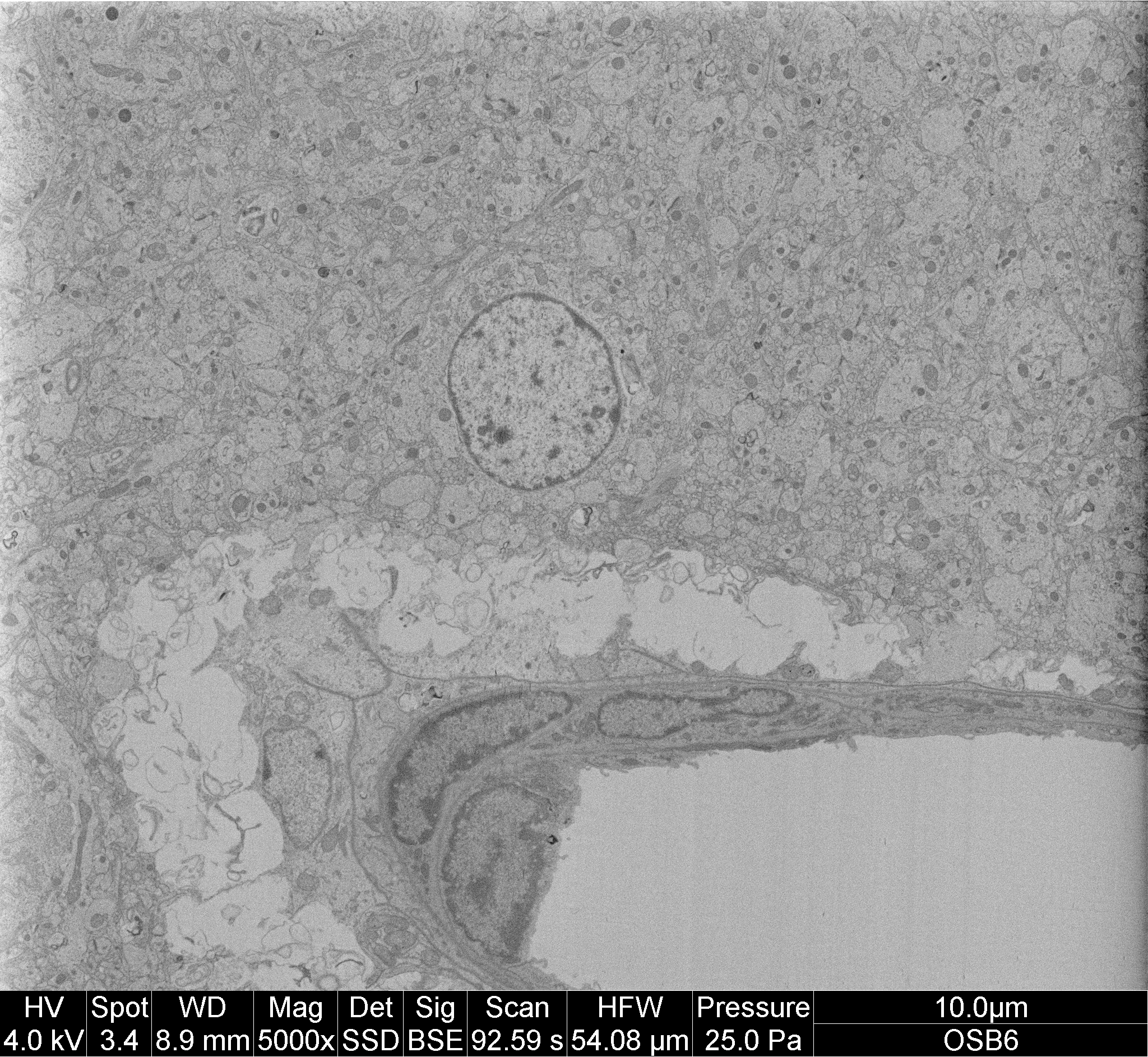

Supplement: Dataset S5 — (251.9 MB ZIP). [file pbio.0020329.sd005.zip › 040604_OS5_st1_488.tif]

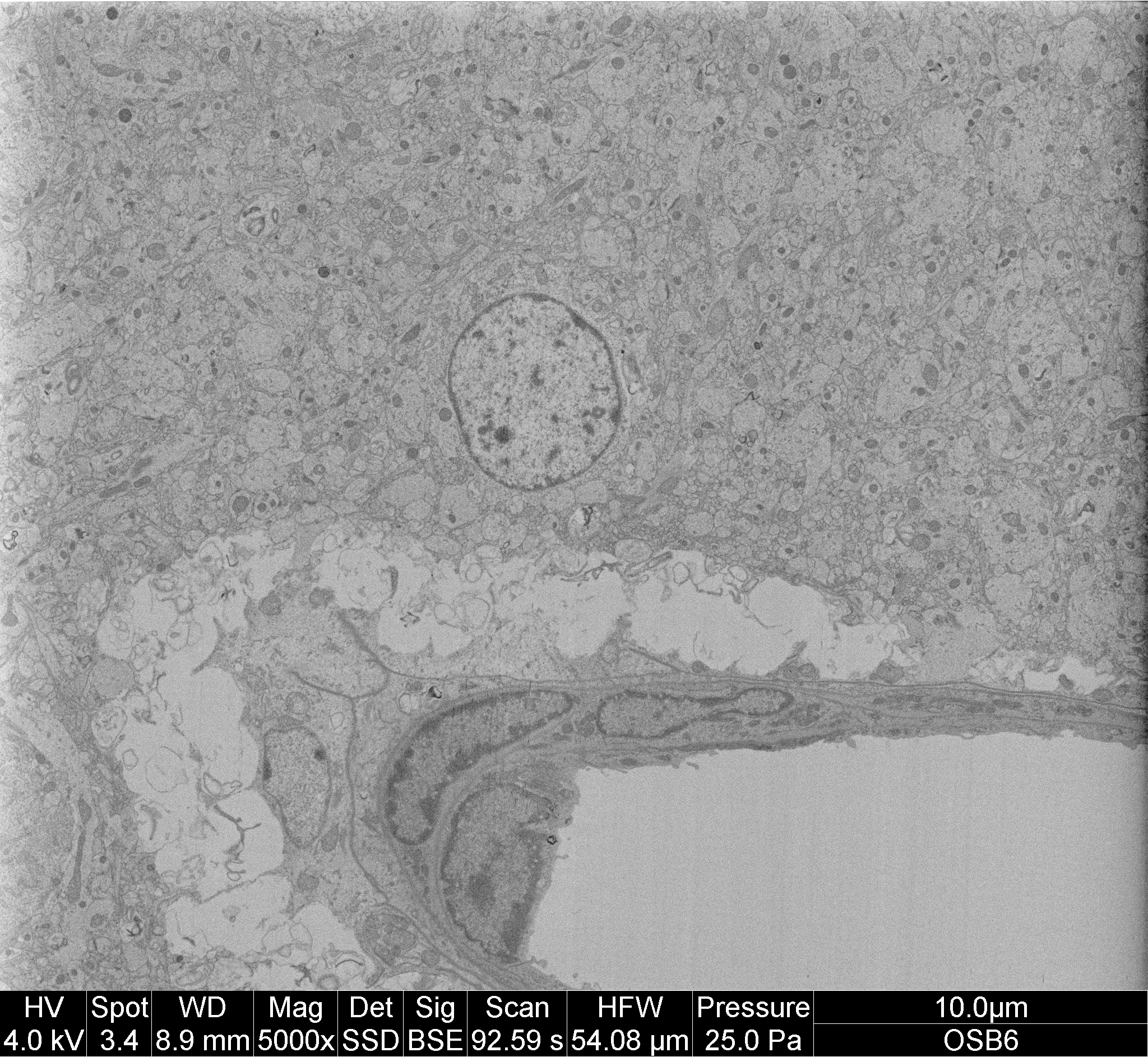

Supplement: Dataset S5 — (251.9 MB ZIP). [file pbio.0020329.sd005.zip › 040604_OS5_st1_489.tif]

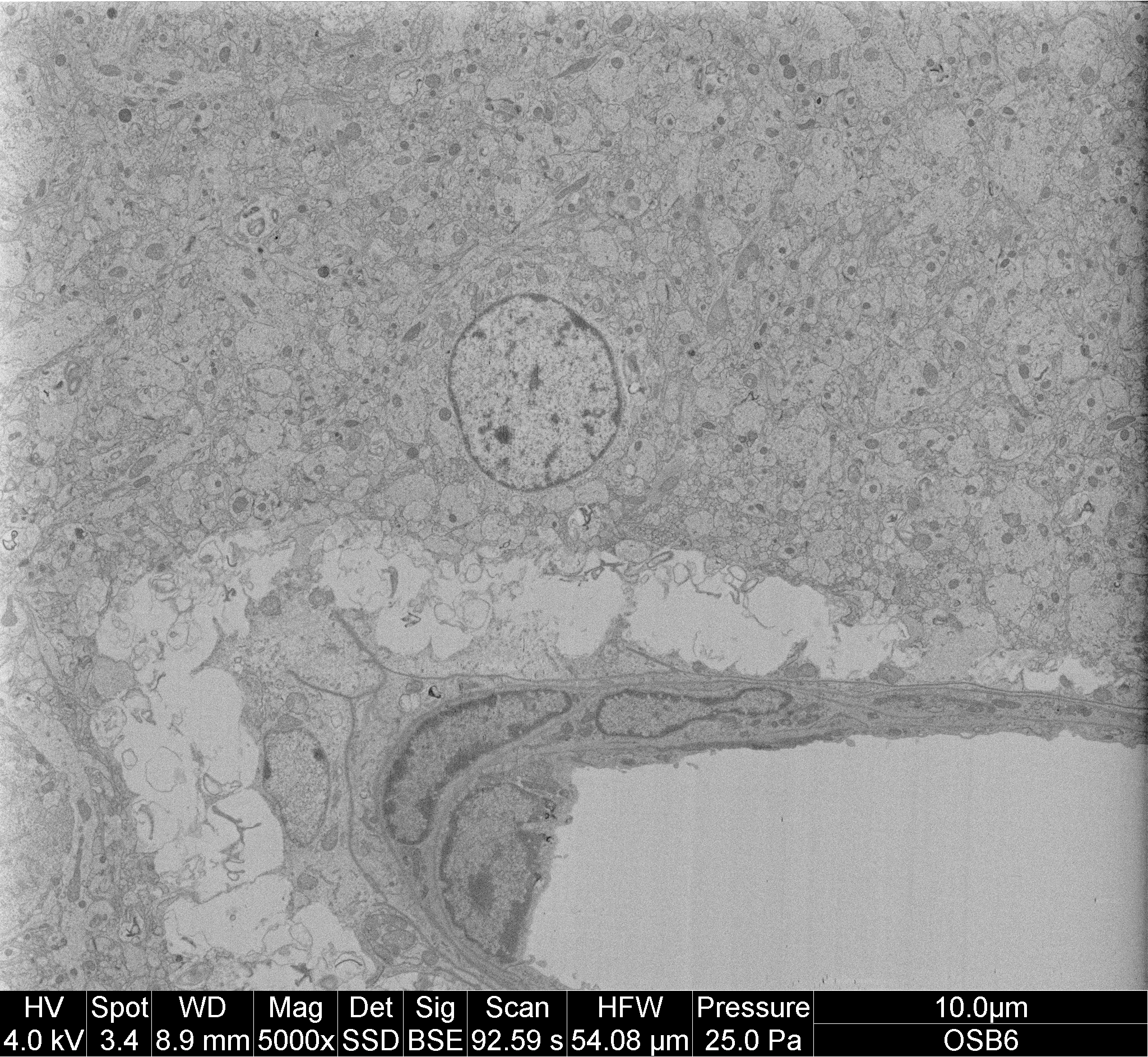

Supplement: Dataset S5 — (251.9 MB ZIP). [file pbio.0020329.sd005.zip › 040604_OS5_st1_490.tif]

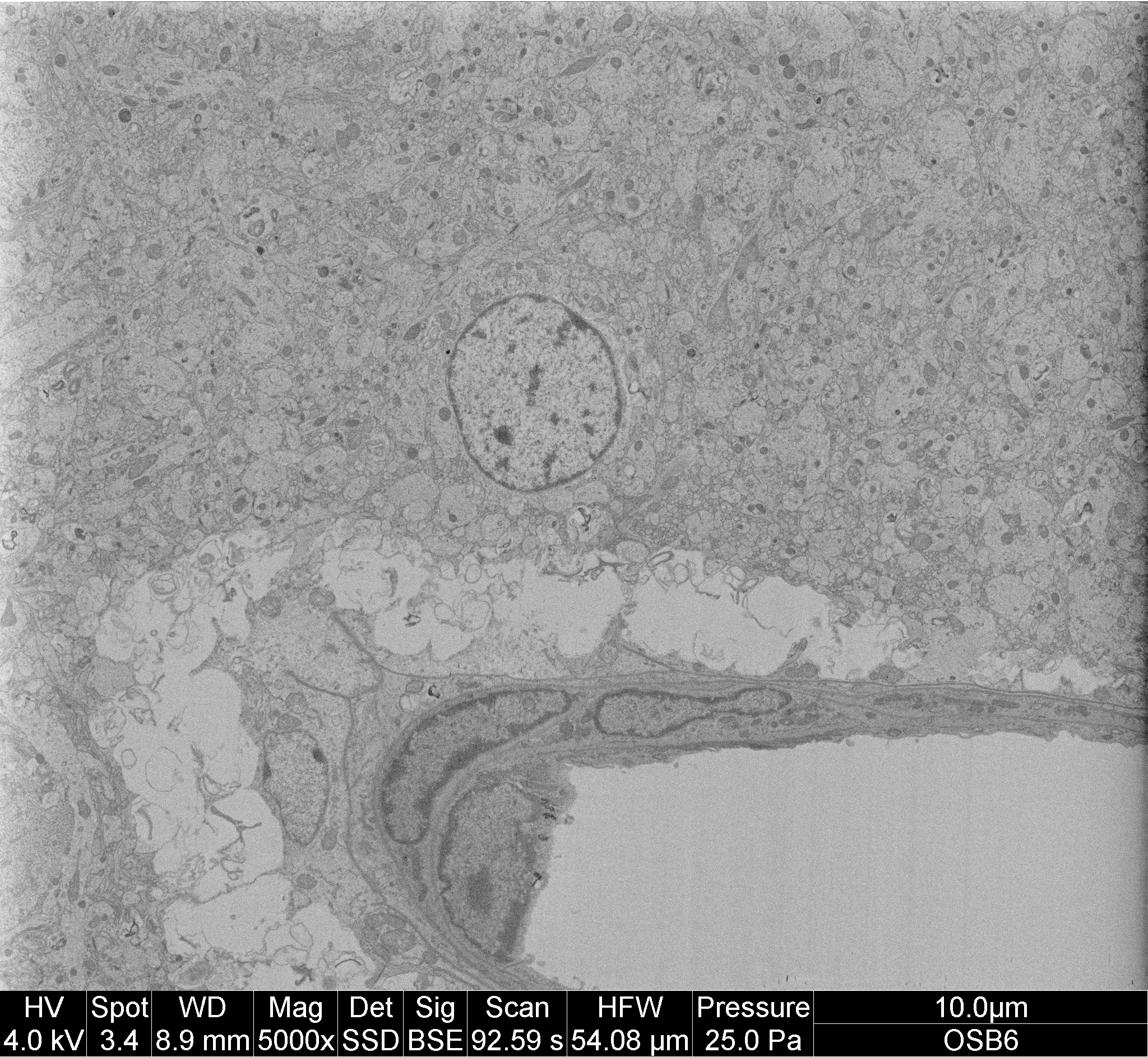

Supplement: Dataset S5 — (251.9 MB ZIP). [file pbio.0020329.sd005.zip › 040604_OS5_st1_491.tif]

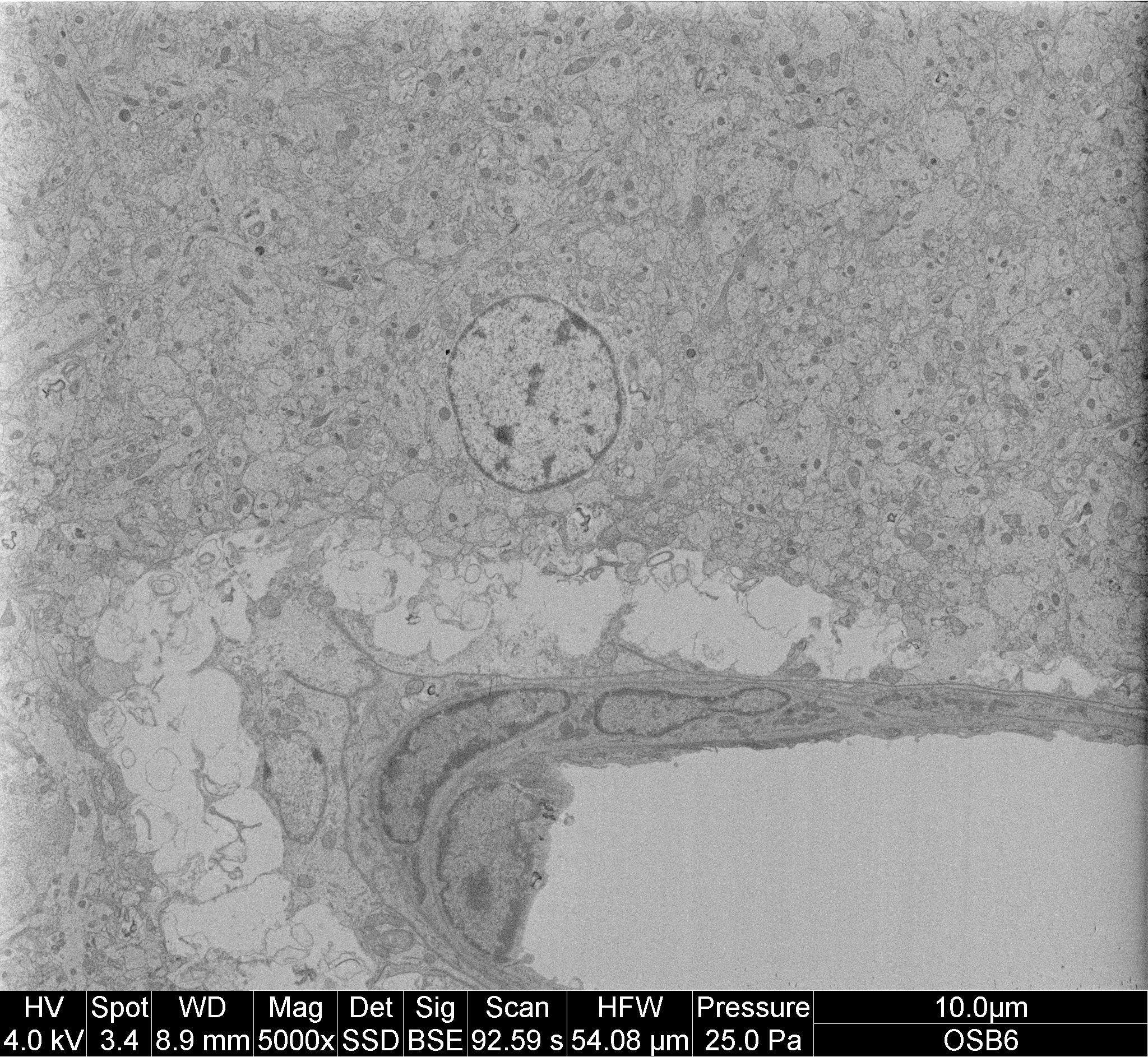

Supplement: Dataset S5 — (251.9 MB ZIP). [file pbio.0020329.sd005.zip › 040604_OS5_st1_492.tif]

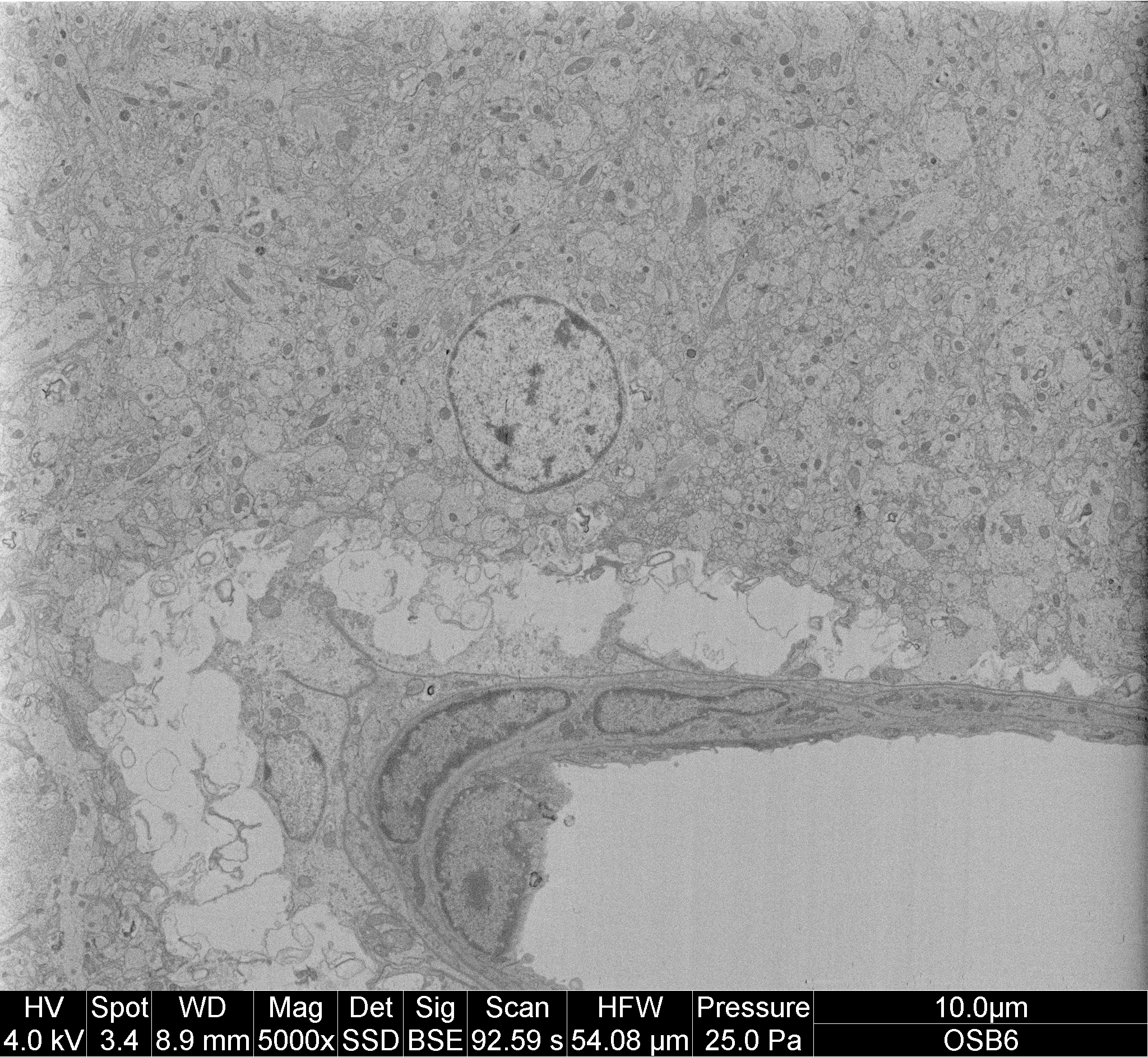

Supplement: Dataset S5 — (251.9 MB ZIP). [file pbio.0020329.sd005.zip › 040604_OS5_st1_493.tif]

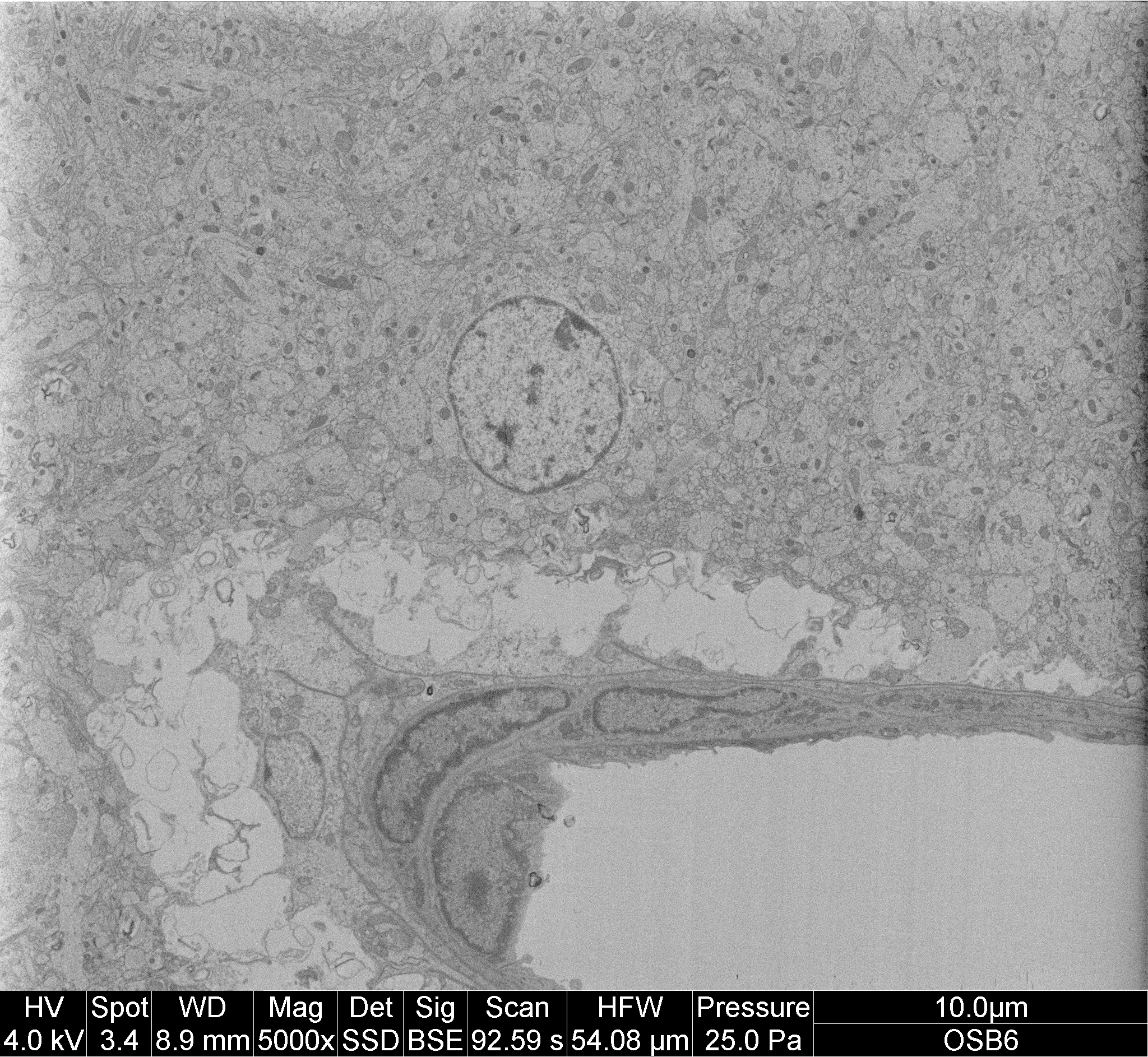

Supplement: Dataset S5 — (251.9 MB ZIP). [file pbio.0020329.sd005.zip › 040604_OS5_st1_494.tif]

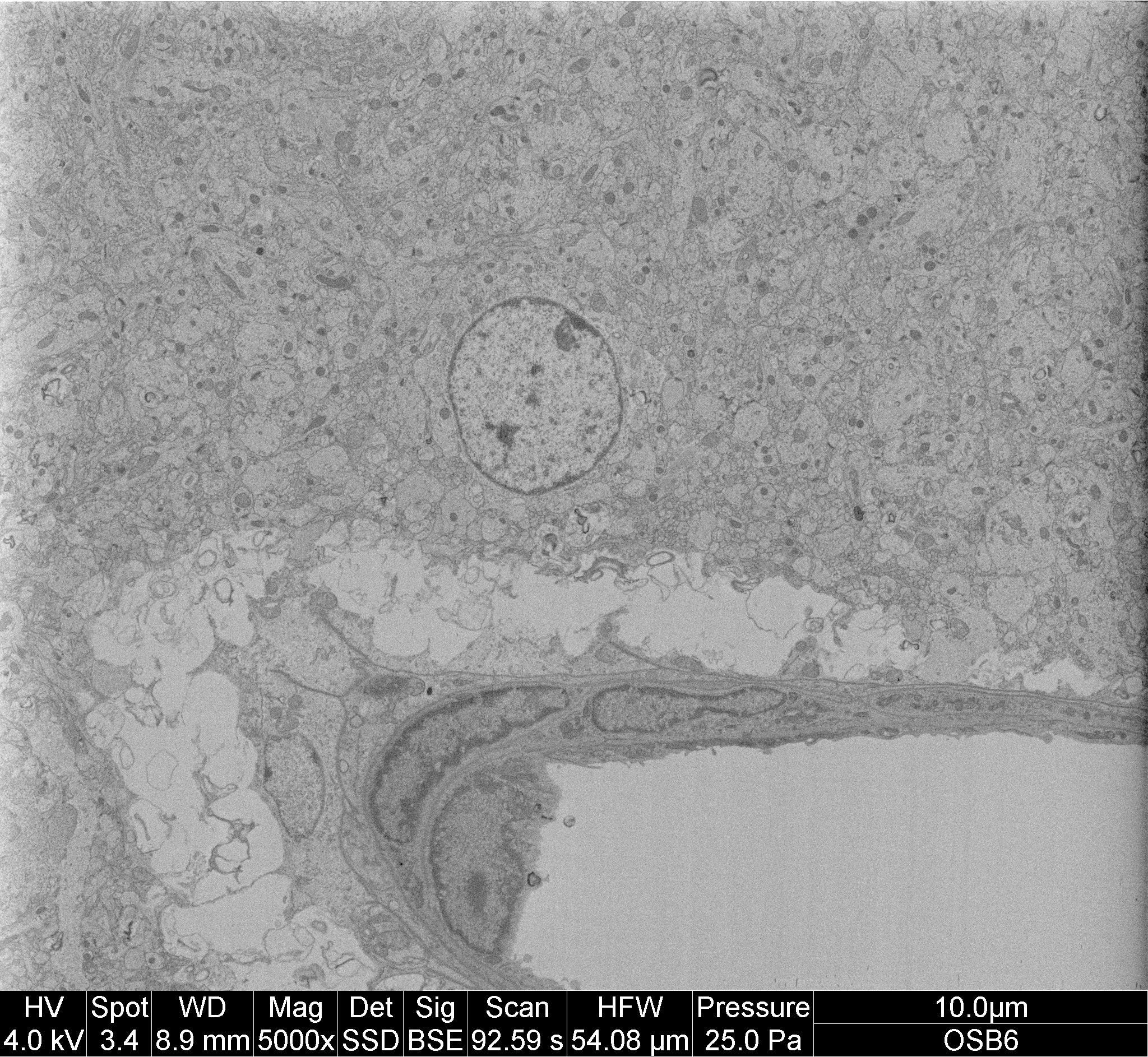

Supplement: Dataset S5 — (251.9 MB ZIP). [file pbio.0020329.sd005.zip › 040604_OS5_st1_495.tif]

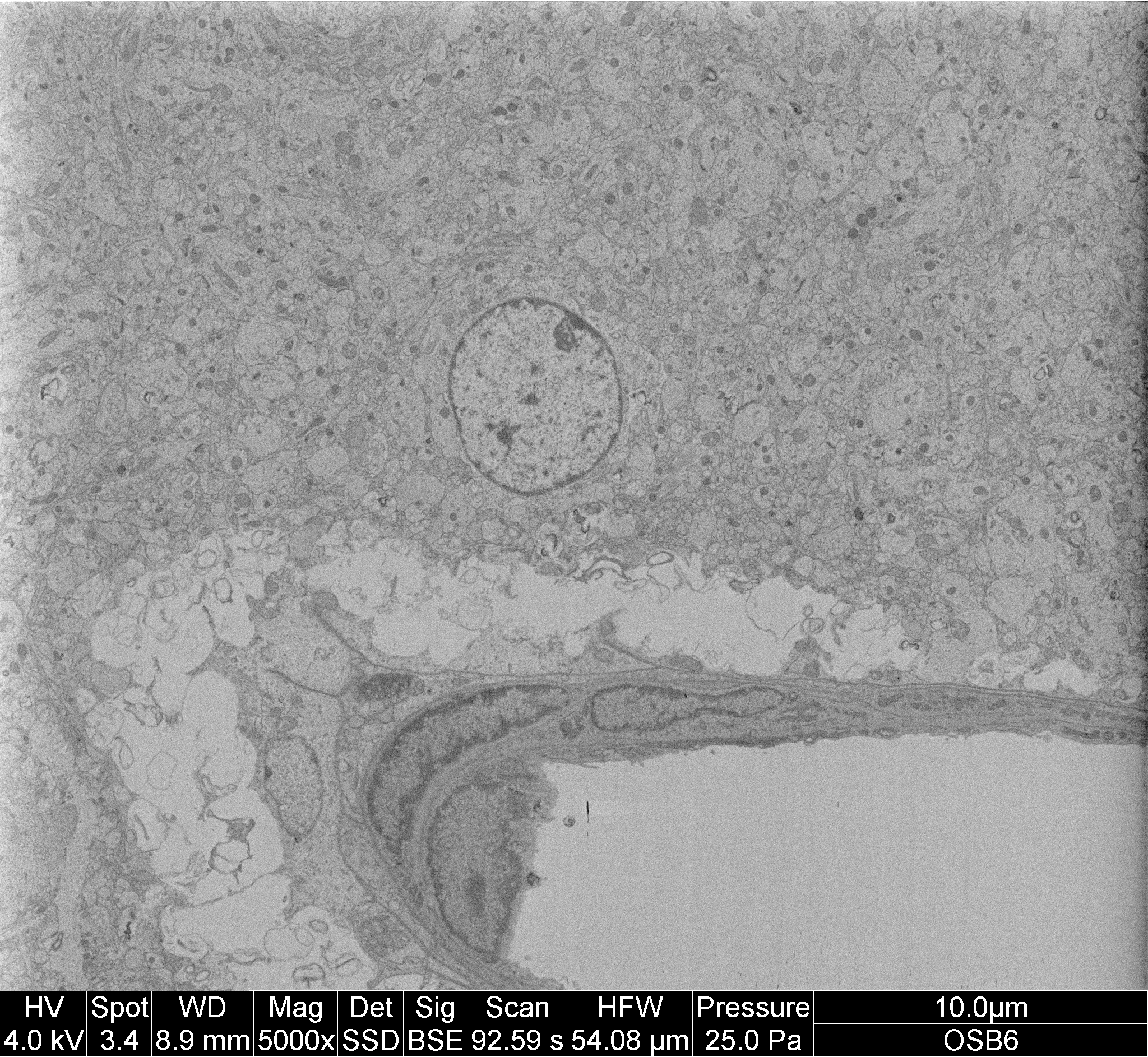

Supplement: Dataset S5 — (251.9 MB ZIP). [file pbio.0020329.sd005.zip › 040604_OS5_st1_496.tif]

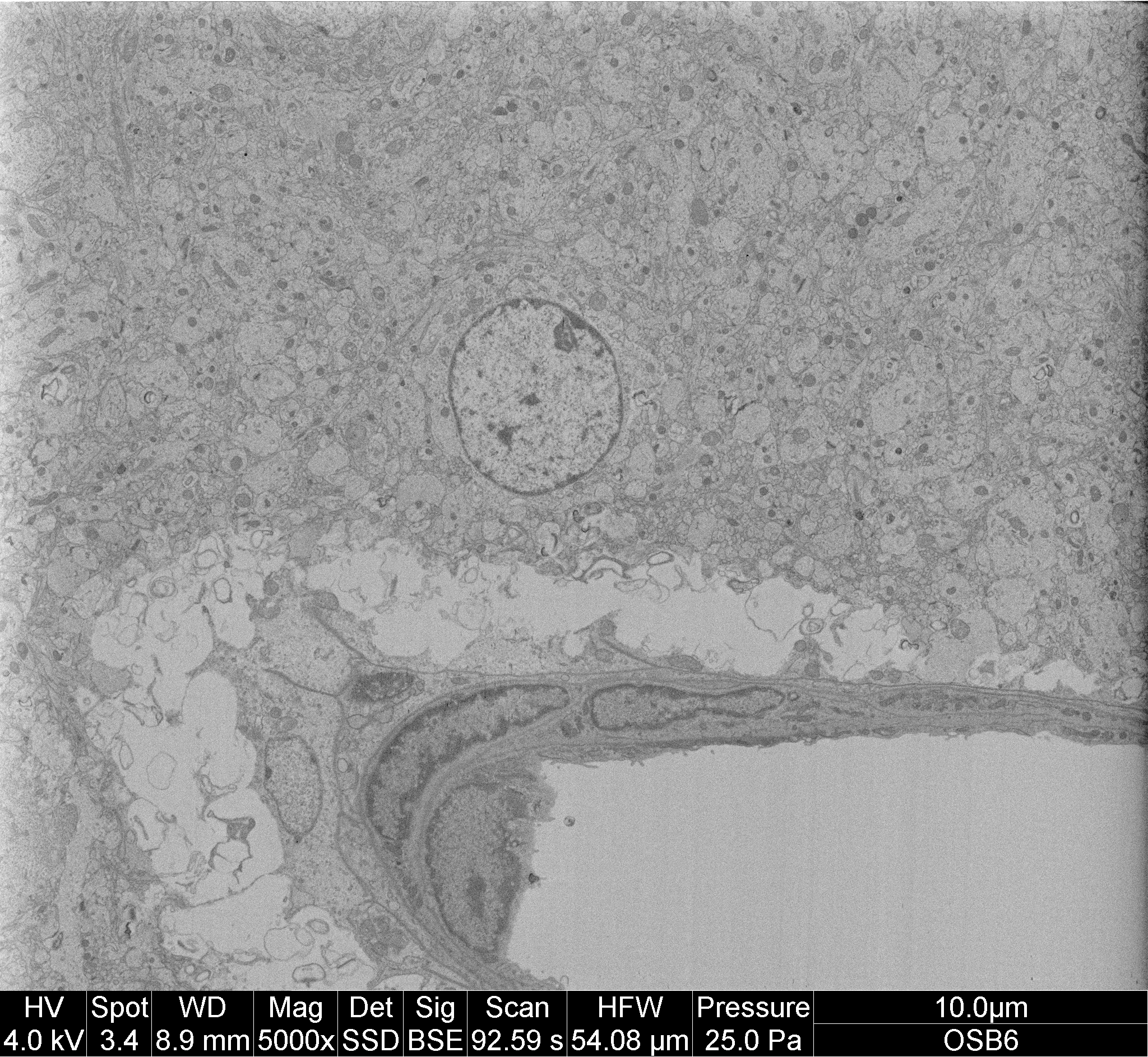

Supplement: Dataset S5 — (251.9 MB ZIP). [file pbio.0020329.sd005.zip › 040604_OS5_st1_497.tif]

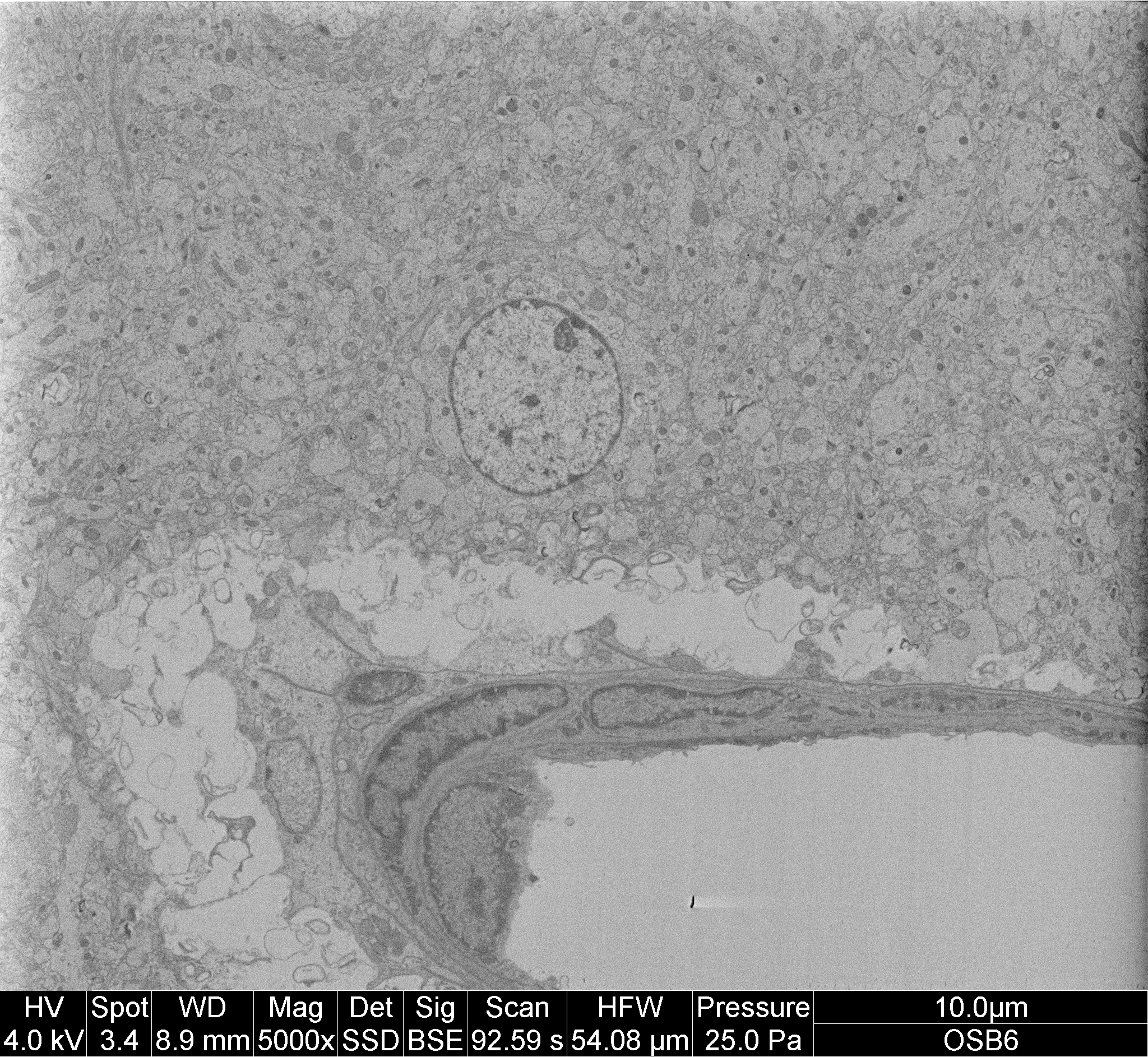

Supplement: Dataset S5 — (251.9 MB ZIP). [file pbio.0020329.sd005.zip › 040604_OS5_st1_498.tif]

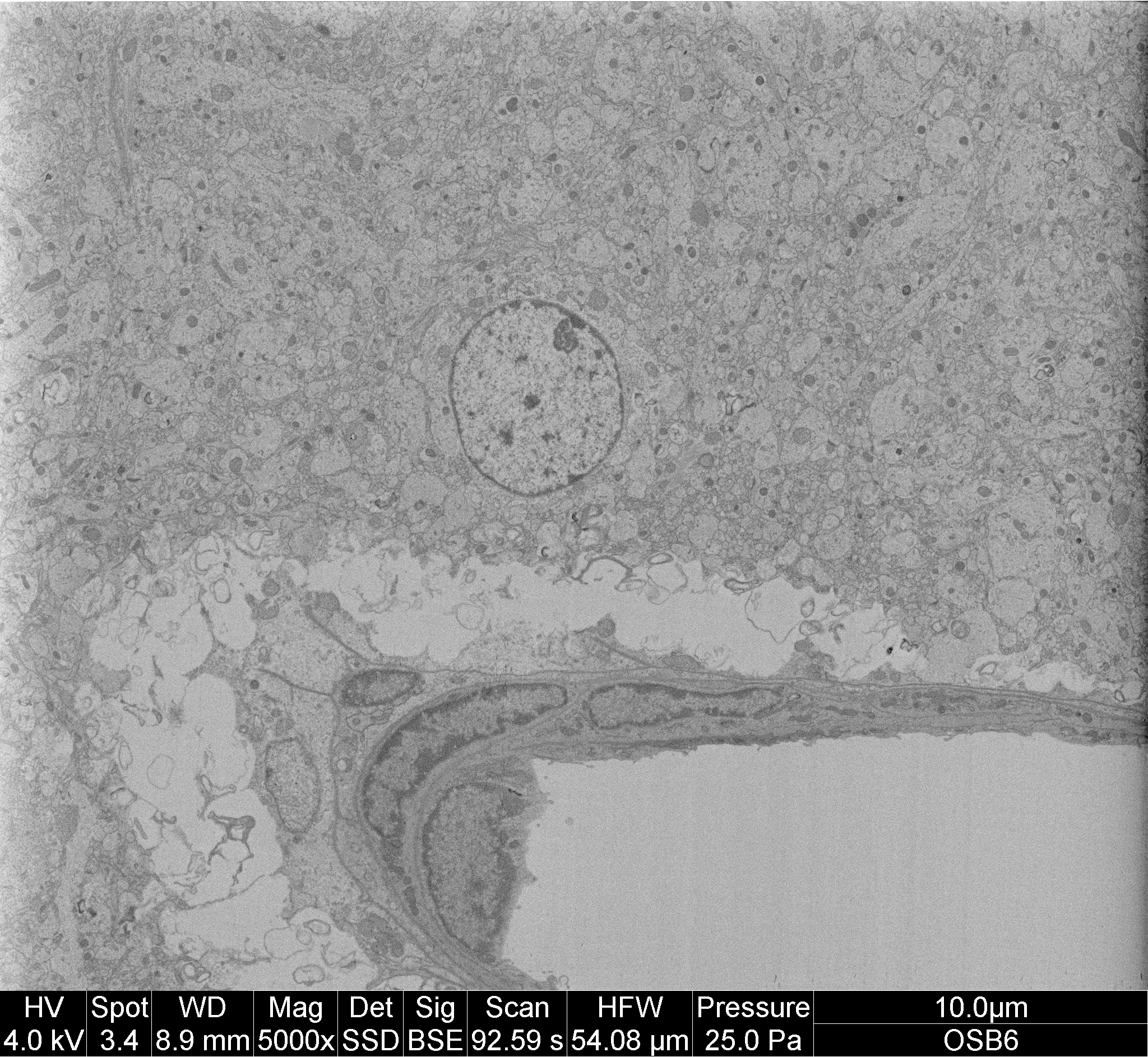

Supplement: Dataset S5 — (251.9 MB ZIP). [file pbio.0020329.sd005.zip › 040604_OS5_st1_499.tif]

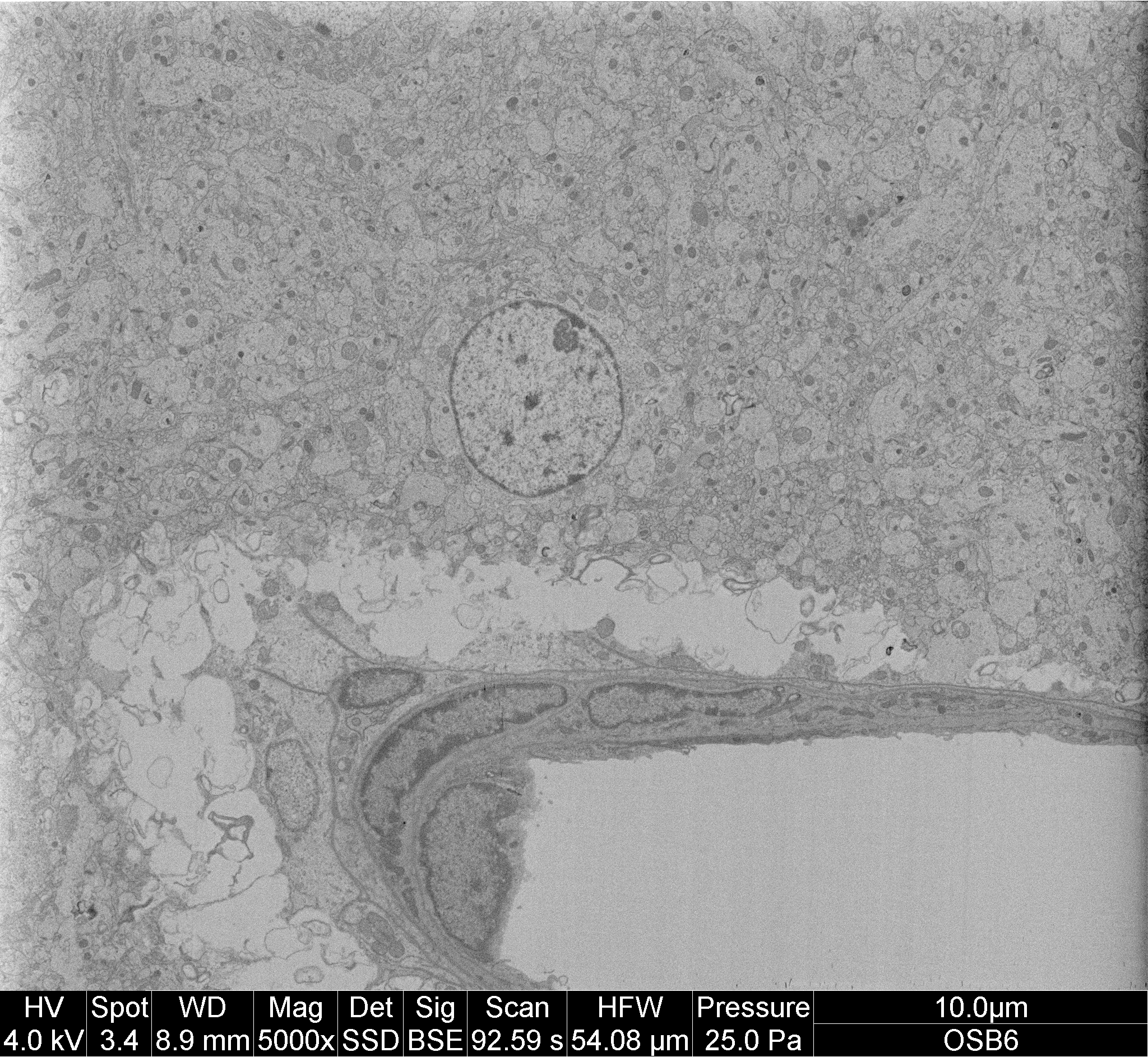

Supplement: Dataset S6 — (252.2 MB ZIP). [file pbio.0020329.sd006.zip › 040604_OS5_st1_500.tif]
