# Supplementary figures and images for: Serial Block-Face Scanning Electron Microscopy to Reconstruct Three-Dimensional Tissue Nanostructure (part 6 of 21)
Source: PLoS Biol. 2004 Oct 19;2(11):e329. doi: 10.1371/journal.pbio.0020329 (PMC524270; doi:10.1371/journal.pbio.0020329)

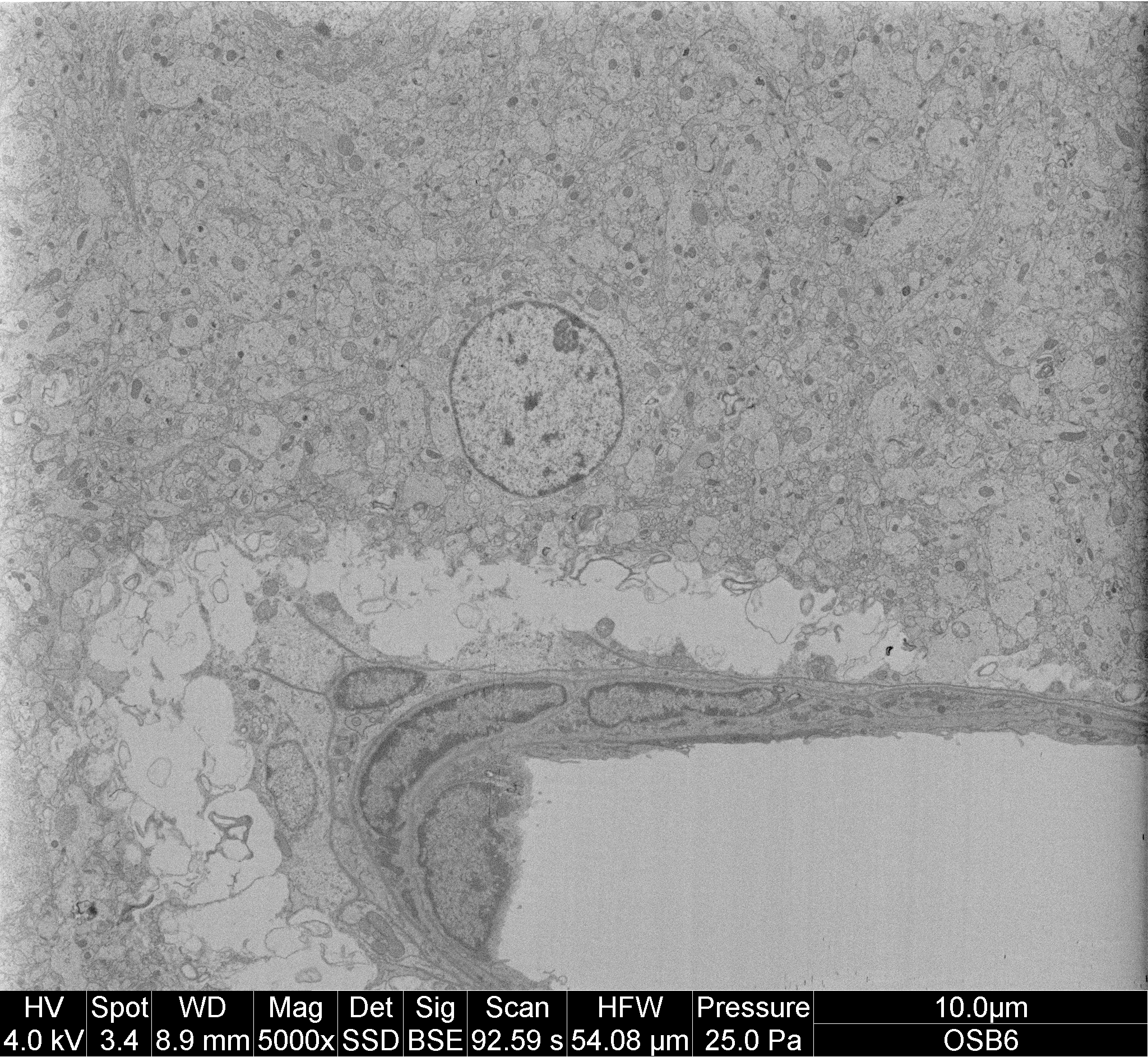

Supplement: Dataset S6 — (252.2 MB ZIP). [file pbio.0020329.sd006.zip › 040604_OS5_st1_501.tif]

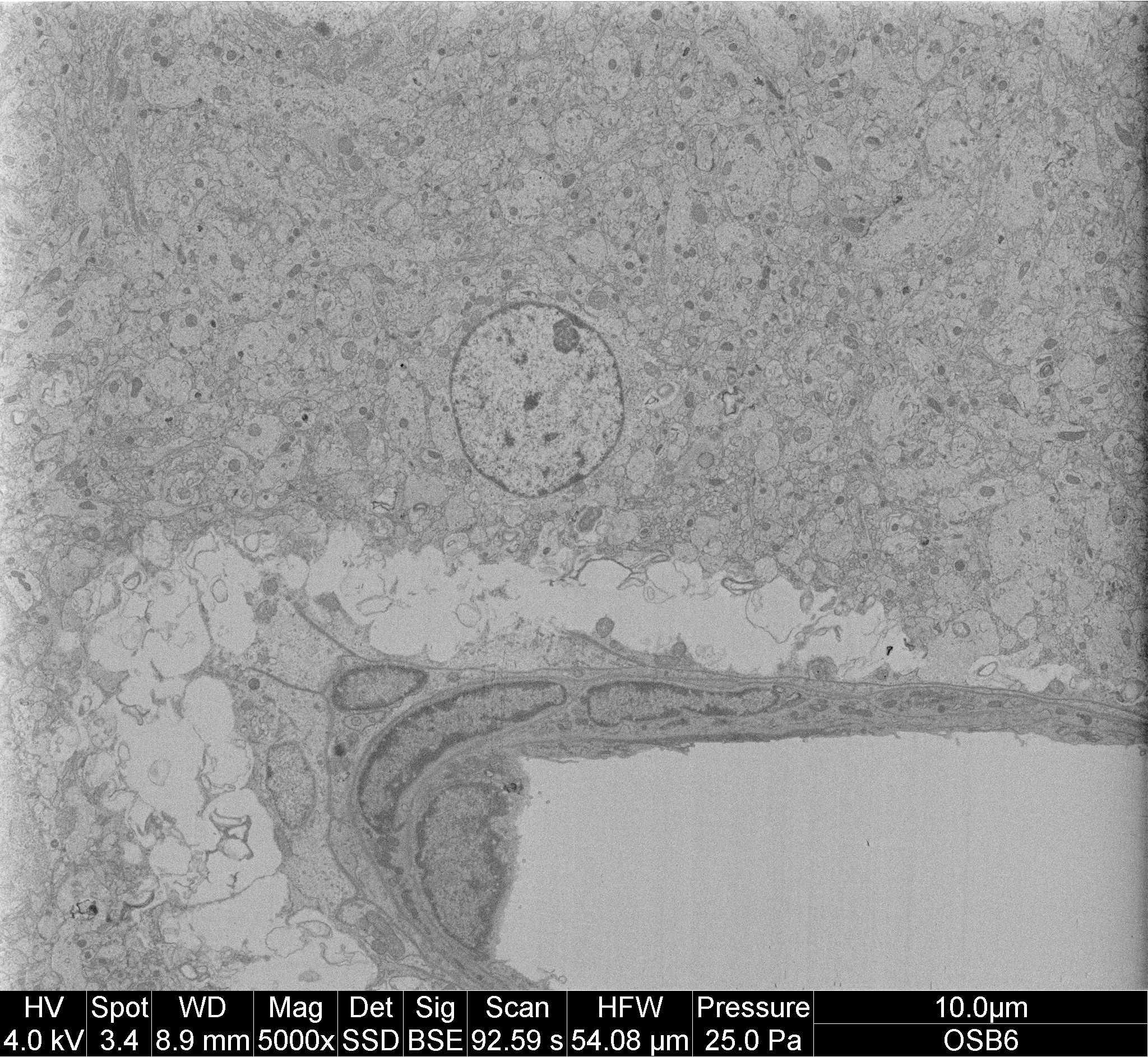

Supplement: Dataset S6 — (252.2 MB ZIP). [file pbio.0020329.sd006.zip › 040604_OS5_st1_502.tif]

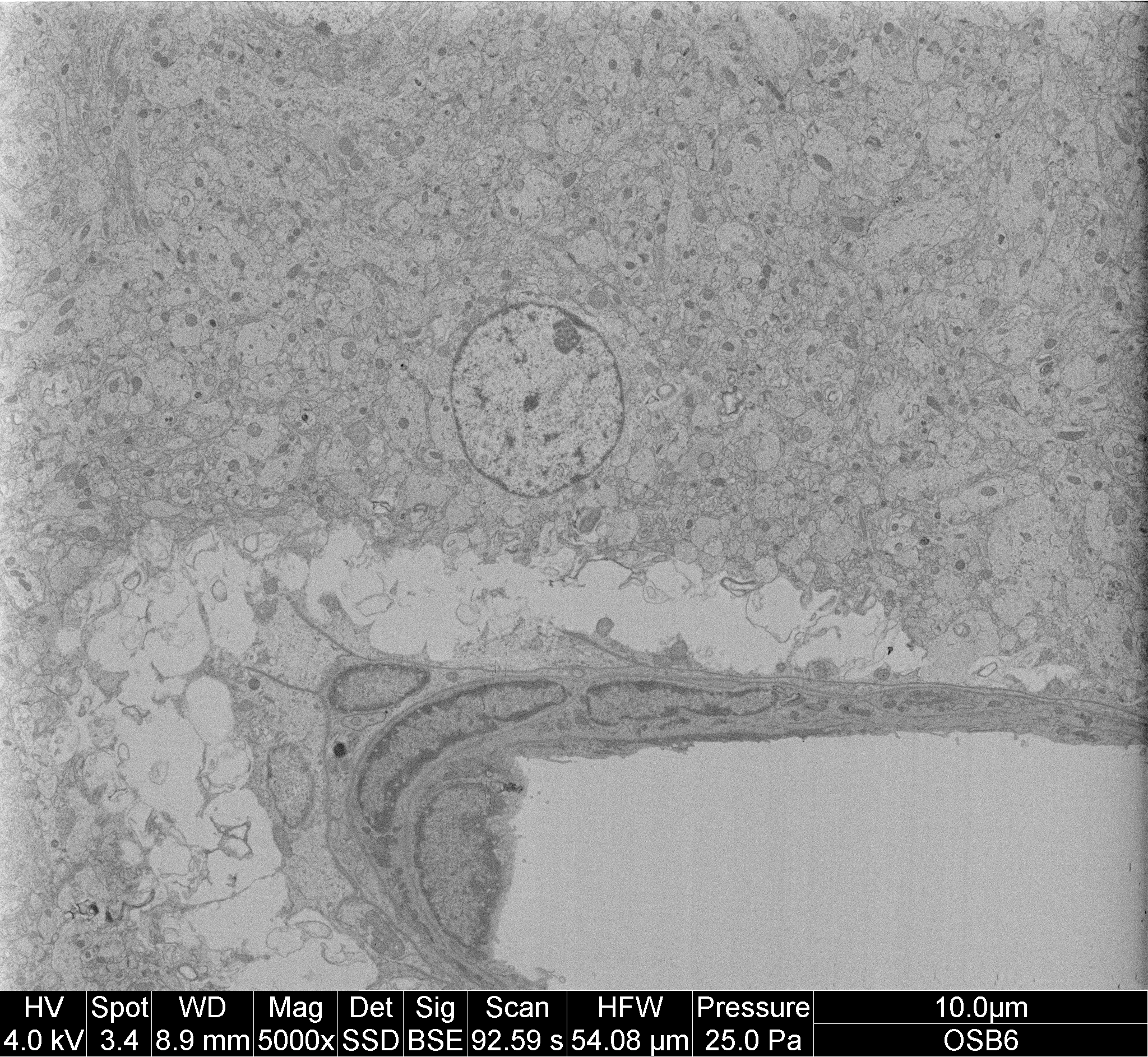

Supplement: Dataset S6 — (252.2 MB ZIP). [file pbio.0020329.sd006.zip › 040604_OS5_st1_503.tif]

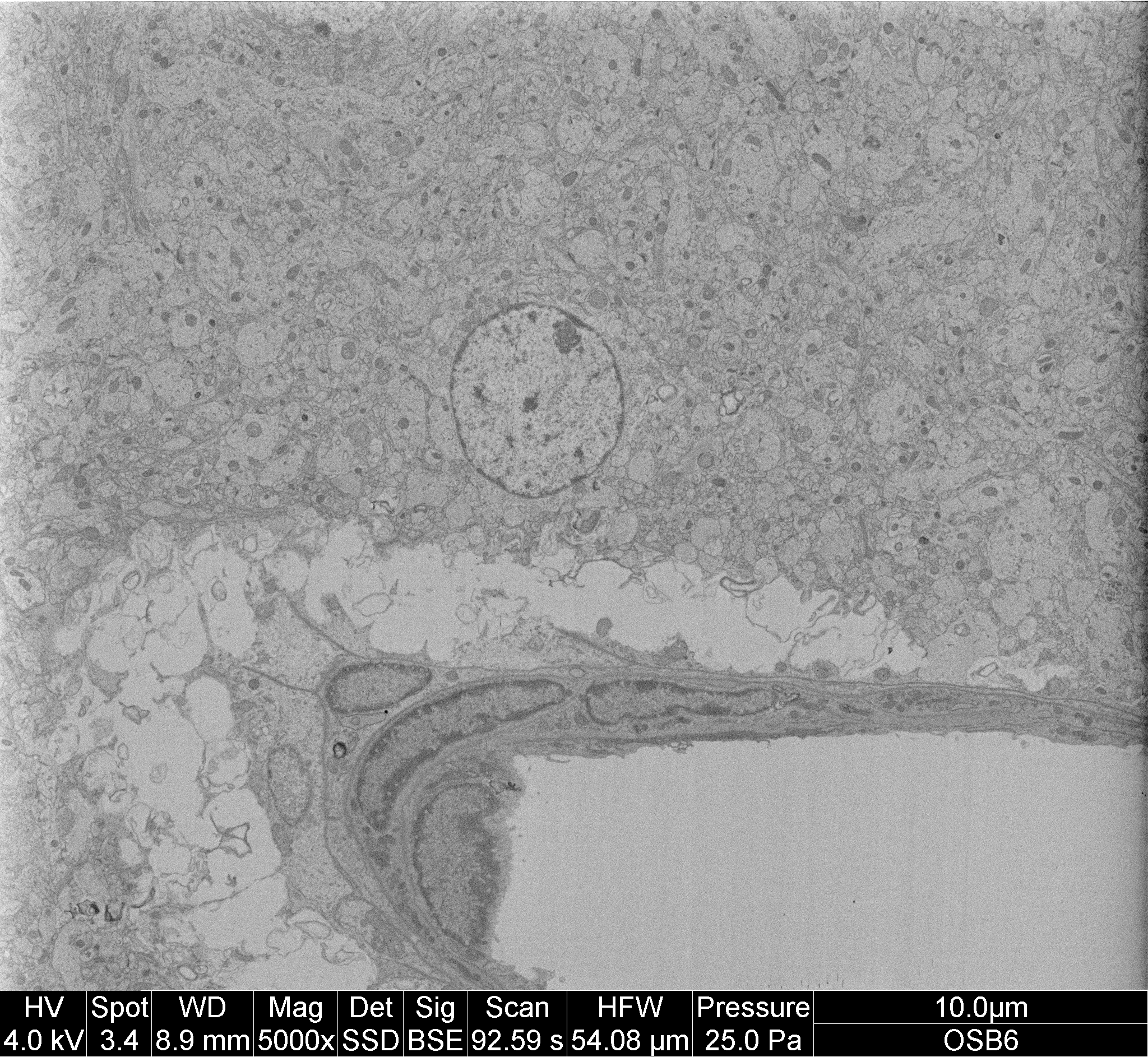

Supplement: Dataset S6 — (252.2 MB ZIP). [file pbio.0020329.sd006.zip › 040604_OS5_st1_504.tif]

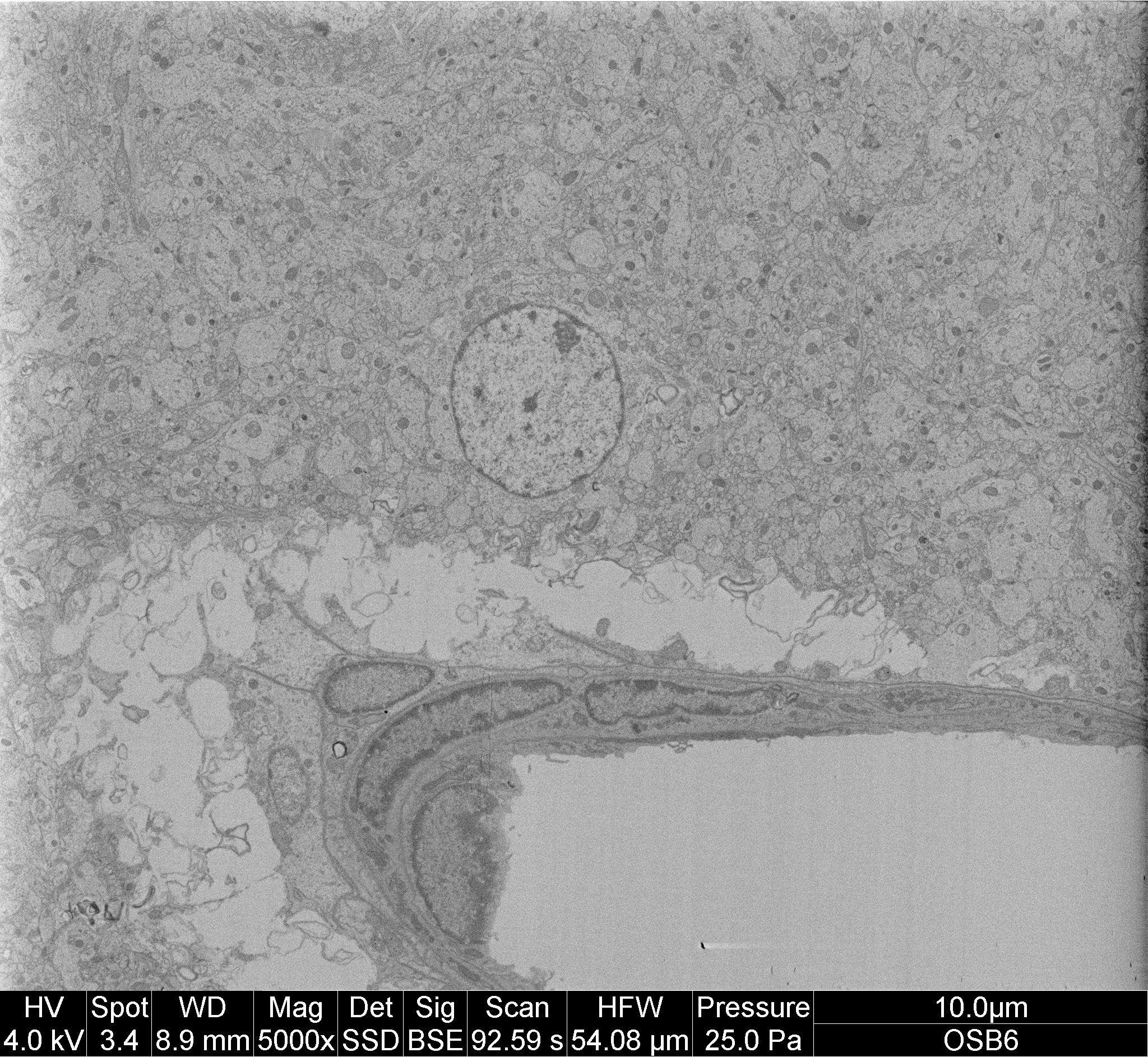

Supplement: Dataset S6 — (252.2 MB ZIP). [file pbio.0020329.sd006.zip › 040604_OS5_st1_505.tif]

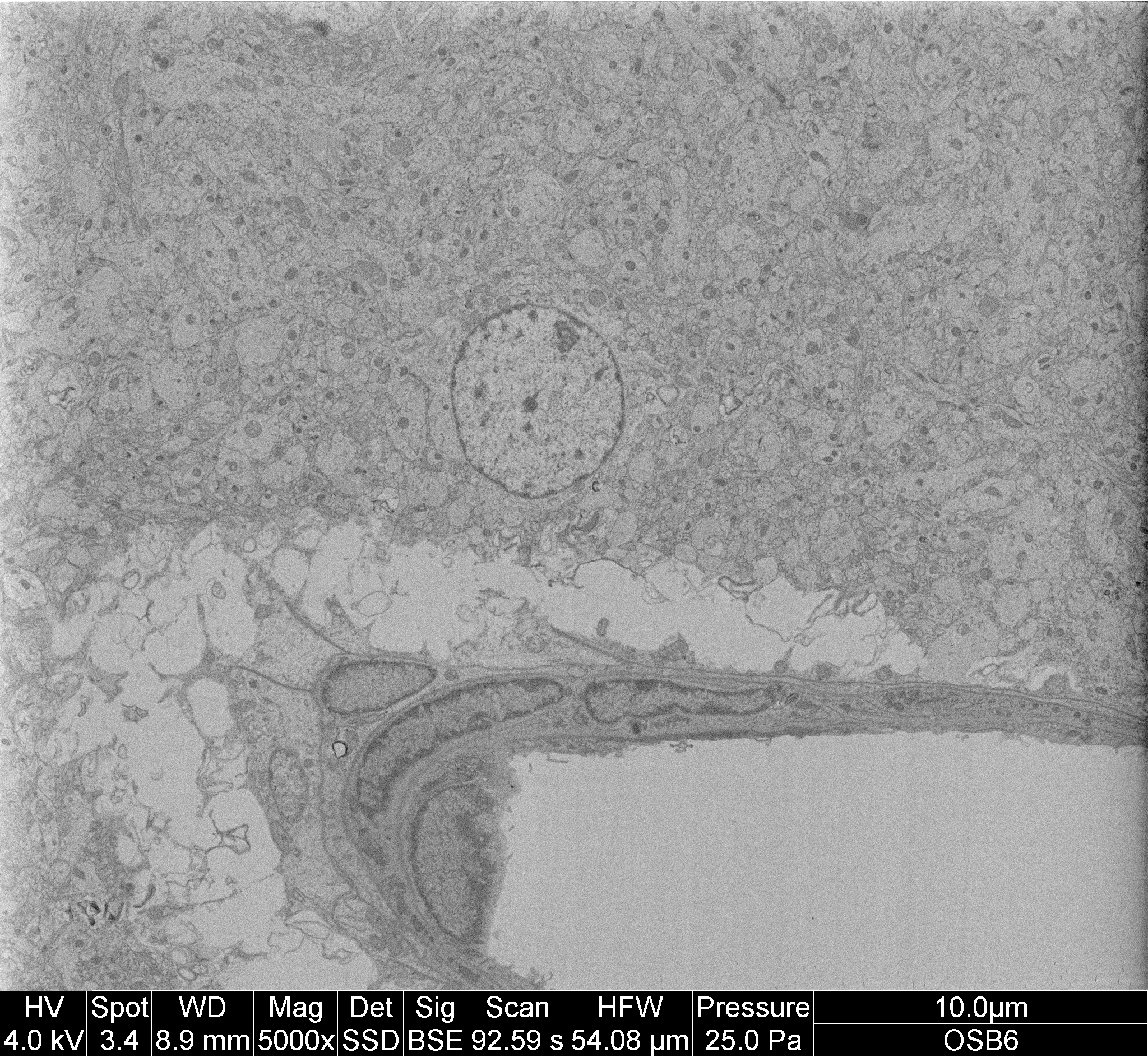

Supplement: Dataset S6 — (252.2 MB ZIP). [file pbio.0020329.sd006.zip › 040604_OS5_st1_506.tif]

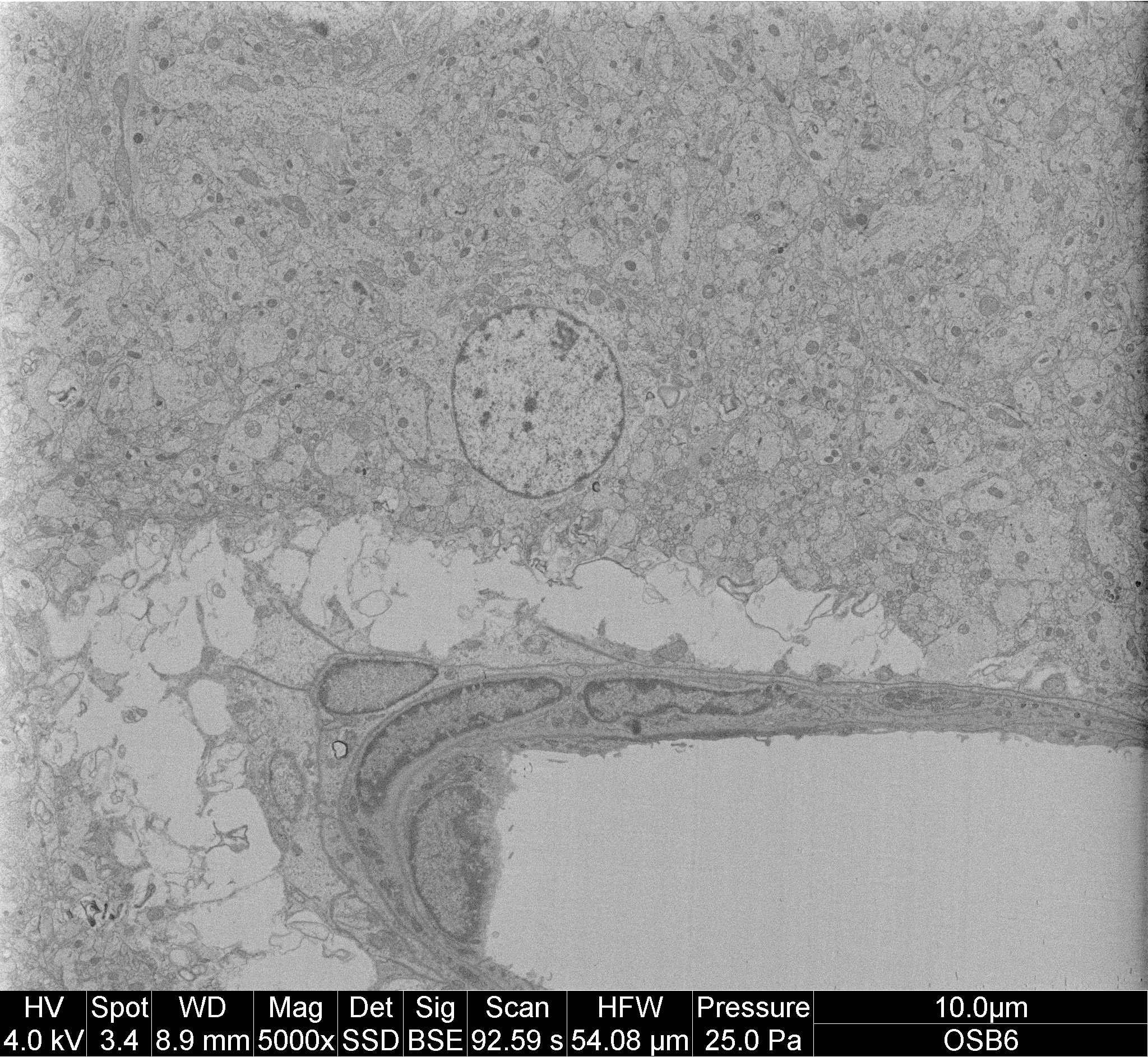

Supplement: Dataset S6 — (252.2 MB ZIP). [file pbio.0020329.sd006.zip › 040604_OS5_st1_507.tif]

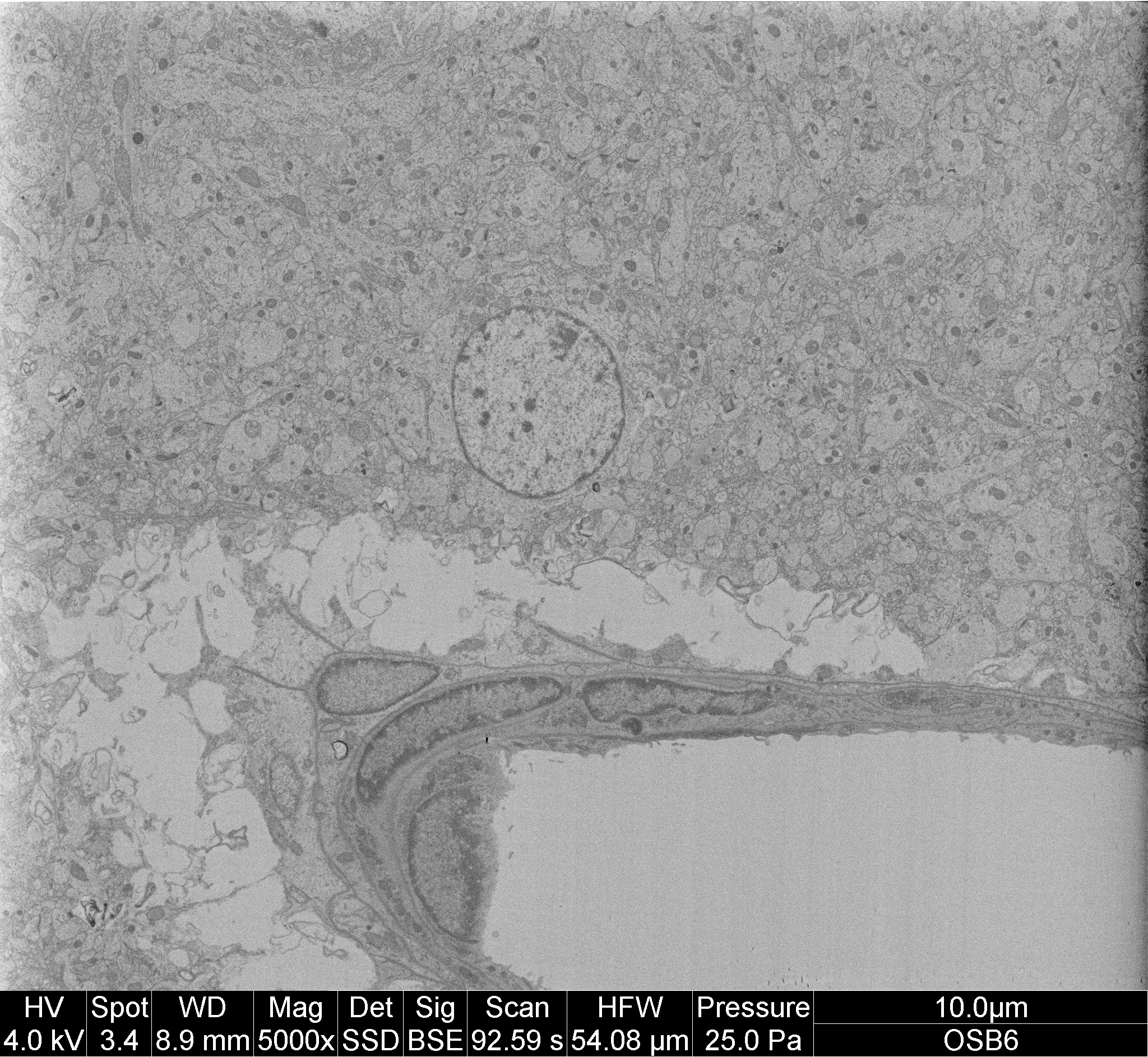

Supplement: Dataset S6 — (252.2 MB ZIP). [file pbio.0020329.sd006.zip › 040604_OS5_st1_508.tif]

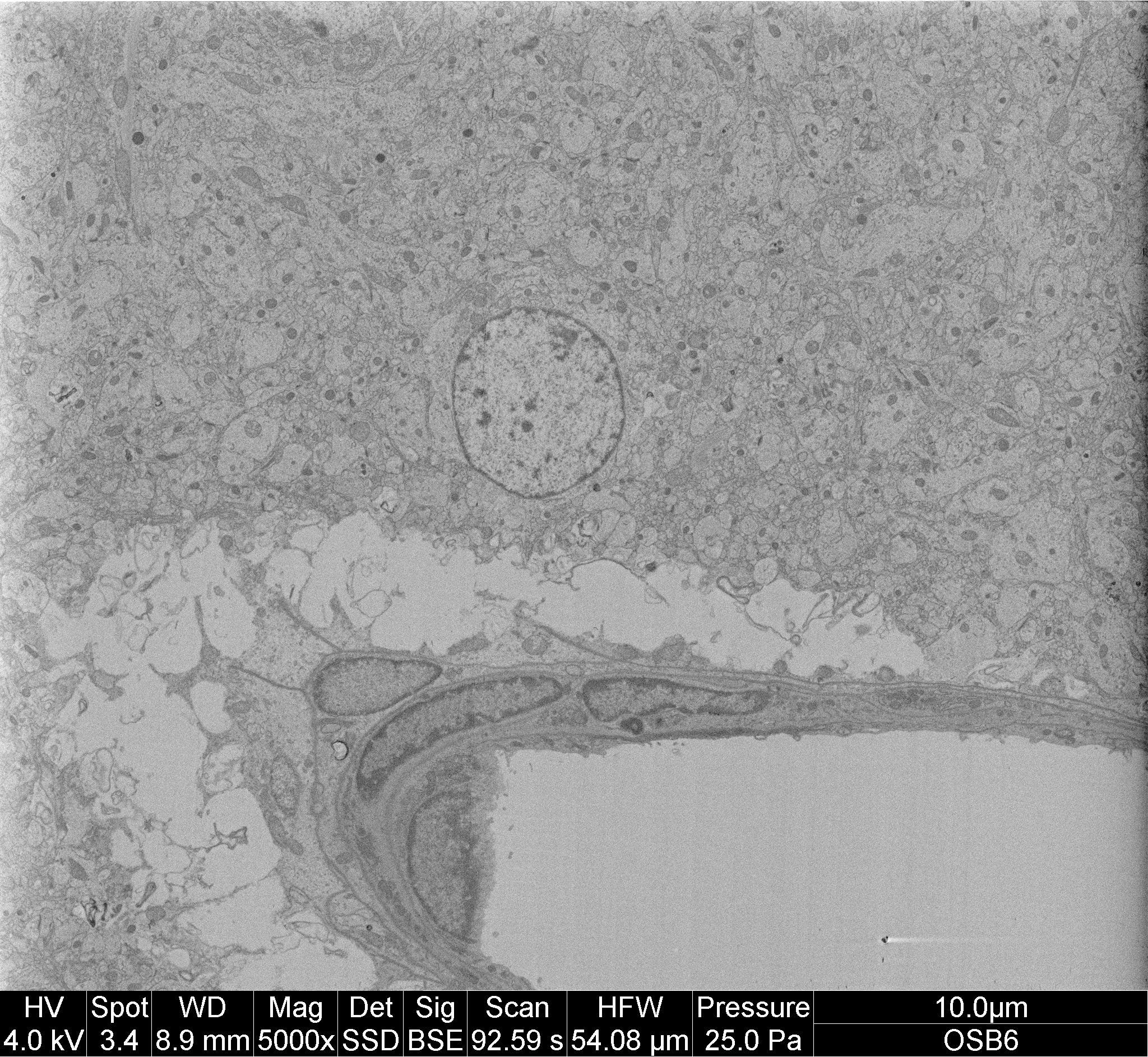

Supplement: Dataset S6 — (252.2 MB ZIP). [file pbio.0020329.sd006.zip › 040604_OS5_st1_509.tif]

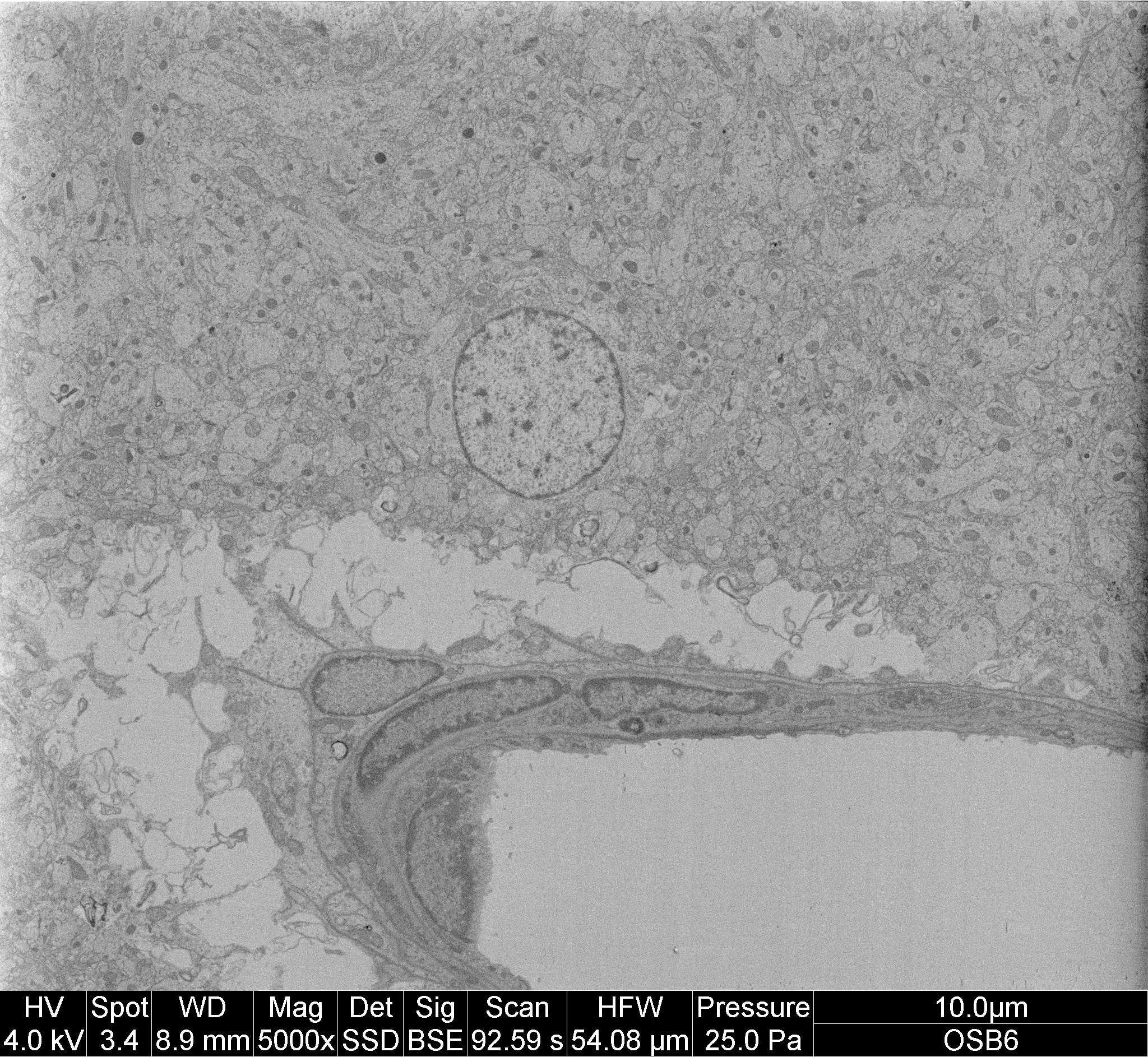

Supplement: Dataset S6 — (252.2 MB ZIP). [file pbio.0020329.sd006.zip › 040604_OS5_st1_510.tif]

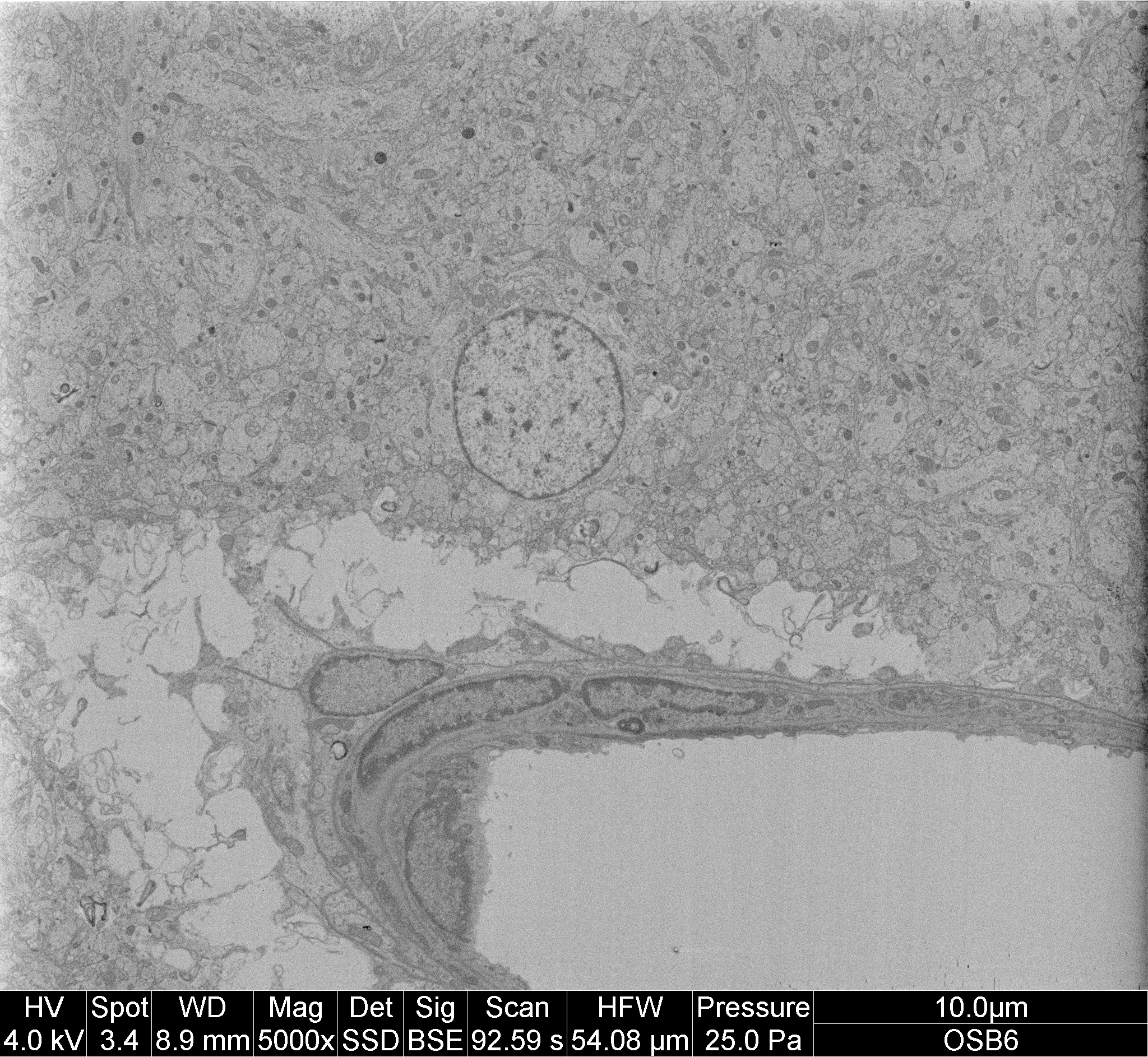

Supplement: Dataset S6 — (252.2 MB ZIP). [file pbio.0020329.sd006.zip › 040604_OS5_st1_511.tif]

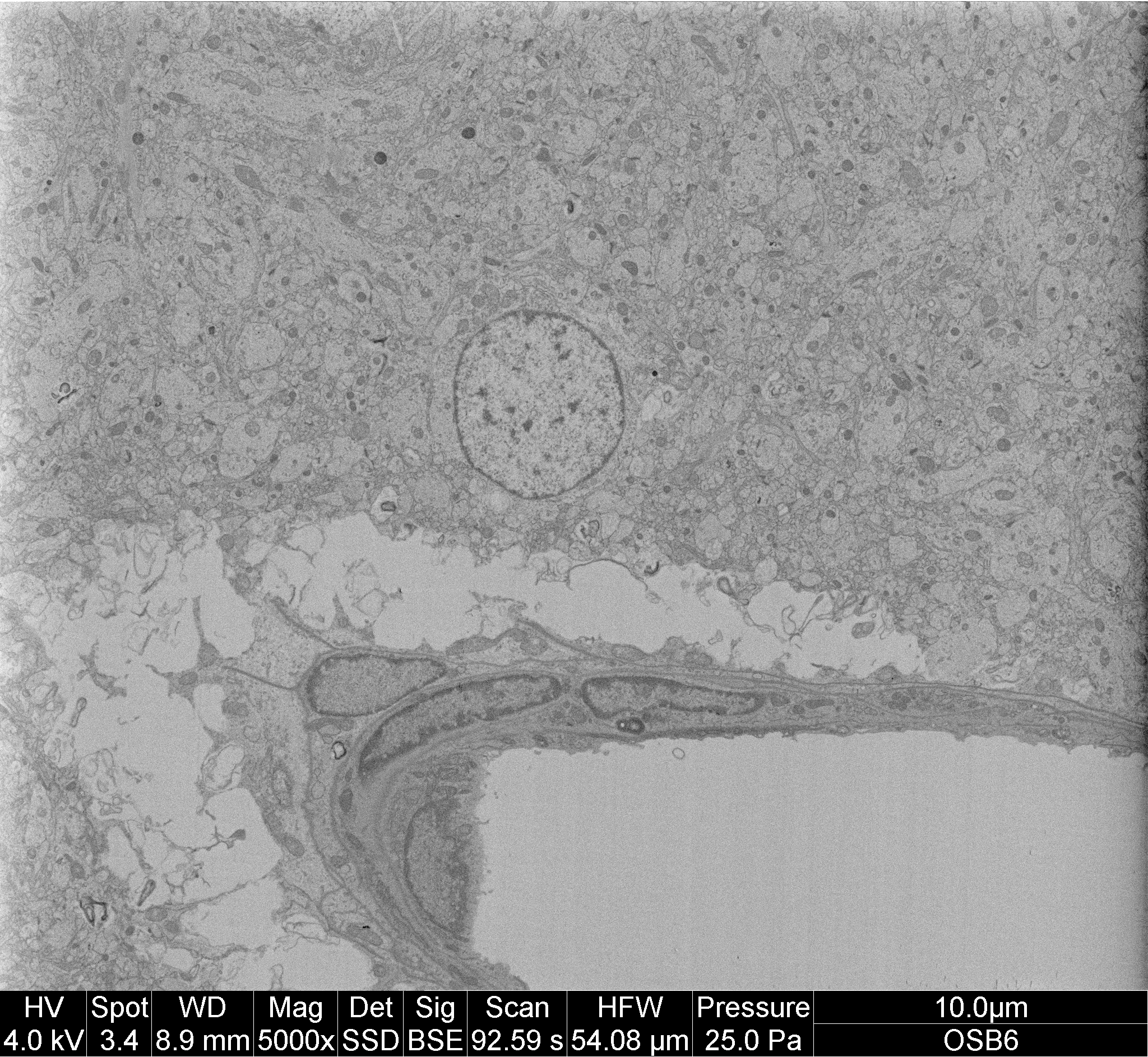

Supplement: Dataset S6 — (252.2 MB ZIP). [file pbio.0020329.sd006.zip › 040604_OS5_st1_512.tif]

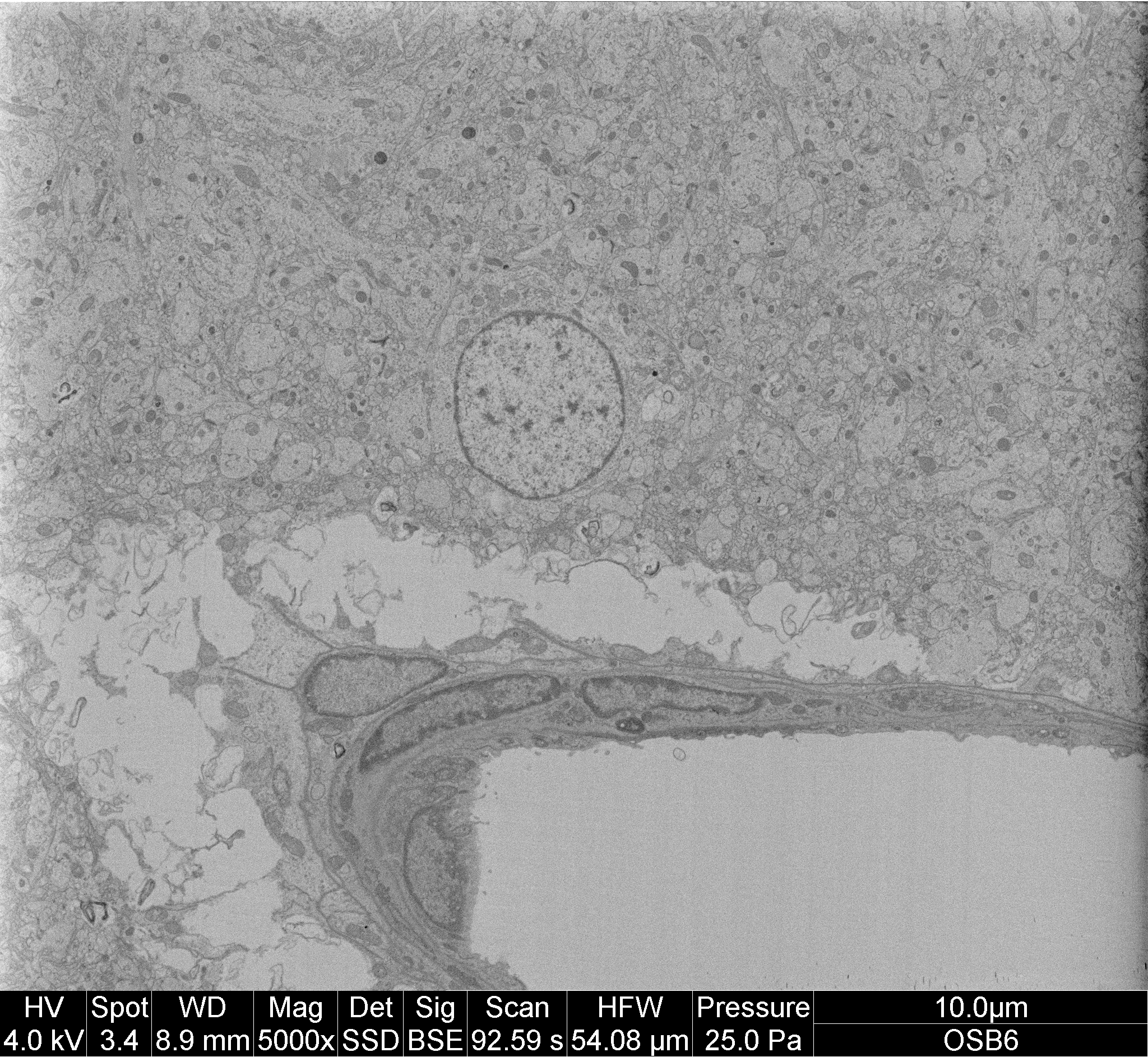

Supplement: Dataset S6 — (252.2 MB ZIP). [file pbio.0020329.sd006.zip › 040604_OS5_st1_513.tif]

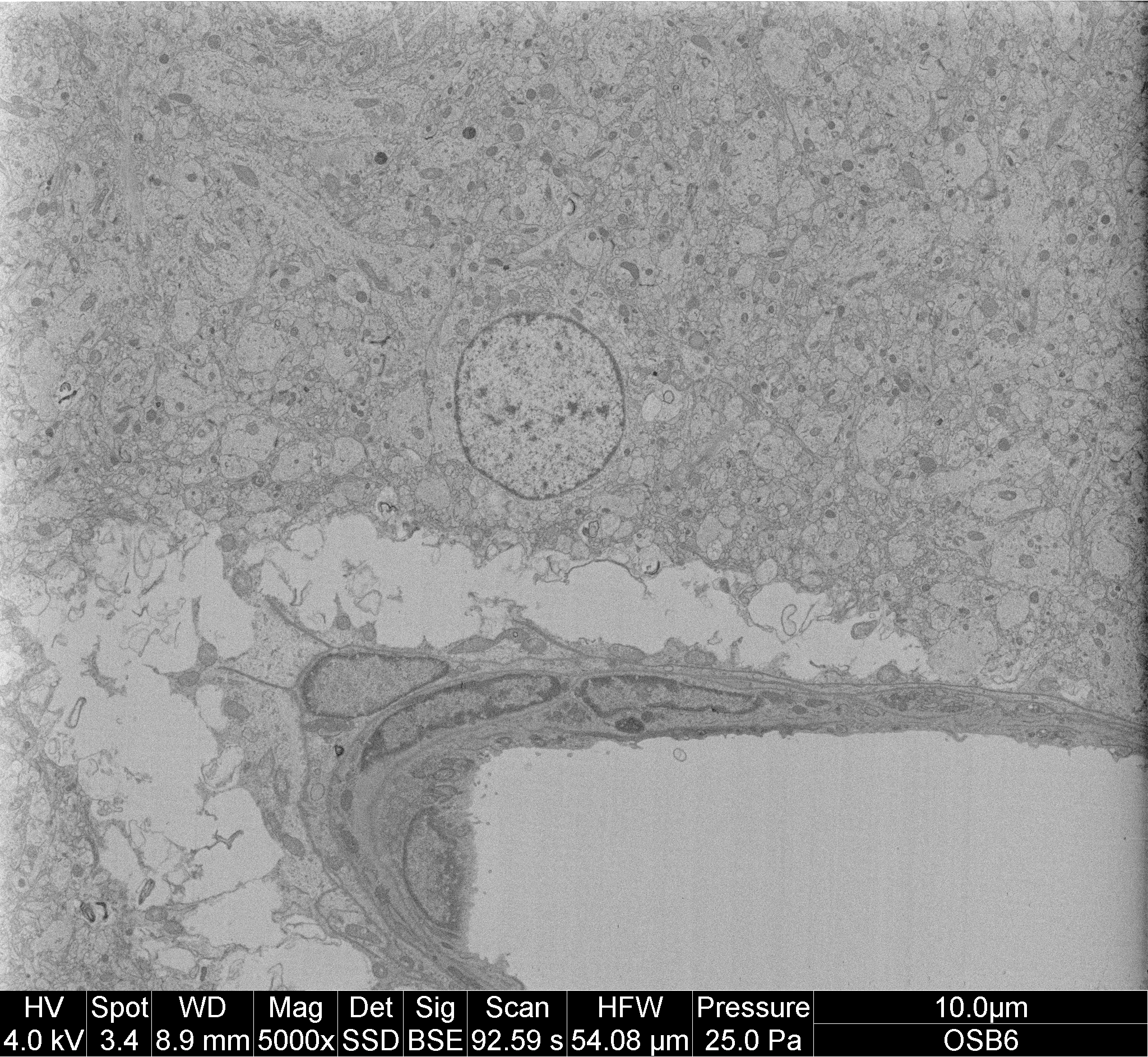

Supplement: Dataset S6 — (252.2 MB ZIP). [file pbio.0020329.sd006.zip › 040604_OS5_st1_514.tif]

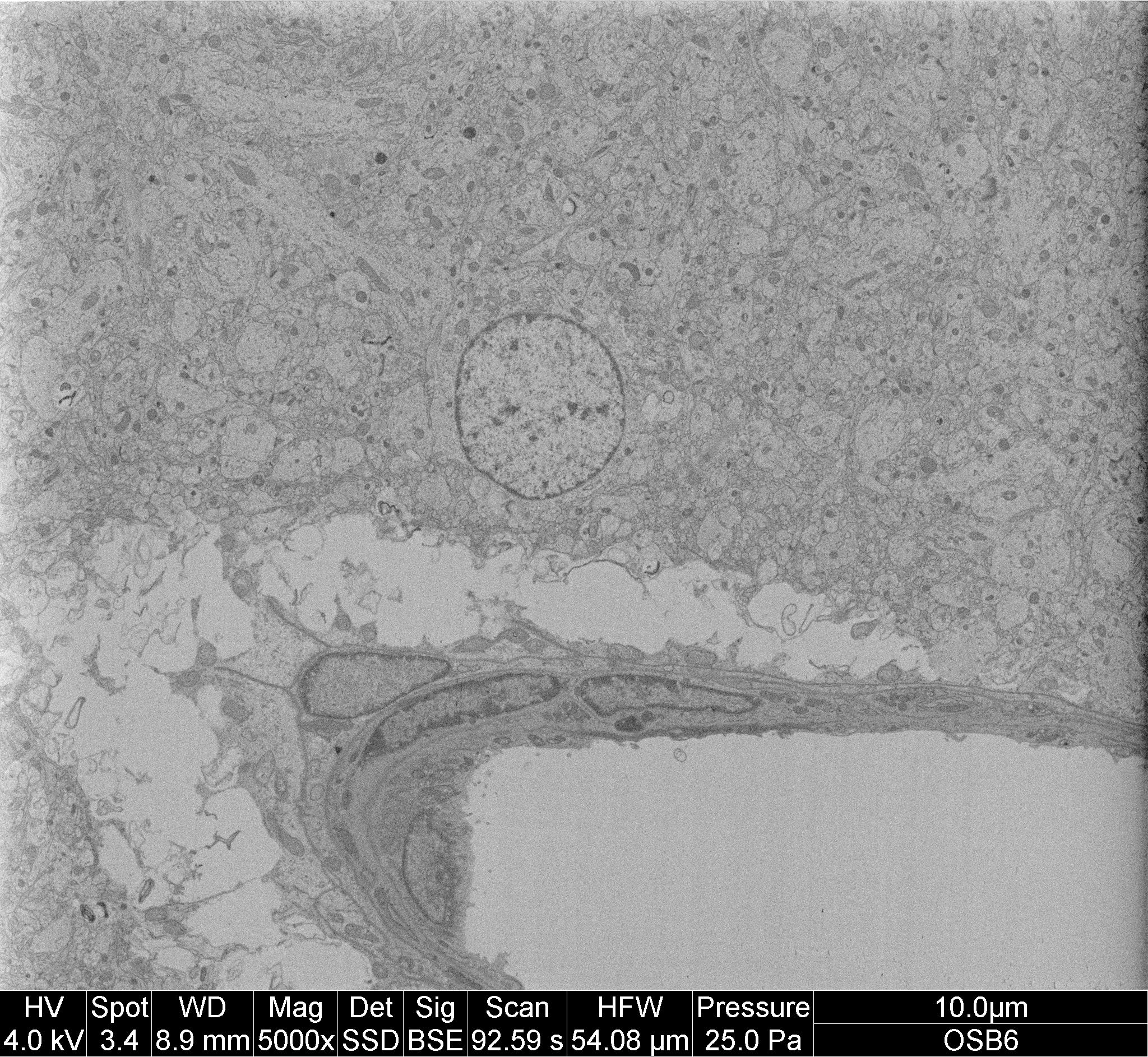

Supplement: Dataset S6 — (252.2 MB ZIP). [file pbio.0020329.sd006.zip › 040604_OS5_st1_515.tif]

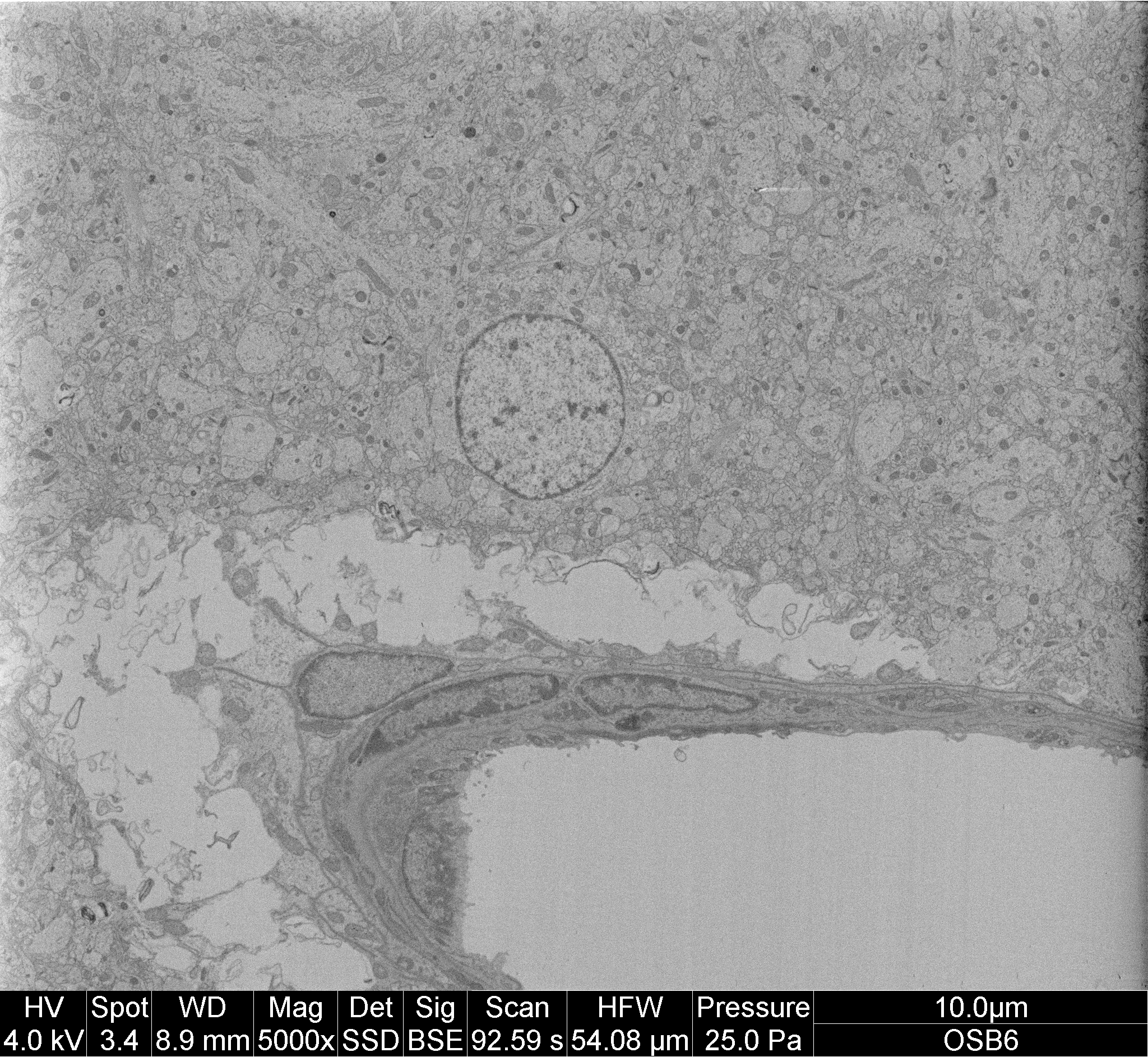

Supplement: Dataset S6 — (252.2 MB ZIP). [file pbio.0020329.sd006.zip › 040604_OS5_st1_516.tif]

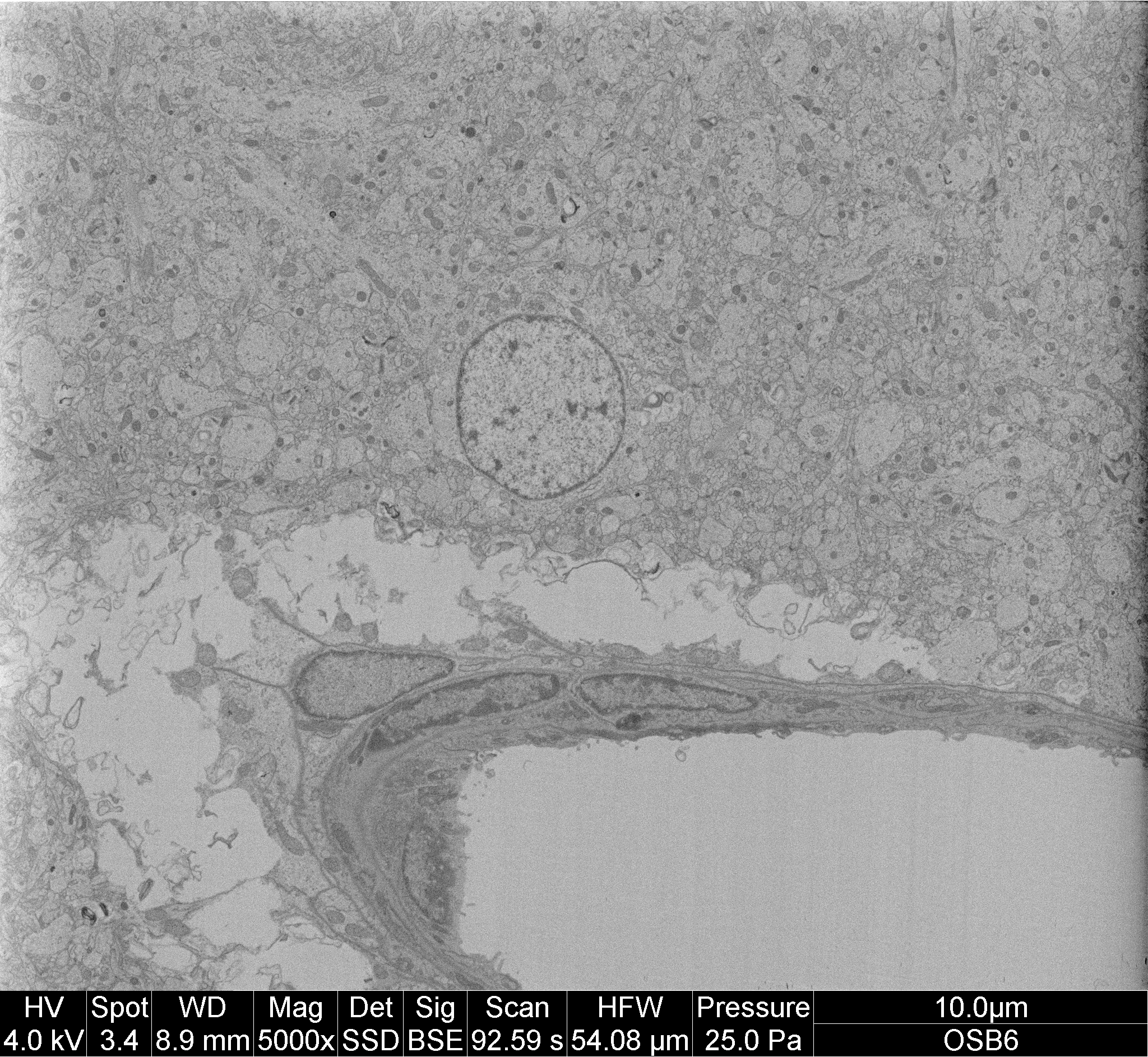

Supplement: Dataset S6 — (252.2 MB ZIP). [file pbio.0020329.sd006.zip › 040604_OS5_st1_517.tif]

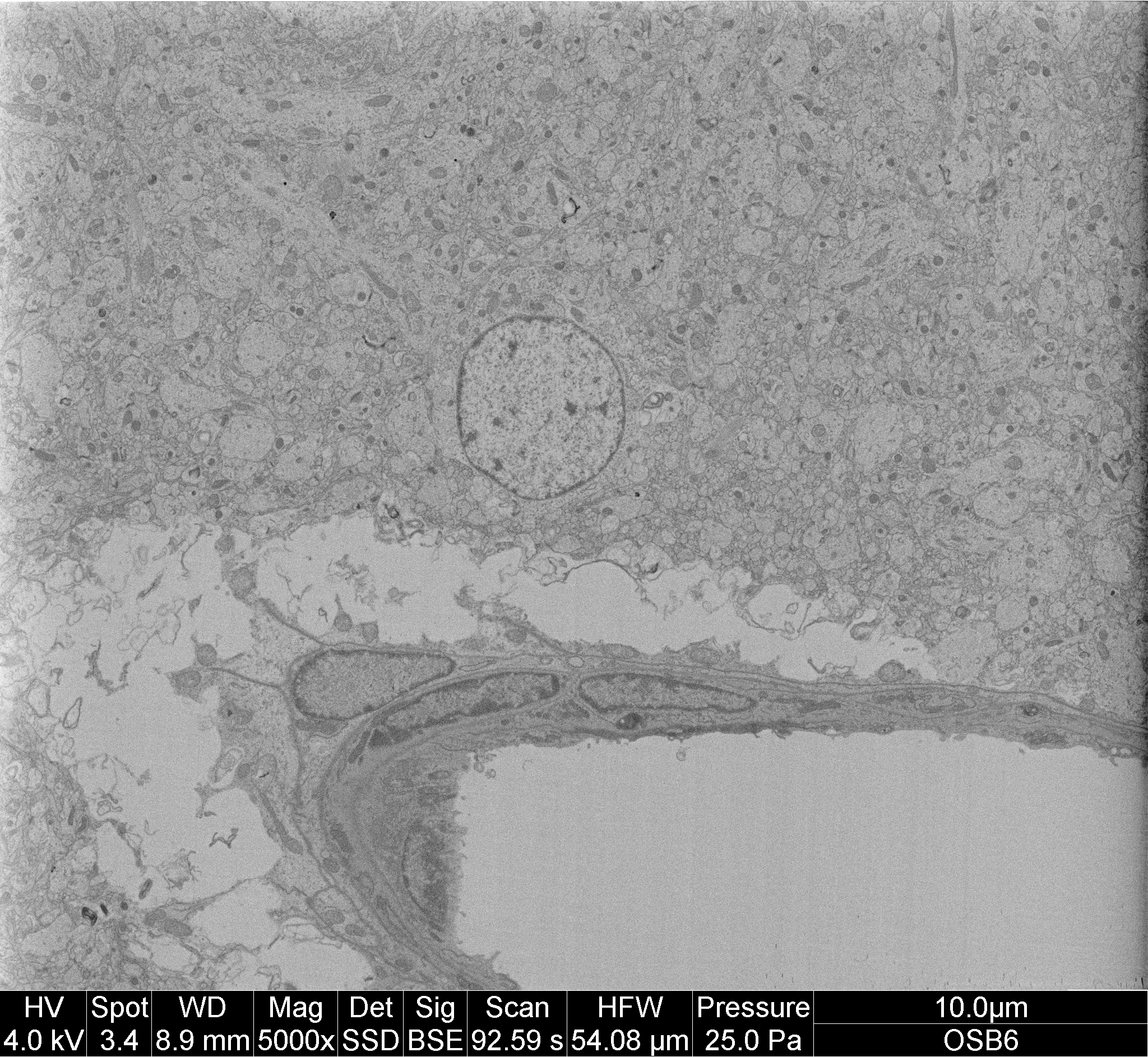

Supplement: Dataset S6 — (252.2 MB ZIP). [file pbio.0020329.sd006.zip › 040604_OS5_st1_518.tif]

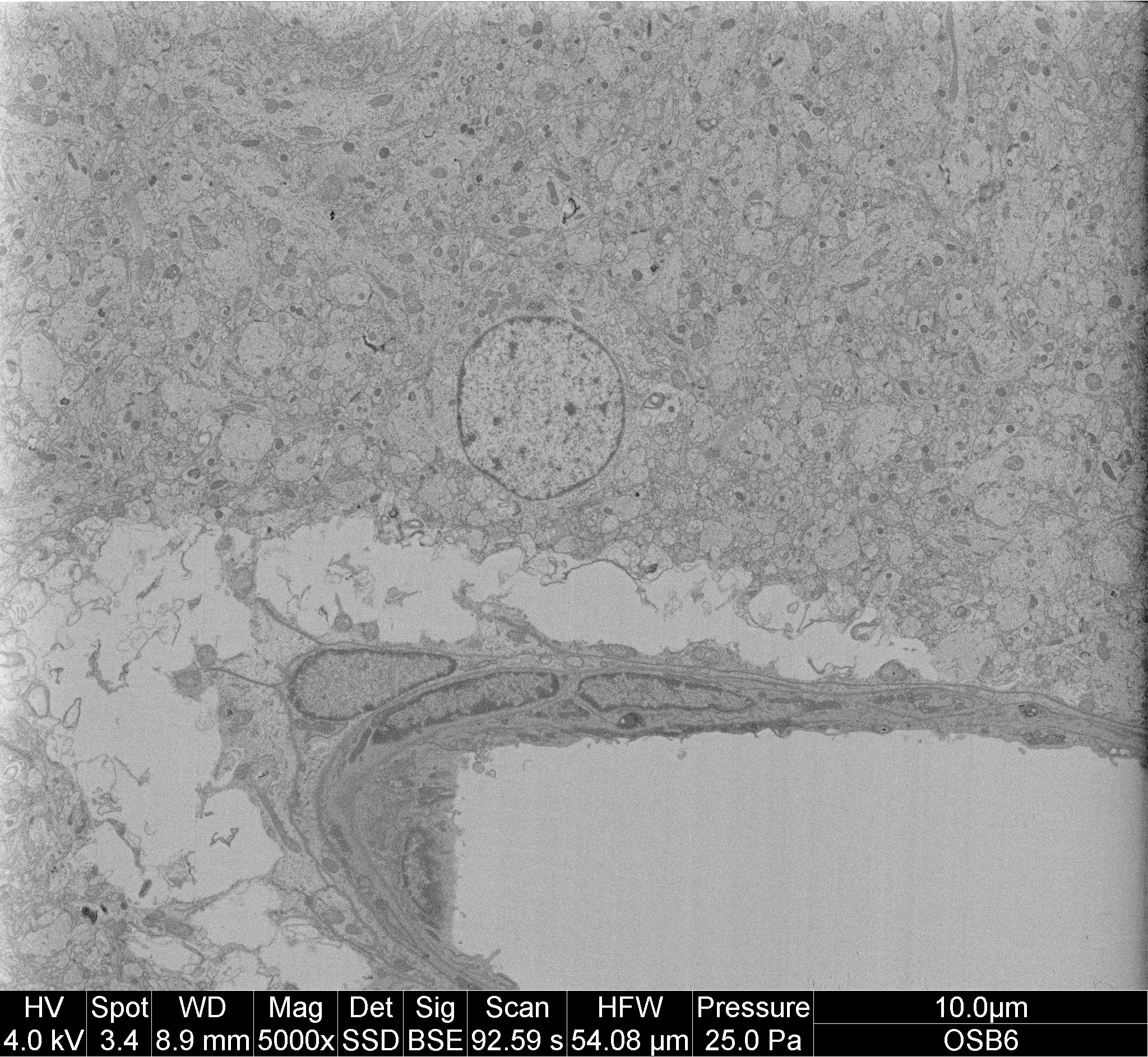

Supplement: Dataset S6 — (252.2 MB ZIP). [file pbio.0020329.sd006.zip › 040604_OS5_st1_519.tif]

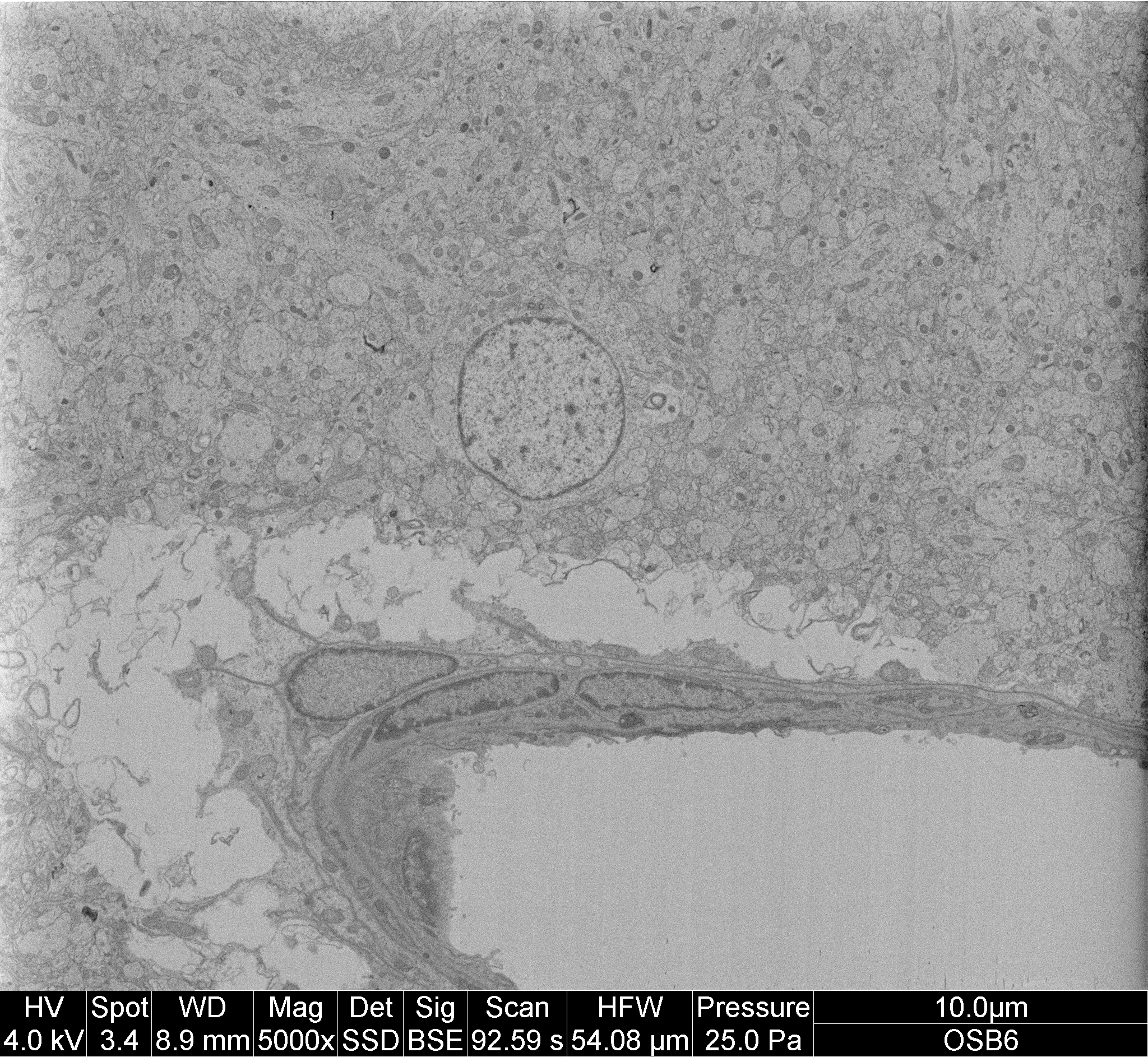

Supplement: Dataset S6 — (252.2 MB ZIP). [file pbio.0020329.sd006.zip › 040604_OS5_st1_520.tif]

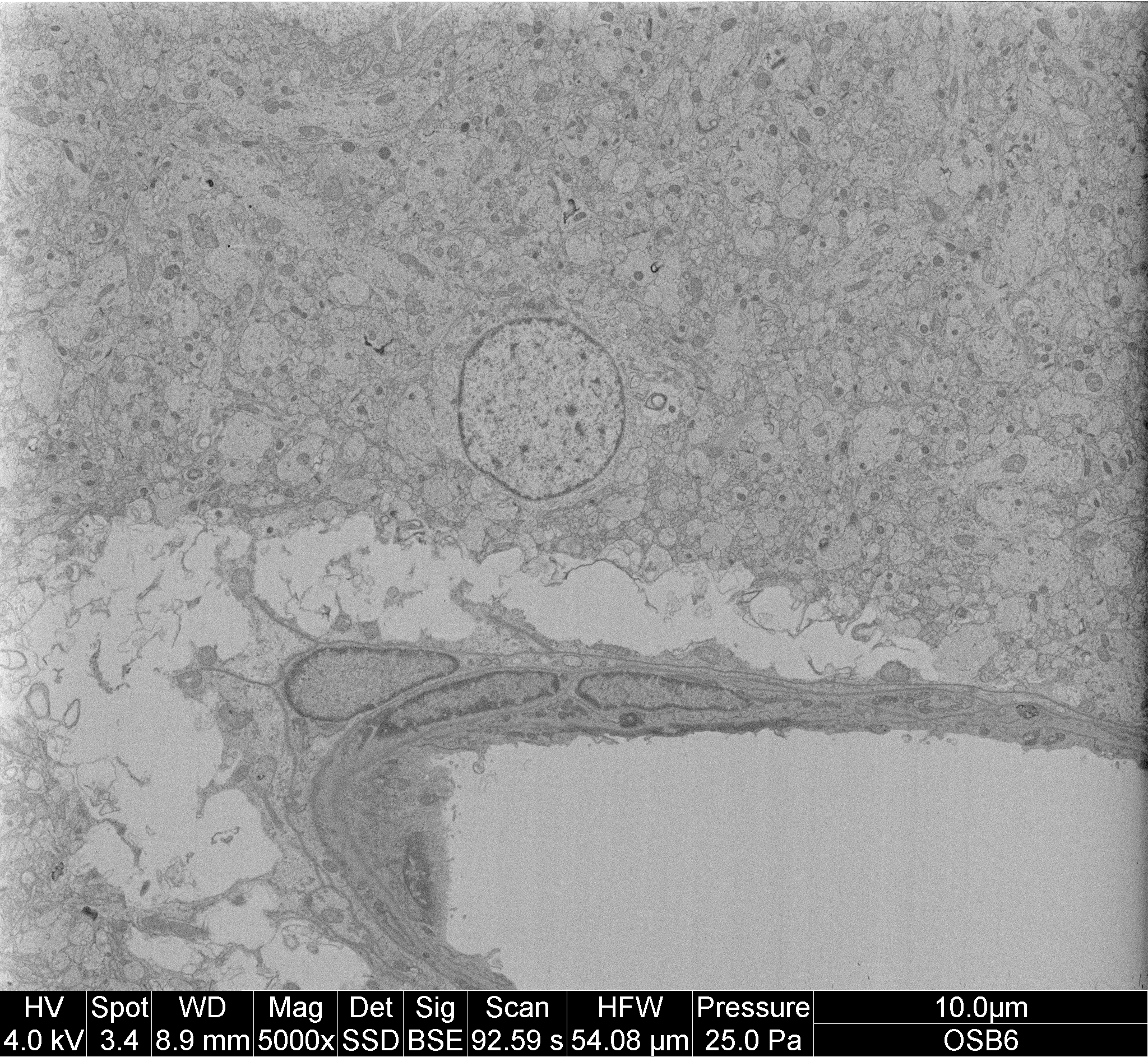

Supplement: Dataset S6 — (252.2 MB ZIP). [file pbio.0020329.sd006.zip › 040604_OS5_st1_521.tif]

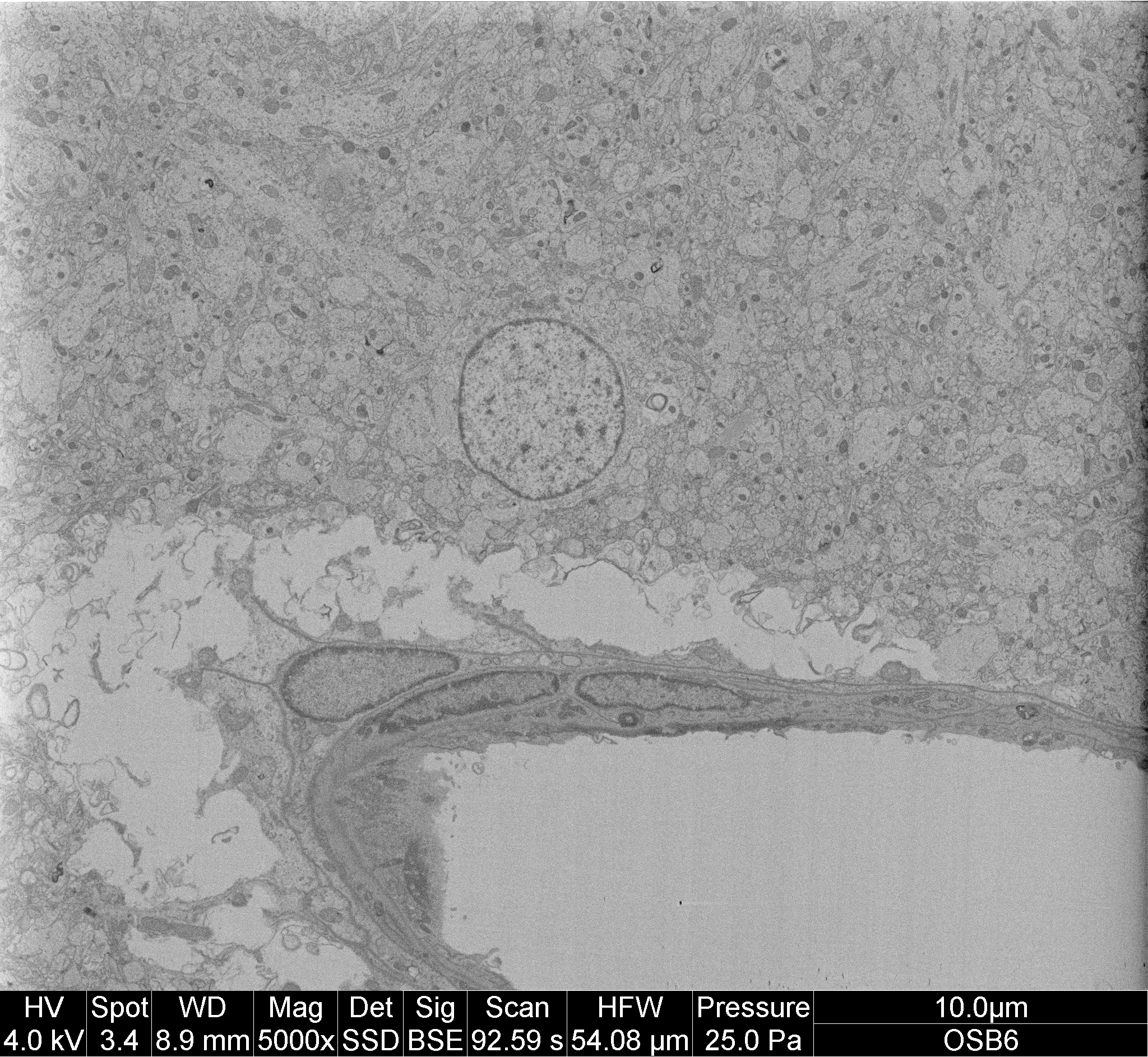

Supplement: Dataset S6 — (252.2 MB ZIP). [file pbio.0020329.sd006.zip › 040604_OS5_st1_522.tif]

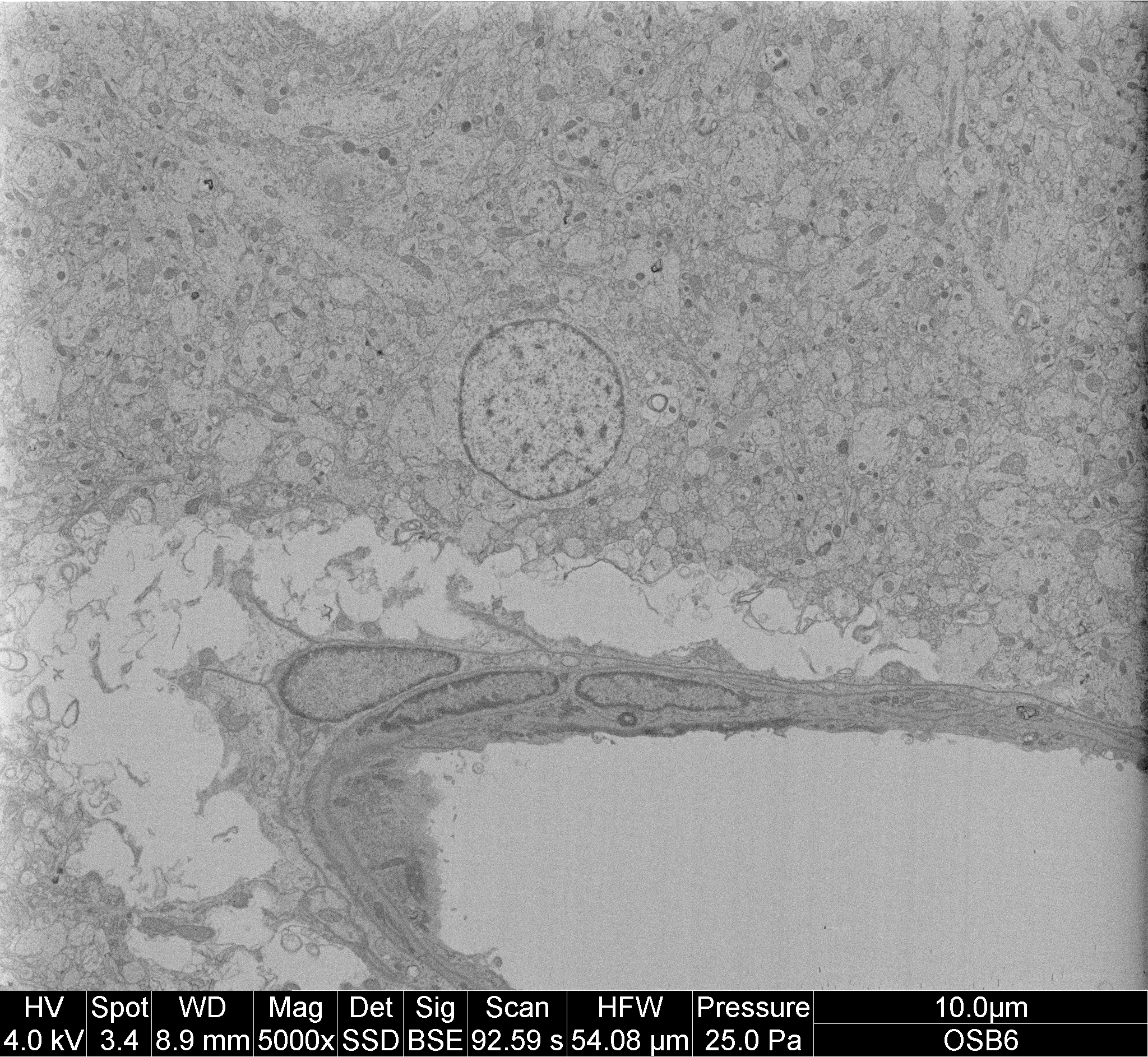

Supplement: Dataset S6 — (252.2 MB ZIP). [file pbio.0020329.sd006.zip › 040604_OS5_st1_523.tif]

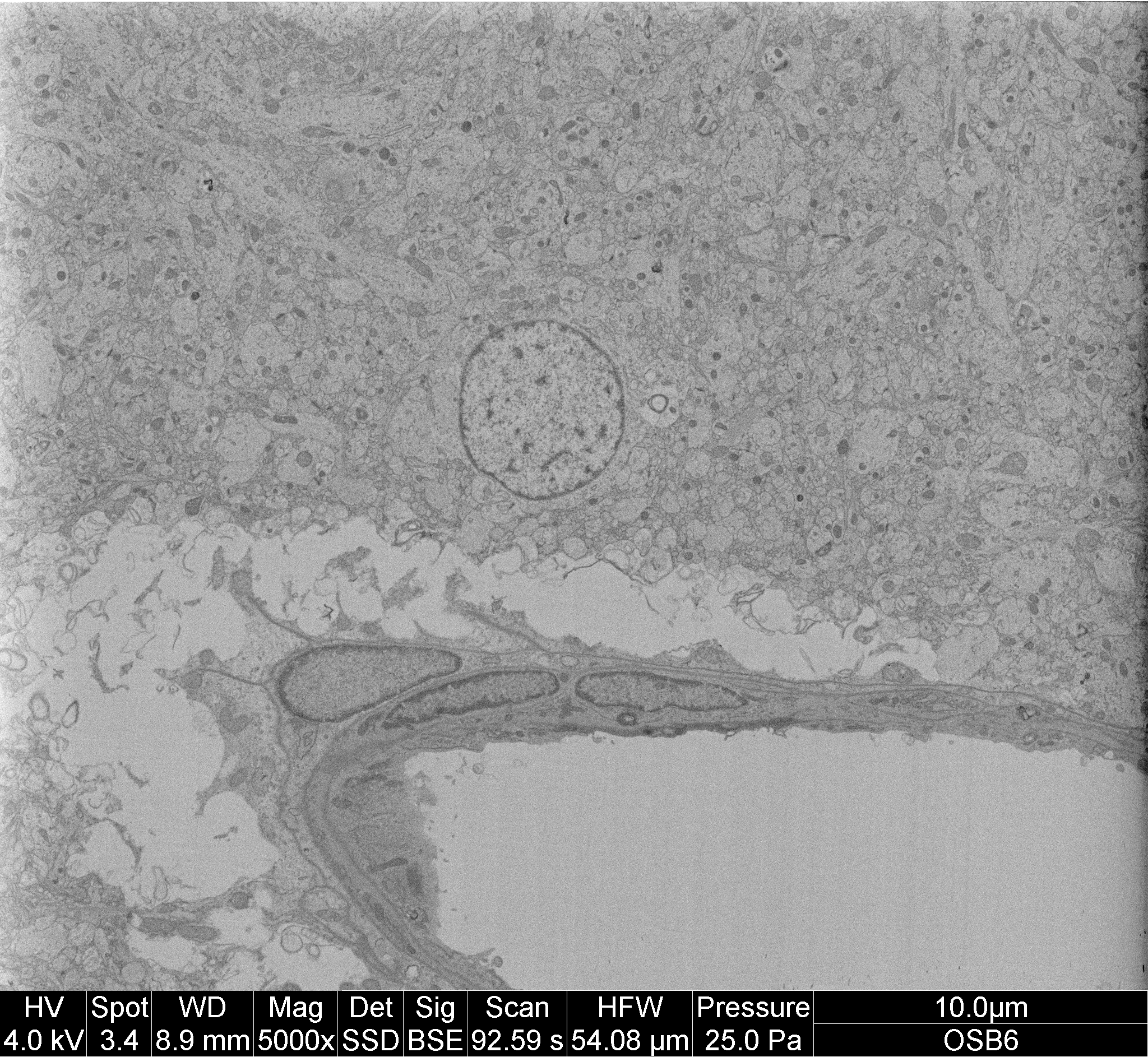

Supplement: Dataset S6 — (252.2 MB ZIP). [file pbio.0020329.sd006.zip › 040604_OS5_st1_524.tif]

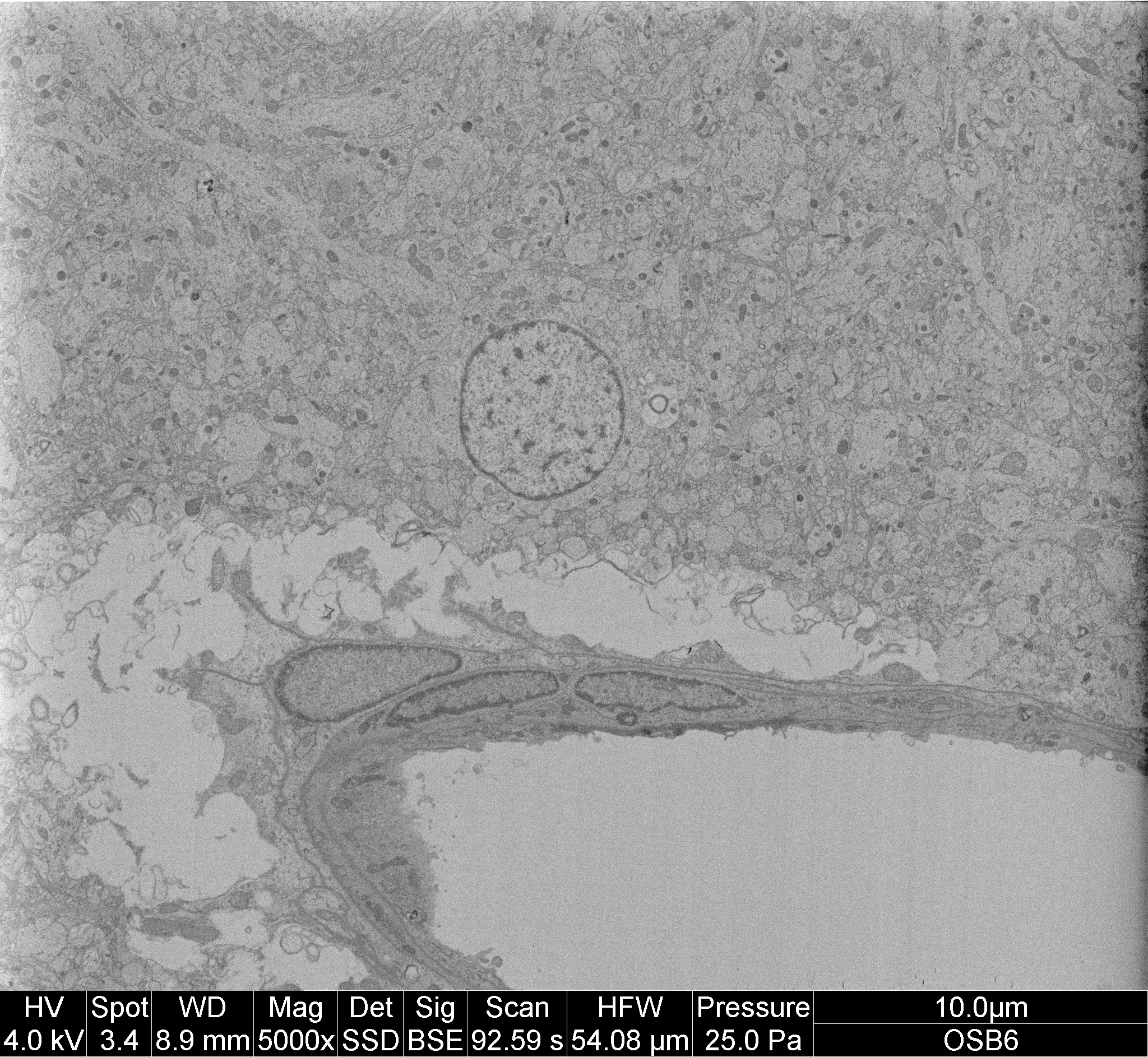

Supplement: Dataset S6 — (252.2 MB ZIP). [file pbio.0020329.sd006.zip › 040604_OS5_st1_525.tif]

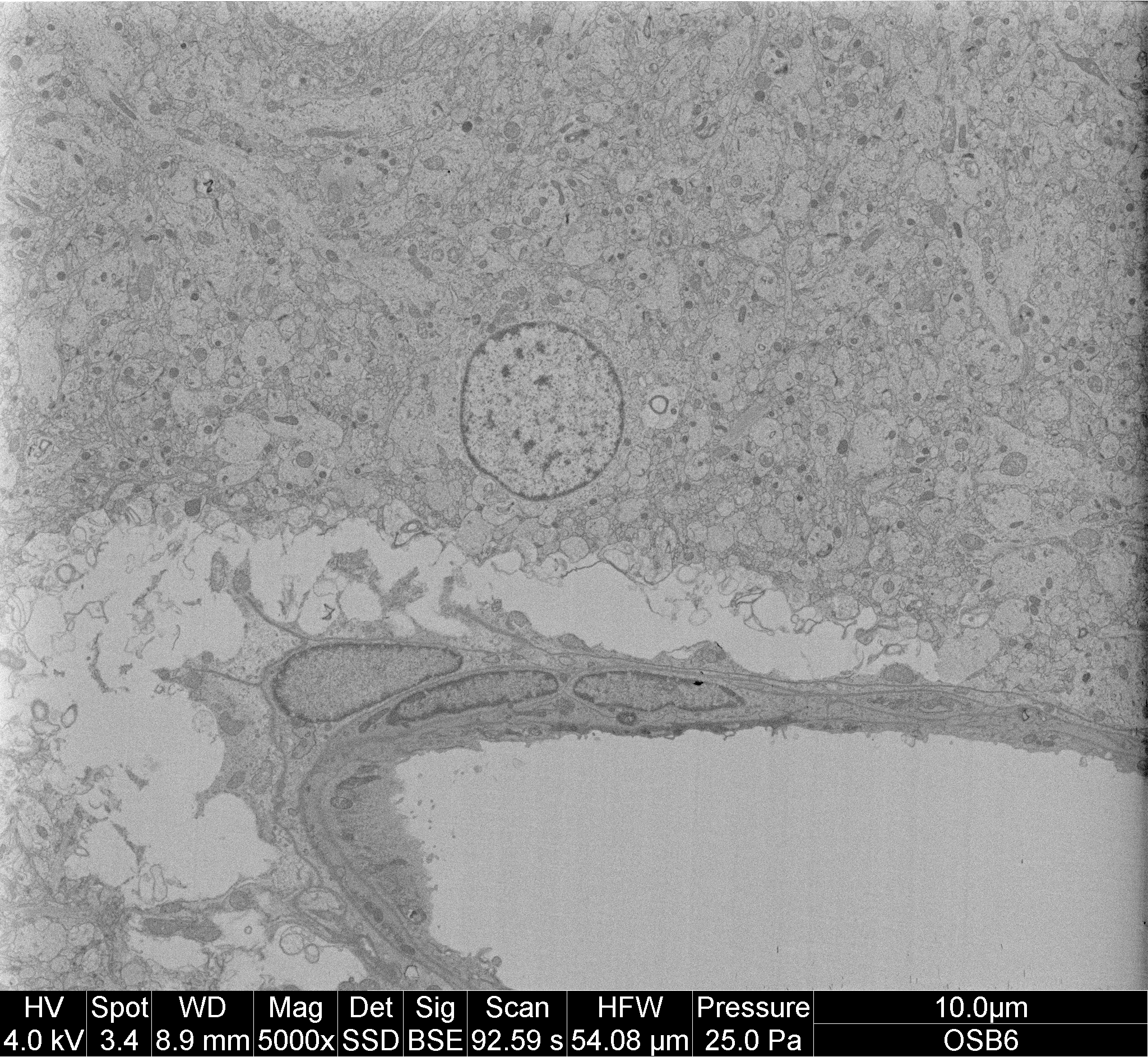

Supplement: Dataset S6 — (252.2 MB ZIP). [file pbio.0020329.sd006.zip › 040604_OS5_st1_526.tif]

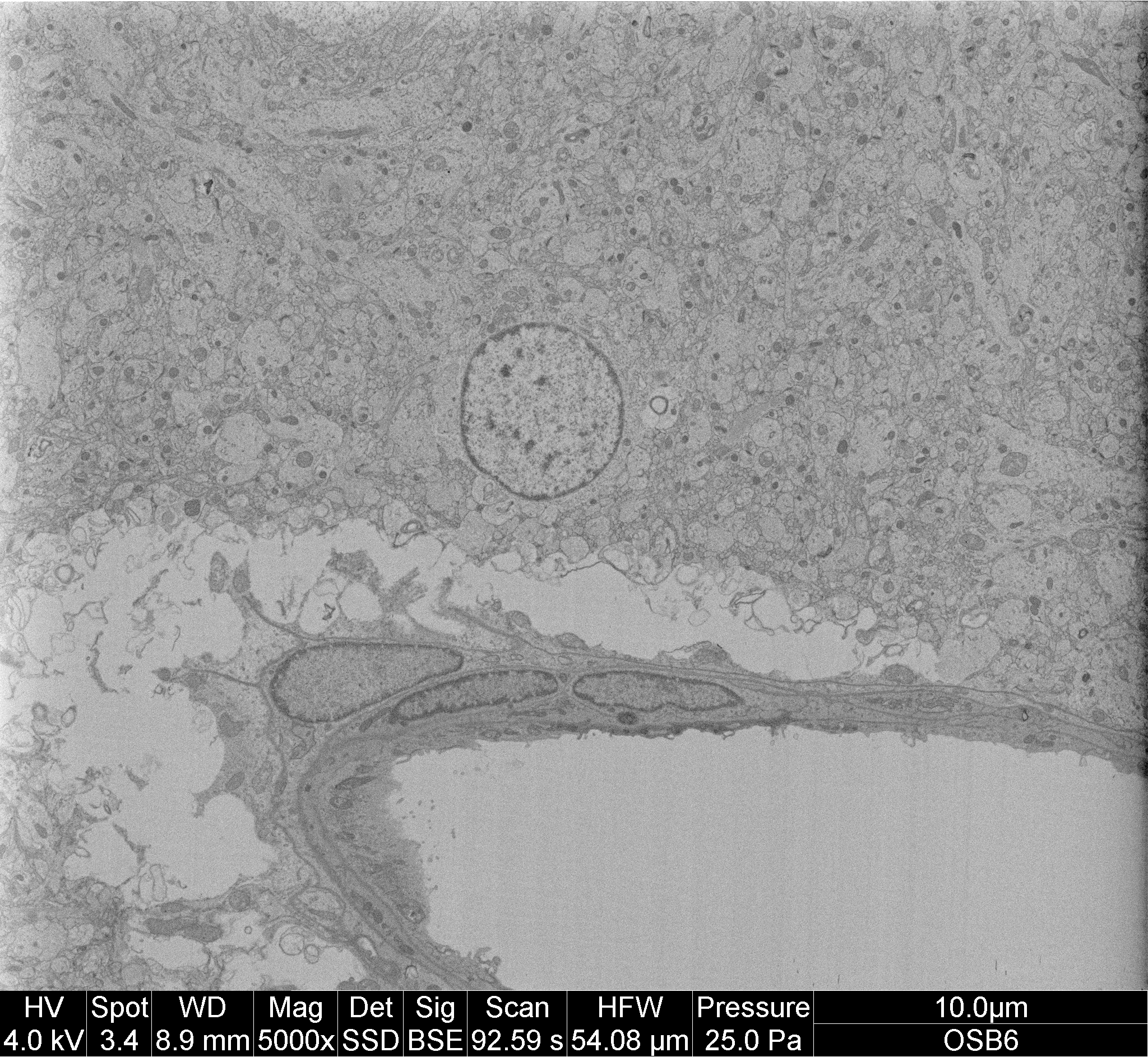

Supplement: Dataset S6 — (252.2 MB ZIP). [file pbio.0020329.sd006.zip › 040604_OS5_st1_527.tif]

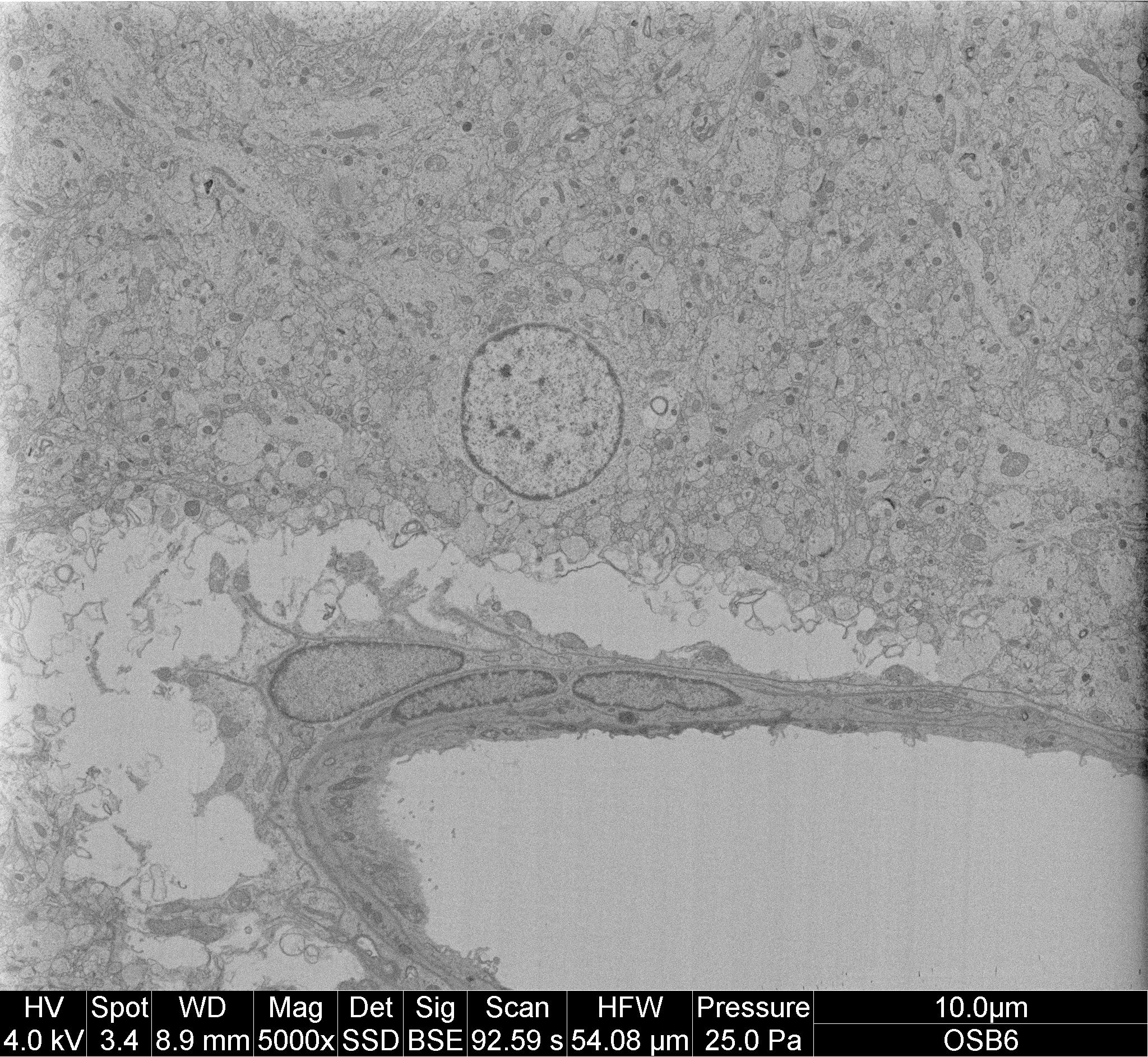

Supplement: Dataset S6 — (252.2 MB ZIP). [file pbio.0020329.sd006.zip › 040604_OS5_st1_528.tif]

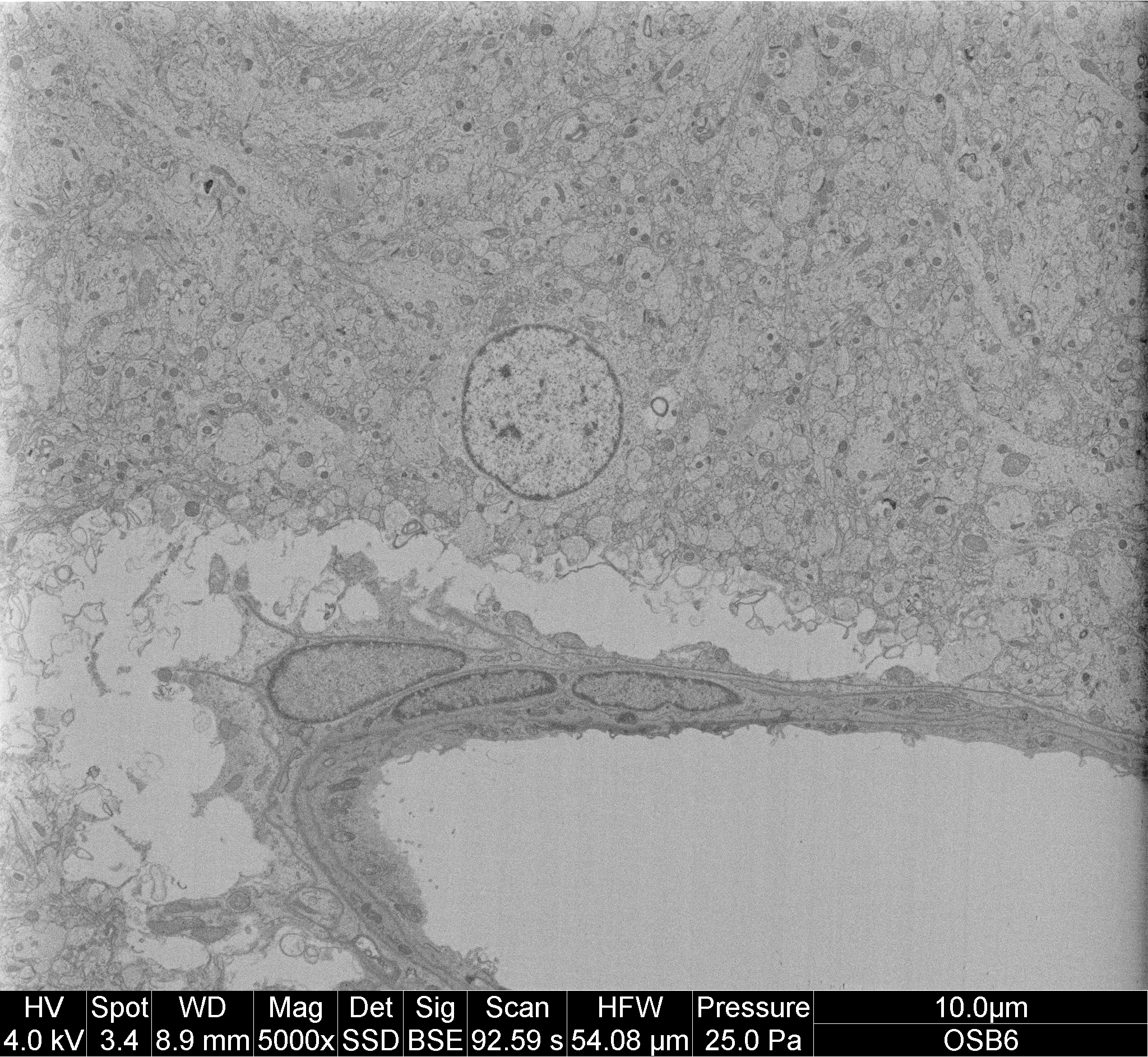

Supplement: Dataset S6 — (252.2 MB ZIP). [file pbio.0020329.sd006.zip › 040604_OS5_st1_529.tif]

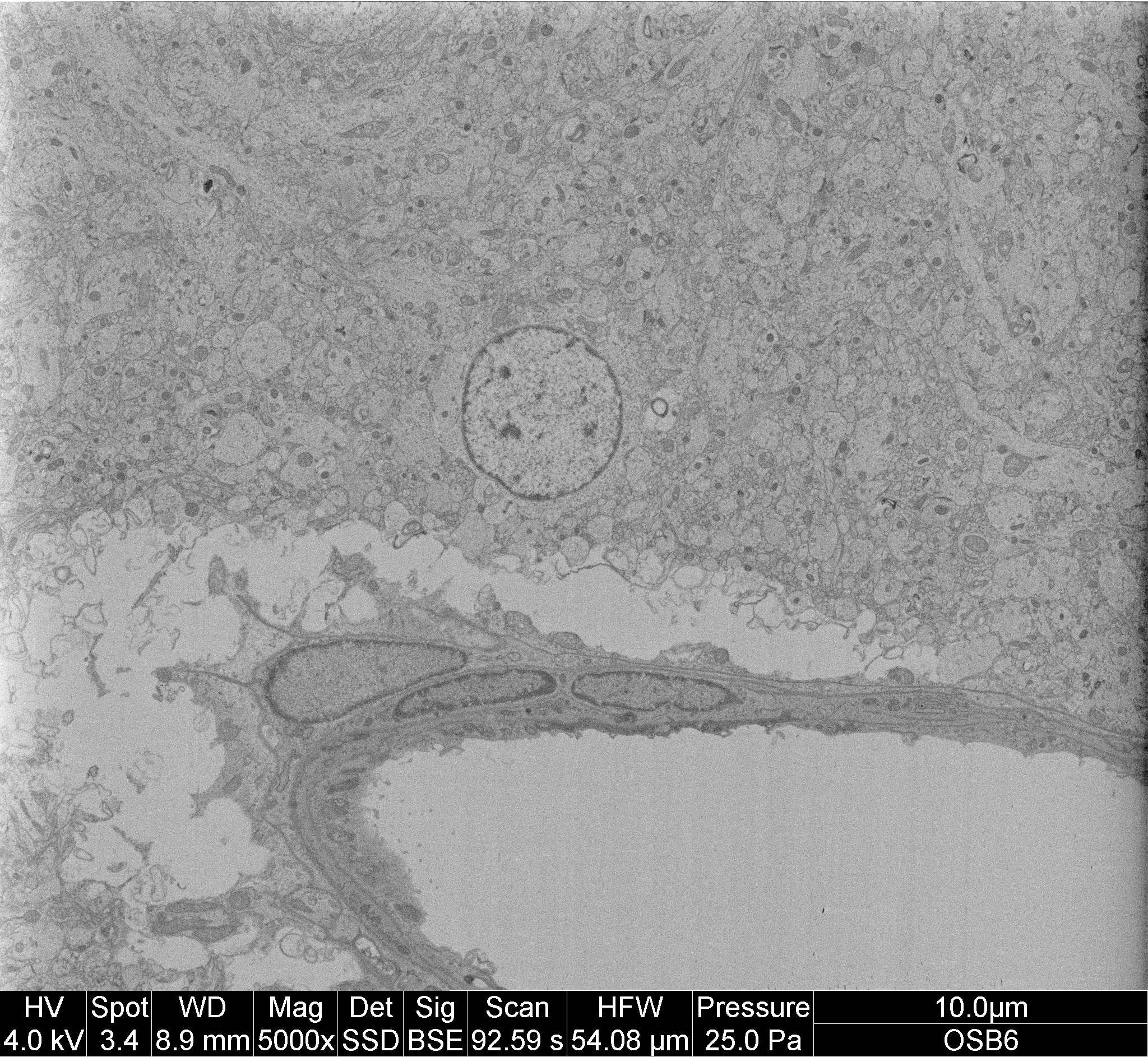

Supplement: Dataset S6 — (252.2 MB ZIP). [file pbio.0020329.sd006.zip › 040604_OS5_st1_530.tif]

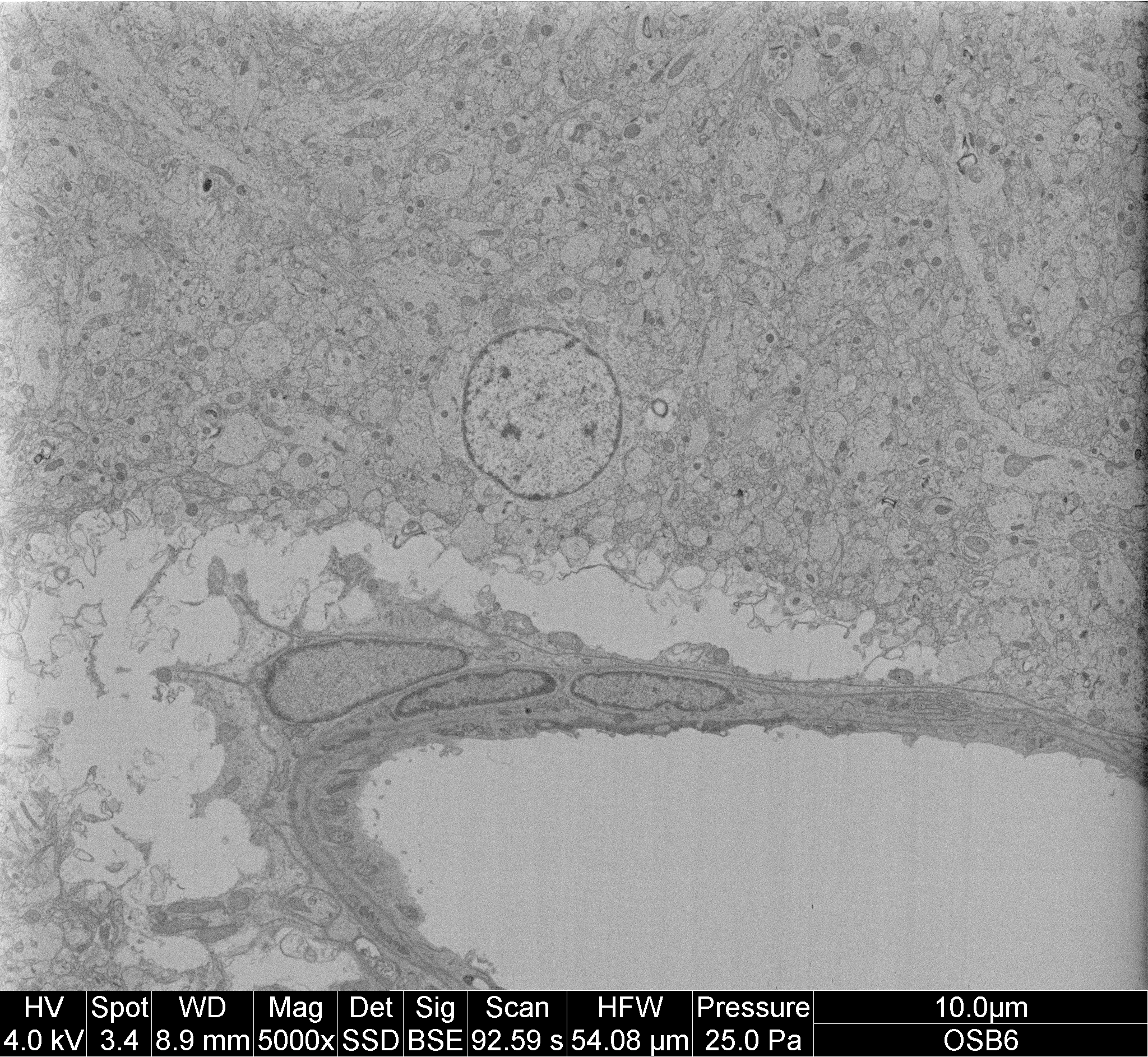

Supplement: Dataset S6 — (252.2 MB ZIP). [file pbio.0020329.sd006.zip › 040604_OS5_st1_531.tif]

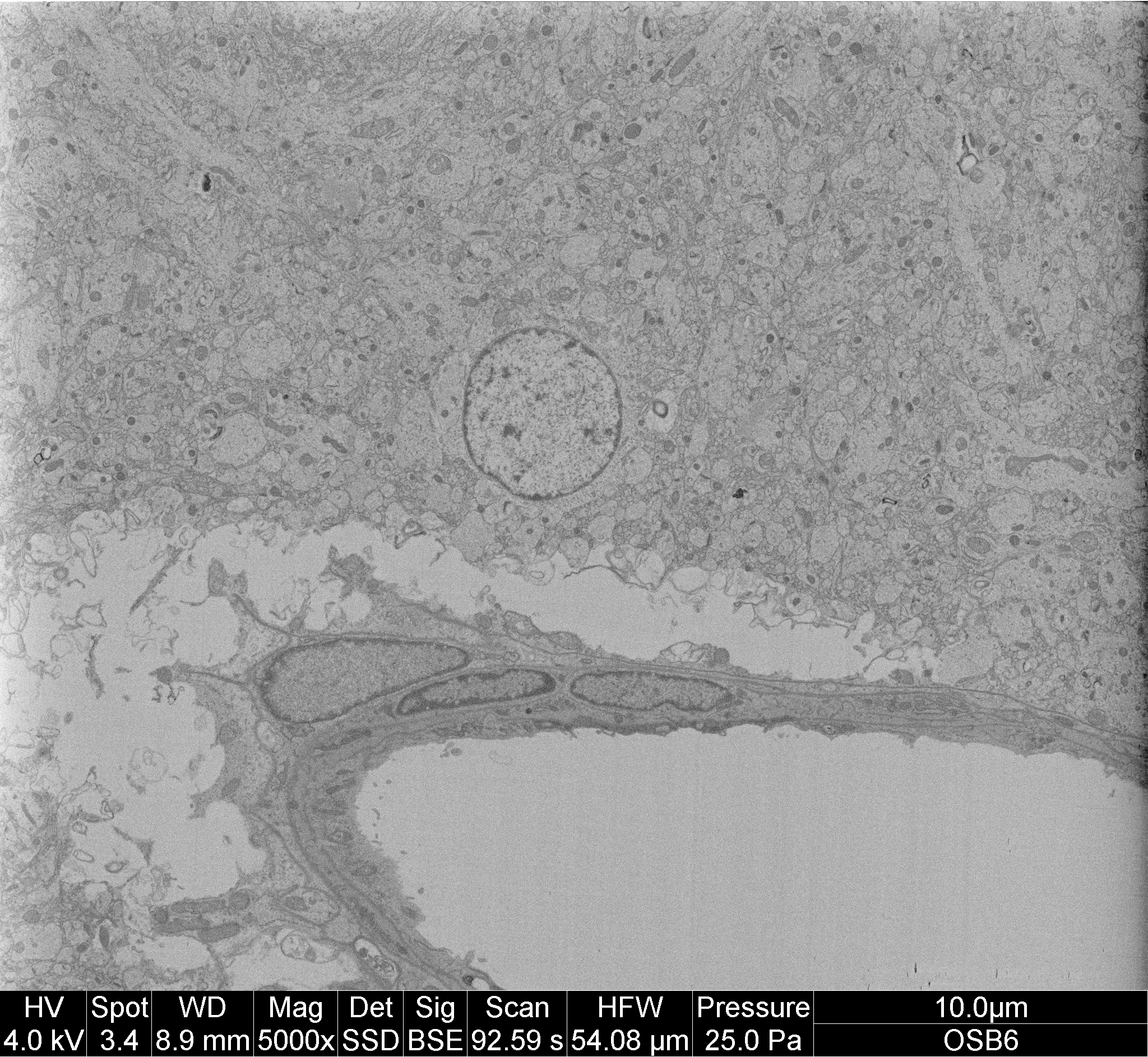

Supplement: Dataset S6 — (252.2 MB ZIP). [file pbio.0020329.sd006.zip › 040604_OS5_st1_532.tif]

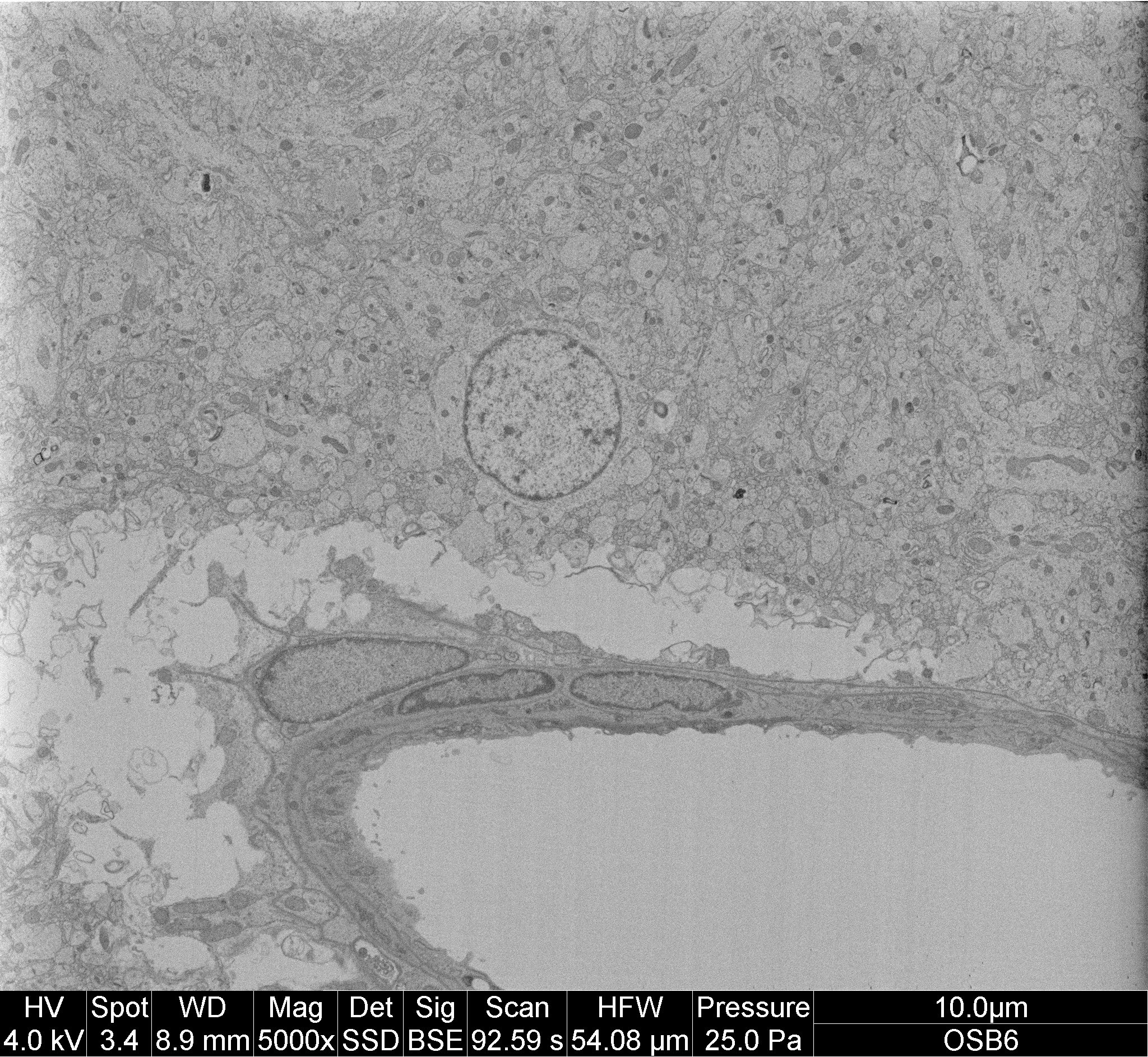

Supplement: Dataset S6 — (252.2 MB ZIP). [file pbio.0020329.sd006.zip › 040604_OS5_st1_533.tif]

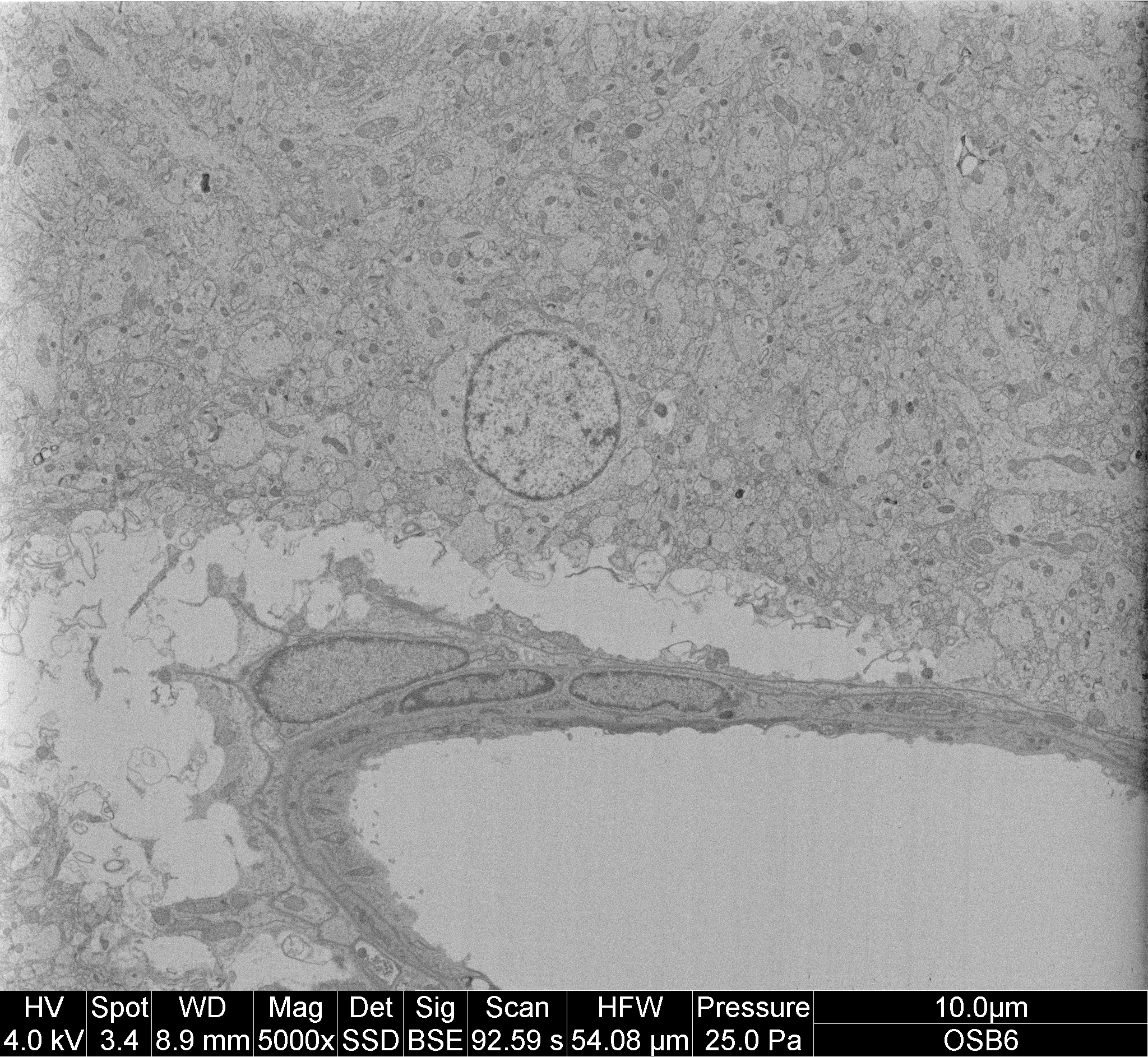

Supplement: Dataset S6 — (252.2 MB ZIP). [file pbio.0020329.sd006.zip › 040604_OS5_st1_534.tif]

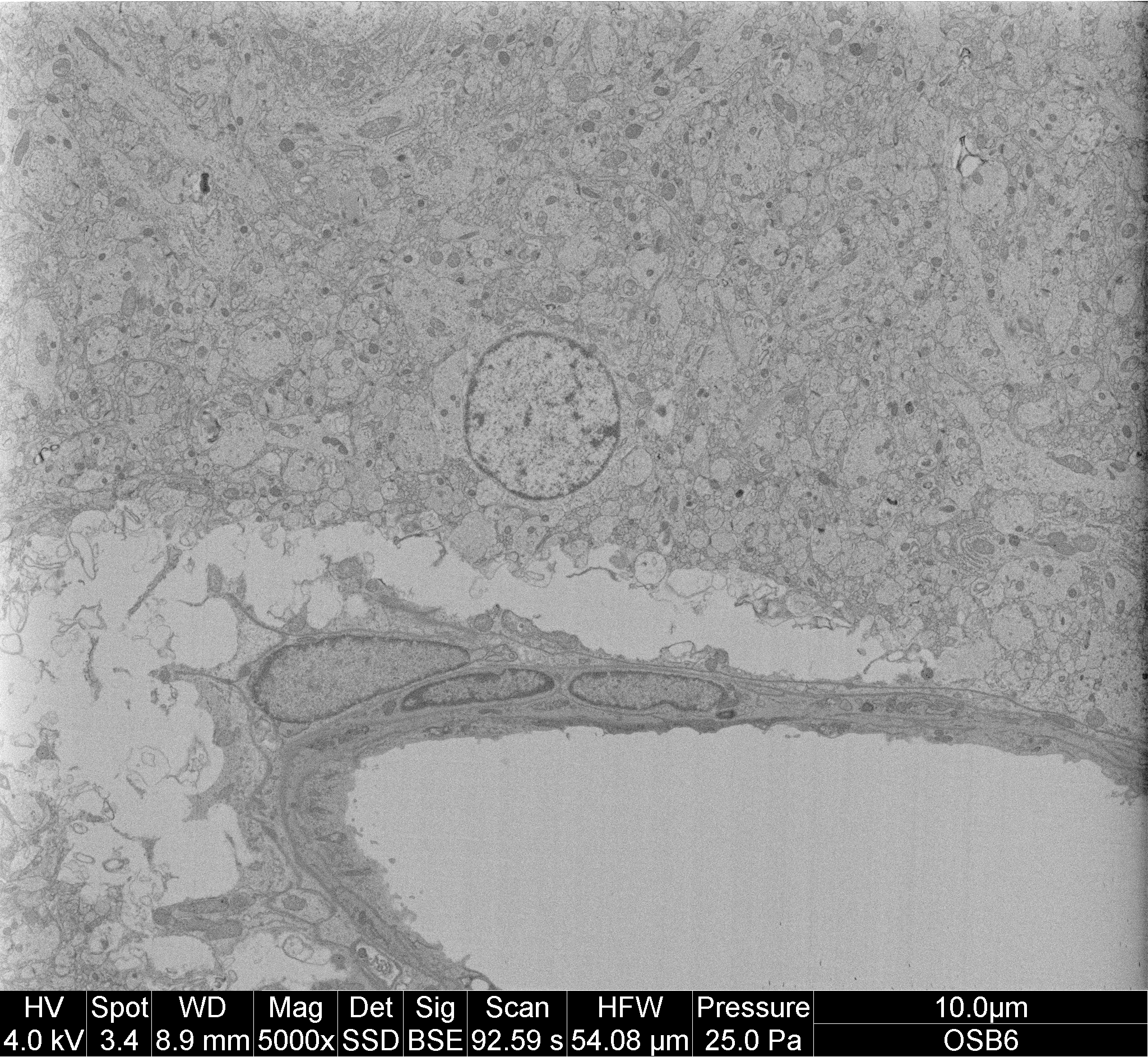

Supplement: Dataset S6 — (252.2 MB ZIP). [file pbio.0020329.sd006.zip › 040604_OS5_st1_535.tif]

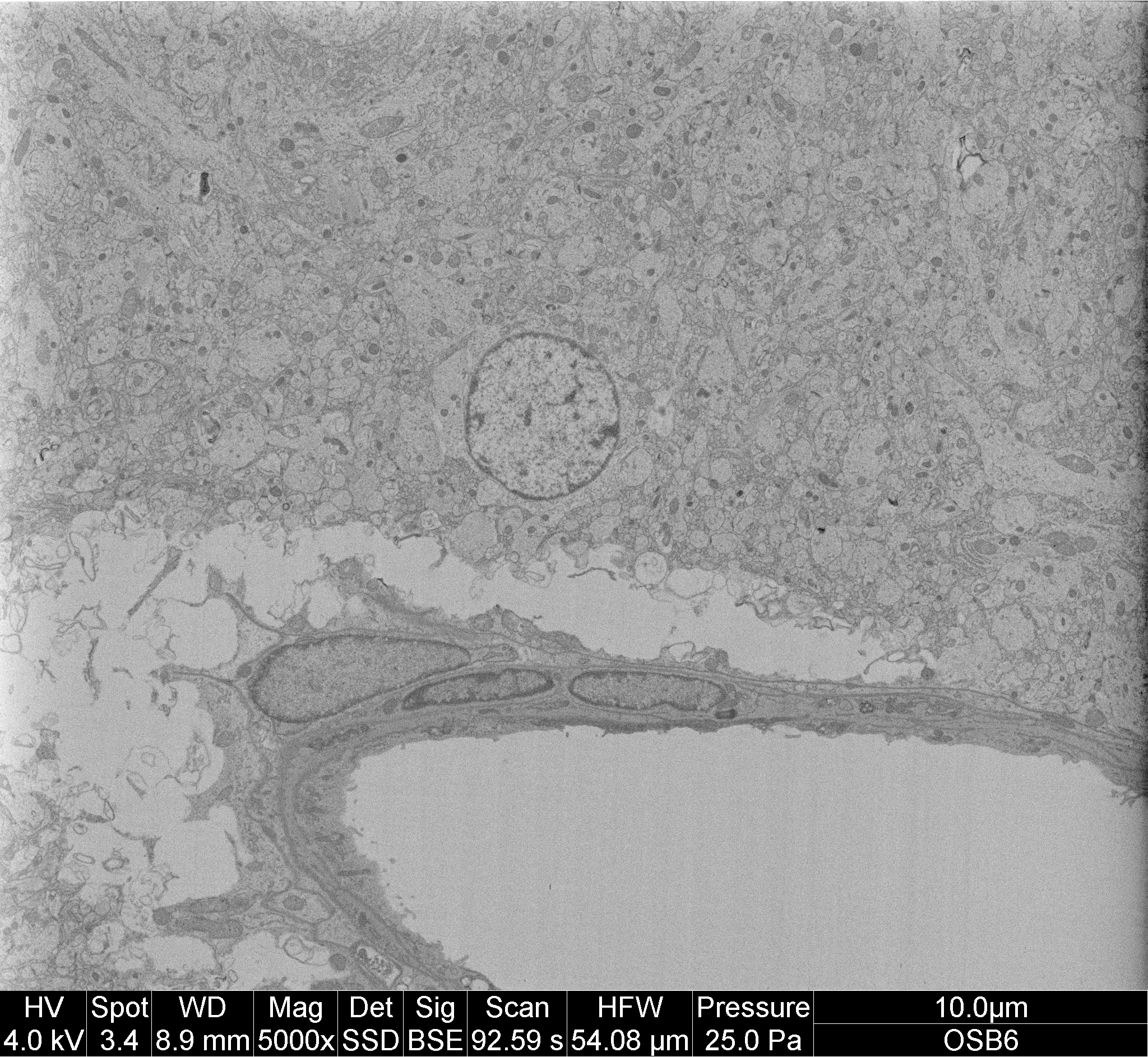

Supplement: Dataset S6 — (252.2 MB ZIP). [file pbio.0020329.sd006.zip › 040604_OS5_st1_536.tif]

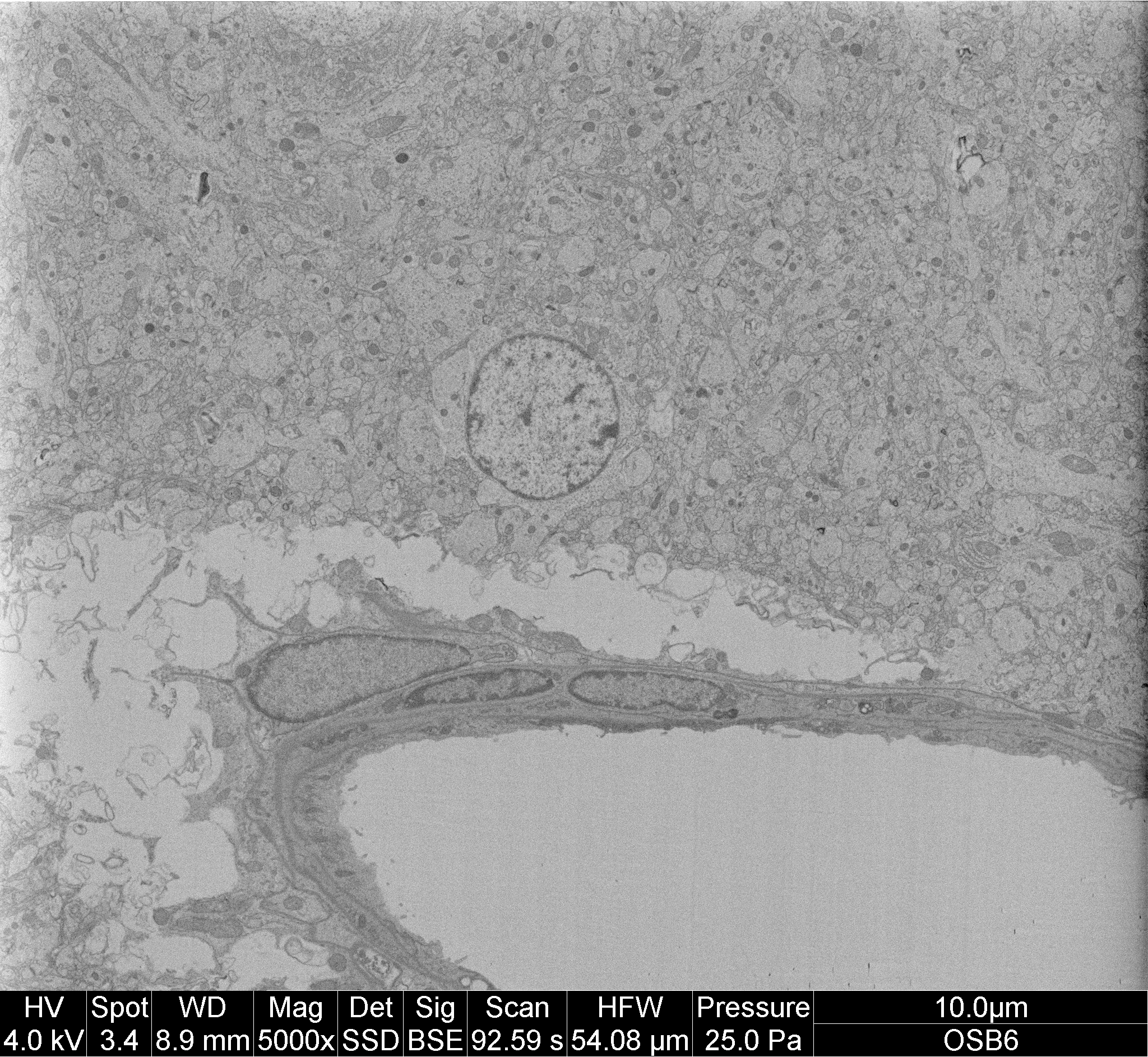

Supplement: Dataset S6 — (252.2 MB ZIP). [file pbio.0020329.sd006.zip › 040604_OS5_st1_537.tif]

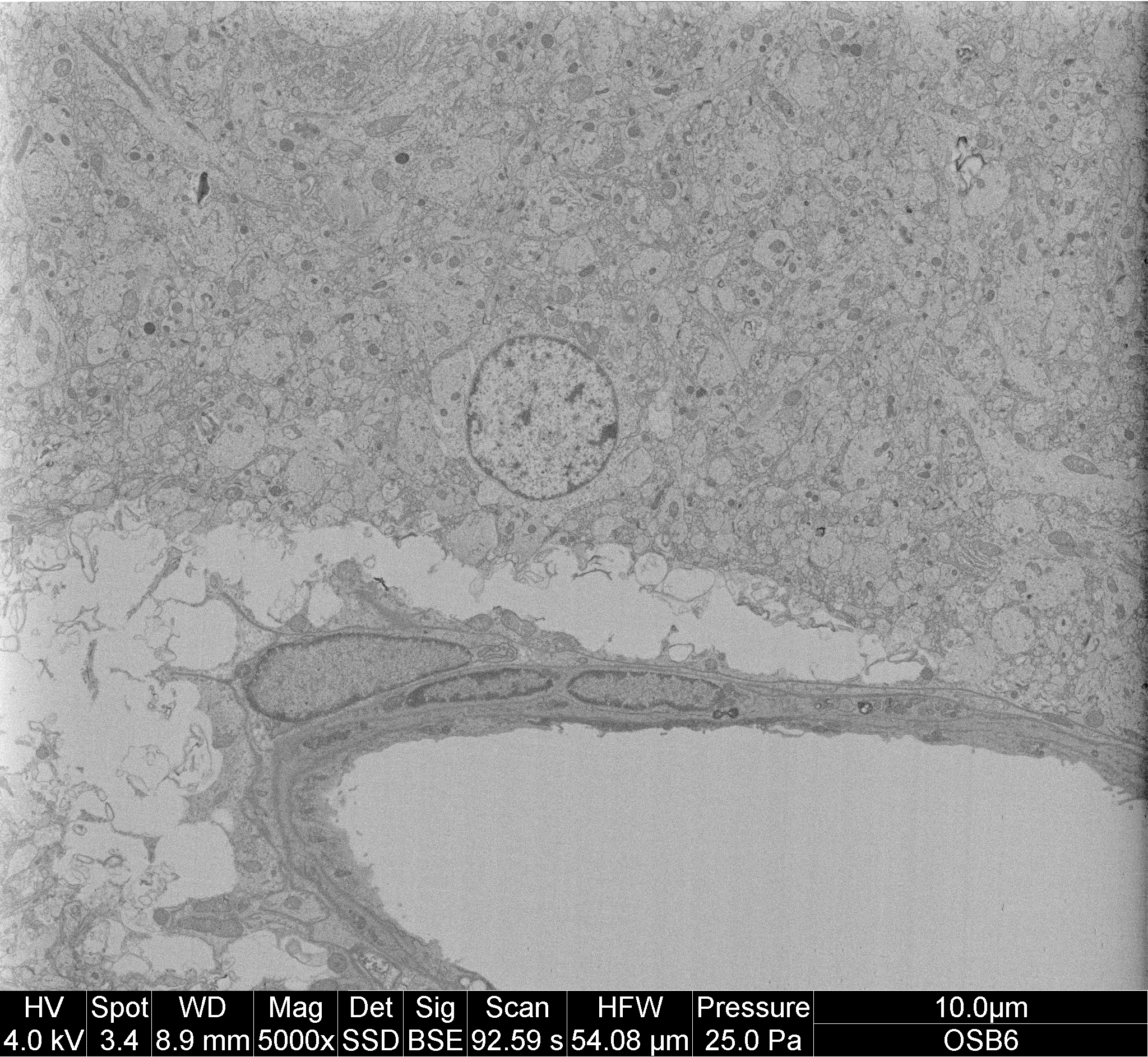

Supplement: Dataset S6 — (252.2 MB ZIP). [file pbio.0020329.sd006.zip › 040604_OS5_st1_538.tif]

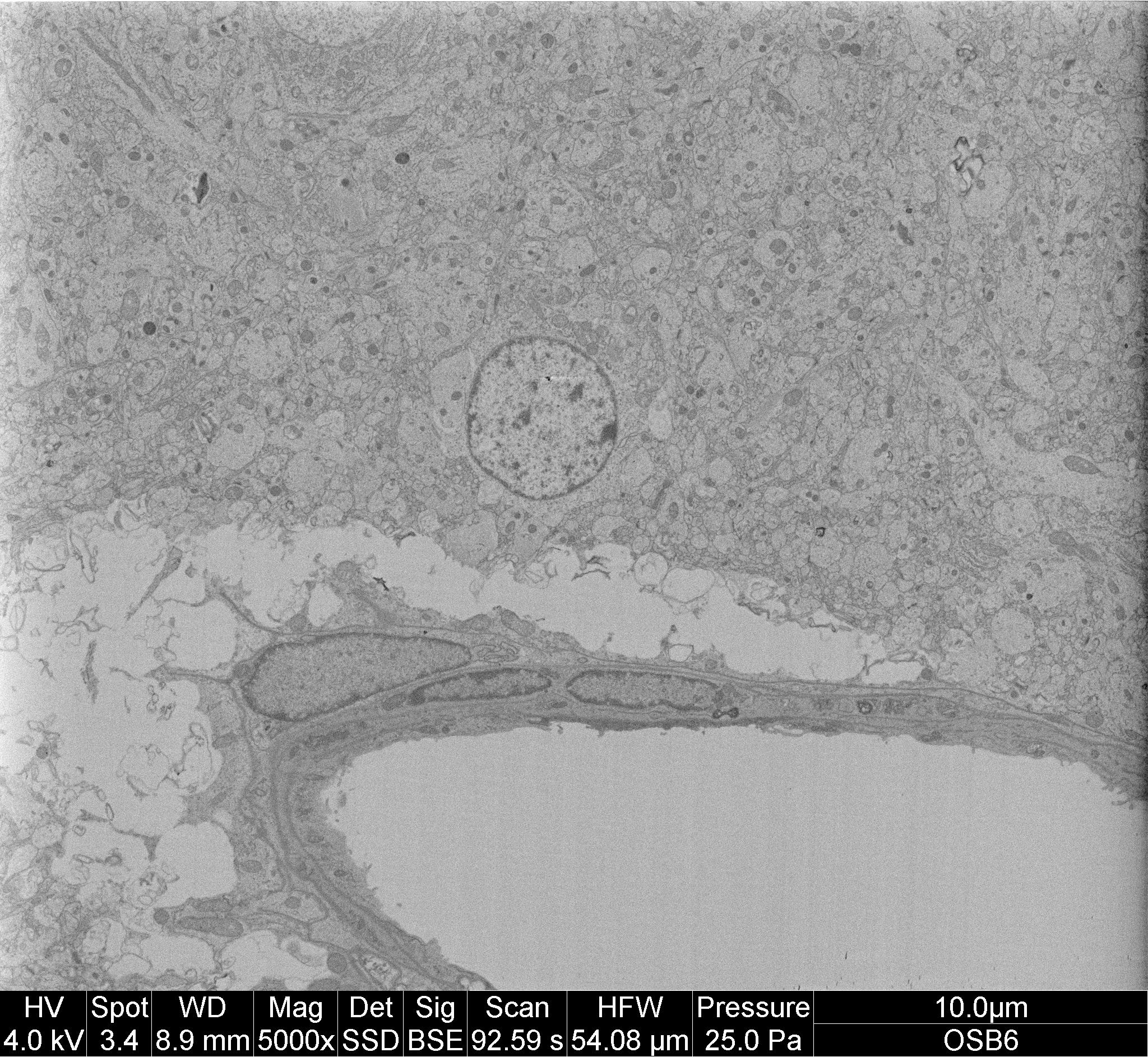

Supplement: Dataset S6 — (252.2 MB ZIP). [file pbio.0020329.sd006.zip › 040604_OS5_st1_539.tif]

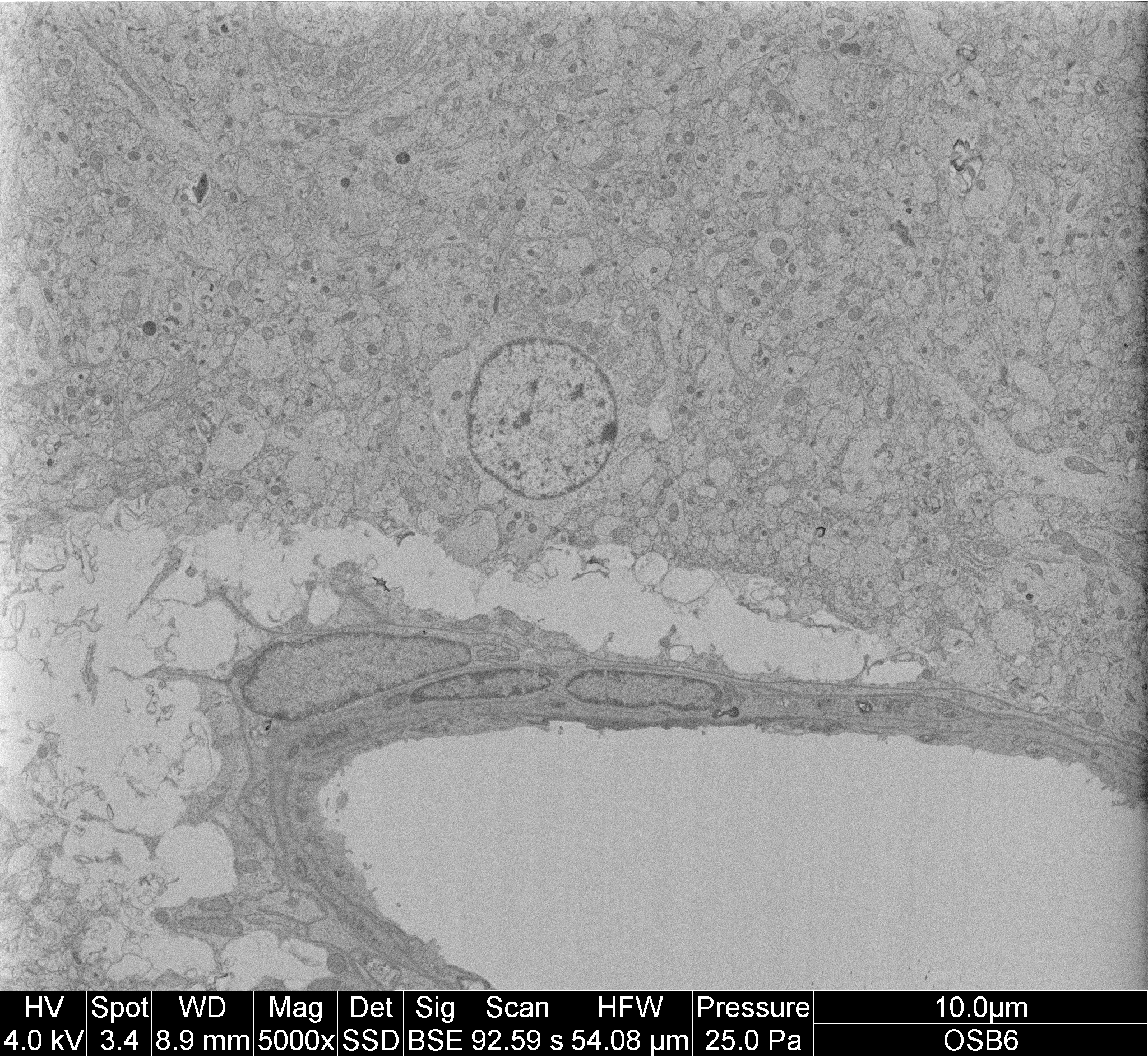

Supplement: Dataset S6 — (252.2 MB ZIP). [file pbio.0020329.sd006.zip › 040604_OS5_st1_540.tif]

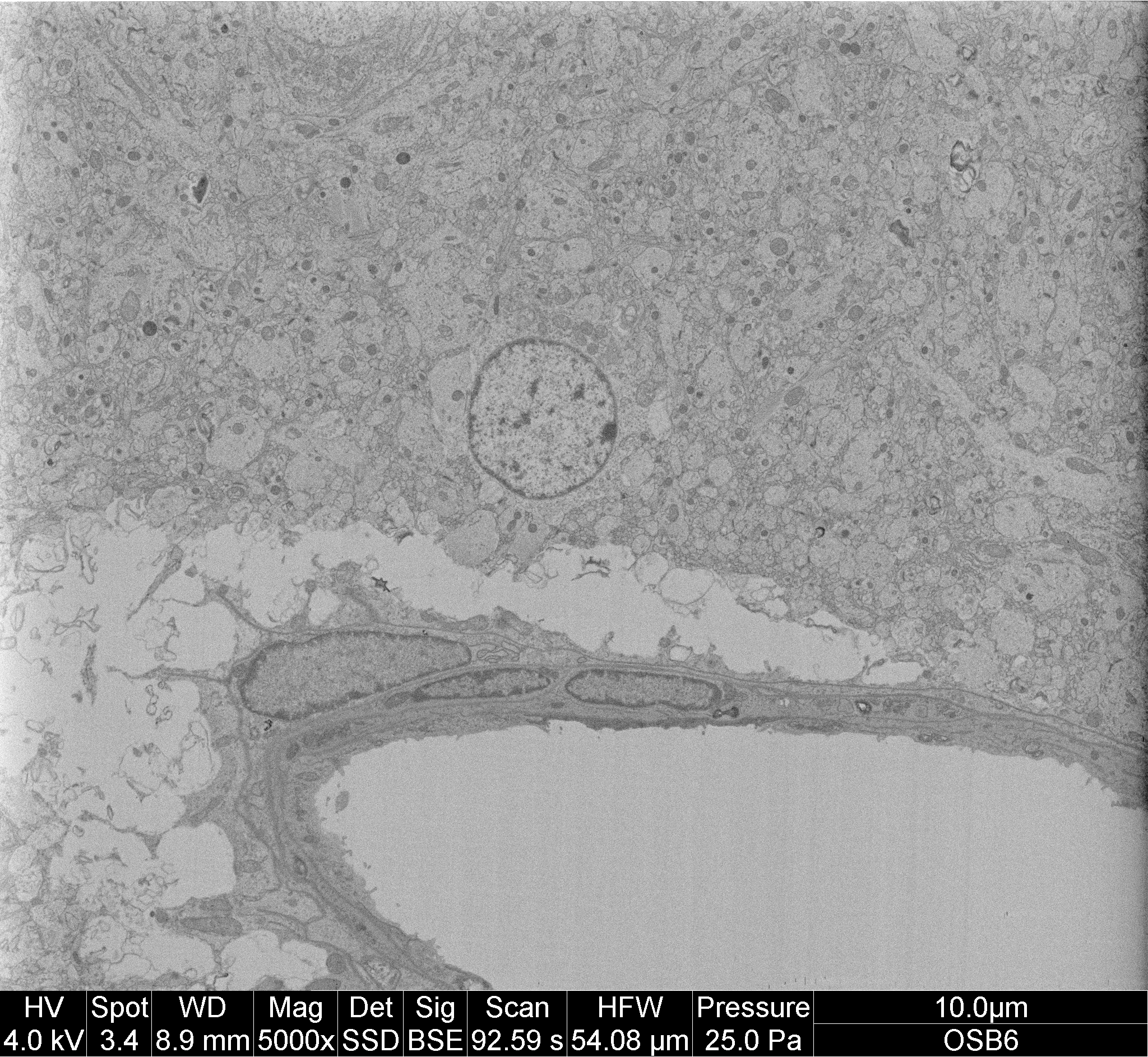

Supplement: Dataset S6 — (252.2 MB ZIP). [file pbio.0020329.sd006.zip › 040604_OS5_st1_541.tif]

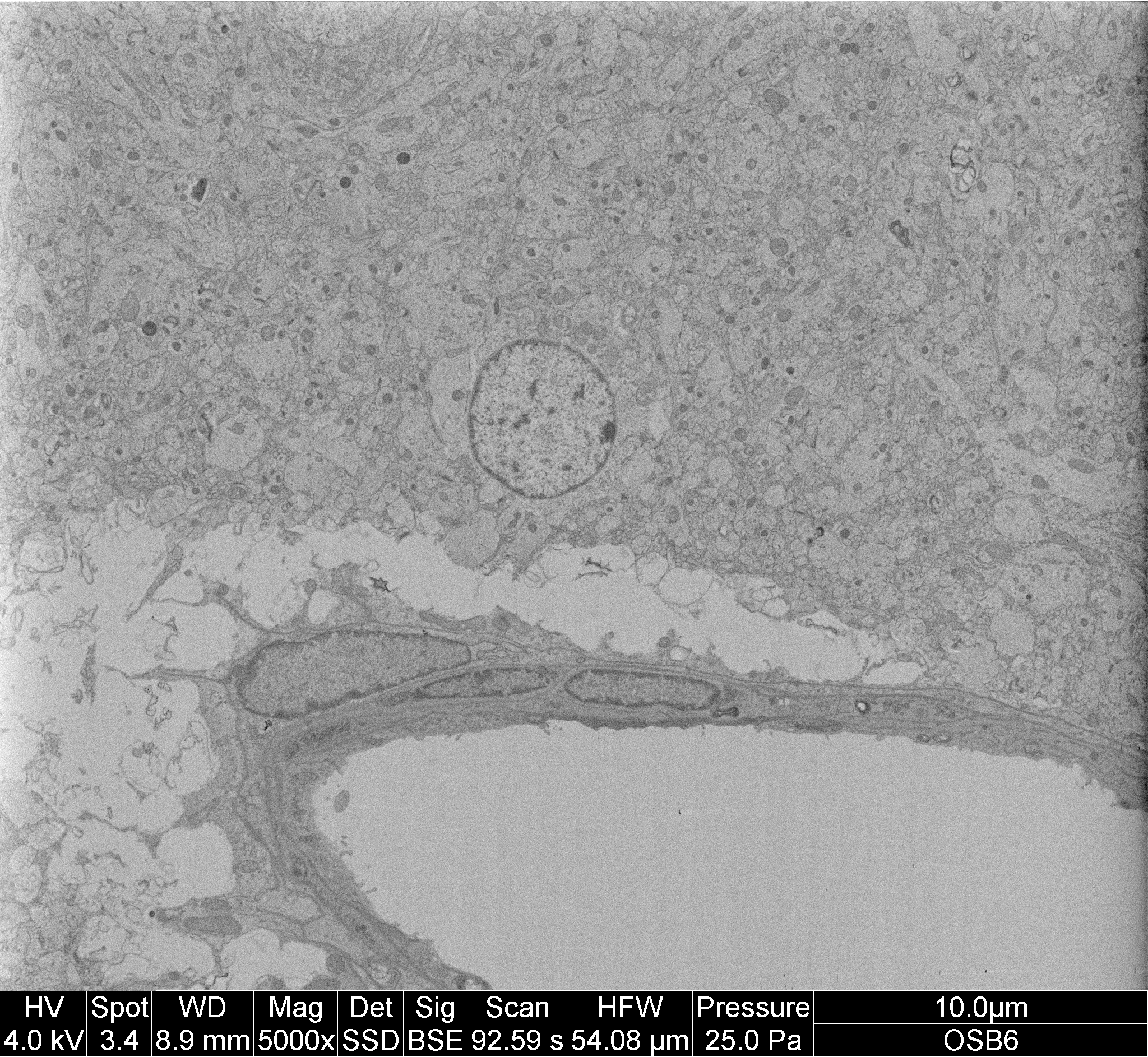

Supplement: Dataset S6 — (252.2 MB ZIP). [file pbio.0020329.sd006.zip › 040604_OS5_st1_542.tif]

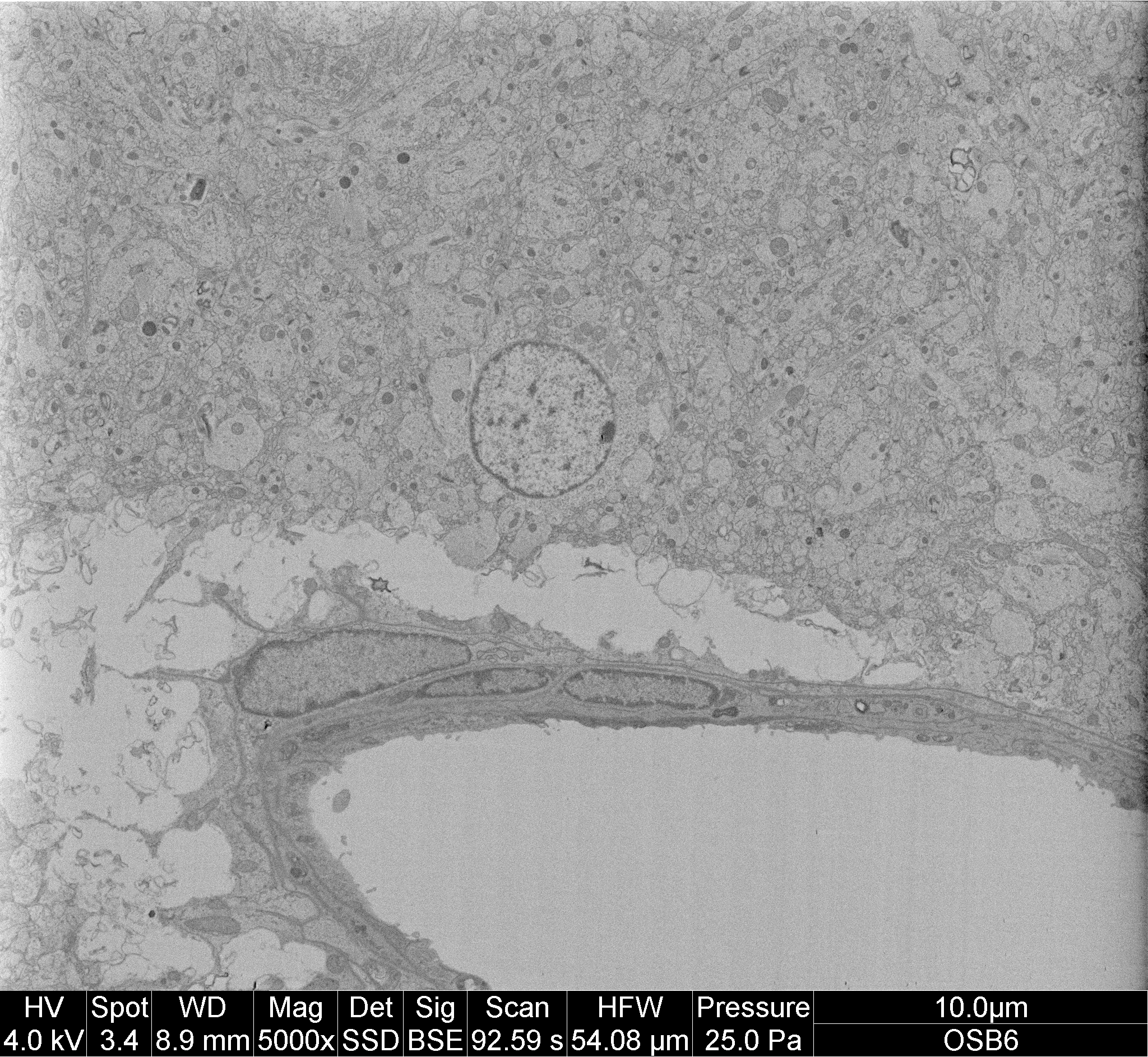

Supplement: Dataset S6 — (252.2 MB ZIP). [file pbio.0020329.sd006.zip › 040604_OS5_st1_543.tif]

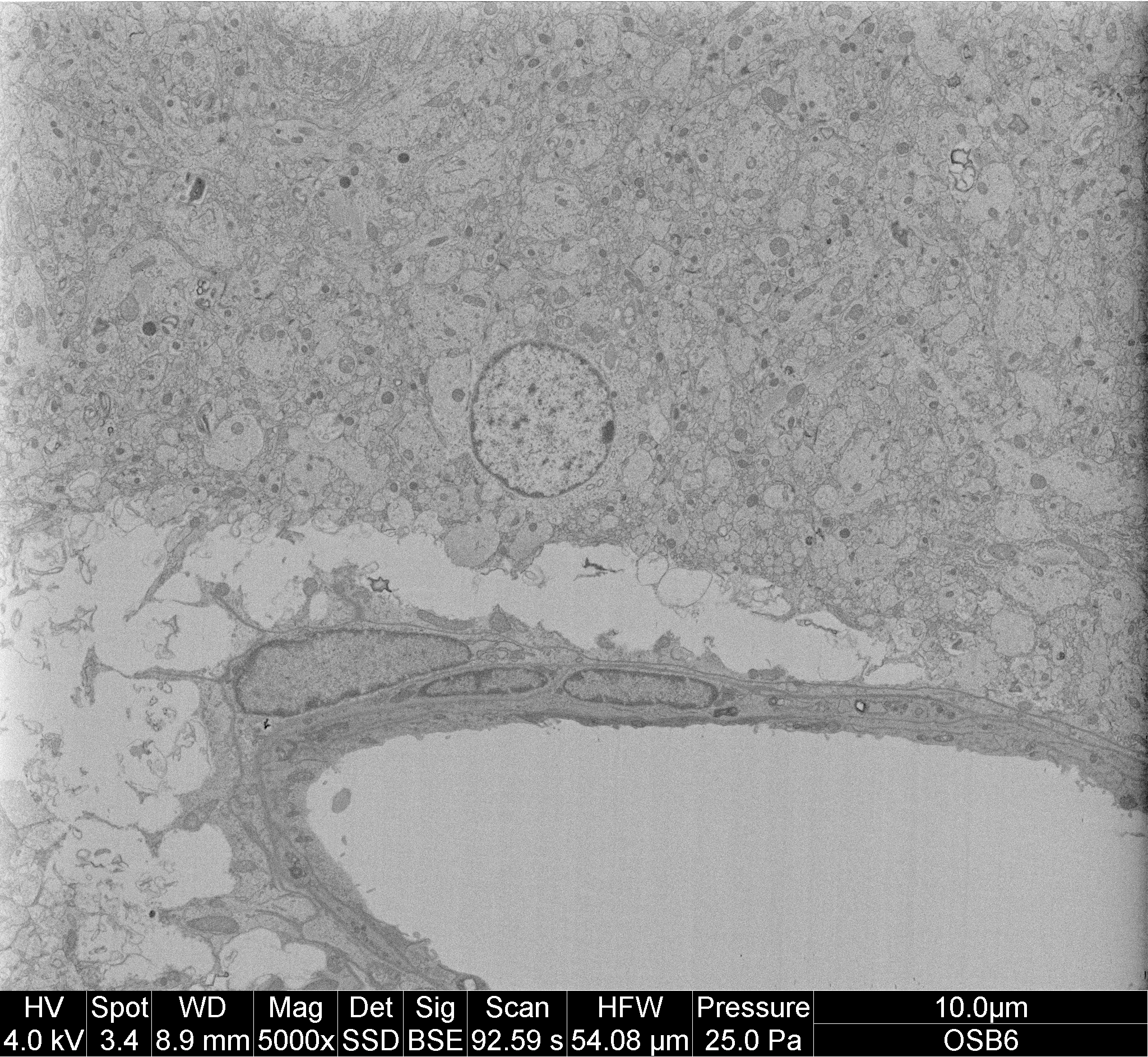

Supplement: Dataset S6 — (252.2 MB ZIP). [file pbio.0020329.sd006.zip › 040604_OS5_st1_544.tif]

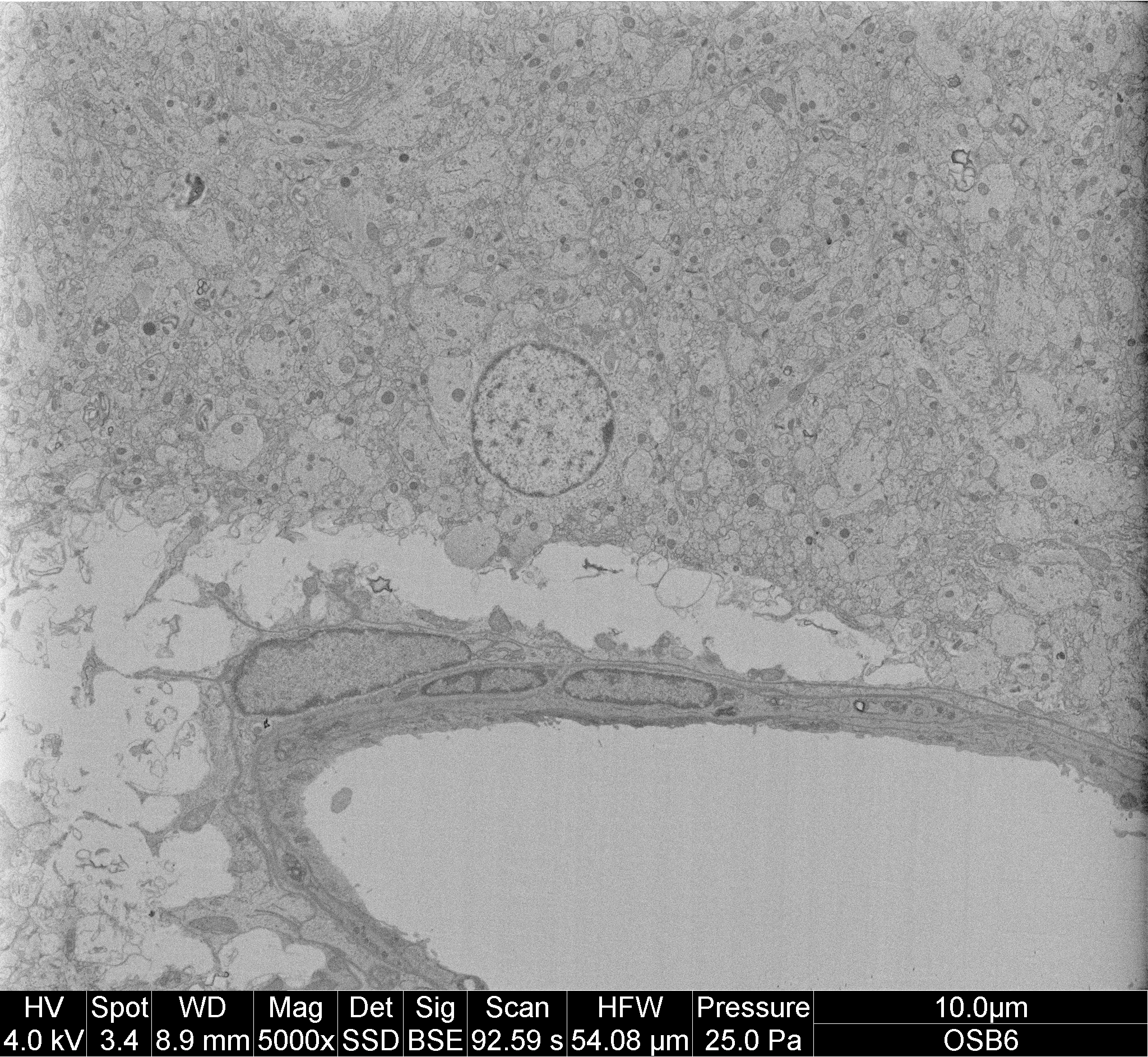

Supplement: Dataset S6 — (252.2 MB ZIP). [file pbio.0020329.sd006.zip › 040604_OS5_st1_545.tif]

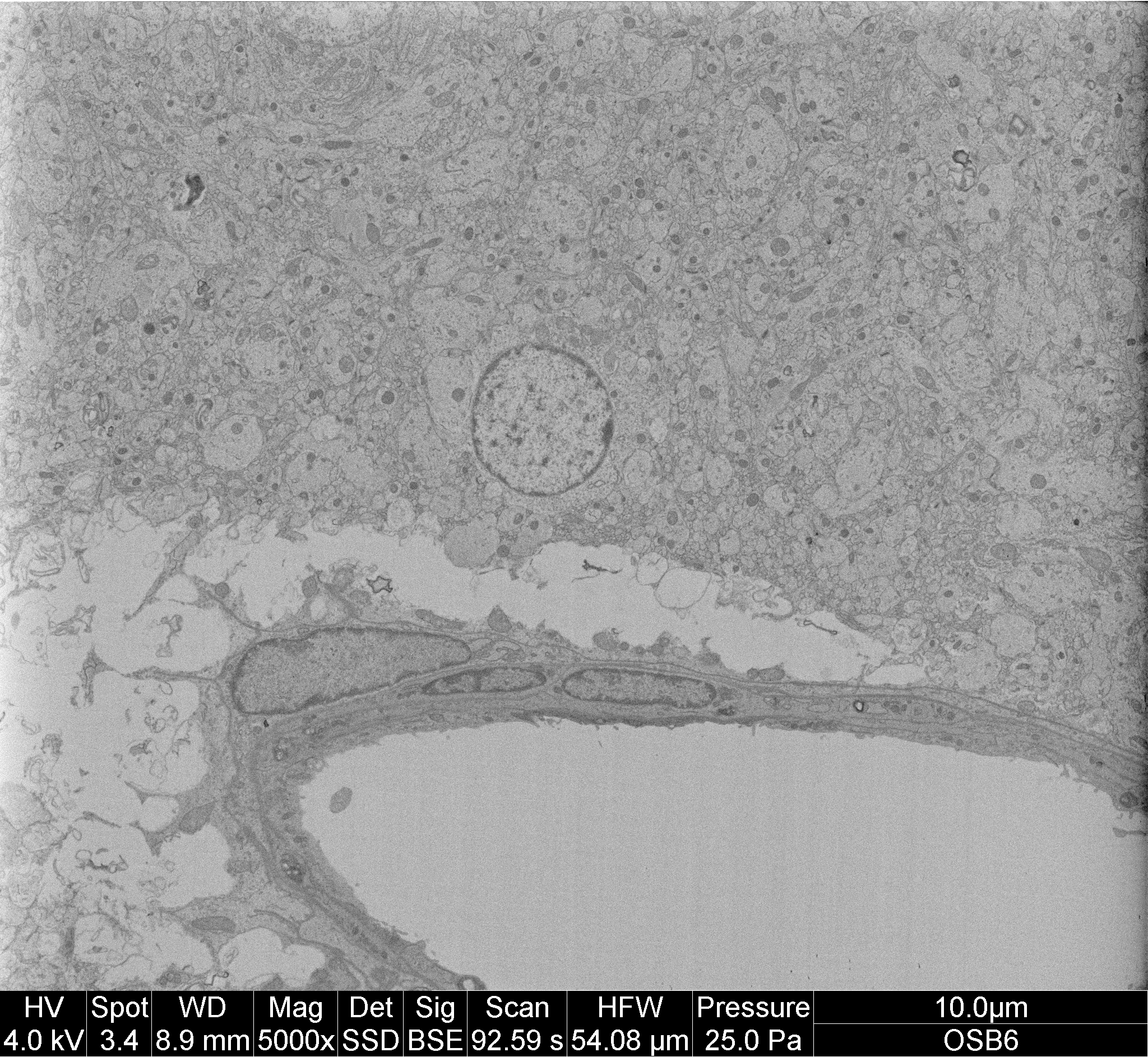

Supplement: Dataset S6 — (252.2 MB ZIP). [file pbio.0020329.sd006.zip › 040604_OS5_st1_546.tif]

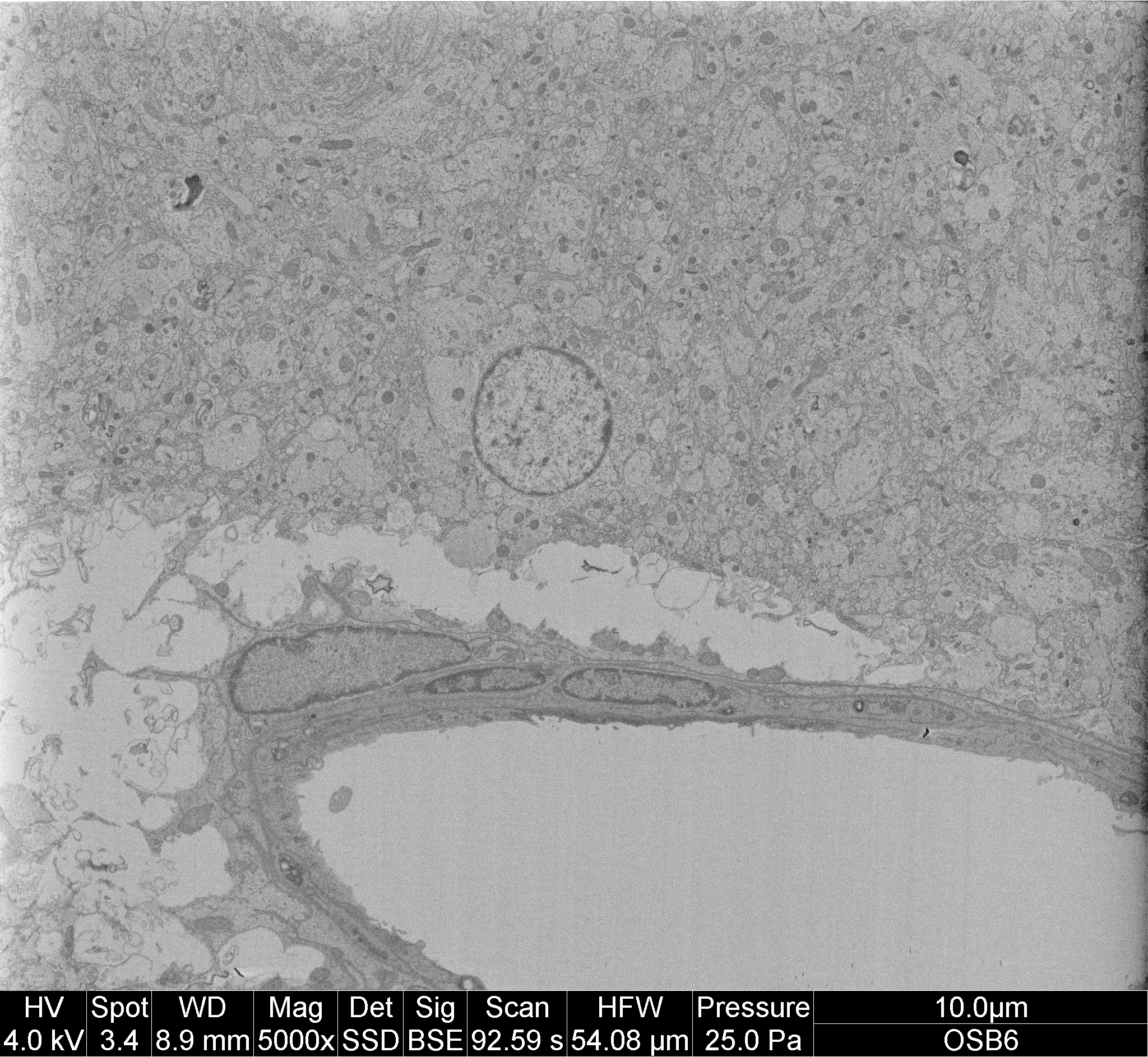

Supplement: Dataset S6 — (252.2 MB ZIP). [file pbio.0020329.sd006.zip › 040604_OS5_st1_547.tif]

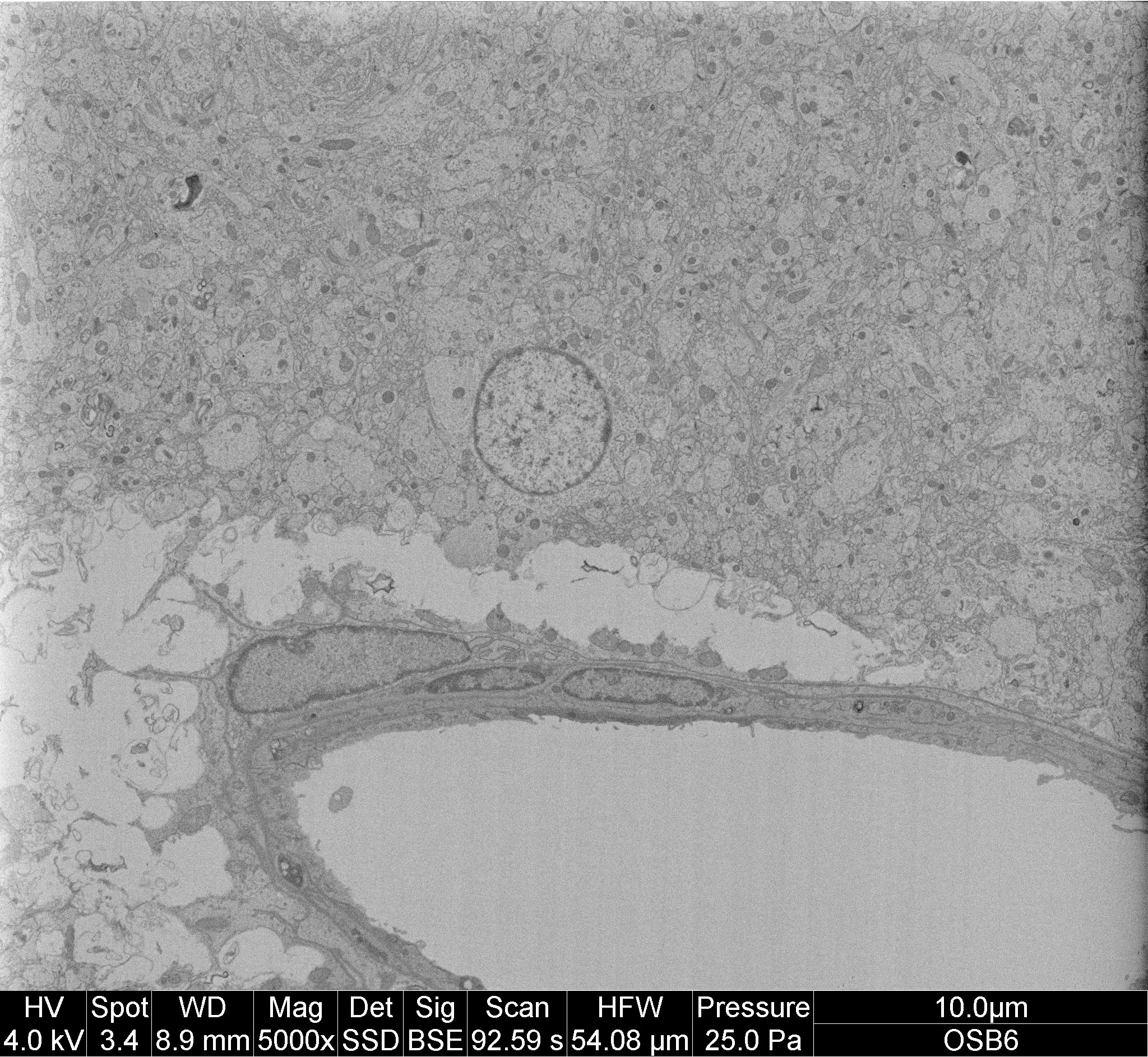

Supplement: Dataset S6 — (252.2 MB ZIP). [file pbio.0020329.sd006.zip › 040604_OS5_st1_548.tif]

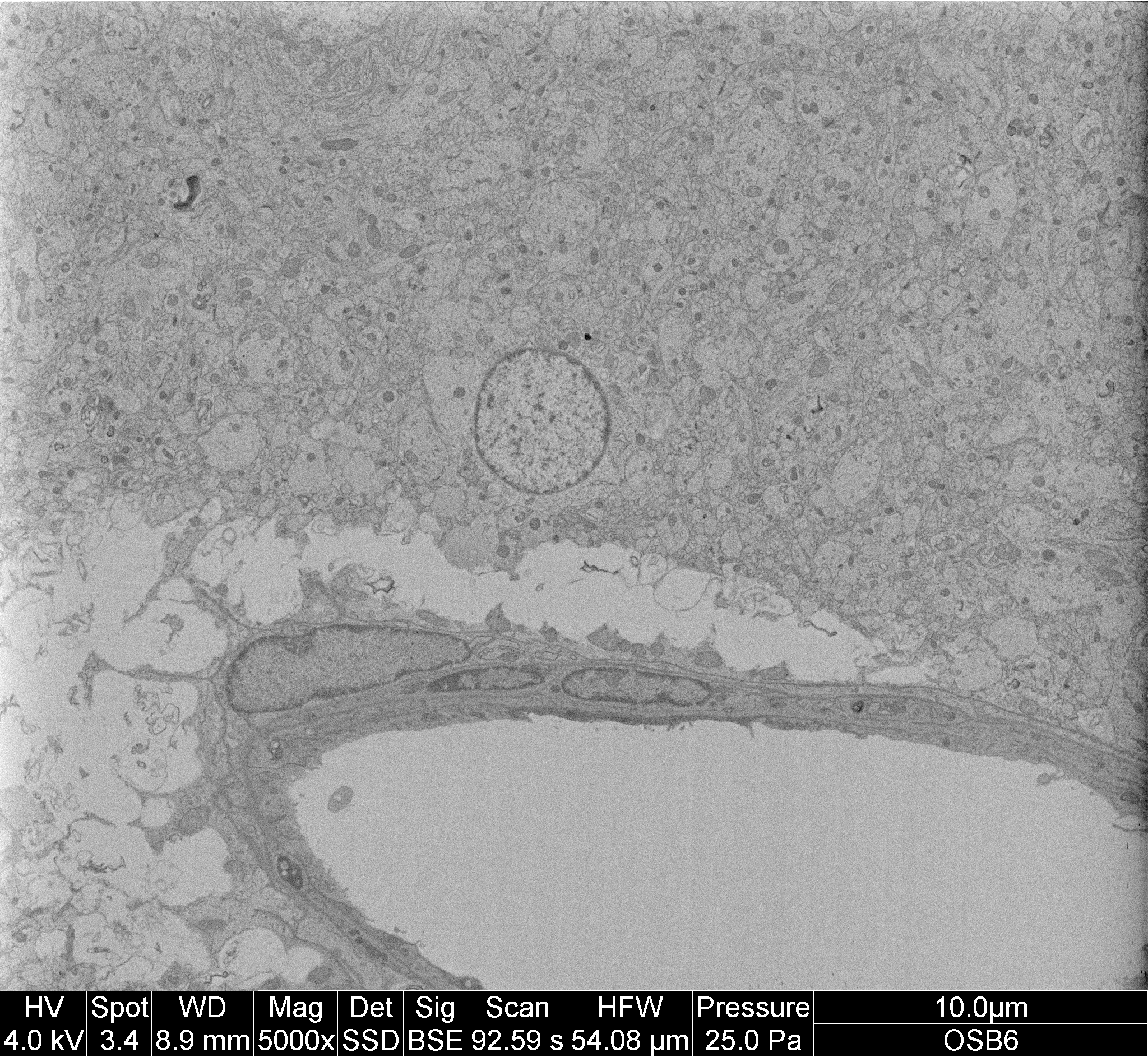

Supplement: Dataset S6 — (252.2 MB ZIP). [file pbio.0020329.sd006.zip › 040604_OS5_st1_549.tif]

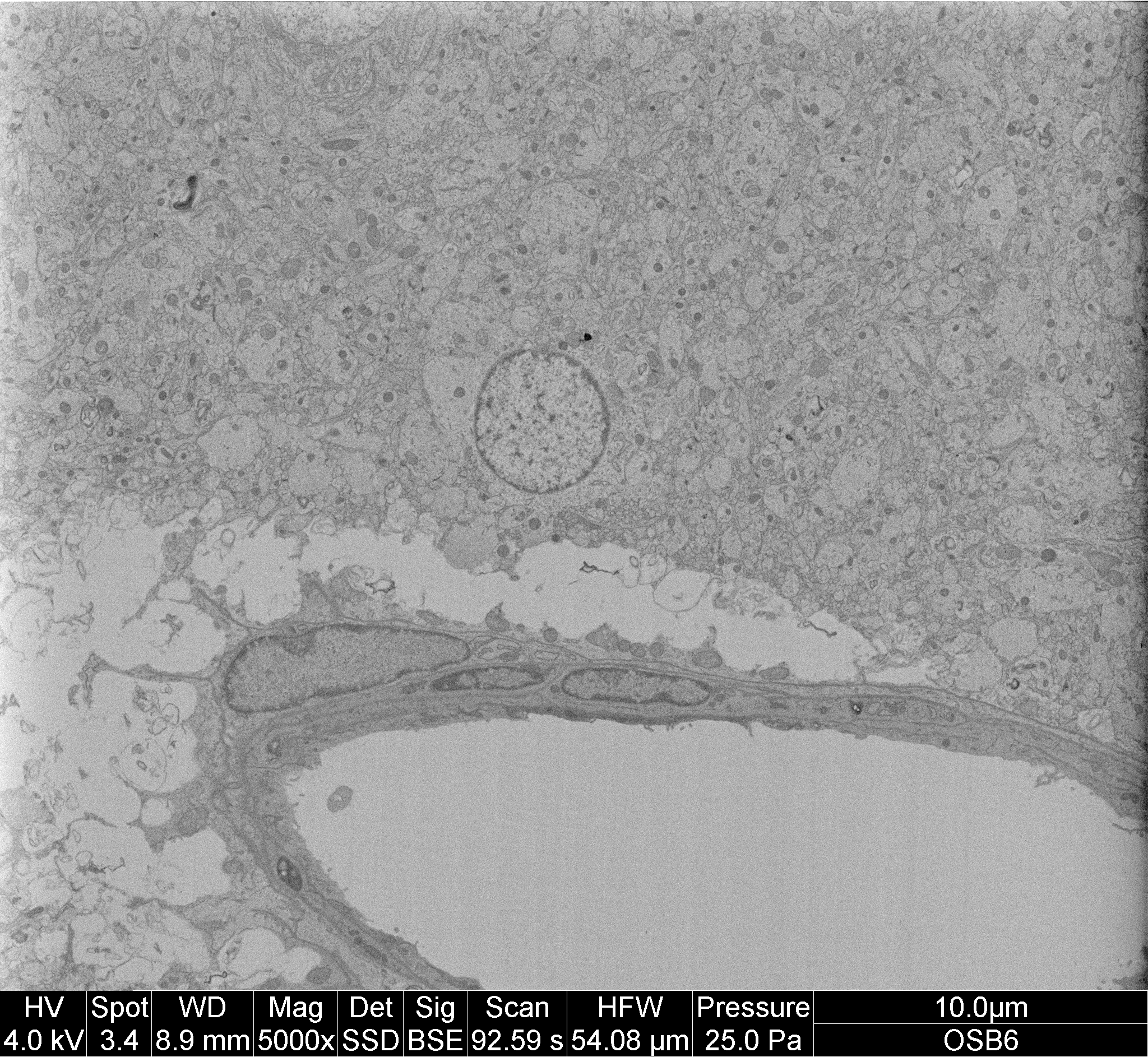

Supplement: Dataset S6 — (252.2 MB ZIP). [file pbio.0020329.sd006.zip › 040604_OS5_st1_550.tif]

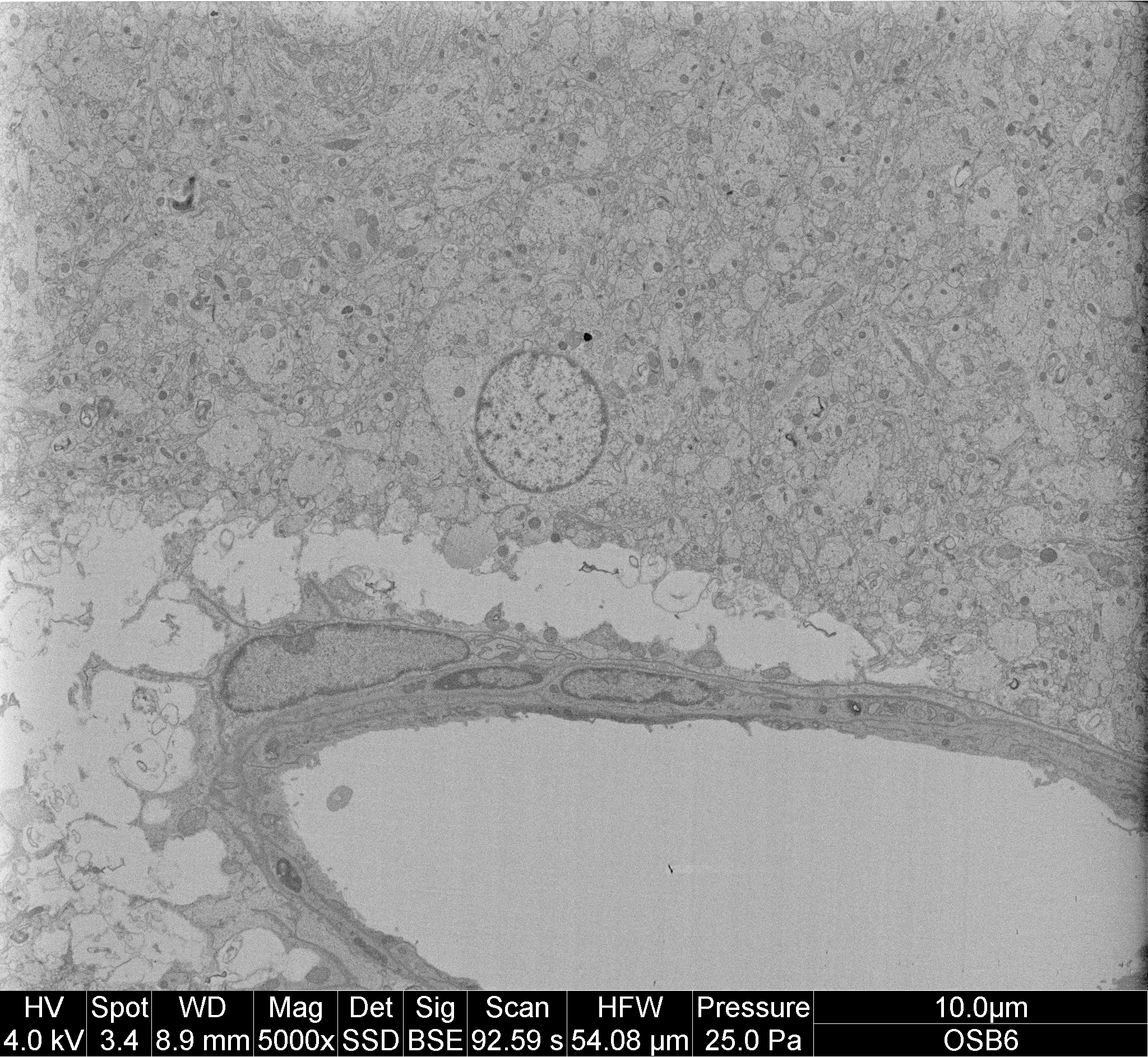

Supplement: Dataset S6 — (252.2 MB ZIP). [file pbio.0020329.sd006.zip › 040604_OS5_st1_551.tif]

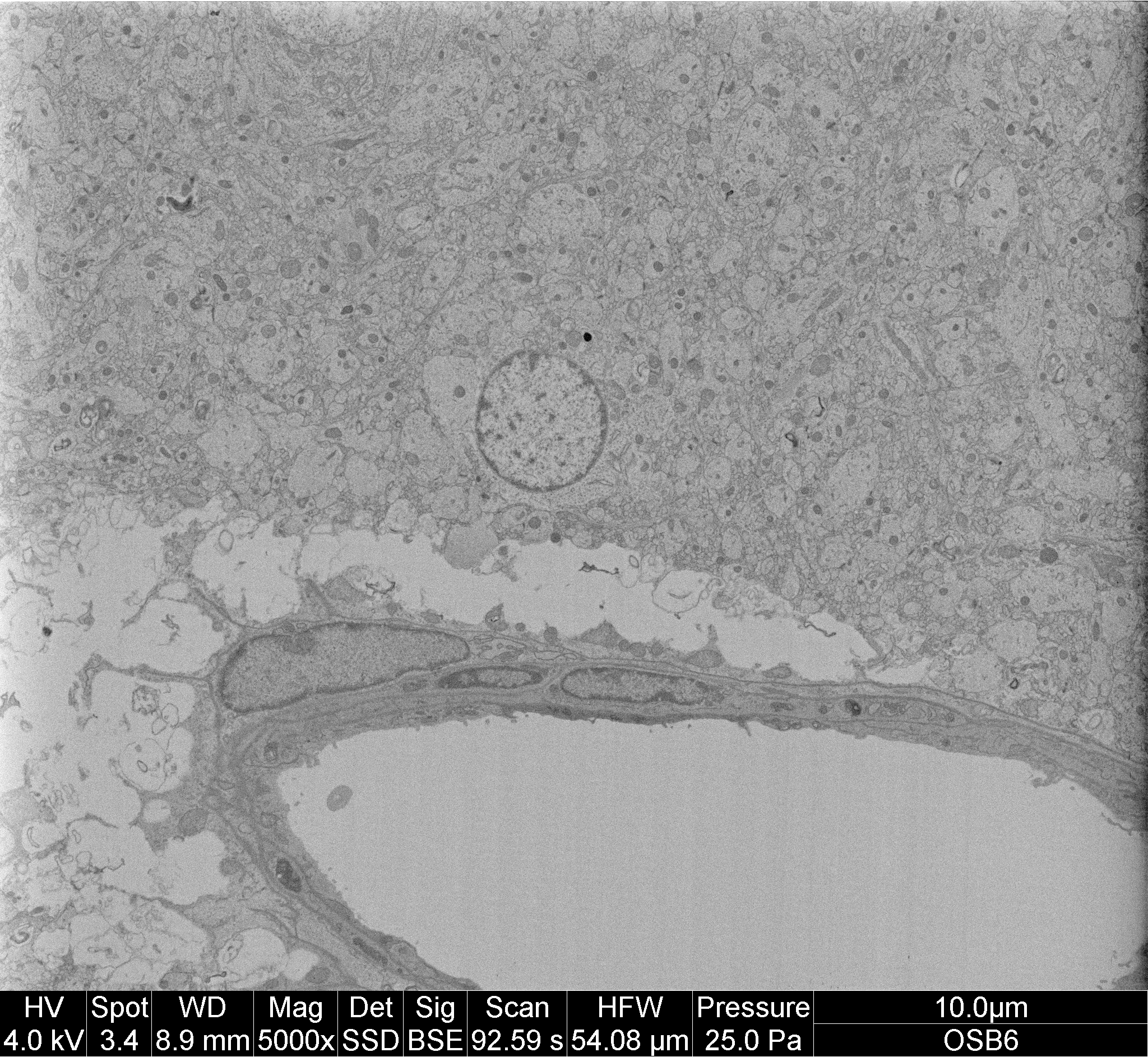

Supplement: Dataset S6 — (252.2 MB ZIP). [file pbio.0020329.sd006.zip › 040604_OS5_st1_552.tif]

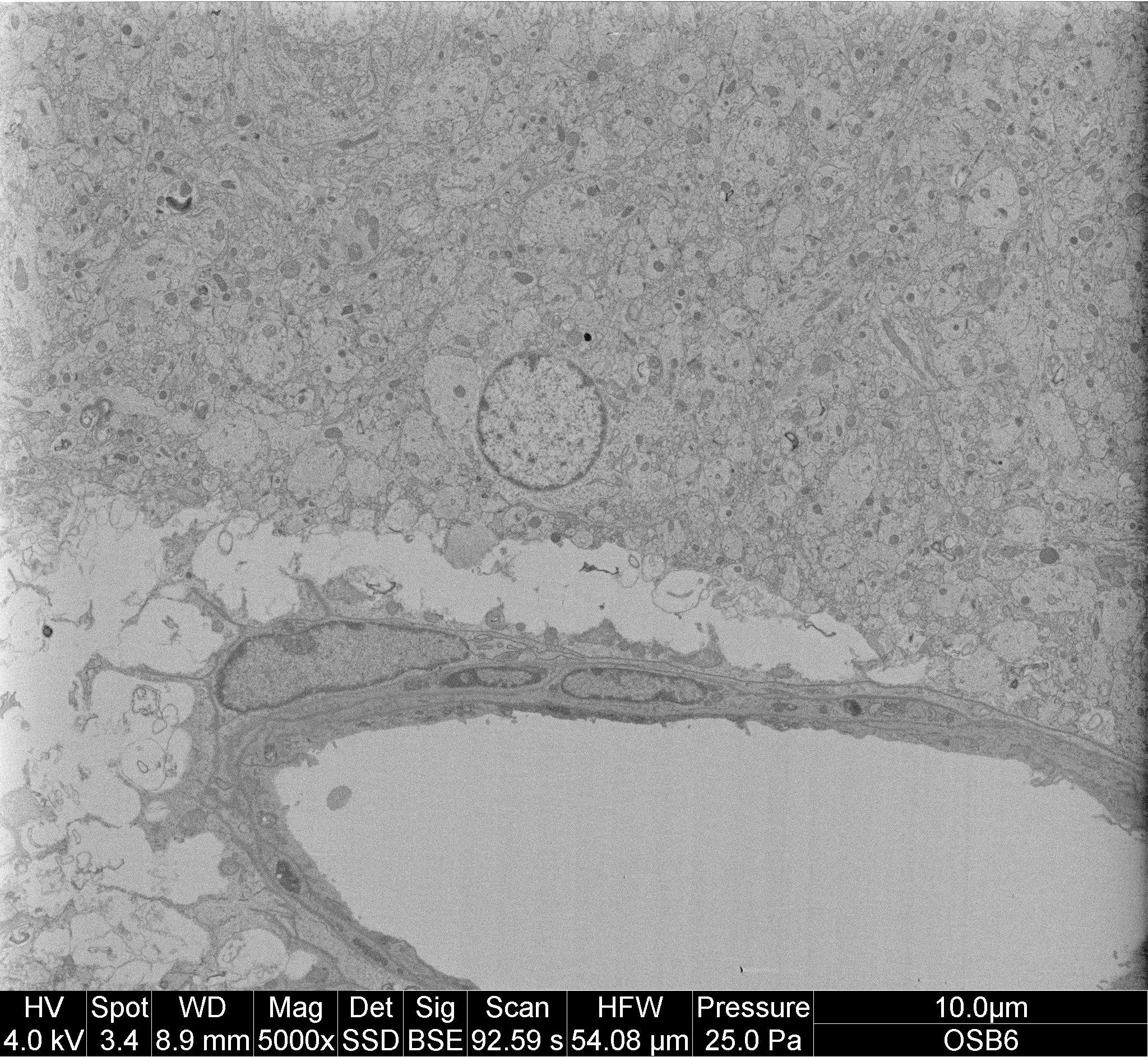

Supplement: Dataset S6 — (252.2 MB ZIP). [file pbio.0020329.sd006.zip › 040604_OS5_st1_553.tif]

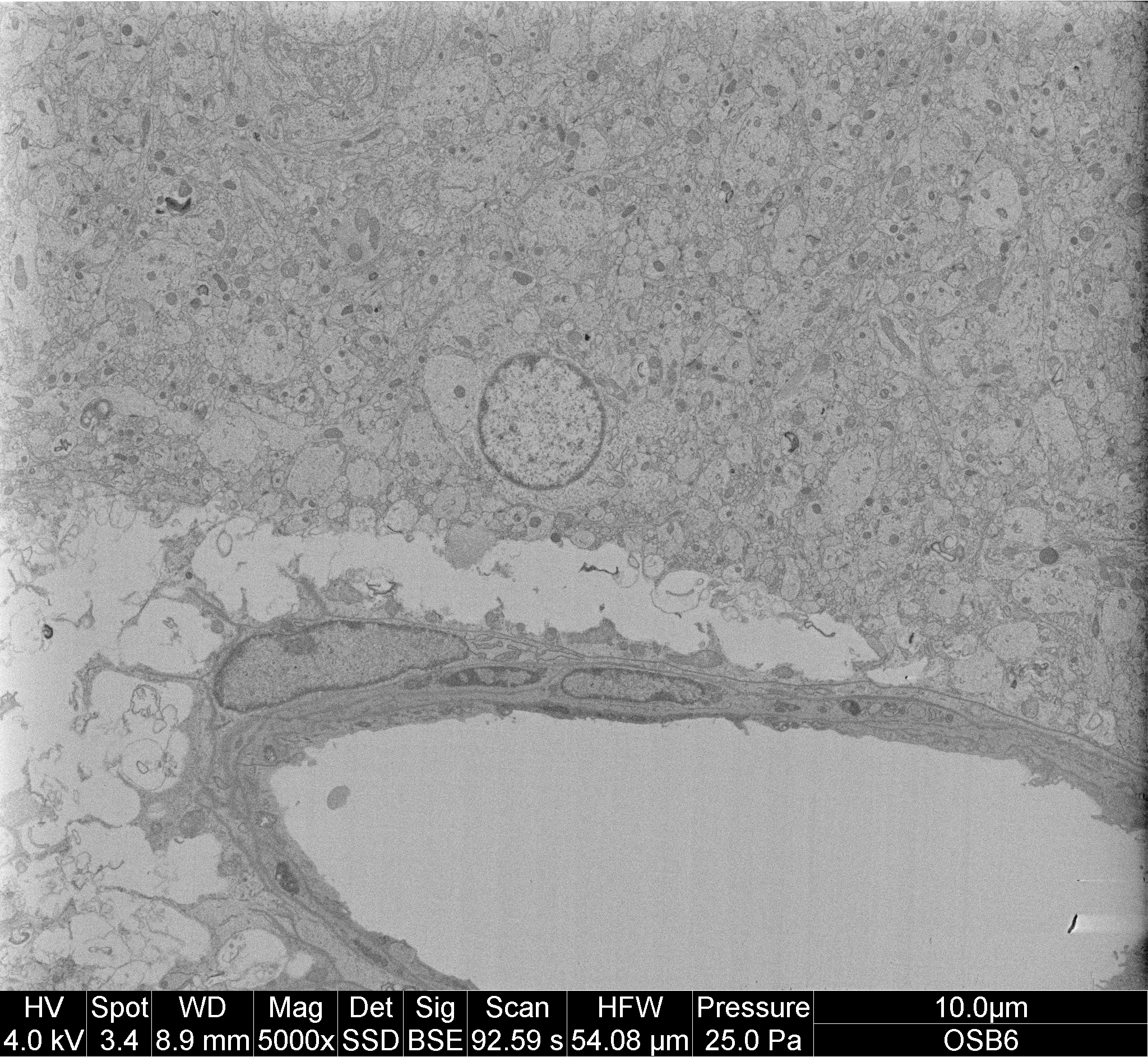

Supplement: Dataset S6 — (252.2 MB ZIP). [file pbio.0020329.sd006.zip › 040604_OS5_st1_554.tif]

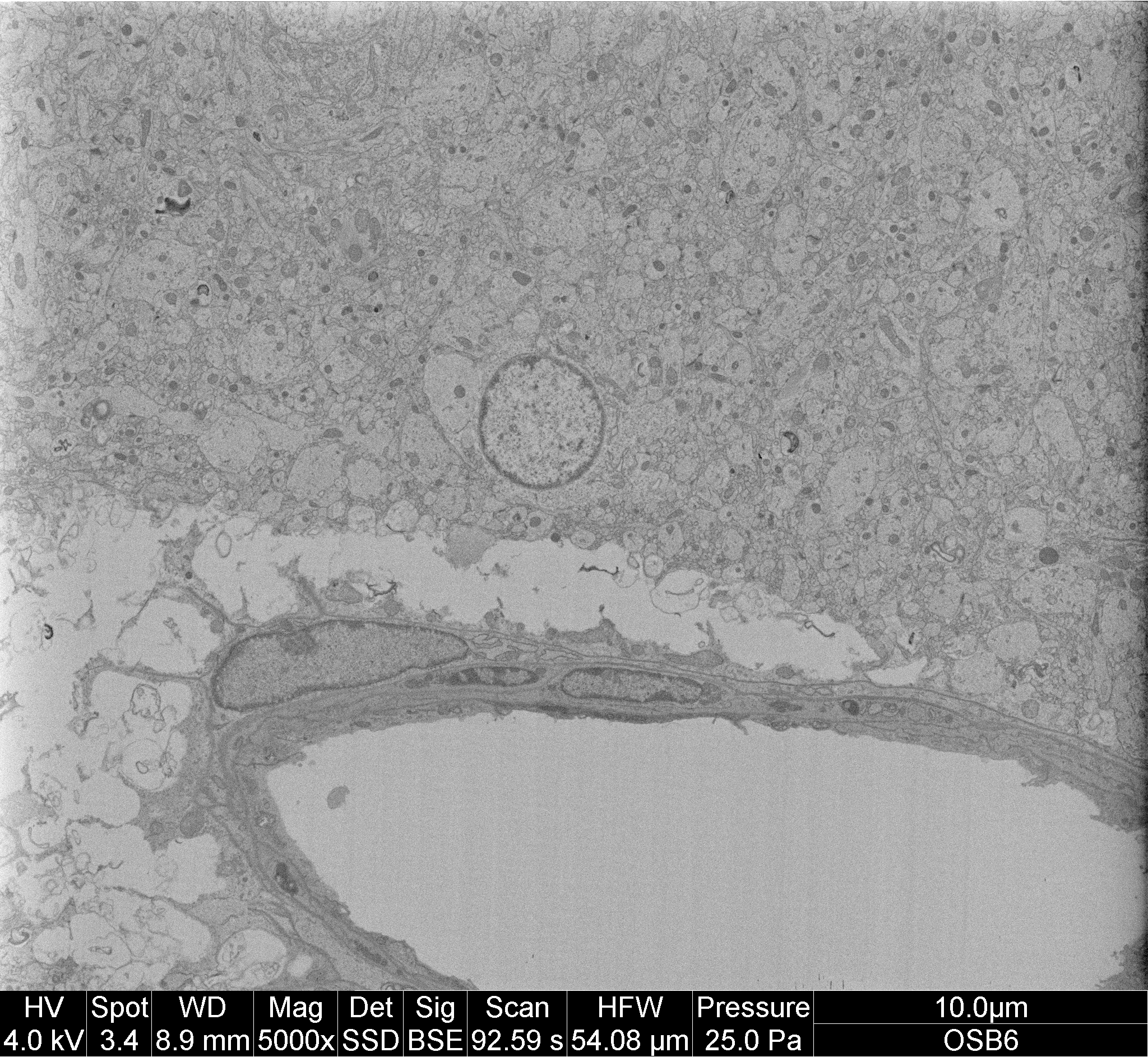

Supplement: Dataset S6 — (252.2 MB ZIP). [file pbio.0020329.sd006.zip › 040604_OS5_st1_555.tif]

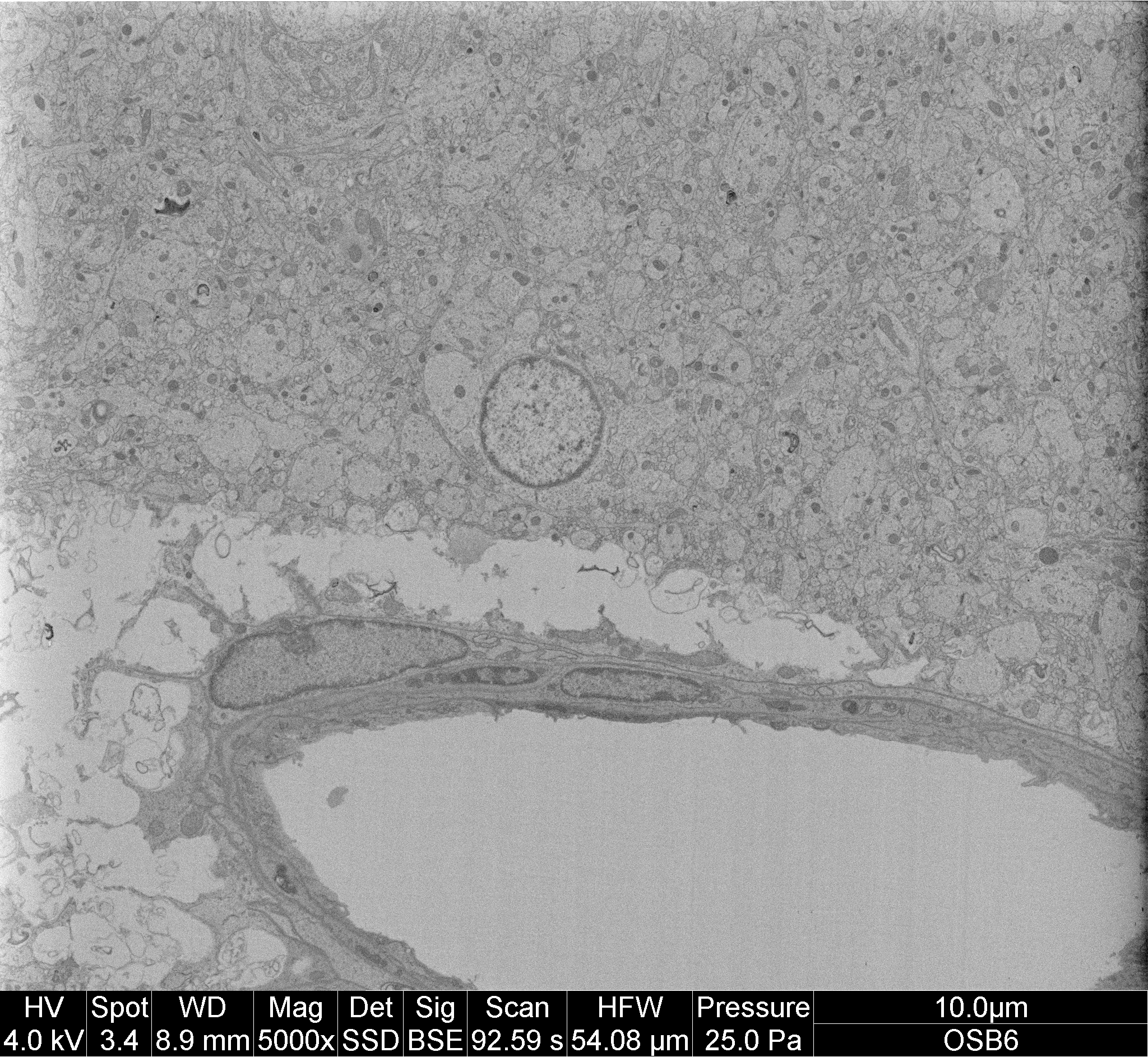

Supplement: Dataset S6 — (252.2 MB ZIP). [file pbio.0020329.sd006.zip › 040604_OS5_st1_556.tif]

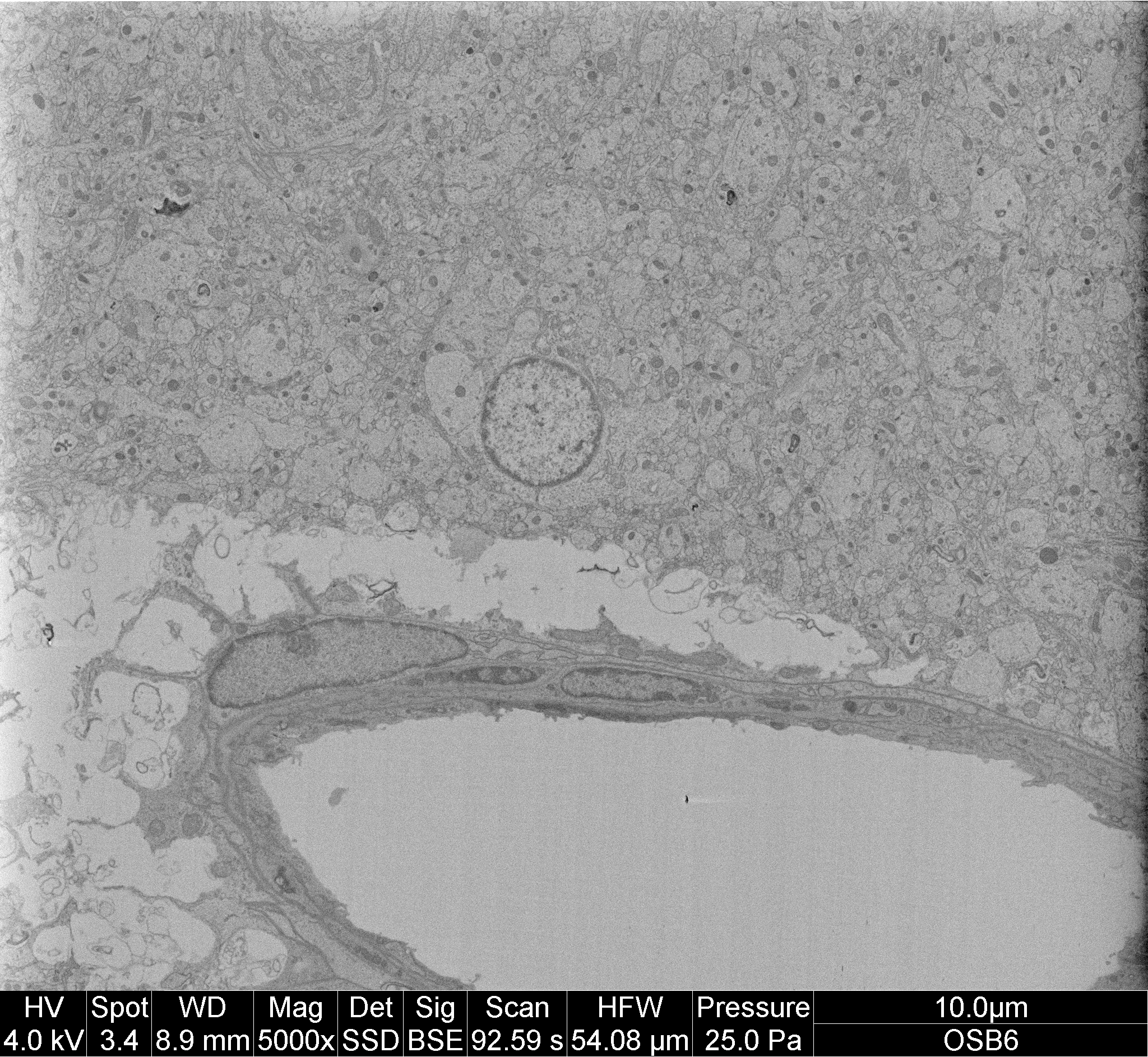

Supplement: Dataset S6 — (252.2 MB ZIP). [file pbio.0020329.sd006.zip › 040604_OS5_st1_557.tif]

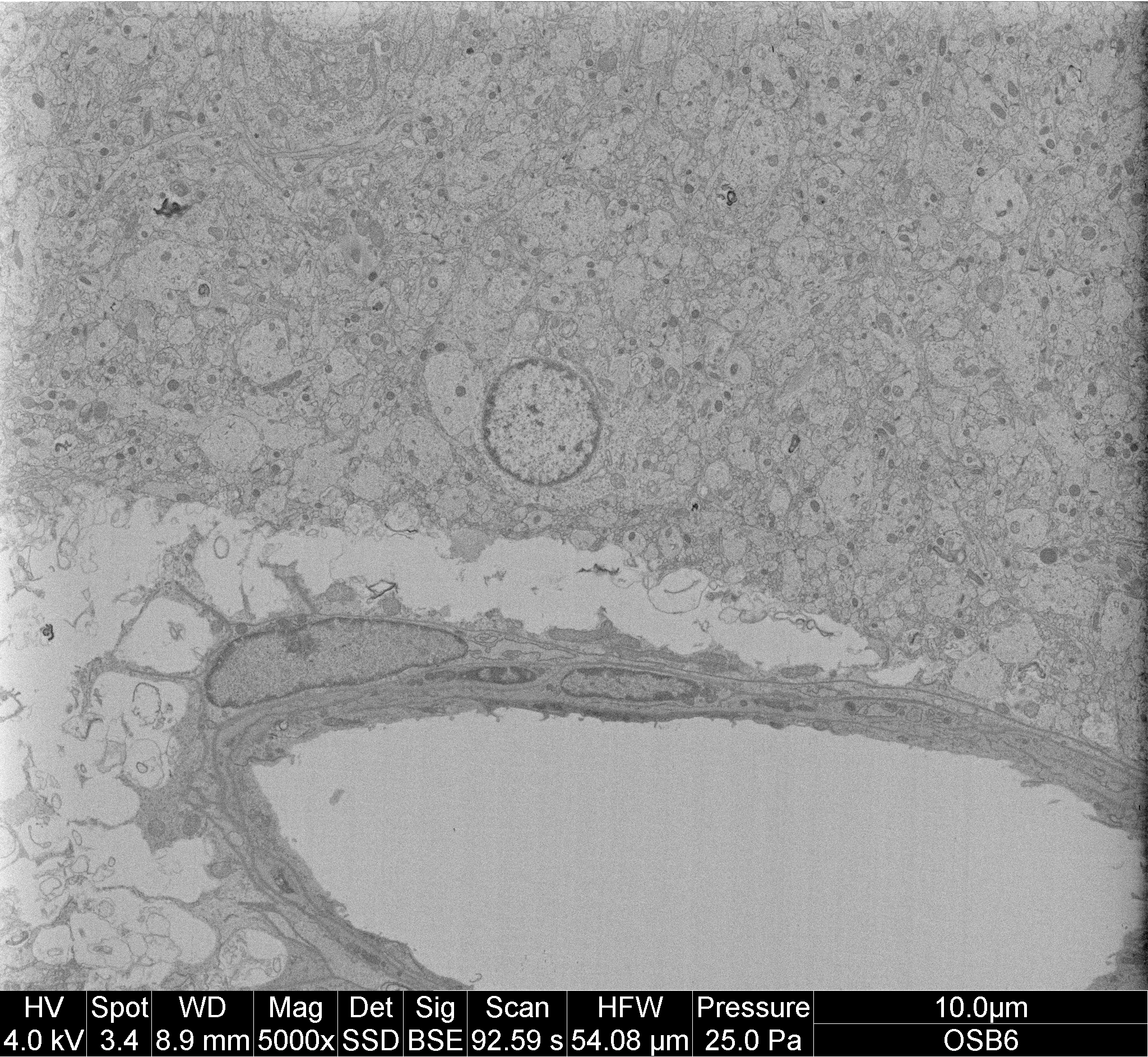

Supplement: Dataset S6 — (252.2 MB ZIP). [file pbio.0020329.sd006.zip › 040604_OS5_st1_558.tif]

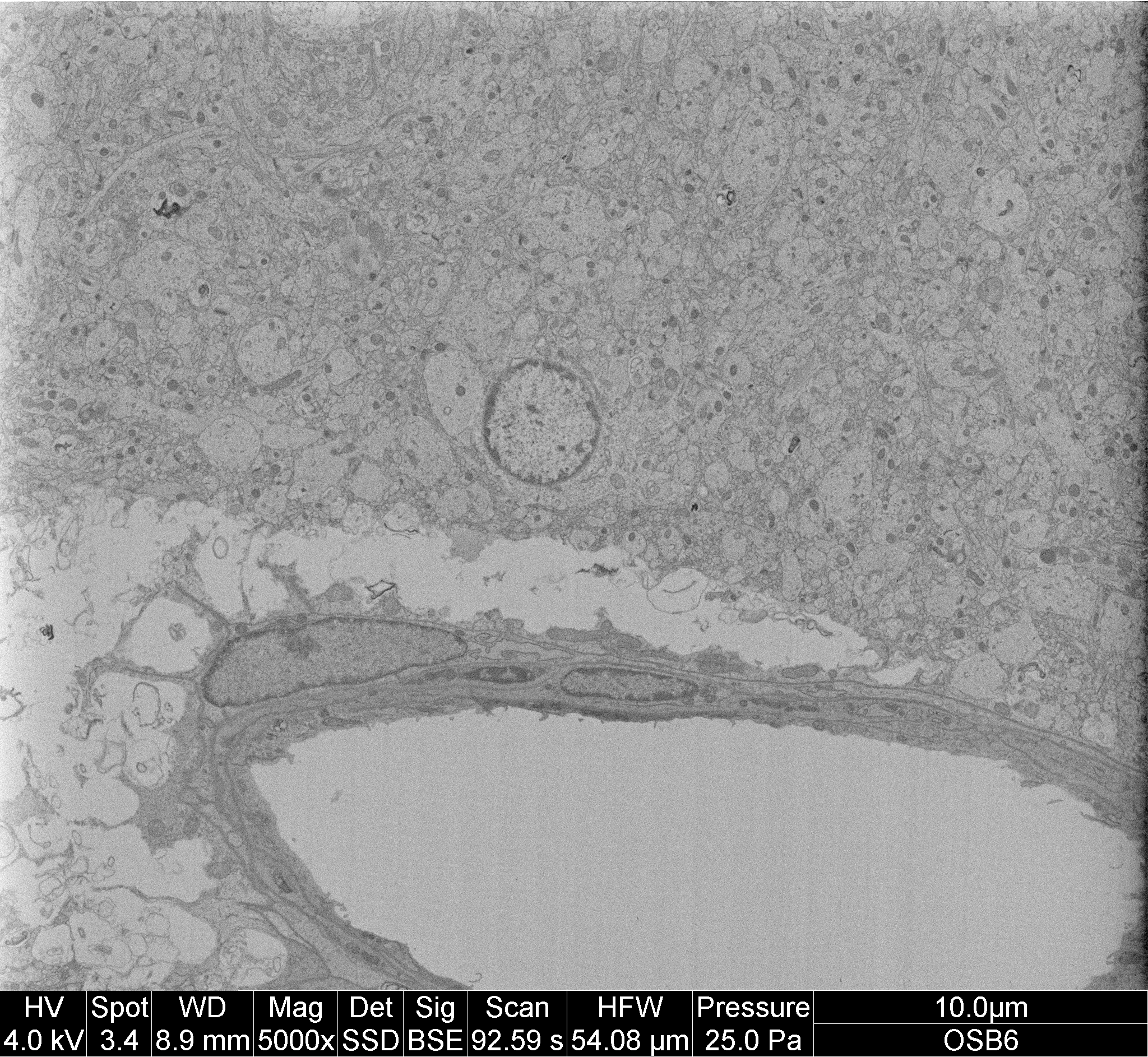

Supplement: Dataset S6 — (252.2 MB ZIP). [file pbio.0020329.sd006.zip › 040604_OS5_st1_559.tif]

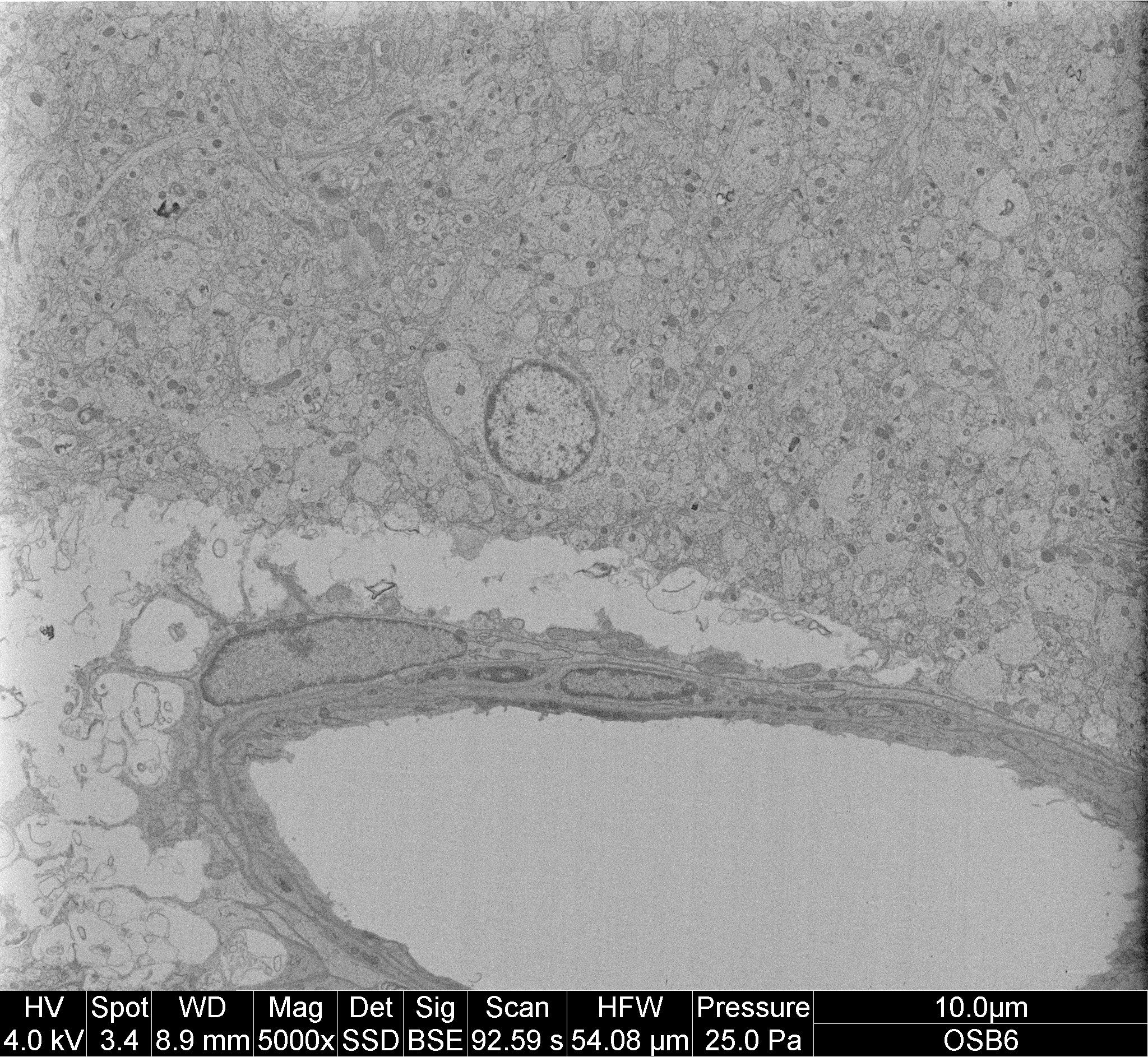

Supplement: Dataset S6 — (252.2 MB ZIP). [file pbio.0020329.sd006.zip › 040604_OS5_st1_560.tif]

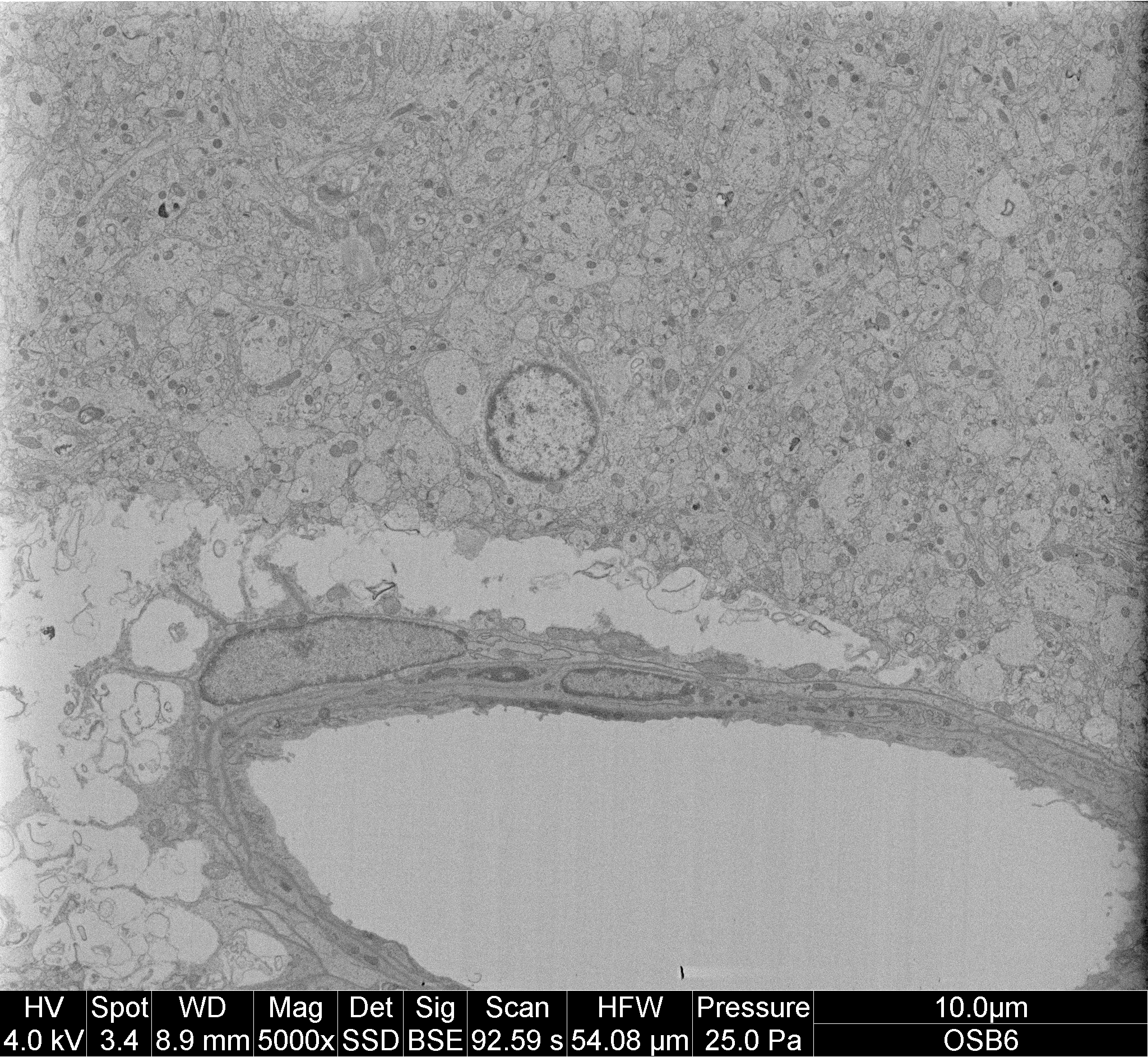

Supplement: Dataset S6 — (252.2 MB ZIP). [file pbio.0020329.sd006.zip › 040604_OS5_st1_561.tif]

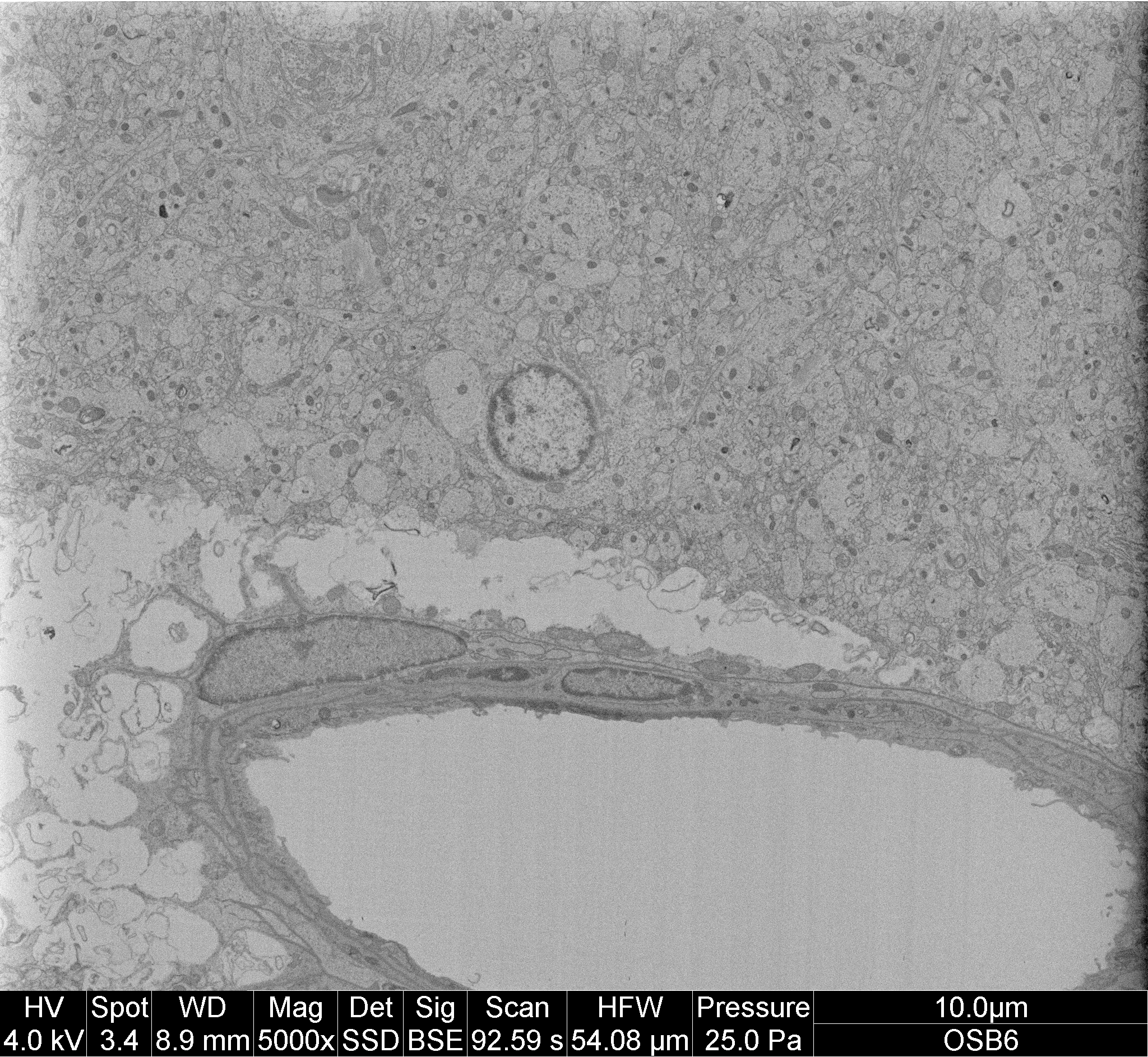

Supplement: Dataset S6 — (252.2 MB ZIP). [file pbio.0020329.sd006.zip › 040604_OS5_st1_562.tif]

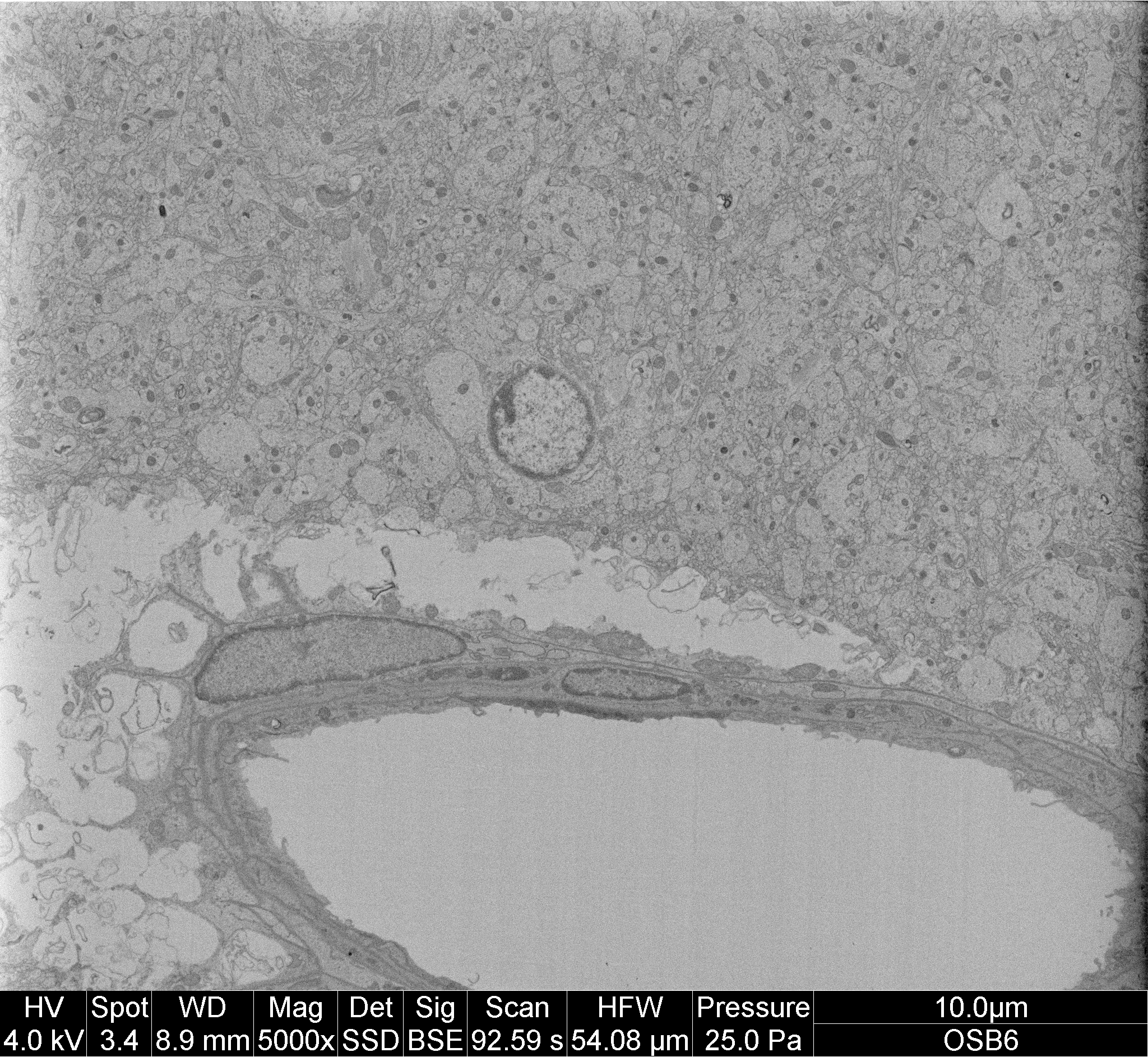

Supplement: Dataset S6 — (252.2 MB ZIP). [file pbio.0020329.sd006.zip › 040604_OS5_st1_563.tif]

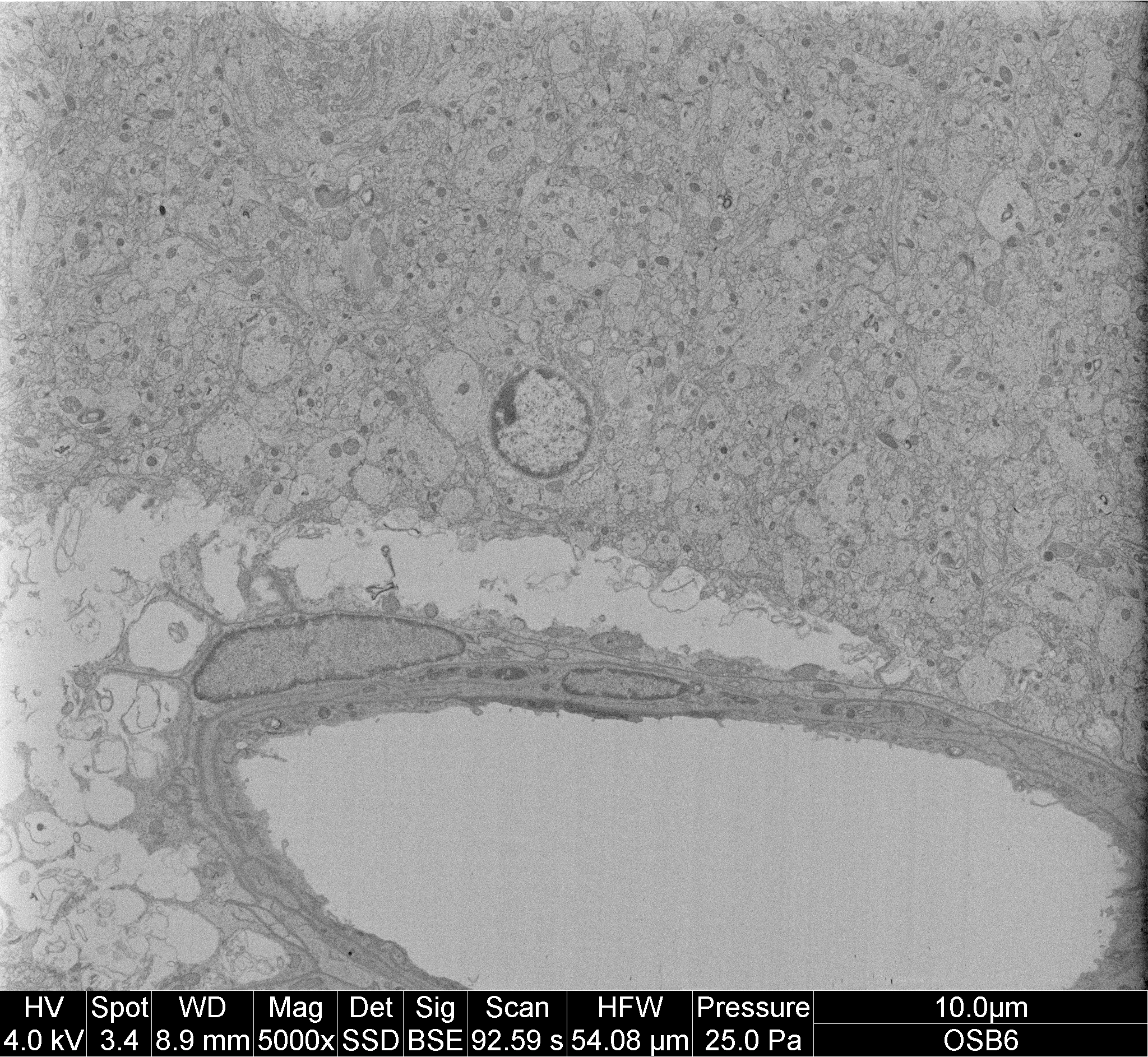

Supplement: Dataset S6 — (252.2 MB ZIP). [file pbio.0020329.sd006.zip › 040604_OS5_st1_564.tif]

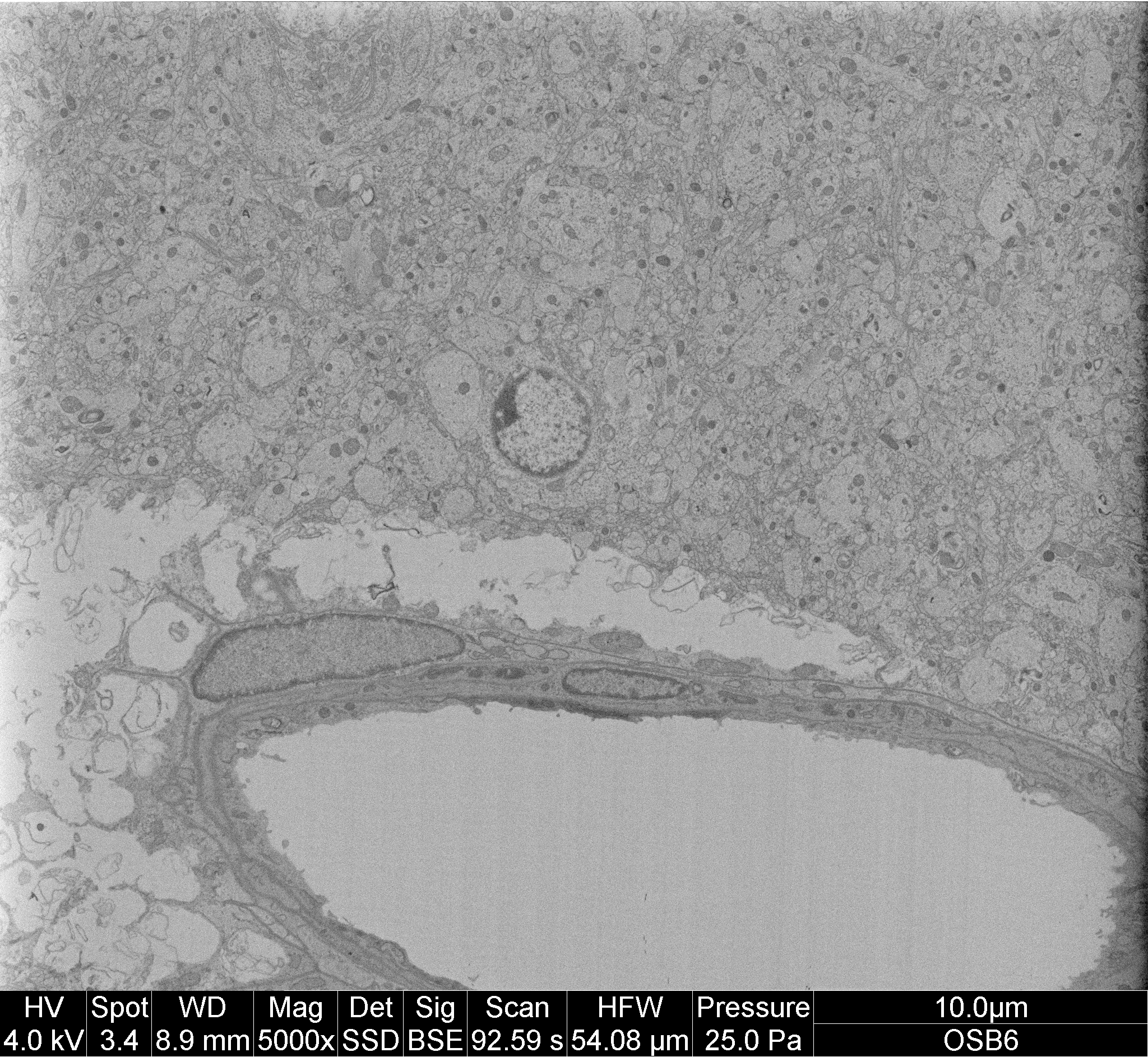

Supplement: Dataset S6 — (252.2 MB ZIP). [file pbio.0020329.sd006.zip › 040604_OS5_st1_565.tif]

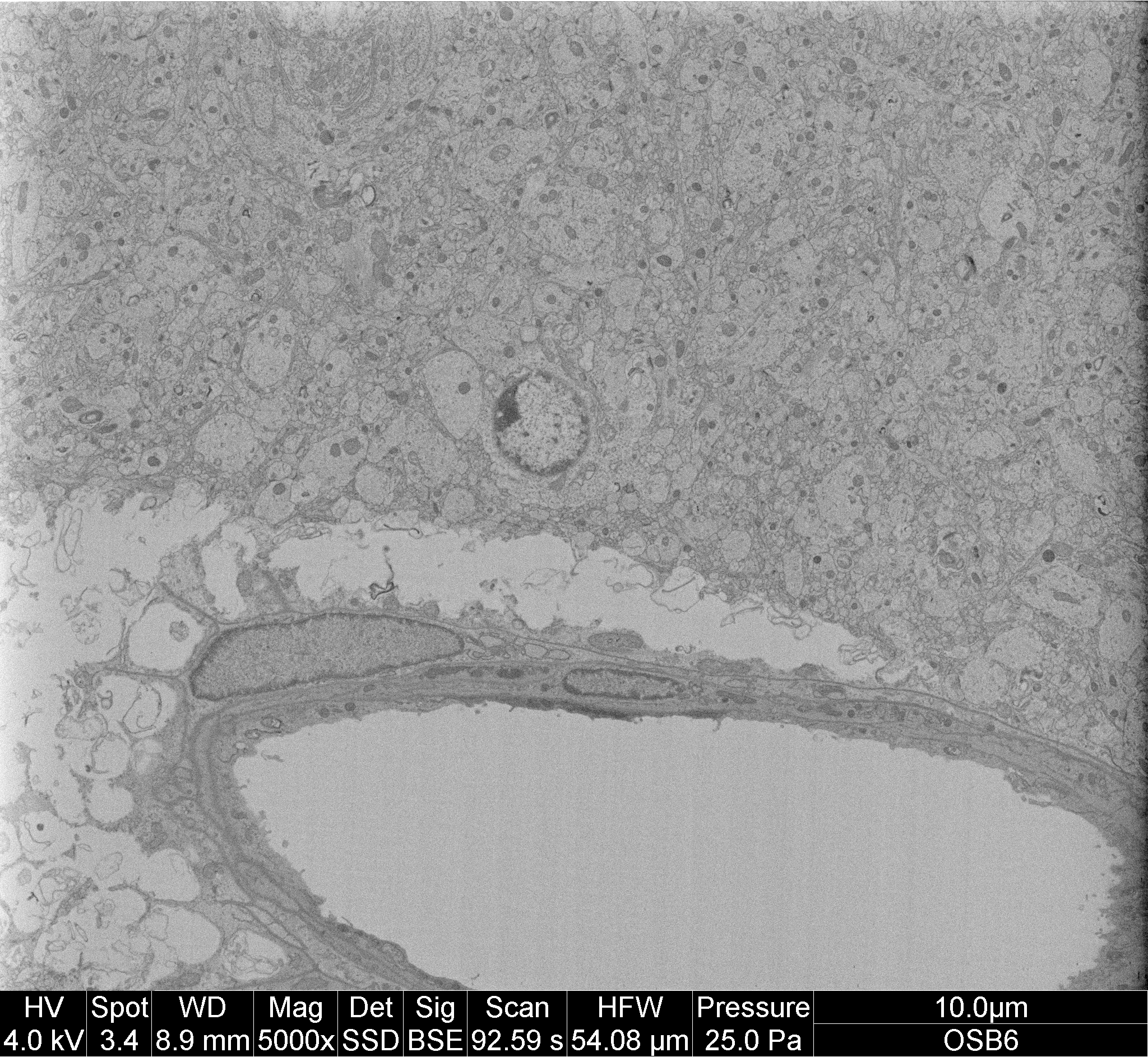

Supplement: Dataset S6 — (252.2 MB ZIP). [file pbio.0020329.sd006.zip › 040604_OS5_st1_566.tif]

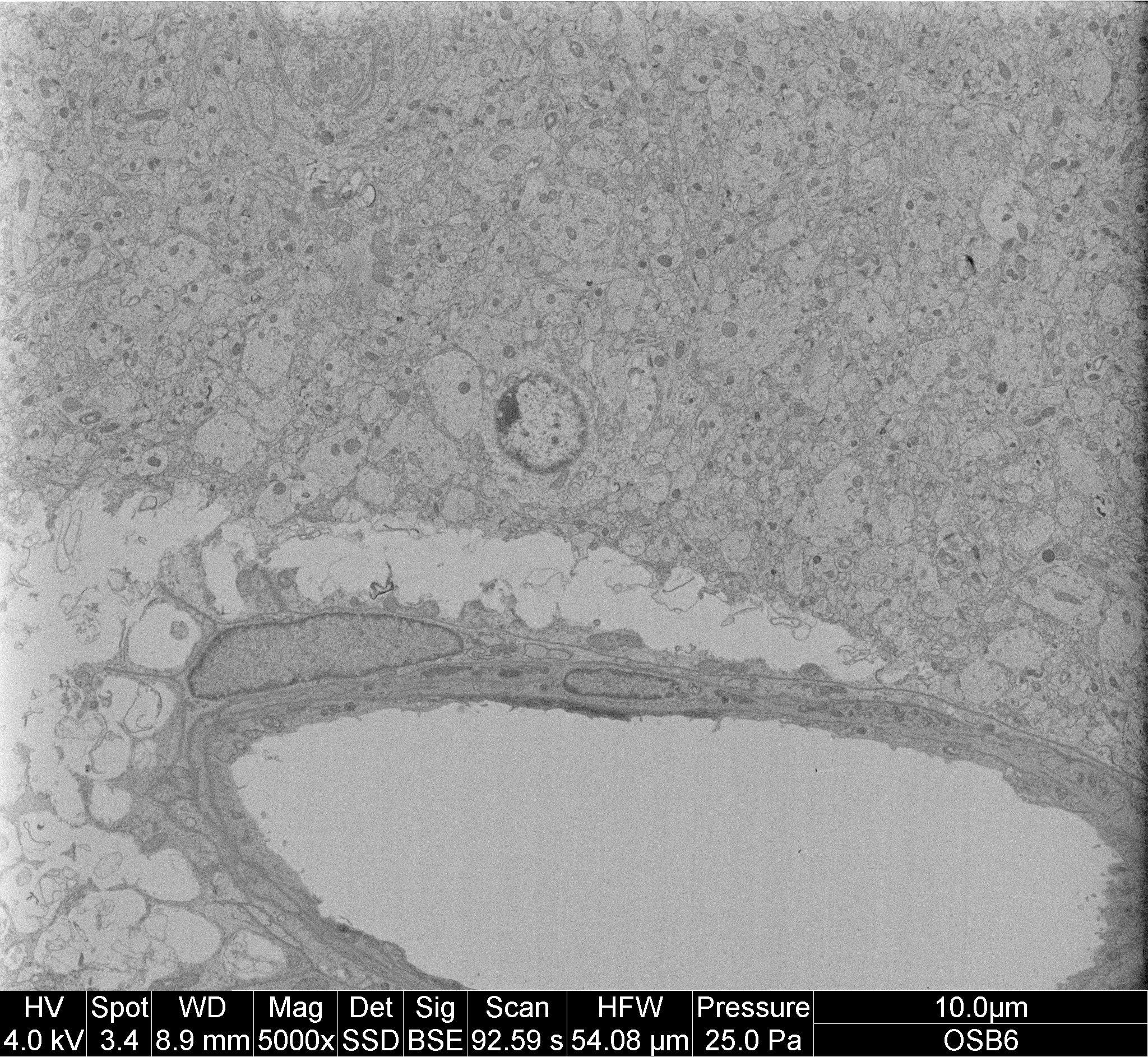

Supplement: Dataset S6 — (252.2 MB ZIP). [file pbio.0020329.sd006.zip › 040604_OS5_st1_567.tif]

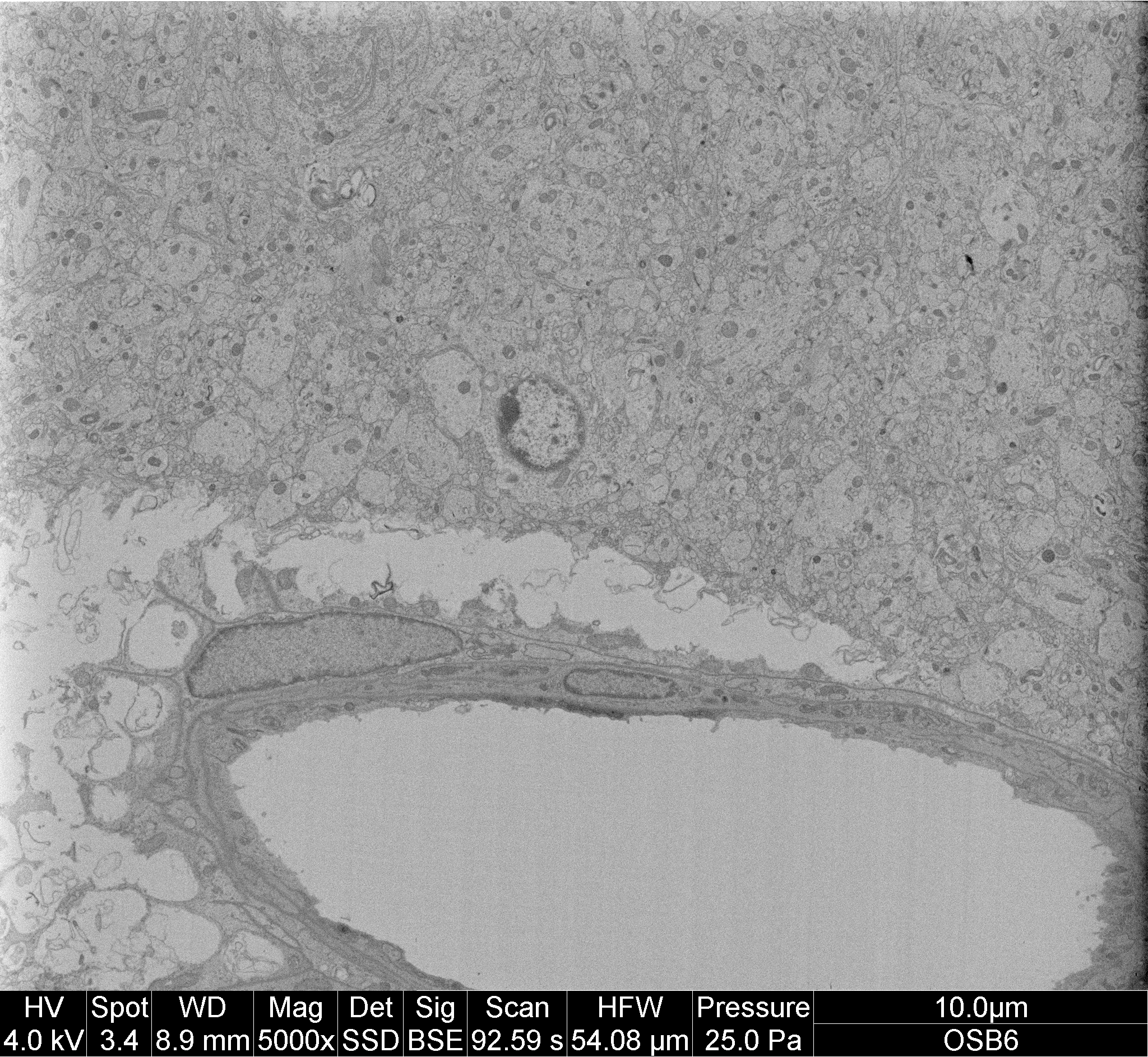

Supplement: Dataset S6 — (252.2 MB ZIP). [file pbio.0020329.sd006.zip › 040604_OS5_st1_568.tif]

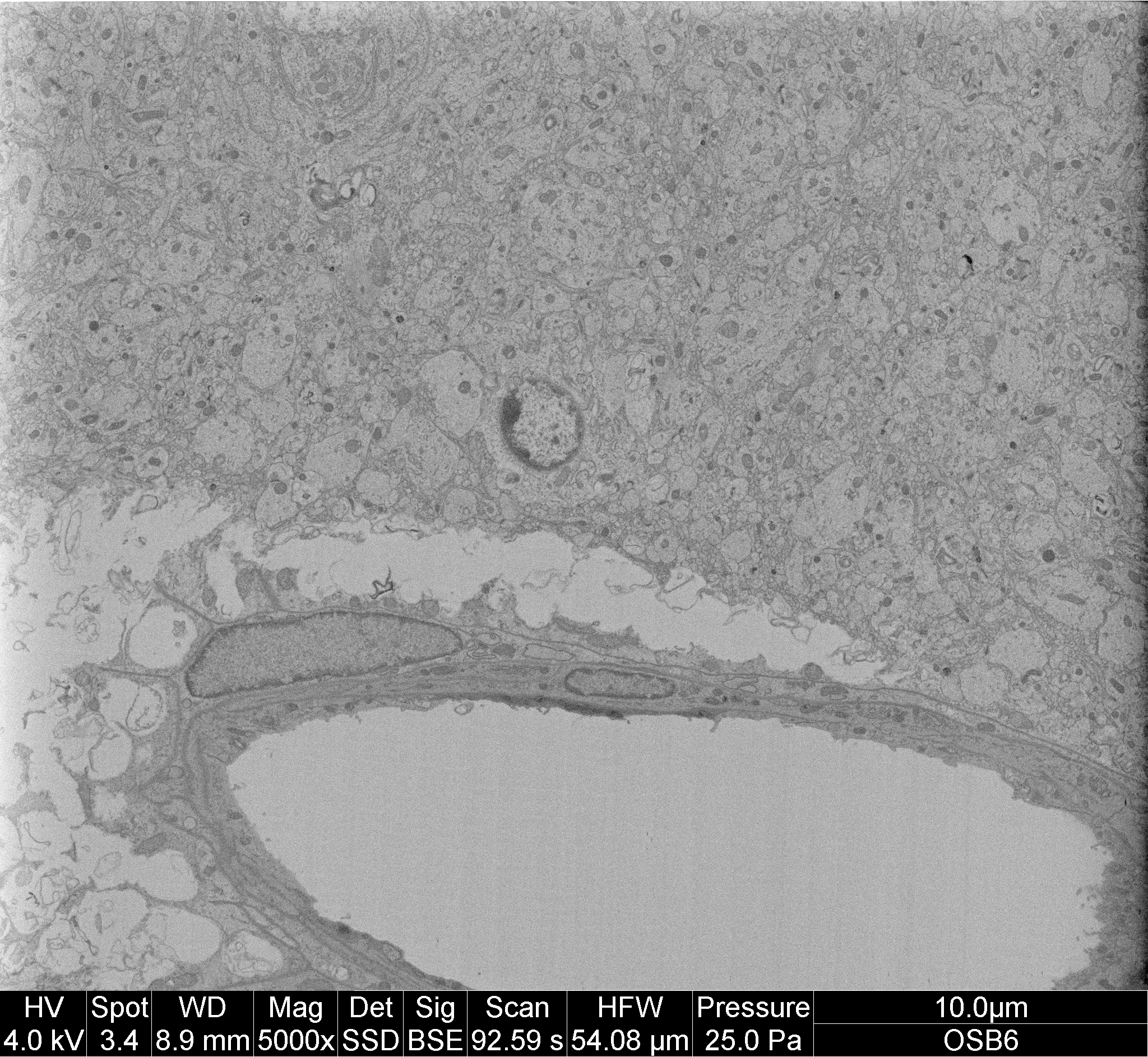

Supplement: Dataset S6 — (252.2 MB ZIP). [file pbio.0020329.sd006.zip › 040604_OS5_st1_569.tif]

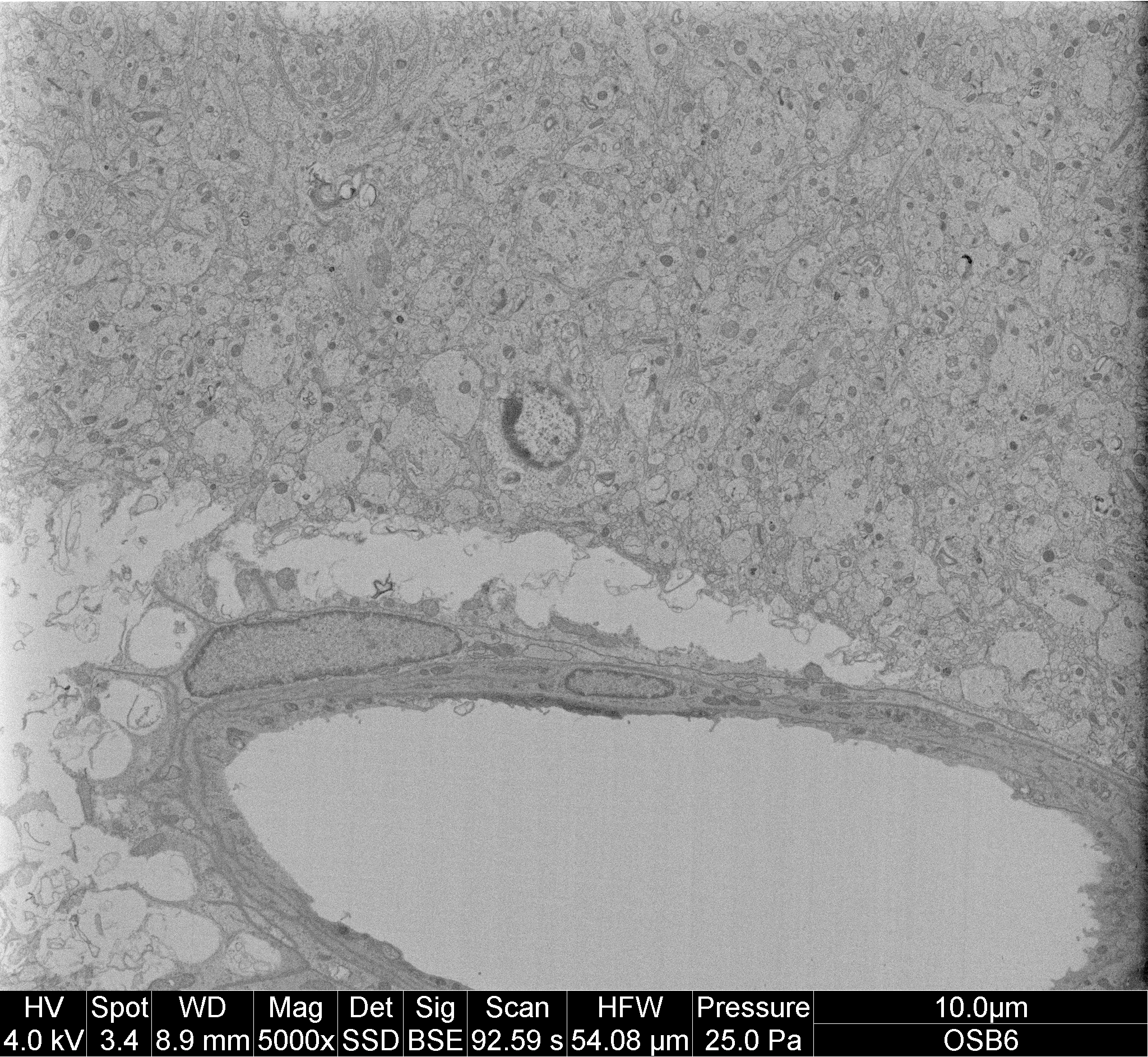

Supplement: Dataset S6 — (252.2 MB ZIP). [file pbio.0020329.sd006.zip › 040604_OS5_st1_570.tif]

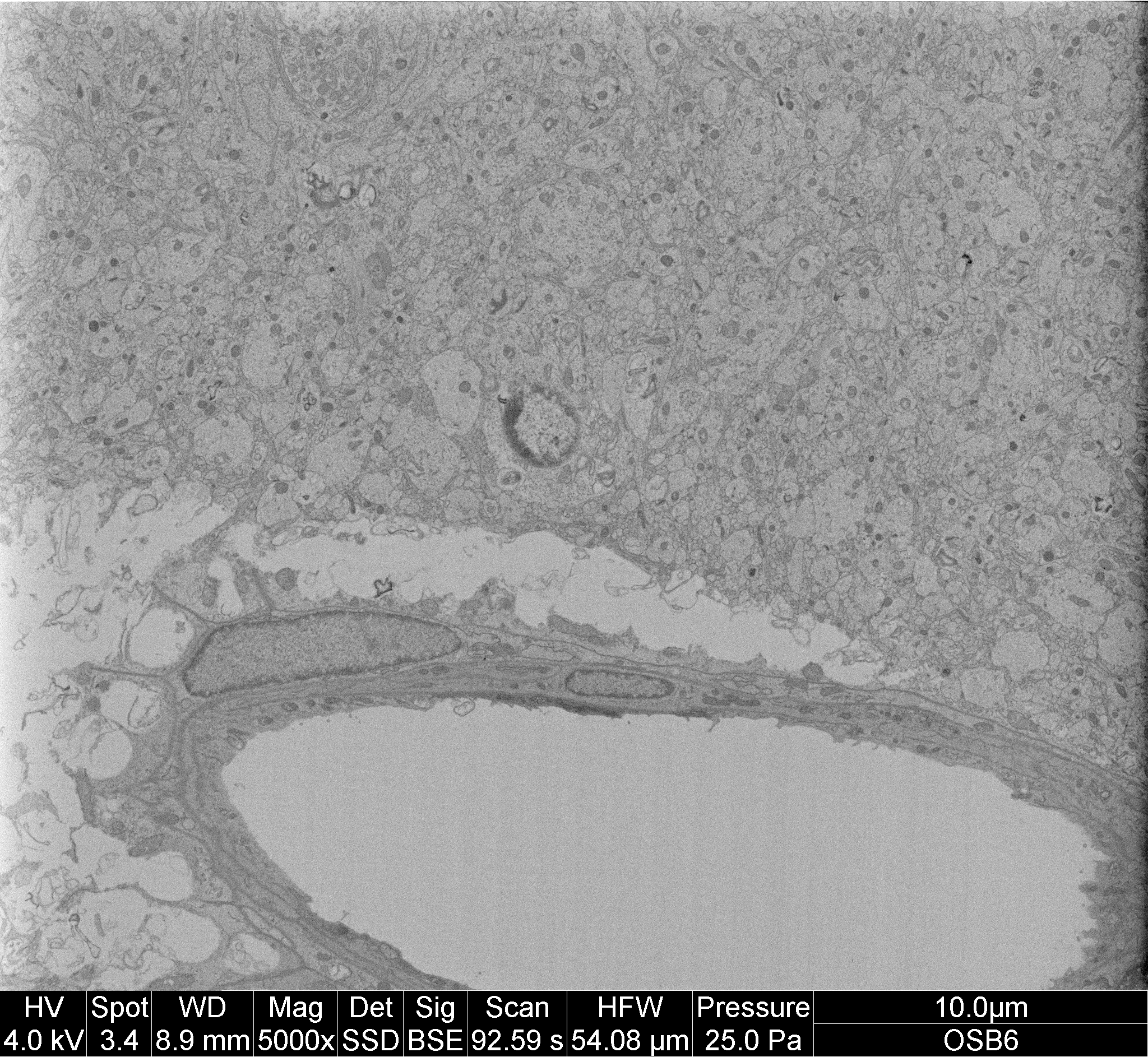

Supplement: Dataset S6 — (252.2 MB ZIP). [file pbio.0020329.sd006.zip › 040604_OS5_st1_571.tif]

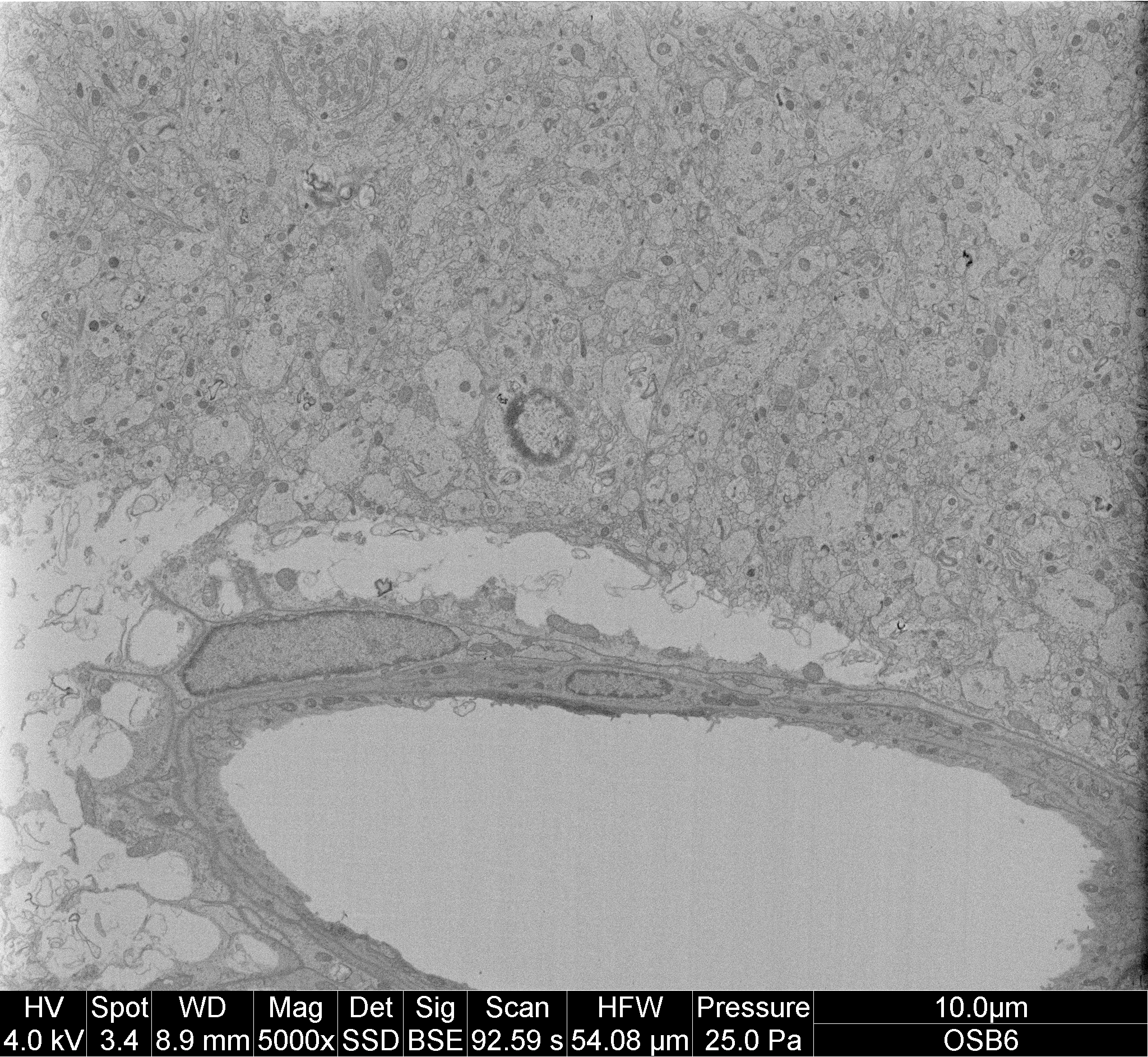

Supplement: Dataset S6 — (252.2 MB ZIP). [file pbio.0020329.sd006.zip › 040604_OS5_st1_572.tif]

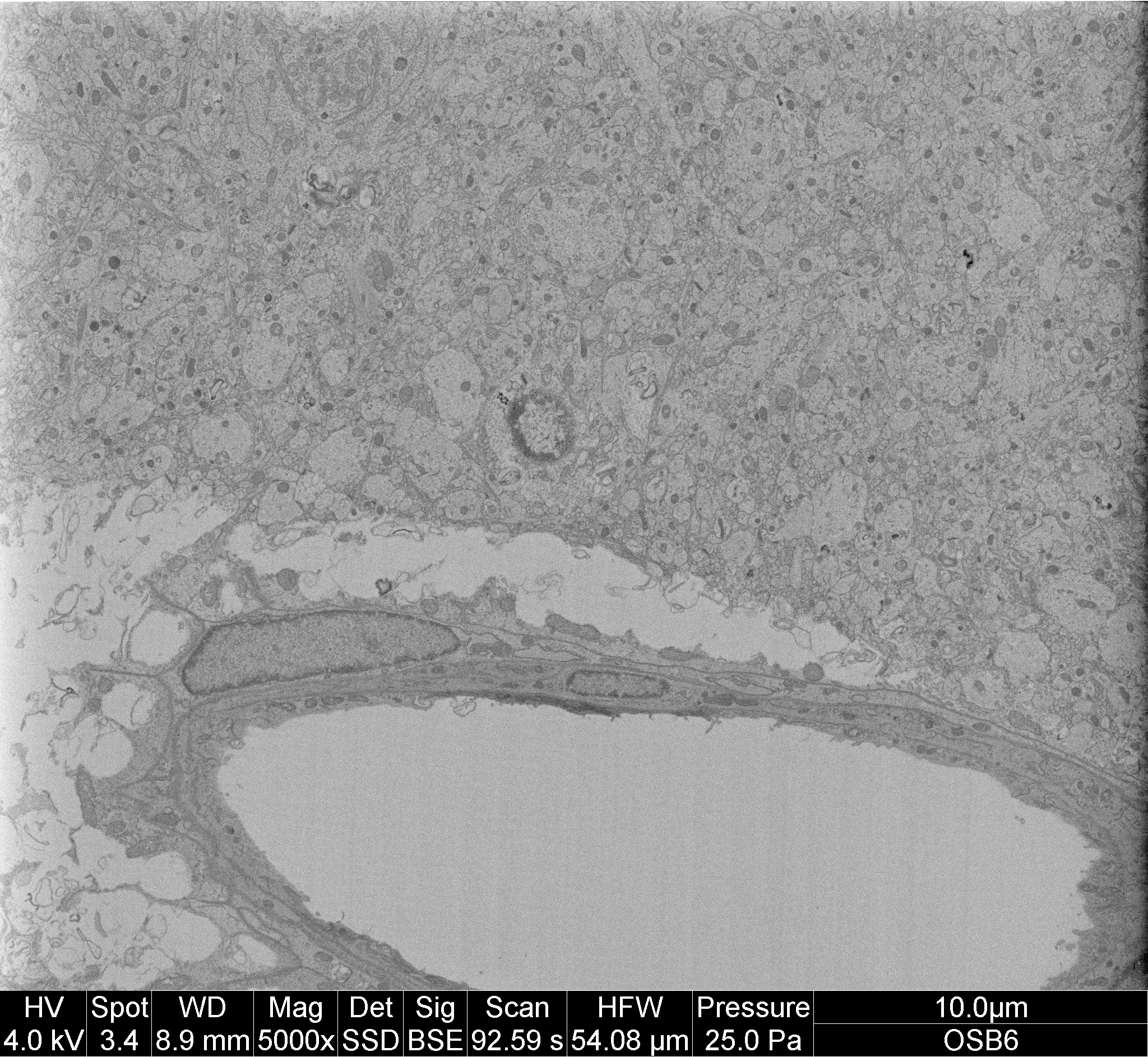

Supplement: Dataset S6 — (252.2 MB ZIP). [file pbio.0020329.sd006.zip › 040604_OS5_st1_573.tif]

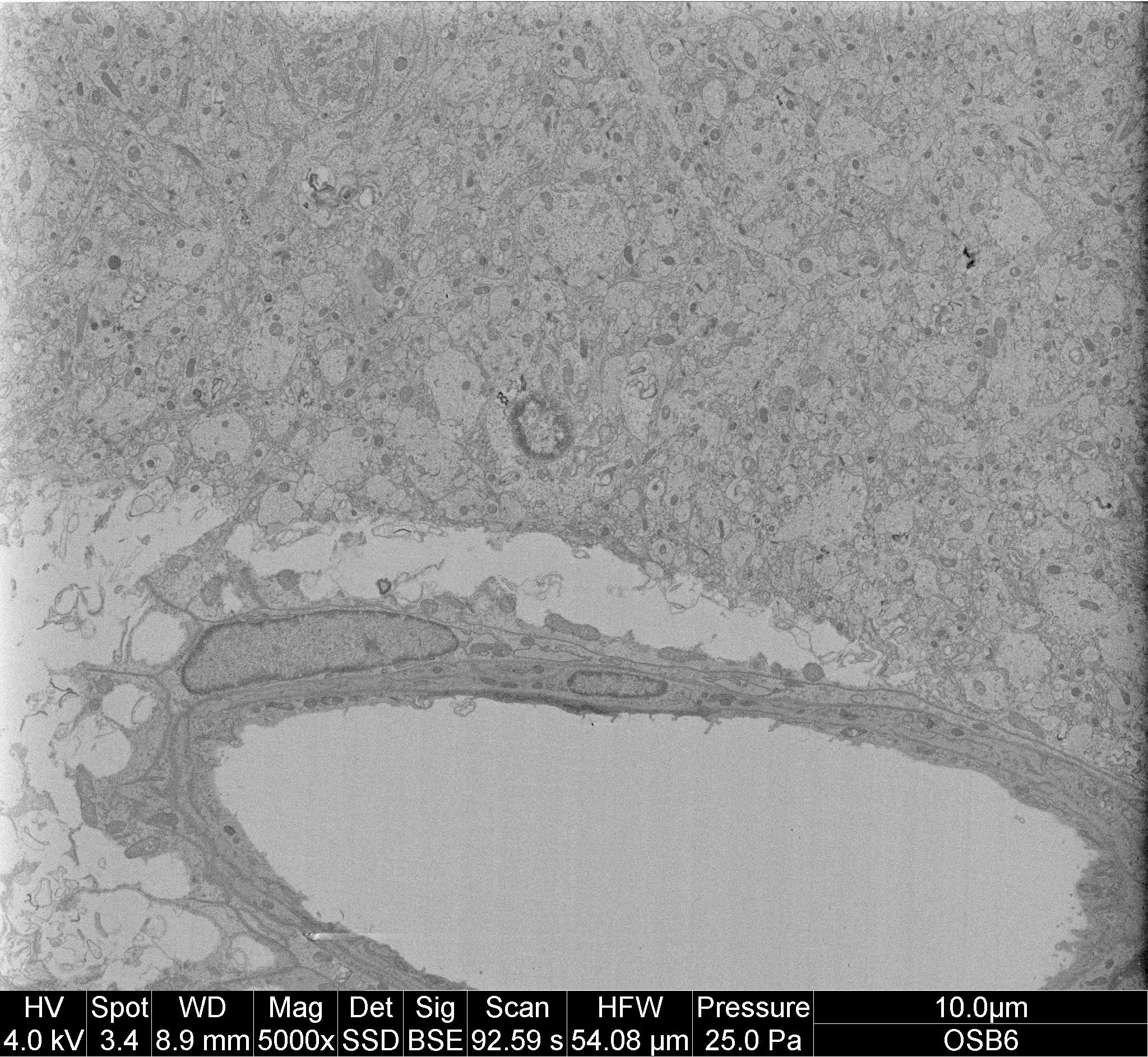

Supplement: Dataset S6 — (252.2 MB ZIP). [file pbio.0020329.sd006.zip › 040604_OS5_st1_574.tif]

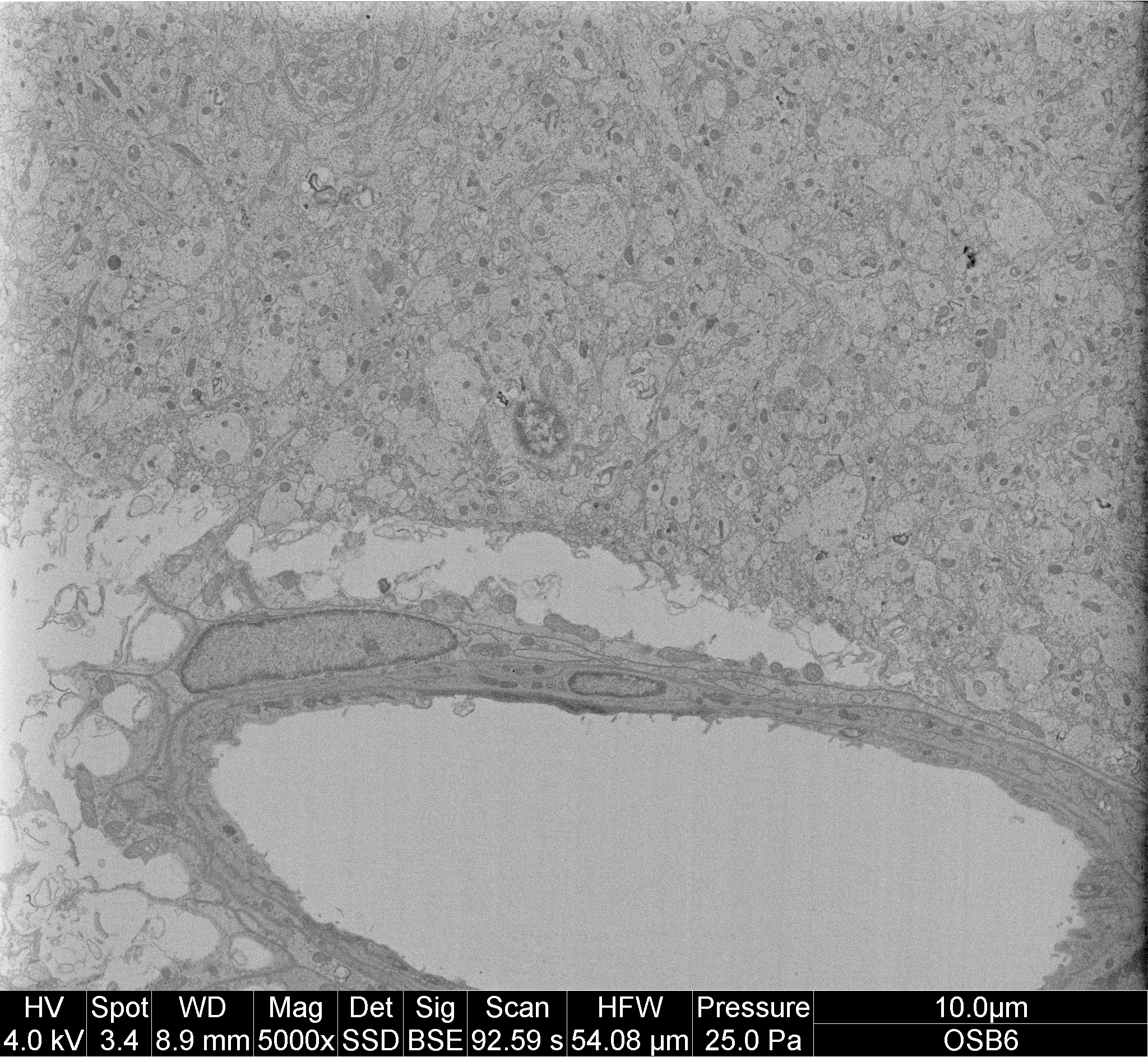

Supplement: Dataset S6 — (252.2 MB ZIP). [file pbio.0020329.sd006.zip › 040604_OS5_st1_575.tif]

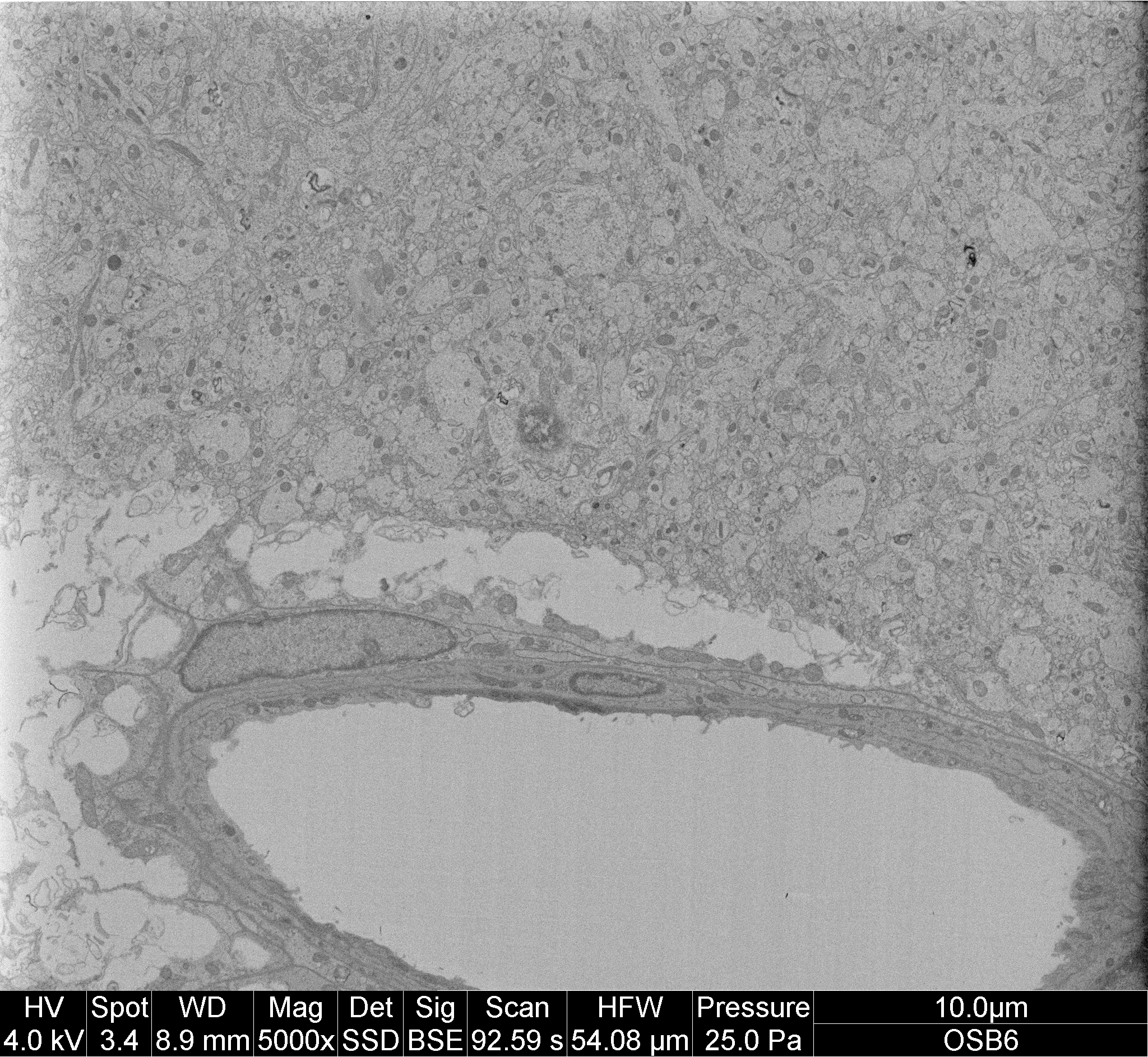

Supplement: Dataset S6 — (252.2 MB ZIP). [file pbio.0020329.sd006.zip › 040604_OS5_st1_576.tif]

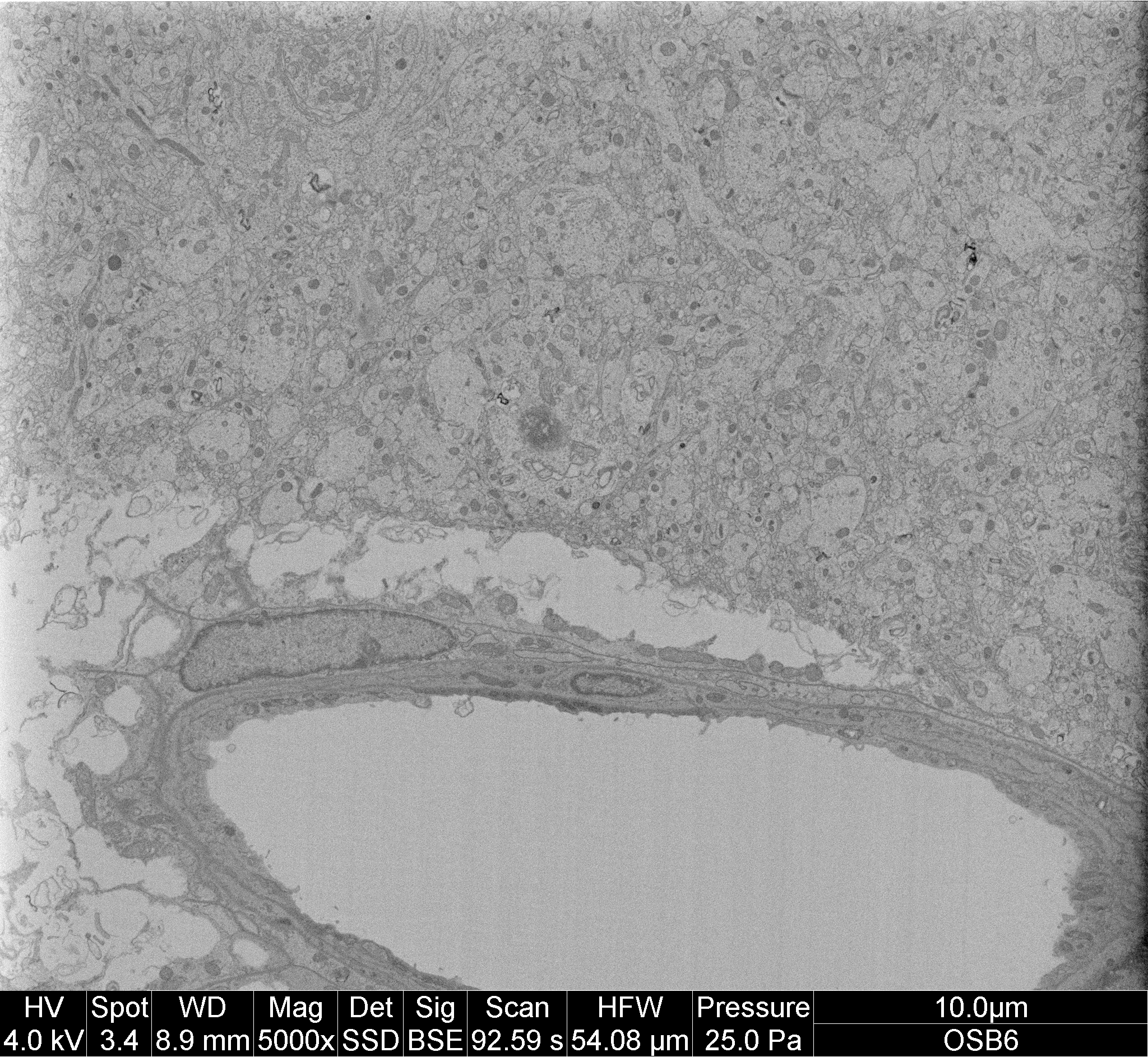

Supplement: Dataset S6 — (252.2 MB ZIP). [file pbio.0020329.sd006.zip › 040604_OS5_st1_577.tif]

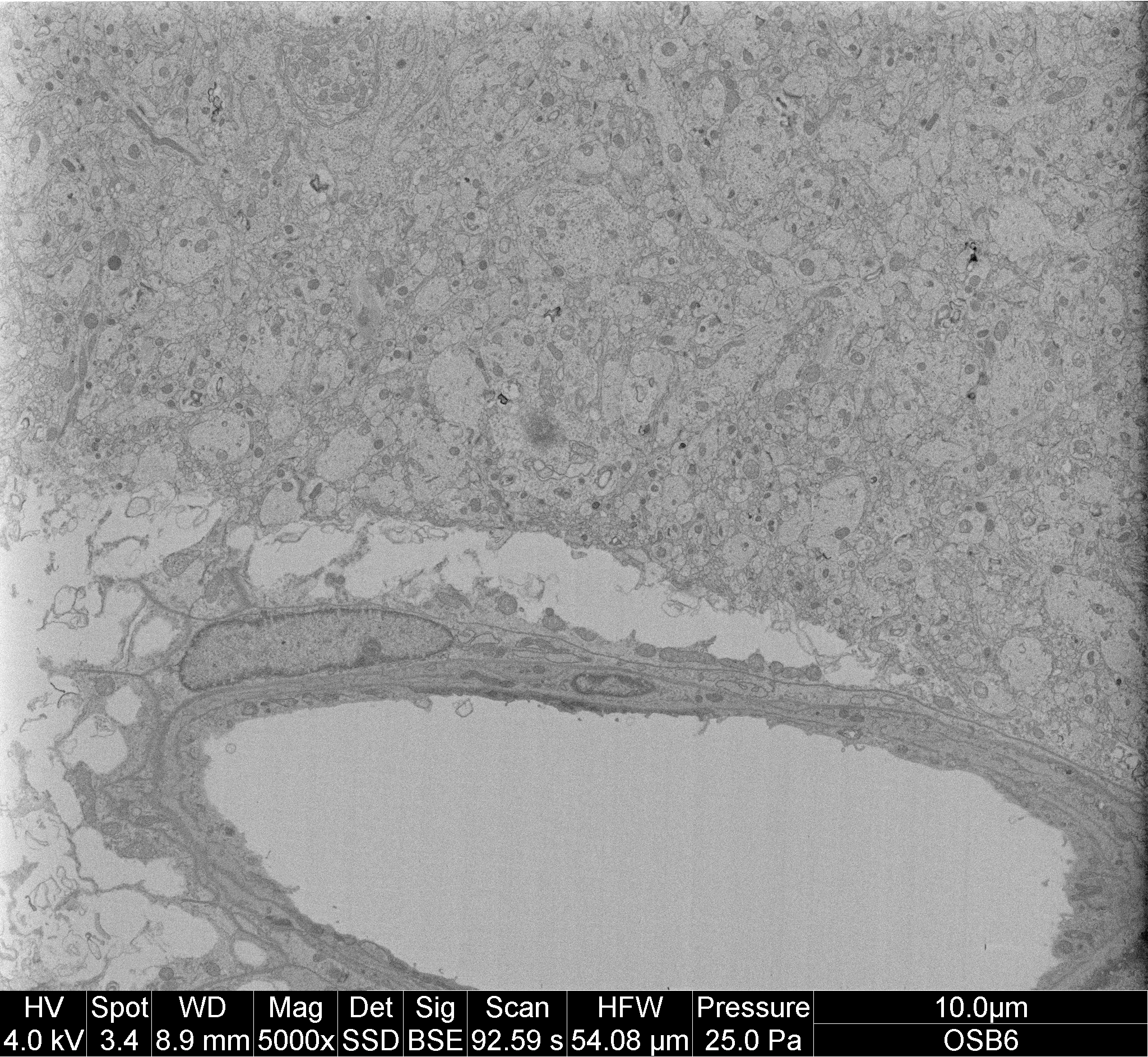

Supplement: Dataset S6 — (252.2 MB ZIP). [file pbio.0020329.sd006.zip › 040604_OS5_st1_578.tif]

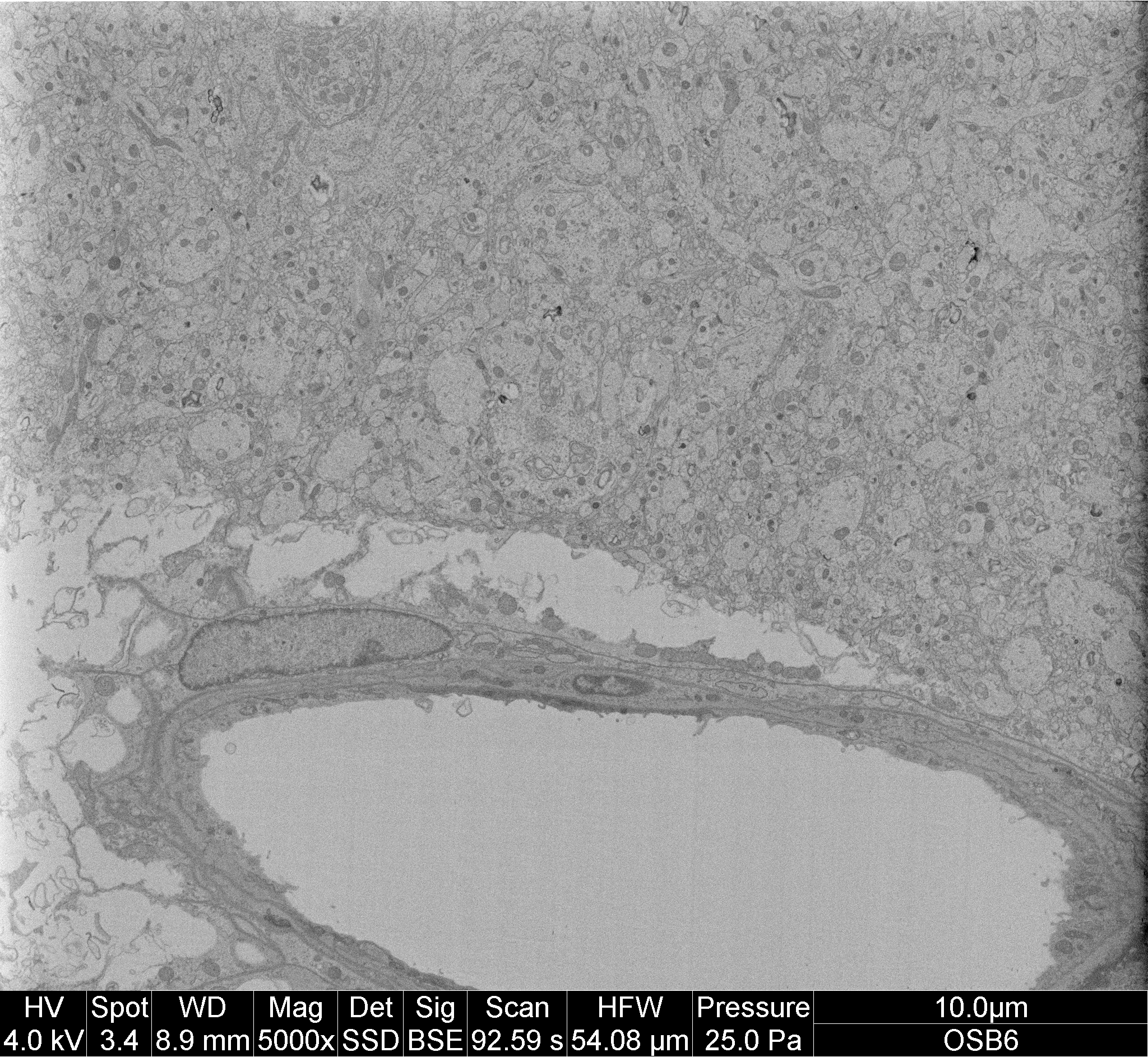

Supplement: Dataset S6 — (252.2 MB ZIP). [file pbio.0020329.sd006.zip › 040604_OS5_st1_579.tif]

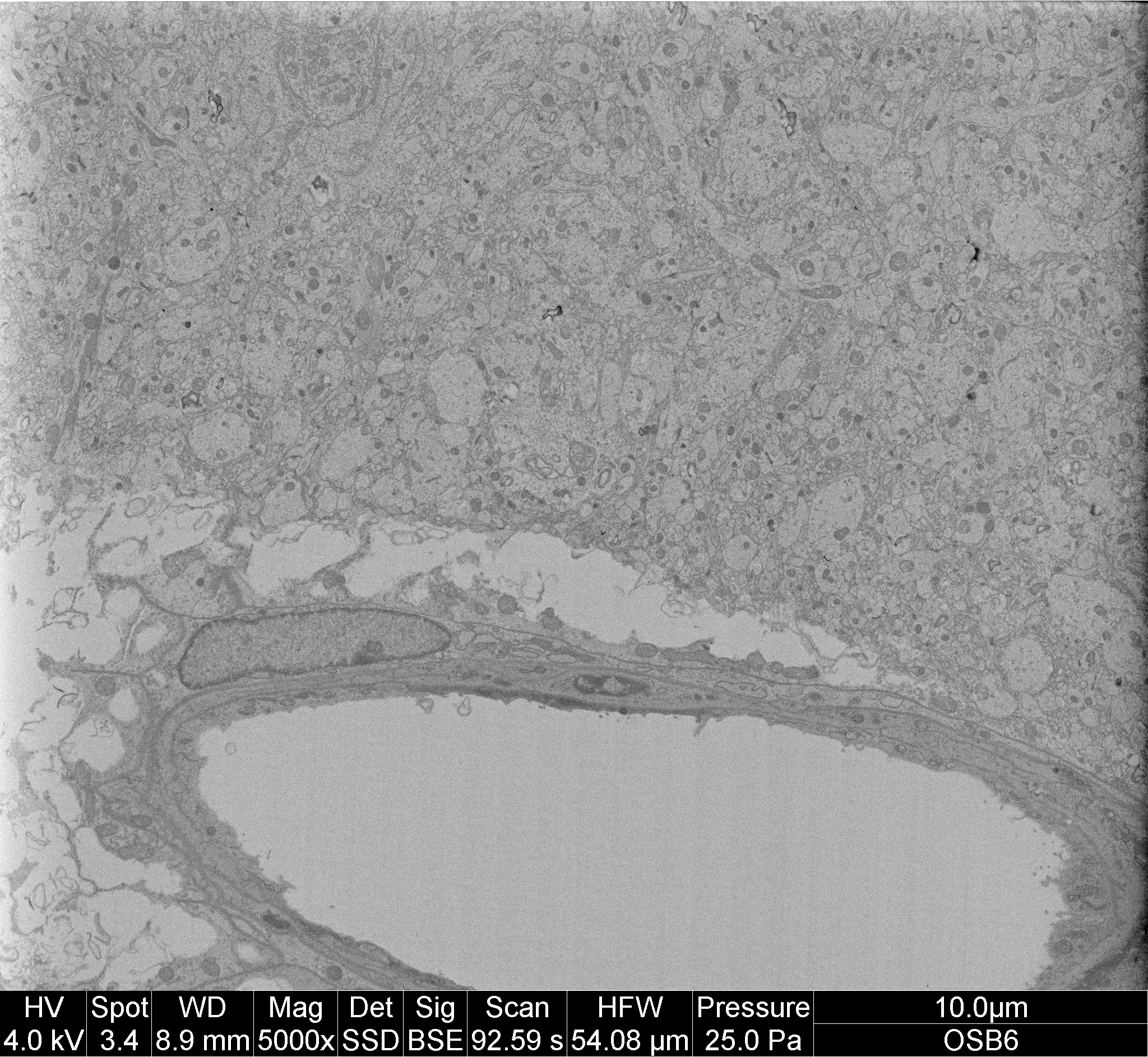

Supplement: Dataset S6 — (252.2 MB ZIP). [file pbio.0020329.sd006.zip › 040604_OS5_st1_580.tif]

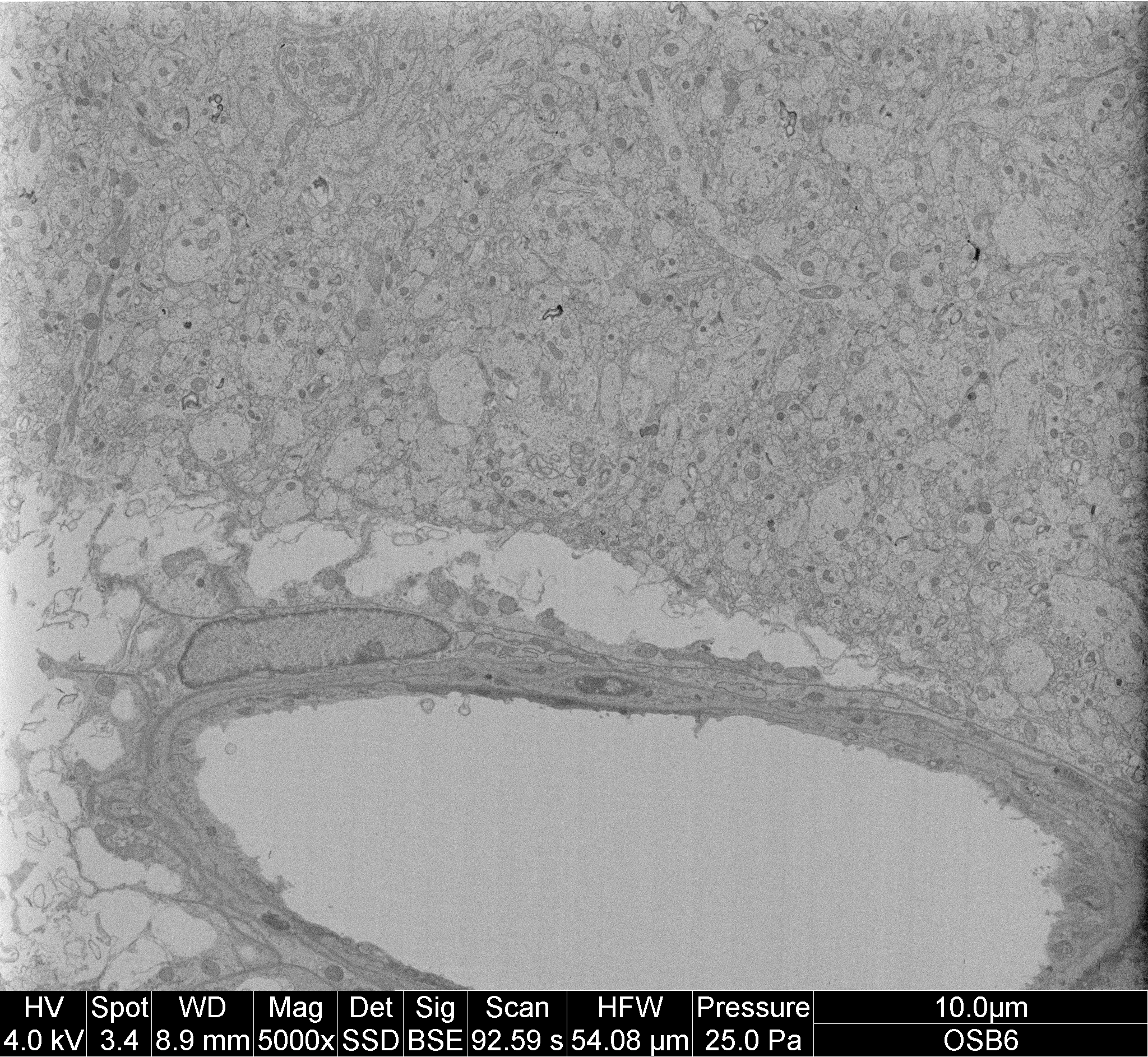

Supplement: Dataset S6 — (252.2 MB ZIP). [file pbio.0020329.sd006.zip › 040604_OS5_st1_581.tif]

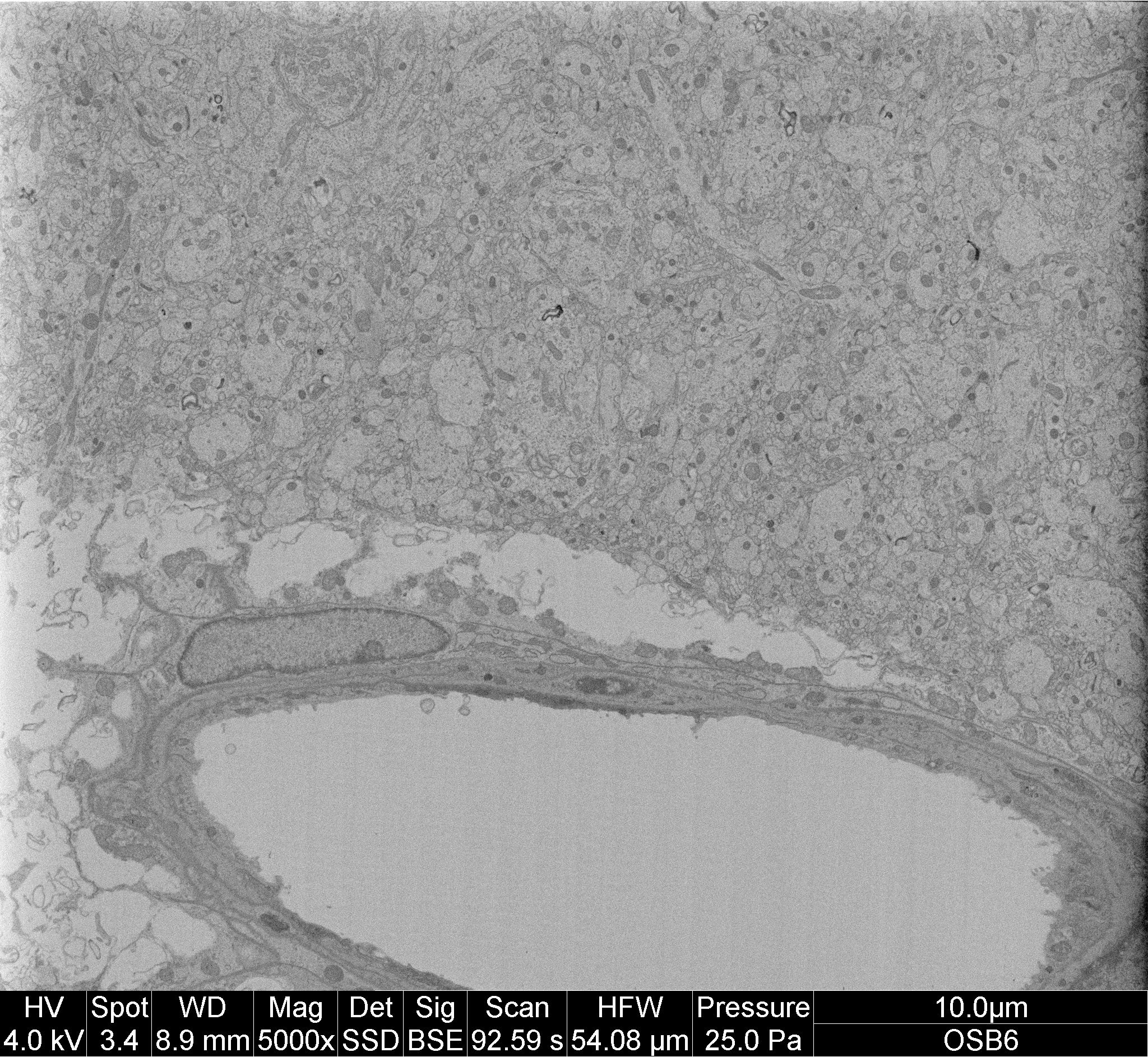

Supplement: Dataset S6 — (252.2 MB ZIP). [file pbio.0020329.sd006.zip › 040604_OS5_st1_582.tif]

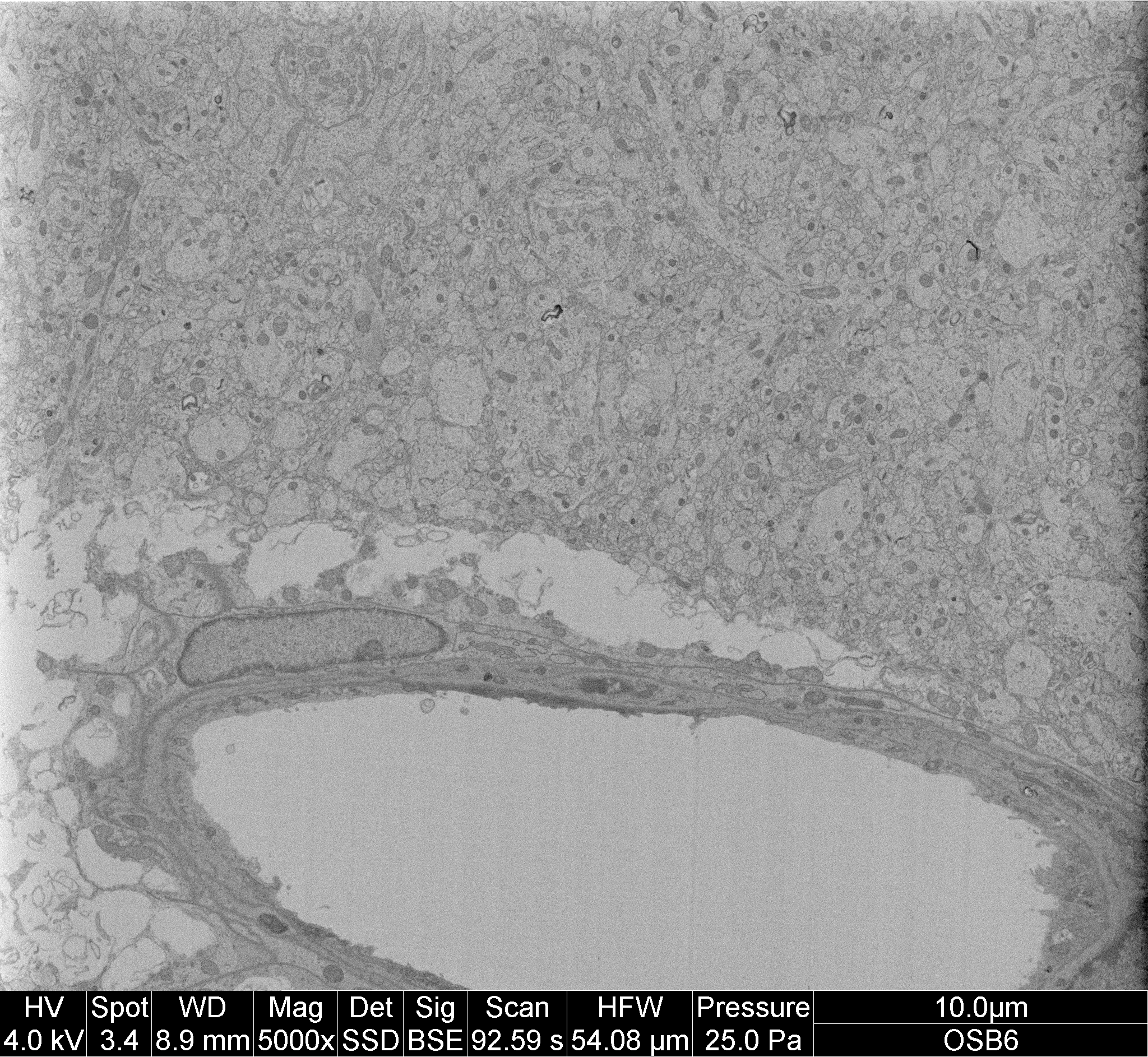

Supplement: Dataset S6 — (252.2 MB ZIP). [file pbio.0020329.sd006.zip › 040604_OS5_st1_583.tif]

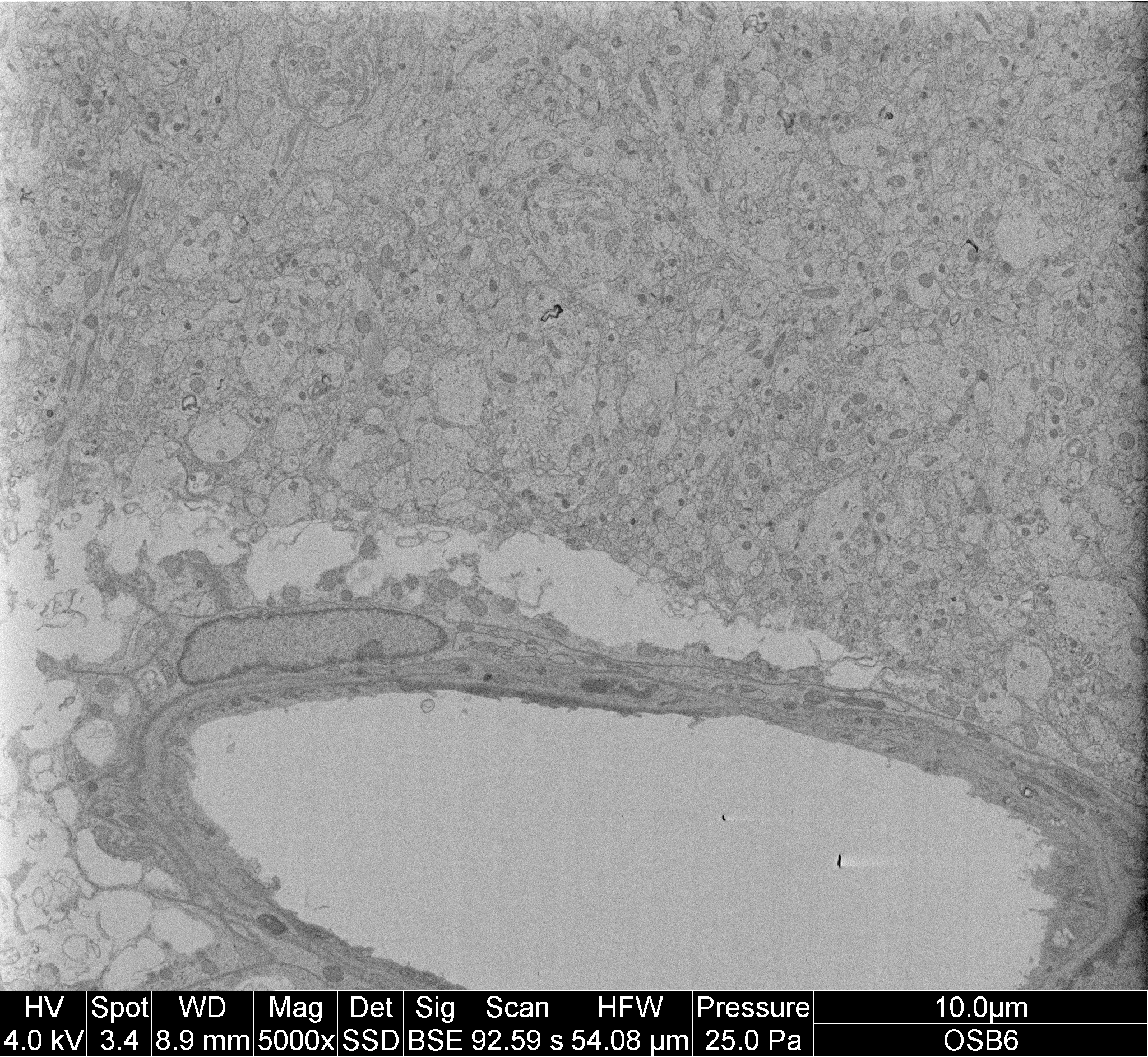

Supplement: Dataset S6 — (252.2 MB ZIP). [file pbio.0020329.sd006.zip › 040604_OS5_st1_584.tif]

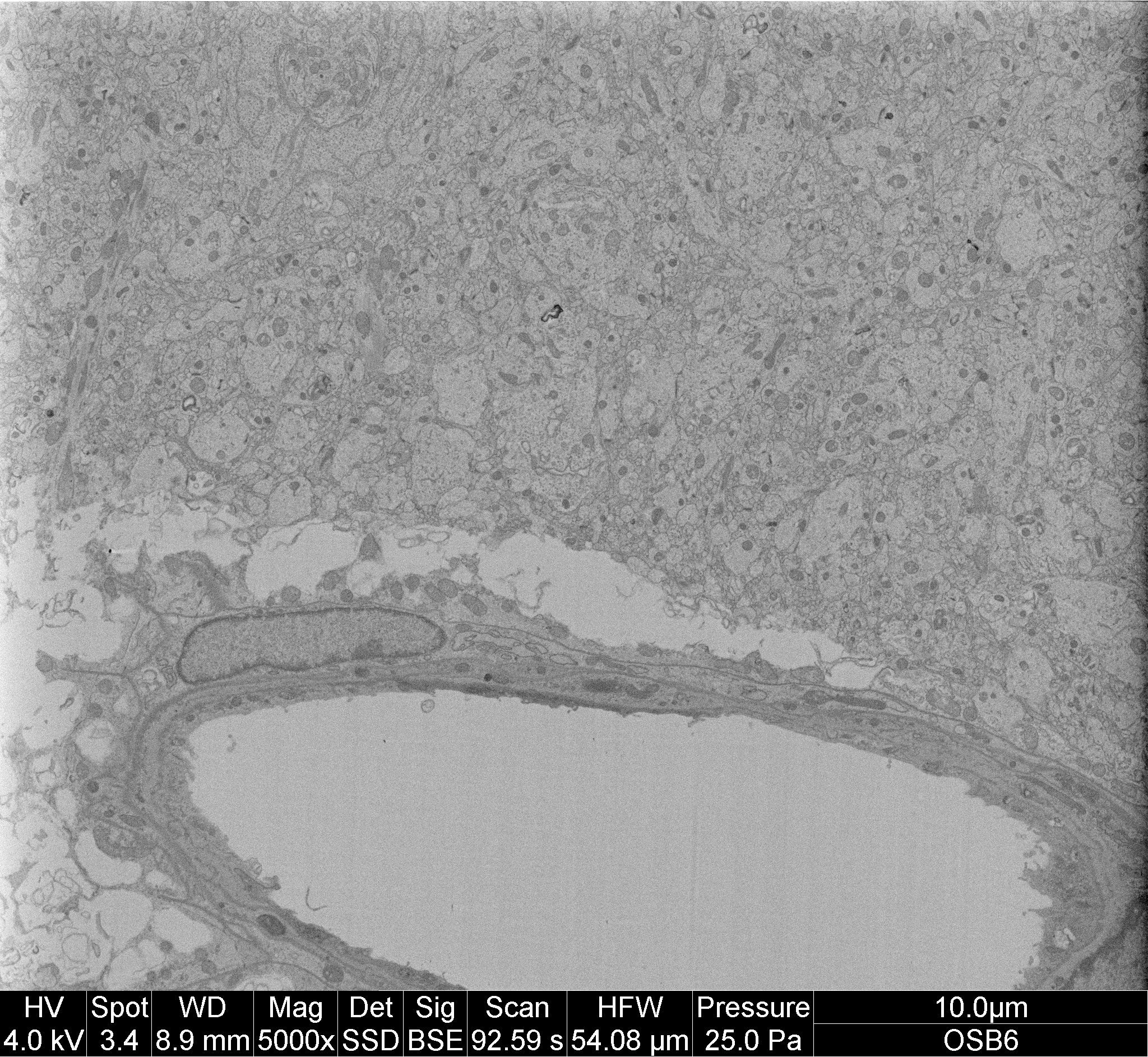

Supplement: Dataset S6 — (252.2 MB ZIP). [file pbio.0020329.sd006.zip › 040604_OS5_st1_585.tif]

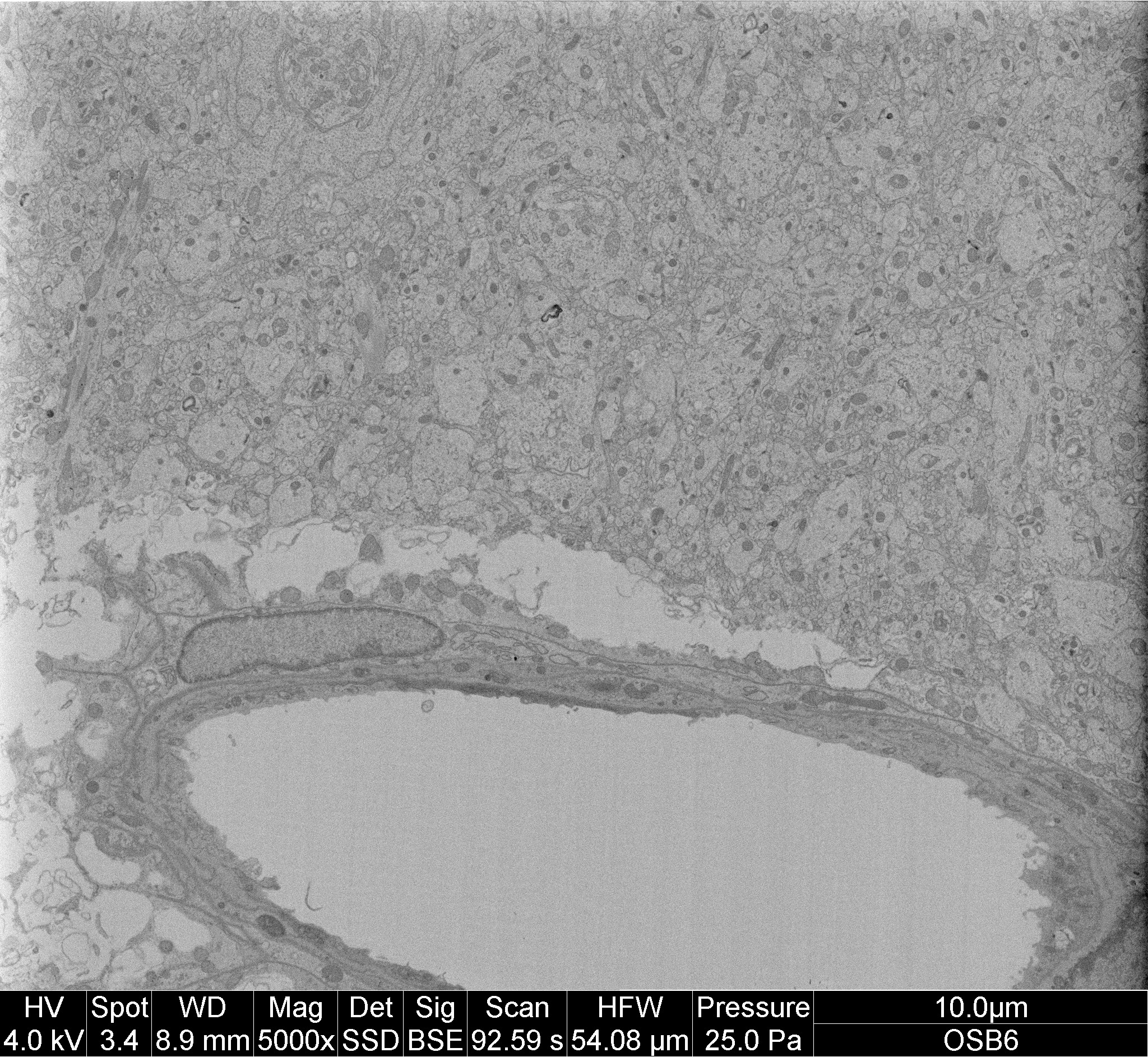

Supplement: Dataset S6 — (252.2 MB ZIP). [file pbio.0020329.sd006.zip › 040604_OS5_st1_586.tif]

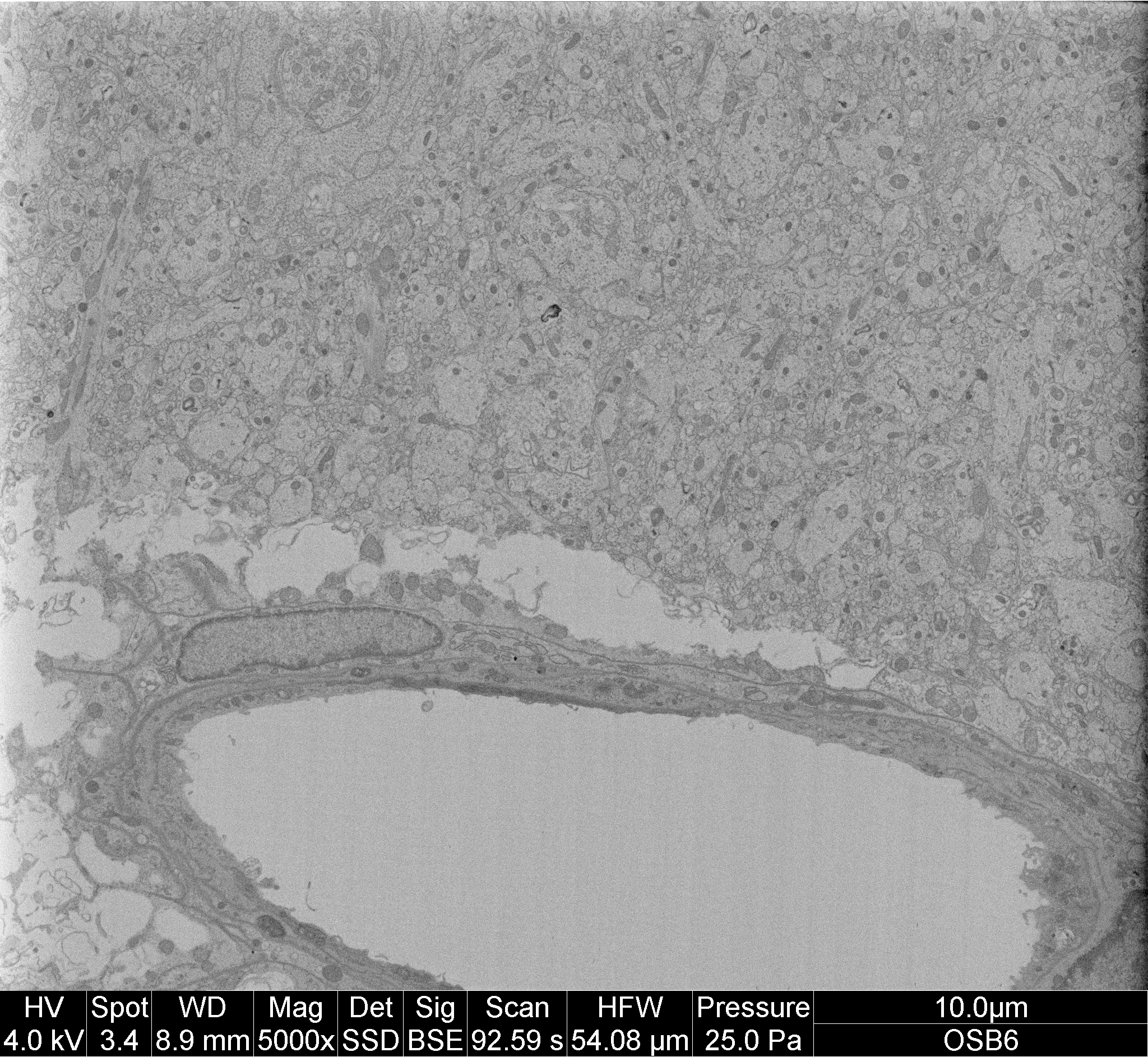

Supplement: Dataset S6 — (252.2 MB ZIP). [file pbio.0020329.sd006.zip › 040604_OS5_st1_587.tif]

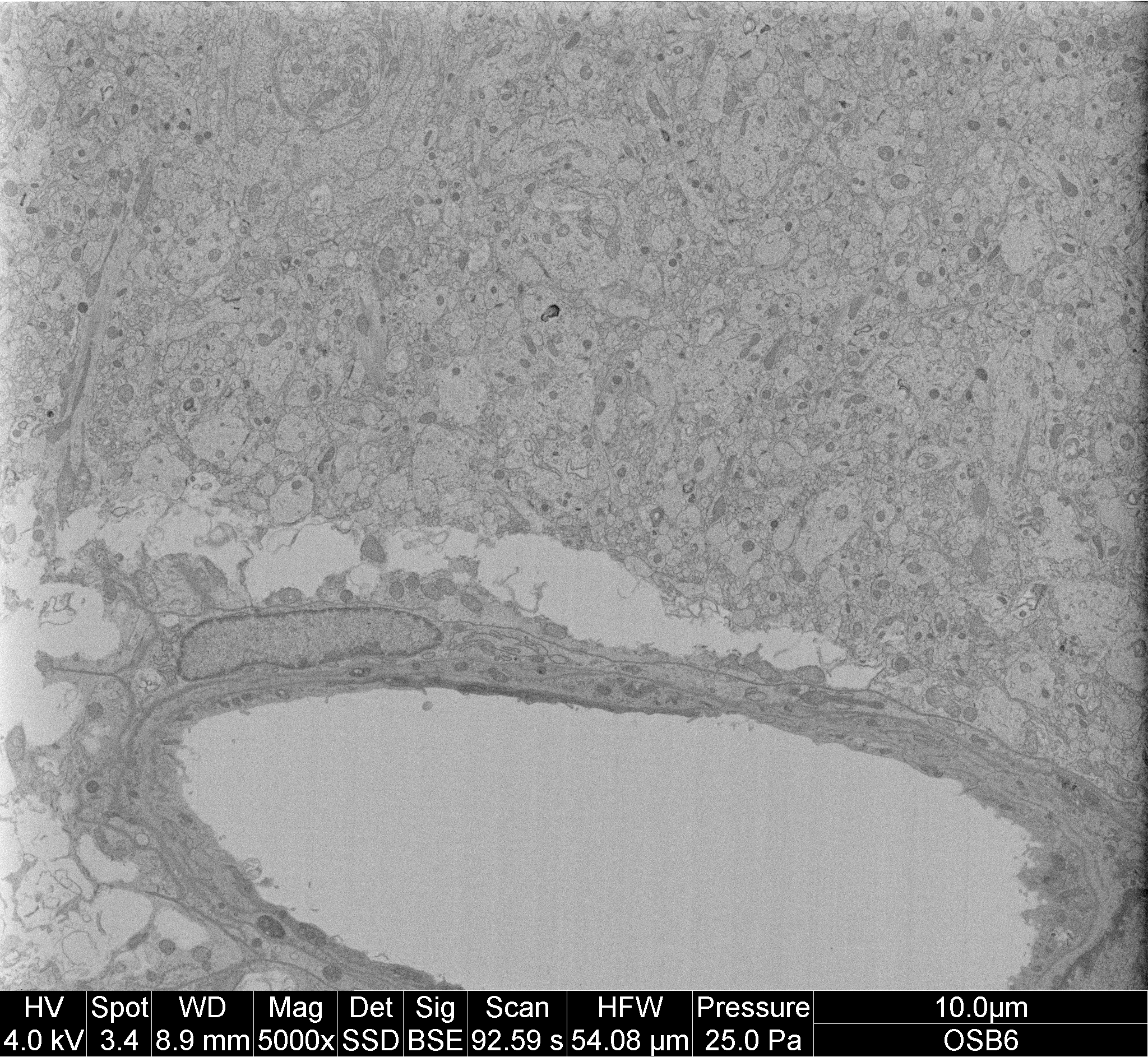

Supplement: Dataset S6 — (252.2 MB ZIP). [file pbio.0020329.sd006.zip › 040604_OS5_st1_588.tif]

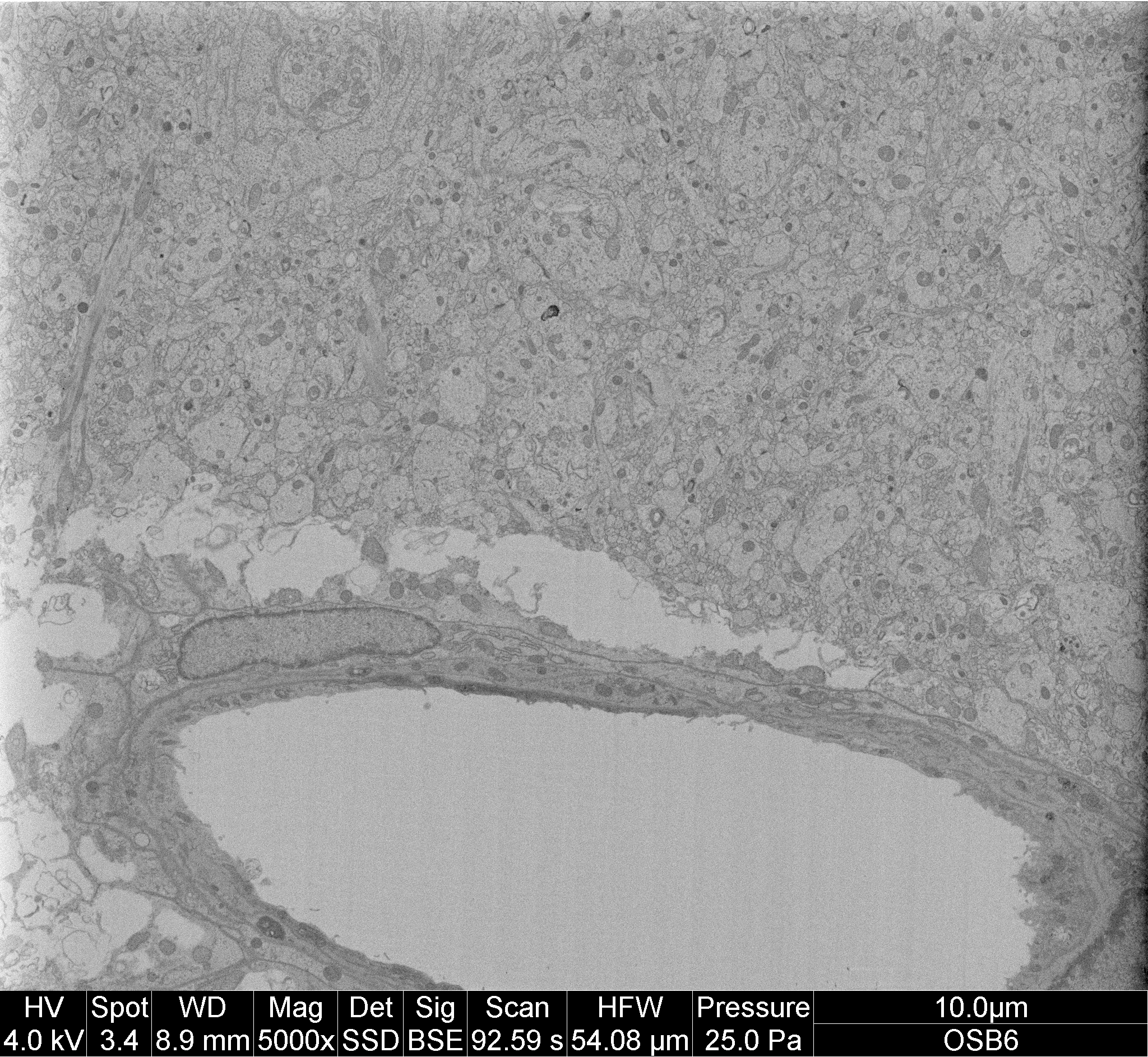

Supplement: Dataset S6 — (252.2 MB ZIP). [file pbio.0020329.sd006.zip › 040604_OS5_st1_589.tif]

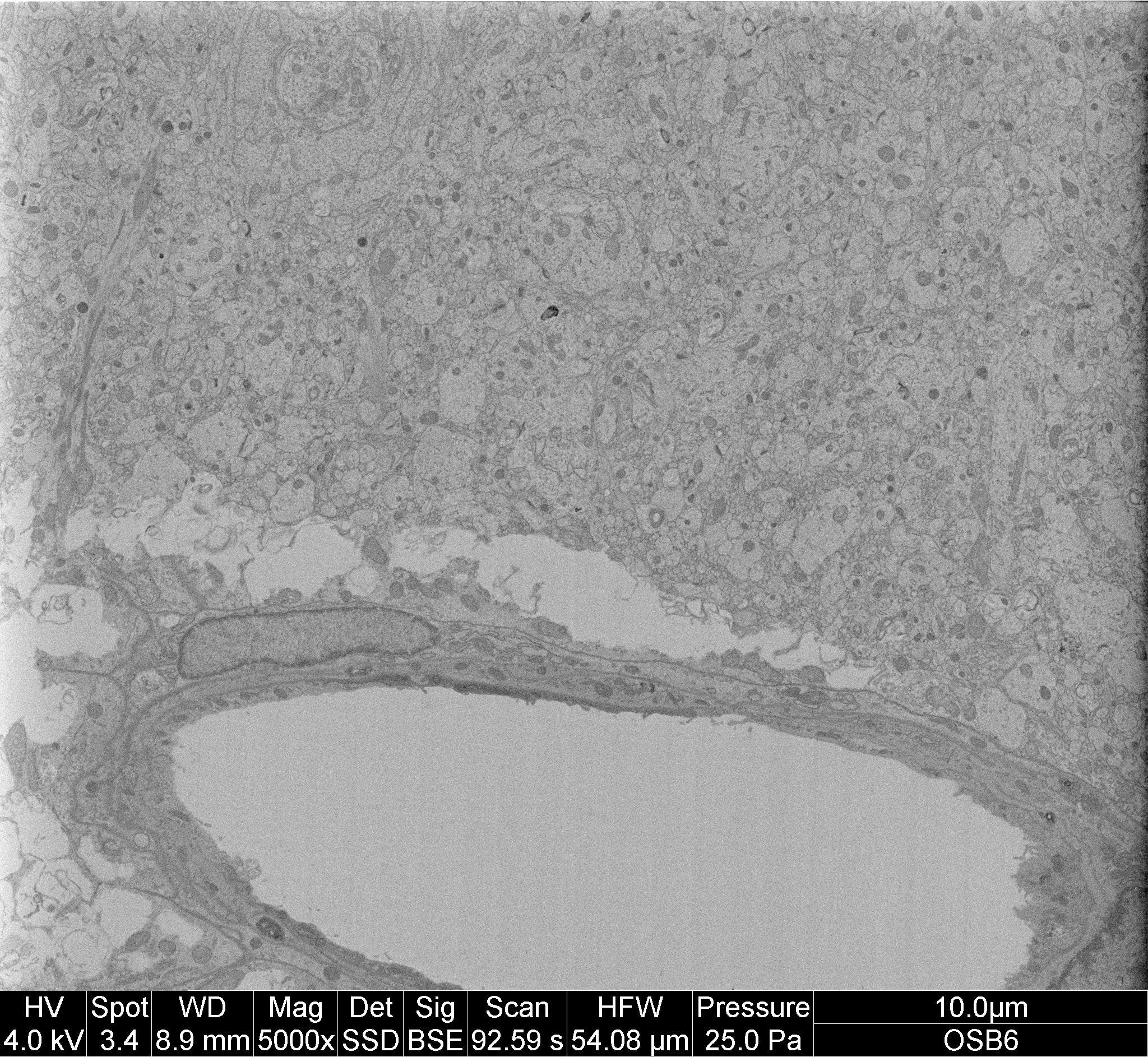

Supplement: Dataset S6 — (252.2 MB ZIP). [file pbio.0020329.sd006.zip › 040604_OS5_st1_590.tif]

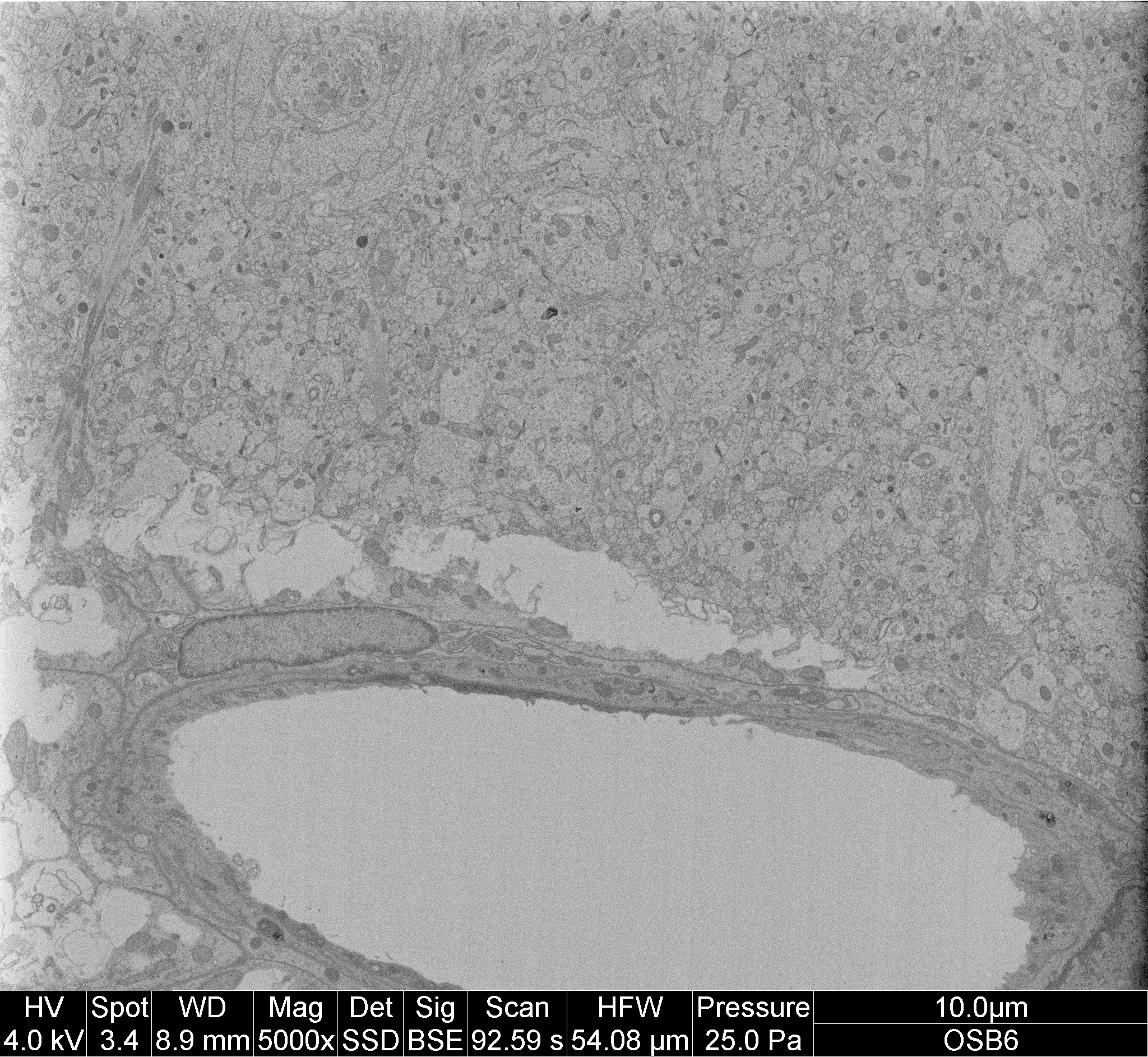

Supplement: Dataset S6 — (252.2 MB ZIP). [file pbio.0020329.sd006.zip › 040604_OS5_st1_591.tif]

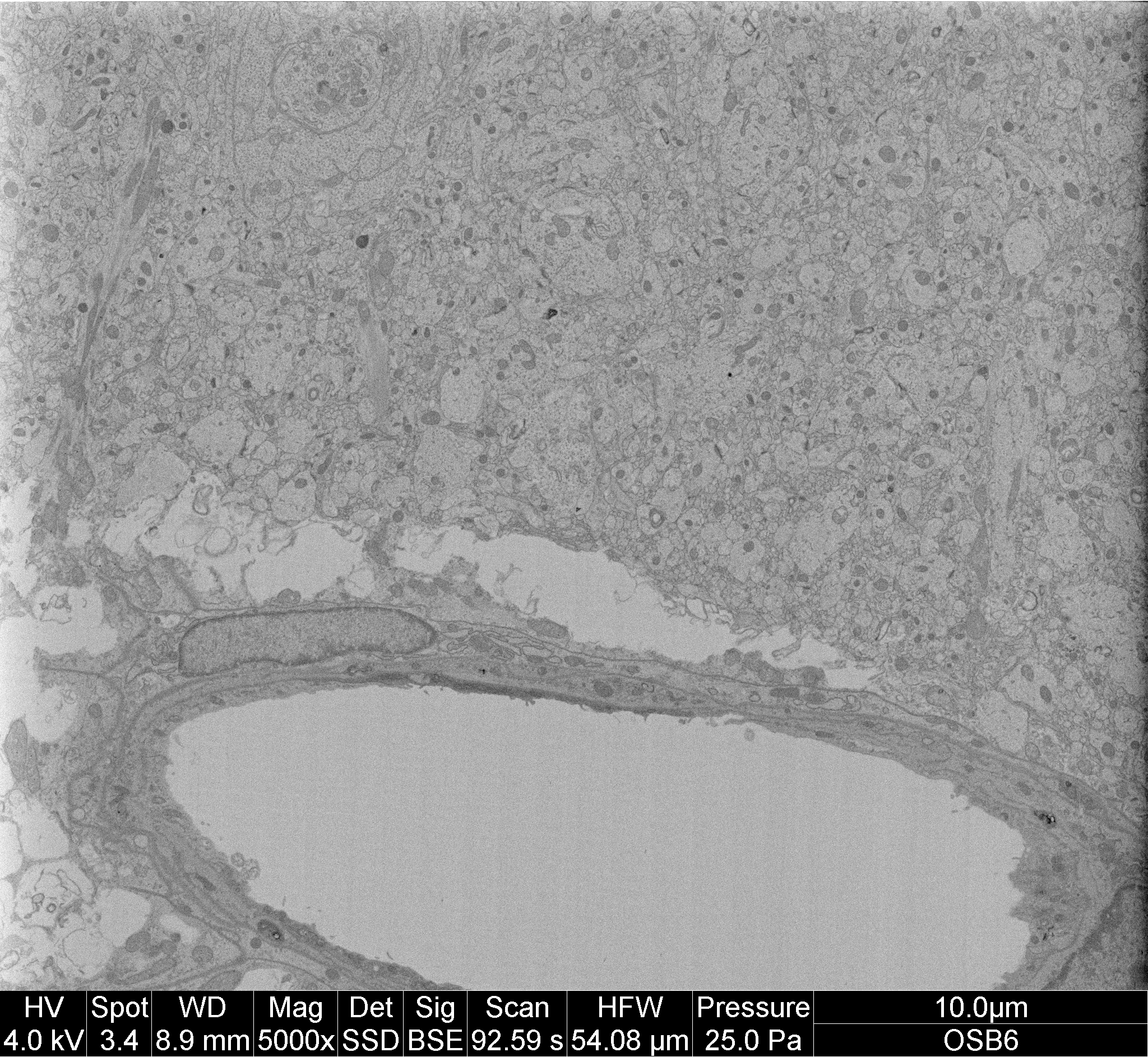

Supplement: Dataset S6 — (252.2 MB ZIP). [file pbio.0020329.sd006.zip › 040604_OS5_st1_592.tif]

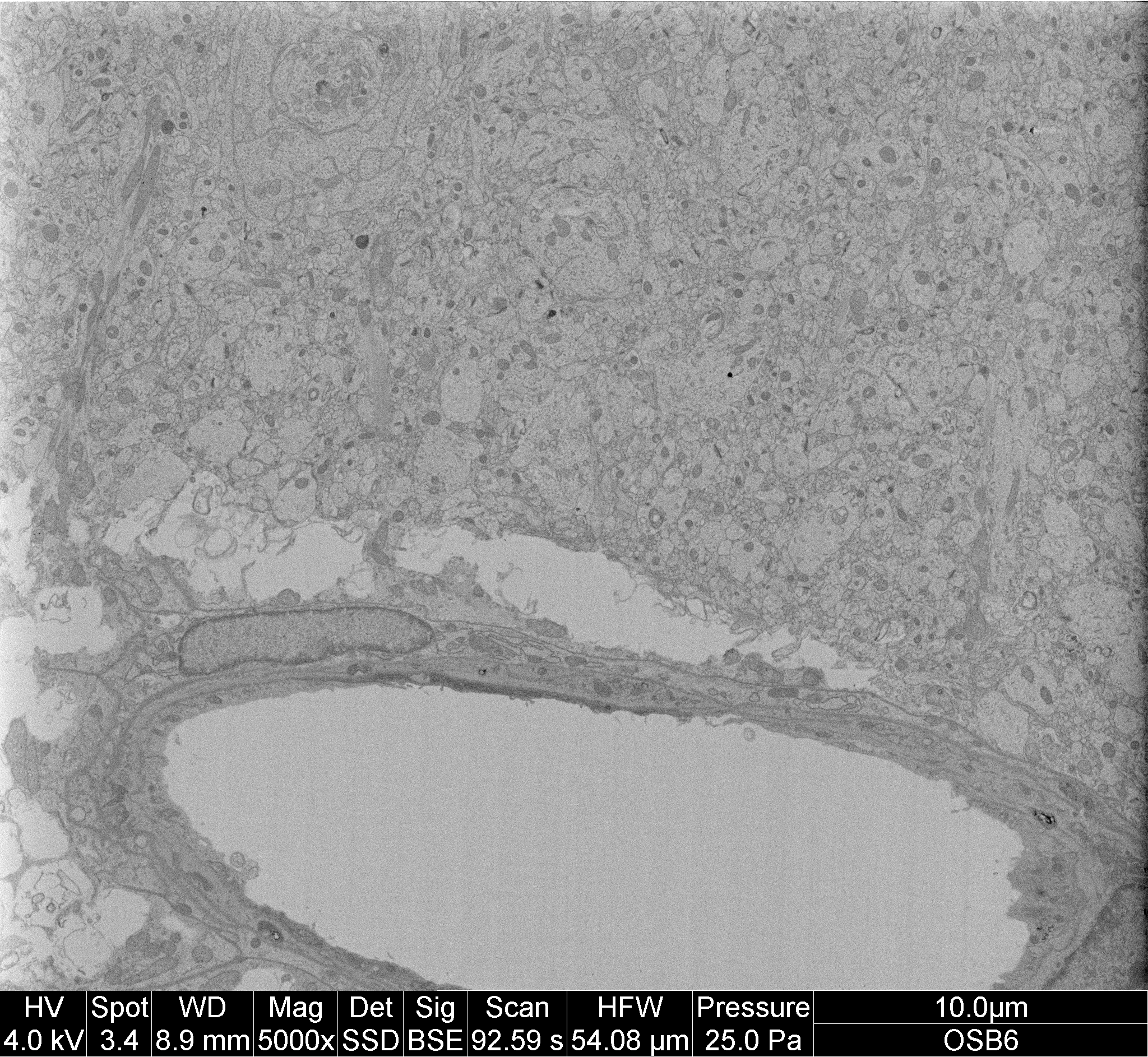

Supplement: Dataset S6 — (252.2 MB ZIP). [file pbio.0020329.sd006.zip › 040604_OS5_st1_593.tif]

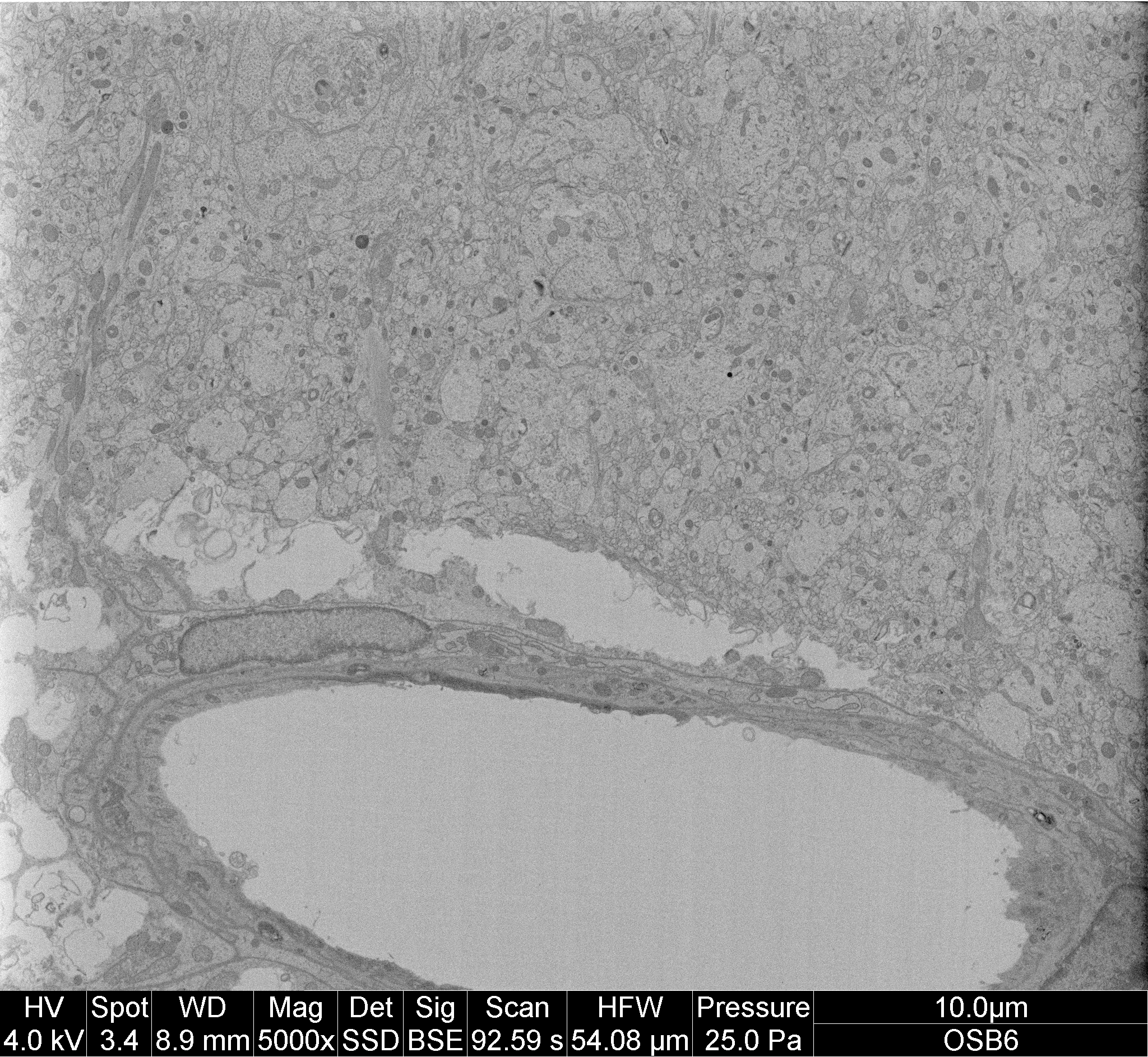

Supplement: Dataset S6 — (252.2 MB ZIP). [file pbio.0020329.sd006.zip › 040604_OS5_st1_594.tif]

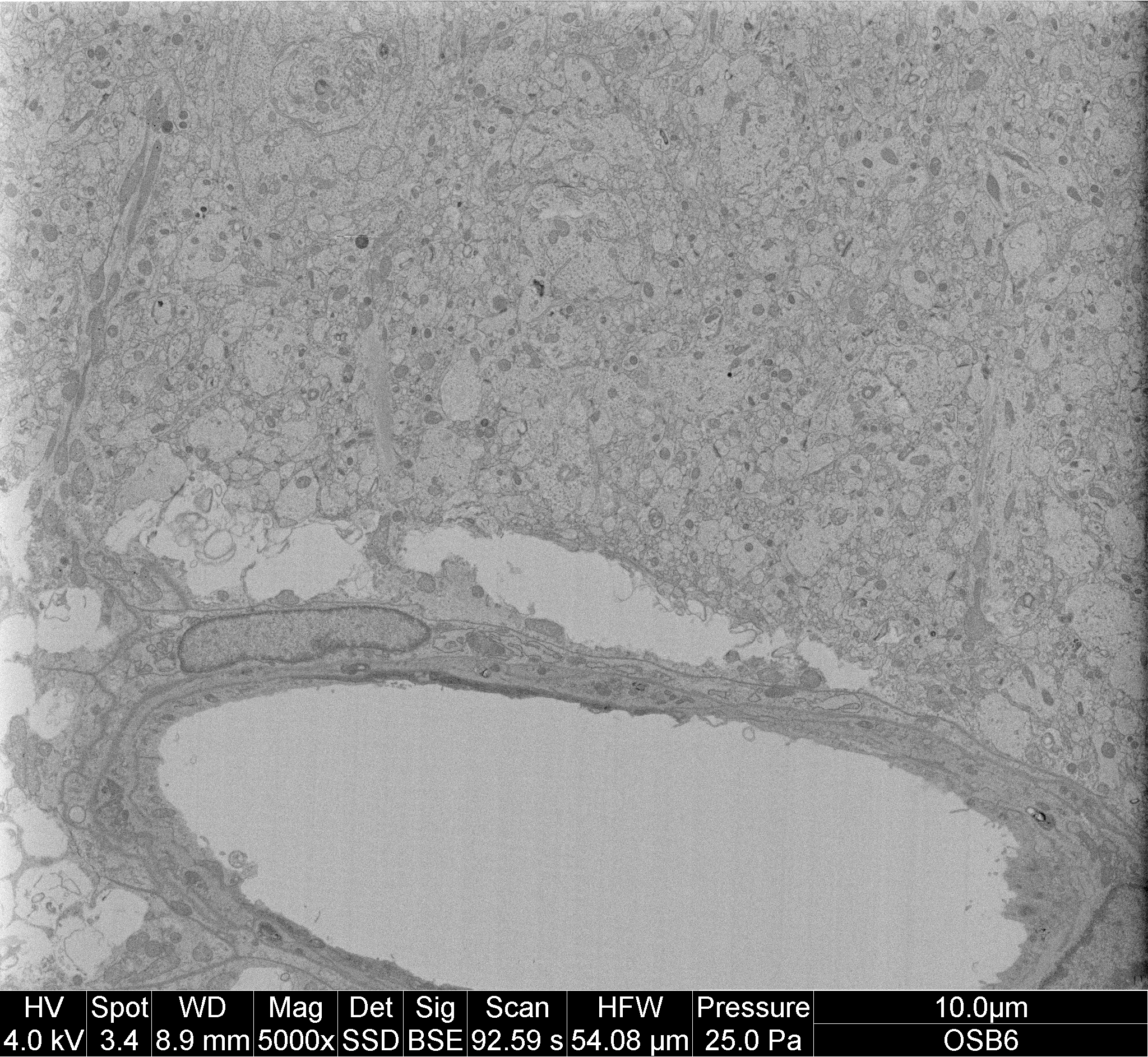

Supplement: Dataset S6 — (252.2 MB ZIP). [file pbio.0020329.sd006.zip › 040604_OS5_st1_595.tif]

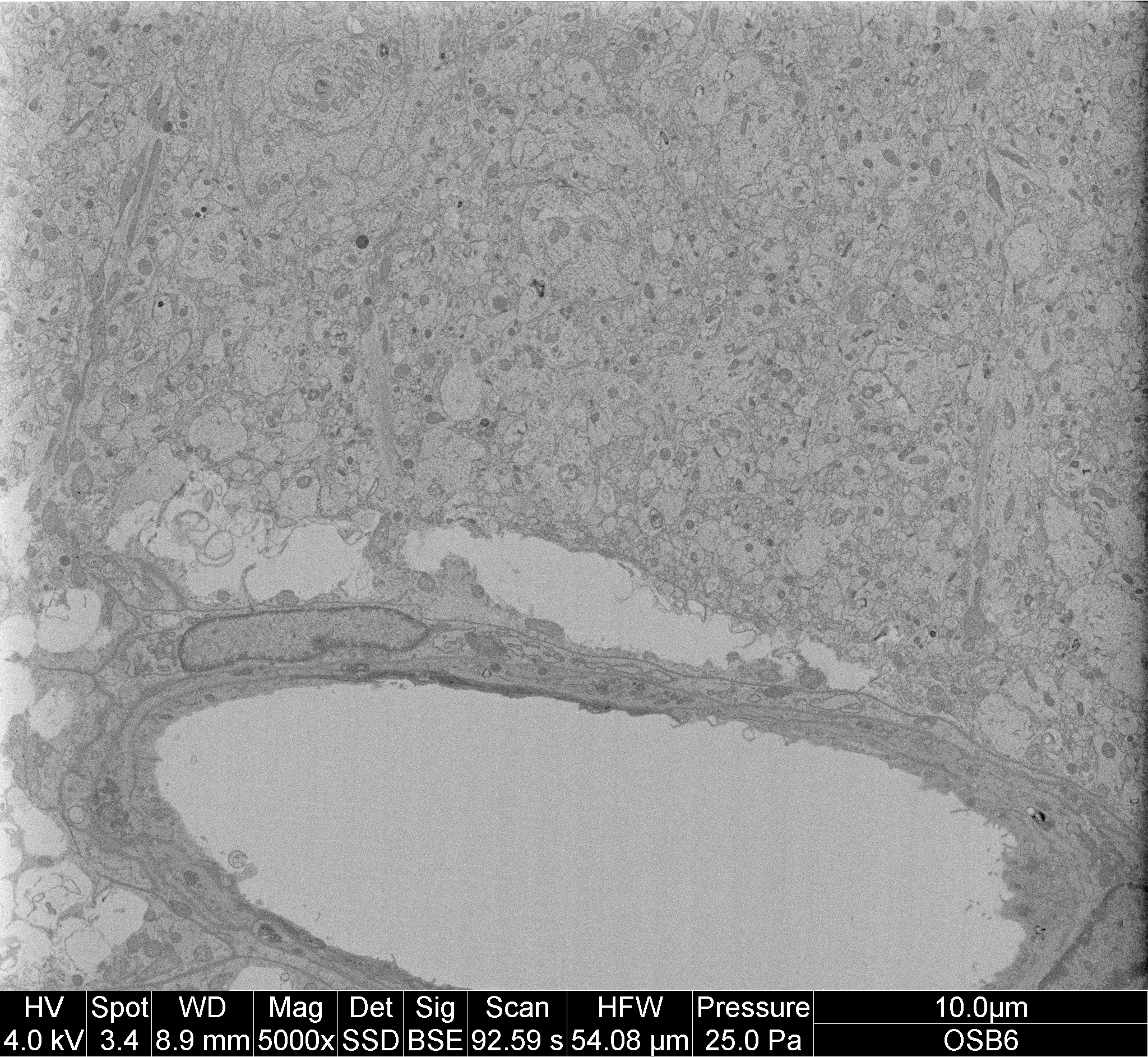

Supplement: Dataset S6 — (252.2 MB ZIP). [file pbio.0020329.sd006.zip › 040604_OS5_st1_596.tif]

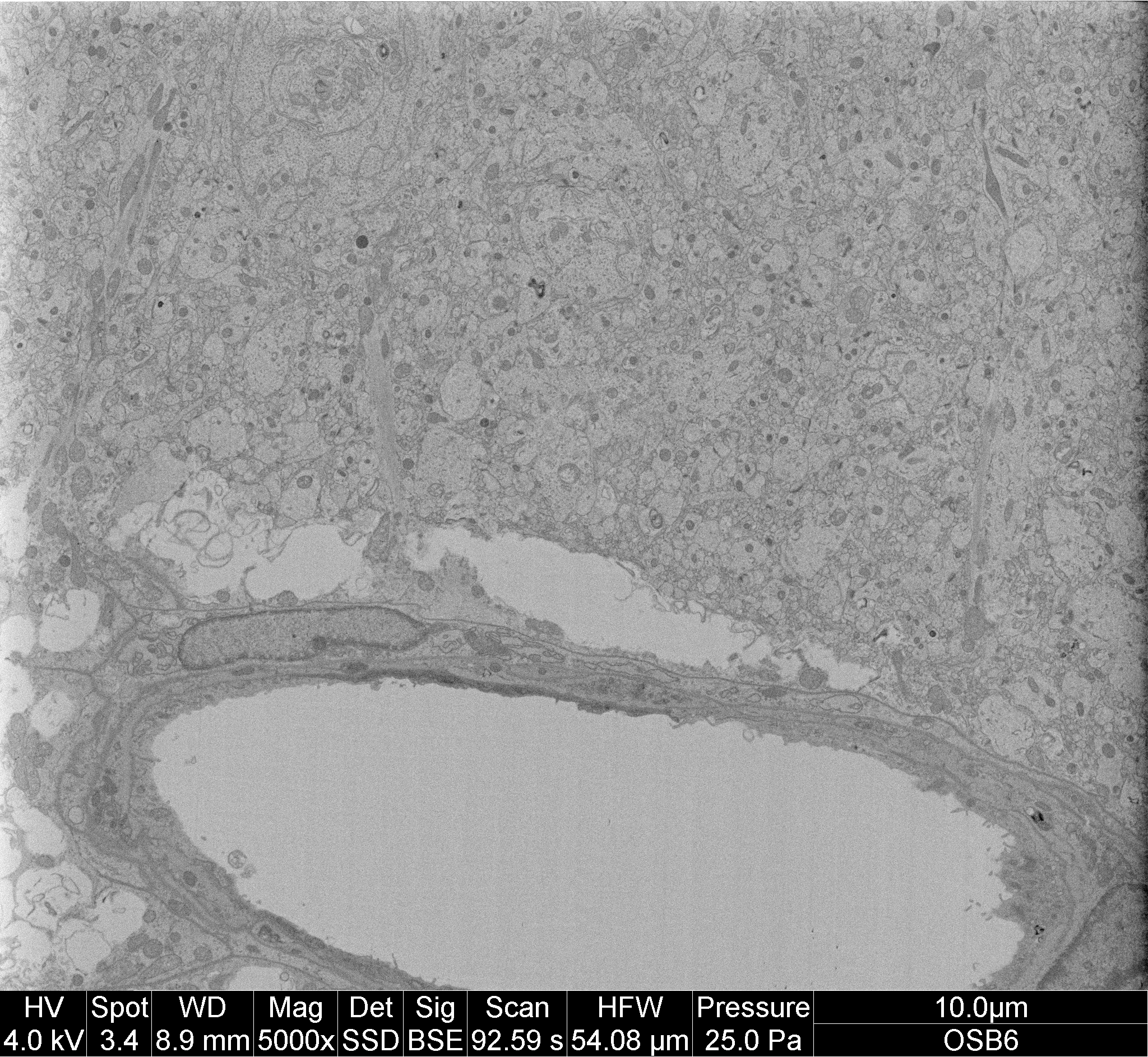

Supplement: Dataset S6 — (252.2 MB ZIP). [file pbio.0020329.sd006.zip › 040604_OS5_st1_597.tif]

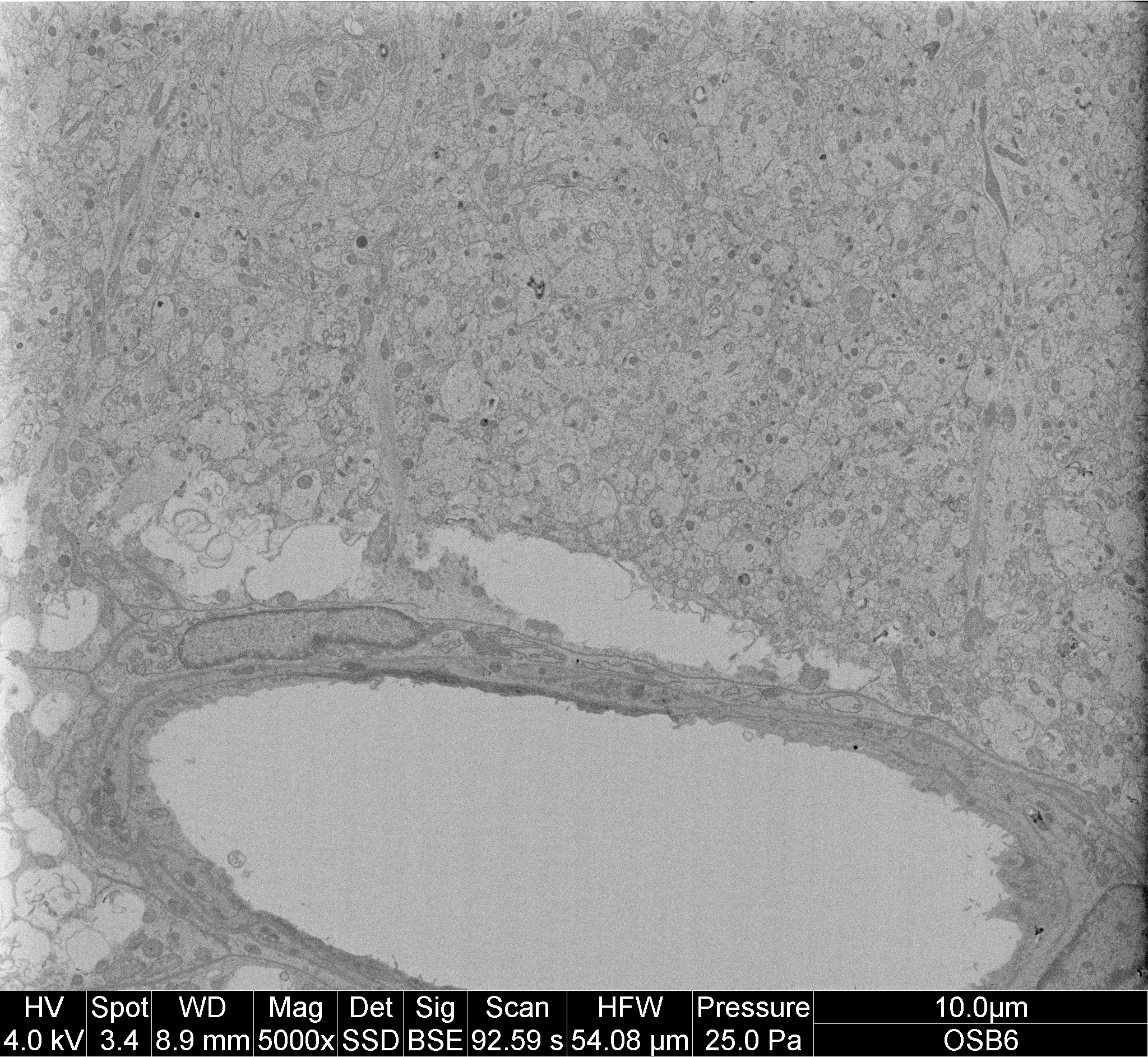

Supplement: Dataset S6 — (252.2 MB ZIP). [file pbio.0020329.sd006.zip › 040604_OS5_st1_598.tif]

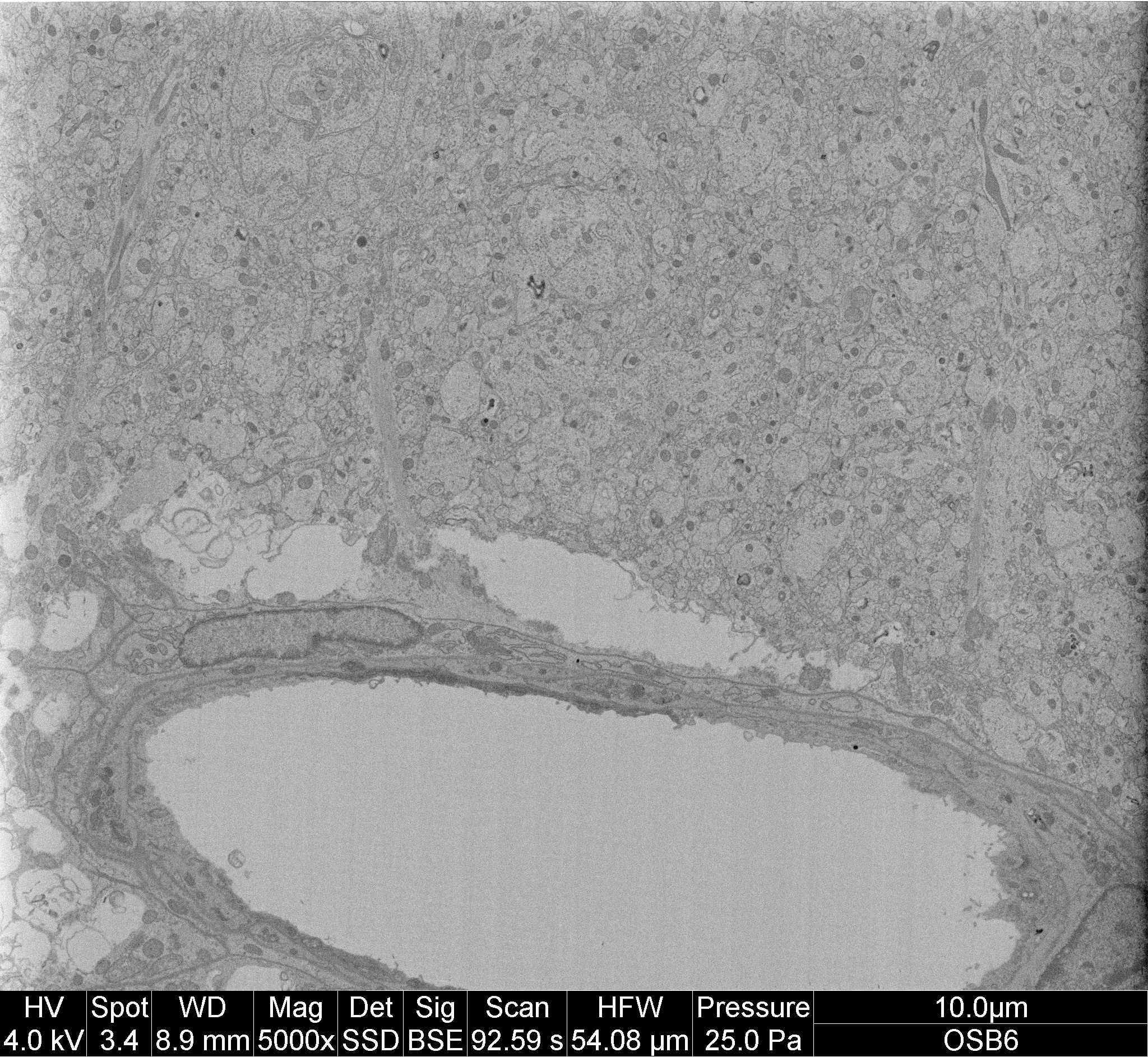

Supplement: Dataset S6 — (252.2 MB ZIP). [file pbio.0020329.sd006.zip › 040604_OS5_st1_599.tif]

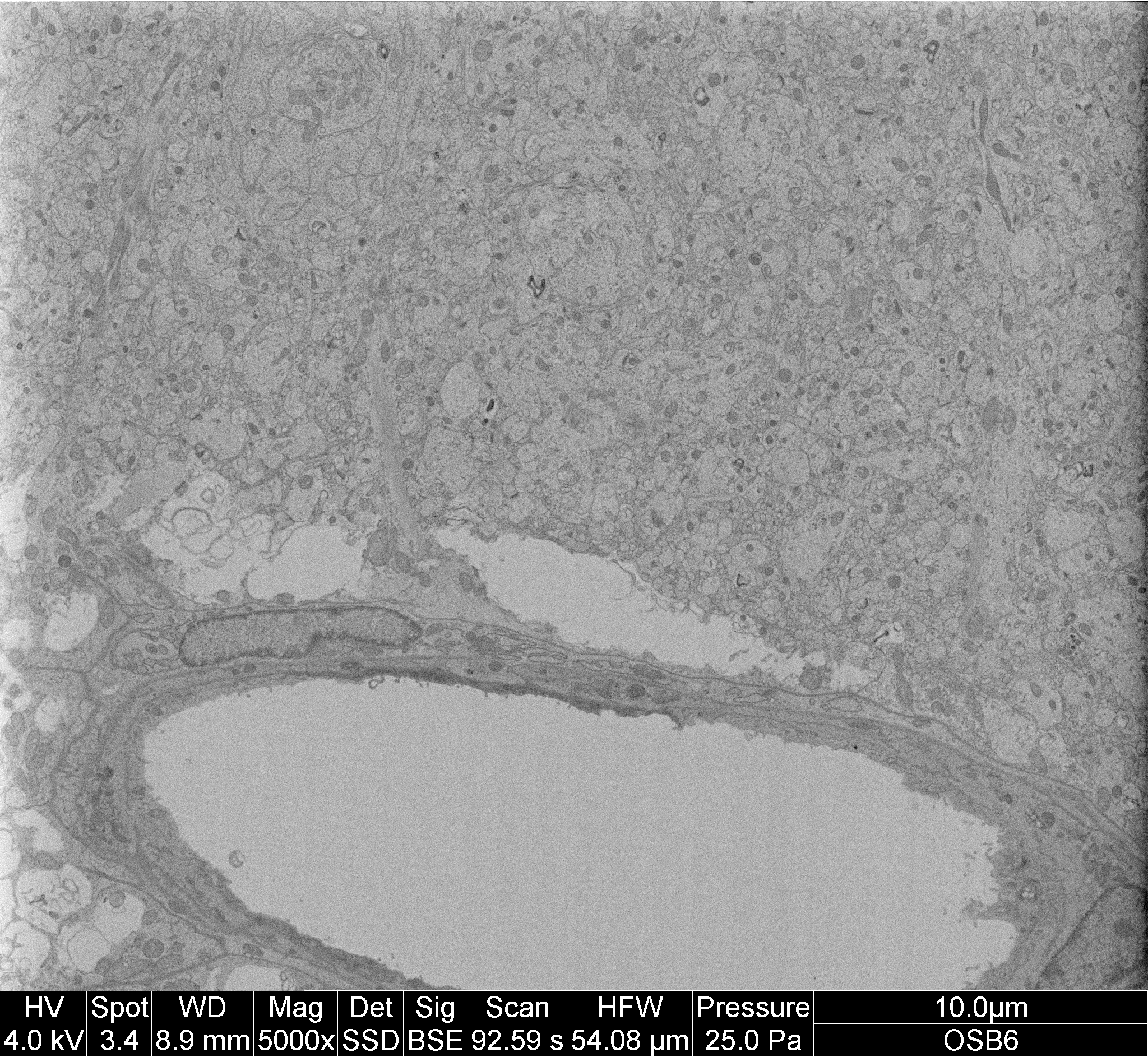

Supplement: Dataset S7 — (253.7 MB ZIP). [file pbio.0020329.sd007.zip › 040604_OS5_st1_600.tif]
