# Supplementary figures and images for: Serial Block-Face Scanning Electron Microscopy to Reconstruct Three-Dimensional Tissue Nanostructure (part 7 of 21)
Source: PLoS Biol. 2004 Oct 19;2(11):e329. doi: 10.1371/journal.pbio.0020329 (PMC524270; doi:10.1371/journal.pbio.0020329)

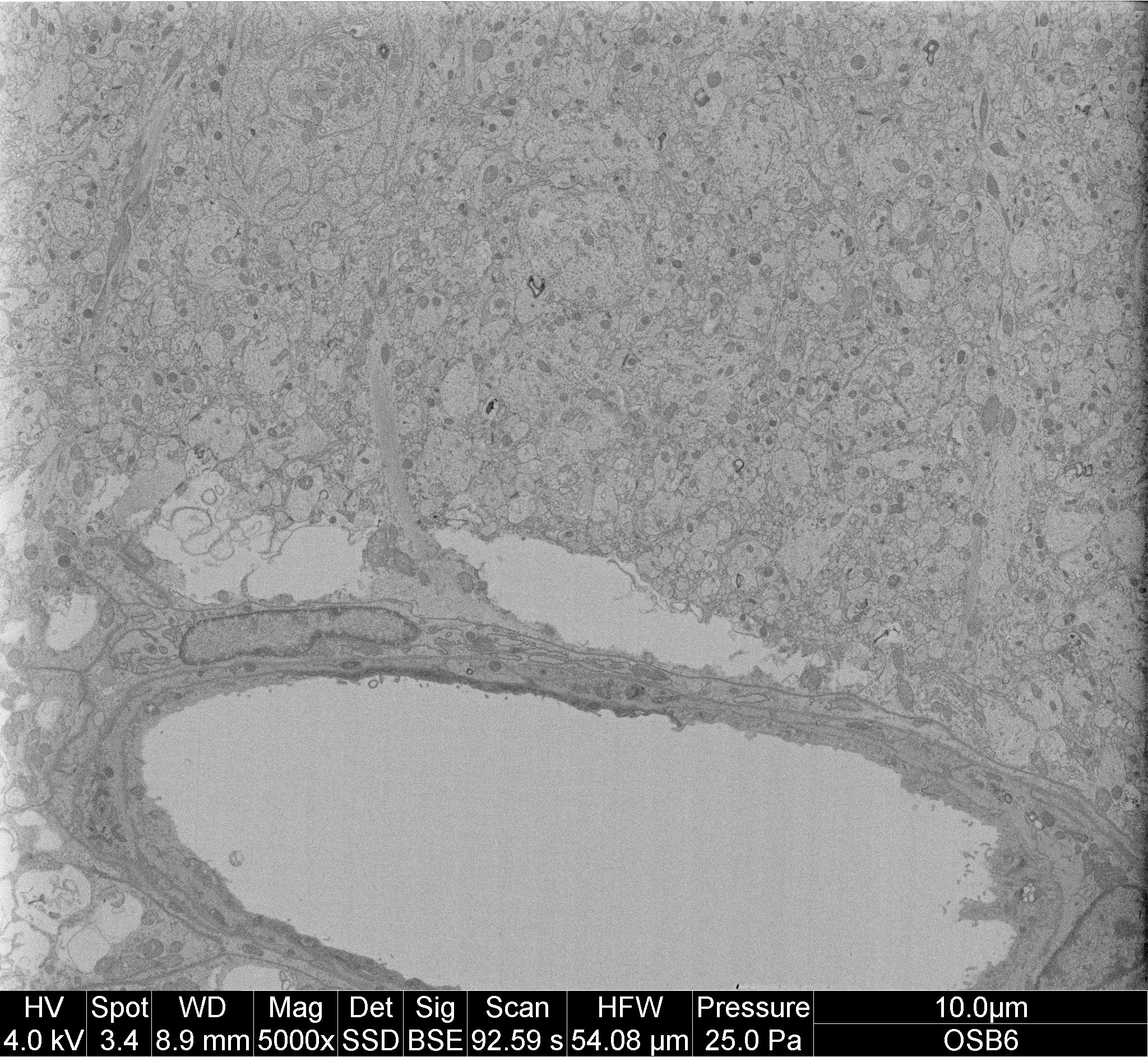

Supplement: Dataset S7 — (253.7 MB ZIP). [file pbio.0020329.sd007.zip › 040604_OS5_st1_601.tif]

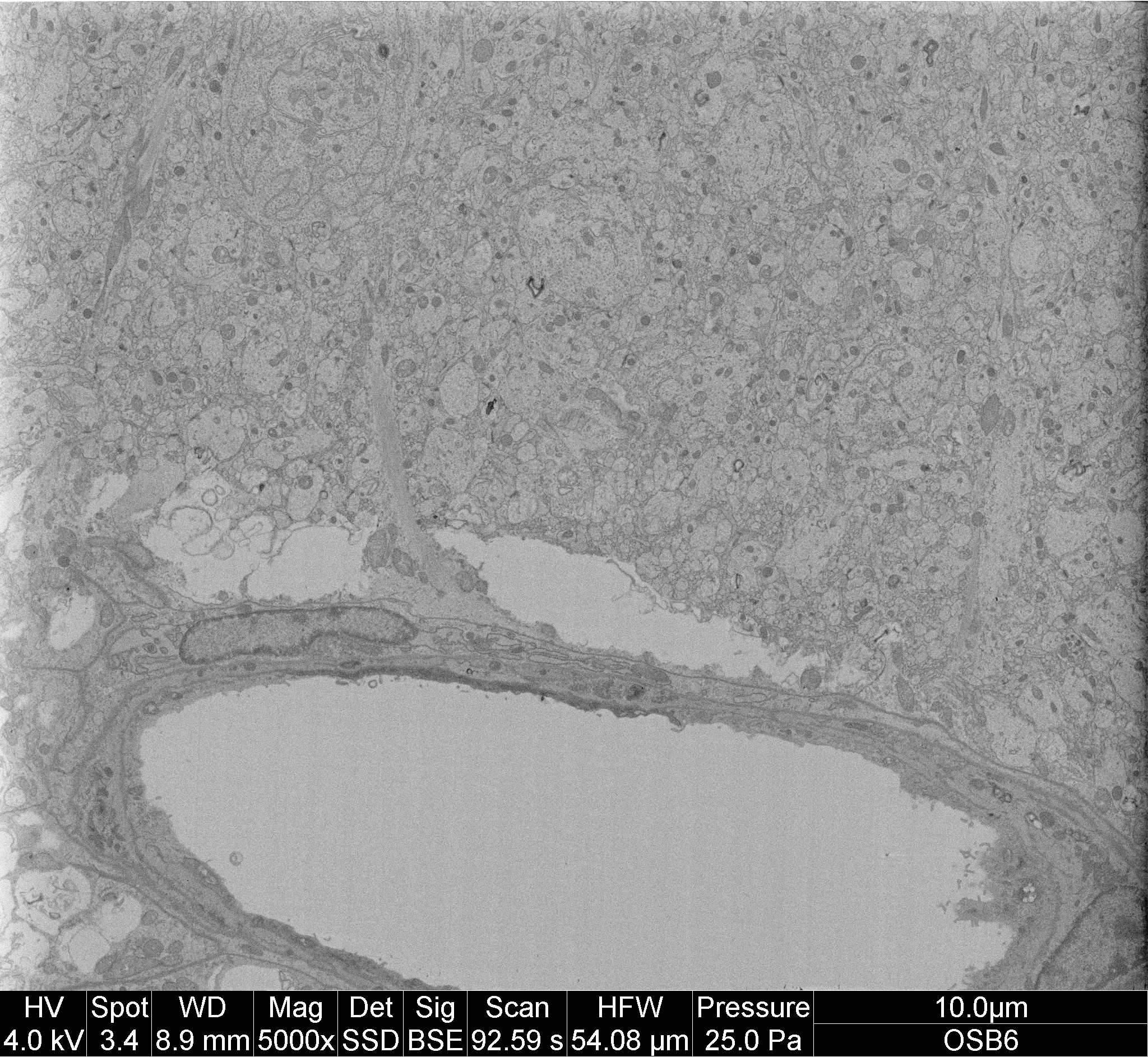

Supplement: Dataset S7 — (253.7 MB ZIP). [file pbio.0020329.sd007.zip › 040604_OS5_st1_602.tif]

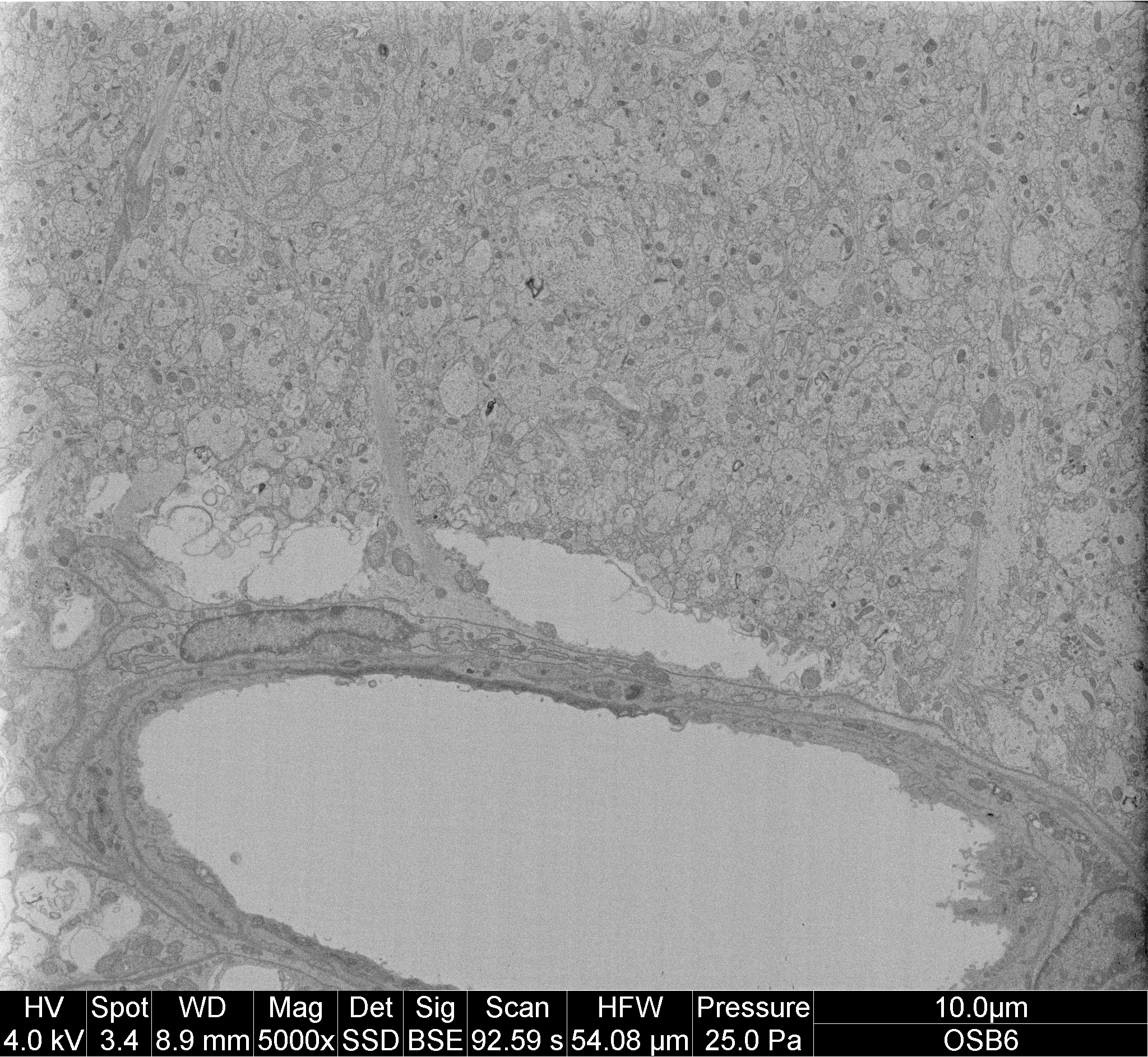

Supplement: Dataset S7 — (253.7 MB ZIP). [file pbio.0020329.sd007.zip › 040604_OS5_st1_603.tif]

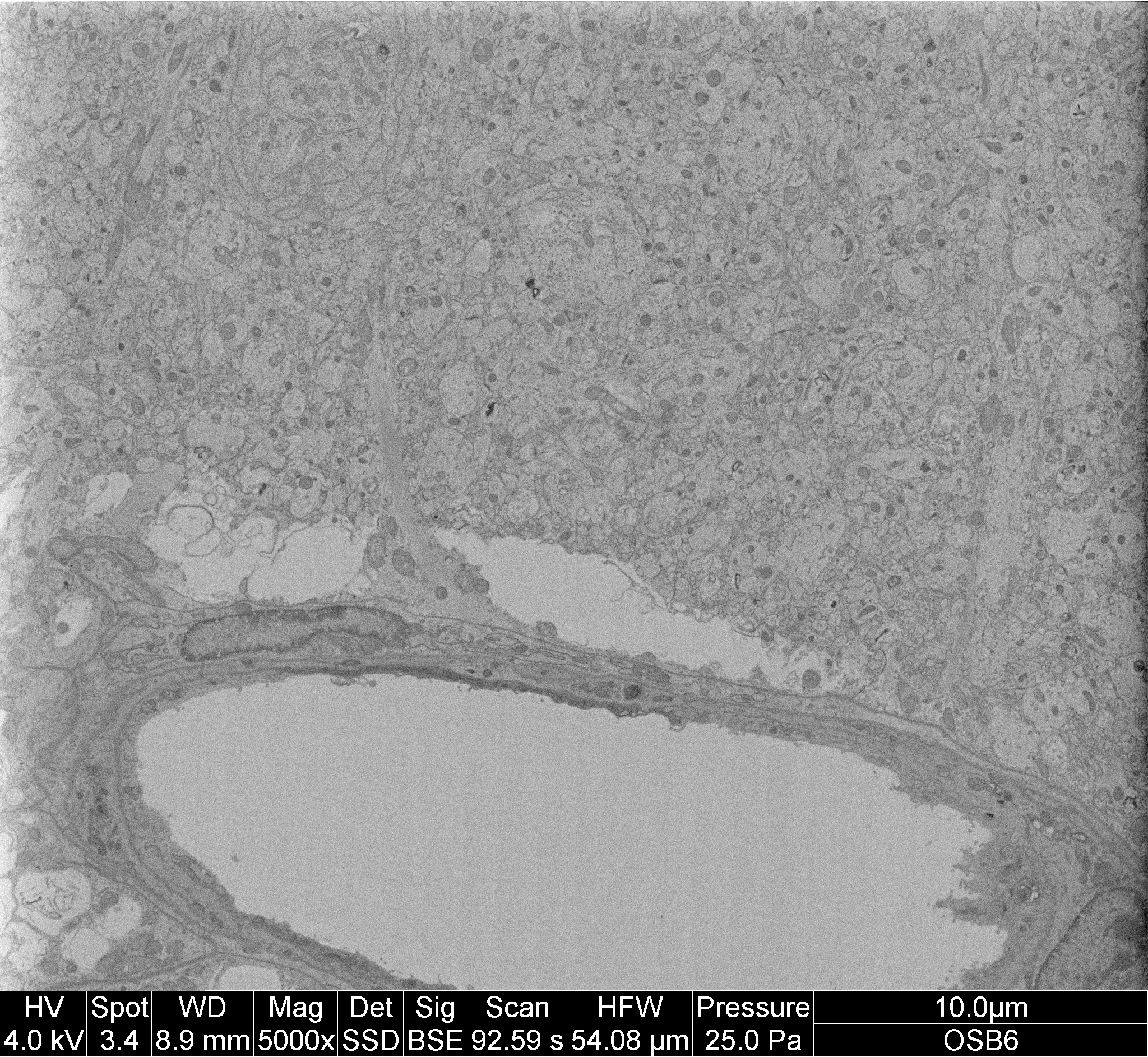

Supplement: Dataset S7 — (253.7 MB ZIP). [file pbio.0020329.sd007.zip › 040604_OS5_st1_604.tif]

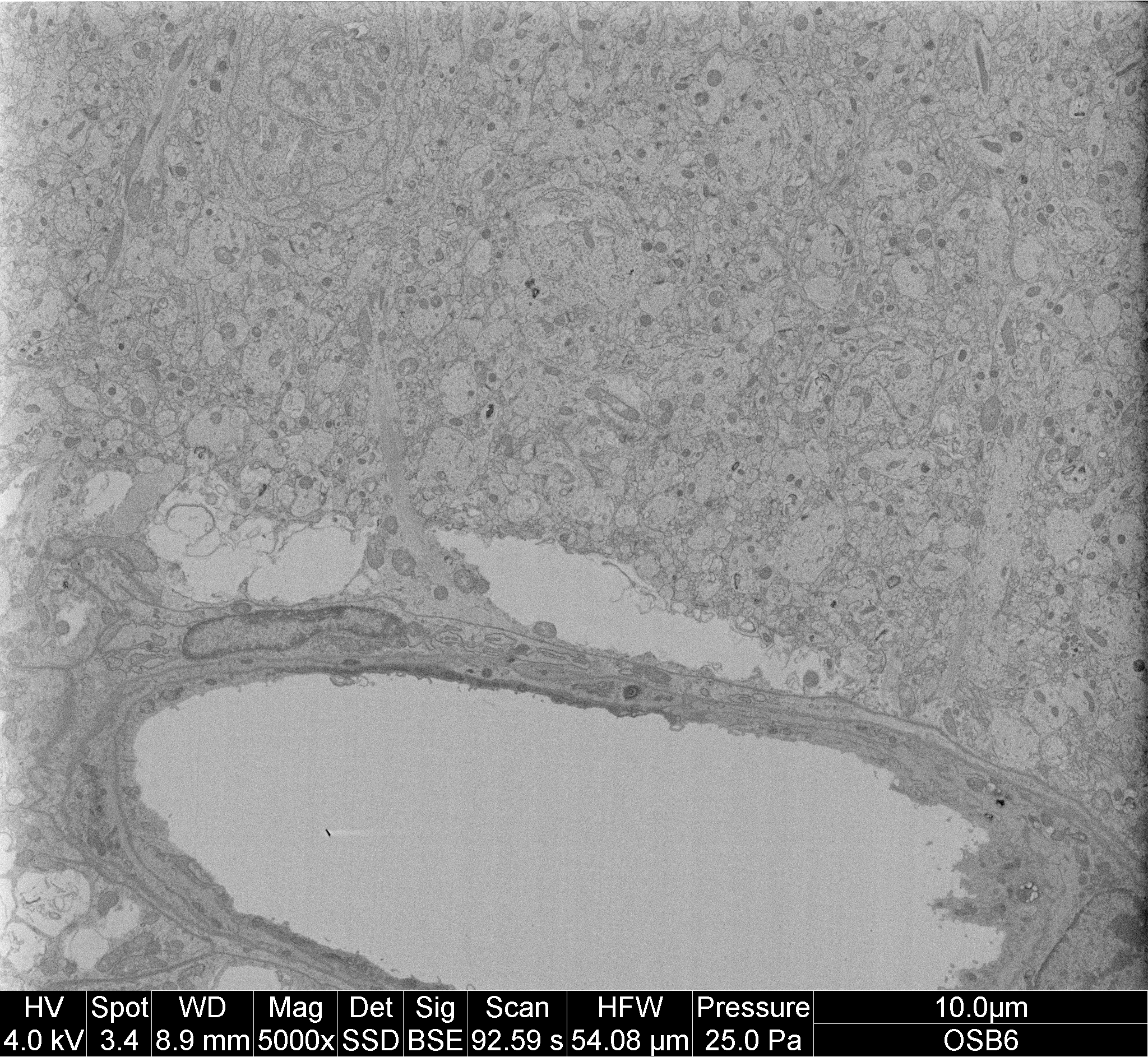

Supplement: Dataset S7 — (253.7 MB ZIP). [file pbio.0020329.sd007.zip › 040604_OS5_st1_605.tif]

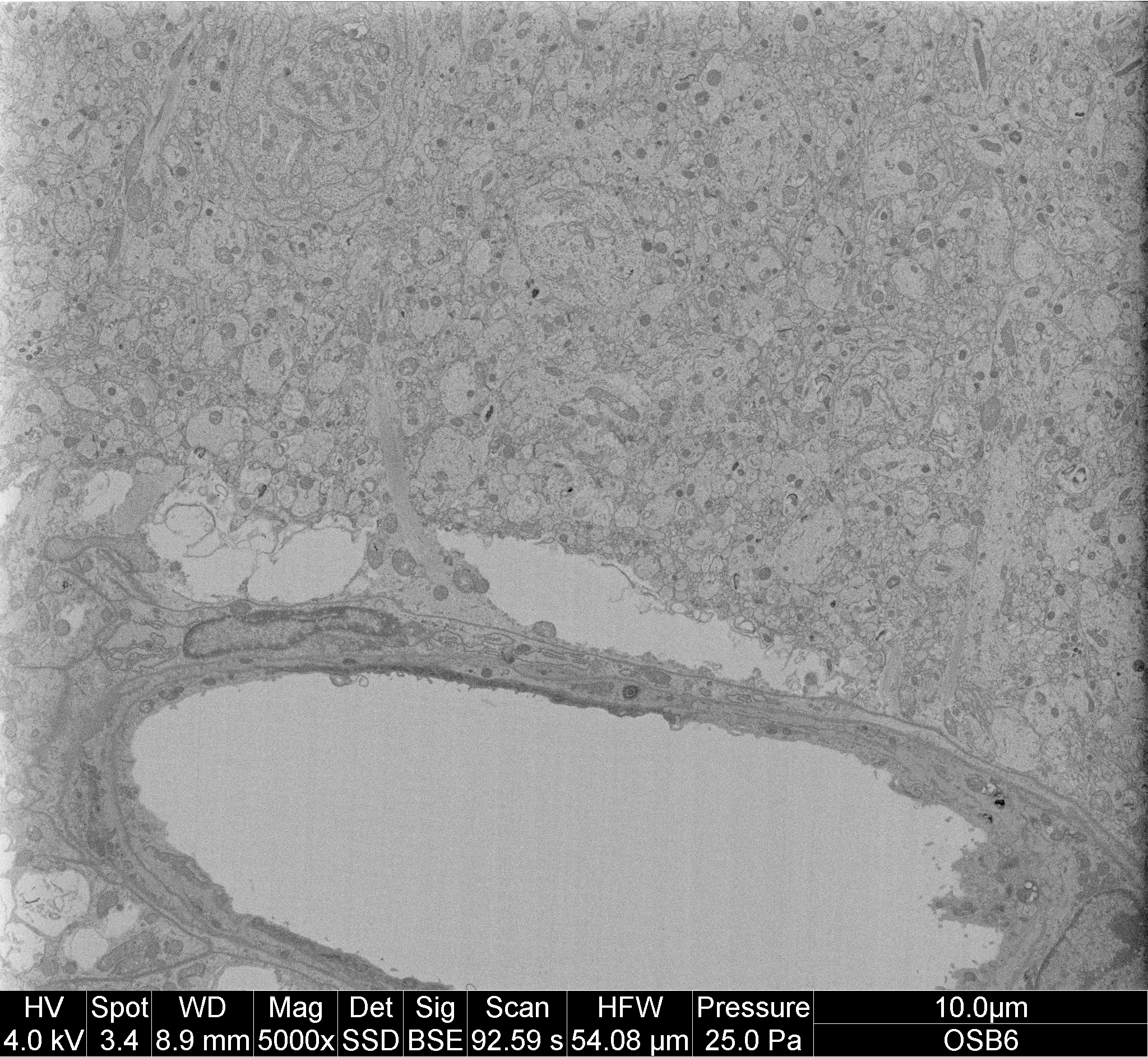

Supplement: Dataset S7 — (253.7 MB ZIP). [file pbio.0020329.sd007.zip › 040604_OS5_st1_606.tif]

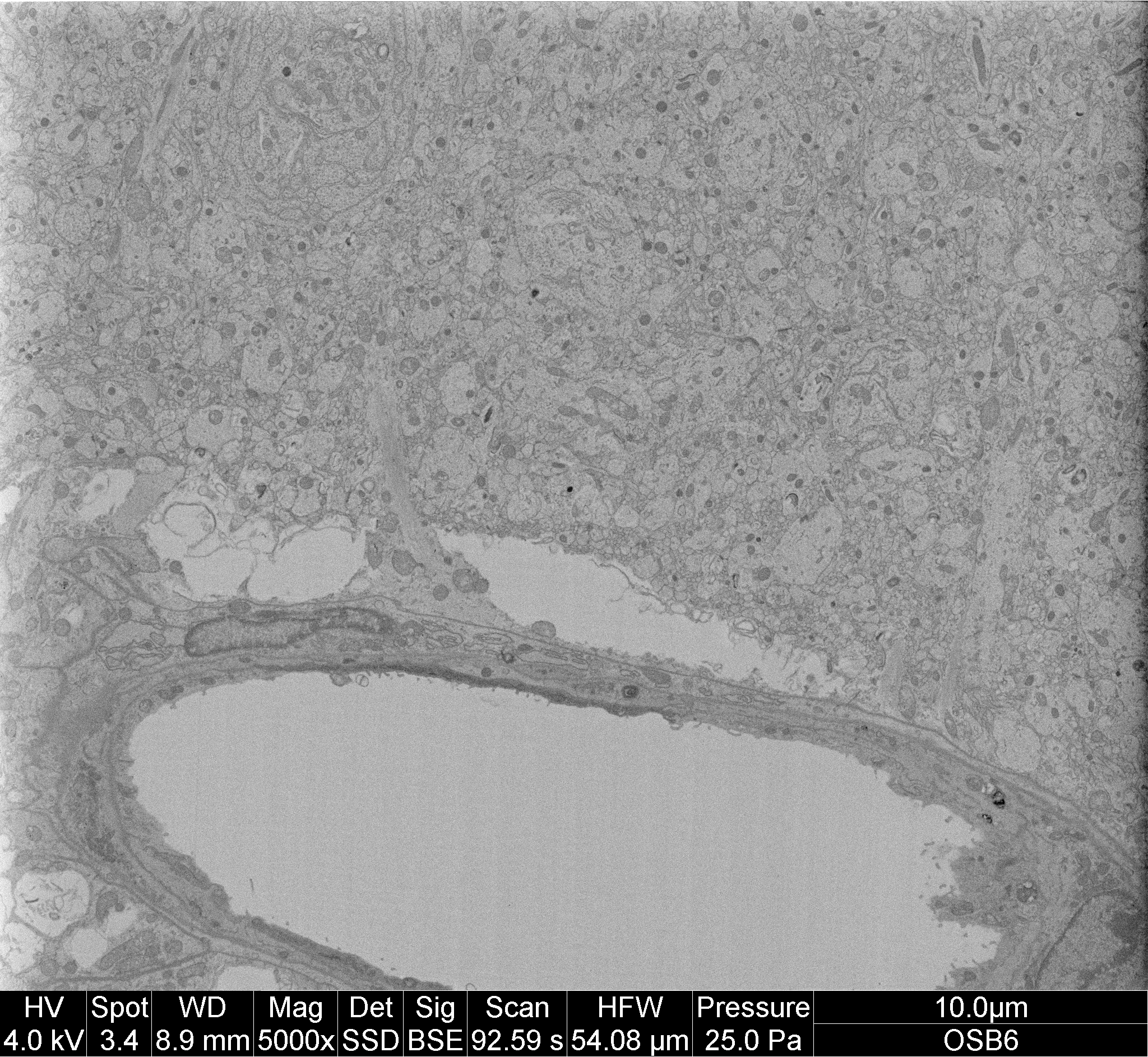

Supplement: Dataset S7 — (253.7 MB ZIP). [file pbio.0020329.sd007.zip › 040604_OS5_st1_607.tif]

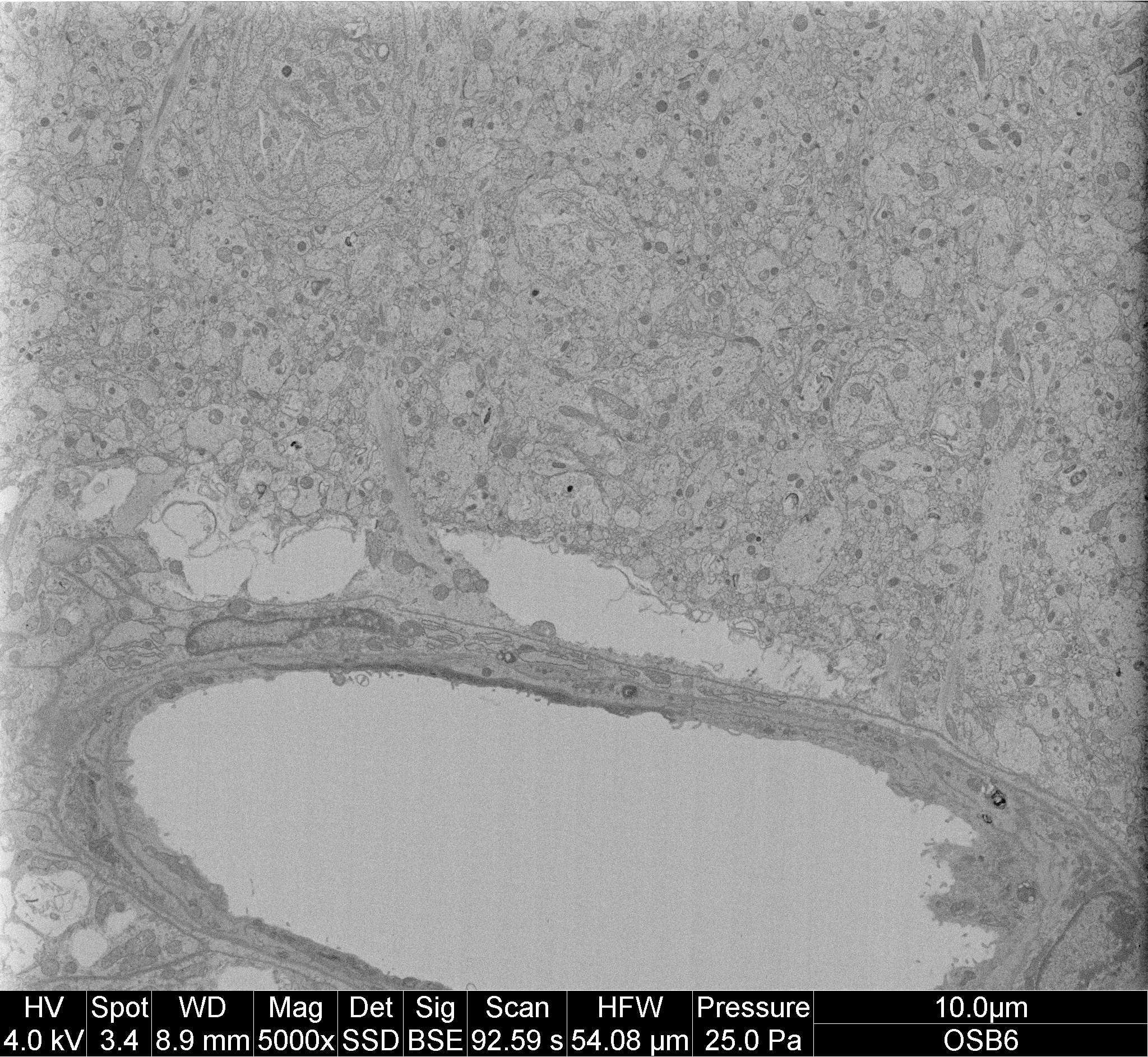

Supplement: Dataset S7 — (253.7 MB ZIP). [file pbio.0020329.sd007.zip › 040604_OS5_st1_608.tif]

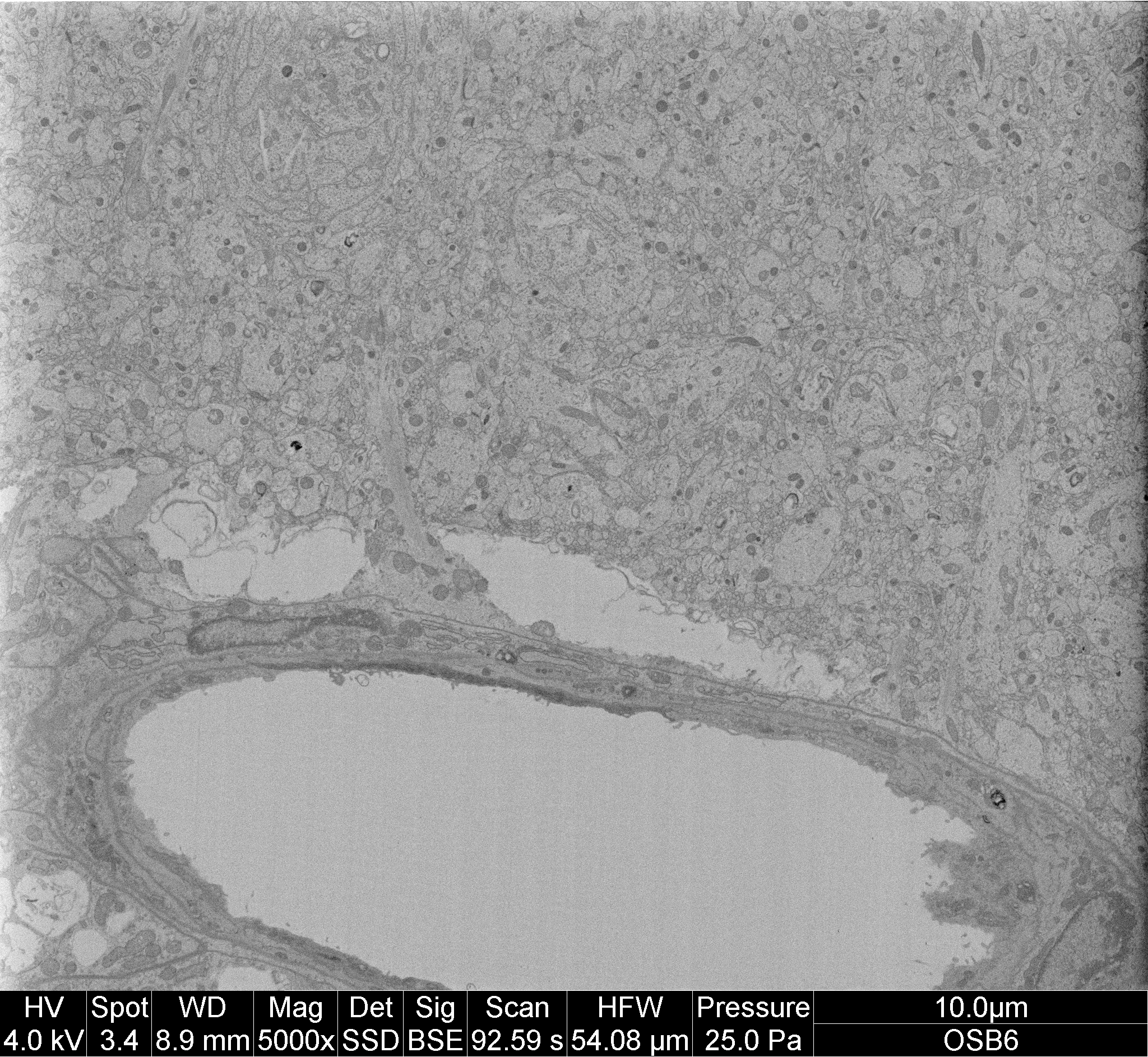

Supplement: Dataset S7 — (253.7 MB ZIP). [file pbio.0020329.sd007.zip › 040604_OS5_st1_609.tif]

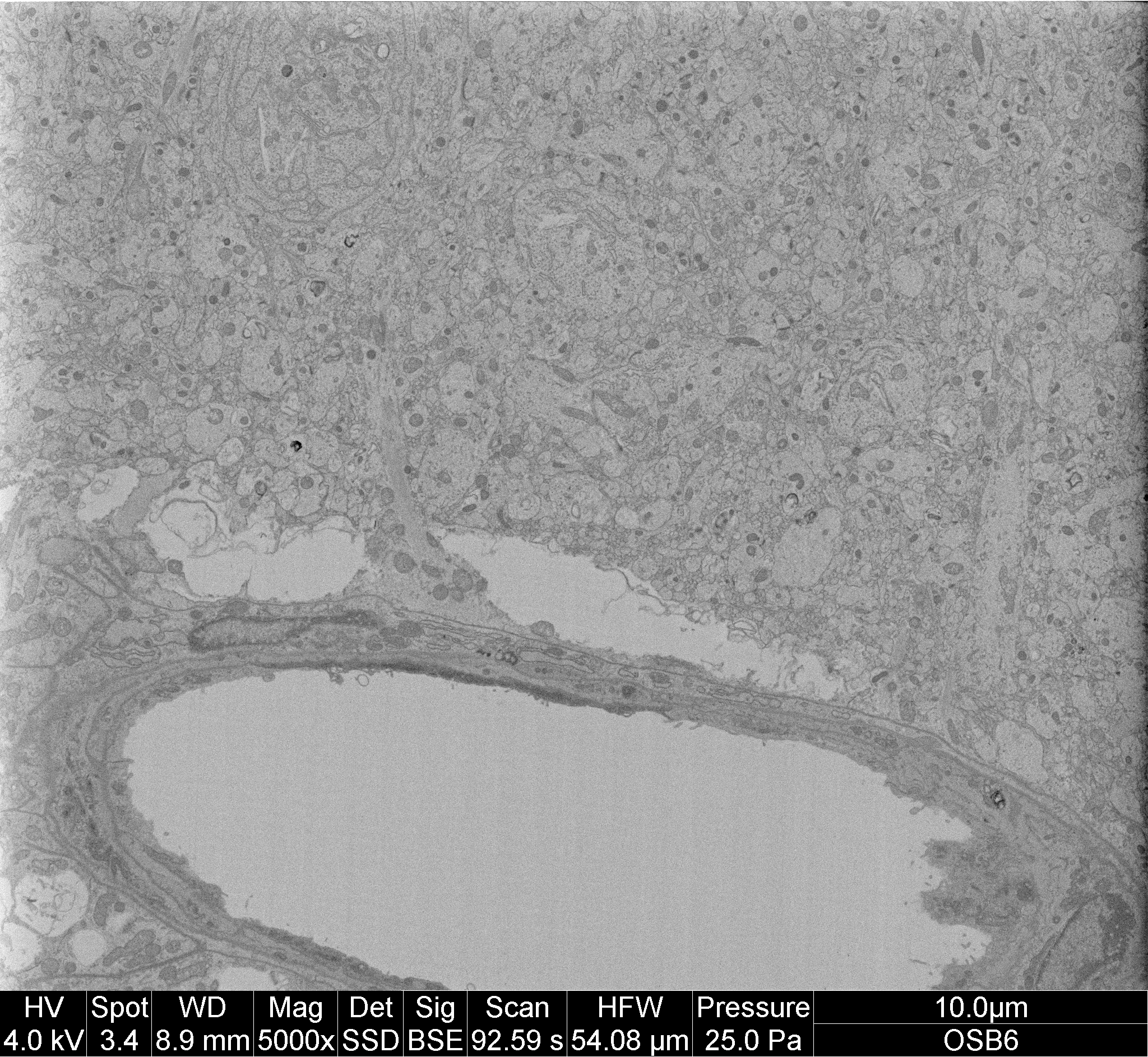

Supplement: Dataset S7 — (253.7 MB ZIP). [file pbio.0020329.sd007.zip › 040604_OS5_st1_610.tif]

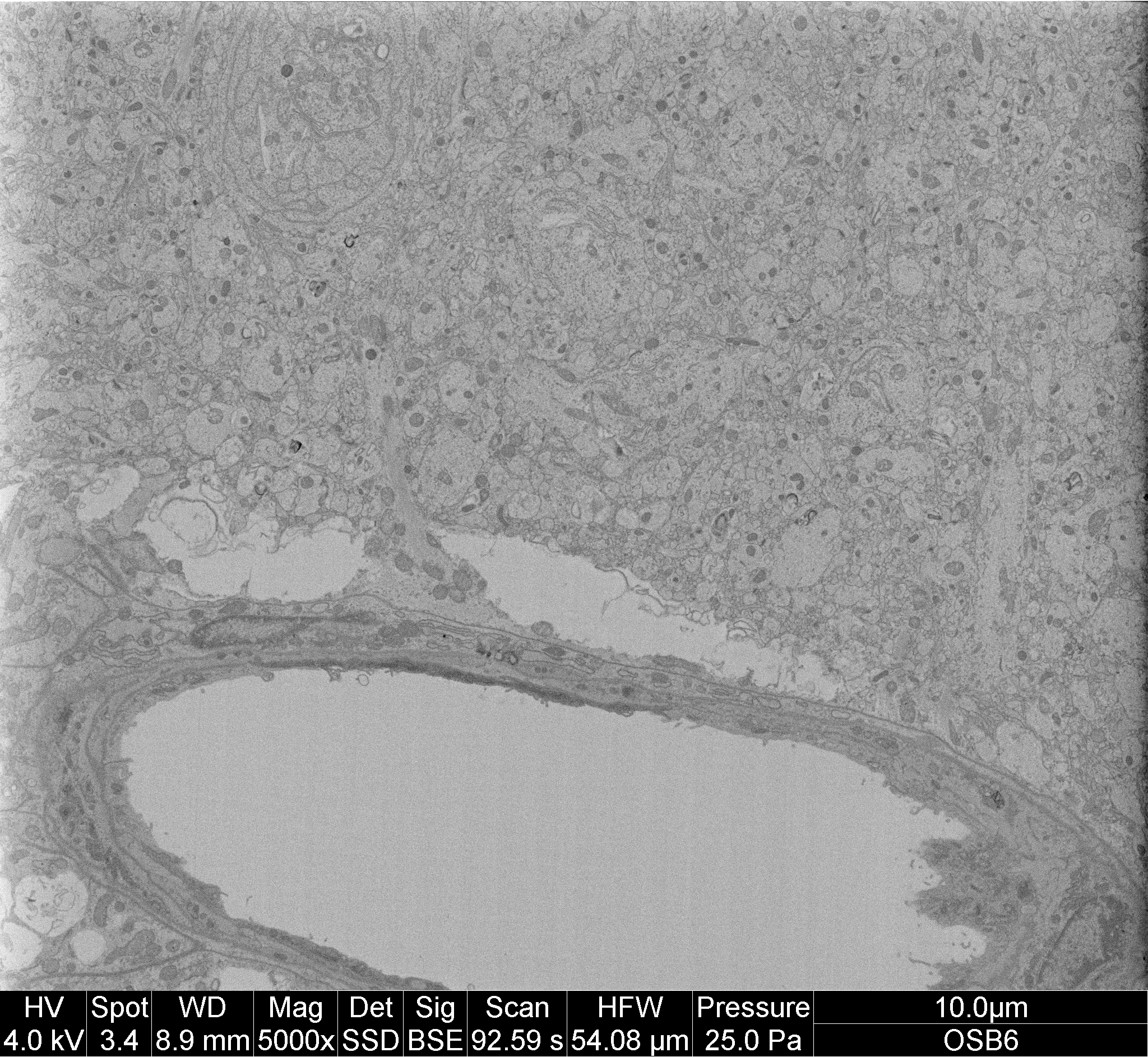

Supplement: Dataset S7 — (253.7 MB ZIP). [file pbio.0020329.sd007.zip › 040604_OS5_st1_611.tif]

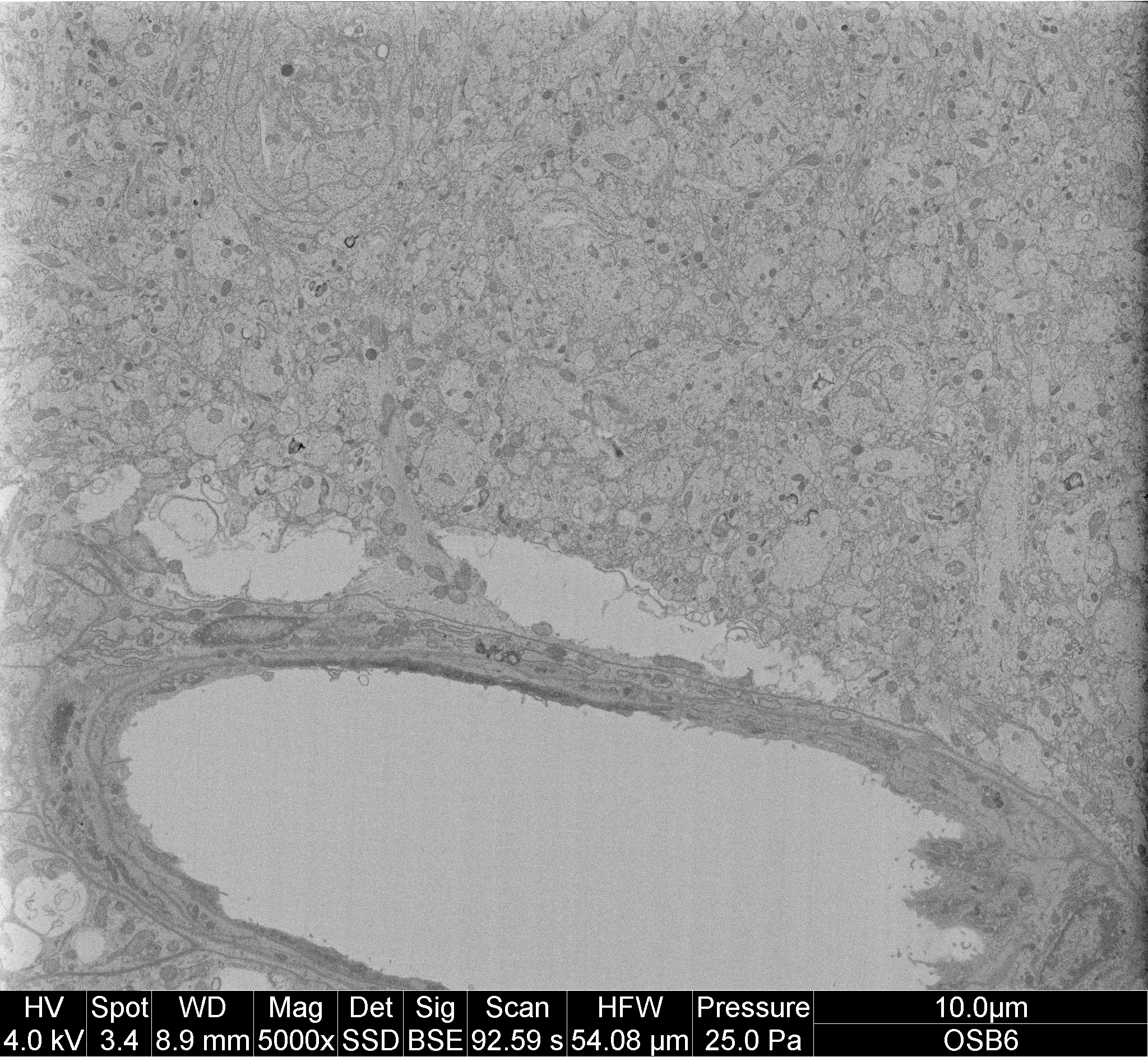

Supplement: Dataset S7 — (253.7 MB ZIP). [file pbio.0020329.sd007.zip › 040604_OS5_st1_612.tif]

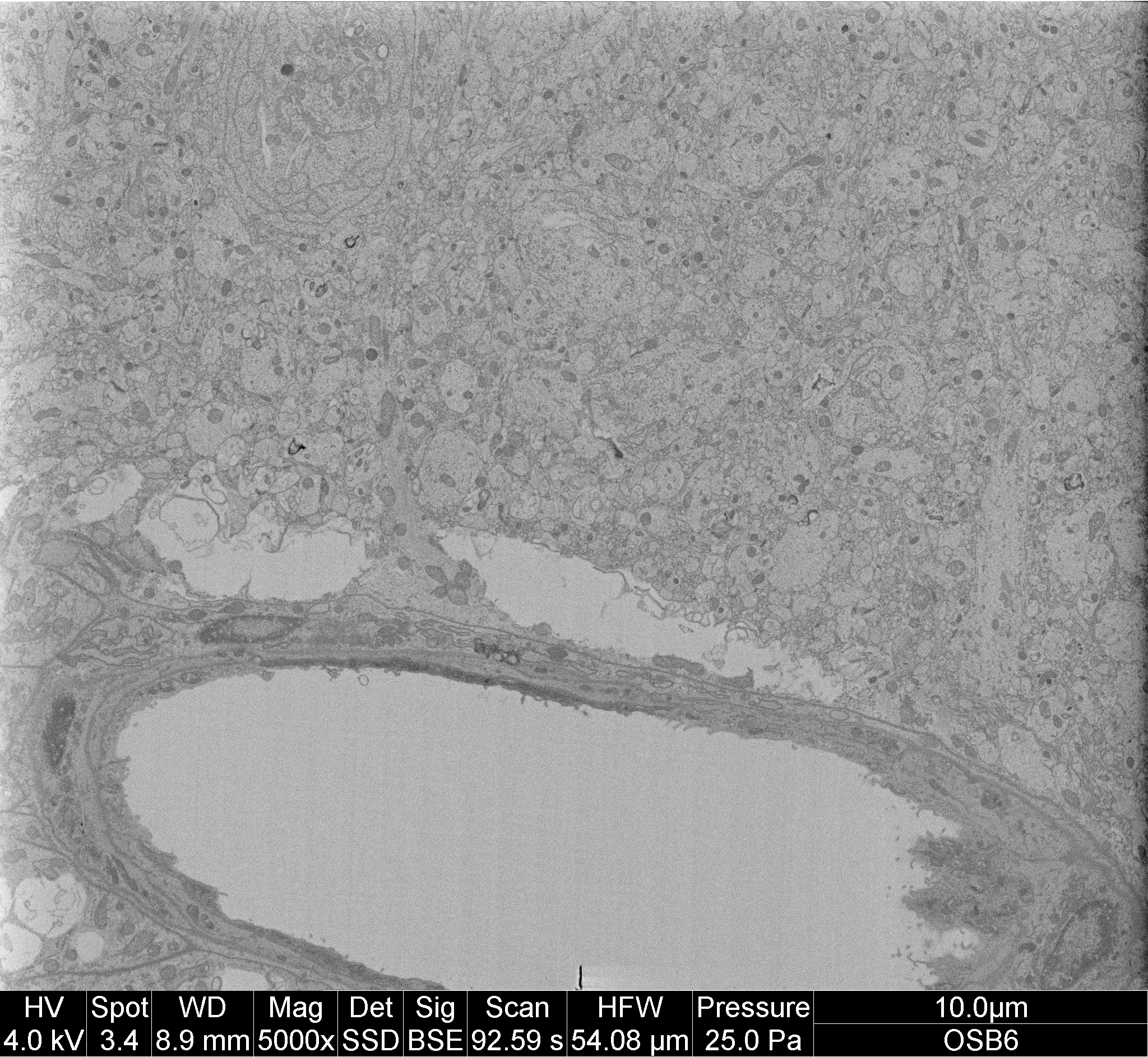

Supplement: Dataset S7 — (253.7 MB ZIP). [file pbio.0020329.sd007.zip › 040604_OS5_st1_613.tif]

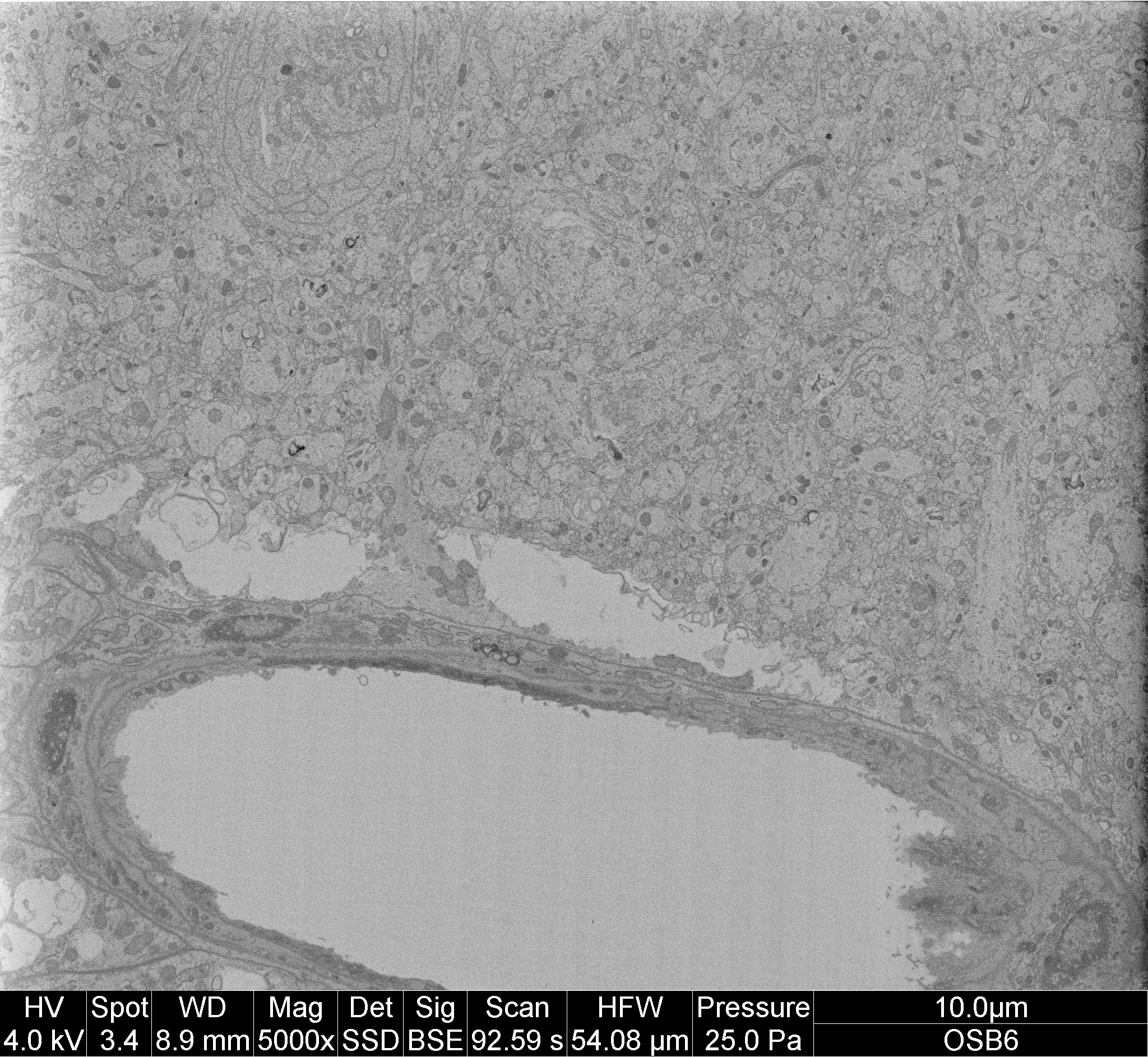

Supplement: Dataset S7 — (253.7 MB ZIP). [file pbio.0020329.sd007.zip › 040604_OS5_st1_614.tif]

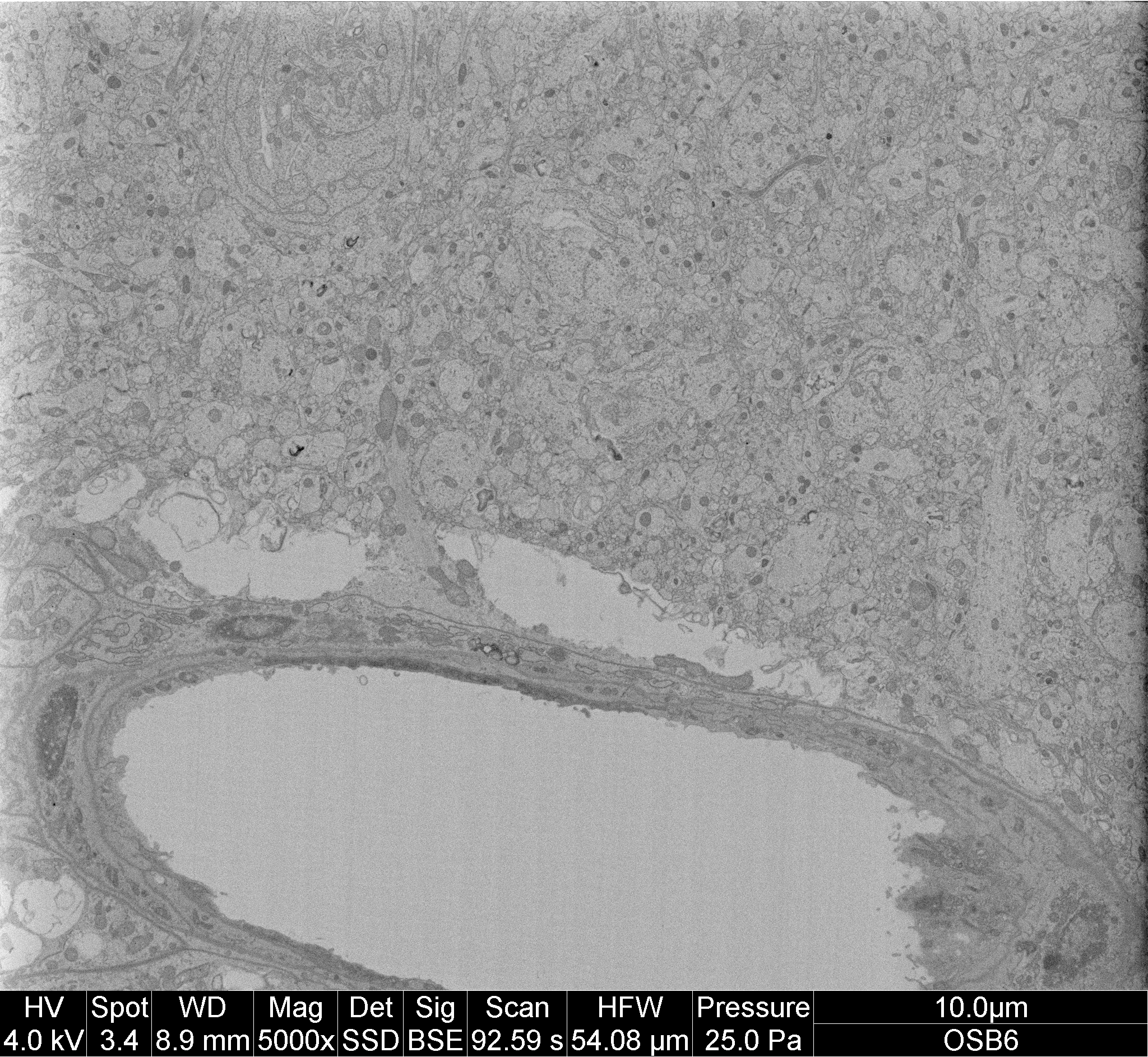

Supplement: Dataset S7 — (253.7 MB ZIP). [file pbio.0020329.sd007.zip › 040604_OS5_st1_615.tif]

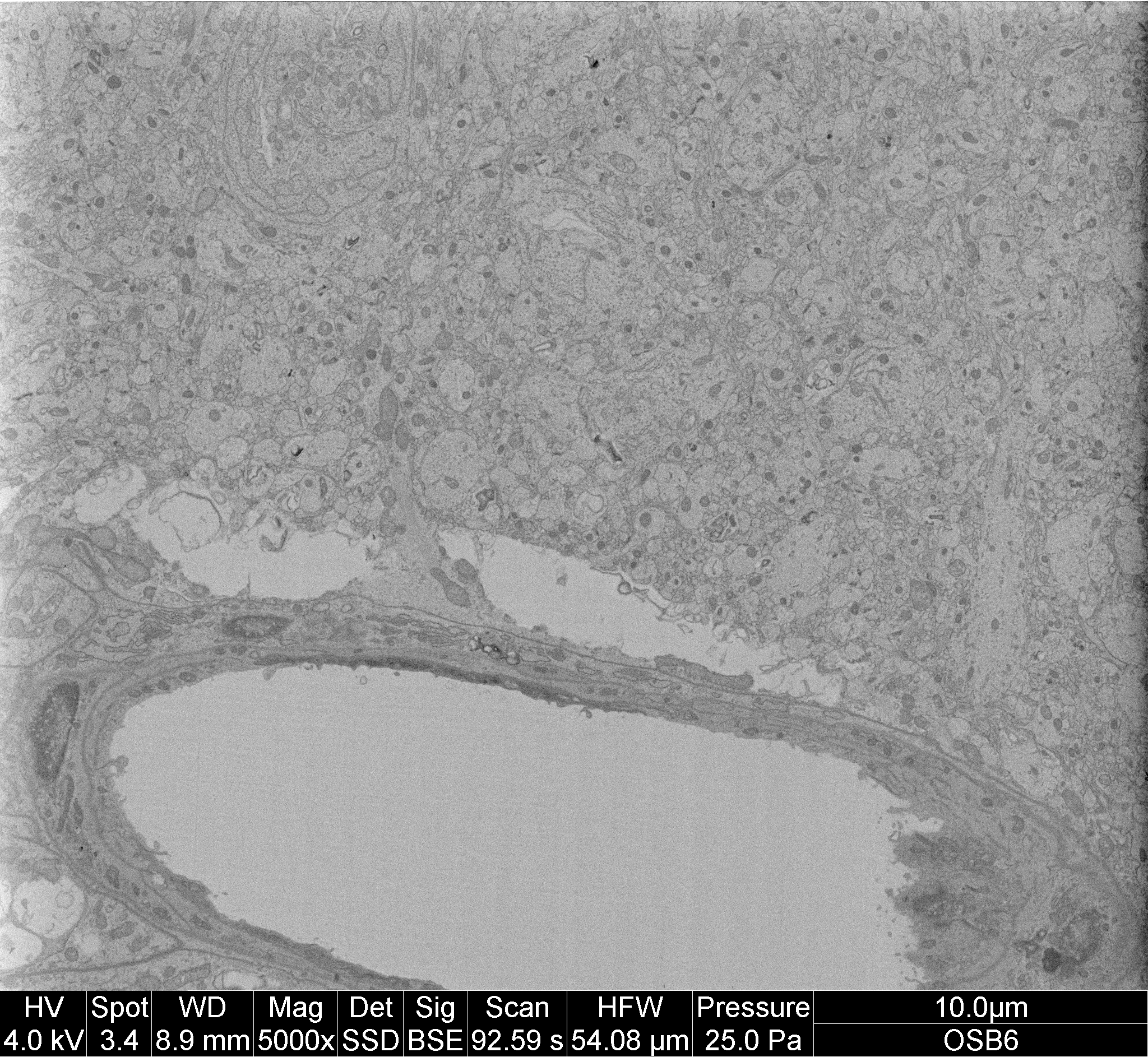

Supplement: Dataset S7 — (253.7 MB ZIP). [file pbio.0020329.sd007.zip › 040604_OS5_st1_616.tif]

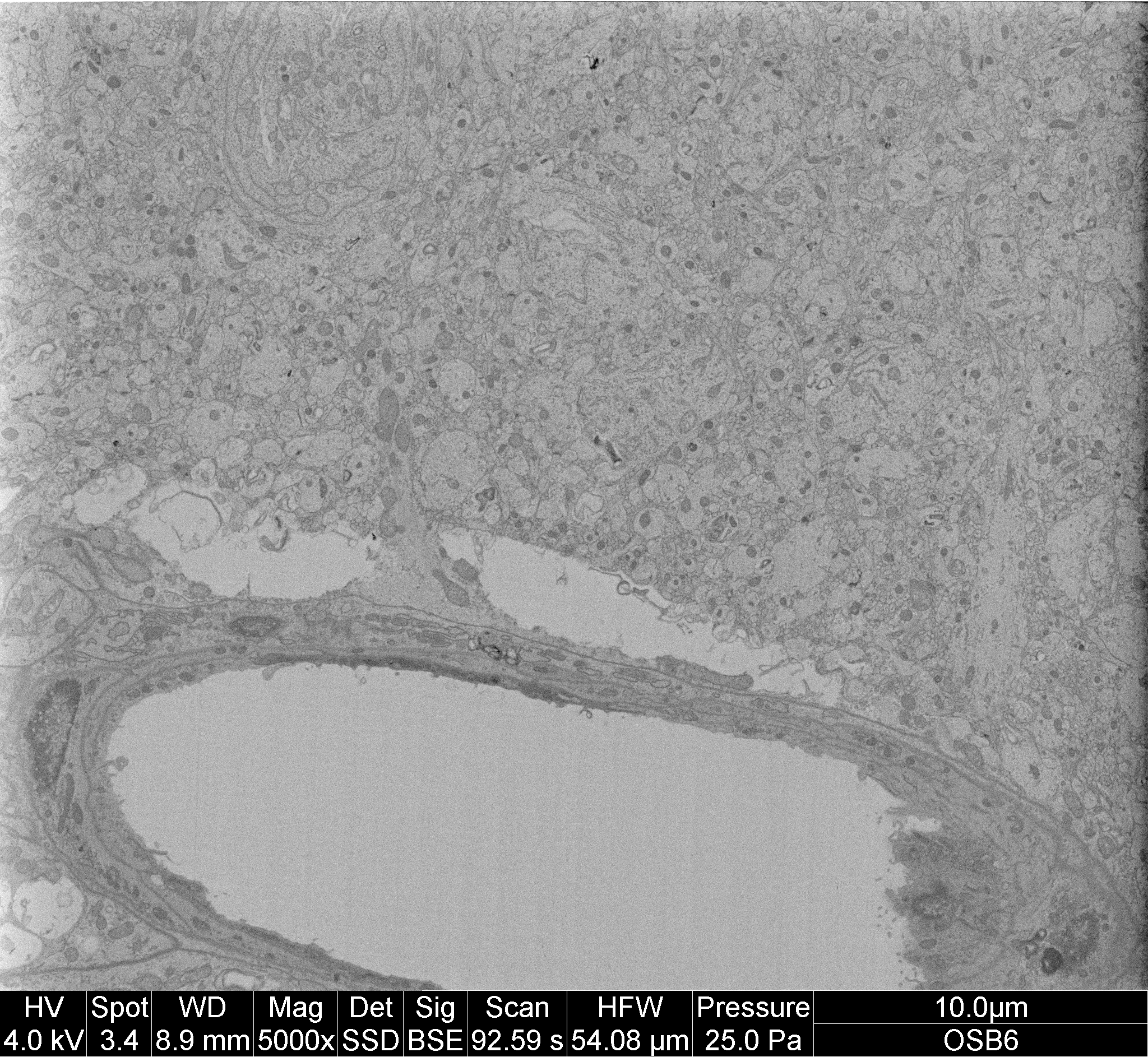

Supplement: Dataset S7 — (253.7 MB ZIP). [file pbio.0020329.sd007.zip › 040604_OS5_st1_617.tif]

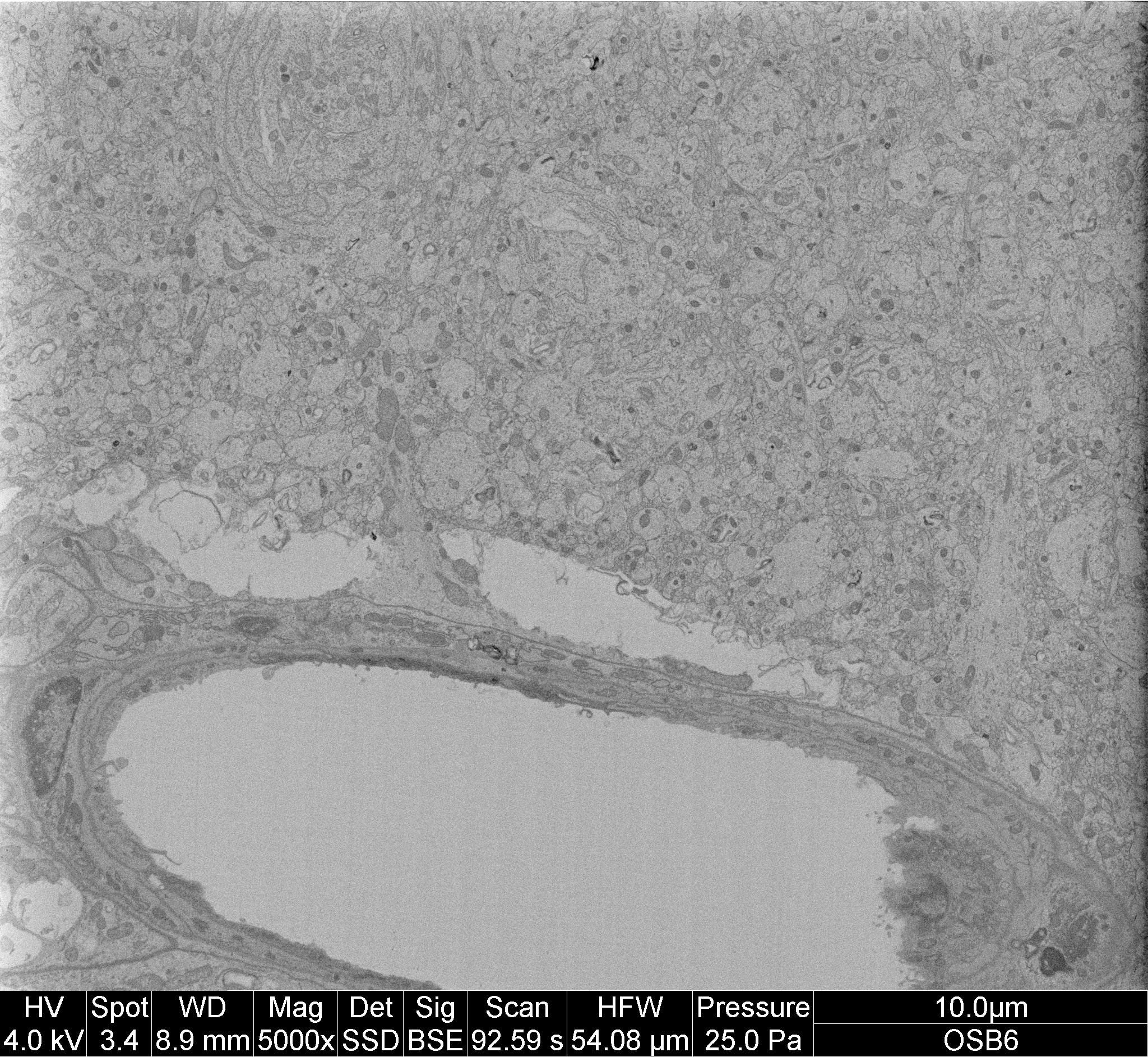

Supplement: Dataset S7 — (253.7 MB ZIP). [file pbio.0020329.sd007.zip › 040604_OS5_st1_618.tif]

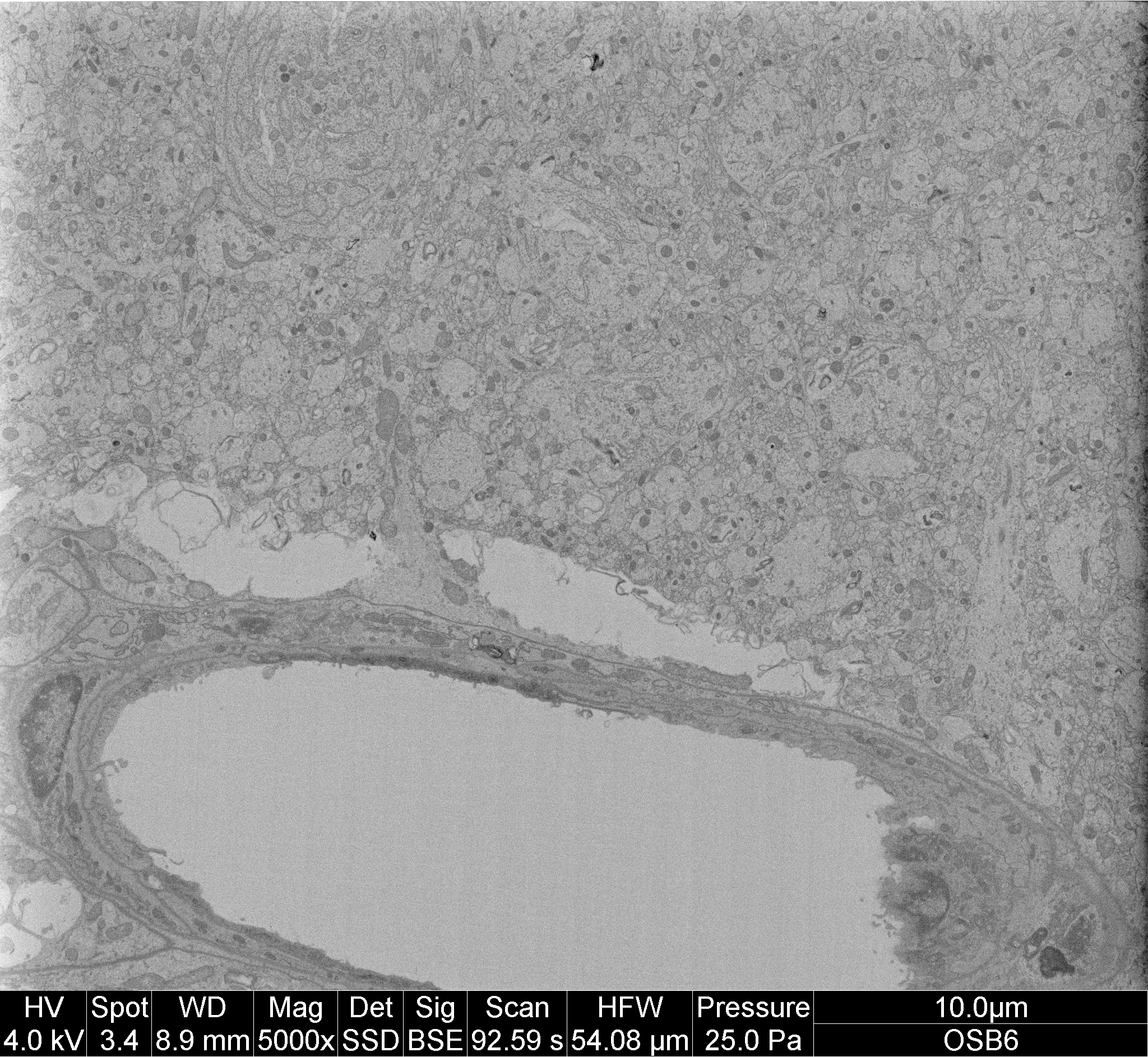

Supplement: Dataset S7 — (253.7 MB ZIP). [file pbio.0020329.sd007.zip › 040604_OS5_st1_619.tif]

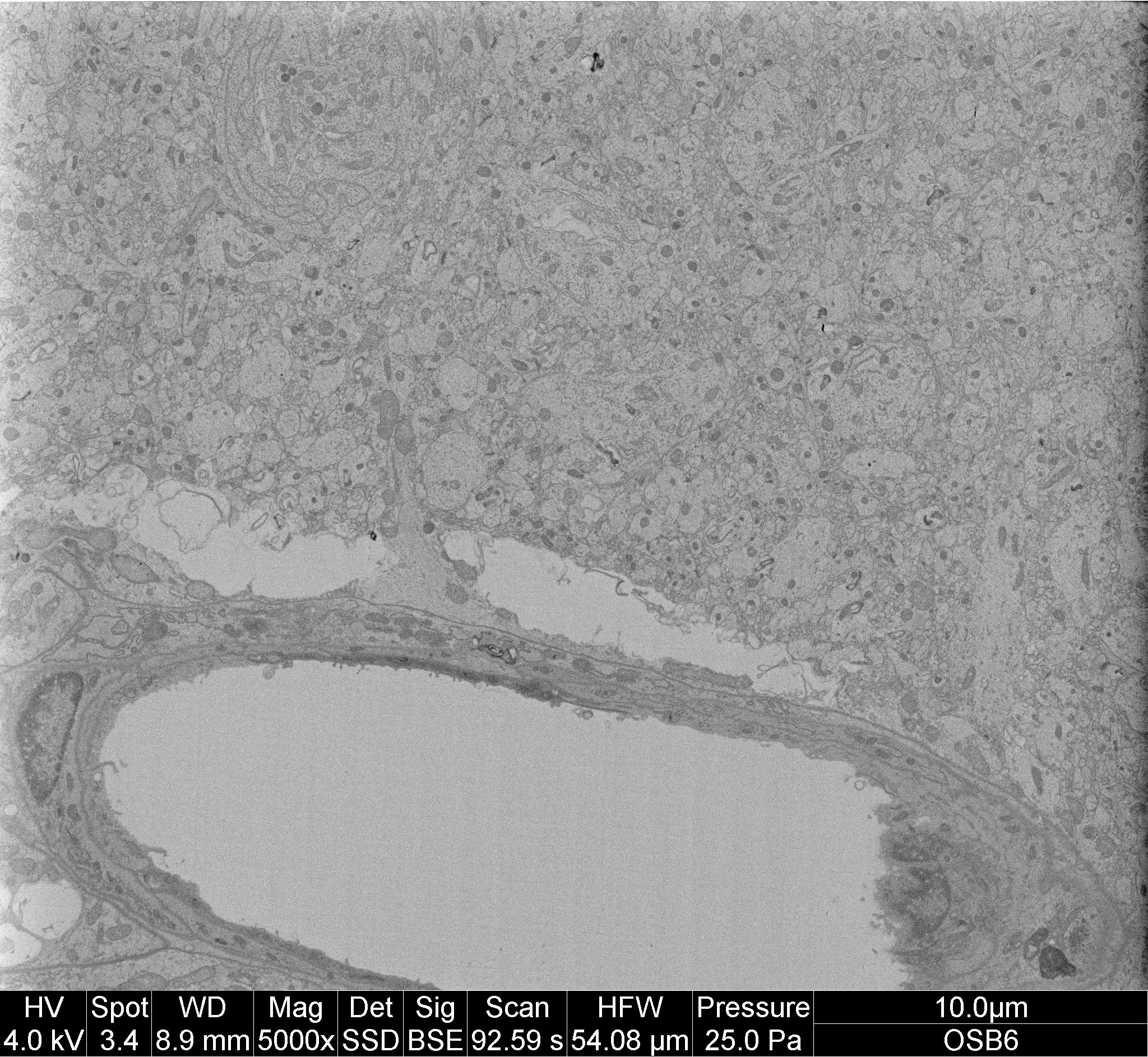

Supplement: Dataset S7 — (253.7 MB ZIP). [file pbio.0020329.sd007.zip › 040604_OS5_st1_620.tif]

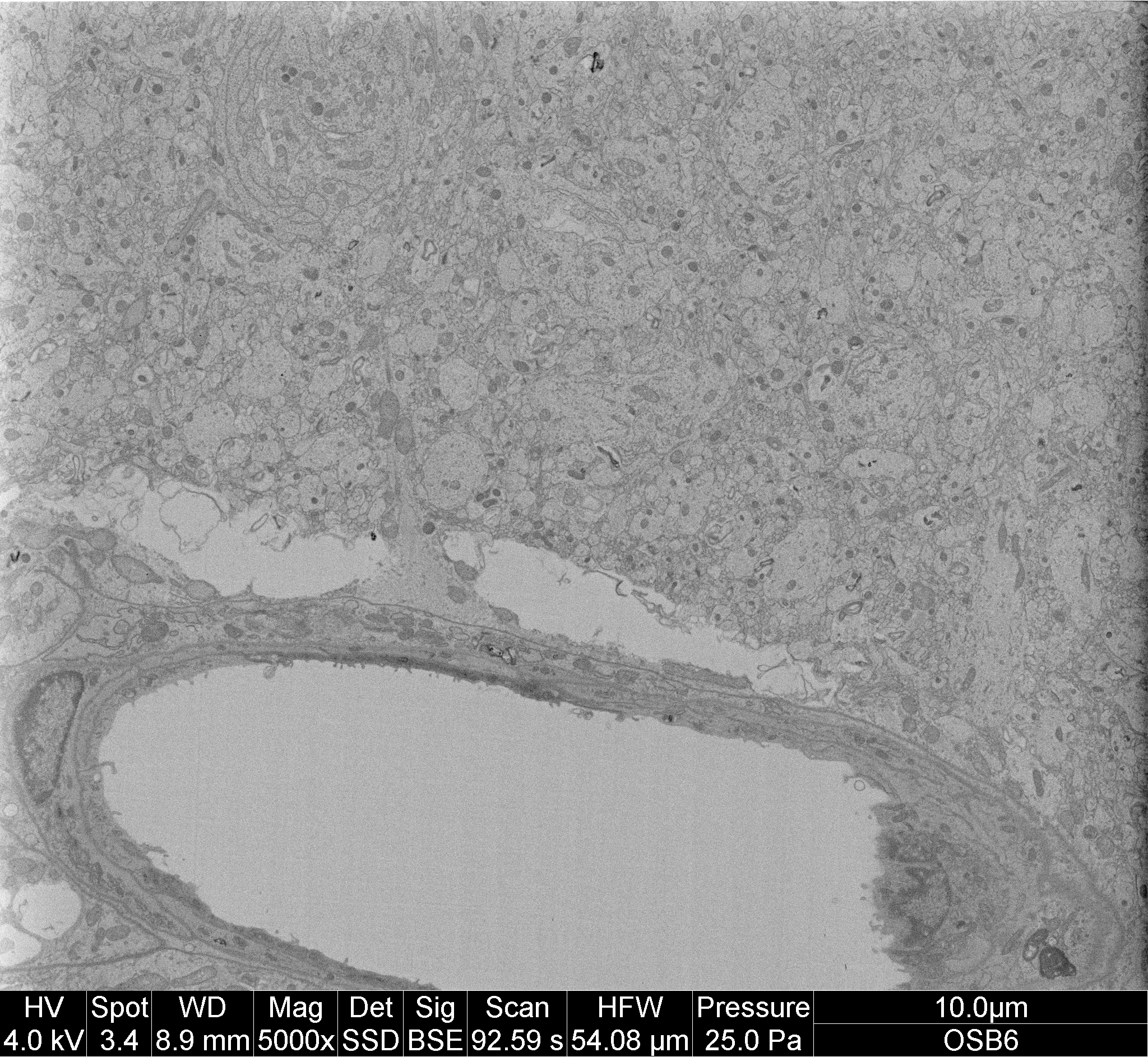

Supplement: Dataset S7 — (253.7 MB ZIP). [file pbio.0020329.sd007.zip › 040604_OS5_st1_621.tif]

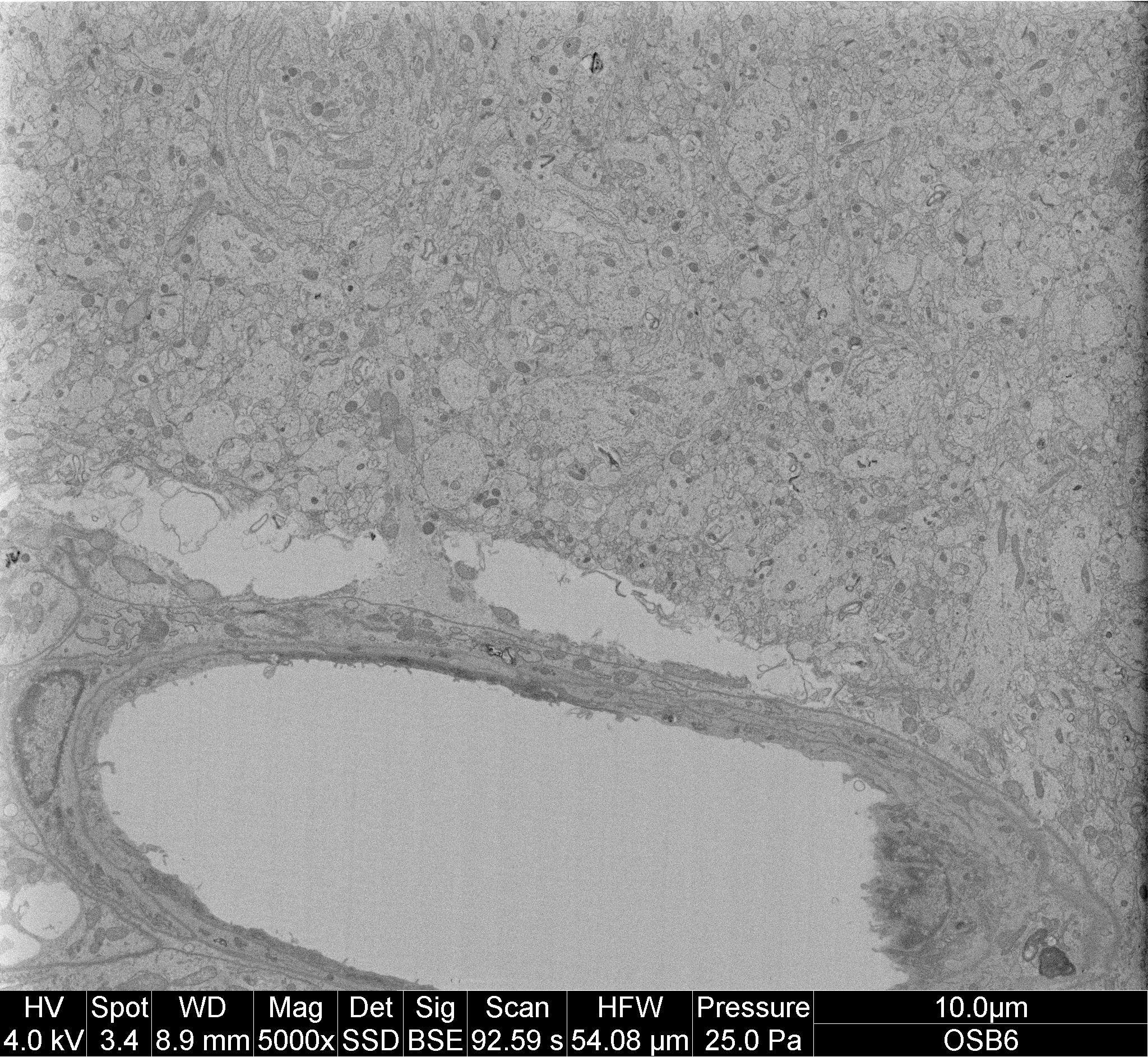

Supplement: Dataset S7 — (253.7 MB ZIP). [file pbio.0020329.sd007.zip › 040604_OS5_st1_622.tif]

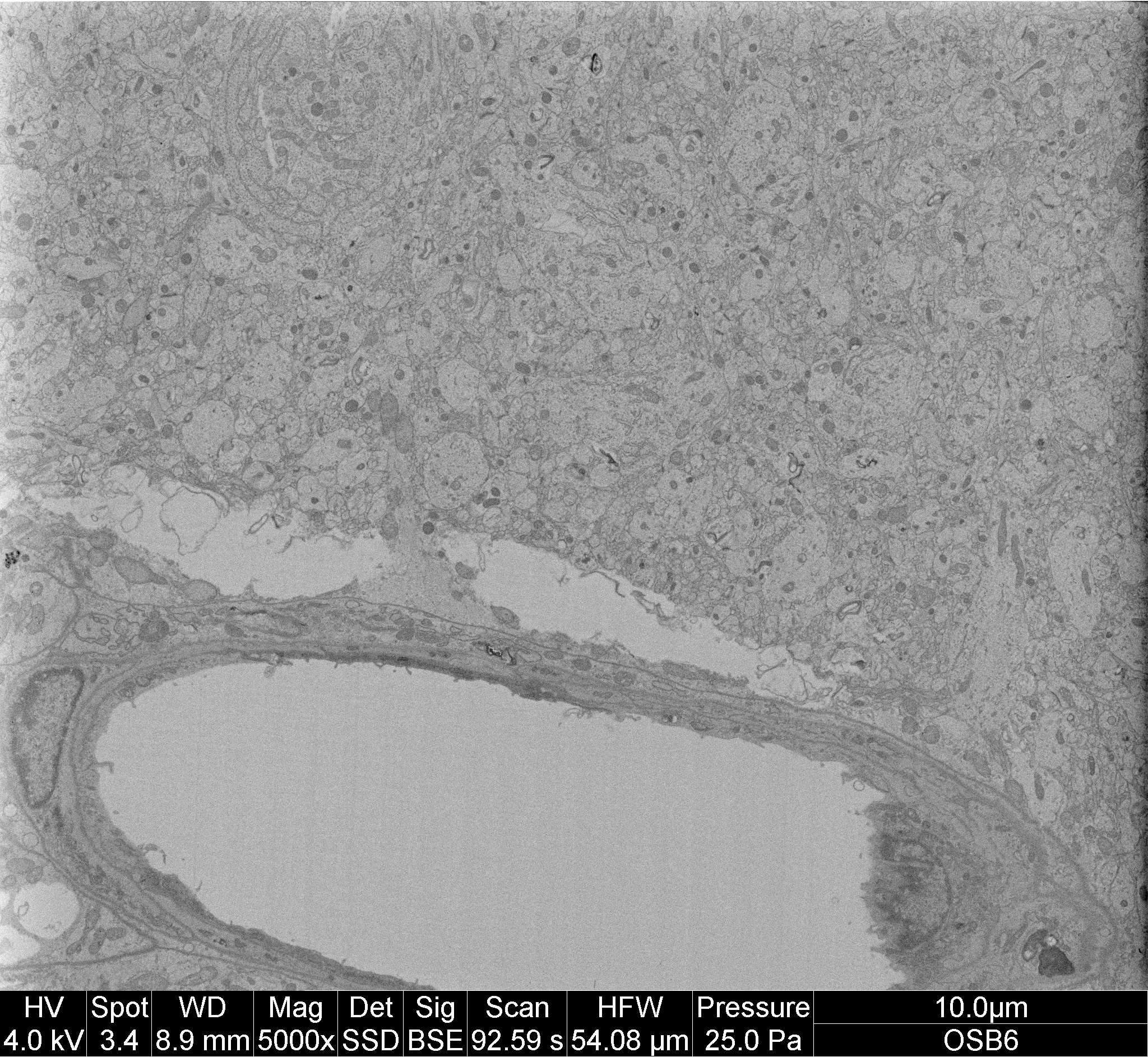

Supplement: Dataset S7 — (253.7 MB ZIP). [file pbio.0020329.sd007.zip › 040604_OS5_st1_623.tif]

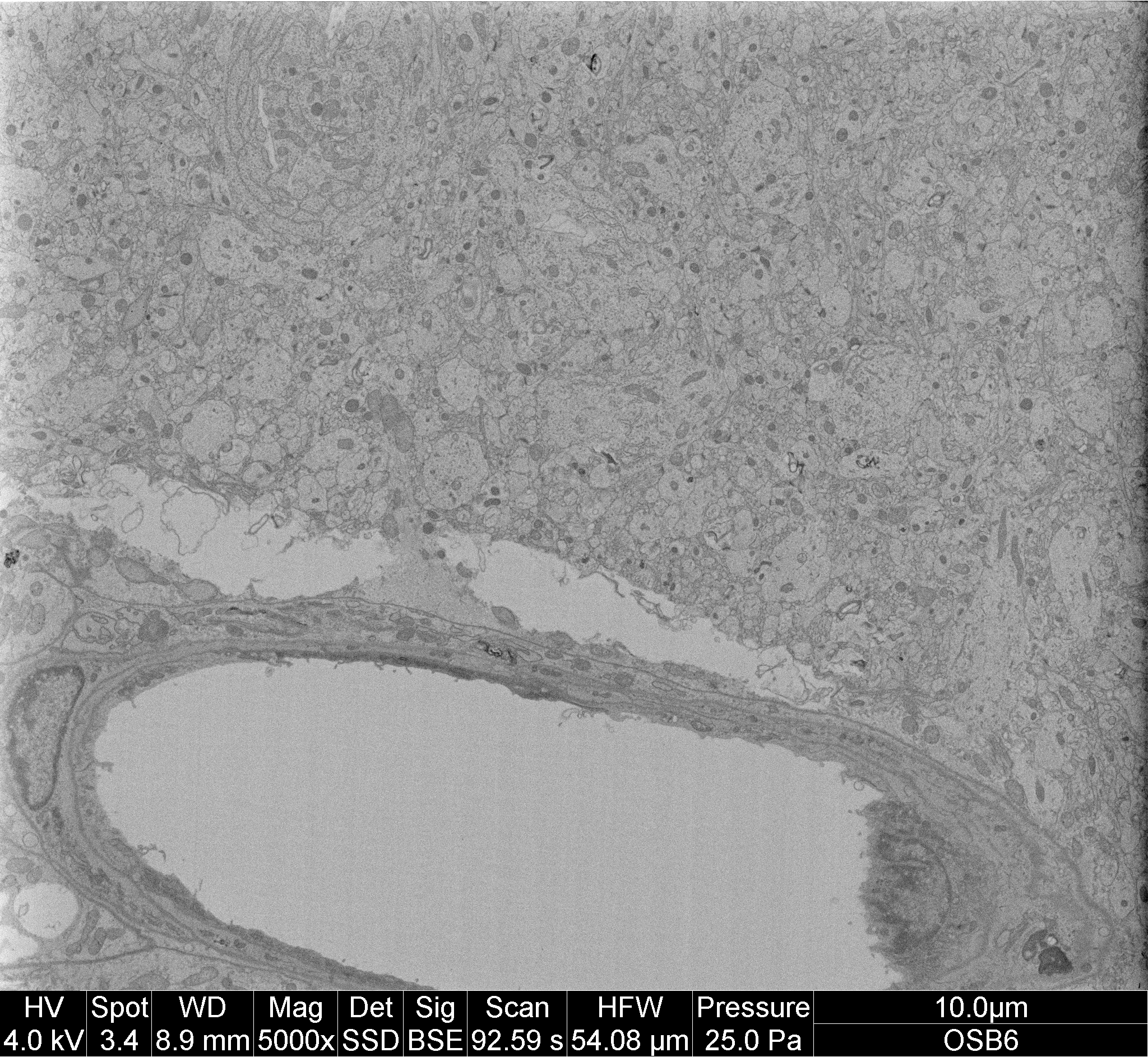

Supplement: Dataset S7 — (253.7 MB ZIP). [file pbio.0020329.sd007.zip › 040604_OS5_st1_624.tif]

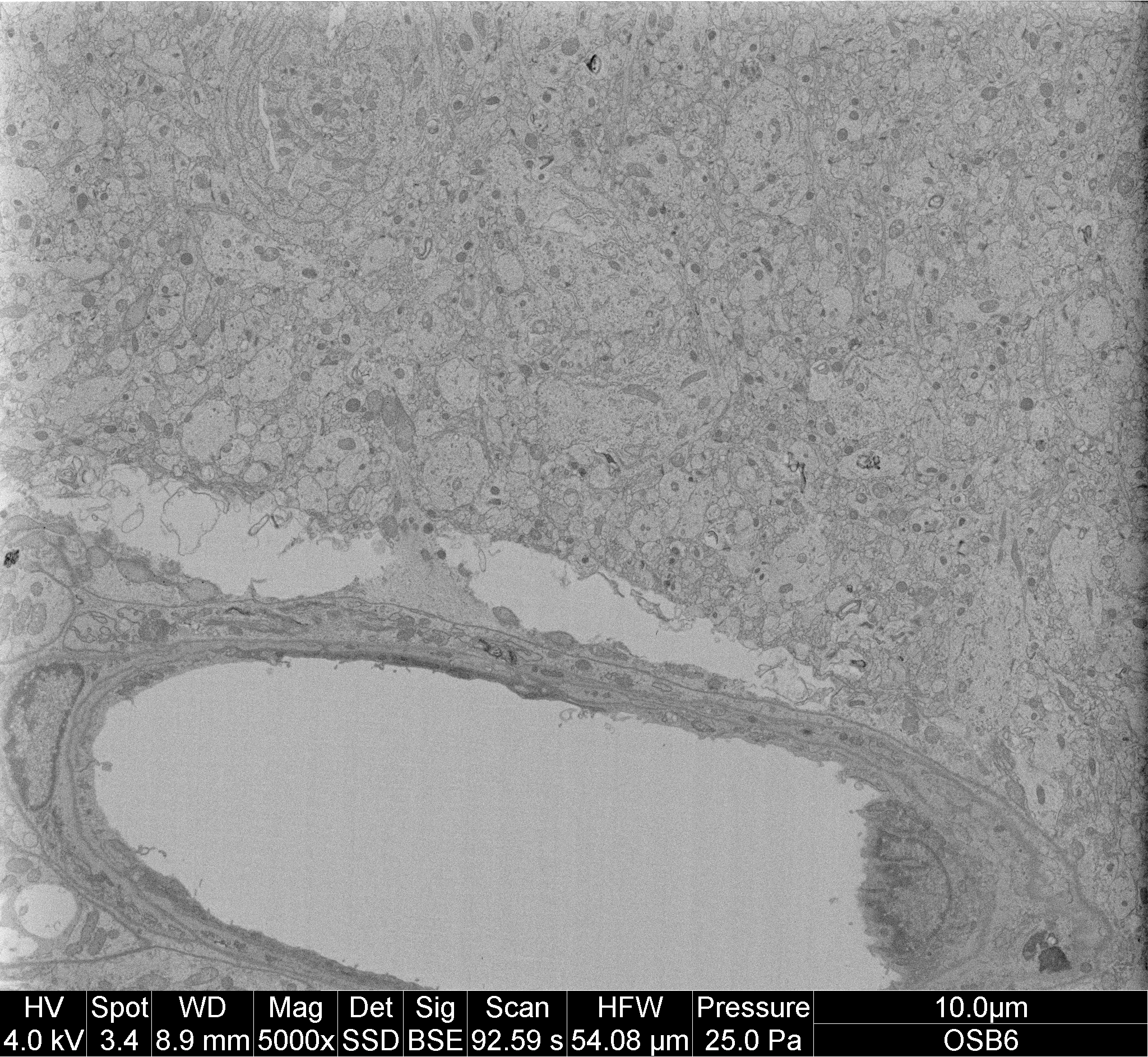

Supplement: Dataset S7 — (253.7 MB ZIP). [file pbio.0020329.sd007.zip › 040604_OS5_st1_625.tif]

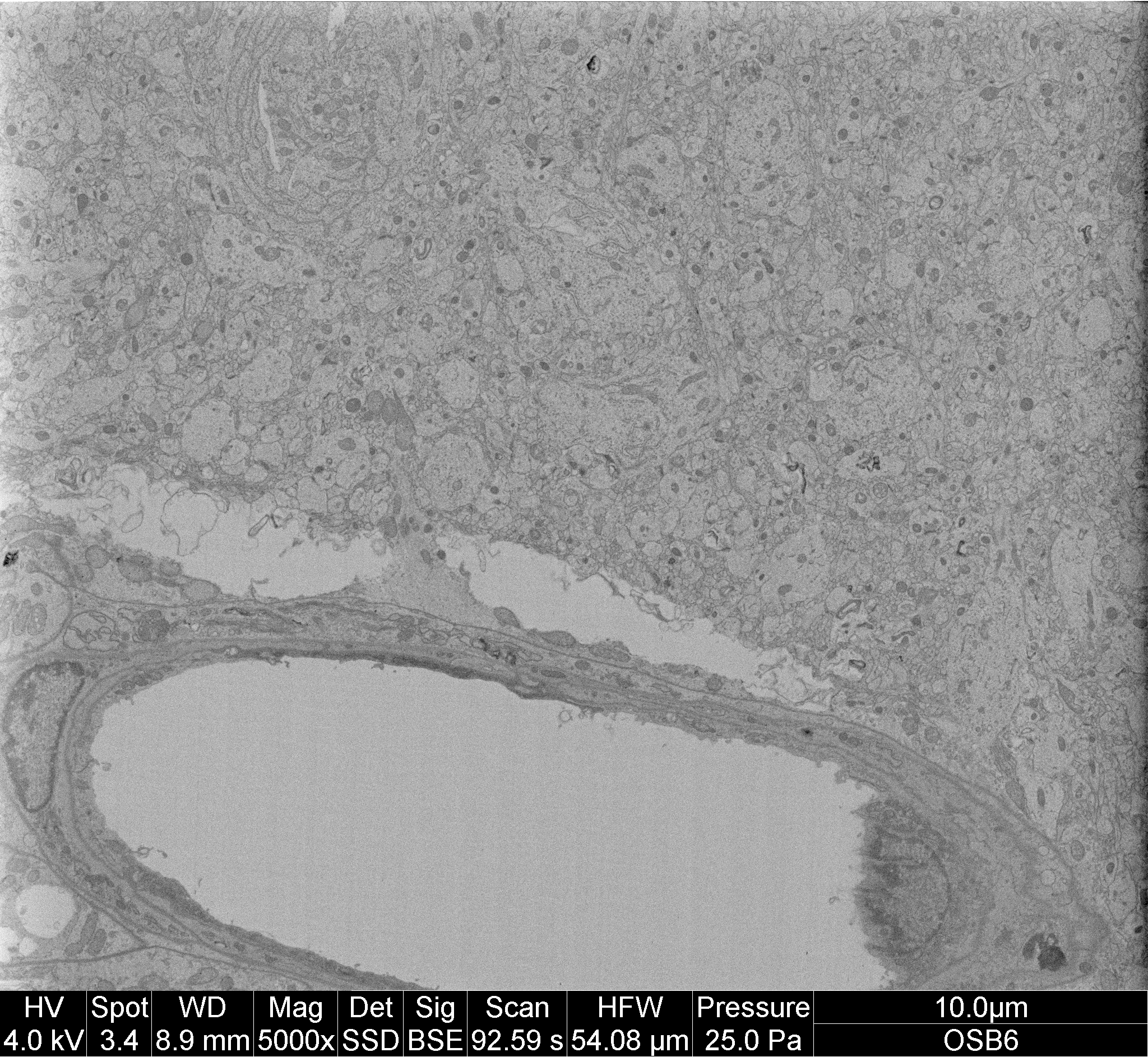

Supplement: Dataset S7 — (253.7 MB ZIP). [file pbio.0020329.sd007.zip › 040604_OS5_st1_626.tif]

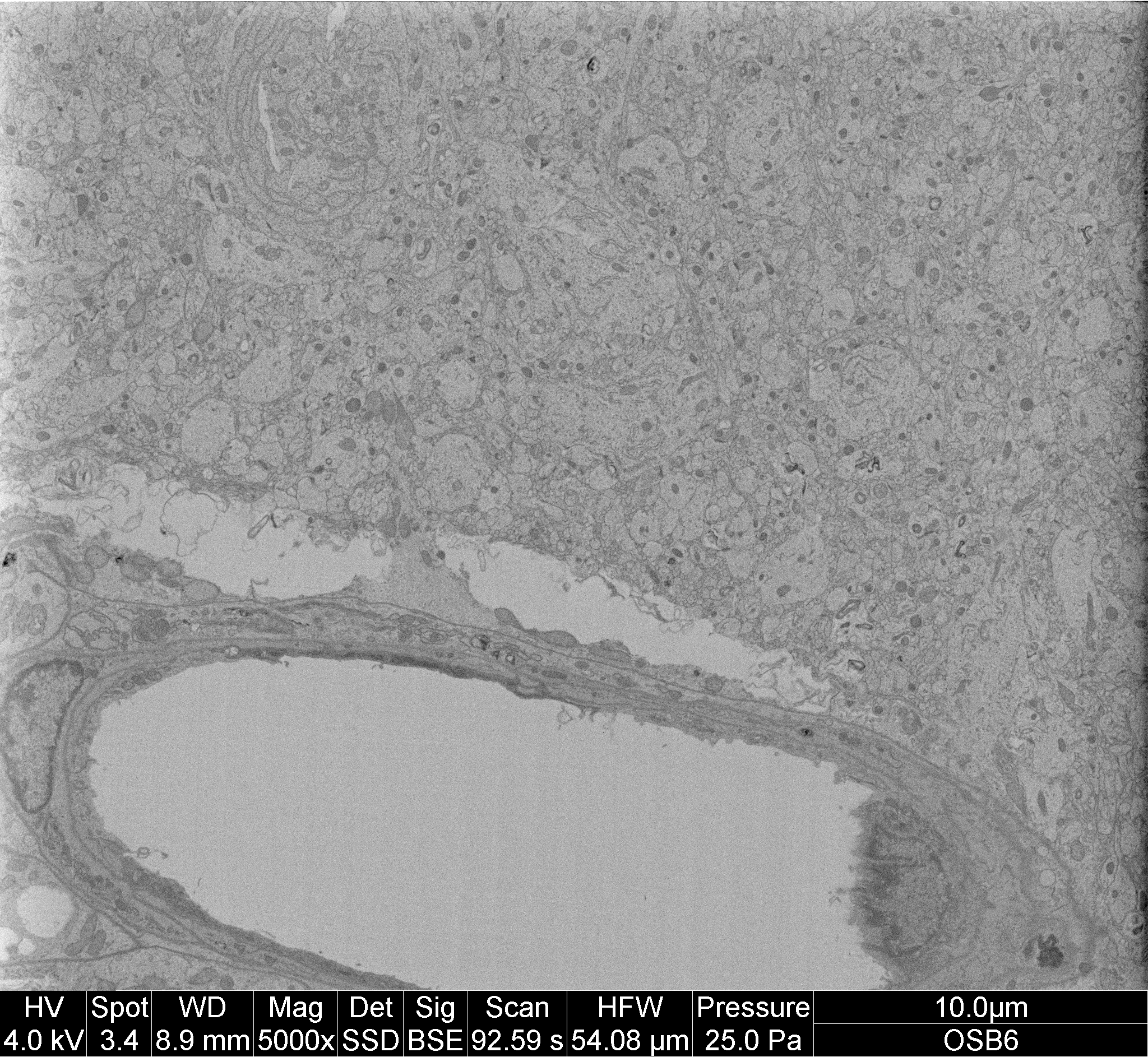

Supplement: Dataset S7 — (253.7 MB ZIP). [file pbio.0020329.sd007.zip › 040604_OS5_st1_627.tif]

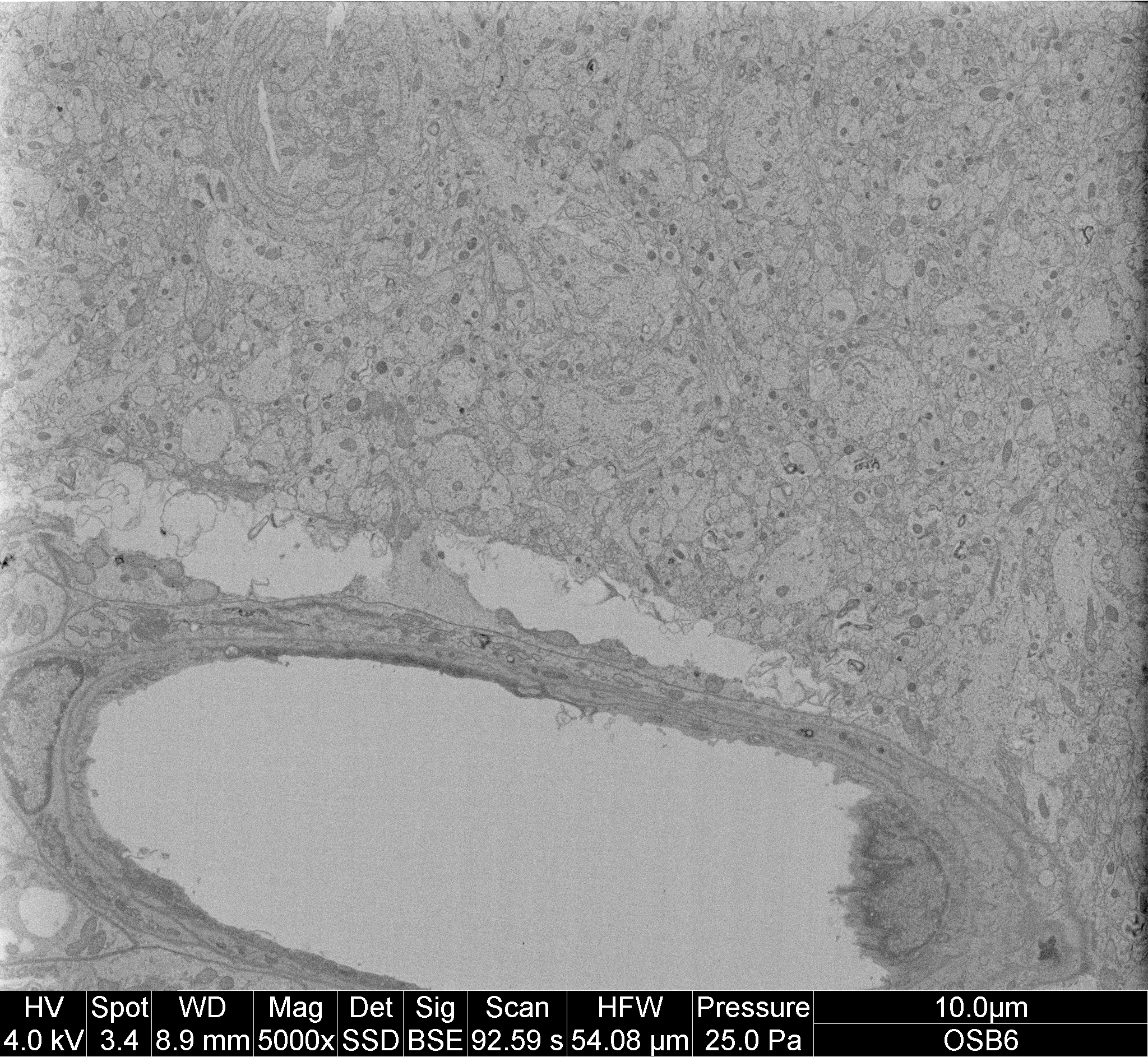

Supplement: Dataset S7 — (253.7 MB ZIP). [file pbio.0020329.sd007.zip › 040604_OS5_st1_628.tif]

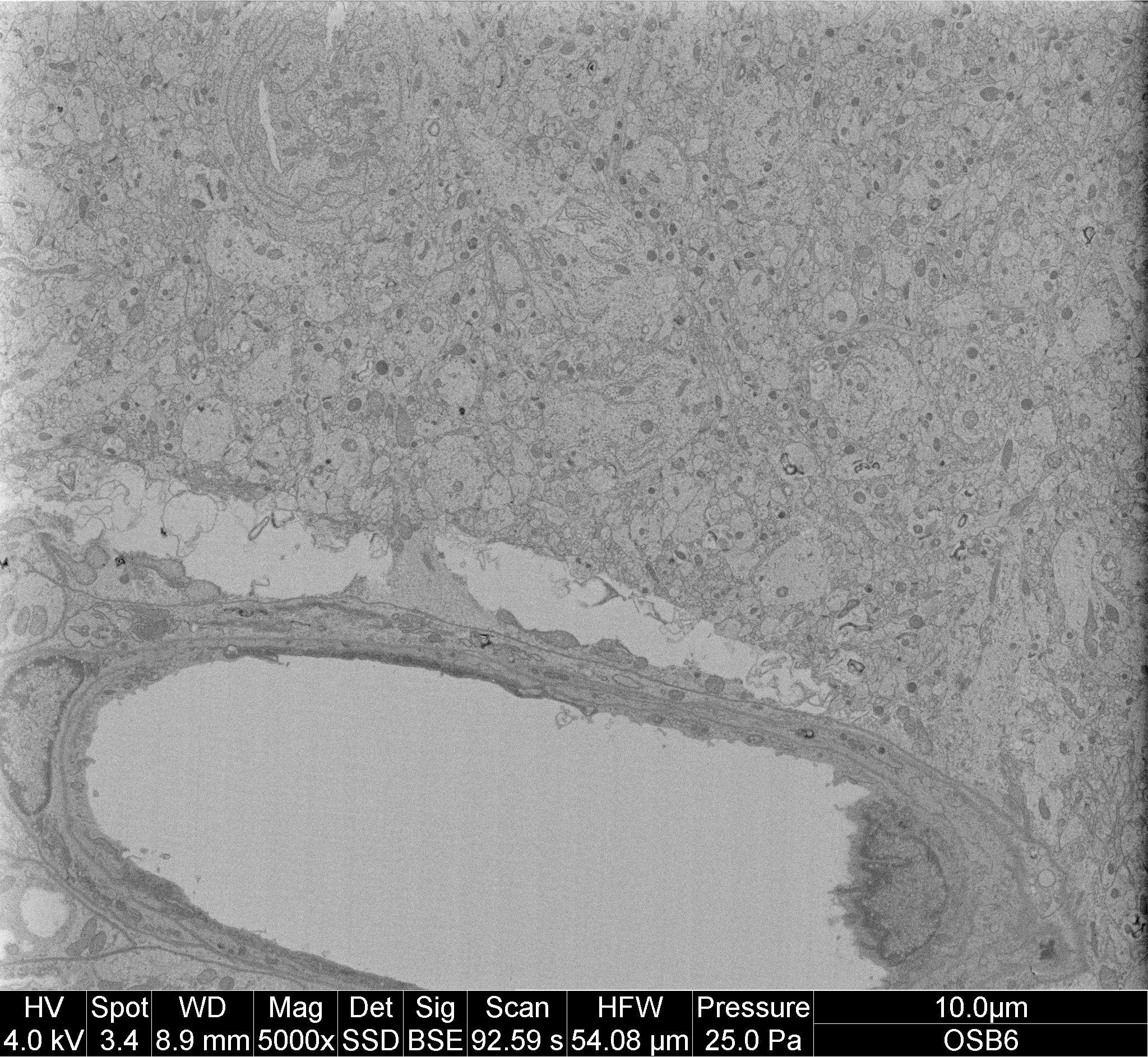

Supplement: Dataset S7 — (253.7 MB ZIP). [file pbio.0020329.sd007.zip › 040604_OS5_st1_629.tif]

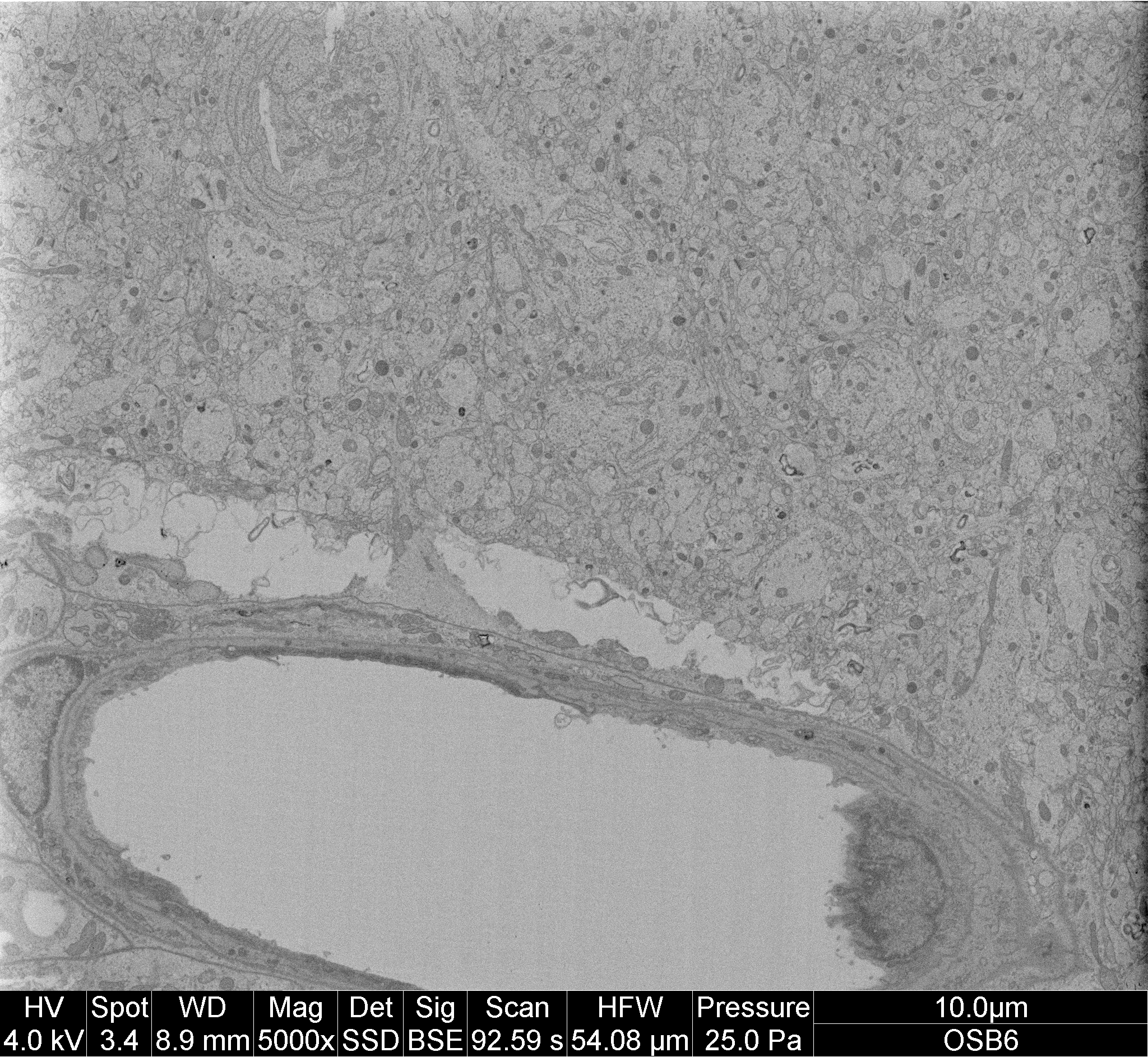

Supplement: Dataset S7 — (253.7 MB ZIP). [file pbio.0020329.sd007.zip › 040604_OS5_st1_630.tif]

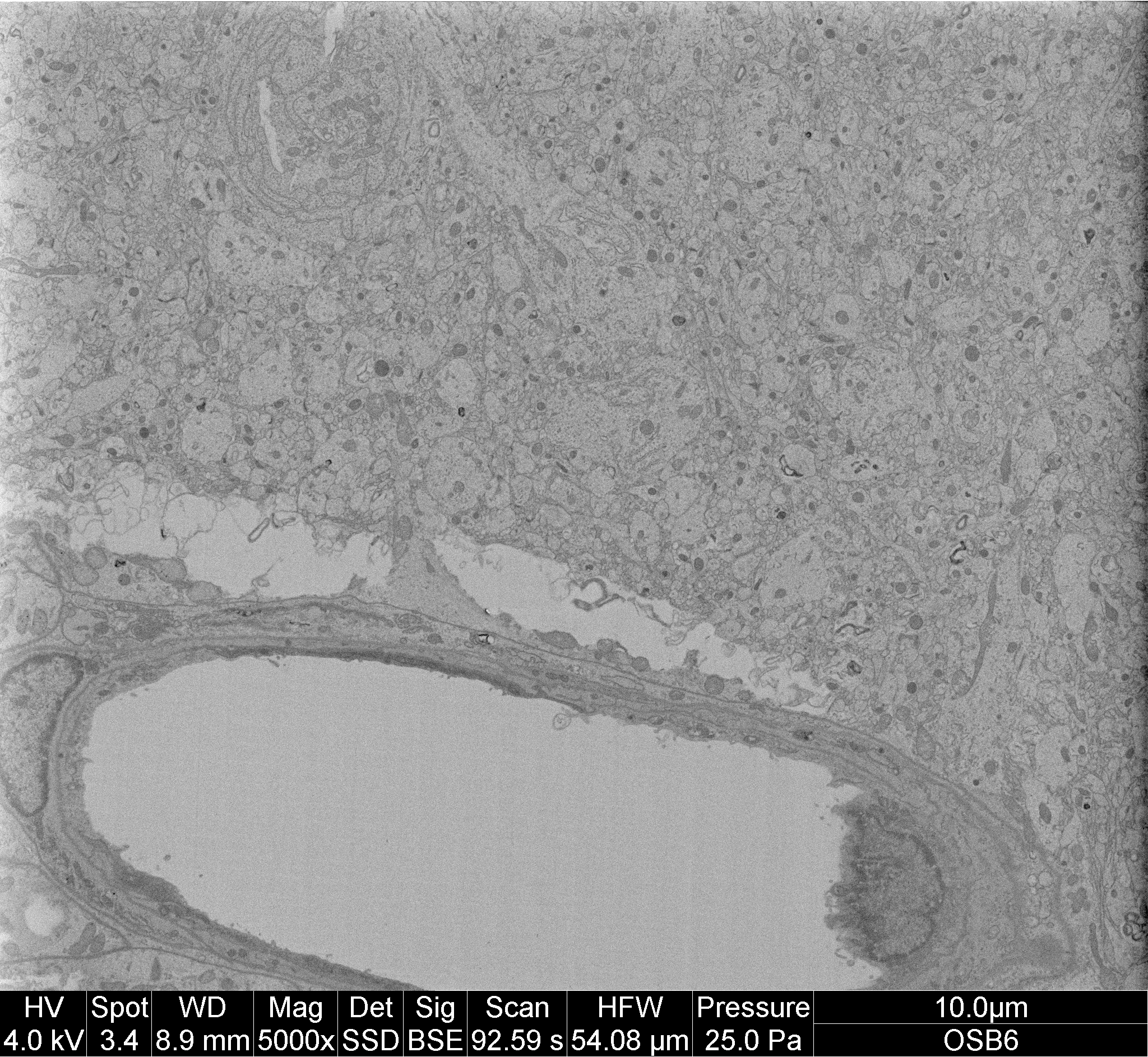

Supplement: Dataset S7 — (253.7 MB ZIP). [file pbio.0020329.sd007.zip › 040604_OS5_st1_631.tif]

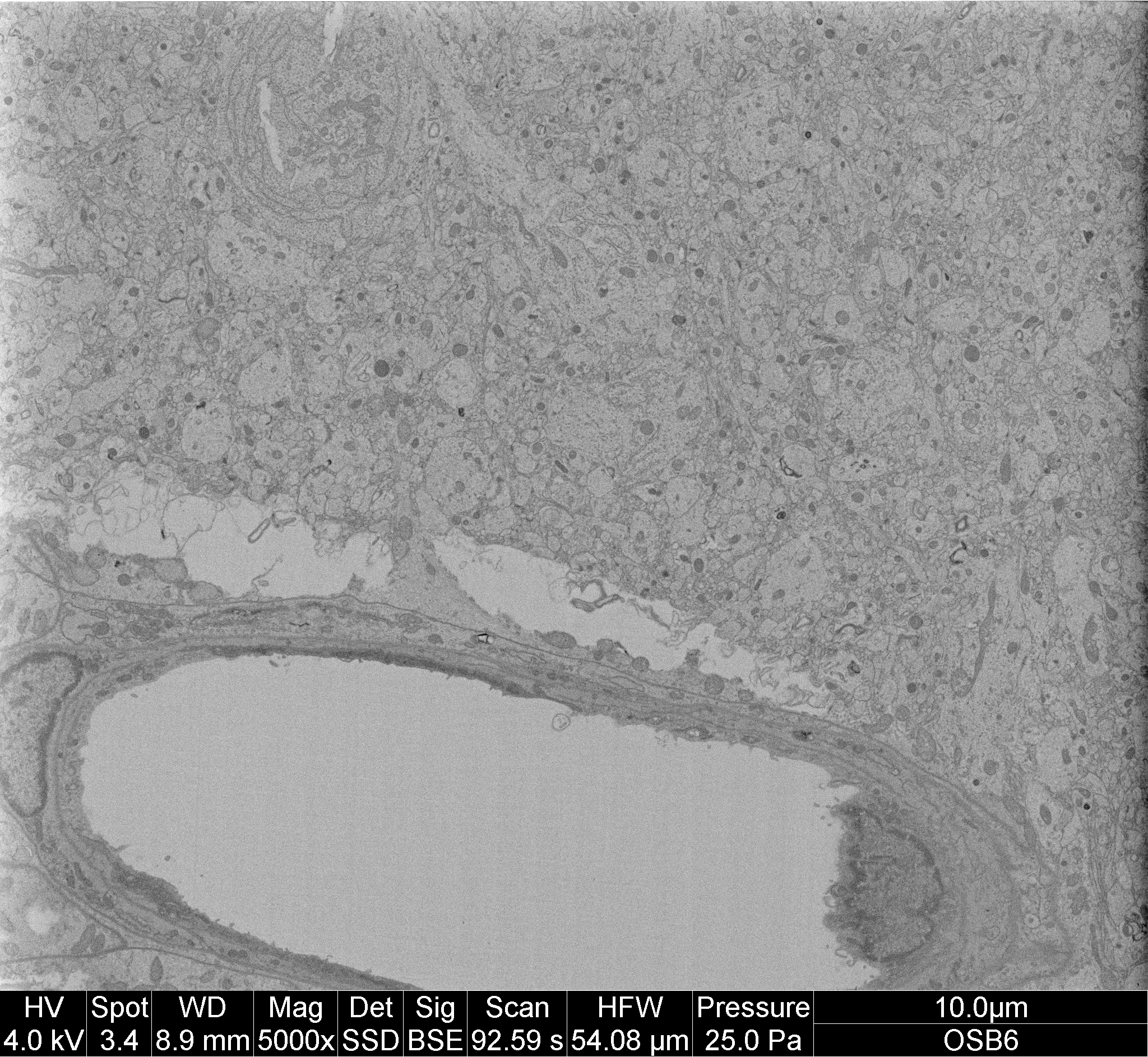

Supplement: Dataset S7 — (253.7 MB ZIP). [file pbio.0020329.sd007.zip › 040604_OS5_st1_632.tif]

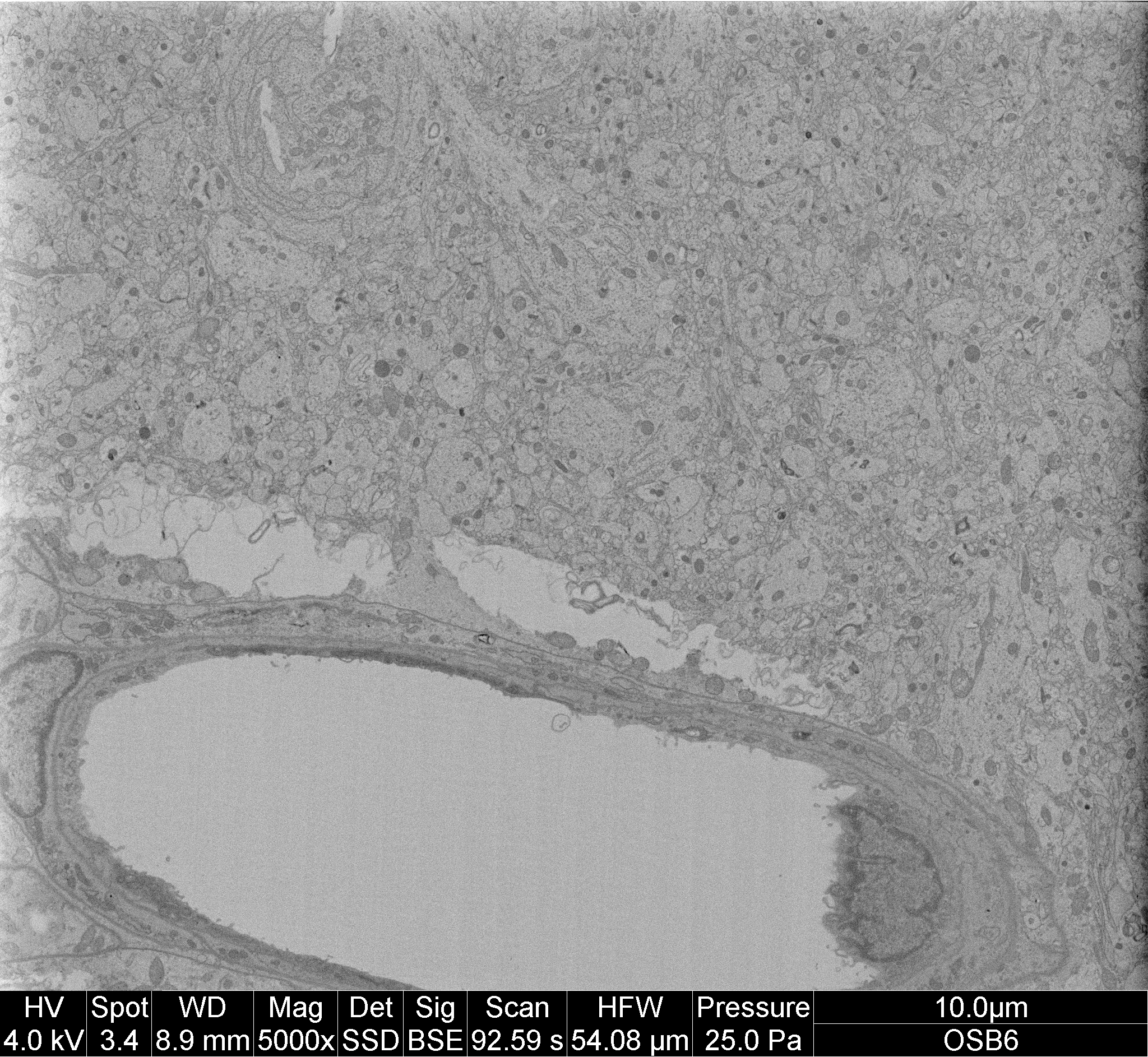

Supplement: Dataset S7 — (253.7 MB ZIP). [file pbio.0020329.sd007.zip › 040604_OS5_st1_633.tif]

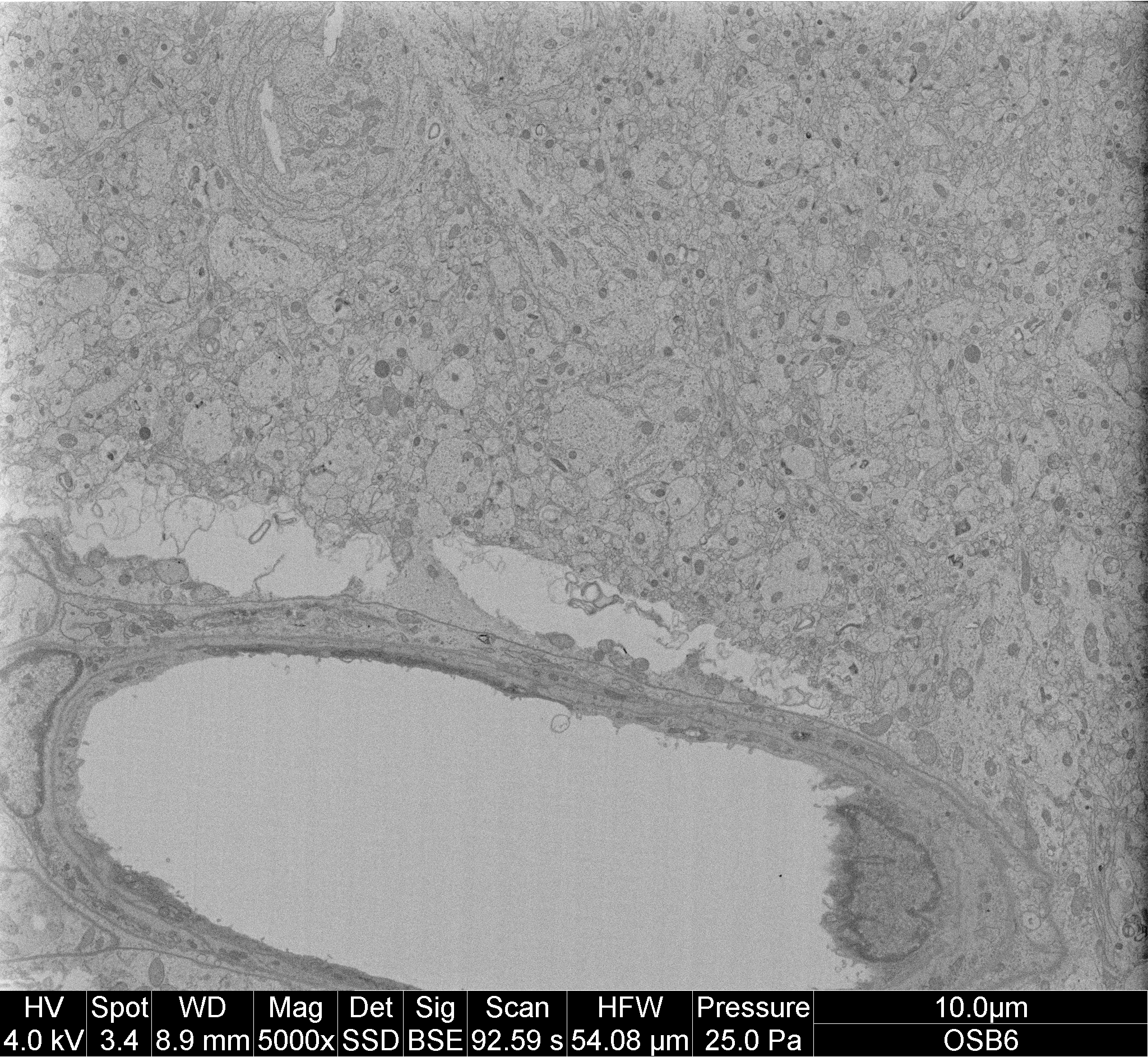

Supplement: Dataset S7 — (253.7 MB ZIP). [file pbio.0020329.sd007.zip › 040604_OS5_st1_634.tif]

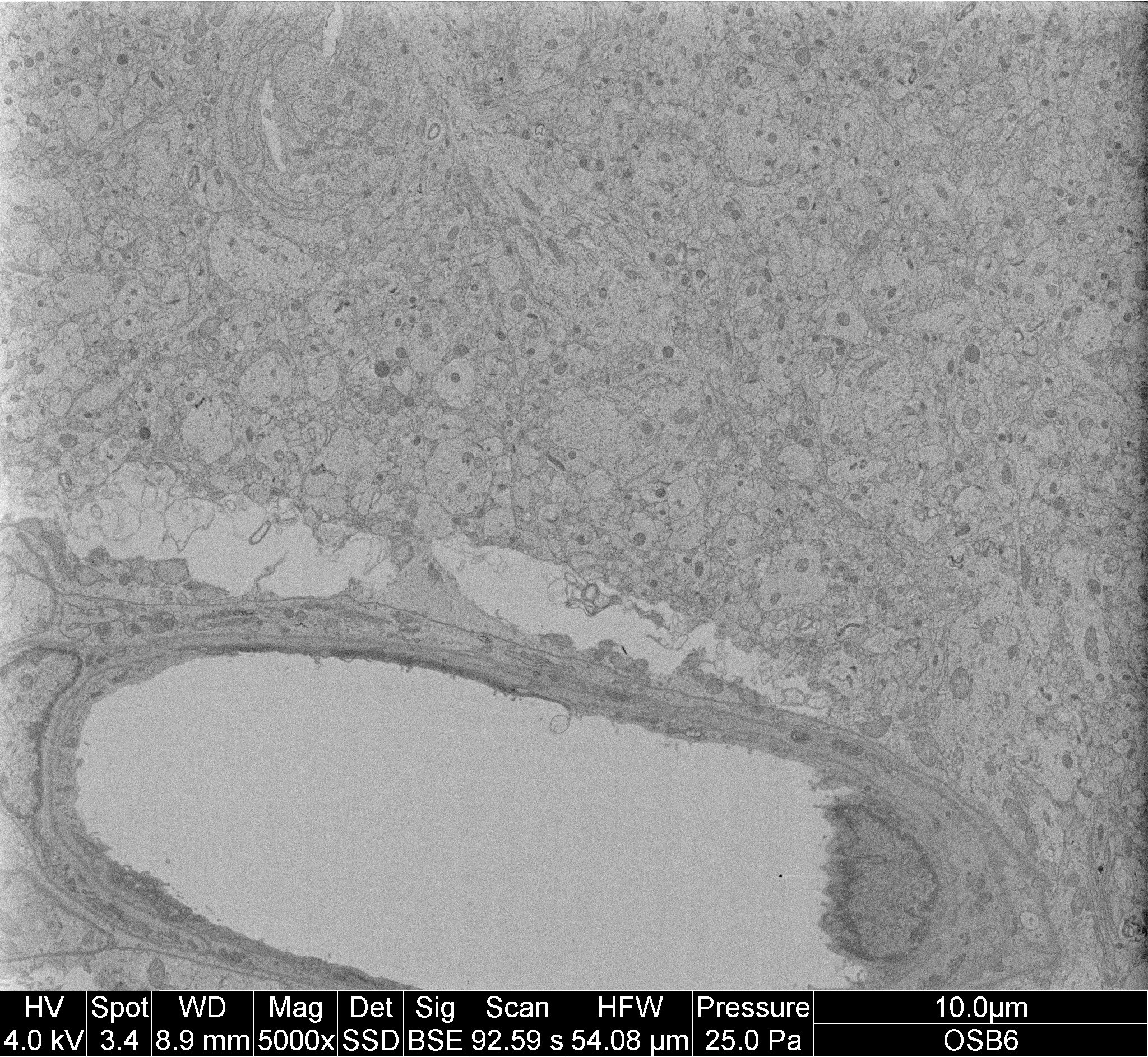

Supplement: Dataset S7 — (253.7 MB ZIP). [file pbio.0020329.sd007.zip › 040604_OS5_st1_635.tif]

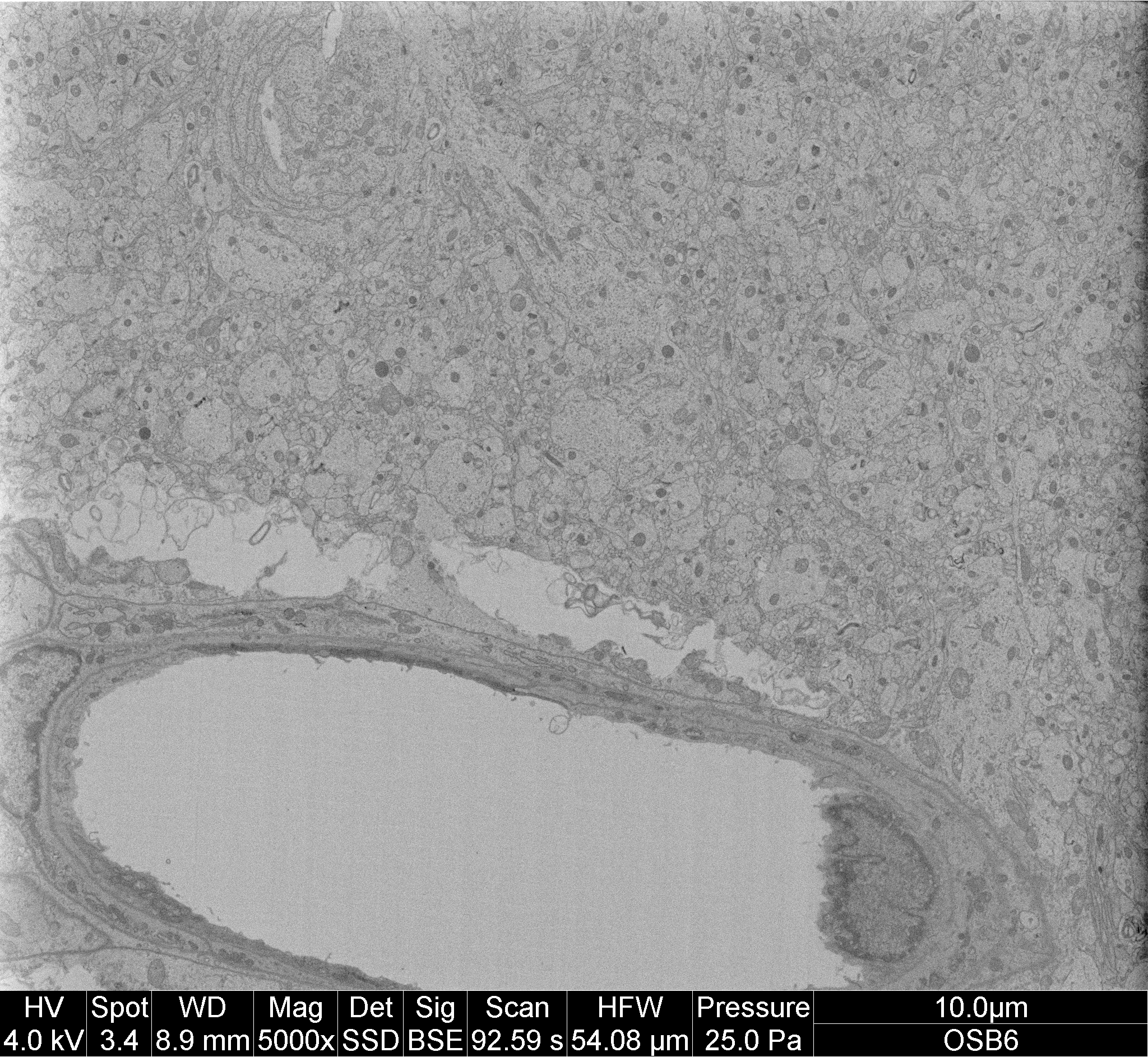

Supplement: Dataset S7 — (253.7 MB ZIP). [file pbio.0020329.sd007.zip › 040604_OS5_st1_636.tif]

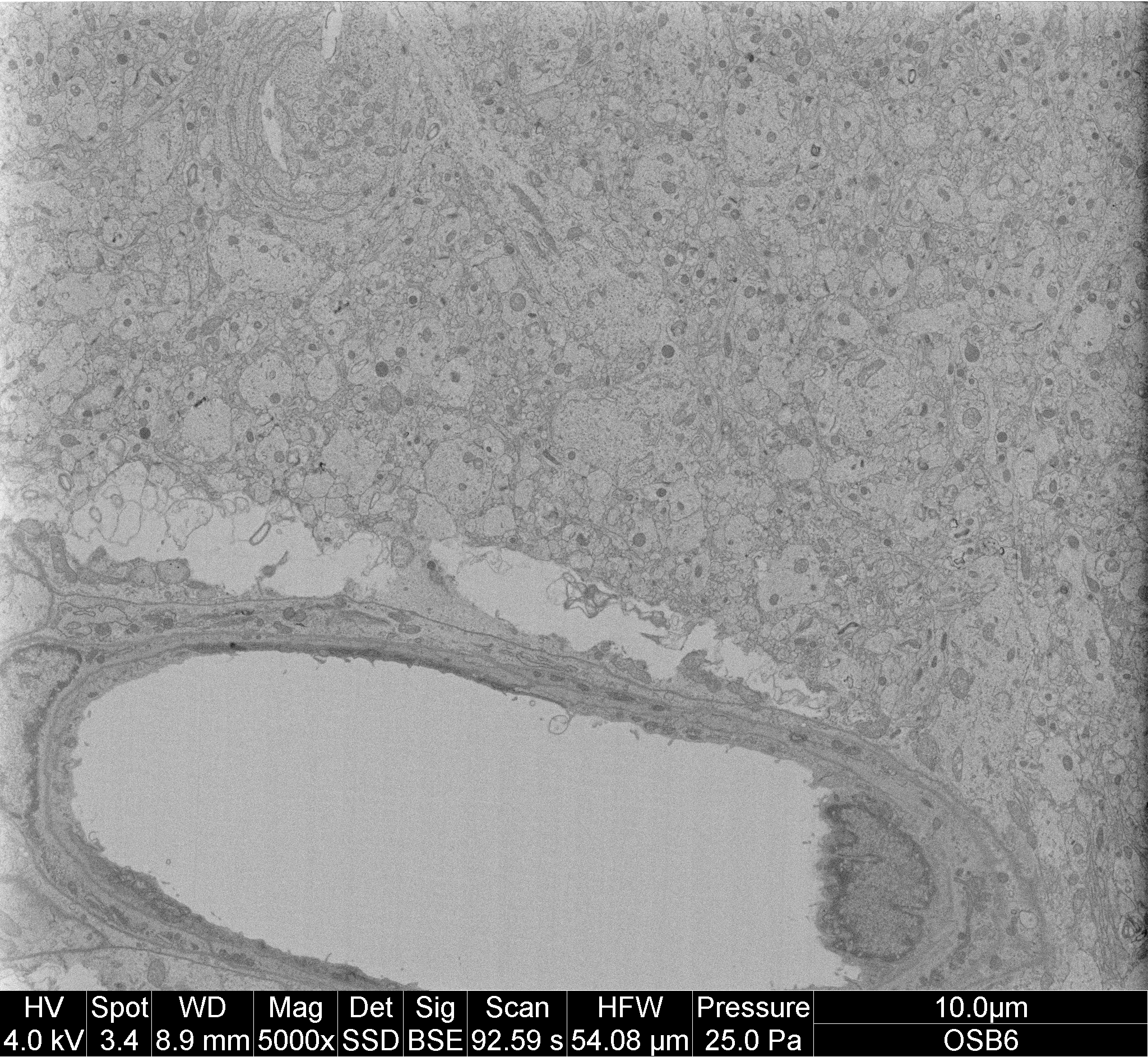

Supplement: Dataset S7 — (253.7 MB ZIP). [file pbio.0020329.sd007.zip › 040604_OS5_st1_637.tif]

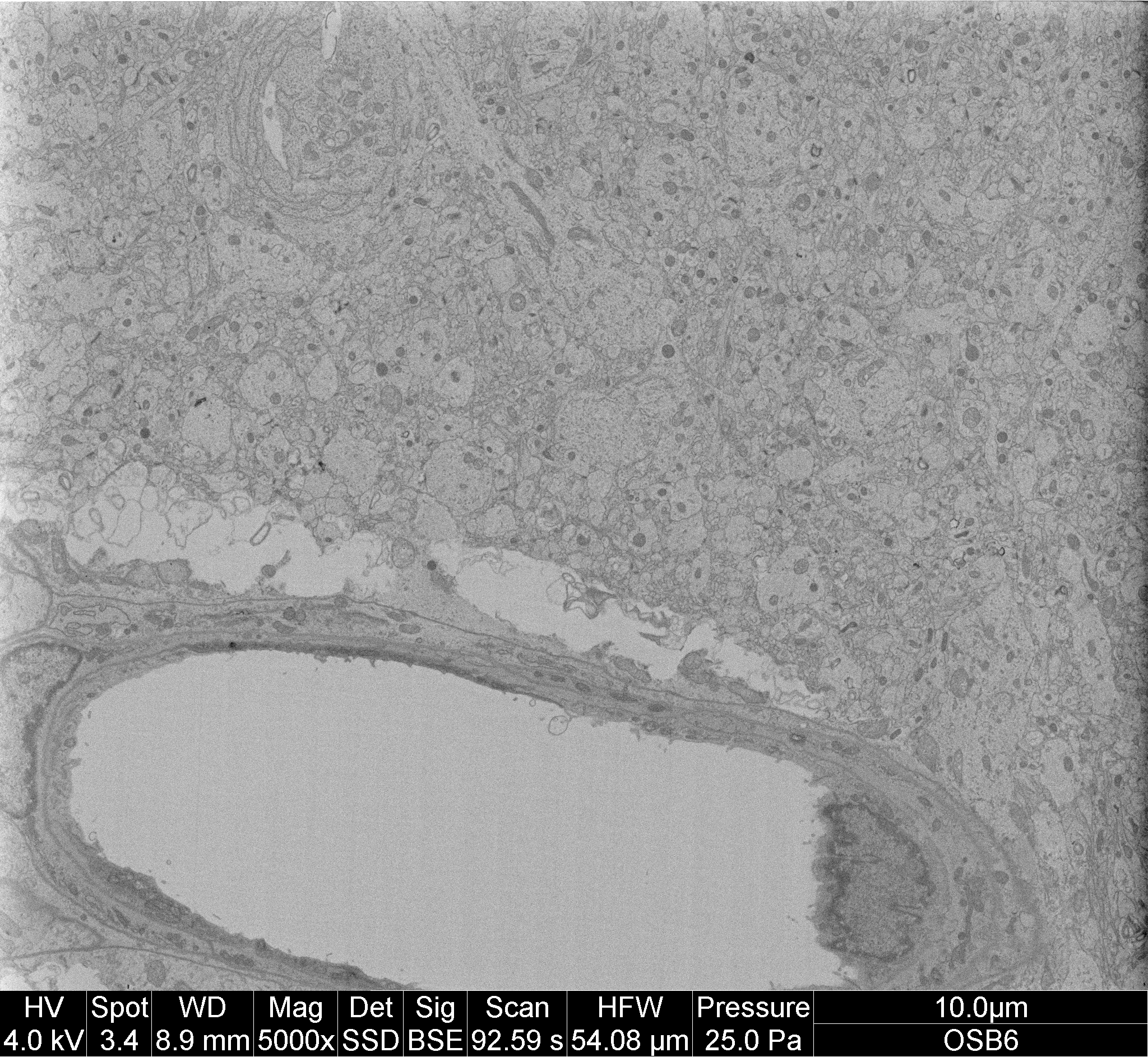

Supplement: Dataset S7 — (253.7 MB ZIP). [file pbio.0020329.sd007.zip › 040604_OS5_st1_638.tif]

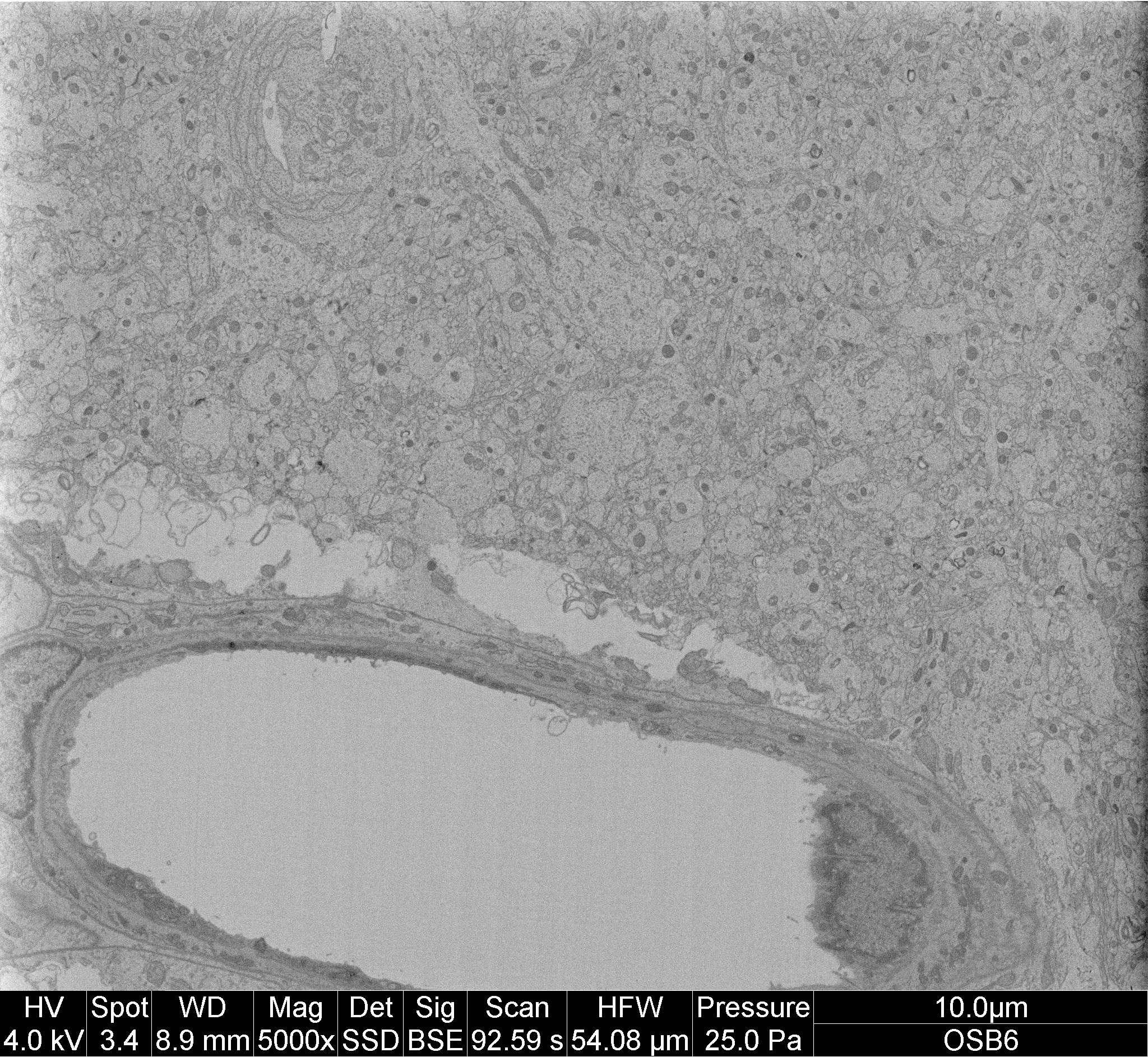

Supplement: Dataset S7 — (253.7 MB ZIP). [file pbio.0020329.sd007.zip › 040604_OS5_st1_639.tif]

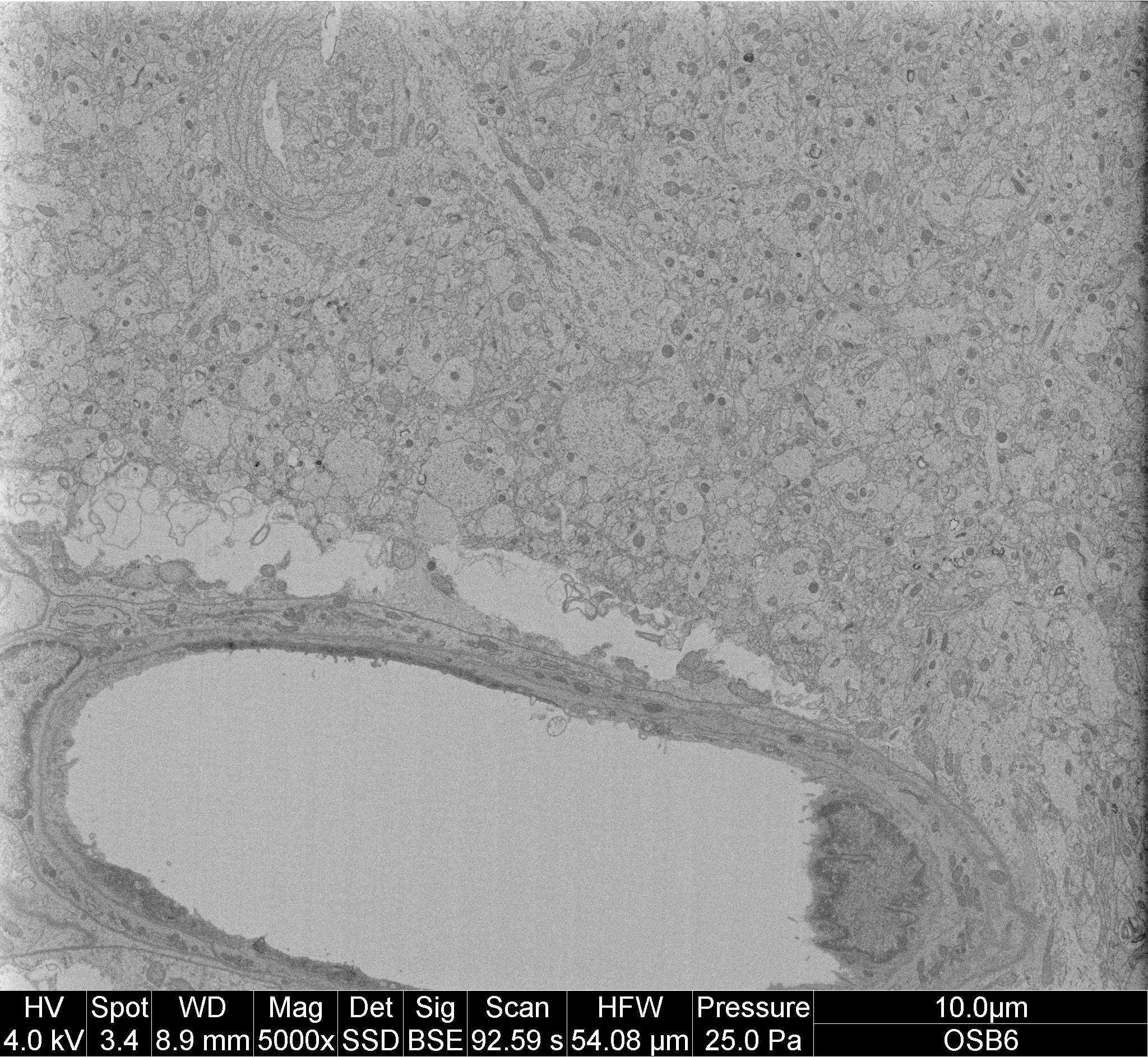

Supplement: Dataset S7 — (253.7 MB ZIP). [file pbio.0020329.sd007.zip › 040604_OS5_st1_640.tif]

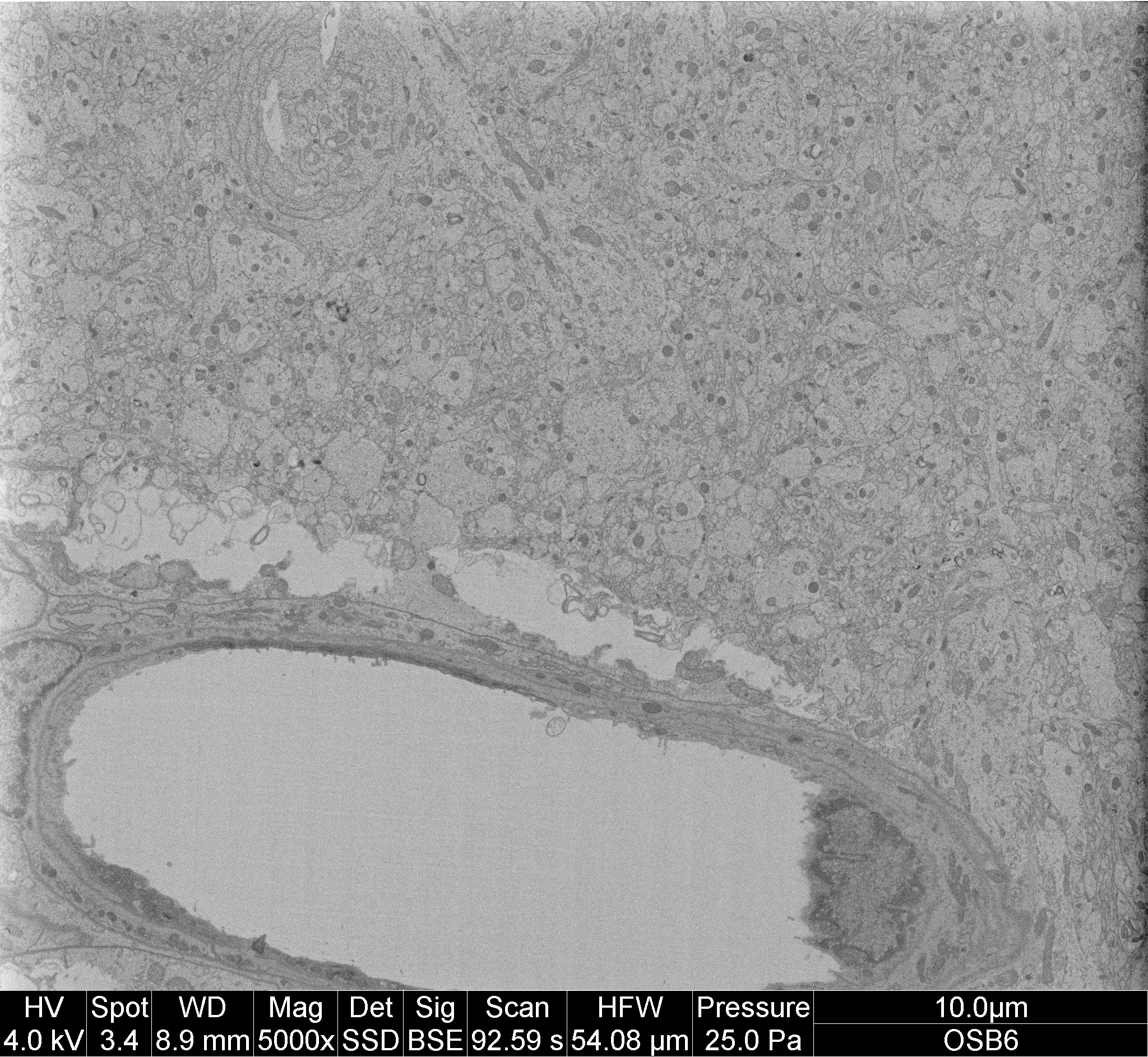

Supplement: Dataset S7 — (253.7 MB ZIP). [file pbio.0020329.sd007.zip › 040604_OS5_st1_641.tif]

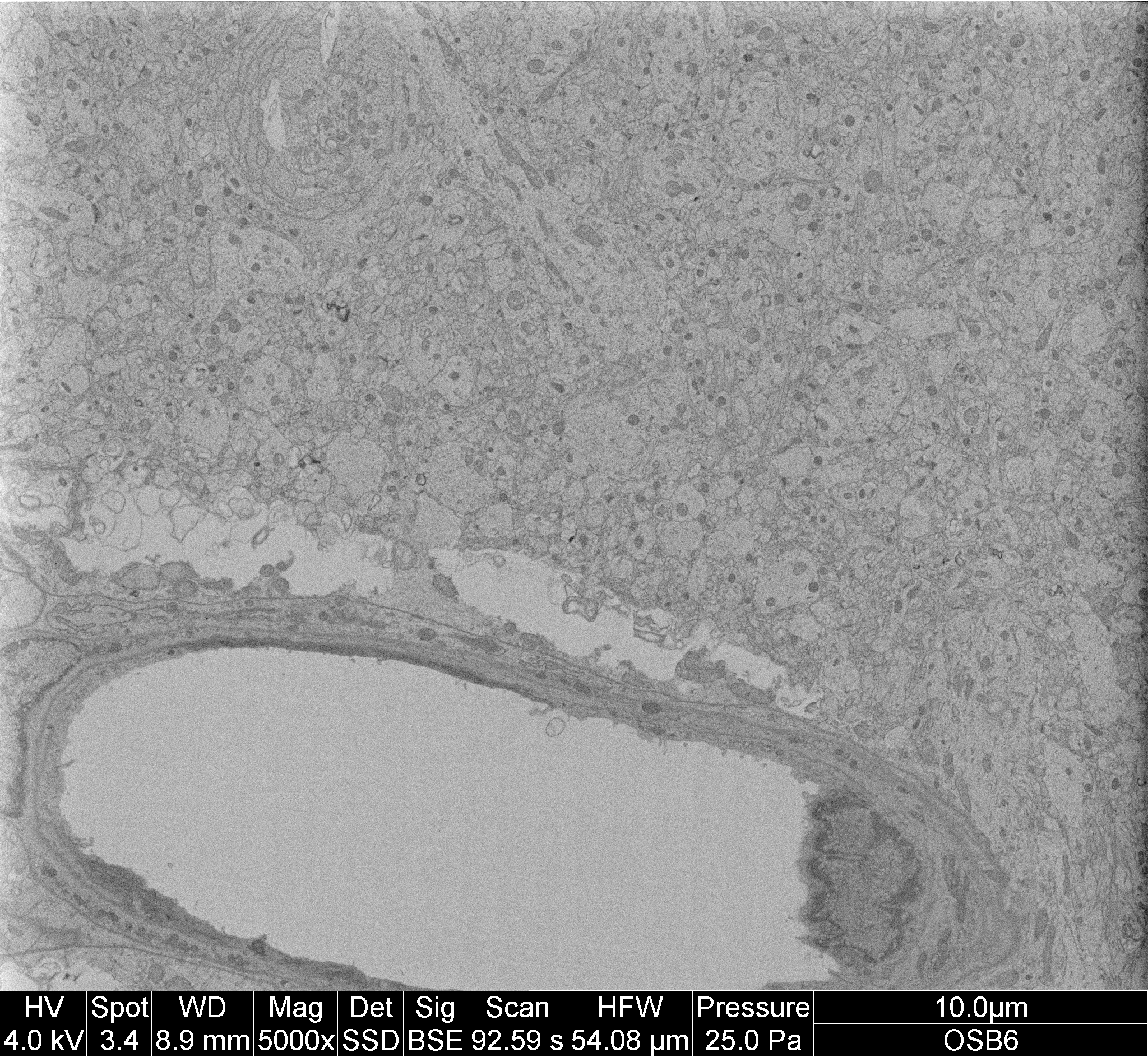

Supplement: Dataset S7 — (253.7 MB ZIP). [file pbio.0020329.sd007.zip › 040604_OS5_st1_642.tif]

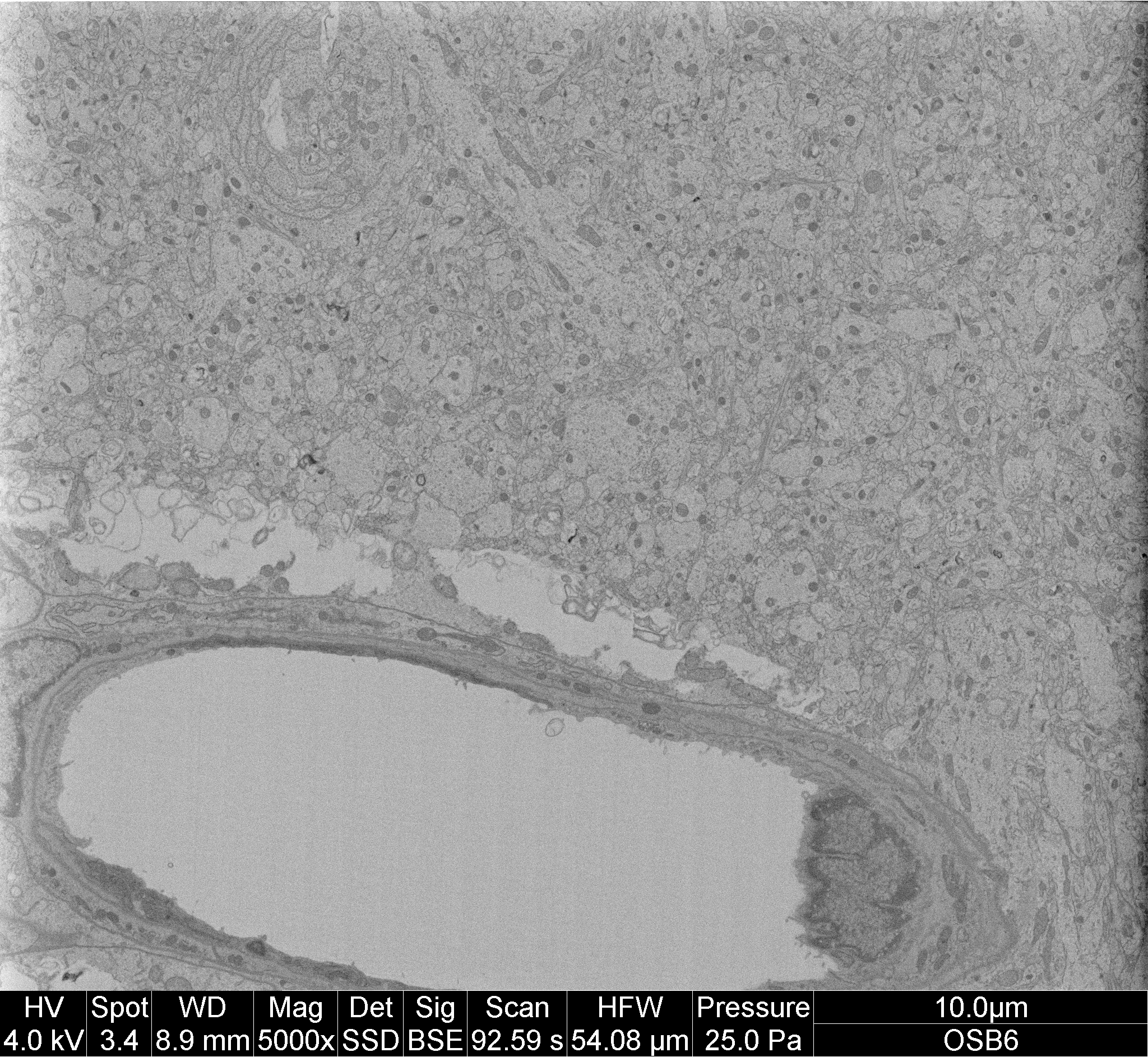

Supplement: Dataset S7 — (253.7 MB ZIP). [file pbio.0020329.sd007.zip › 040604_OS5_st1_643.tif]

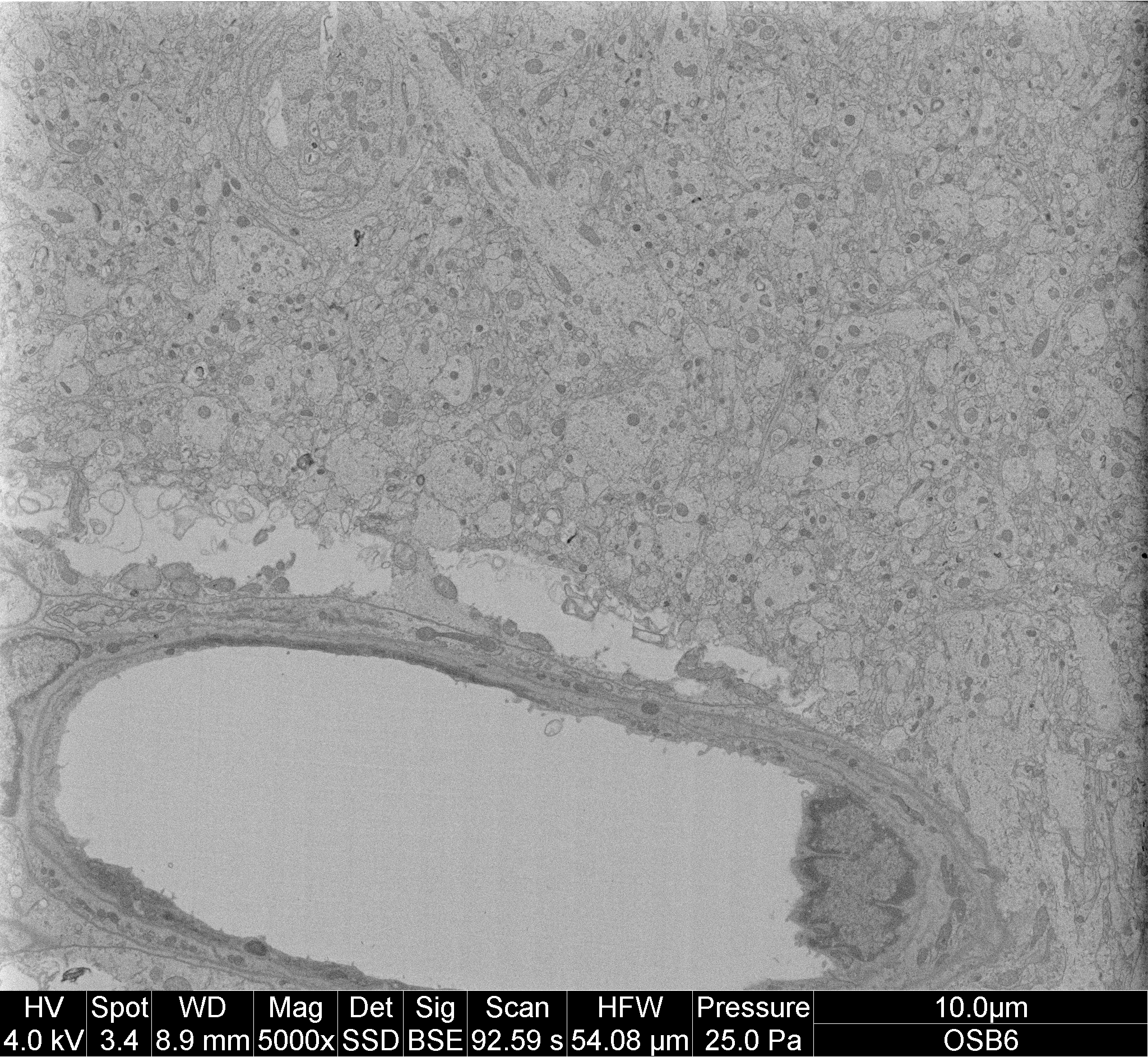

Supplement: Dataset S7 — (253.7 MB ZIP). [file pbio.0020329.sd007.zip › 040604_OS5_st1_644.tif]

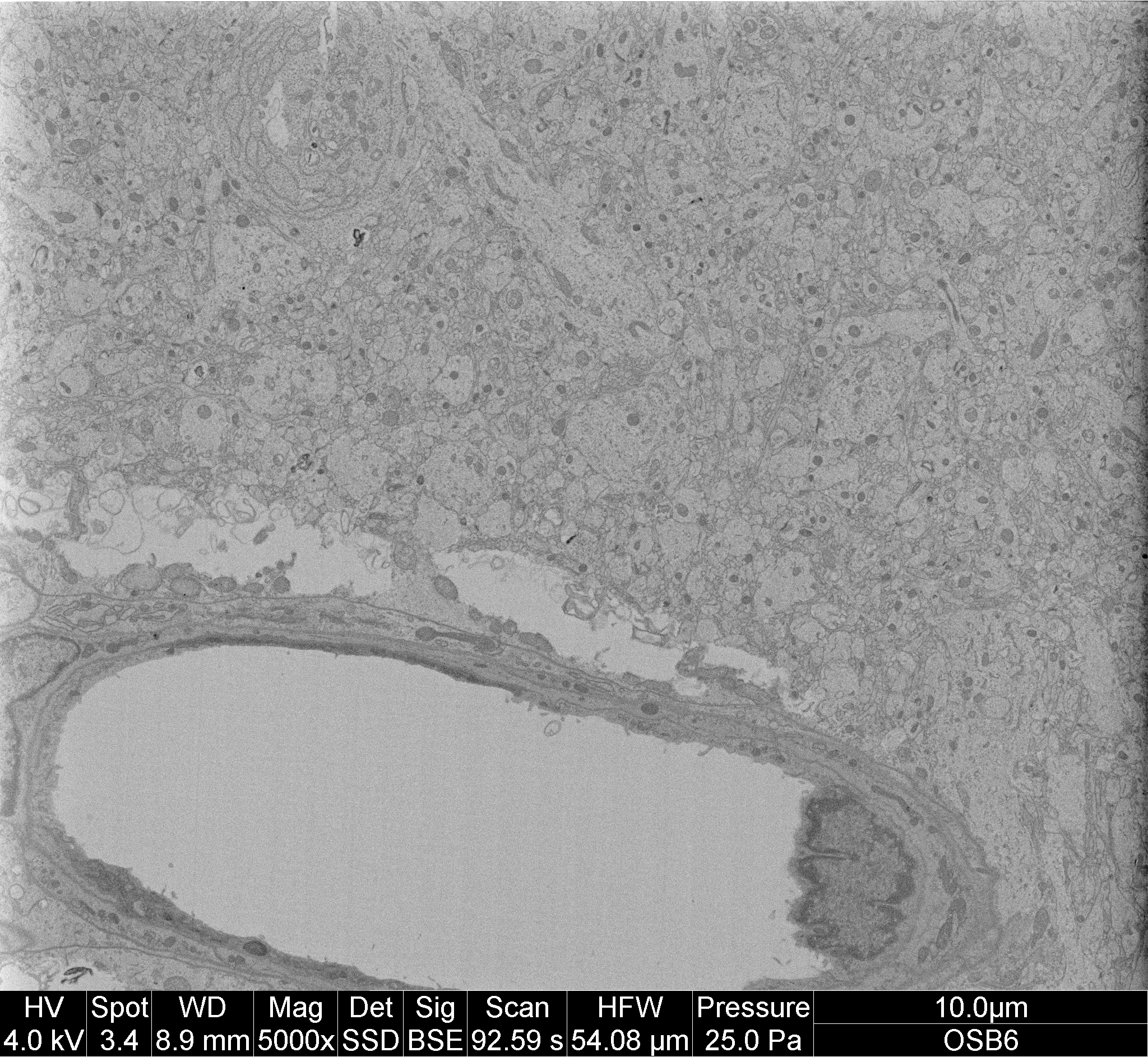

Supplement: Dataset S7 — (253.7 MB ZIP). [file pbio.0020329.sd007.zip › 040604_OS5_st1_645.tif]

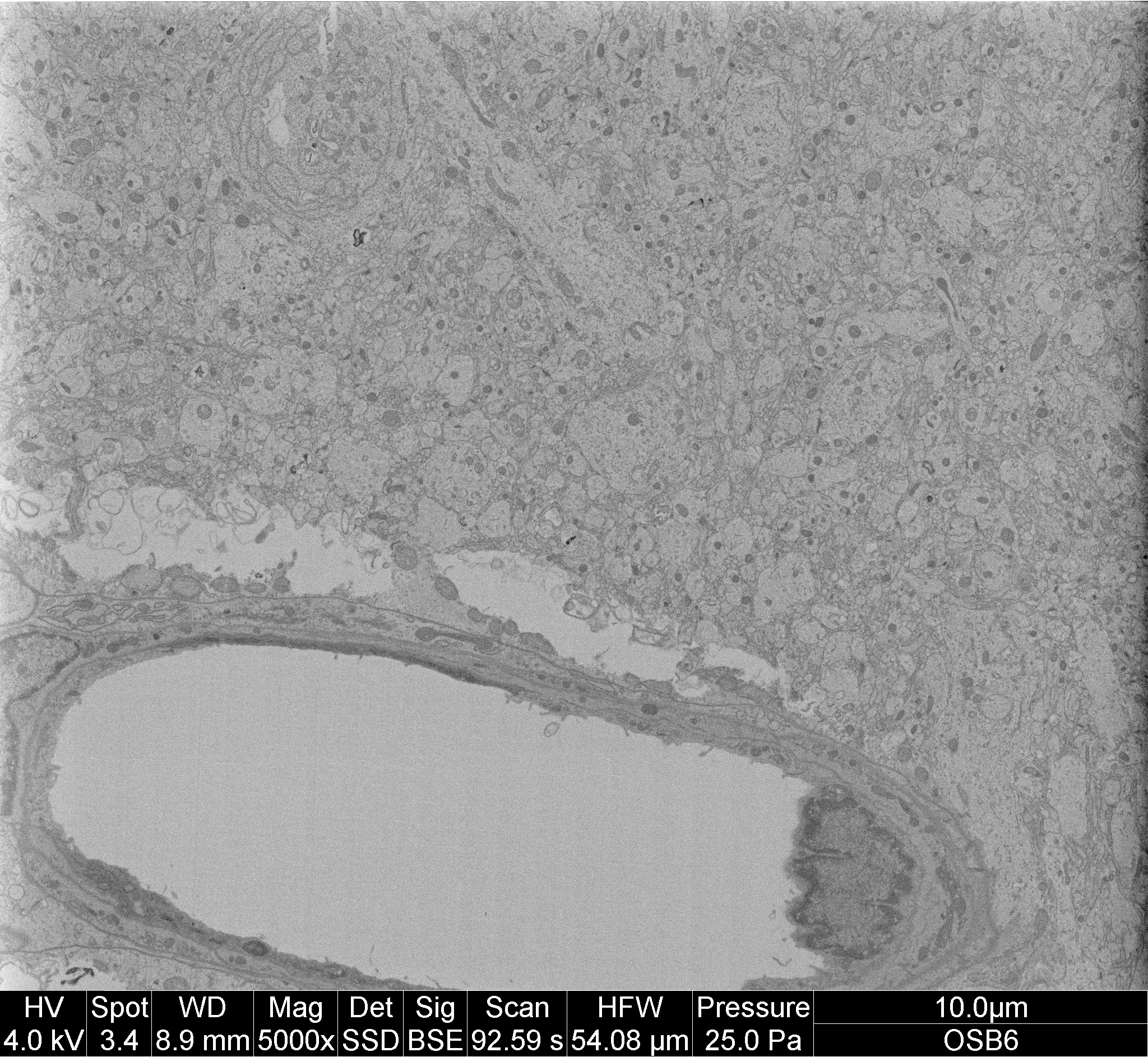

Supplement: Dataset S7 — (253.7 MB ZIP). [file pbio.0020329.sd007.zip › 040604_OS5_st1_646.tif]

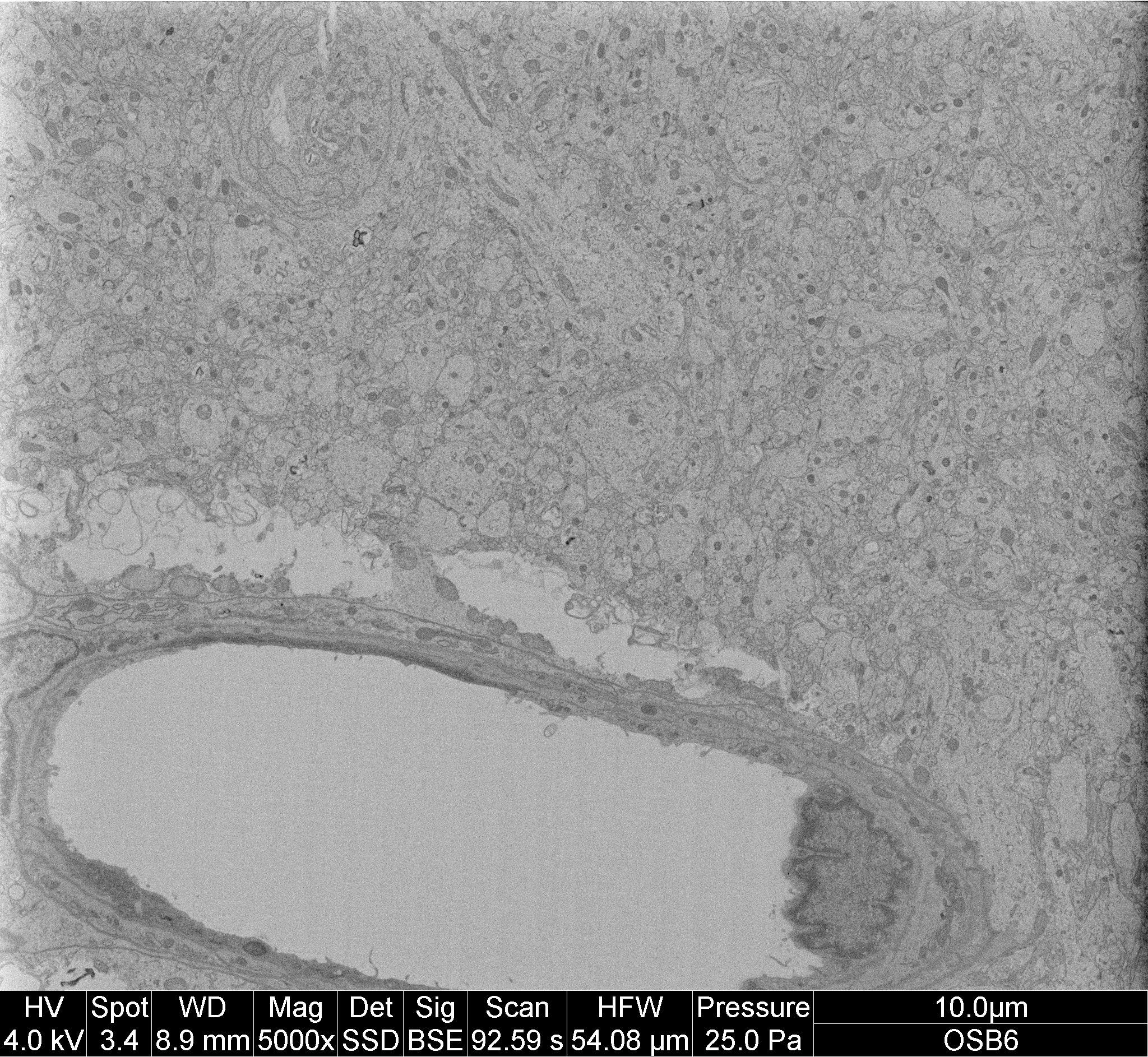

Supplement: Dataset S7 — (253.7 MB ZIP). [file pbio.0020329.sd007.zip › 040604_OS5_st1_647.tif]

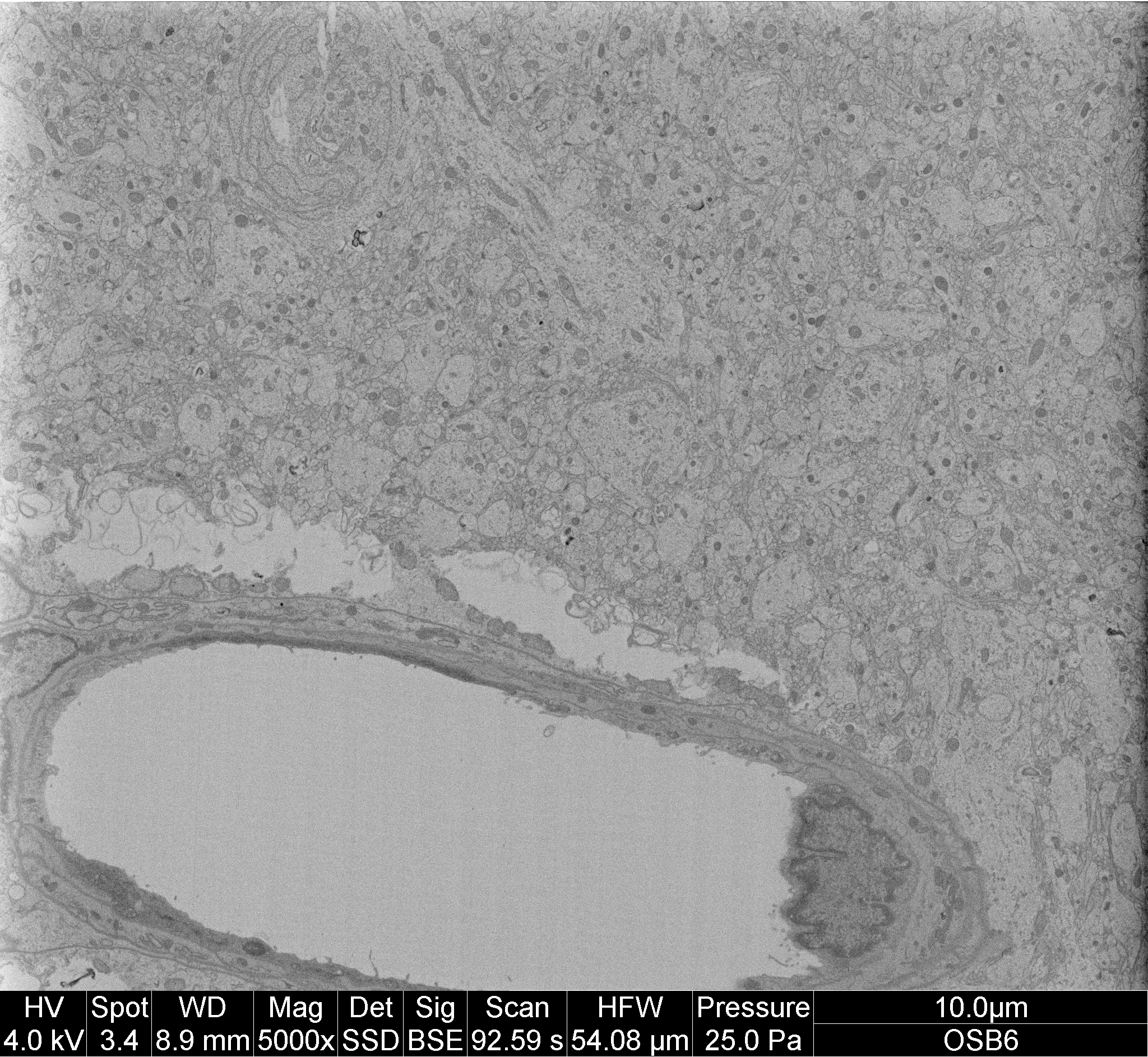

Supplement: Dataset S7 — (253.7 MB ZIP). [file pbio.0020329.sd007.zip › 040604_OS5_st1_648.tif]

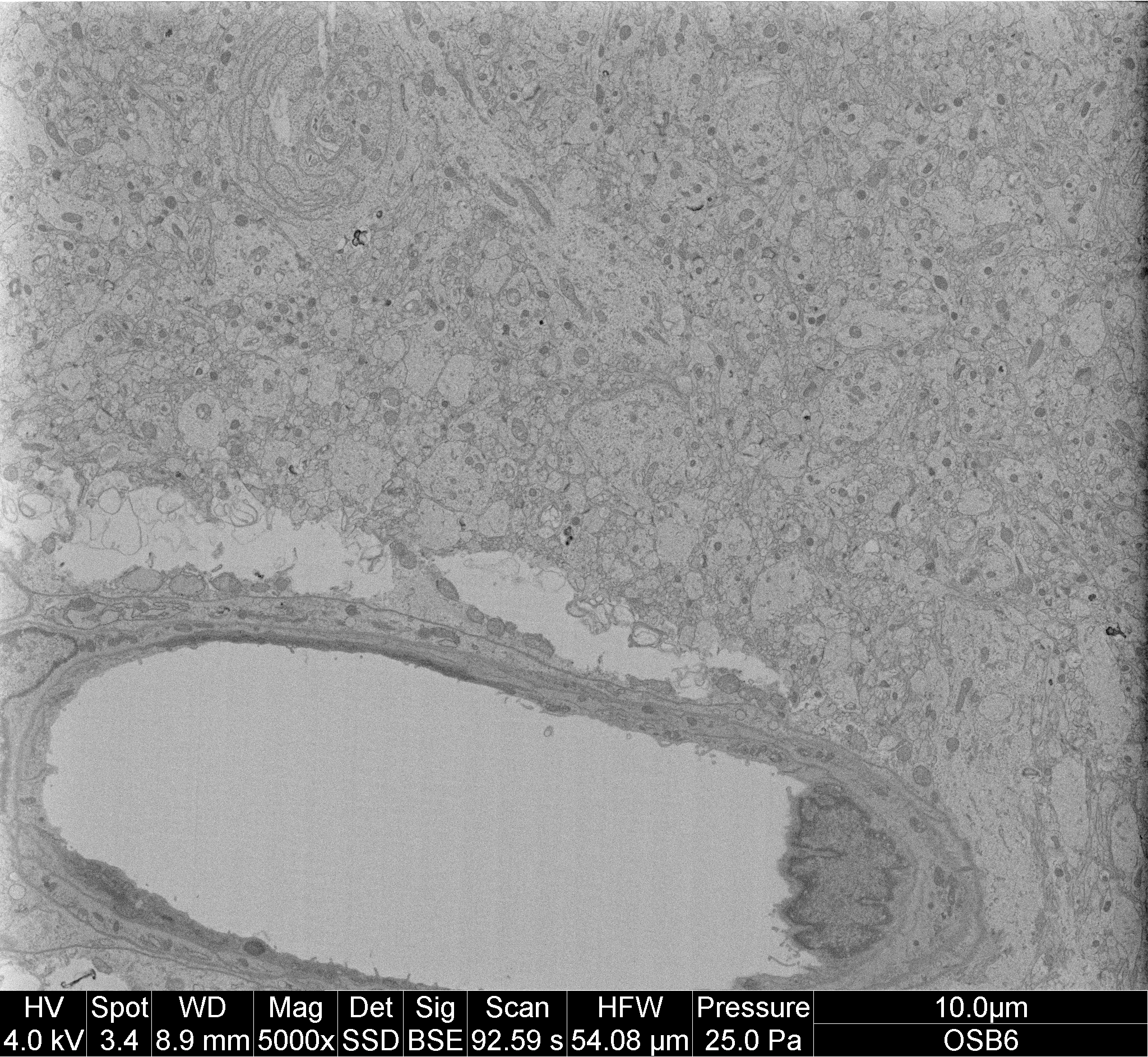

Supplement: Dataset S7 — (253.7 MB ZIP). [file pbio.0020329.sd007.zip › 040604_OS5_st1_649.tif]

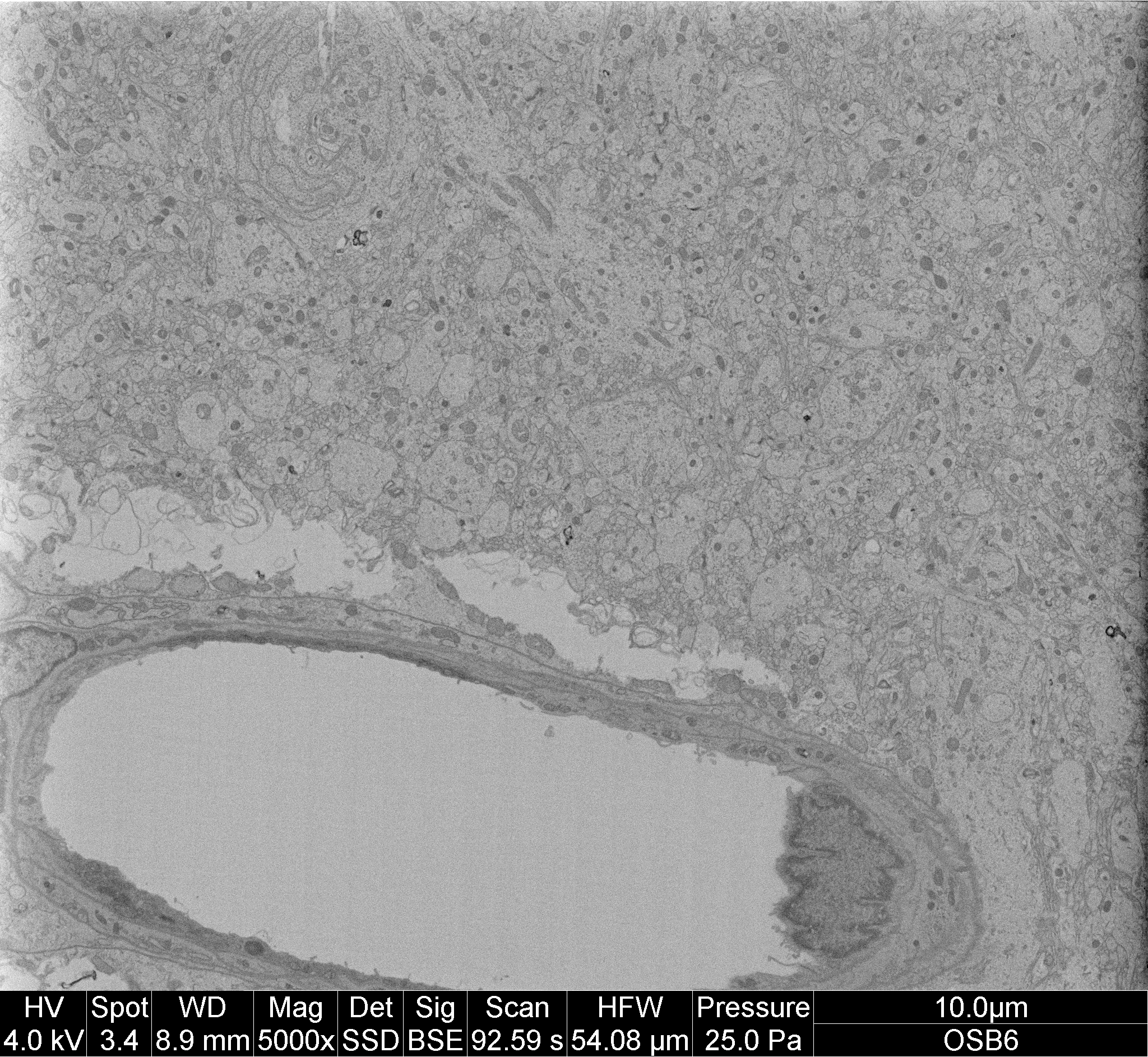

Supplement: Dataset S7 — (253.7 MB ZIP). [file pbio.0020329.sd007.zip › 040604_OS5_st1_650.tif]

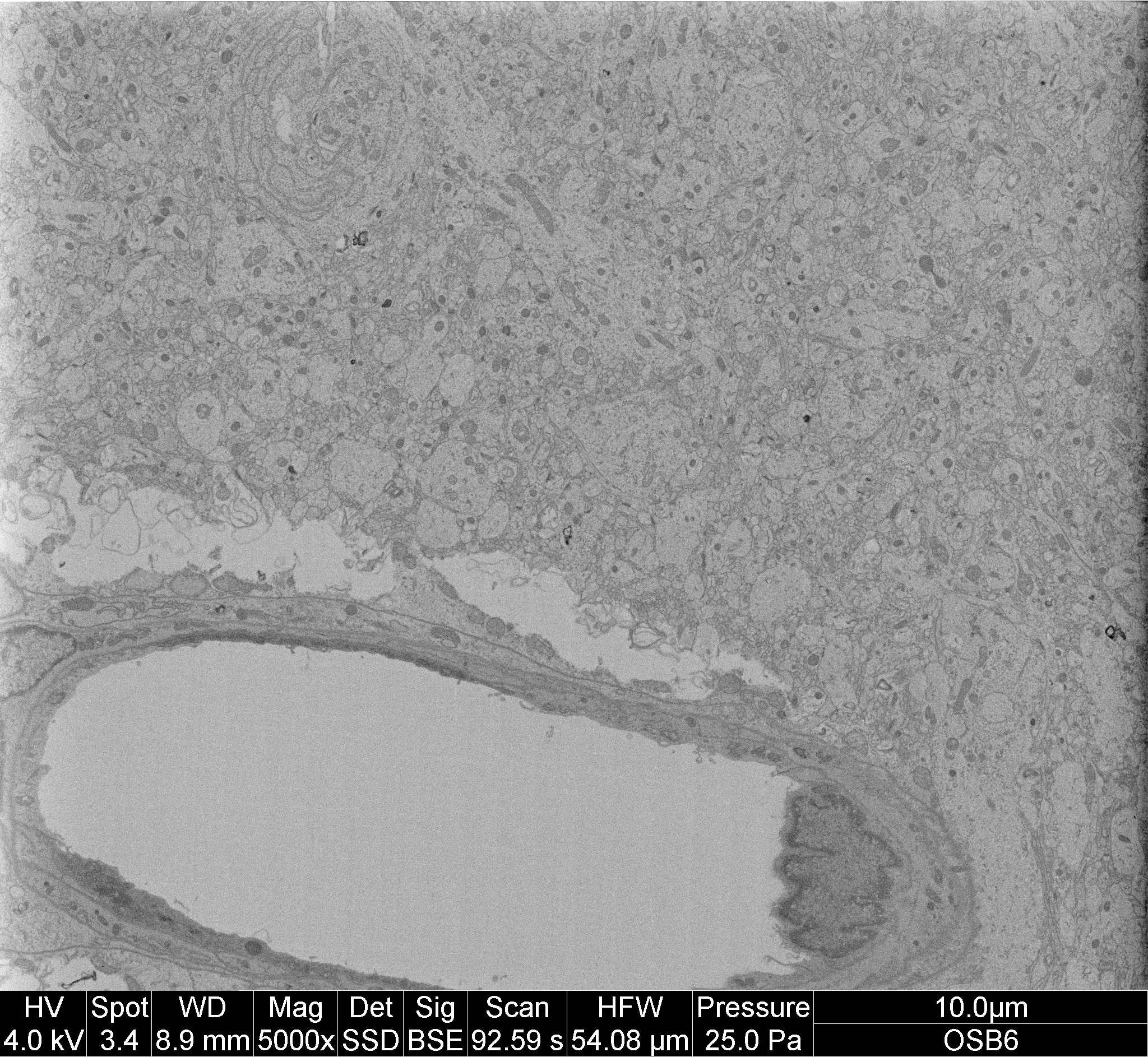

Supplement: Dataset S7 — (253.7 MB ZIP). [file pbio.0020329.sd007.zip › 040604_OS5_st1_651.tif]

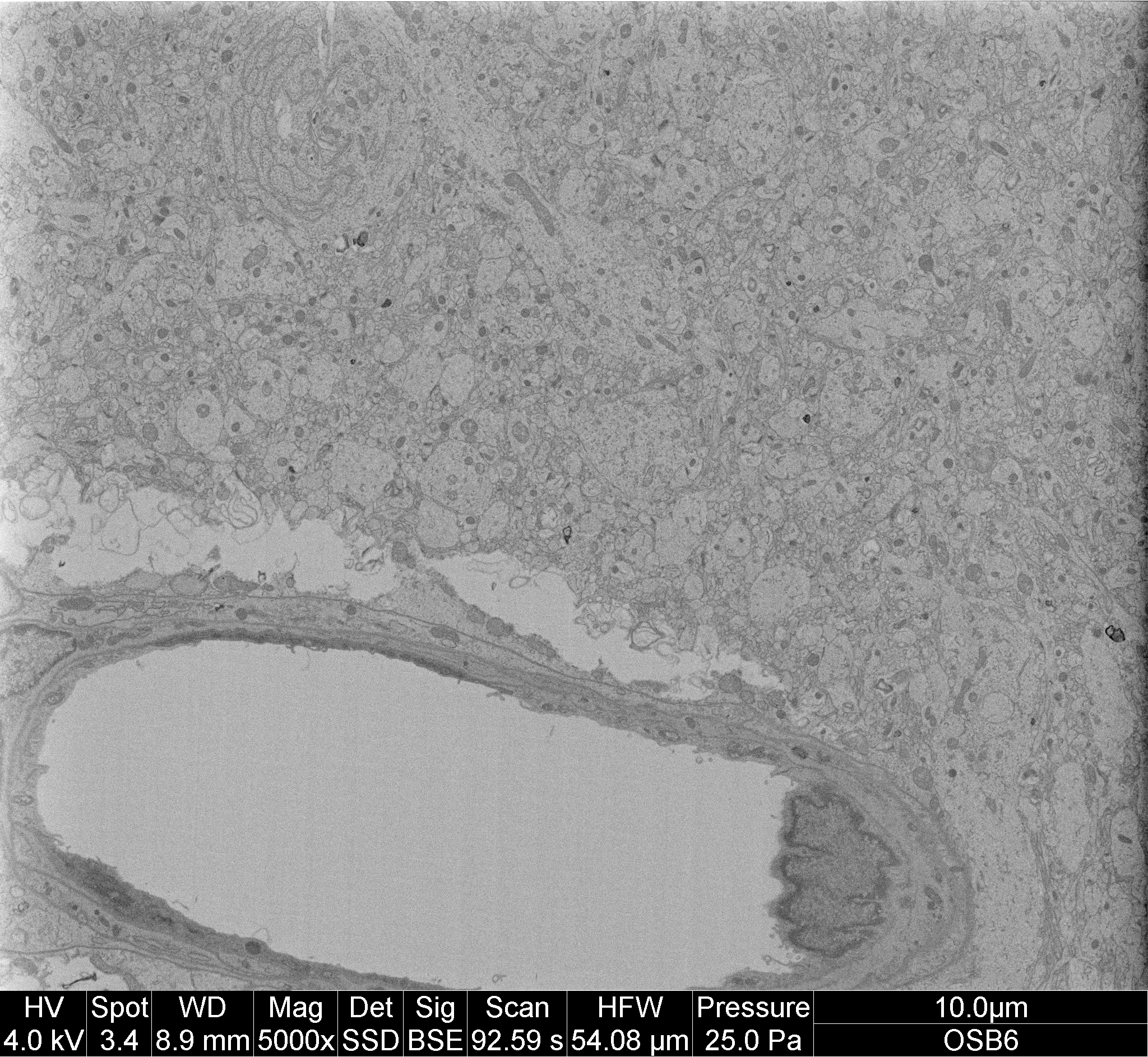

Supplement: Dataset S7 — (253.7 MB ZIP). [file pbio.0020329.sd007.zip › 040604_OS5_st1_652.tif]

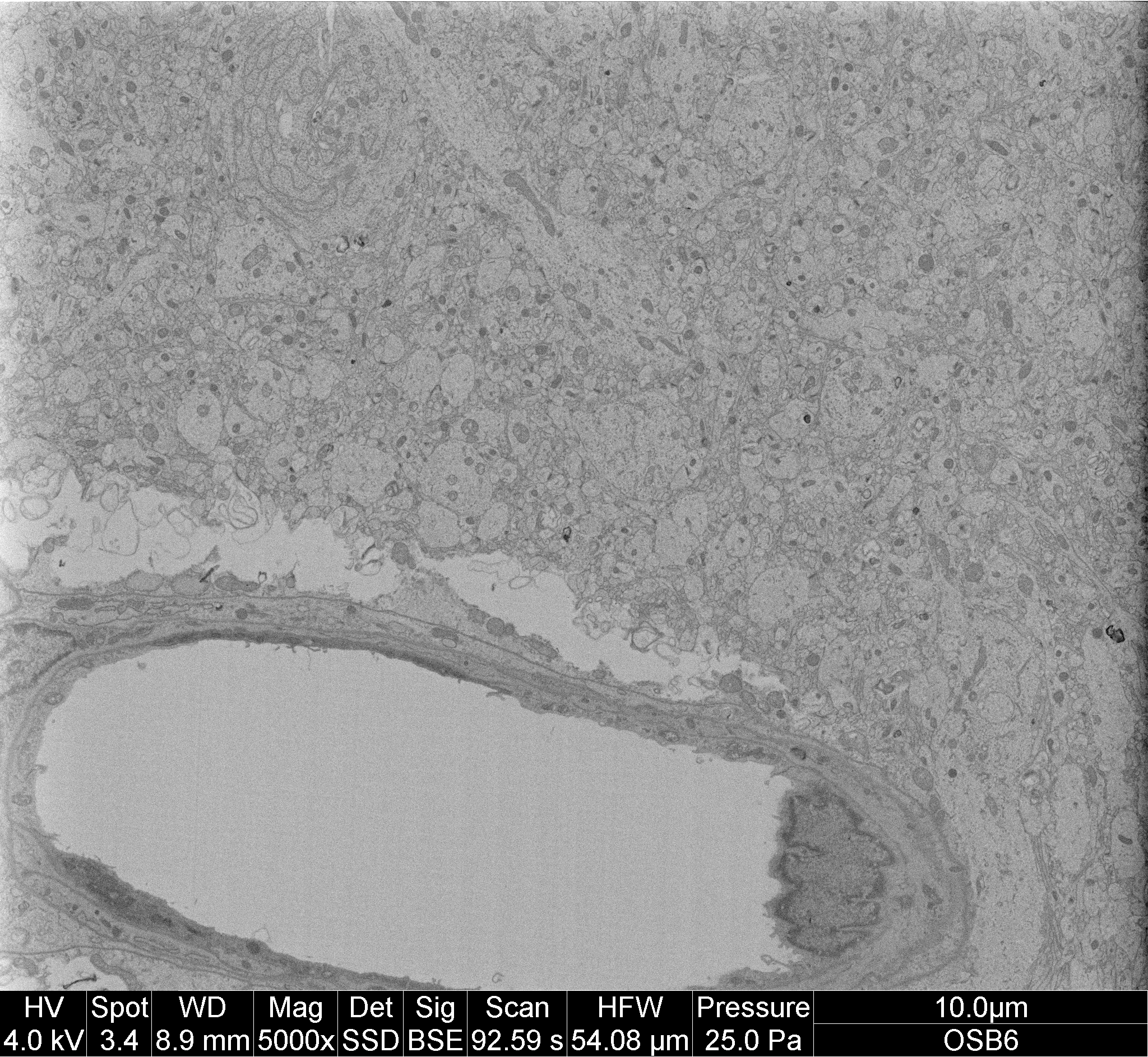

Supplement: Dataset S7 — (253.7 MB ZIP). [file pbio.0020329.sd007.zip › 040604_OS5_st1_653.tif]

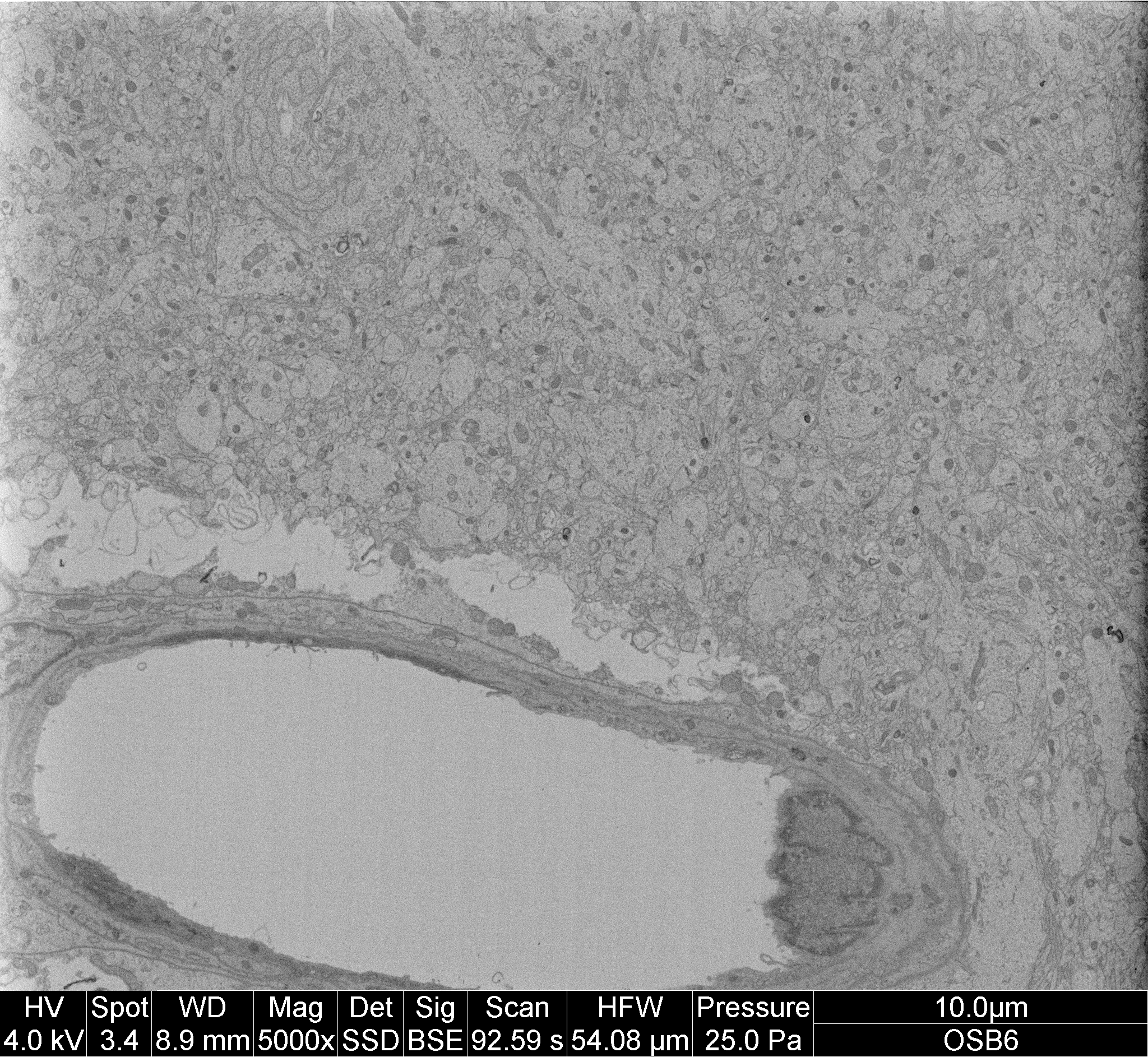

Supplement: Dataset S7 — (253.7 MB ZIP). [file pbio.0020329.sd007.zip › 040604_OS5_st1_654.tif]

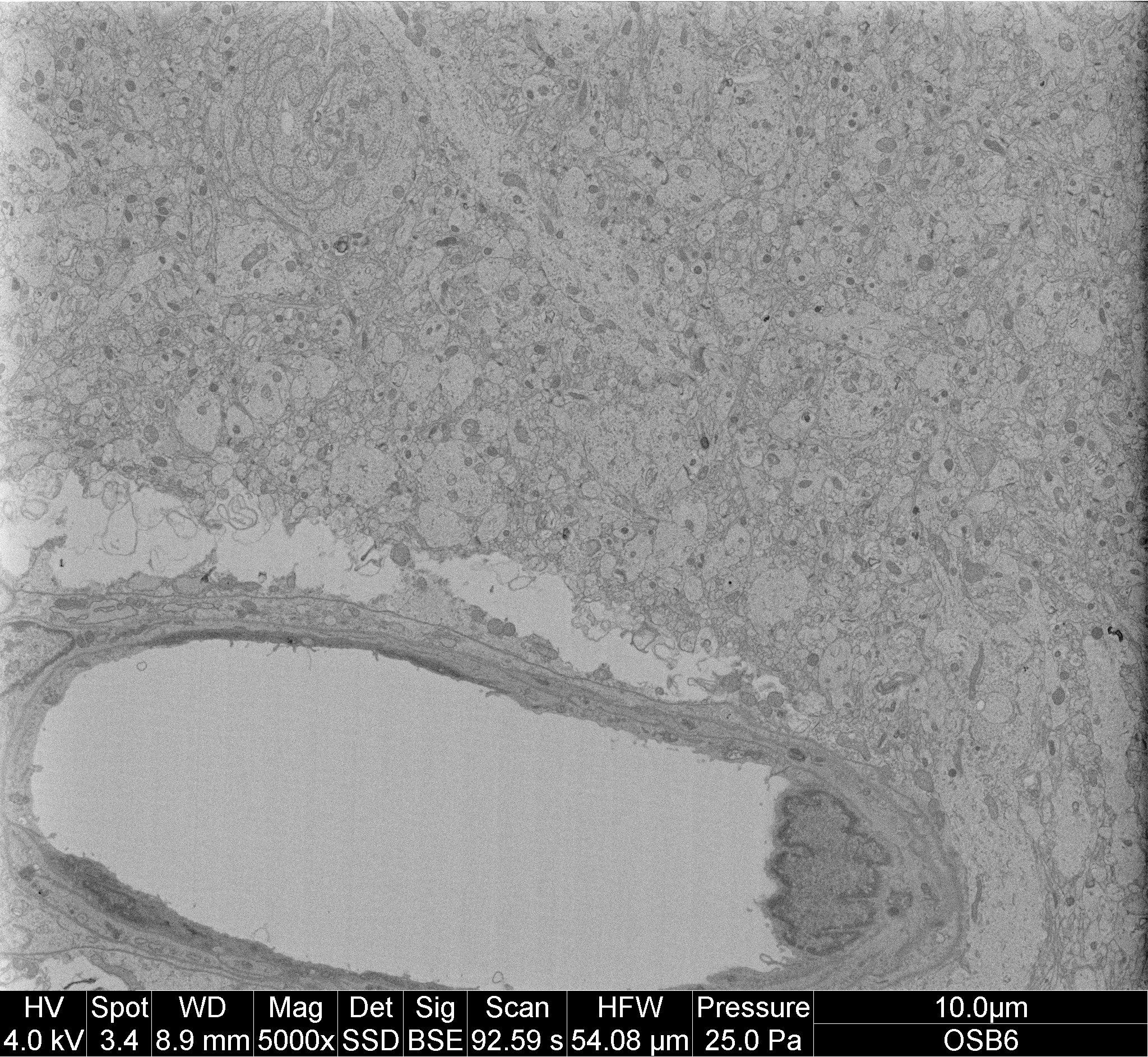

Supplement: Dataset S7 — (253.7 MB ZIP). [file pbio.0020329.sd007.zip › 040604_OS5_st1_655.tif]

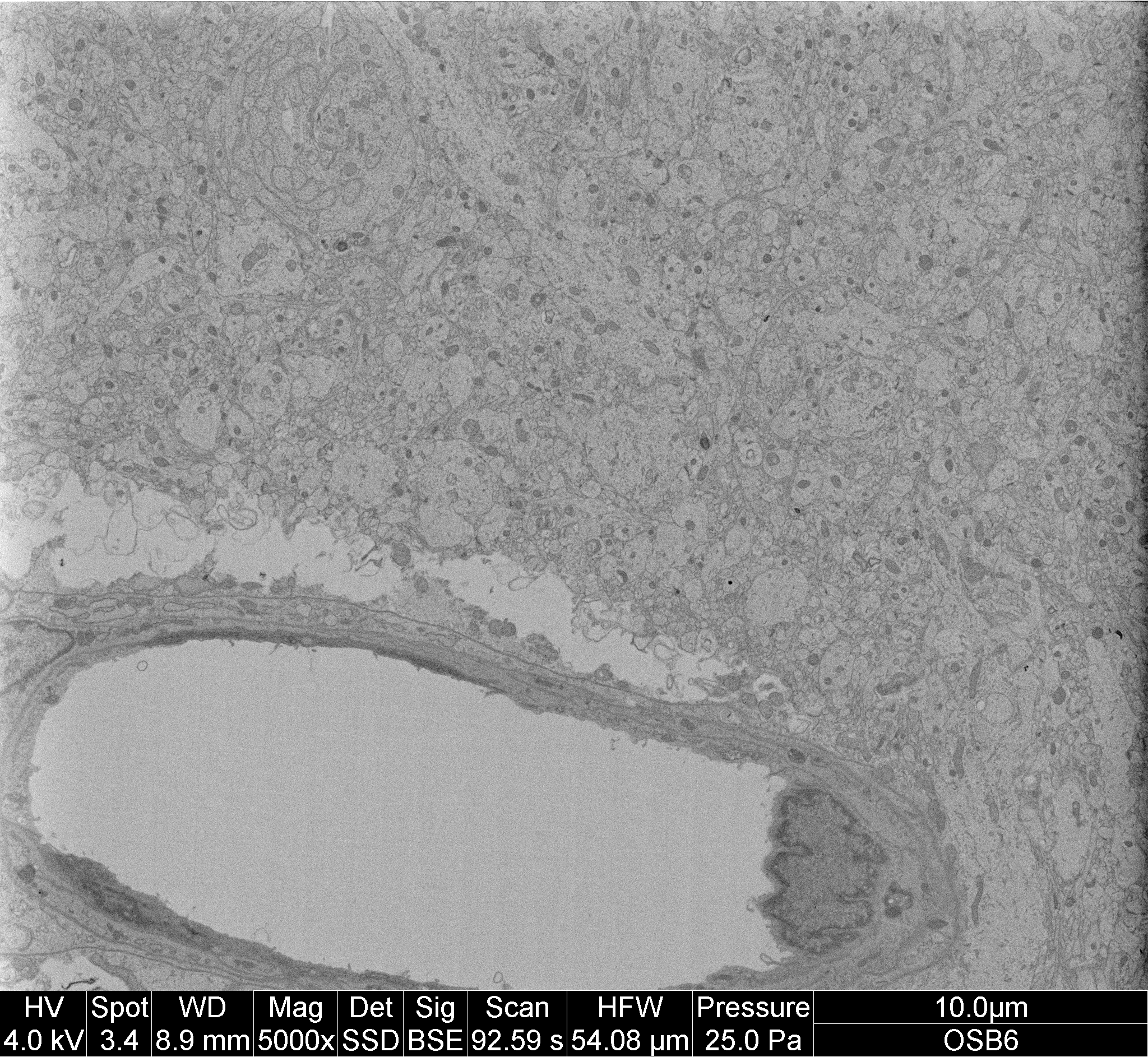

Supplement: Dataset S7 — (253.7 MB ZIP). [file pbio.0020329.sd007.zip › 040604_OS5_st1_656.tif]

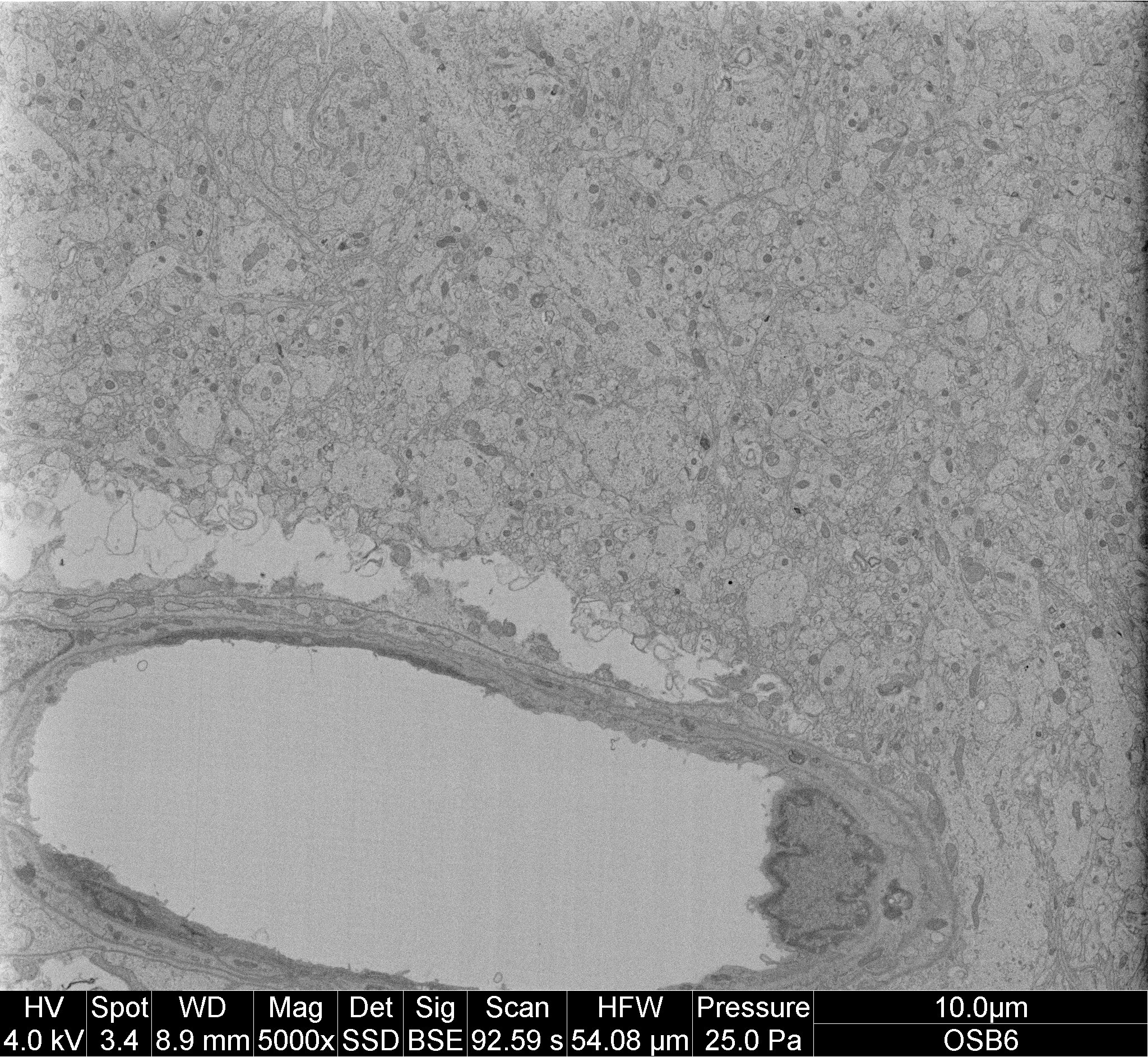

Supplement: Dataset S7 — (253.7 MB ZIP). [file pbio.0020329.sd007.zip › 040604_OS5_st1_657.tif]

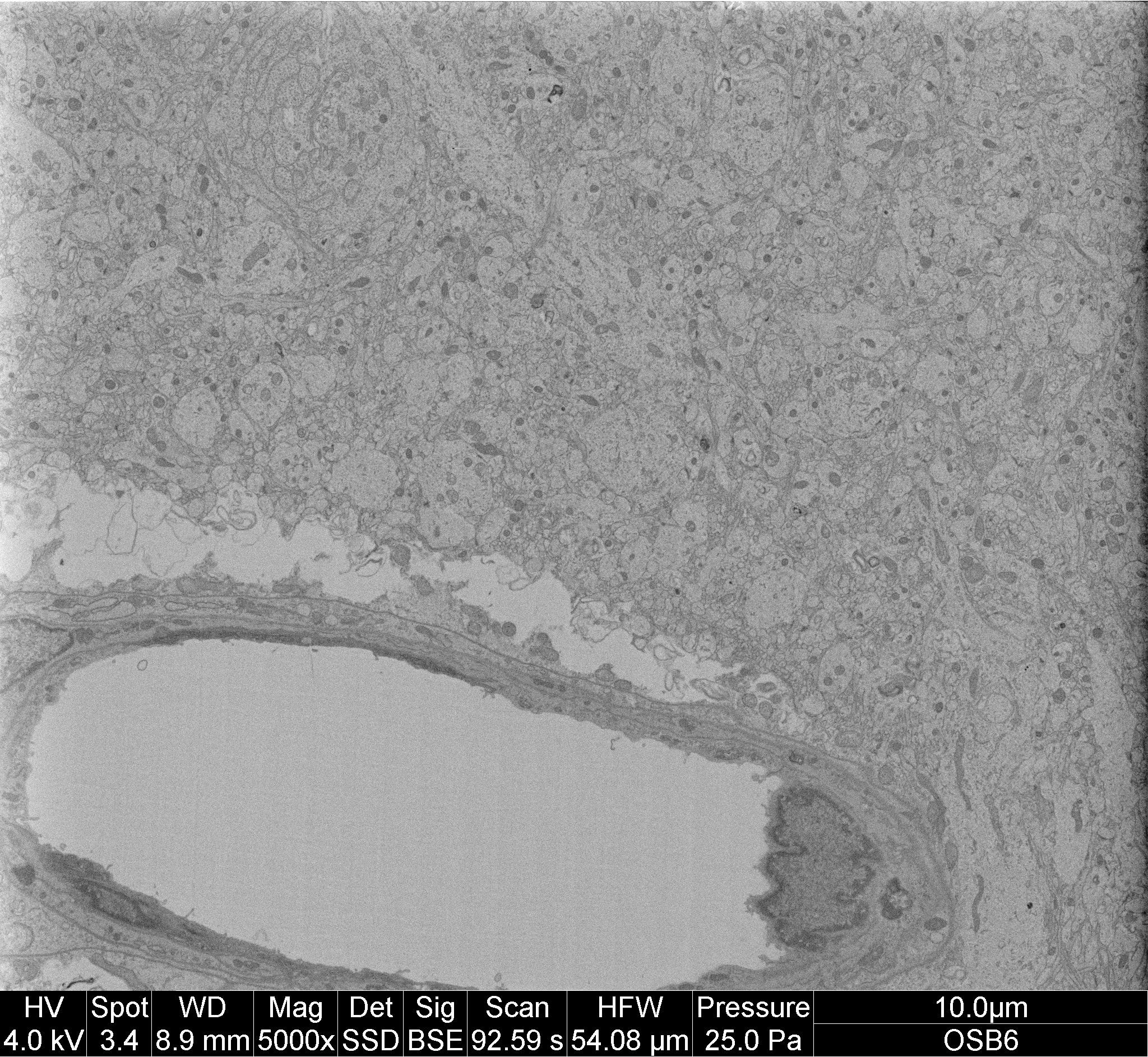

Supplement: Dataset S7 — (253.7 MB ZIP). [file pbio.0020329.sd007.zip › 040604_OS5_st1_658.tif]

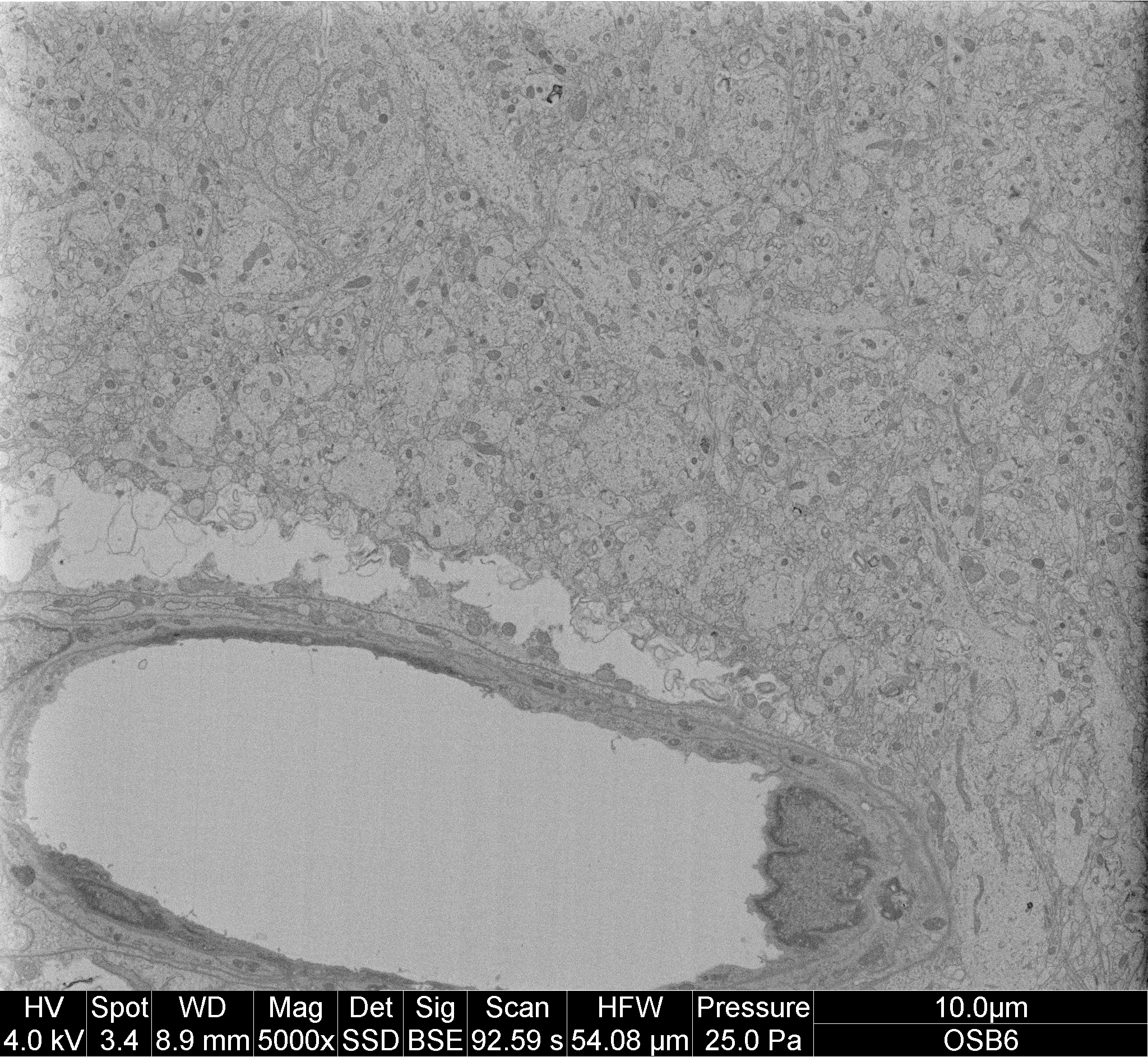

Supplement: Dataset S7 — (253.7 MB ZIP). [file pbio.0020329.sd007.zip › 040604_OS5_st1_659.tif]

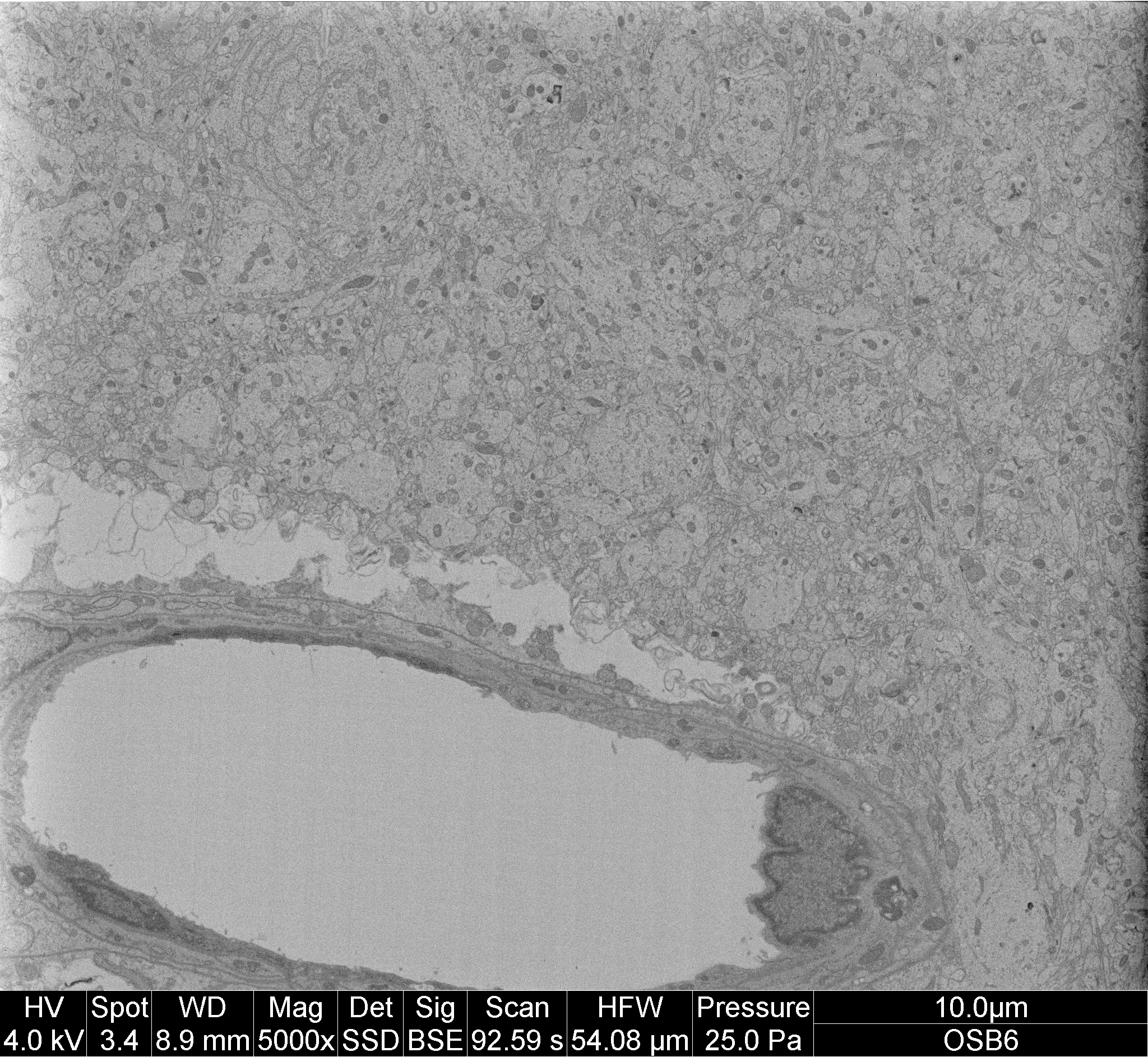

Supplement: Dataset S7 — (253.7 MB ZIP). [file pbio.0020329.sd007.zip › 040604_OS5_st1_660.tif]

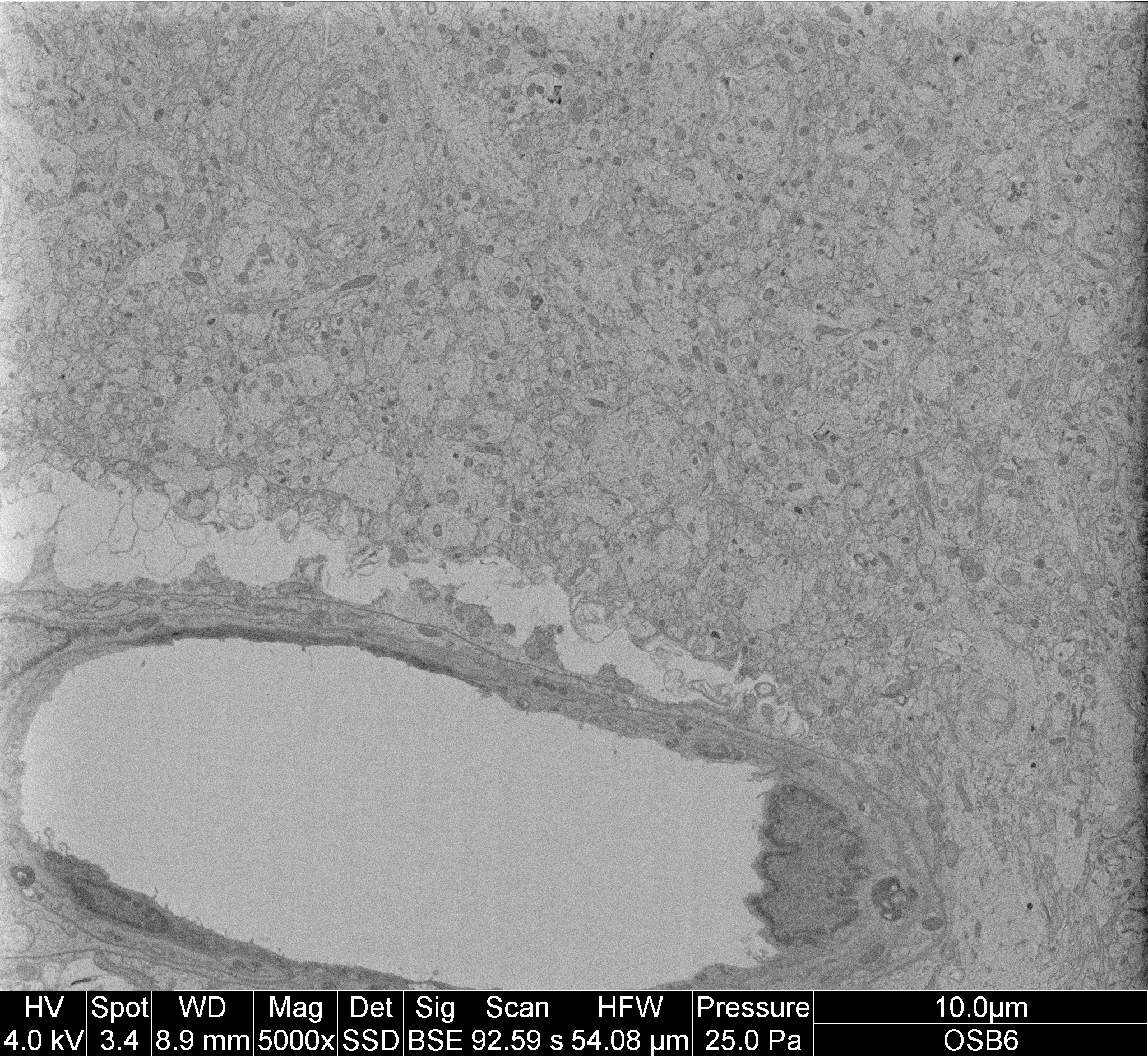

Supplement: Dataset S7 — (253.7 MB ZIP). [file pbio.0020329.sd007.zip › 040604_OS5_st1_661.tif]

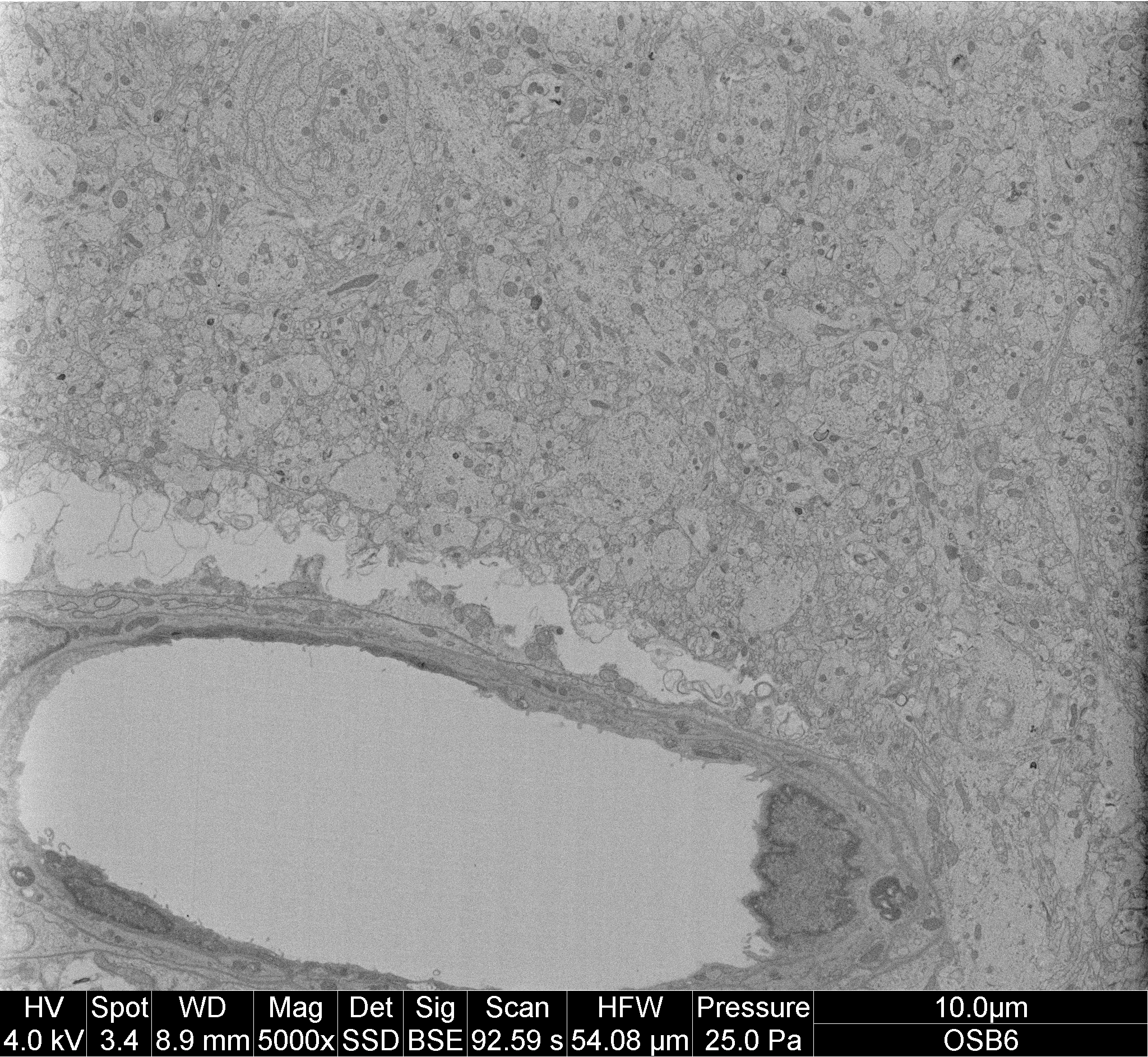

Supplement: Dataset S7 — (253.7 MB ZIP). [file pbio.0020329.sd007.zip › 040604_OS5_st1_662.tif]

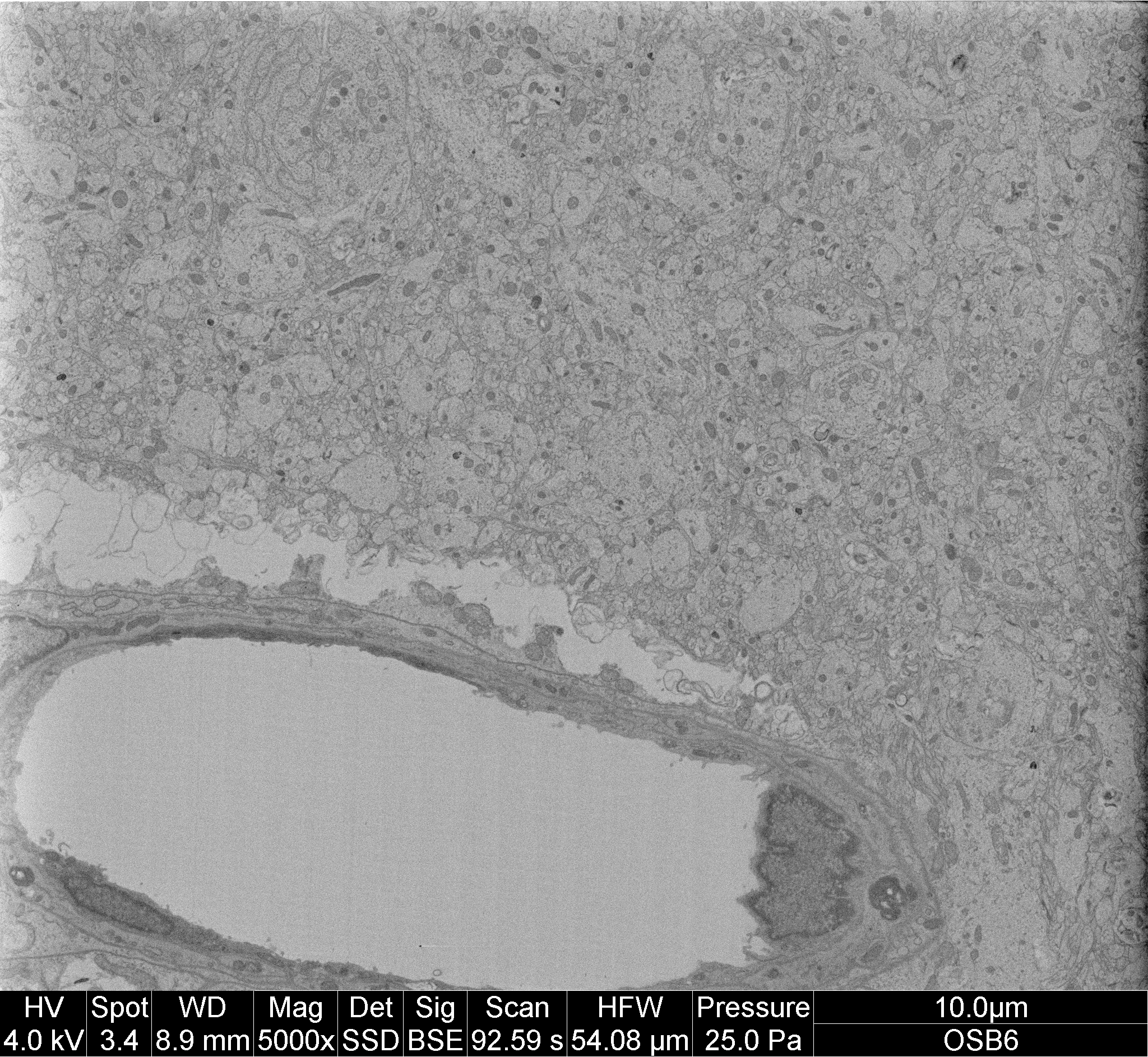

Supplement: Dataset S7 — (253.7 MB ZIP). [file pbio.0020329.sd007.zip › 040604_OS5_st1_663.tif]

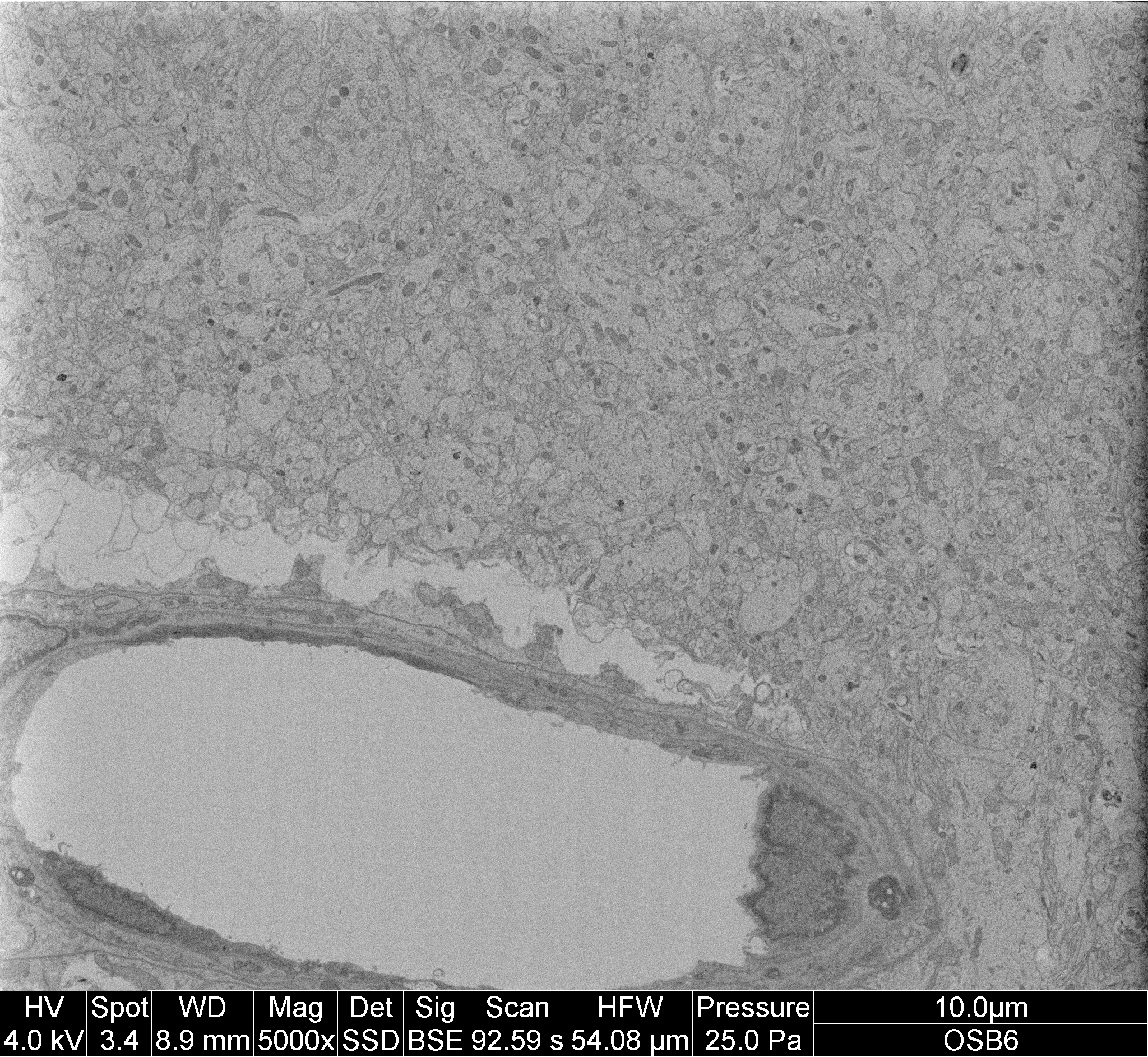

Supplement: Dataset S7 — (253.7 MB ZIP). [file pbio.0020329.sd007.zip › 040604_OS5_st1_664.tif]

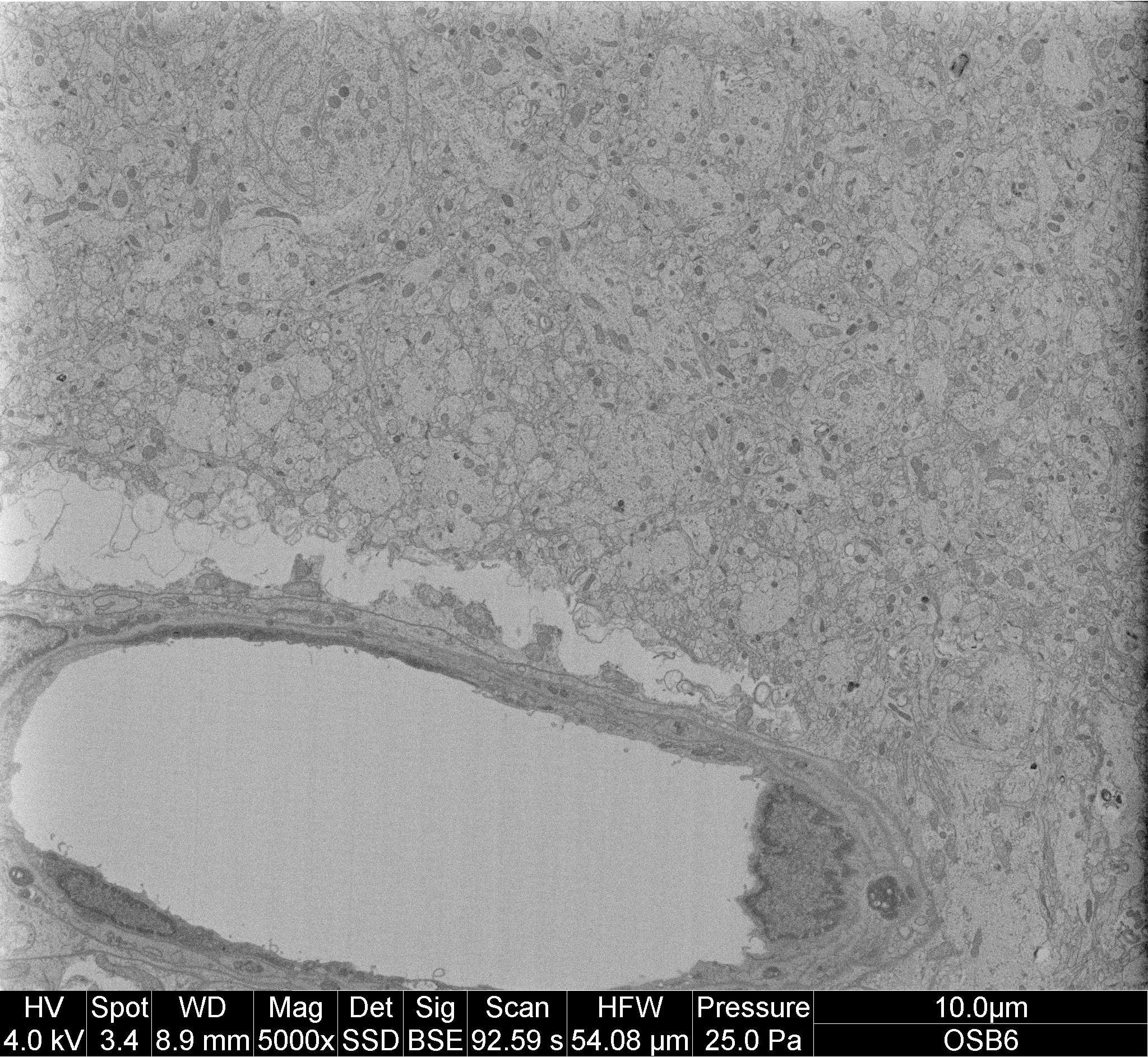

Supplement: Dataset S7 — (253.7 MB ZIP). [file pbio.0020329.sd007.zip › 040604_OS5_st1_665.tif]

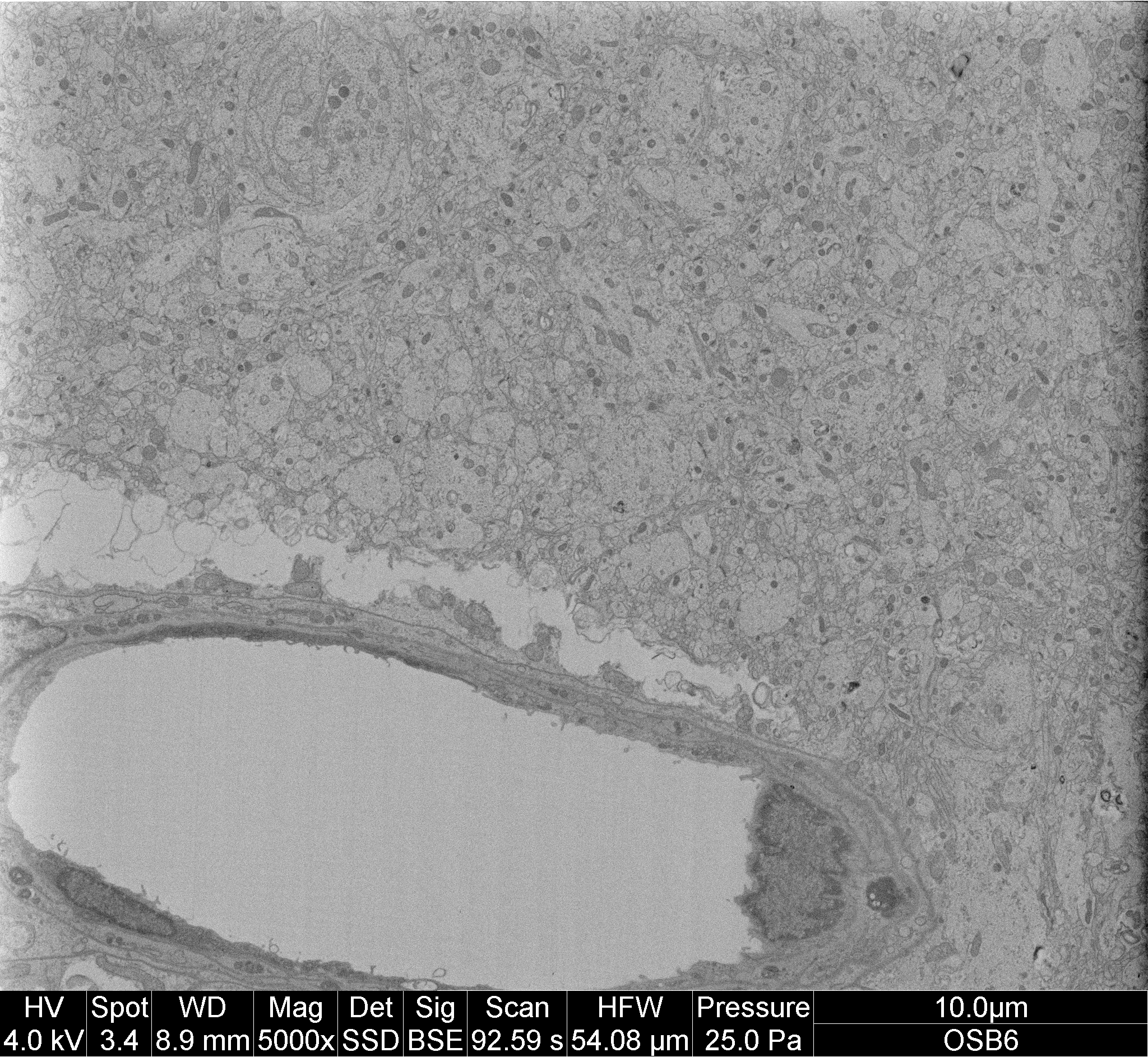

Supplement: Dataset S7 — (253.7 MB ZIP). [file pbio.0020329.sd007.zip › 040604_OS5_st1_666.tif]

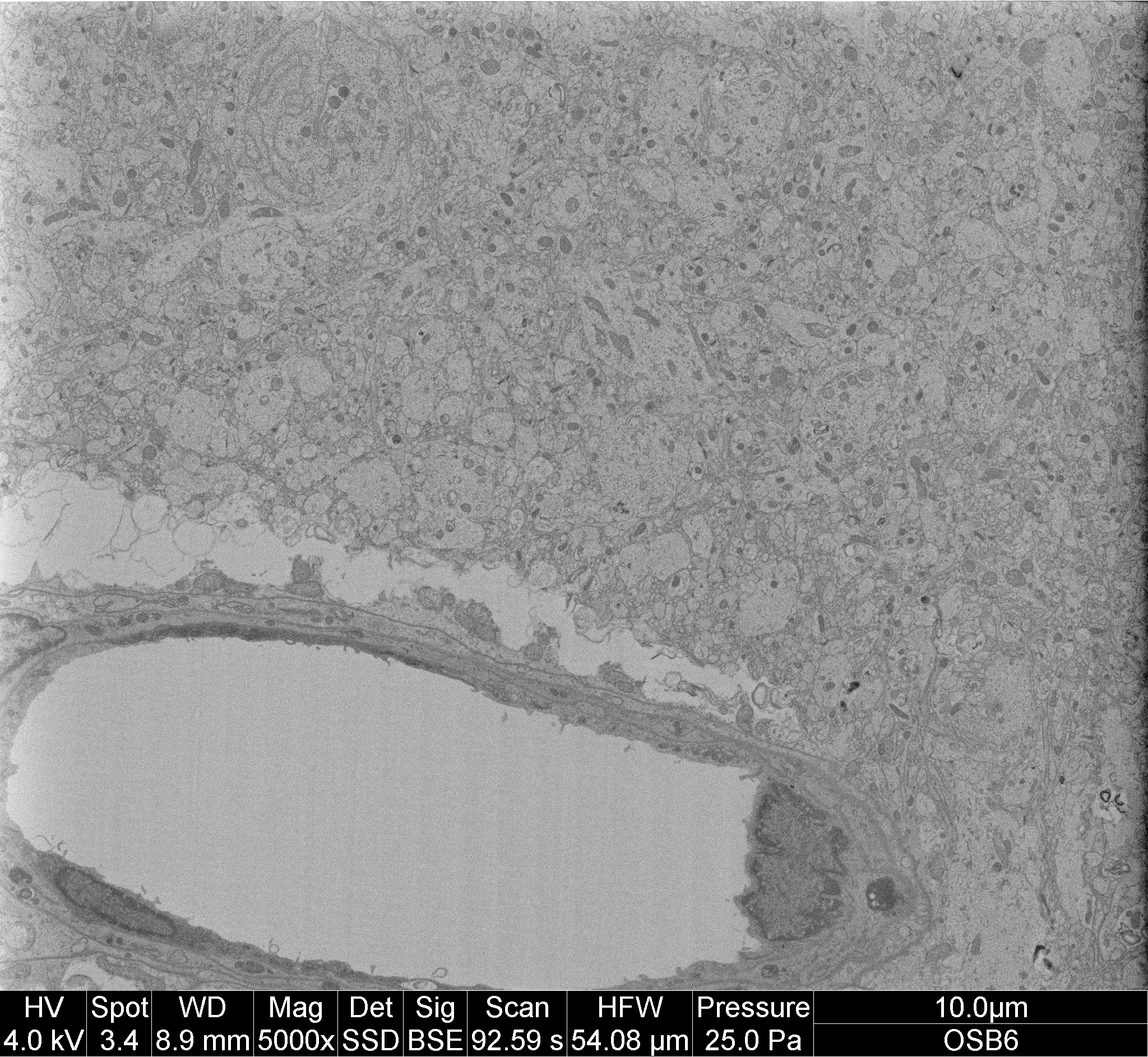

Supplement: Dataset S7 — (253.7 MB ZIP). [file pbio.0020329.sd007.zip › 040604_OS5_st1_667.tif]

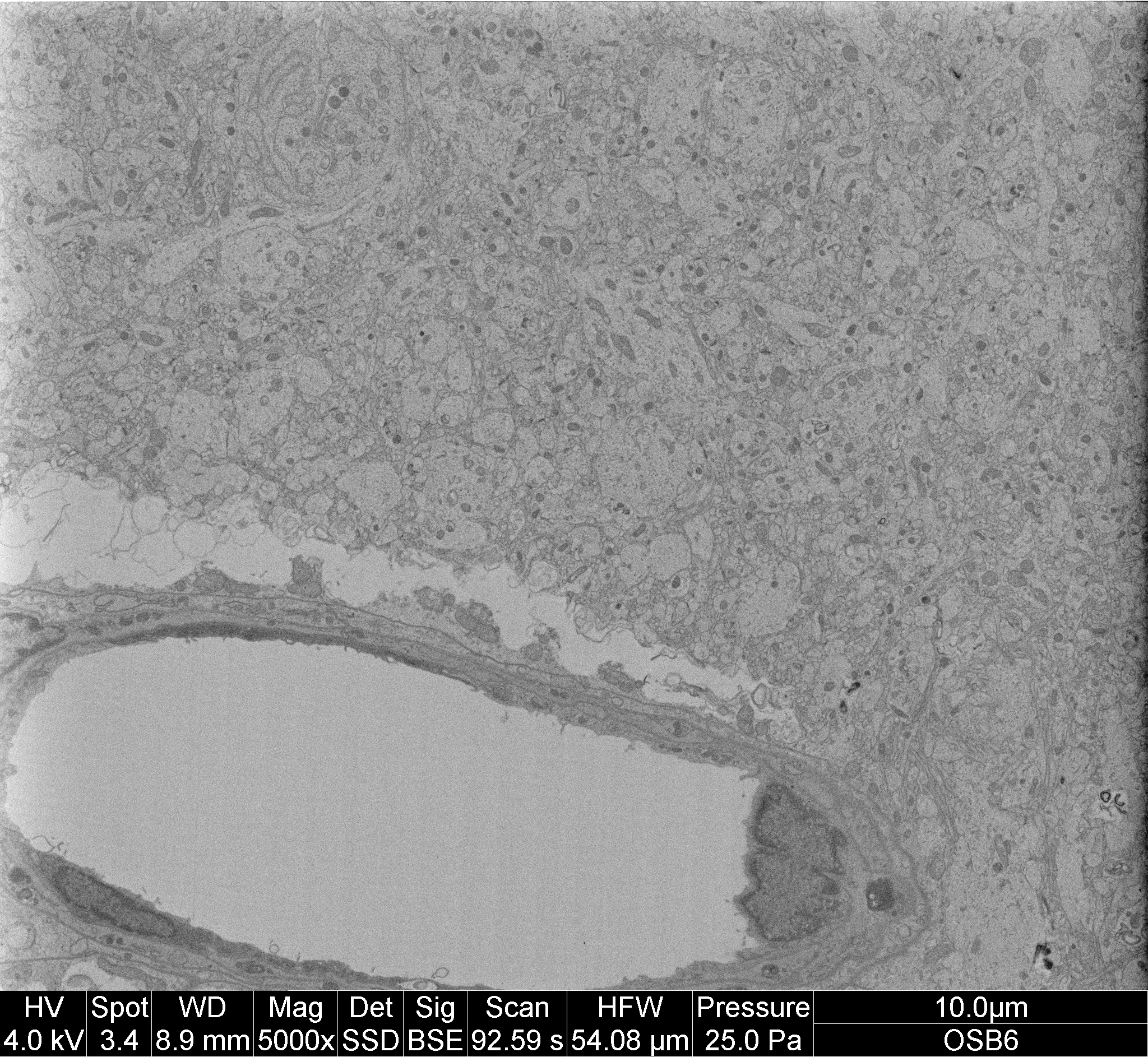

Supplement: Dataset S7 — (253.7 MB ZIP). [file pbio.0020329.sd007.zip › 040604_OS5_st1_668.tif]

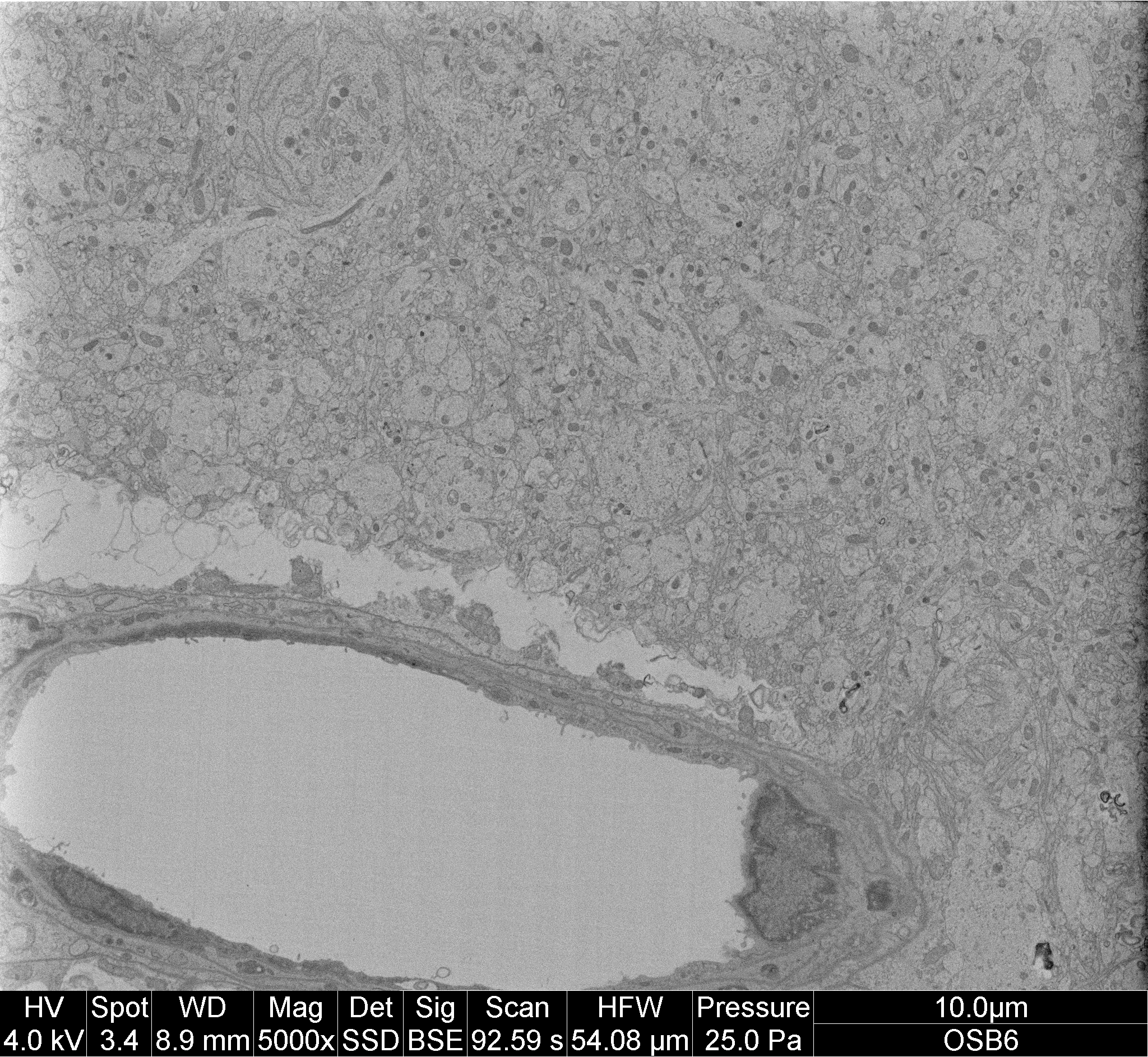

Supplement: Dataset S7 — (253.7 MB ZIP). [file pbio.0020329.sd007.zip › 040604_OS5_st1_669.tif]

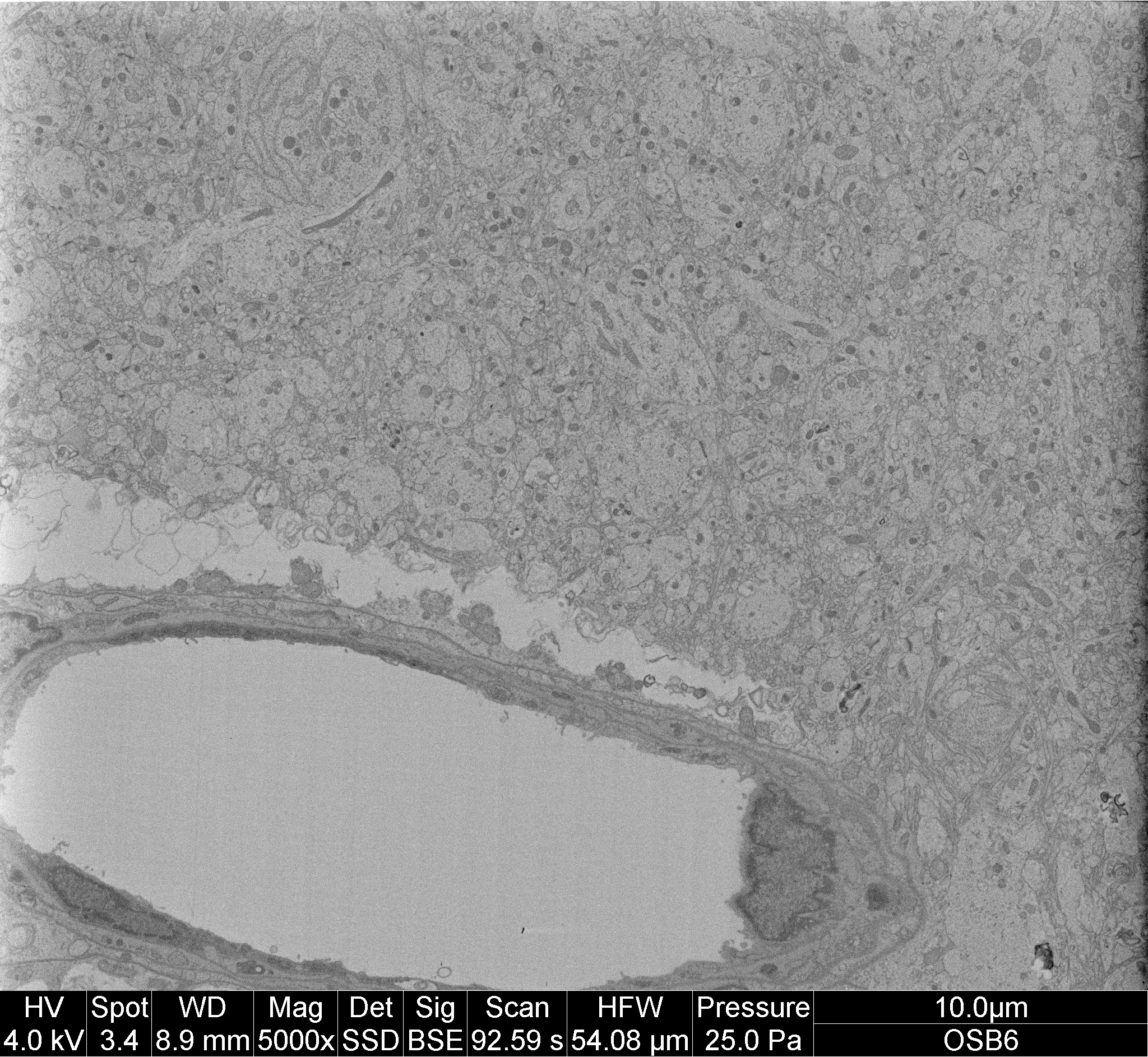

Supplement: Dataset S7 — (253.7 MB ZIP). [file pbio.0020329.sd007.zip › 040604_OS5_st1_670.tif]

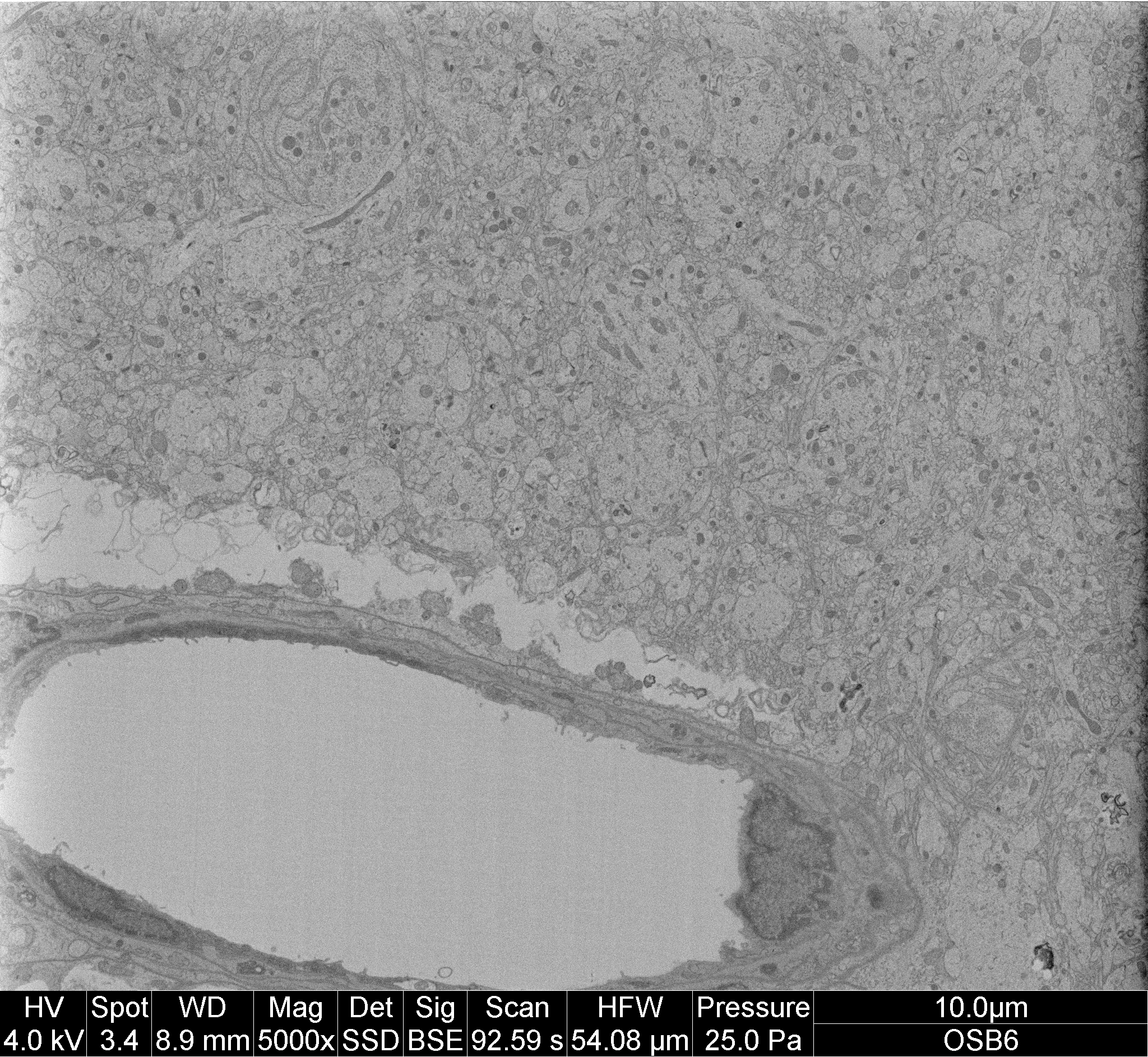

Supplement: Dataset S7 — (253.7 MB ZIP). [file pbio.0020329.sd007.zip › 040604_OS5_st1_671.tif]

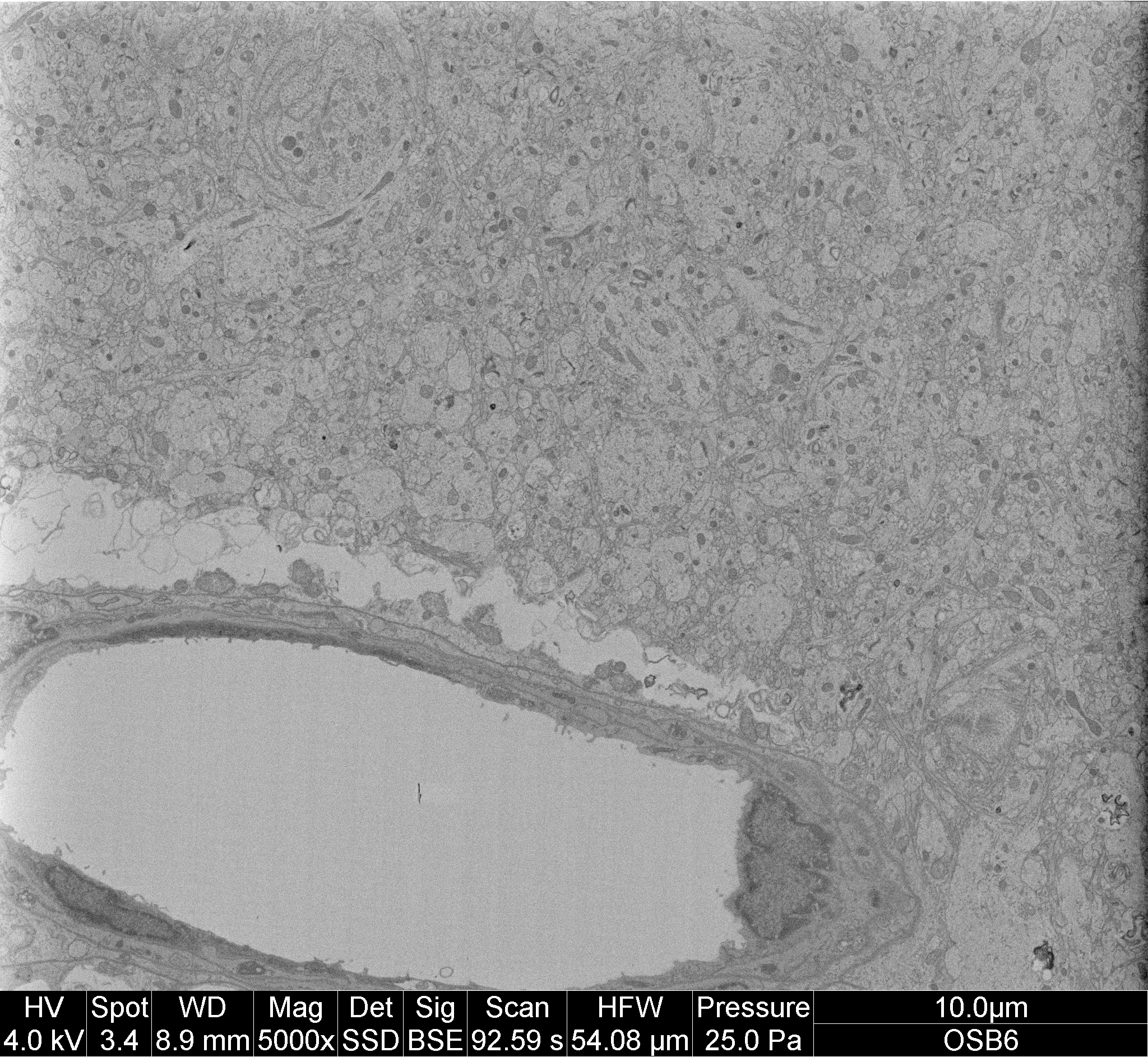

Supplement: Dataset S7 — (253.7 MB ZIP). [file pbio.0020329.sd007.zip › 040604_OS5_st1_672.tif]

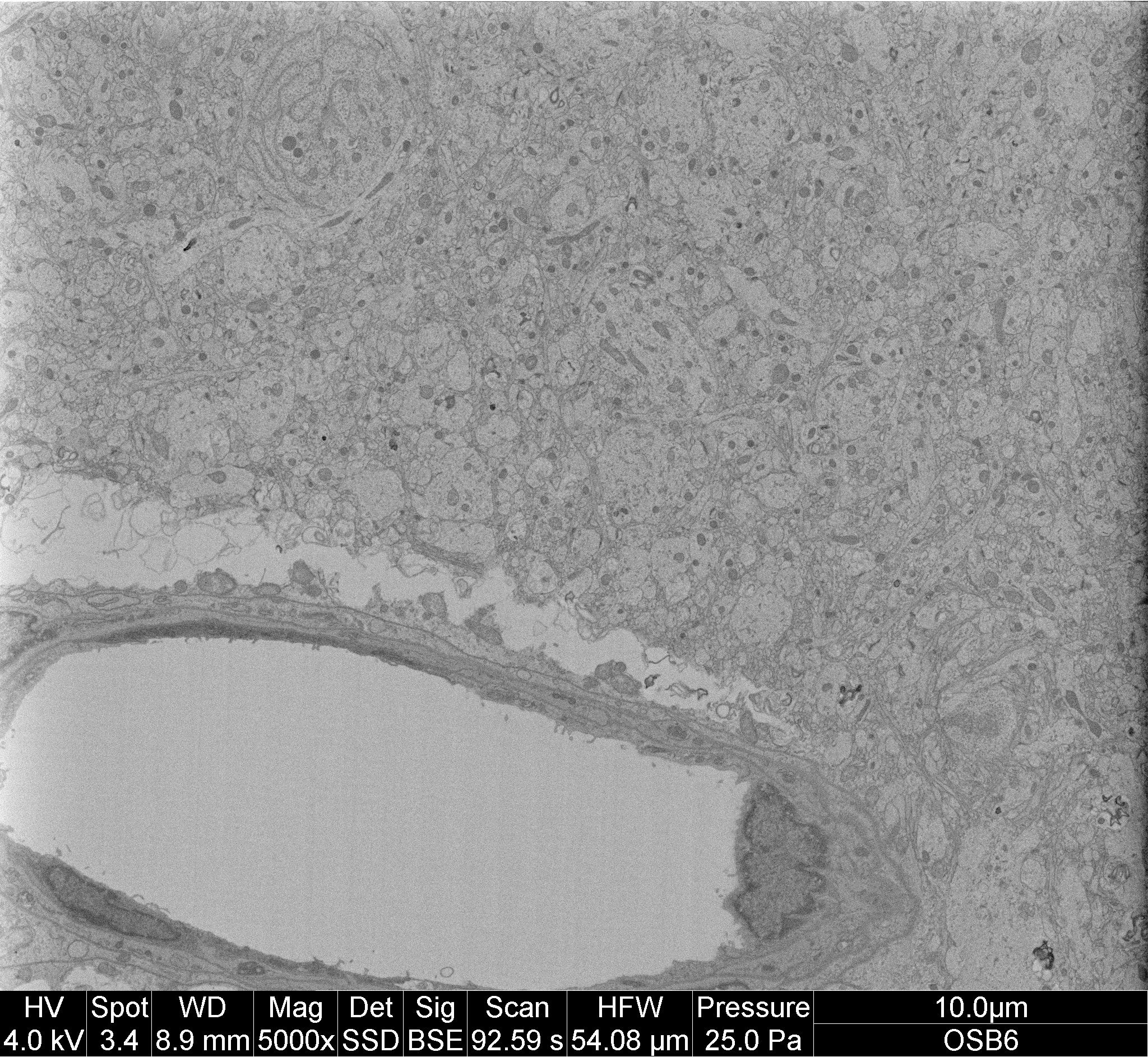

Supplement: Dataset S7 — (253.7 MB ZIP). [file pbio.0020329.sd007.zip › 040604_OS5_st1_673.tif]

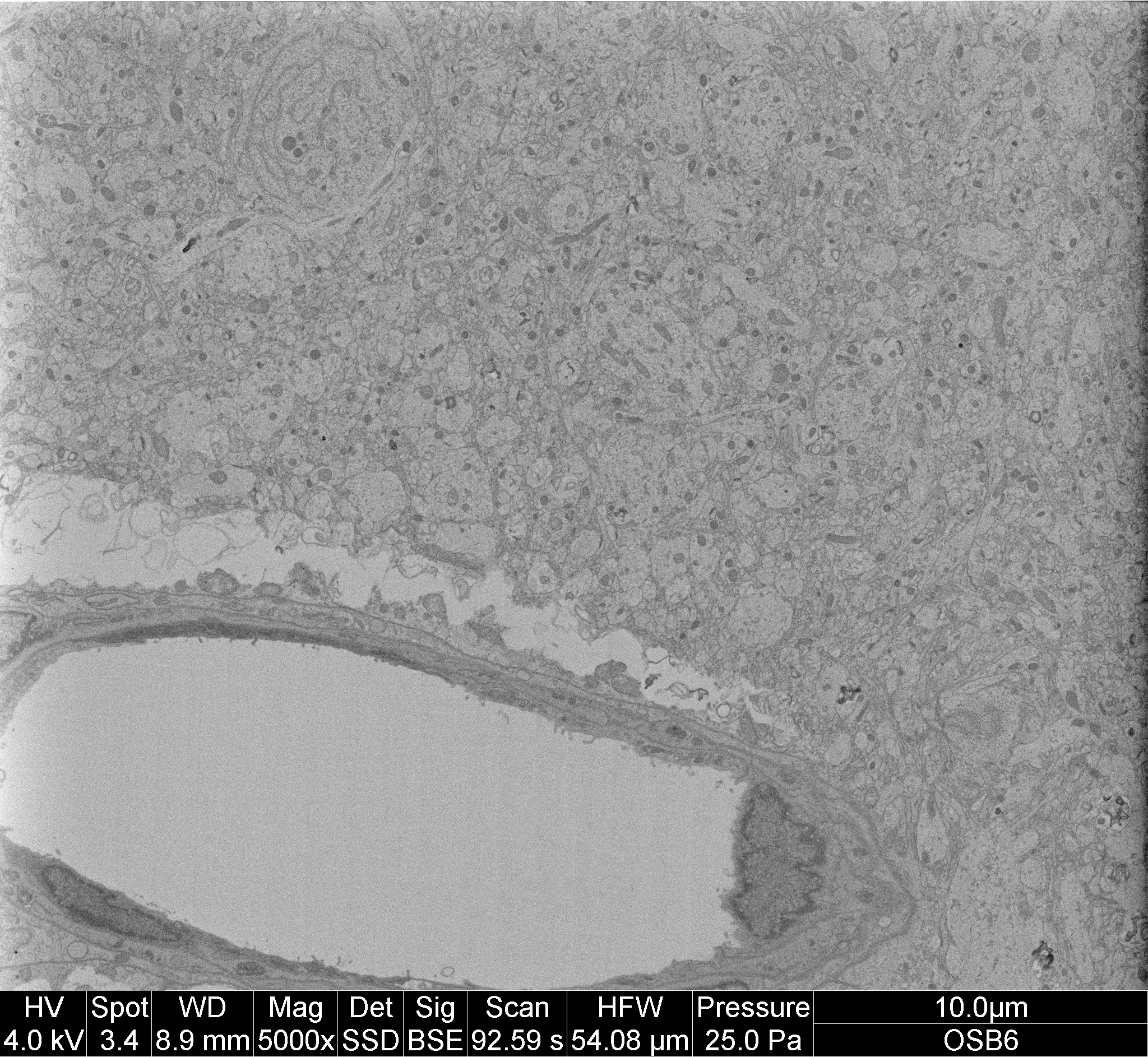

Supplement: Dataset S7 — (253.7 MB ZIP). [file pbio.0020329.sd007.zip › 040604_OS5_st1_674.tif]

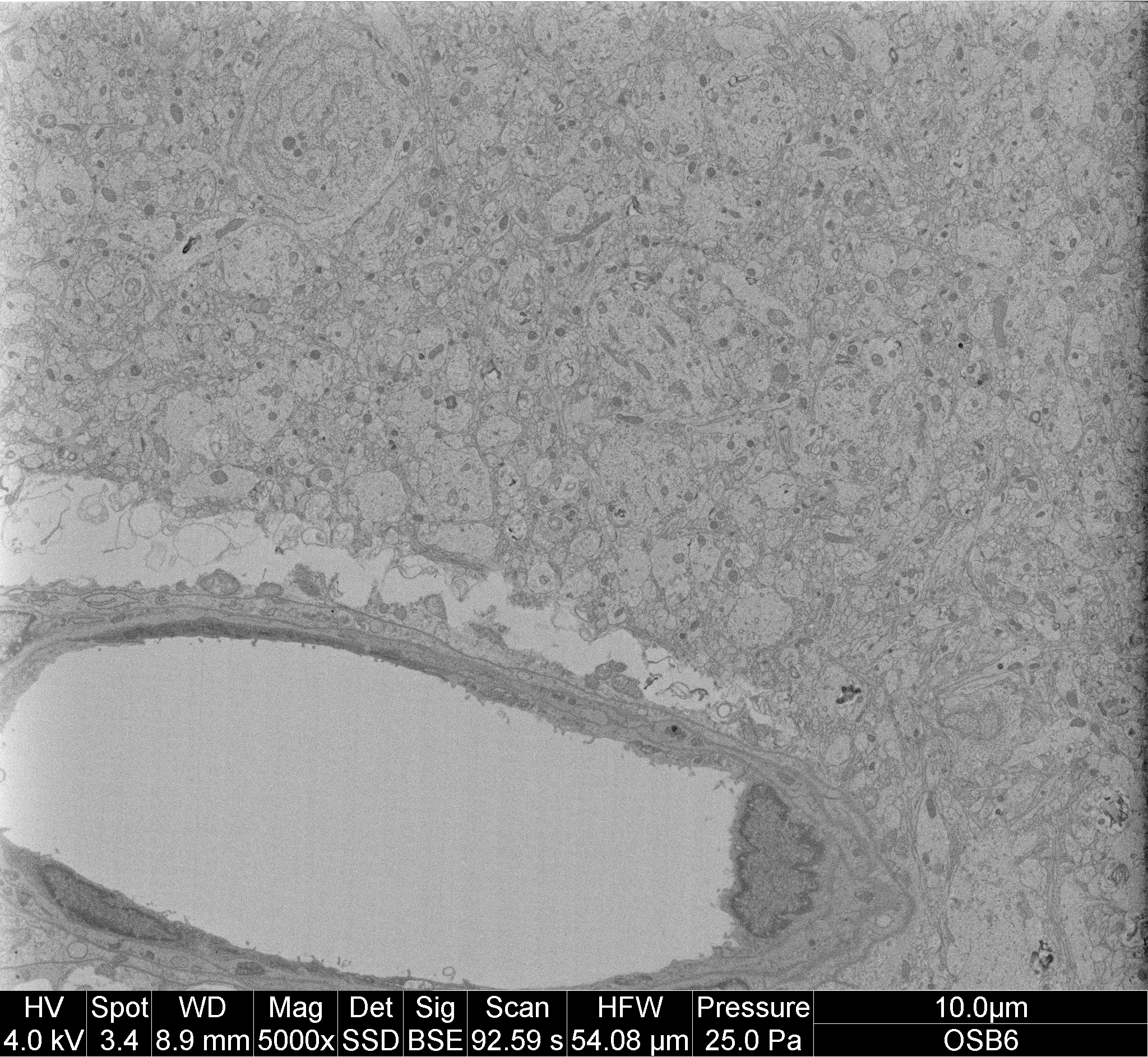

Supplement: Dataset S7 — (253.7 MB ZIP). [file pbio.0020329.sd007.zip › 040604_OS5_st1_675.tif]

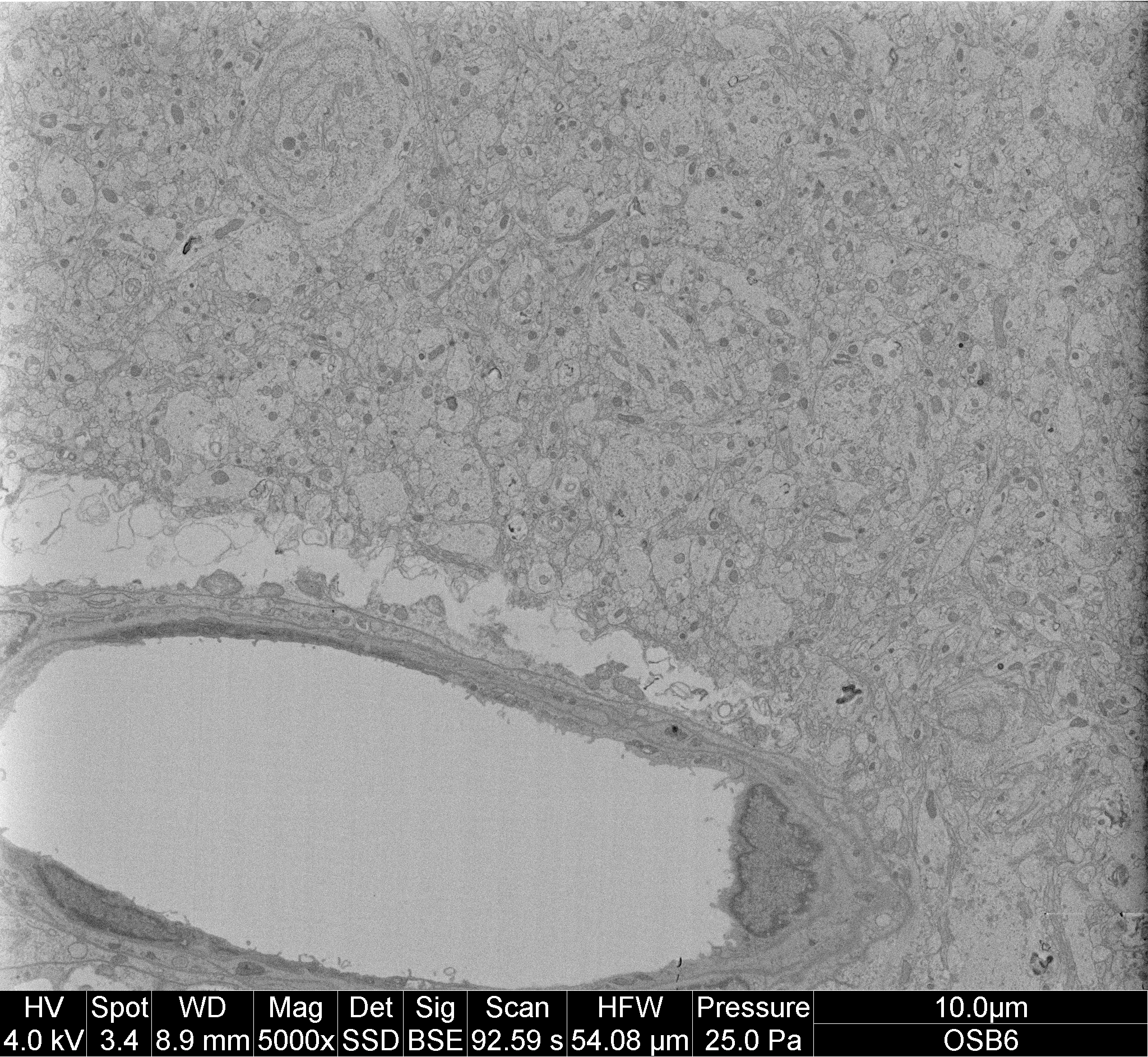

Supplement: Dataset S7 — (253.7 MB ZIP). [file pbio.0020329.sd007.zip › 040604_OS5_st1_676.tif]

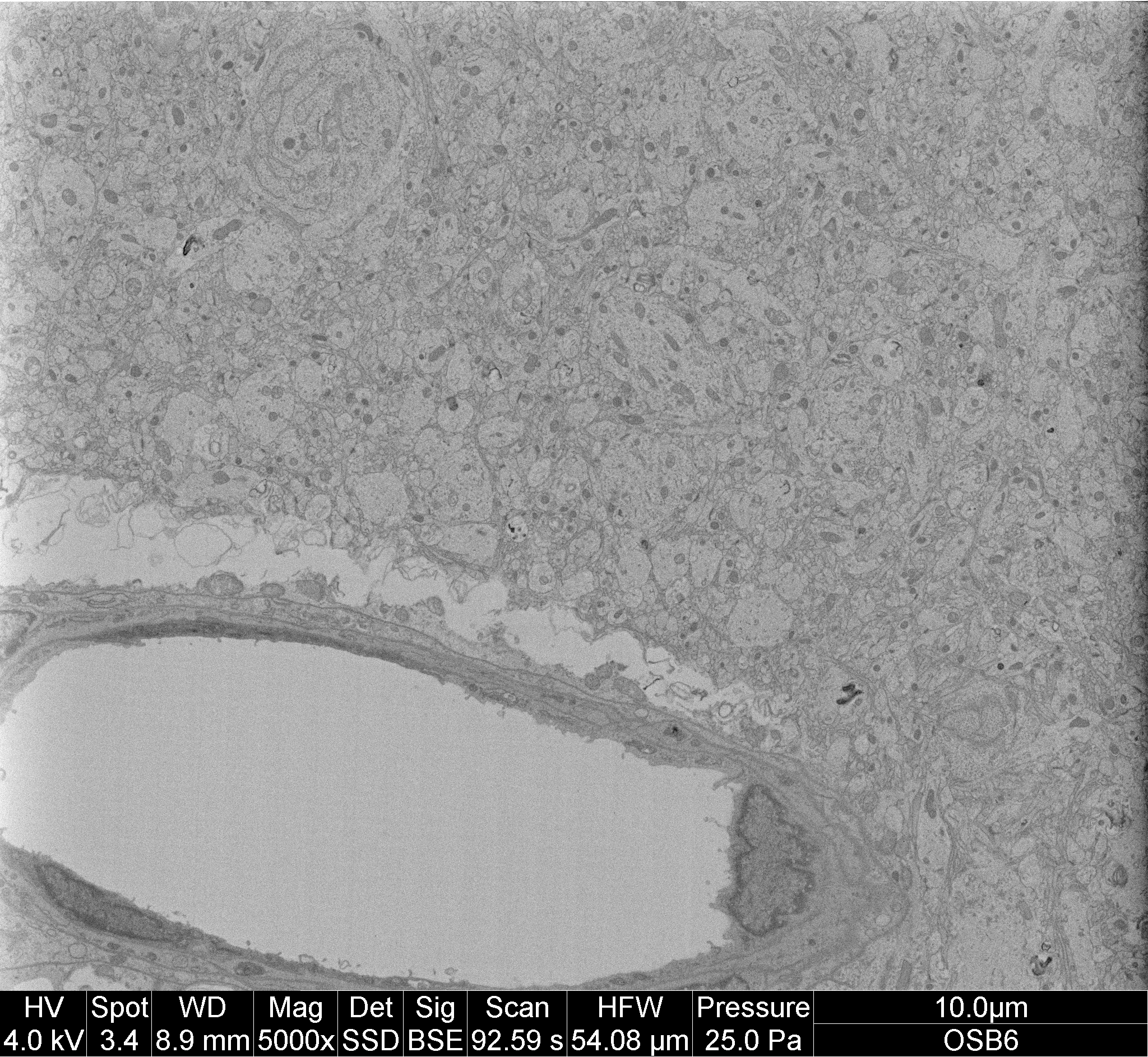

Supplement: Dataset S7 — (253.7 MB ZIP). [file pbio.0020329.sd007.zip › 040604_OS5_st1_677.tif]

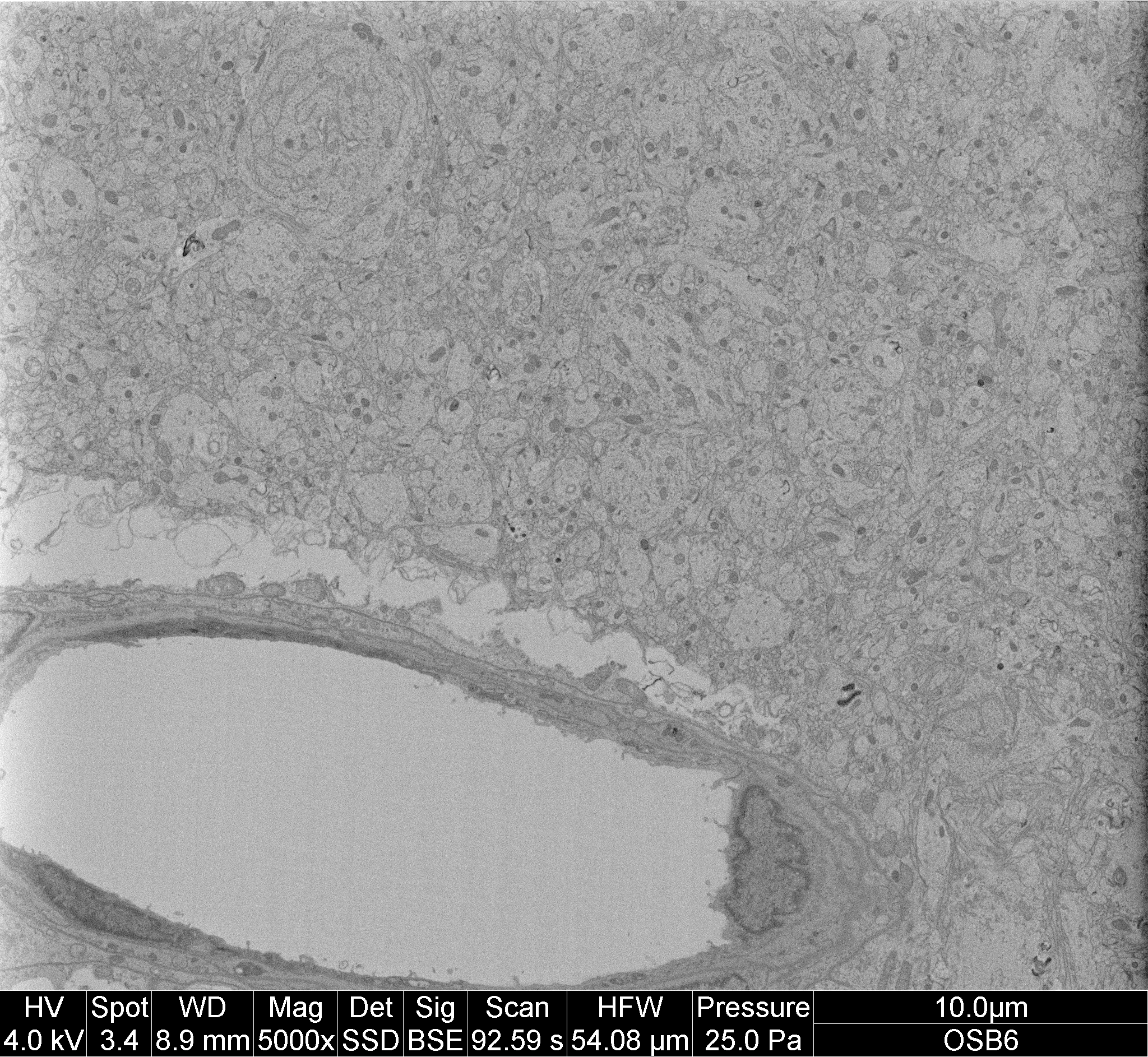

Supplement: Dataset S7 — (253.7 MB ZIP). [file pbio.0020329.sd007.zip › 040604_OS5_st1_678.tif]

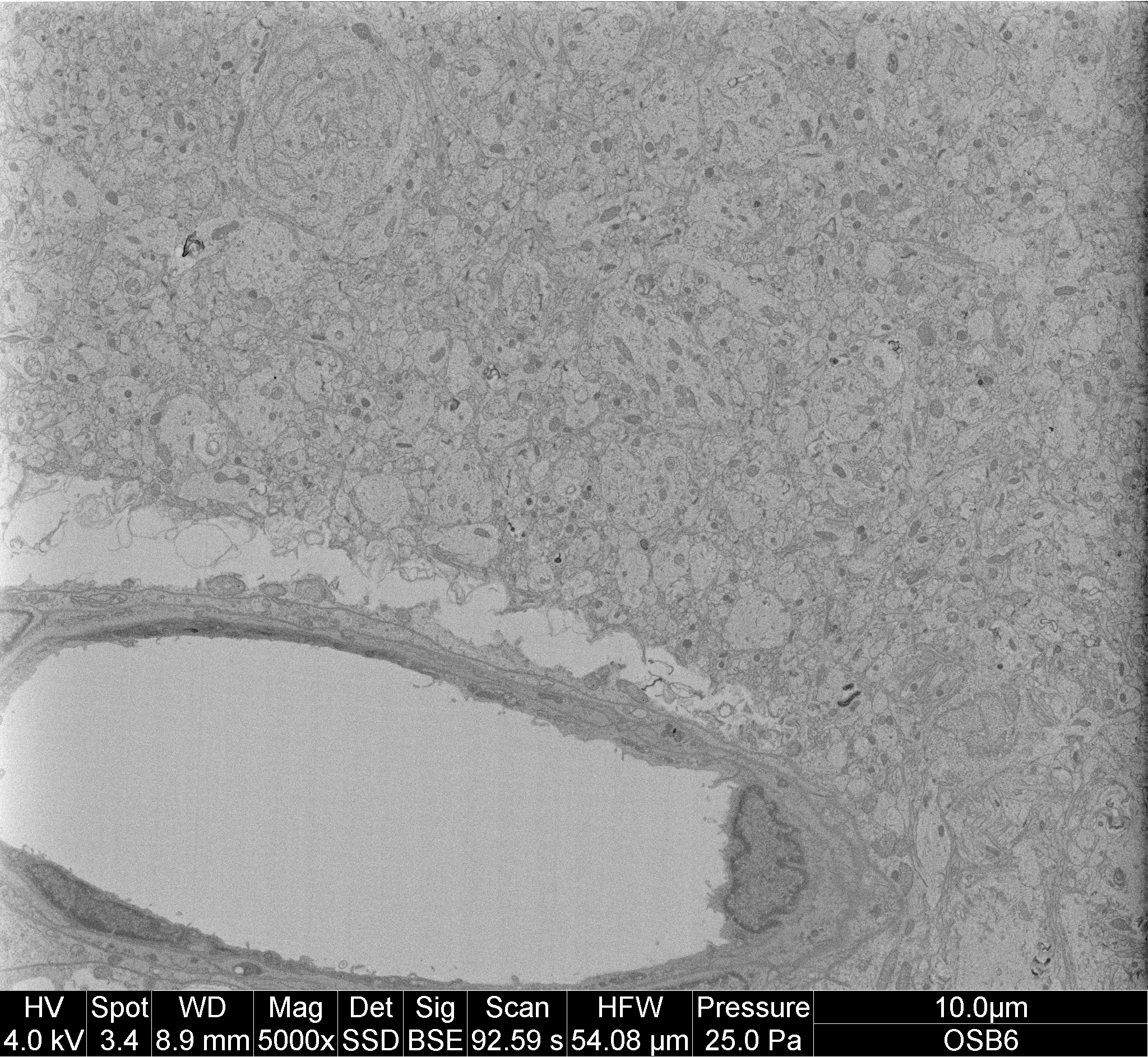

Supplement: Dataset S7 — (253.7 MB ZIP). [file pbio.0020329.sd007.zip › 040604_OS5_st1_679.tif]

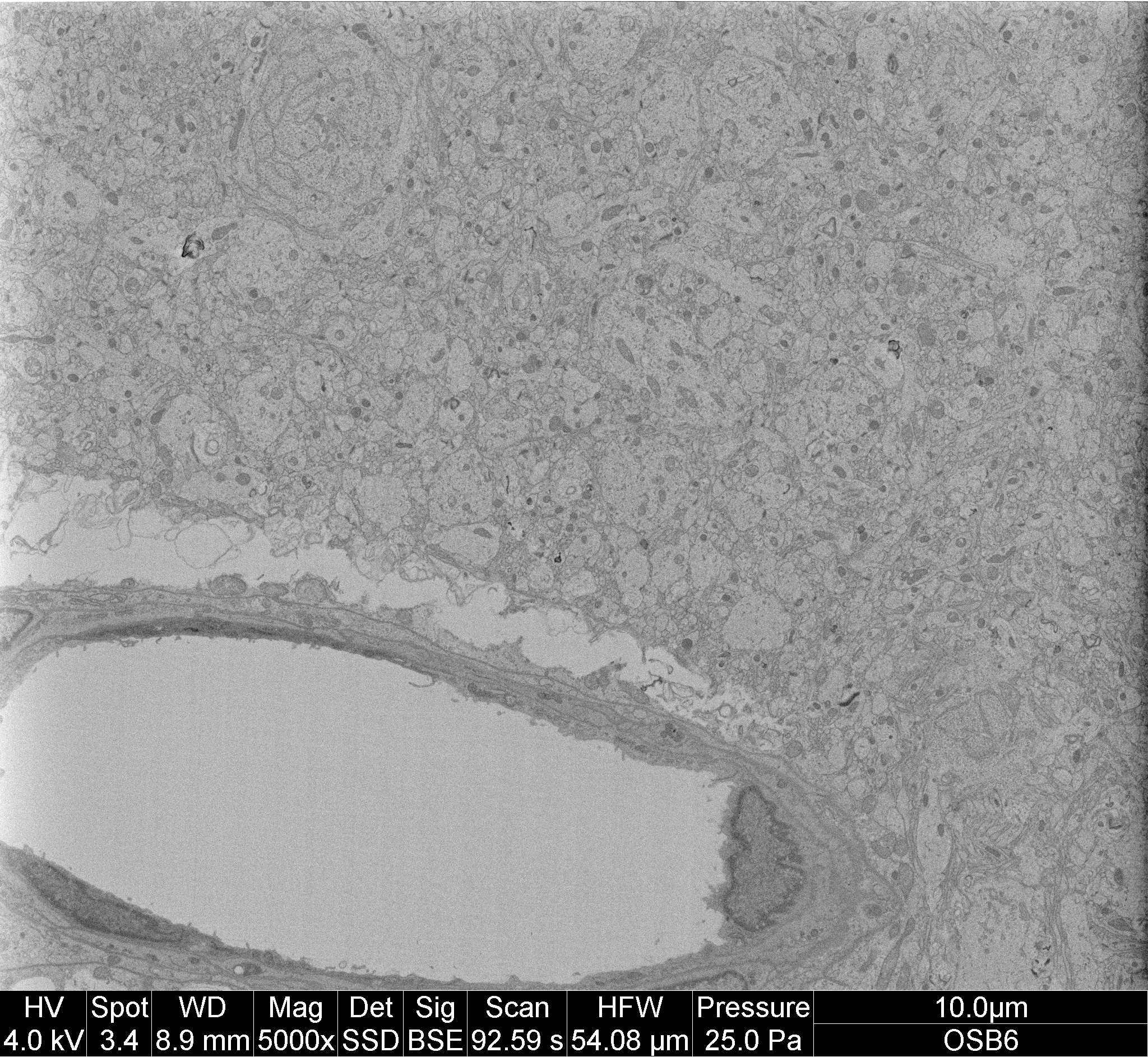

Supplement: Dataset S7 — (253.7 MB ZIP). [file pbio.0020329.sd007.zip › 040604_OS5_st1_680.tif]

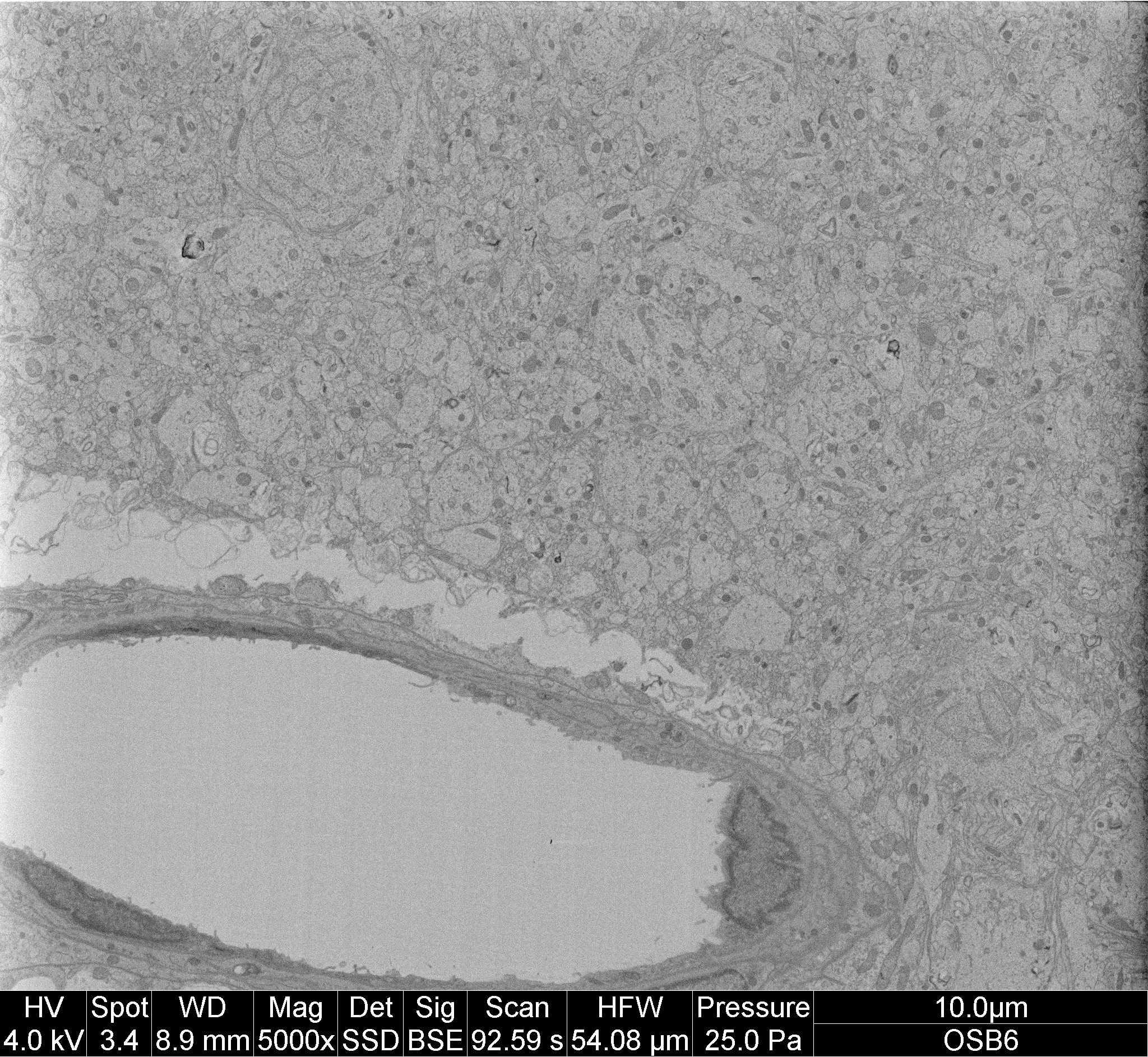

Supplement: Dataset S7 — (253.7 MB ZIP). [file pbio.0020329.sd007.zip › 040604_OS5_st1_681.tif]

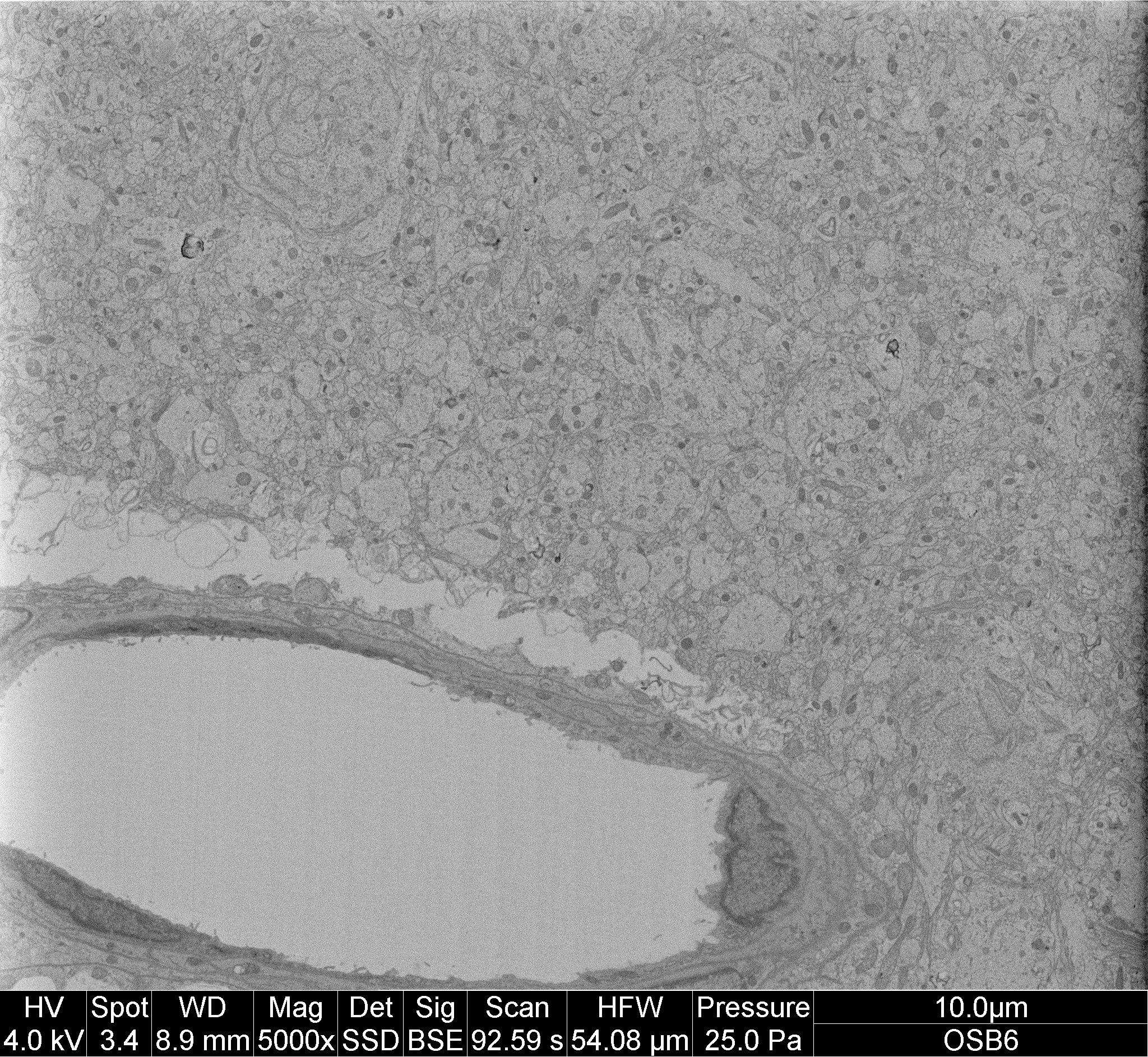

Supplement: Dataset S7 — (253.7 MB ZIP). [file pbio.0020329.sd007.zip › 040604_OS5_st1_682.tif]

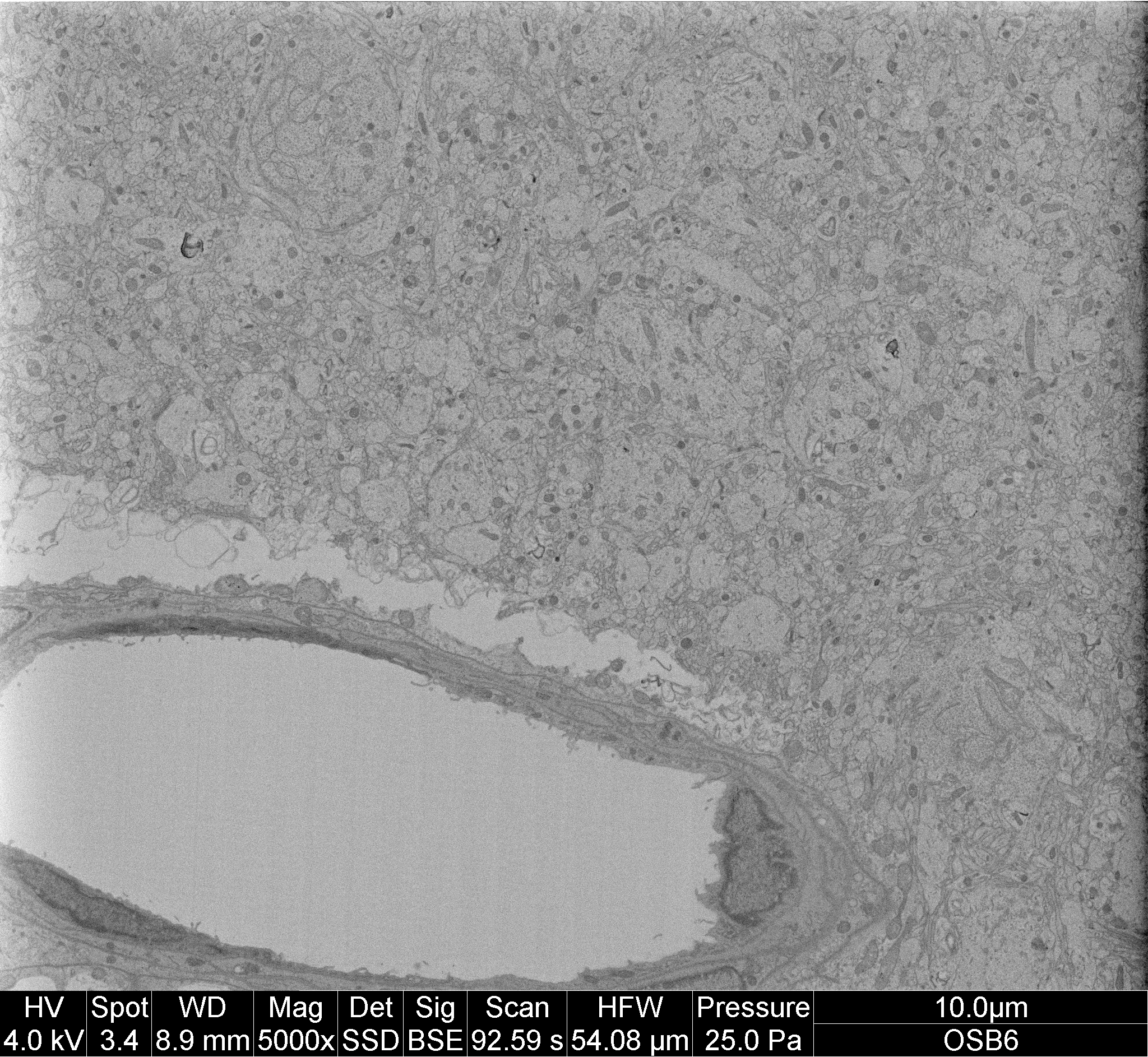

Supplement: Dataset S7 — (253.7 MB ZIP). [file pbio.0020329.sd007.zip › 040604_OS5_st1_683.tif]

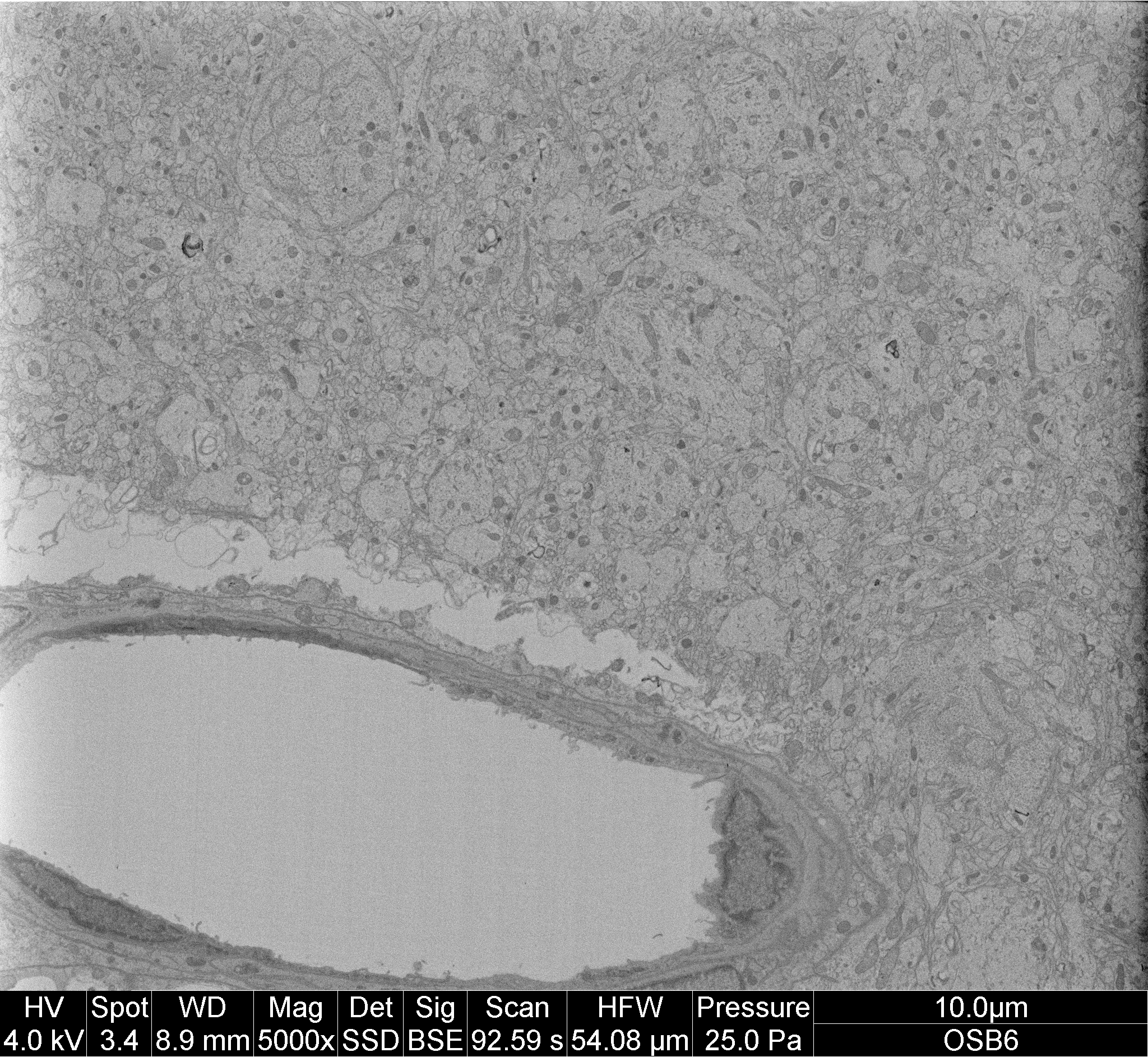

Supplement: Dataset S7 — (253.7 MB ZIP). [file pbio.0020329.sd007.zip › 040604_OS5_st1_684.tif]

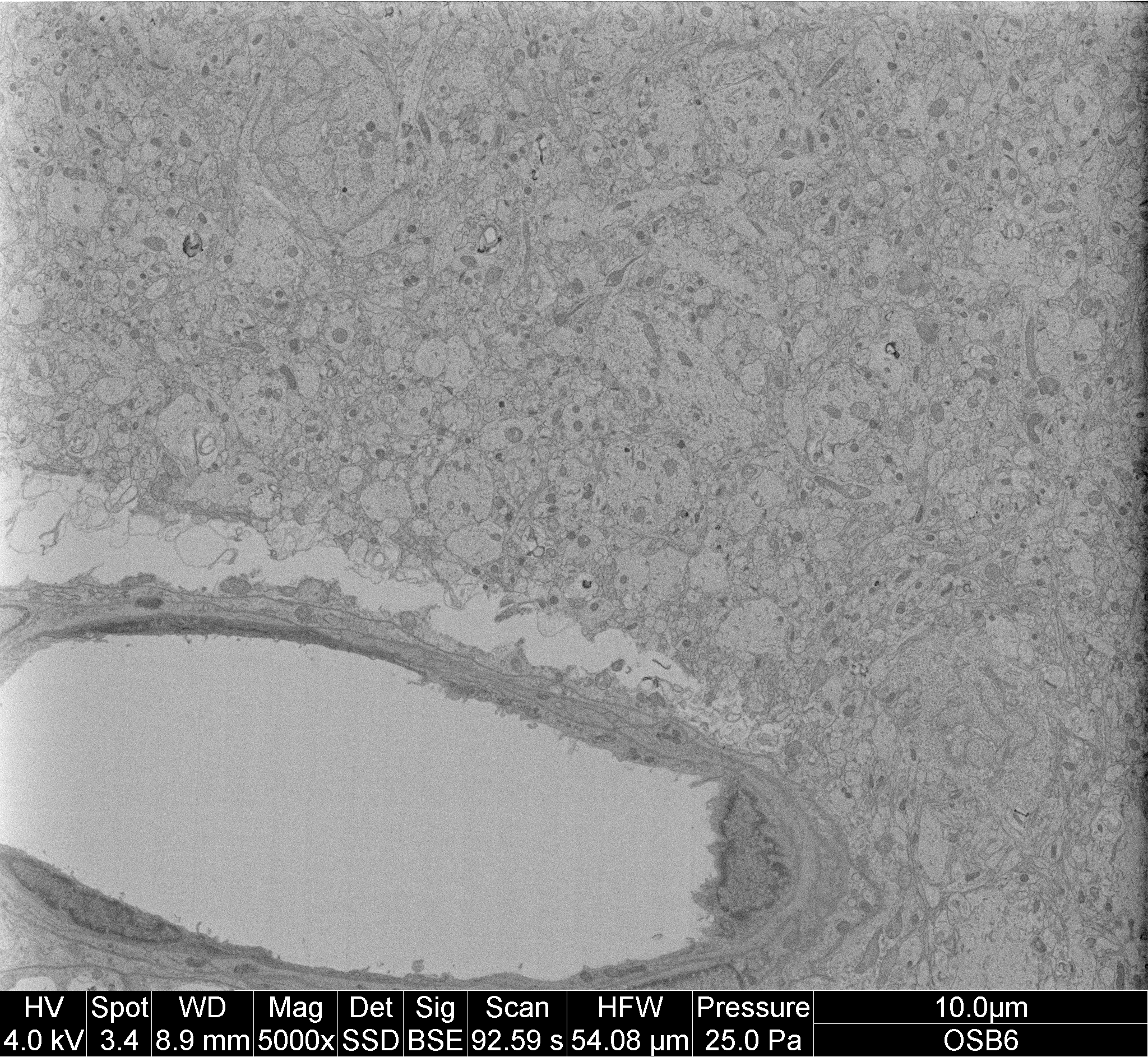

Supplement: Dataset S7 — (253.7 MB ZIP). [file pbio.0020329.sd007.zip › 040604_OS5_st1_685.tif]

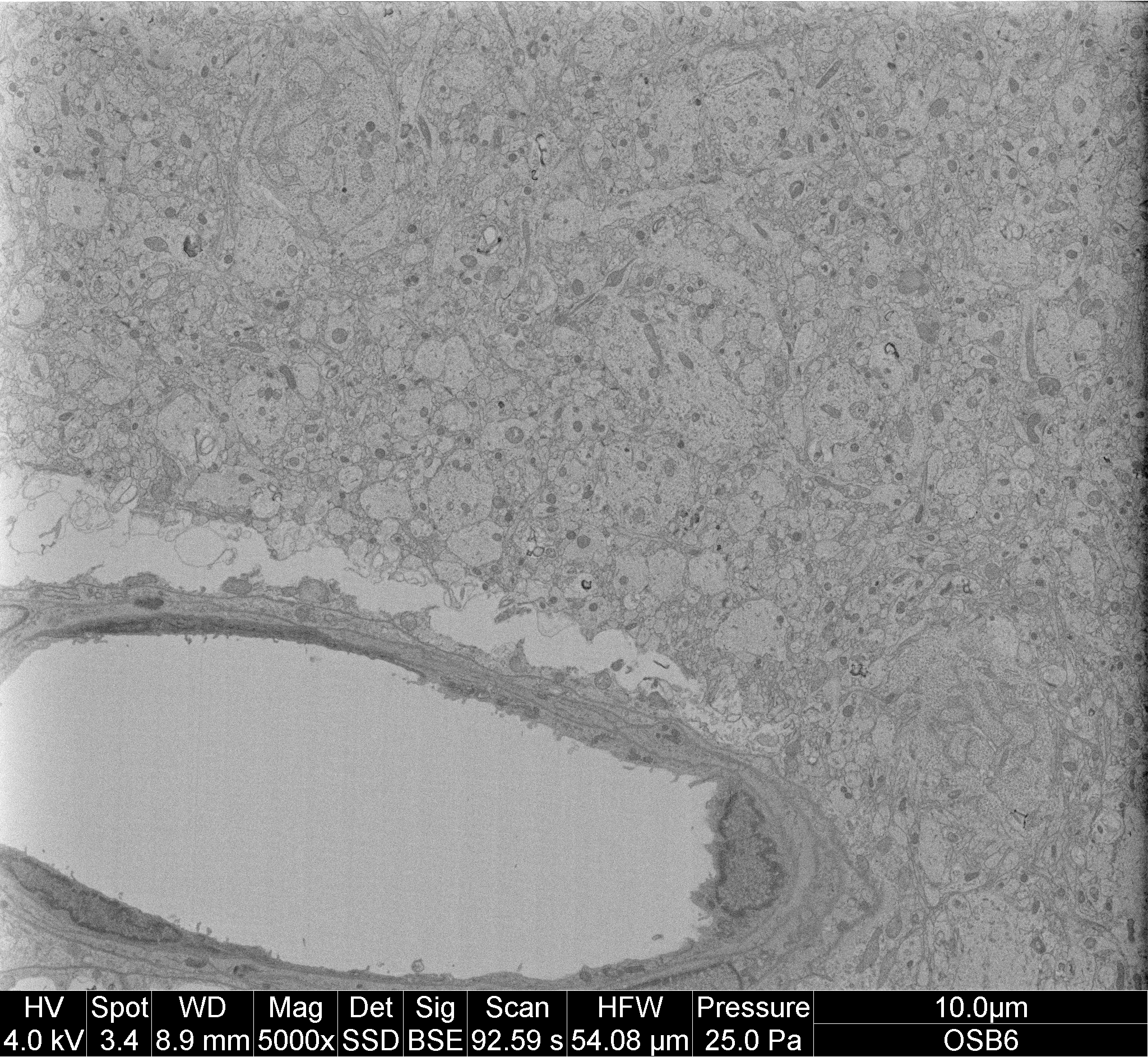

Supplement: Dataset S7 — (253.7 MB ZIP). [file pbio.0020329.sd007.zip › 040604_OS5_st1_686.tif]

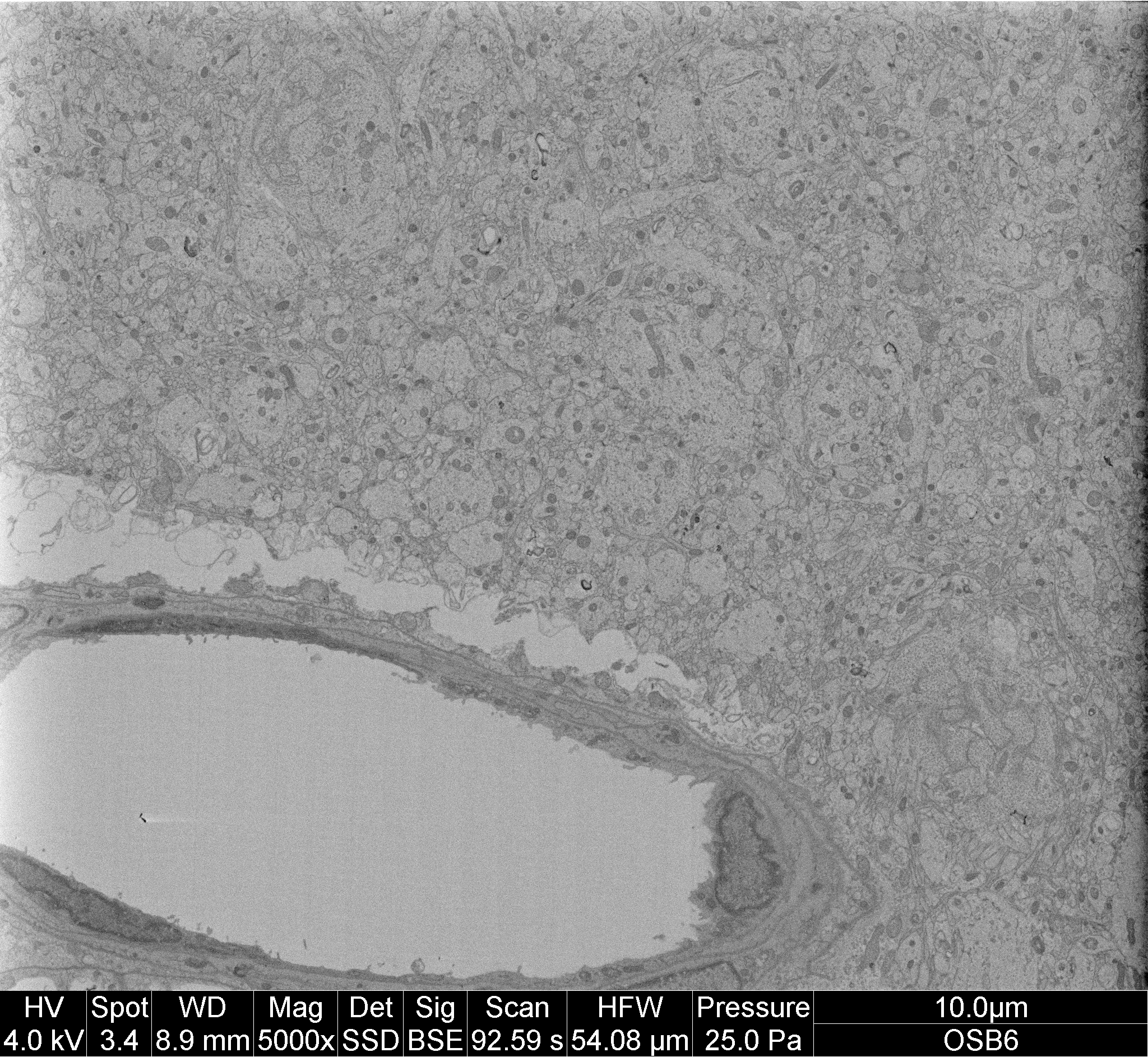

Supplement: Dataset S7 — (253.7 MB ZIP). [file pbio.0020329.sd007.zip › 040604_OS5_st1_687.tif]

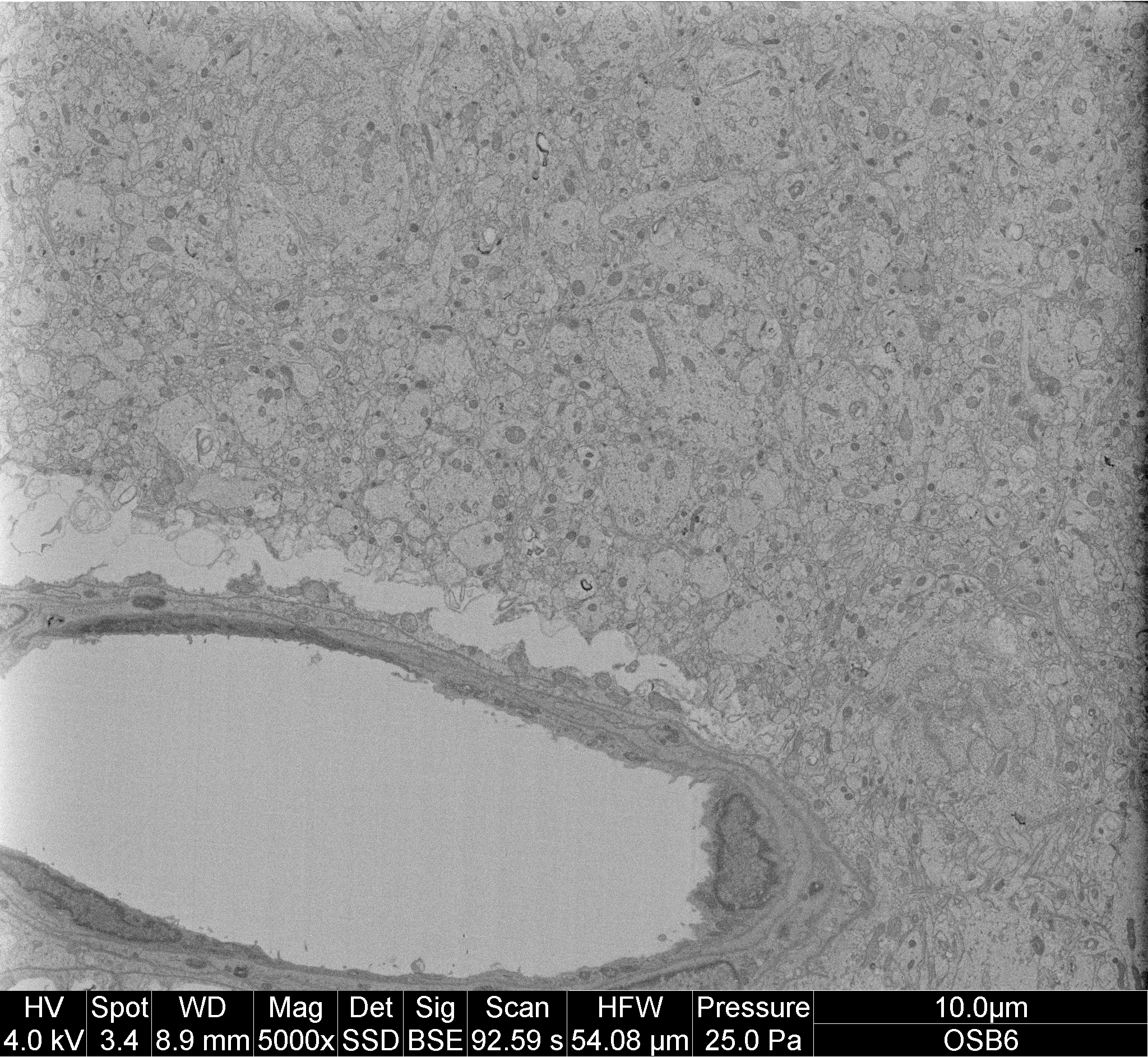

Supplement: Dataset S7 — (253.7 MB ZIP). [file pbio.0020329.sd007.zip › 040604_OS5_st1_688.tif]

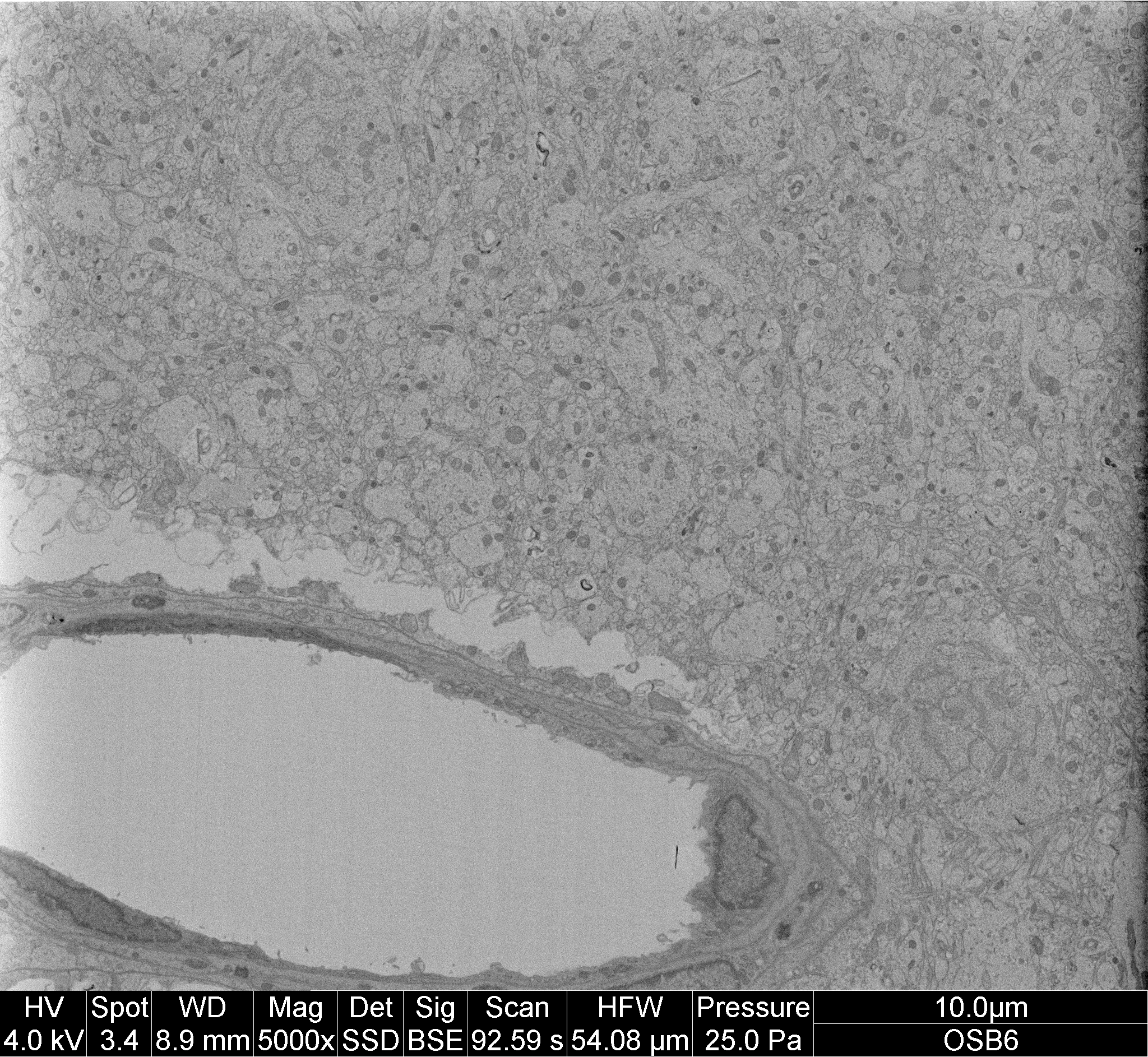

Supplement: Dataset S7 — (253.7 MB ZIP). [file pbio.0020329.sd007.zip › 040604_OS5_st1_689.tif]

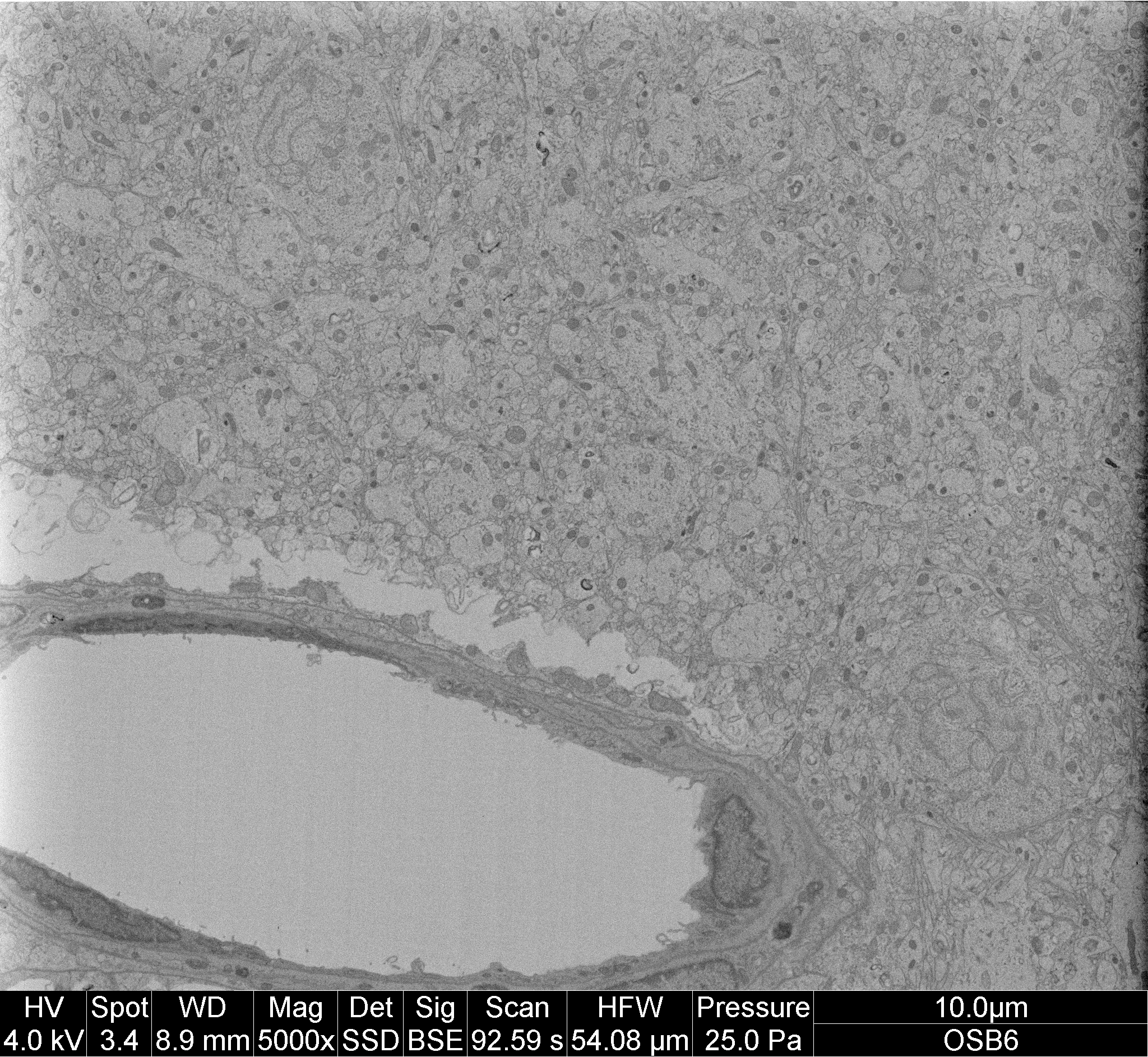

Supplement: Dataset S7 — (253.7 MB ZIP). [file pbio.0020329.sd007.zip › 040604_OS5_st1_690.tif]

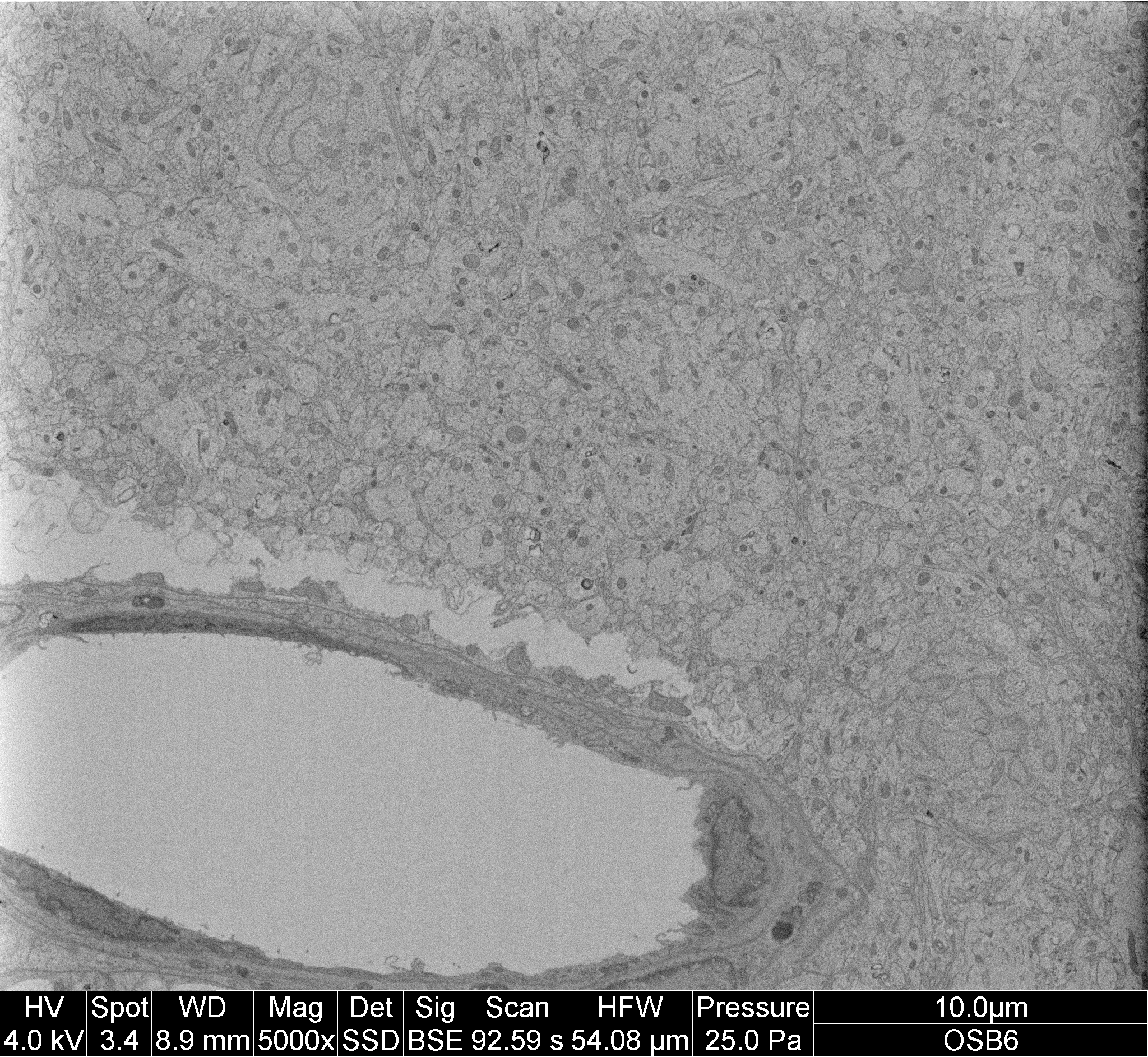

Supplement: Dataset S7 — (253.7 MB ZIP). [file pbio.0020329.sd007.zip › 040604_OS5_st1_691.tif]

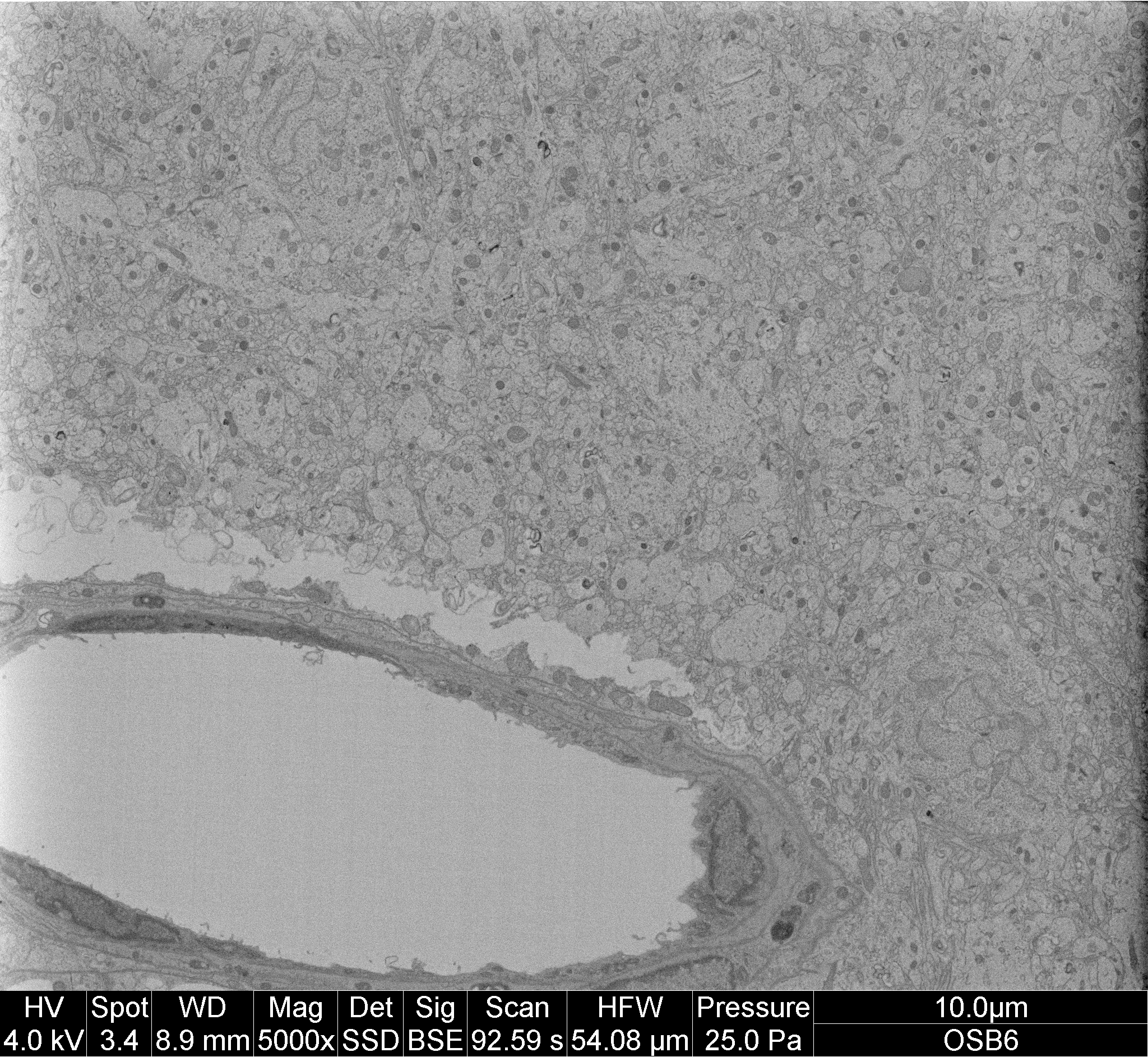

Supplement: Dataset S7 — (253.7 MB ZIP). [file pbio.0020329.sd007.zip › 040604_OS5_st1_692.tif]

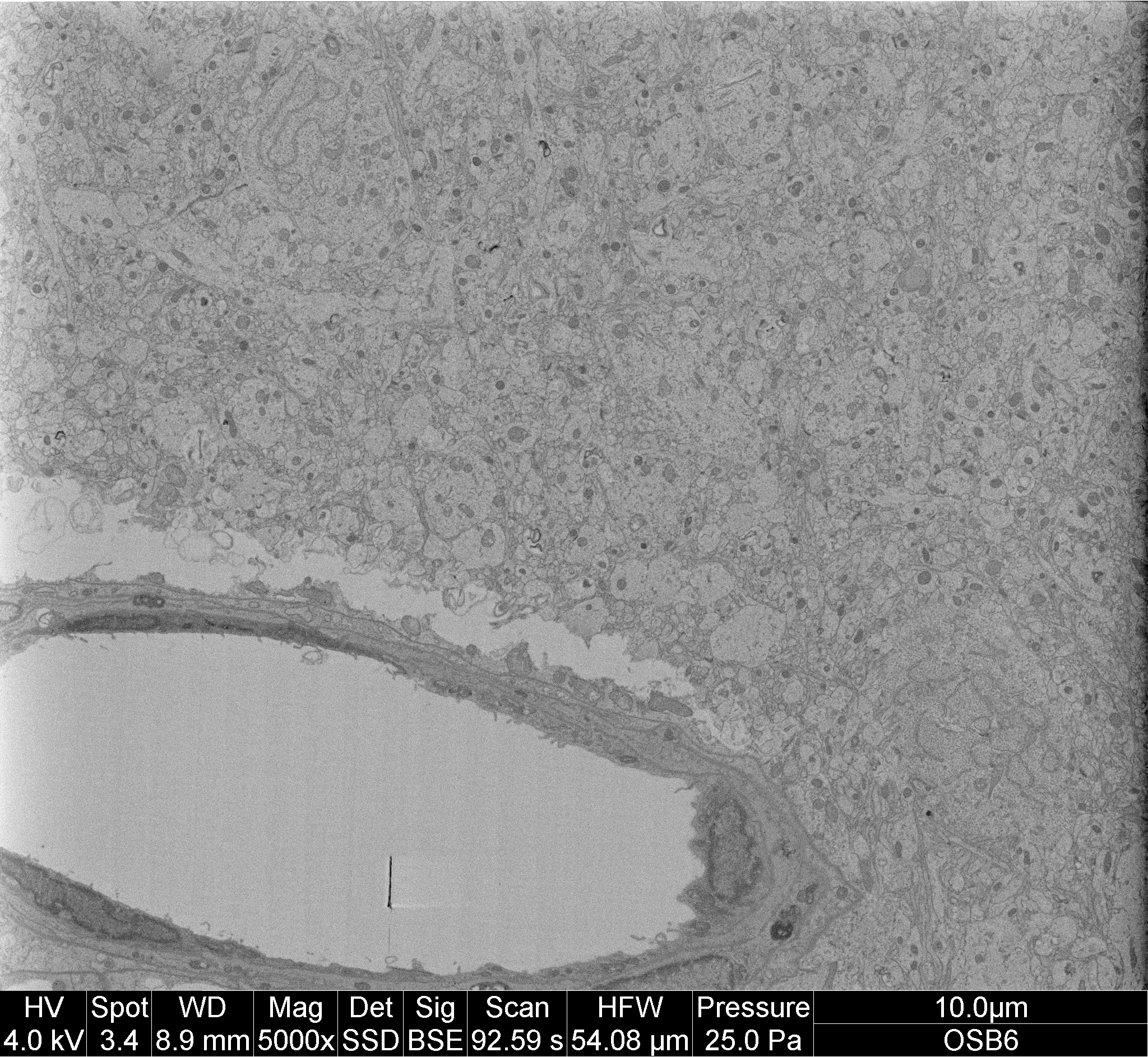

Supplement: Dataset S7 — (253.7 MB ZIP). [file pbio.0020329.sd007.zip › 040604_OS5_st1_693.tif]

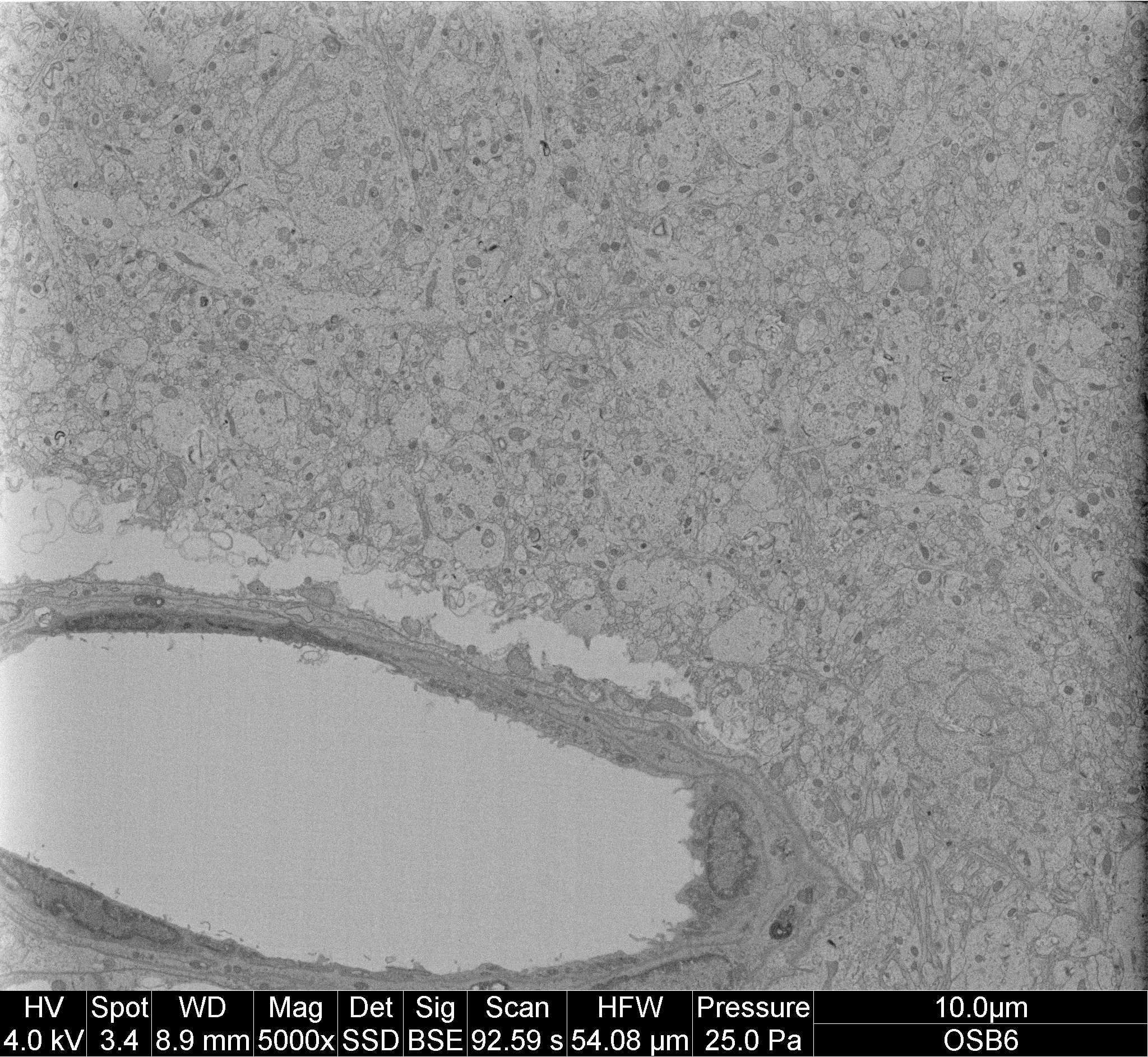

Supplement: Dataset S7 — (253.7 MB ZIP). [file pbio.0020329.sd007.zip › 040604_OS5_st1_694.tif]

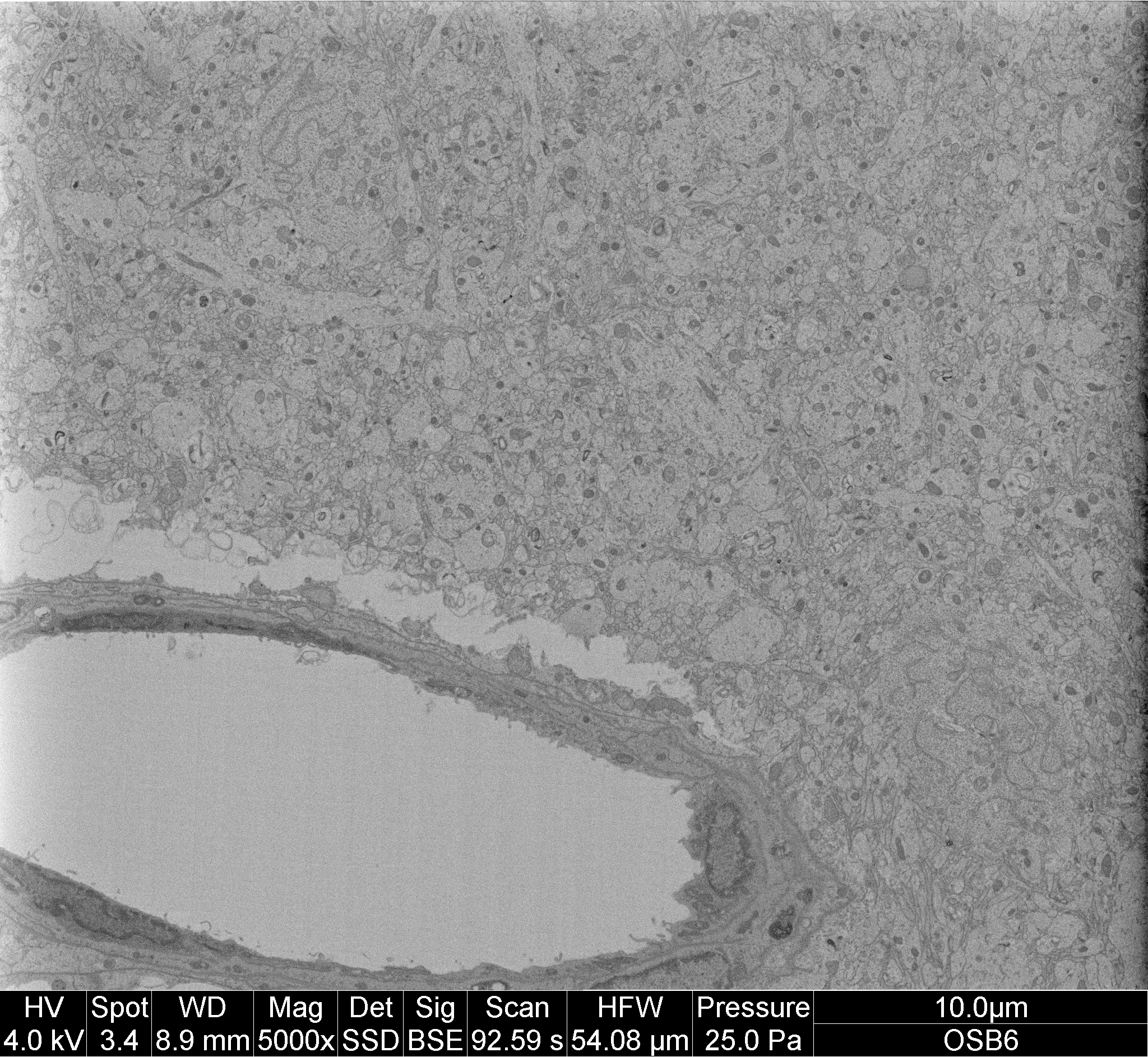

Supplement: Dataset S7 — (253.7 MB ZIP). [file pbio.0020329.sd007.zip › 040604_OS5_st1_695.tif]

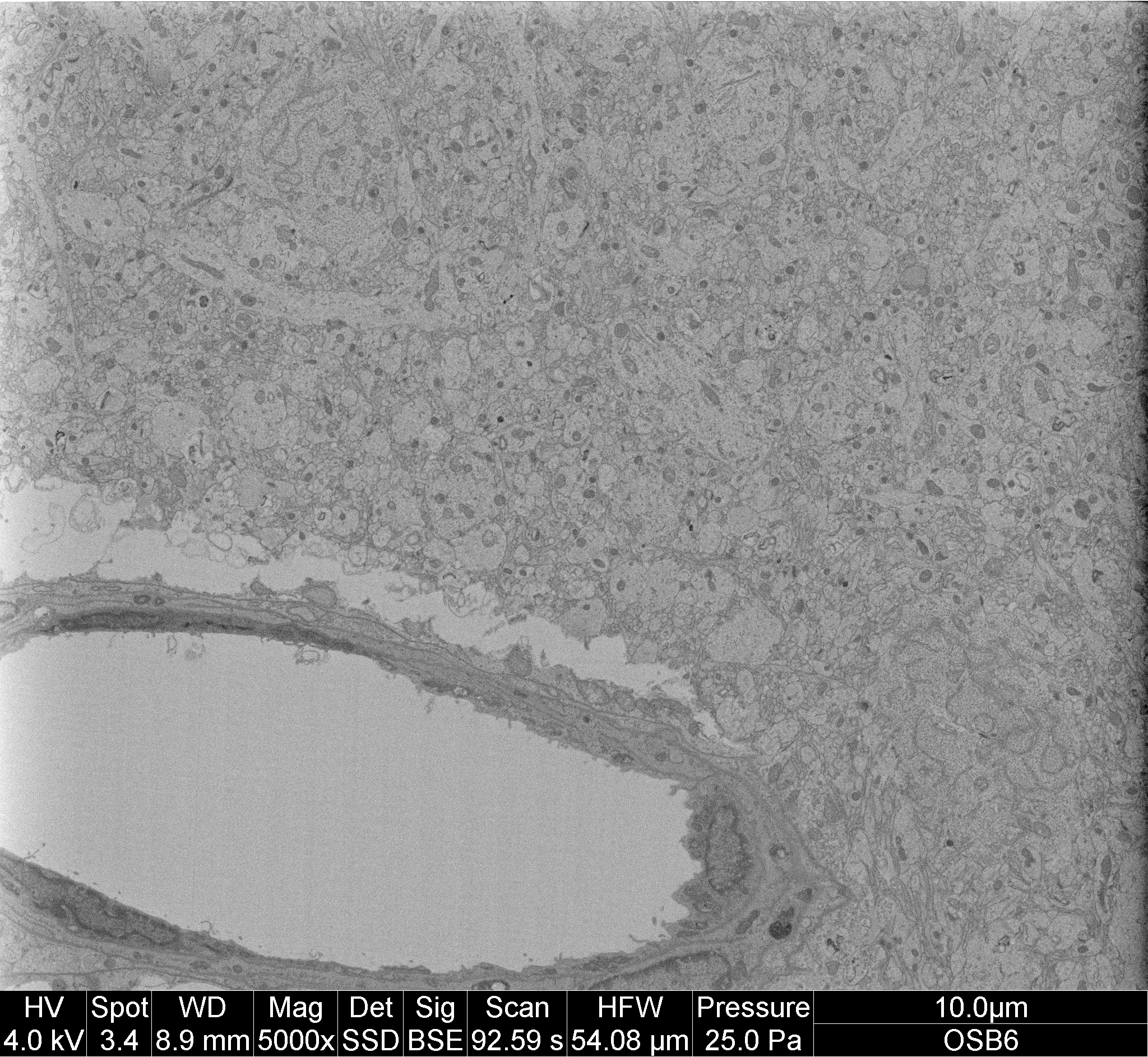

Supplement: Dataset S7 — (253.7 MB ZIP). [file pbio.0020329.sd007.zip › 040604_OS5_st1_696.tif]

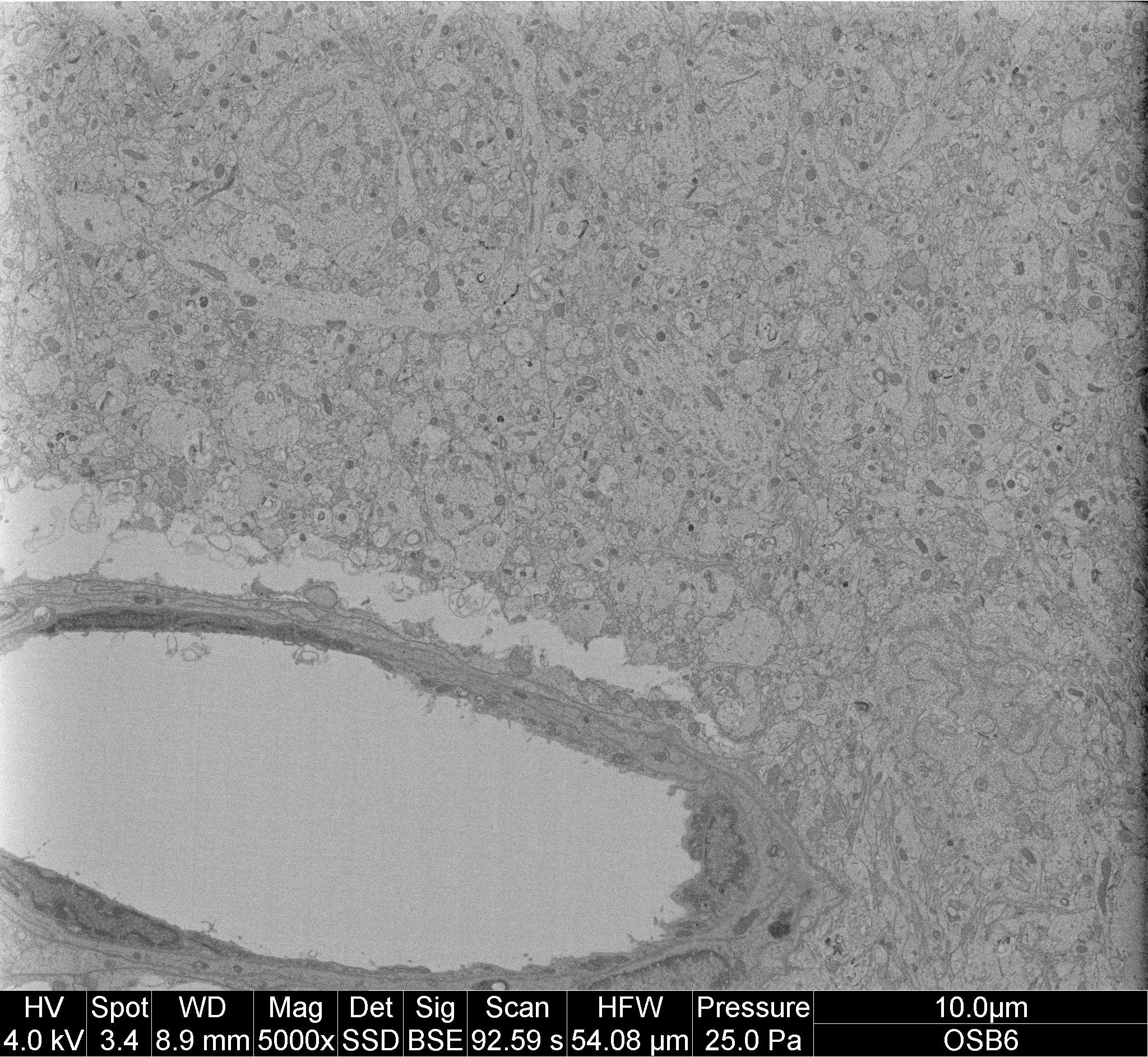

Supplement: Dataset S7 — (253.7 MB ZIP). [file pbio.0020329.sd007.zip › 040604_OS5_st1_697.tif]

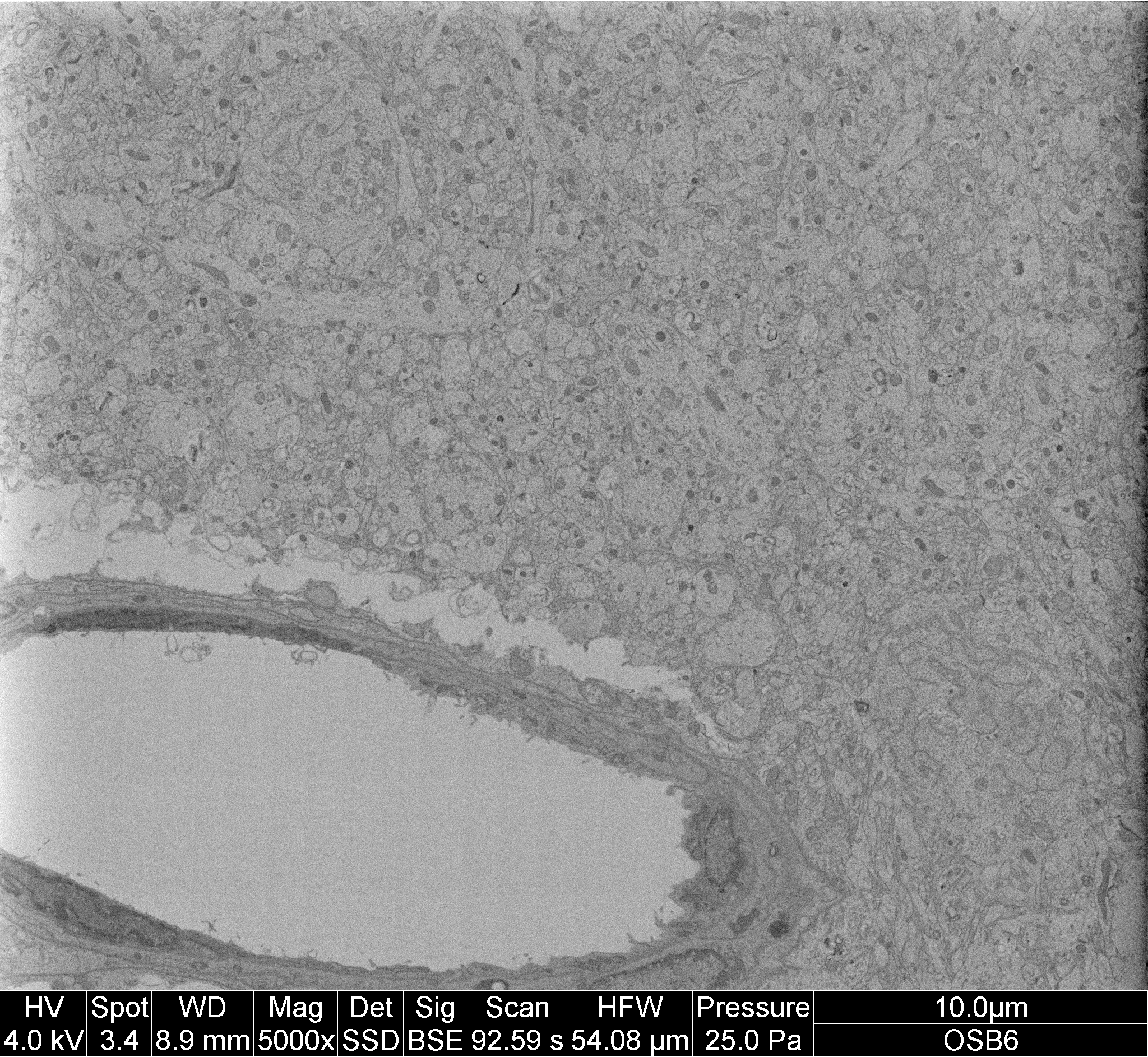

Supplement: Dataset S7 — (253.7 MB ZIP). [file pbio.0020329.sd007.zip › 040604_OS5_st1_698.tif]

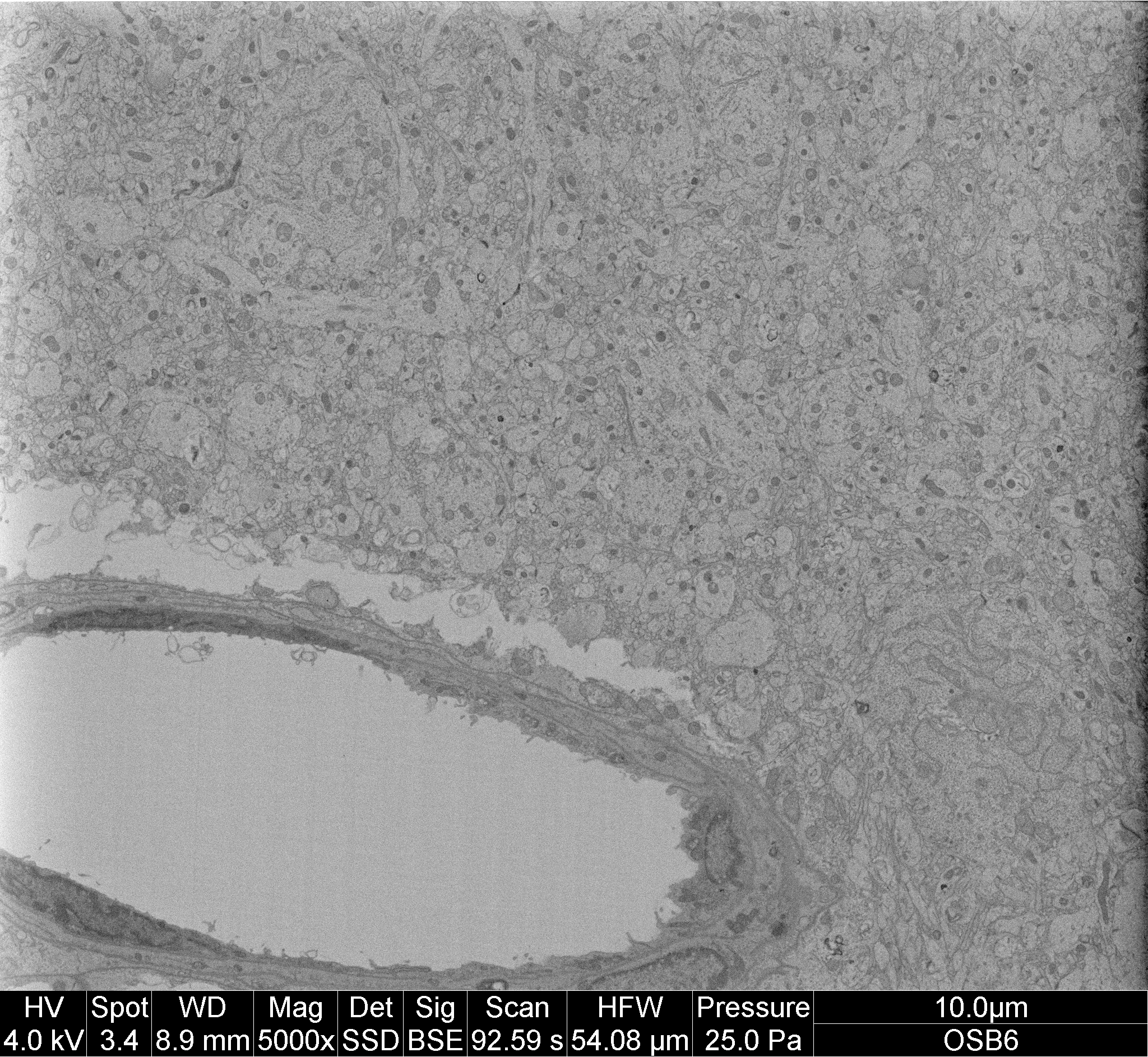

Supplement: Dataset S7 — (253.7 MB ZIP). [file pbio.0020329.sd007.zip › 040604_OS5_st1_699.tif]

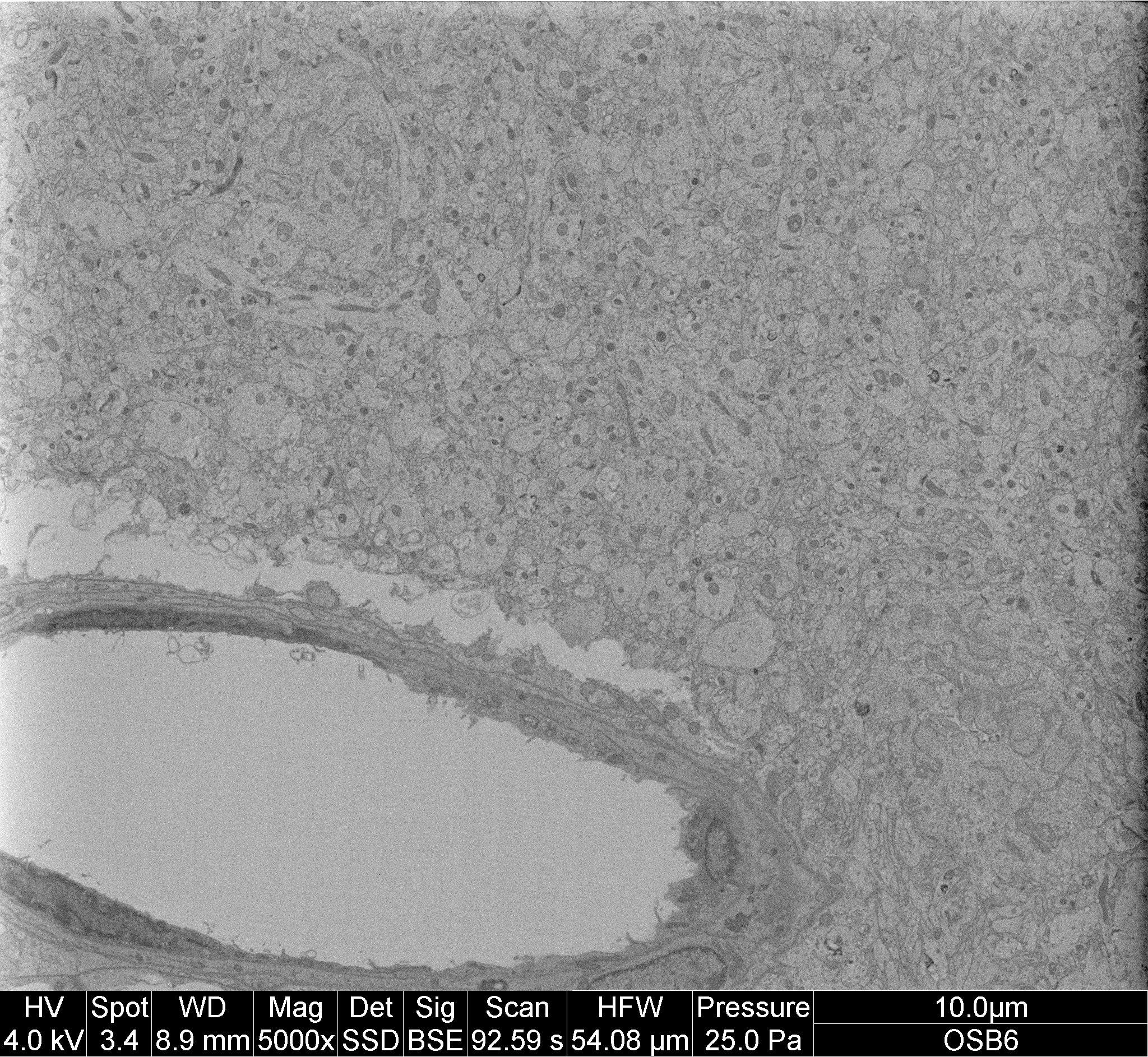

Supplement: Dataset S8 — (255.9 MB ZIP). [file pbio.0020329.sd008.zip › 040604_OS5_st1_700.tif]
