# Supplementary figures and images for: Serial Block-Face Scanning Electron Microscopy to Reconstruct Three-Dimensional Tissue Nanostructure (part 8 of 21)
Source: PLoS Biol. 2004 Oct 19;2(11):e329. doi: 10.1371/journal.pbio.0020329 (PMC524270; doi:10.1371/journal.pbio.0020329)

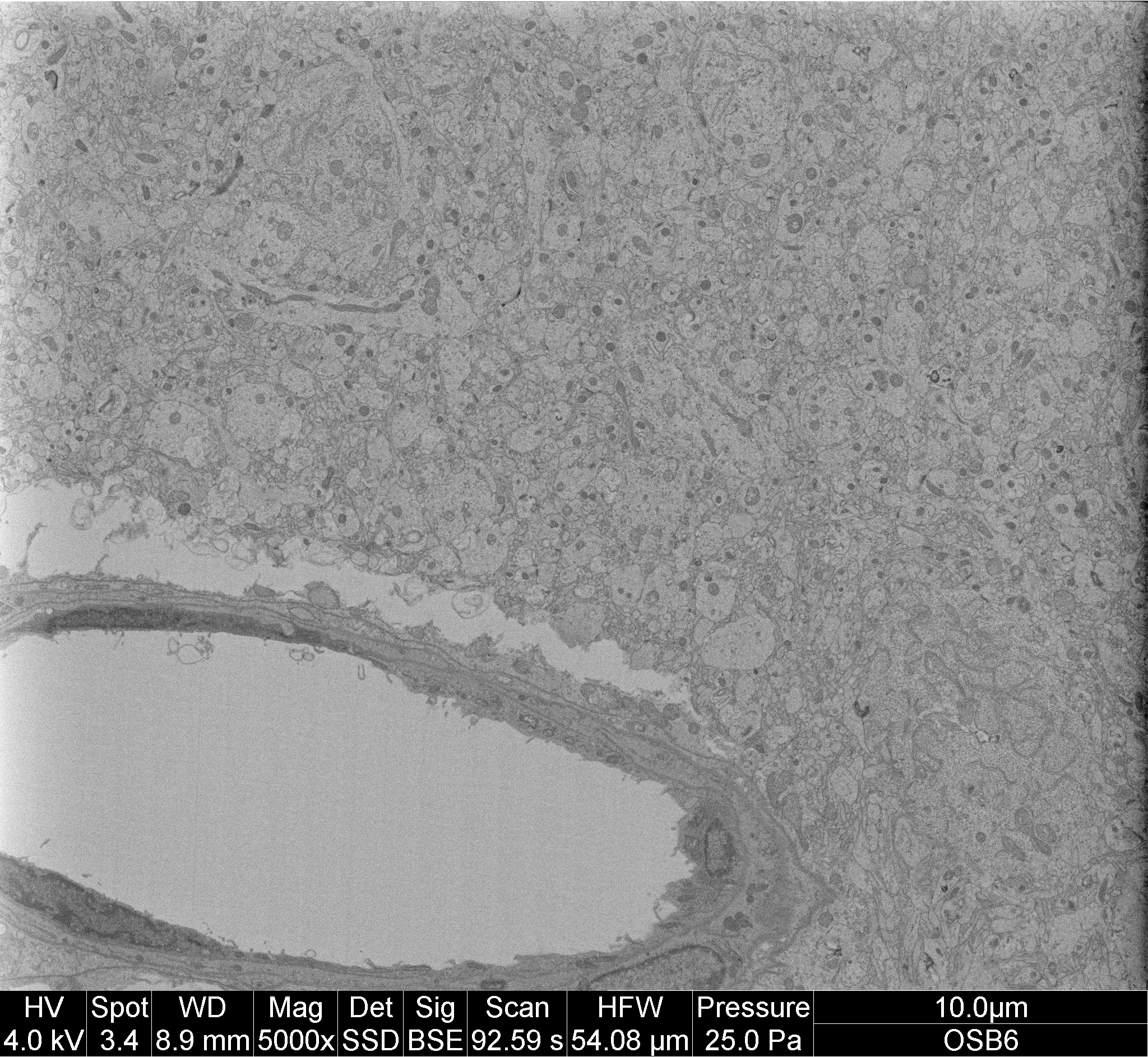

Supplement: Dataset S8 — (255.9 MB ZIP). [file pbio.0020329.sd008.zip › 040604_OS5_st1_701.tif]

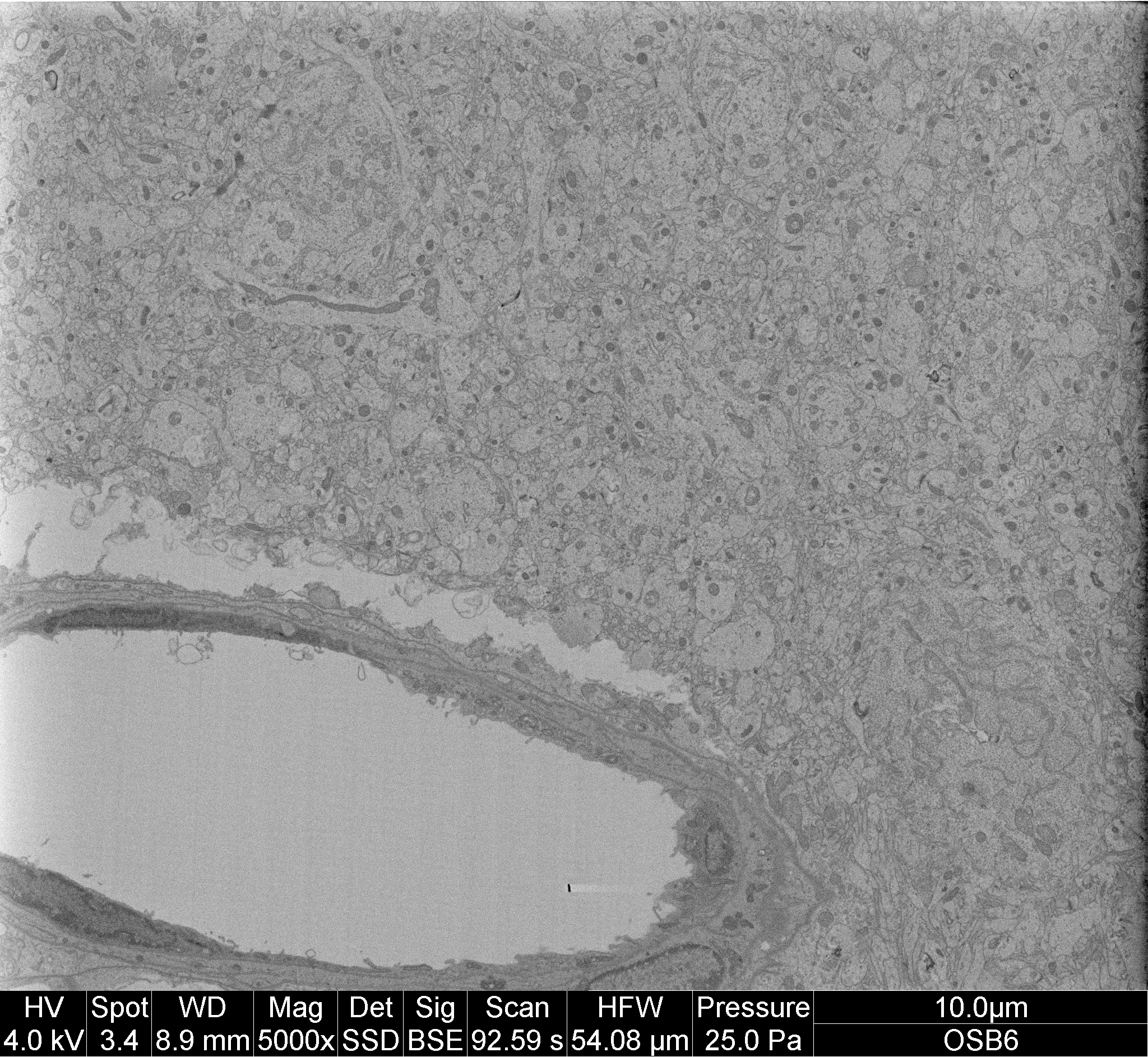

Supplement: Dataset S8 — (255.9 MB ZIP). [file pbio.0020329.sd008.zip › 040604_OS5_st1_702.tif]

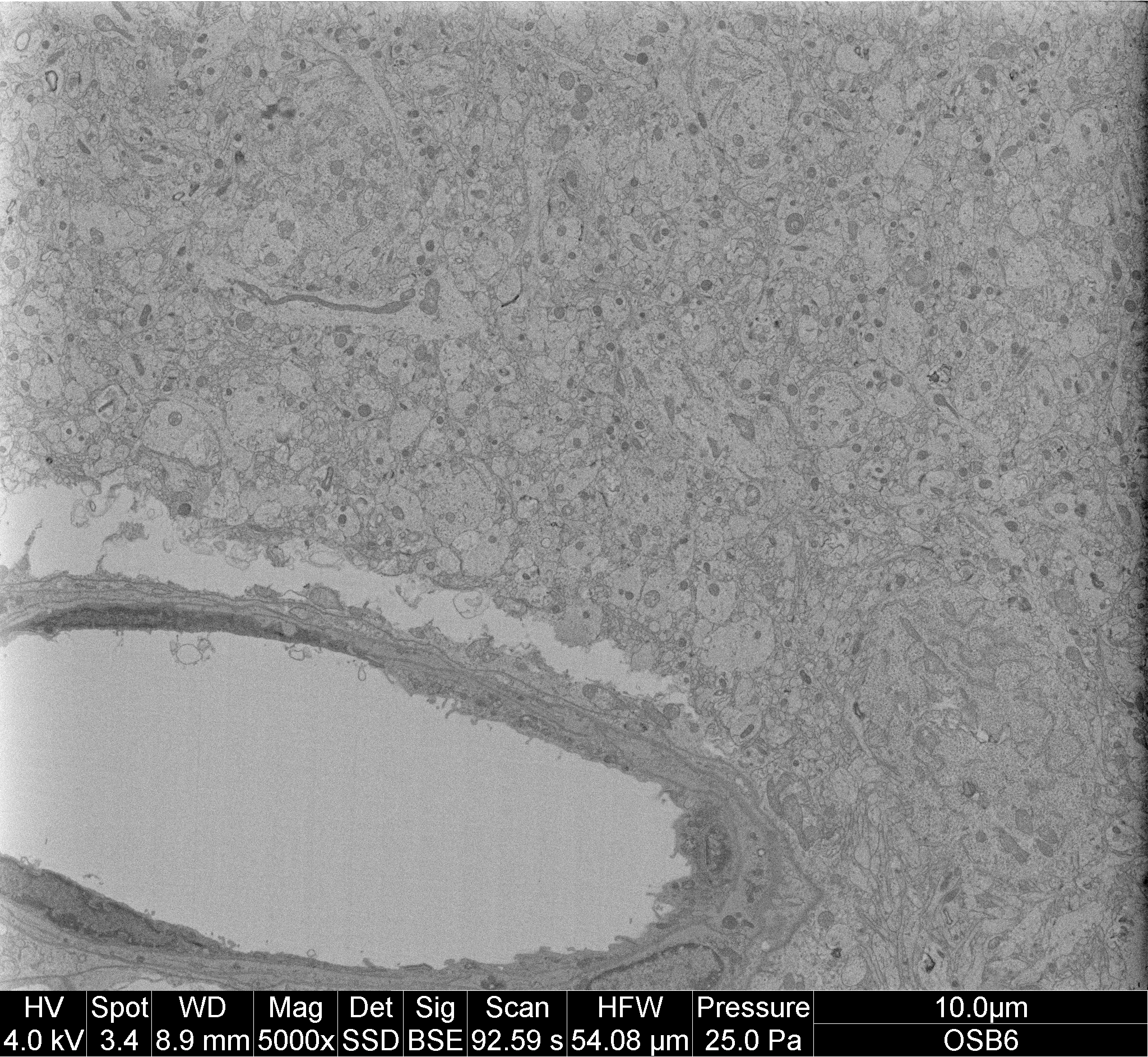

Supplement: Dataset S8 — (255.9 MB ZIP). [file pbio.0020329.sd008.zip › 040604_OS5_st1_703.tif]

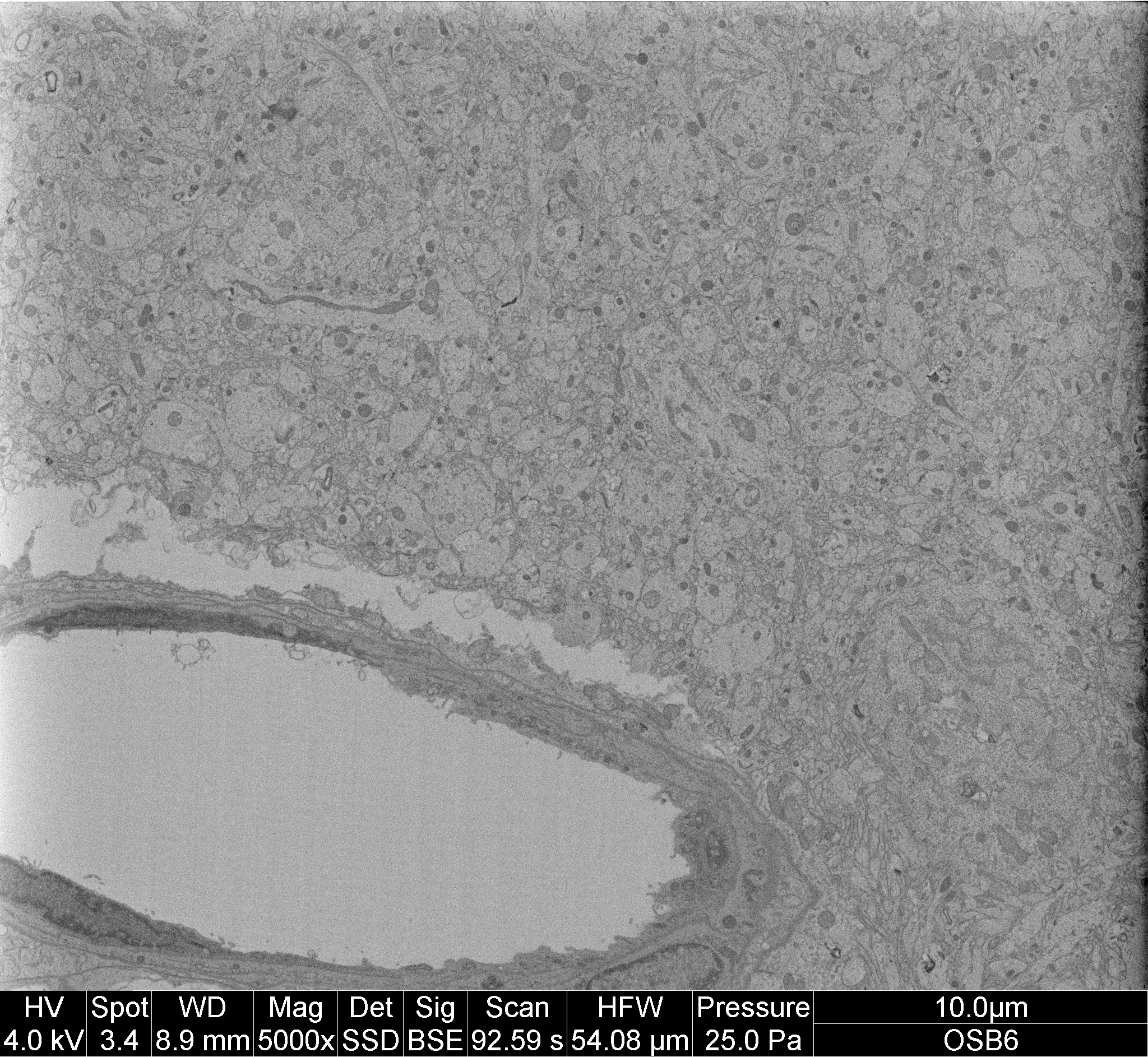

Supplement: Dataset S8 — (255.9 MB ZIP). [file pbio.0020329.sd008.zip › 040604_OS5_st1_704.tif]

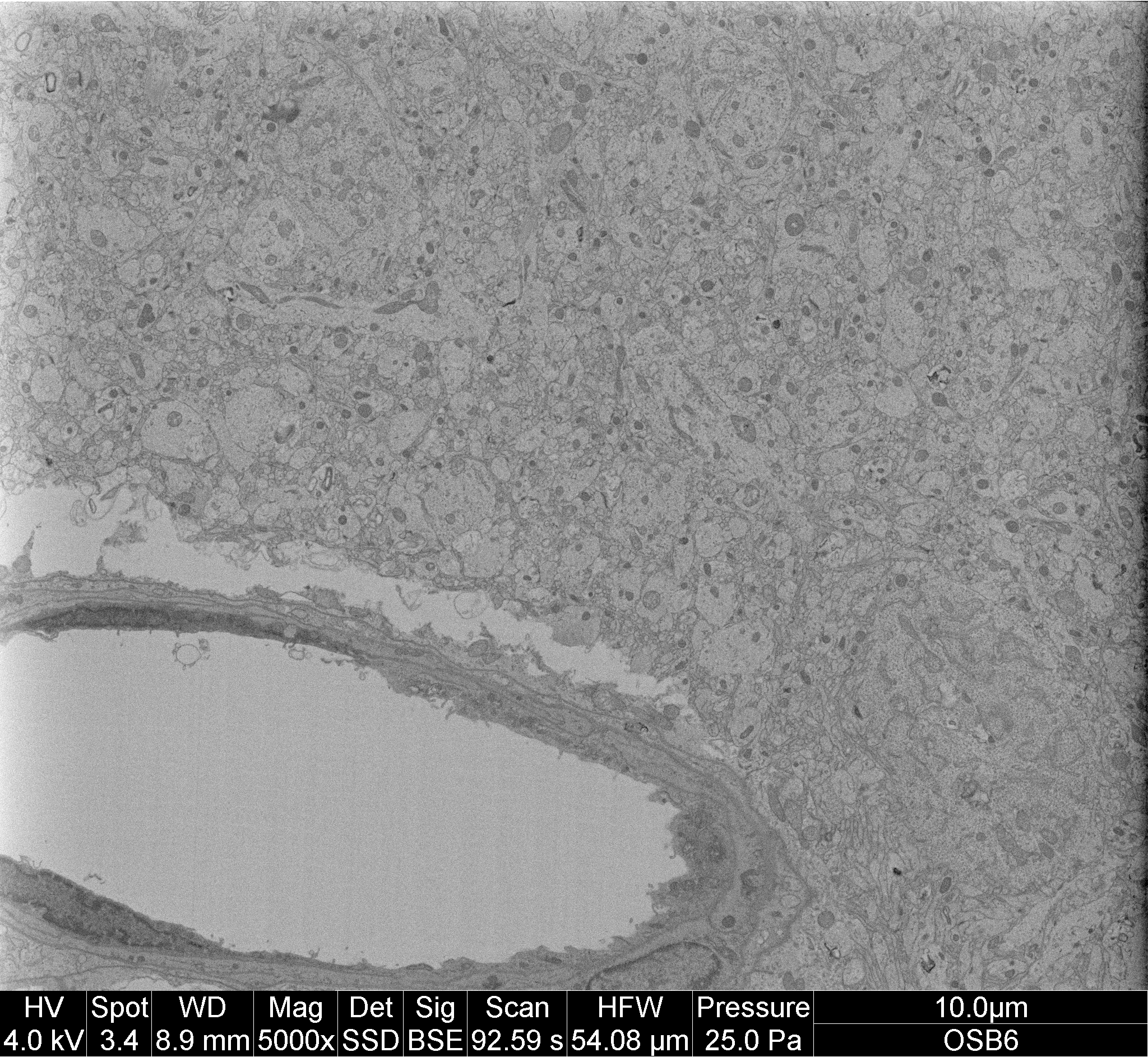

Supplement: Dataset S8 — (255.9 MB ZIP). [file pbio.0020329.sd008.zip › 040604_OS5_st1_705.tif]

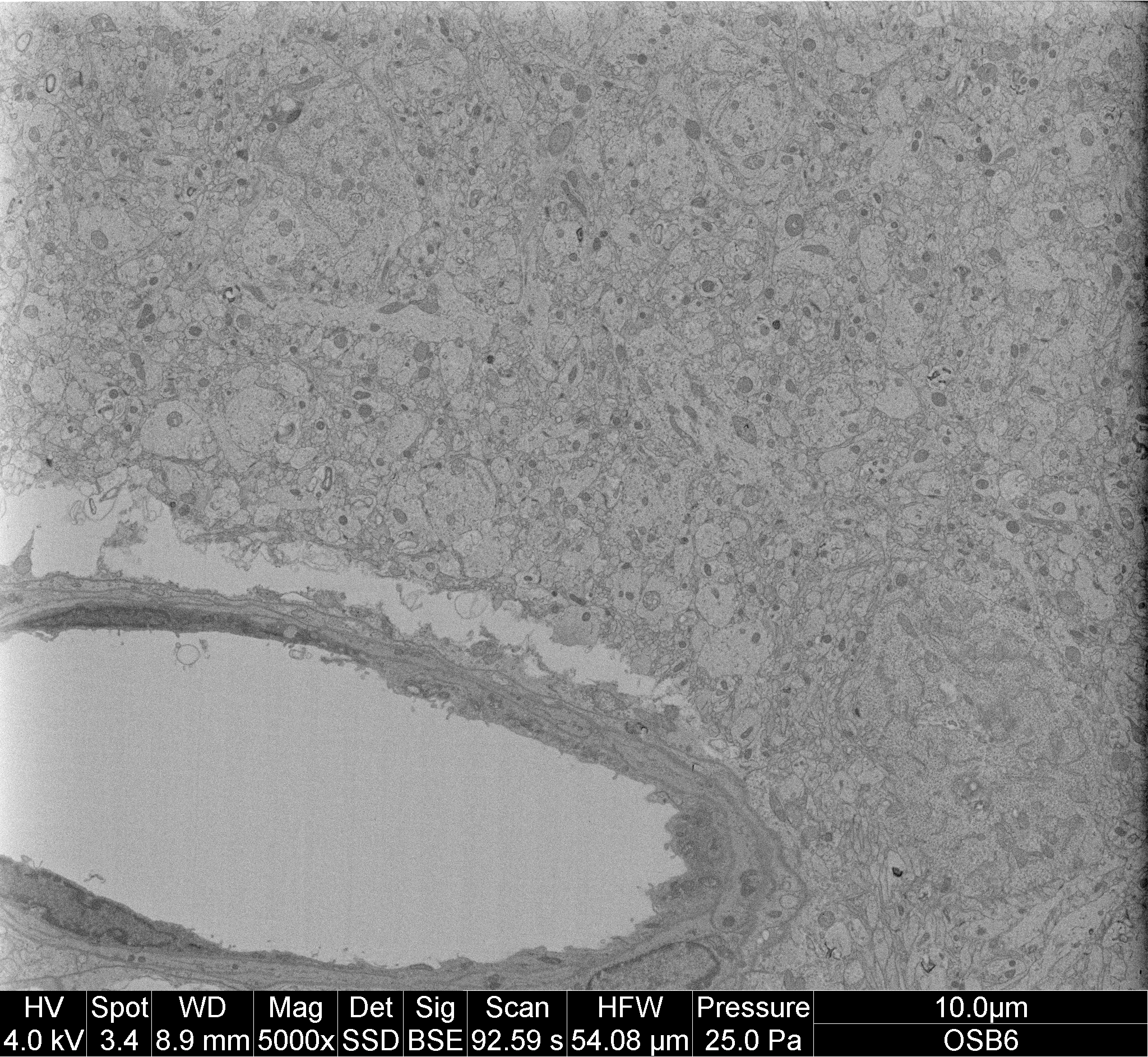

Supplement: Dataset S8 — (255.9 MB ZIP). [file pbio.0020329.sd008.zip › 040604_OS5_st1_706.tif]

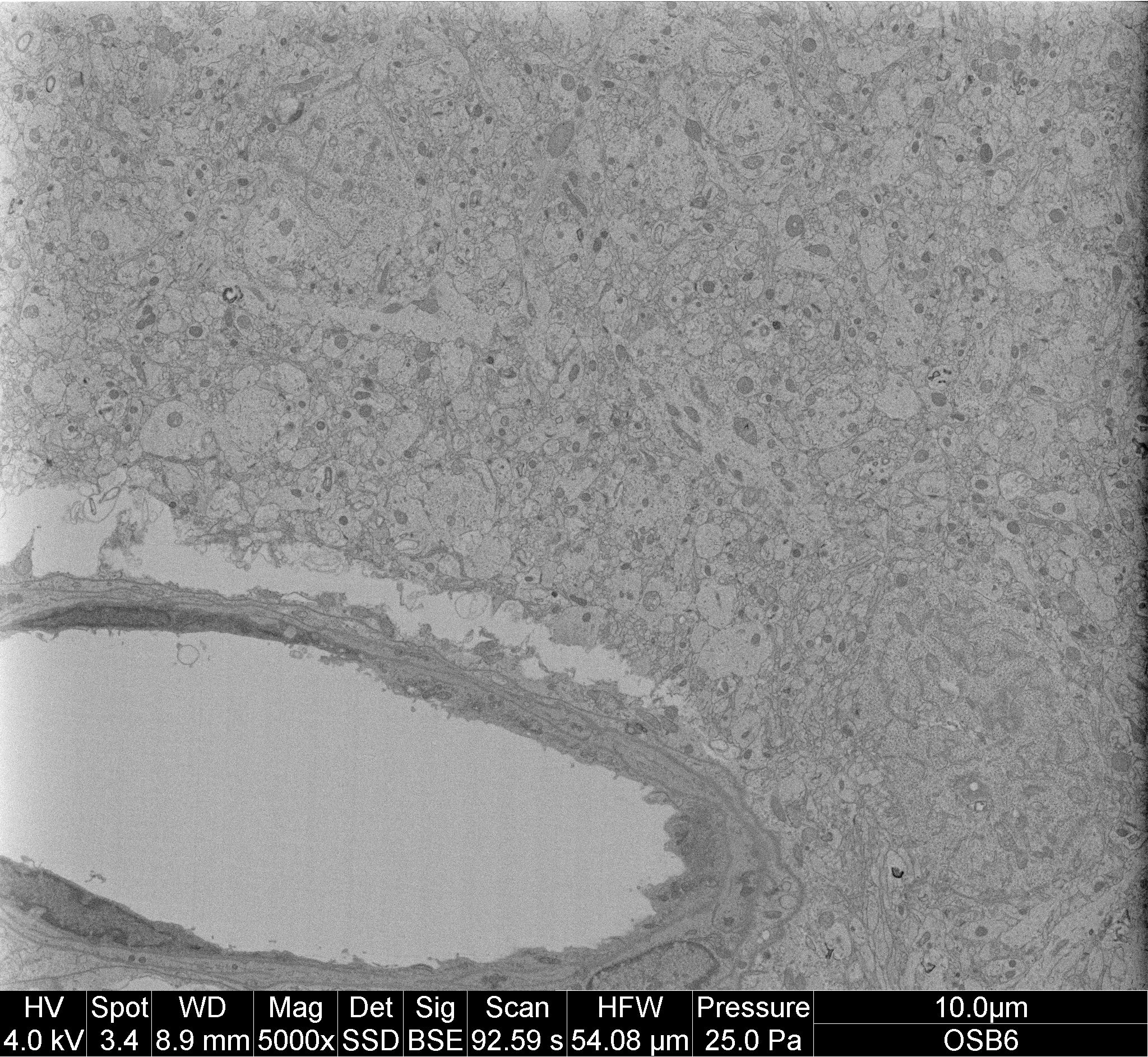

Supplement: Dataset S8 — (255.9 MB ZIP). [file pbio.0020329.sd008.zip › 040604_OS5_st1_707.tif]

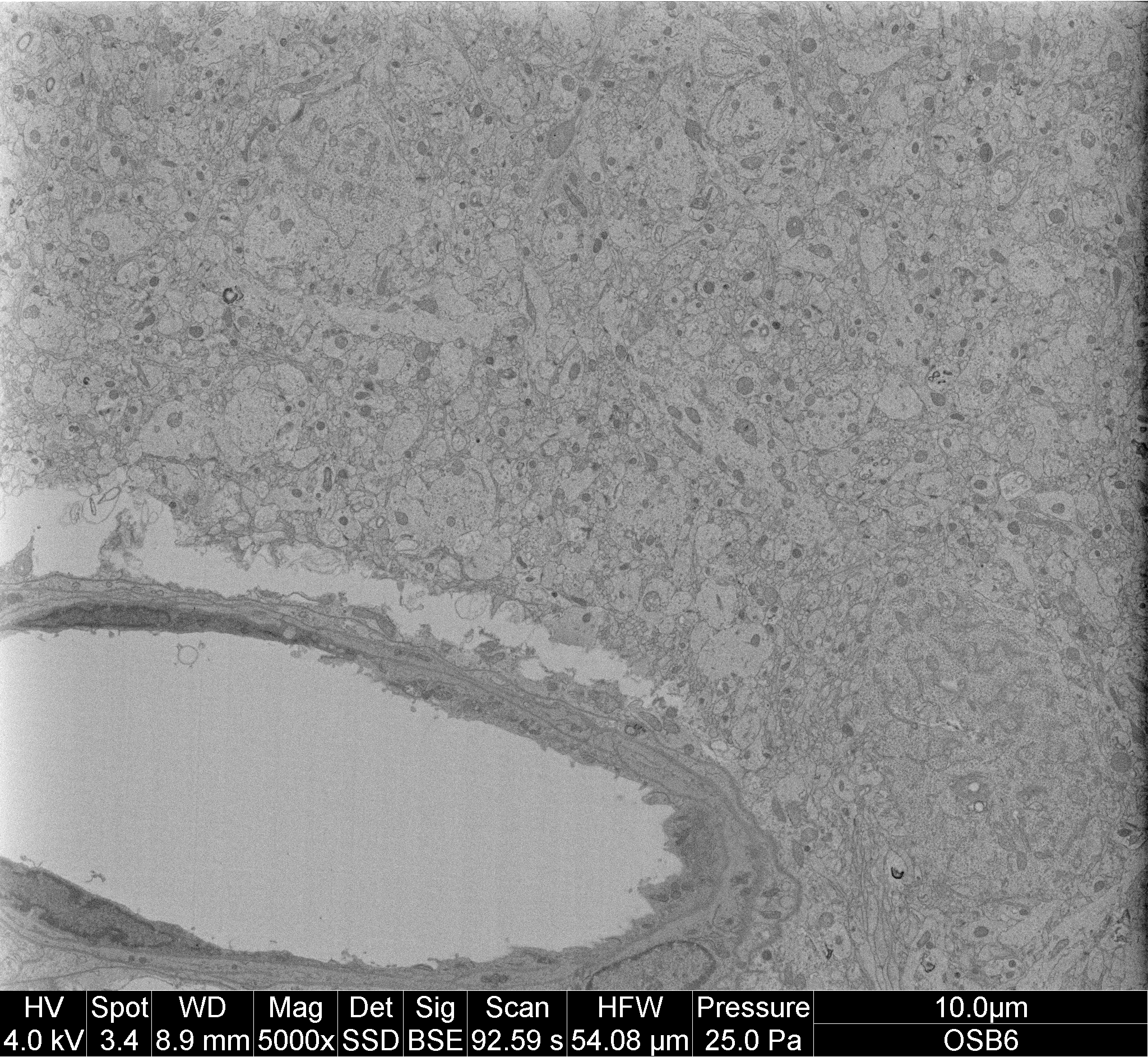

Supplement: Dataset S8 — (255.9 MB ZIP). [file pbio.0020329.sd008.zip › 040604_OS5_st1_708.tif]

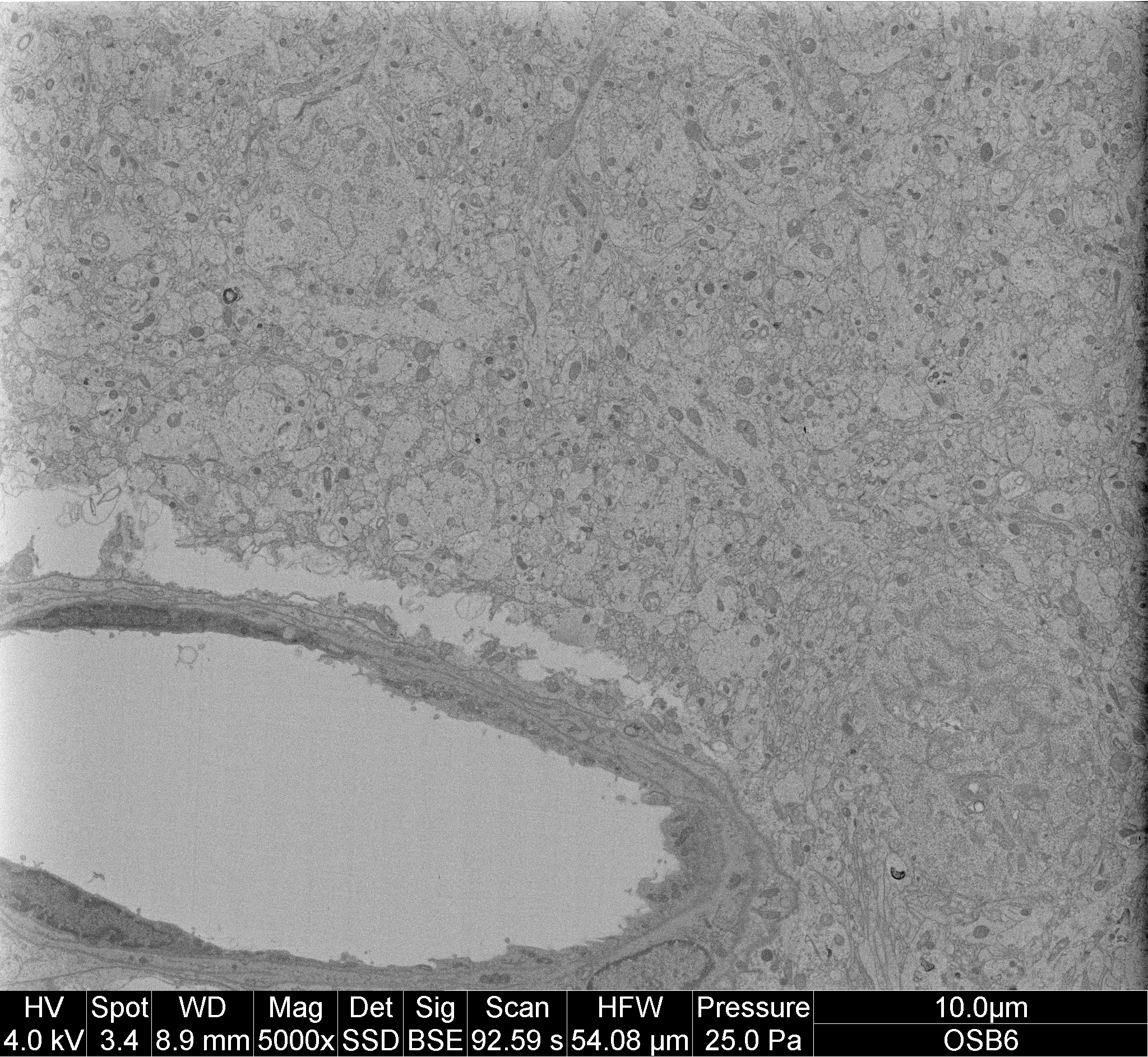

Supplement: Dataset S8 — (255.9 MB ZIP). [file pbio.0020329.sd008.zip › 040604_OS5_st1_709.tif]

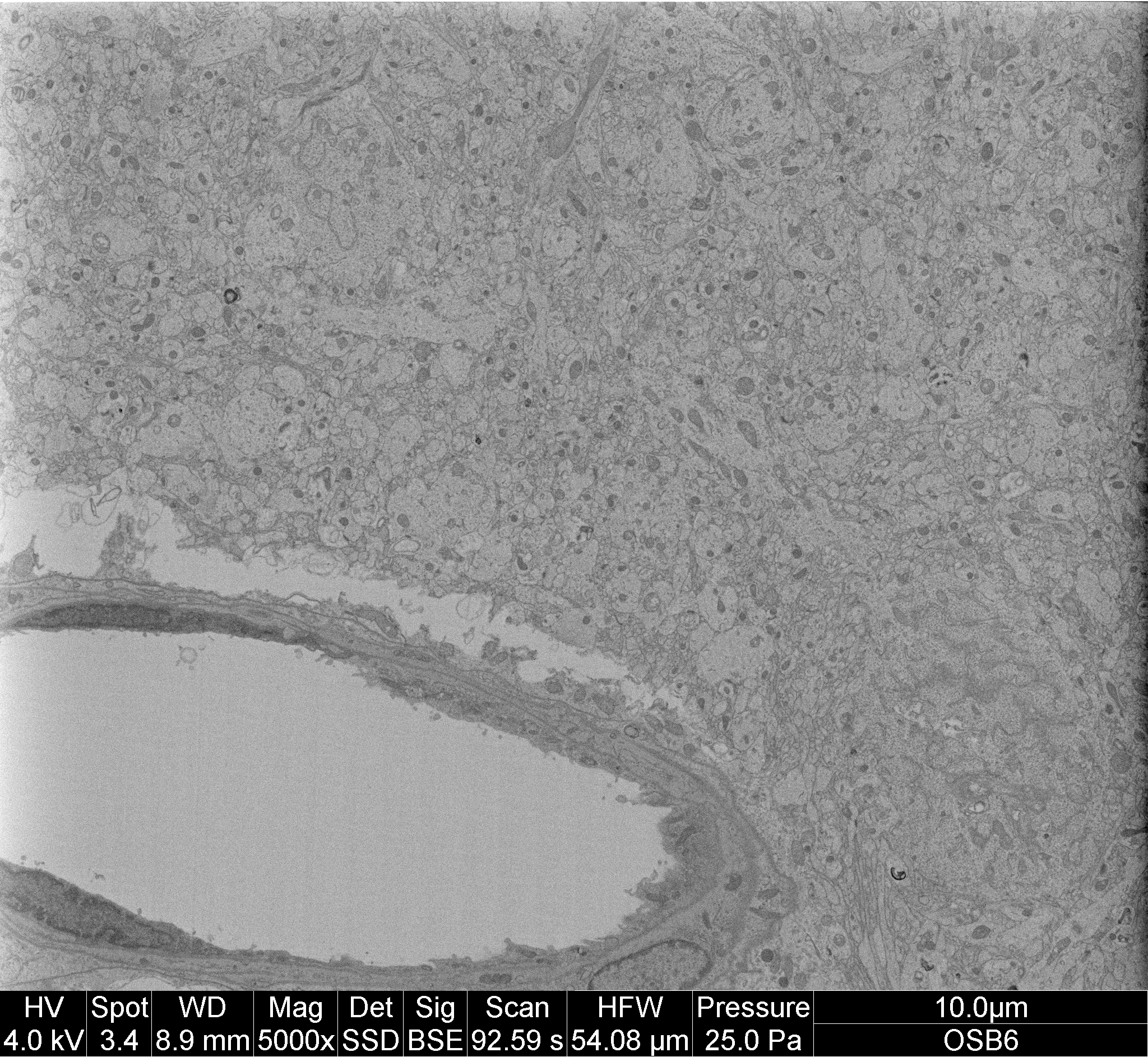

Supplement: Dataset S8 — (255.9 MB ZIP). [file pbio.0020329.sd008.zip › 040604_OS5_st1_710.tif]

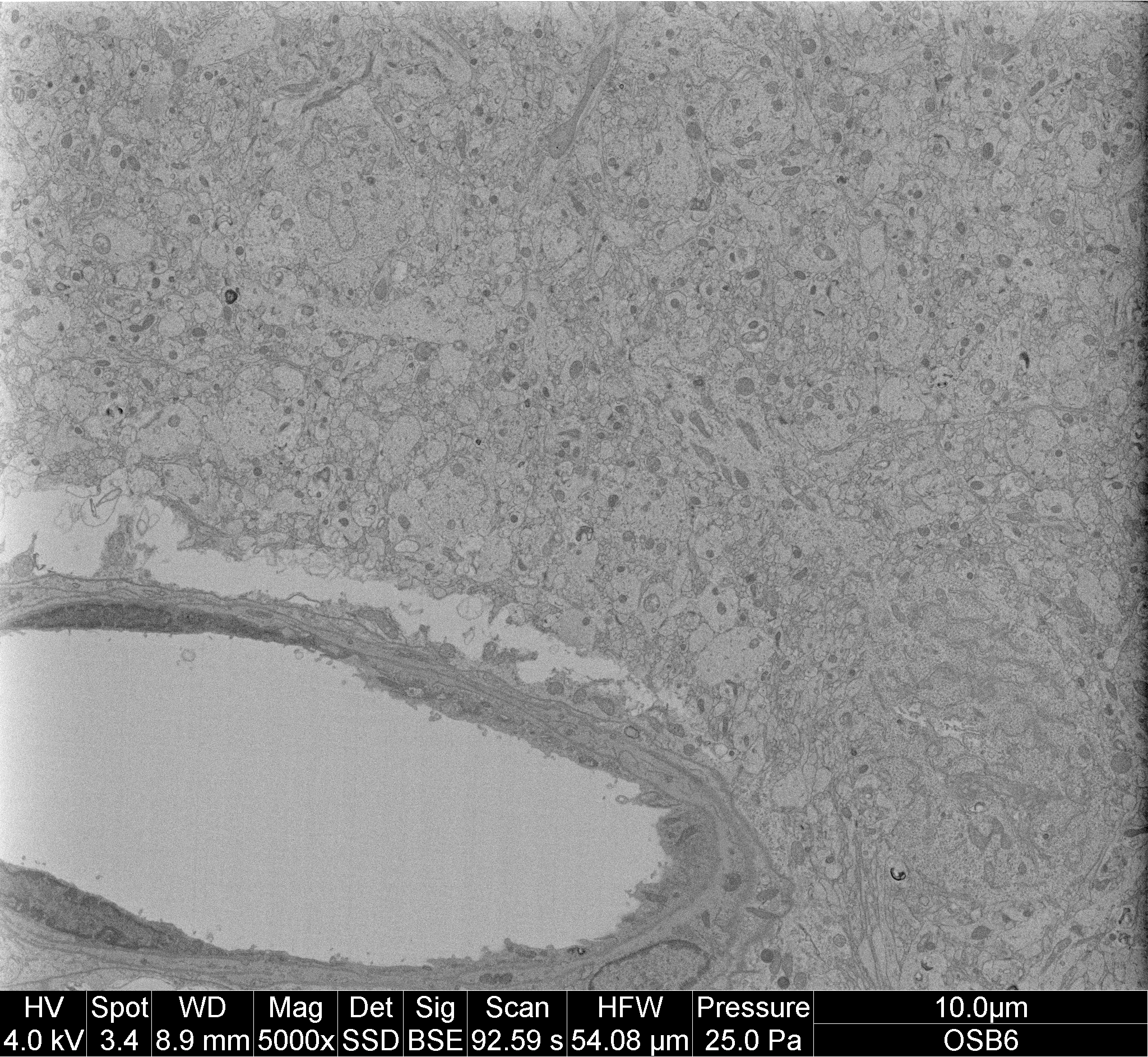

Supplement: Dataset S8 — (255.9 MB ZIP). [file pbio.0020329.sd008.zip › 040604_OS5_st1_711.tif]

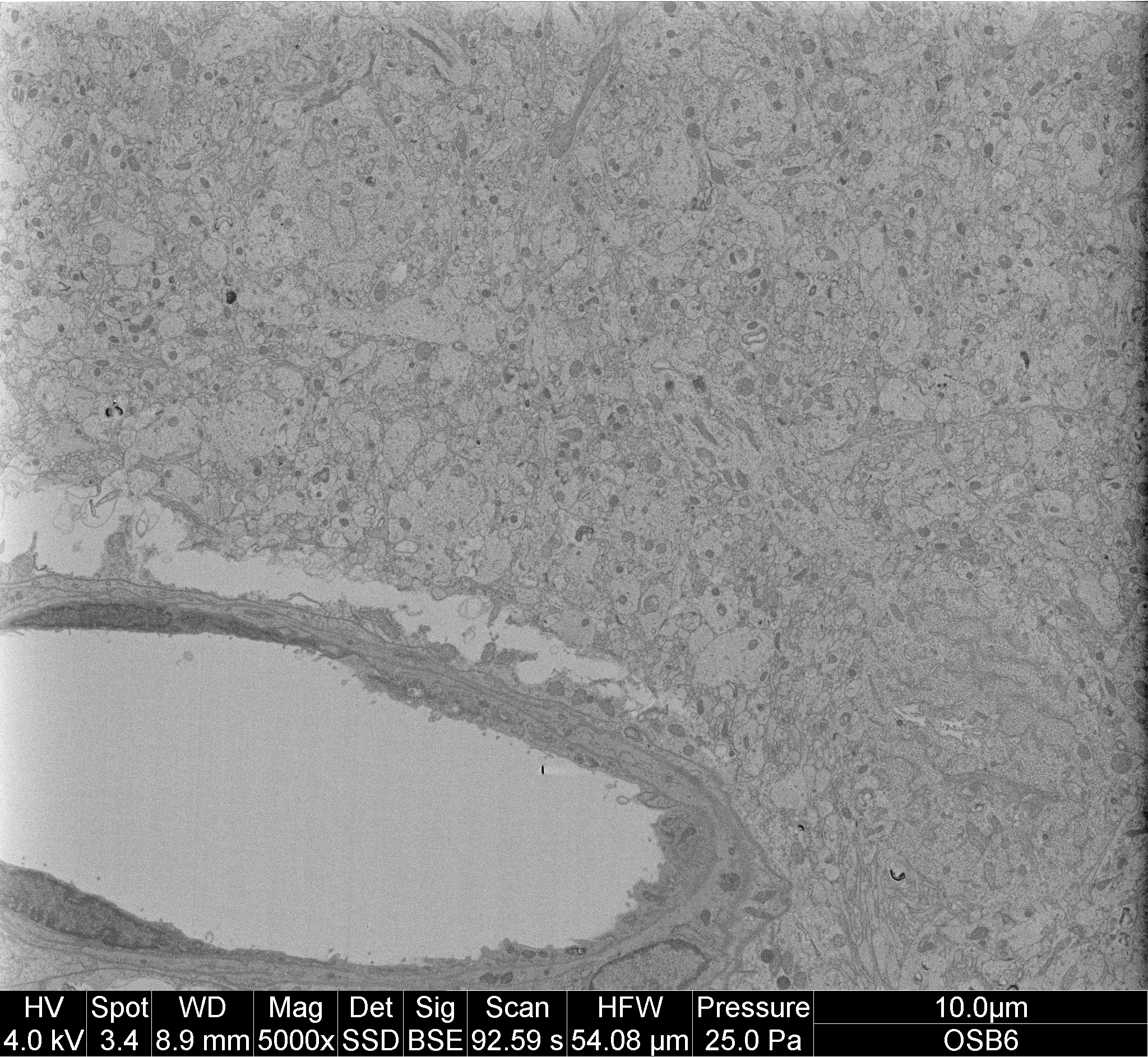

Supplement: Dataset S8 — (255.9 MB ZIP). [file pbio.0020329.sd008.zip › 040604_OS5_st1_712.tif]

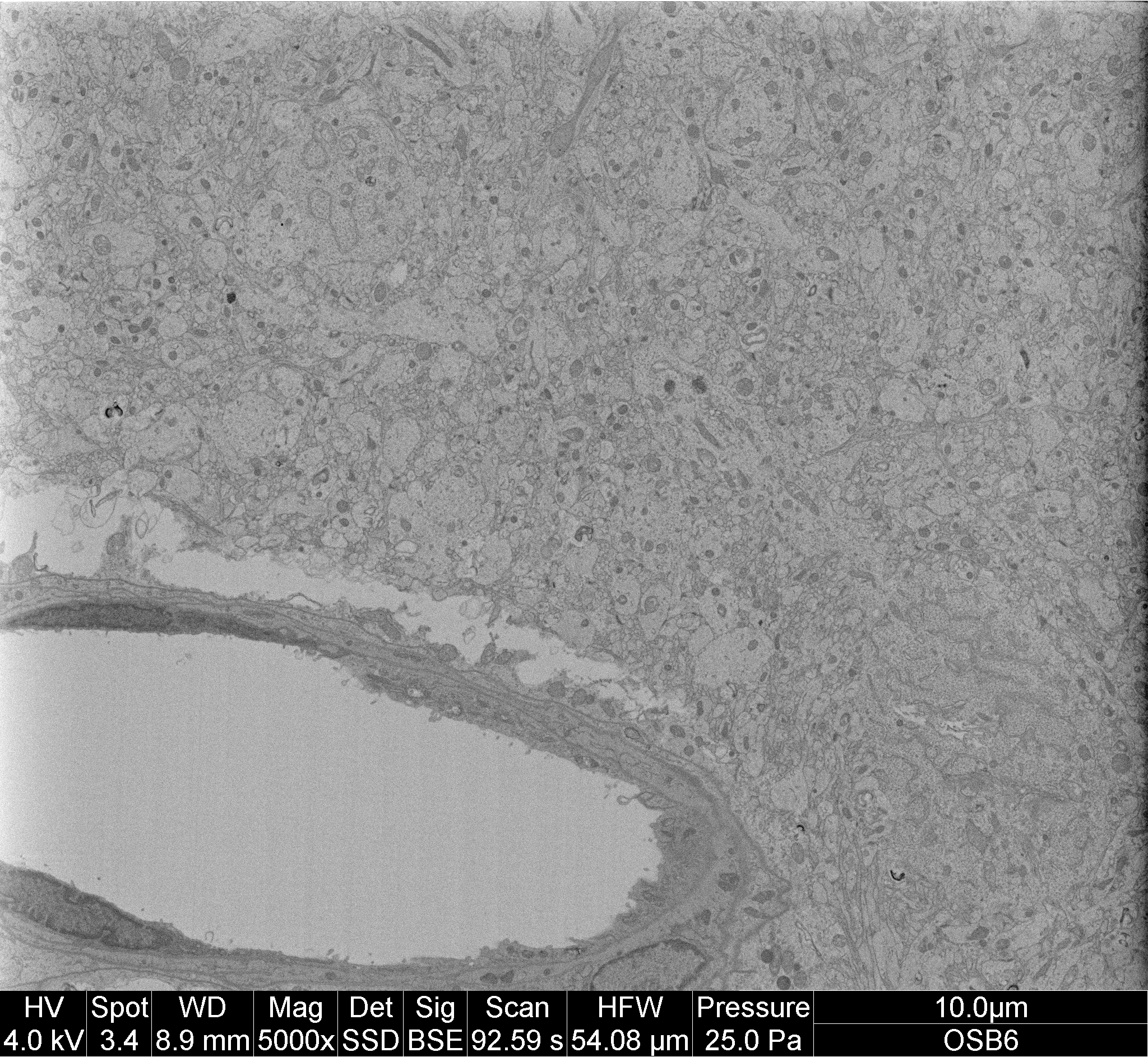

Supplement: Dataset S8 — (255.9 MB ZIP). [file pbio.0020329.sd008.zip › 040604_OS5_st1_713.tif]

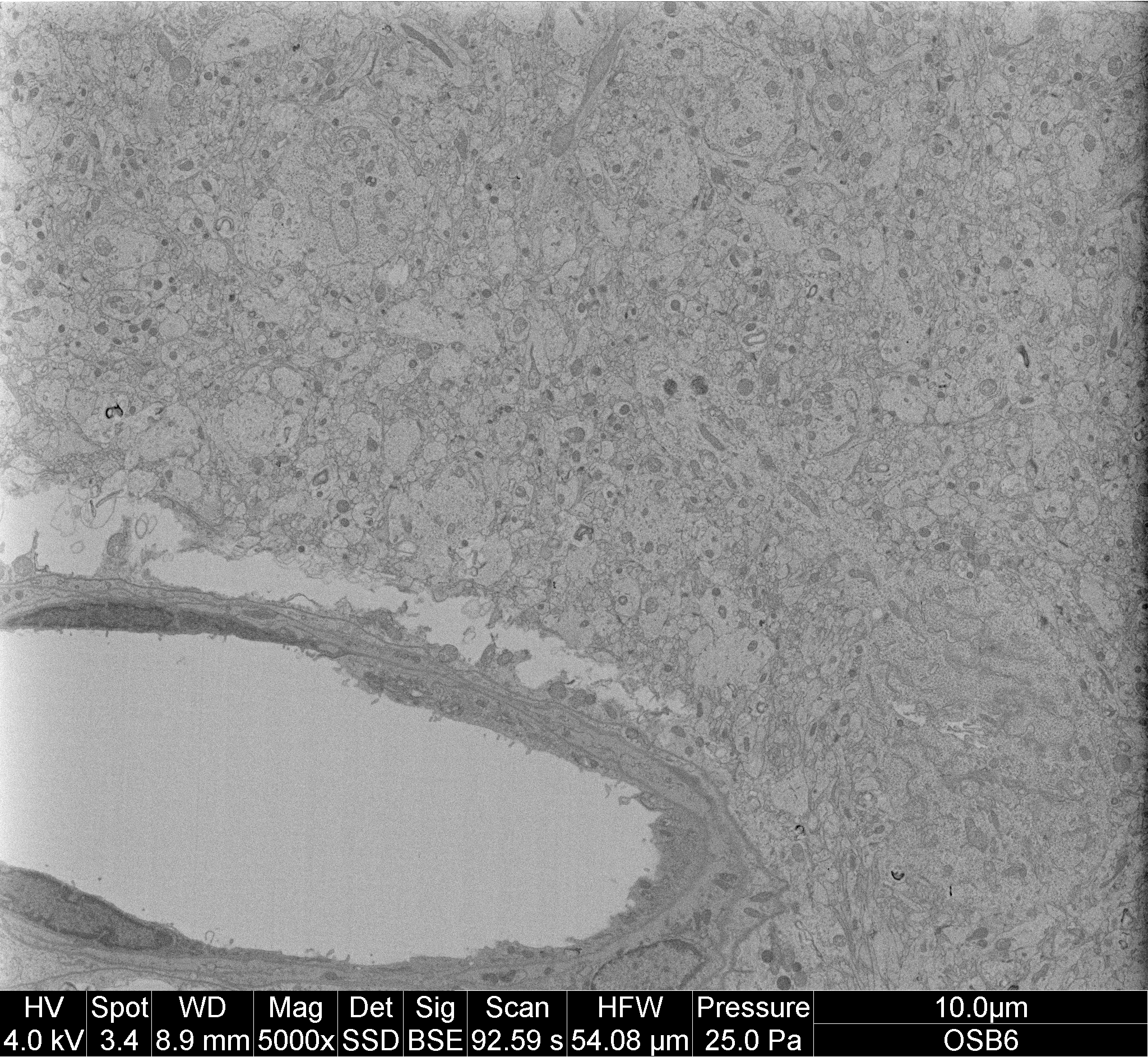

Supplement: Dataset S8 — (255.9 MB ZIP). [file pbio.0020329.sd008.zip › 040604_OS5_st1_714.tif]

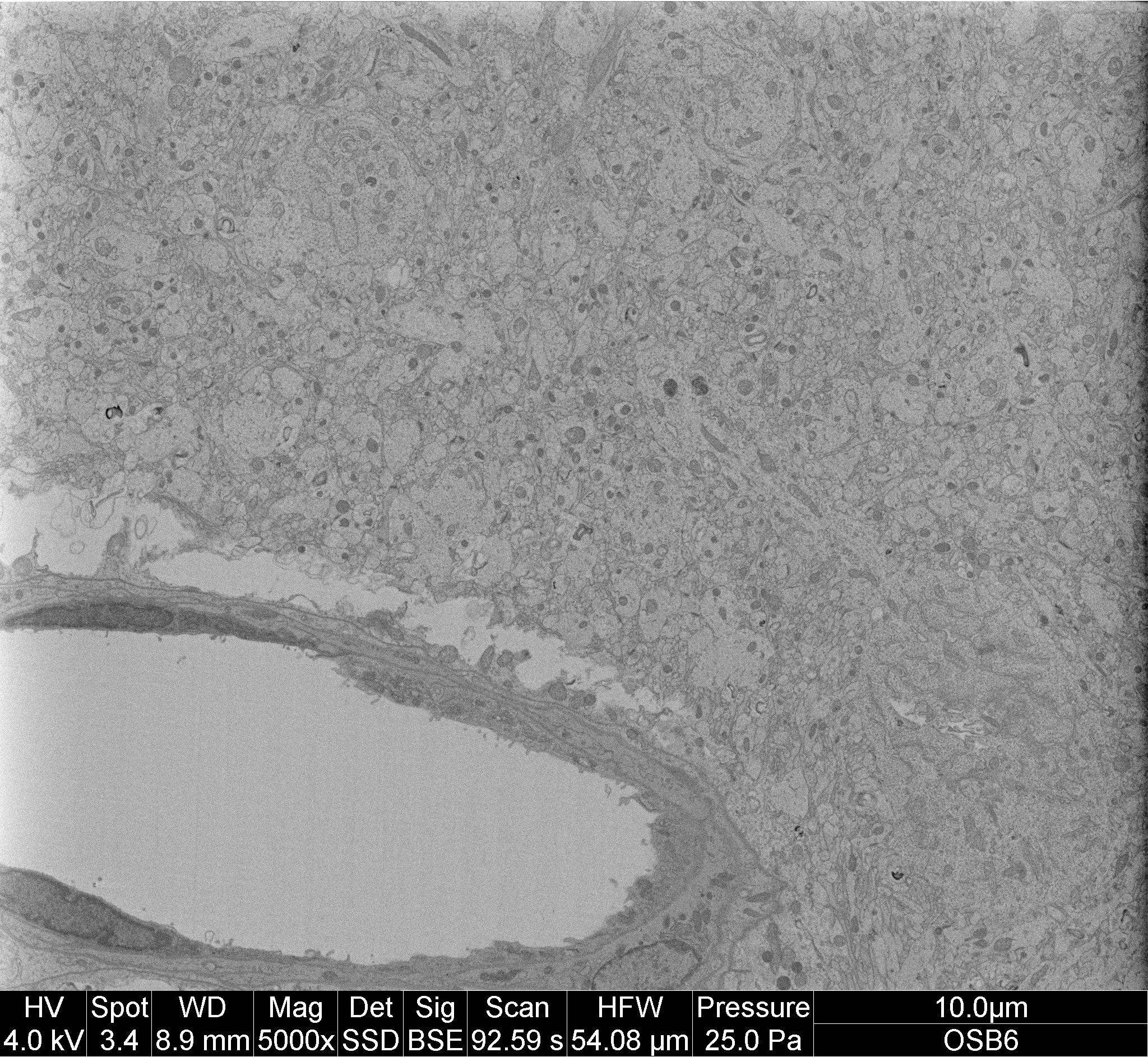

Supplement: Dataset S8 — (255.9 MB ZIP). [file pbio.0020329.sd008.zip › 040604_OS5_st1_715.tif]

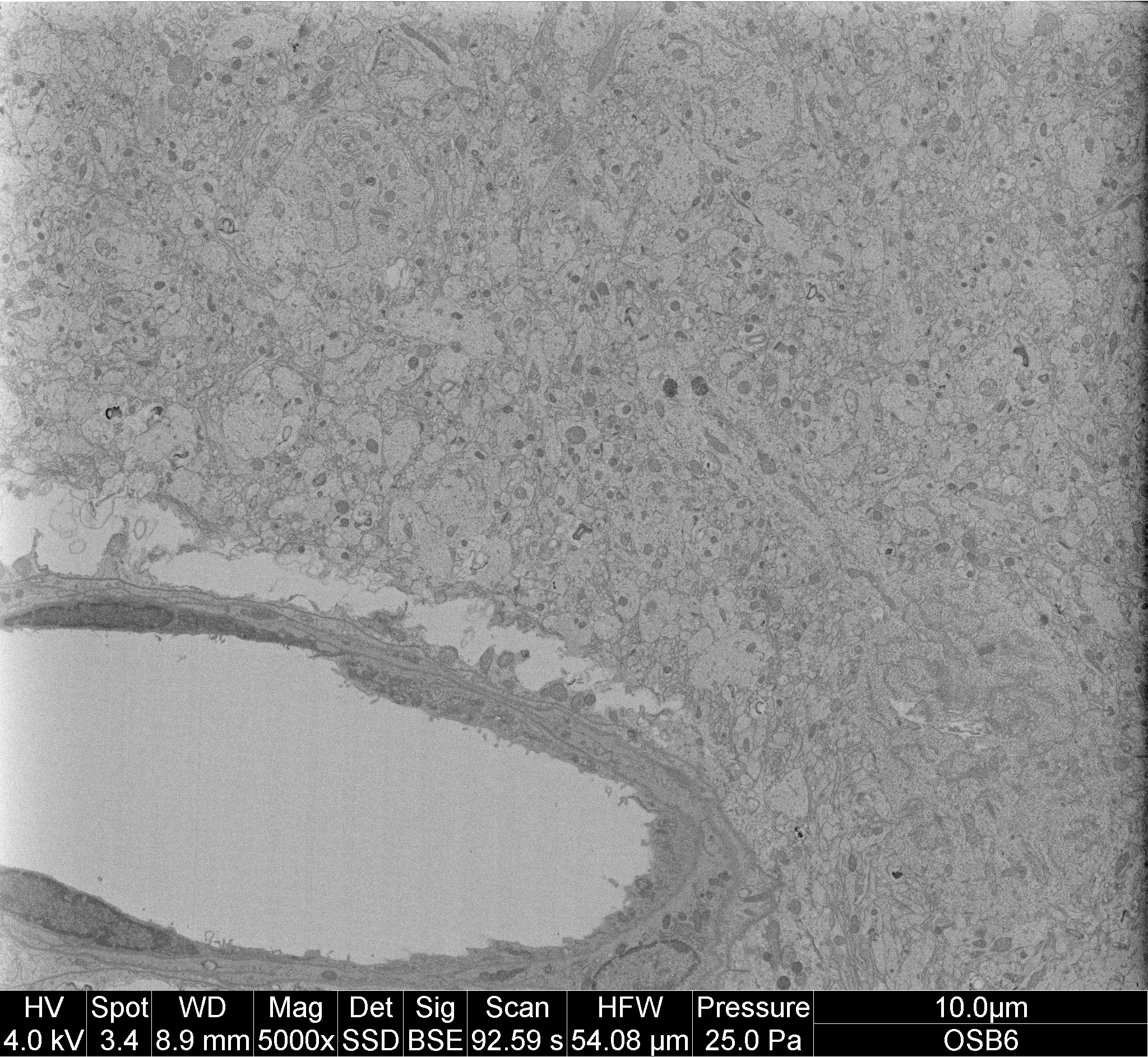

Supplement: Dataset S8 — (255.9 MB ZIP). [file pbio.0020329.sd008.zip › 040604_OS5_st1_716.tif]

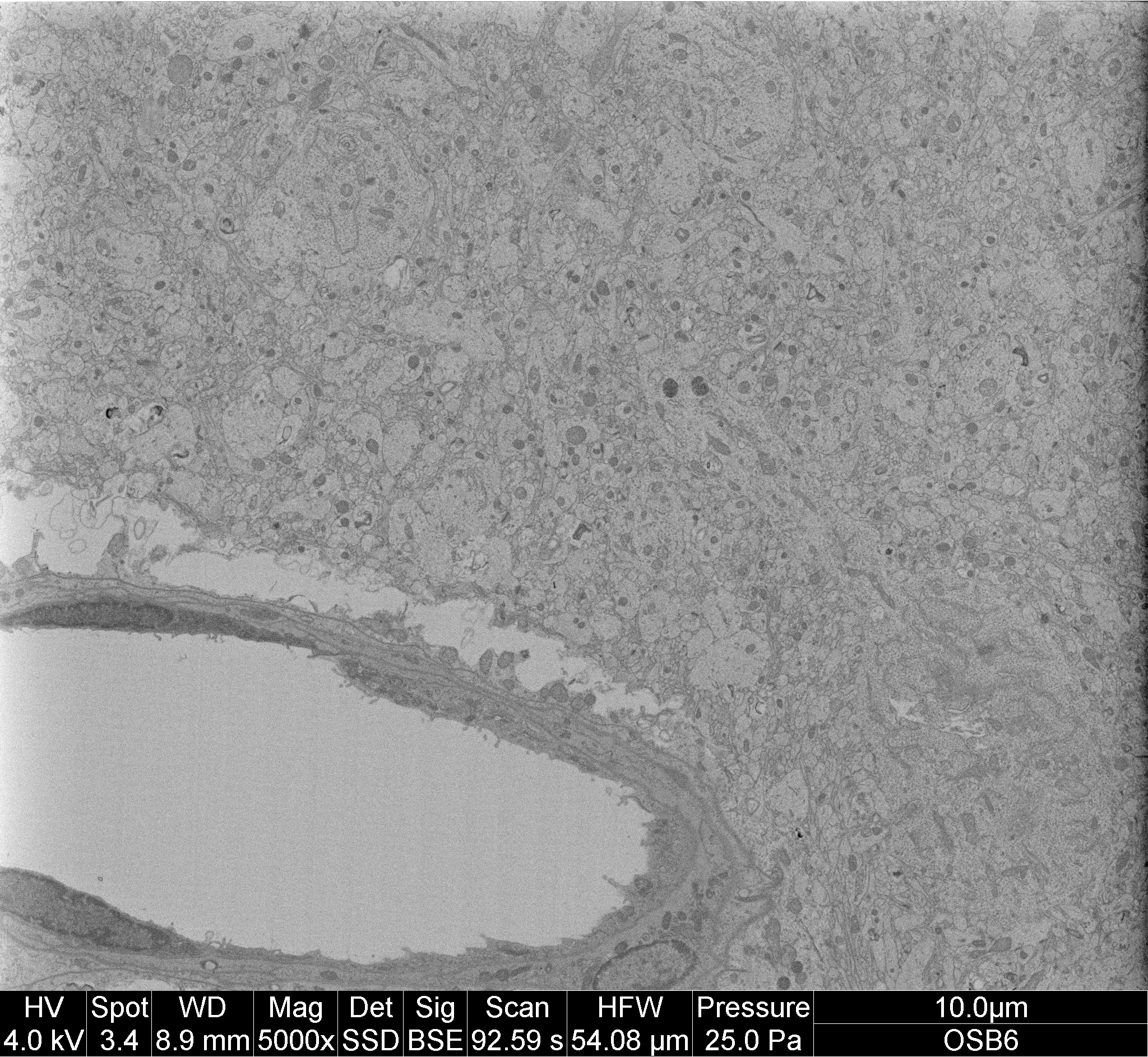

Supplement: Dataset S8 — (255.9 MB ZIP). [file pbio.0020329.sd008.zip › 040604_OS5_st1_717.tif]

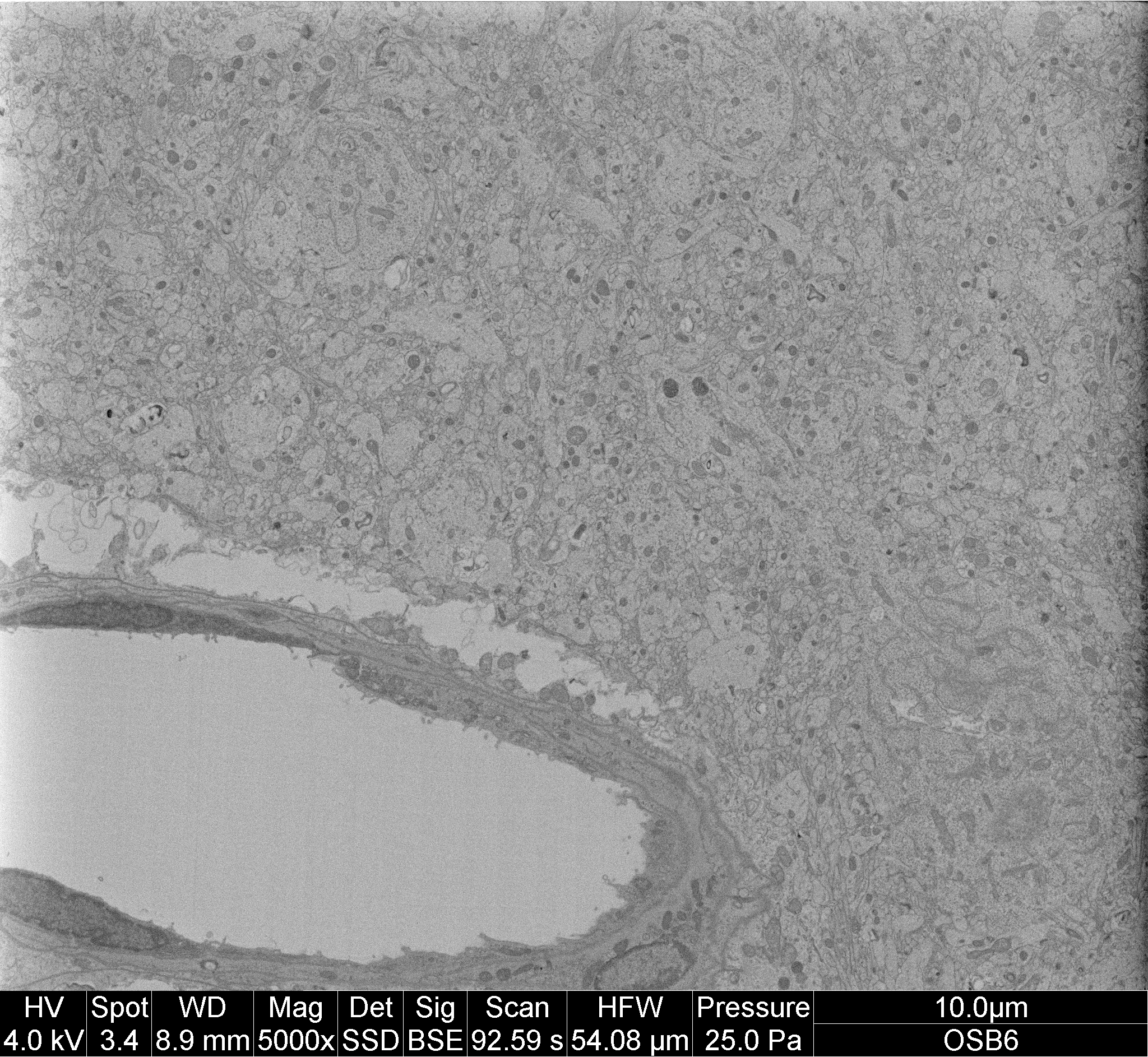

Supplement: Dataset S8 — (255.9 MB ZIP). [file pbio.0020329.sd008.zip › 040604_OS5_st1_718.tif]

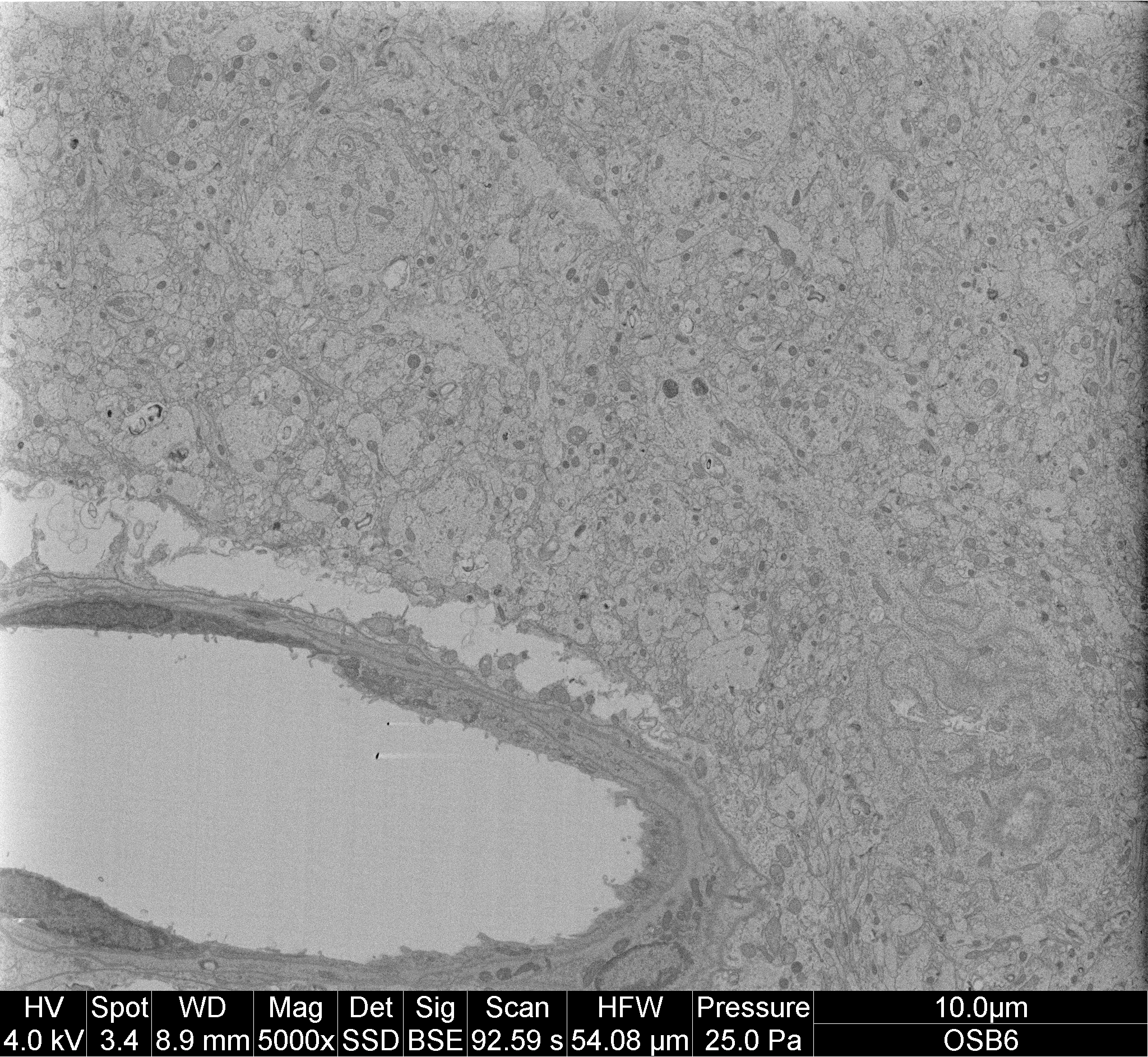

Supplement: Dataset S8 — (255.9 MB ZIP). [file pbio.0020329.sd008.zip › 040604_OS5_st1_719.tif]

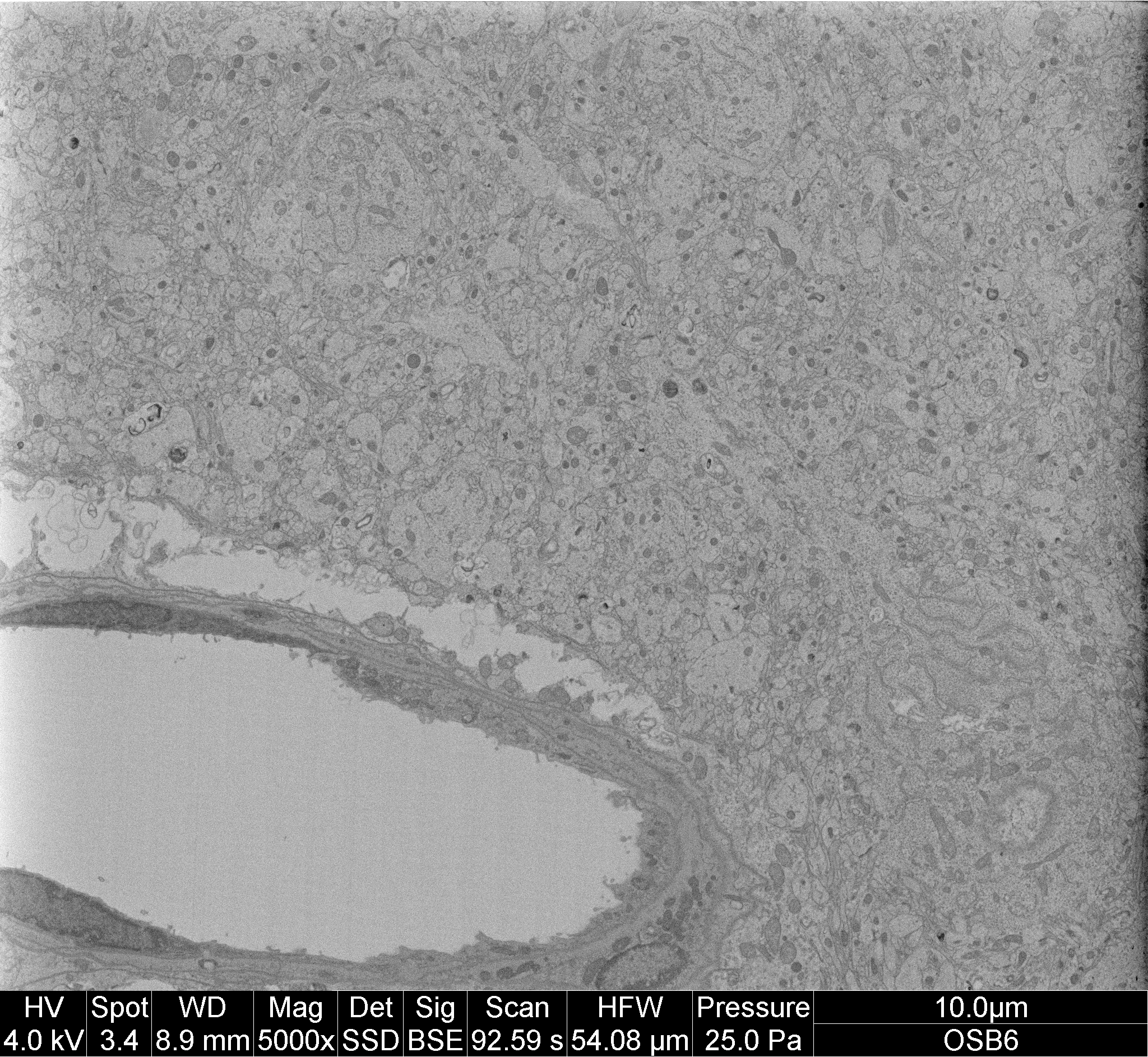

Supplement: Dataset S8 — (255.9 MB ZIP). [file pbio.0020329.sd008.zip › 040604_OS5_st1_720.tif]

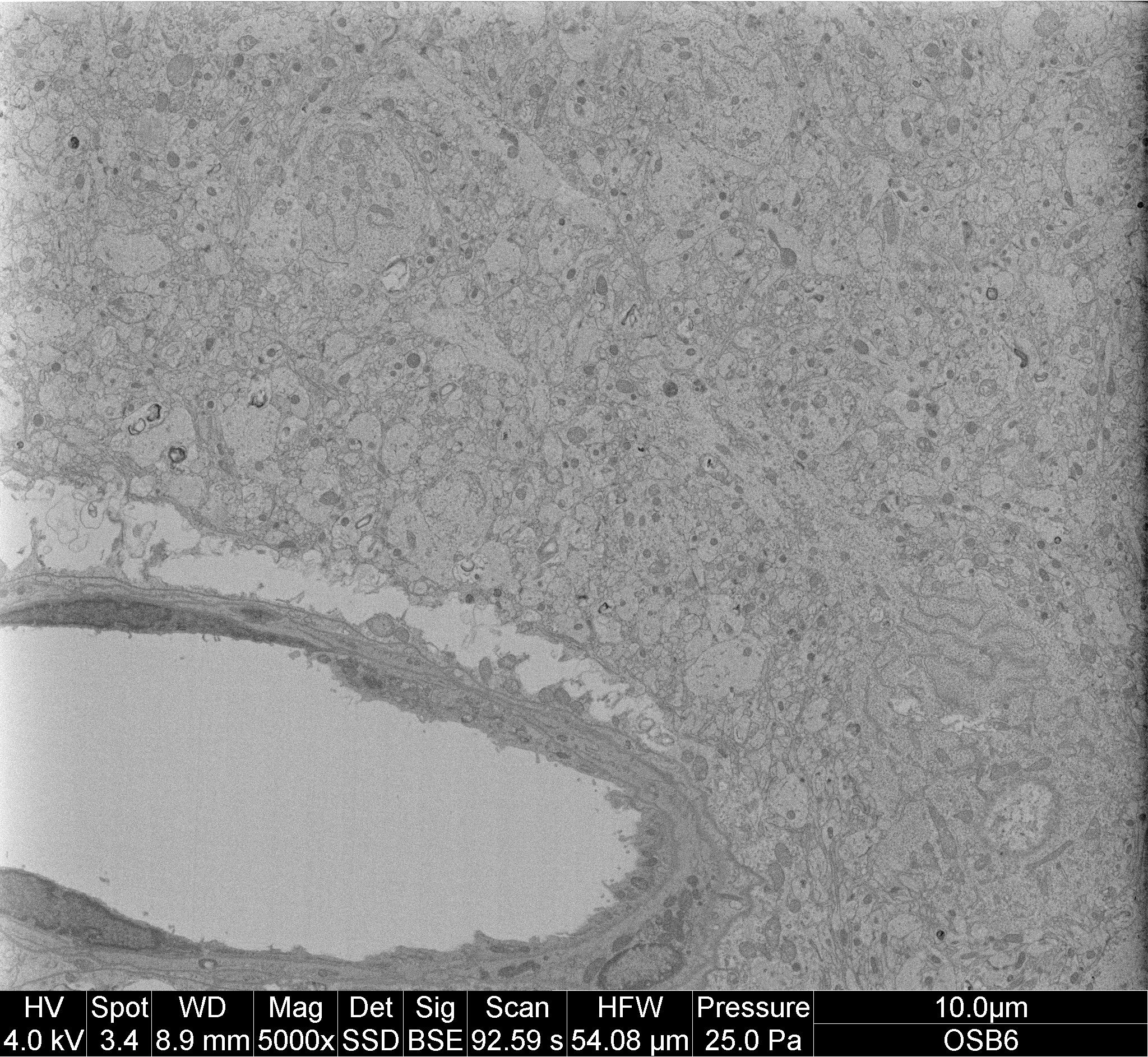

Supplement: Dataset S8 — (255.9 MB ZIP). [file pbio.0020329.sd008.zip › 040604_OS5_st1_721.tif]

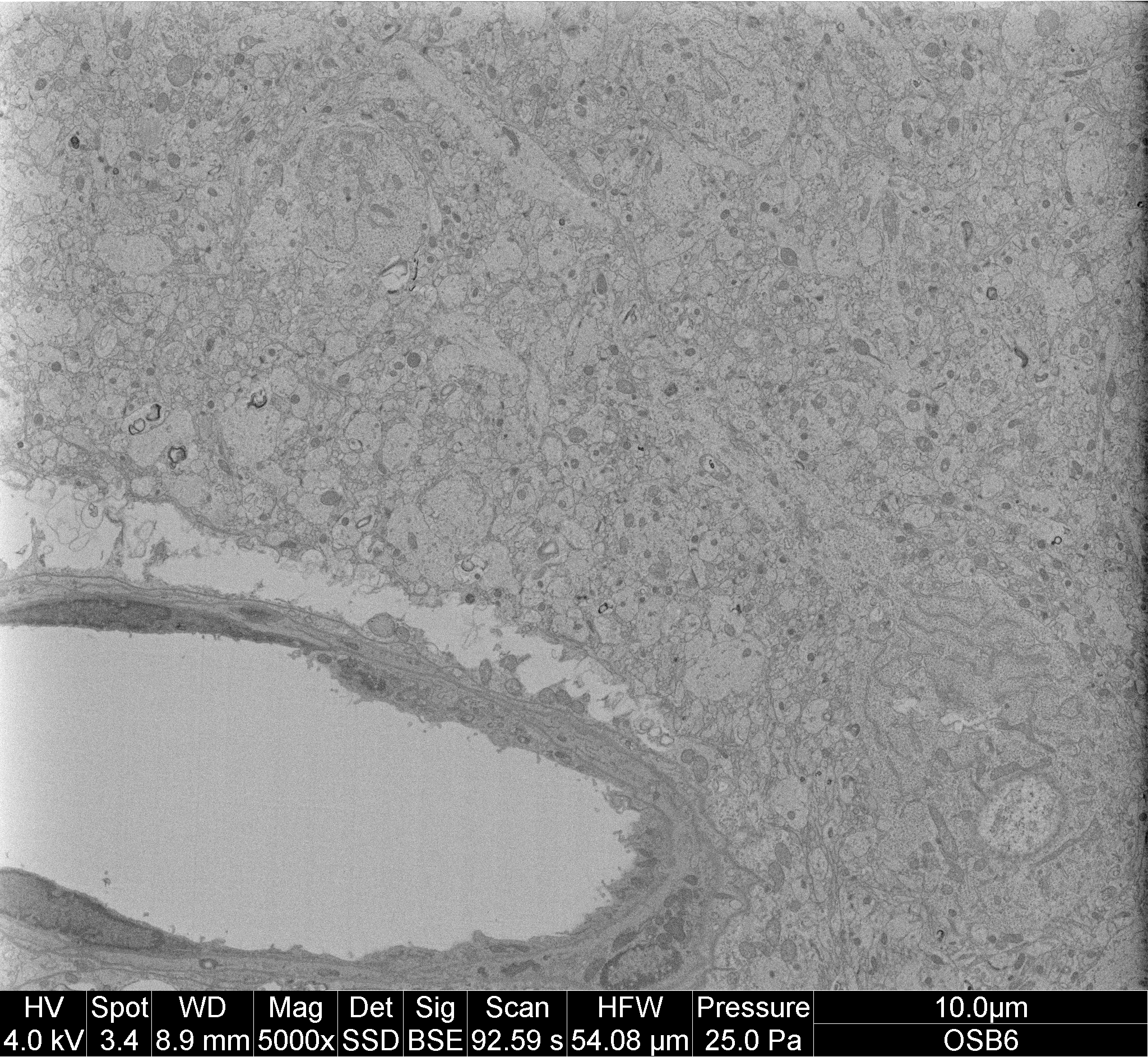

Supplement: Dataset S8 — (255.9 MB ZIP). [file pbio.0020329.sd008.zip › 040604_OS5_st1_722.tif]

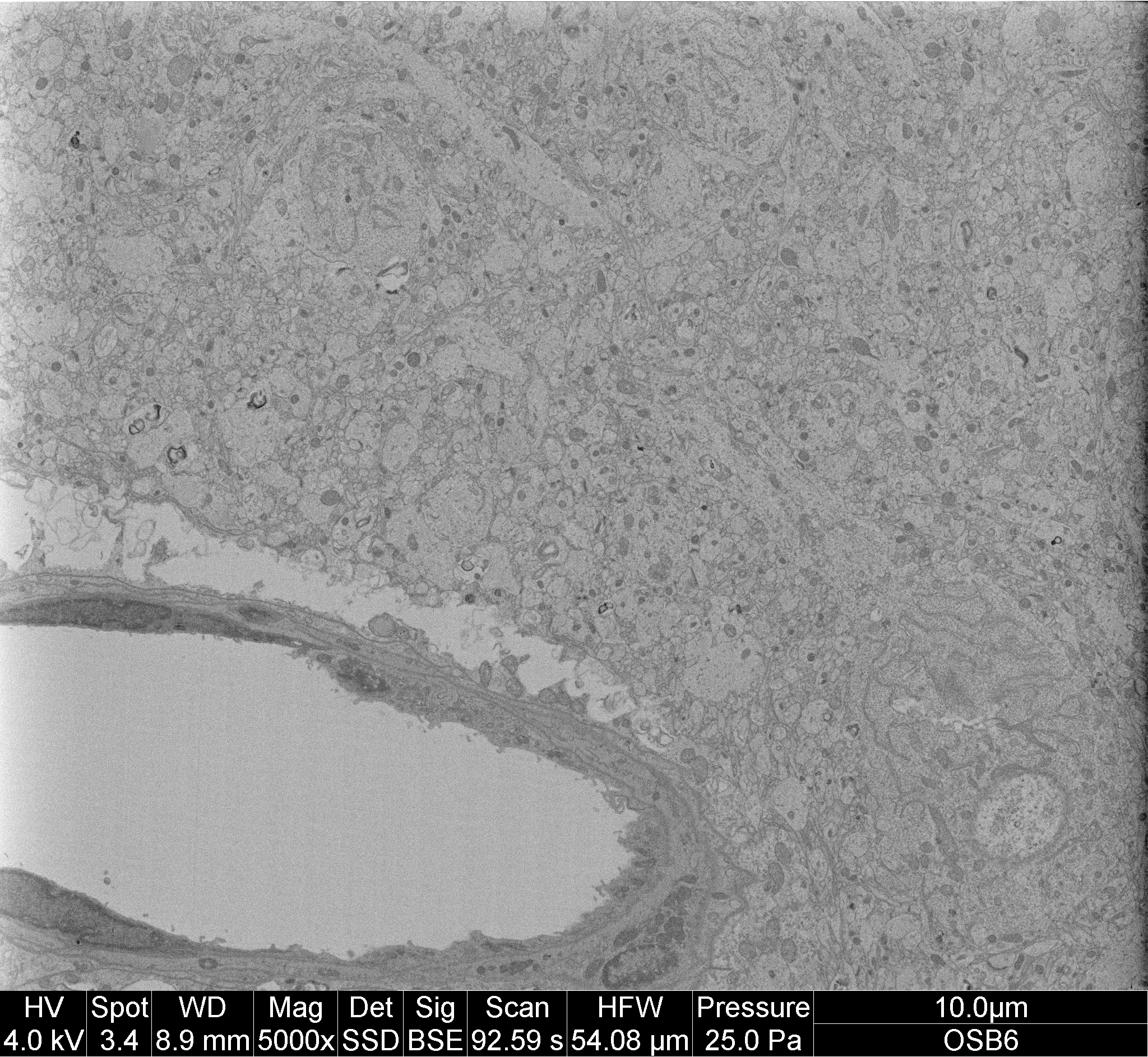

Supplement: Dataset S8 — (255.9 MB ZIP). [file pbio.0020329.sd008.zip › 040604_OS5_st1_723.tif]

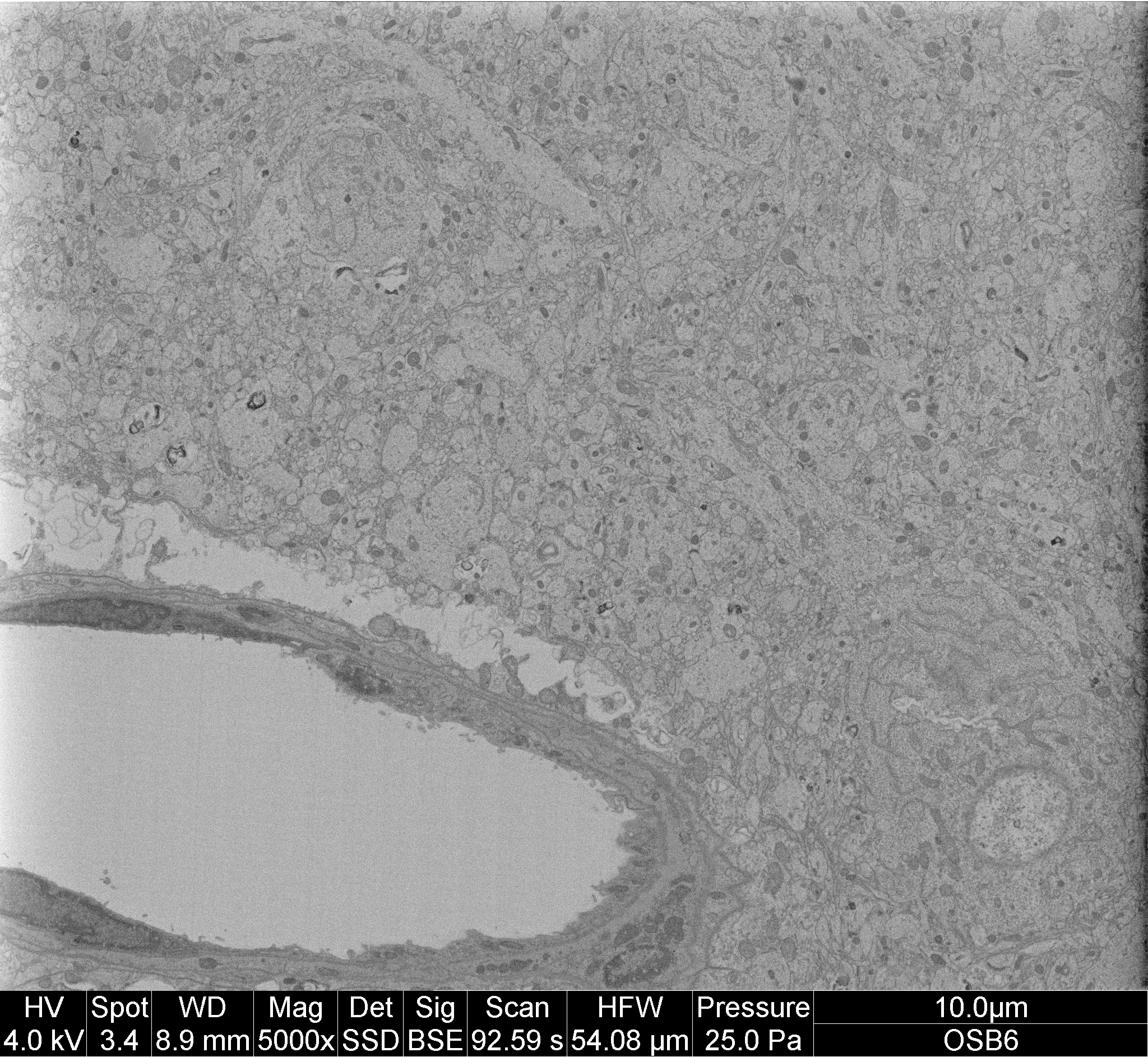

Supplement: Dataset S8 — (255.9 MB ZIP). [file pbio.0020329.sd008.zip › 040604_OS5_st1_724.tif]

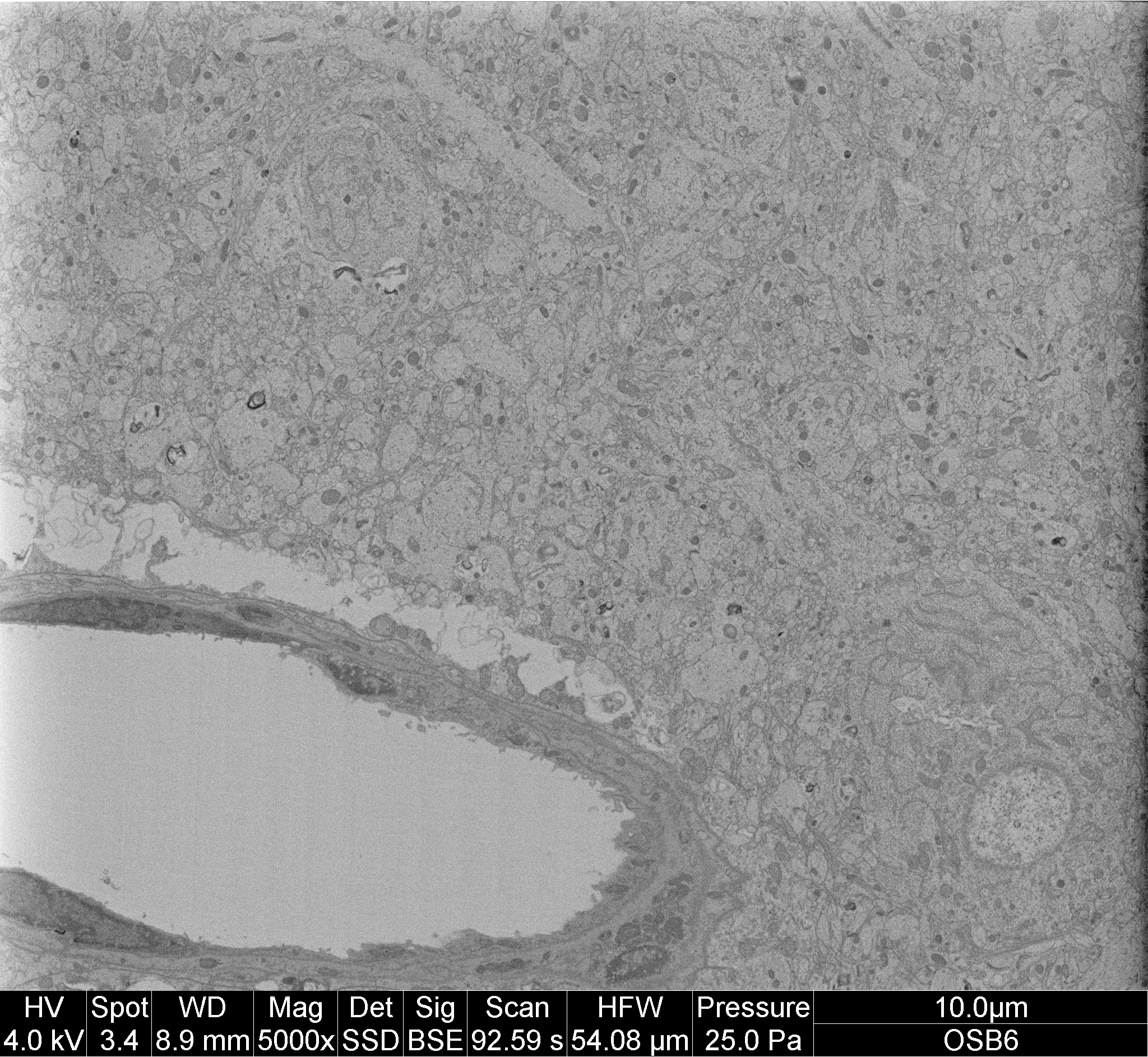

Supplement: Dataset S8 — (255.9 MB ZIP). [file pbio.0020329.sd008.zip › 040604_OS5_st1_725.tif]

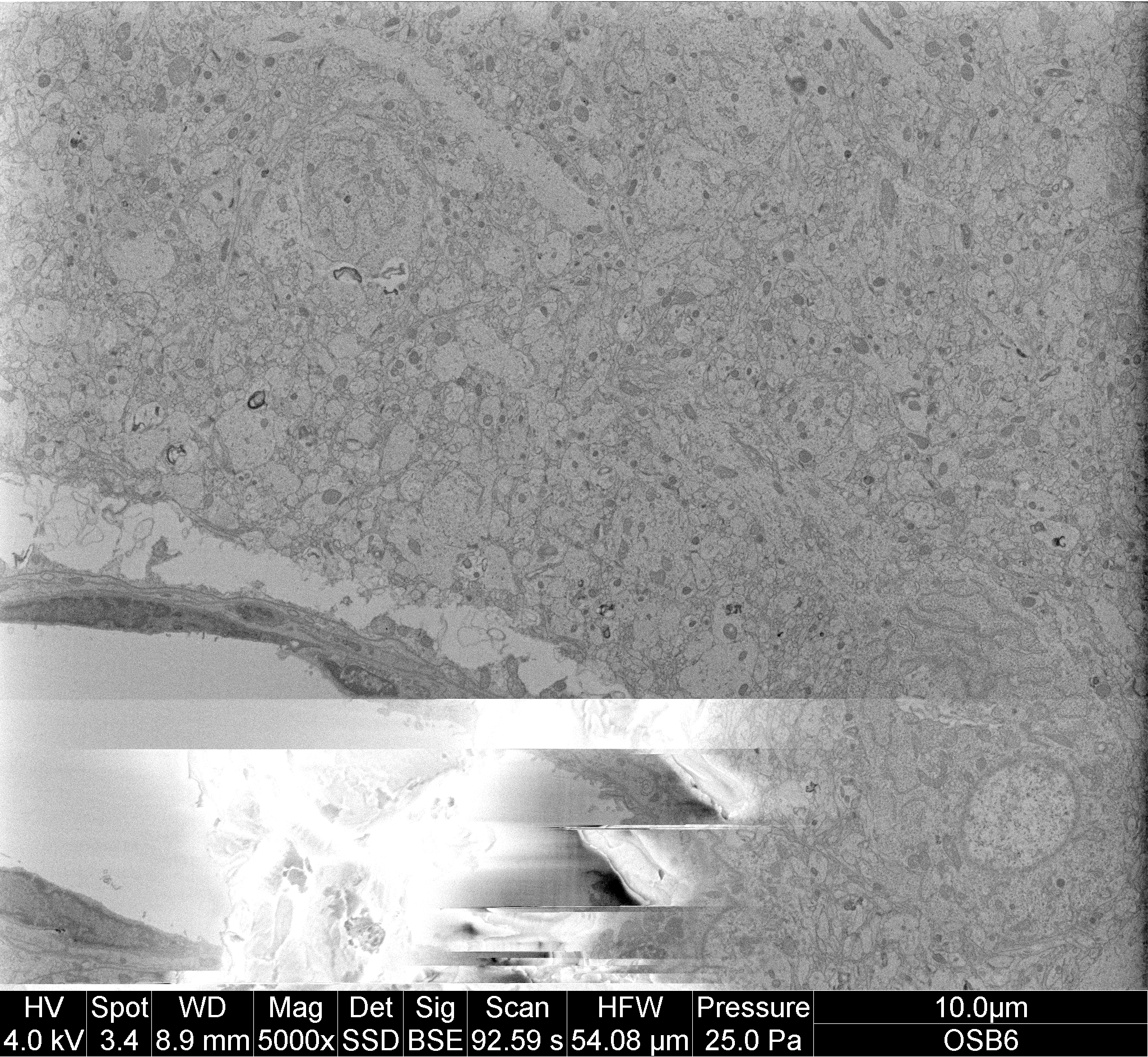

Supplement: Dataset S8 — (255.9 MB ZIP). [file pbio.0020329.sd008.zip › 040604_OS5_st1_726.tif]

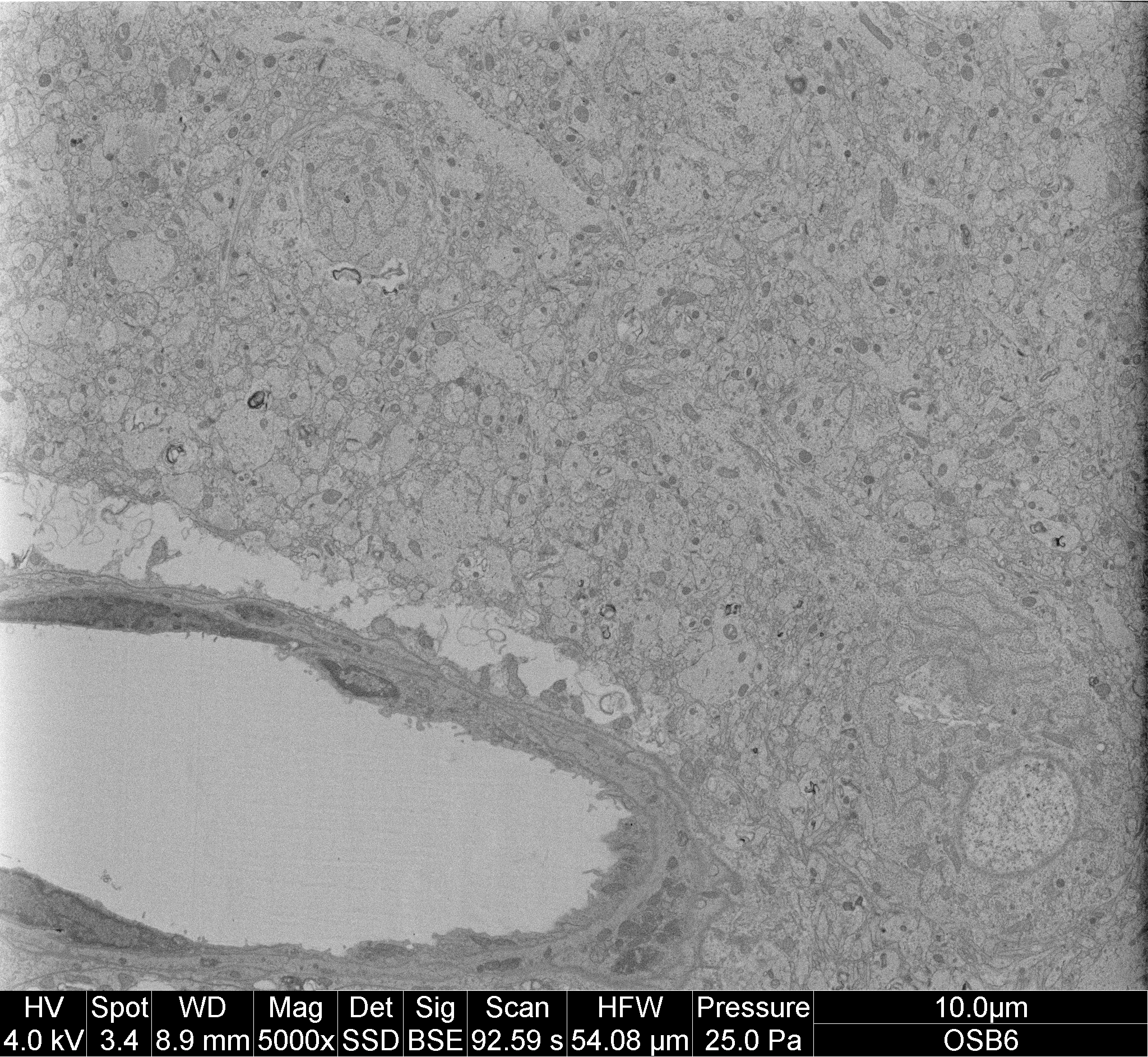

Supplement: Dataset S8 — (255.9 MB ZIP). [file pbio.0020329.sd008.zip › 040604_OS5_st1_727.tif]

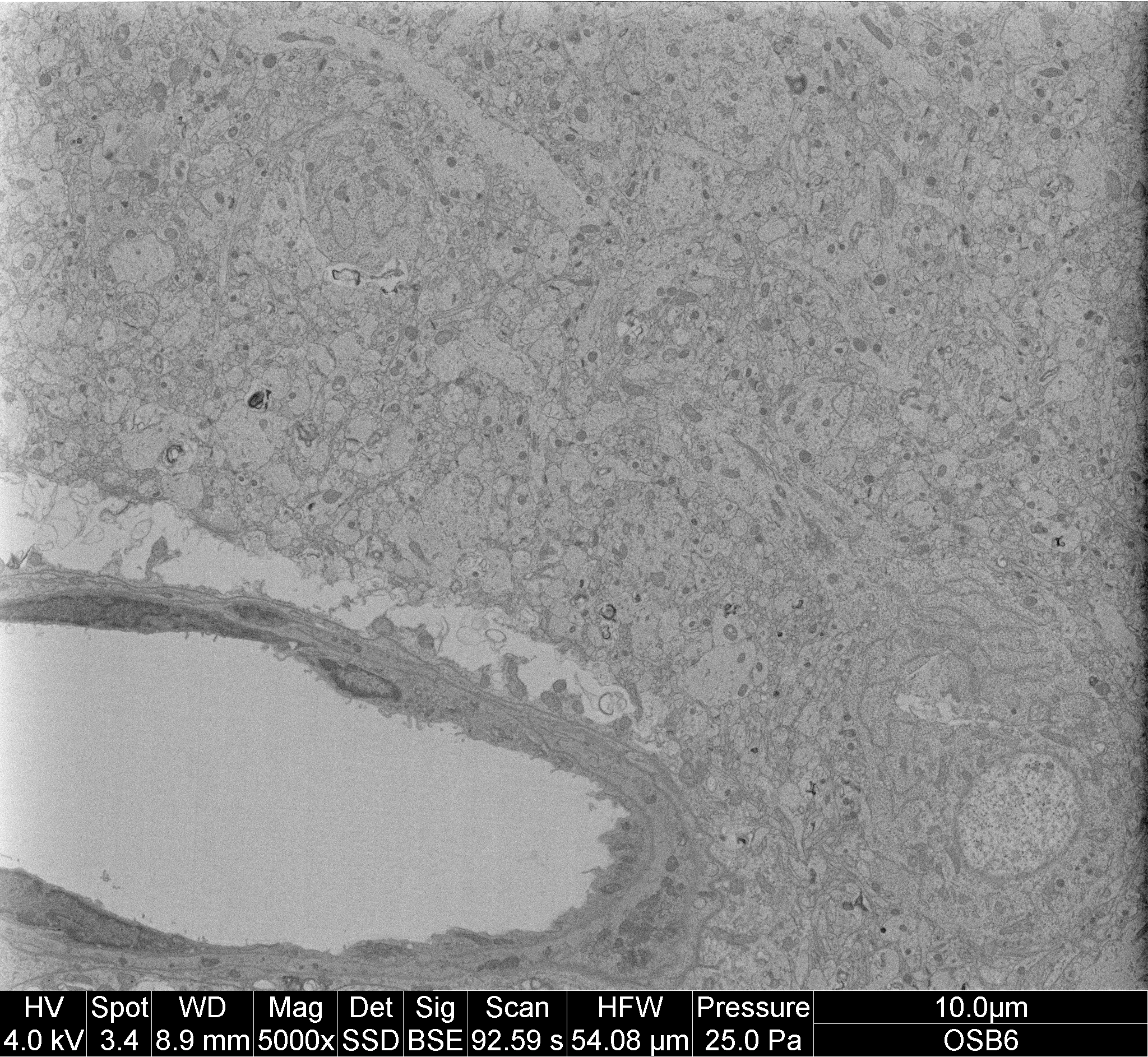

Supplement: Dataset S8 — (255.9 MB ZIP). [file pbio.0020329.sd008.zip › 040604_OS5_st1_728.tif]

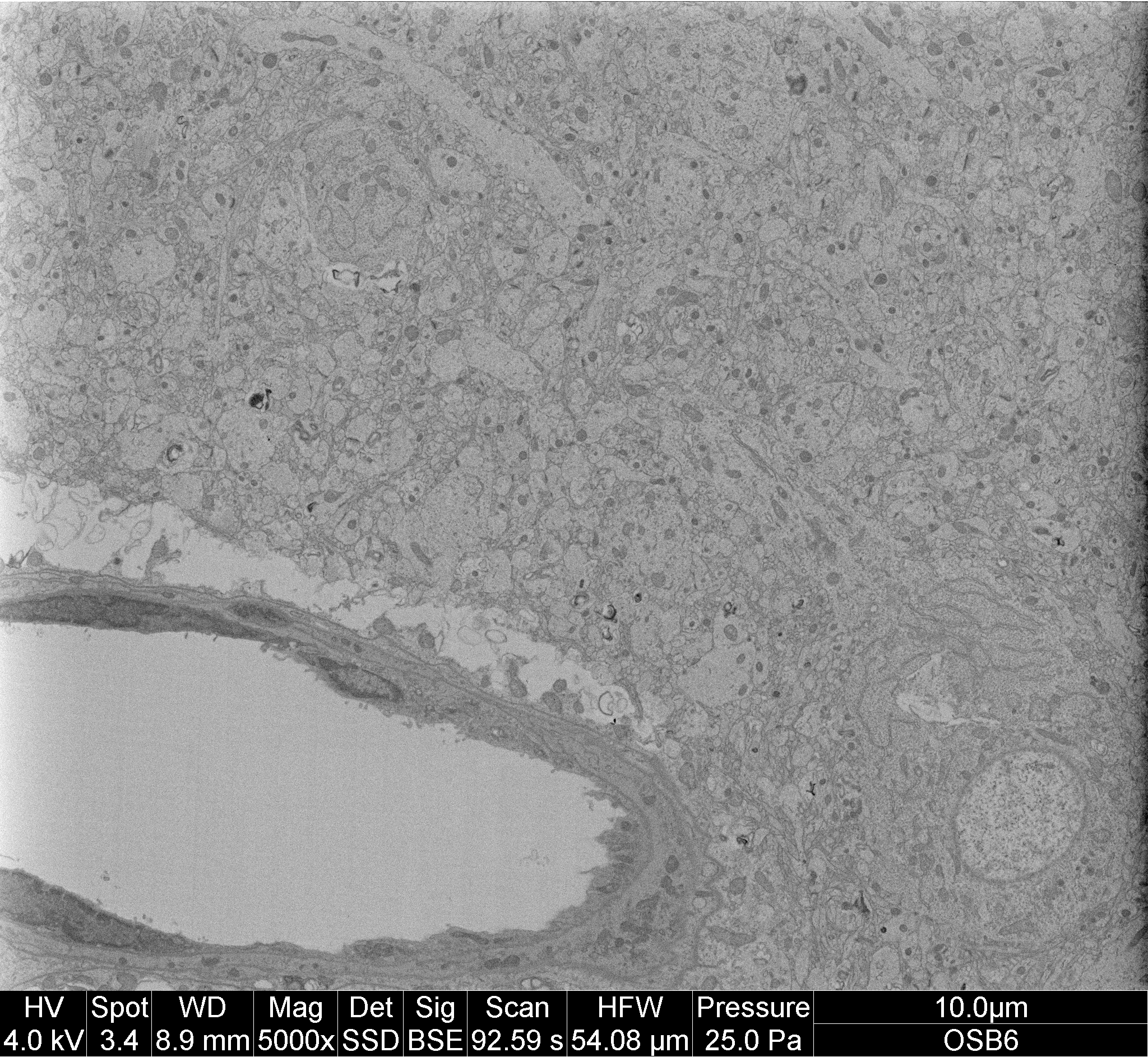

Supplement: Dataset S8 — (255.9 MB ZIP). [file pbio.0020329.sd008.zip › 040604_OS5_st1_729.tif]

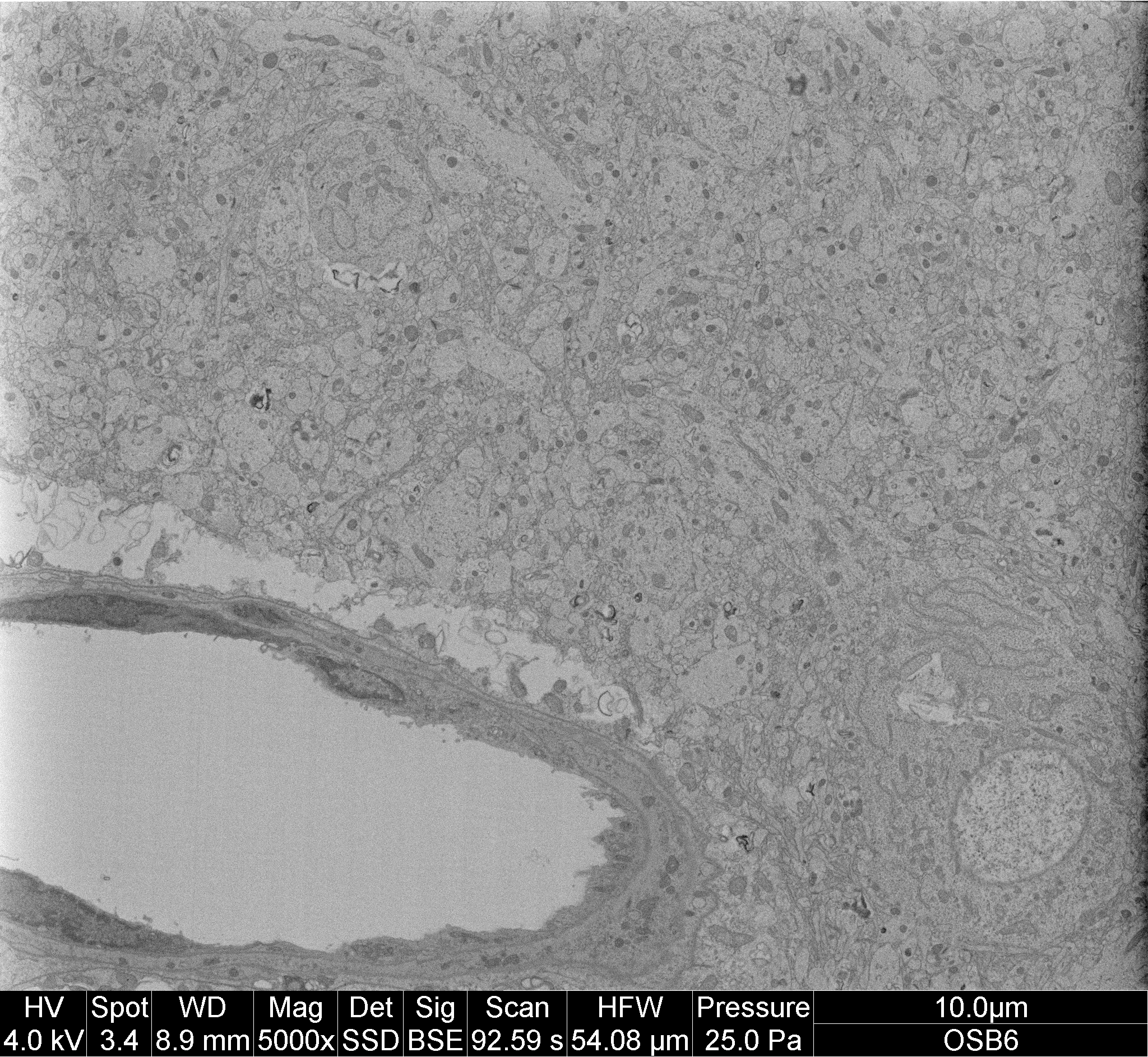

Supplement: Dataset S8 — (255.9 MB ZIP). [file pbio.0020329.sd008.zip › 040604_OS5_st1_730.tif]

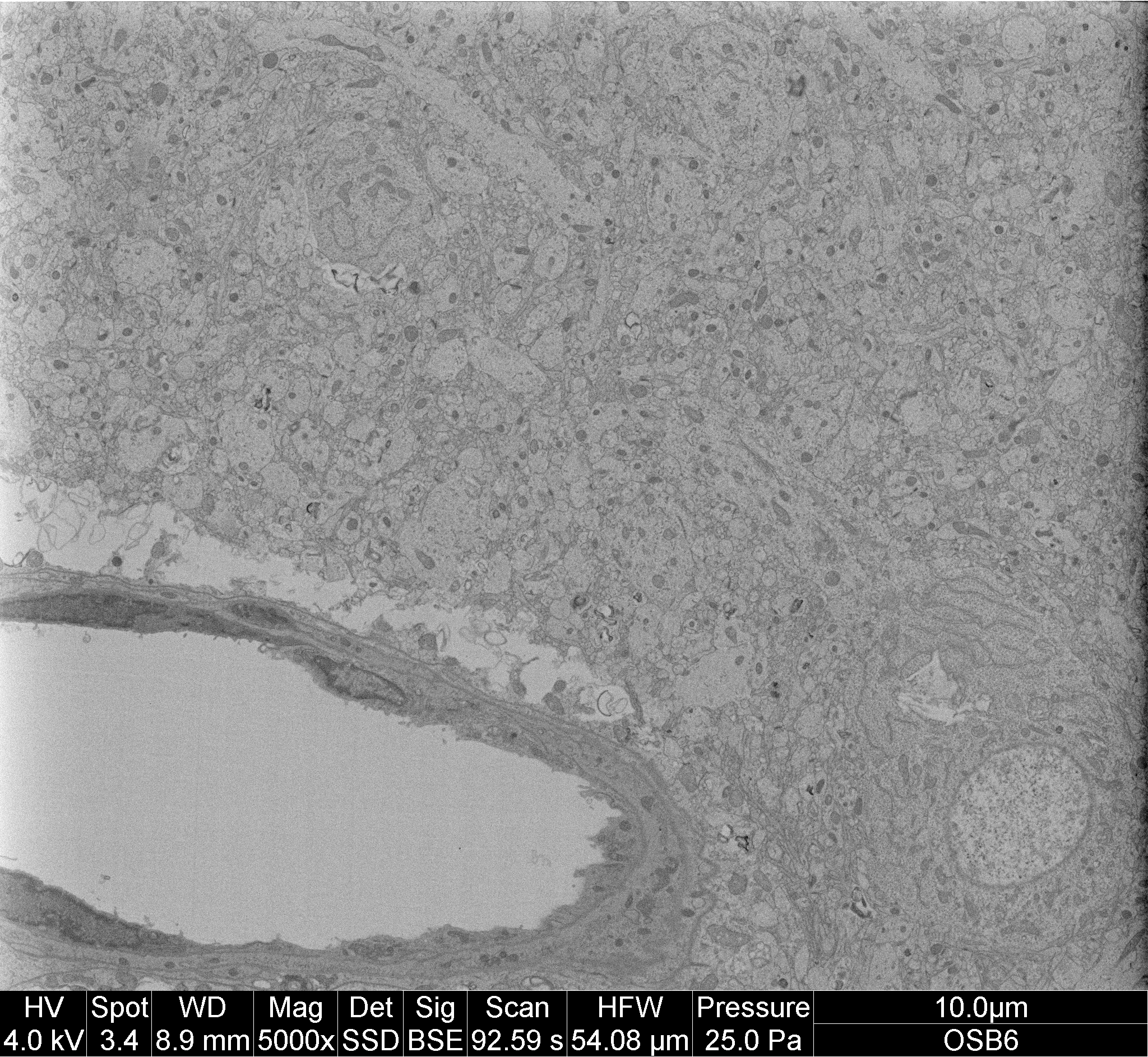

Supplement: Dataset S8 — (255.9 MB ZIP). [file pbio.0020329.sd008.zip › 040604_OS5_st1_731.tif]

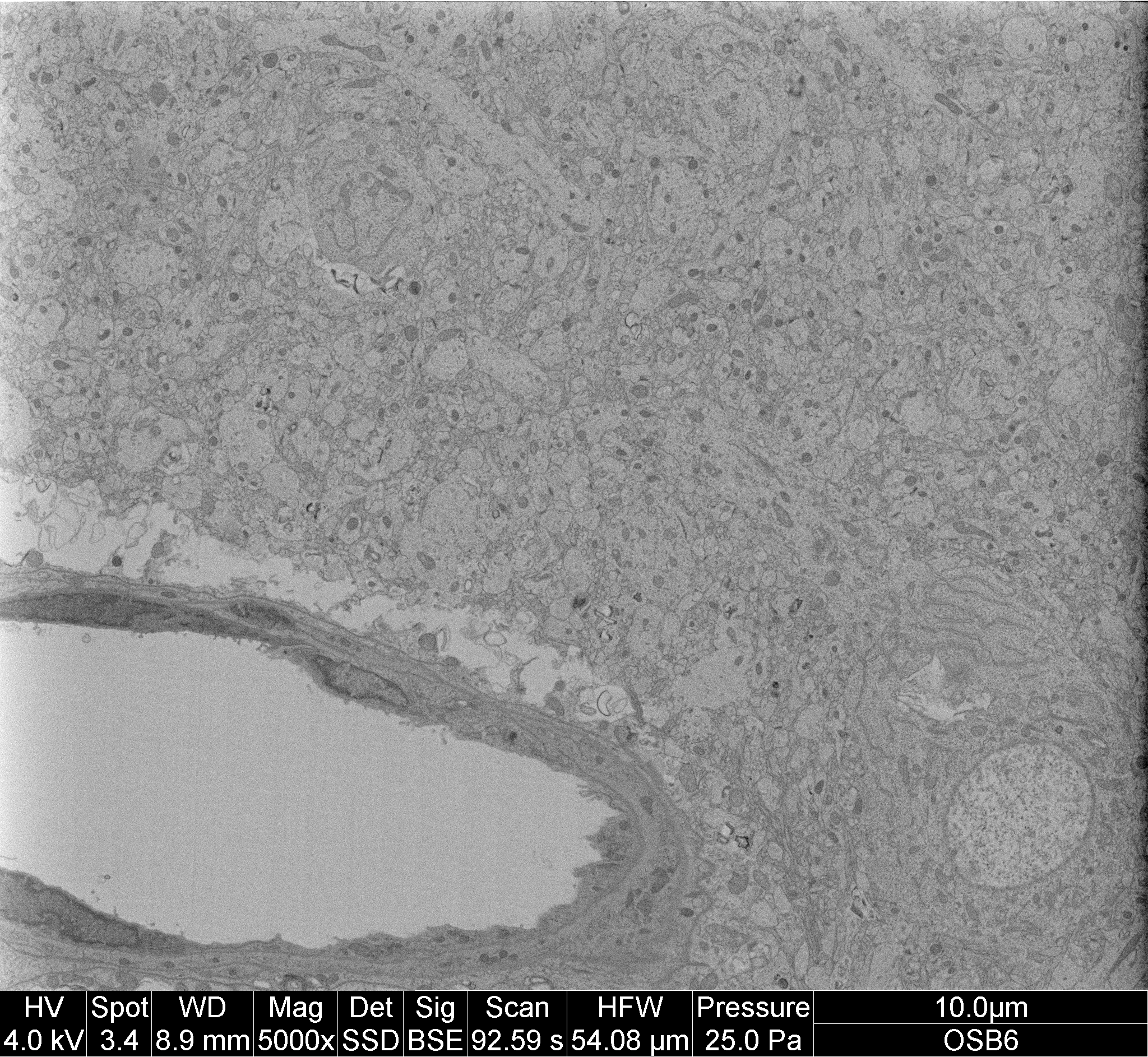

Supplement: Dataset S8 — (255.9 MB ZIP). [file pbio.0020329.sd008.zip › 040604_OS5_st1_732.tif]

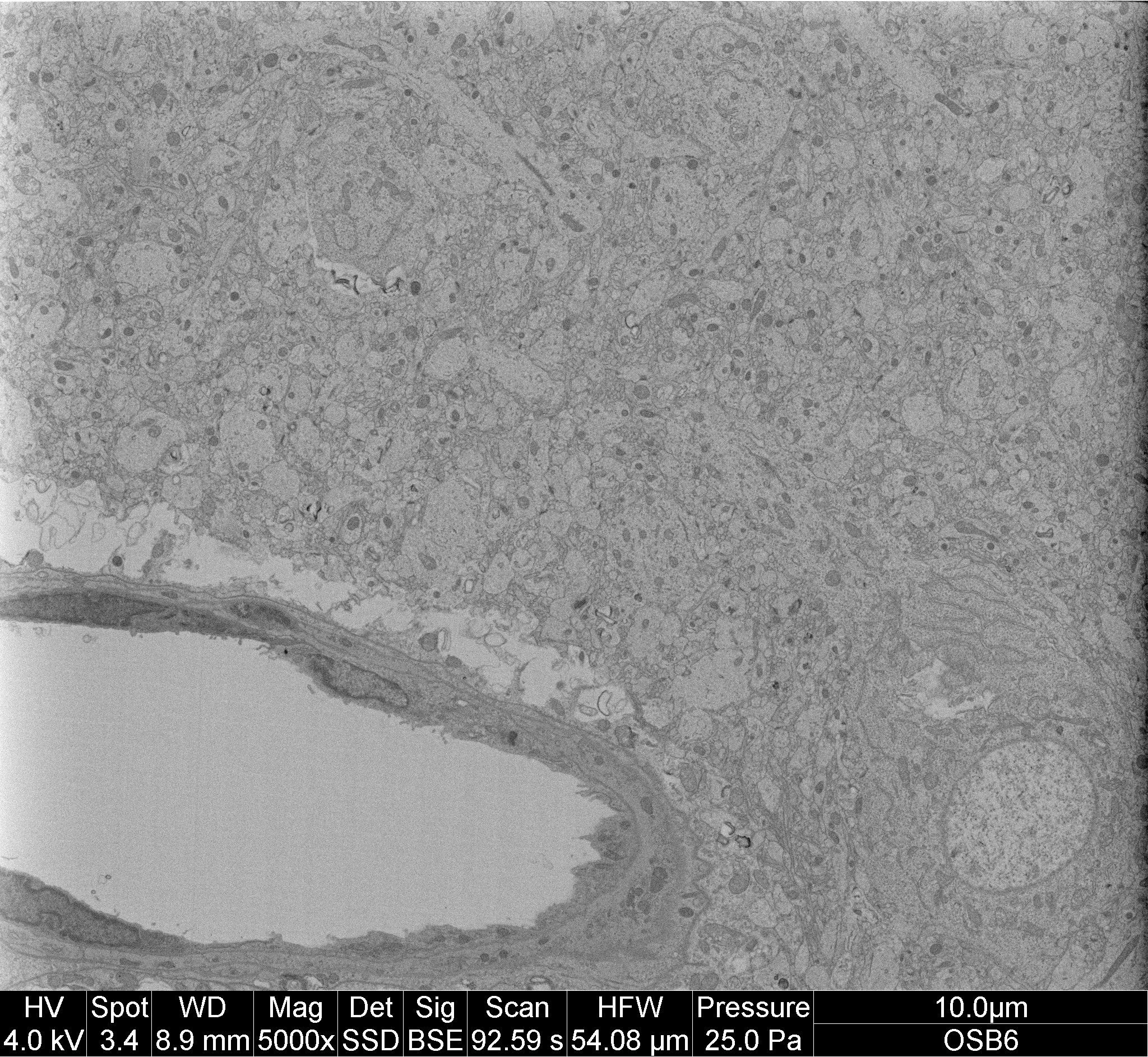

Supplement: Dataset S8 — (255.9 MB ZIP). [file pbio.0020329.sd008.zip › 040604_OS5_st1_733.tif]

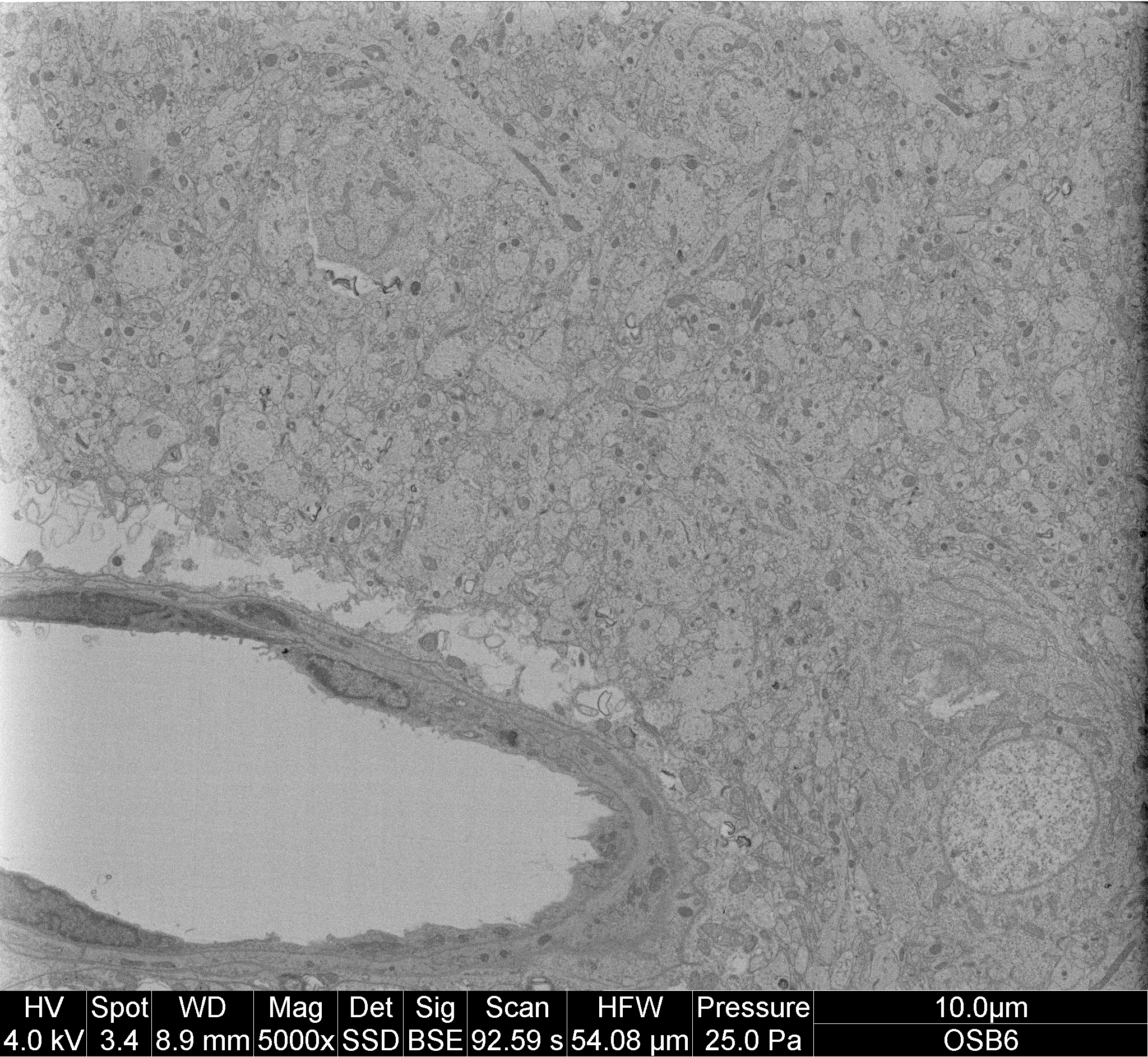

Supplement: Dataset S8 — (255.9 MB ZIP). [file pbio.0020329.sd008.zip › 040604_OS5_st1_734.tif]

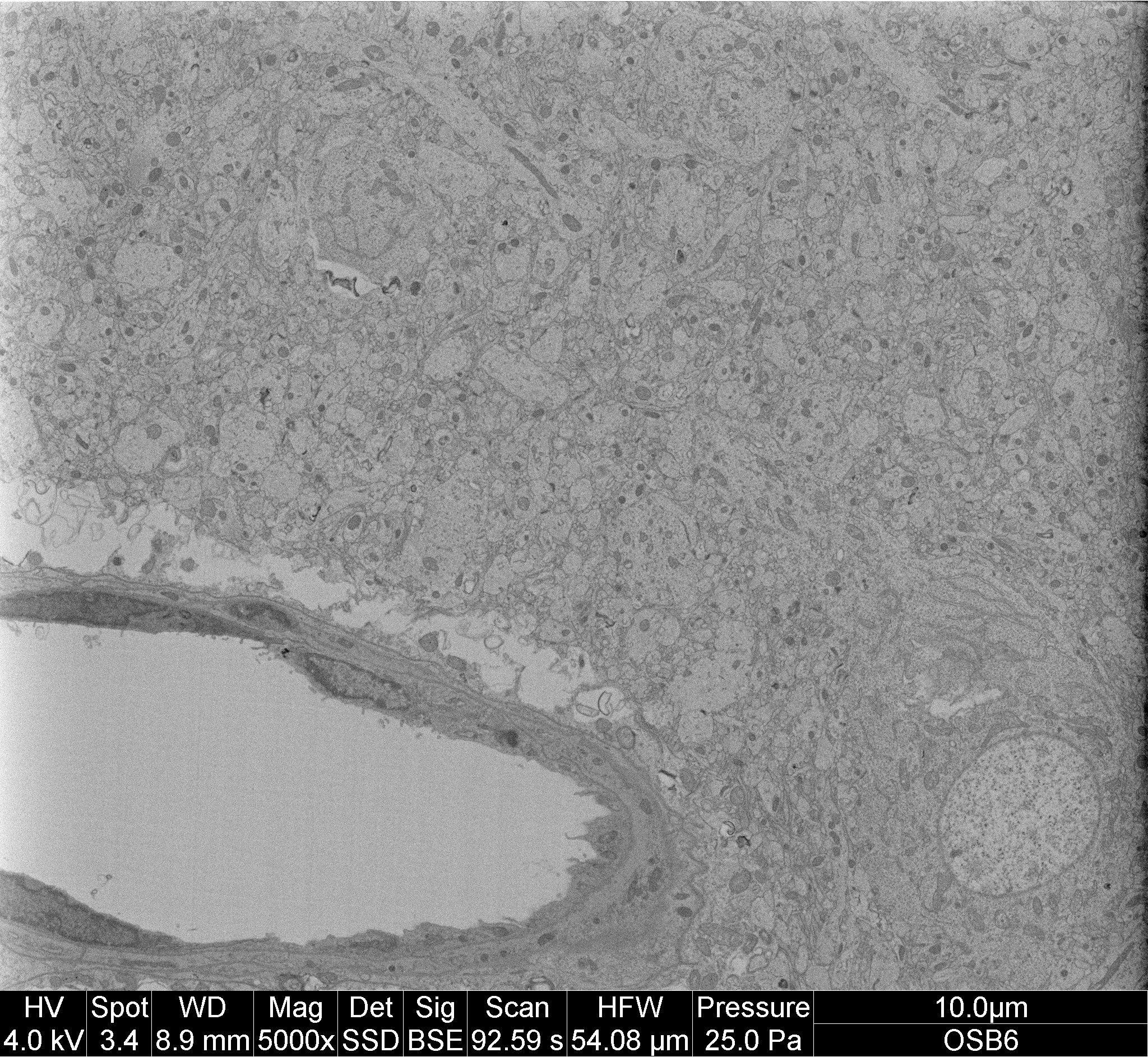

Supplement: Dataset S8 — (255.9 MB ZIP). [file pbio.0020329.sd008.zip › 040604_OS5_st1_735.tif]

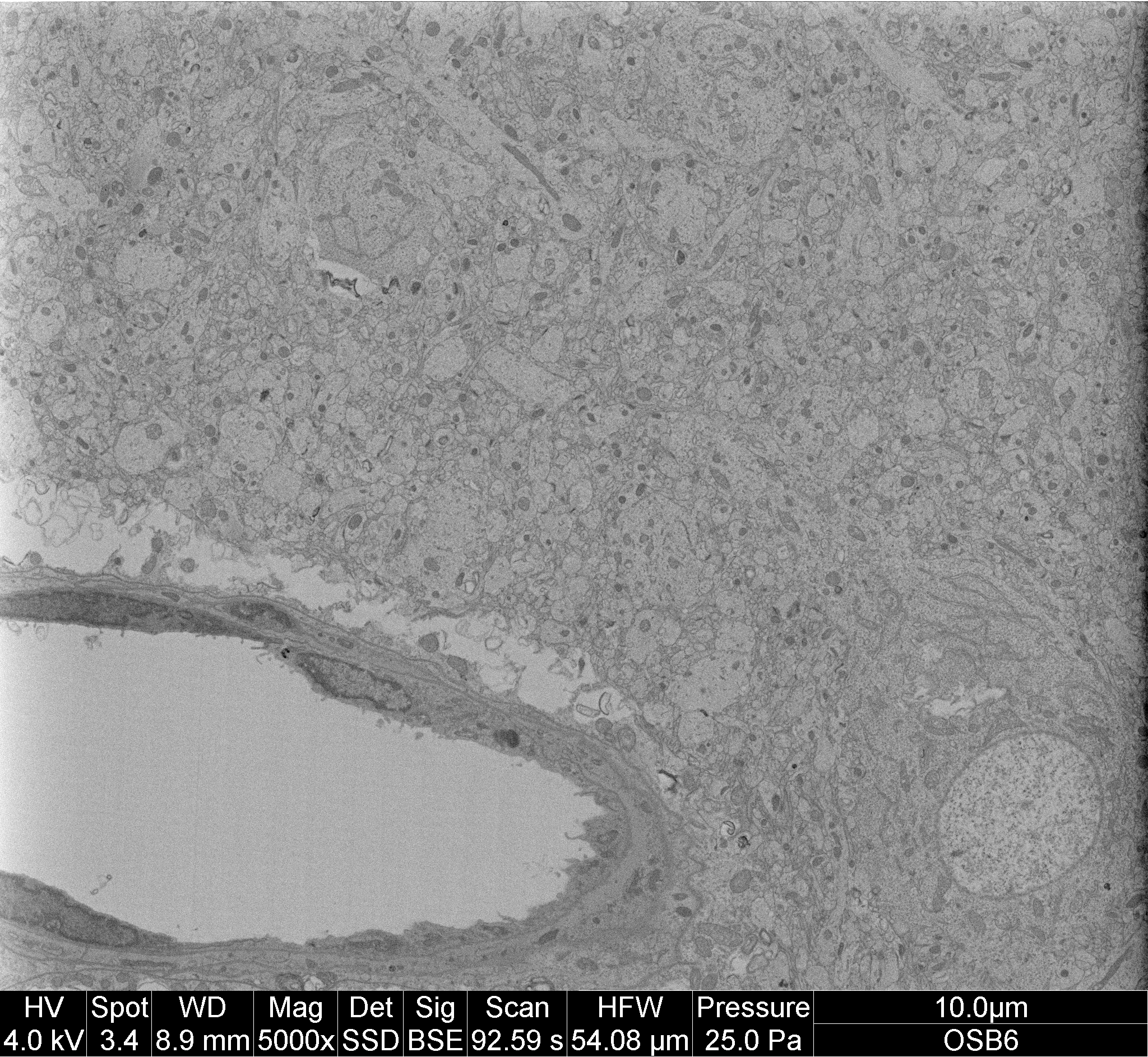

Supplement: Dataset S8 — (255.9 MB ZIP). [file pbio.0020329.sd008.zip › 040604_OS5_st1_736.tif]

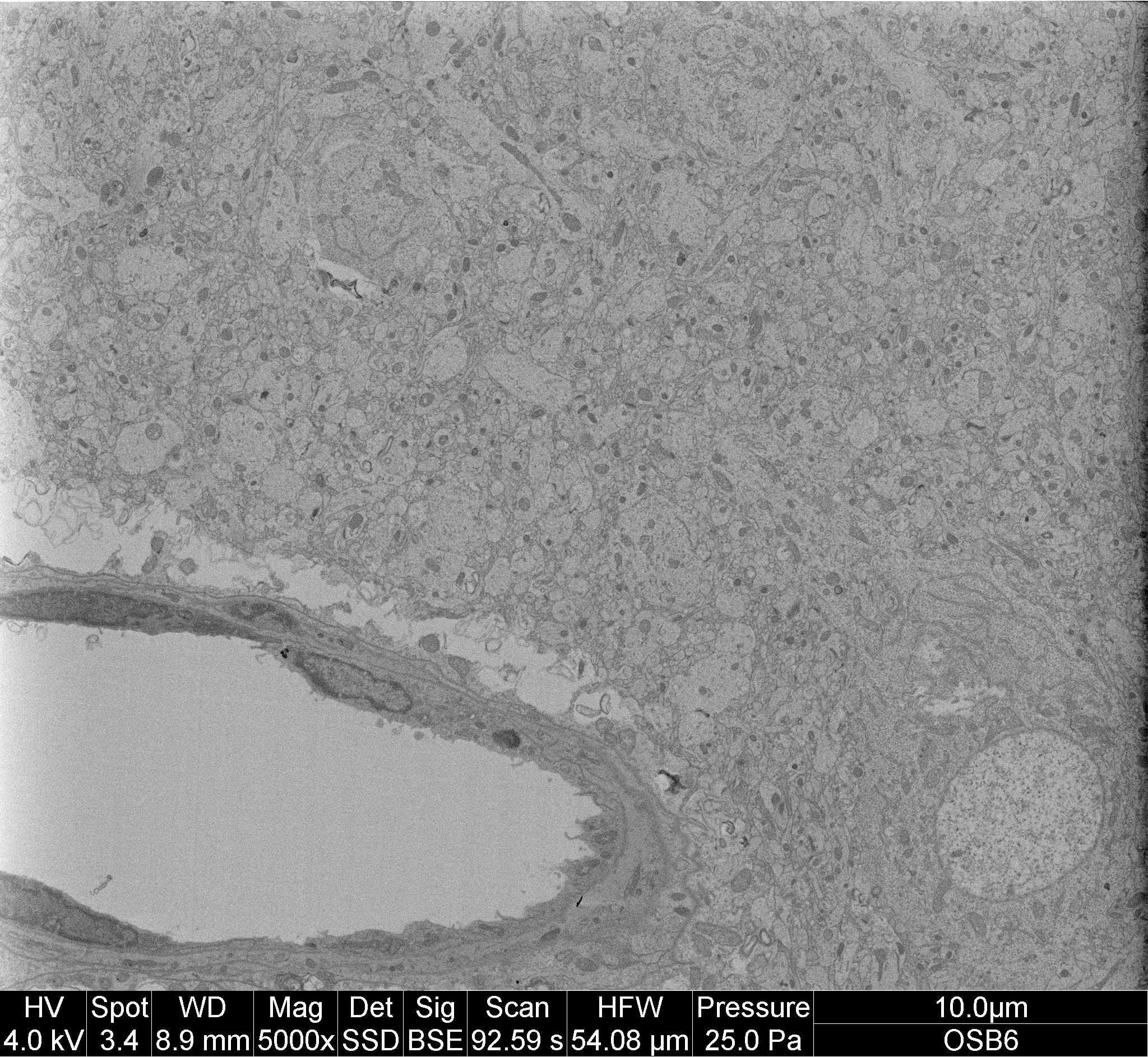

Supplement: Dataset S8 — (255.9 MB ZIP). [file pbio.0020329.sd008.zip › 040604_OS5_st1_737.tif]

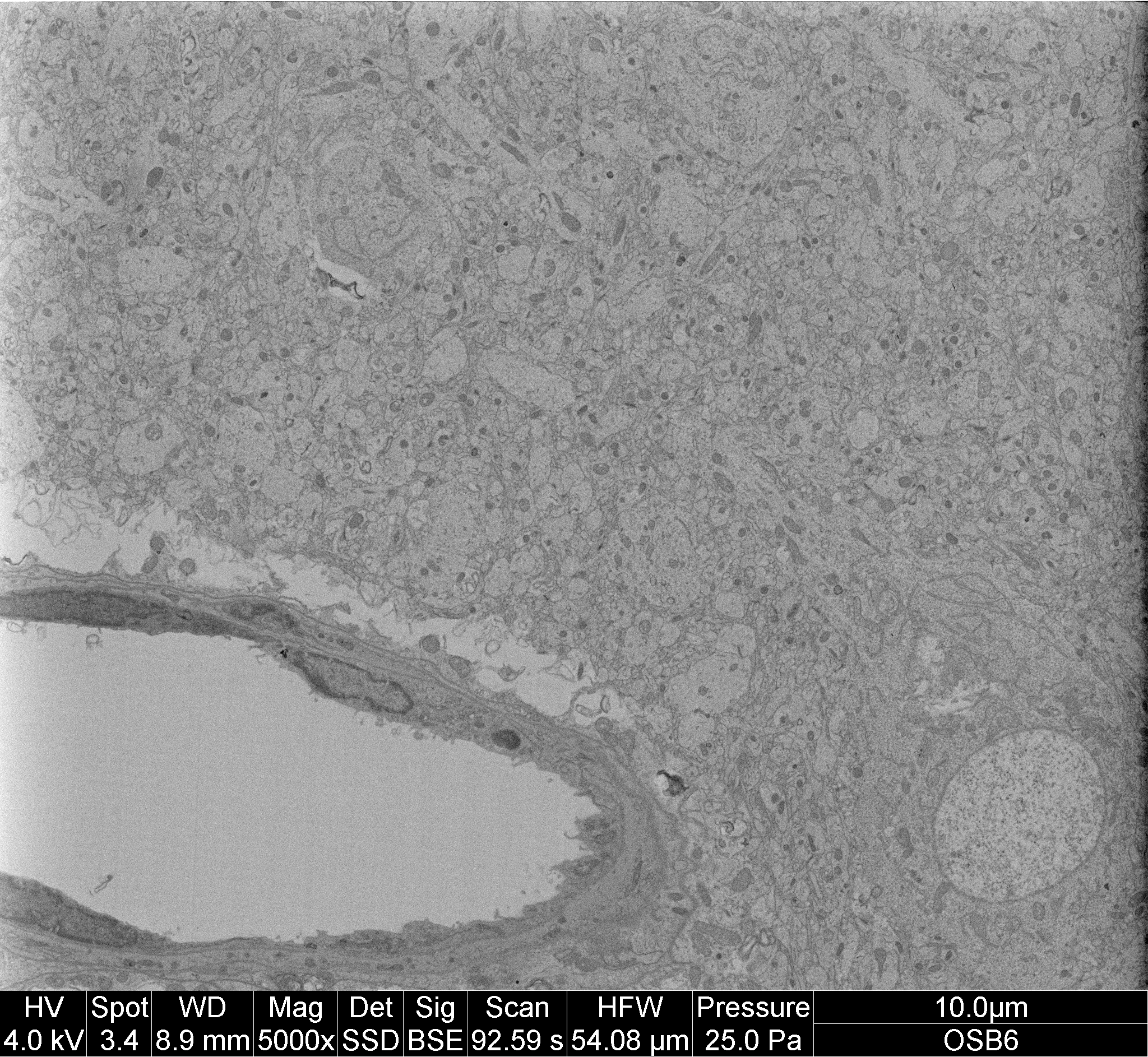

Supplement: Dataset S8 — (255.9 MB ZIP). [file pbio.0020329.sd008.zip › 040604_OS5_st1_738.tif]

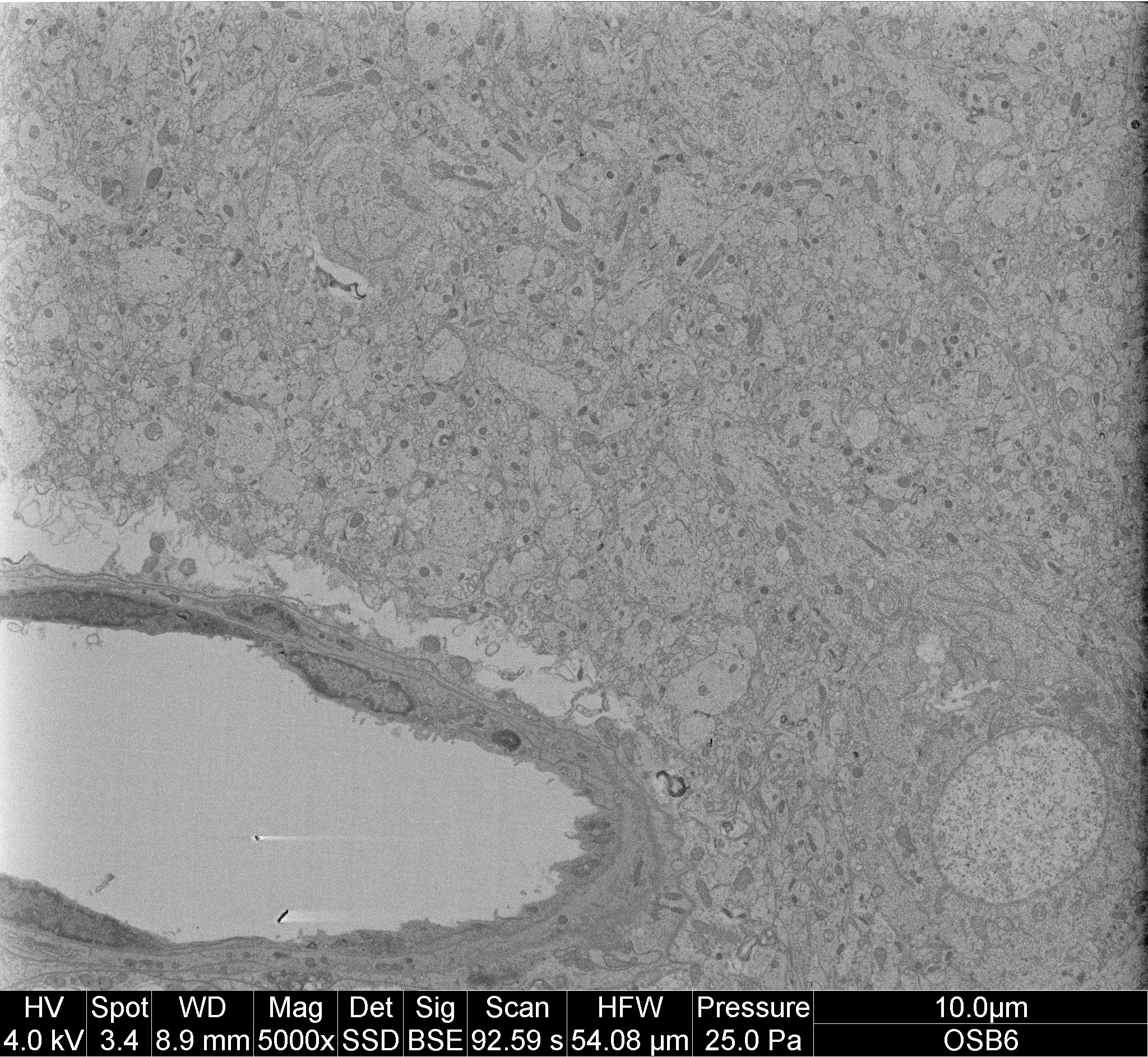

Supplement: Dataset S8 — (255.9 MB ZIP). [file pbio.0020329.sd008.zip › 040604_OS5_st1_739.tif]

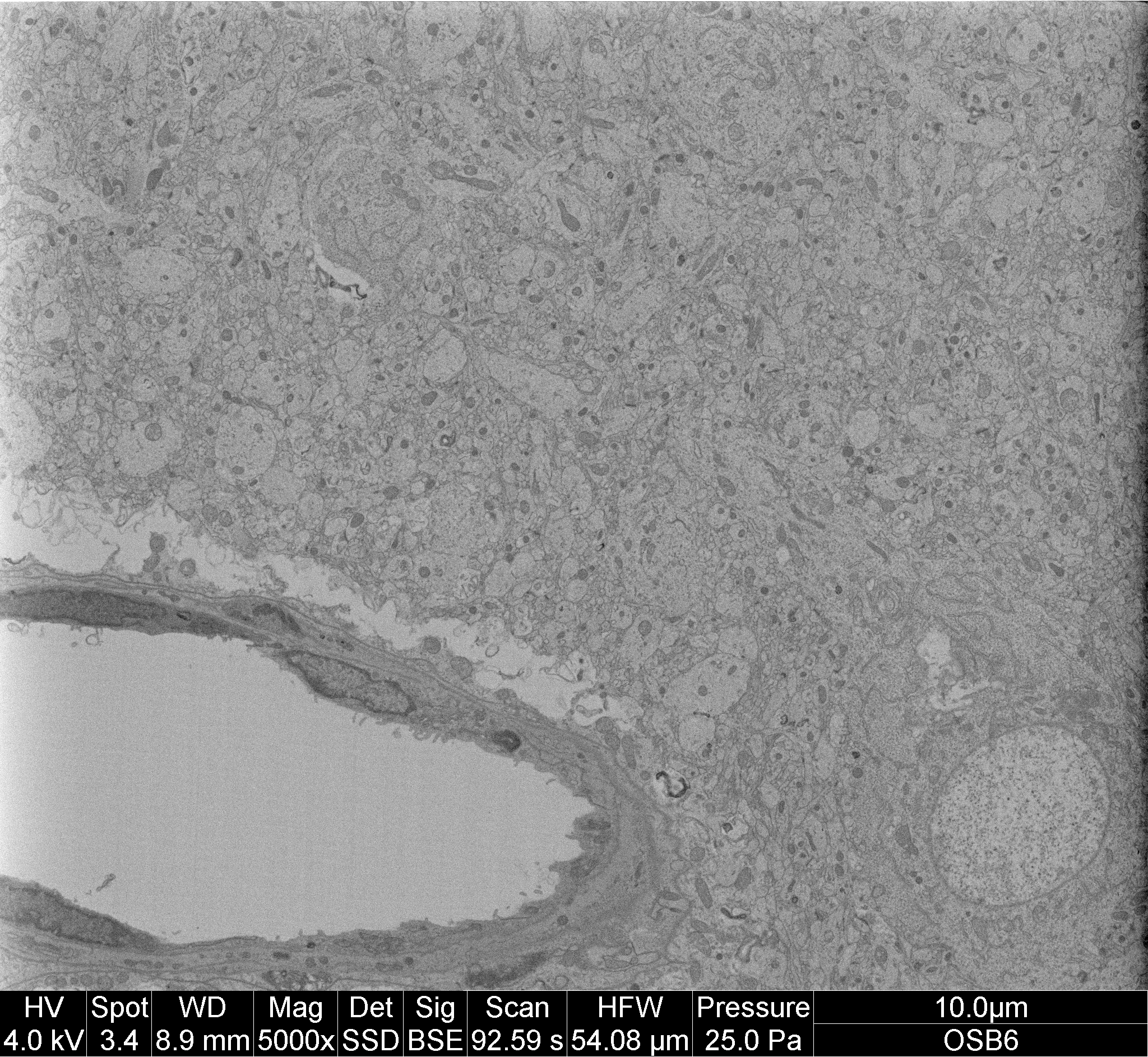

Supplement: Dataset S8 — (255.9 MB ZIP). [file pbio.0020329.sd008.zip › 040604_OS5_st1_740.tif]

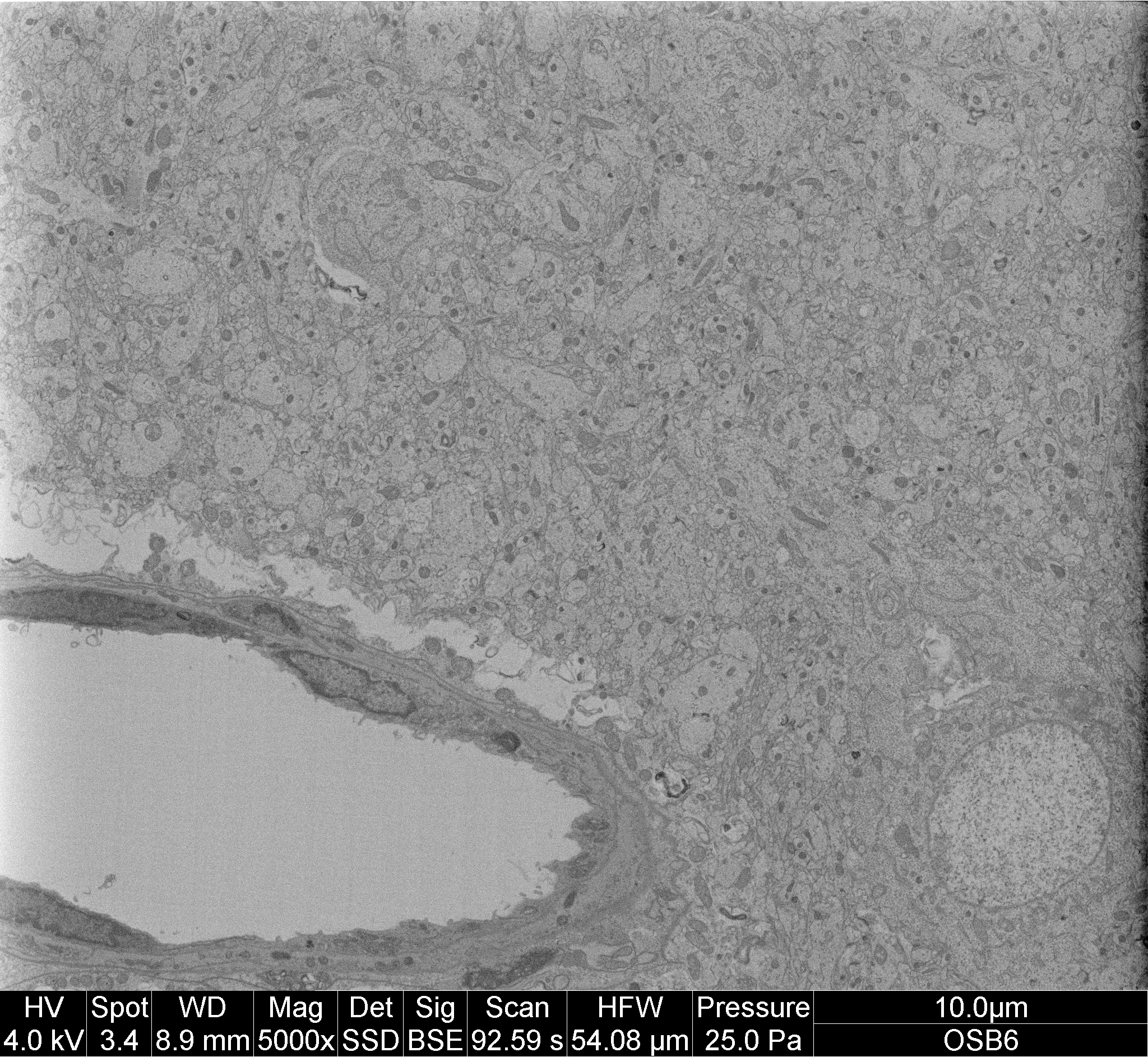

Supplement: Dataset S8 — (255.9 MB ZIP). [file pbio.0020329.sd008.zip › 040604_OS5_st1_741.tif]

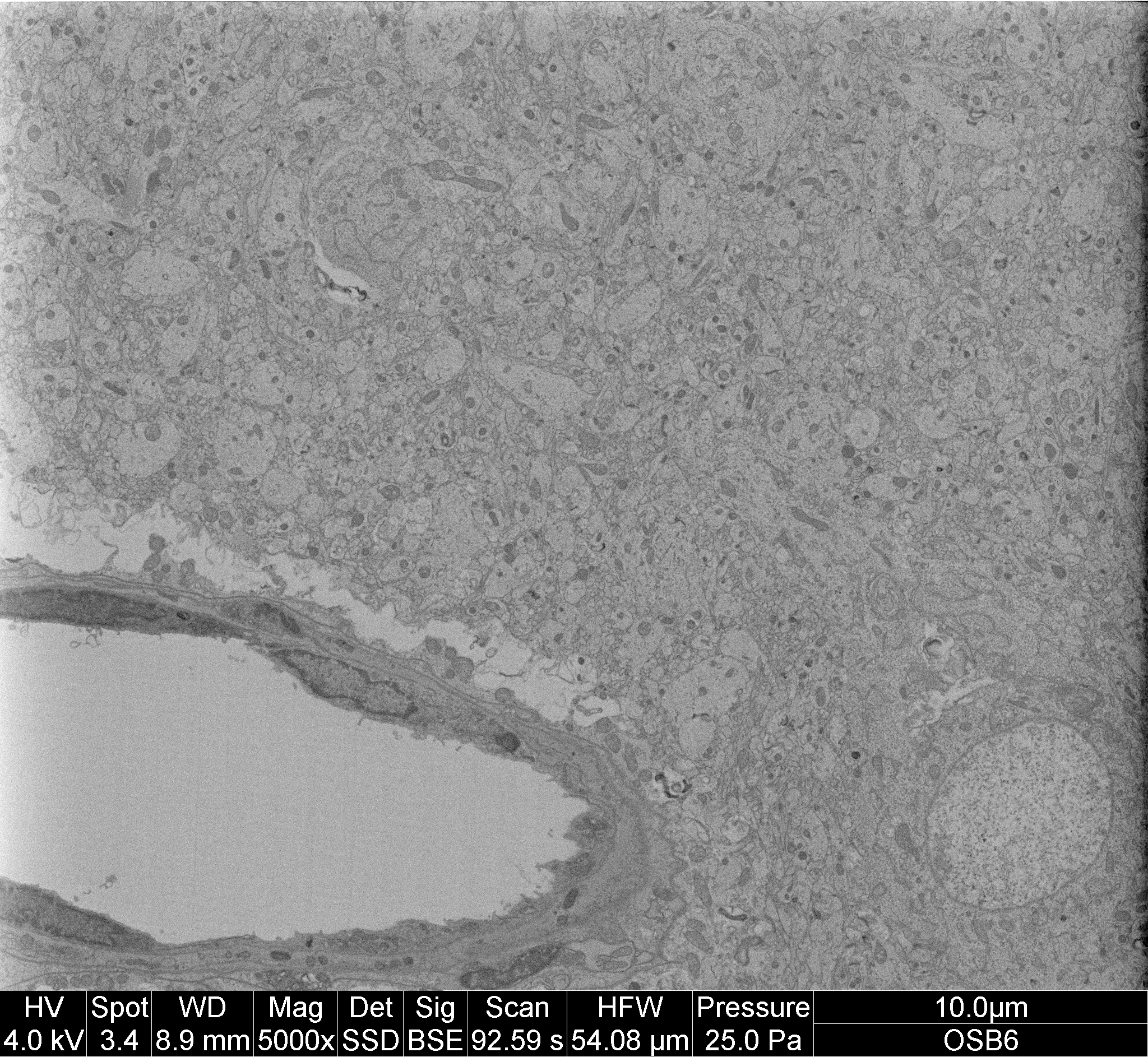

Supplement: Dataset S8 — (255.9 MB ZIP). [file pbio.0020329.sd008.zip › 040604_OS5_st1_742.tif]

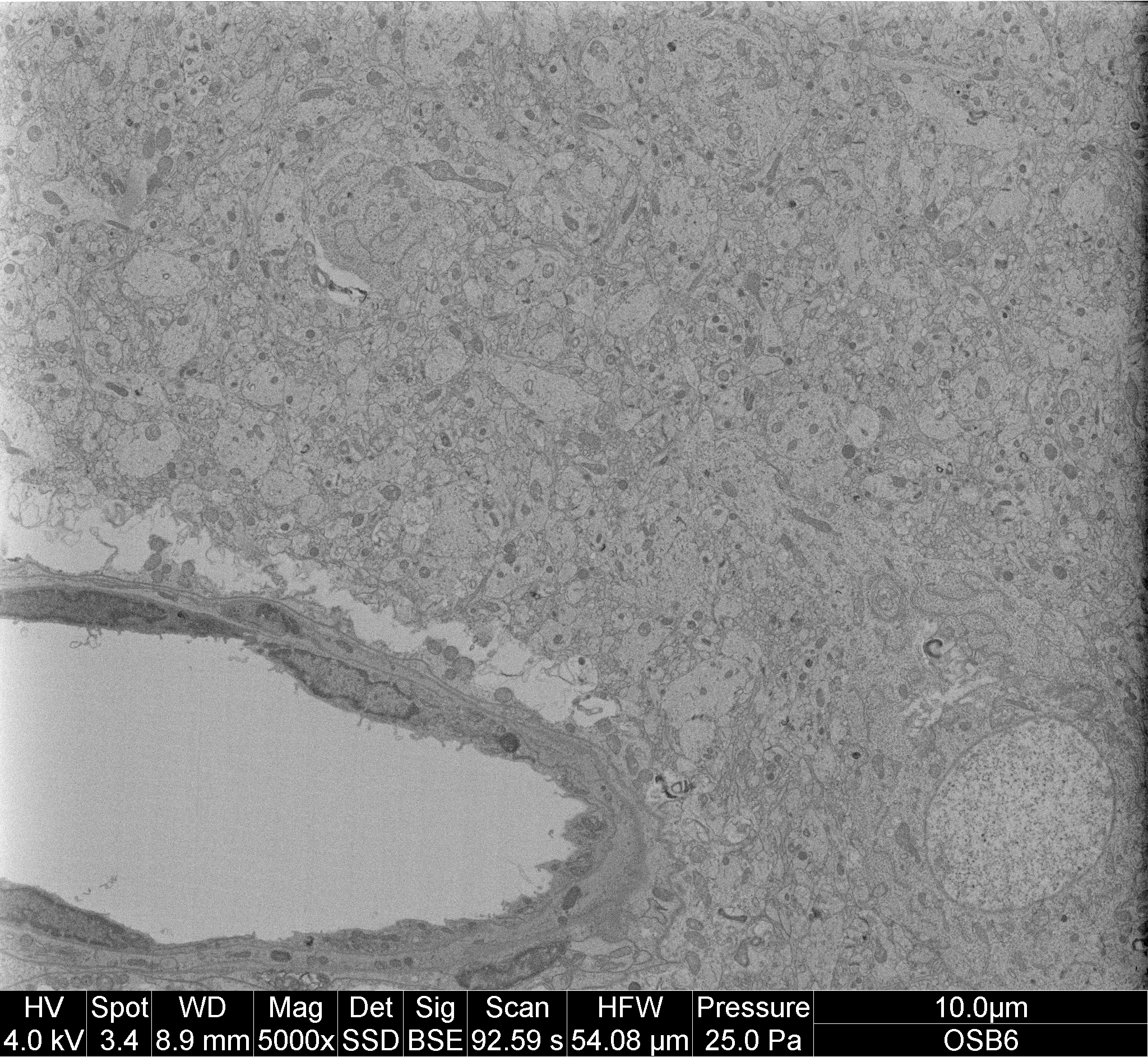

Supplement: Dataset S8 — (255.9 MB ZIP). [file pbio.0020329.sd008.zip › 040604_OS5_st1_743.tif]

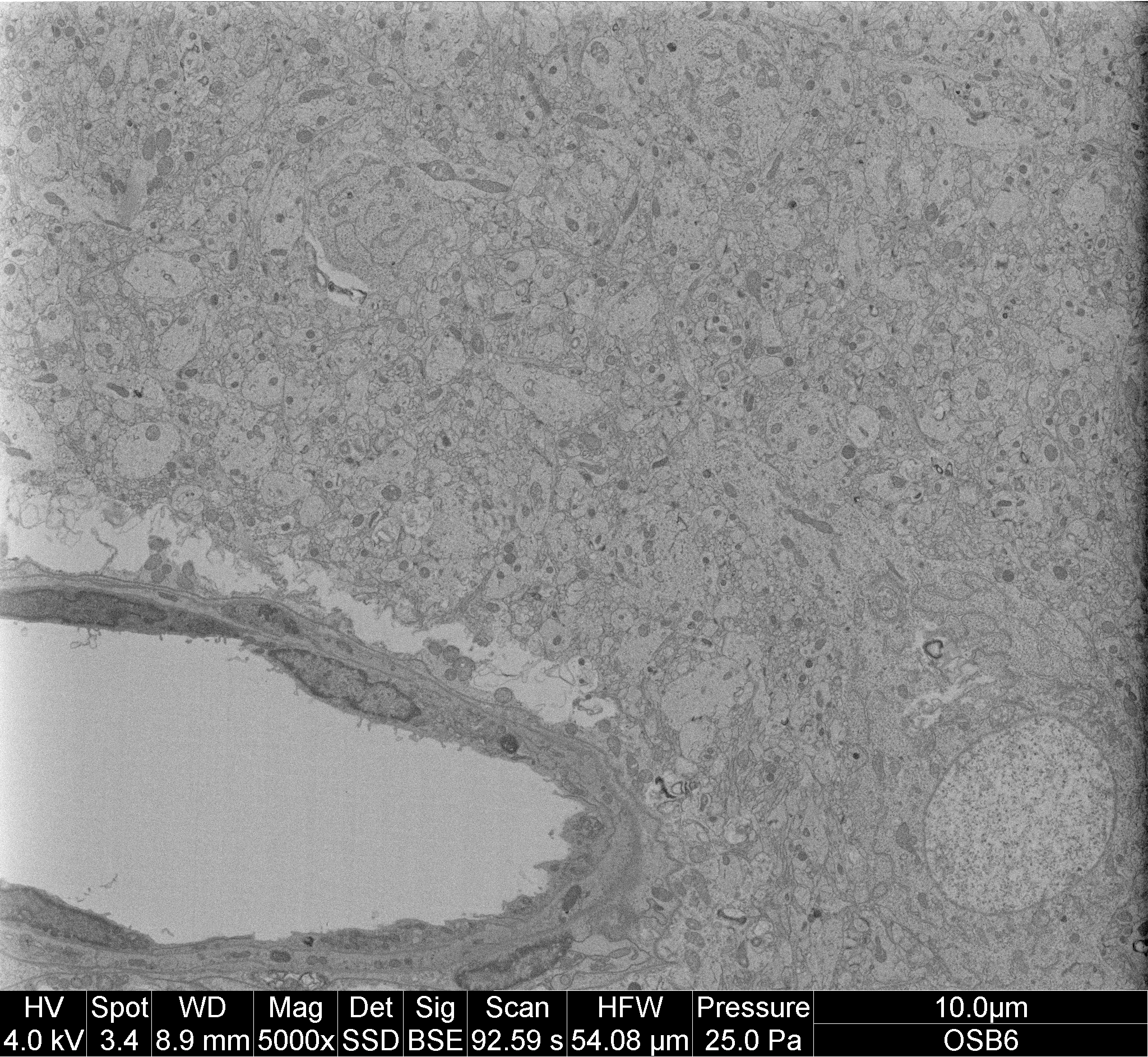

Supplement: Dataset S8 — (255.9 MB ZIP). [file pbio.0020329.sd008.zip › 040604_OS5_st1_744.tif]

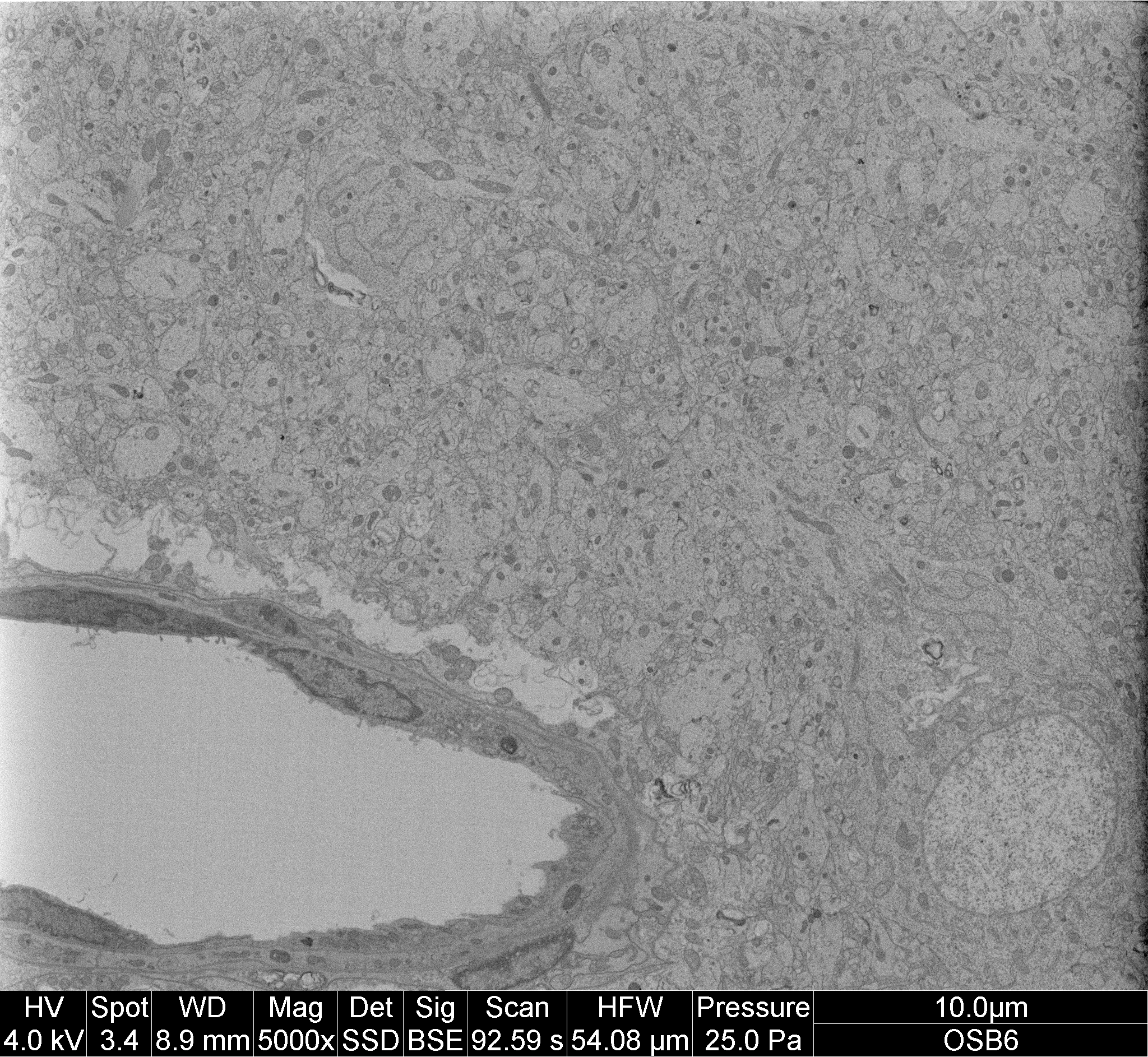

Supplement: Dataset S8 — (255.9 MB ZIP). [file pbio.0020329.sd008.zip › 040604_OS5_st1_745.tif]

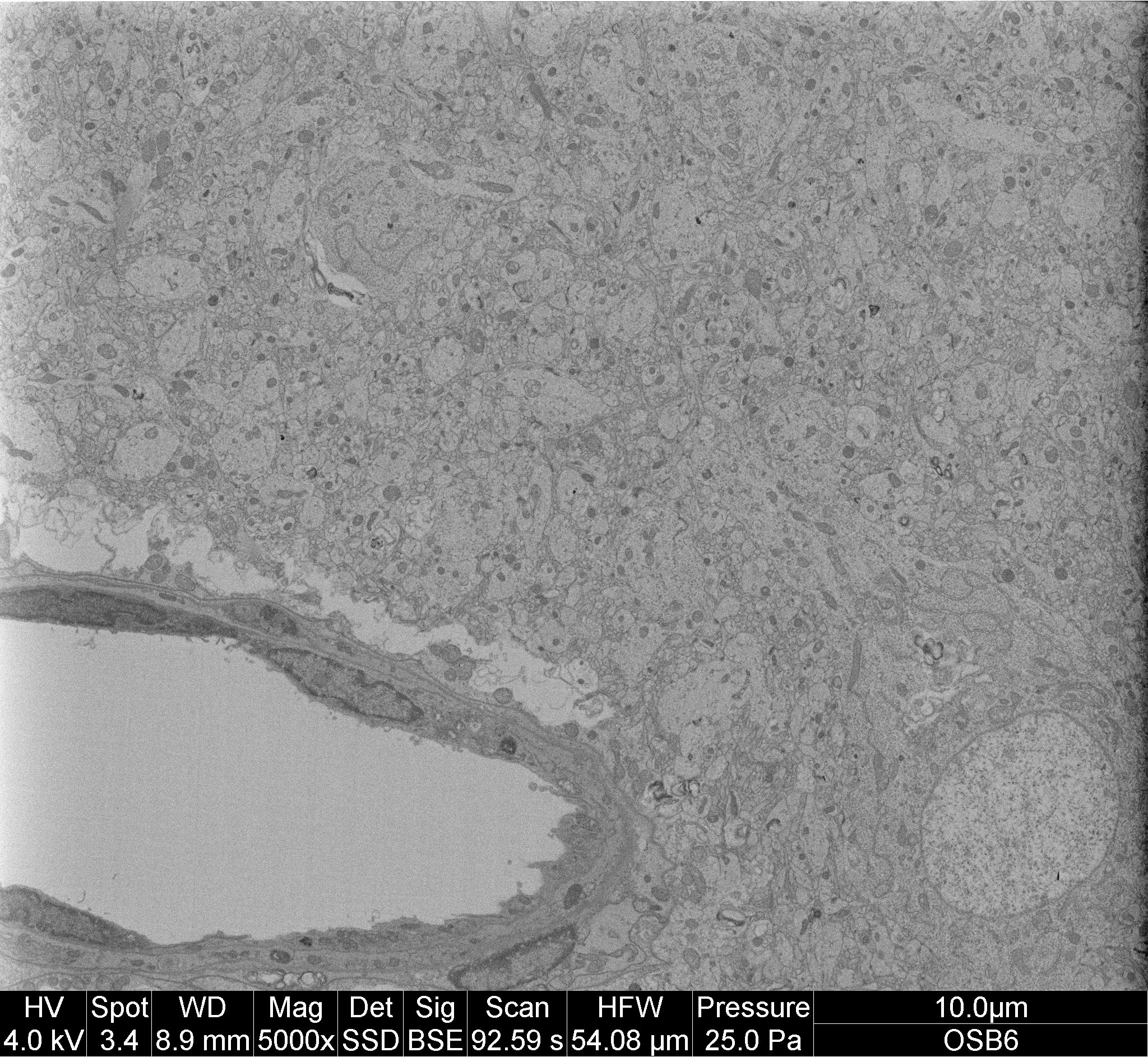

Supplement: Dataset S8 — (255.9 MB ZIP). [file pbio.0020329.sd008.zip › 040604_OS5_st1_746.tif]

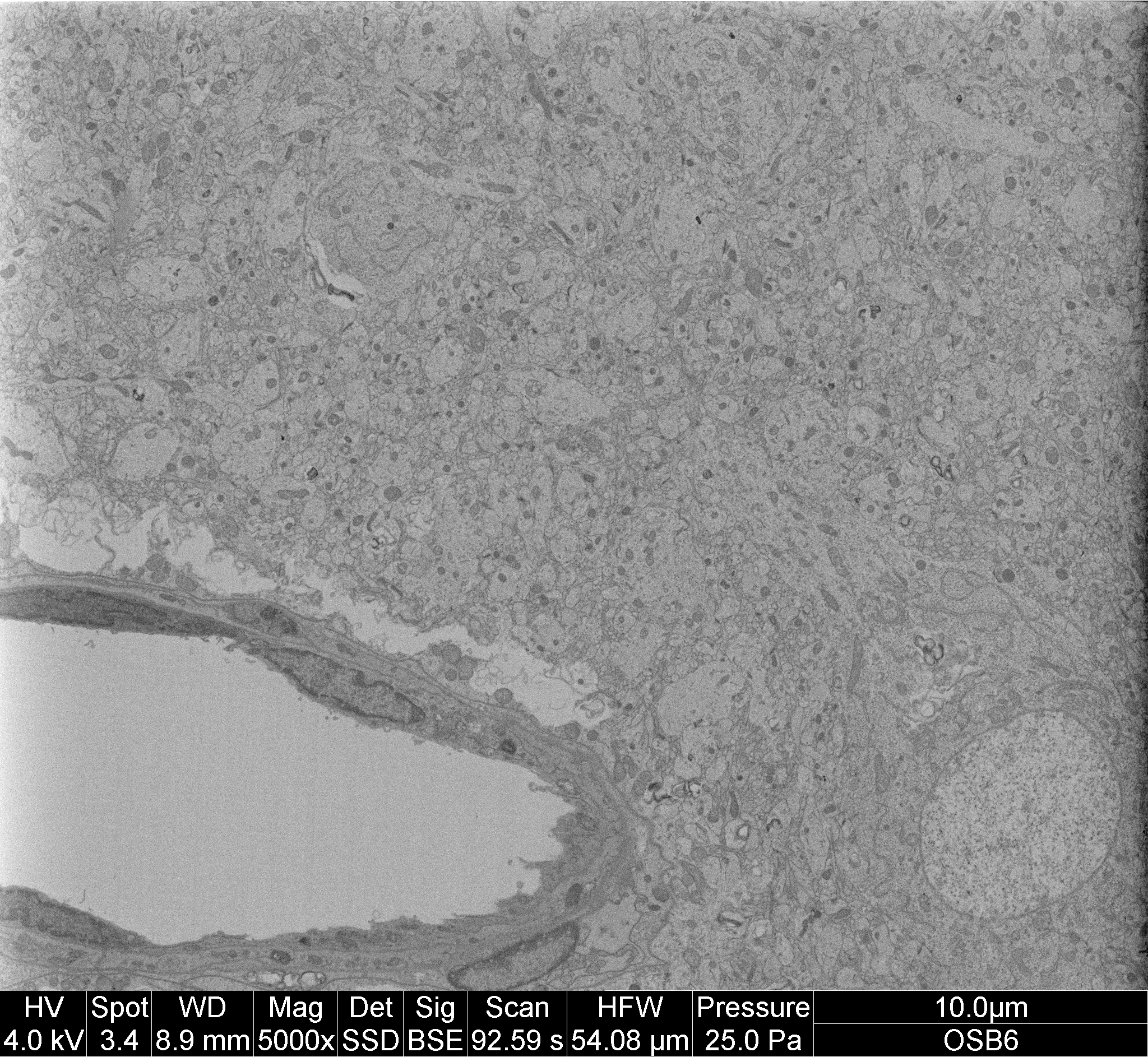

Supplement: Dataset S8 — (255.9 MB ZIP). [file pbio.0020329.sd008.zip › 040604_OS5_st1_747.tif]

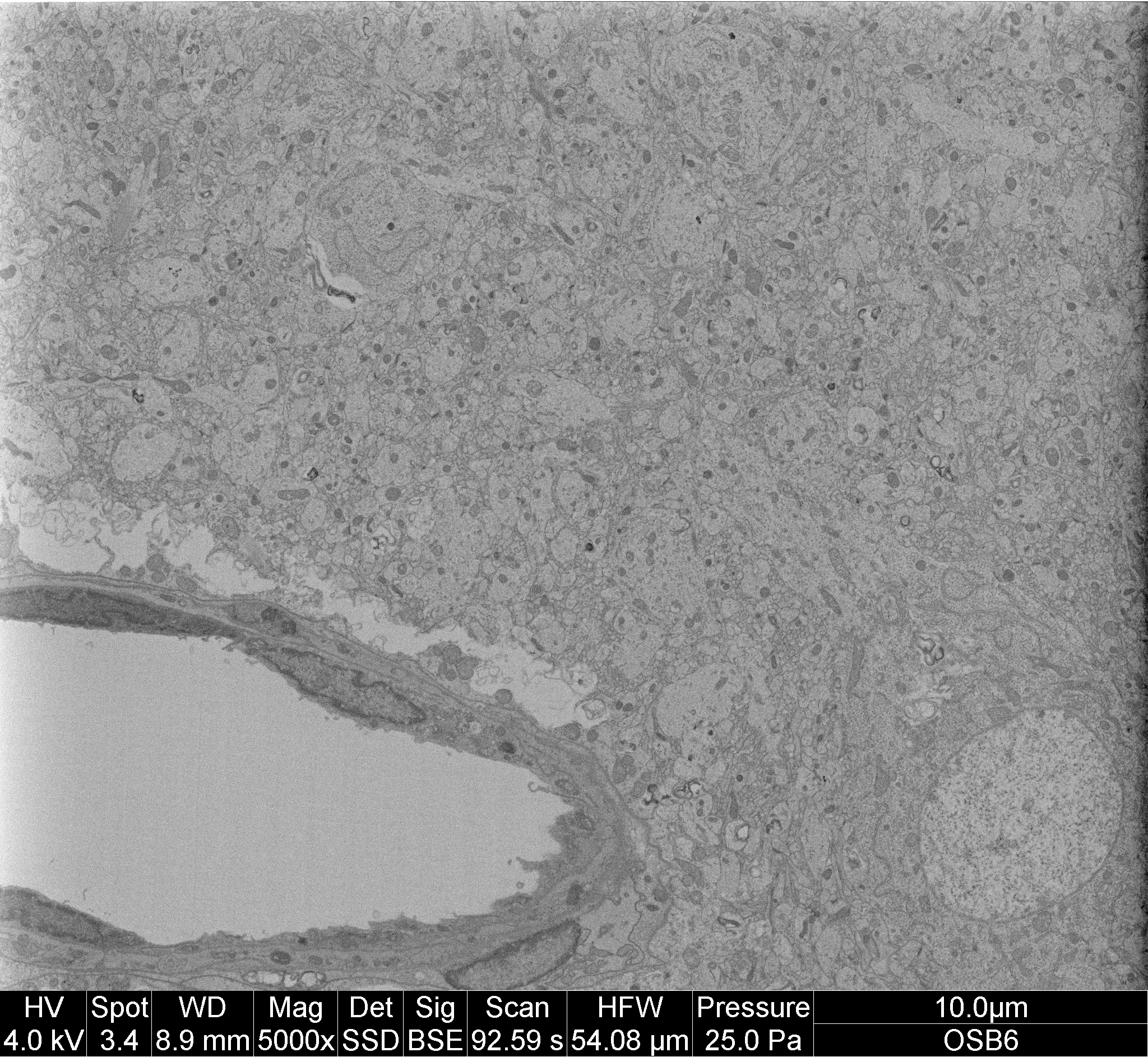

Supplement: Dataset S8 — (255.9 MB ZIP). [file pbio.0020329.sd008.zip › 040604_OS5_st1_748.tif]

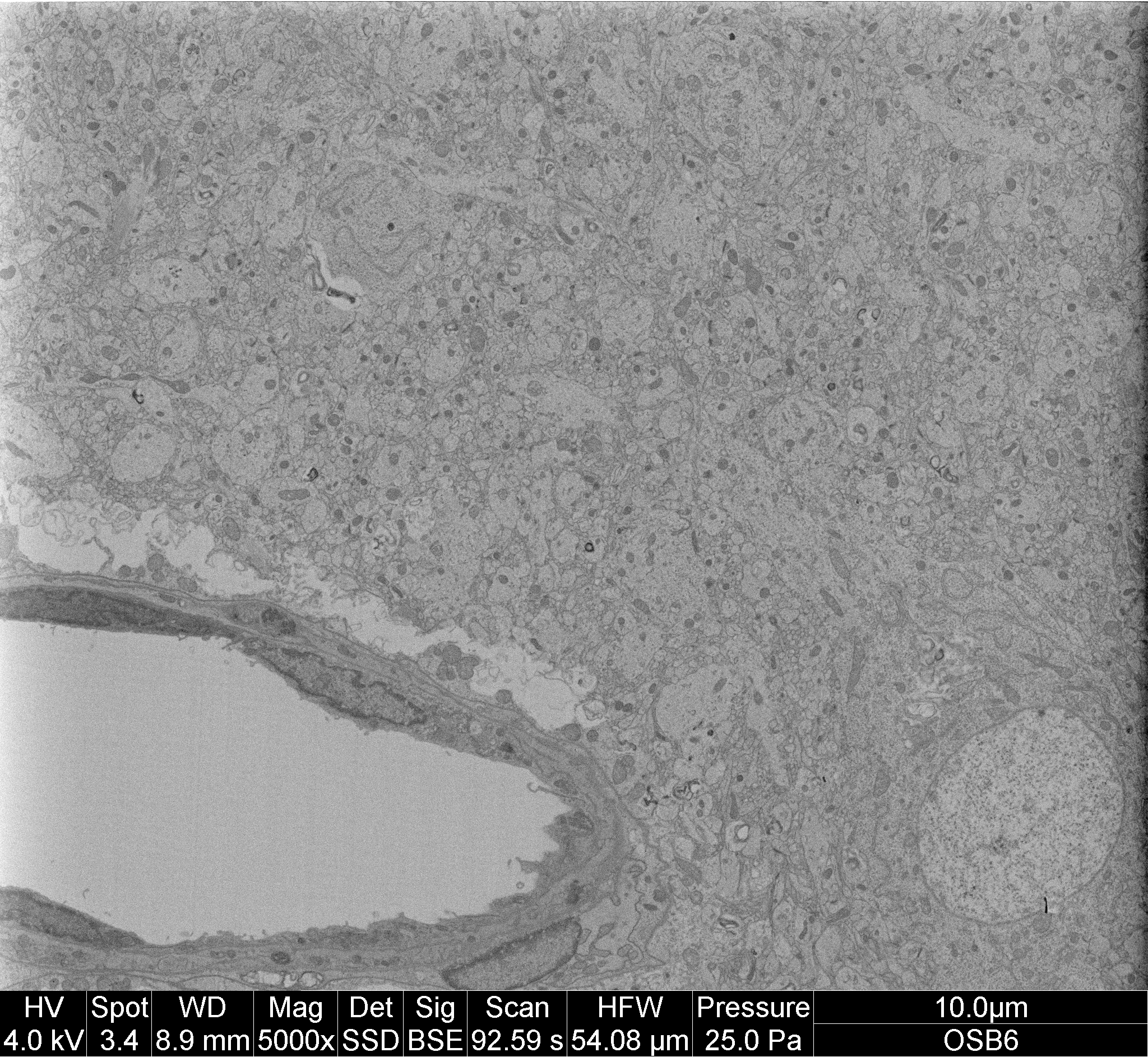

Supplement: Dataset S8 — (255.9 MB ZIP). [file pbio.0020329.sd008.zip › 040604_OS5_st1_749.tif]

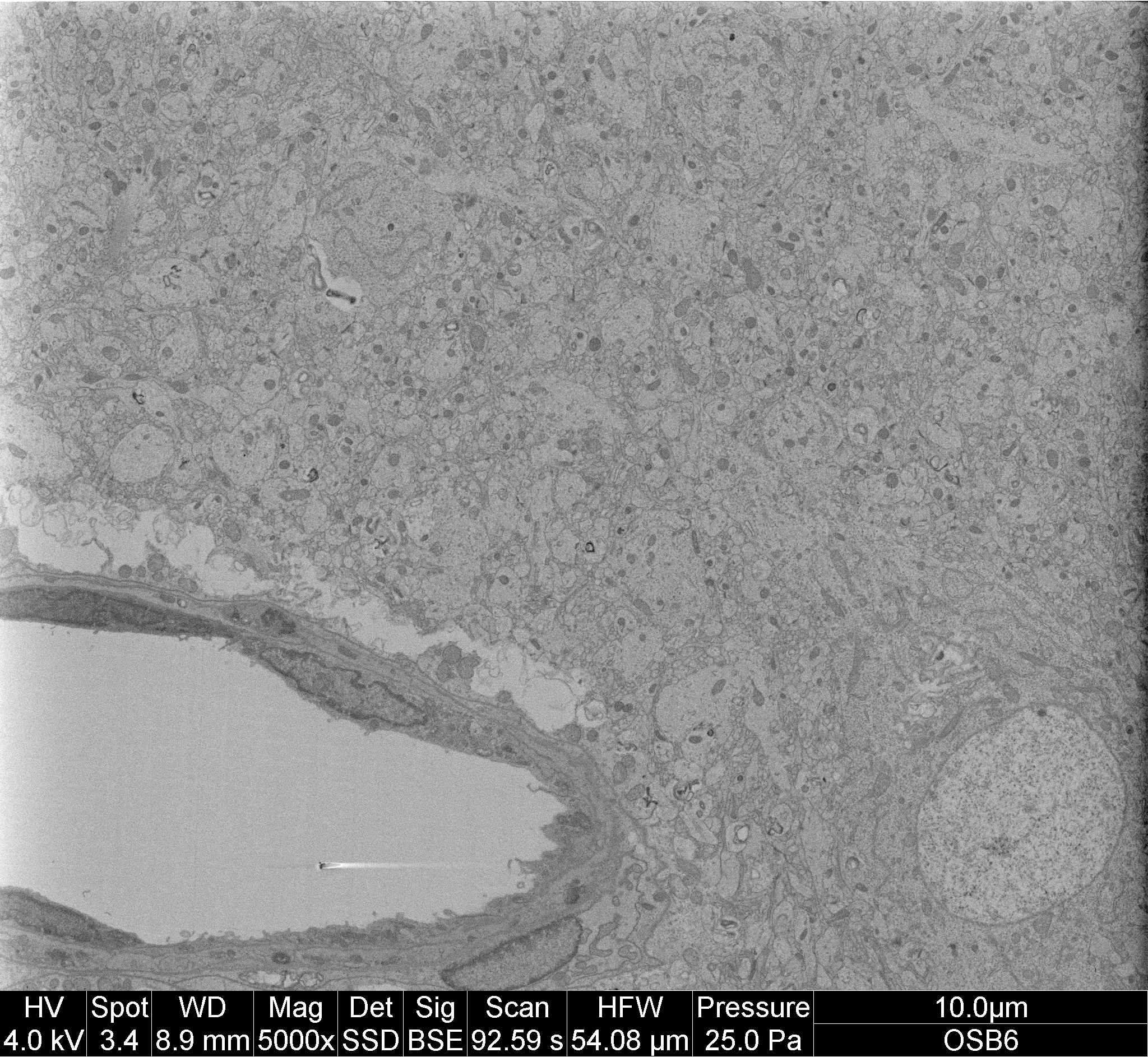

Supplement: Dataset S8 — (255.9 MB ZIP). [file pbio.0020329.sd008.zip › 040604_OS5_st1_750.tif]

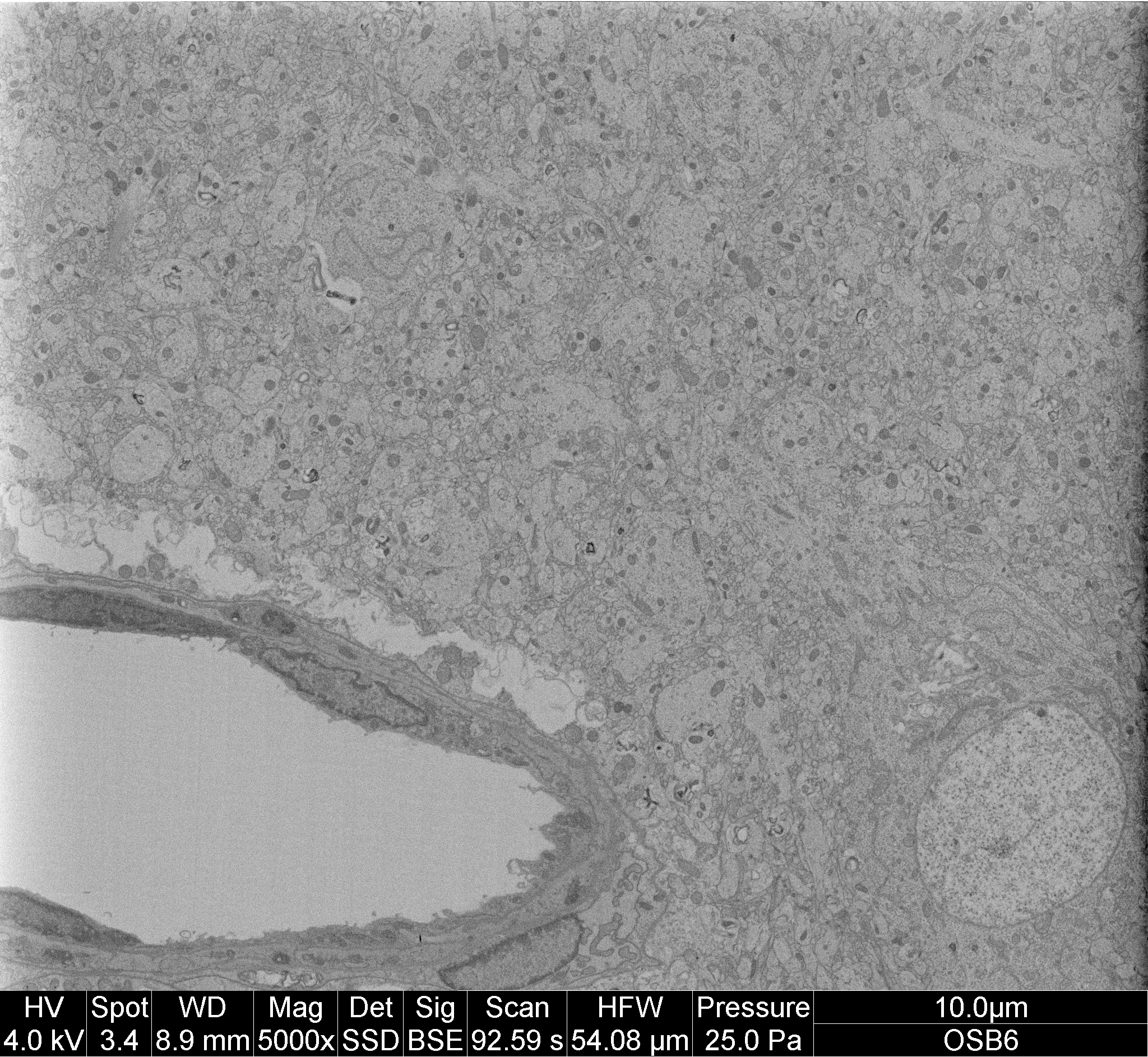

Supplement: Dataset S8 — (255.9 MB ZIP). [file pbio.0020329.sd008.zip › 040604_OS5_st1_751.tif]

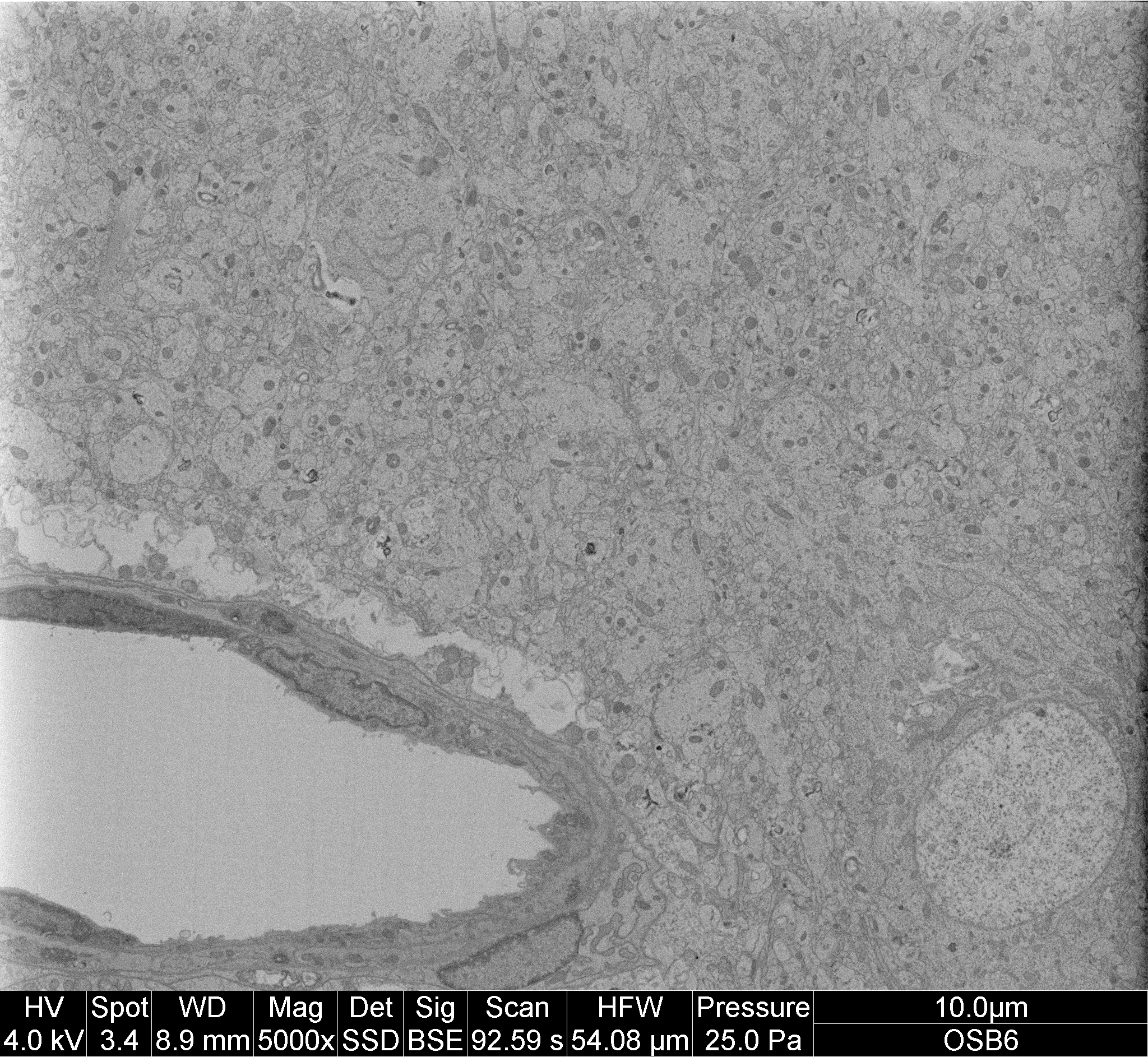

Supplement: Dataset S8 — (255.9 MB ZIP). [file pbio.0020329.sd008.zip › 040604_OS5_st1_752.tif]

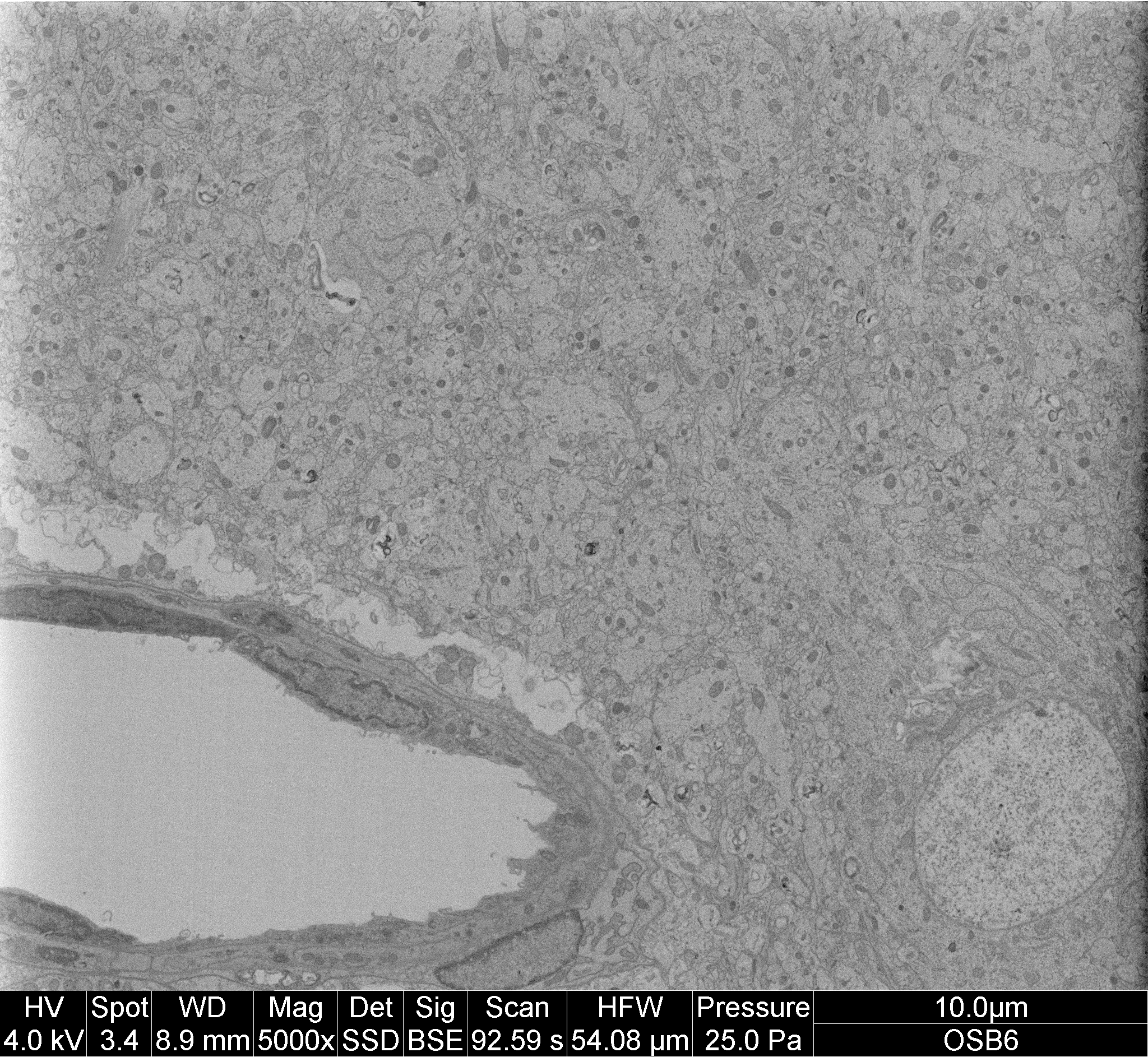

Supplement: Dataset S8 — (255.9 MB ZIP). [file pbio.0020329.sd008.zip › 040604_OS5_st1_753.tif]

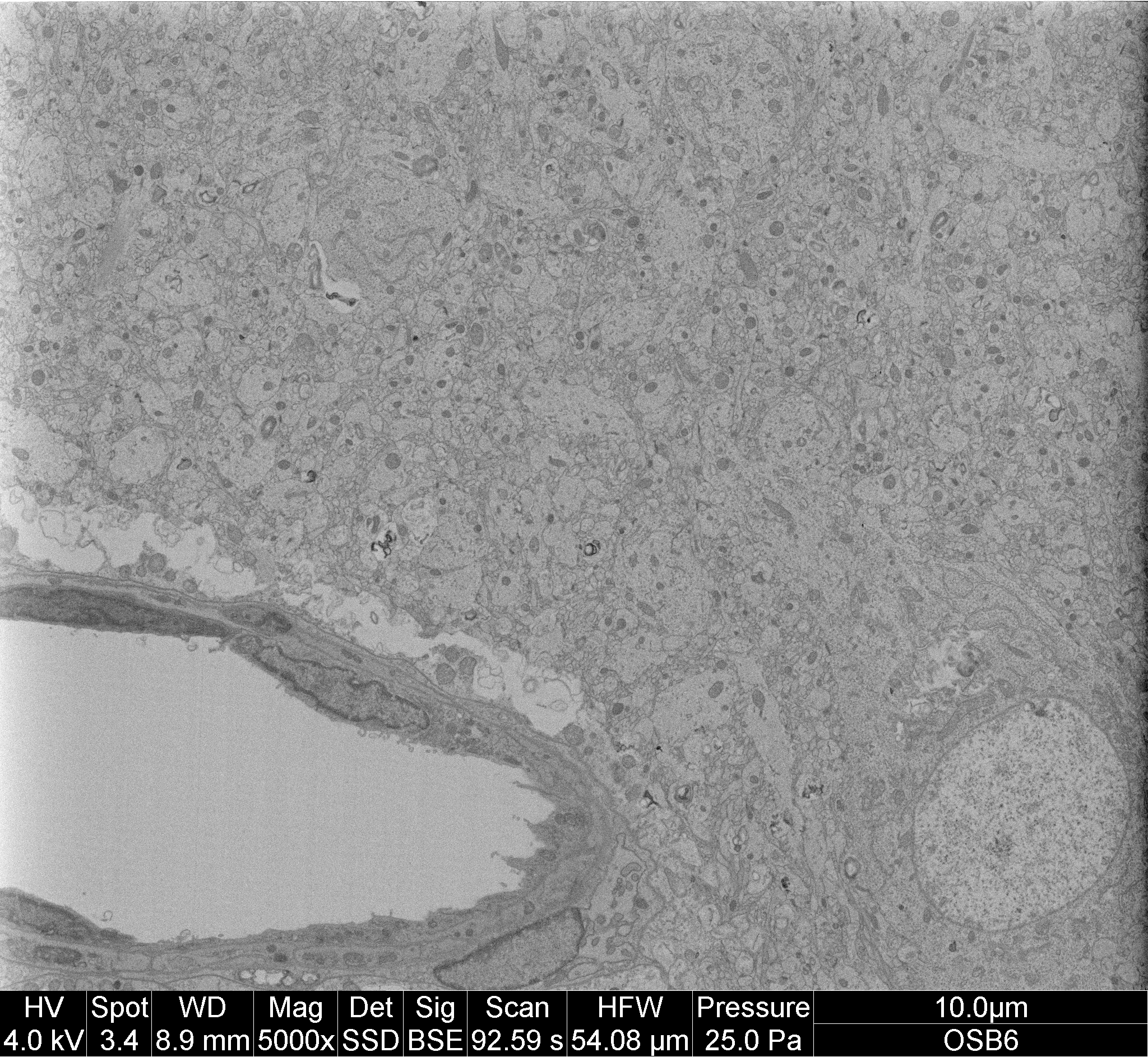

Supplement: Dataset S8 — (255.9 MB ZIP). [file pbio.0020329.sd008.zip › 040604_OS5_st1_754.tif]

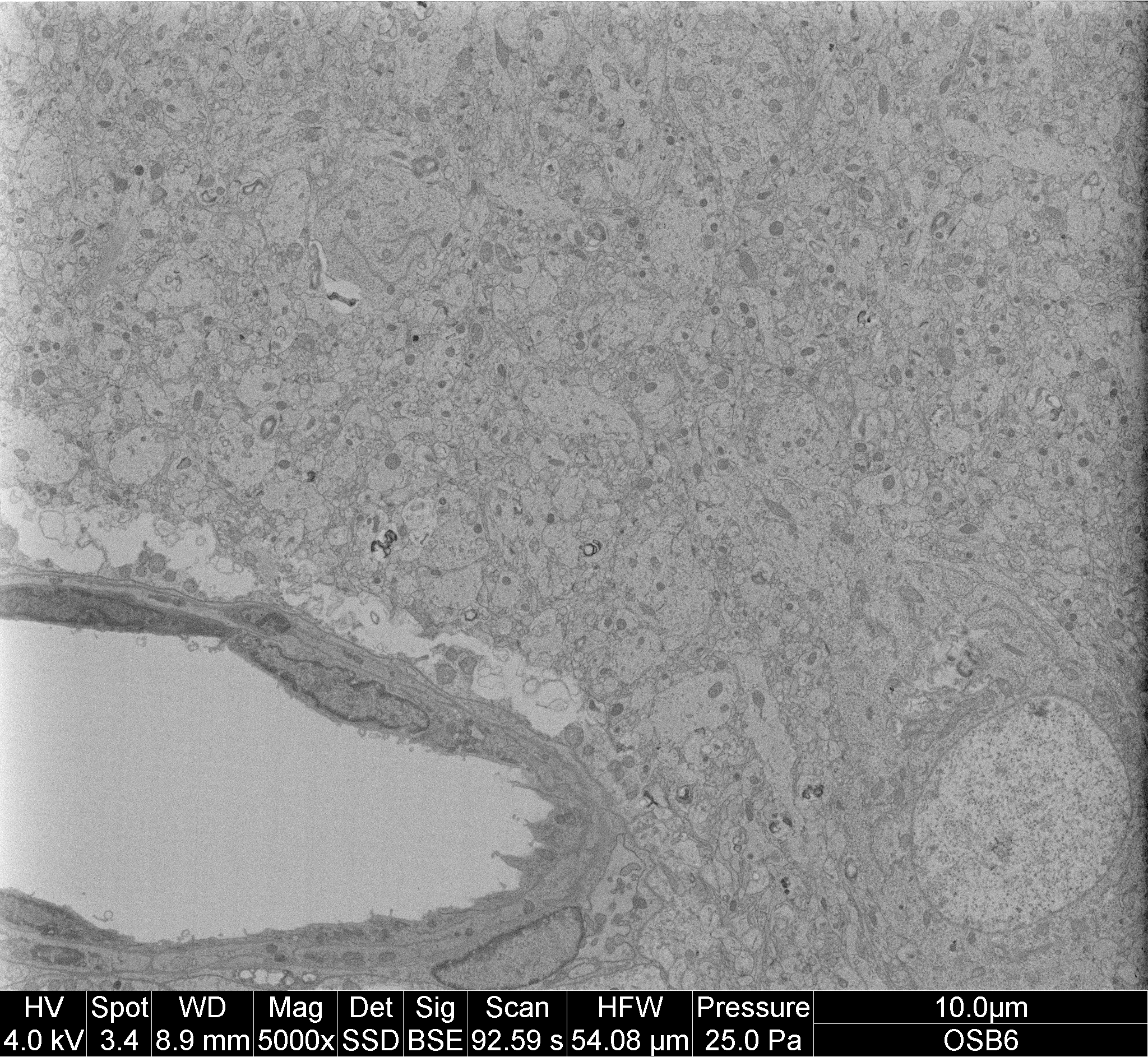

Supplement: Dataset S8 — (255.9 MB ZIP). [file pbio.0020329.sd008.zip › 040604_OS5_st1_755.tif]

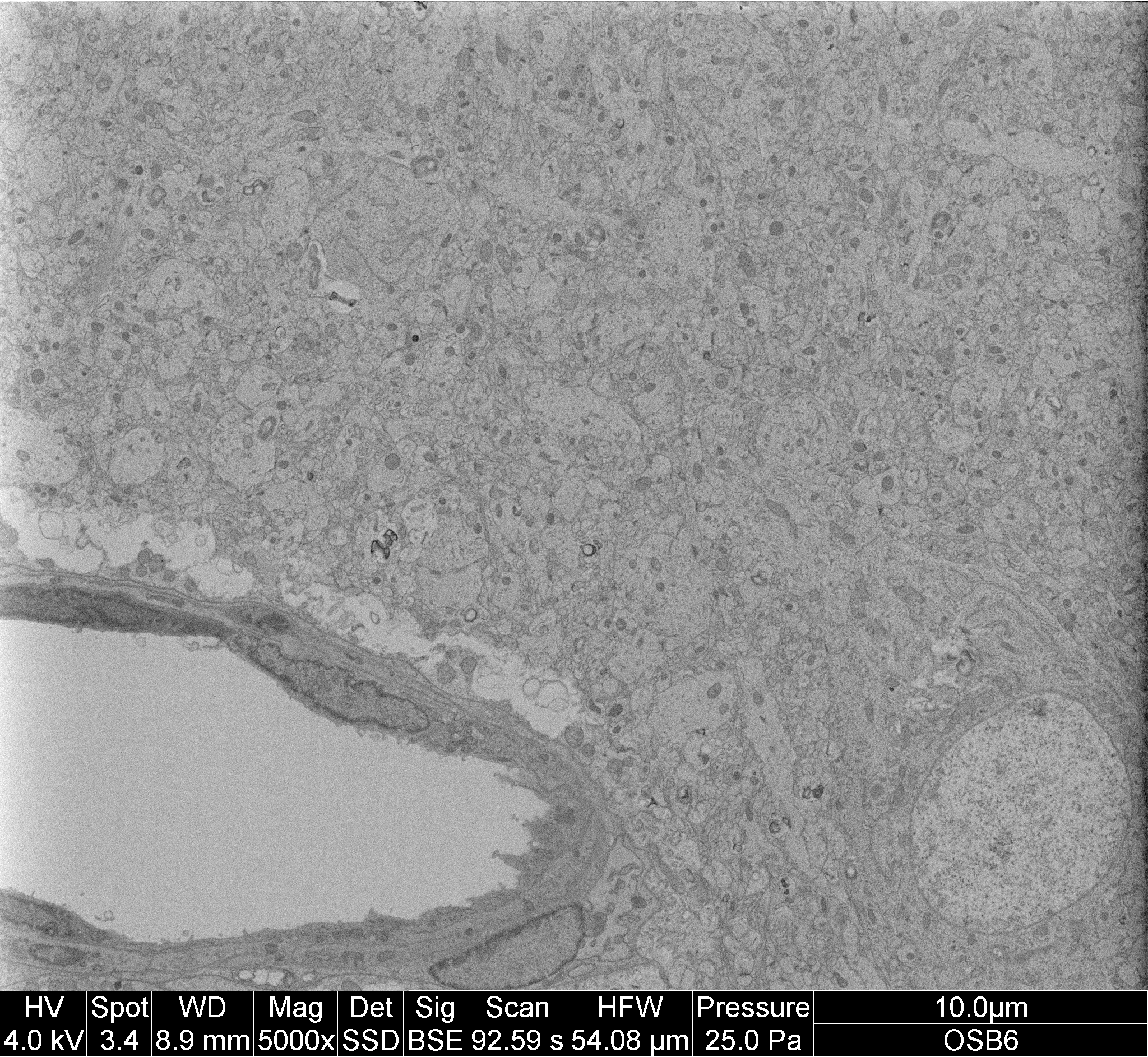

Supplement: Dataset S8 — (255.9 MB ZIP). [file pbio.0020329.sd008.zip › 040604_OS5_st1_756.tif]

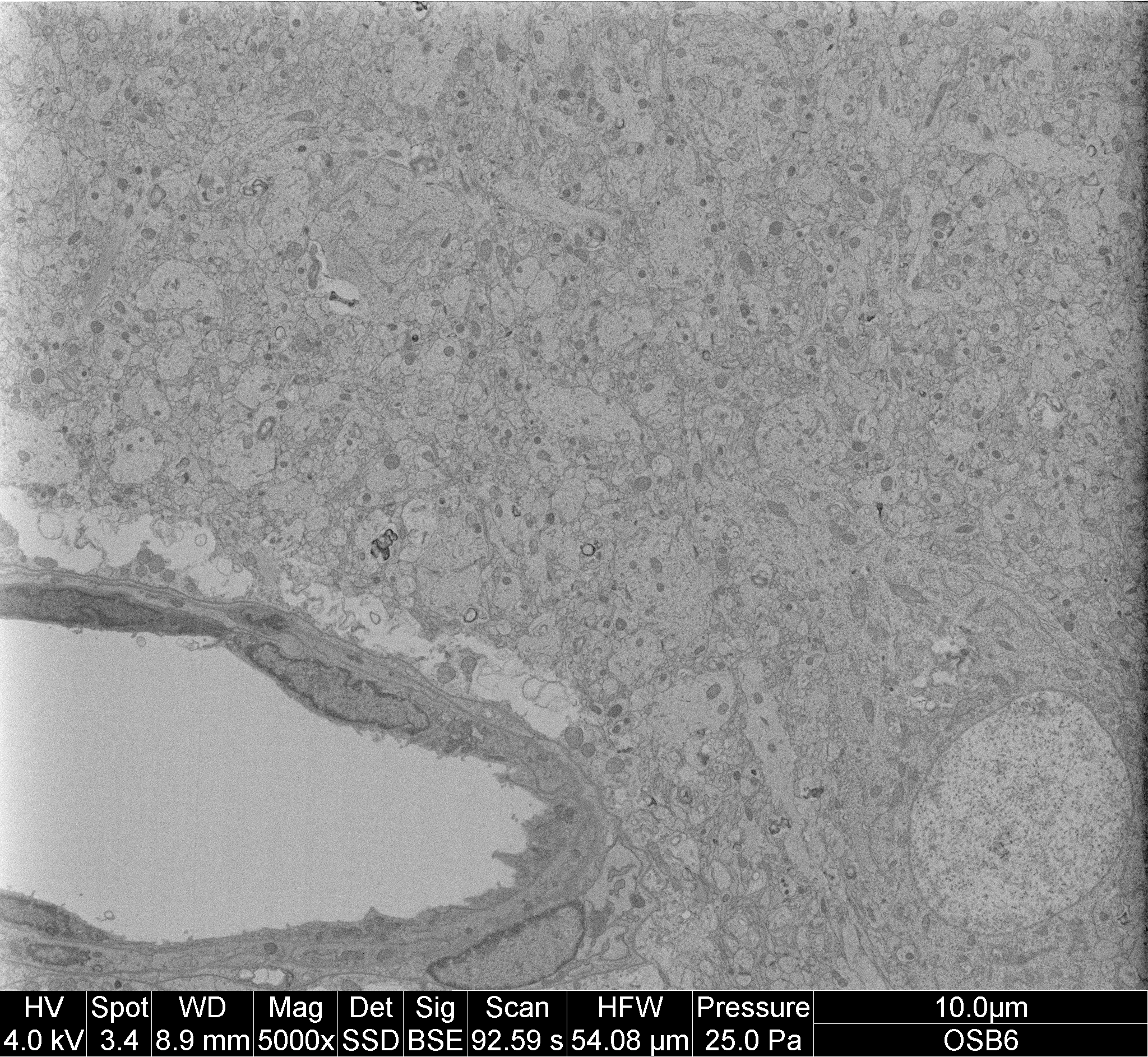

Supplement: Dataset S8 — (255.9 MB ZIP). [file pbio.0020329.sd008.zip › 040604_OS5_st1_757.tif]

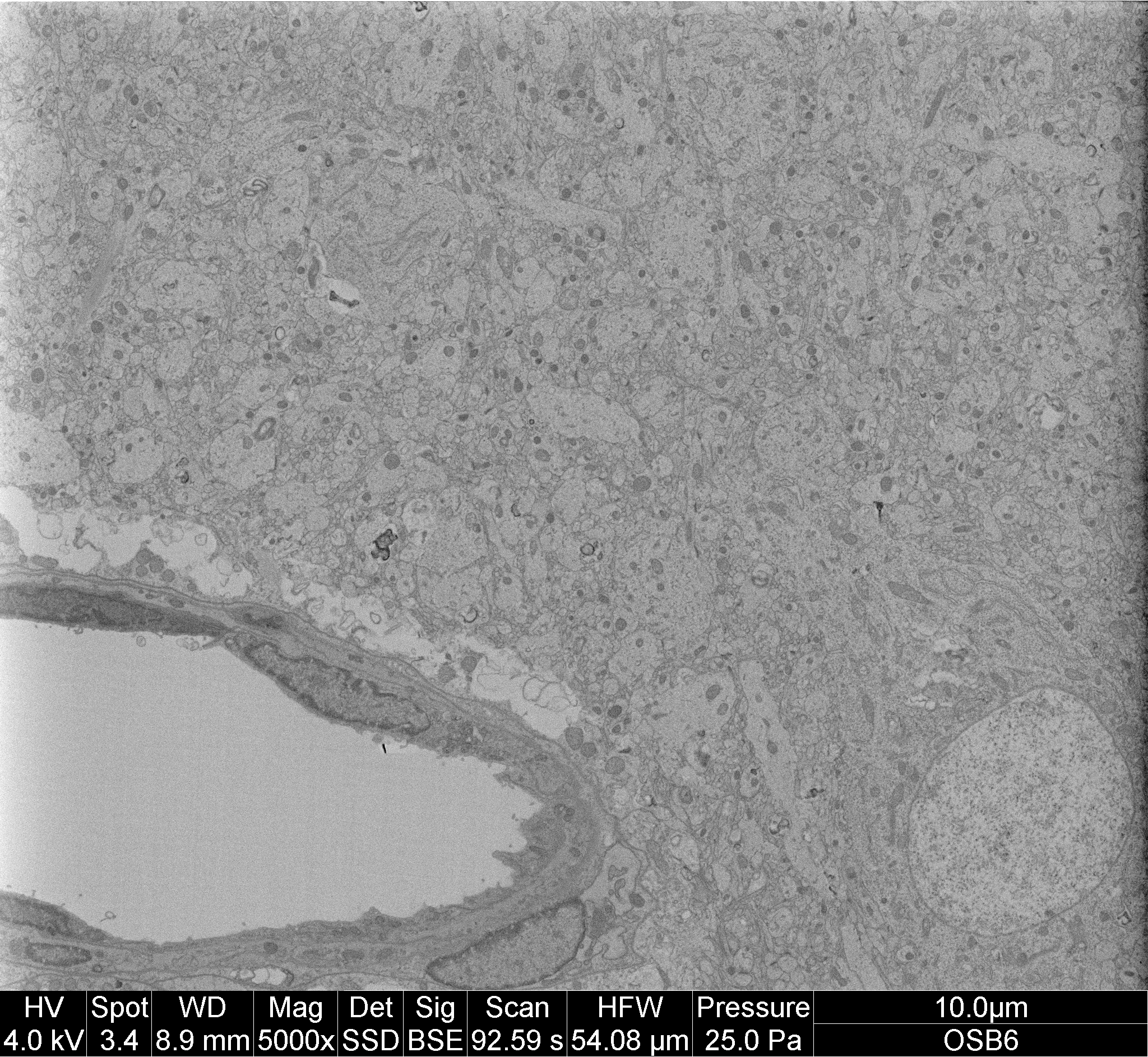

Supplement: Dataset S8 — (255.9 MB ZIP). [file pbio.0020329.sd008.zip › 040604_OS5_st1_758.tif]

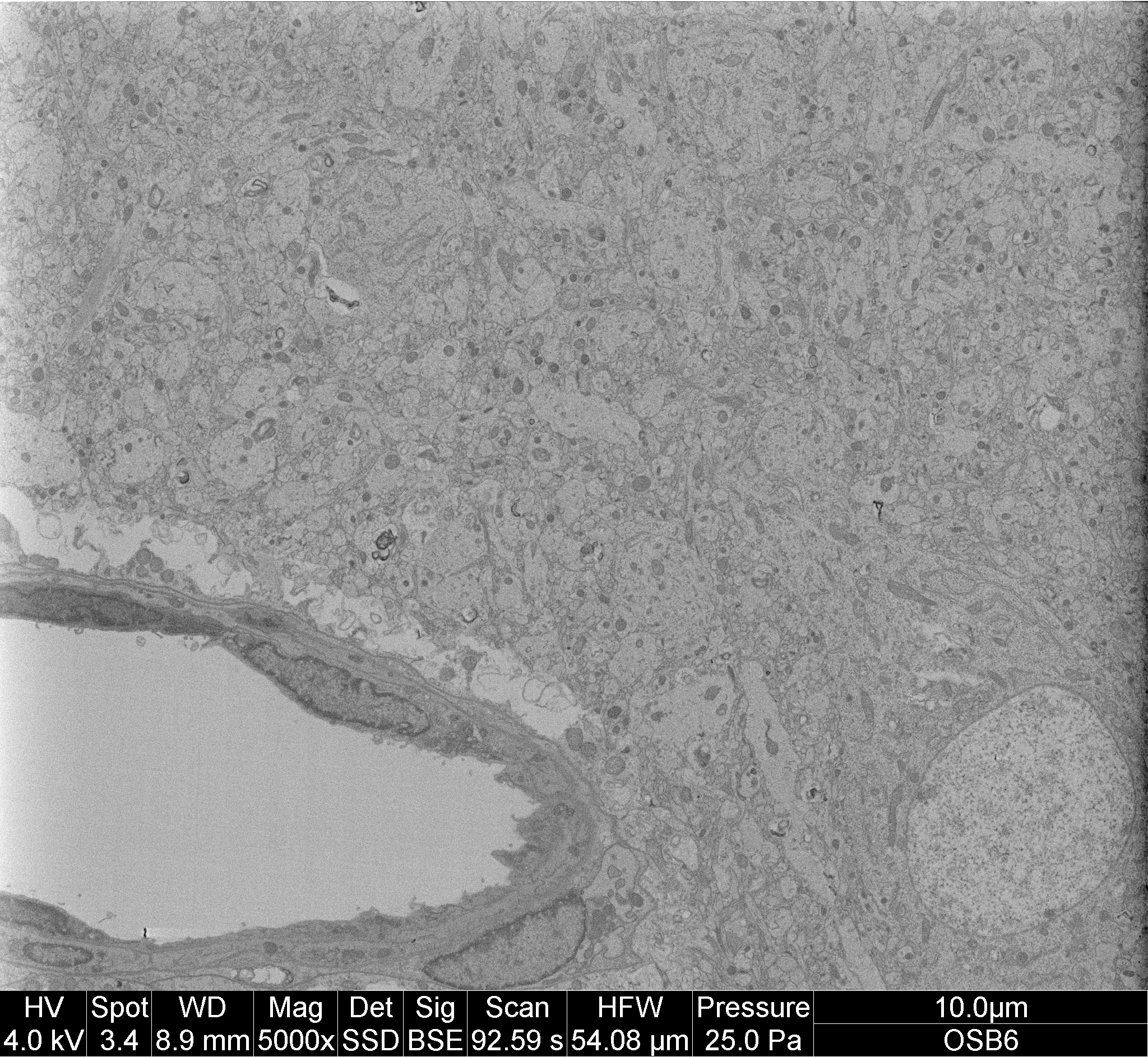

Supplement: Dataset S8 — (255.9 MB ZIP). [file pbio.0020329.sd008.zip › 040604_OS5_st1_759.tif]

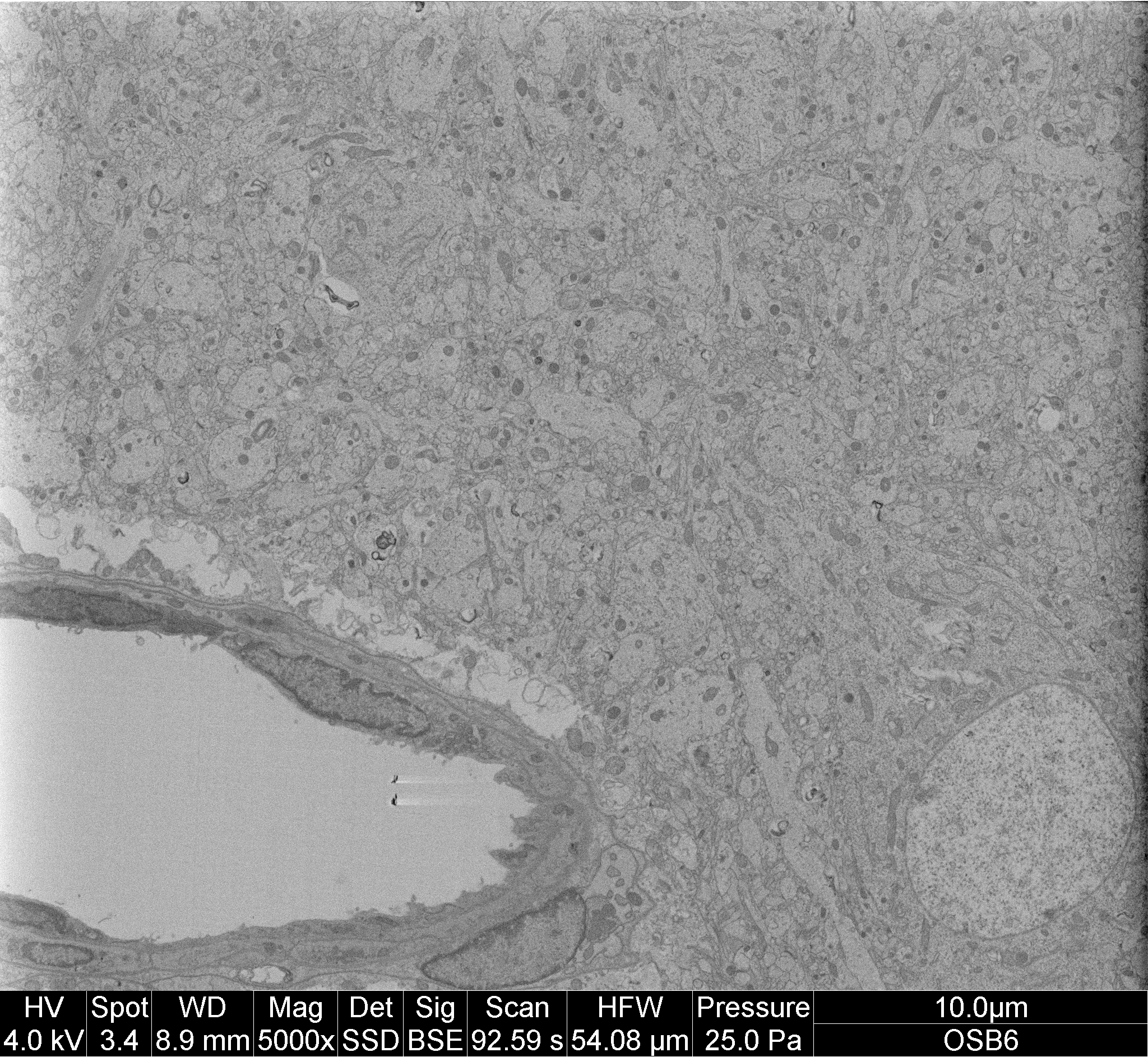

Supplement: Dataset S8 — (255.9 MB ZIP). [file pbio.0020329.sd008.zip › 040604_OS5_st1_760.tif]

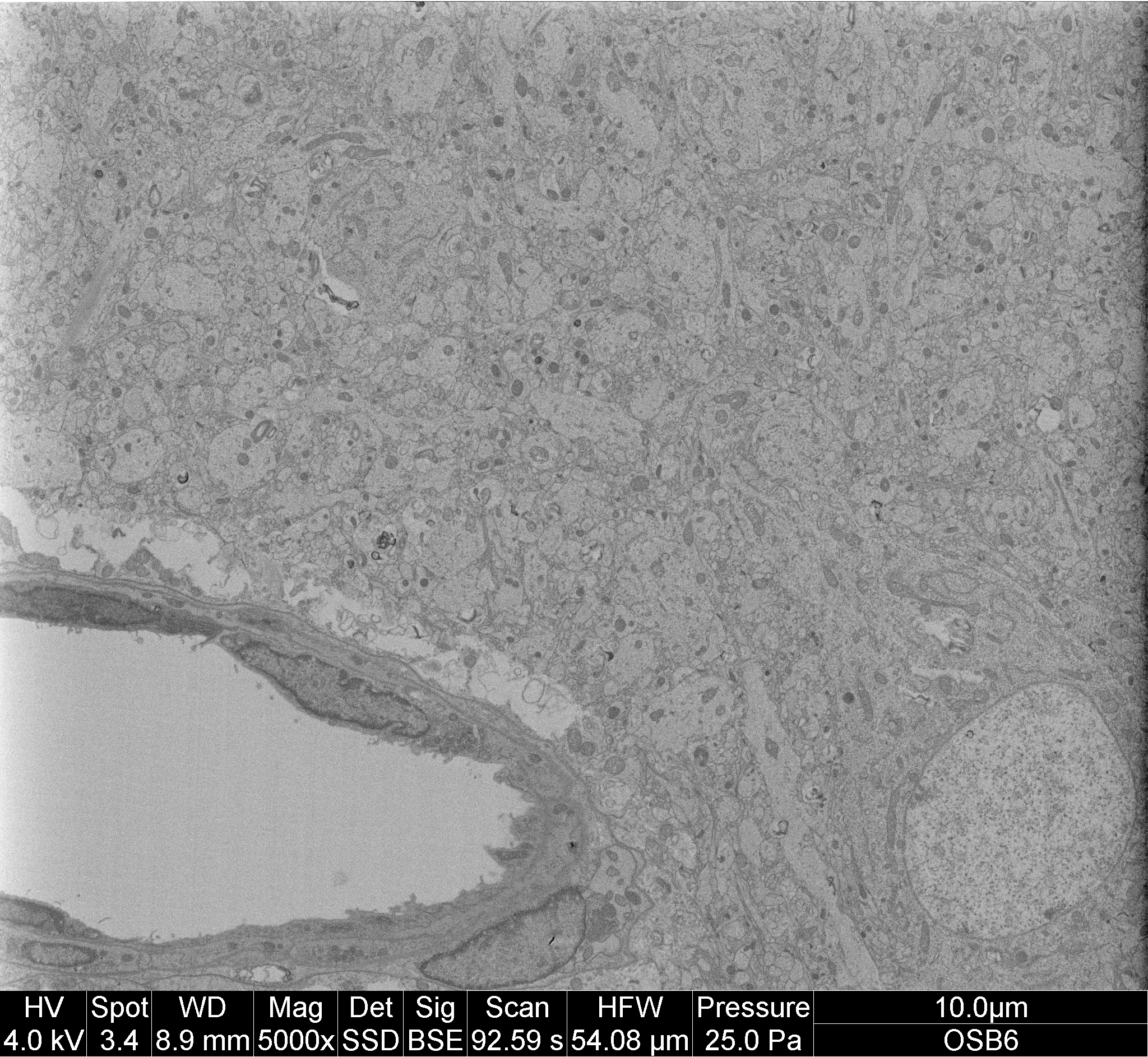

Supplement: Dataset S8 — (255.9 MB ZIP). [file pbio.0020329.sd008.zip › 040604_OS5_st1_761.tif]

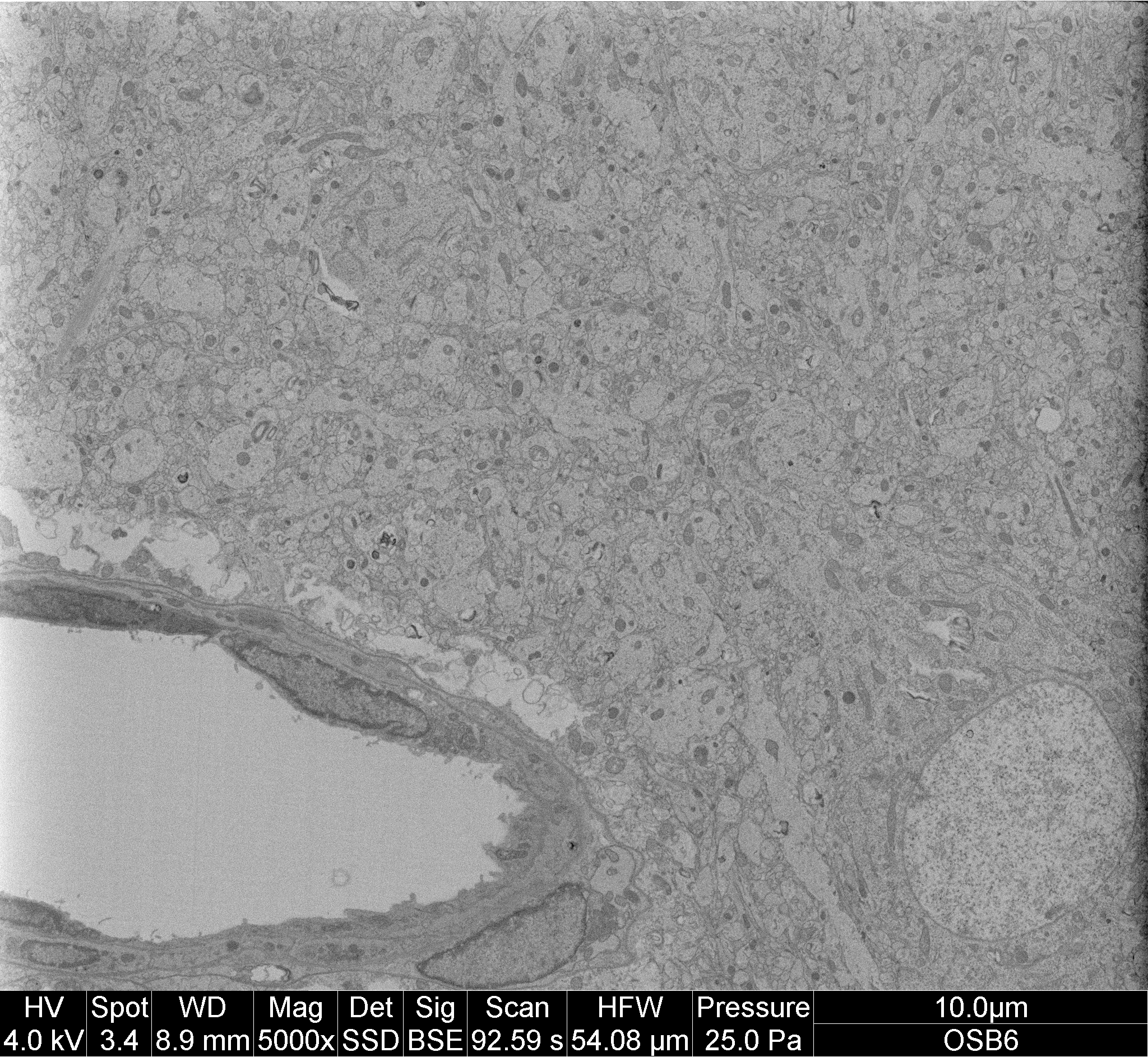

Supplement: Dataset S8 — (255.9 MB ZIP). [file pbio.0020329.sd008.zip › 040604_OS5_st1_762.tif]

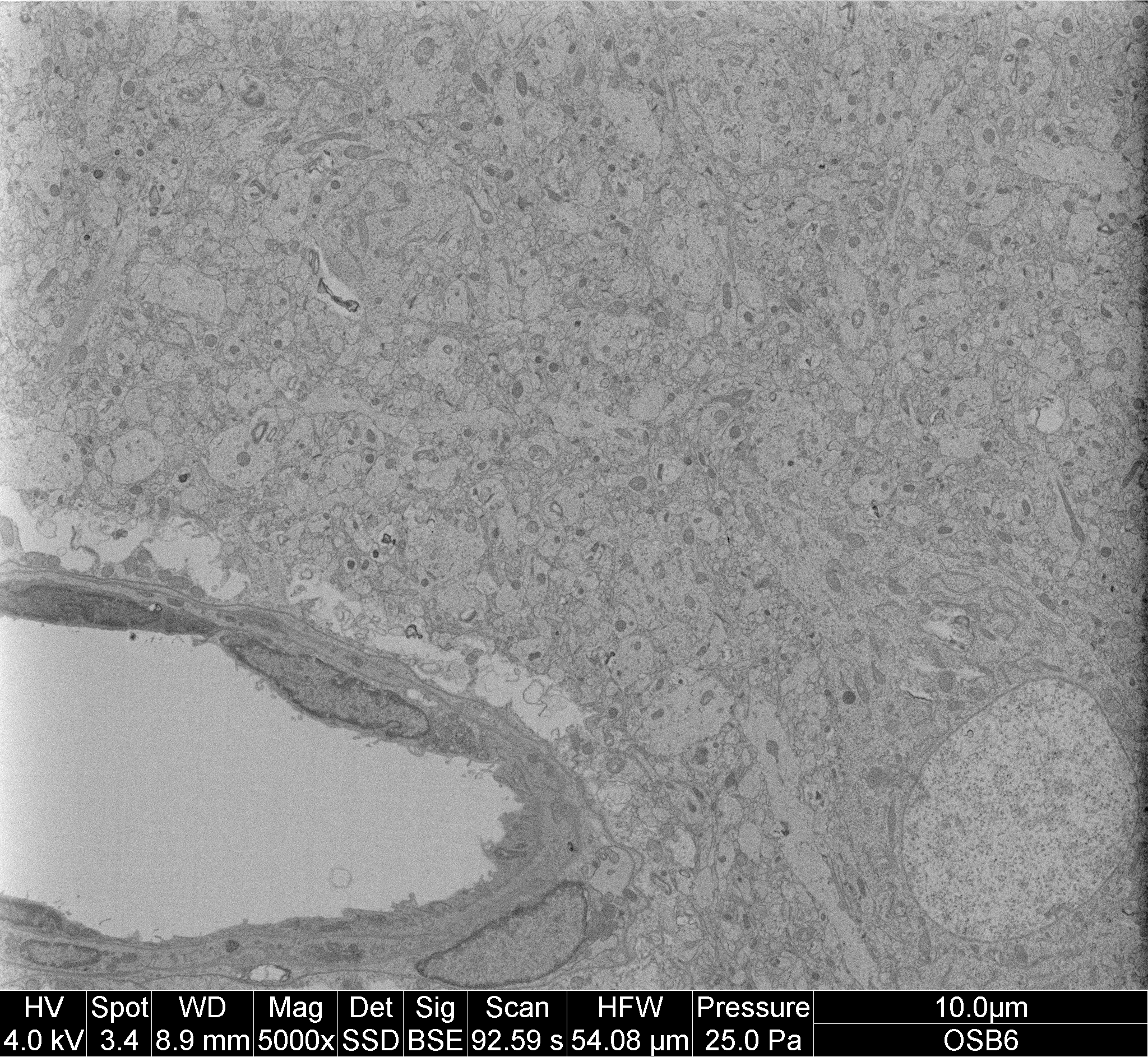

Supplement: Dataset S8 — (255.9 MB ZIP). [file pbio.0020329.sd008.zip › 040604_OS5_st1_763.tif]

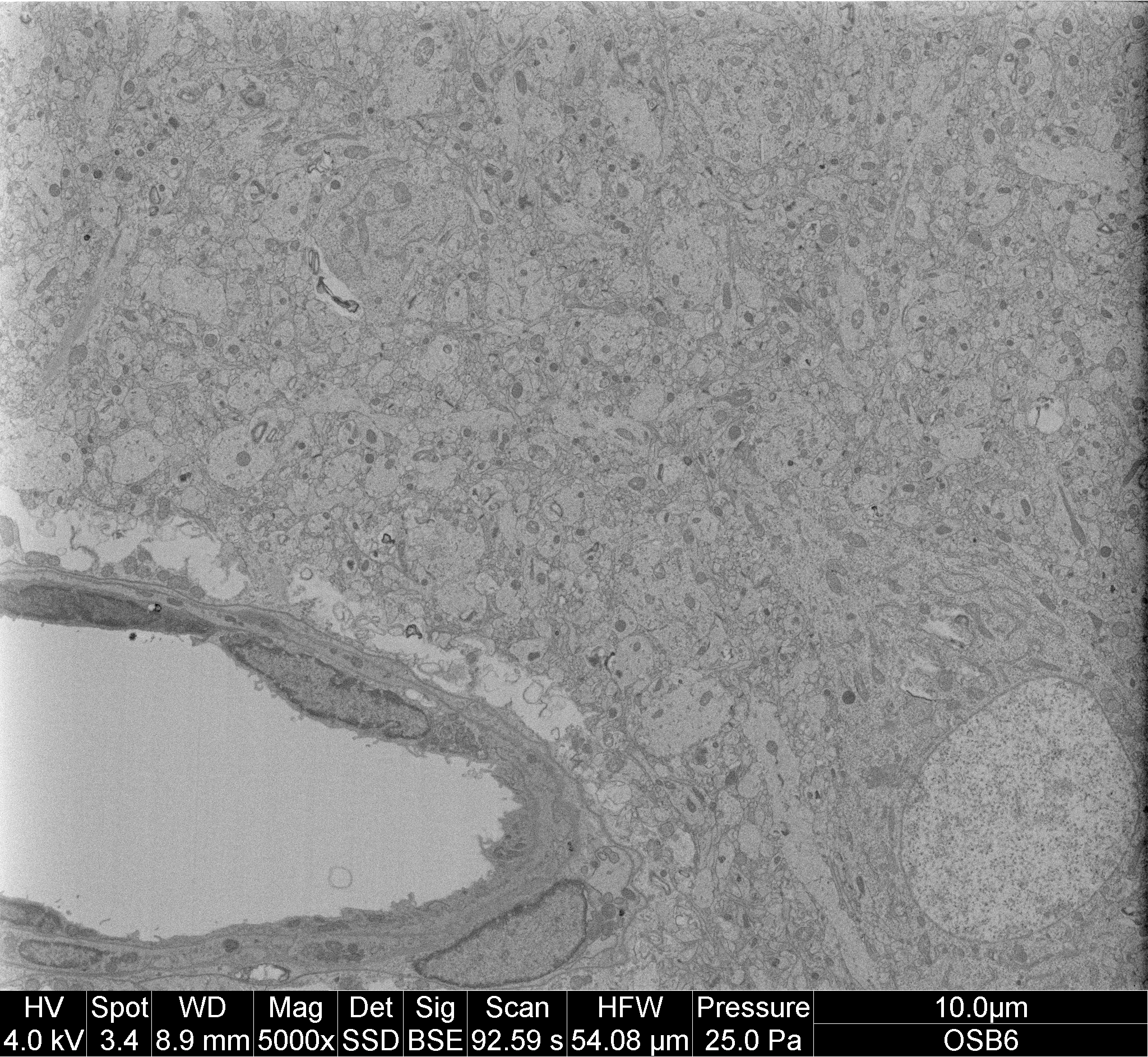

Supplement: Dataset S8 — (255.9 MB ZIP). [file pbio.0020329.sd008.zip › 040604_OS5_st1_764.tif]

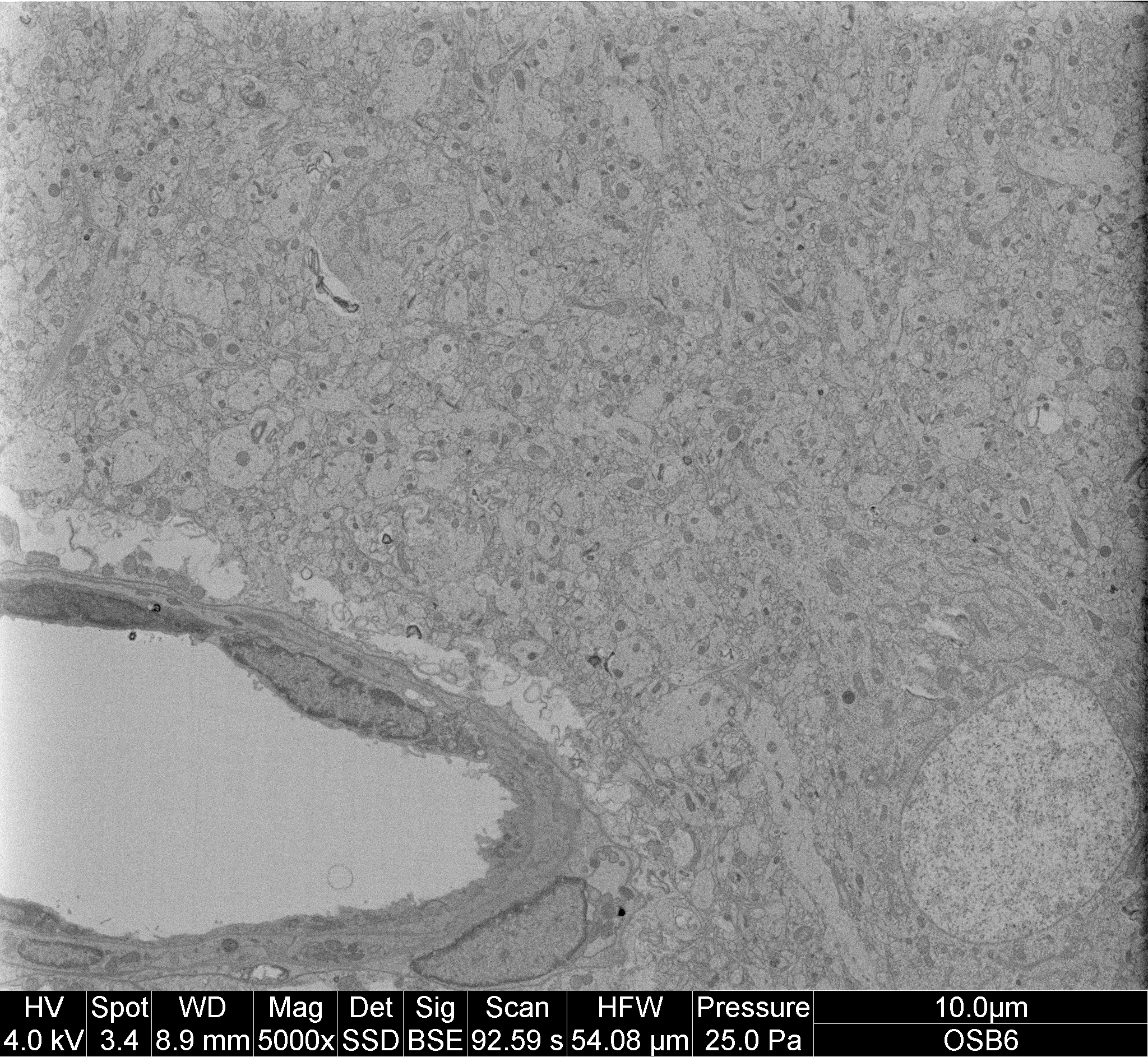

Supplement: Dataset S8 — (255.9 MB ZIP). [file pbio.0020329.sd008.zip › 040604_OS5_st1_765.tif]

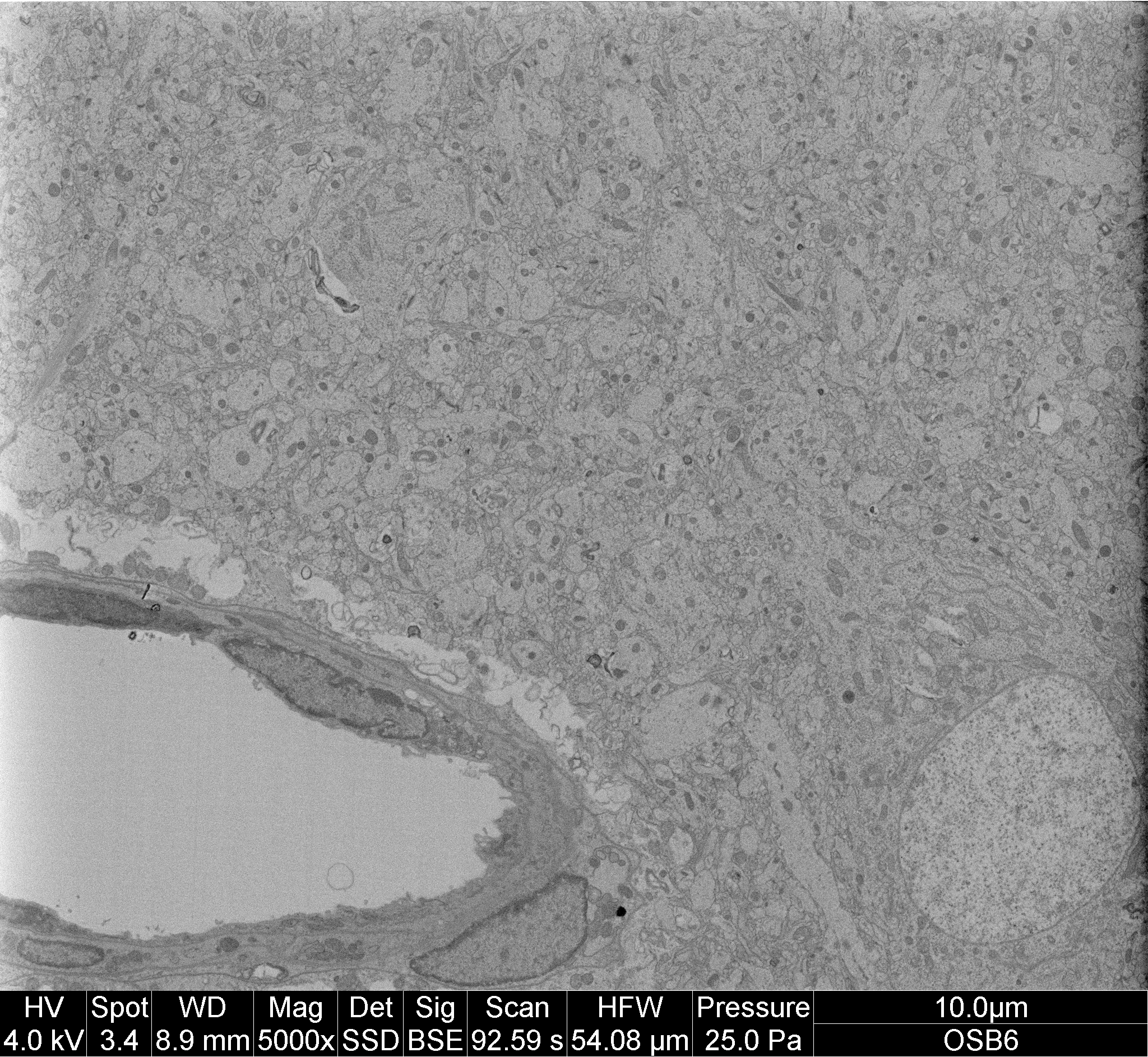

Supplement: Dataset S8 — (255.9 MB ZIP). [file pbio.0020329.sd008.zip › 040604_OS5_st1_766.tif]

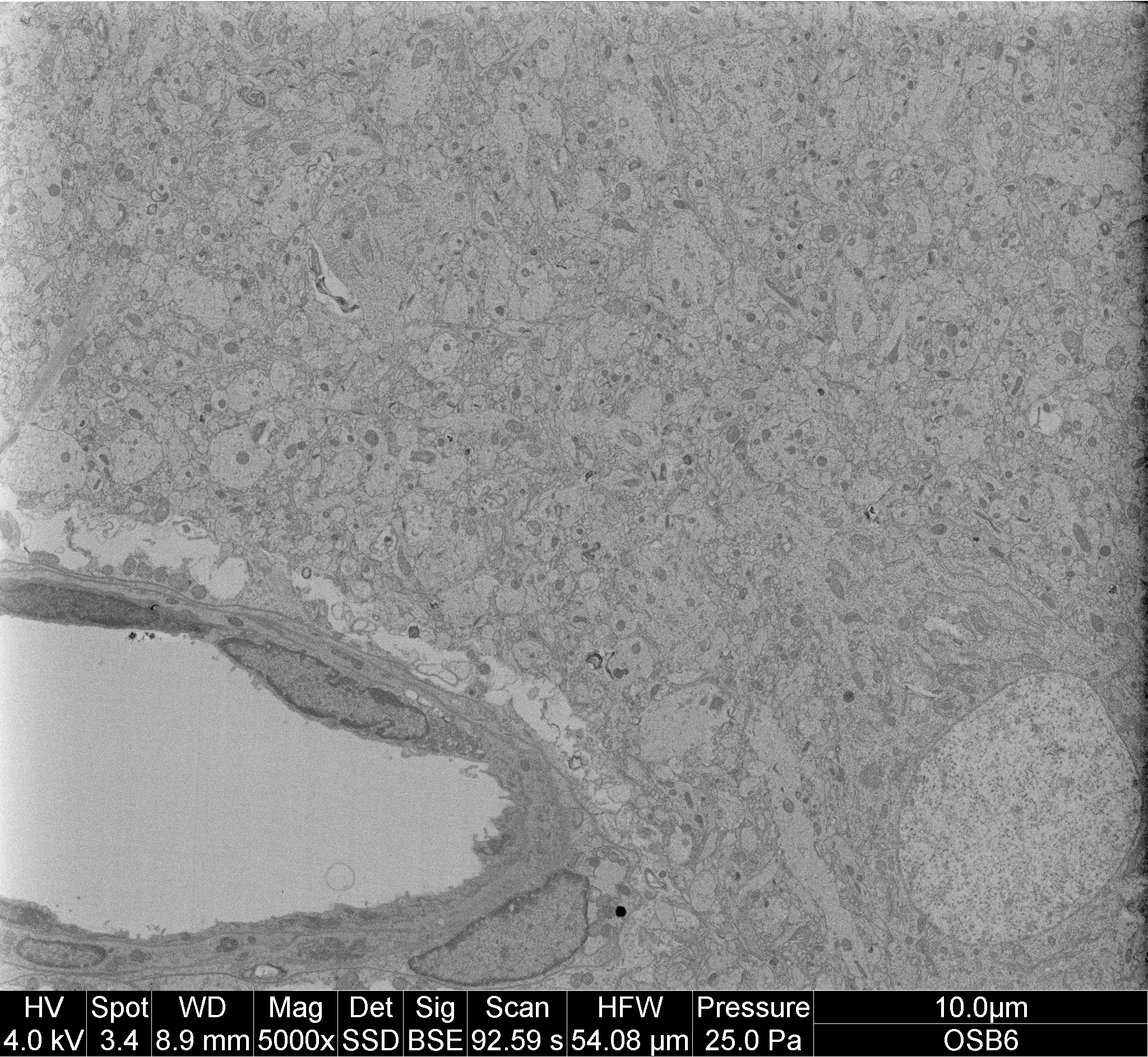

Supplement: Dataset S8 — (255.9 MB ZIP). [file pbio.0020329.sd008.zip › 040604_OS5_st1_767.tif]

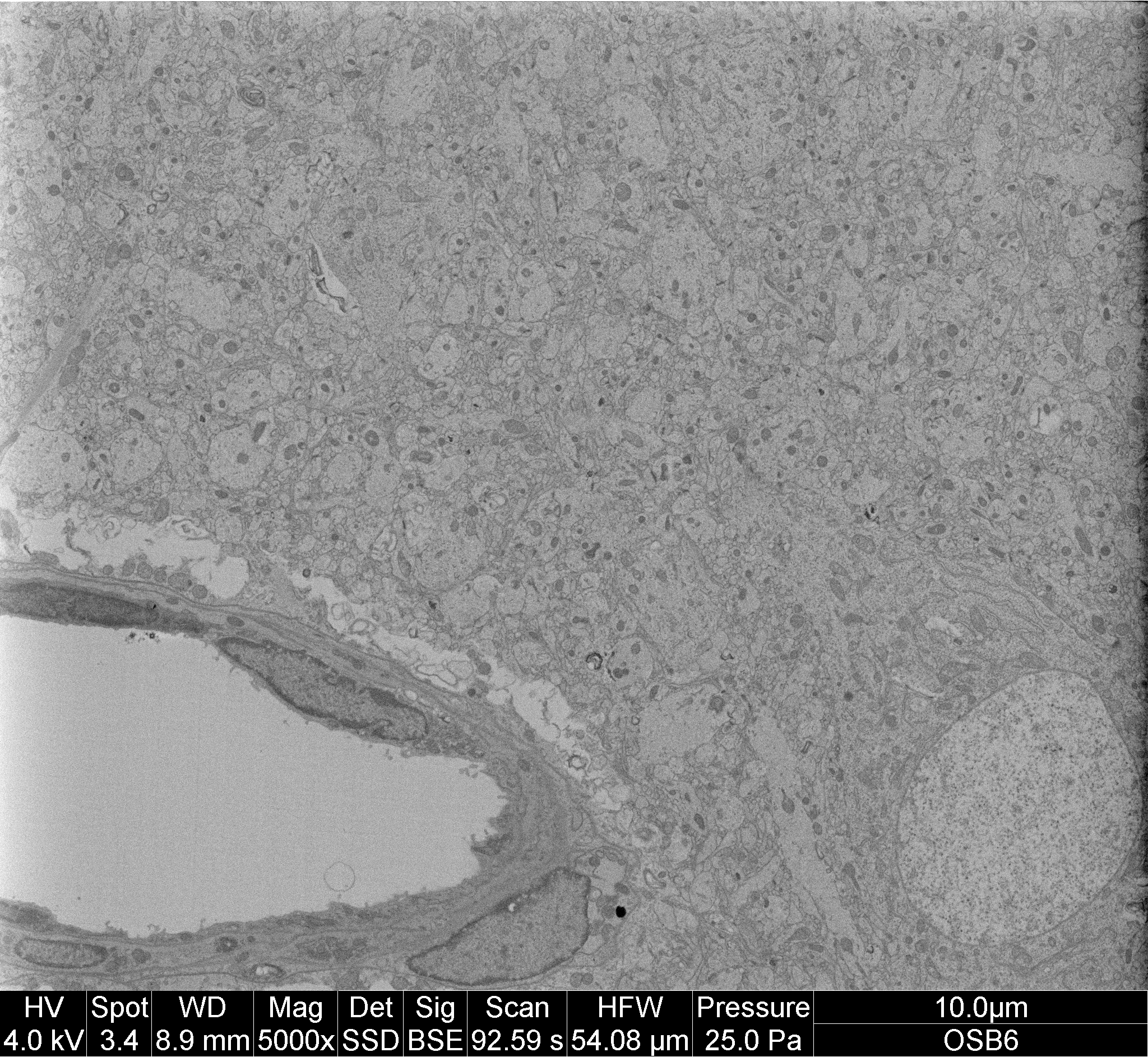

Supplement: Dataset S8 — (255.9 MB ZIP). [file pbio.0020329.sd008.zip › 040604_OS5_st1_768.tif]

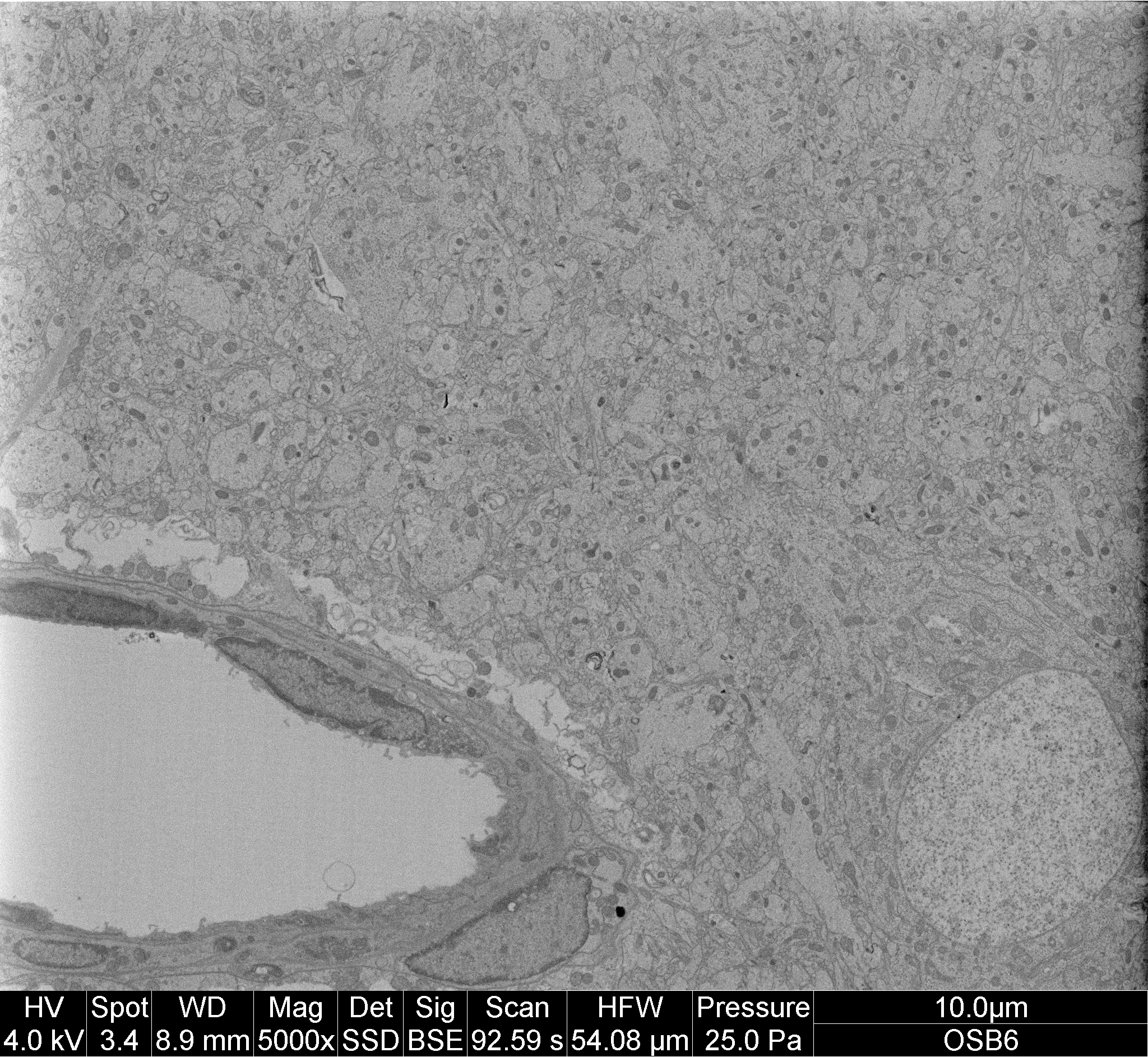

Supplement: Dataset S8 — (255.9 MB ZIP). [file pbio.0020329.sd008.zip › 040604_OS5_st1_769.tif]

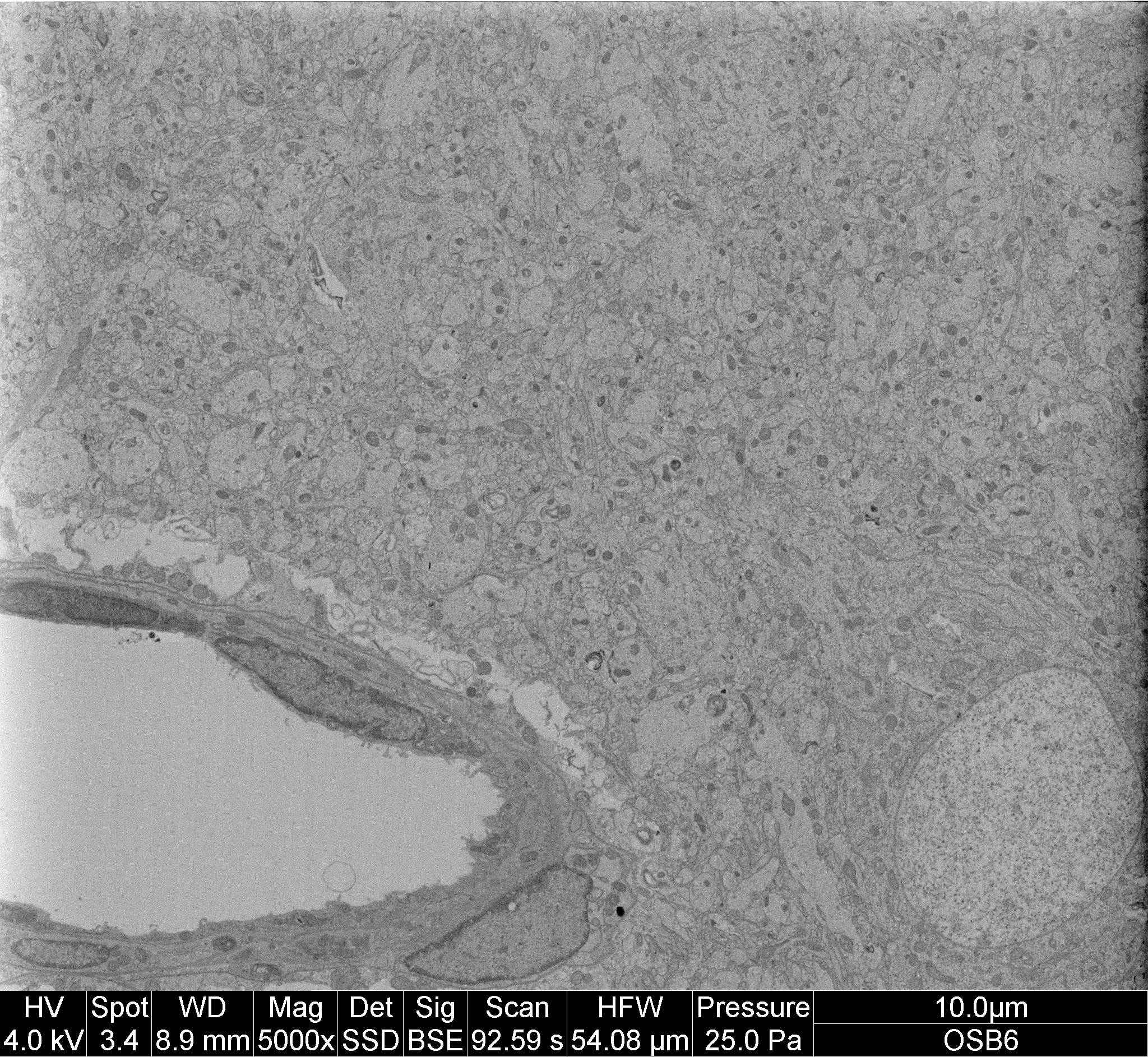

Supplement: Dataset S8 — (255.9 MB ZIP). [file pbio.0020329.sd008.zip › 040604_OS5_st1_770.tif]

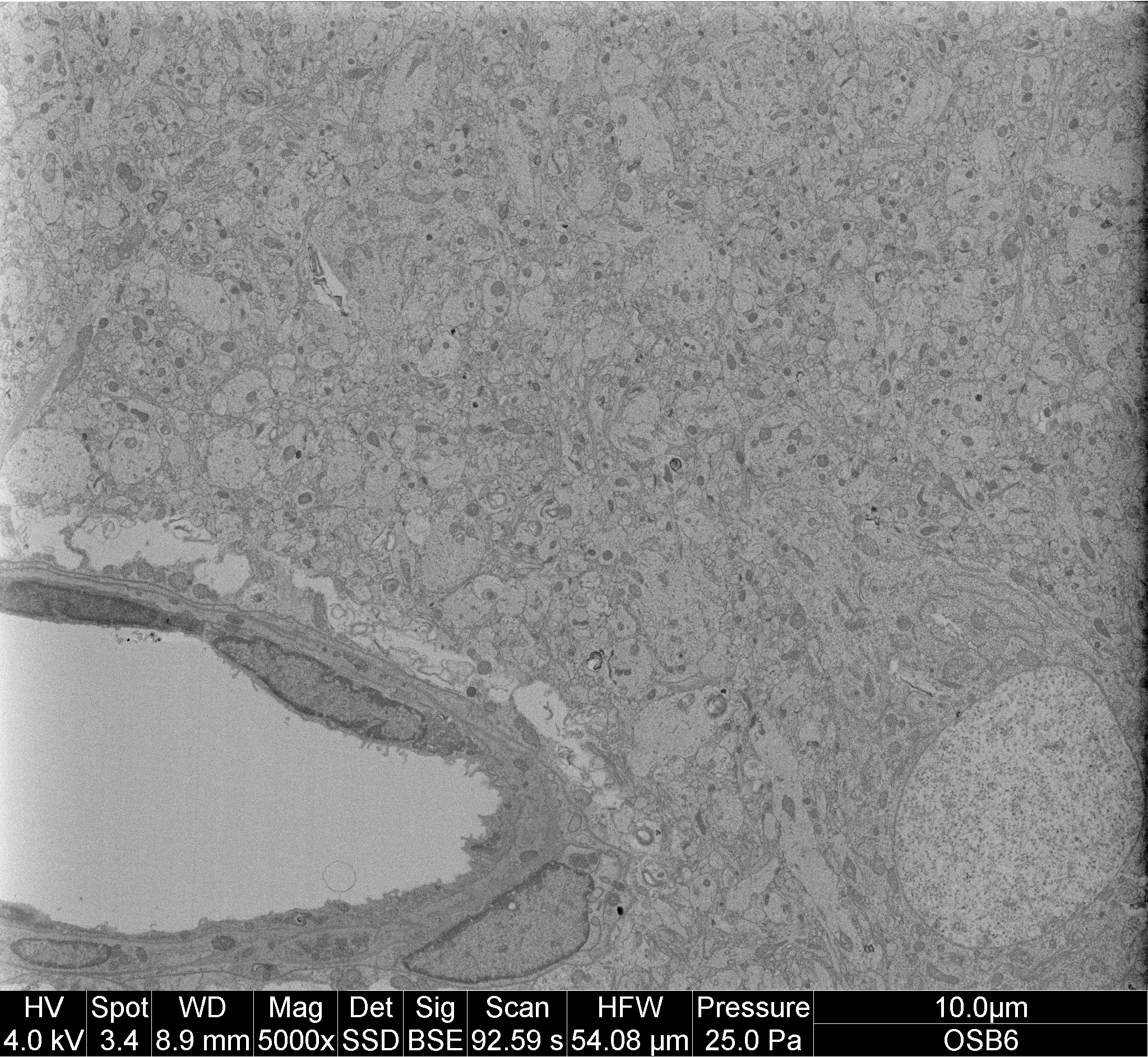

Supplement: Dataset S8 — (255.9 MB ZIP). [file pbio.0020329.sd008.zip › 040604_OS5_st1_771.tif]

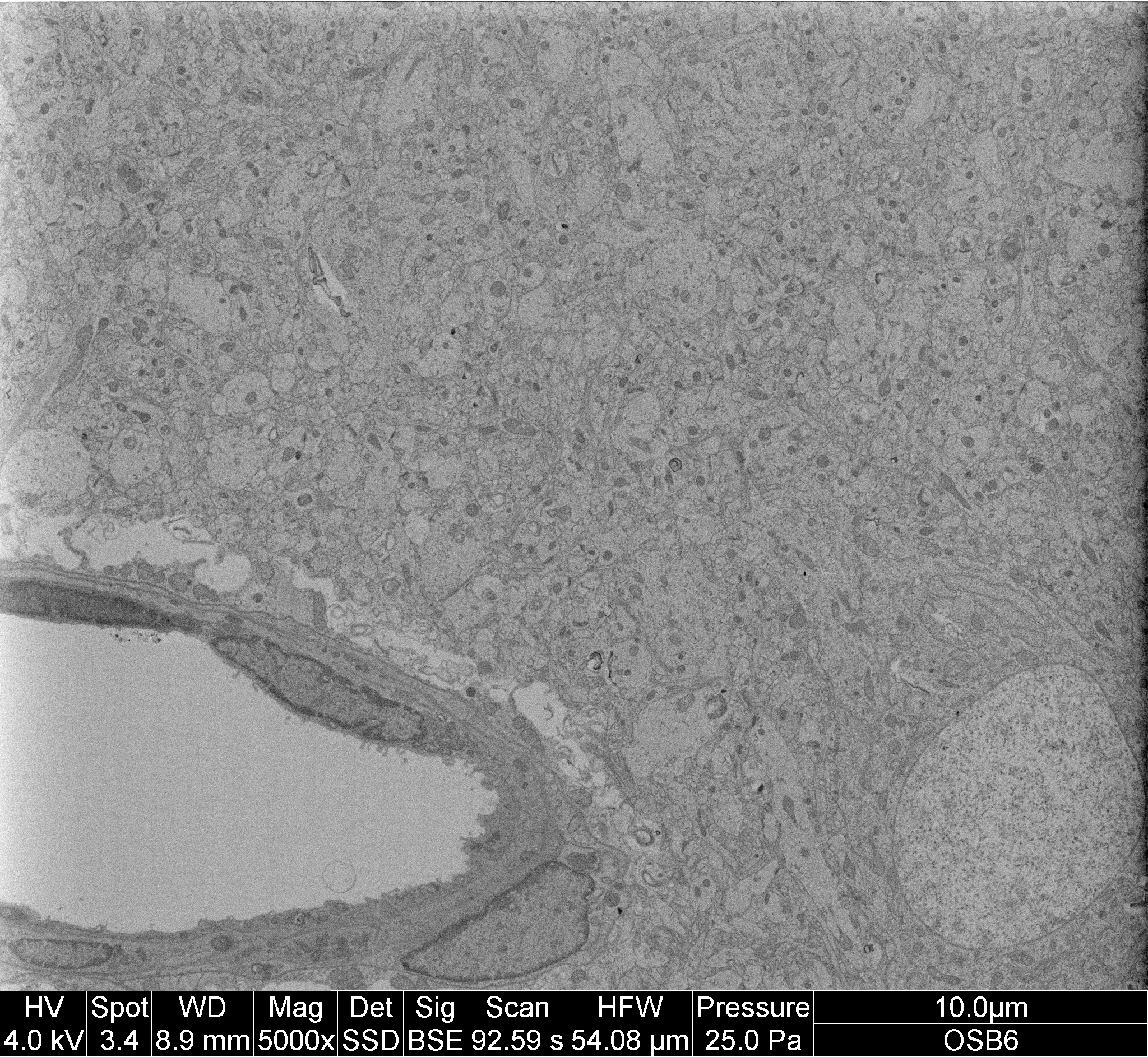

Supplement: Dataset S8 — (255.9 MB ZIP). [file pbio.0020329.sd008.zip › 040604_OS5_st1_772.tif]

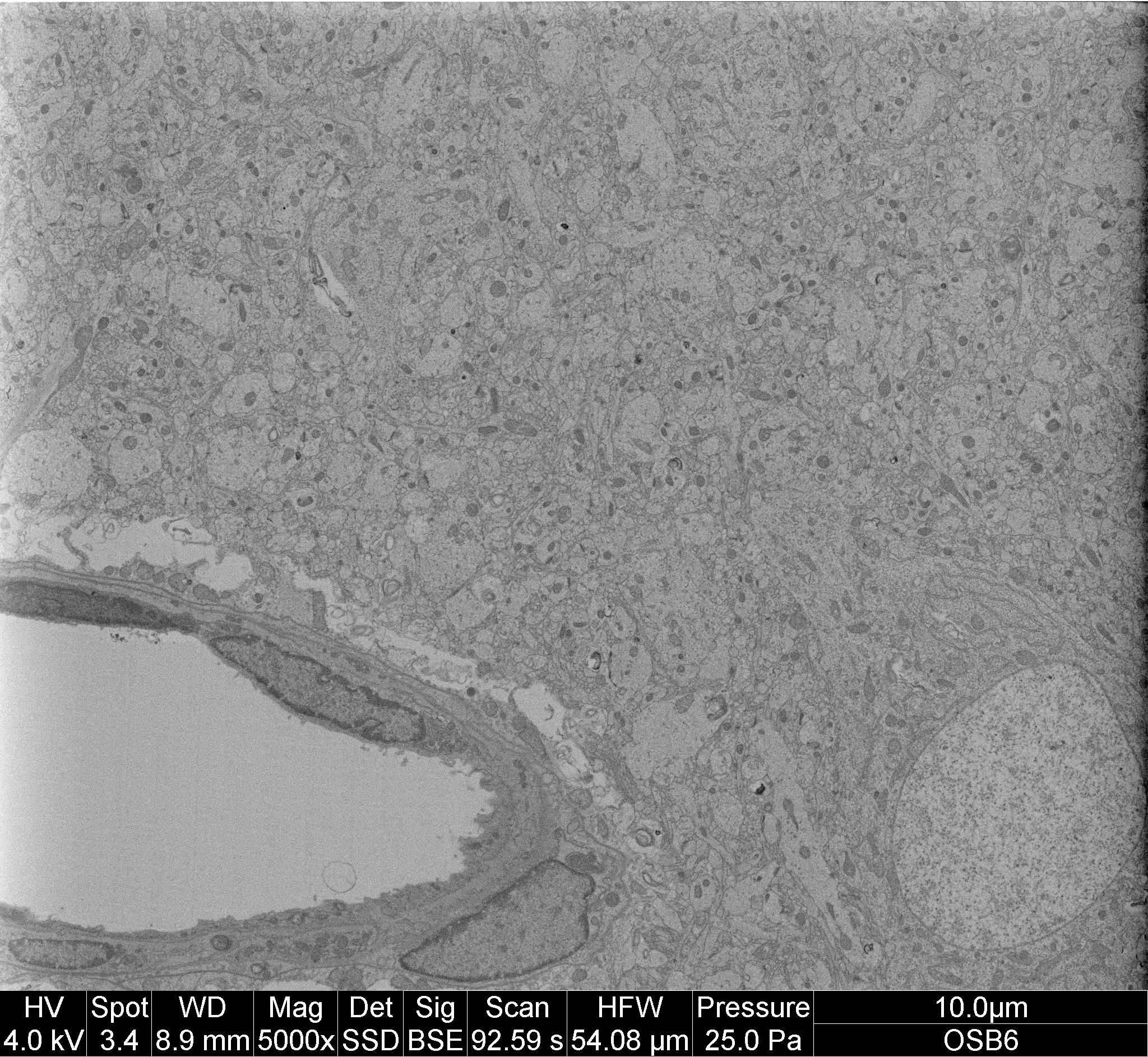

Supplement: Dataset S8 — (255.9 MB ZIP). [file pbio.0020329.sd008.zip › 040604_OS5_st1_773.tif]

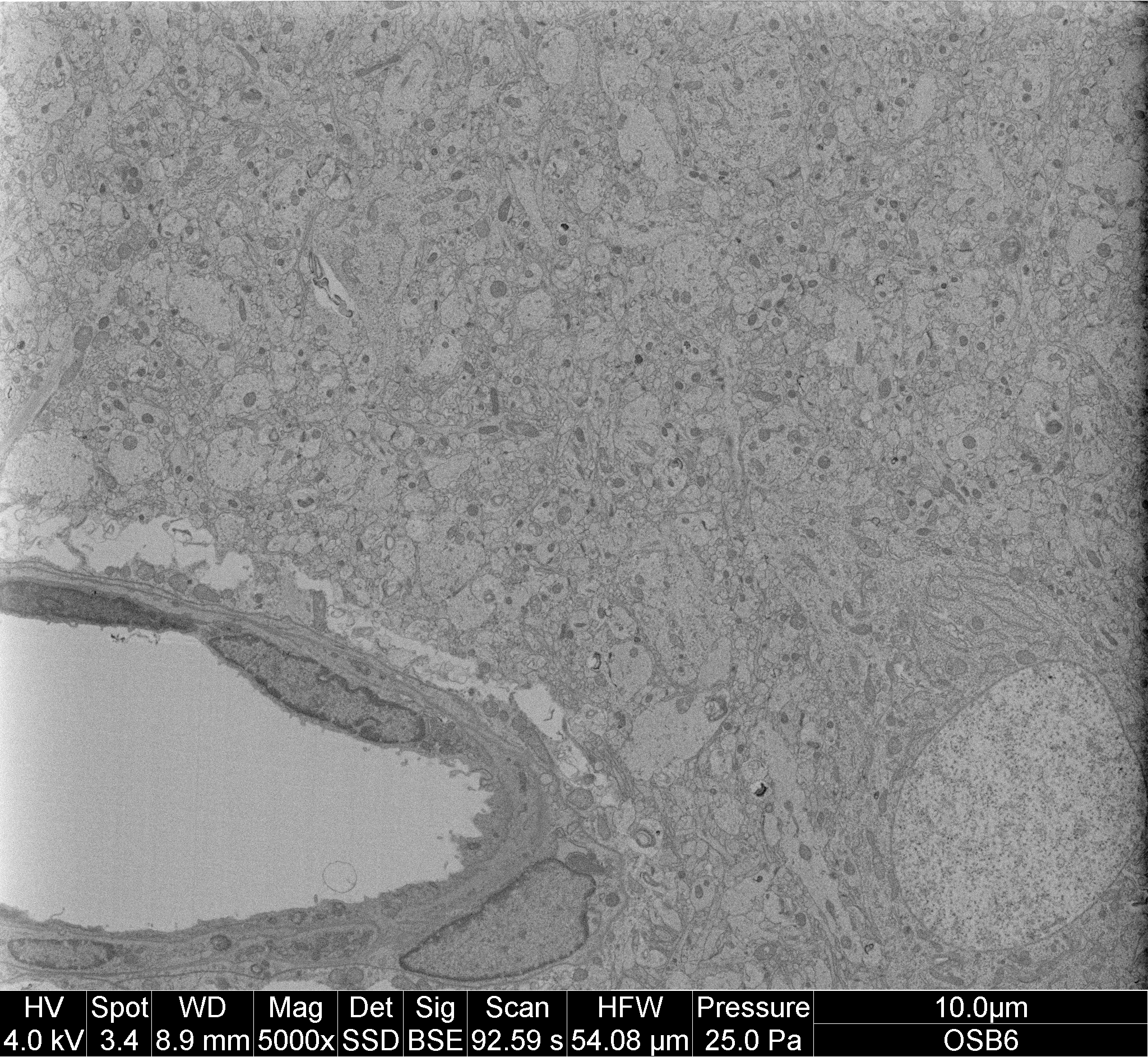

Supplement: Dataset S8 — (255.9 MB ZIP). [file pbio.0020329.sd008.zip › 040604_OS5_st1_774.tif]

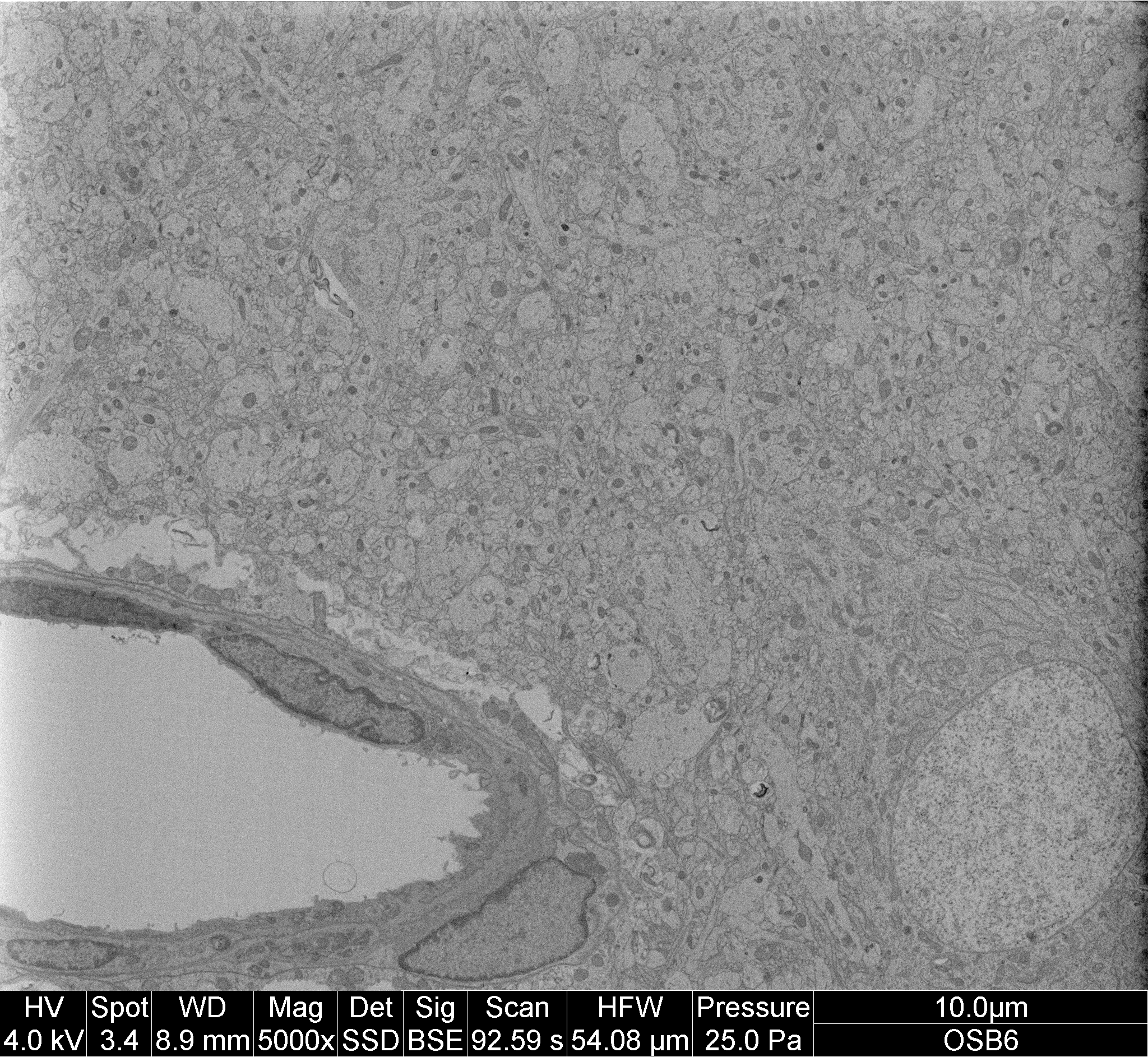

Supplement: Dataset S8 — (255.9 MB ZIP). [file pbio.0020329.sd008.zip › 040604_OS5_st1_775.tif]

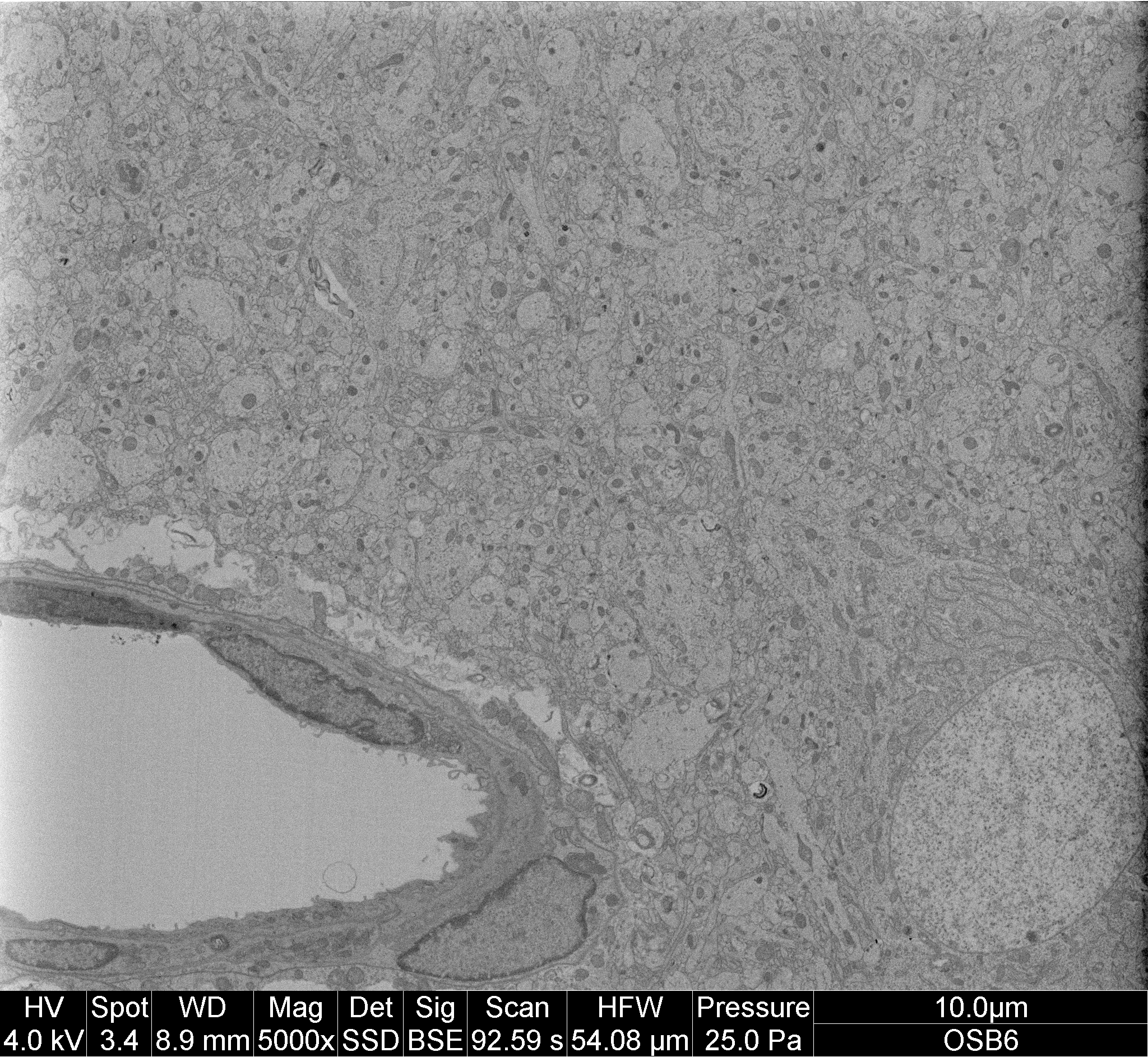

Supplement: Dataset S8 — (255.9 MB ZIP). [file pbio.0020329.sd008.zip › 040604_OS5_st1_776.tif]

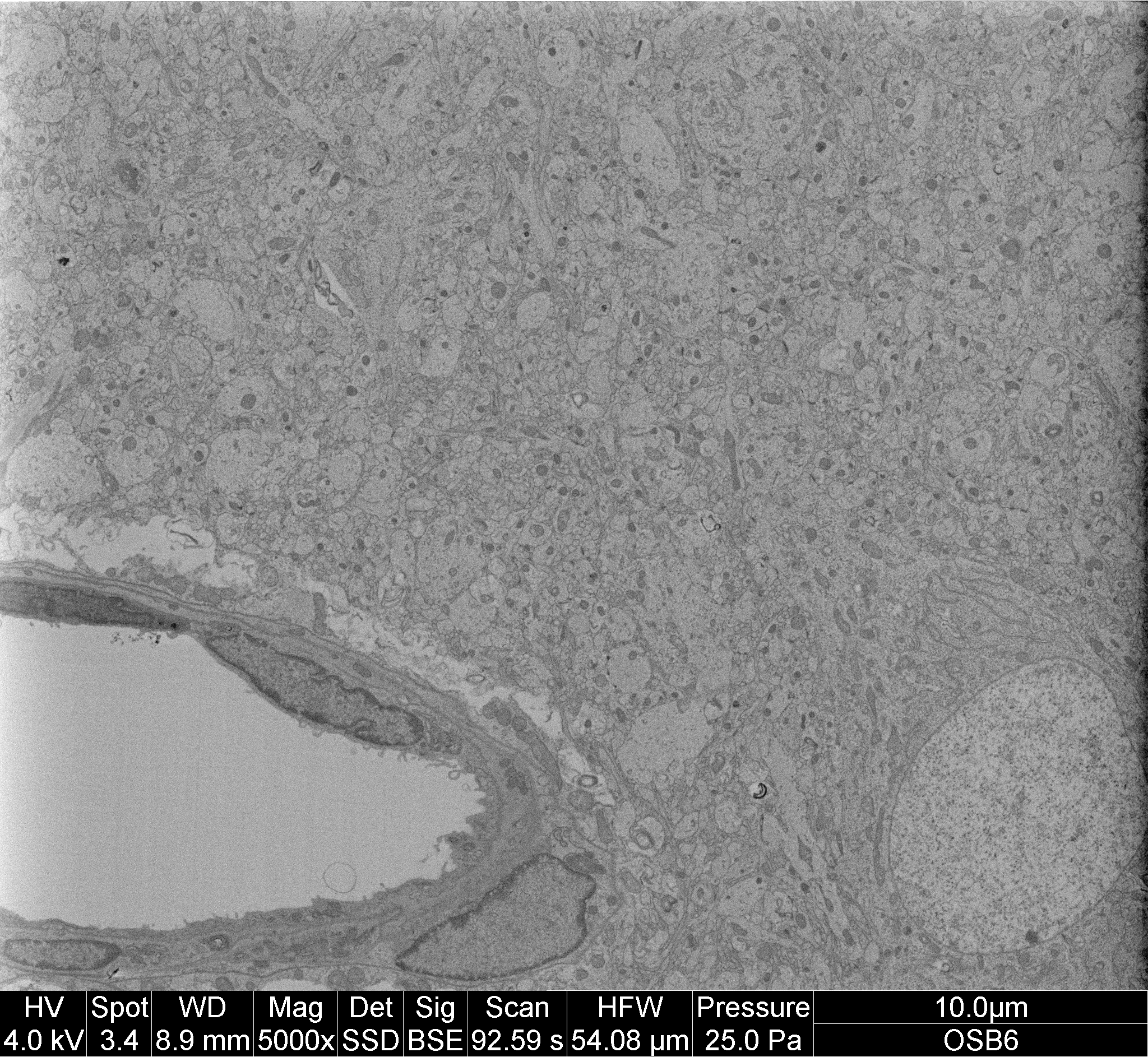

Supplement: Dataset S8 — (255.9 MB ZIP). [file pbio.0020329.sd008.zip › 040604_OS5_st1_777.tif]

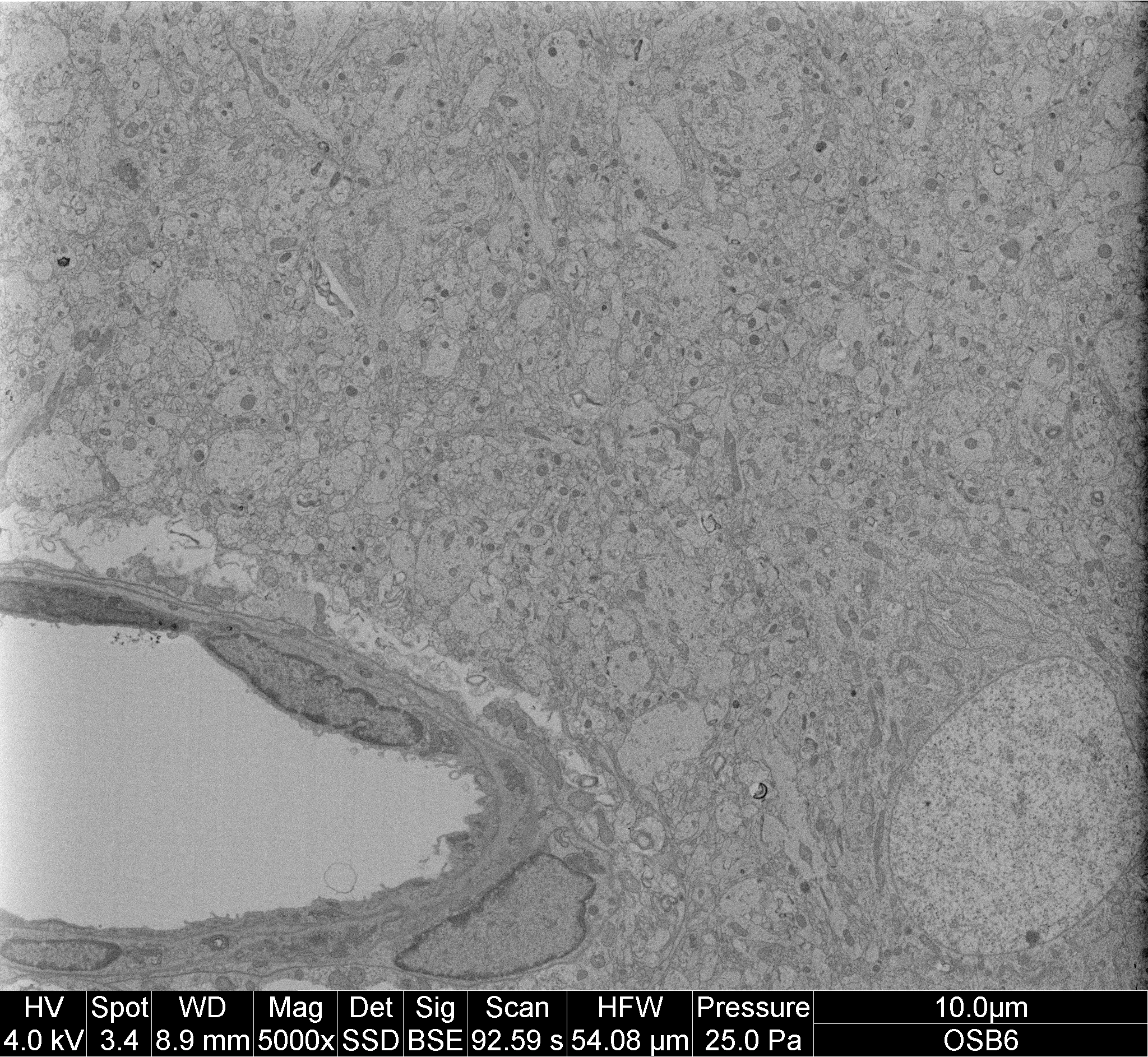

Supplement: Dataset S8 — (255.9 MB ZIP). [file pbio.0020329.sd008.zip › 040604_OS5_st1_778.tif]

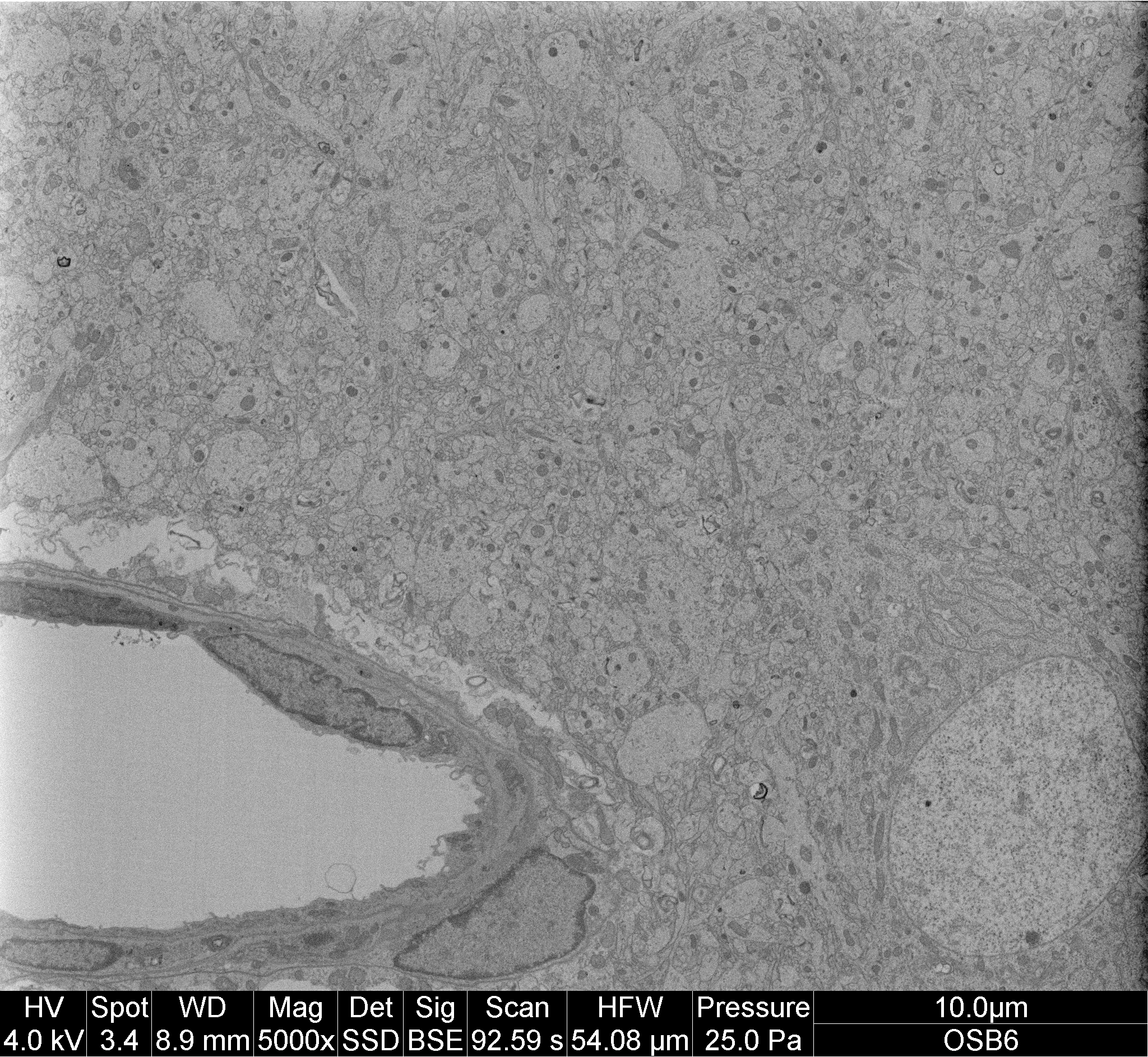

Supplement: Dataset S8 — (255.9 MB ZIP). [file pbio.0020329.sd008.zip › 040604_OS5_st1_779.tif]

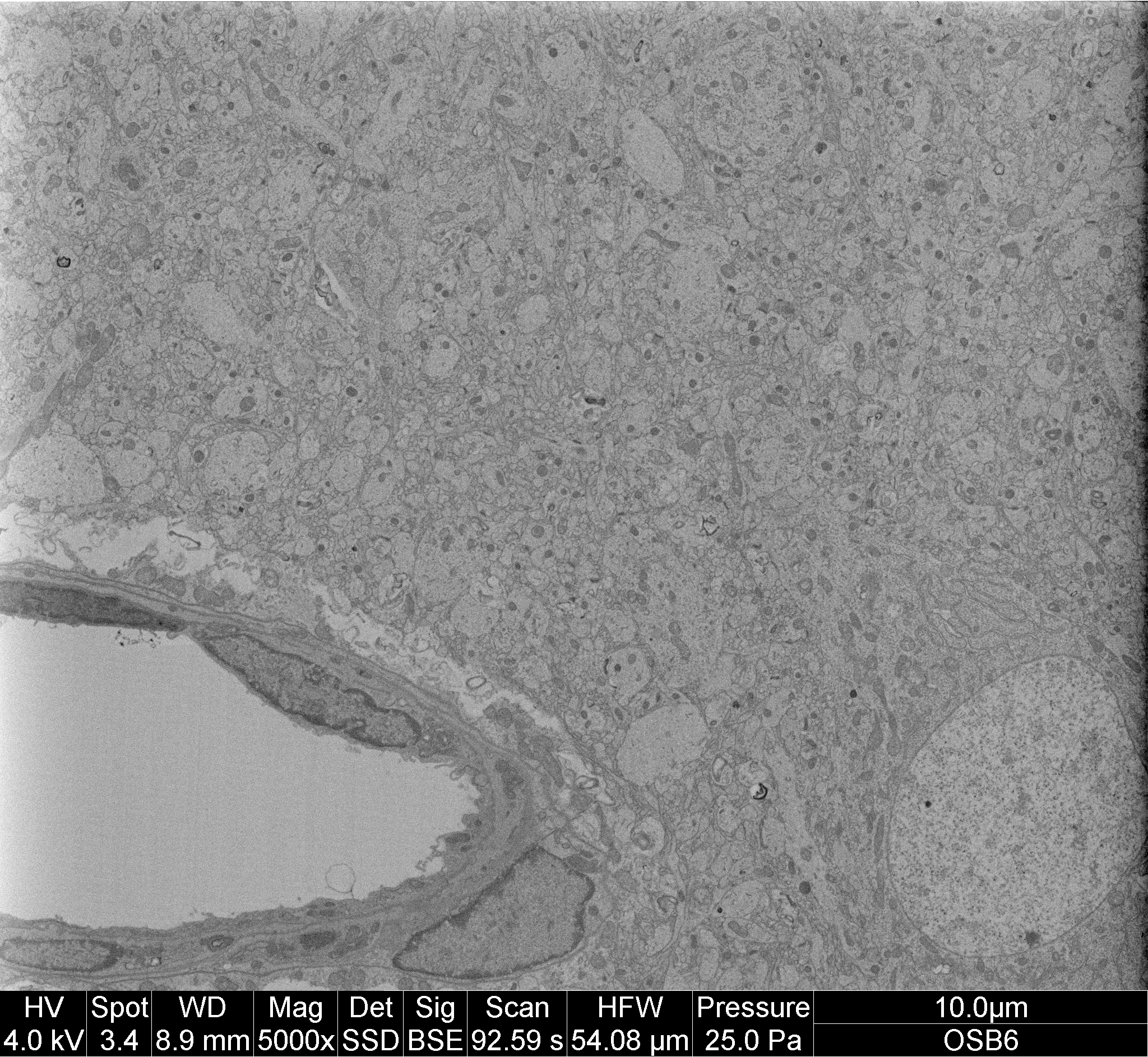

Supplement: Dataset S8 — (255.9 MB ZIP). [file pbio.0020329.sd008.zip › 040604_OS5_st1_780.tif]

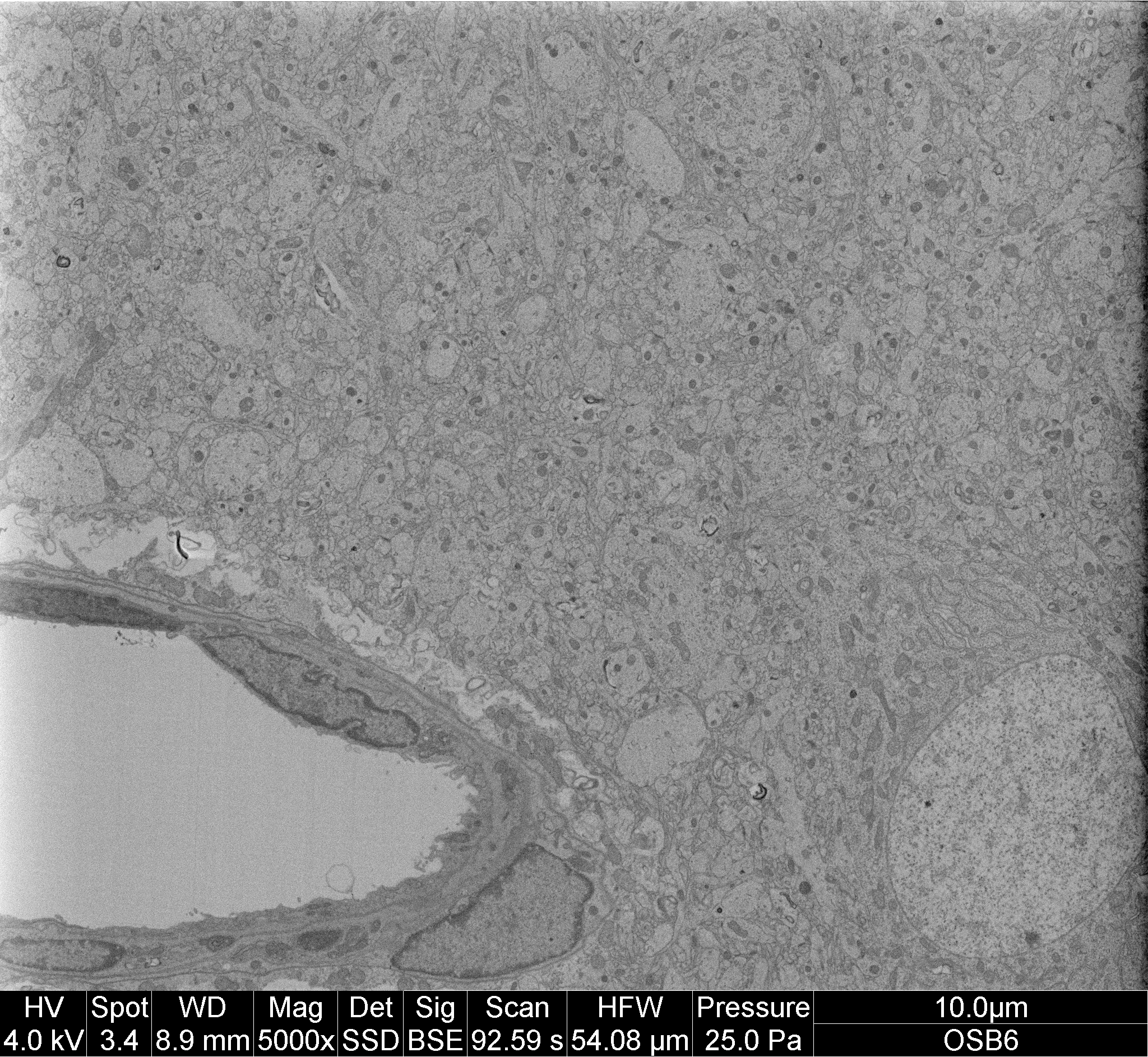

Supplement: Dataset S8 — (255.9 MB ZIP). [file pbio.0020329.sd008.zip › 040604_OS5_st1_781.tif]

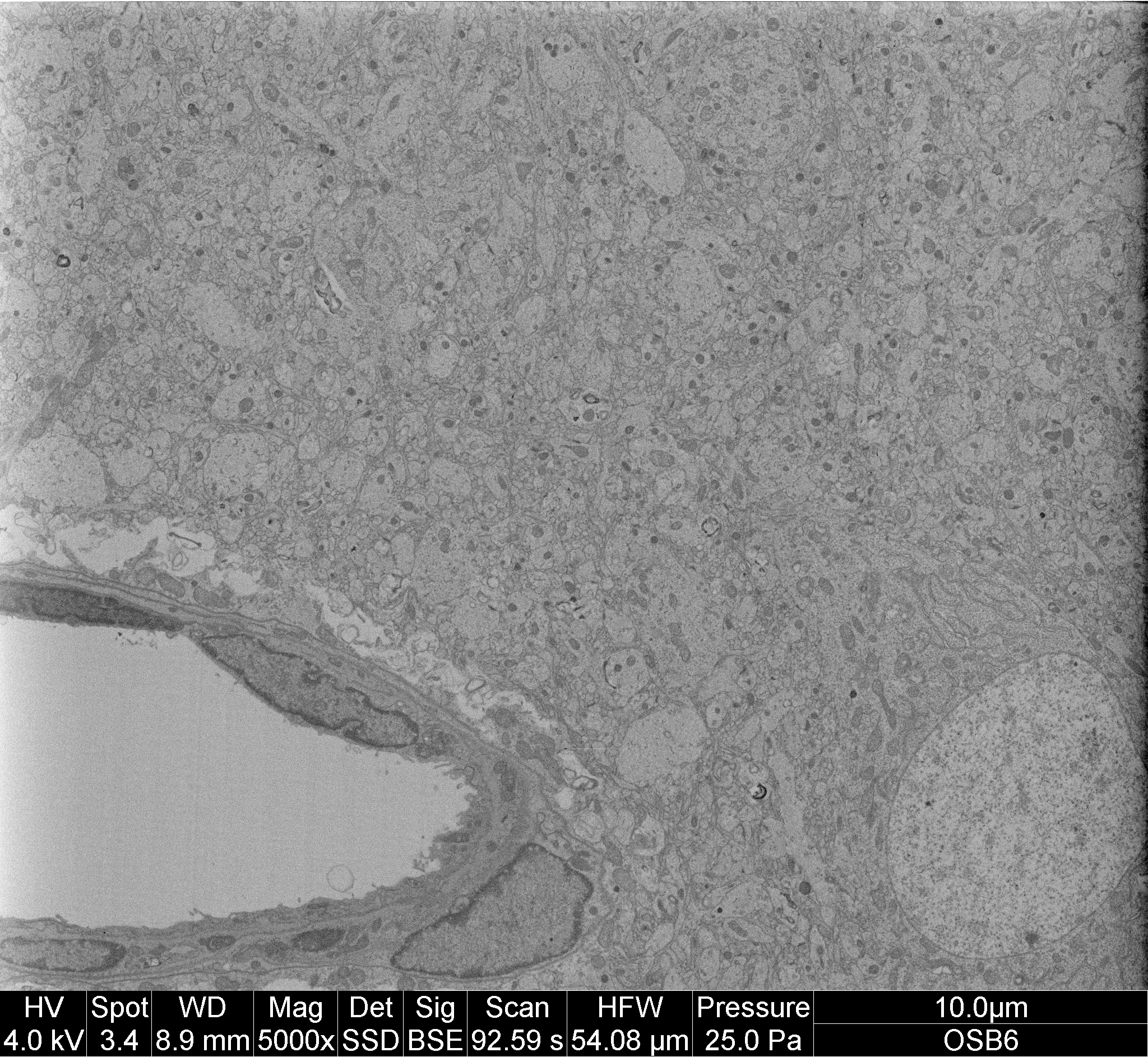

Supplement: Dataset S8 — (255.9 MB ZIP). [file pbio.0020329.sd008.zip › 040604_OS5_st1_782.tif]

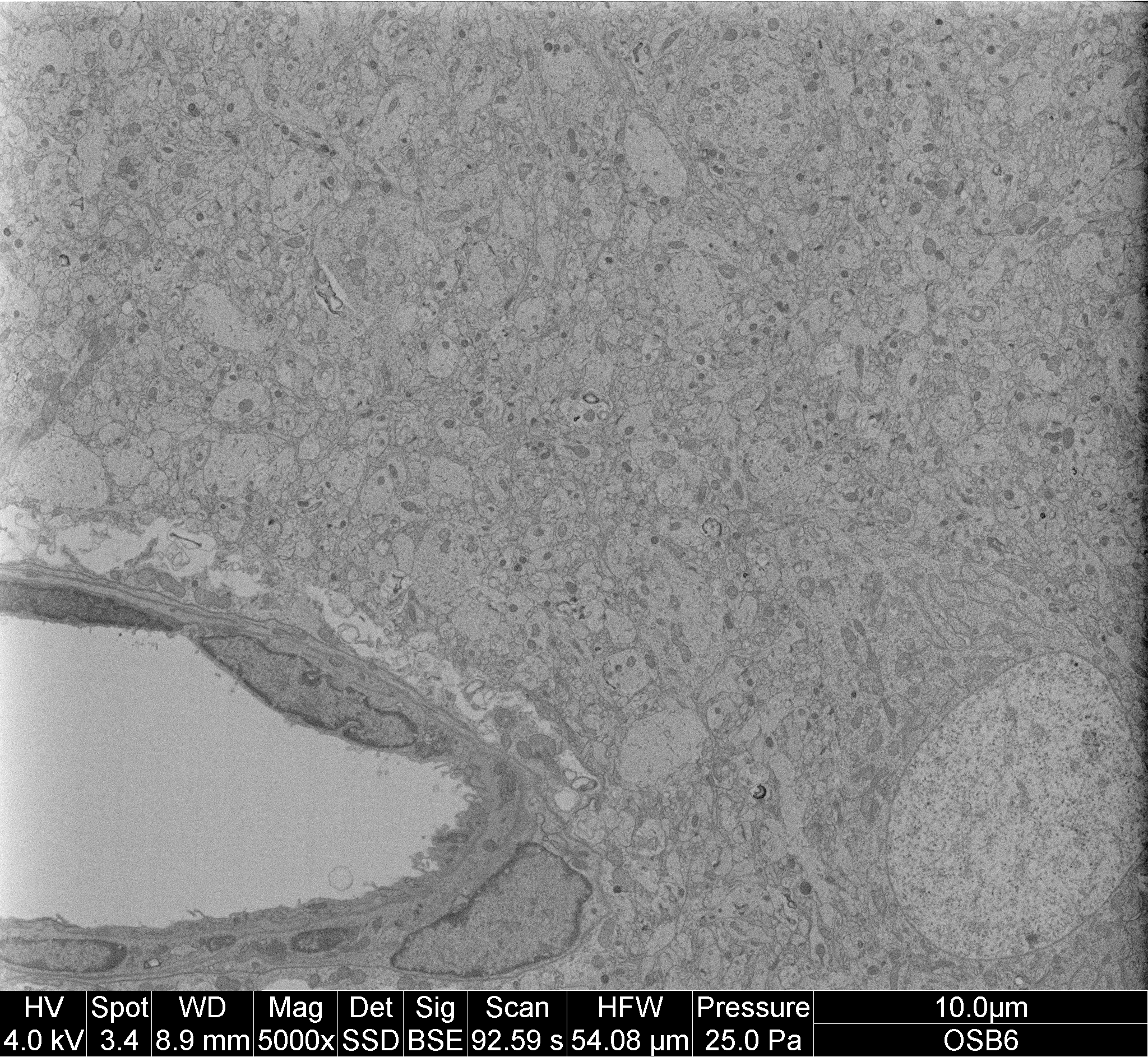

Supplement: Dataset S8 — (255.9 MB ZIP). [file pbio.0020329.sd008.zip › 040604_OS5_st1_783.tif]

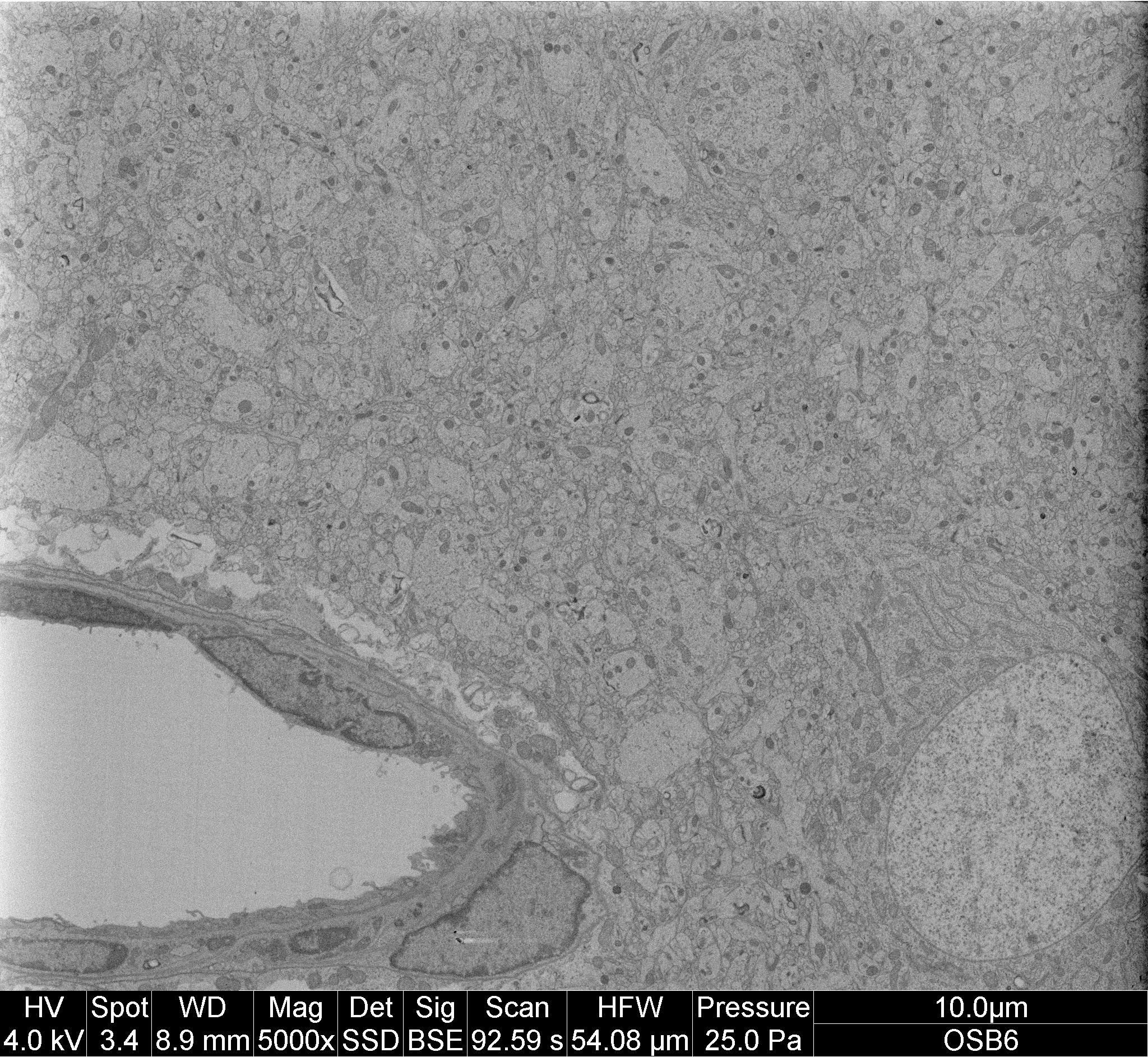

Supplement: Dataset S8 — (255.9 MB ZIP). [file pbio.0020329.sd008.zip › 040604_OS5_st1_784.tif]

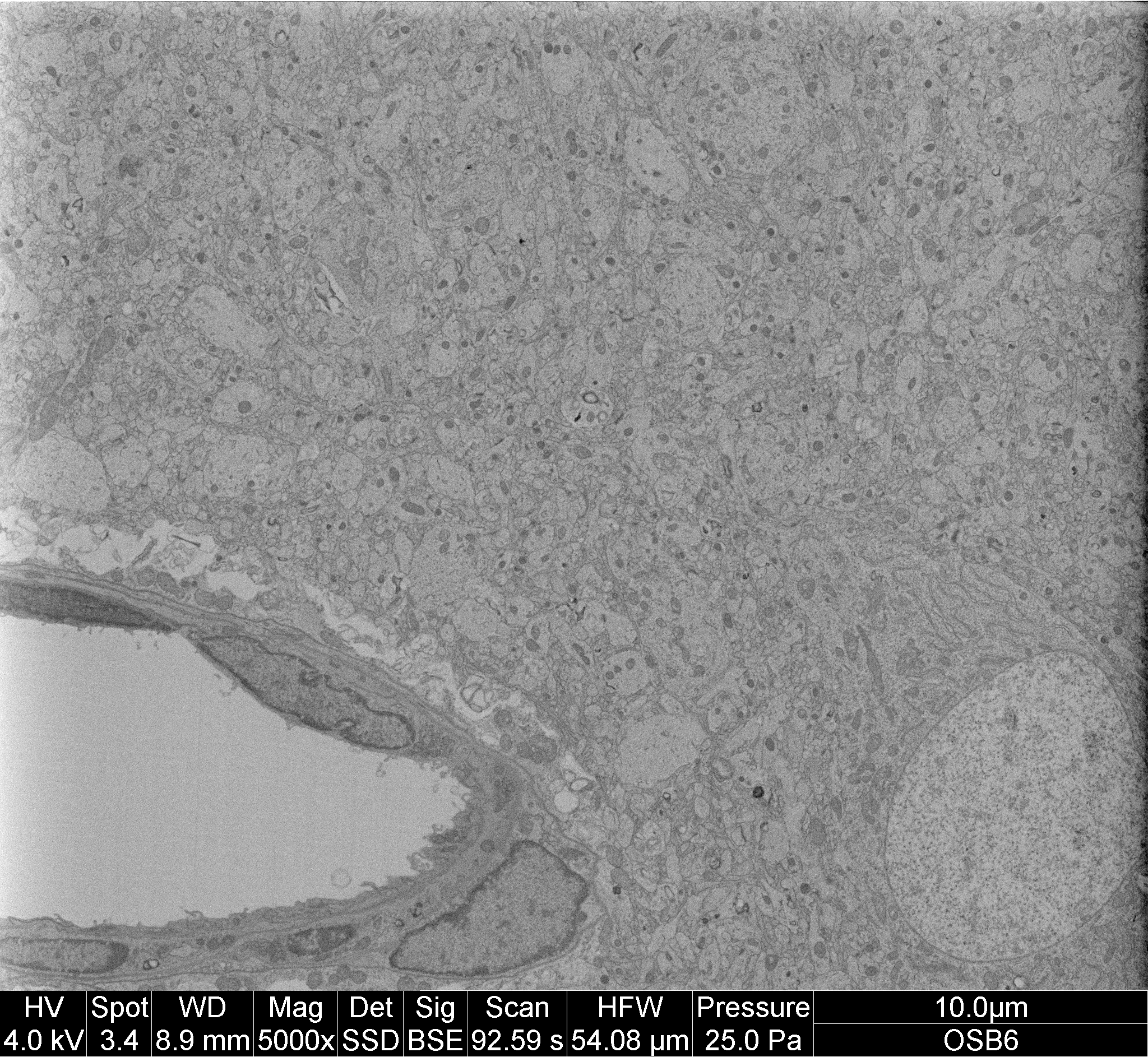

Supplement: Dataset S8 — (255.9 MB ZIP). [file pbio.0020329.sd008.zip › 040604_OS5_st1_785.tif]

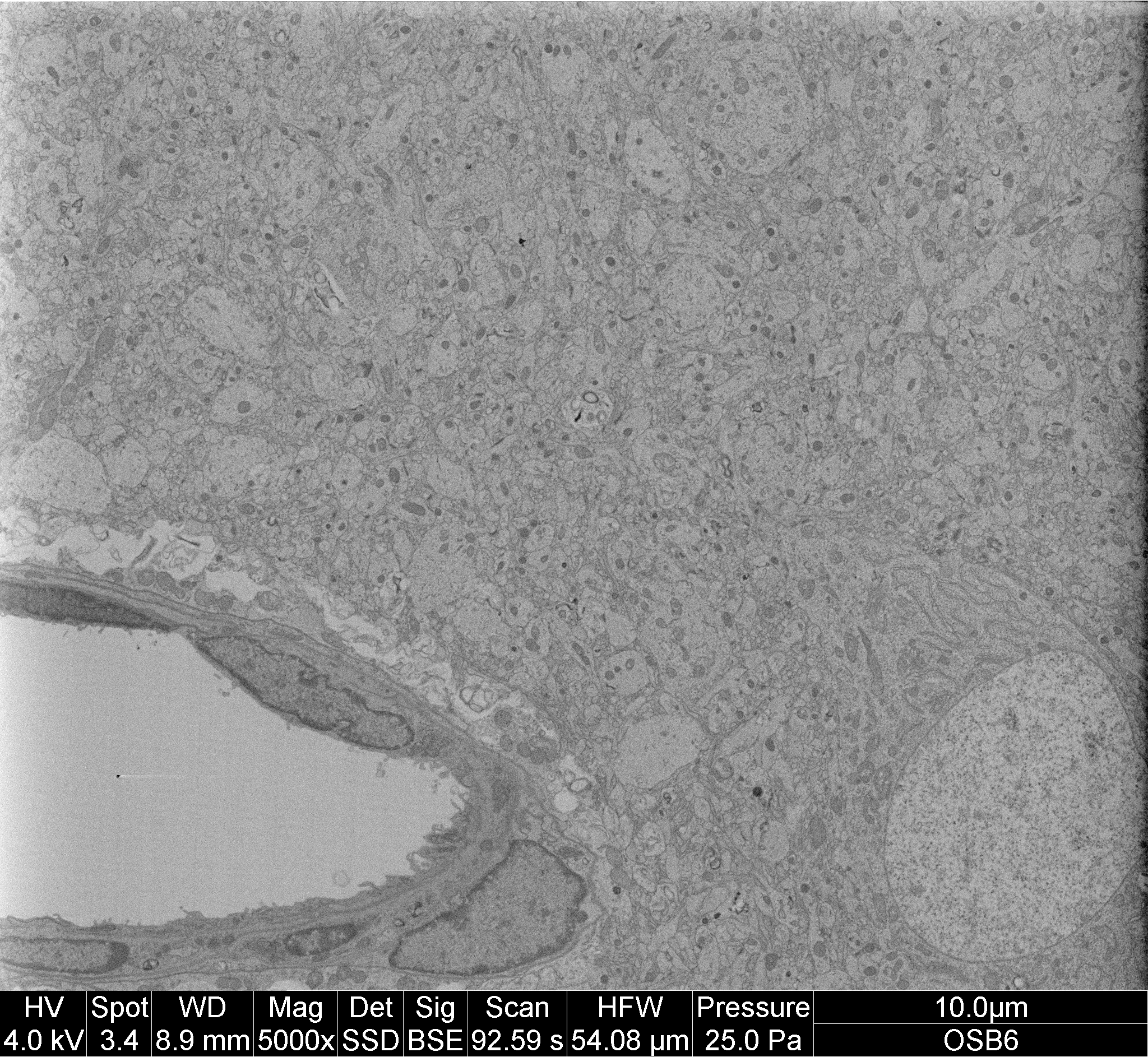

Supplement: Dataset S8 — (255.9 MB ZIP). [file pbio.0020329.sd008.zip › 040604_OS5_st1_786.tif]

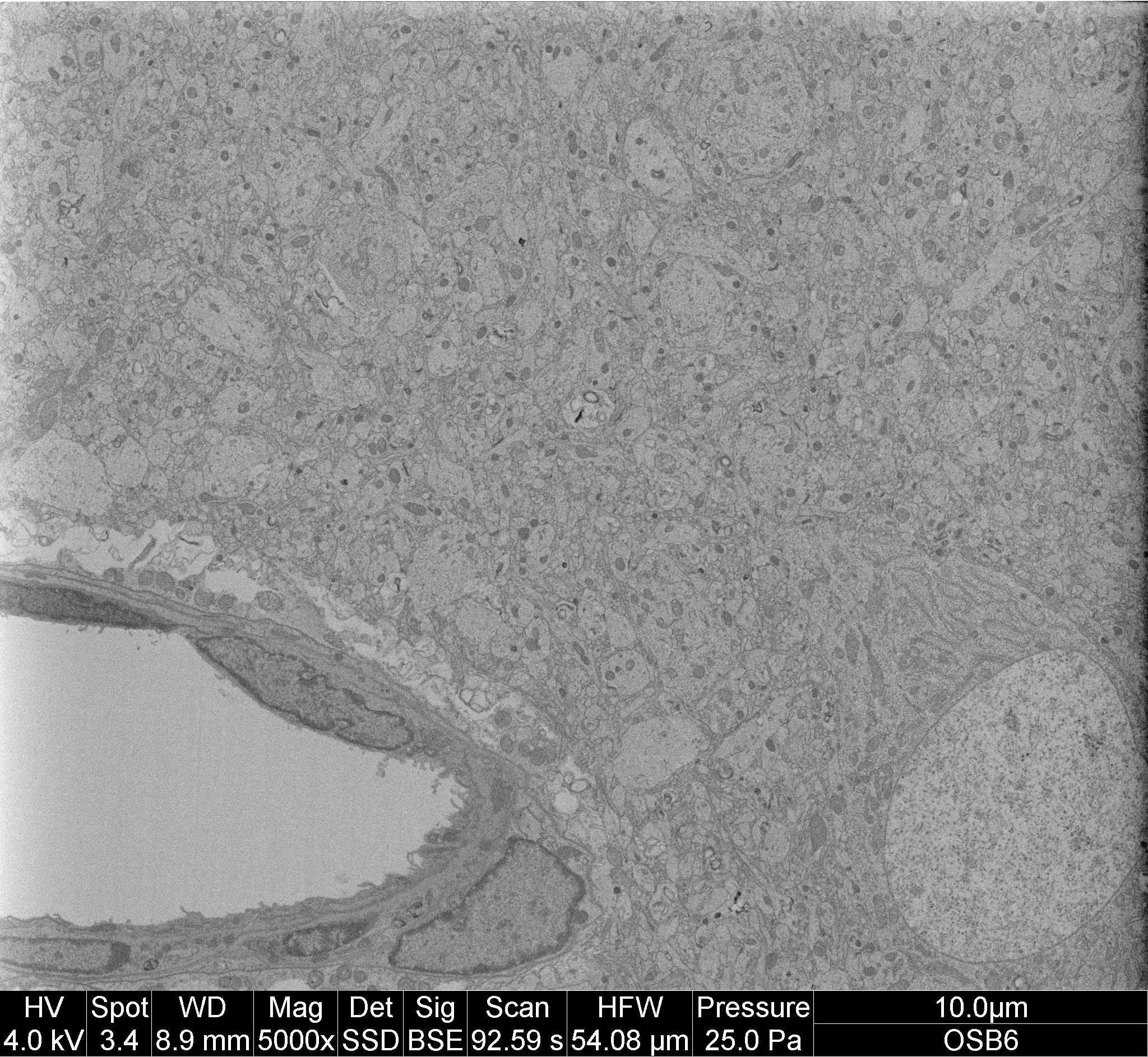

Supplement: Dataset S8 — (255.9 MB ZIP). [file pbio.0020329.sd008.zip › 040604_OS5_st1_787.tif]

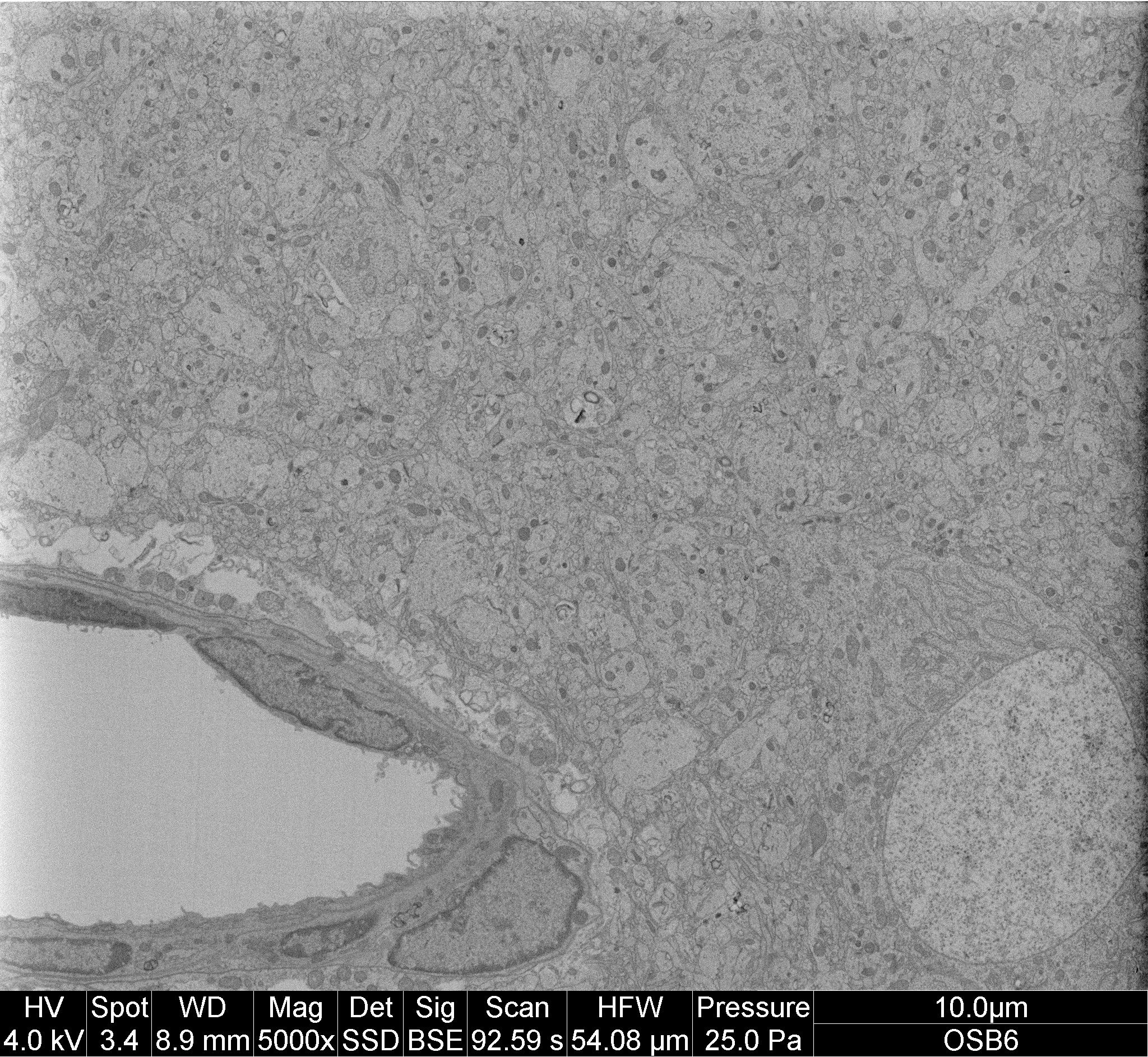

Supplement: Dataset S8 — (255.9 MB ZIP). [file pbio.0020329.sd008.zip › 040604_OS5_st1_788.tif]

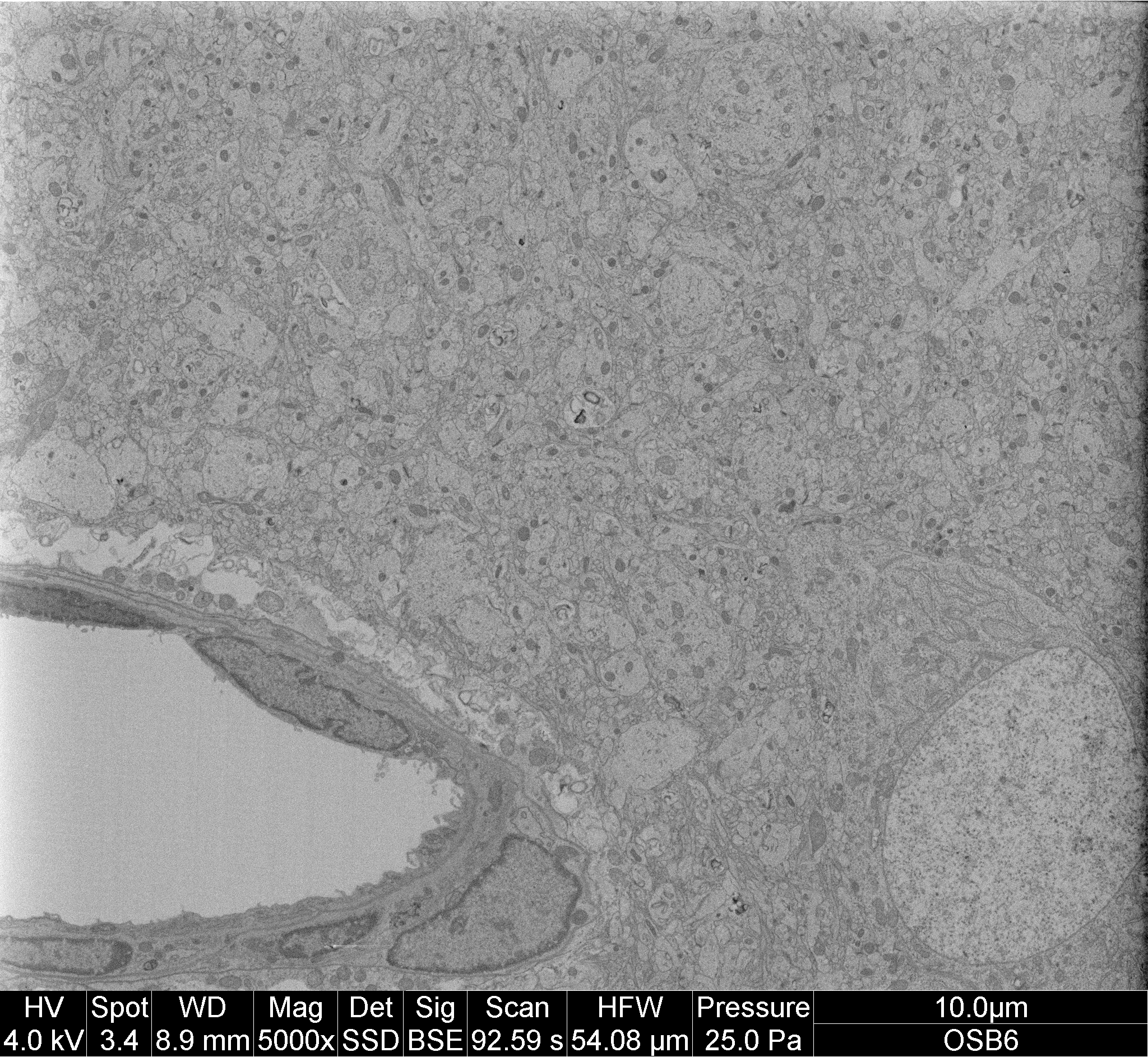

Supplement: Dataset S8 — (255.9 MB ZIP). [file pbio.0020329.sd008.zip › 040604_OS5_st1_789.tif]

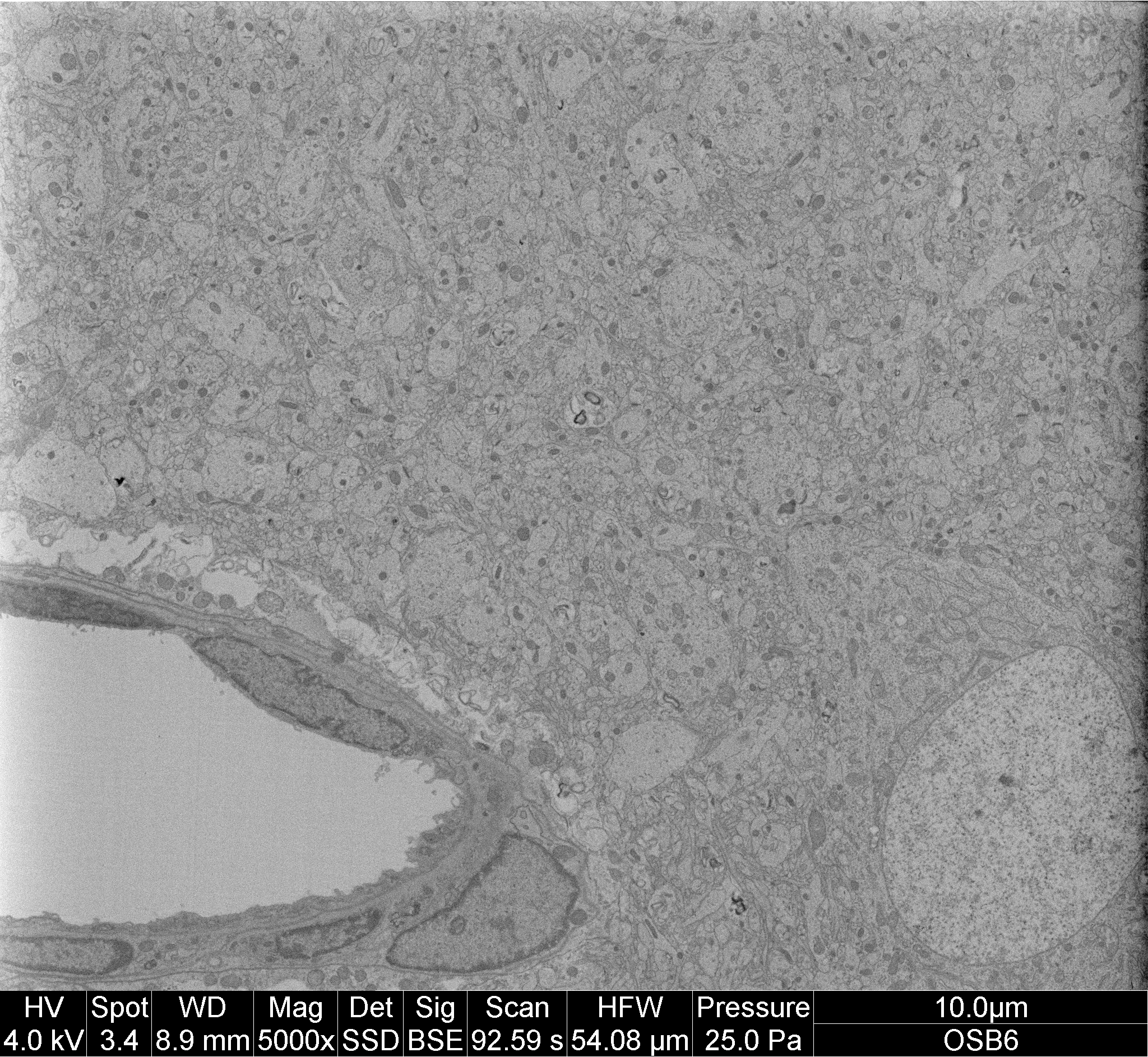

Supplement: Dataset S8 — (255.9 MB ZIP). [file pbio.0020329.sd008.zip › 040604_OS5_st1_790.tif]

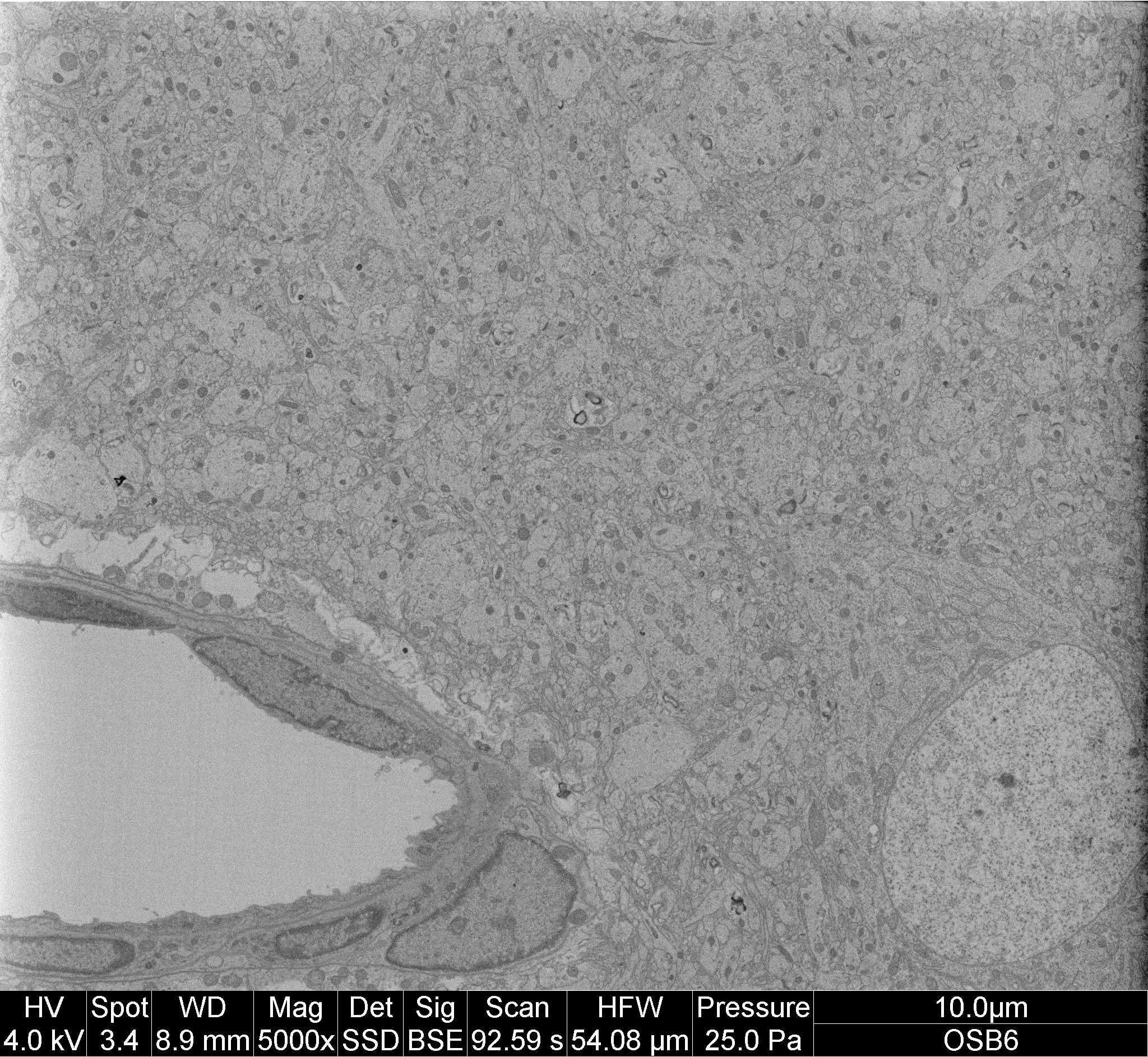

Supplement: Dataset S8 — (255.9 MB ZIP). [file pbio.0020329.sd008.zip › 040604_OS5_st1_791.tif]

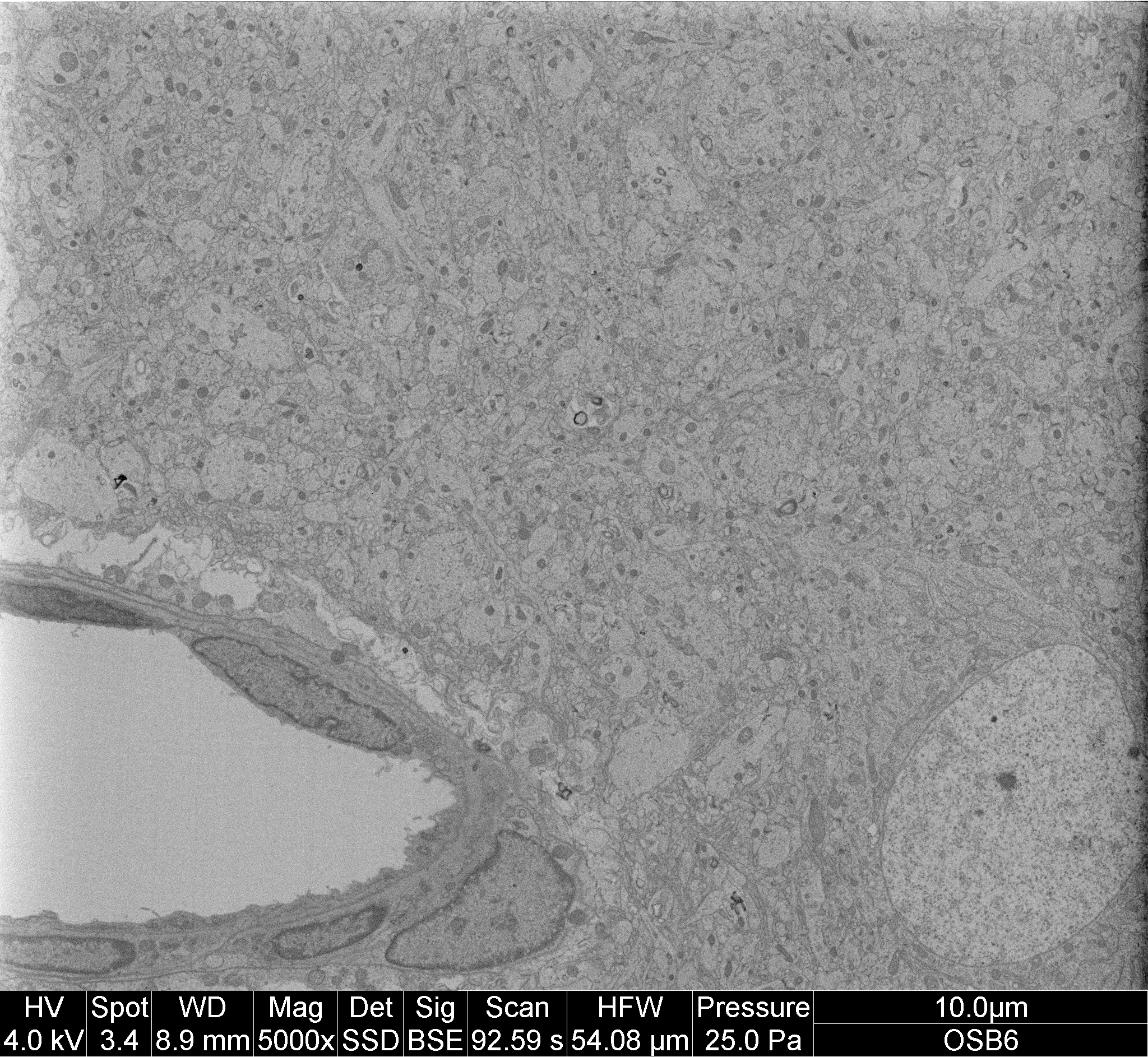

Supplement: Dataset S8 — (255.9 MB ZIP). [file pbio.0020329.sd008.zip › 040604_OS5_st1_792.tif]

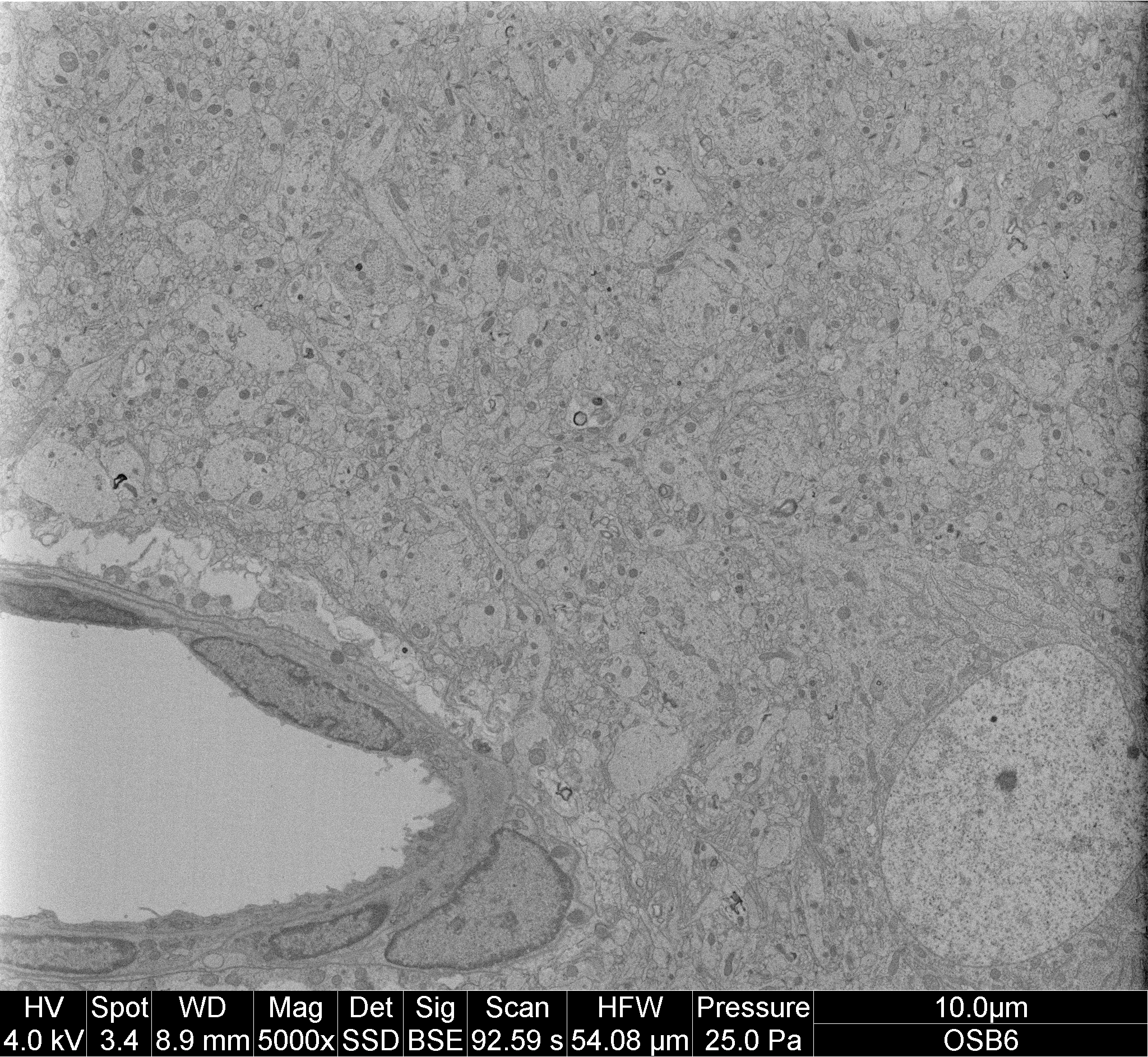

Supplement: Dataset S8 — (255.9 MB ZIP). [file pbio.0020329.sd008.zip › 040604_OS5_st1_793.tif]

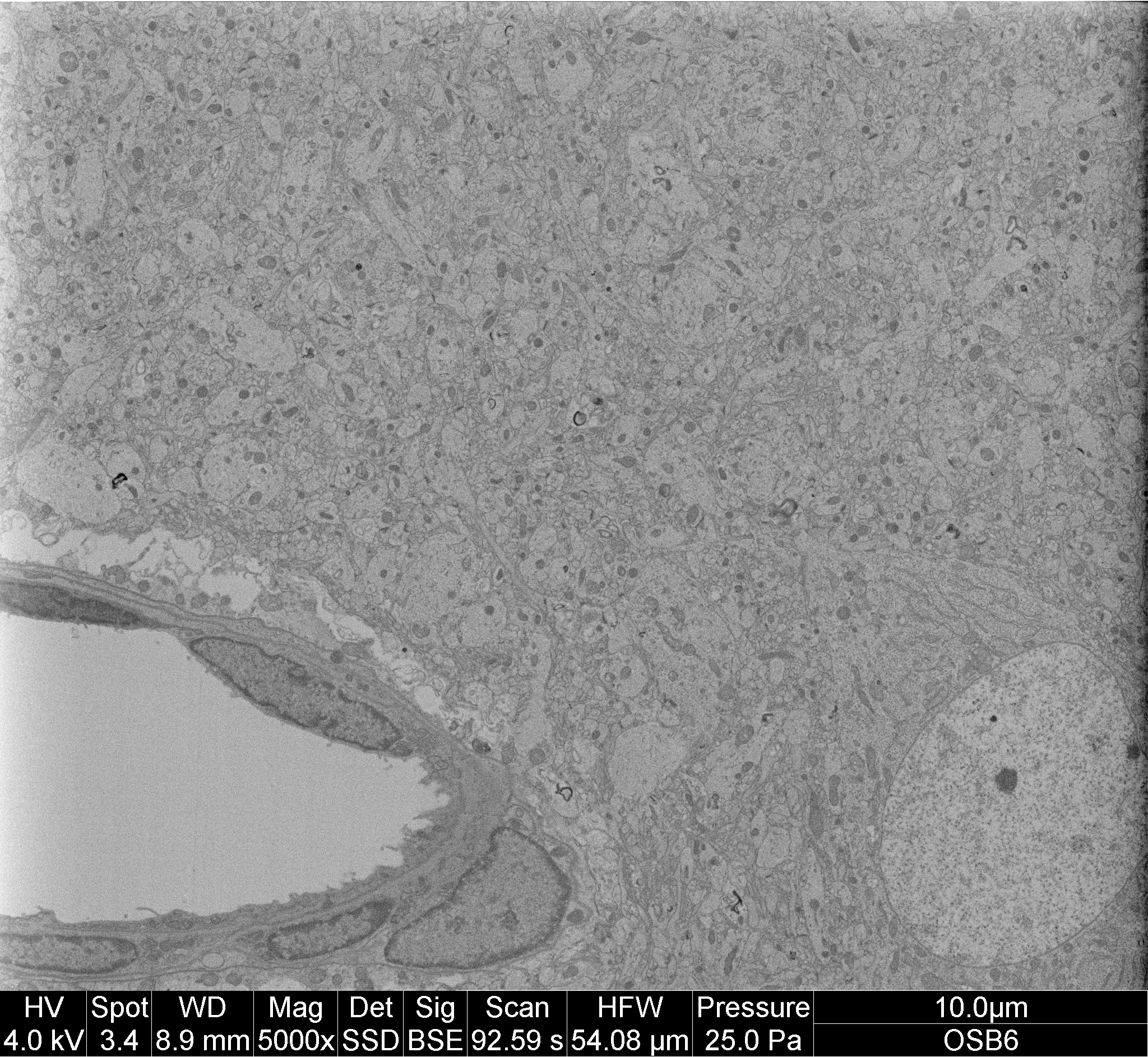

Supplement: Dataset S8 — (255.9 MB ZIP). [file pbio.0020329.sd008.zip › 040604_OS5_st1_794.tif]

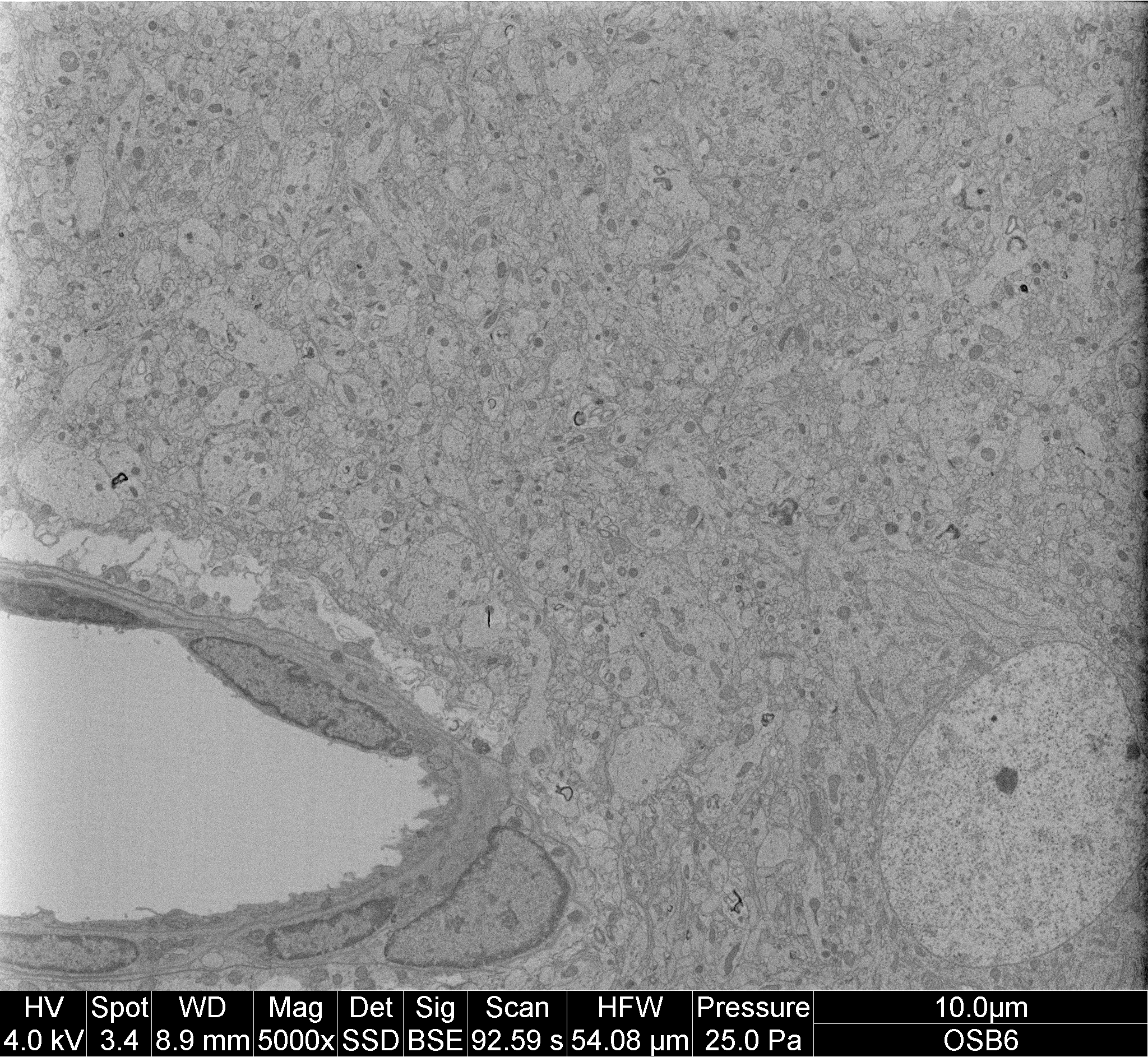

Supplement: Dataset S8 — (255.9 MB ZIP). [file pbio.0020329.sd008.zip › 040604_OS5_st1_795.tif]

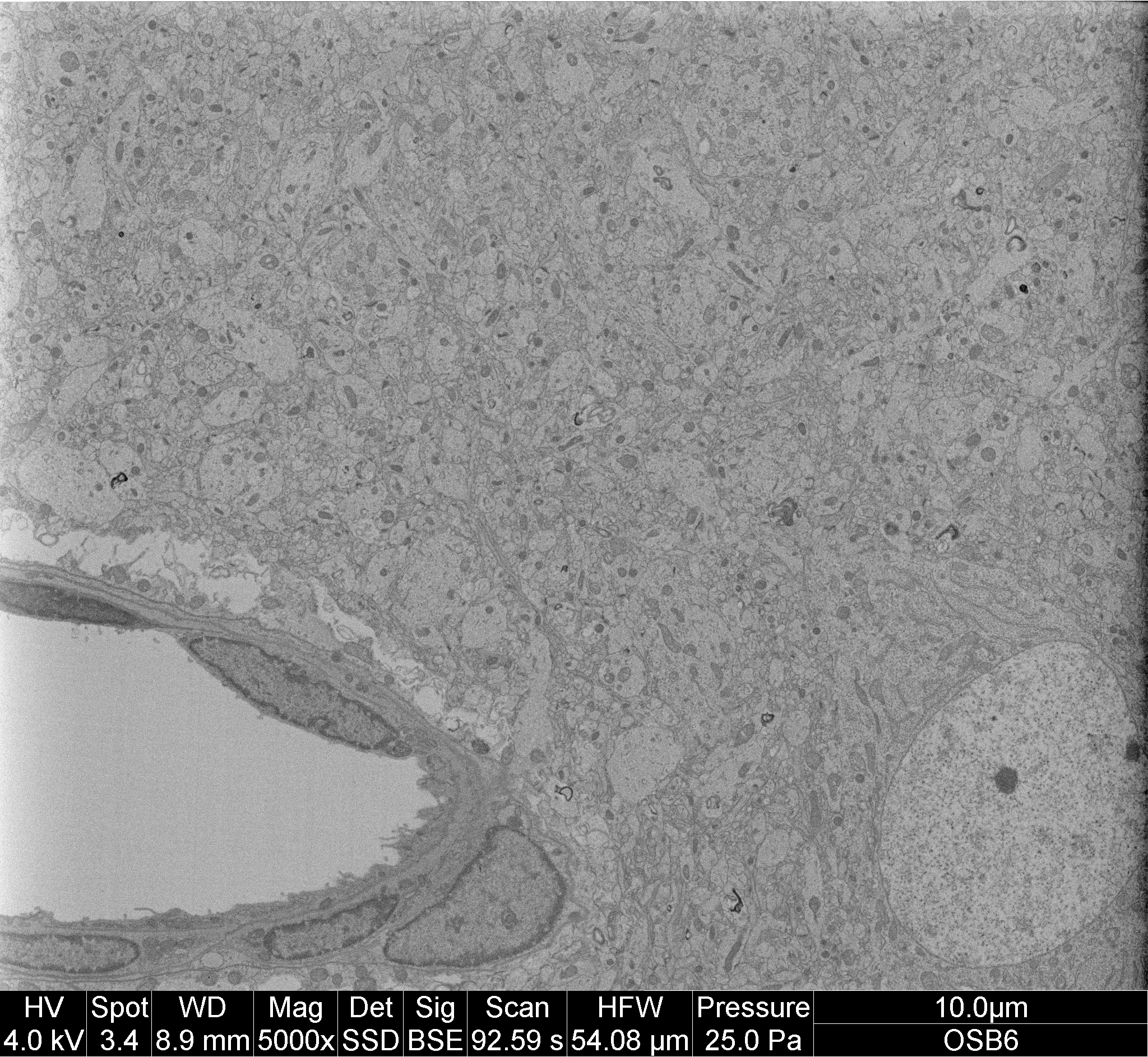

Supplement: Dataset S8 — (255.9 MB ZIP). [file pbio.0020329.sd008.zip › 040604_OS5_st1_796.tif]

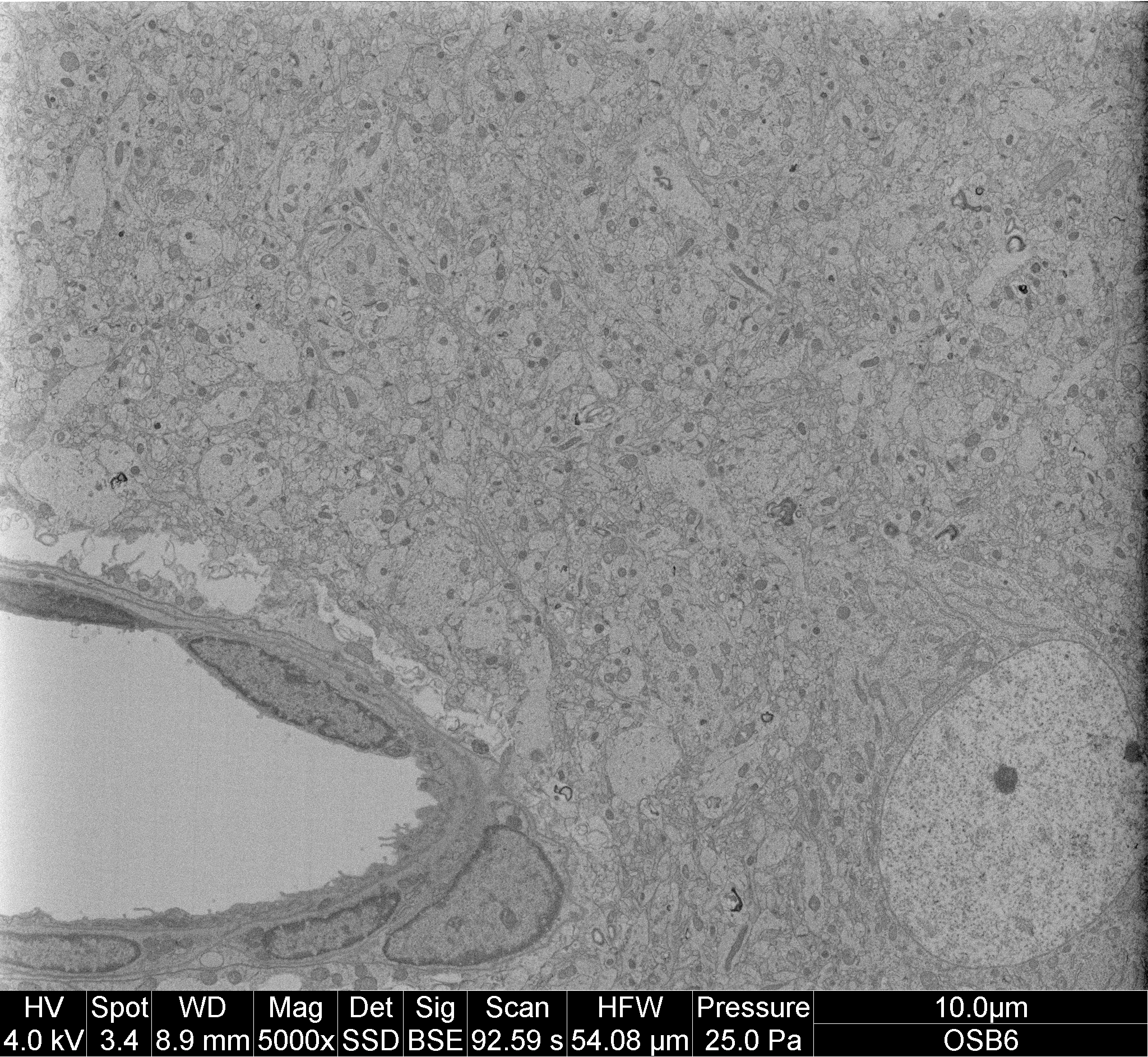

Supplement: Dataset S8 — (255.9 MB ZIP). [file pbio.0020329.sd008.zip › 040604_OS5_st1_797.tif]

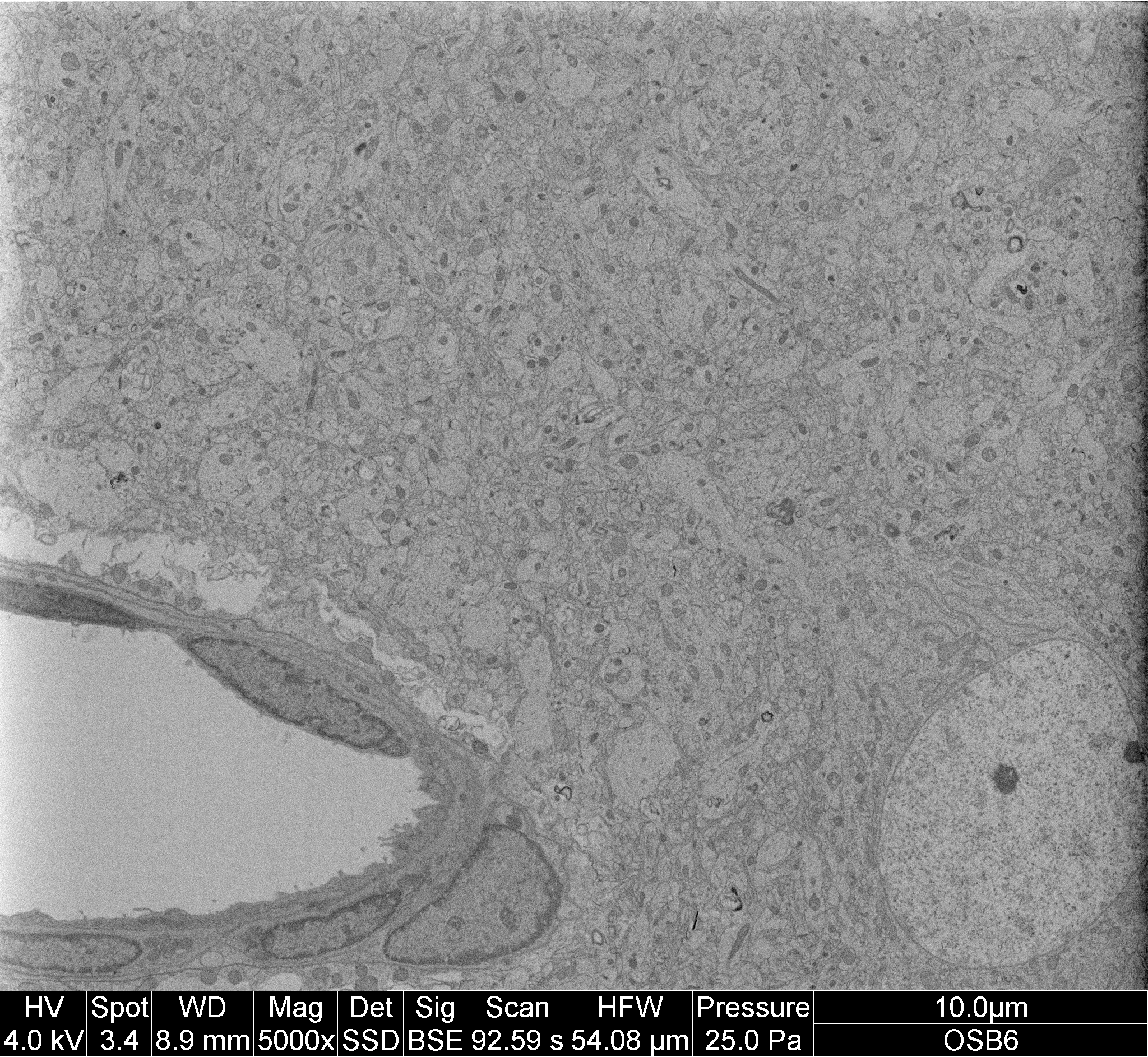

Supplement: Dataset S8 — (255.9 MB ZIP). [file pbio.0020329.sd008.zip › 040604_OS5_st1_798.tif]

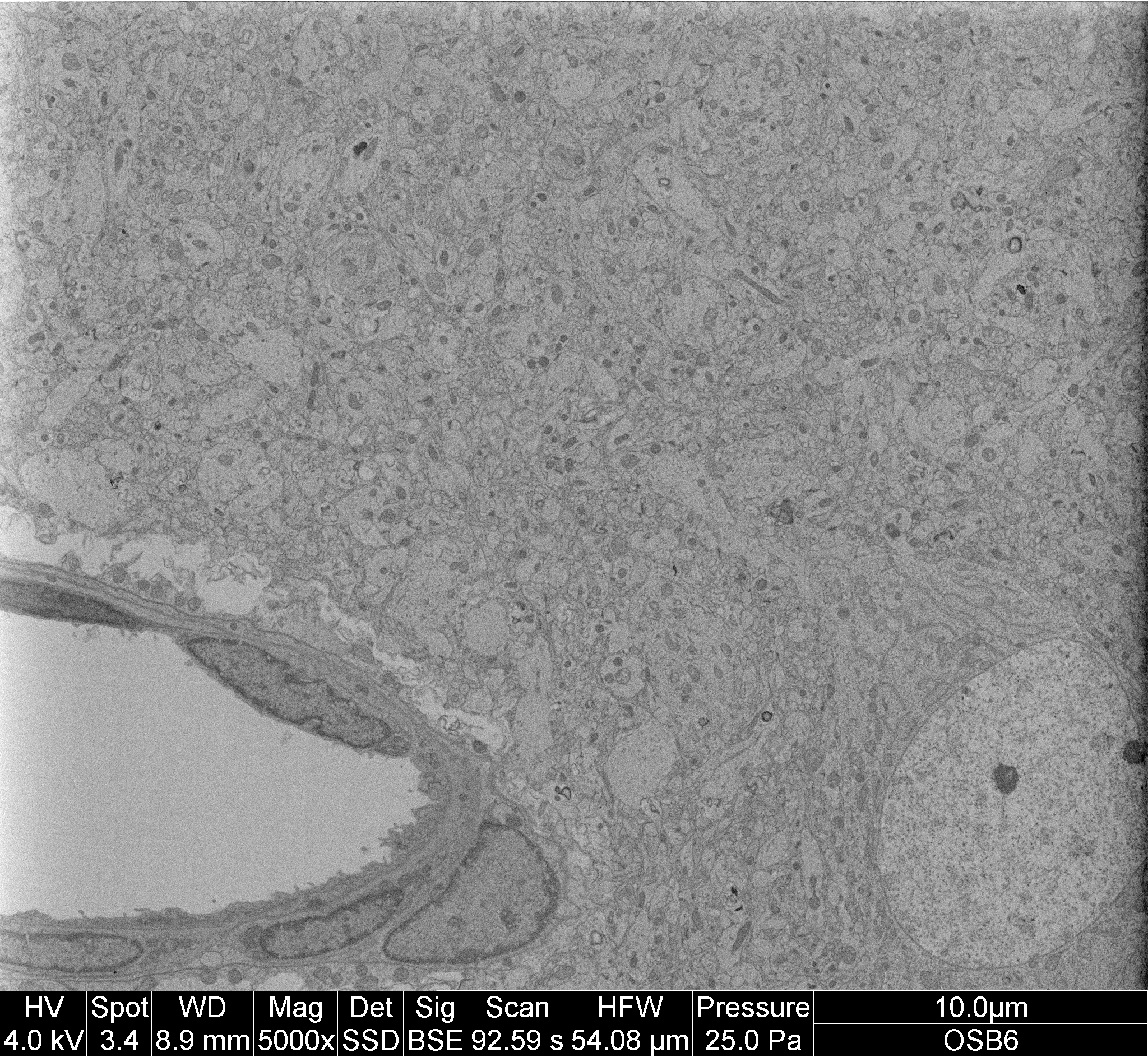

Supplement: Dataset S8 — (255.9 MB ZIP). [file pbio.0020329.sd008.zip › 040604_OS5_st1_799.tif]

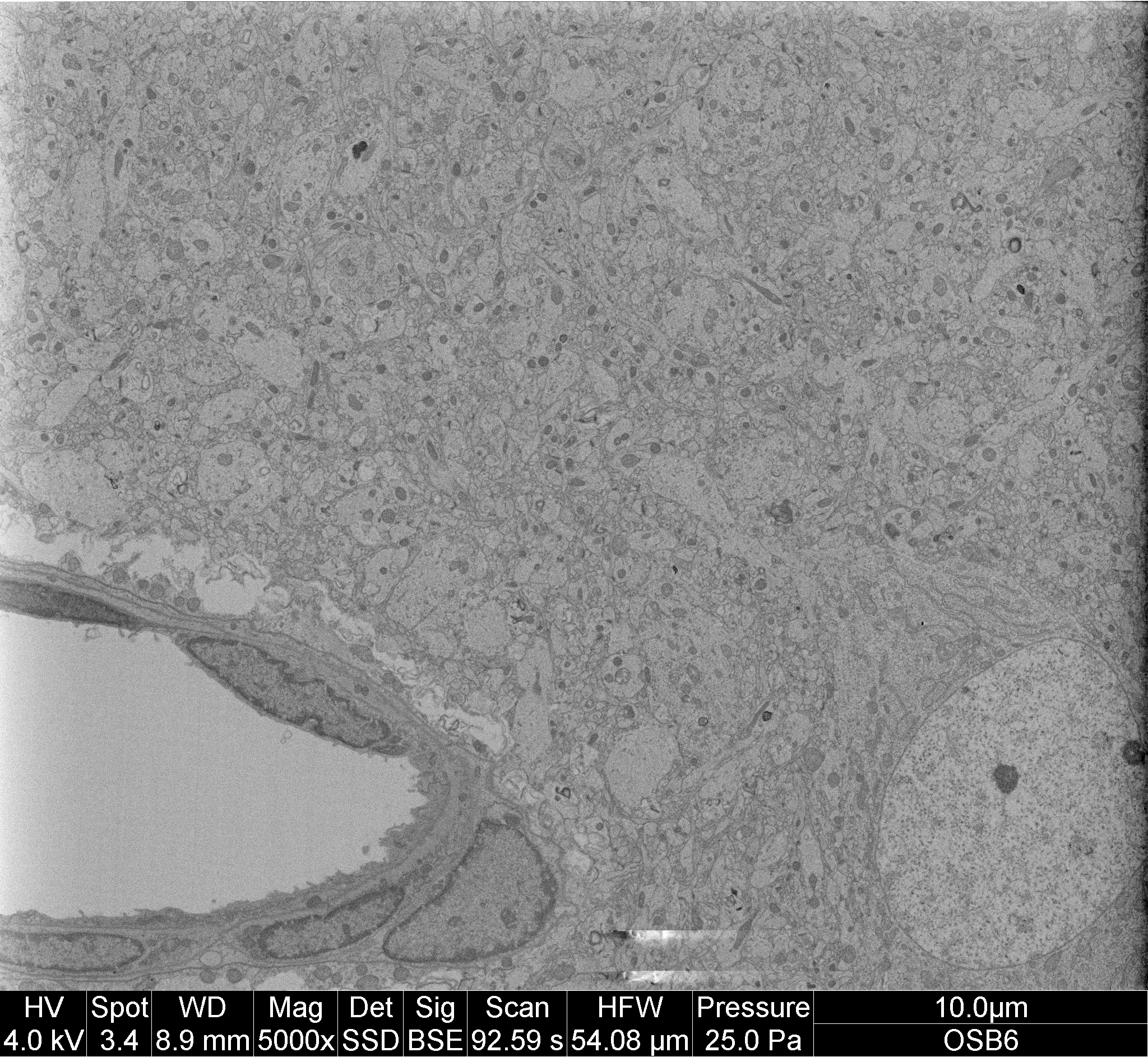

Supplement: Dataset S9 — (256.1 MB ZIP). [file pbio.0020329.sd009.zip › 040604_OS5_st1_800.tif]
