# Supplementary figures and images for: Serial Block-Face Scanning Electron Microscopy to Reconstruct Three-Dimensional Tissue Nanostructure (part 9 of 21)
Source: PLoS Biol. 2004 Oct 19;2(11):e329. doi: 10.1371/journal.pbio.0020329 (PMC524270; doi:10.1371/journal.pbio.0020329)

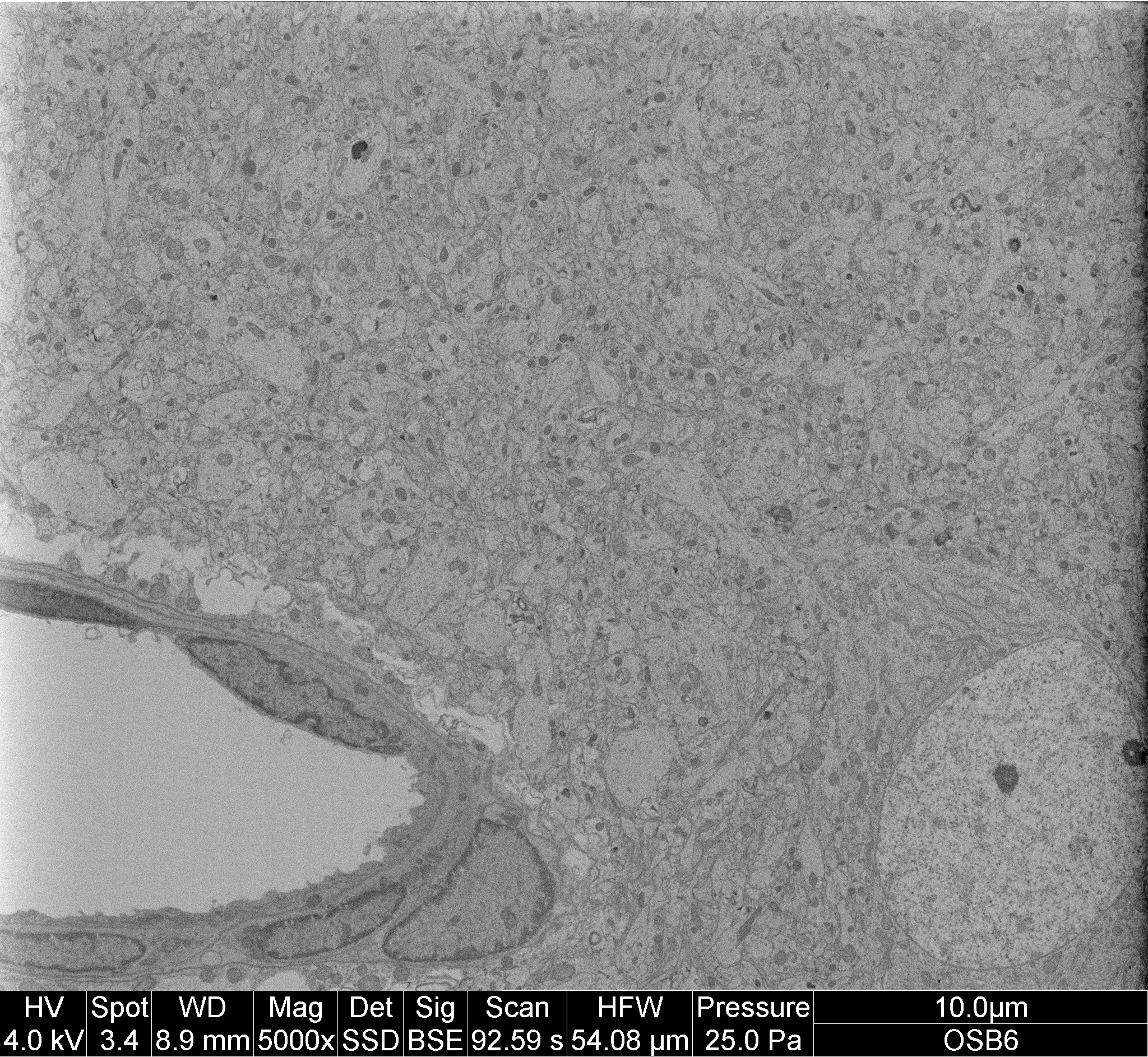

Supplement: Dataset S9 — (256.1 MB ZIP). [file pbio.0020329.sd009.zip › 040604_OS5_st1_801.tif]

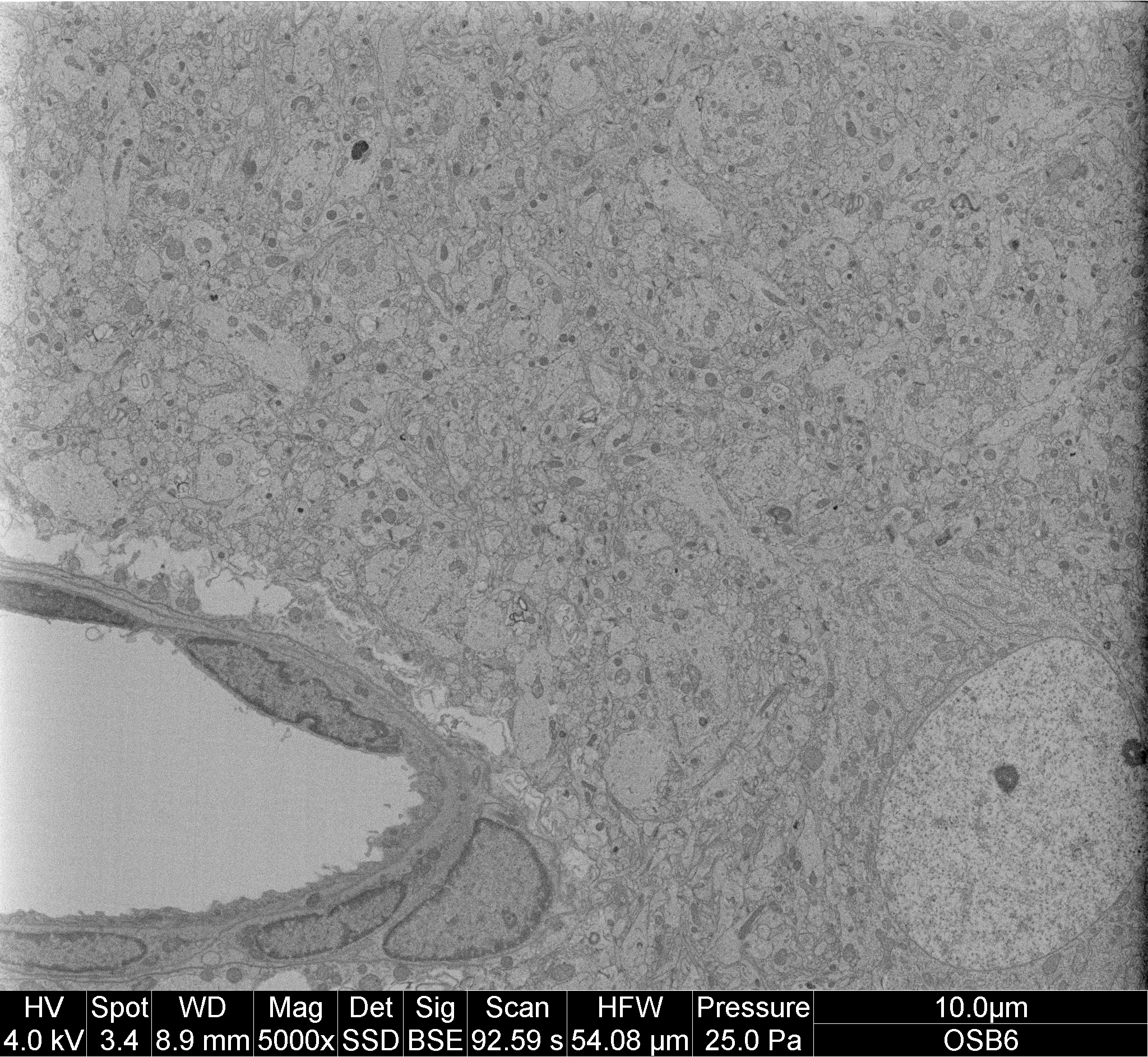

Supplement: Dataset S9 — (256.1 MB ZIP). [file pbio.0020329.sd009.zip › 040604_OS5_st1_802.tif]

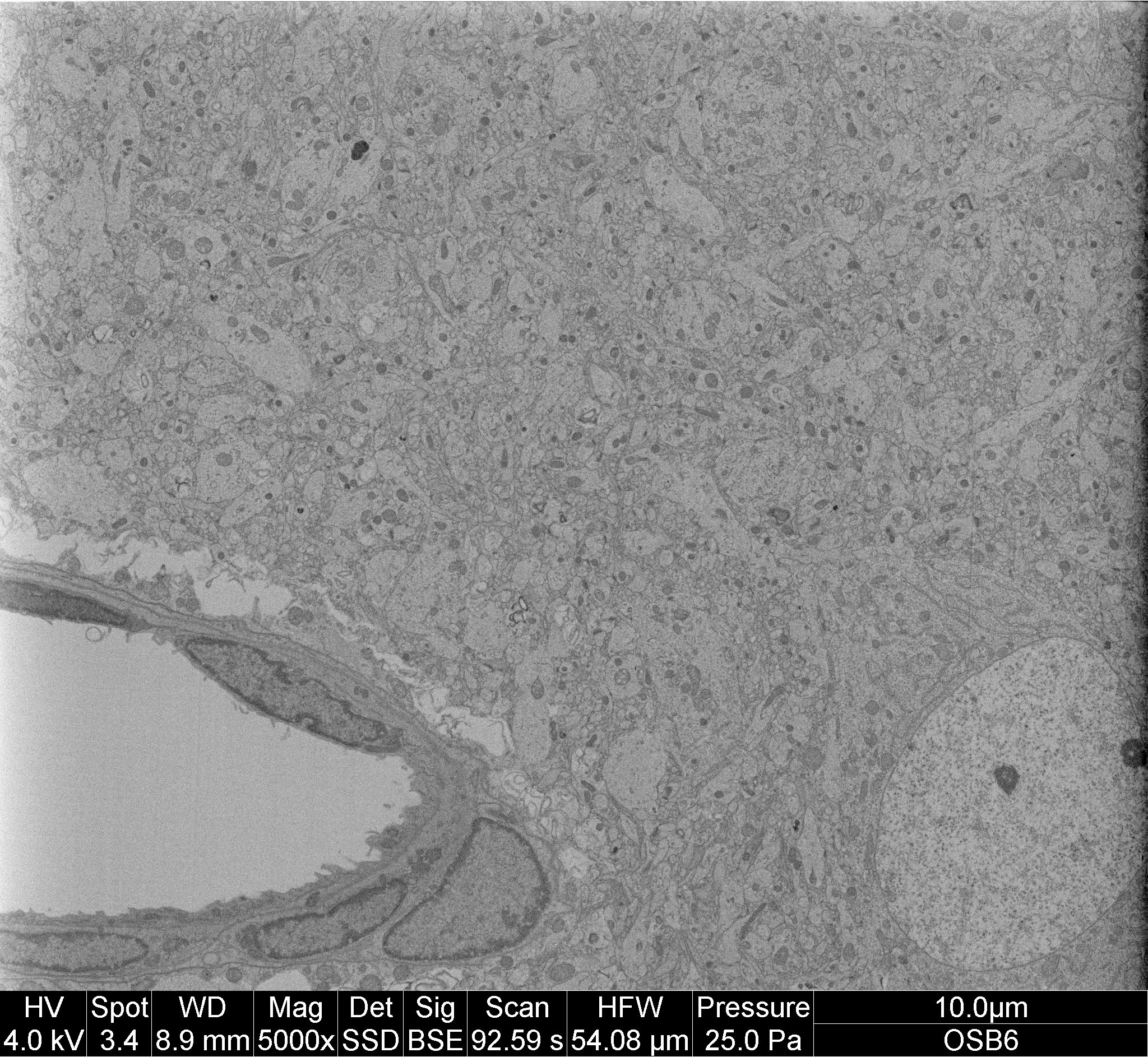

Supplement: Dataset S9 — (256.1 MB ZIP). [file pbio.0020329.sd009.zip › 040604_OS5_st1_803.tif]

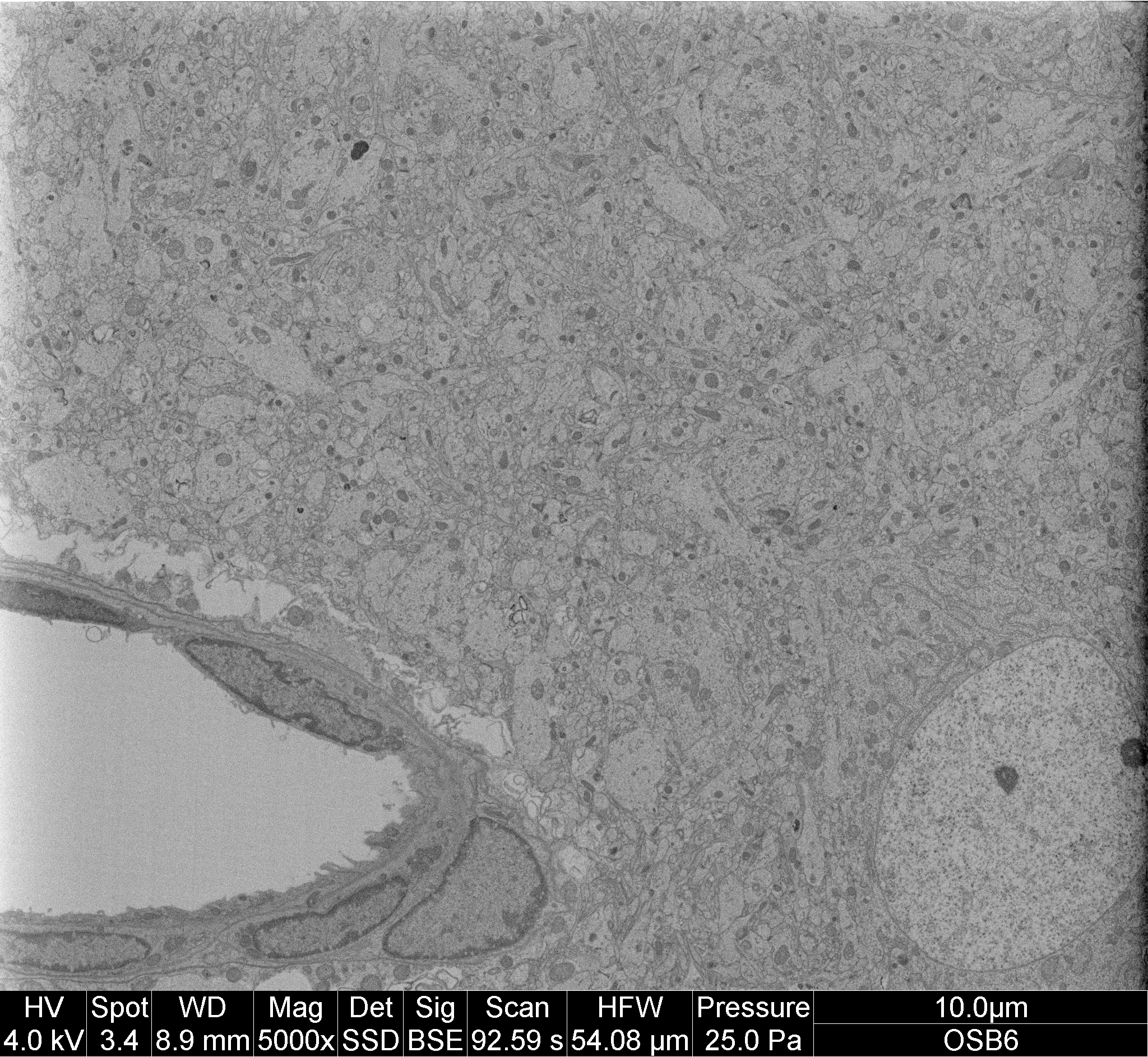

Supplement: Dataset S9 — (256.1 MB ZIP). [file pbio.0020329.sd009.zip › 040604_OS5_st1_804.tif]

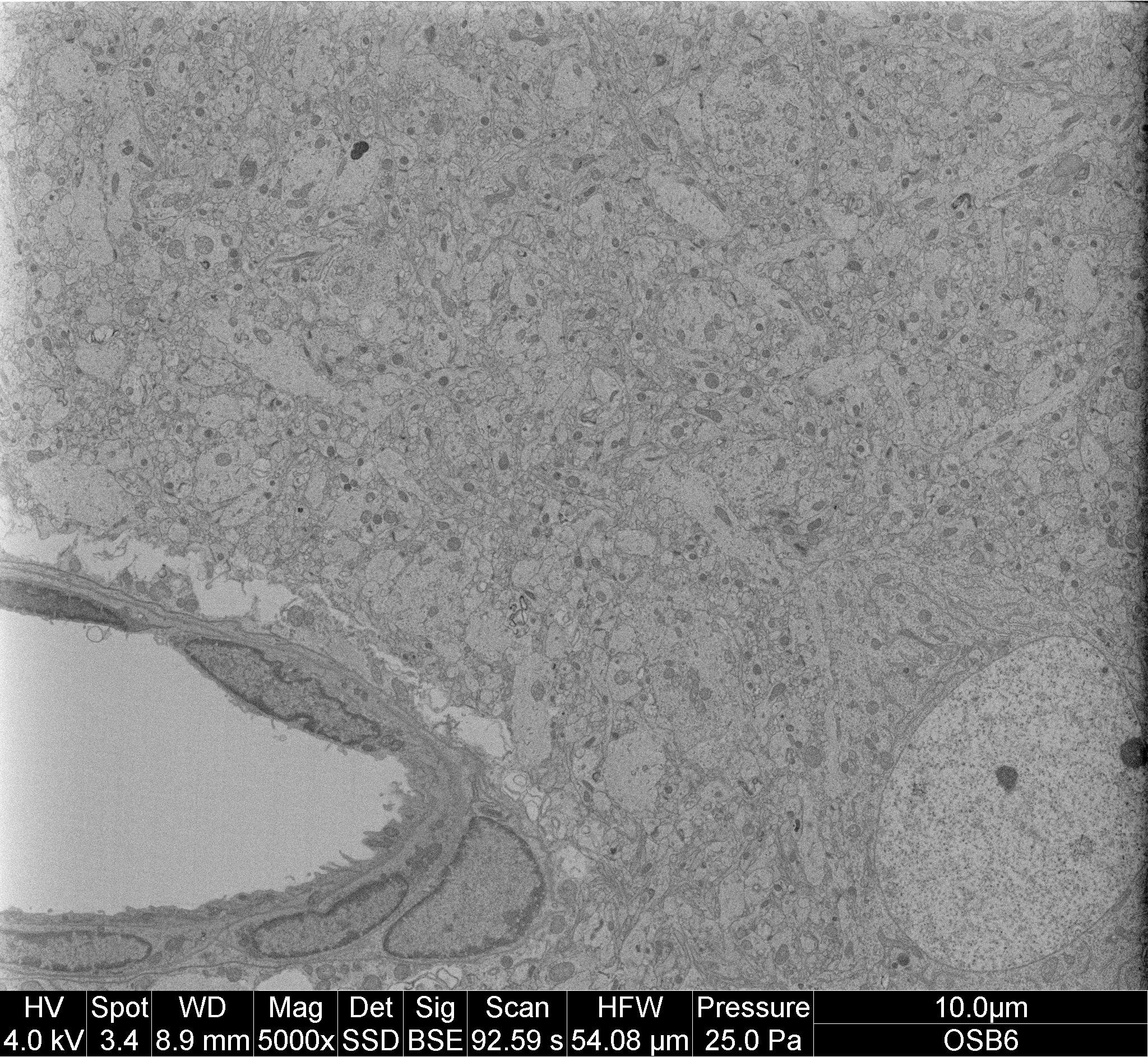

Supplement: Dataset S9 — (256.1 MB ZIP). [file pbio.0020329.sd009.zip › 040604_OS5_st1_805.tif]

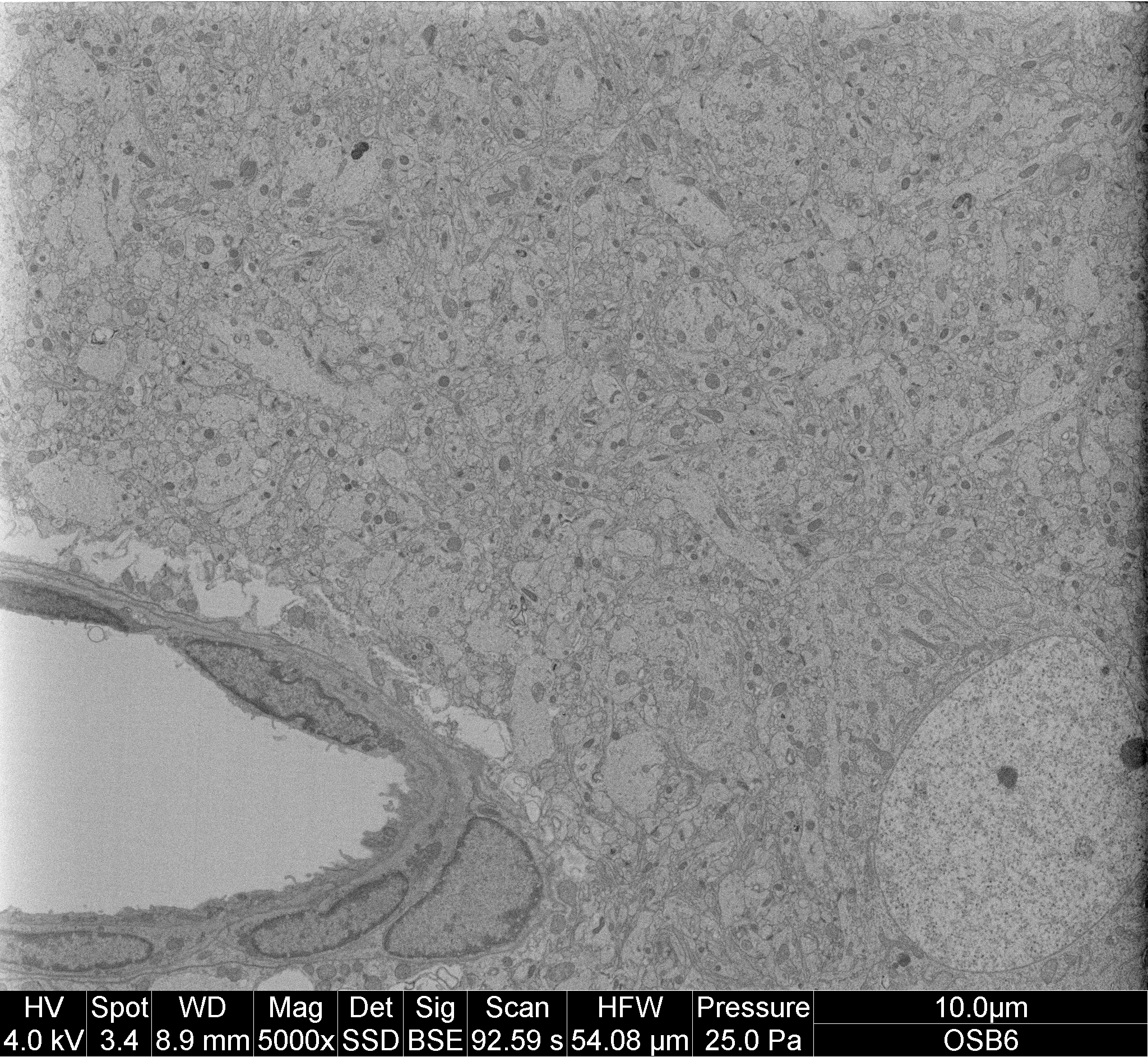

Supplement: Dataset S9 — (256.1 MB ZIP). [file pbio.0020329.sd009.zip › 040604_OS5_st1_806.tif]

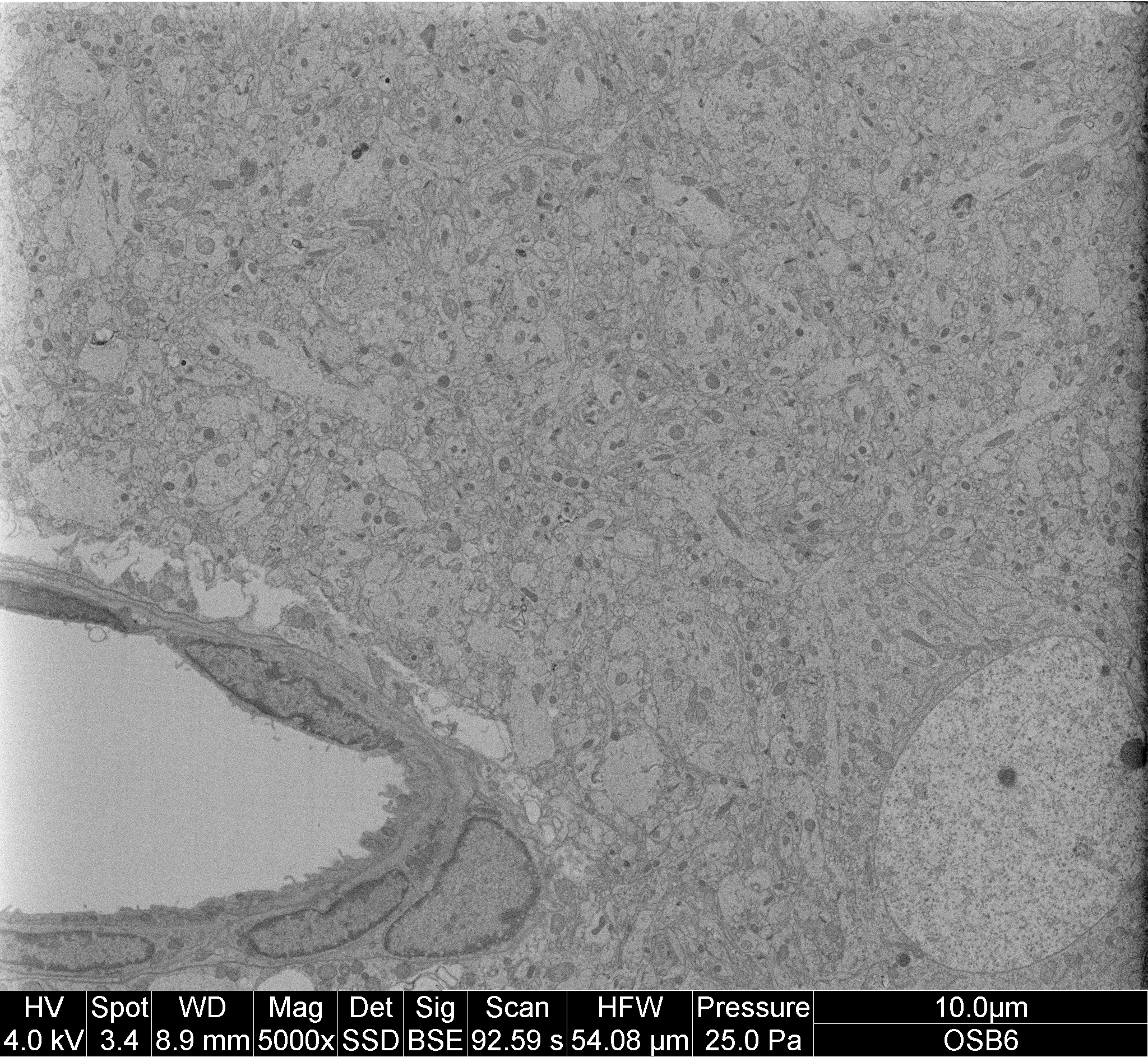

Supplement: Dataset S9 — (256.1 MB ZIP). [file pbio.0020329.sd009.zip › 040604_OS5_st1_807.tif]

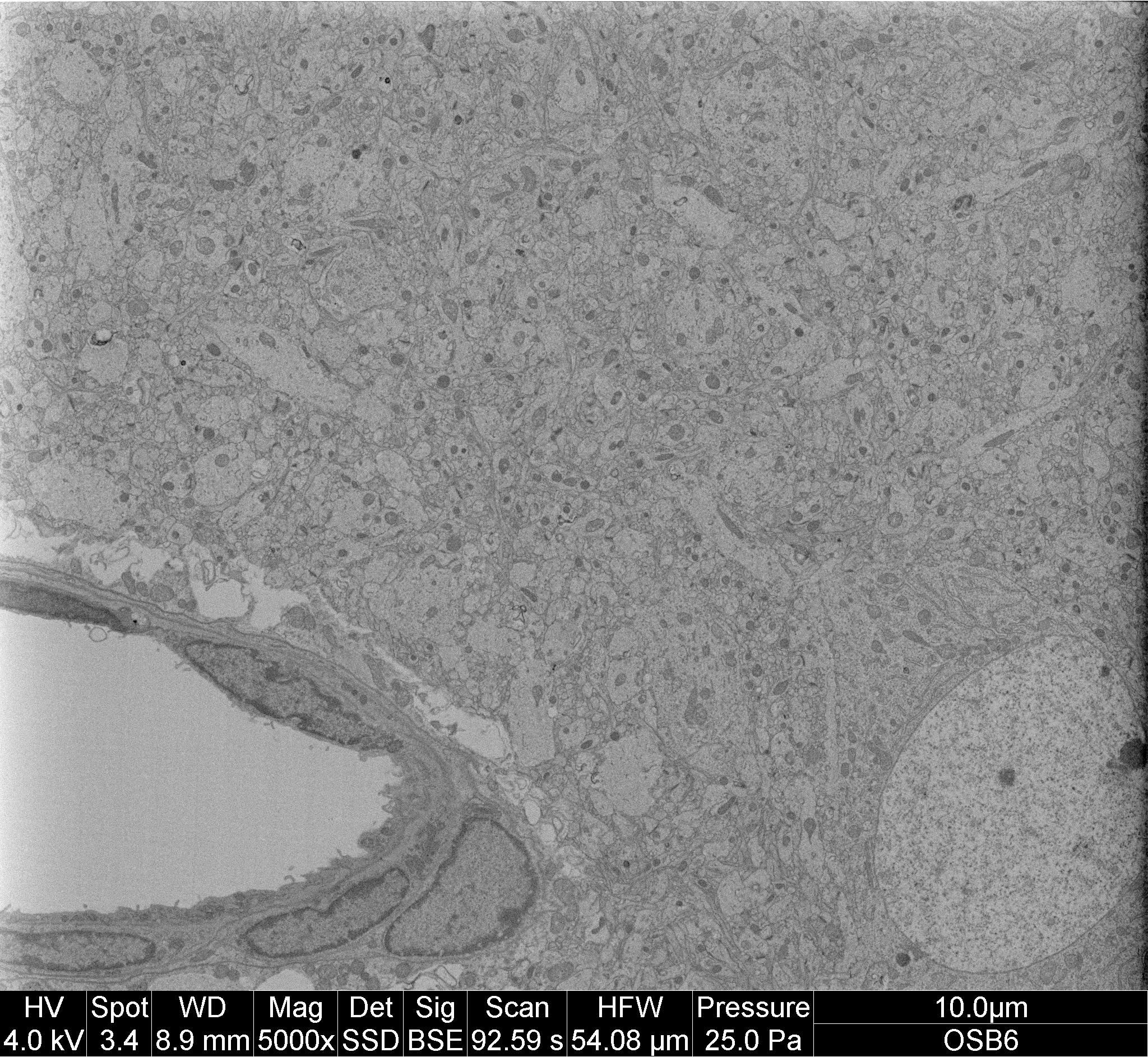

Supplement: Dataset S9 — (256.1 MB ZIP). [file pbio.0020329.sd009.zip › 040604_OS5_st1_808.tif]

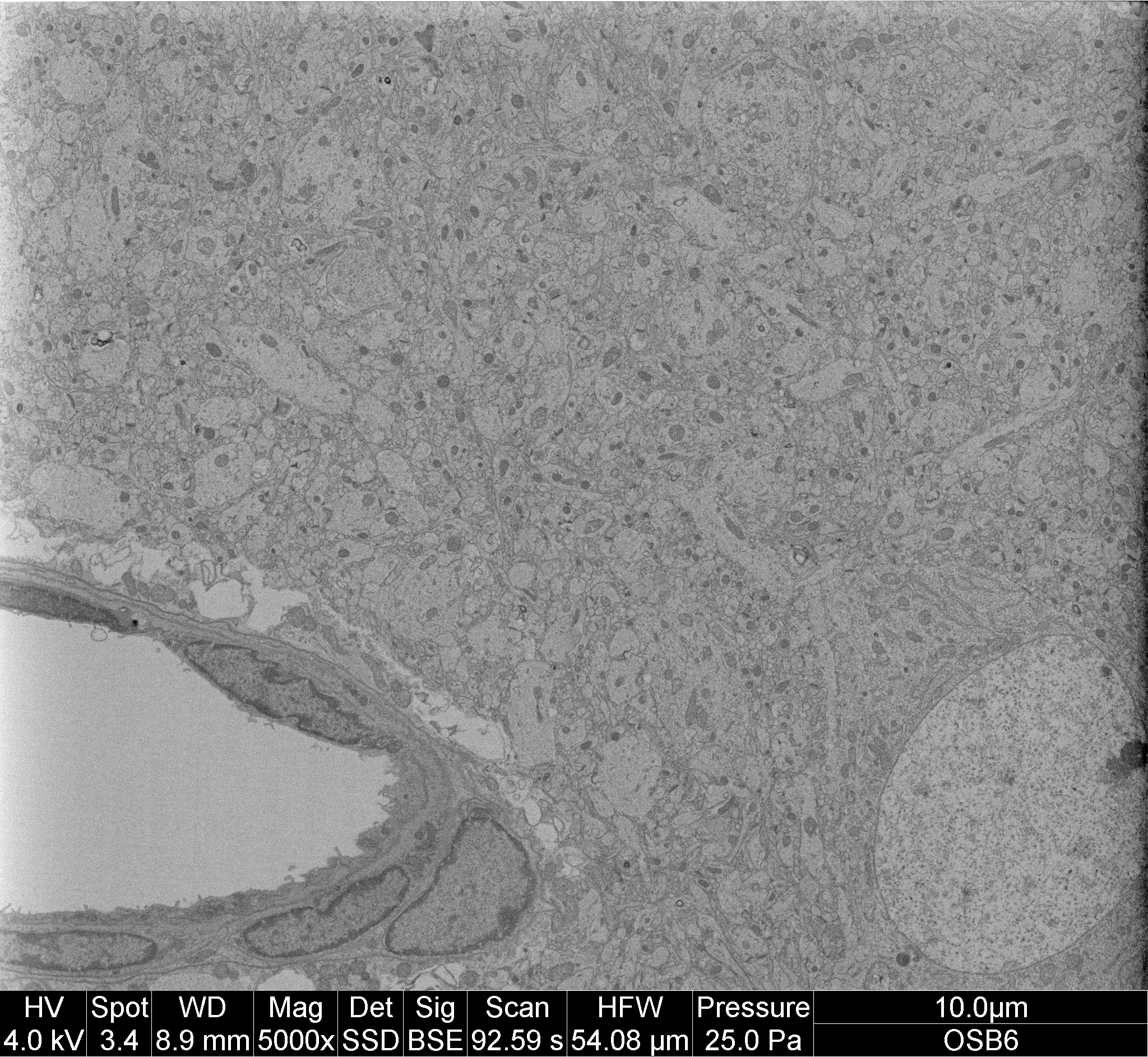

Supplement: Dataset S9 — (256.1 MB ZIP). [file pbio.0020329.sd009.zip › 040604_OS5_st1_809.tif]

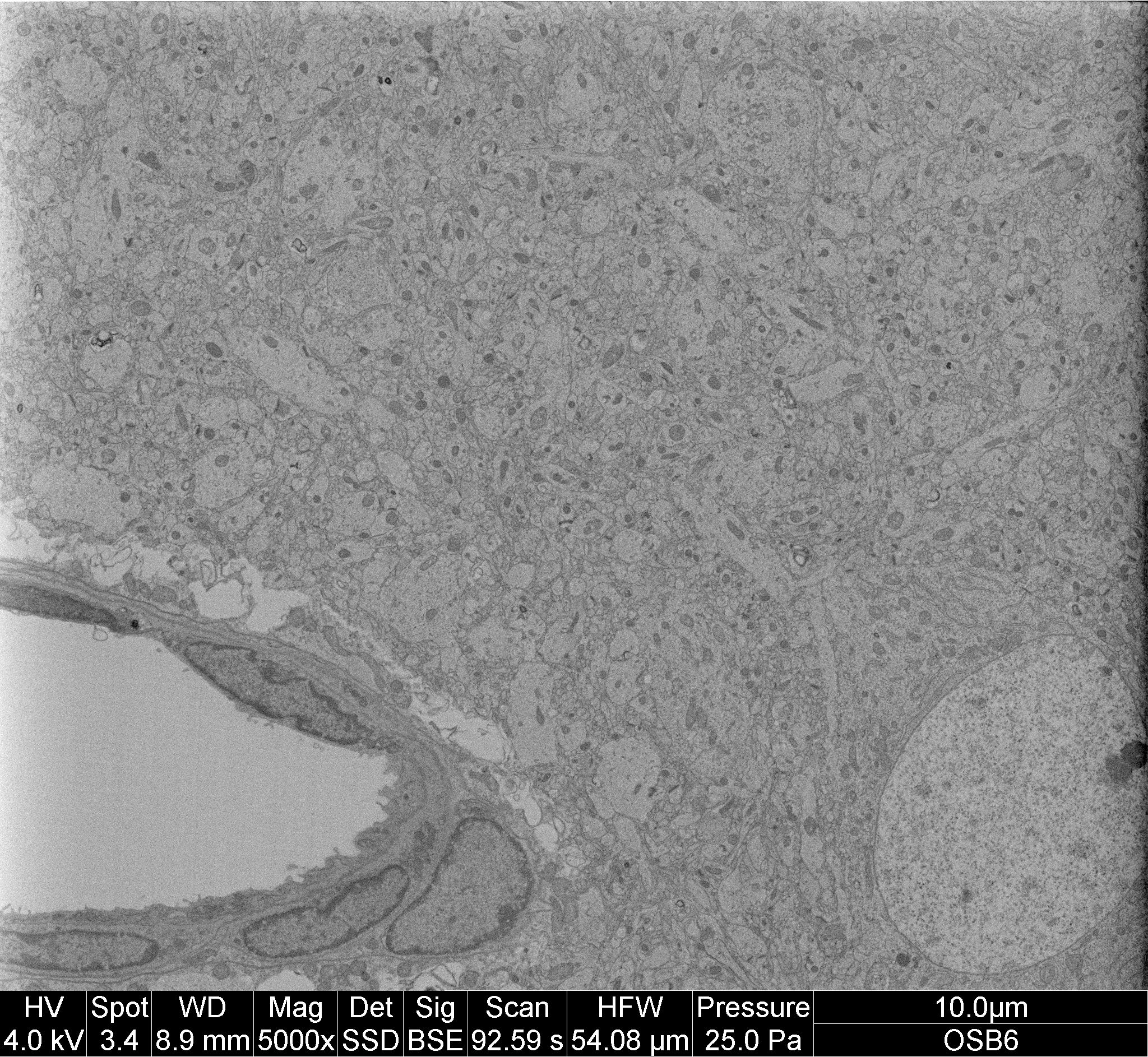

Supplement: Dataset S9 — (256.1 MB ZIP). [file pbio.0020329.sd009.zip › 040604_OS5_st1_810.tif]

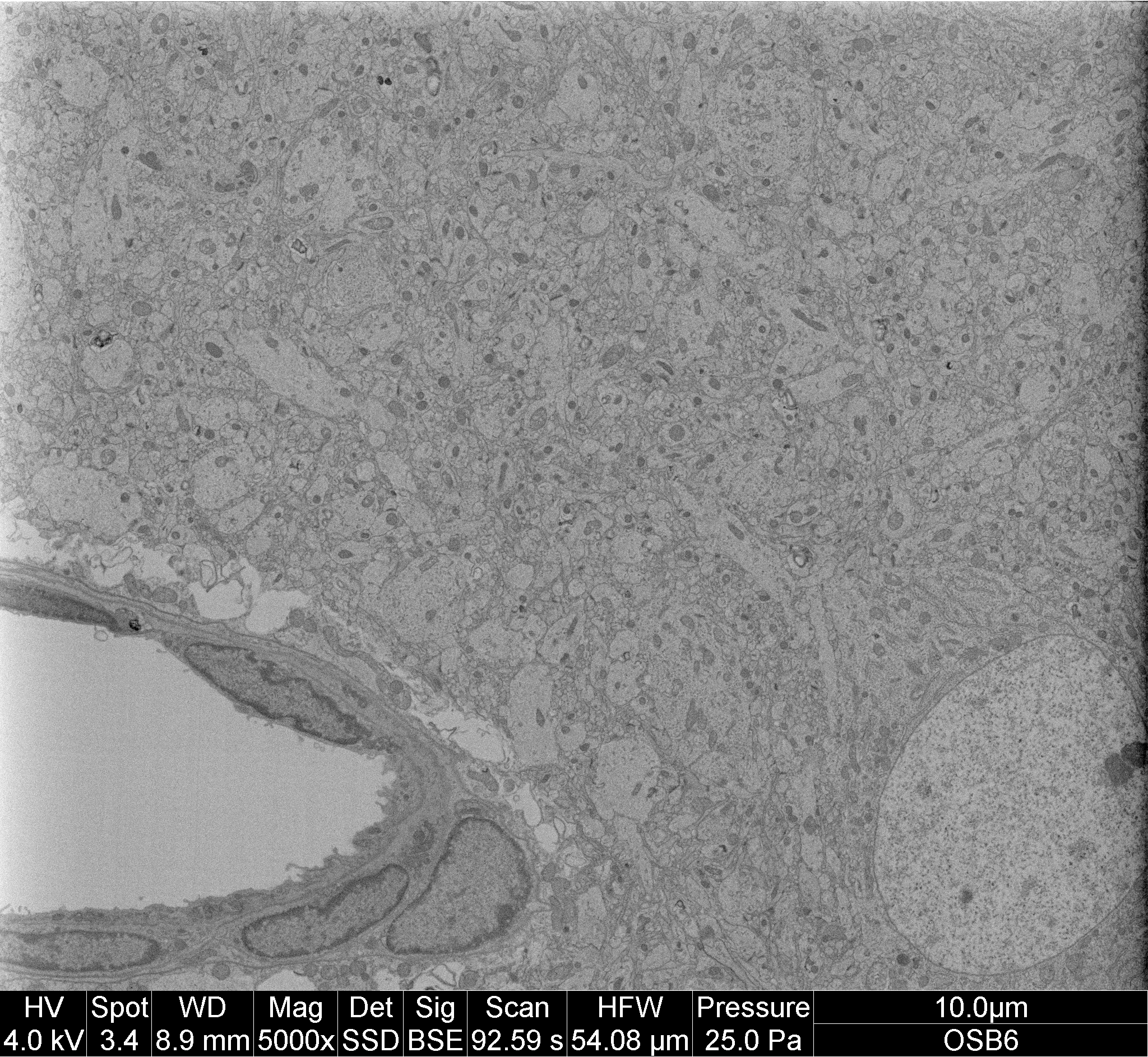

Supplement: Dataset S9 — (256.1 MB ZIP). [file pbio.0020329.sd009.zip › 040604_OS5_st1_811.tif]

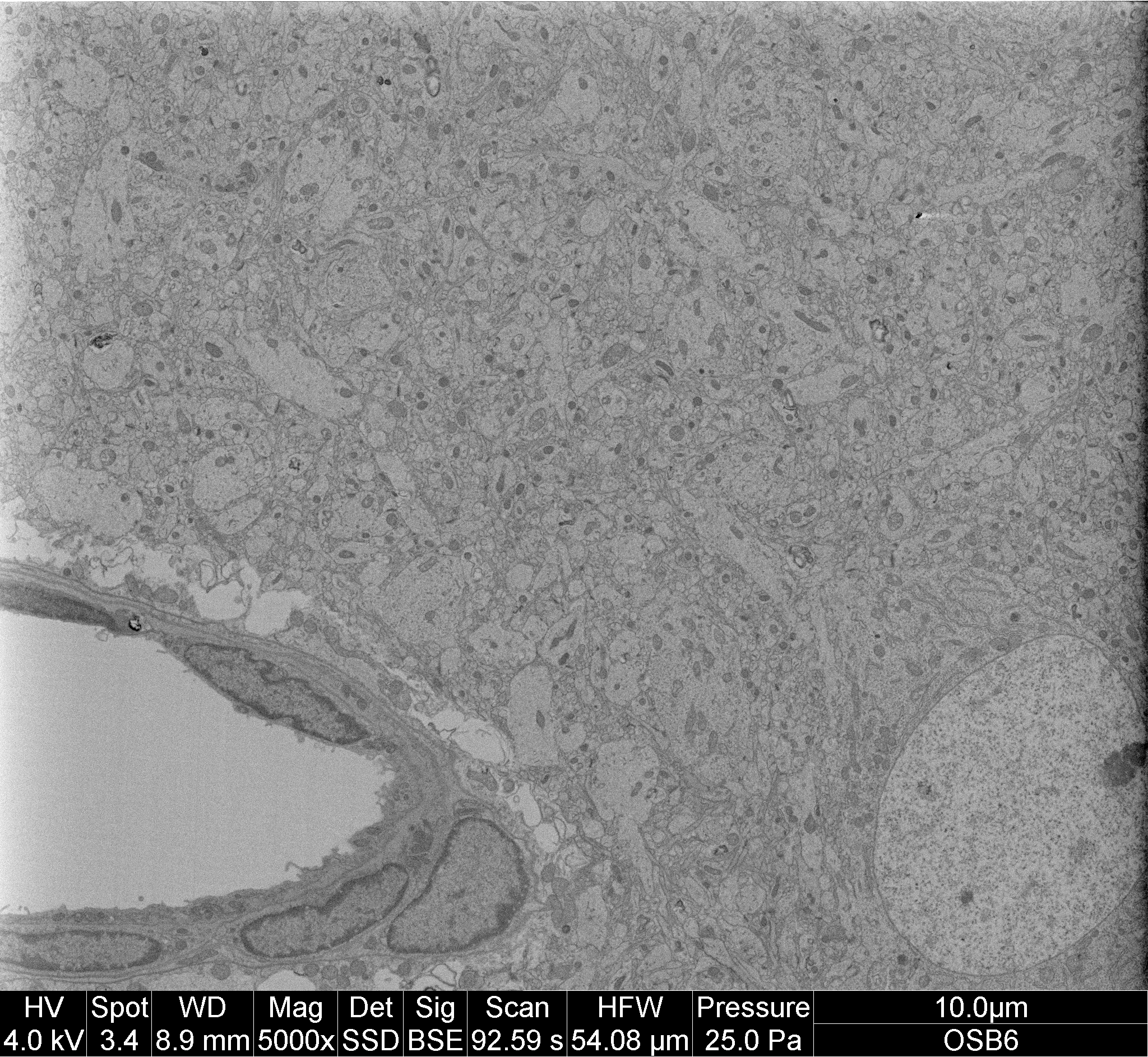

Supplement: Dataset S9 — (256.1 MB ZIP). [file pbio.0020329.sd009.zip › 040604_OS5_st1_812.tif]

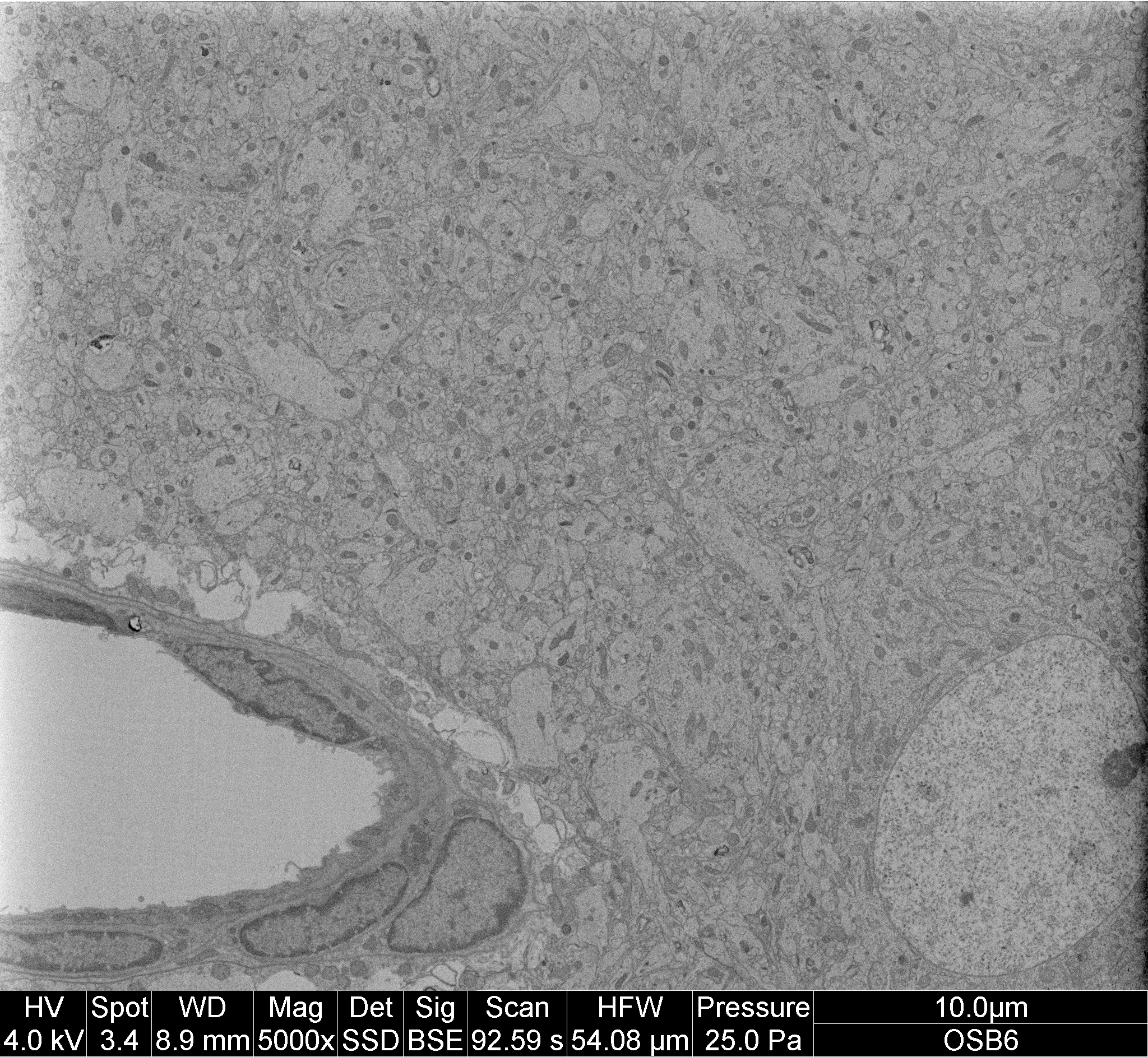

Supplement: Dataset S9 — (256.1 MB ZIP). [file pbio.0020329.sd009.zip › 040604_OS5_st1_813.tif]

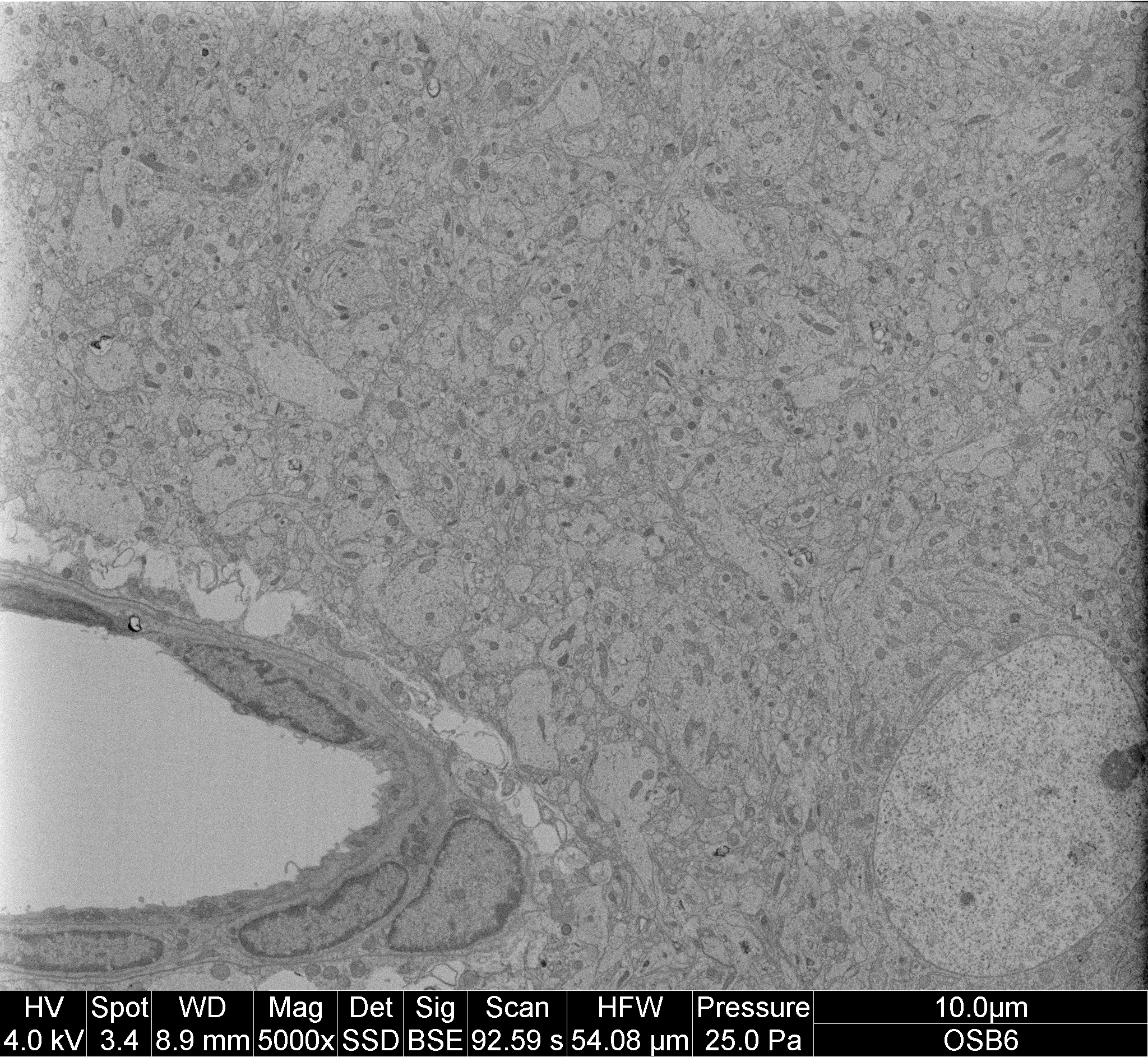

Supplement: Dataset S9 — (256.1 MB ZIP). [file pbio.0020329.sd009.zip › 040604_OS5_st1_814.tif]

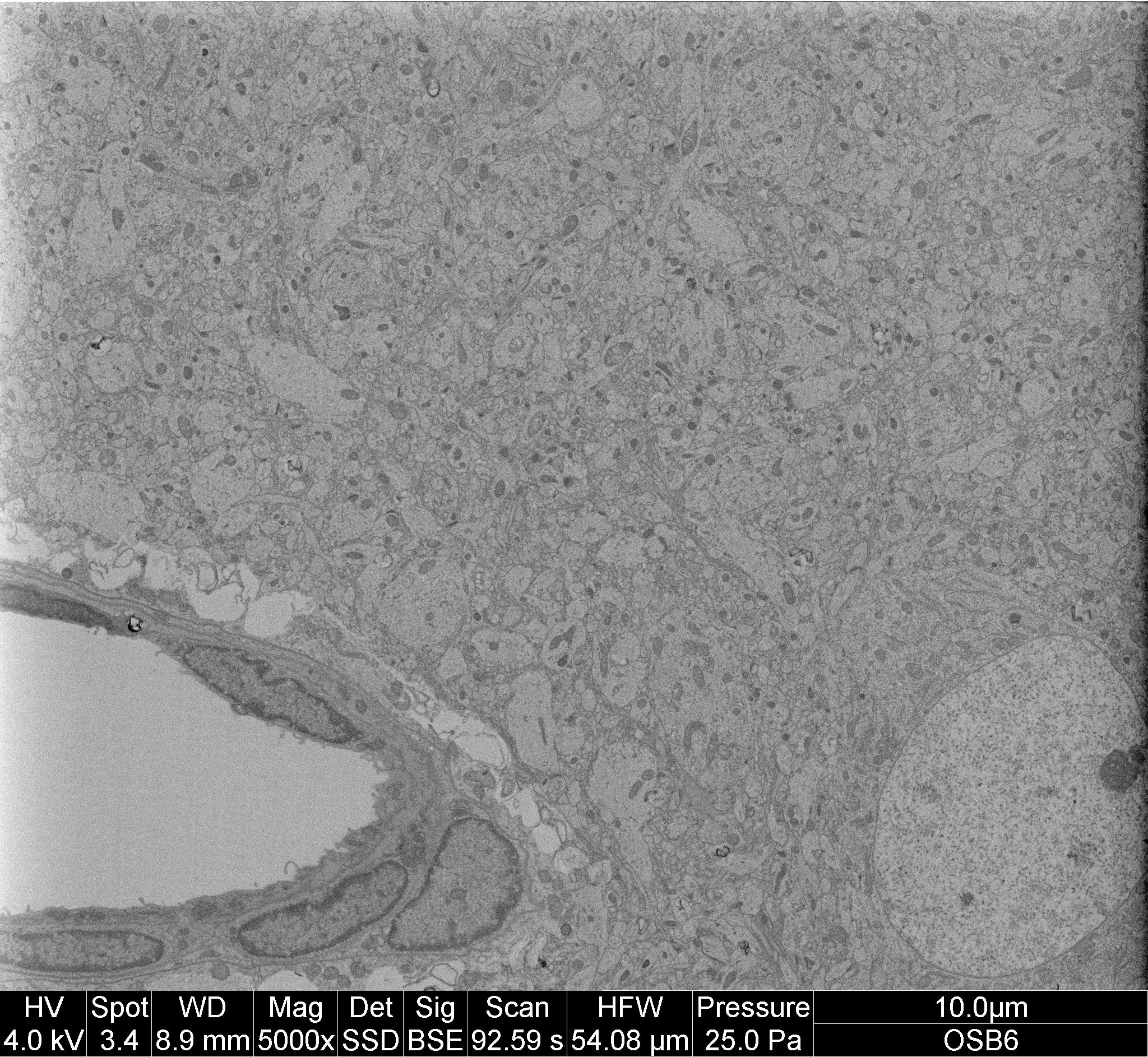

Supplement: Dataset S9 — (256.1 MB ZIP). [file pbio.0020329.sd009.zip › 040604_OS5_st1_815.tif]

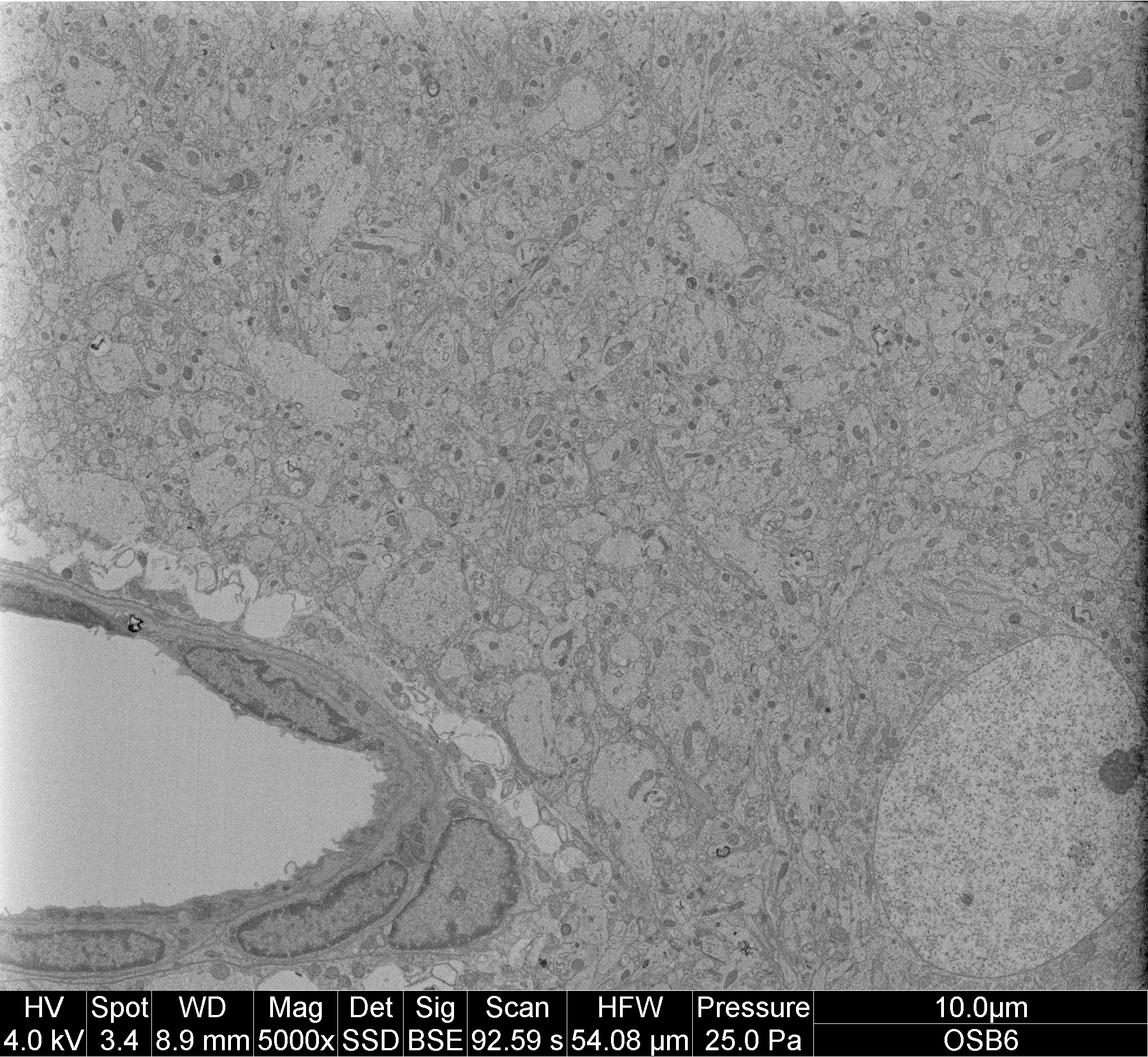

Supplement: Dataset S9 — (256.1 MB ZIP). [file pbio.0020329.sd009.zip › 040604_OS5_st1_816.tif]

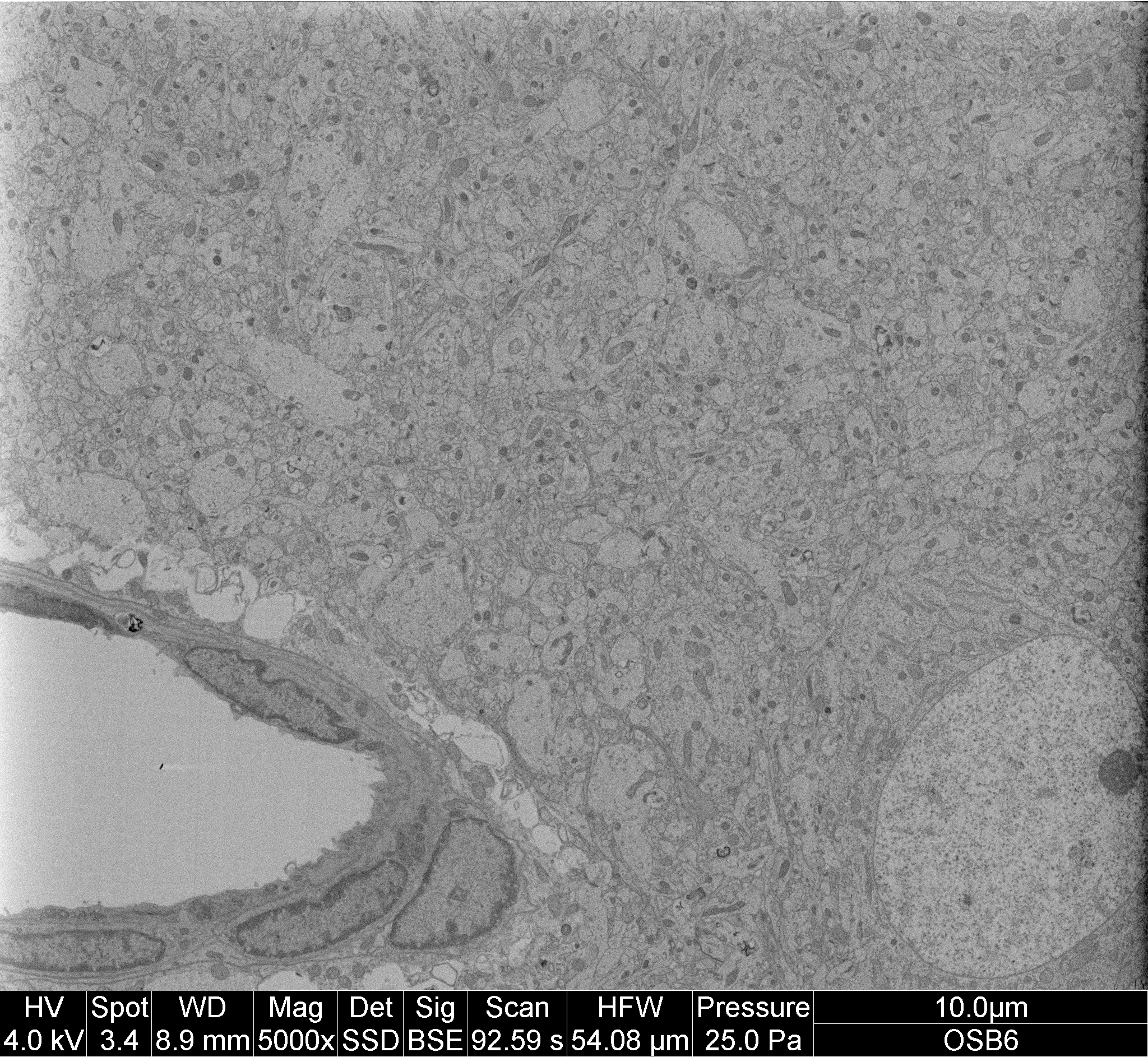

Supplement: Dataset S9 — (256.1 MB ZIP). [file pbio.0020329.sd009.zip › 040604_OS5_st1_817.tif]

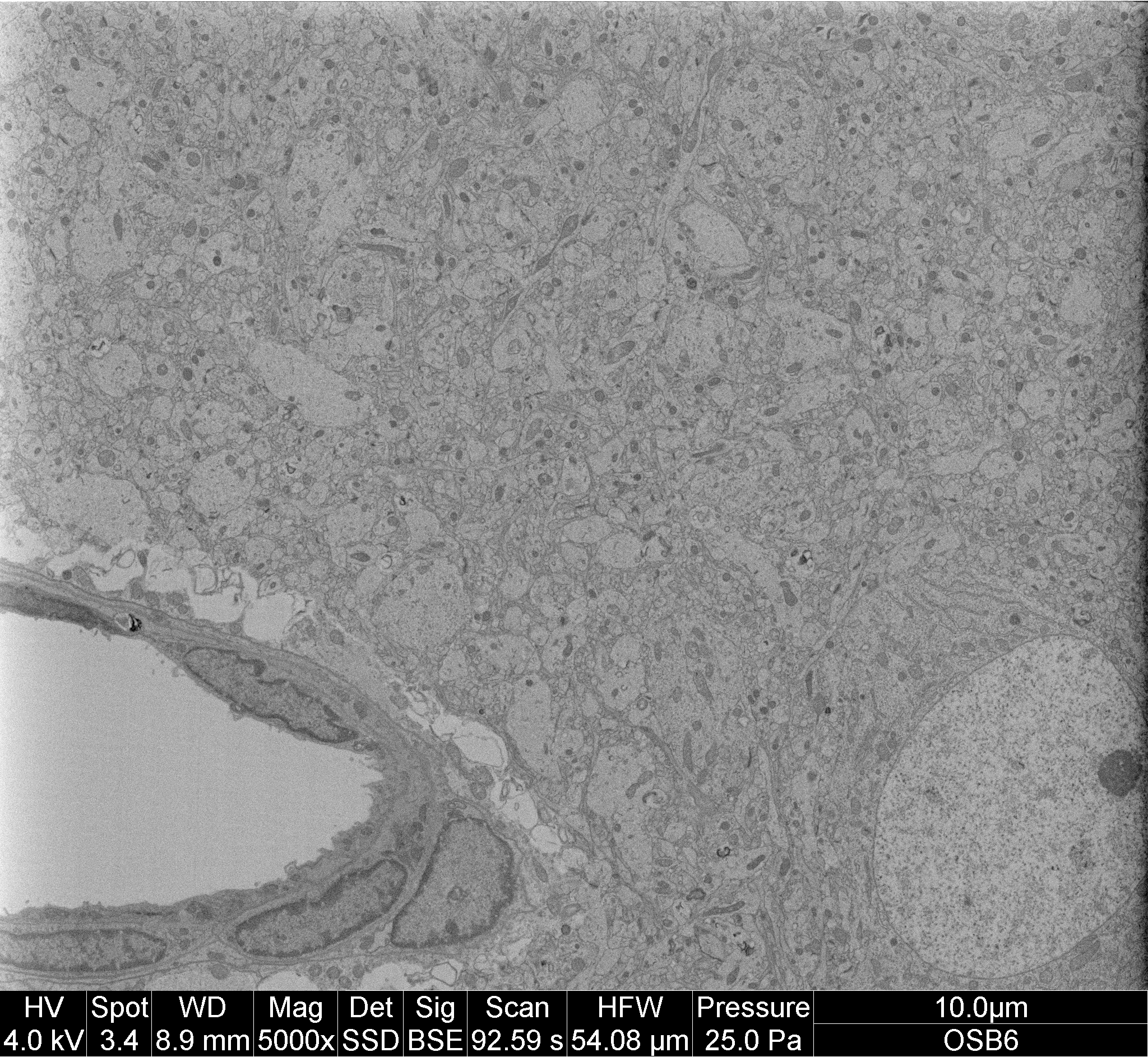

Supplement: Dataset S9 — (256.1 MB ZIP). [file pbio.0020329.sd009.zip › 040604_OS5_st1_818.tif]

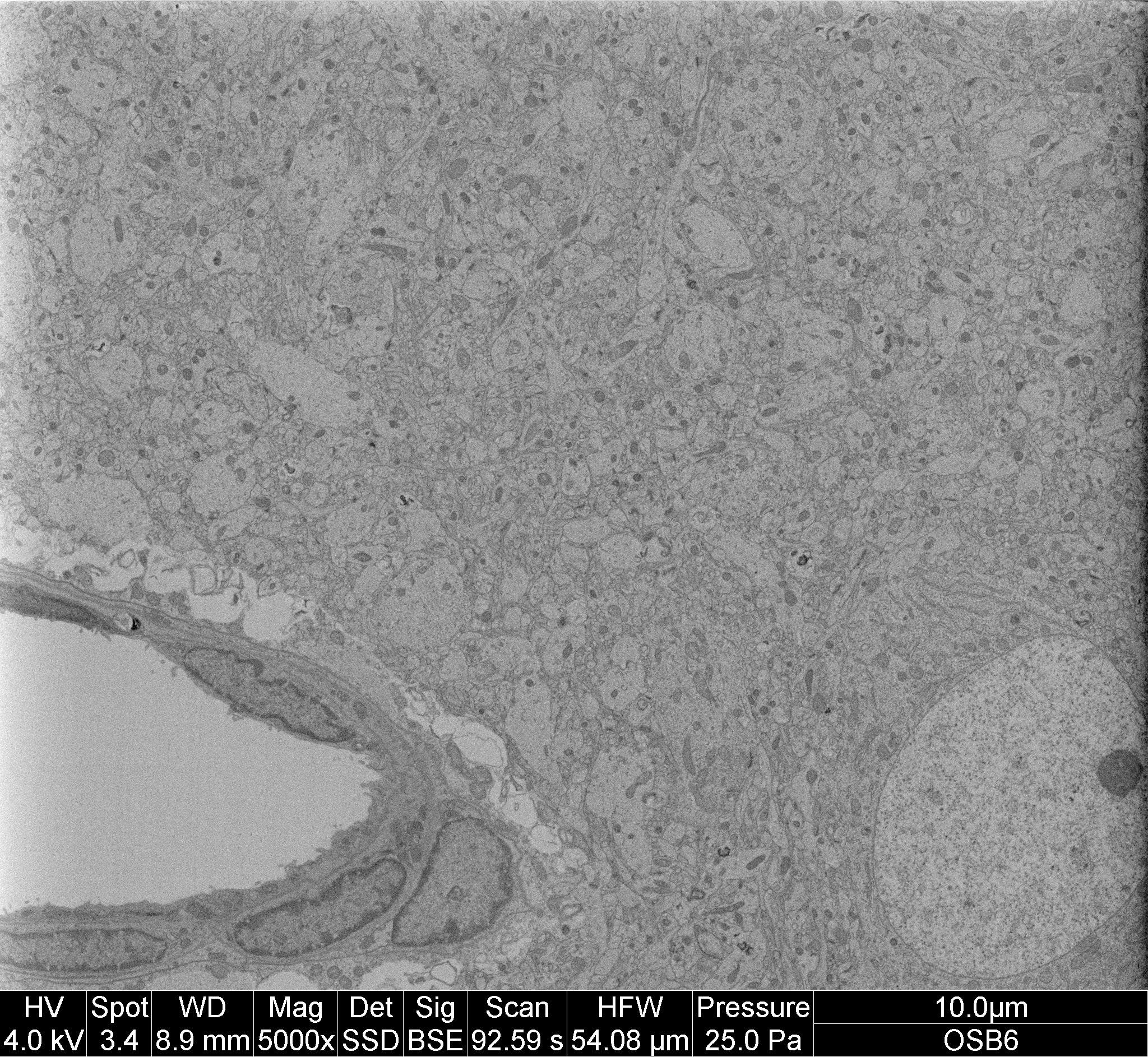

Supplement: Dataset S9 — (256.1 MB ZIP). [file pbio.0020329.sd009.zip › 040604_OS5_st1_819.tif]

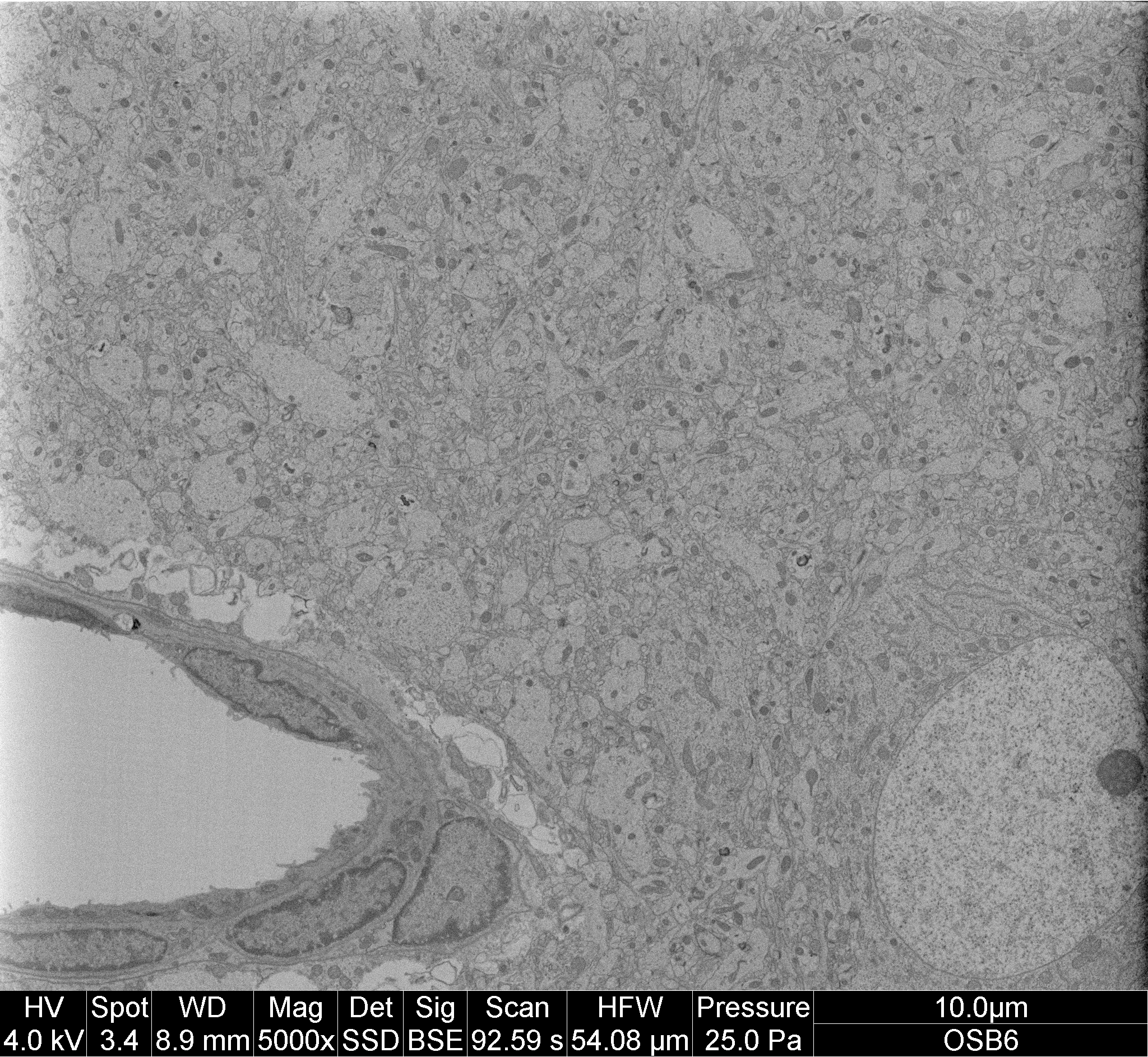

Supplement: Dataset S9 — (256.1 MB ZIP). [file pbio.0020329.sd009.zip › 040604_OS5_st1_820.tif]

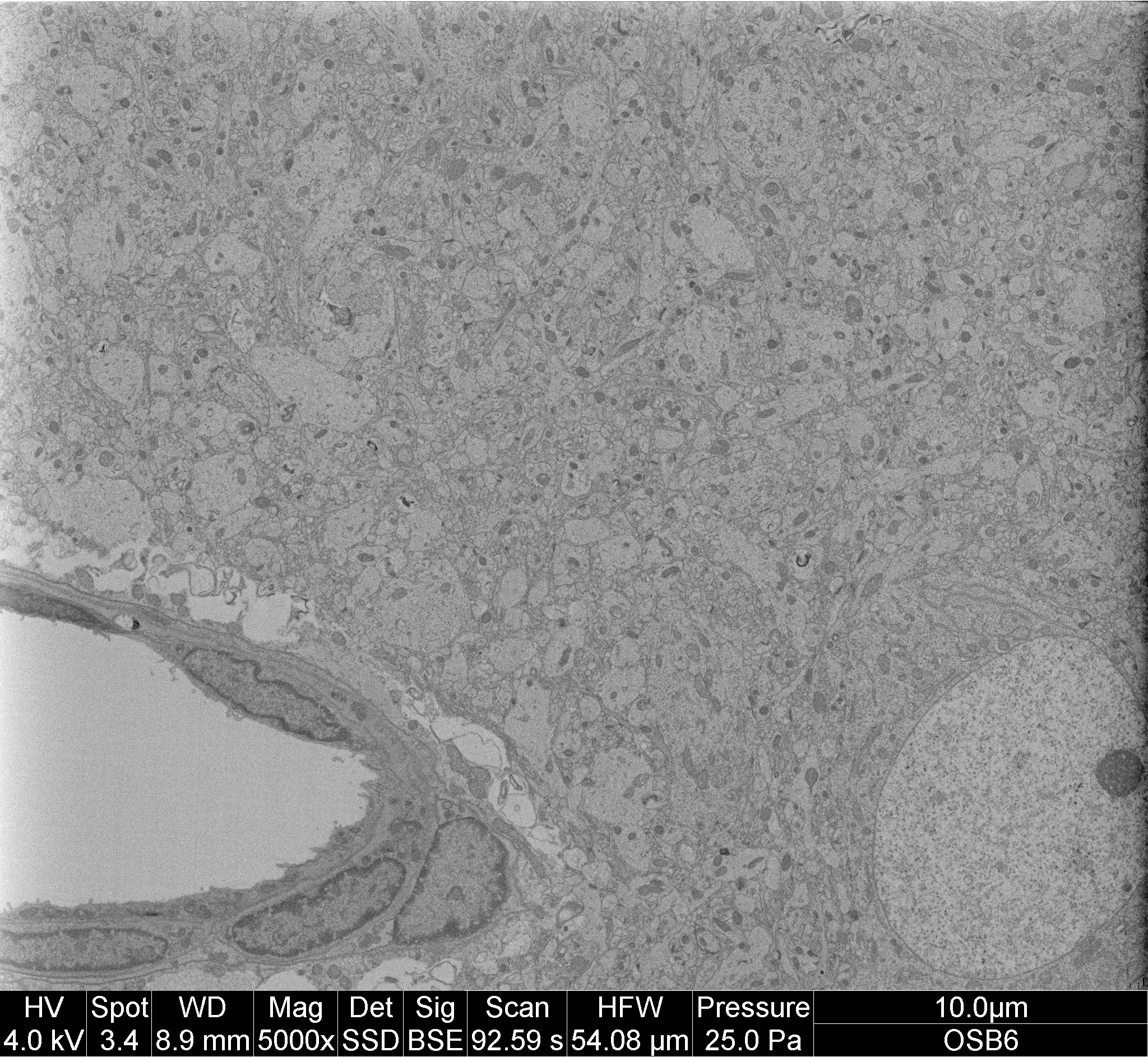

Supplement: Dataset S9 — (256.1 MB ZIP). [file pbio.0020329.sd009.zip › 040604_OS5_st1_821.tif]

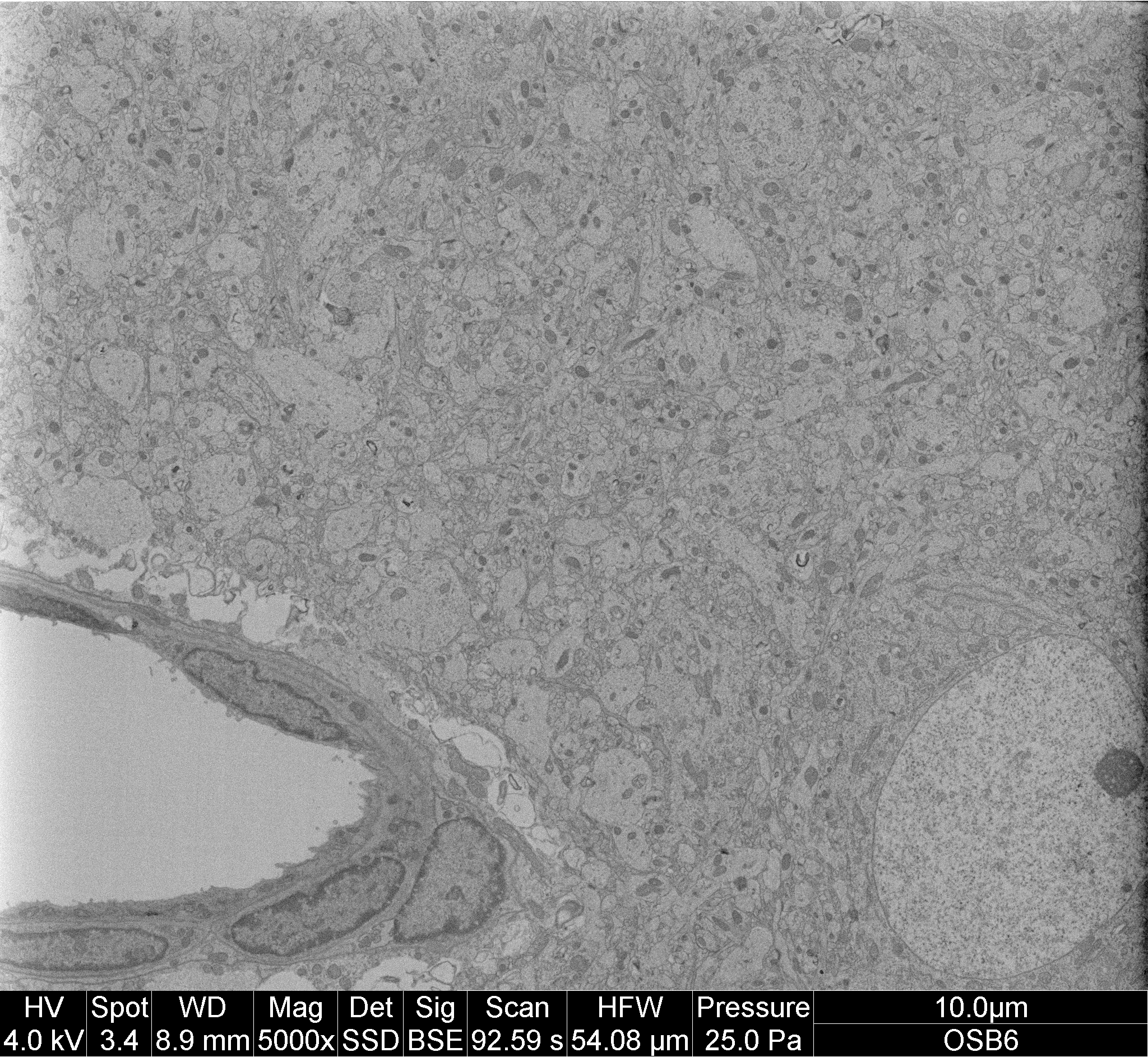

Supplement: Dataset S9 — (256.1 MB ZIP). [file pbio.0020329.sd009.zip › 040604_OS5_st1_822.tif]

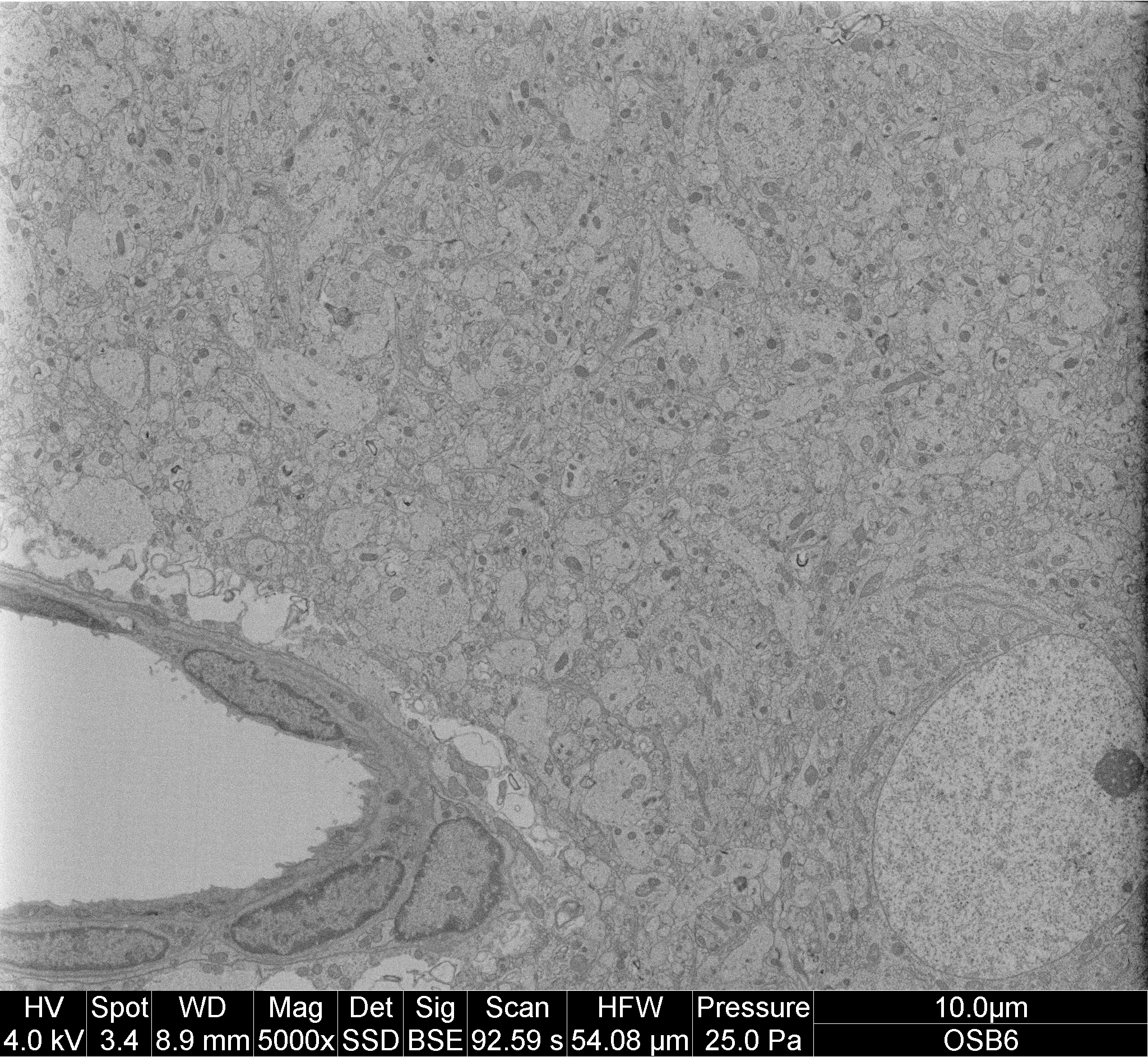

Supplement: Dataset S9 — (256.1 MB ZIP). [file pbio.0020329.sd009.zip › 040604_OS5_st1_823.tif]

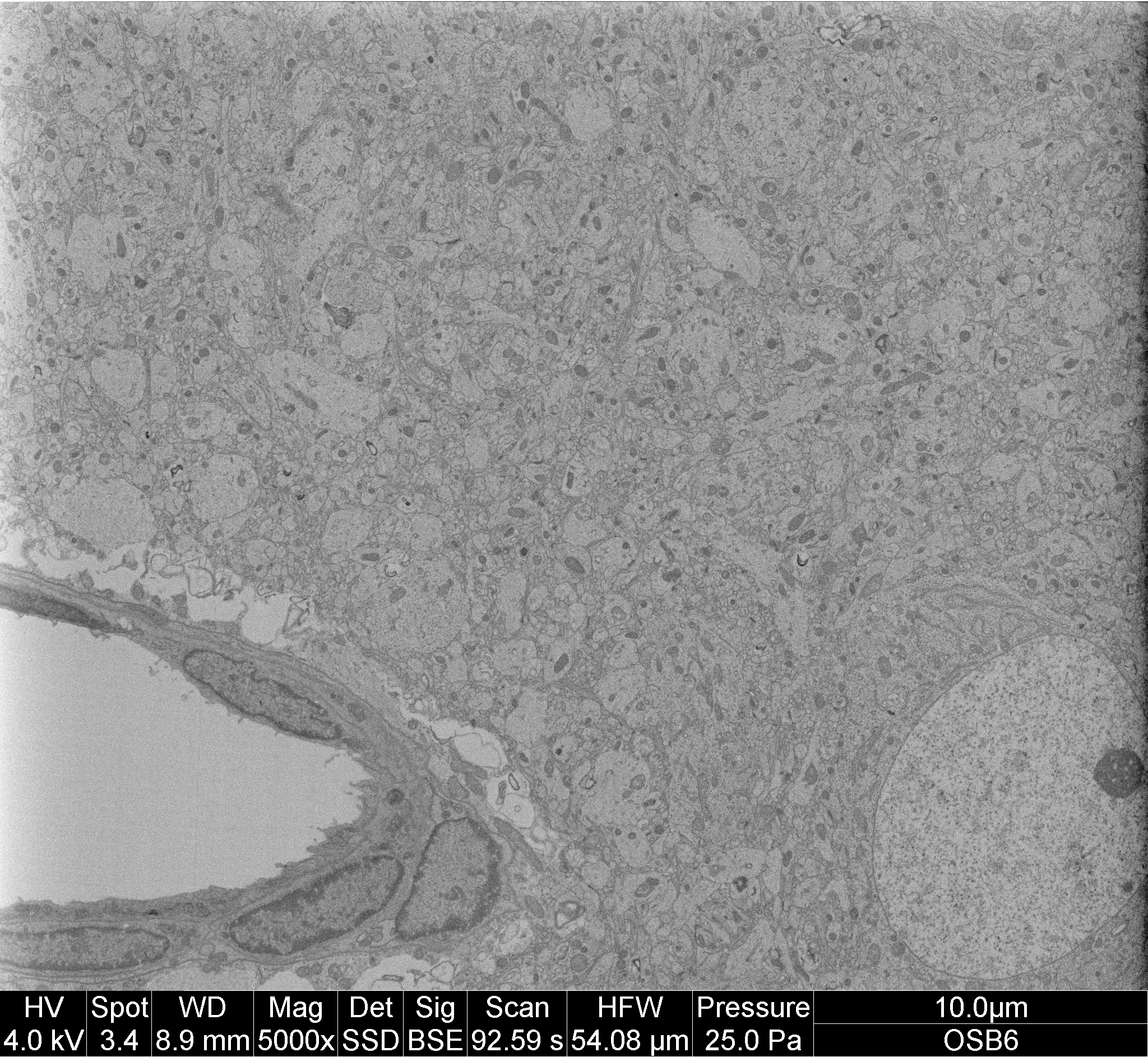

Supplement: Dataset S9 — (256.1 MB ZIP). [file pbio.0020329.sd009.zip › 040604_OS5_st1_824.tif]

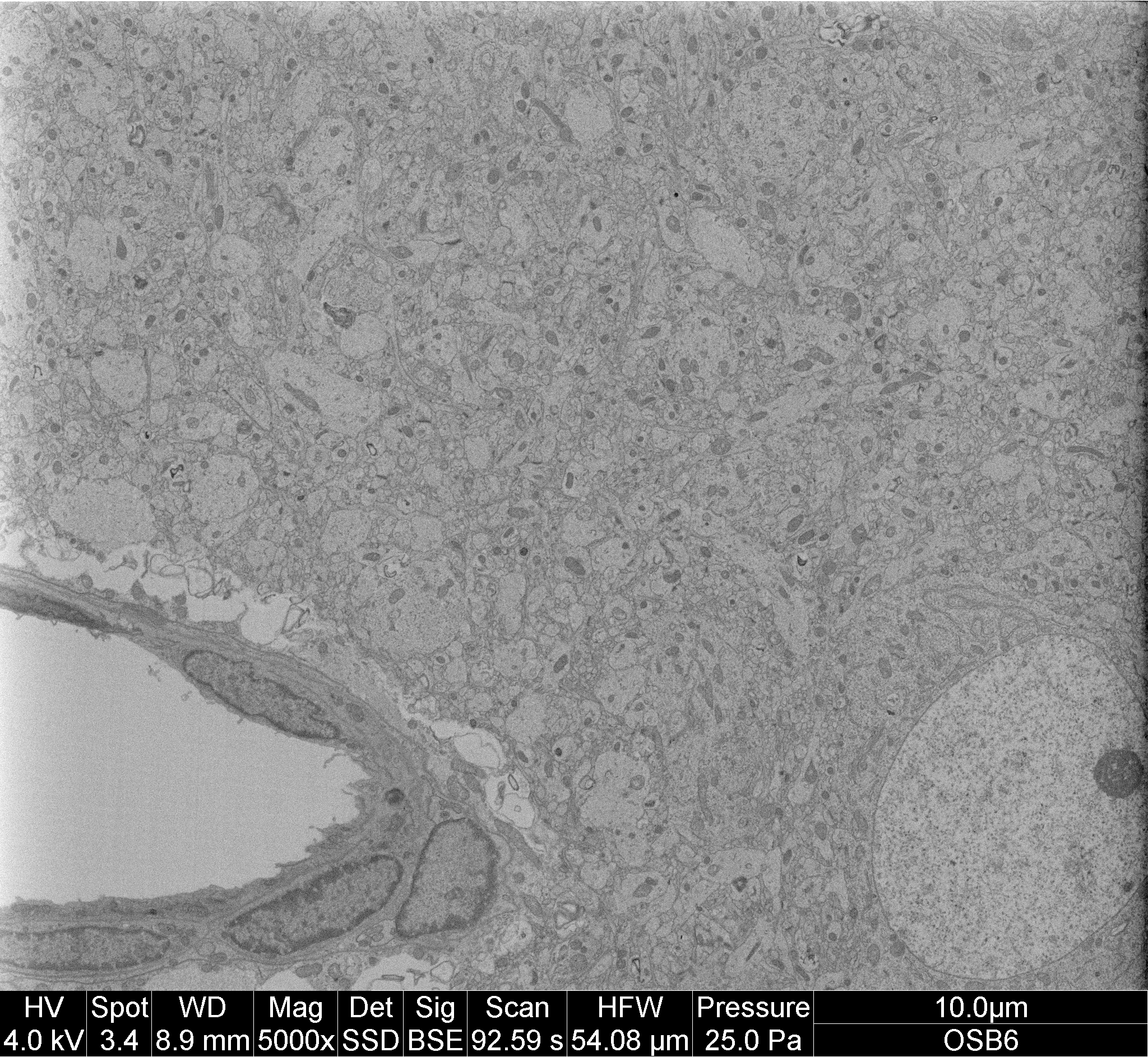

Supplement: Dataset S9 — (256.1 MB ZIP). [file pbio.0020329.sd009.zip › 040604_OS5_st1_825.tif]

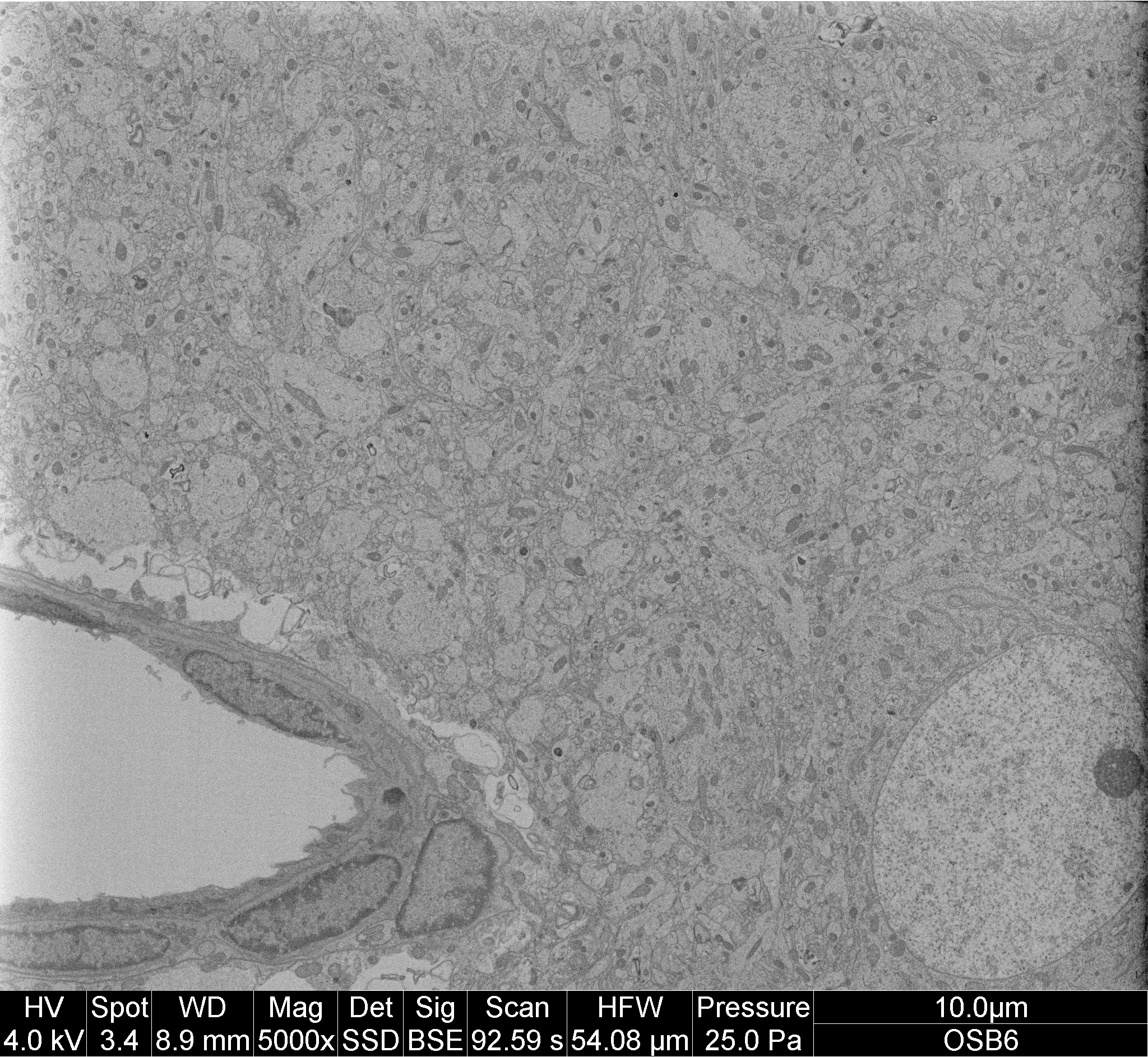

Supplement: Dataset S9 — (256.1 MB ZIP). [file pbio.0020329.sd009.zip › 040604_OS5_st1_826.tif]

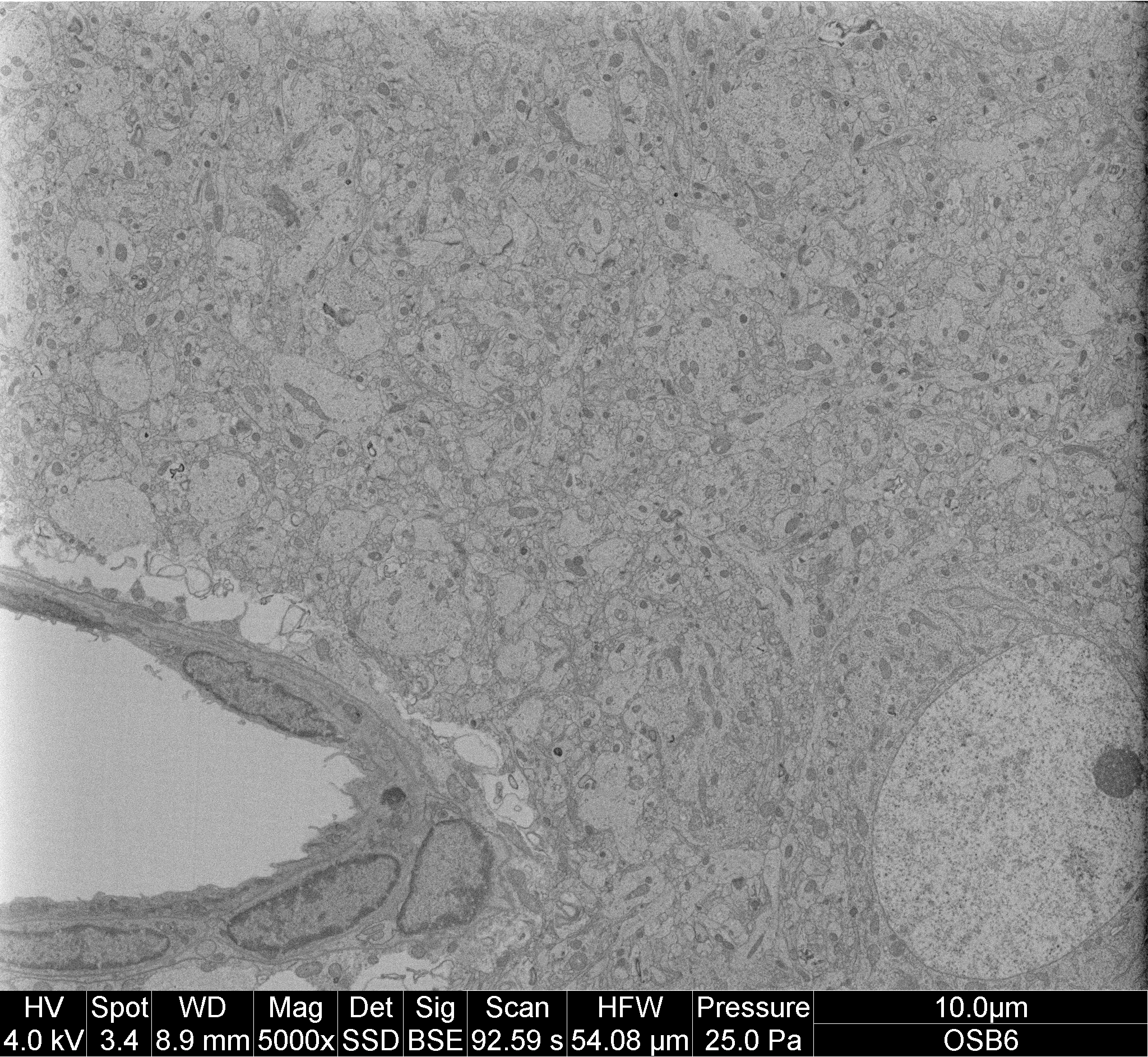

Supplement: Dataset S9 — (256.1 MB ZIP). [file pbio.0020329.sd009.zip › 040604_OS5_st1_827.tif]

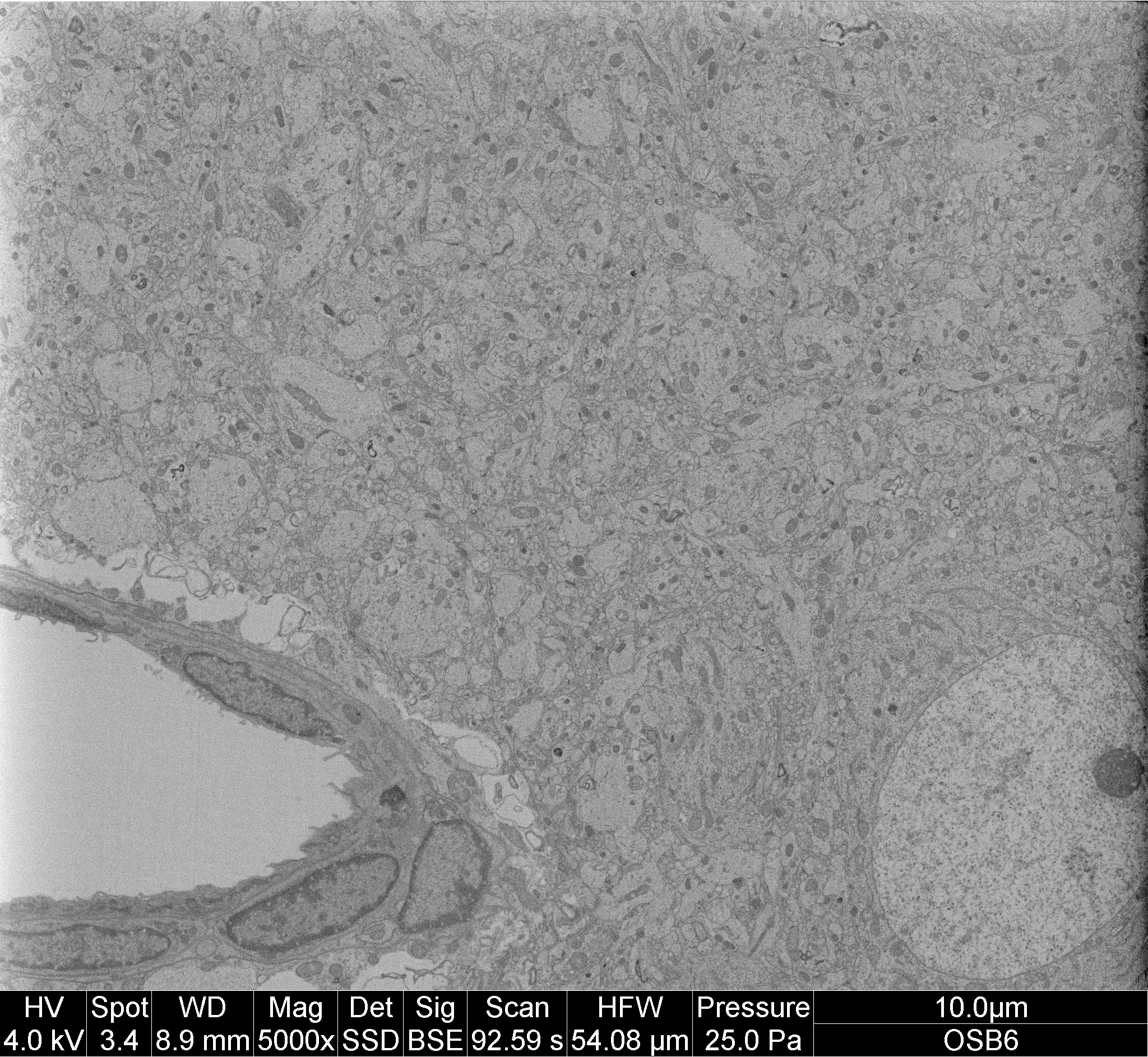

Supplement: Dataset S9 — (256.1 MB ZIP). [file pbio.0020329.sd009.zip › 040604_OS5_st1_828.tif]

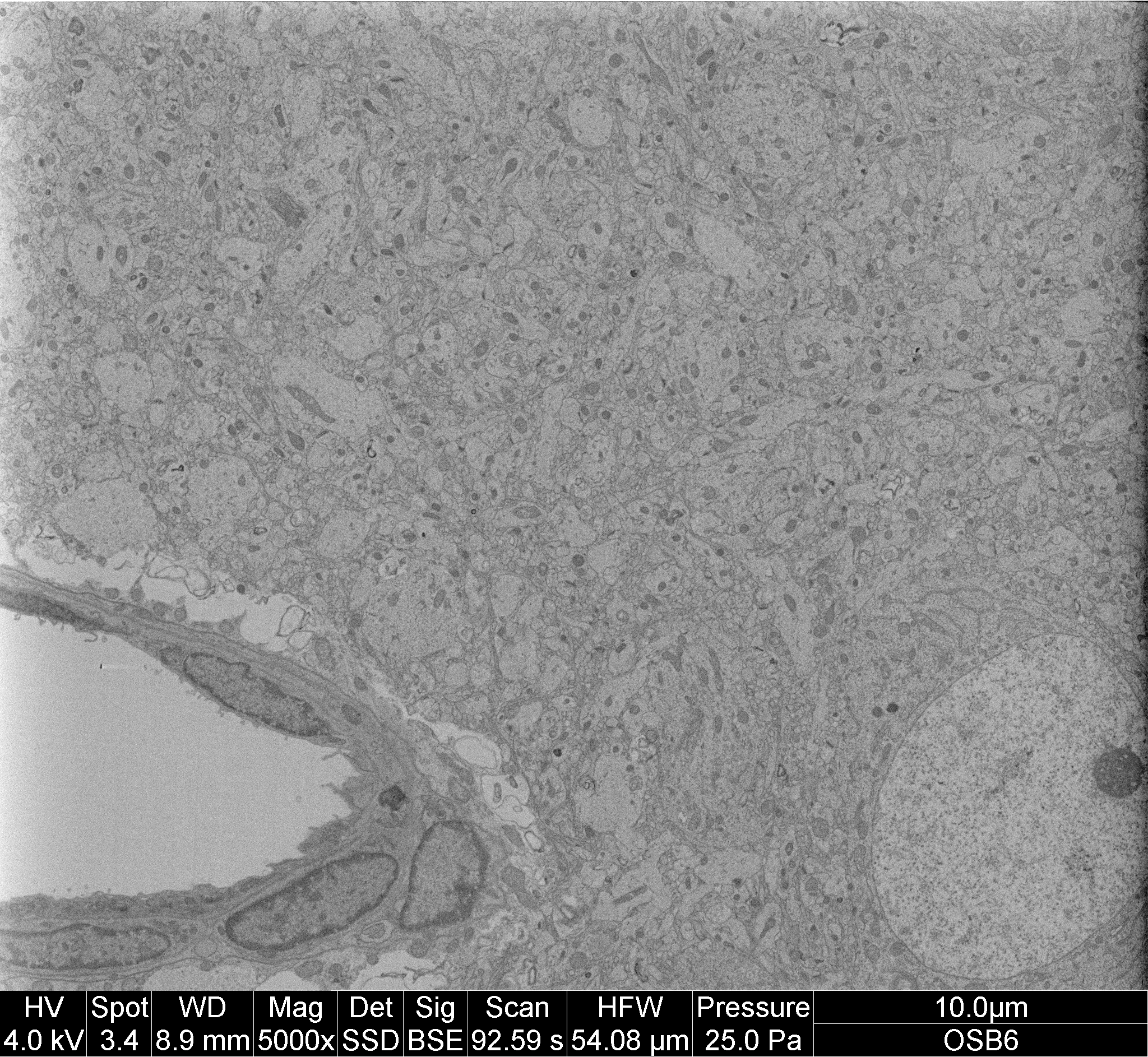

Supplement: Dataset S9 — (256.1 MB ZIP). [file pbio.0020329.sd009.zip › 040604_OS5_st1_829.tif]

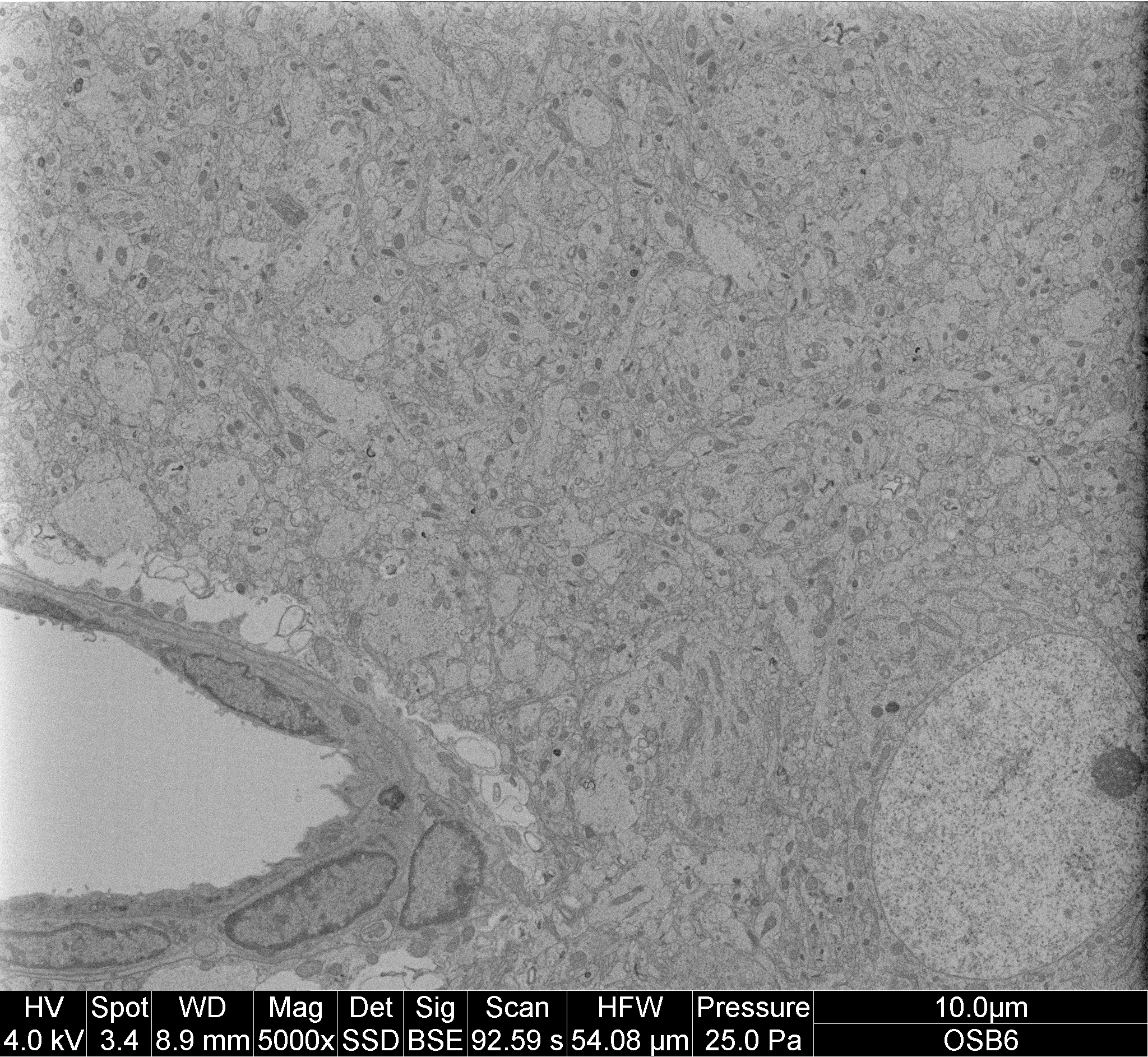

Supplement: Dataset S9 — (256.1 MB ZIP). [file pbio.0020329.sd009.zip › 040604_OS5_st1_830.tif]

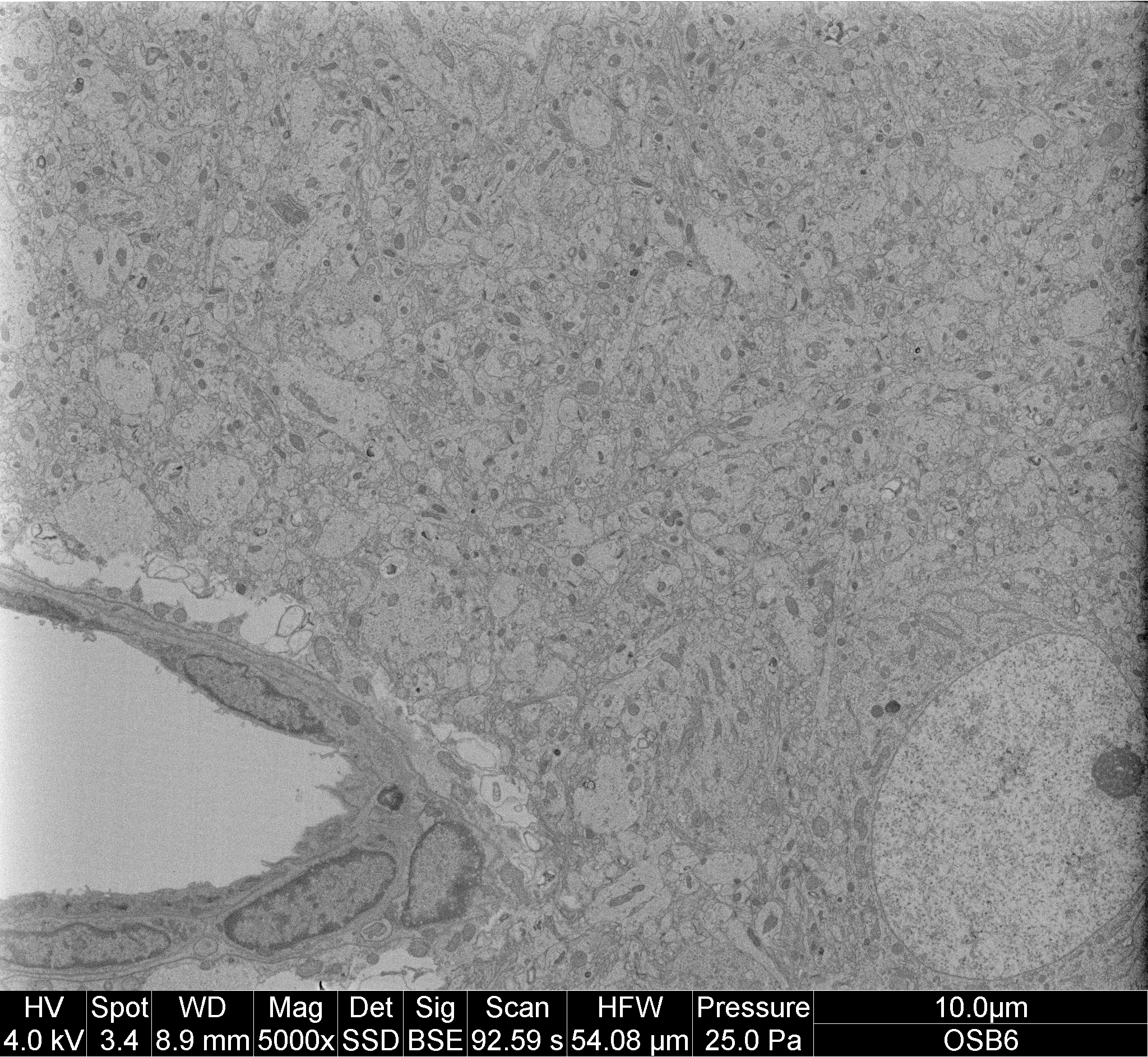

Supplement: Dataset S9 — (256.1 MB ZIP). [file pbio.0020329.sd009.zip › 040604_OS5_st1_831.tif]

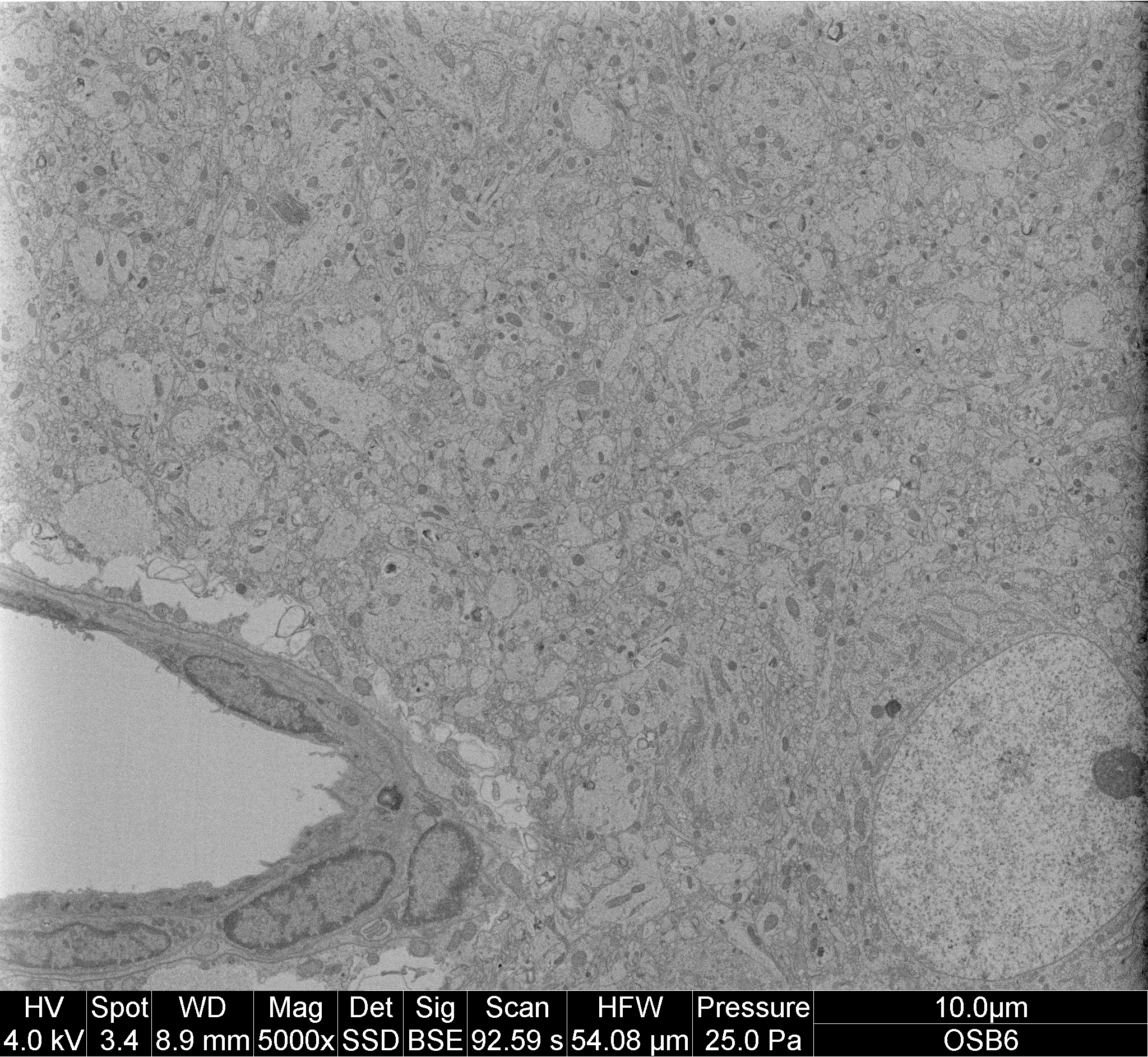

Supplement: Dataset S9 — (256.1 MB ZIP). [file pbio.0020329.sd009.zip › 040604_OS5_st1_832.tif]

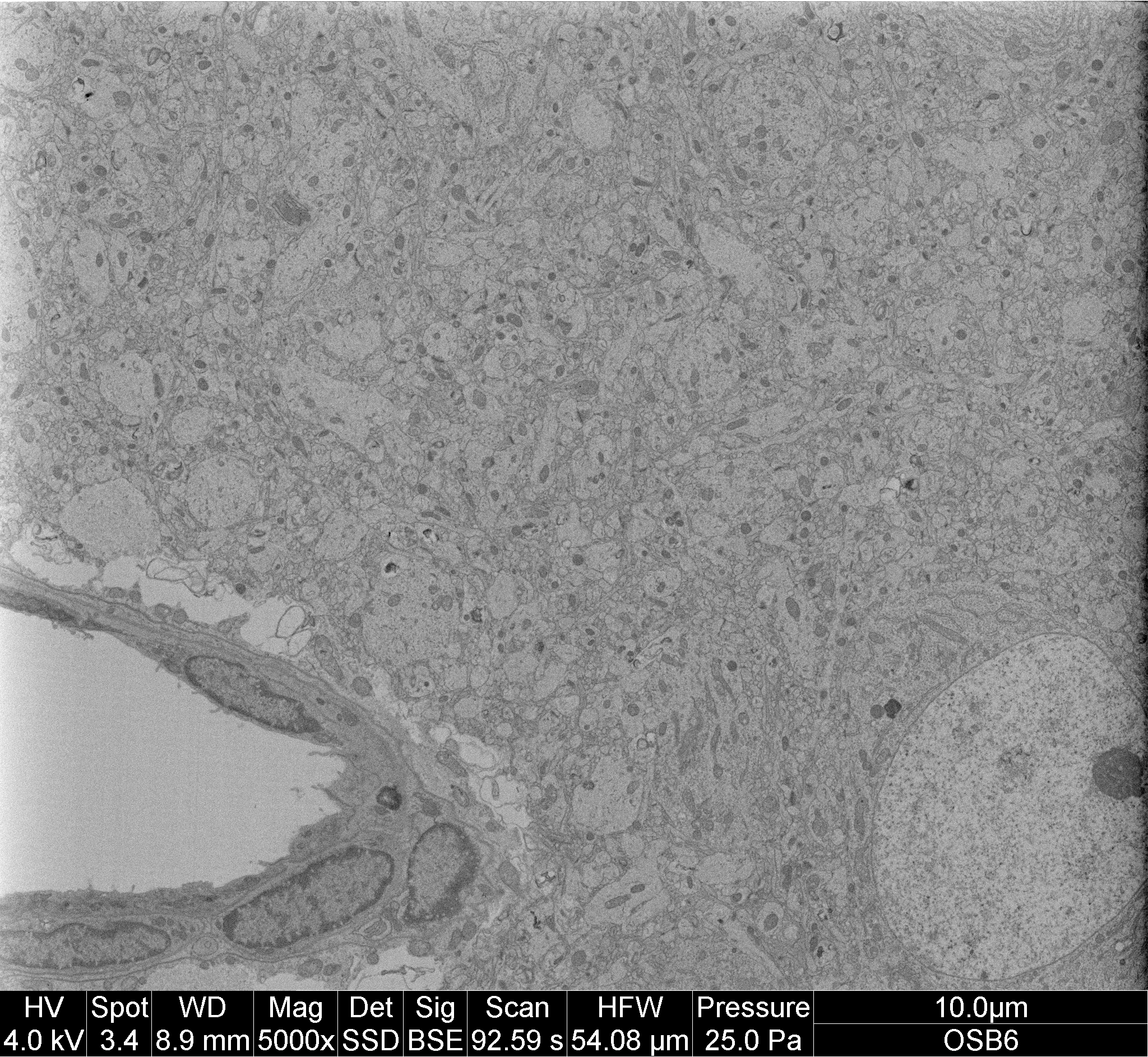

Supplement: Dataset S9 — (256.1 MB ZIP). [file pbio.0020329.sd009.zip › 040604_OS5_st1_833.tif]

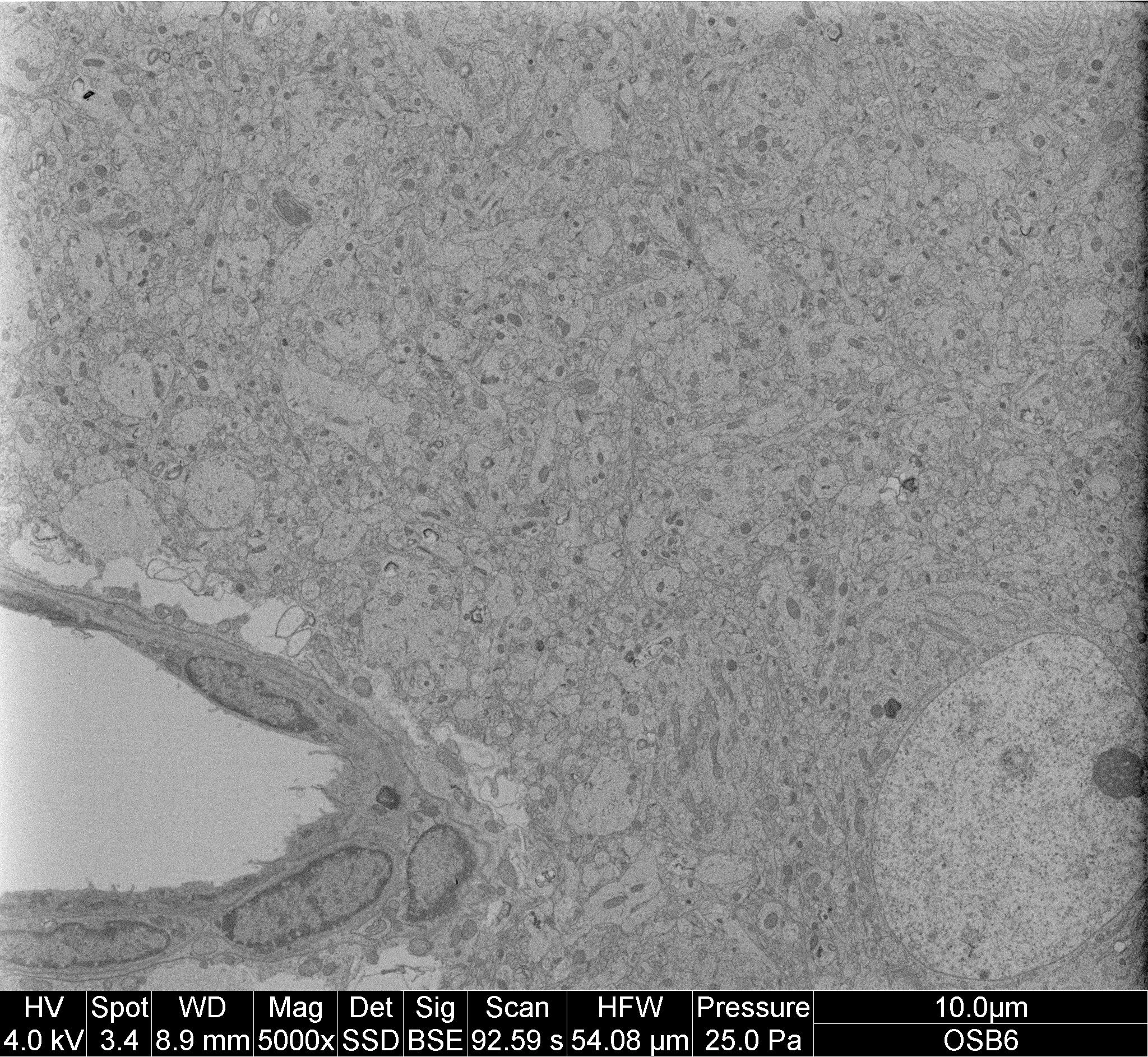

Supplement: Dataset S9 — (256.1 MB ZIP). [file pbio.0020329.sd009.zip › 040604_OS5_st1_834.tif]

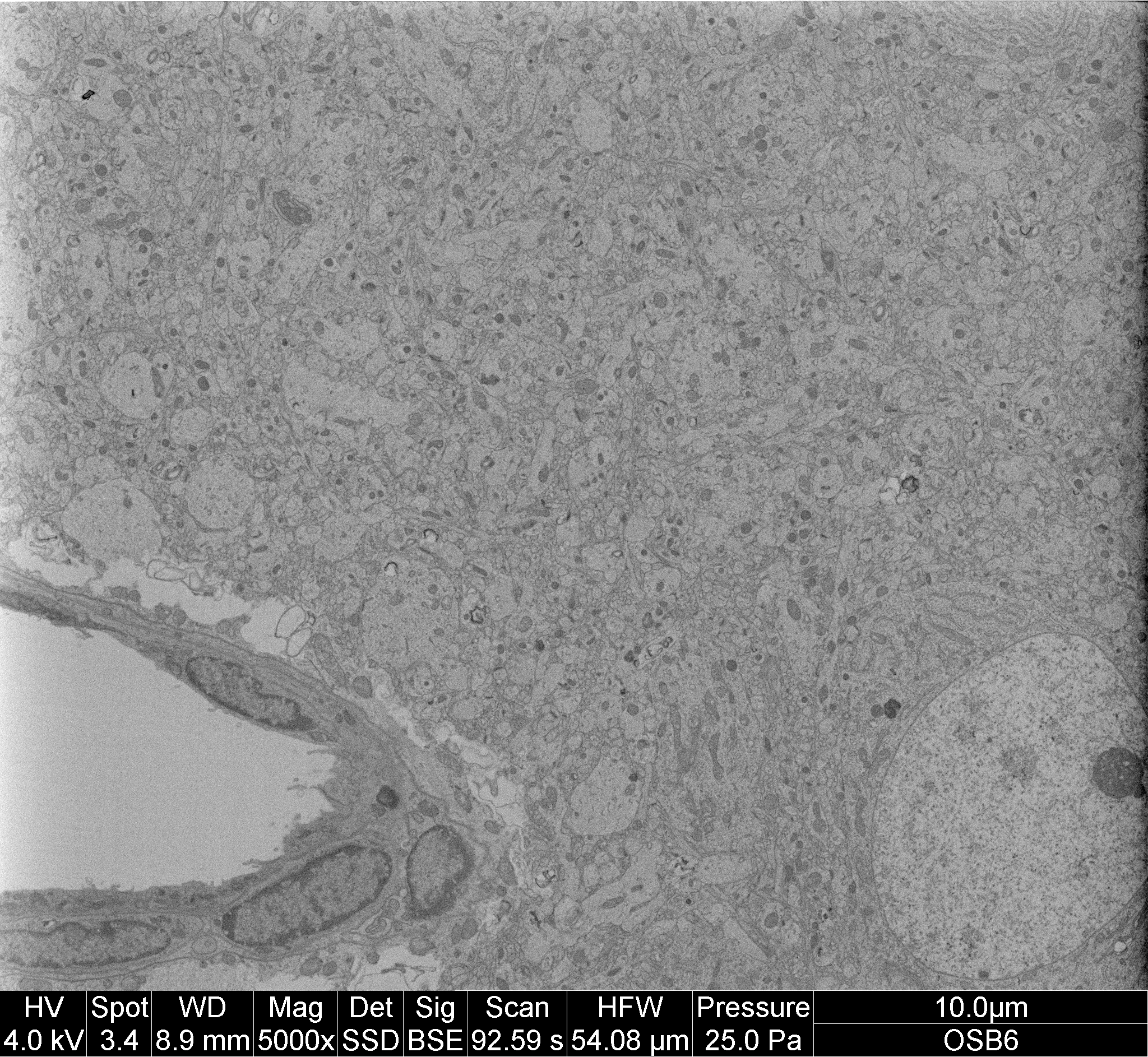

Supplement: Dataset S9 — (256.1 MB ZIP). [file pbio.0020329.sd009.zip › 040604_OS5_st1_835.tif]

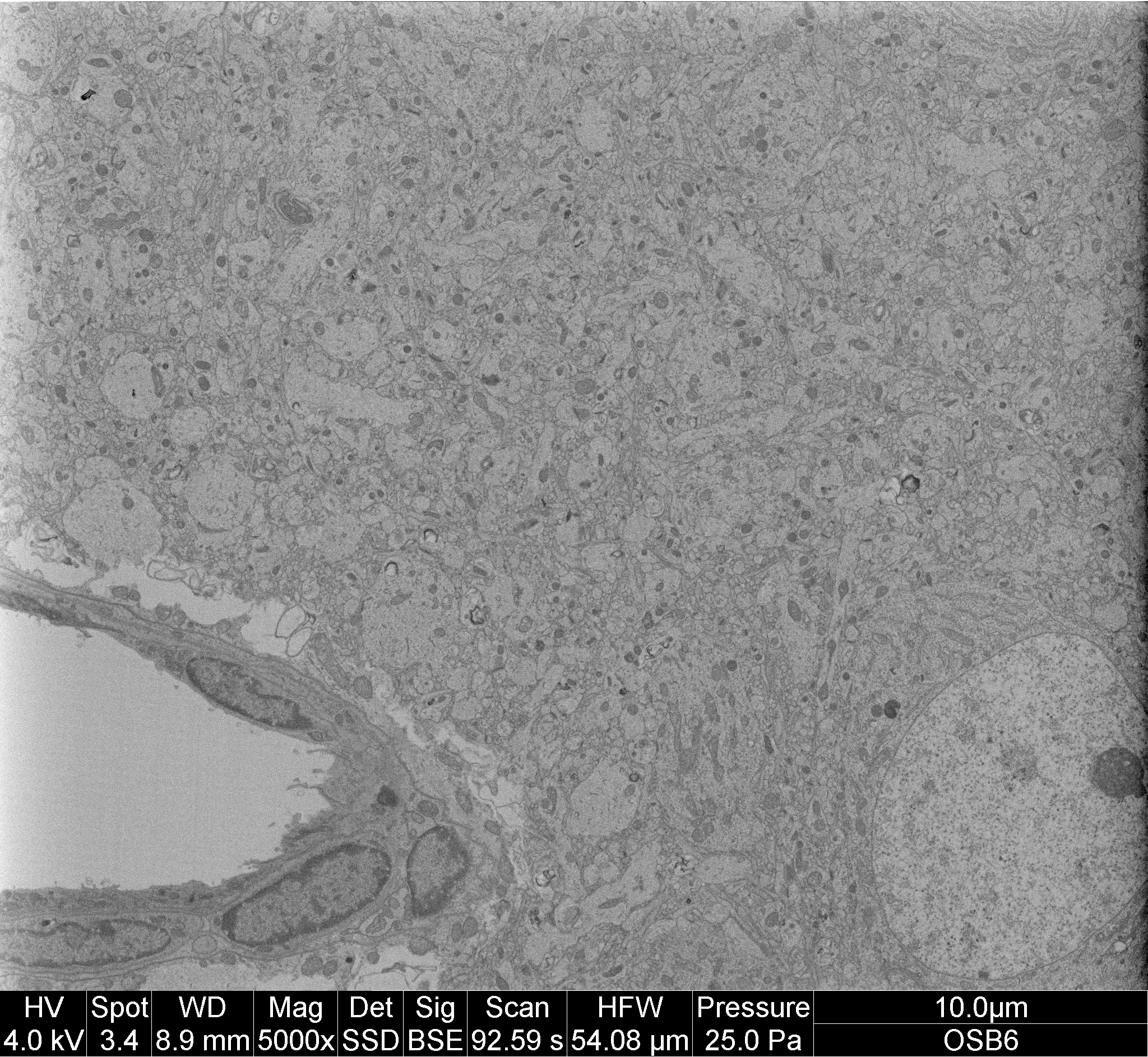

Supplement: Dataset S9 — (256.1 MB ZIP). [file pbio.0020329.sd009.zip › 040604_OS5_st1_836.tif]

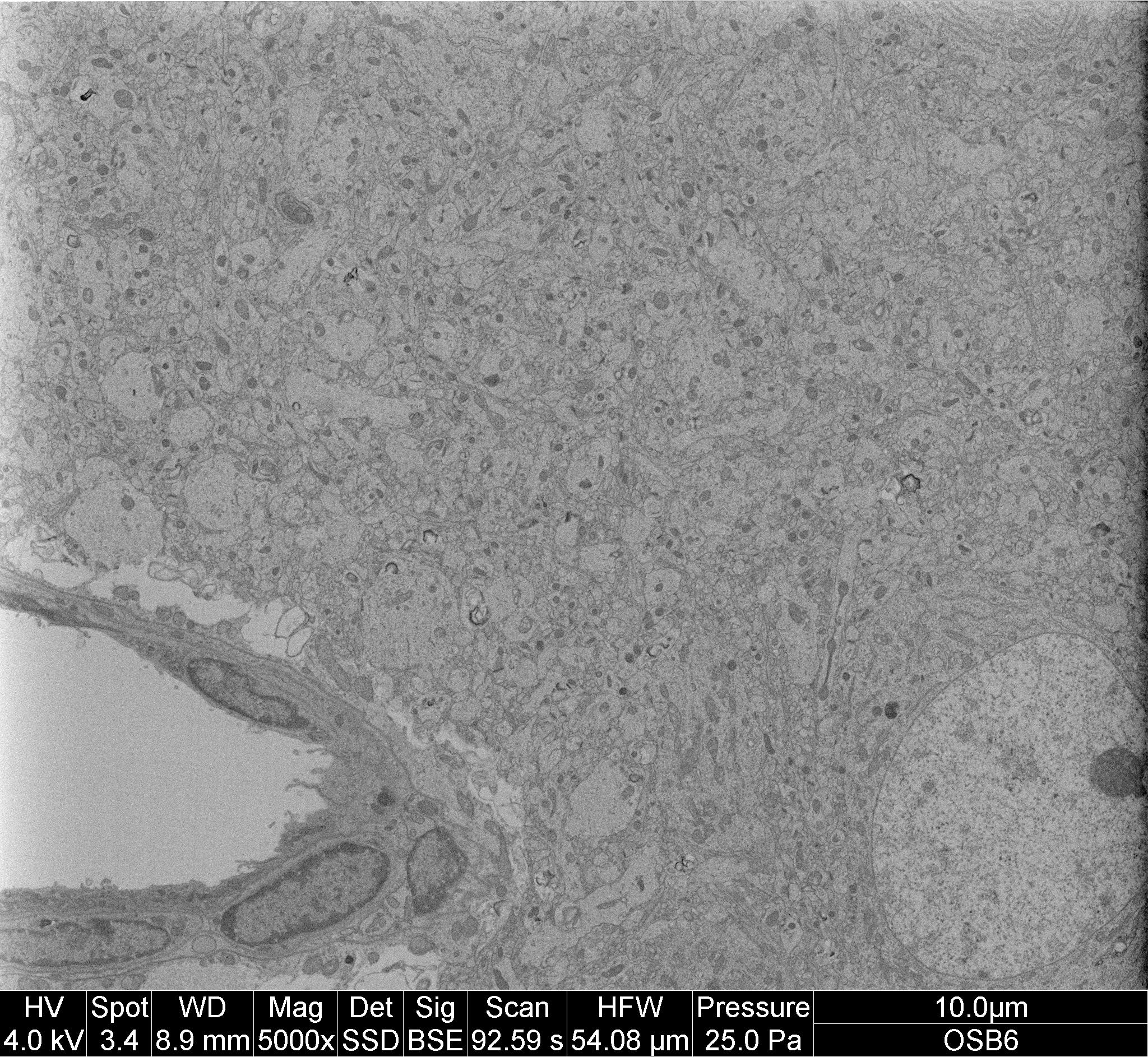

Supplement: Dataset S9 — (256.1 MB ZIP). [file pbio.0020329.sd009.zip › 040604_OS5_st1_837.tif]

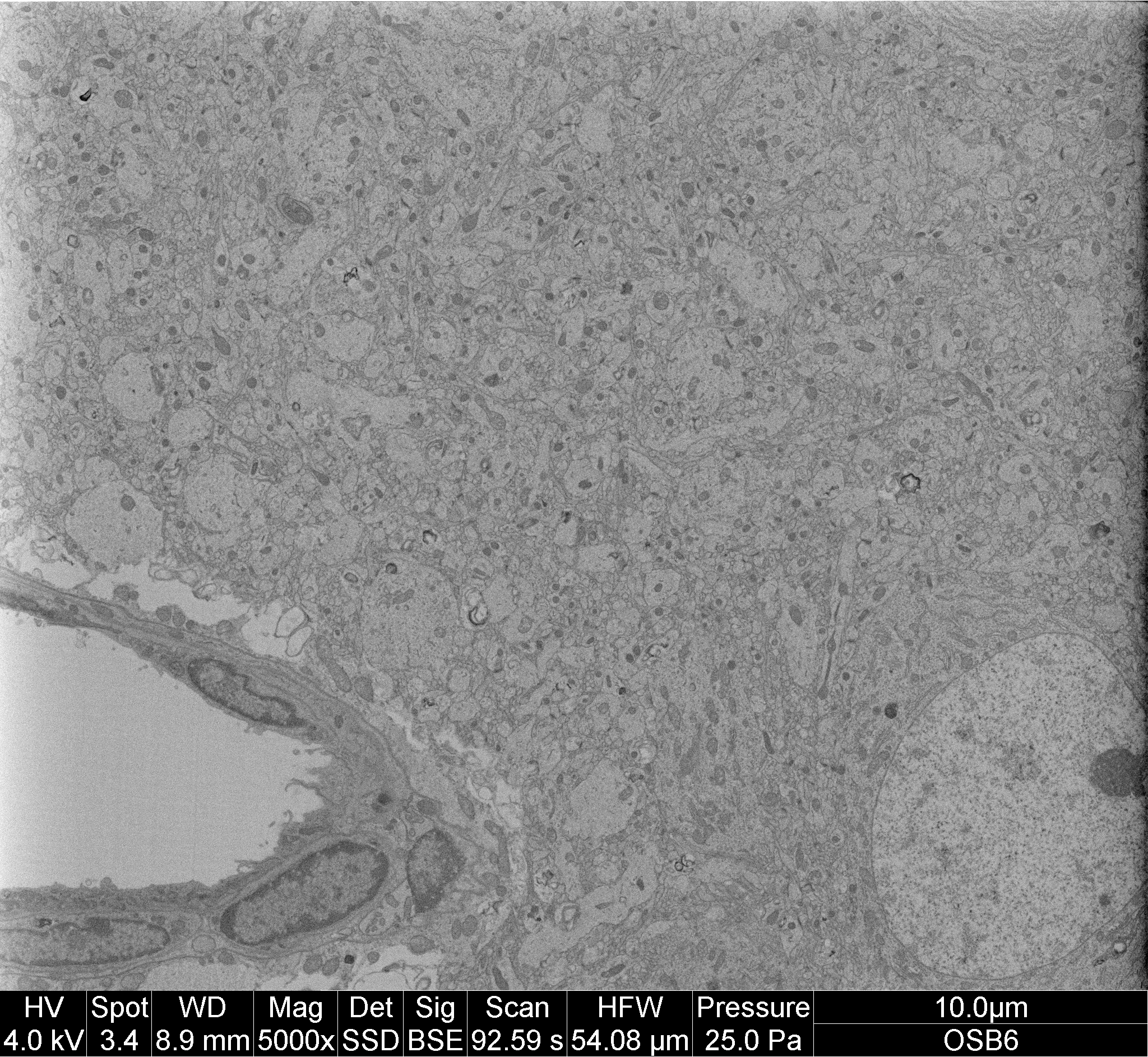

Supplement: Dataset S9 — (256.1 MB ZIP). [file pbio.0020329.sd009.zip › 040604_OS5_st1_838.tif]

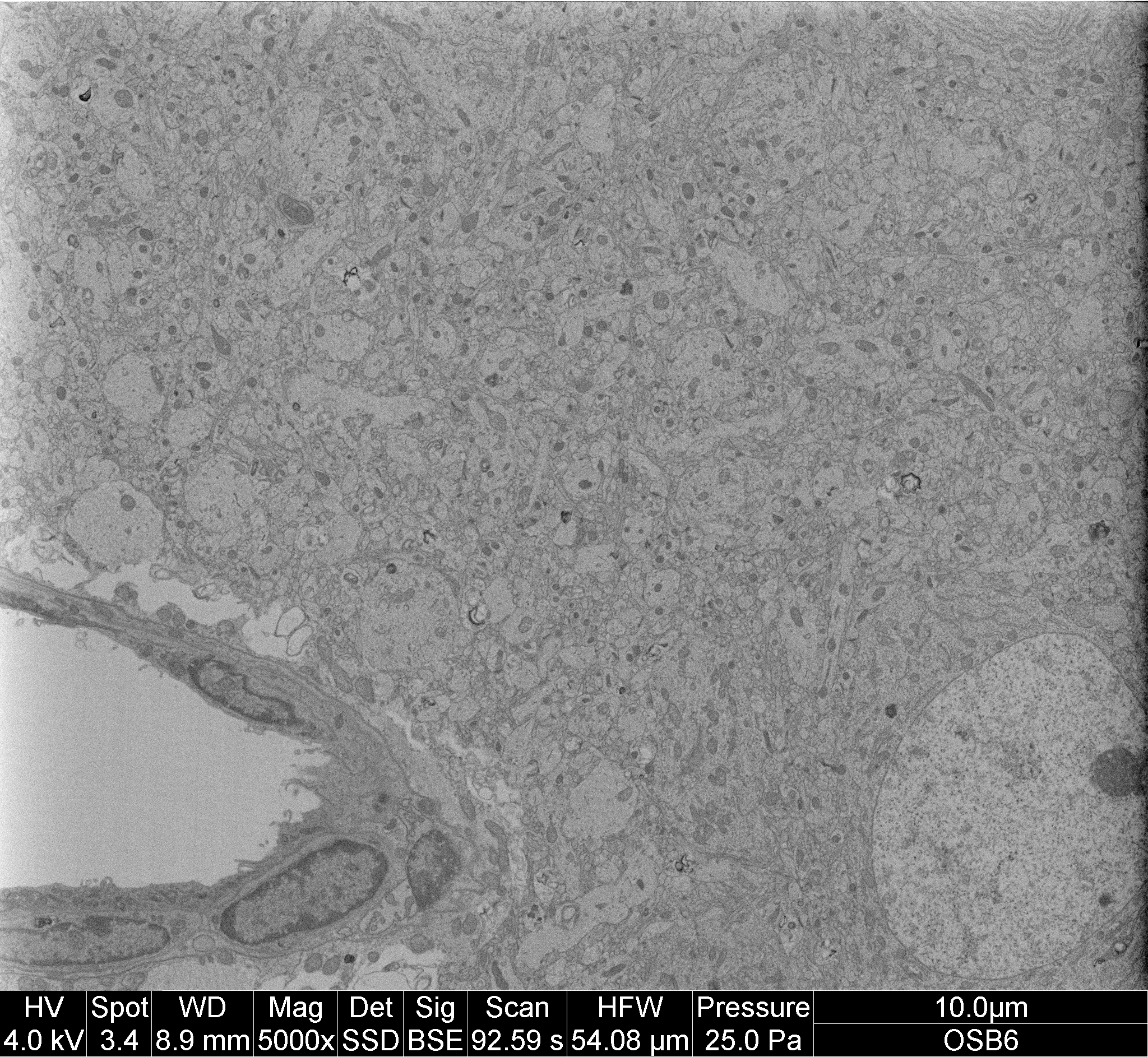

Supplement: Dataset S9 — (256.1 MB ZIP). [file pbio.0020329.sd009.zip › 040604_OS5_st1_839.tif]

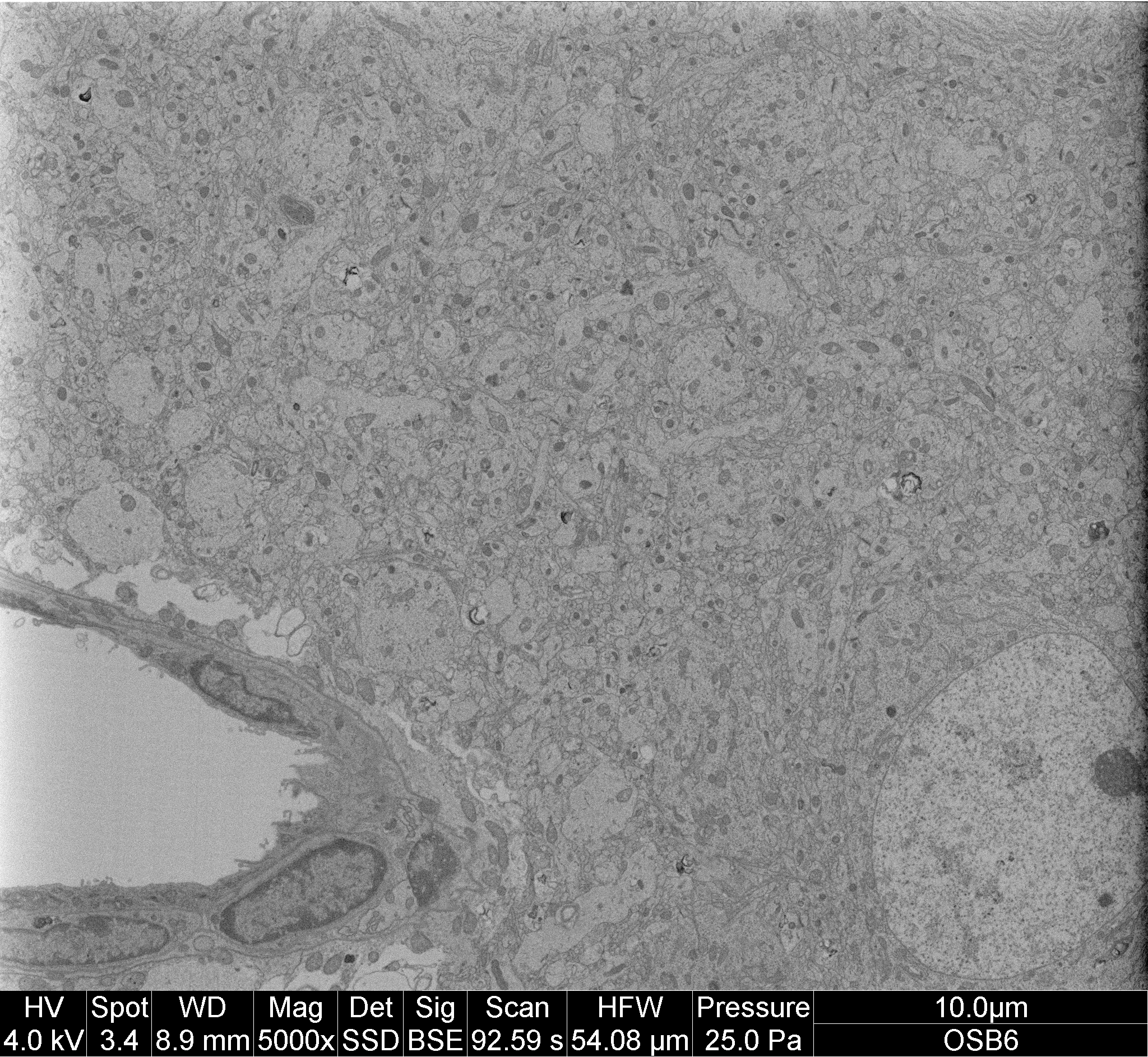

Supplement: Dataset S9 — (256.1 MB ZIP). [file pbio.0020329.sd009.zip › 040604_OS5_st1_840.tif]

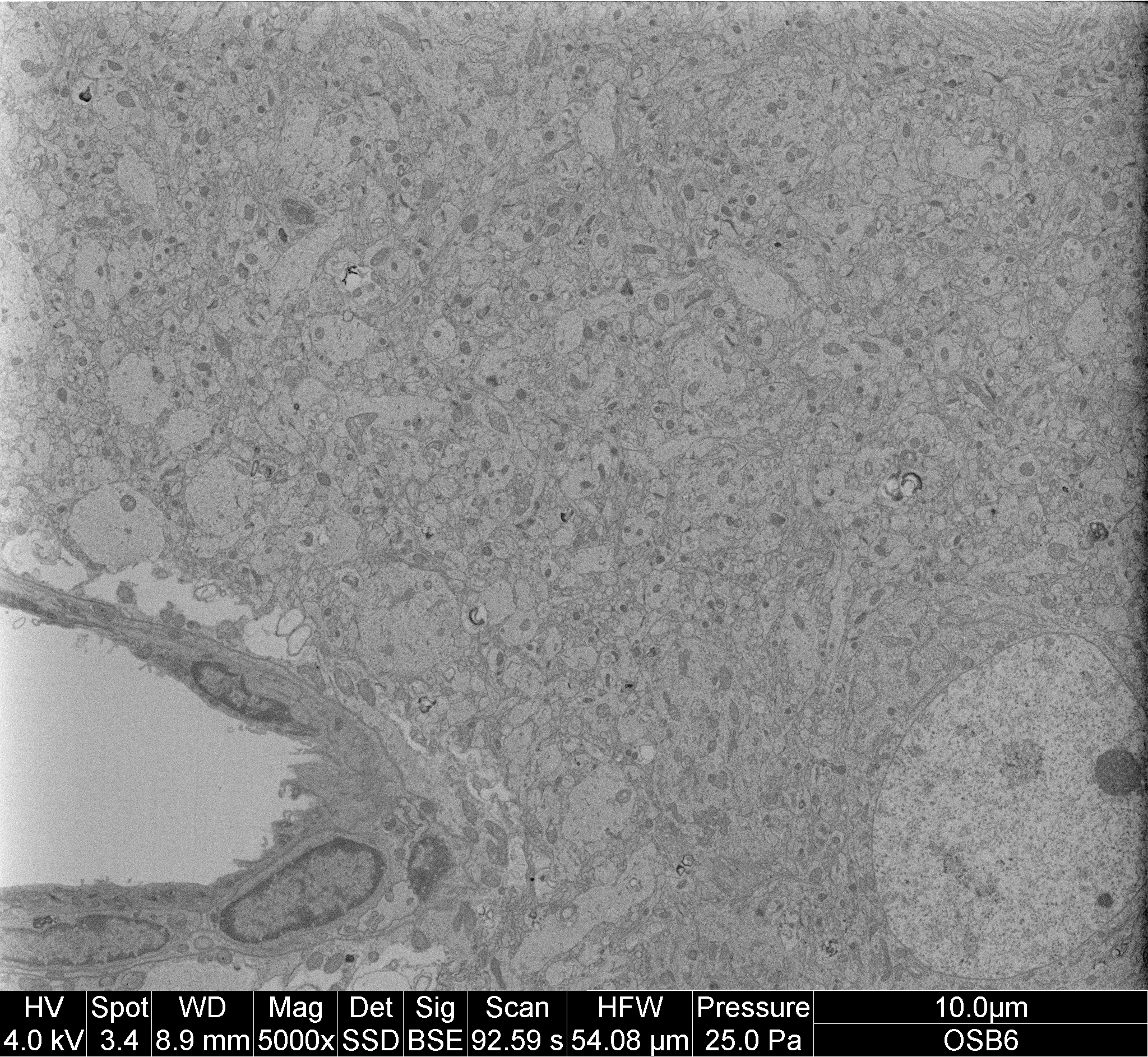

Supplement: Dataset S9 — (256.1 MB ZIP). [file pbio.0020329.sd009.zip › 040604_OS5_st1_841.tif]

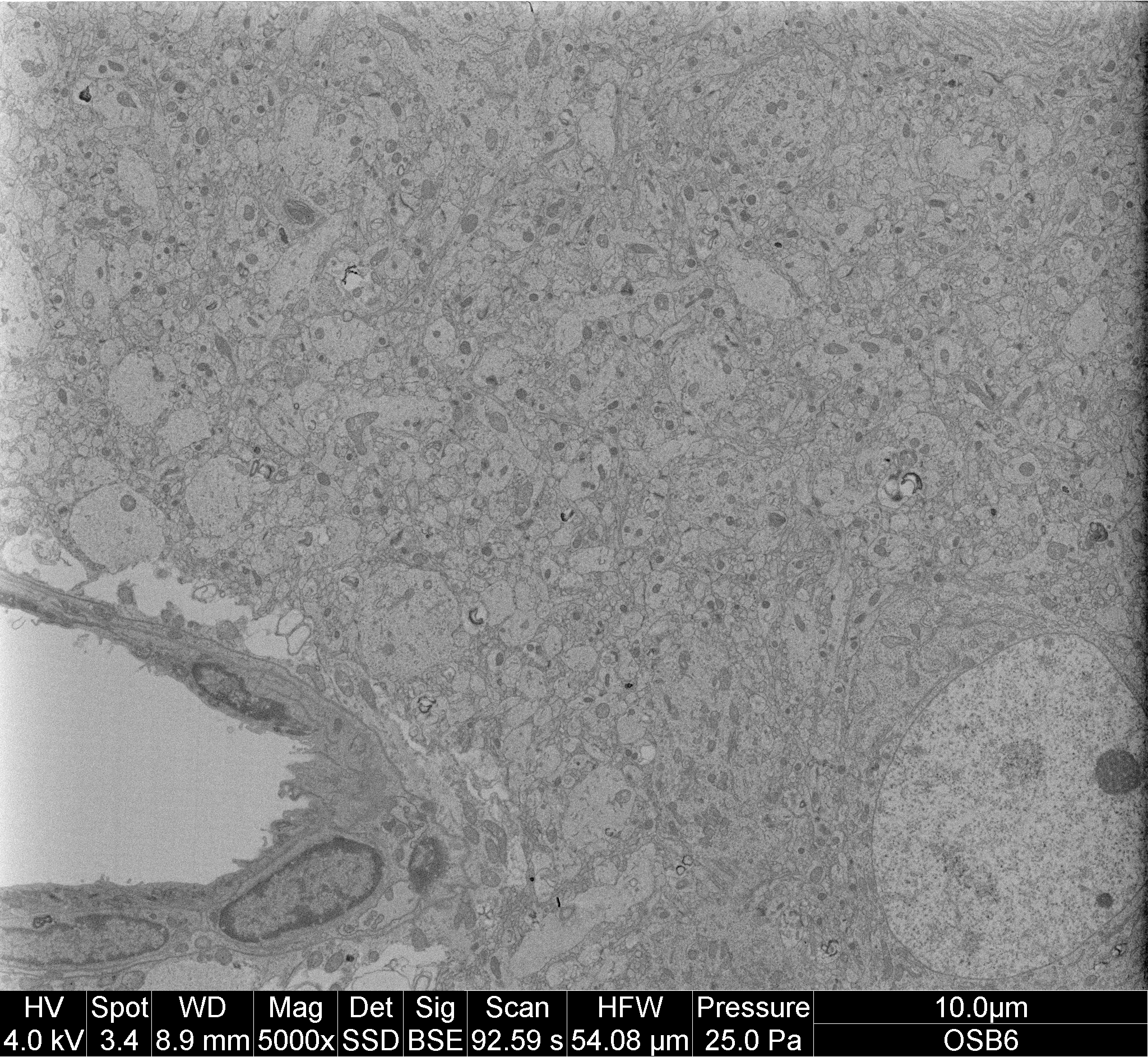

Supplement: Dataset S9 — (256.1 MB ZIP). [file pbio.0020329.sd009.zip › 040604_OS5_st1_842.tif]

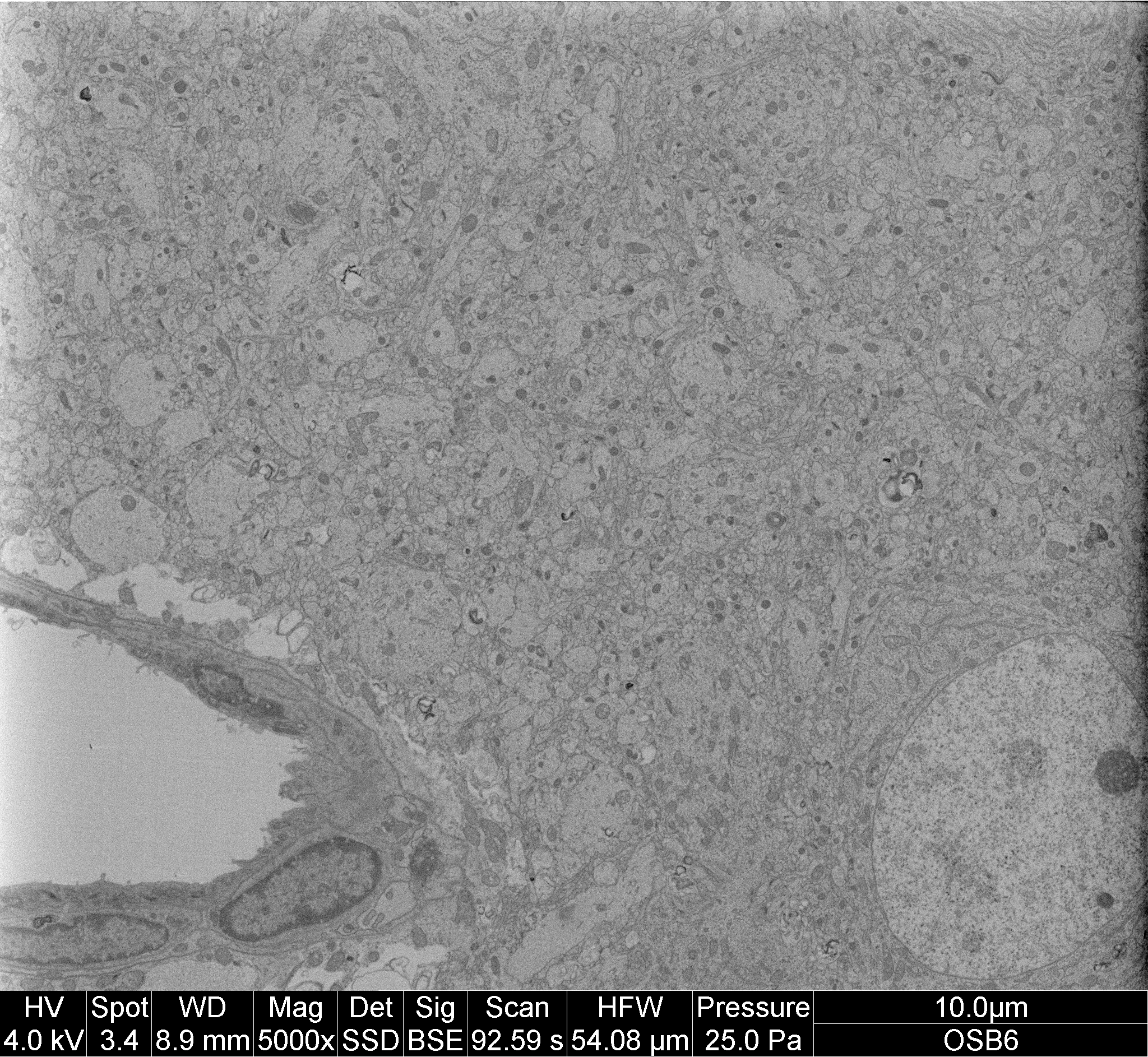

Supplement: Dataset S9 — (256.1 MB ZIP). [file pbio.0020329.sd009.zip › 040604_OS5_st1_843.tif]

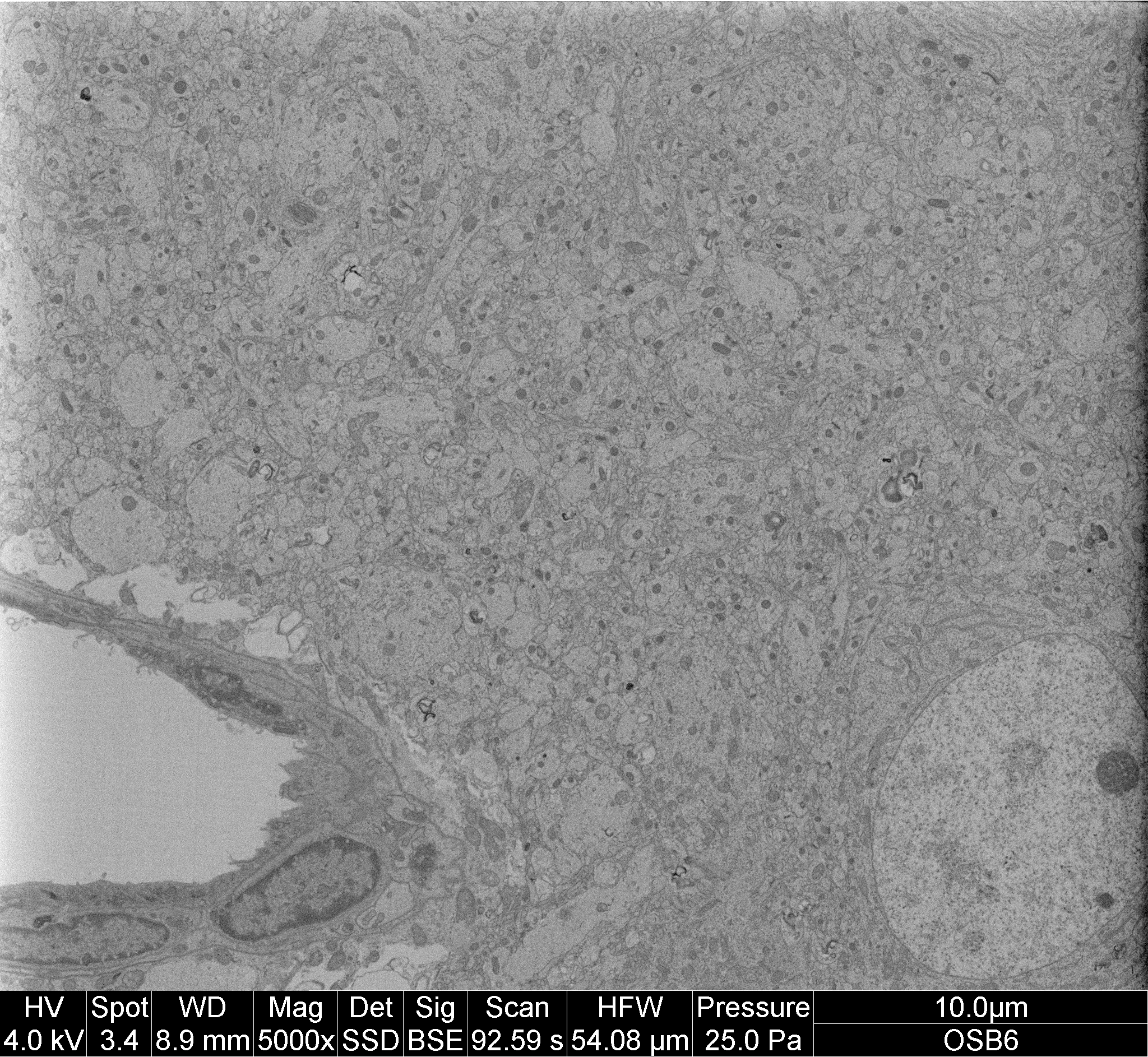

Supplement: Dataset S9 — (256.1 MB ZIP). [file pbio.0020329.sd009.zip › 040604_OS5_st1_844.tif]

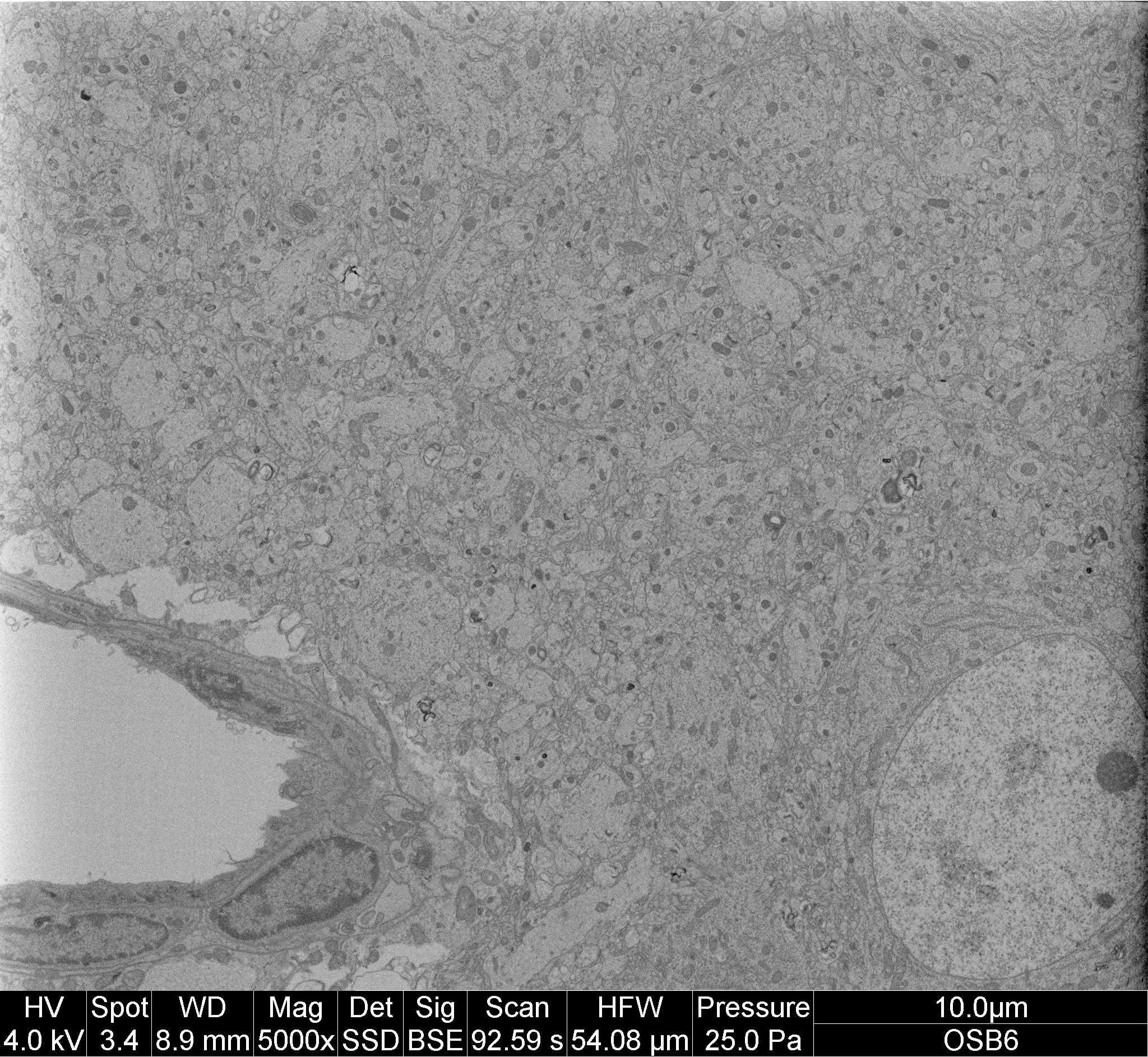

Supplement: Dataset S9 — (256.1 MB ZIP). [file pbio.0020329.sd009.zip › 040604_OS5_st1_845.tif]

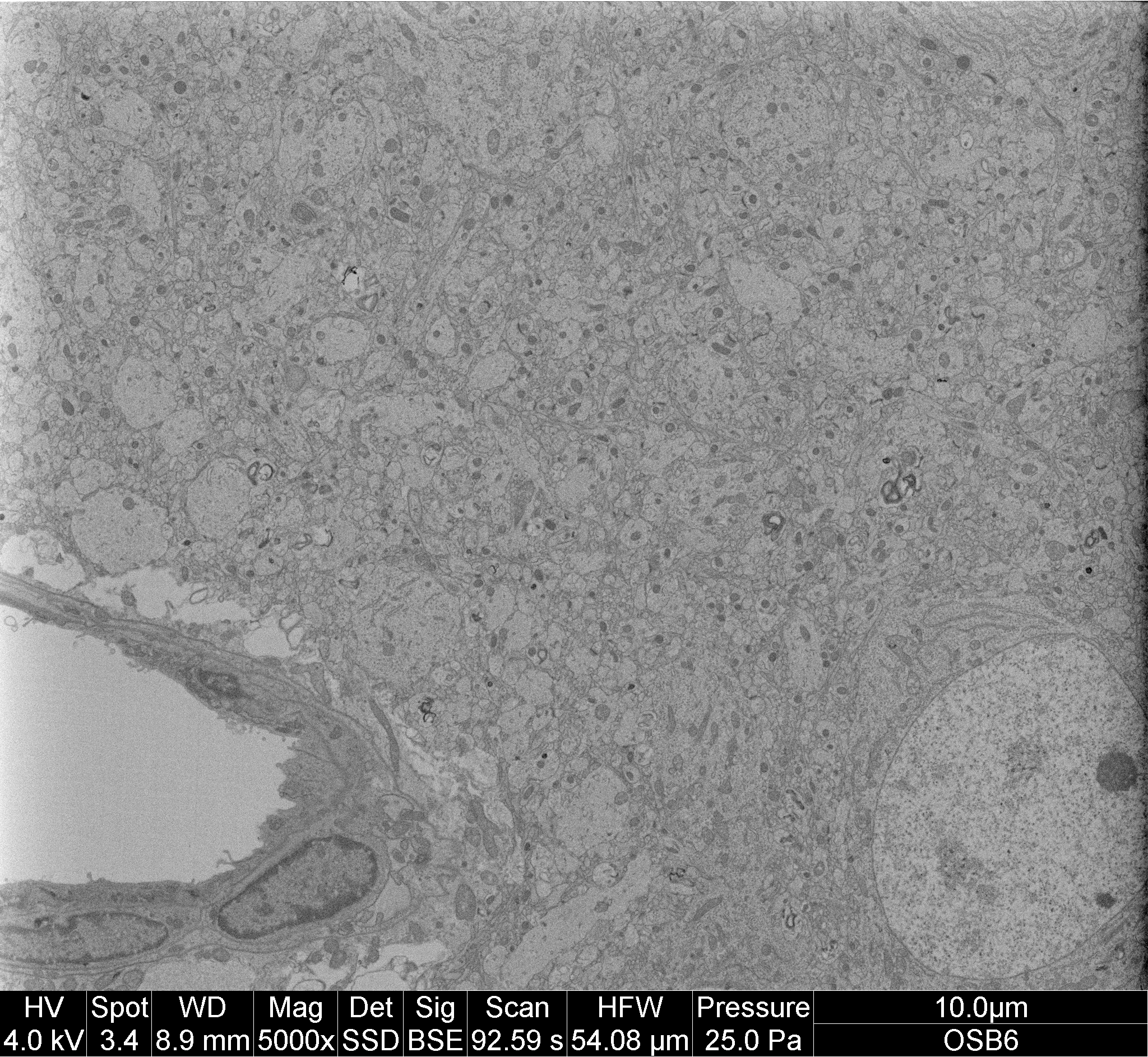

Supplement: Dataset S9 — (256.1 MB ZIP). [file pbio.0020329.sd009.zip › 040604_OS5_st1_846.tif]

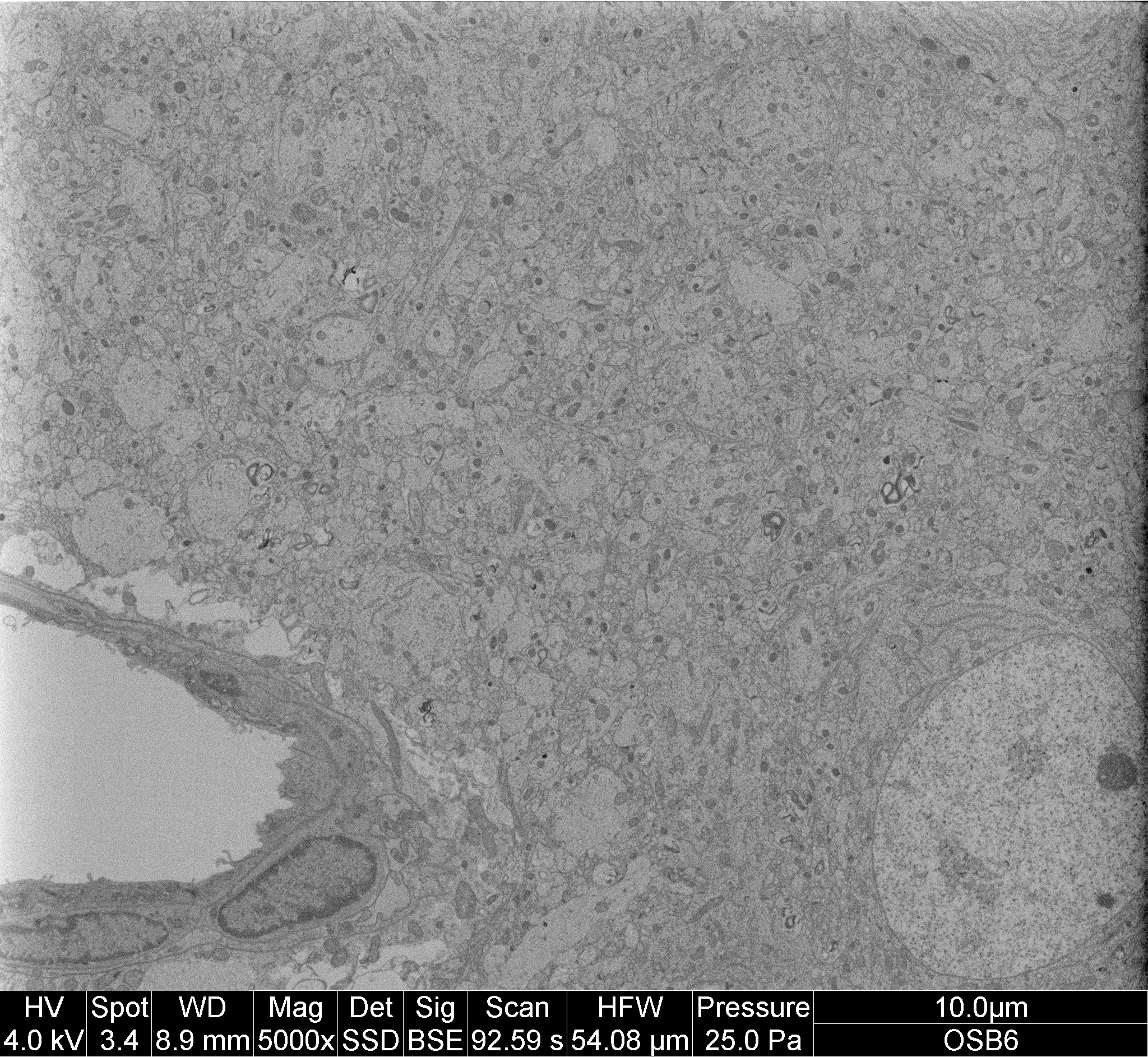

Supplement: Dataset S9 — (256.1 MB ZIP). [file pbio.0020329.sd009.zip › 040604_OS5_st1_847.tif]

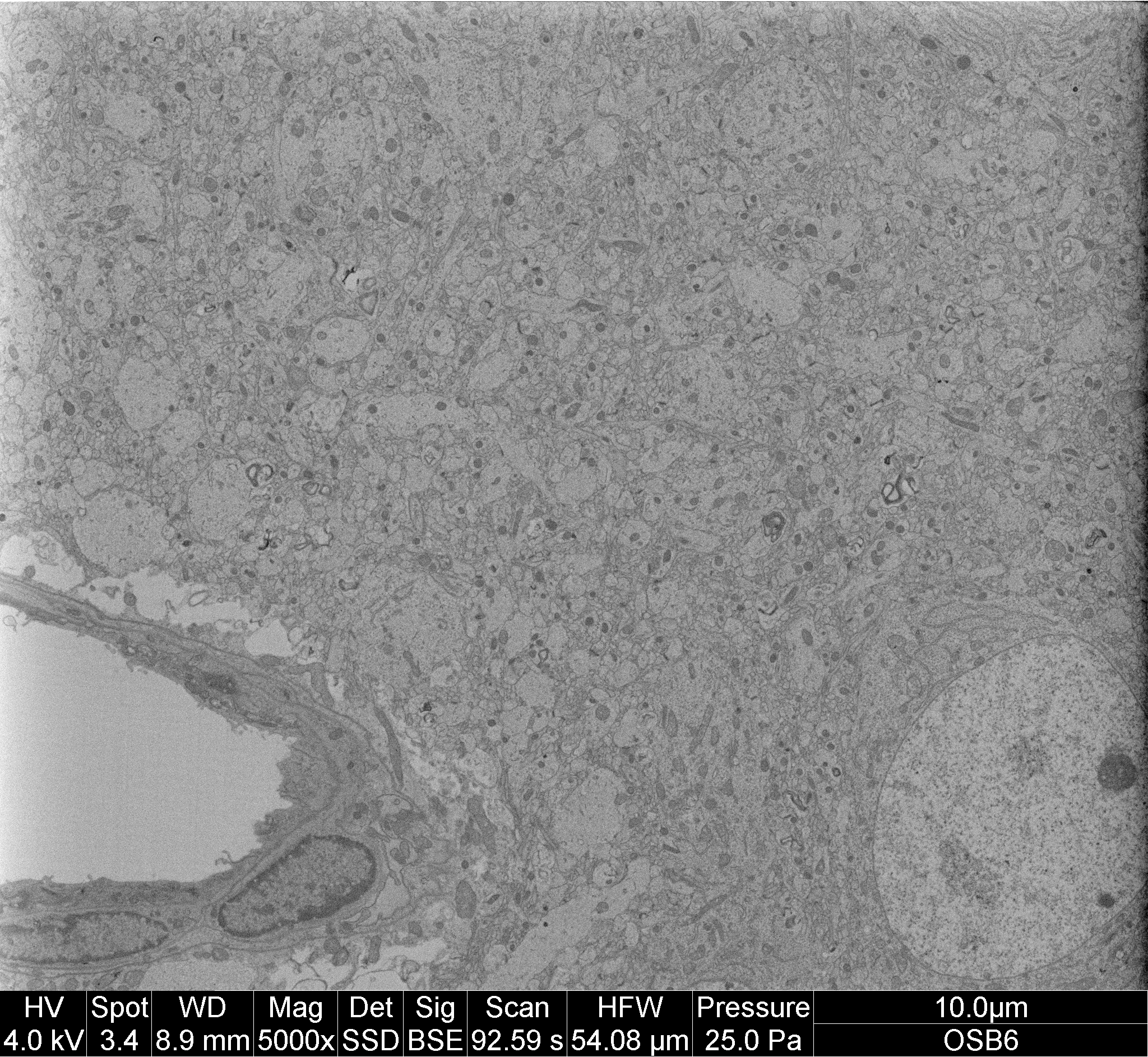

Supplement: Dataset S9 — (256.1 MB ZIP). [file pbio.0020329.sd009.zip › 040604_OS5_st1_848.tif]

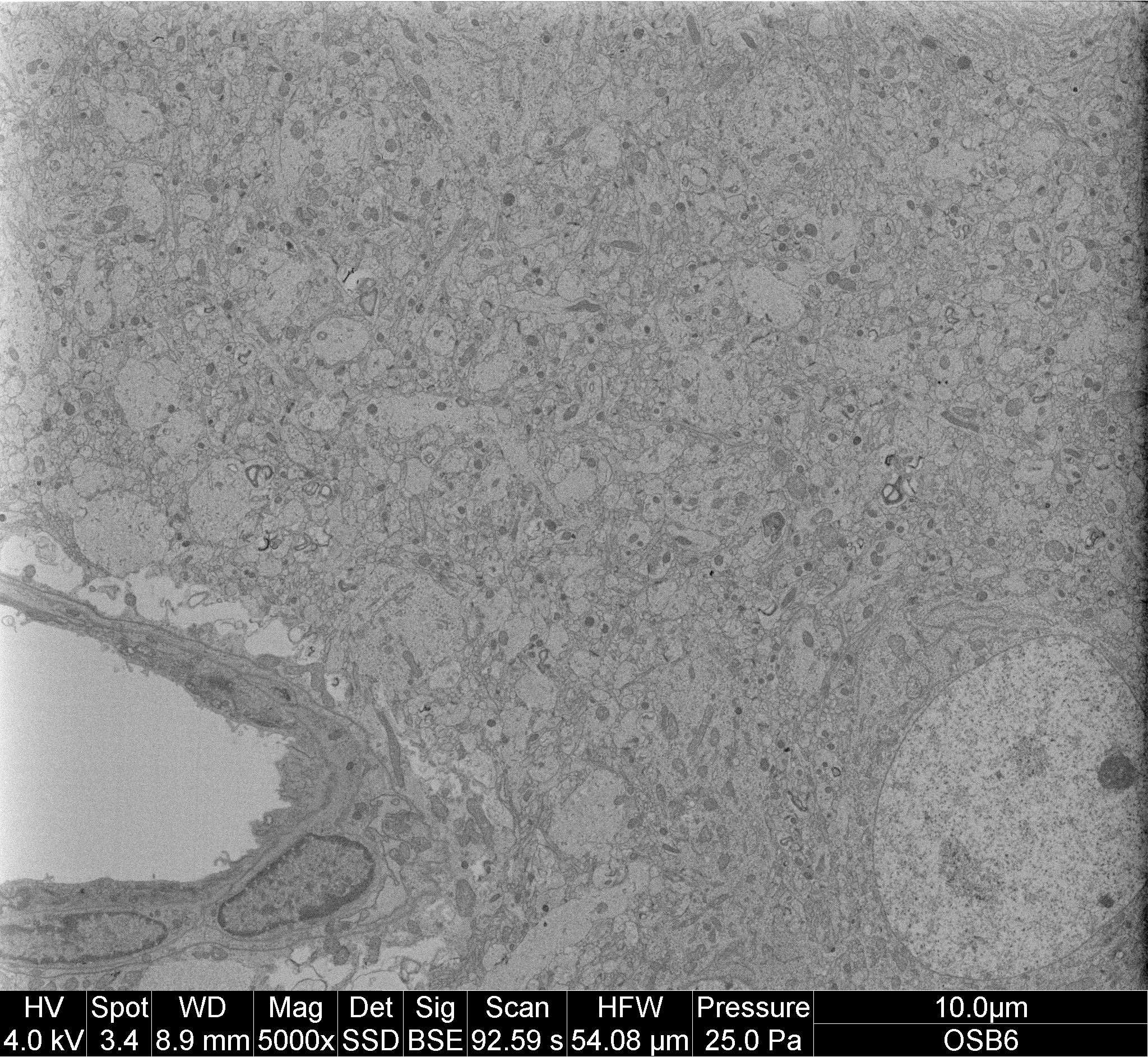

Supplement: Dataset S9 — (256.1 MB ZIP). [file pbio.0020329.sd009.zip › 040604_OS5_st1_849.tif]

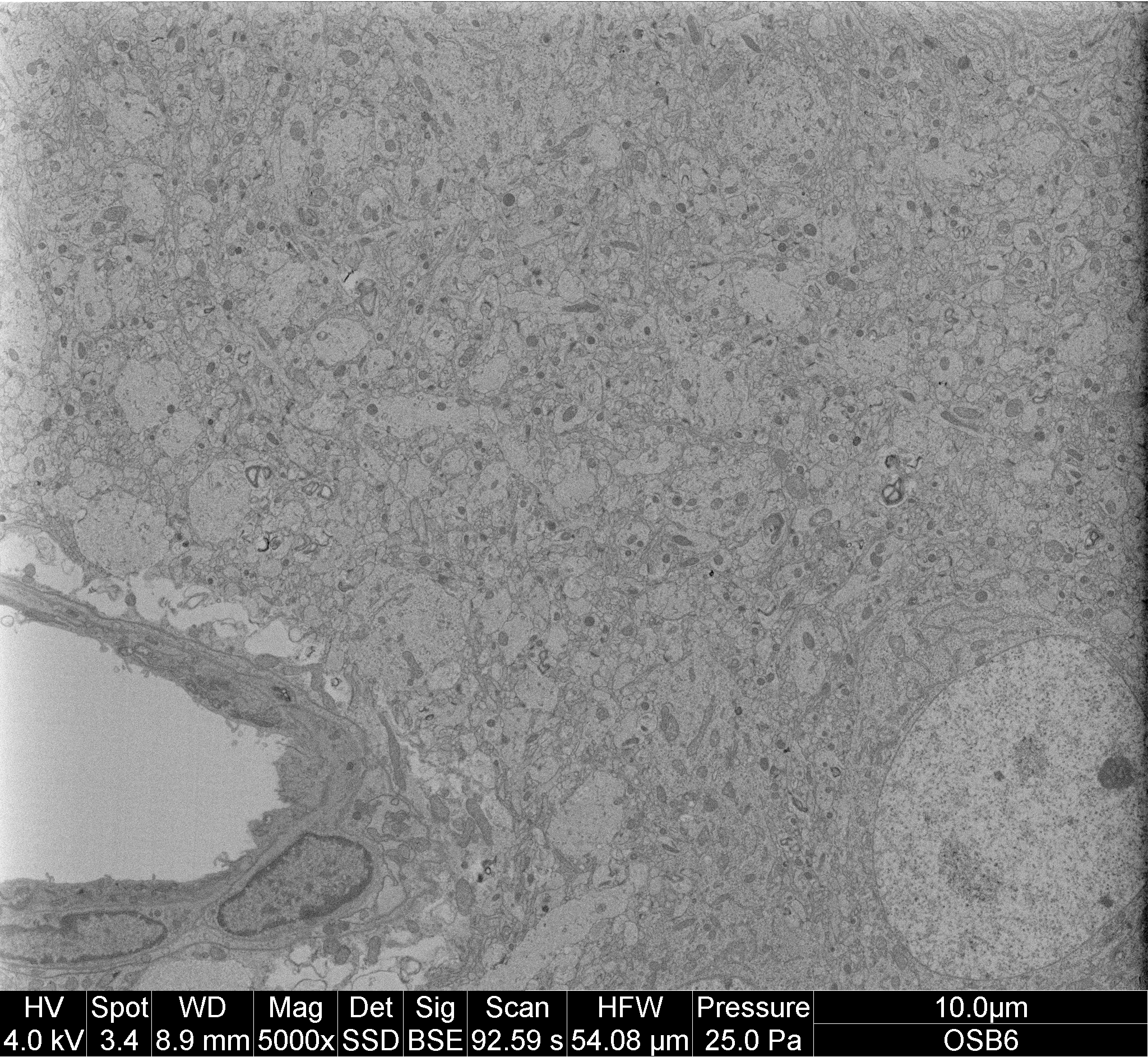

Supplement: Dataset S9 — (256.1 MB ZIP). [file pbio.0020329.sd009.zip › 040604_OS5_st1_850.tif]

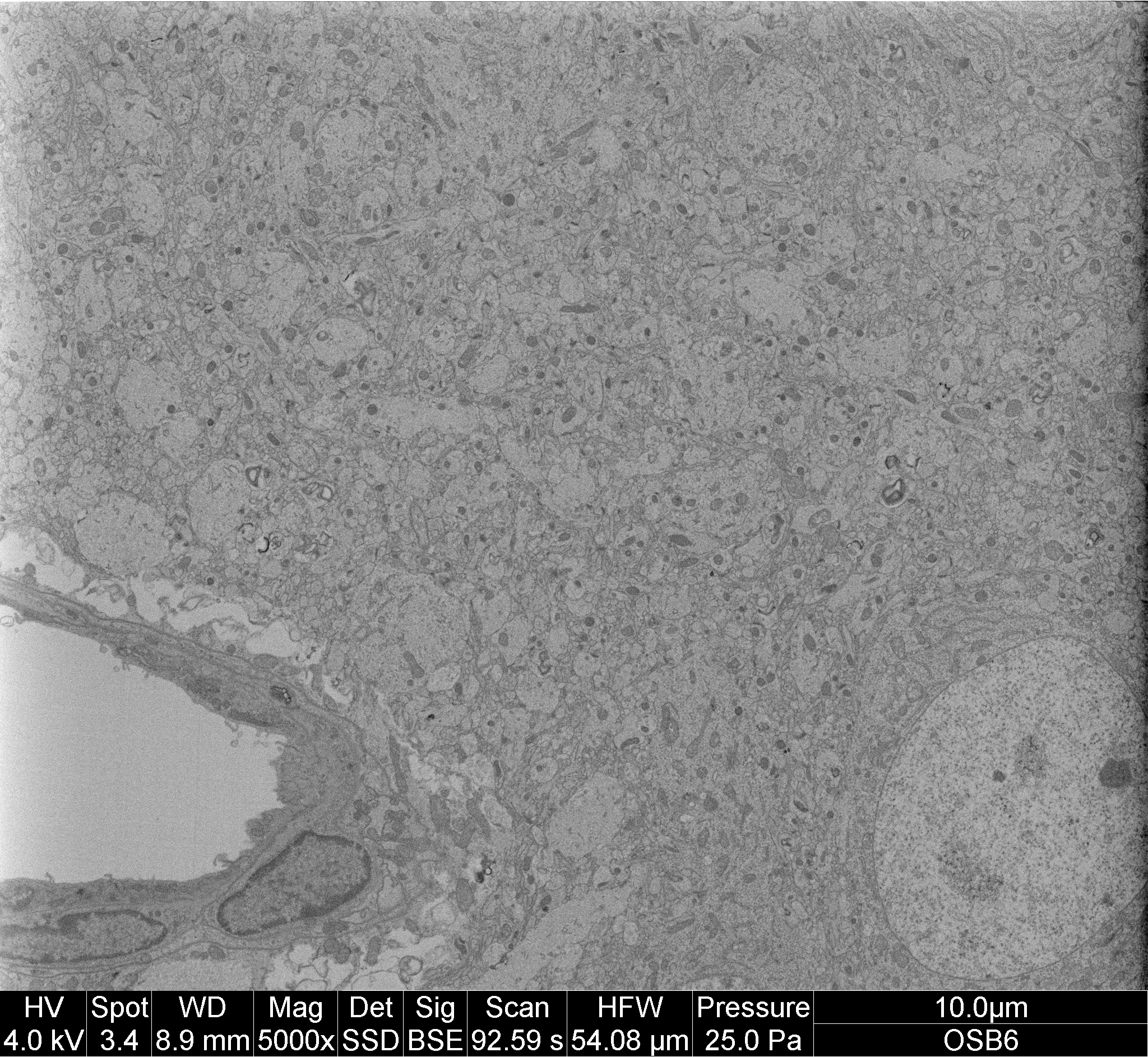

Supplement: Dataset S9 — (256.1 MB ZIP). [file pbio.0020329.sd009.zip › 040604_OS5_st1_851.tif]

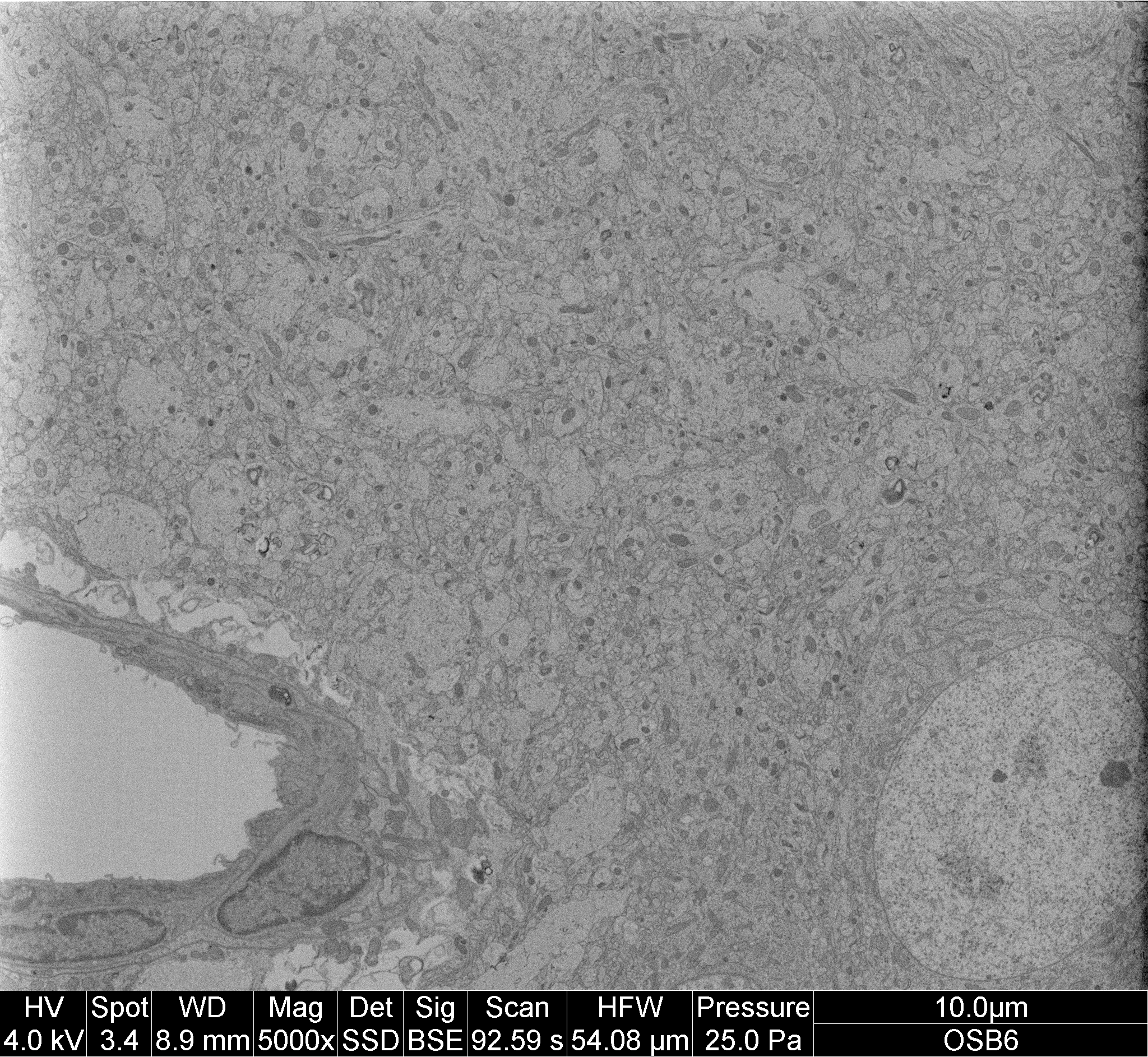

Supplement: Dataset S9 — (256.1 MB ZIP). [file pbio.0020329.sd009.zip › 040604_OS5_st1_852.tif]

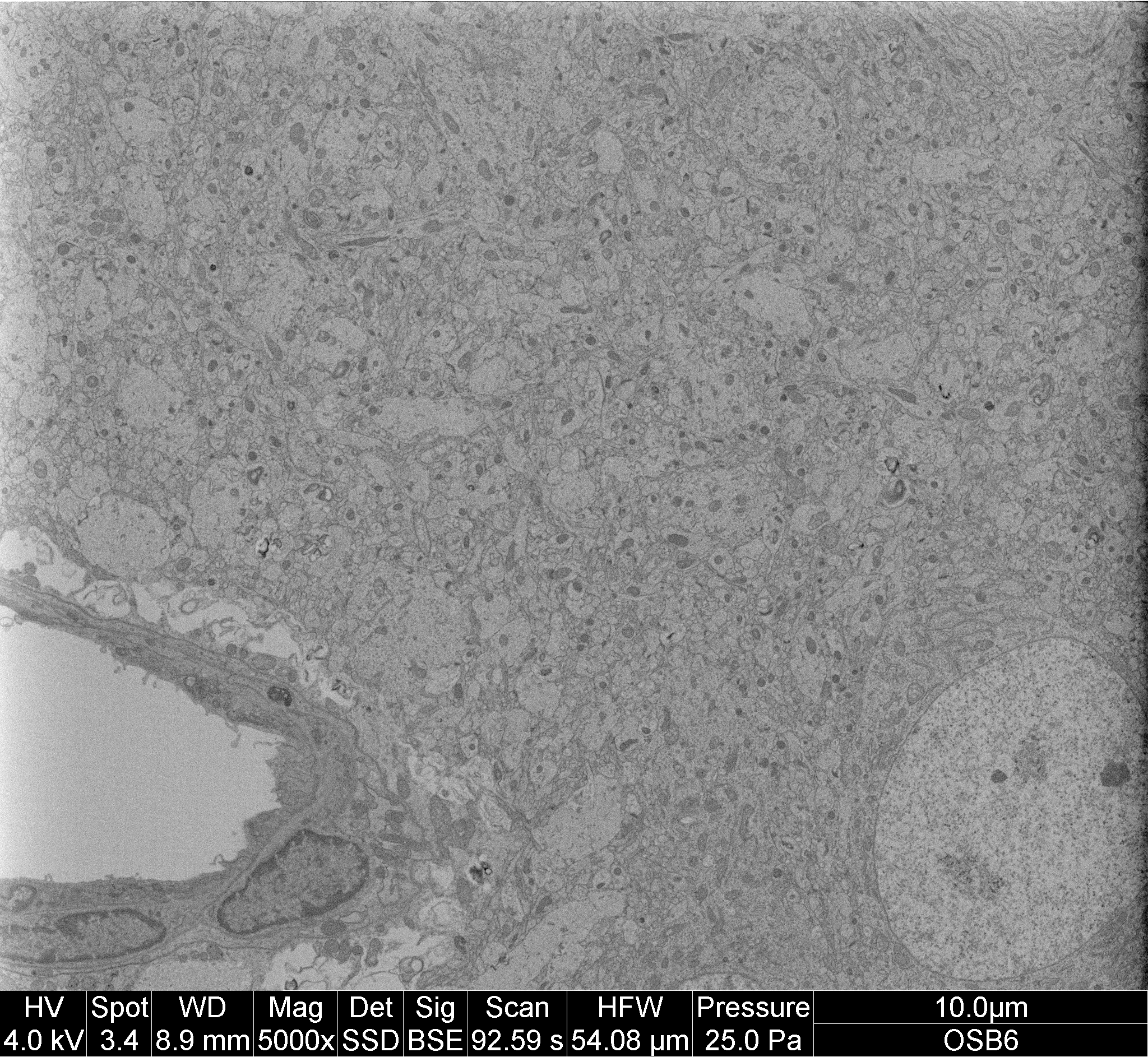

Supplement: Dataset S9 — (256.1 MB ZIP). [file pbio.0020329.sd009.zip › 040604_OS5_st1_853.tif]

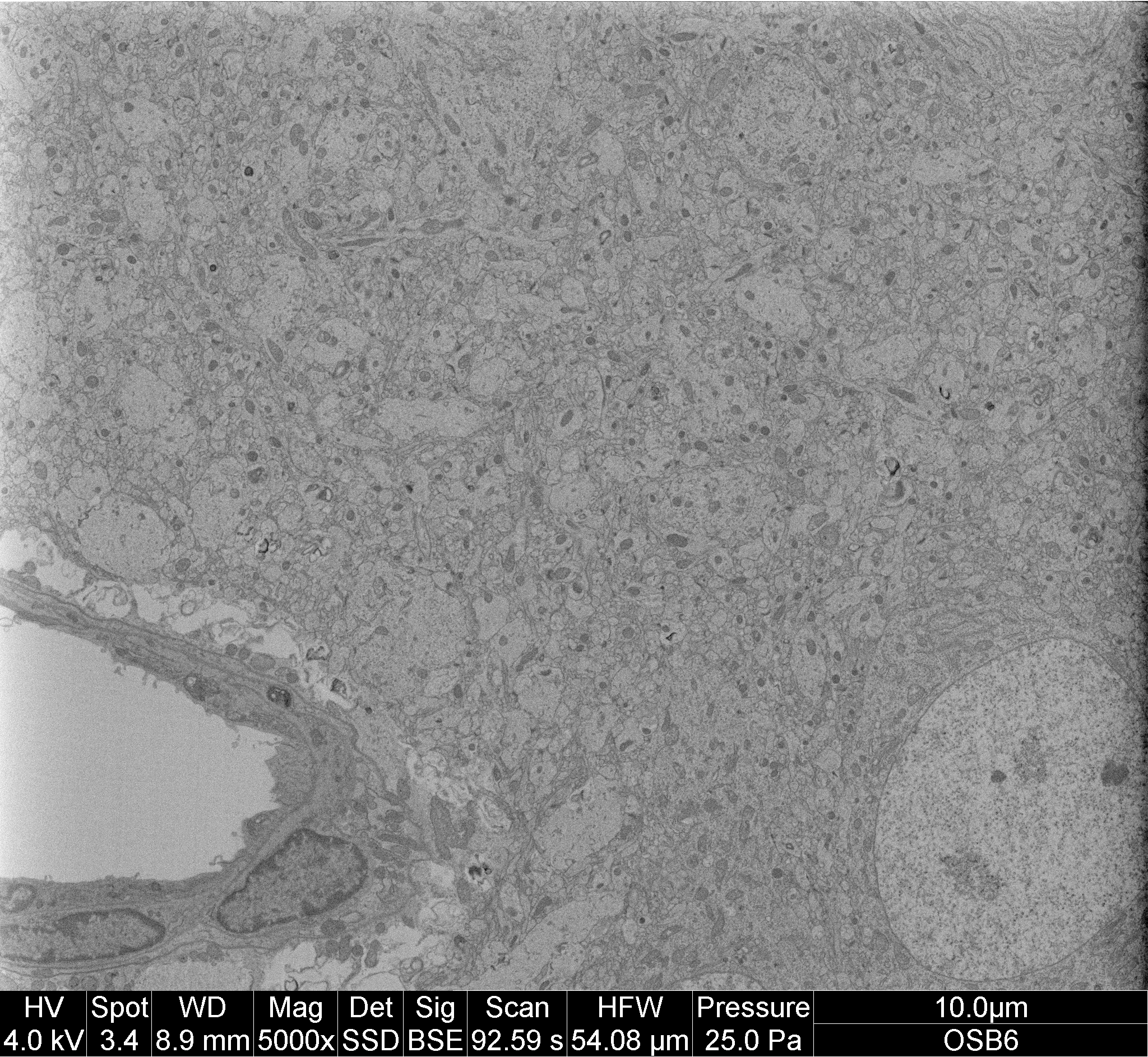

Supplement: Dataset S9 — (256.1 MB ZIP). [file pbio.0020329.sd009.zip › 040604_OS5_st1_854.tif]

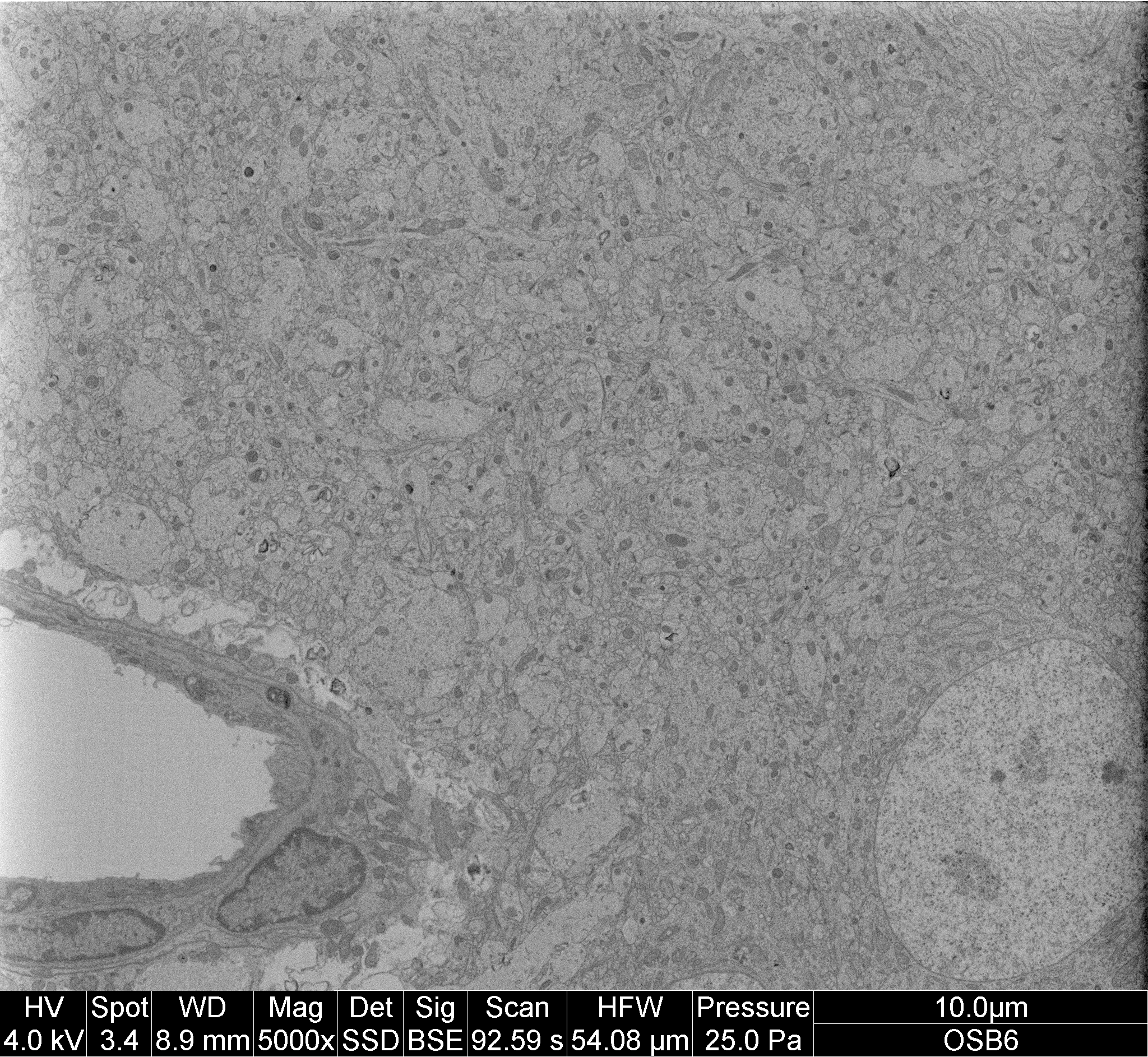

Supplement: Dataset S9 — (256.1 MB ZIP). [file pbio.0020329.sd009.zip › 040604_OS5_st1_855.tif]

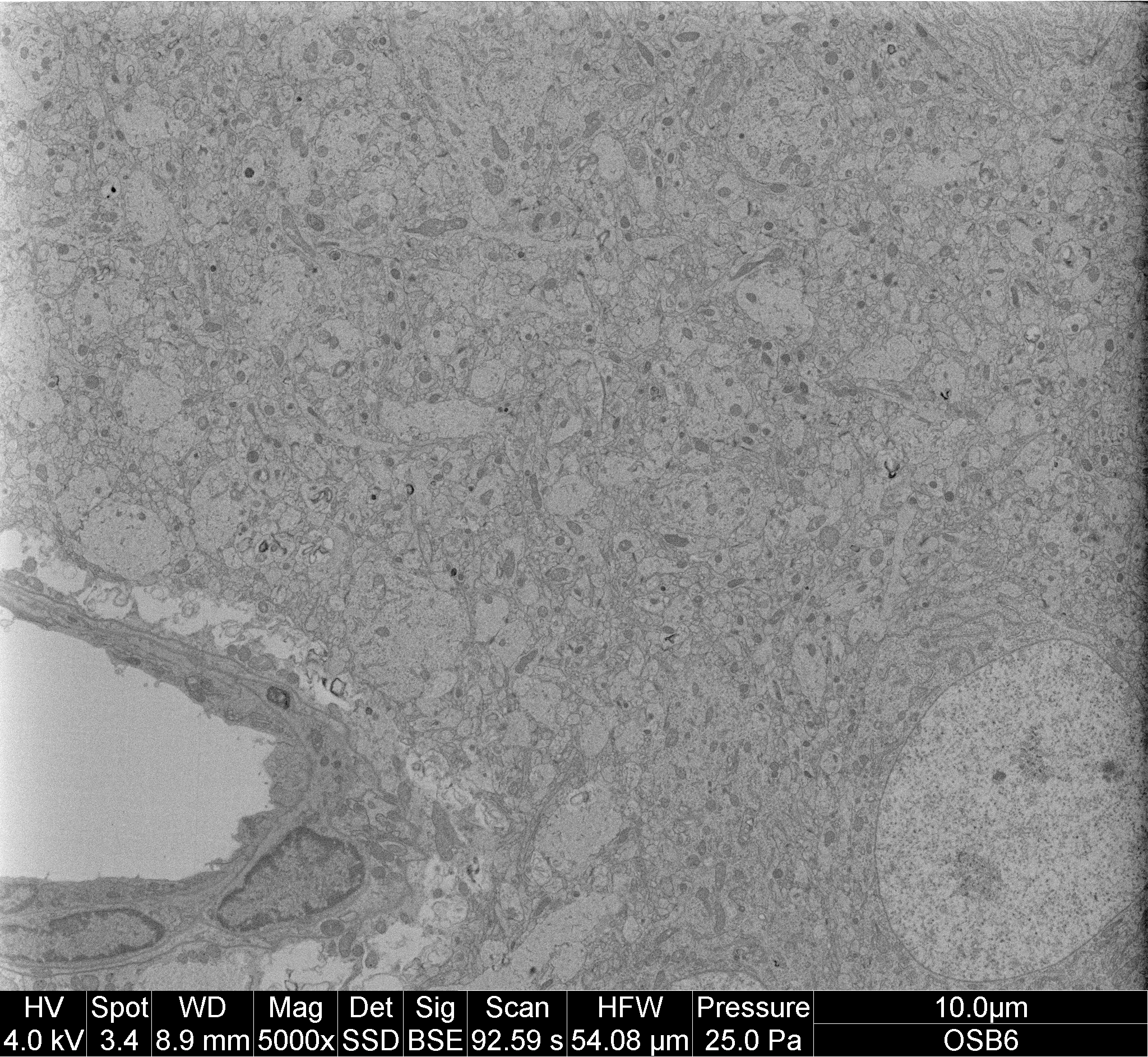

Supplement: Dataset S9 — (256.1 MB ZIP). [file pbio.0020329.sd009.zip › 040604_OS5_st1_856.tif]

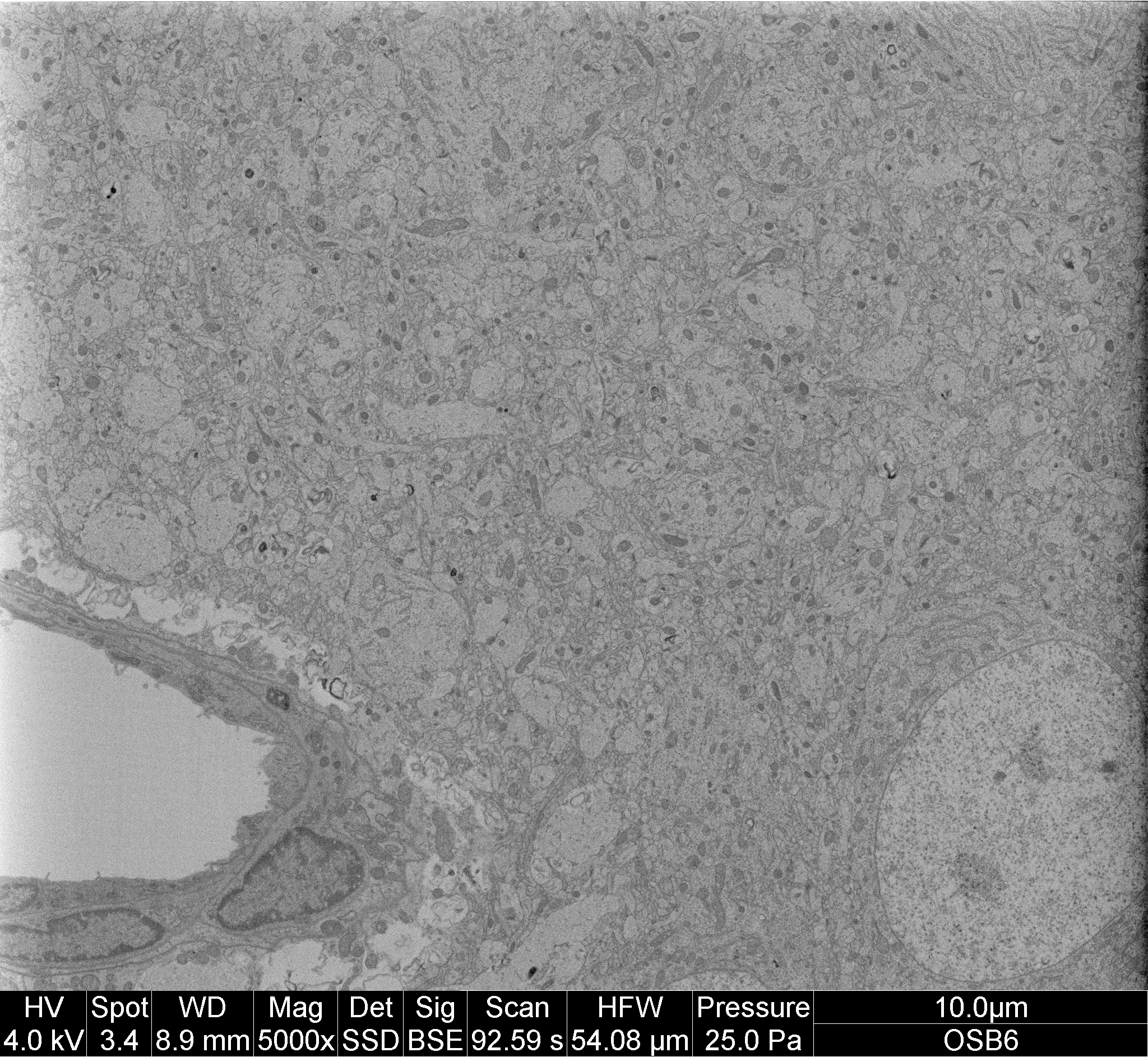

Supplement: Dataset S9 — (256.1 MB ZIP). [file pbio.0020329.sd009.zip › 040604_OS5_st1_857.tif]

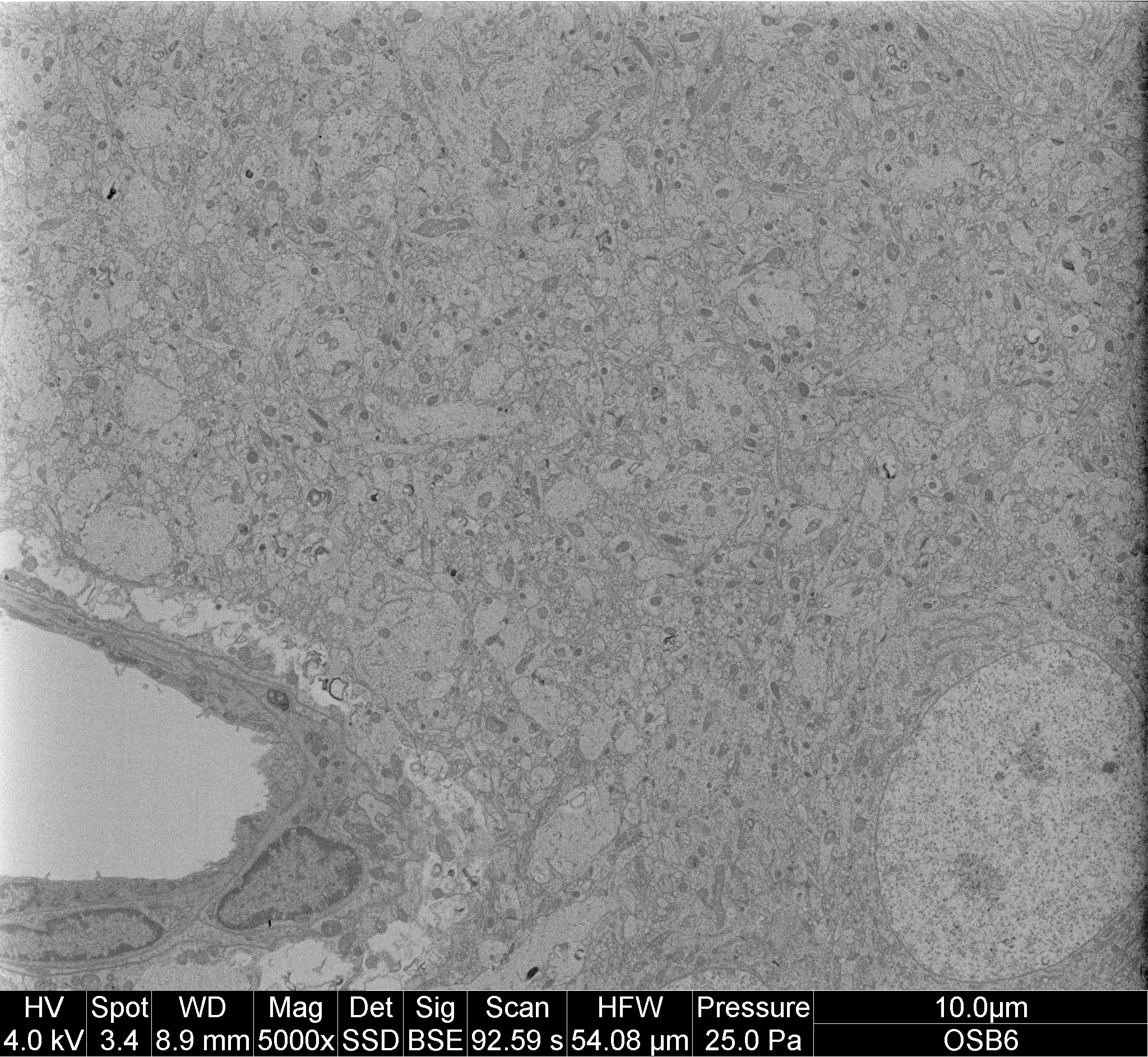

Supplement: Dataset S9 — (256.1 MB ZIP). [file pbio.0020329.sd009.zip › 040604_OS5_st1_858.tif]

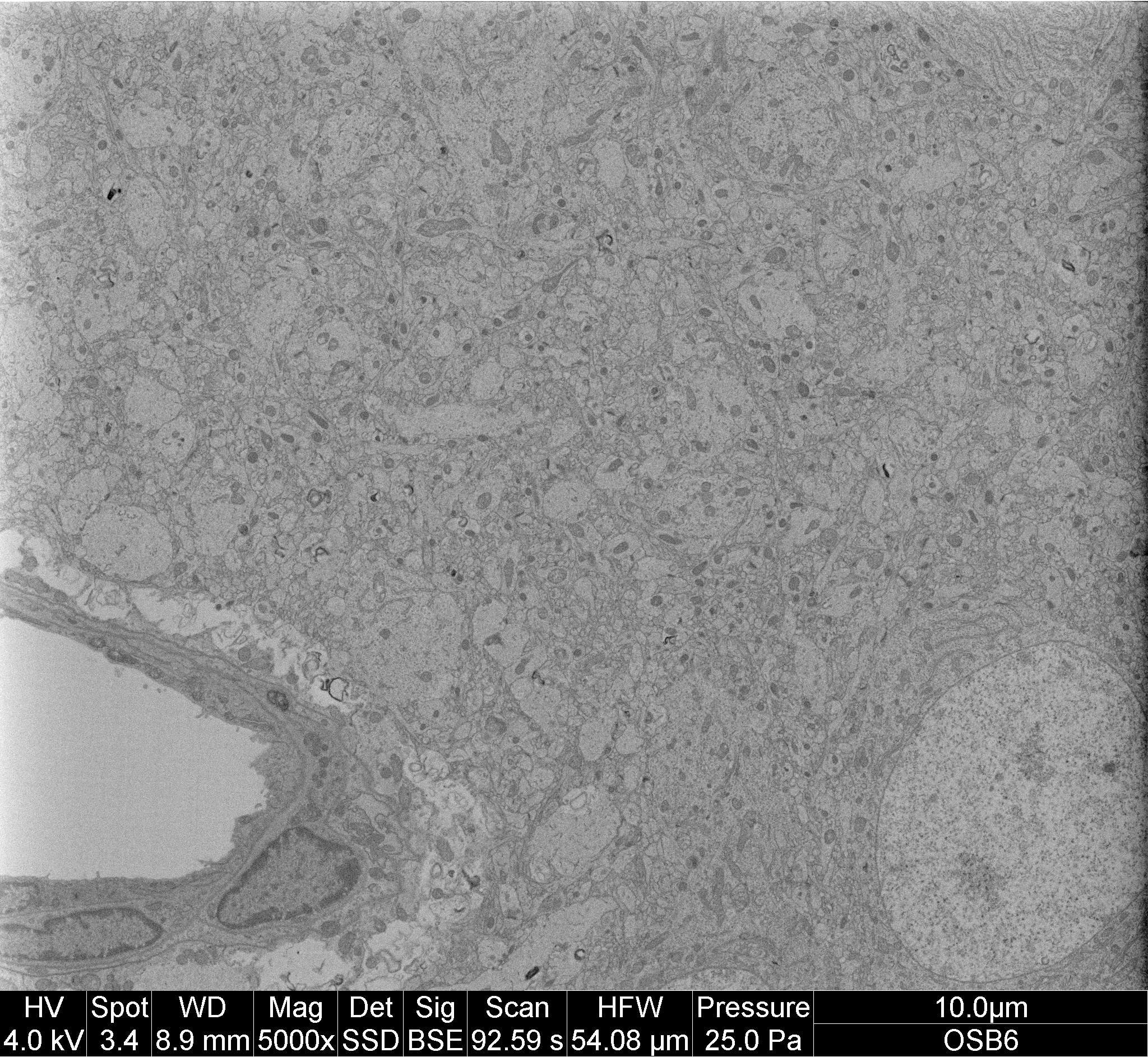

Supplement: Dataset S9 — (256.1 MB ZIP). [file pbio.0020329.sd009.zip › 040604_OS5_st1_859.tif]

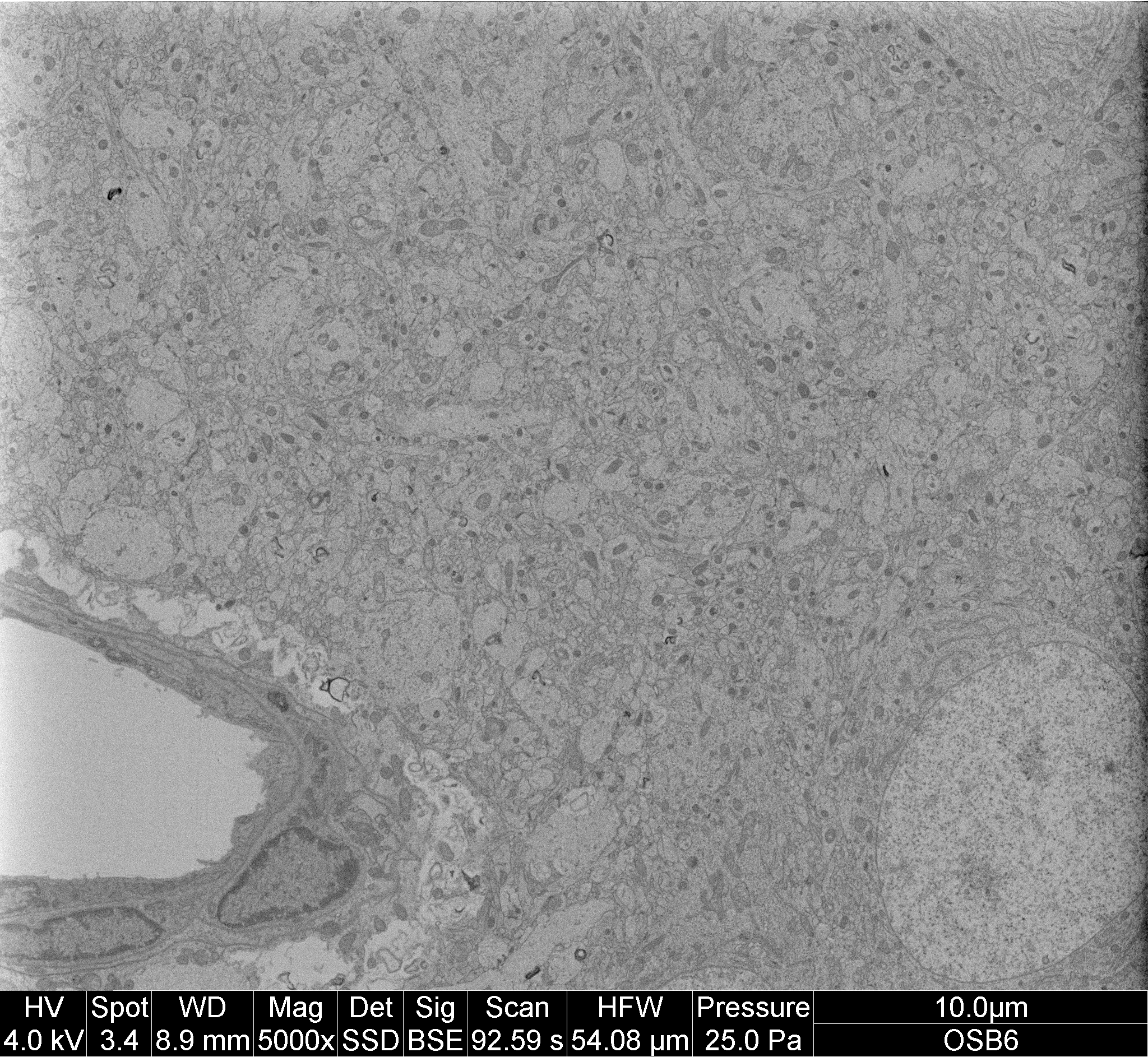

Supplement: Dataset S9 — (256.1 MB ZIP). [file pbio.0020329.sd009.zip › 040604_OS5_st1_860.tif]

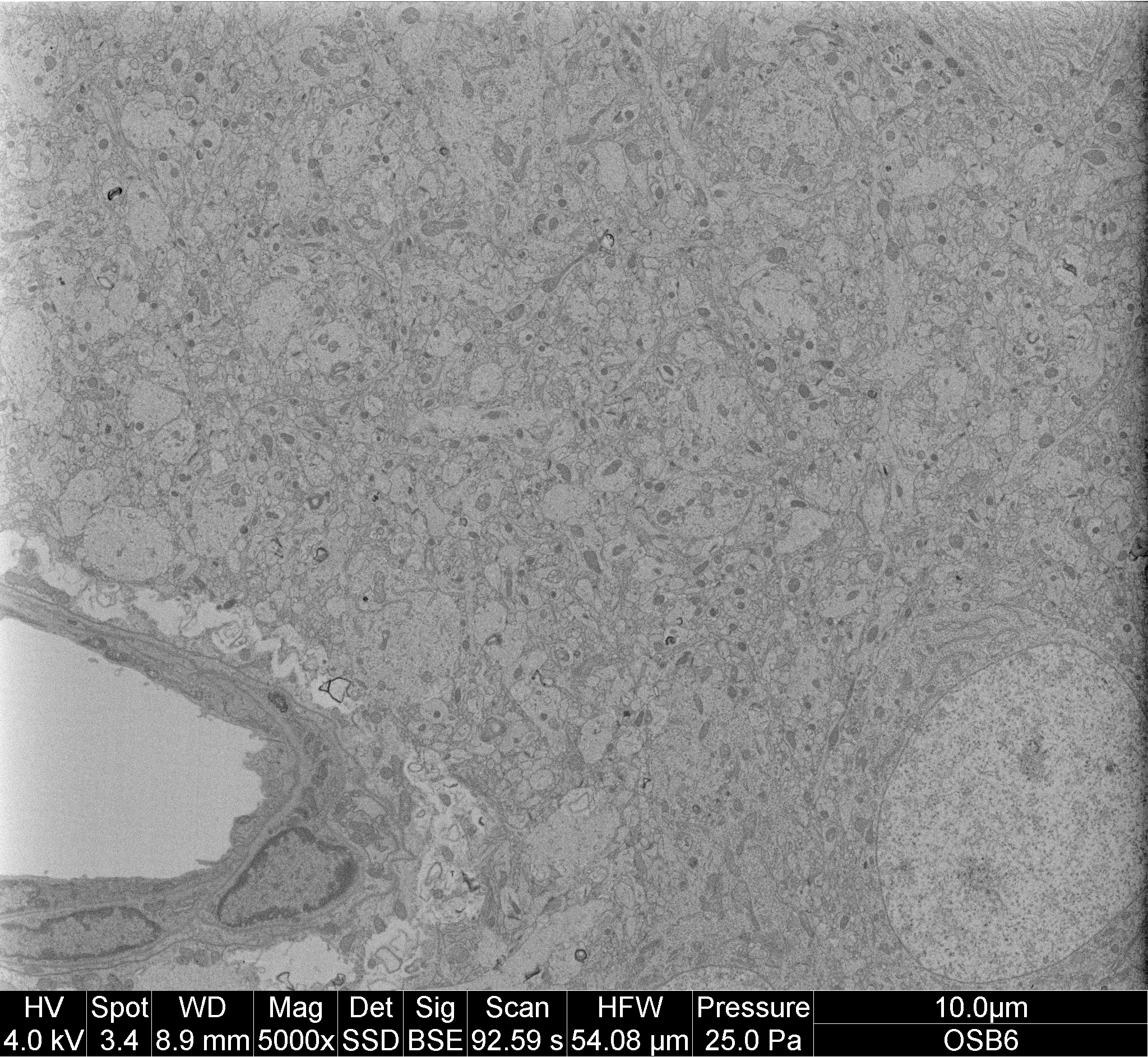

Supplement: Dataset S9 — (256.1 MB ZIP). [file pbio.0020329.sd009.zip › 040604_OS5_st1_861.tif]

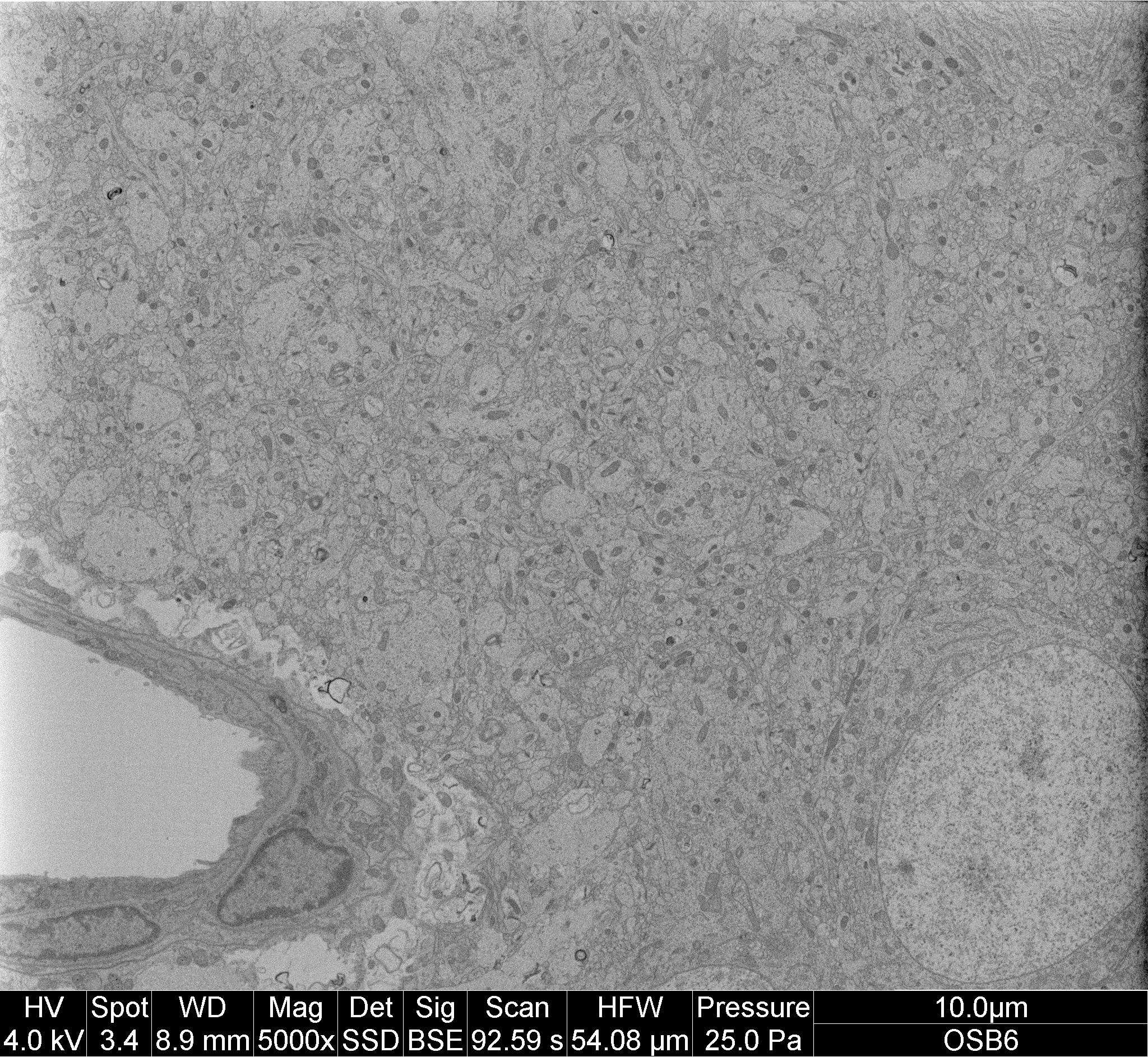

Supplement: Dataset S9 — (256.1 MB ZIP). [file pbio.0020329.sd009.zip › 040604_OS5_st1_862.tif]

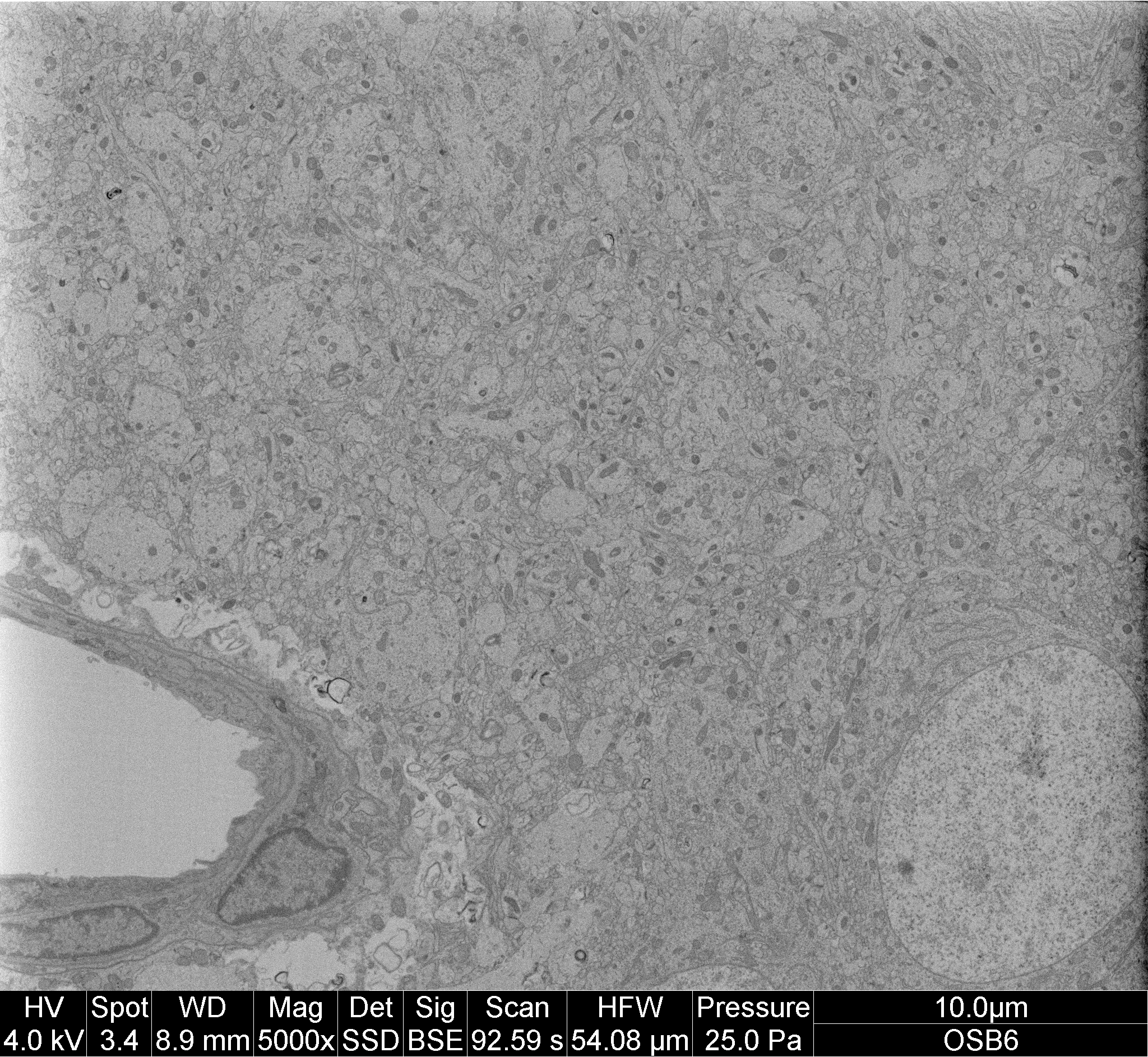

Supplement: Dataset S9 — (256.1 MB ZIP). [file pbio.0020329.sd009.zip › 040604_OS5_st1_863.tif]

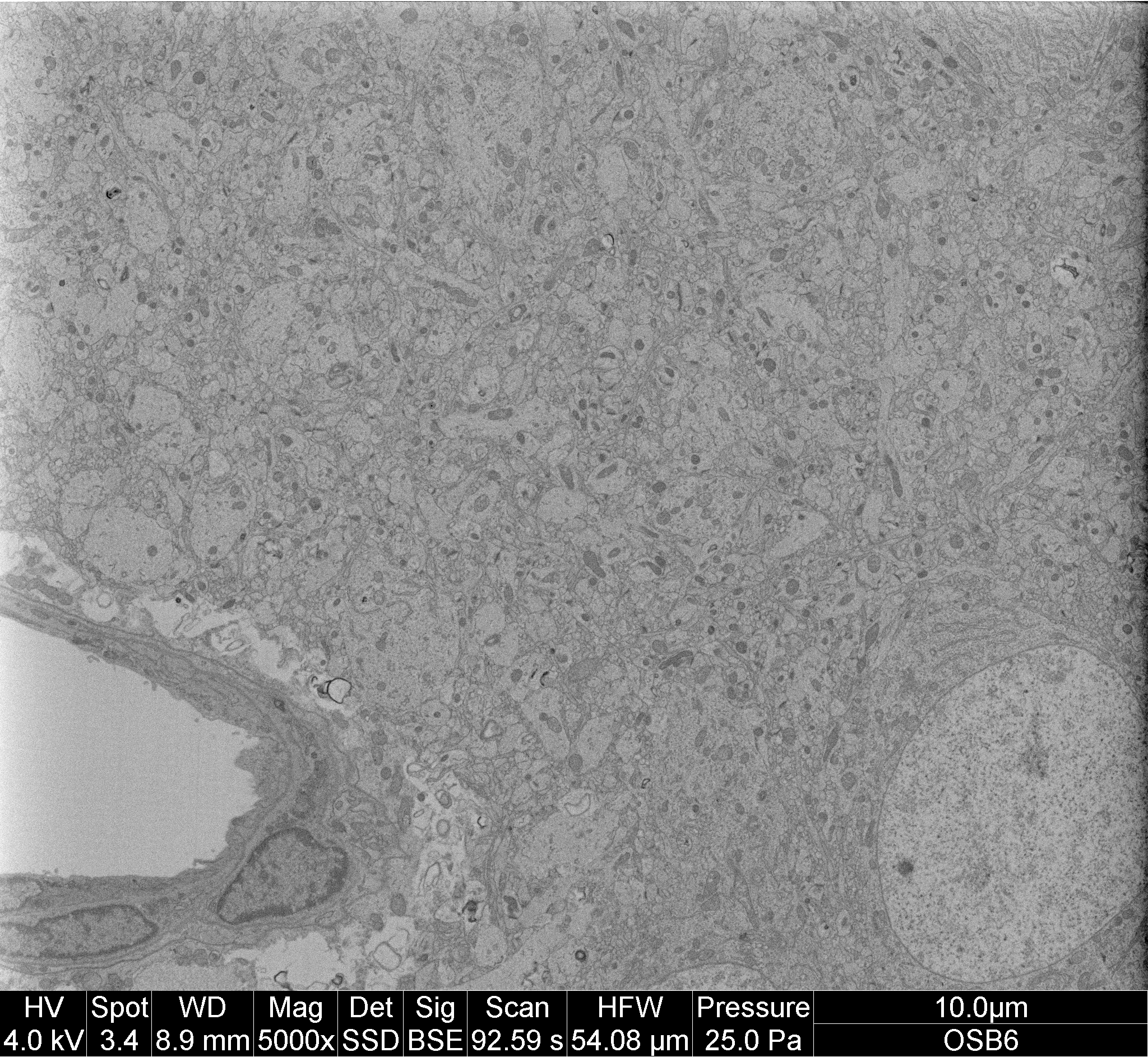

Supplement: Dataset S9 — (256.1 MB ZIP). [file pbio.0020329.sd009.zip › 040604_OS5_st1_864.tif]

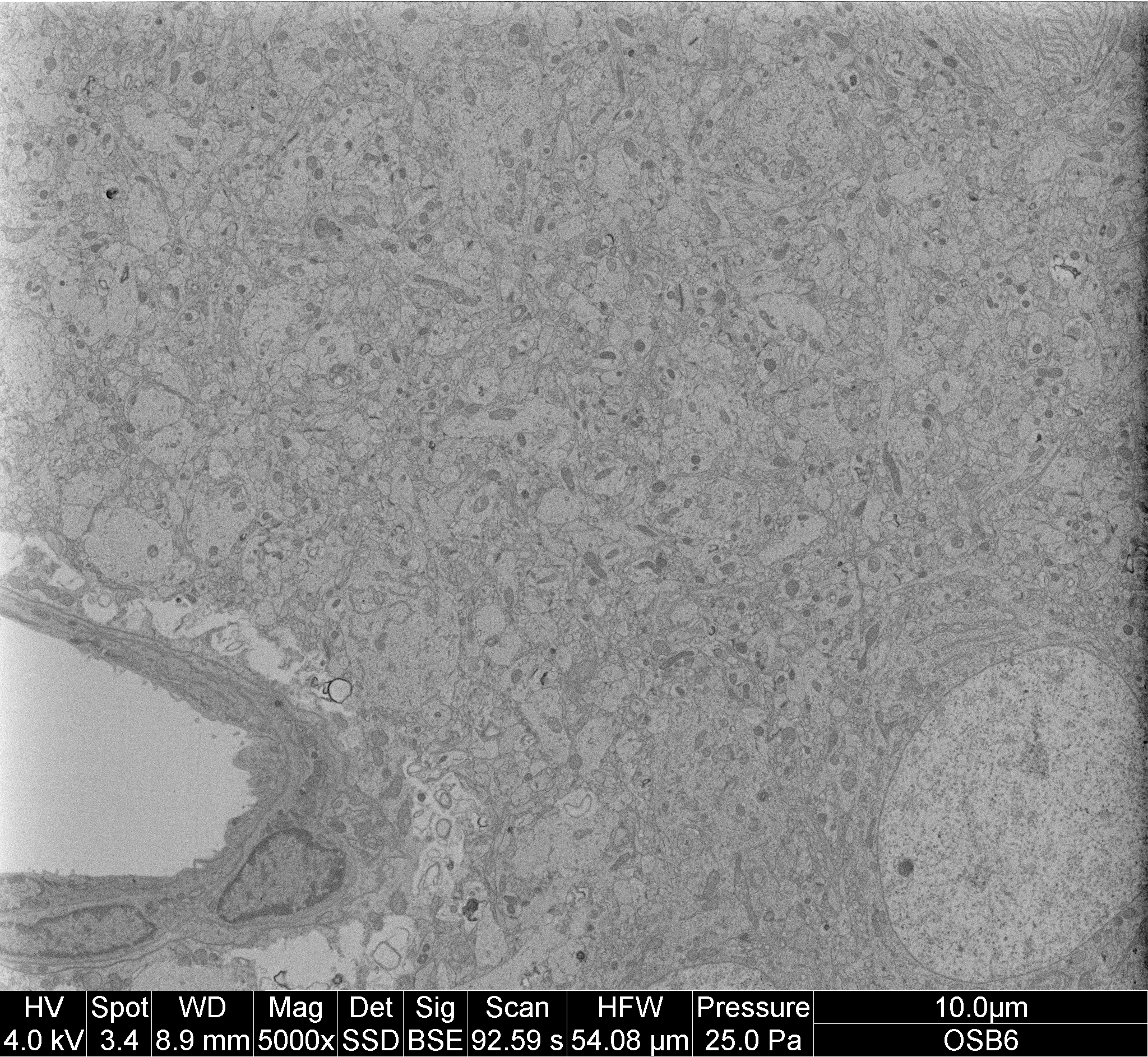

Supplement: Dataset S9 — (256.1 MB ZIP). [file pbio.0020329.sd009.zip › 040604_OS5_st1_865.tif]

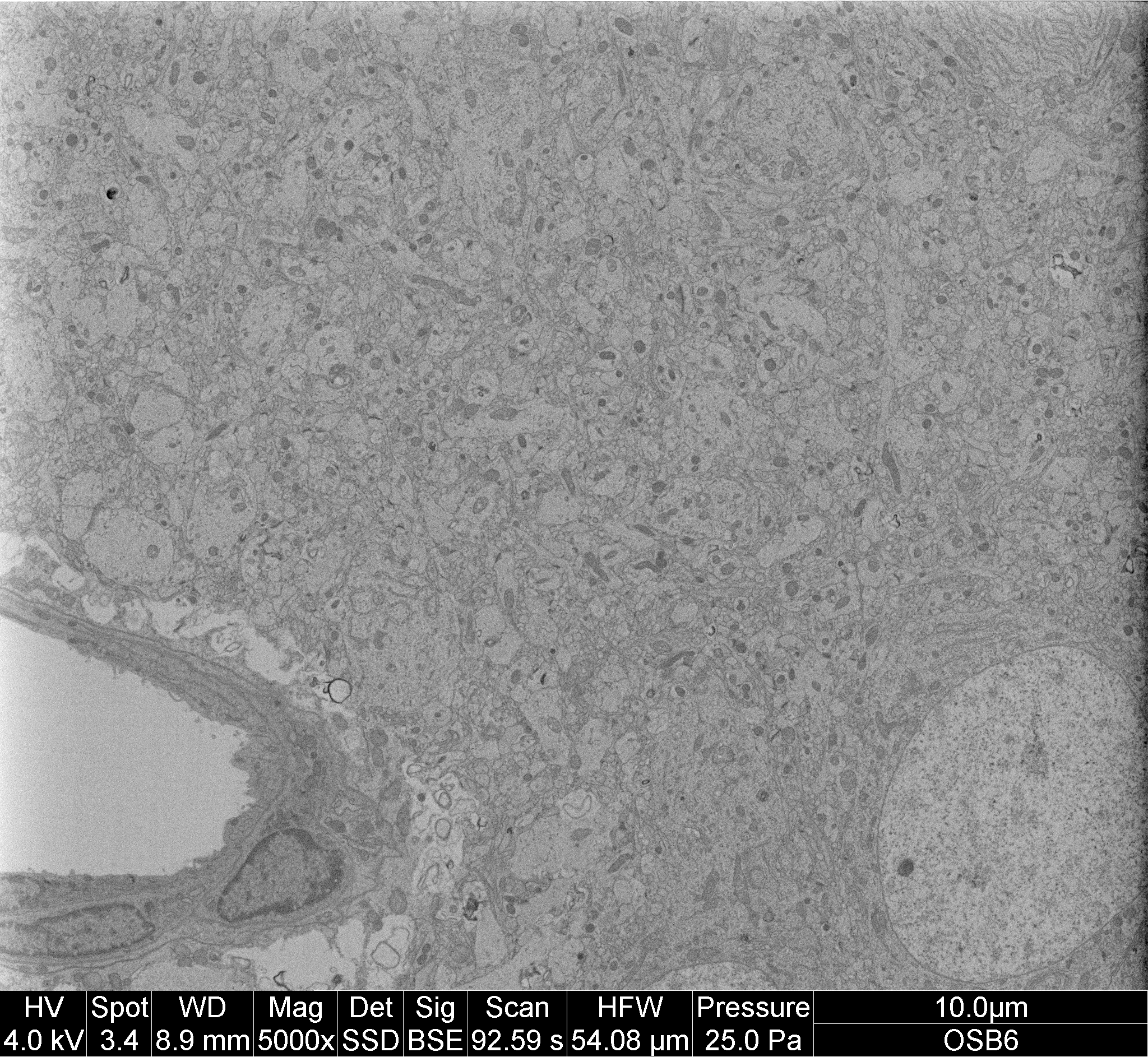

Supplement: Dataset S9 — (256.1 MB ZIP). [file pbio.0020329.sd009.zip › 040604_OS5_st1_866.tif]

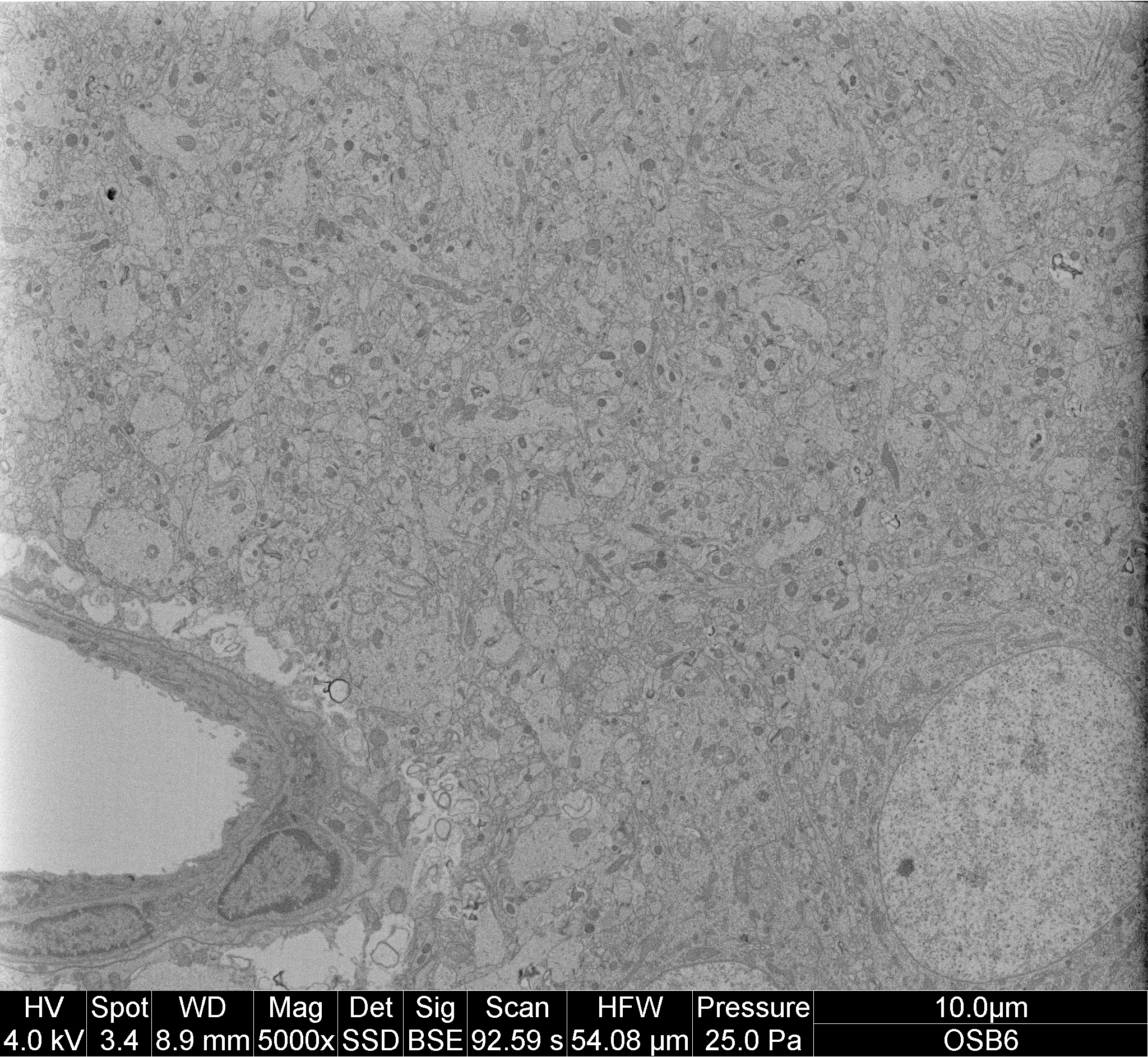

Supplement: Dataset S9 — (256.1 MB ZIP). [file pbio.0020329.sd009.zip › 040604_OS5_st1_867.tif]

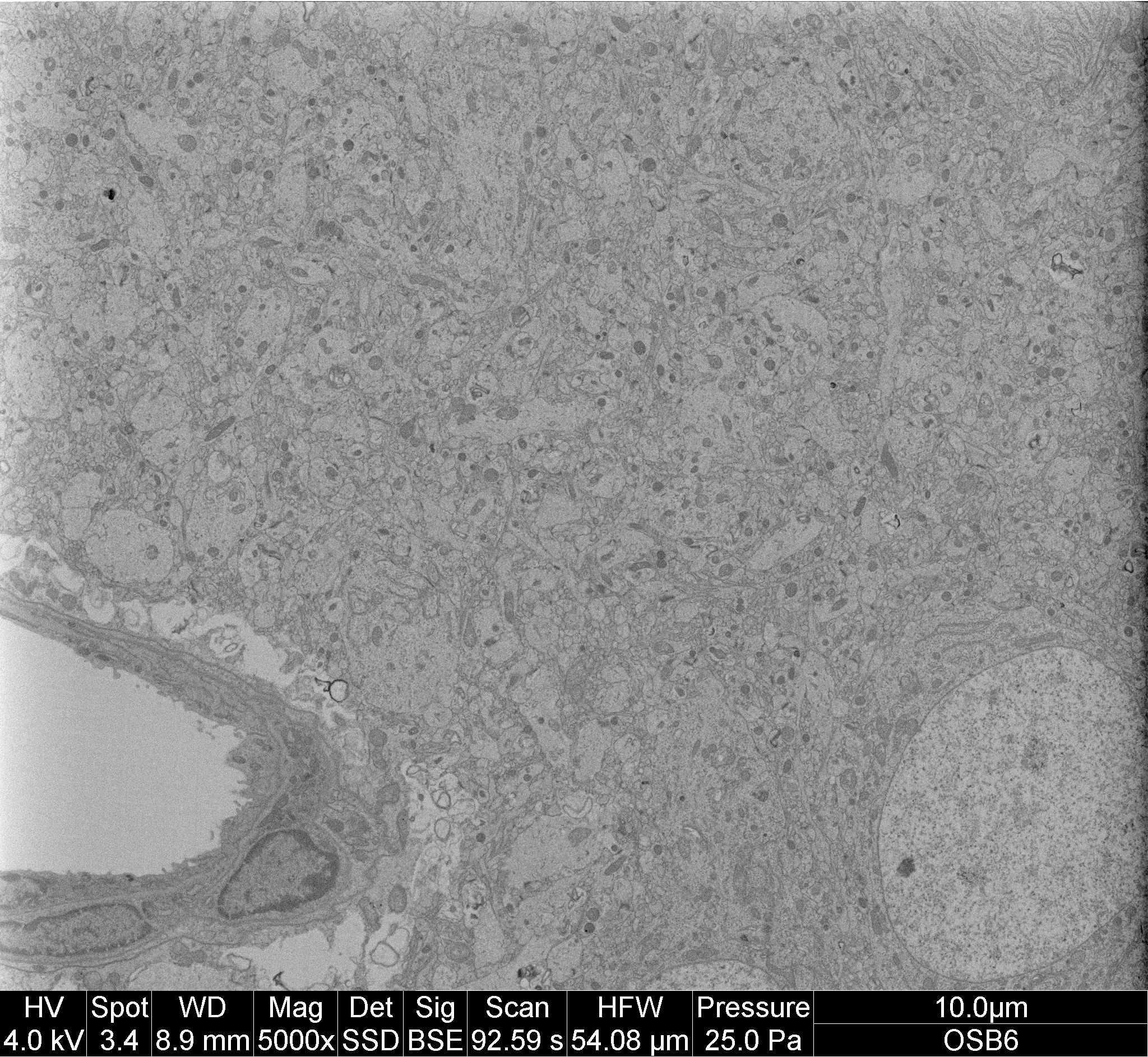

Supplement: Dataset S9 — (256.1 MB ZIP). [file pbio.0020329.sd009.zip › 040604_OS5_st1_868.tif]

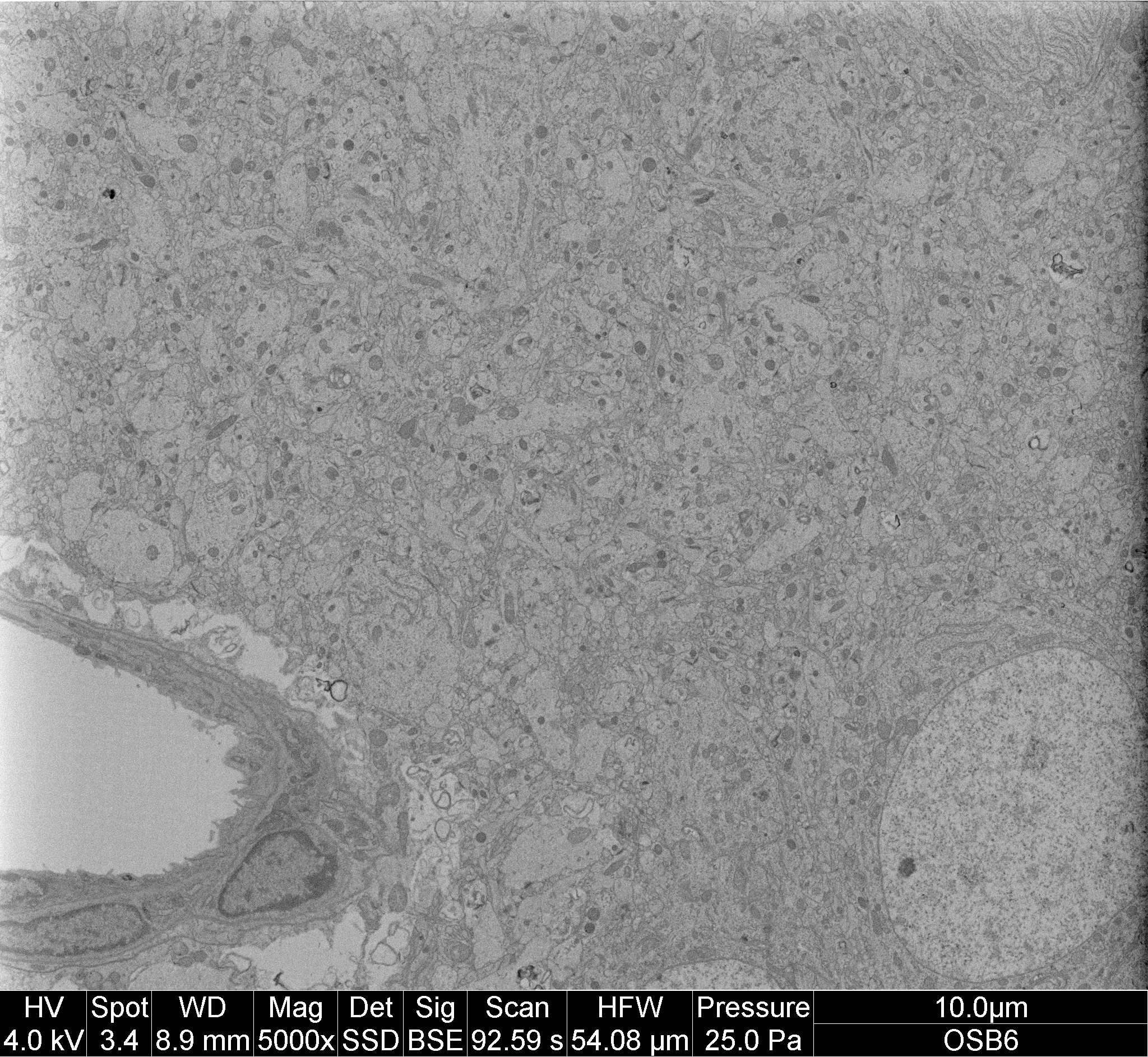

Supplement: Dataset S9 — (256.1 MB ZIP). [file pbio.0020329.sd009.zip › 040604_OS5_st1_869.tif]

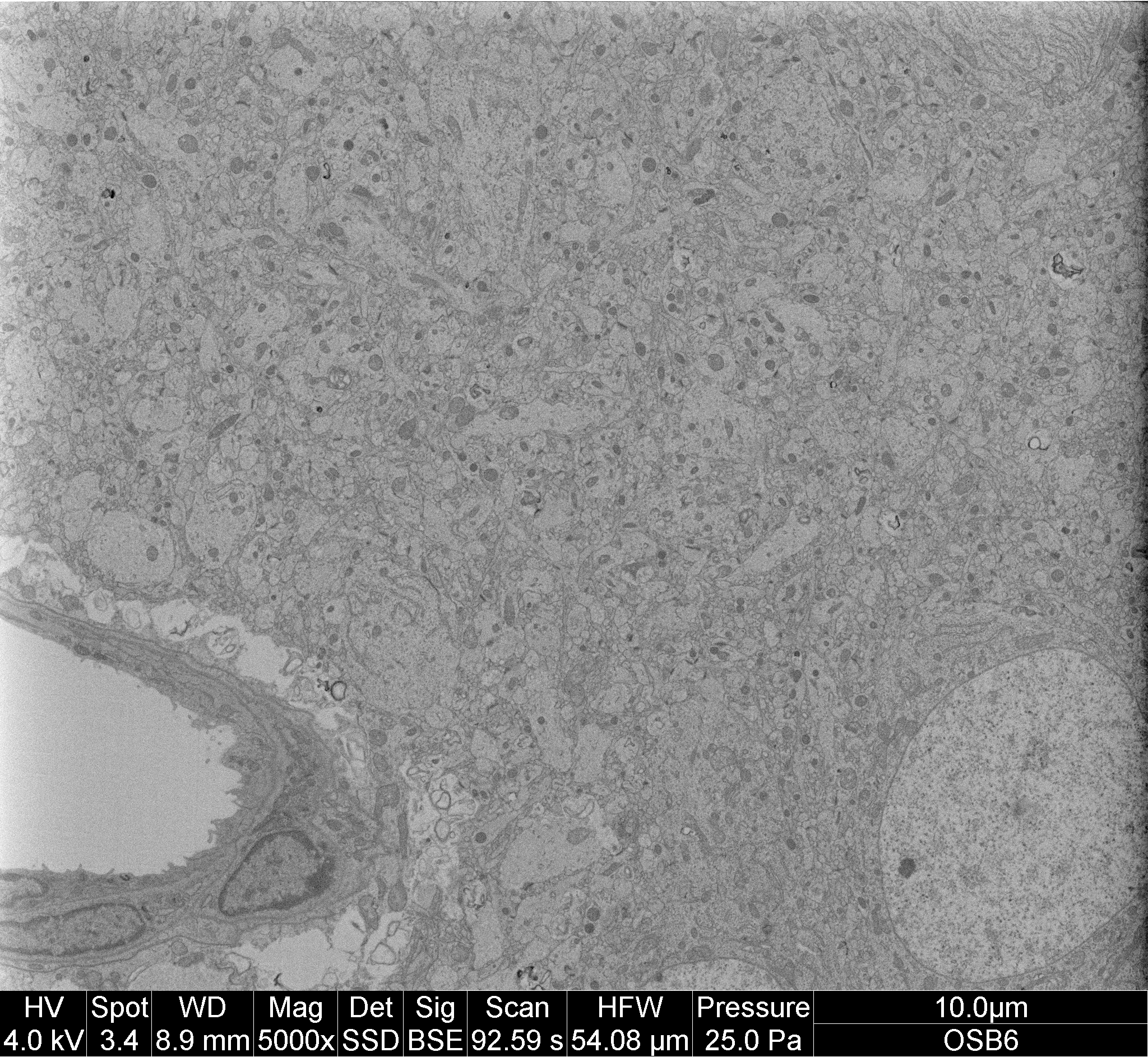

Supplement: Dataset S9 — (256.1 MB ZIP). [file pbio.0020329.sd009.zip › 040604_OS5_st1_870.tif]

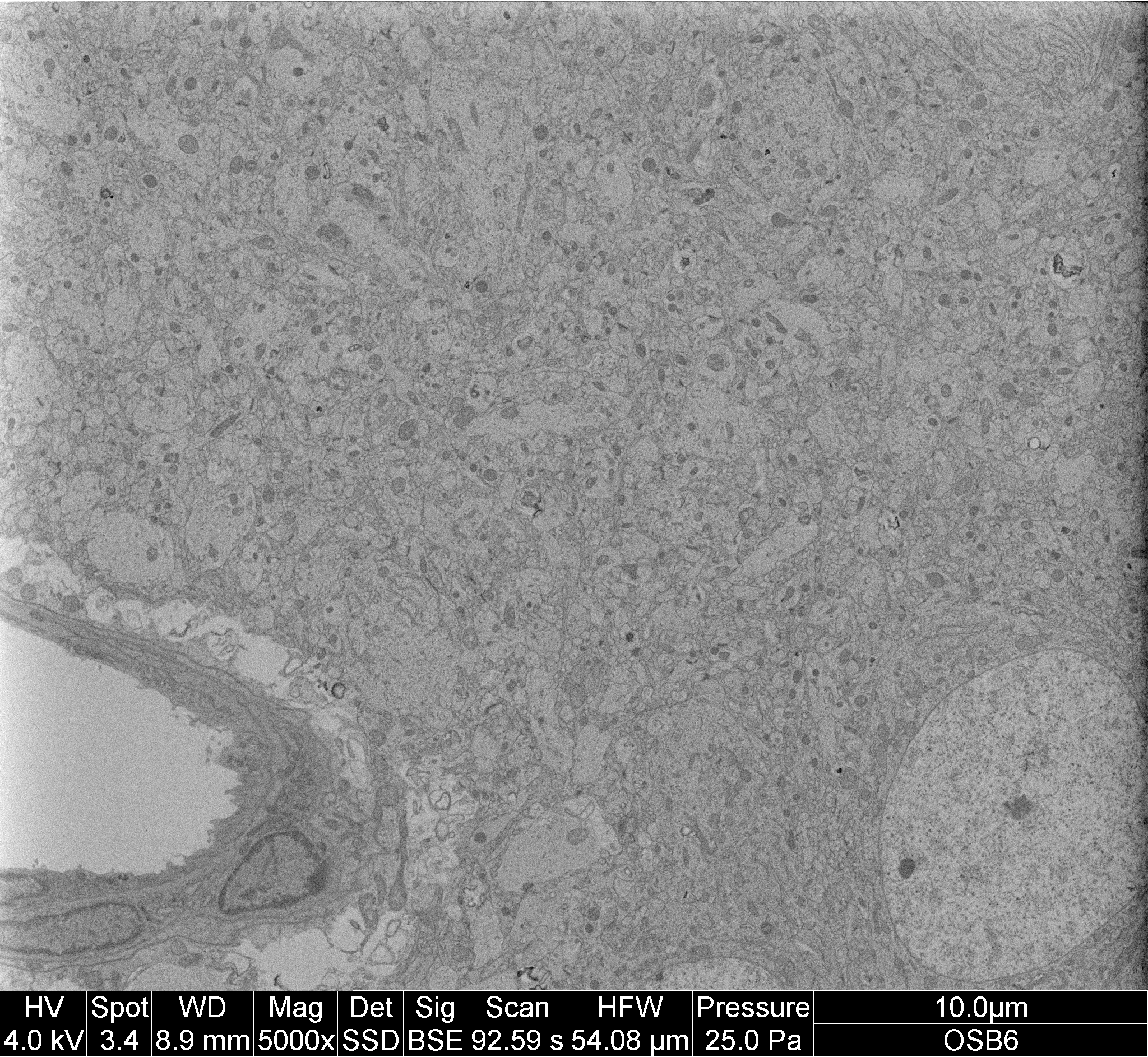

Supplement: Dataset S9 — (256.1 MB ZIP). [file pbio.0020329.sd009.zip › 040604_OS5_st1_871.tif]

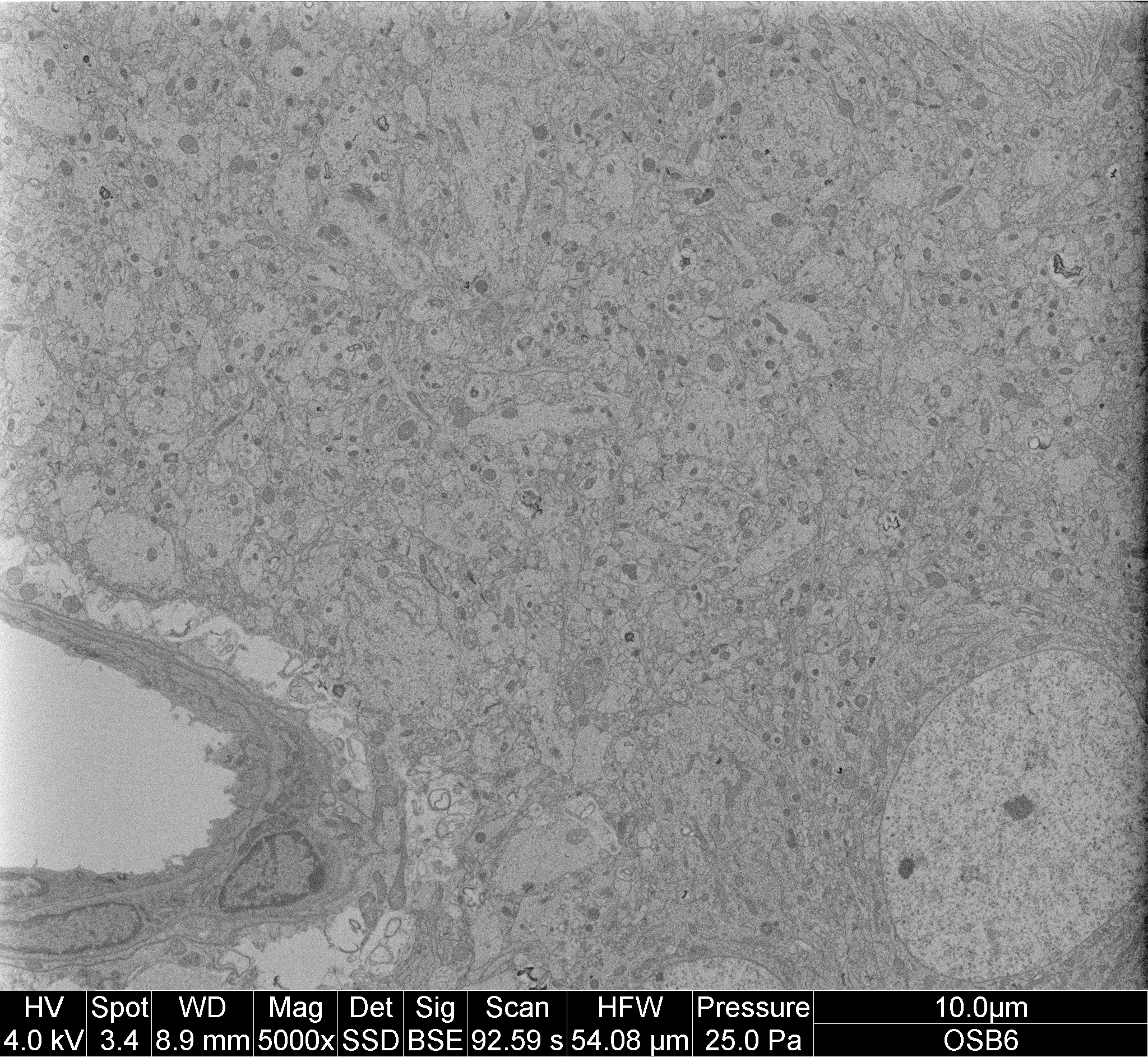

Supplement: Dataset S9 — (256.1 MB ZIP). [file pbio.0020329.sd009.zip › 040604_OS5_st1_872.tif]

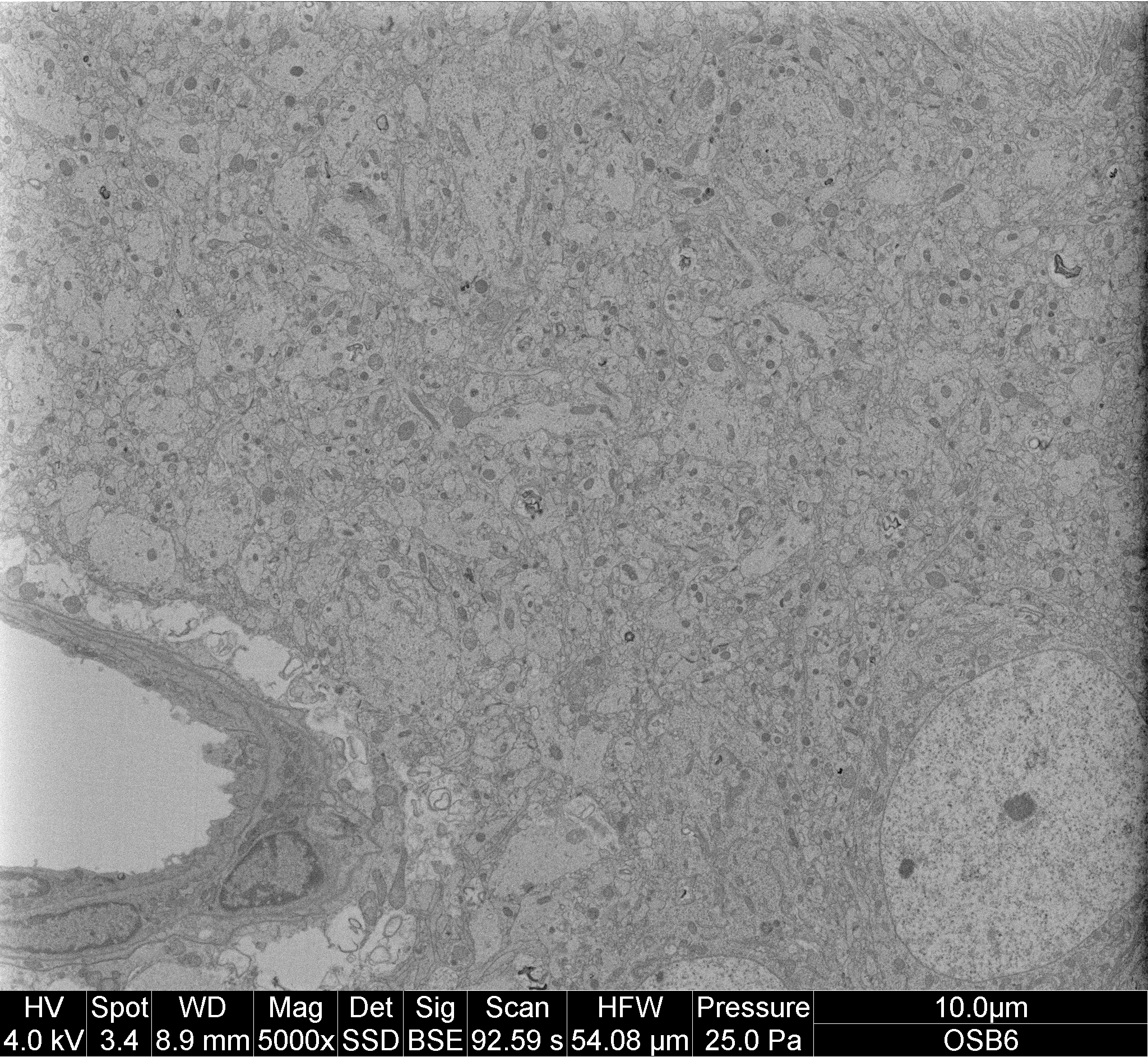

Supplement: Dataset S9 — (256.1 MB ZIP). [file pbio.0020329.sd009.zip › 040604_OS5_st1_873.tif]

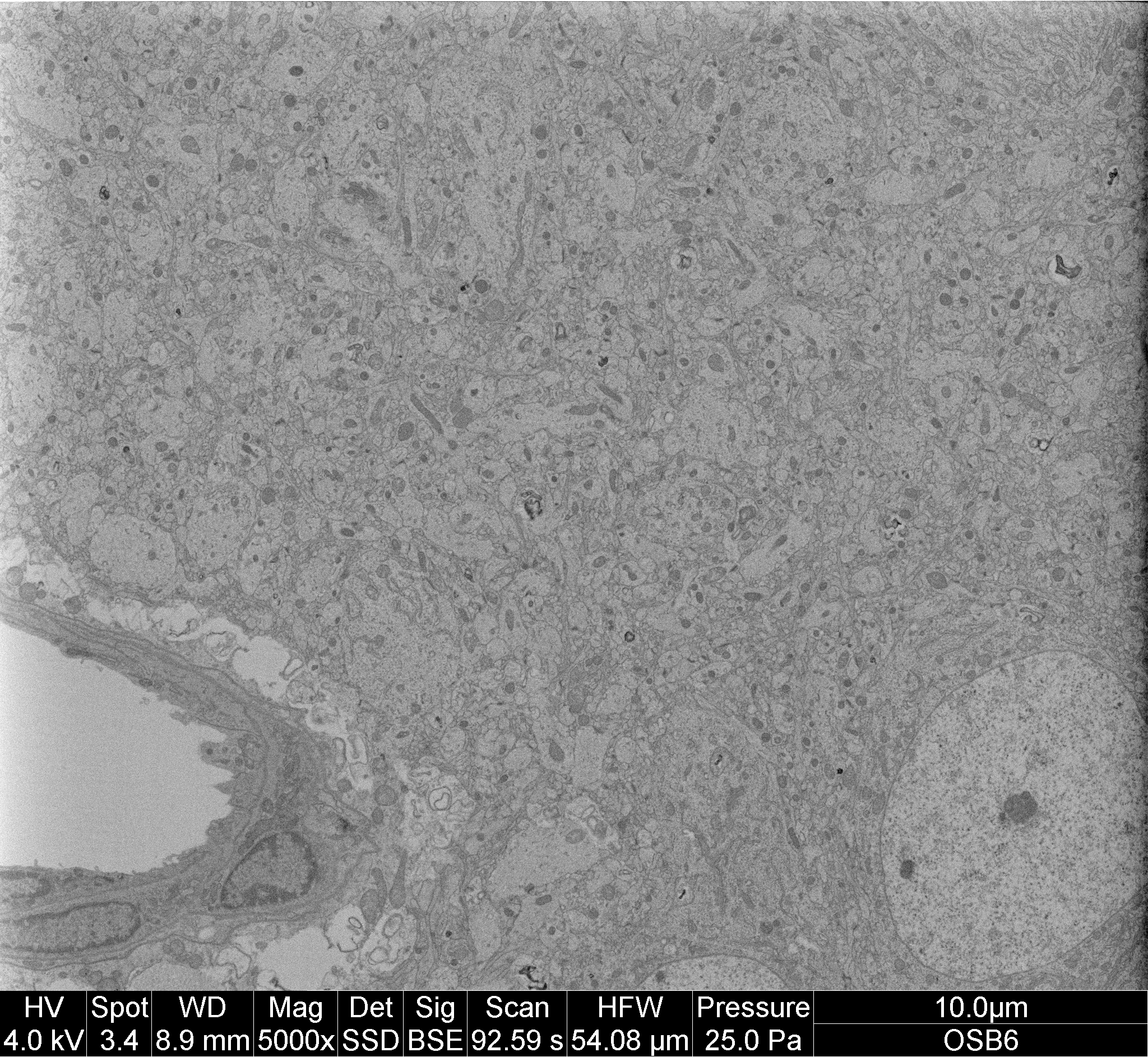

Supplement: Dataset S9 — (256.1 MB ZIP). [file pbio.0020329.sd009.zip › 040604_OS5_st1_874.tif]

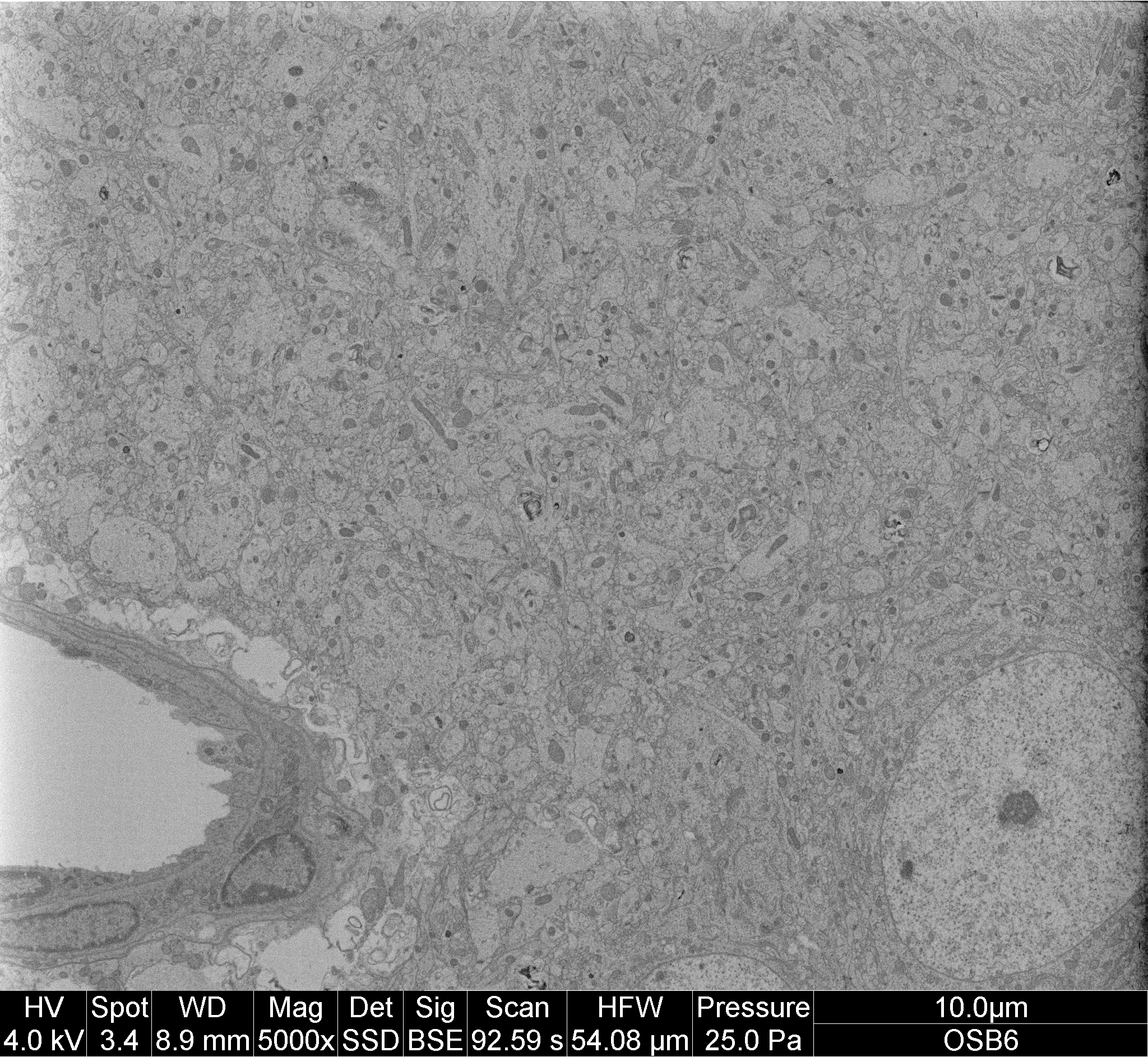

Supplement: Dataset S9 — (256.1 MB ZIP). [file pbio.0020329.sd009.zip › 040604_OS5_st1_875.tif]

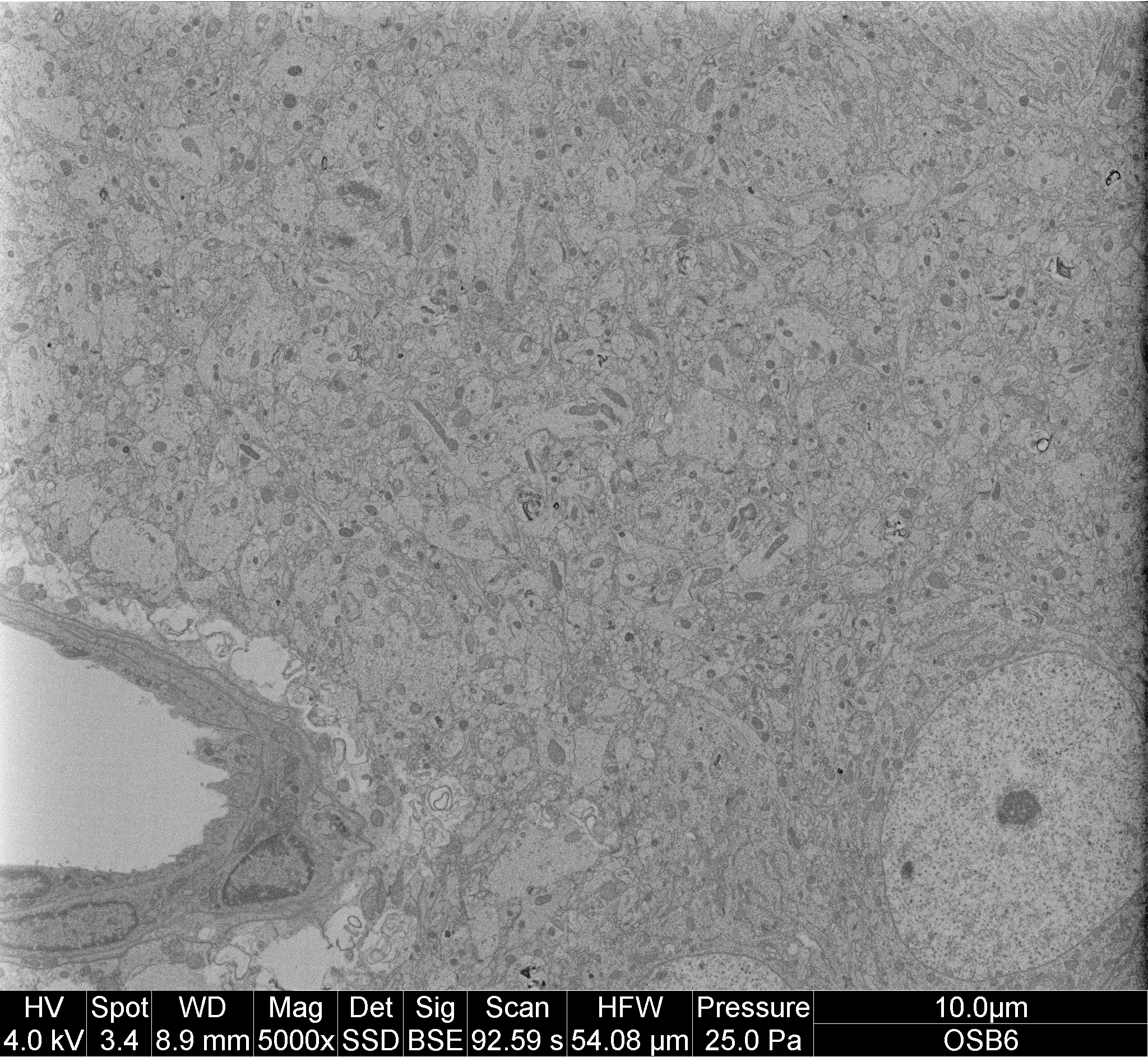

Supplement: Dataset S9 — (256.1 MB ZIP). [file pbio.0020329.sd009.zip › 040604_OS5_st1_876.tif]

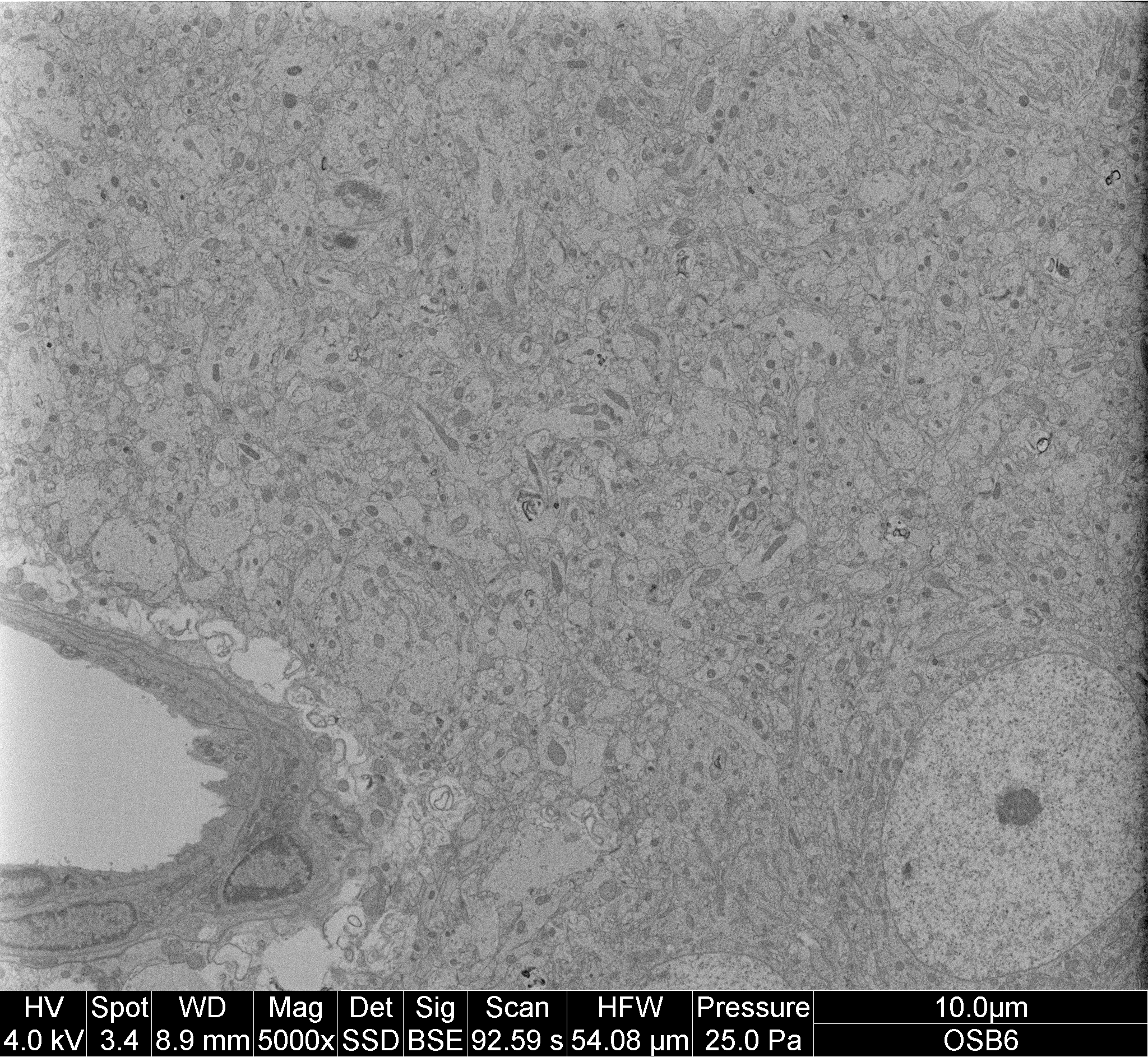

Supplement: Dataset S9 — (256.1 MB ZIP). [file pbio.0020329.sd009.zip › 040604_OS5_st1_877.tif]

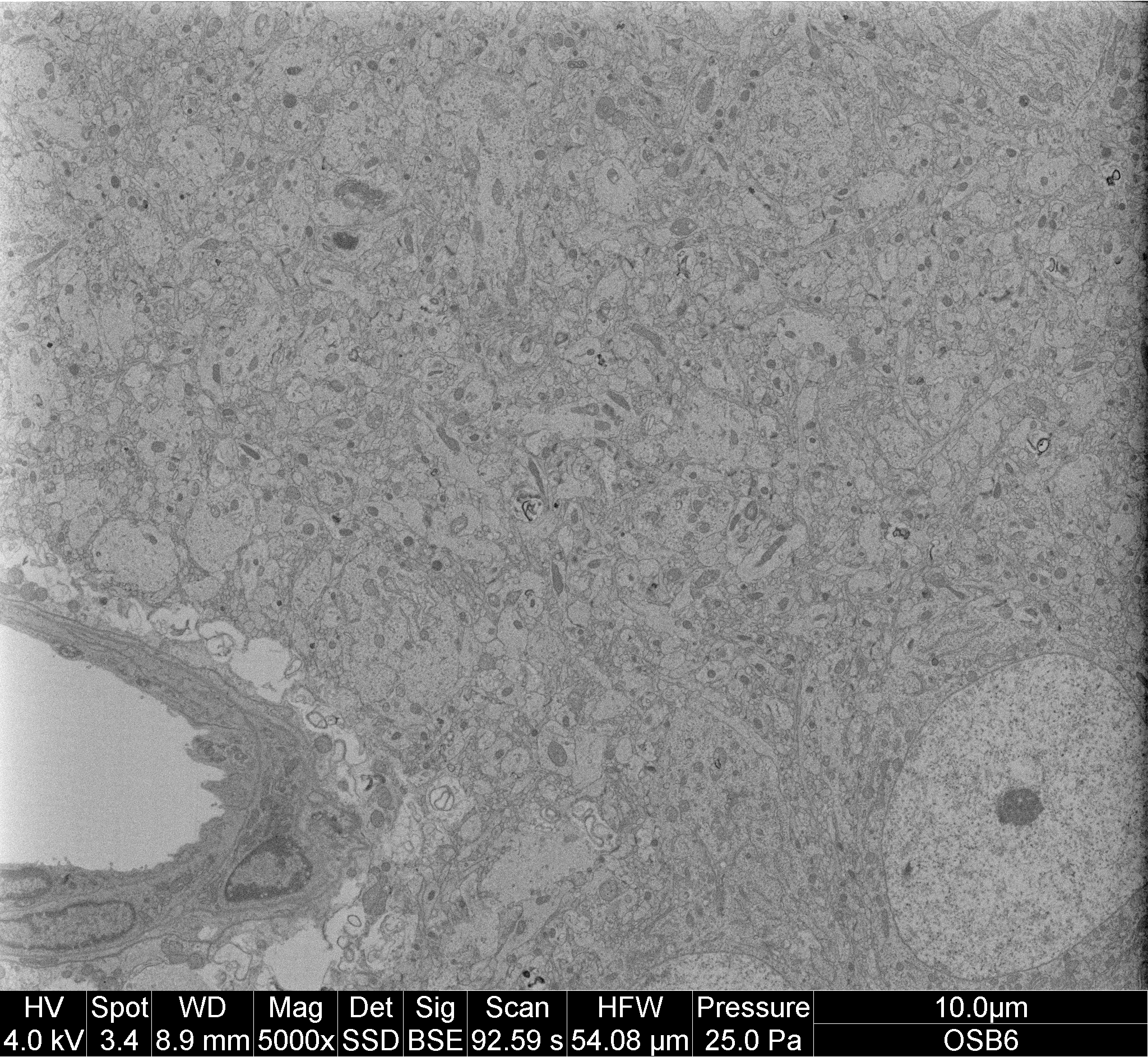

Supplement: Dataset S9 — (256.1 MB ZIP). [file pbio.0020329.sd009.zip › 040604_OS5_st1_878.tif]

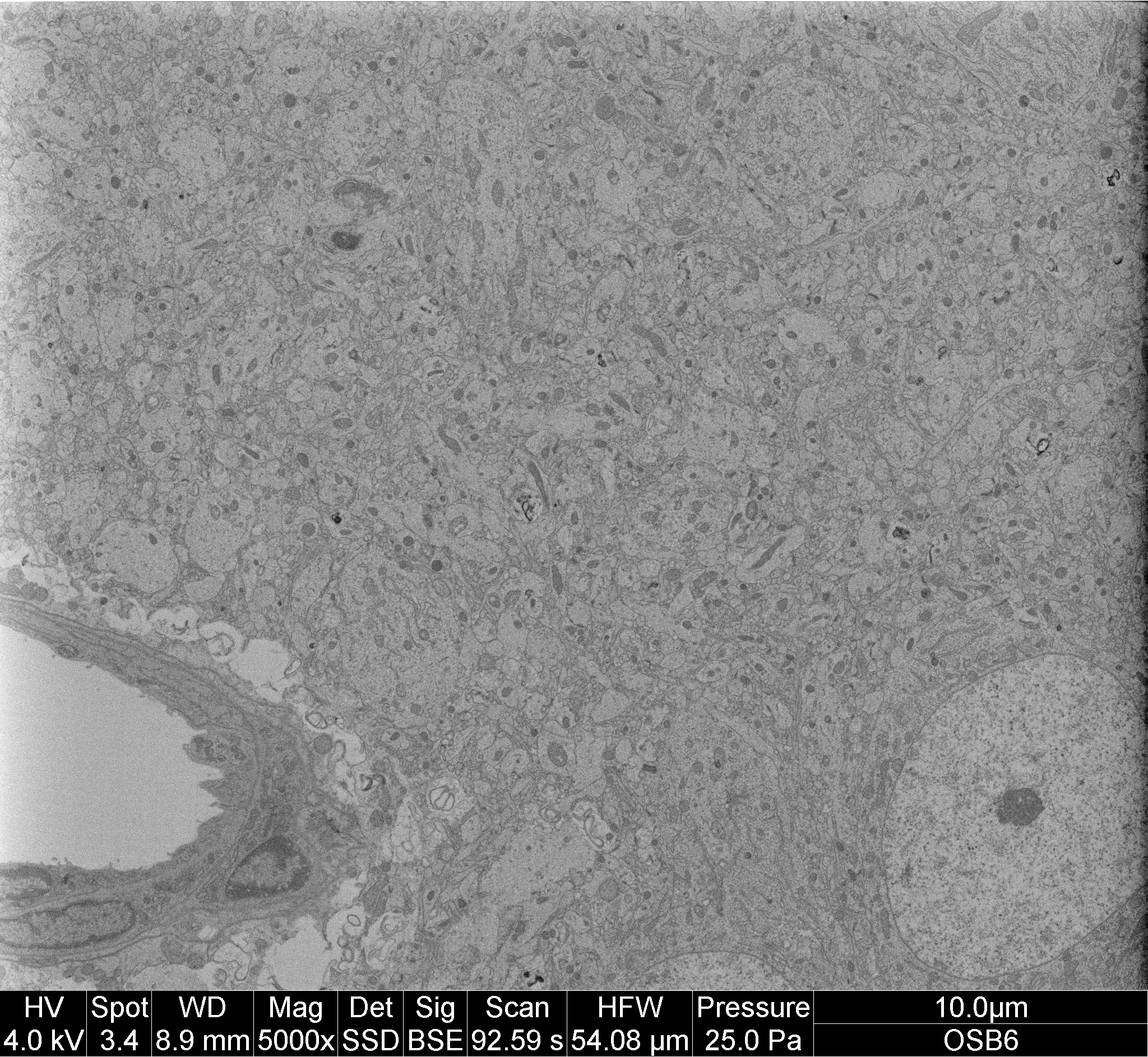

Supplement: Dataset S9 — (256.1 MB ZIP). [file pbio.0020329.sd009.zip › 040604_OS5_st1_879.tif]

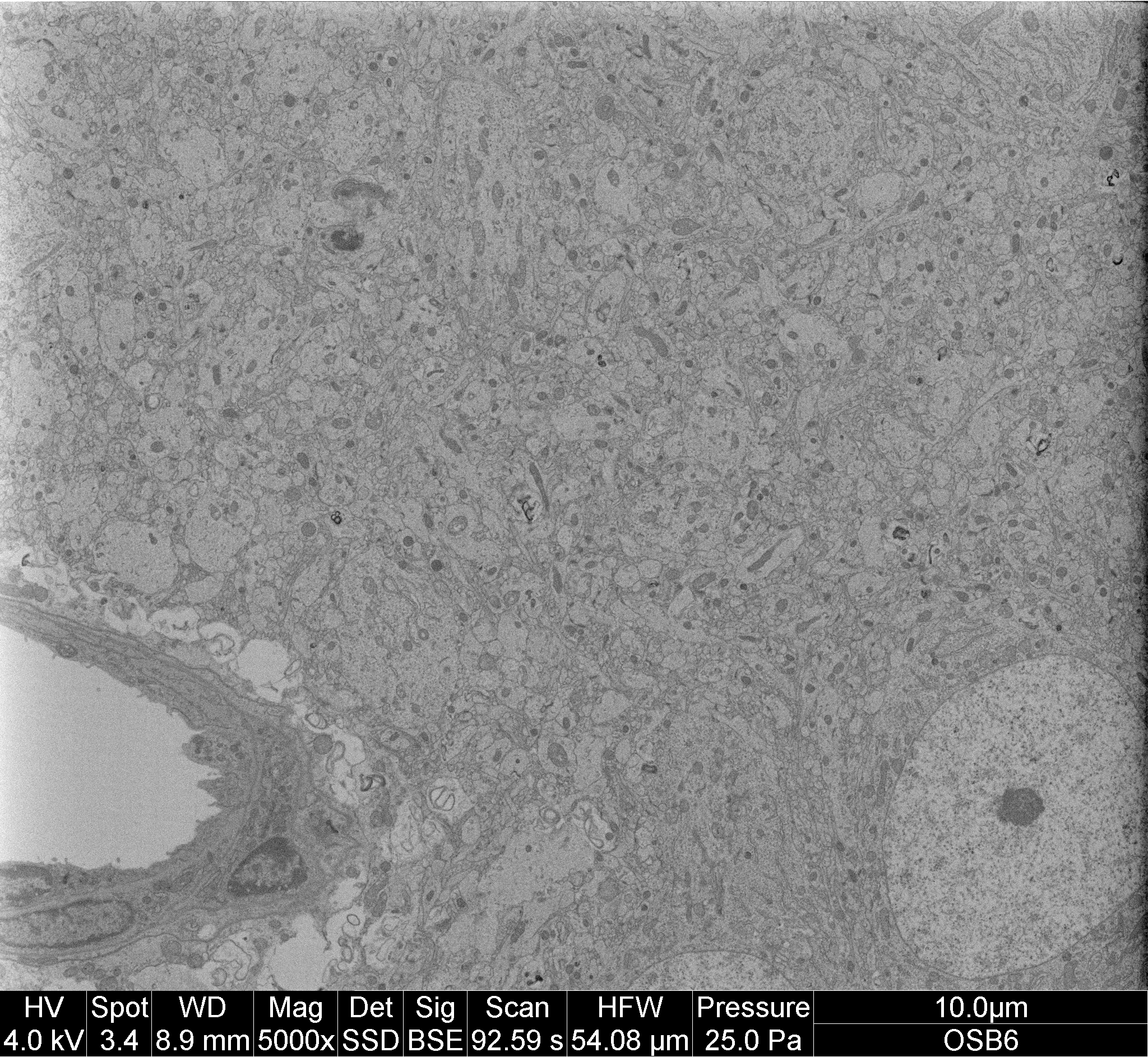

Supplement: Dataset S9 — (256.1 MB ZIP). [file pbio.0020329.sd009.zip › 040604_OS5_st1_880.tif]

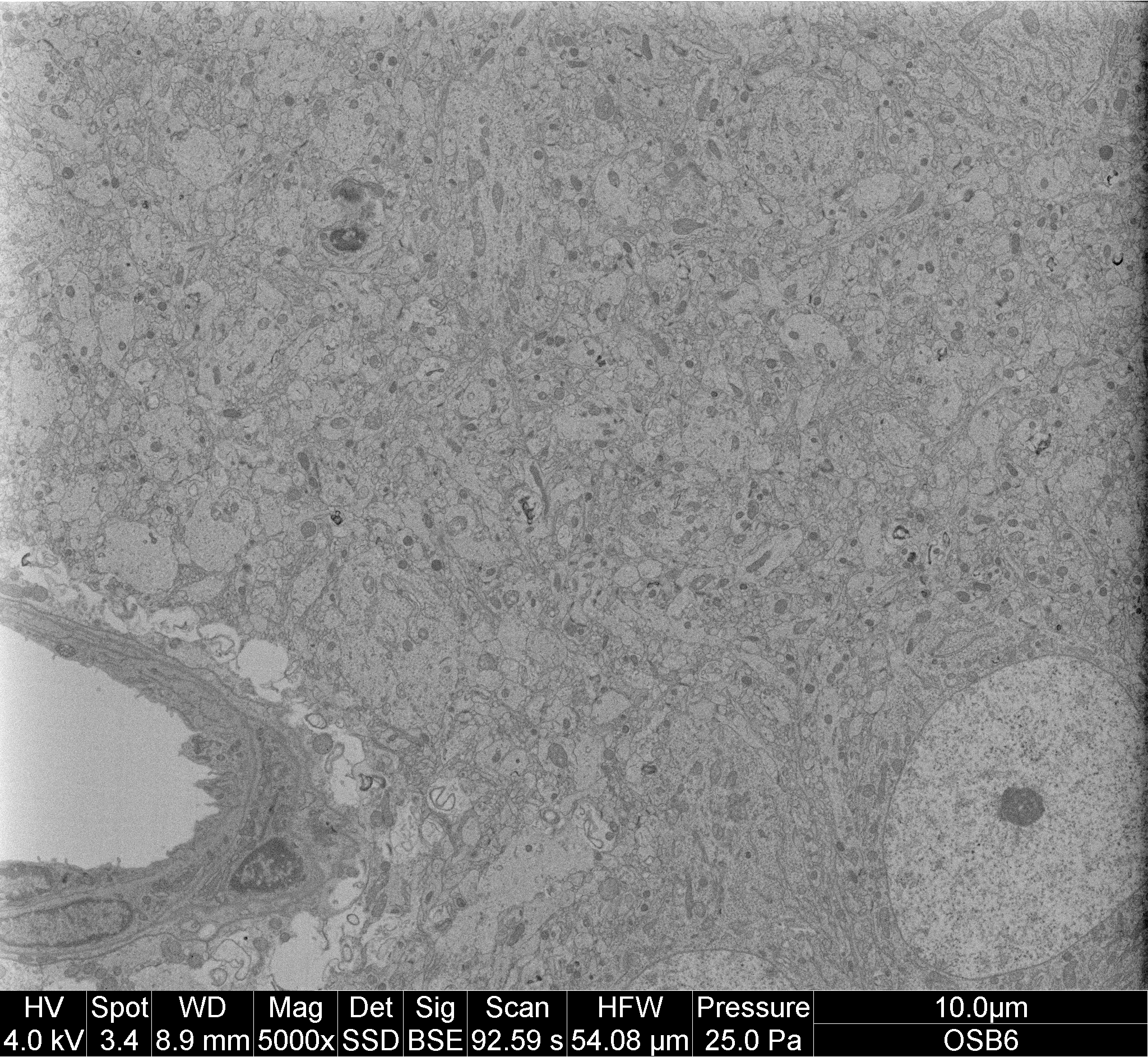

Supplement: Dataset S9 — (256.1 MB ZIP). [file pbio.0020329.sd009.zip › 040604_OS5_st1_881.tif]

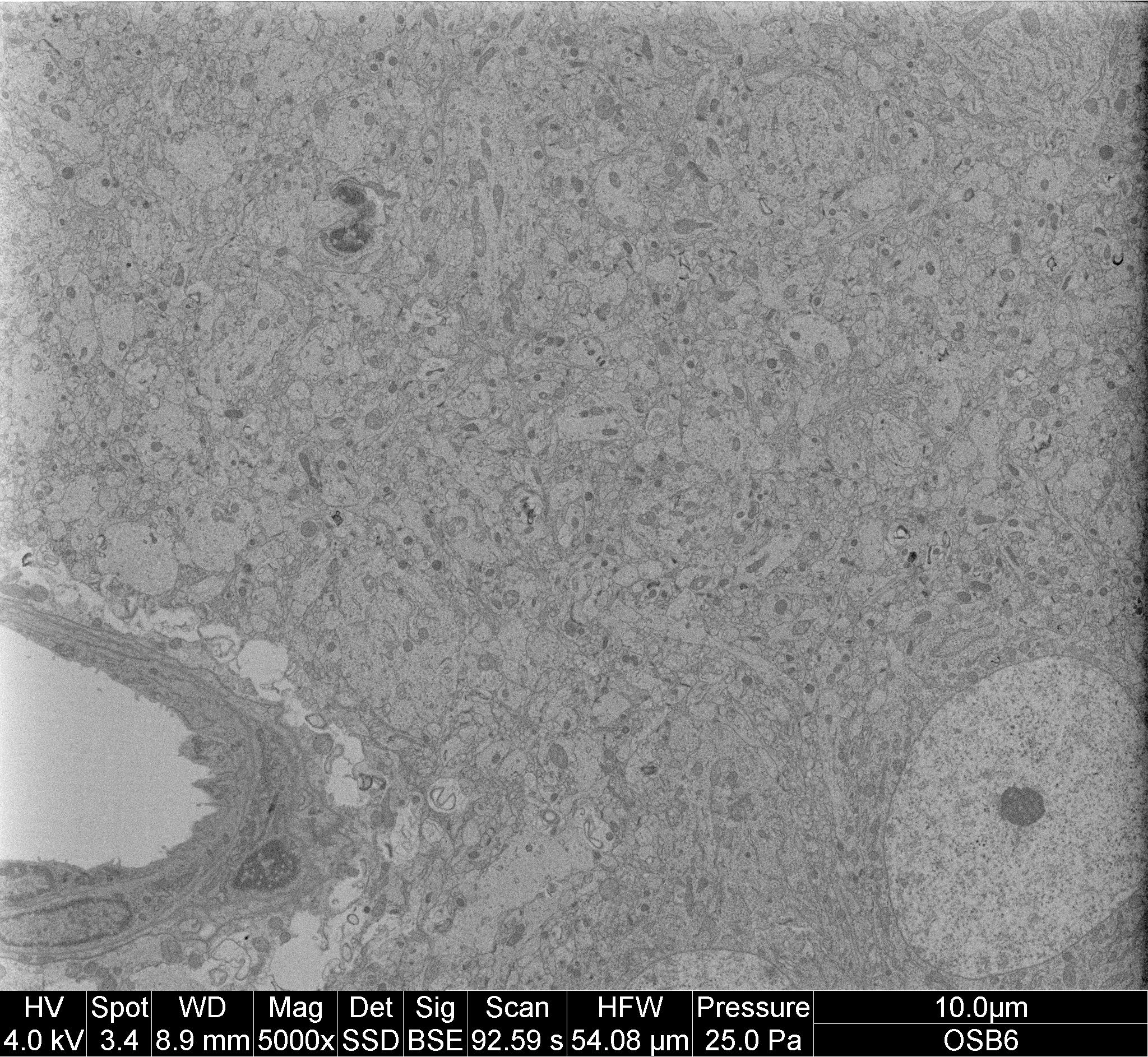

Supplement: Dataset S9 — (256.1 MB ZIP). [file pbio.0020329.sd009.zip › 040604_OS5_st1_882.tif]

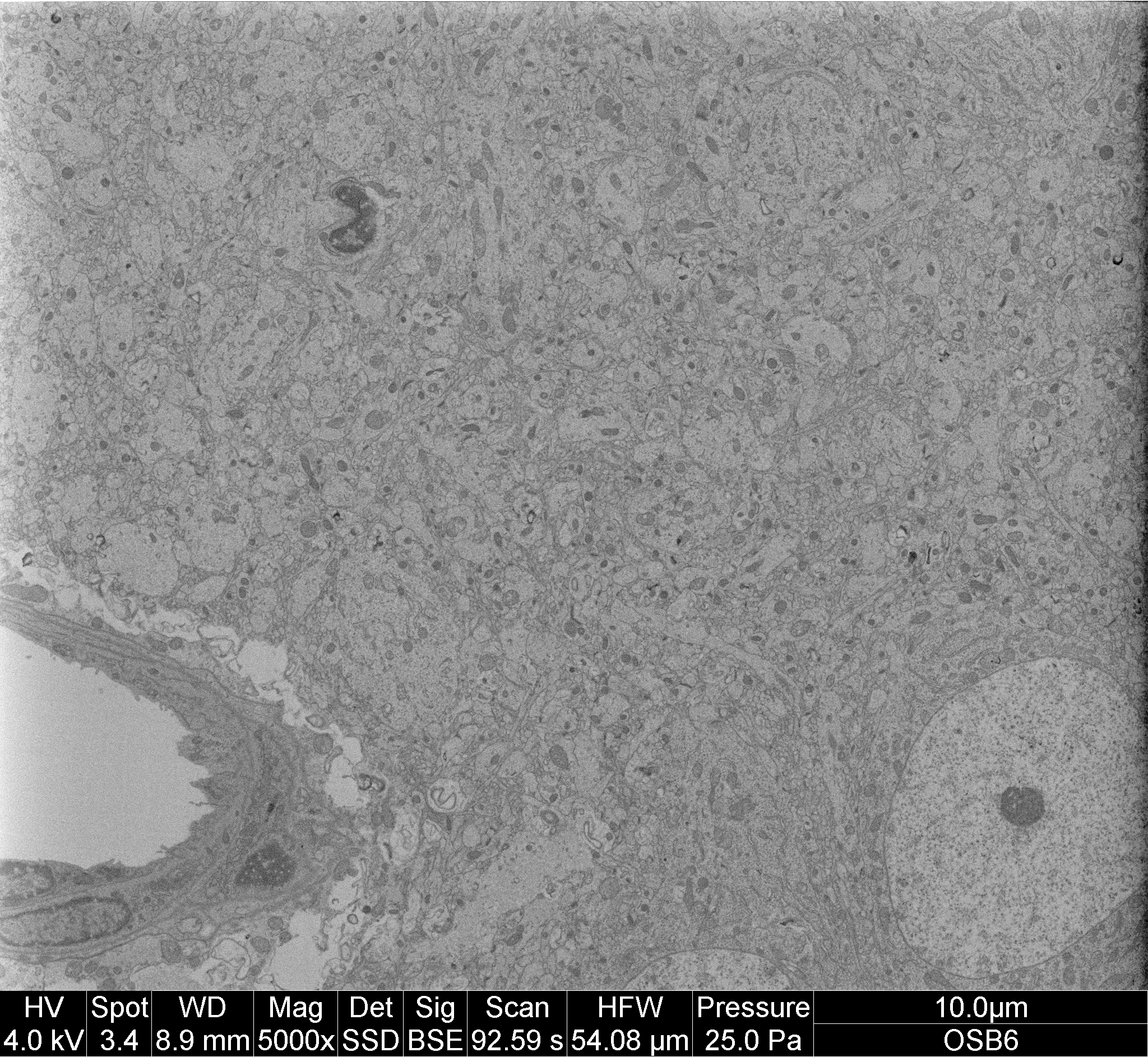

Supplement: Dataset S9 — (256.1 MB ZIP). [file pbio.0020329.sd009.zip › 040604_OS5_st1_883.tif]

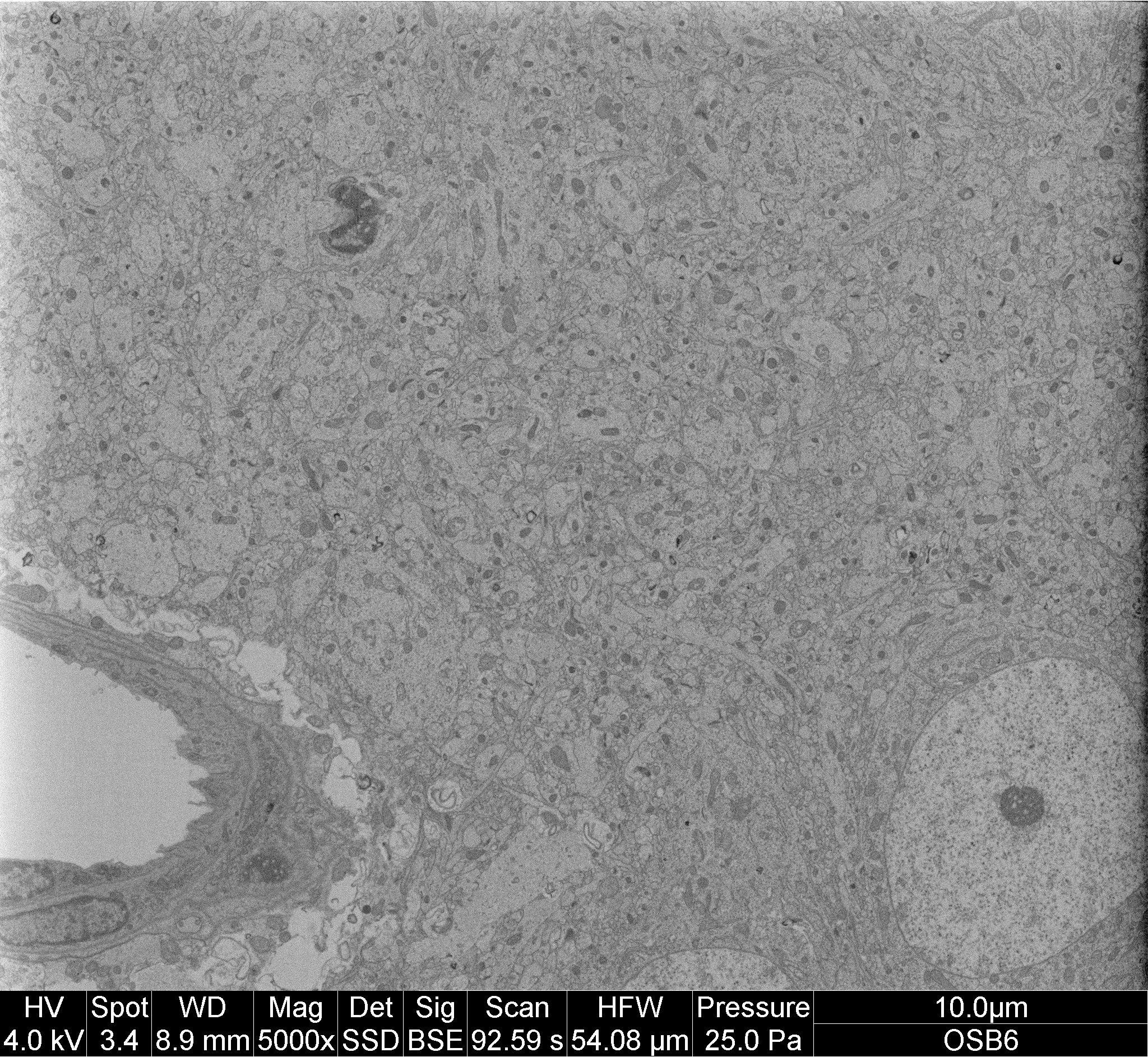

Supplement: Dataset S9 — (256.1 MB ZIP). [file pbio.0020329.sd009.zip › 040604_OS5_st1_884.tif]

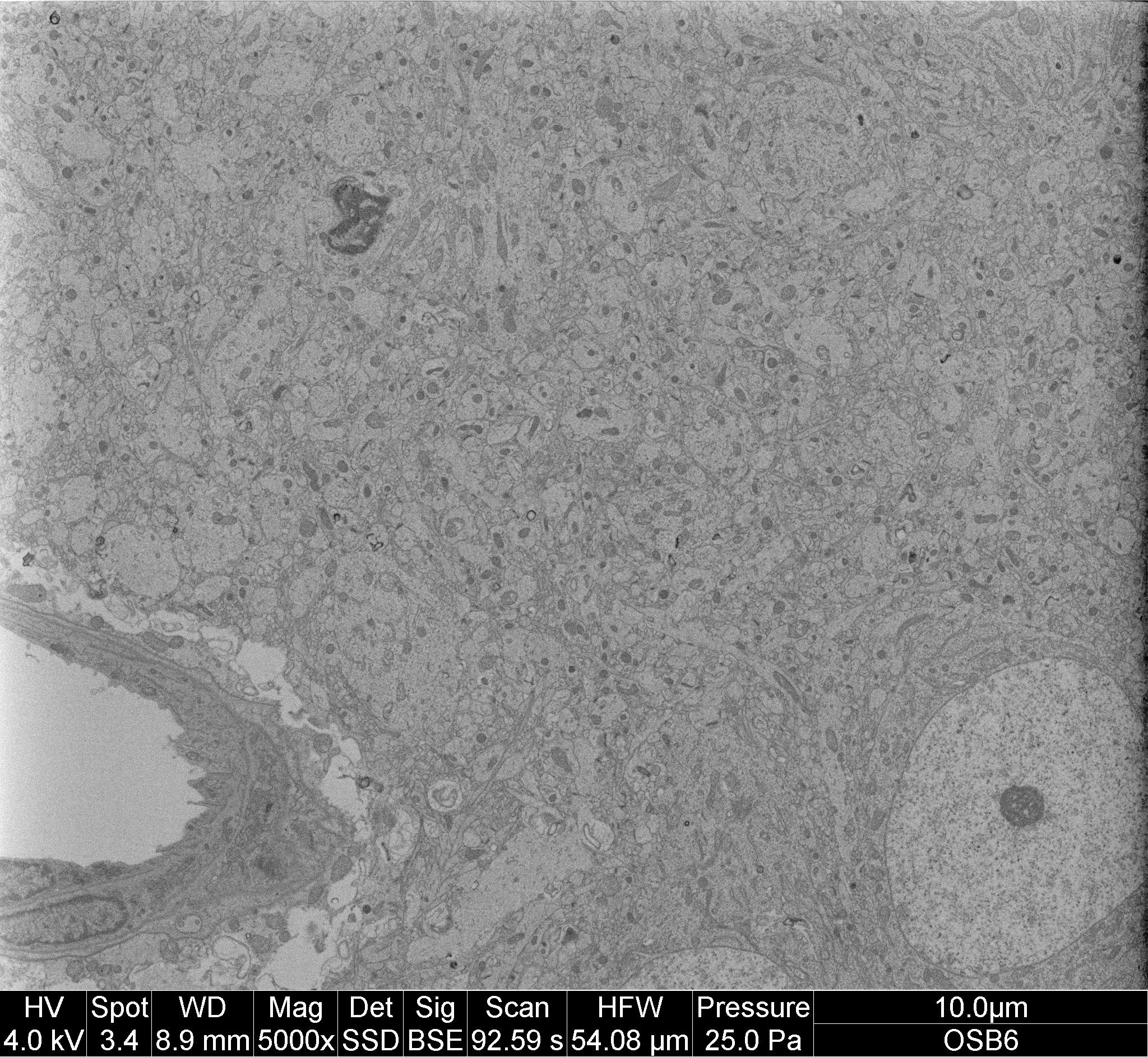

Supplement: Dataset S9 — (256.1 MB ZIP). [file pbio.0020329.sd009.zip › 040604_OS5_st1_885.tif]

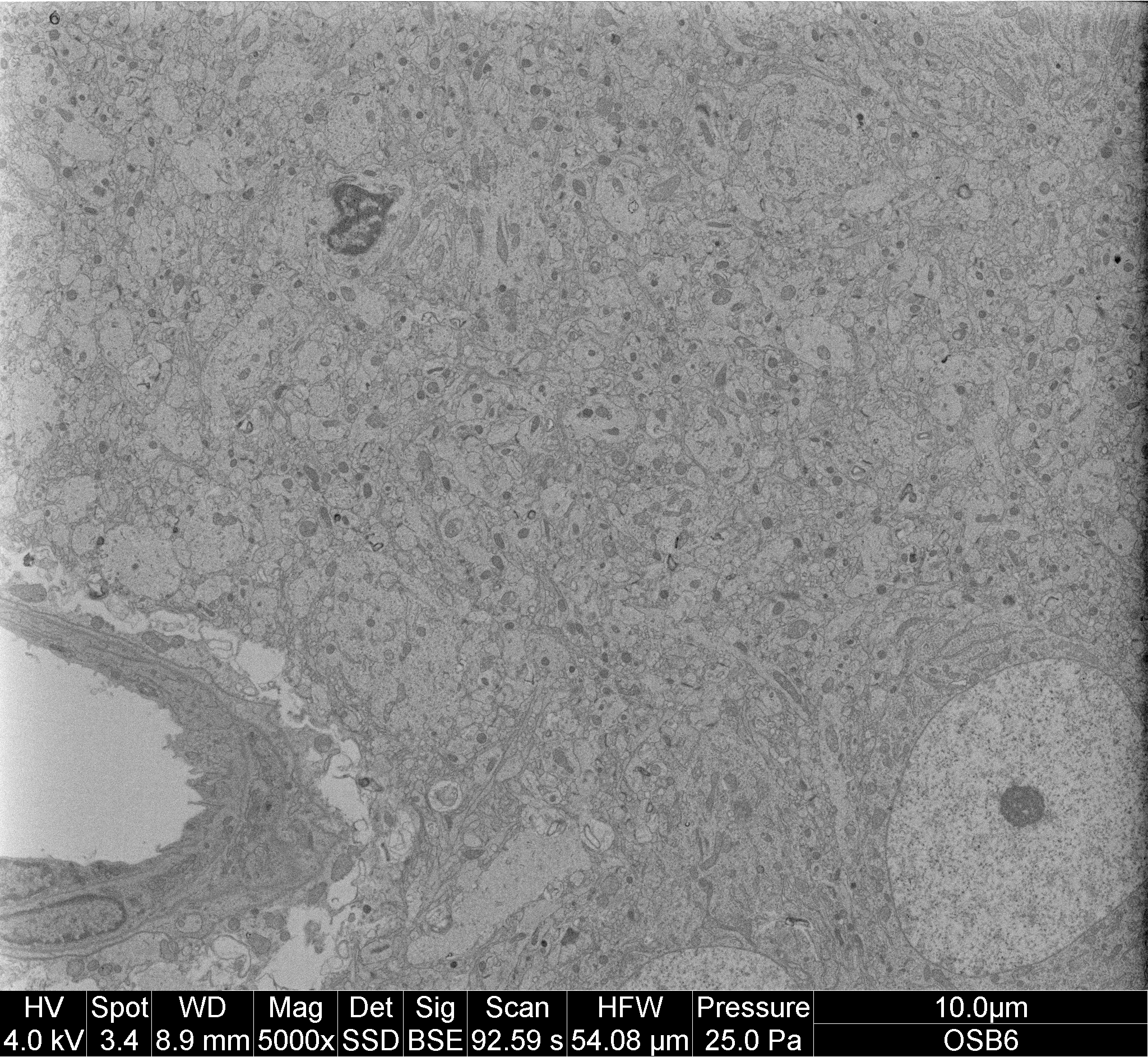

Supplement: Dataset S9 — (256.1 MB ZIP). [file pbio.0020329.sd009.zip › 040604_OS5_st1_886.tif]

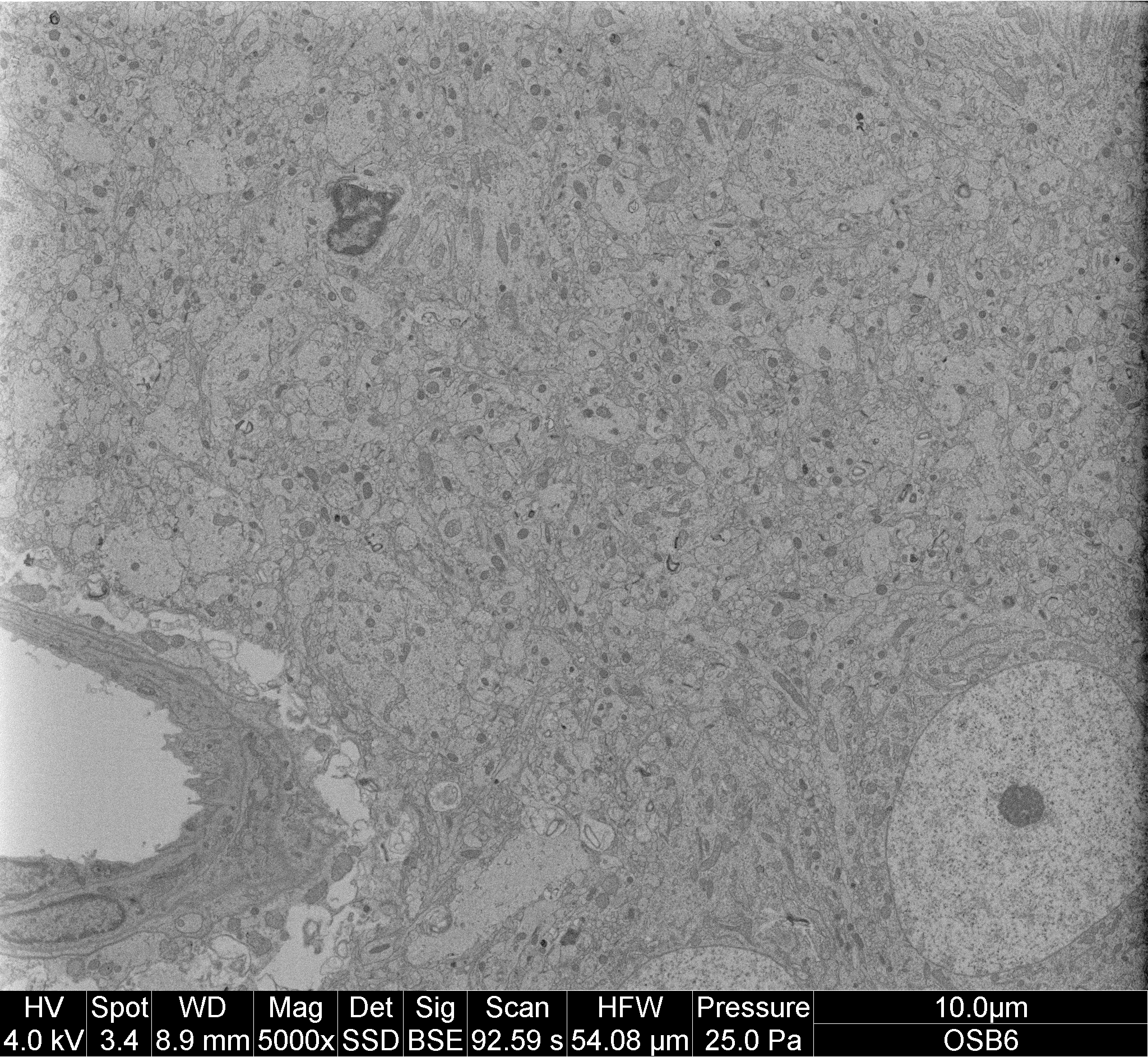

Supplement: Dataset S9 — (256.1 MB ZIP). [file pbio.0020329.sd009.zip › 040604_OS5_st1_887.tif]

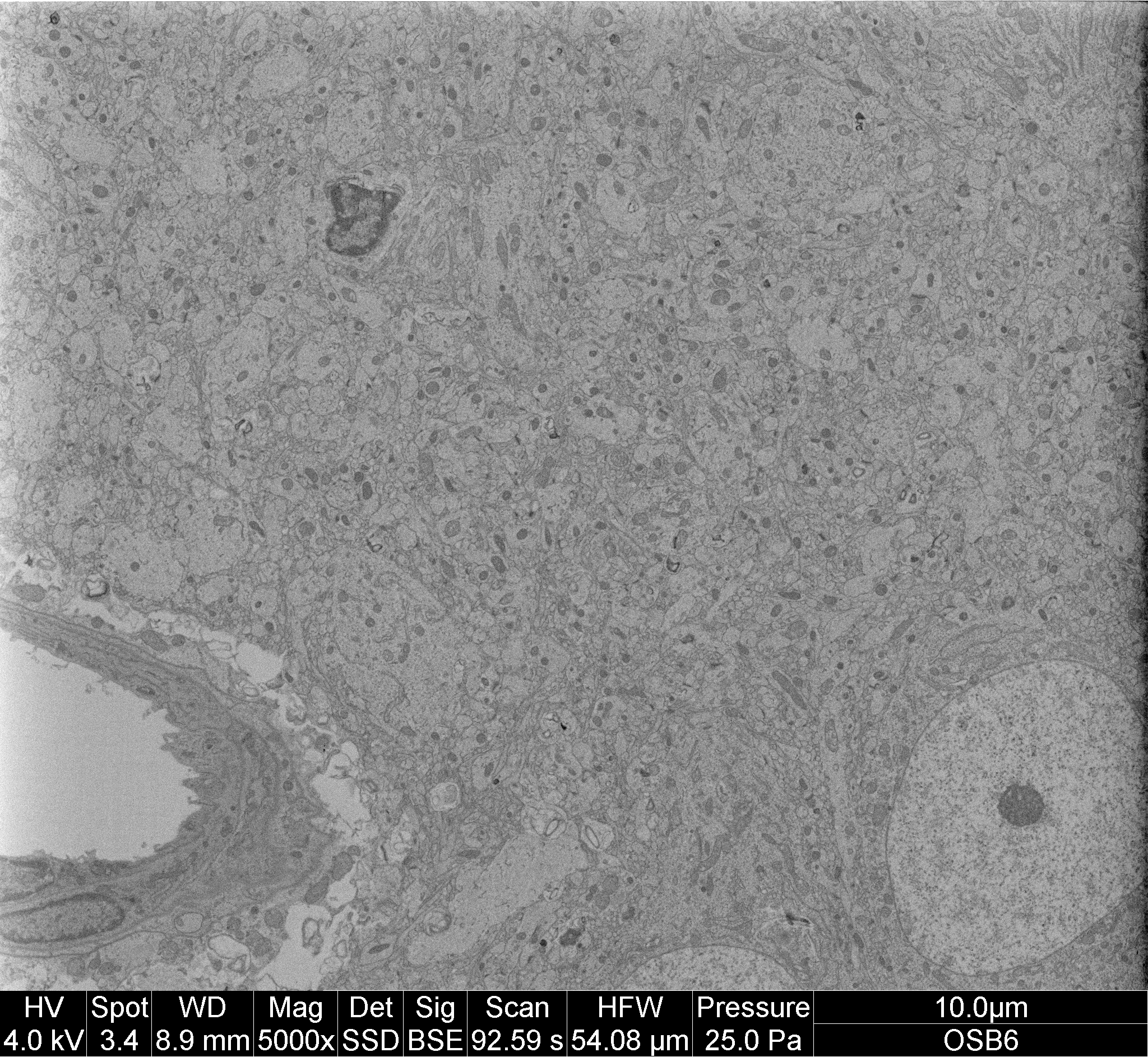

Supplement: Dataset S9 — (256.1 MB ZIP). [file pbio.0020329.sd009.zip › 040604_OS5_st1_888.tif]

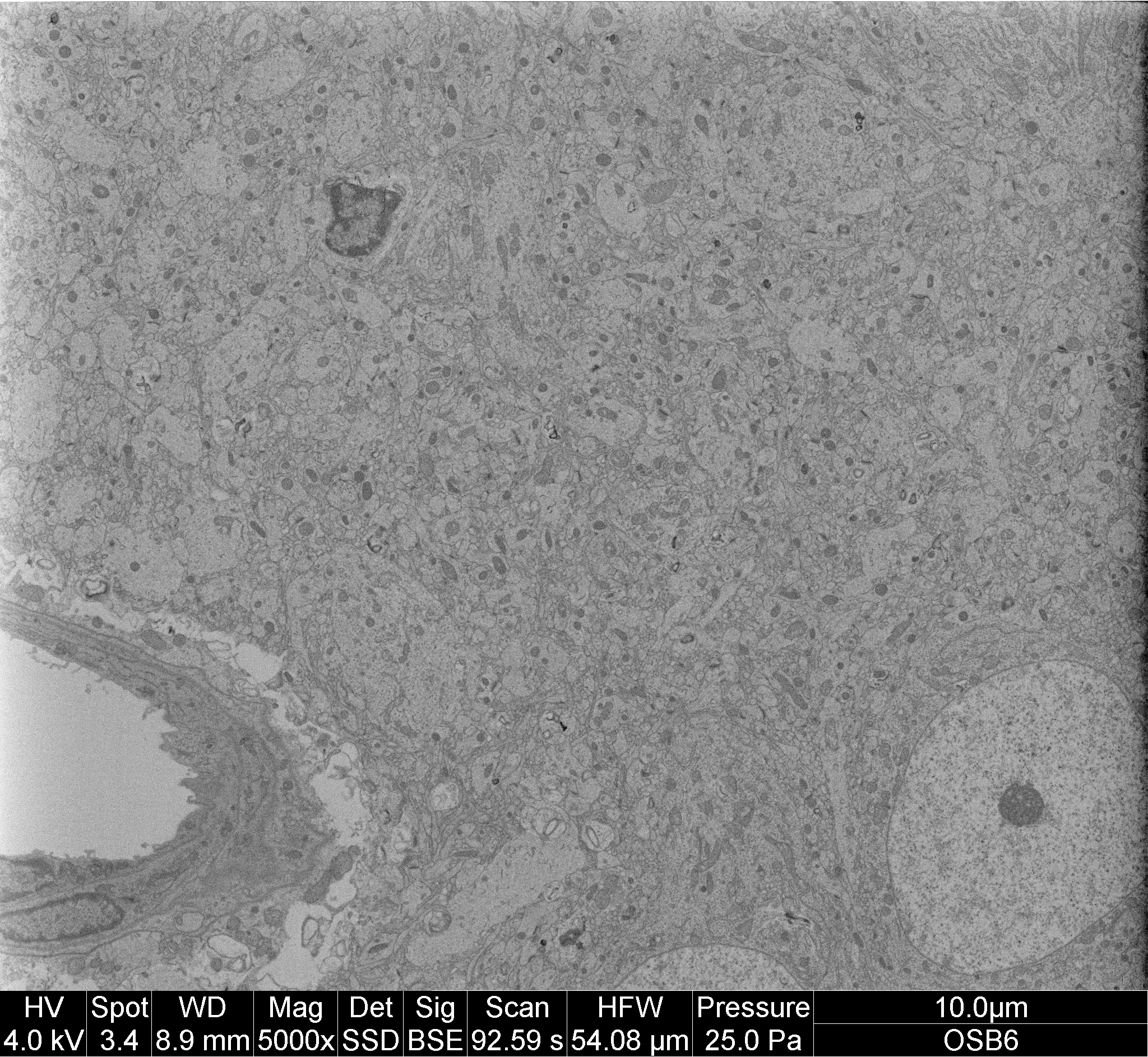

Supplement: Dataset S9 — (256.1 MB ZIP). [file pbio.0020329.sd009.zip › 040604_OS5_st1_889.tif]

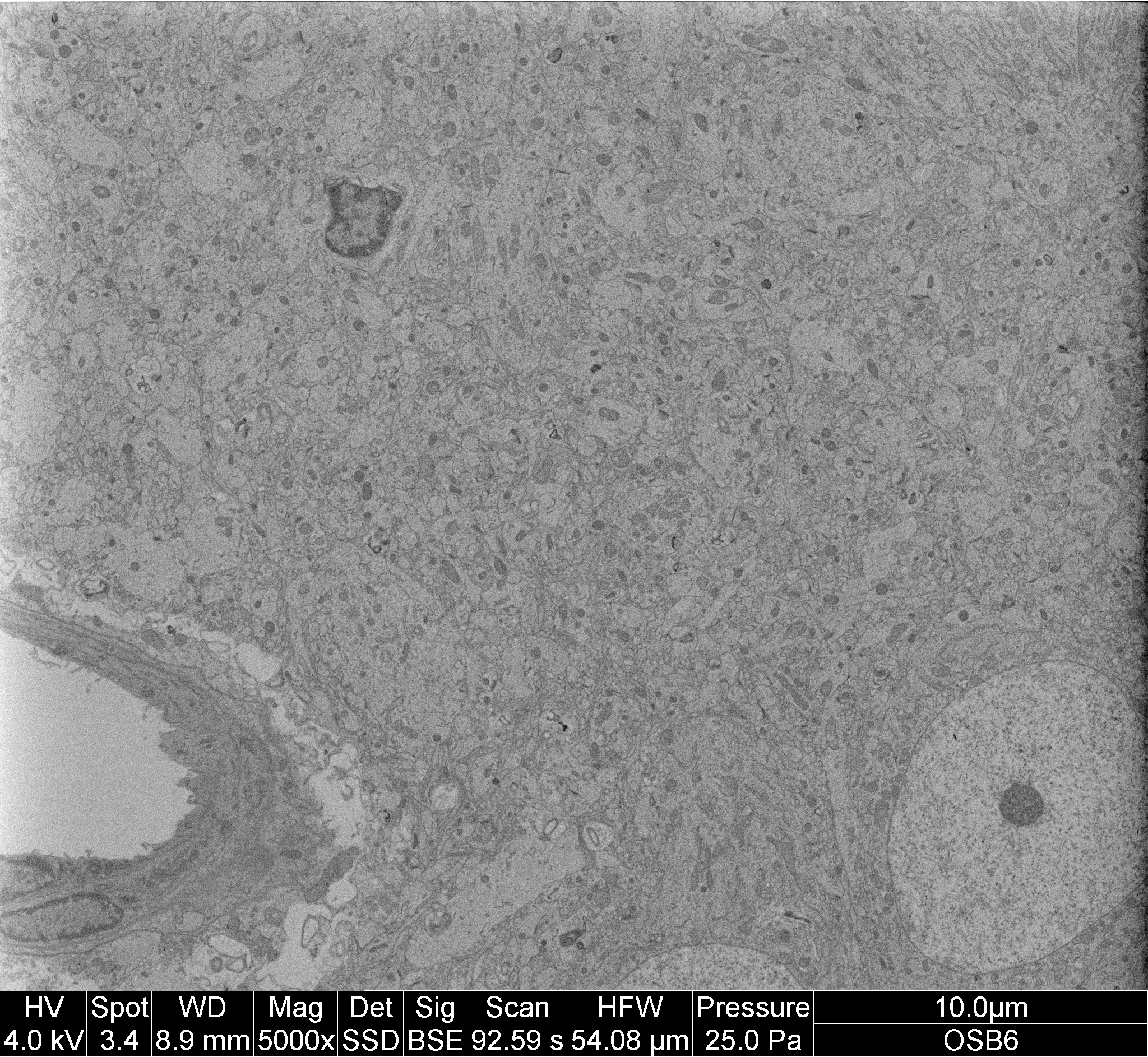

Supplement: Dataset S9 — (256.1 MB ZIP). [file pbio.0020329.sd009.zip › 040604_OS5_st1_890.tif]

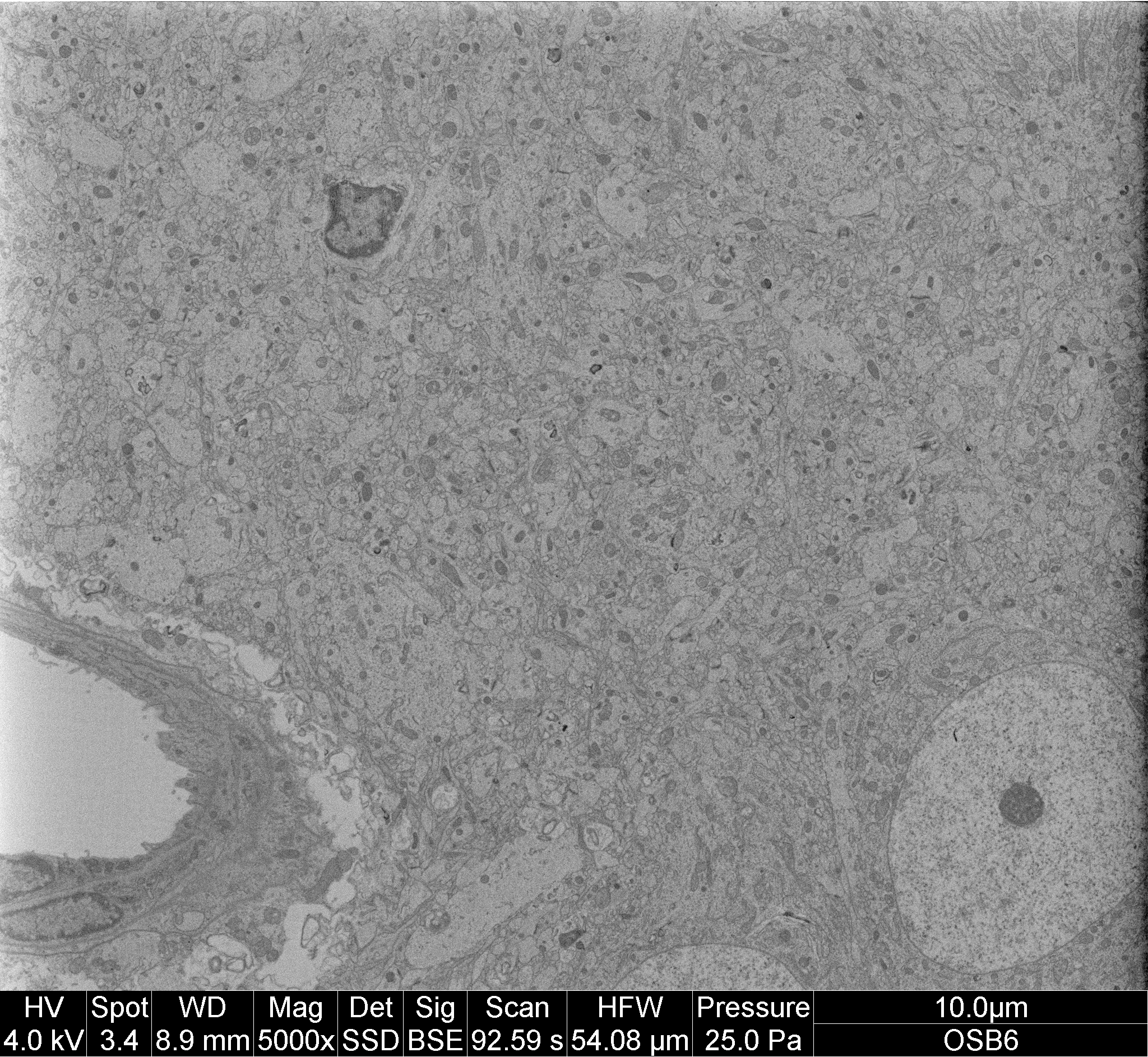

Supplement: Dataset S9 — (256.1 MB ZIP). [file pbio.0020329.sd009.zip › 040604_OS5_st1_891.tif]

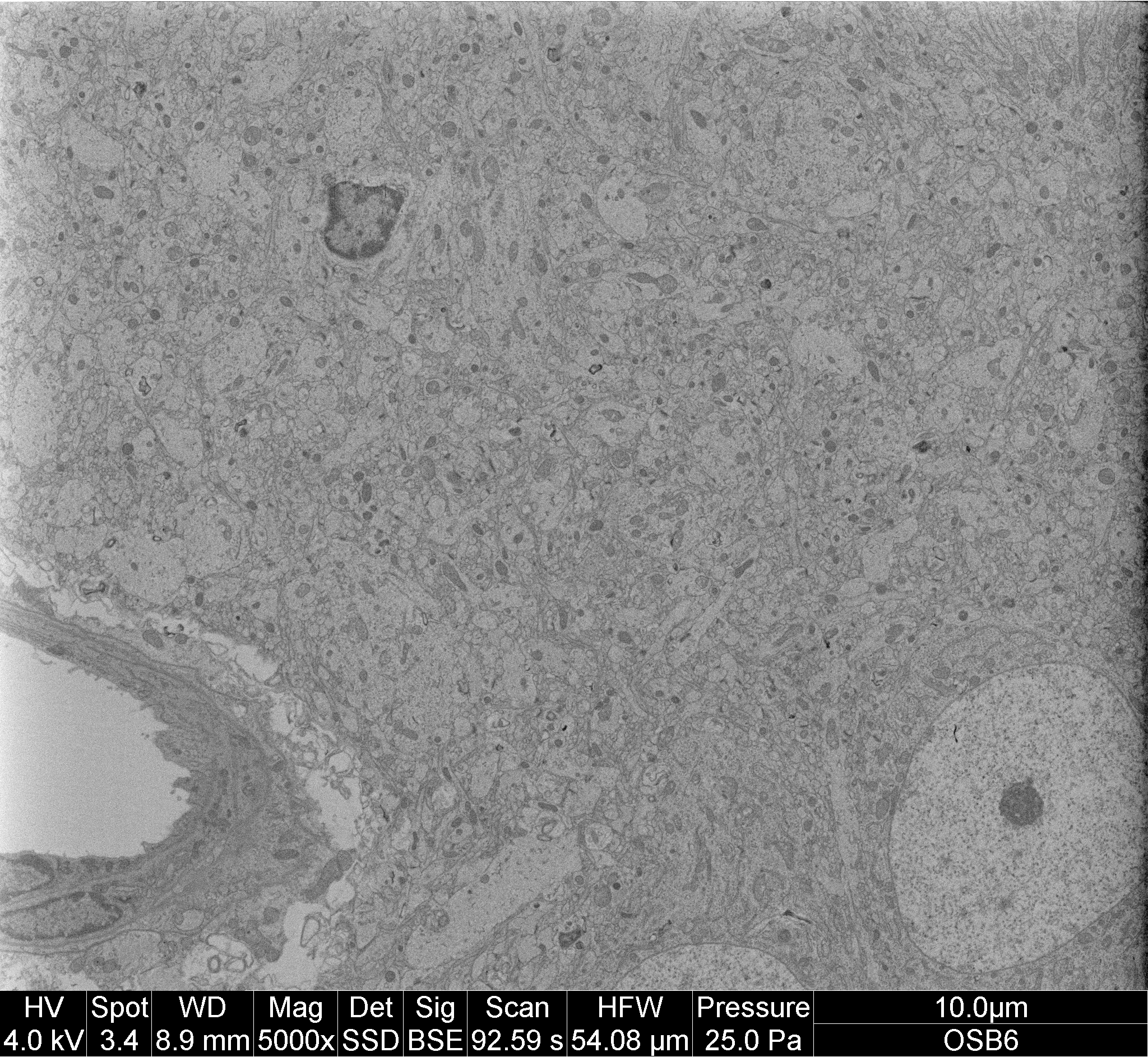

Supplement: Dataset S9 — (256.1 MB ZIP). [file pbio.0020329.sd009.zip › 040604_OS5_st1_892.tif]

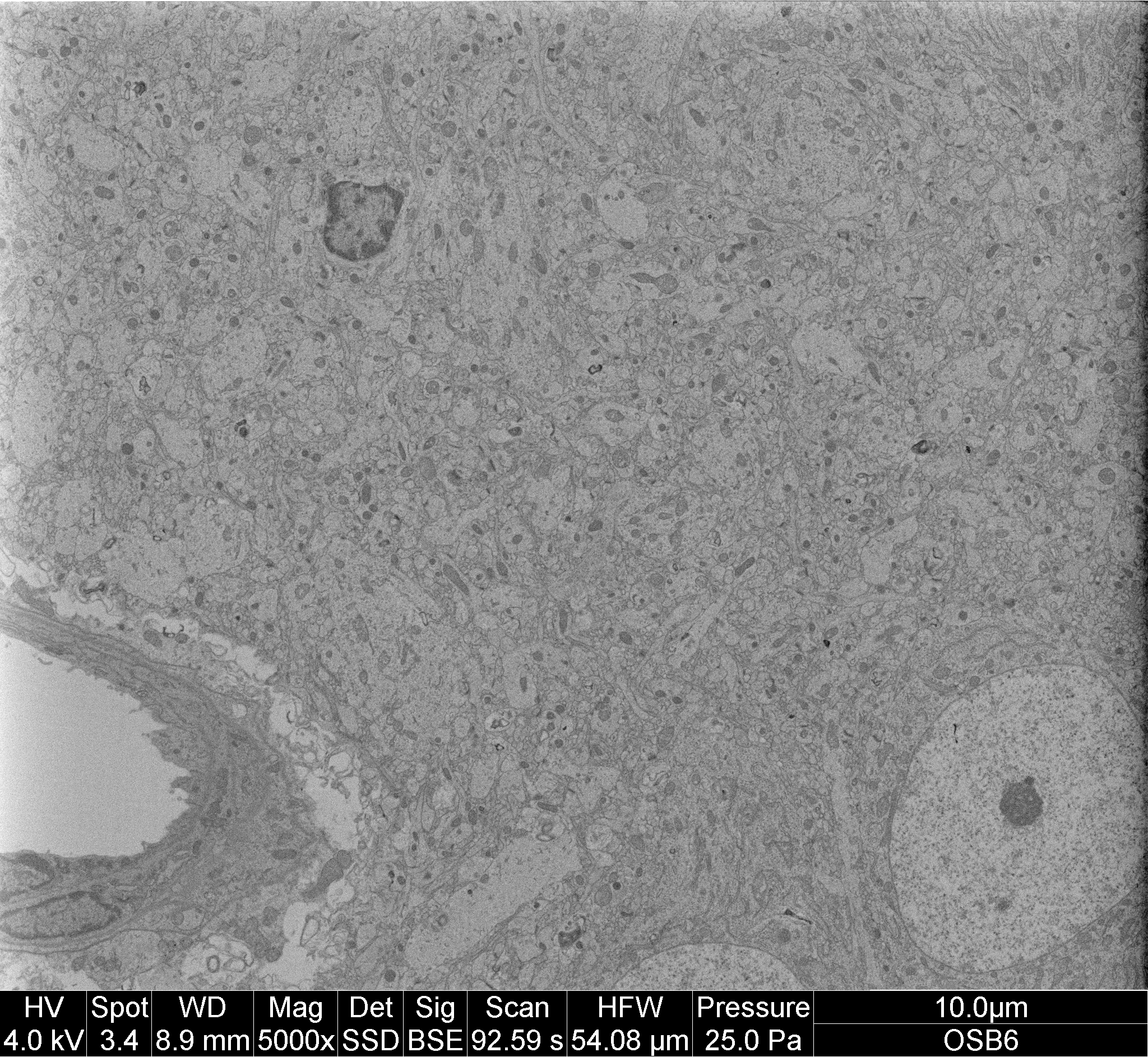

Supplement: Dataset S9 — (256.1 MB ZIP). [file pbio.0020329.sd009.zip › 040604_OS5_st1_893.tif]

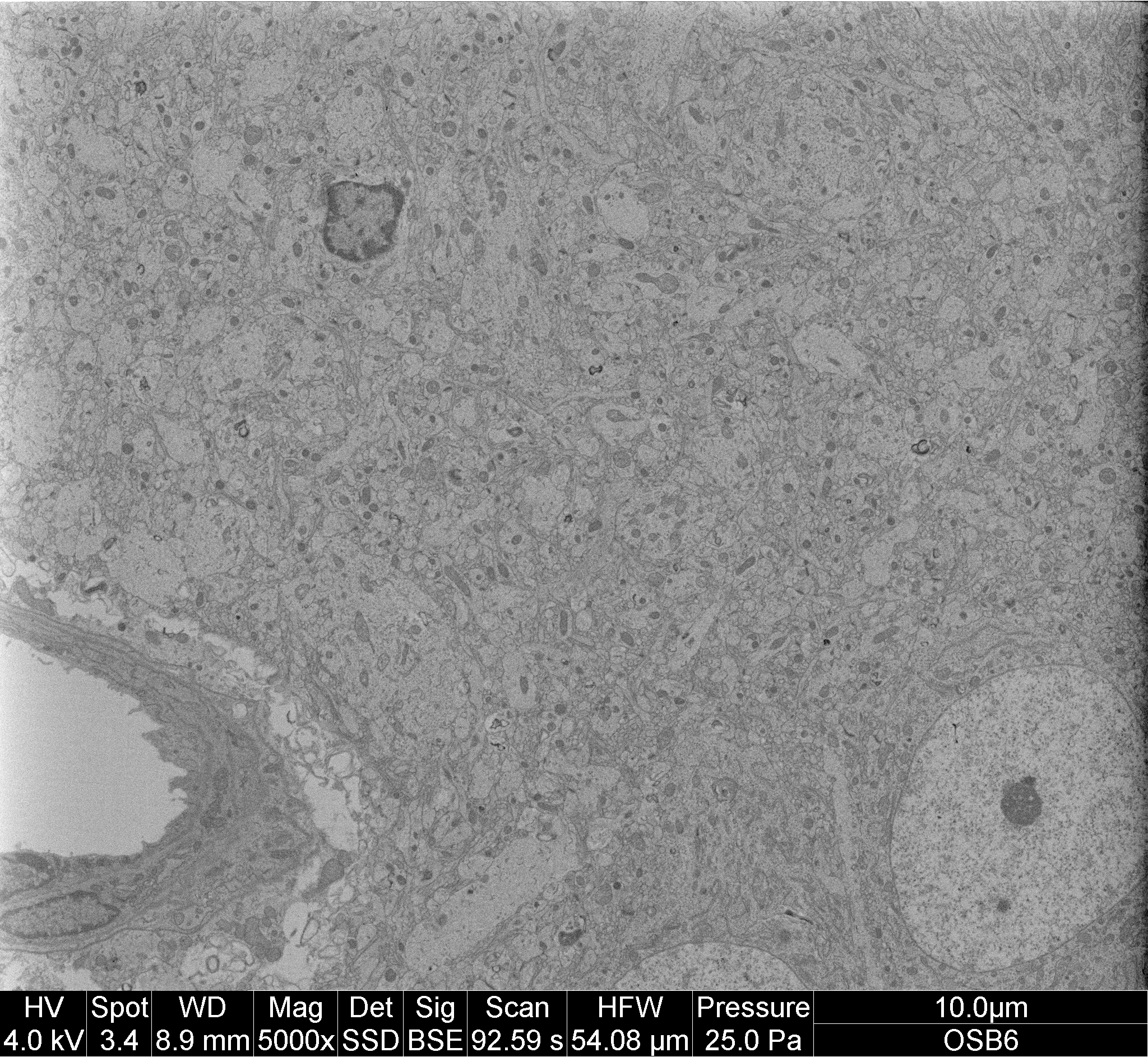

Supplement: Dataset S9 — (256.1 MB ZIP). [file pbio.0020329.sd009.zip › 040604_OS5_st1_894.tif]

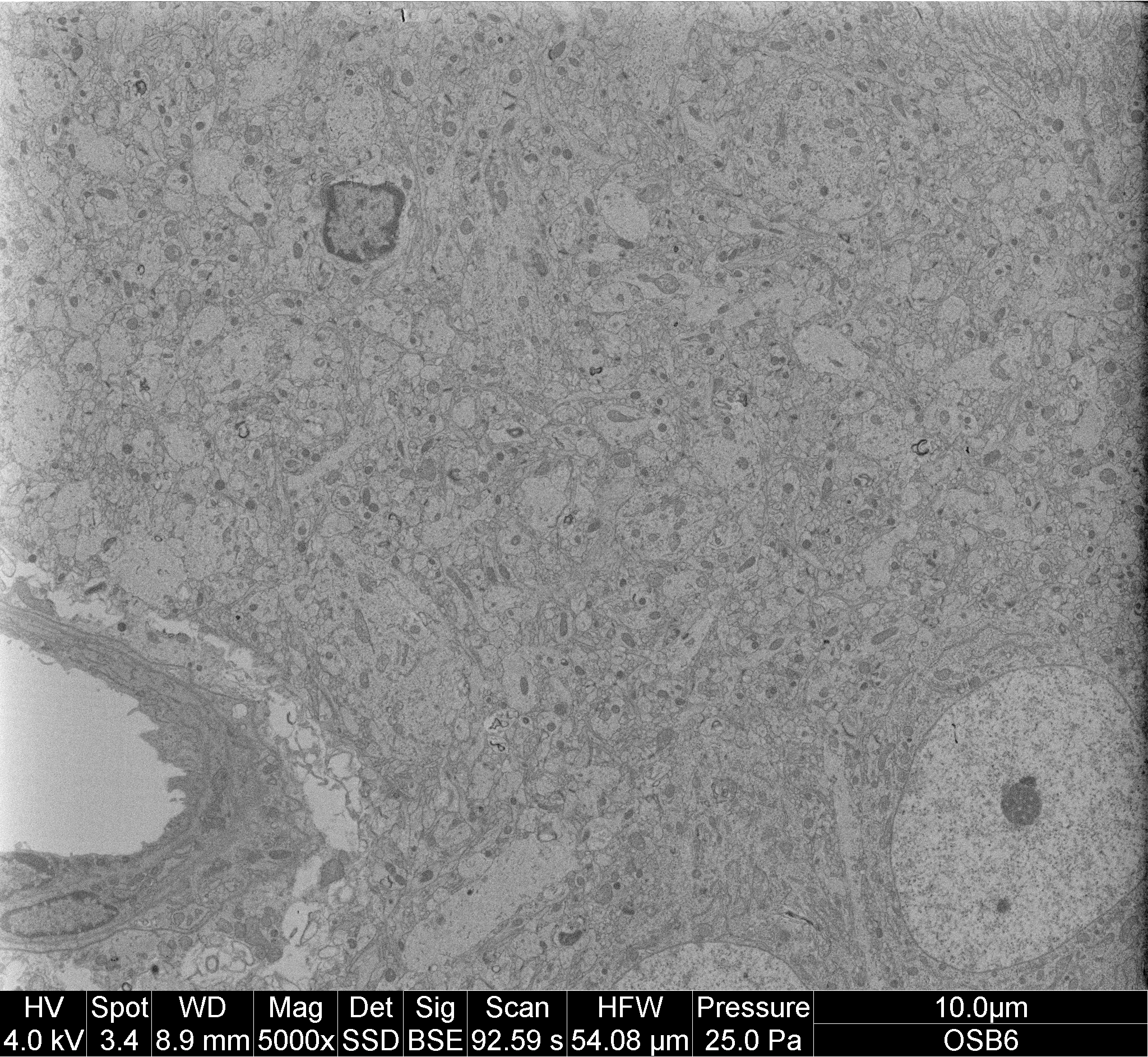

Supplement: Dataset S9 — (256.1 MB ZIP). [file pbio.0020329.sd009.zip › 040604_OS5_st1_895.tif]

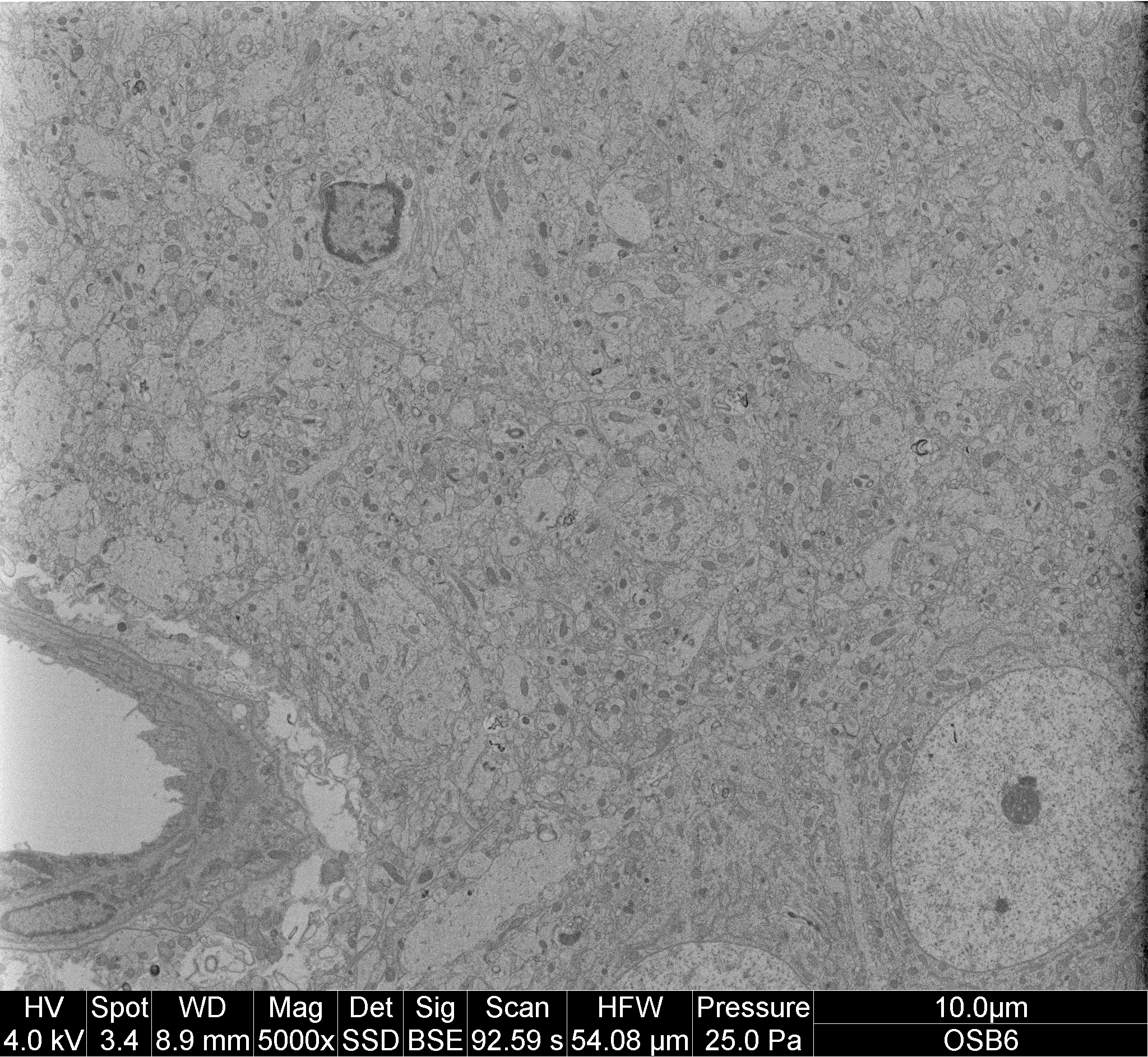

Supplement: Dataset S9 — (256.1 MB ZIP). [file pbio.0020329.sd009.zip › 040604_OS5_st1_896.tif]

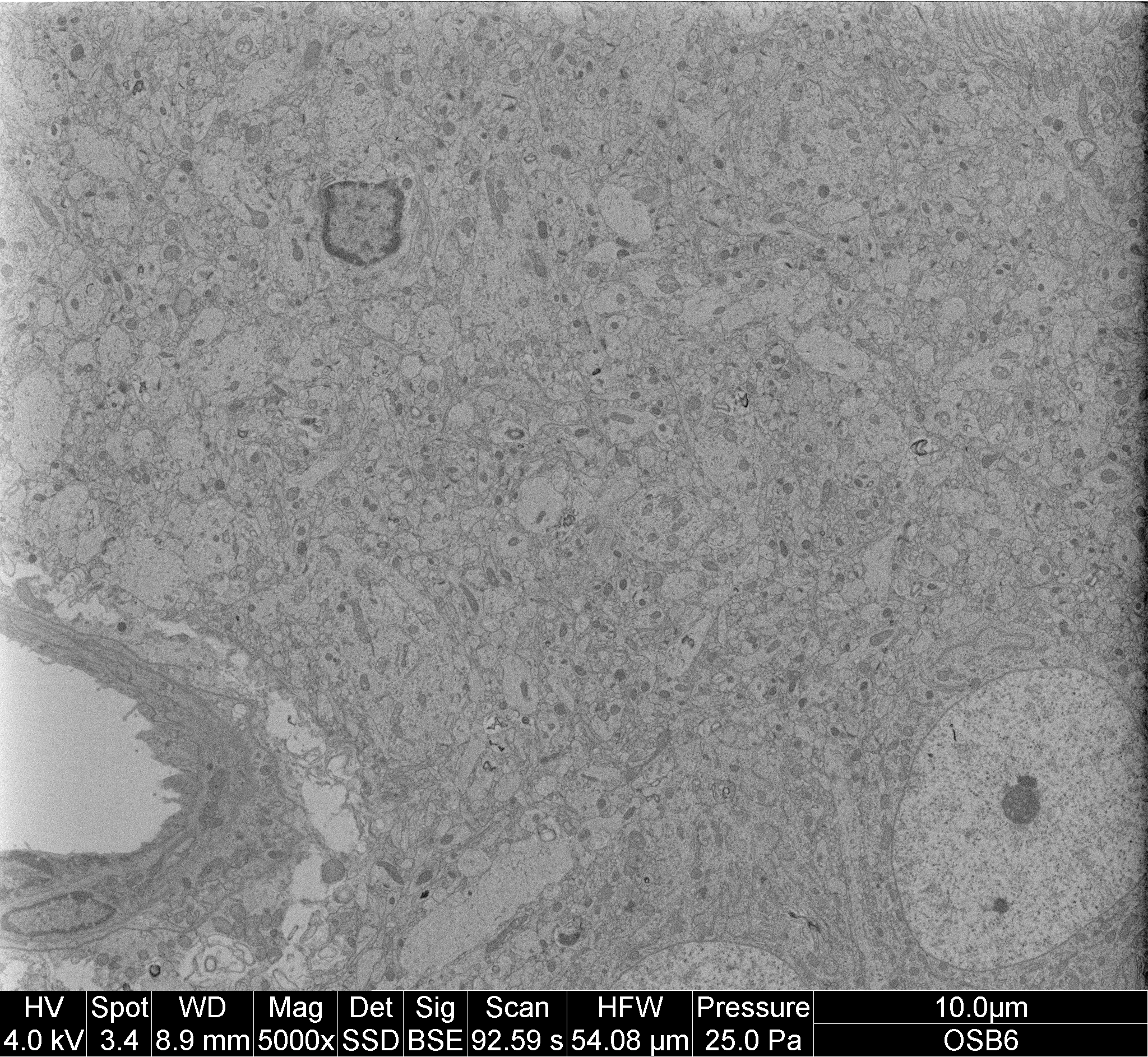

Supplement: Dataset S9 — (256.1 MB ZIP). [file pbio.0020329.sd009.zip › 040604_OS5_st1_897.tif]

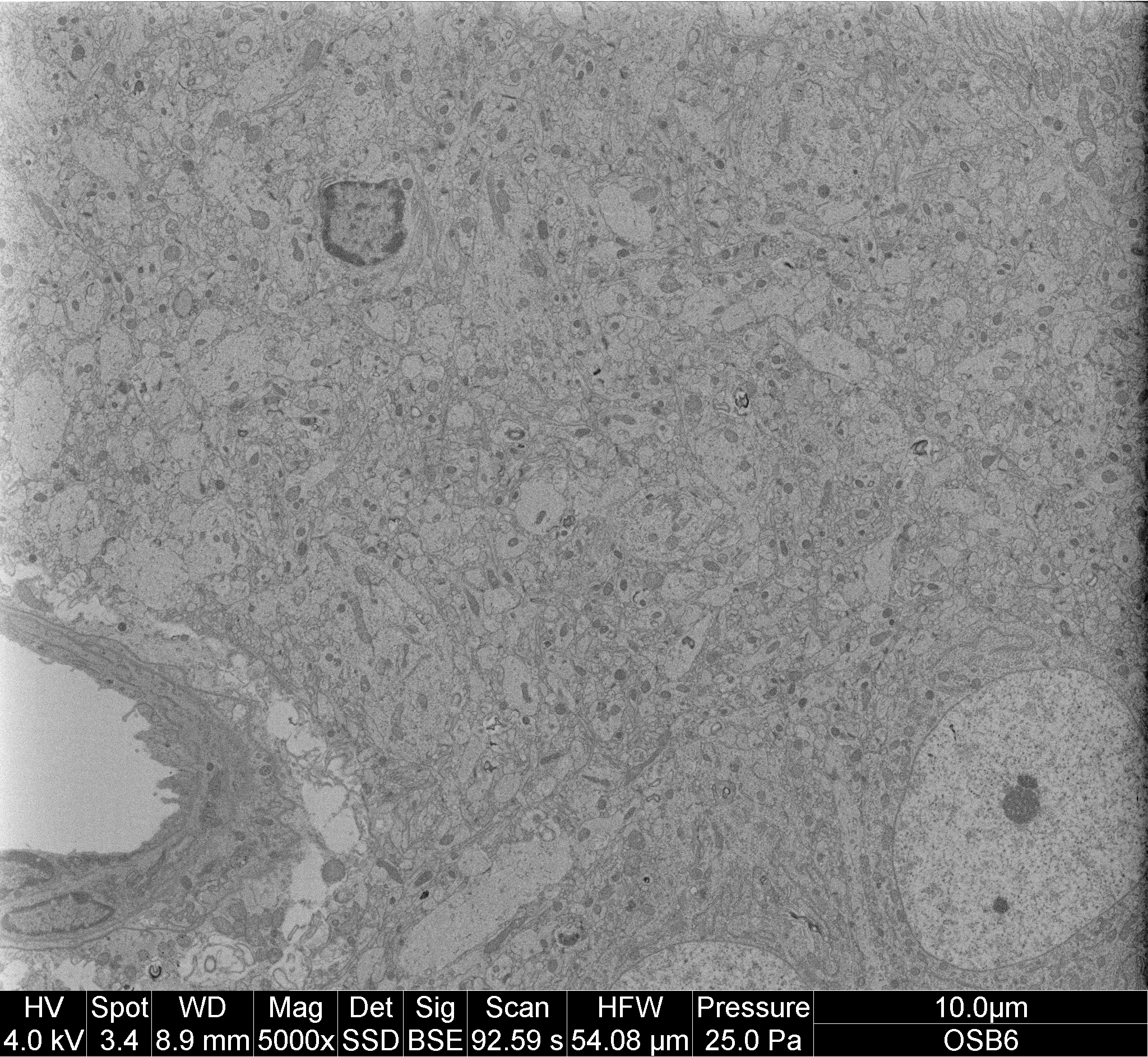

Supplement: Dataset S9 — (256.1 MB ZIP). [file pbio.0020329.sd009.zip › 040604_OS5_st1_898.tif]

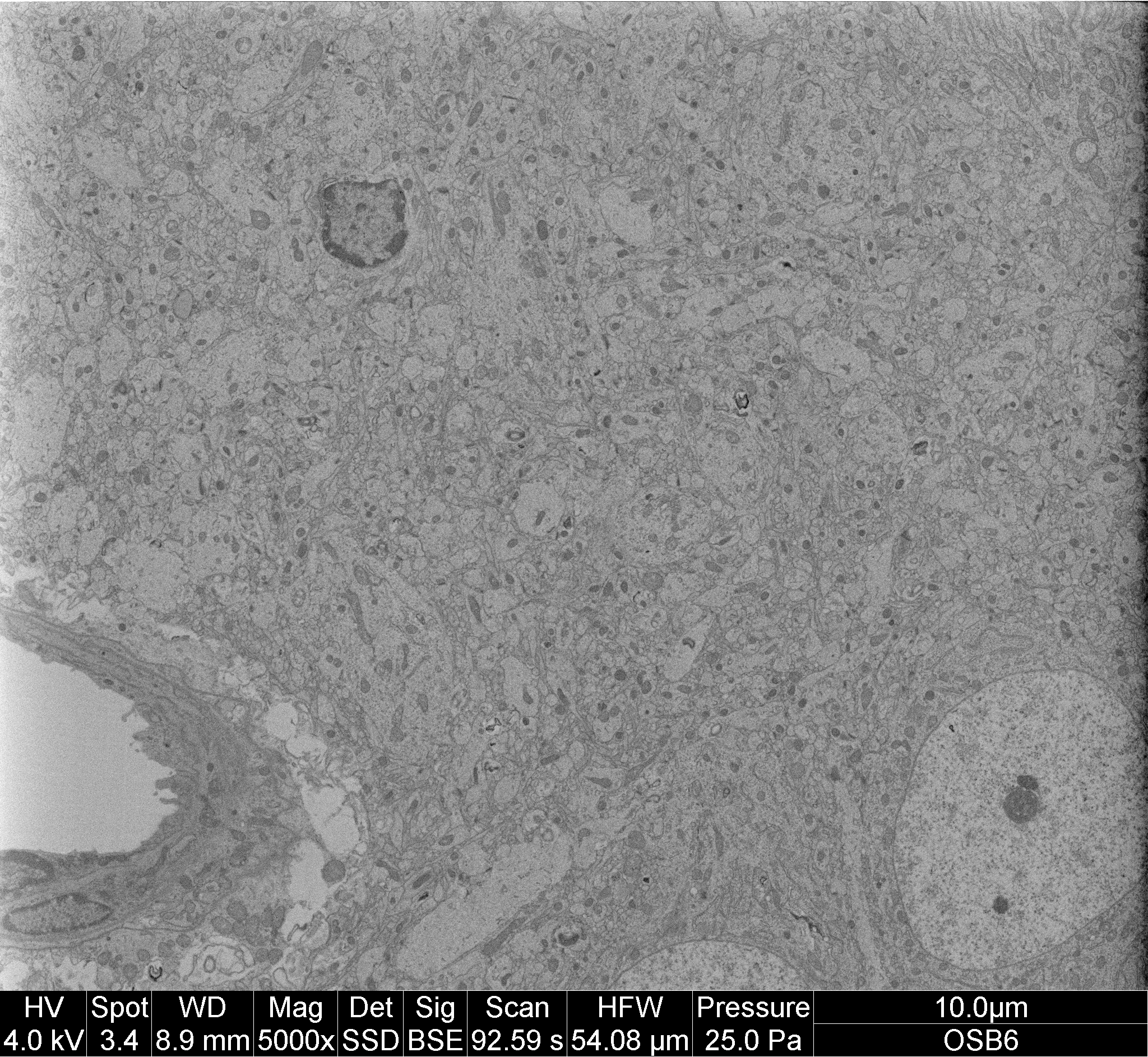

Supplement: Dataset S9 — (256.1 MB ZIP). [file pbio.0020329.sd009.zip › 040604_OS5_st1_899.tif]

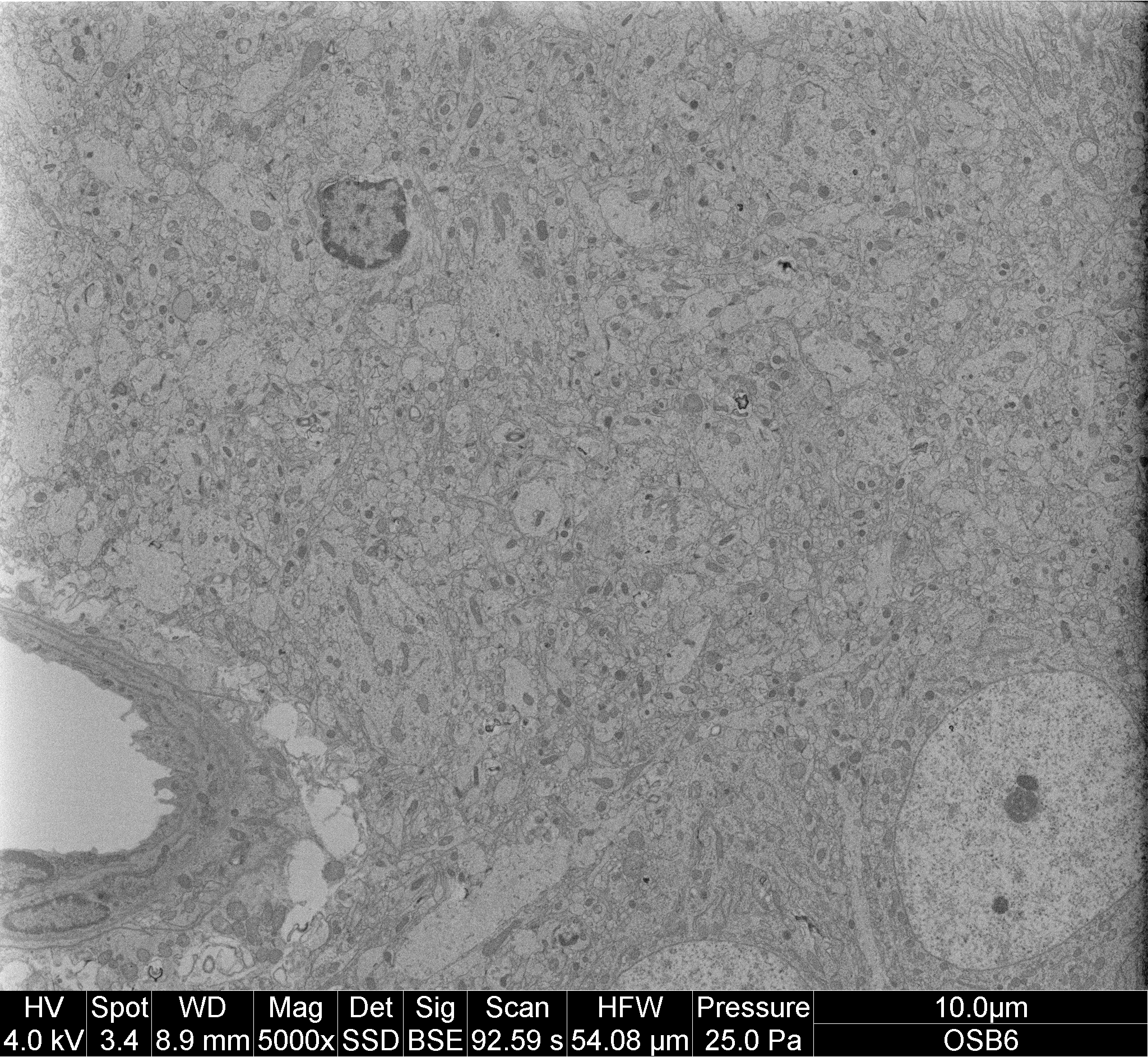

Supplement: Dataset S10 — (253.8 MB ZIP). [file pbio.0020329.sd010.zip › 040604_OS5_st1_900.tif]
