# Supplementary figures and images for: Serial Block-Face Scanning Electron Microscopy to Reconstruct Three-Dimensional Tissue Nanostructure (part 10 of 21)
Source: PLoS Biol. 2004 Oct 19;2(11):e329. doi: 10.1371/journal.pbio.0020329 (PMC524270; doi:10.1371/journal.pbio.0020329)

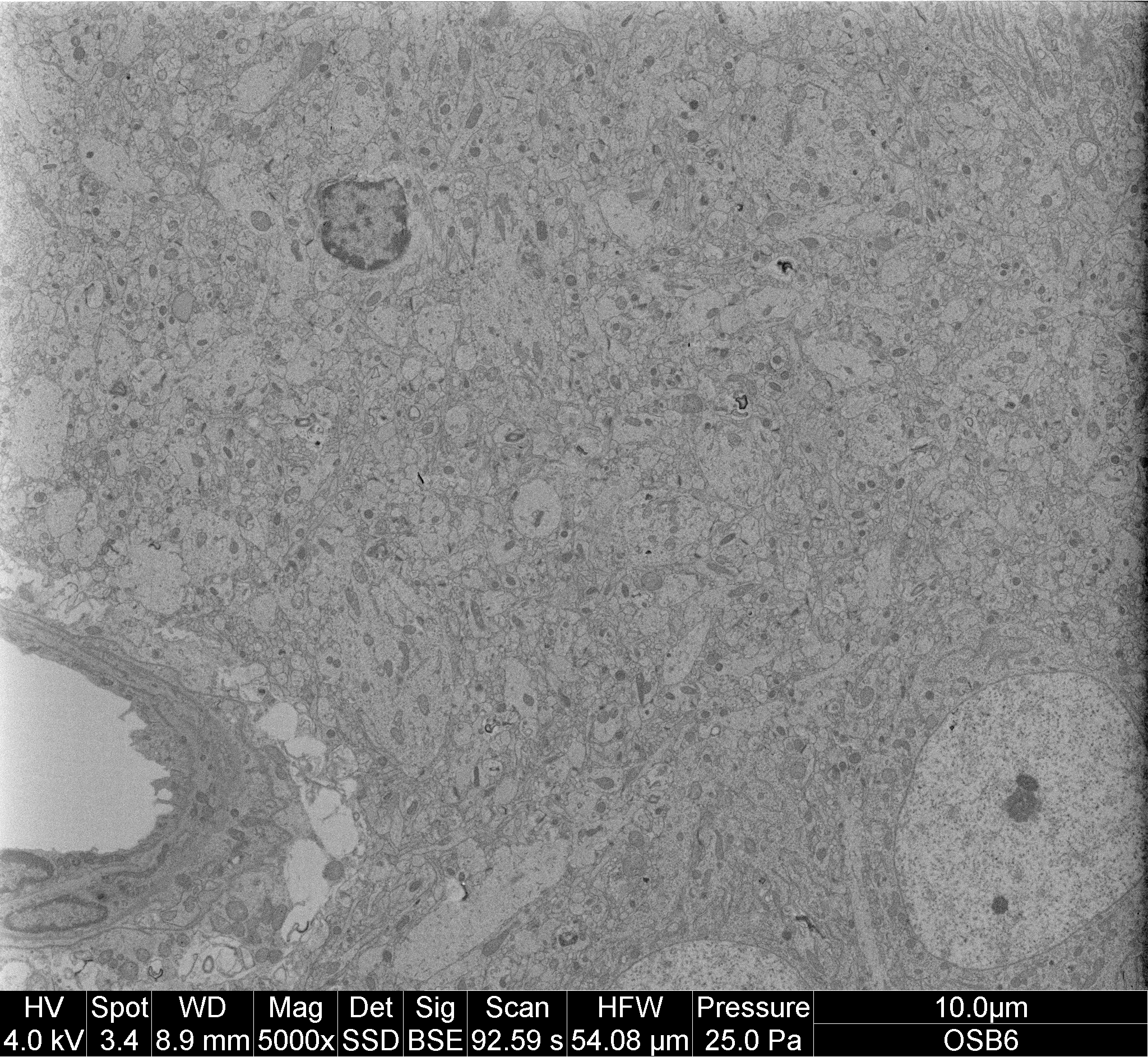

Supplement: Dataset S10 — (253.8 MB ZIP). [file pbio.0020329.sd010.zip › 040604_OS5_st1_901.tif]

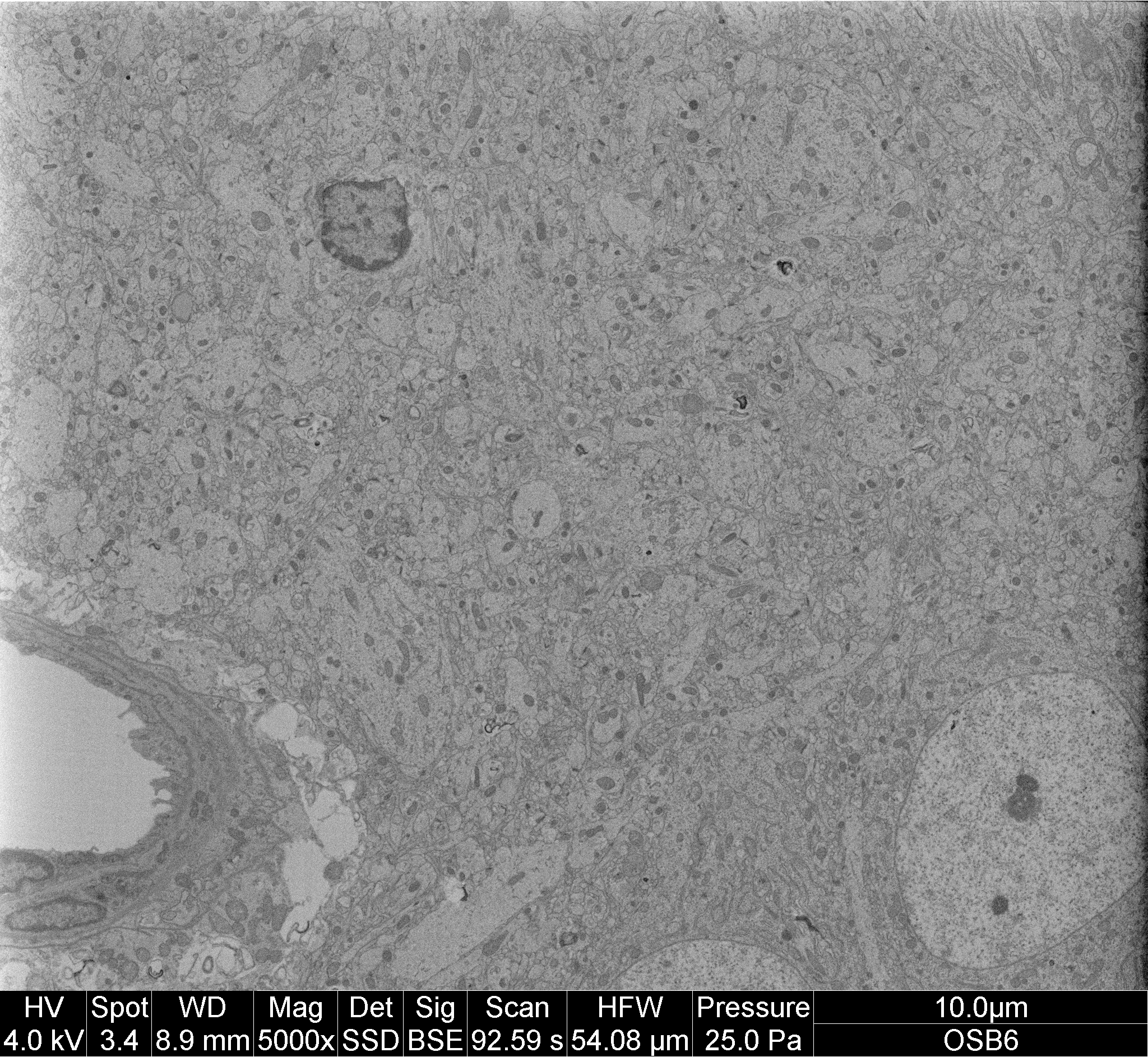

Supplement: Dataset S10 — (253.8 MB ZIP). [file pbio.0020329.sd010.zip › 040604_OS5_st1_902.tif]

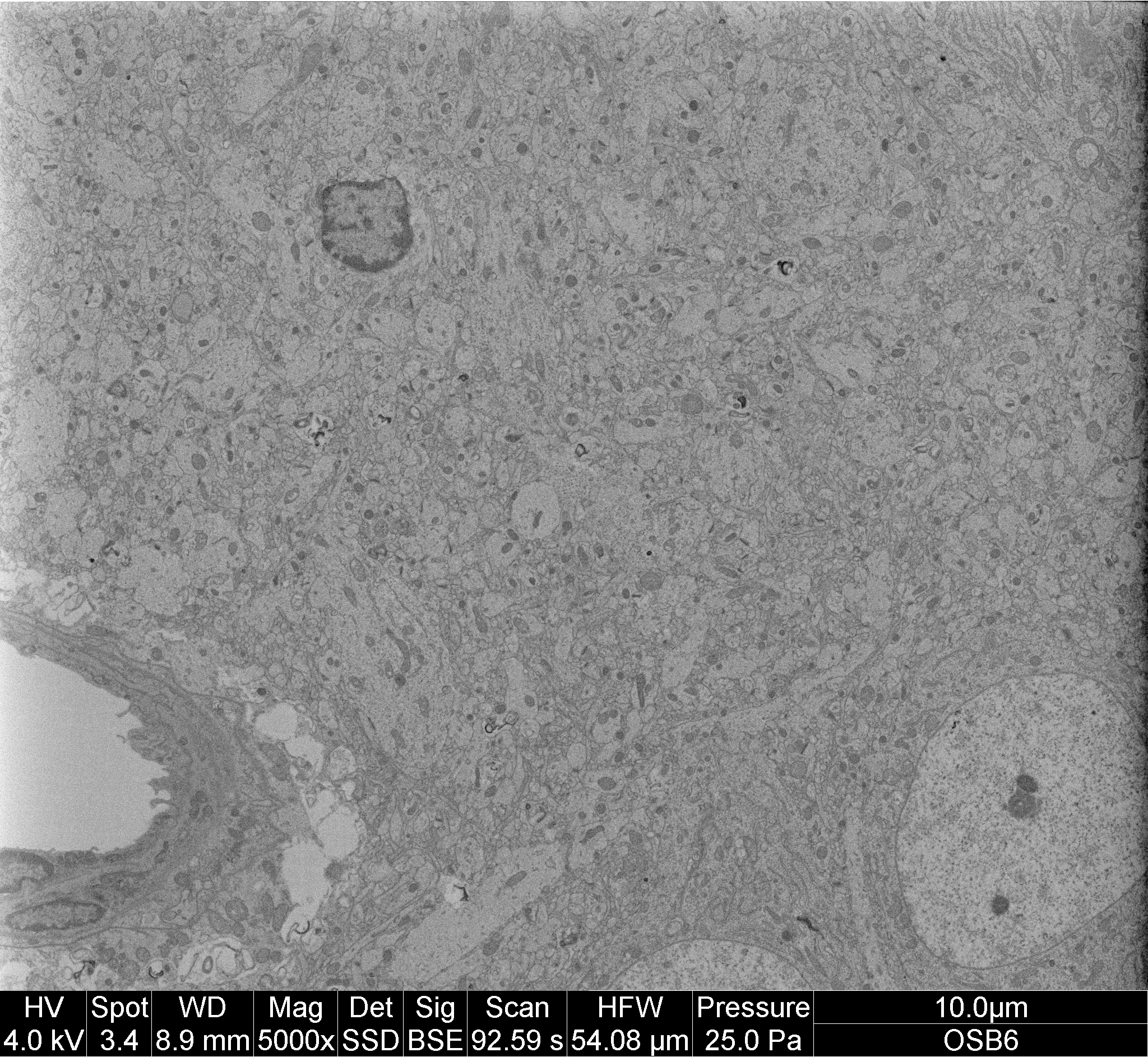

Supplement: Dataset S10 — (253.8 MB ZIP). [file pbio.0020329.sd010.zip › 040604_OS5_st1_903.tif]

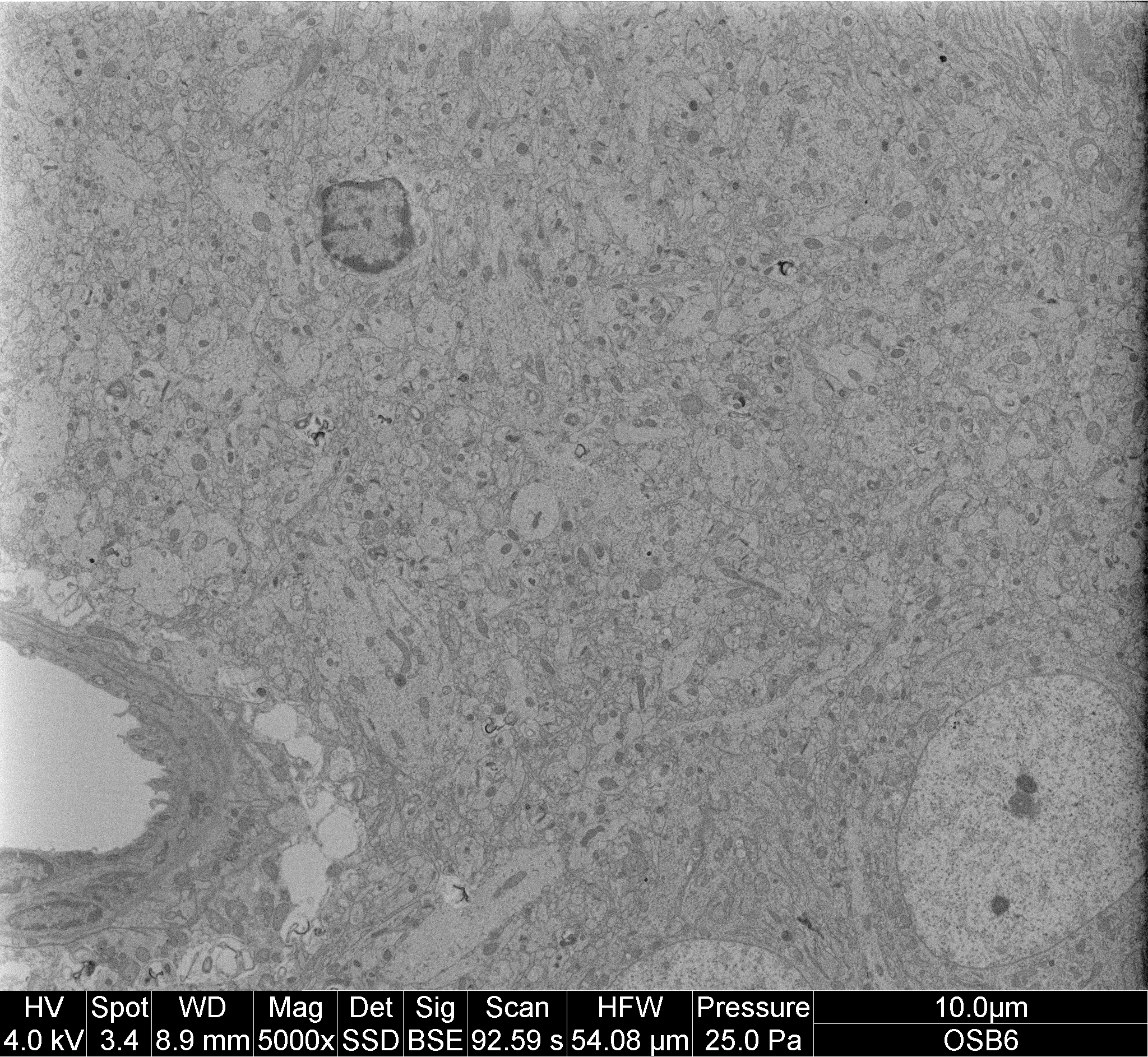

Supplement: Dataset S10 — (253.8 MB ZIP). [file pbio.0020329.sd010.zip › 040604_OS5_st1_904.tif]

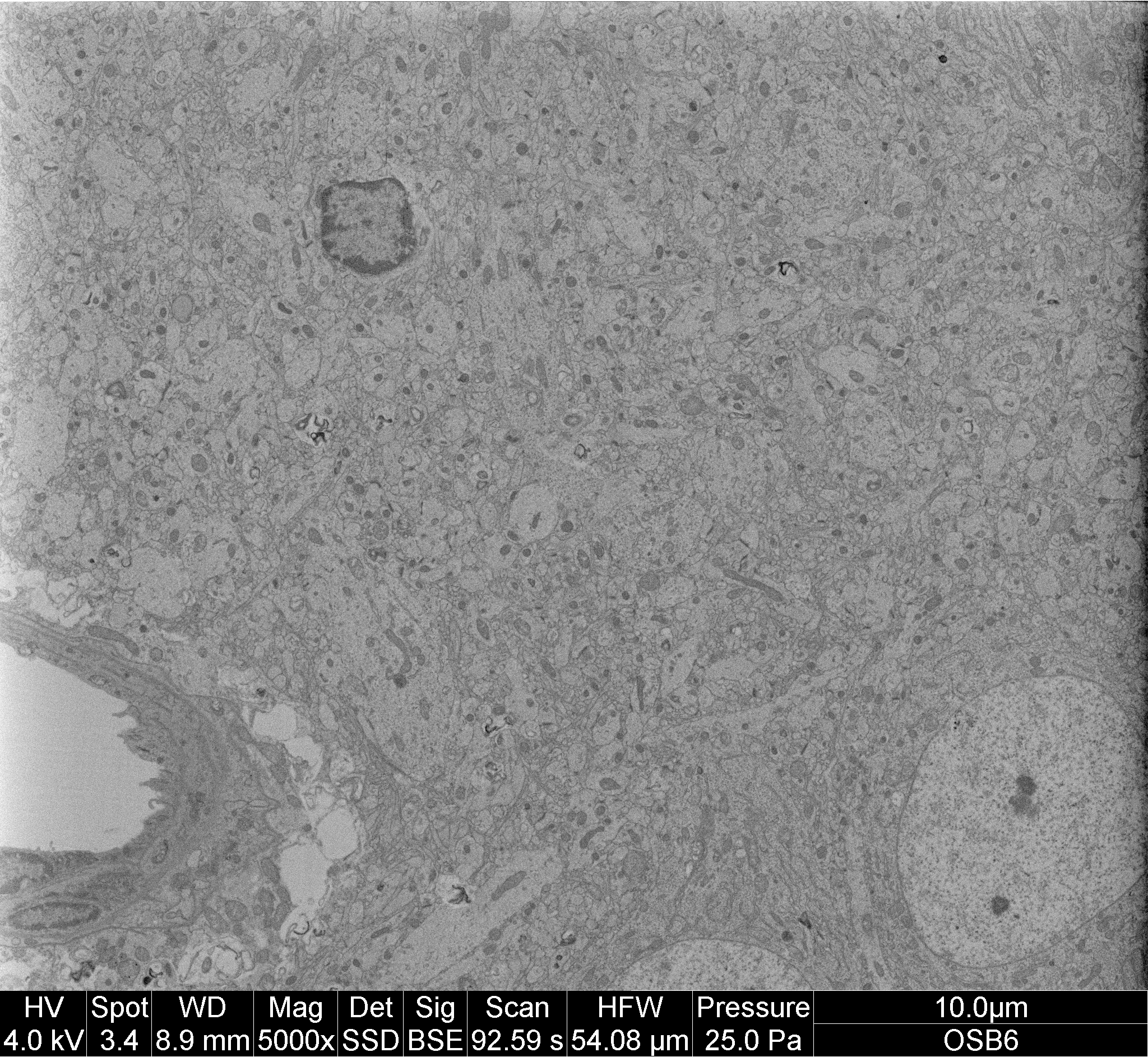

Supplement: Dataset S10 — (253.8 MB ZIP). [file pbio.0020329.sd010.zip › 040604_OS5_st1_905.tif]

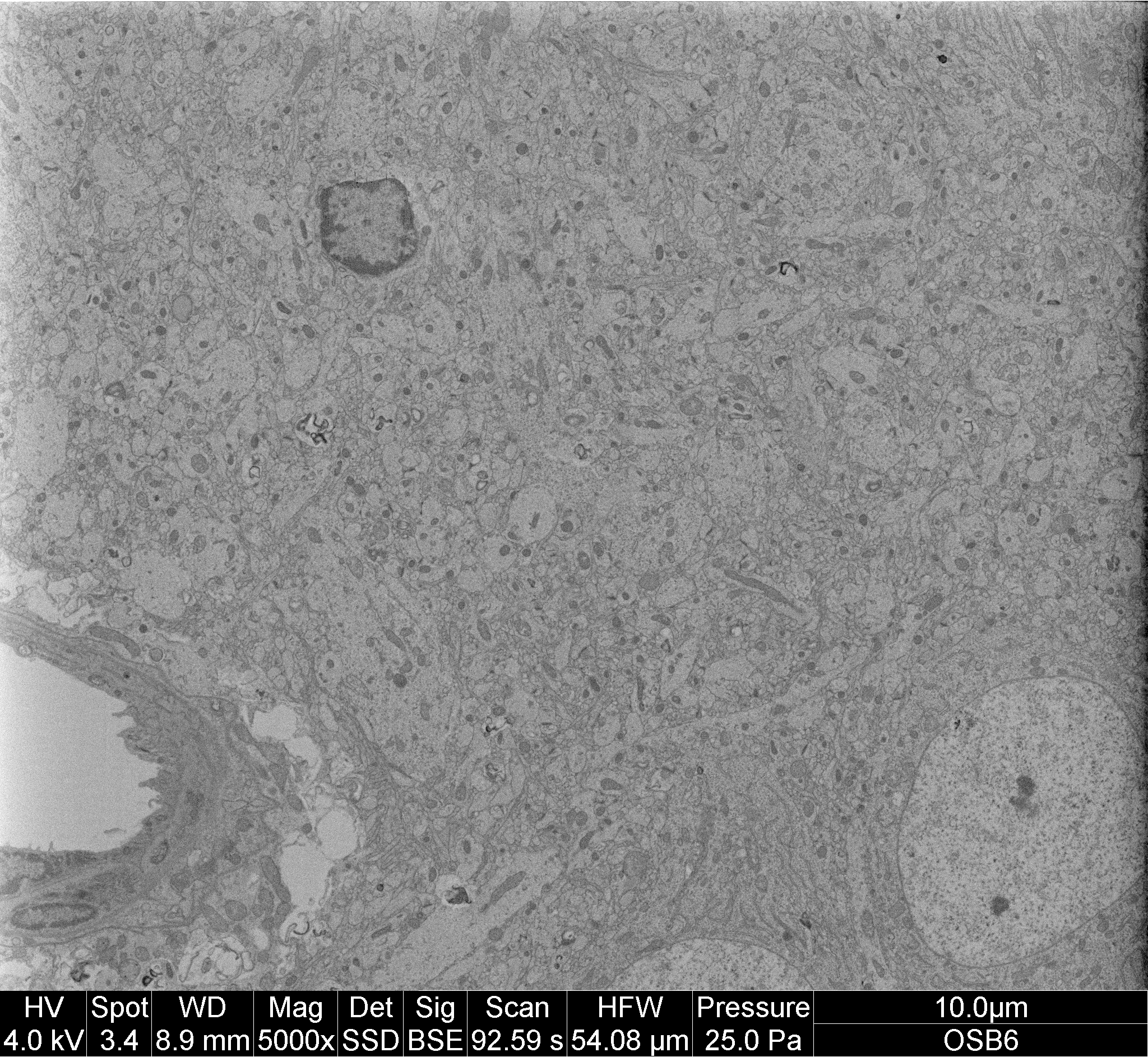

Supplement: Dataset S10 — (253.8 MB ZIP). [file pbio.0020329.sd010.zip › 040604_OS5_st1_906.tif]

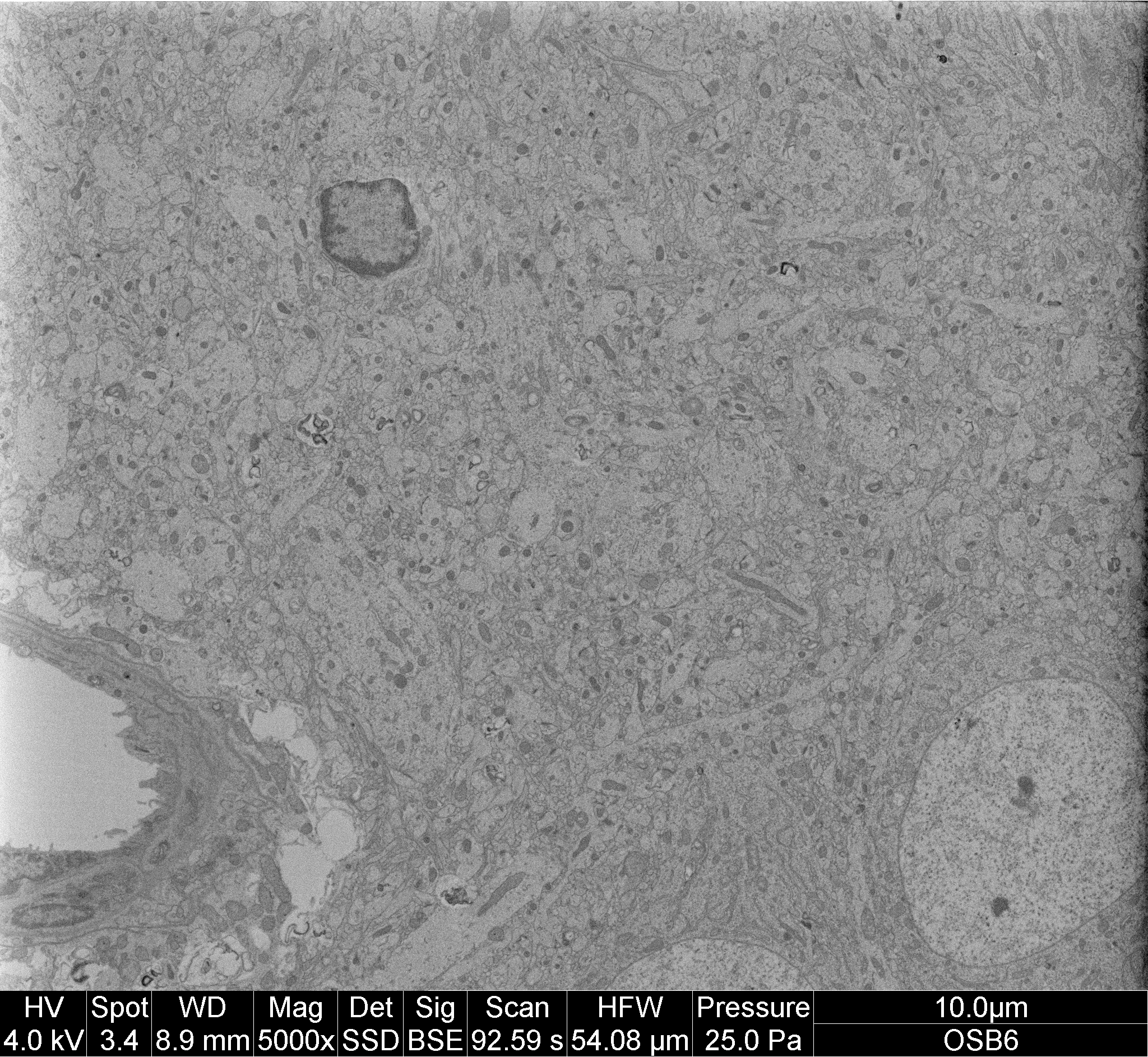

Supplement: Dataset S10 — (253.8 MB ZIP). [file pbio.0020329.sd010.zip › 040604_OS5_st1_907.tif]

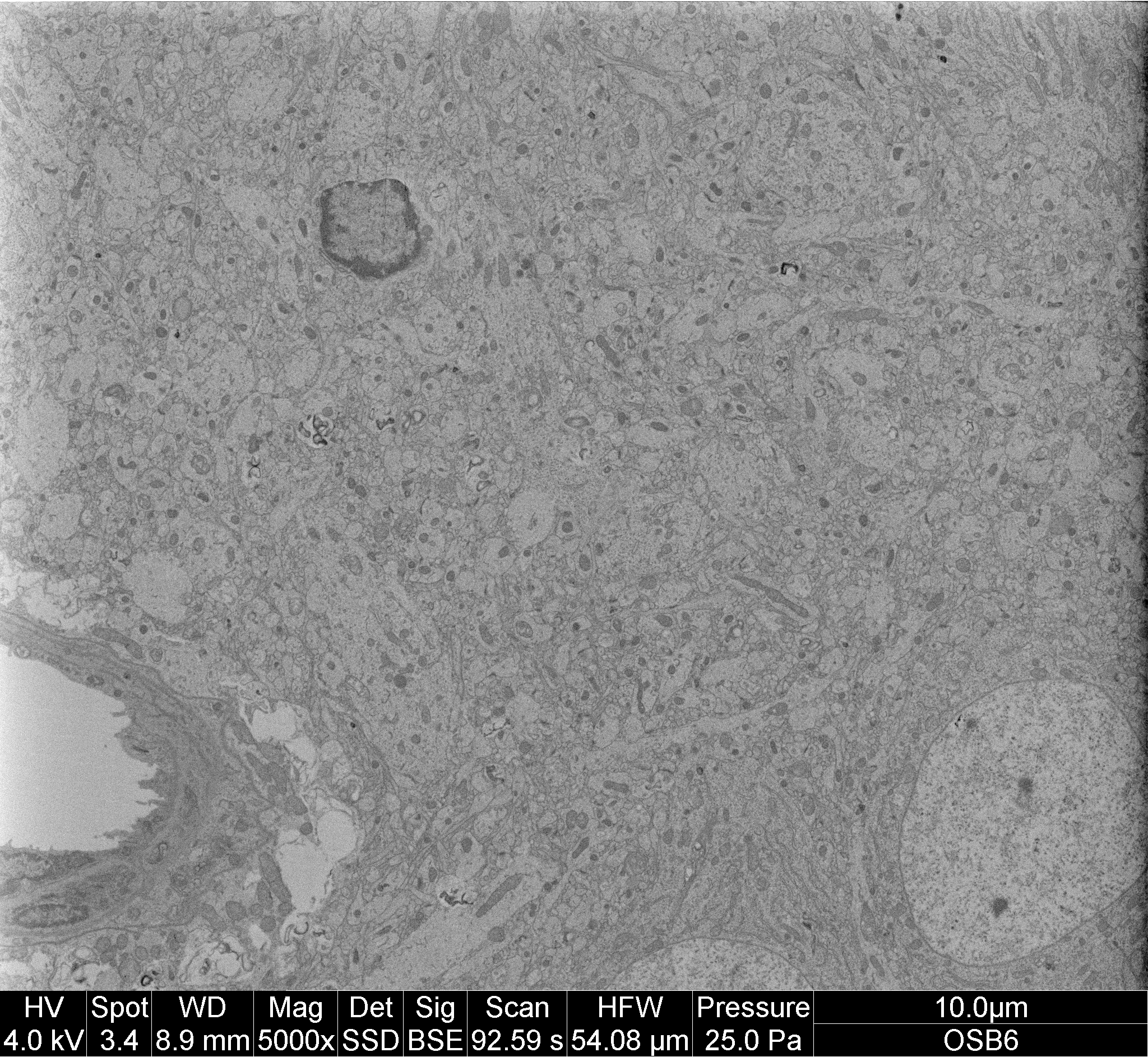

Supplement: Dataset S10 — (253.8 MB ZIP). [file pbio.0020329.sd010.zip › 040604_OS5_st1_908.tif]

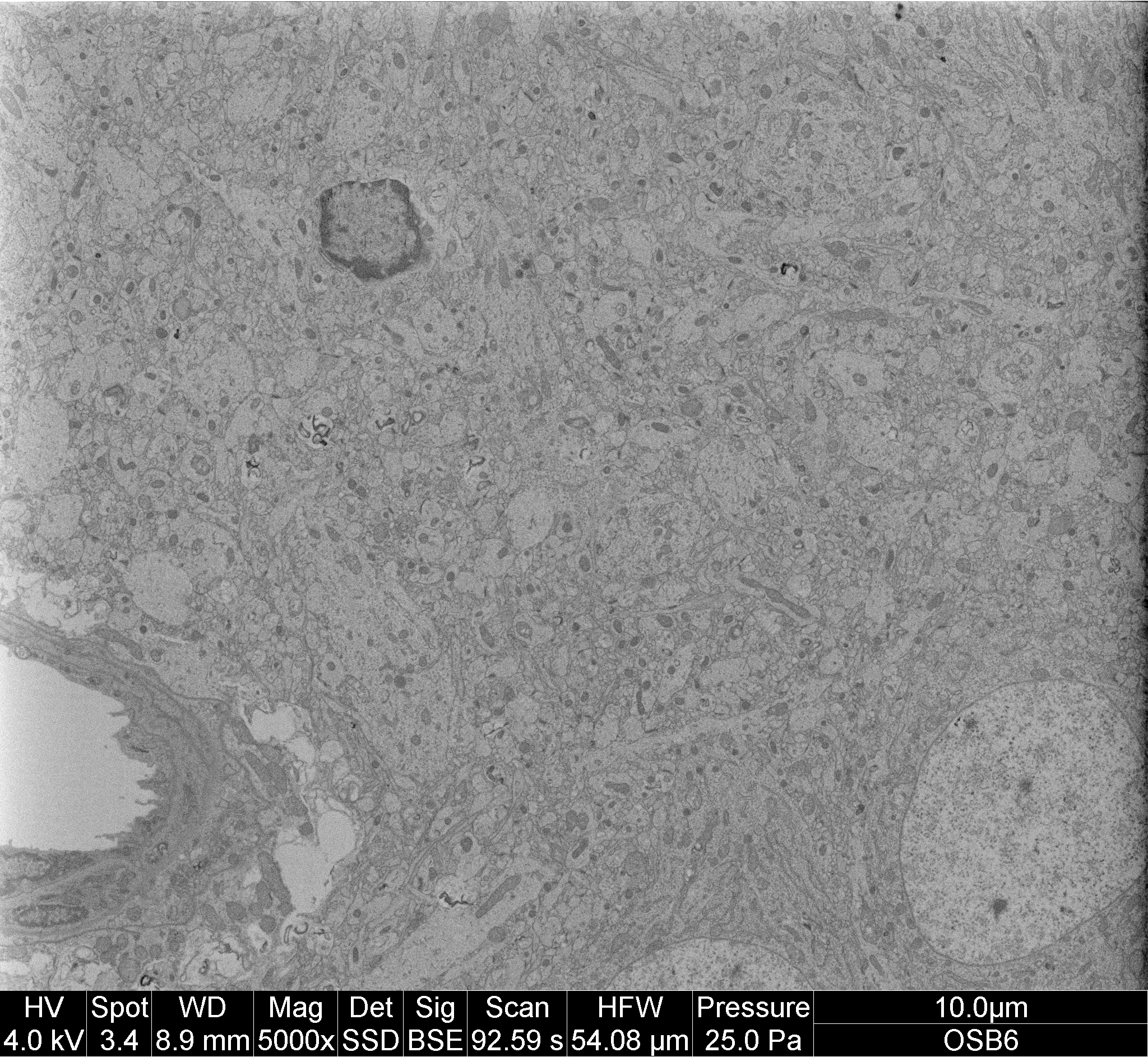

Supplement: Dataset S10 — (253.8 MB ZIP). [file pbio.0020329.sd010.zip › 040604_OS5_st1_909.tif]

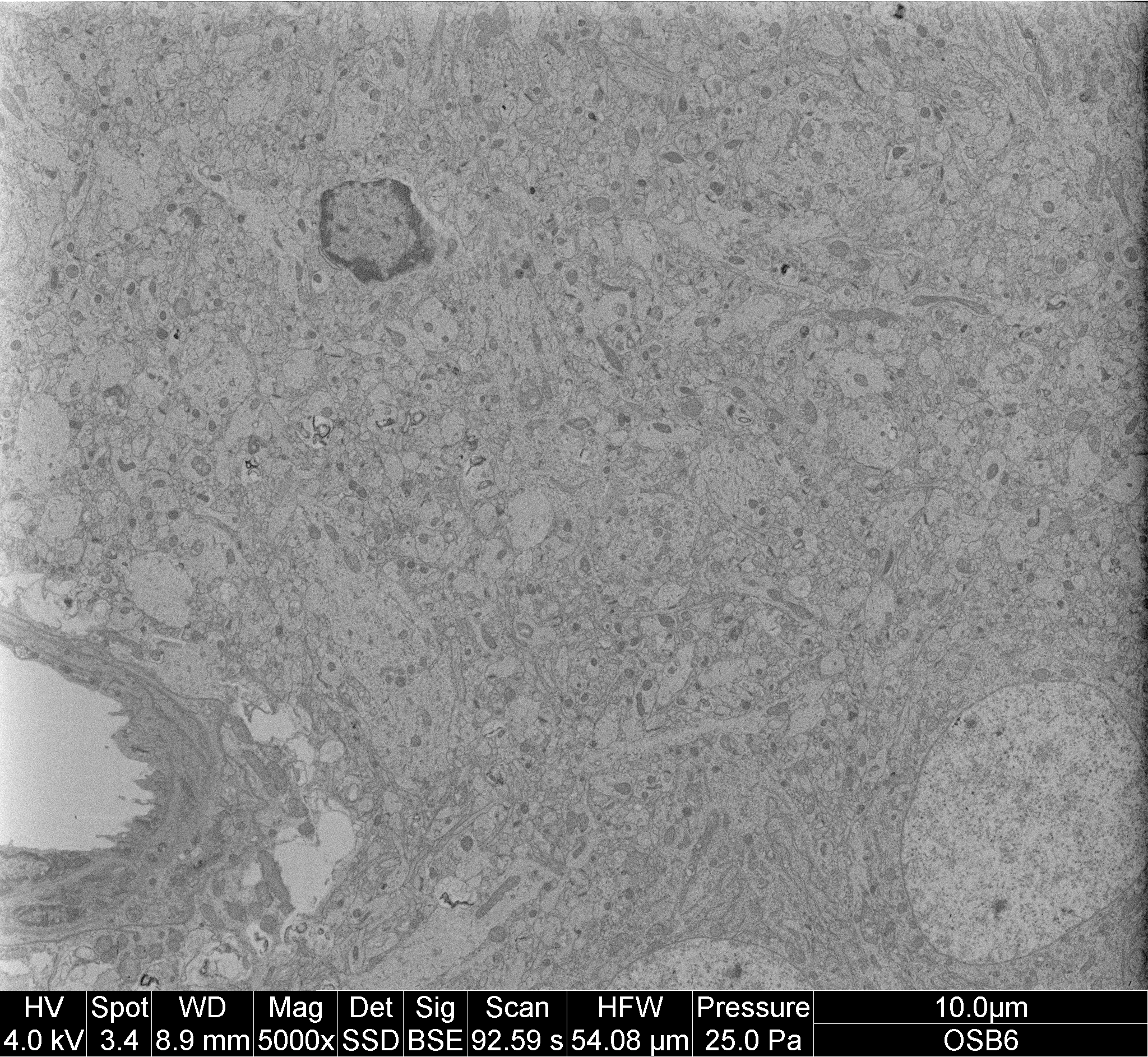

Supplement: Dataset S10 — (253.8 MB ZIP). [file pbio.0020329.sd010.zip › 040604_OS5_st1_910.tif]

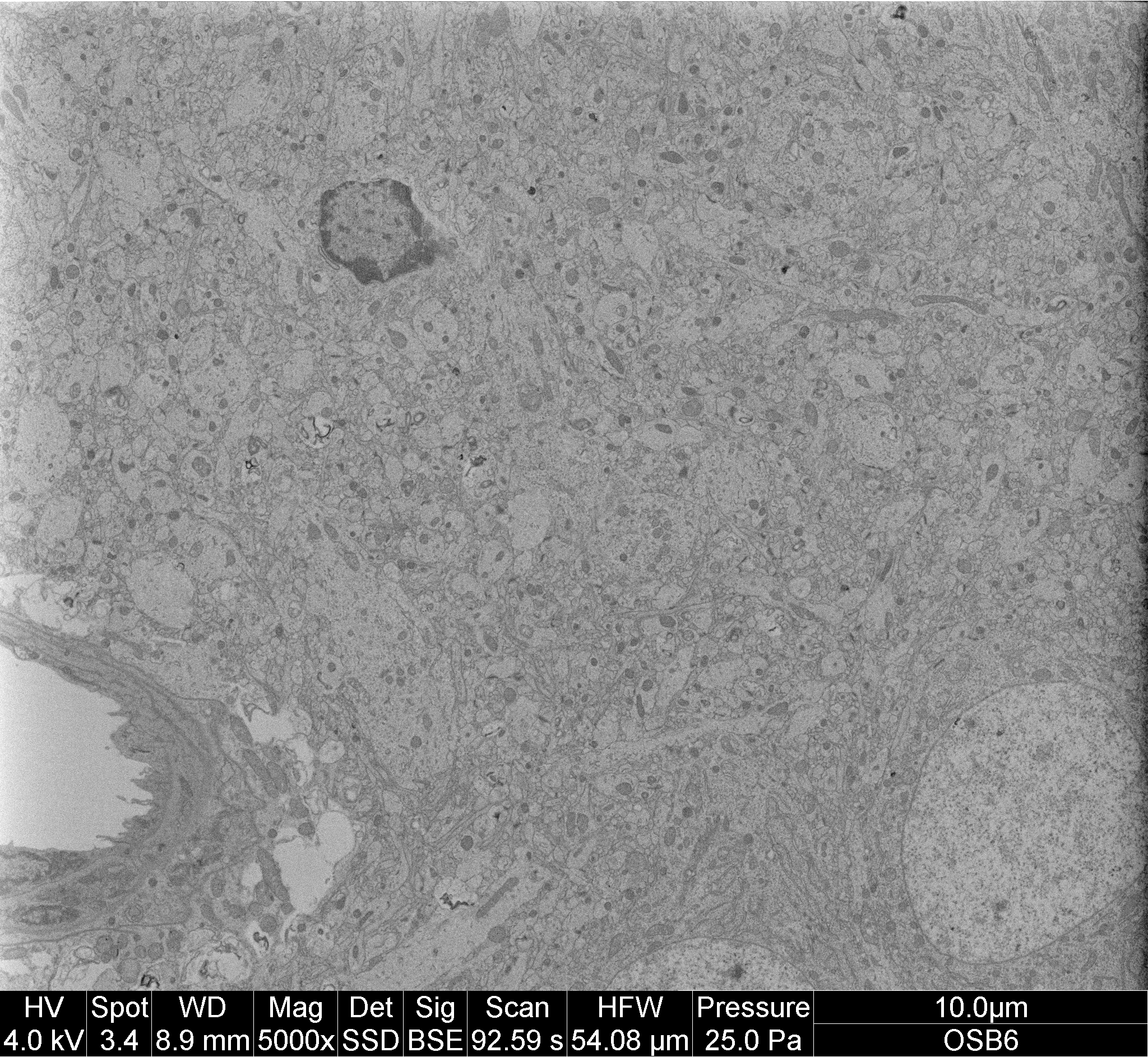

Supplement: Dataset S10 — (253.8 MB ZIP). [file pbio.0020329.sd010.zip › 040604_OS5_st1_911.tif]

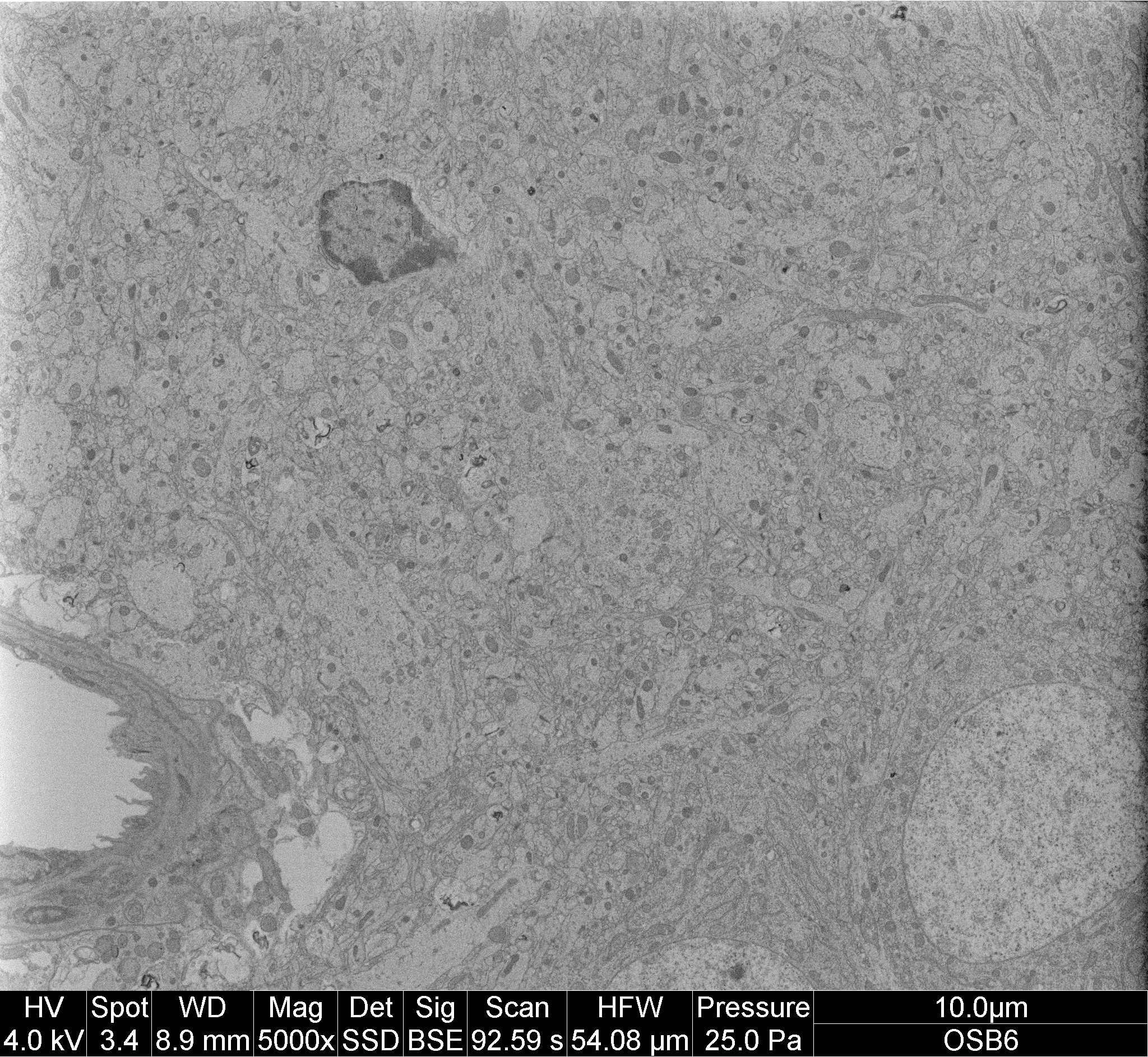

Supplement: Dataset S10 — (253.8 MB ZIP). [file pbio.0020329.sd010.zip › 040604_OS5_st1_912.tif]

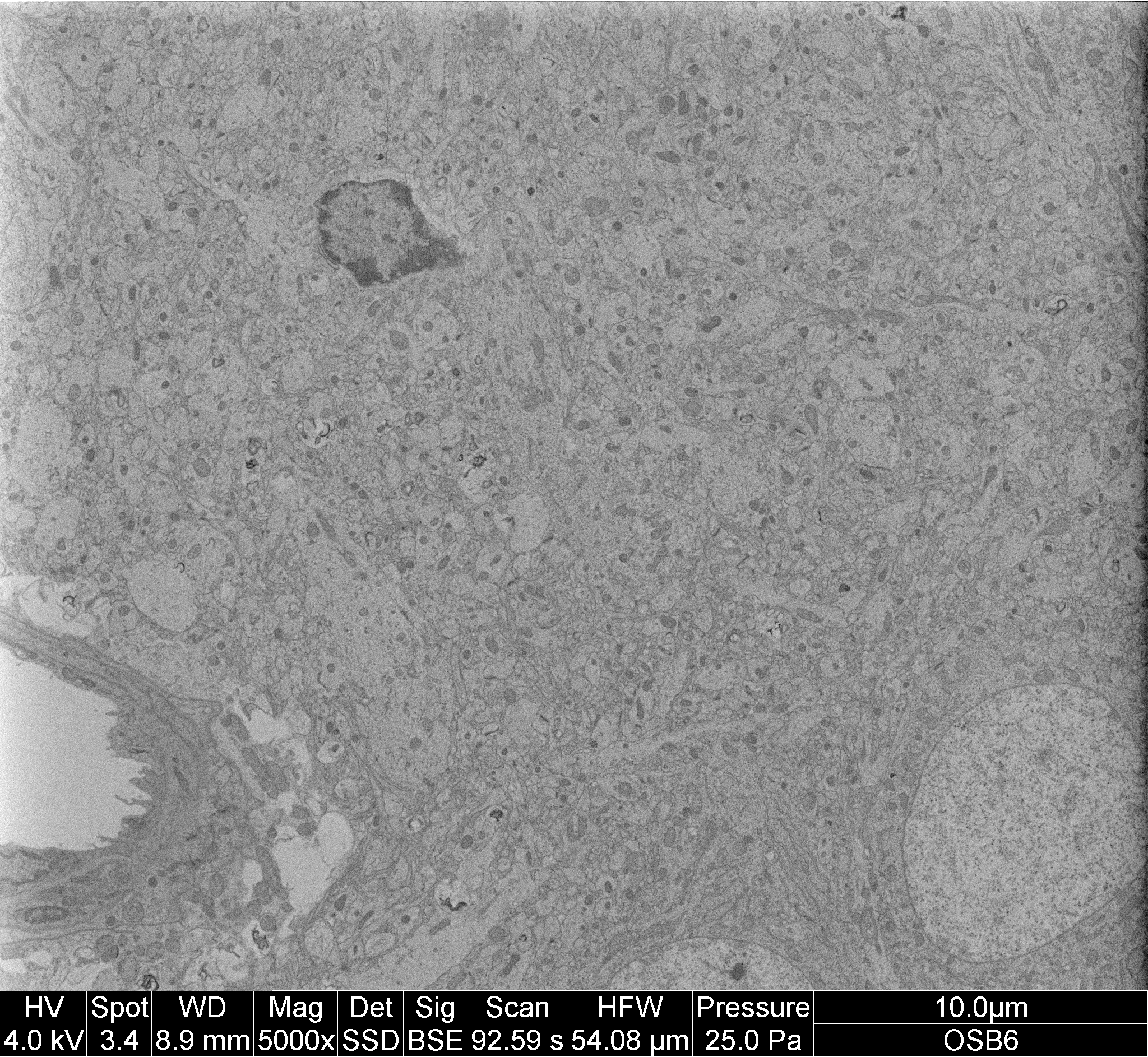

Supplement: Dataset S10 — (253.8 MB ZIP). [file pbio.0020329.sd010.zip › 040604_OS5_st1_913.tif]

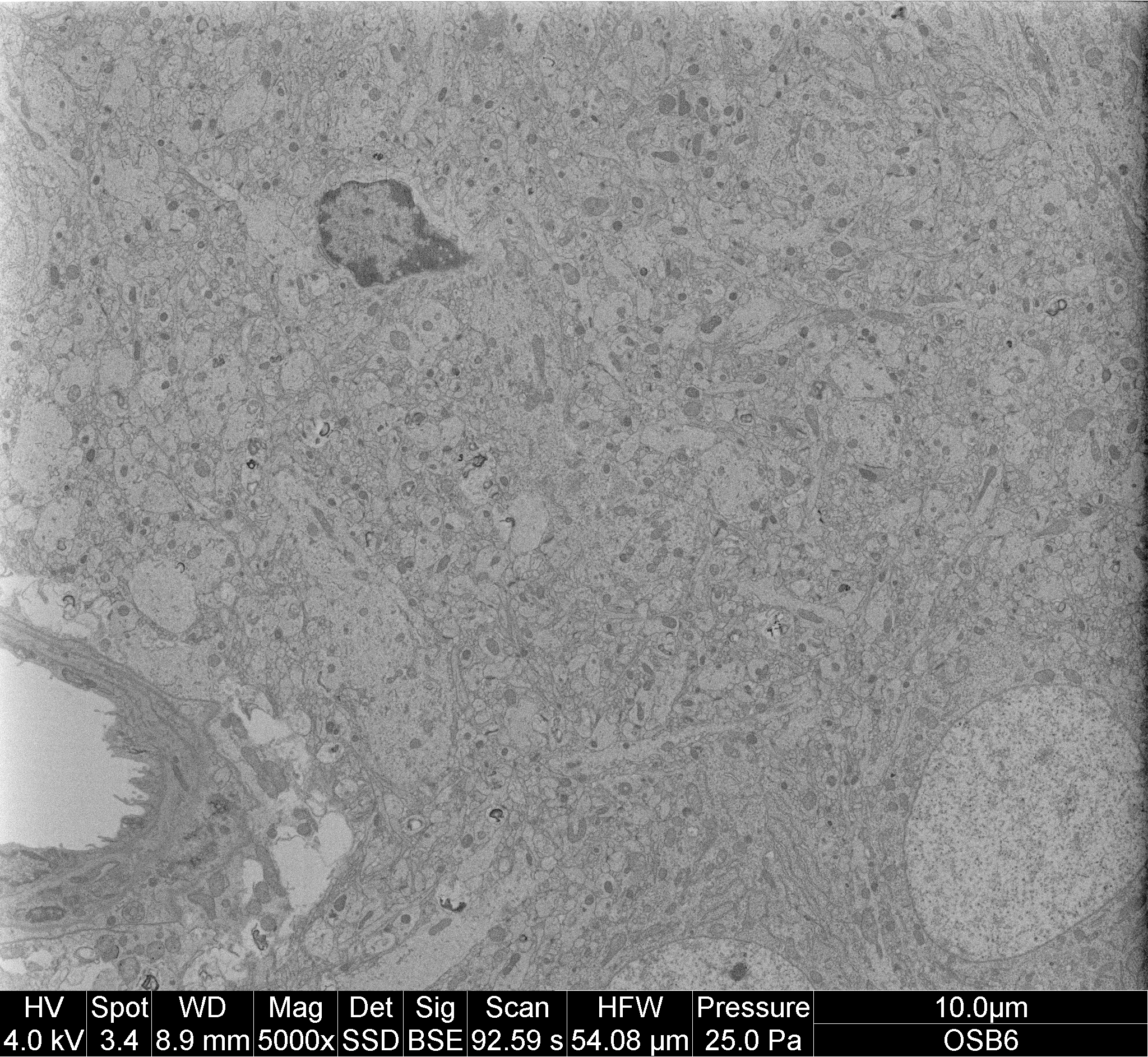

Supplement: Dataset S10 — (253.8 MB ZIP). [file pbio.0020329.sd010.zip › 040604_OS5_st1_914.tif]

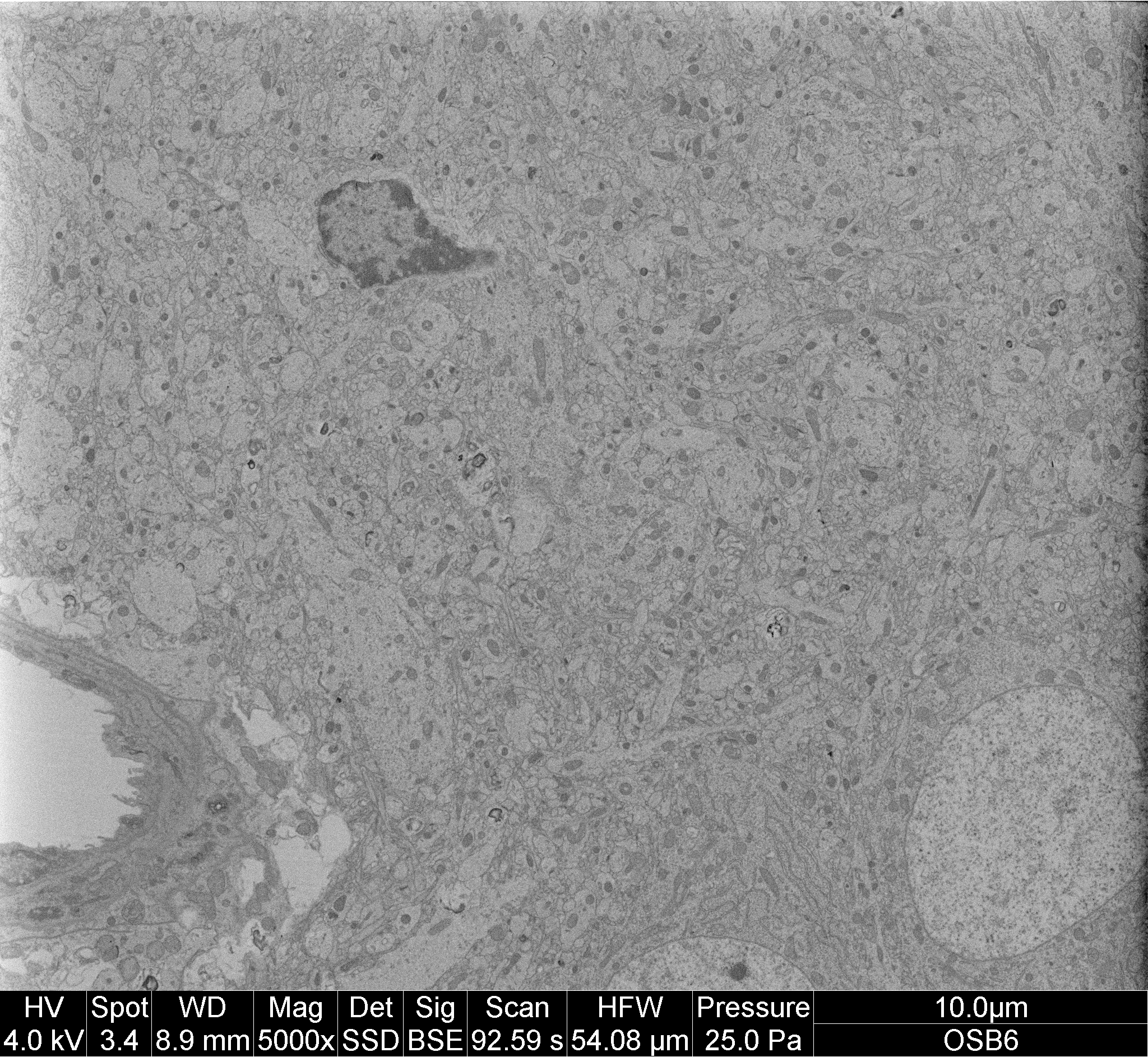

Supplement: Dataset S10 — (253.8 MB ZIP). [file pbio.0020329.sd010.zip › 040604_OS5_st1_915.tif]

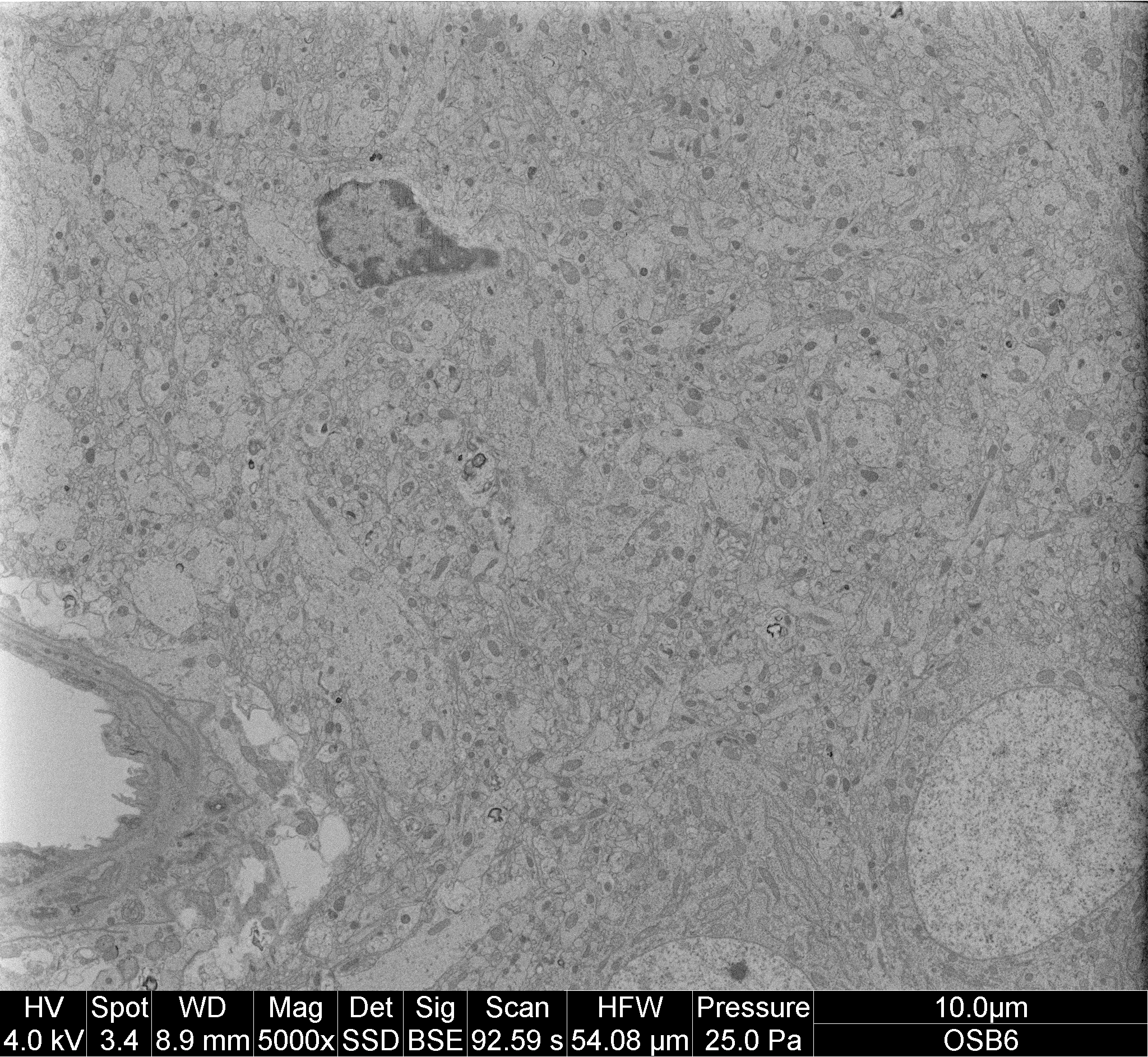

Supplement: Dataset S10 — (253.8 MB ZIP). [file pbio.0020329.sd010.zip › 040604_OS5_st1_916.tif]

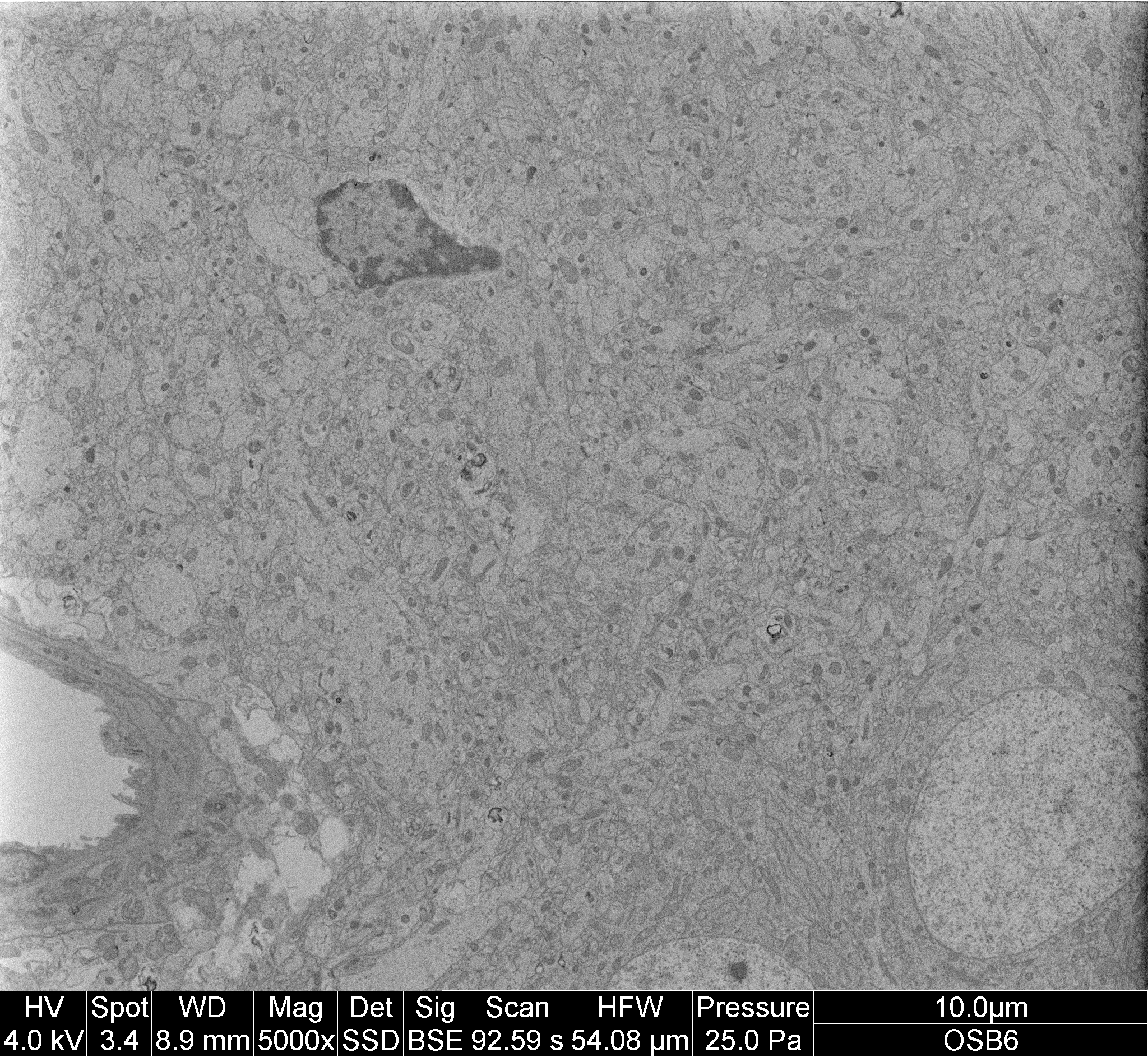

Supplement: Dataset S10 — (253.8 MB ZIP). [file pbio.0020329.sd010.zip › 040604_OS5_st1_917.tif]

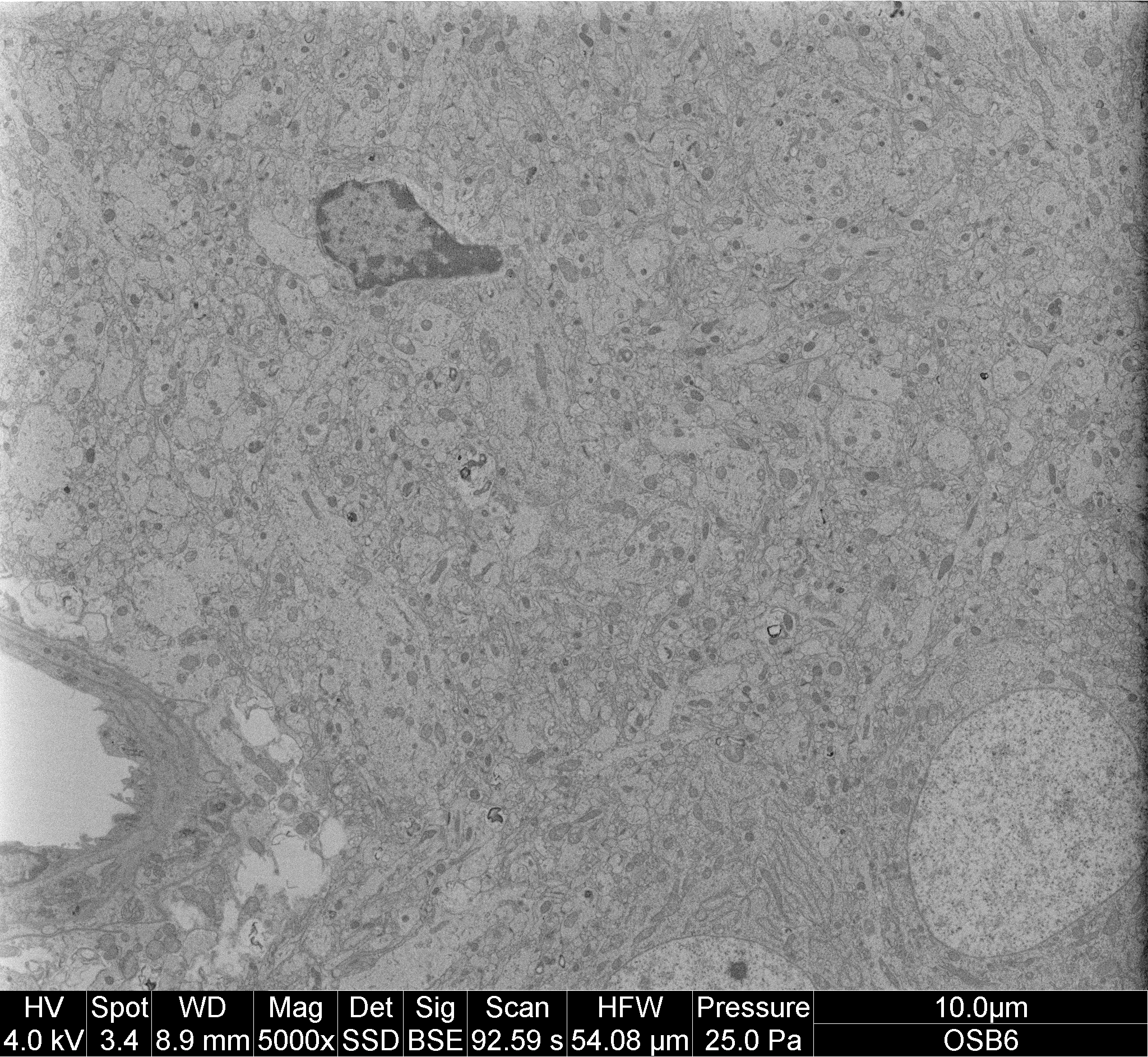

Supplement: Dataset S10 — (253.8 MB ZIP). [file pbio.0020329.sd010.zip › 040604_OS5_st1_918.tif]

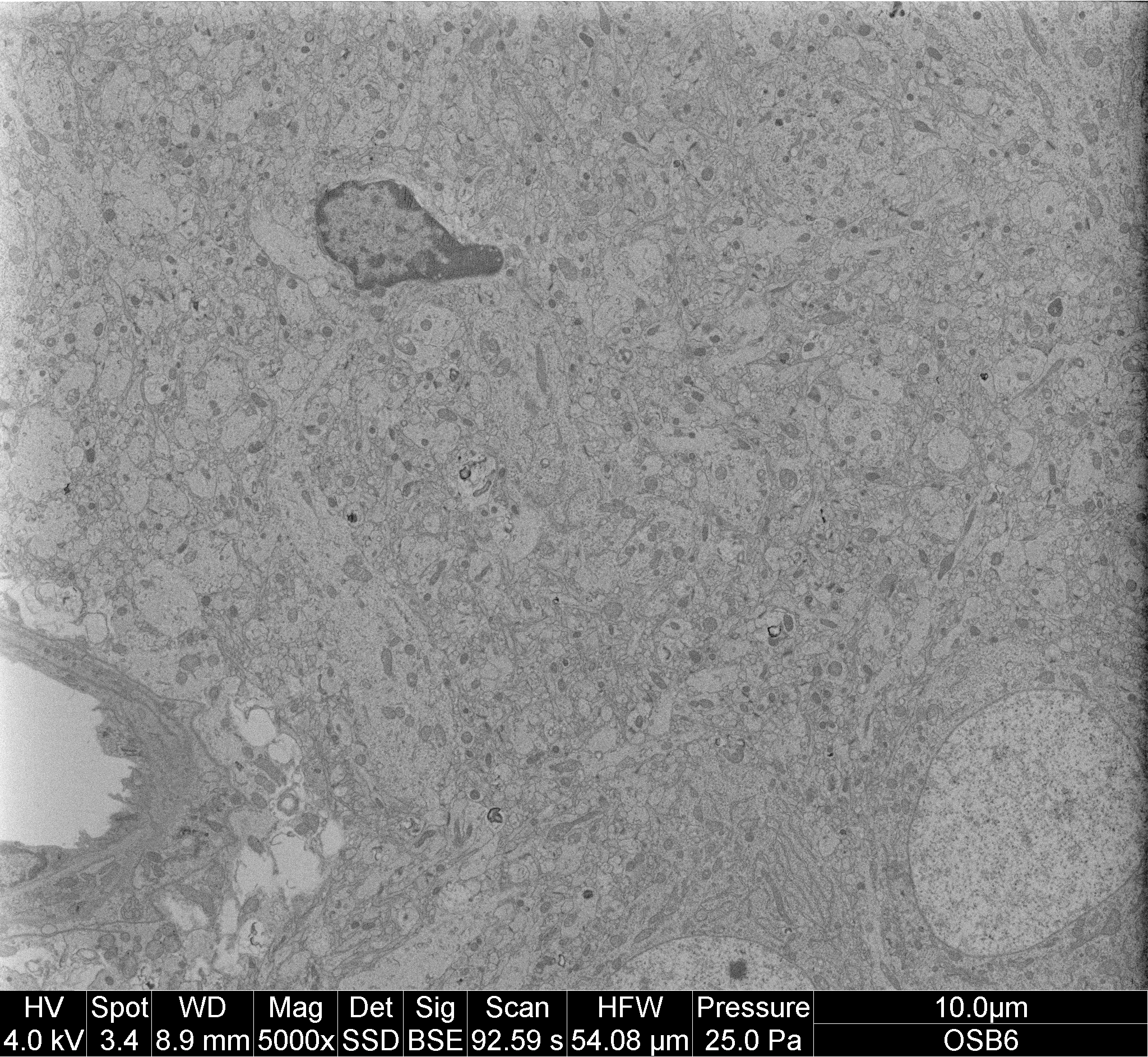

Supplement: Dataset S10 — (253.8 MB ZIP). [file pbio.0020329.sd010.zip › 040604_OS5_st1_919.tif]

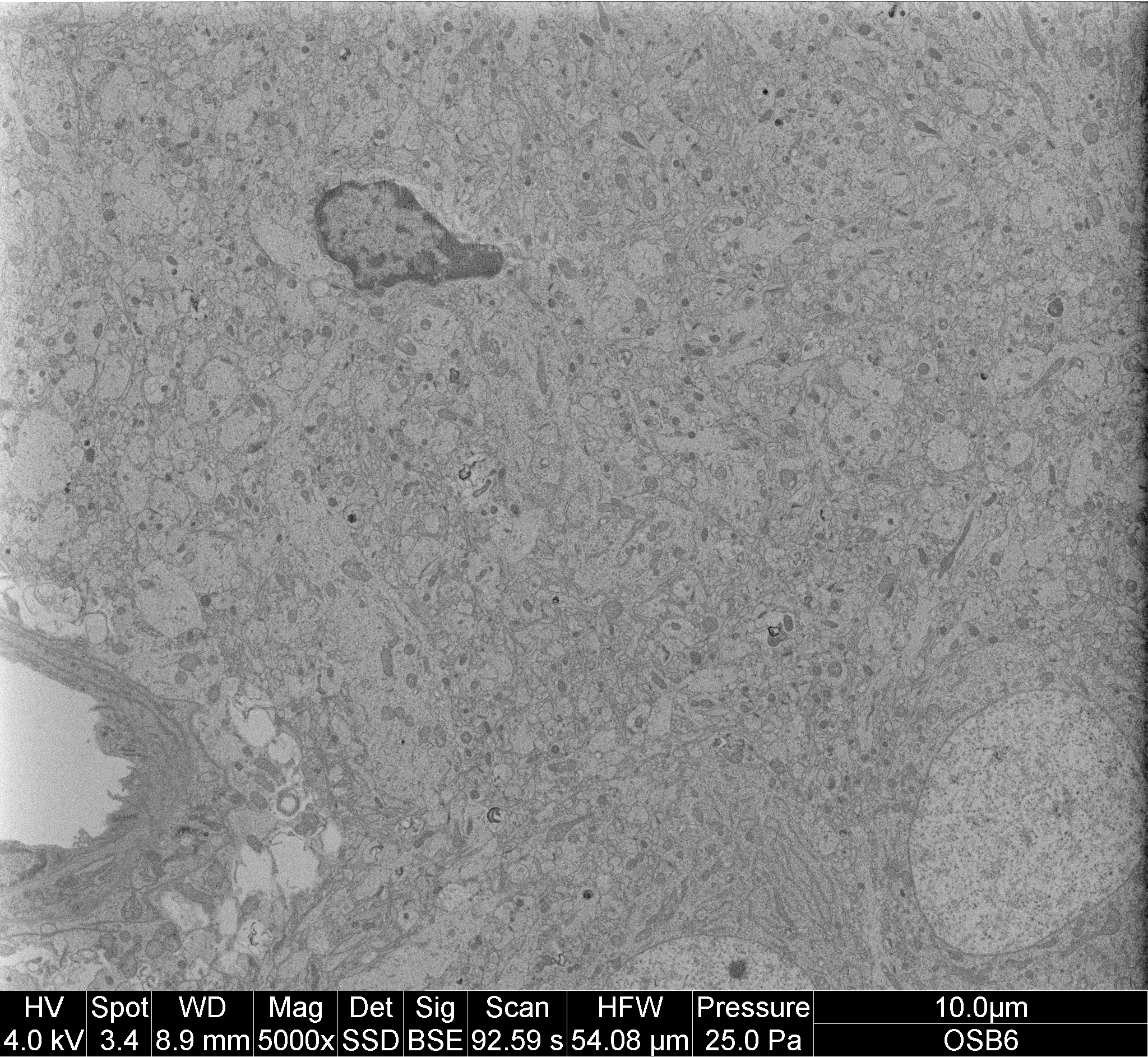

Supplement: Dataset S10 — (253.8 MB ZIP). [file pbio.0020329.sd010.zip › 040604_OS5_st1_920.tif]

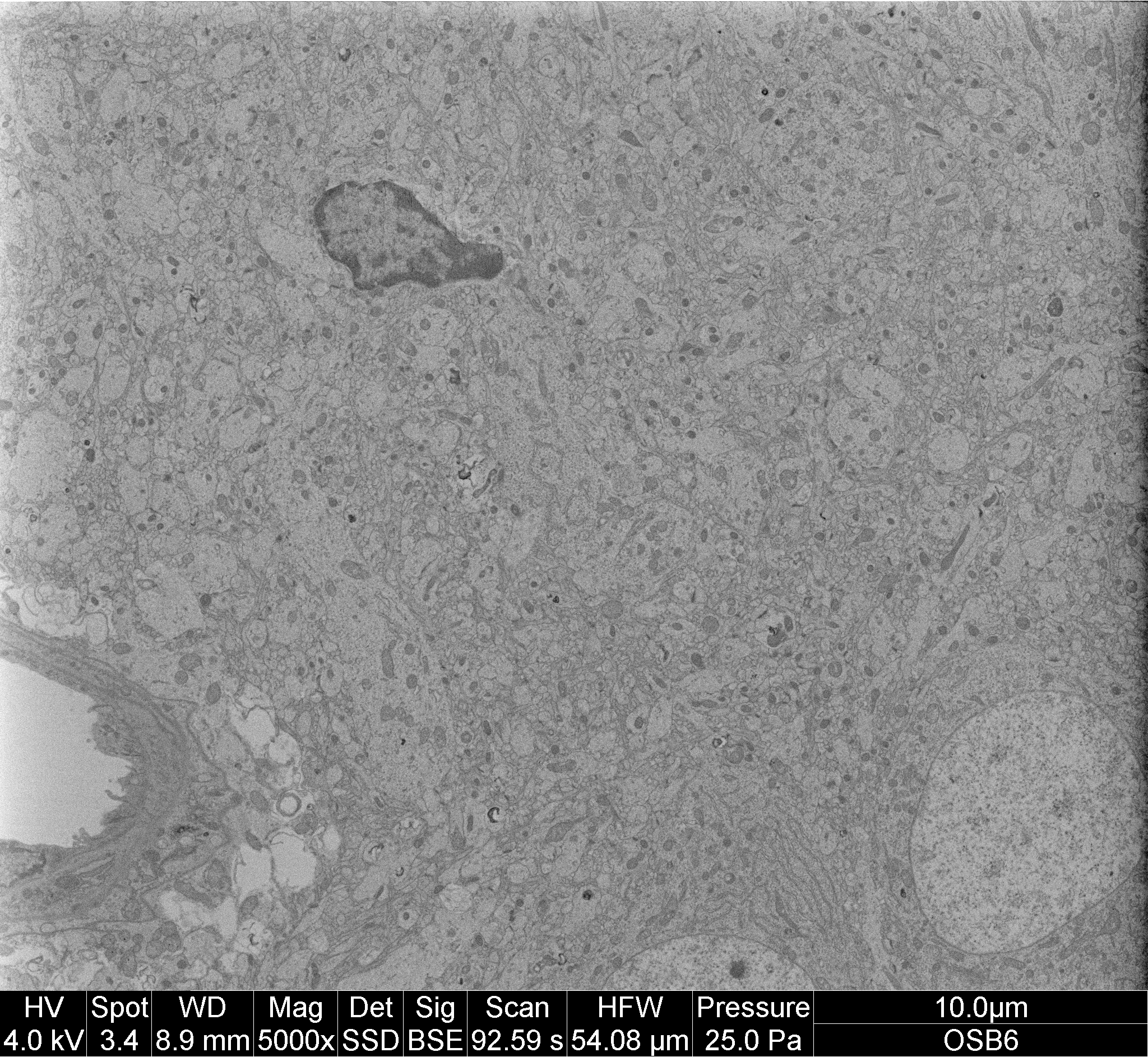

Supplement: Dataset S10 — (253.8 MB ZIP). [file pbio.0020329.sd010.zip › 040604_OS5_st1_921.tif]

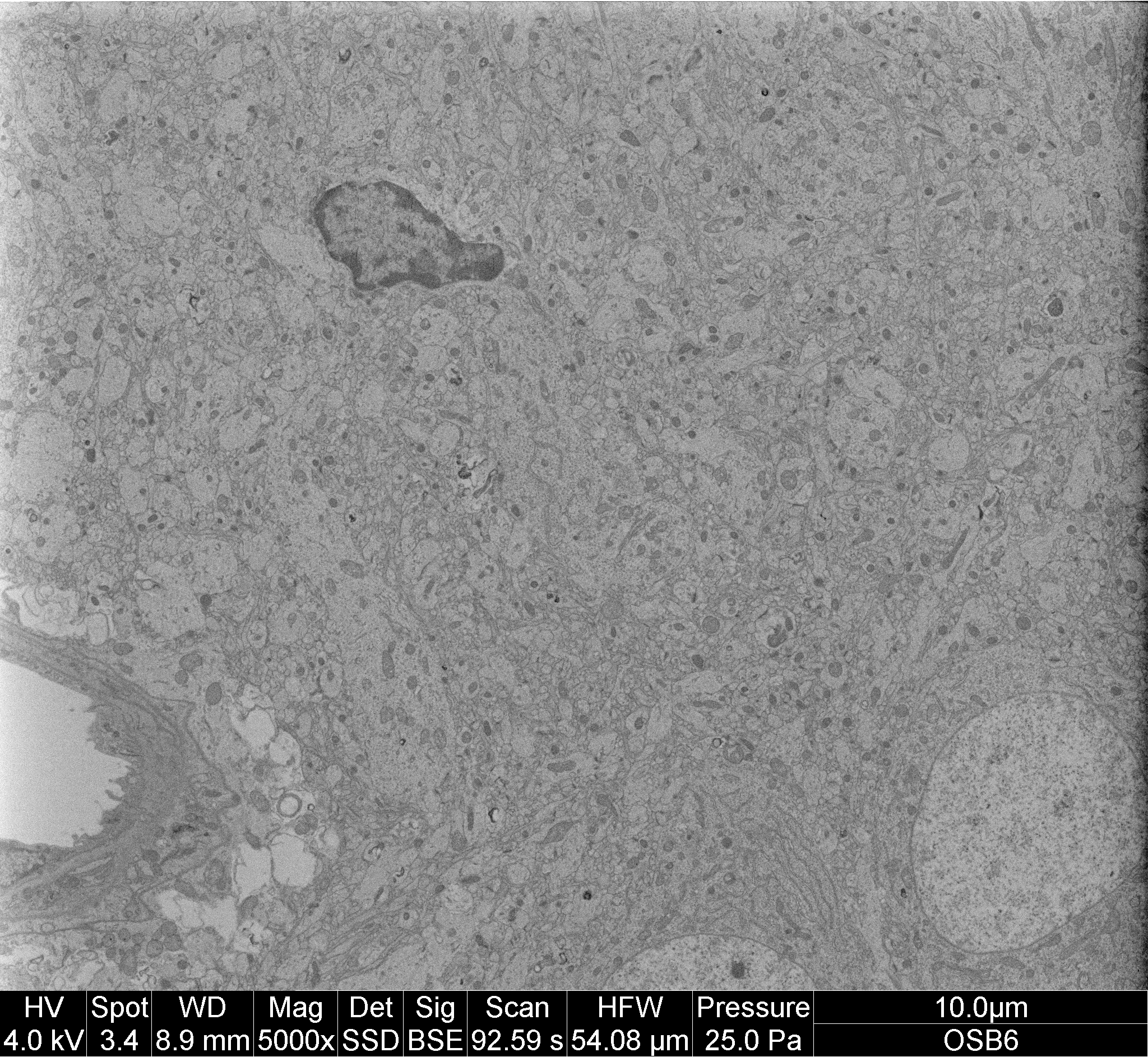

Supplement: Dataset S10 — (253.8 MB ZIP). [file pbio.0020329.sd010.zip › 040604_OS5_st1_922.tif]

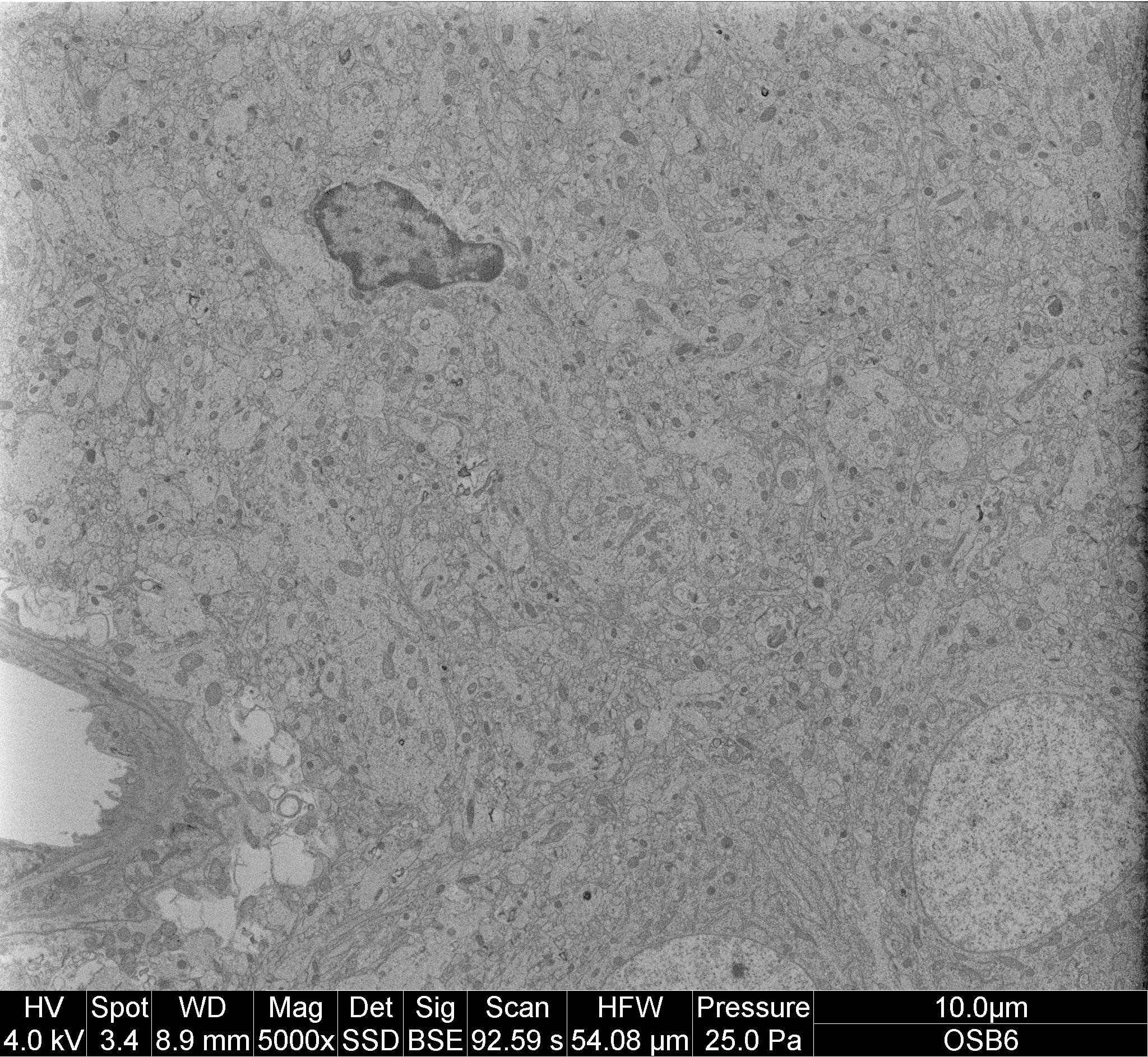

Supplement: Dataset S10 — (253.8 MB ZIP). [file pbio.0020329.sd010.zip › 040604_OS5_st1_923.tif]

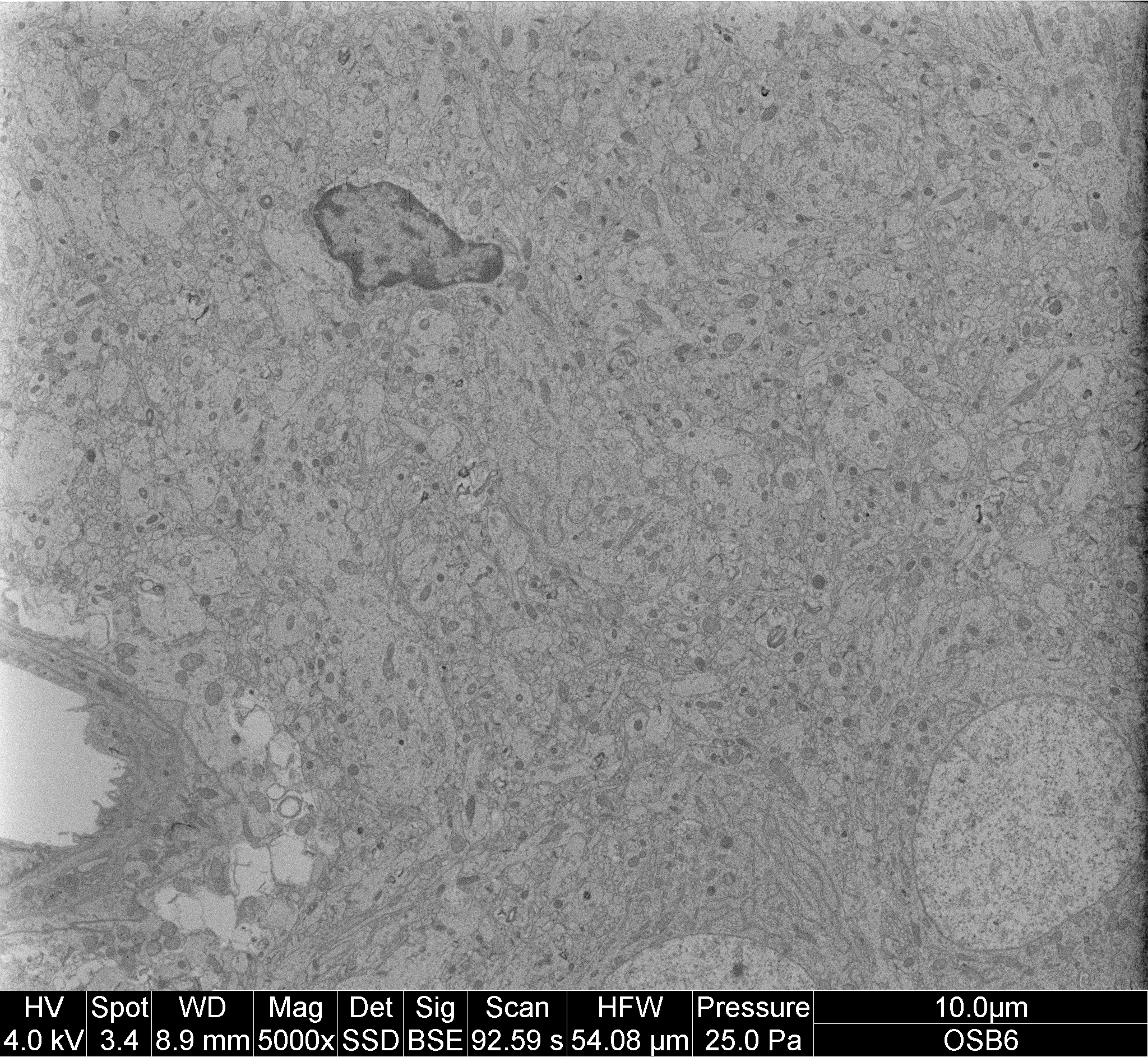

Supplement: Dataset S10 — (253.8 MB ZIP). [file pbio.0020329.sd010.zip › 040604_OS5_st1_924.tif]

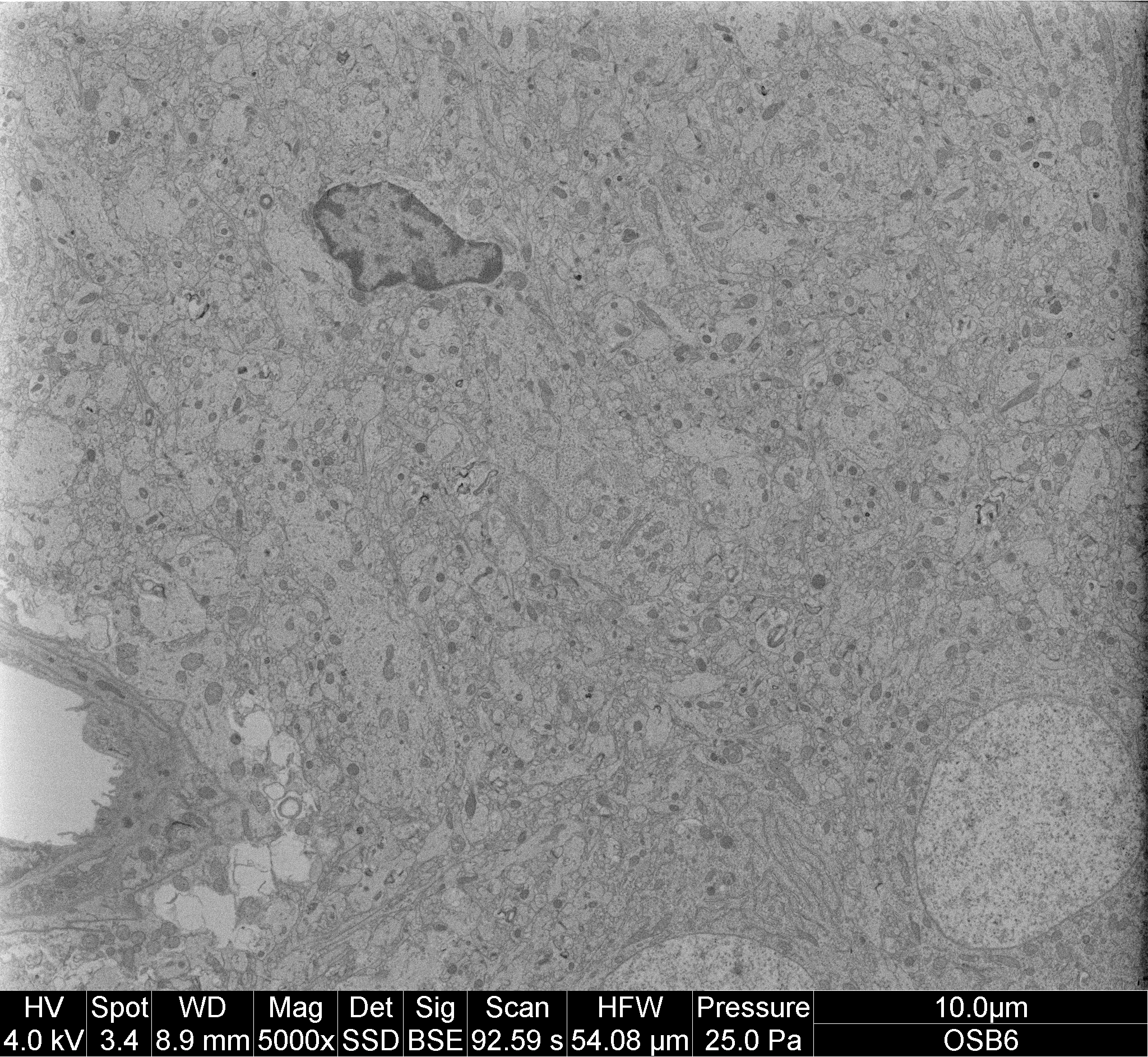

Supplement: Dataset S10 — (253.8 MB ZIP). [file pbio.0020329.sd010.zip › 040604_OS5_st1_925.tif]

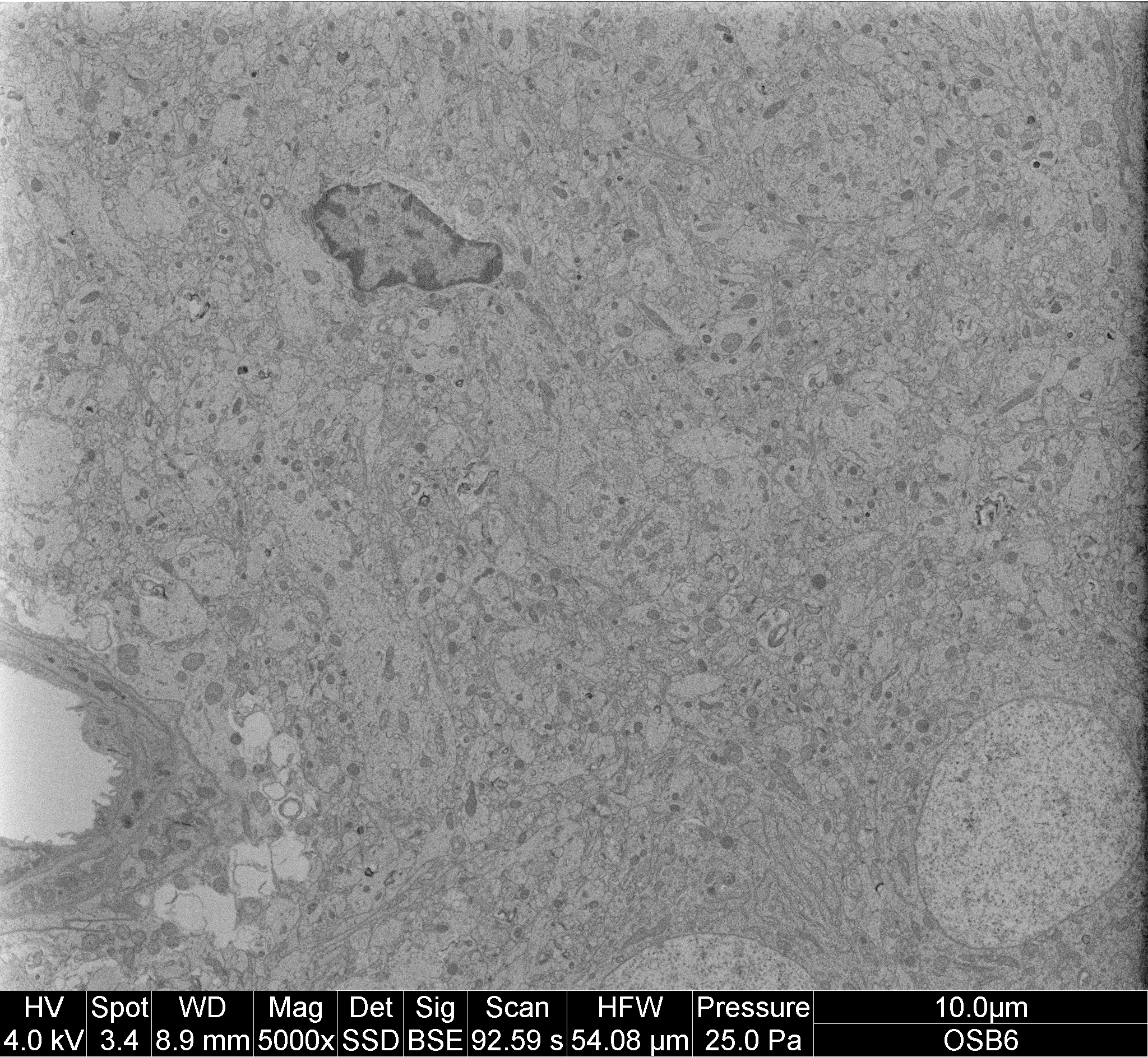

Supplement: Dataset S10 — (253.8 MB ZIP). [file pbio.0020329.sd010.zip › 040604_OS5_st1_926.tif]

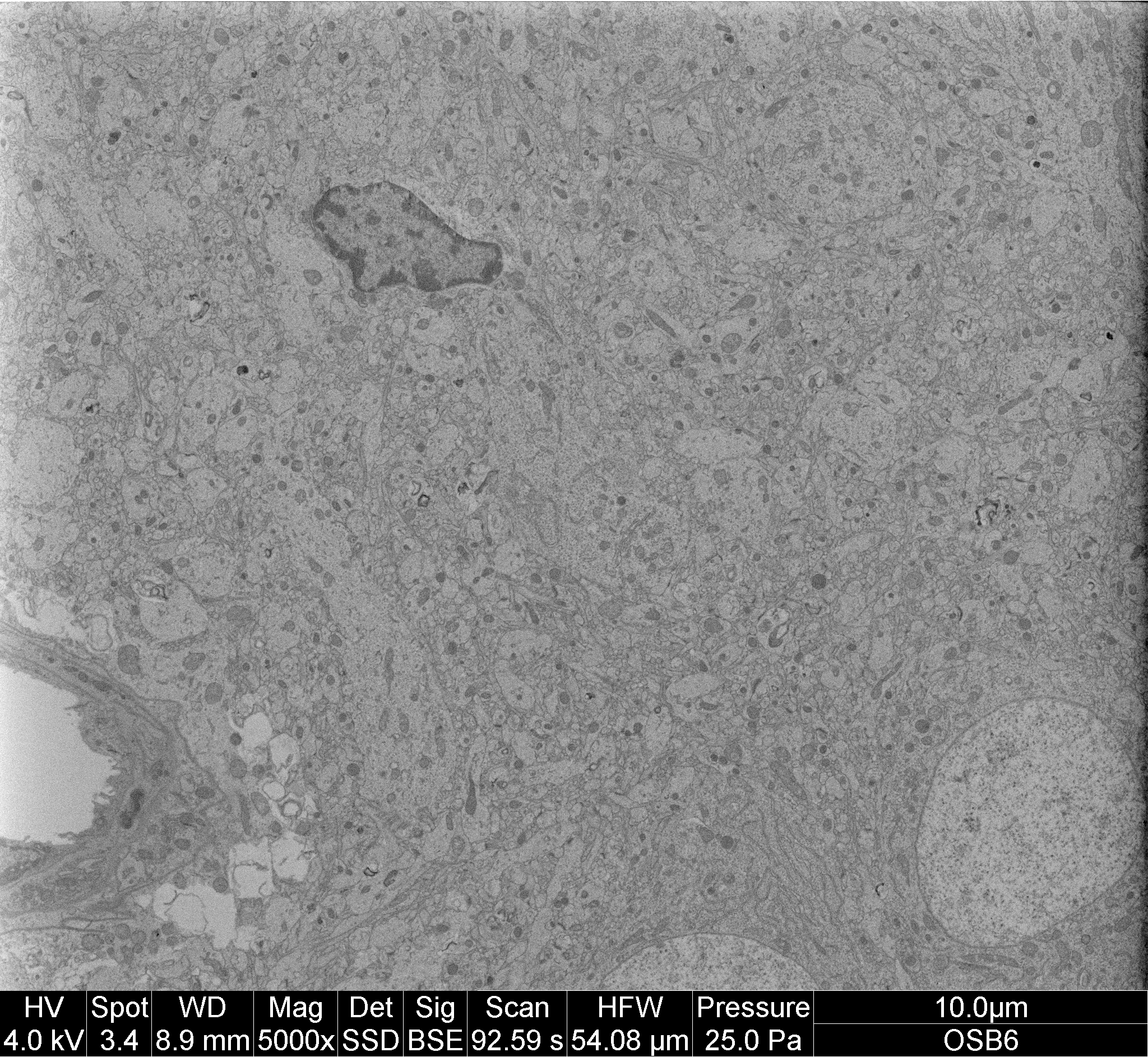

Supplement: Dataset S10 — (253.8 MB ZIP). [file pbio.0020329.sd010.zip › 040604_OS5_st1_927.tif]

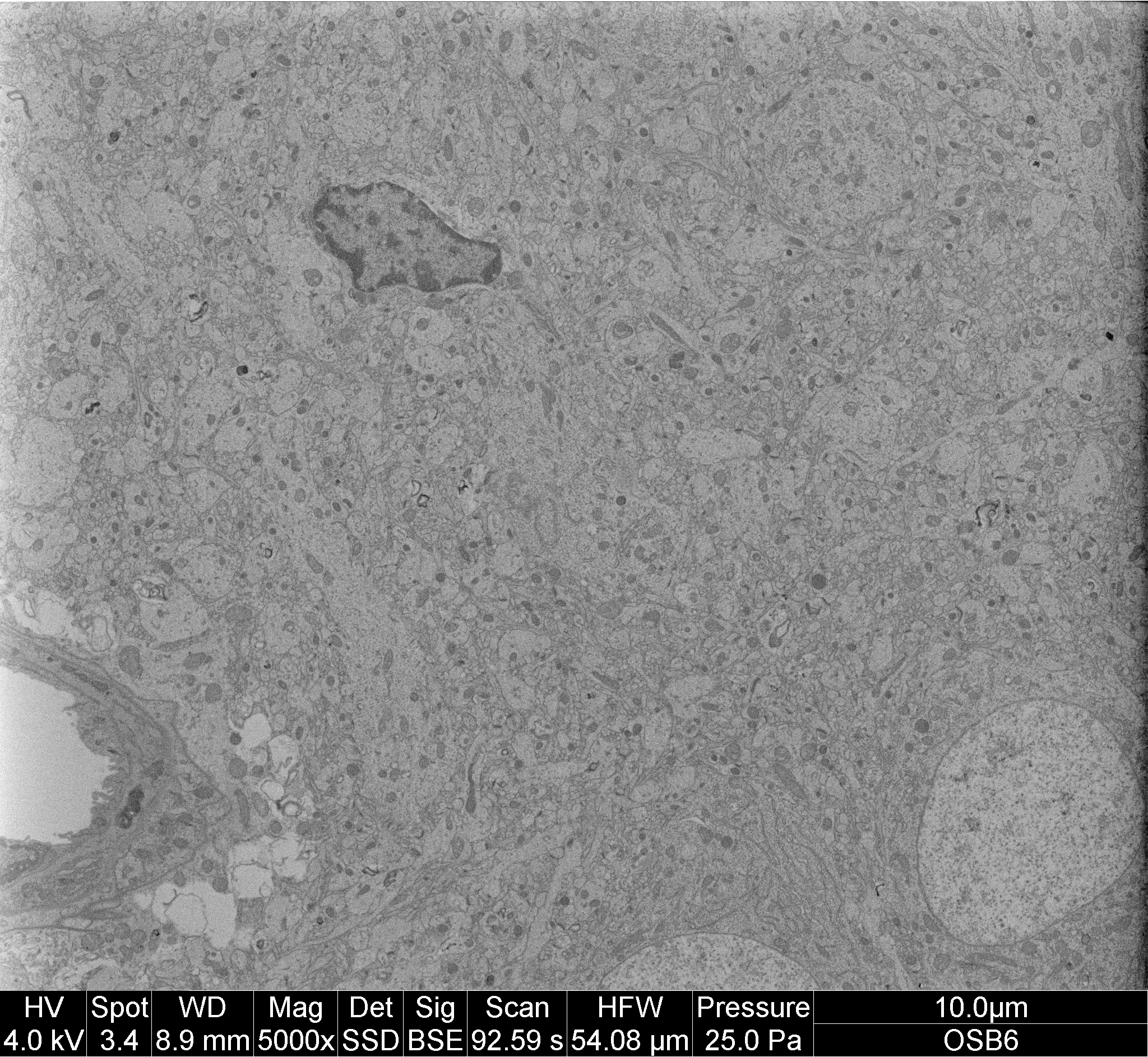

Supplement: Dataset S10 — (253.8 MB ZIP). [file pbio.0020329.sd010.zip › 040604_OS5_st1_928.tif]

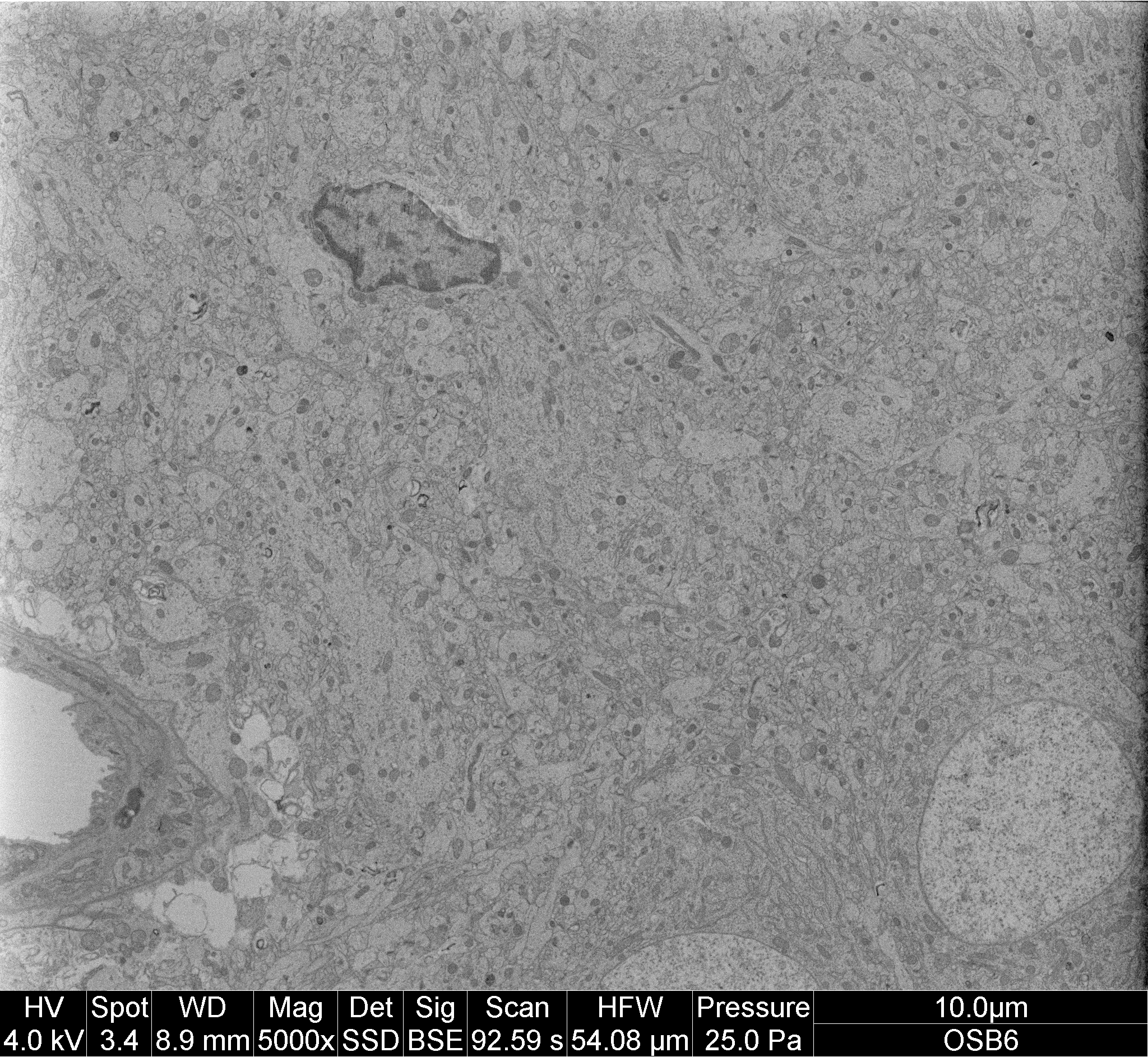

Supplement: Dataset S10 — (253.8 MB ZIP). [file pbio.0020329.sd010.zip › 040604_OS5_st1_929.tif]

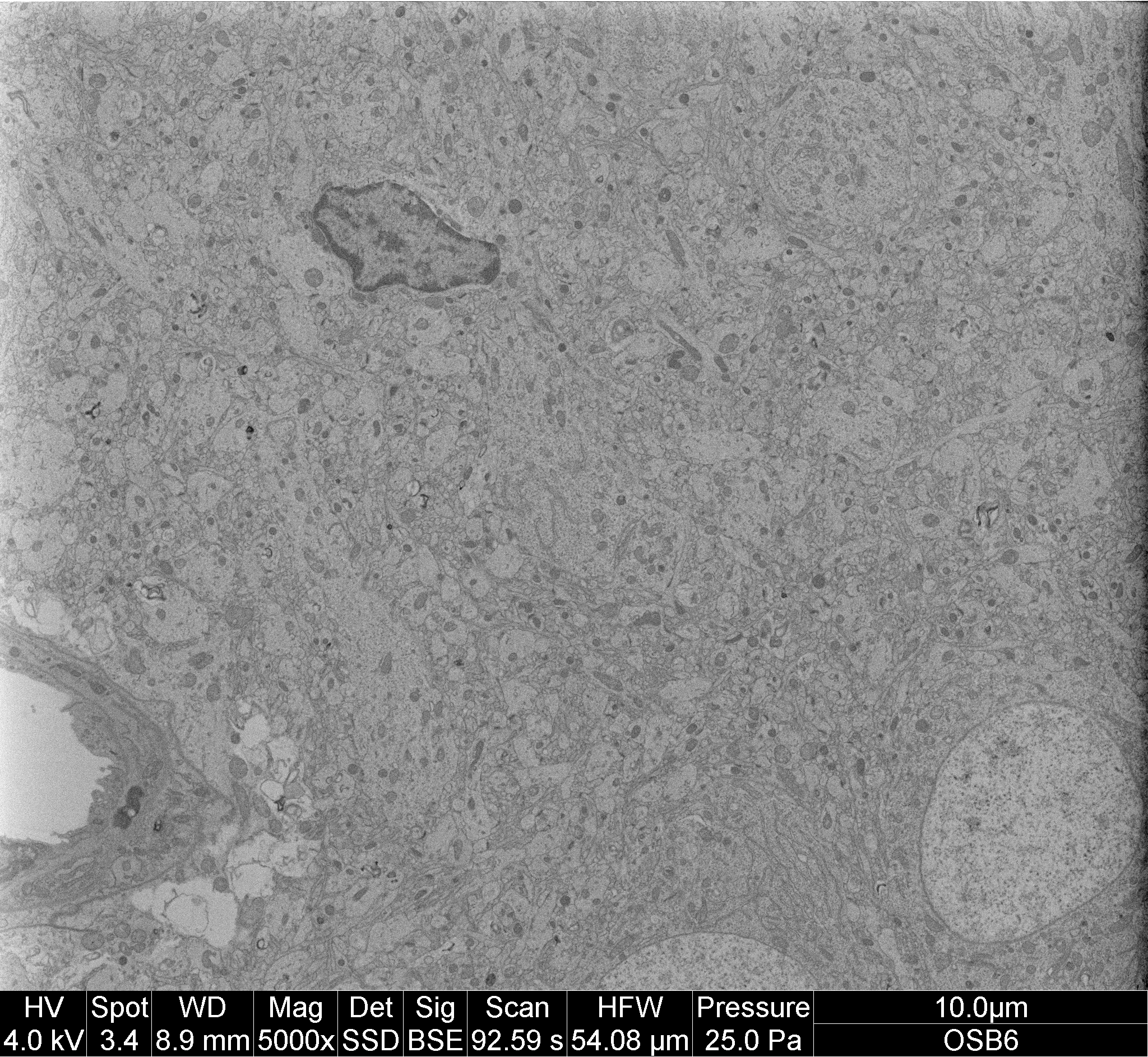

Supplement: Dataset S10 — (253.8 MB ZIP). [file pbio.0020329.sd010.zip › 040604_OS5_st1_930.tif]

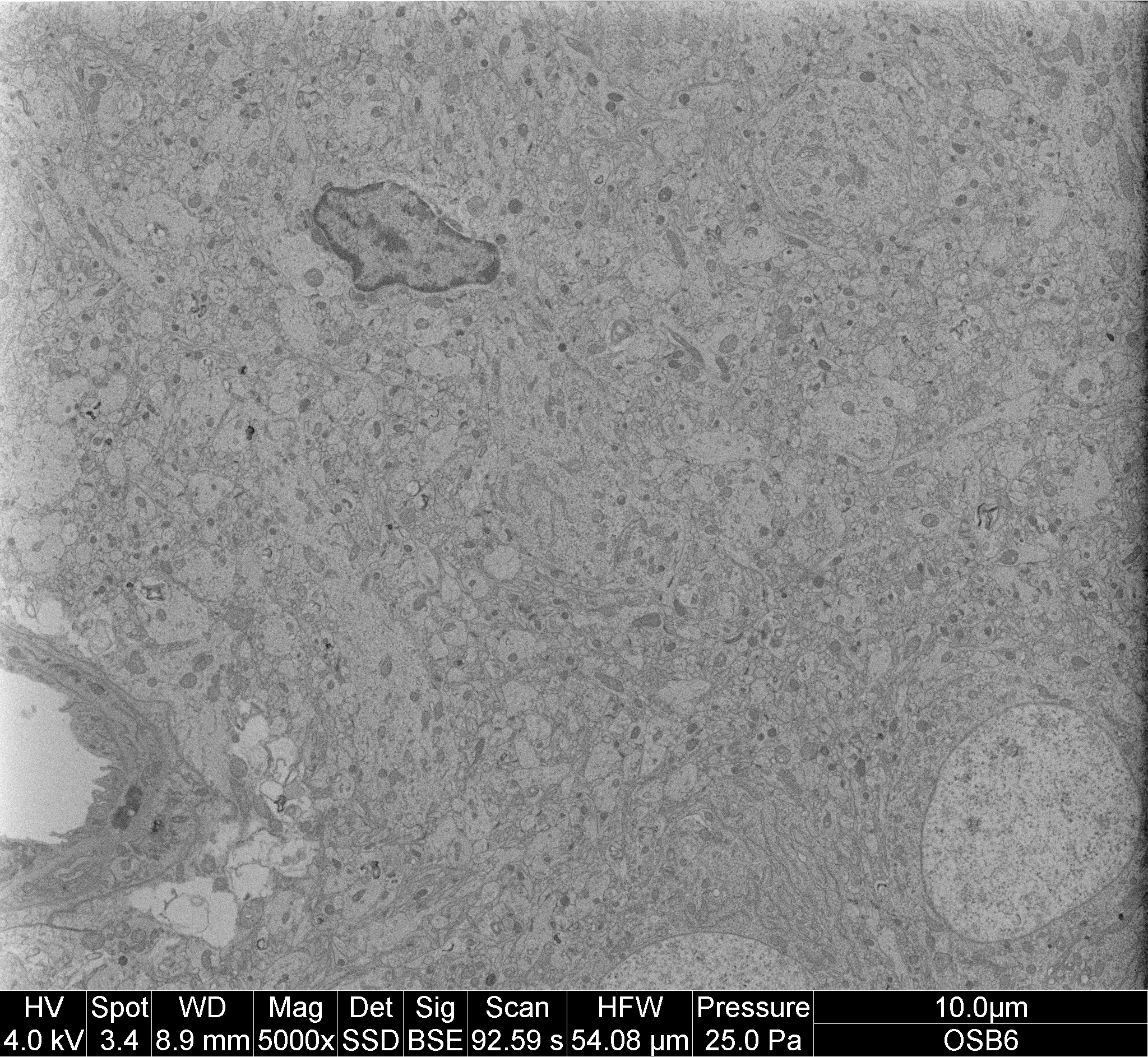

Supplement: Dataset S10 — (253.8 MB ZIP). [file pbio.0020329.sd010.zip › 040604_OS5_st1_931.tif]

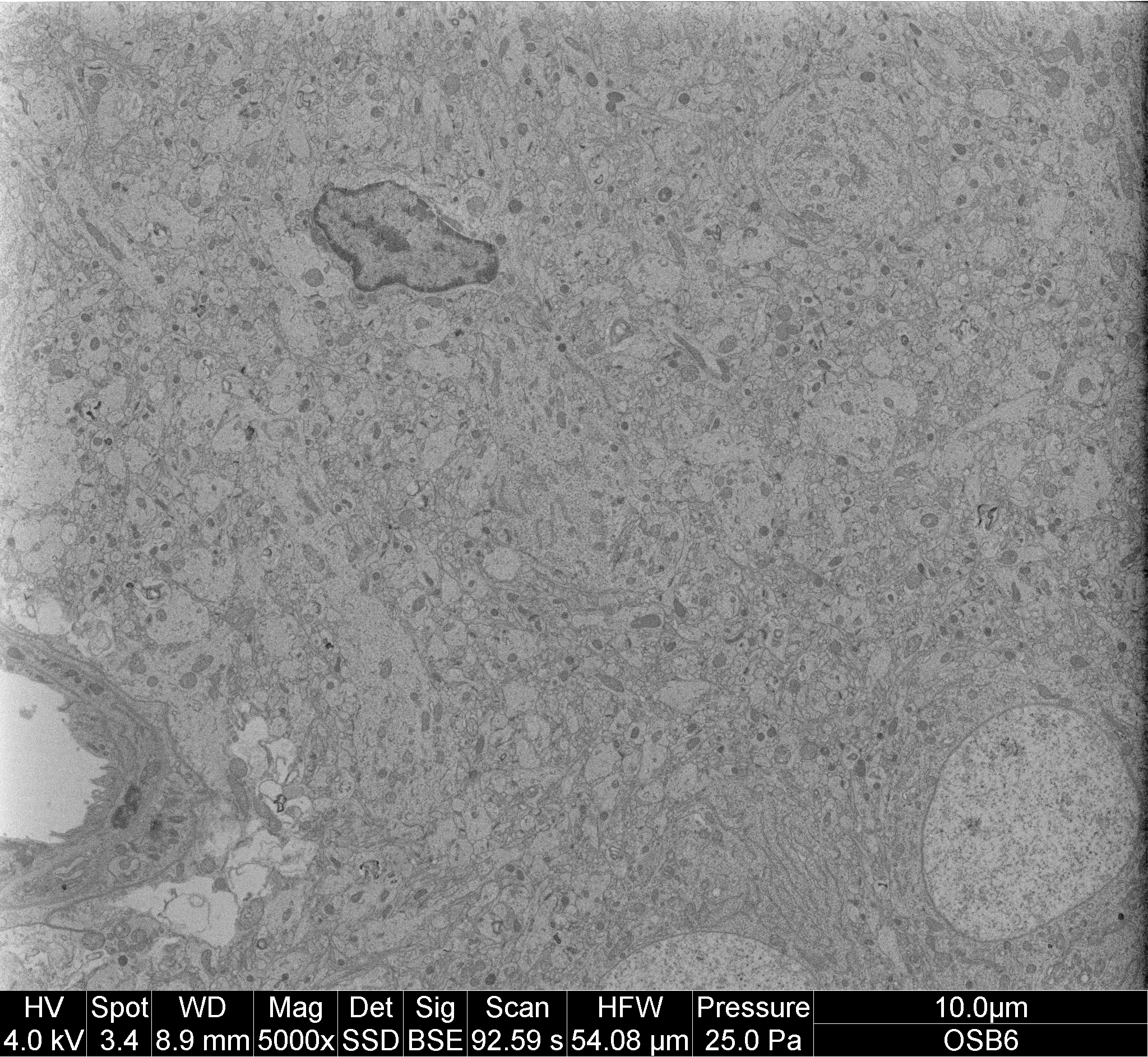

Supplement: Dataset S10 — (253.8 MB ZIP). [file pbio.0020329.sd010.zip › 040604_OS5_st1_932.tif]

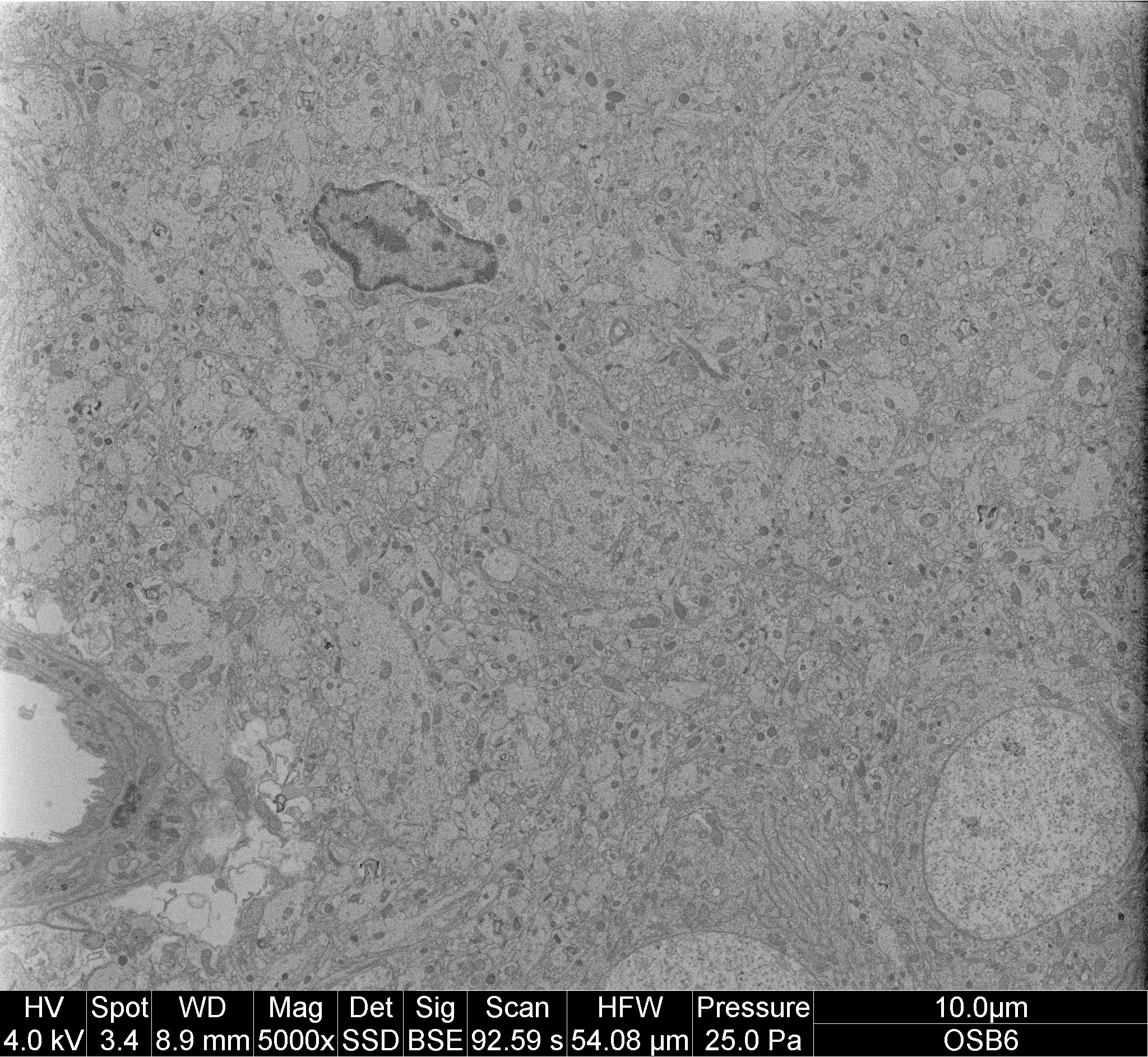

Supplement: Dataset S10 — (253.8 MB ZIP). [file pbio.0020329.sd010.zip › 040604_OS5_st1_933.tif]

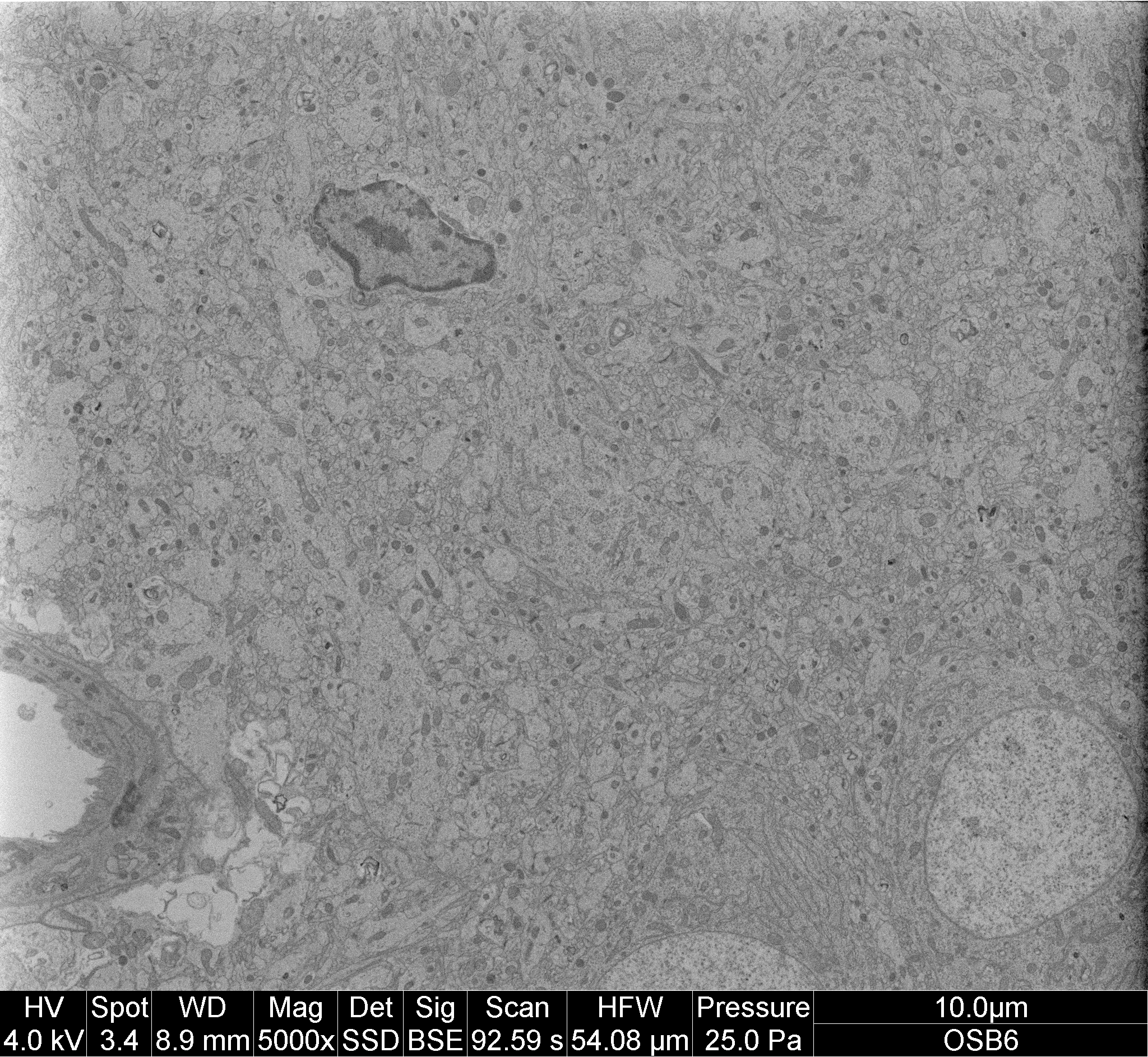

Supplement: Dataset S10 — (253.8 MB ZIP). [file pbio.0020329.sd010.zip › 040604_OS5_st1_934.tif]

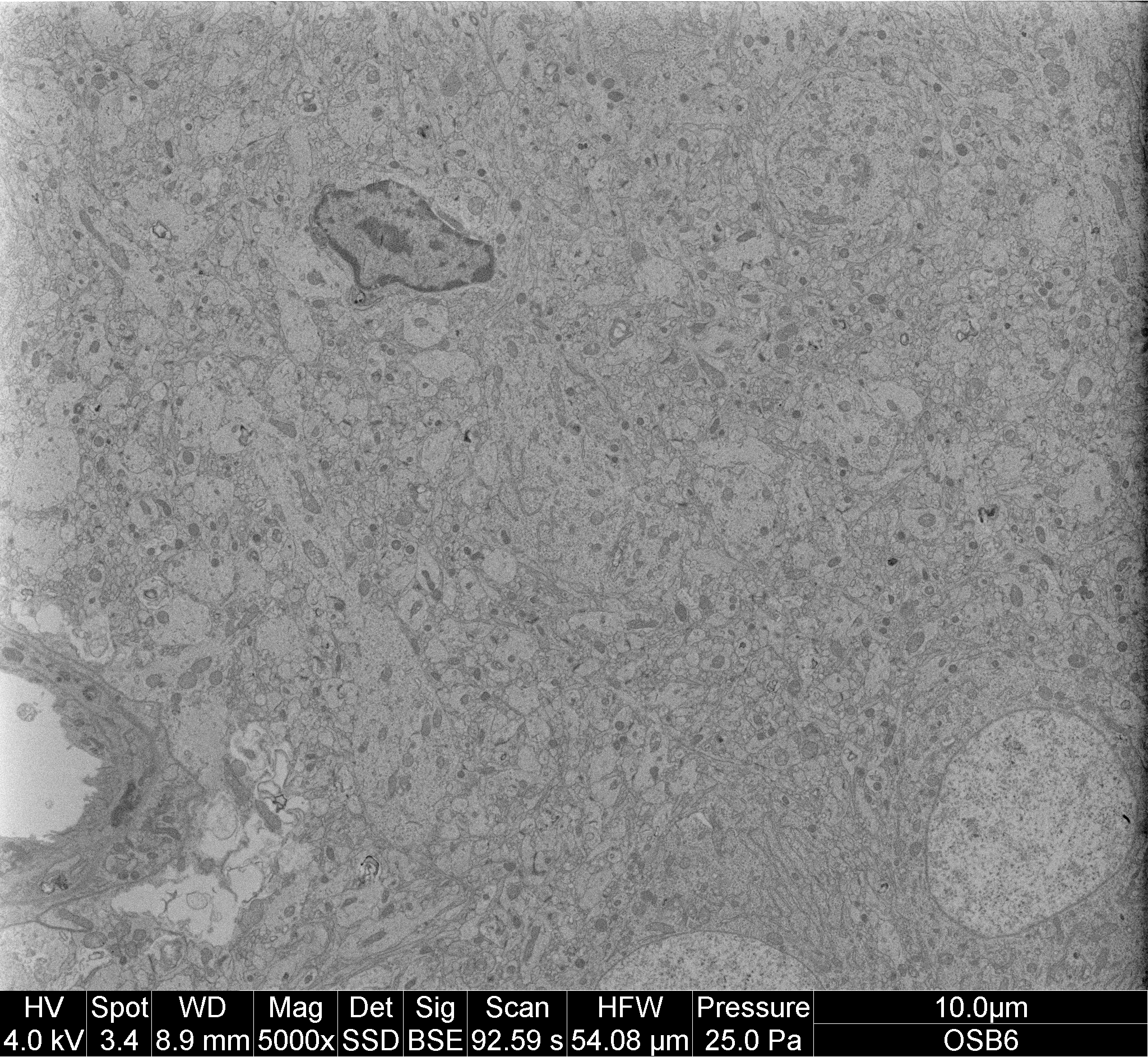

Supplement: Dataset S10 — (253.8 MB ZIP). [file pbio.0020329.sd010.zip › 040604_OS5_st1_935.tif]

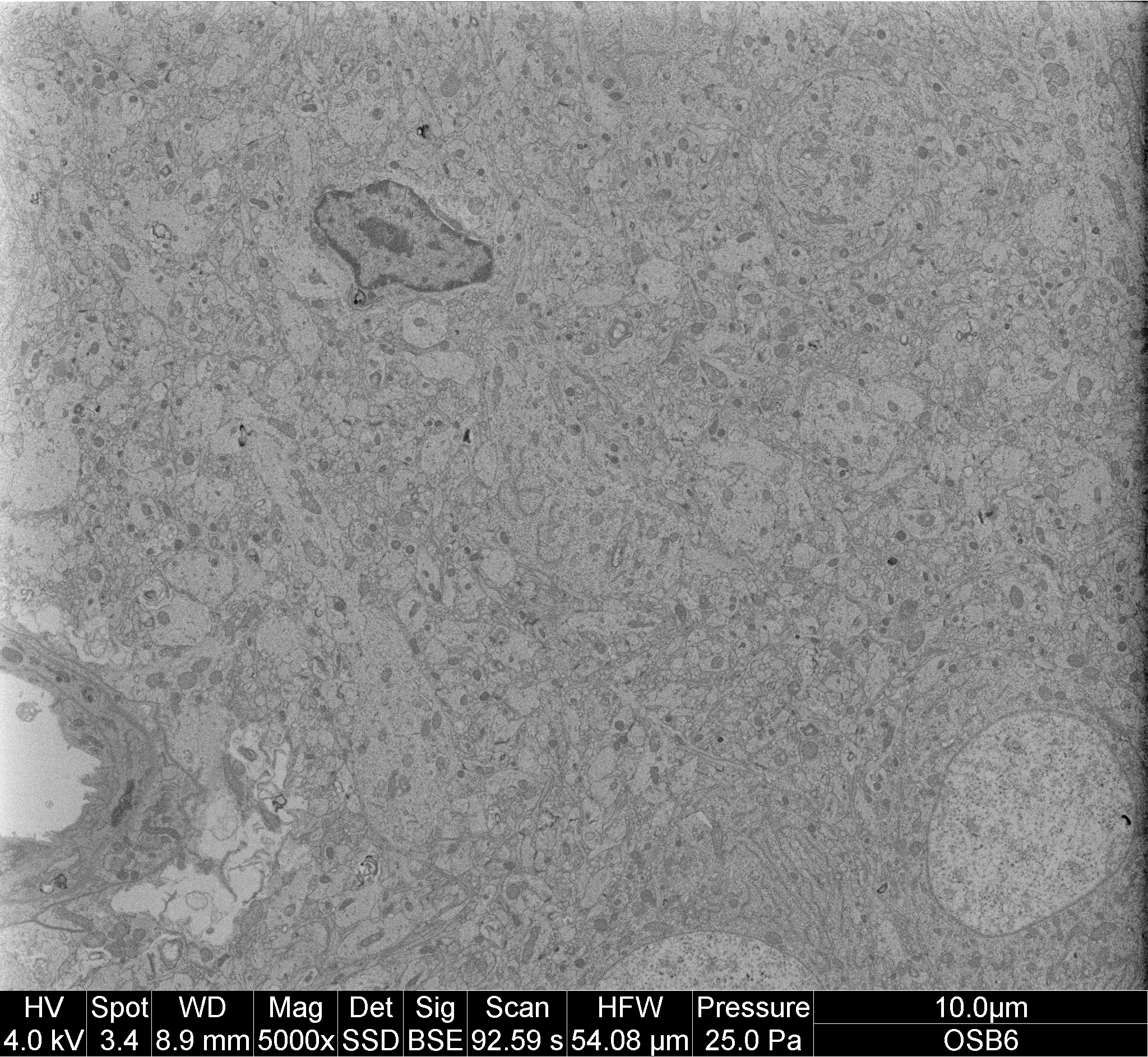

Supplement: Dataset S10 — (253.8 MB ZIP). [file pbio.0020329.sd010.zip › 040604_OS5_st1_936.tif]

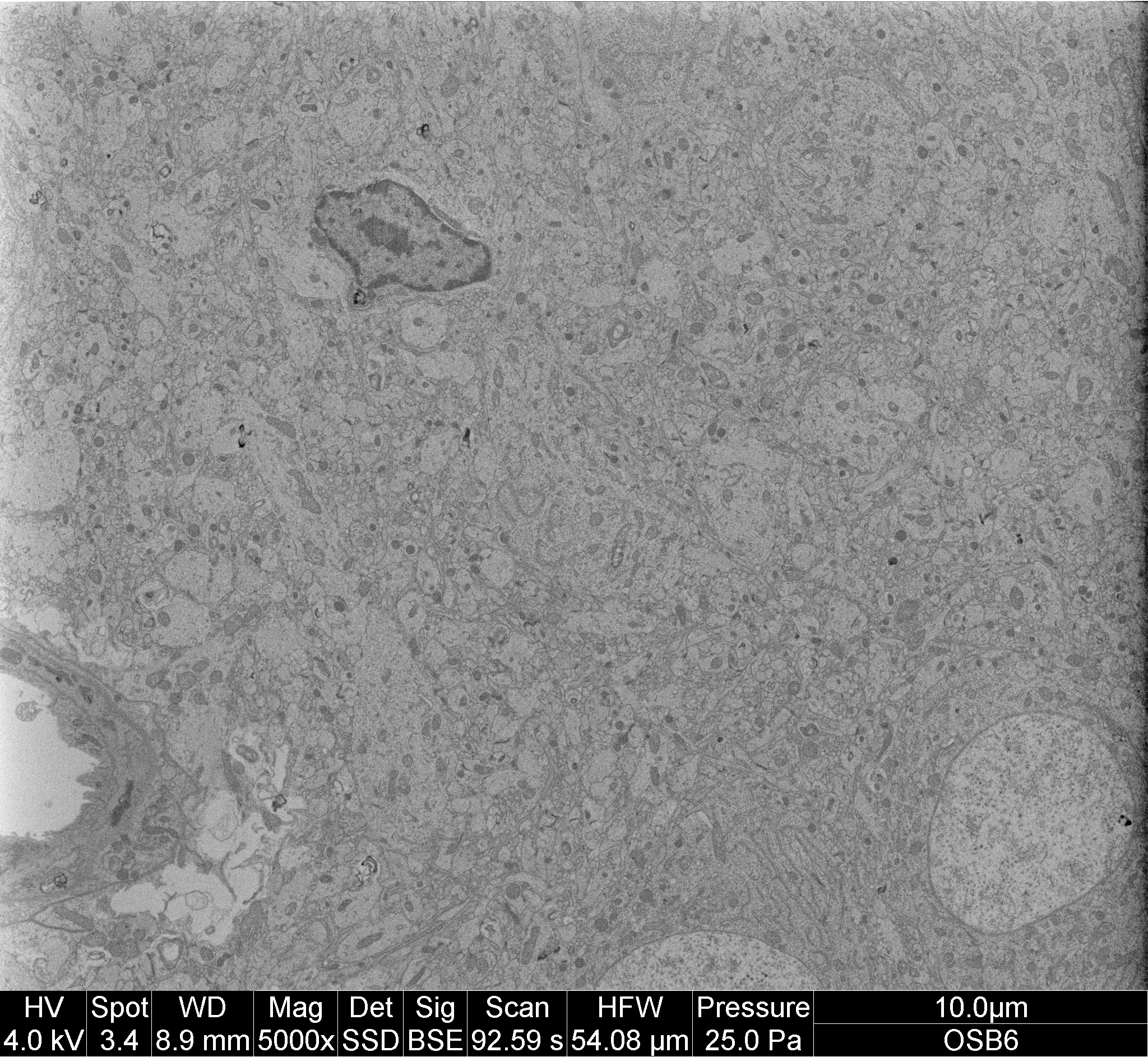

Supplement: Dataset S10 — (253.8 MB ZIP). [file pbio.0020329.sd010.zip › 040604_OS5_st1_937.tif]

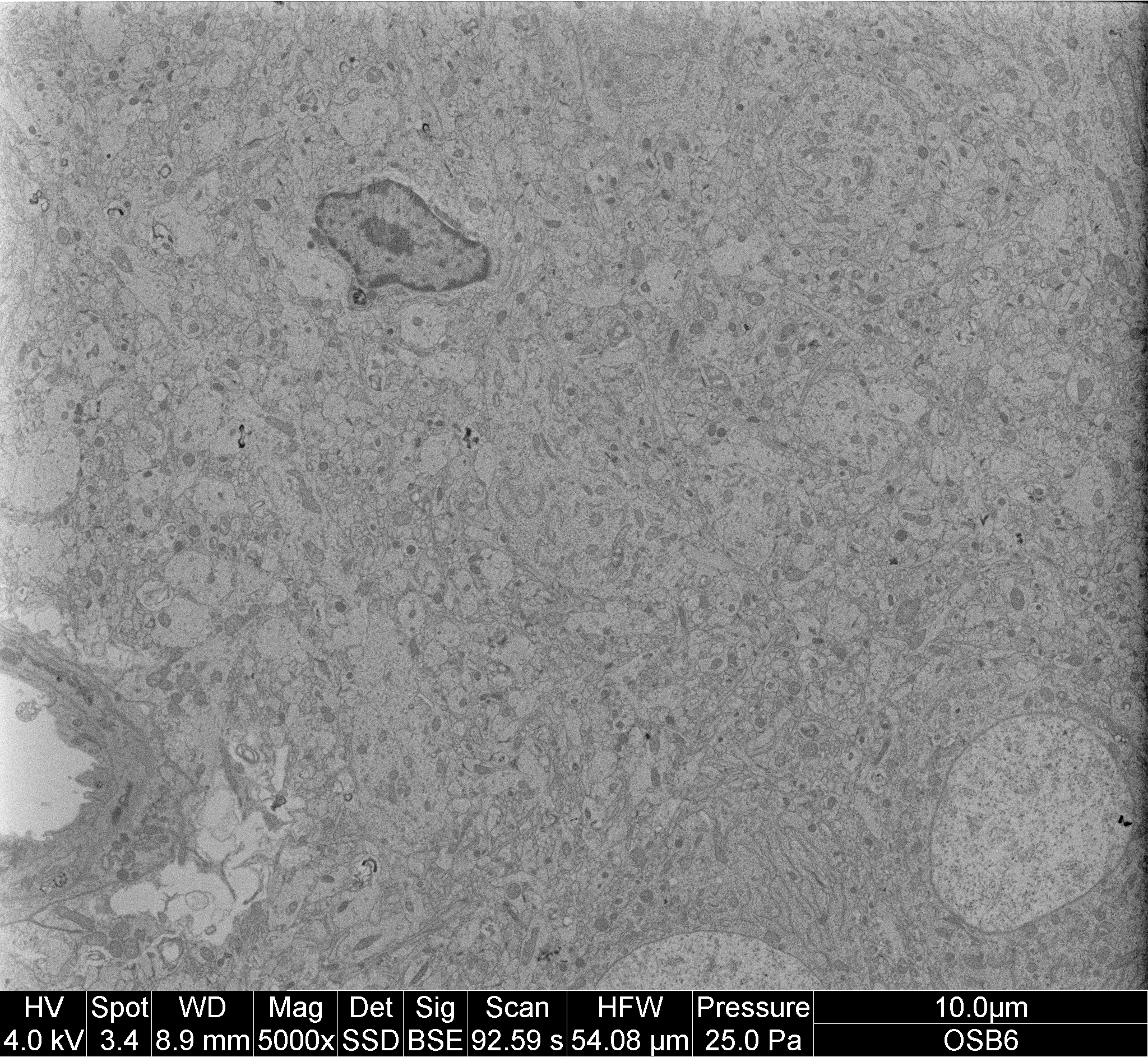

Supplement: Dataset S10 — (253.8 MB ZIP). [file pbio.0020329.sd010.zip › 040604_OS5_st1_938.tif]

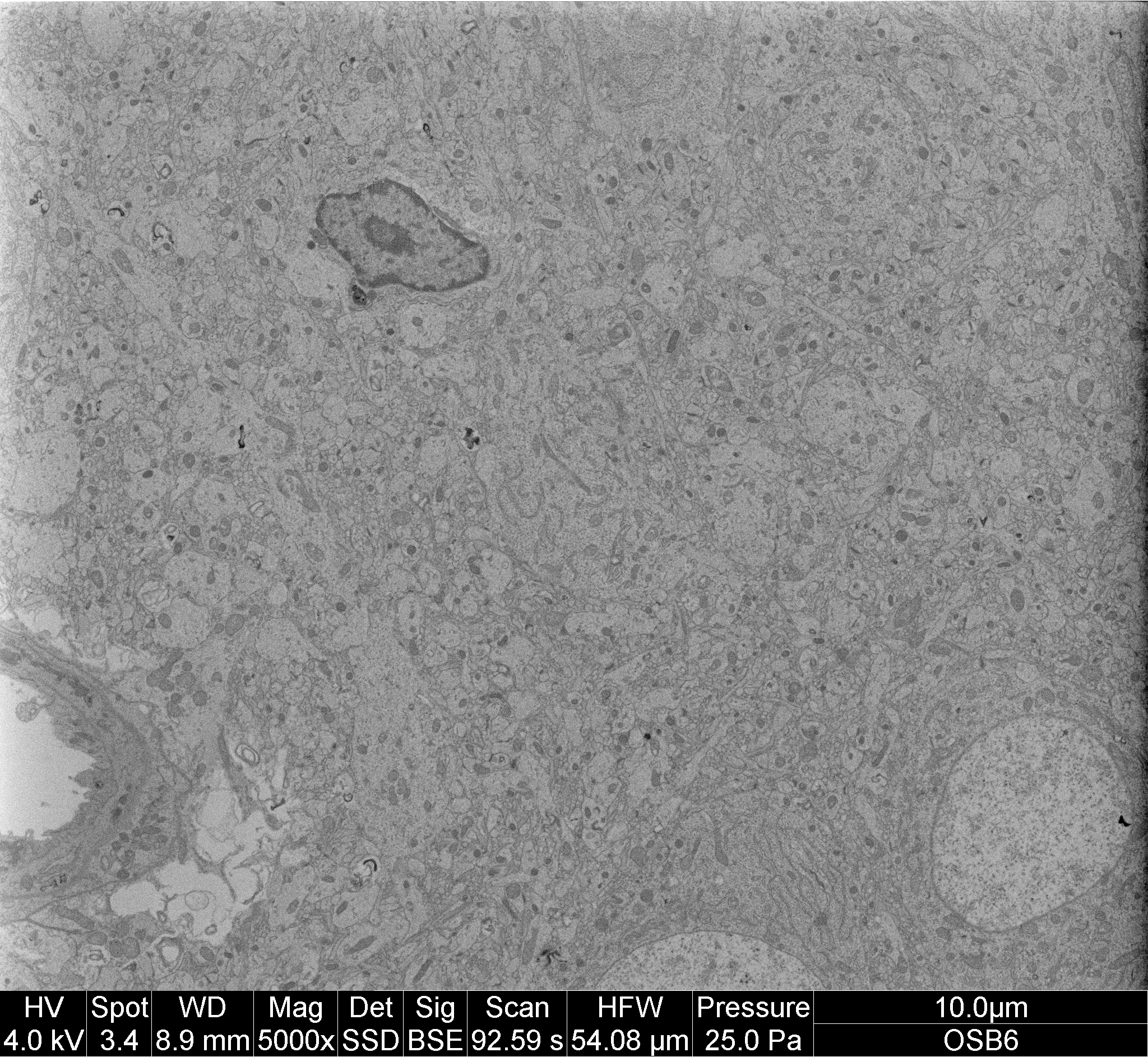

Supplement: Dataset S10 — (253.8 MB ZIP). [file pbio.0020329.sd010.zip › 040604_OS5_st1_939.tif]

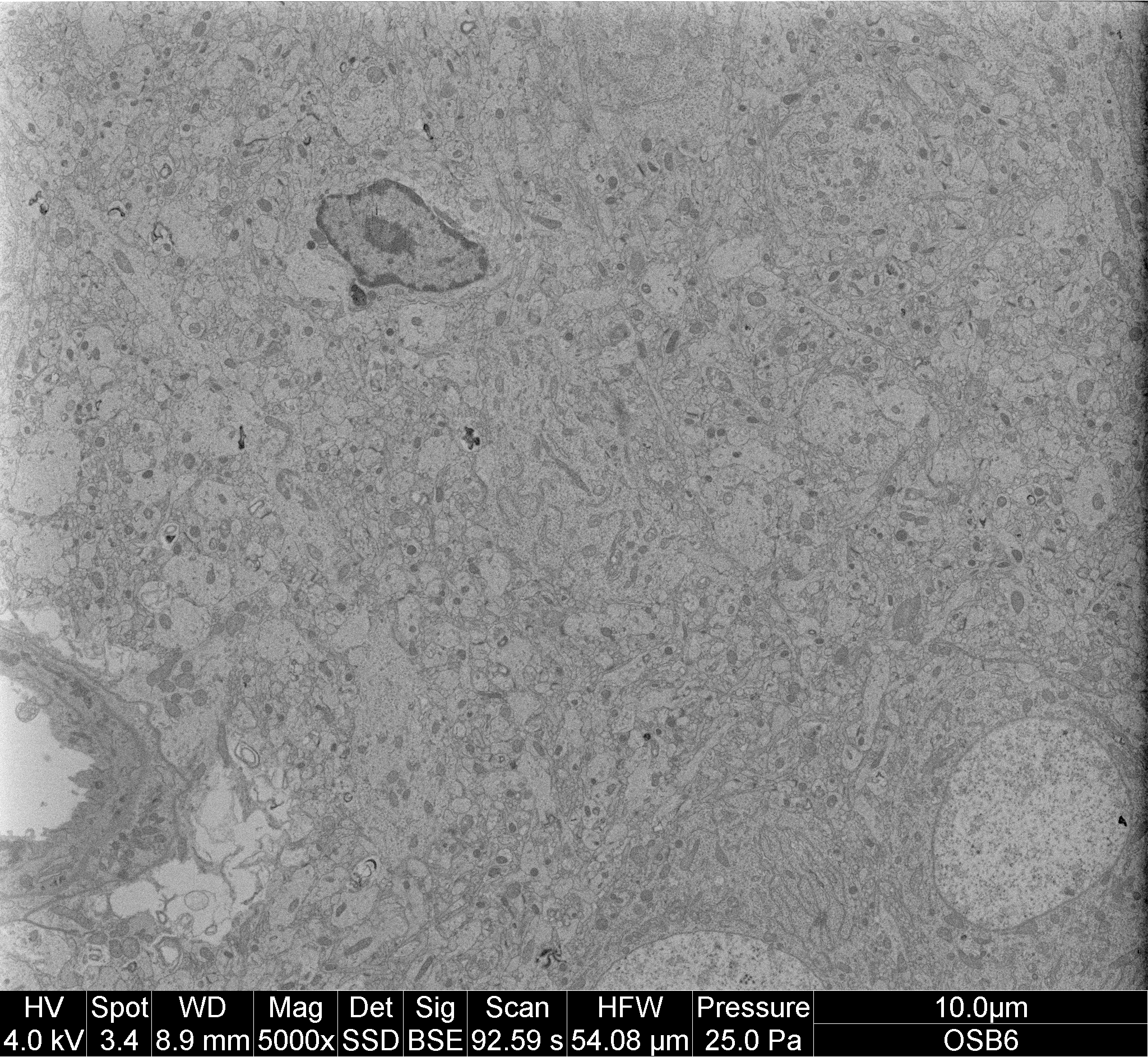

Supplement: Dataset S10 — (253.8 MB ZIP). [file pbio.0020329.sd010.zip › 040604_OS5_st1_940.tif]

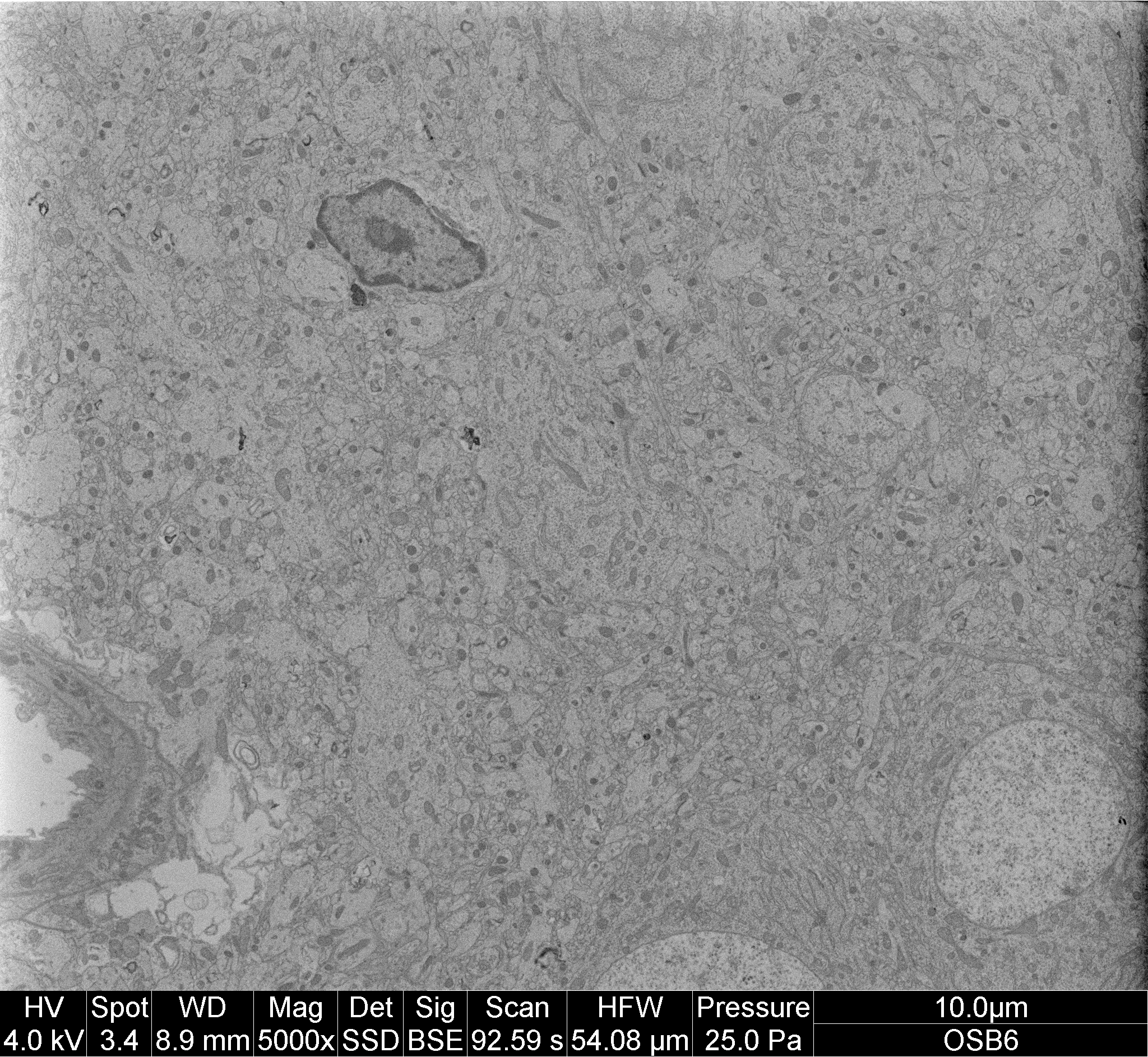

Supplement: Dataset S10 — (253.8 MB ZIP). [file pbio.0020329.sd010.zip › 040604_OS5_st1_941.tif]

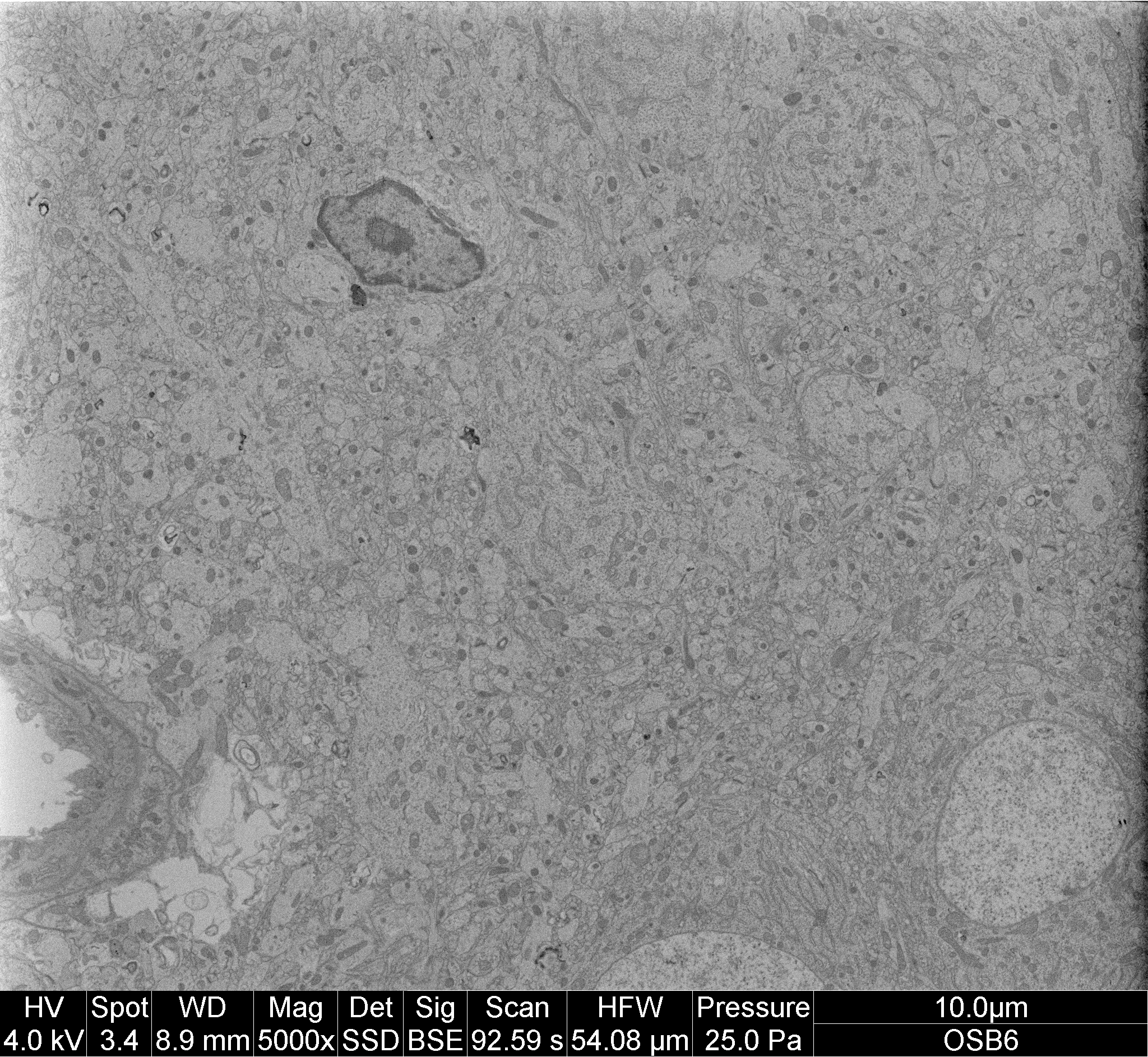

Supplement: Dataset S10 — (253.8 MB ZIP). [file pbio.0020329.sd010.zip › 040604_OS5_st1_942.tif]

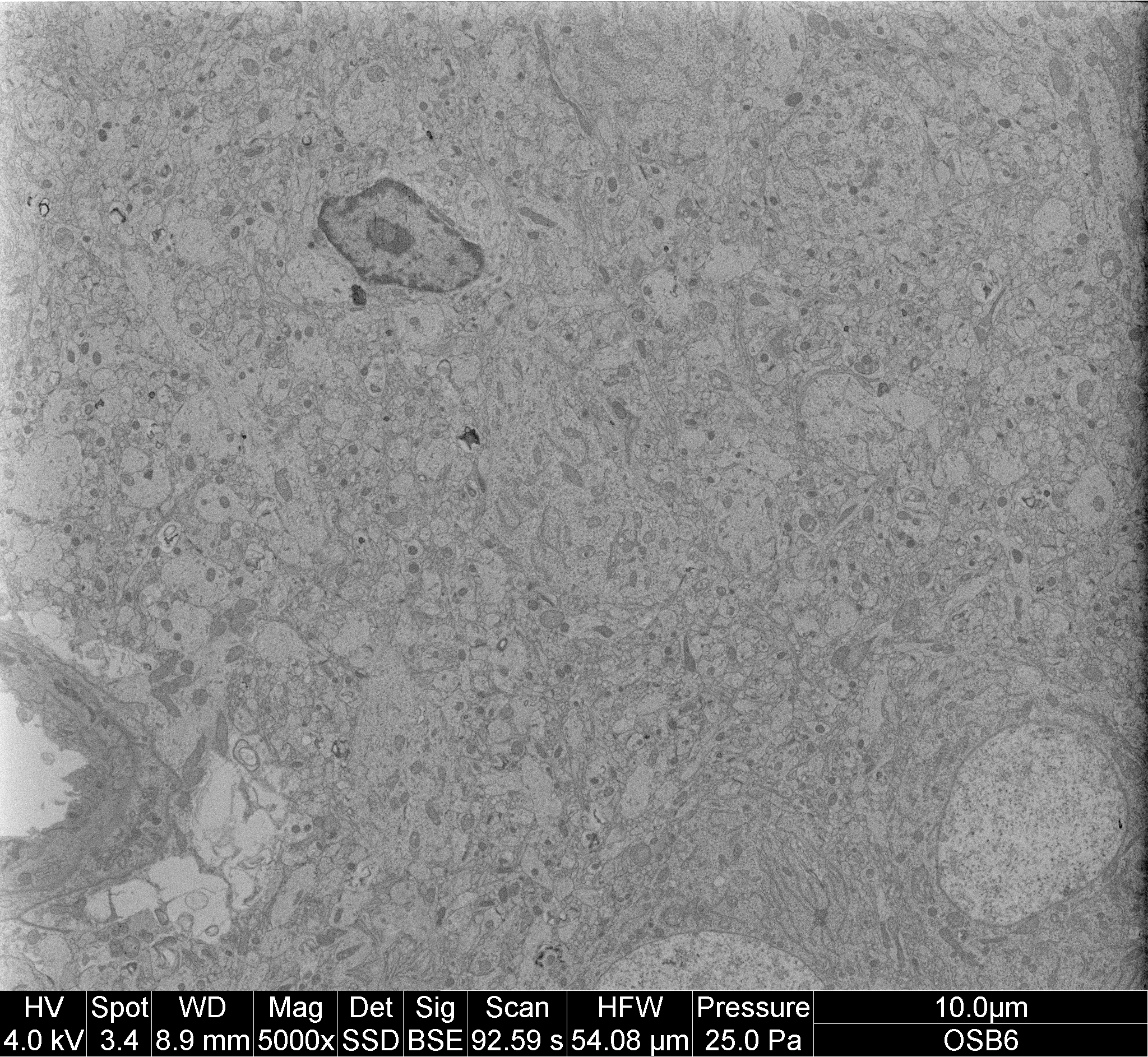

Supplement: Dataset S10 — (253.8 MB ZIP). [file pbio.0020329.sd010.zip › 040604_OS5_st1_943.tif]

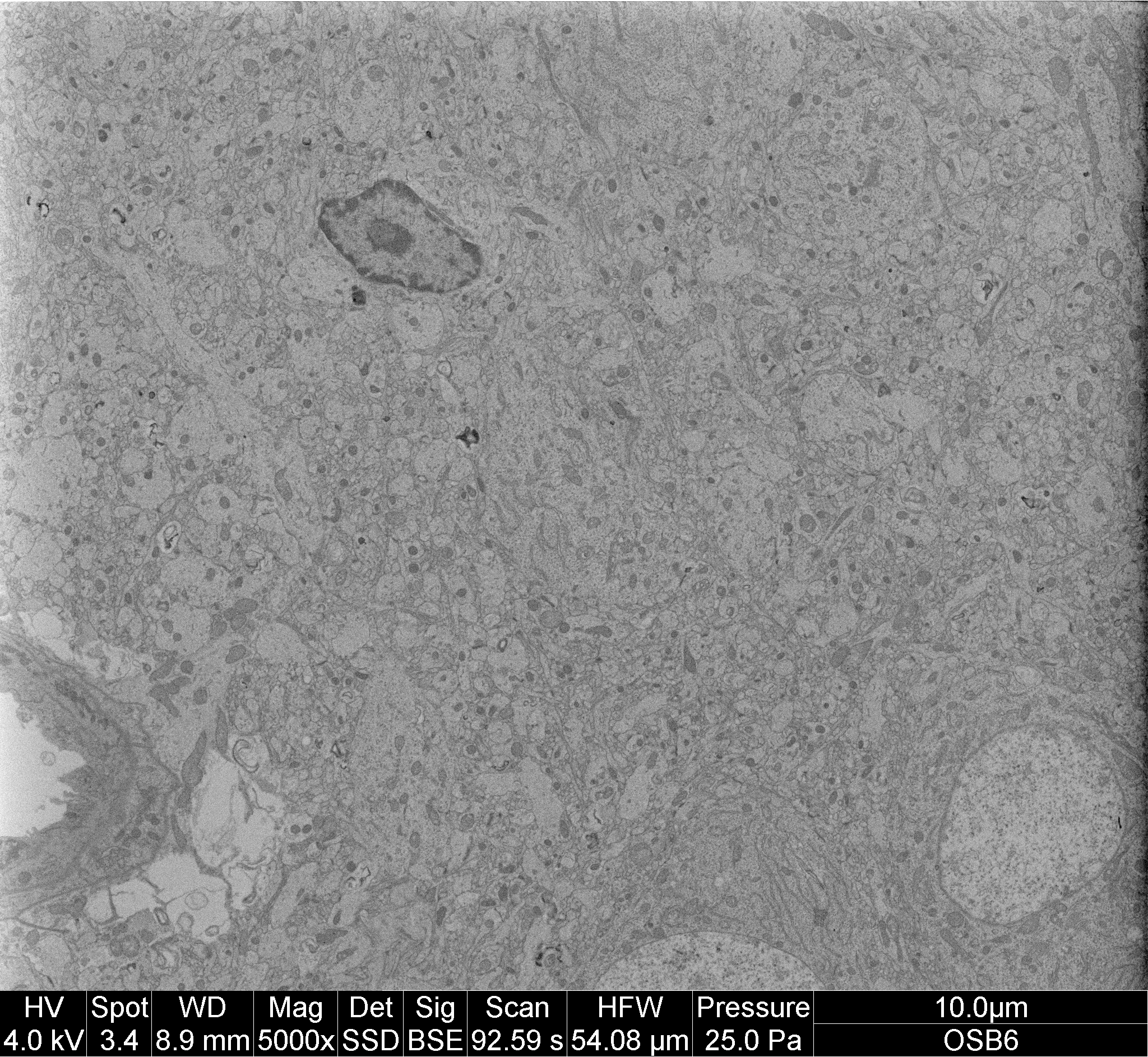

Supplement: Dataset S10 — (253.8 MB ZIP). [file pbio.0020329.sd010.zip › 040604_OS5_st1_944.tif]

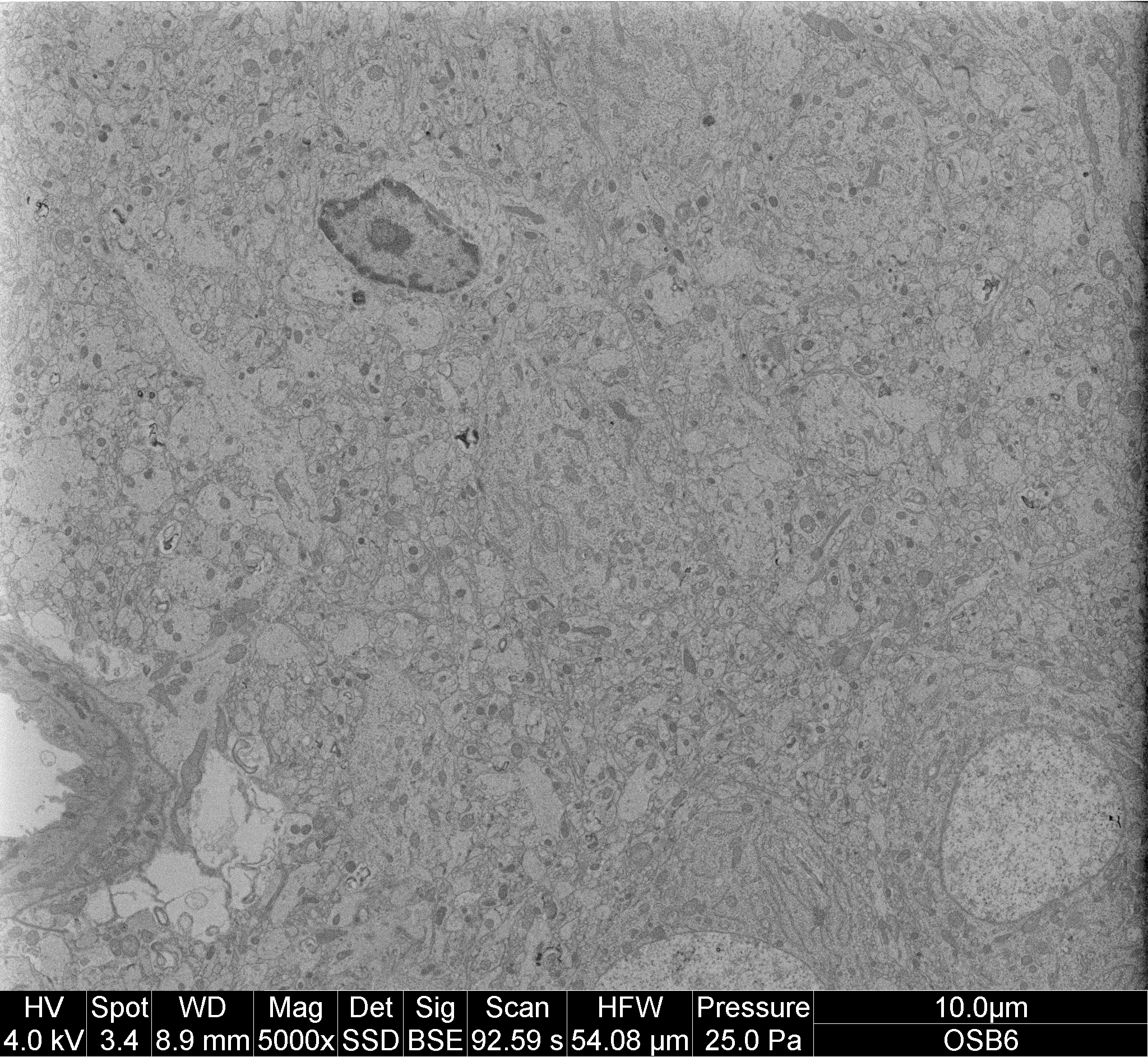

Supplement: Dataset S10 — (253.8 MB ZIP). [file pbio.0020329.sd010.zip › 040604_OS5_st1_945.tif]

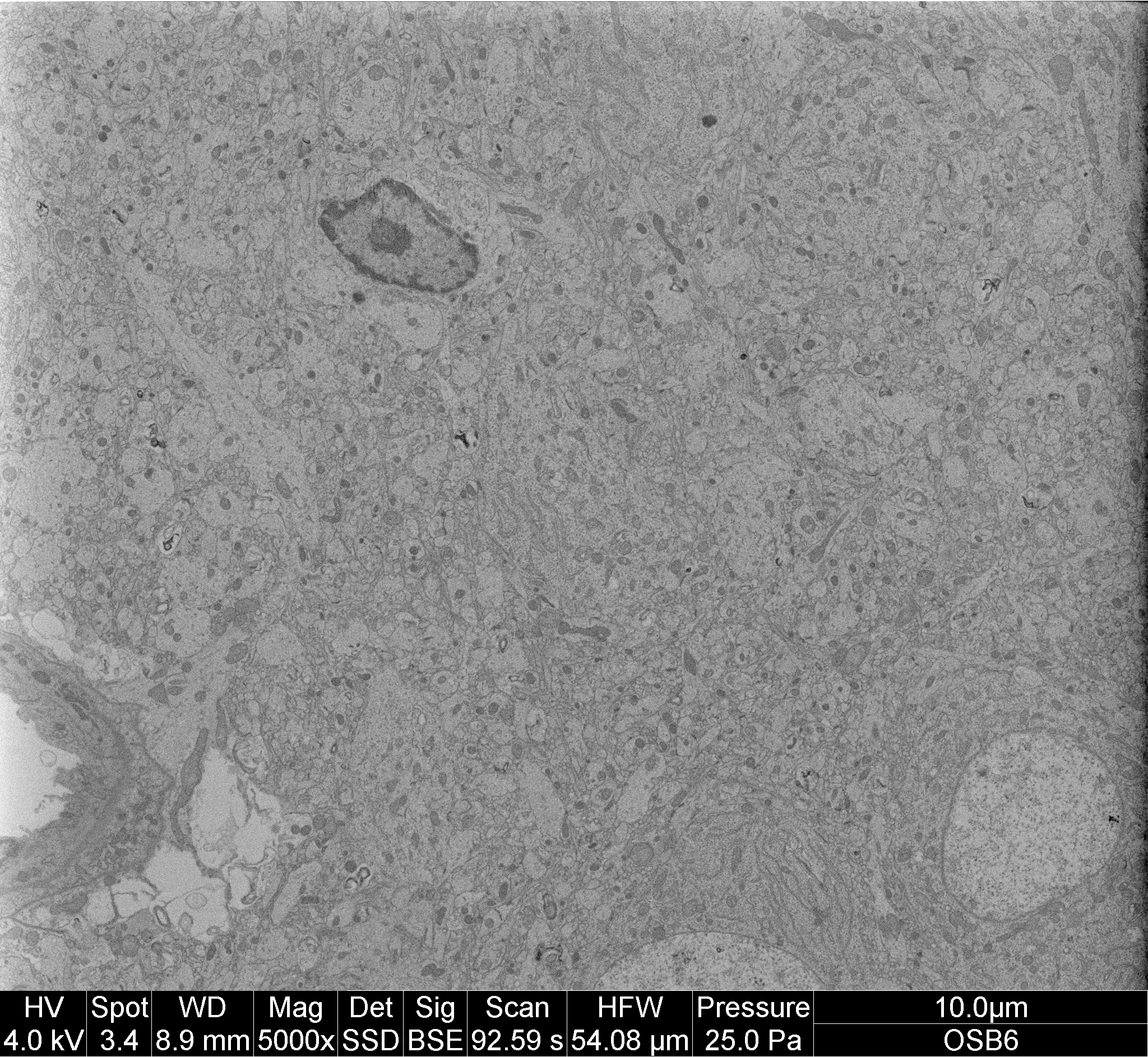

Supplement: Dataset S10 — (253.8 MB ZIP). [file pbio.0020329.sd010.zip › 040604_OS5_st1_946.tif]

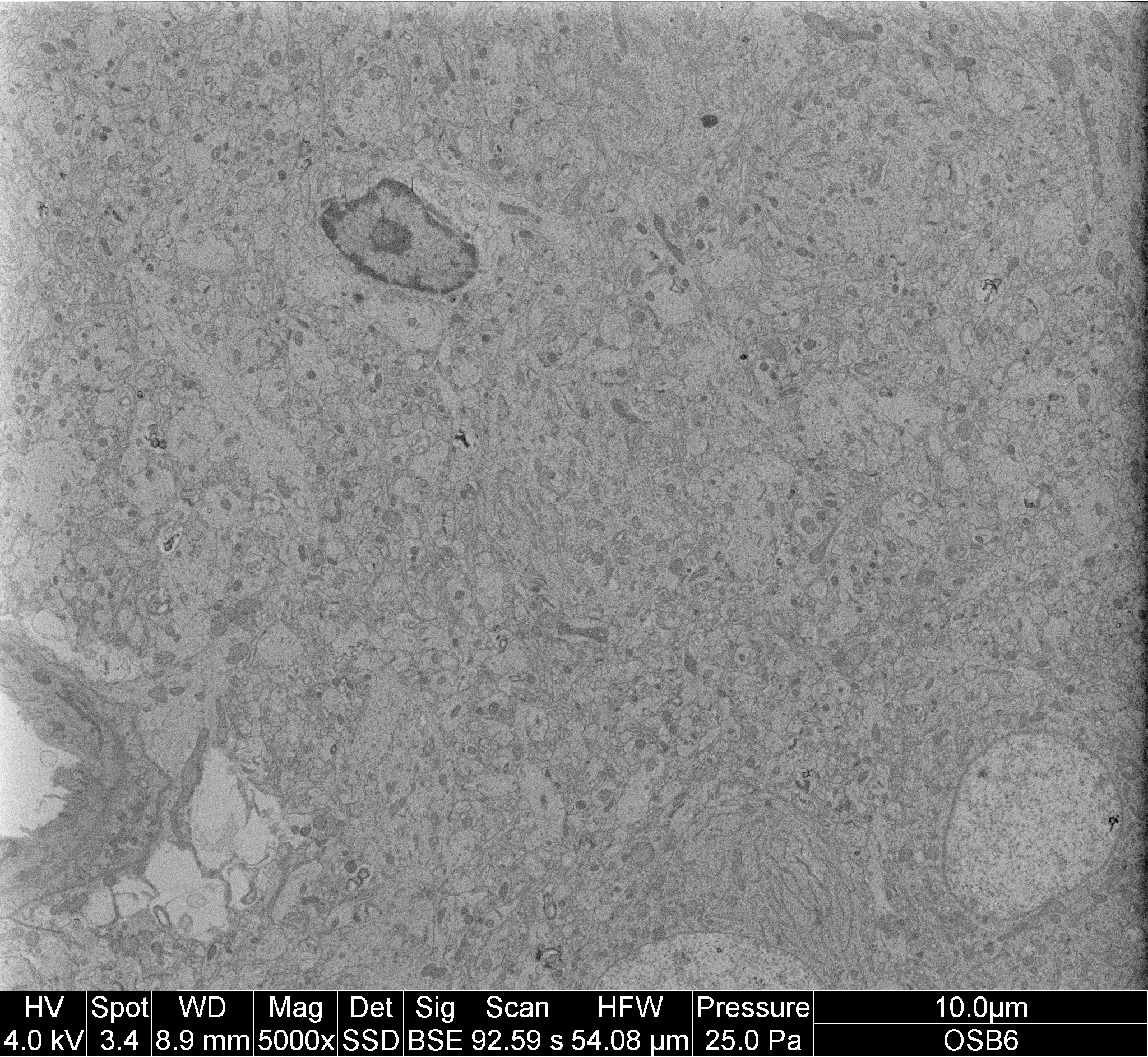

Supplement: Dataset S10 — (253.8 MB ZIP). [file pbio.0020329.sd010.zip › 040604_OS5_st1_947.tif]

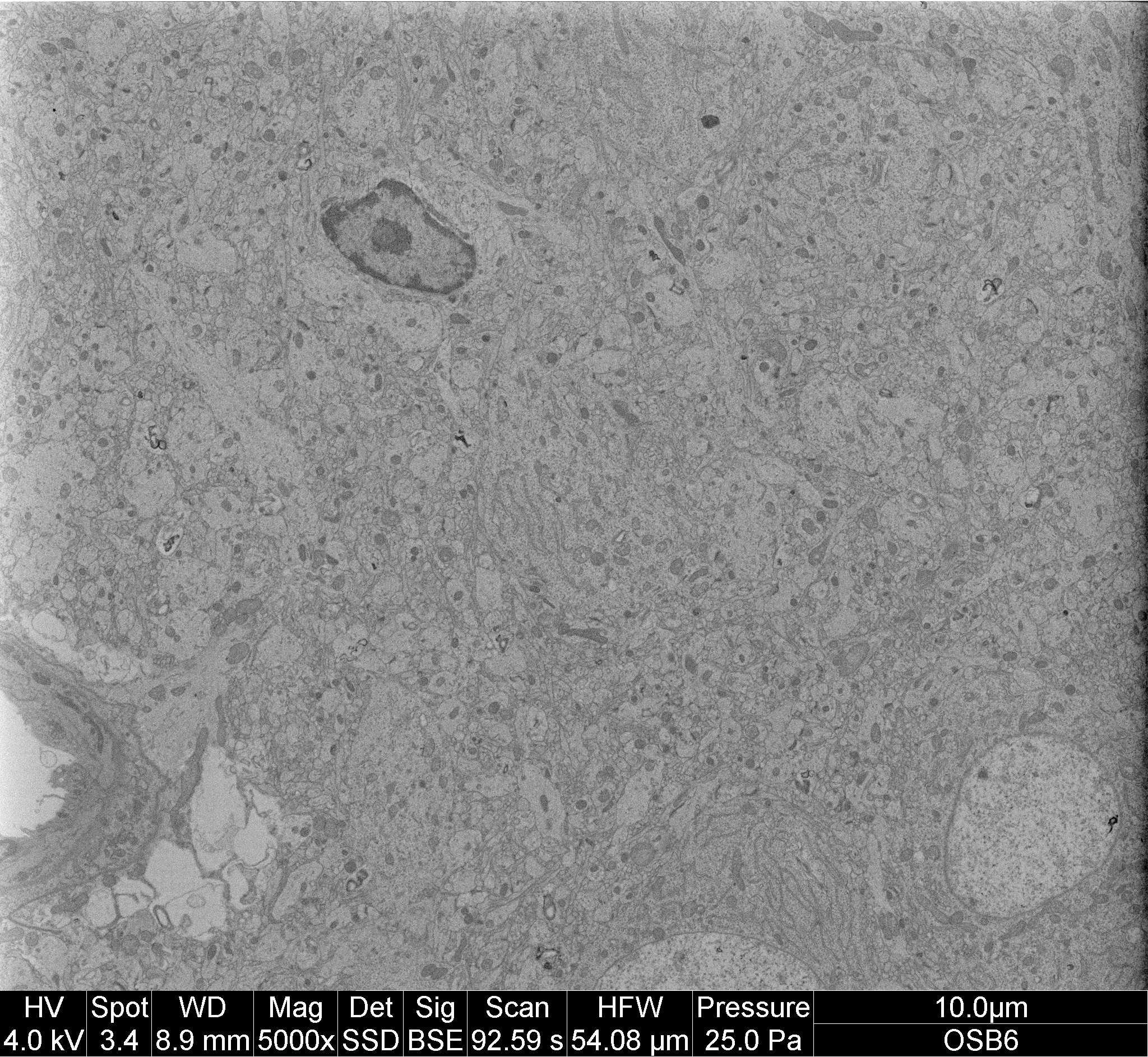

Supplement: Dataset S10 — (253.8 MB ZIP). [file pbio.0020329.sd010.zip › 040604_OS5_st1_948.tif]

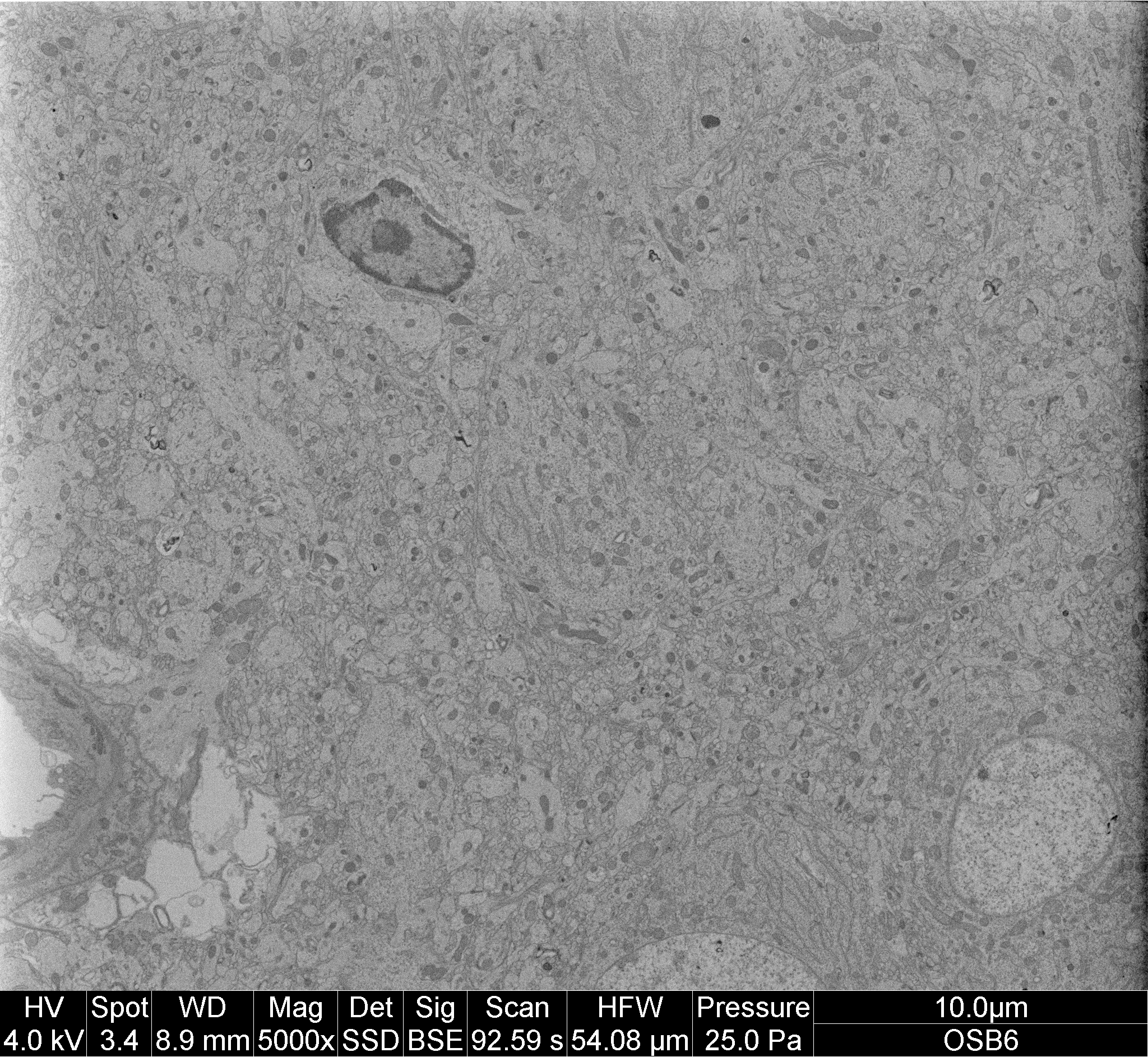

Supplement: Dataset S10 — (253.8 MB ZIP). [file pbio.0020329.sd010.zip › 040604_OS5_st1_949.tif]

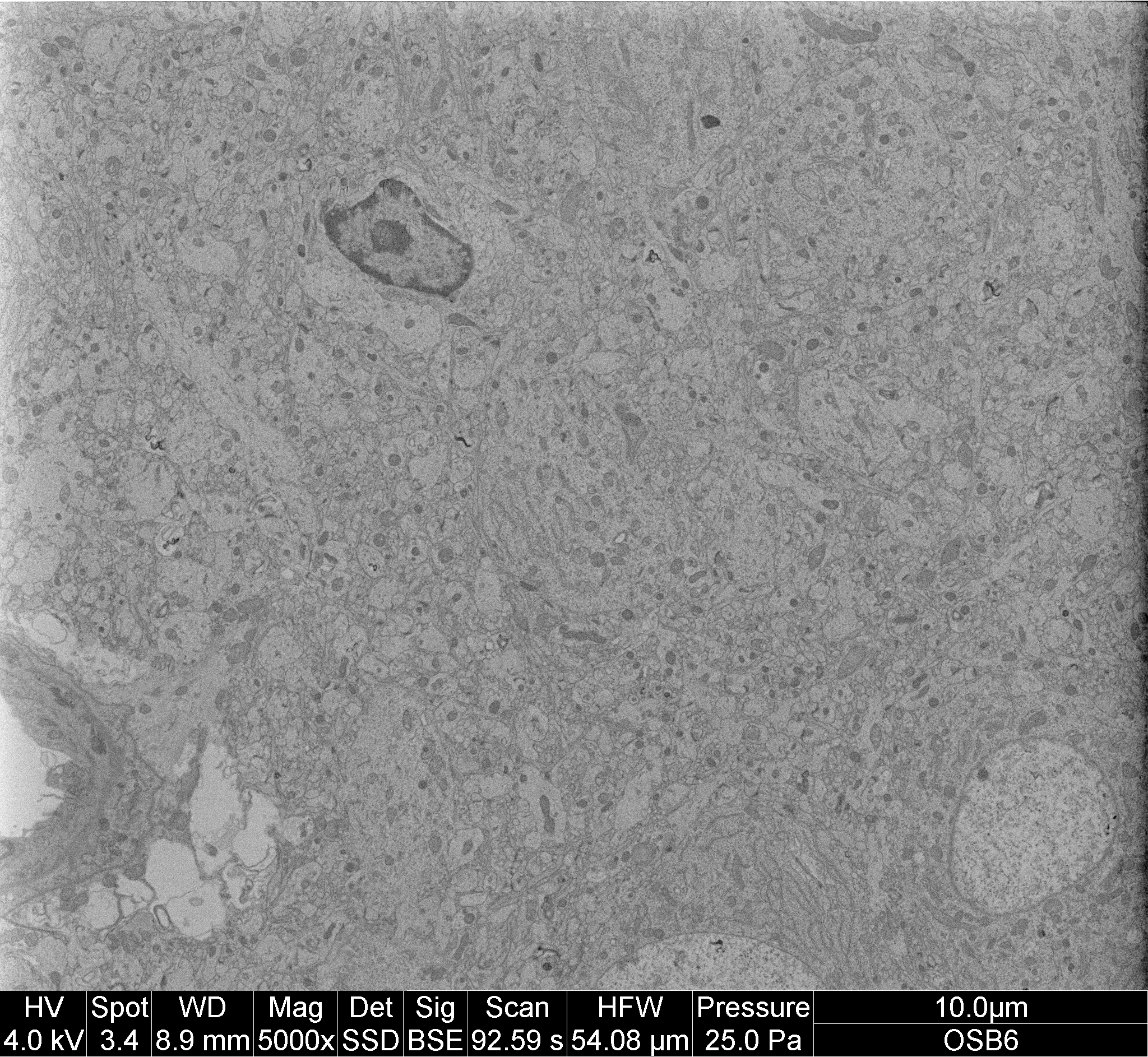

Supplement: Dataset S10 — (253.8 MB ZIP). [file pbio.0020329.sd010.zip › 040604_OS5_st1_950.tif]

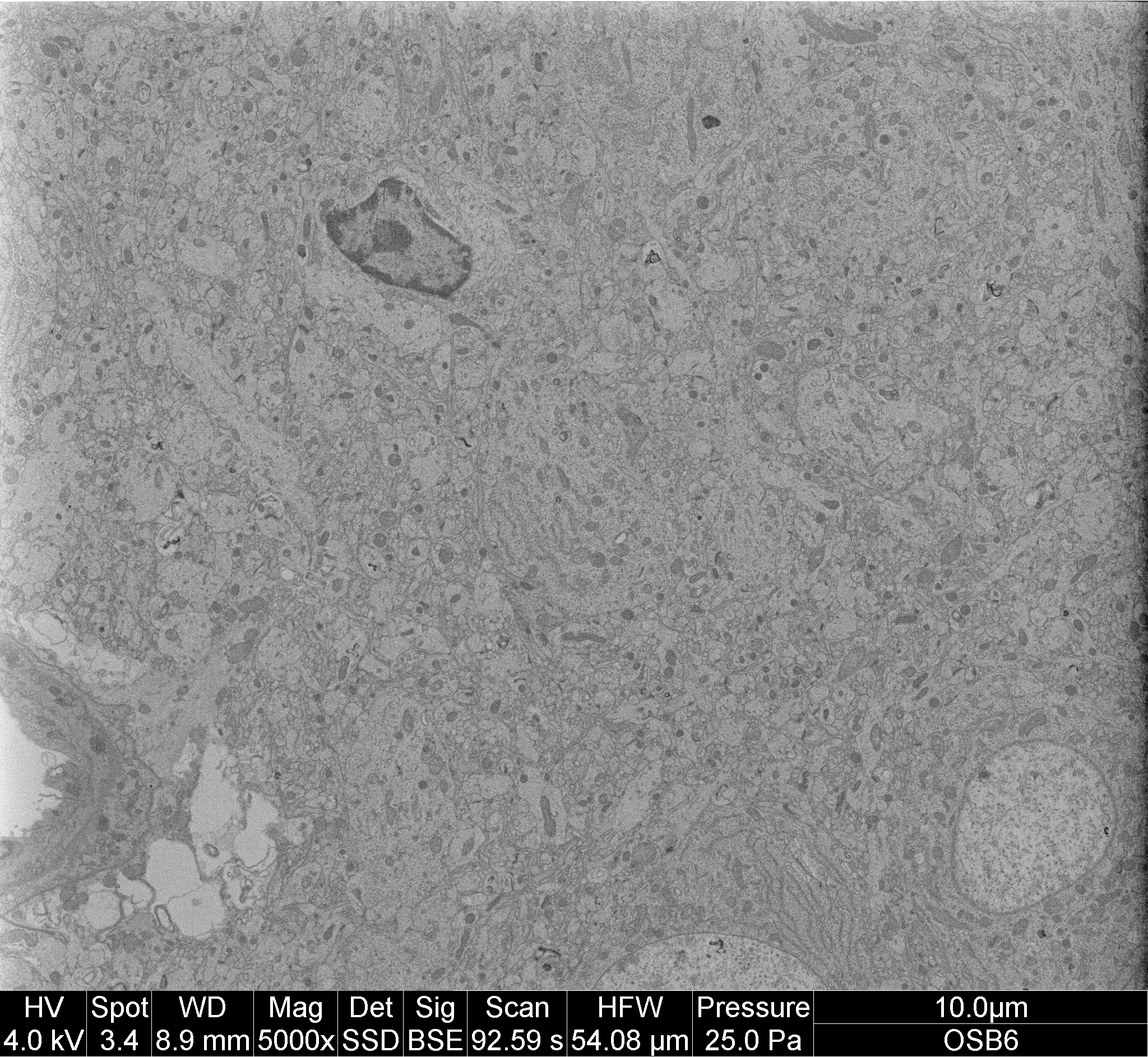

Supplement: Dataset S10 — (253.8 MB ZIP). [file pbio.0020329.sd010.zip › 040604_OS5_st1_951.tif]

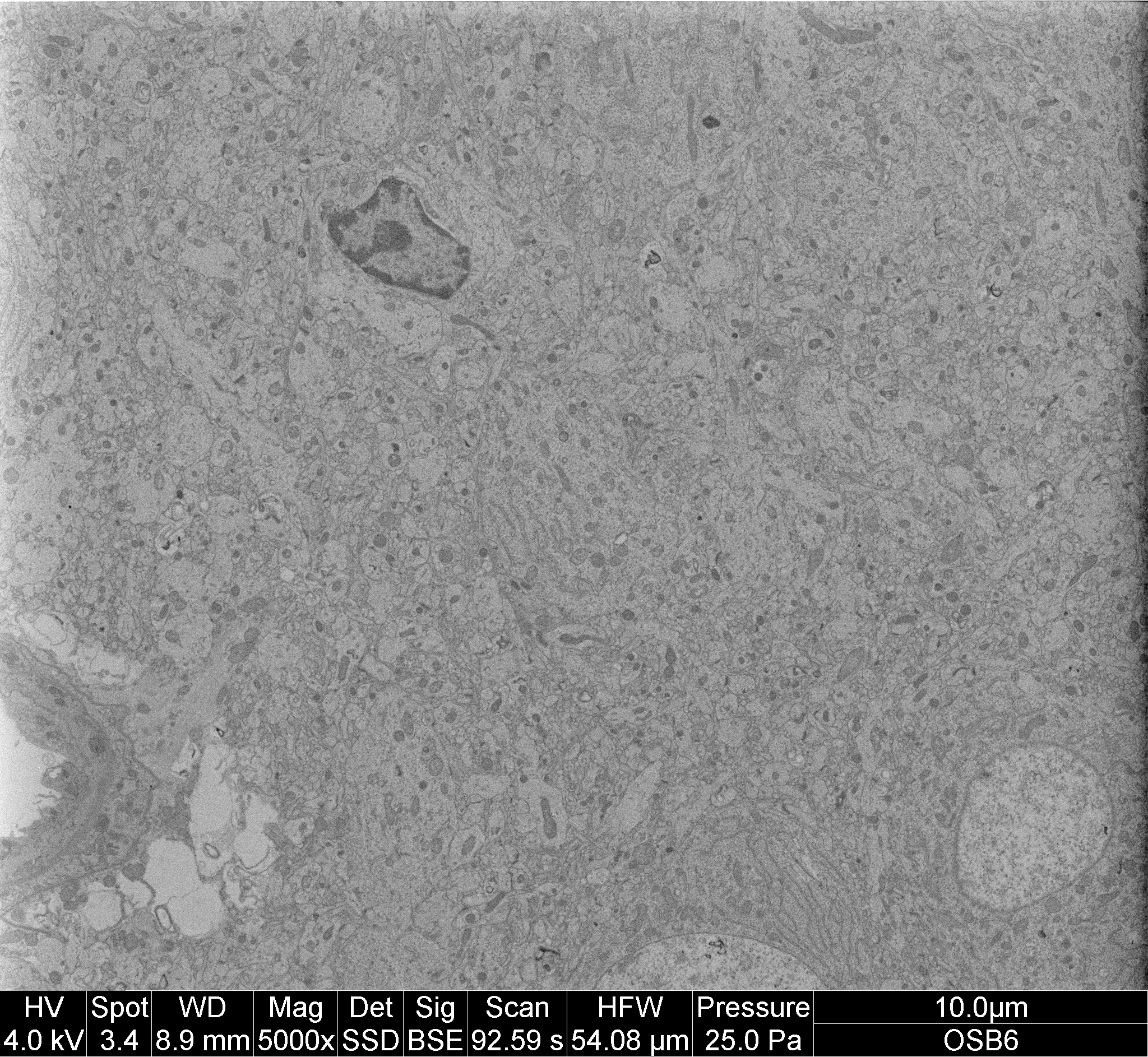

Supplement: Dataset S10 — (253.8 MB ZIP). [file pbio.0020329.sd010.zip › 040604_OS5_st1_952.tif]

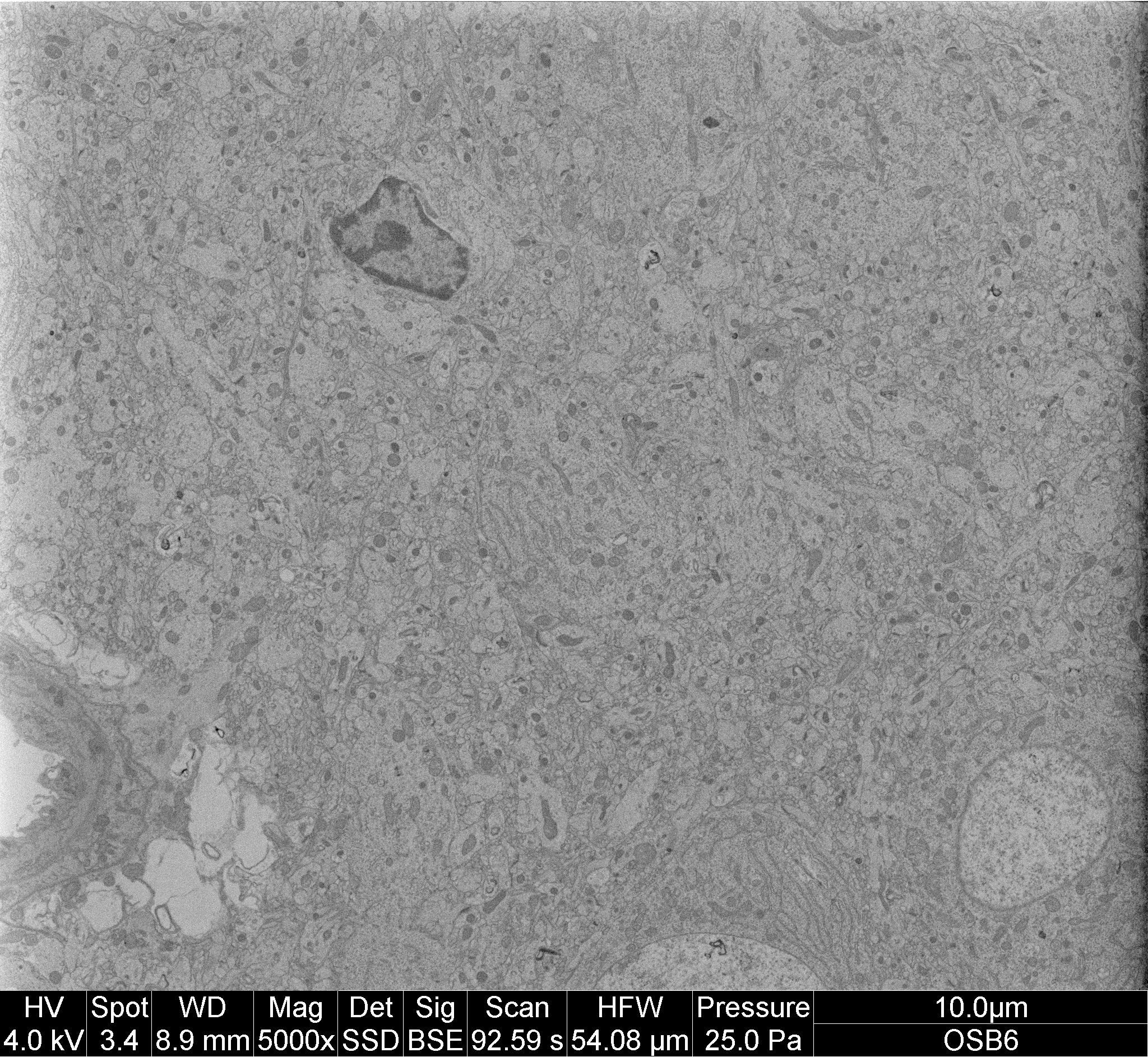

Supplement: Dataset S10 — (253.8 MB ZIP). [file pbio.0020329.sd010.zip › 040604_OS5_st1_953.tif]

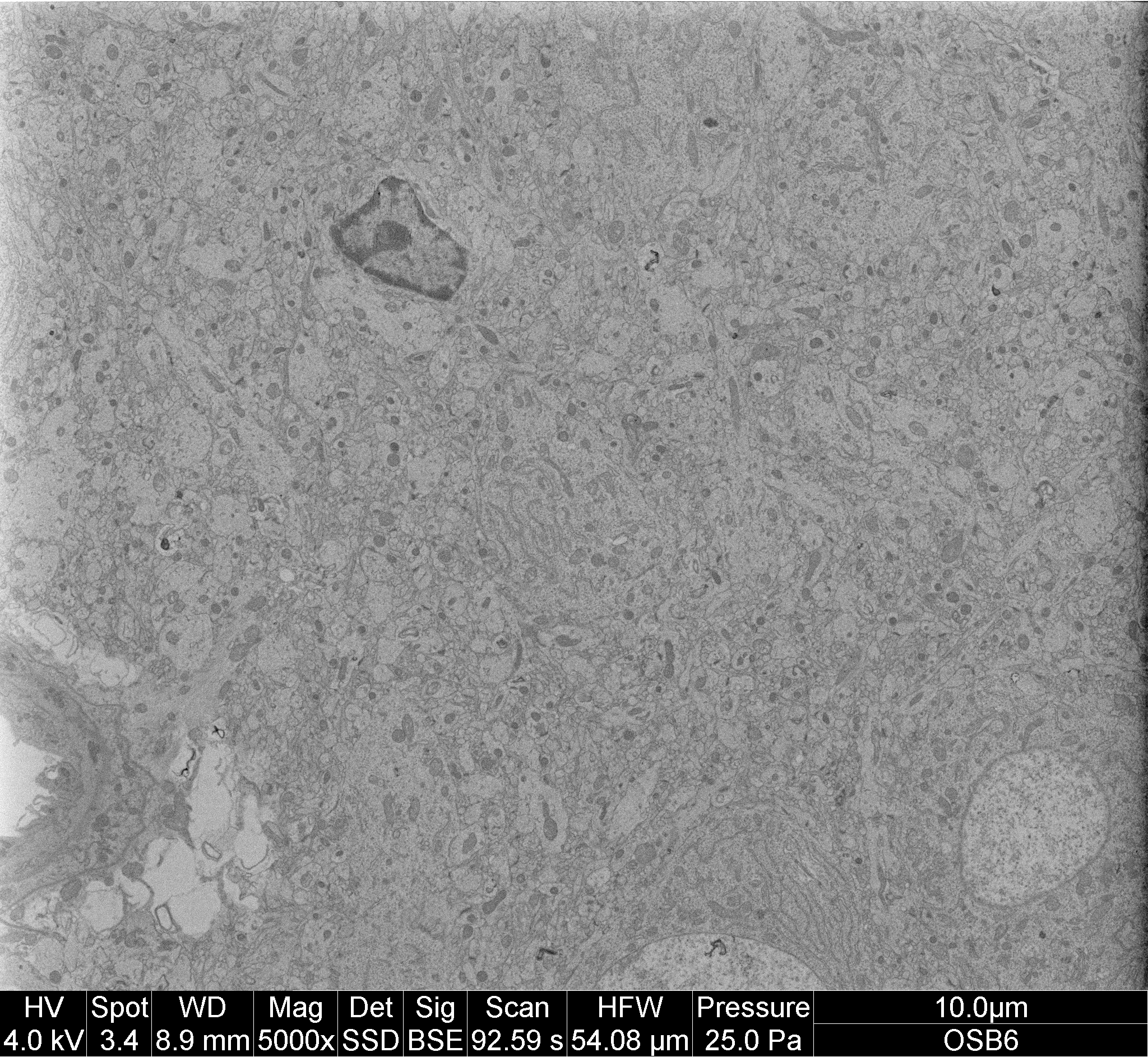

Supplement: Dataset S10 — (253.8 MB ZIP). [file pbio.0020329.sd010.zip › 040604_OS5_st1_954.tif]

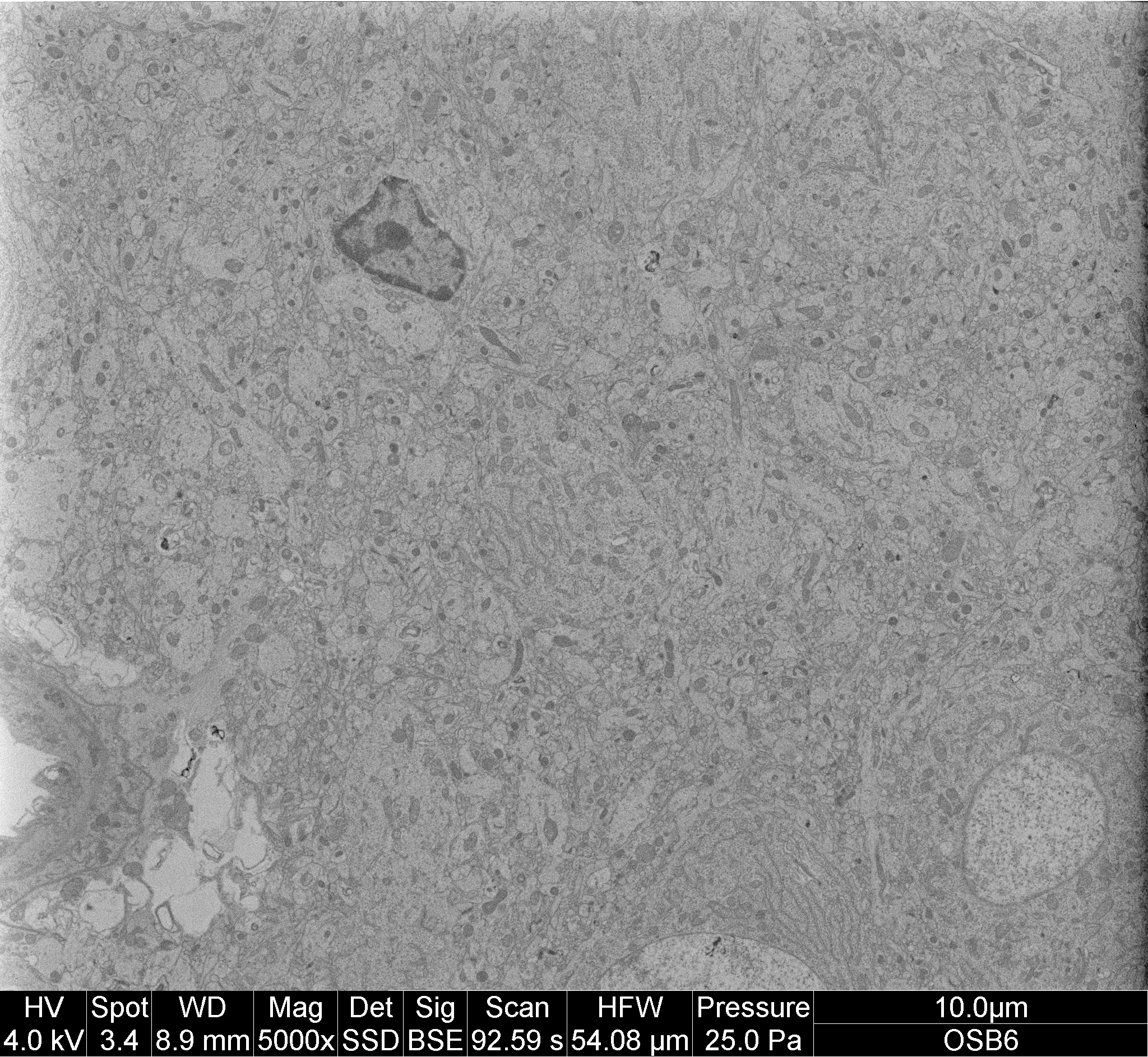

Supplement: Dataset S10 — (253.8 MB ZIP). [file pbio.0020329.sd010.zip › 040604_OS5_st1_955.tif]

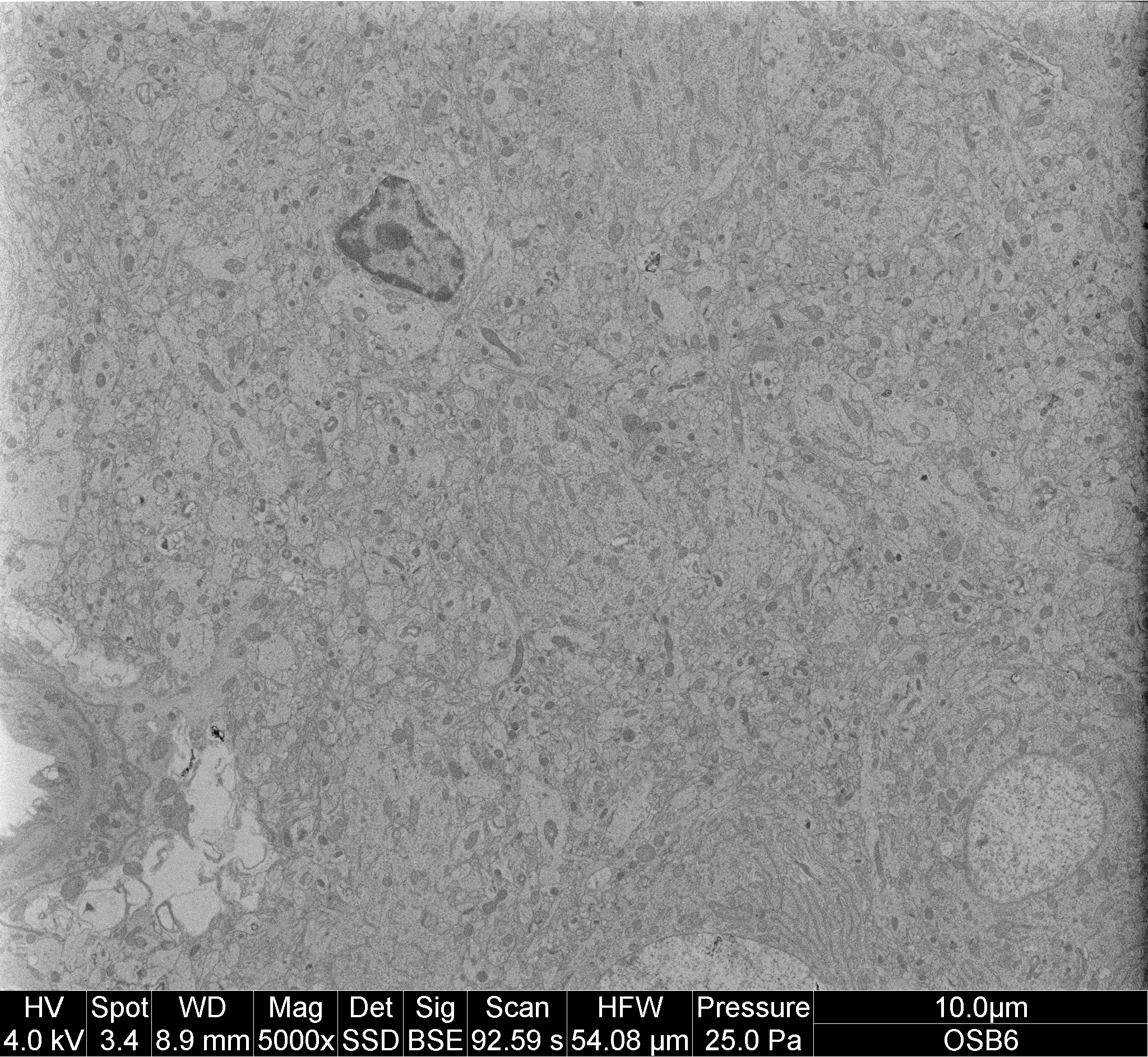

Supplement: Dataset S10 — (253.8 MB ZIP). [file pbio.0020329.sd010.zip › 040604_OS5_st1_956.tif]

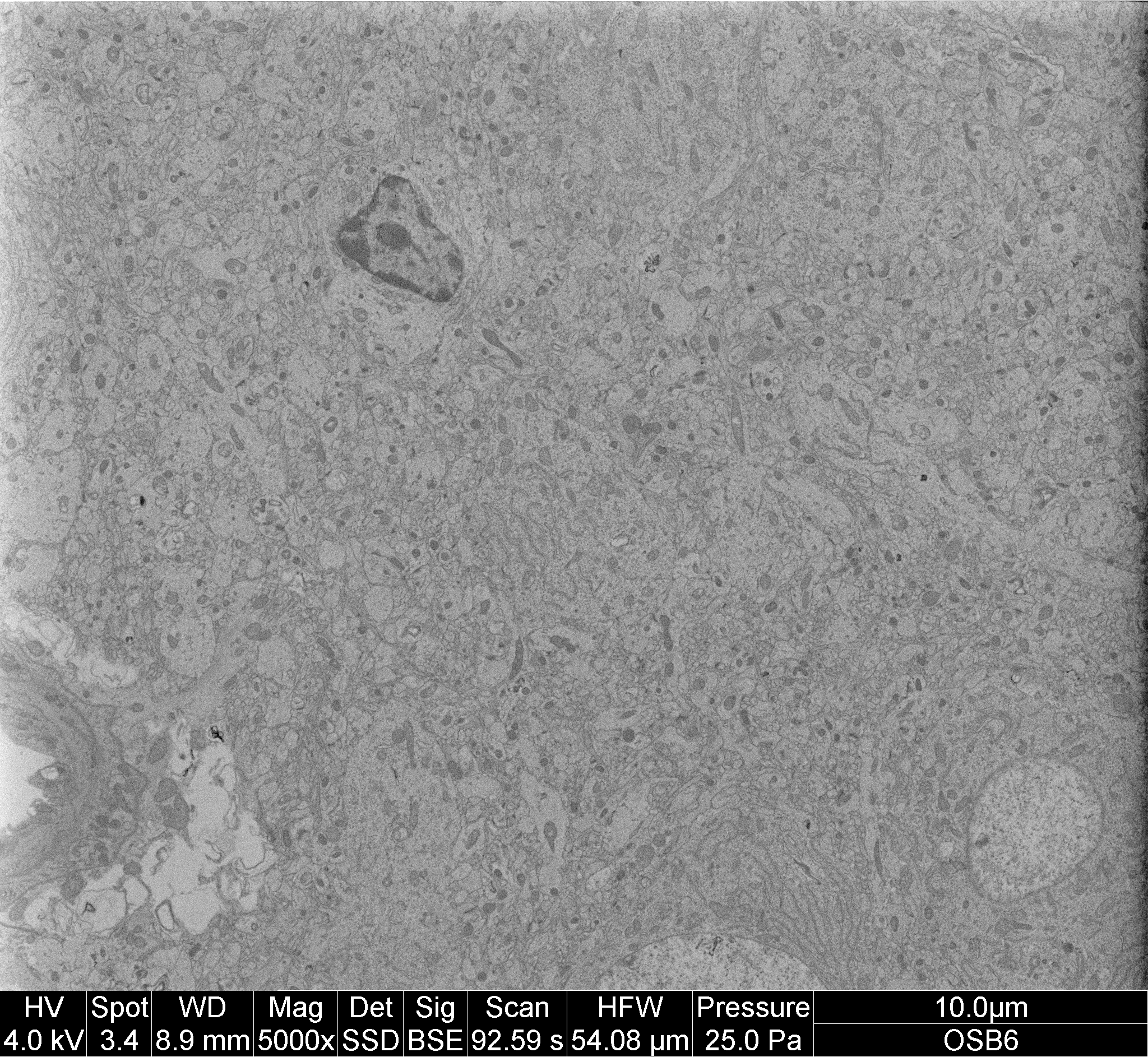

Supplement: Dataset S10 — (253.8 MB ZIP). [file pbio.0020329.sd010.zip › 040604_OS5_st1_957.tif]

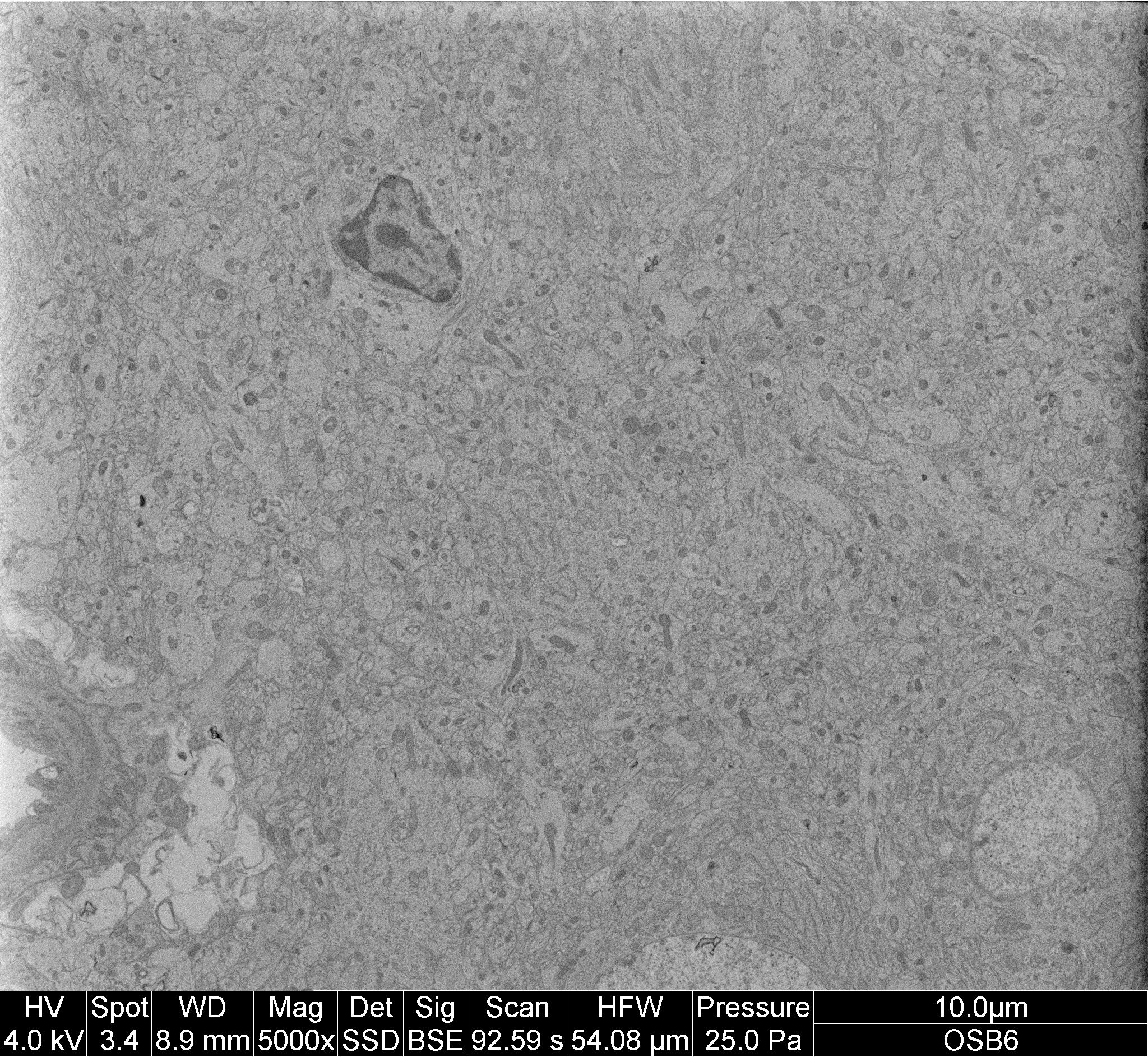

Supplement: Dataset S10 — (253.8 MB ZIP). [file pbio.0020329.sd010.zip › 040604_OS5_st1_958.tif]

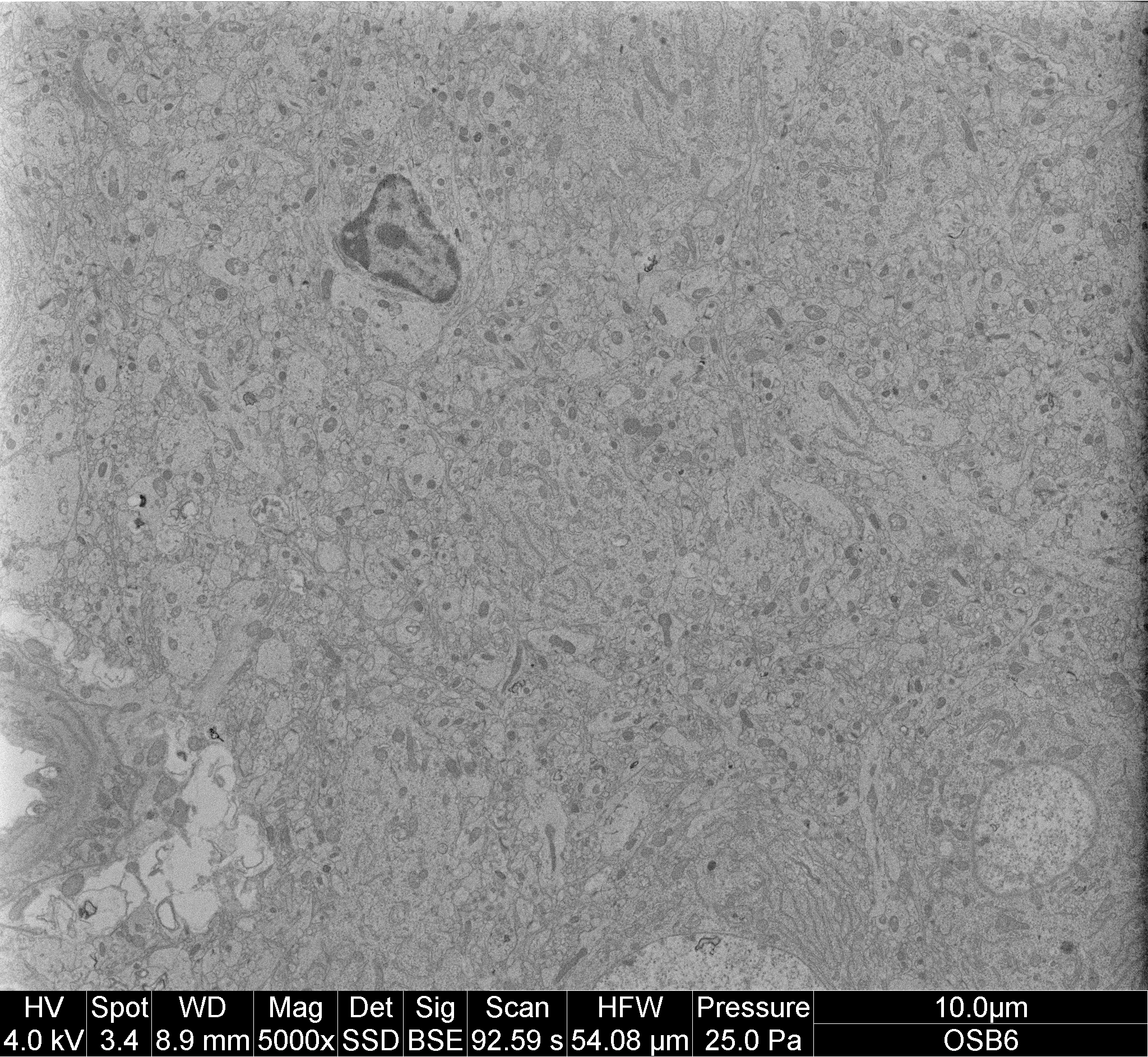

Supplement: Dataset S10 — (253.8 MB ZIP). [file pbio.0020329.sd010.zip › 040604_OS5_st1_959.tif]

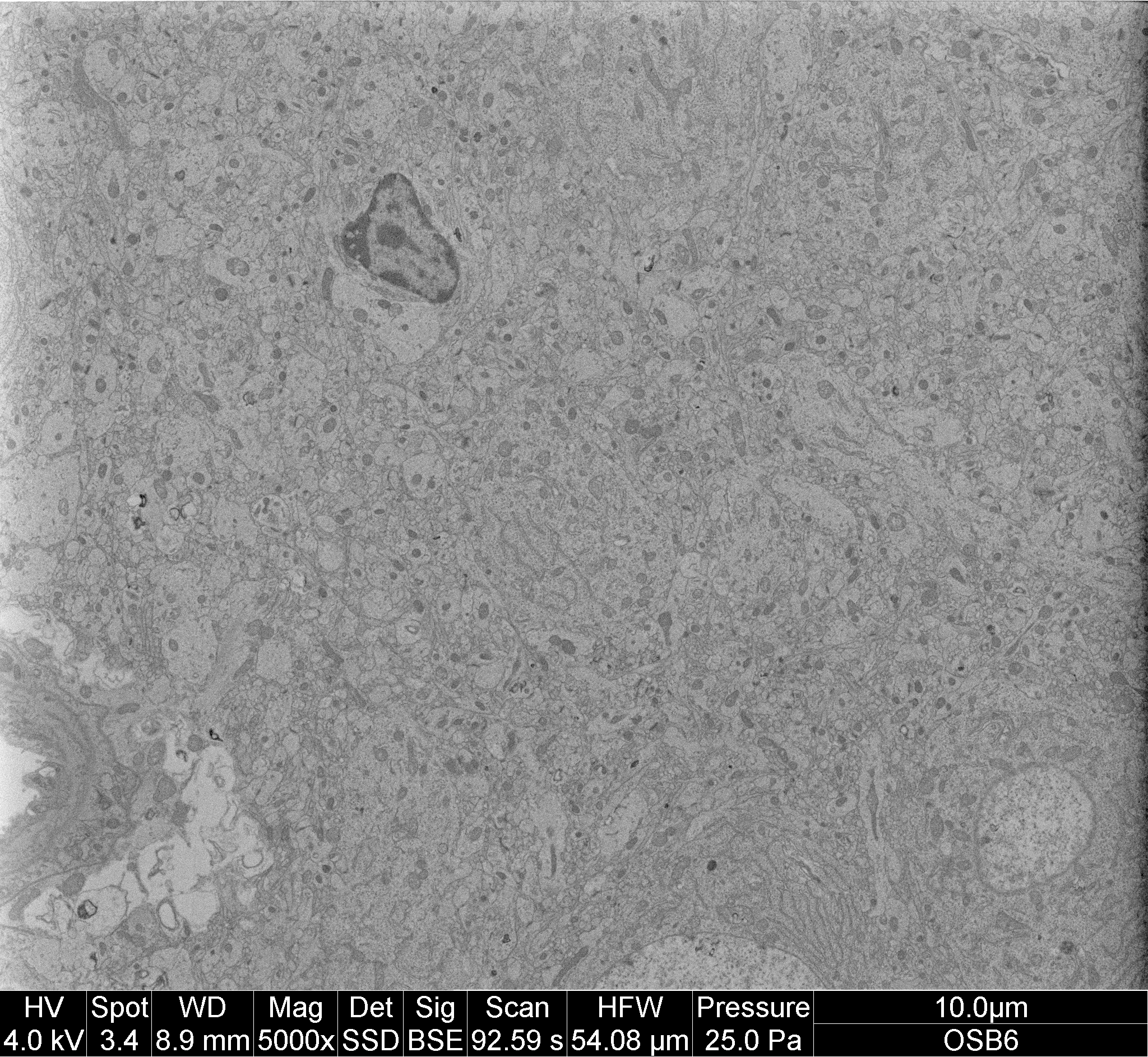

Supplement: Dataset S10 — (253.8 MB ZIP). [file pbio.0020329.sd010.zip › 040604_OS5_st1_960.tif]

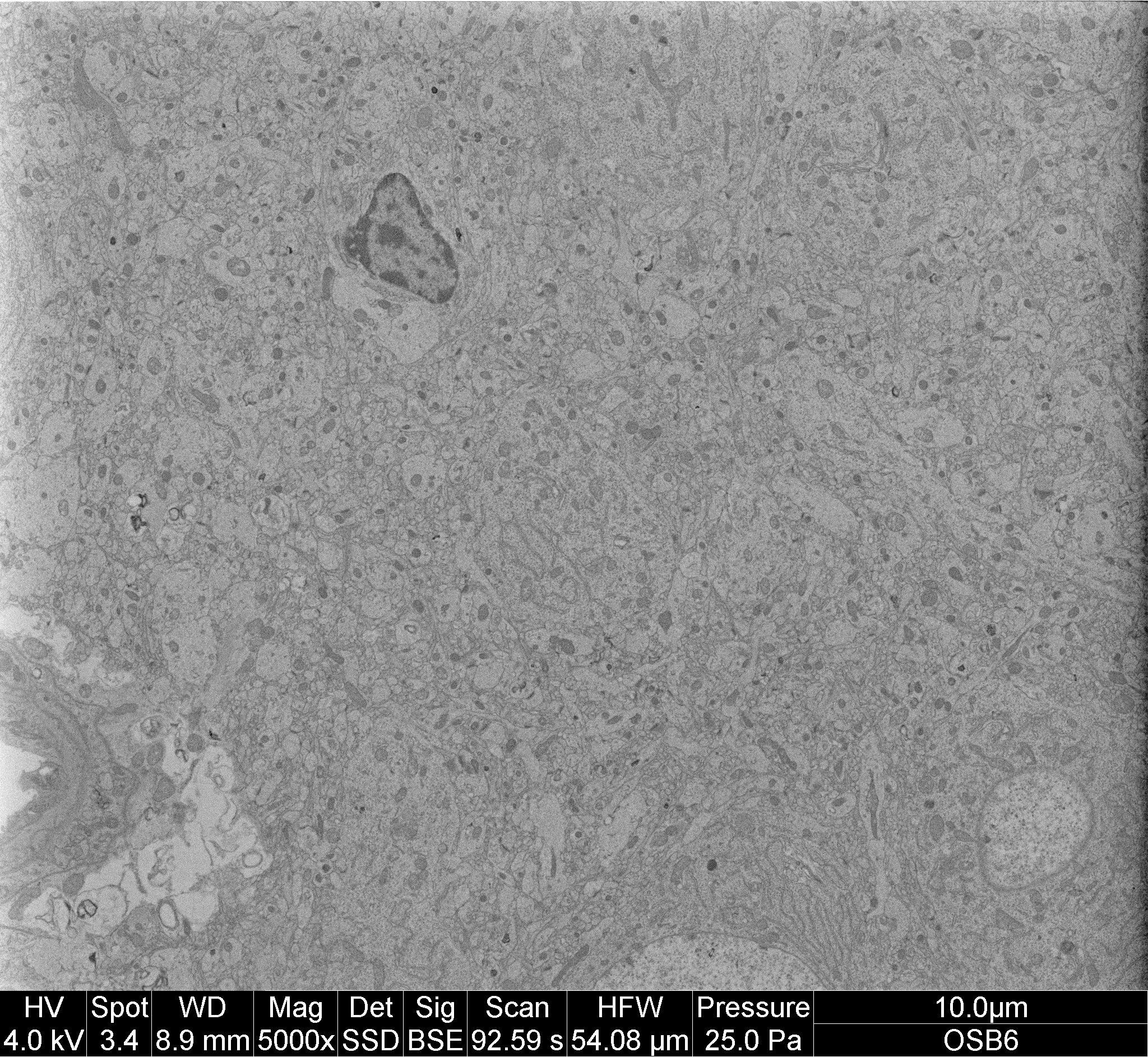

Supplement: Dataset S10 — (253.8 MB ZIP). [file pbio.0020329.sd010.zip › 040604_OS5_st1_961.tif]

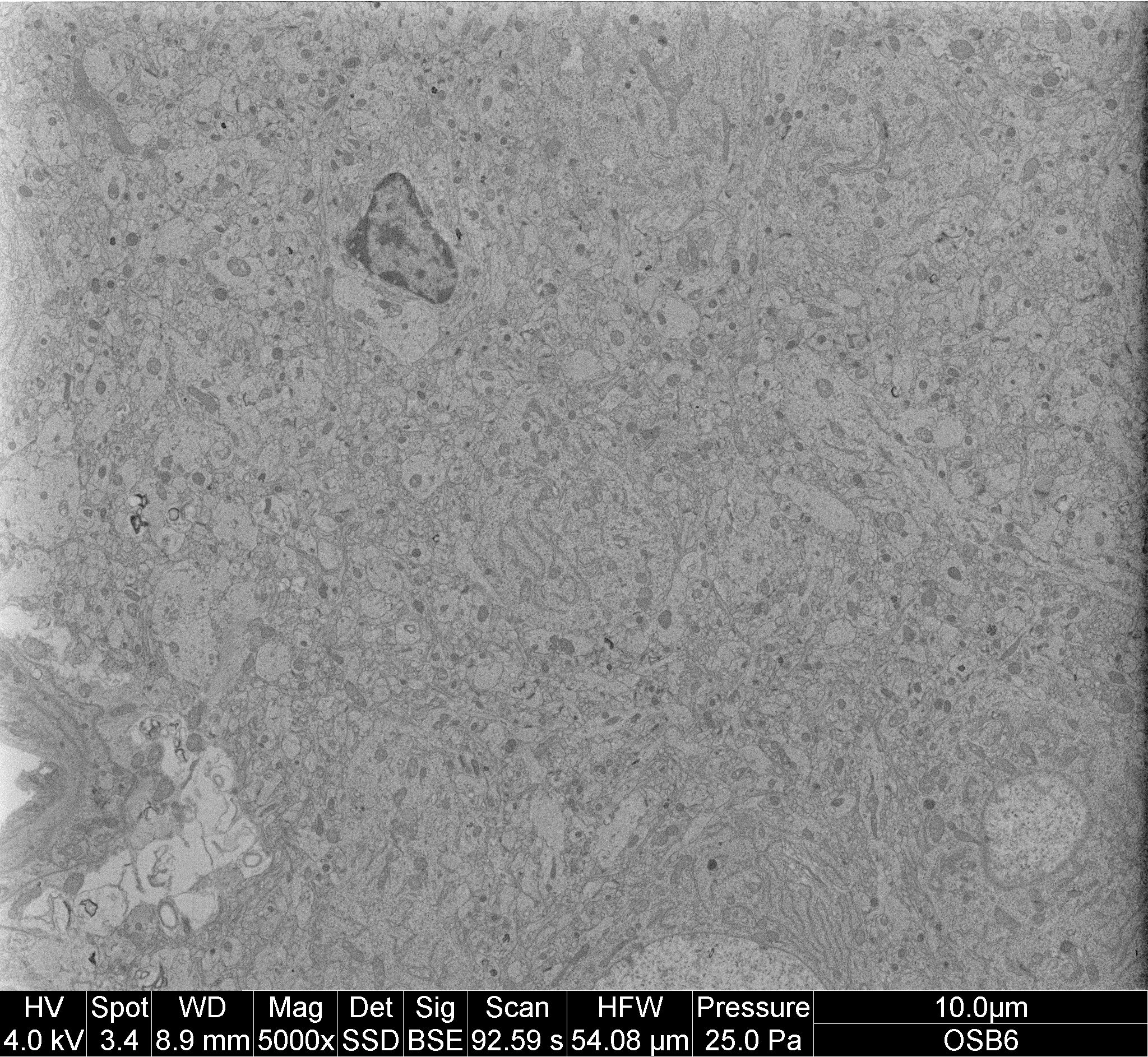

Supplement: Dataset S10 — (253.8 MB ZIP). [file pbio.0020329.sd010.zip › 040604_OS5_st1_962.tif]

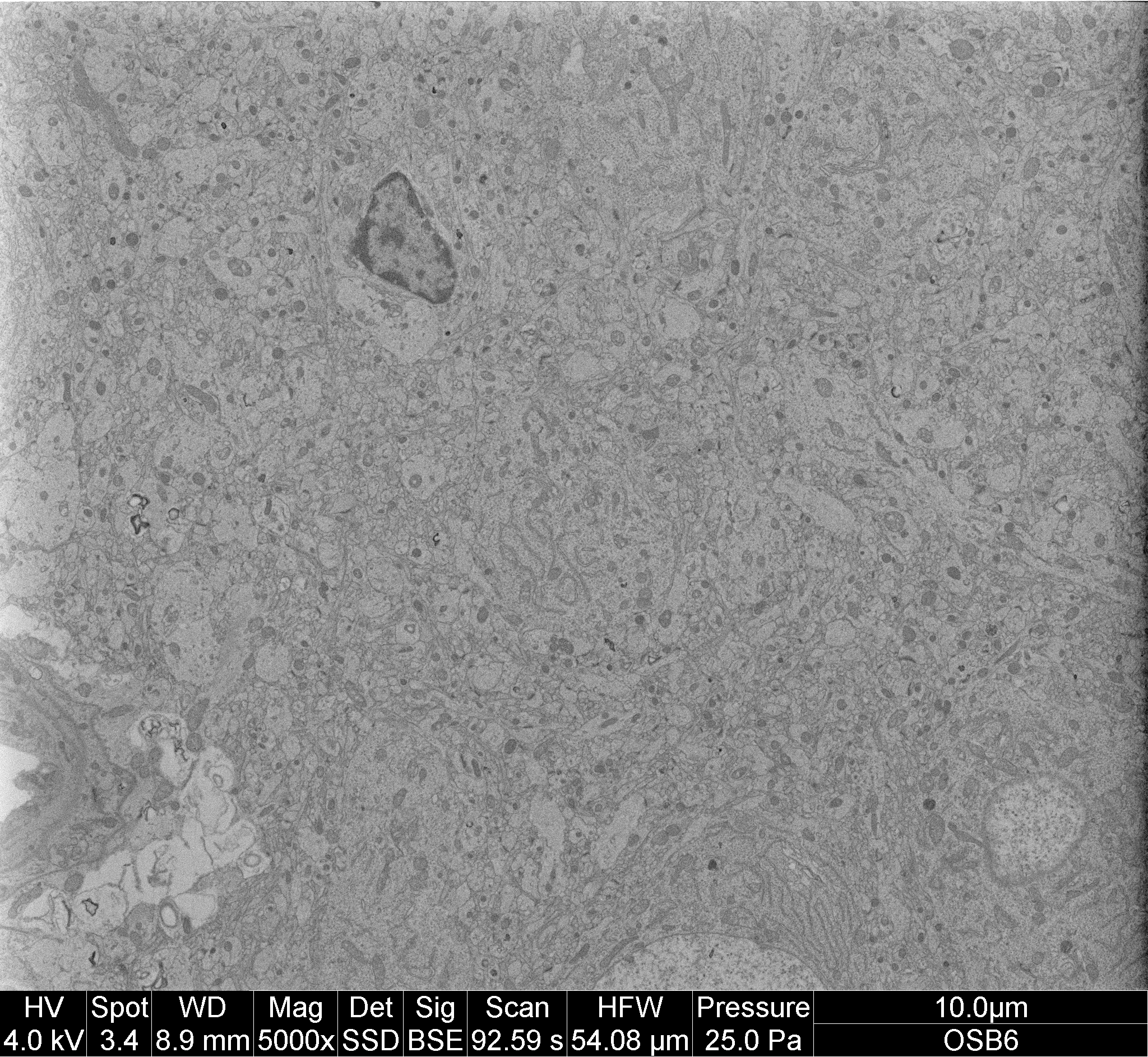

Supplement: Dataset S10 — (253.8 MB ZIP). [file pbio.0020329.sd010.zip › 040604_OS5_st1_963.tif]

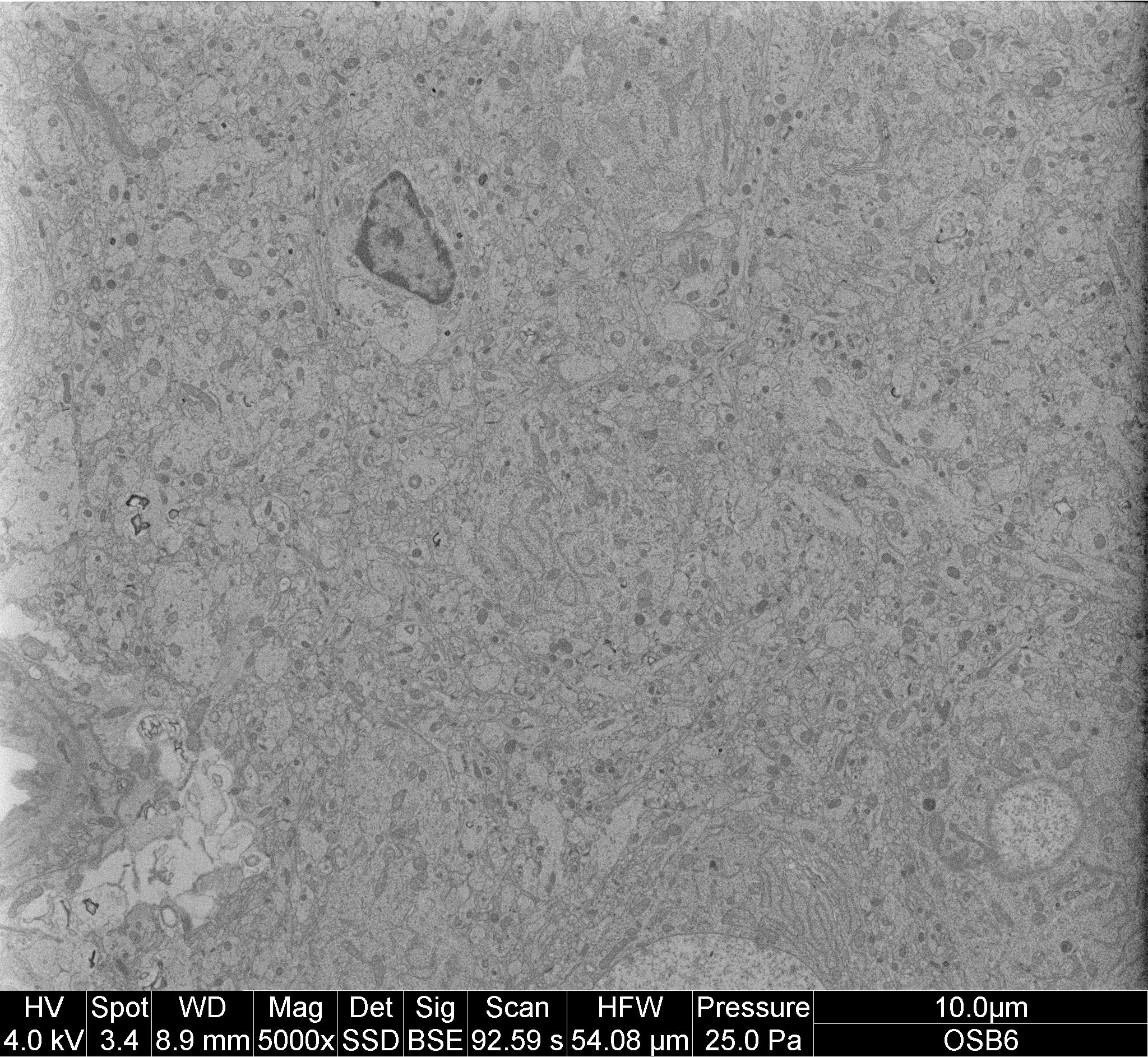

Supplement: Dataset S10 — (253.8 MB ZIP). [file pbio.0020329.sd010.zip › 040604_OS5_st1_964.tif]

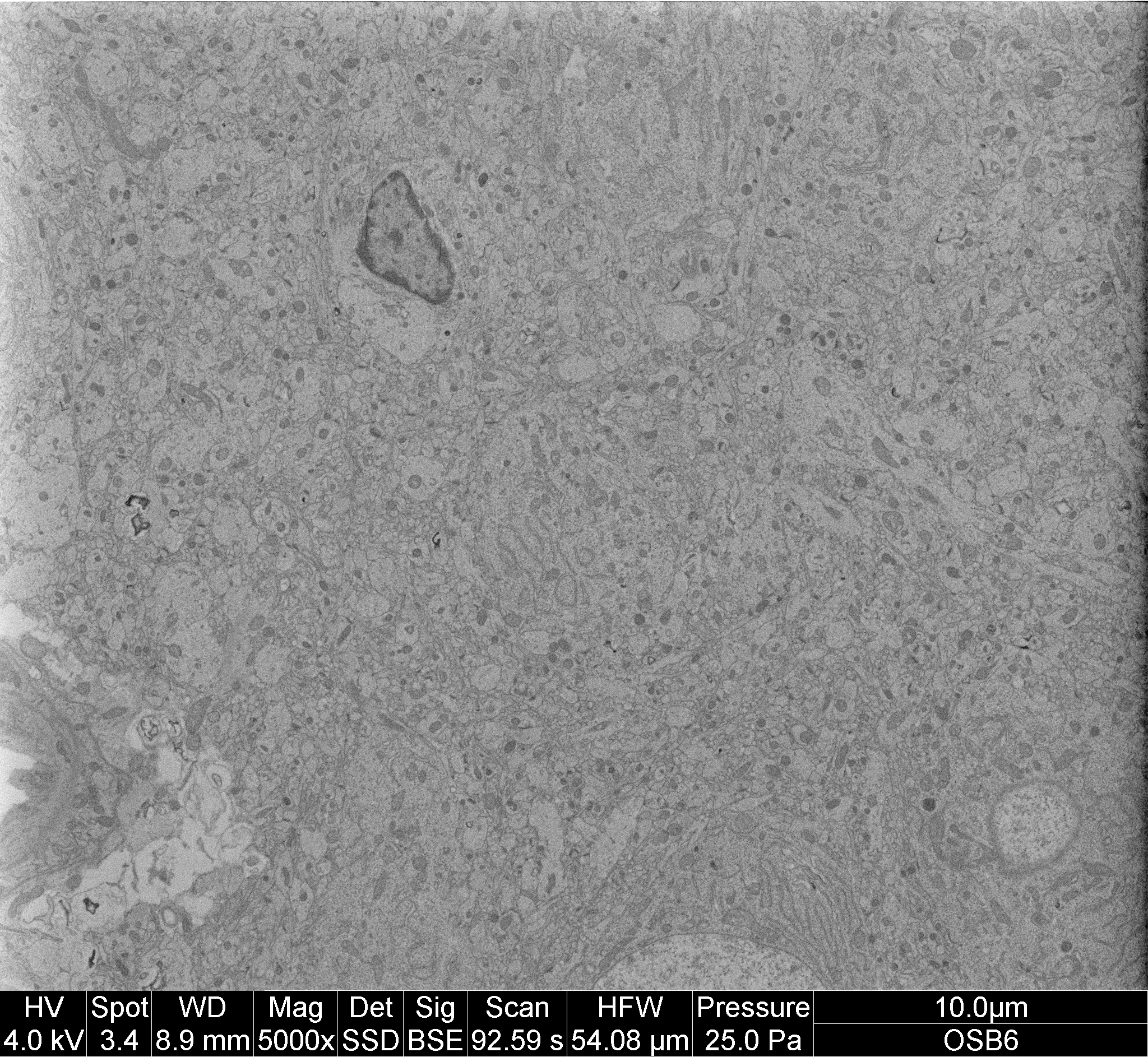

Supplement: Dataset S10 — (253.8 MB ZIP). [file pbio.0020329.sd010.zip › 040604_OS5_st1_965.tif]

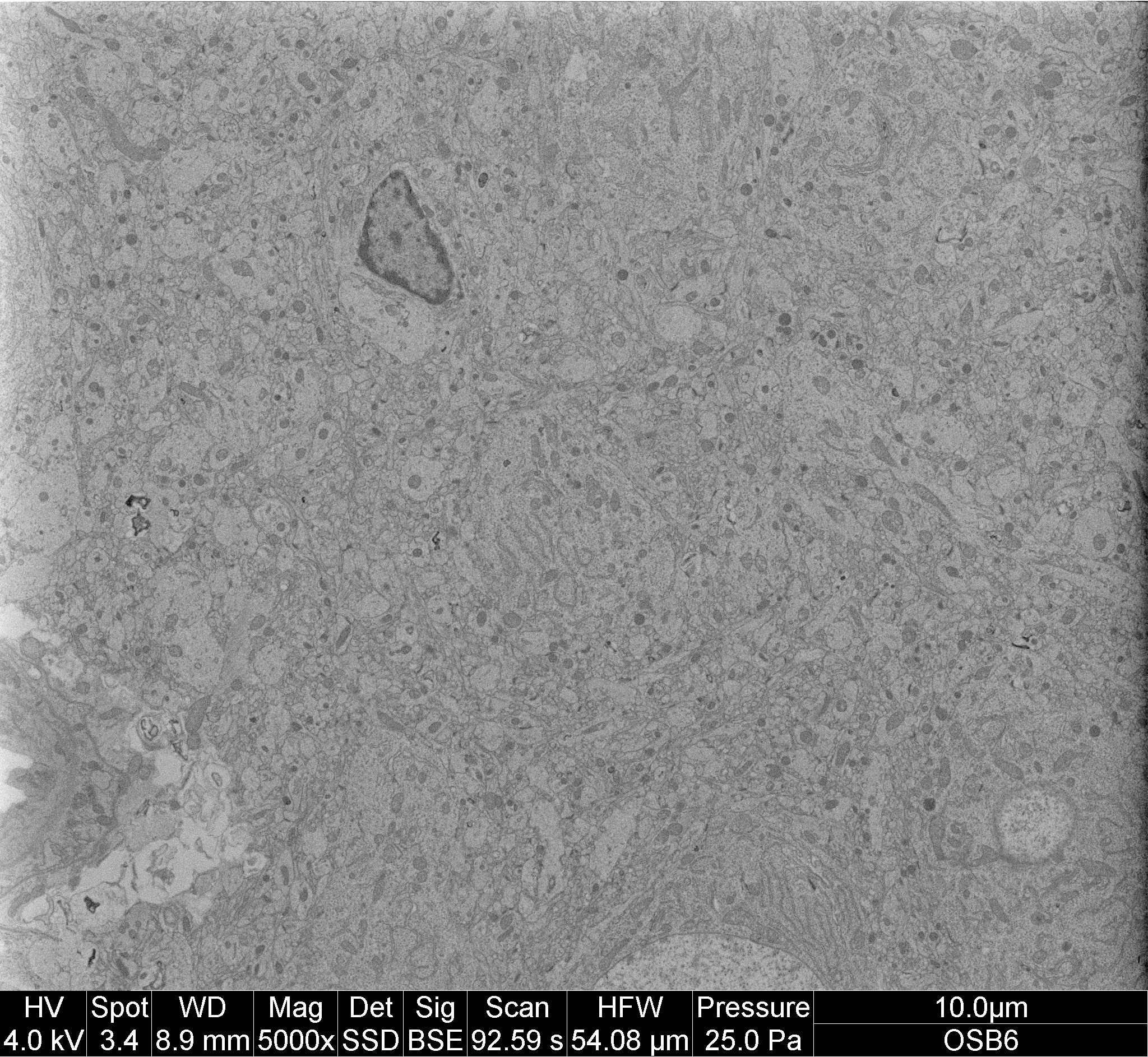

Supplement: Dataset S10 — (253.8 MB ZIP). [file pbio.0020329.sd010.zip › 040604_OS5_st1_966.tif]

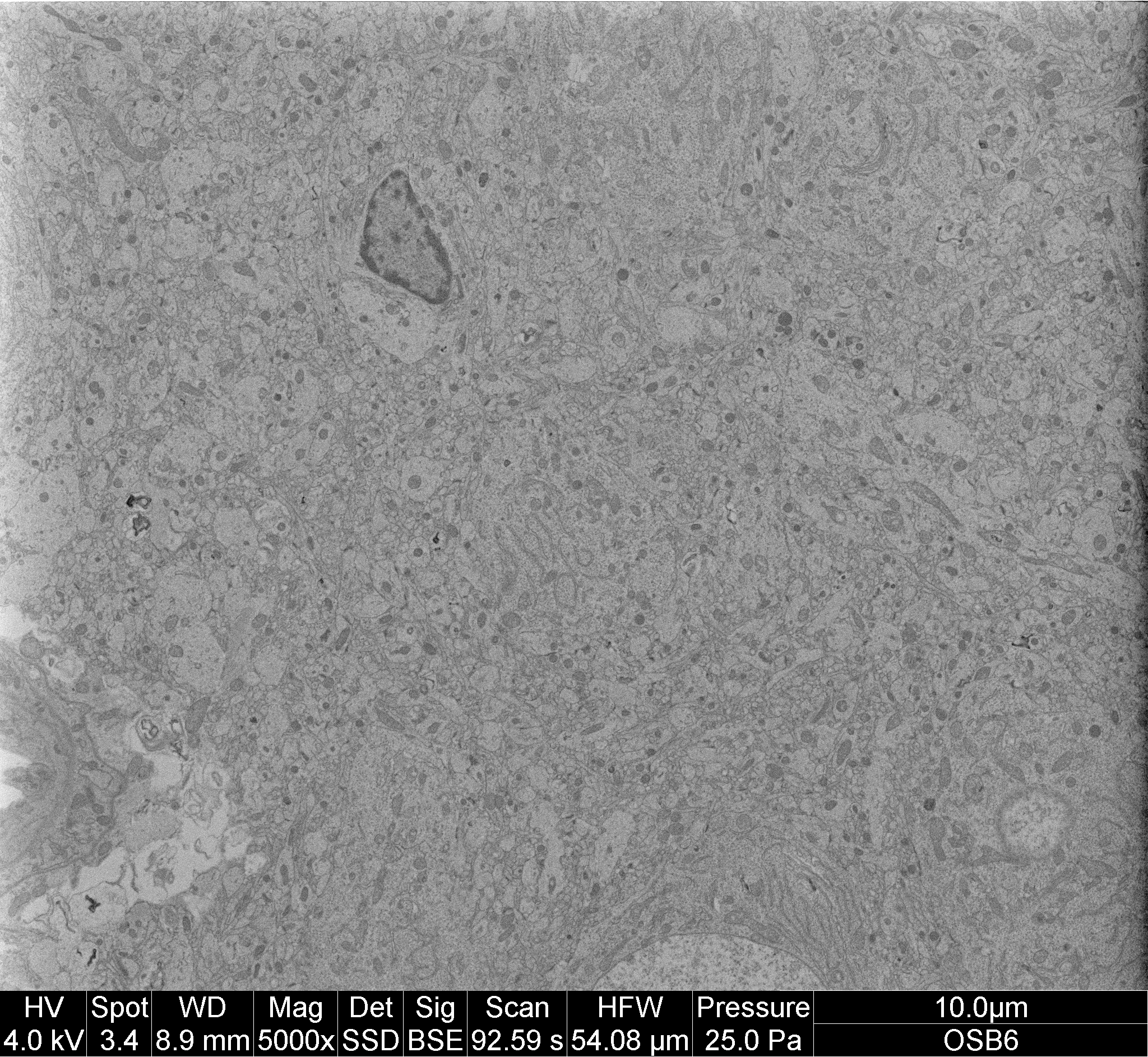

Supplement: Dataset S10 — (253.8 MB ZIP). [file pbio.0020329.sd010.zip › 040604_OS5_st1_967.tif]

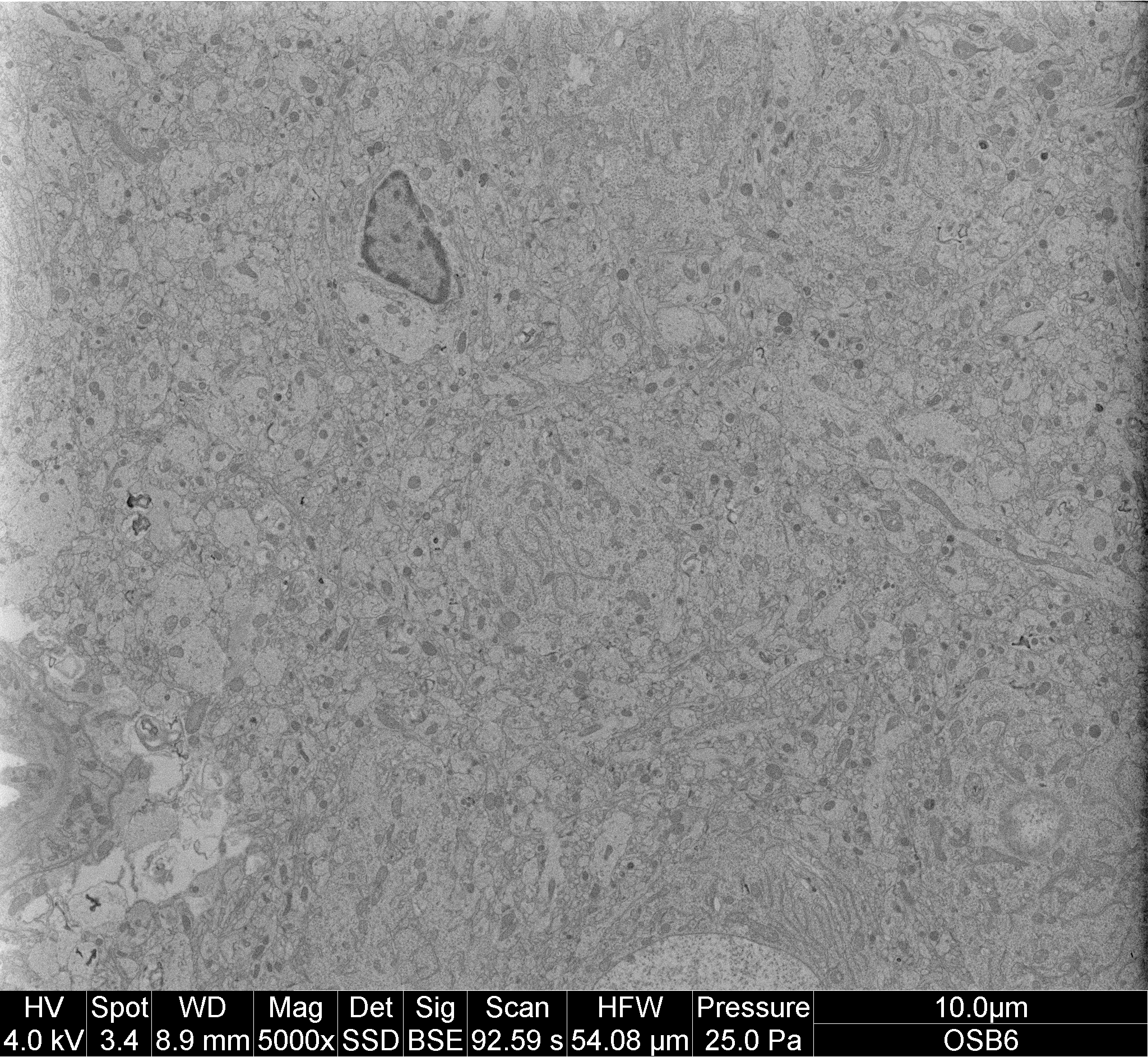

Supplement: Dataset S10 — (253.8 MB ZIP). [file pbio.0020329.sd010.zip › 040604_OS5_st1_968.tif]

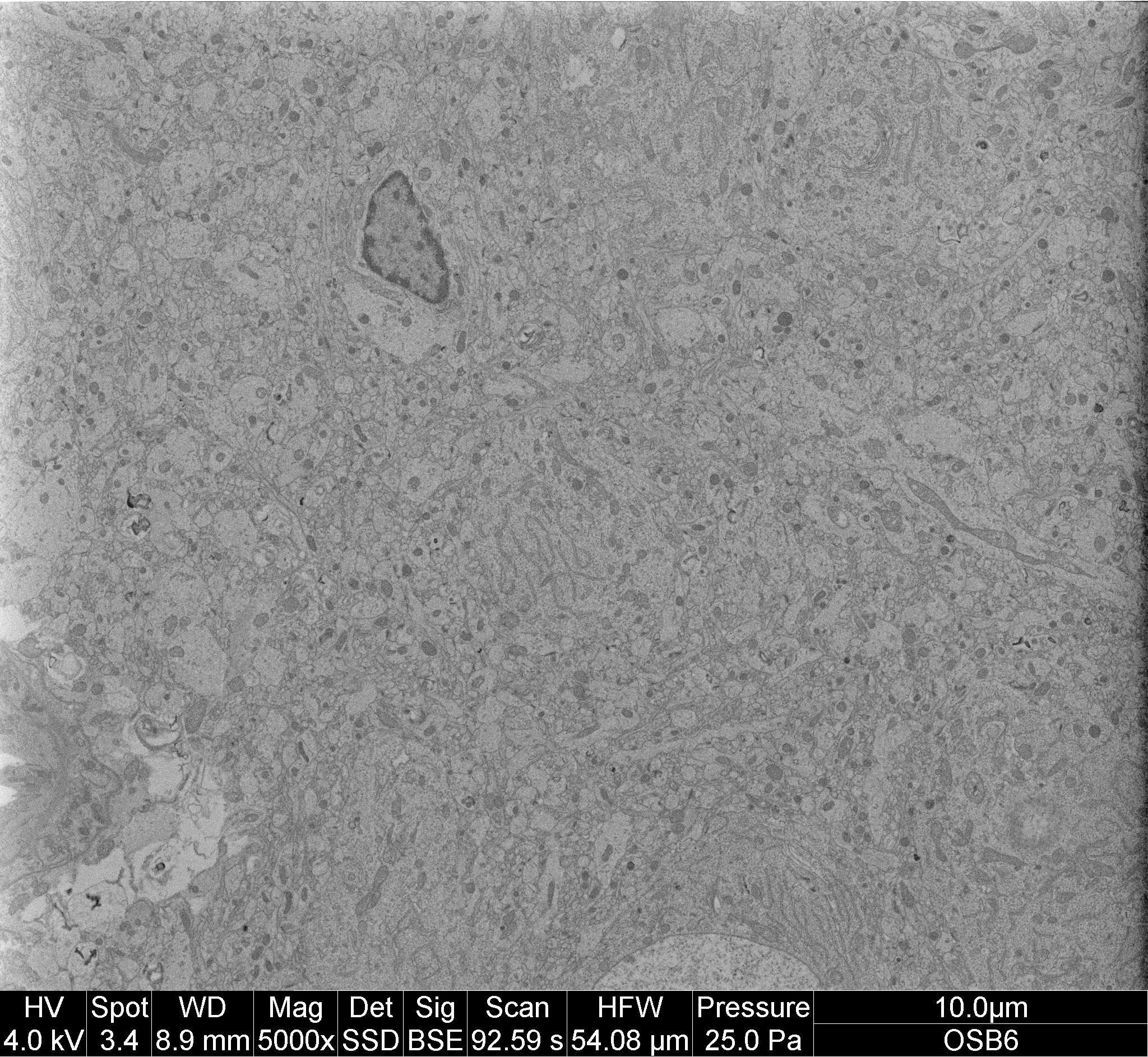

Supplement: Dataset S10 — (253.8 MB ZIP). [file pbio.0020329.sd010.zip › 040604_OS5_st1_969.tif]

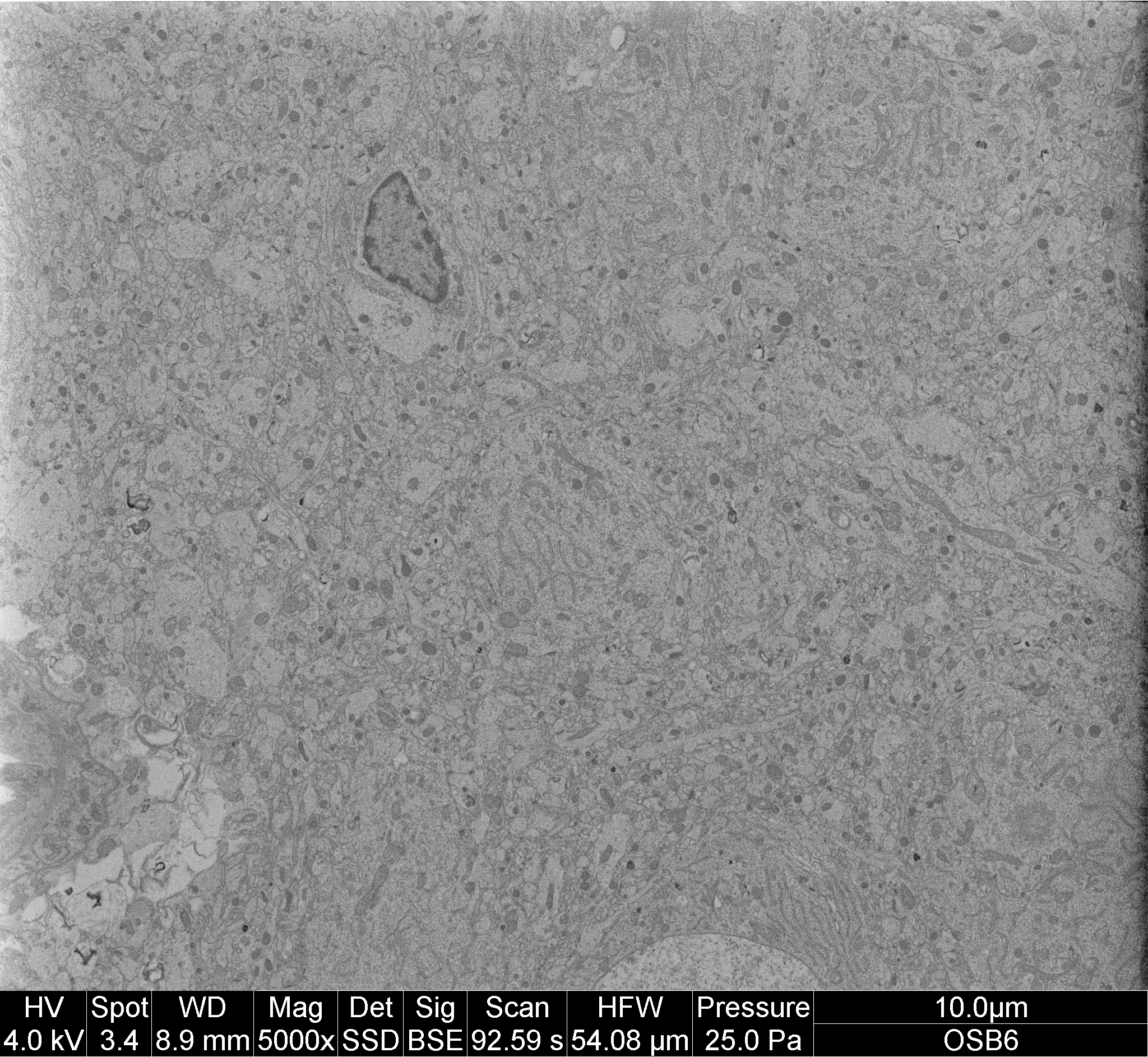

Supplement: Dataset S10 — (253.8 MB ZIP). [file pbio.0020329.sd010.zip › 040604_OS5_st1_970.tif]

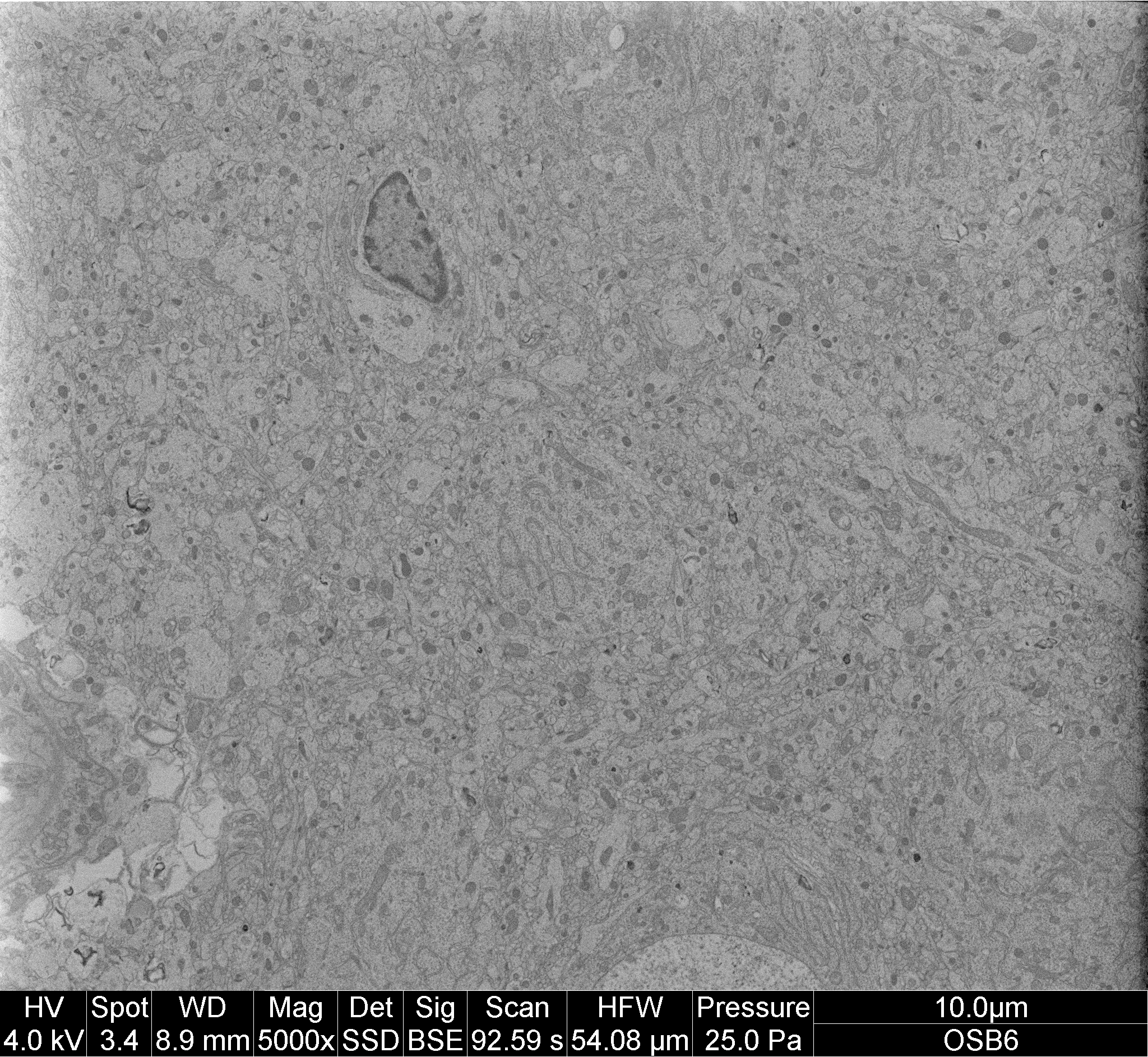

Supplement: Dataset S10 — (253.8 MB ZIP). [file pbio.0020329.sd010.zip › 040604_OS5_st1_971.tif]

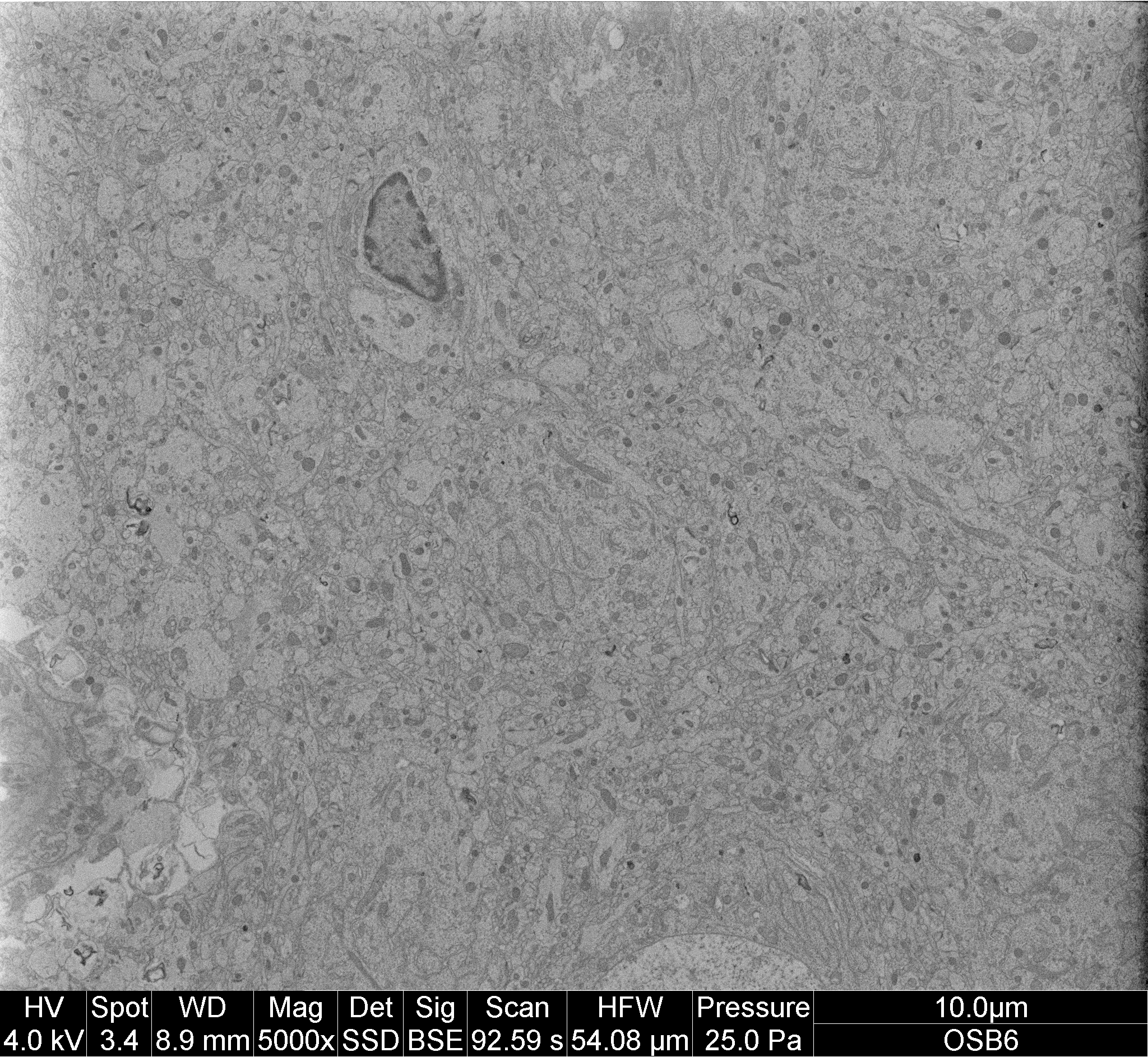

Supplement: Dataset S10 — (253.8 MB ZIP). [file pbio.0020329.sd010.zip › 040604_OS5_st1_972.tif]

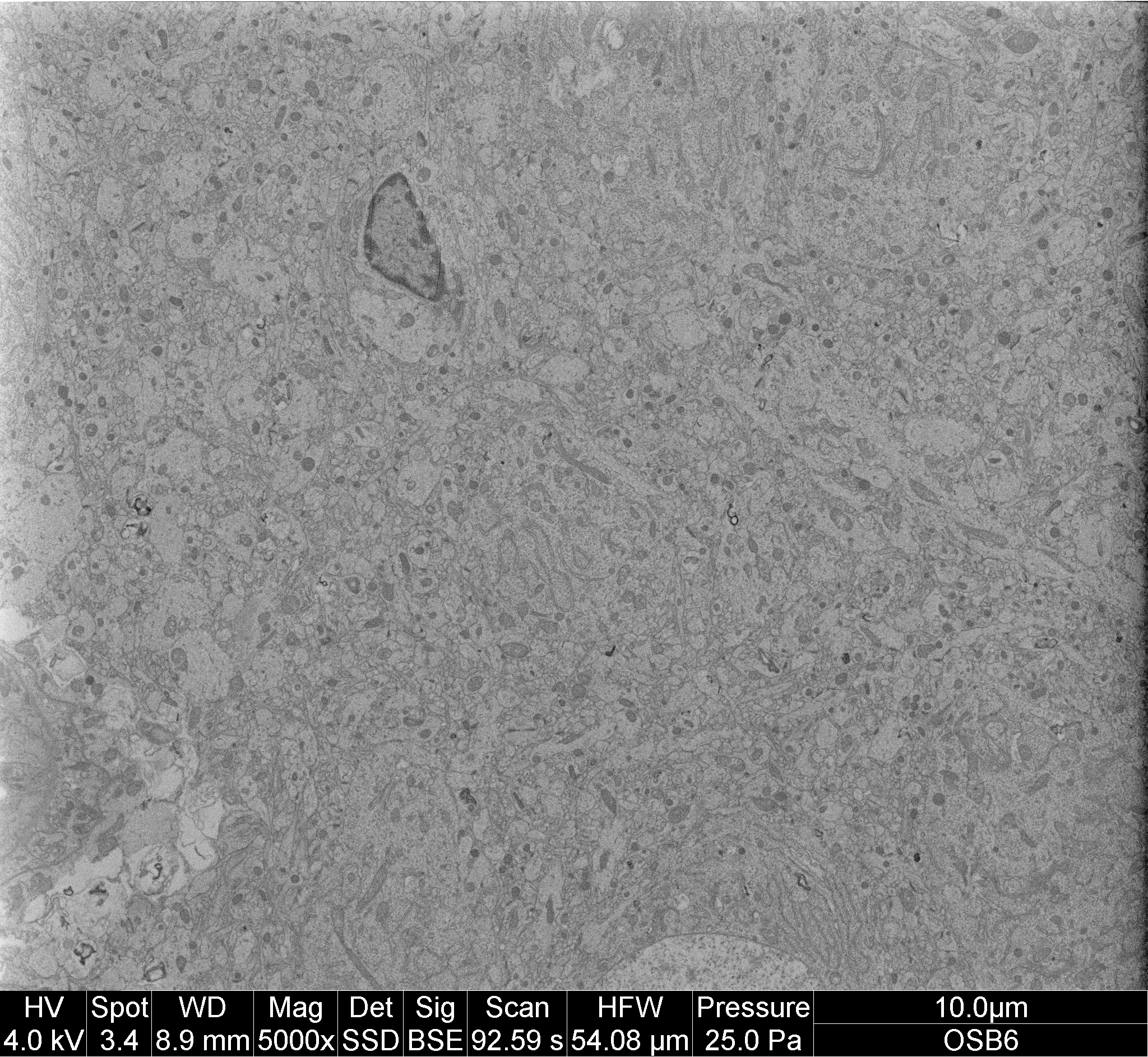

Supplement: Dataset S10 — (253.8 MB ZIP). [file pbio.0020329.sd010.zip › 040604_OS5_st1_973.tif]

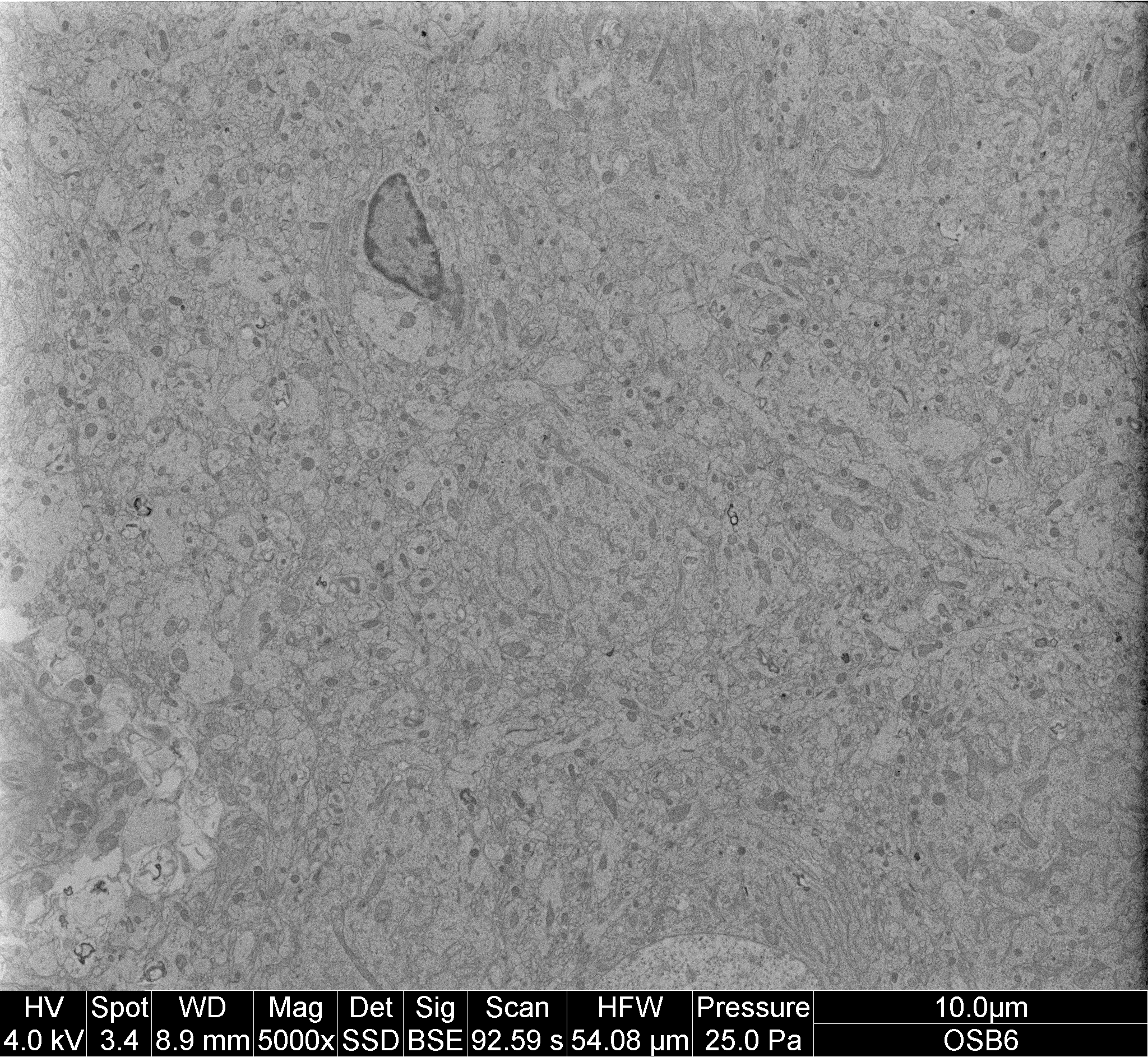

Supplement: Dataset S10 — (253.8 MB ZIP). [file pbio.0020329.sd010.zip › 040604_OS5_st1_974.tif]

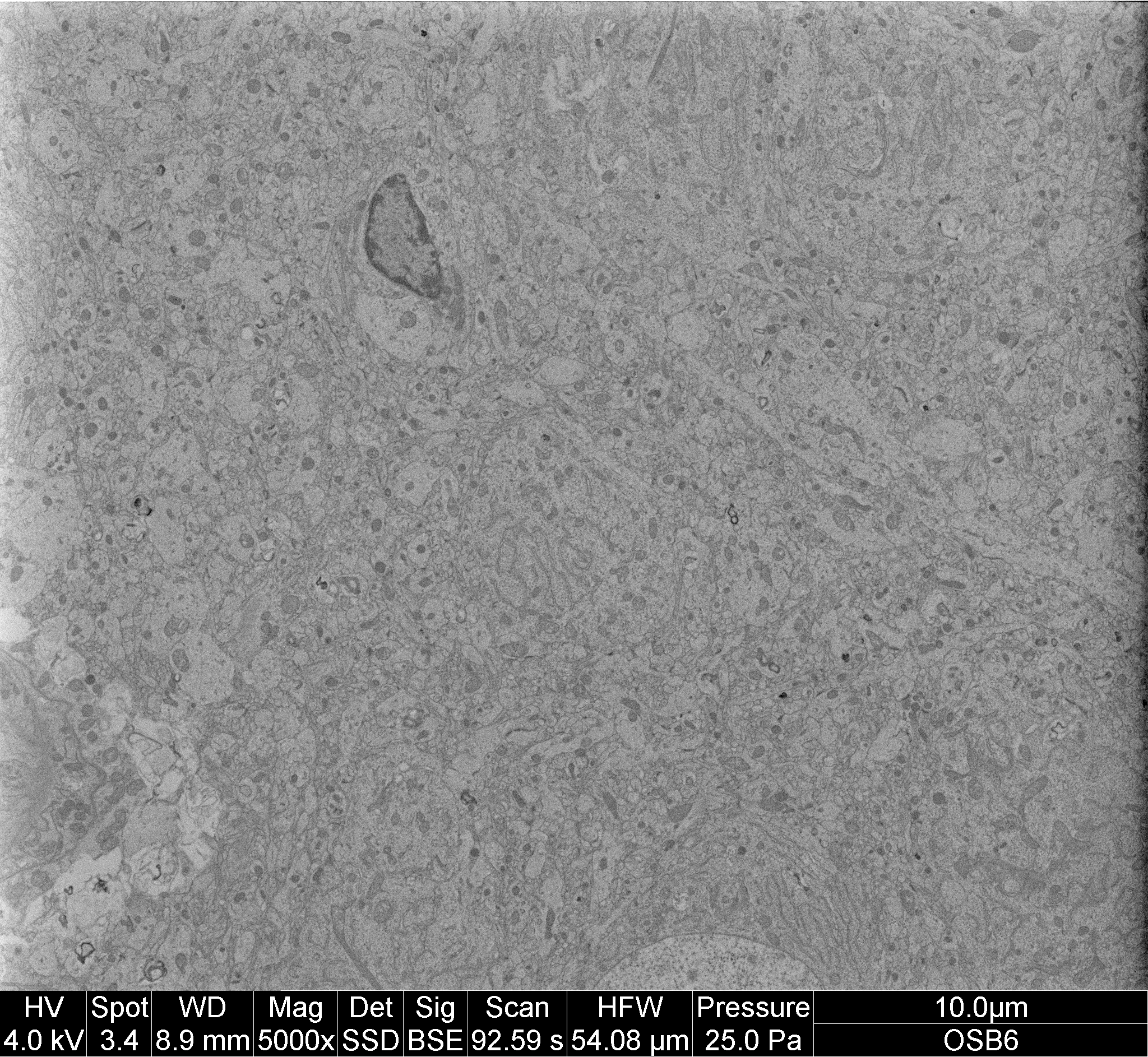

Supplement: Dataset S10 — (253.8 MB ZIP). [file pbio.0020329.sd010.zip › 040604_OS5_st1_975.tif]

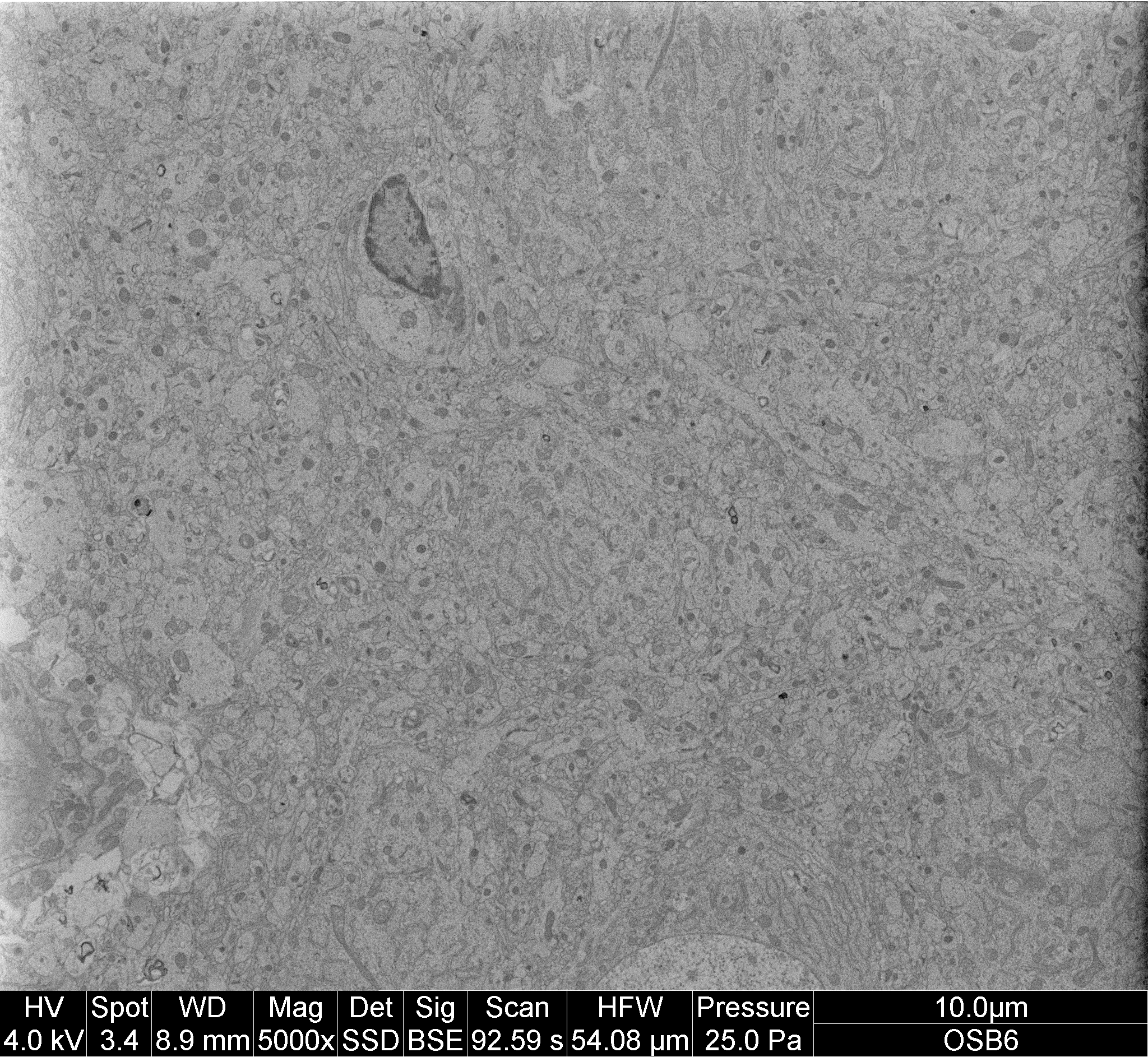

Supplement: Dataset S10 — (253.8 MB ZIP). [file pbio.0020329.sd010.zip › 040604_OS5_st1_976.tif]

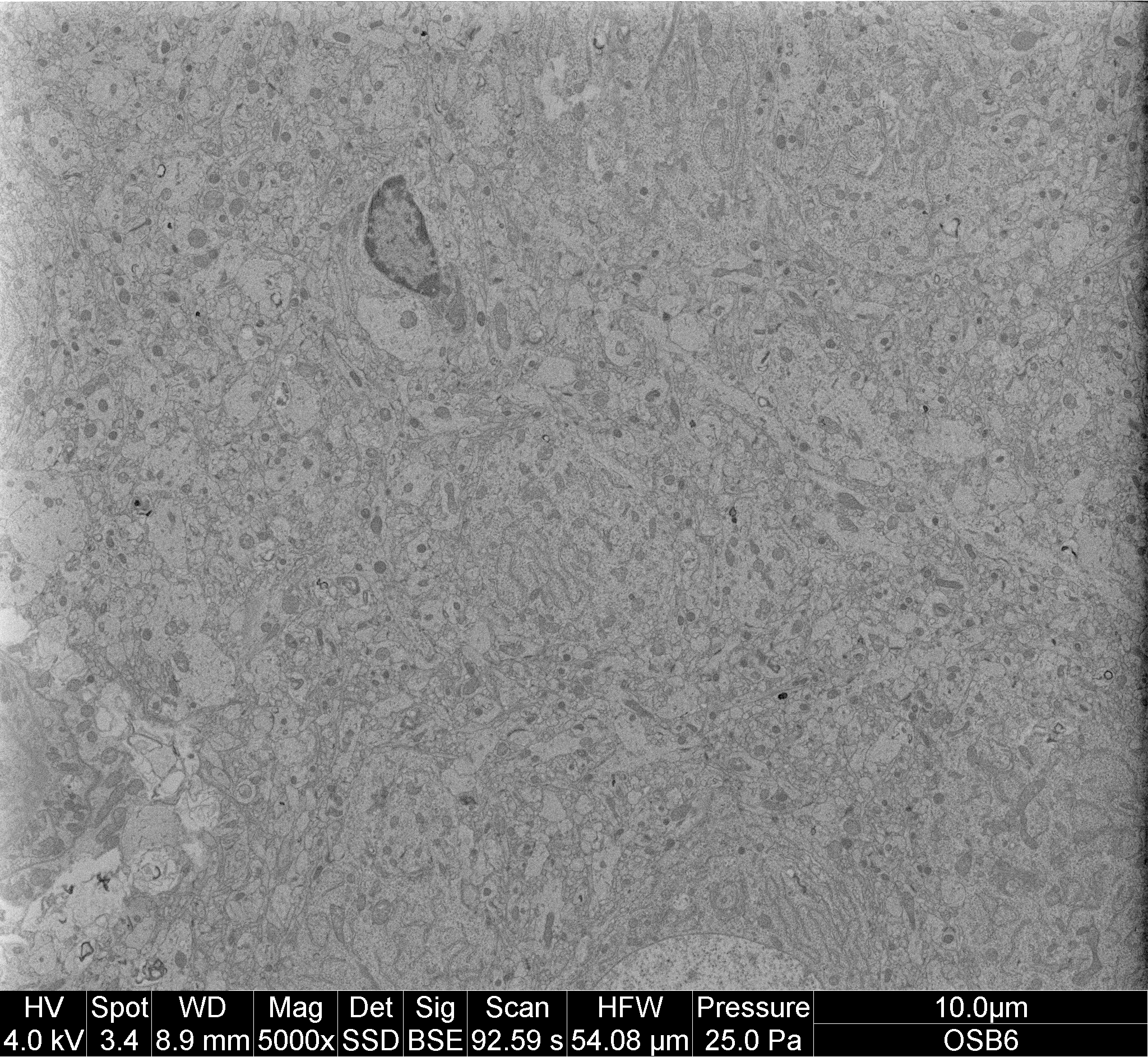

Supplement: Dataset S10 — (253.8 MB ZIP). [file pbio.0020329.sd010.zip › 040604_OS5_st1_977.tif]

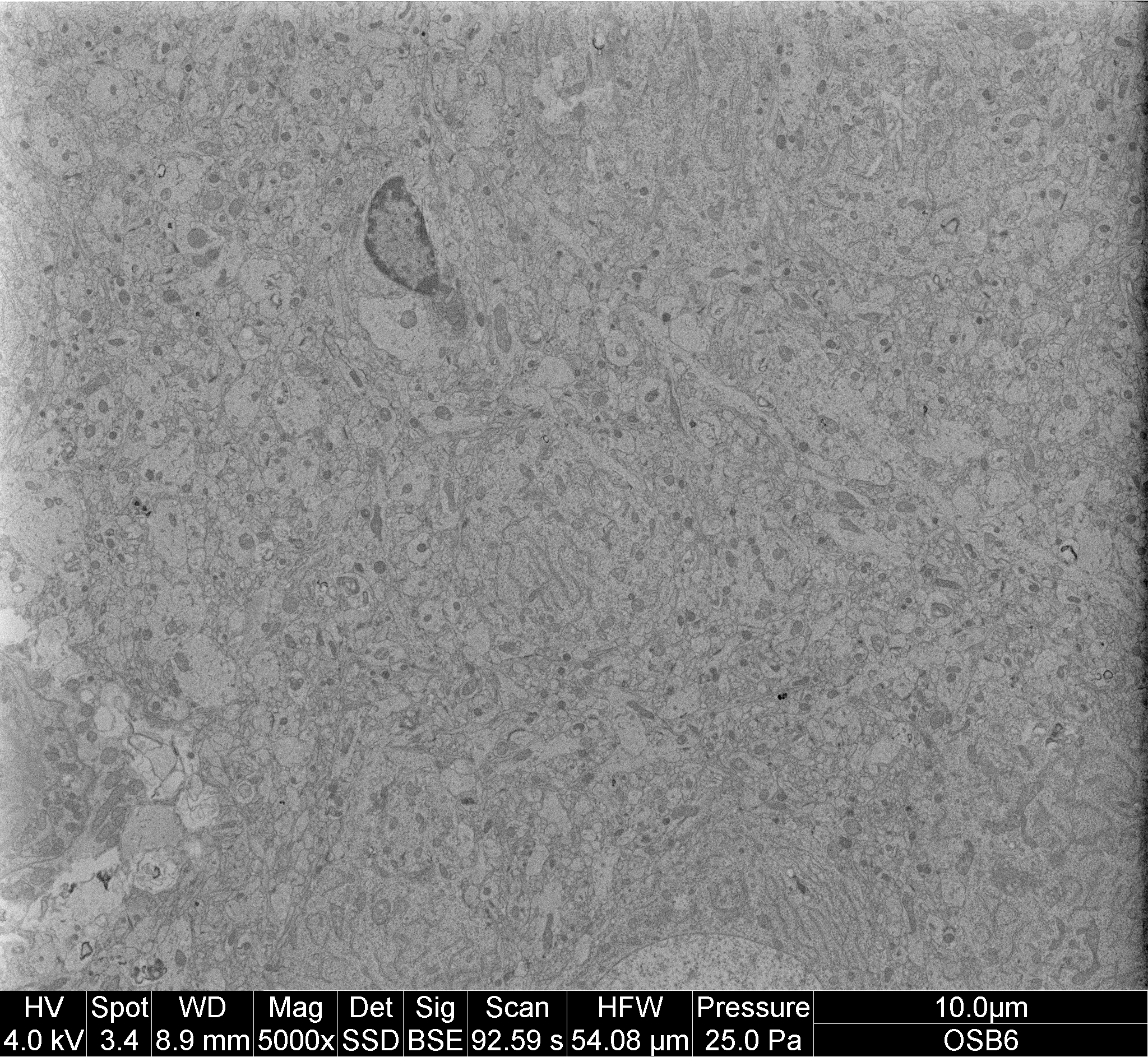

Supplement: Dataset S10 — (253.8 MB ZIP). [file pbio.0020329.sd010.zip › 040604_OS5_st1_978.tif]

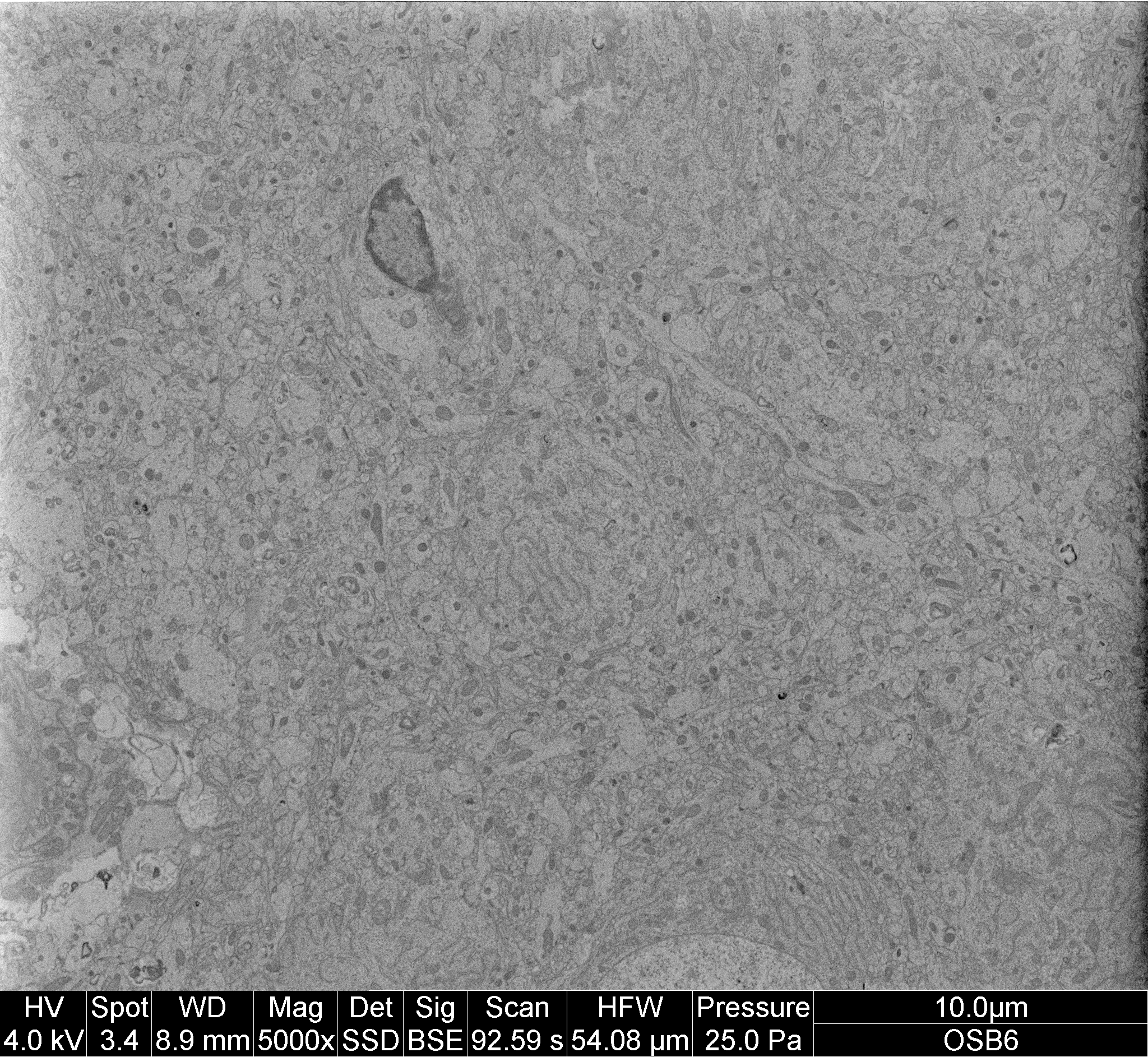

Supplement: Dataset S10 — (253.8 MB ZIP). [file pbio.0020329.sd010.zip › 040604_OS5_st1_979.tif]

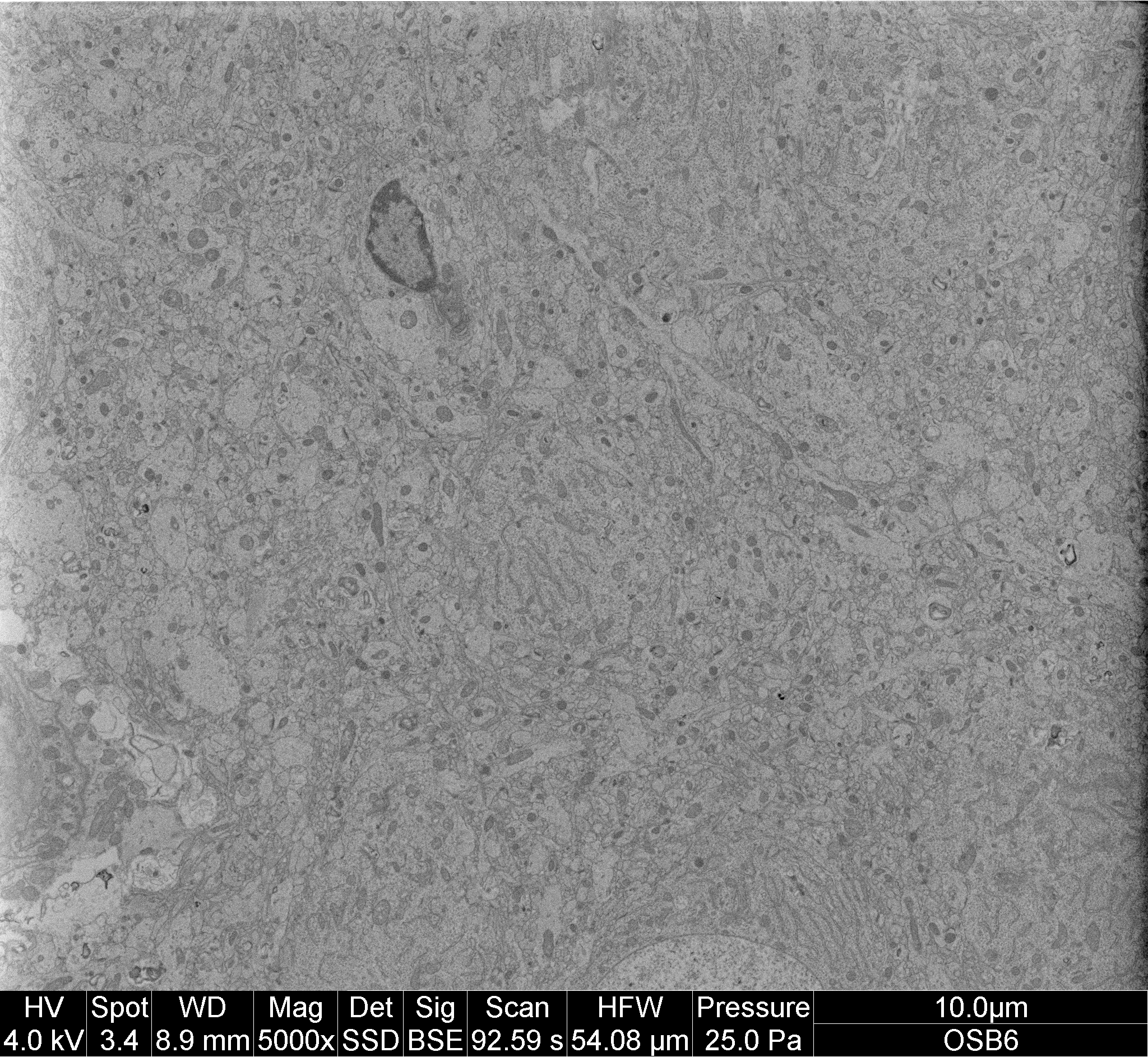

Supplement: Dataset S10 — (253.8 MB ZIP). [file pbio.0020329.sd010.zip › 040604_OS5_st1_980.tif]

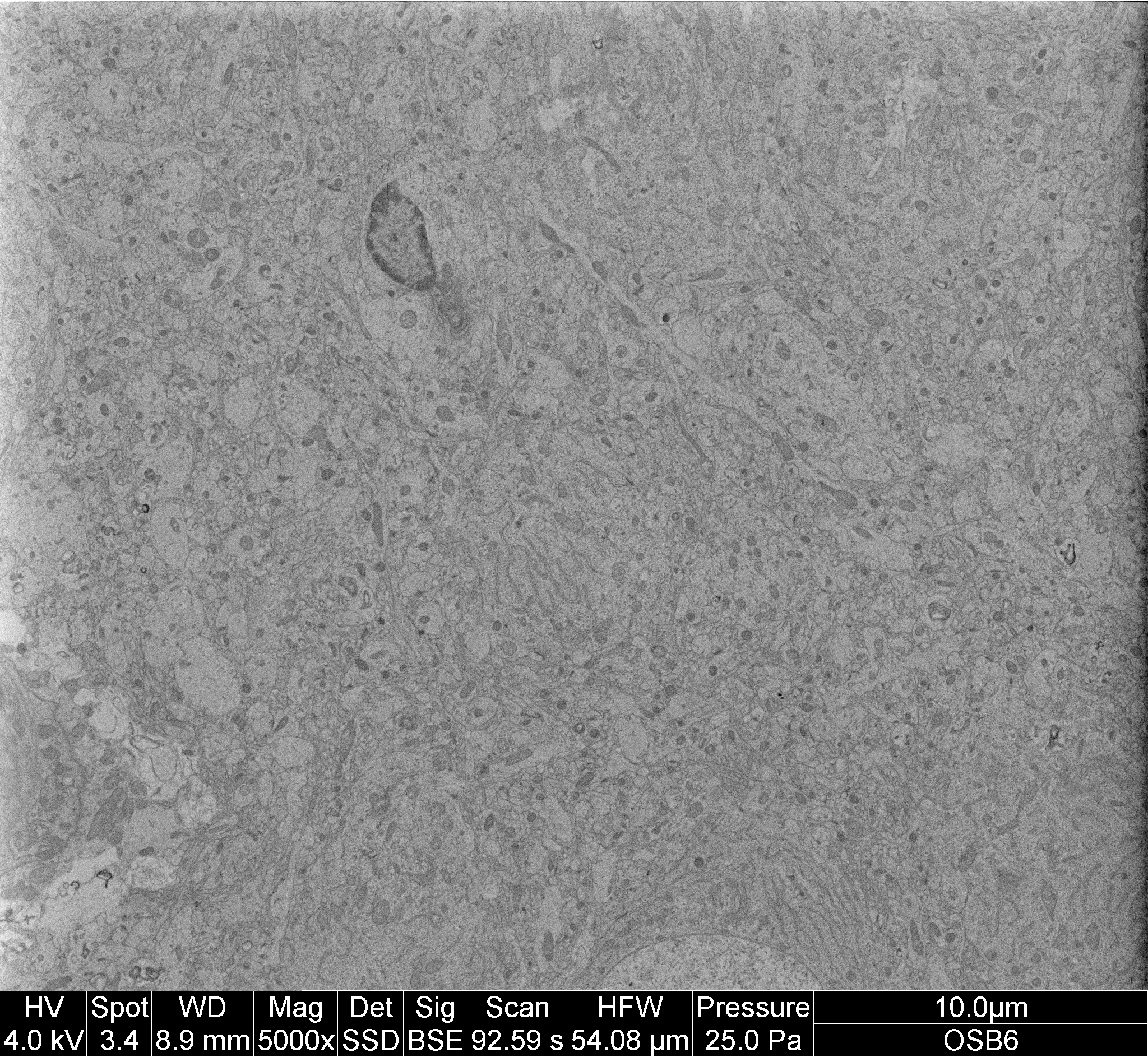

Supplement: Dataset S10 — (253.8 MB ZIP). [file pbio.0020329.sd010.zip › 040604_OS5_st1_981.tif]

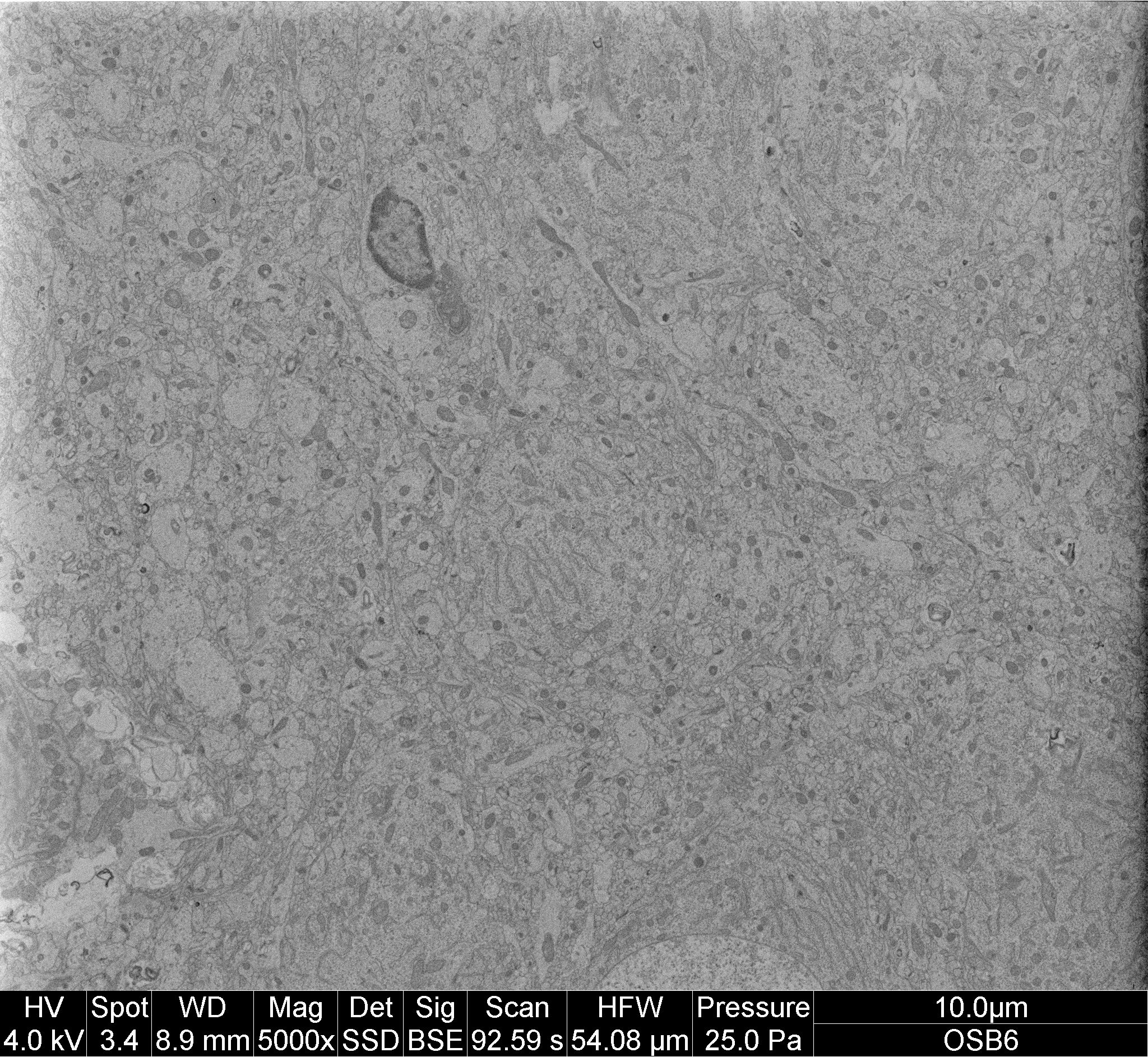

Supplement: Dataset S10 — (253.8 MB ZIP). [file pbio.0020329.sd010.zip › 040604_OS5_st1_982.tif]

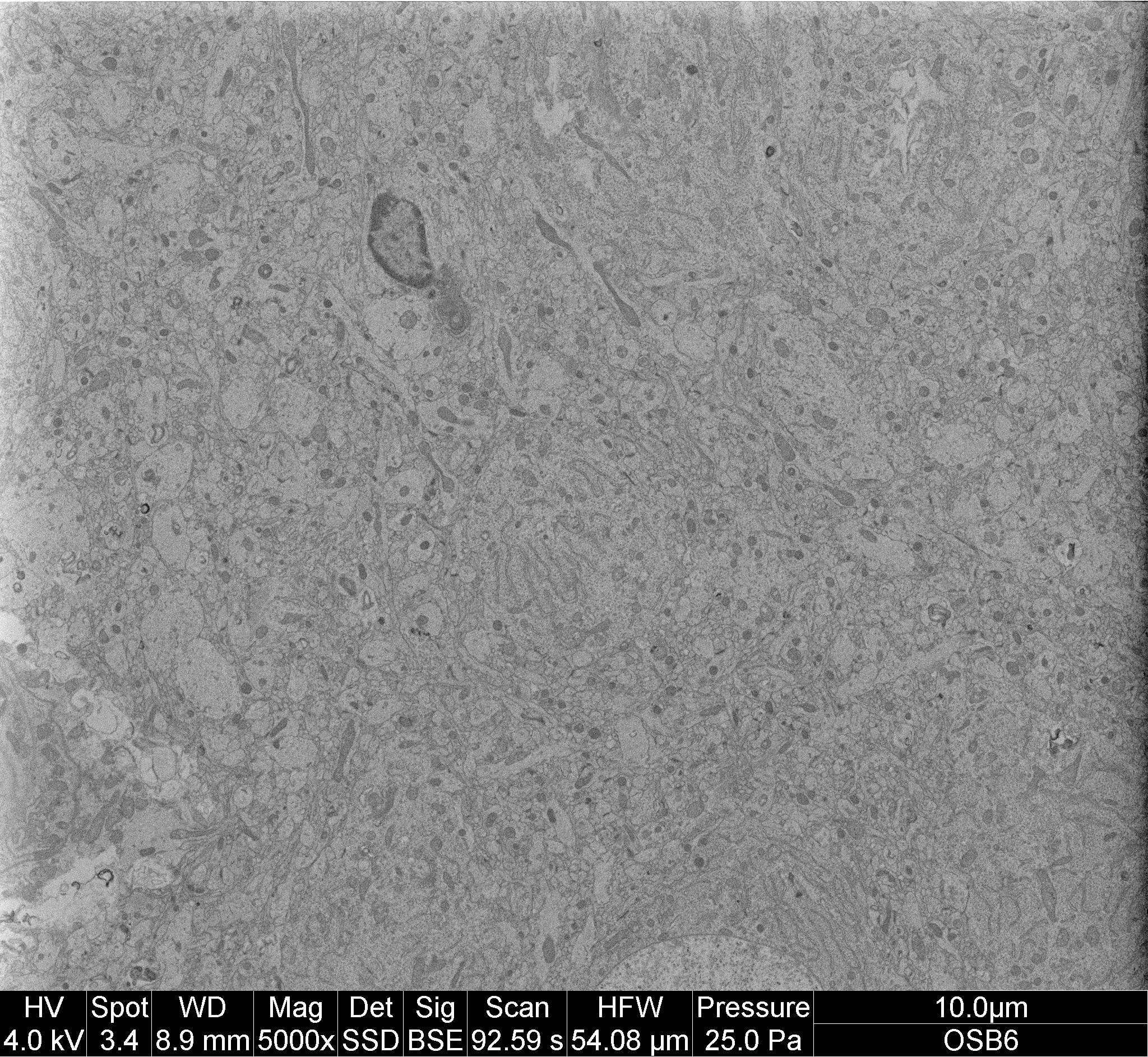

Supplement: Dataset S10 — (253.8 MB ZIP). [file pbio.0020329.sd010.zip › 040604_OS5_st1_983.tif]

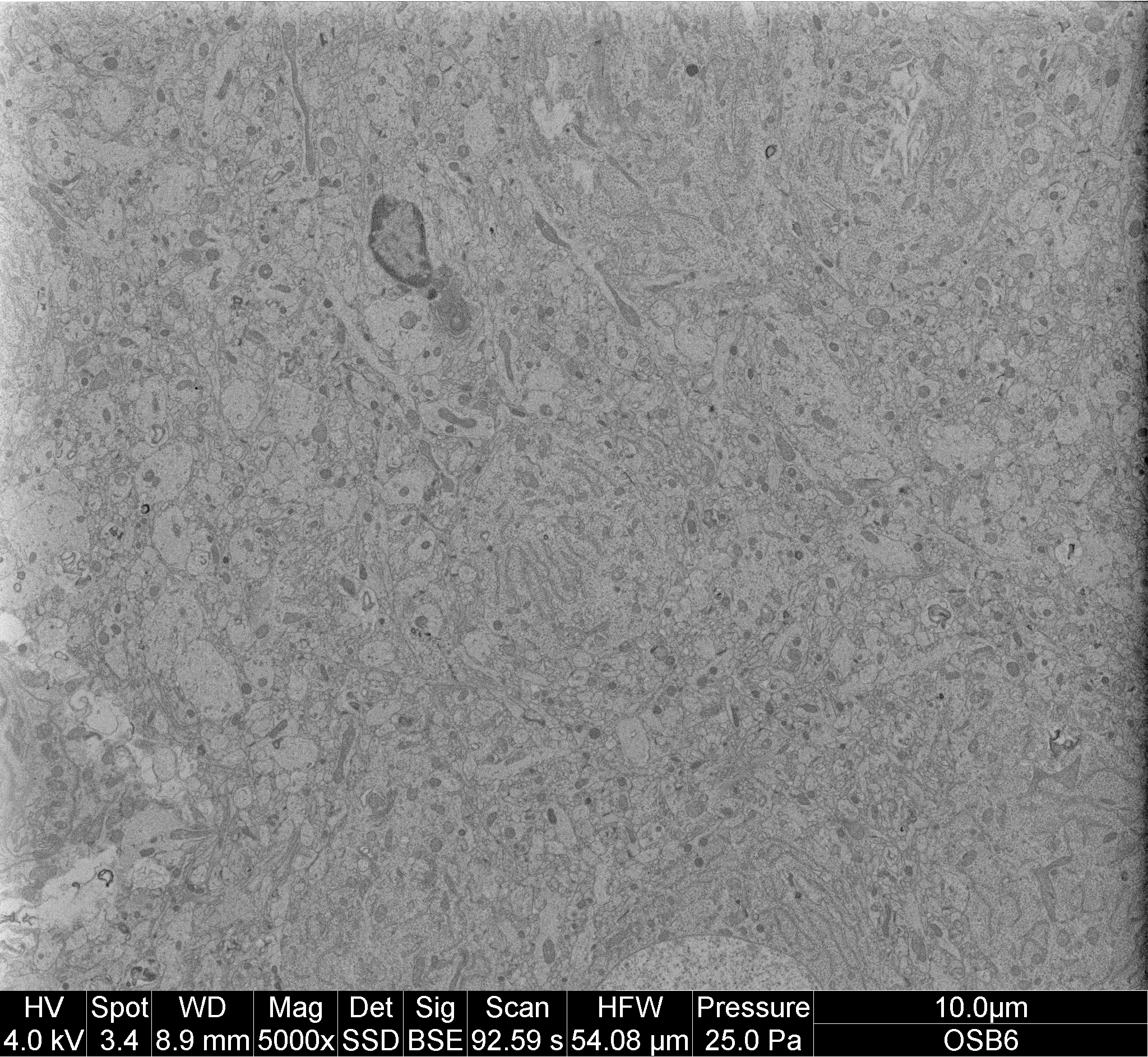

Supplement: Dataset S10 — (253.8 MB ZIP). [file pbio.0020329.sd010.zip › 040604_OS5_st1_984.tif]

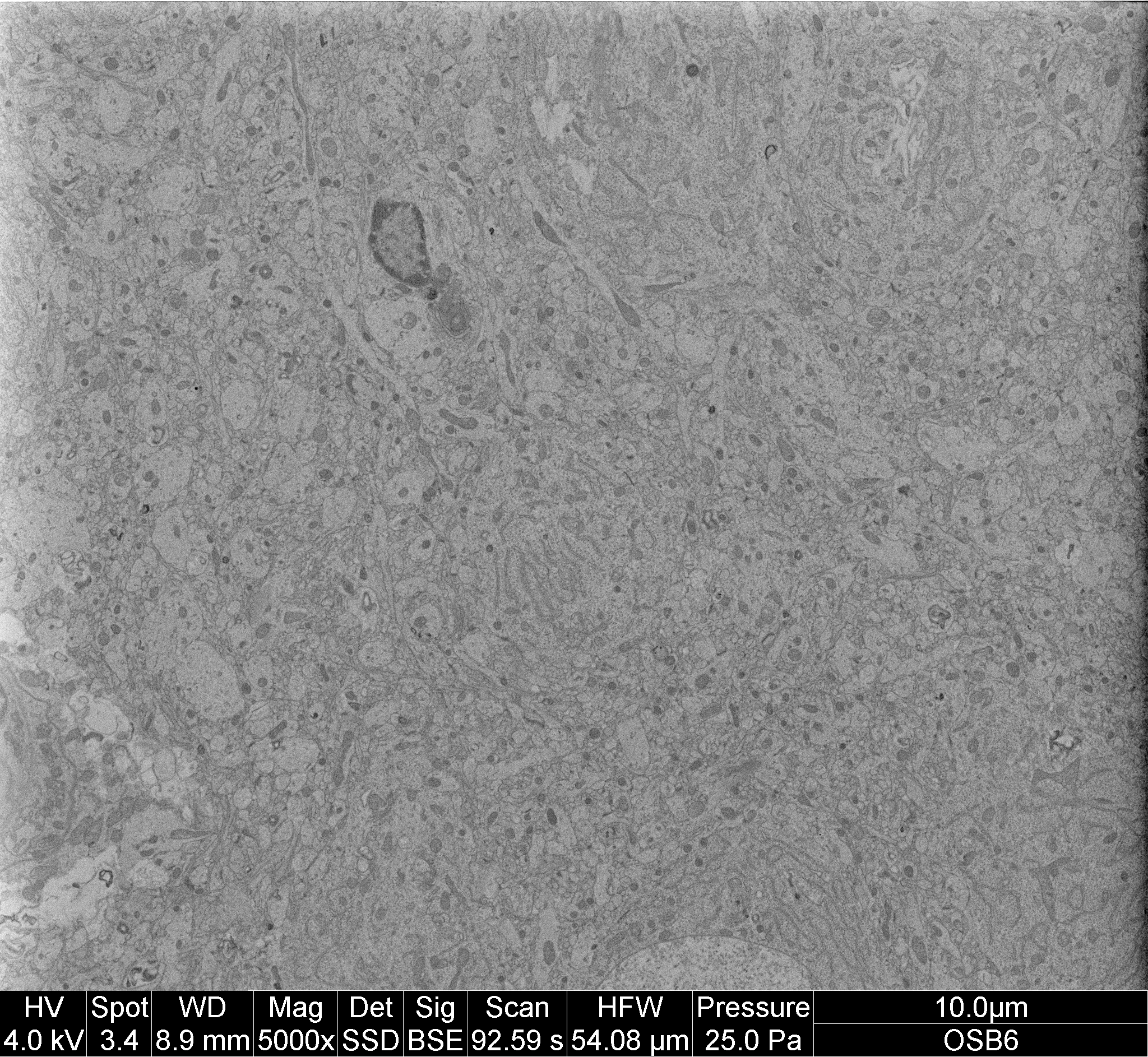

Supplement: Dataset S10 — (253.8 MB ZIP). [file pbio.0020329.sd010.zip › 040604_OS5_st1_985.tif]

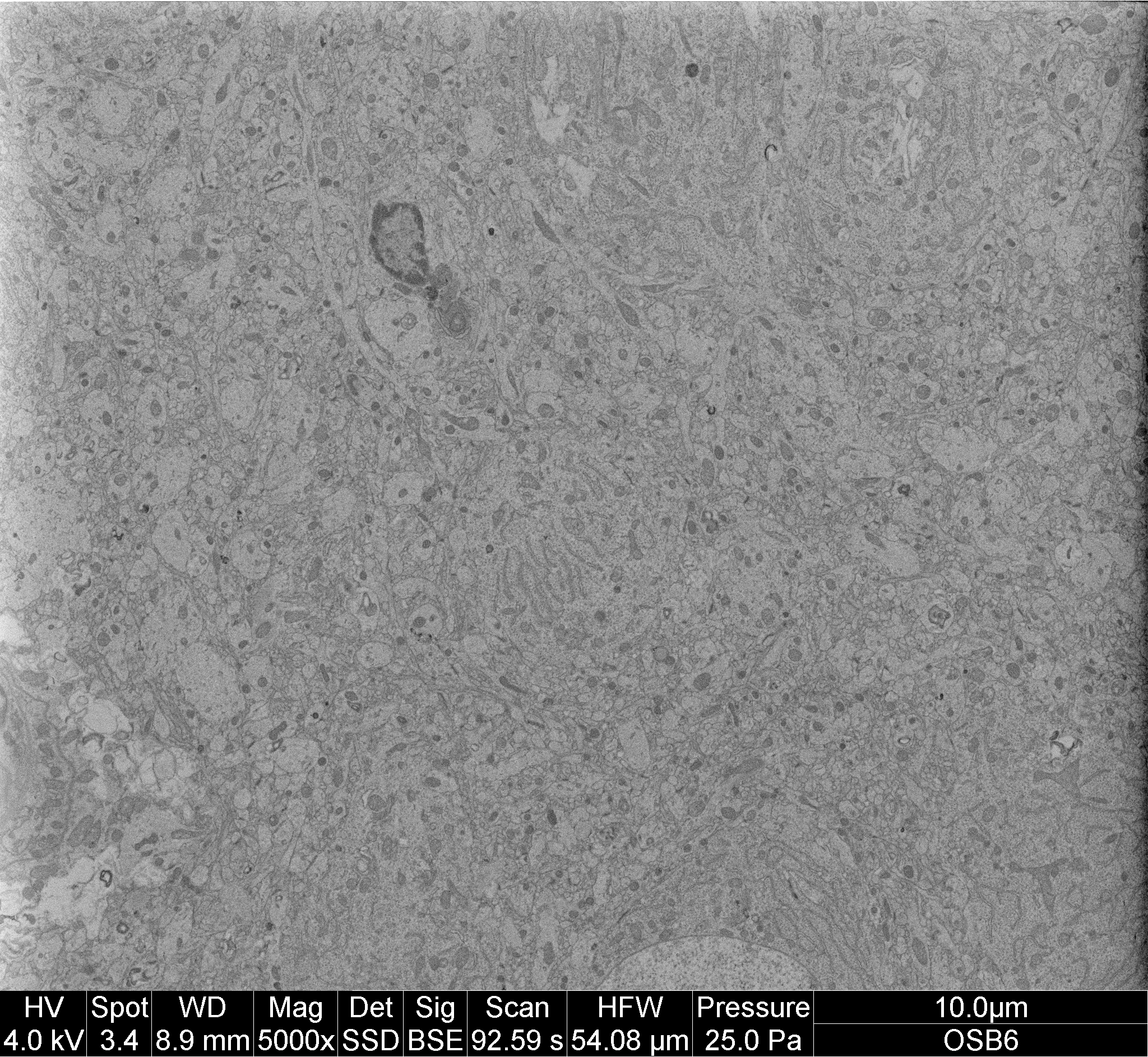

Supplement: Dataset S10 — (253.8 MB ZIP). [file pbio.0020329.sd010.zip › 040604_OS5_st1_986.tif]

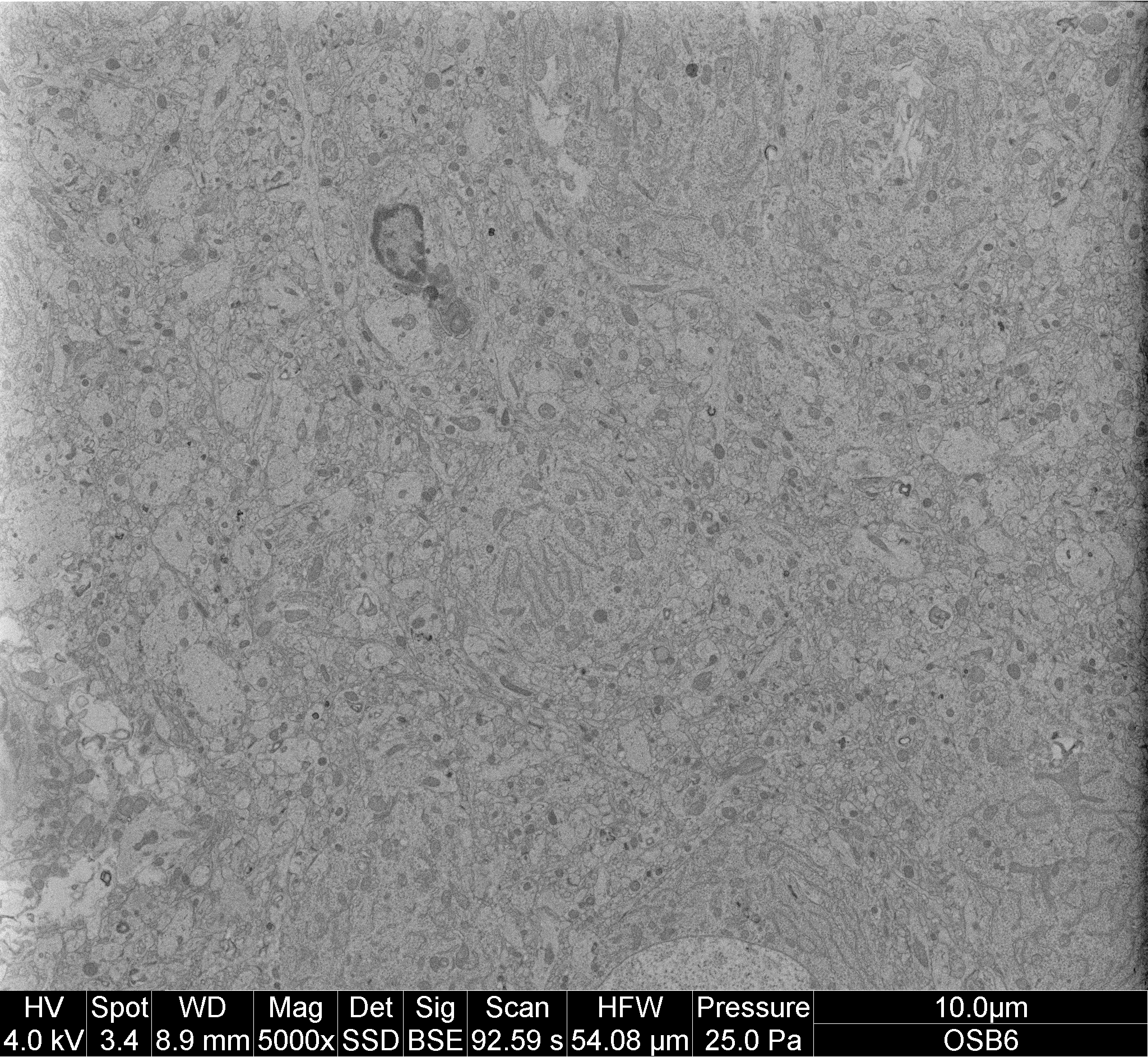

Supplement: Dataset S10 — (253.8 MB ZIP). [file pbio.0020329.sd010.zip › 040604_OS5_st1_987.tif]

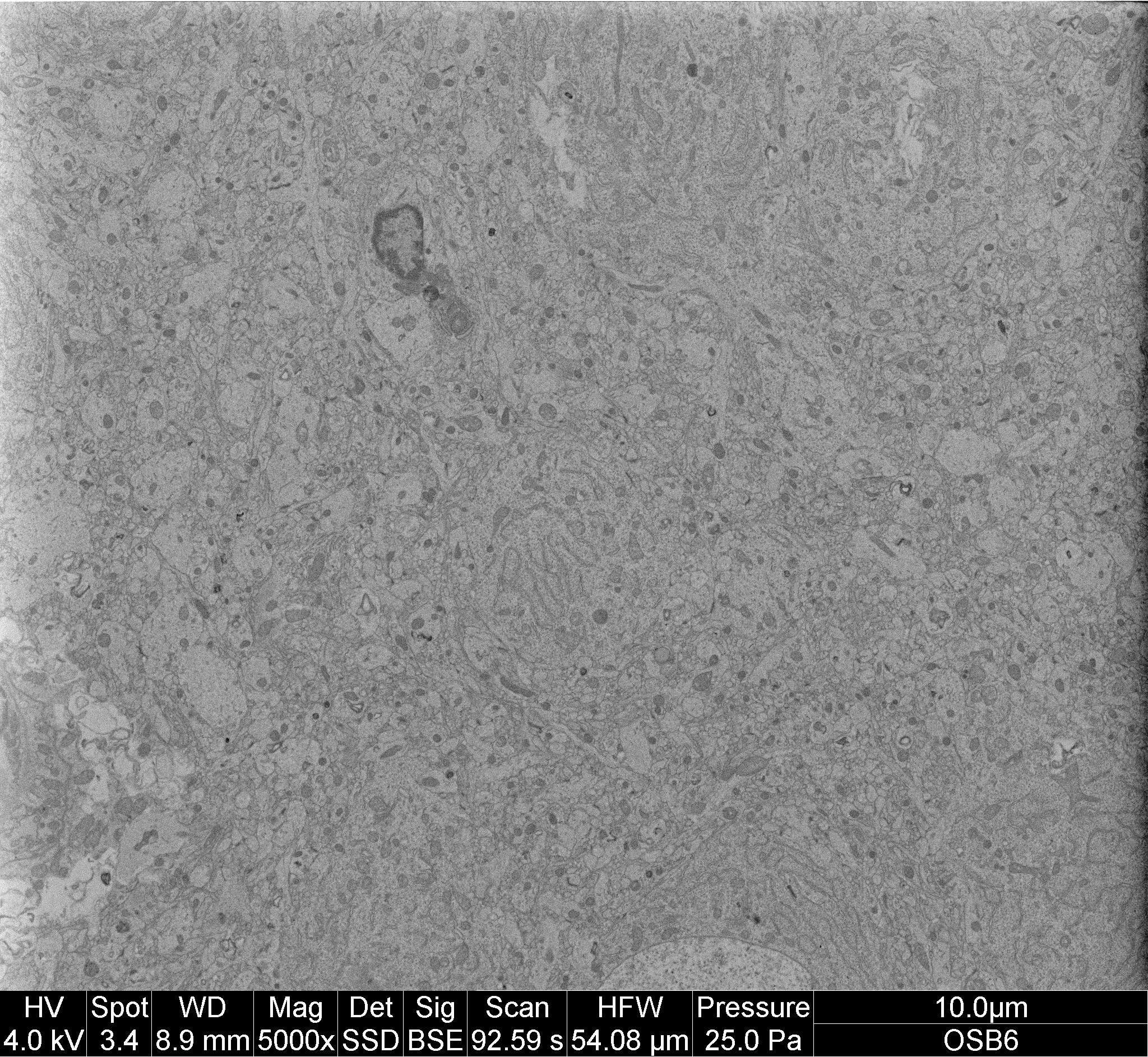

Supplement: Dataset S10 — (253.8 MB ZIP). [file pbio.0020329.sd010.zip › 040604_OS5_st1_988.tif]

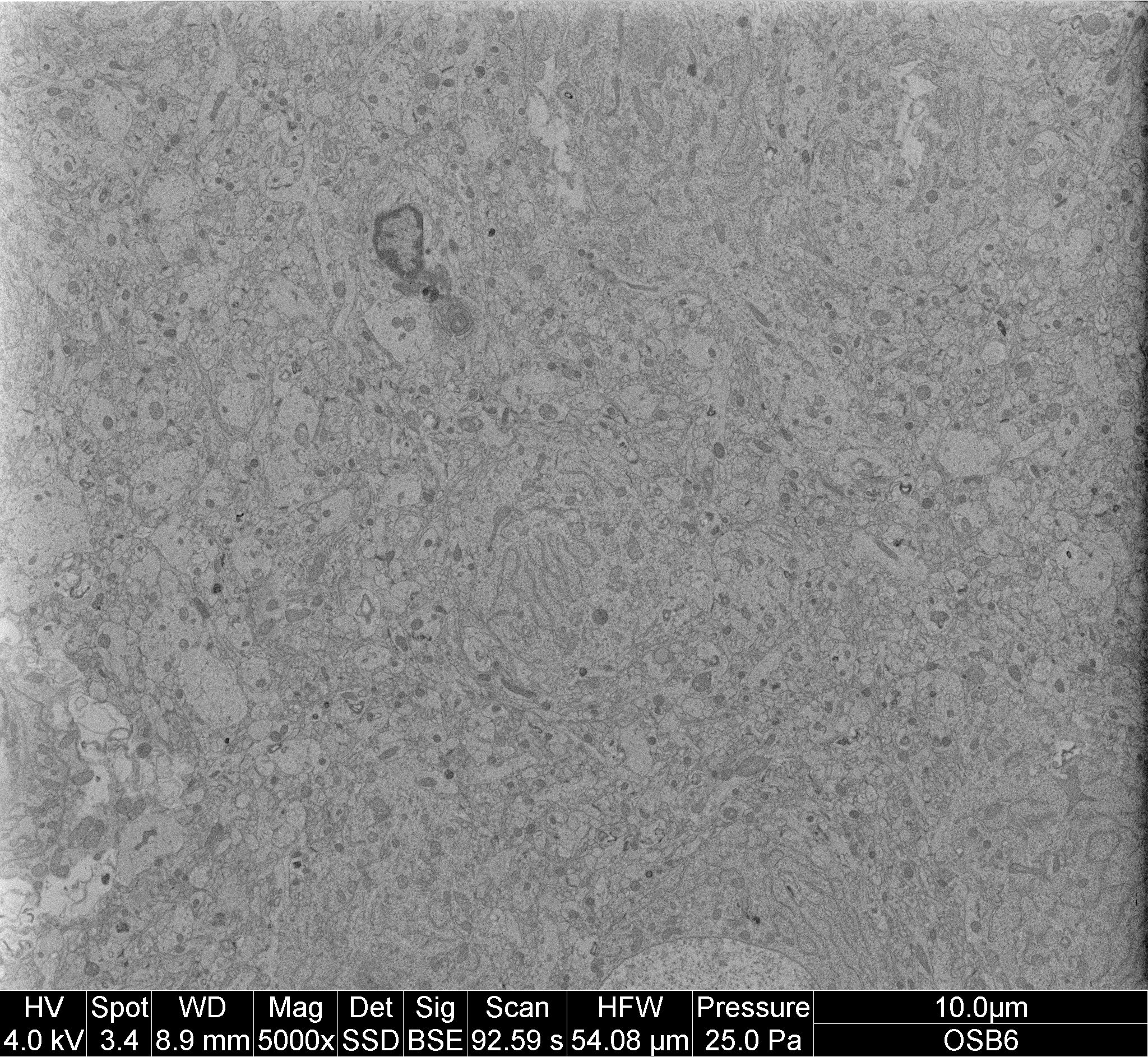

Supplement: Dataset S10 — (253.8 MB ZIP). [file pbio.0020329.sd010.zip › 040604_OS5_st1_989.tif]

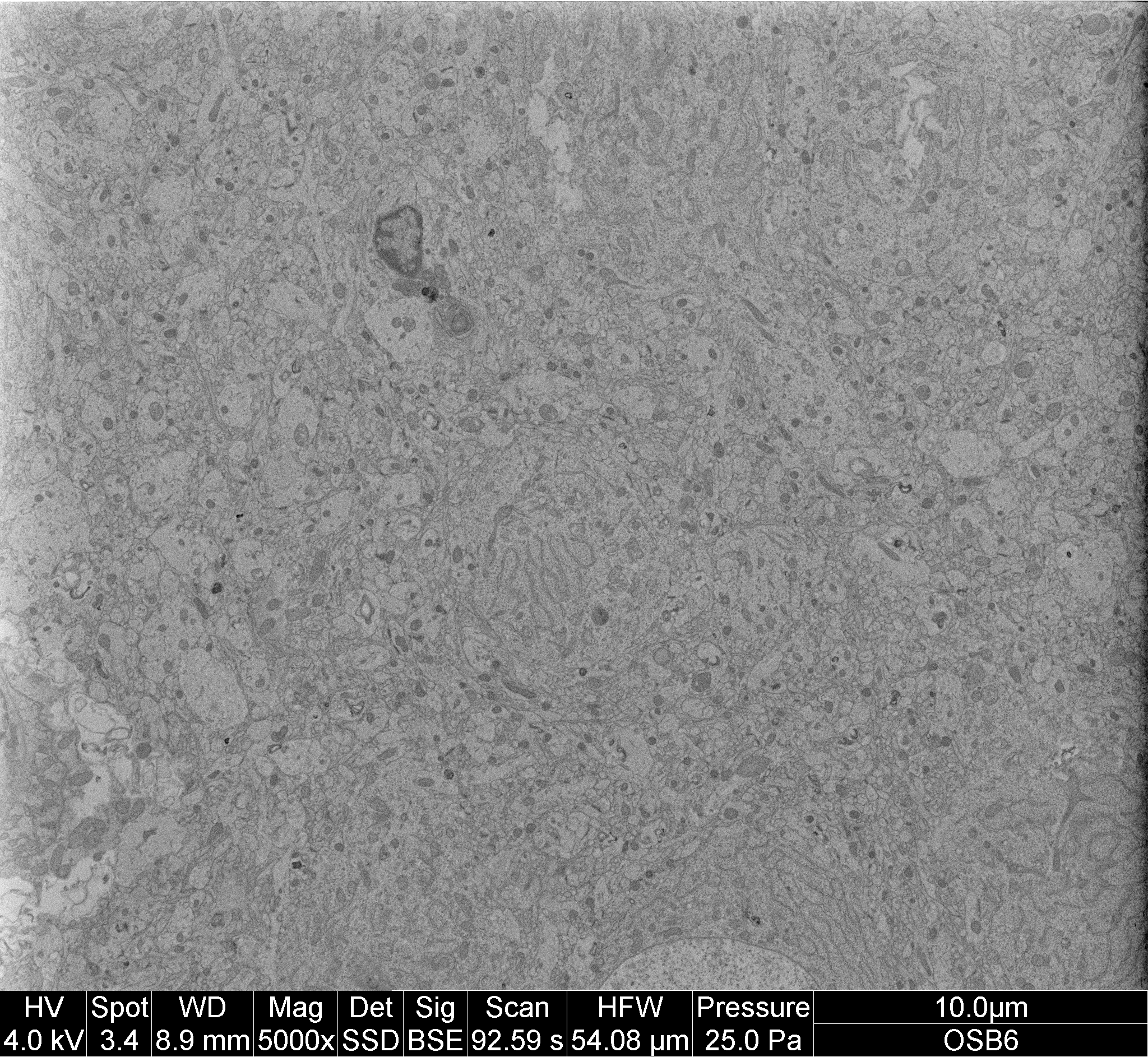

Supplement: Dataset S10 — (253.8 MB ZIP). [file pbio.0020329.sd010.zip › 040604_OS5_st1_990.tif]

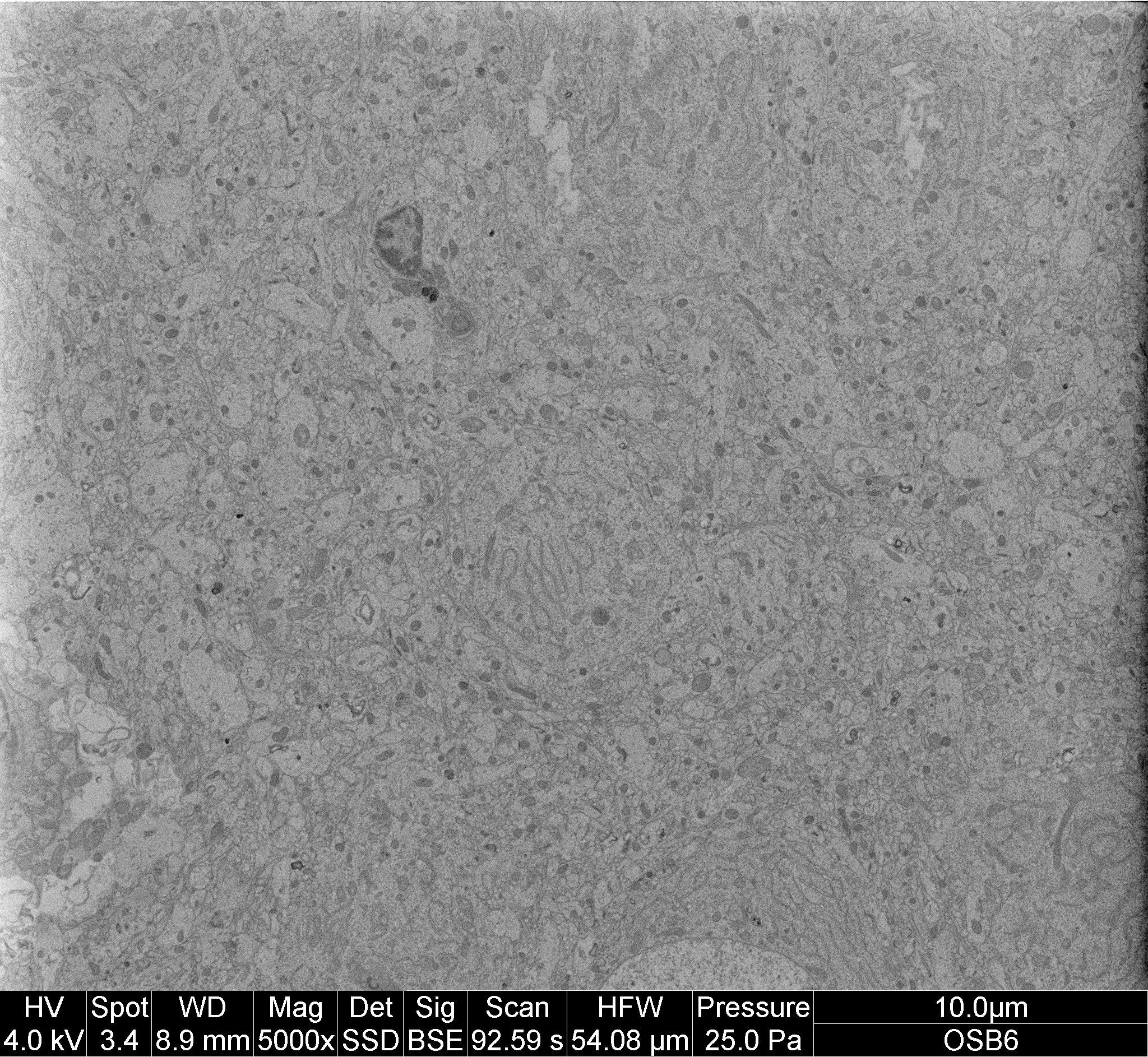

Supplement: Dataset S10 — (253.8 MB ZIP). [file pbio.0020329.sd010.zip › 040604_OS5_st1_991.tif]

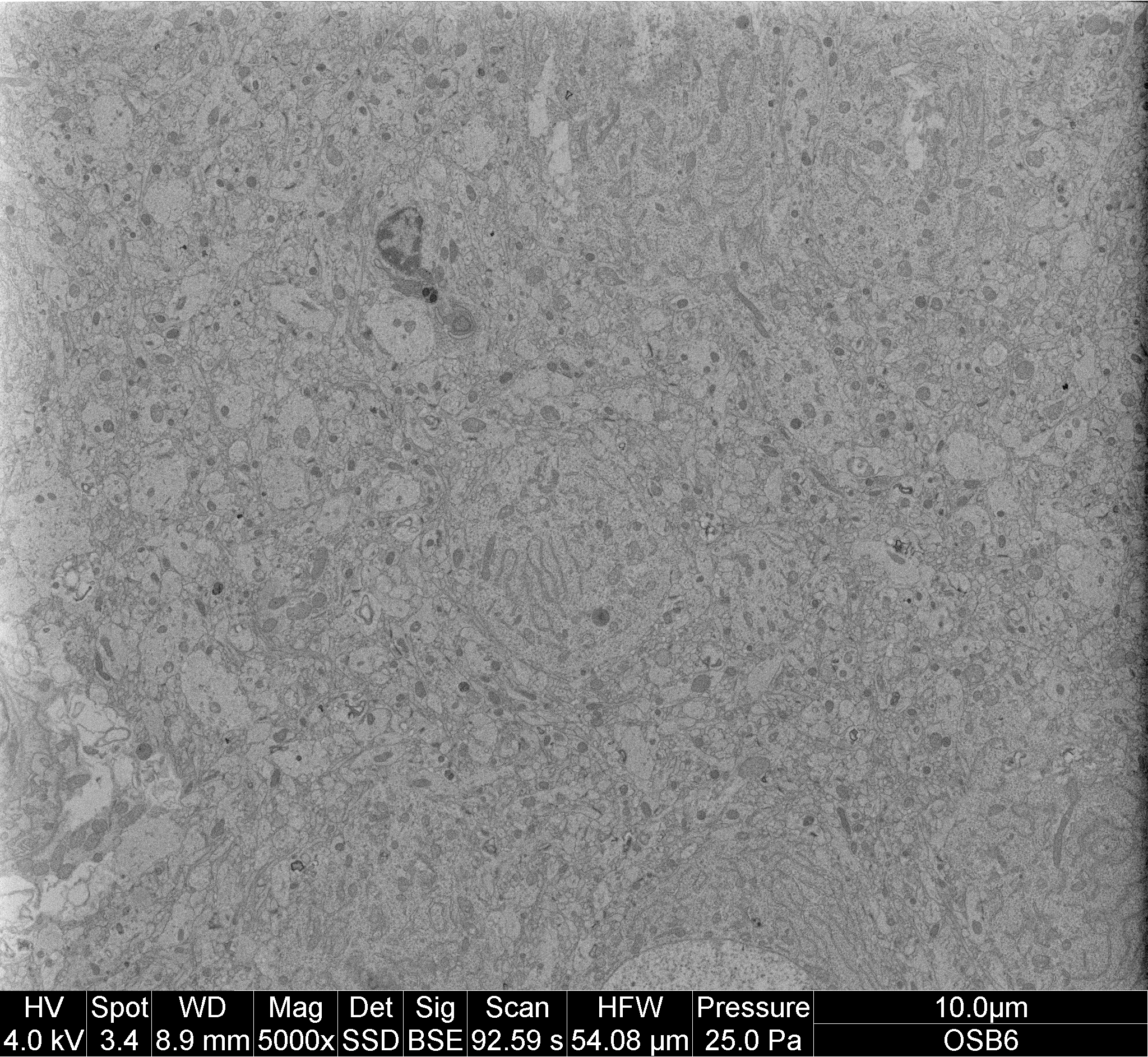

Supplement: Dataset S10 — (253.8 MB ZIP). [file pbio.0020329.sd010.zip › 040604_OS5_st1_992.tif]

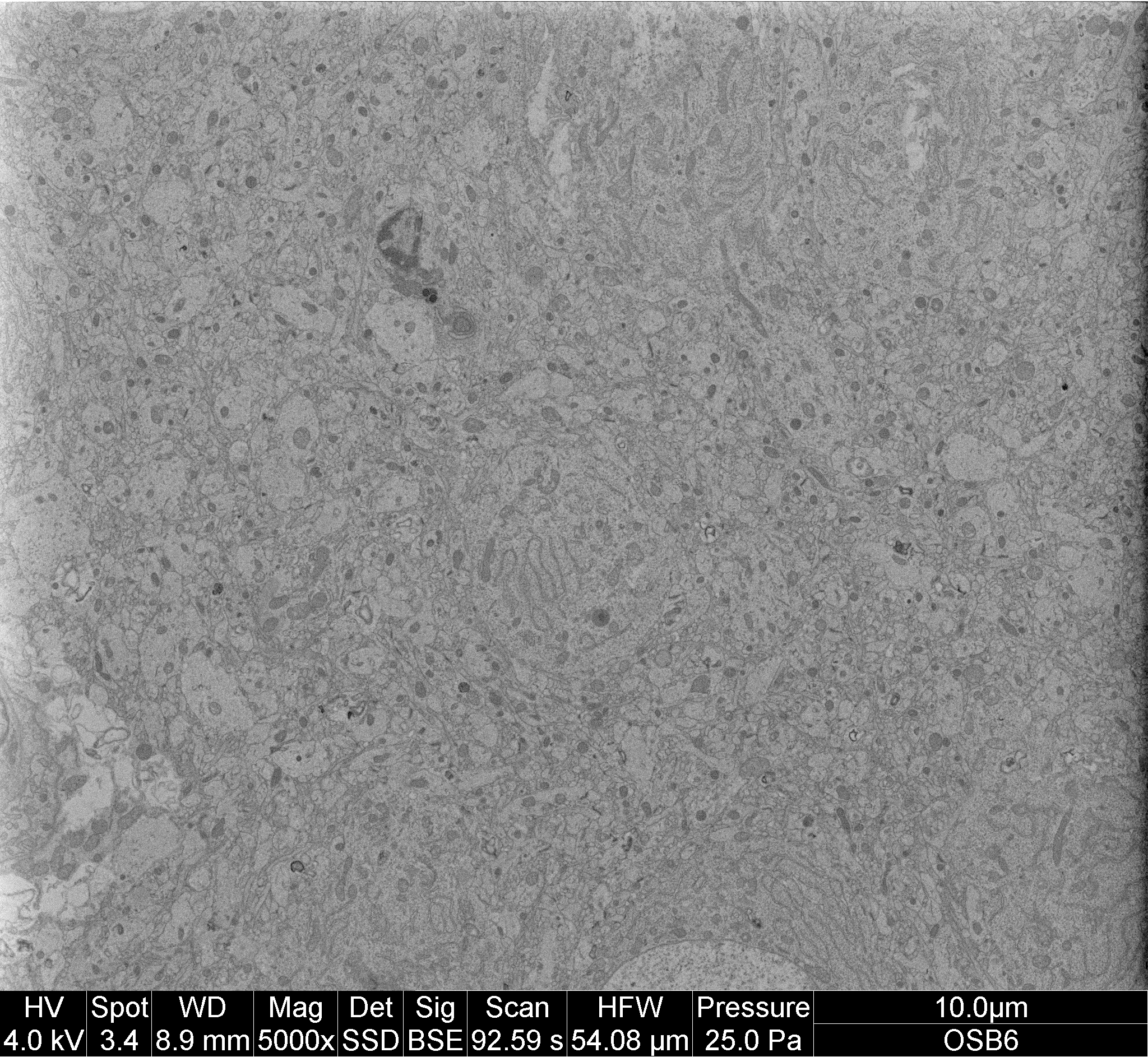

Supplement: Dataset S10 — (253.8 MB ZIP). [file pbio.0020329.sd010.zip › 040604_OS5_st1_993.tif]

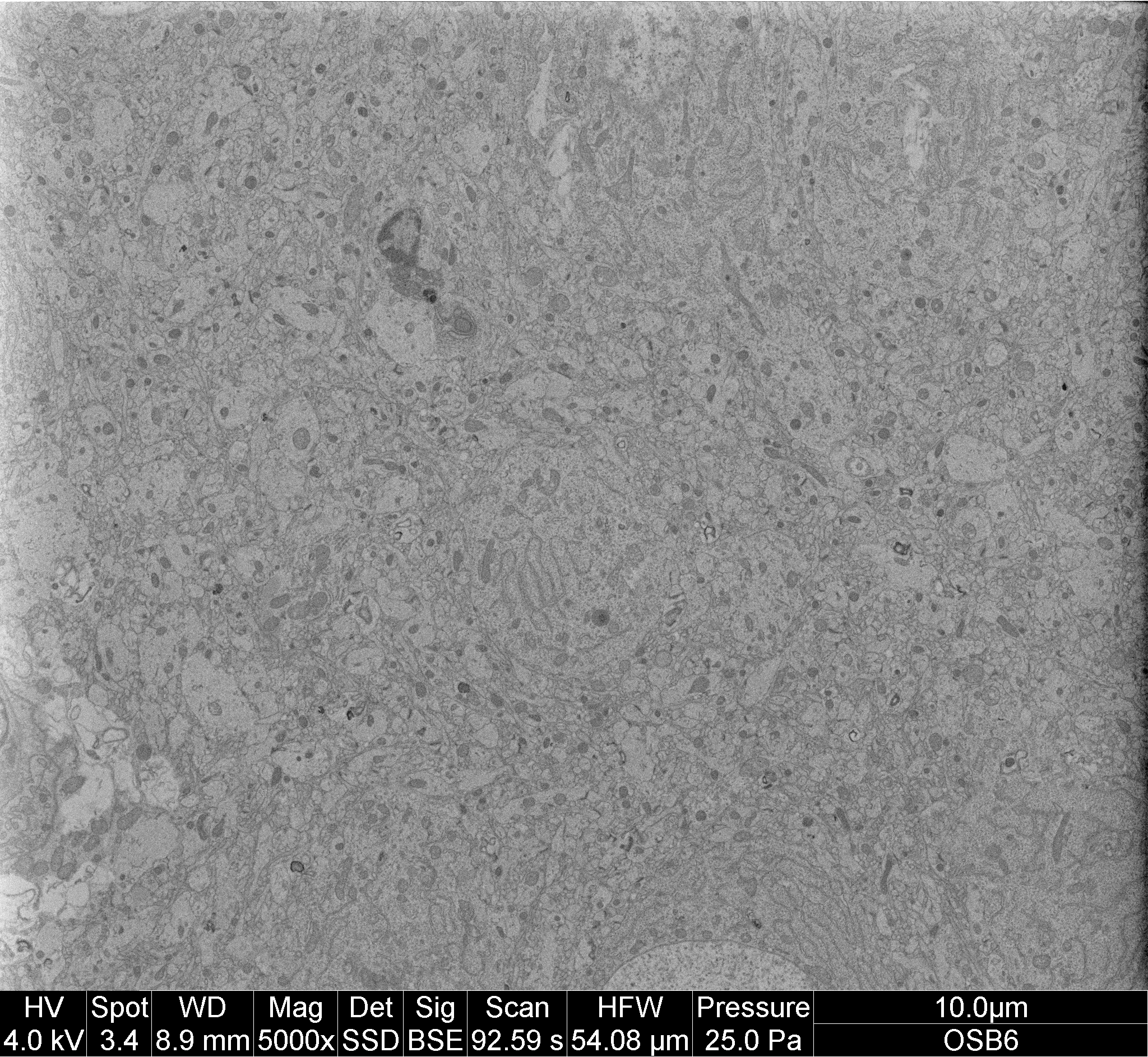

Supplement: Dataset S10 — (253.8 MB ZIP). [file pbio.0020329.sd010.zip › 040604_OS5_st1_994.tif]

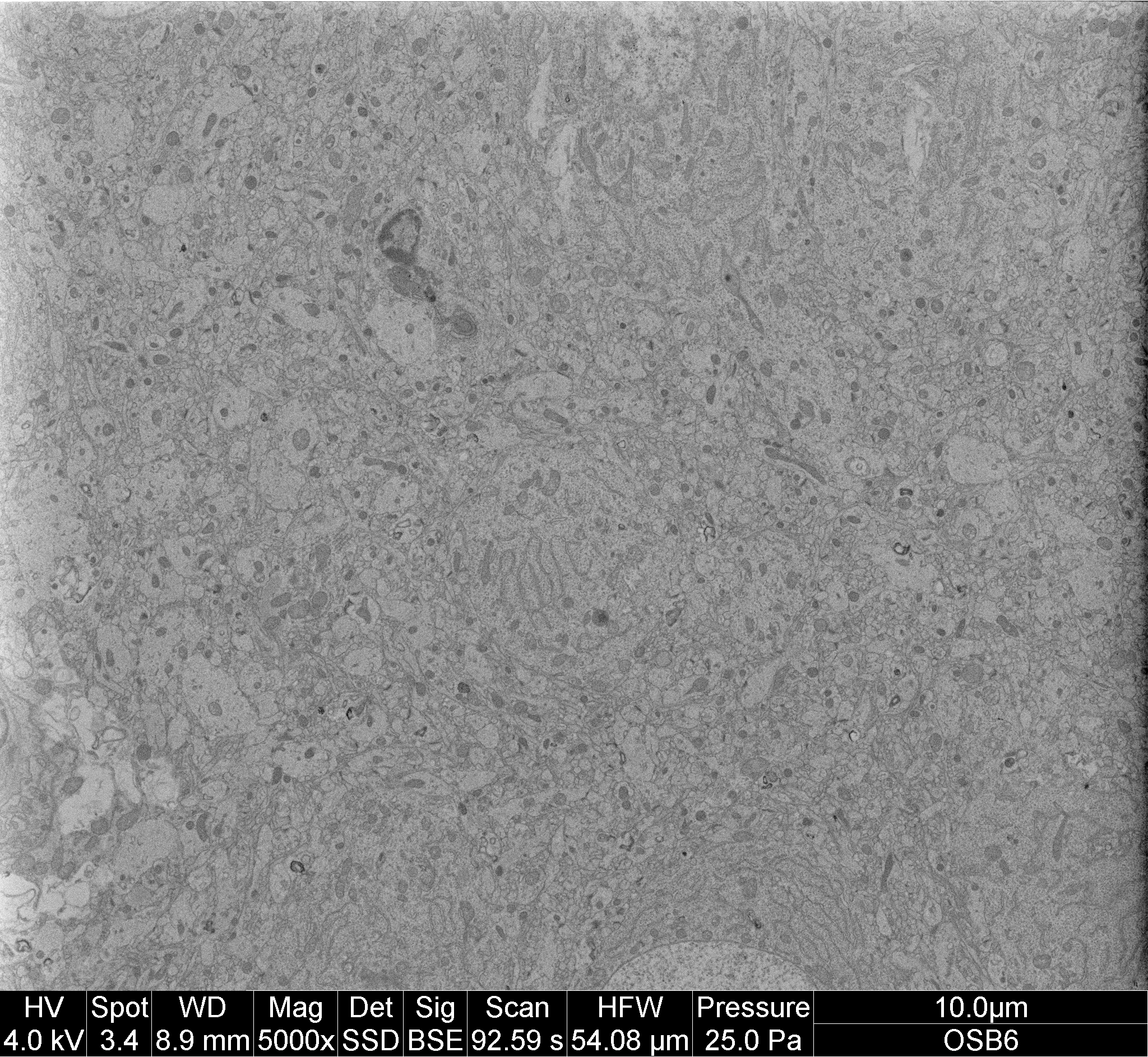

Supplement: Dataset S10 — (253.8 MB ZIP). [file pbio.0020329.sd010.zip › 040604_OS5_st1_995.tif]

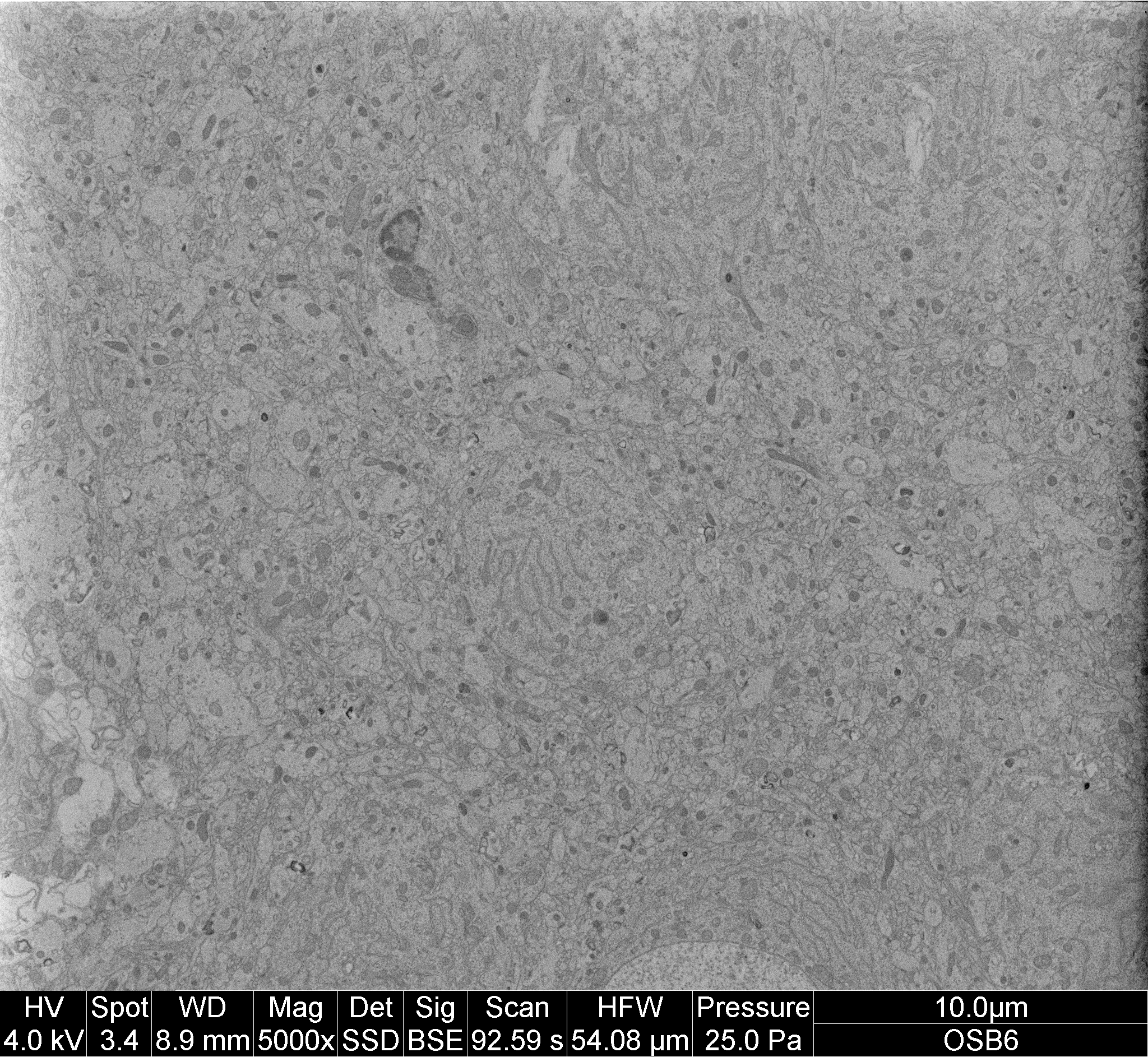

Supplement: Dataset S10 — (253.8 MB ZIP). [file pbio.0020329.sd010.zip › 040604_OS5_st1_996.tif]

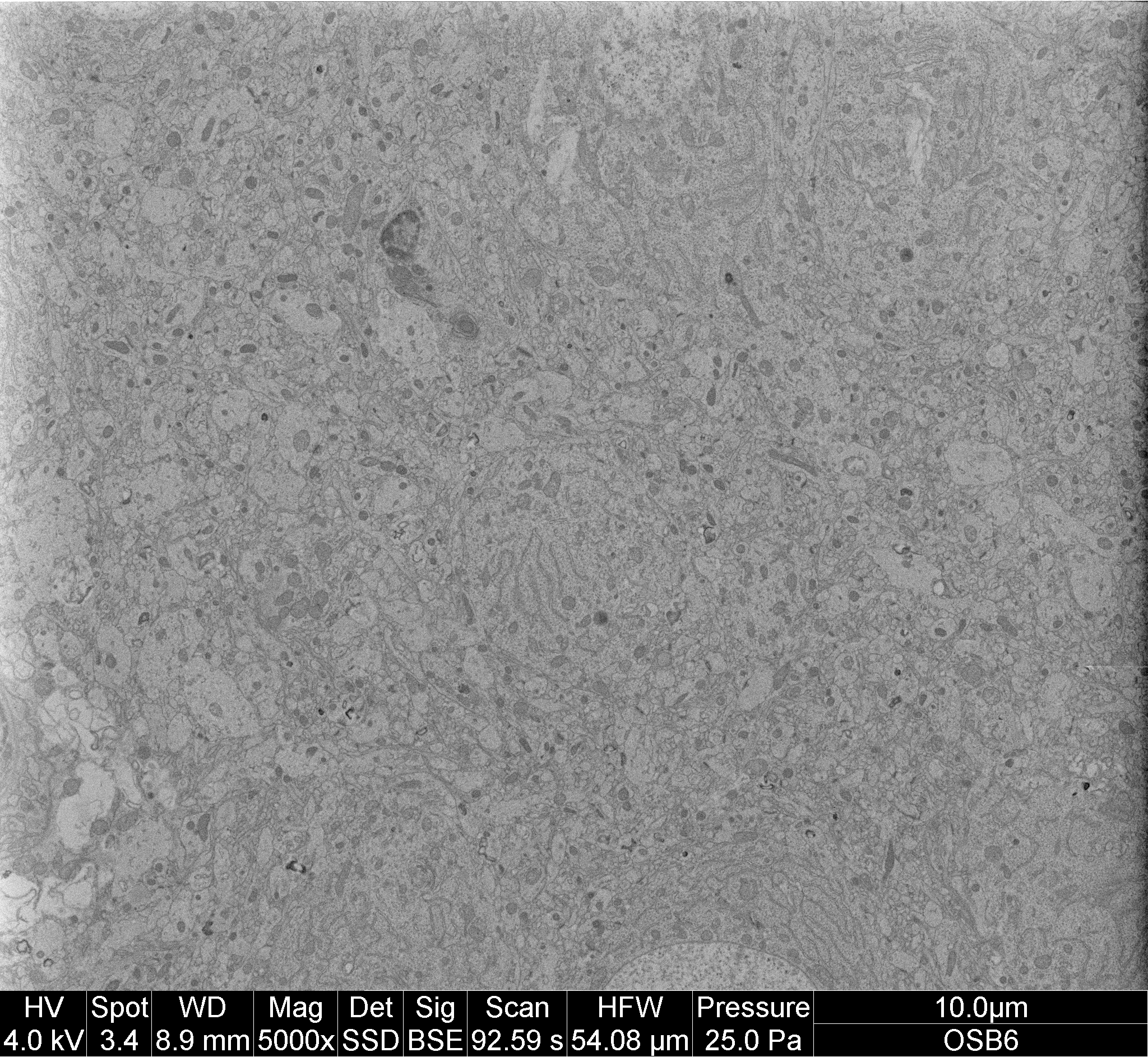

Supplement: Dataset S10 — (253.8 MB ZIP). [file pbio.0020329.sd010.zip › 040604_OS5_st1_997.tif]

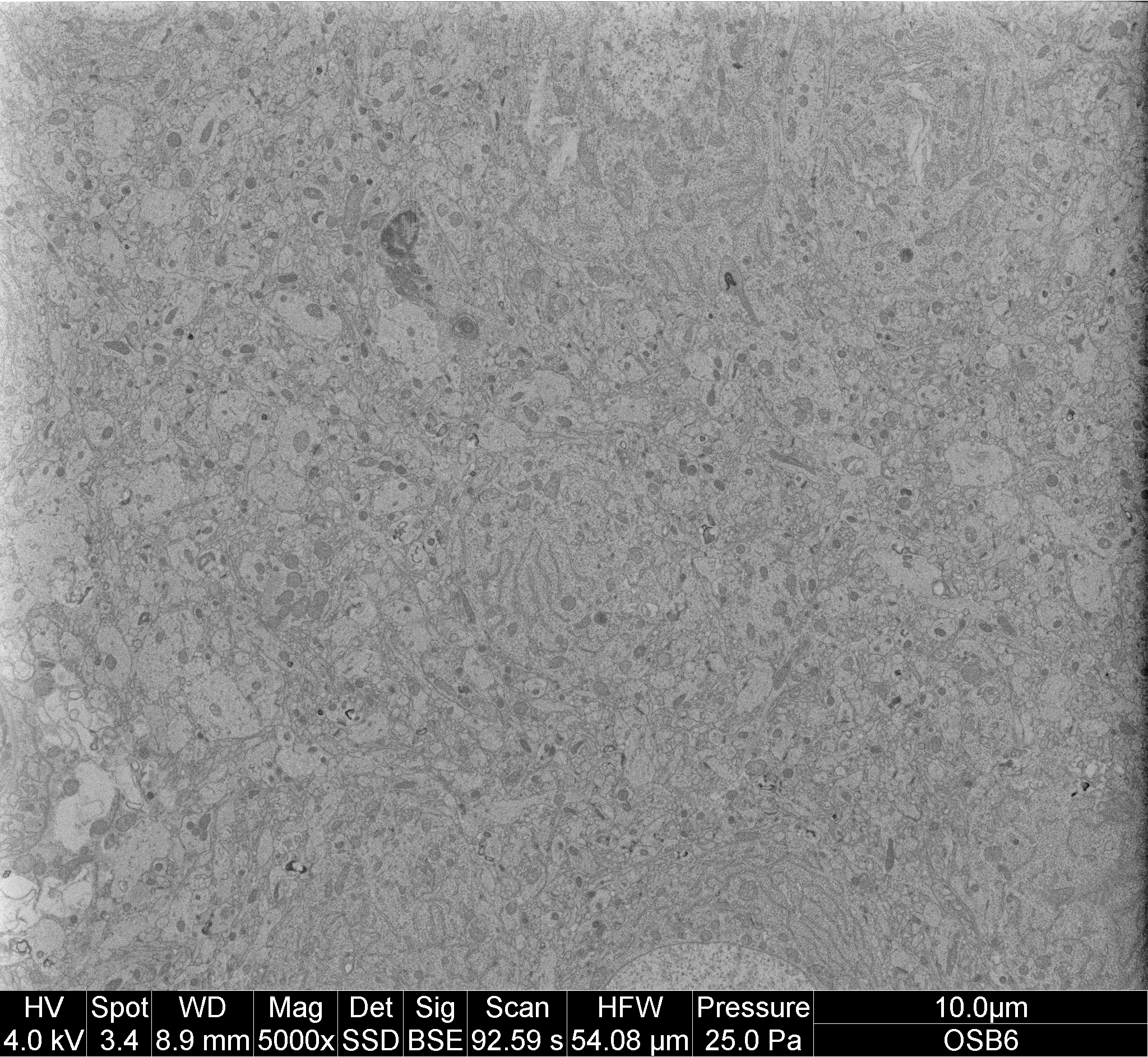

Supplement: Dataset S10 — (253.8 MB ZIP). [file pbio.0020329.sd010.zip › 040604_OS5_st1_998.tif]

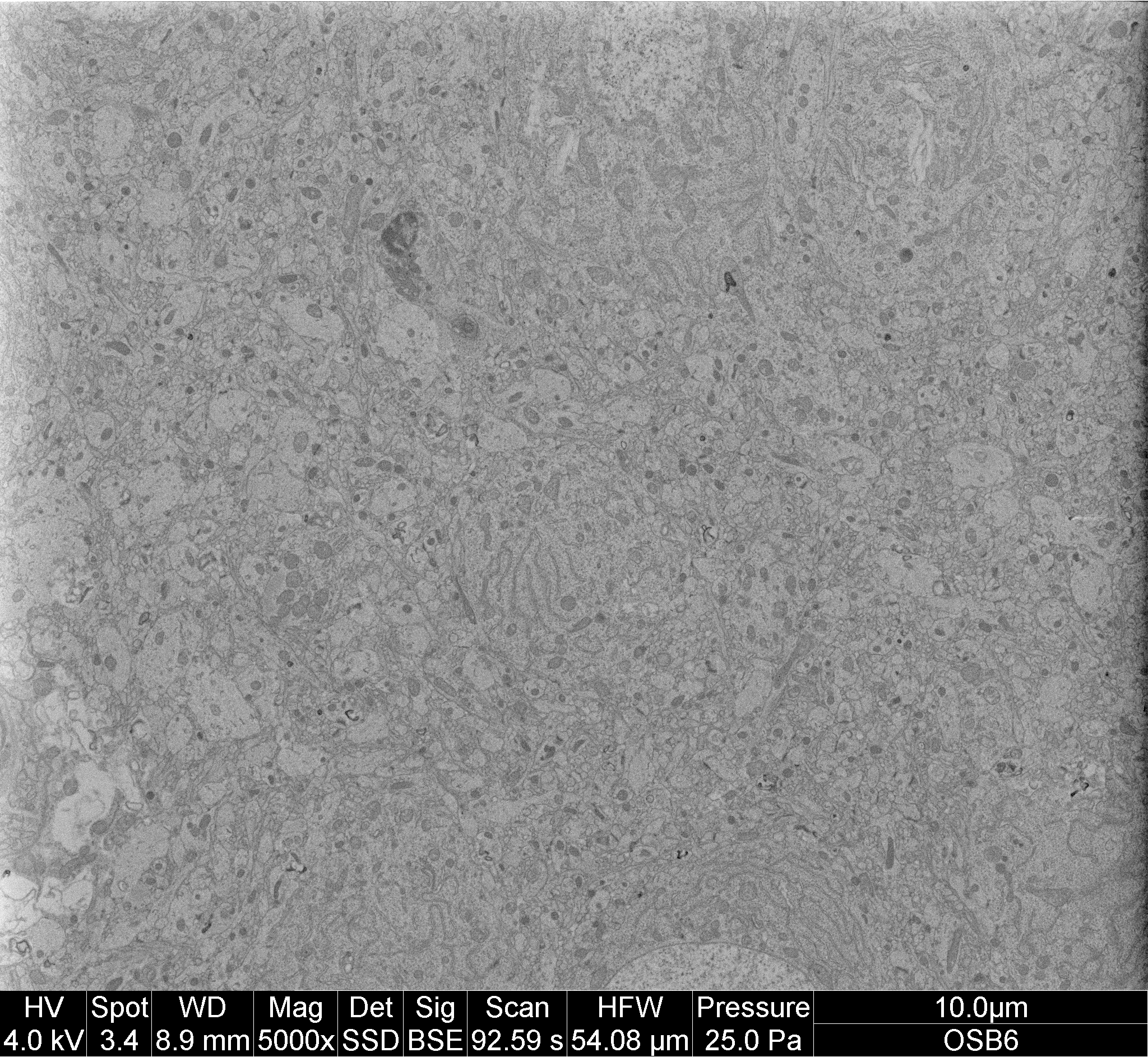

Supplement: Dataset S10 — (253.8 MB ZIP). [file pbio.0020329.sd010.zip › 040604_OS5_st1_999.tif]

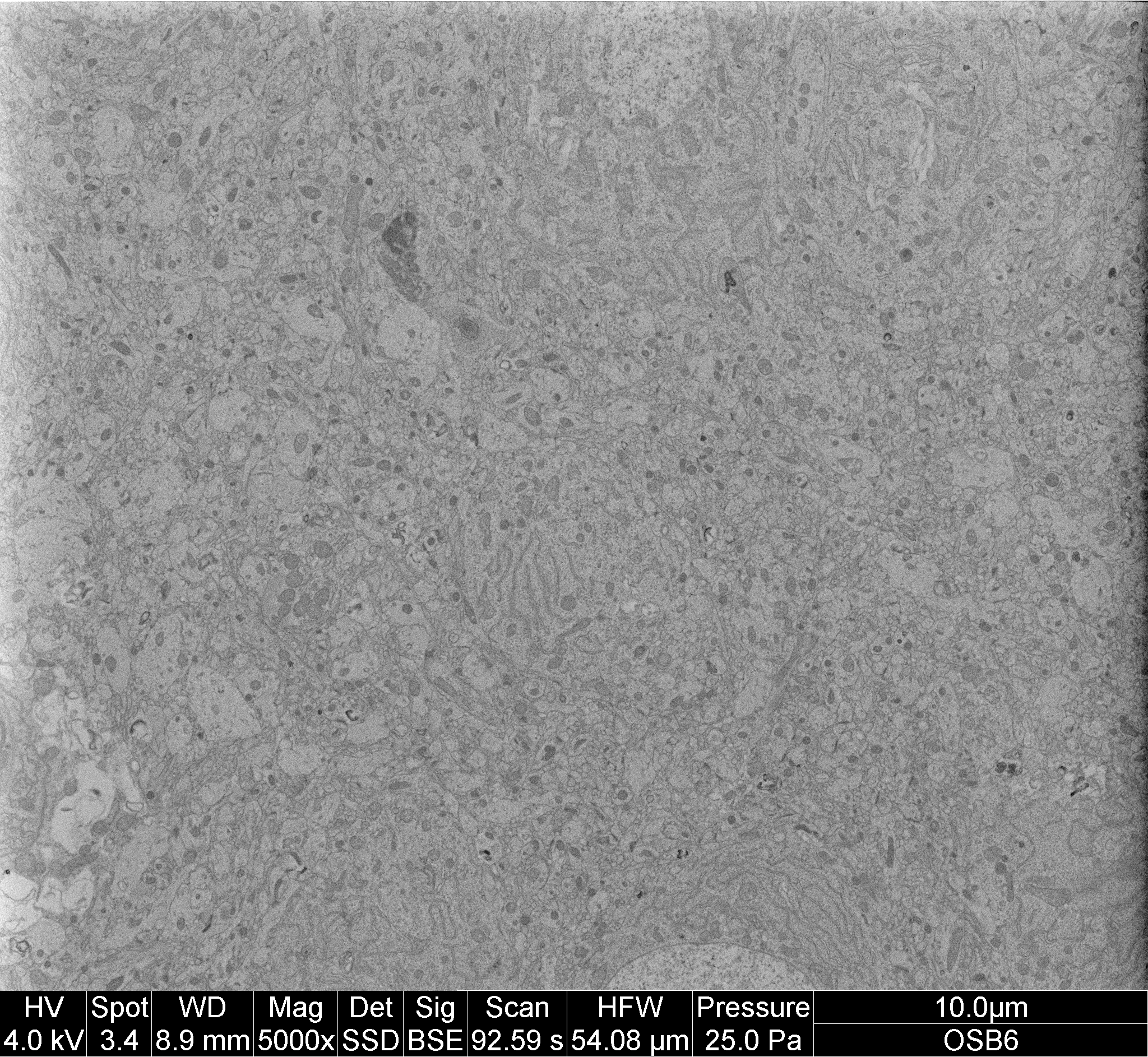

Supplement: Dataset S11 — (252.6 MB ZIP). [file pbio.0020329.sd011.zip › 040604_OS5_st1_1000.tif]
