# Supplementary figures and images for: Serial Block-Face Scanning Electron Microscopy to Reconstruct Three-Dimensional Tissue Nanostructure (part 11 of 21)
Source: PLoS Biol. 2004 Oct 19;2(11):e329. doi: 10.1371/journal.pbio.0020329 (PMC524270; doi:10.1371/journal.pbio.0020329)

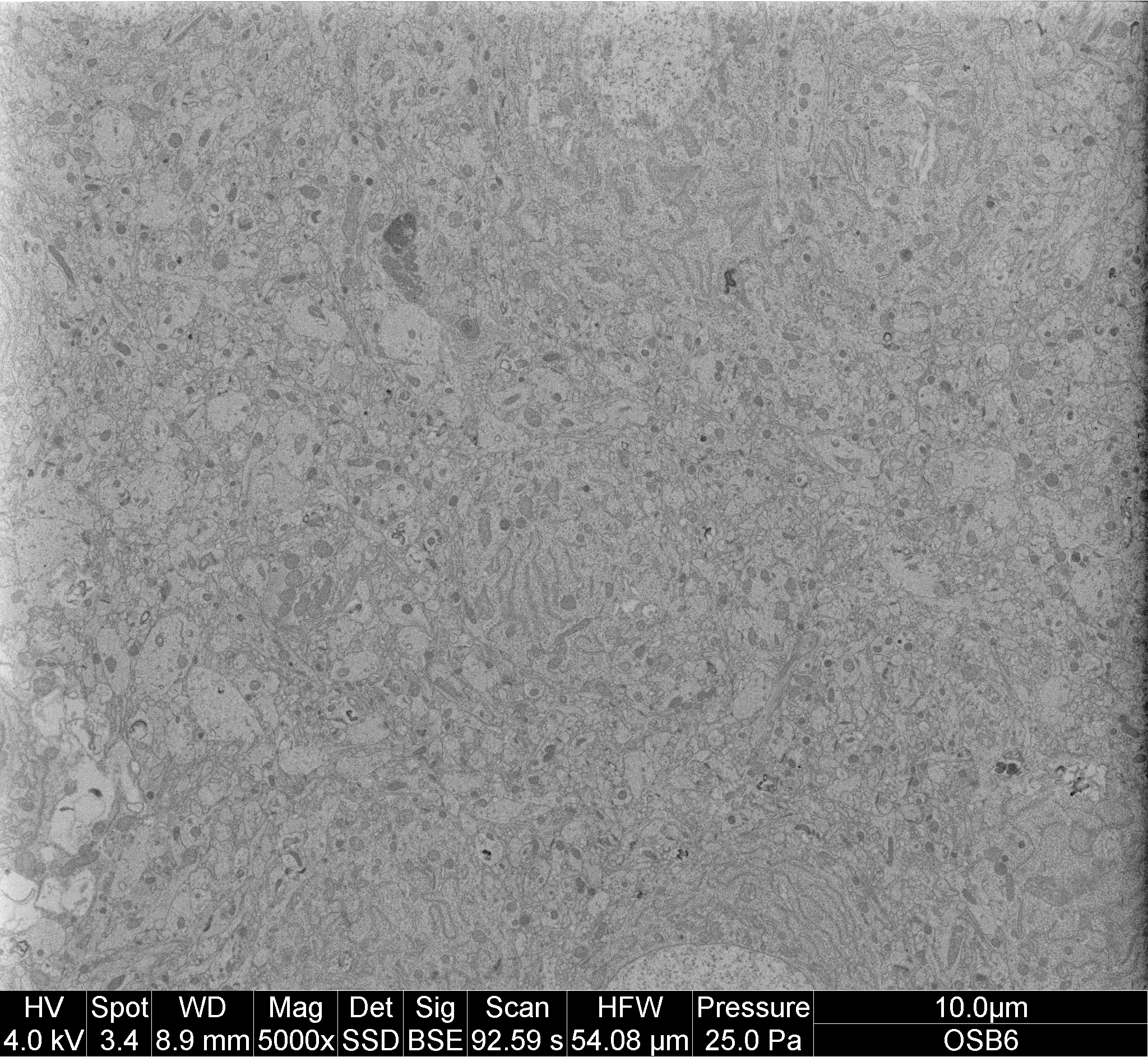

Supplement: Dataset S11 — (252.6 MB ZIP). [file pbio.0020329.sd011.zip › 040604_OS5_st1_1001.tif]

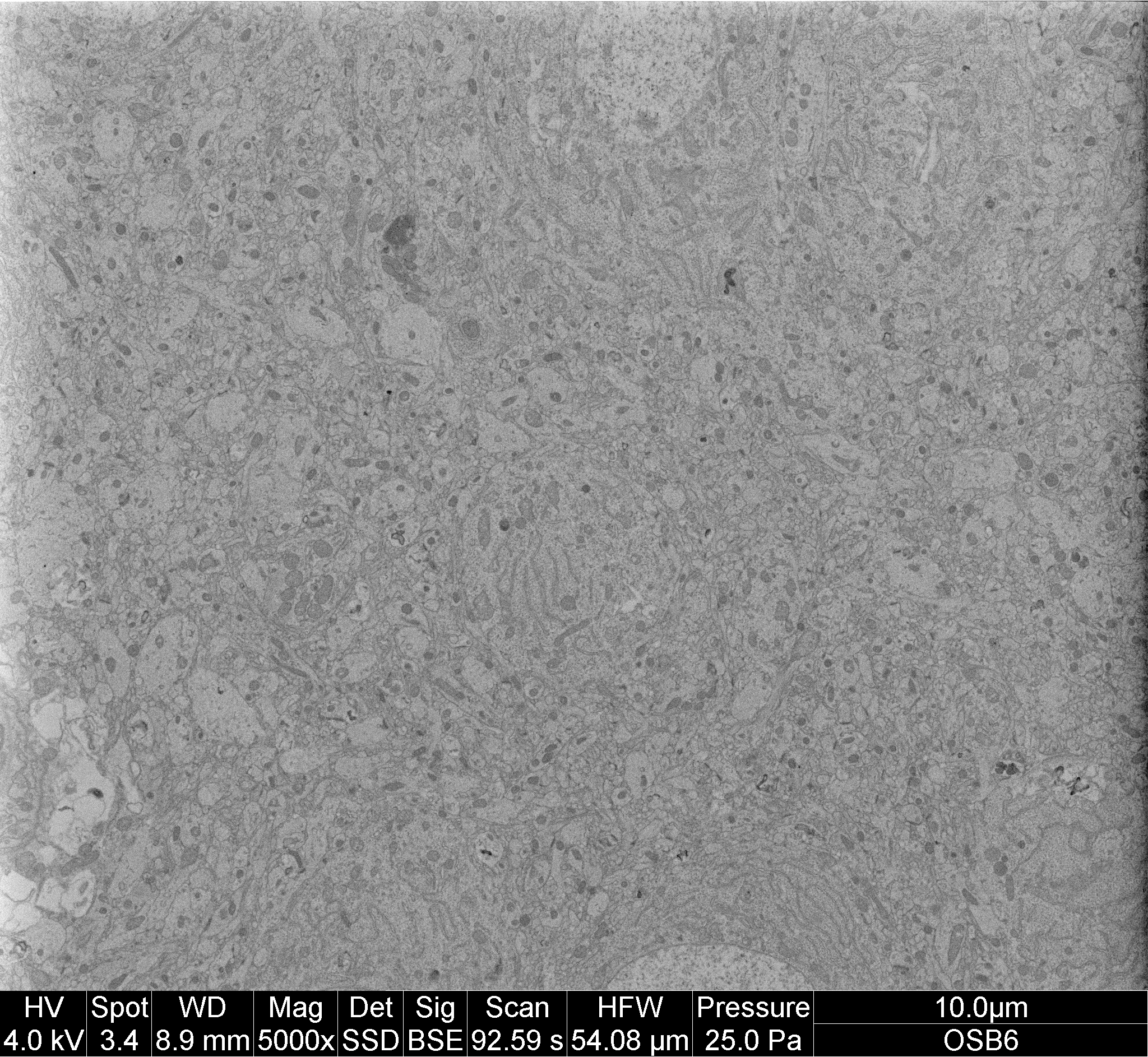

Supplement: Dataset S11 — (252.6 MB ZIP). [file pbio.0020329.sd011.zip › 040604_OS5_st1_1002.tif]

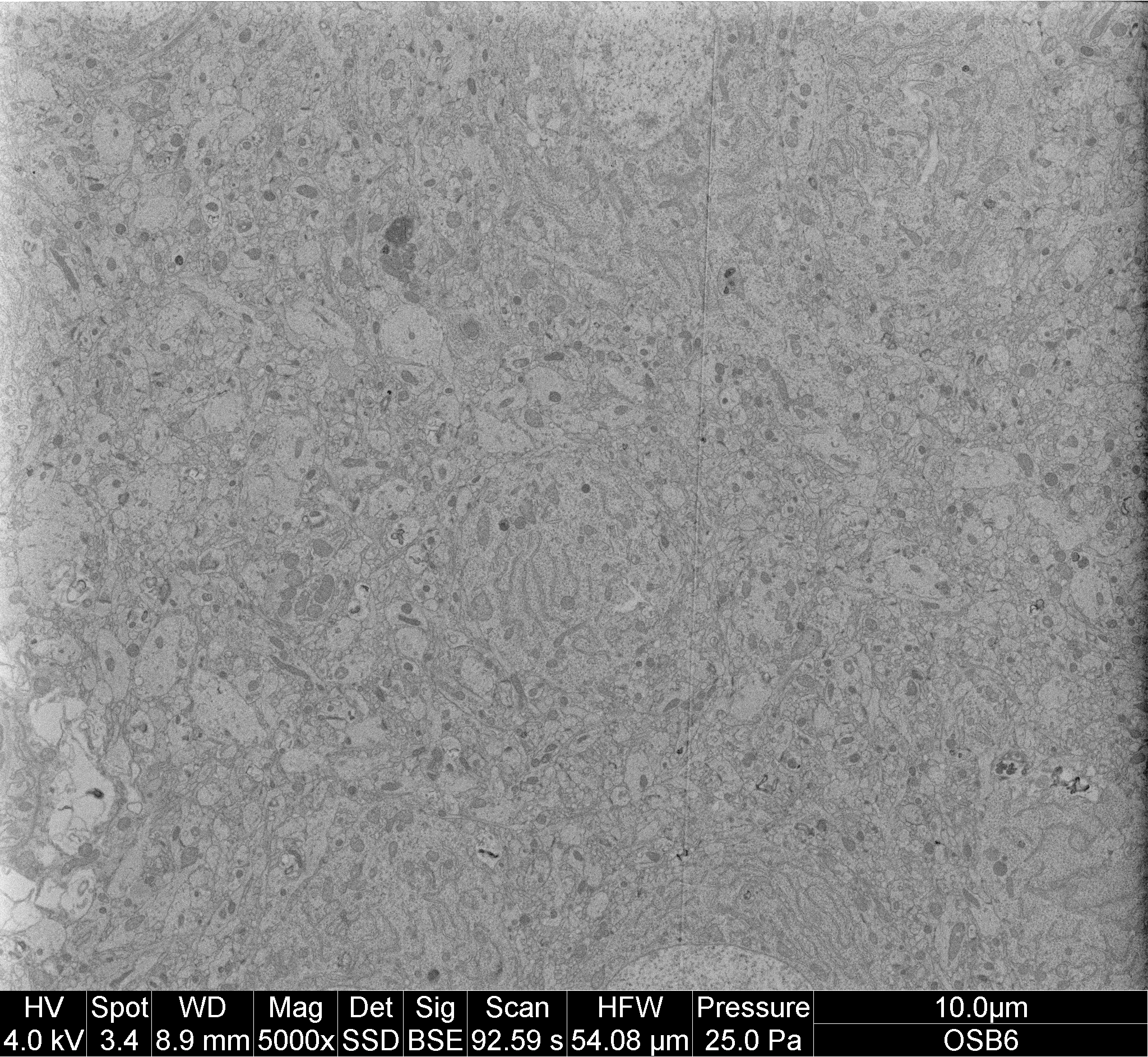

Supplement: Dataset S11 — (252.6 MB ZIP). [file pbio.0020329.sd011.zip › 040604_OS5_st1_1003.tif]

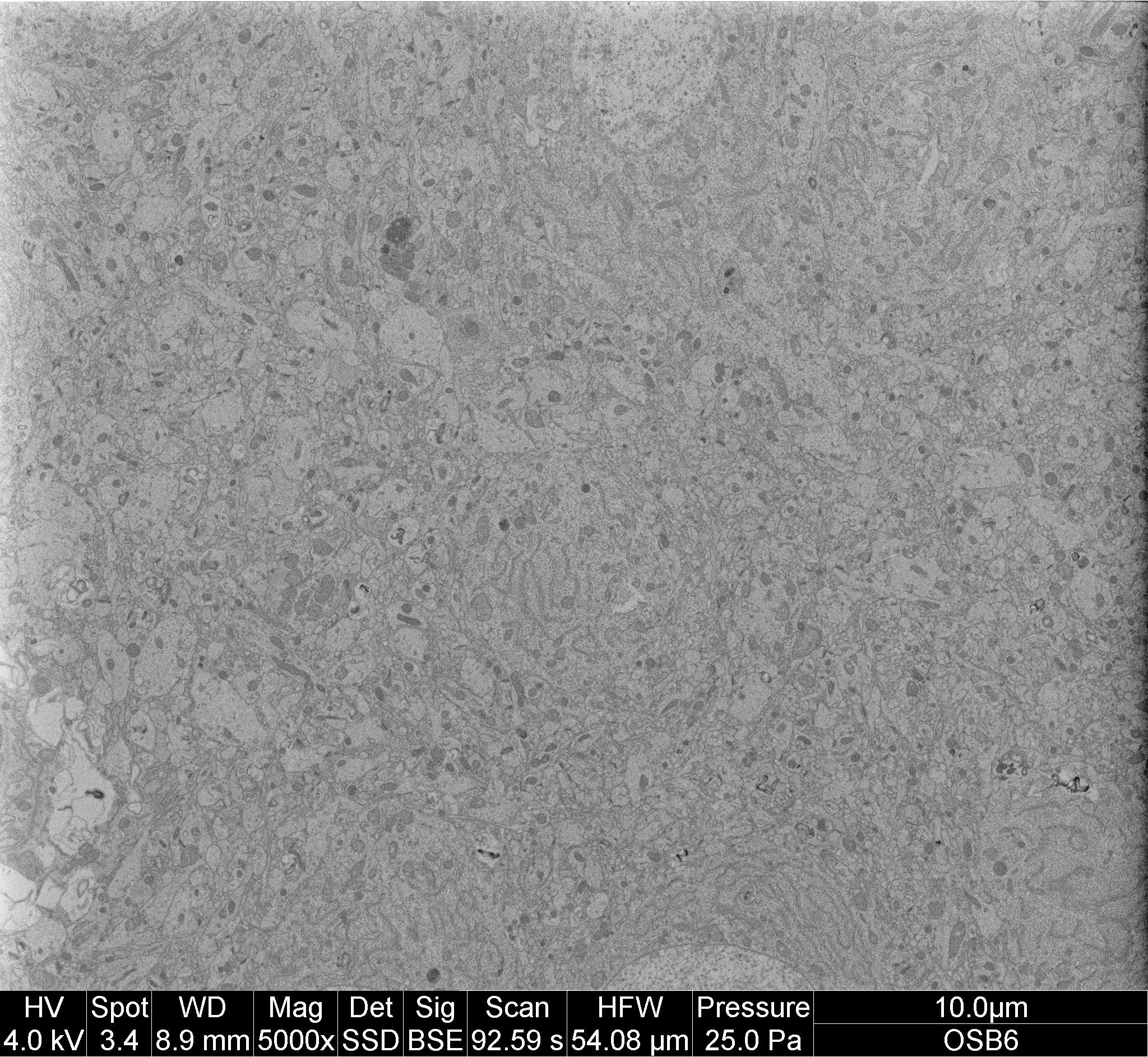

Supplement: Dataset S11 — (252.6 MB ZIP). [file pbio.0020329.sd011.zip › 040604_OS5_st1_1004.tif]

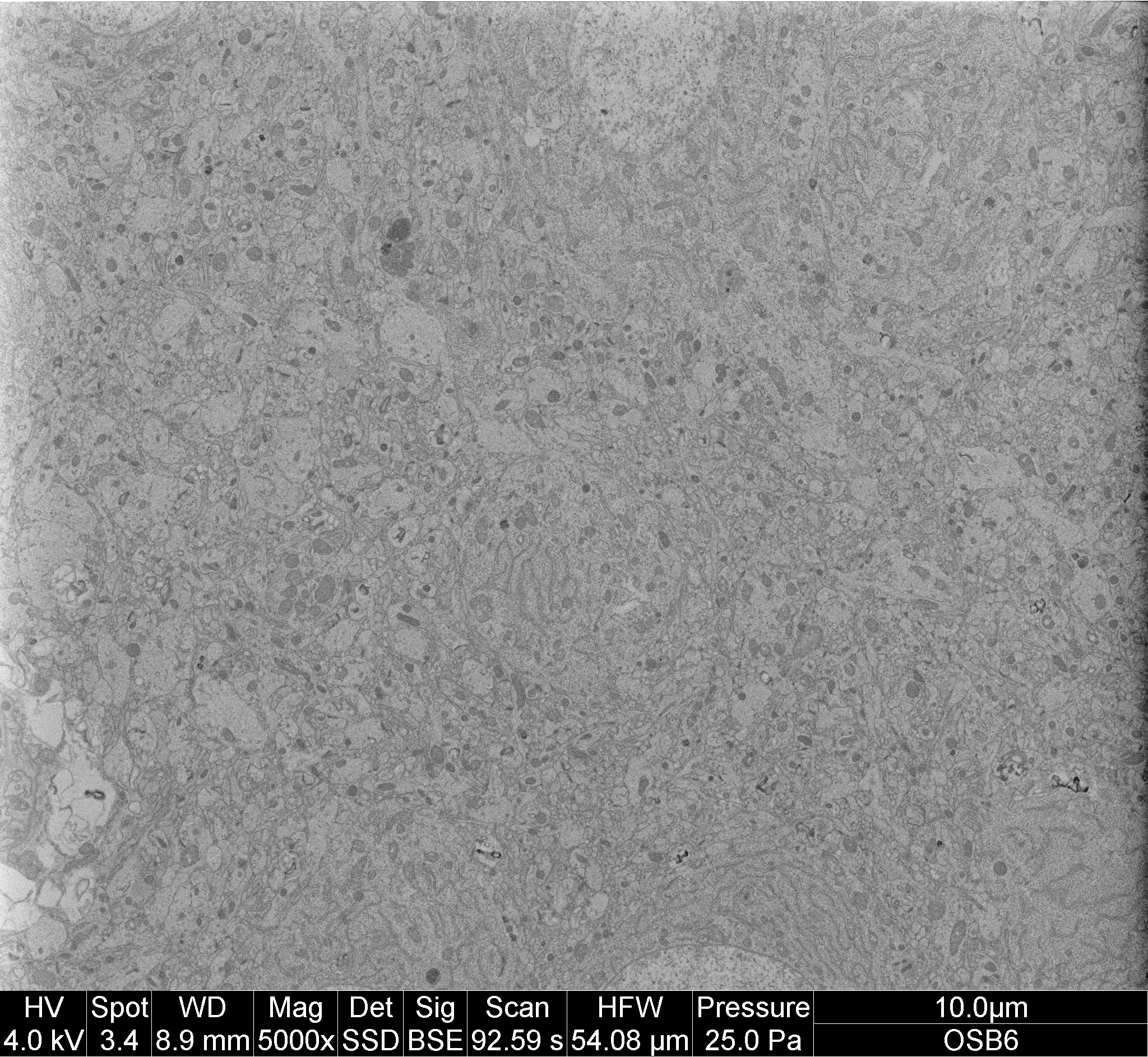

Supplement: Dataset S11 — (252.6 MB ZIP). [file pbio.0020329.sd011.zip › 040604_OS5_st1_1005.tif]

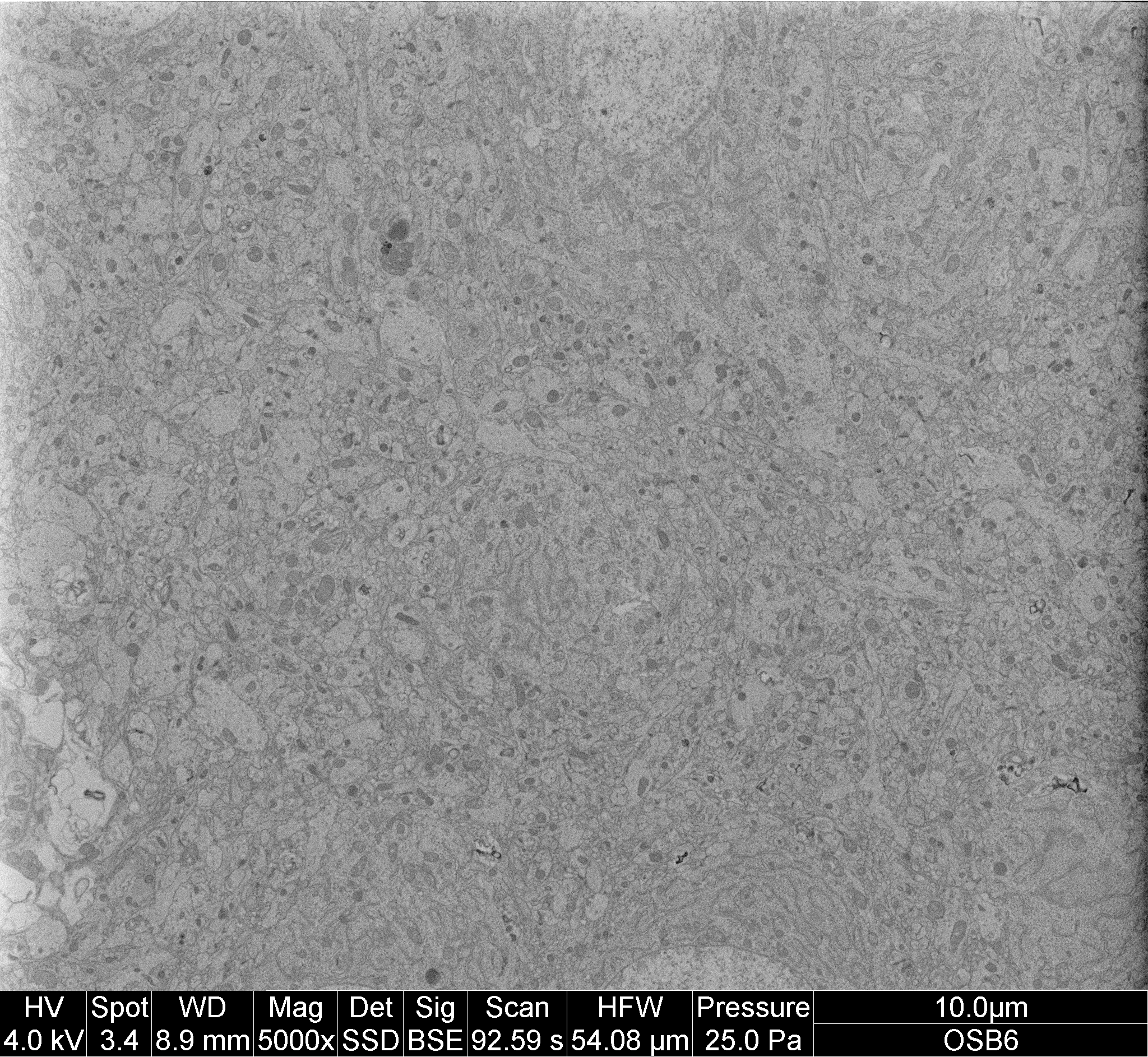

Supplement: Dataset S11 — (252.6 MB ZIP). [file pbio.0020329.sd011.zip › 040604_OS5_st1_1006.tif]

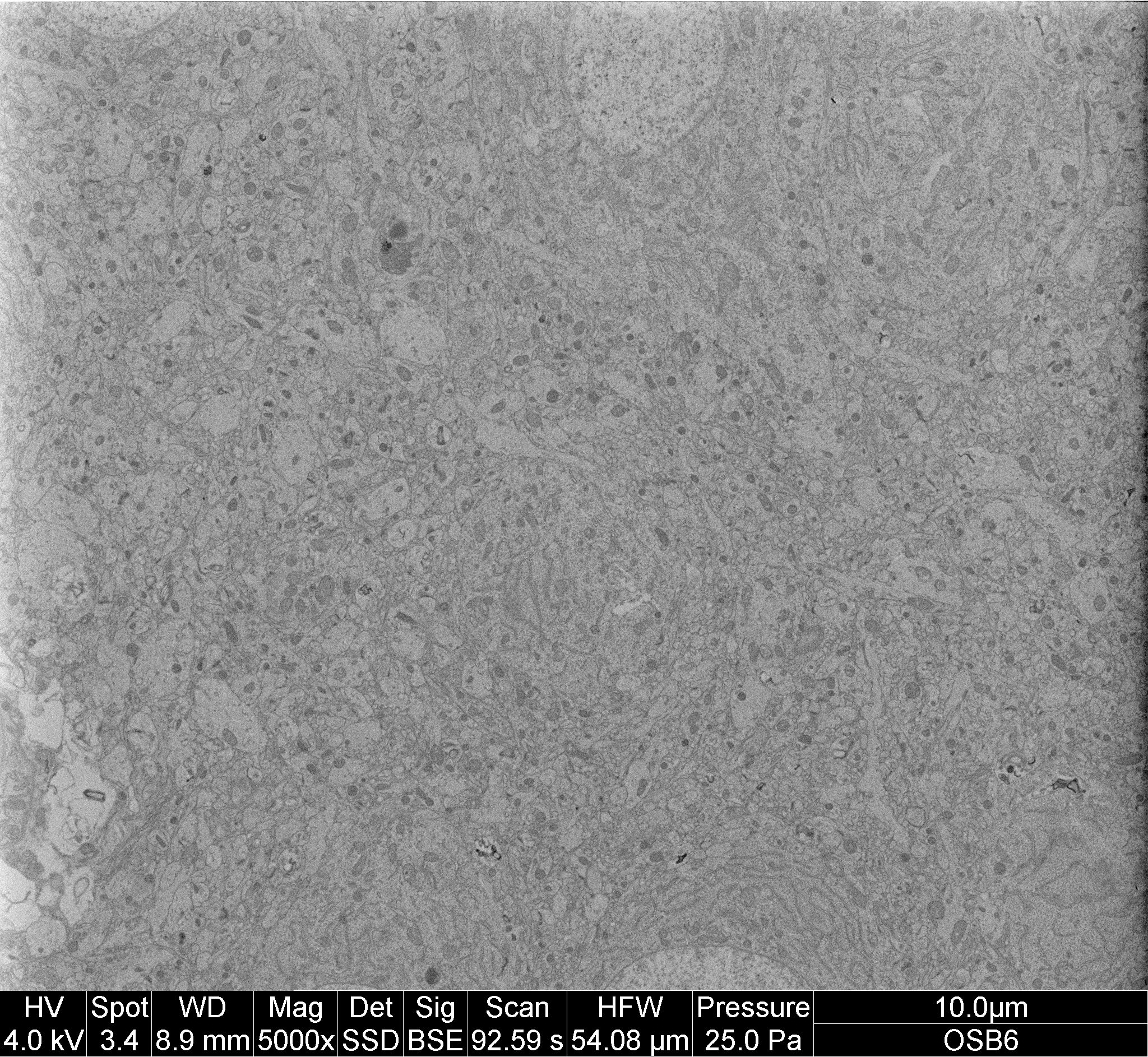

Supplement: Dataset S11 — (252.6 MB ZIP). [file pbio.0020329.sd011.zip › 040604_OS5_st1_1007.tif]

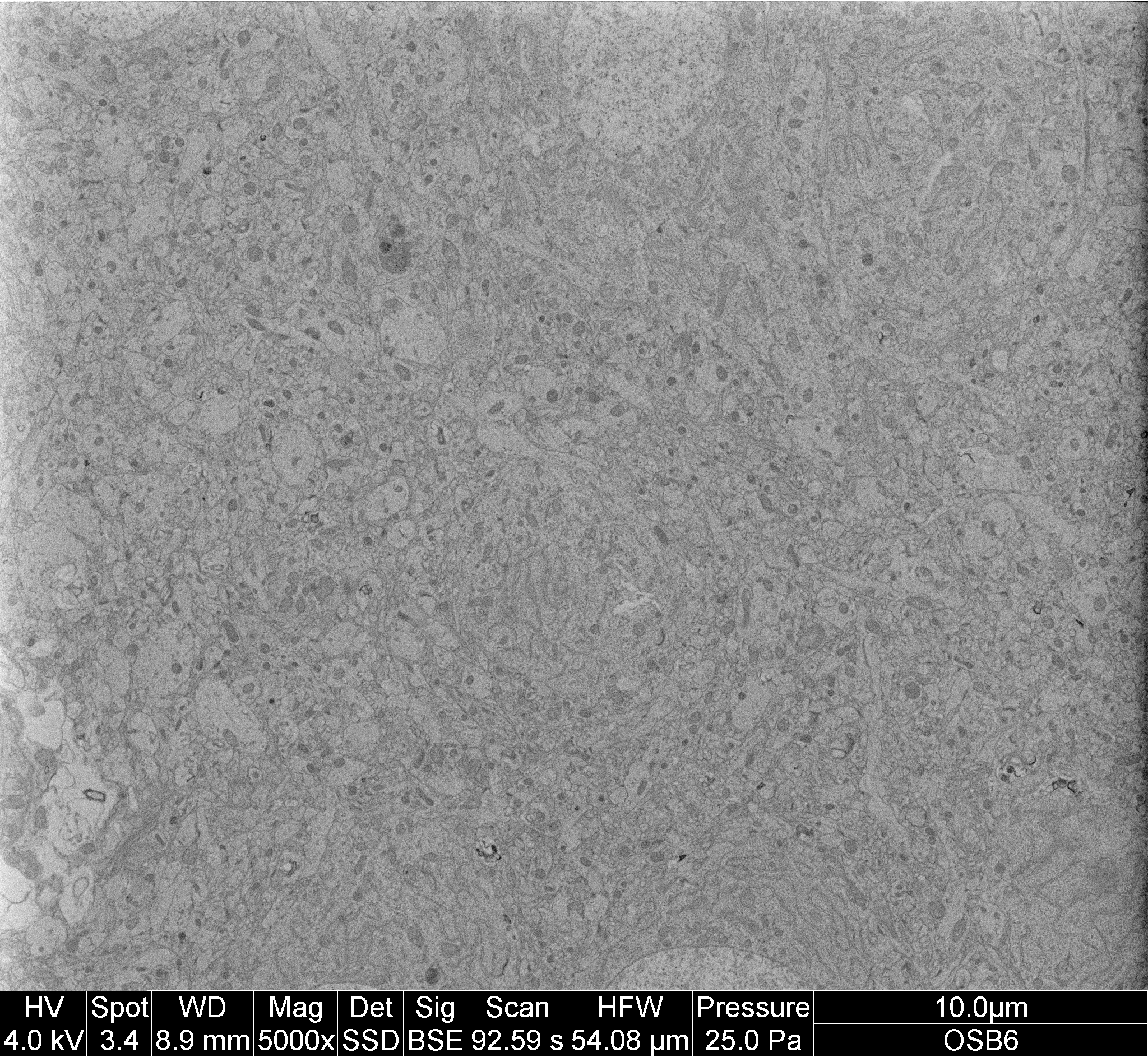

Supplement: Dataset S11 — (252.6 MB ZIP). [file pbio.0020329.sd011.zip › 040604_OS5_st1_1008.tif]

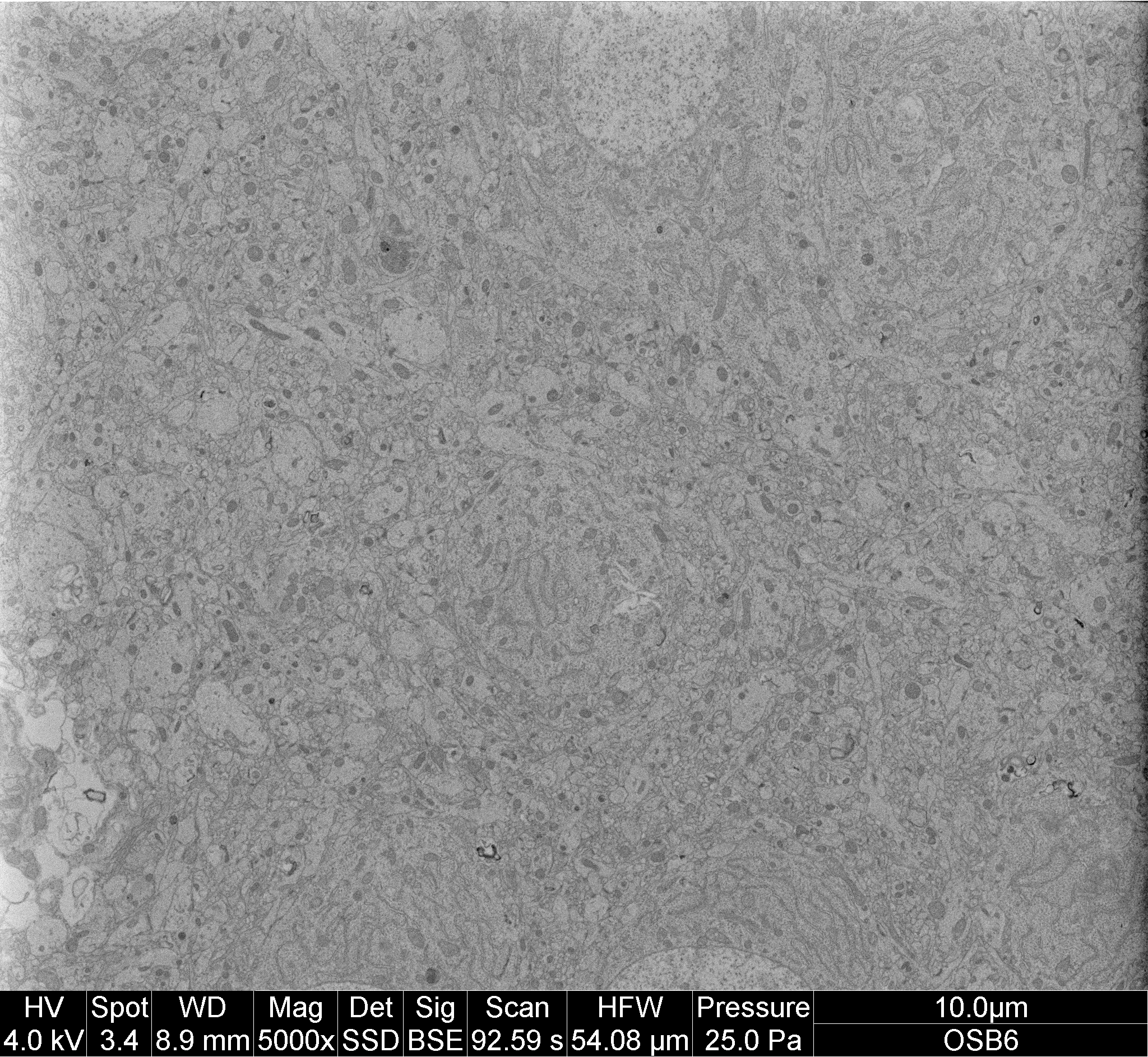

Supplement: Dataset S11 — (252.6 MB ZIP). [file pbio.0020329.sd011.zip › 040604_OS5_st1_1009.tif]

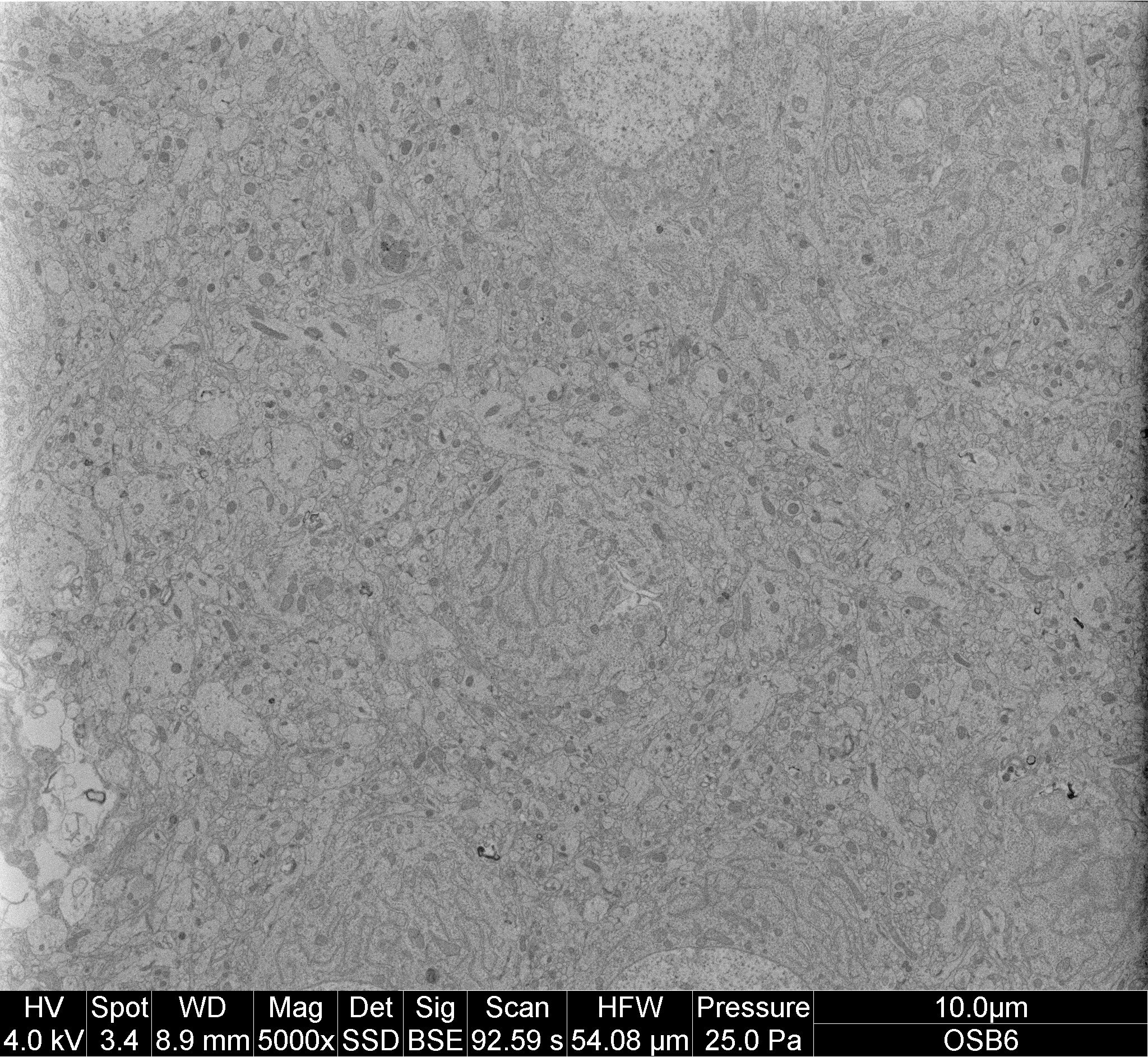

Supplement: Dataset S11 — (252.6 MB ZIP). [file pbio.0020329.sd011.zip › 040604_OS5_st1_1010.tif]

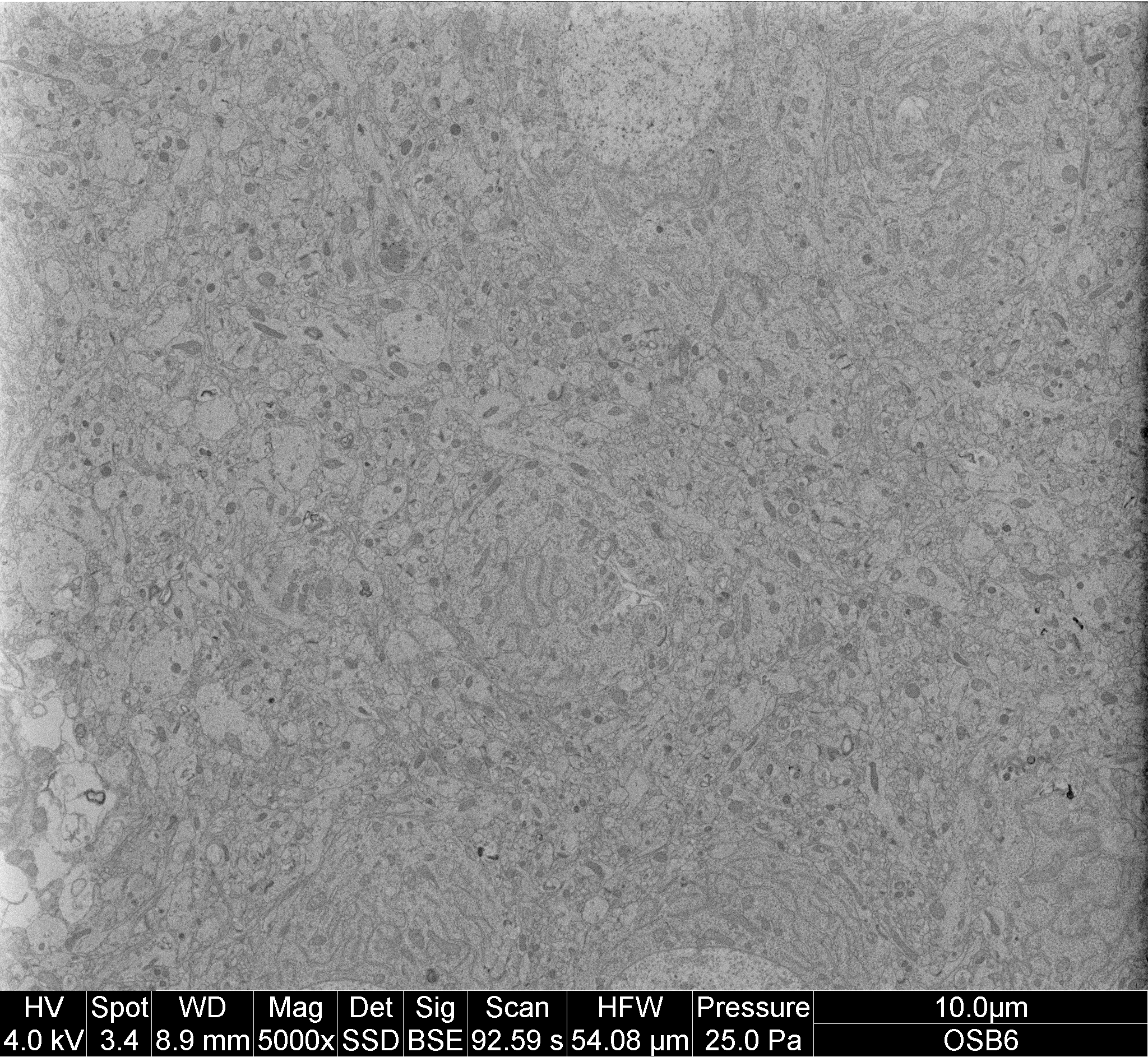

Supplement: Dataset S11 — (252.6 MB ZIP). [file pbio.0020329.sd011.zip › 040604_OS5_st1_1011.tif]

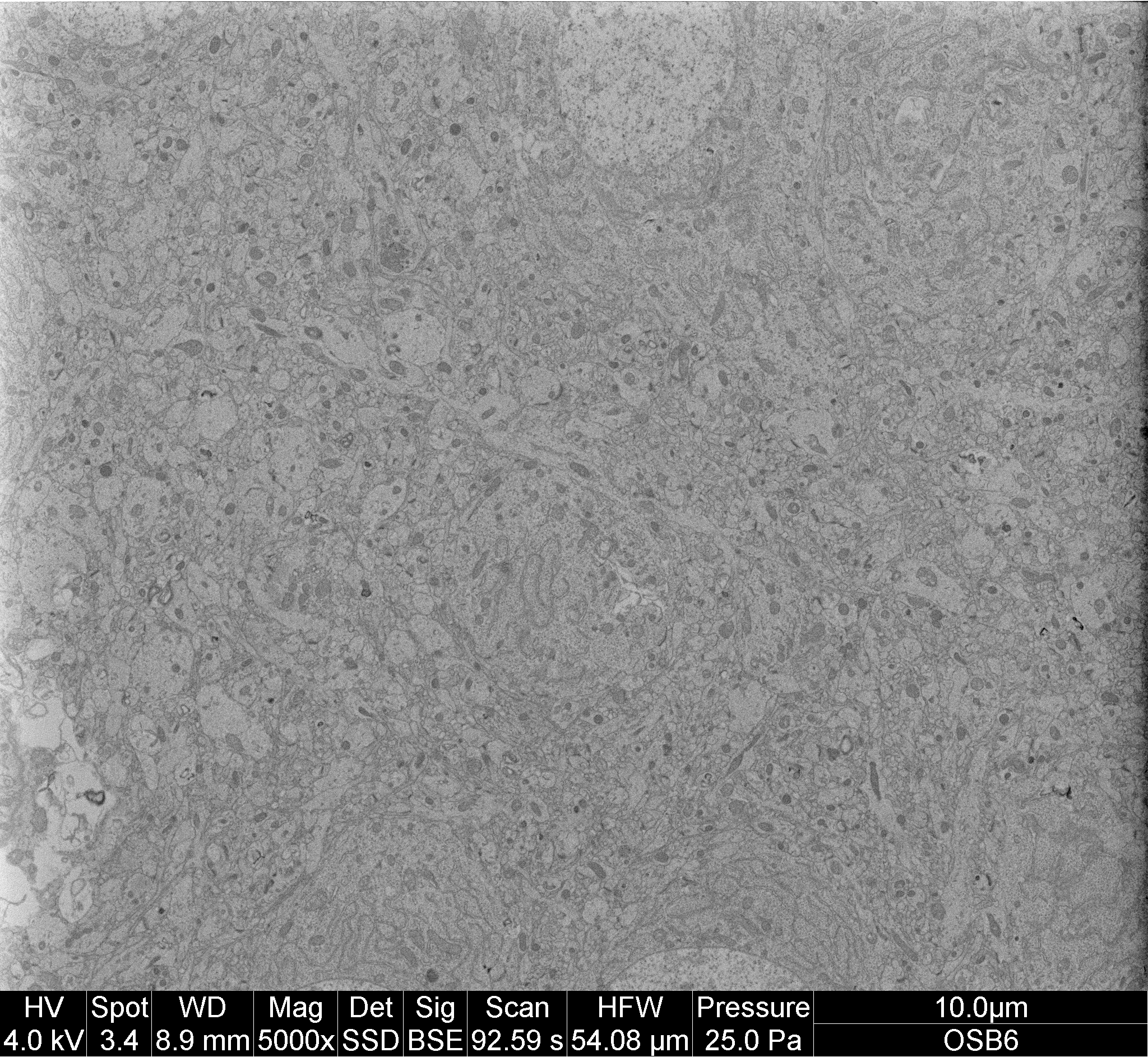

Supplement: Dataset S11 — (252.6 MB ZIP). [file pbio.0020329.sd011.zip › 040604_OS5_st1_1012.tif]

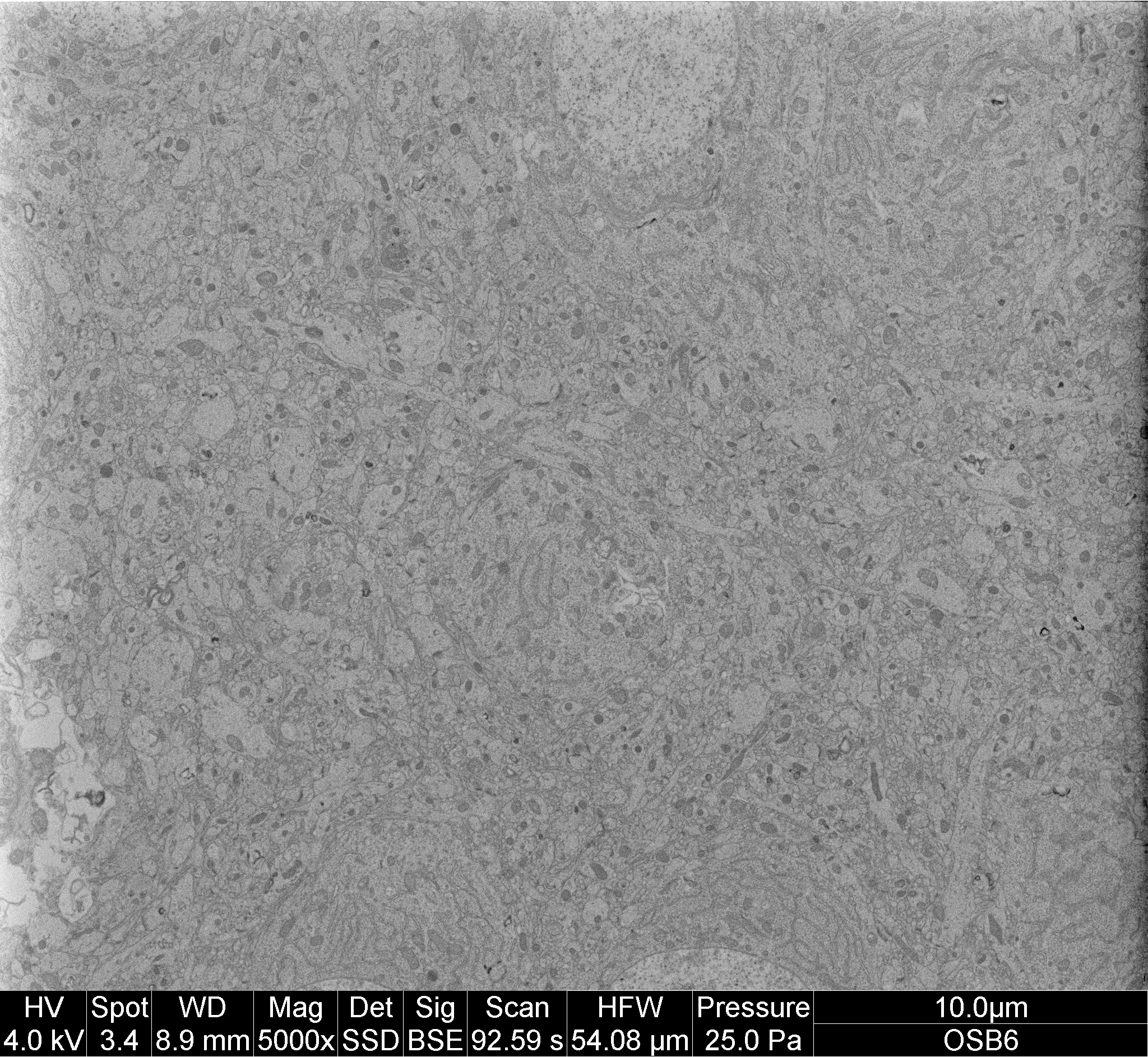

Supplement: Dataset S11 — (252.6 MB ZIP). [file pbio.0020329.sd011.zip › 040604_OS5_st1_1013.tif]

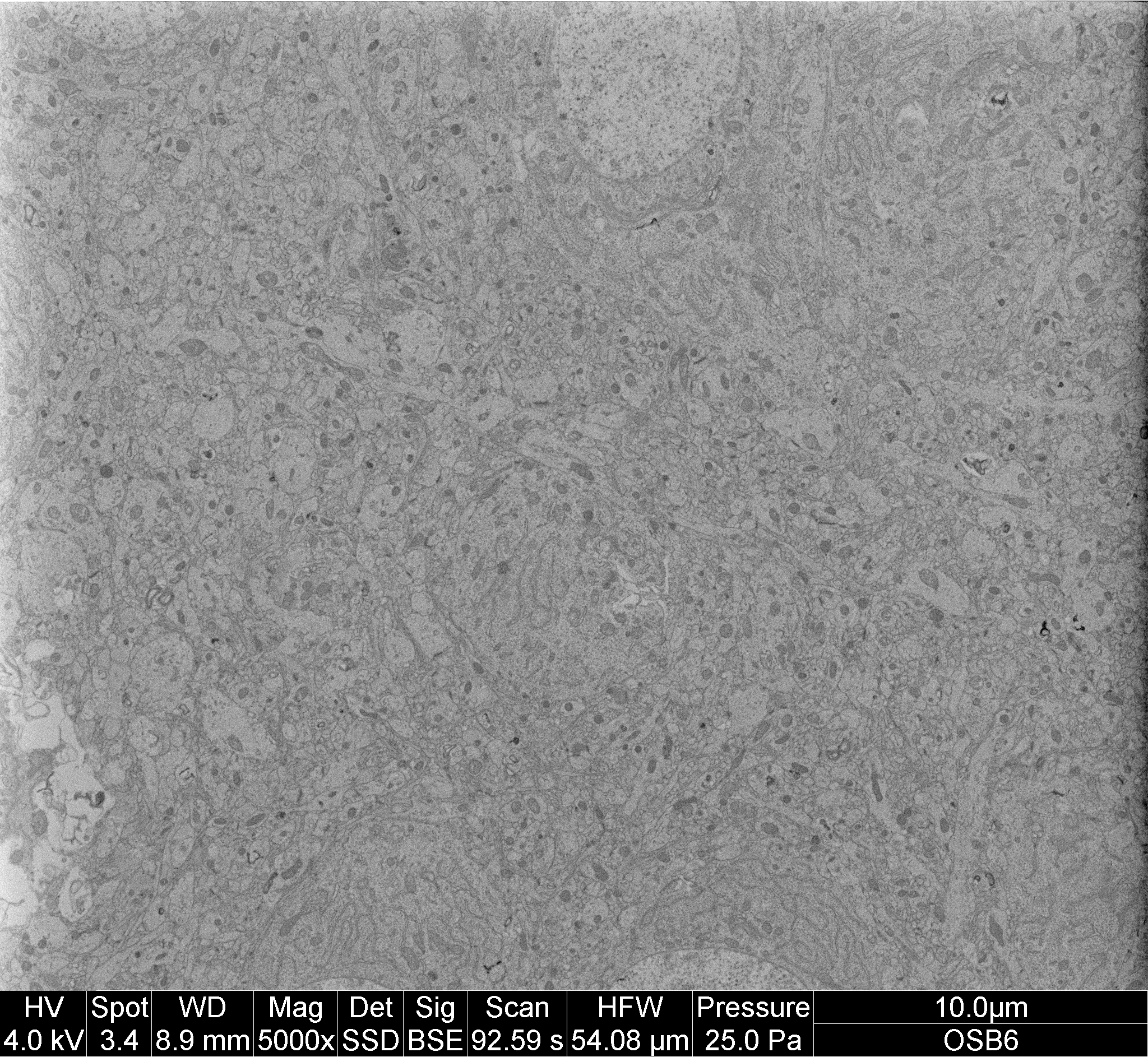

Supplement: Dataset S11 — (252.6 MB ZIP). [file pbio.0020329.sd011.zip › 040604_OS5_st1_1014.tif]

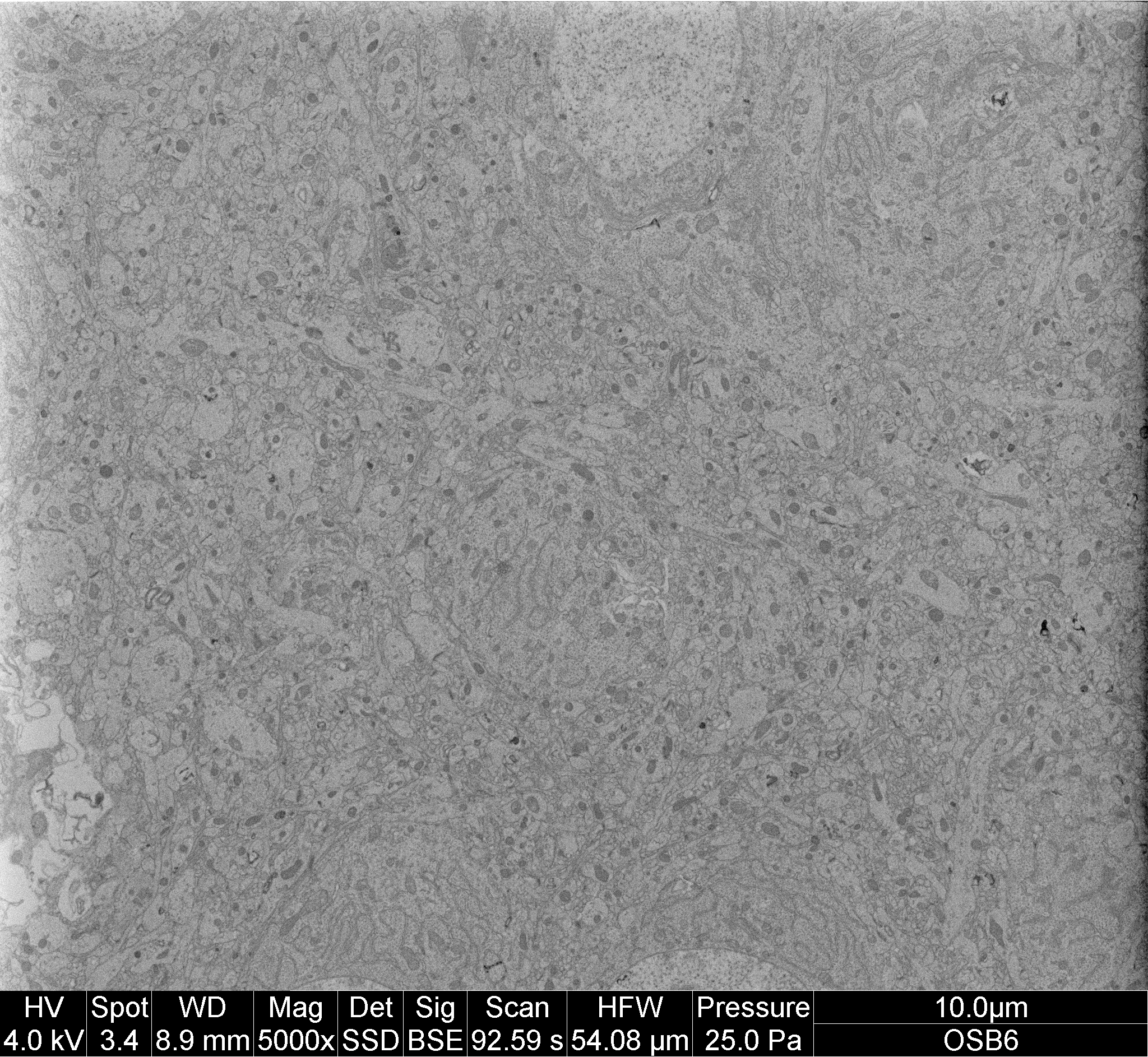

Supplement: Dataset S11 — (252.6 MB ZIP). [file pbio.0020329.sd011.zip › 040604_OS5_st1_1015.tif]

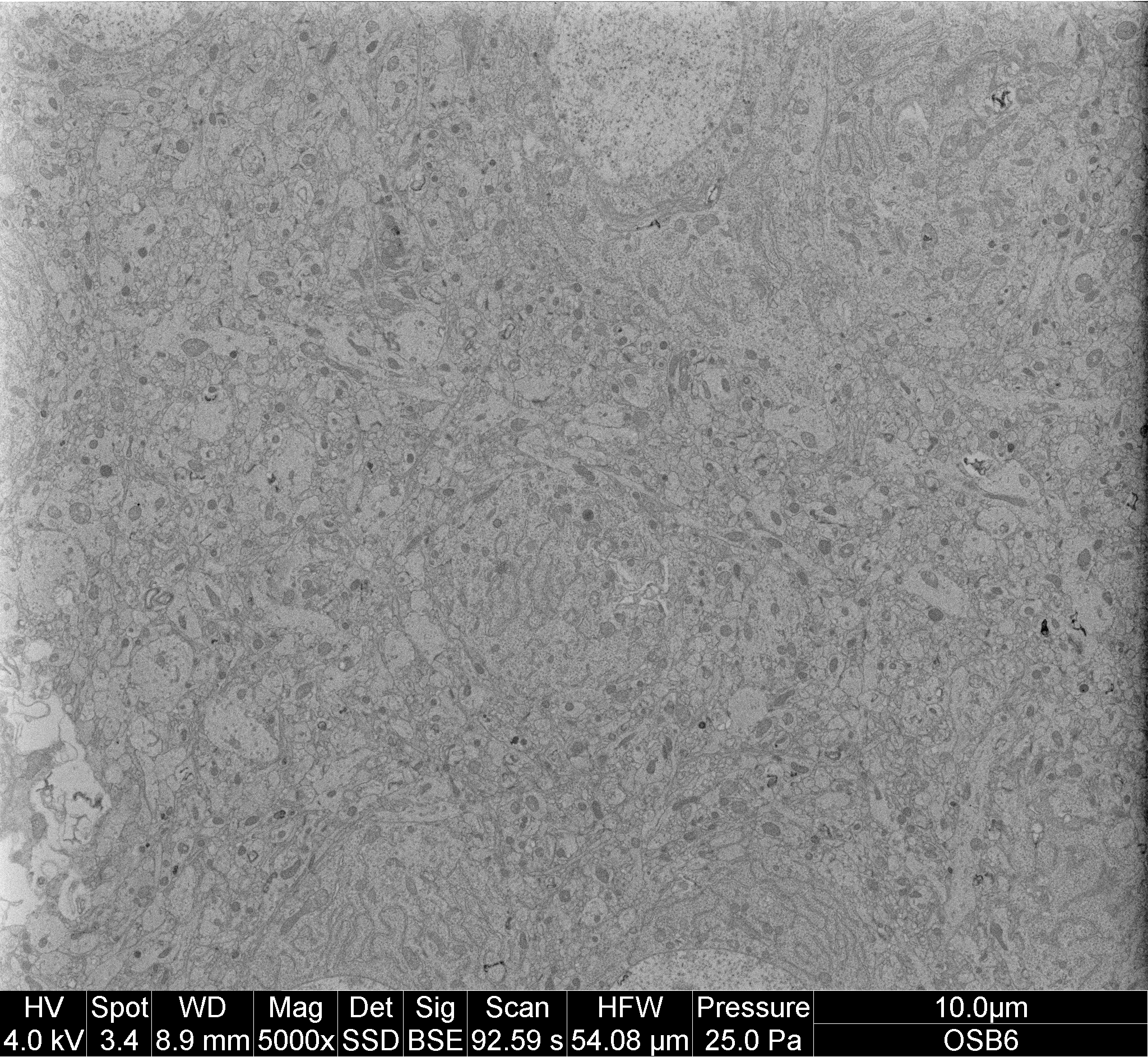

Supplement: Dataset S11 — (252.6 MB ZIP). [file pbio.0020329.sd011.zip › 040604_OS5_st1_1016.tif]

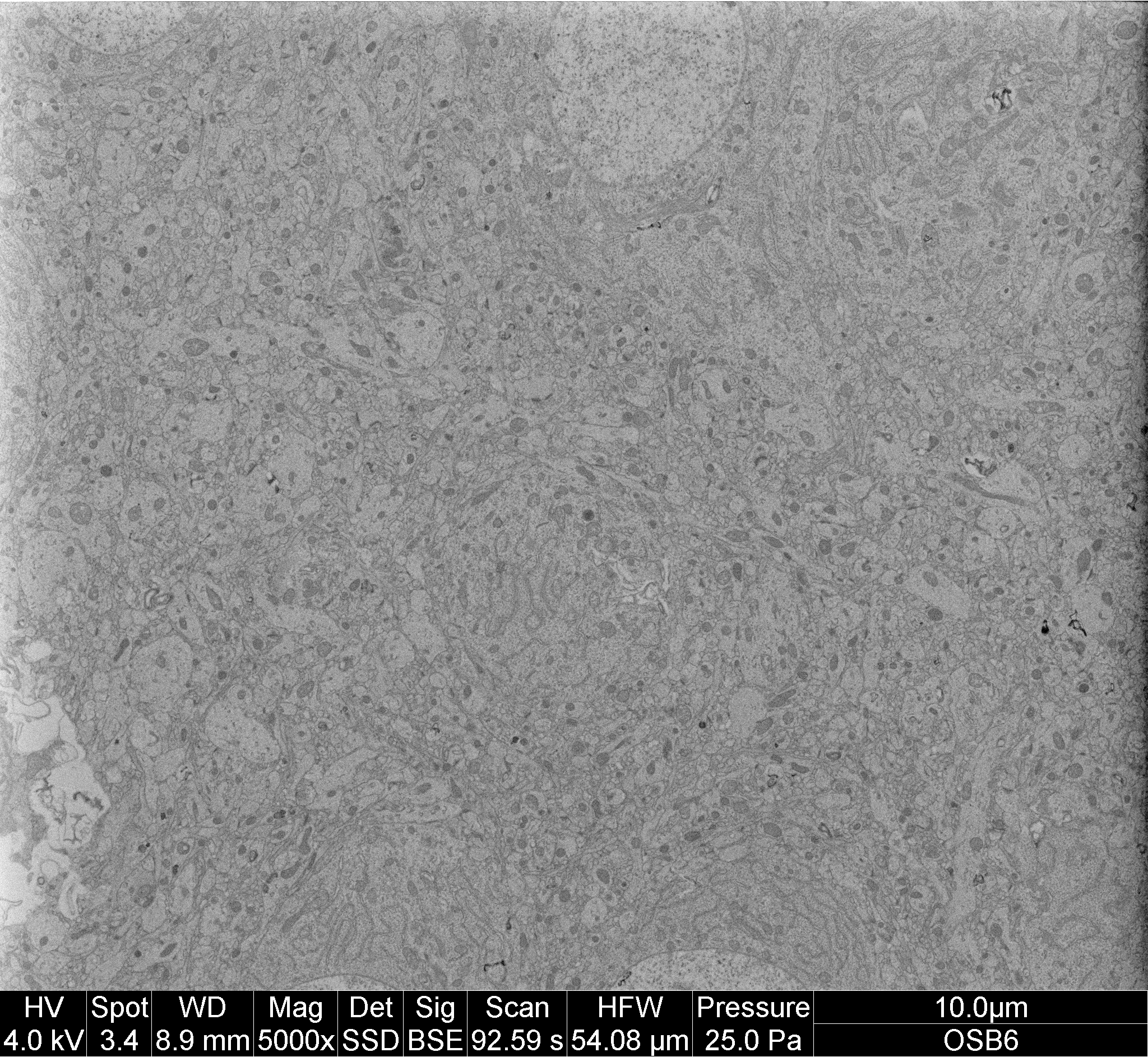

Supplement: Dataset S11 — (252.6 MB ZIP). [file pbio.0020329.sd011.zip › 040604_OS5_st1_1017.tif]

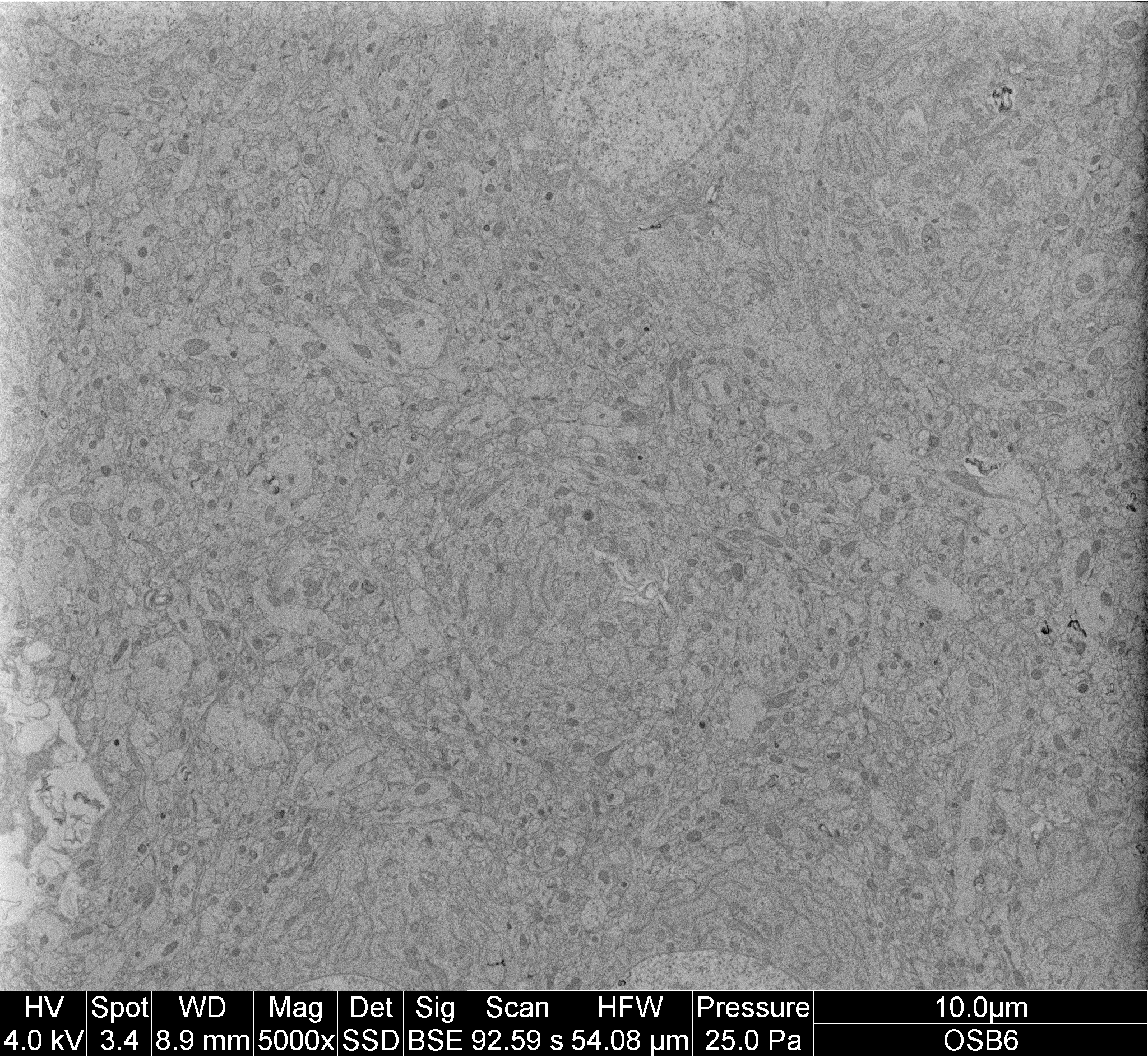

Supplement: Dataset S11 — (252.6 MB ZIP). [file pbio.0020329.sd011.zip › 040604_OS5_st1_1018.tif]

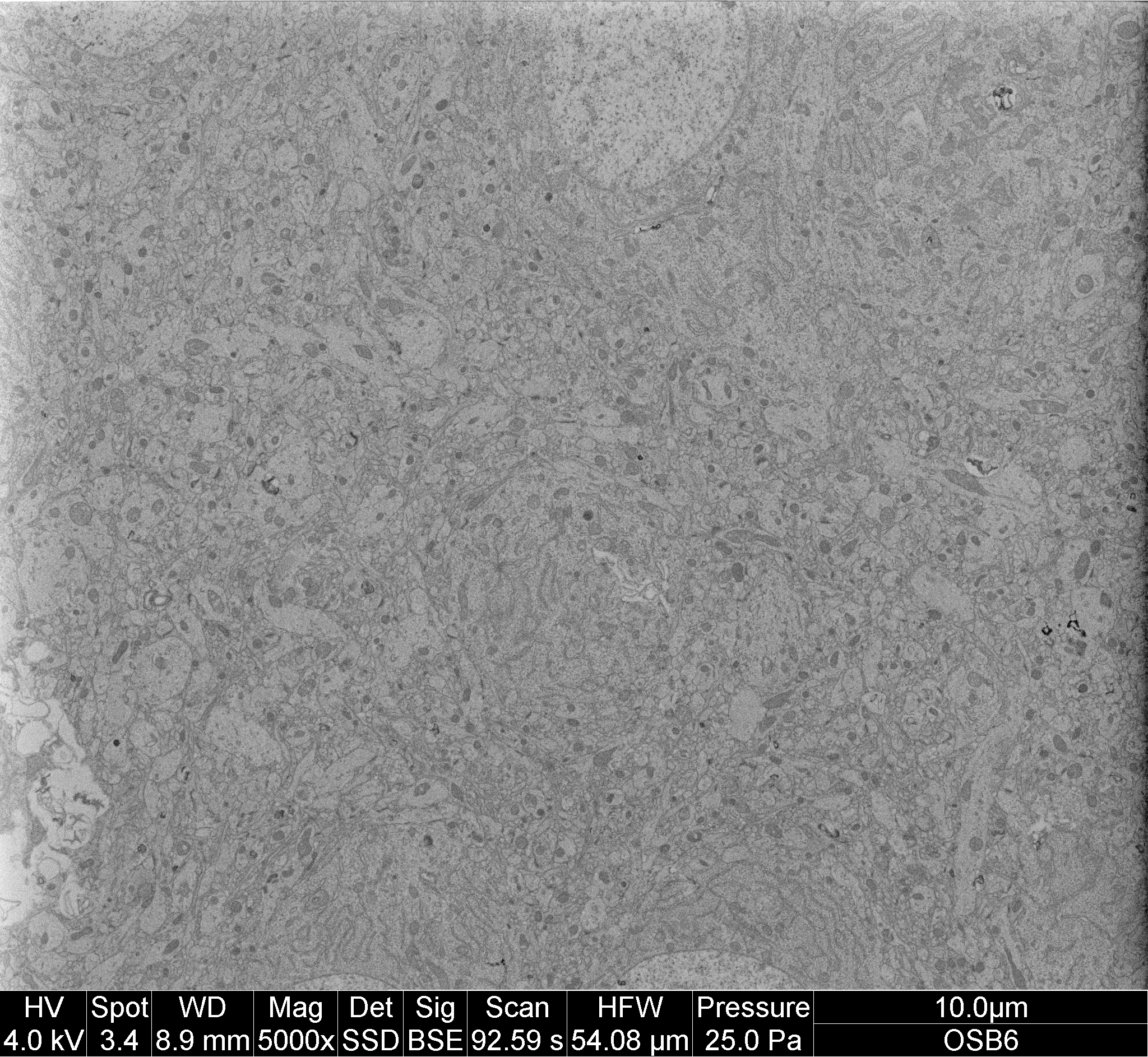

Supplement: Dataset S11 — (252.6 MB ZIP). [file pbio.0020329.sd011.zip › 040604_OS5_st1_1019.tif]

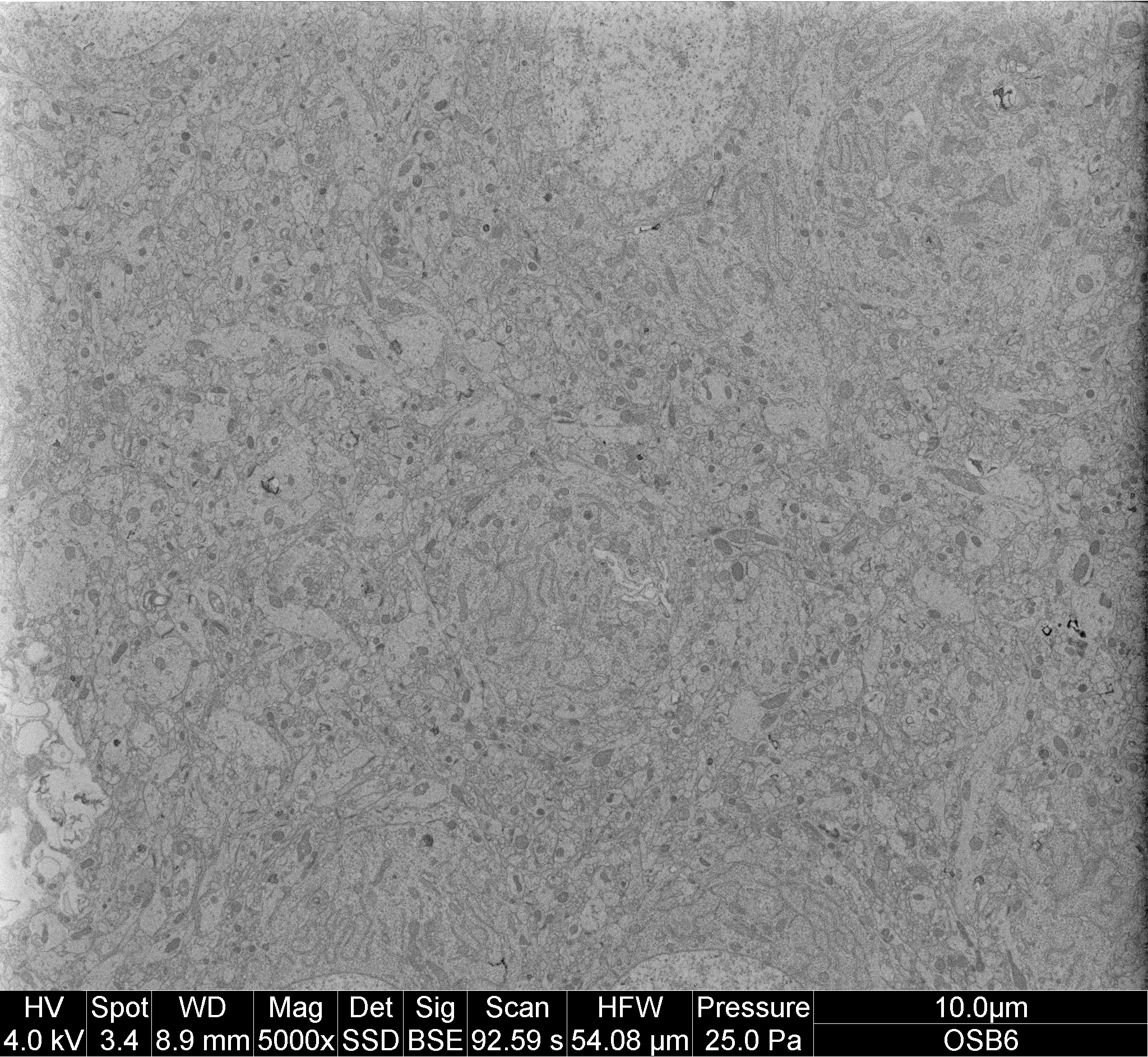

Supplement: Dataset S11 — (252.6 MB ZIP). [file pbio.0020329.sd011.zip › 040604_OS5_st1_1020.tif]

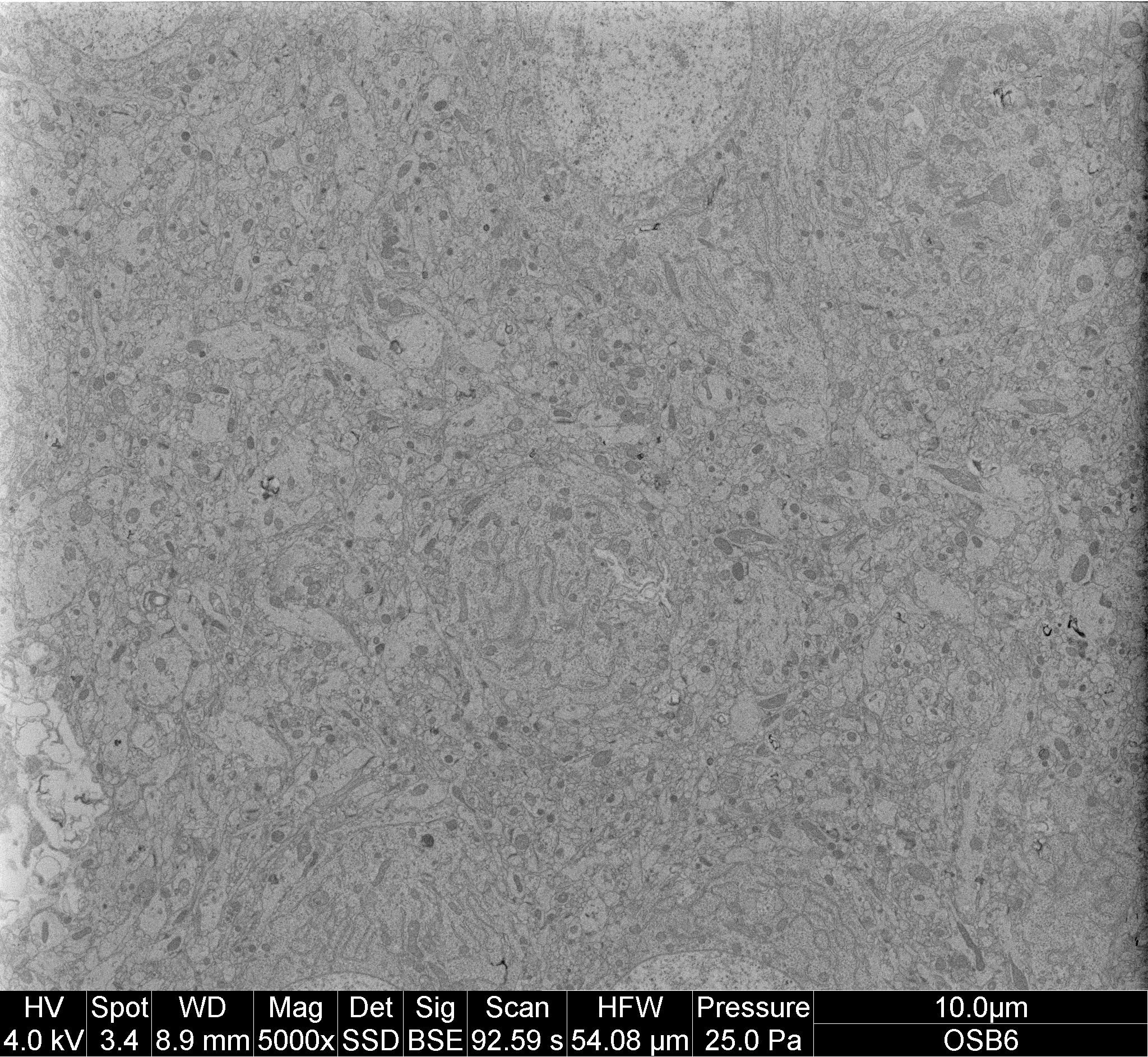

Supplement: Dataset S11 — (252.6 MB ZIP). [file pbio.0020329.sd011.zip › 040604_OS5_st1_1021.tif]

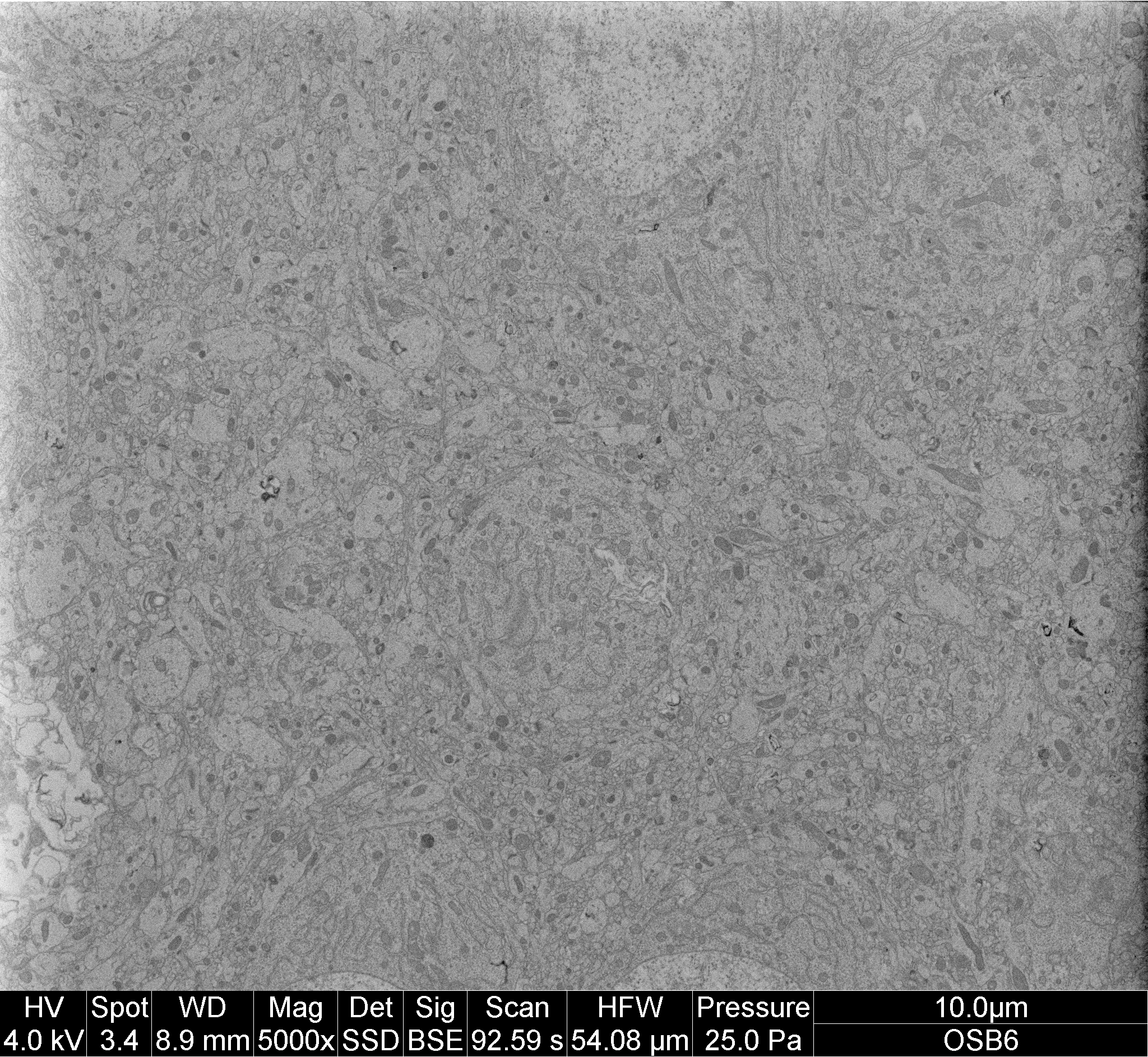

Supplement: Dataset S11 — (252.6 MB ZIP). [file pbio.0020329.sd011.zip › 040604_OS5_st1_1022.tif]

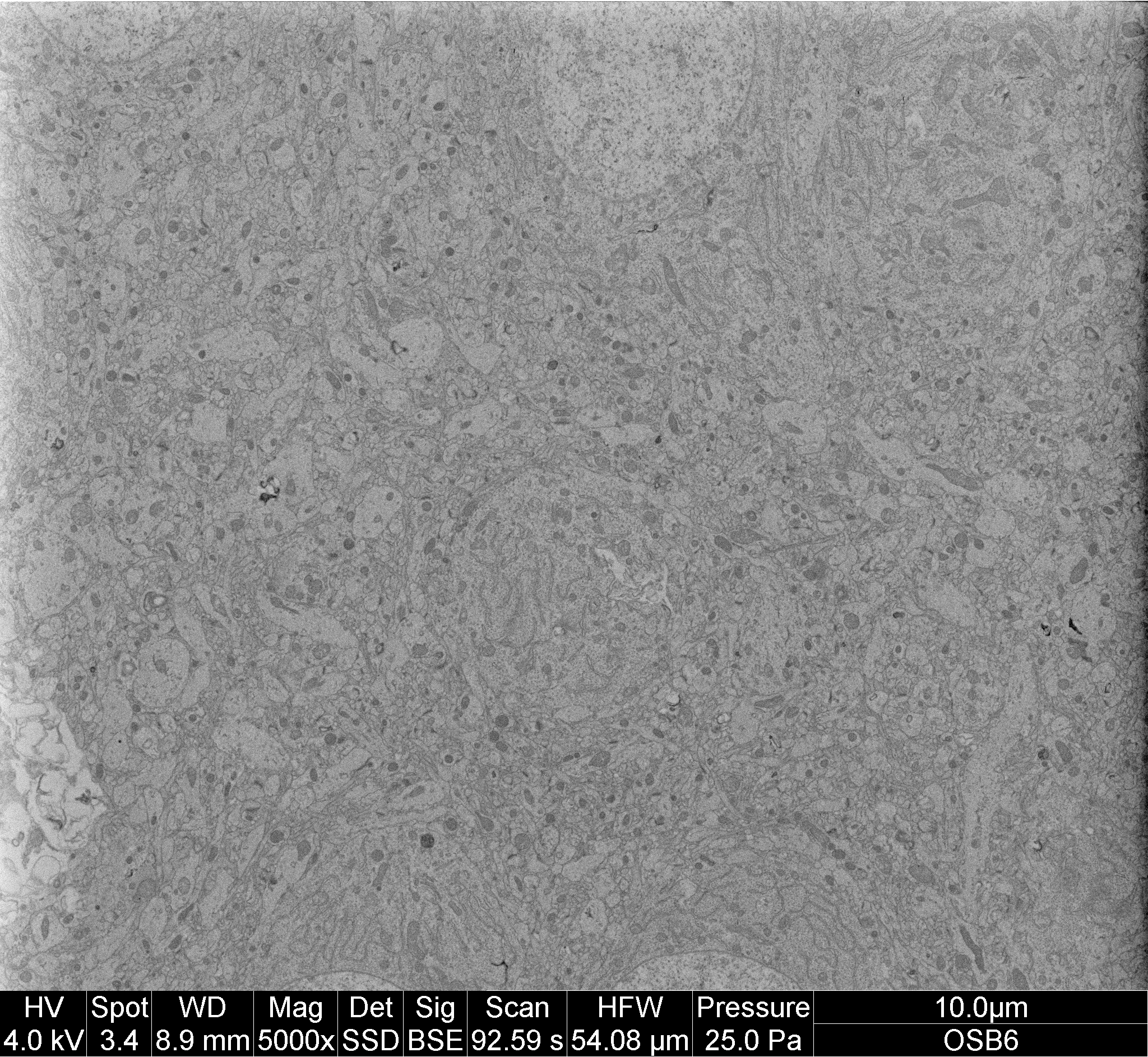

Supplement: Dataset S11 — (252.6 MB ZIP). [file pbio.0020329.sd011.zip › 040604_OS5_st1_1023.tif]

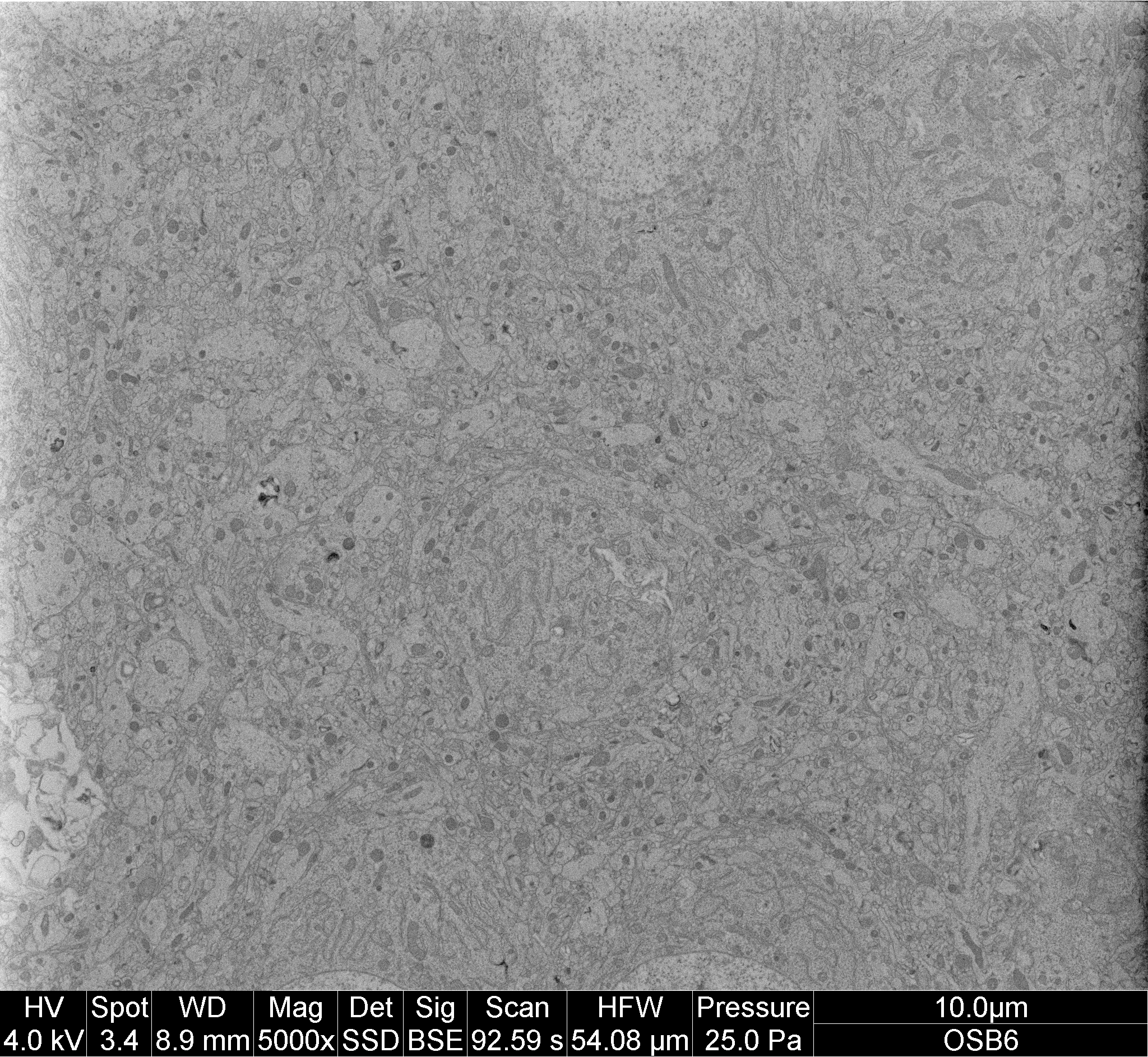

Supplement: Dataset S11 — (252.6 MB ZIP). [file pbio.0020329.sd011.zip › 040604_OS5_st1_1024.tif]

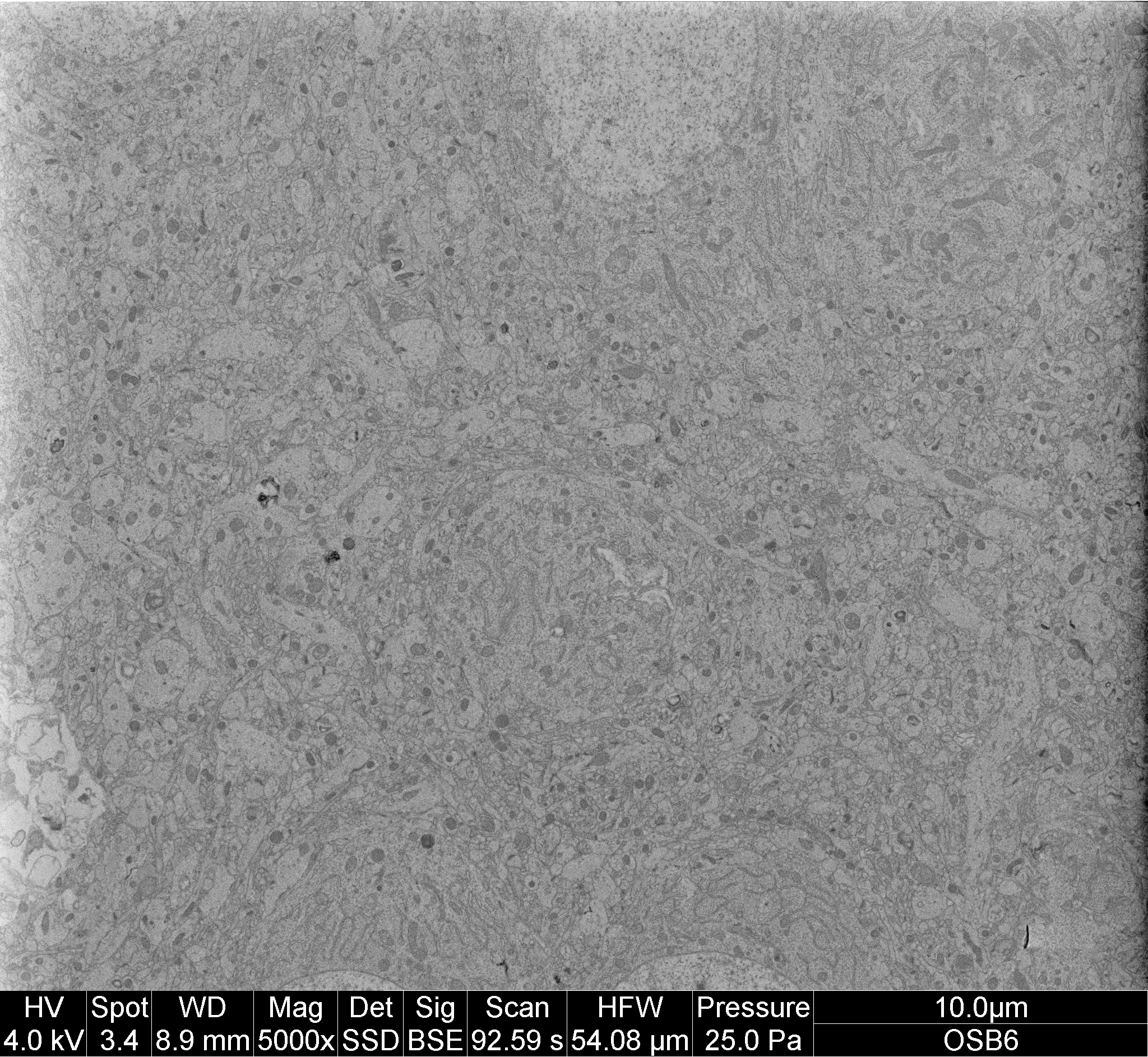

Supplement: Dataset S11 — (252.6 MB ZIP). [file pbio.0020329.sd011.zip › 040604_OS5_st1_1025.tif]

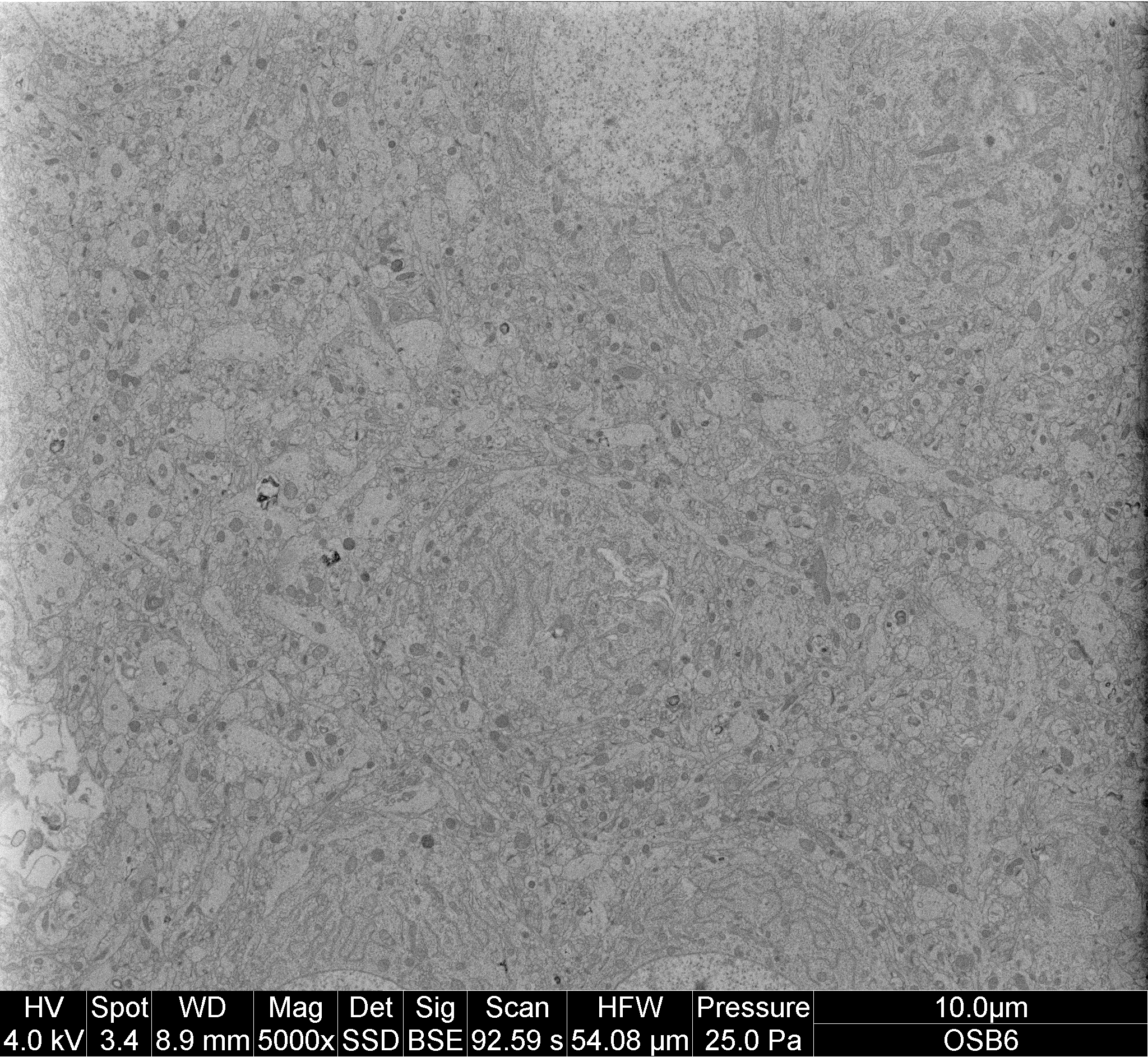

Supplement: Dataset S11 — (252.6 MB ZIP). [file pbio.0020329.sd011.zip › 040604_OS5_st1_1026.tif]

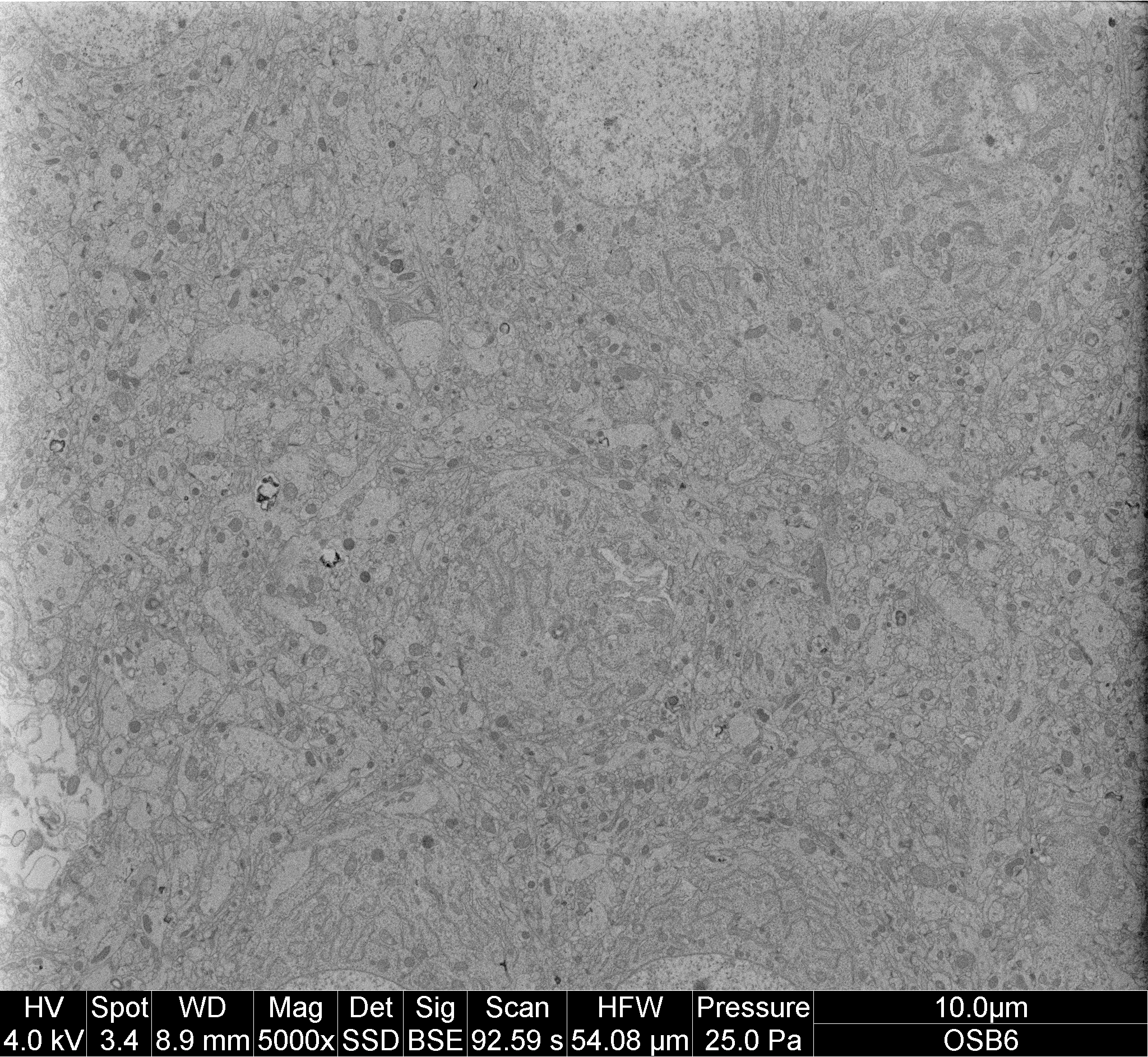

Supplement: Dataset S11 — (252.6 MB ZIP). [file pbio.0020329.sd011.zip › 040604_OS5_st1_1027.tif]

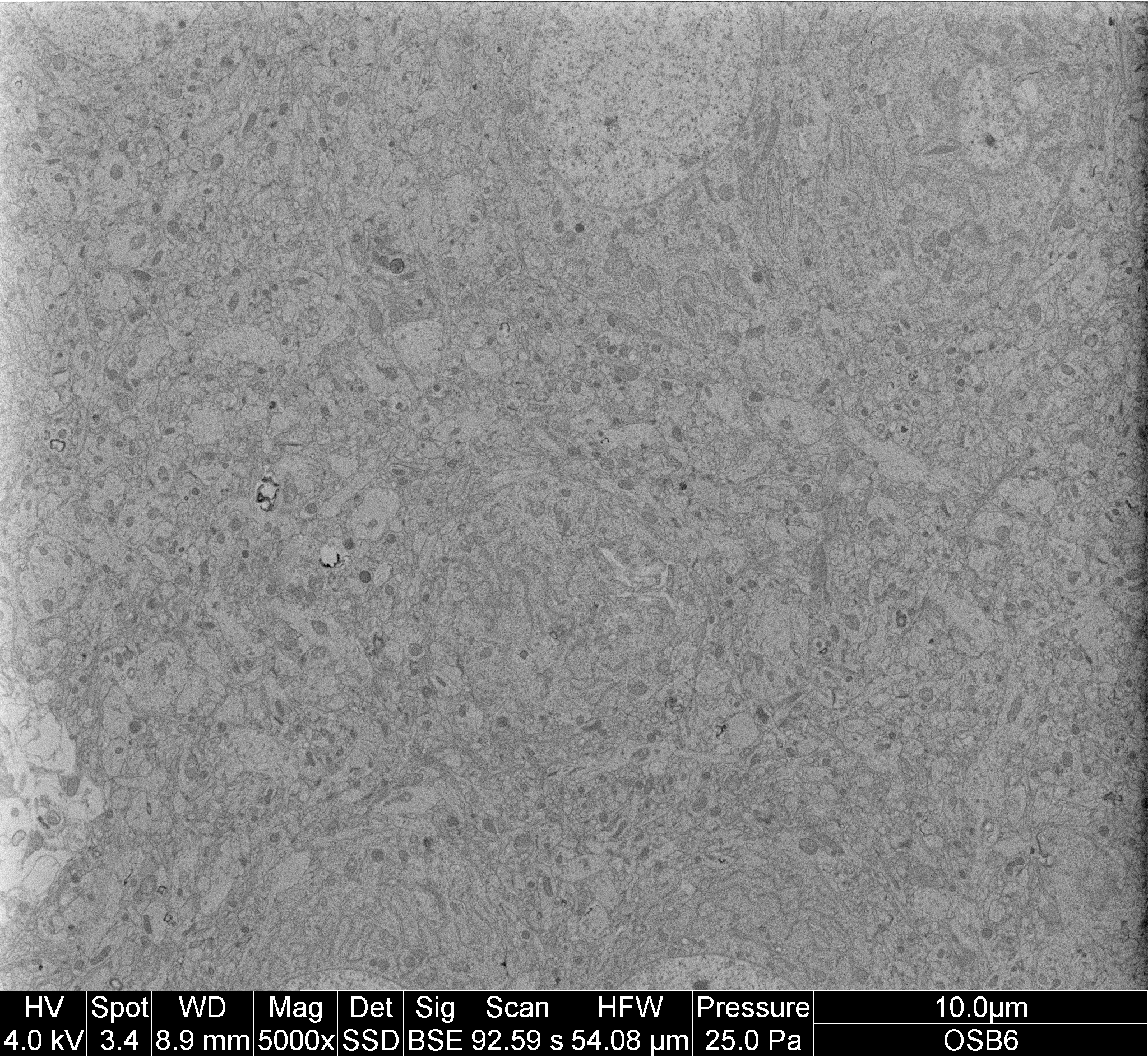

Supplement: Dataset S11 — (252.6 MB ZIP). [file pbio.0020329.sd011.zip › 040604_OS5_st1_1028.tif]

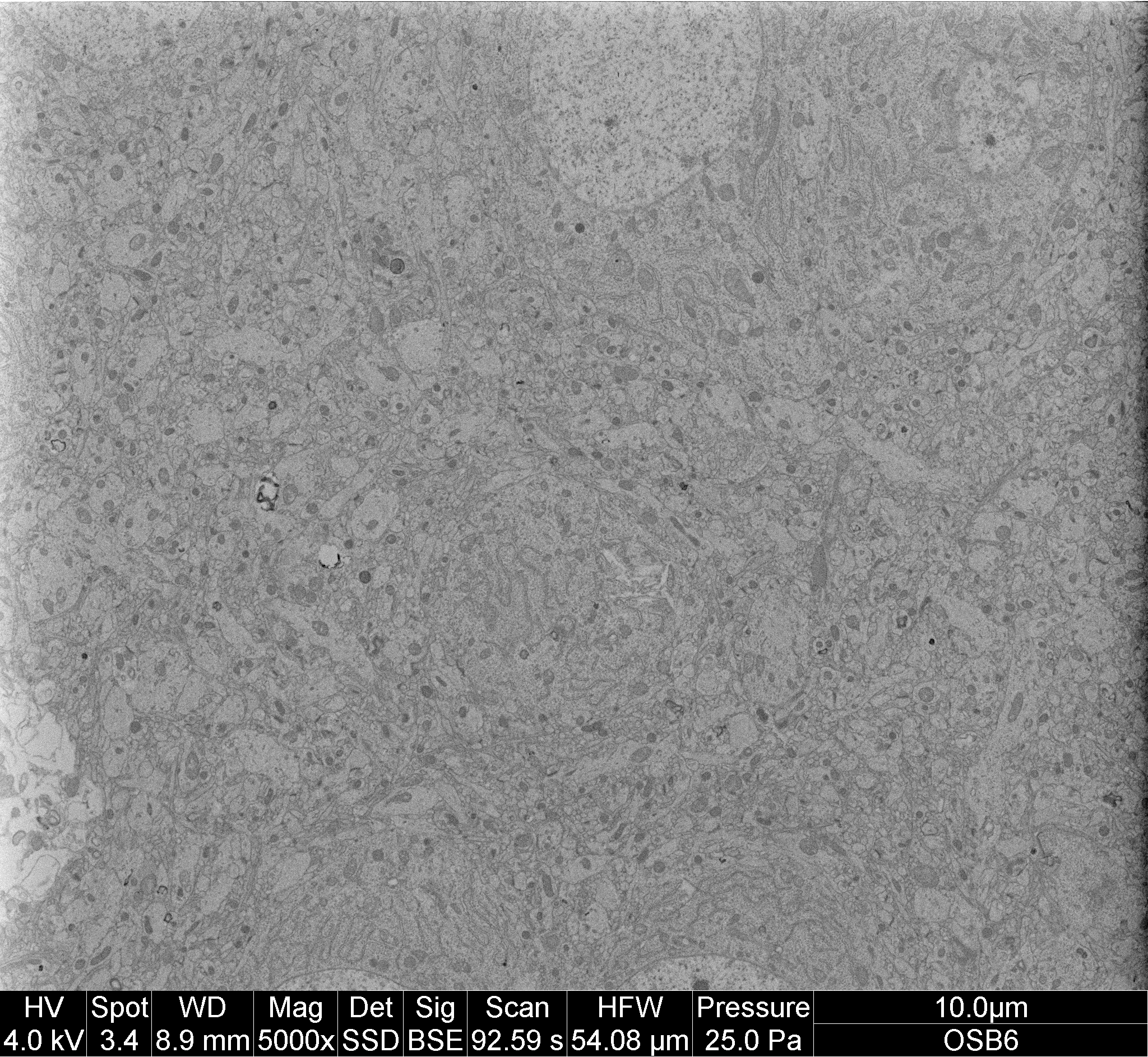

Supplement: Dataset S11 — (252.6 MB ZIP). [file pbio.0020329.sd011.zip › 040604_OS5_st1_1029.tif]

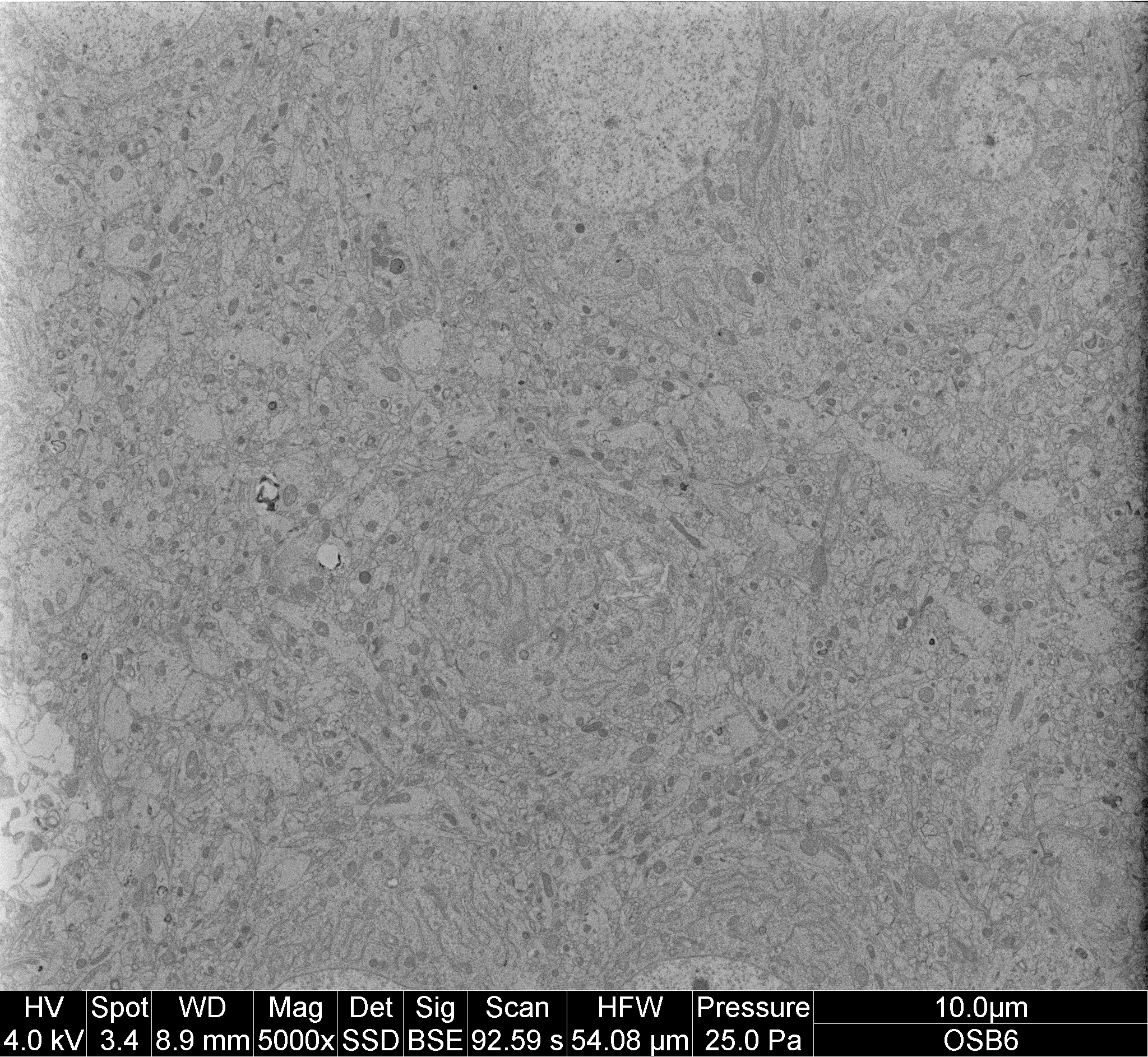

Supplement: Dataset S11 — (252.6 MB ZIP). [file pbio.0020329.sd011.zip › 040604_OS5_st1_1030.tif]

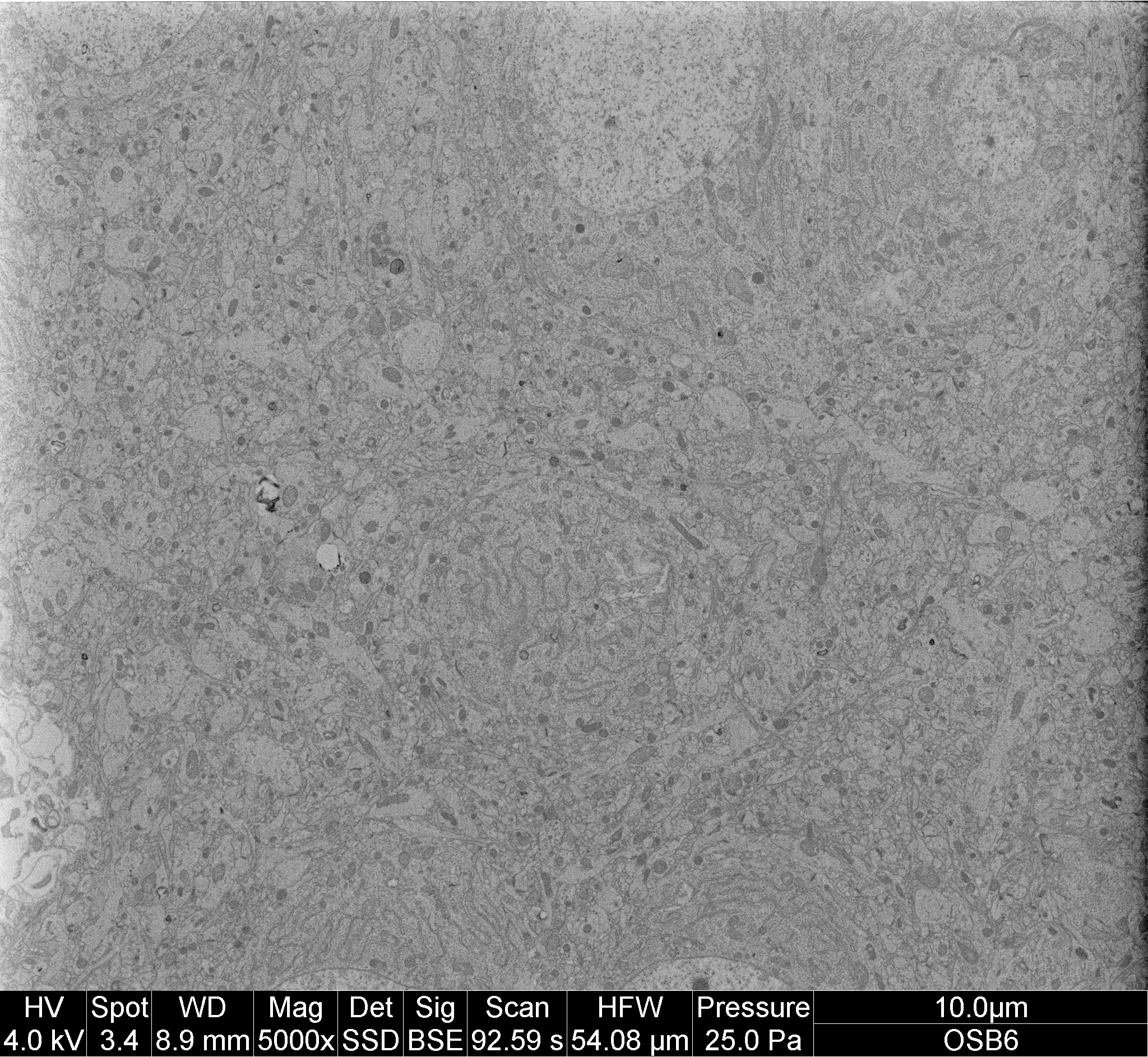

Supplement: Dataset S11 — (252.6 MB ZIP). [file pbio.0020329.sd011.zip › 040604_OS5_st1_1031.tif]

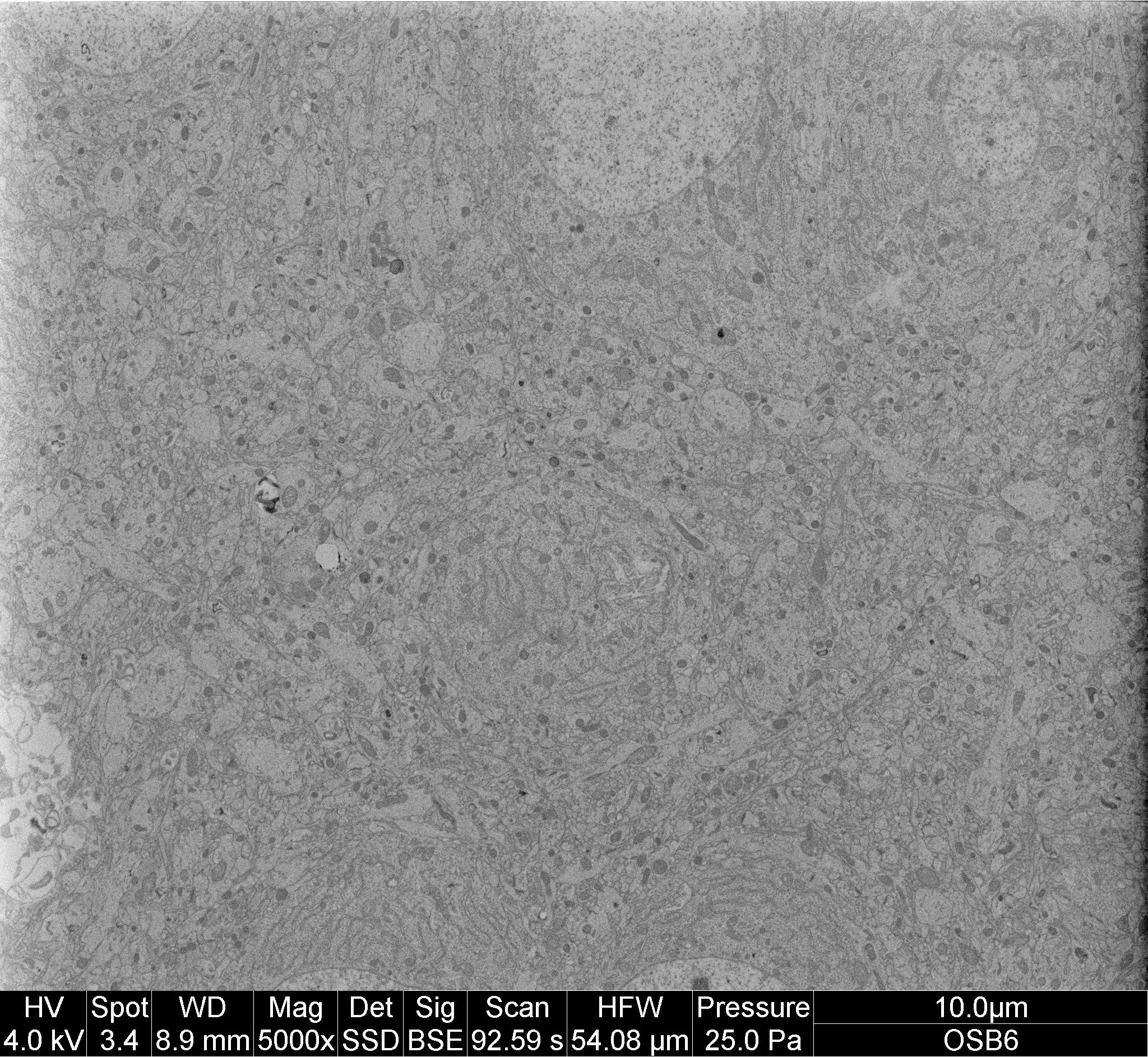

Supplement: Dataset S11 — (252.6 MB ZIP). [file pbio.0020329.sd011.zip › 040604_OS5_st1_1032.tif]

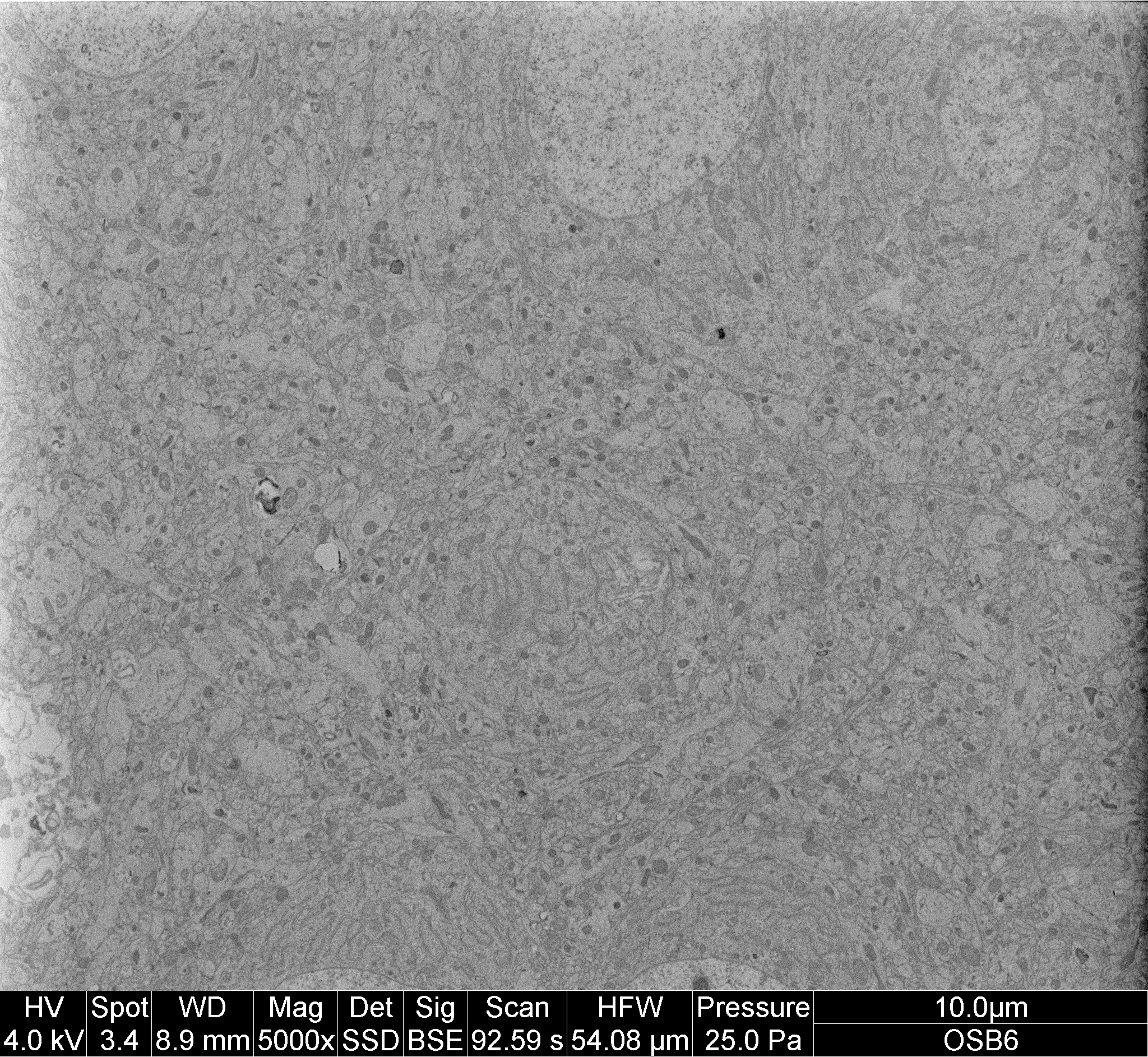

Supplement: Dataset S11 — (252.6 MB ZIP). [file pbio.0020329.sd011.zip › 040604_OS5_st1_1033.tif]

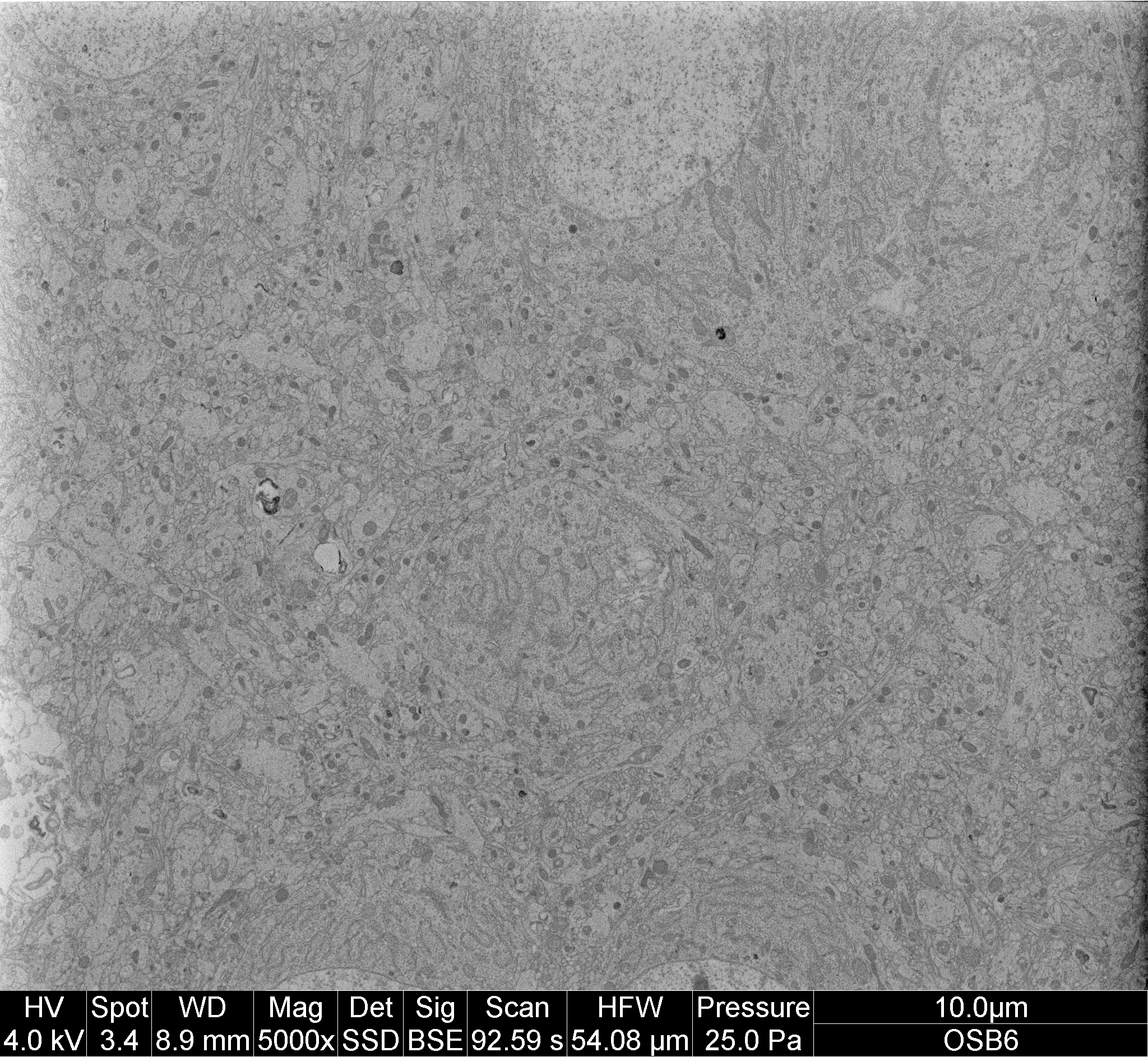

Supplement: Dataset S11 — (252.6 MB ZIP). [file pbio.0020329.sd011.zip › 040604_OS5_st1_1034.tif]

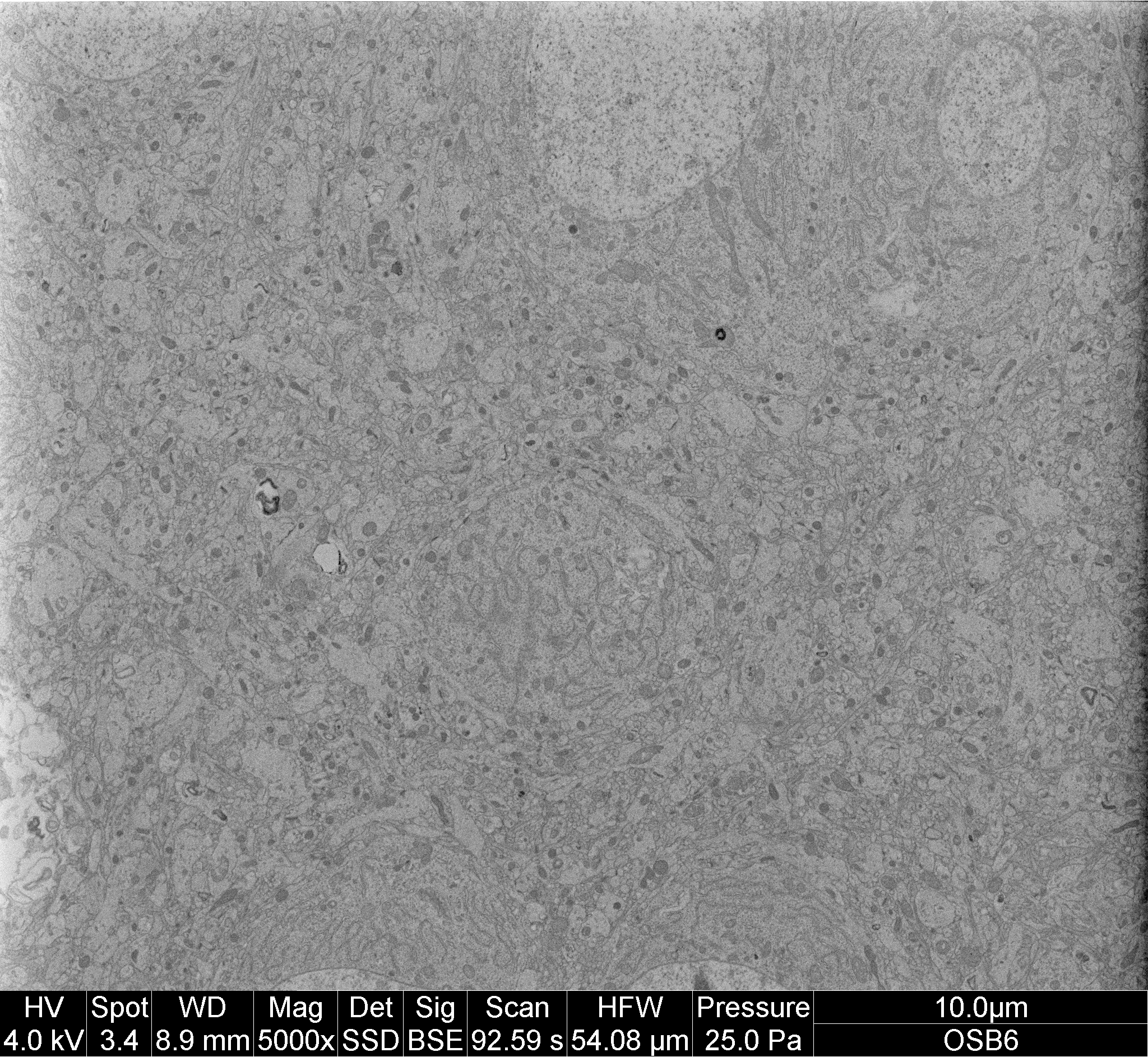

Supplement: Dataset S11 — (252.6 MB ZIP). [file pbio.0020329.sd011.zip › 040604_OS5_st1_1035.tif]

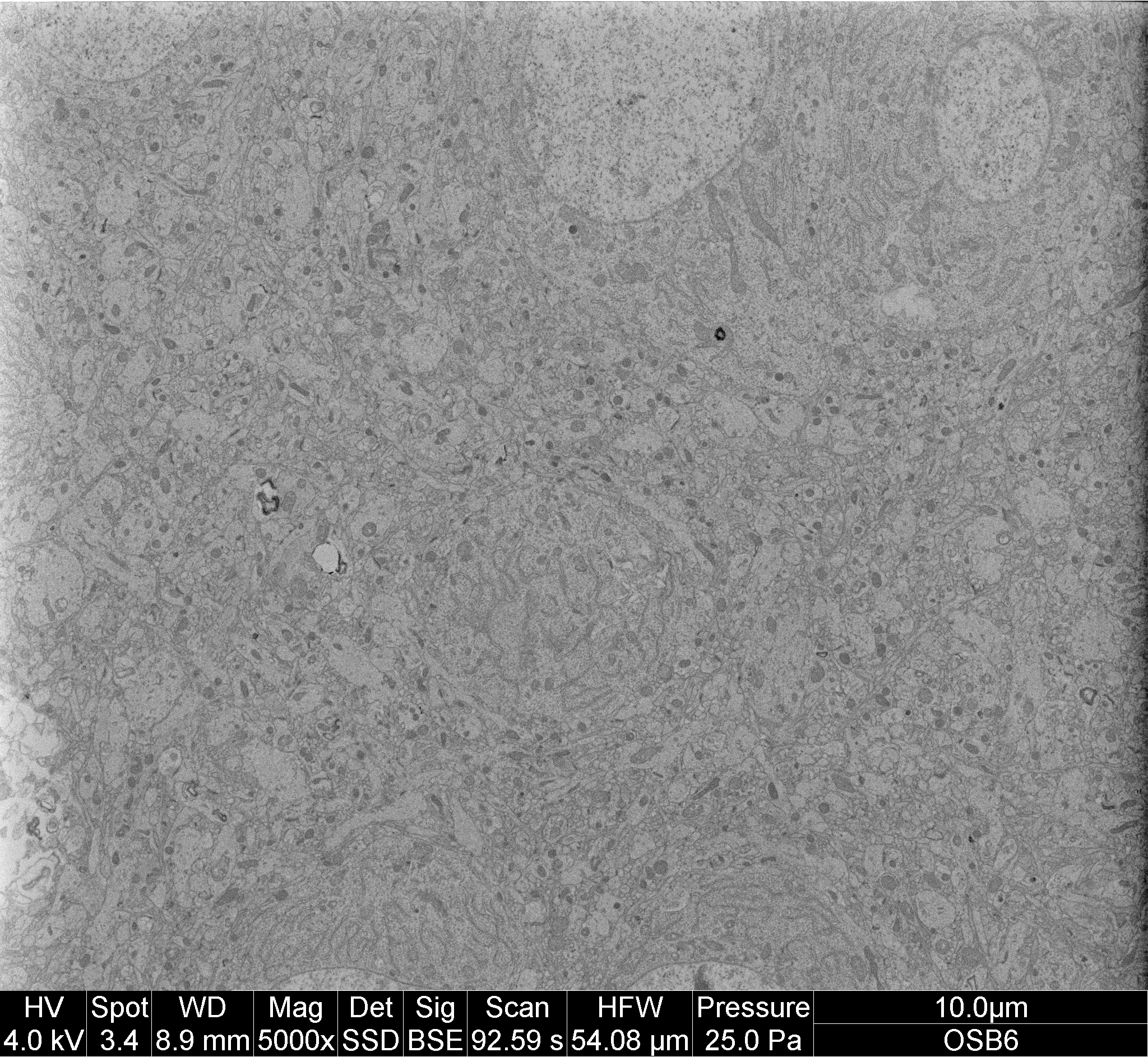

Supplement: Dataset S11 — (252.6 MB ZIP). [file pbio.0020329.sd011.zip › 040604_OS5_st1_1036.tif]

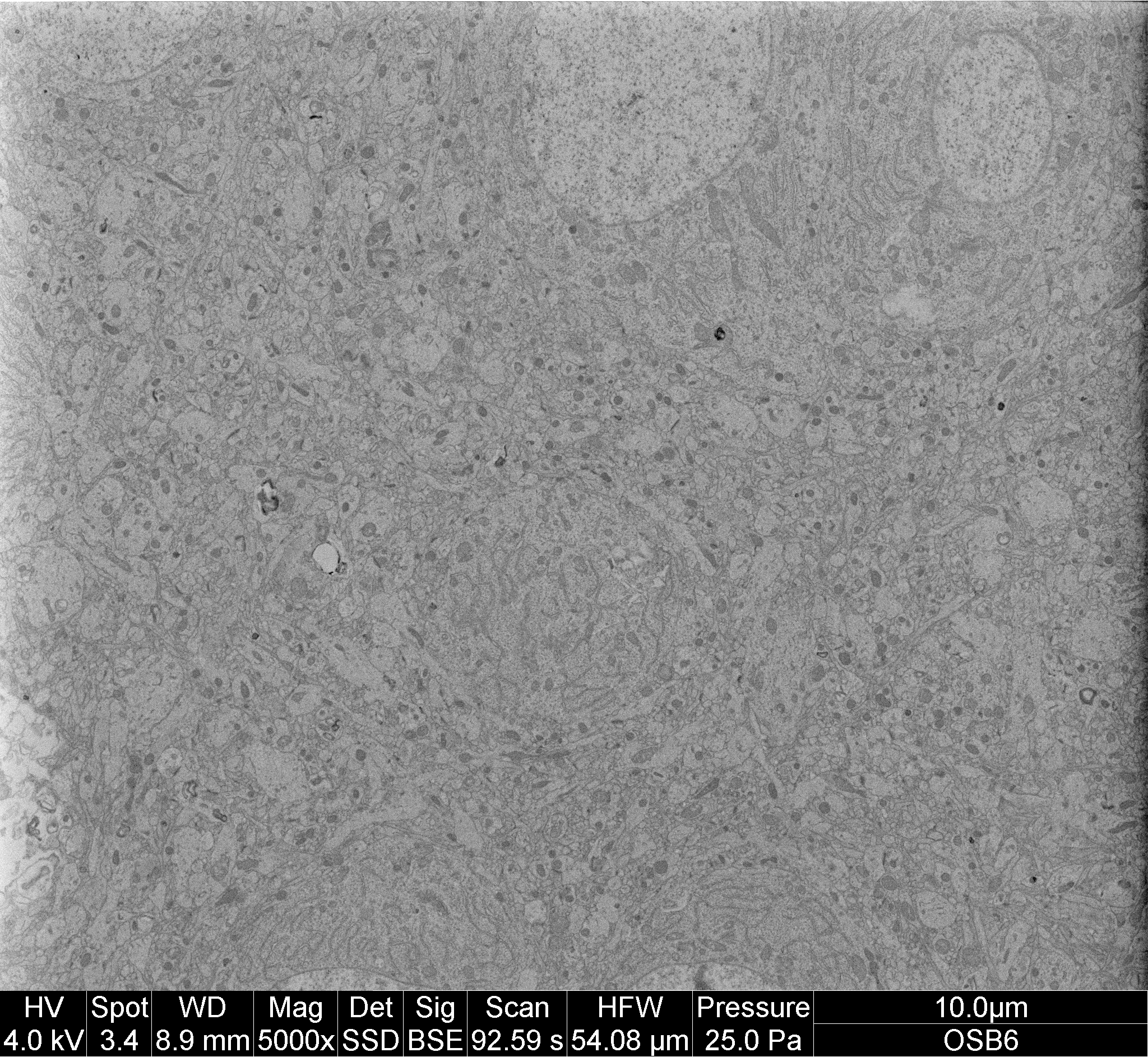

Supplement: Dataset S11 — (252.6 MB ZIP). [file pbio.0020329.sd011.zip › 040604_OS5_st1_1037.tif]

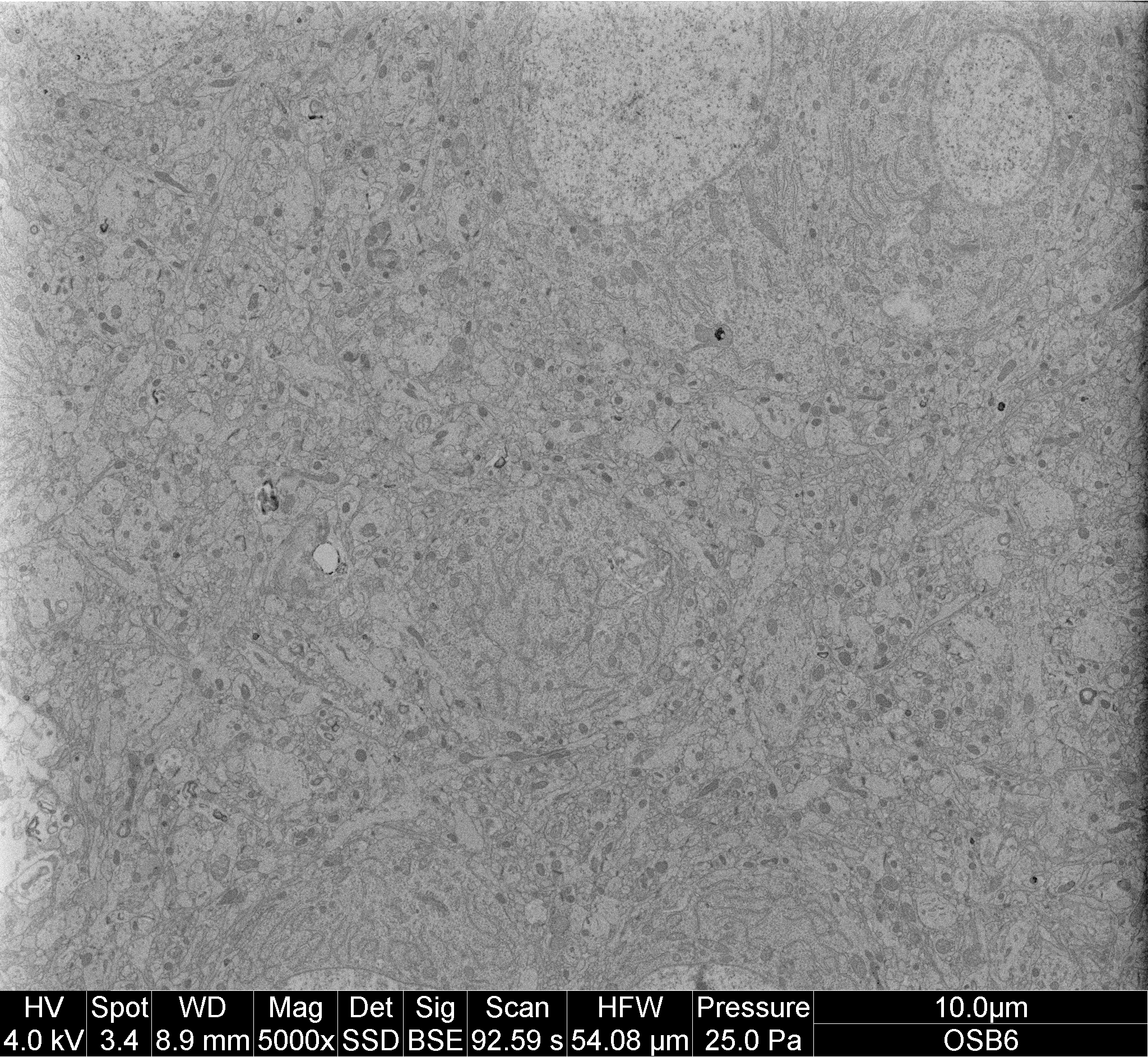

Supplement: Dataset S11 — (252.6 MB ZIP). [file pbio.0020329.sd011.zip › 040604_OS5_st1_1038.tif]

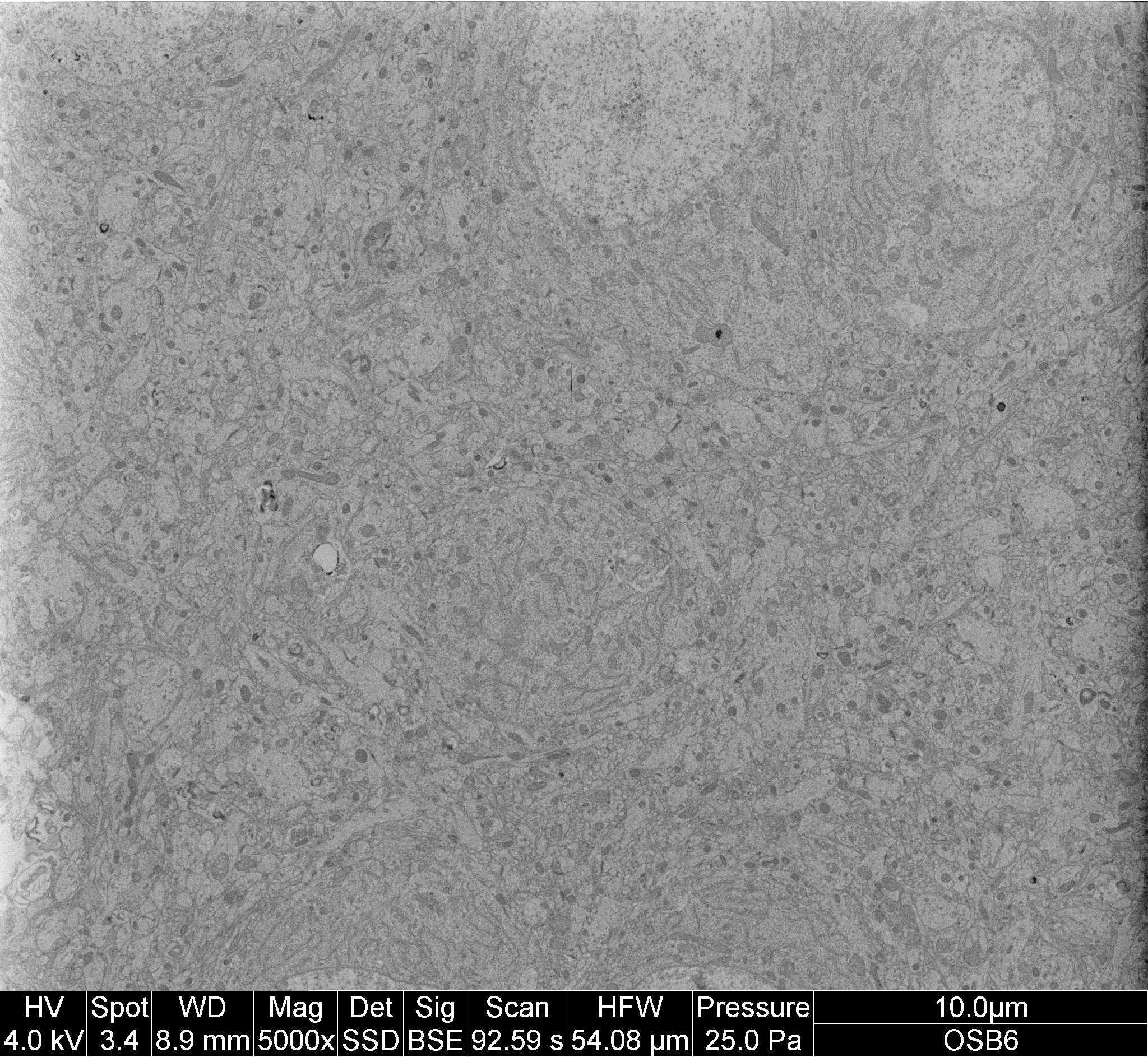

Supplement: Dataset S11 — (252.6 MB ZIP). [file pbio.0020329.sd011.zip › 040604_OS5_st1_1039.tif]

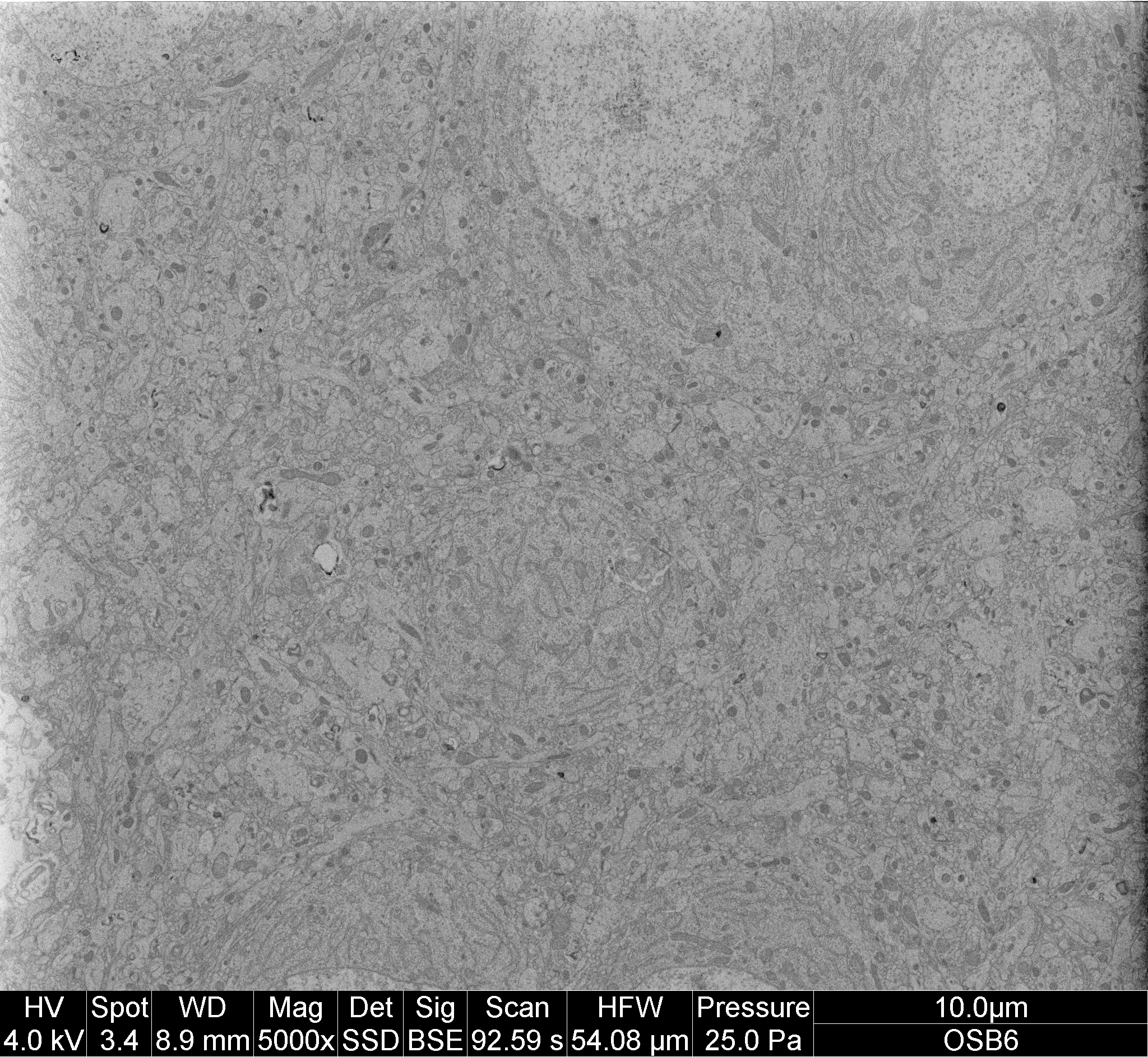

Supplement: Dataset S11 — (252.6 MB ZIP). [file pbio.0020329.sd011.zip › 040604_OS5_st1_1040.tif]

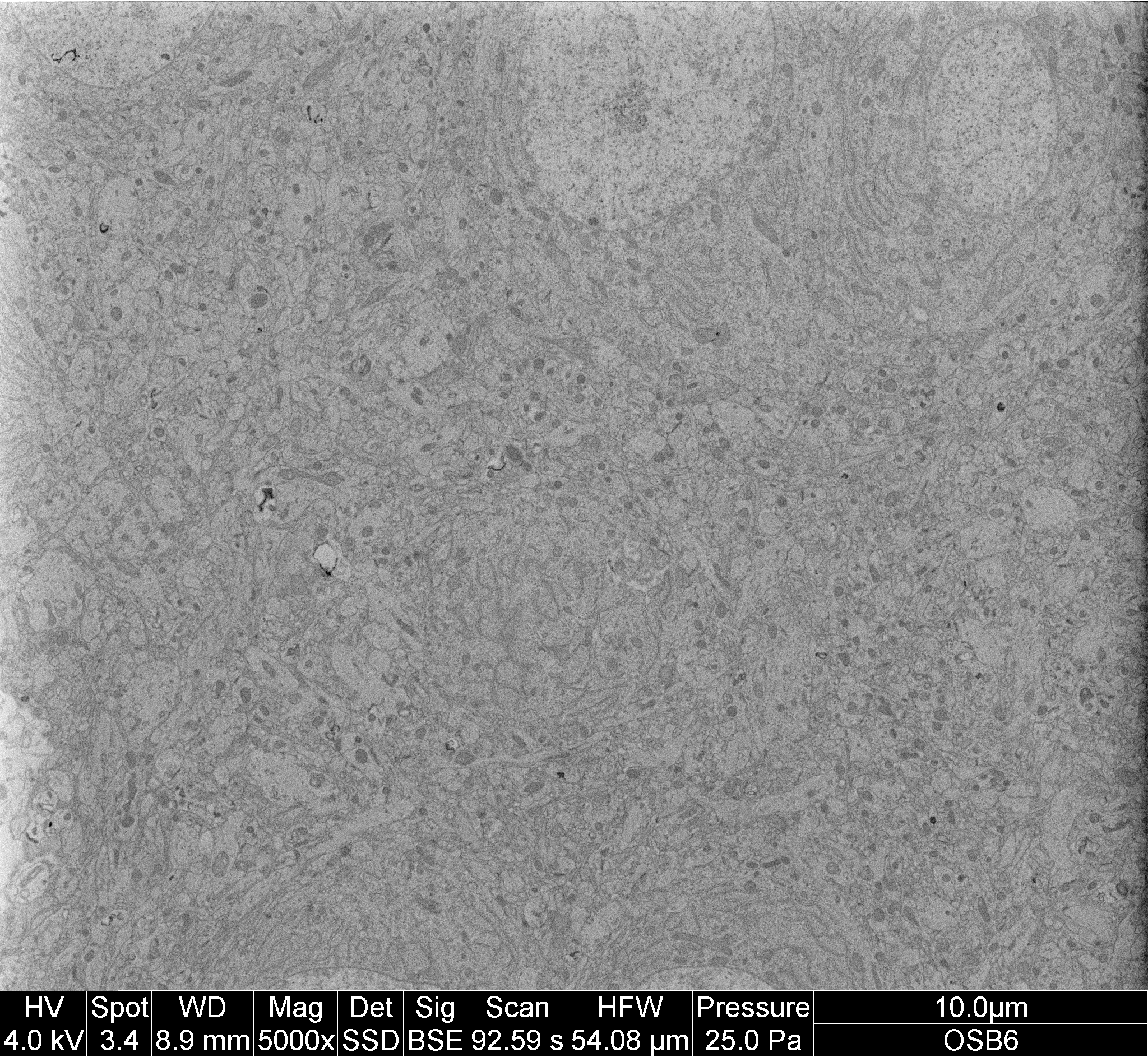

Supplement: Dataset S11 — (252.6 MB ZIP). [file pbio.0020329.sd011.zip › 040604_OS5_st1_1041.tif]

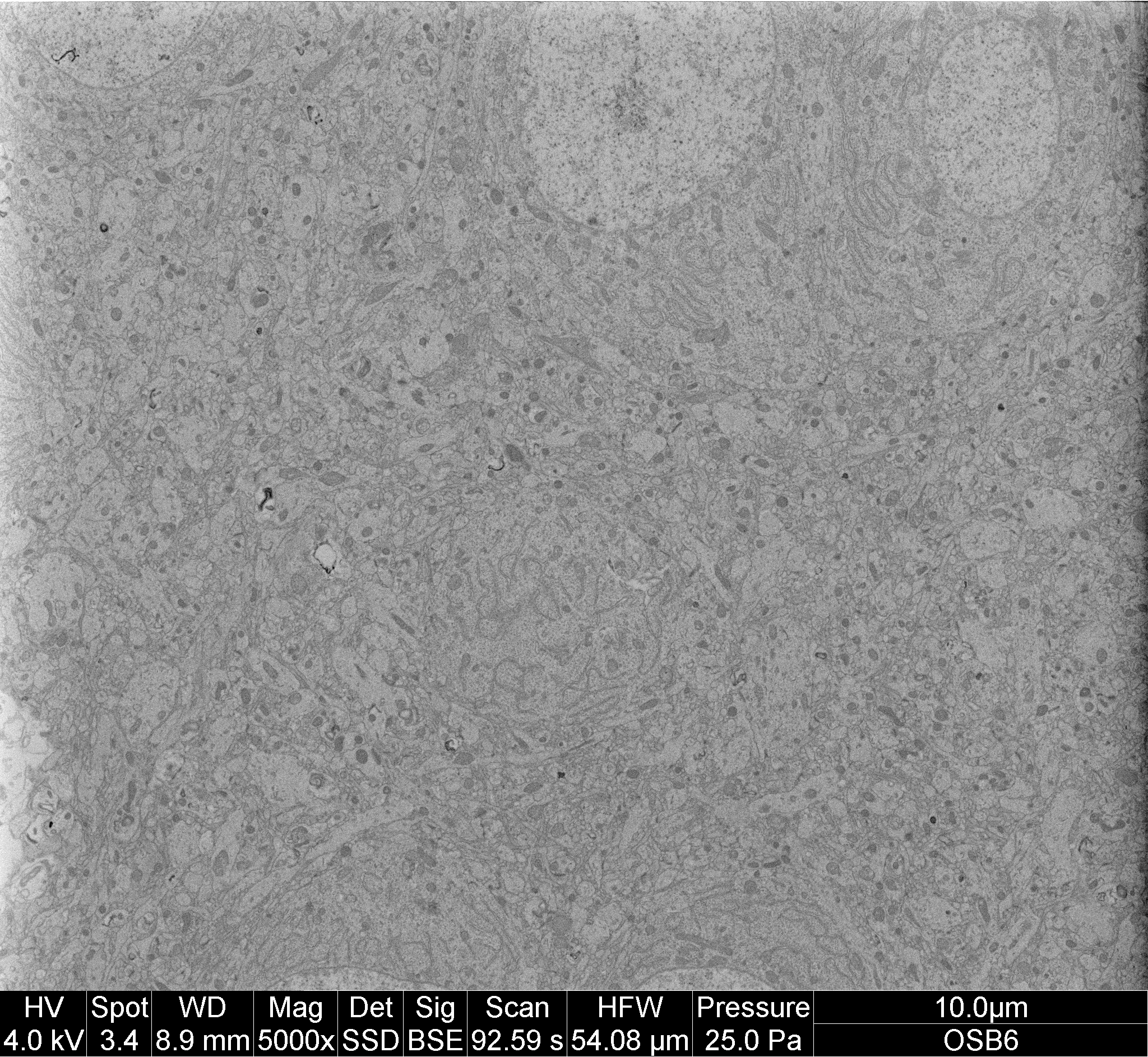

Supplement: Dataset S11 — (252.6 MB ZIP). [file pbio.0020329.sd011.zip › 040604_OS5_st1_1042.tif]

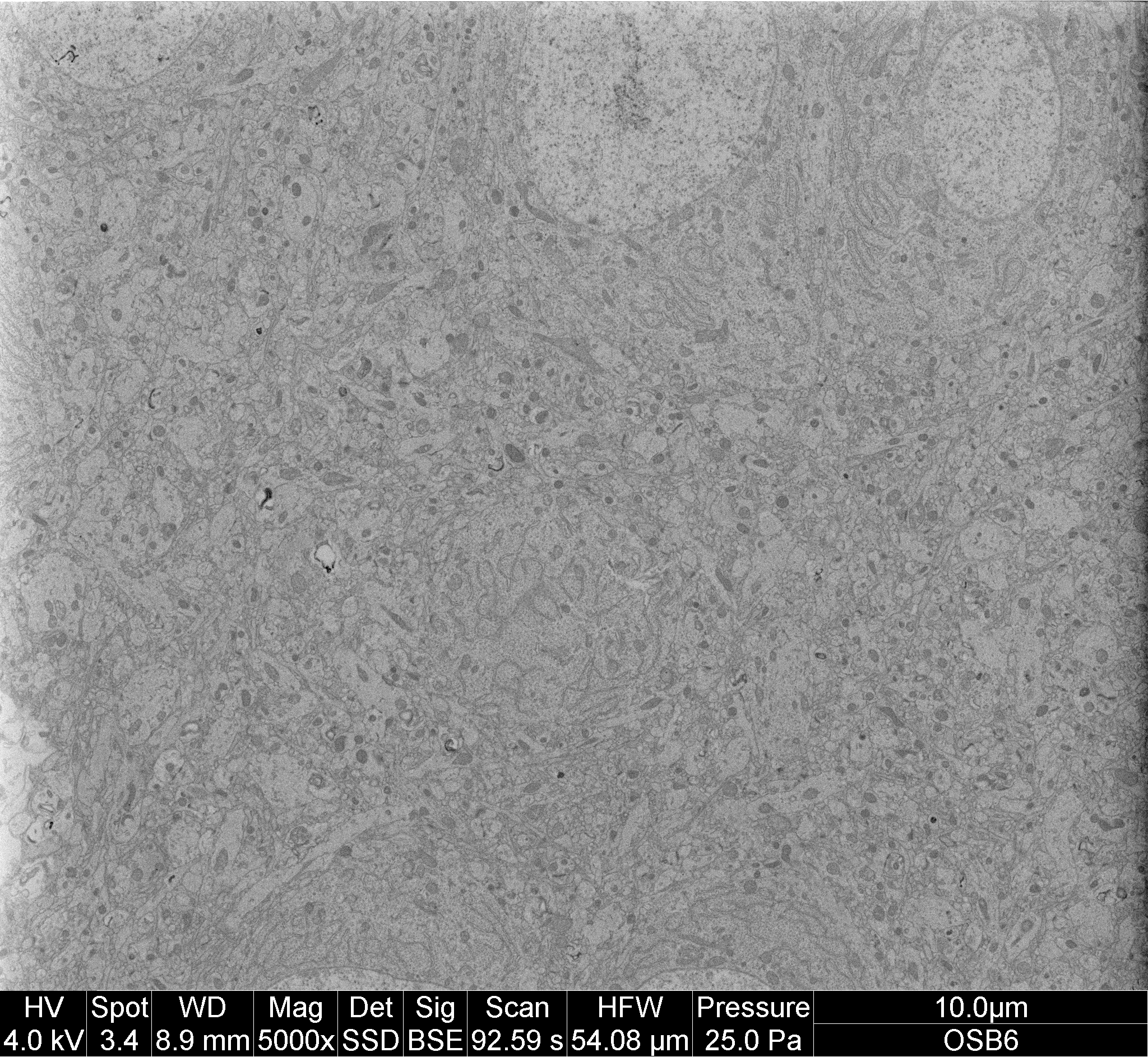

Supplement: Dataset S11 — (252.6 MB ZIP). [file pbio.0020329.sd011.zip › 040604_OS5_st1_1043.tif]

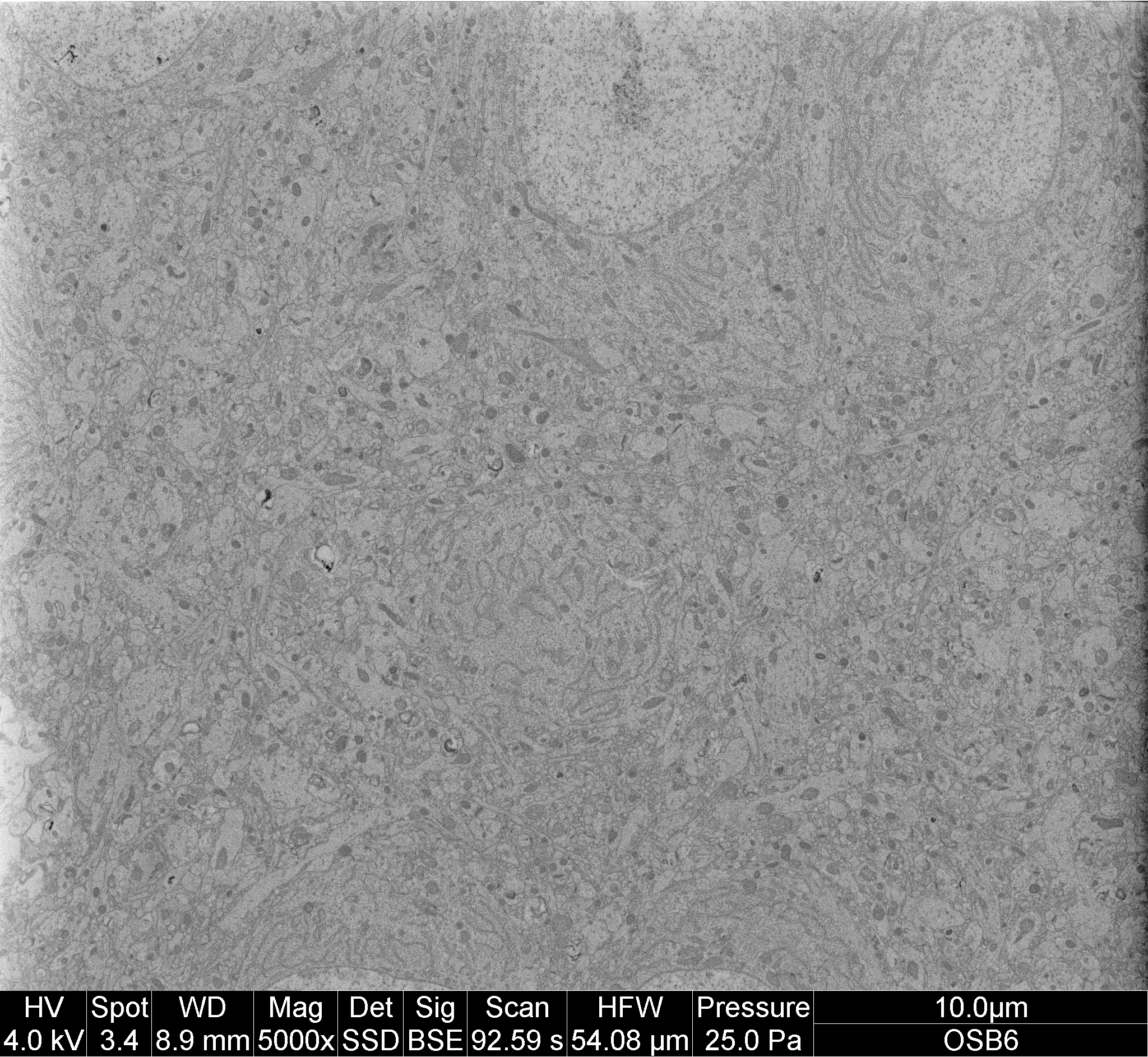

Supplement: Dataset S11 — (252.6 MB ZIP). [file pbio.0020329.sd011.zip › 040604_OS5_st1_1044.tif]

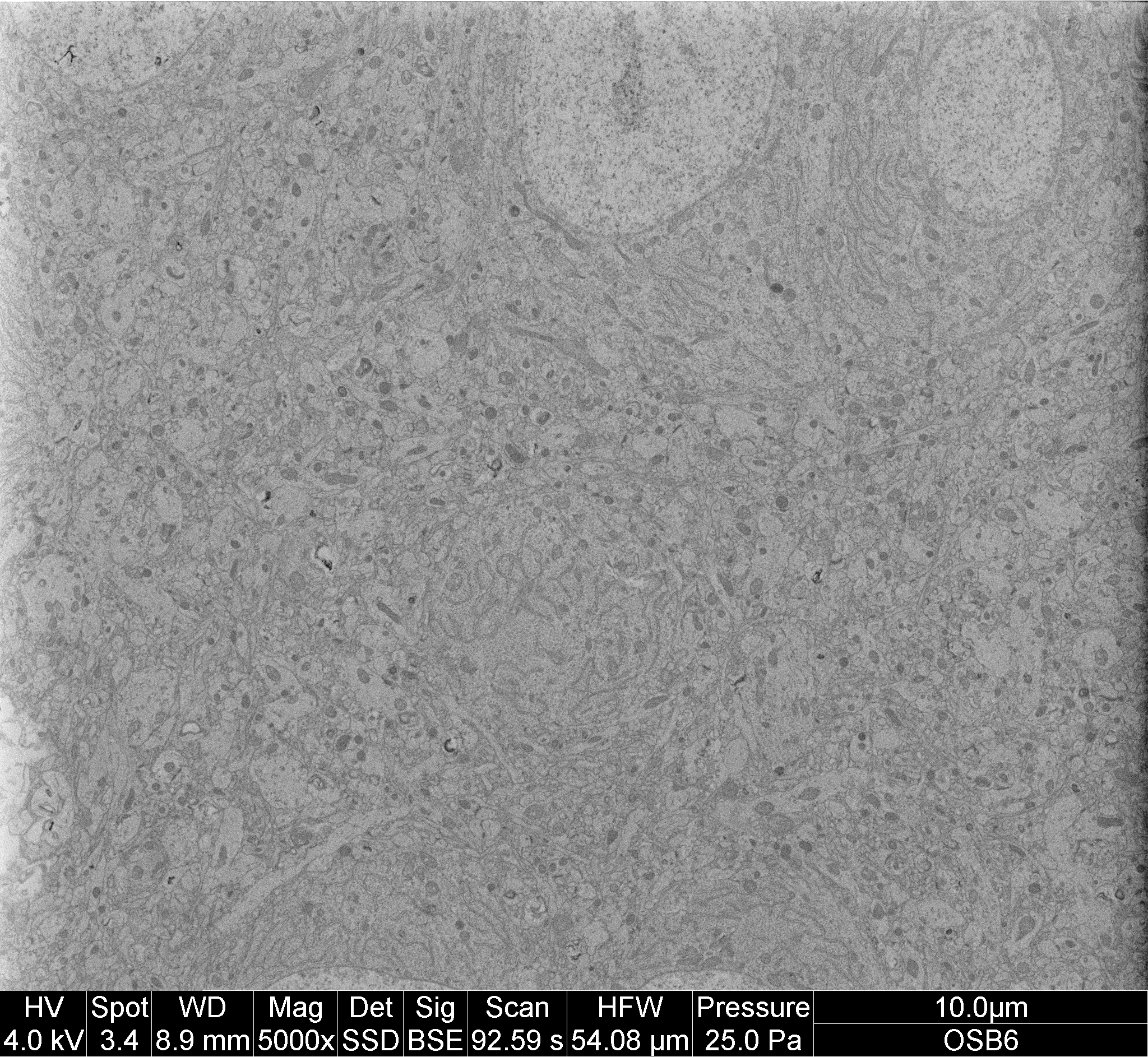

Supplement: Dataset S11 — (252.6 MB ZIP). [file pbio.0020329.sd011.zip › 040604_OS5_st1_1045.tif]

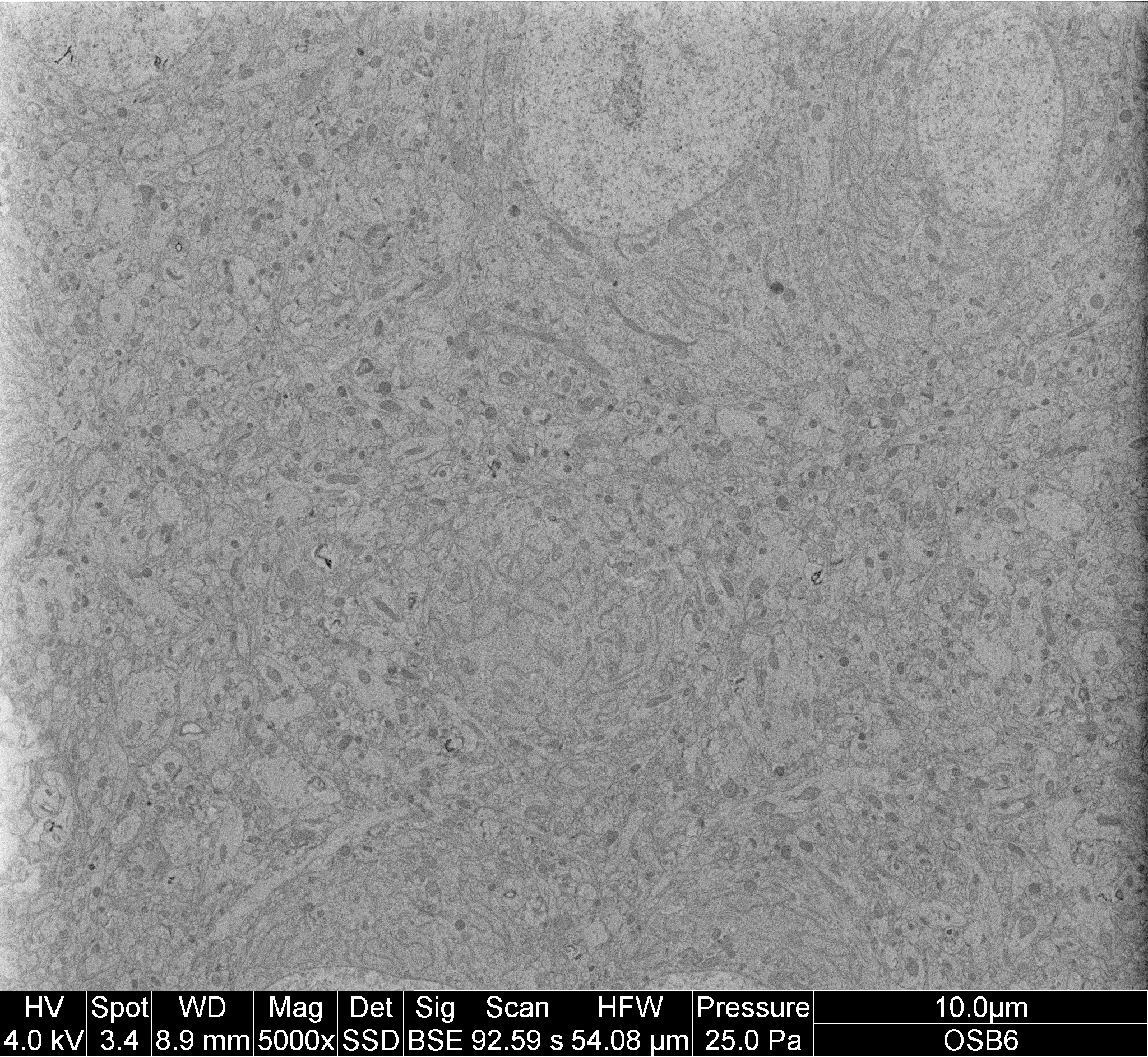

Supplement: Dataset S11 — (252.6 MB ZIP). [file pbio.0020329.sd011.zip › 040604_OS5_st1_1046.tif]

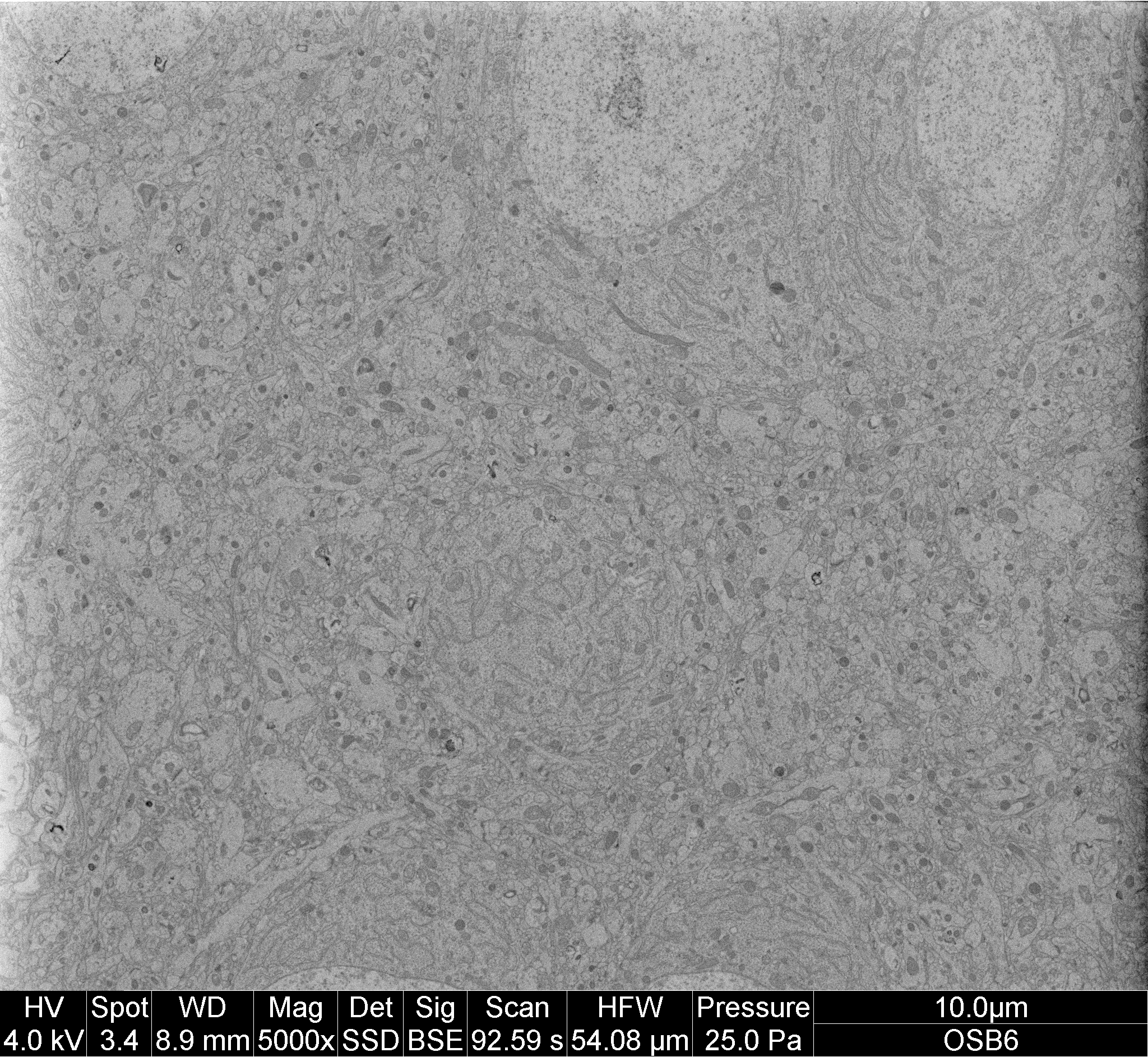

Supplement: Dataset S11 — (252.6 MB ZIP). [file pbio.0020329.sd011.zip › 040604_OS5_st1_1047.tif]

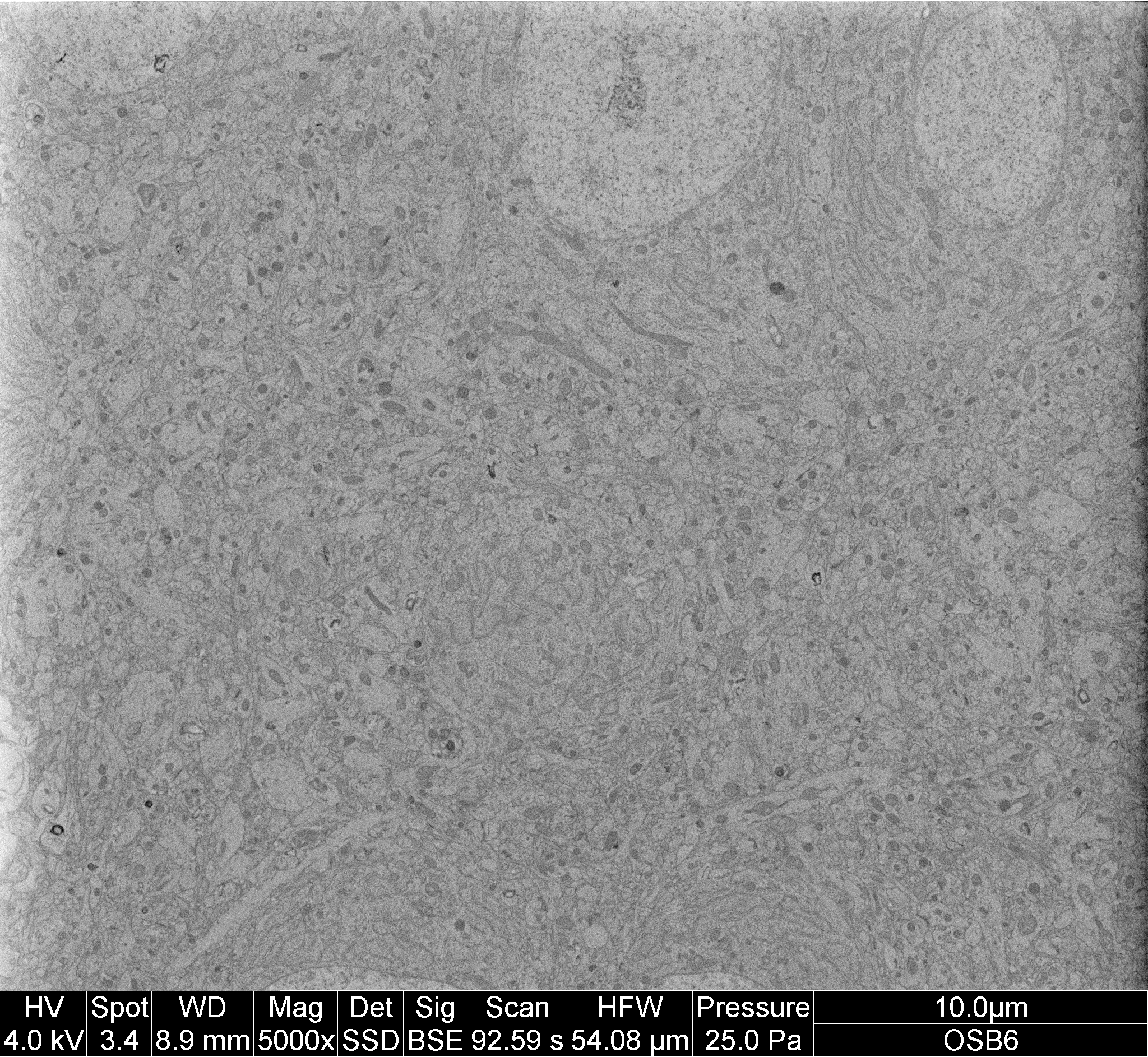

Supplement: Dataset S11 — (252.6 MB ZIP). [file pbio.0020329.sd011.zip › 040604_OS5_st1_1048.tif]

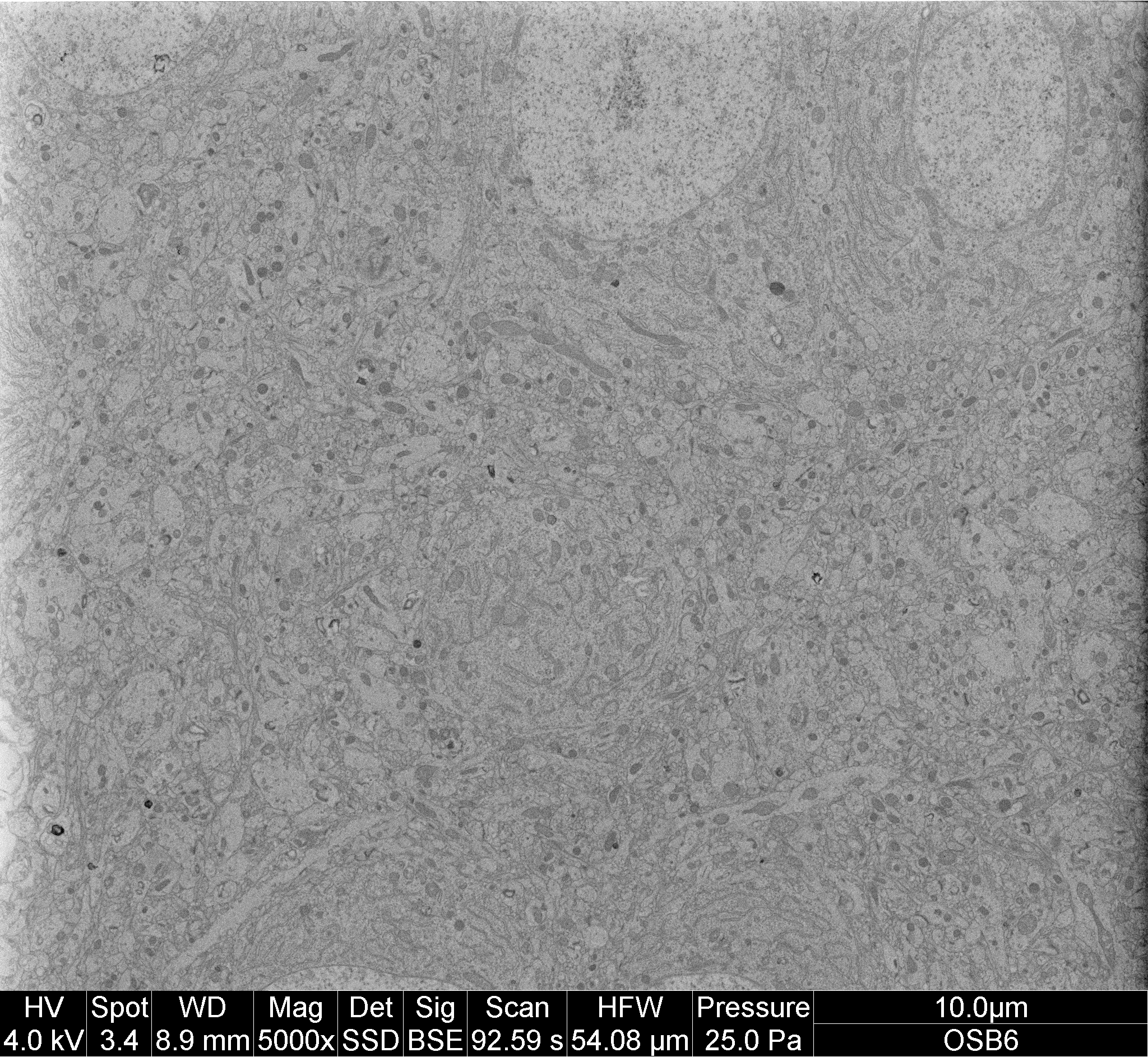

Supplement: Dataset S11 — (252.6 MB ZIP). [file pbio.0020329.sd011.zip › 040604_OS5_st1_1049.tif]

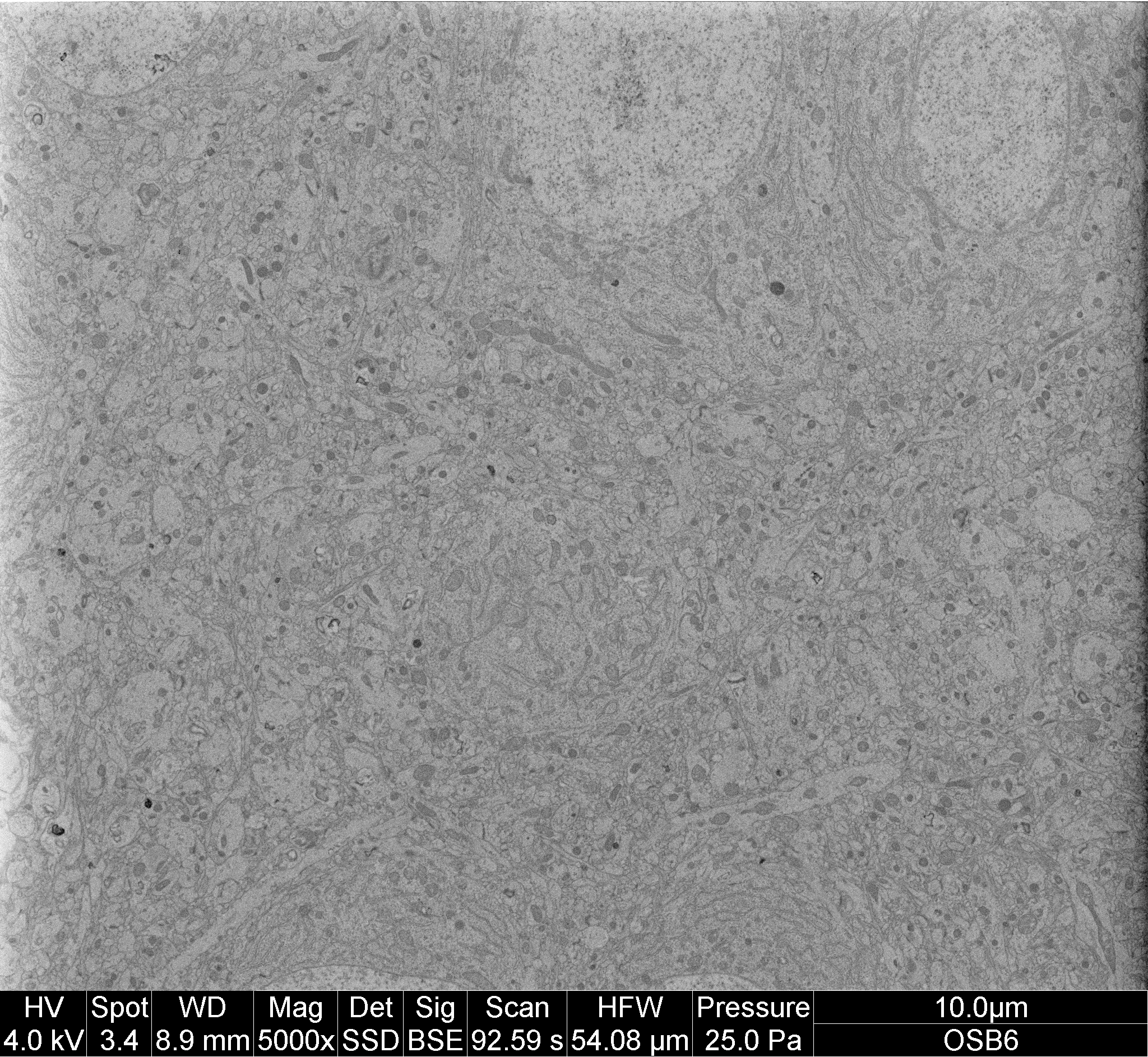

Supplement: Dataset S11 — (252.6 MB ZIP). [file pbio.0020329.sd011.zip › 040604_OS5_st1_1050.tif]

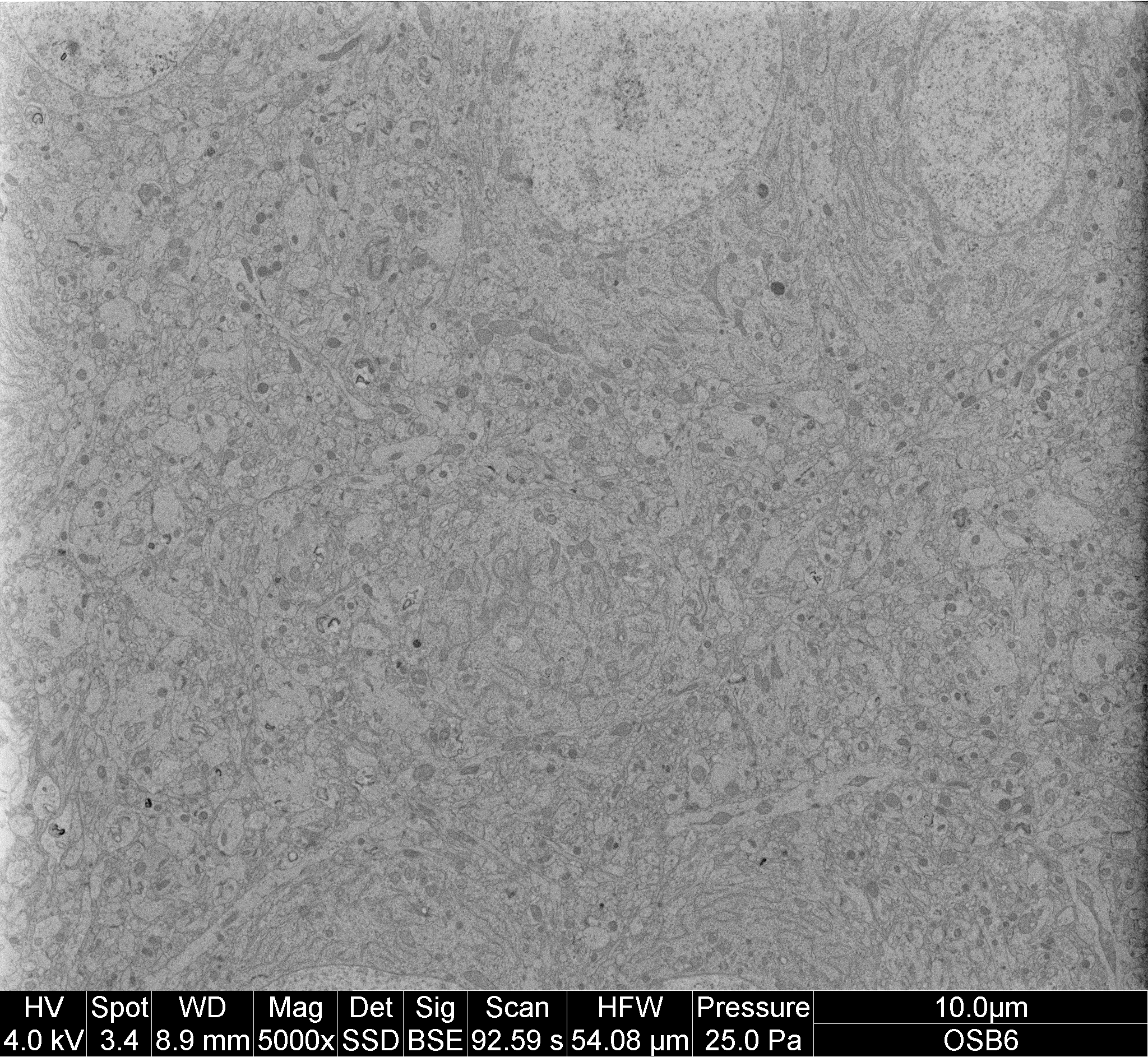

Supplement: Dataset S11 — (252.6 MB ZIP). [file pbio.0020329.sd011.zip › 040604_OS5_st1_1051.tif]

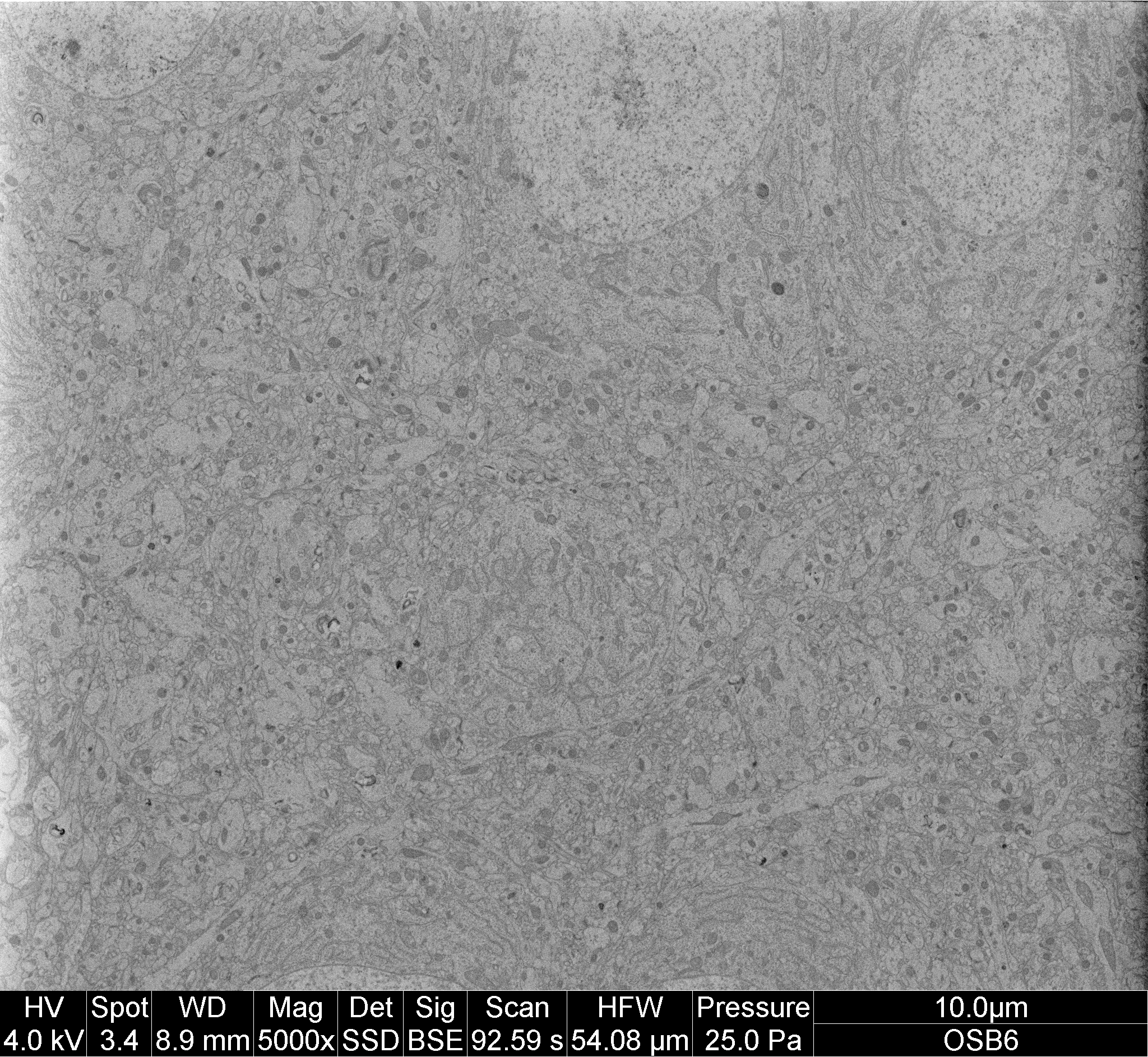

Supplement: Dataset S11 — (252.6 MB ZIP). [file pbio.0020329.sd011.zip › 040604_OS5_st1_1052.tif]

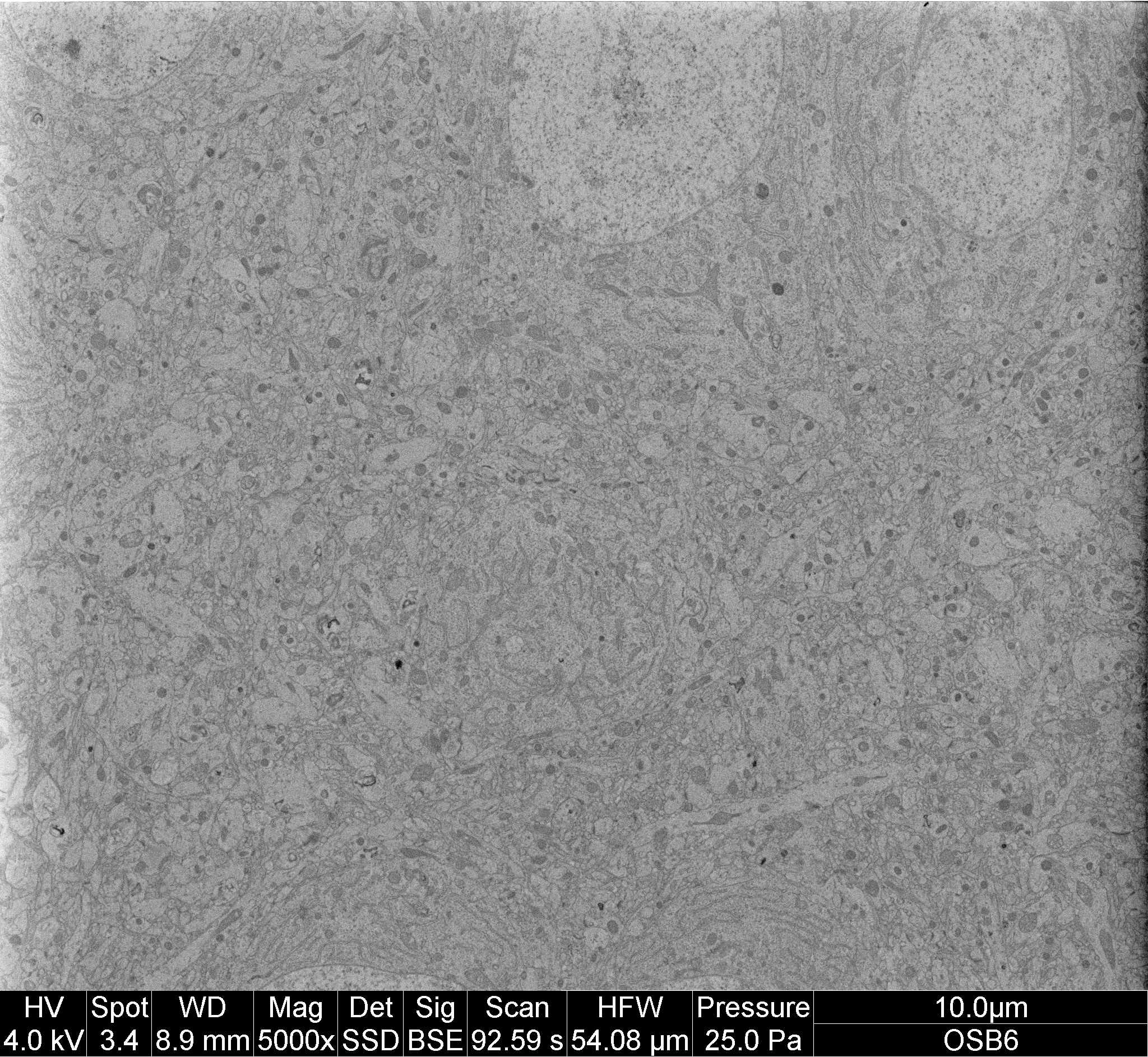

Supplement: Dataset S11 — (252.6 MB ZIP). [file pbio.0020329.sd011.zip › 040604_OS5_st1_1053.tif]

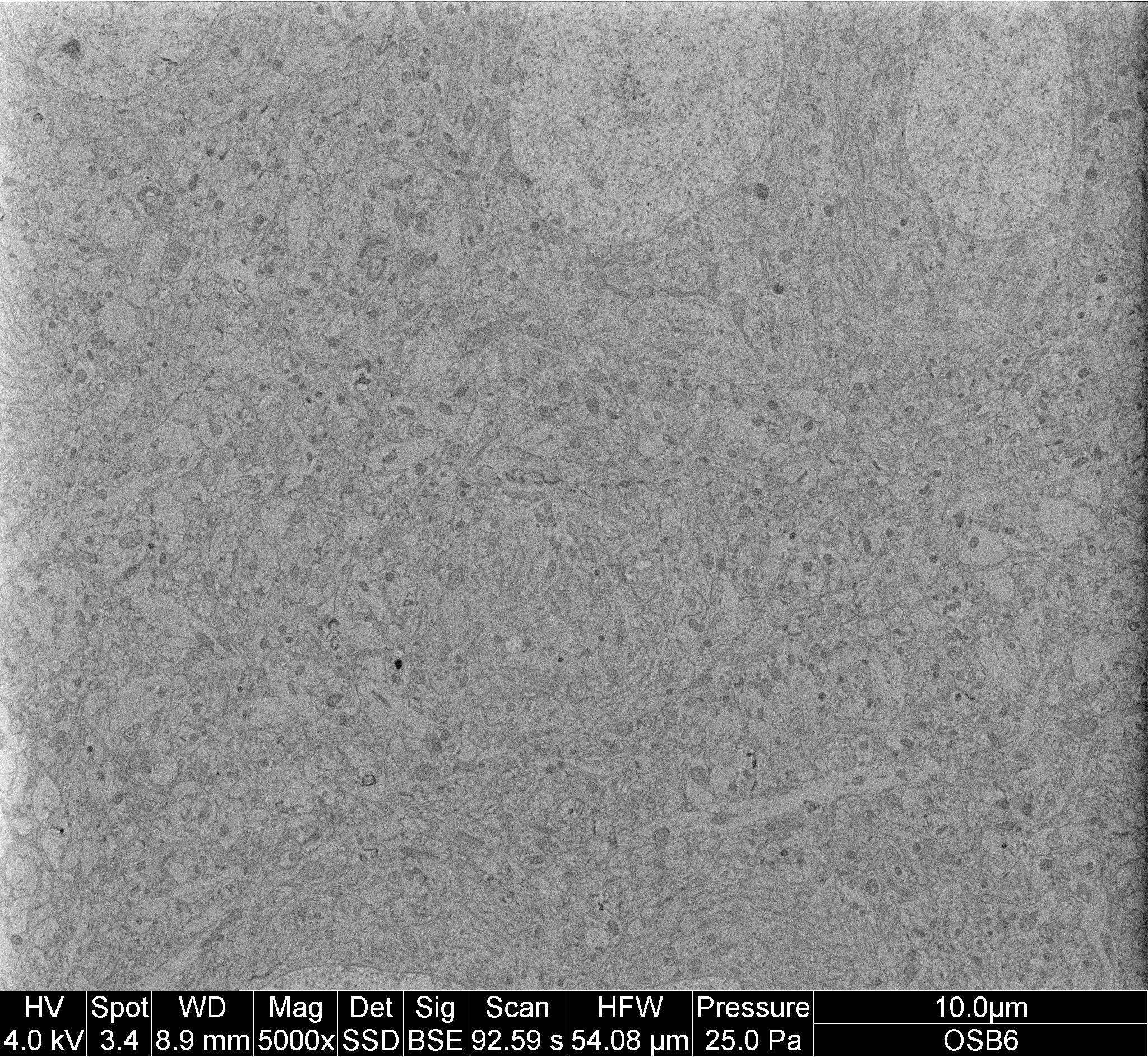

Supplement: Dataset S11 — (252.6 MB ZIP). [file pbio.0020329.sd011.zip › 040604_OS5_st1_1054.tif]

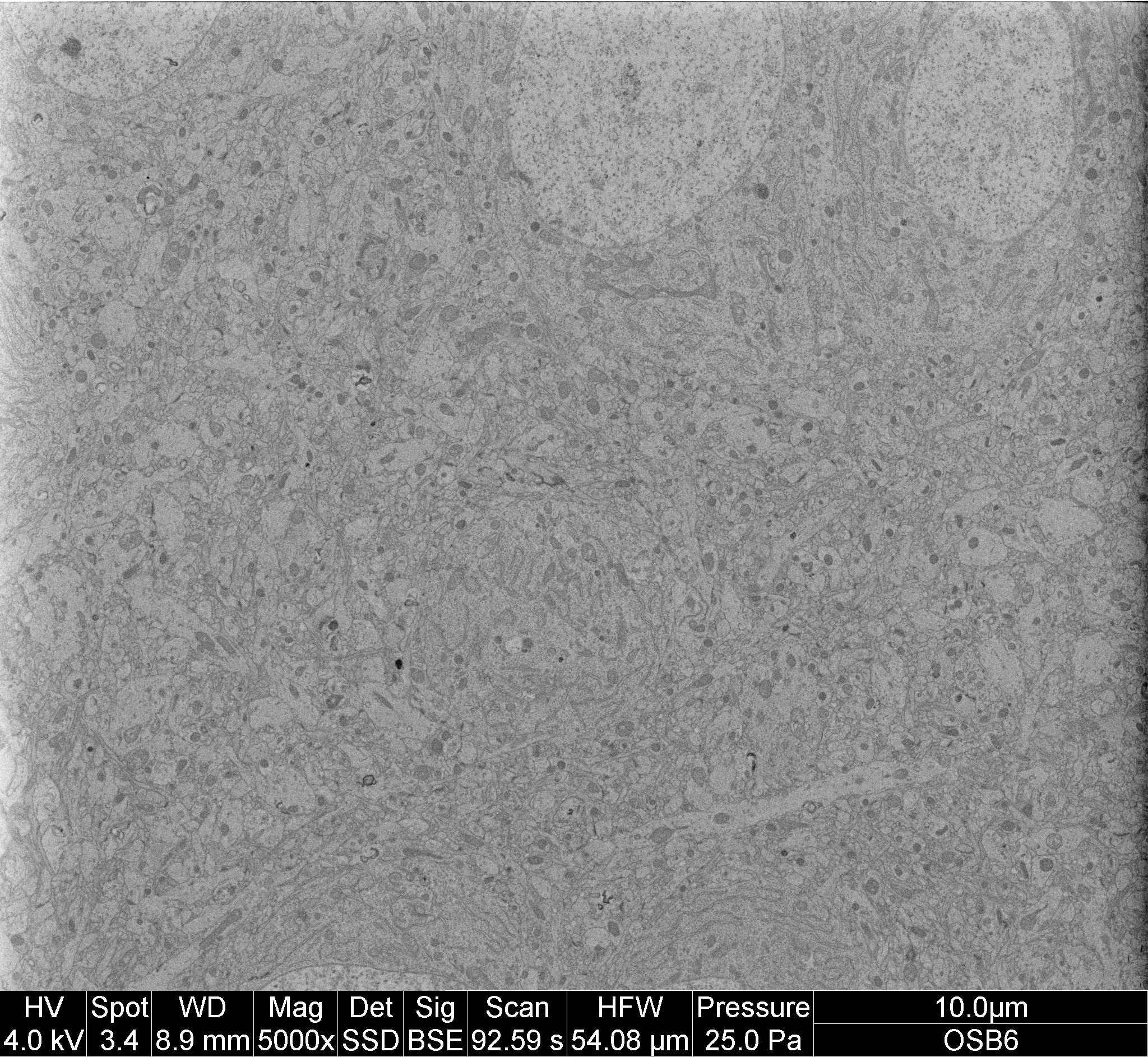

Supplement: Dataset S11 — (252.6 MB ZIP). [file pbio.0020329.sd011.zip › 040604_OS5_st1_1055.tif]

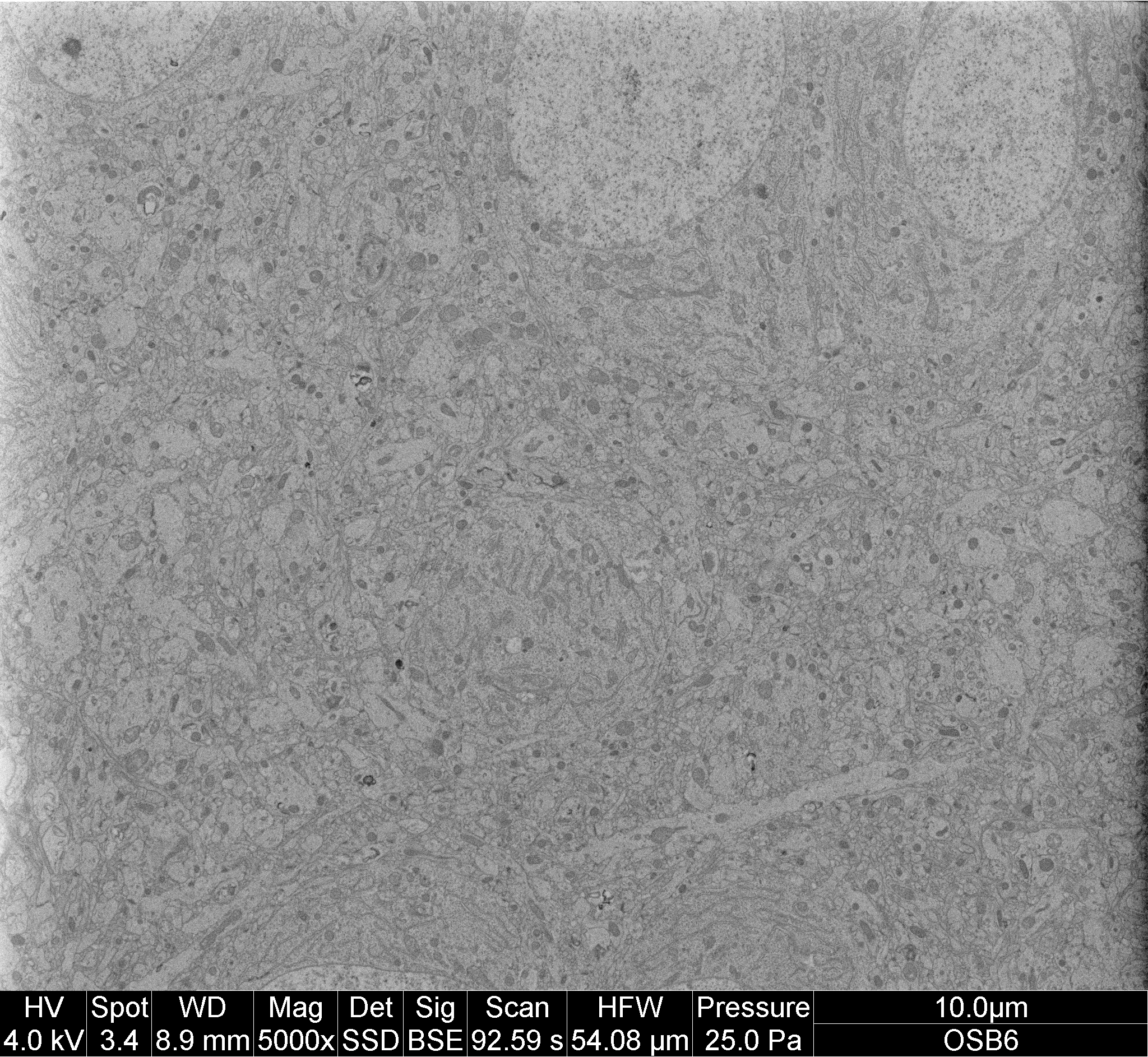

Supplement: Dataset S11 — (252.6 MB ZIP). [file pbio.0020329.sd011.zip › 040604_OS5_st1_1056.tif]

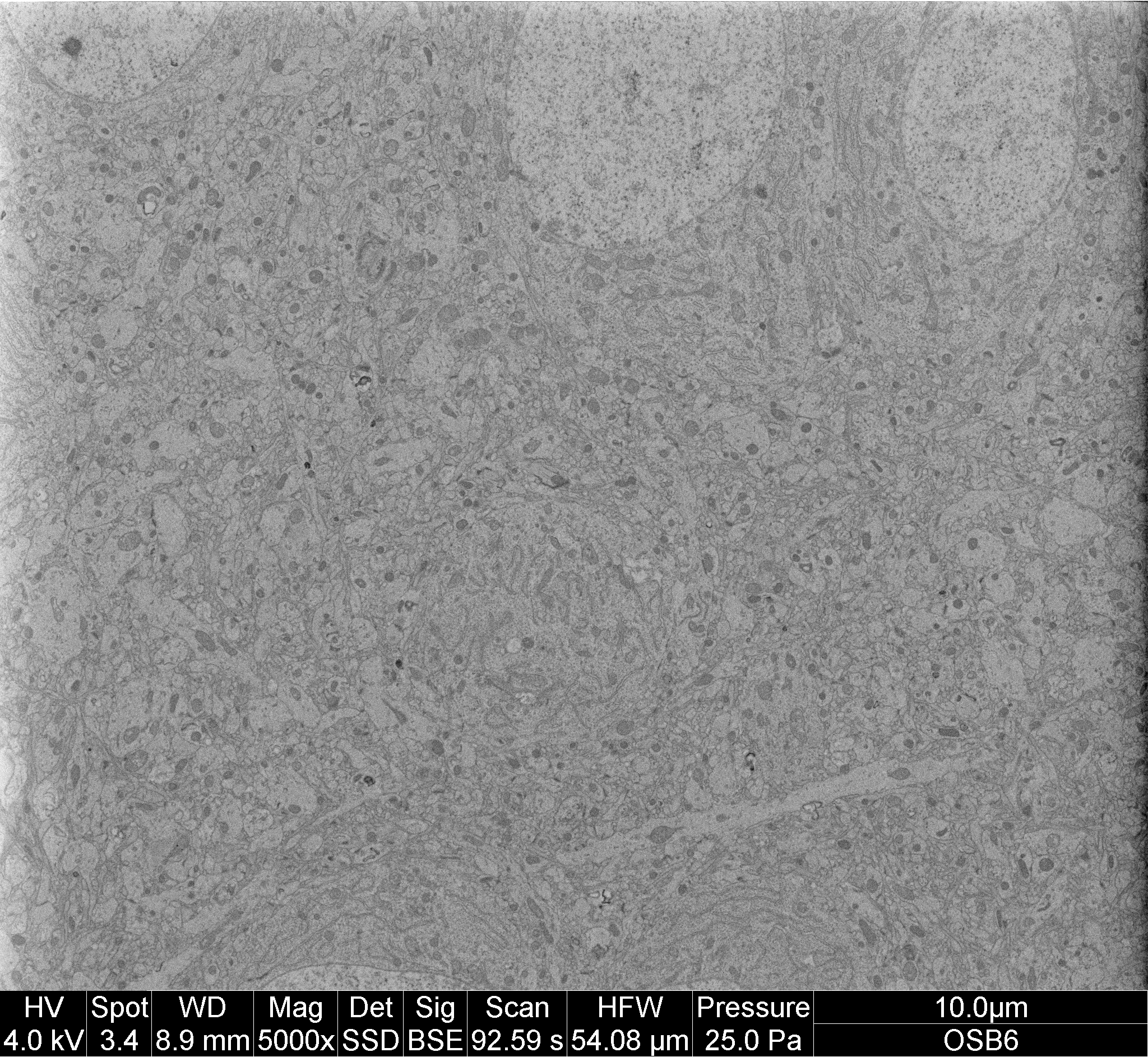

Supplement: Dataset S11 — (252.6 MB ZIP). [file pbio.0020329.sd011.zip › 040604_OS5_st1_1057.tif]

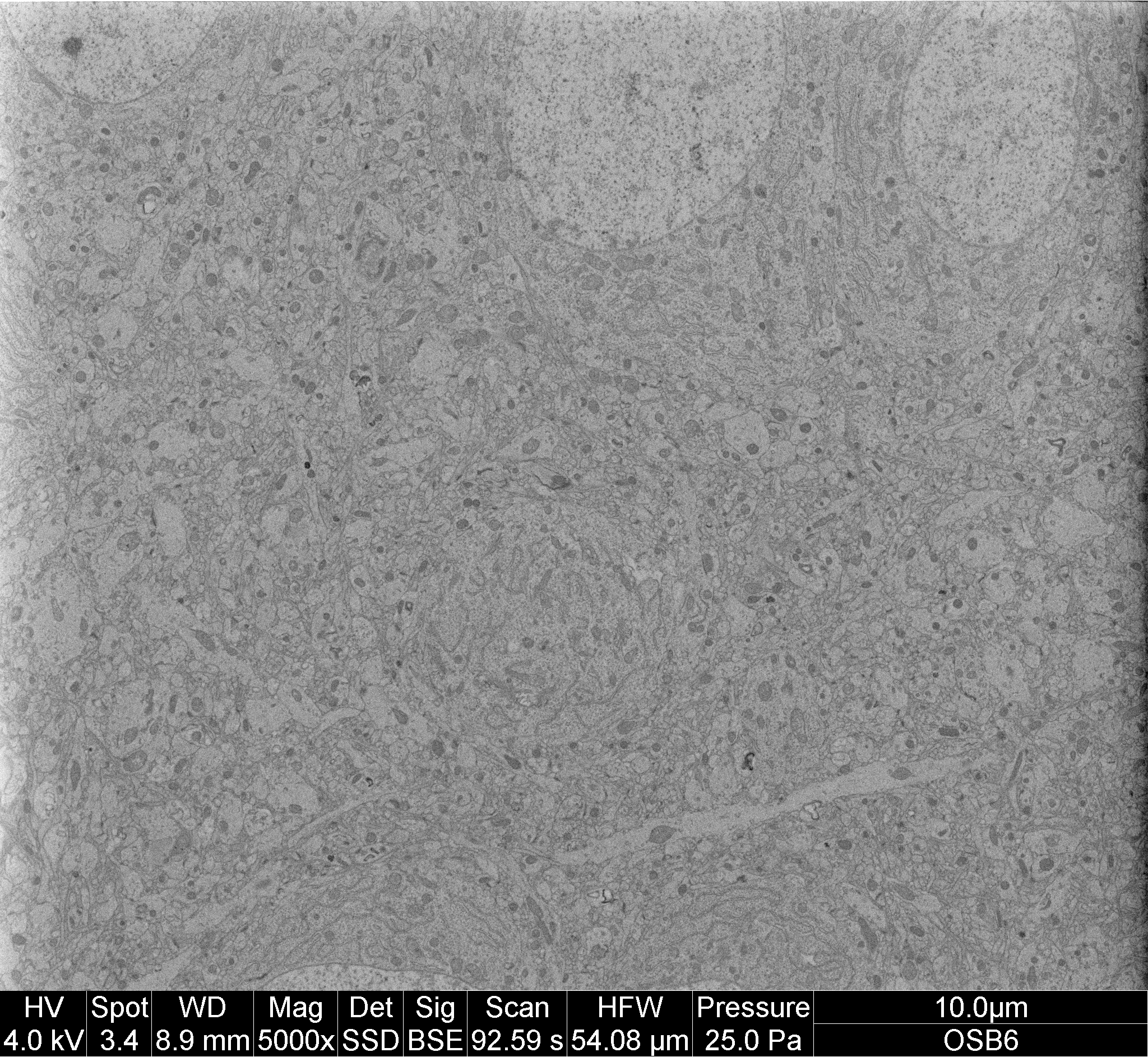

Supplement: Dataset S11 — (252.6 MB ZIP). [file pbio.0020329.sd011.zip › 040604_OS5_st1_1058.tif]

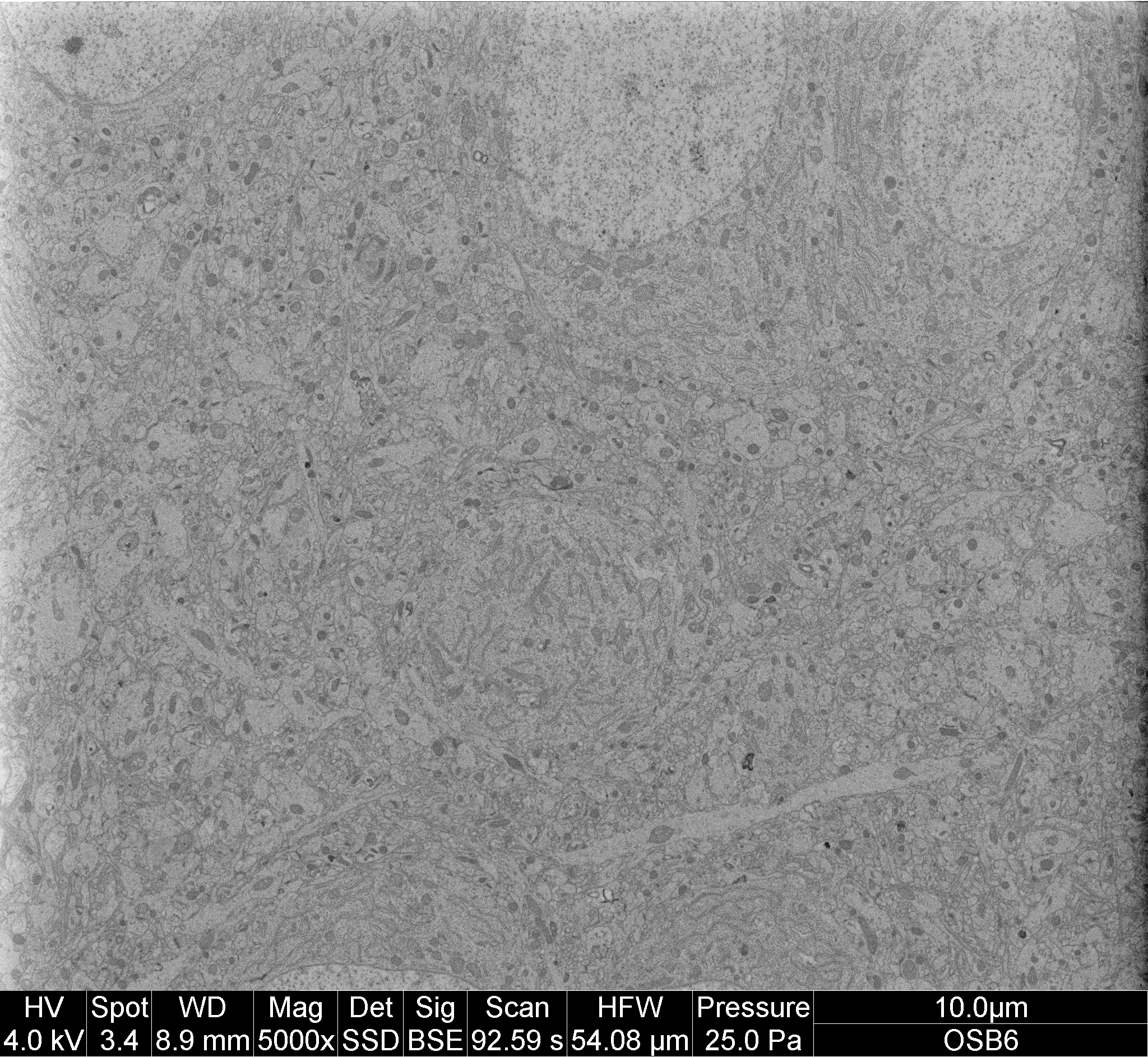

Supplement: Dataset S11 — (252.6 MB ZIP). [file pbio.0020329.sd011.zip › 040604_OS5_st1_1059.tif]

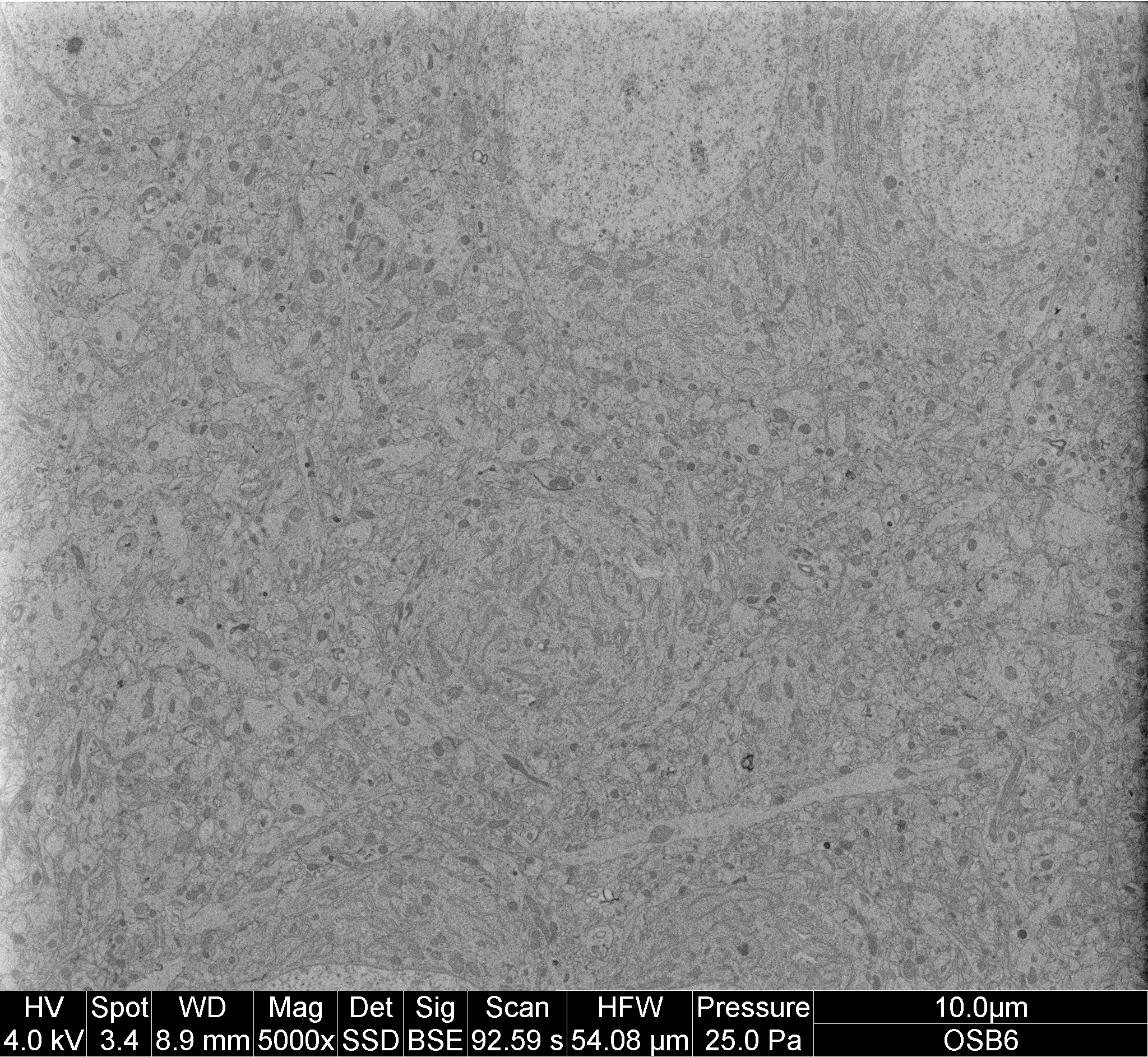

Supplement: Dataset S11 — (252.6 MB ZIP). [file pbio.0020329.sd011.zip › 040604_OS5_st1_1060.tif]

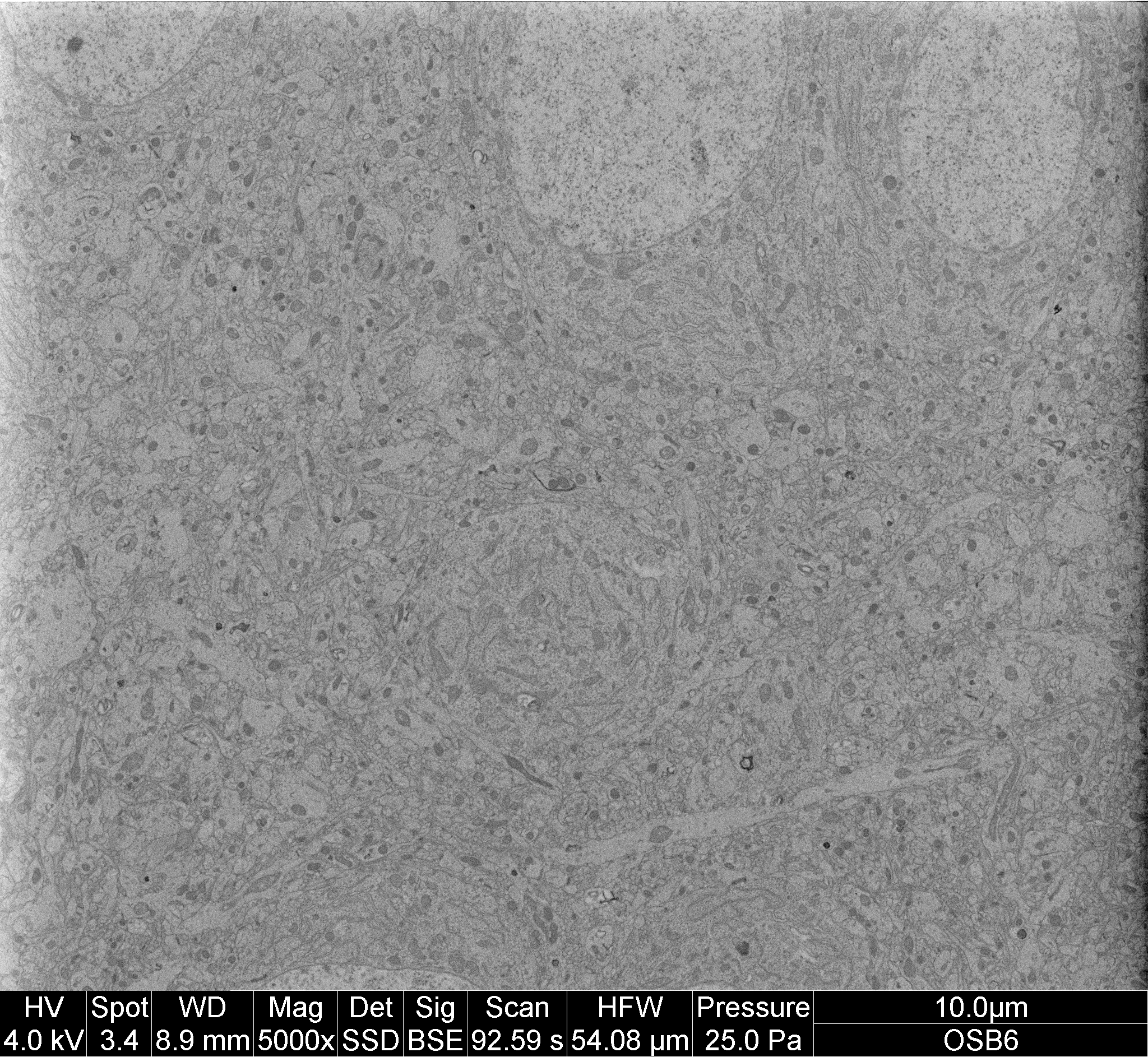

Supplement: Dataset S11 — (252.6 MB ZIP). [file pbio.0020329.sd011.zip › 040604_OS5_st1_1061.tif]

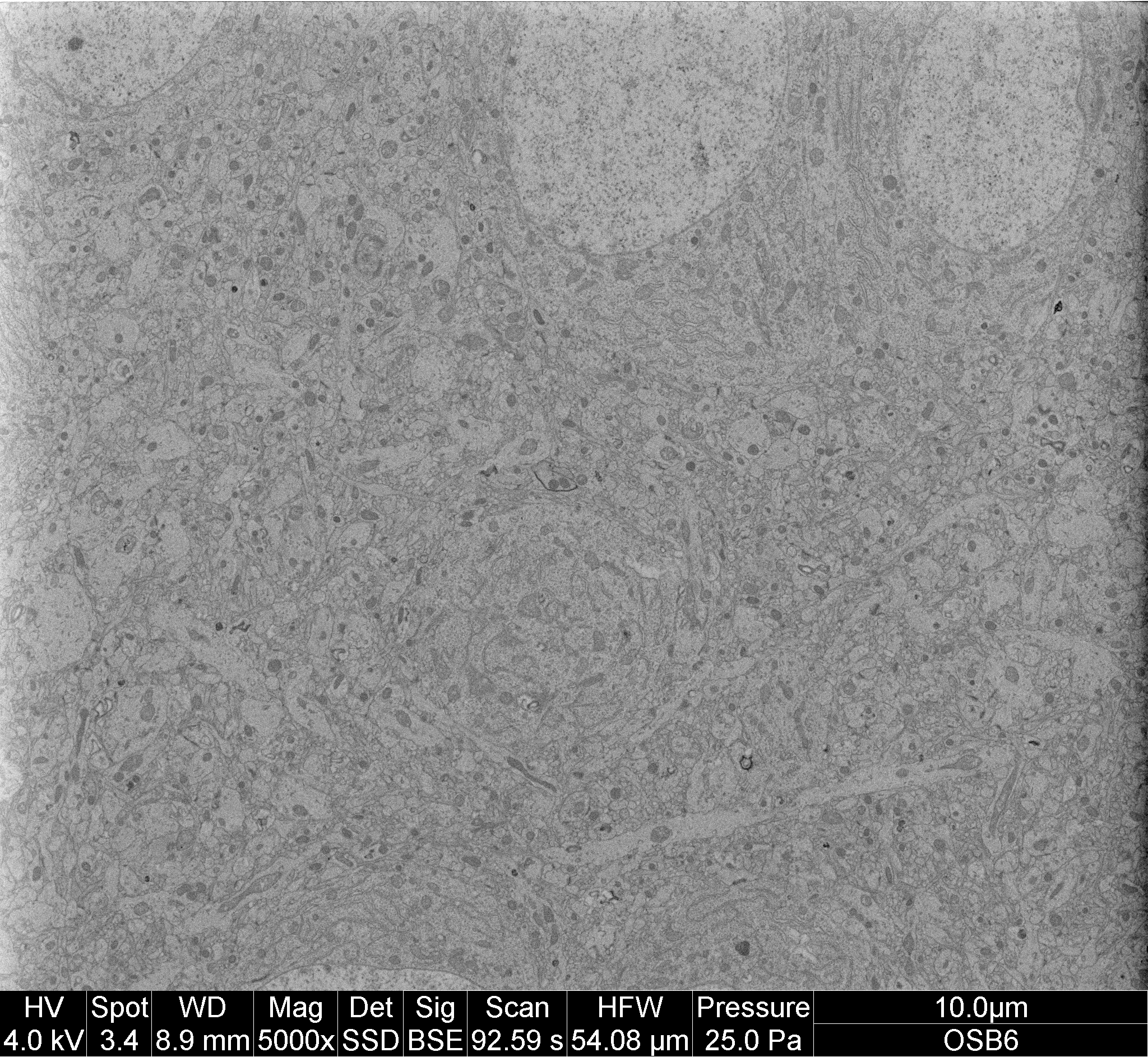

Supplement: Dataset S11 — (252.6 MB ZIP). [file pbio.0020329.sd011.zip › 040604_OS5_st1_1062.tif]

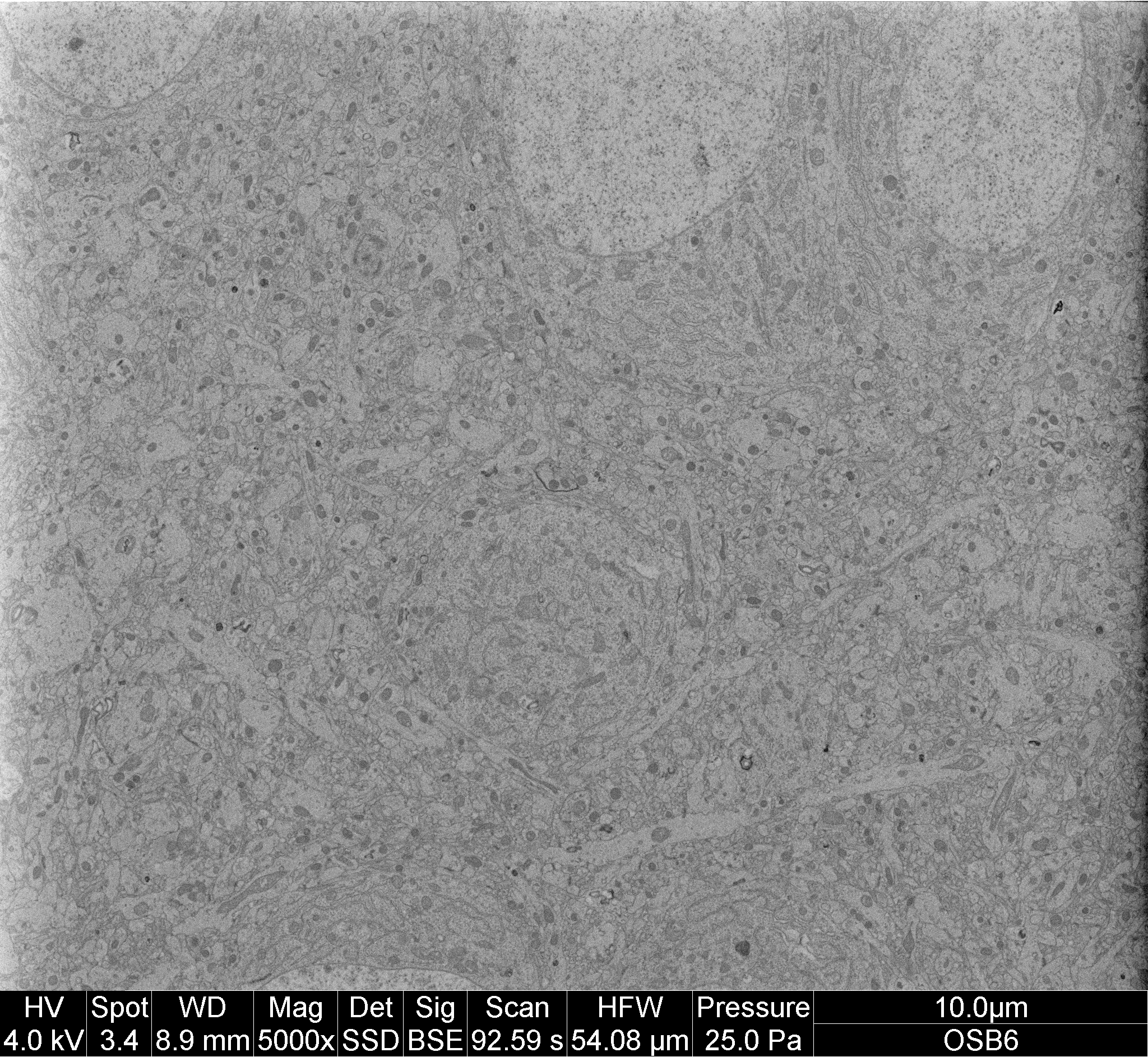

Supplement: Dataset S11 — (252.6 MB ZIP). [file pbio.0020329.sd011.zip › 040604_OS5_st1_1063.tif]

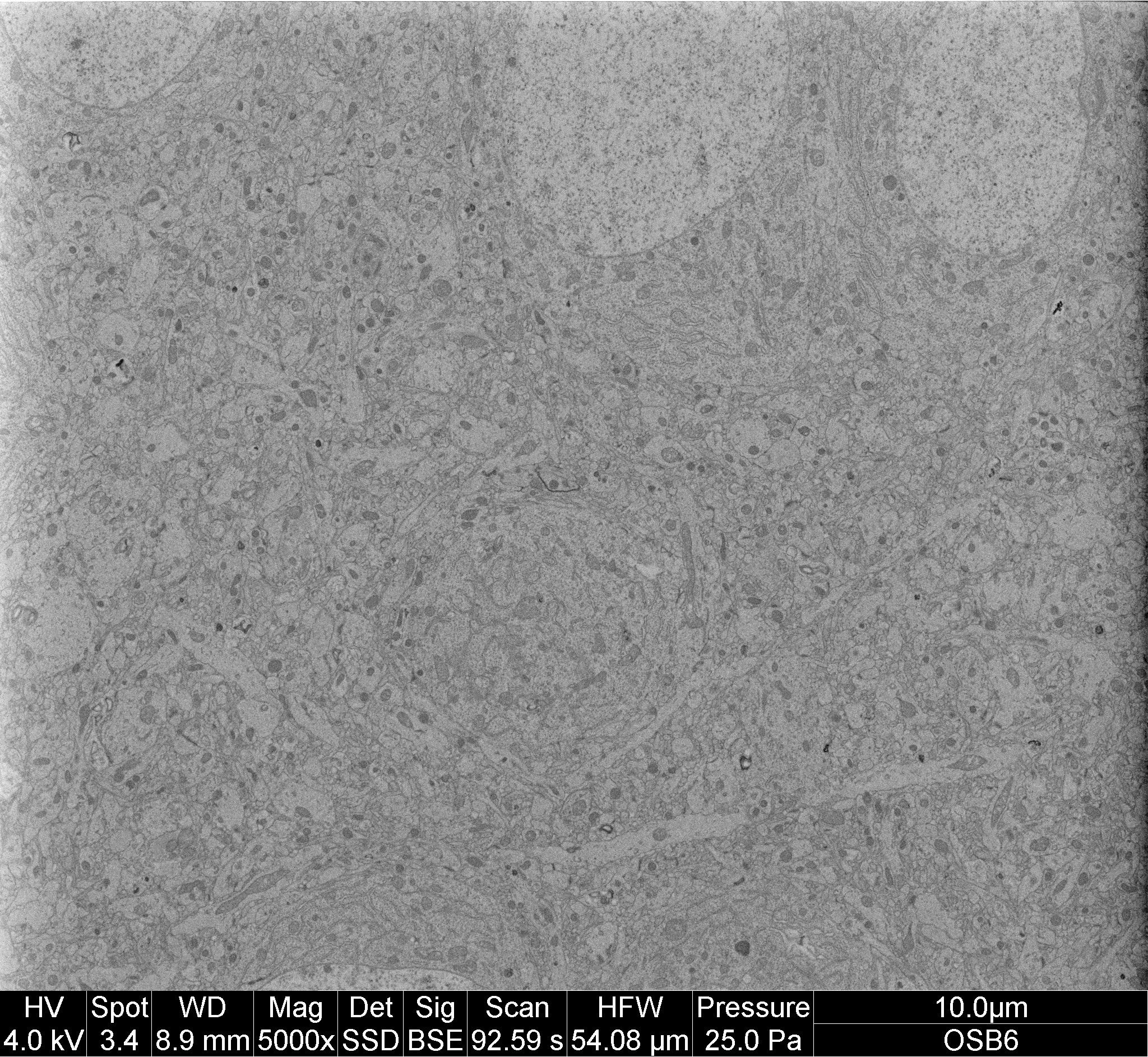

Supplement: Dataset S11 — (252.6 MB ZIP). [file pbio.0020329.sd011.zip › 040604_OS5_st1_1064.tif]

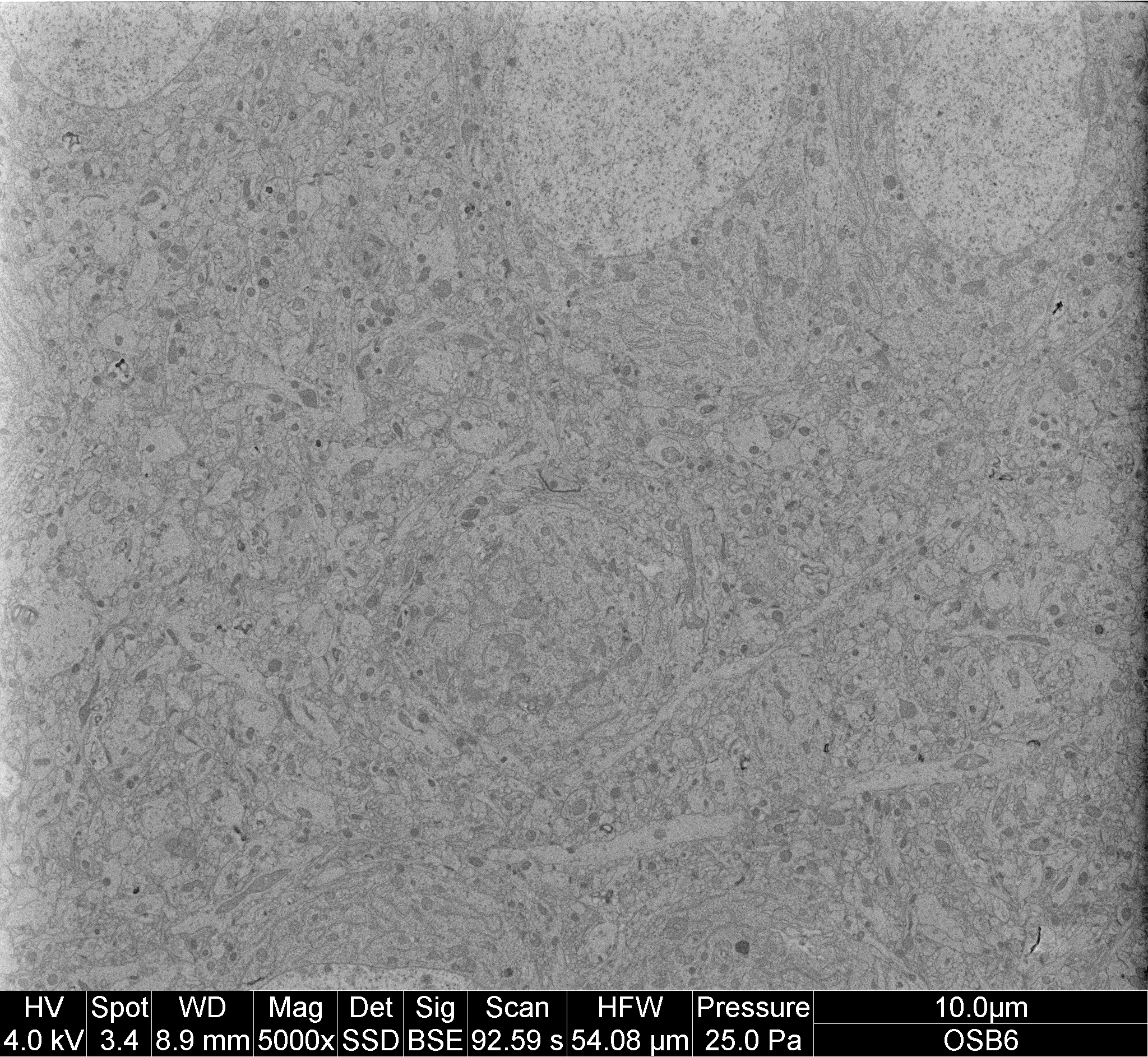

Supplement: Dataset S11 — (252.6 MB ZIP). [file pbio.0020329.sd011.zip › 040604_OS5_st1_1065.tif]

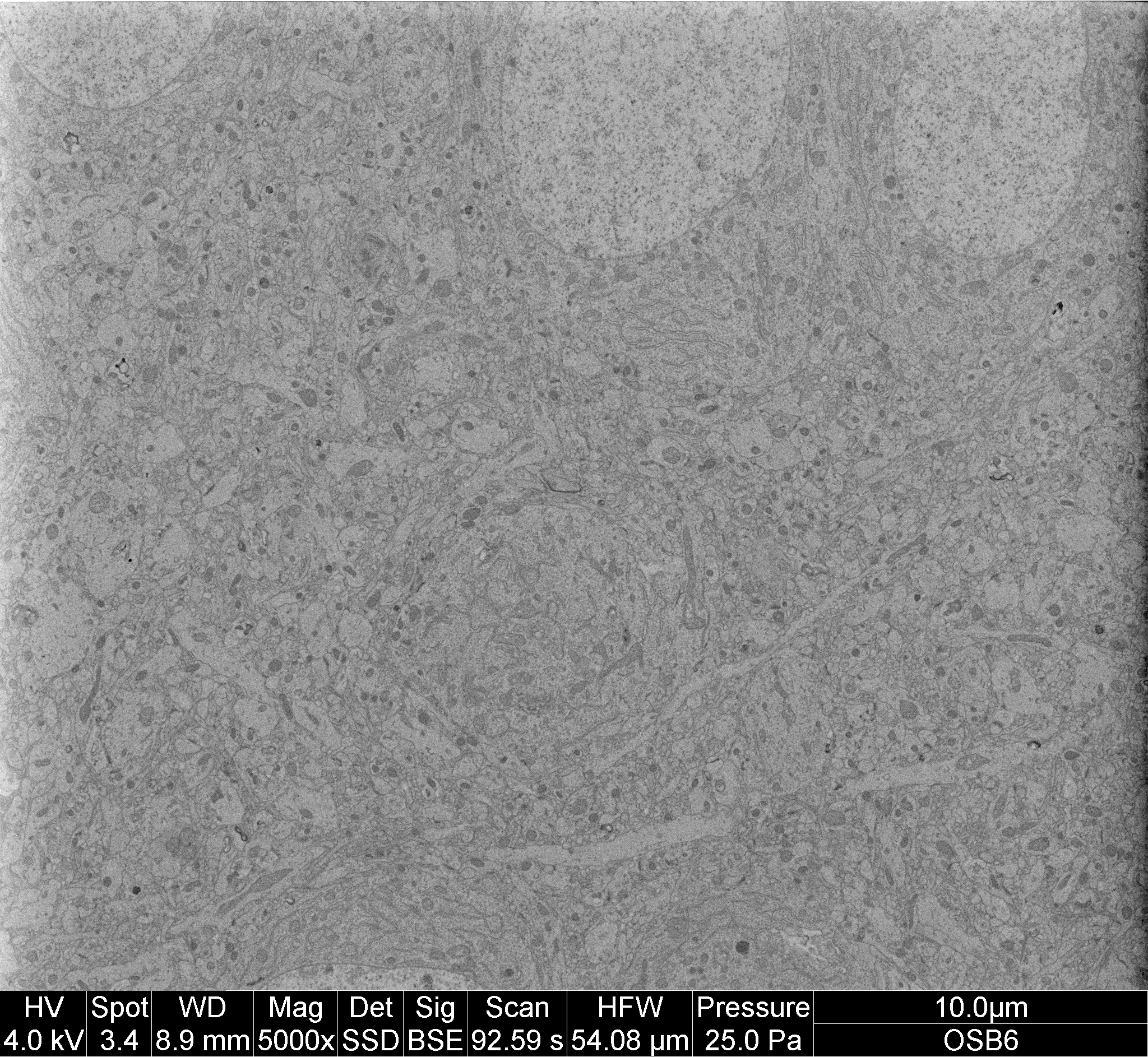

Supplement: Dataset S11 — (252.6 MB ZIP). [file pbio.0020329.sd011.zip › 040604_OS5_st1_1066.tif]

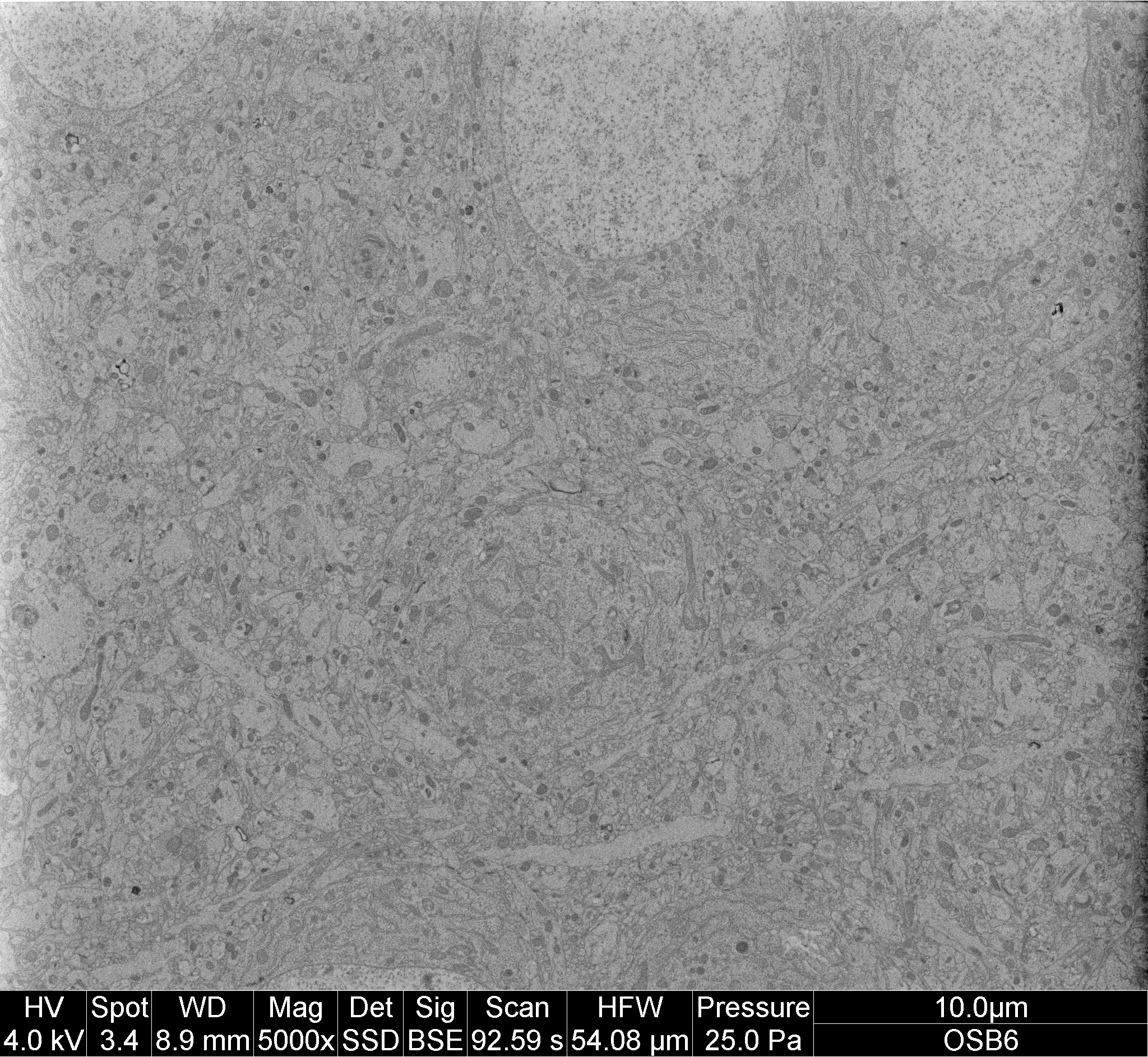

Supplement: Dataset S11 — (252.6 MB ZIP). [file pbio.0020329.sd011.zip › 040604_OS5_st1_1067.tif]

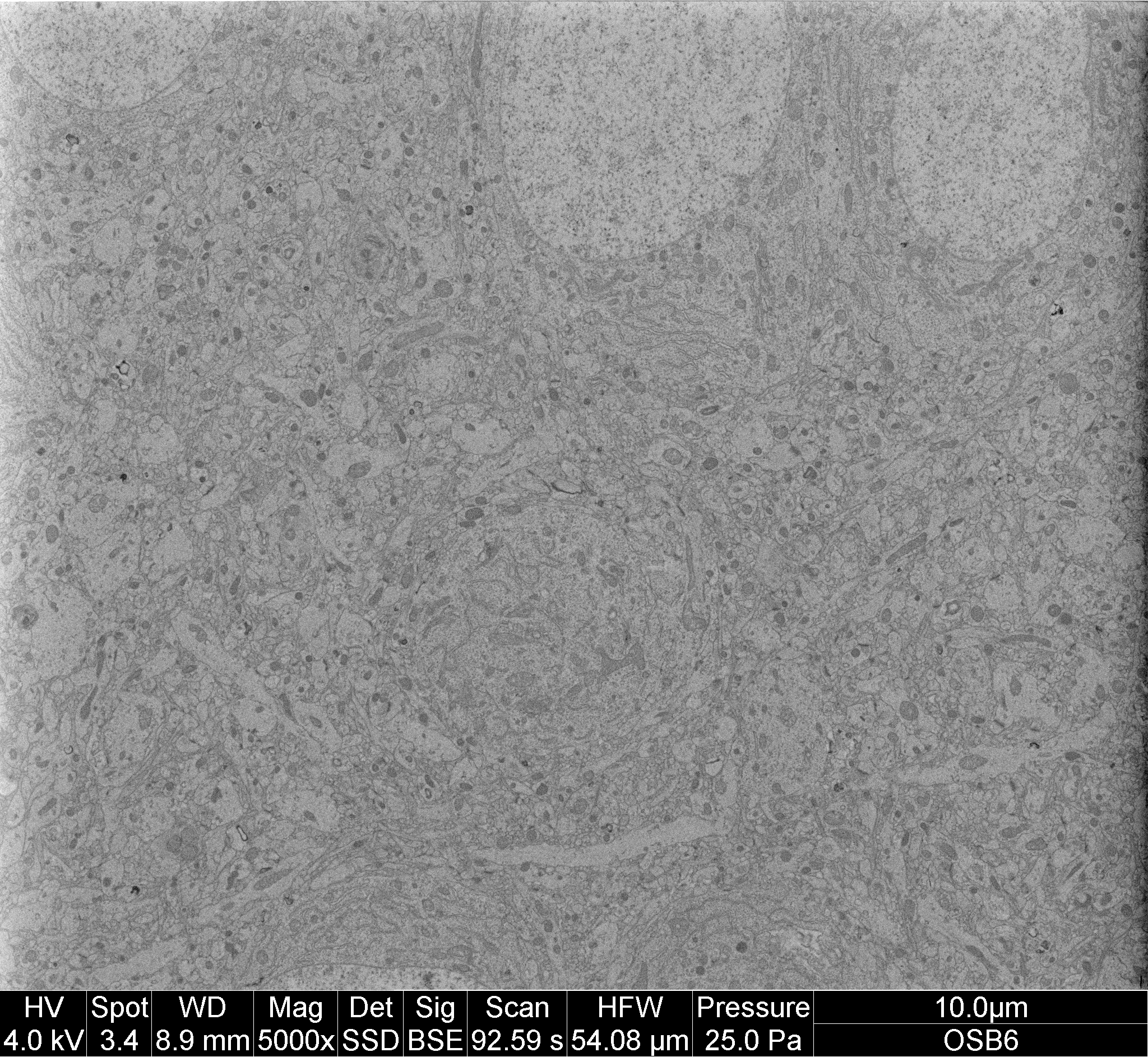

Supplement: Dataset S11 — (252.6 MB ZIP). [file pbio.0020329.sd011.zip › 040604_OS5_st1_1068.tif]

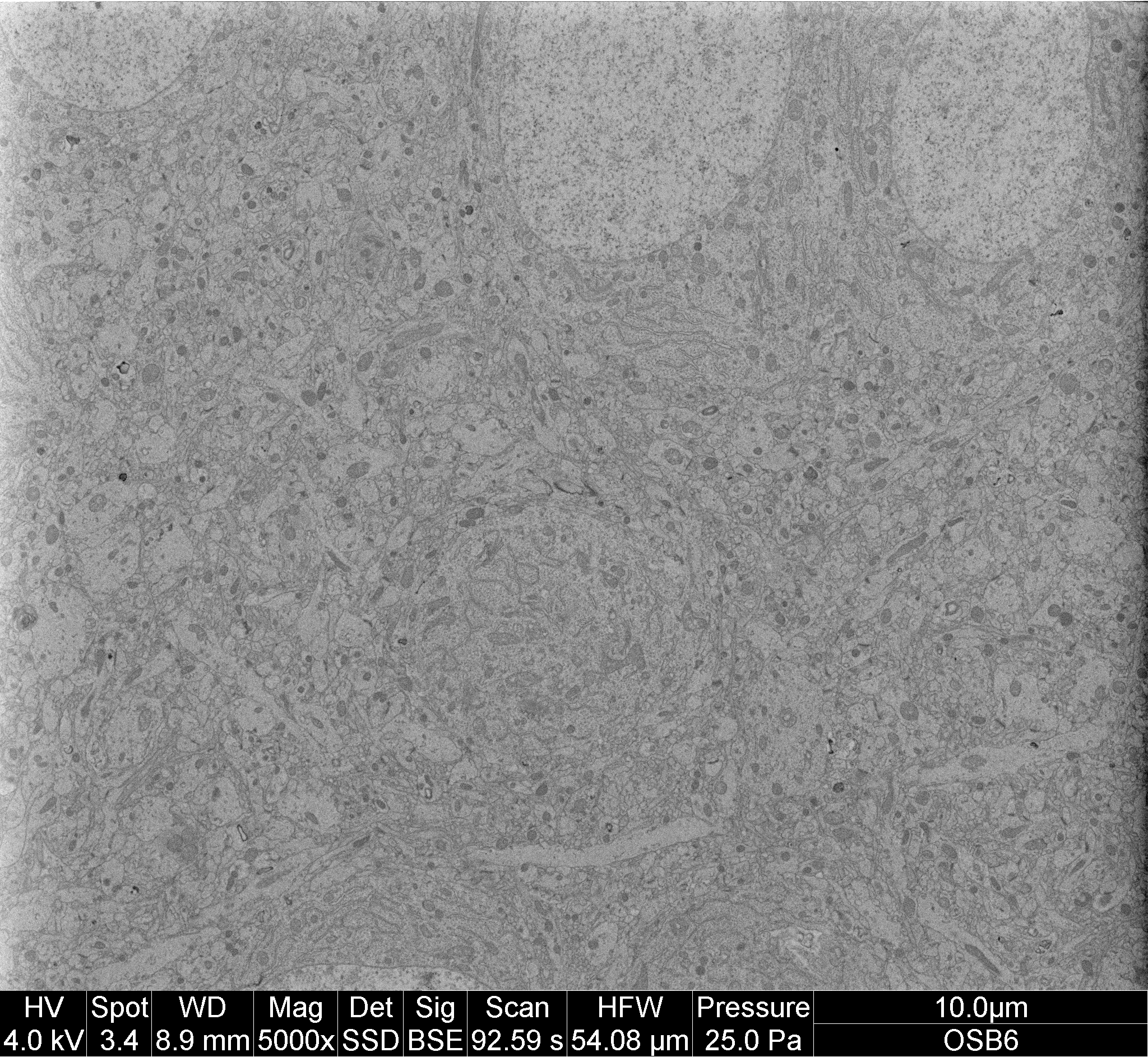

Supplement: Dataset S11 — (252.6 MB ZIP). [file pbio.0020329.sd011.zip › 040604_OS5_st1_1069.tif]

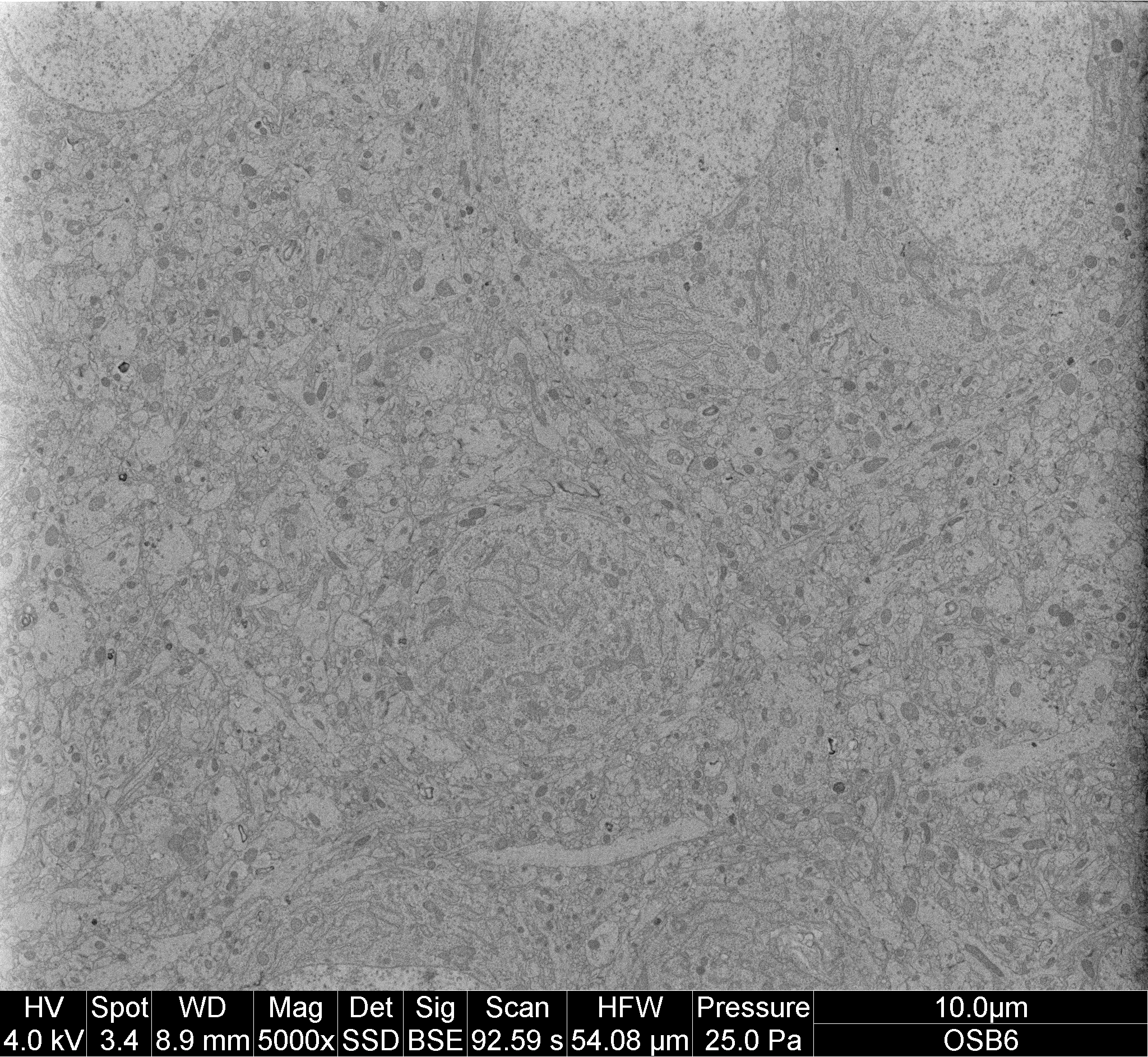

Supplement: Dataset S11 — (252.6 MB ZIP). [file pbio.0020329.sd011.zip › 040604_OS5_st1_1070.tif]

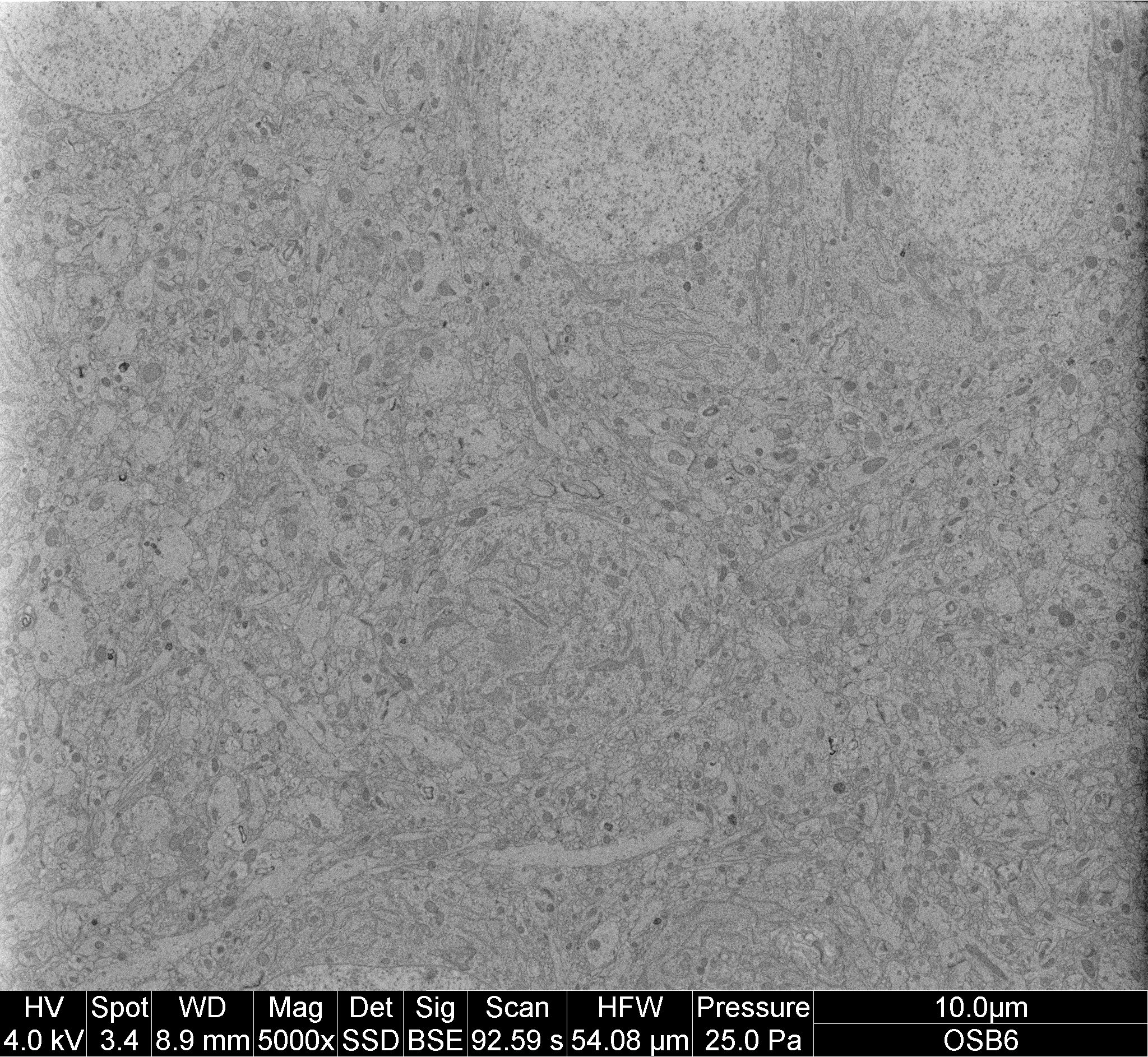

Supplement: Dataset S11 — (252.6 MB ZIP). [file pbio.0020329.sd011.zip › 040604_OS5_st1_1071.tif]

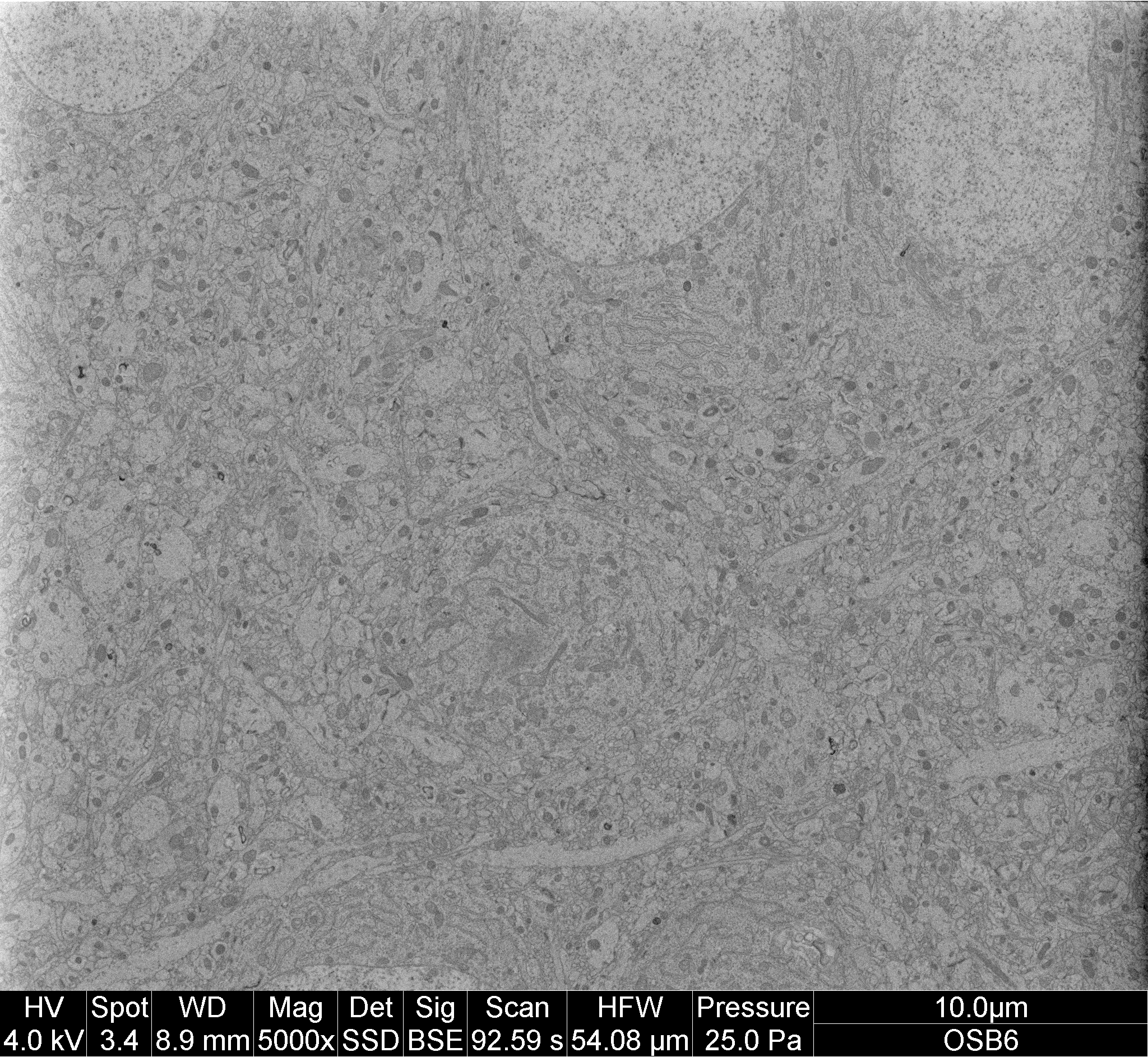

Supplement: Dataset S11 — (252.6 MB ZIP). [file pbio.0020329.sd011.zip › 040604_OS5_st1_1072.tif]

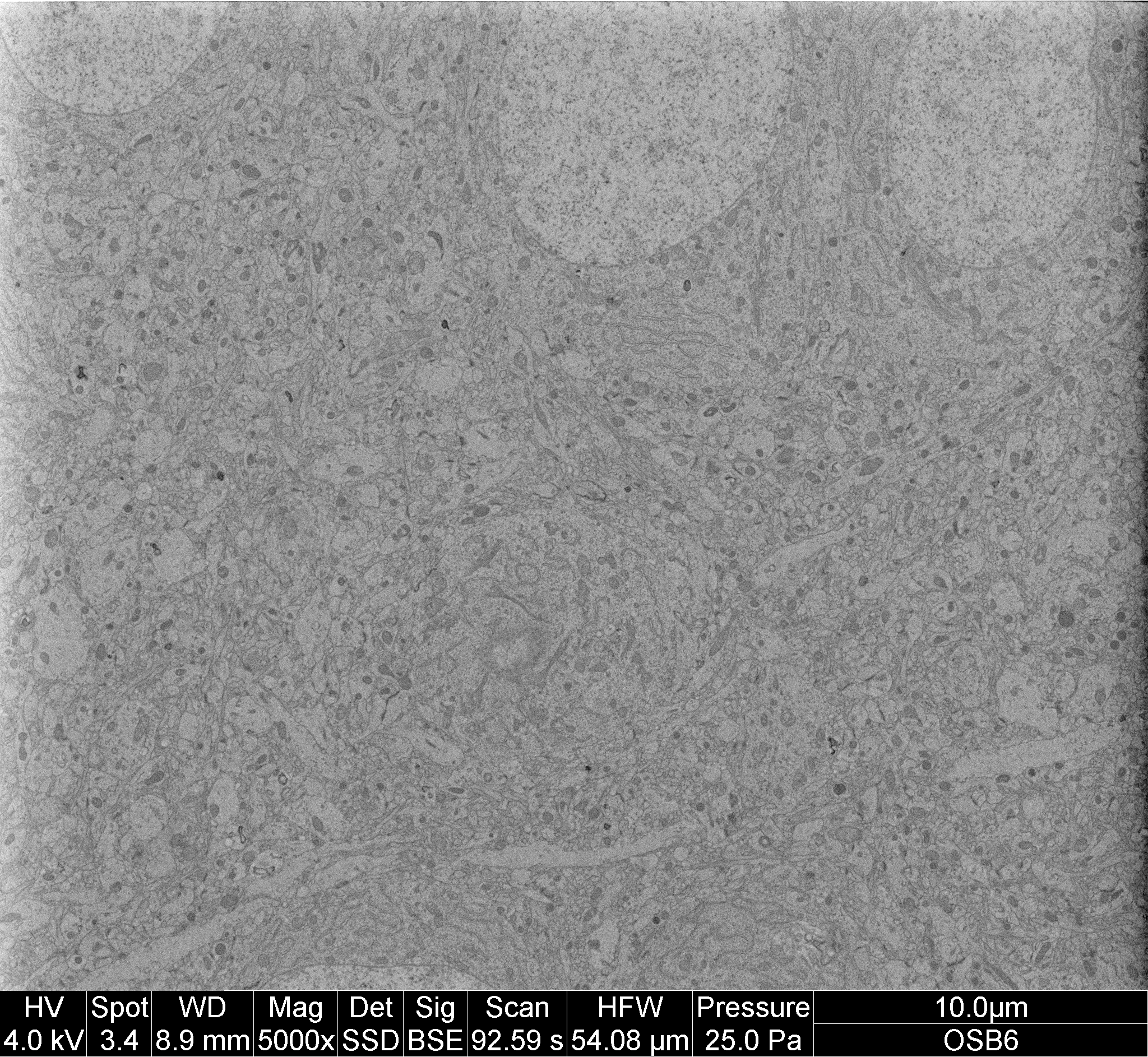

Supplement: Dataset S11 — (252.6 MB ZIP). [file pbio.0020329.sd011.zip › 040604_OS5_st1_1073.tif]

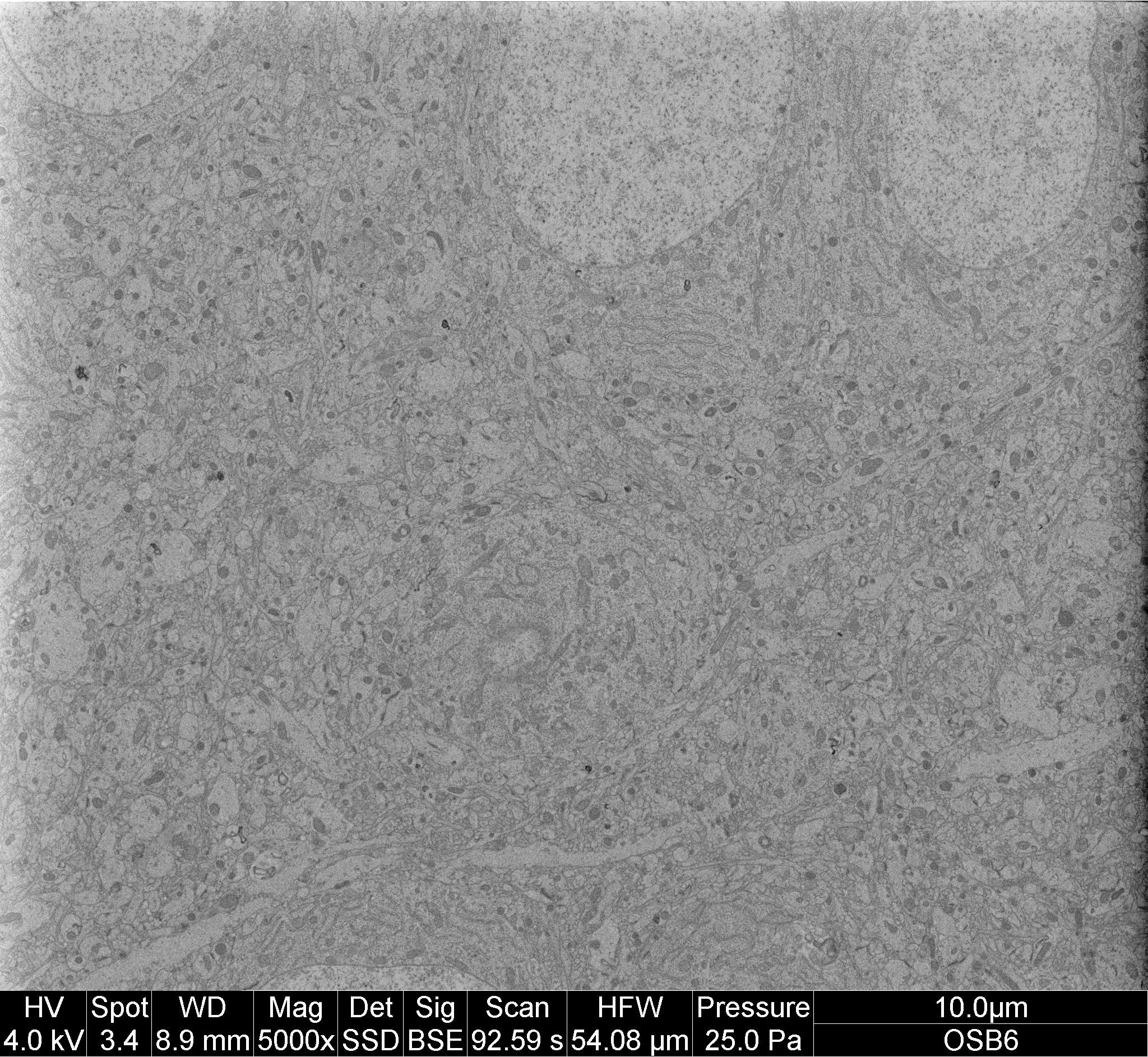

Supplement: Dataset S11 — (252.6 MB ZIP). [file pbio.0020329.sd011.zip › 040604_OS5_st1_1074.tif]

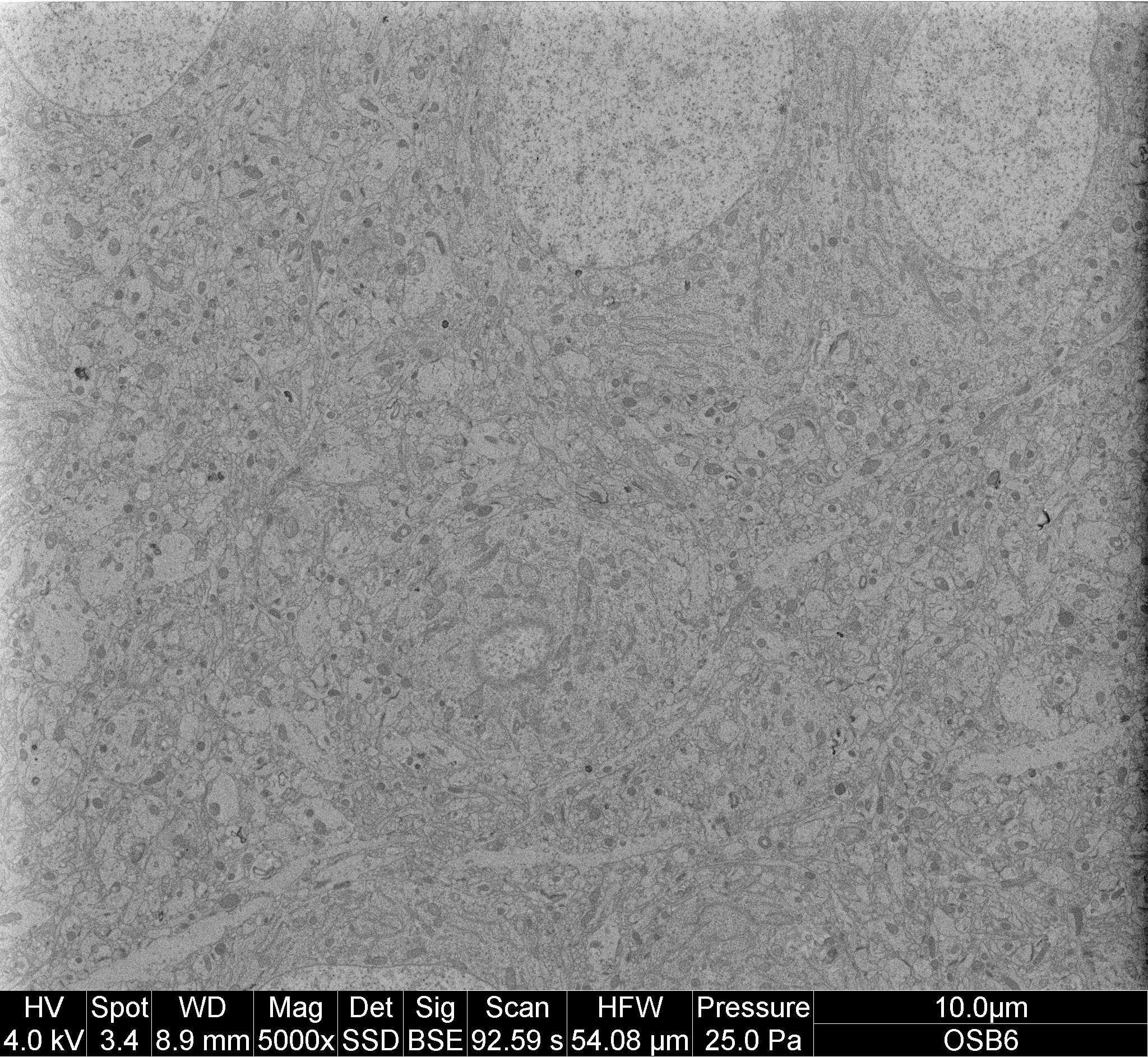

Supplement: Dataset S11 — (252.6 MB ZIP). [file pbio.0020329.sd011.zip › 040604_OS5_st1_1075.tif]

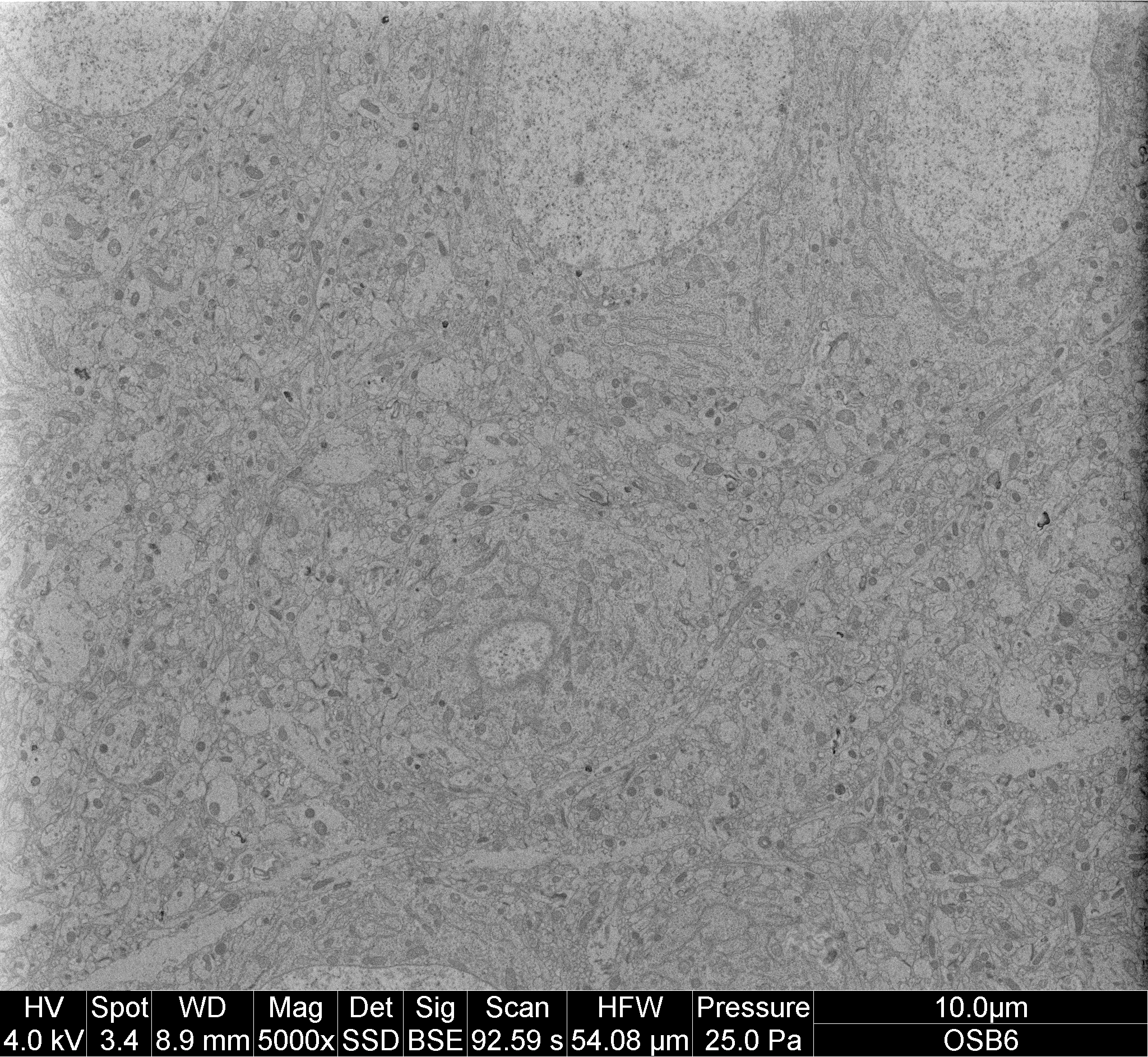

Supplement: Dataset S11 — (252.6 MB ZIP). [file pbio.0020329.sd011.zip › 040604_OS5_st1_1076.tif]

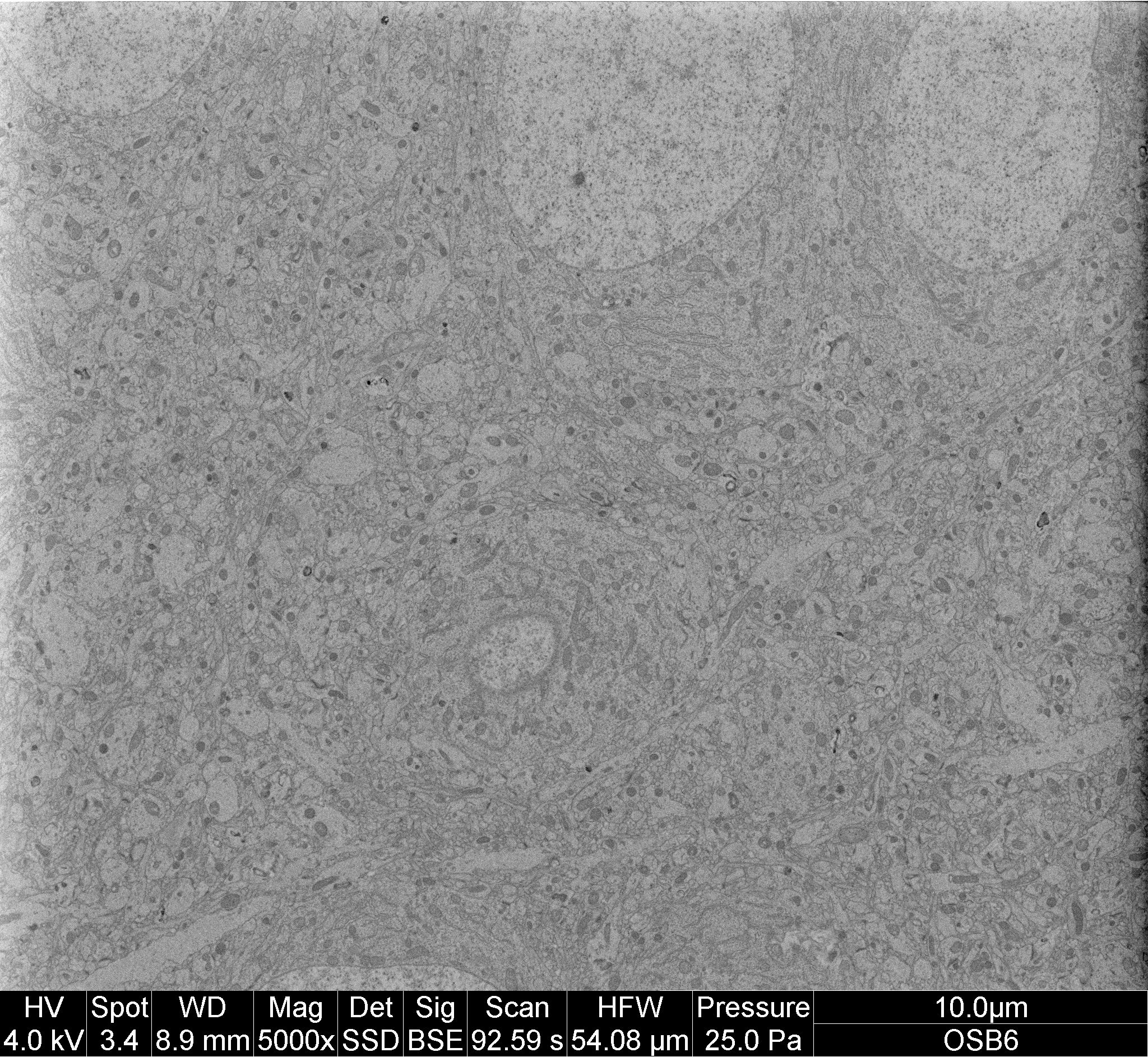

Supplement: Dataset S11 — (252.6 MB ZIP). [file pbio.0020329.sd011.zip › 040604_OS5_st1_1077.tif]

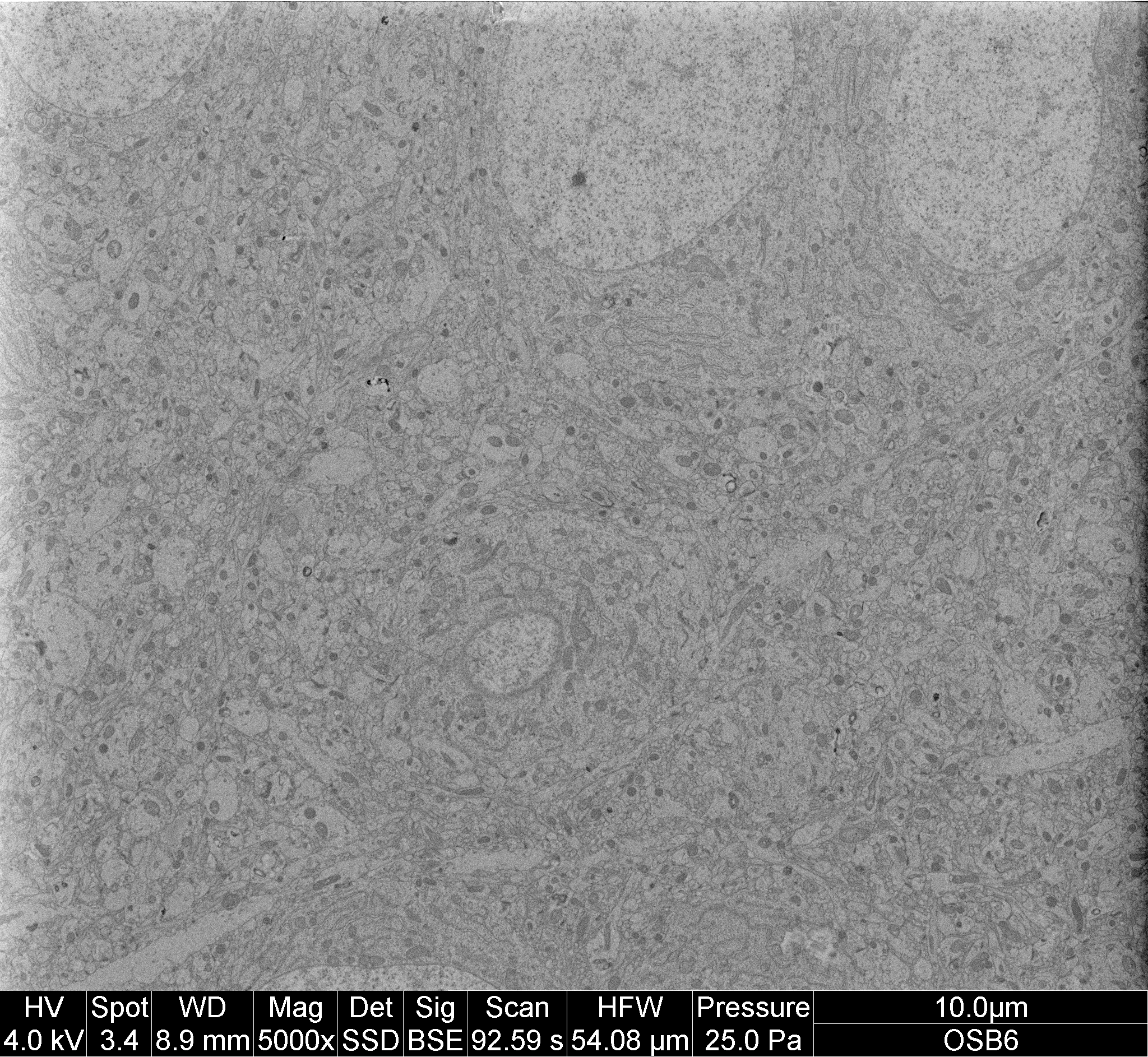

Supplement: Dataset S11 — (252.6 MB ZIP). [file pbio.0020329.sd011.zip › 040604_OS5_st1_1078.tif]

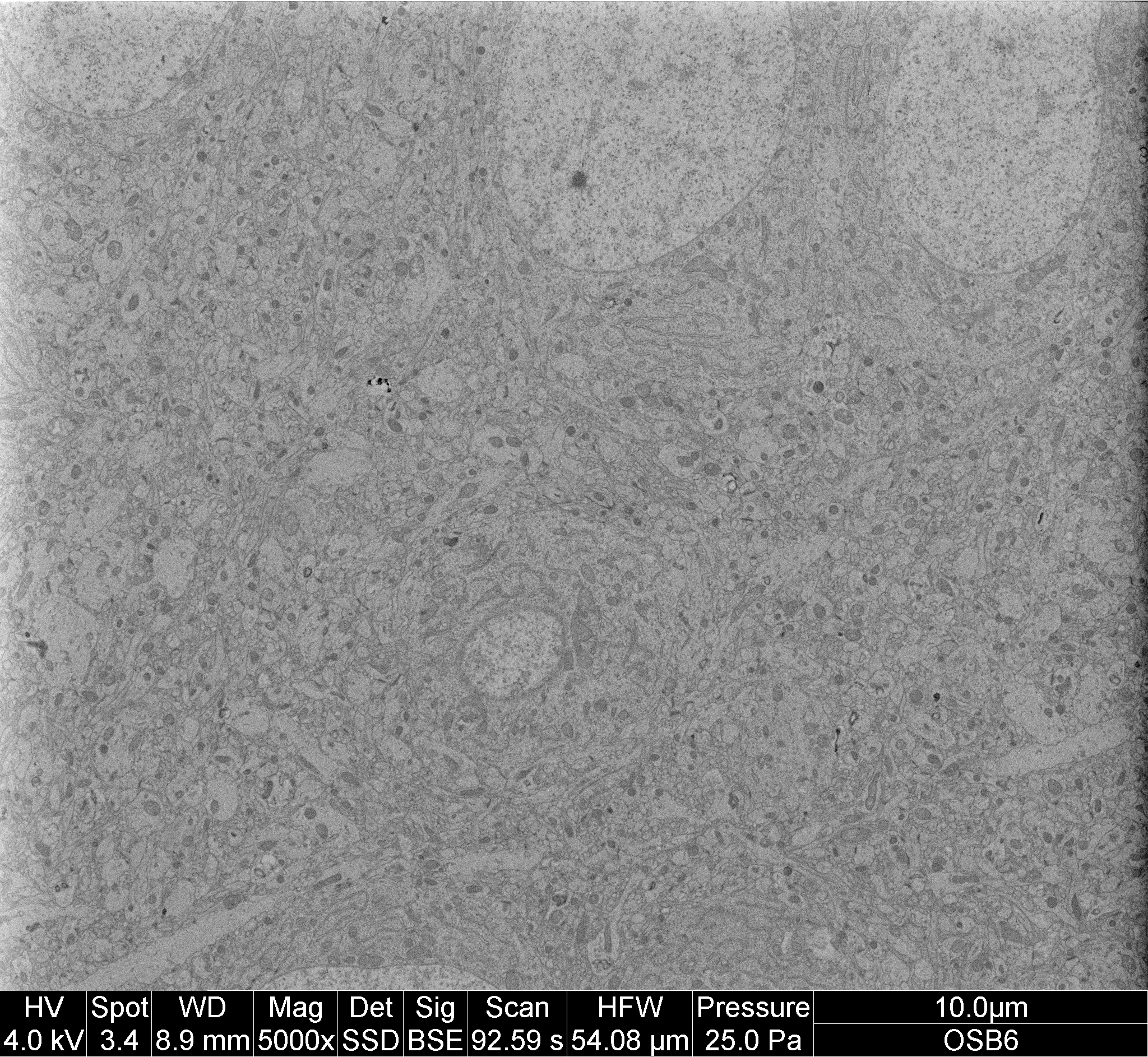

Supplement: Dataset S11 — (252.6 MB ZIP). [file pbio.0020329.sd011.zip › 040604_OS5_st1_1079.tif]

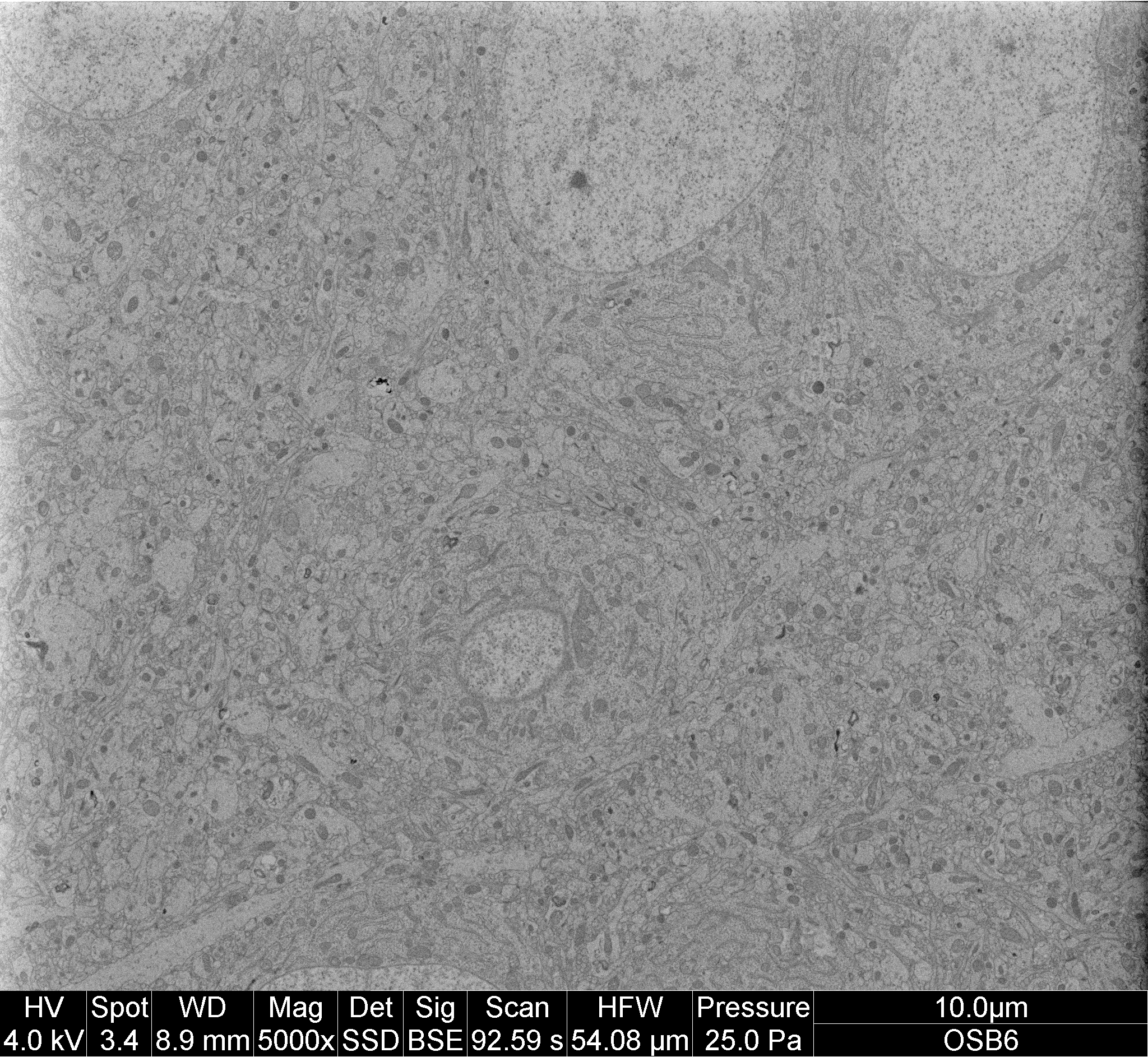

Supplement: Dataset S11 — (252.6 MB ZIP). [file pbio.0020329.sd011.zip › 040604_OS5_st1_1080.tif]

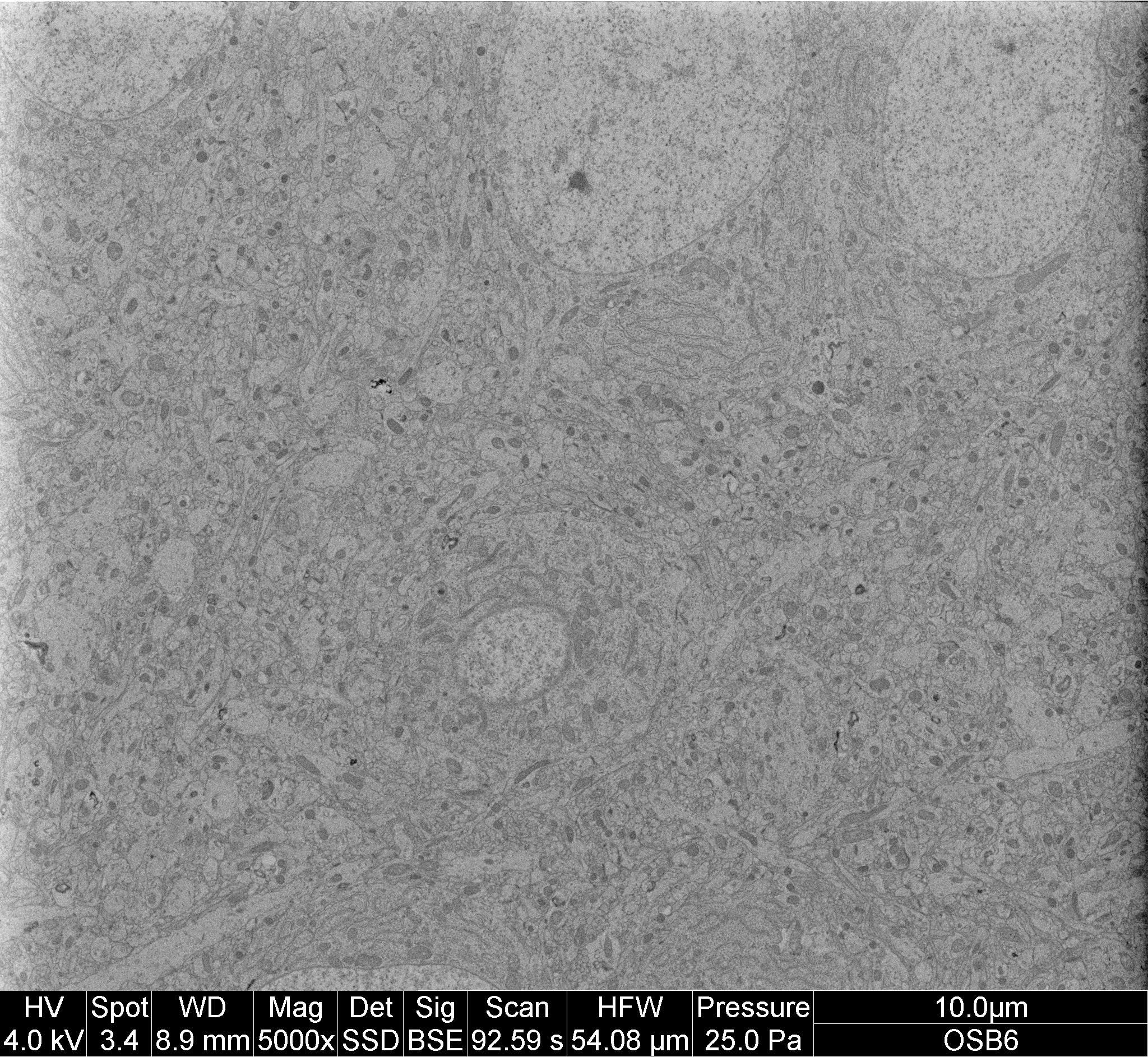

Supplement: Dataset S11 — (252.6 MB ZIP). [file pbio.0020329.sd011.zip › 040604_OS5_st1_1081.tif]

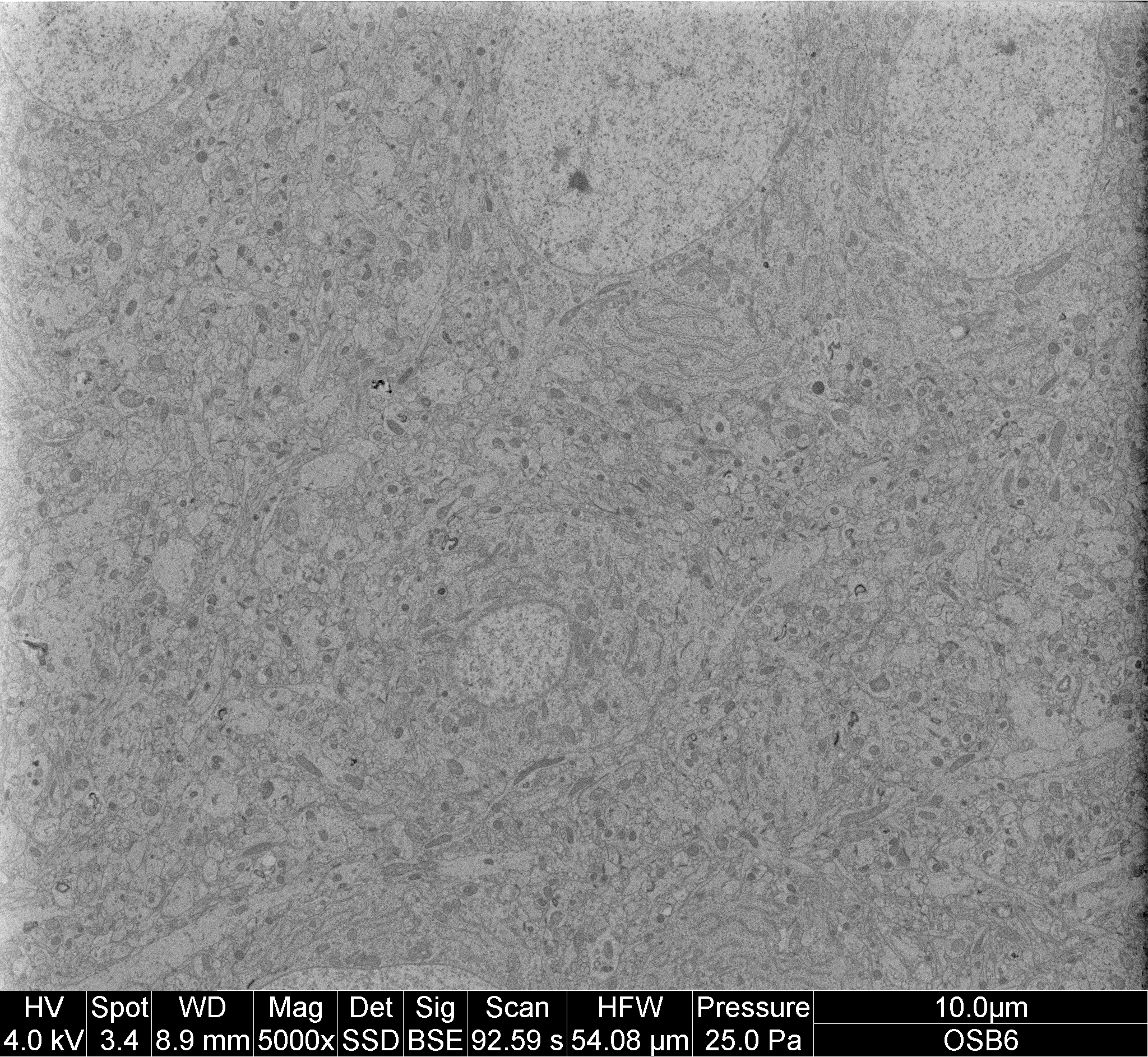

Supplement: Dataset S11 — (252.6 MB ZIP). [file pbio.0020329.sd011.zip › 040604_OS5_st1_1082.tif]

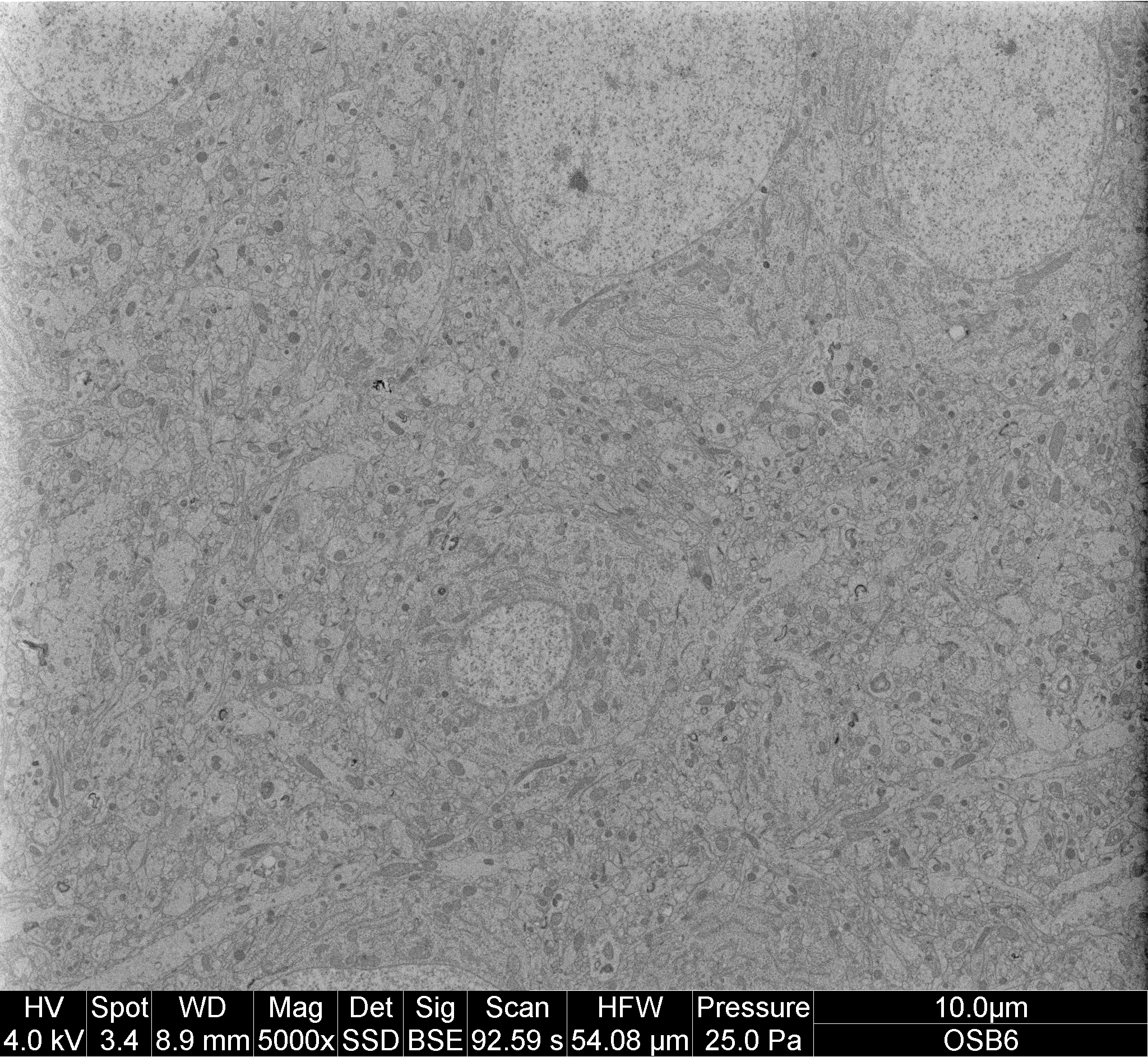

Supplement: Dataset S11 — (252.6 MB ZIP). [file pbio.0020329.sd011.zip › 040604_OS5_st1_1083.tif]

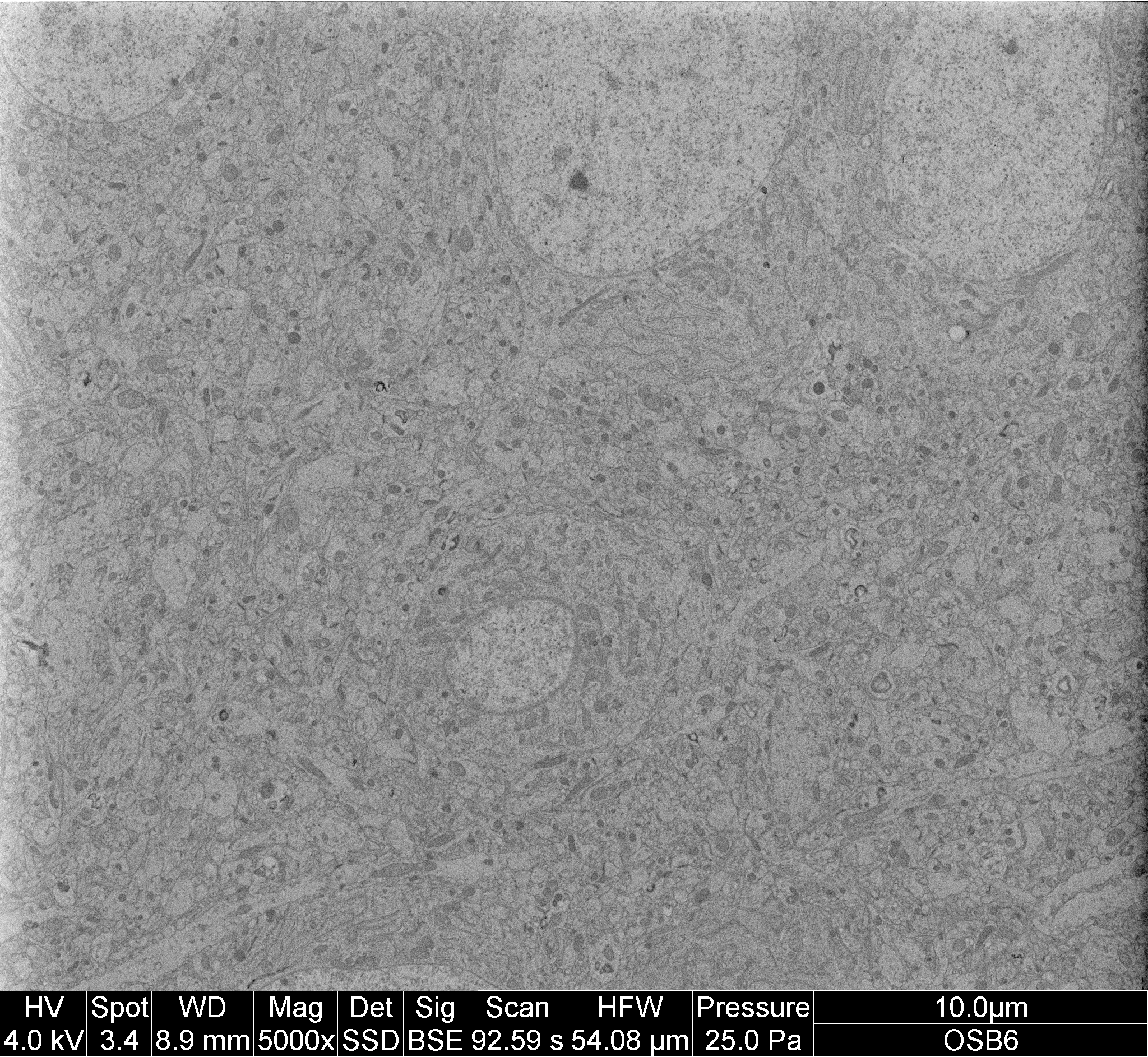

Supplement: Dataset S11 — (252.6 MB ZIP). [file pbio.0020329.sd011.zip › 040604_OS5_st1_1084.tif]

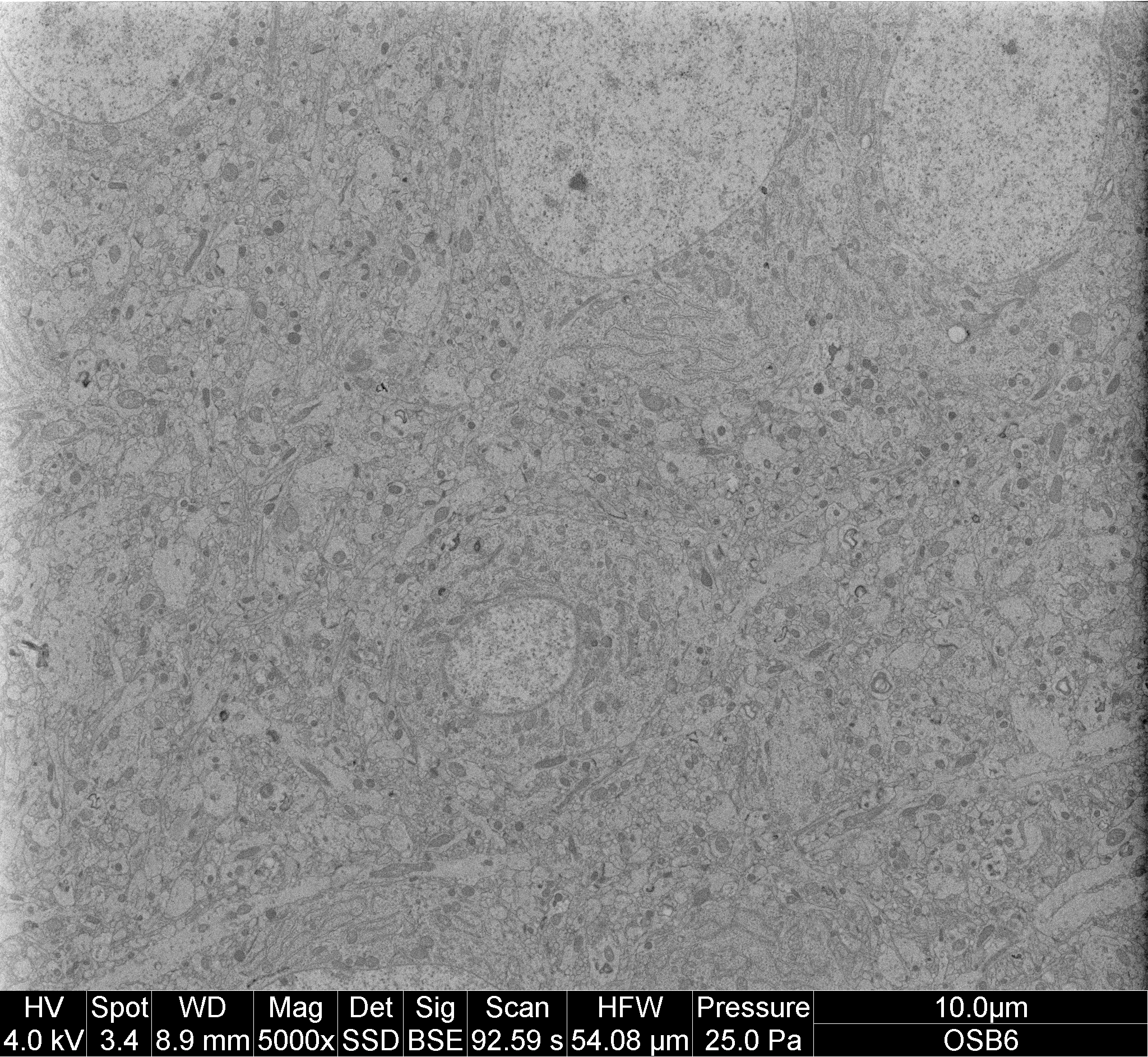

Supplement: Dataset S11 — (252.6 MB ZIP). [file pbio.0020329.sd011.zip › 040604_OS5_st1_1085.tif]

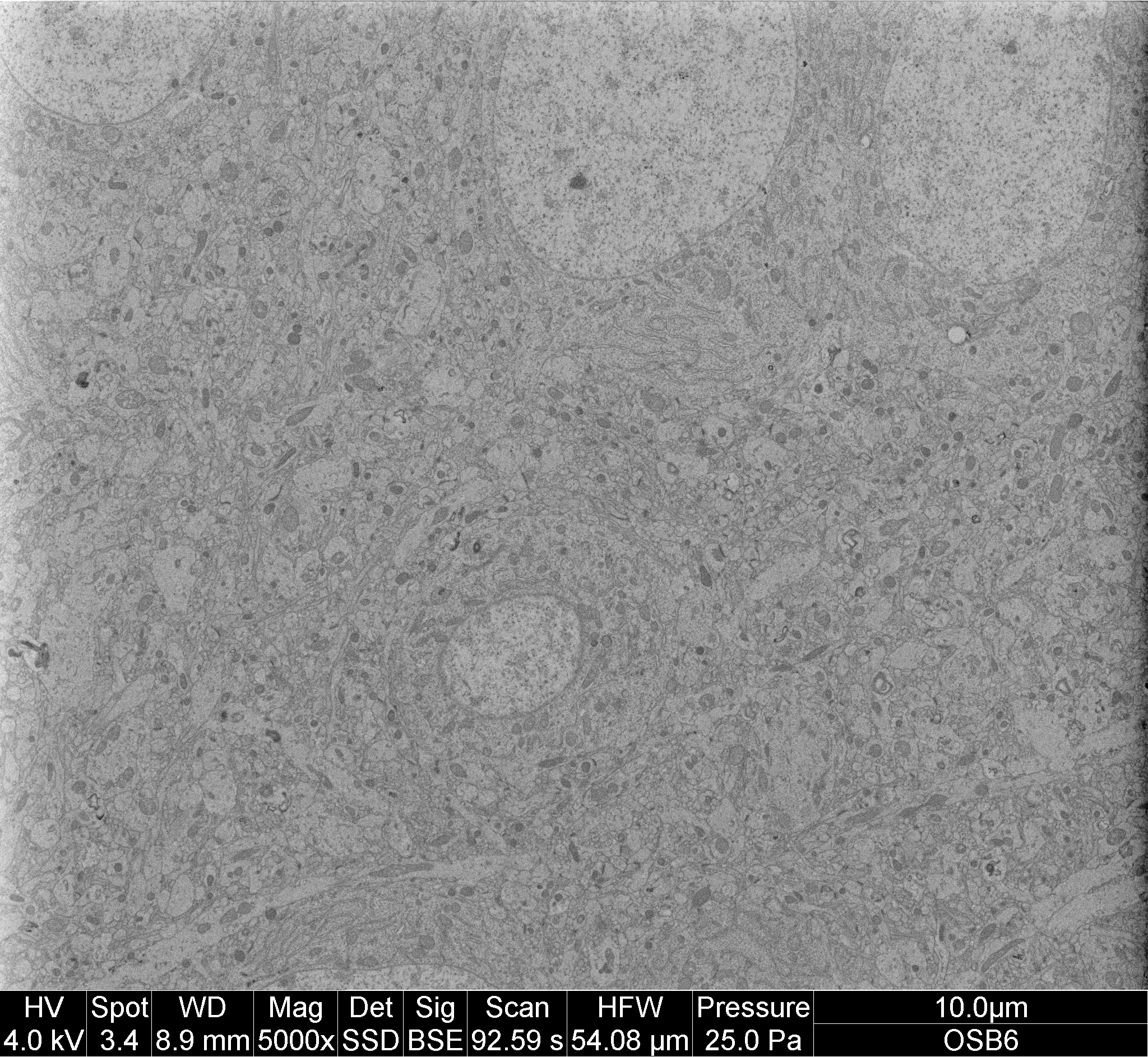

Supplement: Dataset S11 — (252.6 MB ZIP). [file pbio.0020329.sd011.zip › 040604_OS5_st1_1086.tif]

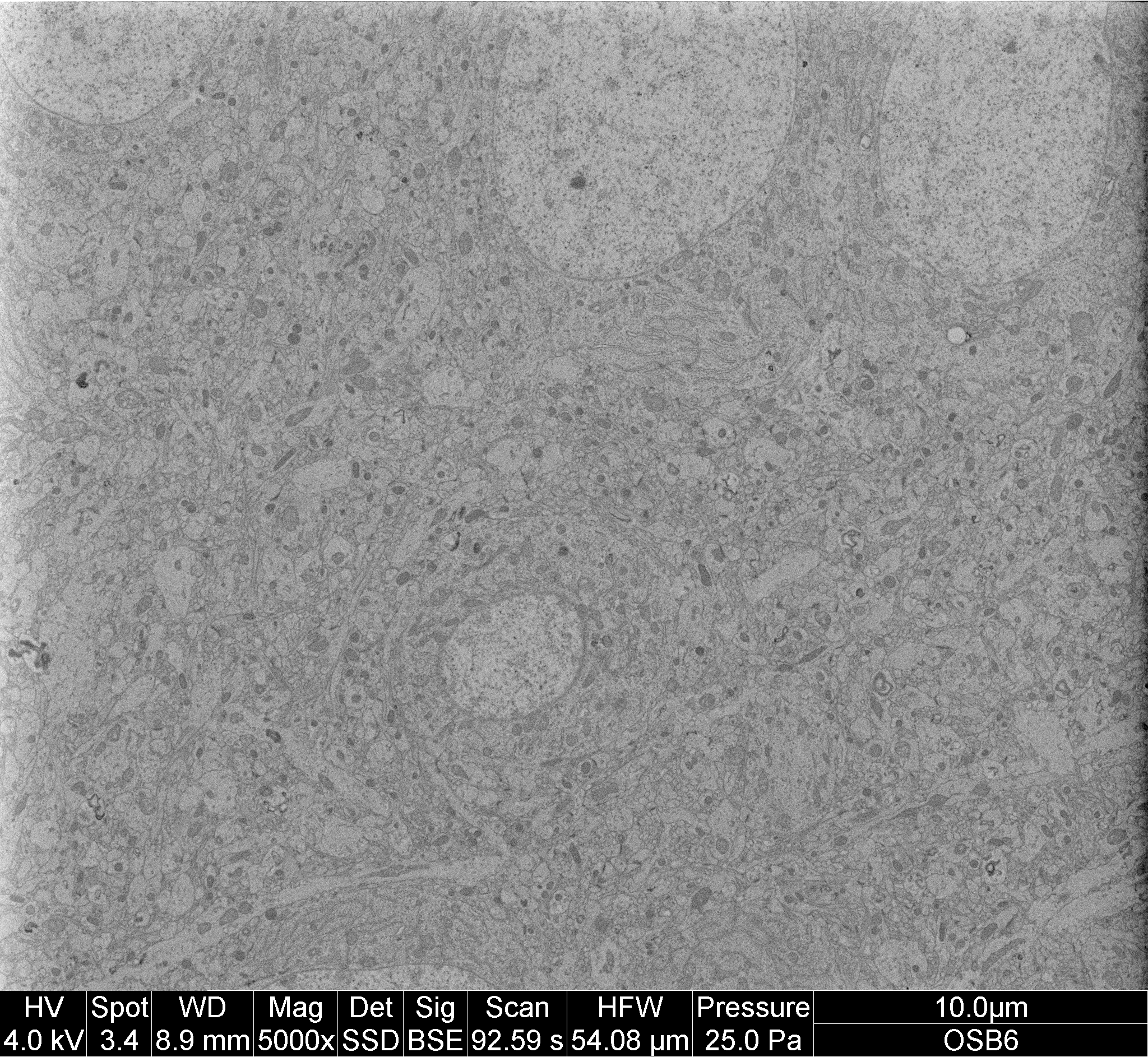

Supplement: Dataset S11 — (252.6 MB ZIP). [file pbio.0020329.sd011.zip › 040604_OS5_st1_1087.tif]

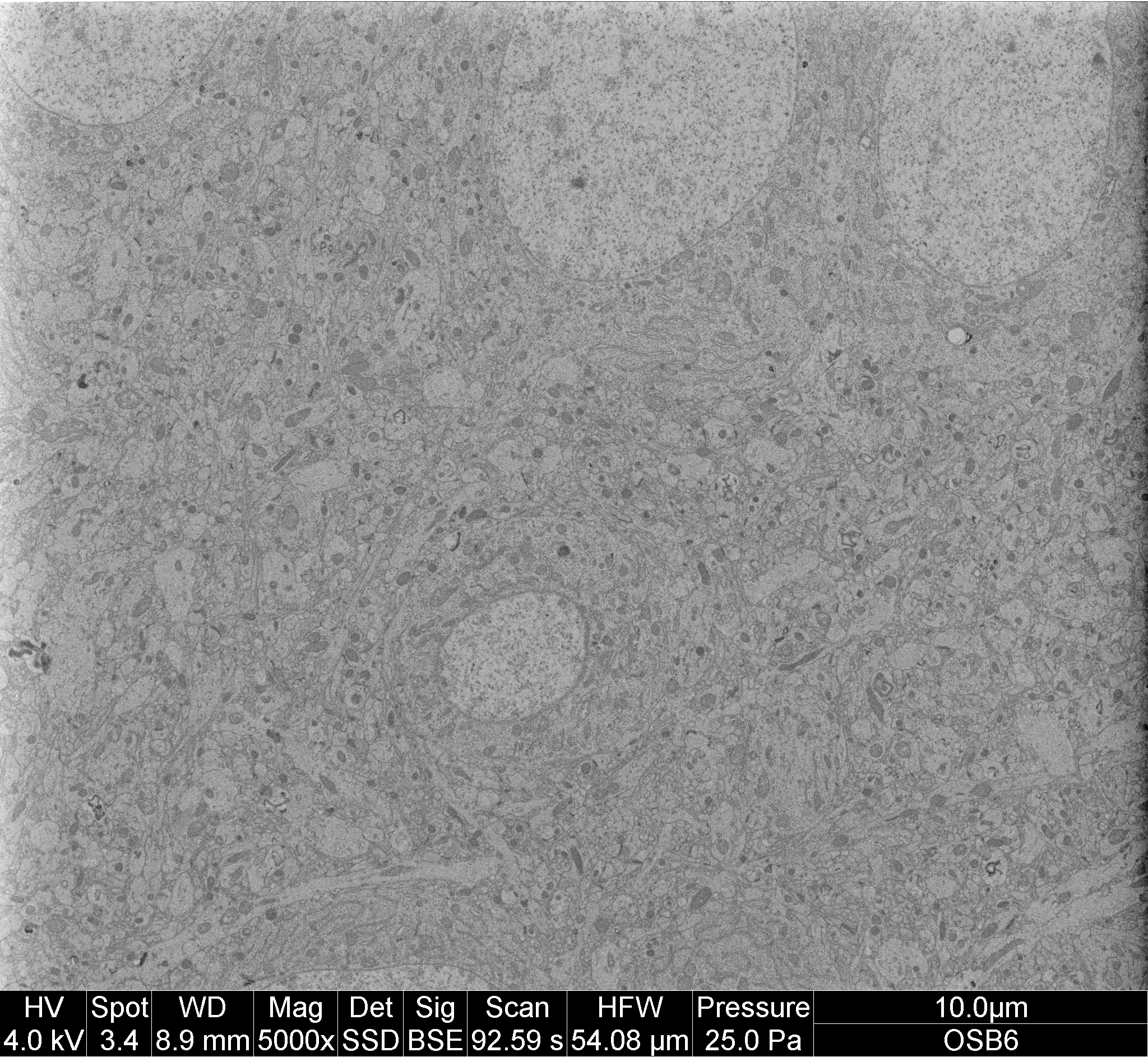

Supplement: Dataset S11 — (252.6 MB ZIP). [file pbio.0020329.sd011.zip › 040604_OS5_st1_1088.tif]

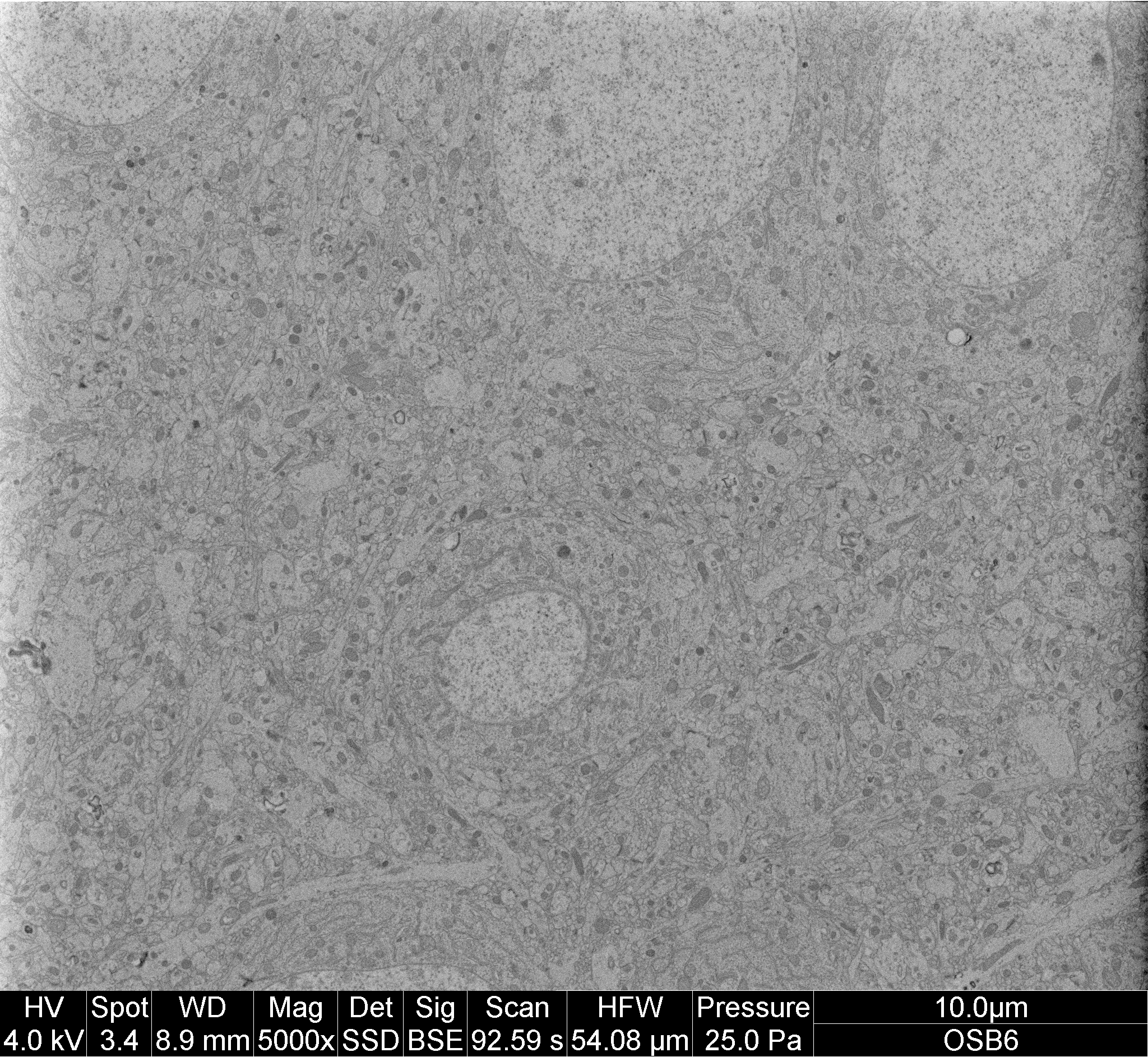

Supplement: Dataset S11 — (252.6 MB ZIP). [file pbio.0020329.sd011.zip › 040604_OS5_st1_1089.tif]

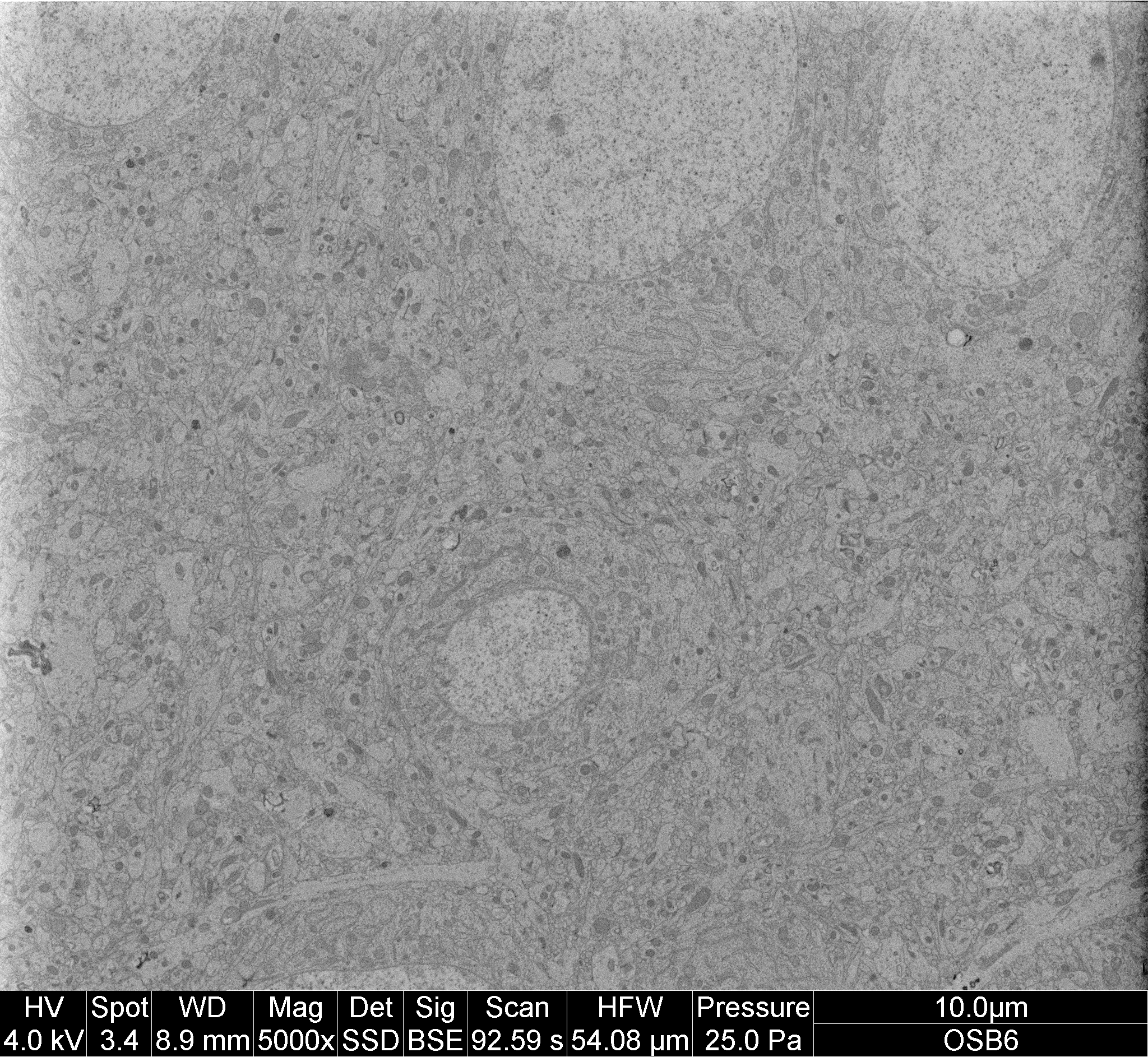

Supplement: Dataset S11 — (252.6 MB ZIP). [file pbio.0020329.sd011.zip › 040604_OS5_st1_1090.tif]

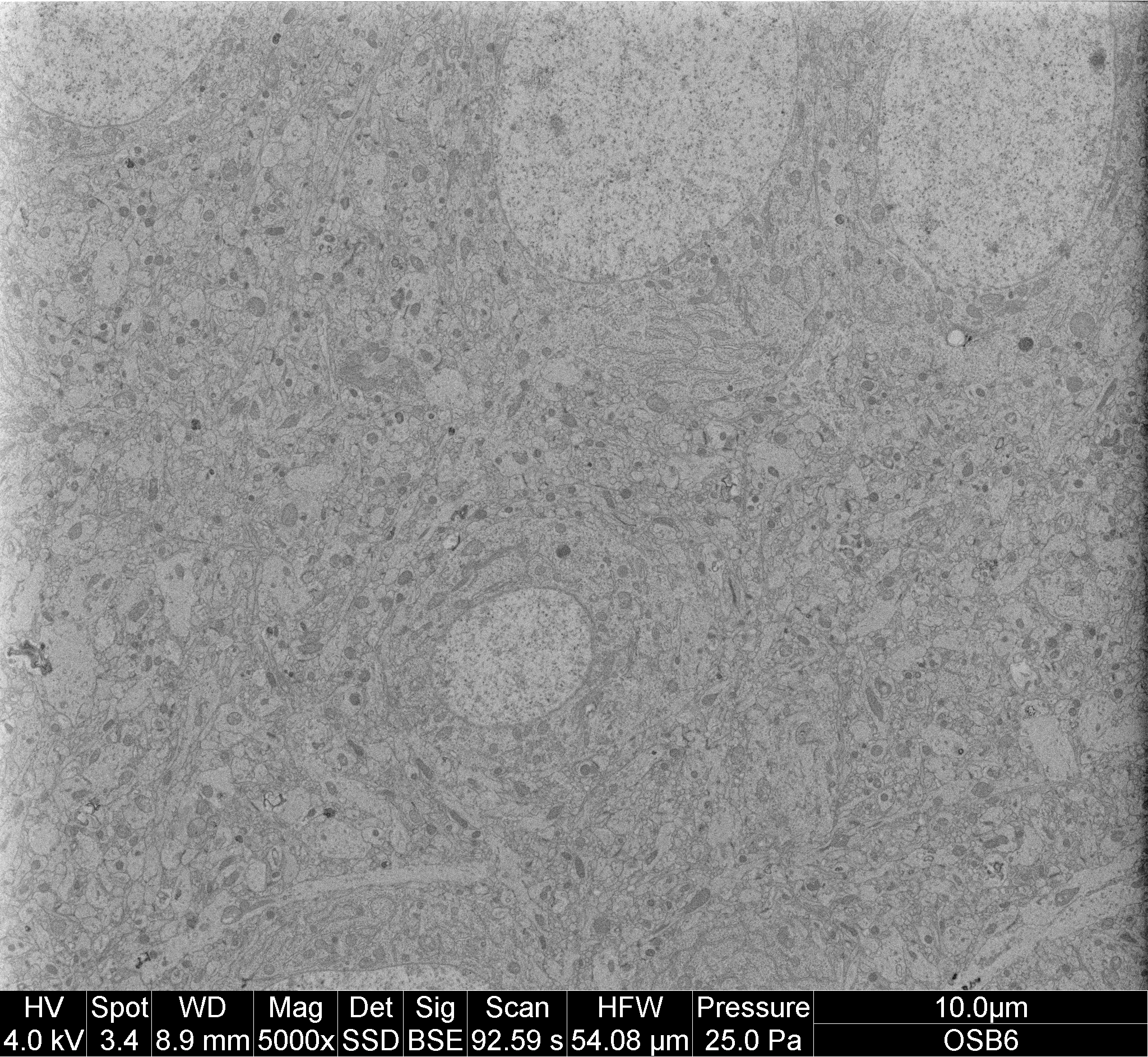

Supplement: Dataset S11 — (252.6 MB ZIP). [file pbio.0020329.sd011.zip › 040604_OS5_st1_1091.tif]

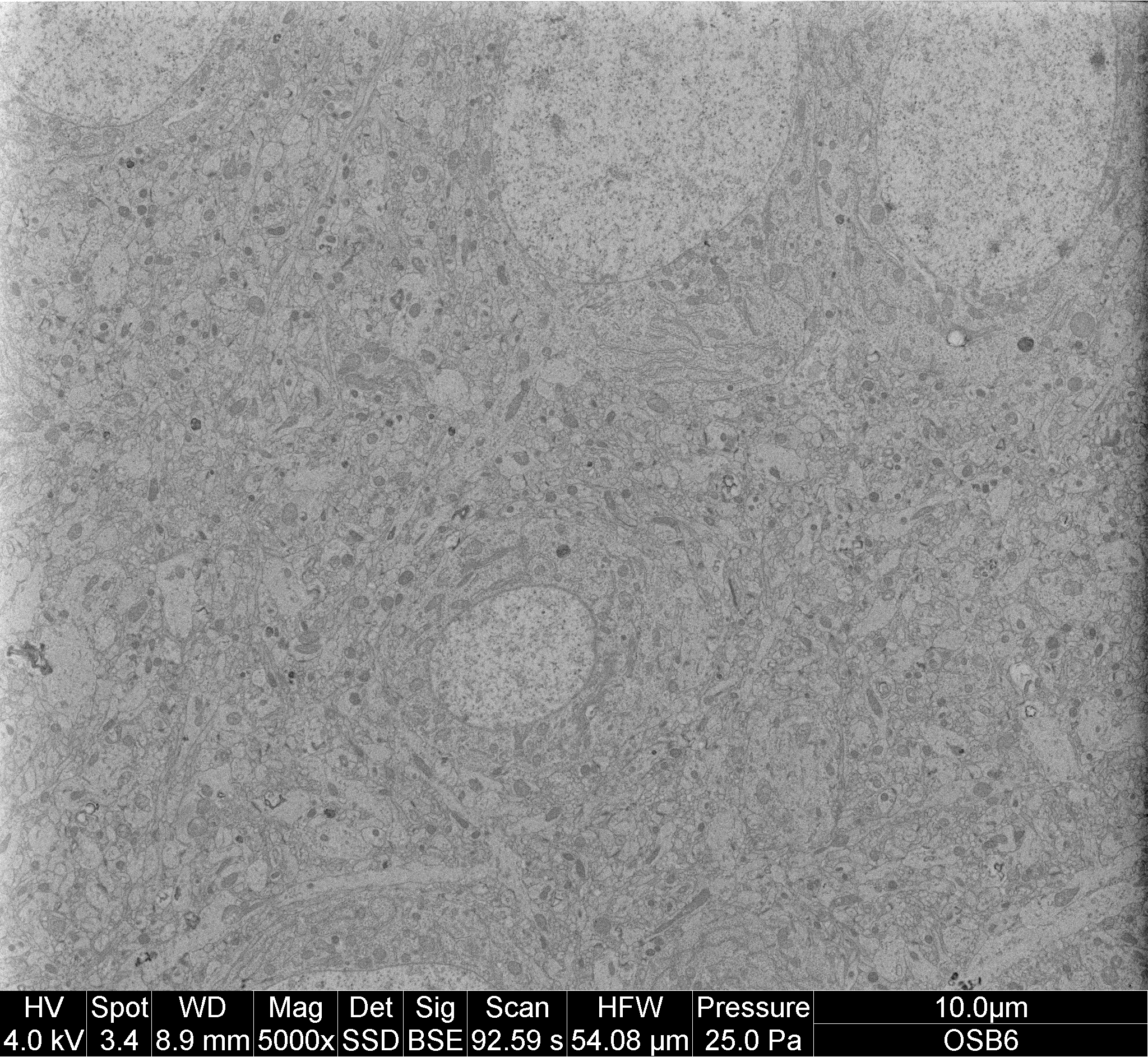

Supplement: Dataset S11 — (252.6 MB ZIP). [file pbio.0020329.sd011.zip › 040604_OS5_st1_1092.tif]

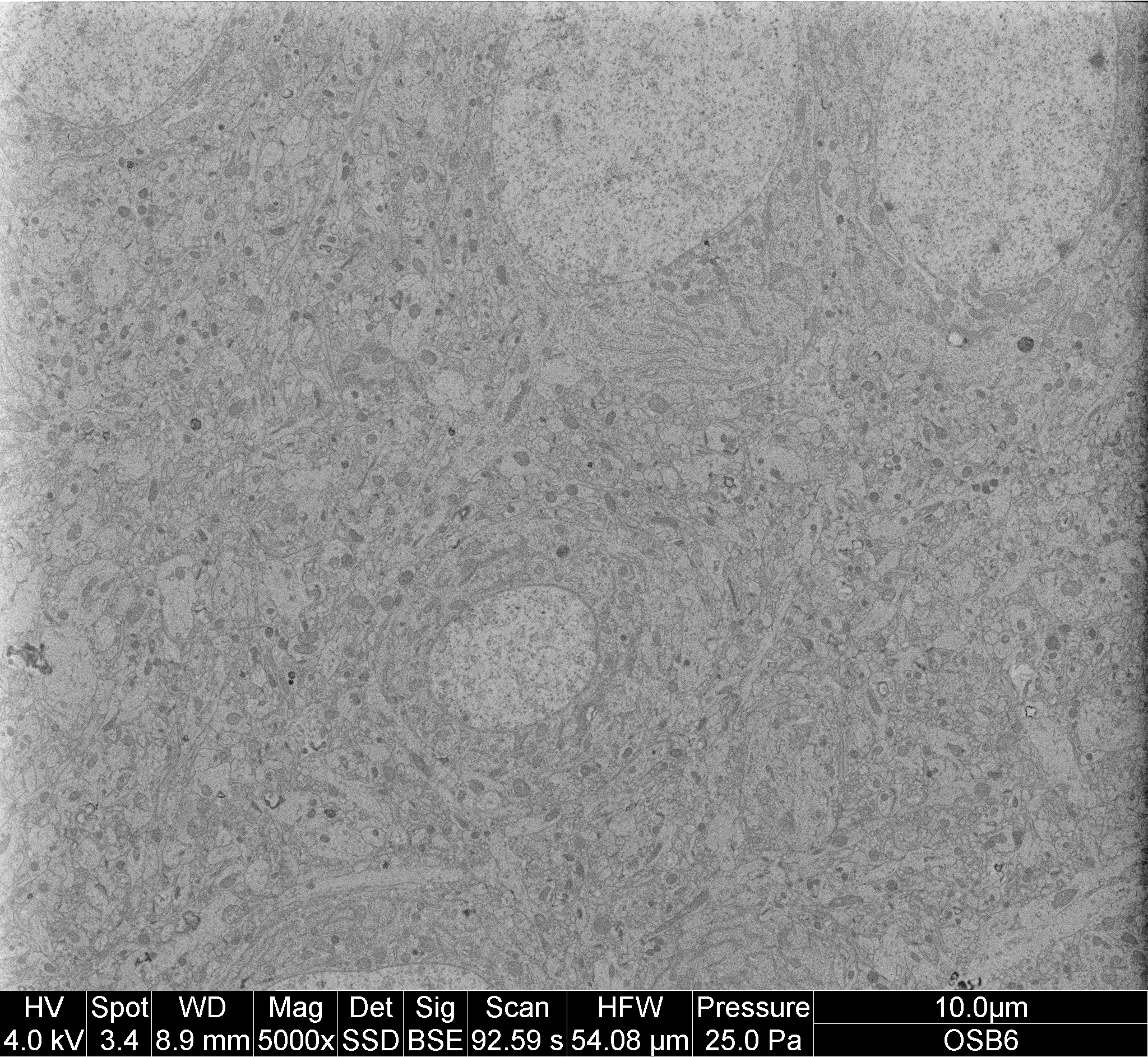

Supplement: Dataset S11 — (252.6 MB ZIP). [file pbio.0020329.sd011.zip › 040604_OS5_st1_1093.tif]

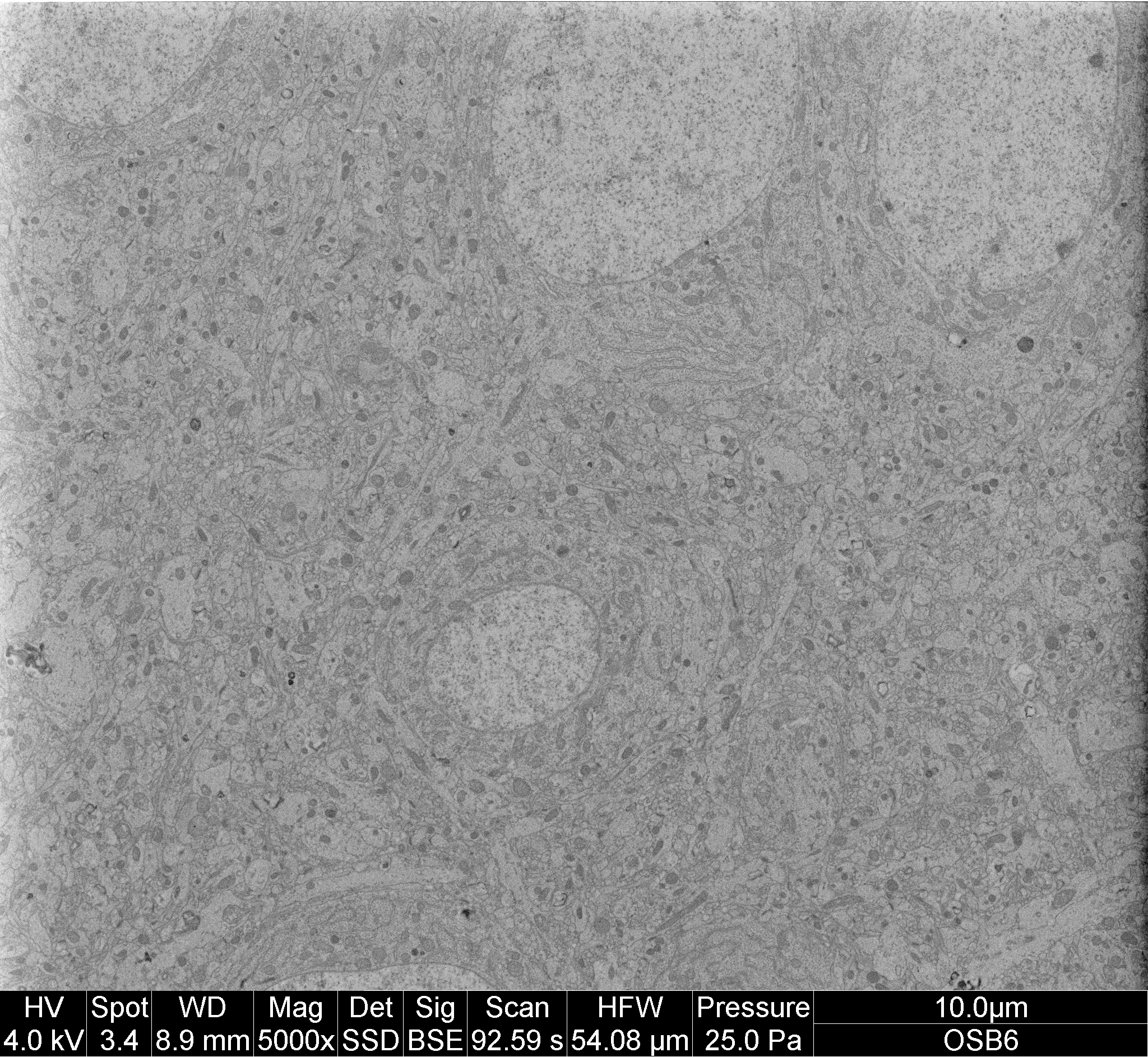

Supplement: Dataset S11 — (252.6 MB ZIP). [file pbio.0020329.sd011.zip › 040604_OS5_st1_1094.tif]

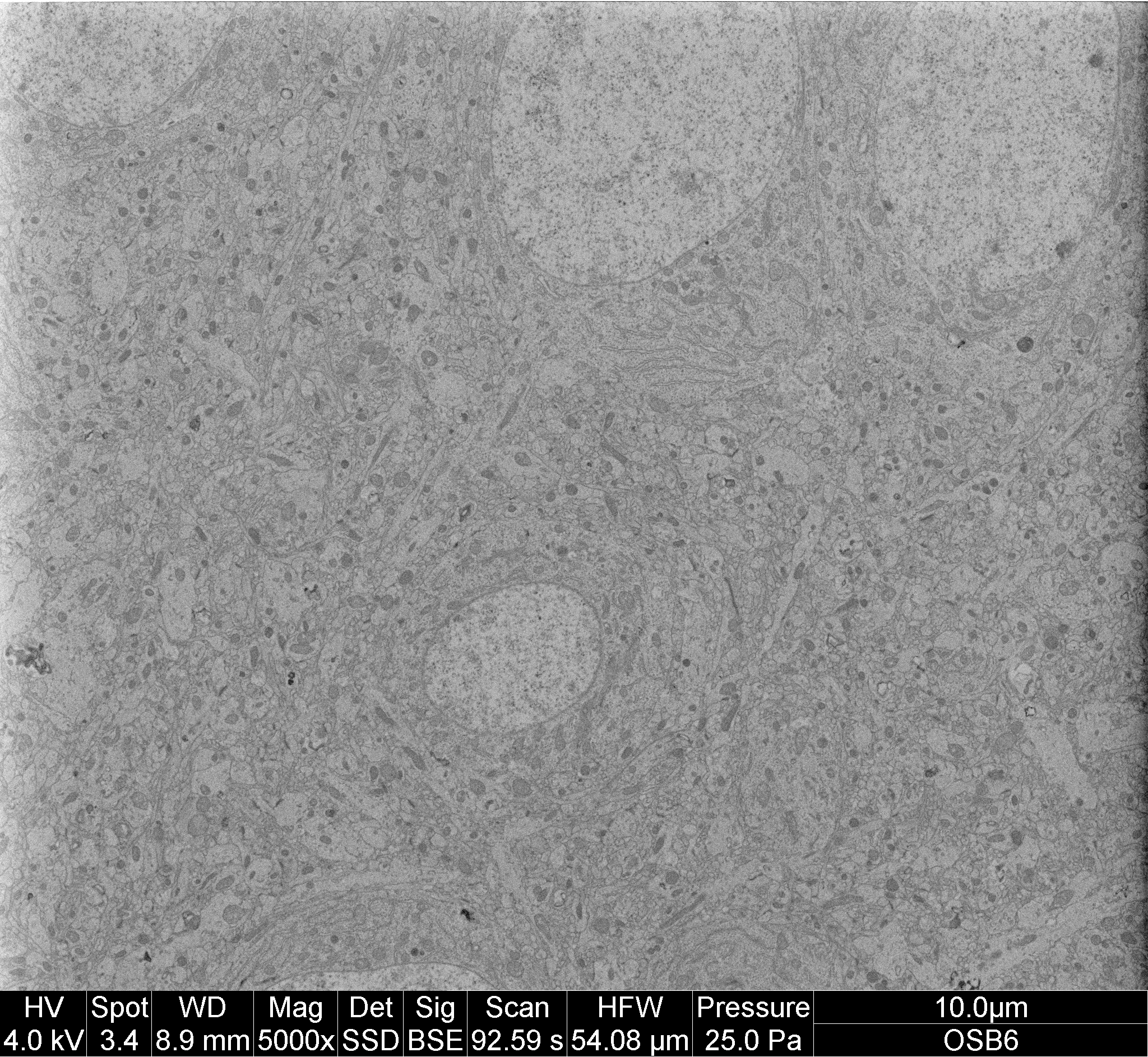

Supplement: Dataset S11 — (252.6 MB ZIP). [file pbio.0020329.sd011.zip › 040604_OS5_st1_1095.tif]

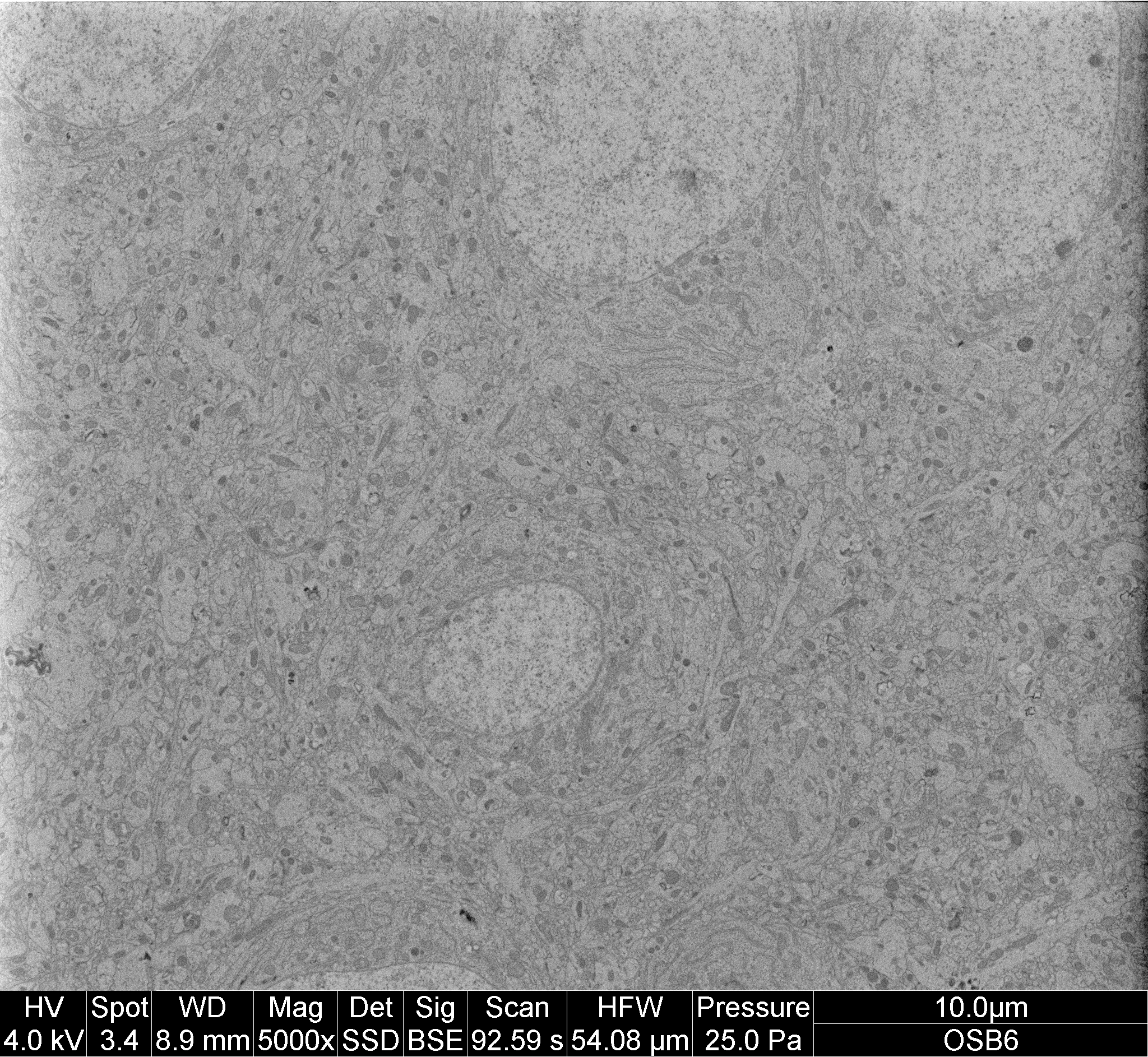

Supplement: Dataset S11 — (252.6 MB ZIP). [file pbio.0020329.sd011.zip › 040604_OS5_st1_1096.tif]

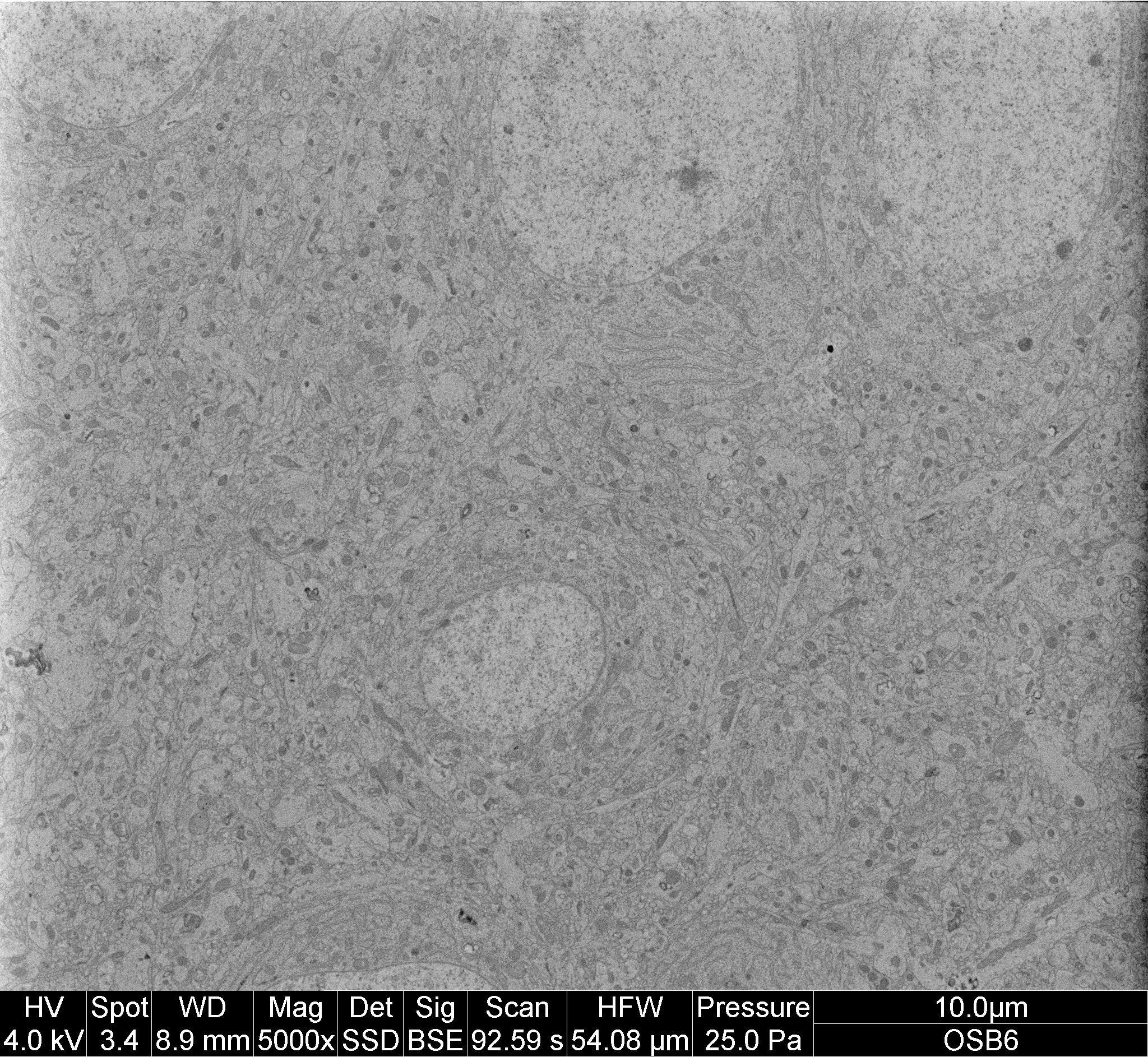

Supplement: Dataset S11 — (252.6 MB ZIP). [file pbio.0020329.sd011.zip › 040604_OS5_st1_1097.tif]

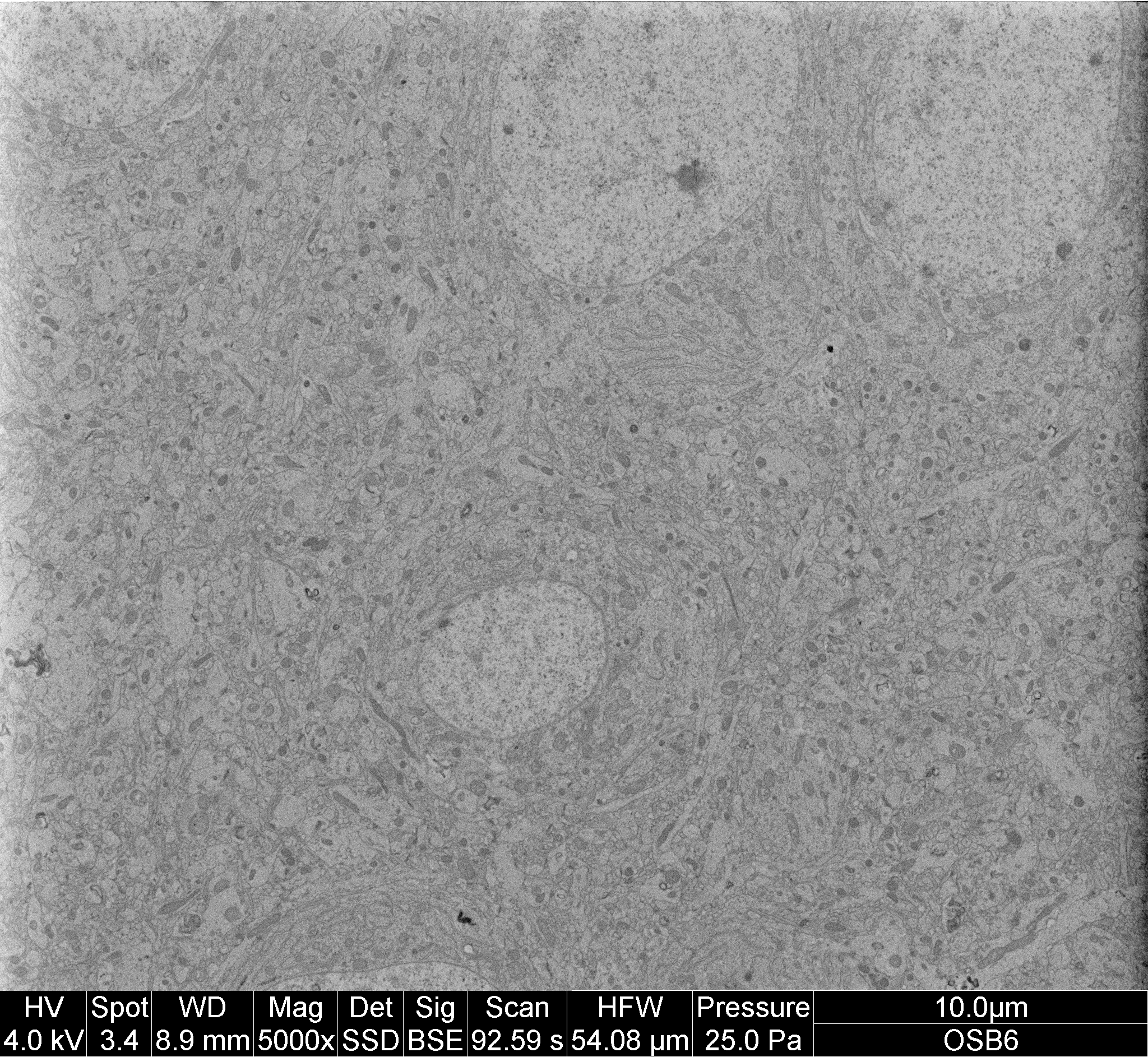

Supplement: Dataset S11 — (252.6 MB ZIP). [file pbio.0020329.sd011.zip › 040604_OS5_st1_1098.tif]

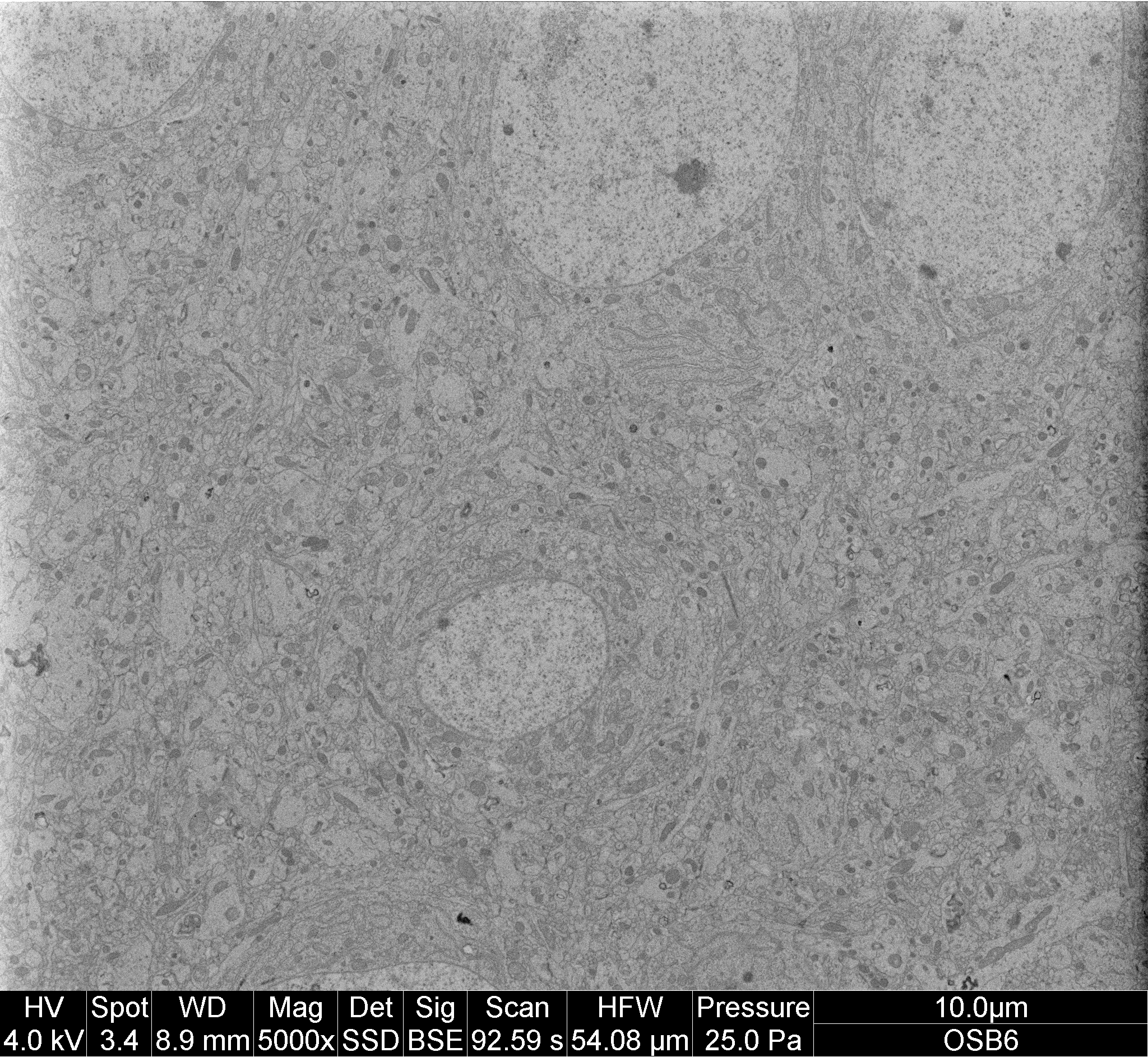

Supplement: Dataset S11 — (252.6 MB ZIP). [file pbio.0020329.sd011.zip › 040604_OS5_st1_1099.tif]

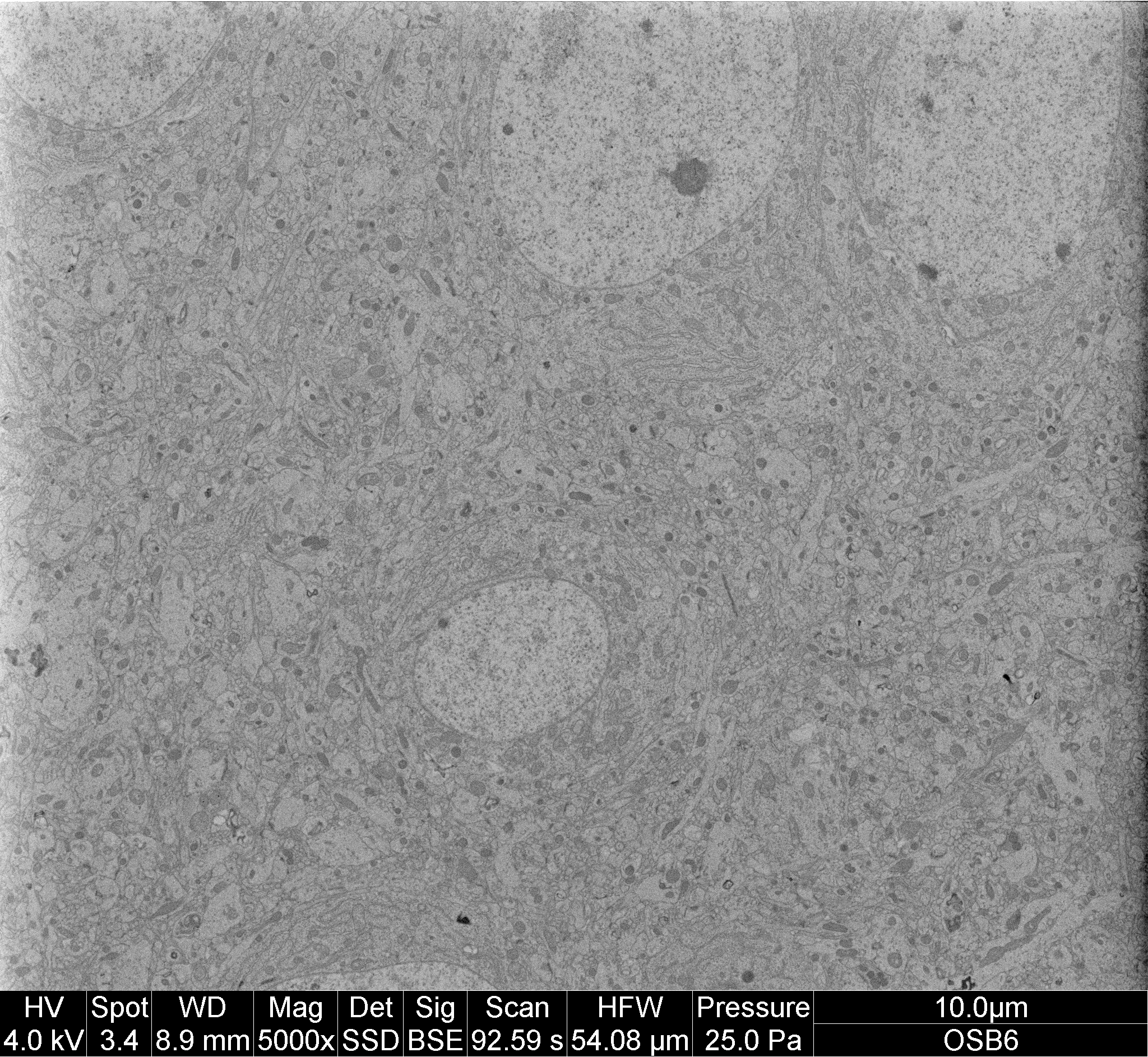

Supplement: Dataset S12 — (252.6 MB ZIP). [file pbio.0020329.sd012.zip › 040604_OS5_st1_1100.tif]
