# Supplementary figures and images for: Serial Block-Face Scanning Electron Microscopy to Reconstruct Three-Dimensional Tissue Nanostructure (part 12 of 21)
Source: PLoS Biol. 2004 Oct 19;2(11):e329. doi: 10.1371/journal.pbio.0020329 (PMC524270; doi:10.1371/journal.pbio.0020329)

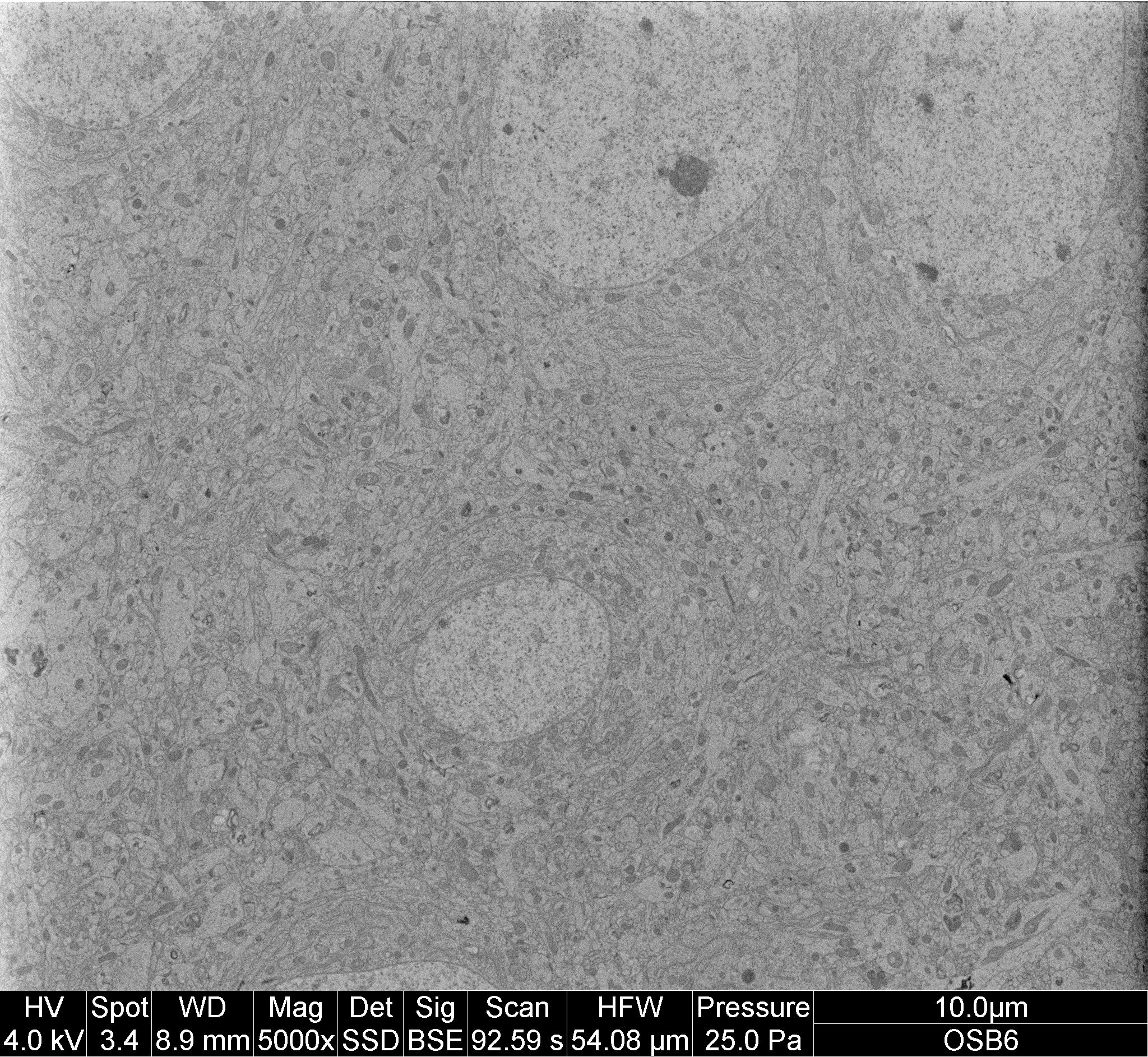

Supplement: Dataset S12 — (252.6 MB ZIP). [file pbio.0020329.sd012.zip › 040604_OS5_st1_1101.tif]

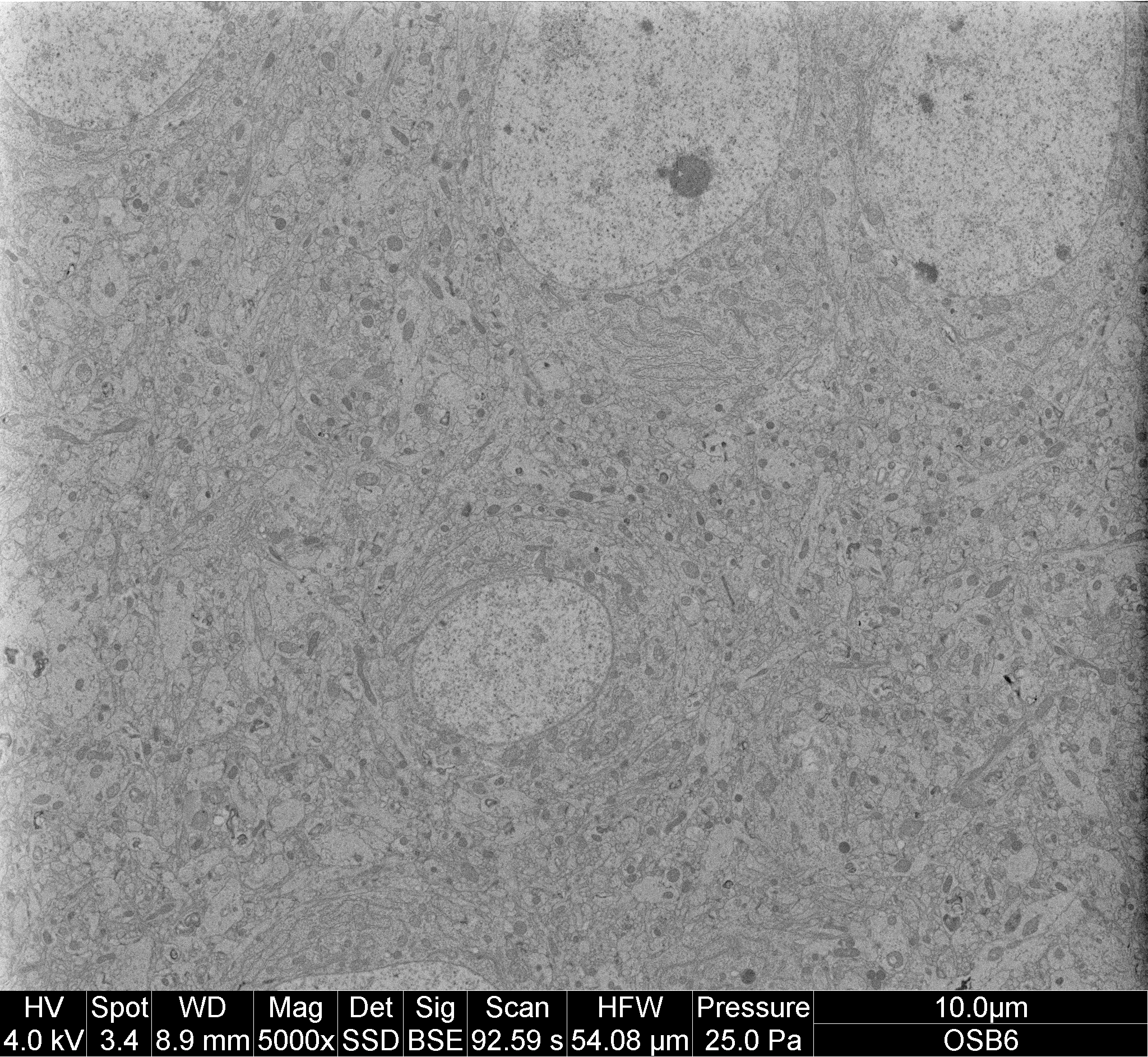

Supplement: Dataset S12 — (252.6 MB ZIP). [file pbio.0020329.sd012.zip › 040604_OS5_st1_1102.tif]

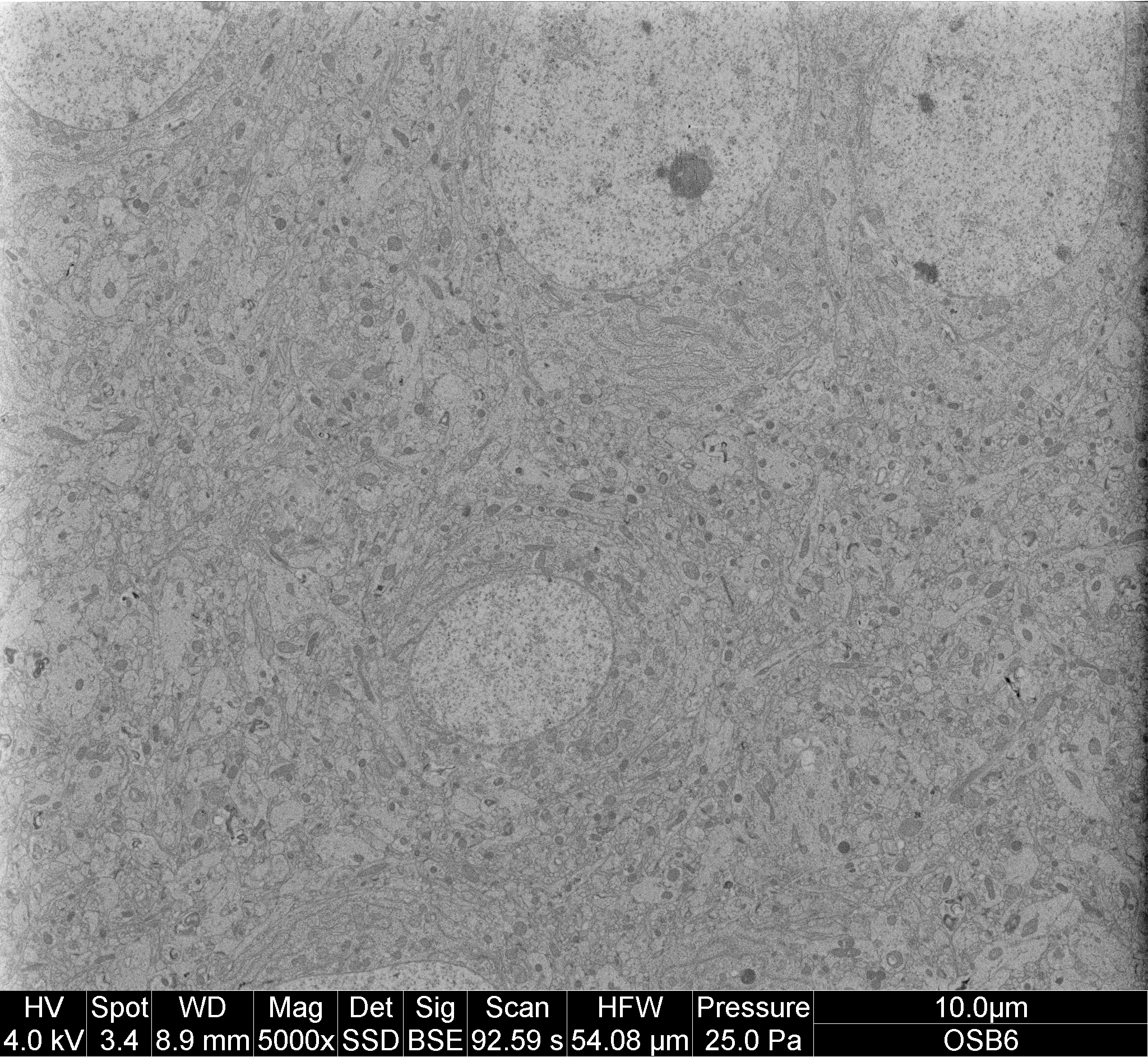

Supplement: Dataset S12 — (252.6 MB ZIP). [file pbio.0020329.sd012.zip › 040604_OS5_st1_1103.tif]

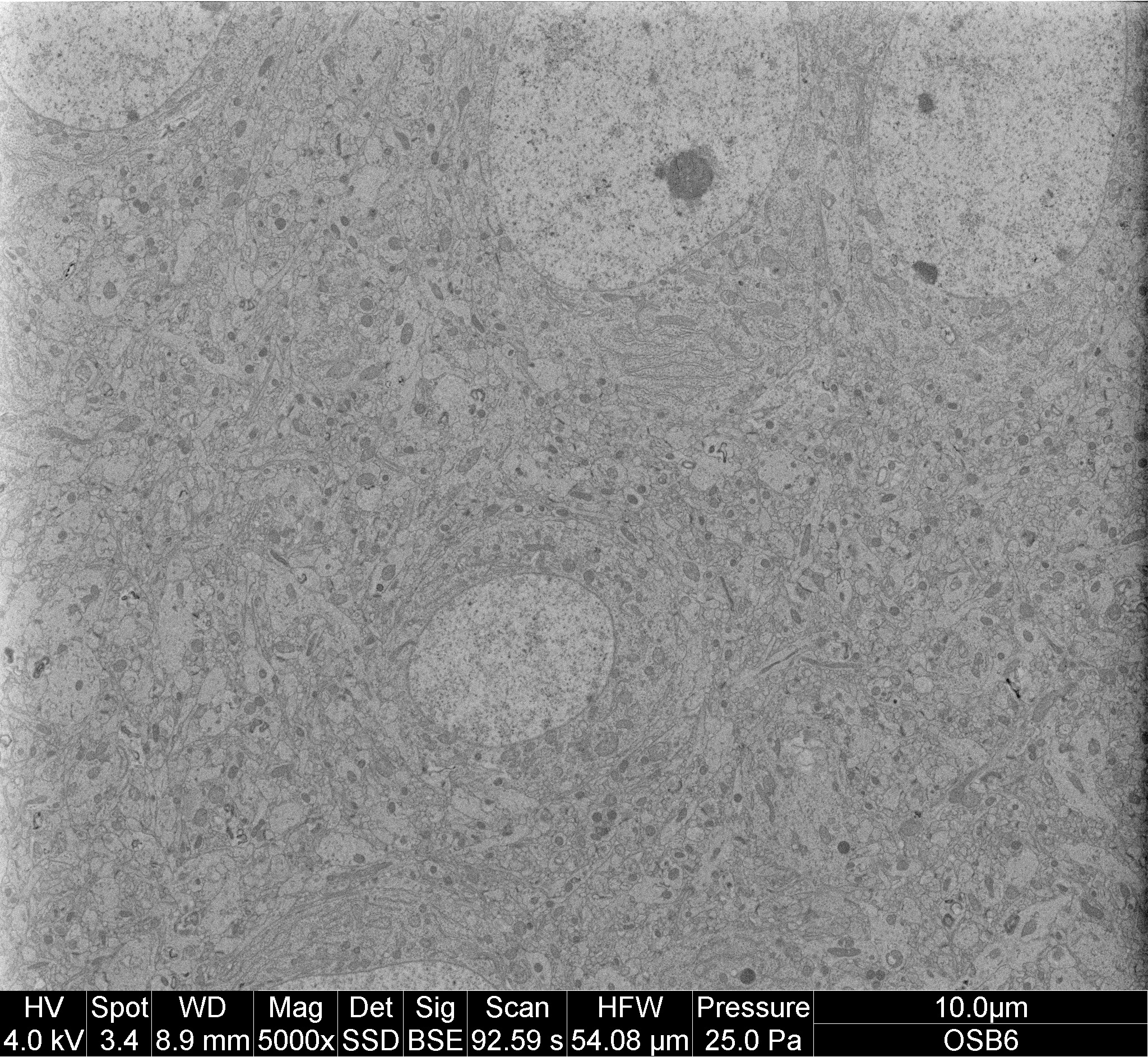

Supplement: Dataset S12 — (252.6 MB ZIP). [file pbio.0020329.sd012.zip › 040604_OS5_st1_1104.tif]

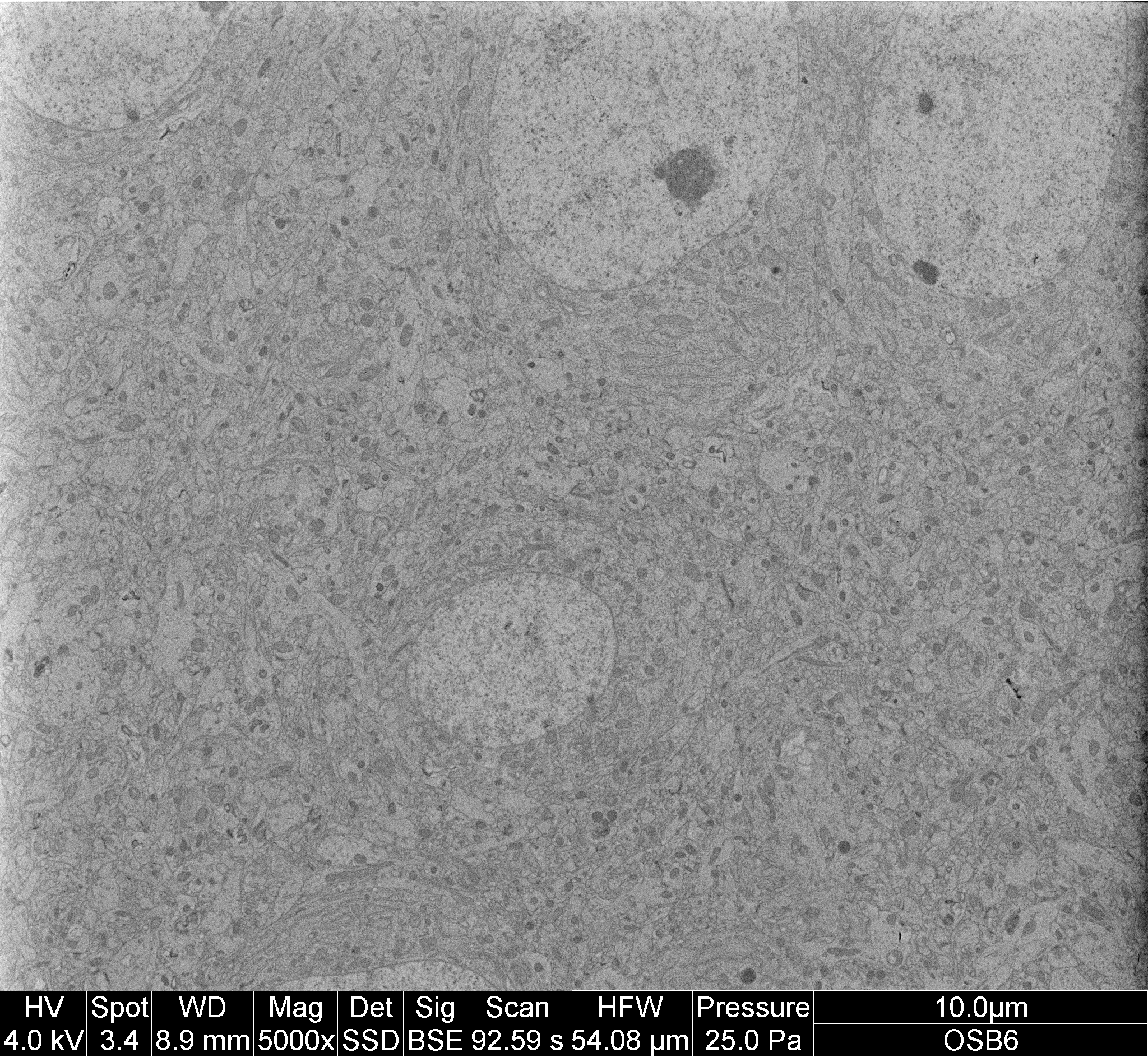

Supplement: Dataset S12 — (252.6 MB ZIP). [file pbio.0020329.sd012.zip › 040604_OS5_st1_1105.tif]

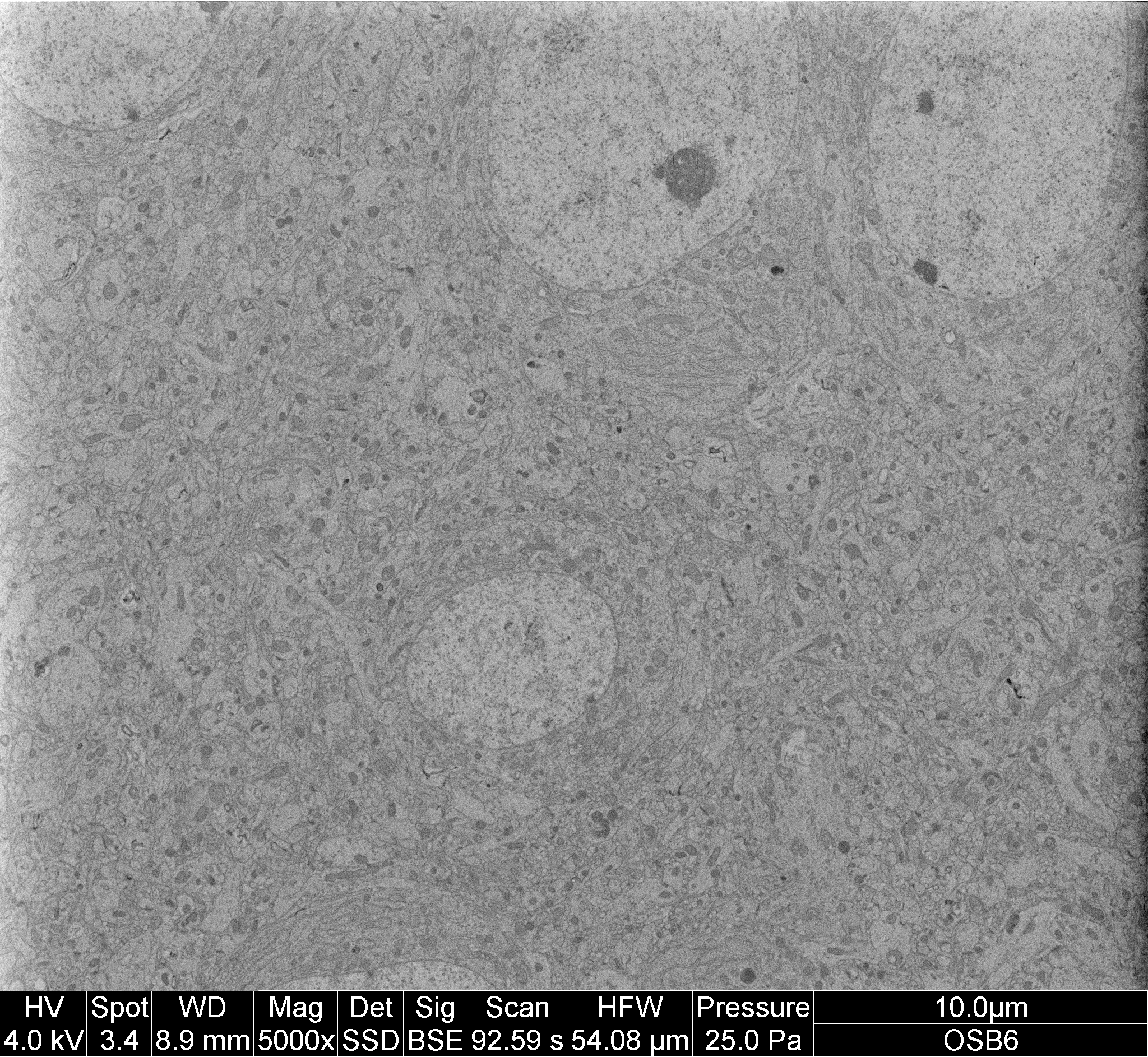

Supplement: Dataset S12 — (252.6 MB ZIP). [file pbio.0020329.sd012.zip › 040604_OS5_st1_1106.tif]

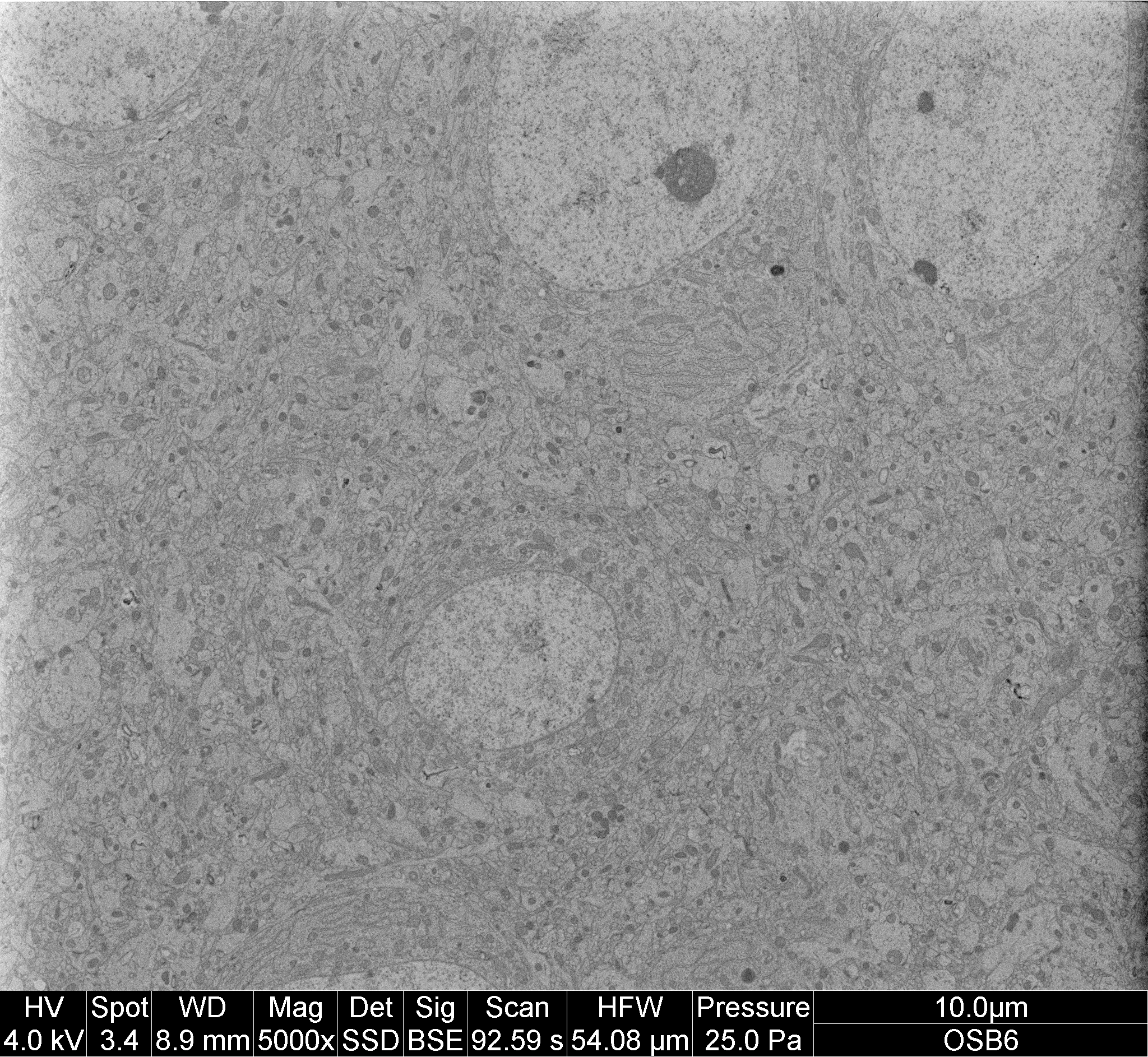

Supplement: Dataset S12 — (252.6 MB ZIP). [file pbio.0020329.sd012.zip › 040604_OS5_st1_1107.tif]

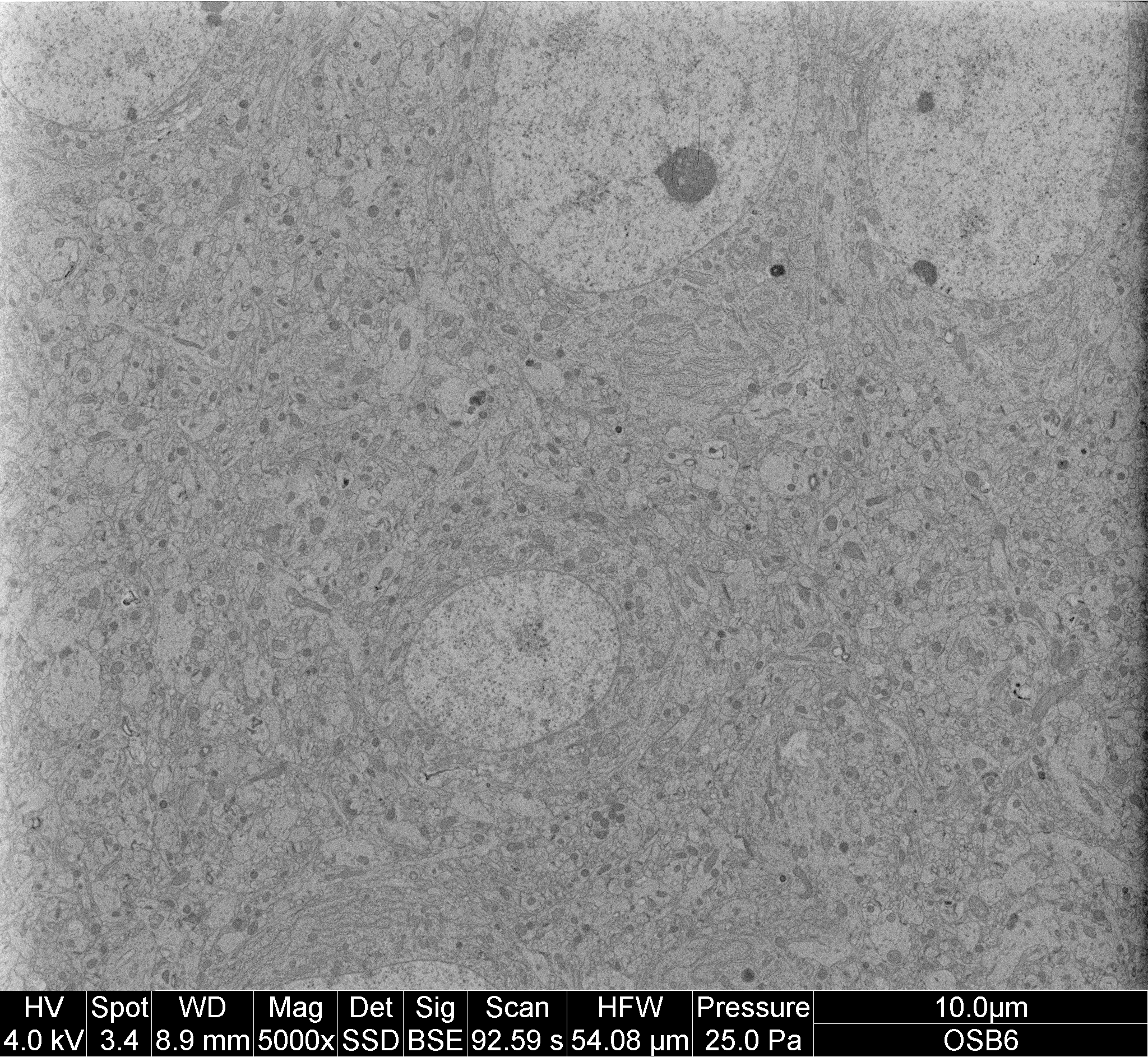

Supplement: Dataset S12 — (252.6 MB ZIP). [file pbio.0020329.sd012.zip › 040604_OS5_st1_1108.tif]

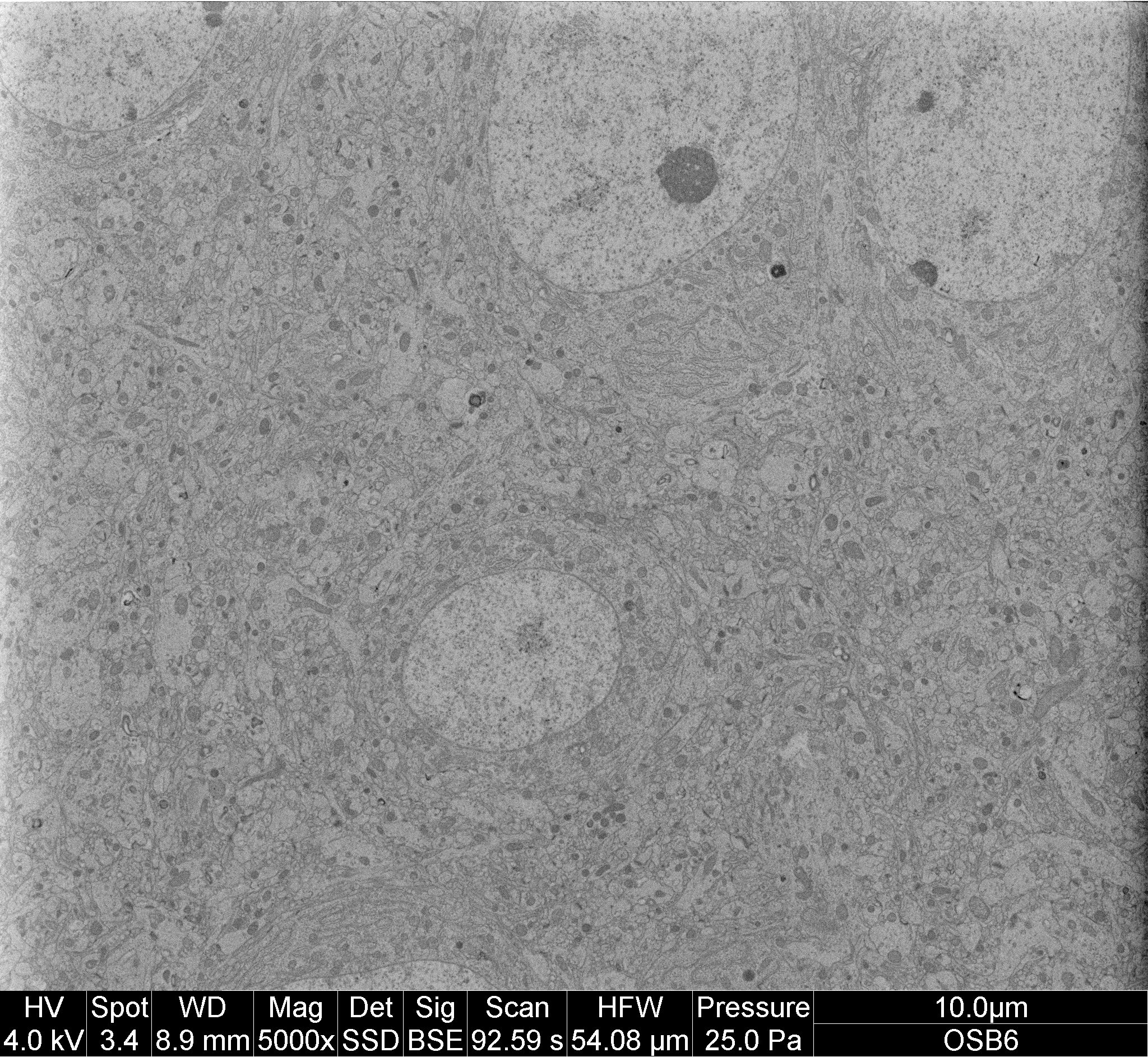

Supplement: Dataset S12 — (252.6 MB ZIP). [file pbio.0020329.sd012.zip › 040604_OS5_st1_1109.tif]

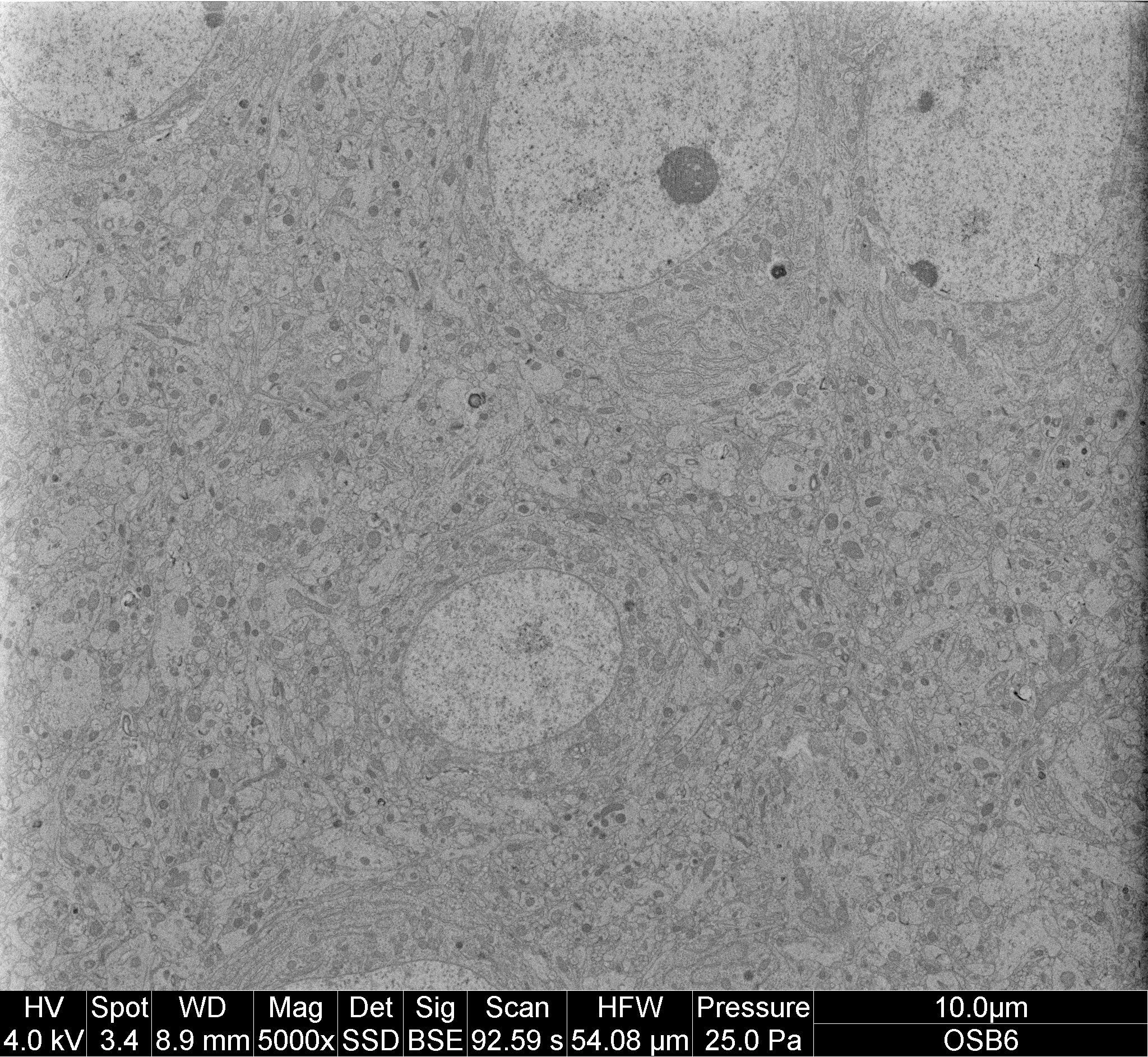

Supplement: Dataset S12 — (252.6 MB ZIP). [file pbio.0020329.sd012.zip › 040604_OS5_st1_1110.tif]

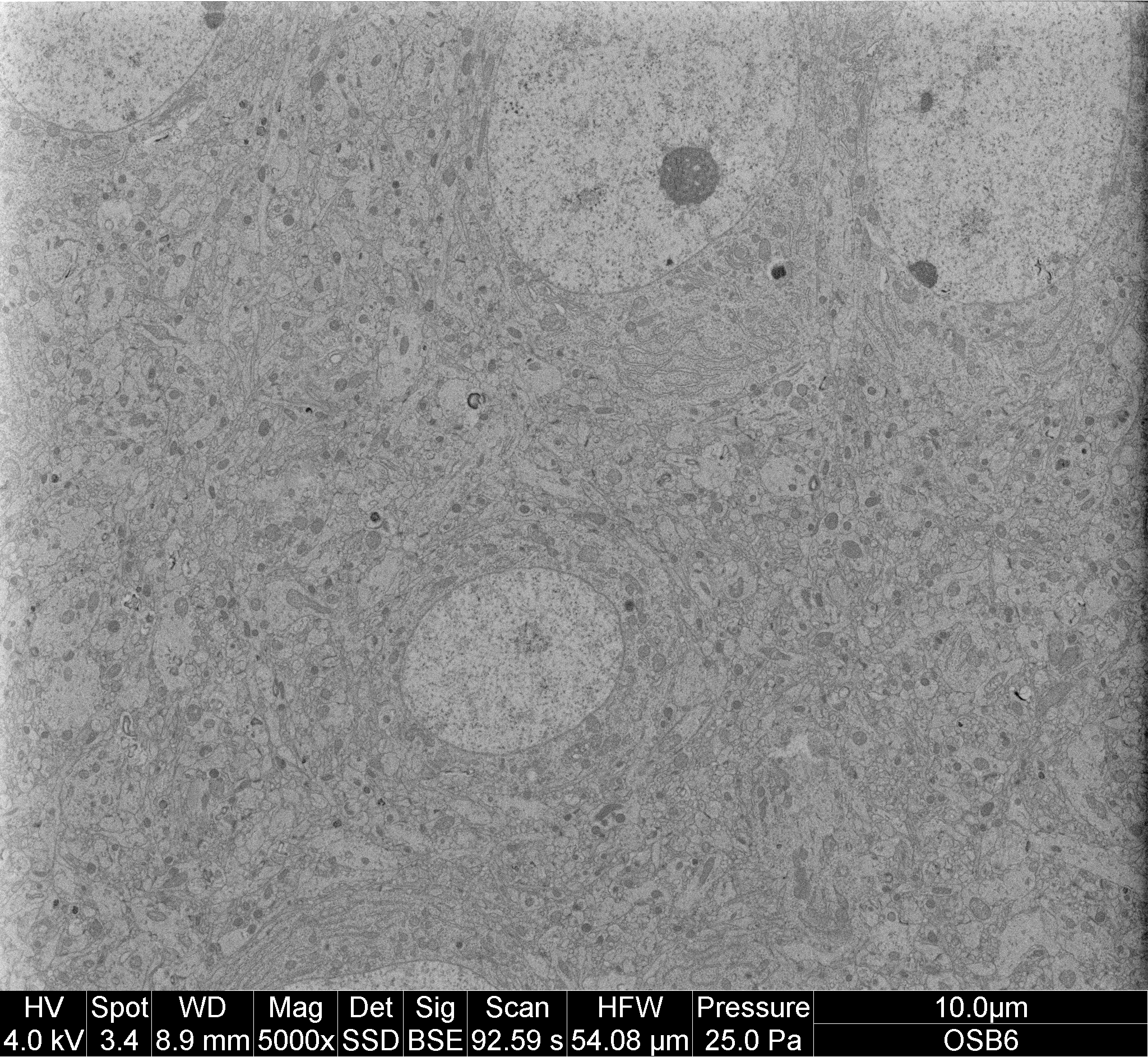

Supplement: Dataset S12 — (252.6 MB ZIP). [file pbio.0020329.sd012.zip › 040604_OS5_st1_1111.tif]

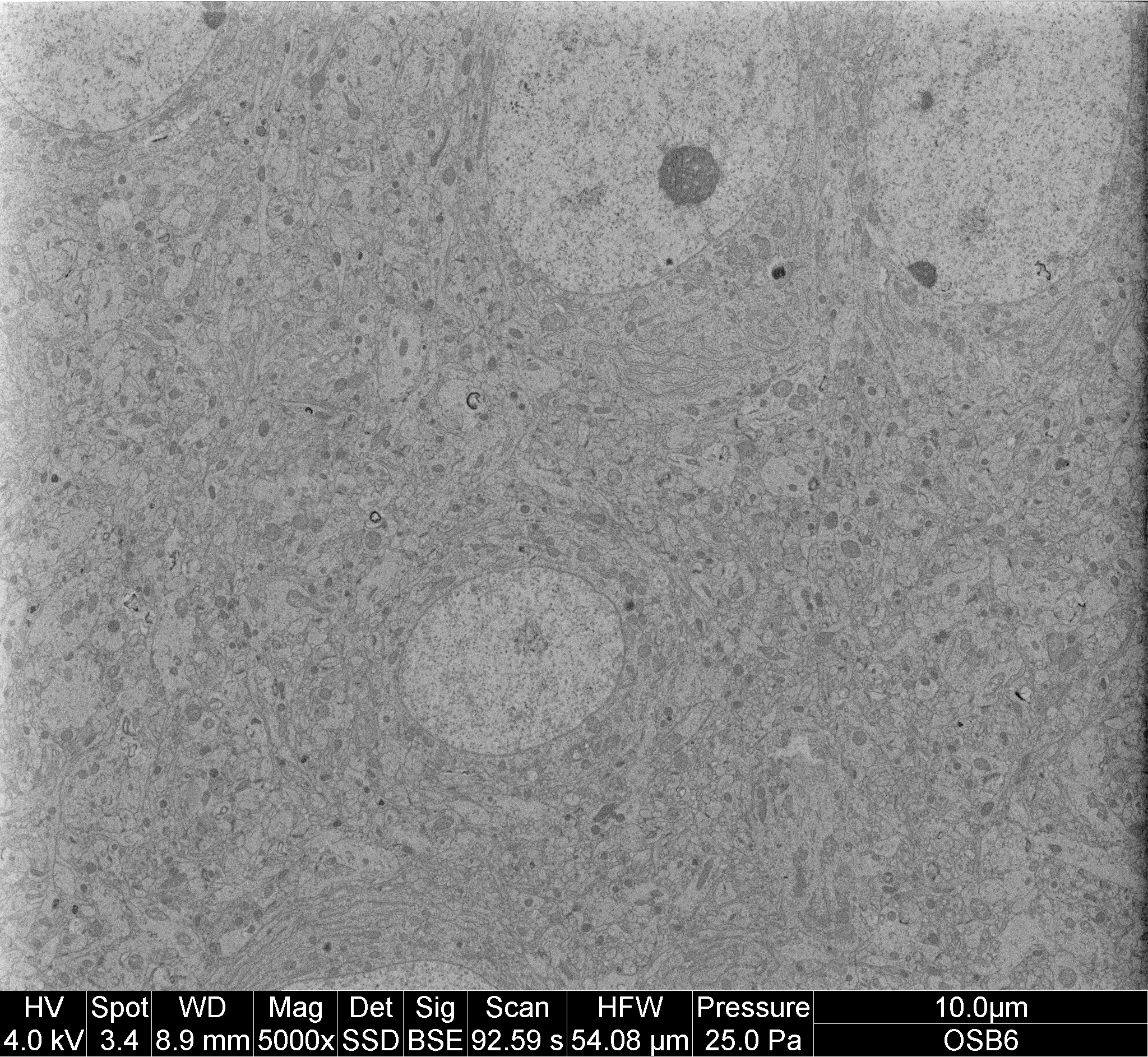

Supplement: Dataset S12 — (252.6 MB ZIP). [file pbio.0020329.sd012.zip › 040604_OS5_st1_1112.tif]

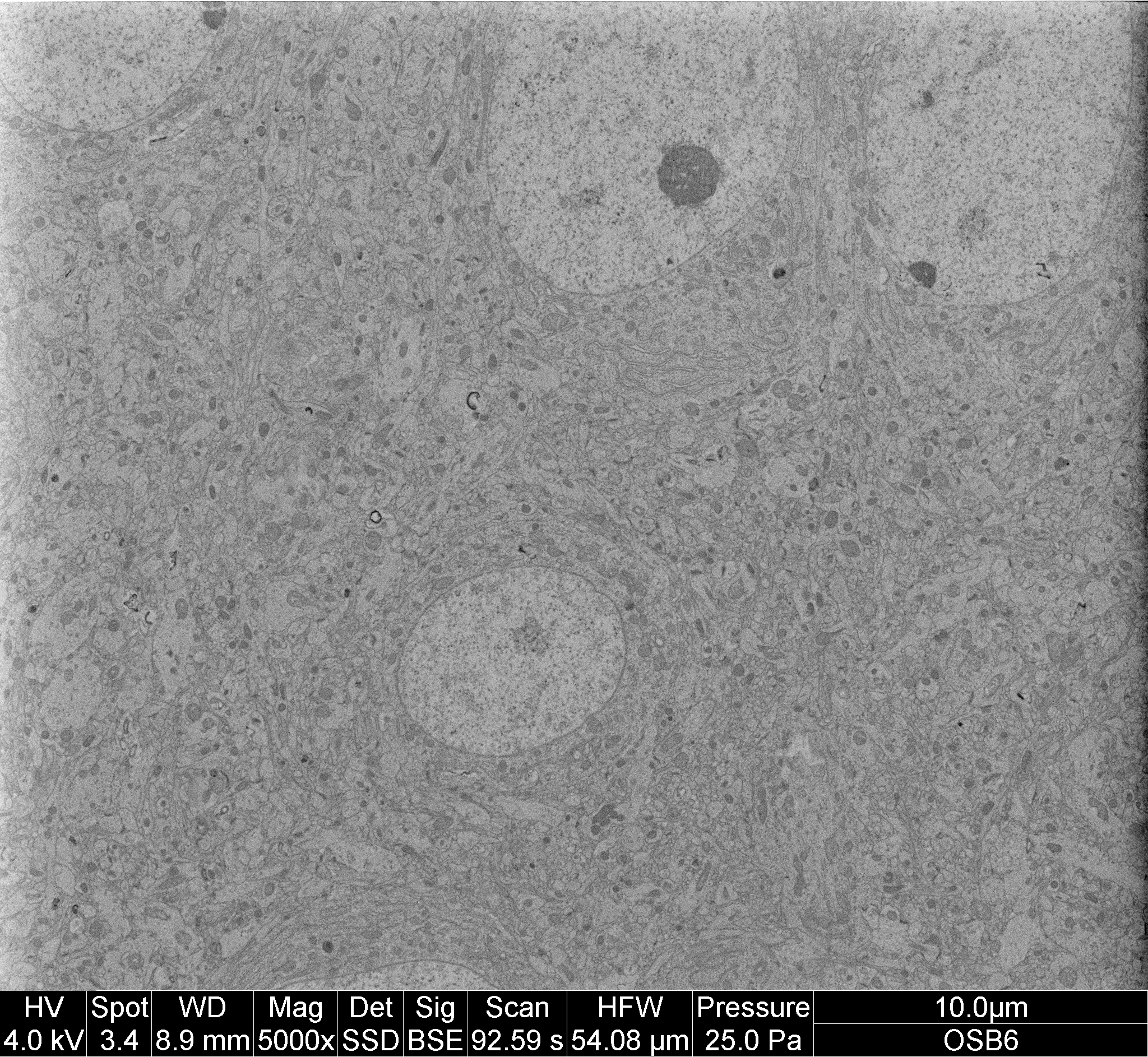

Supplement: Dataset S12 — (252.6 MB ZIP). [file pbio.0020329.sd012.zip › 040604_OS5_st1_1113.tif]

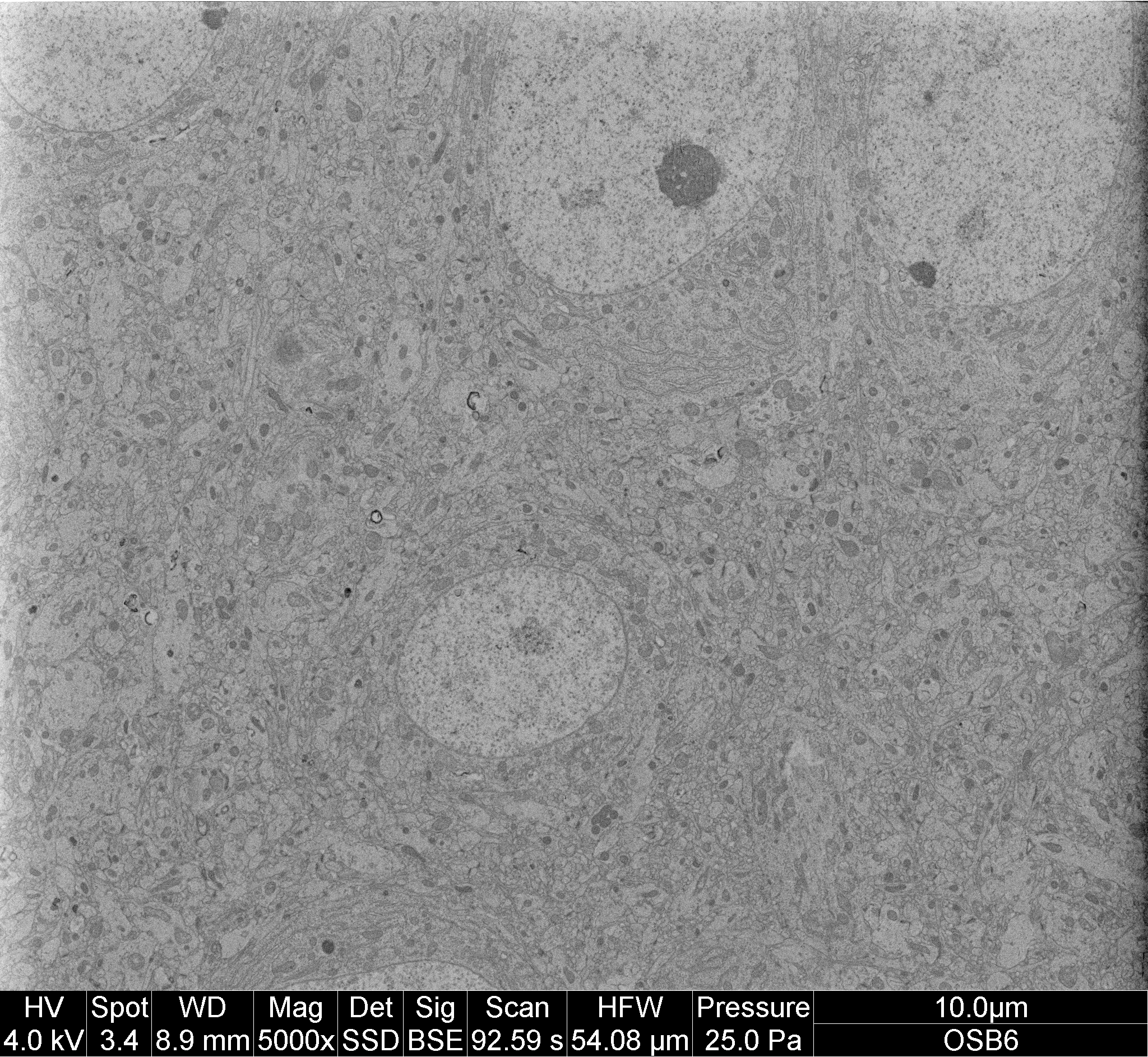

Supplement: Dataset S12 — (252.6 MB ZIP). [file pbio.0020329.sd012.zip › 040604_OS5_st1_1114.tif]

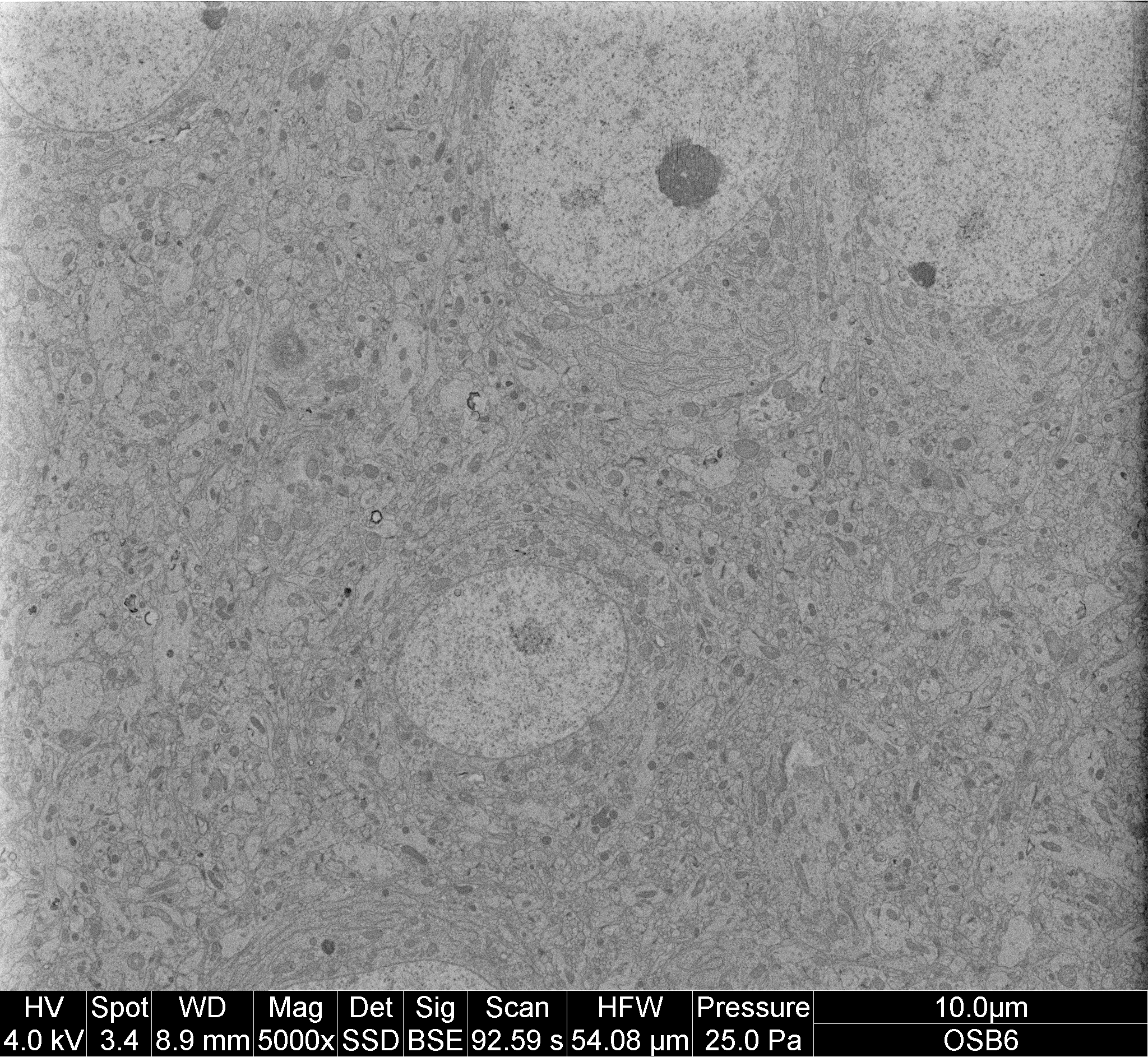

Supplement: Dataset S12 — (252.6 MB ZIP). [file pbio.0020329.sd012.zip › 040604_OS5_st1_1115.tif]

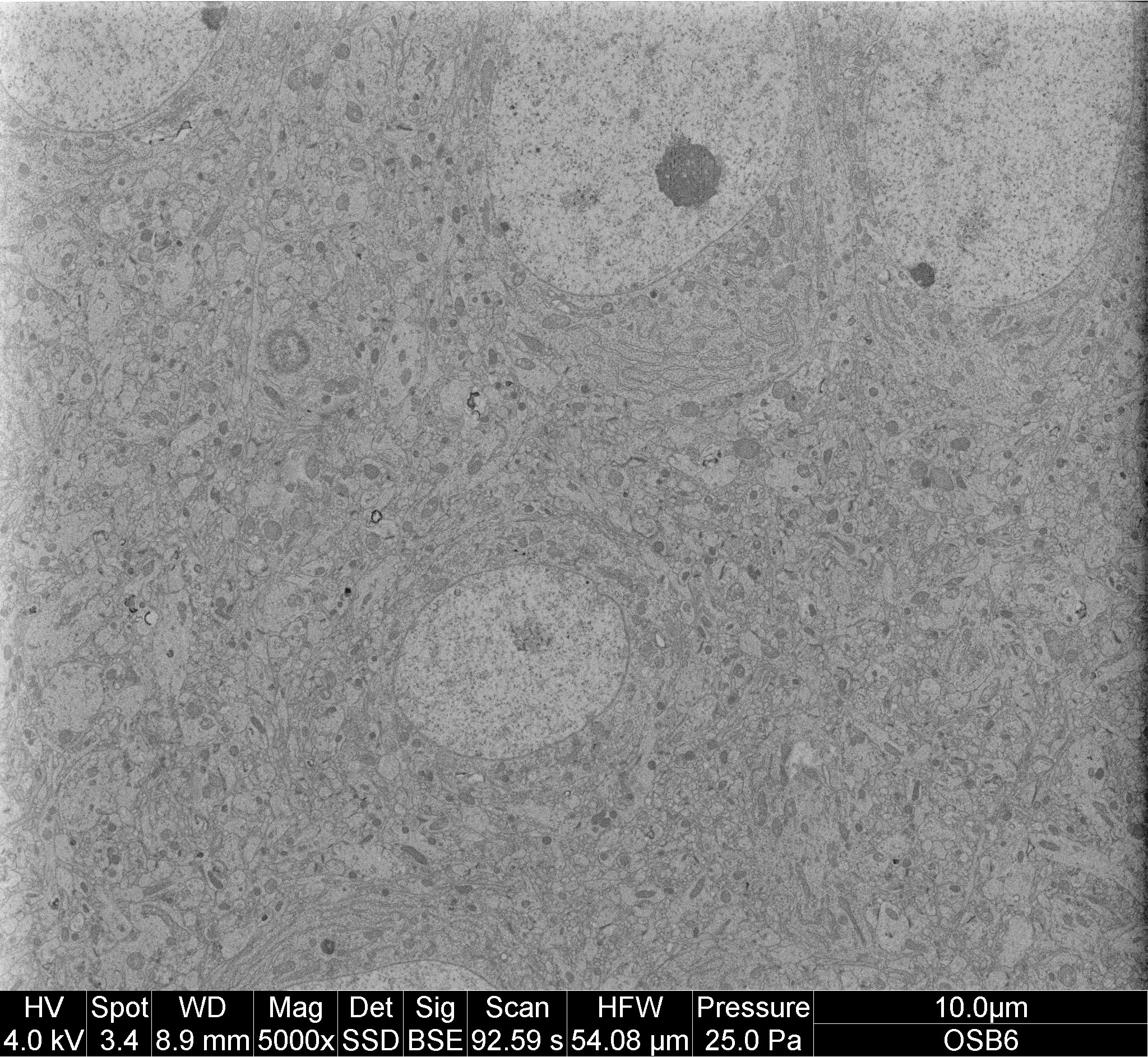

Supplement: Dataset S12 — (252.6 MB ZIP). [file pbio.0020329.sd012.zip › 040604_OS5_st1_1116.tif]

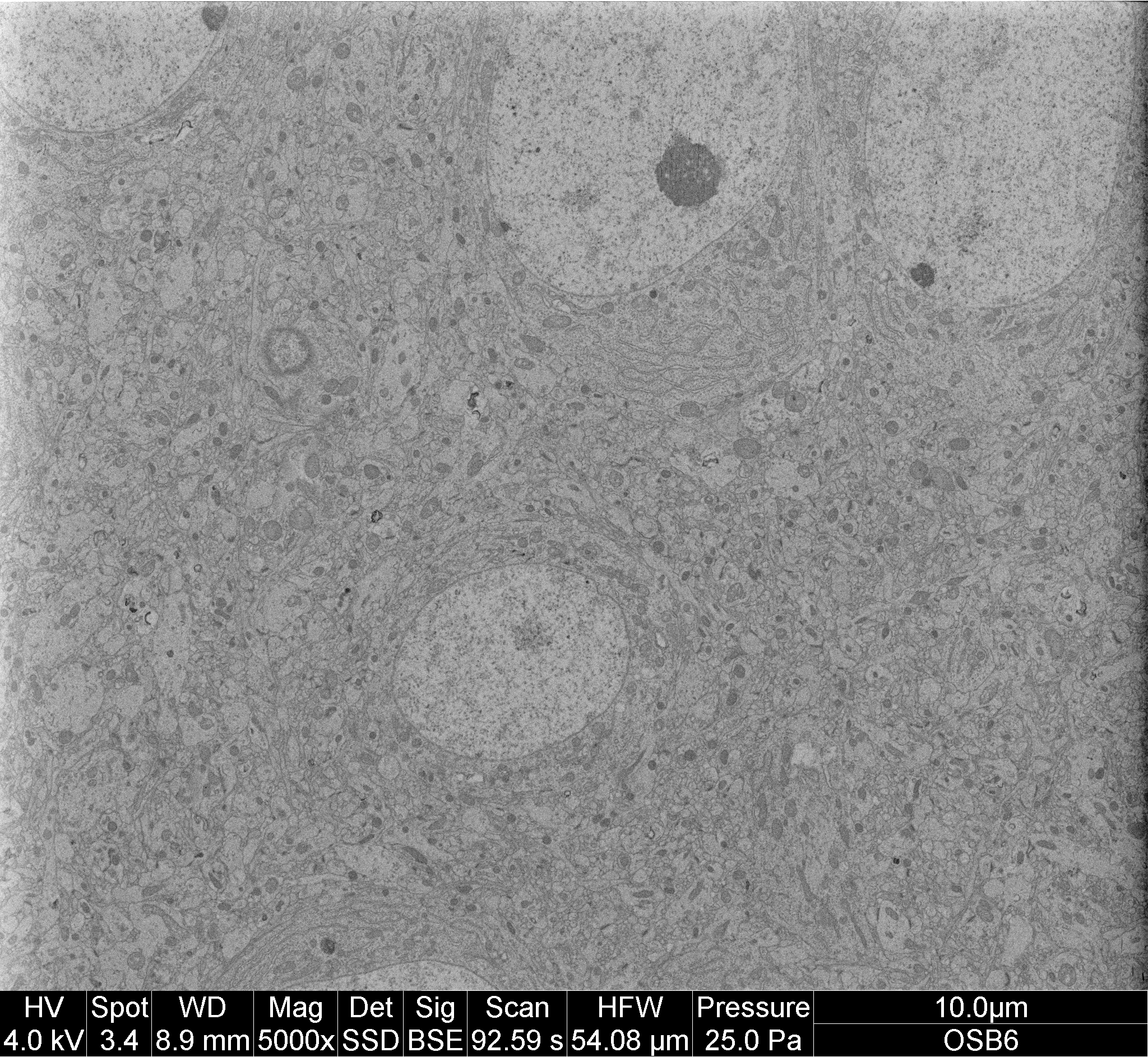

Supplement: Dataset S12 — (252.6 MB ZIP). [file pbio.0020329.sd012.zip › 040604_OS5_st1_1117.tif]

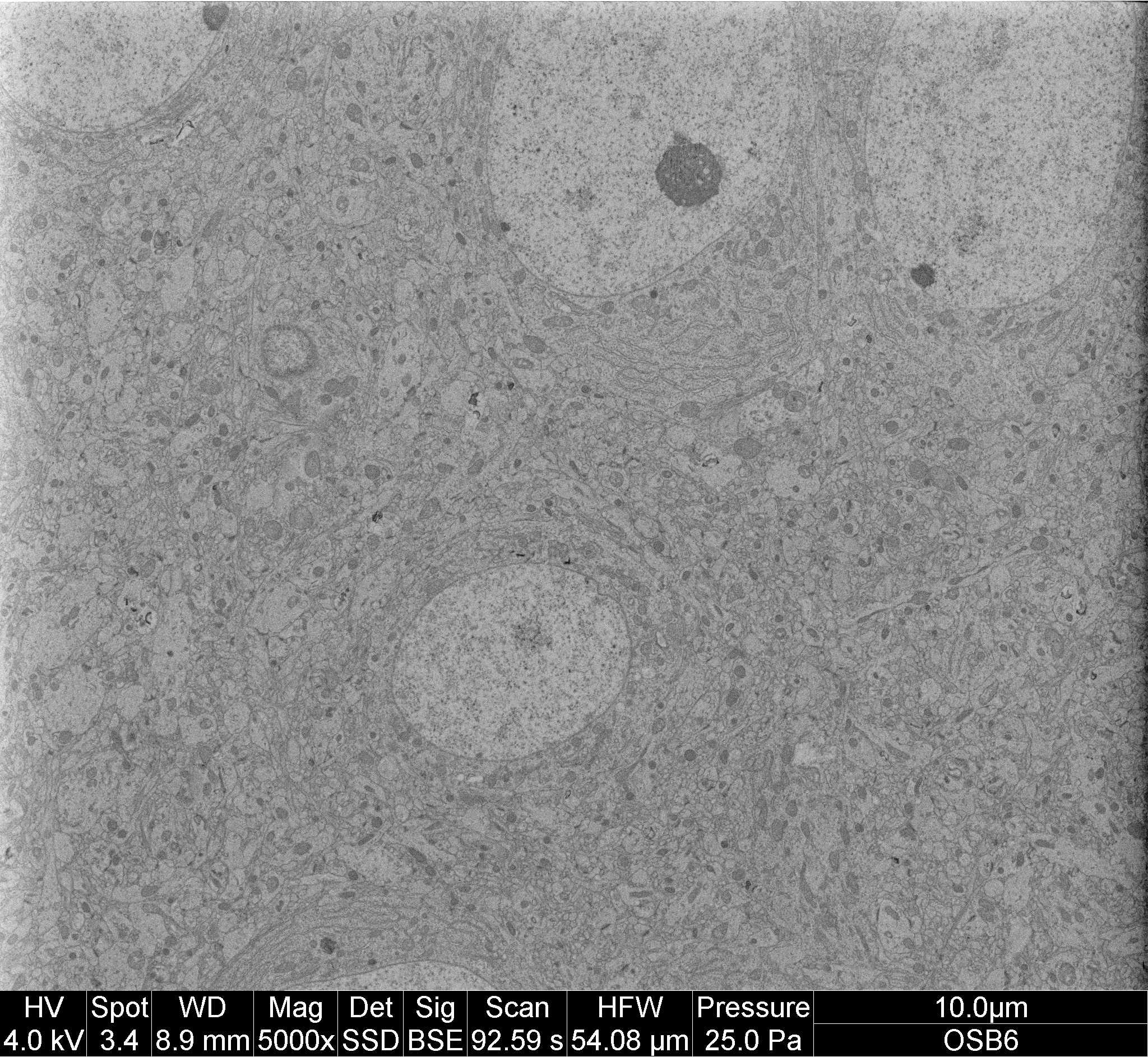

Supplement: Dataset S12 — (252.6 MB ZIP). [file pbio.0020329.sd012.zip › 040604_OS5_st1_1118.tif]

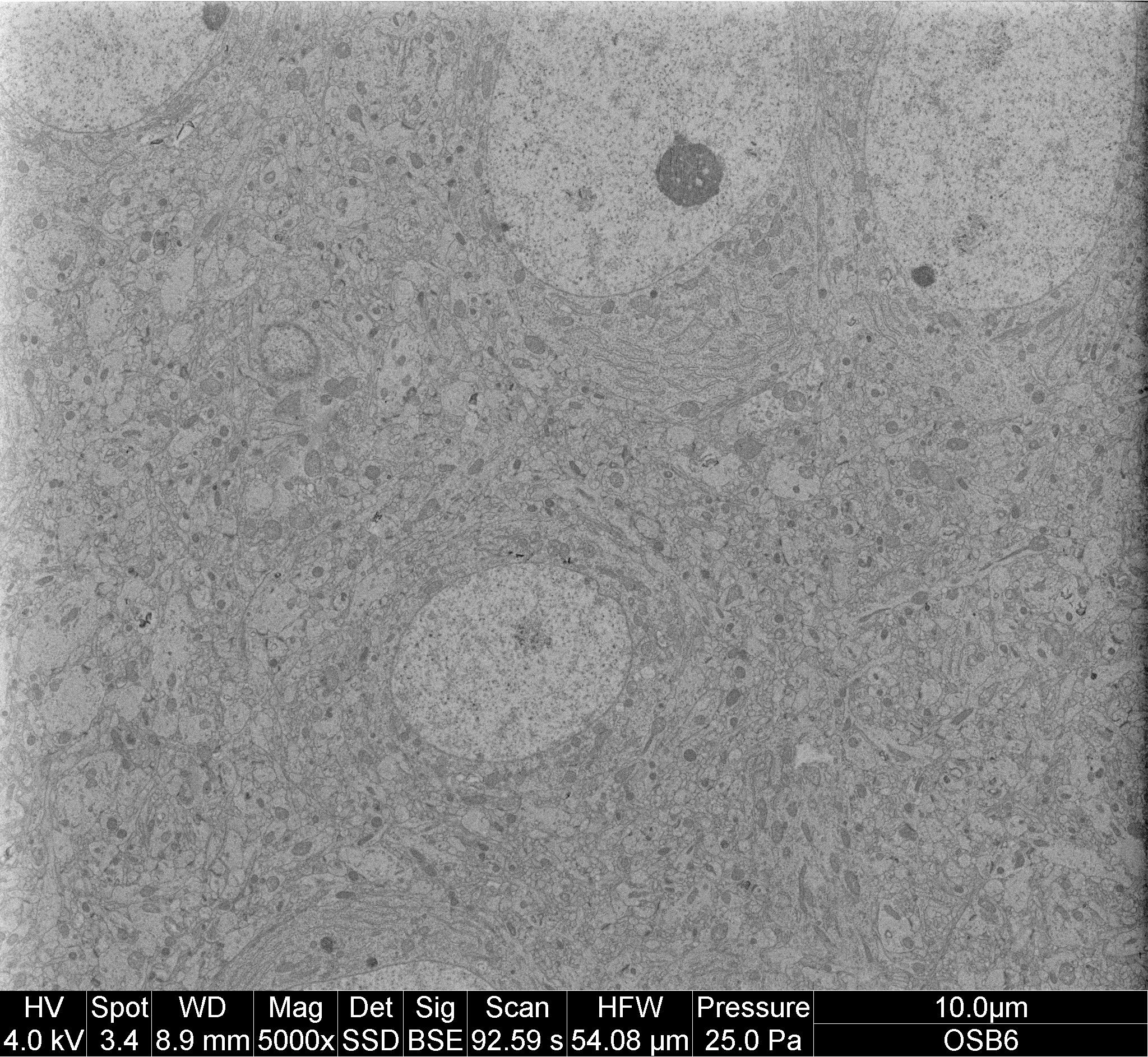

Supplement: Dataset S12 — (252.6 MB ZIP). [file pbio.0020329.sd012.zip › 040604_OS5_st1_1119.tif]

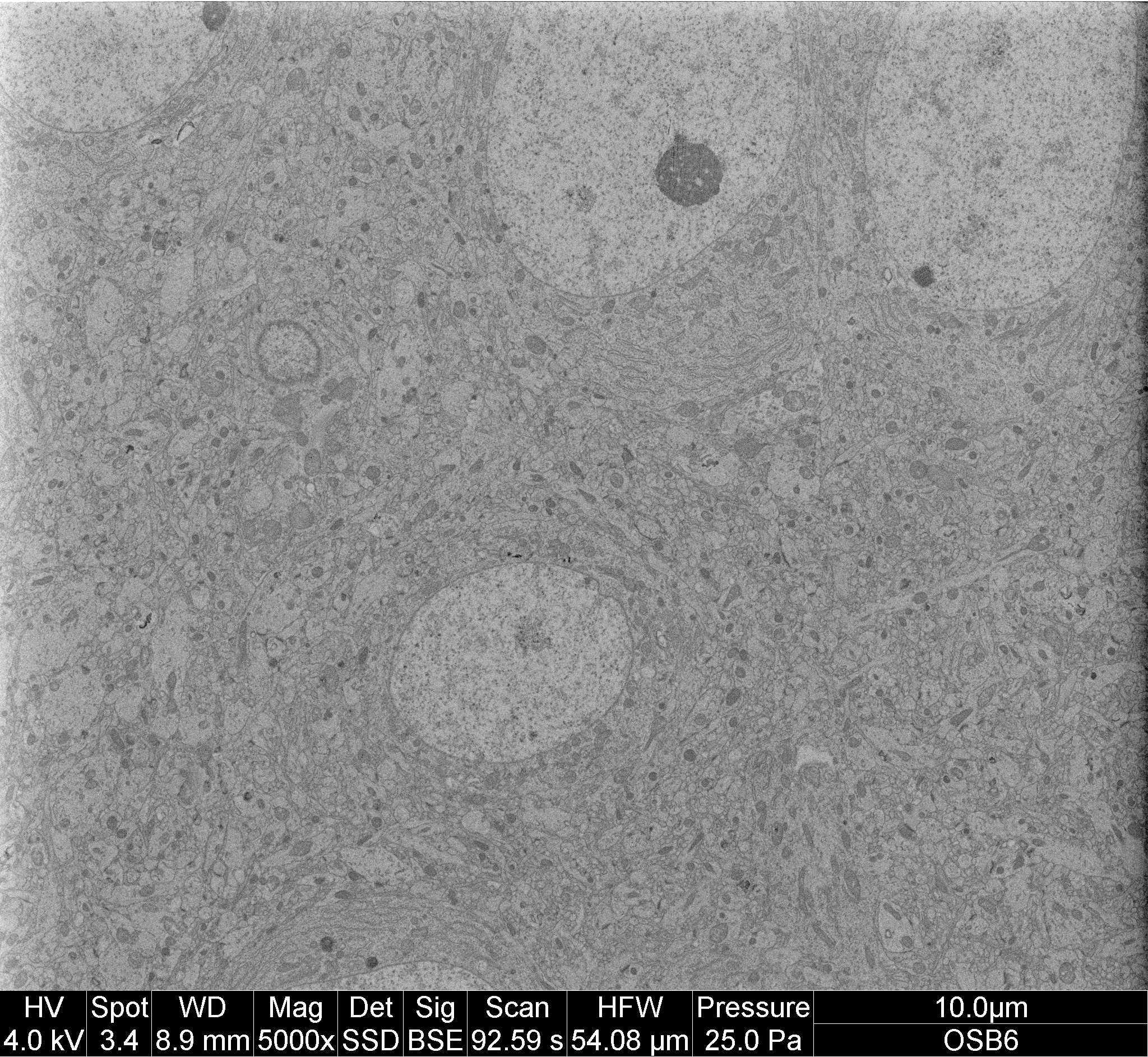

Supplement: Dataset S12 — (252.6 MB ZIP). [file pbio.0020329.sd012.zip › 040604_OS5_st1_1120.tif]

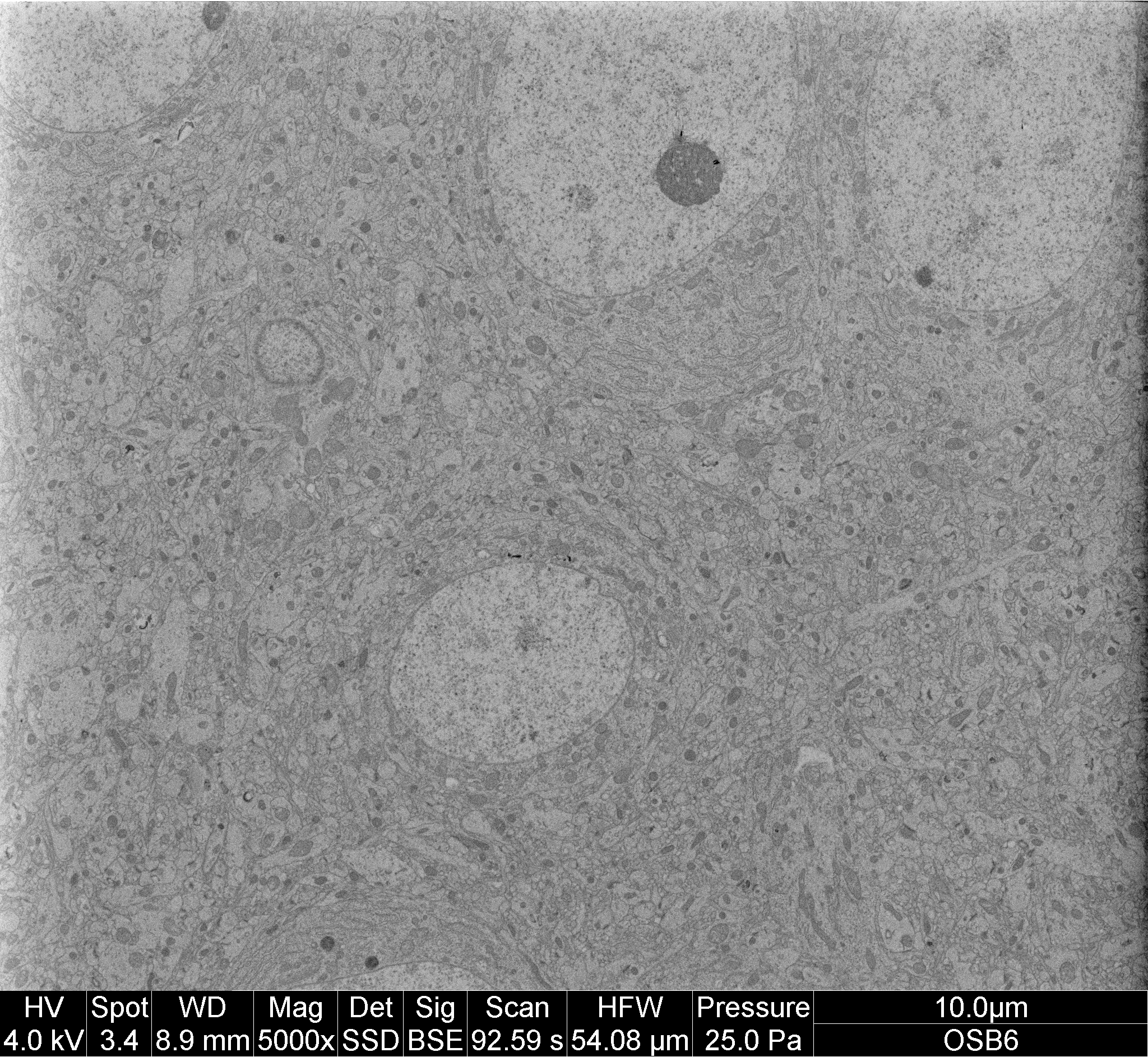

Supplement: Dataset S12 — (252.6 MB ZIP). [file pbio.0020329.sd012.zip › 040604_OS5_st1_1121.tif]

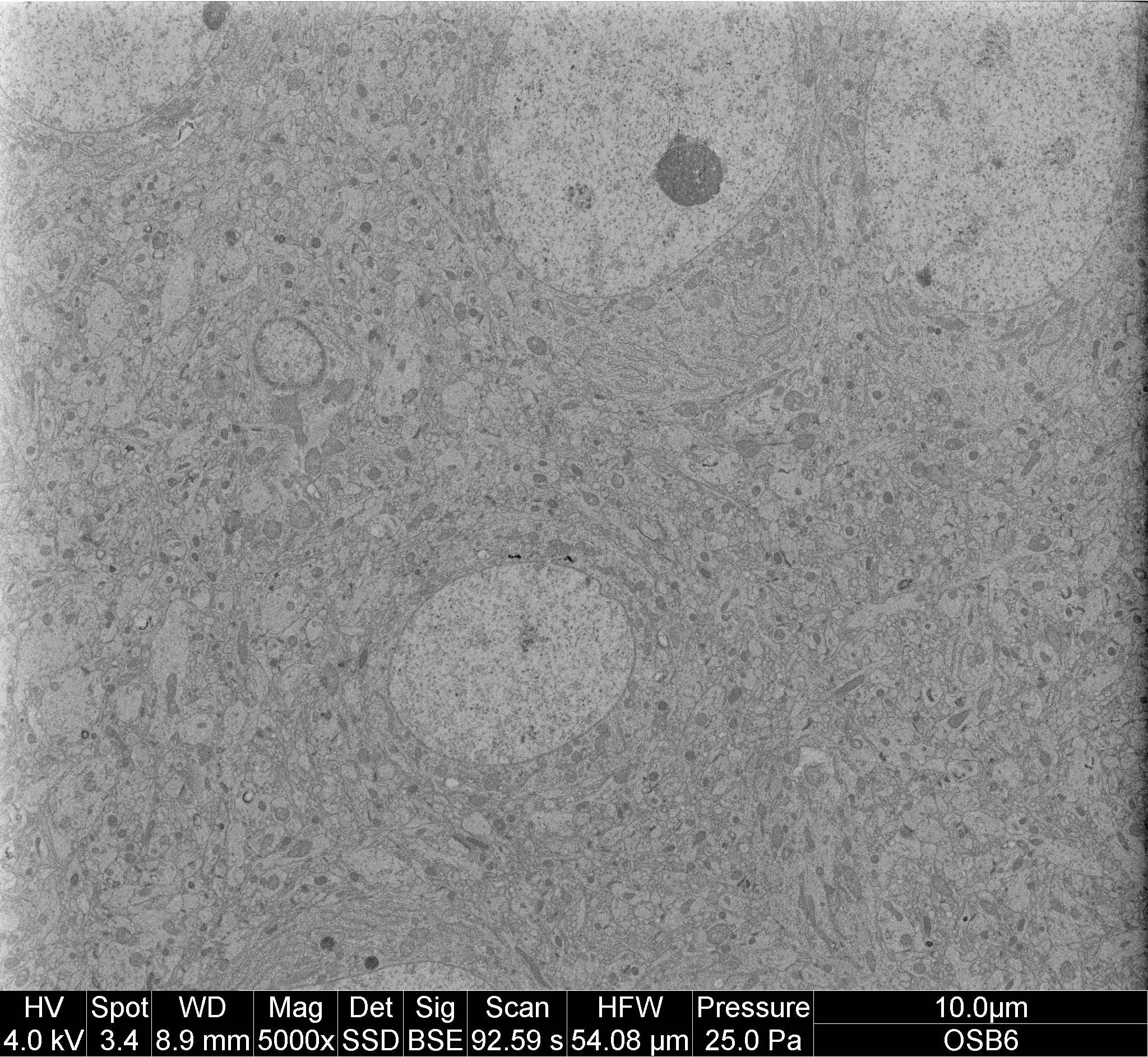

Supplement: Dataset S12 — (252.6 MB ZIP). [file pbio.0020329.sd012.zip › 040604_OS5_st1_1122.tif]

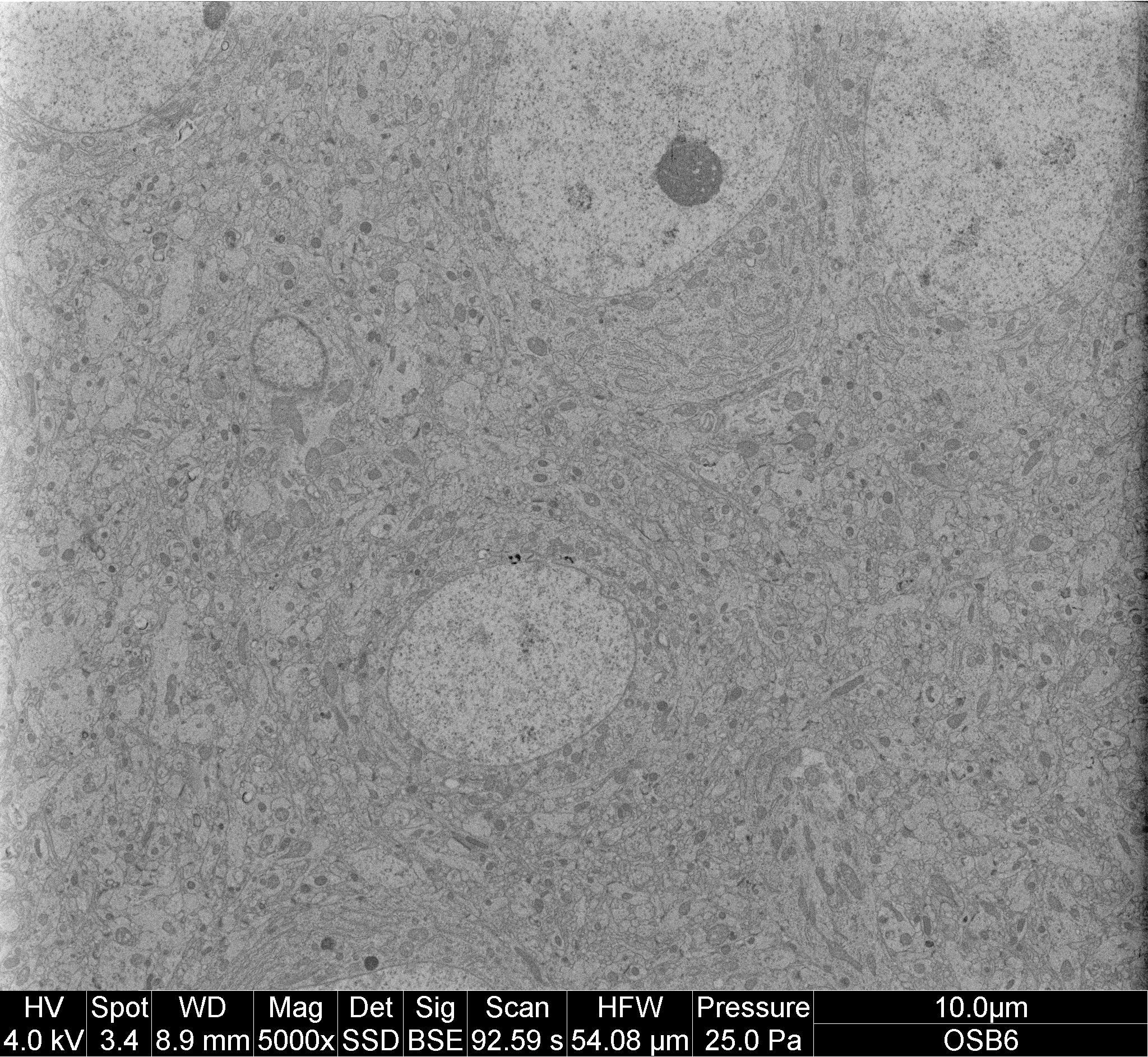

Supplement: Dataset S12 — (252.6 MB ZIP). [file pbio.0020329.sd012.zip › 040604_OS5_st1_1123.tif]

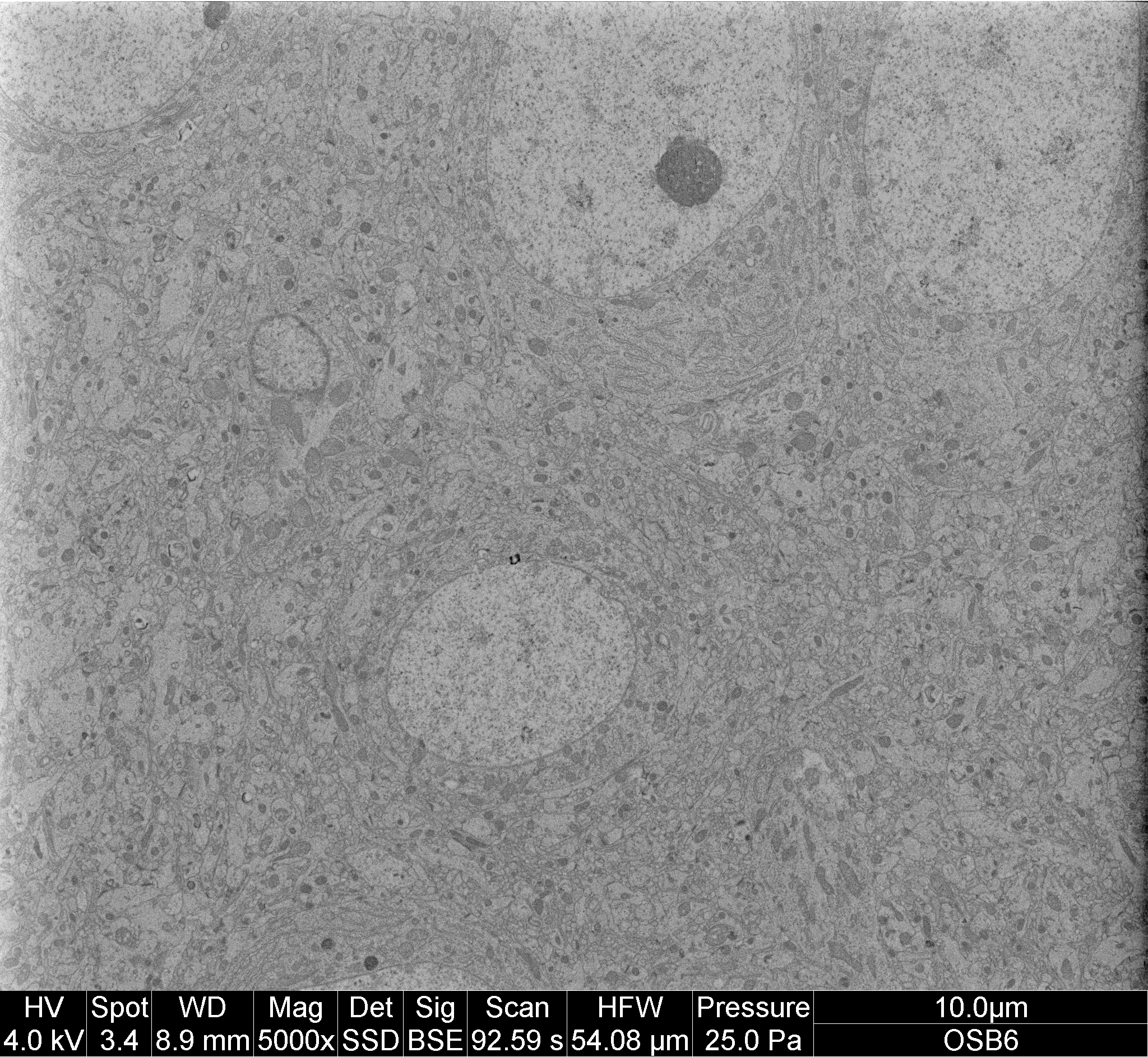

Supplement: Dataset S12 — (252.6 MB ZIP). [file pbio.0020329.sd012.zip › 040604_OS5_st1_1124.tif]

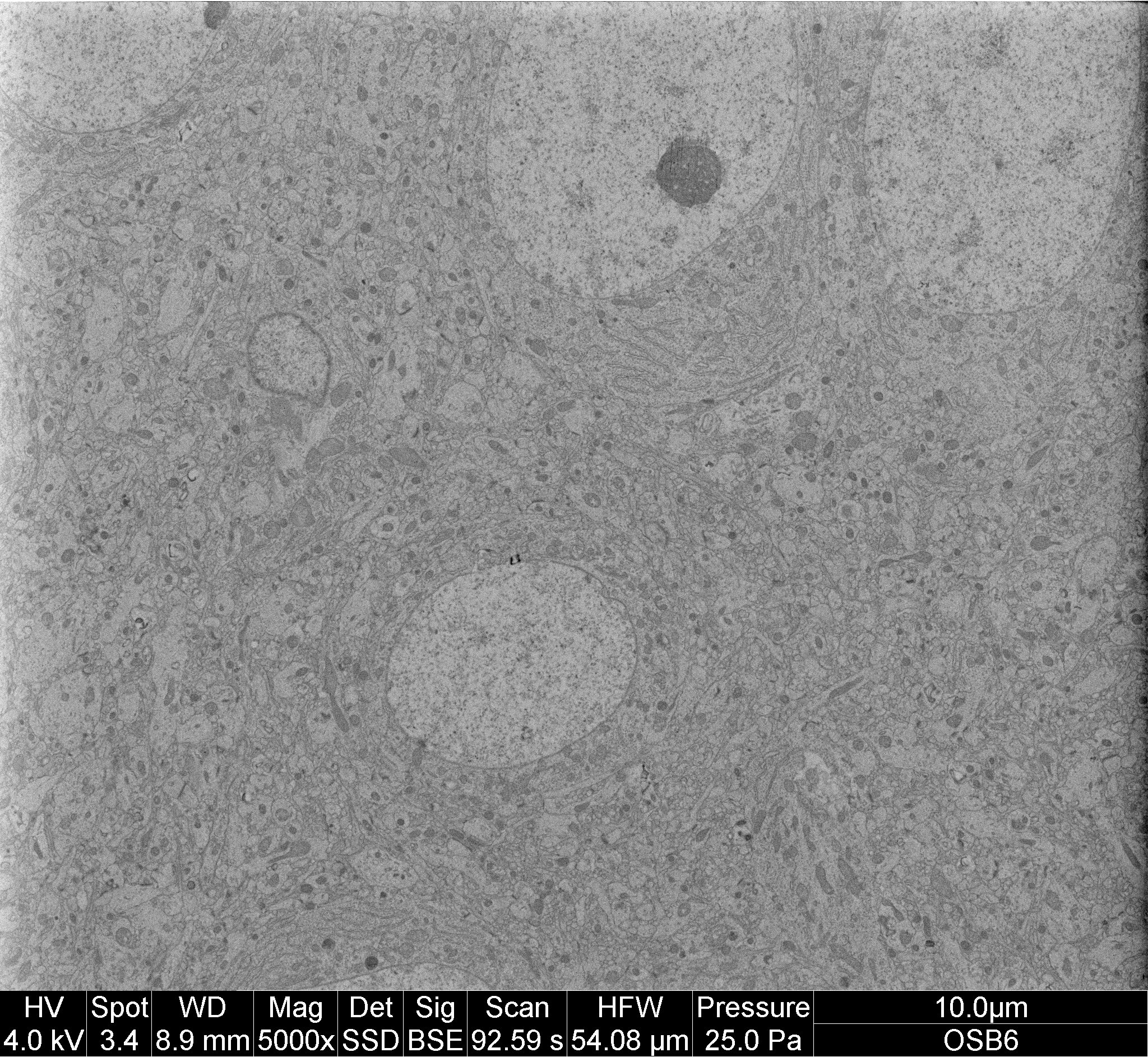

Supplement: Dataset S12 — (252.6 MB ZIP). [file pbio.0020329.sd012.zip › 040604_OS5_st1_1125.tif]

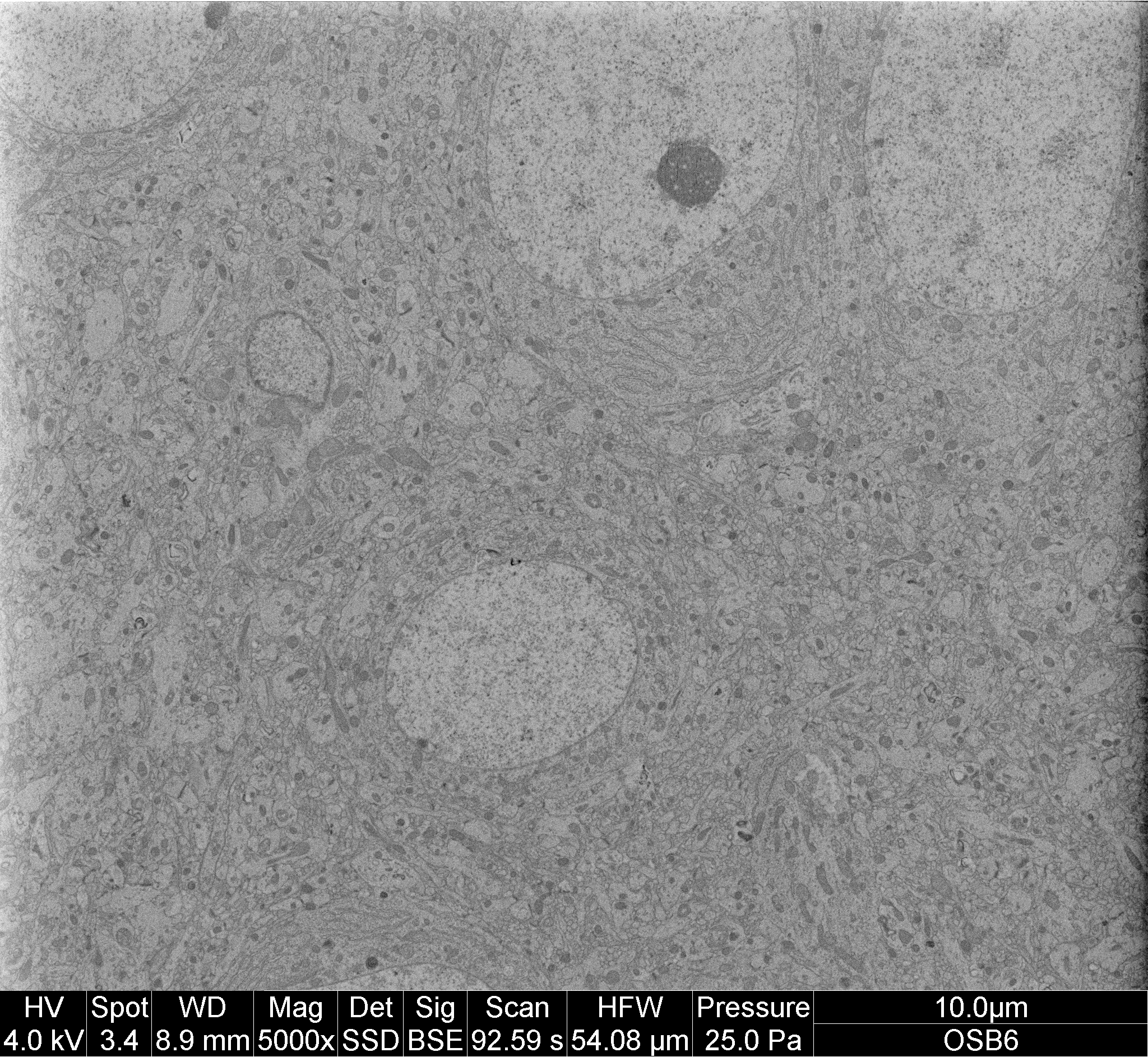

Supplement: Dataset S12 — (252.6 MB ZIP). [file pbio.0020329.sd012.zip › 040604_OS5_st1_1126.tif]

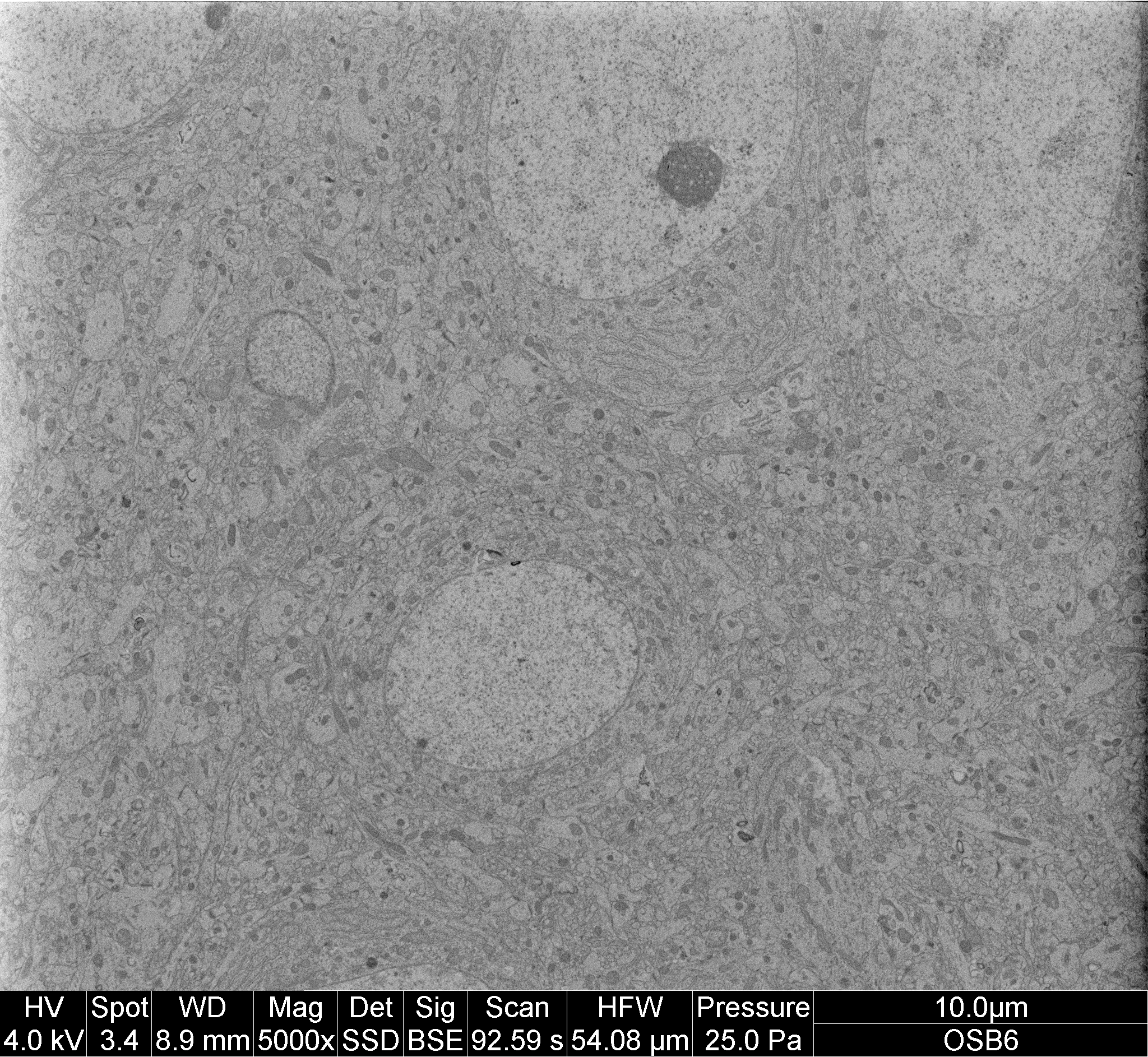

Supplement: Dataset S12 — (252.6 MB ZIP). [file pbio.0020329.sd012.zip › 040604_OS5_st1_1127.tif]

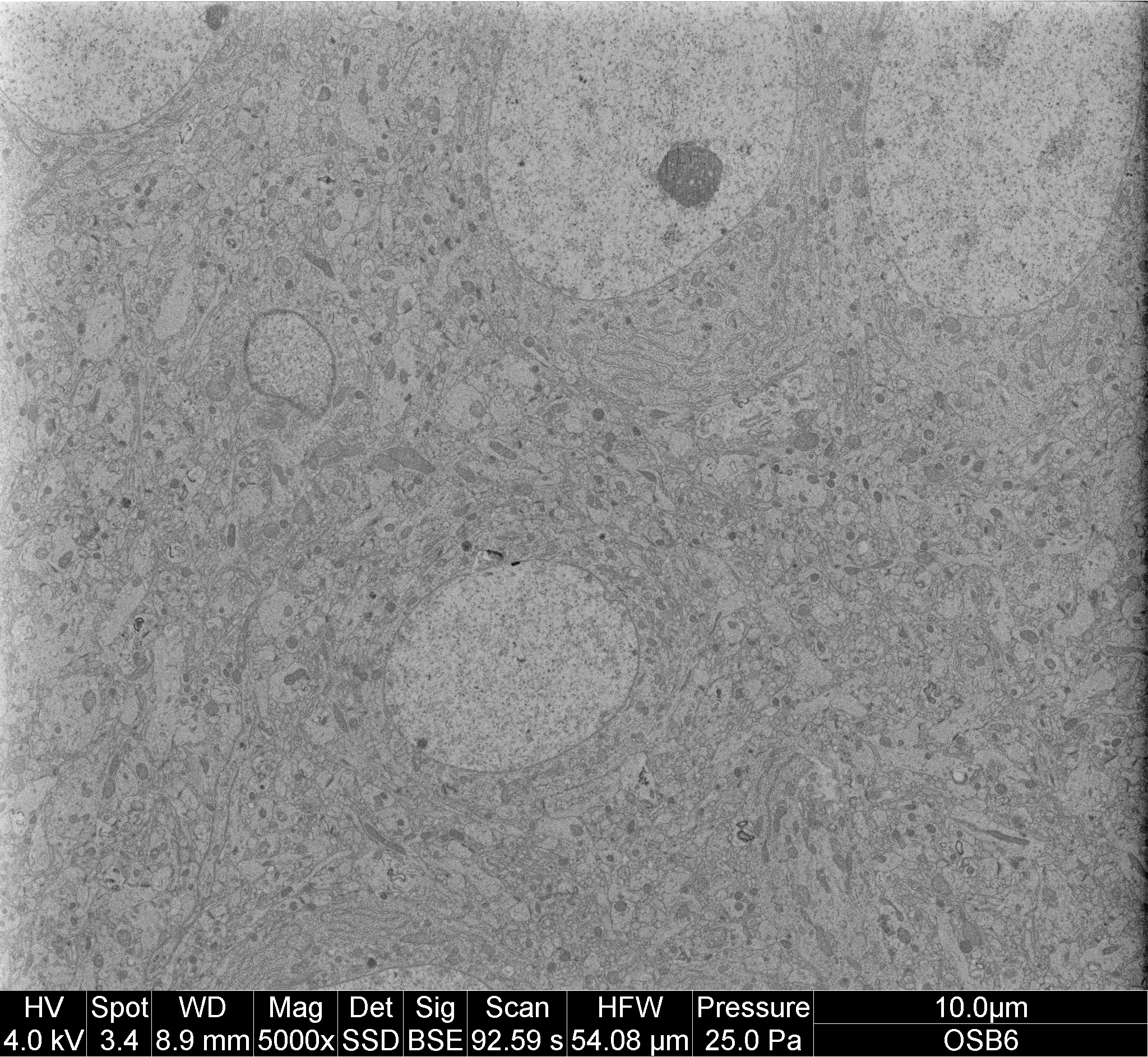

Supplement: Dataset S12 — (252.6 MB ZIP). [file pbio.0020329.sd012.zip › 040604_OS5_st1_1128.tif]

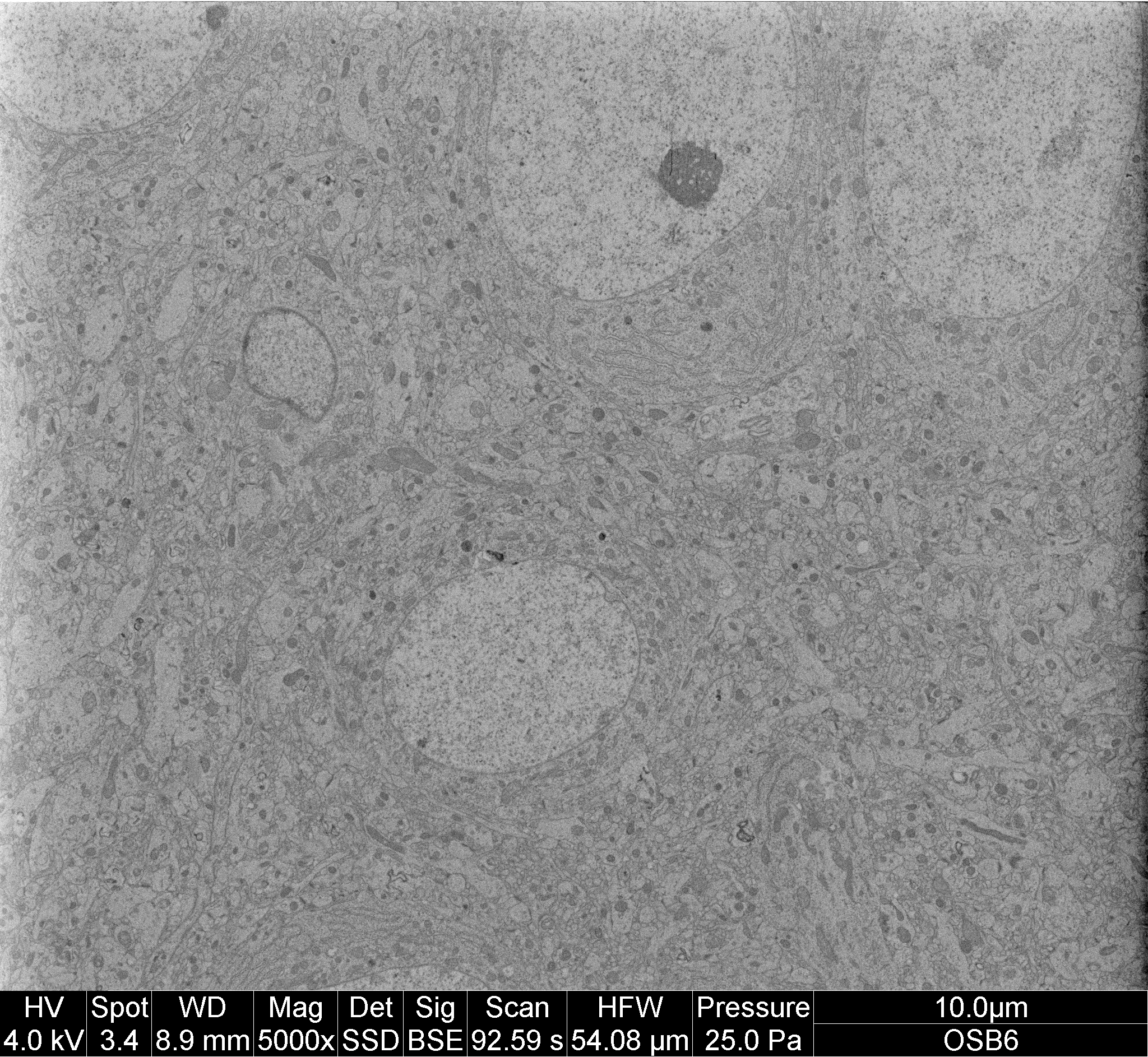

Supplement: Dataset S12 — (252.6 MB ZIP). [file pbio.0020329.sd012.zip › 040604_OS5_st1_1129.tif]

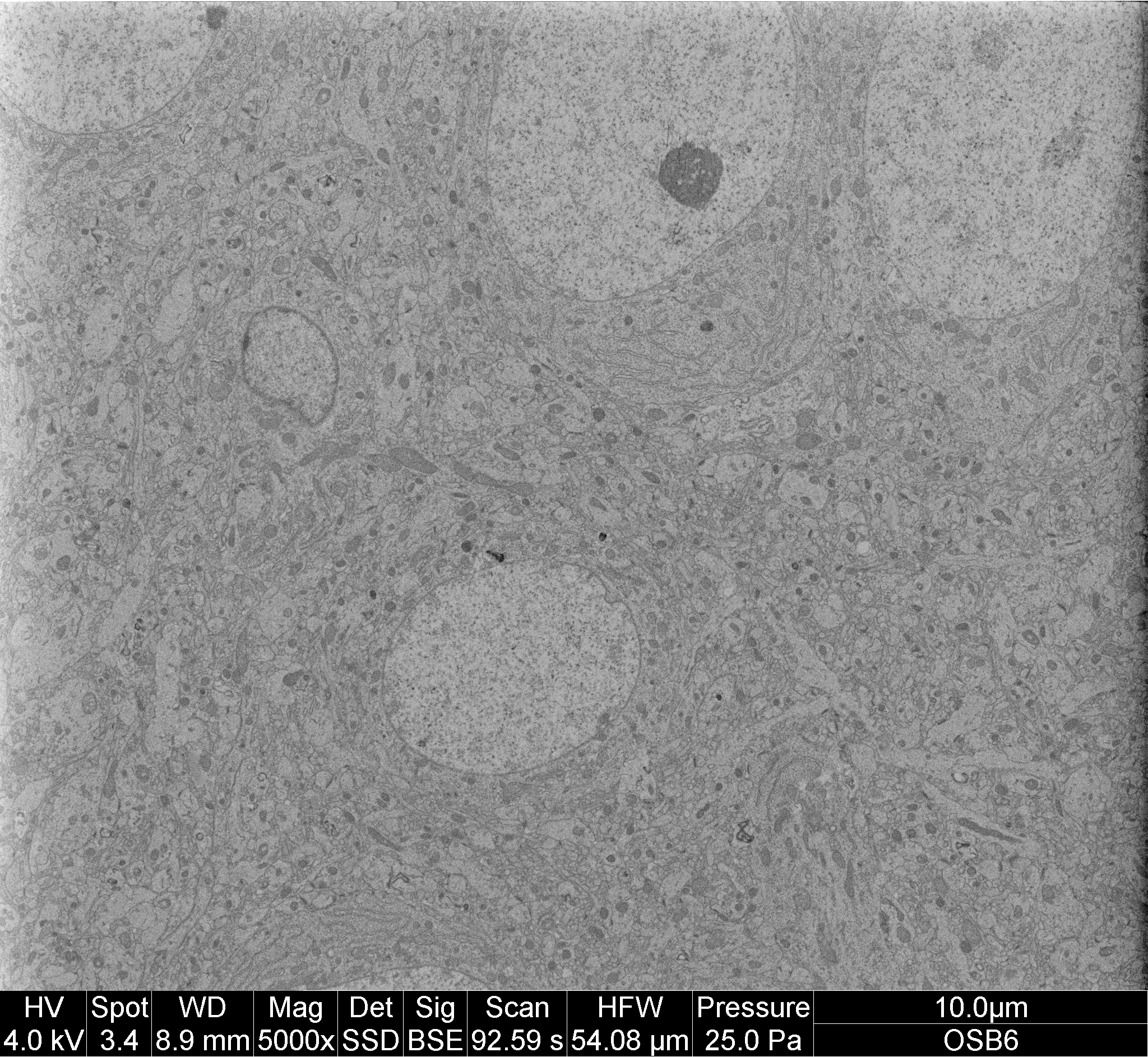

Supplement: Dataset S12 — (252.6 MB ZIP). [file pbio.0020329.sd012.zip › 040604_OS5_st1_1130.tif]

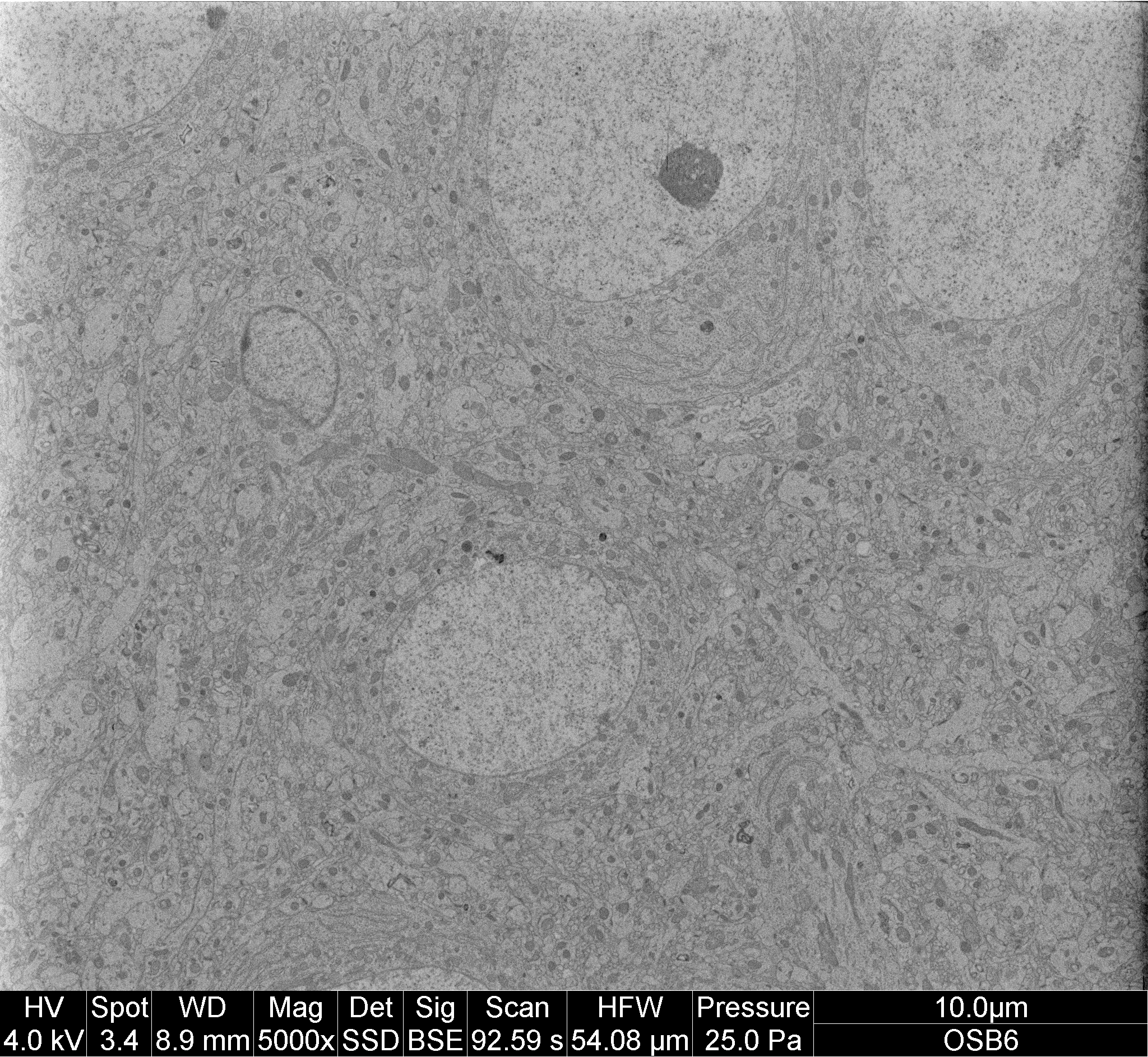

Supplement: Dataset S12 — (252.6 MB ZIP). [file pbio.0020329.sd012.zip › 040604_OS5_st1_1131.tif]

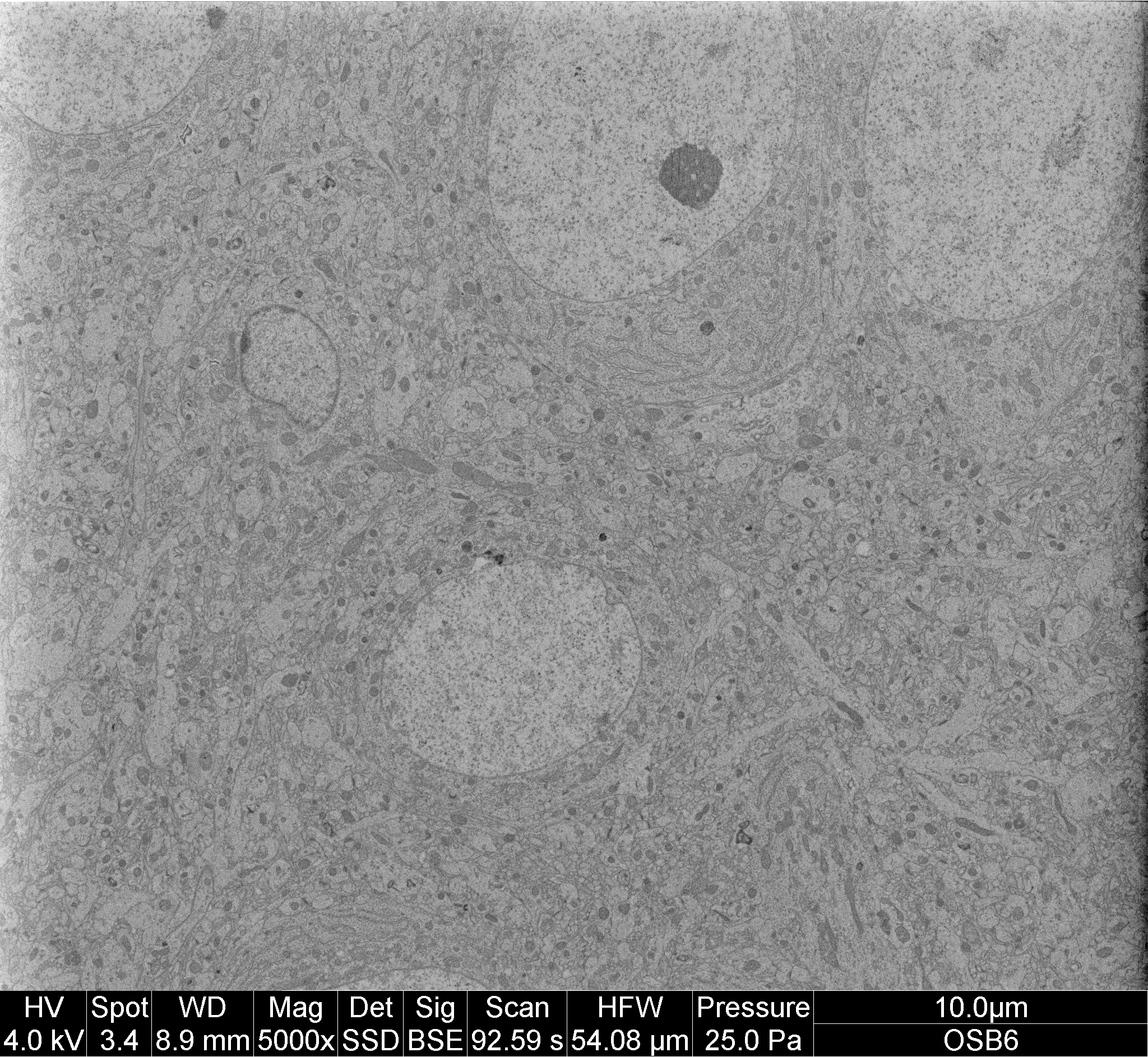

Supplement: Dataset S12 — (252.6 MB ZIP). [file pbio.0020329.sd012.zip › 040604_OS5_st1_1132.tif]

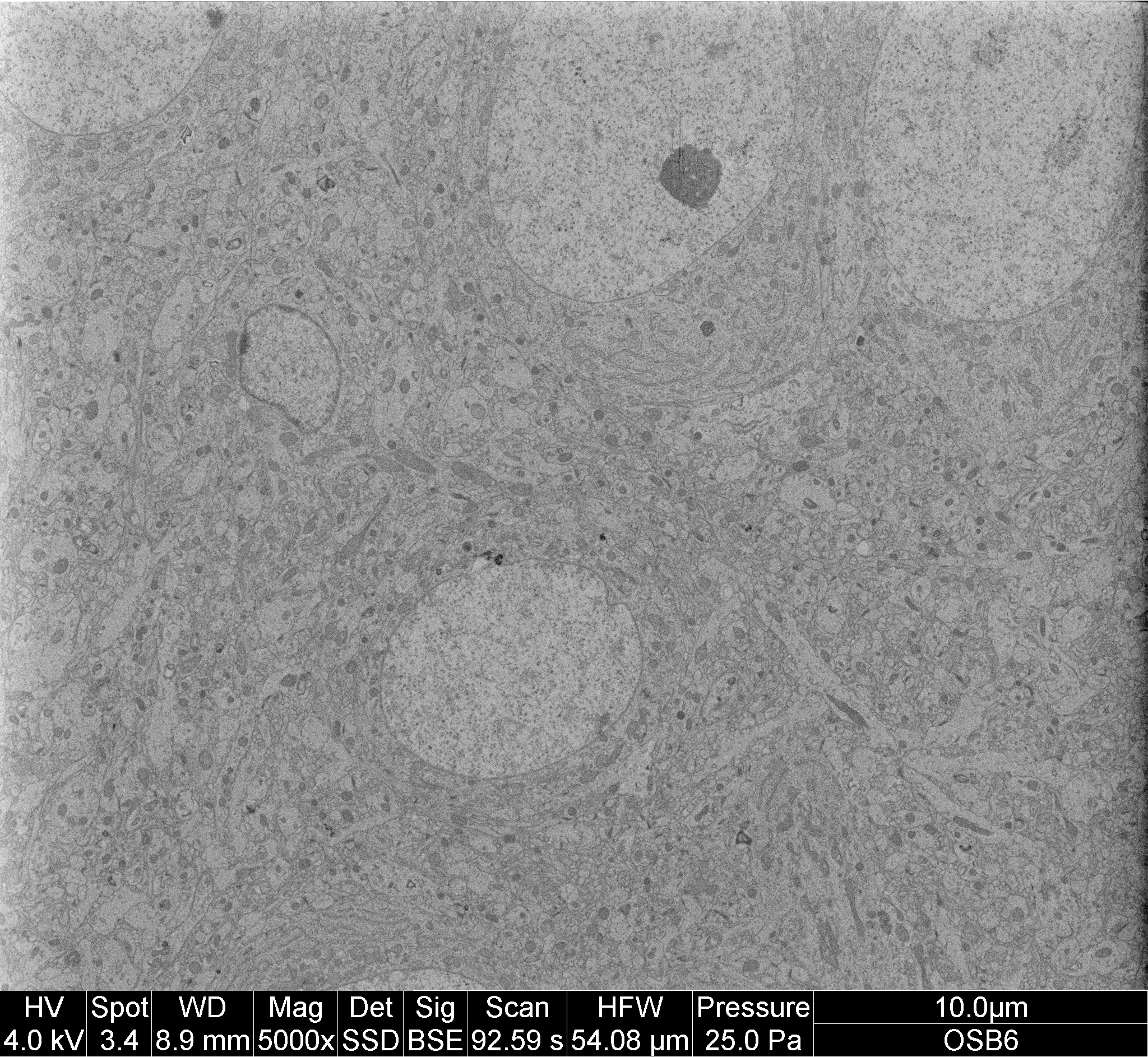

Supplement: Dataset S12 — (252.6 MB ZIP). [file pbio.0020329.sd012.zip › 040604_OS5_st1_1133.tif]

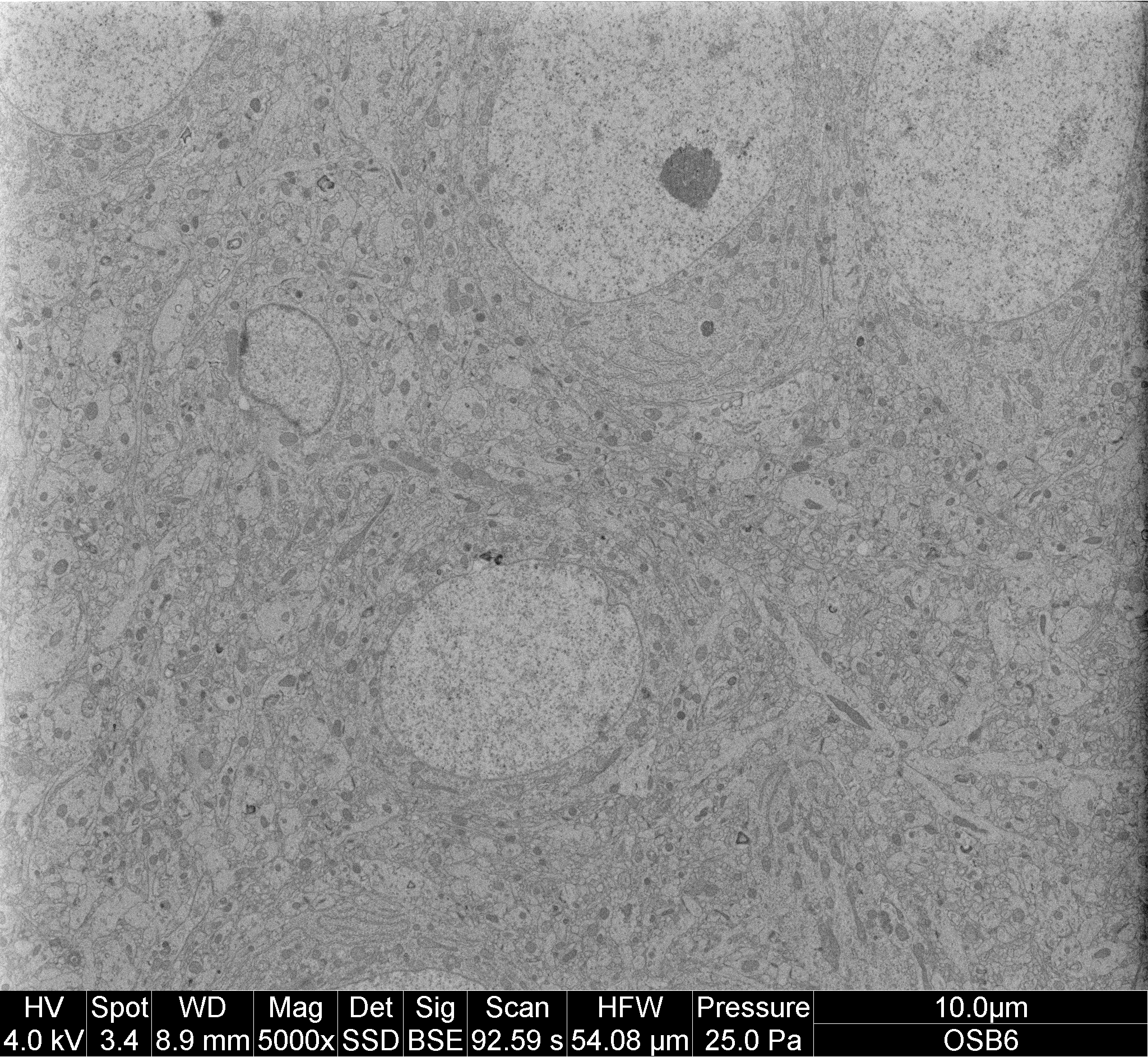

Supplement: Dataset S12 — (252.6 MB ZIP). [file pbio.0020329.sd012.zip › 040604_OS5_st1_1134.tif]

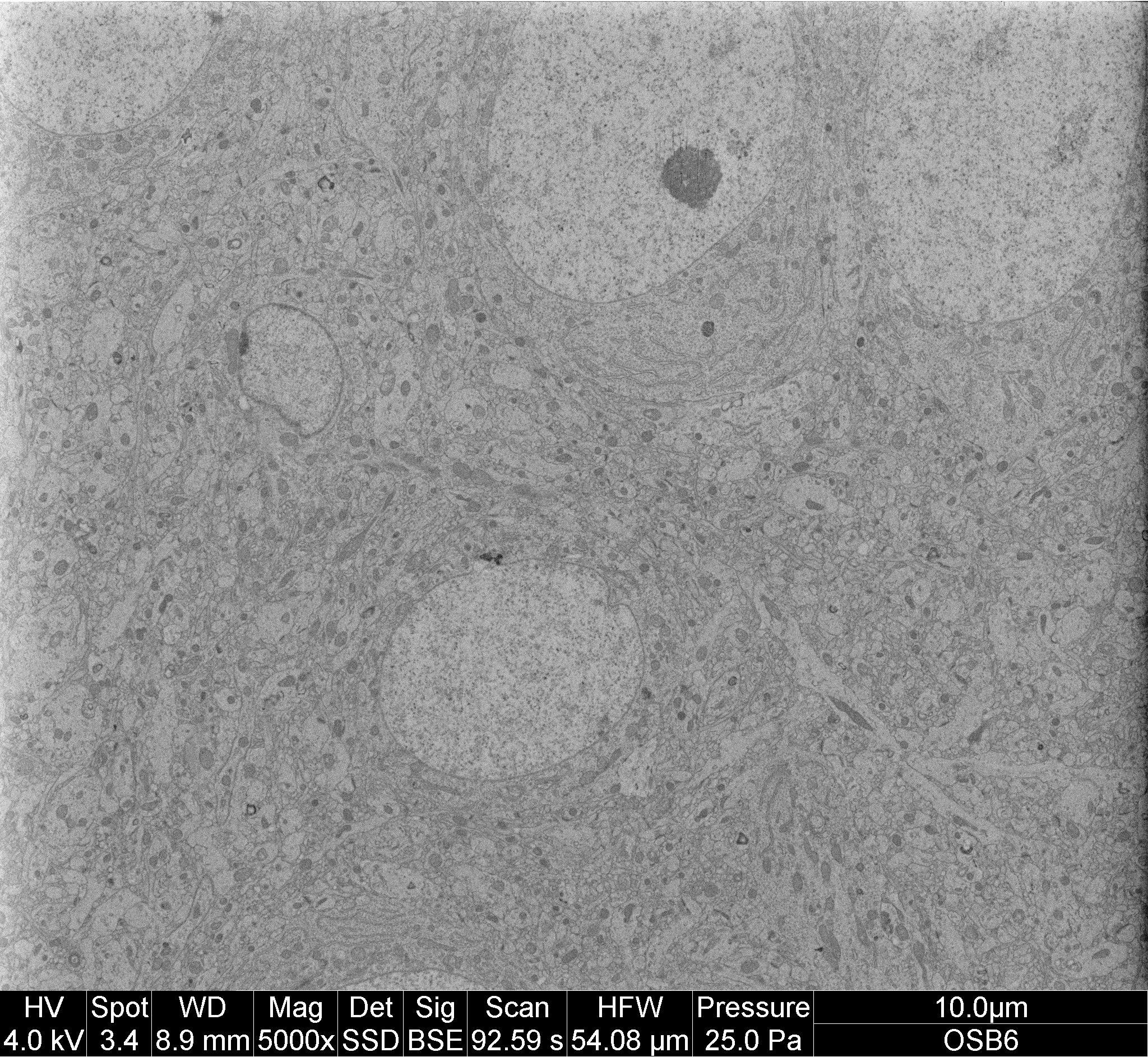

Supplement: Dataset S12 — (252.6 MB ZIP). [file pbio.0020329.sd012.zip › 040604_OS5_st1_1135.tif]

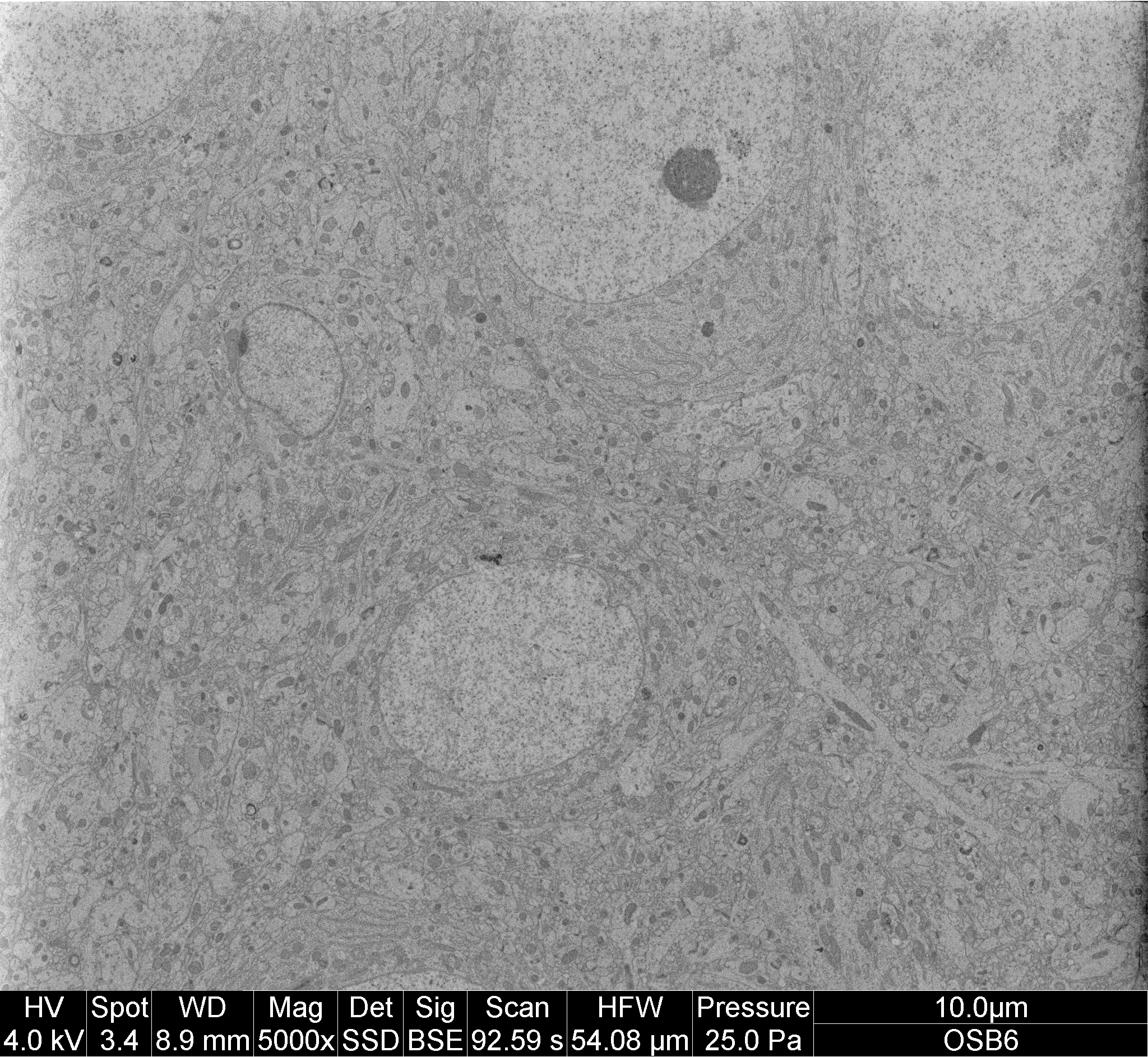

Supplement: Dataset S12 — (252.6 MB ZIP). [file pbio.0020329.sd012.zip › 040604_OS5_st1_1136.tif]

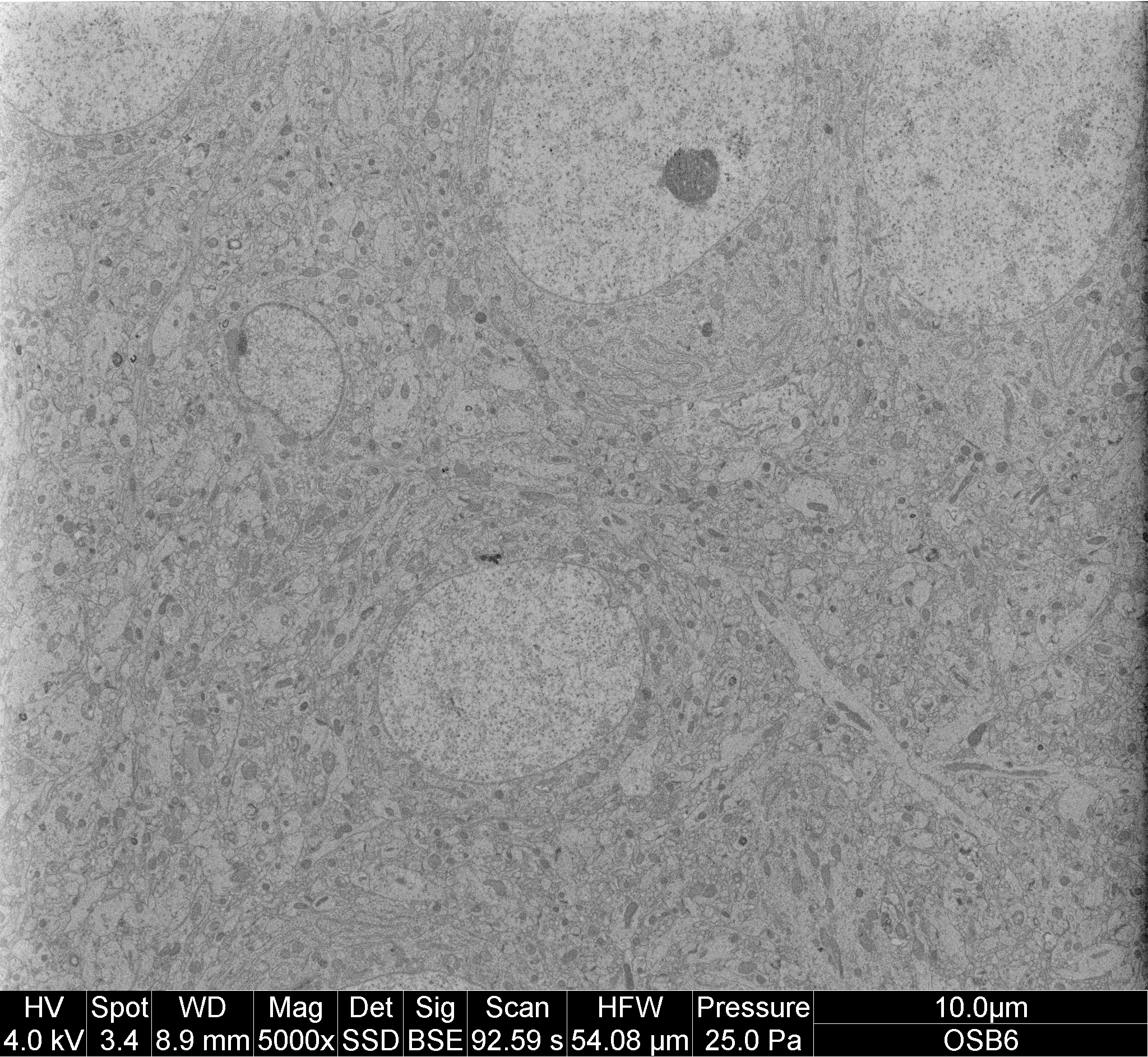

Supplement: Dataset S12 — (252.6 MB ZIP). [file pbio.0020329.sd012.zip › 040604_OS5_st1_1137.tif]

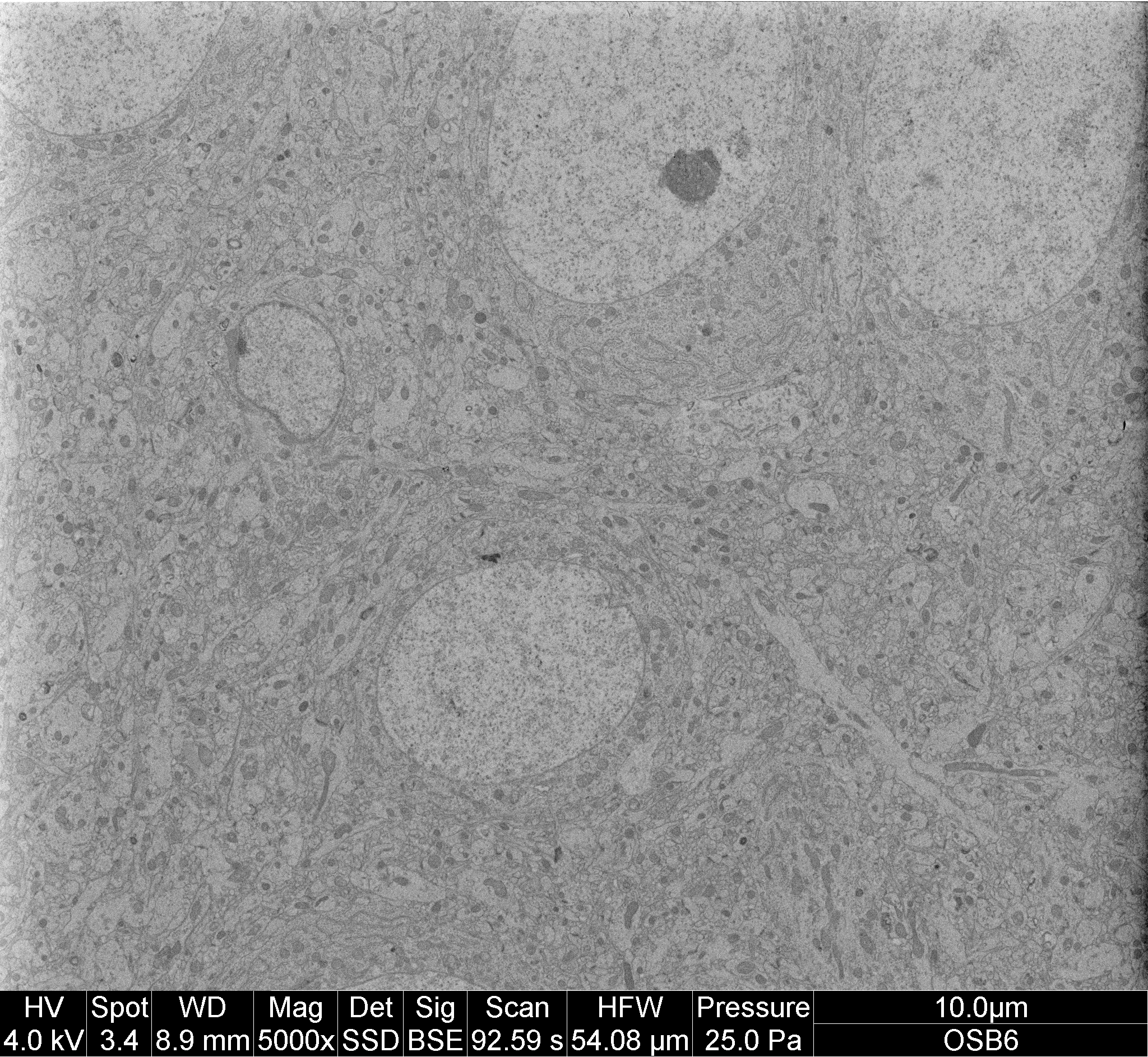

Supplement: Dataset S12 — (252.6 MB ZIP). [file pbio.0020329.sd012.zip › 040604_OS5_st1_1138.tif]

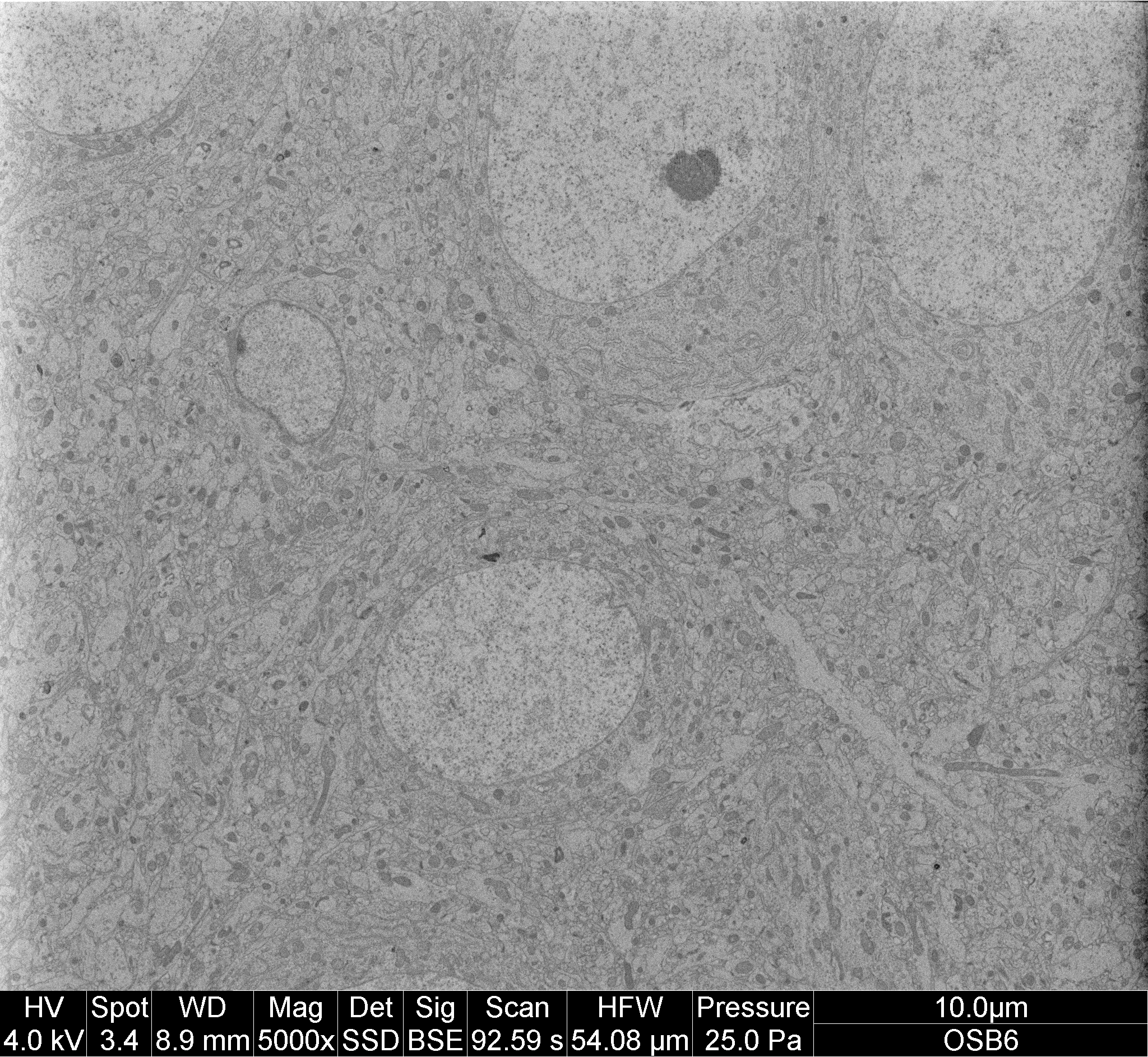

Supplement: Dataset S12 — (252.6 MB ZIP). [file pbio.0020329.sd012.zip › 040604_OS5_st1_1139.tif]

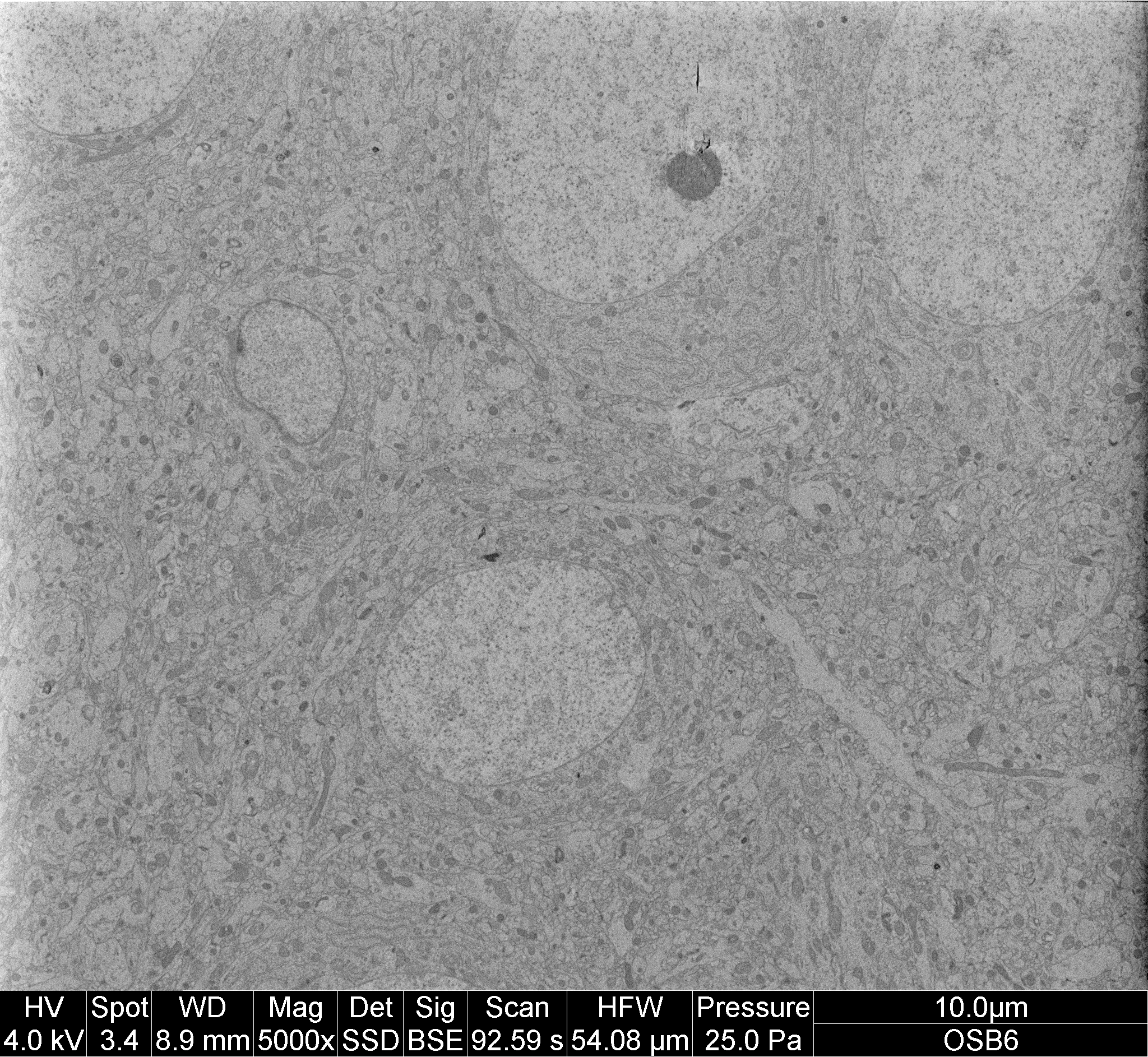

Supplement: Dataset S12 — (252.6 MB ZIP). [file pbio.0020329.sd012.zip › 040604_OS5_st1_1140.tif]

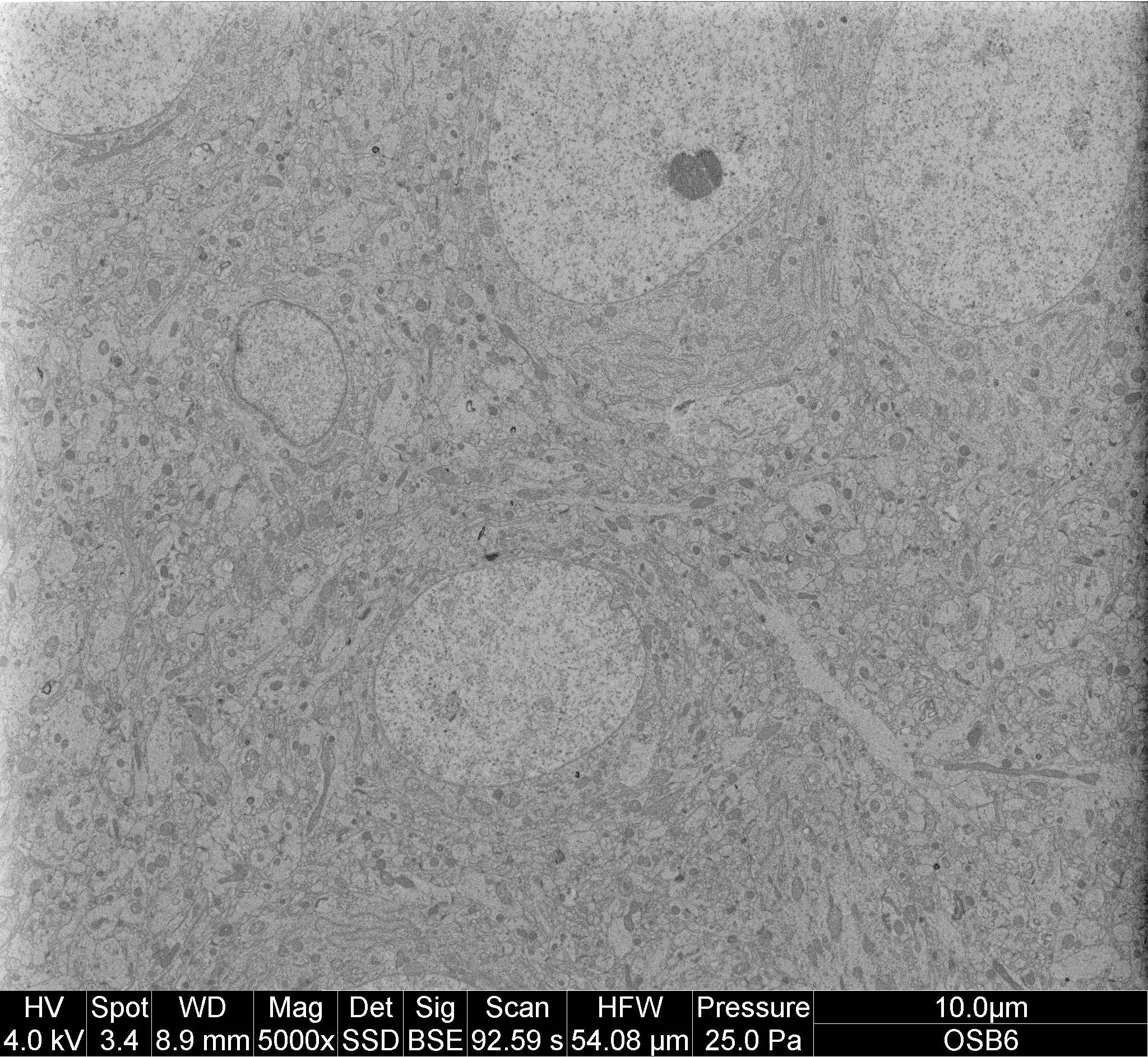

Supplement: Dataset S12 — (252.6 MB ZIP). [file pbio.0020329.sd012.zip › 040604_OS5_st1_1141.tif]

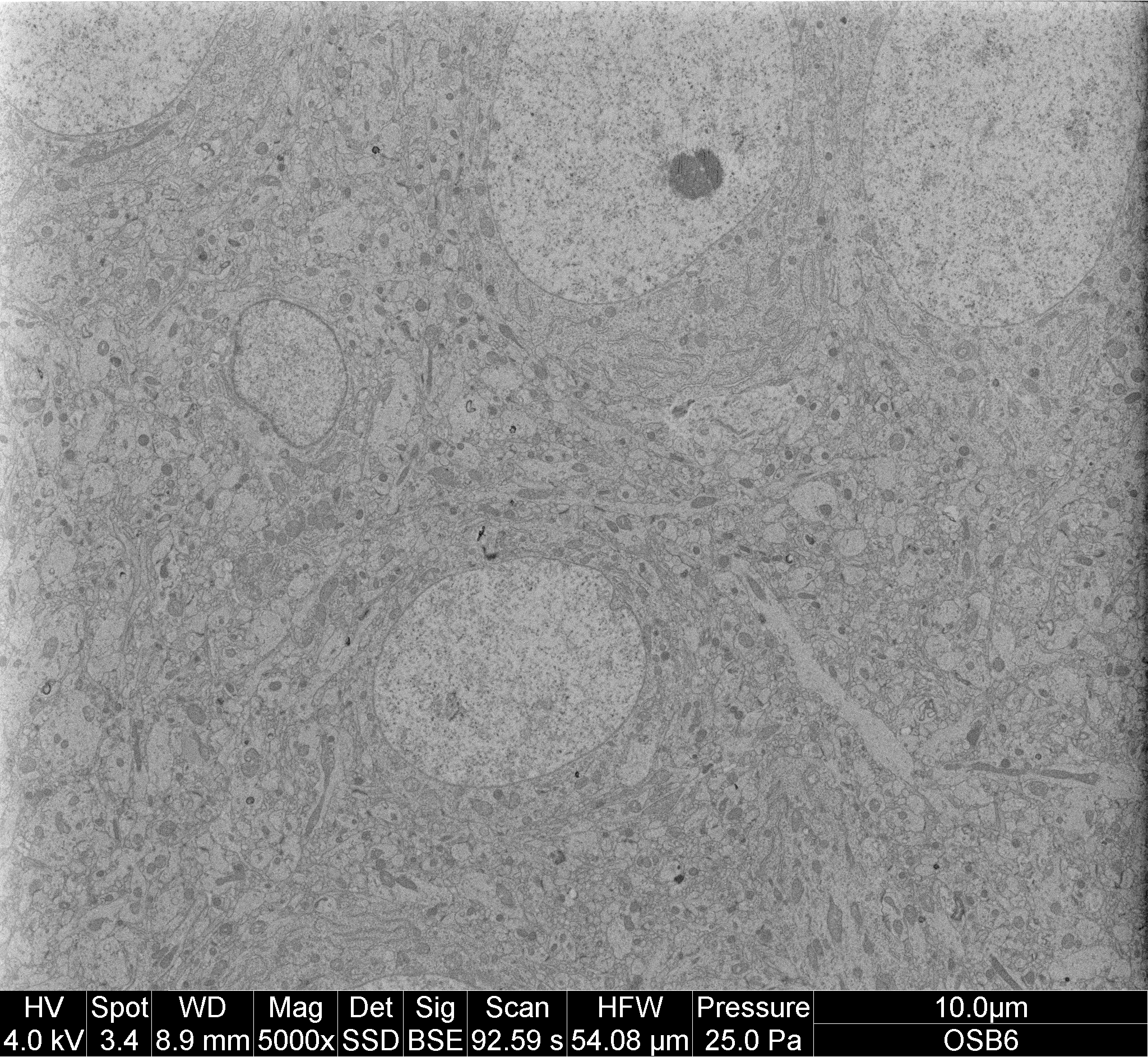

Supplement: Dataset S12 — (252.6 MB ZIP). [file pbio.0020329.sd012.zip › 040604_OS5_st1_1142.tif]

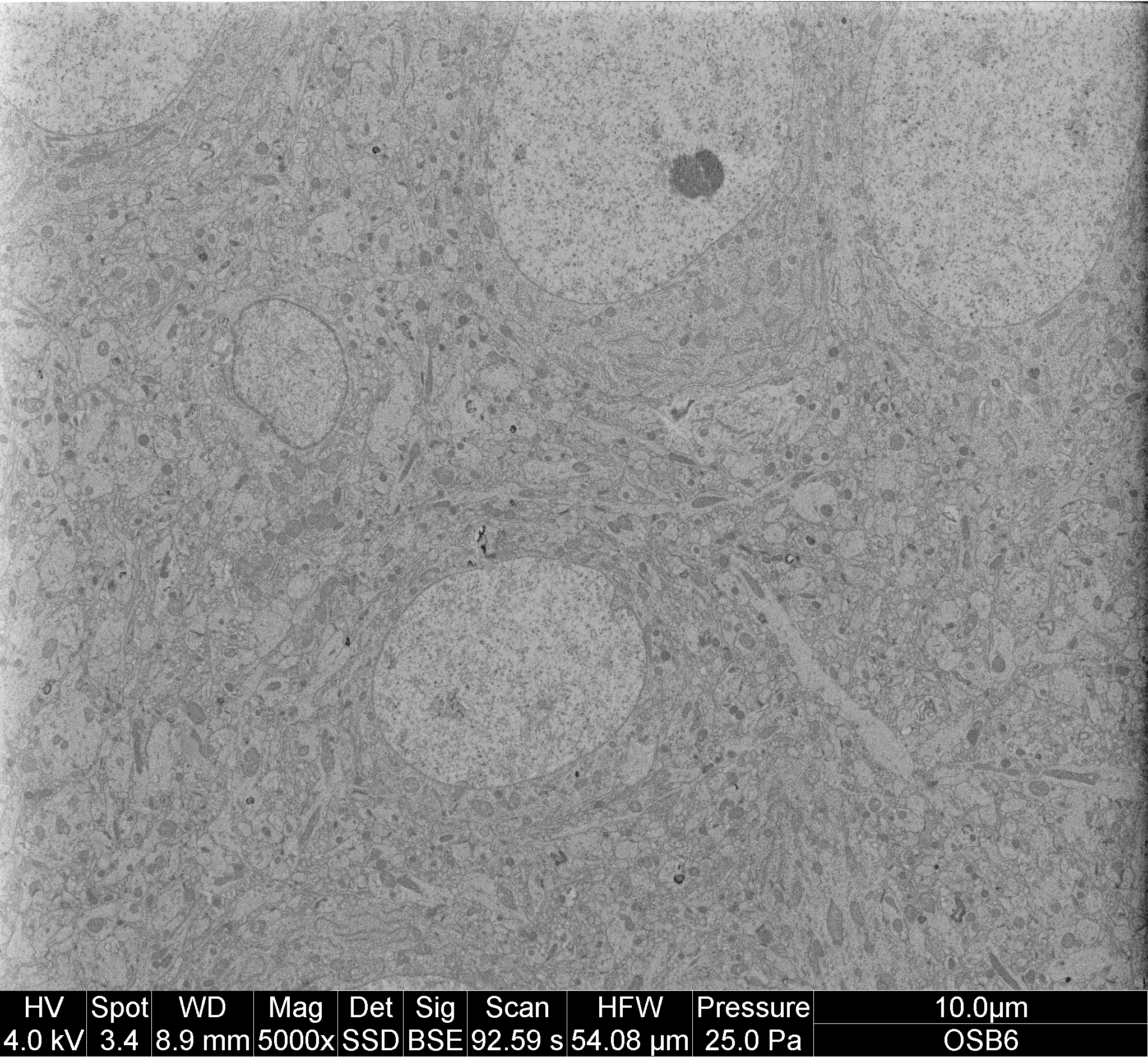

Supplement: Dataset S12 — (252.6 MB ZIP). [file pbio.0020329.sd012.zip › 040604_OS5_st1_1143.tif]

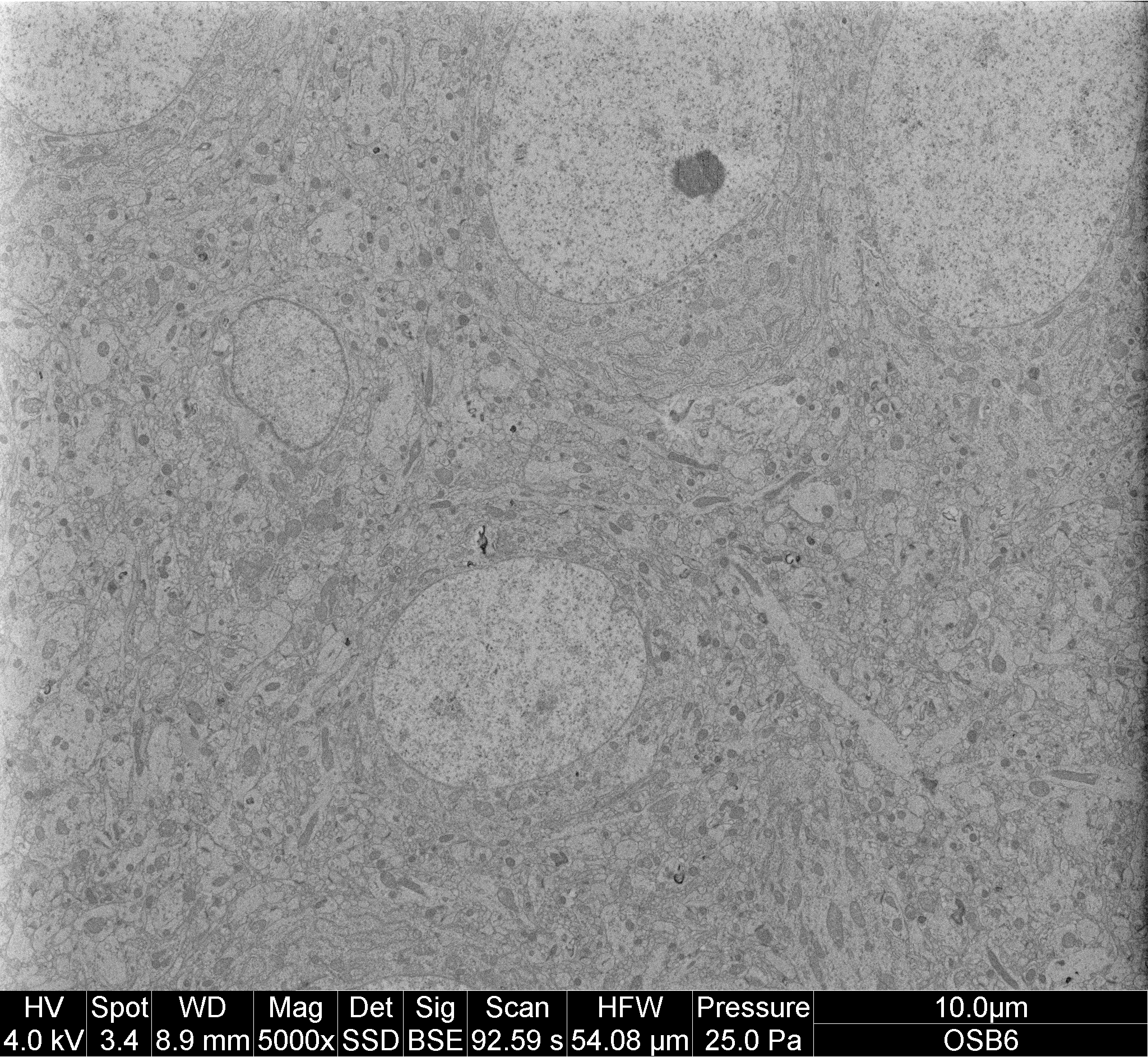

Supplement: Dataset S12 — (252.6 MB ZIP). [file pbio.0020329.sd012.zip › 040604_OS5_st1_1144.tif]

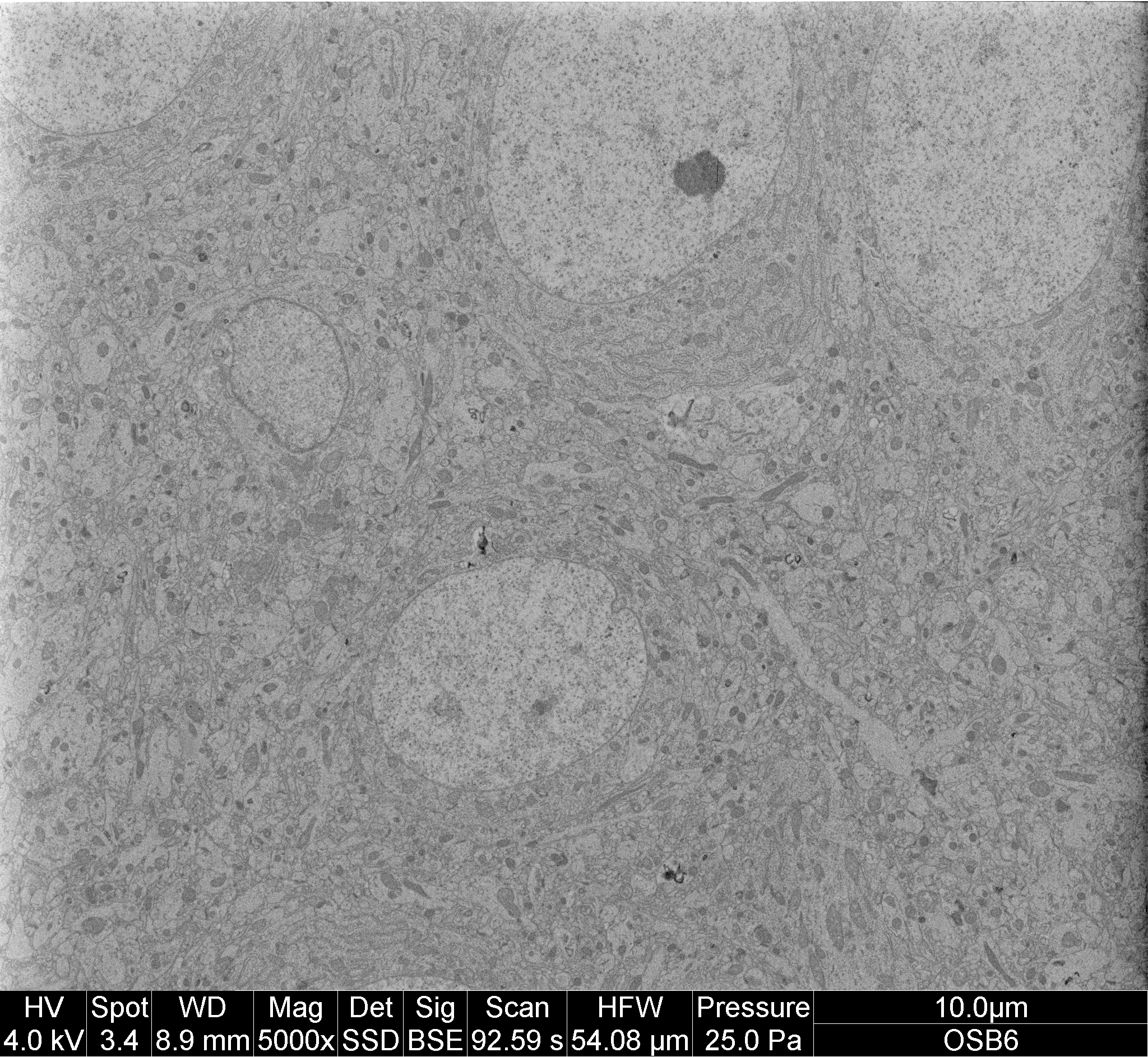

Supplement: Dataset S12 — (252.6 MB ZIP). [file pbio.0020329.sd012.zip › 040604_OS5_st1_1145.tif]

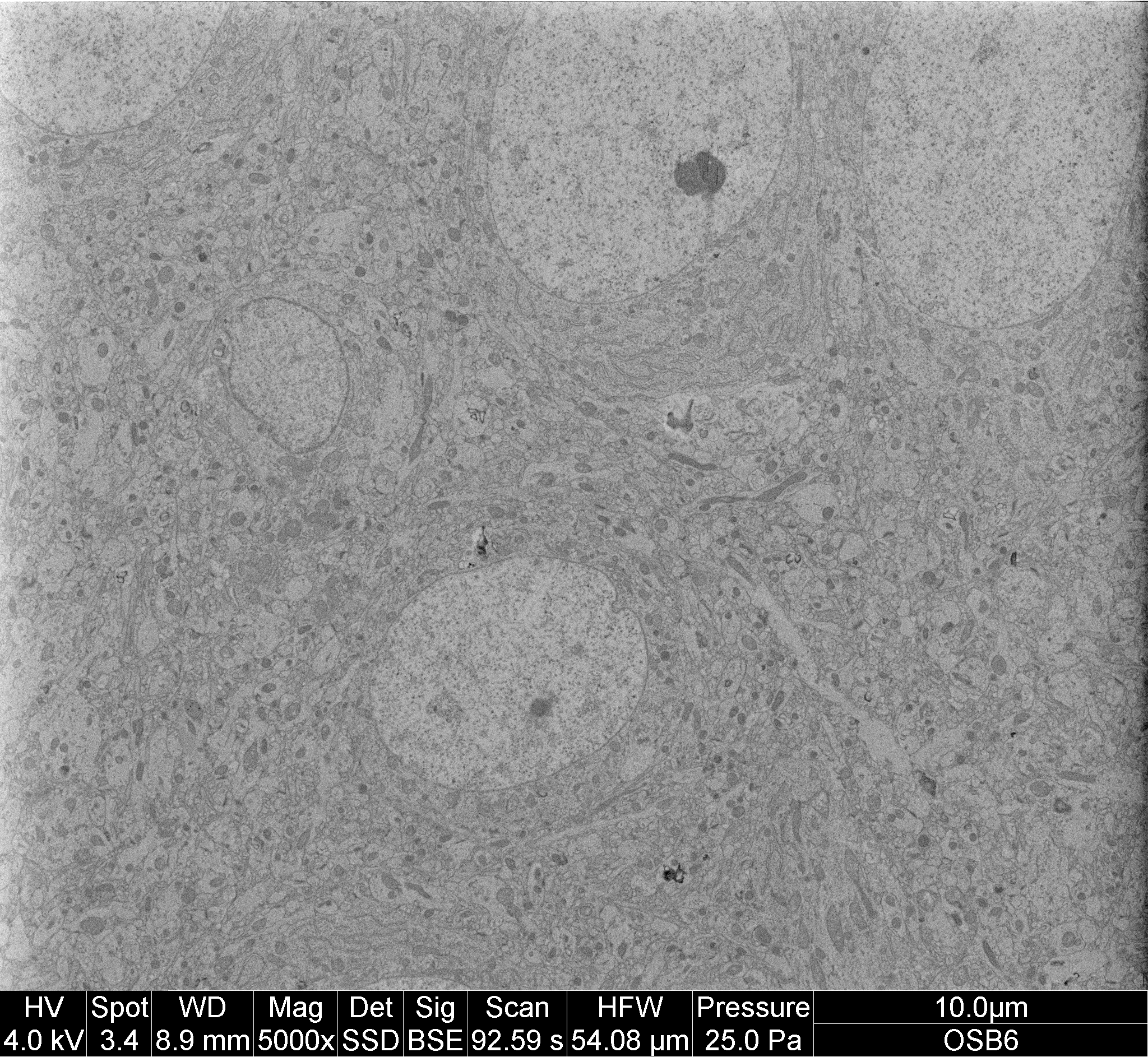

Supplement: Dataset S12 — (252.6 MB ZIP). [file pbio.0020329.sd012.zip › 040604_OS5_st1_1146.tif]

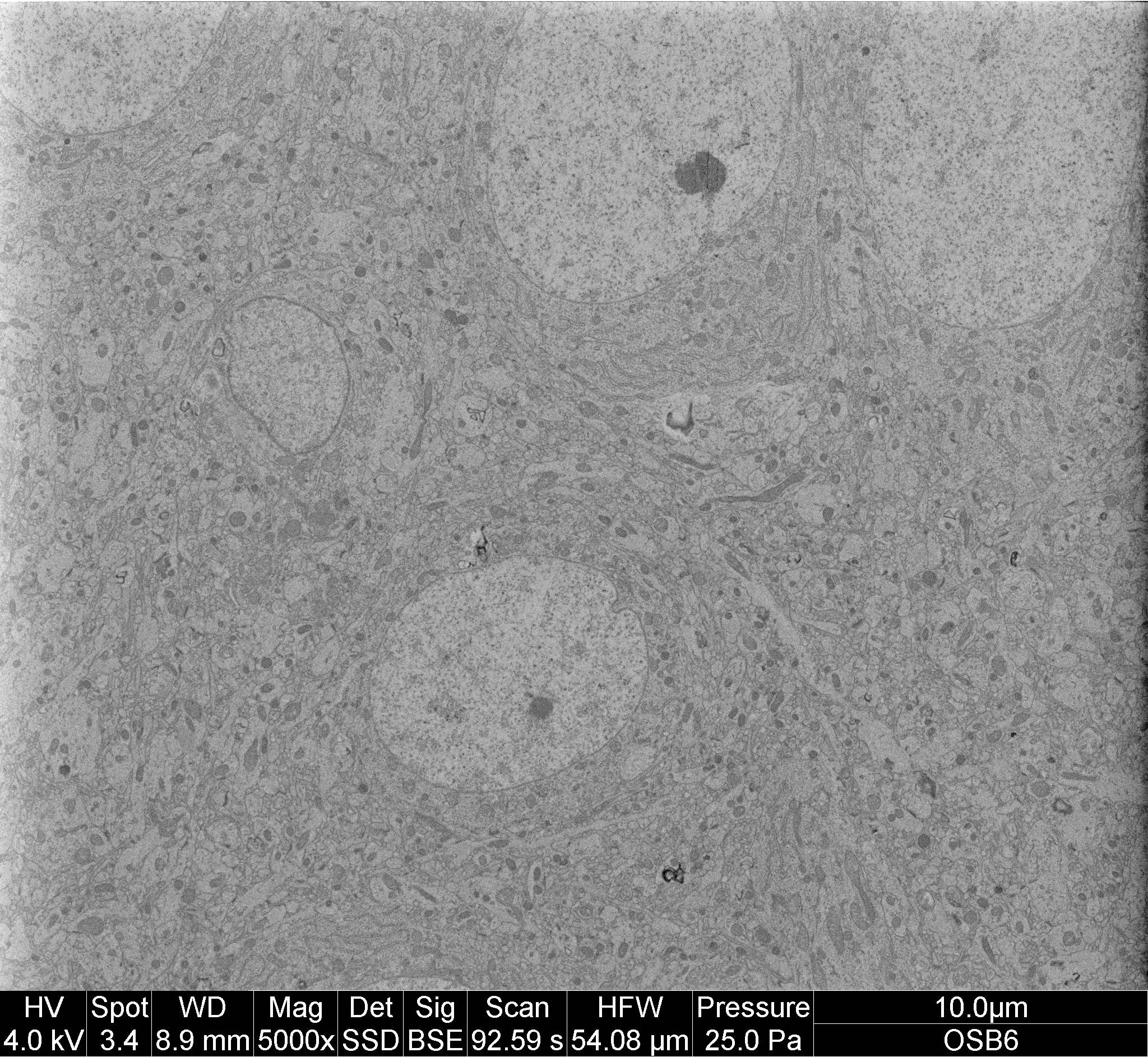

Supplement: Dataset S12 — (252.6 MB ZIP). [file pbio.0020329.sd012.zip › 040604_OS5_st1_1147.tif]

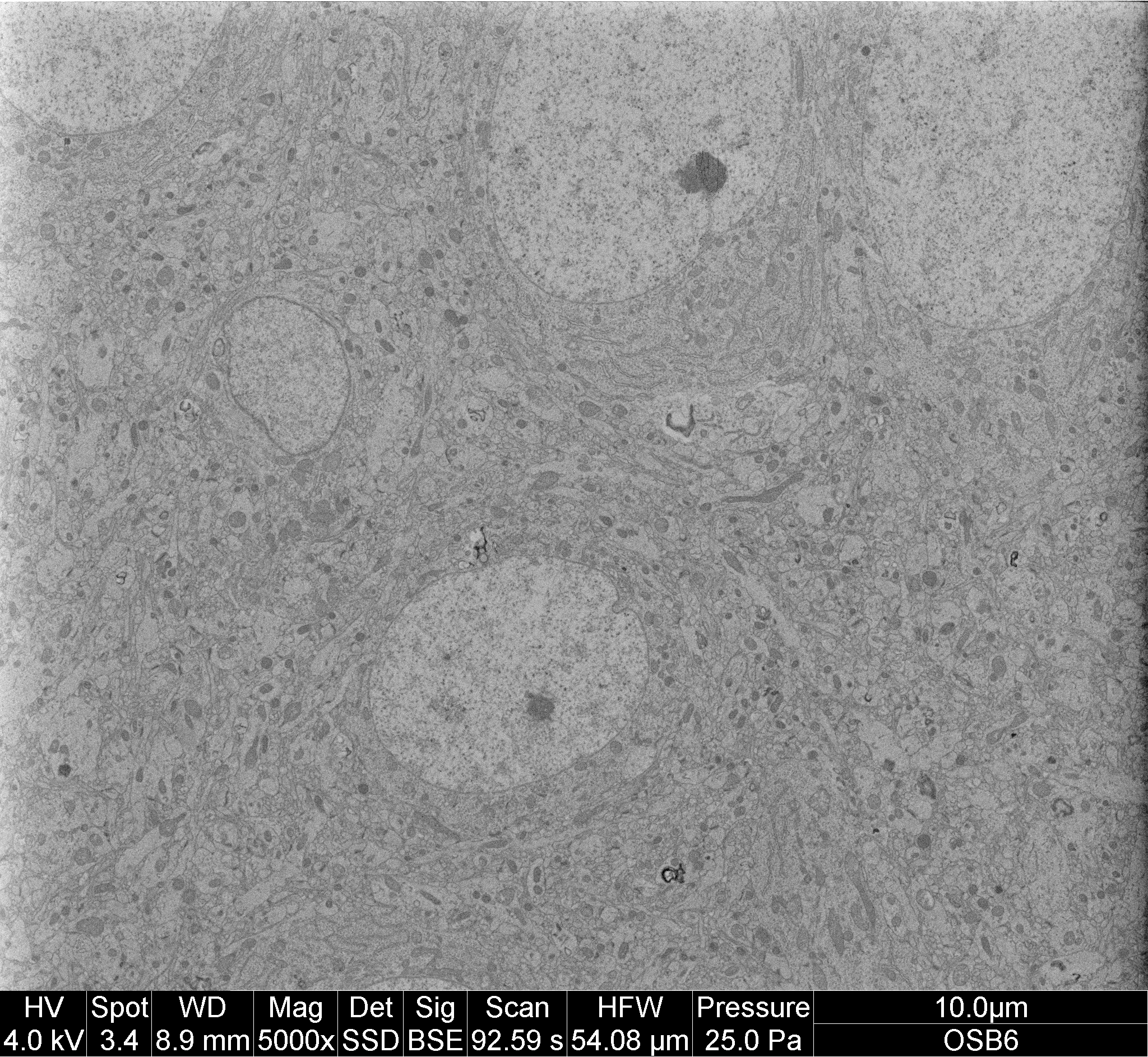

Supplement: Dataset S12 — (252.6 MB ZIP). [file pbio.0020329.sd012.zip › 040604_OS5_st1_1148.tif]

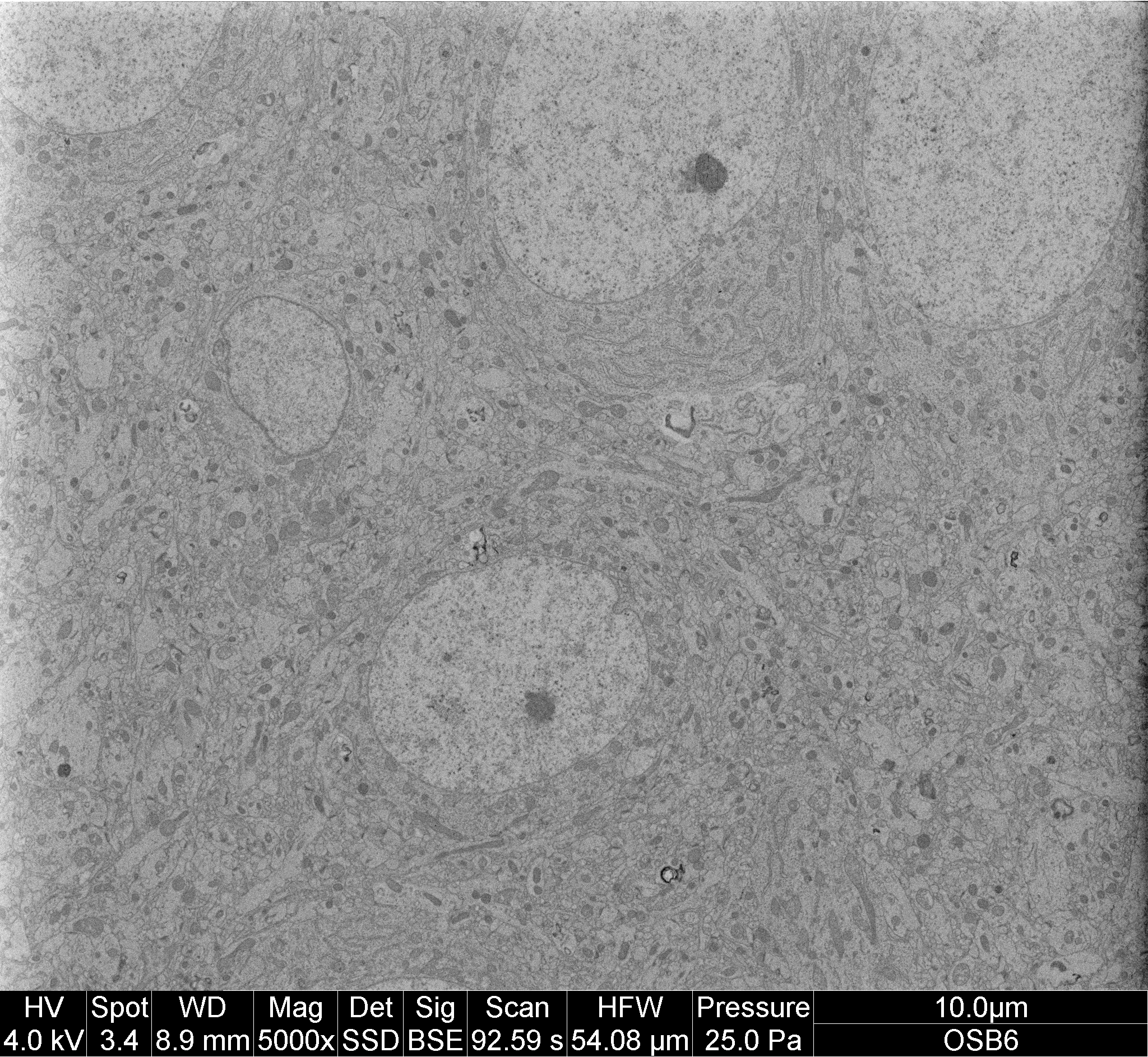

Supplement: Dataset S12 — (252.6 MB ZIP). [file pbio.0020329.sd012.zip › 040604_OS5_st1_1149.tif]

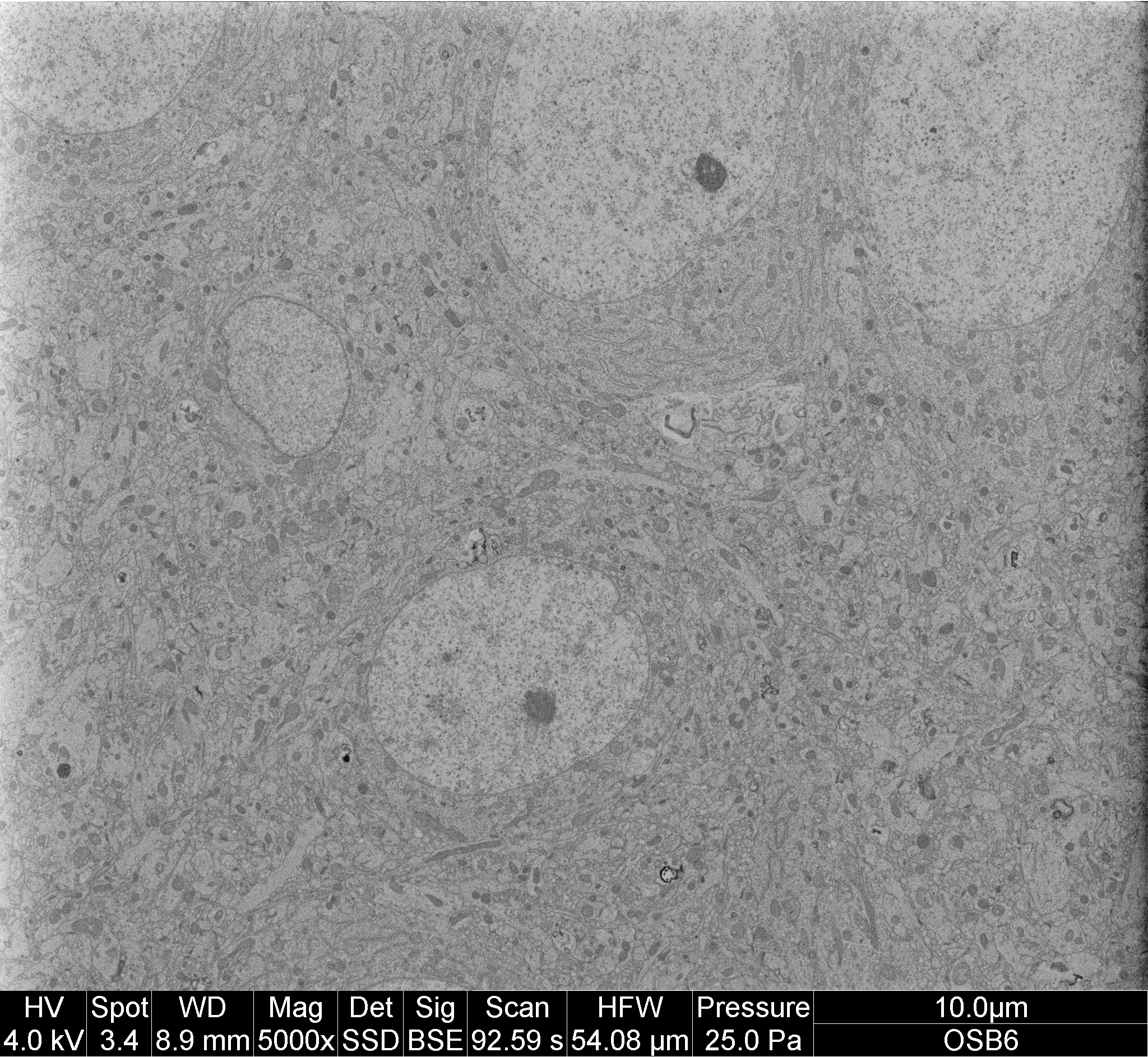

Supplement: Dataset S12 — (252.6 MB ZIP). [file pbio.0020329.sd012.zip › 040604_OS5_st1_1150.tif]

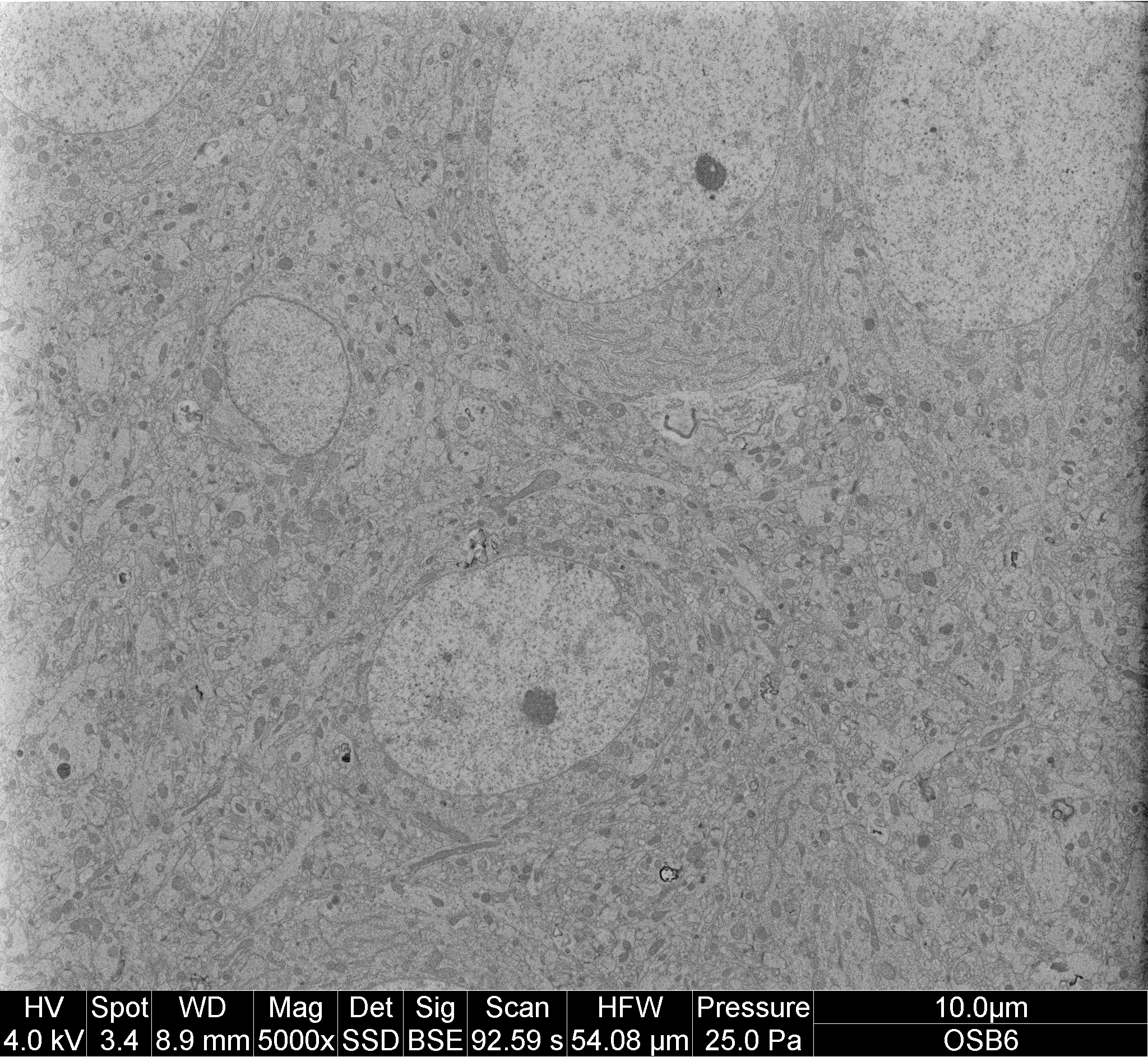

Supplement: Dataset S12 — (252.6 MB ZIP). [file pbio.0020329.sd012.zip › 040604_OS5_st1_1151.tif]

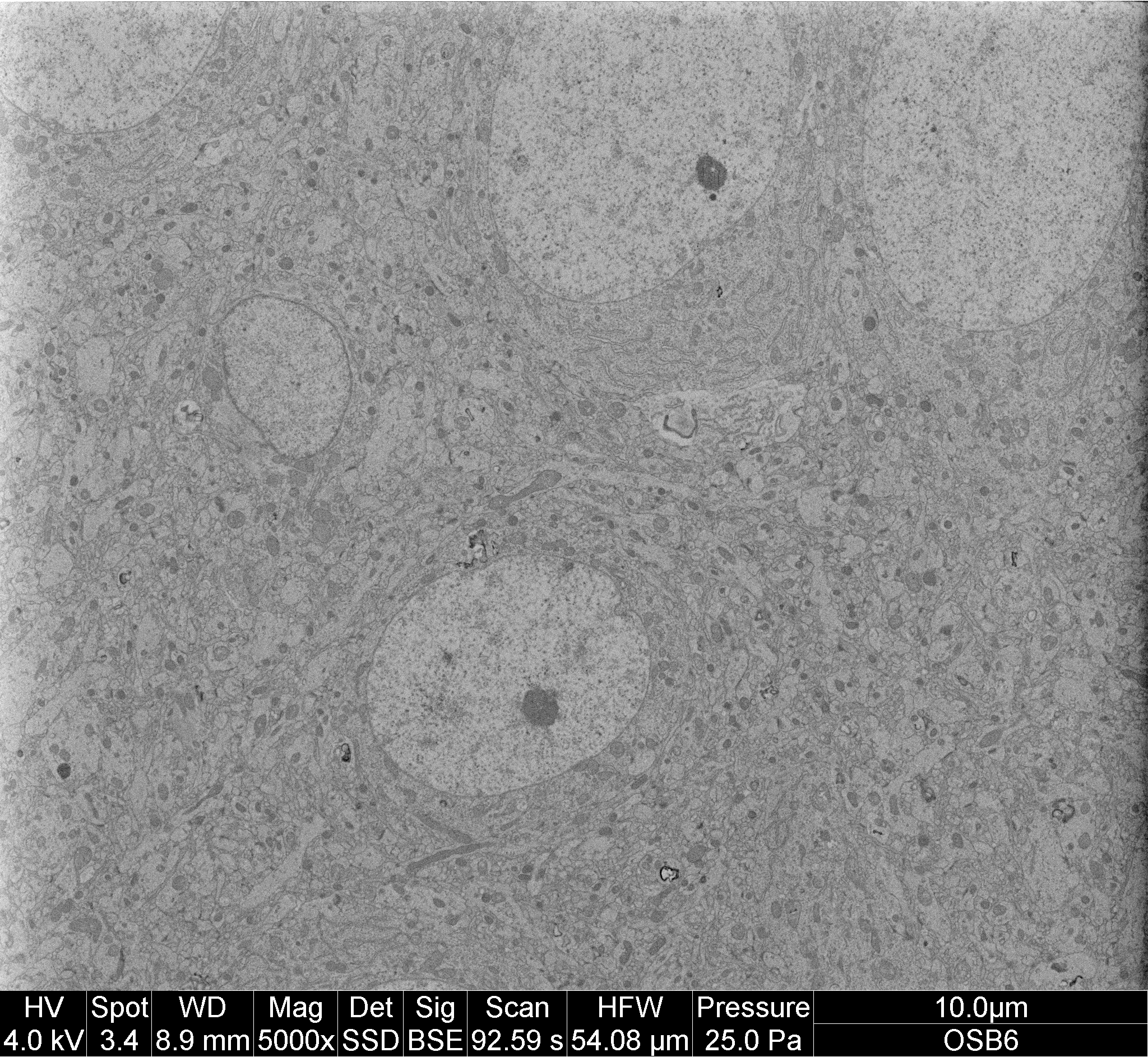

Supplement: Dataset S12 — (252.6 MB ZIP). [file pbio.0020329.sd012.zip › 040604_OS5_st1_1152.tif]

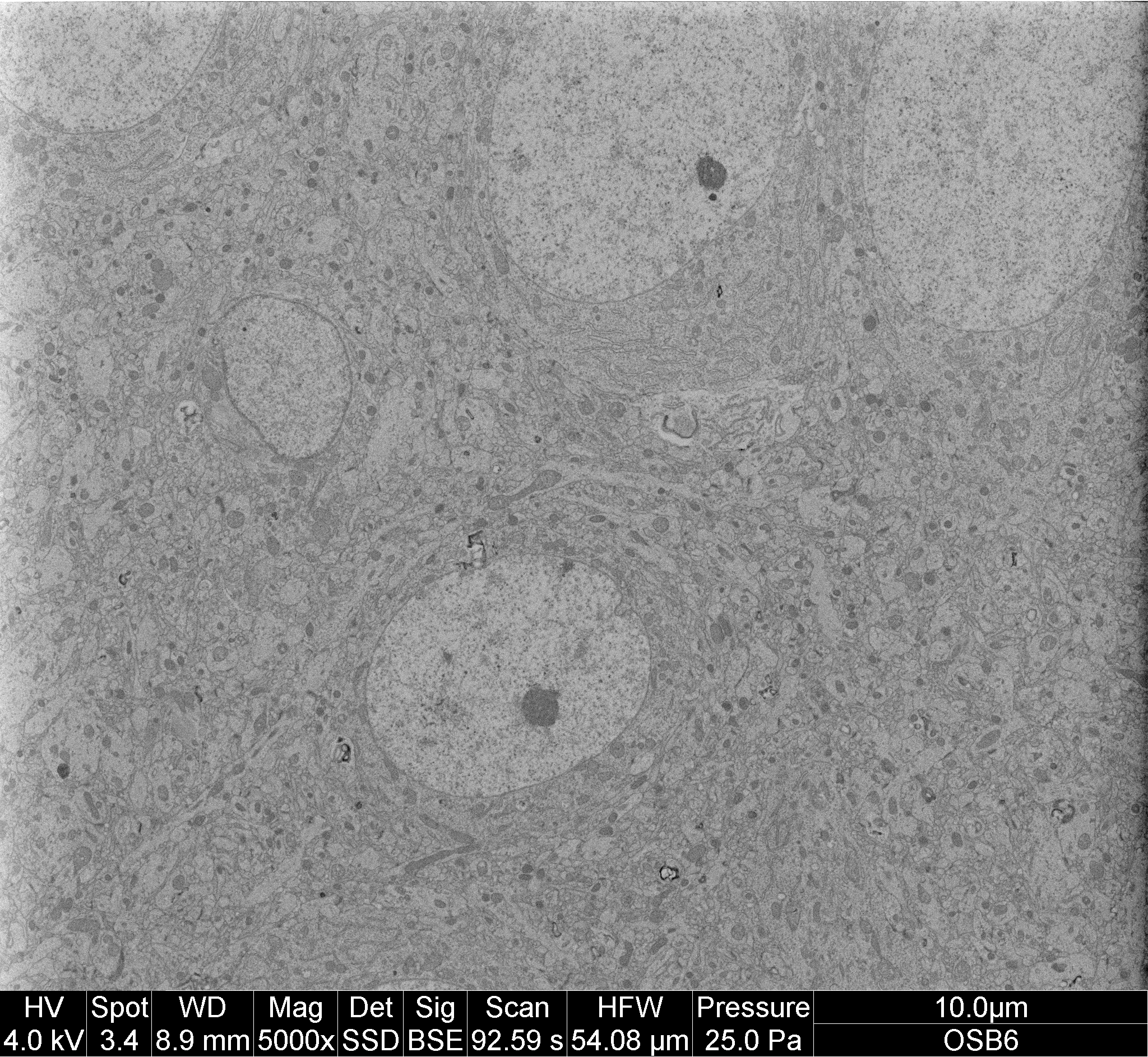

Supplement: Dataset S12 — (252.6 MB ZIP). [file pbio.0020329.sd012.zip › 040604_OS5_st1_1153.tif]

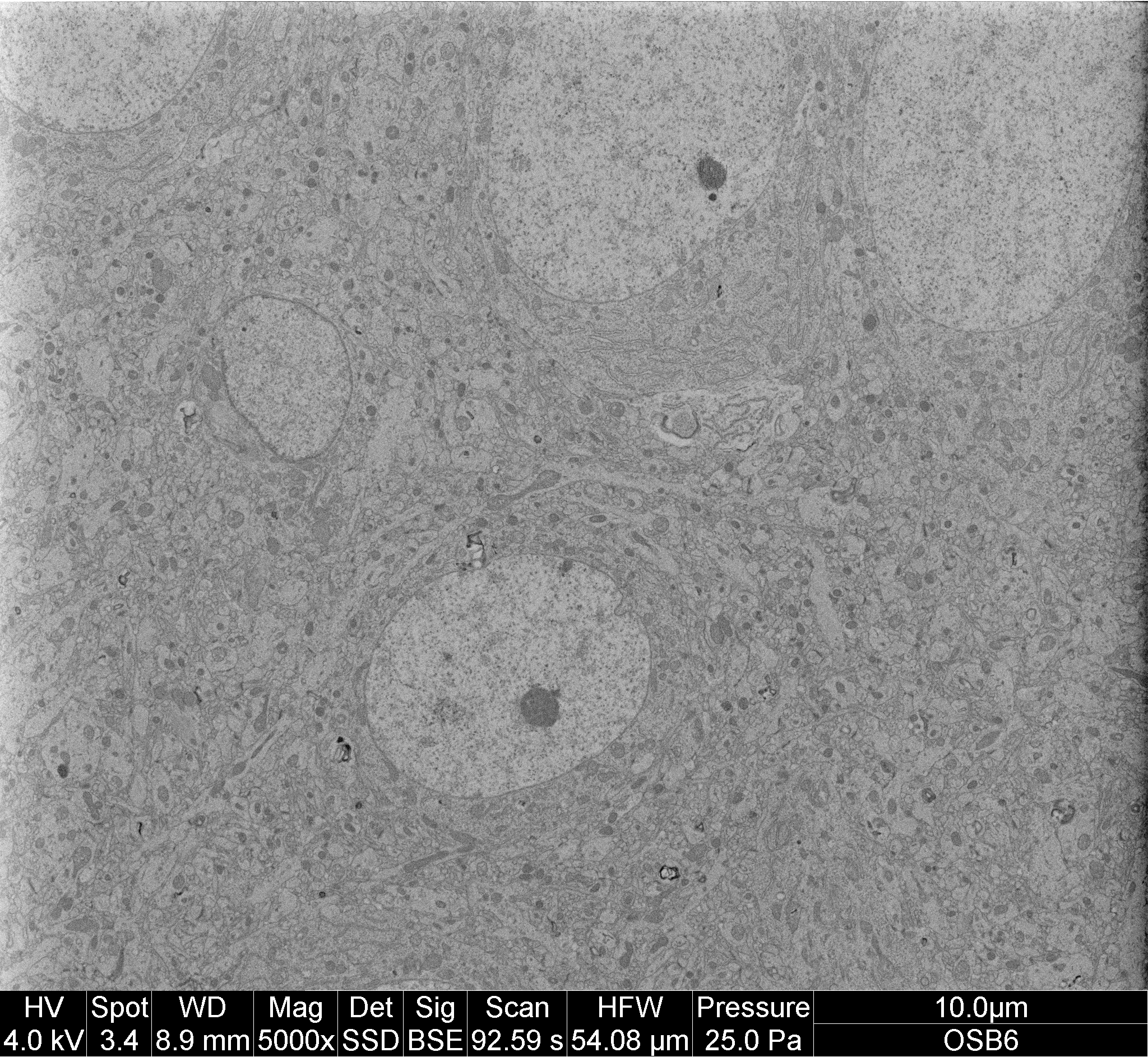

Supplement: Dataset S12 — (252.6 MB ZIP). [file pbio.0020329.sd012.zip › 040604_OS5_st1_1154.tif]

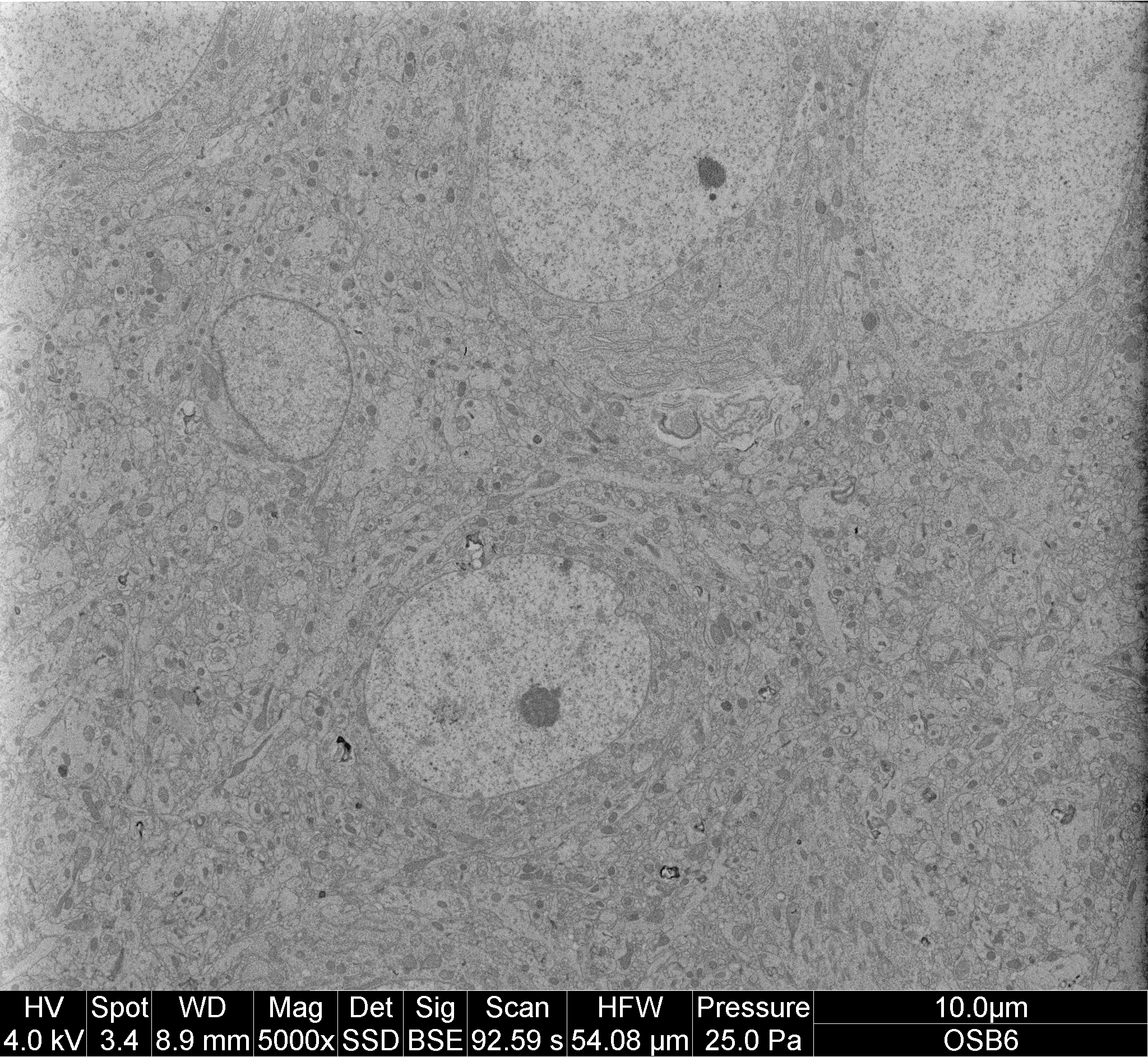

Supplement: Dataset S12 — (252.6 MB ZIP). [file pbio.0020329.sd012.zip › 040604_OS5_st1_1155.tif]

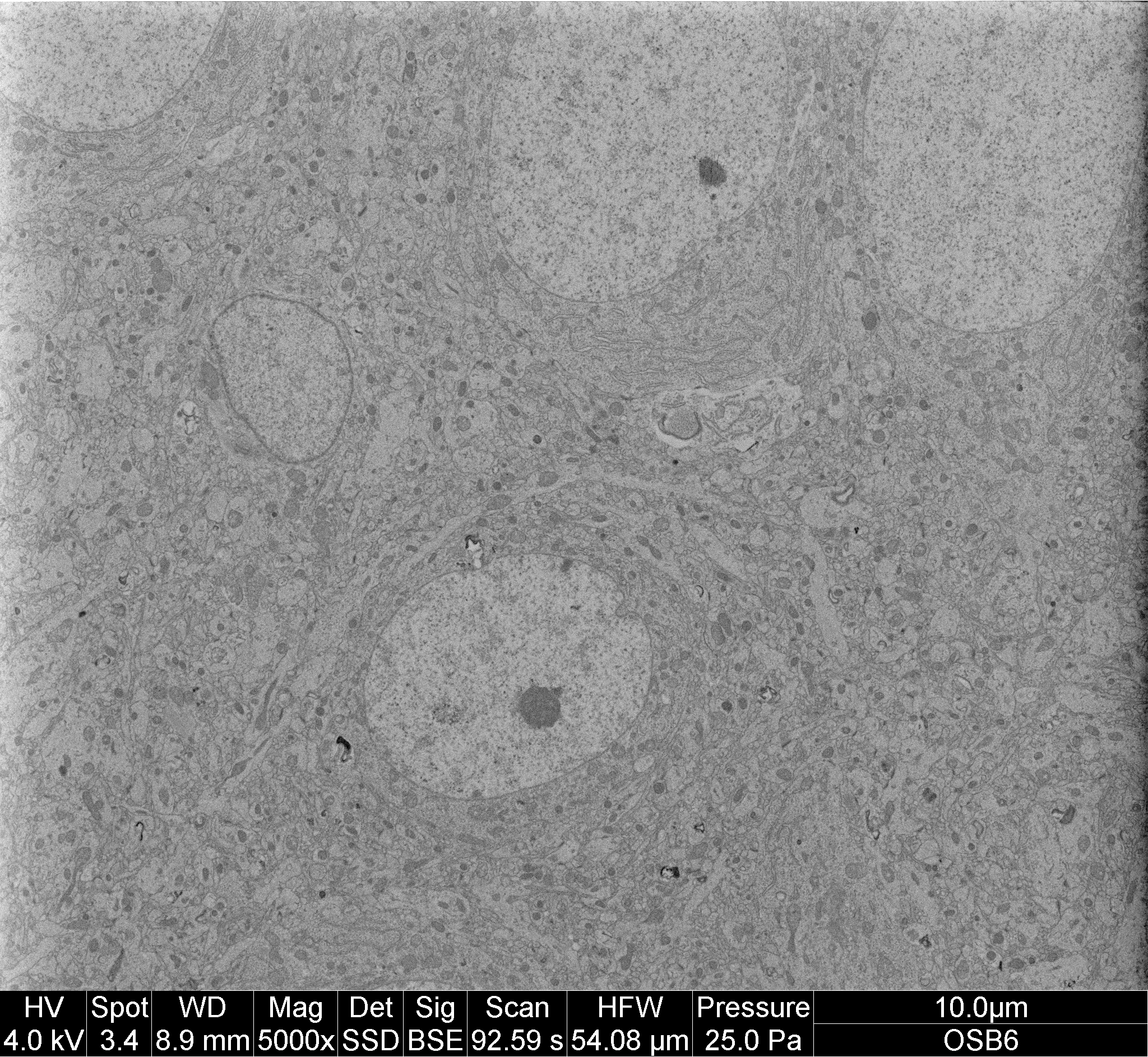

Supplement: Dataset S12 — (252.6 MB ZIP). [file pbio.0020329.sd012.zip › 040604_OS5_st1_1156.tif]

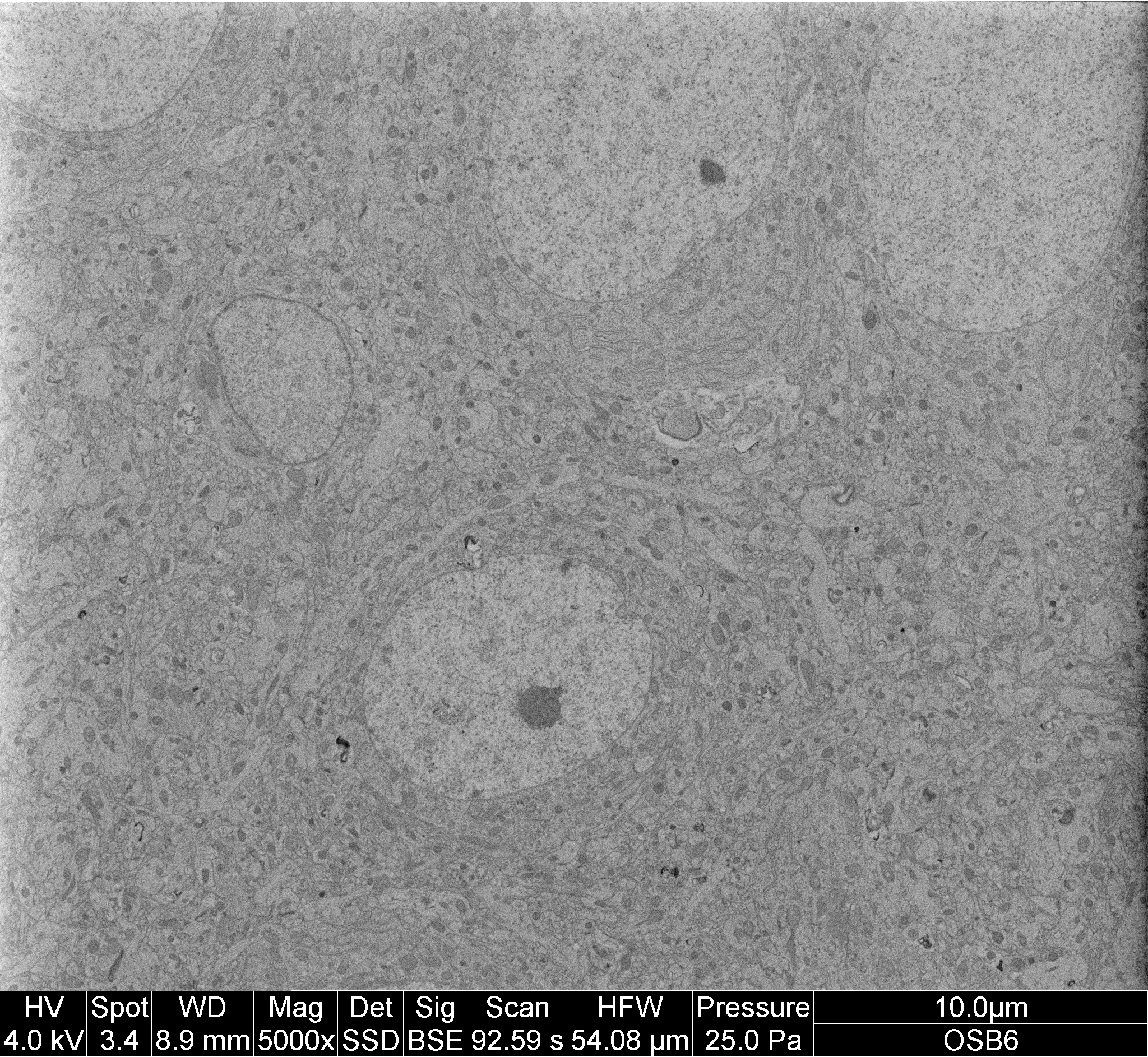

Supplement: Dataset S12 — (252.6 MB ZIP). [file pbio.0020329.sd012.zip › 040604_OS5_st1_1157.tif]

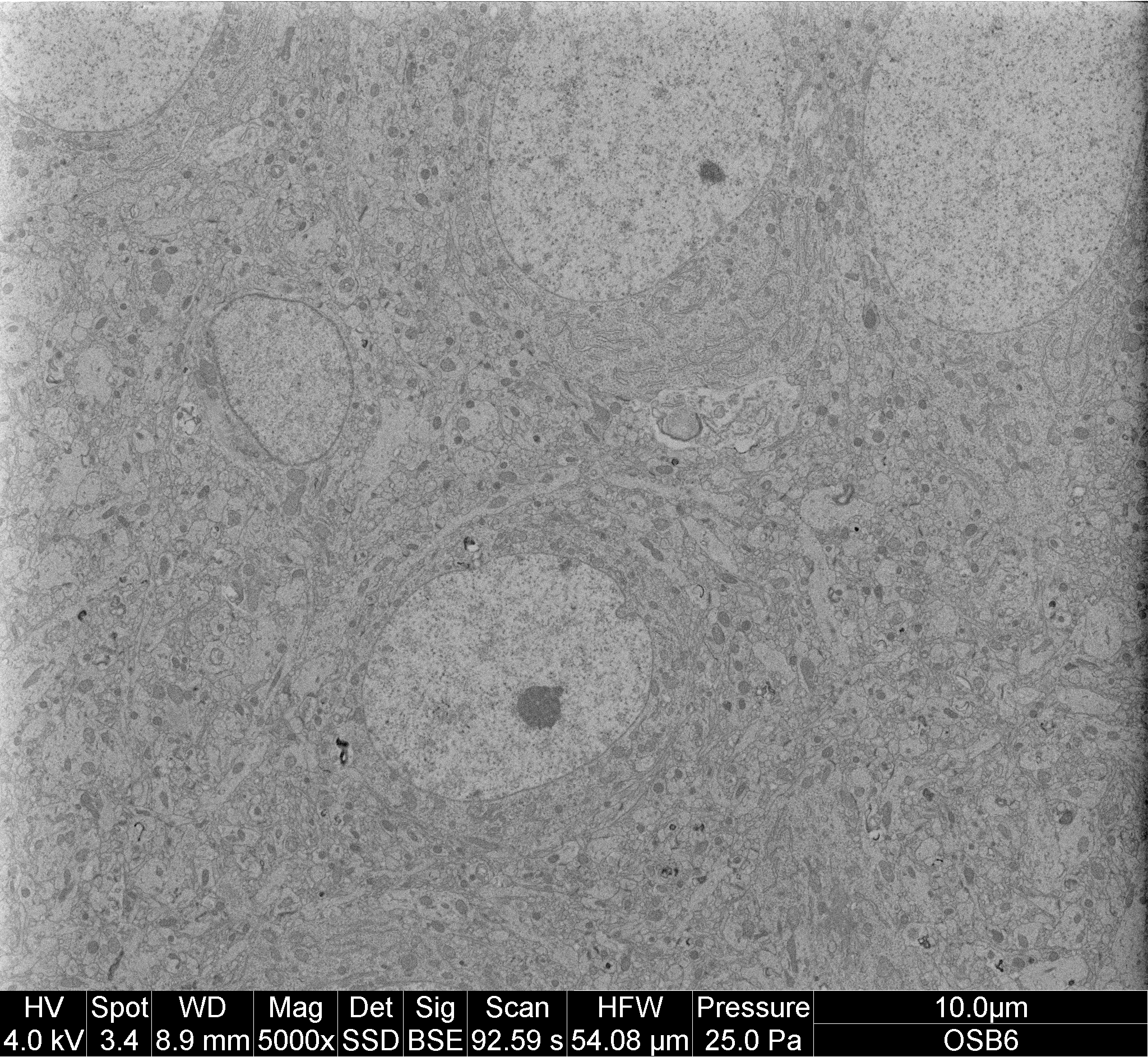

Supplement: Dataset S12 — (252.6 MB ZIP). [file pbio.0020329.sd012.zip › 040604_OS5_st1_1158.tif]

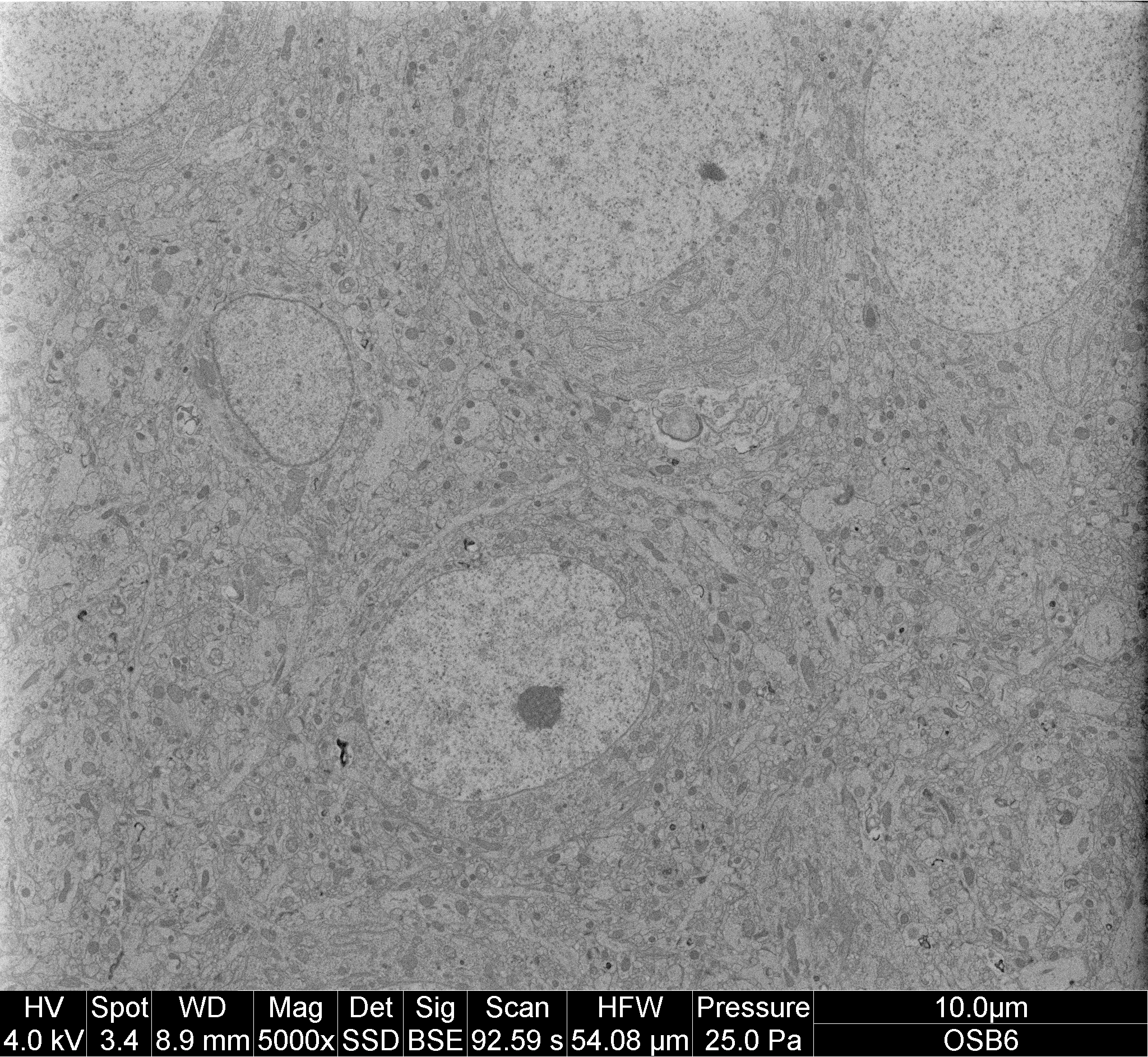

Supplement: Dataset S12 — (252.6 MB ZIP). [file pbio.0020329.sd012.zip › 040604_OS5_st1_1159.tif]

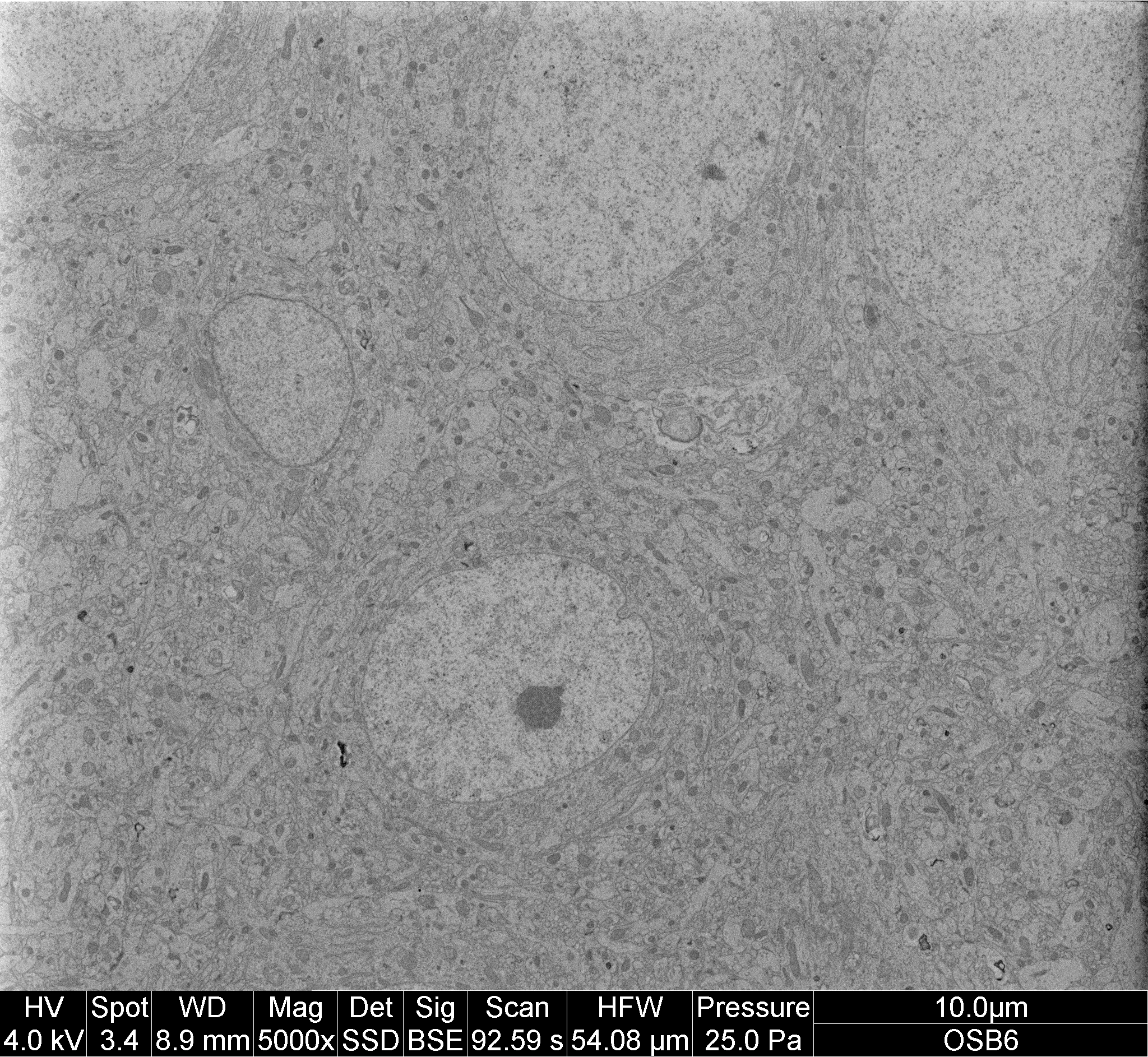

Supplement: Dataset S12 — (252.6 MB ZIP). [file pbio.0020329.sd012.zip › 040604_OS5_st1_1160.tif]

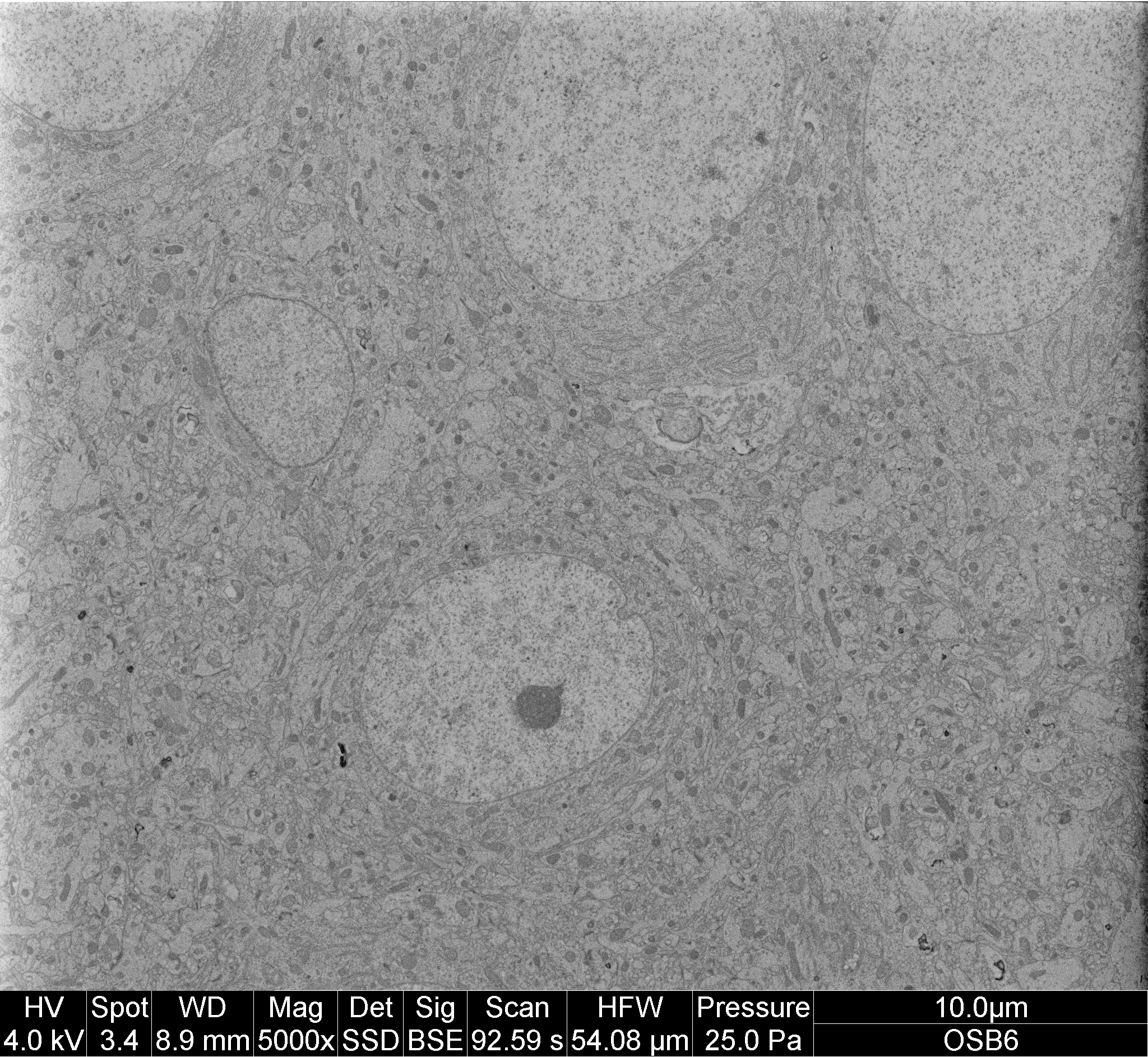

Supplement: Dataset S12 — (252.6 MB ZIP). [file pbio.0020329.sd012.zip › 040604_OS5_st1_1161.tif]

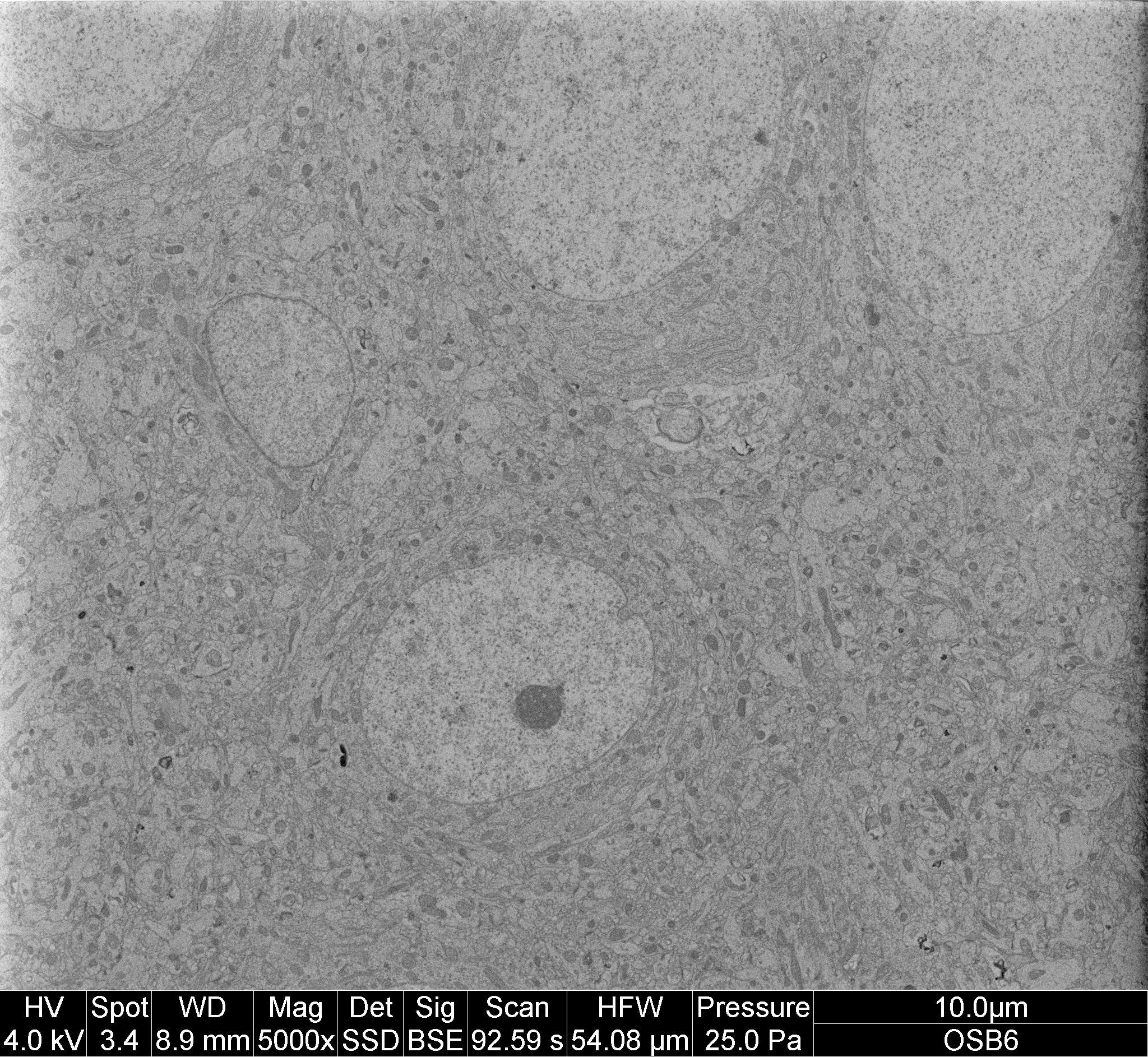

Supplement: Dataset S12 — (252.6 MB ZIP). [file pbio.0020329.sd012.zip › 040604_OS5_st1_1162.tif]

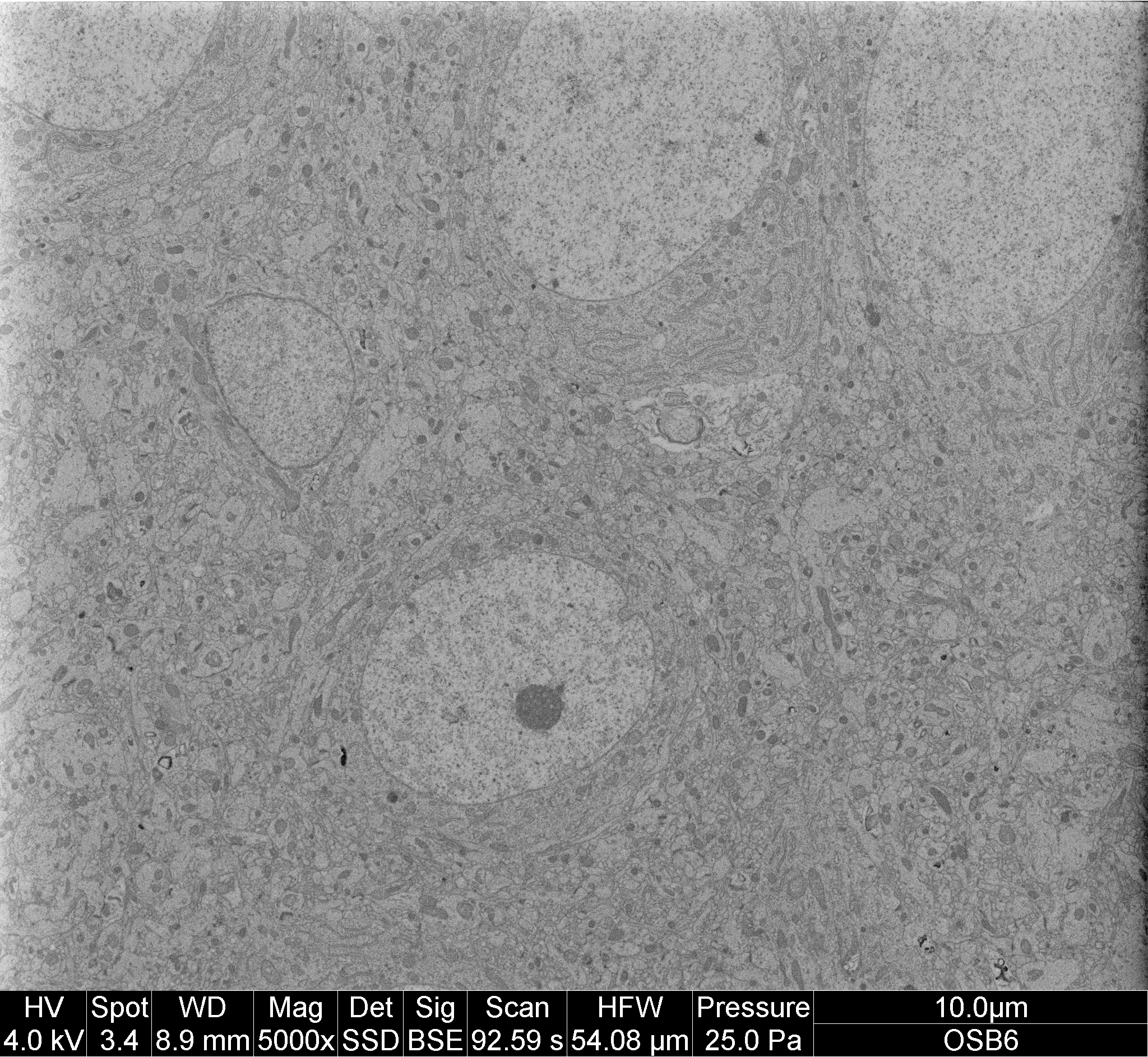

Supplement: Dataset S12 — (252.6 MB ZIP). [file pbio.0020329.sd012.zip › 040604_OS5_st1_1163.tif]

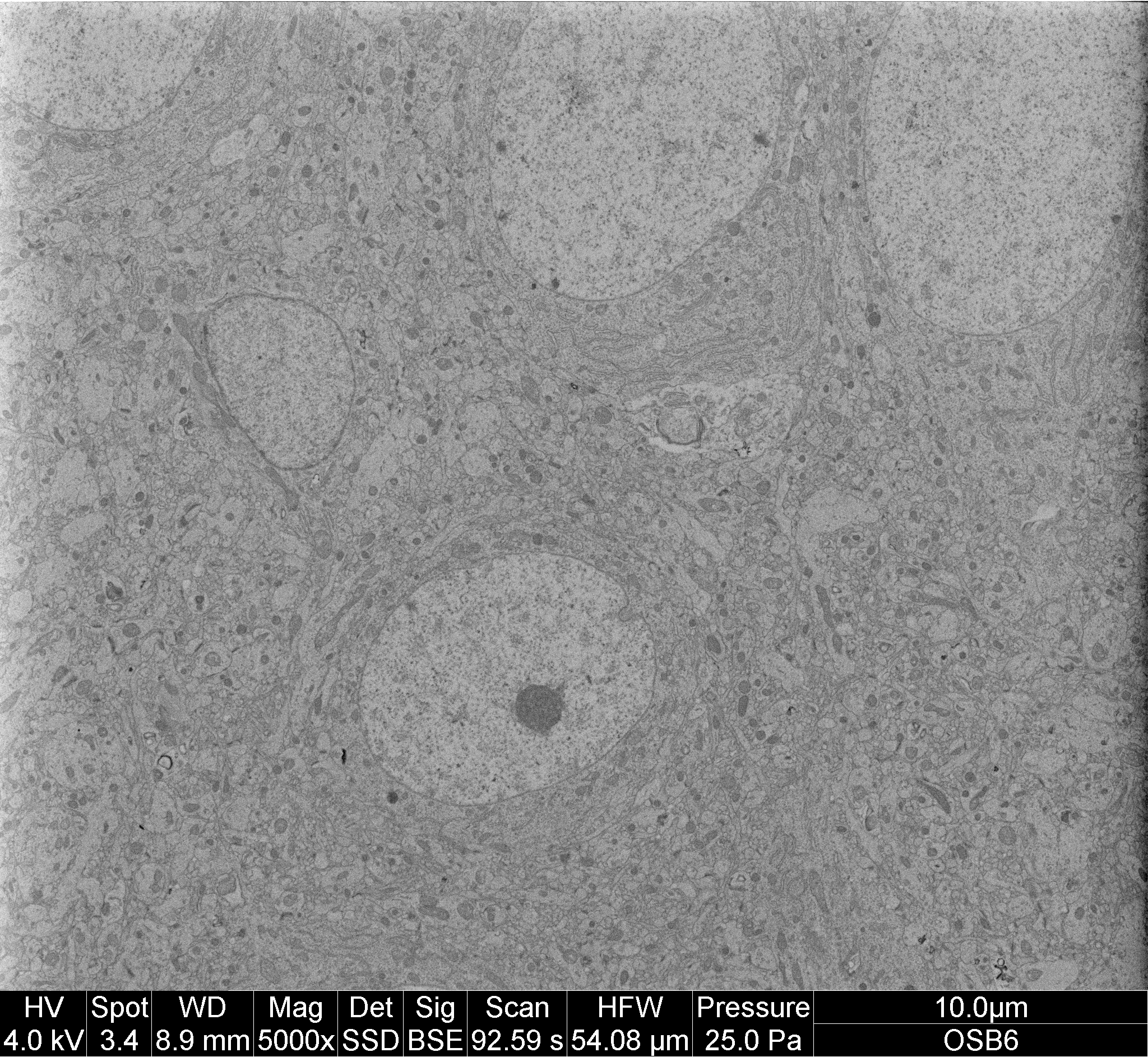

Supplement: Dataset S12 — (252.6 MB ZIP). [file pbio.0020329.sd012.zip › 040604_OS5_st1_1164.tif]

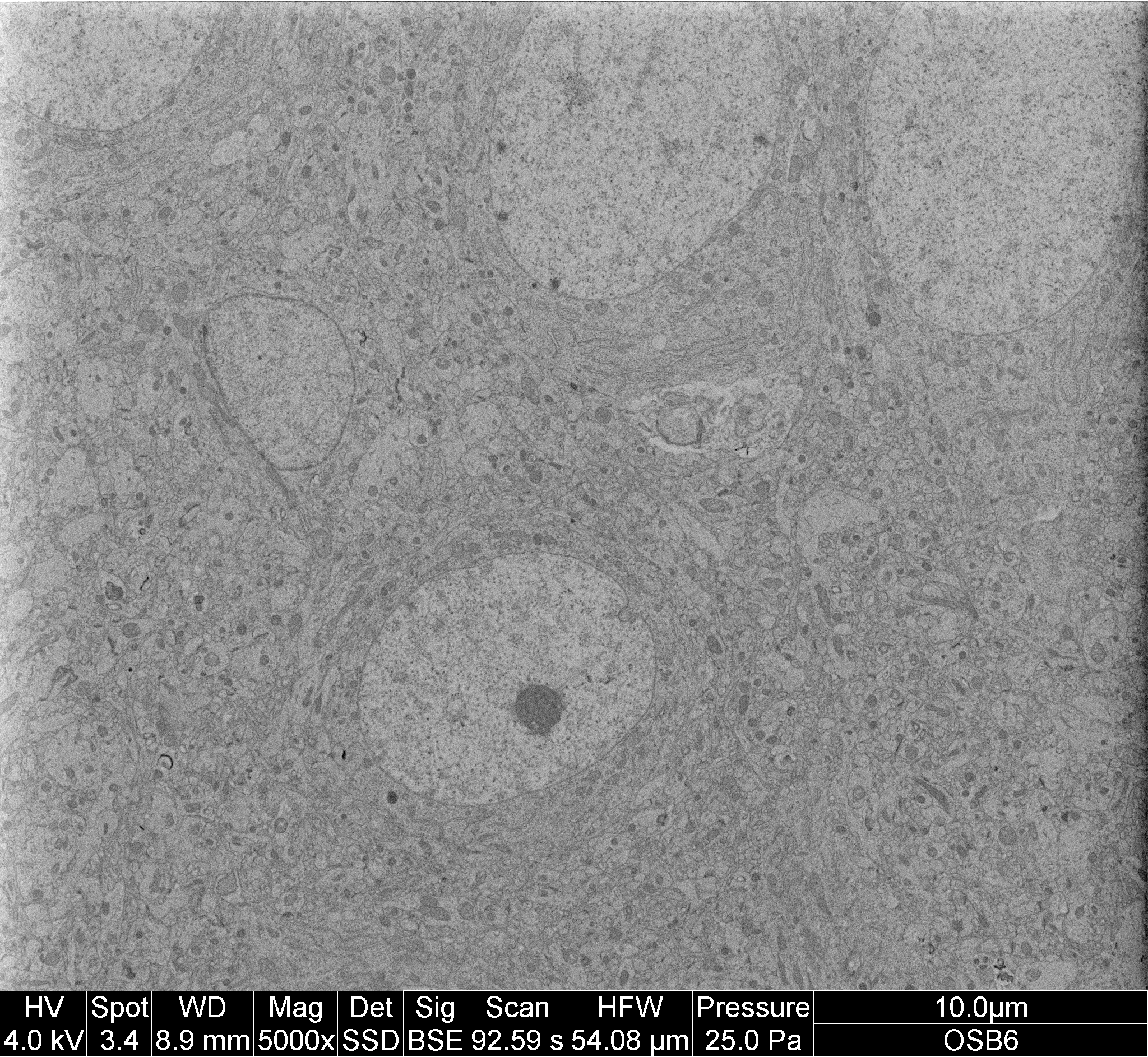

Supplement: Dataset S12 — (252.6 MB ZIP). [file pbio.0020329.sd012.zip › 040604_OS5_st1_1165.tif]

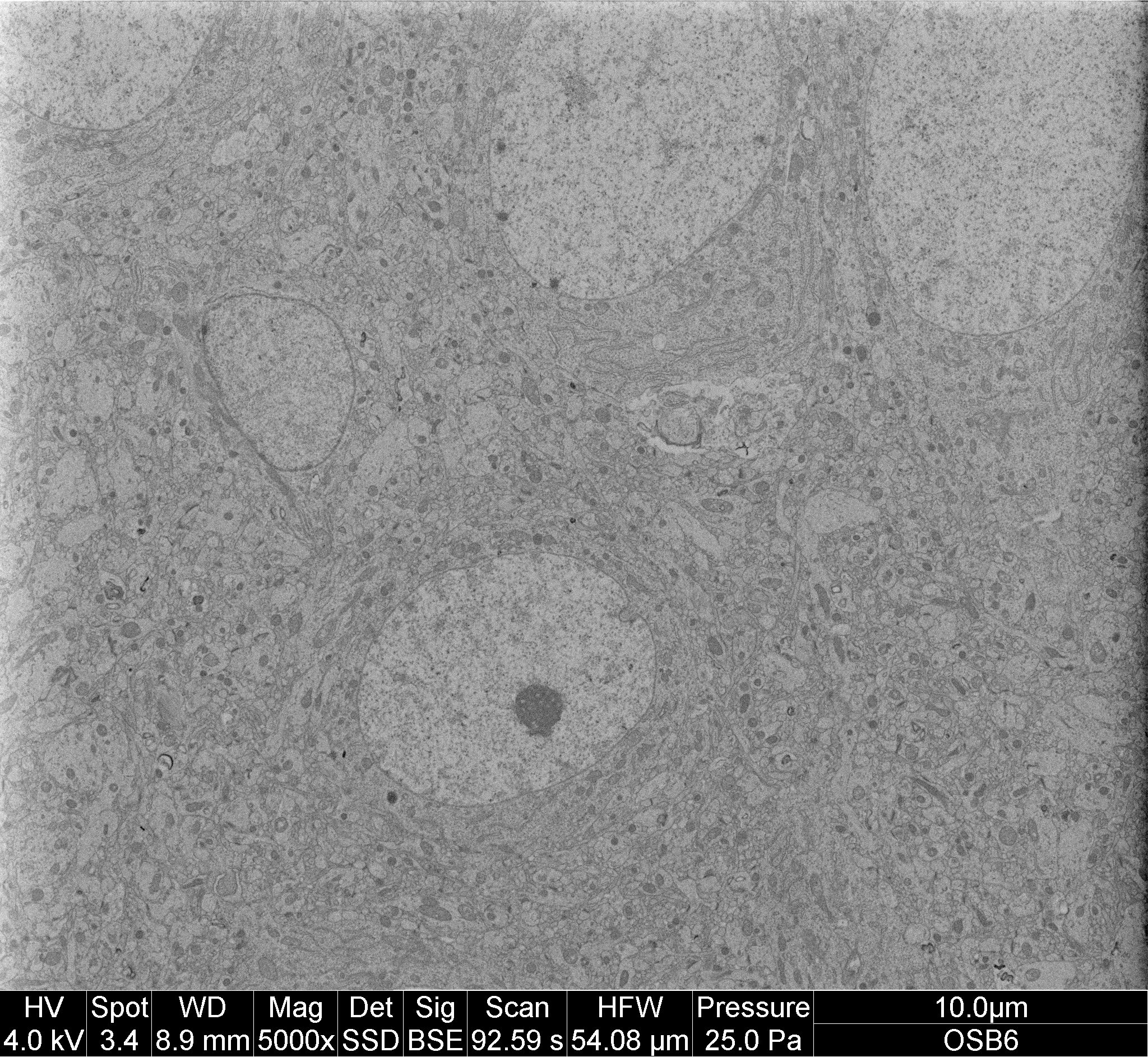

Supplement: Dataset S12 — (252.6 MB ZIP). [file pbio.0020329.sd012.zip › 040604_OS5_st1_1166.tif]

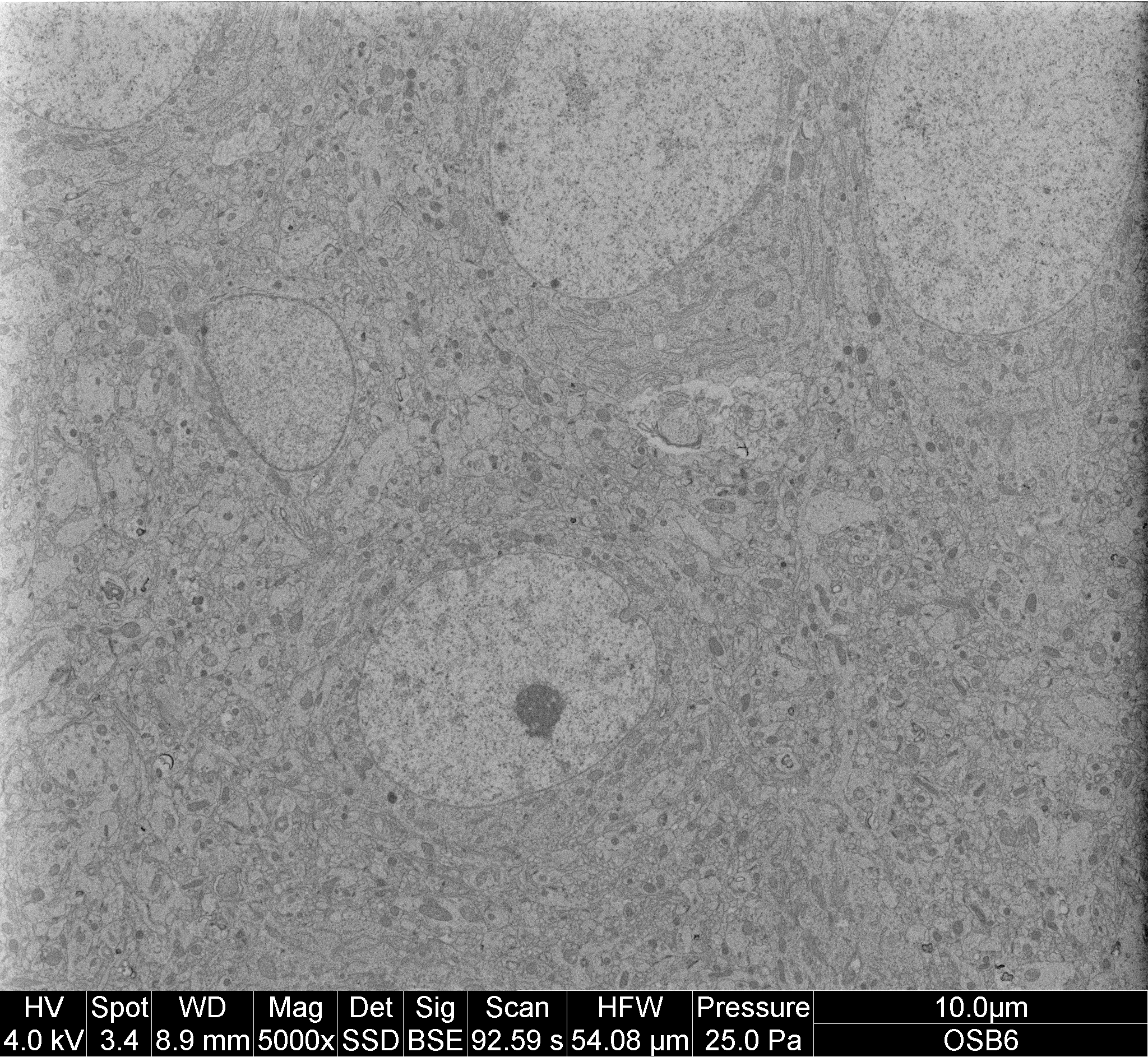

Supplement: Dataset S12 — (252.6 MB ZIP). [file pbio.0020329.sd012.zip › 040604_OS5_st1_1167.tif]

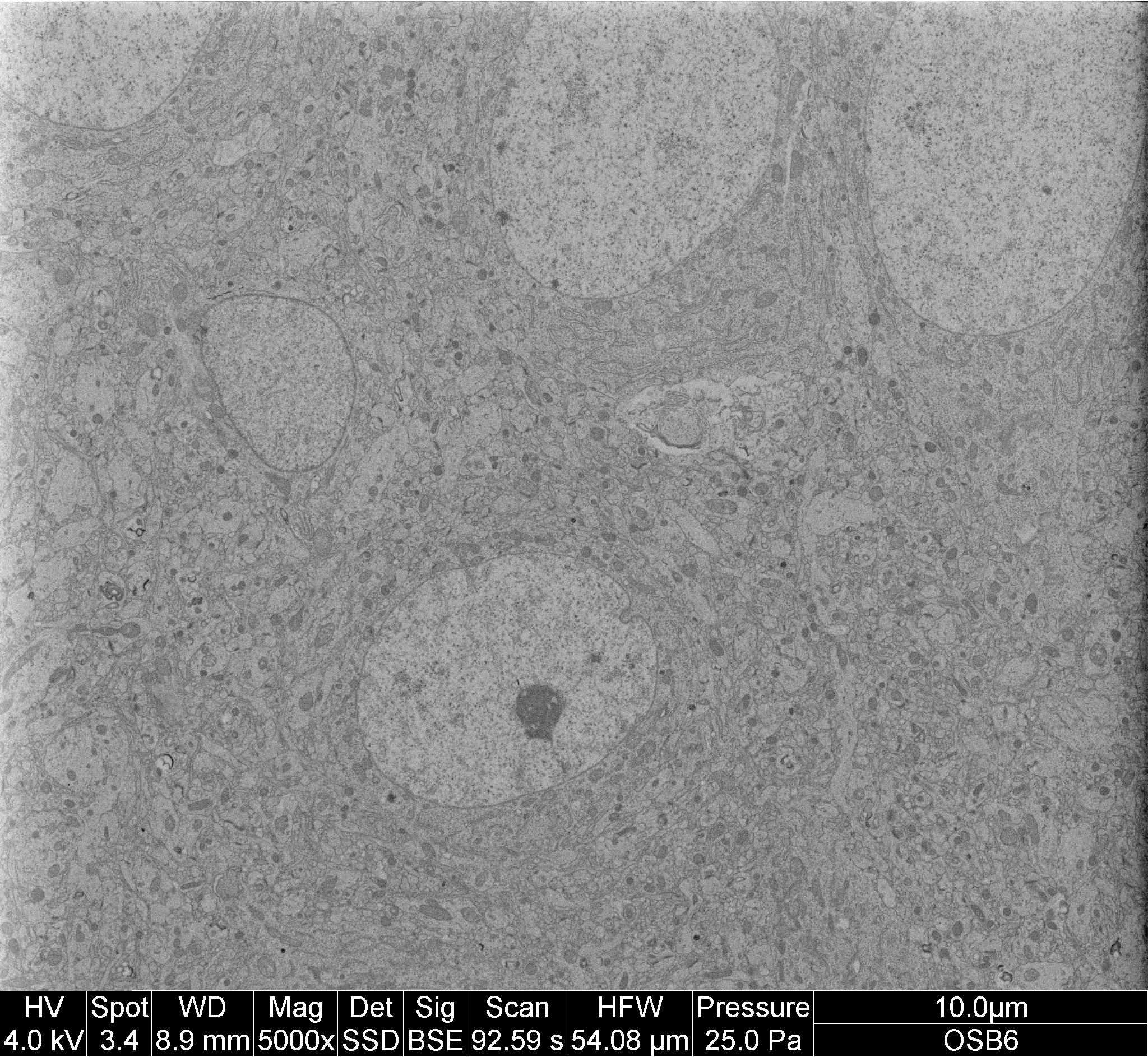

Supplement: Dataset S12 — (252.6 MB ZIP). [file pbio.0020329.sd012.zip › 040604_OS5_st1_1168.tif]

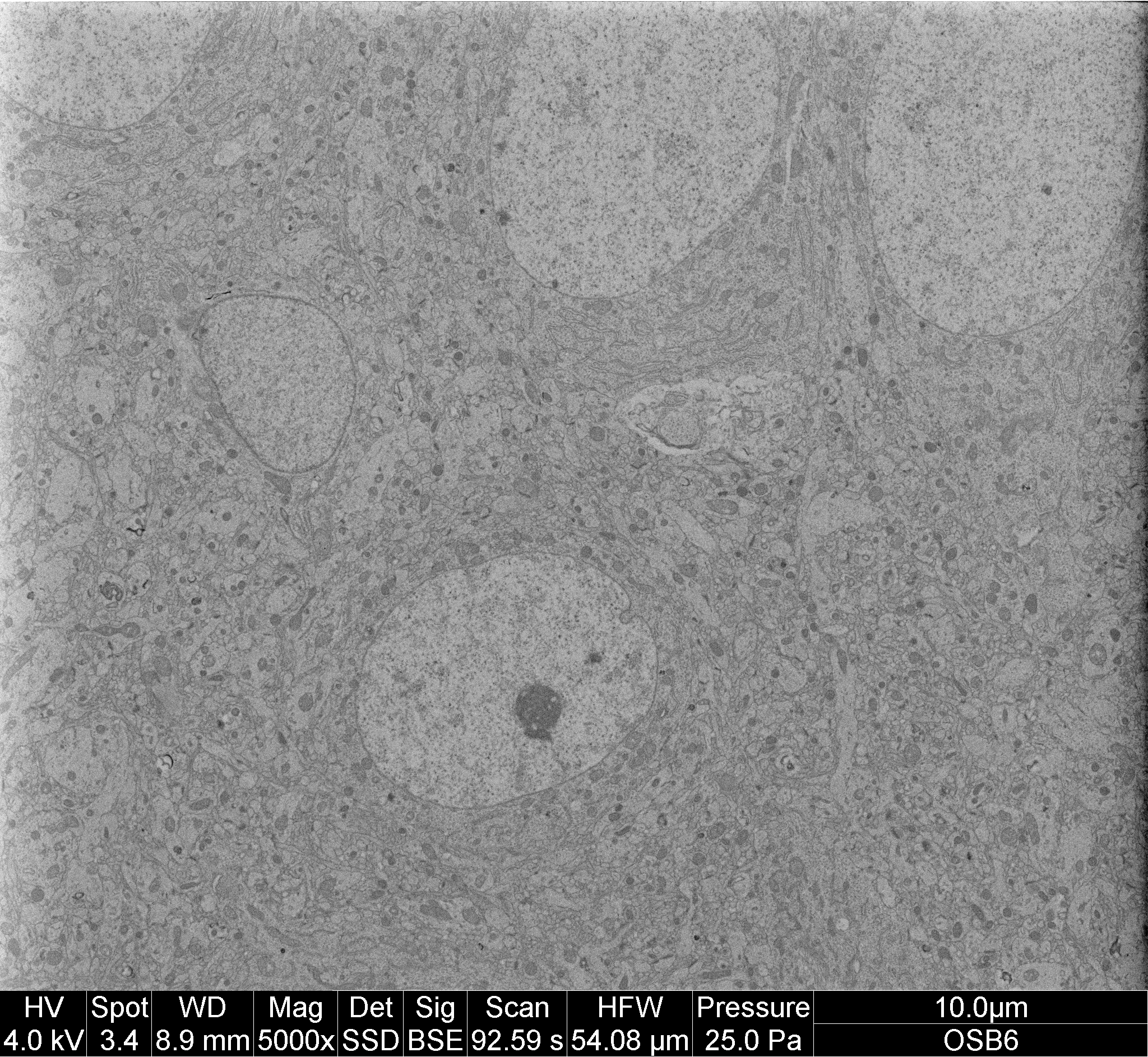

Supplement: Dataset S12 — (252.6 MB ZIP). [file pbio.0020329.sd012.zip › 040604_OS5_st1_1169.tif]

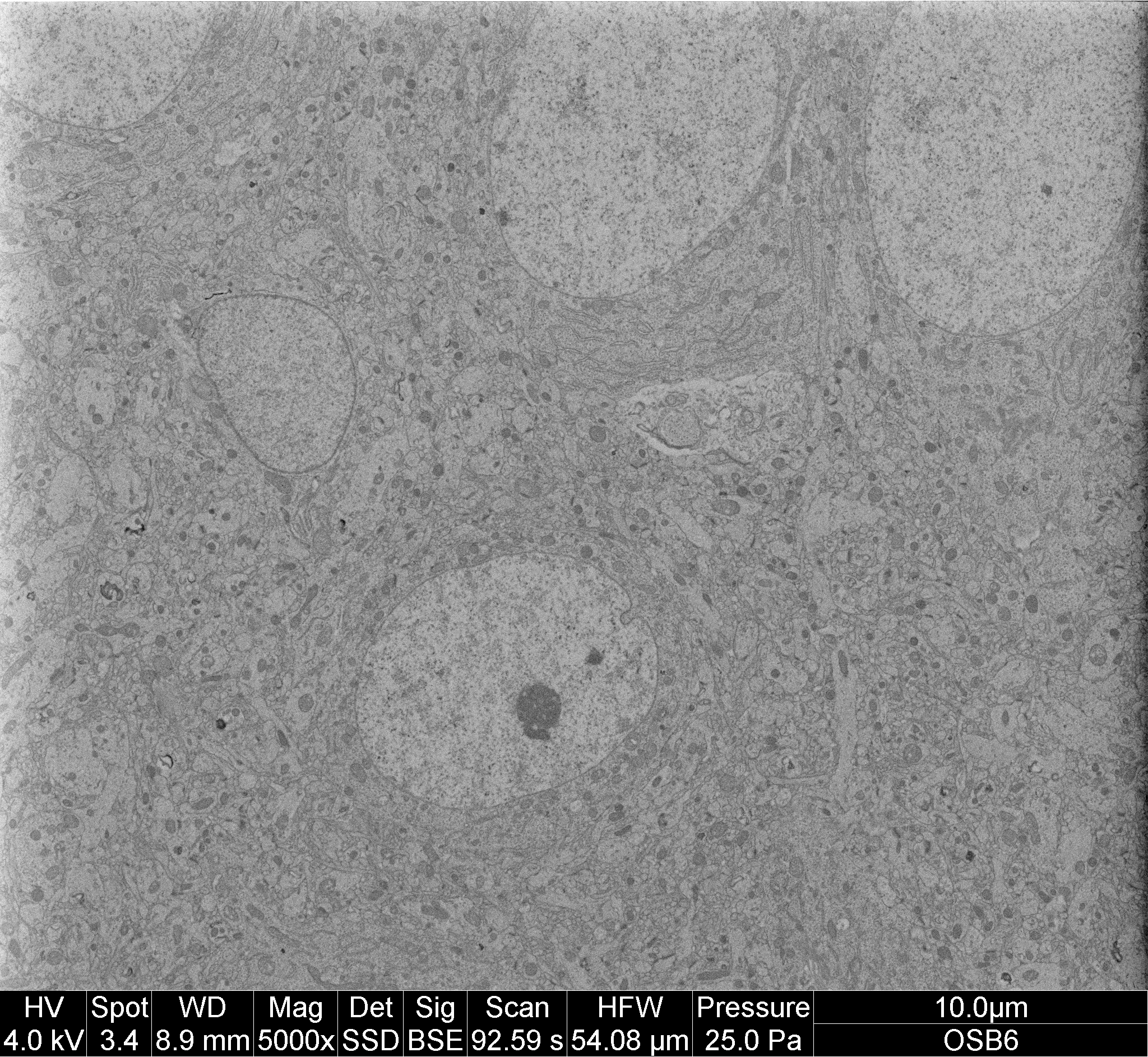

Supplement: Dataset S12 — (252.6 MB ZIP). [file pbio.0020329.sd012.zip › 040604_OS5_st1_1170.tif]

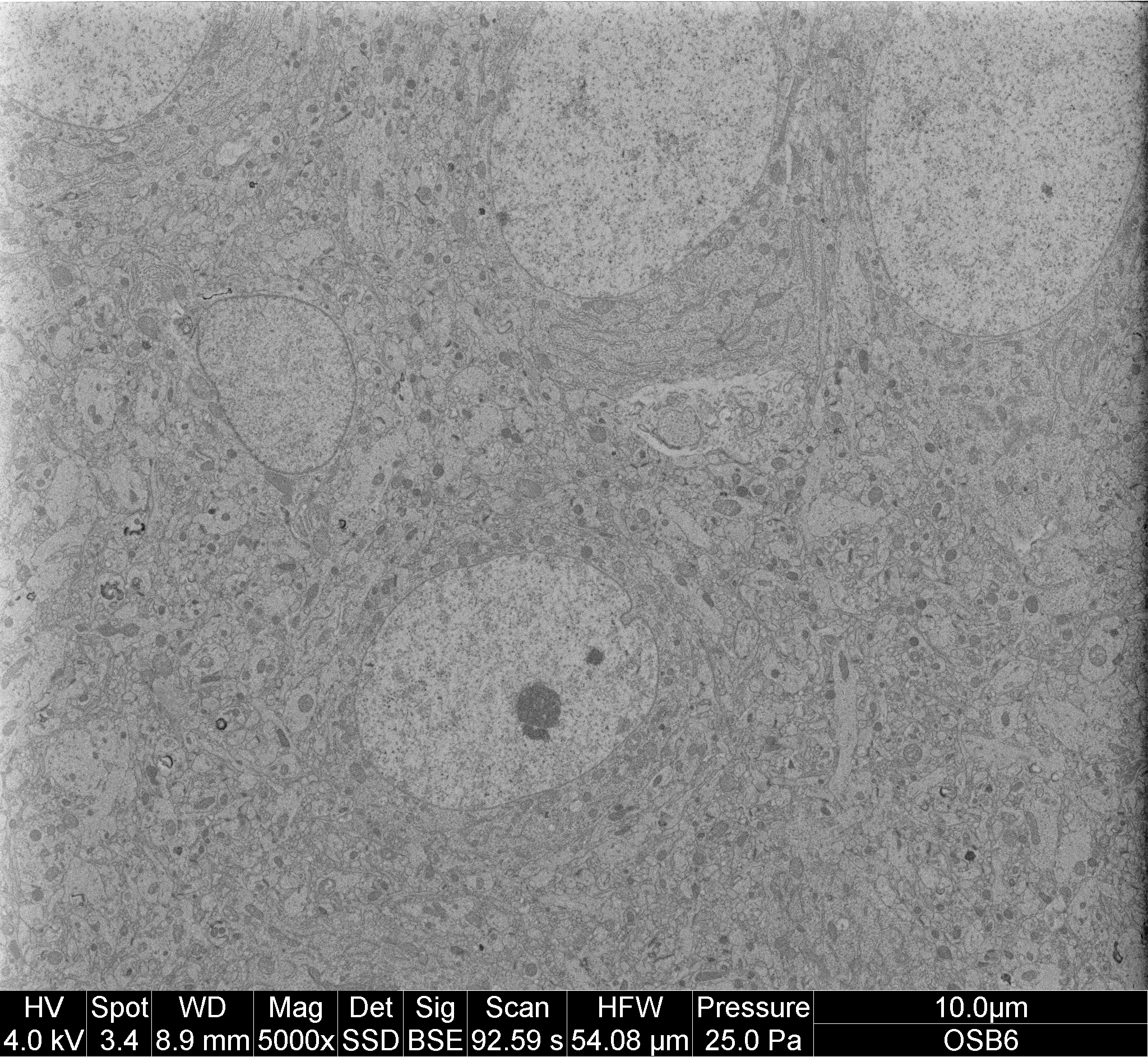

Supplement: Dataset S12 — (252.6 MB ZIP). [file pbio.0020329.sd012.zip › 040604_OS5_st1_1171.tif]

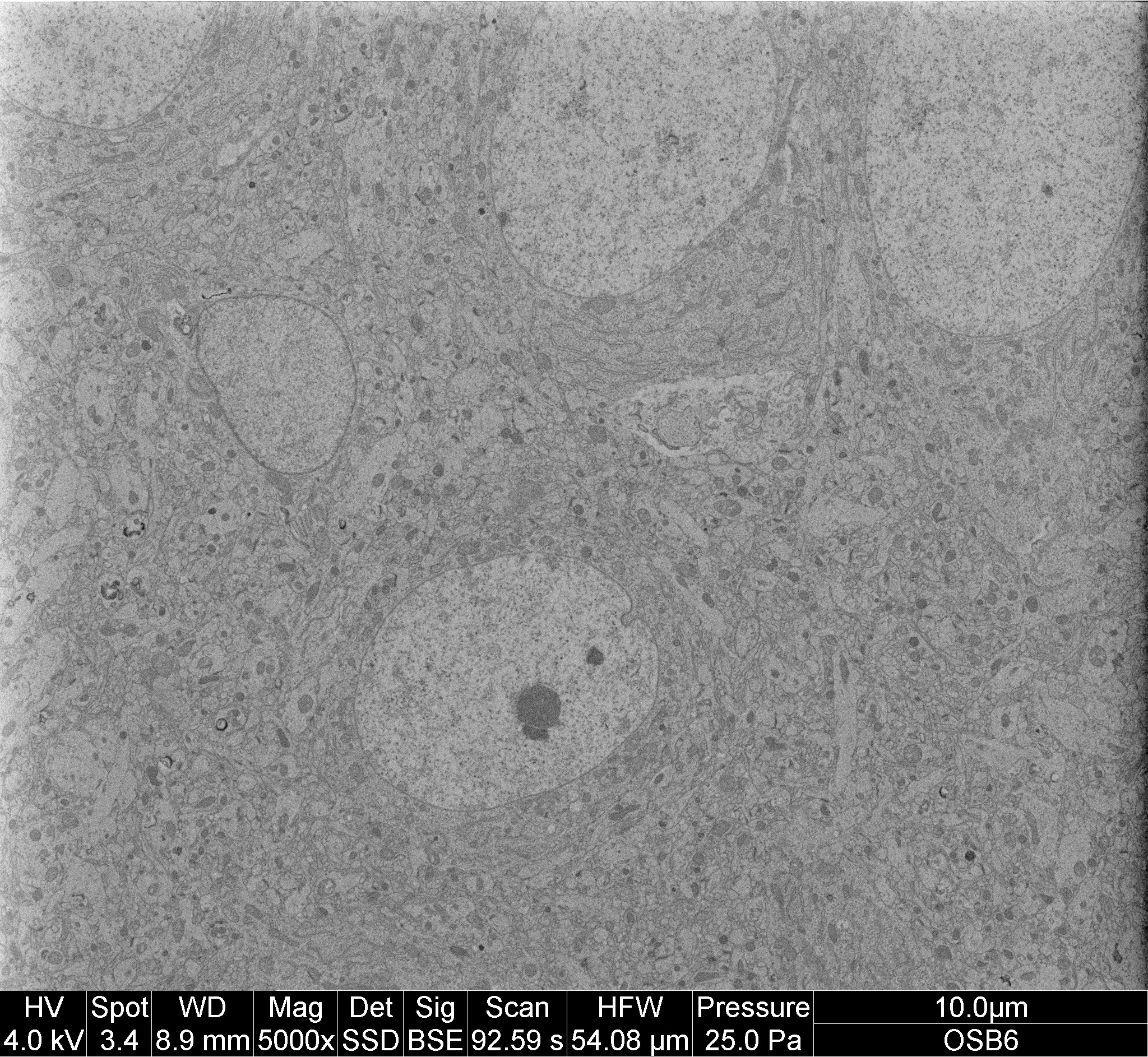

Supplement: Dataset S12 — (252.6 MB ZIP). [file pbio.0020329.sd012.zip › 040604_OS5_st1_1172.tif]

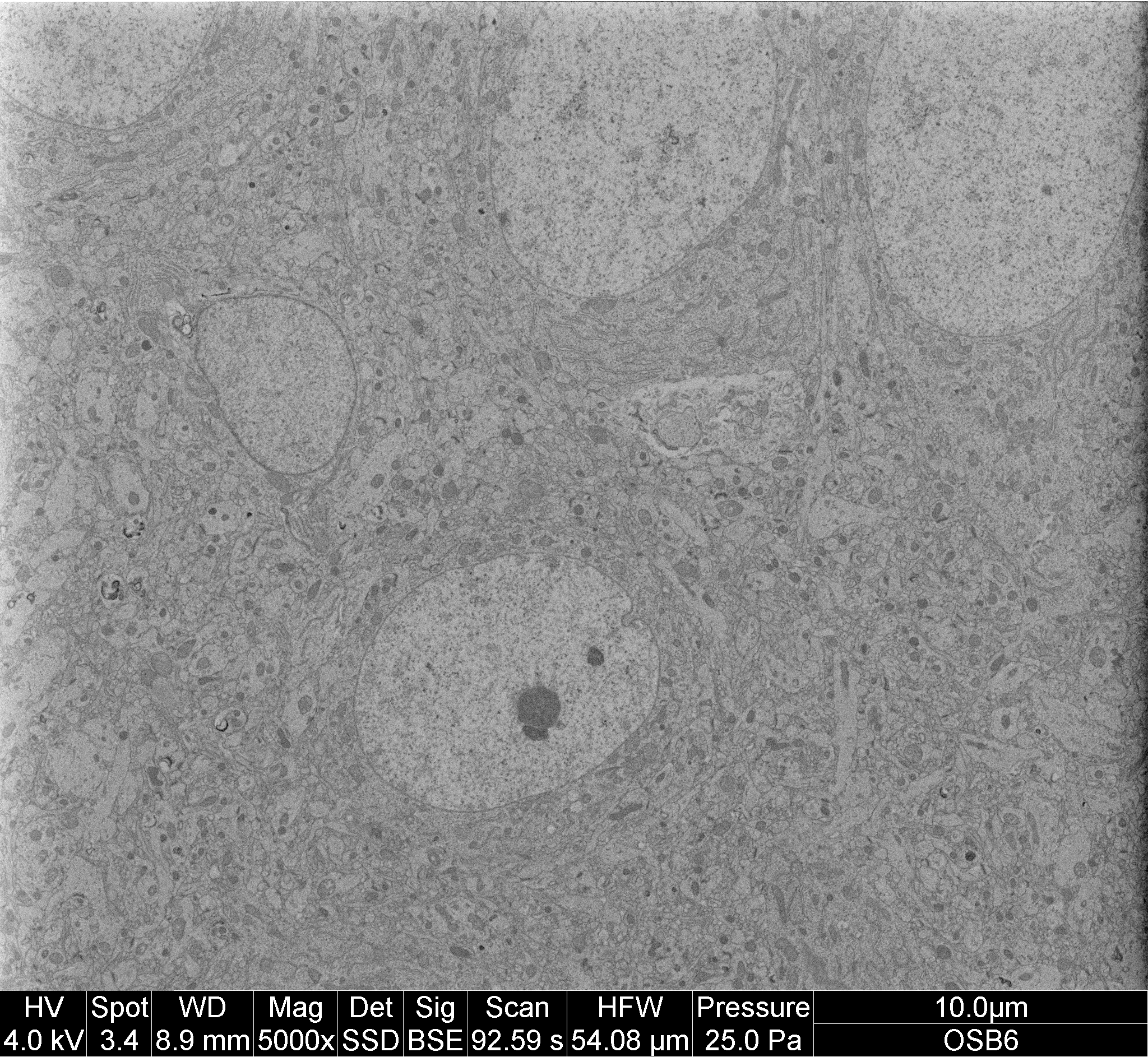

Supplement: Dataset S12 — (252.6 MB ZIP). [file pbio.0020329.sd012.zip › 040604_OS5_st1_1173.tif]

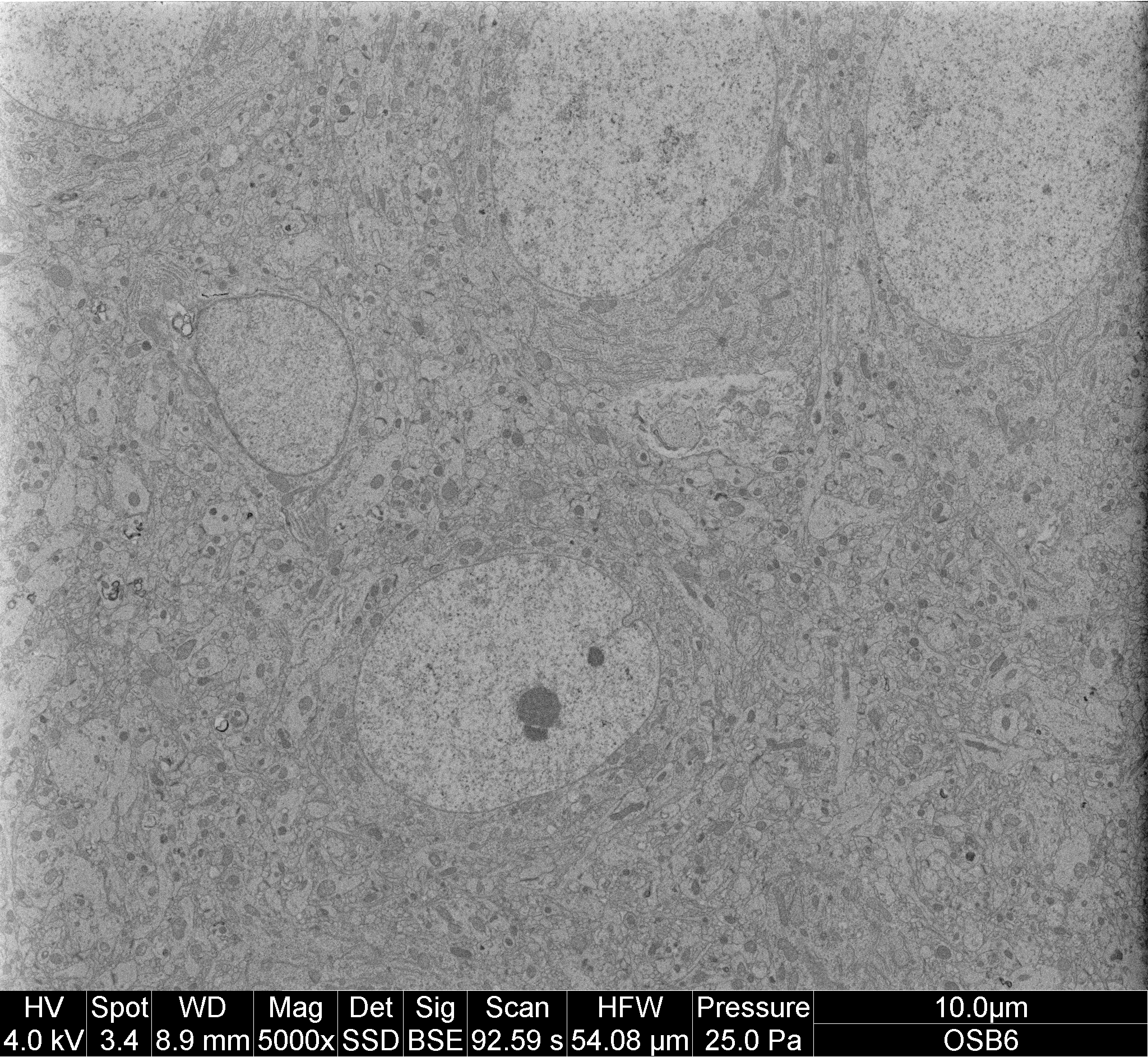

Supplement: Dataset S12 — (252.6 MB ZIP). [file pbio.0020329.sd012.zip › 040604_OS5_st1_1174.tif]

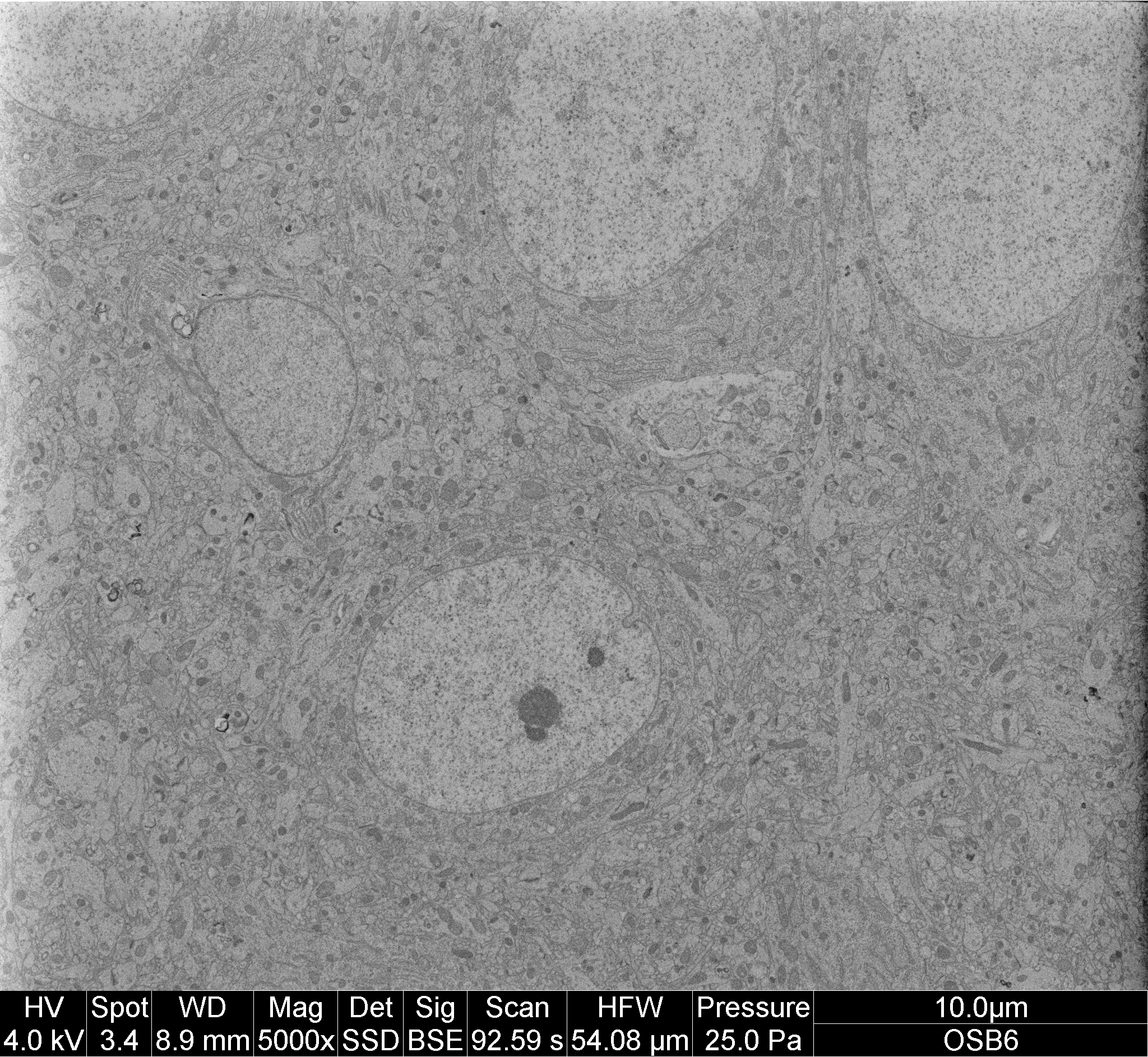

Supplement: Dataset S12 — (252.6 MB ZIP). [file pbio.0020329.sd012.zip › 040604_OS5_st1_1175.tif]

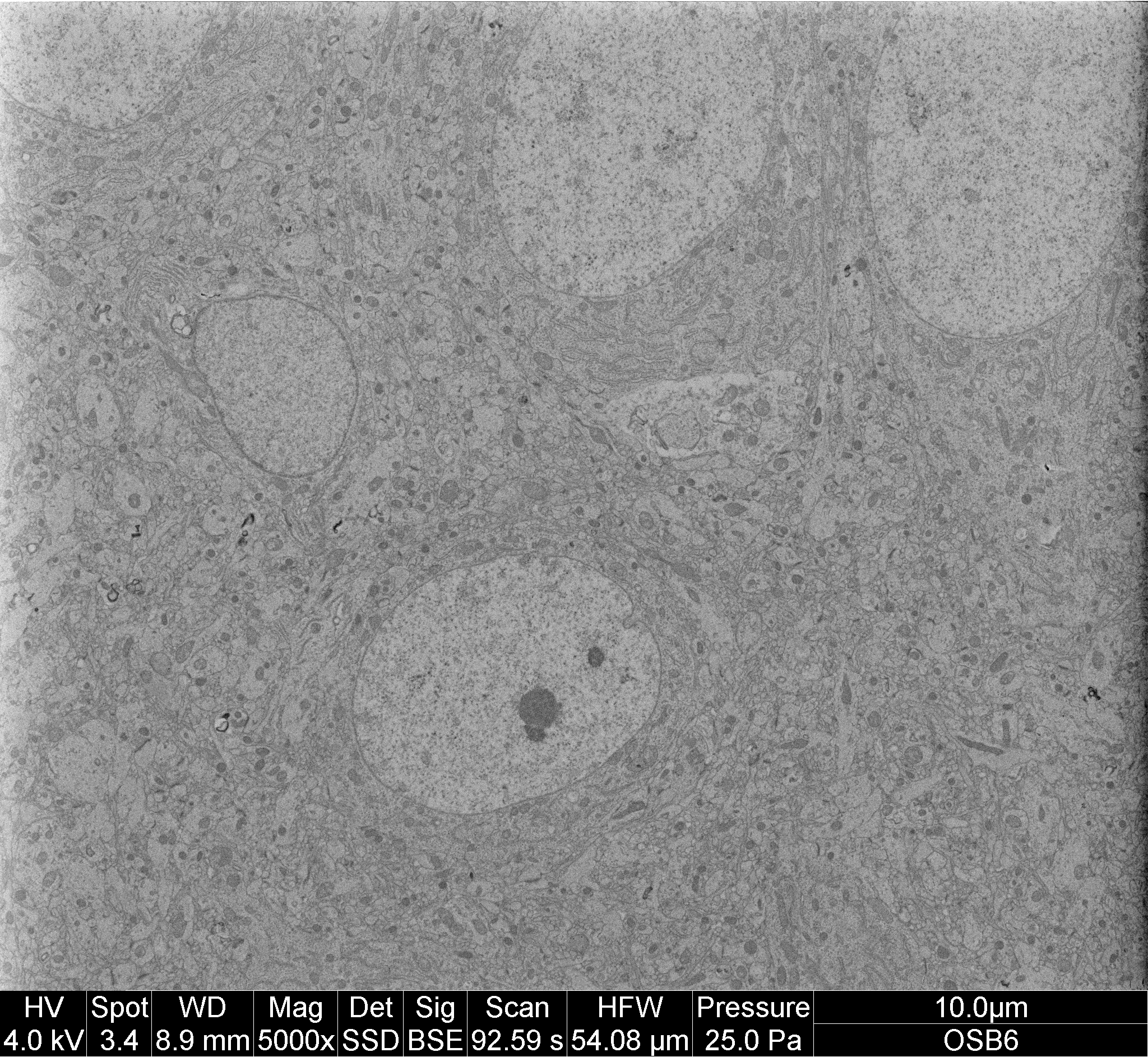

Supplement: Dataset S12 — (252.6 MB ZIP). [file pbio.0020329.sd012.zip › 040604_OS5_st1_1176.tif]

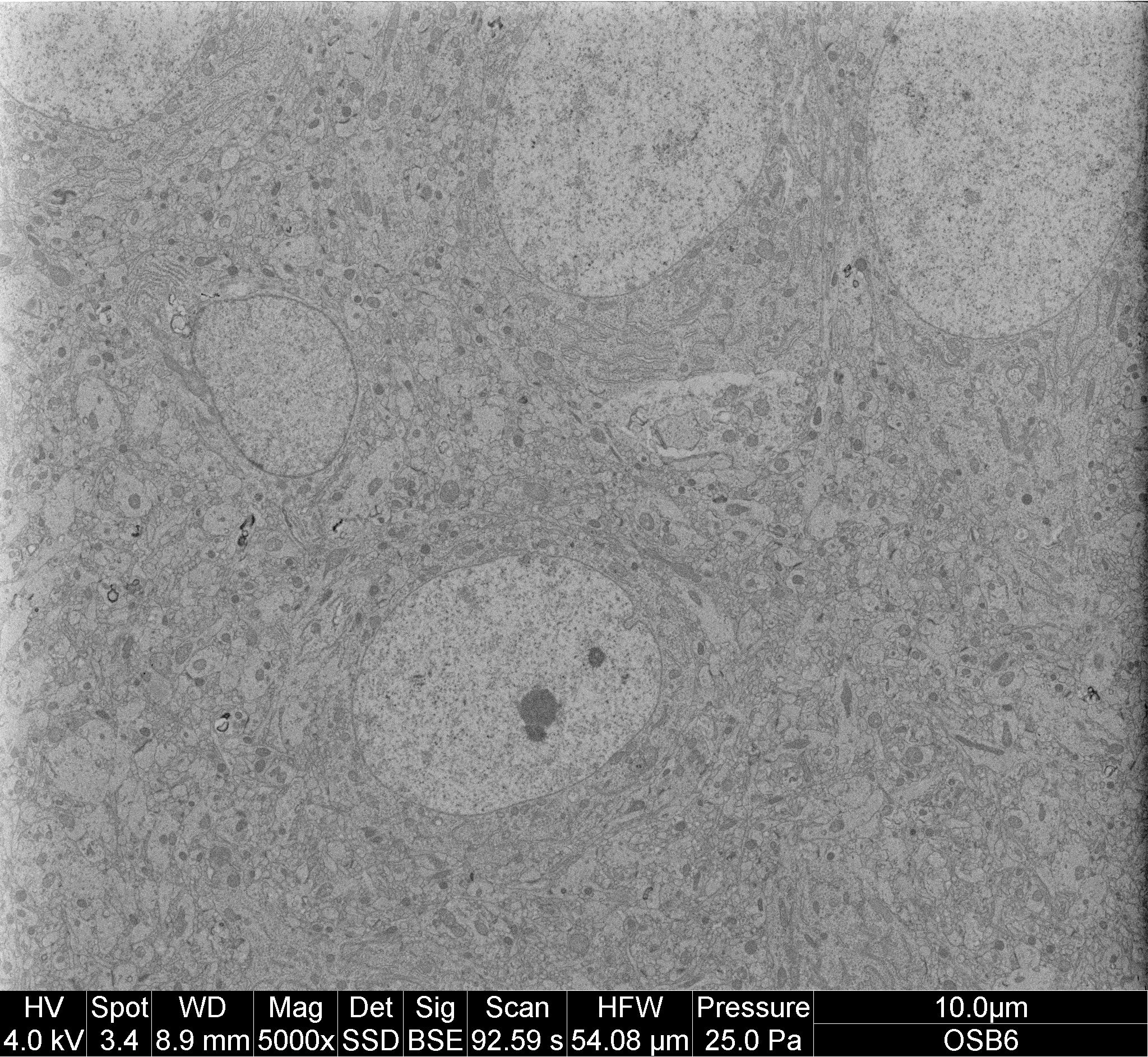

Supplement: Dataset S12 — (252.6 MB ZIP). [file pbio.0020329.sd012.zip › 040604_OS5_st1_1177.tif]

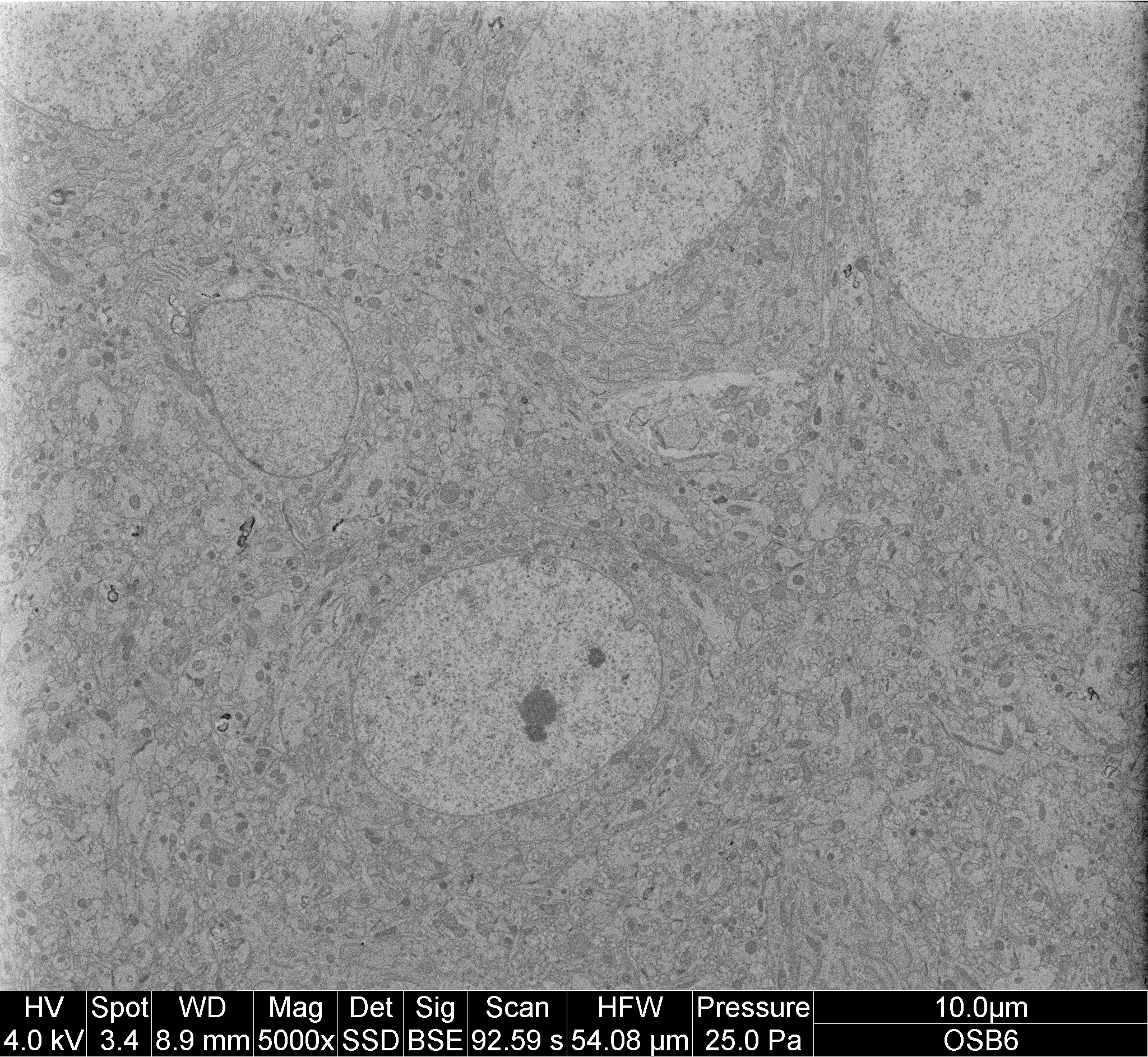

Supplement: Dataset S12 — (252.6 MB ZIP). [file pbio.0020329.sd012.zip › 040604_OS5_st1_1178.tif]

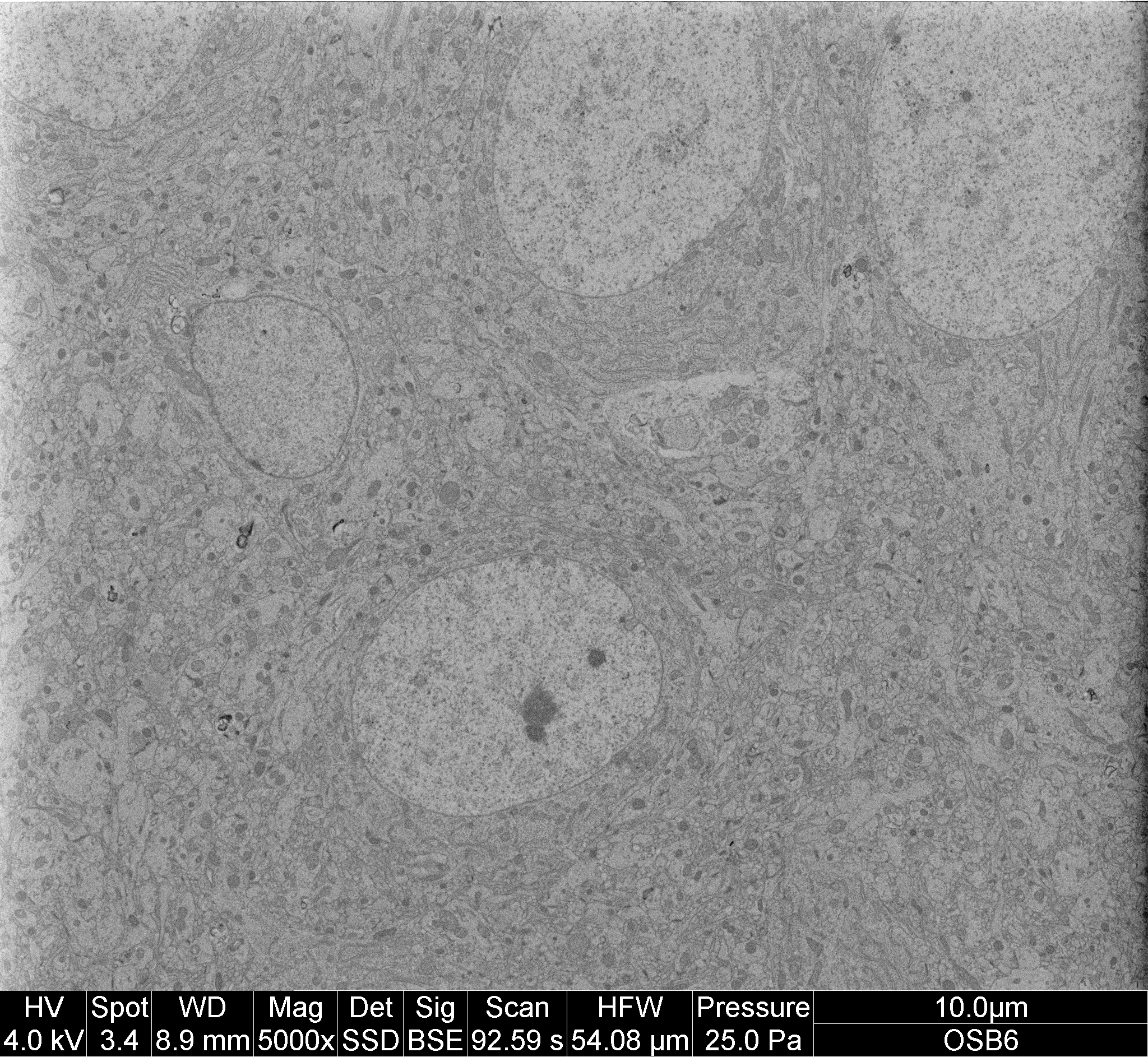

Supplement: Dataset S12 — (252.6 MB ZIP). [file pbio.0020329.sd012.zip › 040604_OS5_st1_1179.tif]

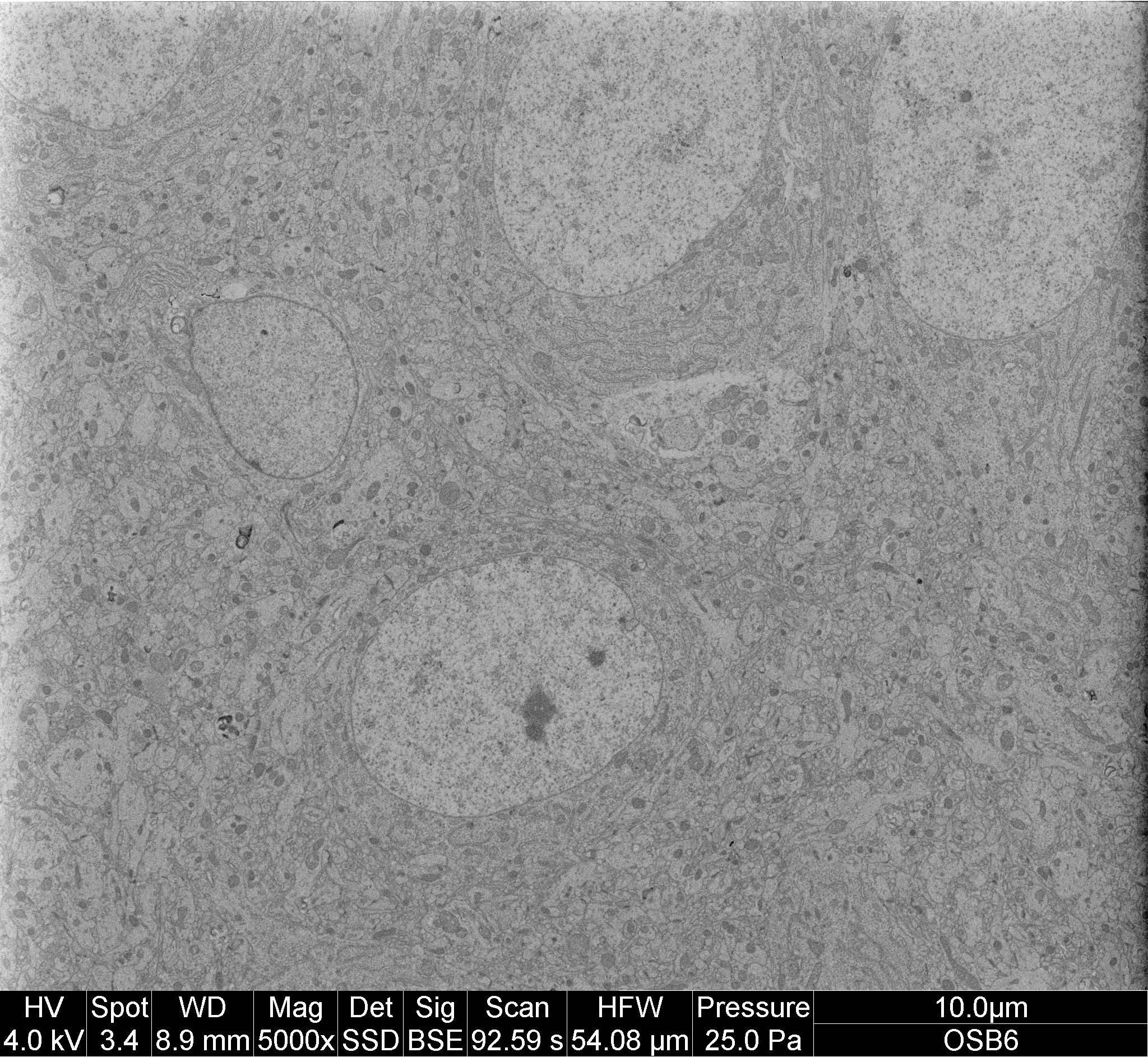

Supplement: Dataset S12 — (252.6 MB ZIP). [file pbio.0020329.sd012.zip › 040604_OS5_st1_1180.tif]

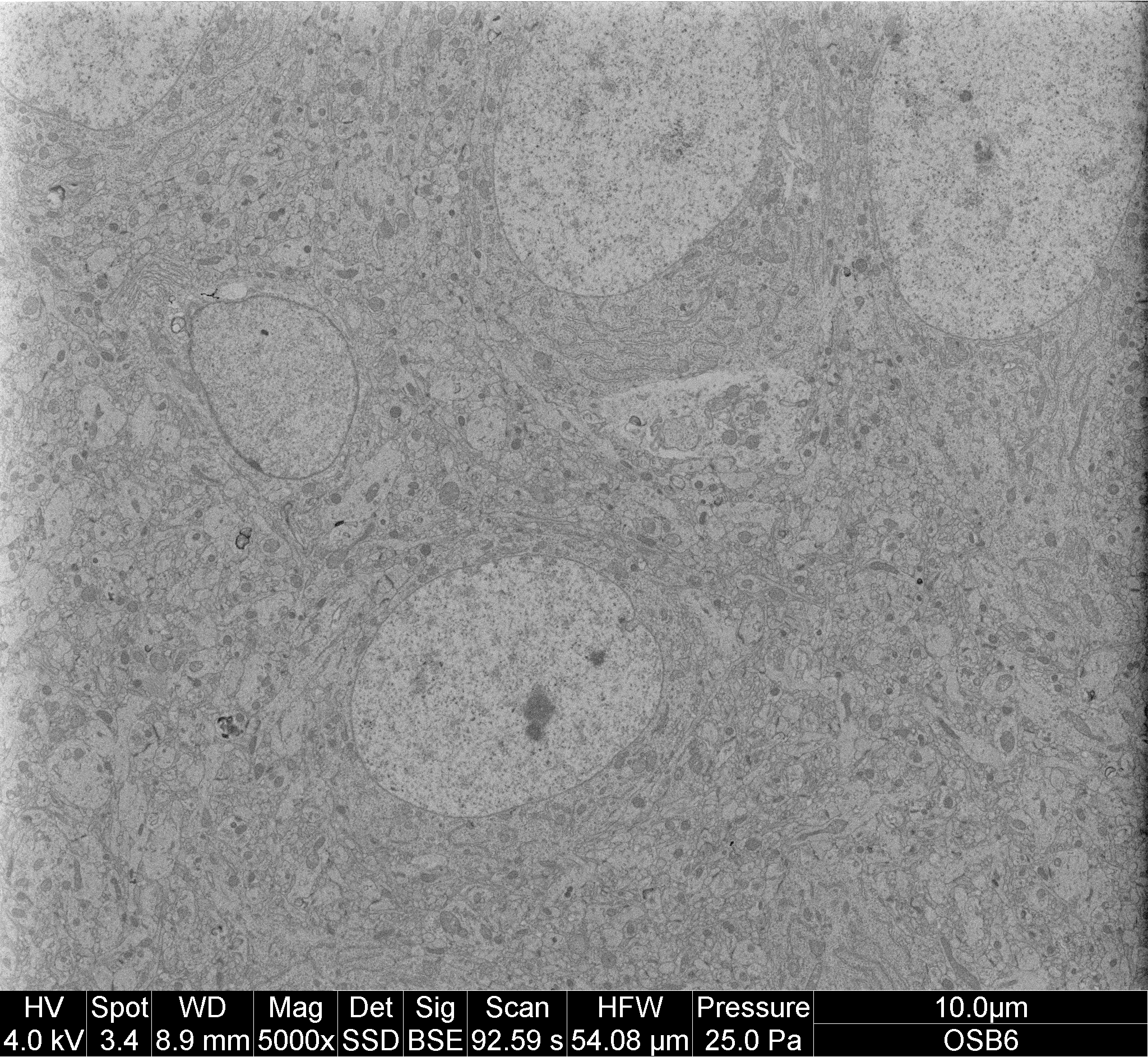

Supplement: Dataset S12 — (252.6 MB ZIP). [file pbio.0020329.sd012.zip › 040604_OS5_st1_1181.tif]

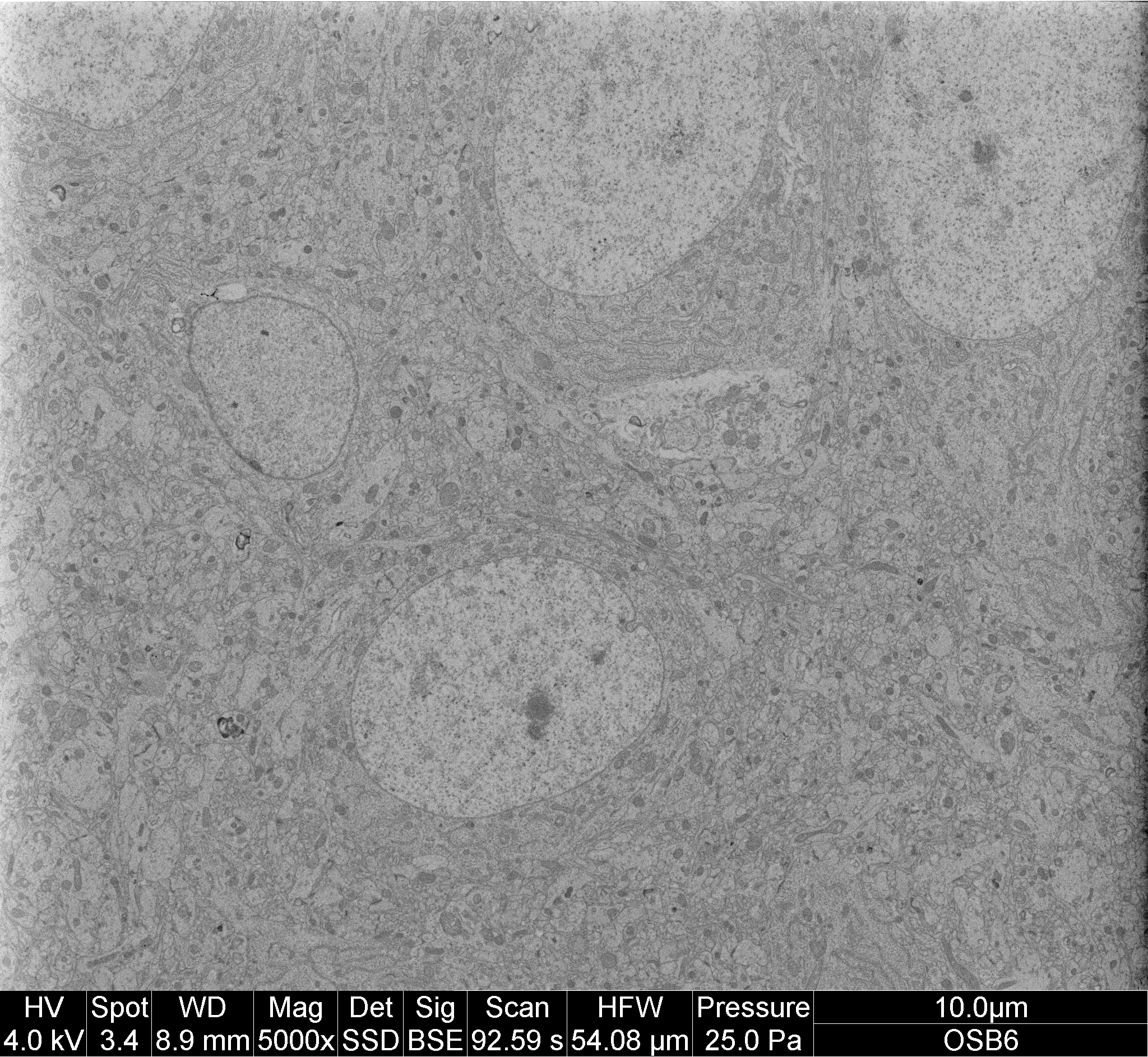

Supplement: Dataset S12 — (252.6 MB ZIP). [file pbio.0020329.sd012.zip › 040604_OS5_st1_1182.tif]

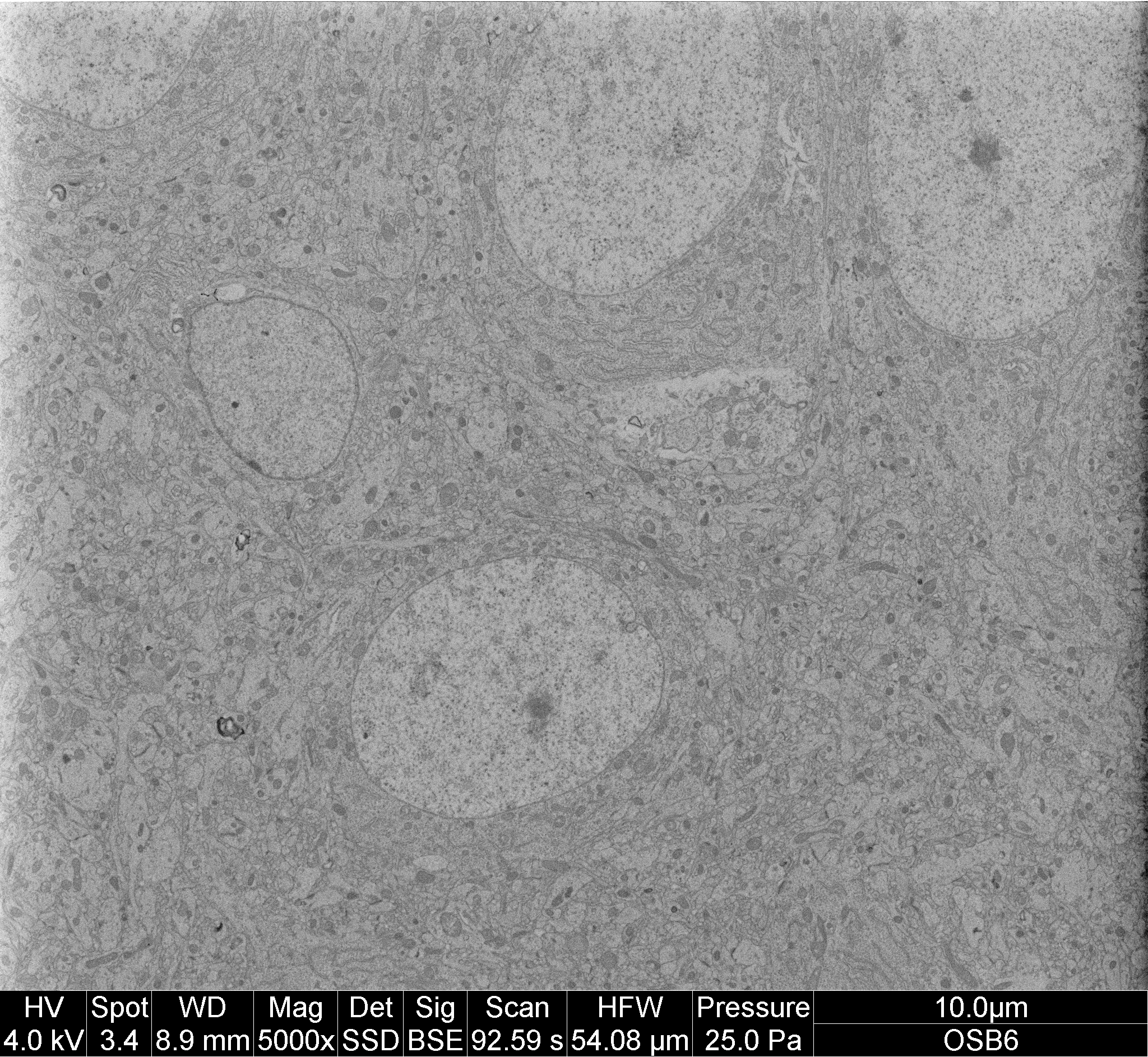

Supplement: Dataset S12 — (252.6 MB ZIP). [file pbio.0020329.sd012.zip › 040604_OS5_st1_1183.tif]

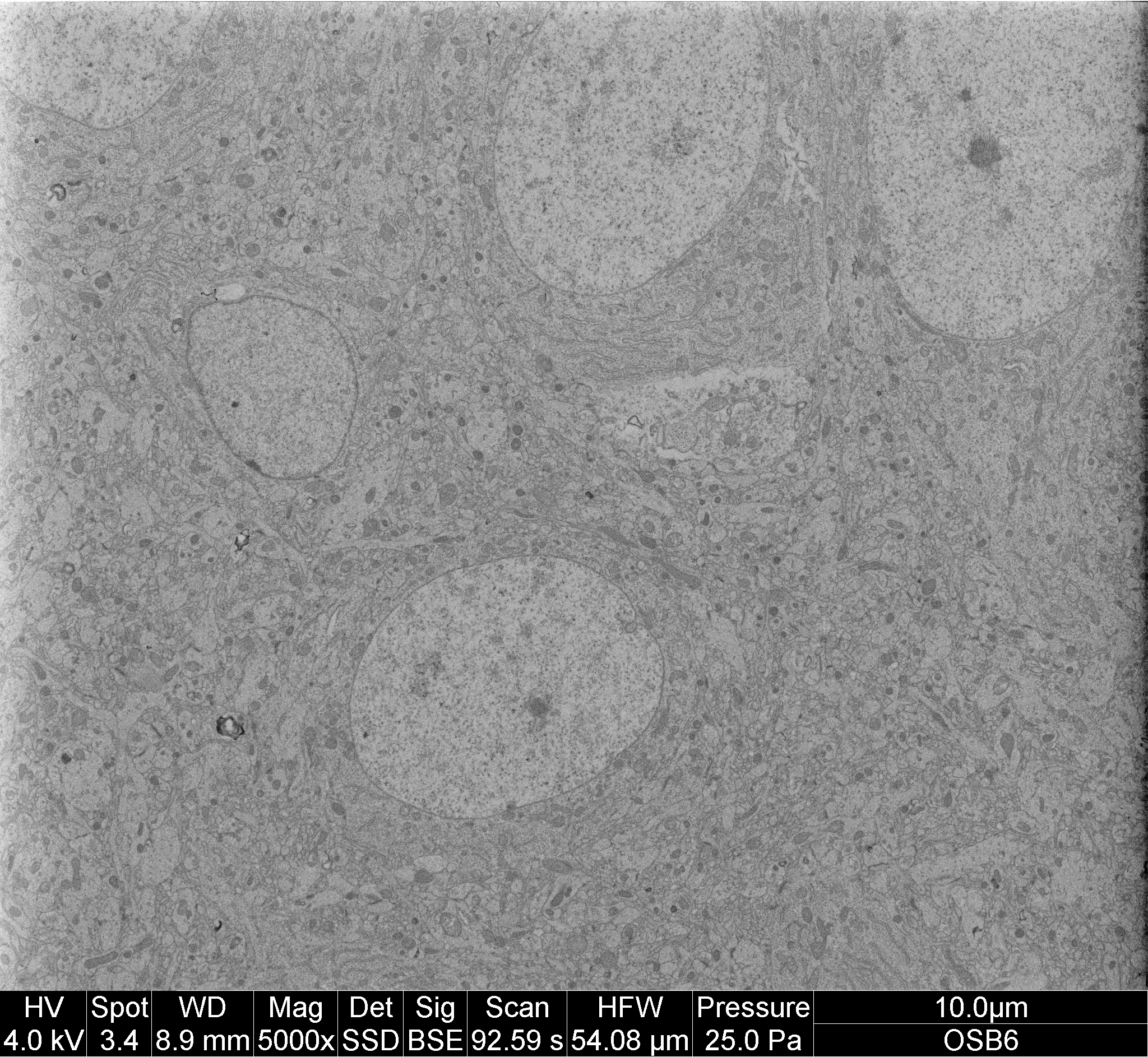

Supplement: Dataset S12 — (252.6 MB ZIP). [file pbio.0020329.sd012.zip › 040604_OS5_st1_1184.tif]

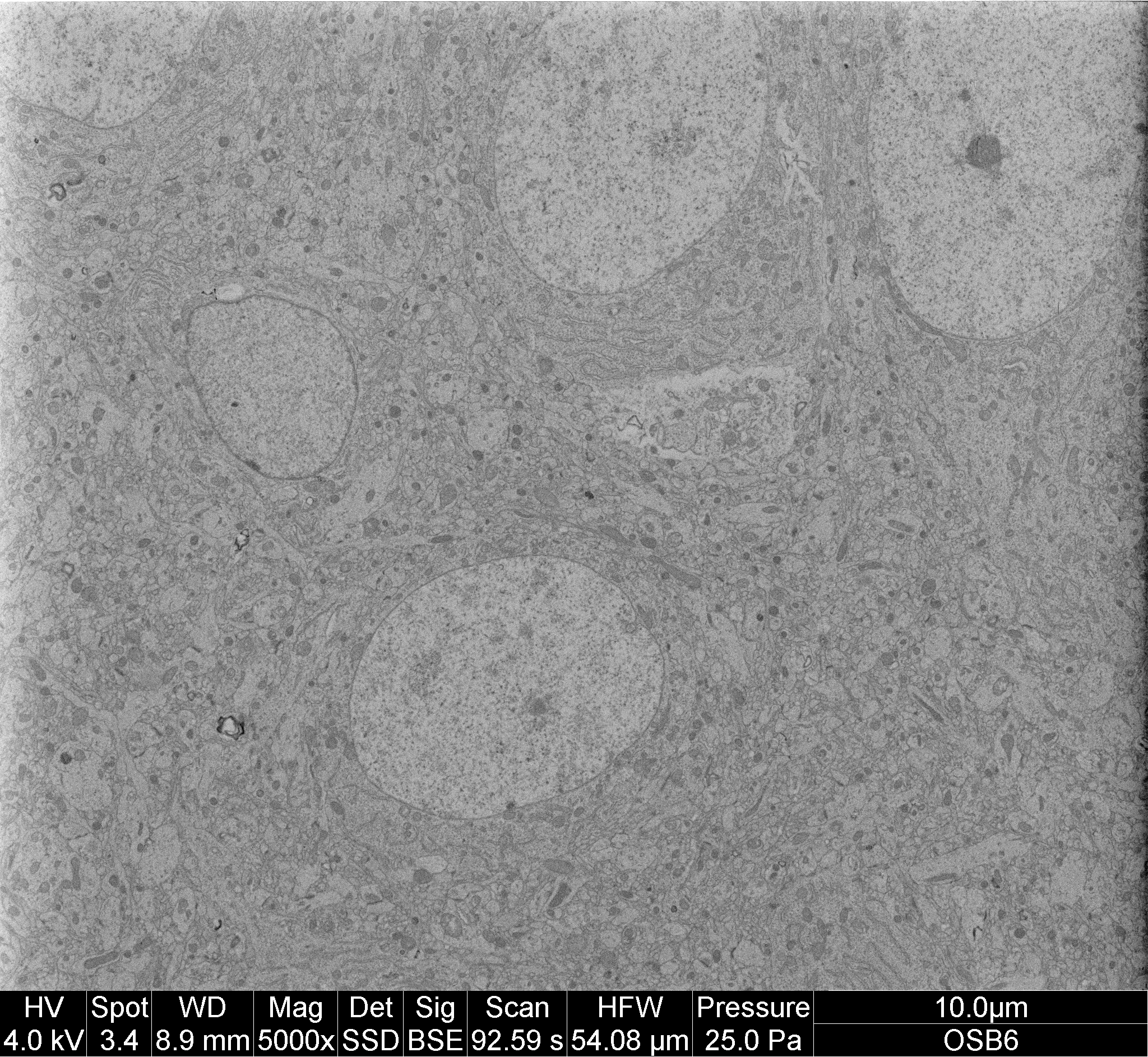

Supplement: Dataset S12 — (252.6 MB ZIP). [file pbio.0020329.sd012.zip › 040604_OS5_st1_1185.tif]

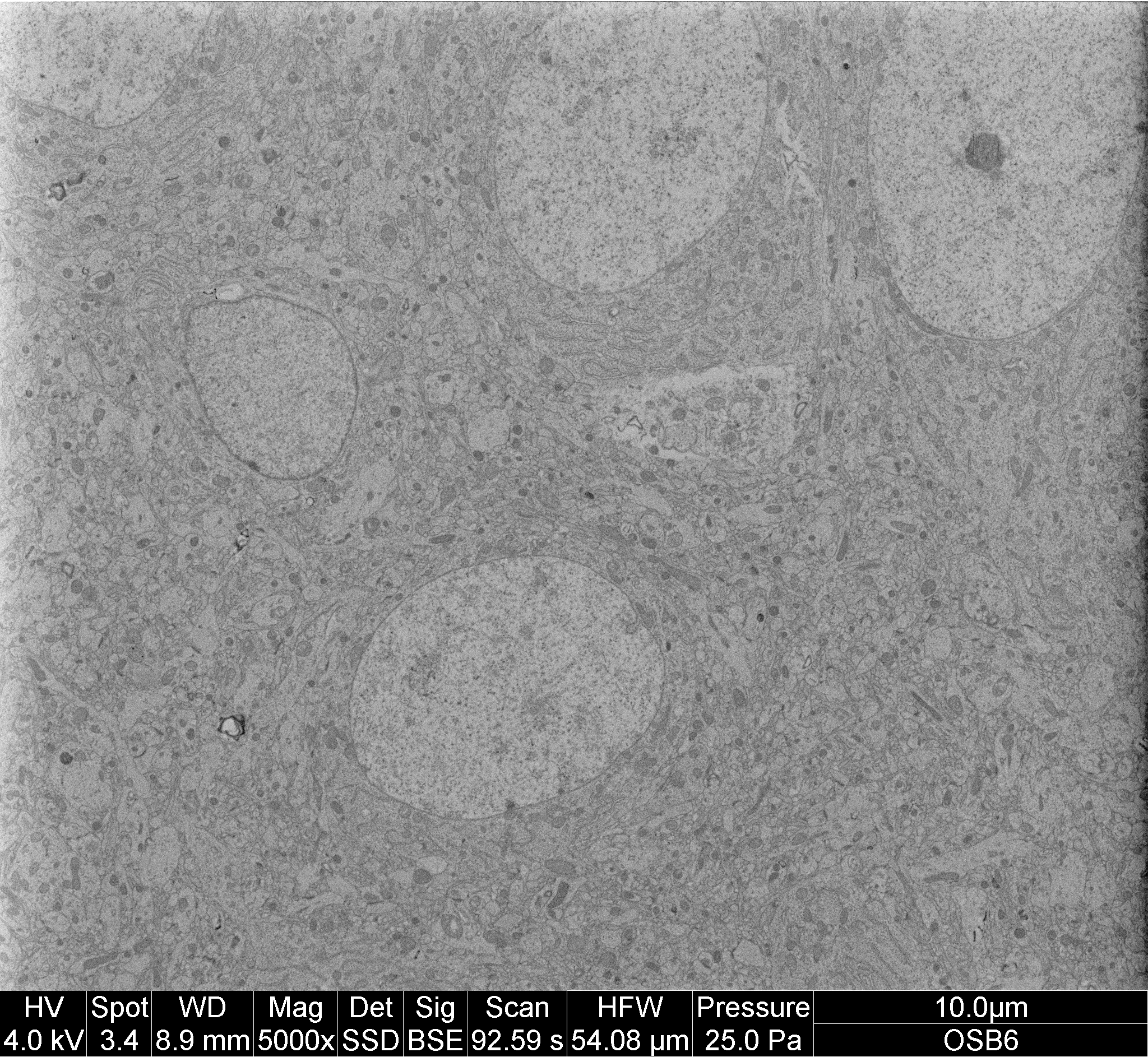

Supplement: Dataset S12 — (252.6 MB ZIP). [file pbio.0020329.sd012.zip › 040604_OS5_st1_1186.tif]

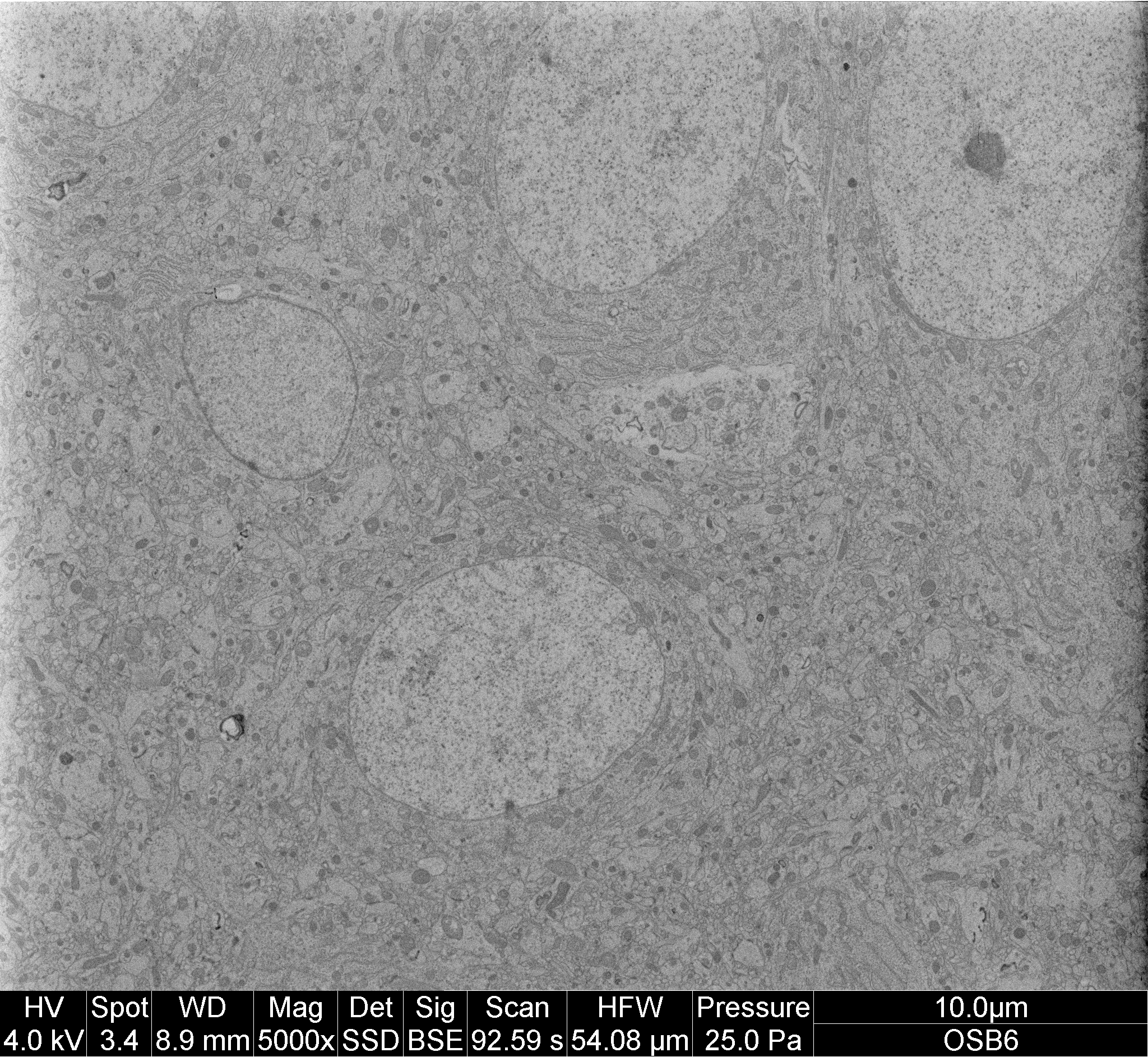

Supplement: Dataset S12 — (252.6 MB ZIP). [file pbio.0020329.sd012.zip › 040604_OS5_st1_1187.tif]

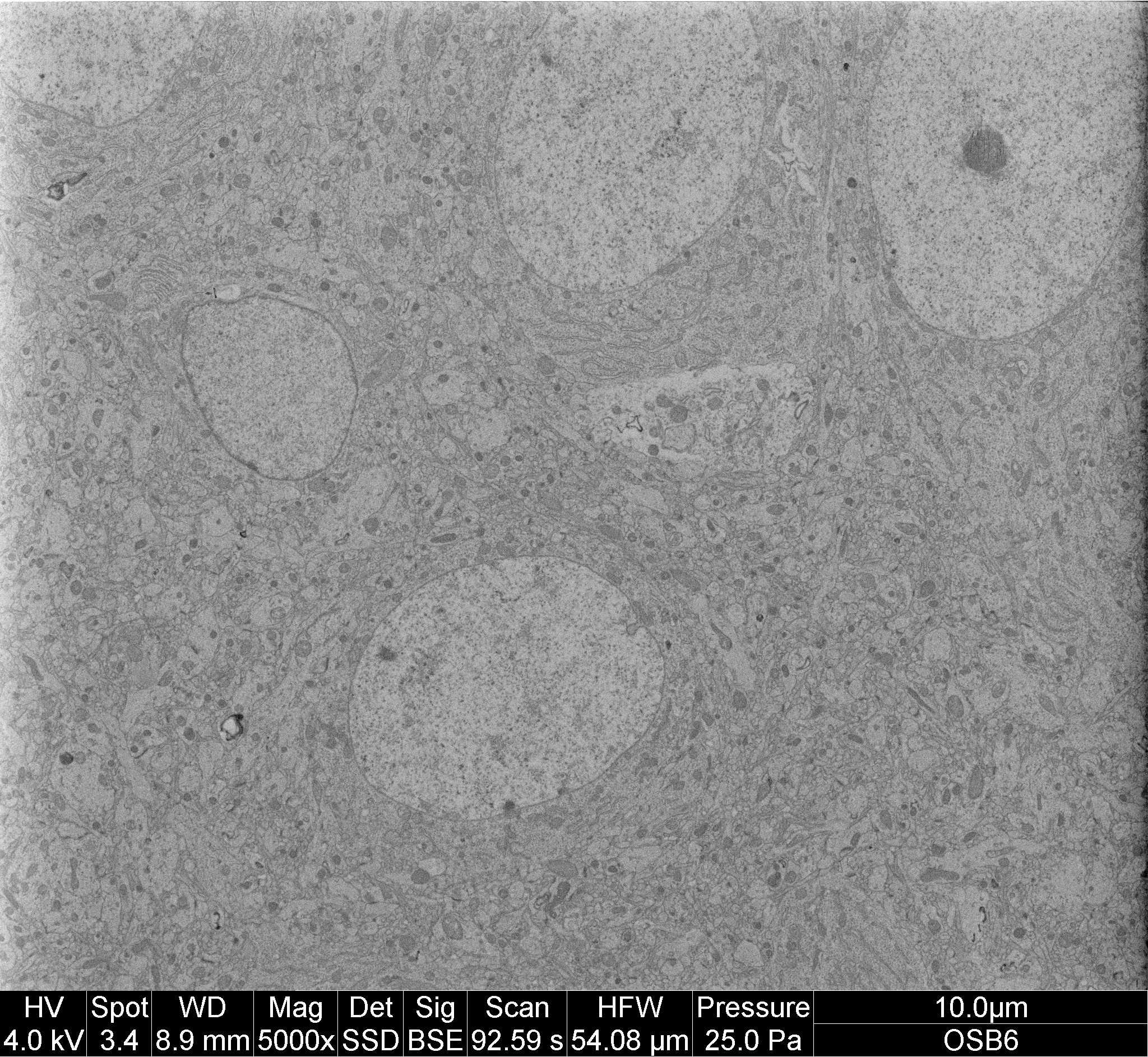

Supplement: Dataset S12 — (252.6 MB ZIP). [file pbio.0020329.sd012.zip › 040604_OS5_st1_1188.tif]

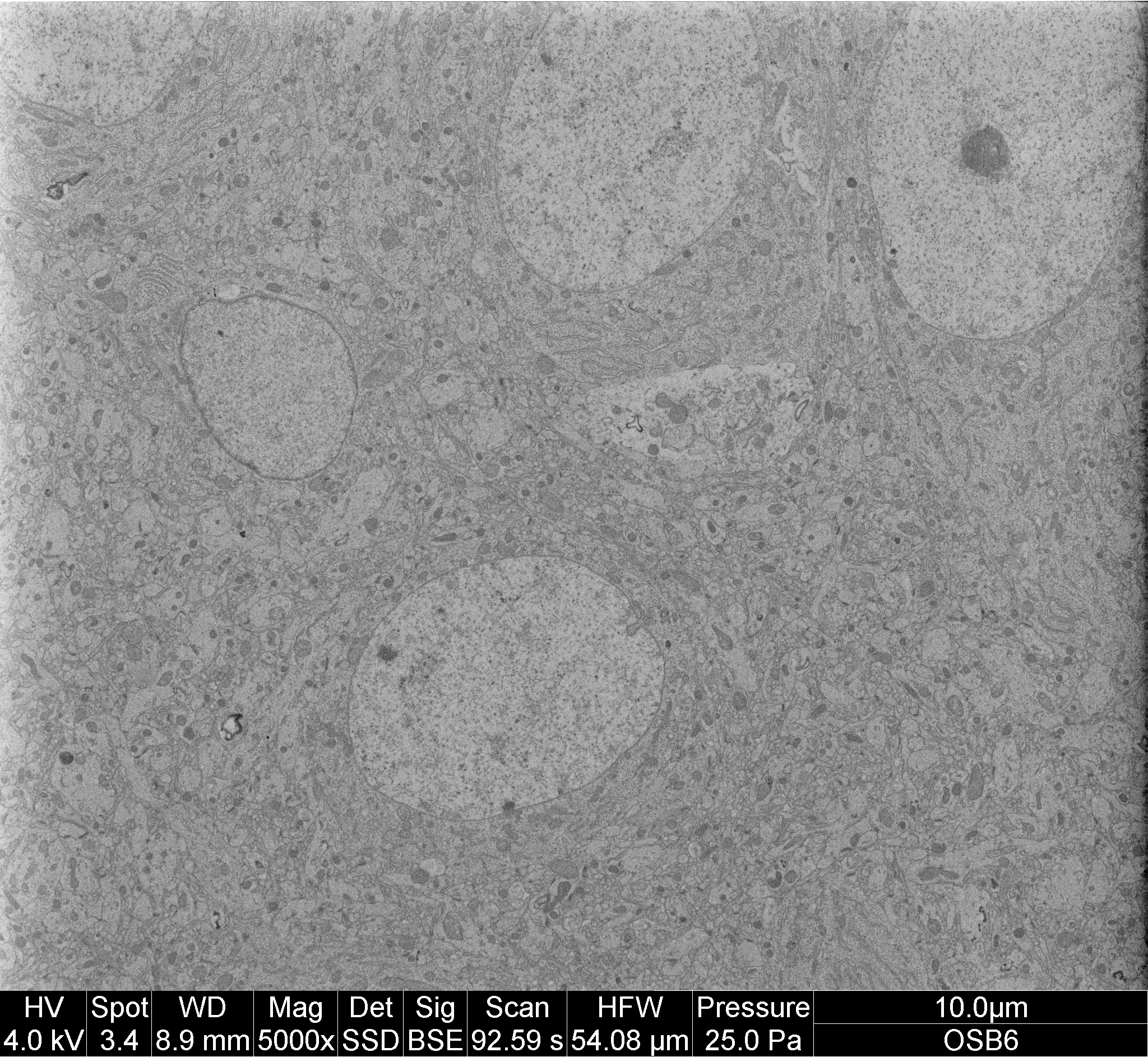

Supplement: Dataset S12 — (252.6 MB ZIP). [file pbio.0020329.sd012.zip › 040604_OS5_st1_1189.tif]

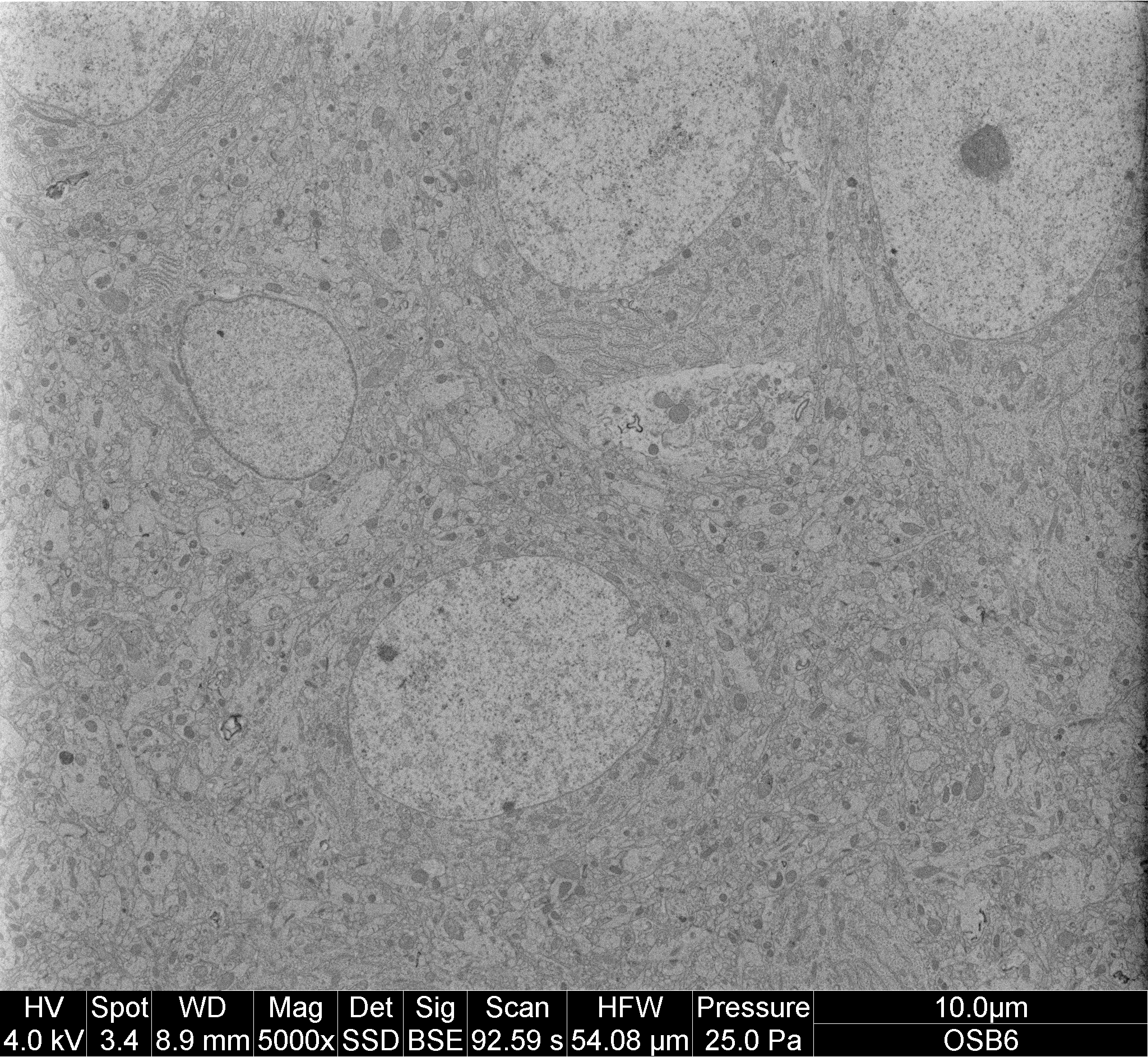

Supplement: Dataset S12 — (252.6 MB ZIP). [file pbio.0020329.sd012.zip › 040604_OS5_st1_1190.tif]

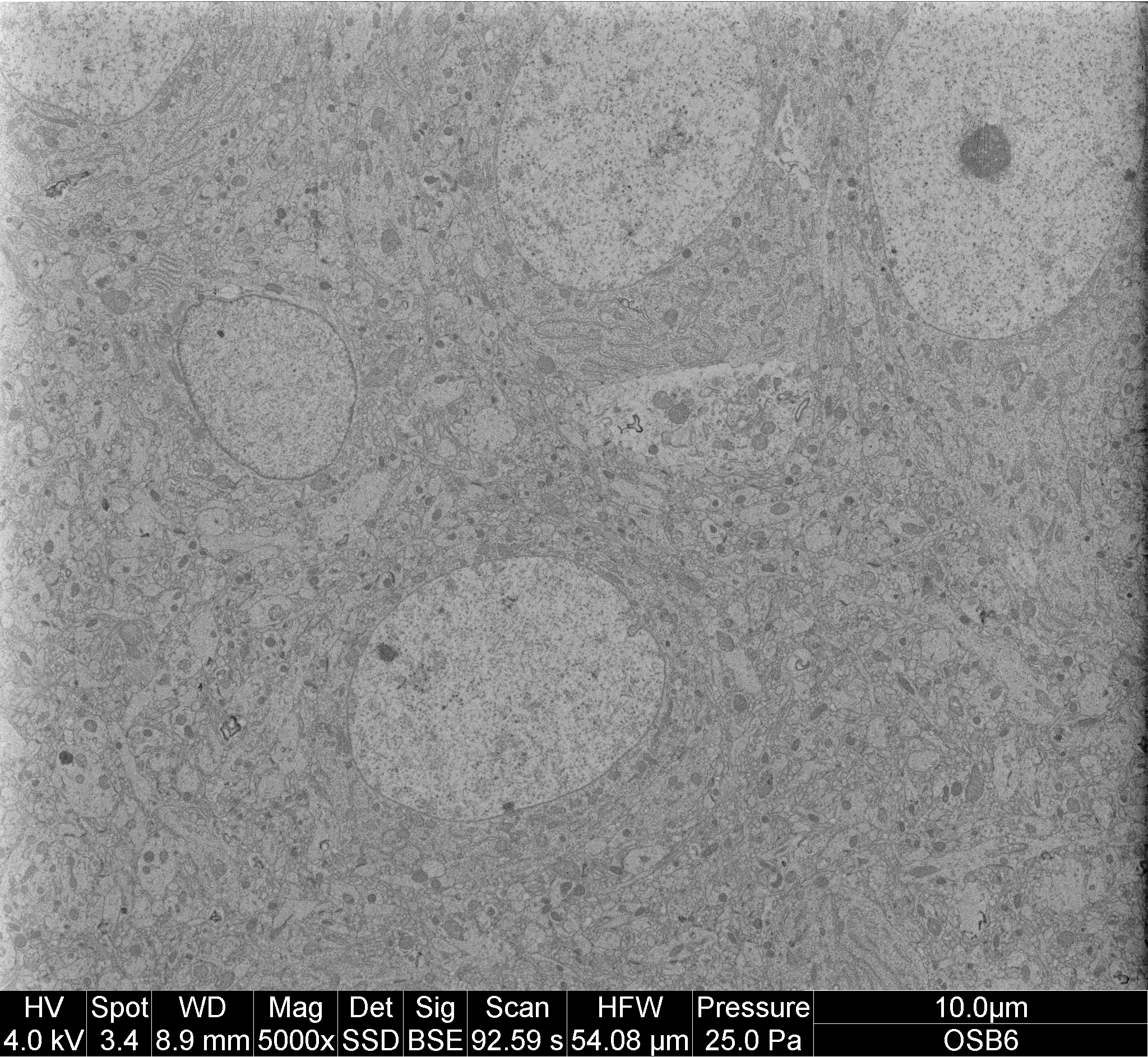

Supplement: Dataset S12 — (252.6 MB ZIP). [file pbio.0020329.sd012.zip › 040604_OS5_st1_1191.tif]

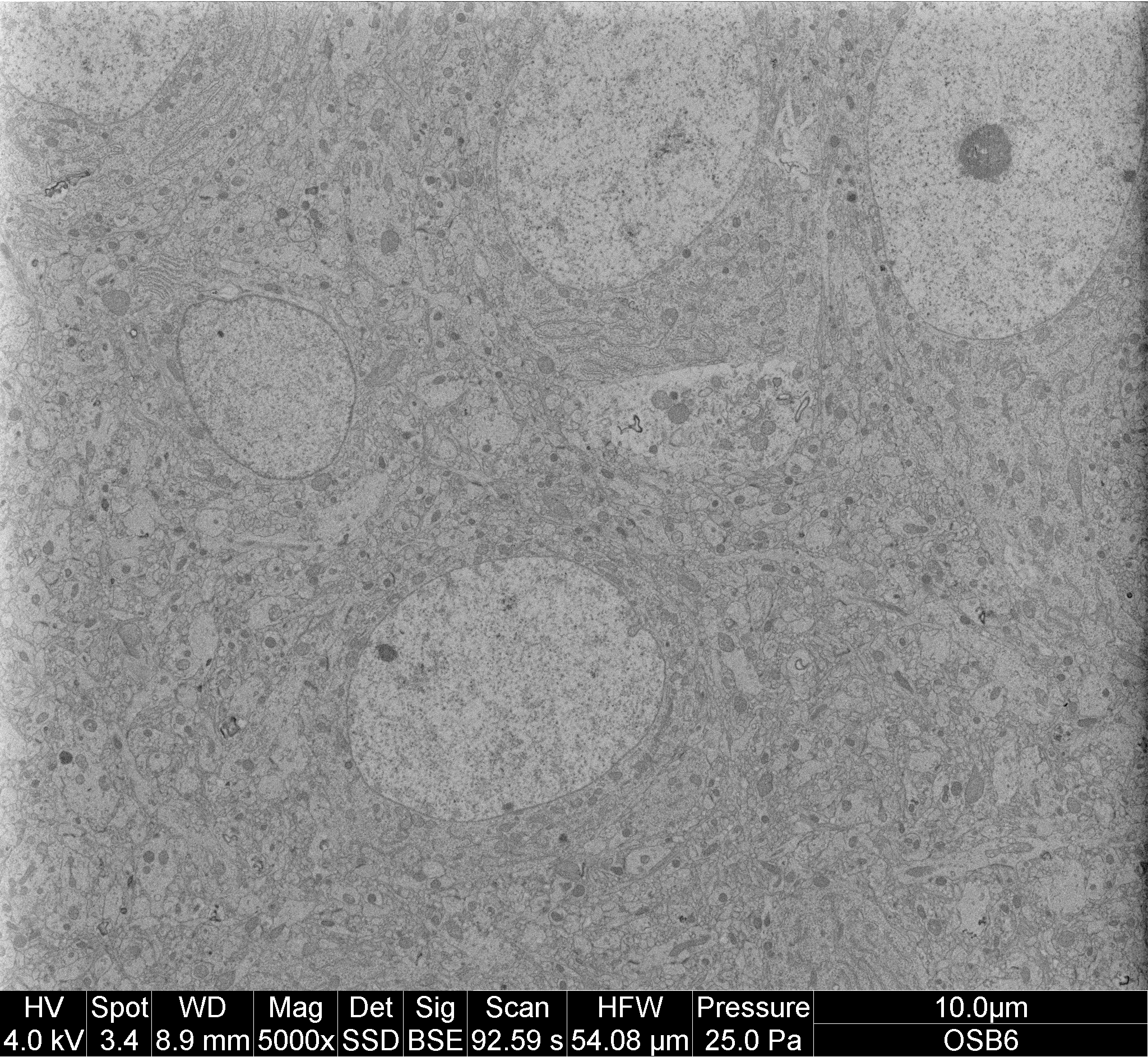

Supplement: Dataset S12 — (252.6 MB ZIP). [file pbio.0020329.sd012.zip › 040604_OS5_st1_1192.tif]

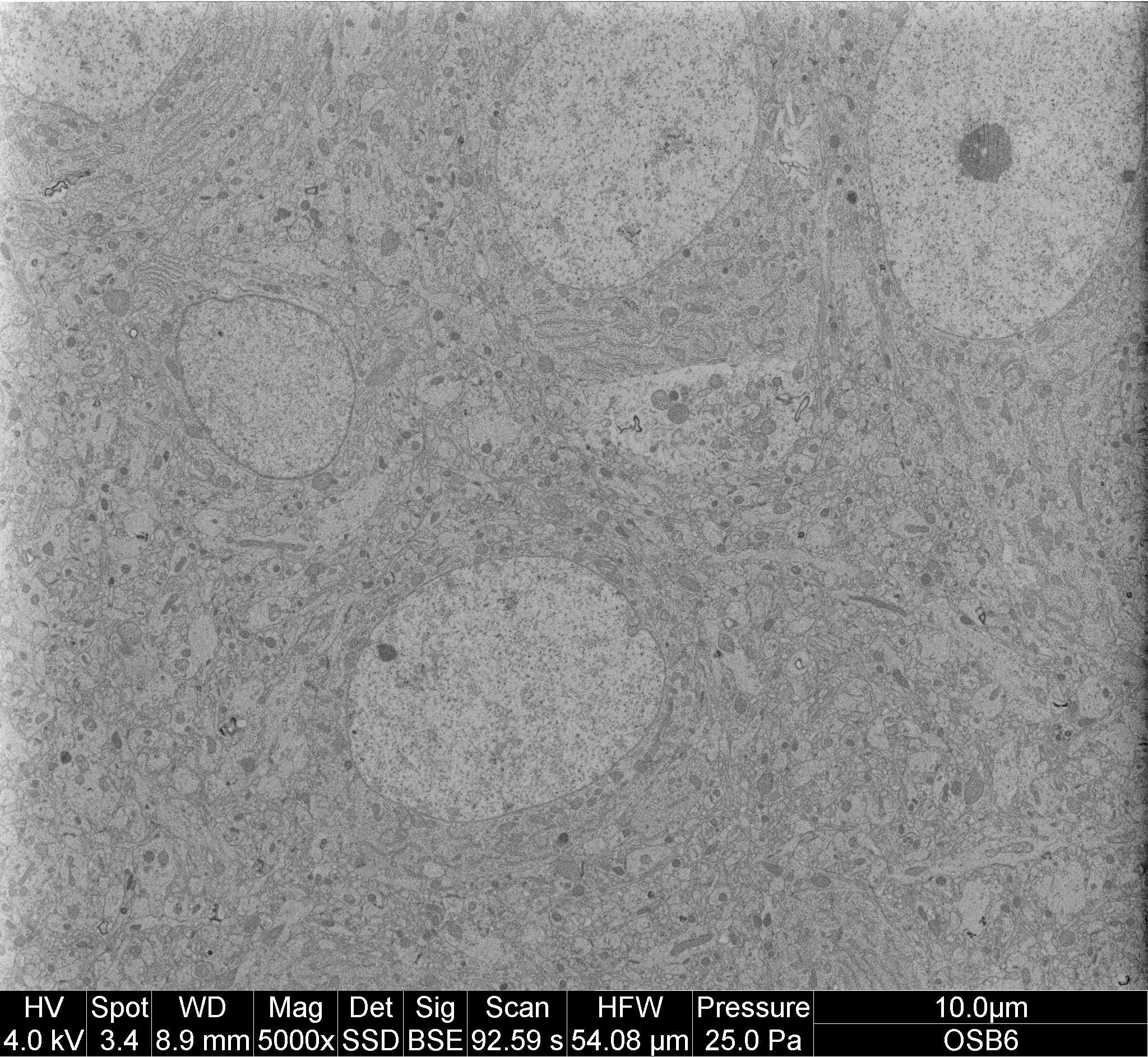

Supplement: Dataset S12 — (252.6 MB ZIP). [file pbio.0020329.sd012.zip › 040604_OS5_st1_1193.tif]

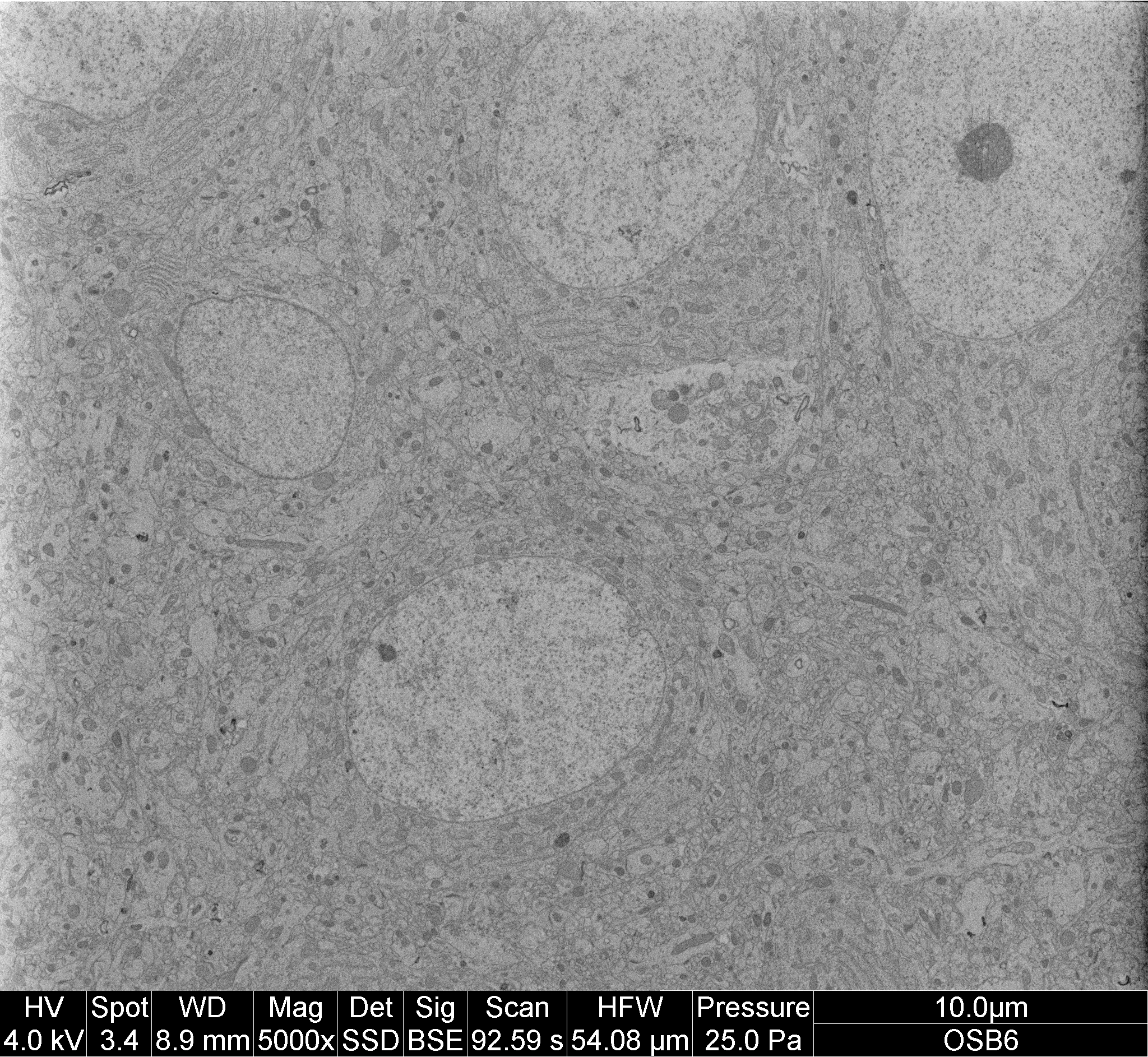

Supplement: Dataset S12 — (252.6 MB ZIP). [file pbio.0020329.sd012.zip › 040604_OS5_st1_1194.tif]

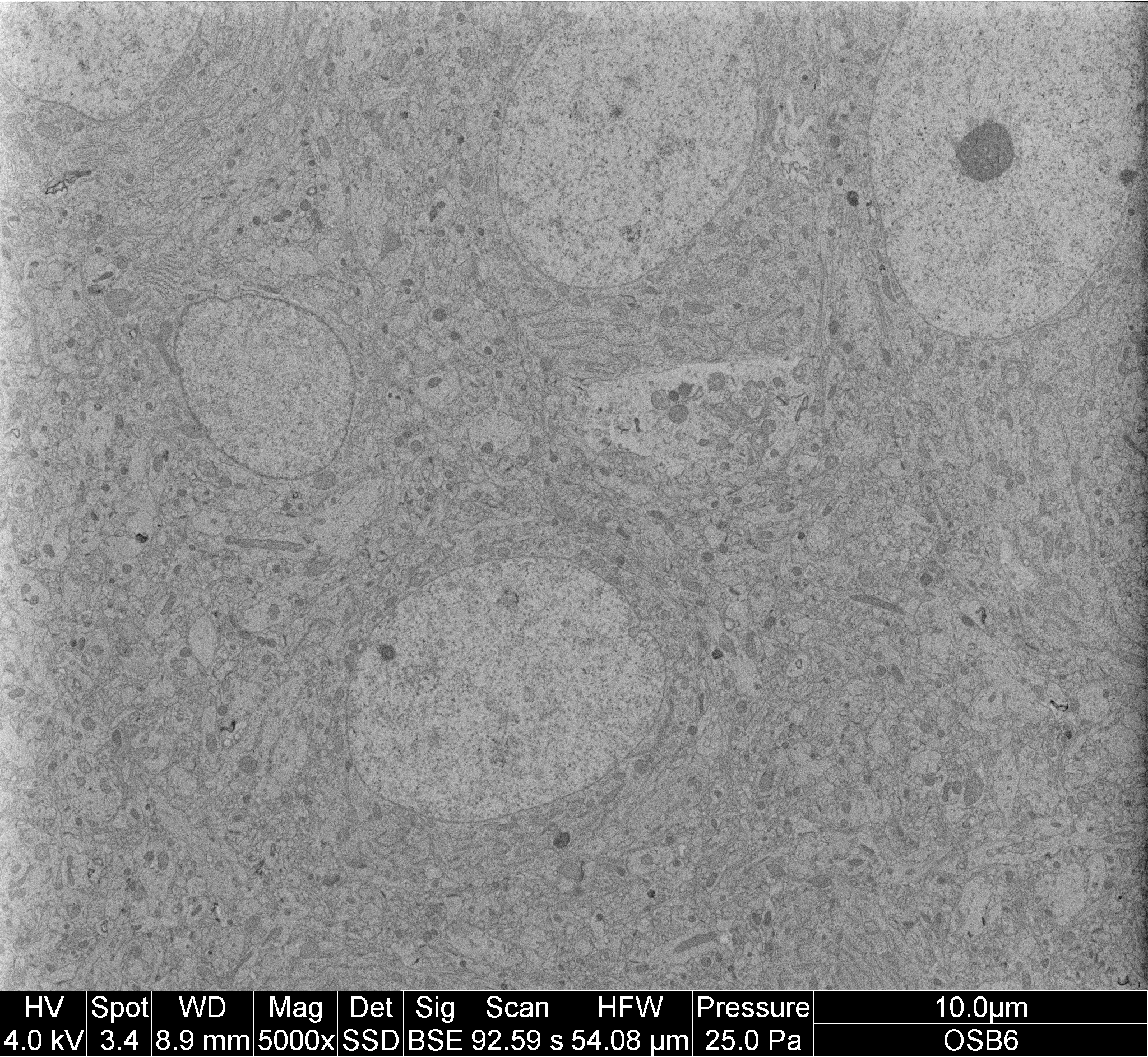

Supplement: Dataset S12 — (252.6 MB ZIP). [file pbio.0020329.sd012.zip › 040604_OS5_st1_1195.tif]

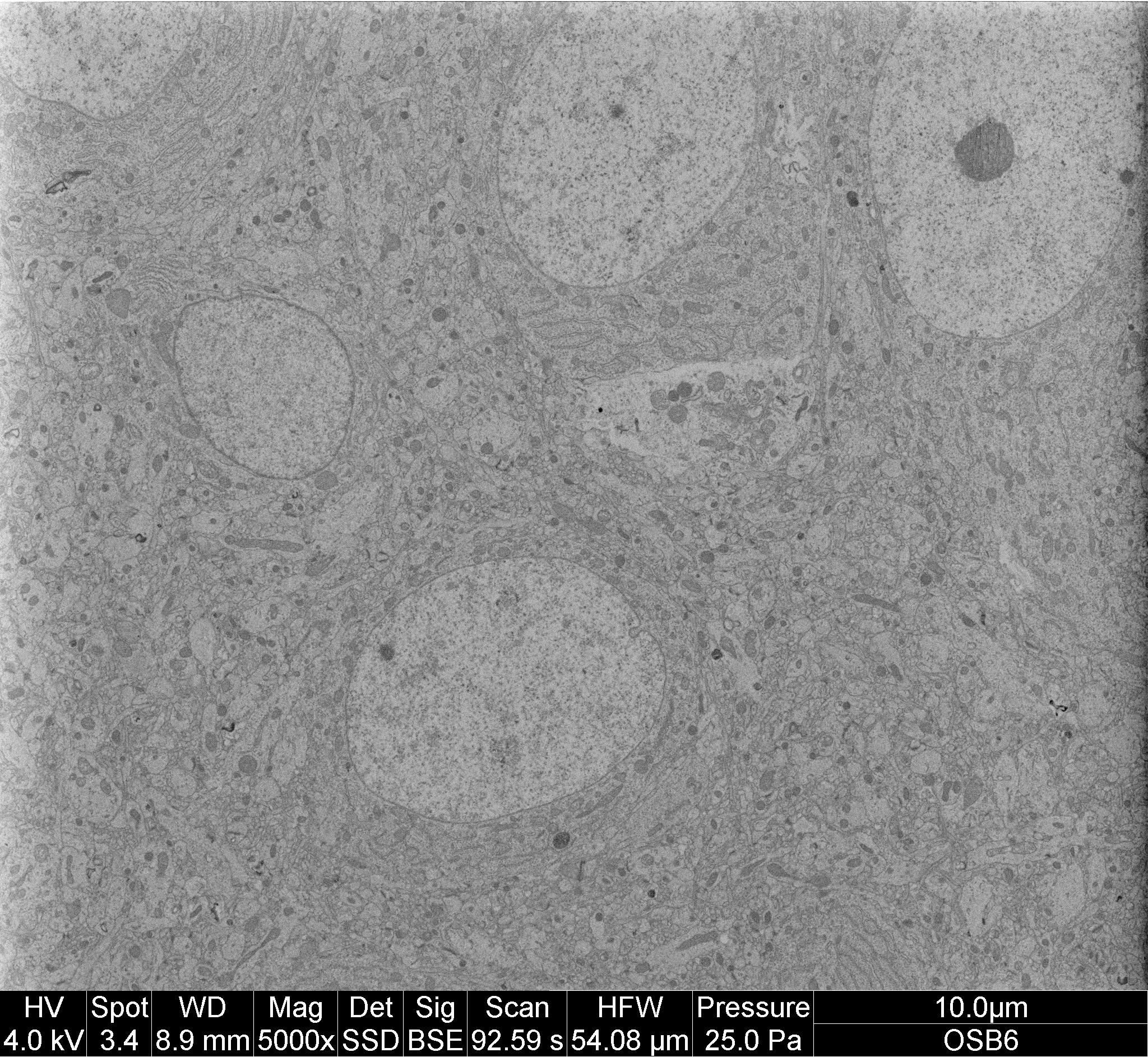

Supplement: Dataset S12 — (252.6 MB ZIP). [file pbio.0020329.sd012.zip › 040604_OS5_st1_1196.tif]

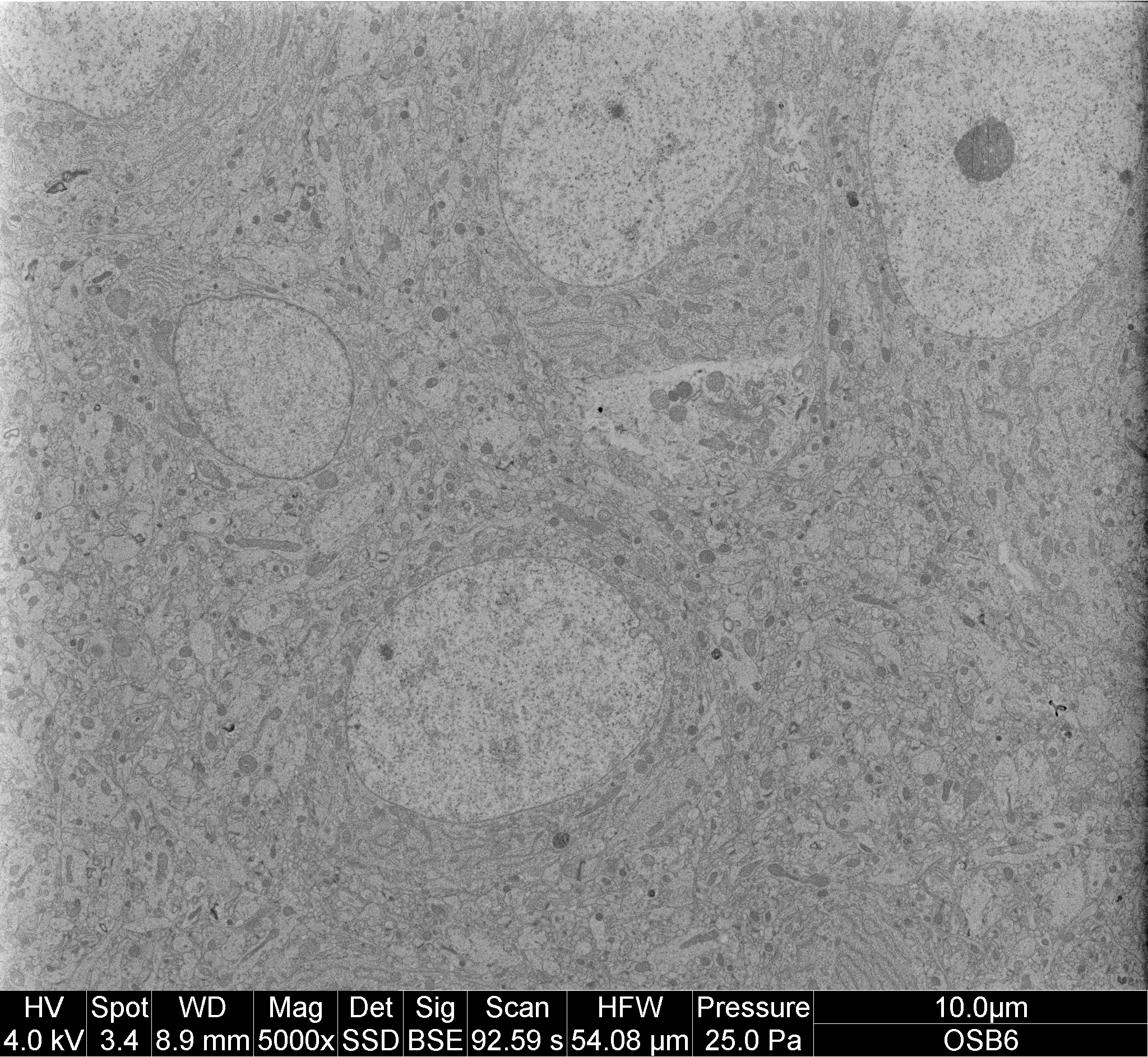

Supplement: Dataset S12 — (252.6 MB ZIP). [file pbio.0020329.sd012.zip › 040604_OS5_st1_1197.tif]

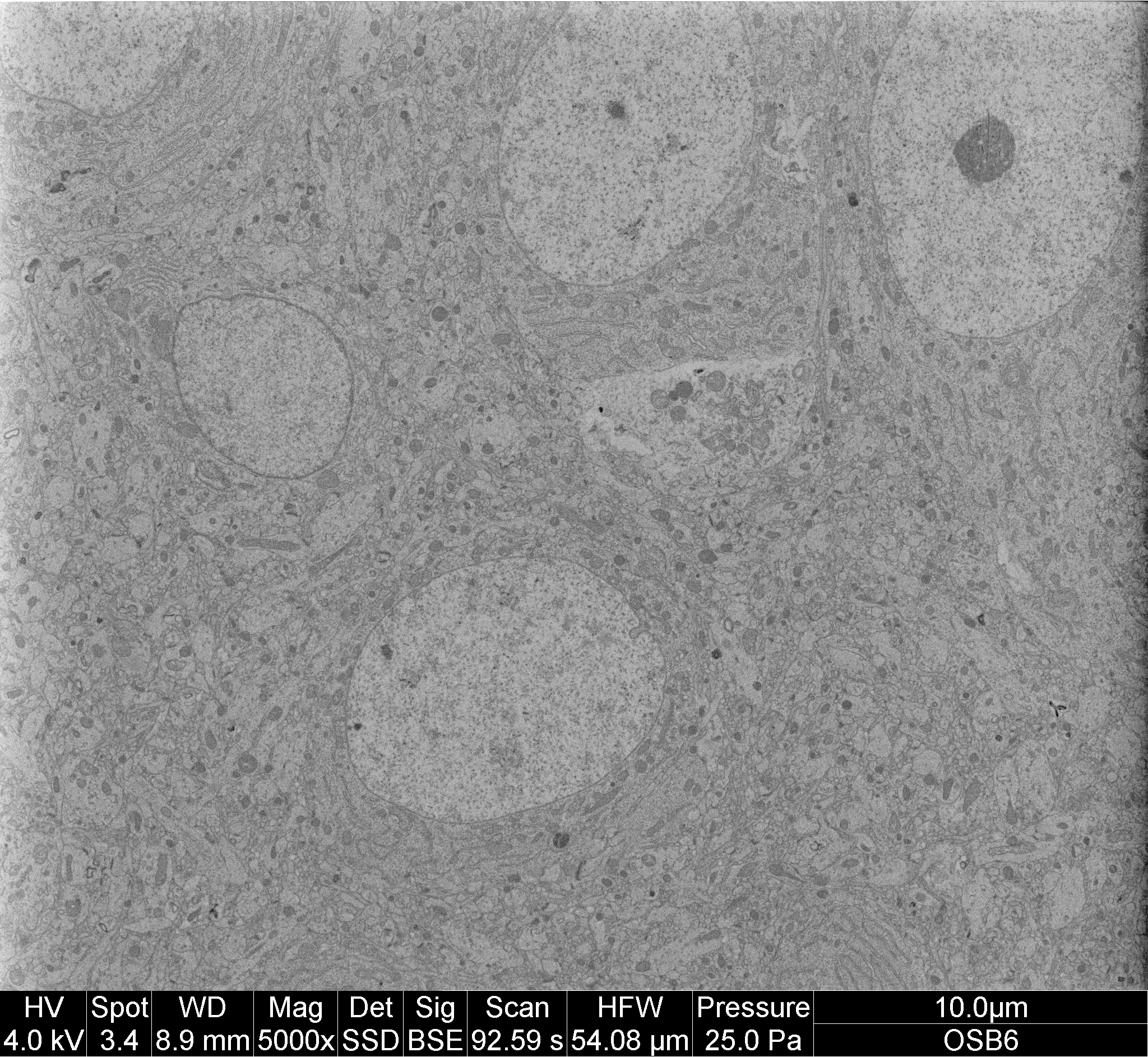

Supplement: Dataset S12 — (252.6 MB ZIP). [file pbio.0020329.sd012.zip › 040604_OS5_st1_1198.tif]

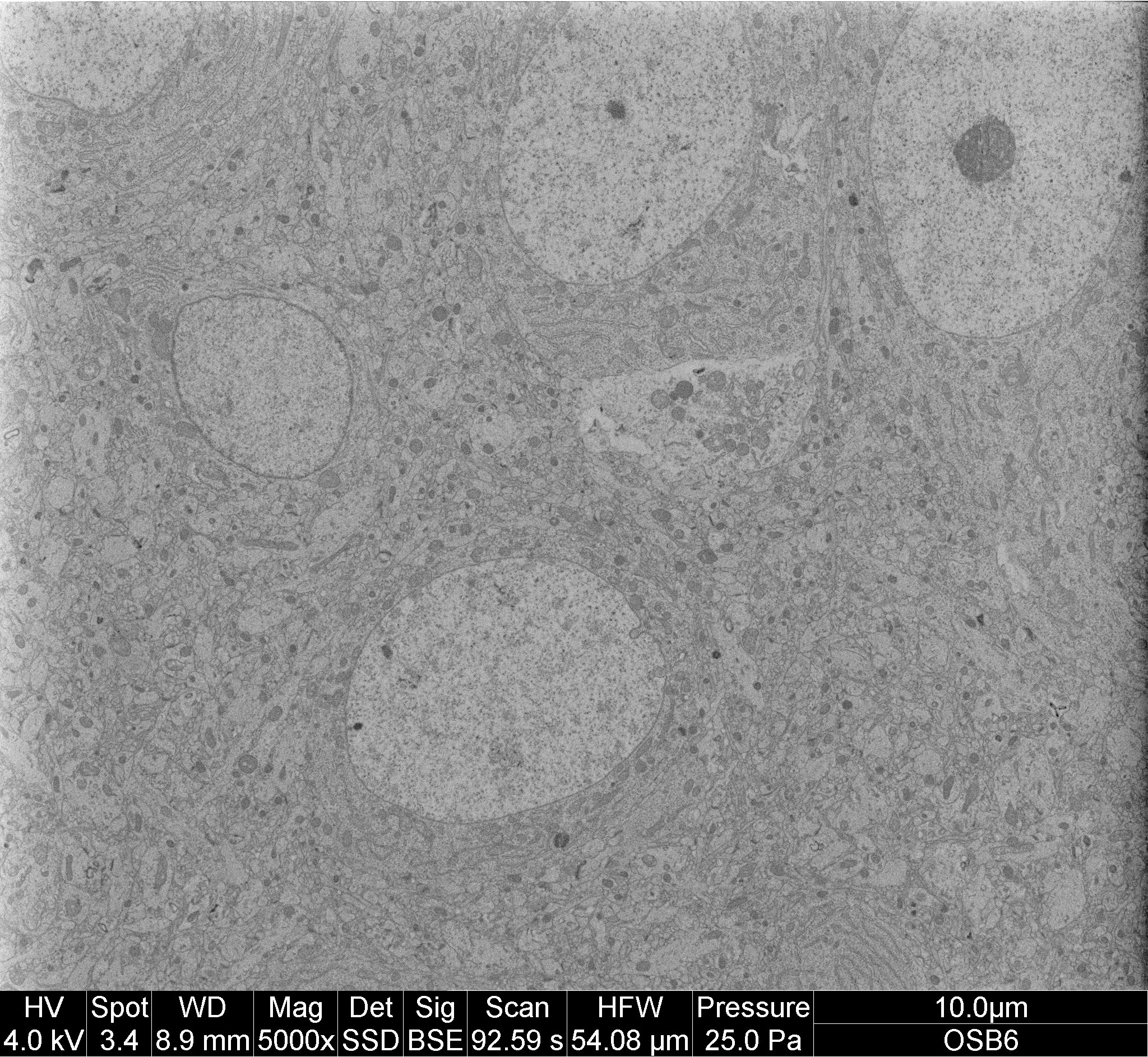

Supplement: Dataset S12 — (252.6 MB ZIP). [file pbio.0020329.sd012.zip › 040604_OS5_st1_1199.tif]

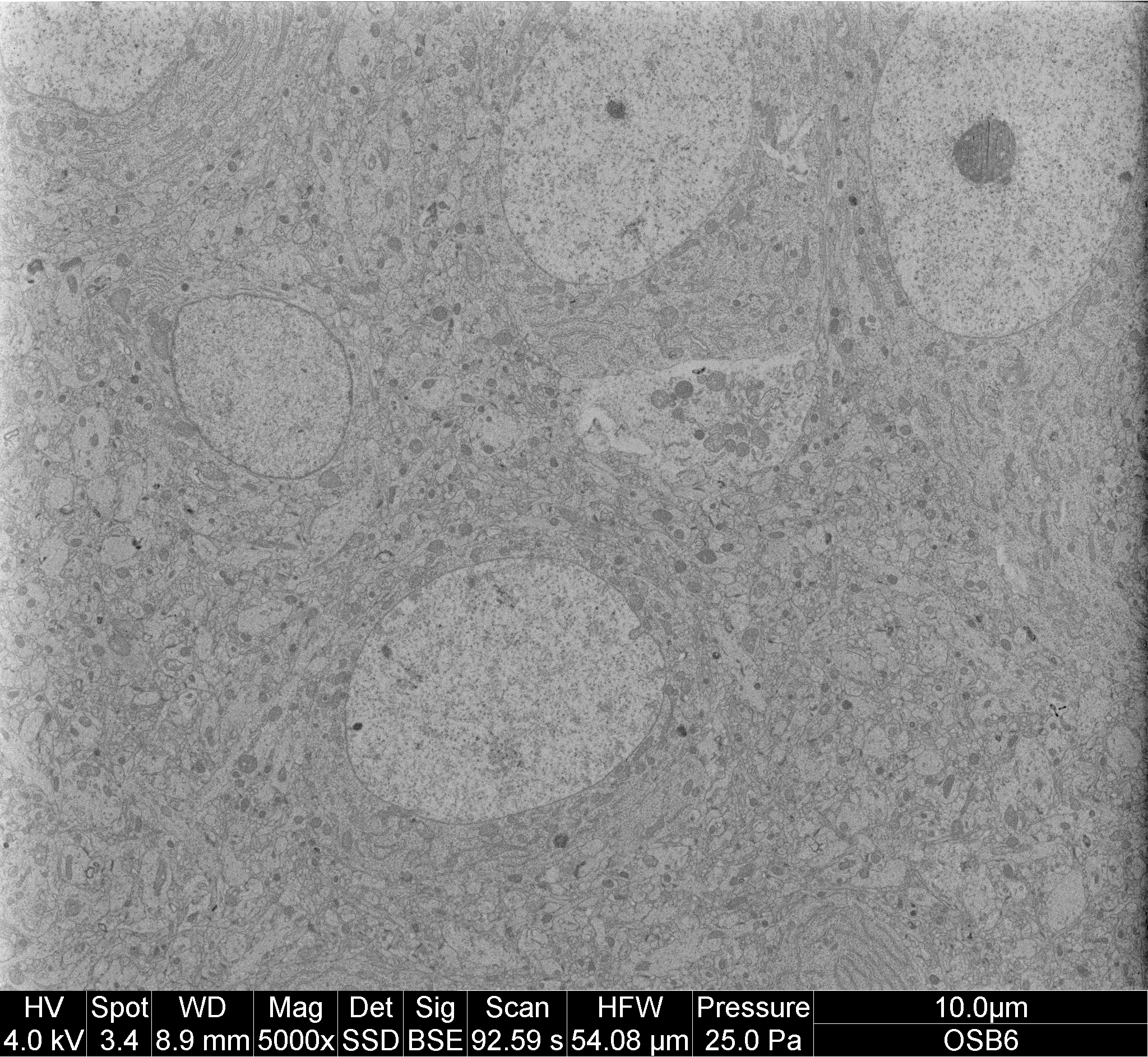

Supplement: Dataset S13 — (251.9 MB ZIP). [file pbio.0020329.sd013.zip › 040604_OS5_st1_1200.tif]
