# Supplementary figures and images for: Serial Block-Face Scanning Electron Microscopy to Reconstruct Three-Dimensional Tissue Nanostructure (part 13 of 21)
Source: PLoS Biol. 2004 Oct 19;2(11):e329. doi: 10.1371/journal.pbio.0020329 (PMC524270; doi:10.1371/journal.pbio.0020329)

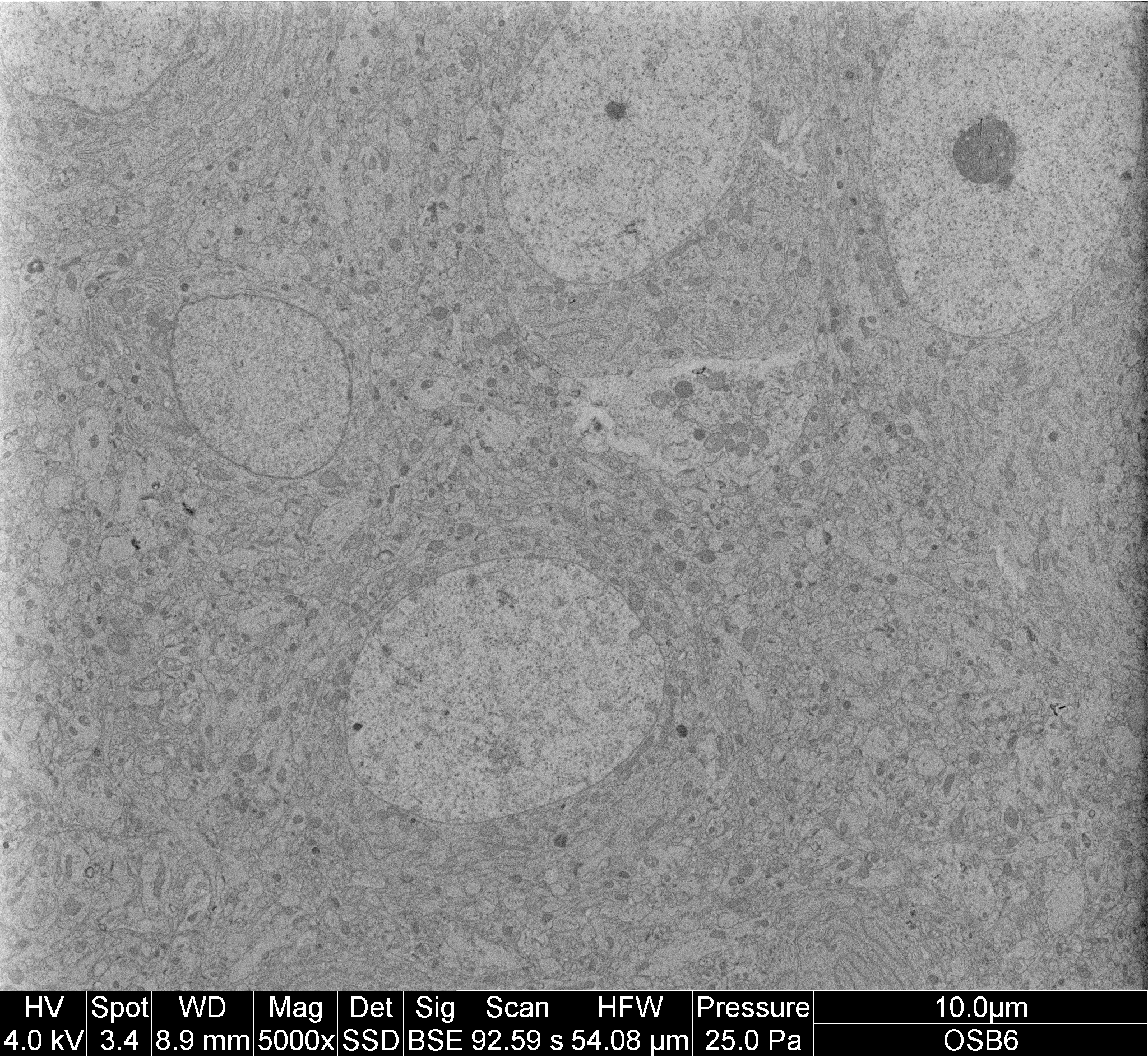

Supplement: Dataset S13 — (251.9 MB ZIP). [file pbio.0020329.sd013.zip › 040604_OS5_st1_1201.tif]

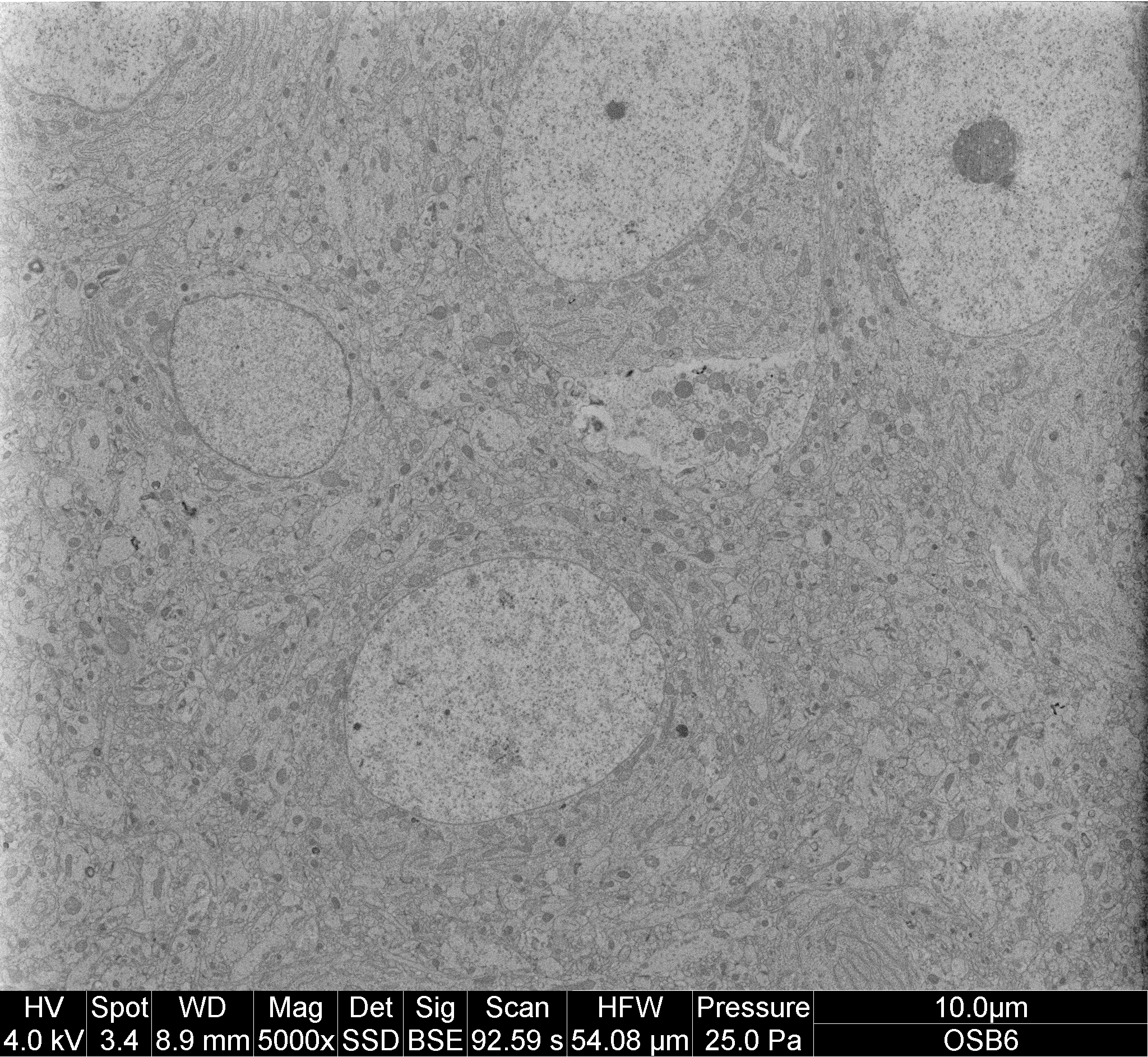

Supplement: Dataset S13 — (251.9 MB ZIP). [file pbio.0020329.sd013.zip › 040604_OS5_st1_1202.tif]

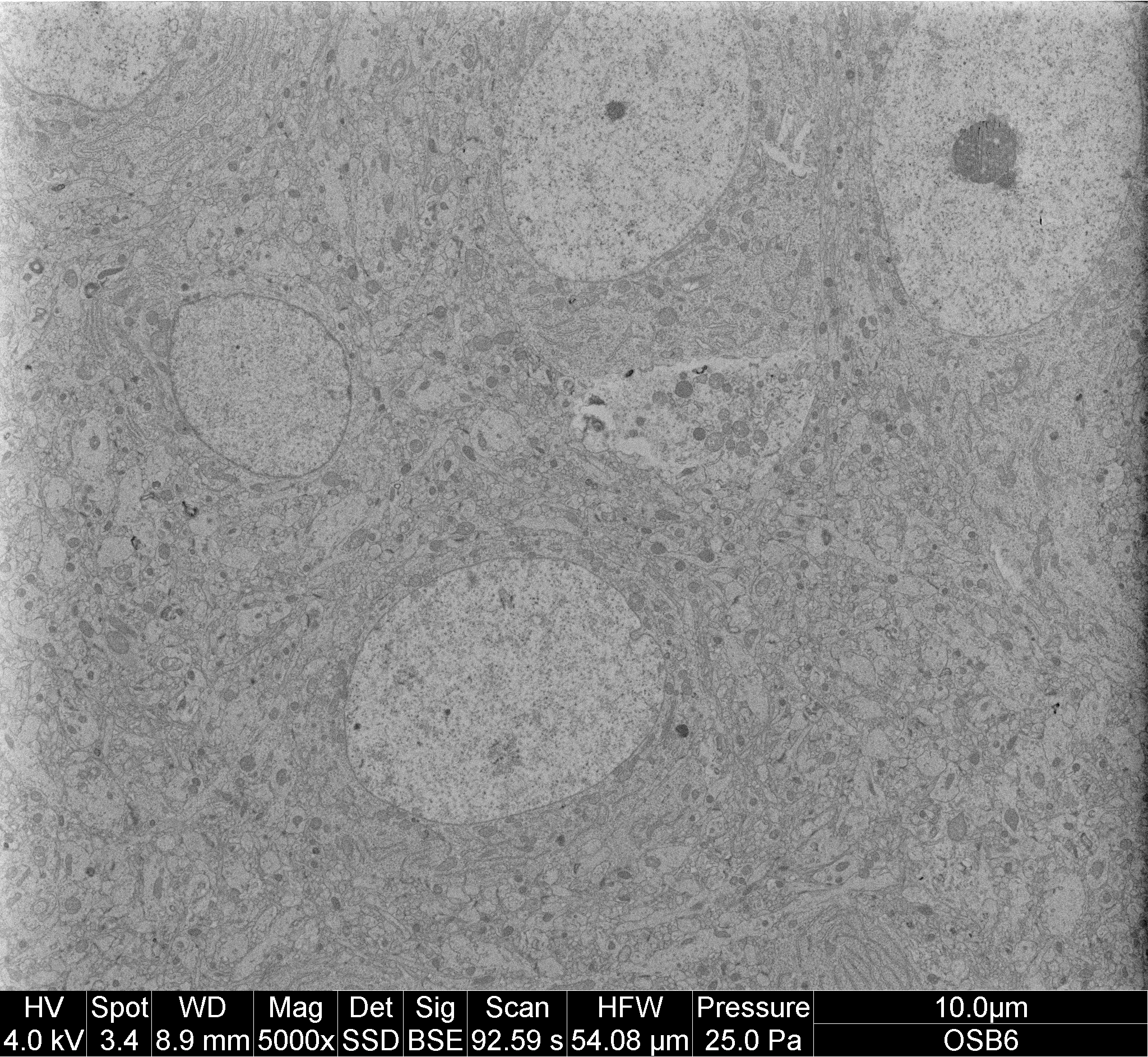

Supplement: Dataset S13 — (251.9 MB ZIP). [file pbio.0020329.sd013.zip › 040604_OS5_st1_1203.tif]

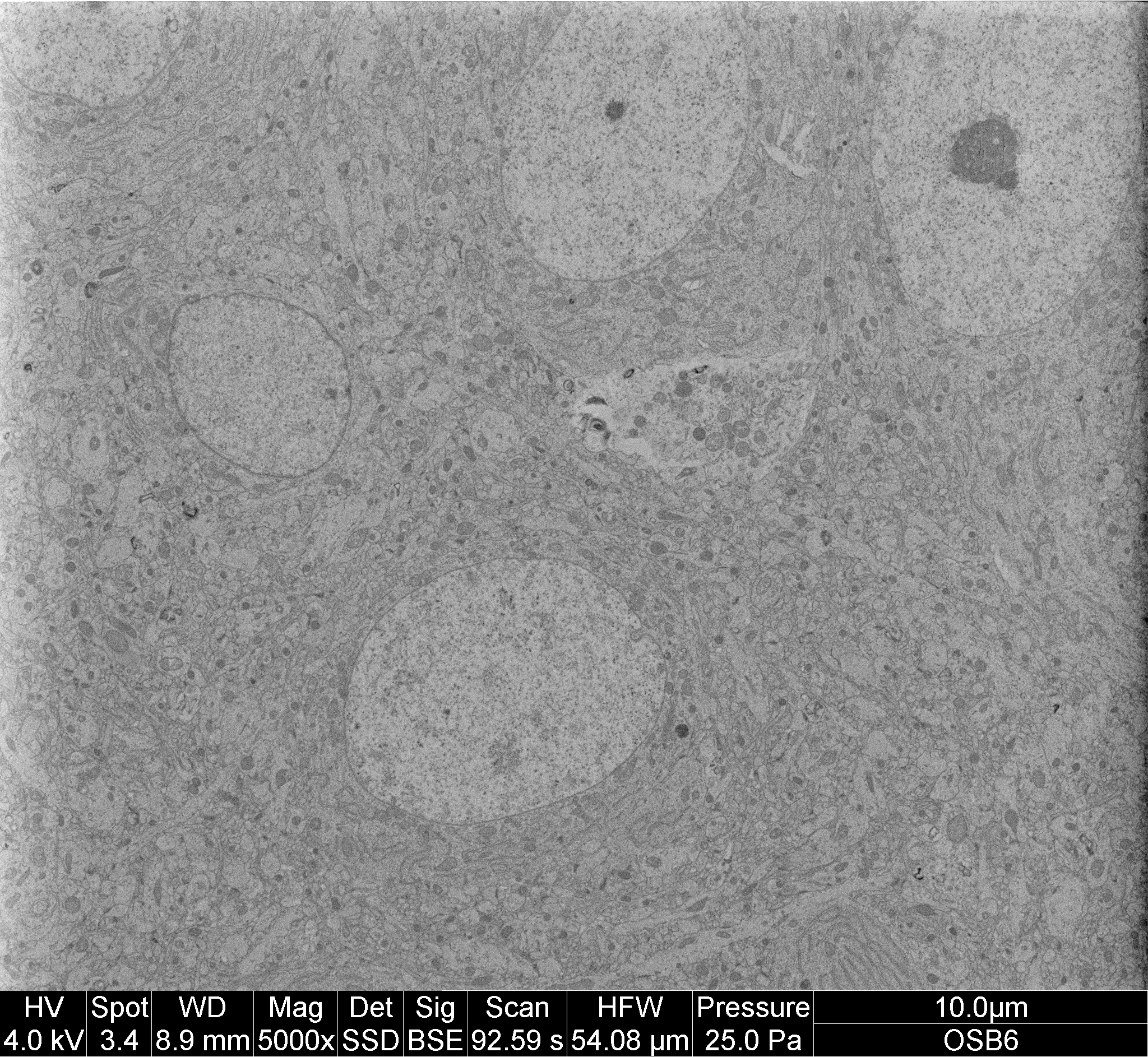

Supplement: Dataset S13 — (251.9 MB ZIP). [file pbio.0020329.sd013.zip › 040604_OS5_st1_1204.tif]

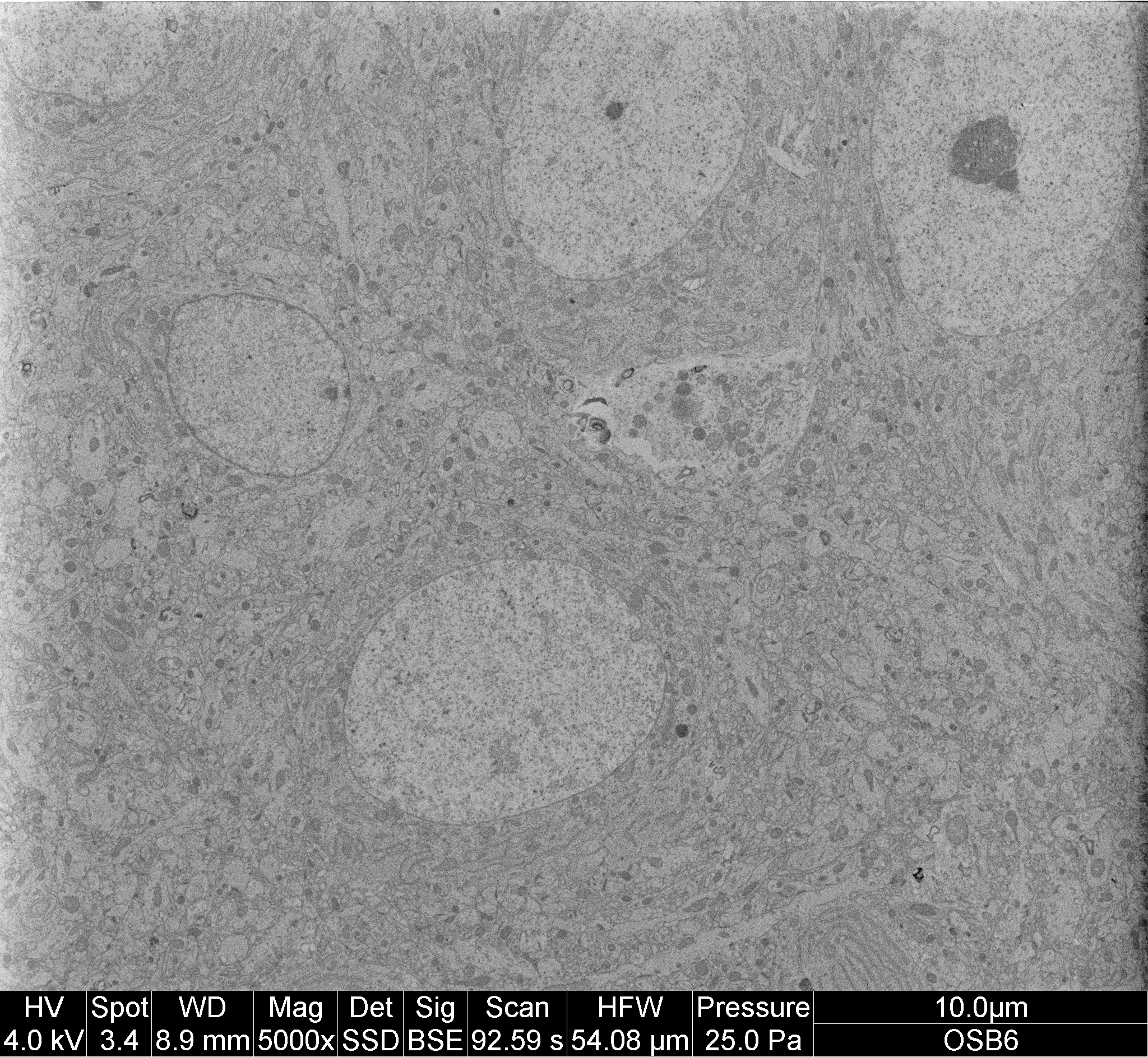

Supplement: Dataset S13 — (251.9 MB ZIP). [file pbio.0020329.sd013.zip › 040604_OS5_st1_1205.tif]

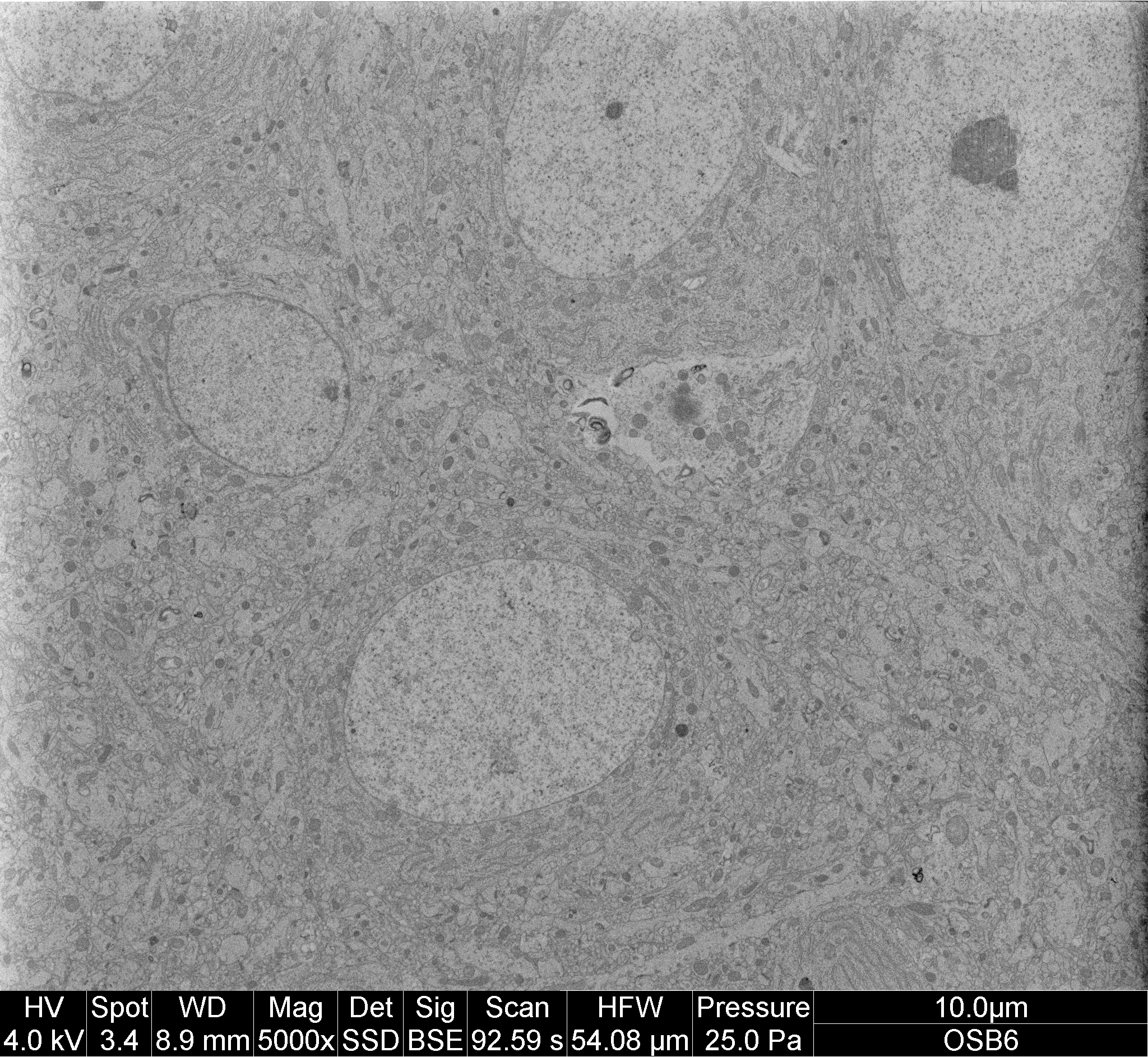

Supplement: Dataset S13 — (251.9 MB ZIP). [file pbio.0020329.sd013.zip › 040604_OS5_st1_1206.tif]

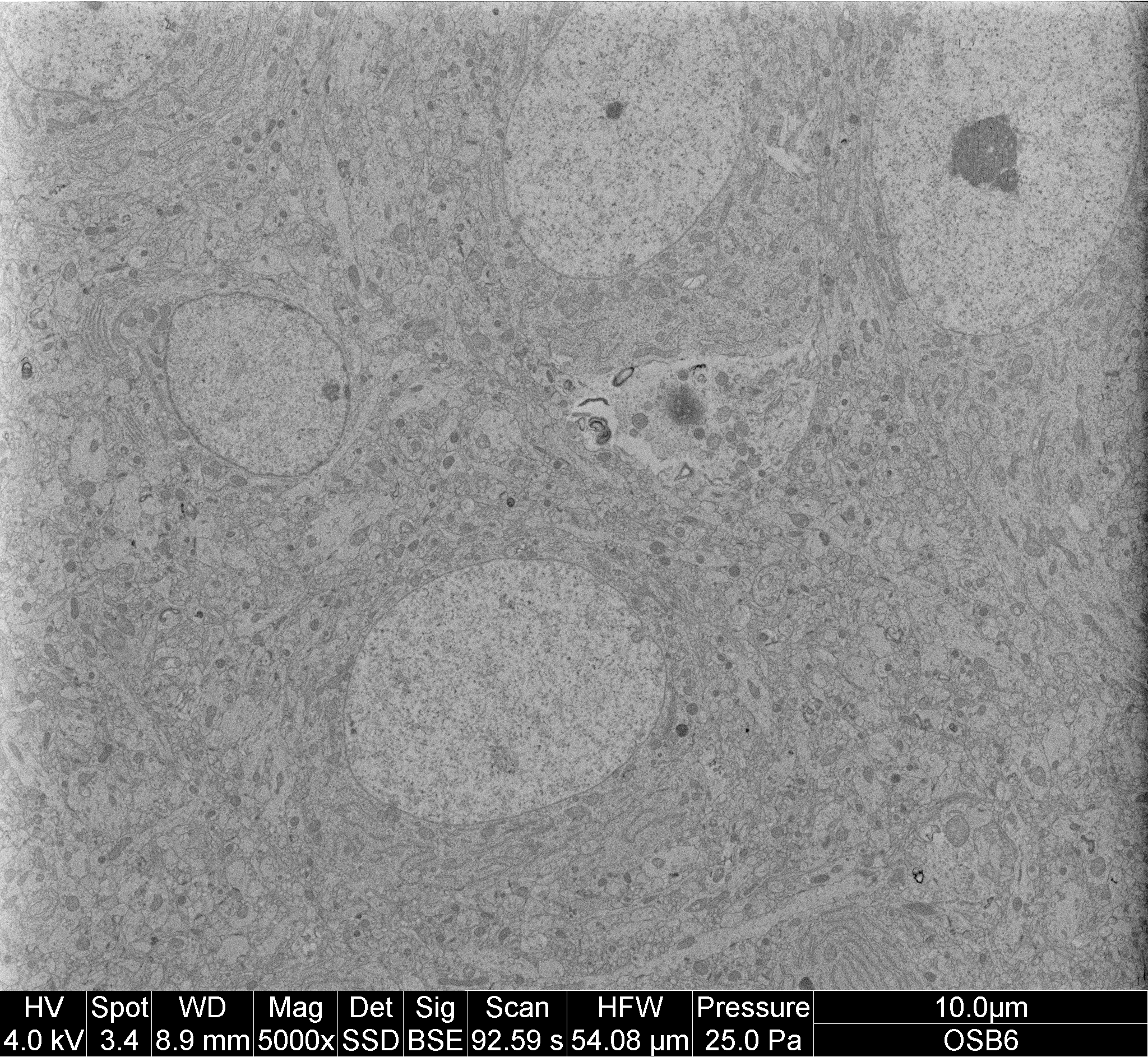

Supplement: Dataset S13 — (251.9 MB ZIP). [file pbio.0020329.sd013.zip › 040604_OS5_st1_1207.tif]

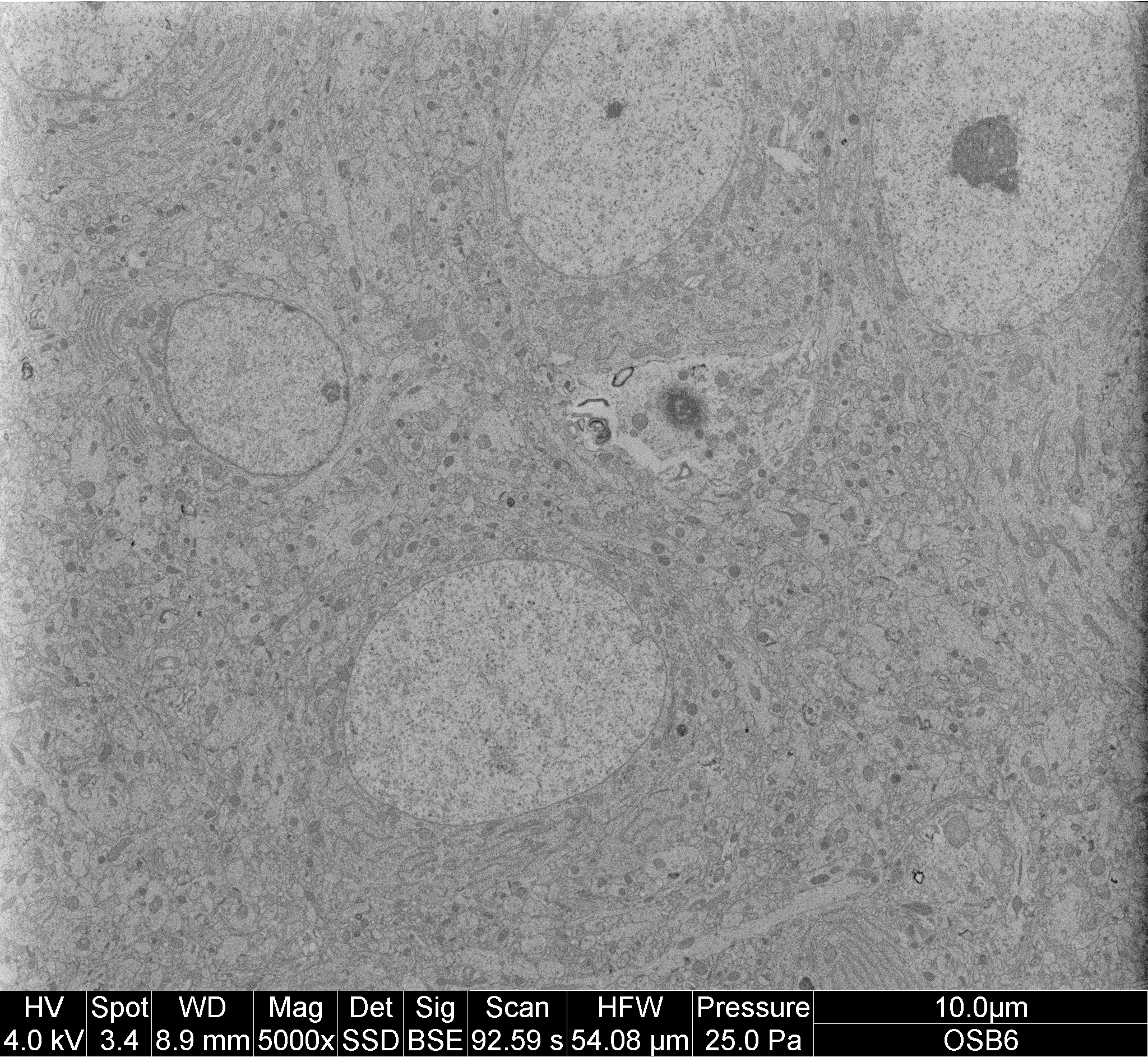

Supplement: Dataset S13 — (251.9 MB ZIP). [file pbio.0020329.sd013.zip › 040604_OS5_st1_1208.tif]

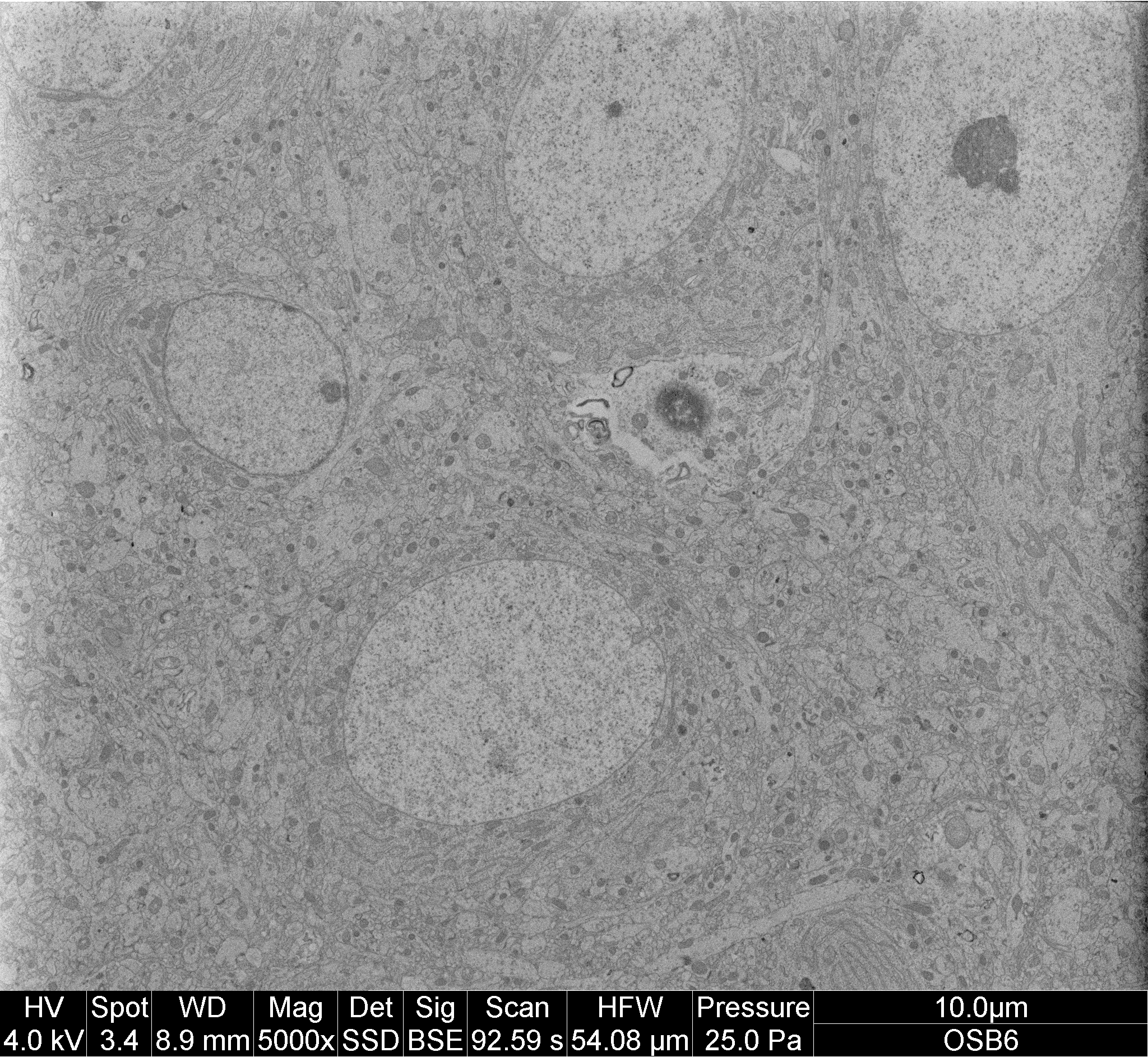

Supplement: Dataset S13 — (251.9 MB ZIP). [file pbio.0020329.sd013.zip › 040604_OS5_st1_1209.tif]

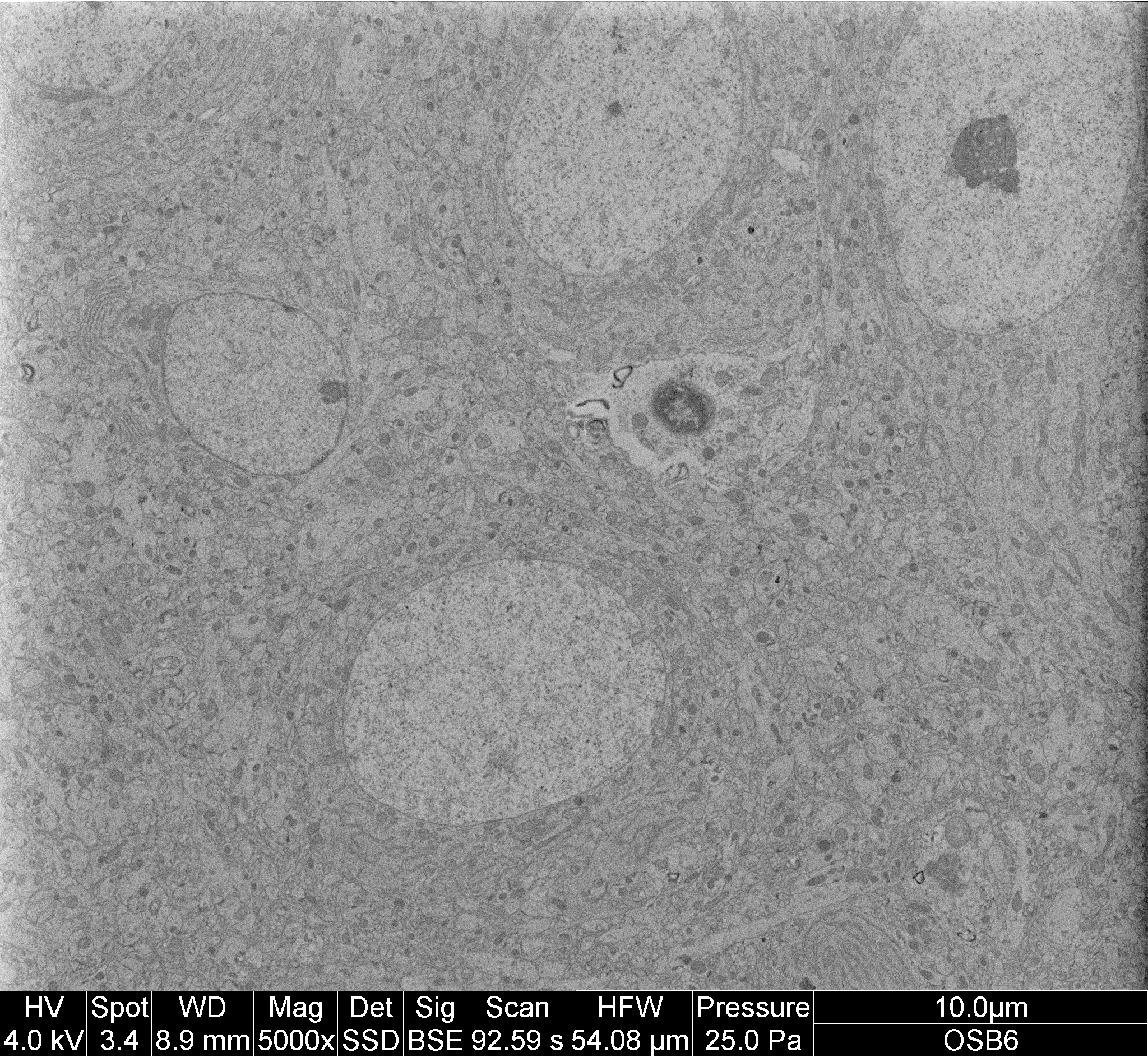

Supplement: Dataset S13 — (251.9 MB ZIP). [file pbio.0020329.sd013.zip › 040604_OS5_st1_1210.tif]

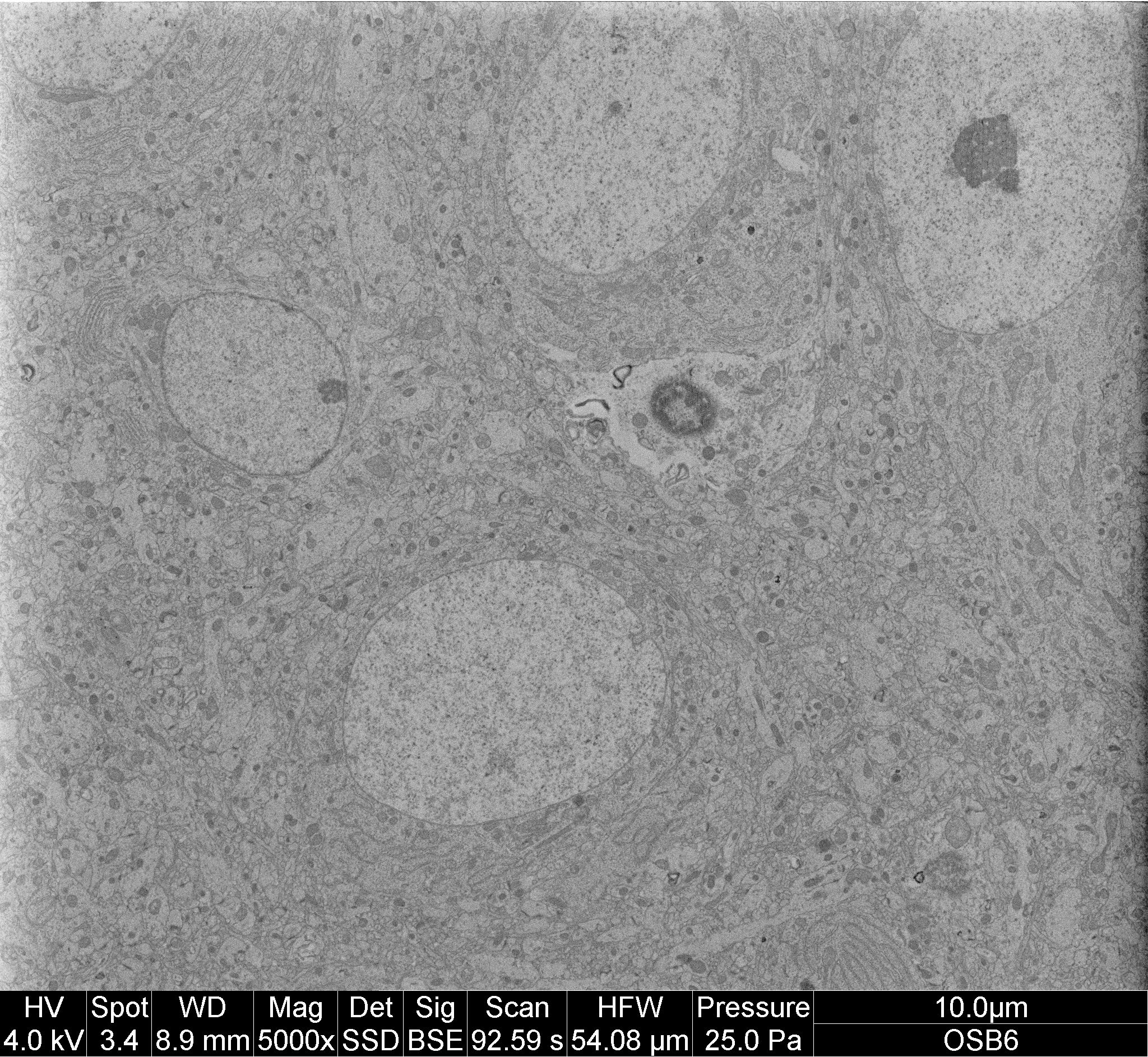

Supplement: Dataset S13 — (251.9 MB ZIP). [file pbio.0020329.sd013.zip › 040604_OS5_st1_1211.tif]

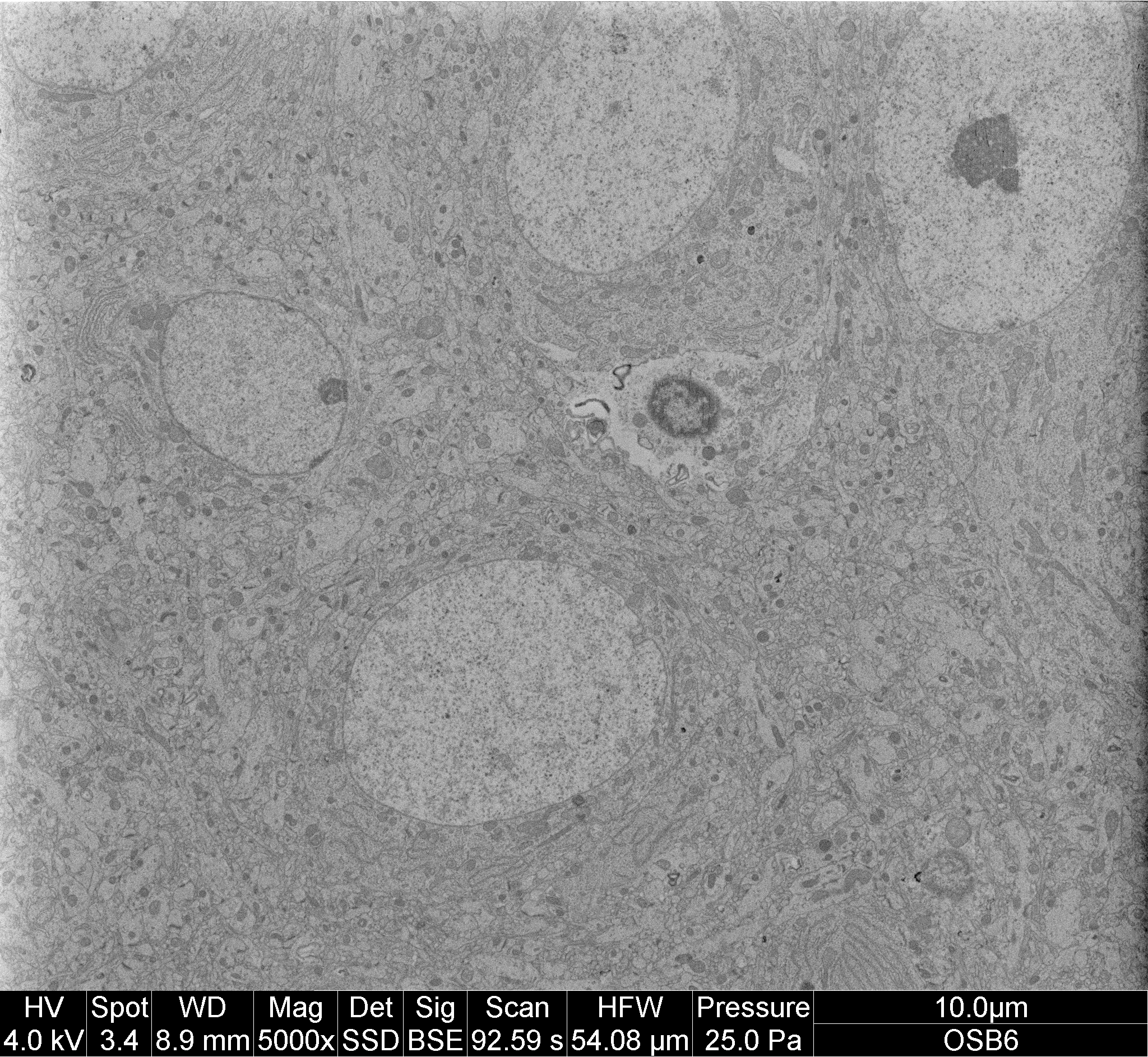

Supplement: Dataset S13 — (251.9 MB ZIP). [file pbio.0020329.sd013.zip › 040604_OS5_st1_1212.tif]

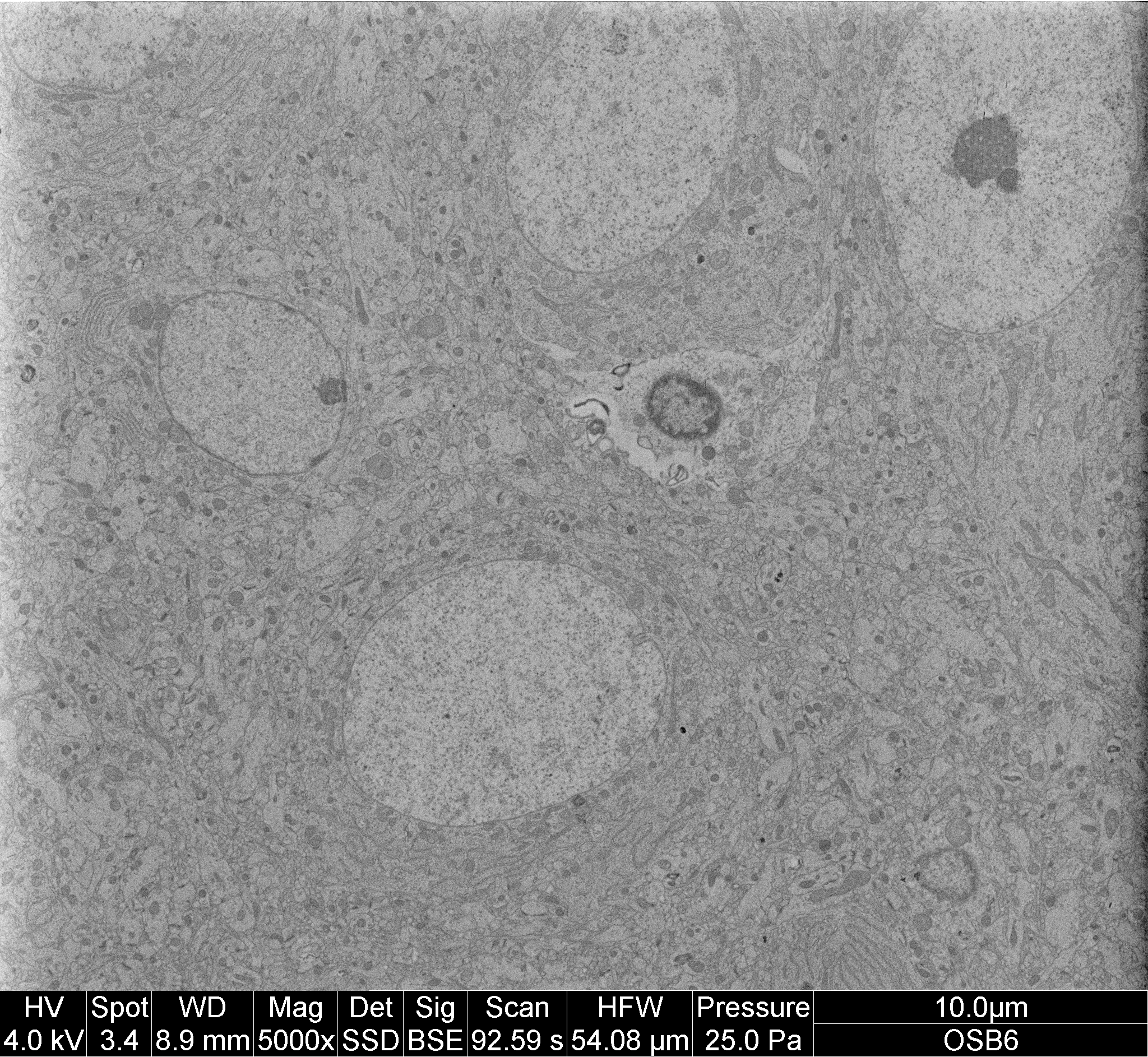

Supplement: Dataset S13 — (251.9 MB ZIP). [file pbio.0020329.sd013.zip › 040604_OS5_st1_1213.tif]

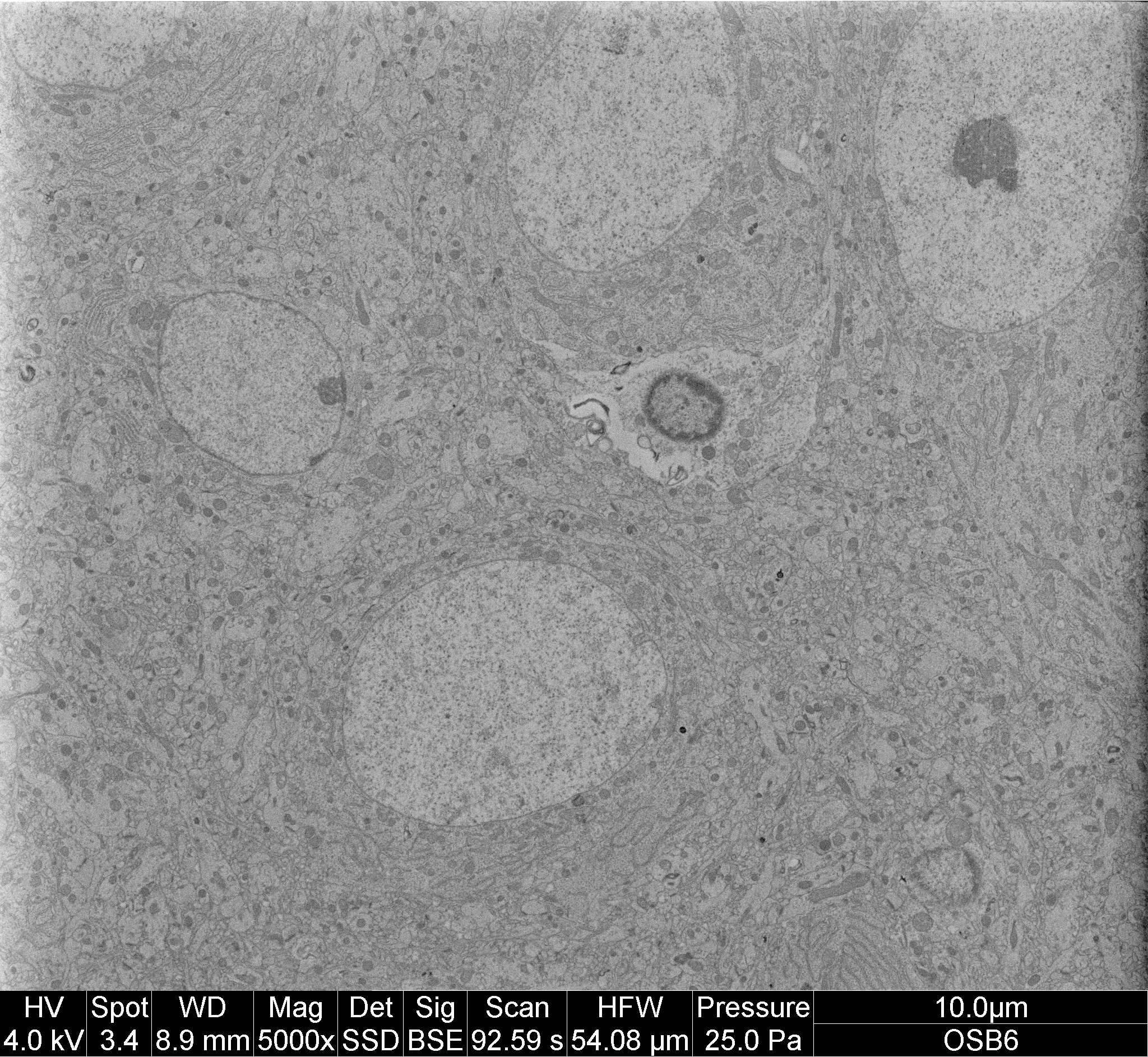

Supplement: Dataset S13 — (251.9 MB ZIP). [file pbio.0020329.sd013.zip › 040604_OS5_st1_1214.tif]

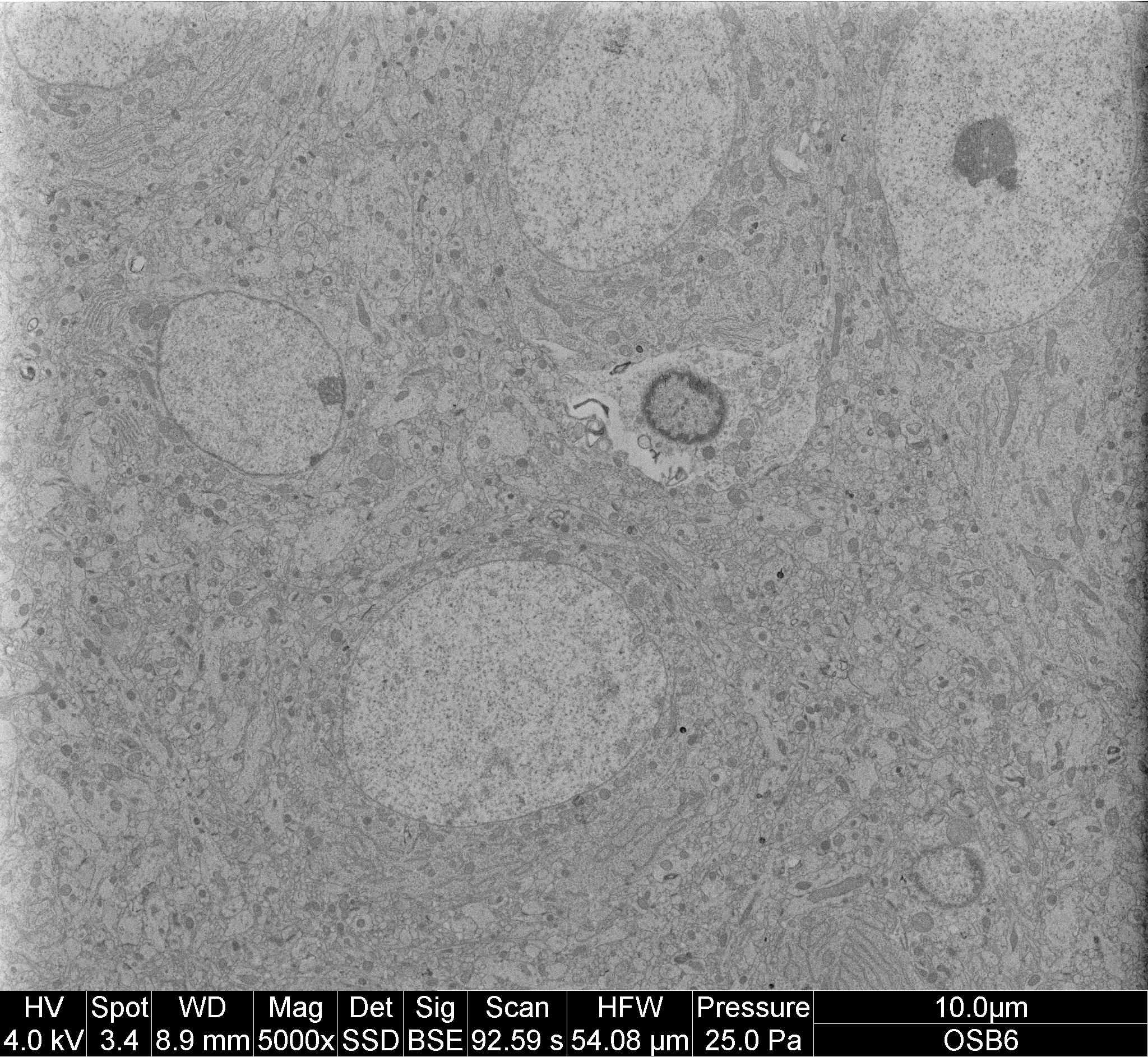

Supplement: Dataset S13 — (251.9 MB ZIP). [file pbio.0020329.sd013.zip › 040604_OS5_st1_1215.tif]

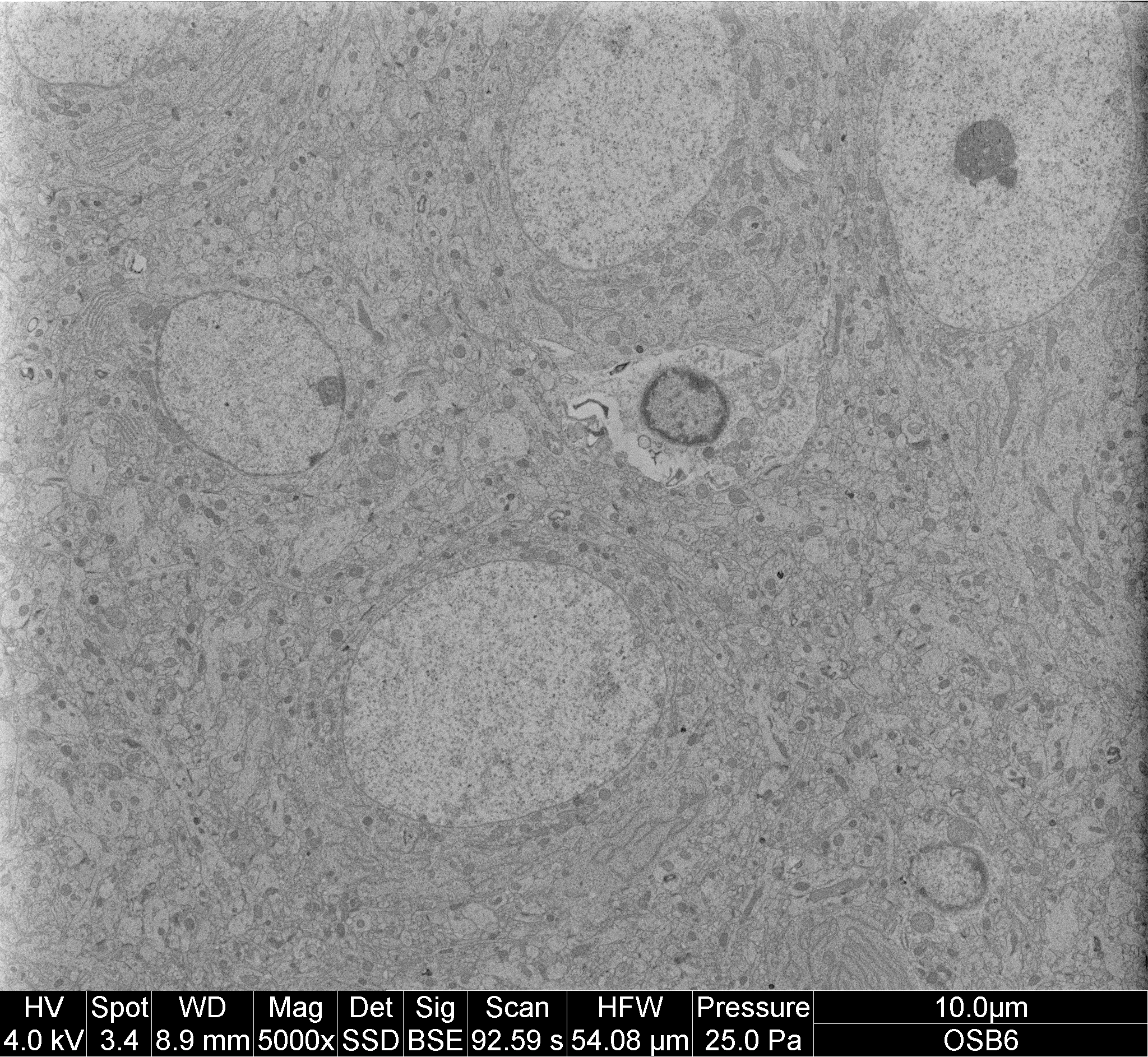

Supplement: Dataset S13 — (251.9 MB ZIP). [file pbio.0020329.sd013.zip › 040604_OS5_st1_1216.tif]

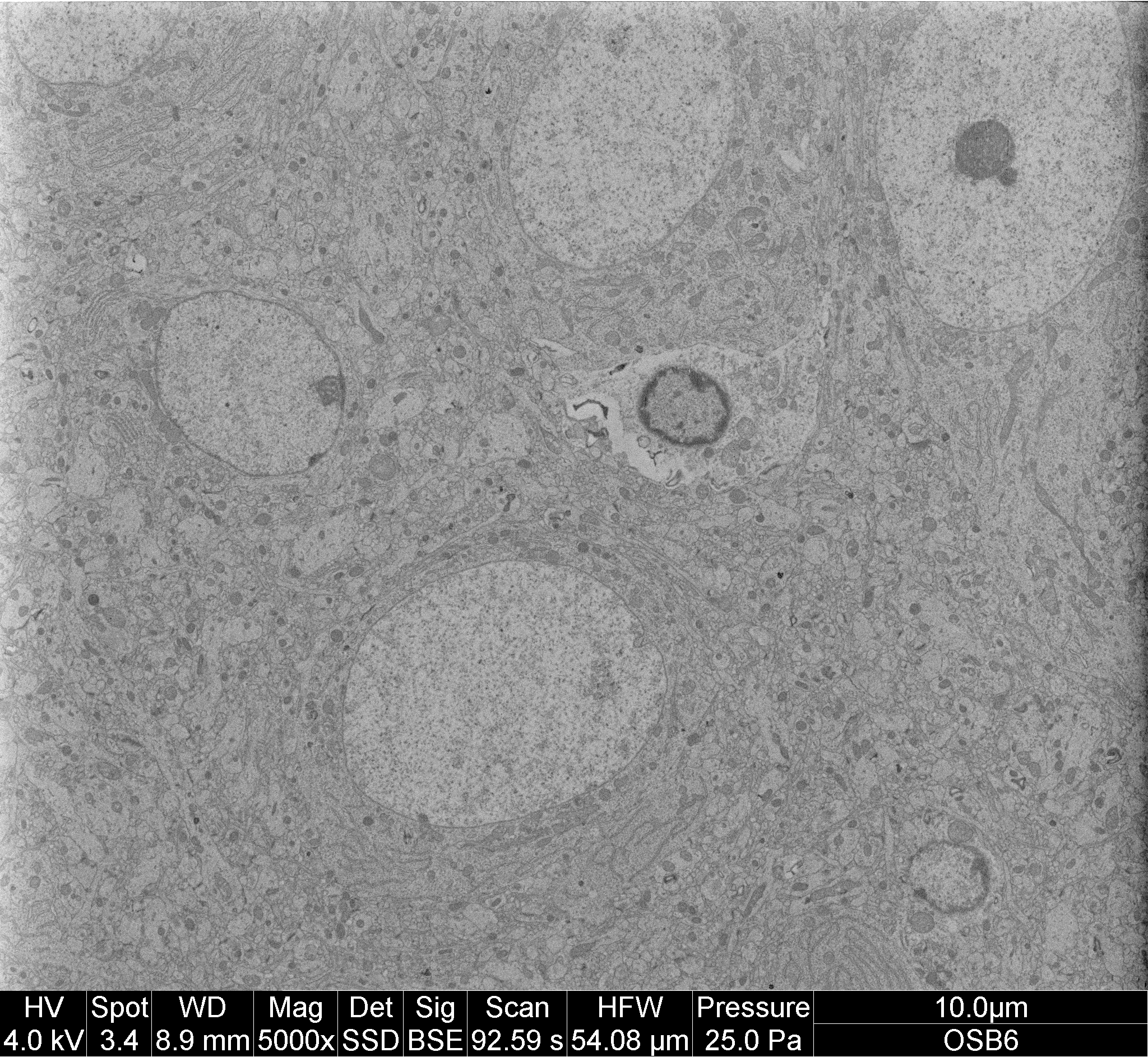

Supplement: Dataset S13 — (251.9 MB ZIP). [file pbio.0020329.sd013.zip › 040604_OS5_st1_1217.tif]

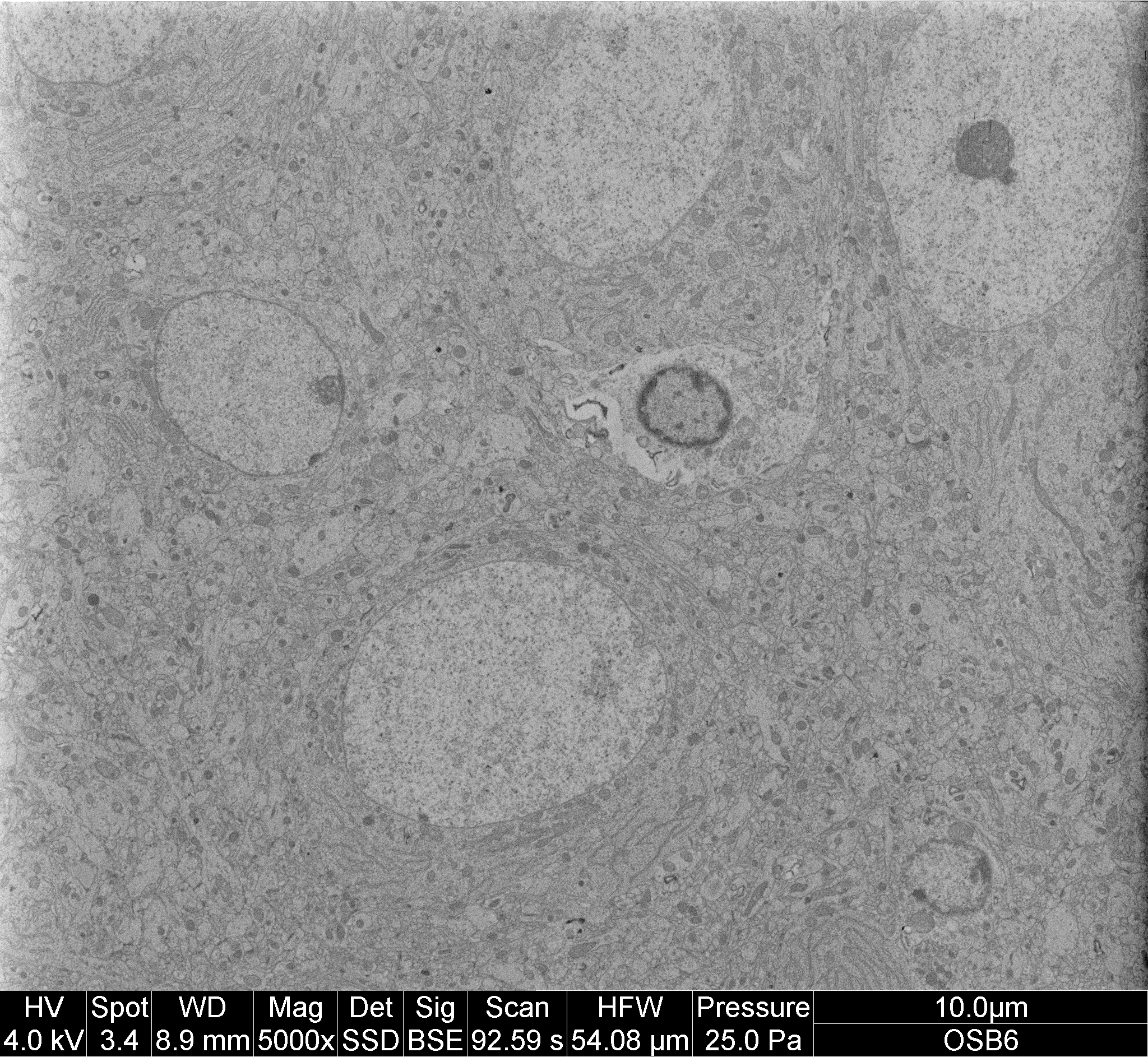

Supplement: Dataset S13 — (251.9 MB ZIP). [file pbio.0020329.sd013.zip › 040604_OS5_st1_1218.tif]

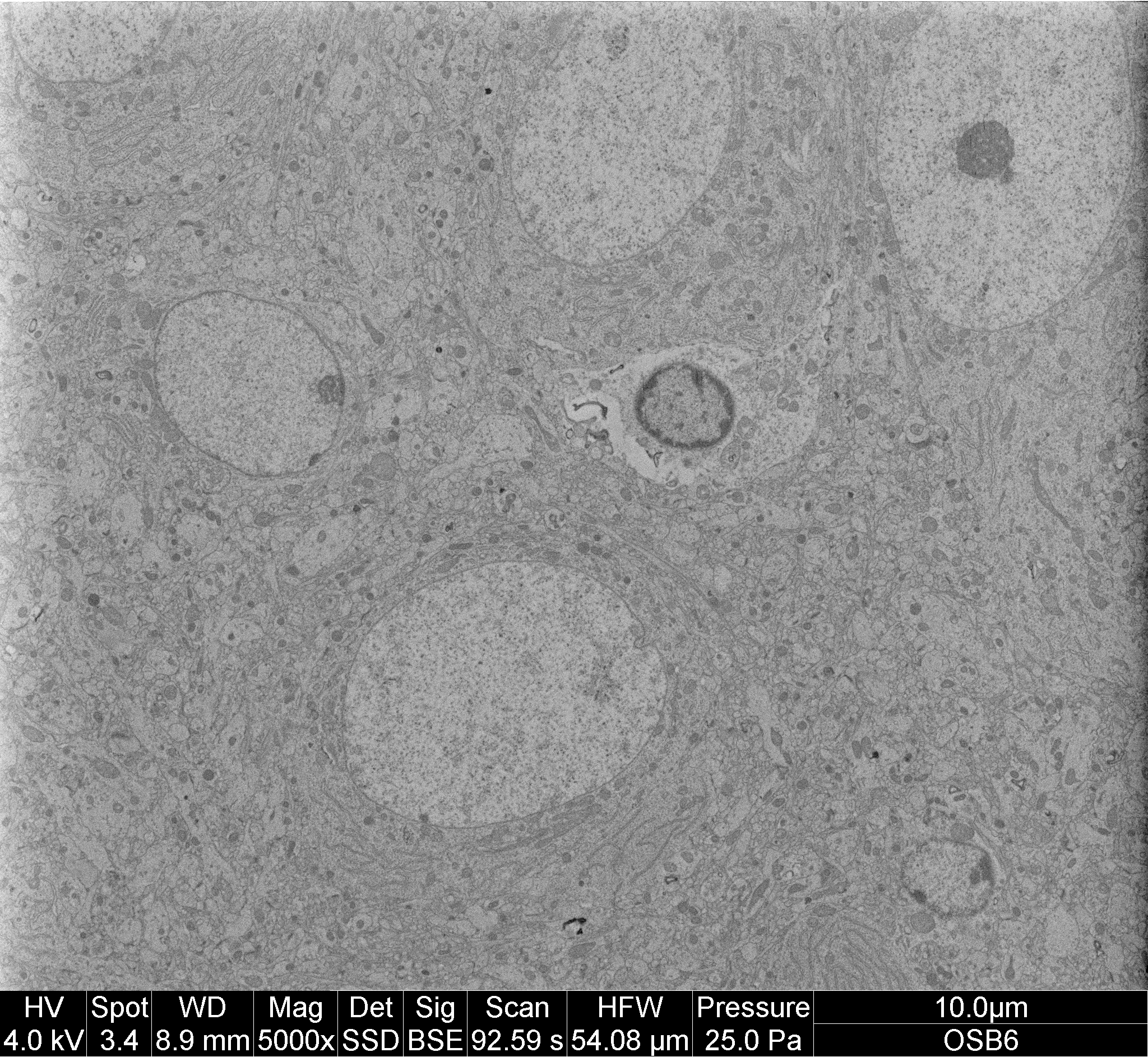

Supplement: Dataset S13 — (251.9 MB ZIP). [file pbio.0020329.sd013.zip › 040604_OS5_st1_1219.tif]

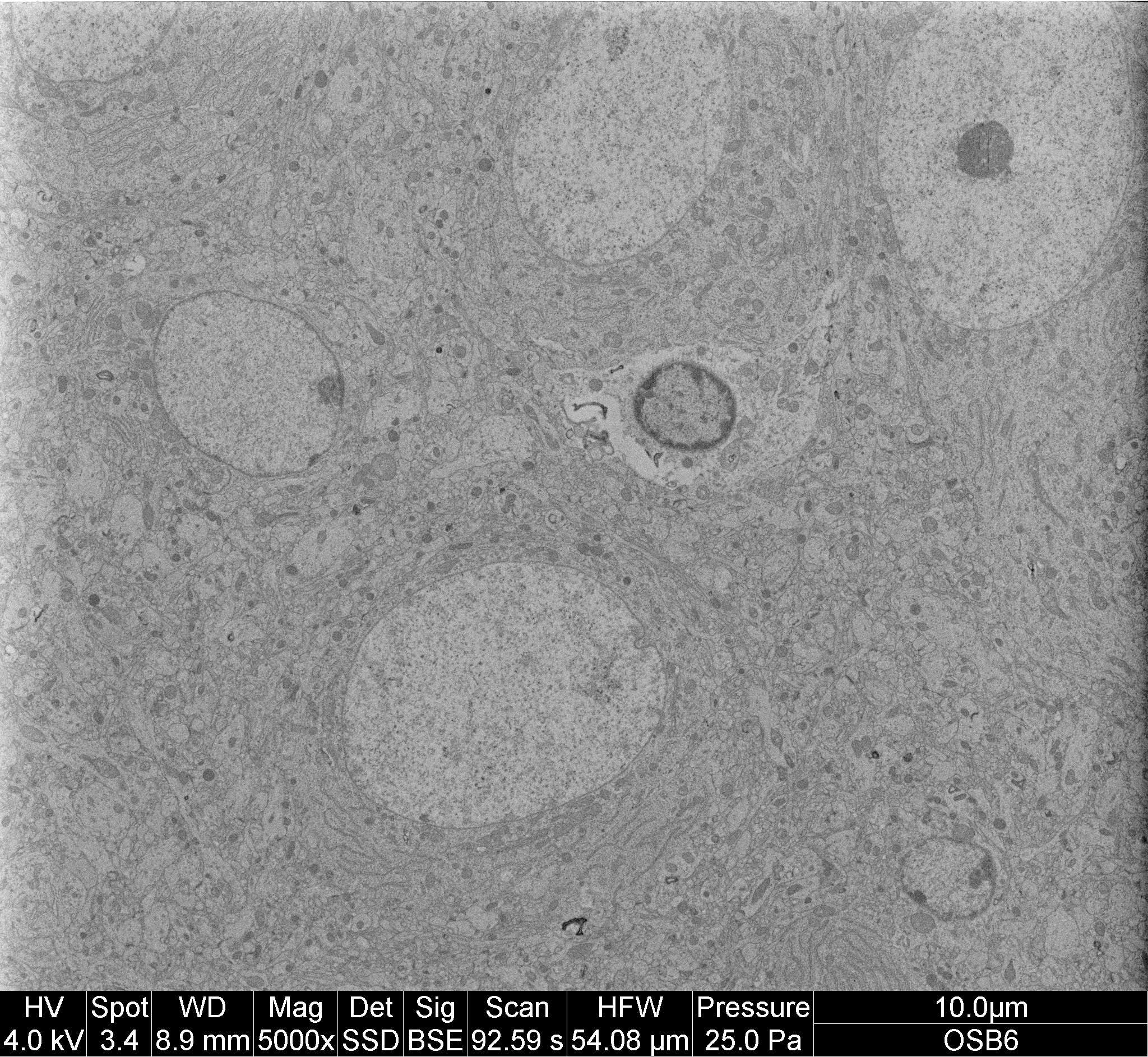

Supplement: Dataset S13 — (251.9 MB ZIP). [file pbio.0020329.sd013.zip › 040604_OS5_st1_1220.tif]

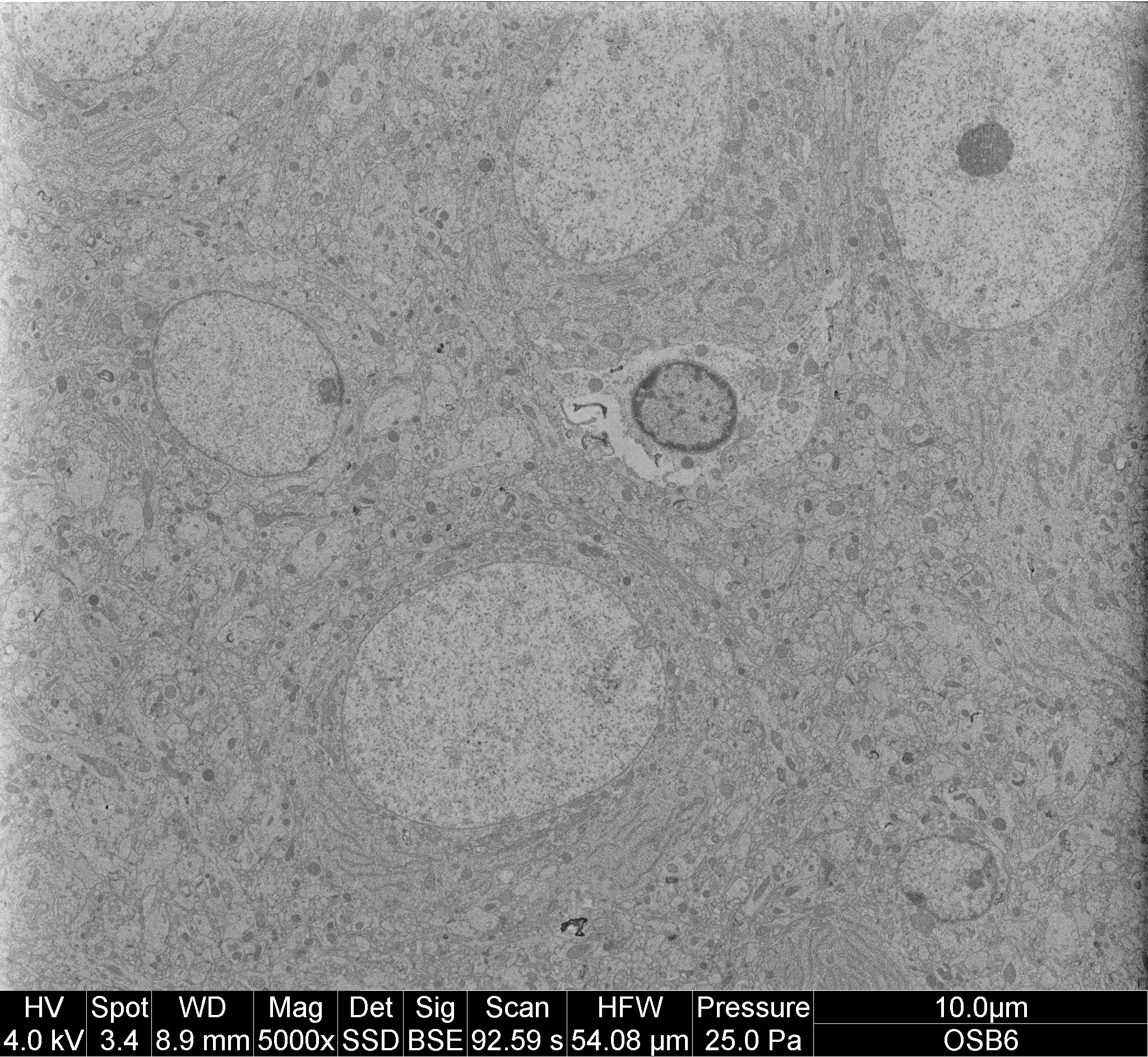

Supplement: Dataset S13 — (251.9 MB ZIP). [file pbio.0020329.sd013.zip › 040604_OS5_st1_1221.tif]

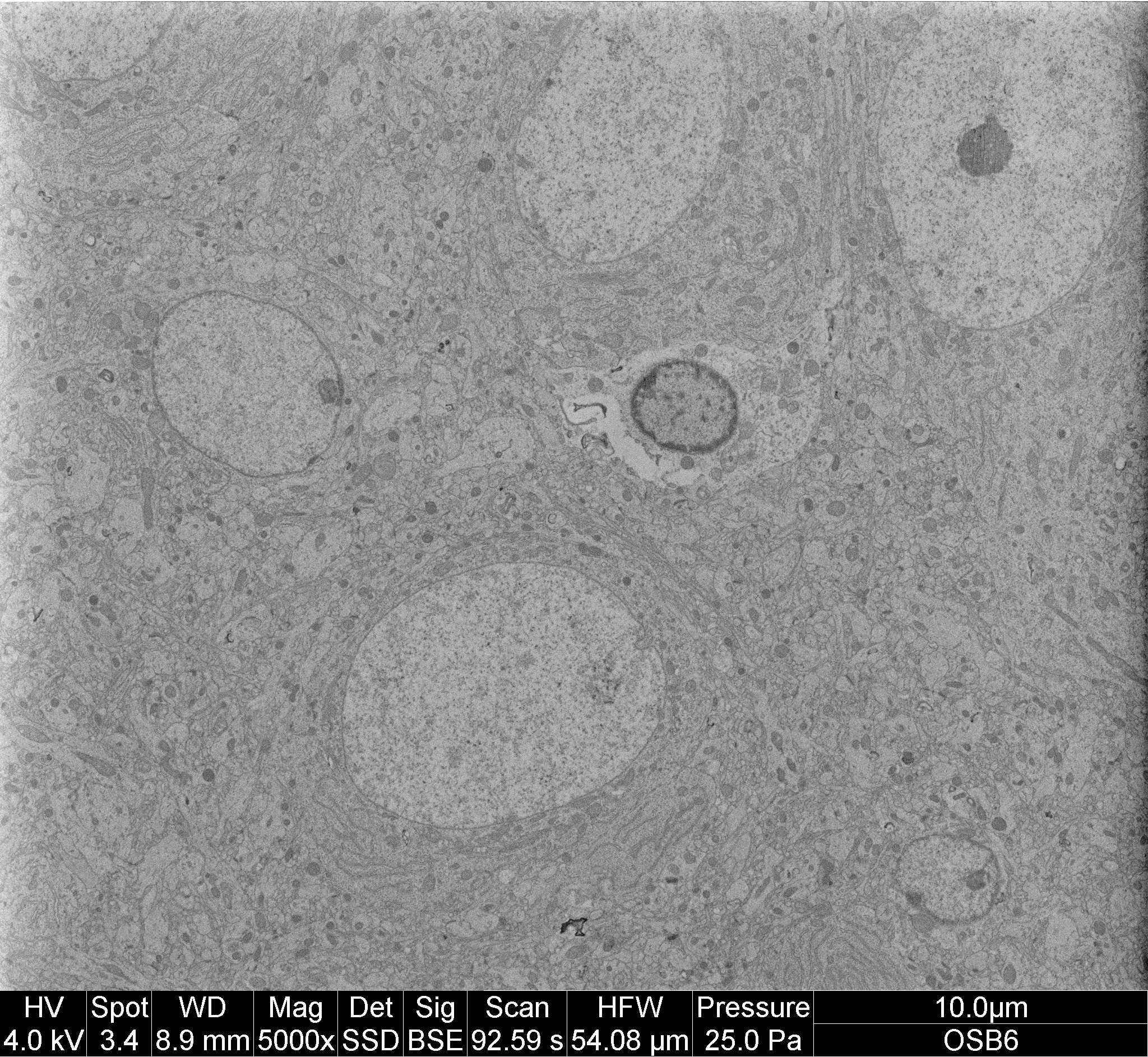

Supplement: Dataset S13 — (251.9 MB ZIP). [file pbio.0020329.sd013.zip › 040604_OS5_st1_1222.tif]

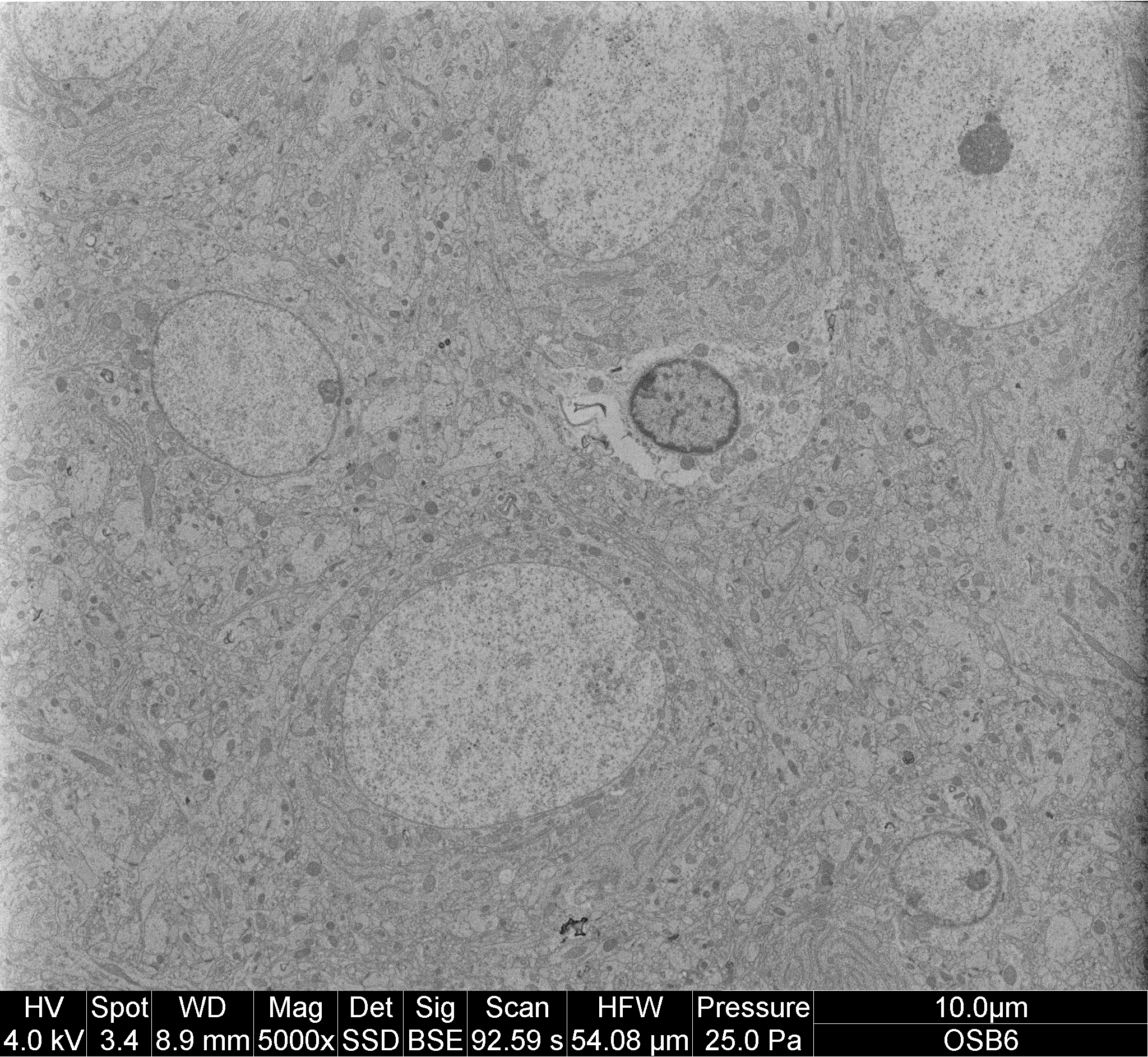

Supplement: Dataset S13 — (251.9 MB ZIP). [file pbio.0020329.sd013.zip › 040604_OS5_st1_1223.tif]

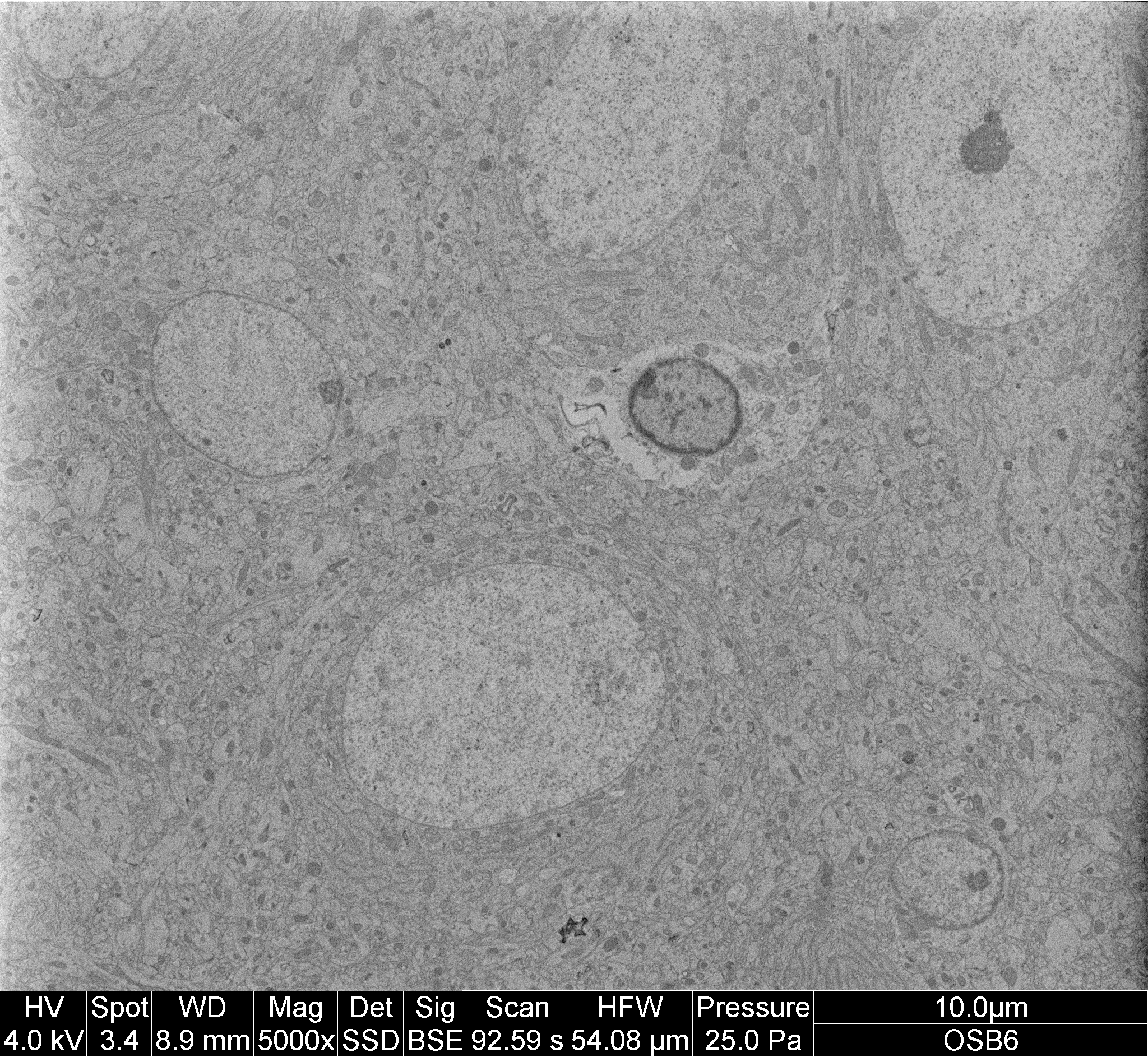

Supplement: Dataset S13 — (251.9 MB ZIP). [file pbio.0020329.sd013.zip › 040604_OS5_st1_1224.tif]

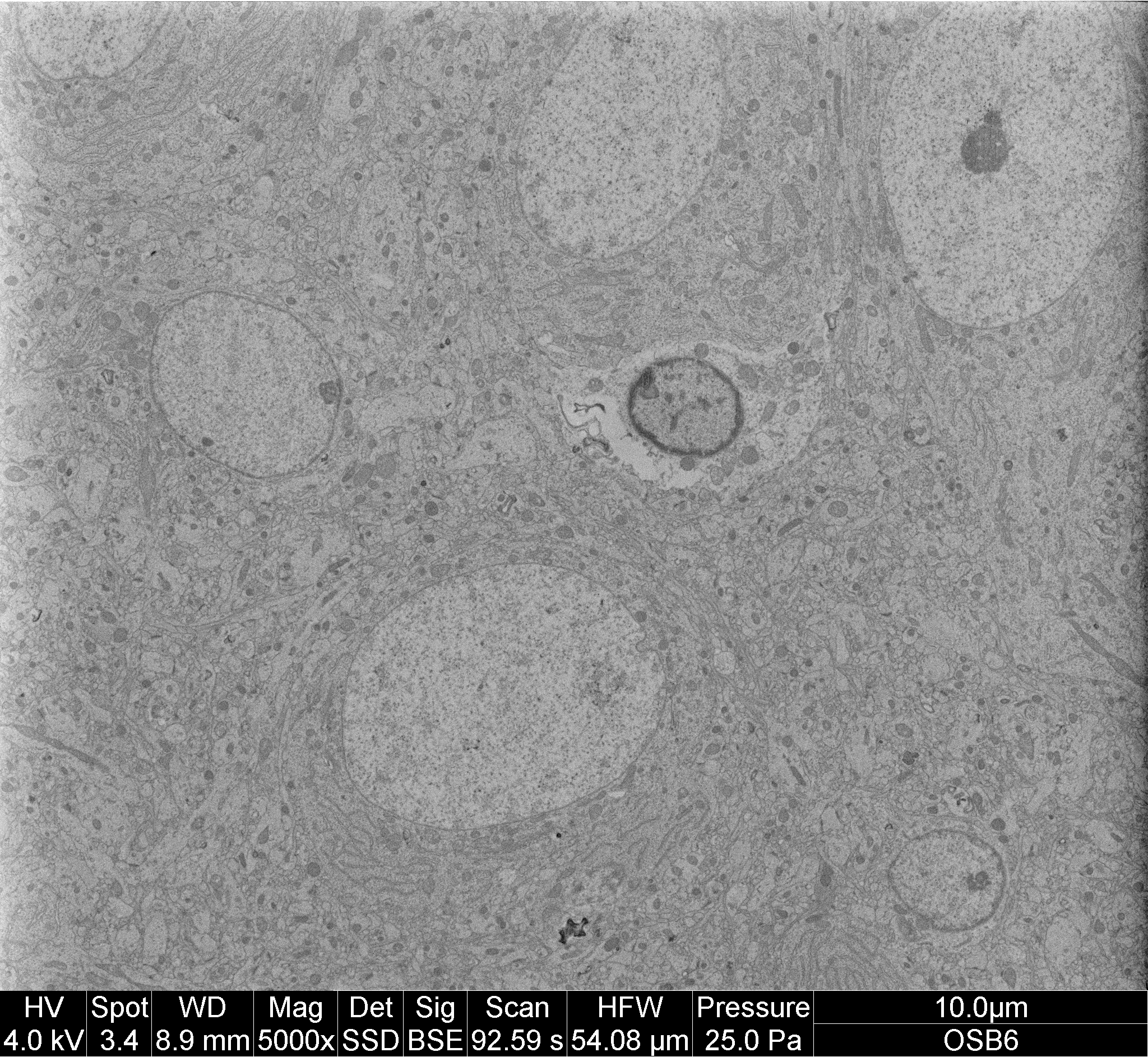

Supplement: Dataset S13 — (251.9 MB ZIP). [file pbio.0020329.sd013.zip › 040604_OS5_st1_1225.tif]

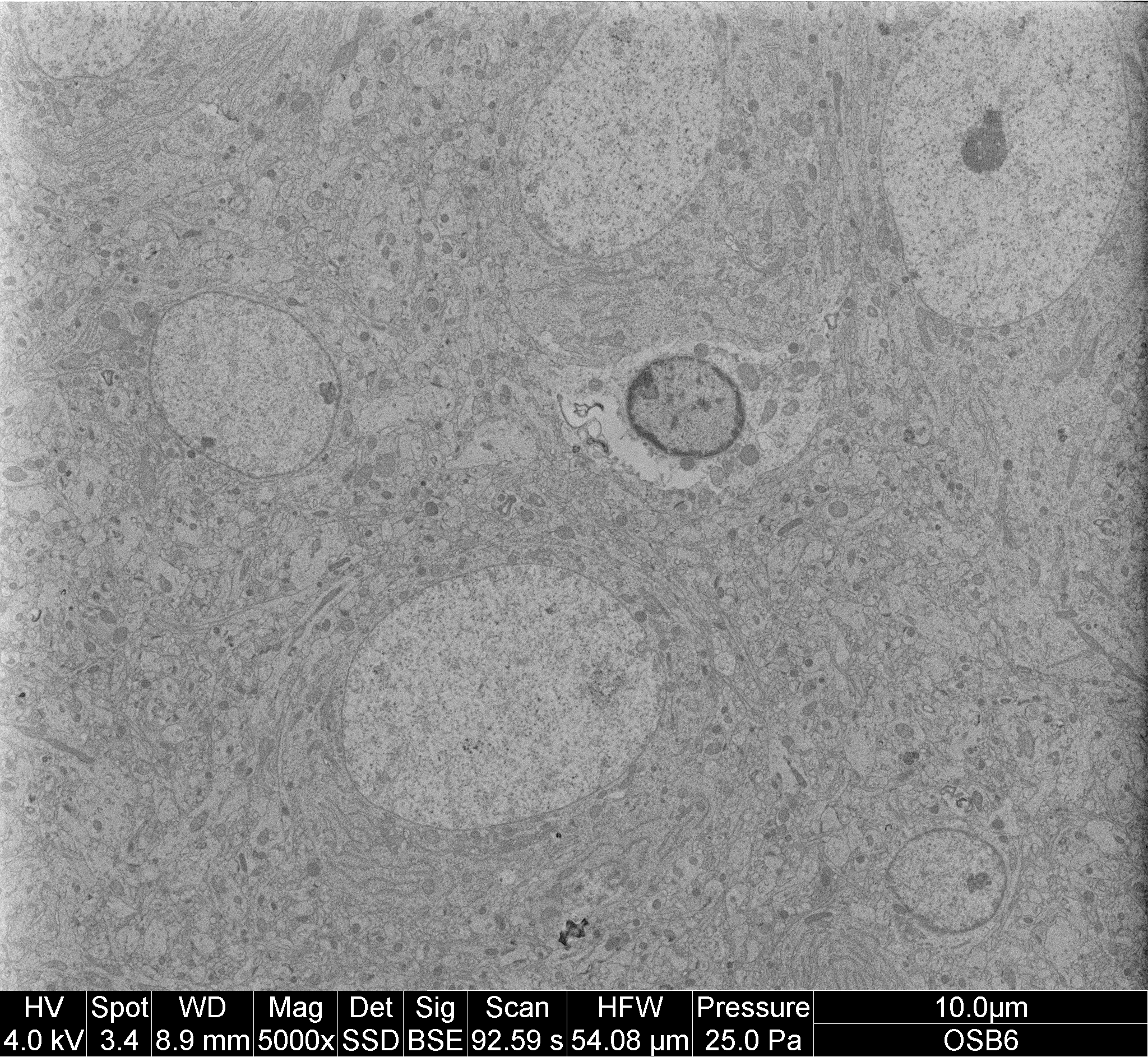

Supplement: Dataset S13 — (251.9 MB ZIP). [file pbio.0020329.sd013.zip › 040604_OS5_st1_1226.tif]

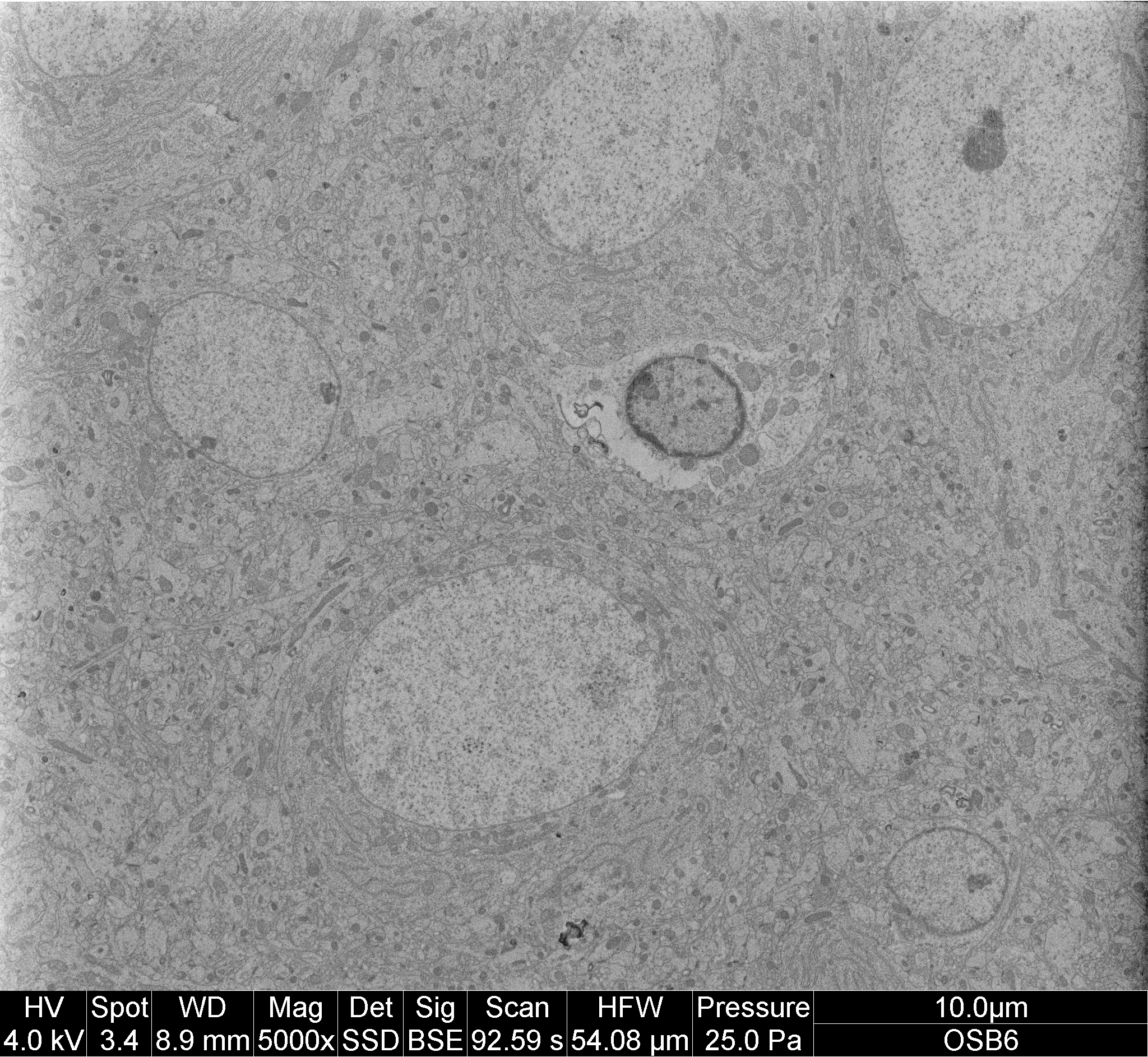

Supplement: Dataset S13 — (251.9 MB ZIP). [file pbio.0020329.sd013.zip › 040604_OS5_st1_1227.tif]

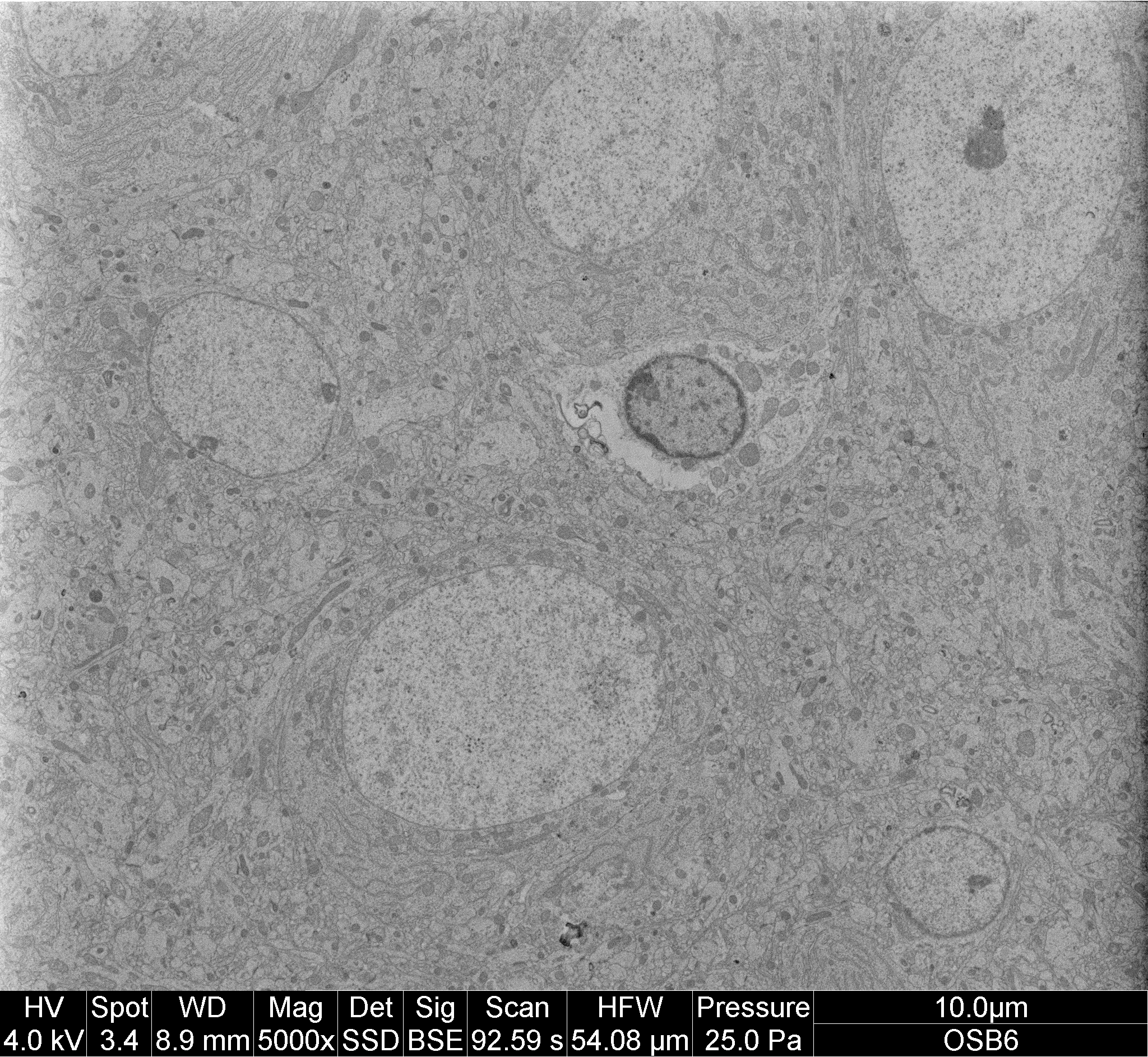

Supplement: Dataset S13 — (251.9 MB ZIP). [file pbio.0020329.sd013.zip › 040604_OS5_st1_1228.tif]

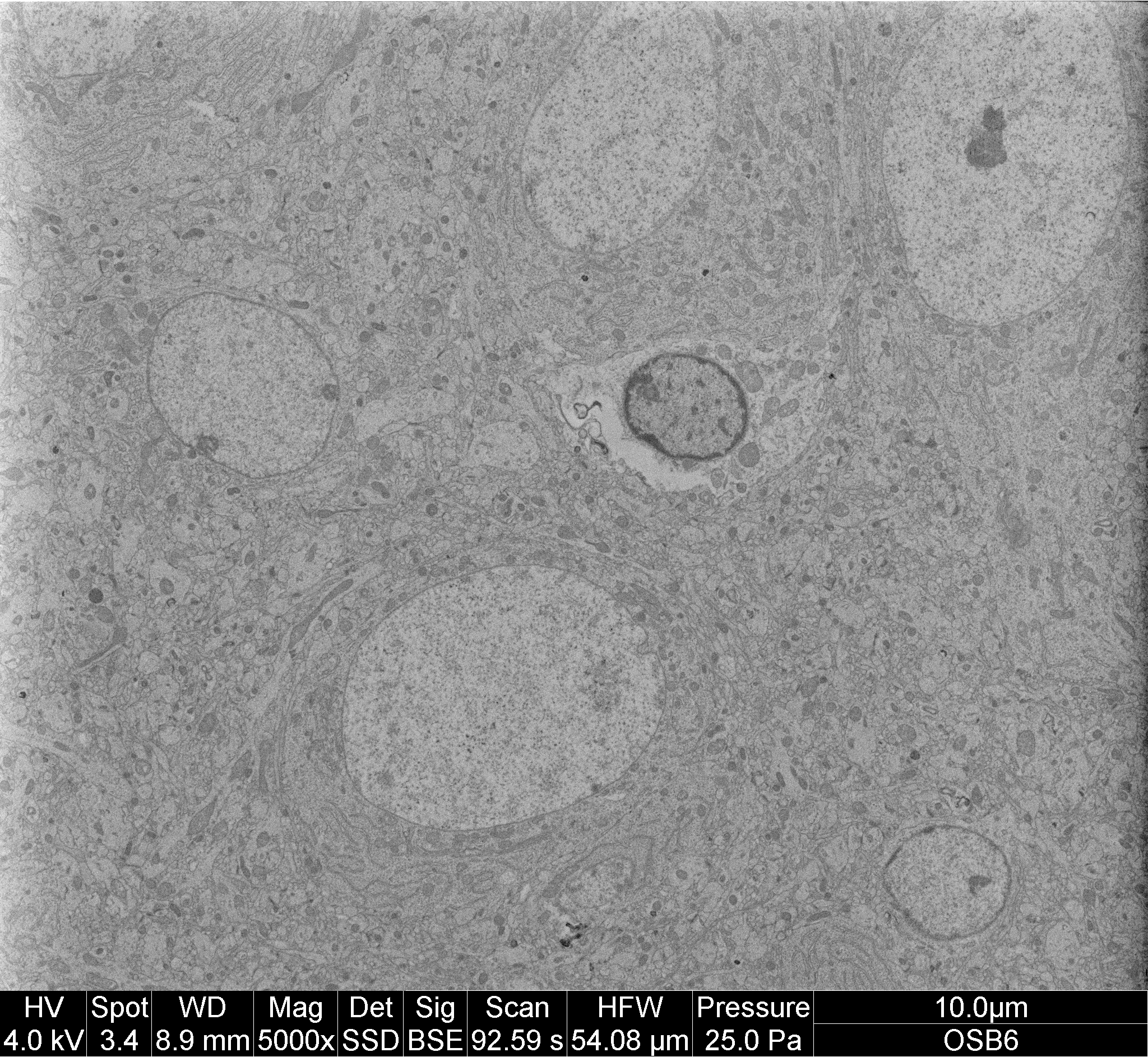

Supplement: Dataset S13 — (251.9 MB ZIP). [file pbio.0020329.sd013.zip › 040604_OS5_st1_1229.tif]

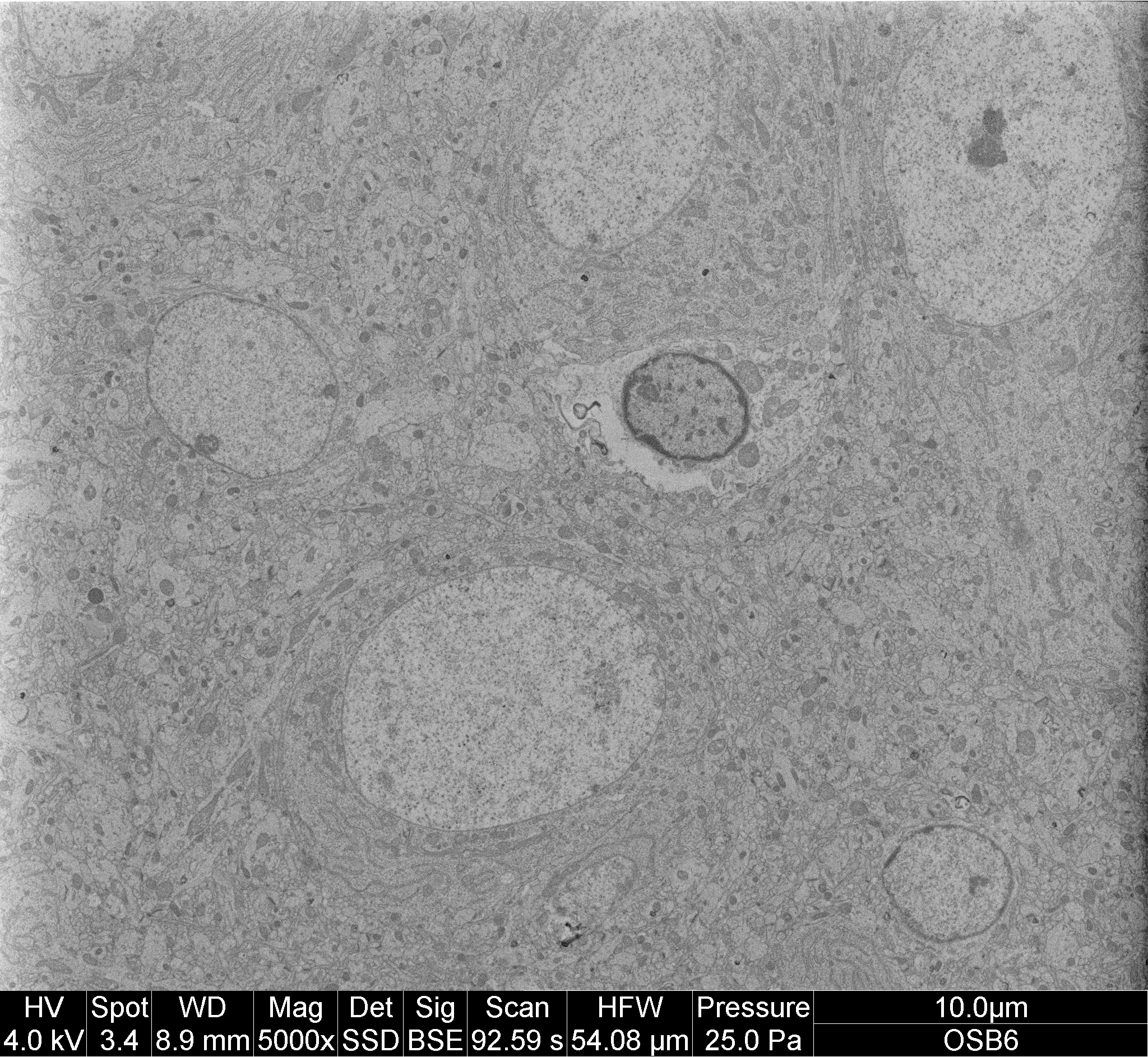

Supplement: Dataset S13 — (251.9 MB ZIP). [file pbio.0020329.sd013.zip › 040604_OS5_st1_1230.tif]

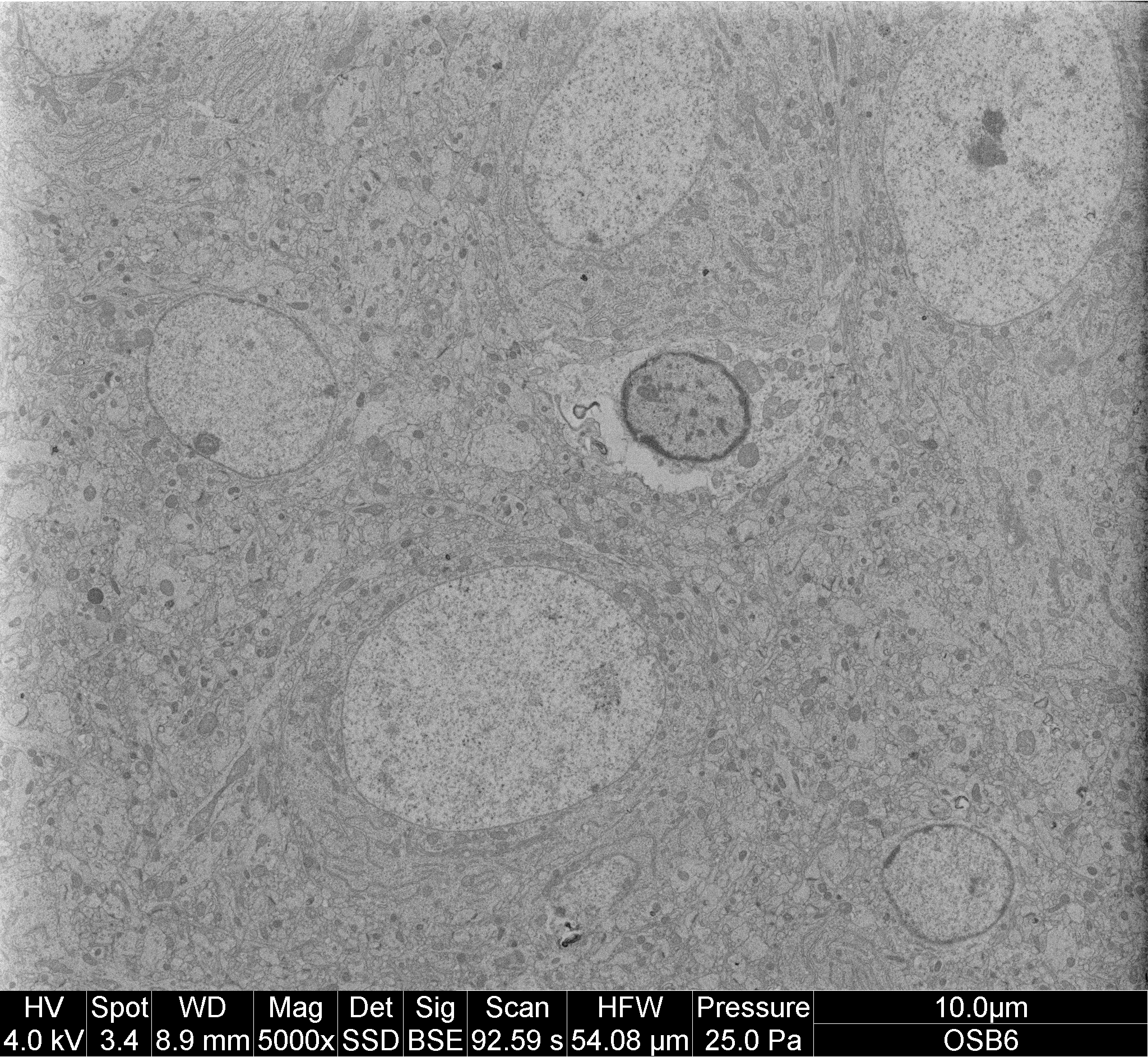

Supplement: Dataset S13 — (251.9 MB ZIP). [file pbio.0020329.sd013.zip › 040604_OS5_st1_1231.tif]

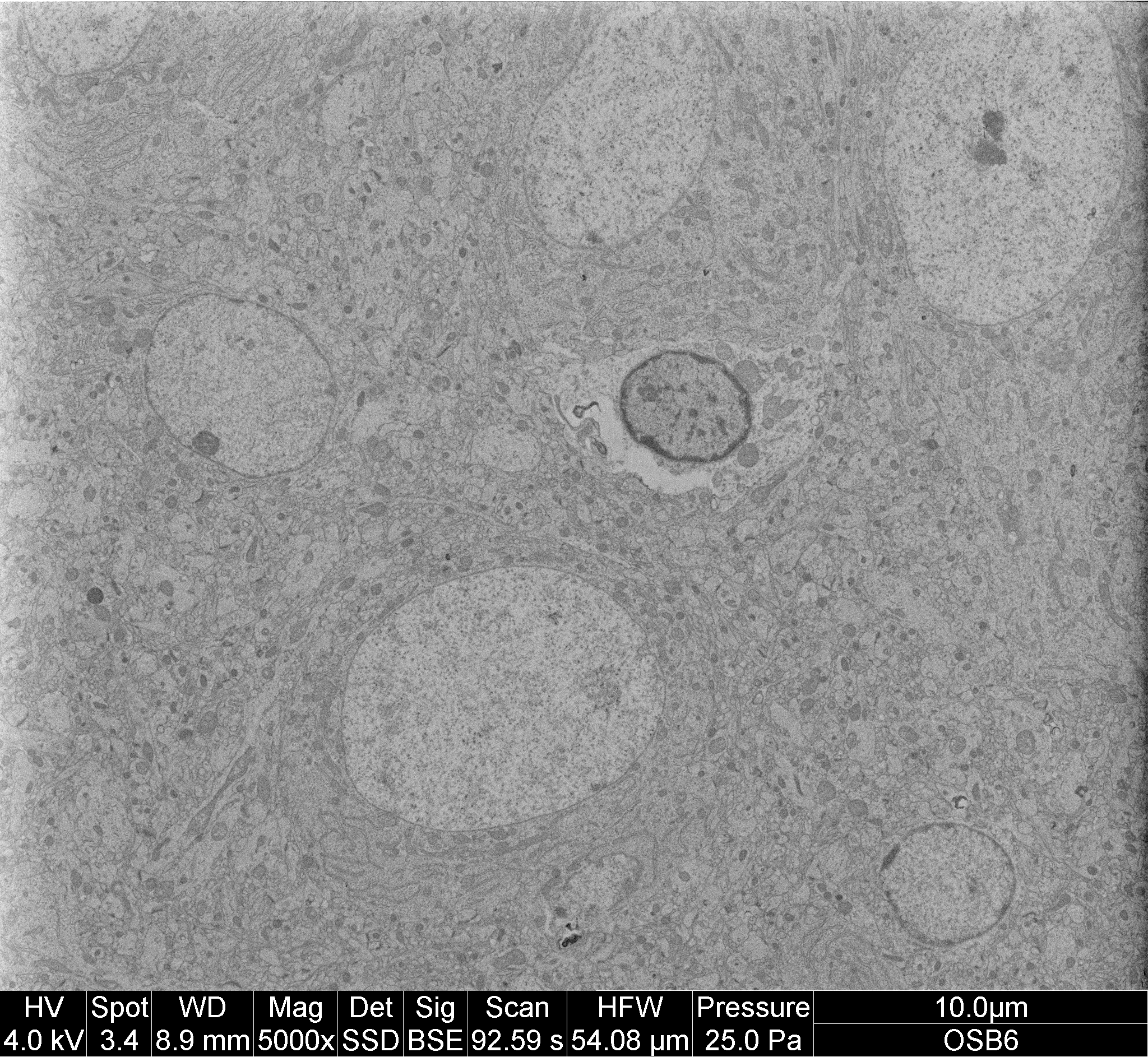

Supplement: Dataset S13 — (251.9 MB ZIP). [file pbio.0020329.sd013.zip › 040604_OS5_st1_1232.tif]

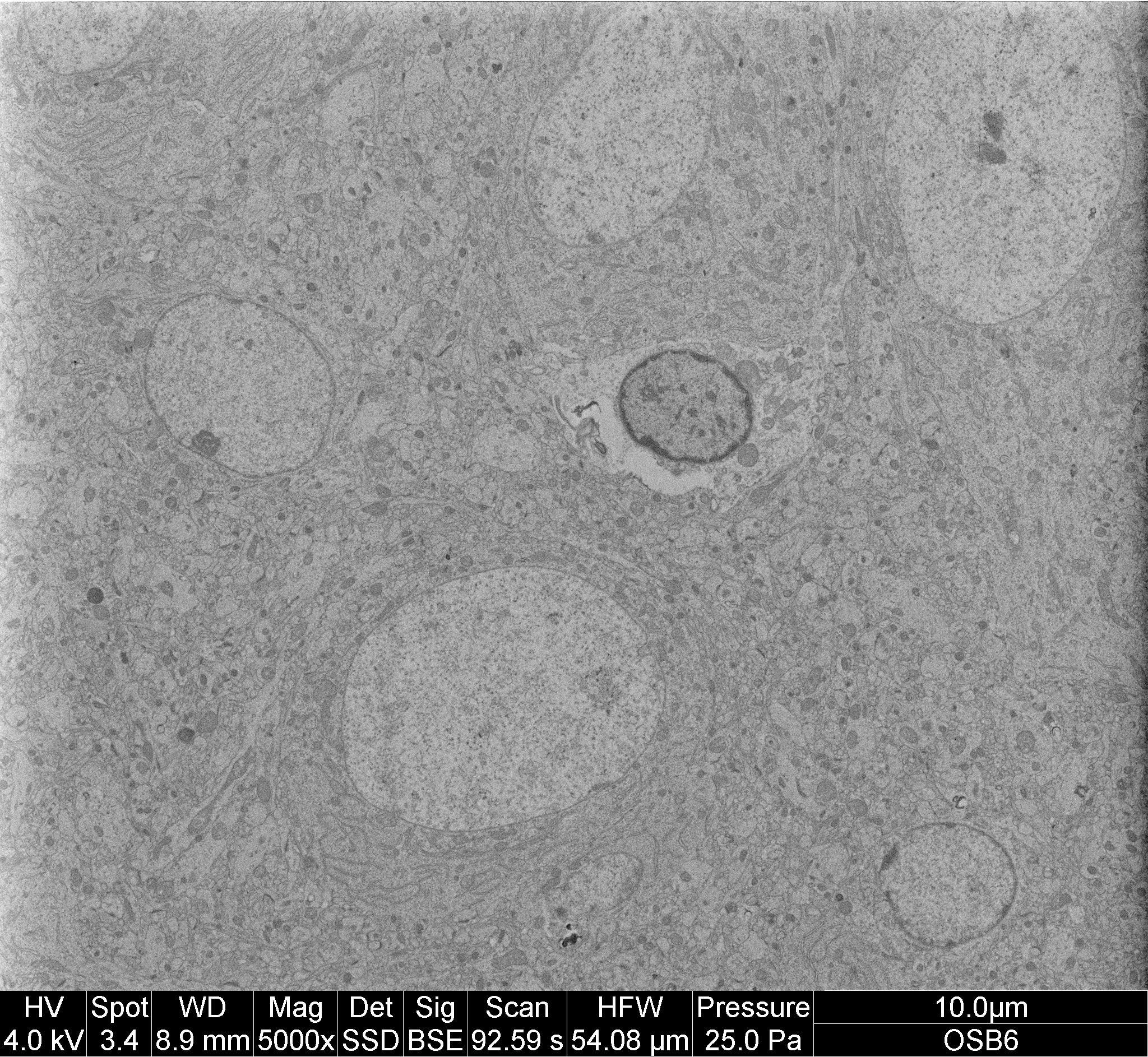

Supplement: Dataset S13 — (251.9 MB ZIP). [file pbio.0020329.sd013.zip › 040604_OS5_st1_1233.tif]

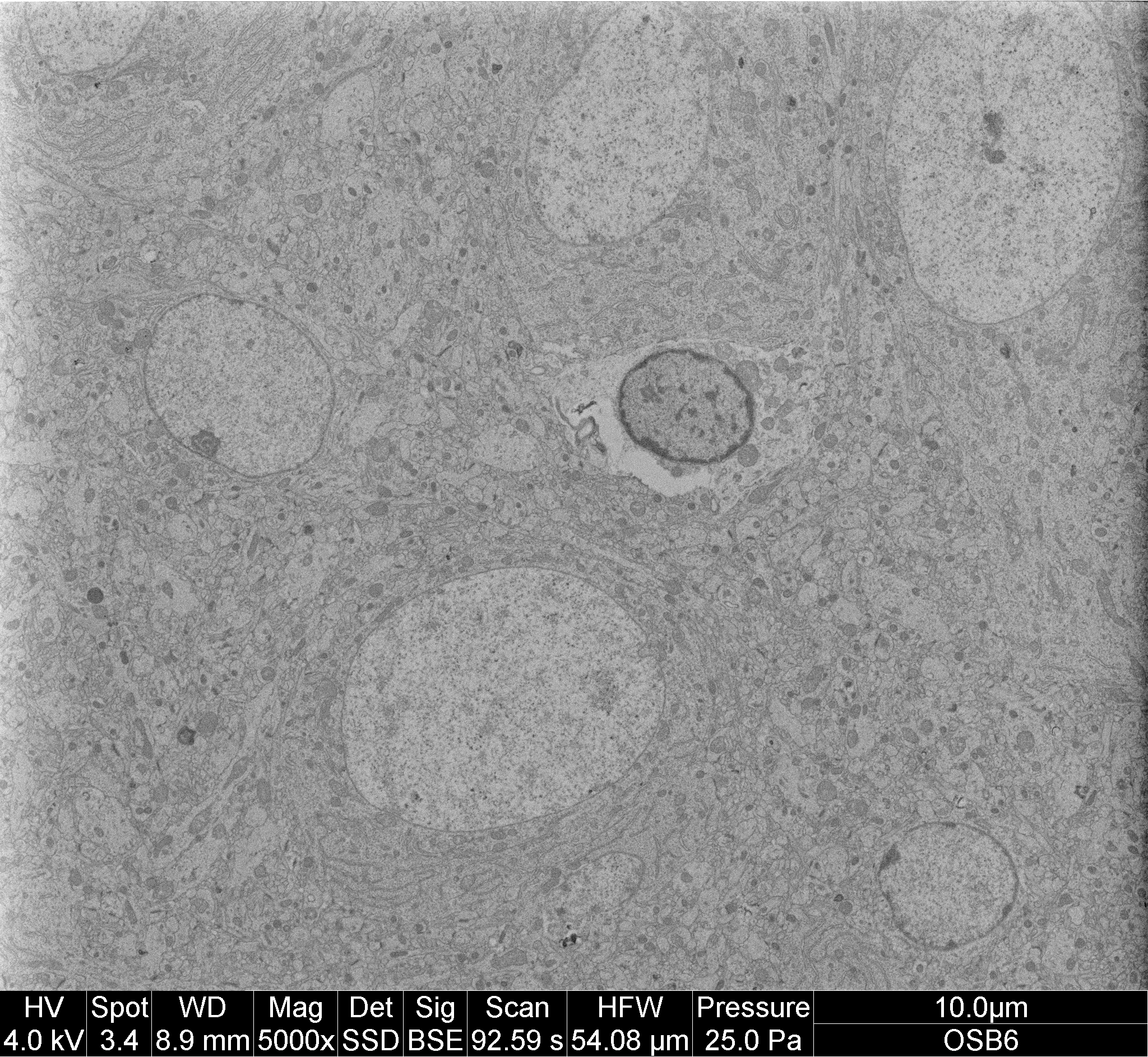

Supplement: Dataset S13 — (251.9 MB ZIP). [file pbio.0020329.sd013.zip › 040604_OS5_st1_1234.tif]

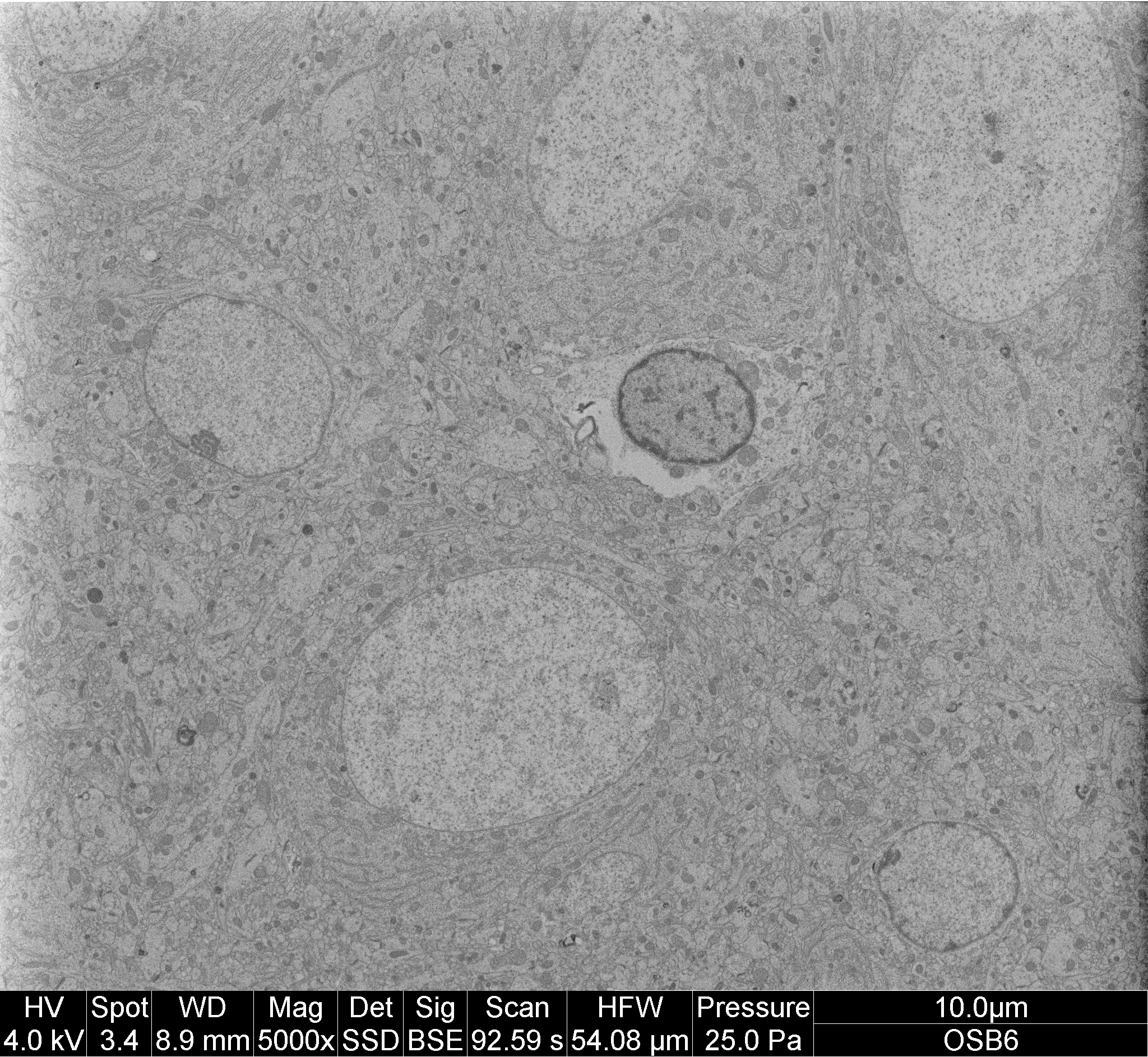

Supplement: Dataset S13 — (251.9 MB ZIP). [file pbio.0020329.sd013.zip › 040604_OS5_st1_1235.tif]

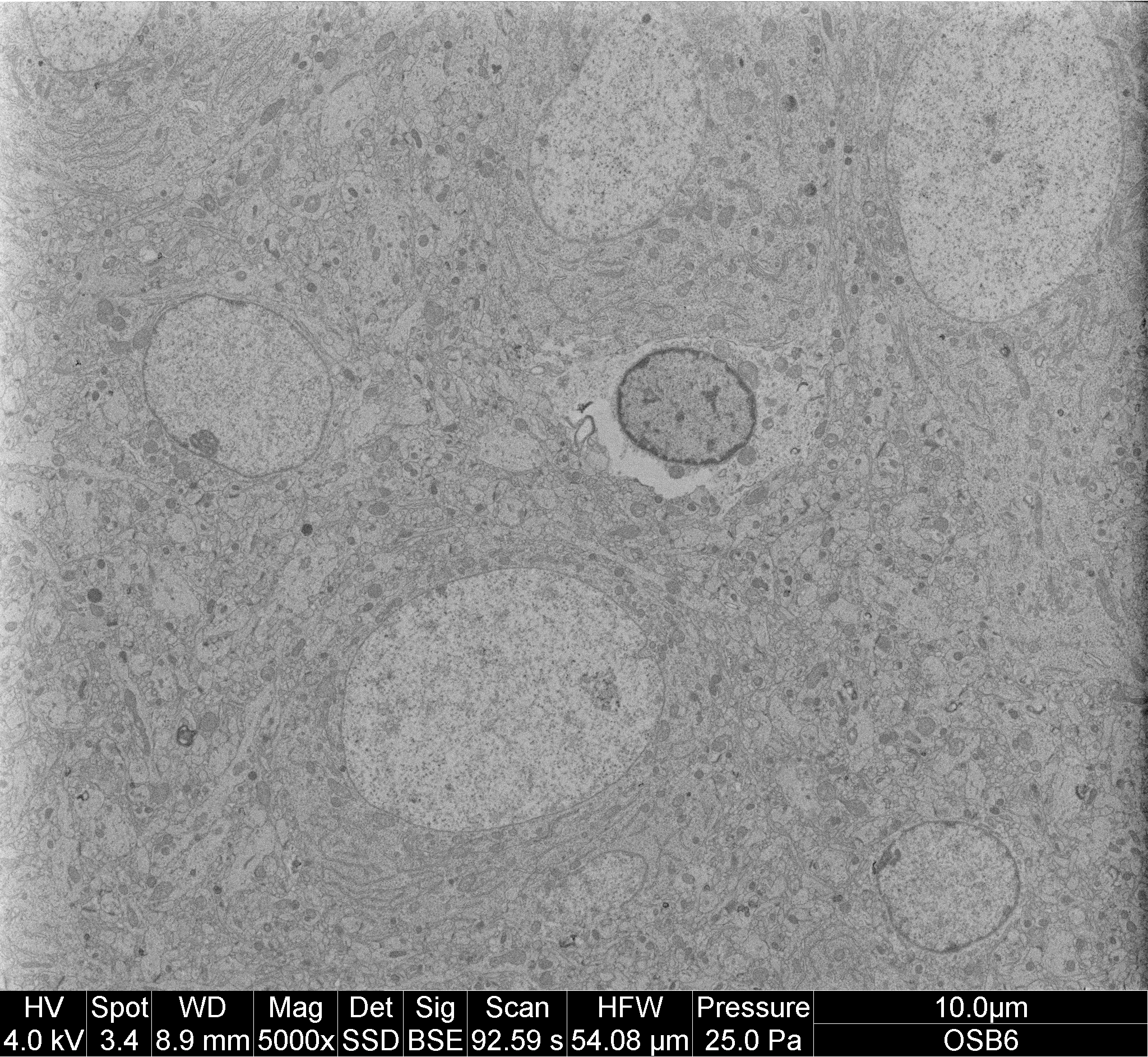

Supplement: Dataset S13 — (251.9 MB ZIP). [file pbio.0020329.sd013.zip › 040604_OS5_st1_1236.tif]

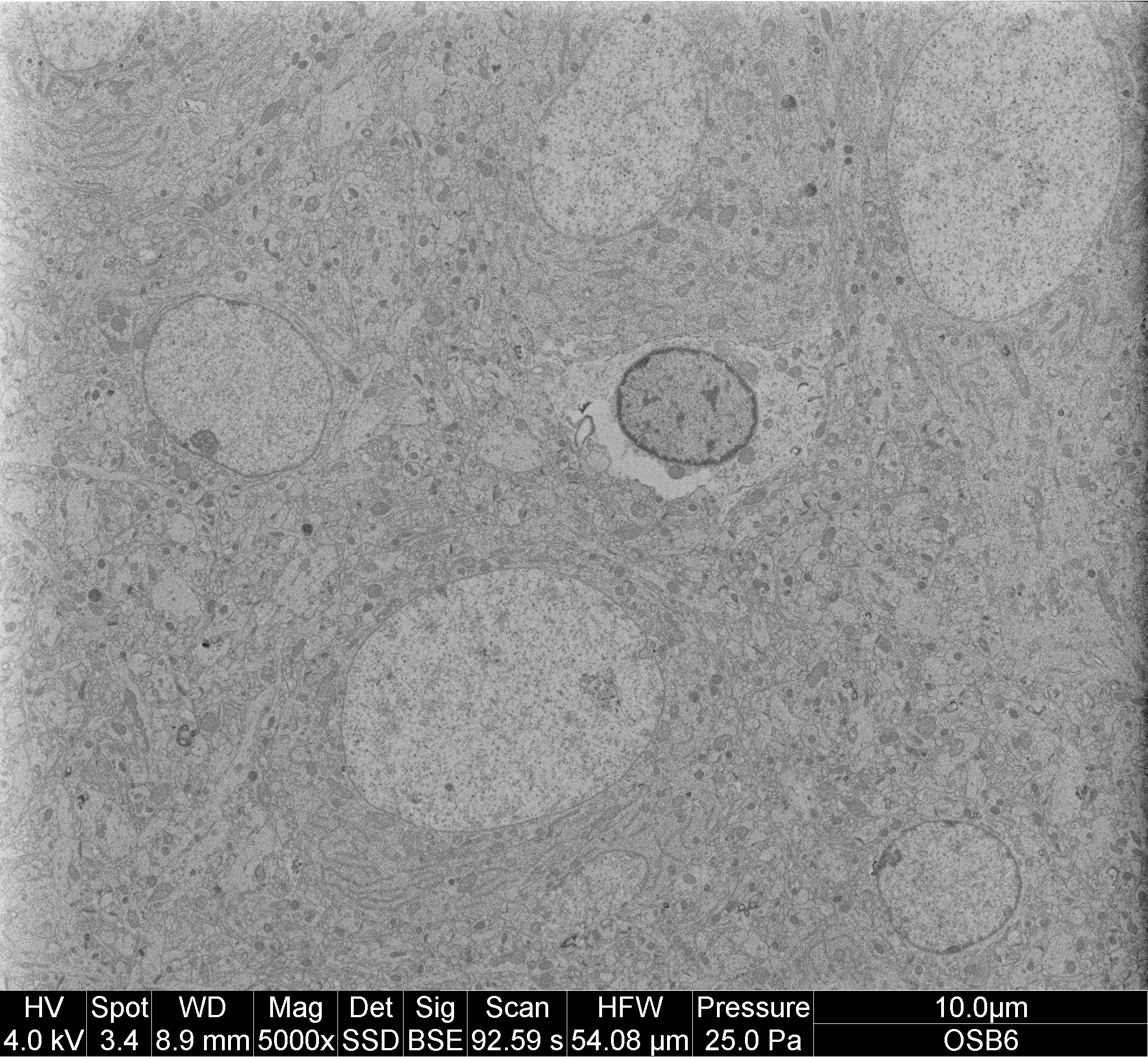

Supplement: Dataset S13 — (251.9 MB ZIP). [file pbio.0020329.sd013.zip › 040604_OS5_st1_1237.tif]

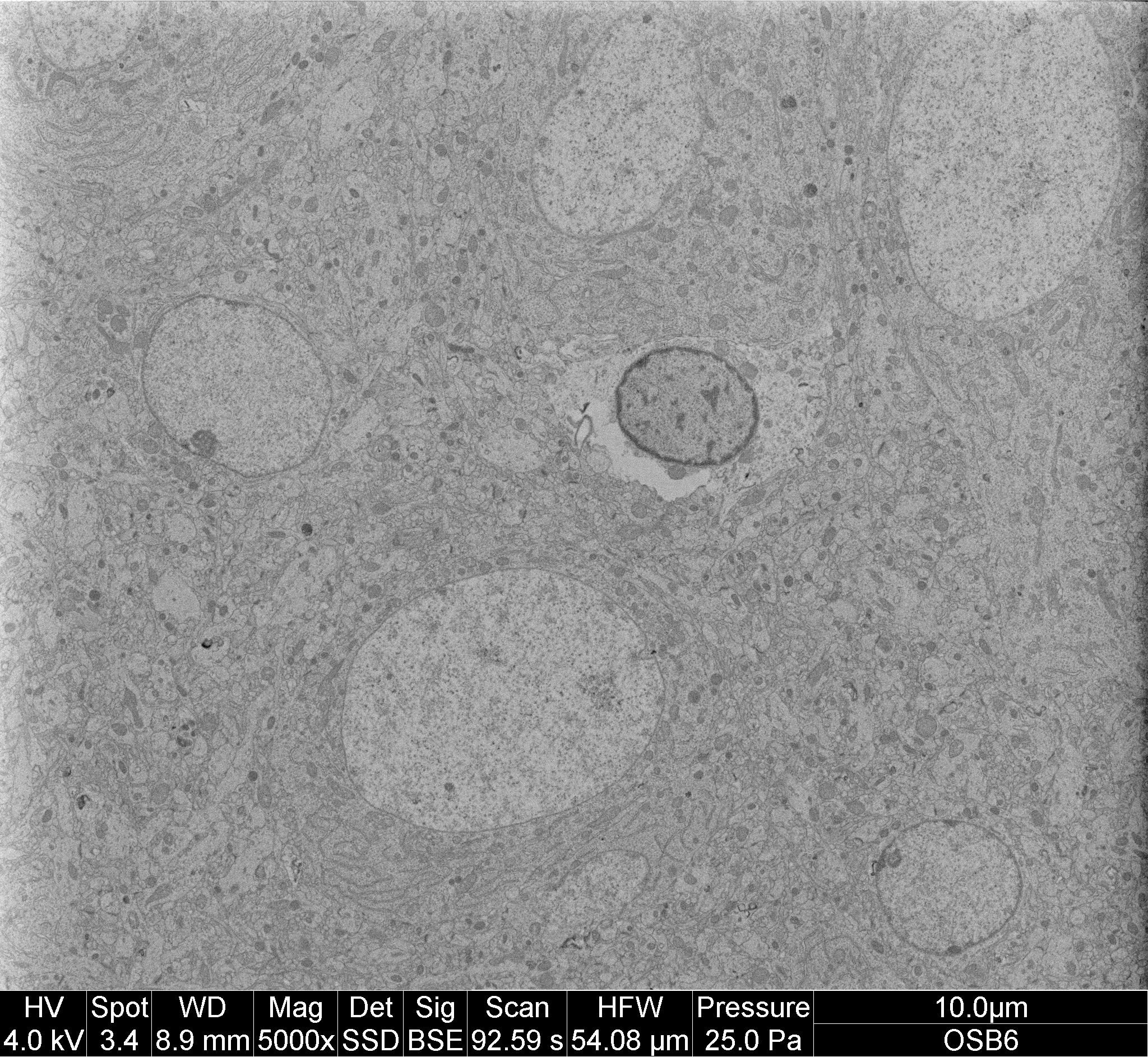

Supplement: Dataset S13 — (251.9 MB ZIP). [file pbio.0020329.sd013.zip › 040604_OS5_st1_1238.tif]

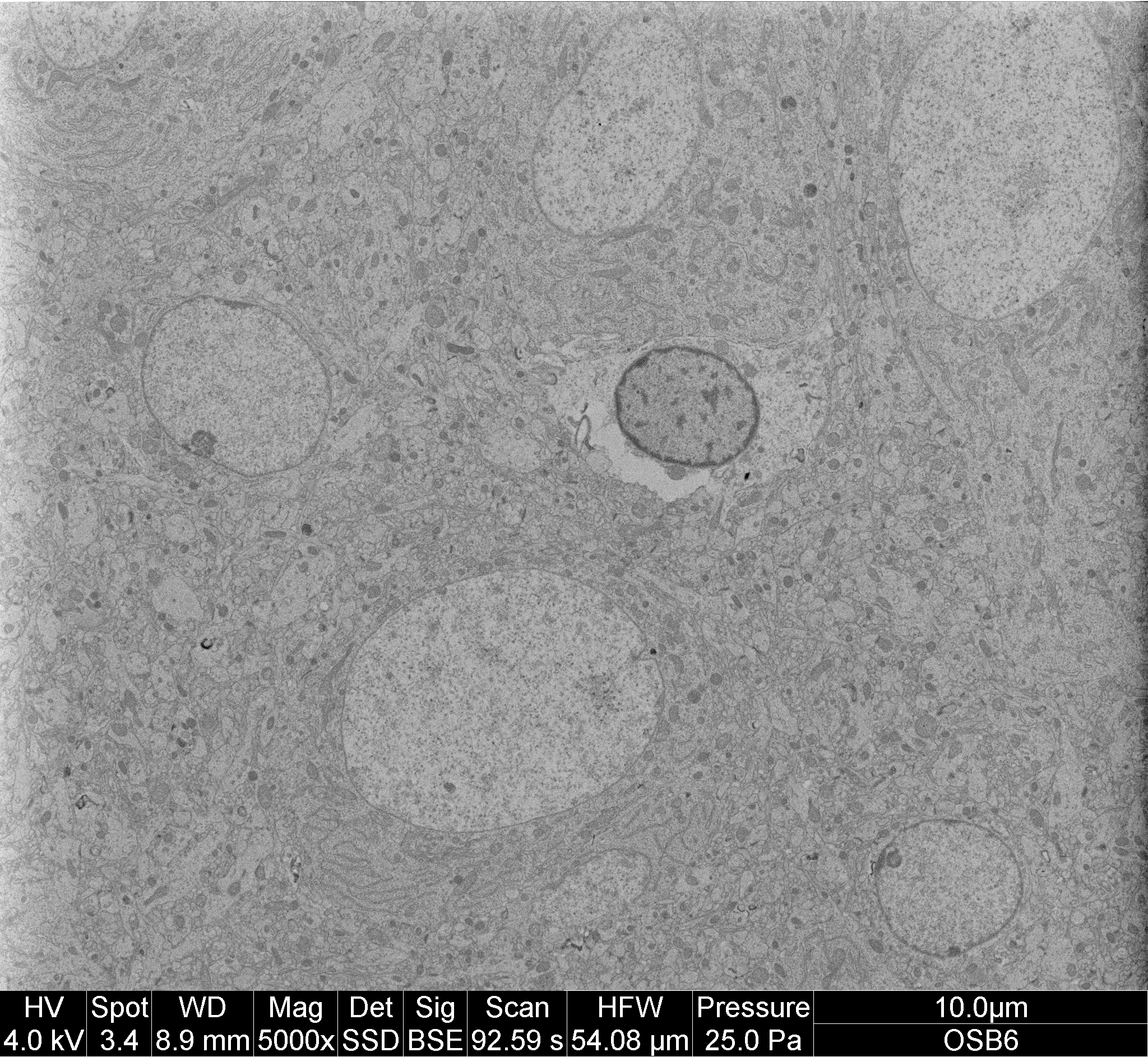

Supplement: Dataset S13 — (251.9 MB ZIP). [file pbio.0020329.sd013.zip › 040604_OS5_st1_1239.tif]

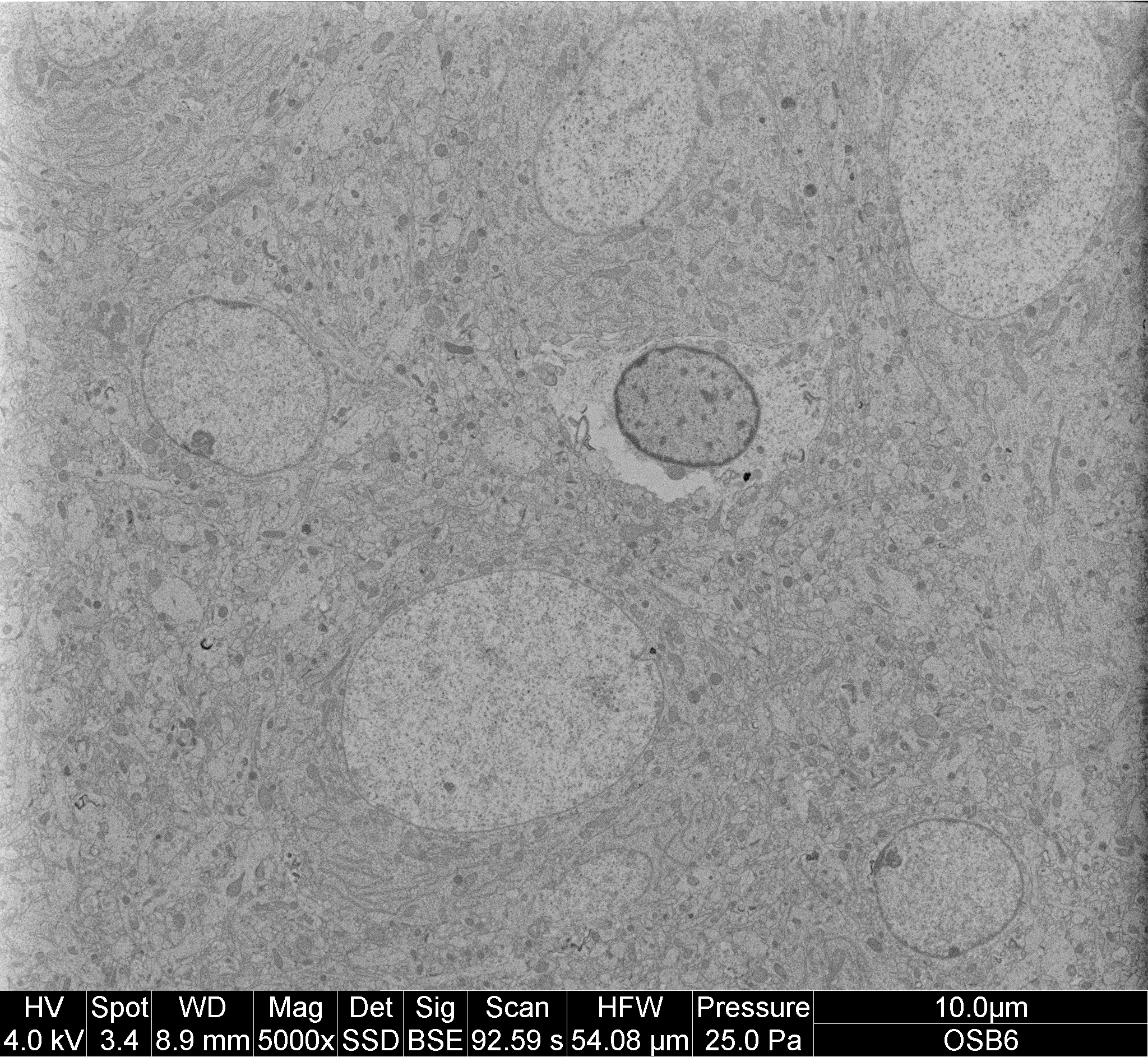

Supplement: Dataset S13 — (251.9 MB ZIP). [file pbio.0020329.sd013.zip › 040604_OS5_st1_1240.tif]

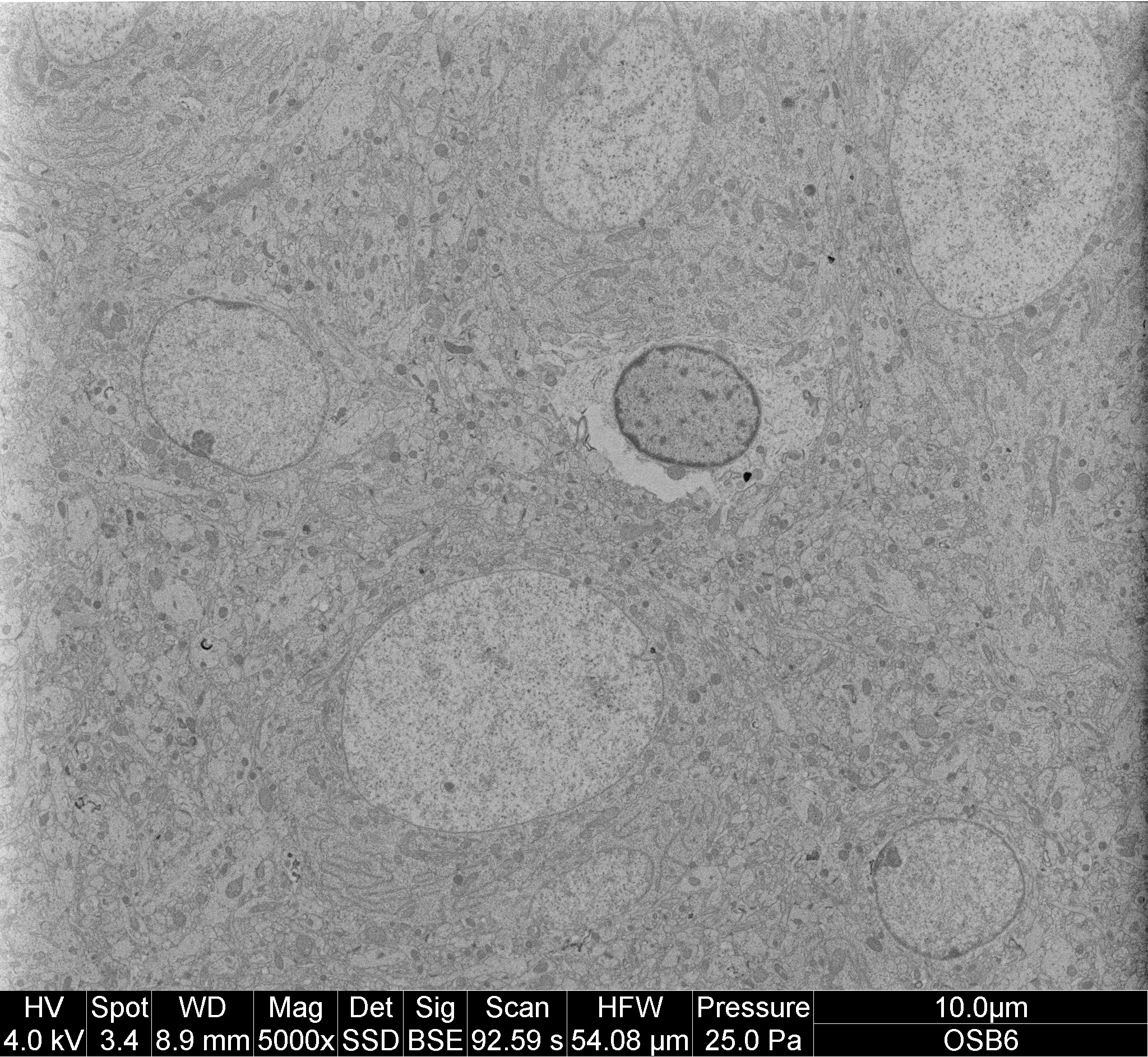

Supplement: Dataset S13 — (251.9 MB ZIP). [file pbio.0020329.sd013.zip › 040604_OS5_st1_1241.tif]

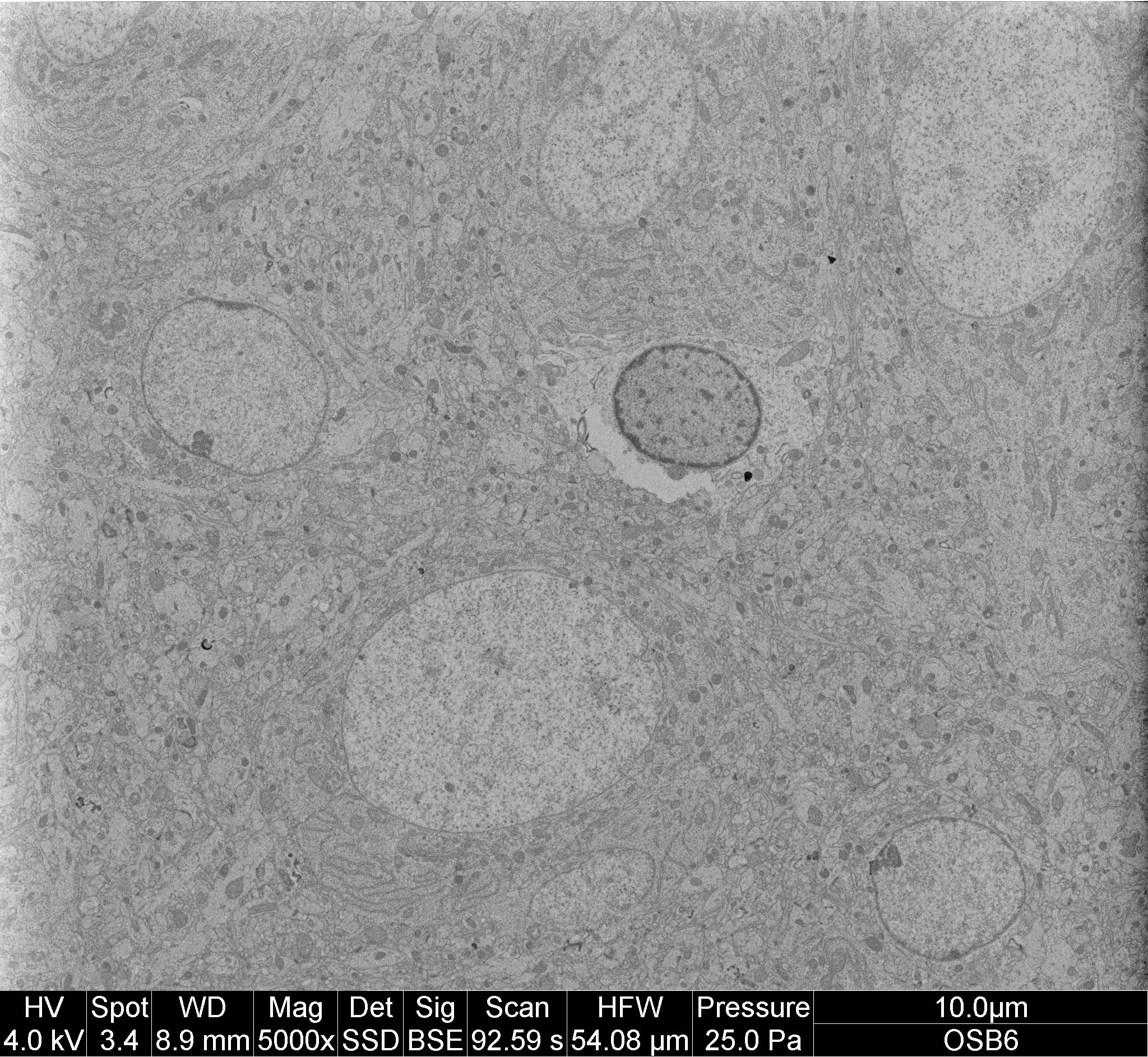

Supplement: Dataset S13 — (251.9 MB ZIP). [file pbio.0020329.sd013.zip › 040604_OS5_st1_1242.tif]

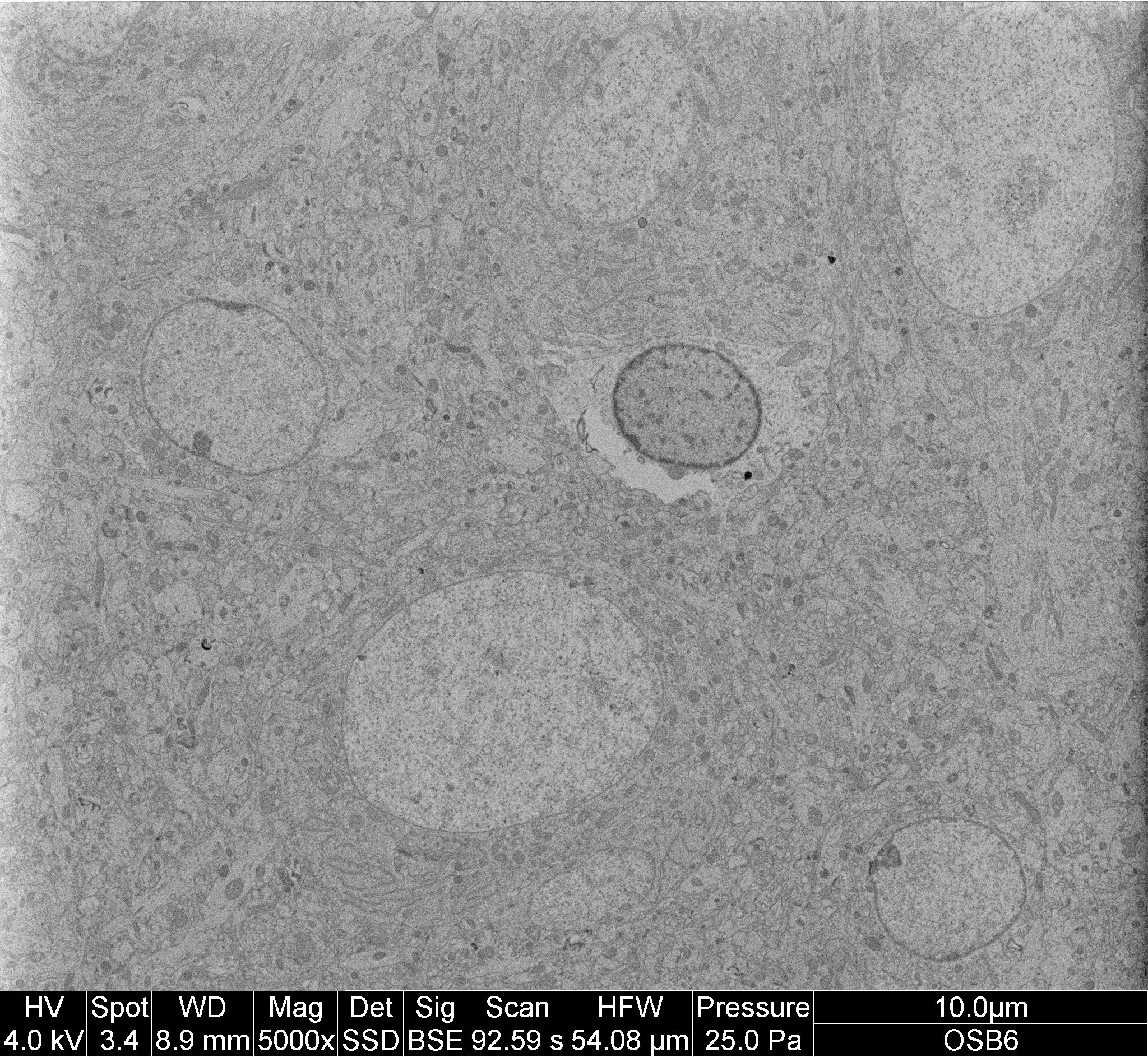

Supplement: Dataset S13 — (251.9 MB ZIP). [file pbio.0020329.sd013.zip › 040604_OS5_st1_1243.tif]

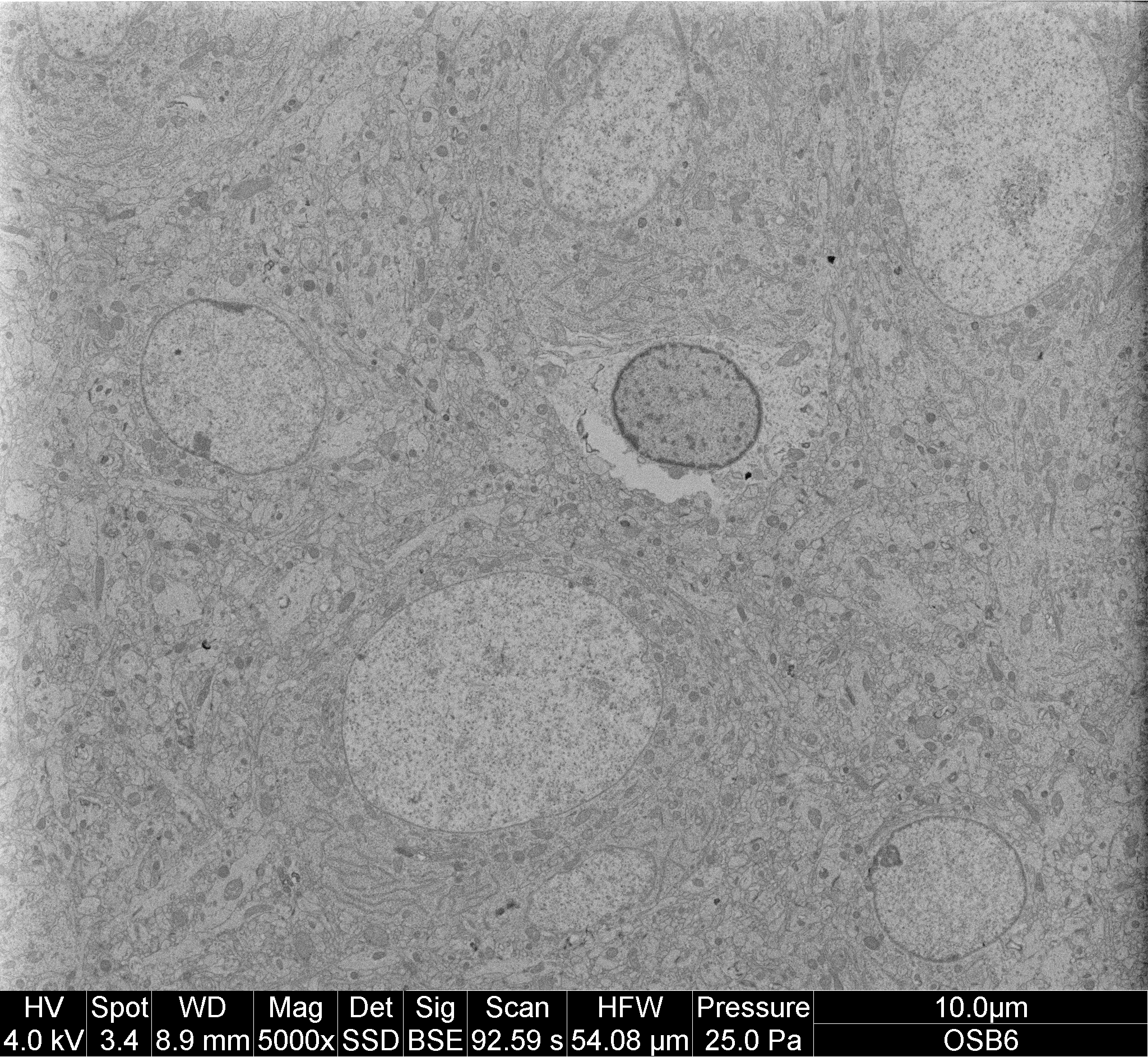

Supplement: Dataset S13 — (251.9 MB ZIP). [file pbio.0020329.sd013.zip › 040604_OS5_st1_1244.tif]

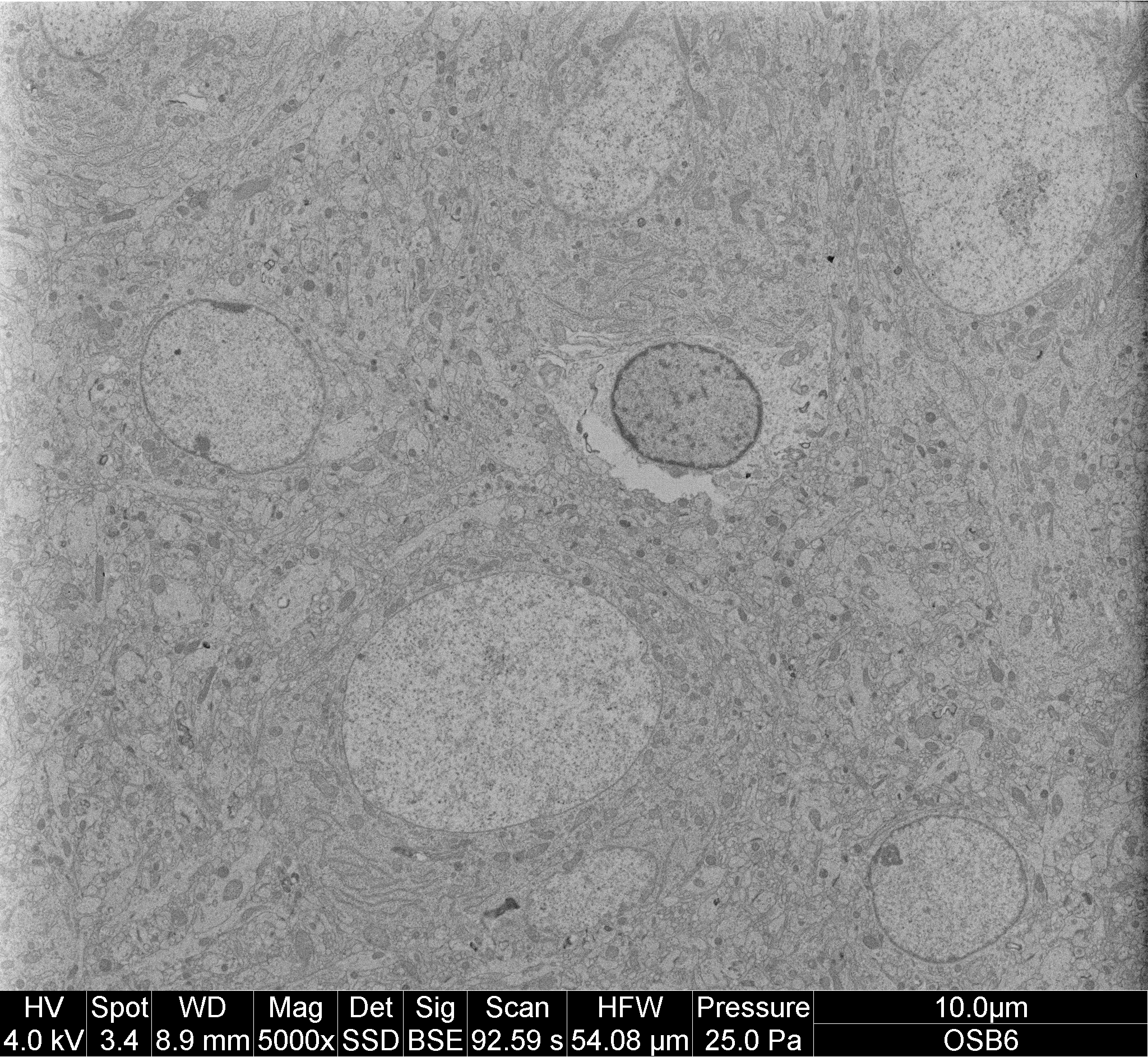

Supplement: Dataset S13 — (251.9 MB ZIP). [file pbio.0020329.sd013.zip › 040604_OS5_st1_1245.tif]

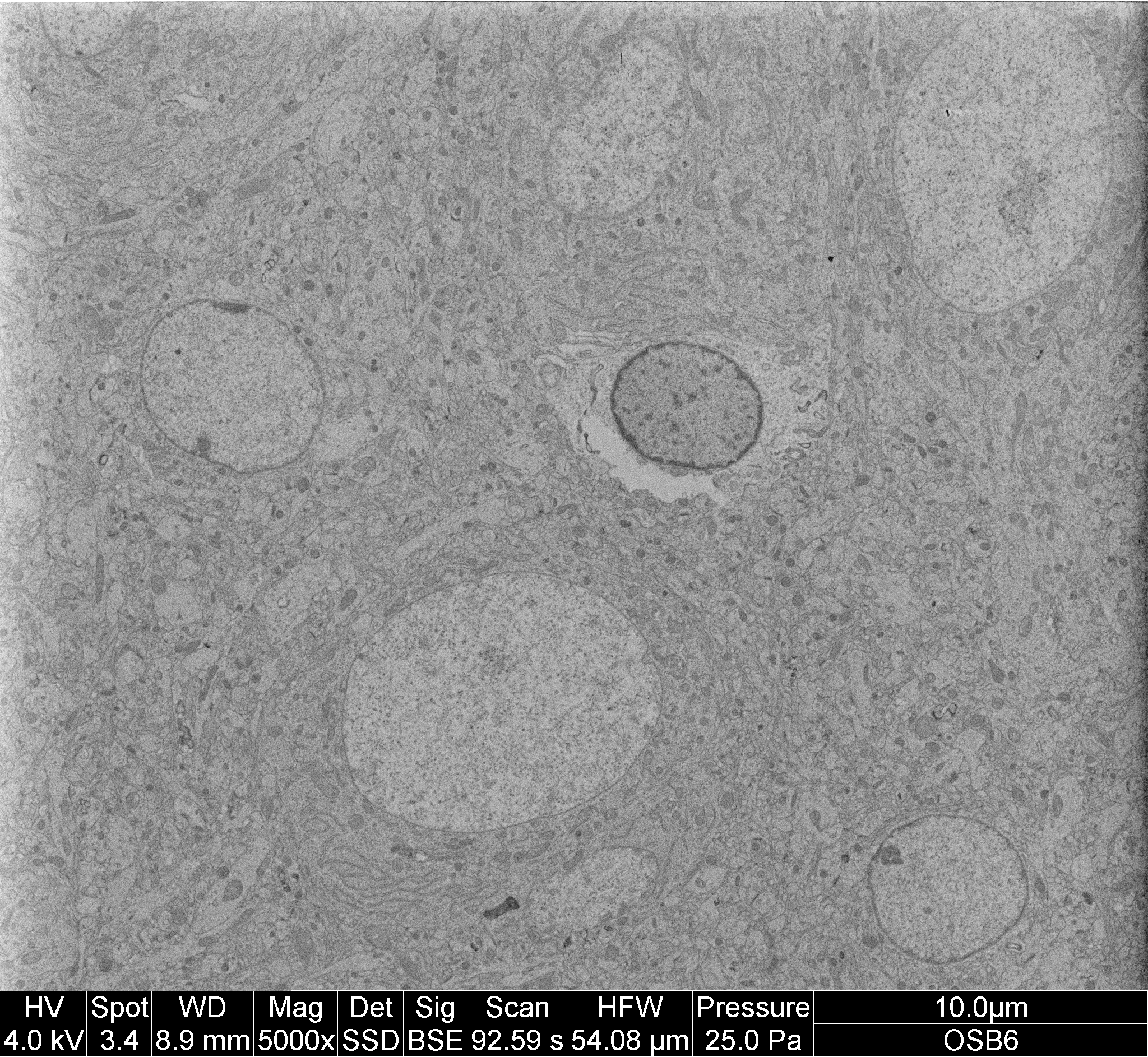

Supplement: Dataset S13 — (251.9 MB ZIP). [file pbio.0020329.sd013.zip › 040604_OS5_st1_1246.tif]

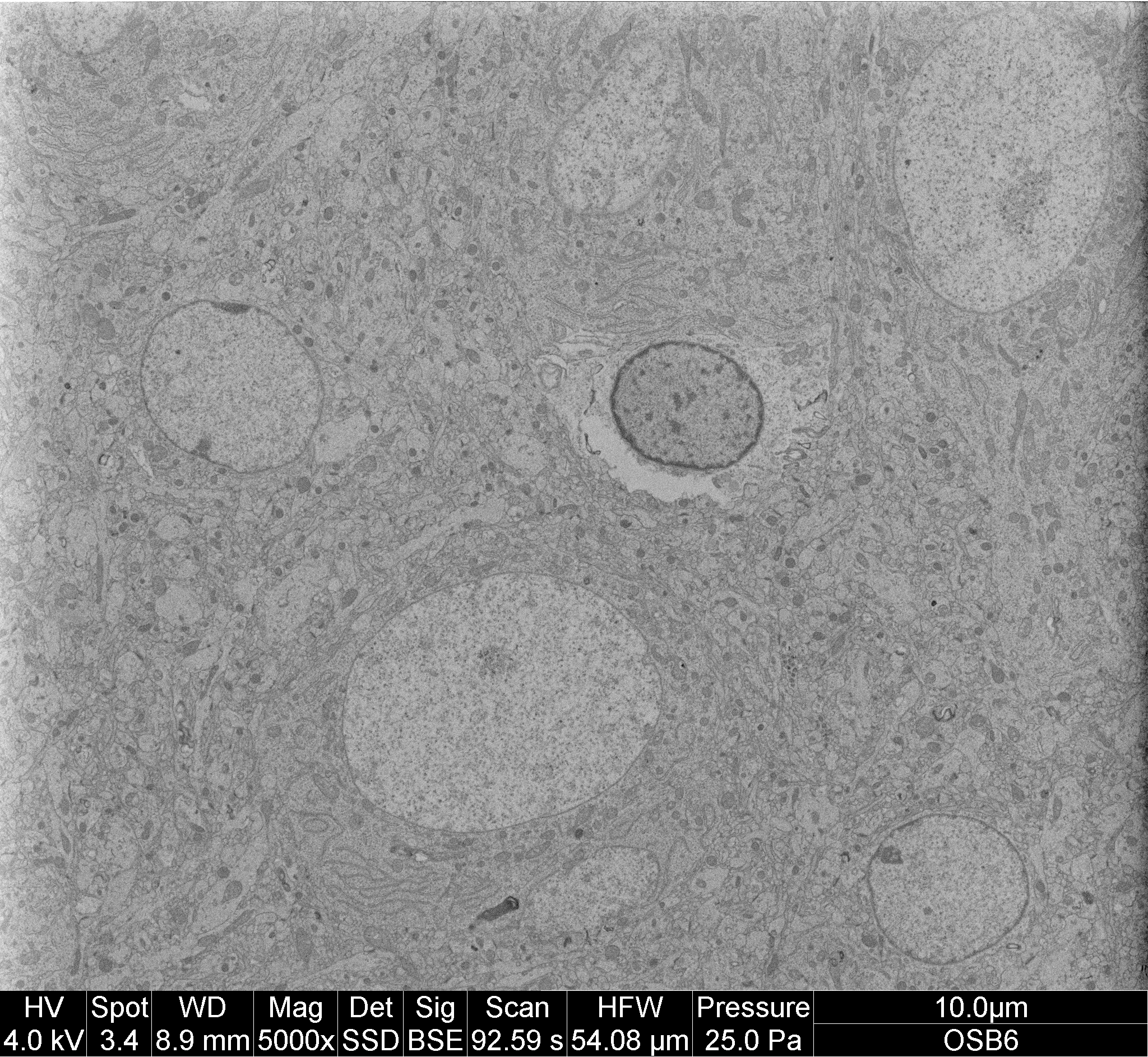

Supplement: Dataset S13 — (251.9 MB ZIP). [file pbio.0020329.sd013.zip › 040604_OS5_st1_1247.tif]

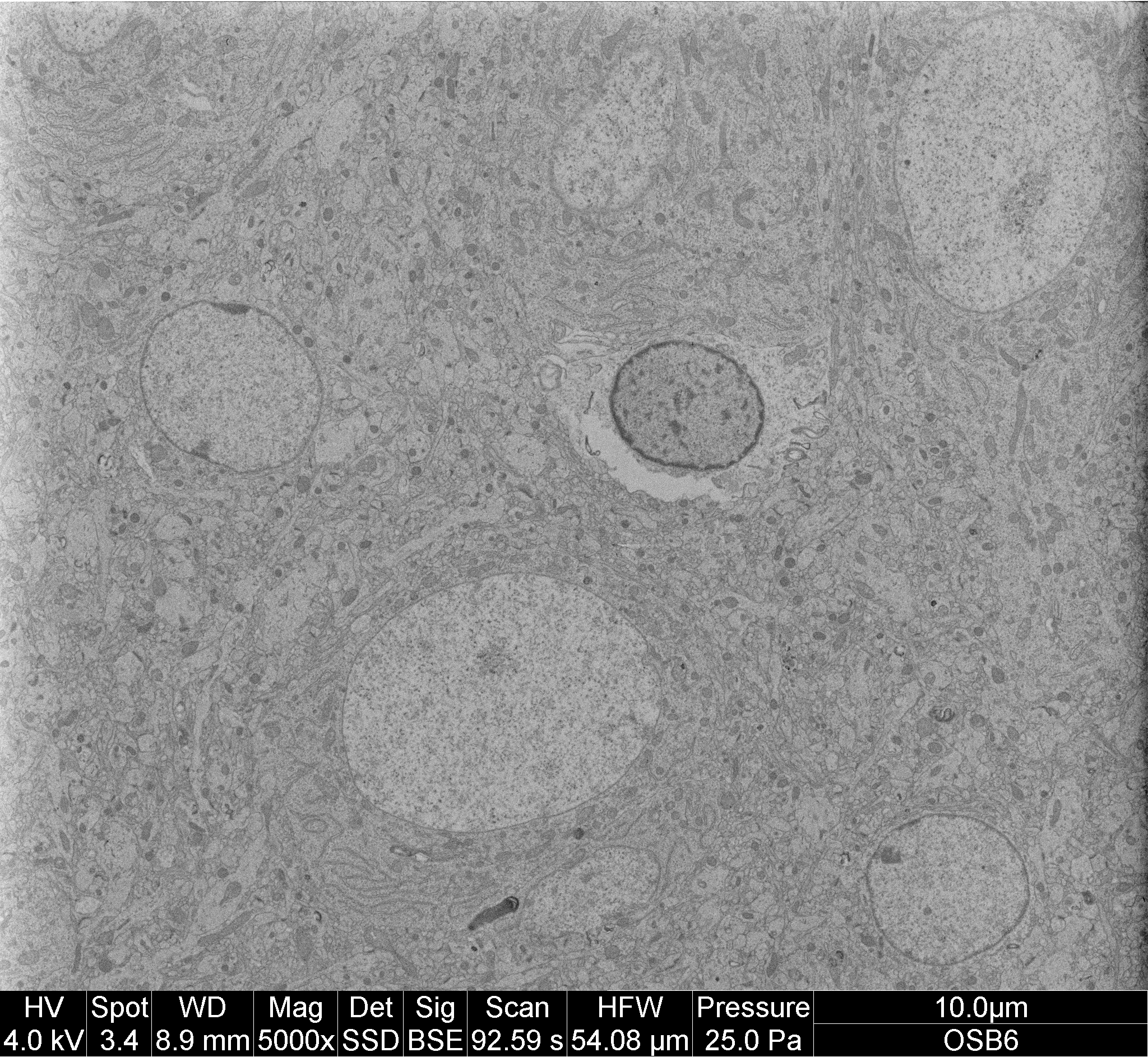

Supplement: Dataset S13 — (251.9 MB ZIP). [file pbio.0020329.sd013.zip › 040604_OS5_st1_1248.tif]

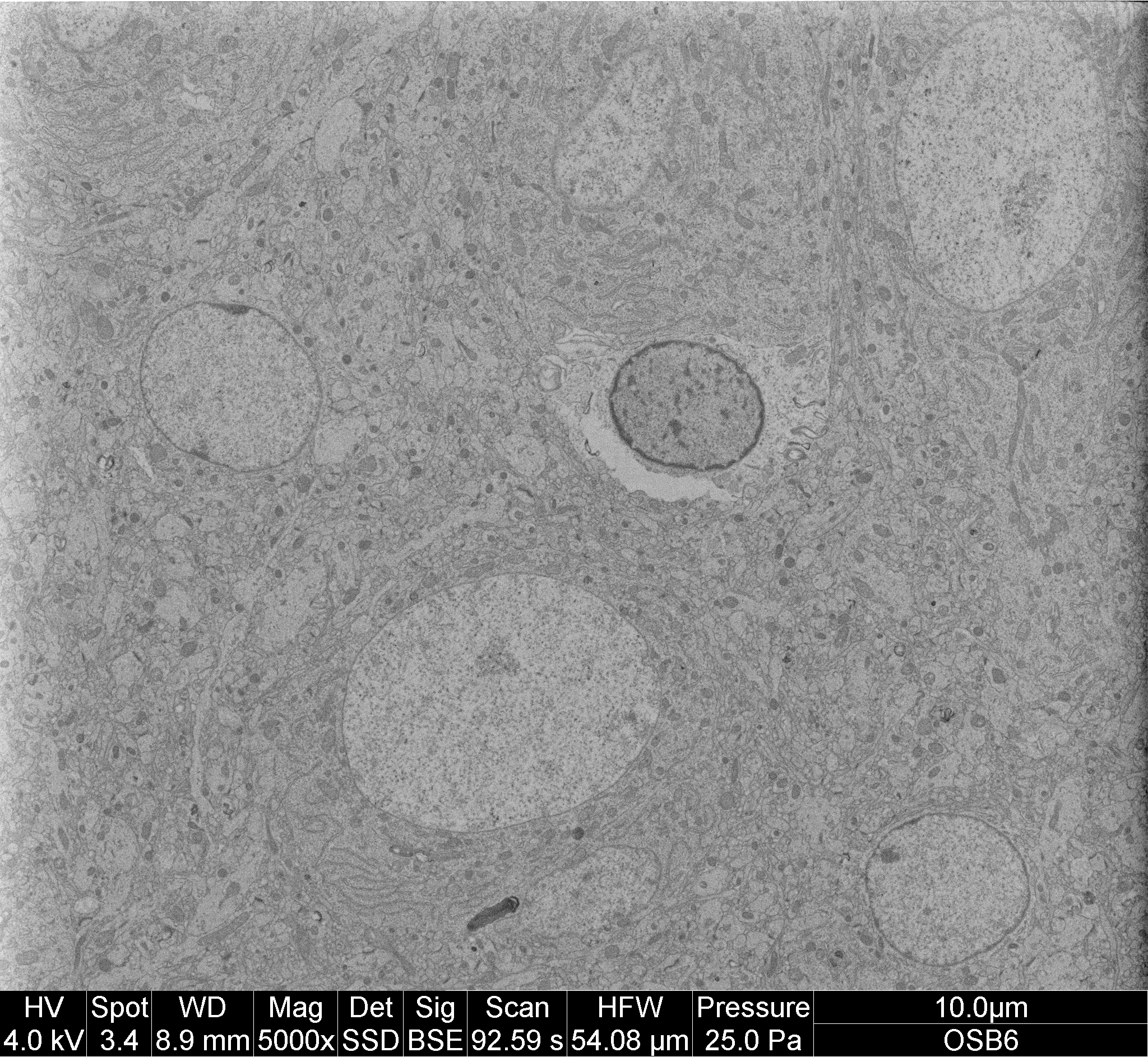

Supplement: Dataset S13 — (251.9 MB ZIP). [file pbio.0020329.sd013.zip › 040604_OS5_st1_1249.tif]

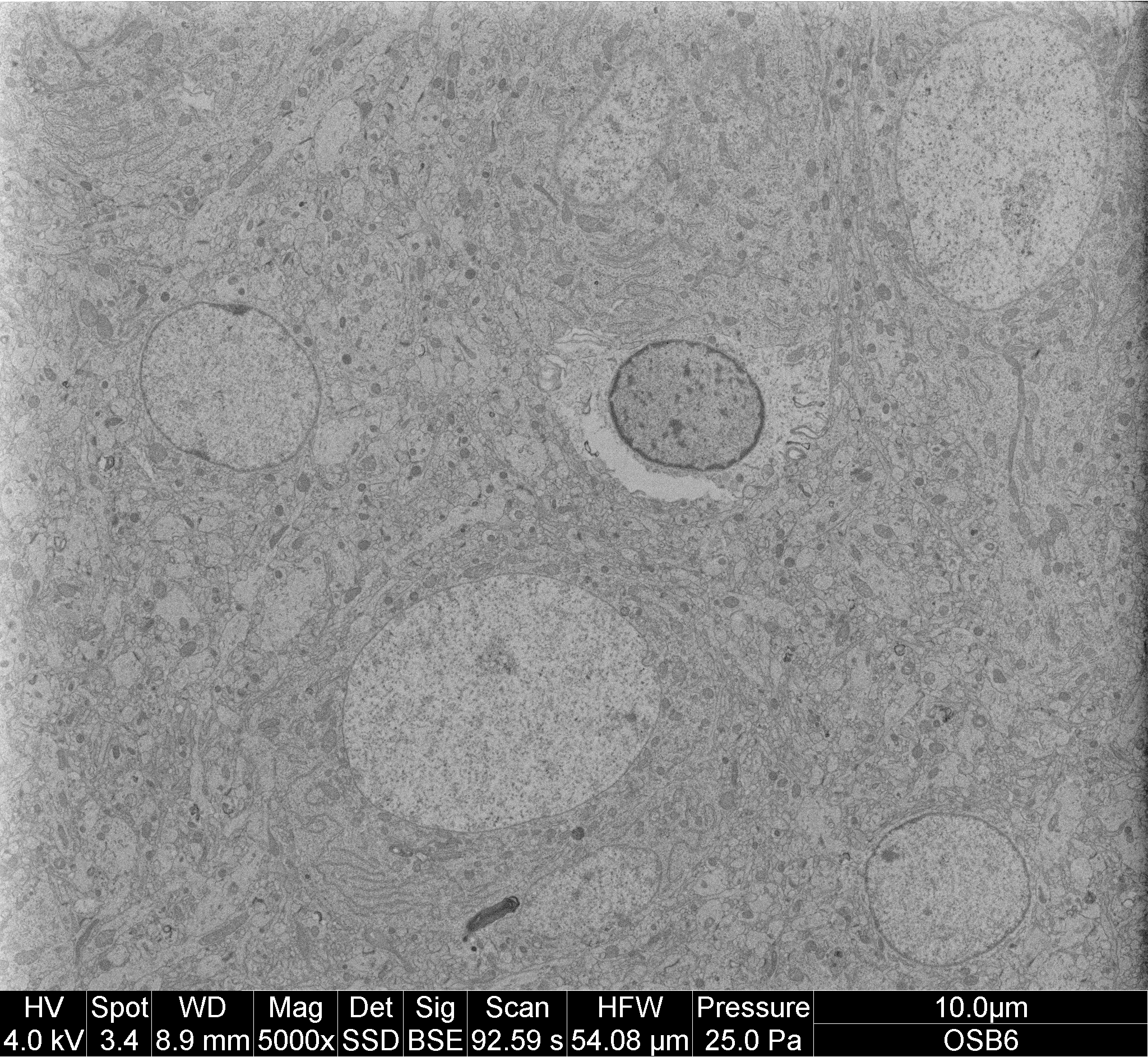

Supplement: Dataset S13 — (251.9 MB ZIP). [file pbio.0020329.sd013.zip › 040604_OS5_st1_1250.tif]

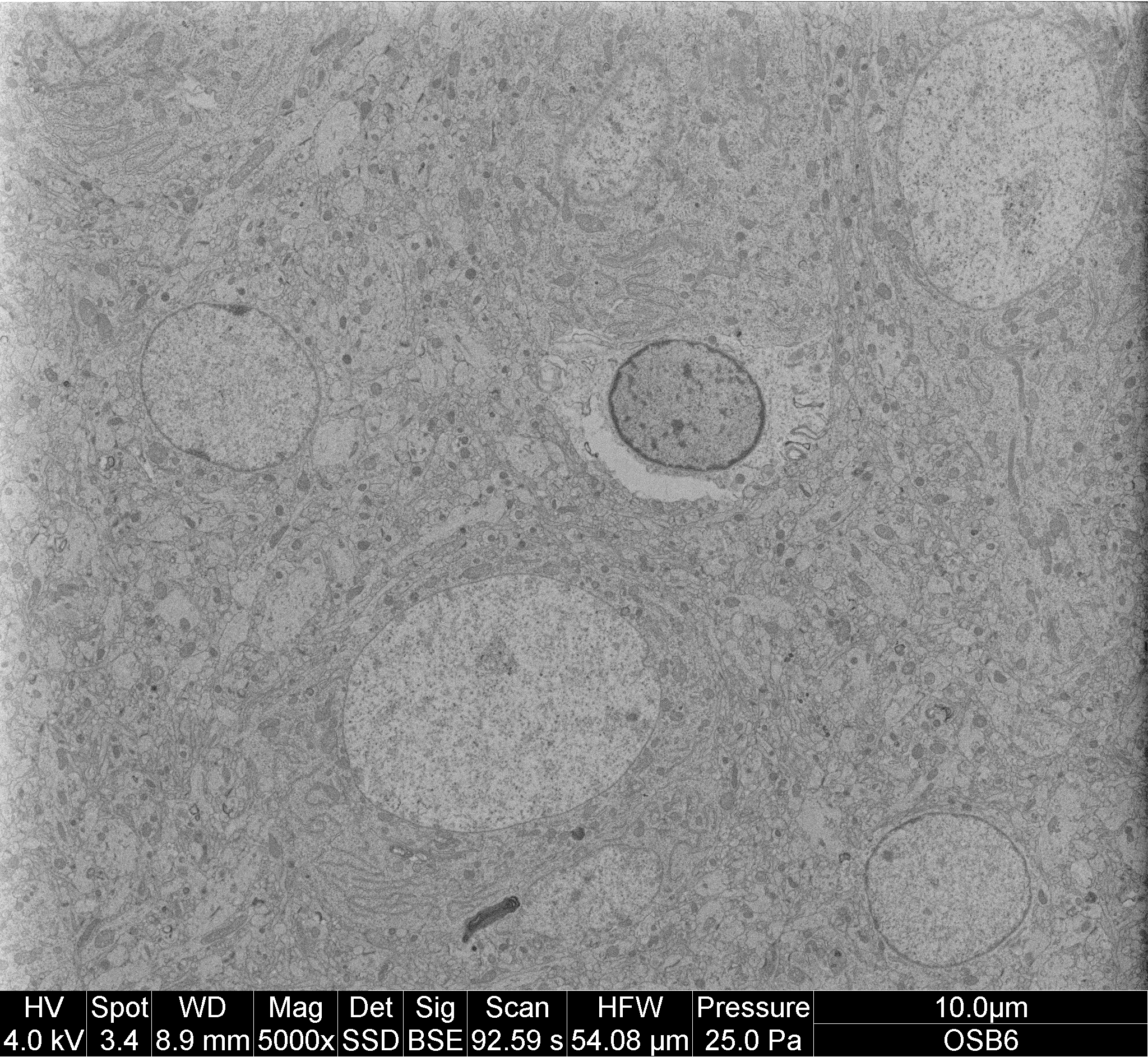

Supplement: Dataset S13 — (251.9 MB ZIP). [file pbio.0020329.sd013.zip › 040604_OS5_st1_1251.tif]

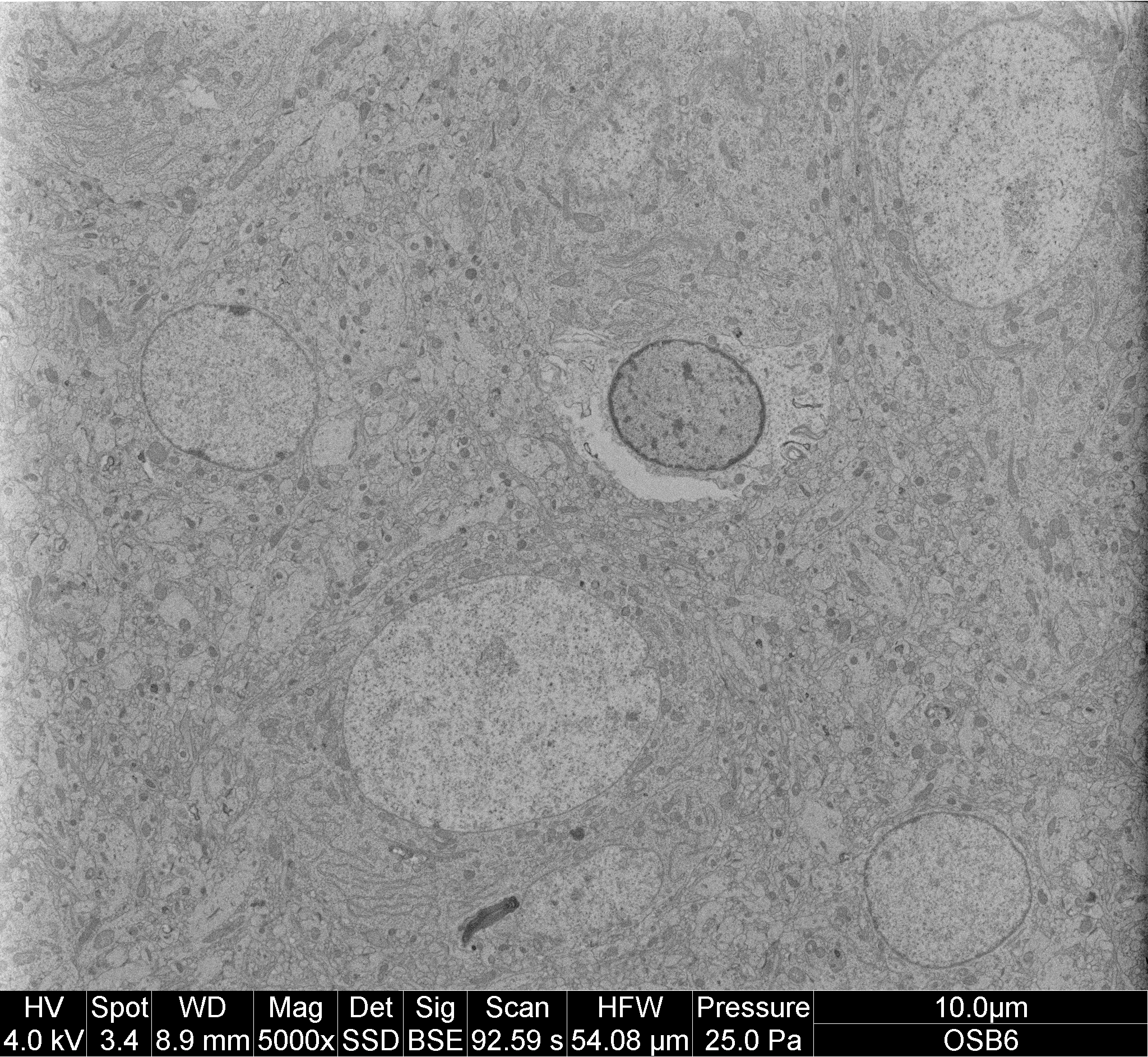

Supplement: Dataset S13 — (251.9 MB ZIP). [file pbio.0020329.sd013.zip › 040604_OS5_st1_1252.tif]

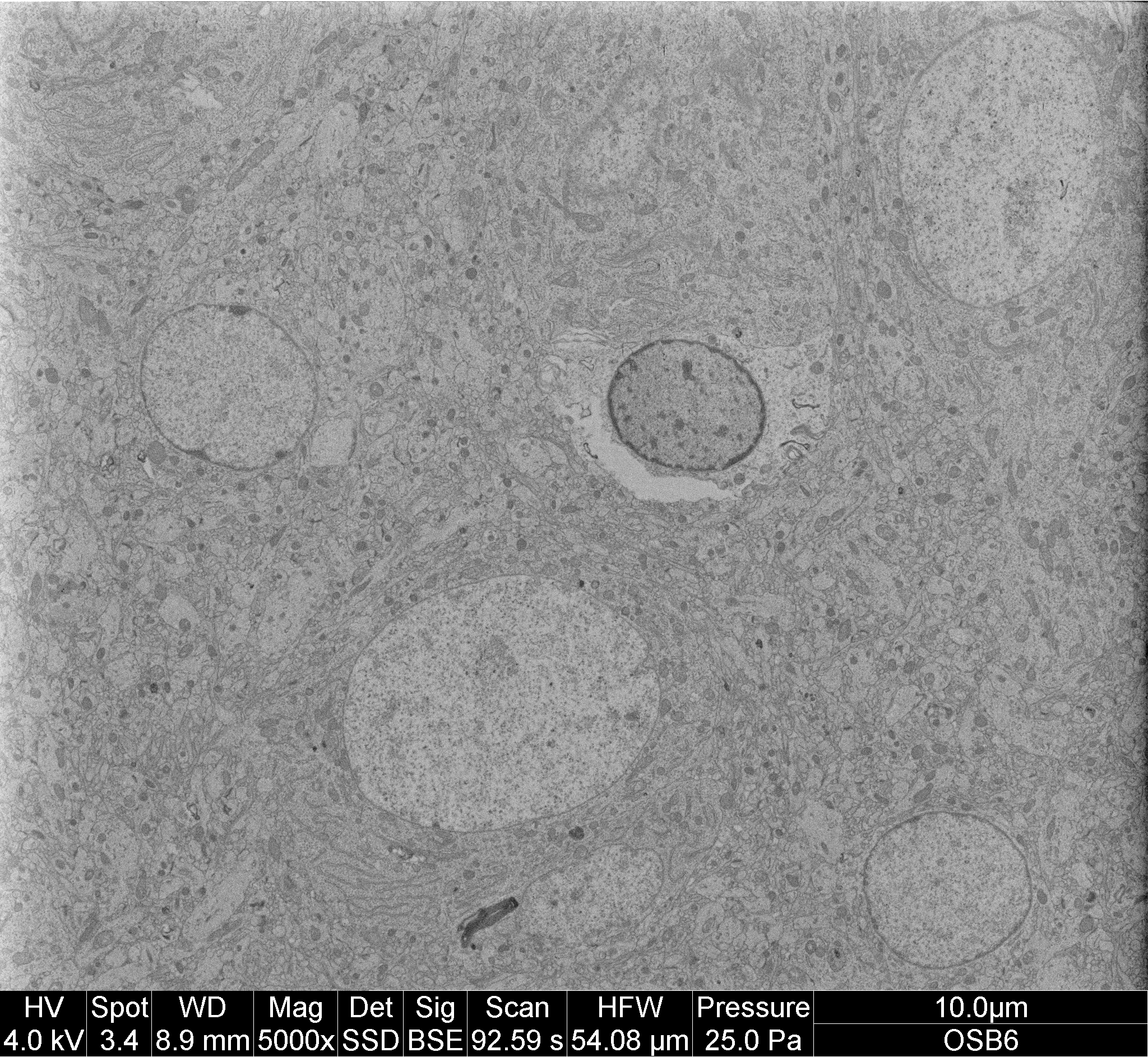

Supplement: Dataset S13 — (251.9 MB ZIP). [file pbio.0020329.sd013.zip › 040604_OS5_st1_1253.tif]

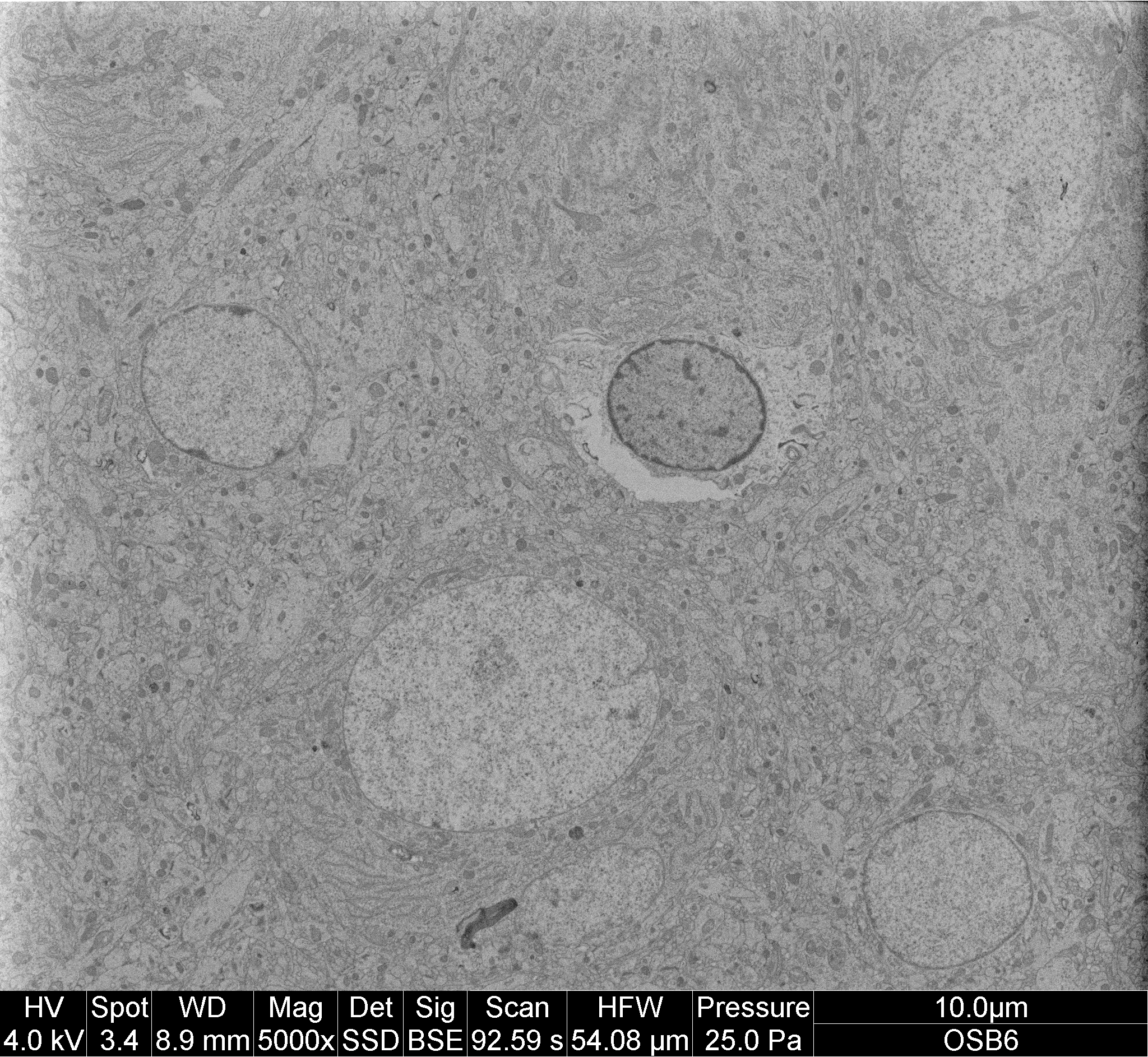

Supplement: Dataset S13 — (251.9 MB ZIP). [file pbio.0020329.sd013.zip › 040604_OS5_st1_1254.tif]

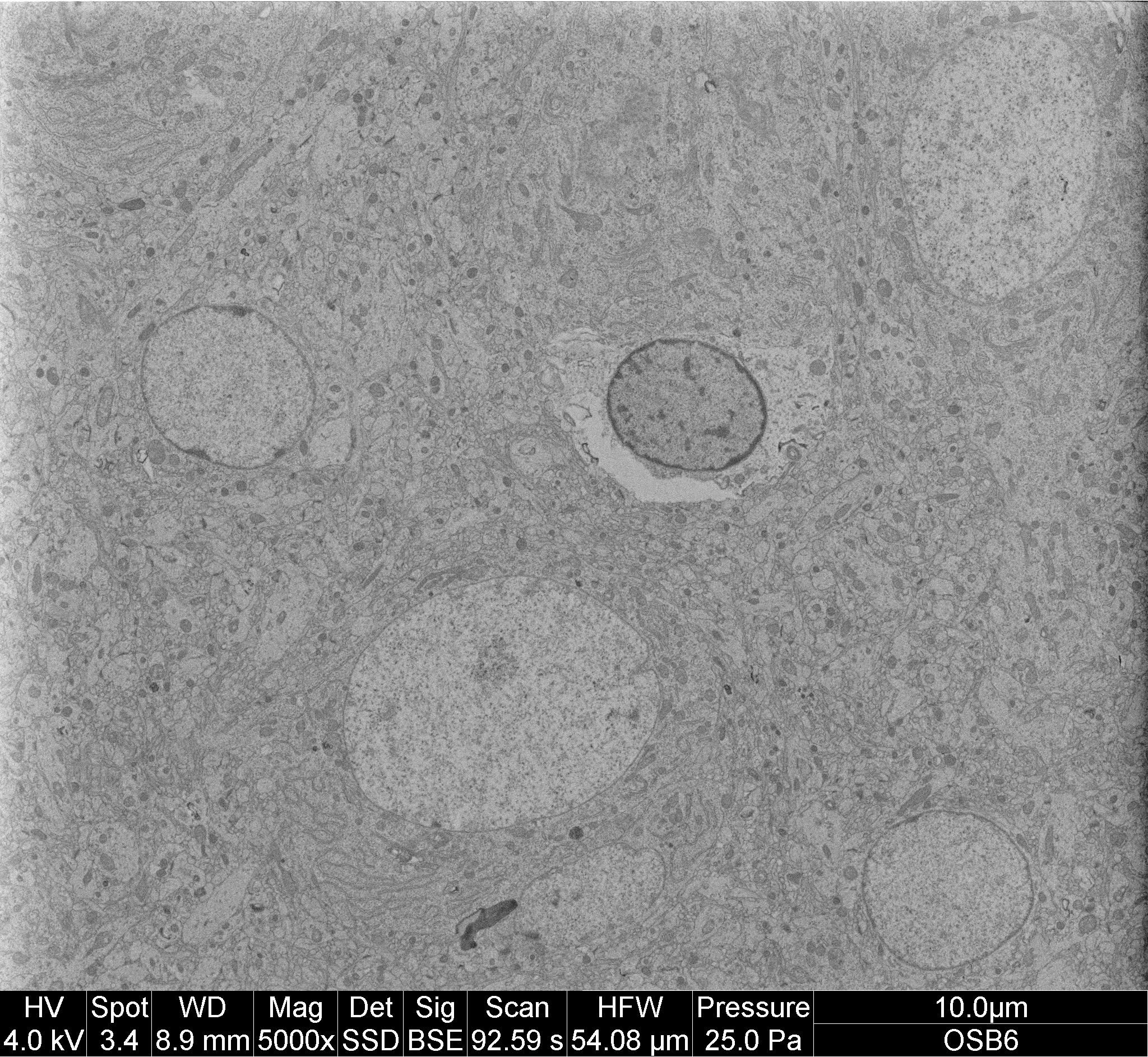

Supplement: Dataset S13 — (251.9 MB ZIP). [file pbio.0020329.sd013.zip › 040604_OS5_st1_1255.tif]

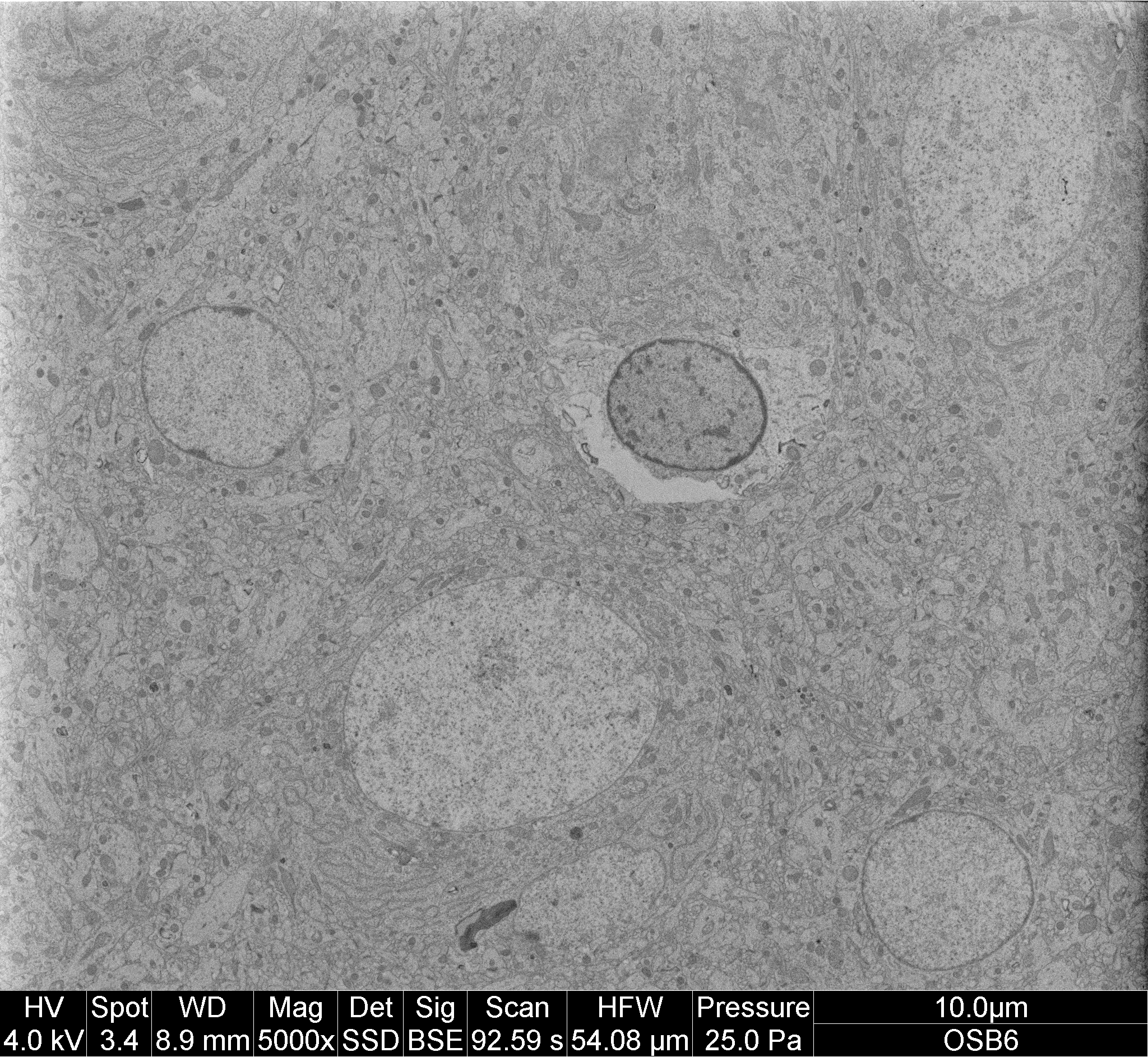

Supplement: Dataset S13 — (251.9 MB ZIP). [file pbio.0020329.sd013.zip › 040604_OS5_st1_1256.tif]

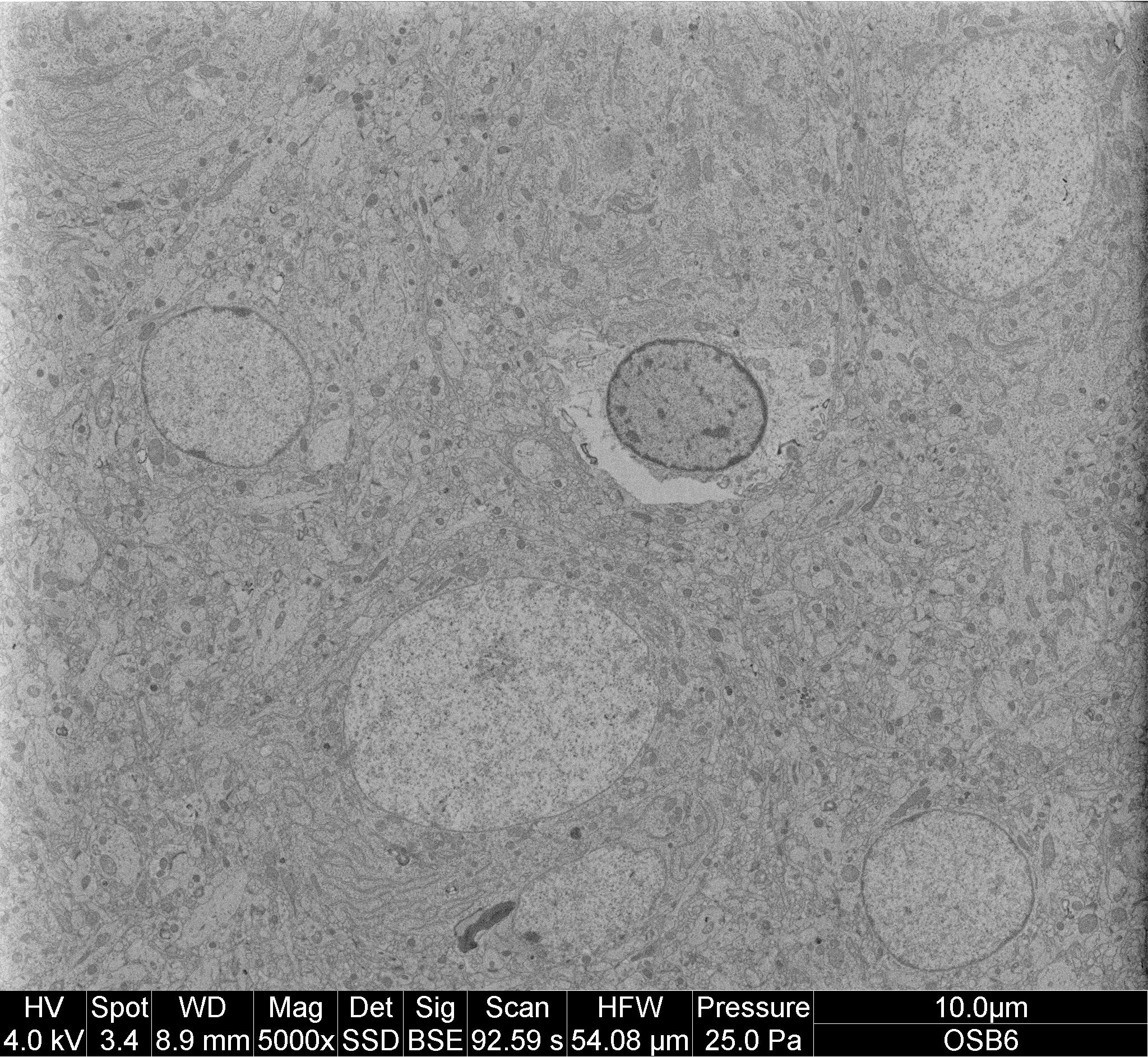

Supplement: Dataset S13 — (251.9 MB ZIP). [file pbio.0020329.sd013.zip › 040604_OS5_st1_1257.tif]

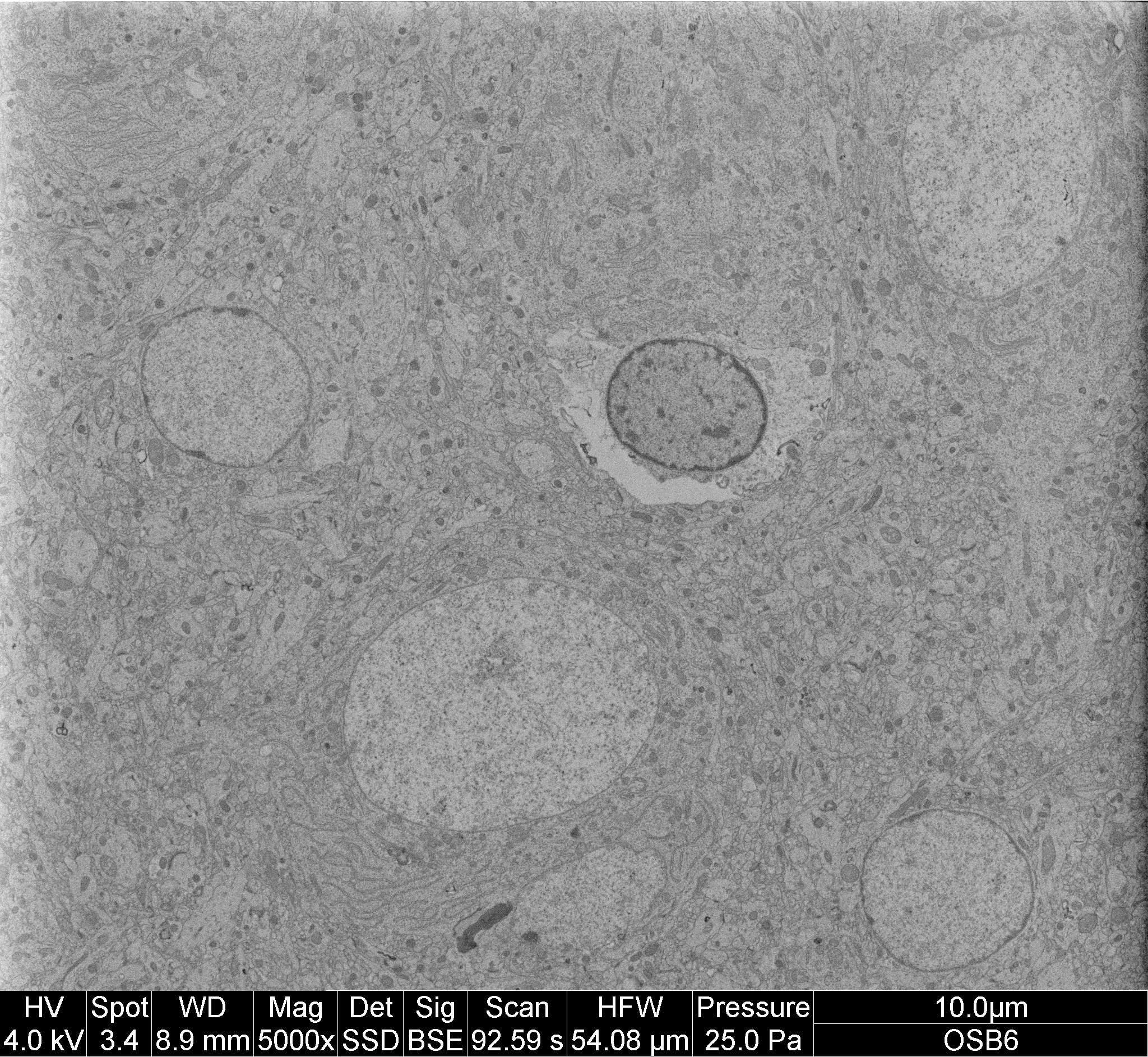

Supplement: Dataset S13 — (251.9 MB ZIP). [file pbio.0020329.sd013.zip › 040604_OS5_st1_1258.tif]

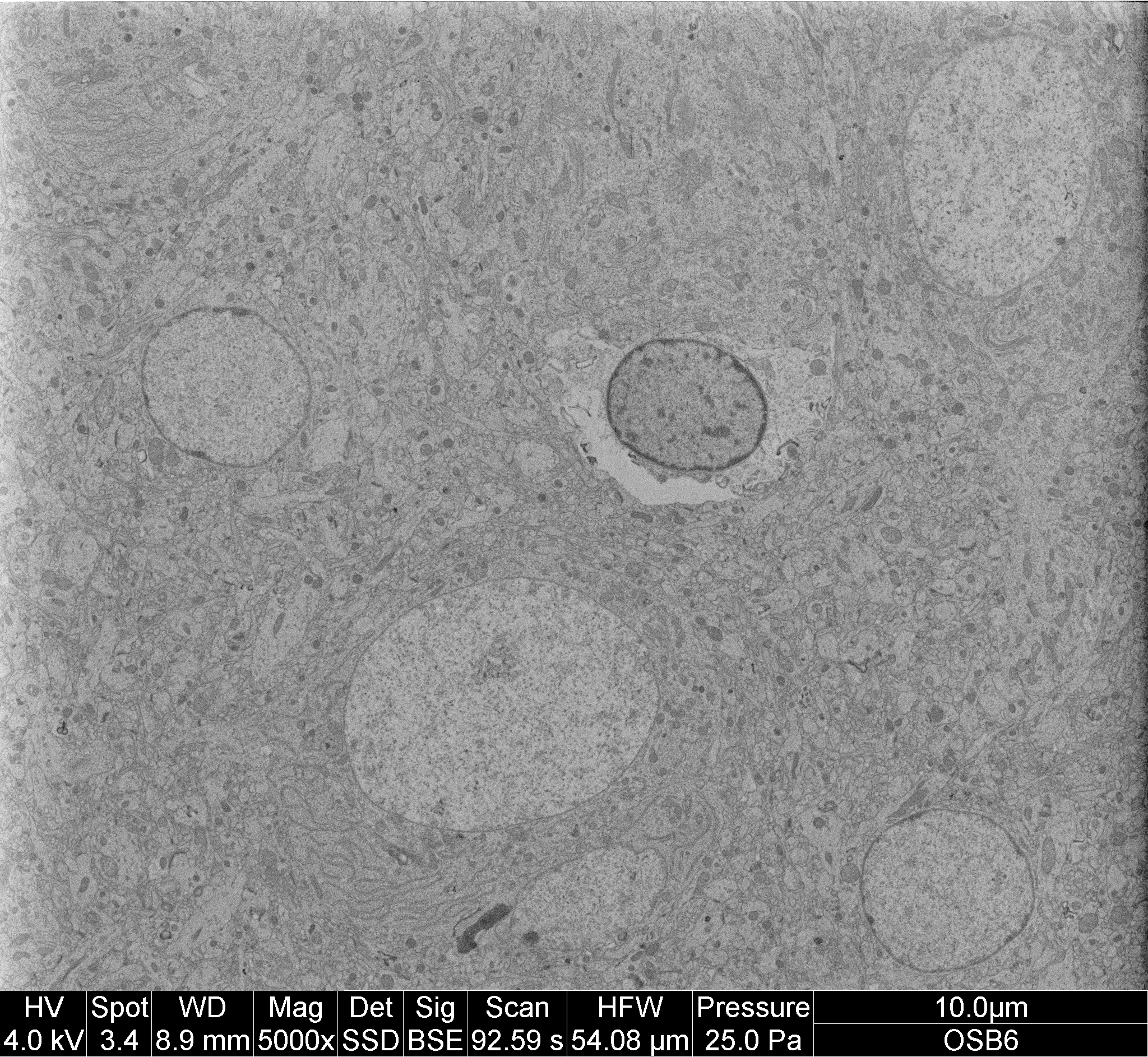

Supplement: Dataset S13 — (251.9 MB ZIP). [file pbio.0020329.sd013.zip › 040604_OS5_st1_1259.tif]

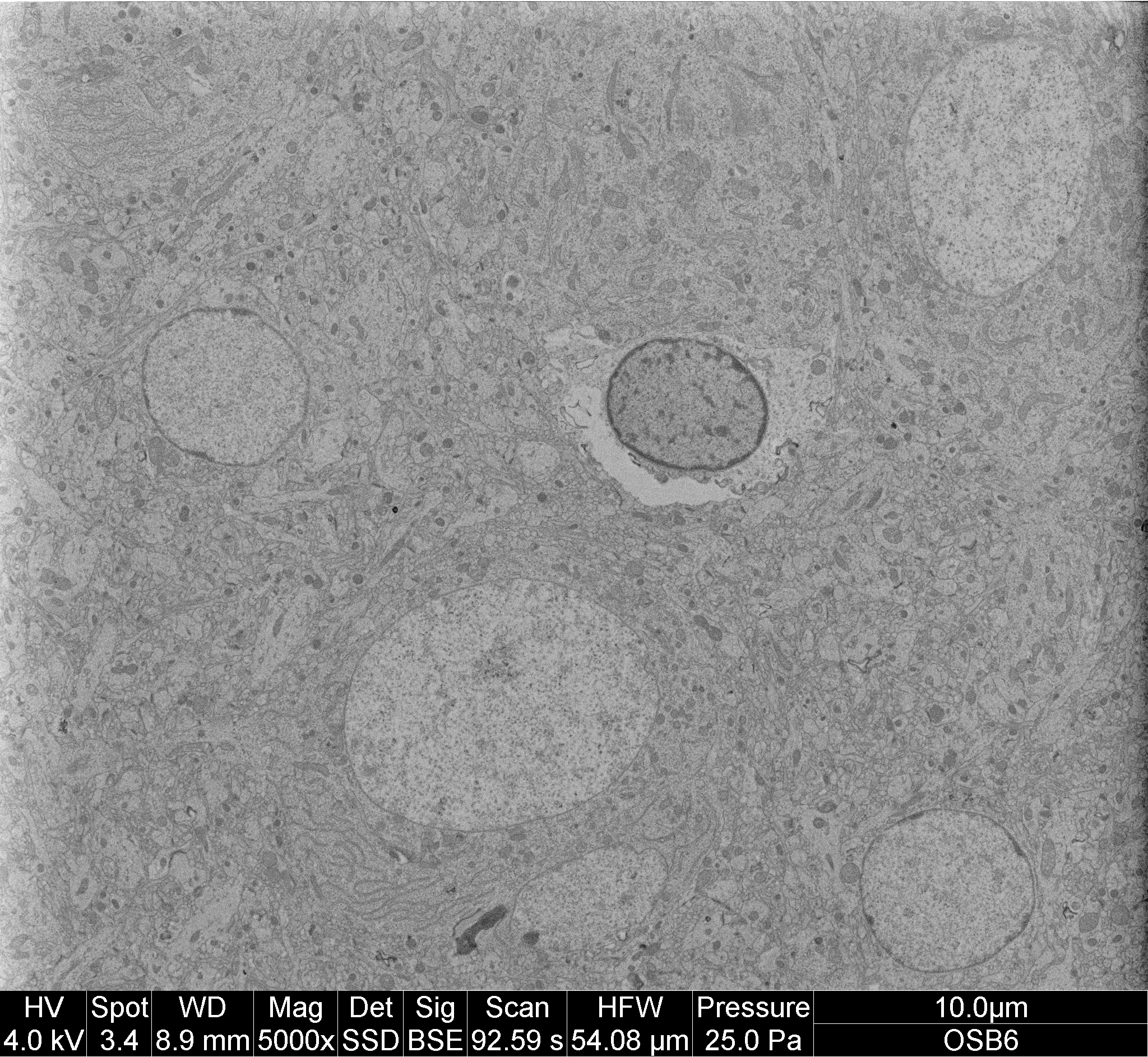

Supplement: Dataset S13 — (251.9 MB ZIP). [file pbio.0020329.sd013.zip › 040604_OS5_st1_1260.tif]

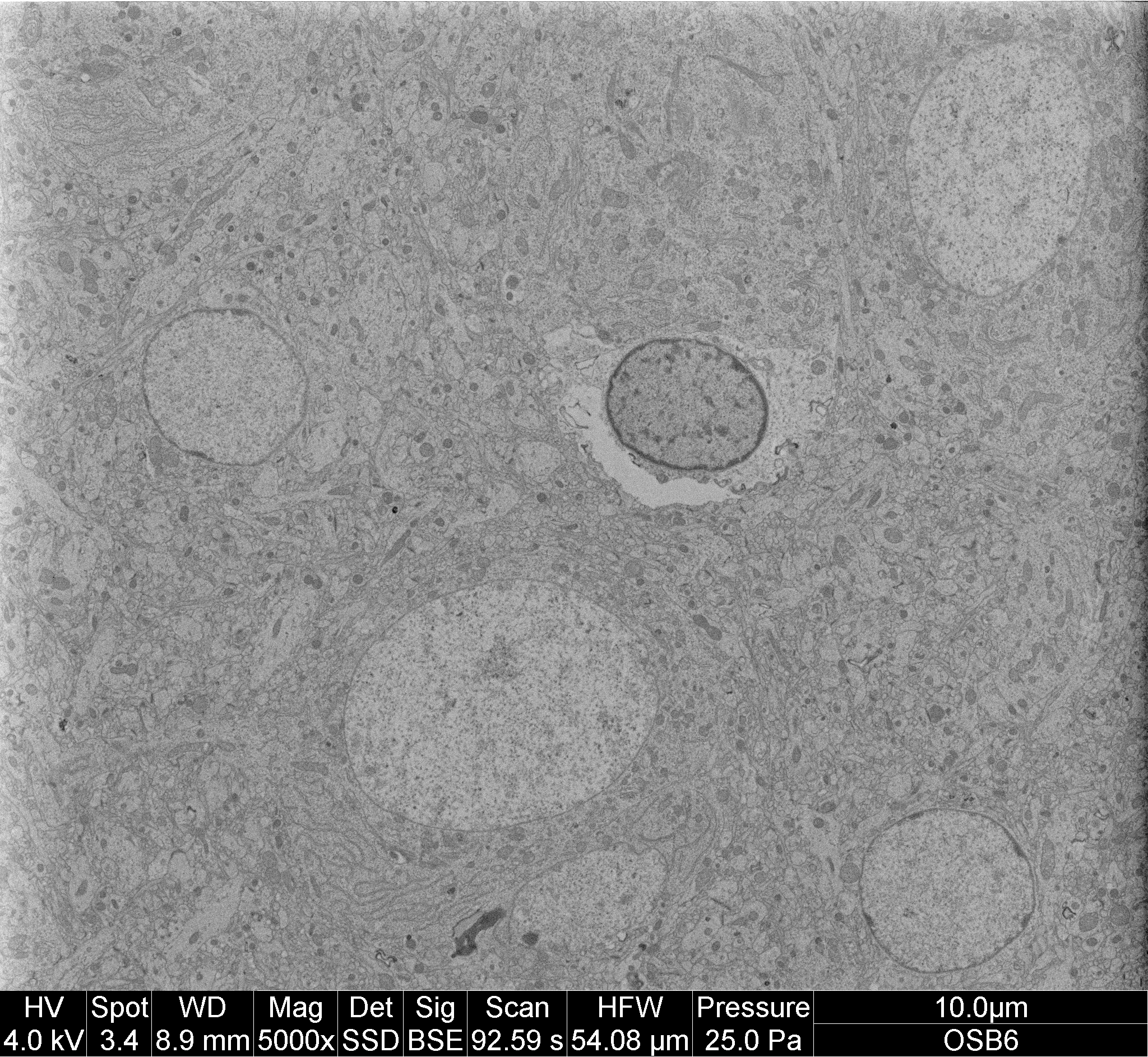

Supplement: Dataset S13 — (251.9 MB ZIP). [file pbio.0020329.sd013.zip › 040604_OS5_st1_1261.tif]

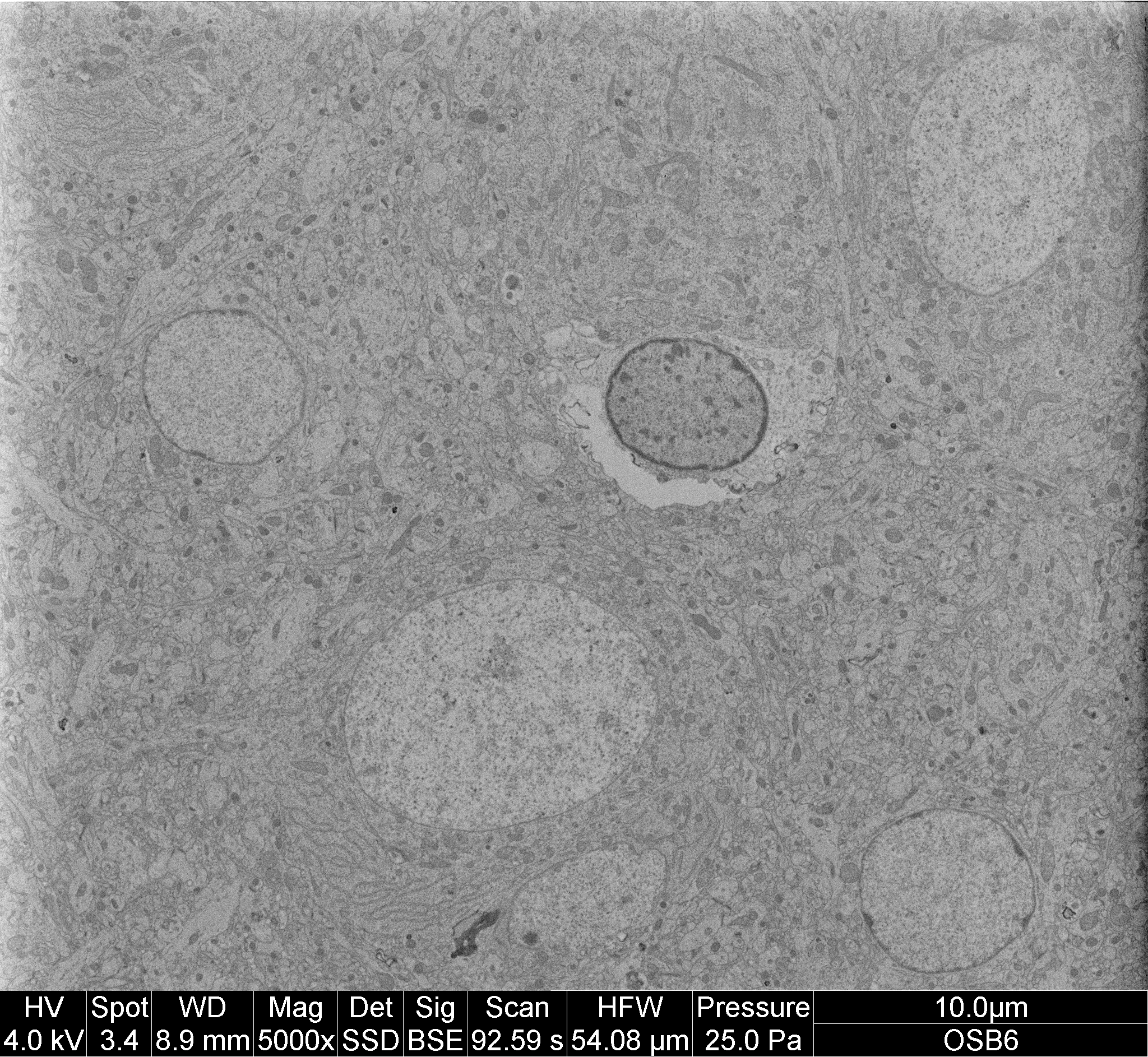

Supplement: Dataset S13 — (251.9 MB ZIP). [file pbio.0020329.sd013.zip › 040604_OS5_st1_1262.tif]

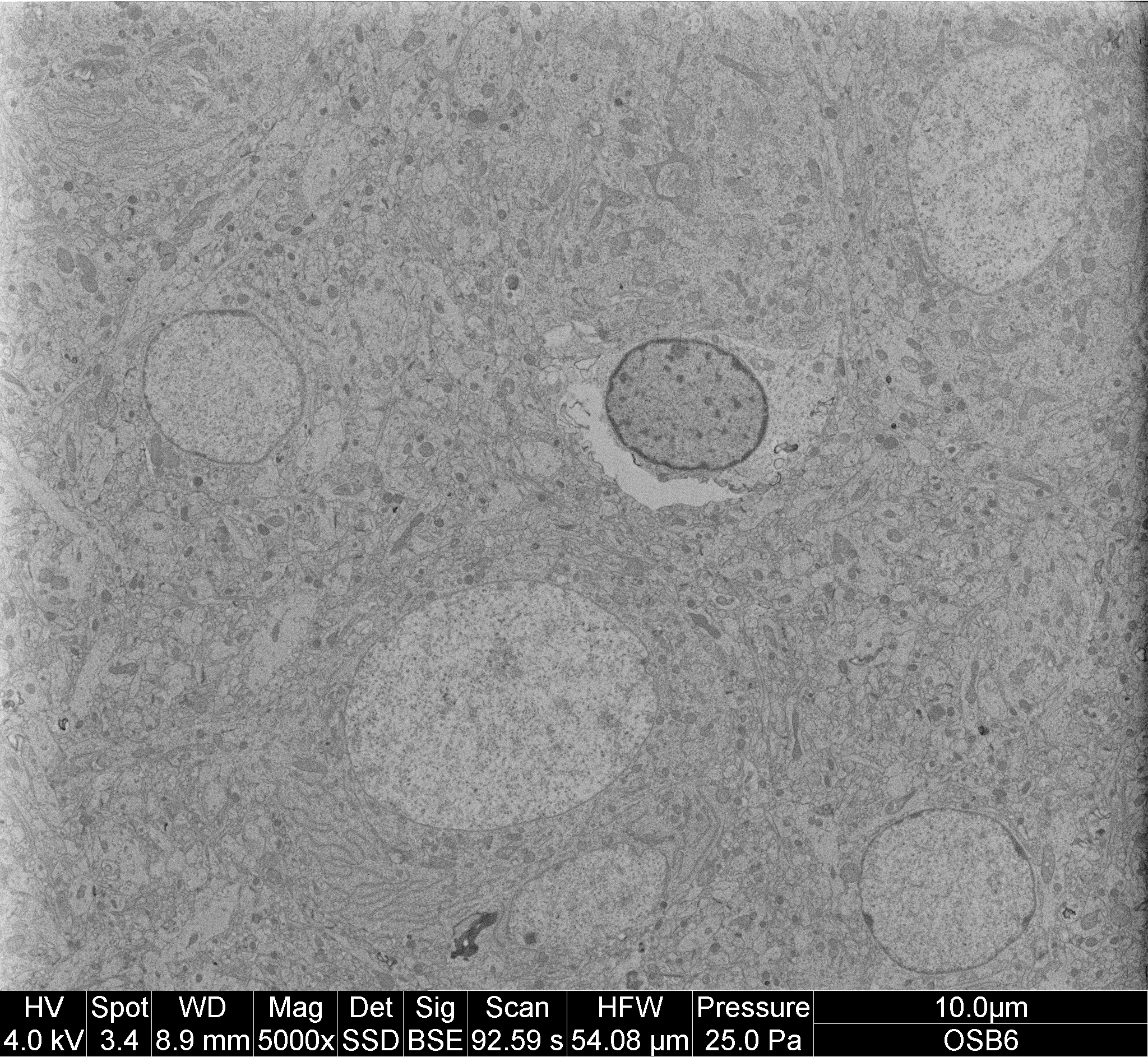

Supplement: Dataset S13 — (251.9 MB ZIP). [file pbio.0020329.sd013.zip › 040604_OS5_st1_1263.tif]

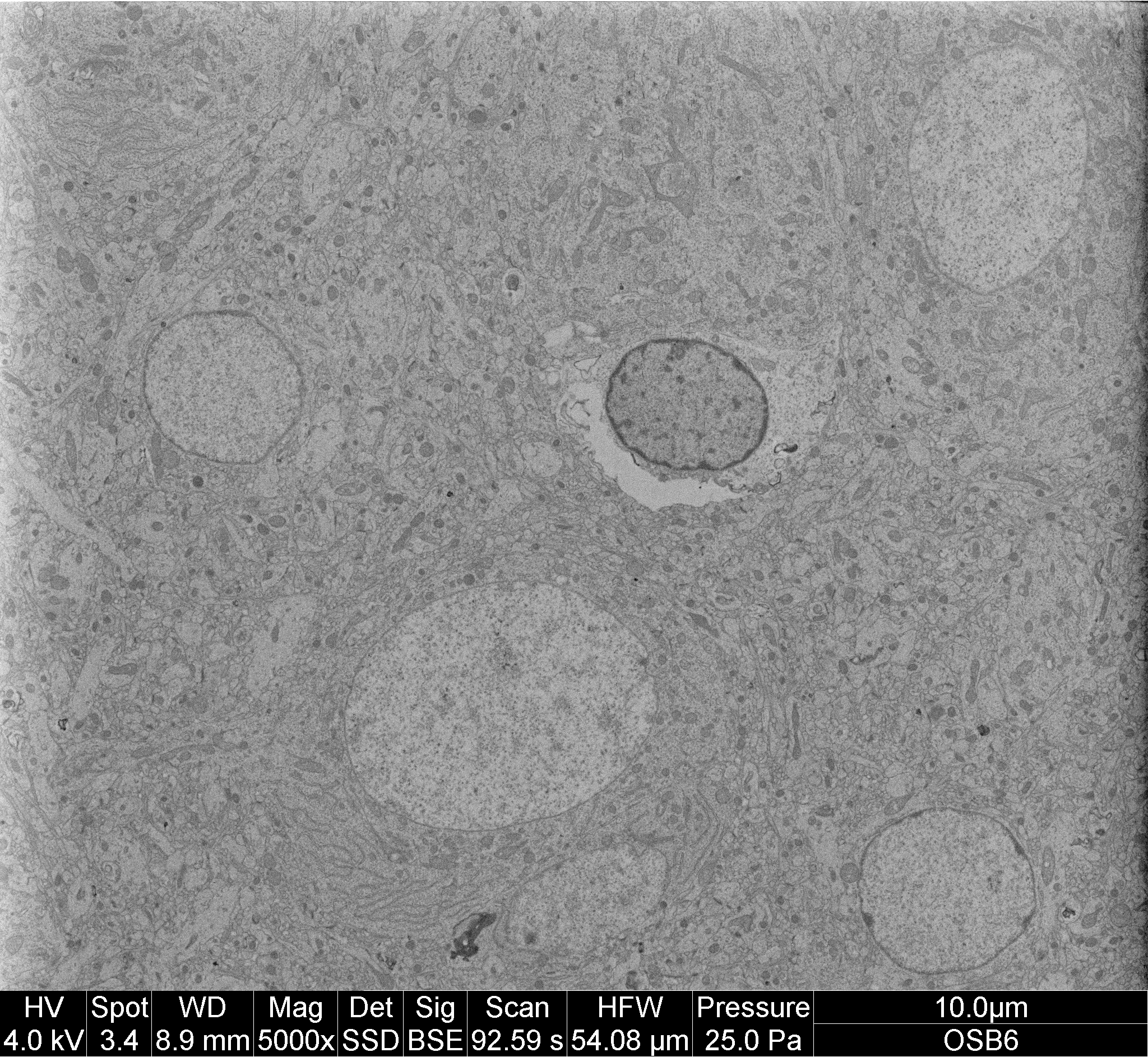

Supplement: Dataset S13 — (251.9 MB ZIP). [file pbio.0020329.sd013.zip › 040604_OS5_st1_1264.tif]

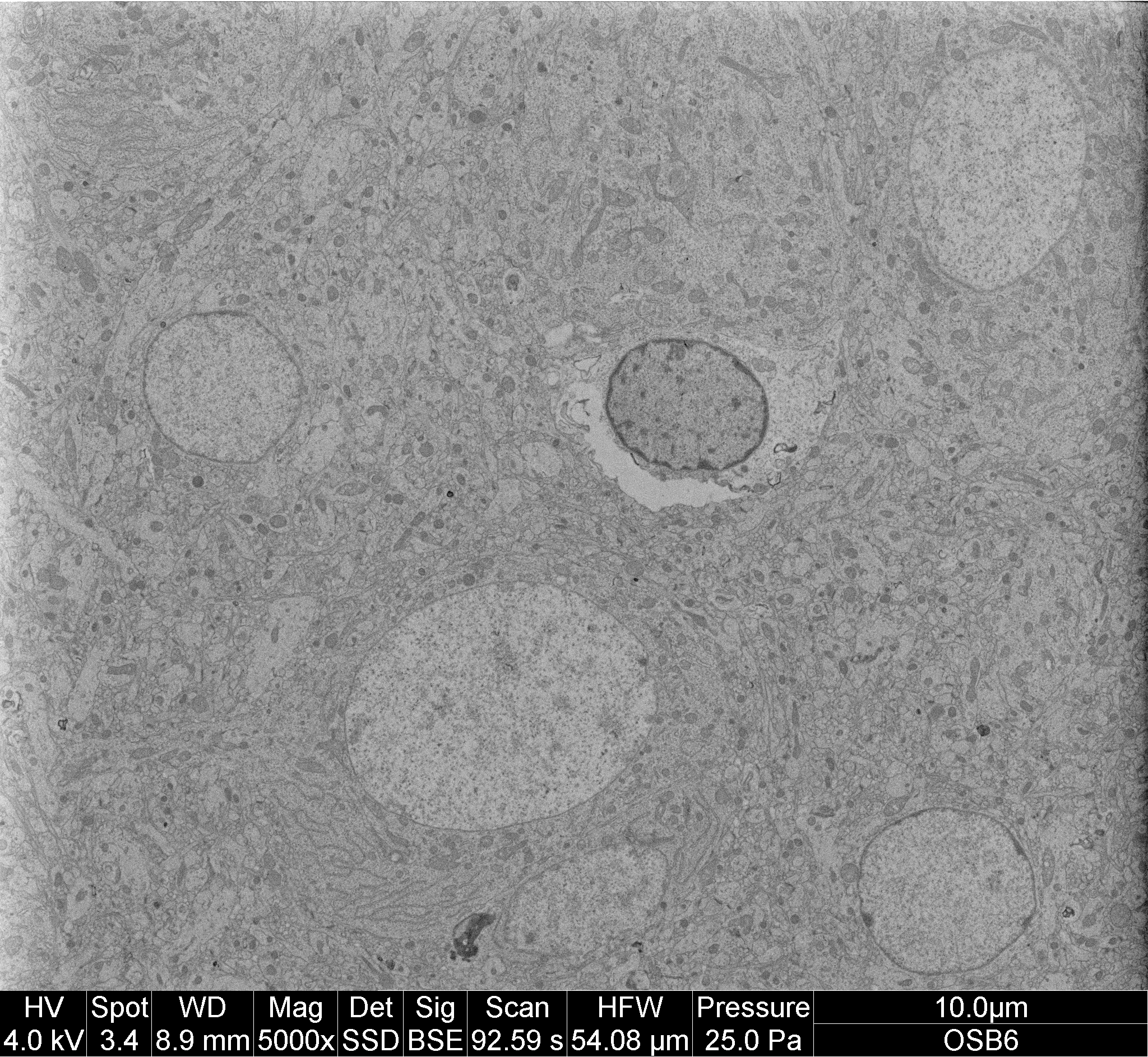

Supplement: Dataset S13 — (251.9 MB ZIP). [file pbio.0020329.sd013.zip › 040604_OS5_st1_1265.tif]

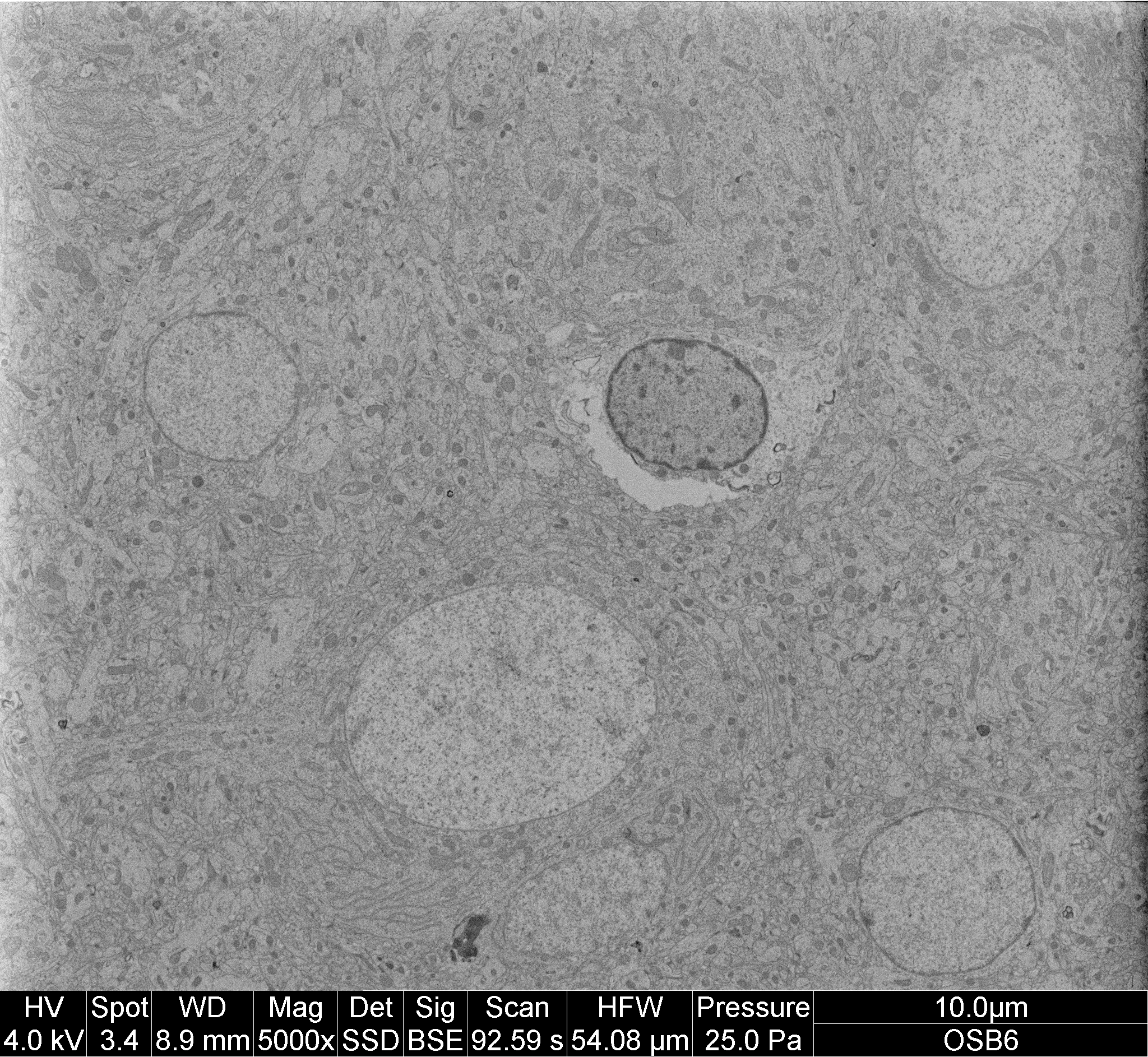

Supplement: Dataset S13 — (251.9 MB ZIP). [file pbio.0020329.sd013.zip › 040604_OS5_st1_1266.tif]

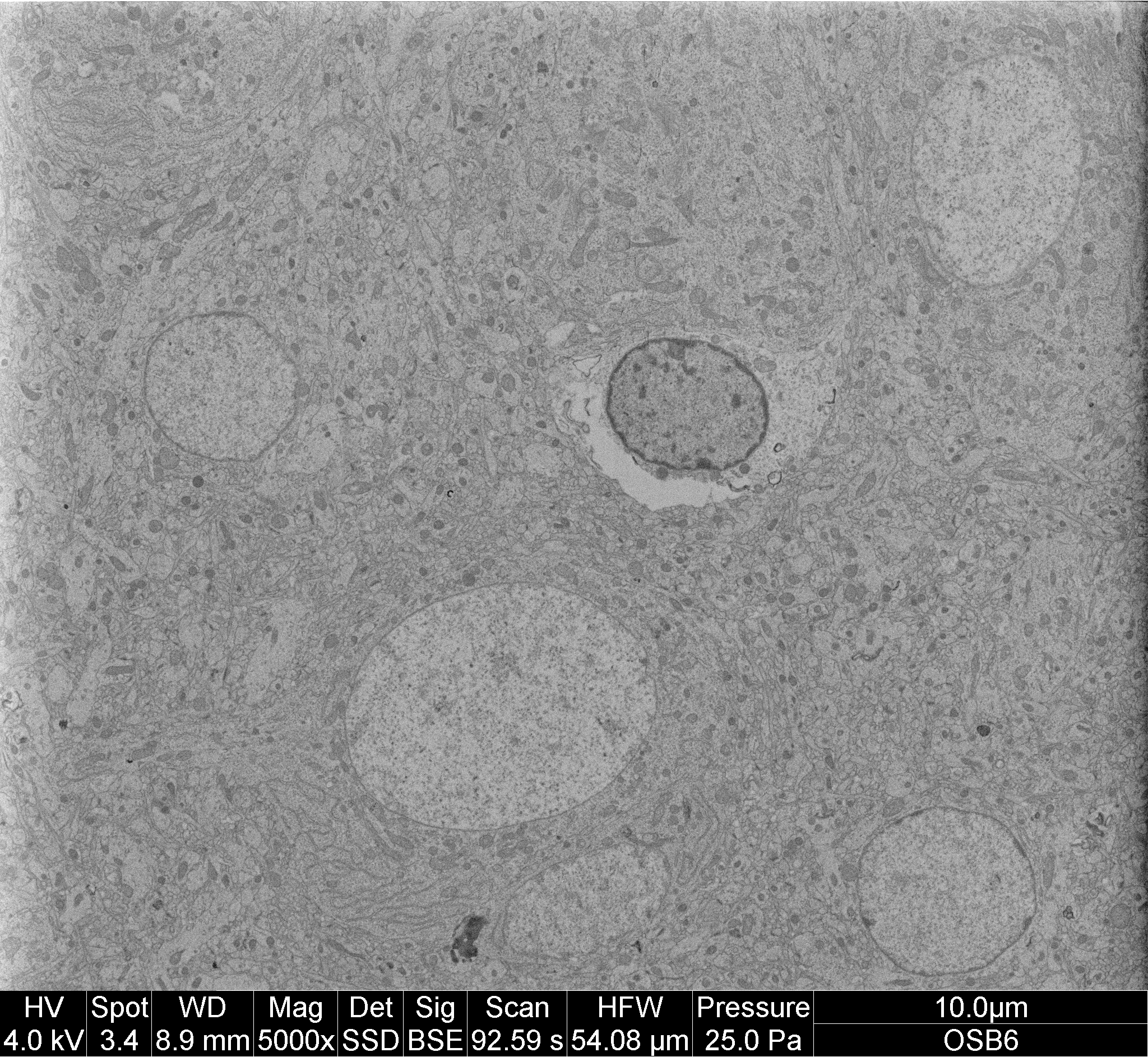

Supplement: Dataset S13 — (251.9 MB ZIP). [file pbio.0020329.sd013.zip › 040604_OS5_st1_1267.tif]

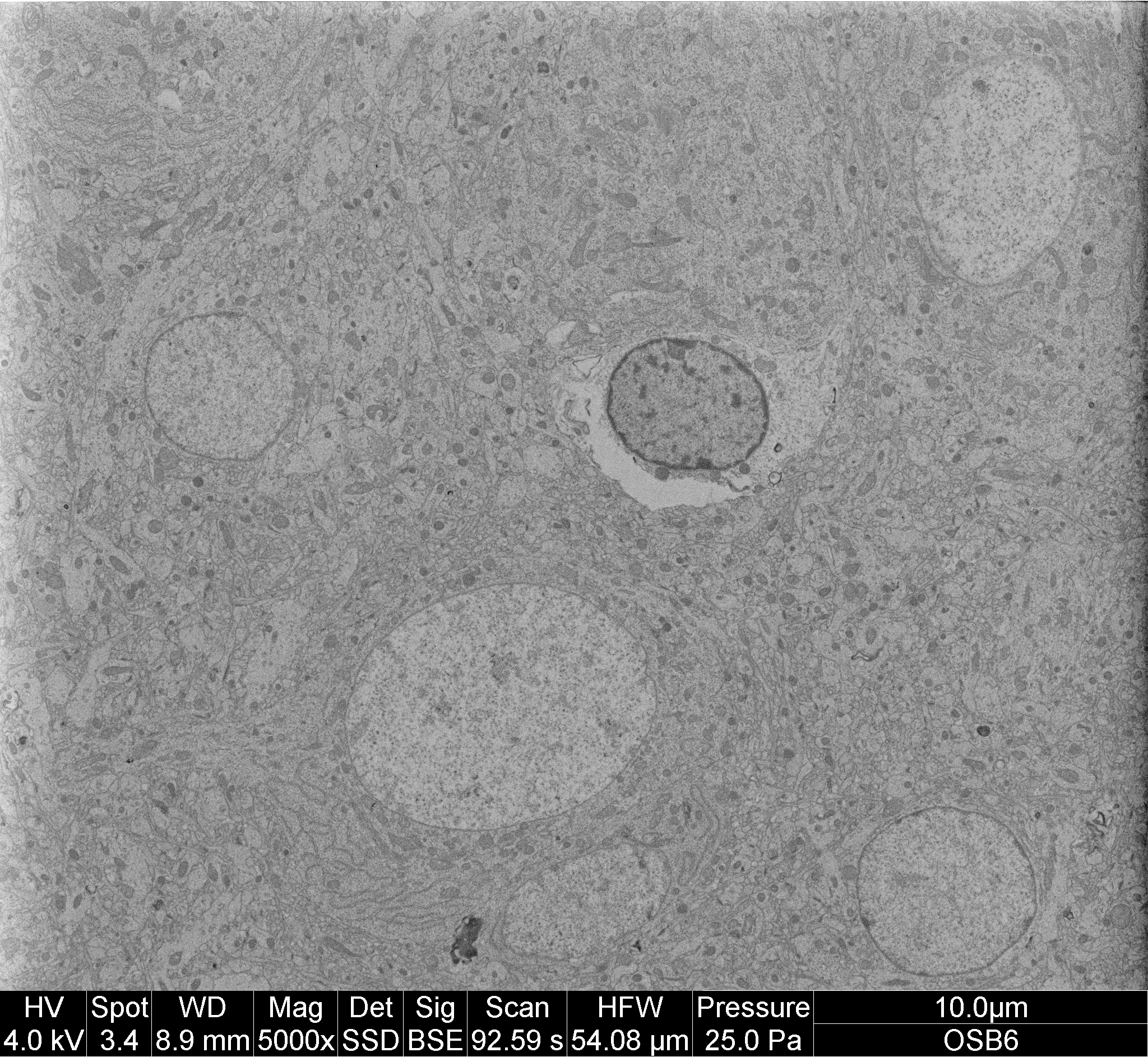

Supplement: Dataset S13 — (251.9 MB ZIP). [file pbio.0020329.sd013.zip › 040604_OS5_st1_1268.tif]

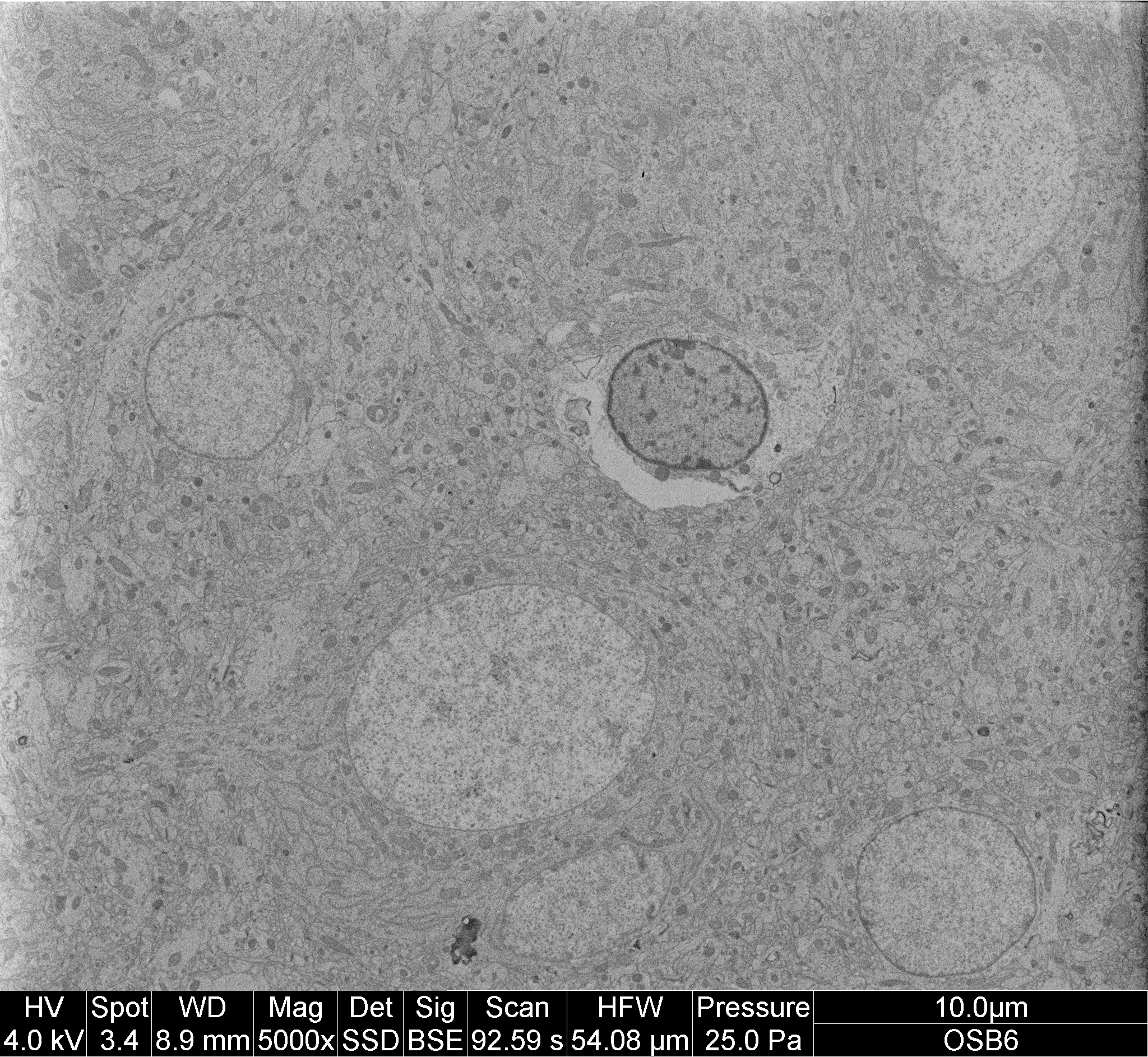

Supplement: Dataset S13 — (251.9 MB ZIP). [file pbio.0020329.sd013.zip › 040604_OS5_st1_1269.tif]

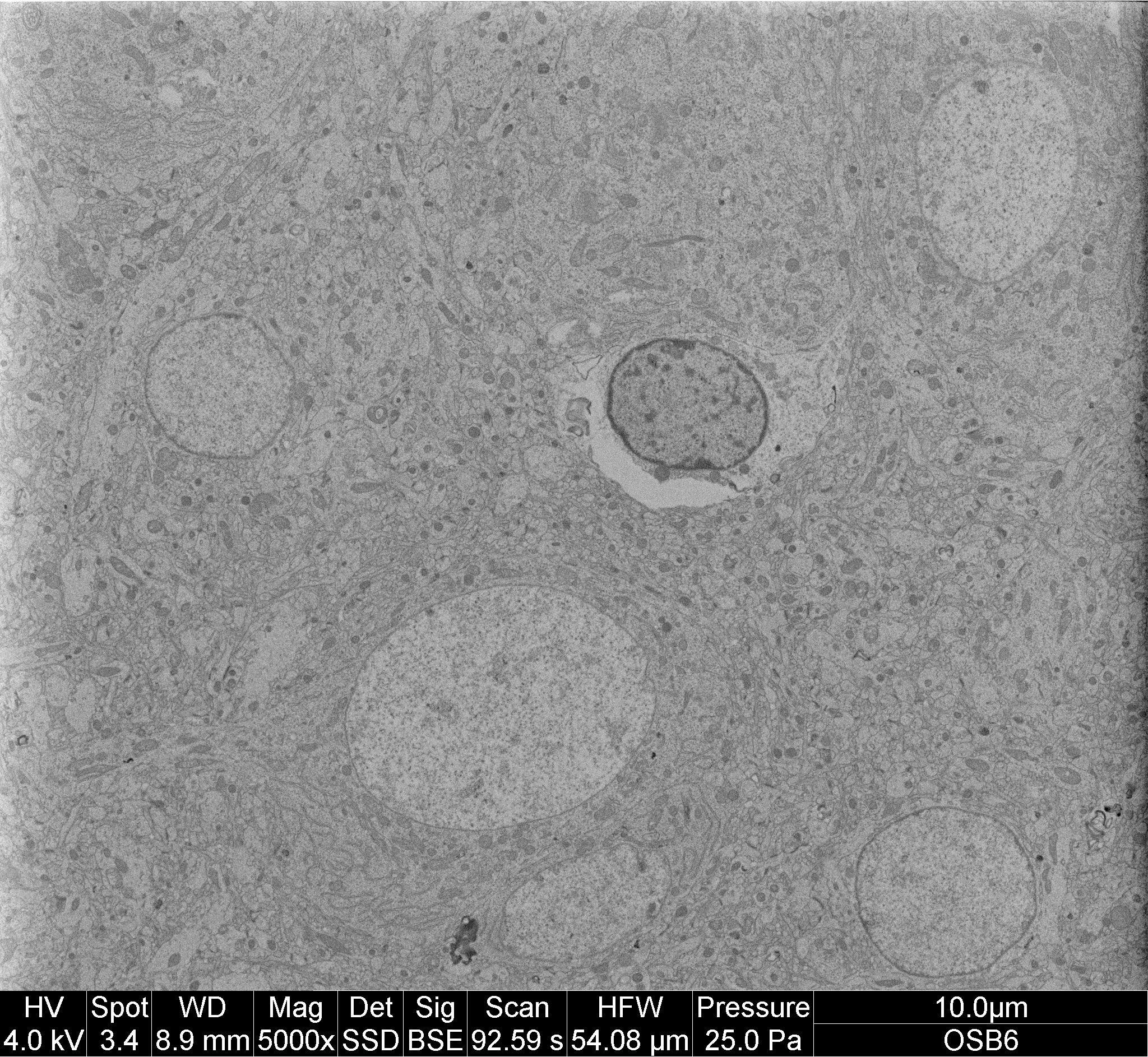

Supplement: Dataset S13 — (251.9 MB ZIP). [file pbio.0020329.sd013.zip › 040604_OS5_st1_1270.tif]

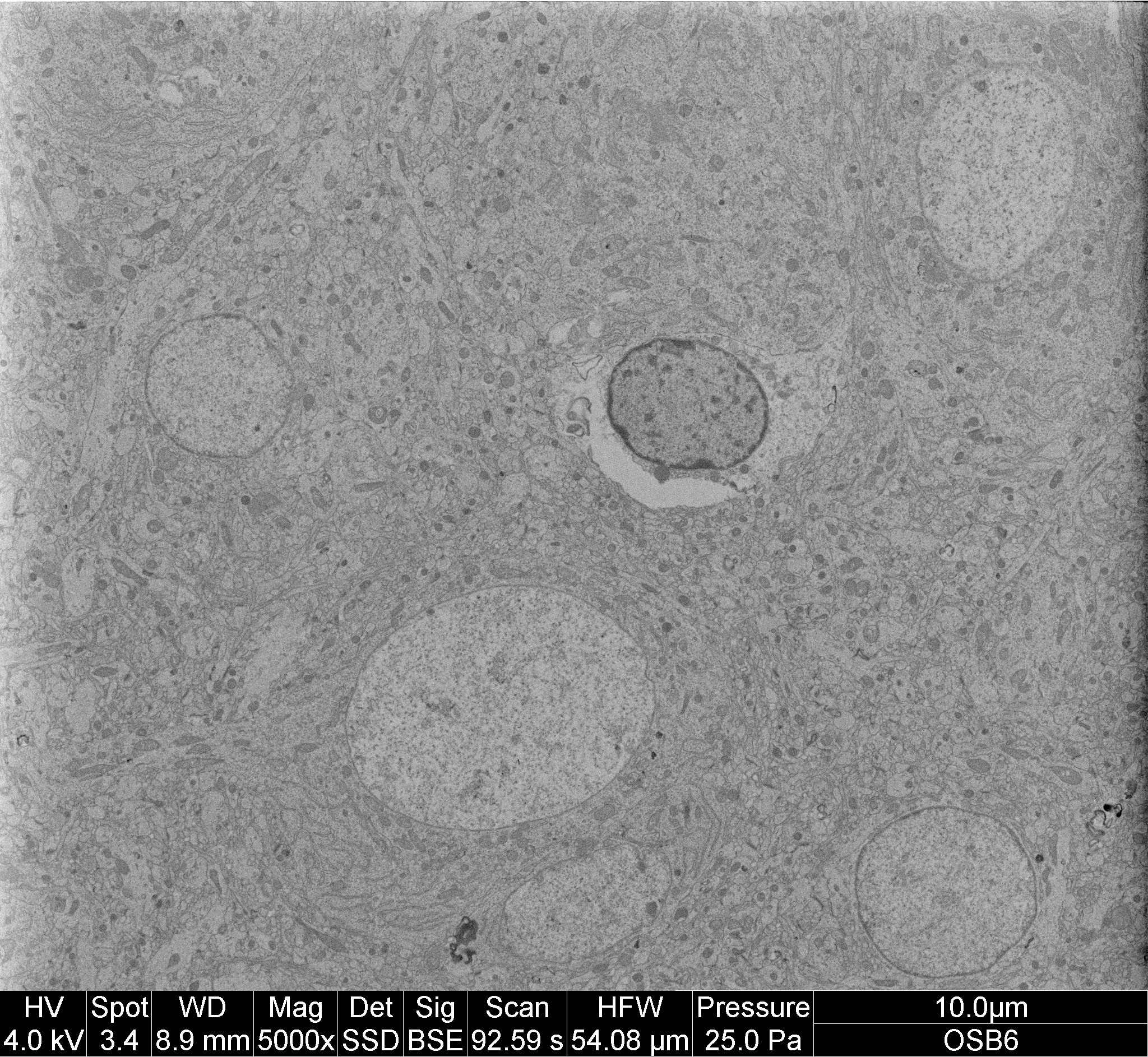

Supplement: Dataset S13 — (251.9 MB ZIP). [file pbio.0020329.sd013.zip › 040604_OS5_st1_1271.tif]

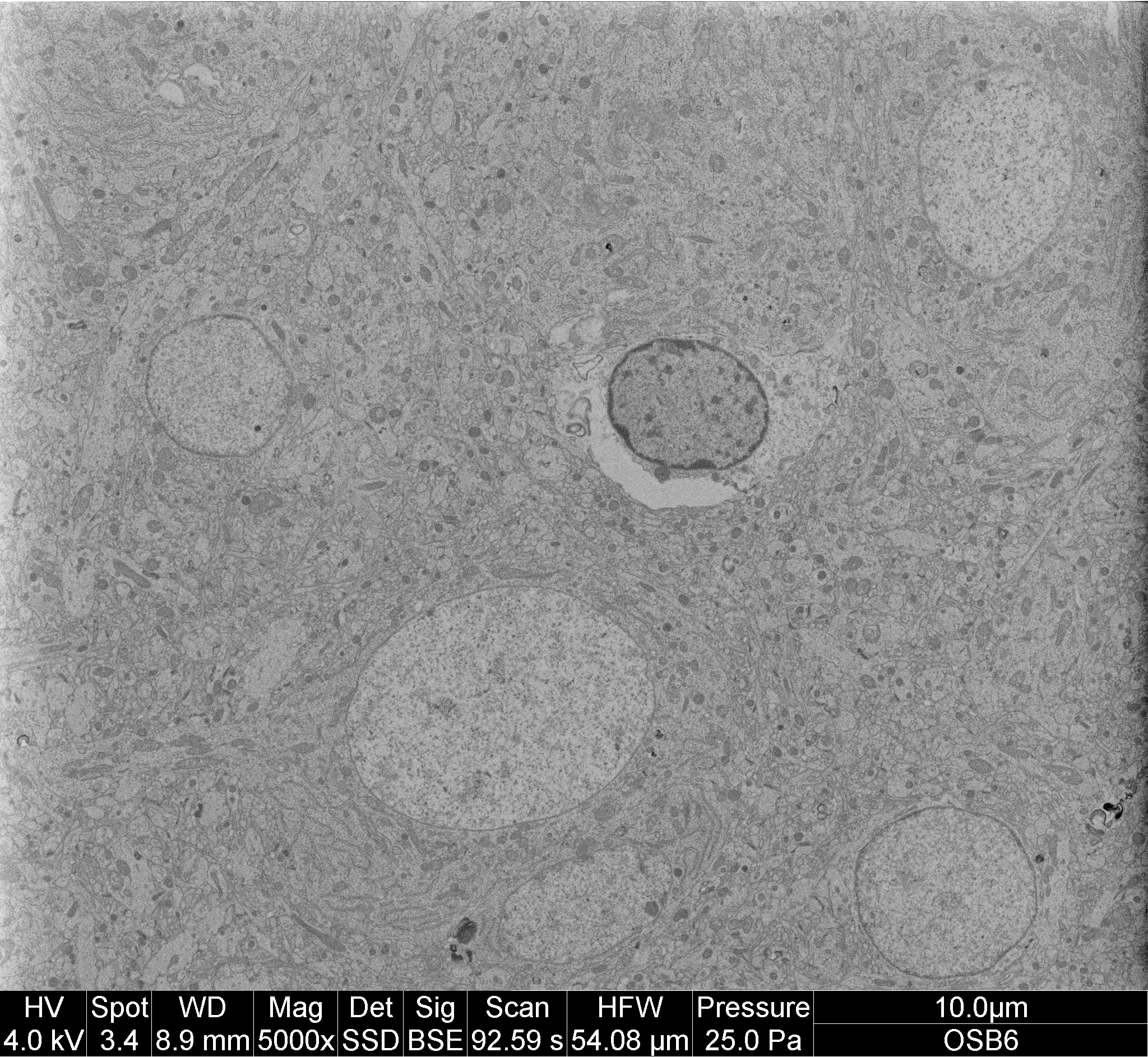

Supplement: Dataset S13 — (251.9 MB ZIP). [file pbio.0020329.sd013.zip › 040604_OS5_st1_1272.tif]

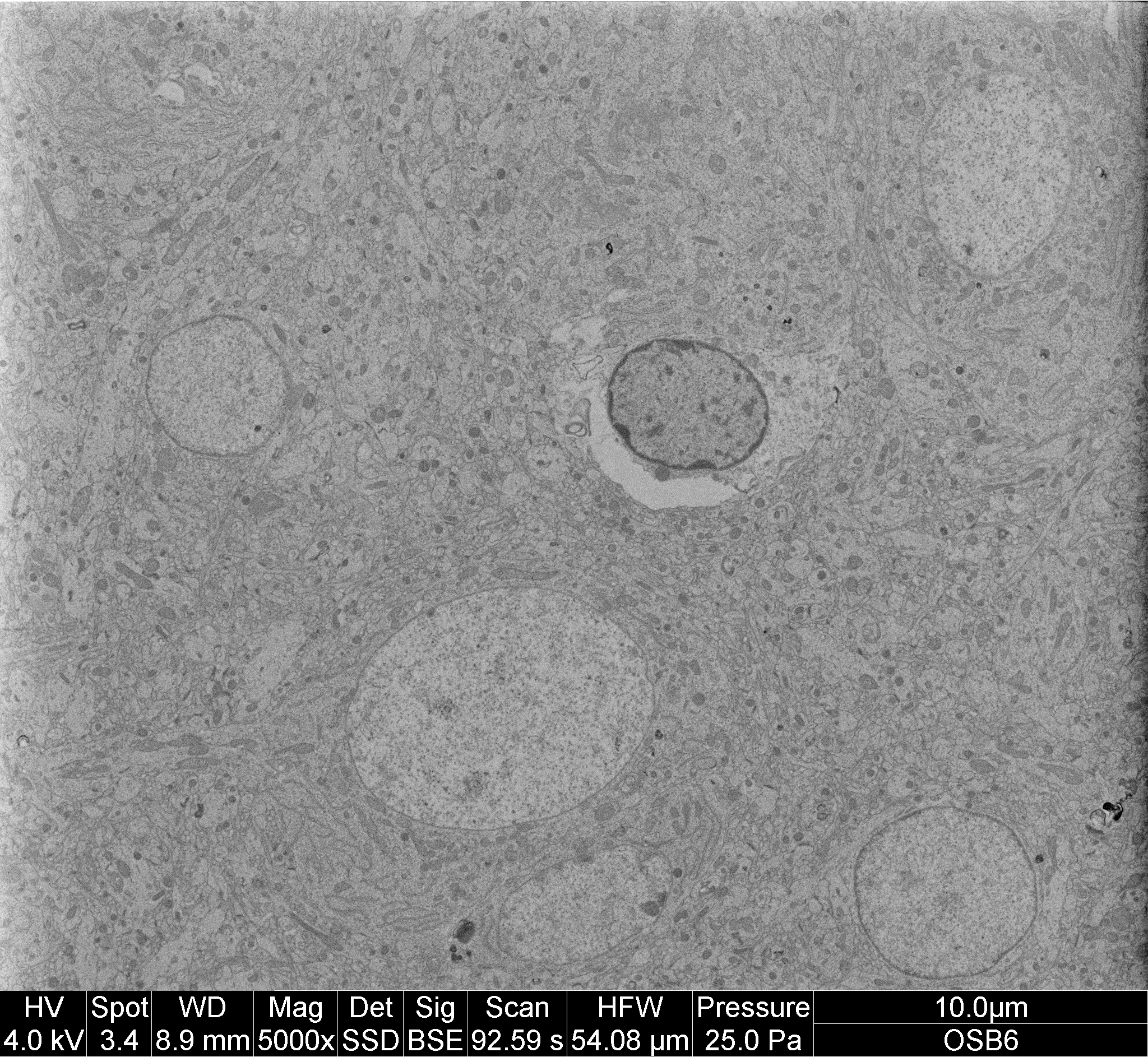

Supplement: Dataset S13 — (251.9 MB ZIP). [file pbio.0020329.sd013.zip › 040604_OS5_st1_1273.tif]

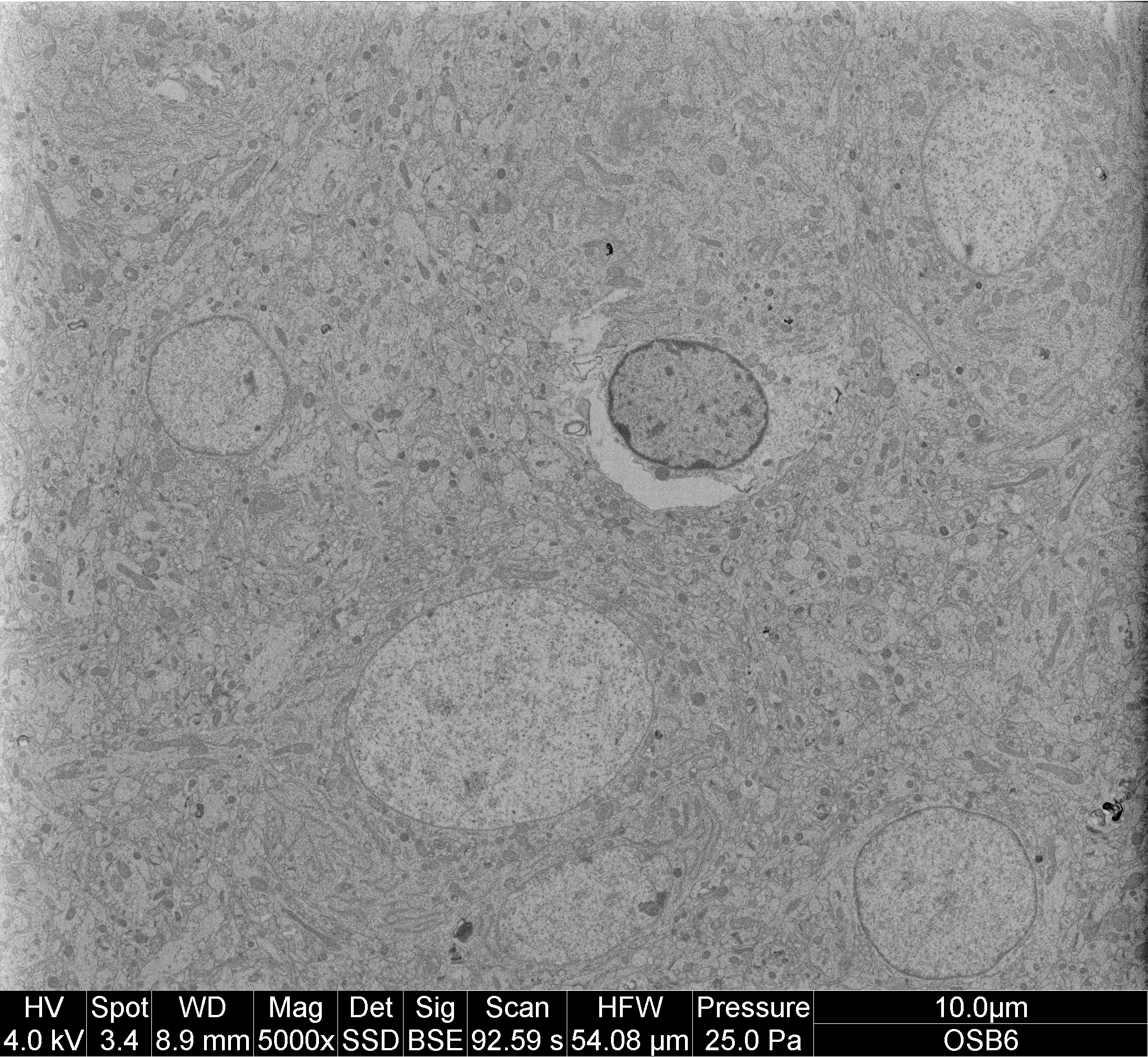

Supplement: Dataset S13 — (251.9 MB ZIP). [file pbio.0020329.sd013.zip › 040604_OS5_st1_1274.tif]

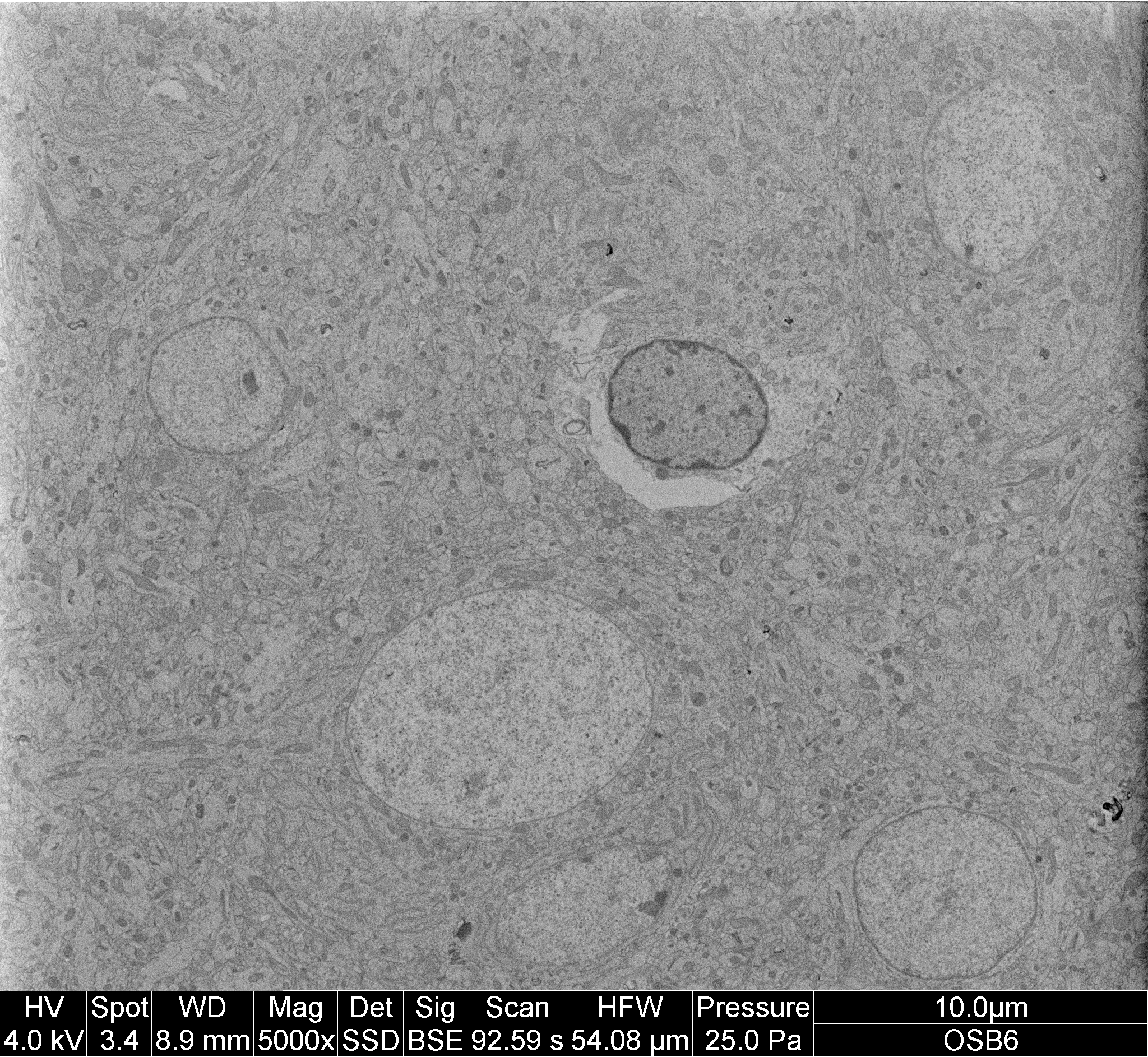

Supplement: Dataset S13 — (251.9 MB ZIP). [file pbio.0020329.sd013.zip › 040604_OS5_st1_1275.tif]

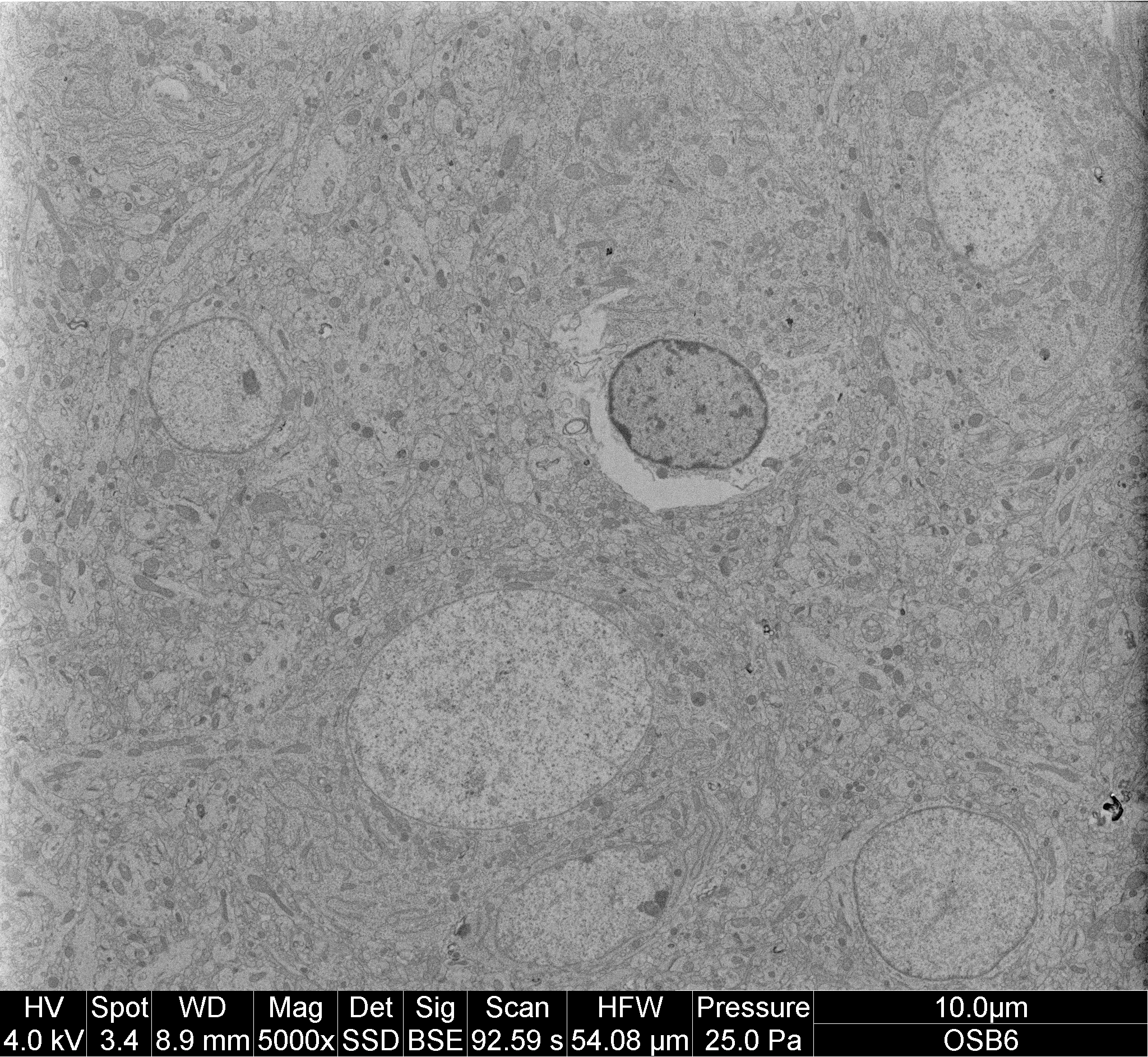

Supplement: Dataset S13 — (251.9 MB ZIP). [file pbio.0020329.sd013.zip › 040604_OS5_st1_1276.tif]

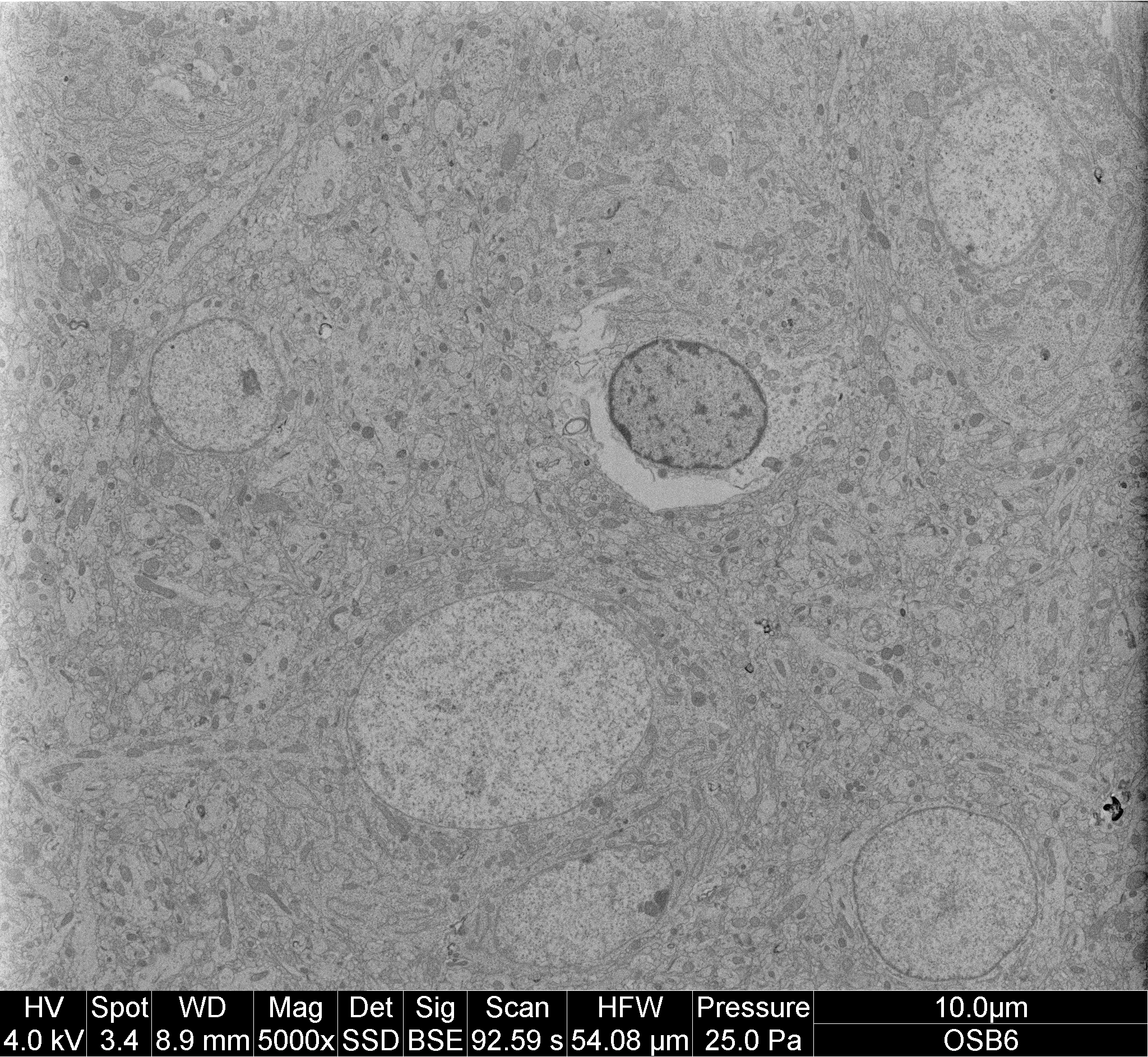

Supplement: Dataset S13 — (251.9 MB ZIP). [file pbio.0020329.sd013.zip › 040604_OS5_st1_1277.tif]

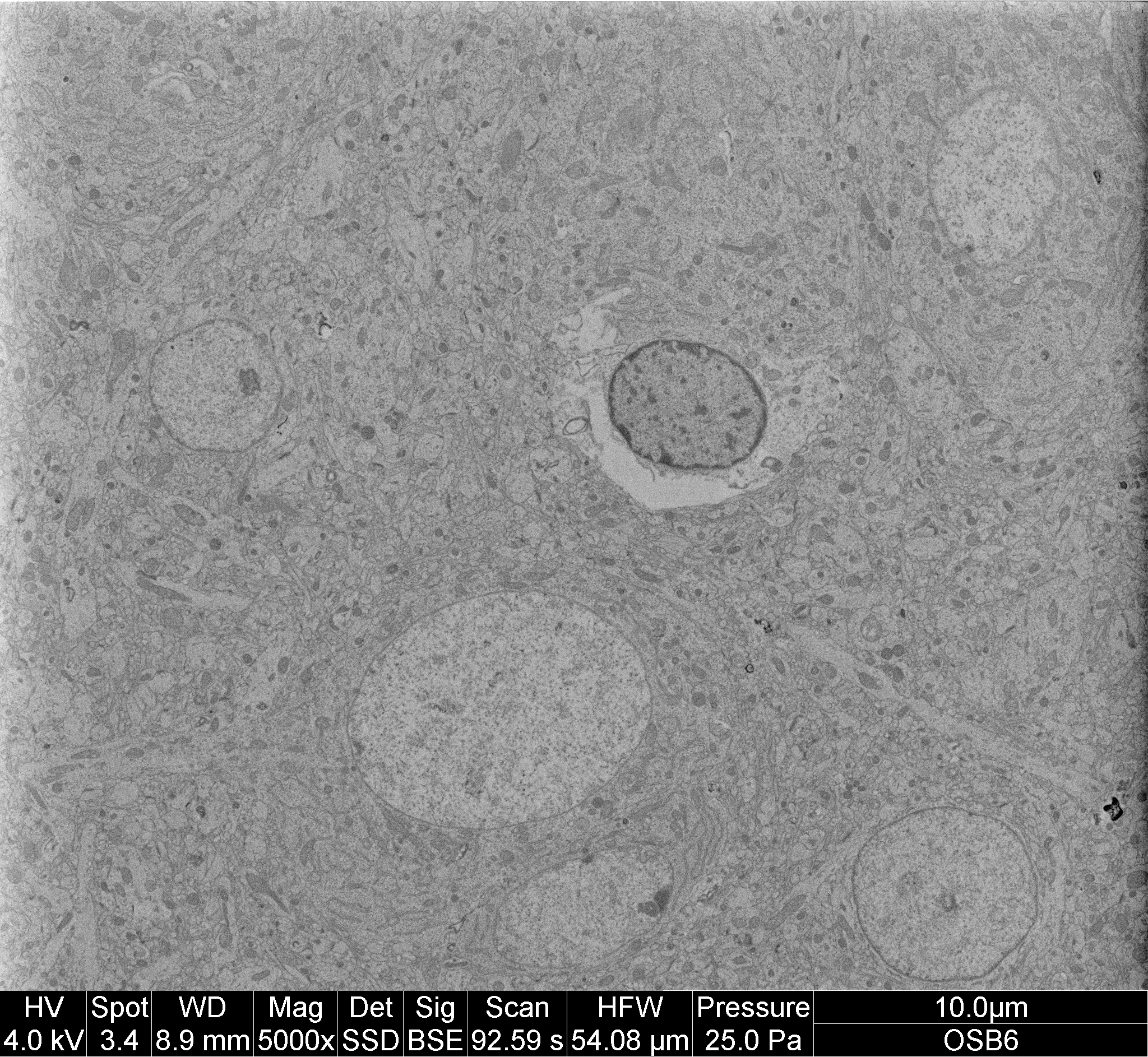

Supplement: Dataset S13 — (251.9 MB ZIP). [file pbio.0020329.sd013.zip › 040604_OS5_st1_1278.tif]

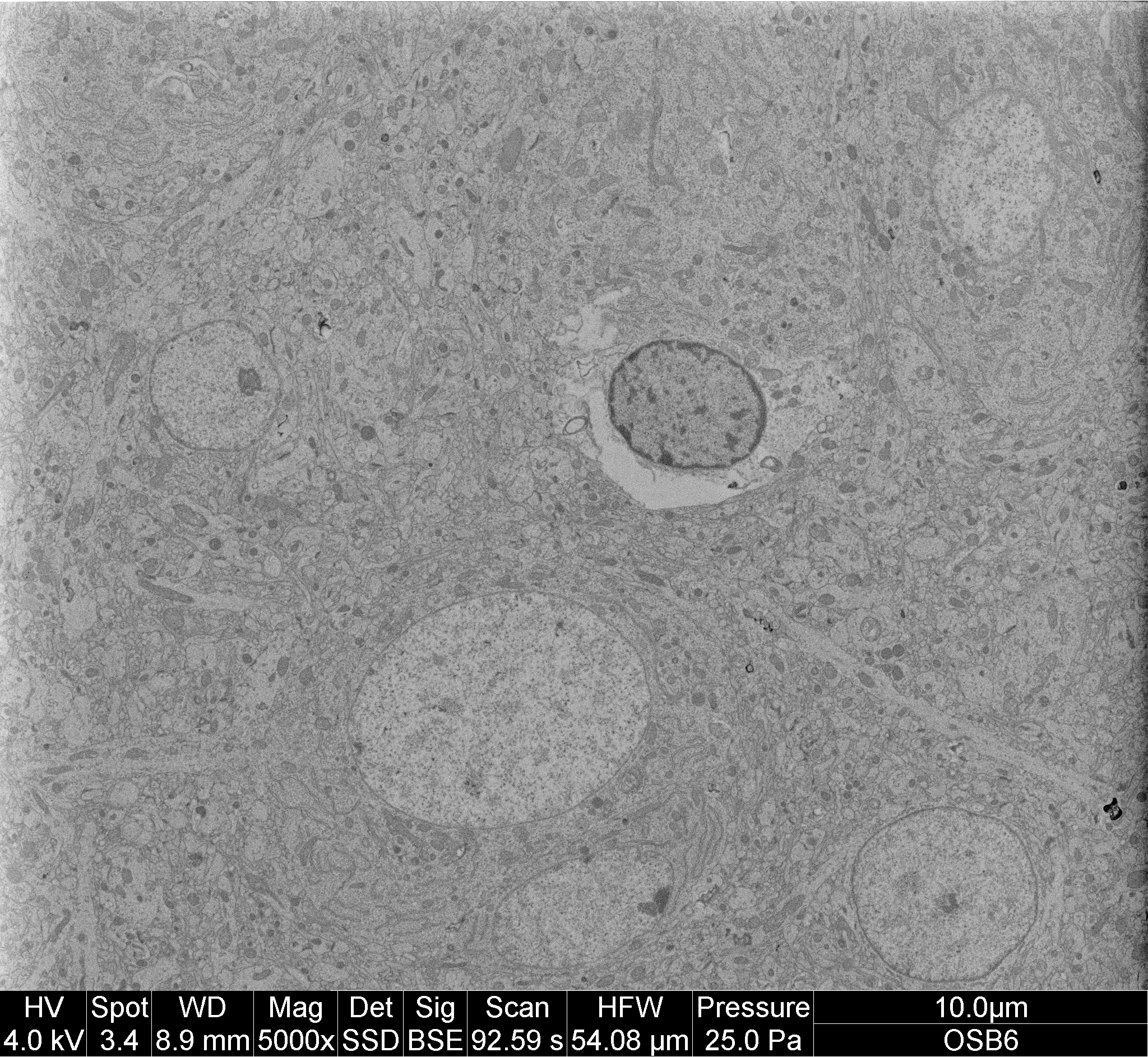

Supplement: Dataset S13 — (251.9 MB ZIP). [file pbio.0020329.sd013.zip › 040604_OS5_st1_1279.tif]

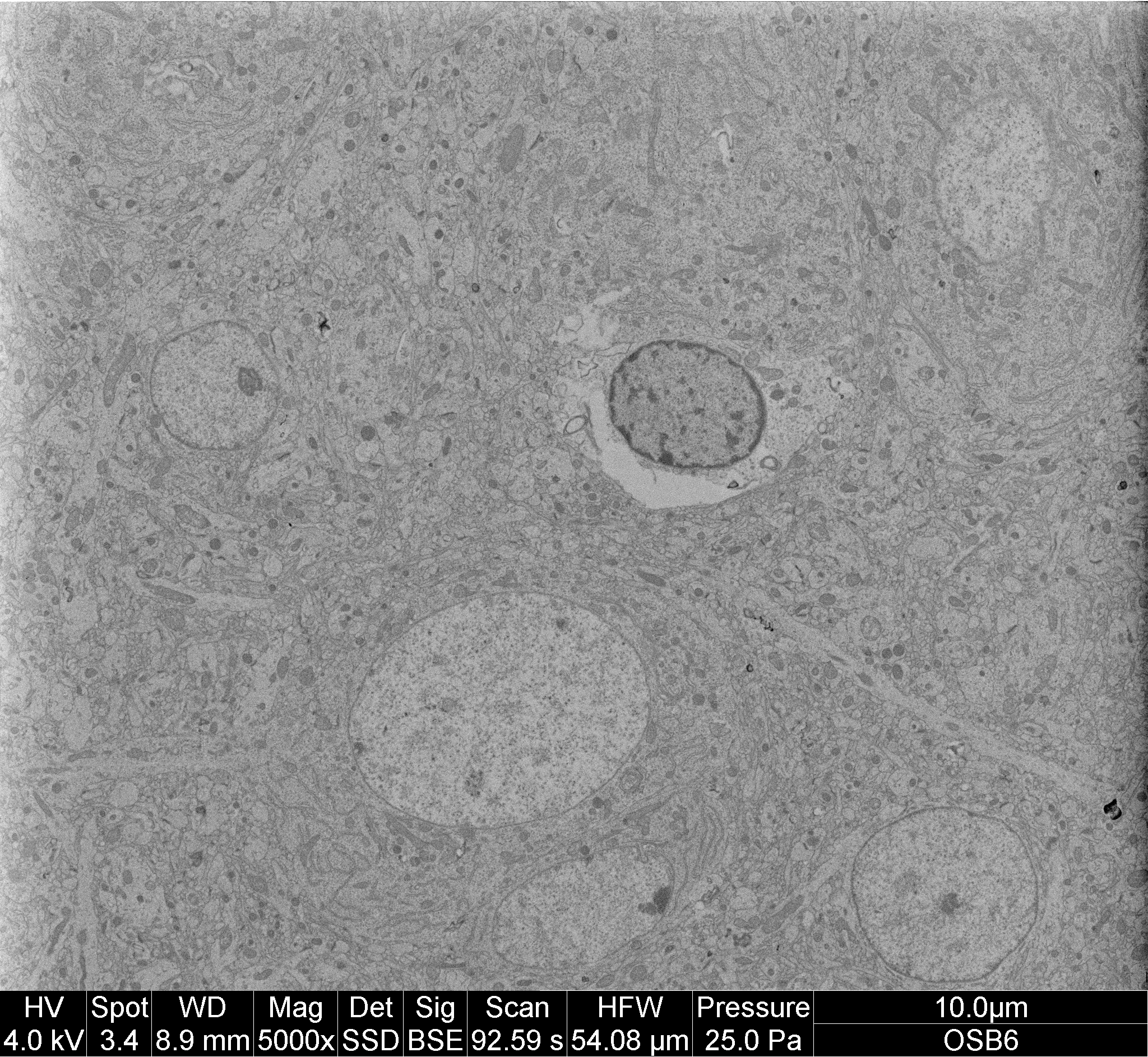

Supplement: Dataset S13 — (251.9 MB ZIP). [file pbio.0020329.sd013.zip › 040604_OS5_st1_1280.tif]

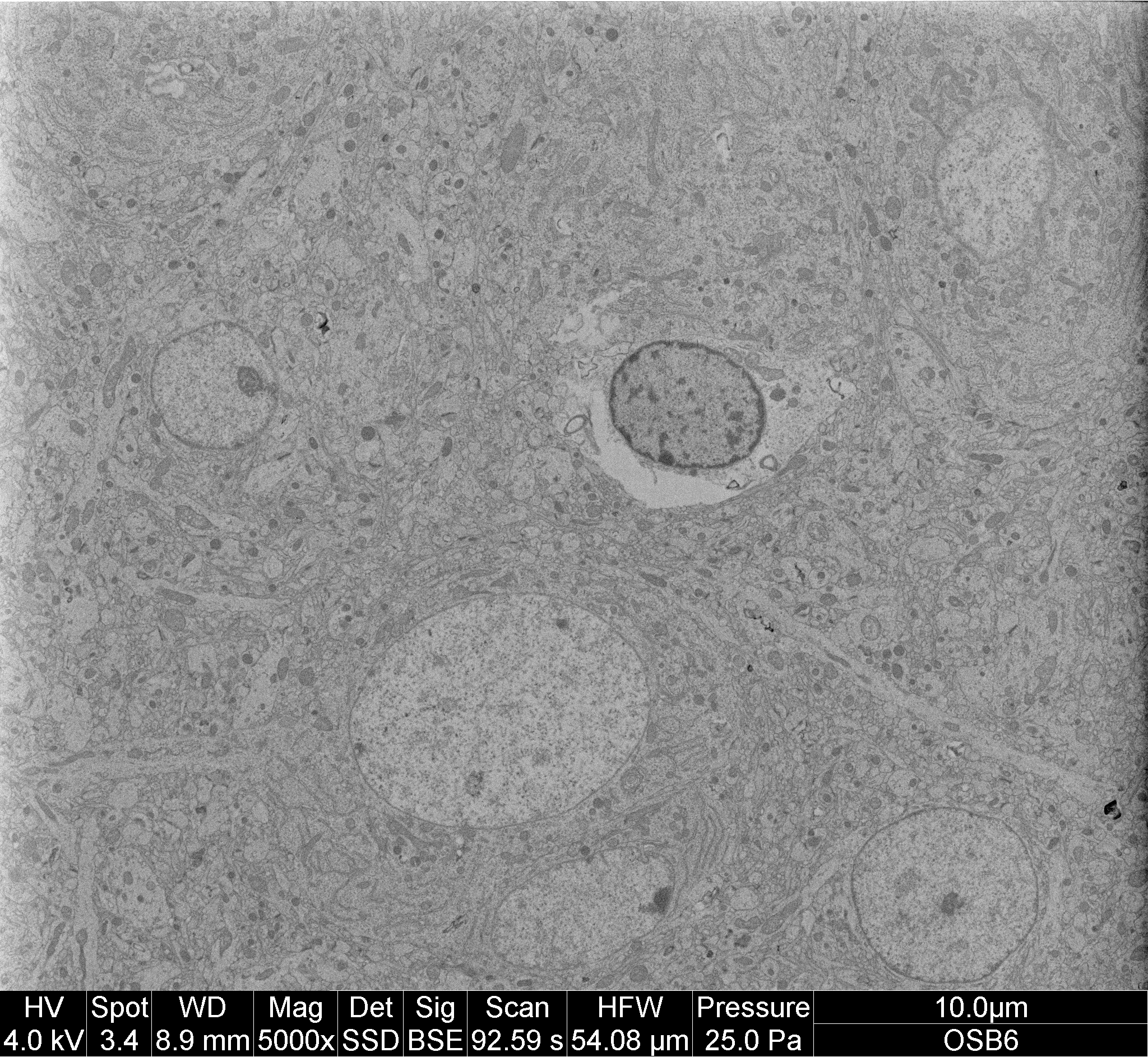

Supplement: Dataset S13 — (251.9 MB ZIP). [file pbio.0020329.sd013.zip › 040604_OS5_st1_1281.tif]

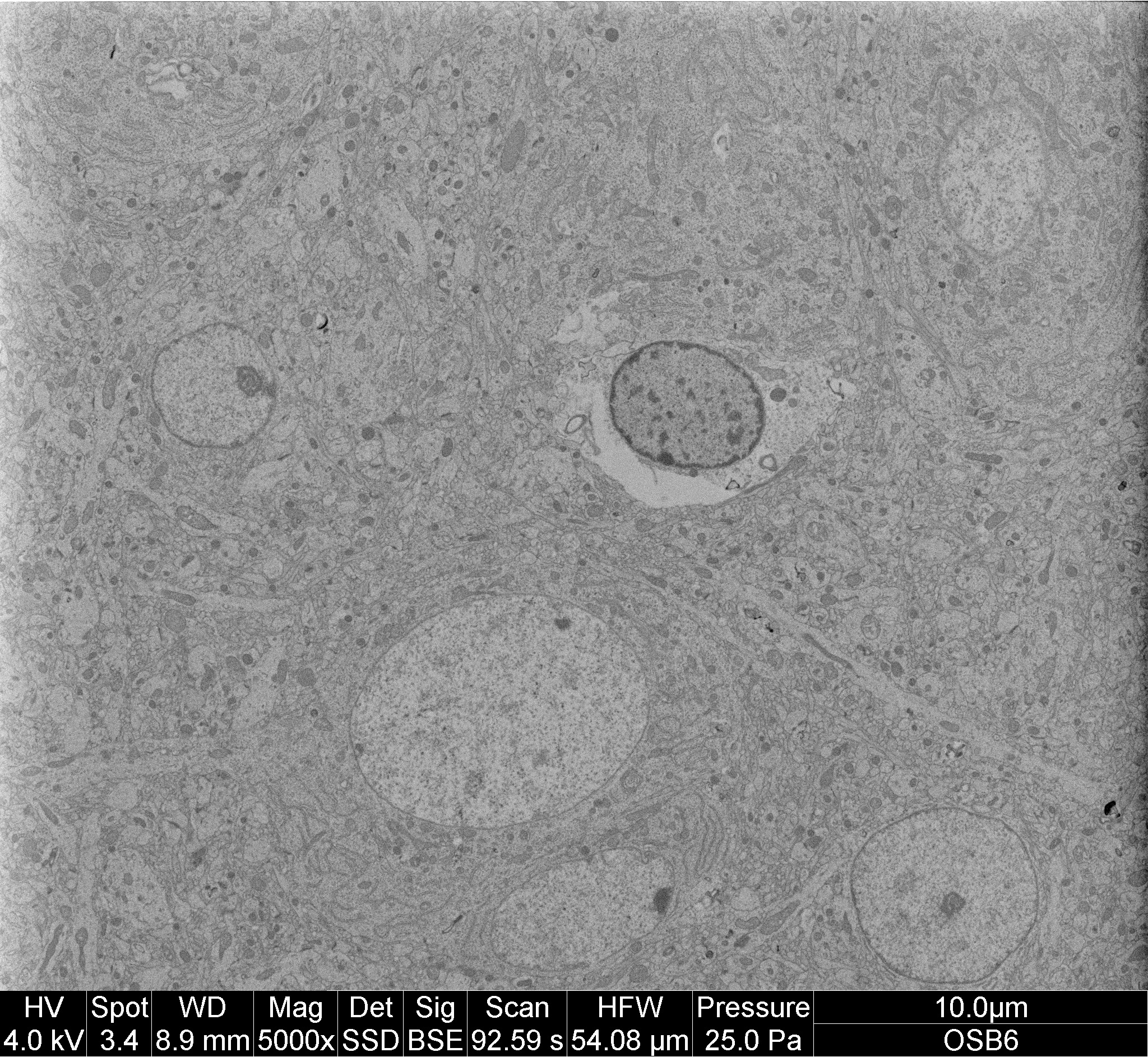

Supplement: Dataset S13 — (251.9 MB ZIP). [file pbio.0020329.sd013.zip › 040604_OS5_st1_1282.tif]

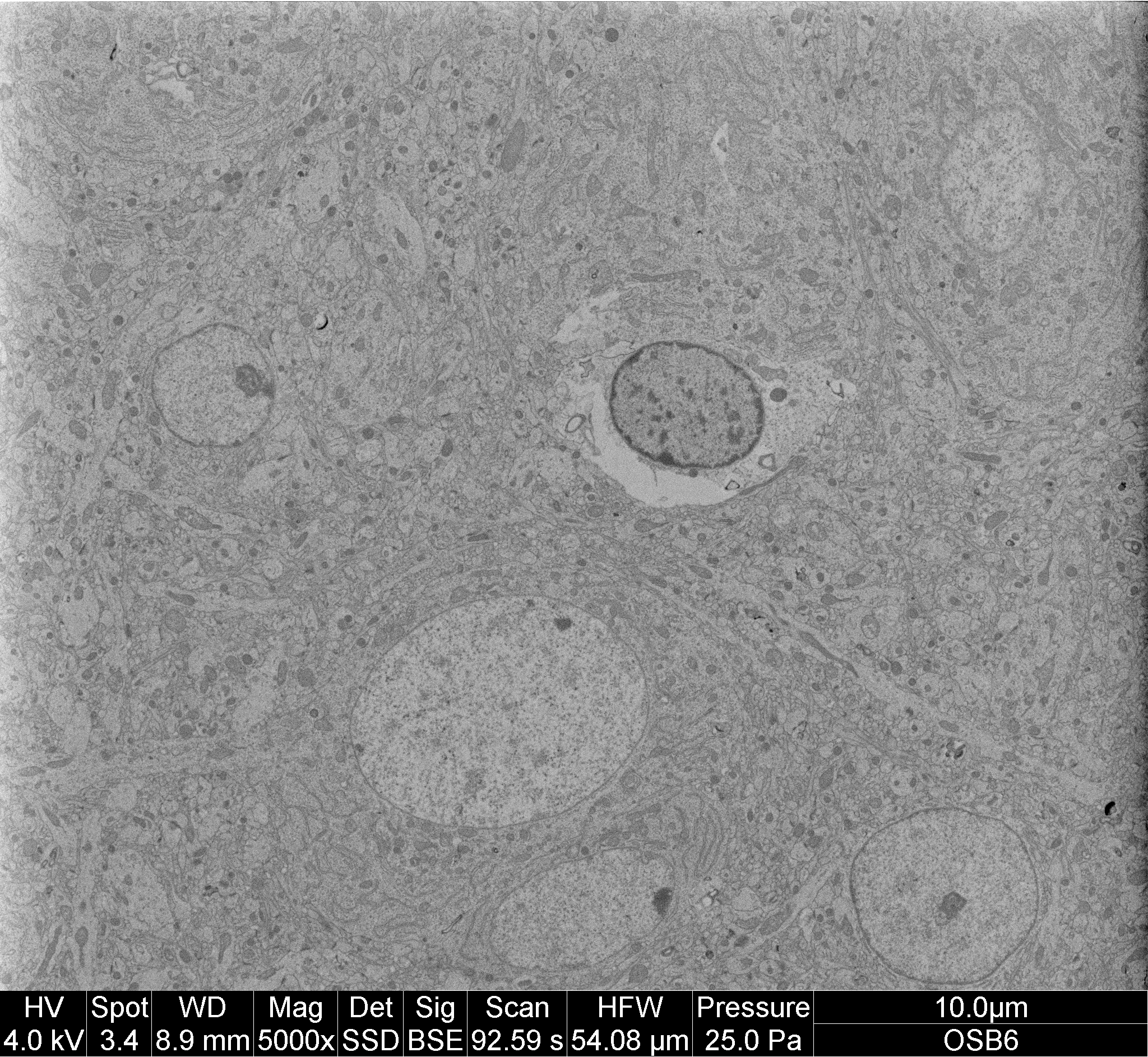

Supplement: Dataset S13 — (251.9 MB ZIP). [file pbio.0020329.sd013.zip › 040604_OS5_st1_1283.tif]

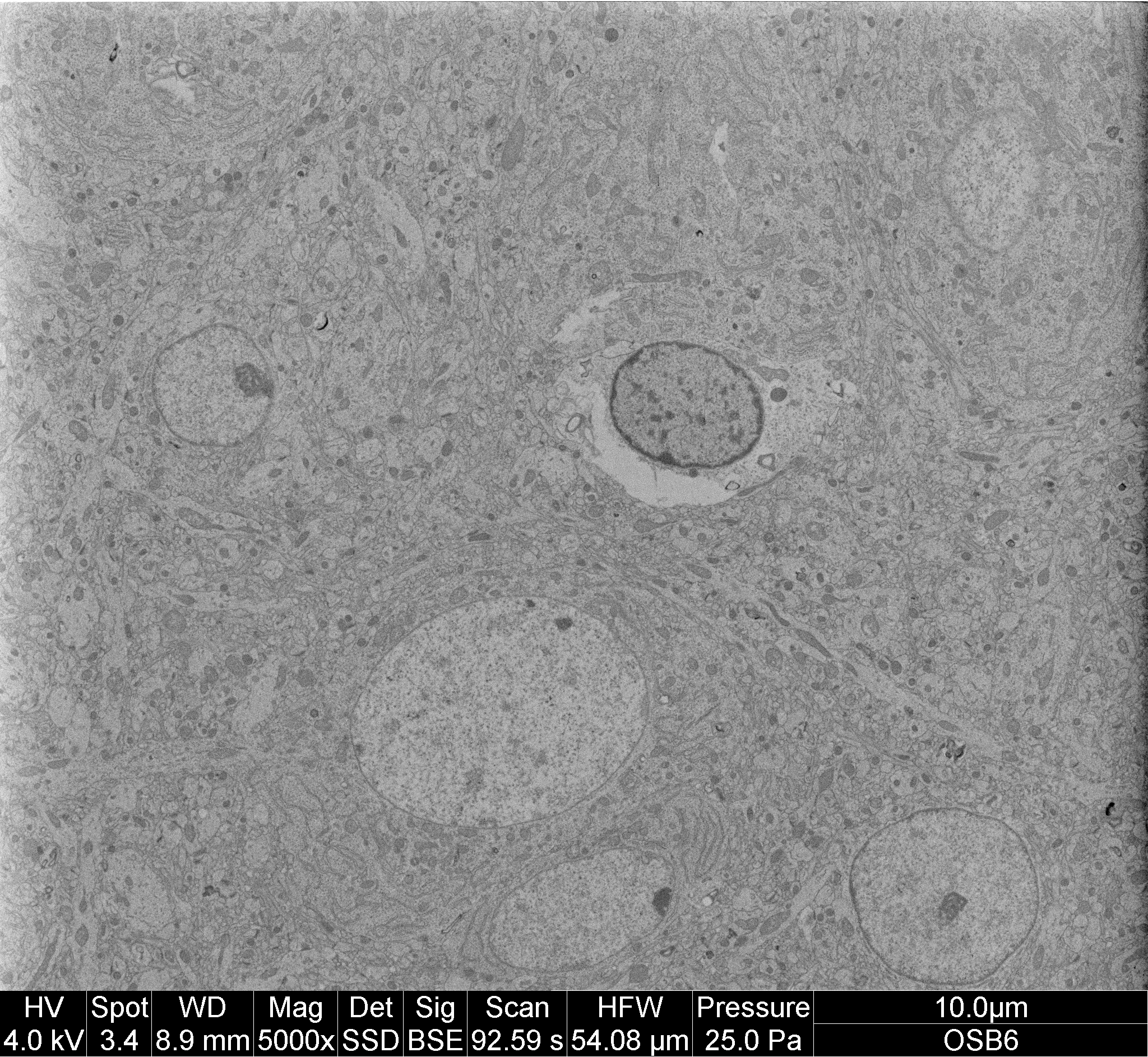

Supplement: Dataset S13 — (251.9 MB ZIP). [file pbio.0020329.sd013.zip › 040604_OS5_st1_1284.tif]

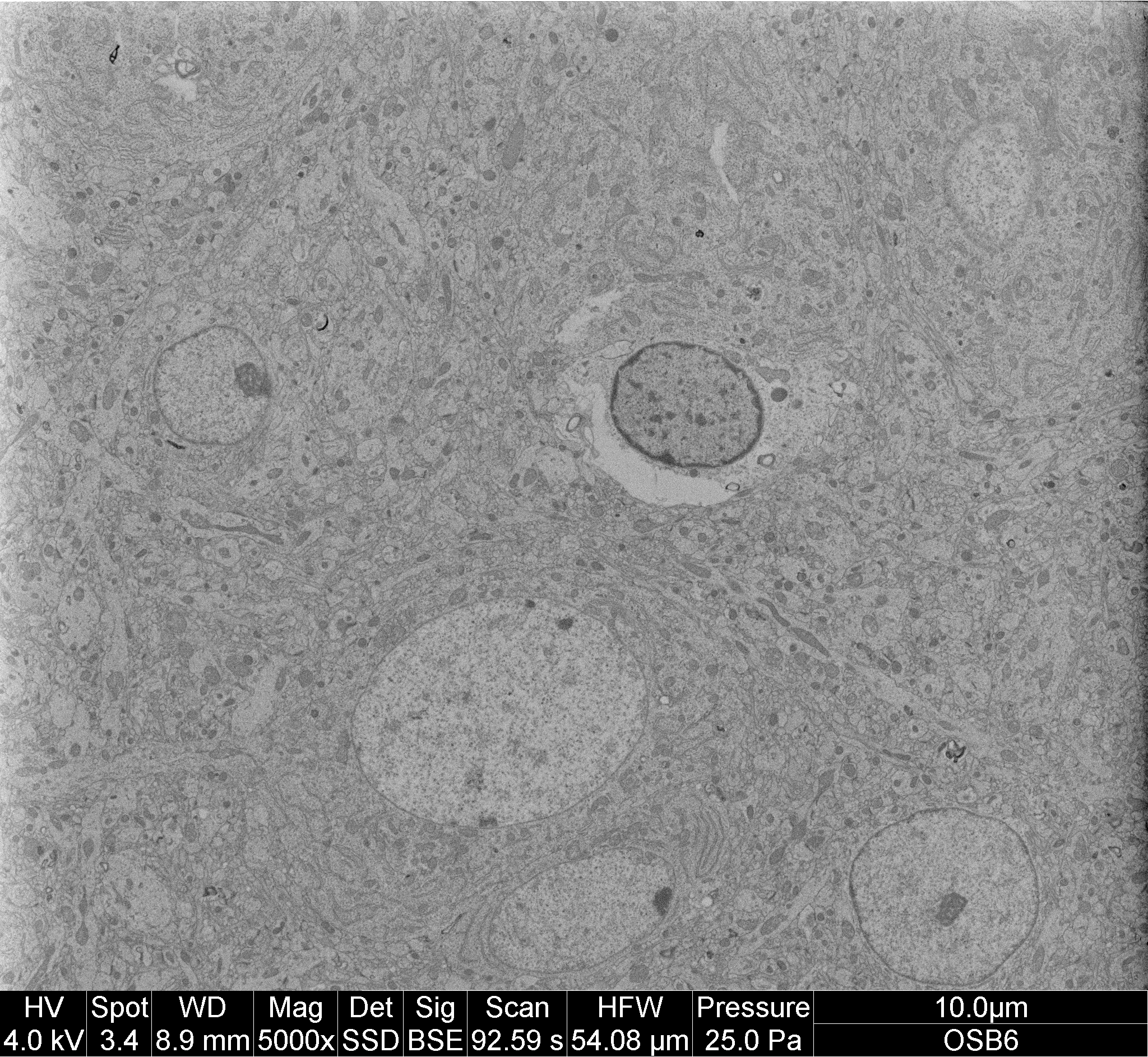

Supplement: Dataset S13 — (251.9 MB ZIP). [file pbio.0020329.sd013.zip › 040604_OS5_st1_1285.tif]

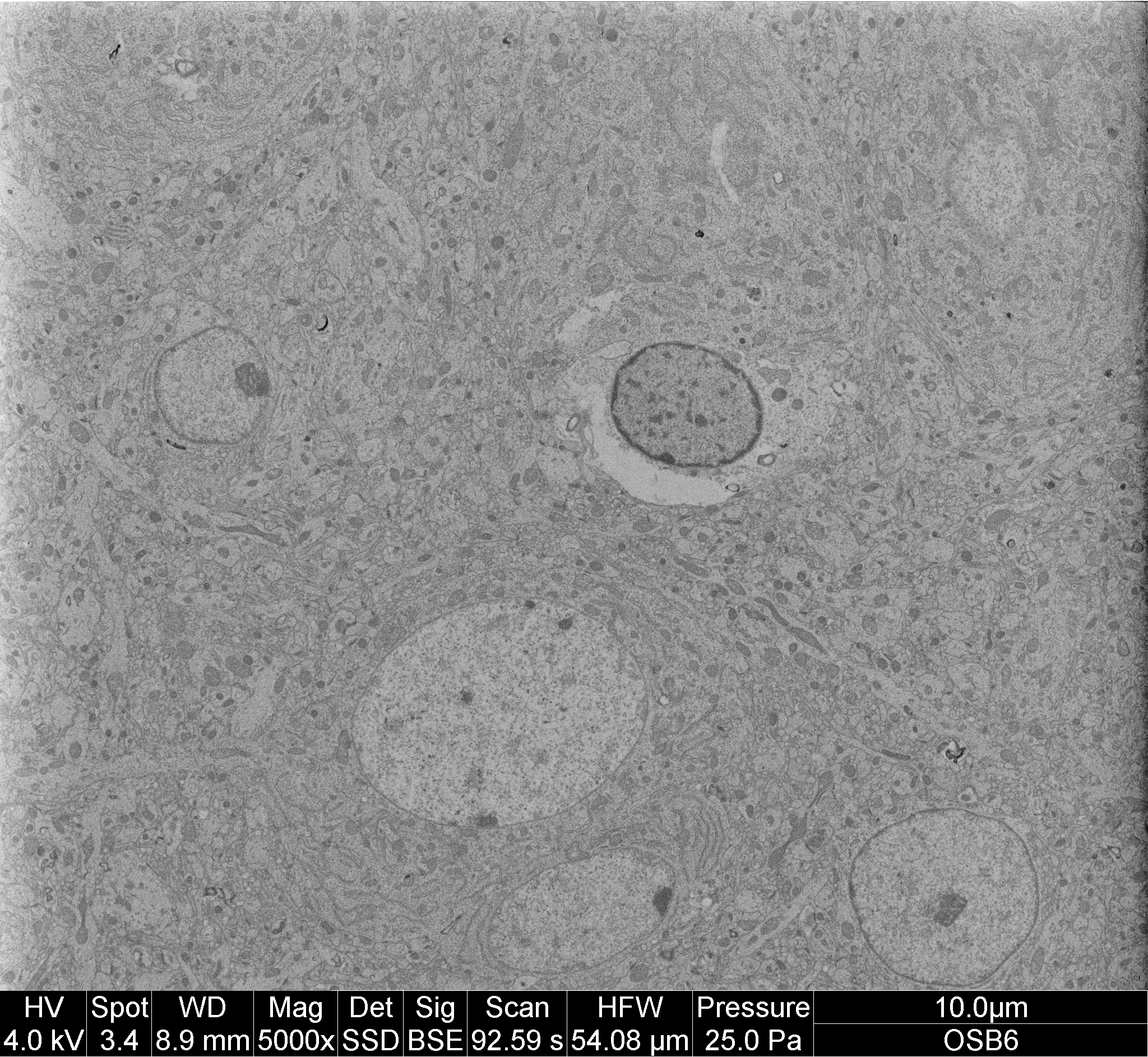

Supplement: Dataset S13 — (251.9 MB ZIP). [file pbio.0020329.sd013.zip › 040604_OS5_st1_1286.tif]

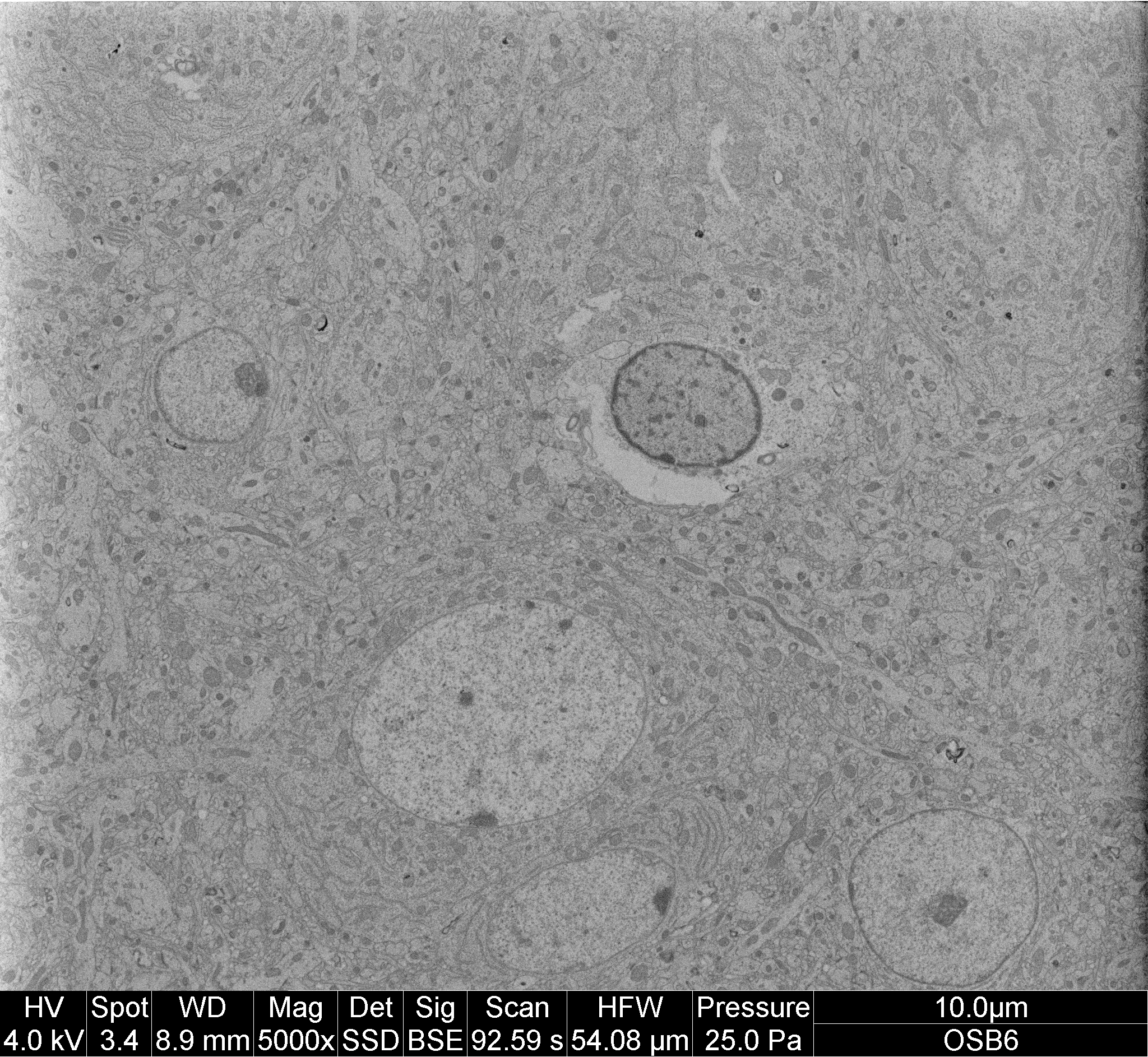

Supplement: Dataset S13 — (251.9 MB ZIP). [file pbio.0020329.sd013.zip › 040604_OS5_st1_1287.tif]

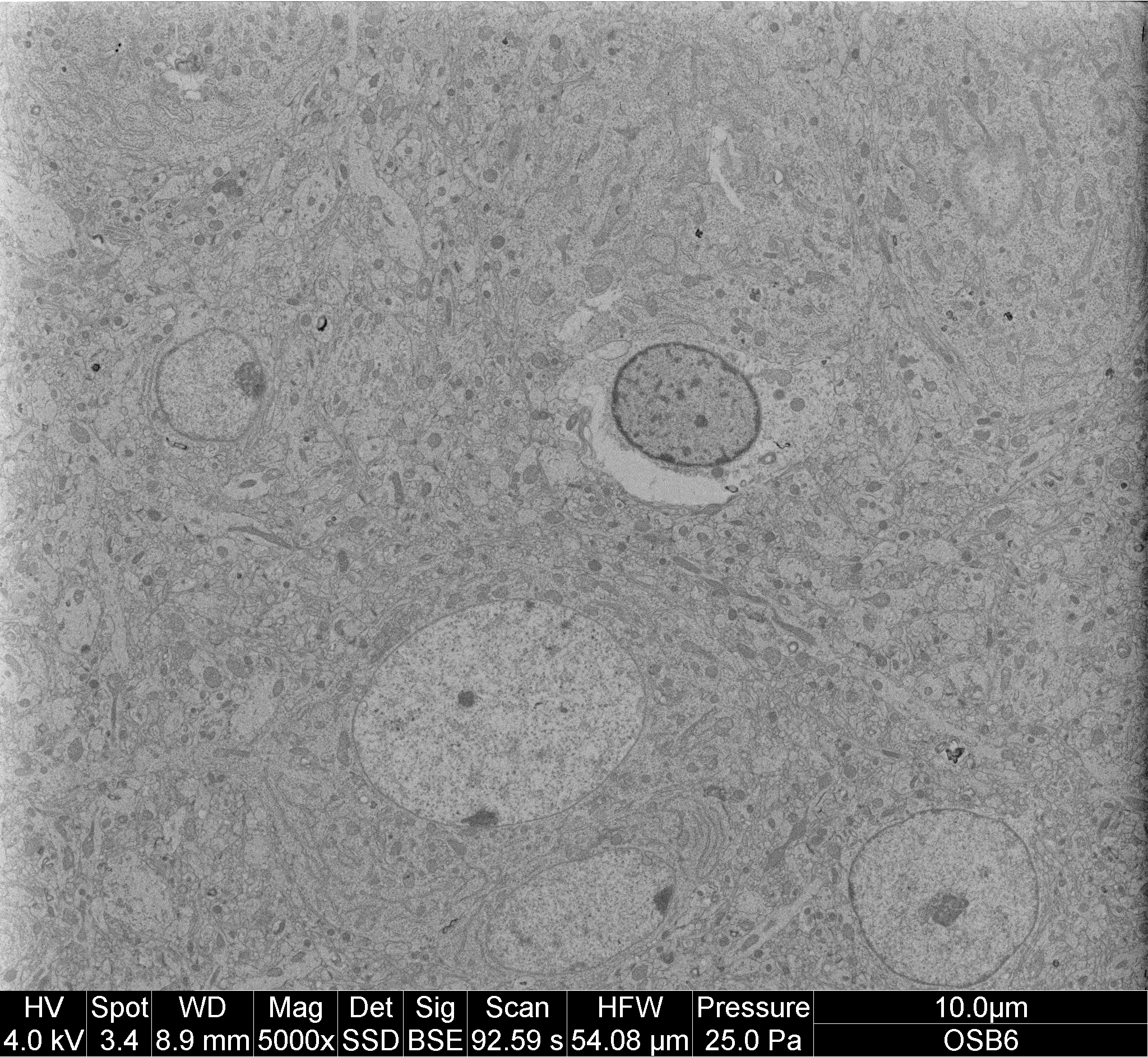

Supplement: Dataset S13 — (251.9 MB ZIP). [file pbio.0020329.sd013.zip › 040604_OS5_st1_1288.tif]

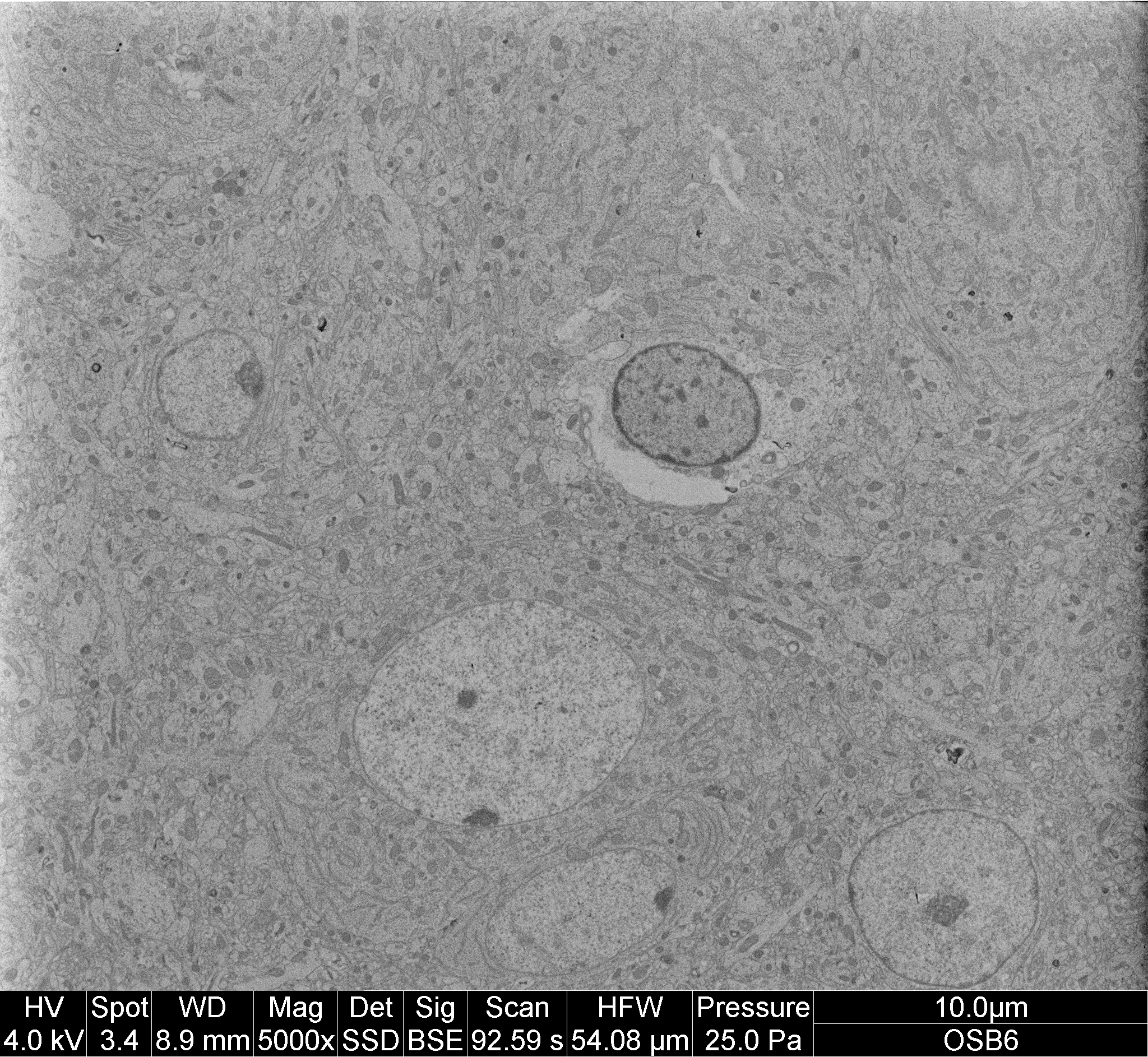

Supplement: Dataset S13 — (251.9 MB ZIP). [file pbio.0020329.sd013.zip › 040604_OS5_st1_1289.tif]

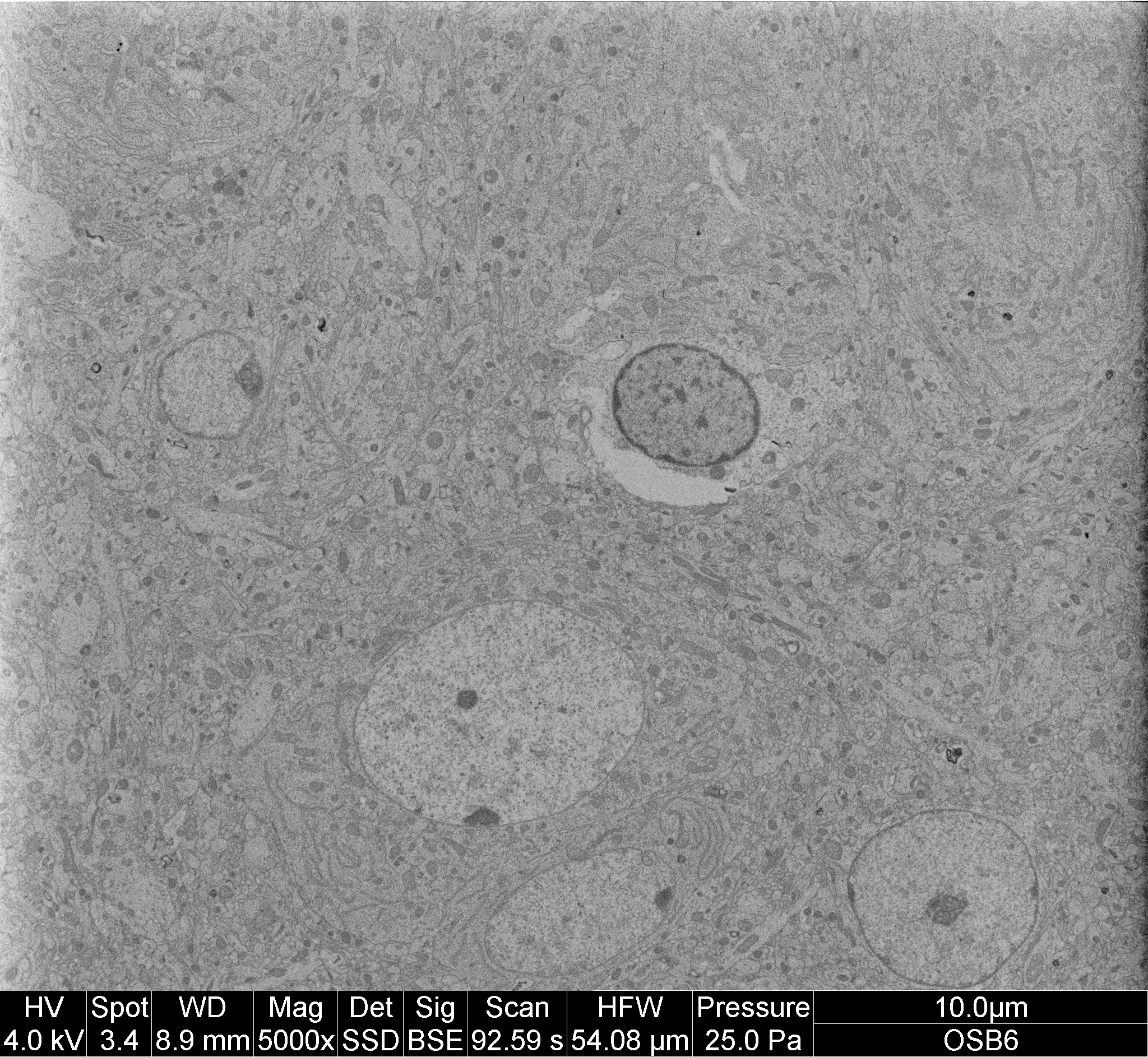

Supplement: Dataset S13 — (251.9 MB ZIP). [file pbio.0020329.sd013.zip › 040604_OS5_st1_1290.tif]

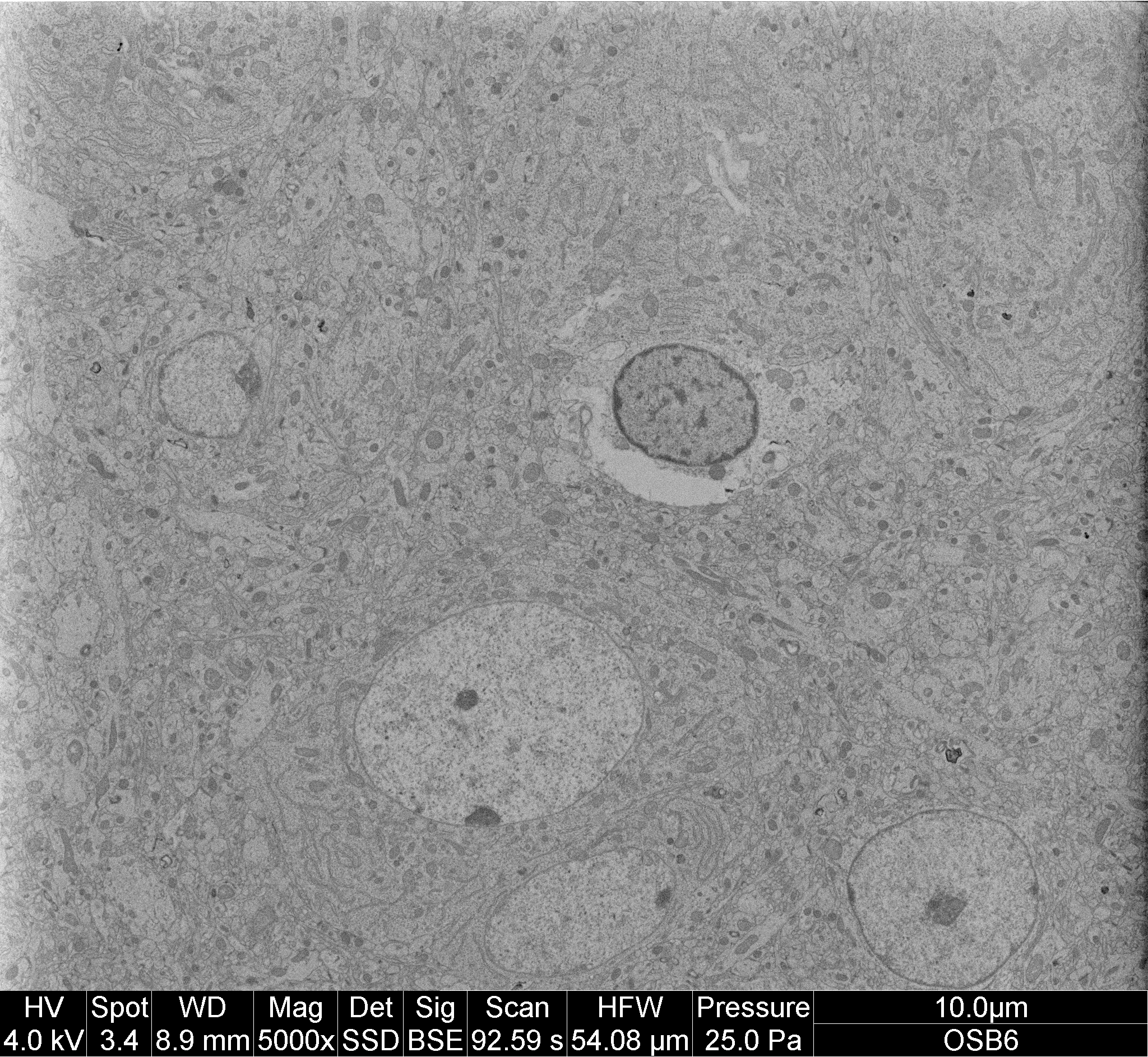

Supplement: Dataset S13 — (251.9 MB ZIP). [file pbio.0020329.sd013.zip › 040604_OS5_st1_1291.tif]

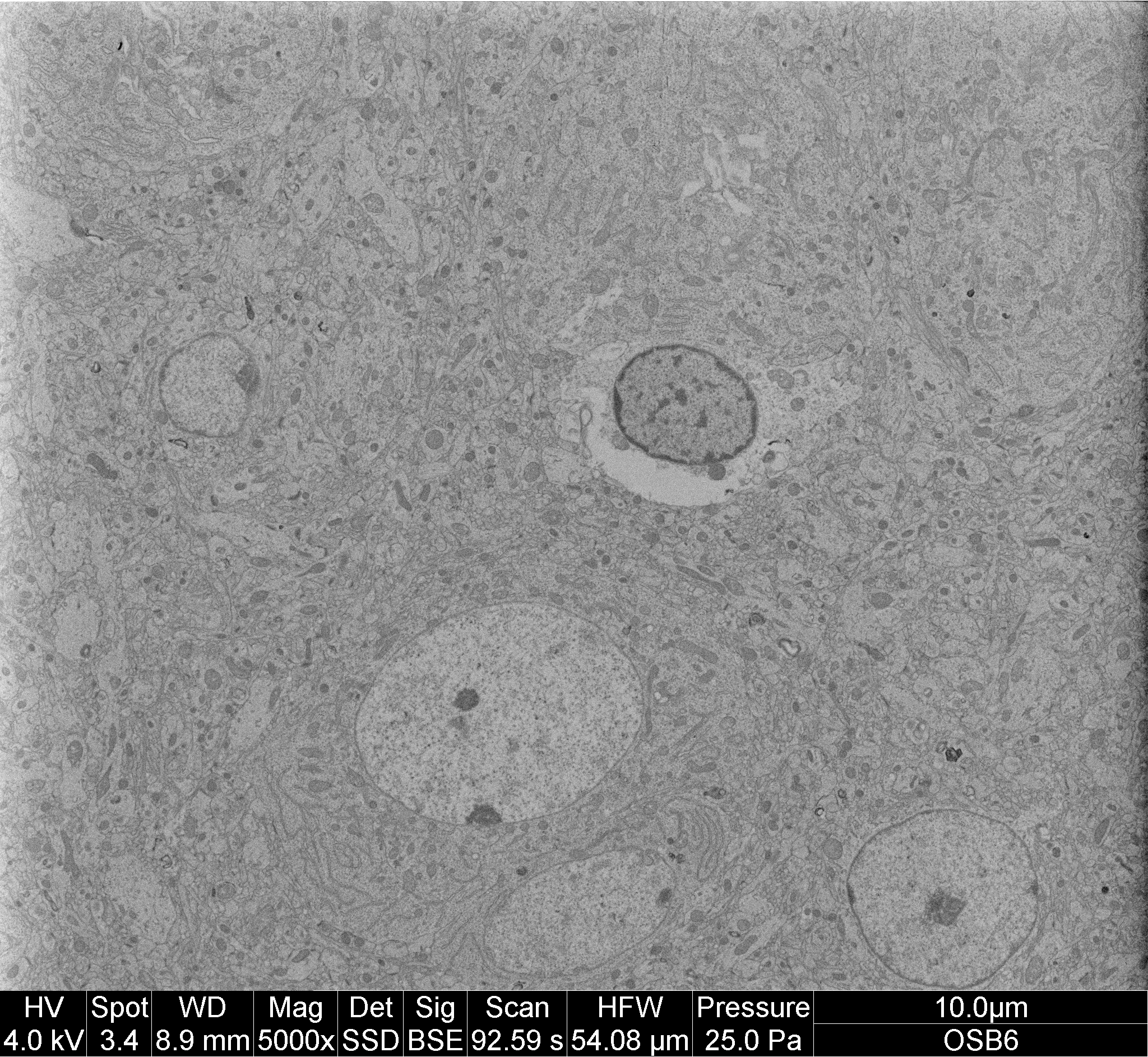

Supplement: Dataset S13 — (251.9 MB ZIP). [file pbio.0020329.sd013.zip › 040604_OS5_st1_1292.tif]

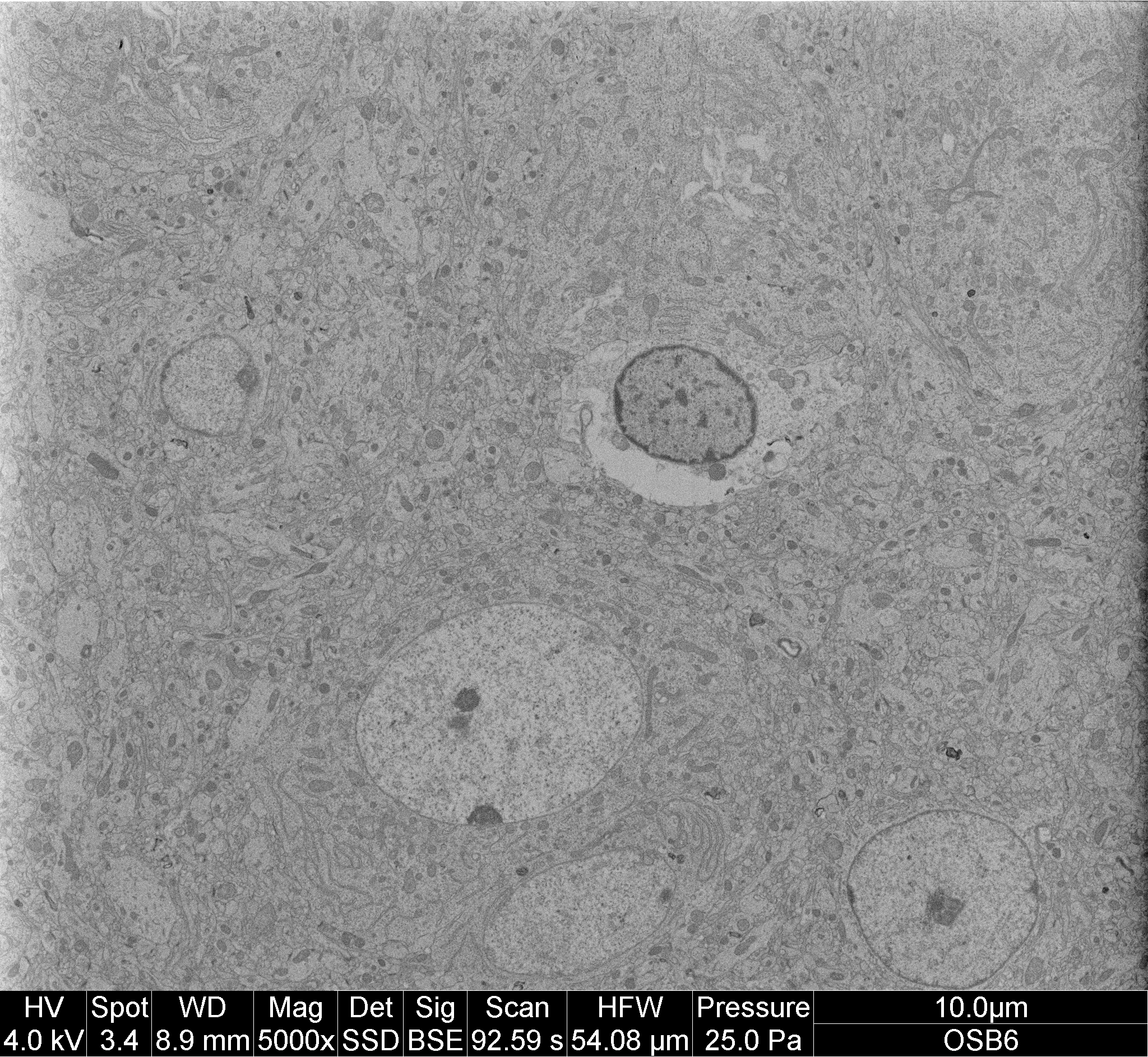

Supplement: Dataset S13 — (251.9 MB ZIP). [file pbio.0020329.sd013.zip › 040604_OS5_st1_1293.tif]

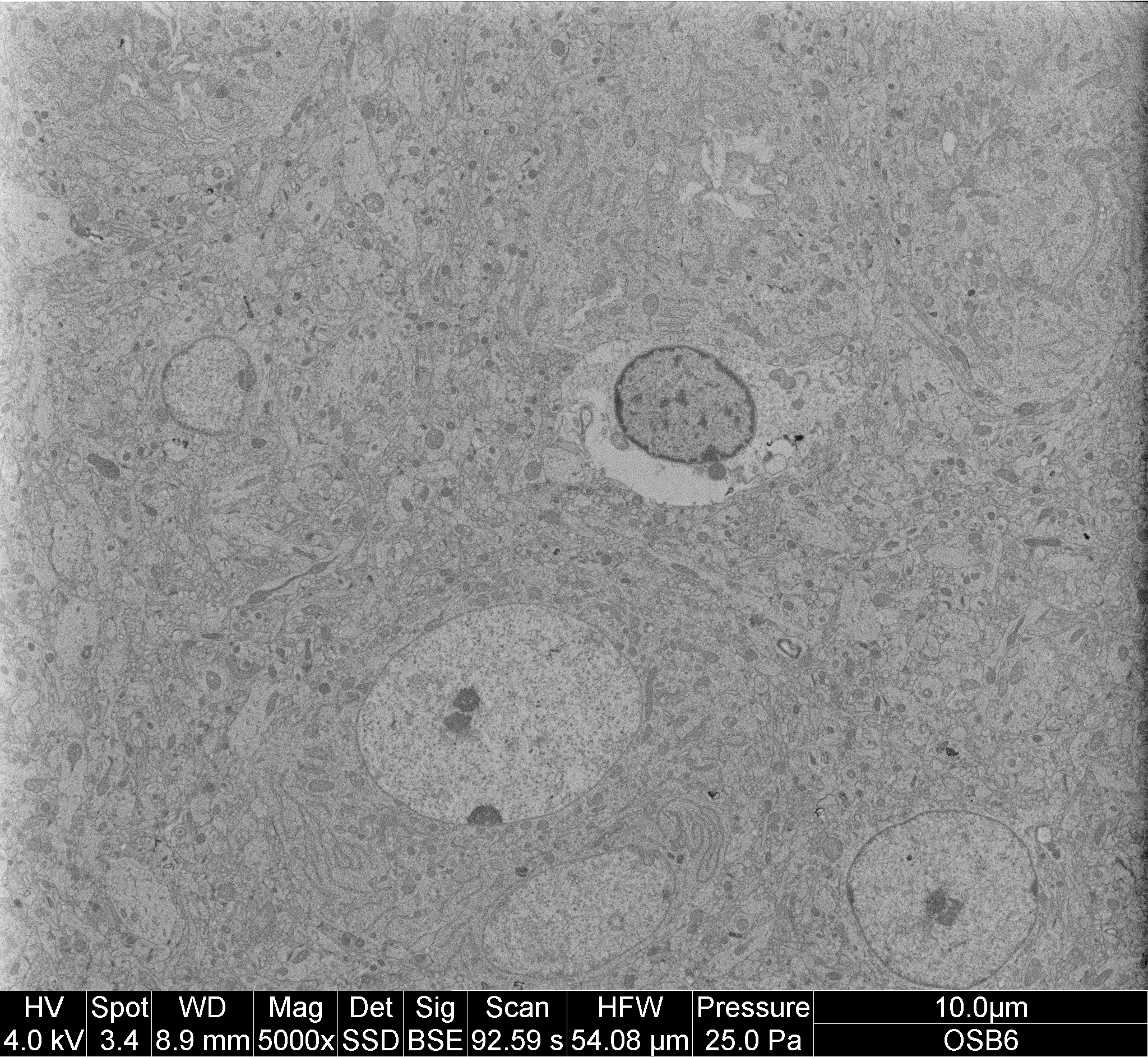

Supplement: Dataset S13 — (251.9 MB ZIP). [file pbio.0020329.sd013.zip › 040604_OS5_st1_1294.tif]

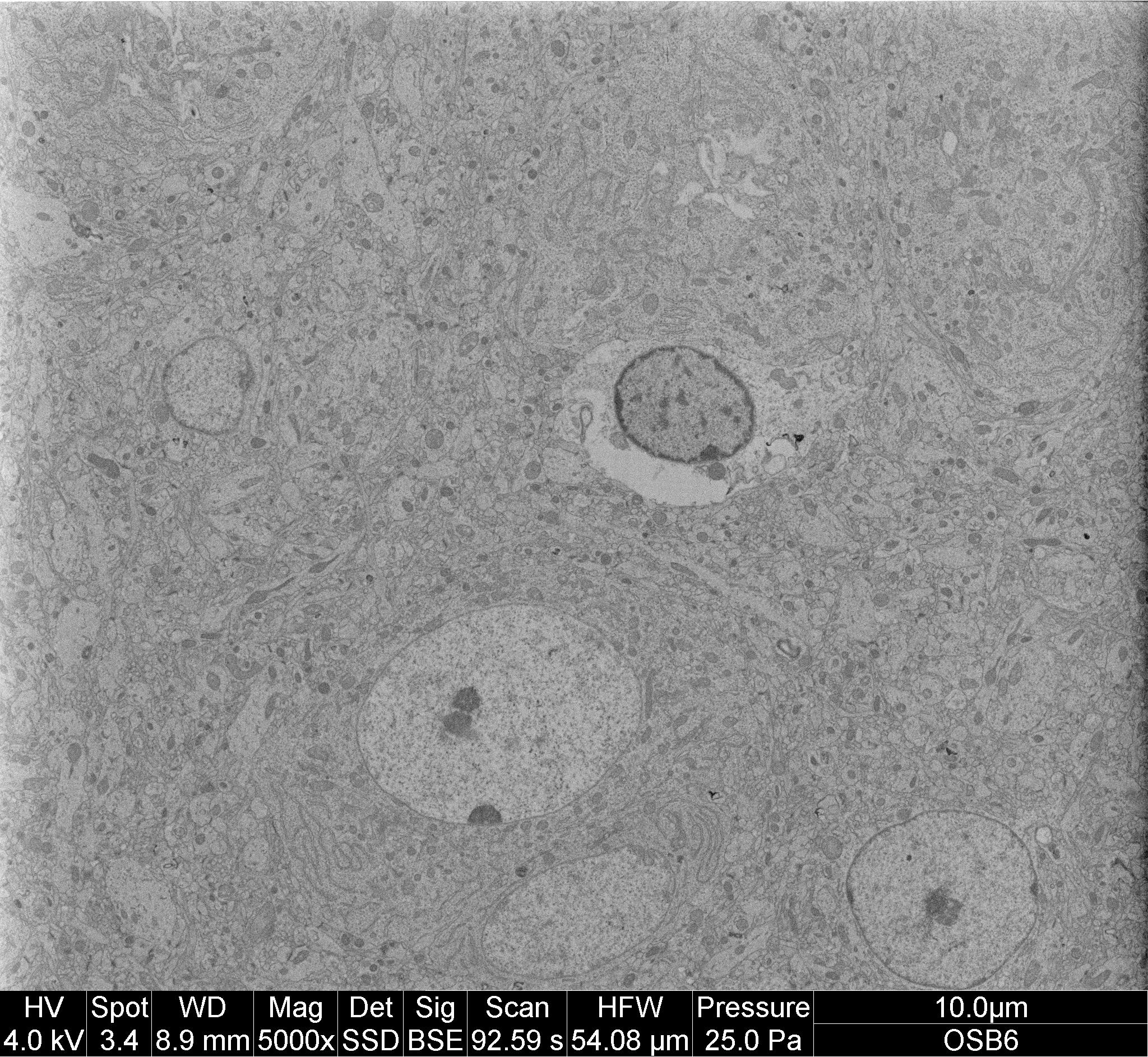

Supplement: Dataset S13 — (251.9 MB ZIP). [file pbio.0020329.sd013.zip › 040604_OS5_st1_1295.tif]

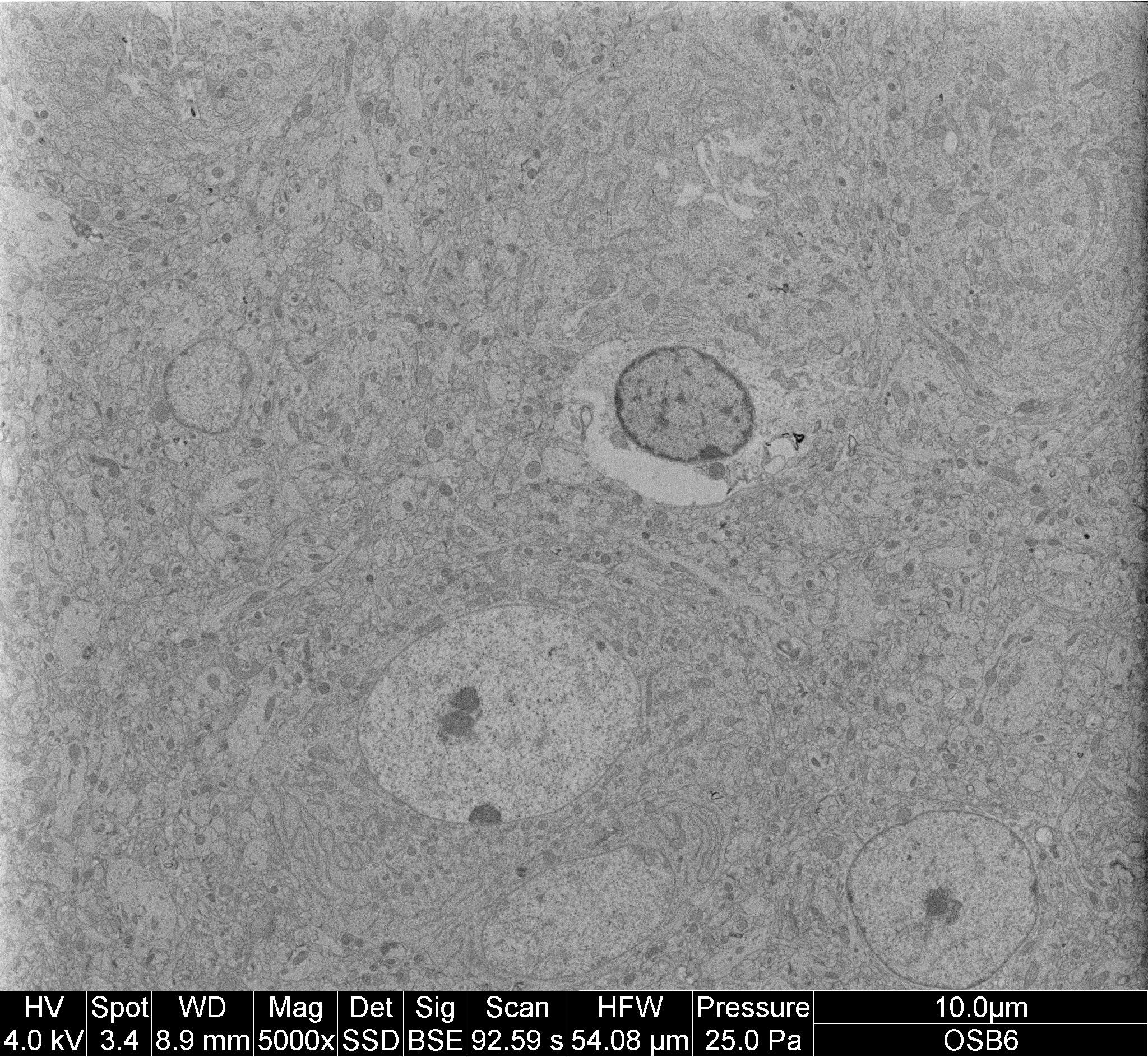

Supplement: Dataset S13 — (251.9 MB ZIP). [file pbio.0020329.sd013.zip › 040604_OS5_st1_1296.tif]

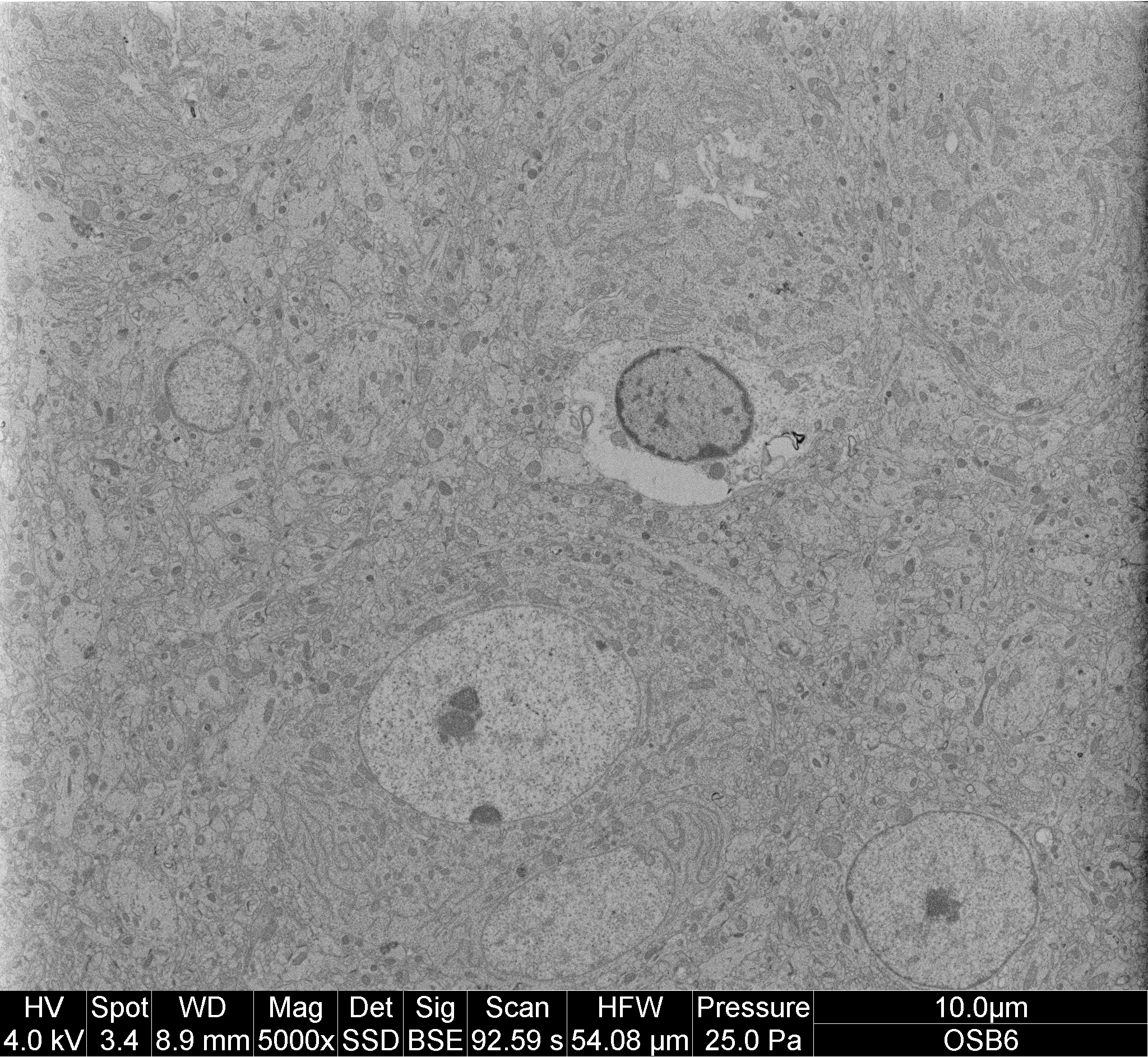

Supplement: Dataset S13 — (251.9 MB ZIP). [file pbio.0020329.sd013.zip › 040604_OS5_st1_1297.tif]

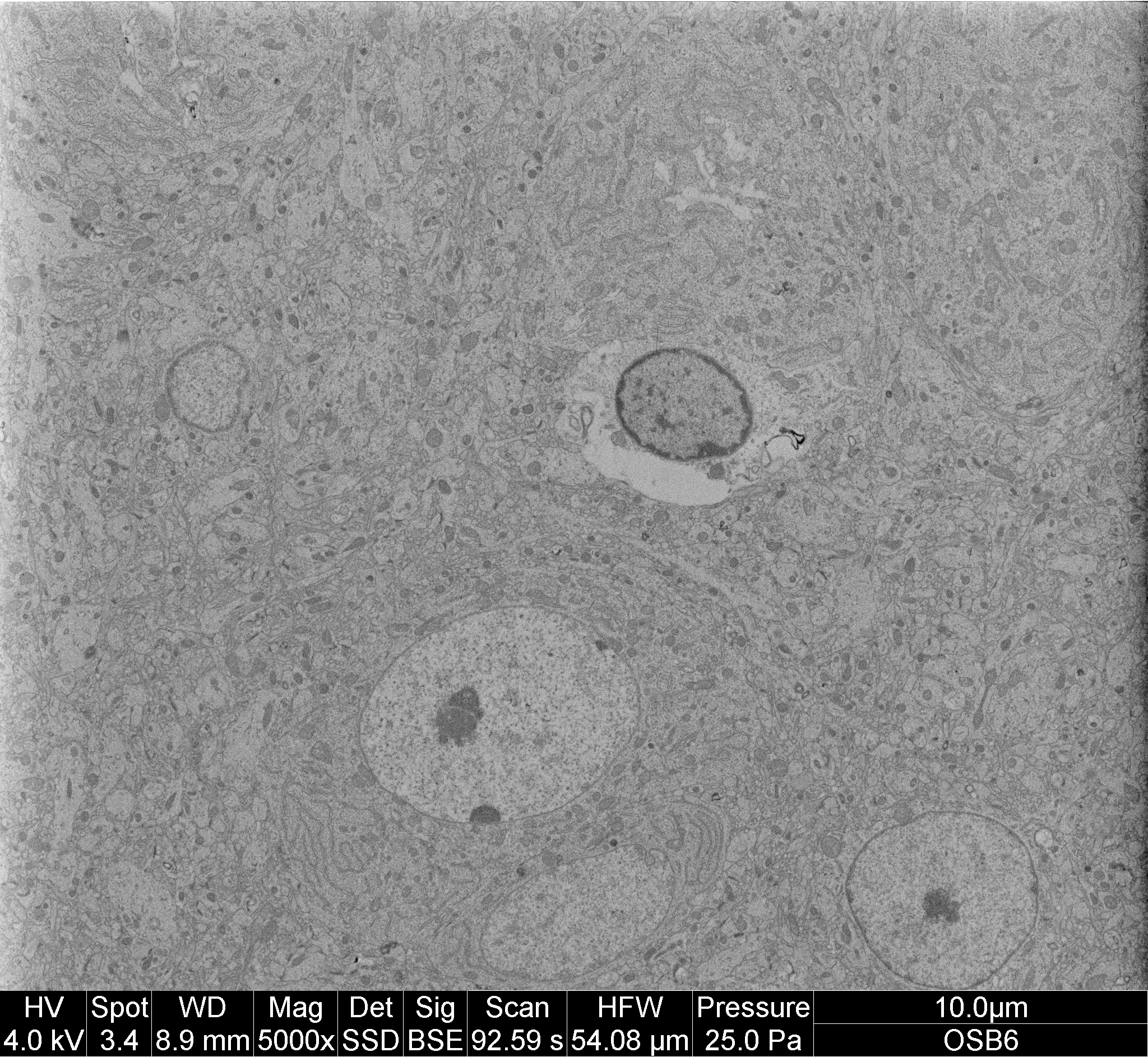

Supplement: Dataset S13 — (251.9 MB ZIP). [file pbio.0020329.sd013.zip › 040604_OS5_st1_1298.tif]

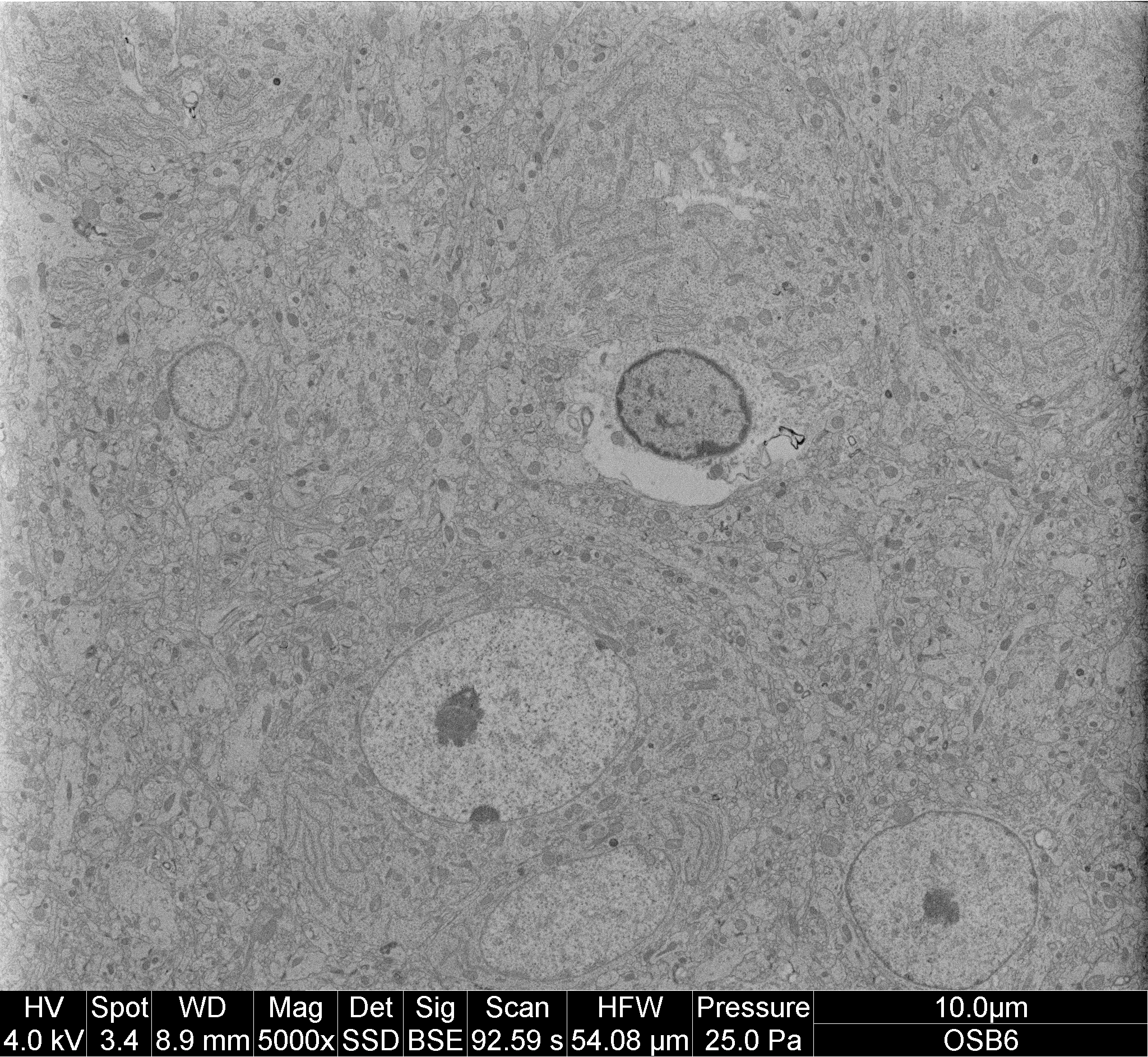

Supplement: Dataset S13 — (251.9 MB ZIP). [file pbio.0020329.sd013.zip › 040604_OS5_st1_1299.tif]

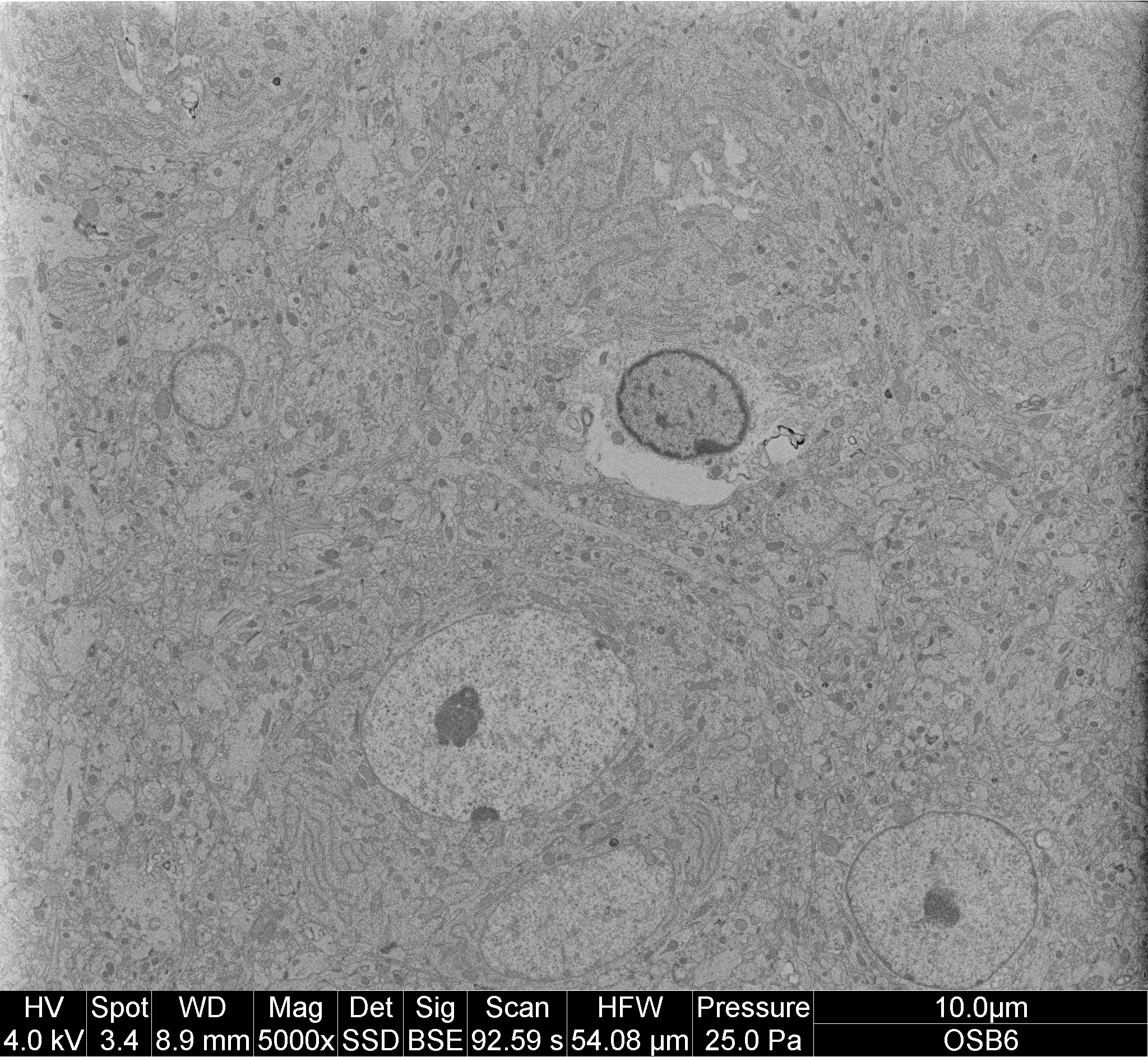

Supplement: Dataset S14 — (251.8 MB ZIP). [file pbio.0020329.sd014.zip › 040604_OS5_st1_1300.tif]
