# Supplementary figures and images for: Serial Block-Face Scanning Electron Microscopy to Reconstruct Three-Dimensional Tissue Nanostructure (part 14 of 21)
Source: PLoS Biol. 2004 Oct 19;2(11):e329. doi: 10.1371/journal.pbio.0020329 (PMC524270; doi:10.1371/journal.pbio.0020329)

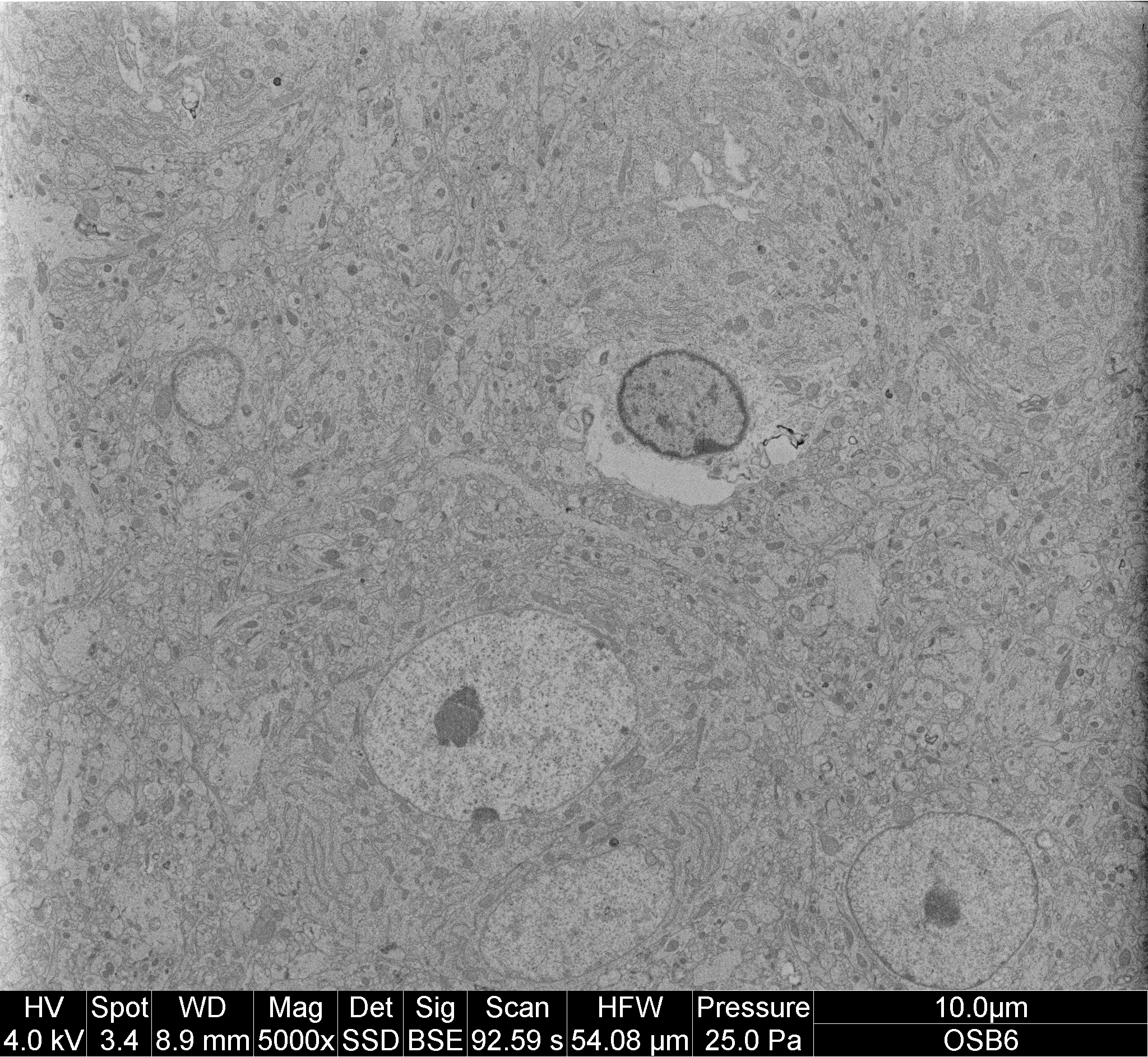

Supplement: Dataset S14 — (251.8 MB ZIP). [file pbio.0020329.sd014.zip › 040604_OS5_st1_1301.tif]

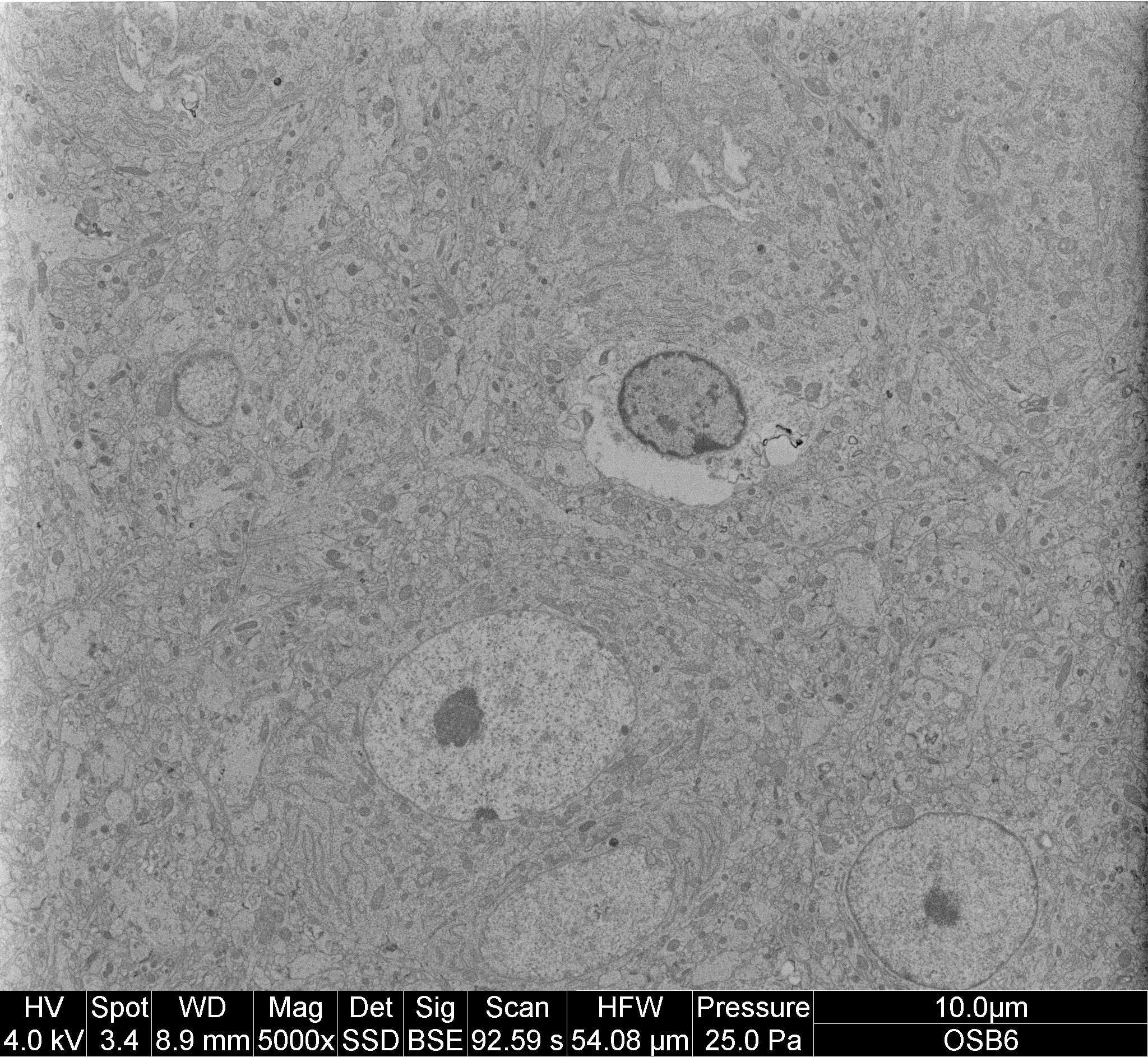

Supplement: Dataset S14 — (251.8 MB ZIP). [file pbio.0020329.sd014.zip › 040604_OS5_st1_1302.tif]

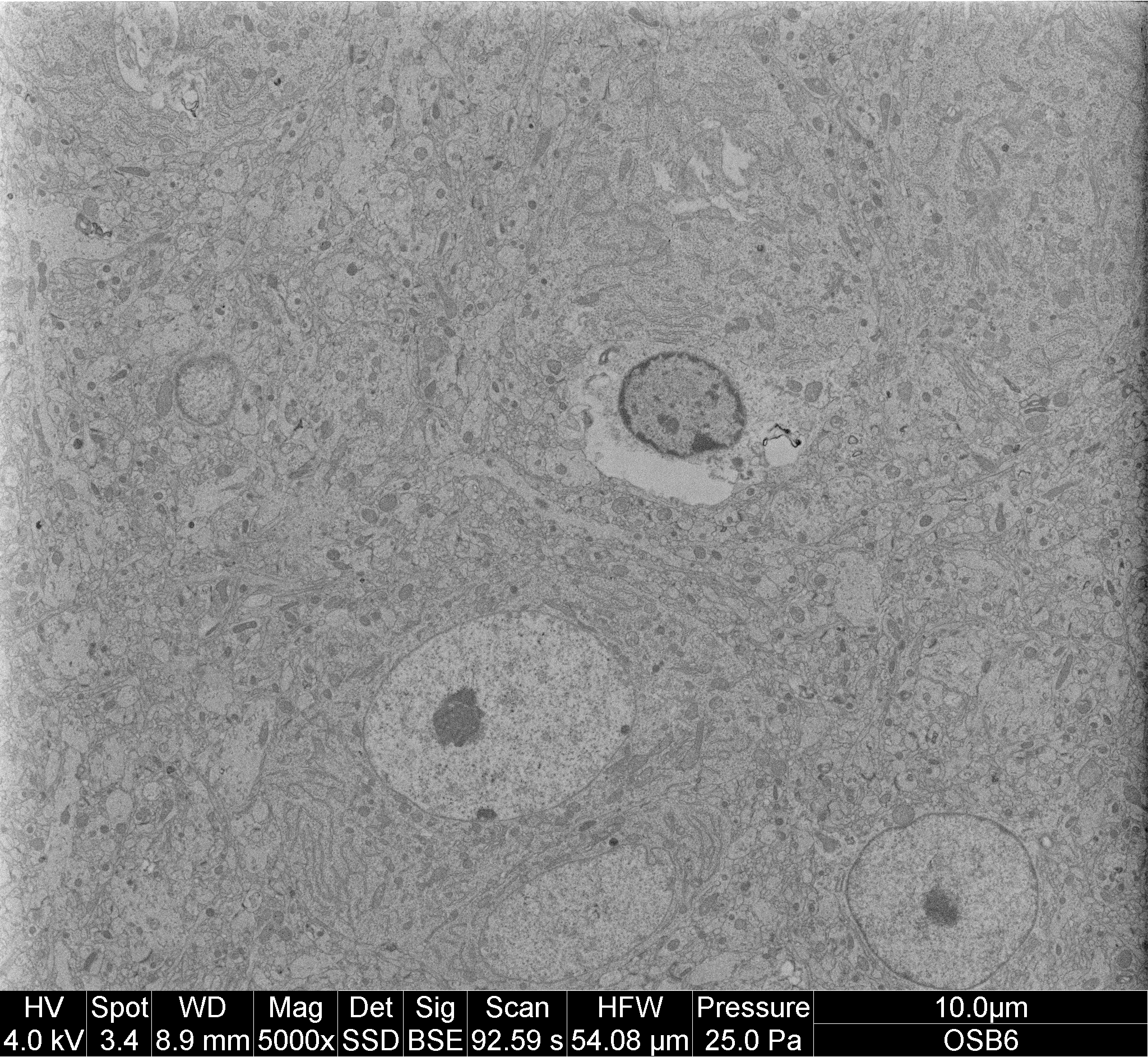

Supplement: Dataset S14 — (251.8 MB ZIP). [file pbio.0020329.sd014.zip › 040604_OS5_st1_1303.tif]

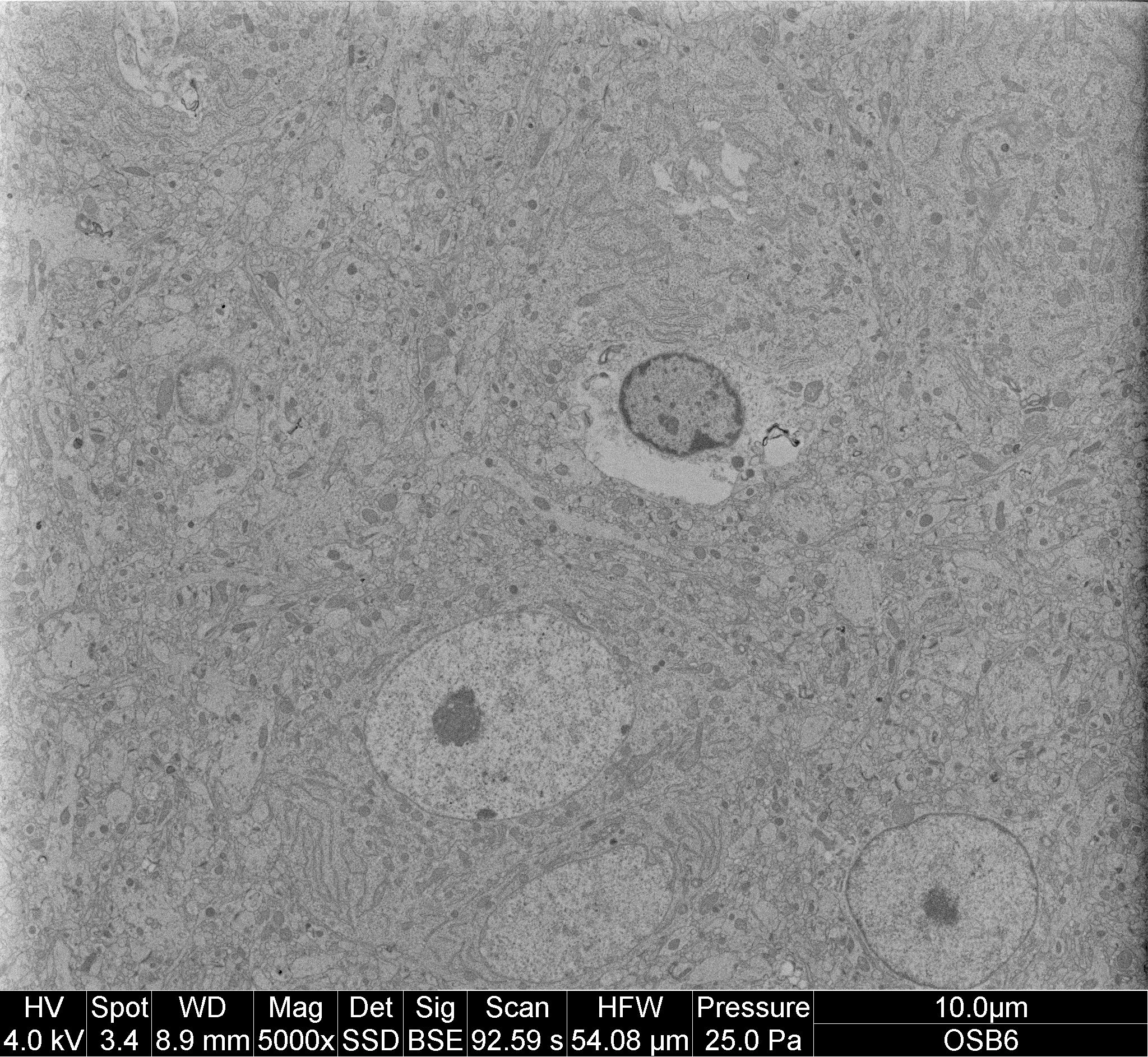

Supplement: Dataset S14 — (251.8 MB ZIP). [file pbio.0020329.sd014.zip › 040604_OS5_st1_1304.tif]

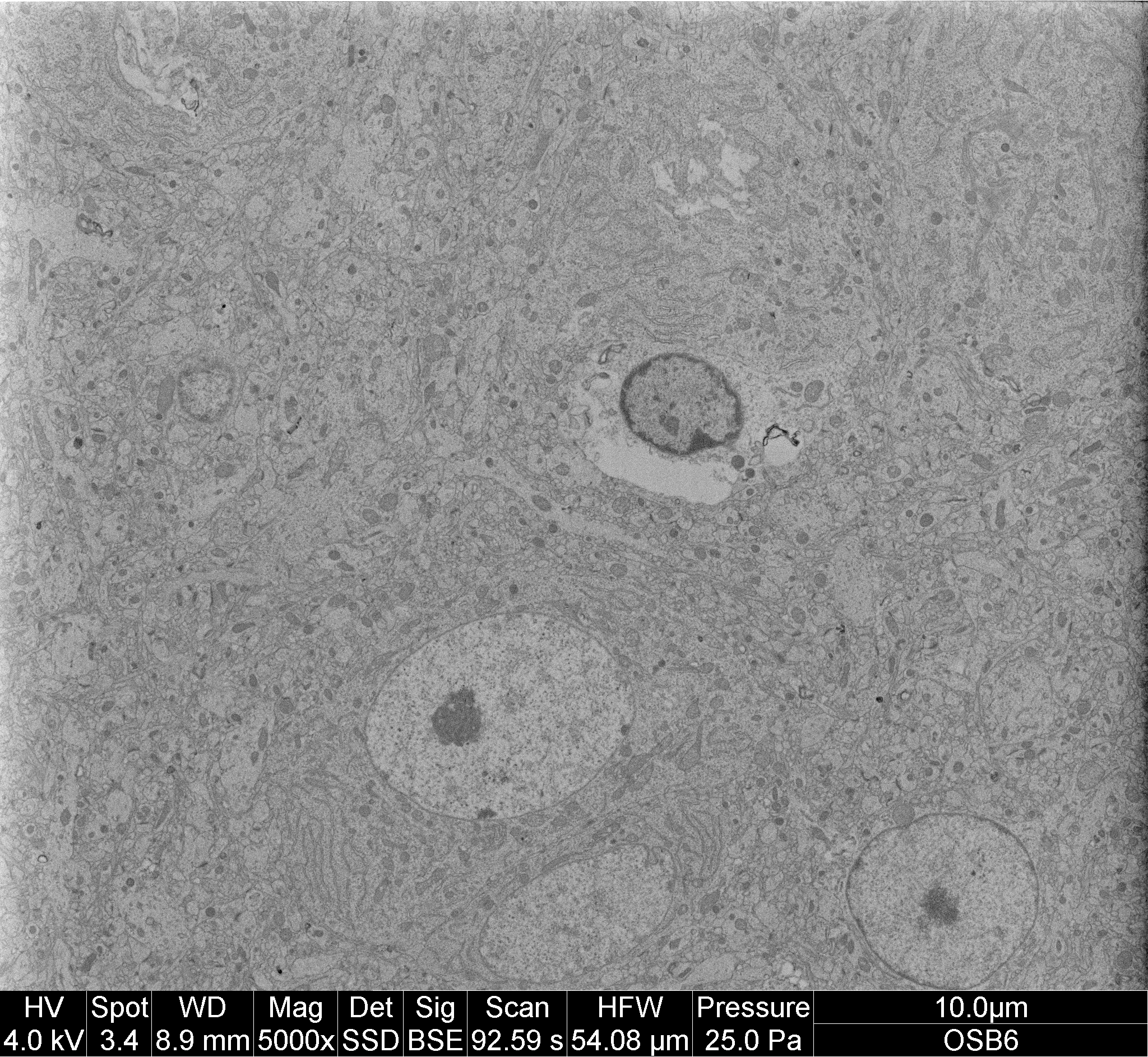

Supplement: Dataset S14 — (251.8 MB ZIP). [file pbio.0020329.sd014.zip › 040604_OS5_st1_1305.tif]

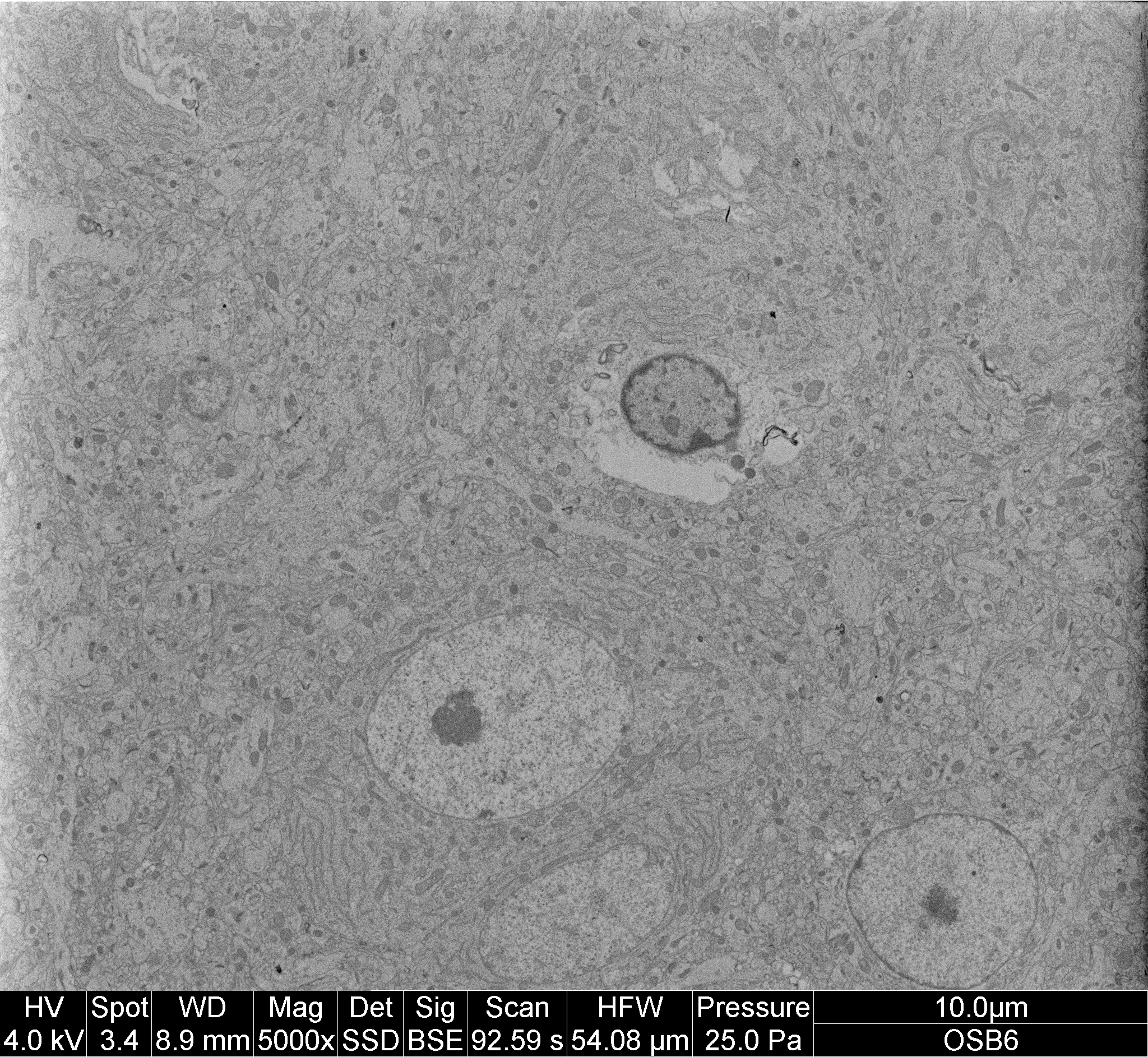

Supplement: Dataset S14 — (251.8 MB ZIP). [file pbio.0020329.sd014.zip › 040604_OS5_st1_1306.tif]

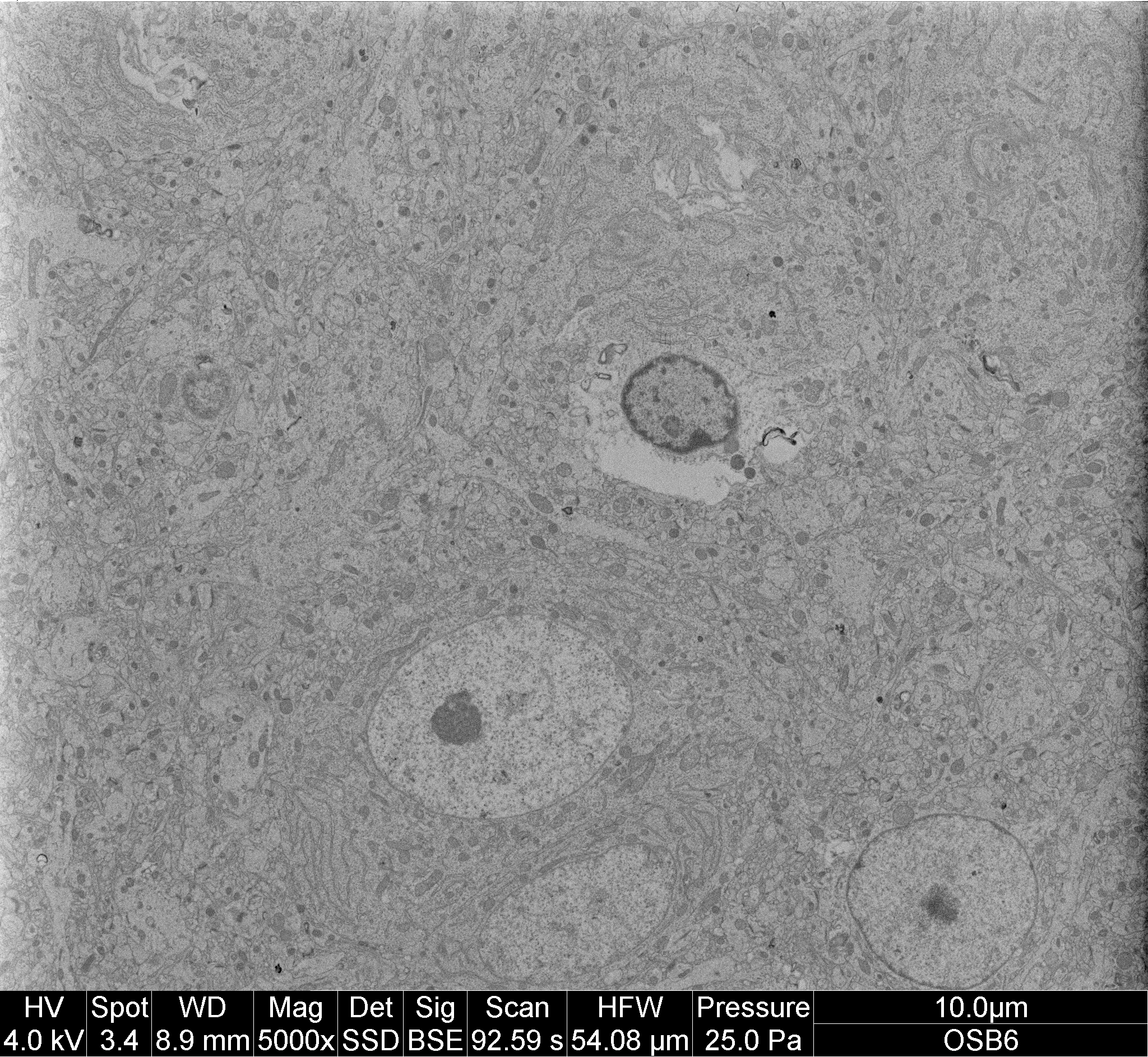

Supplement: Dataset S14 — (251.8 MB ZIP). [file pbio.0020329.sd014.zip › 040604_OS5_st1_1307.tif]

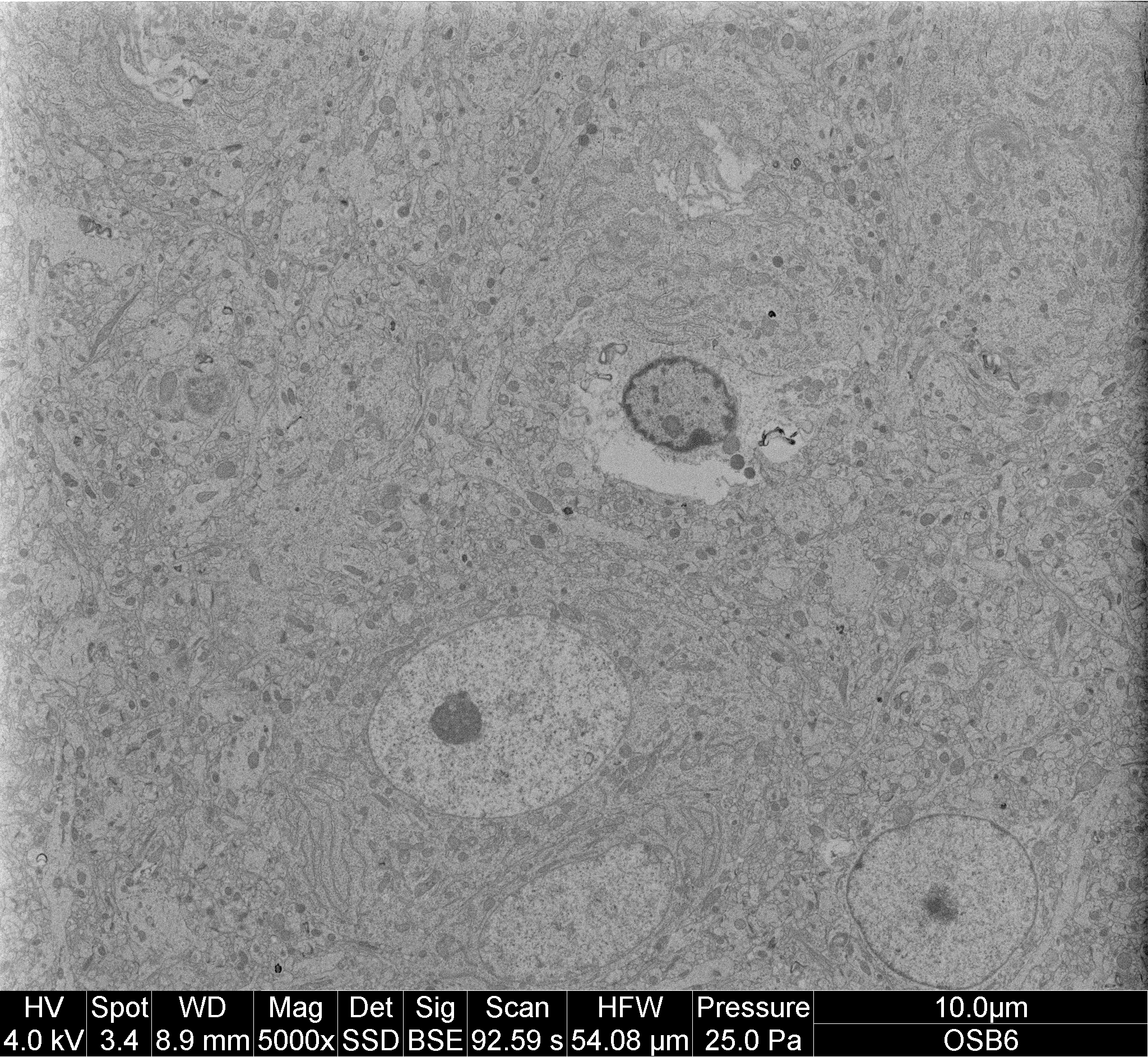

Supplement: Dataset S14 — (251.8 MB ZIP). [file pbio.0020329.sd014.zip › 040604_OS5_st1_1308.tif]

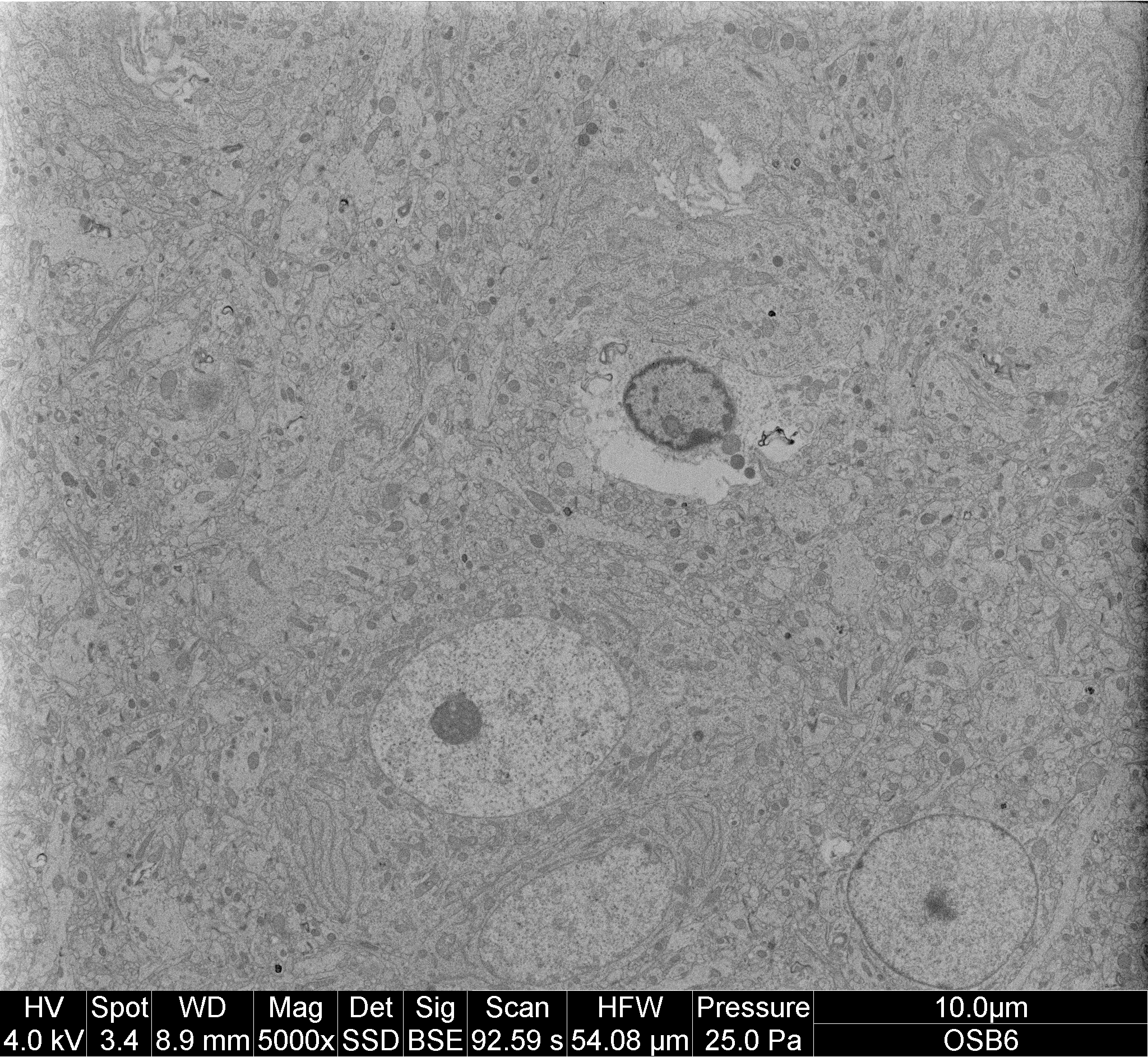

Supplement: Dataset S14 — (251.8 MB ZIP). [file pbio.0020329.sd014.zip › 040604_OS5_st1_1309.tif]

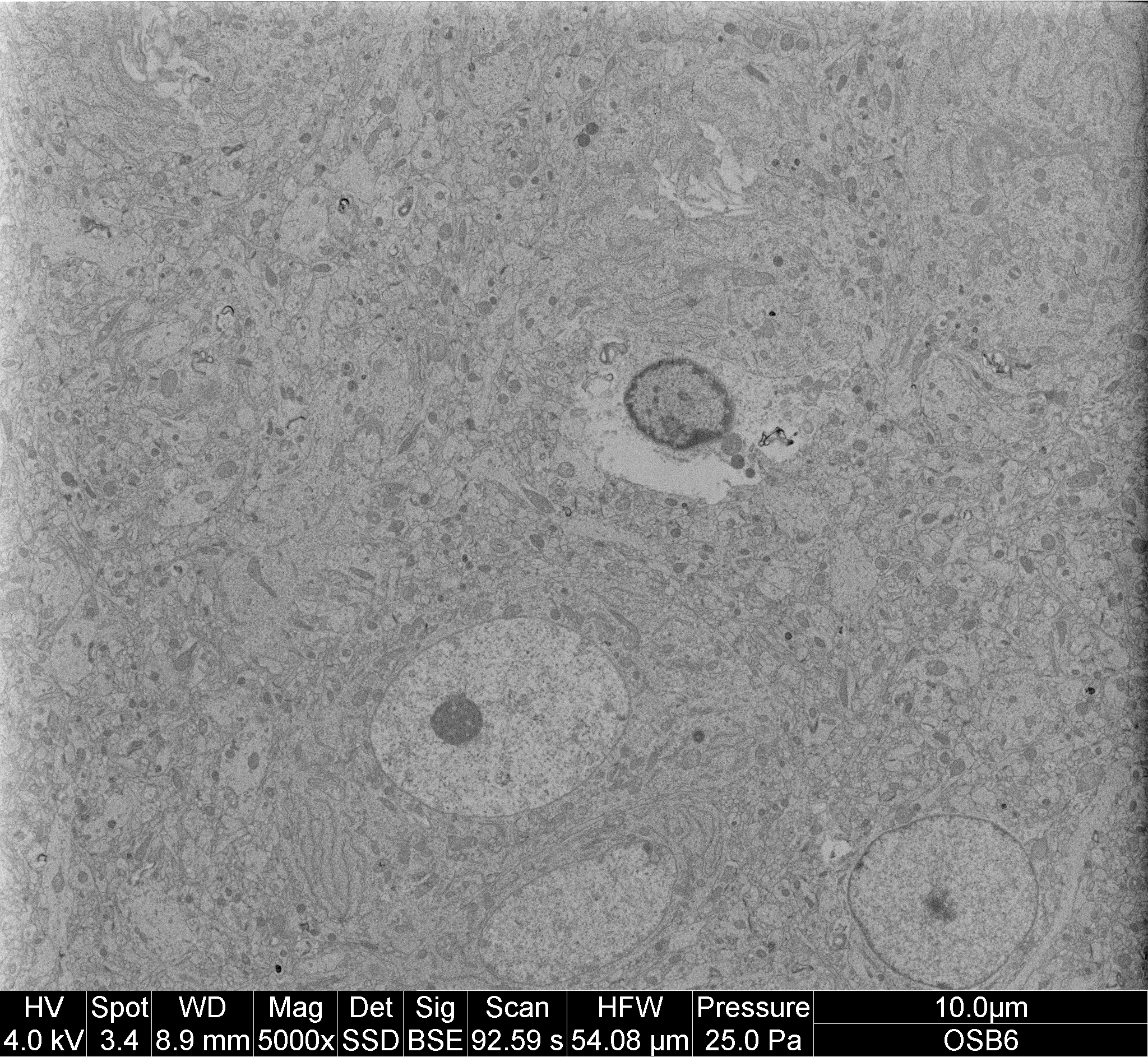

Supplement: Dataset S14 — (251.8 MB ZIP). [file pbio.0020329.sd014.zip › 040604_OS5_st1_1310.tif]

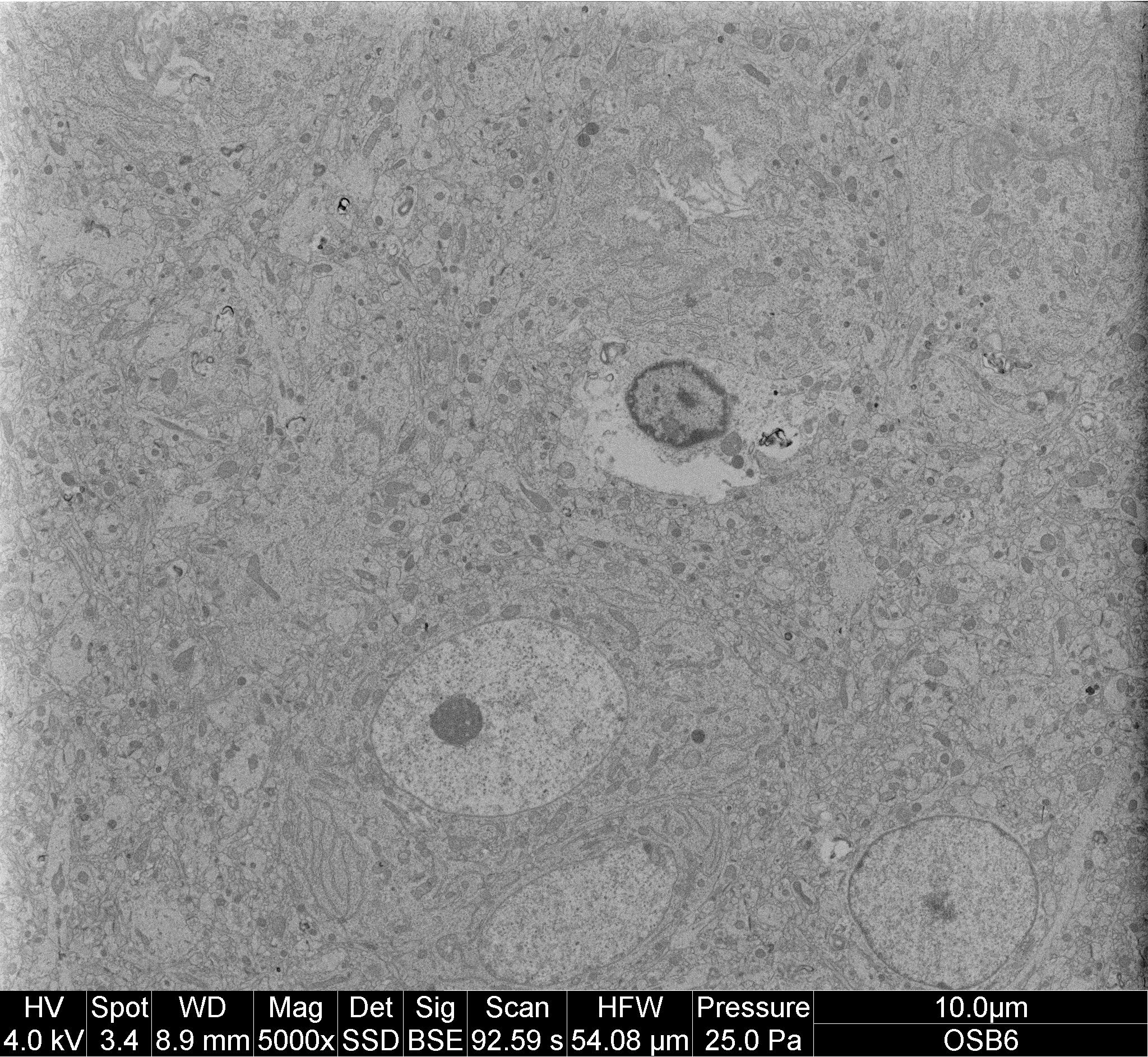

Supplement: Dataset S14 — (251.8 MB ZIP). [file pbio.0020329.sd014.zip › 040604_OS5_st1_1311.tif]

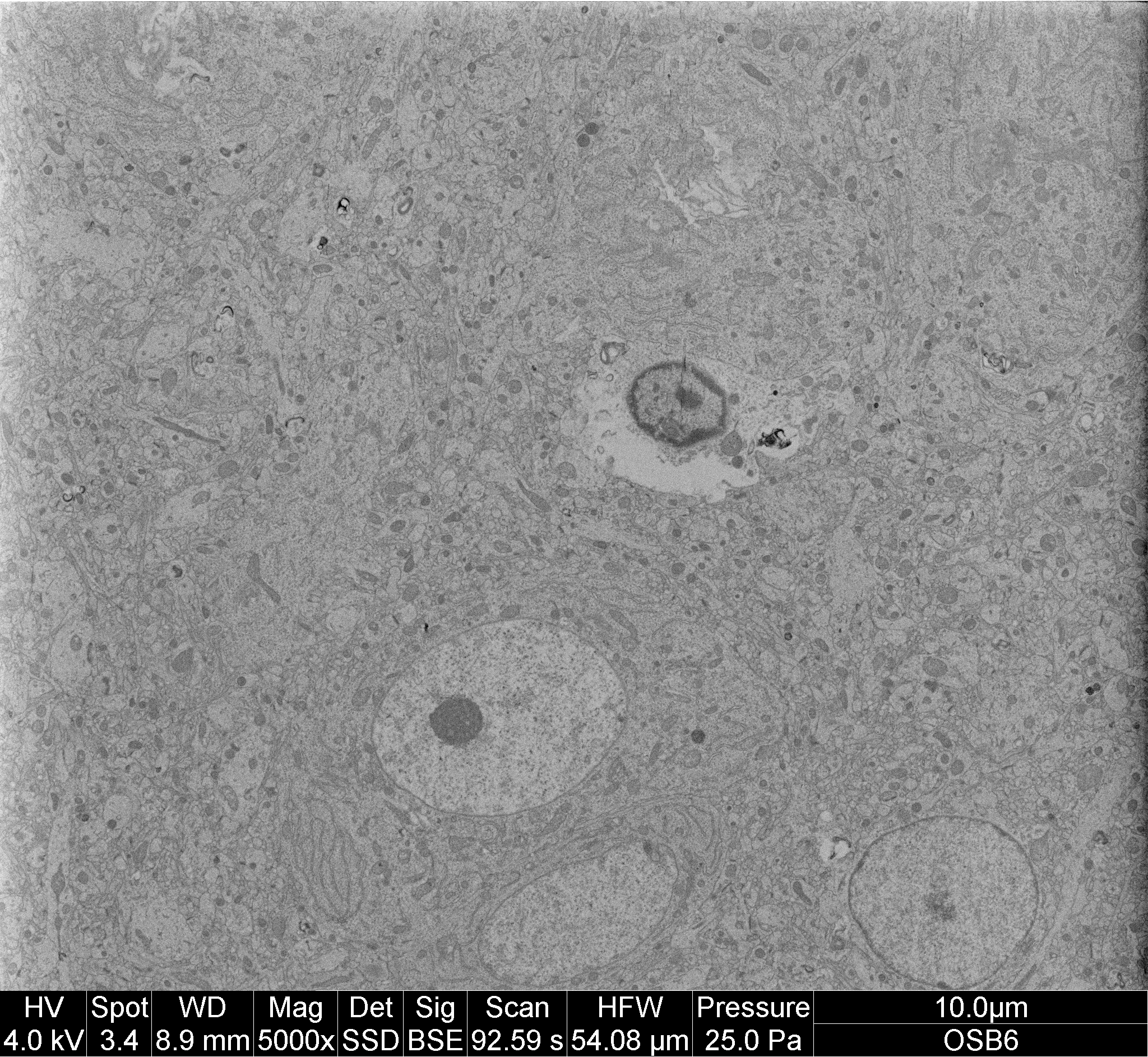

Supplement: Dataset S14 — (251.8 MB ZIP). [file pbio.0020329.sd014.zip › 040604_OS5_st1_1312.tif]

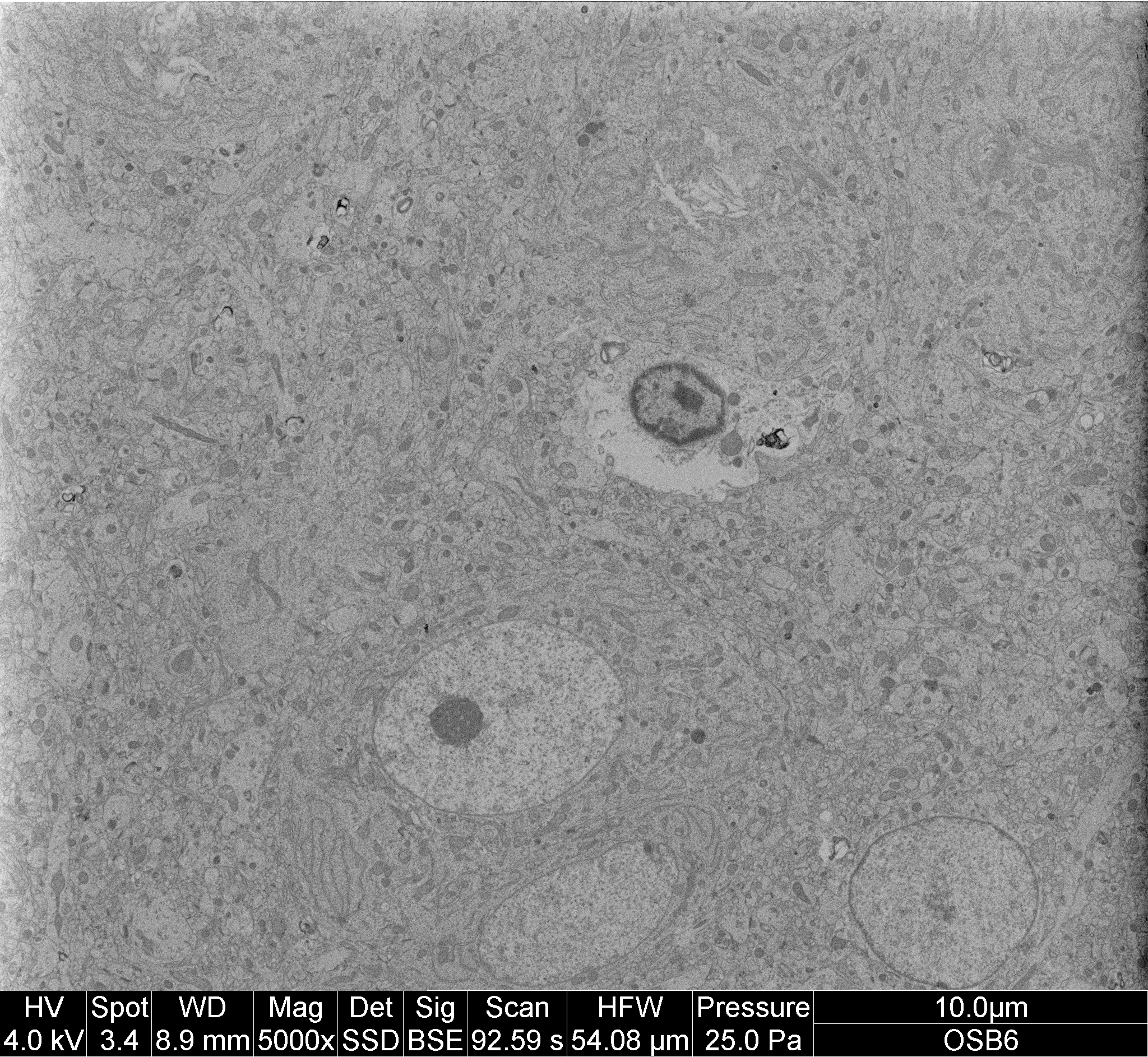

Supplement: Dataset S14 — (251.8 MB ZIP). [file pbio.0020329.sd014.zip › 040604_OS5_st1_1313.tif]

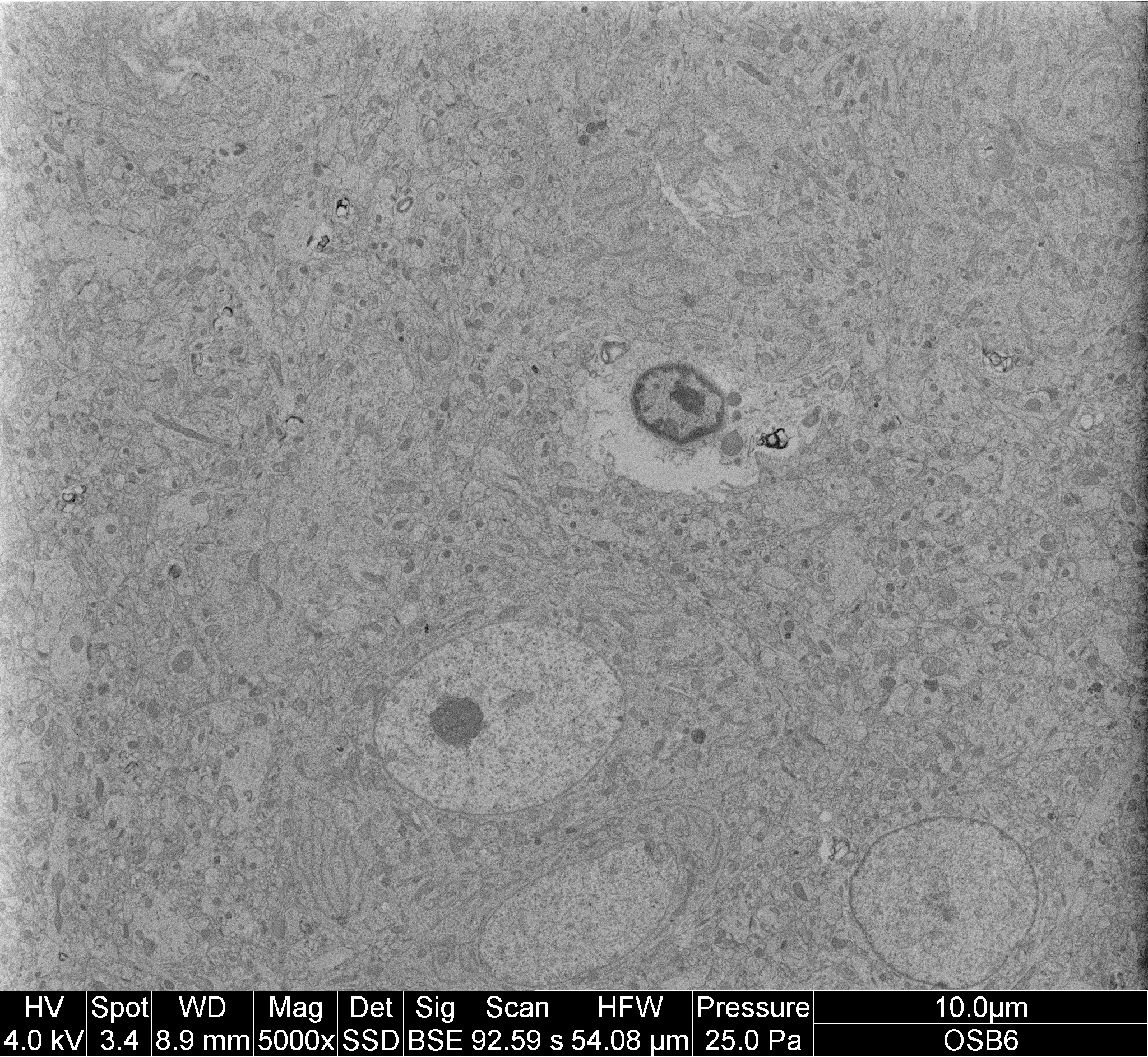

Supplement: Dataset S14 — (251.8 MB ZIP). [file pbio.0020329.sd014.zip › 040604_OS5_st1_1314.tif]

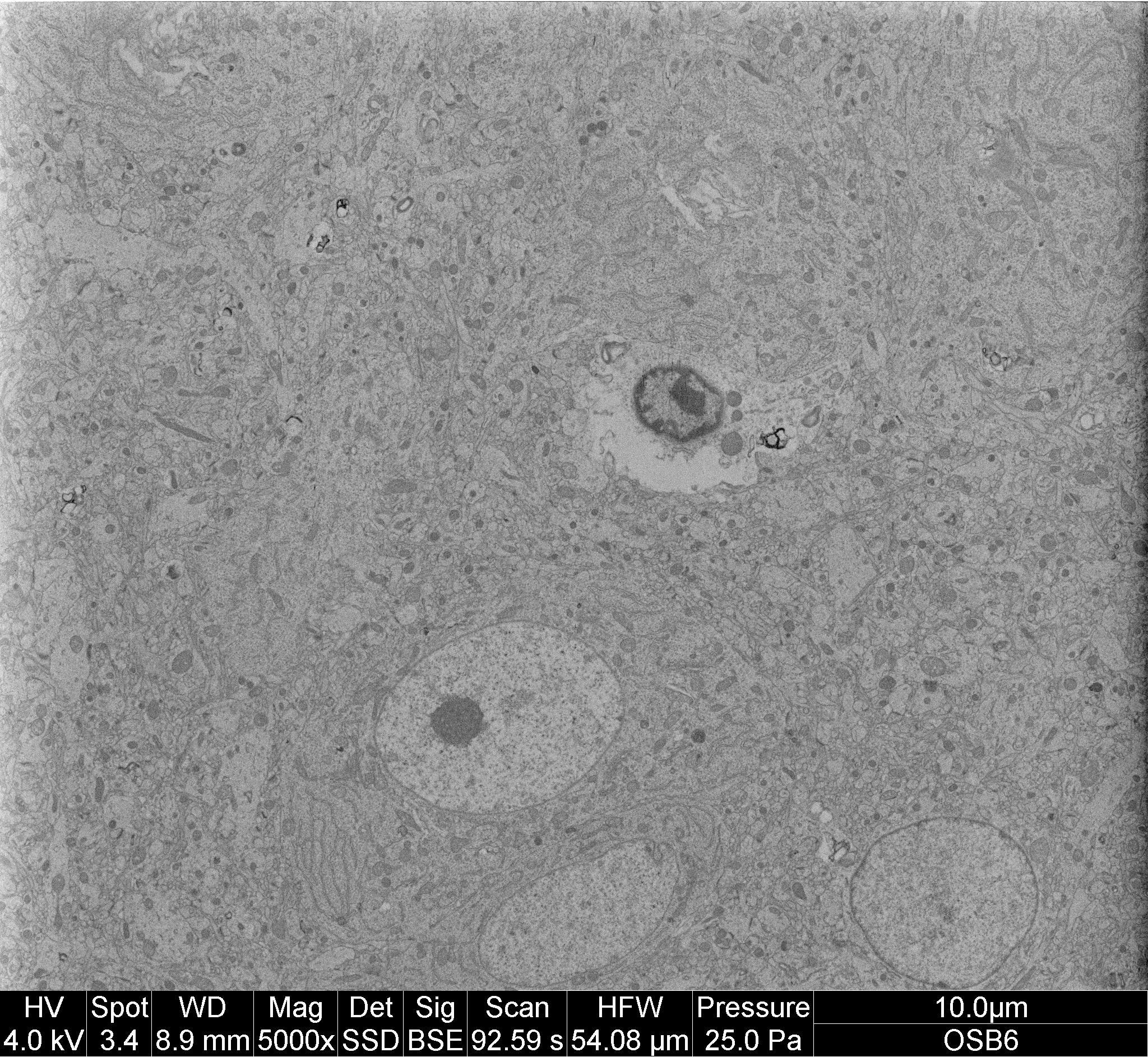

Supplement: Dataset S14 — (251.8 MB ZIP). [file pbio.0020329.sd014.zip › 040604_OS5_st1_1315.tif]

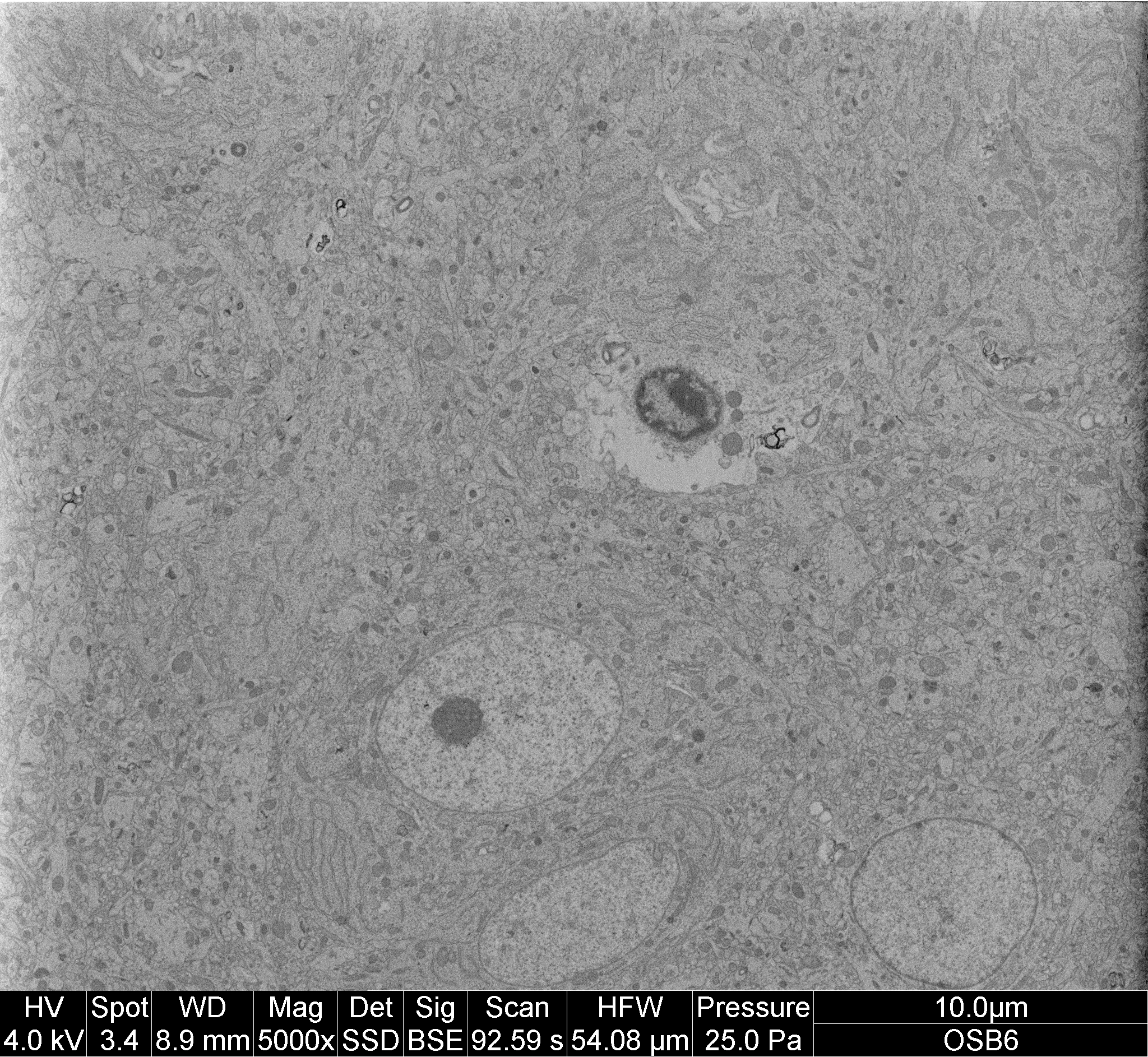

Supplement: Dataset S14 — (251.8 MB ZIP). [file pbio.0020329.sd014.zip › 040604_OS5_st1_1316.tif]

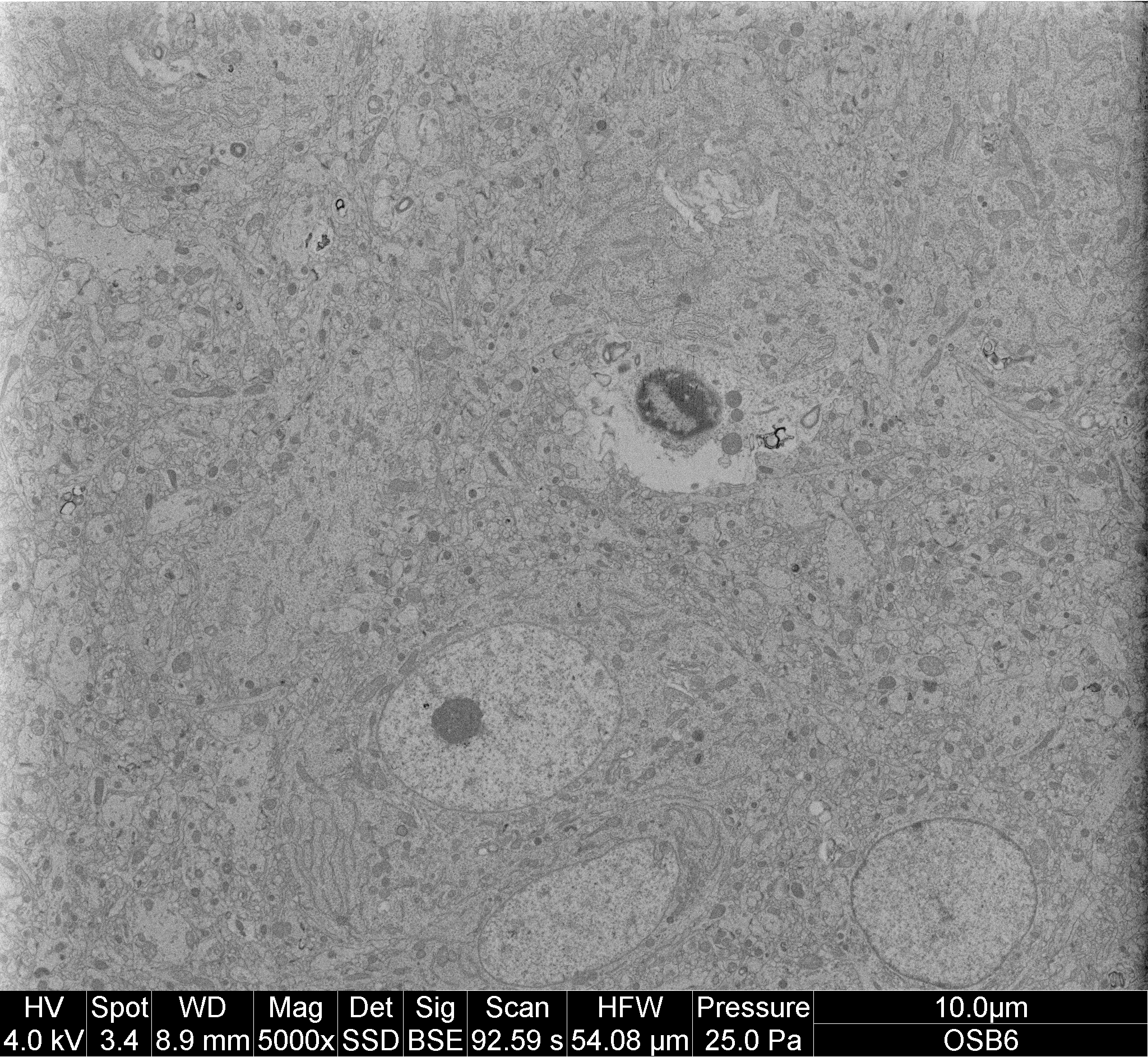

Supplement: Dataset S14 — (251.8 MB ZIP). [file pbio.0020329.sd014.zip › 040604_OS5_st1_1317.tif]

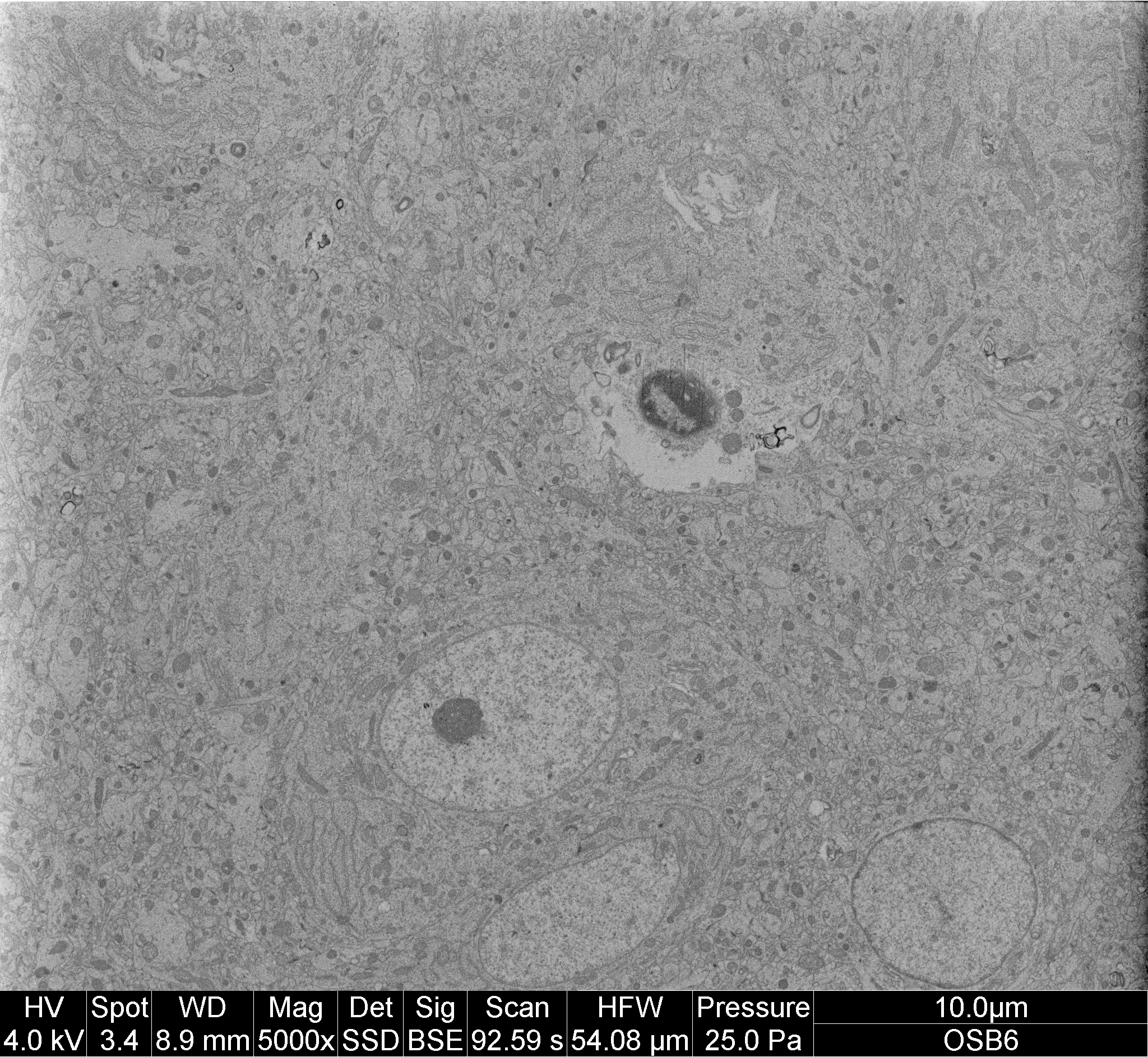

Supplement: Dataset S14 — (251.8 MB ZIP). [file pbio.0020329.sd014.zip › 040604_OS5_st1_1318.tif]

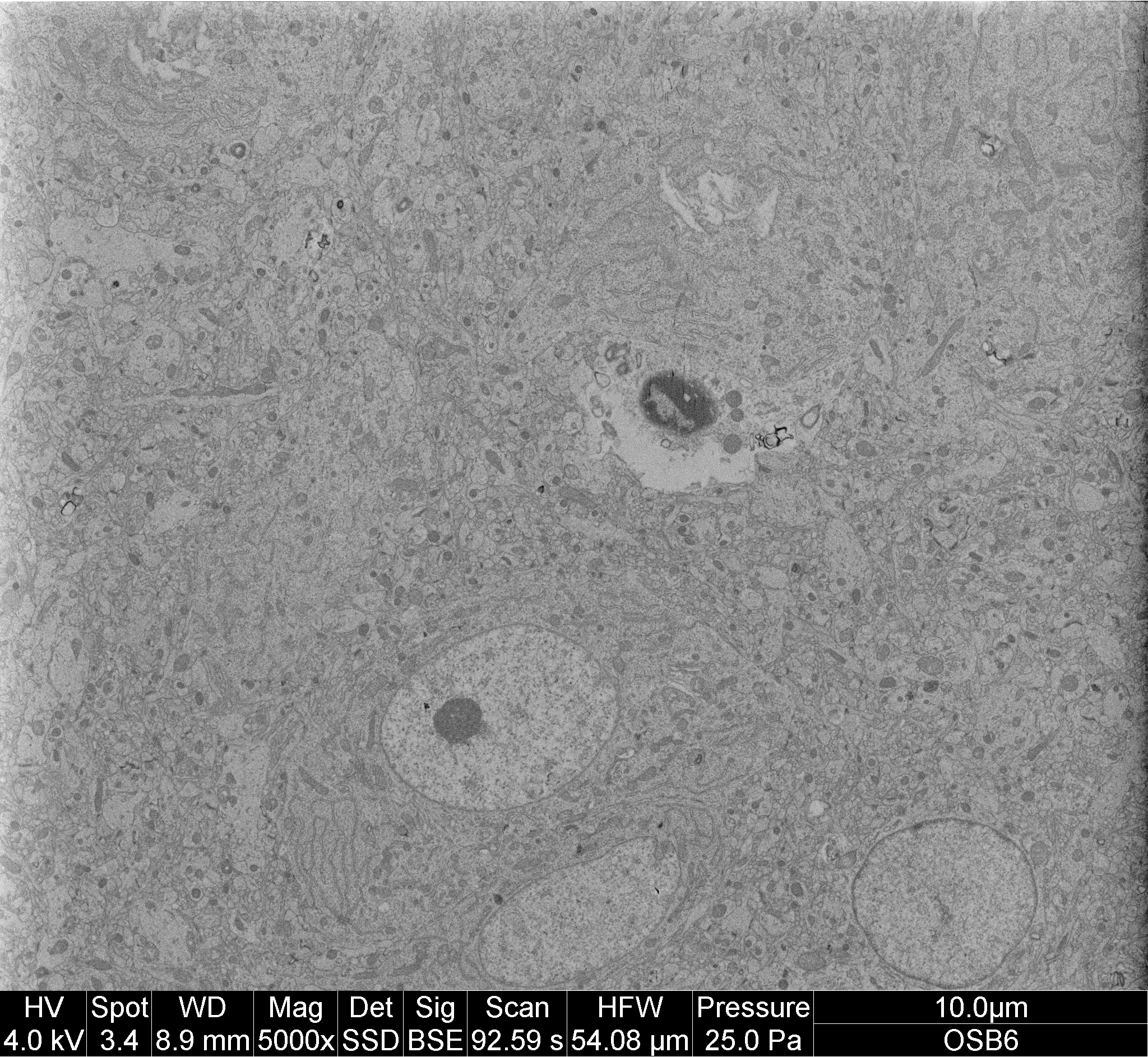

Supplement: Dataset S14 — (251.8 MB ZIP). [file pbio.0020329.sd014.zip › 040604_OS5_st1_1319.tif]

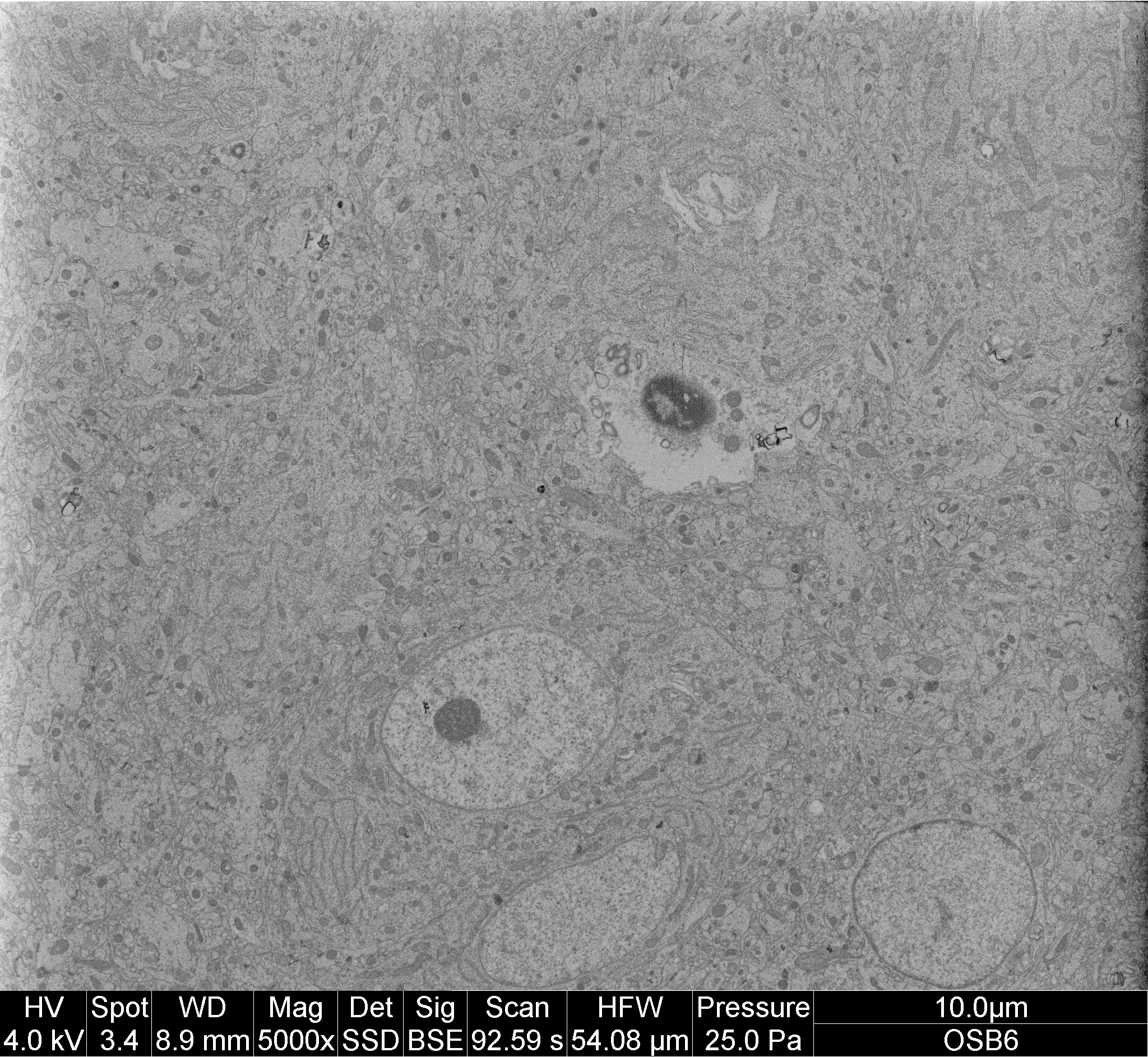

Supplement: Dataset S14 — (251.8 MB ZIP). [file pbio.0020329.sd014.zip › 040604_OS5_st1_1320.tif]

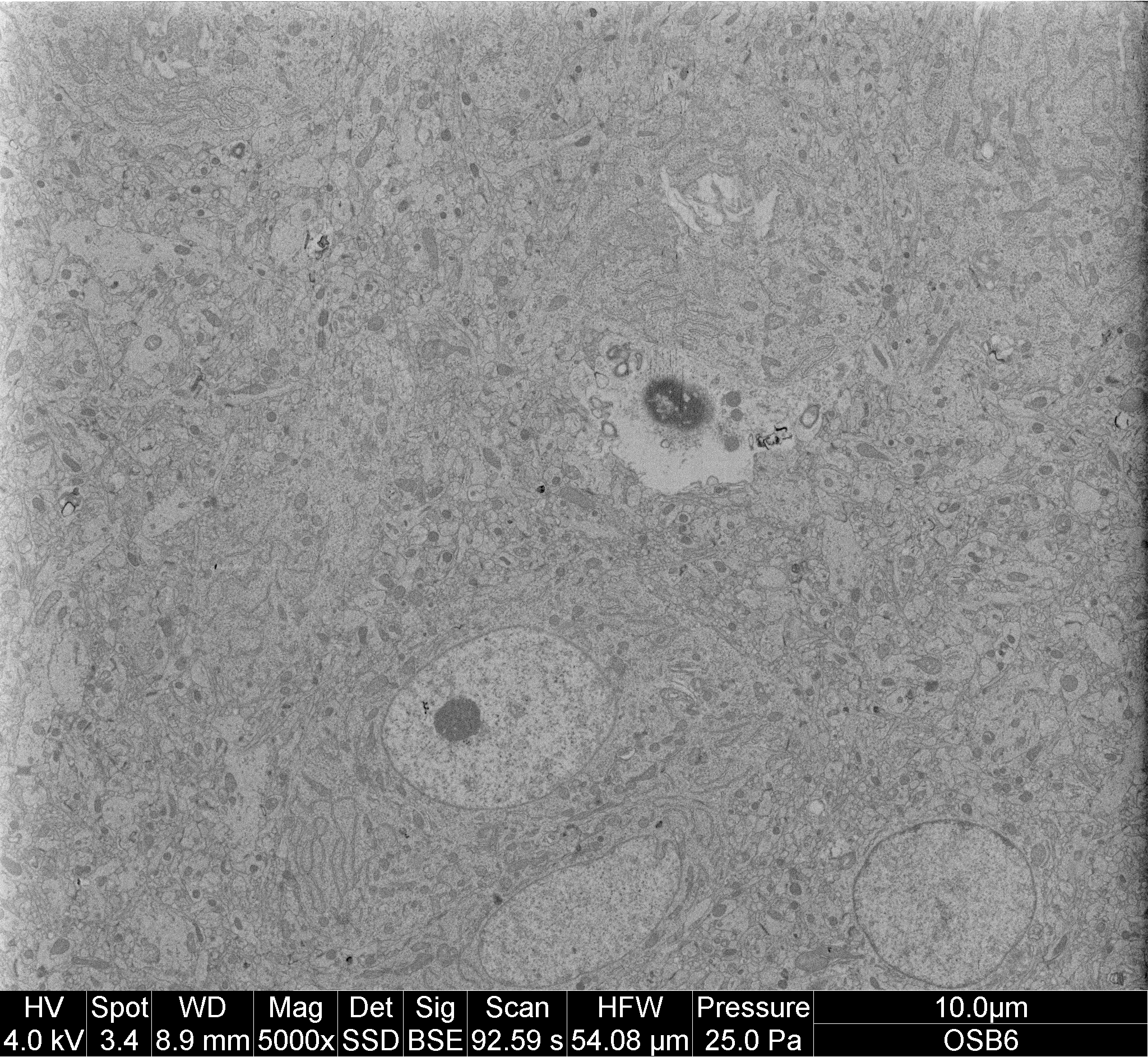

Supplement: Dataset S14 — (251.8 MB ZIP). [file pbio.0020329.sd014.zip › 040604_OS5_st1_1321.tif]

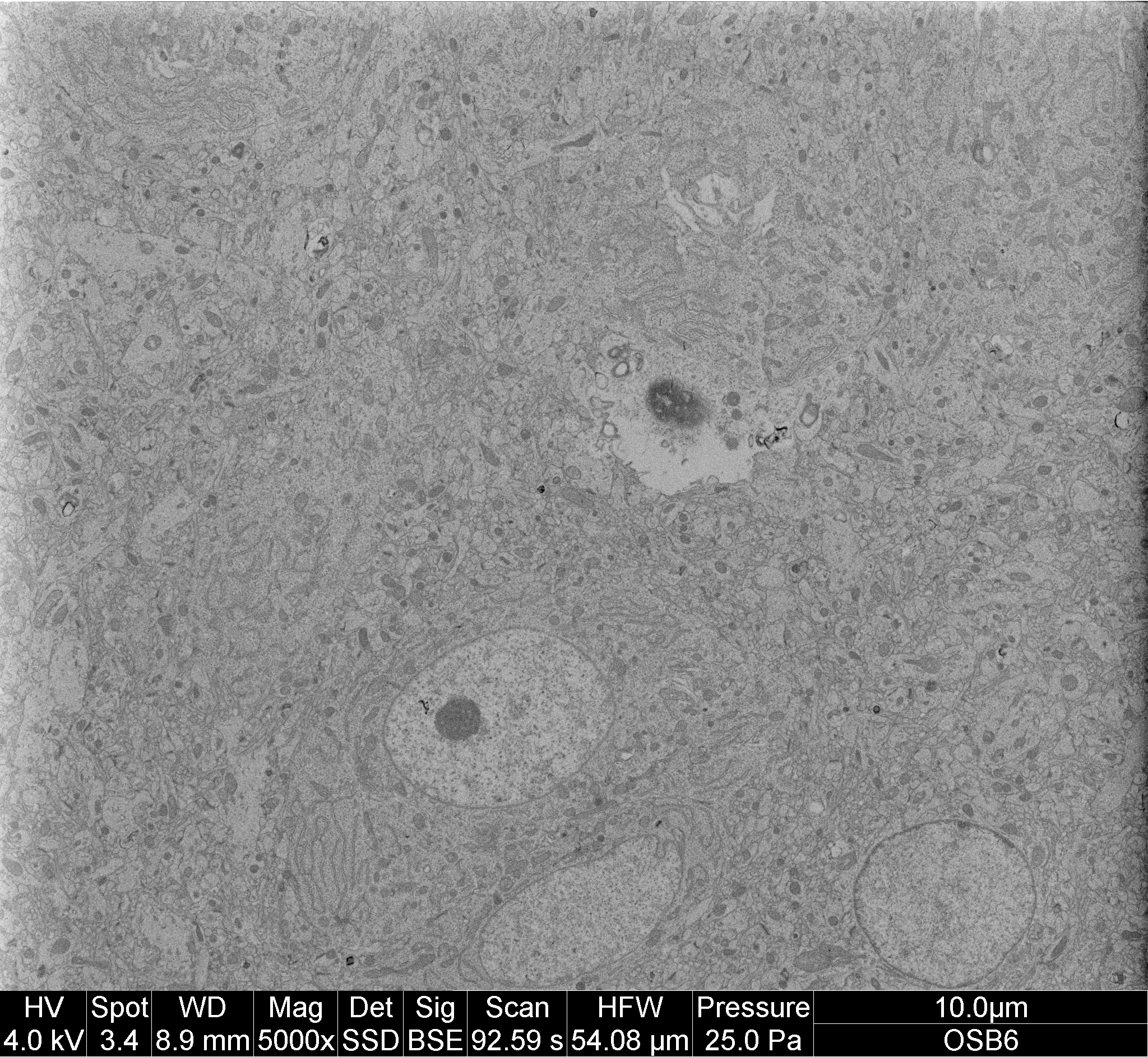

Supplement: Dataset S14 — (251.8 MB ZIP). [file pbio.0020329.sd014.zip › 040604_OS5_st1_1322.tif]

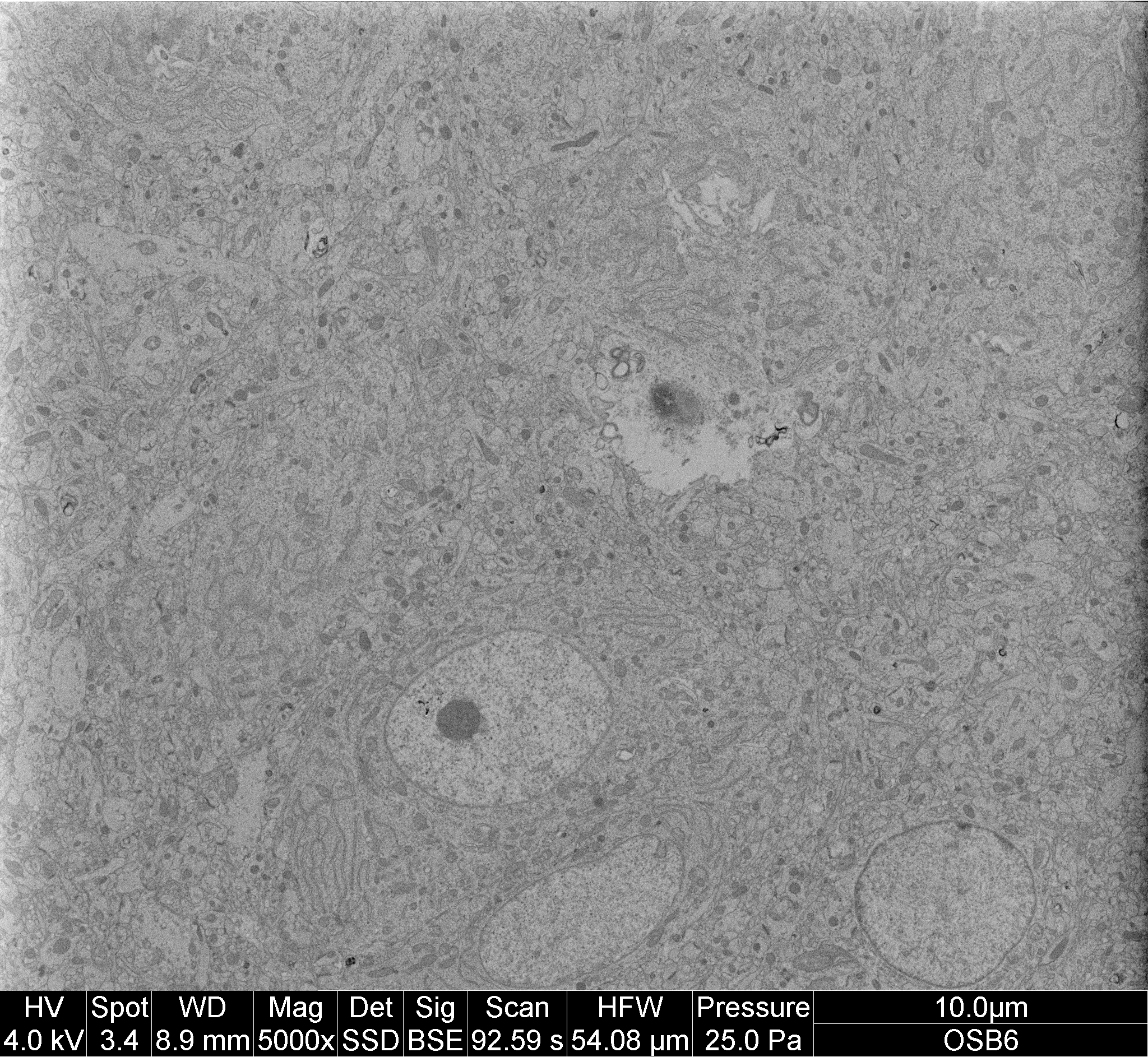

Supplement: Dataset S14 — (251.8 MB ZIP). [file pbio.0020329.sd014.zip › 040604_OS5_st1_1323.tif]

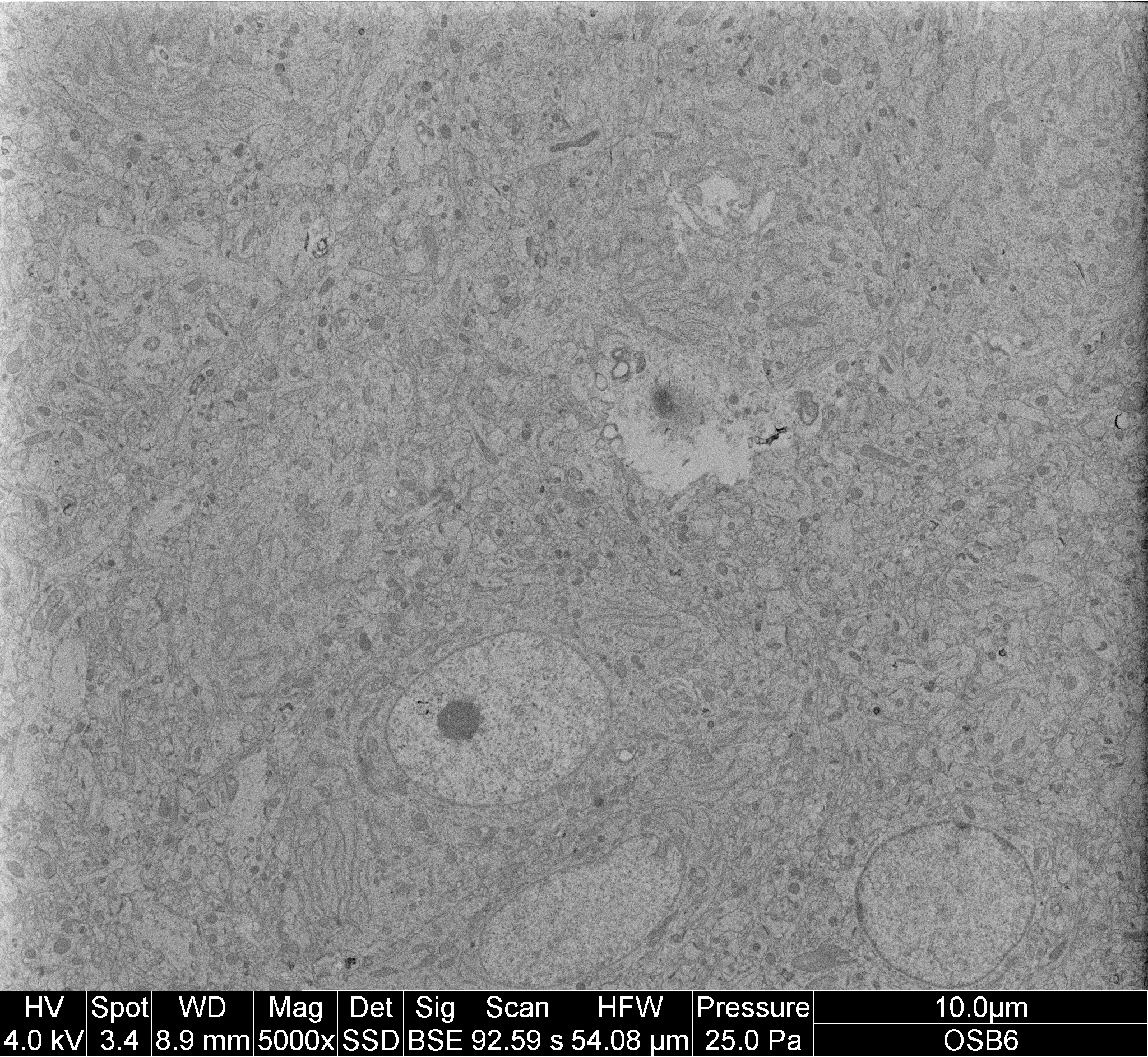

Supplement: Dataset S14 — (251.8 MB ZIP). [file pbio.0020329.sd014.zip › 040604_OS5_st1_1324.tif]

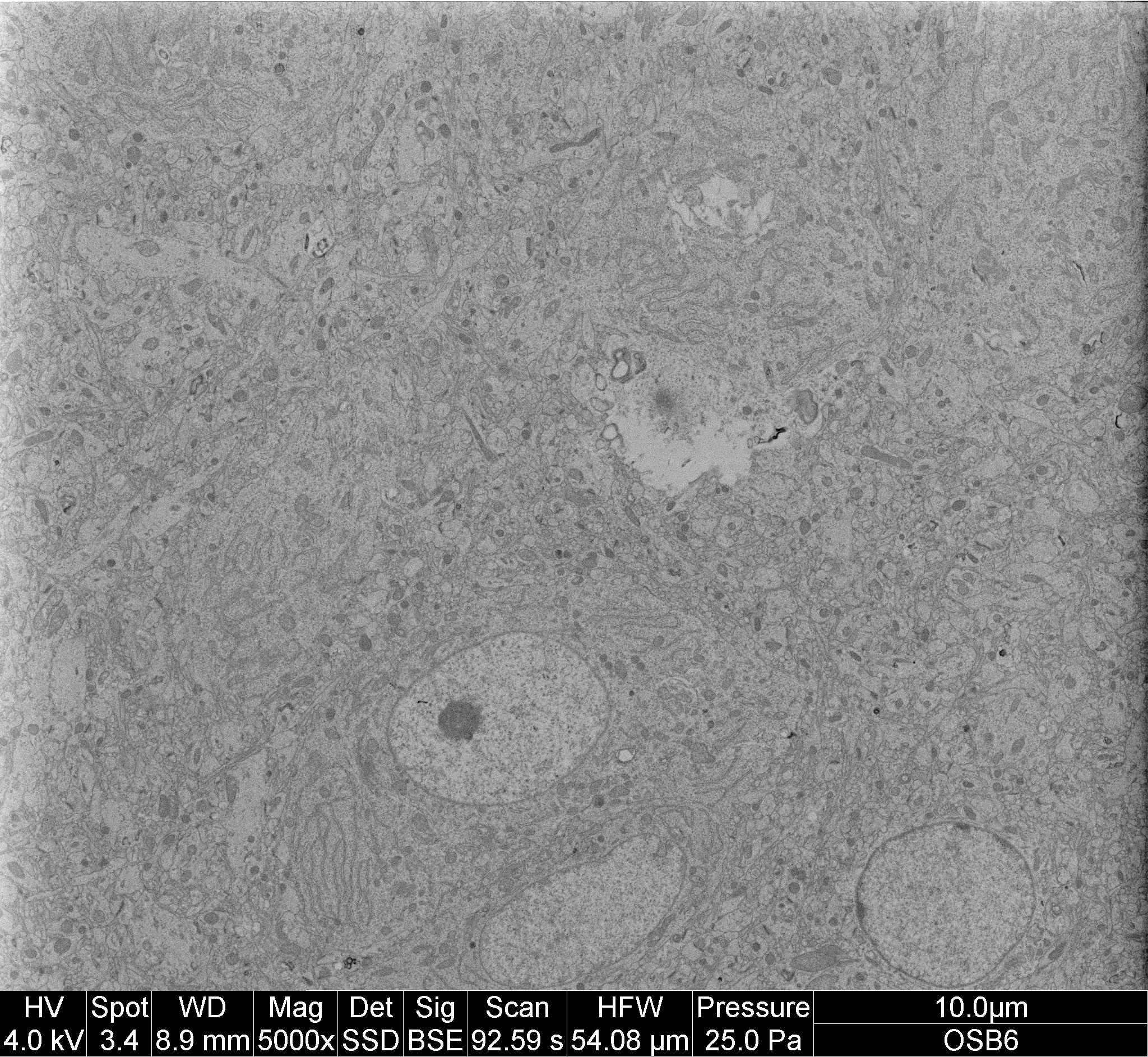

Supplement: Dataset S14 — (251.8 MB ZIP). [file pbio.0020329.sd014.zip › 040604_OS5_st1_1325.tif]

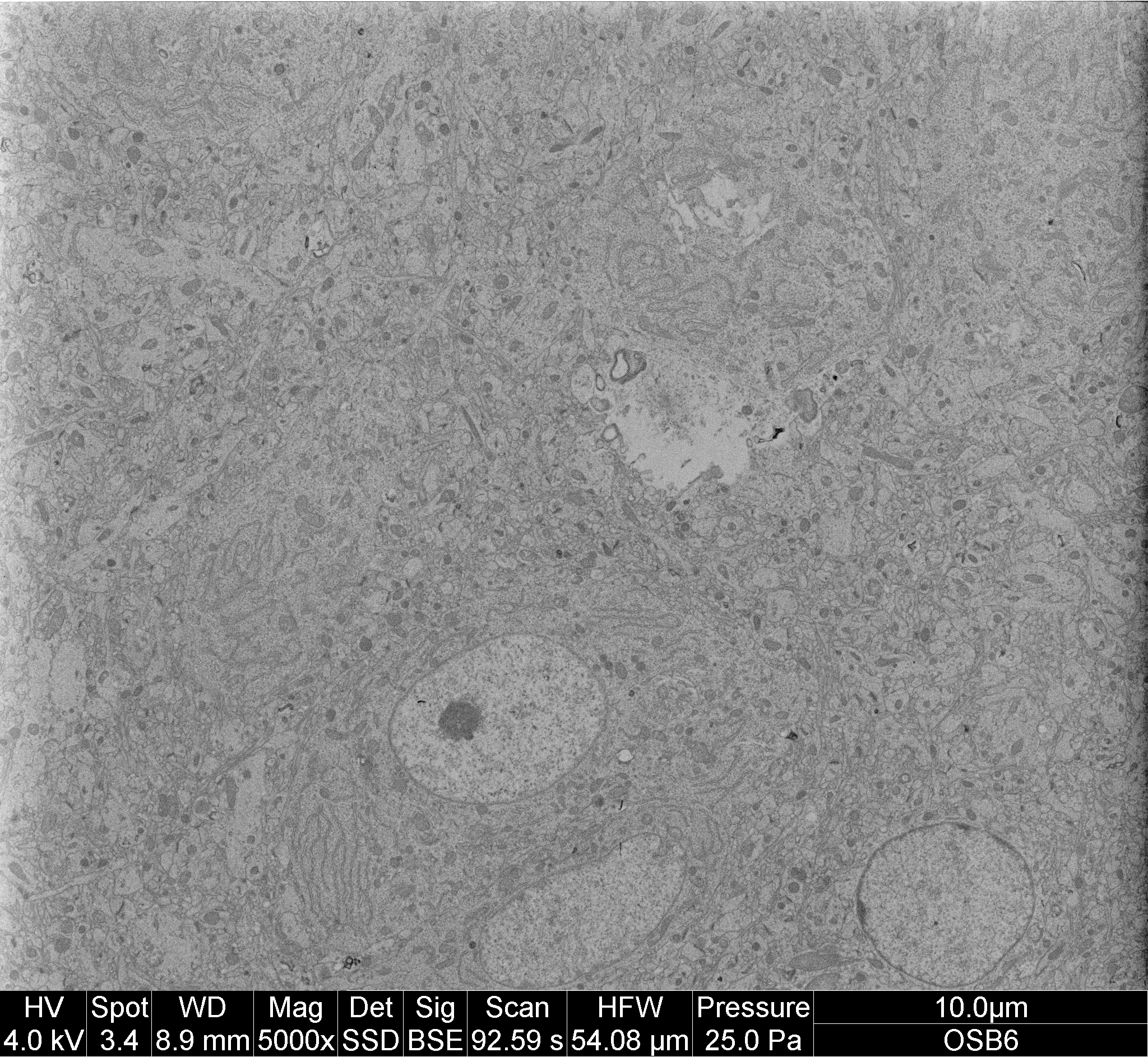

Supplement: Dataset S14 — (251.8 MB ZIP). [file pbio.0020329.sd014.zip › 040604_OS5_st1_1326.tif]

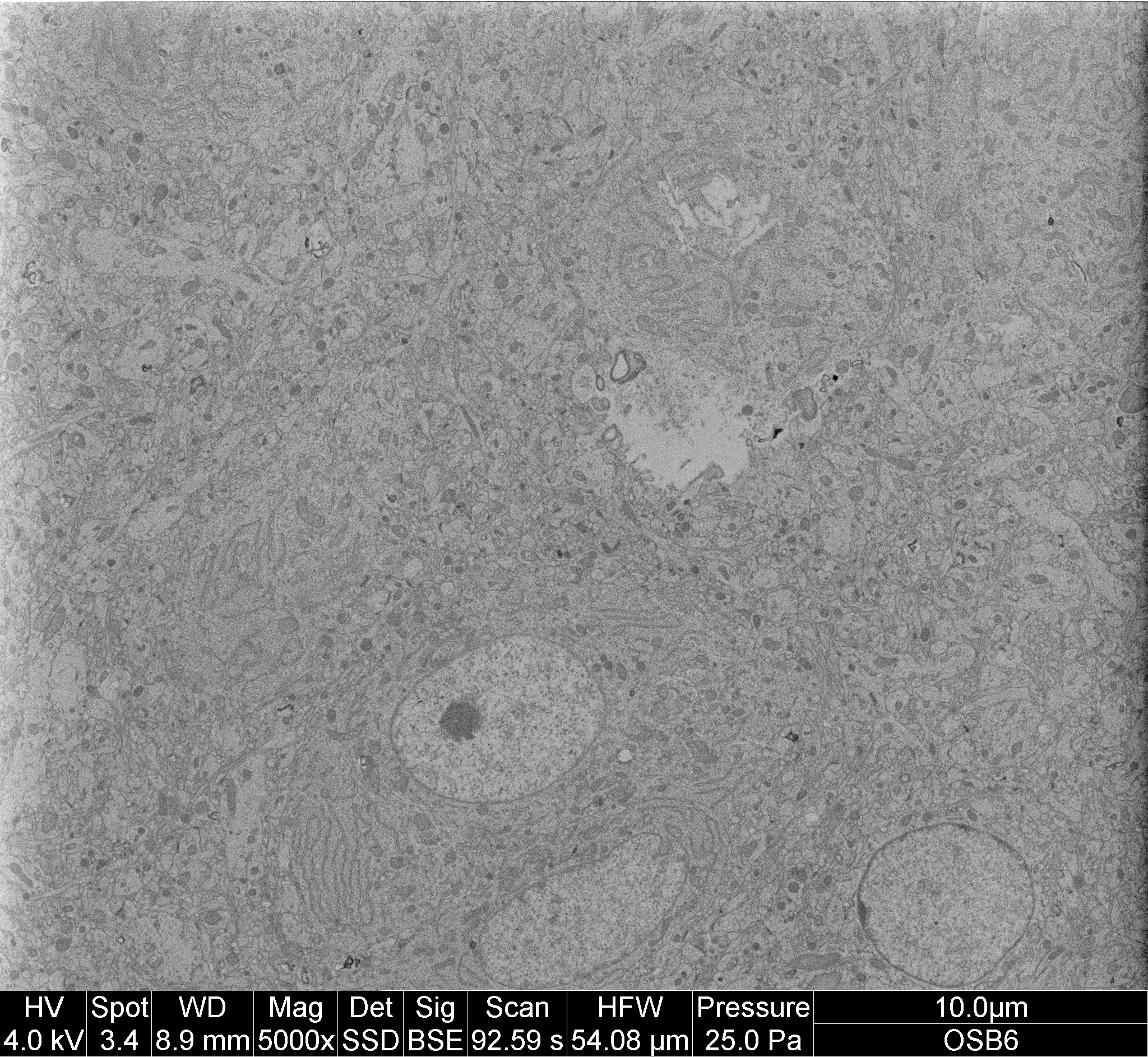

Supplement: Dataset S14 — (251.8 MB ZIP). [file pbio.0020329.sd014.zip › 040604_OS5_st1_1327.tif]

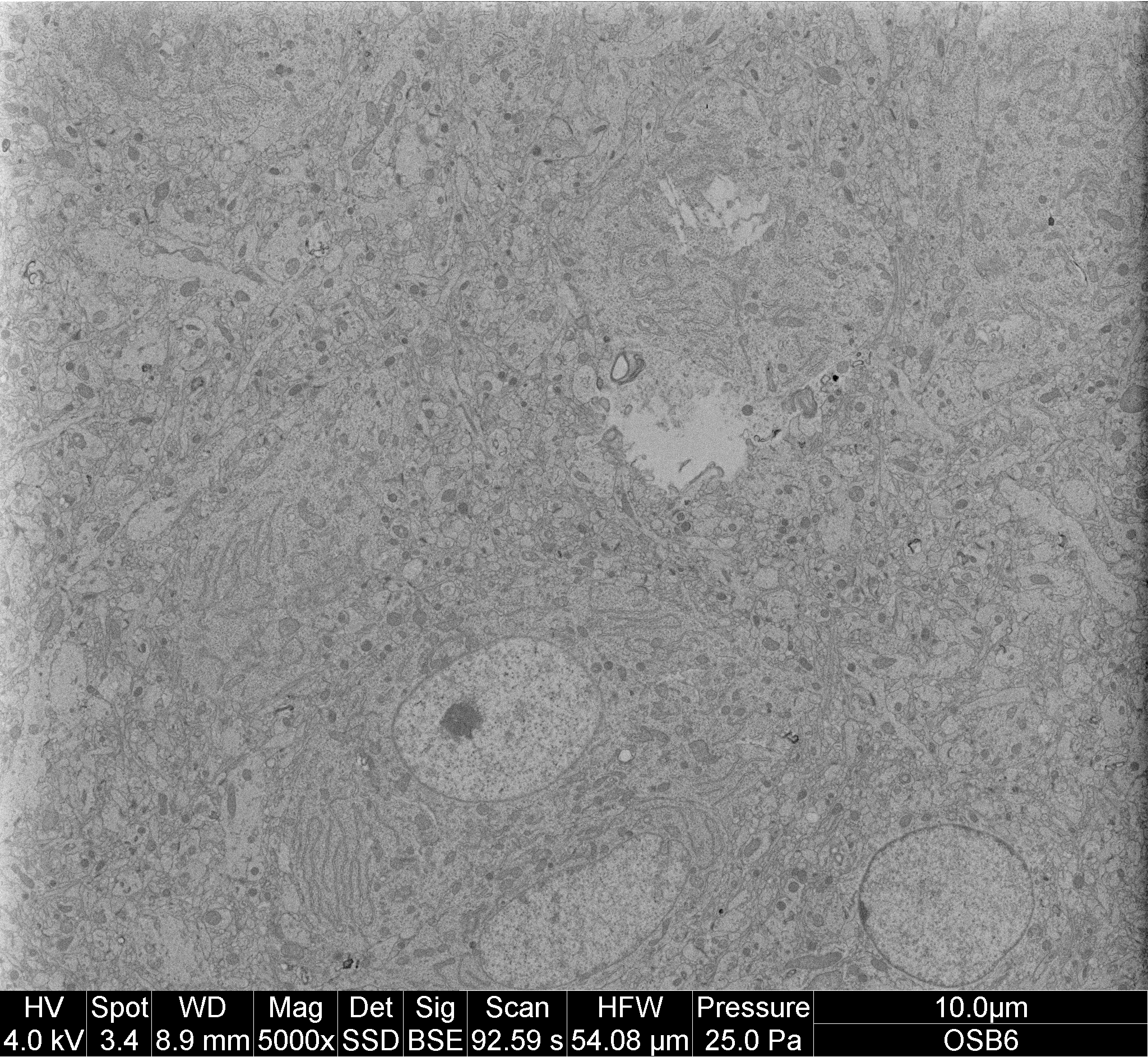

Supplement: Dataset S14 — (251.8 MB ZIP). [file pbio.0020329.sd014.zip › 040604_OS5_st1_1328.tif]

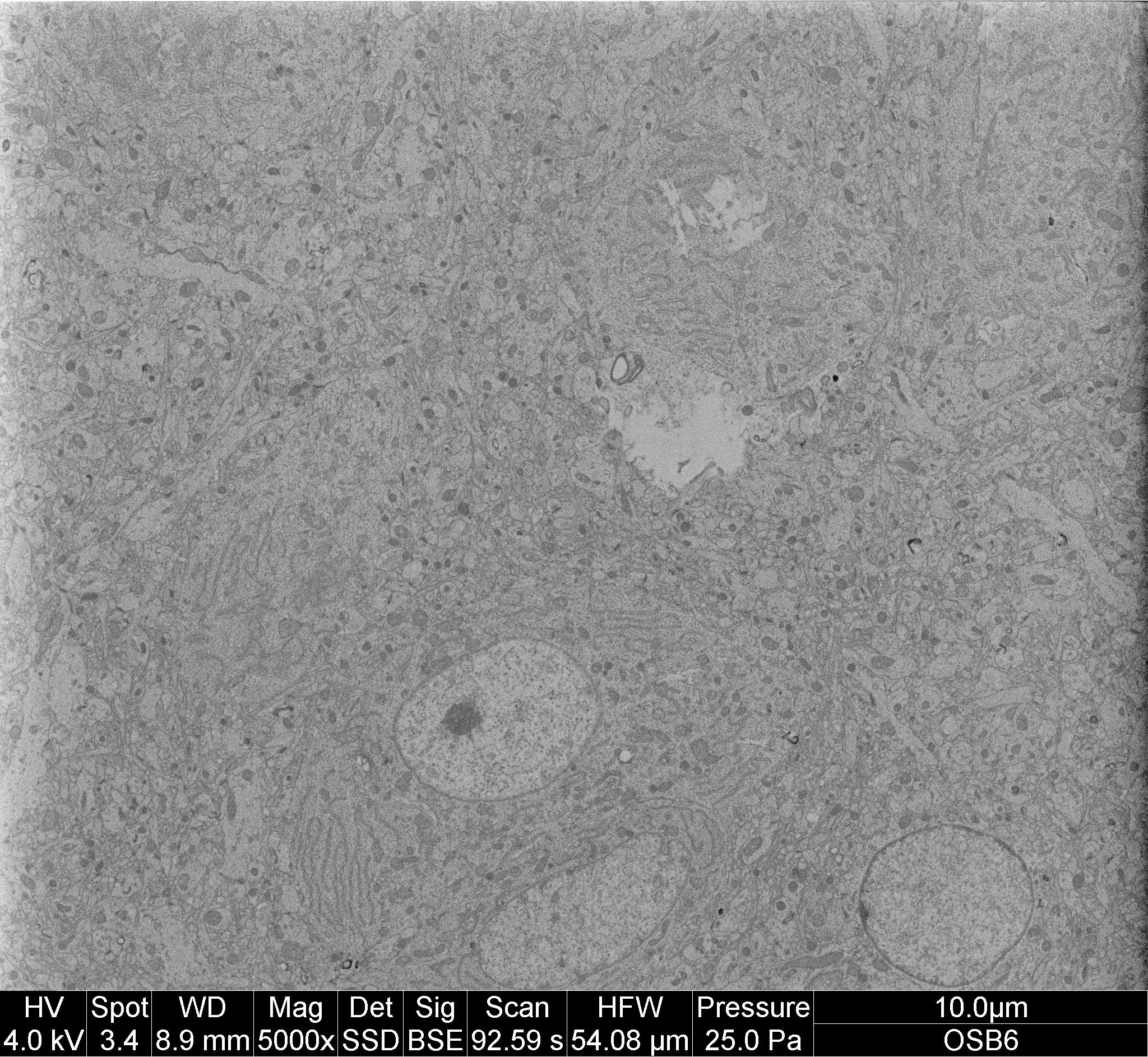

Supplement: Dataset S14 — (251.8 MB ZIP). [file pbio.0020329.sd014.zip › 040604_OS5_st1_1329.tif]

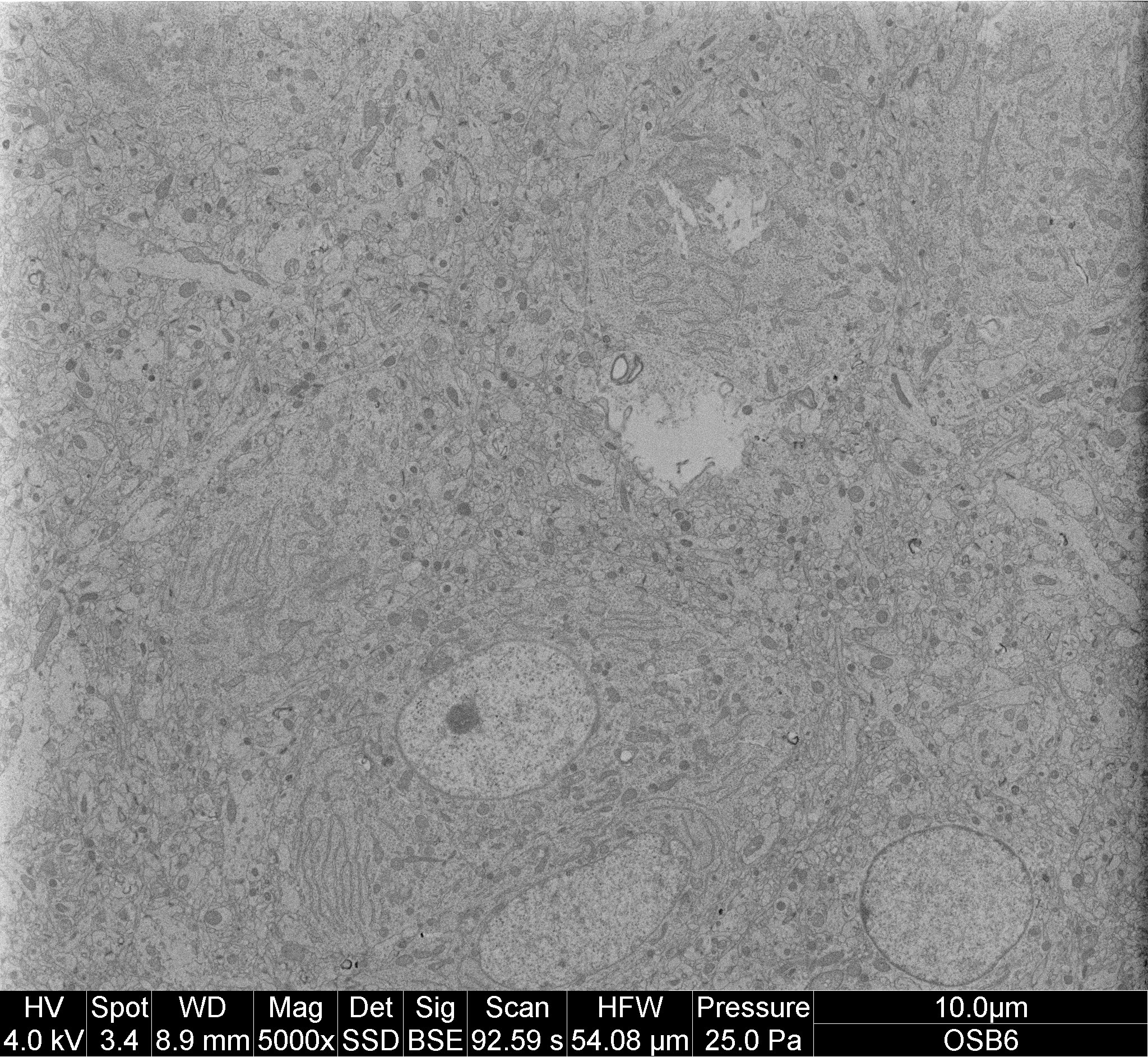

Supplement: Dataset S14 — (251.8 MB ZIP). [file pbio.0020329.sd014.zip › 040604_OS5_st1_1330.tif]

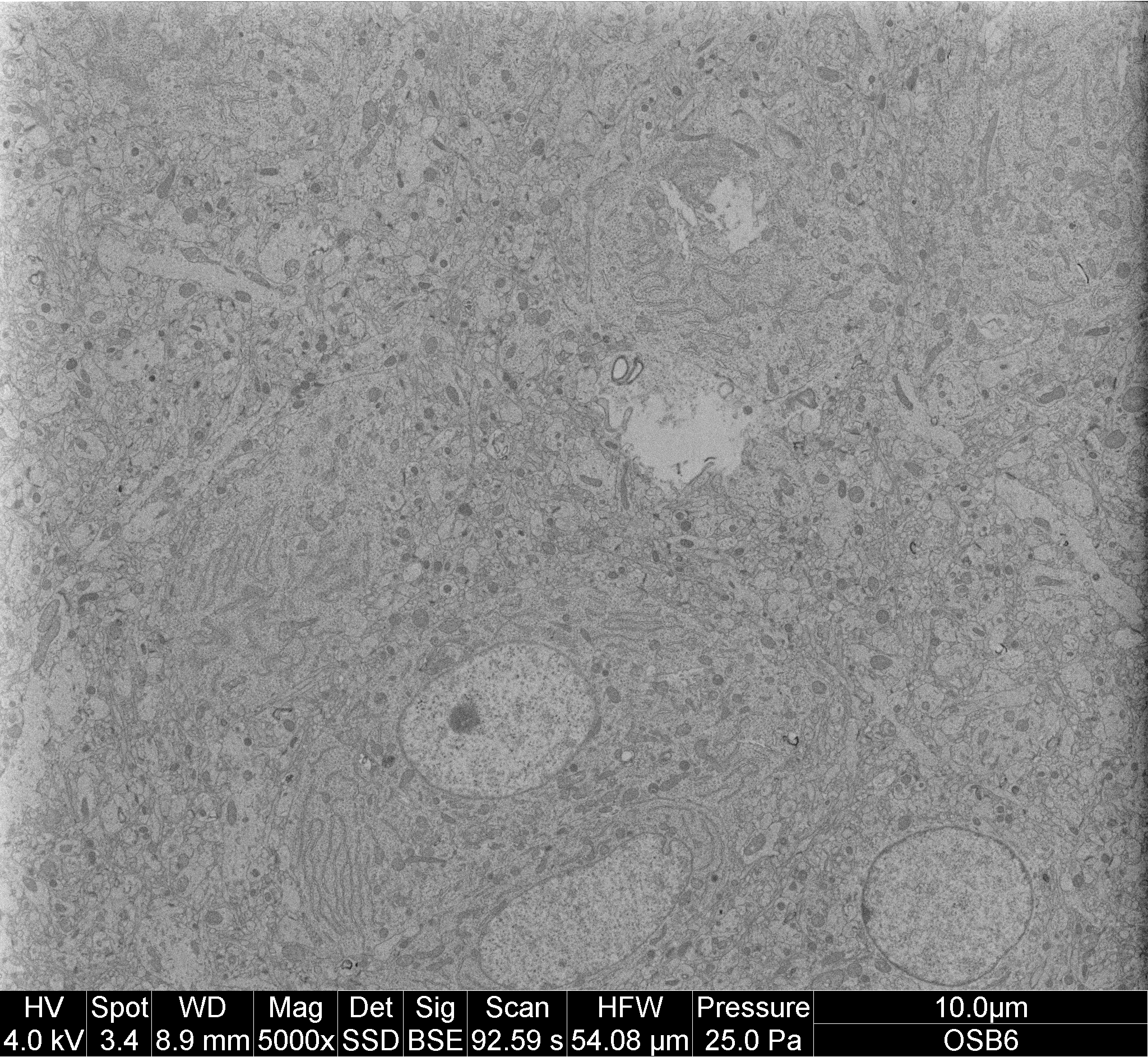

Supplement: Dataset S14 — (251.8 MB ZIP). [file pbio.0020329.sd014.zip › 040604_OS5_st1_1331.tif]

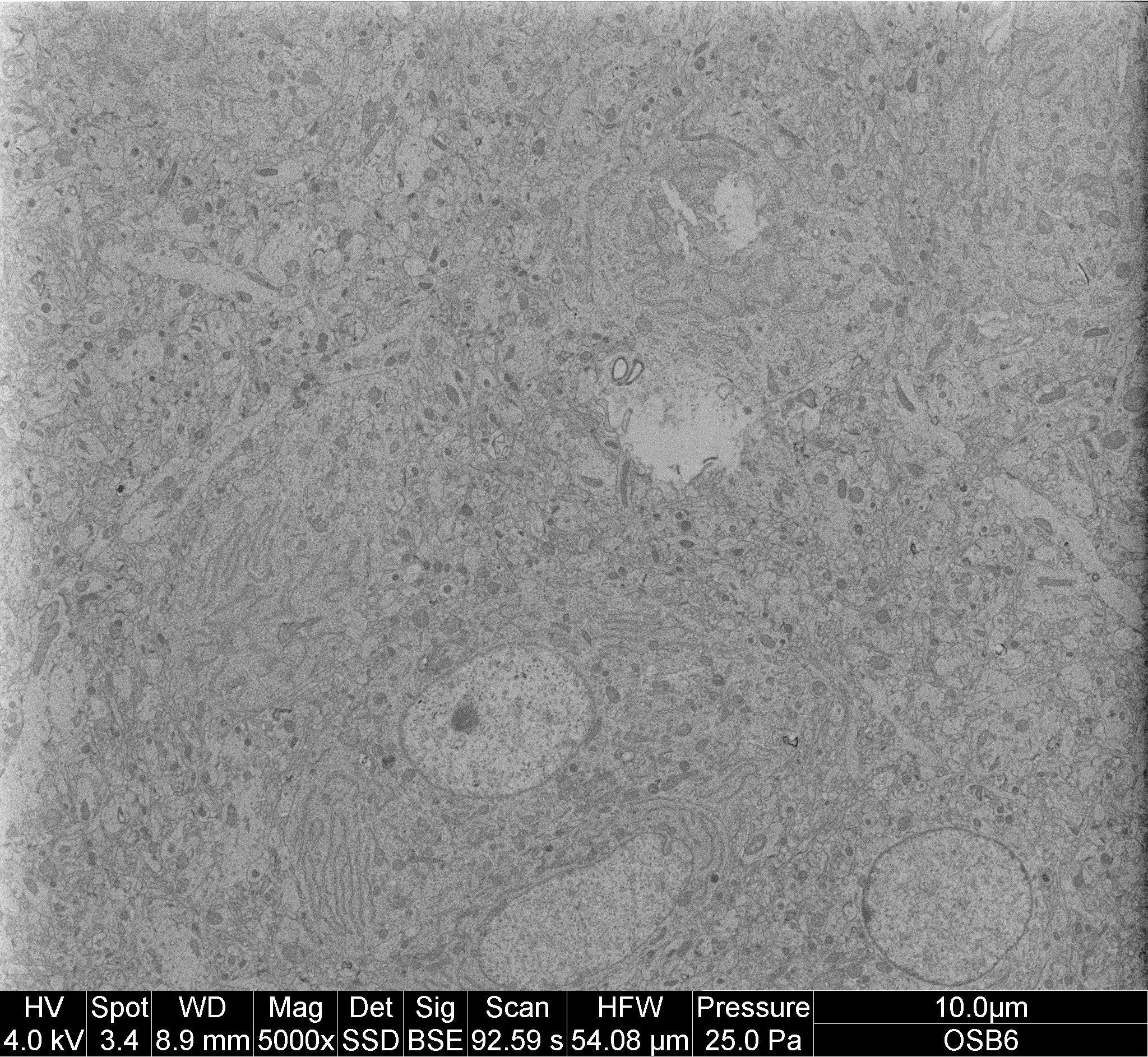

Supplement: Dataset S14 — (251.8 MB ZIP). [file pbio.0020329.sd014.zip › 040604_OS5_st1_1332.tif]

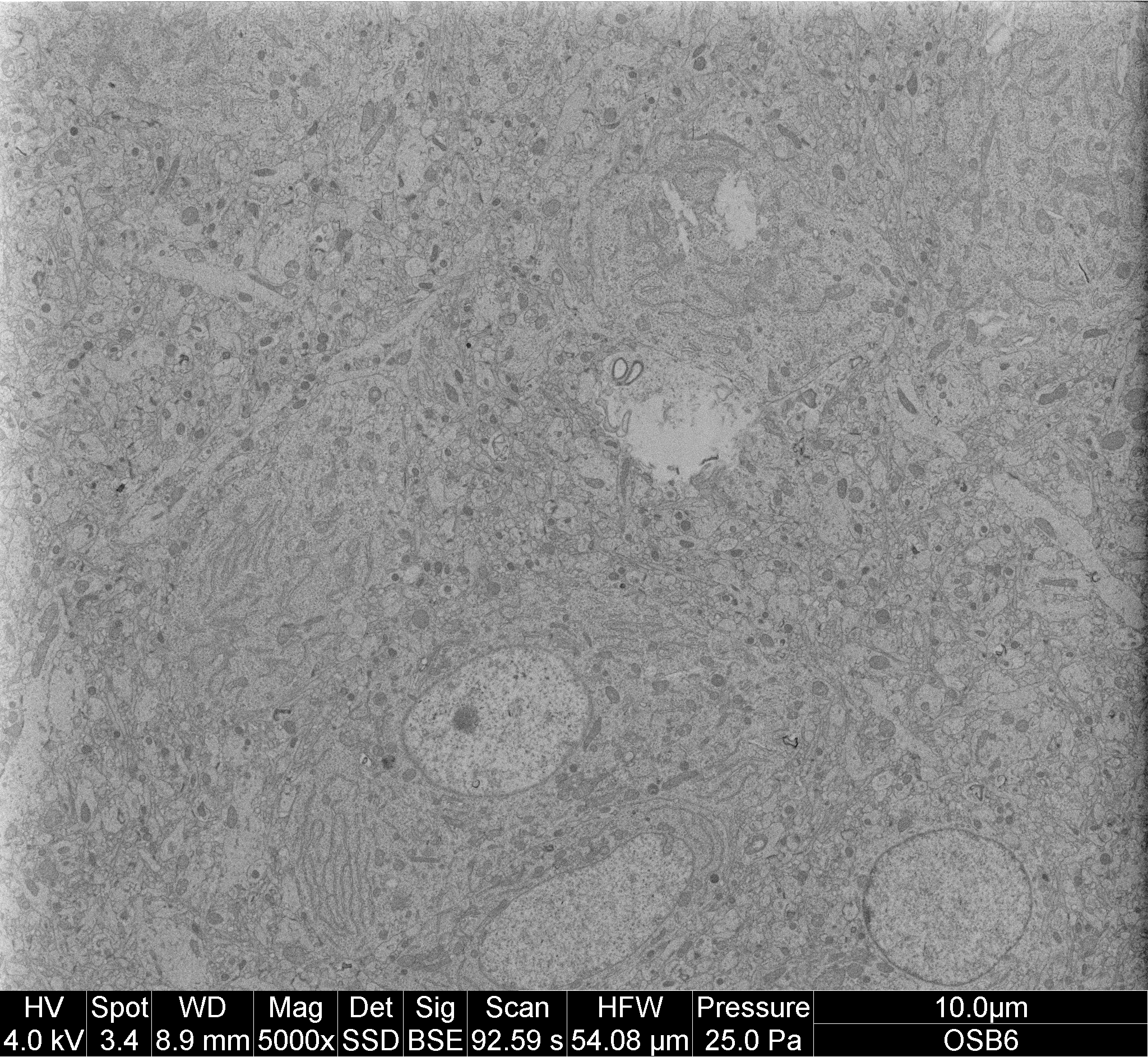

Supplement: Dataset S14 — (251.8 MB ZIP). [file pbio.0020329.sd014.zip › 040604_OS5_st1_1333.tif]

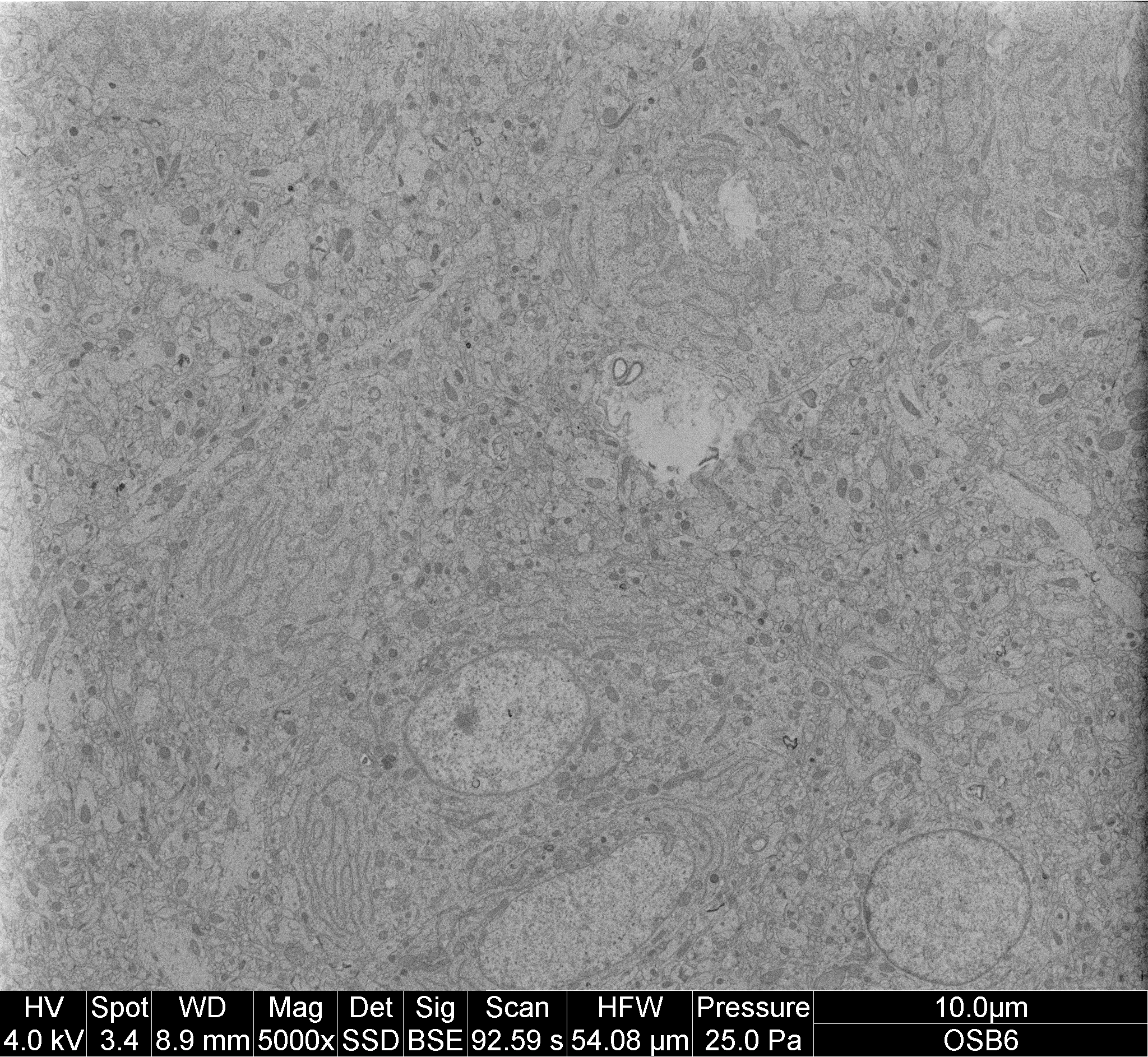

Supplement: Dataset S14 — (251.8 MB ZIP). [file pbio.0020329.sd014.zip › 040604_OS5_st1_1334.tif]

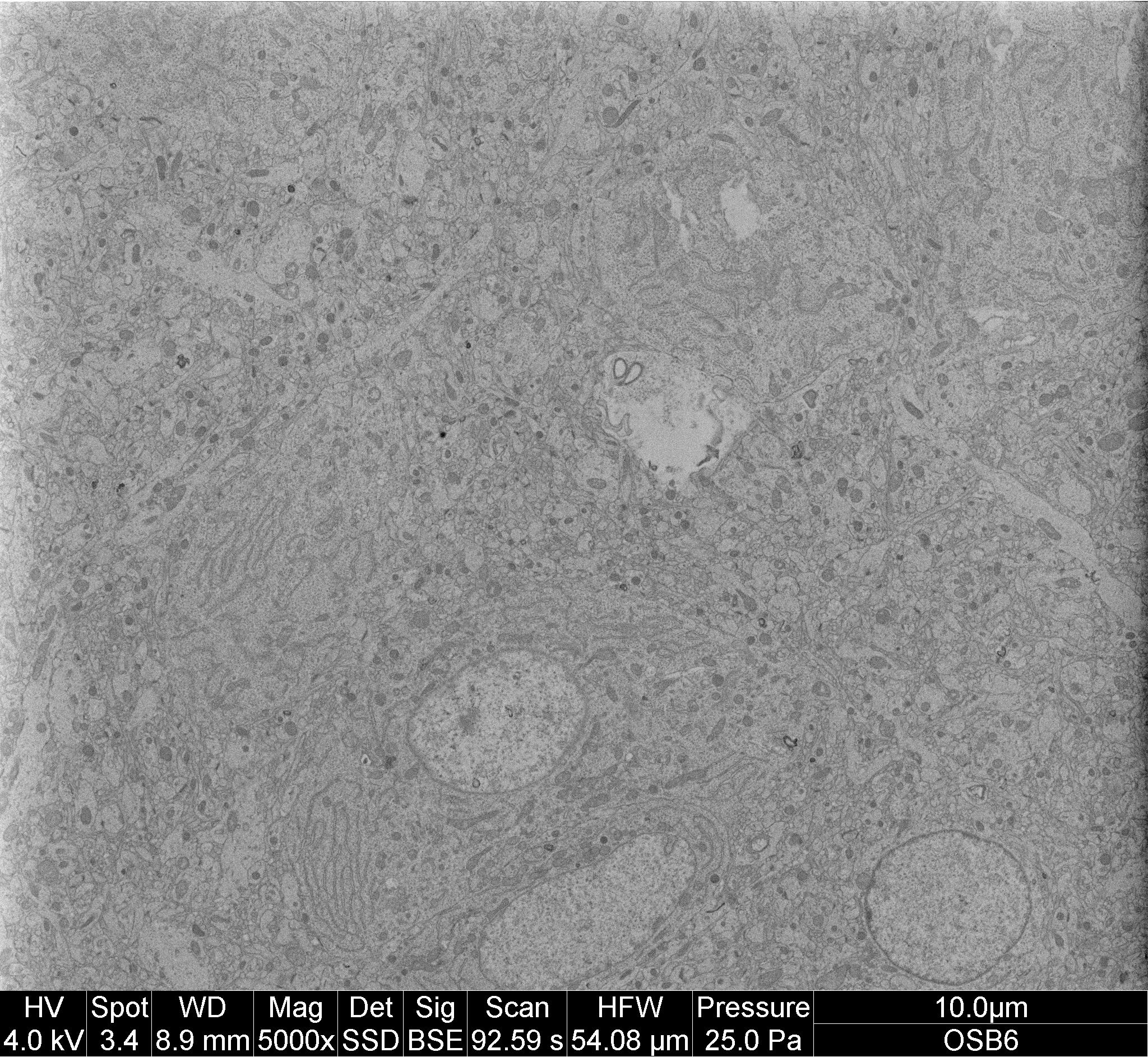

Supplement: Dataset S14 — (251.8 MB ZIP). [file pbio.0020329.sd014.zip › 040604_OS5_st1_1335.tif]

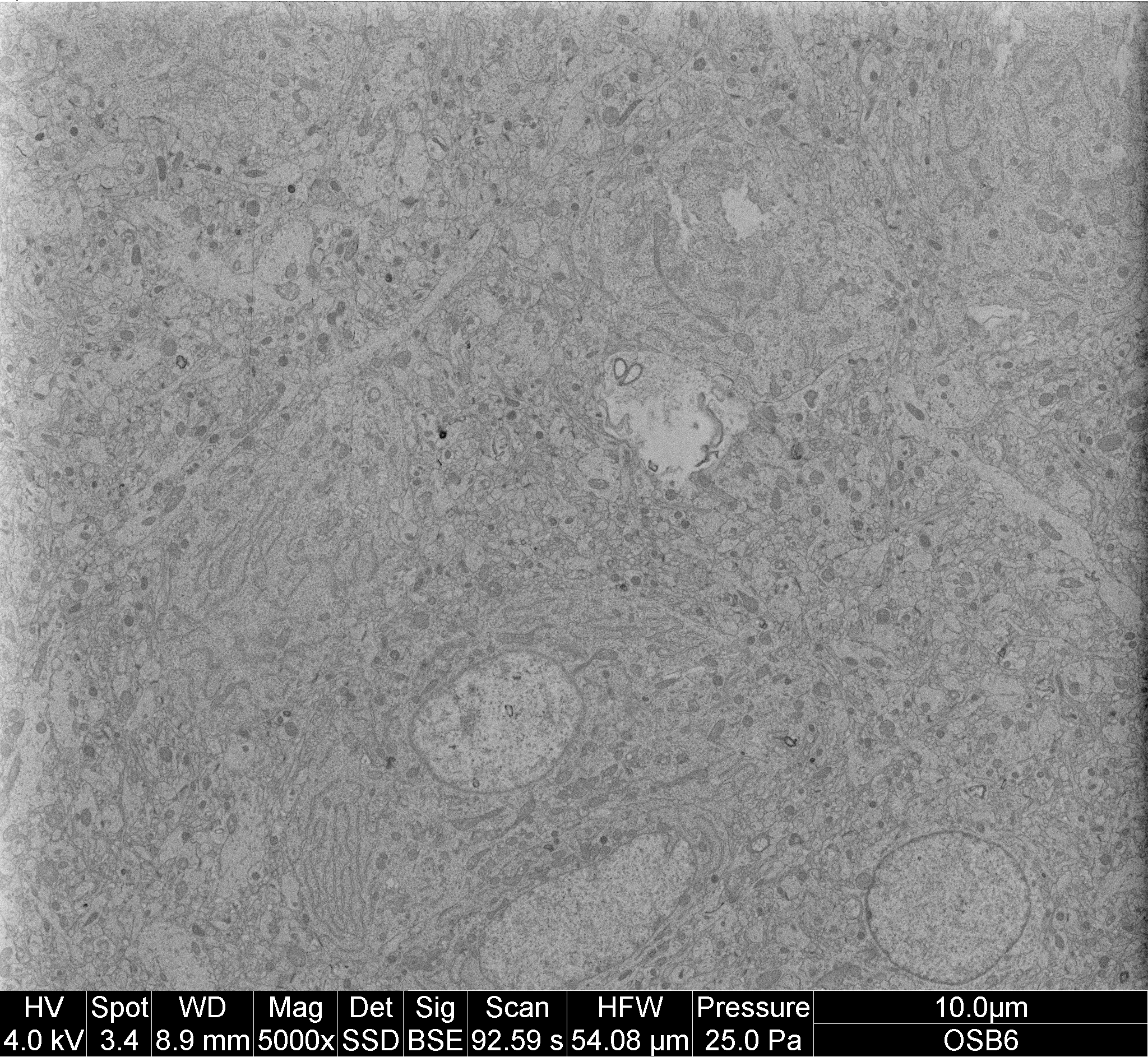

Supplement: Dataset S14 — (251.8 MB ZIP). [file pbio.0020329.sd014.zip › 040604_OS5_st1_1336.tif]

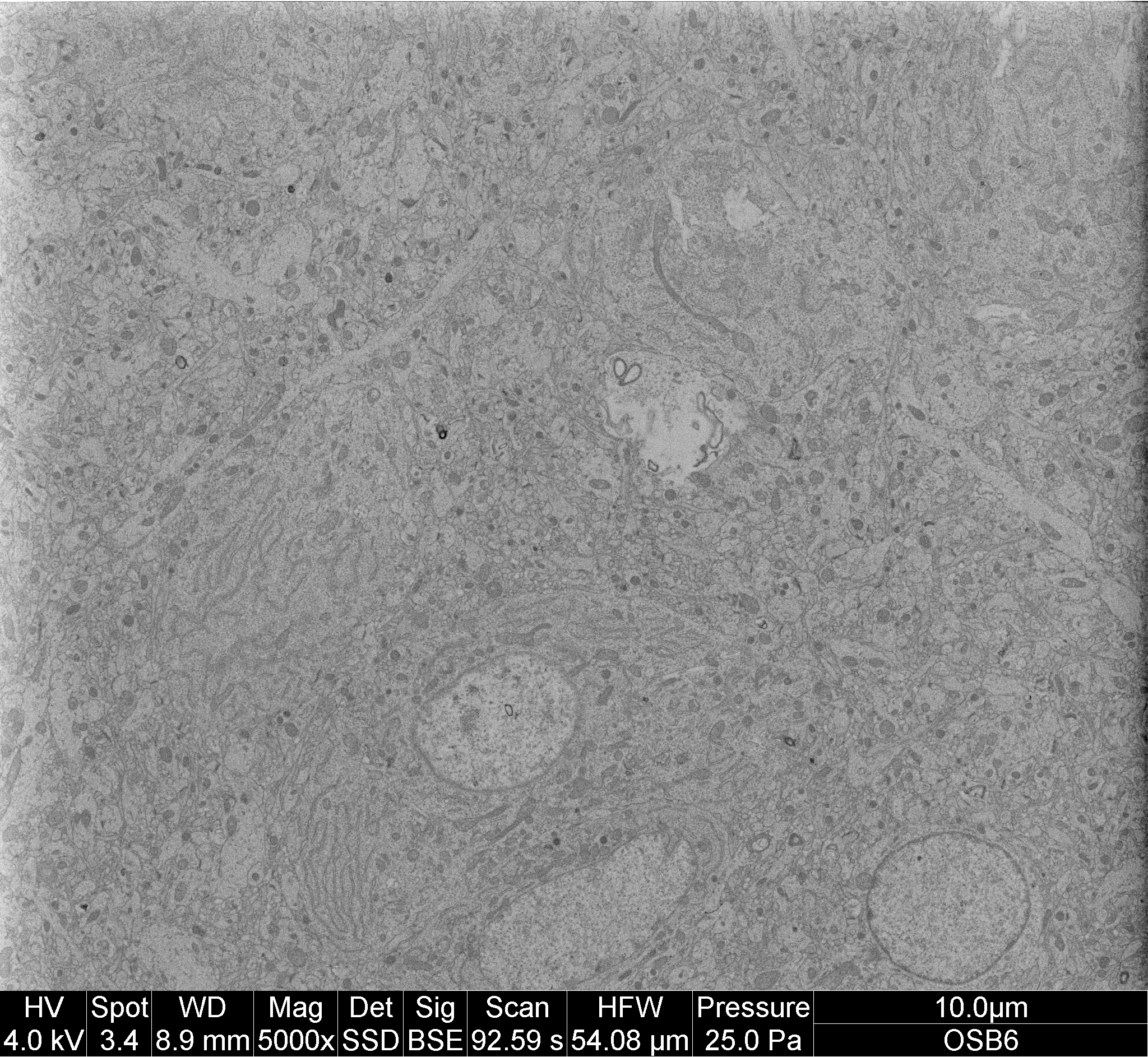

Supplement: Dataset S14 — (251.8 MB ZIP). [file pbio.0020329.sd014.zip › 040604_OS5_st1_1337.tif]

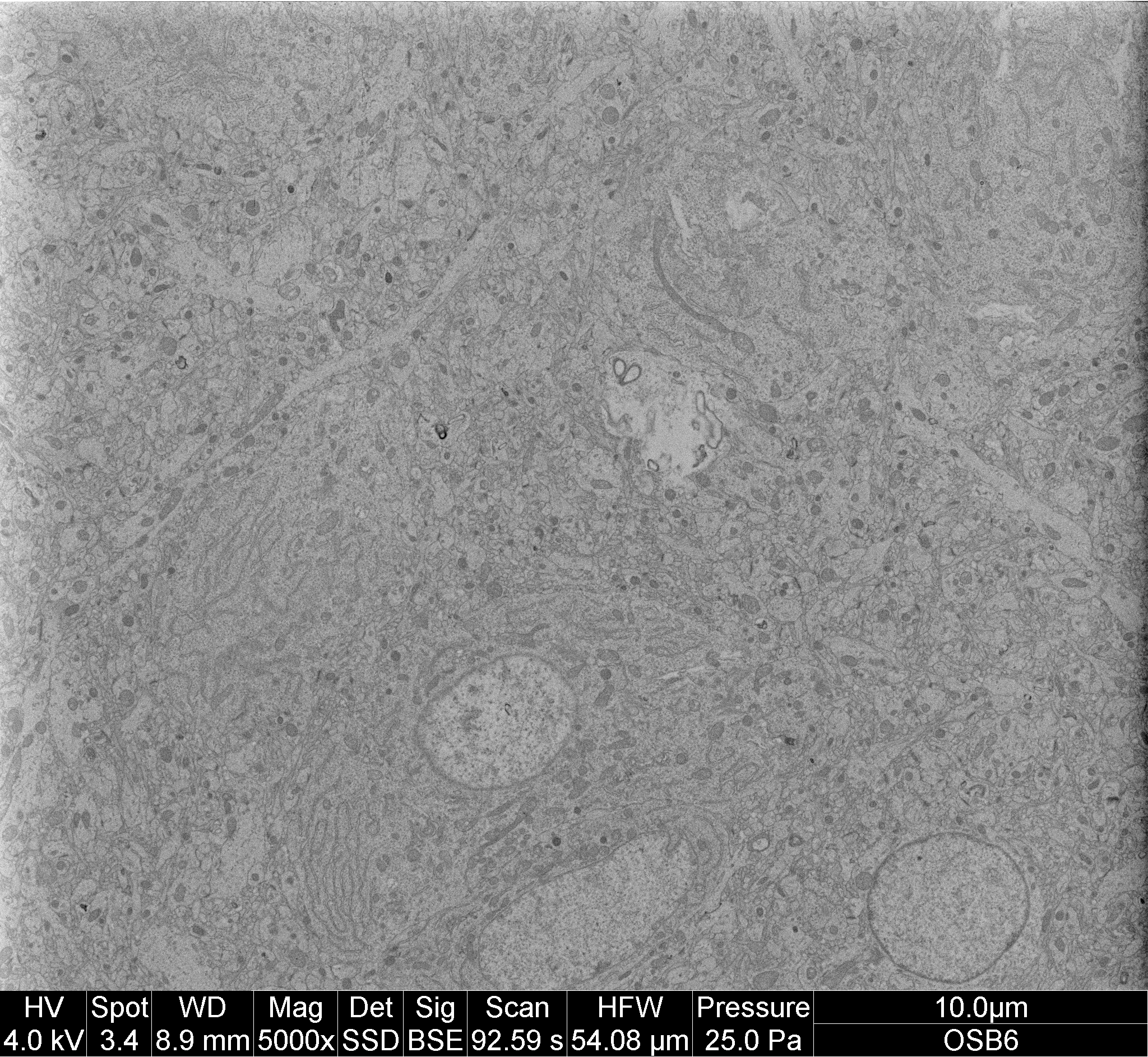

Supplement: Dataset S14 — (251.8 MB ZIP). [file pbio.0020329.sd014.zip › 040604_OS5_st1_1338.tif]

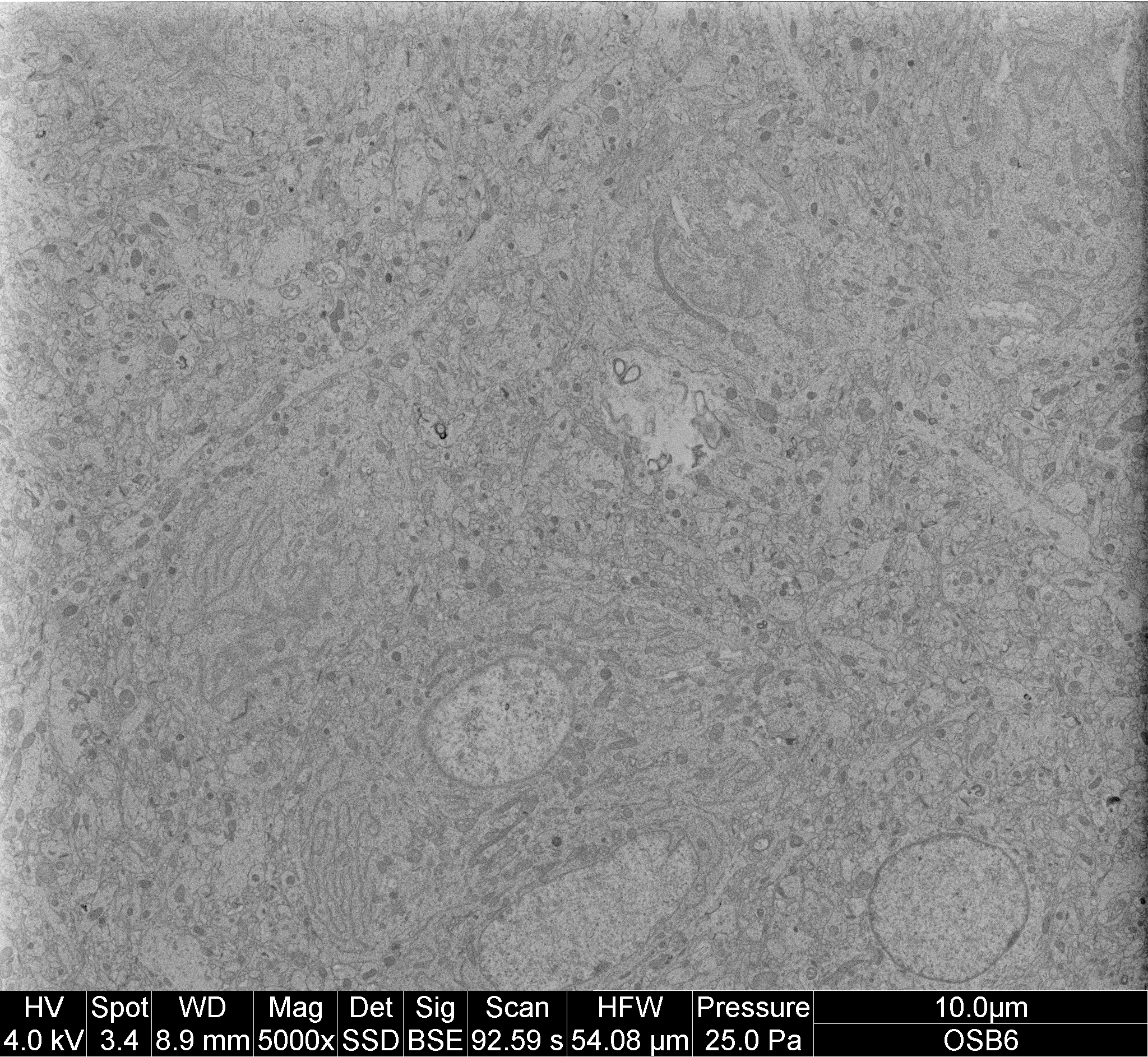

Supplement: Dataset S14 — (251.8 MB ZIP). [file pbio.0020329.sd014.zip › 040604_OS5_st1_1339.tif]

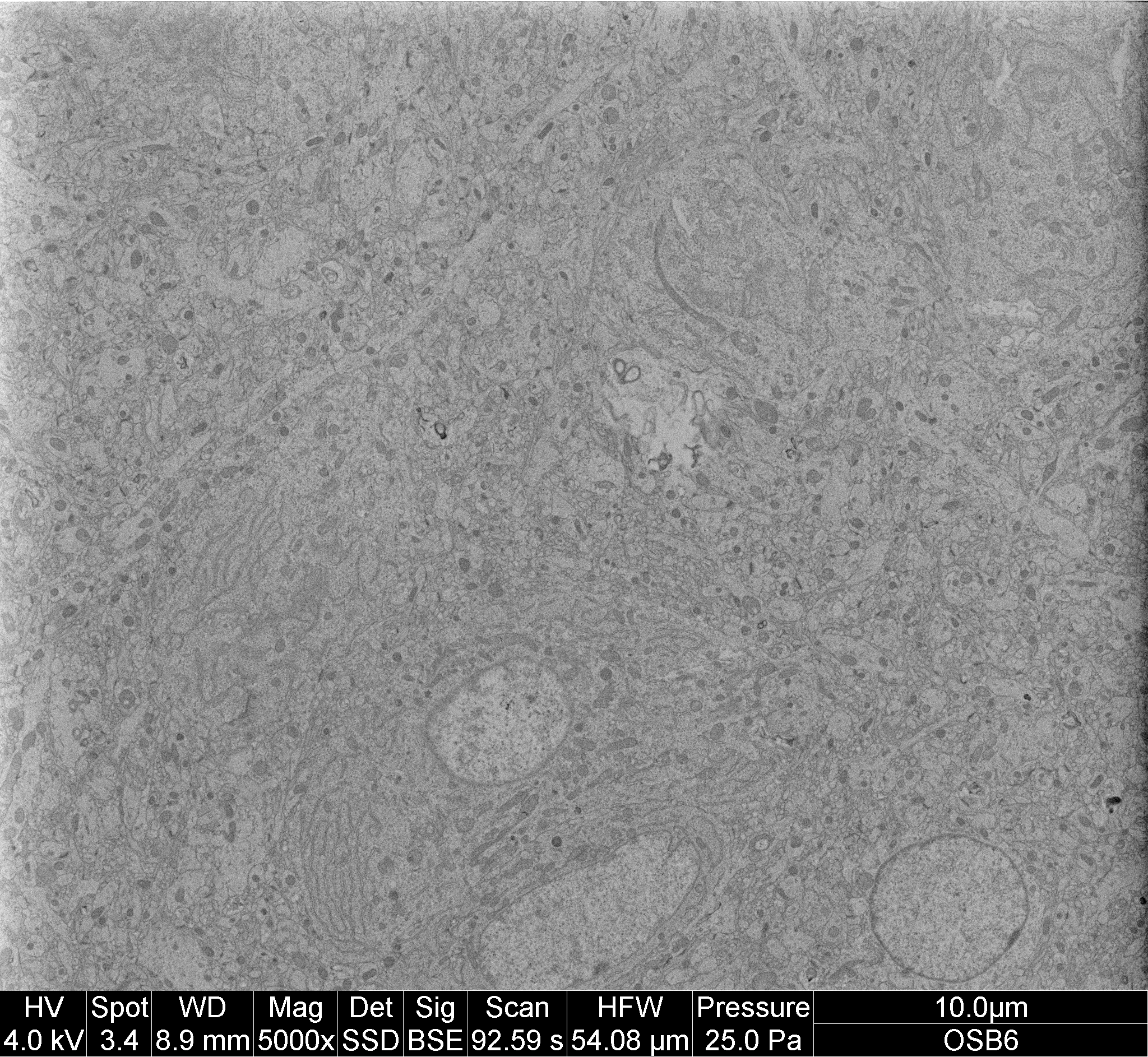

Supplement: Dataset S14 — (251.8 MB ZIP). [file pbio.0020329.sd014.zip › 040604_OS5_st1_1340.tif]

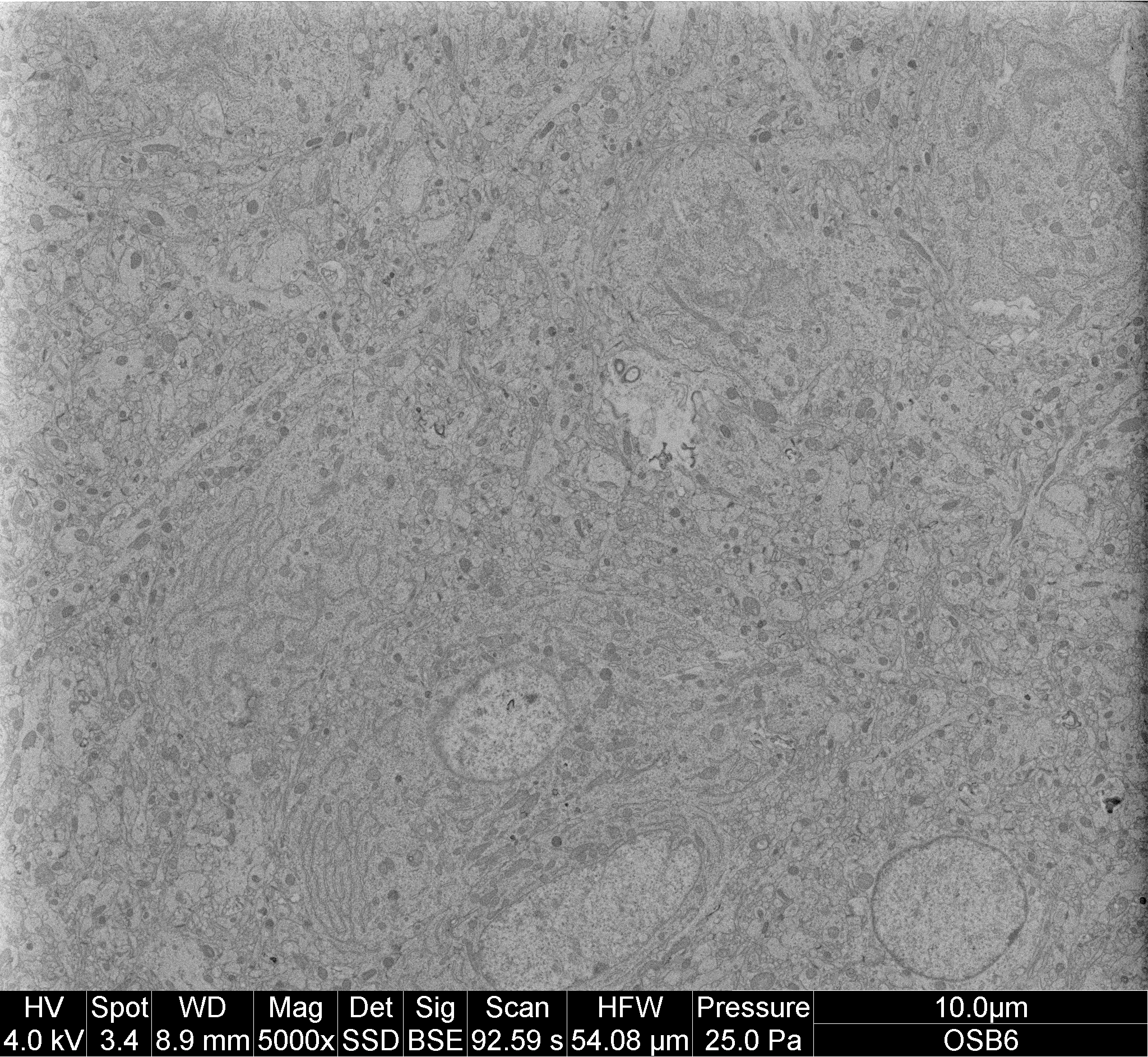

Supplement: Dataset S14 — (251.8 MB ZIP). [file pbio.0020329.sd014.zip › 040604_OS5_st1_1341.tif]

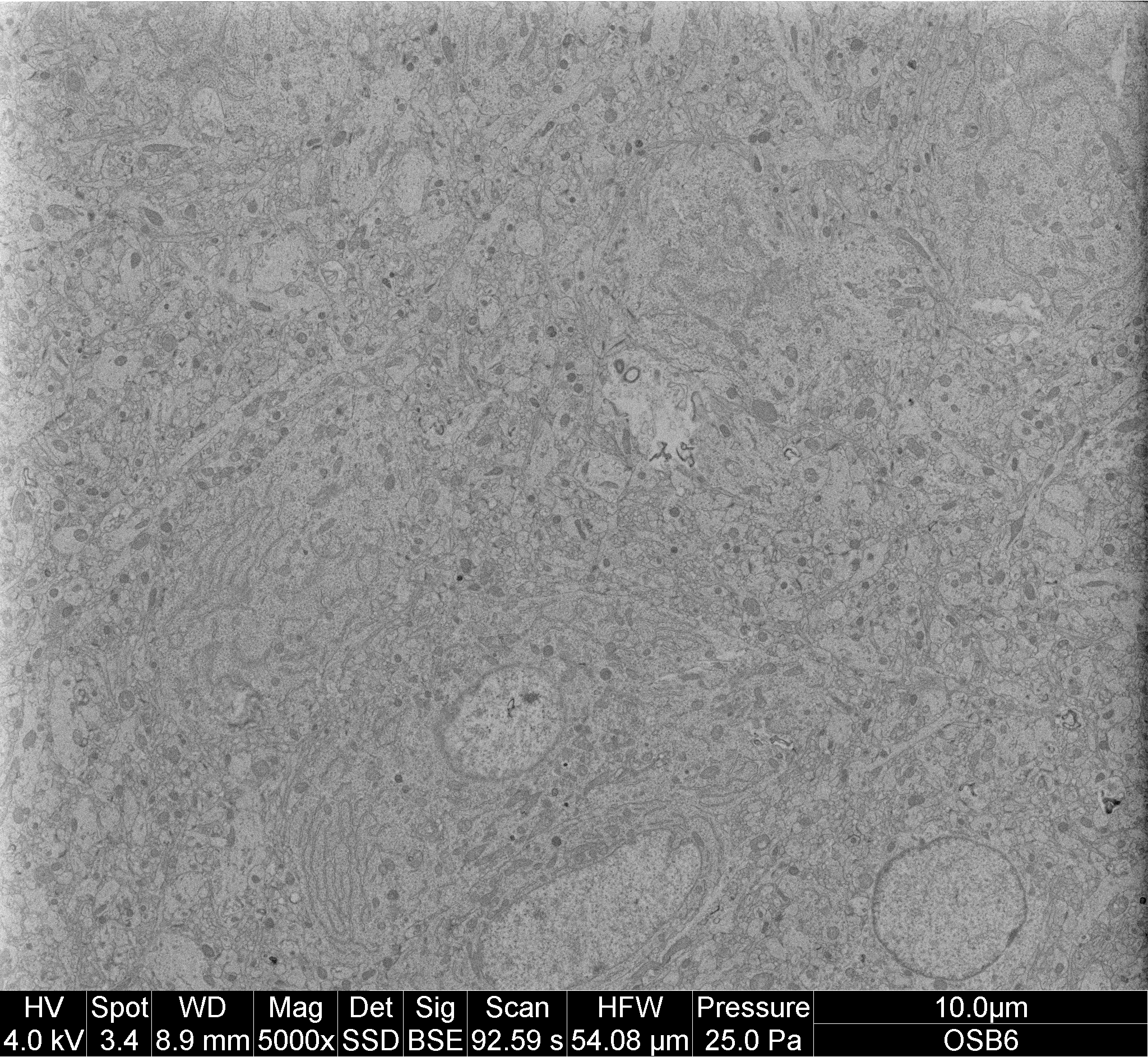

Supplement: Dataset S14 — (251.8 MB ZIP). [file pbio.0020329.sd014.zip › 040604_OS5_st1_1342.tif]

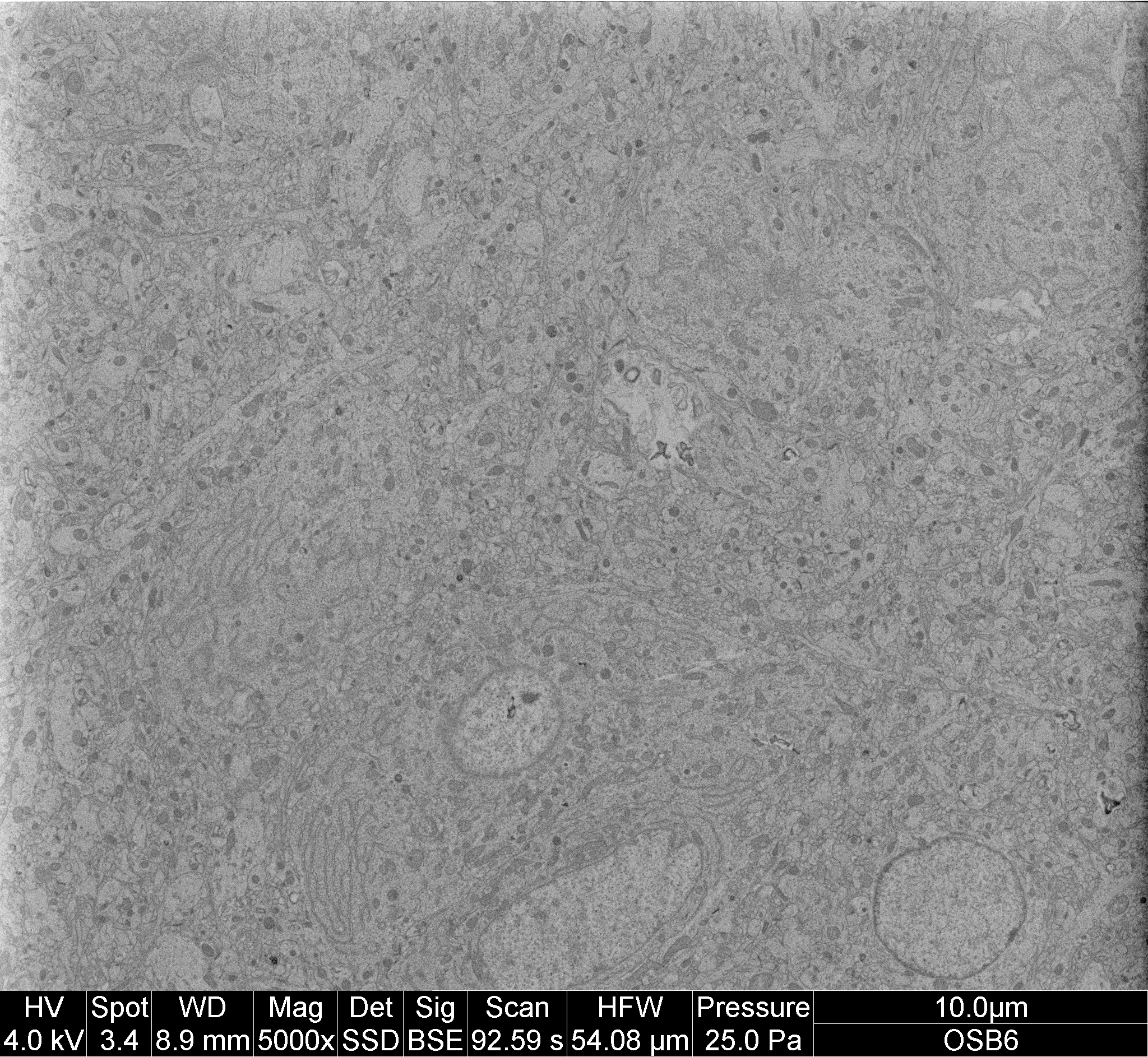

Supplement: Dataset S14 — (251.8 MB ZIP). [file pbio.0020329.sd014.zip › 040604_OS5_st1_1343.tif]

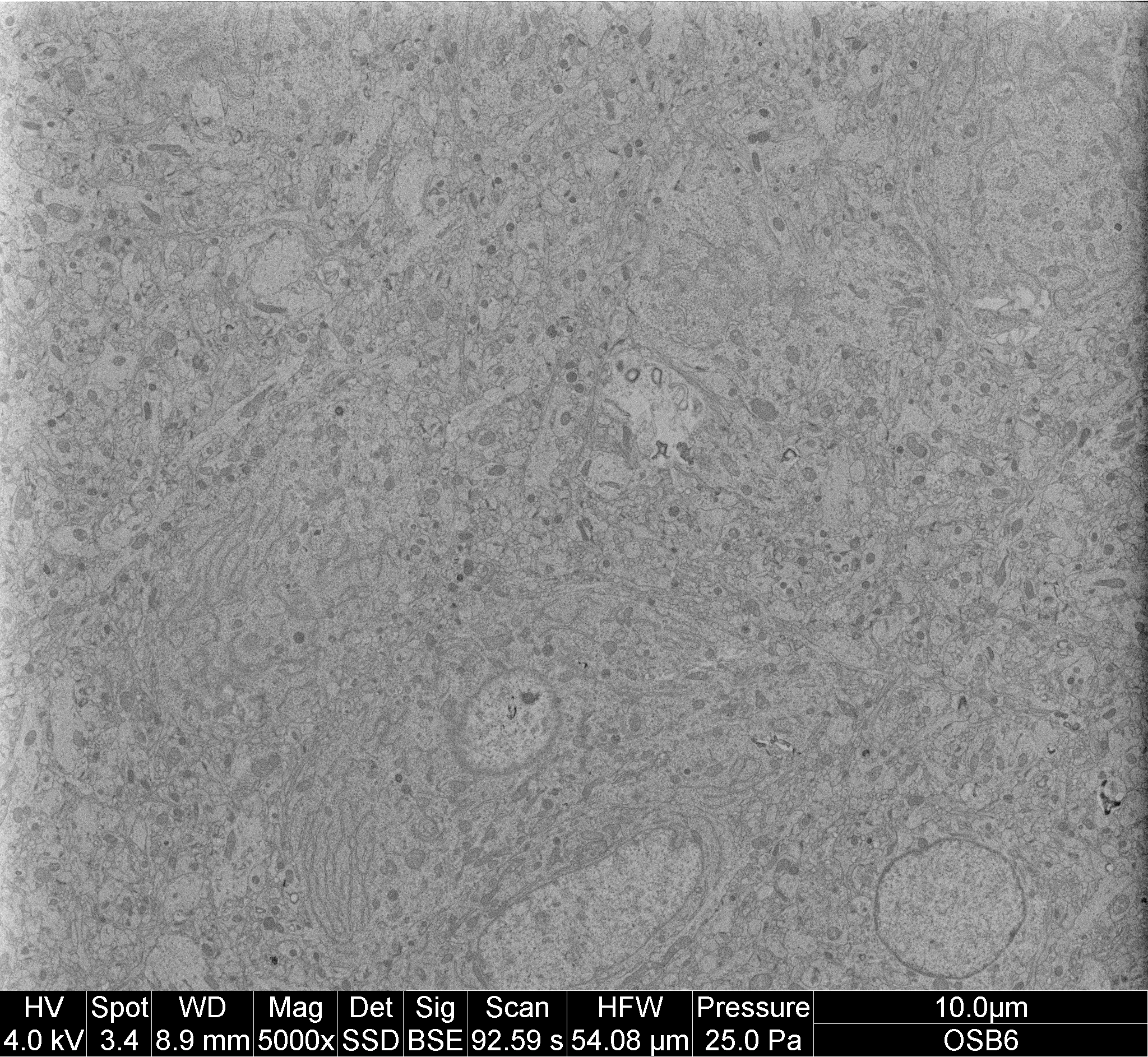

Supplement: Dataset S14 — (251.8 MB ZIP). [file pbio.0020329.sd014.zip › 040604_OS5_st1_1344.tif]

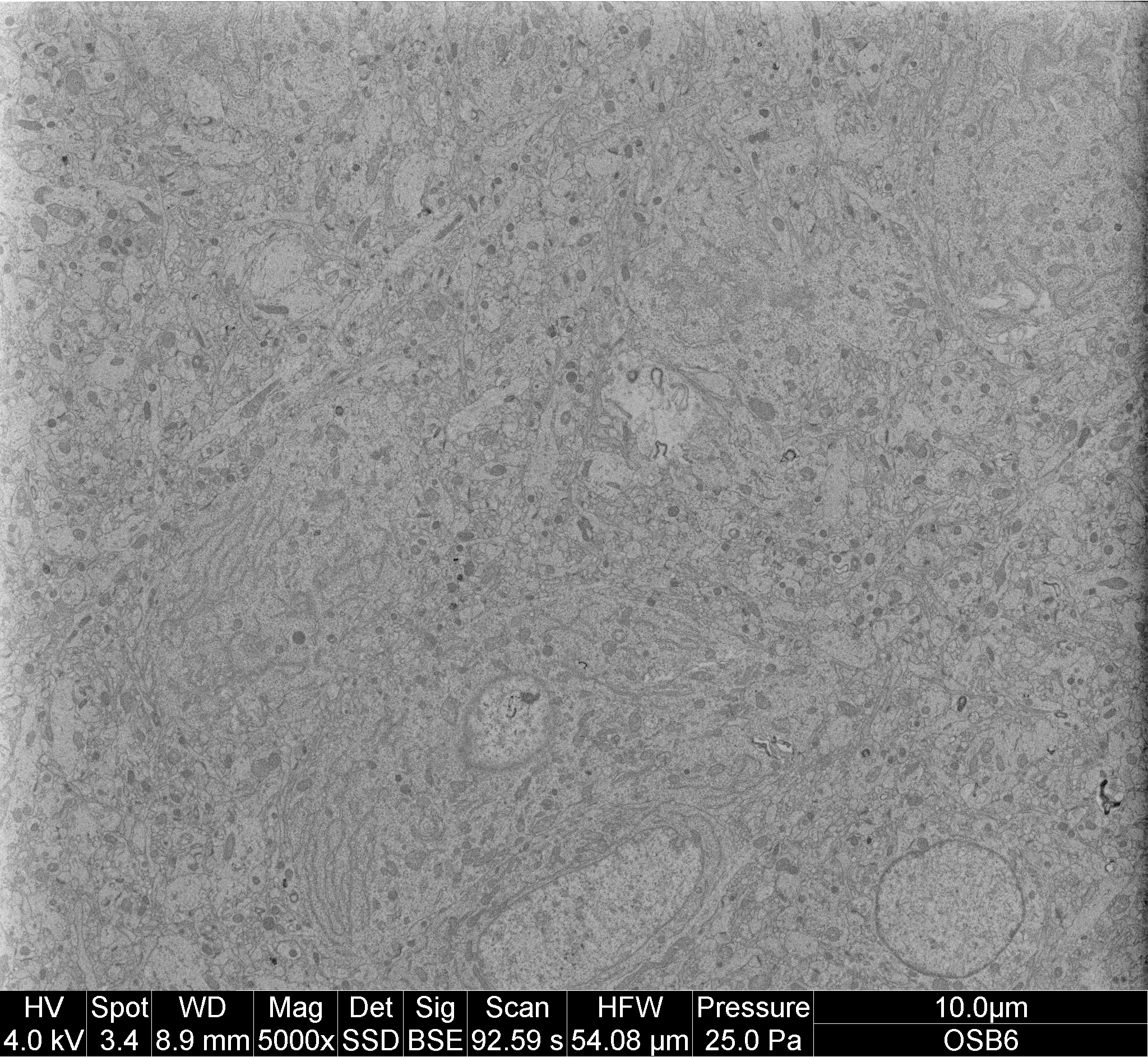

Supplement: Dataset S14 — (251.8 MB ZIP). [file pbio.0020329.sd014.zip › 040604_OS5_st1_1345.tif]

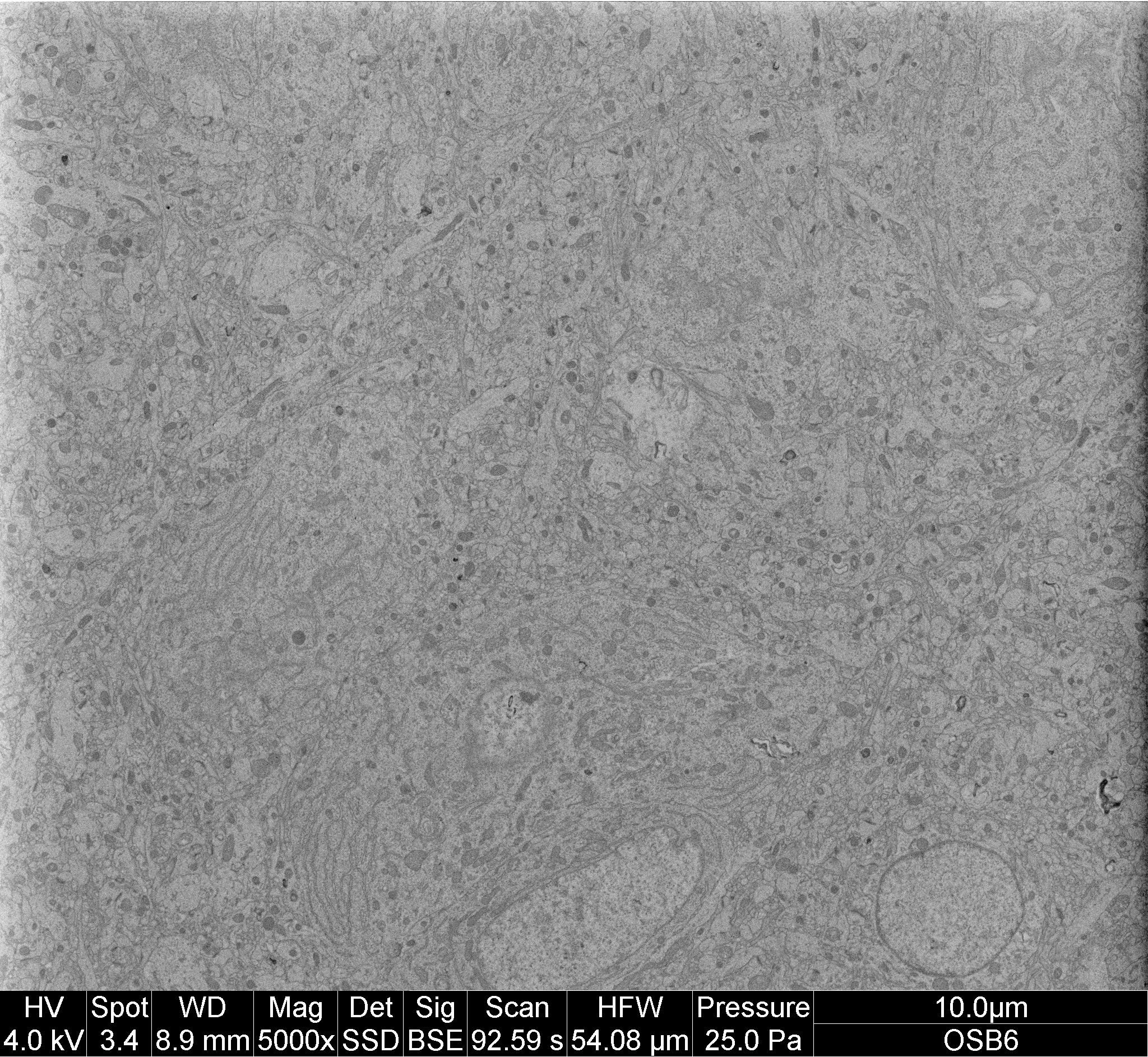

Supplement: Dataset S14 — (251.8 MB ZIP). [file pbio.0020329.sd014.zip › 040604_OS5_st1_1346.tif]

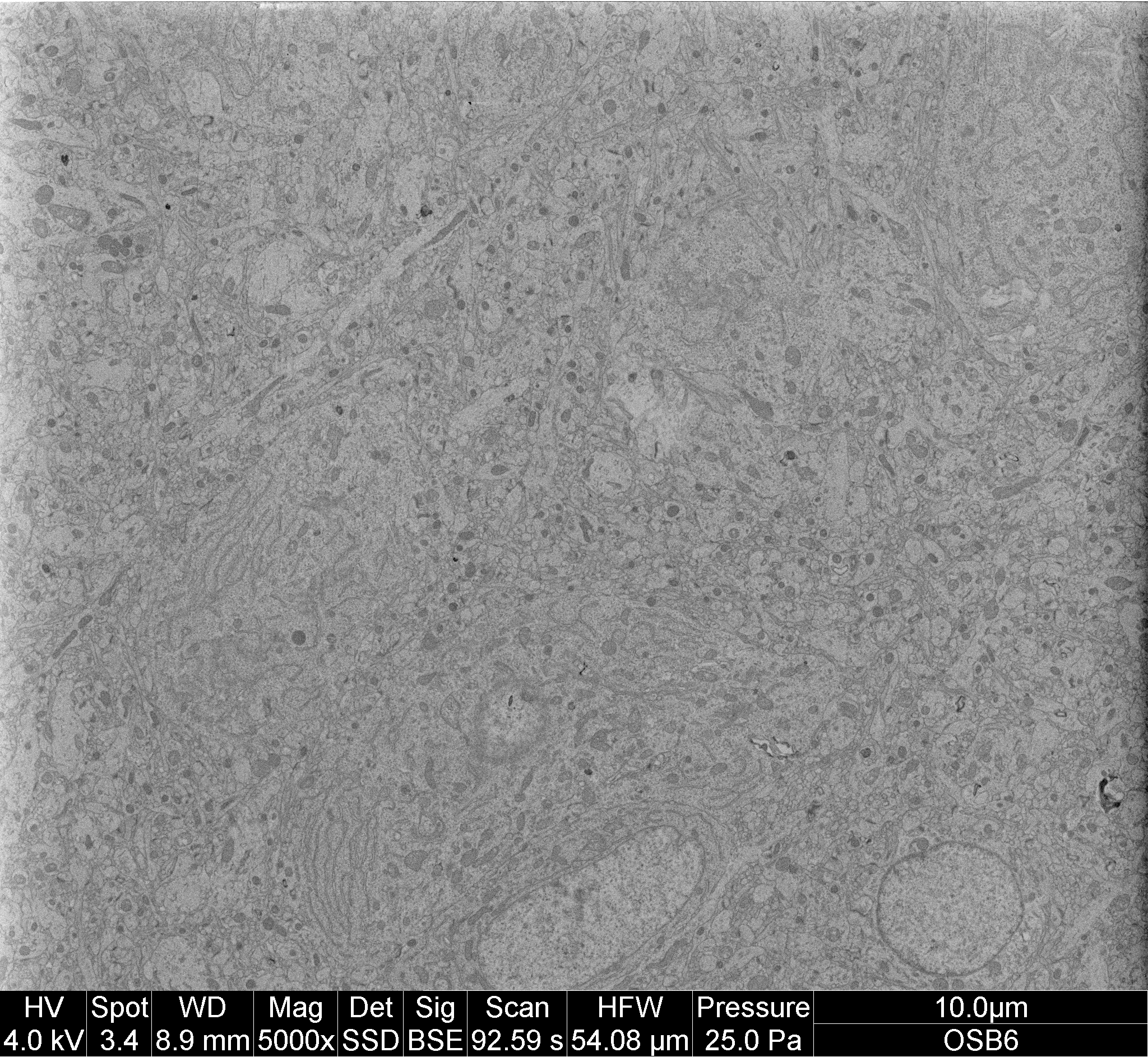

Supplement: Dataset S14 — (251.8 MB ZIP). [file pbio.0020329.sd014.zip › 040604_OS5_st1_1347.tif]

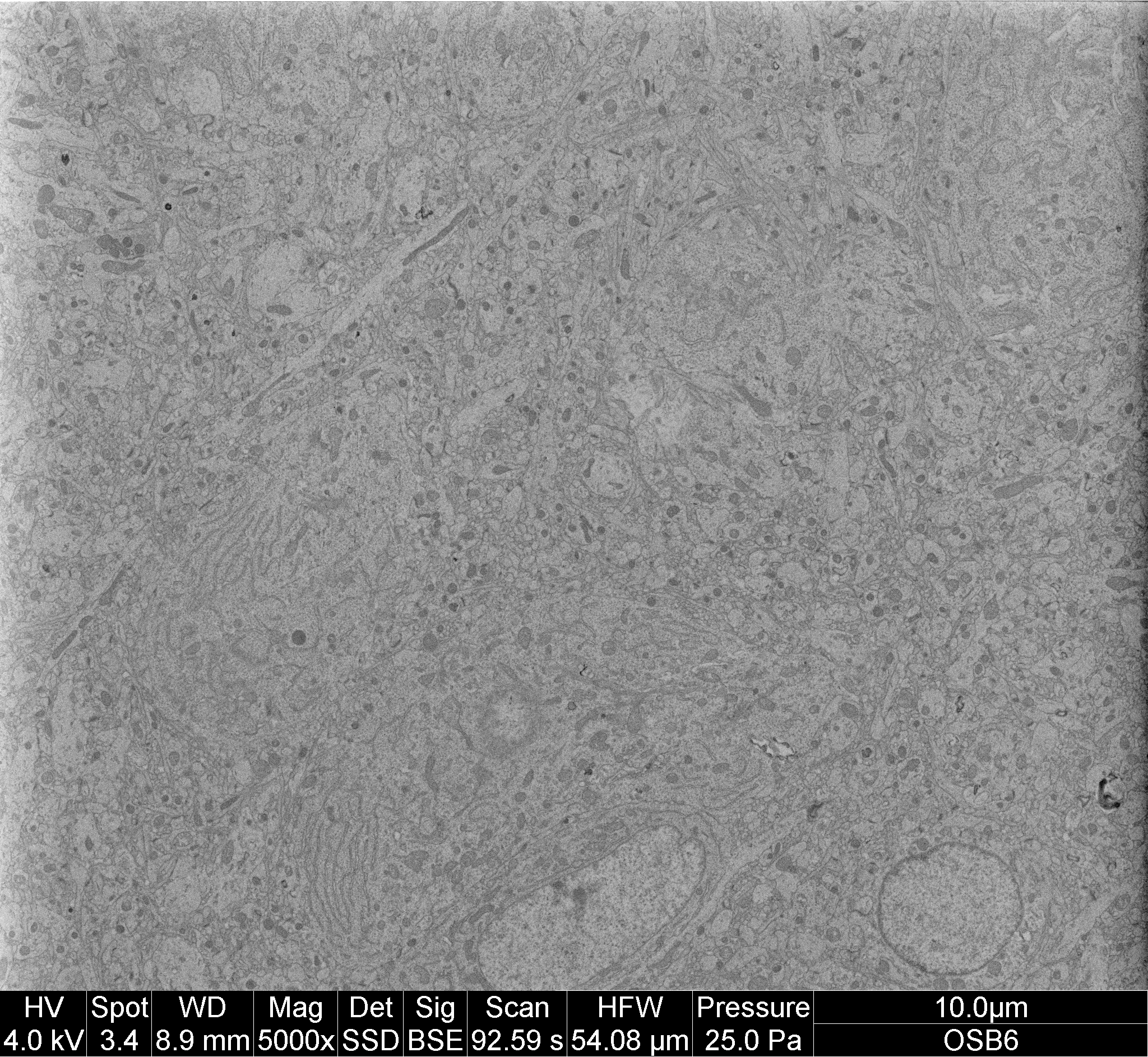

Supplement: Dataset S14 — (251.8 MB ZIP). [file pbio.0020329.sd014.zip › 040604_OS5_st1_1348.tif]

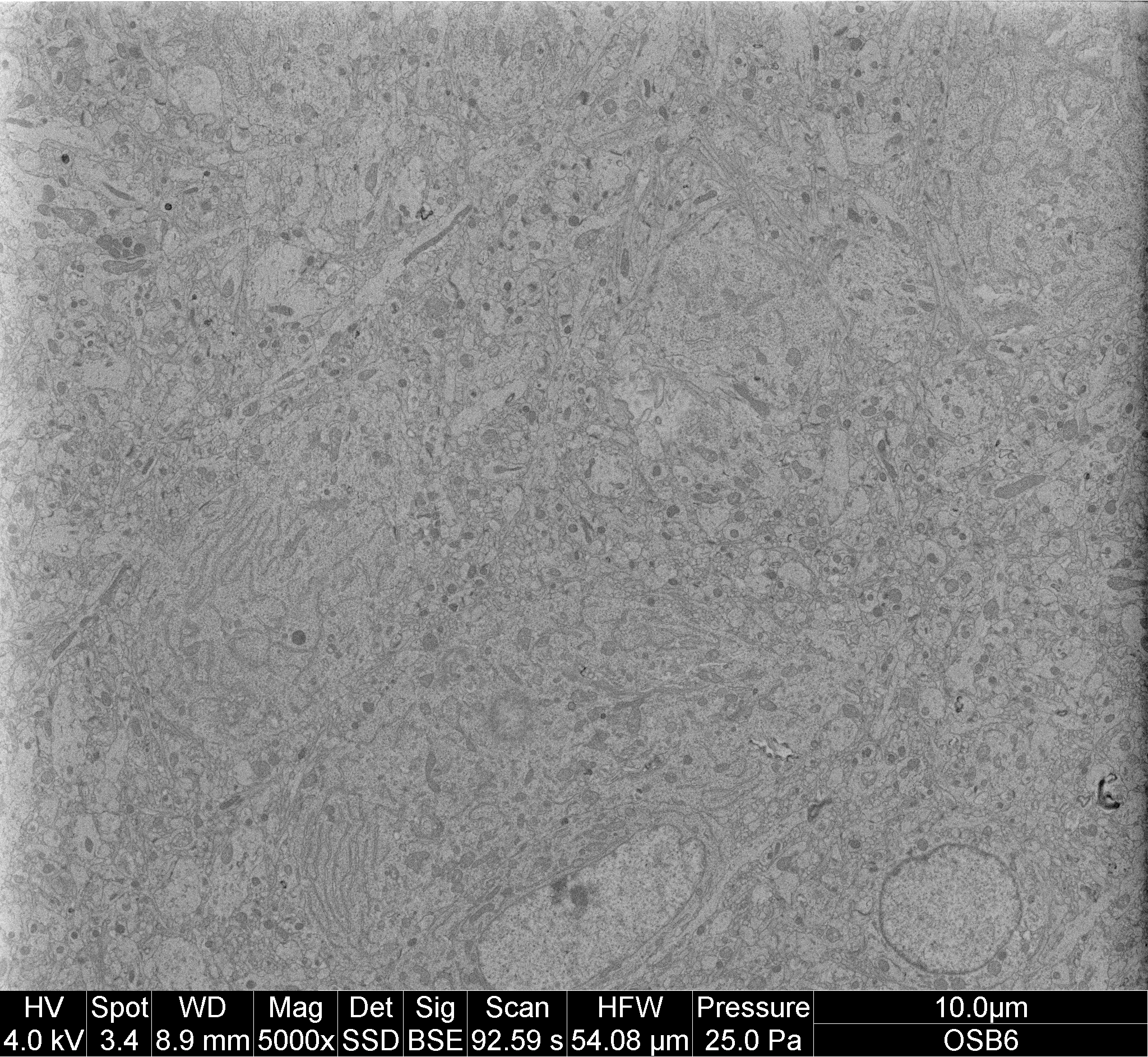

Supplement: Dataset S14 — (251.8 MB ZIP). [file pbio.0020329.sd014.zip › 040604_OS5_st1_1349.tif]

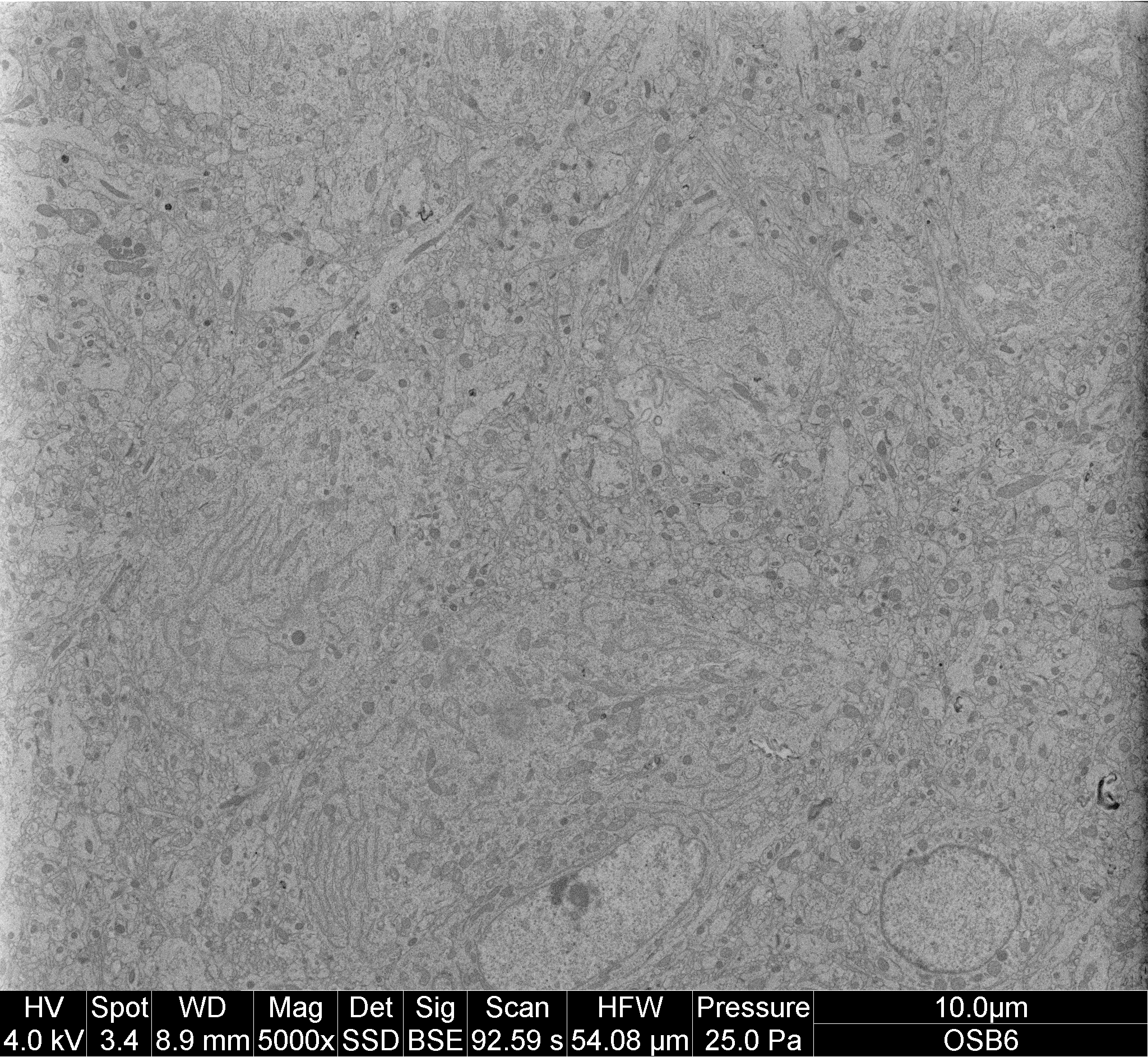

Supplement: Dataset S14 — (251.8 MB ZIP). [file pbio.0020329.sd014.zip › 040604_OS5_st1_1350.tif]

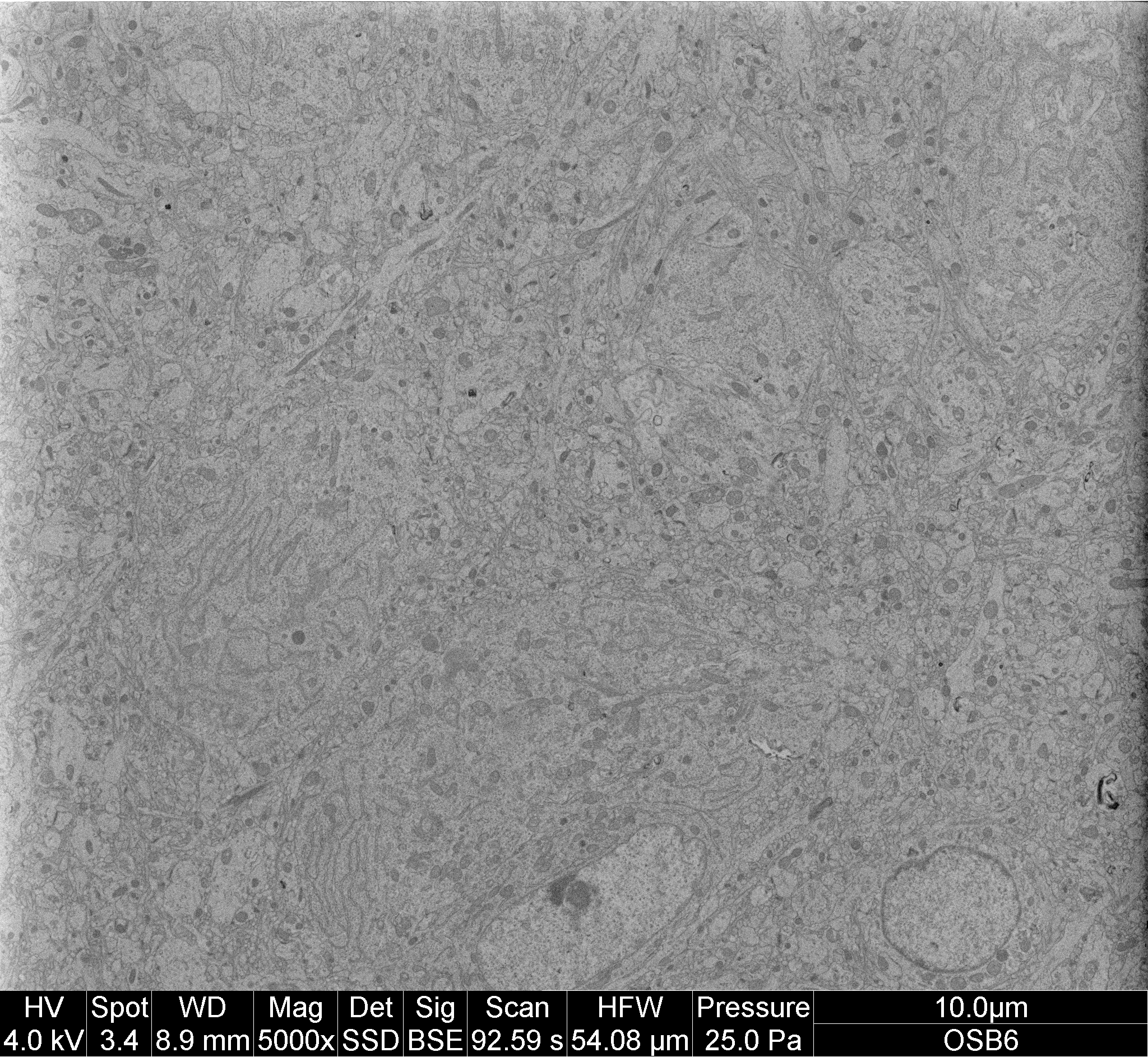

Supplement: Dataset S14 — (251.8 MB ZIP). [file pbio.0020329.sd014.zip › 040604_OS5_st1_1351.tif]

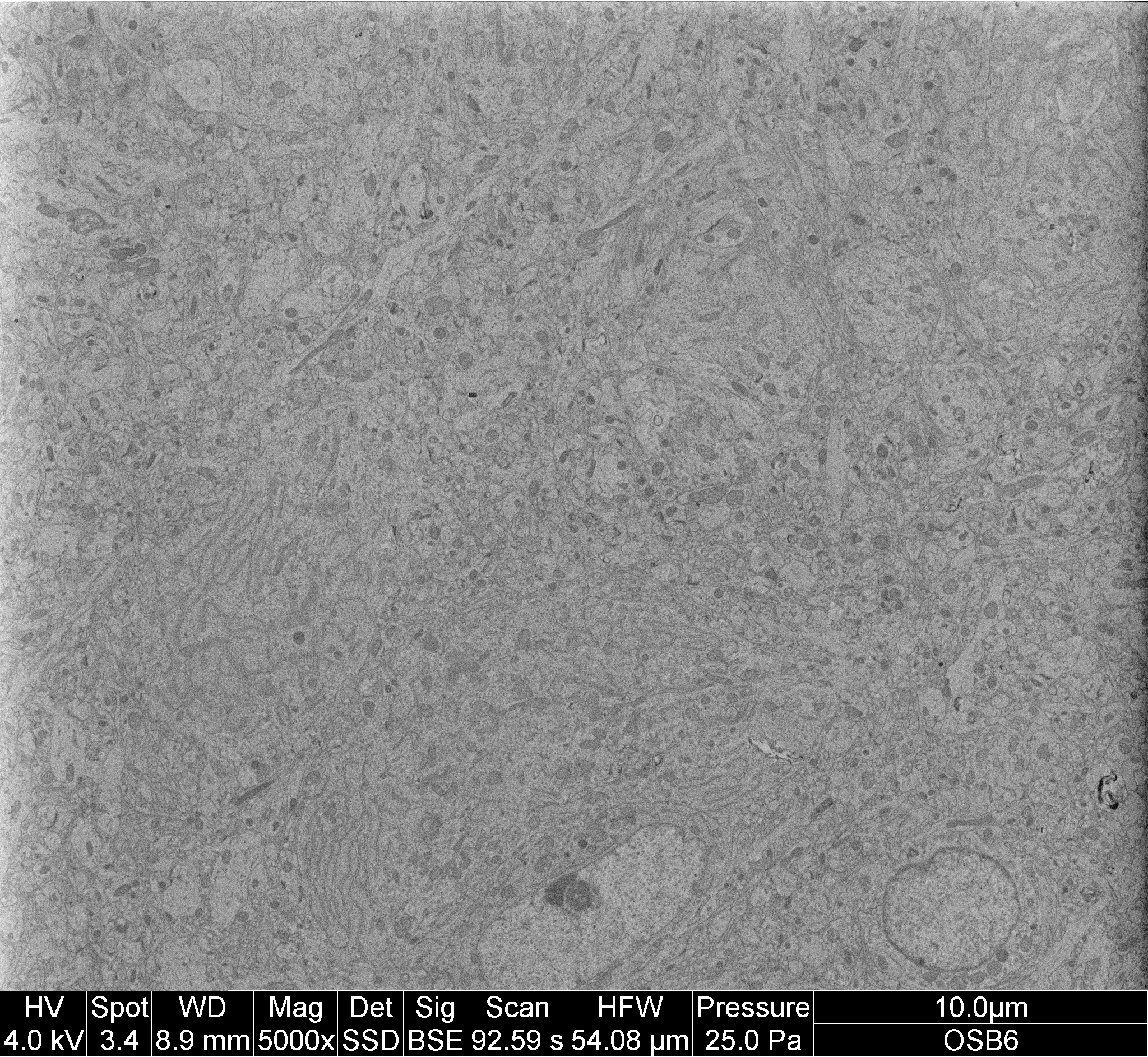

Supplement: Dataset S14 — (251.8 MB ZIP). [file pbio.0020329.sd014.zip › 040604_OS5_st1_1352.tif]

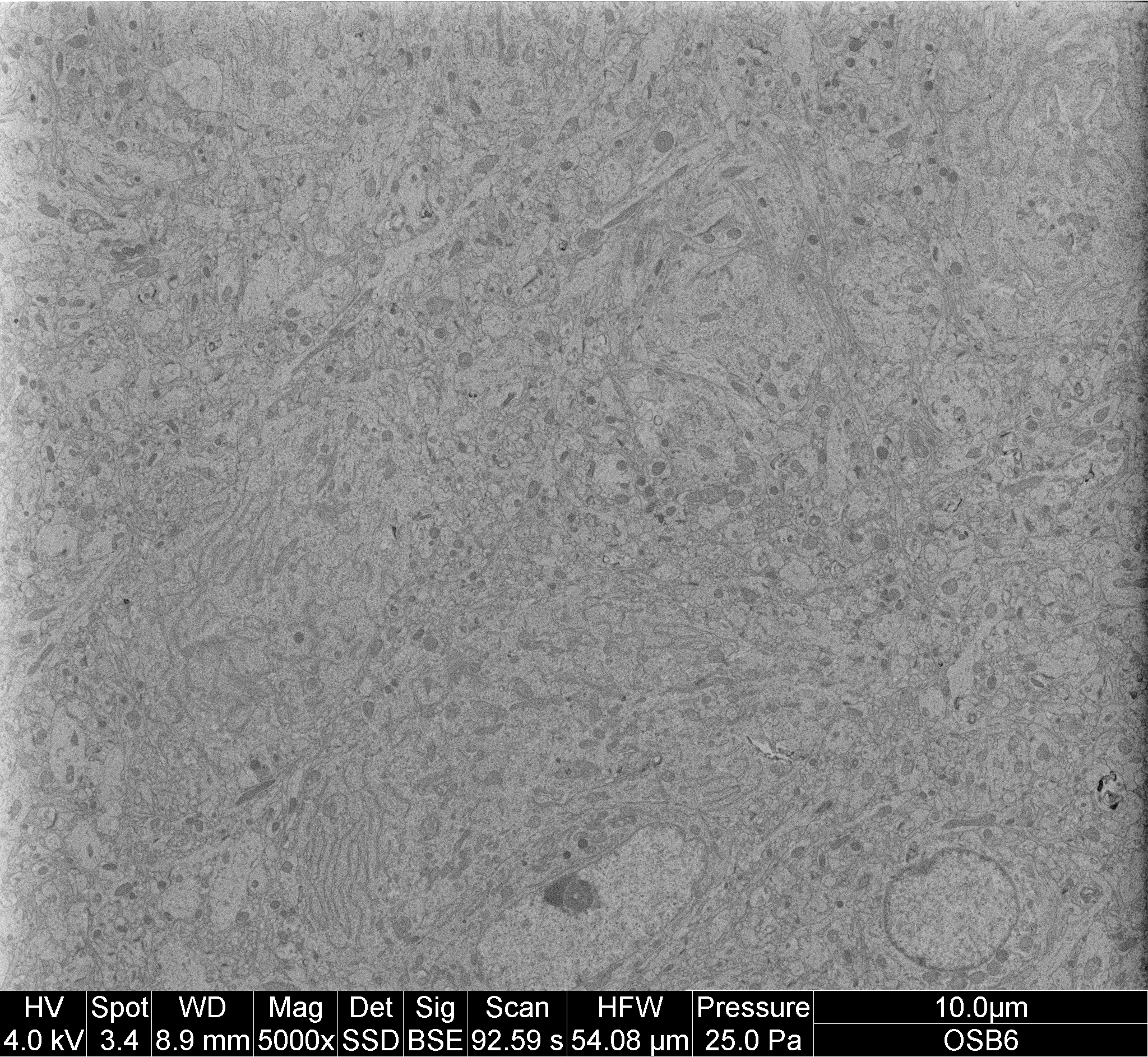

Supplement: Dataset S14 — (251.8 MB ZIP). [file pbio.0020329.sd014.zip › 040604_OS5_st1_1353.tif]

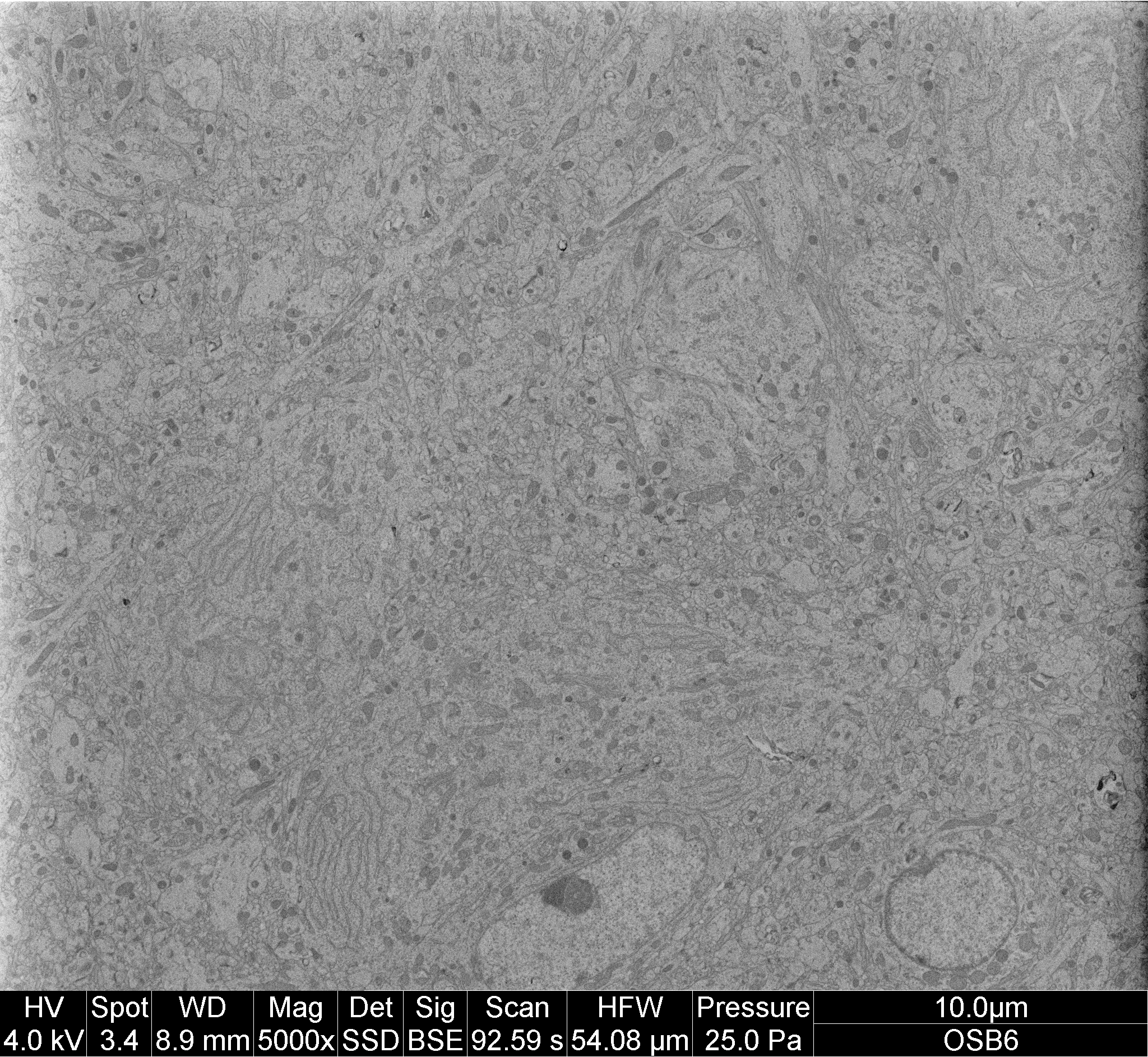

Supplement: Dataset S14 — (251.8 MB ZIP). [file pbio.0020329.sd014.zip › 040604_OS5_st1_1354.tif]

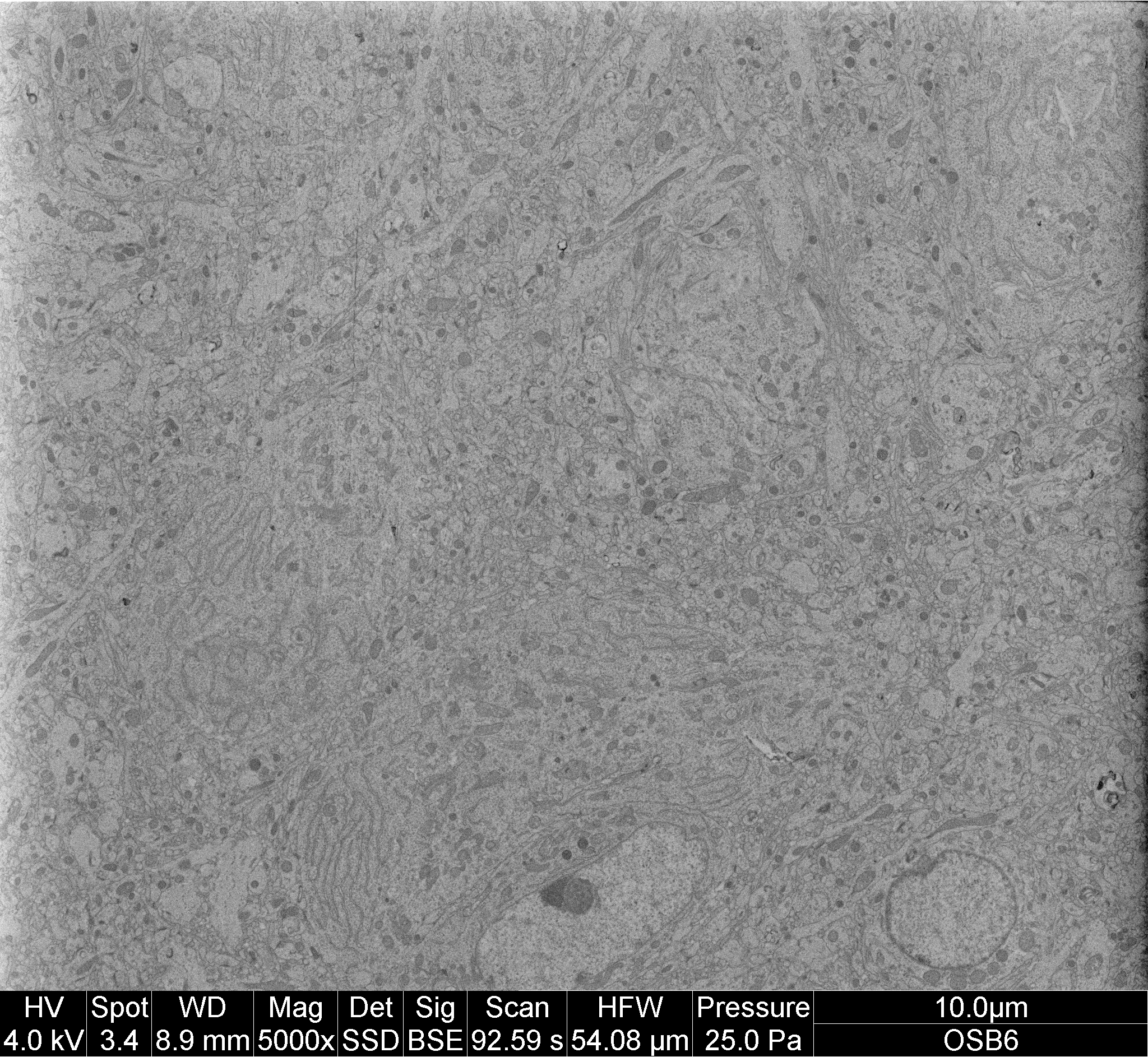

Supplement: Dataset S14 — (251.8 MB ZIP). [file pbio.0020329.sd014.zip › 040604_OS5_st1_1355.tif]

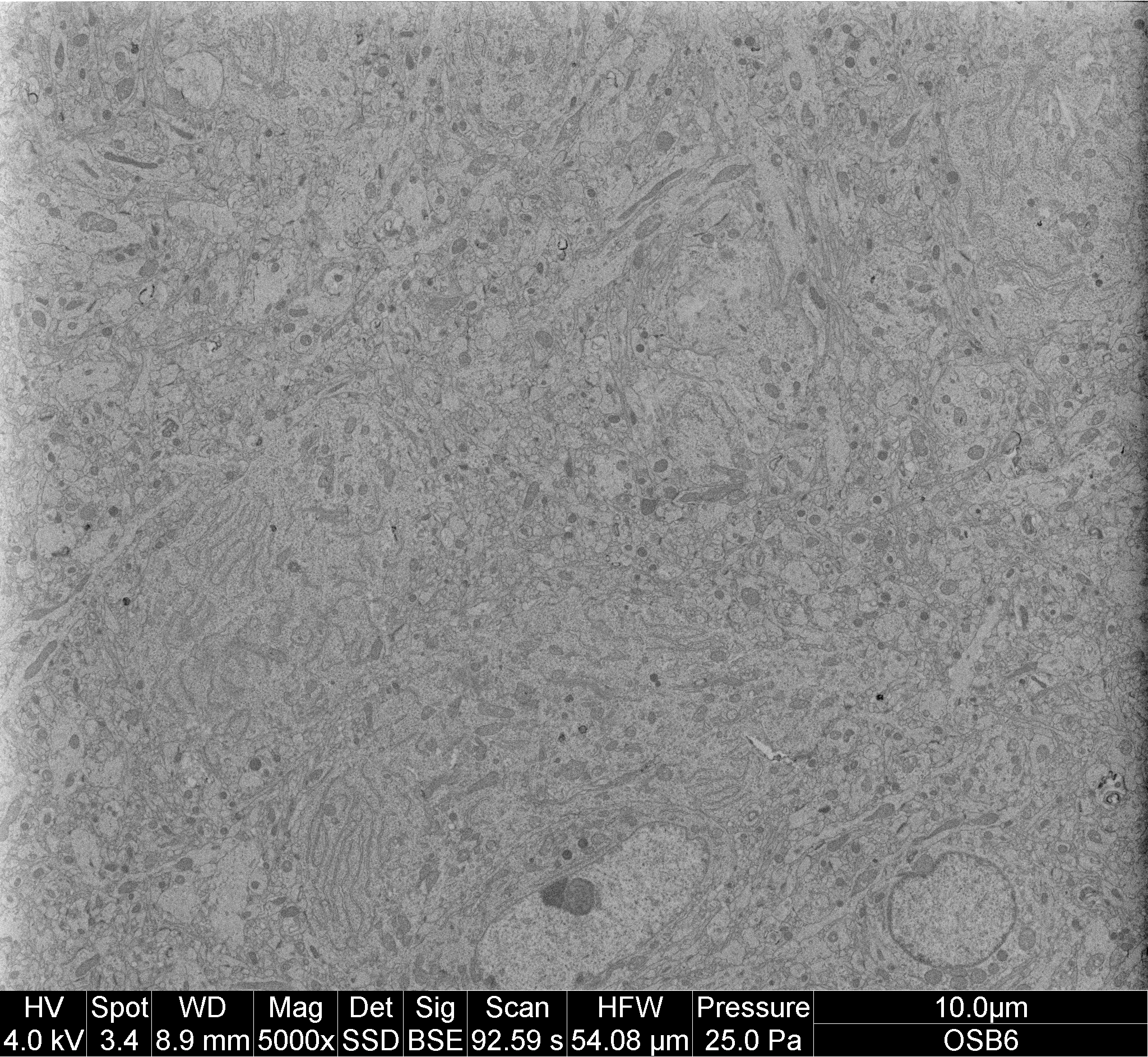

Supplement: Dataset S14 — (251.8 MB ZIP). [file pbio.0020329.sd014.zip › 040604_OS5_st1_1356.tif]

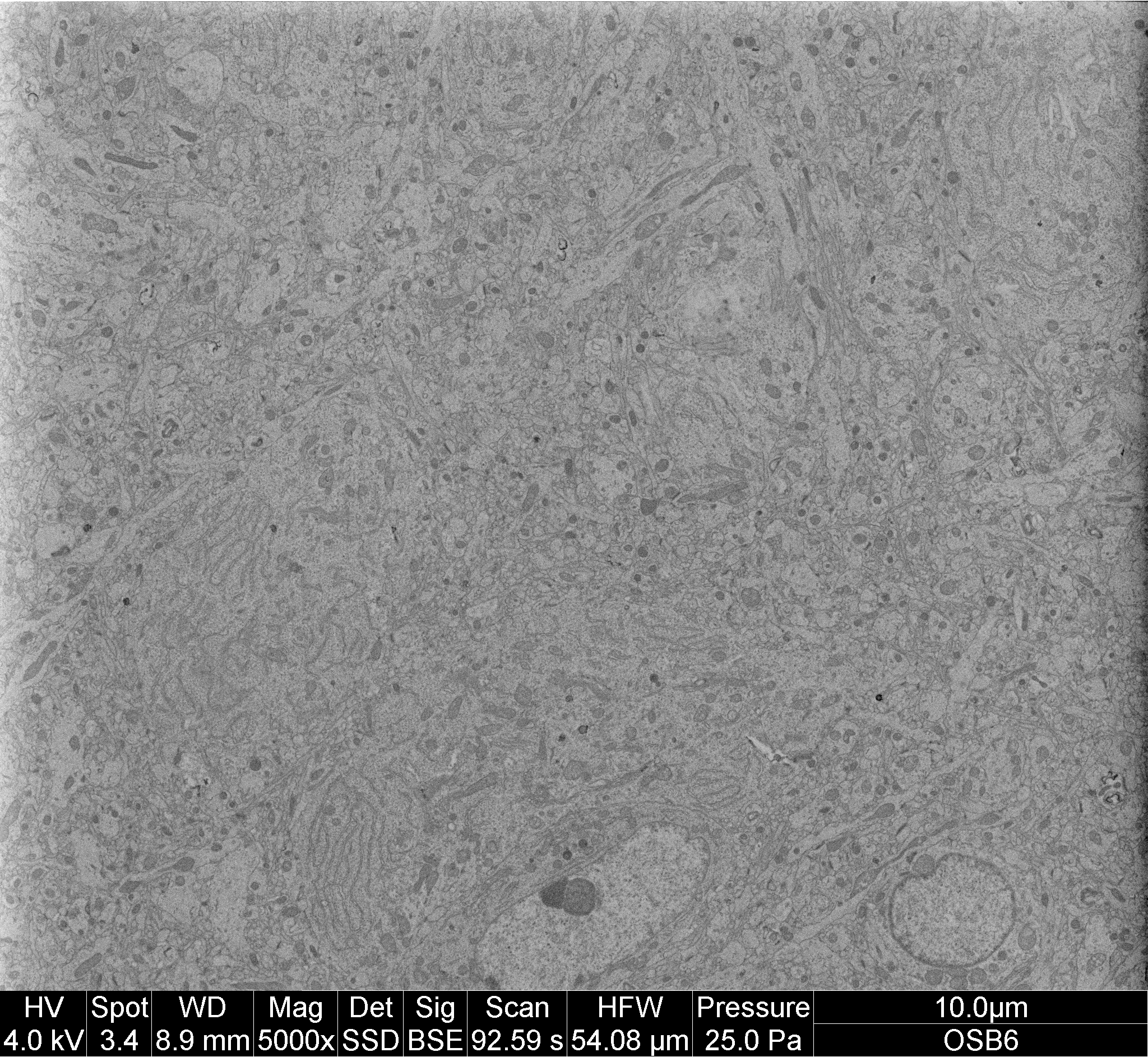

Supplement: Dataset S14 — (251.8 MB ZIP). [file pbio.0020329.sd014.zip › 040604_OS5_st1_1357.tif]

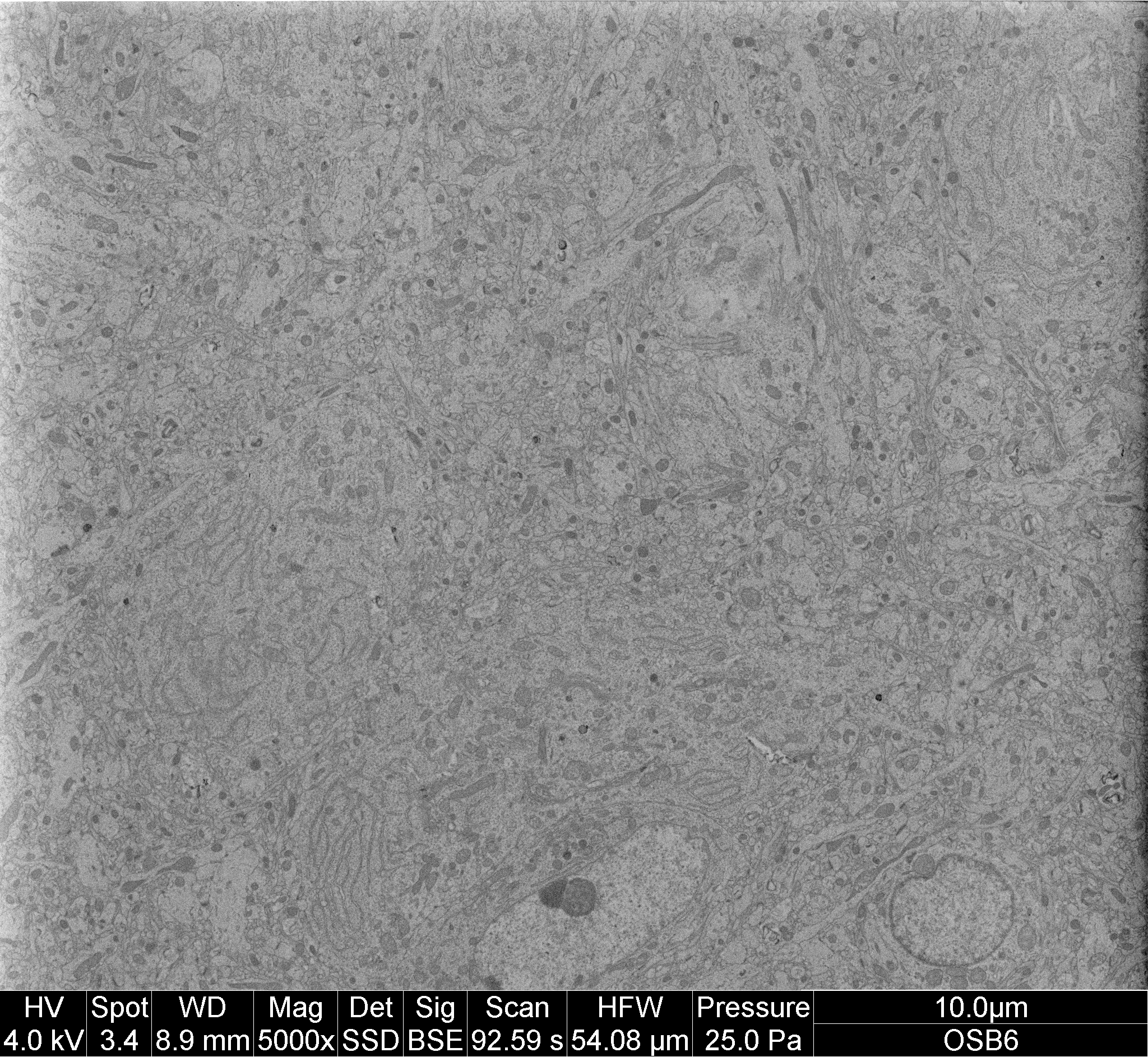

Supplement: Dataset S14 — (251.8 MB ZIP). [file pbio.0020329.sd014.zip › 040604_OS5_st1_1358.tif]

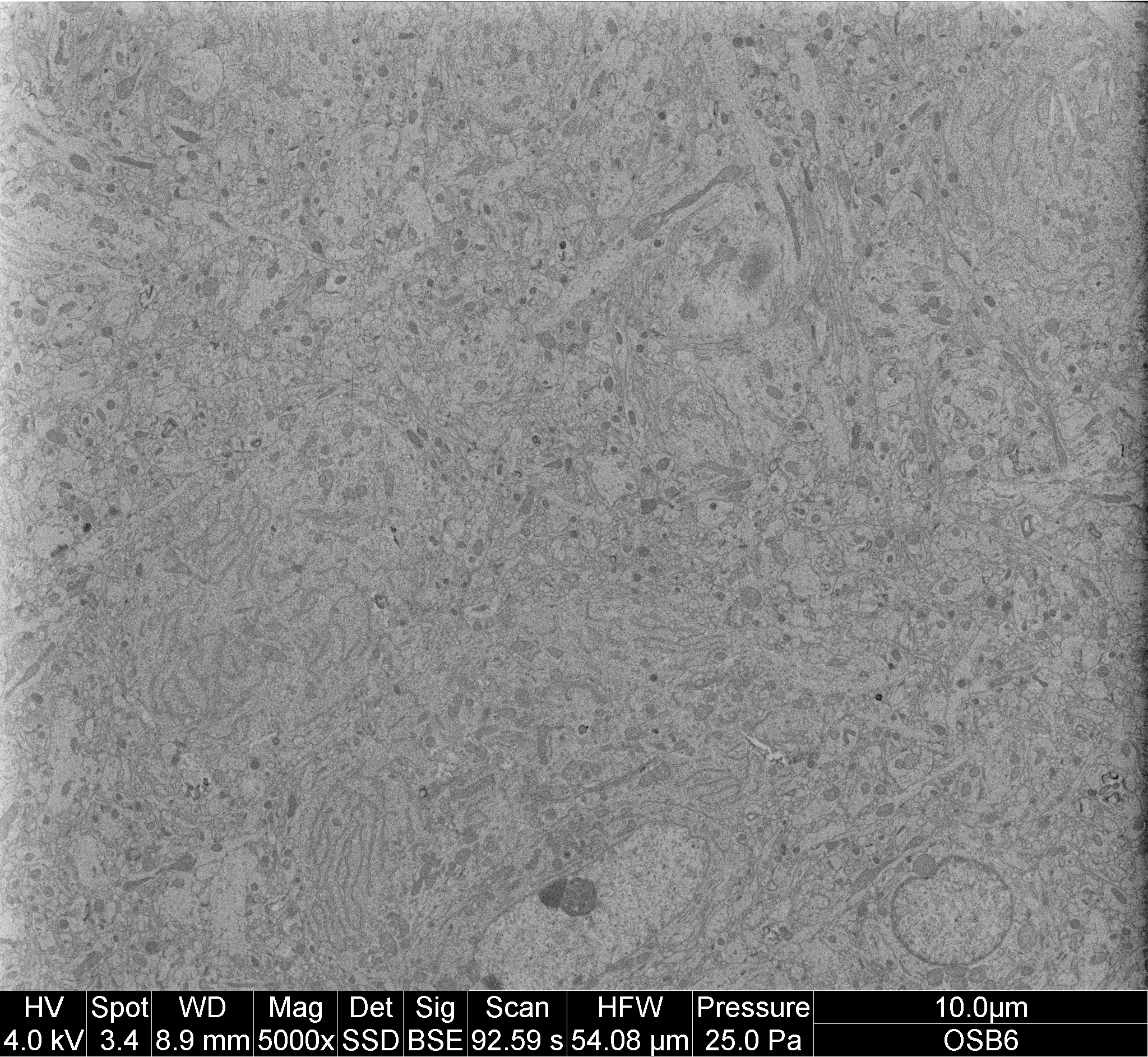

Supplement: Dataset S14 — (251.8 MB ZIP). [file pbio.0020329.sd014.zip › 040604_OS5_st1_1359.tif]

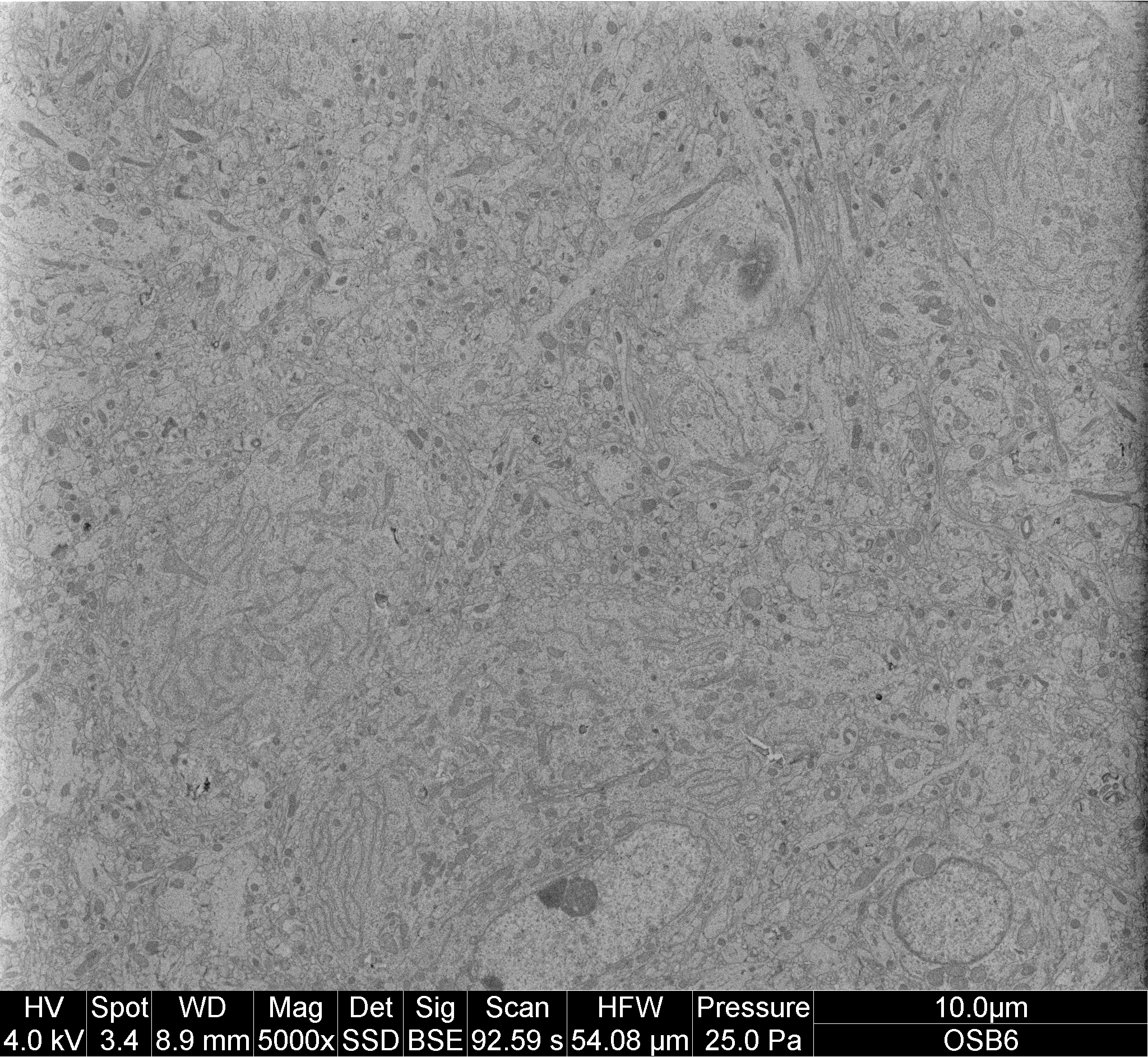

Supplement: Dataset S14 — (251.8 MB ZIP). [file pbio.0020329.sd014.zip › 040604_OS5_st1_1360.tif]

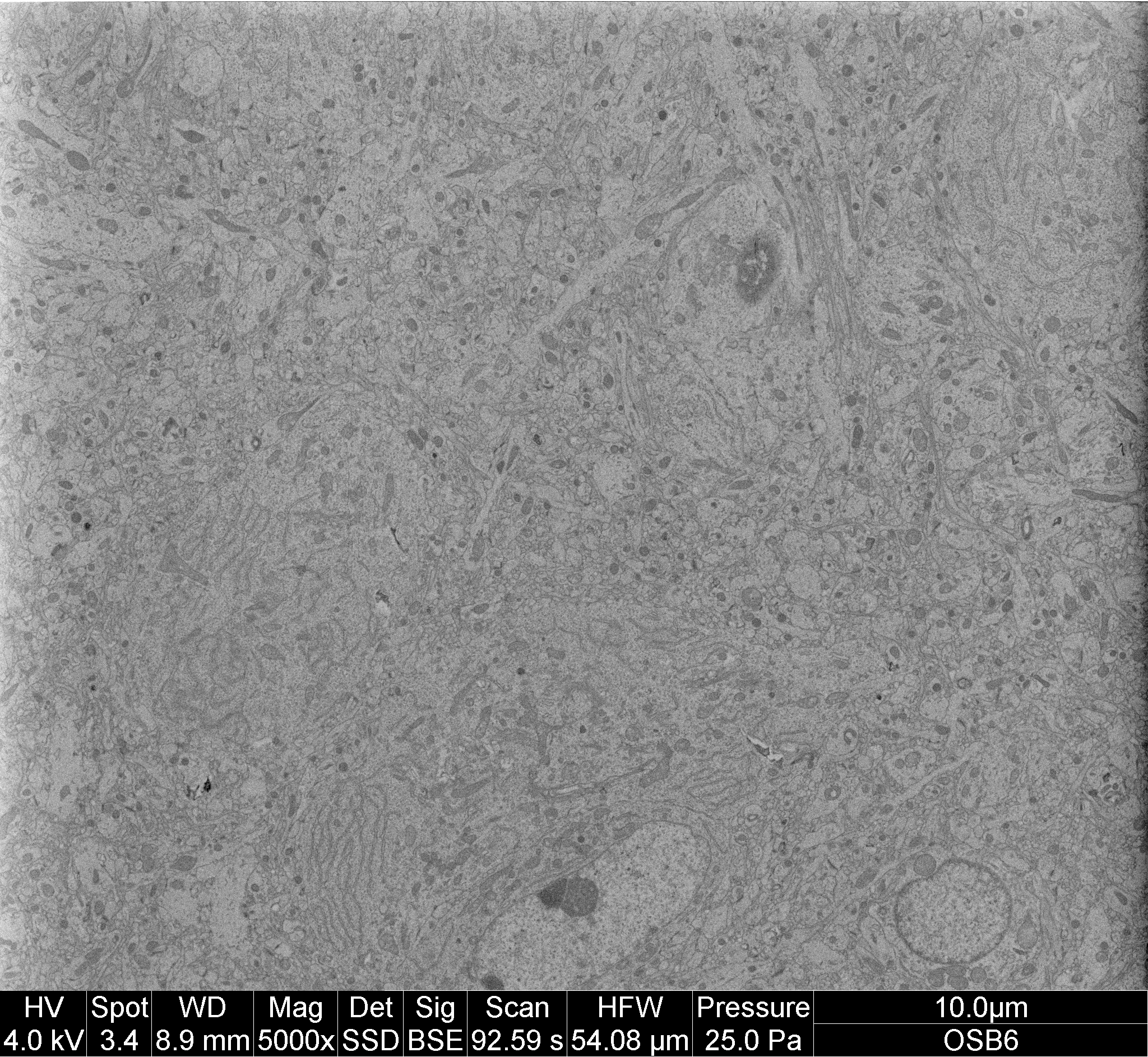

Supplement: Dataset S14 — (251.8 MB ZIP). [file pbio.0020329.sd014.zip › 040604_OS5_st1_1361.tif]

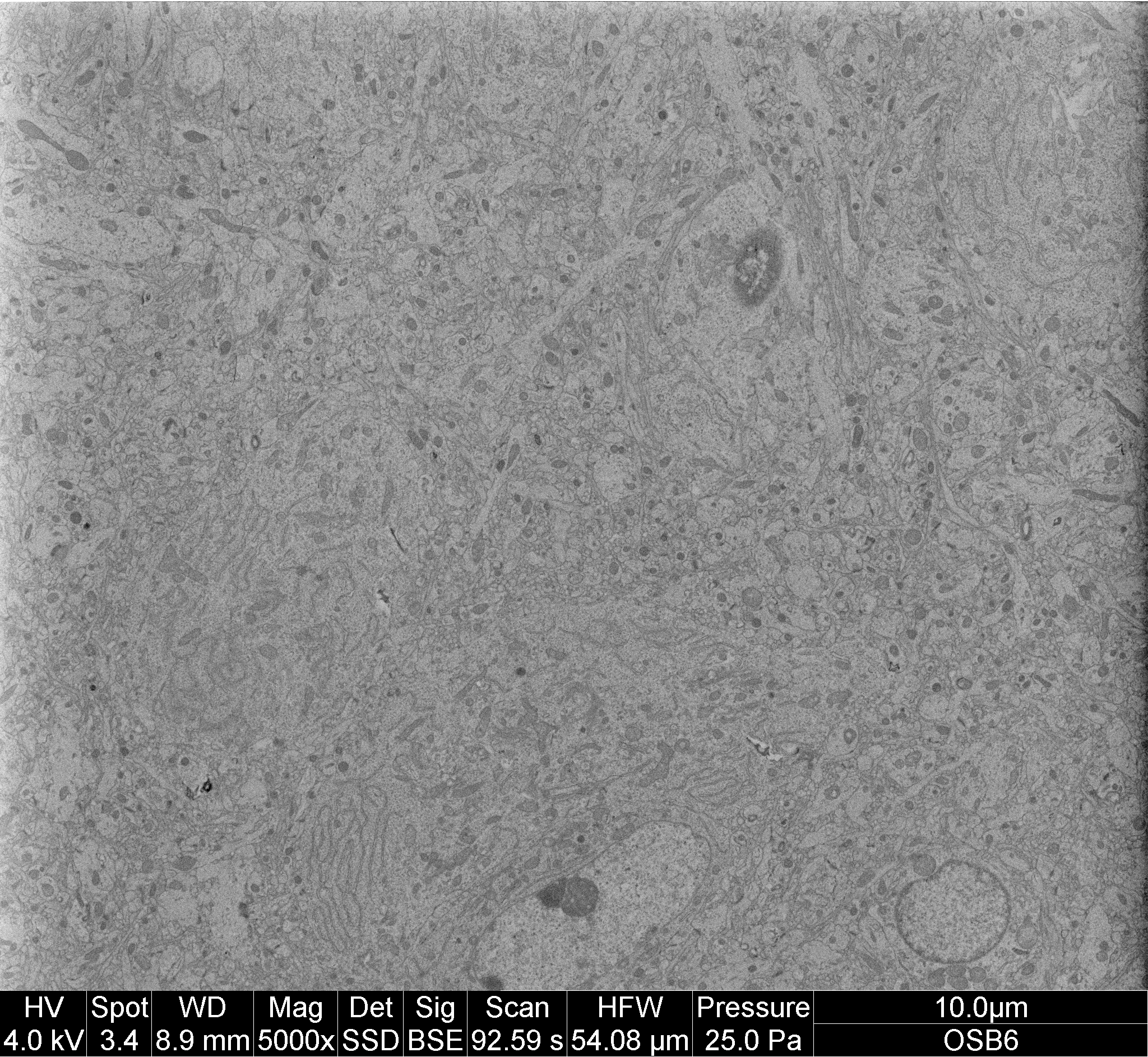

Supplement: Dataset S14 — (251.8 MB ZIP). [file pbio.0020329.sd014.zip › 040604_OS5_st1_1362.tif]

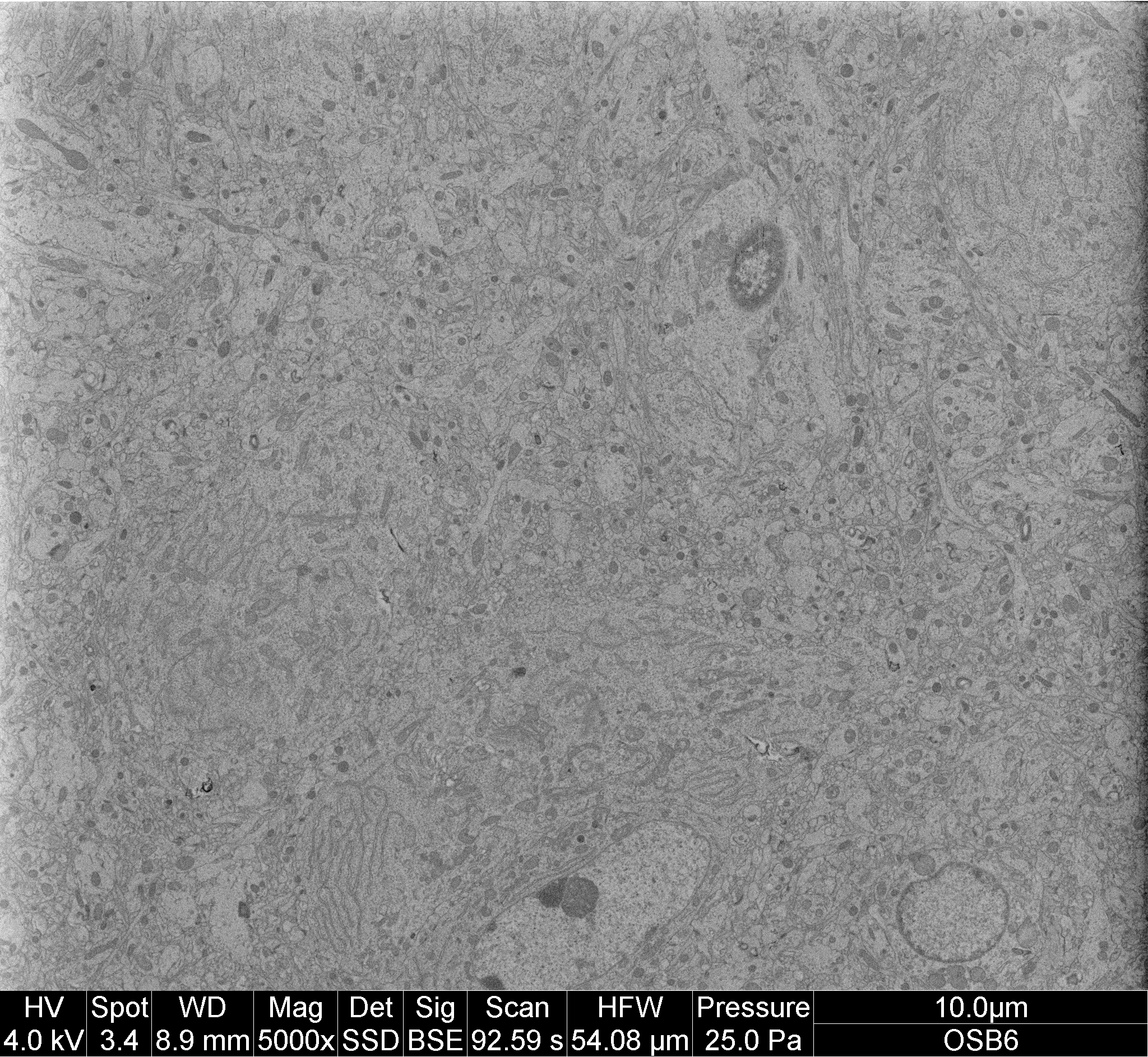

Supplement: Dataset S14 — (251.8 MB ZIP). [file pbio.0020329.sd014.zip › 040604_OS5_st1_1363.tif]

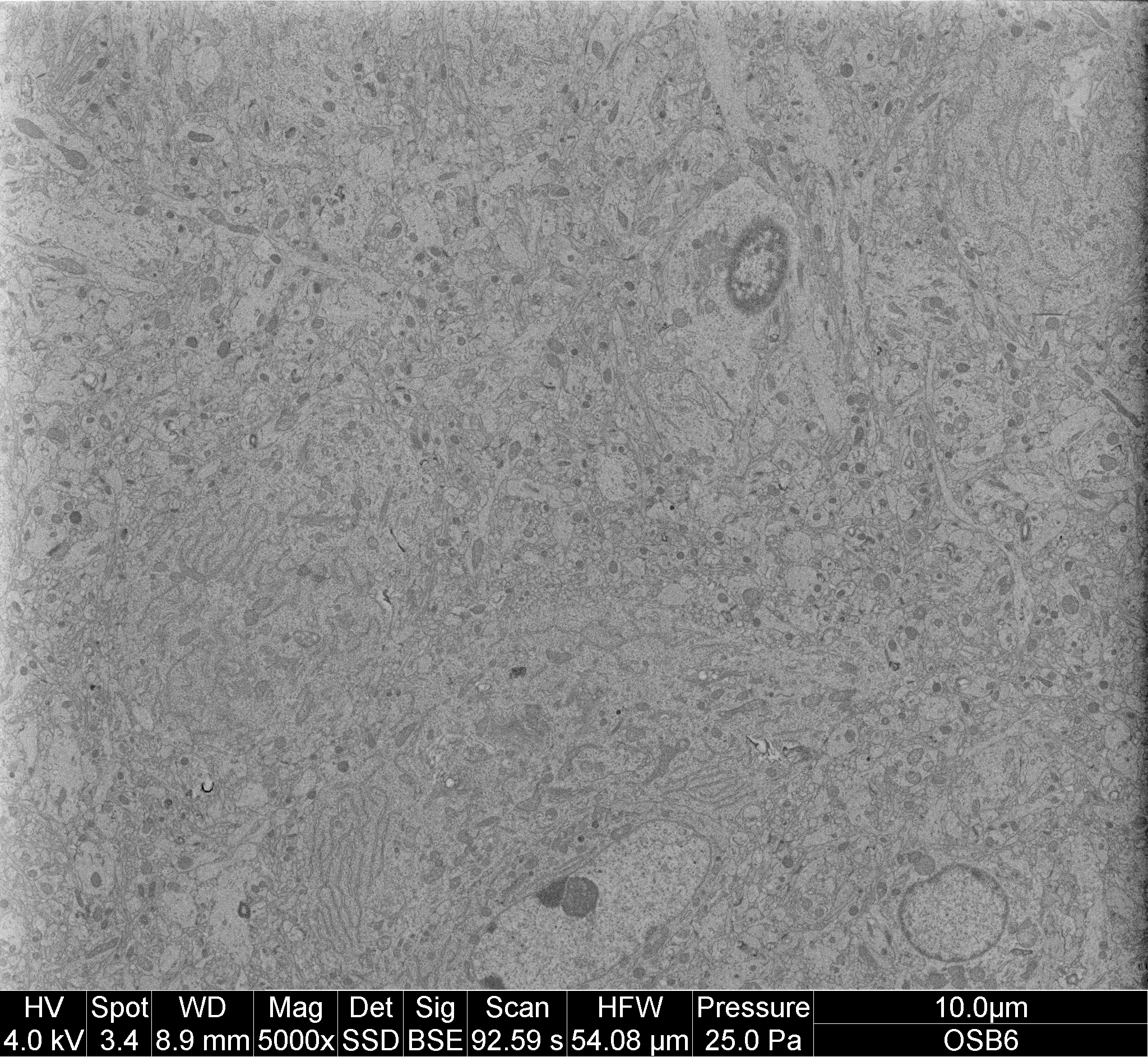

Supplement: Dataset S14 — (251.8 MB ZIP). [file pbio.0020329.sd014.zip › 040604_OS5_st1_1364.tif]

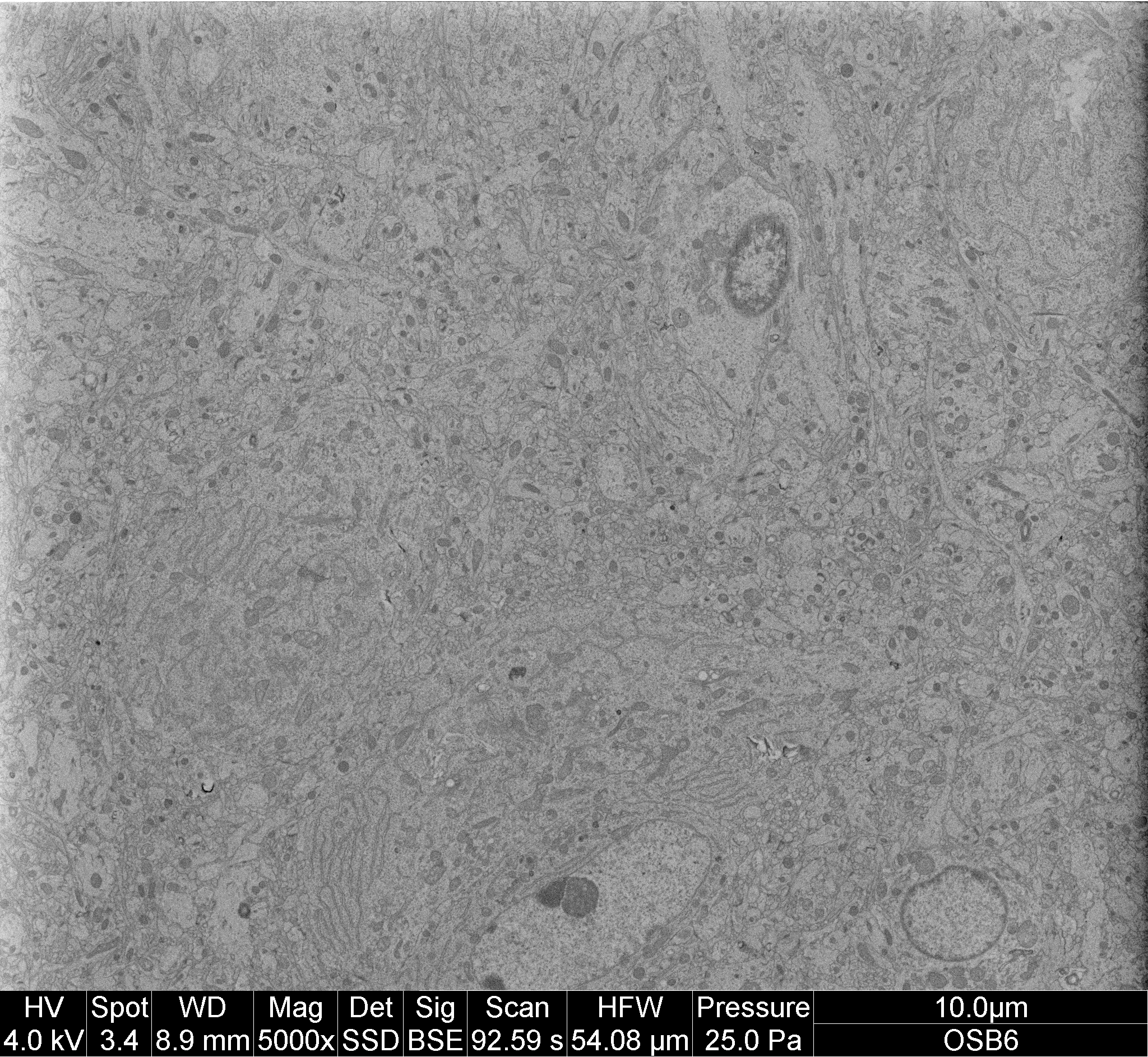

Supplement: Dataset S14 — (251.8 MB ZIP). [file pbio.0020329.sd014.zip › 040604_OS5_st1_1365.tif]

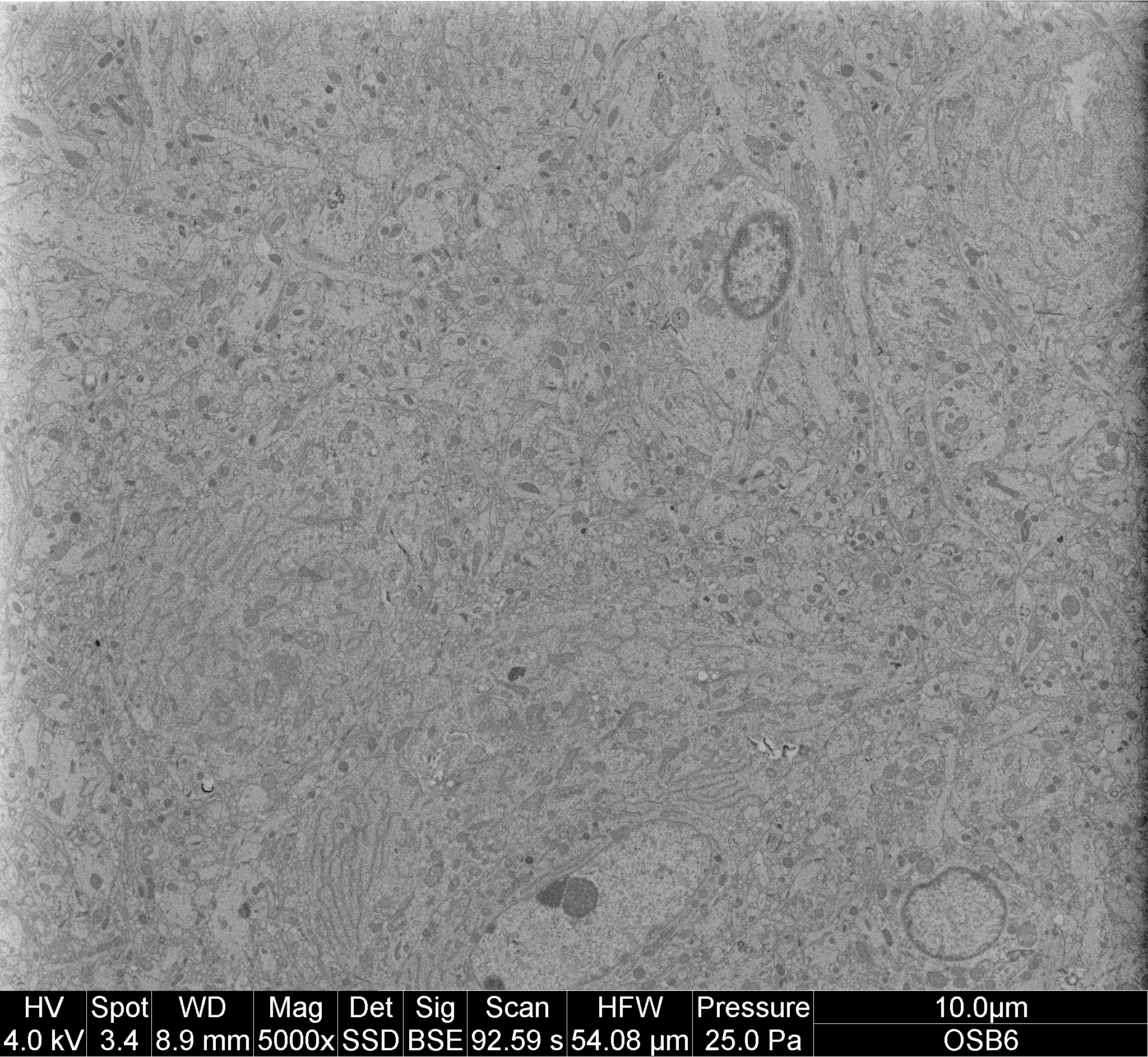

Supplement: Dataset S14 — (251.8 MB ZIP). [file pbio.0020329.sd014.zip › 040604_OS5_st1_1366.tif]

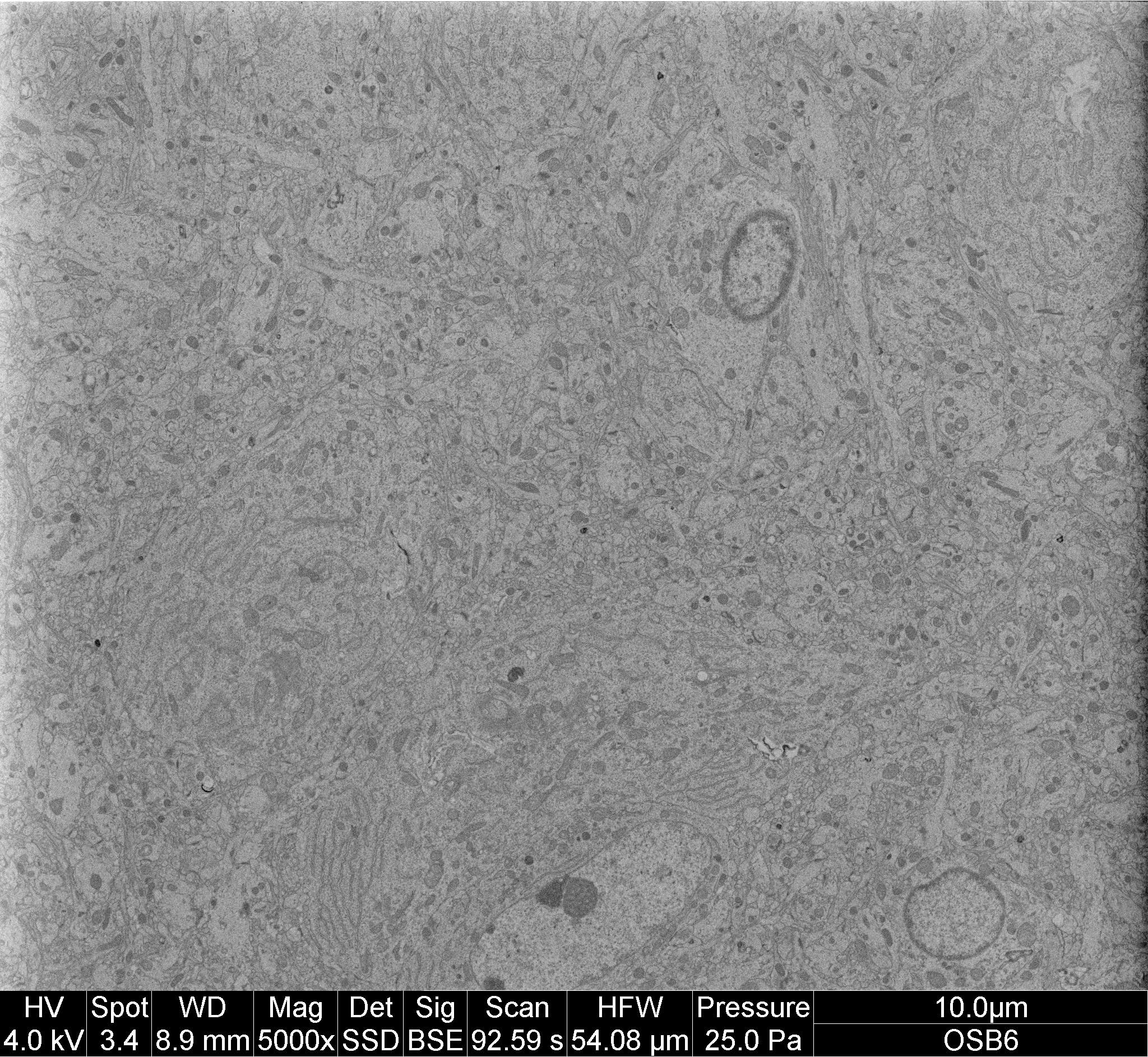

Supplement: Dataset S14 — (251.8 MB ZIP). [file pbio.0020329.sd014.zip › 040604_OS5_st1_1367.tif]

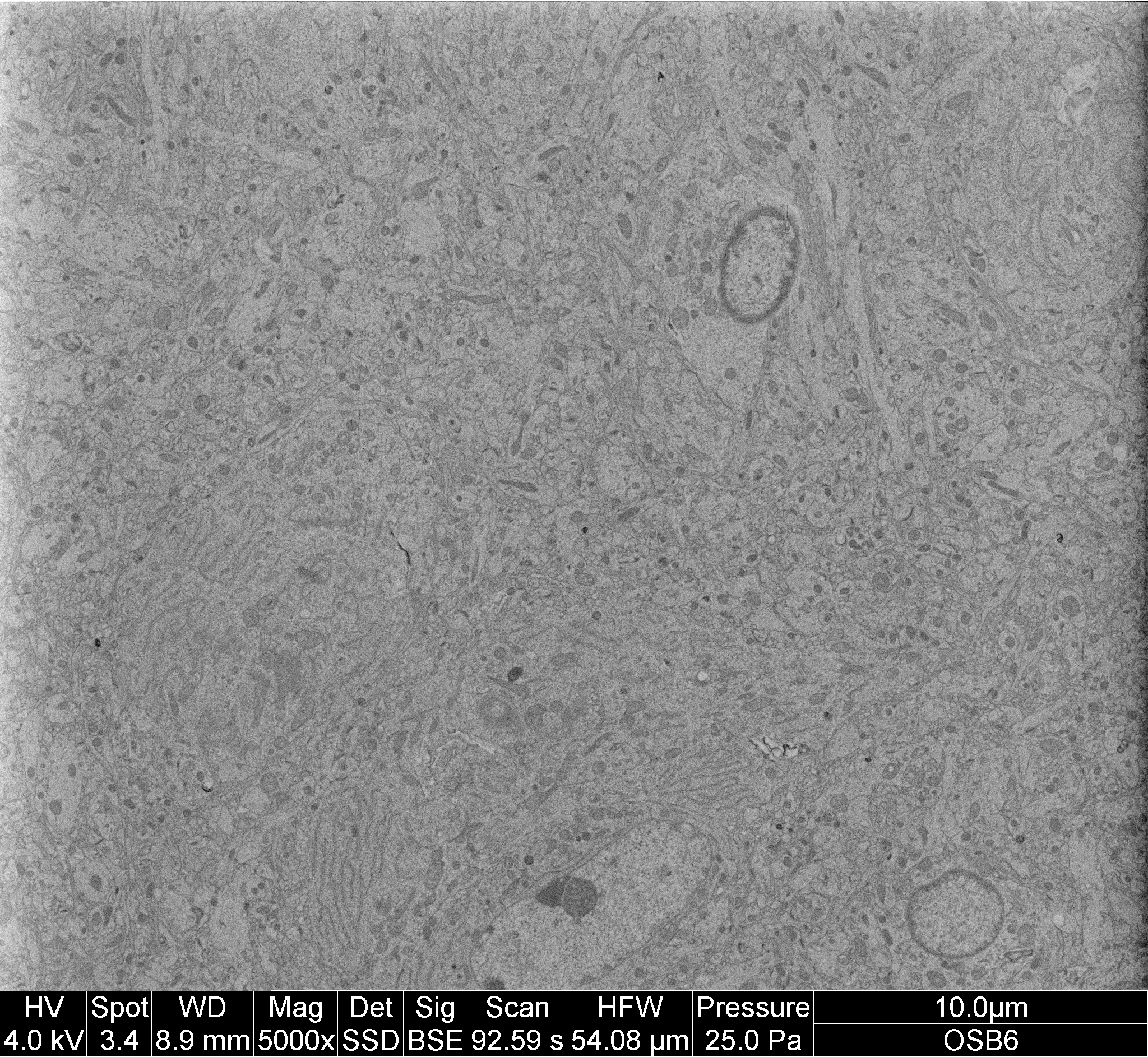

Supplement: Dataset S14 — (251.8 MB ZIP). [file pbio.0020329.sd014.zip › 040604_OS5_st1_1368.tif]

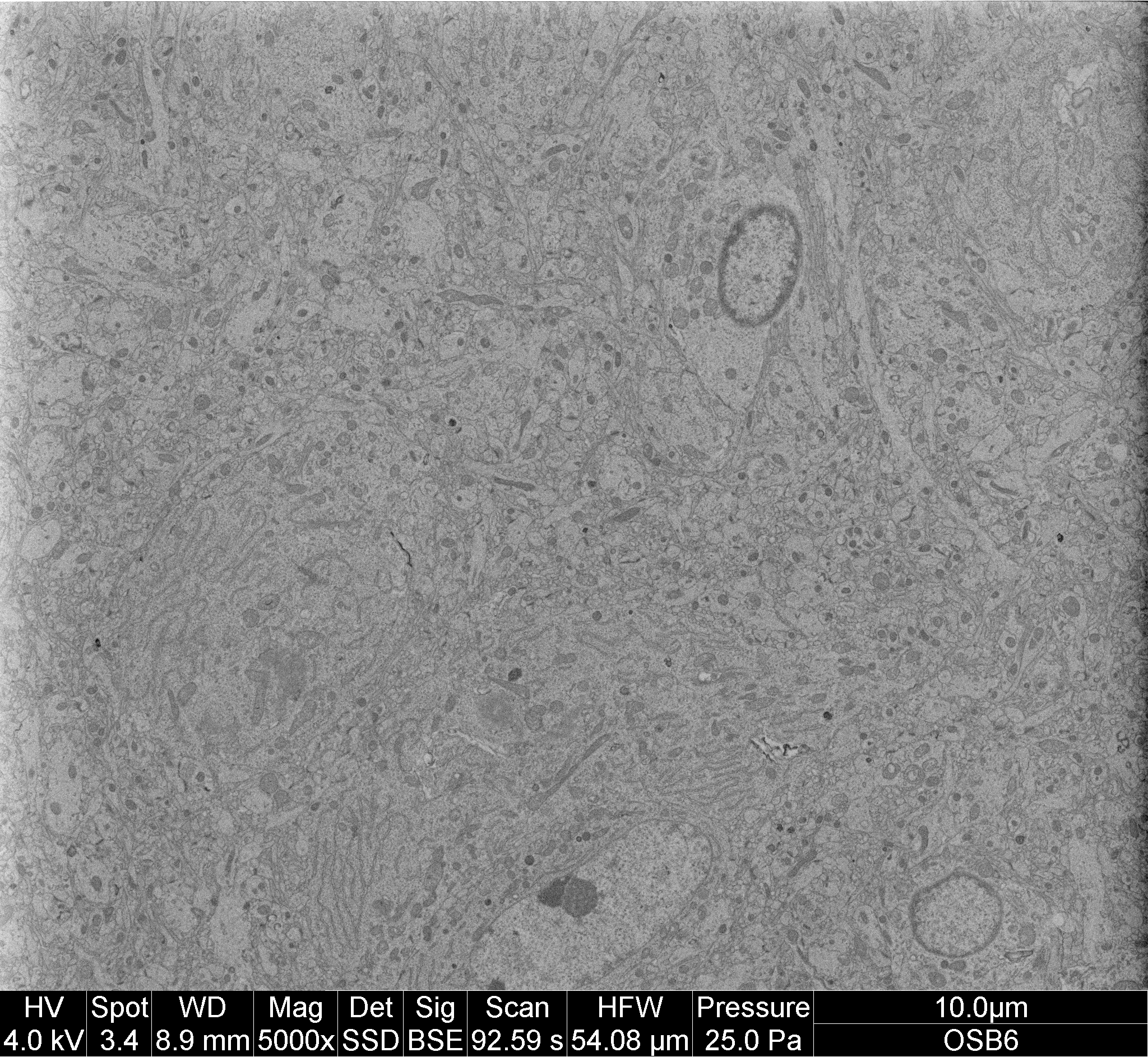

Supplement: Dataset S14 — (251.8 MB ZIP). [file pbio.0020329.sd014.zip › 040604_OS5_st1_1369.tif]

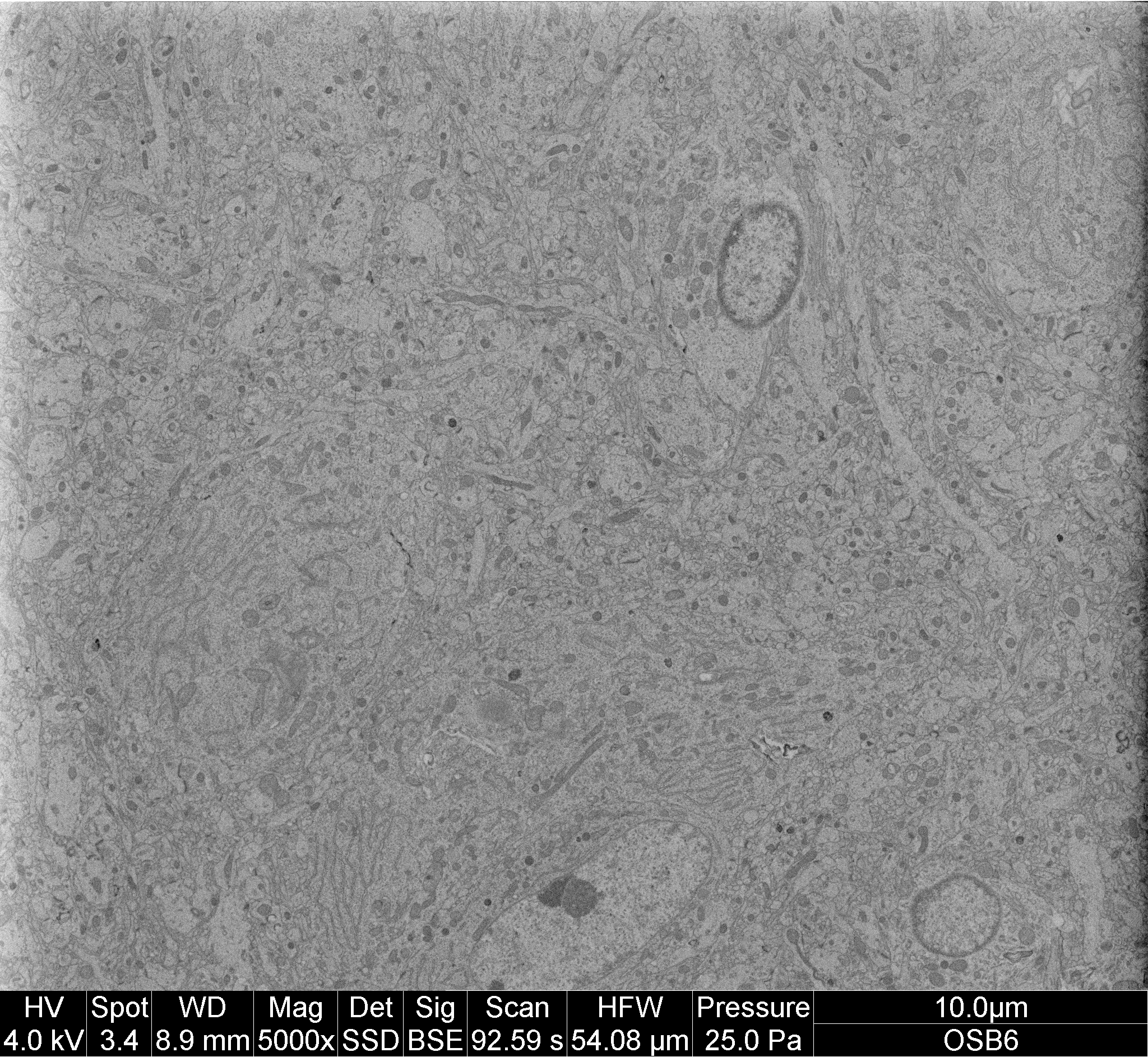

Supplement: Dataset S14 — (251.8 MB ZIP). [file pbio.0020329.sd014.zip › 040604_OS5_st1_1370.tif]

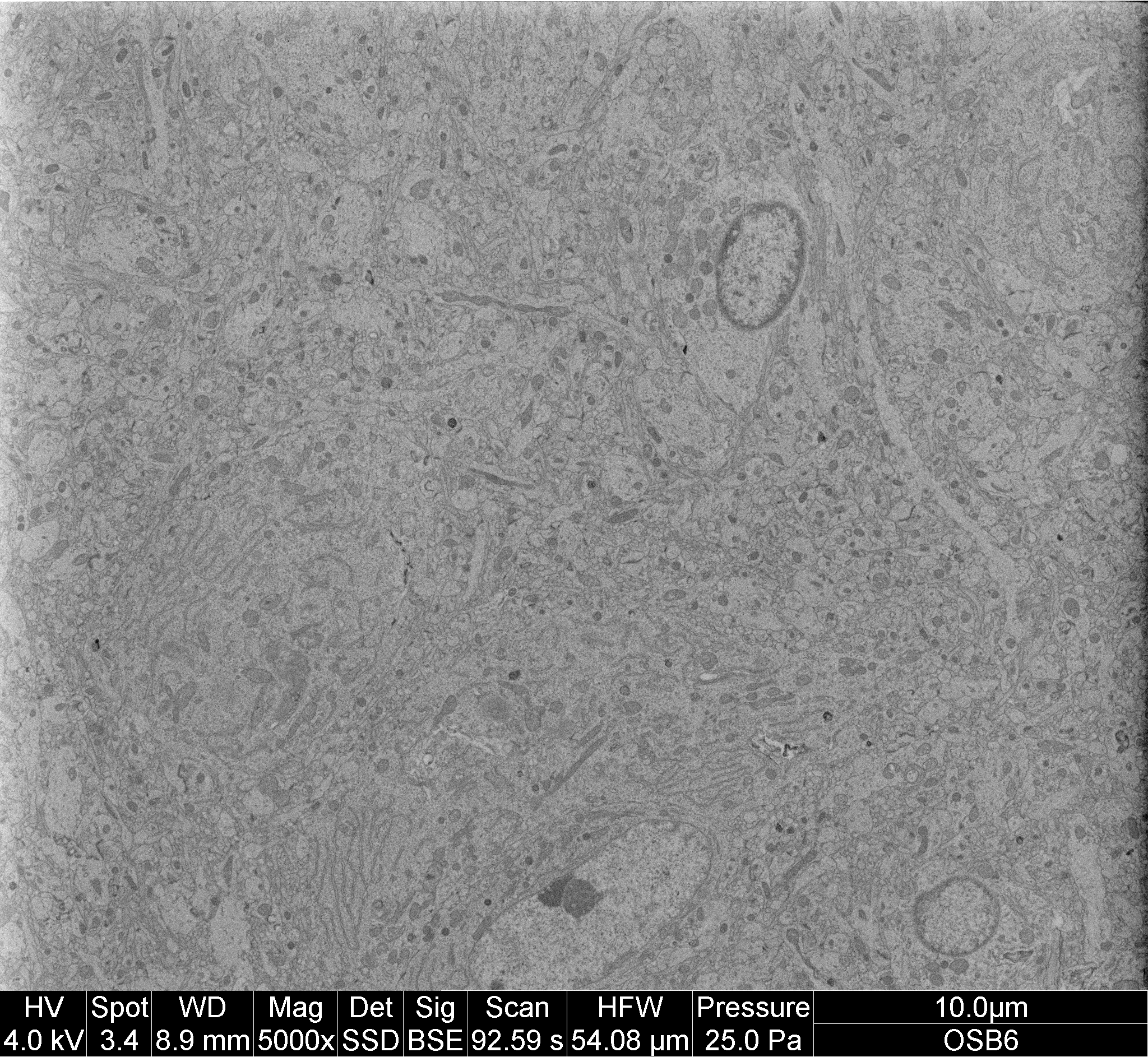

Supplement: Dataset S14 — (251.8 MB ZIP). [file pbio.0020329.sd014.zip › 040604_OS5_st1_1371.tif]

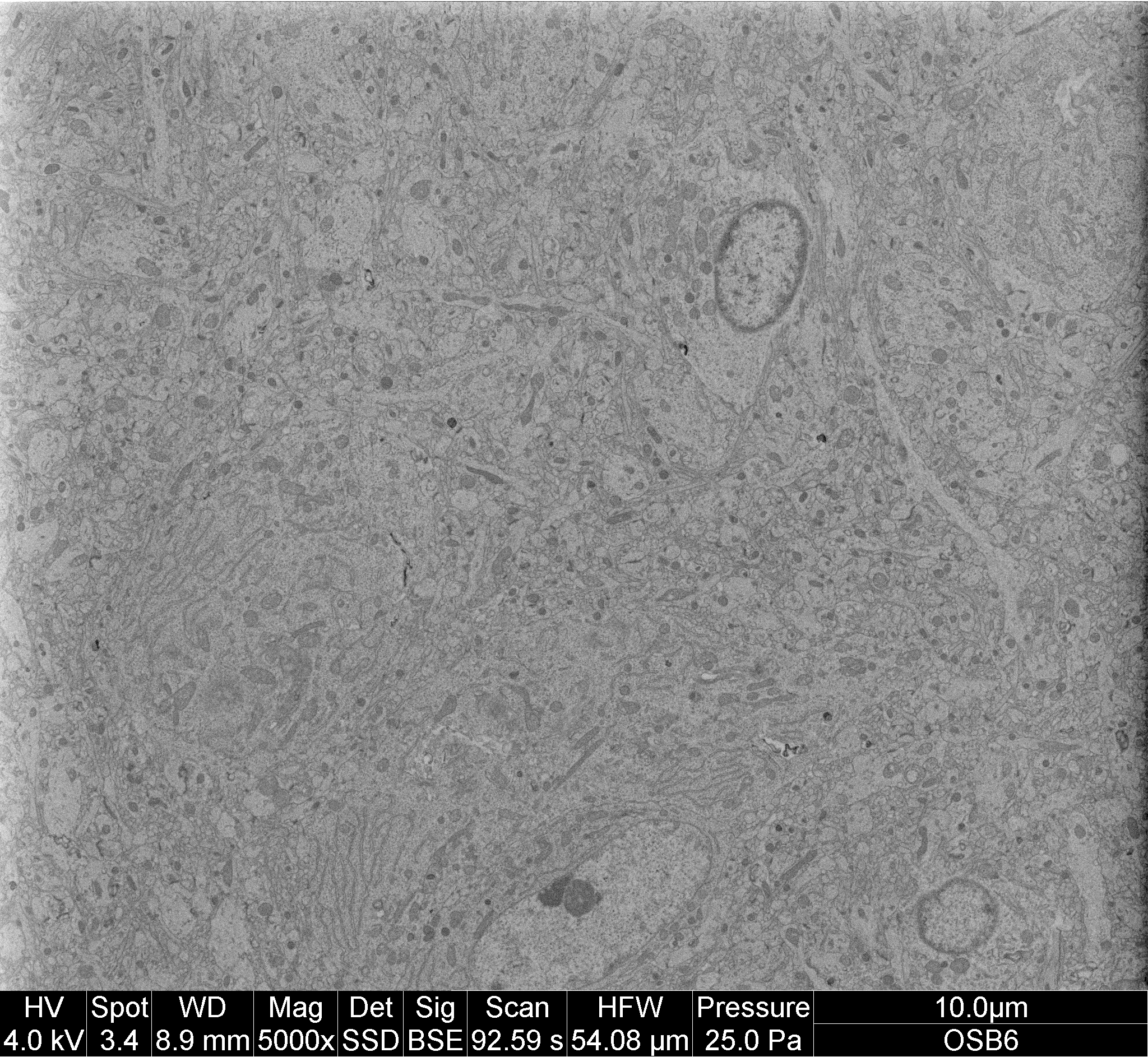

Supplement: Dataset S14 — (251.8 MB ZIP). [file pbio.0020329.sd014.zip › 040604_OS5_st1_1372.tif]

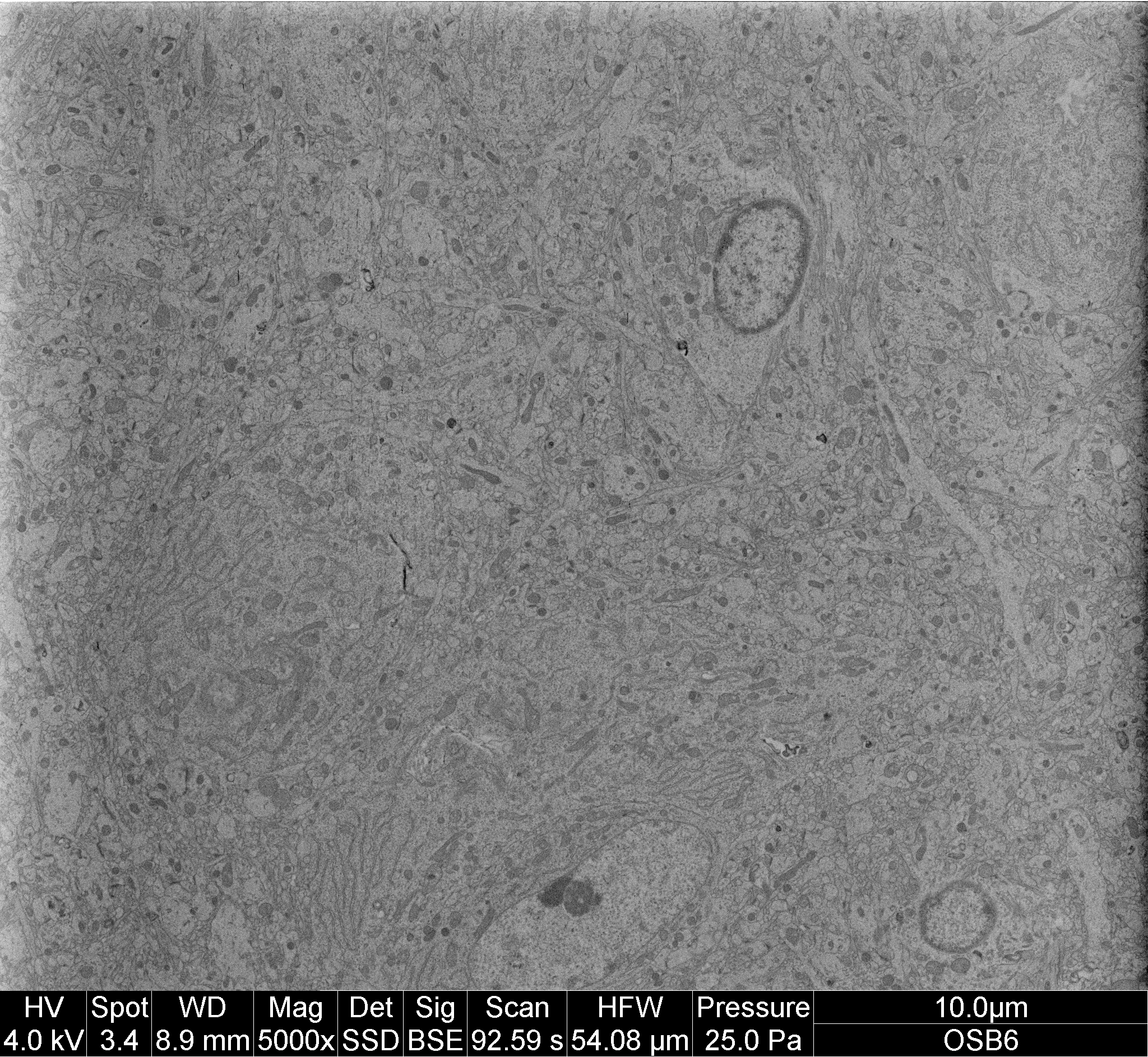

Supplement: Dataset S14 — (251.8 MB ZIP). [file pbio.0020329.sd014.zip › 040604_OS5_st1_1373.tif]

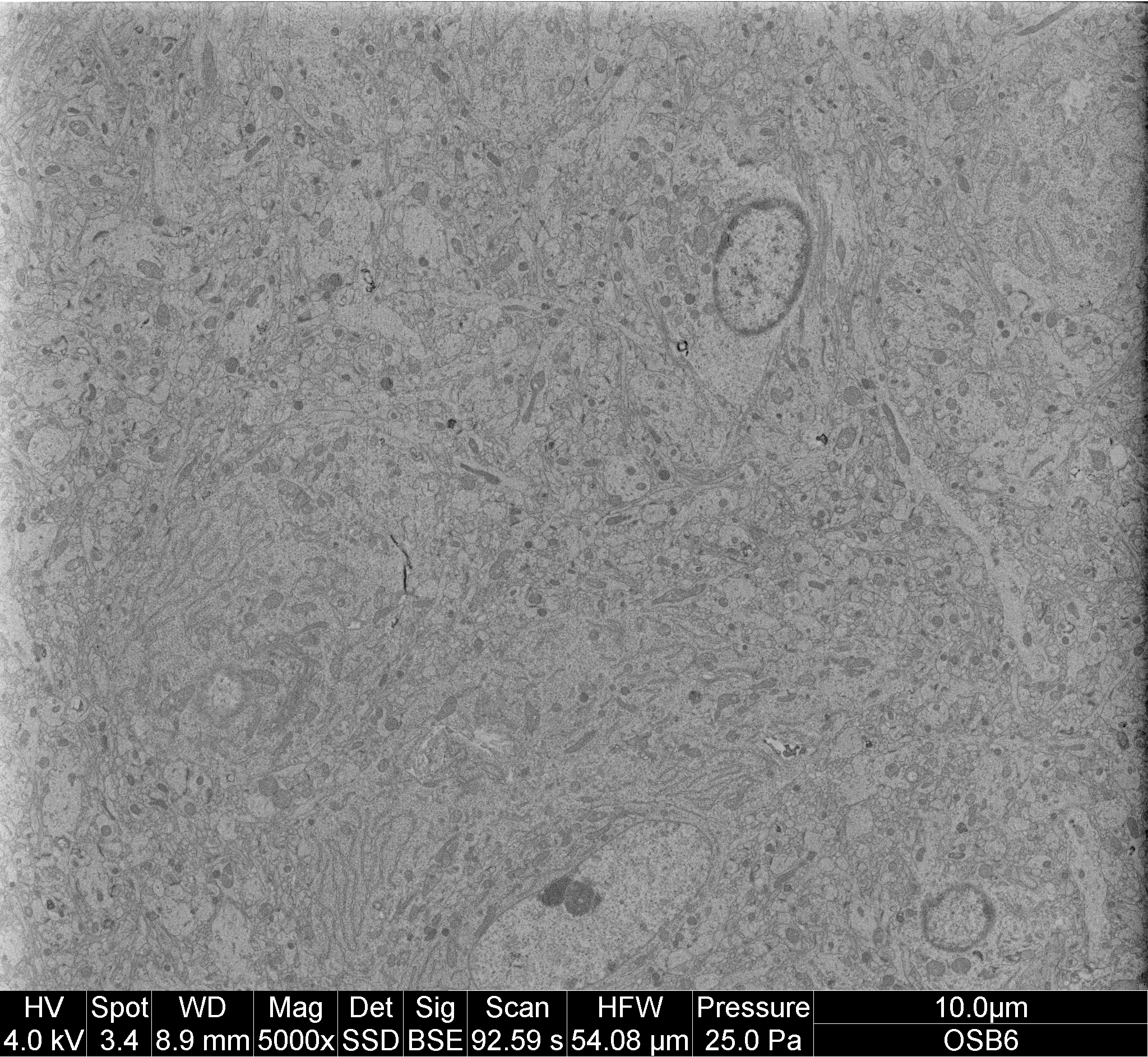

Supplement: Dataset S14 — (251.8 MB ZIP). [file pbio.0020329.sd014.zip › 040604_OS5_st1_1374.tif]

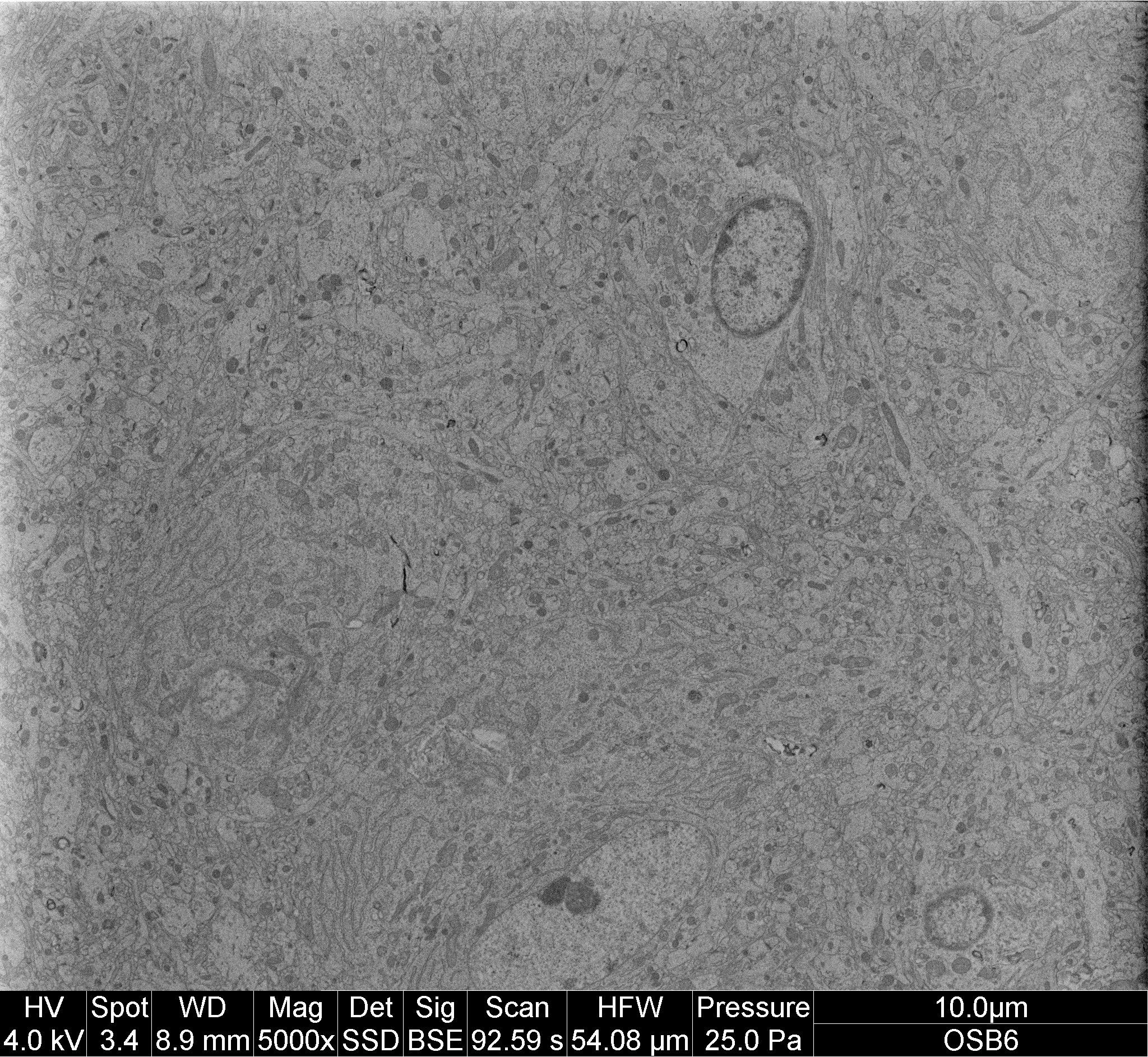

Supplement: Dataset S14 — (251.8 MB ZIP). [file pbio.0020329.sd014.zip › 040604_OS5_st1_1375.tif]

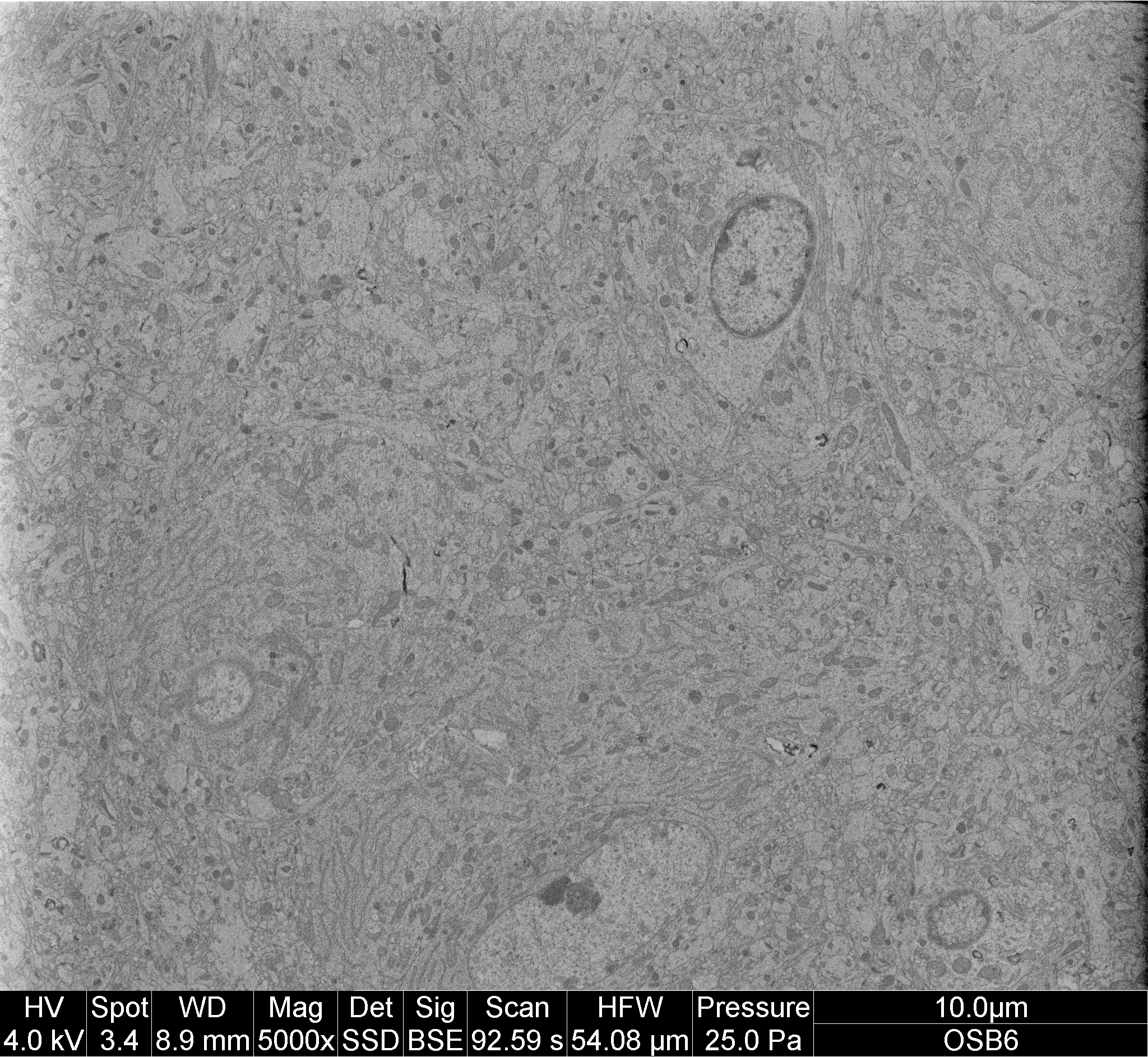

Supplement: Dataset S14 — (251.8 MB ZIP). [file pbio.0020329.sd014.zip › 040604_OS5_st1_1376.tif]

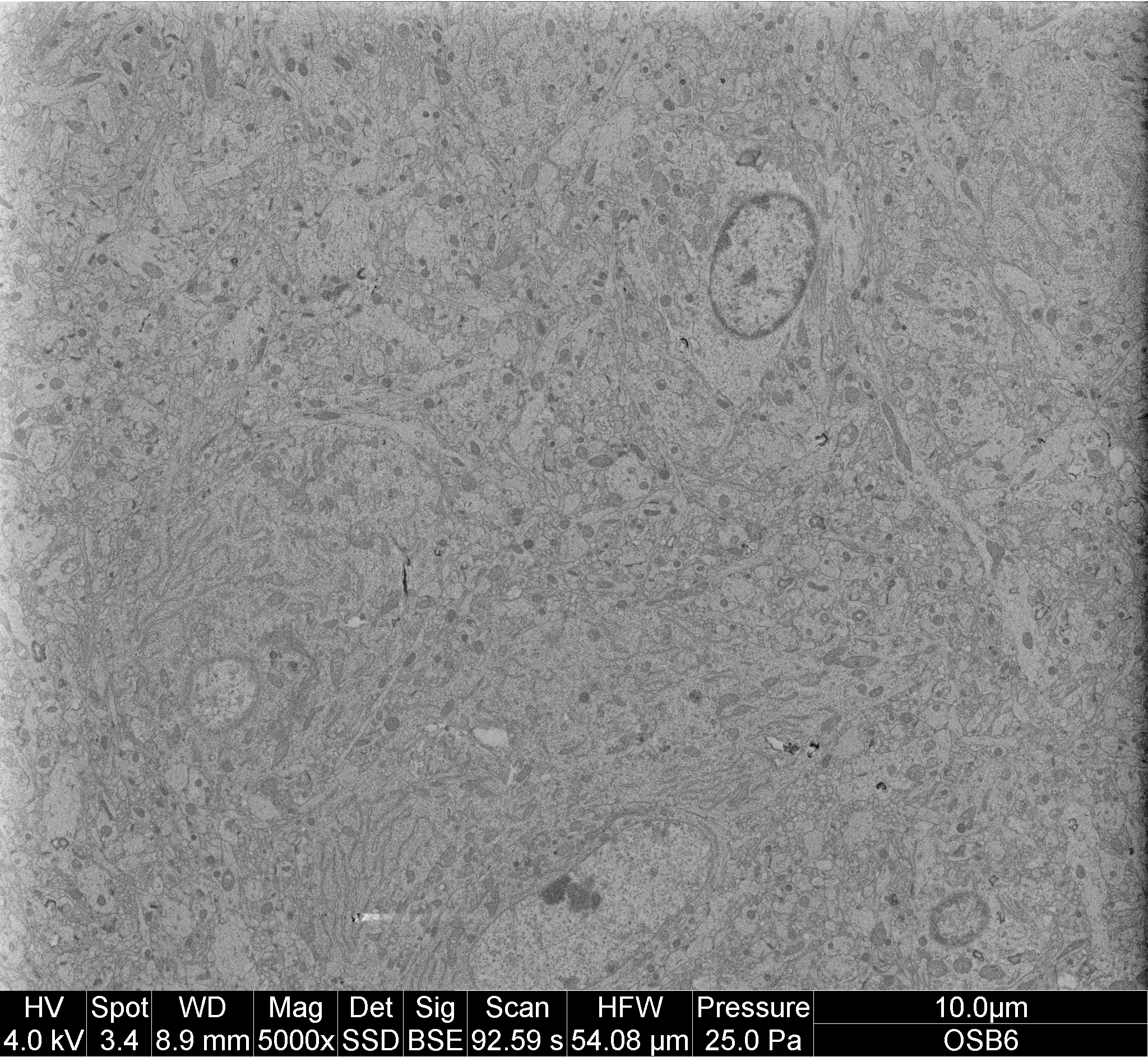

Supplement: Dataset S14 — (251.8 MB ZIP). [file pbio.0020329.sd014.zip › 040604_OS5_st1_1377.tif]

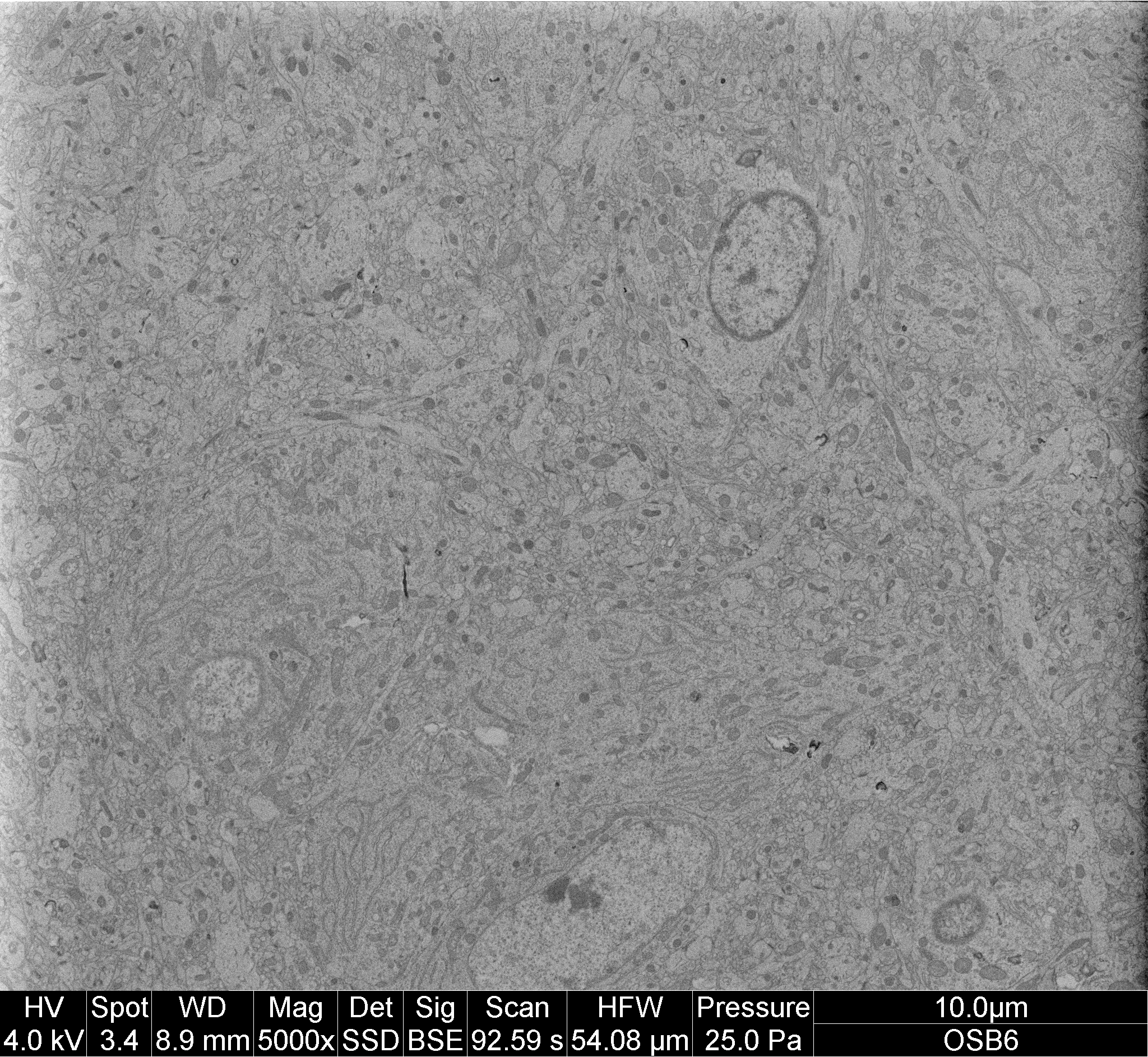

Supplement: Dataset S14 — (251.8 MB ZIP). [file pbio.0020329.sd014.zip › 040604_OS5_st1_1378.tif]

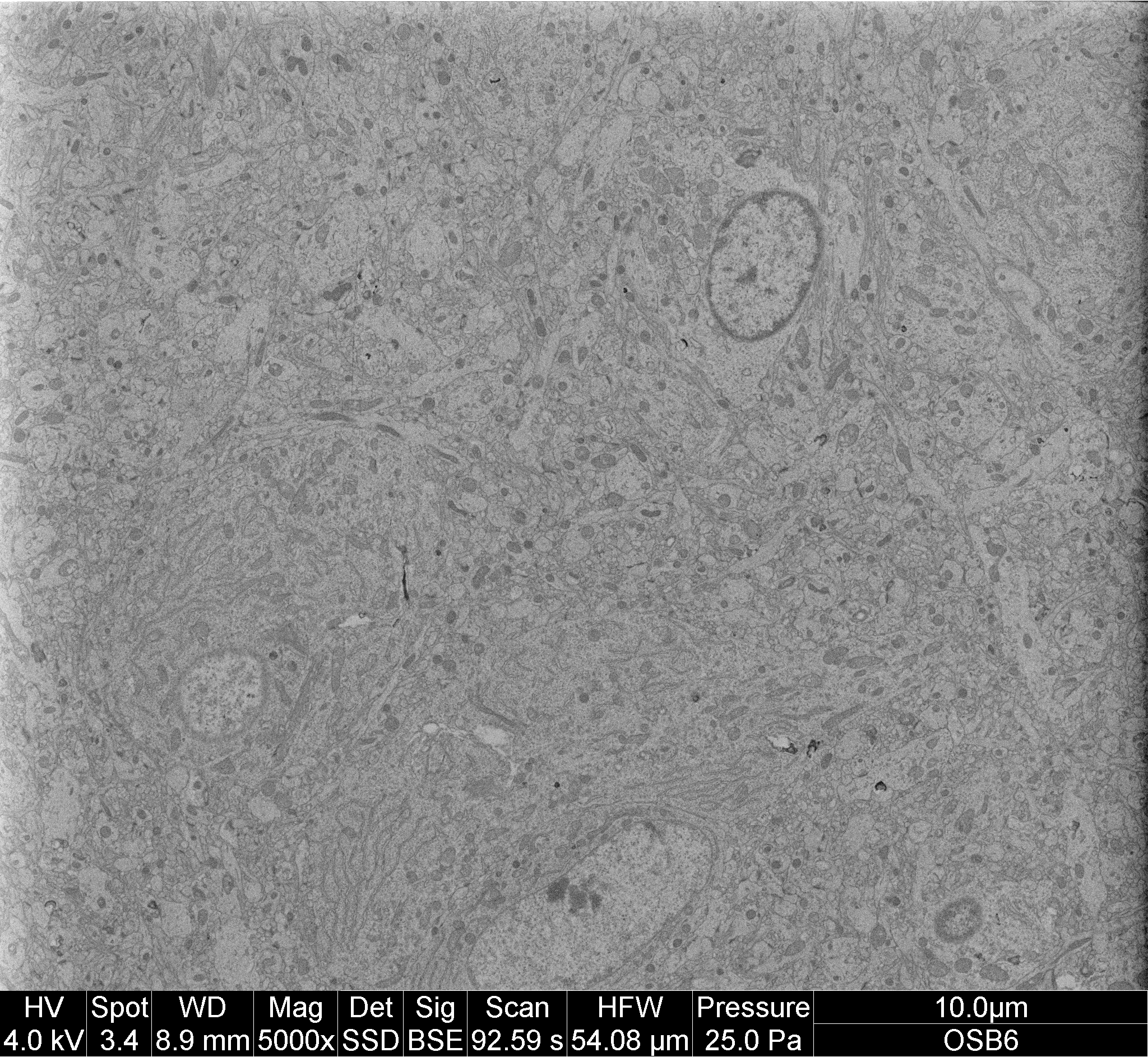

Supplement: Dataset S14 — (251.8 MB ZIP). [file pbio.0020329.sd014.zip › 040604_OS5_st1_1379.tif]

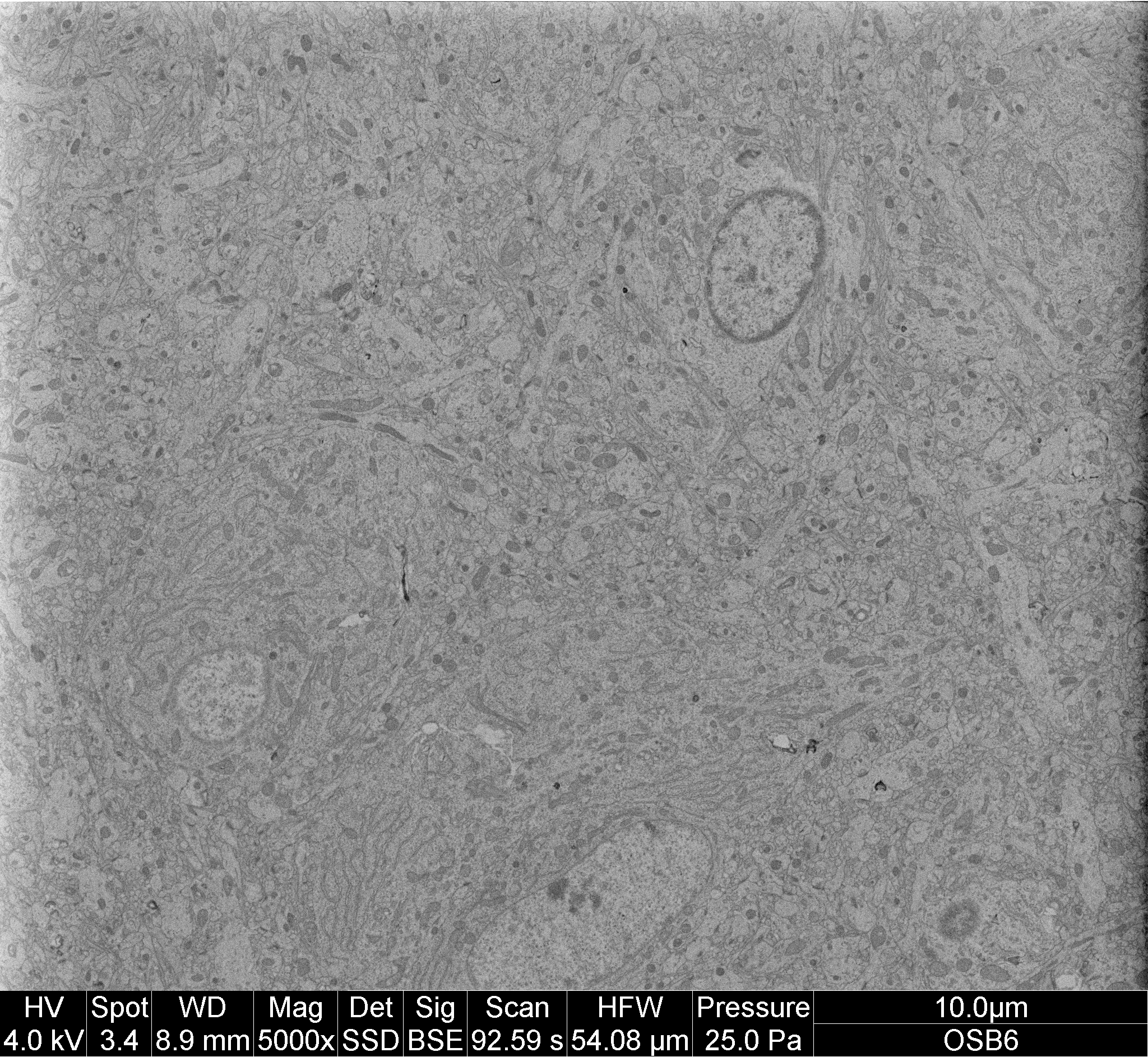

Supplement: Dataset S14 — (251.8 MB ZIP). [file pbio.0020329.sd014.zip › 040604_OS5_st1_1380.tif]

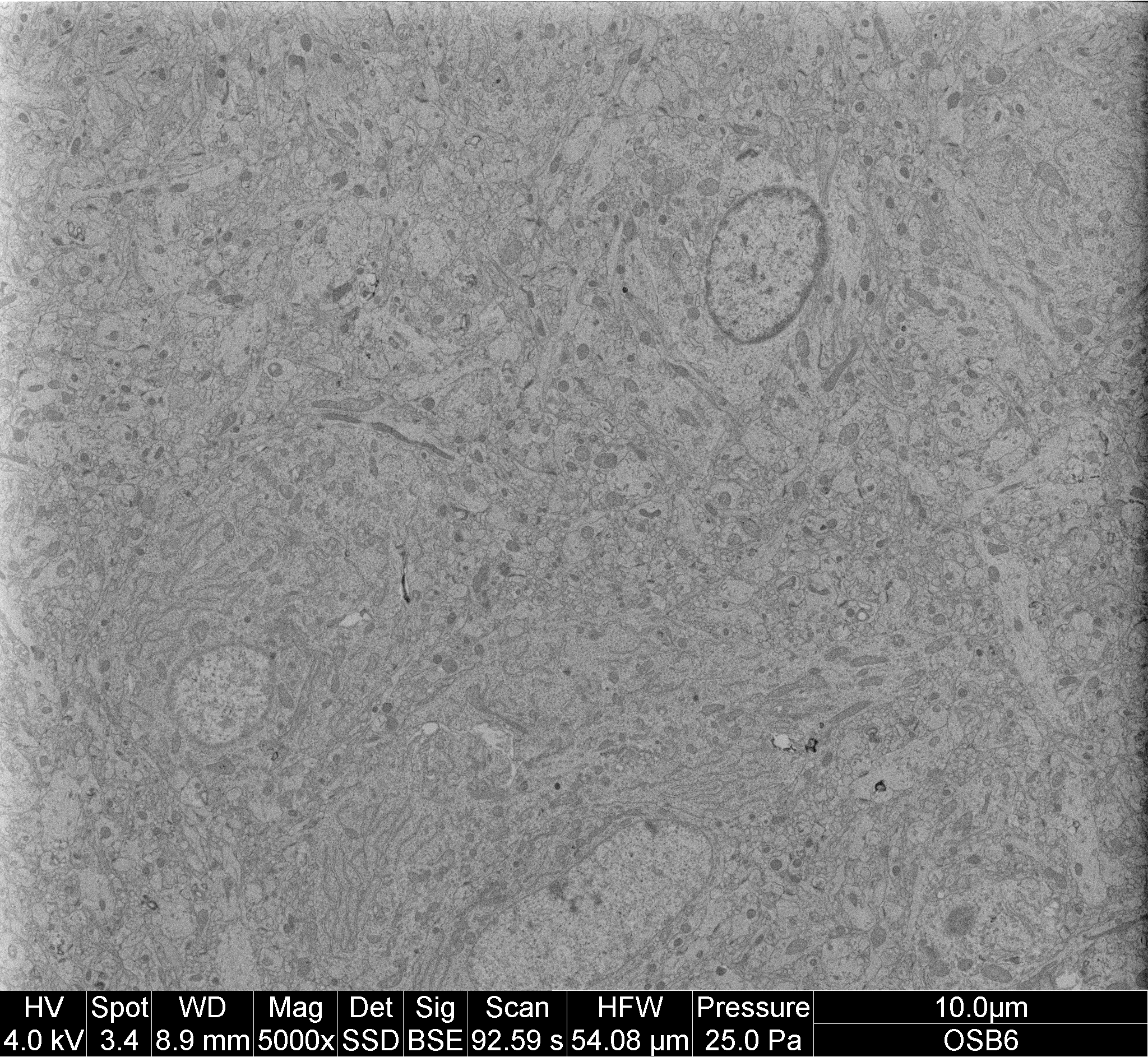

Supplement: Dataset S14 — (251.8 MB ZIP). [file pbio.0020329.sd014.zip › 040604_OS5_st1_1381.tif]

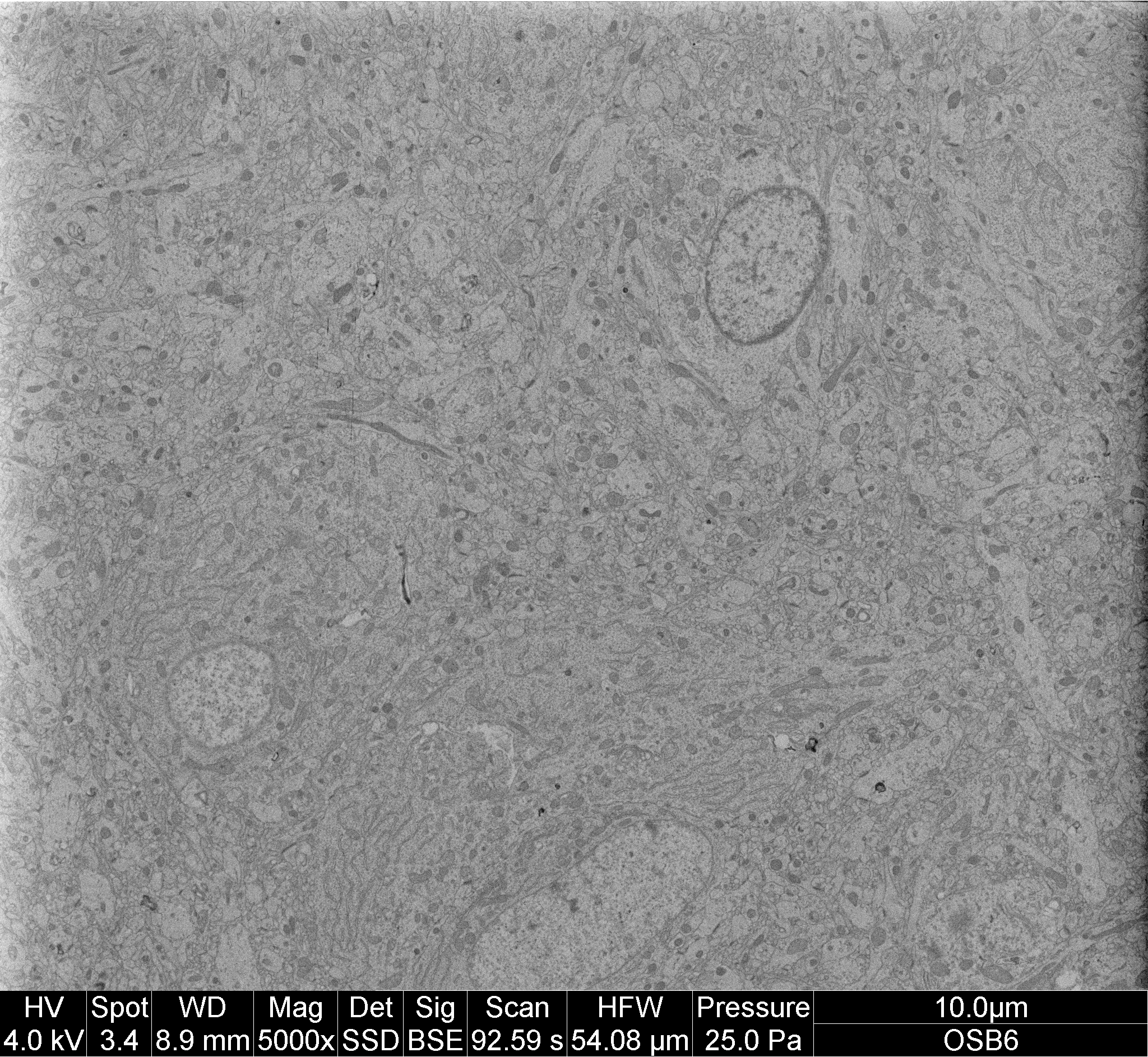

Supplement: Dataset S14 — (251.8 MB ZIP). [file pbio.0020329.sd014.zip › 040604_OS5_st1_1382.tif]

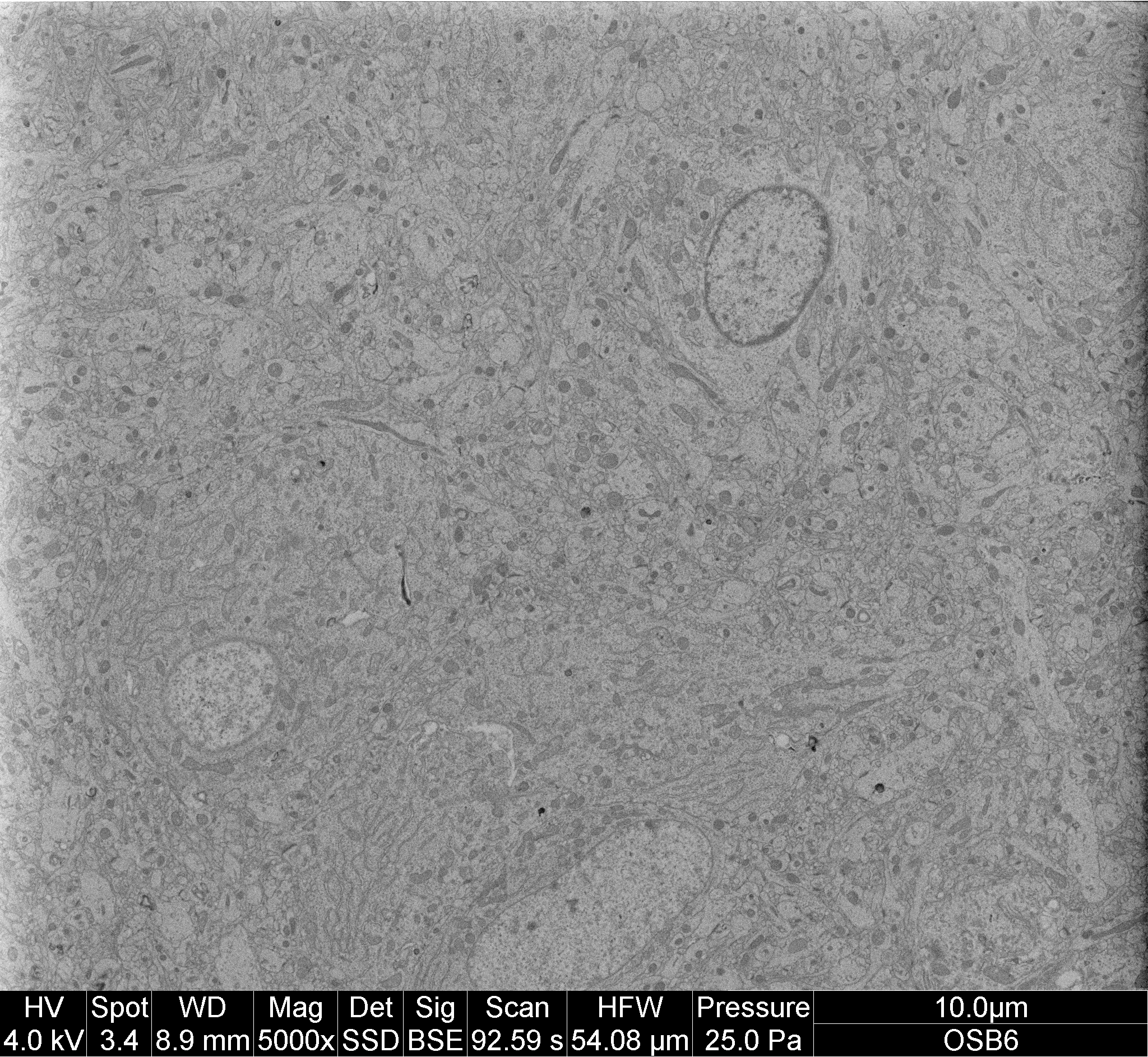

Supplement: Dataset S14 — (251.8 MB ZIP). [file pbio.0020329.sd014.zip › 040604_OS5_st1_1383.tif]

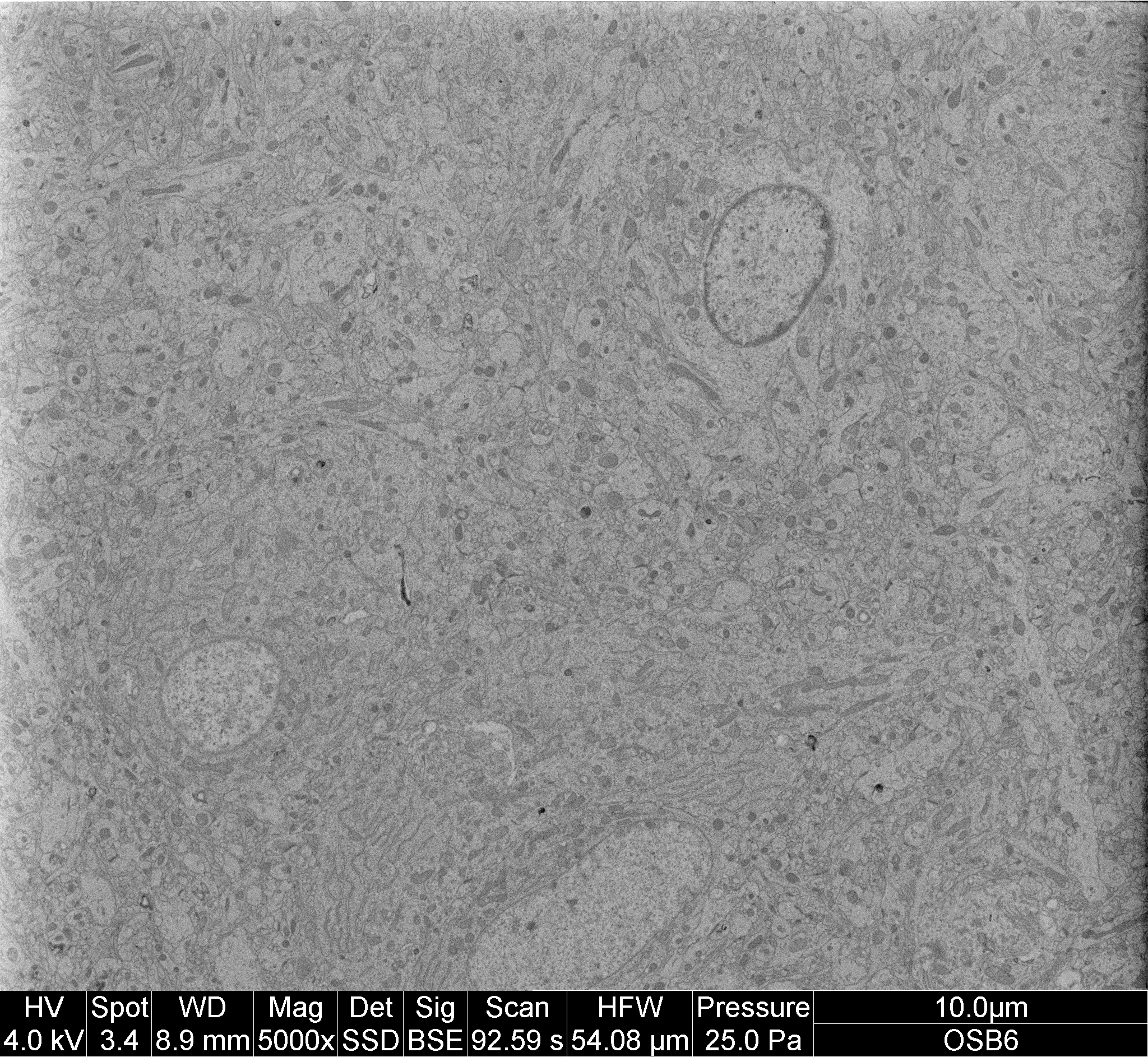

Supplement: Dataset S14 — (251.8 MB ZIP). [file pbio.0020329.sd014.zip › 040604_OS5_st1_1384.tif]

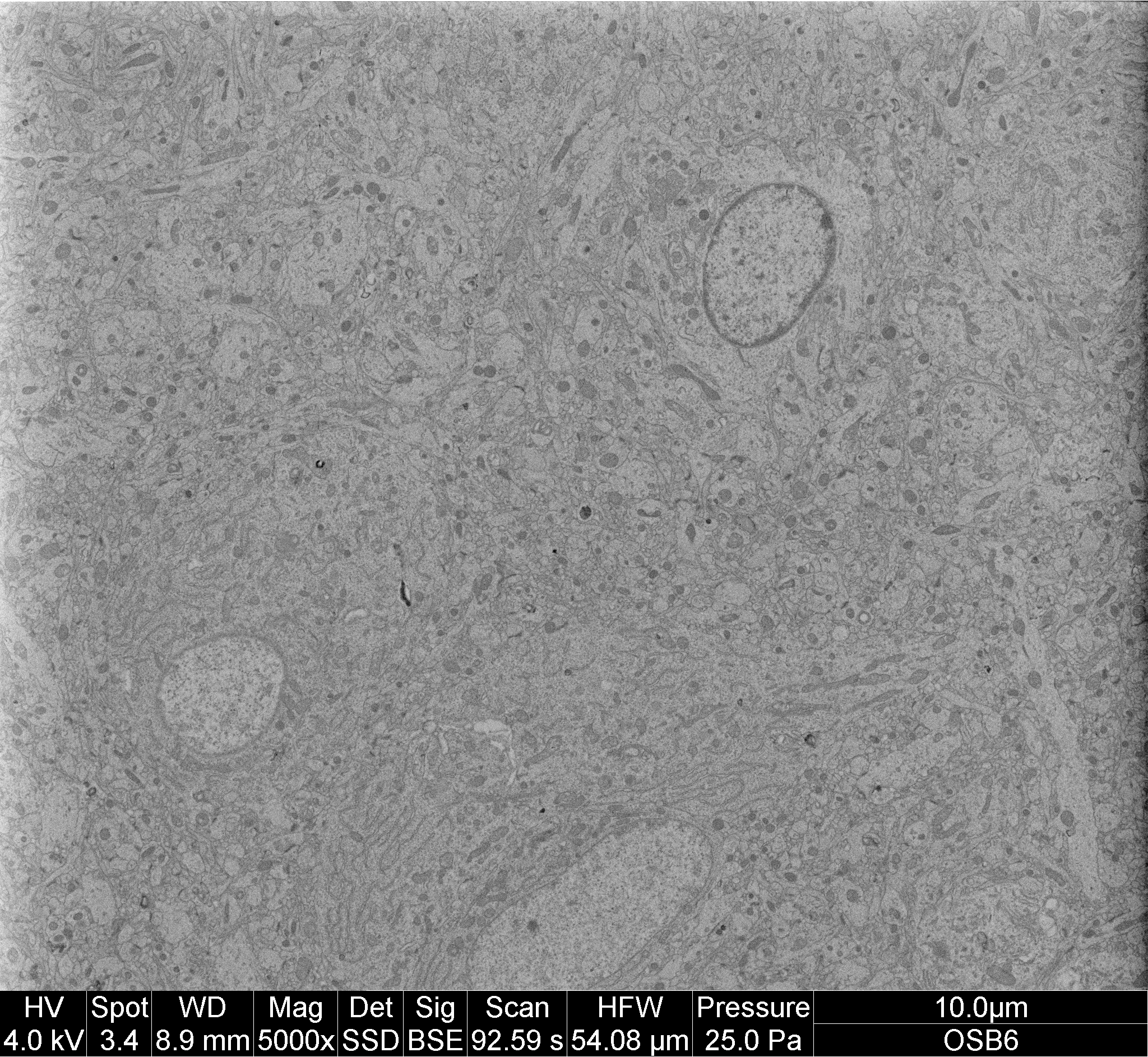

Supplement: Dataset S14 — (251.8 MB ZIP). [file pbio.0020329.sd014.zip › 040604_OS5_st1_1385.tif]

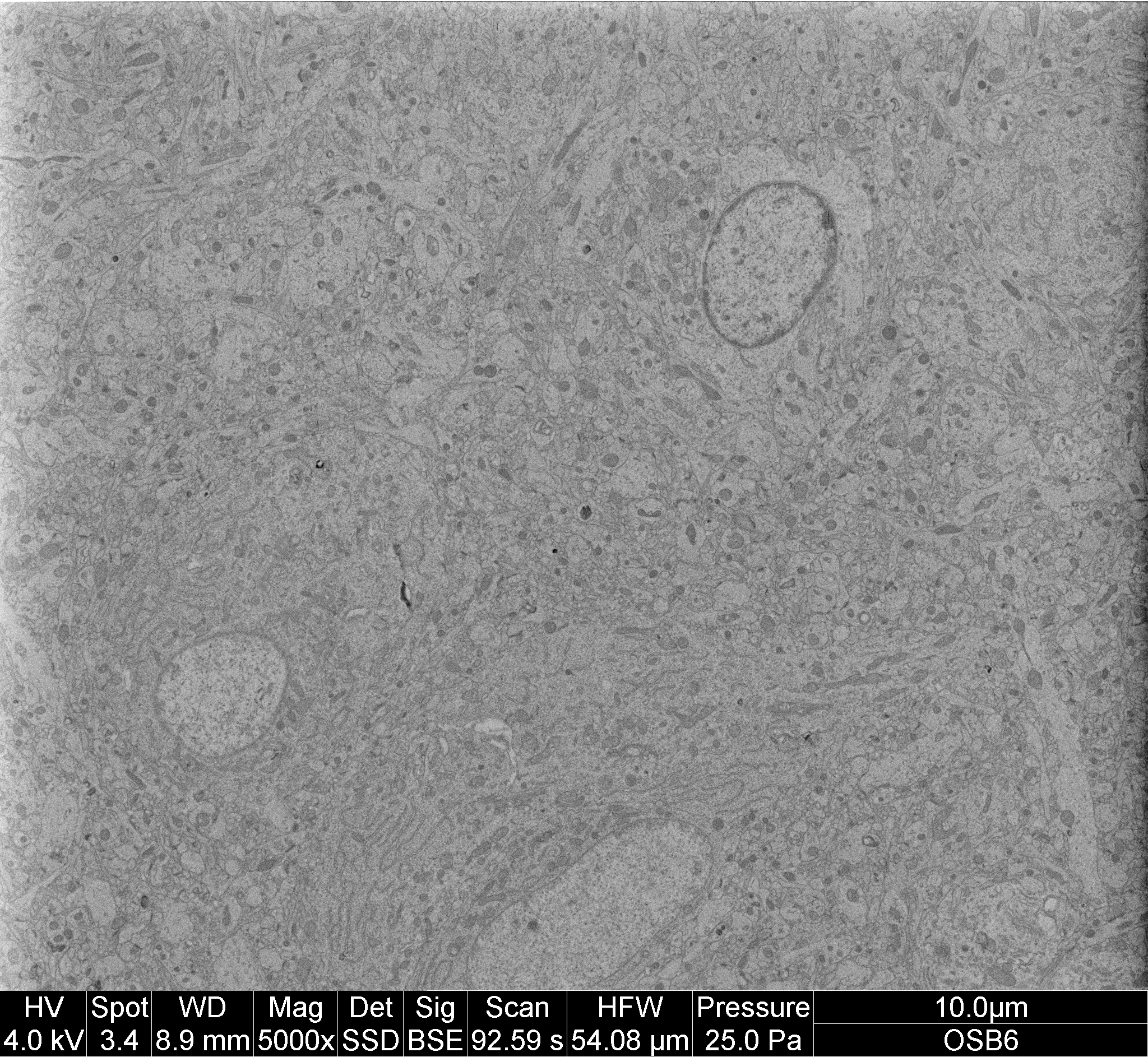

Supplement: Dataset S14 — (251.8 MB ZIP). [file pbio.0020329.sd014.zip › 040604_OS5_st1_1386.tif]

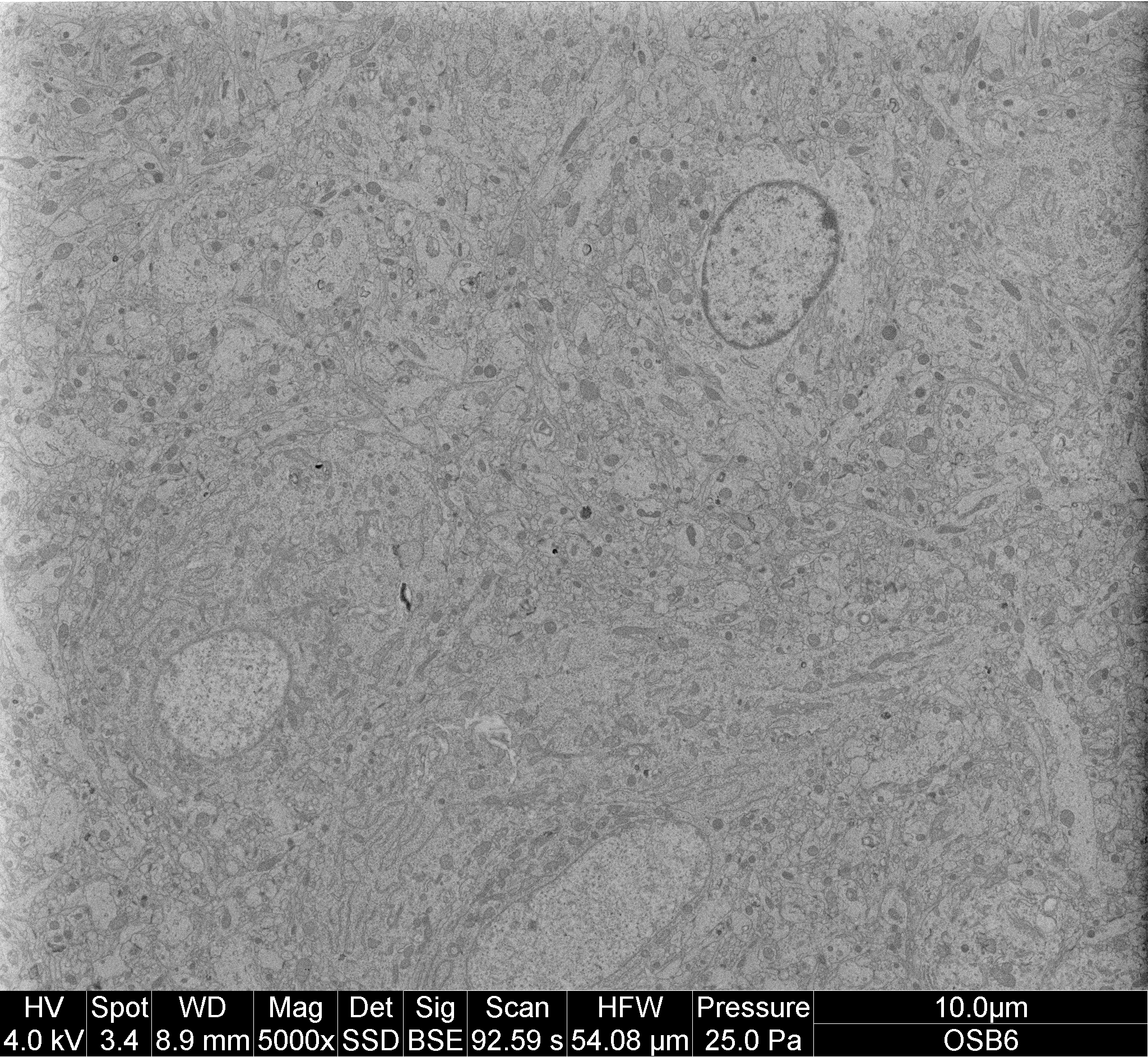

Supplement: Dataset S14 — (251.8 MB ZIP). [file pbio.0020329.sd014.zip › 040604_OS5_st1_1387.tif]

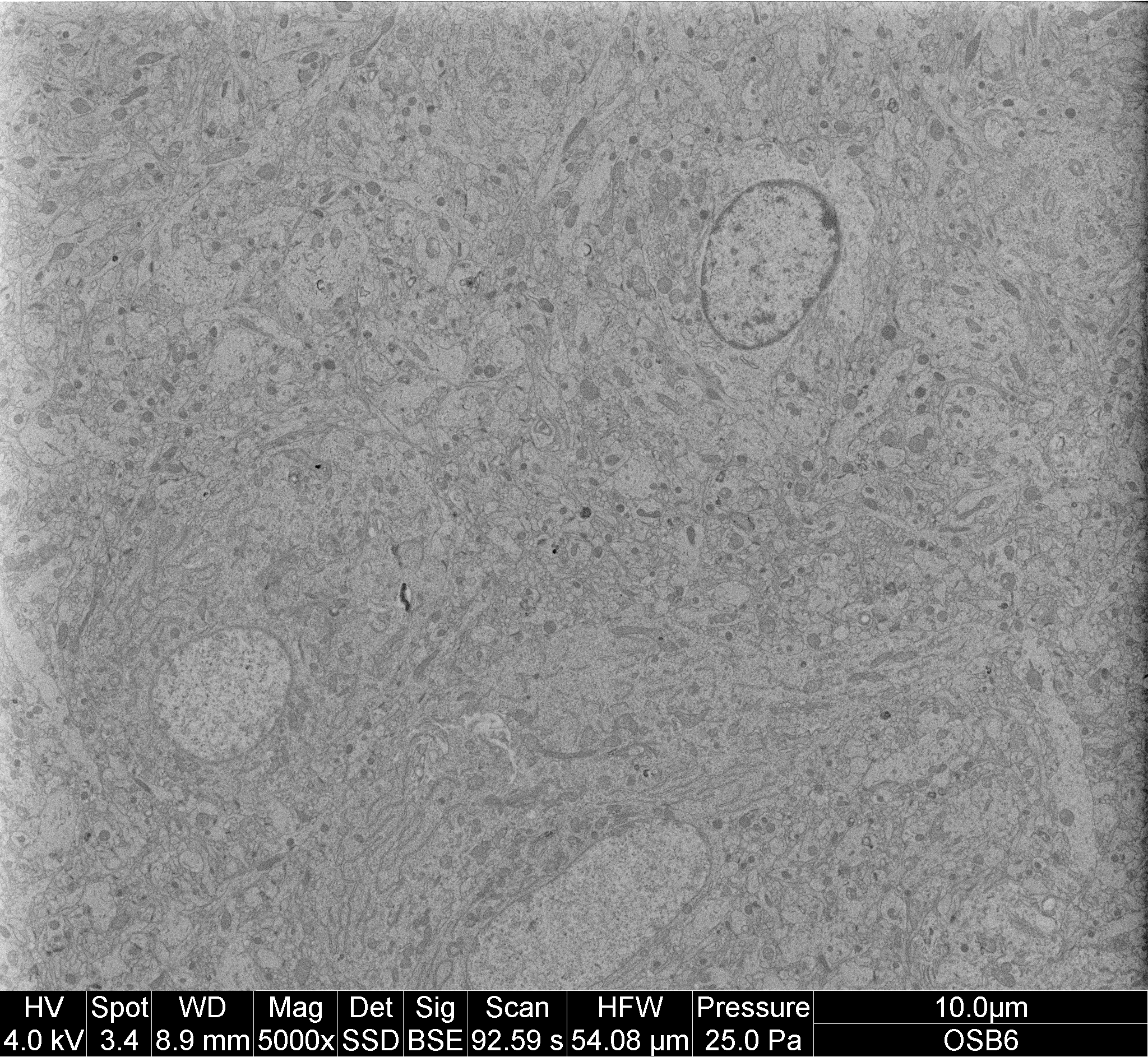

Supplement: Dataset S14 — (251.8 MB ZIP). [file pbio.0020329.sd014.zip › 040604_OS5_st1_1388.tif]

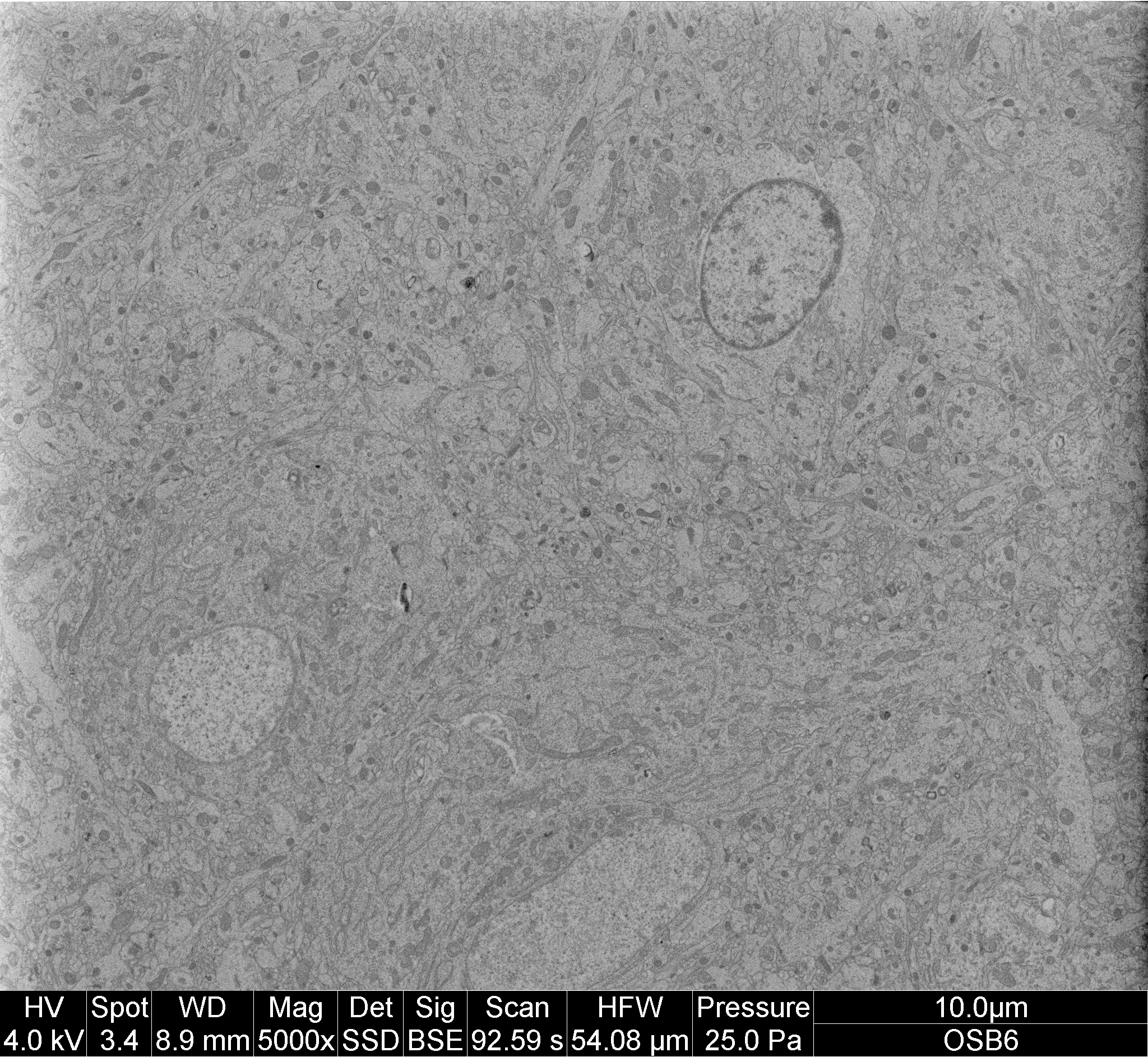

Supplement: Dataset S14 — (251.8 MB ZIP). [file pbio.0020329.sd014.zip › 040604_OS5_st1_1389.tif]

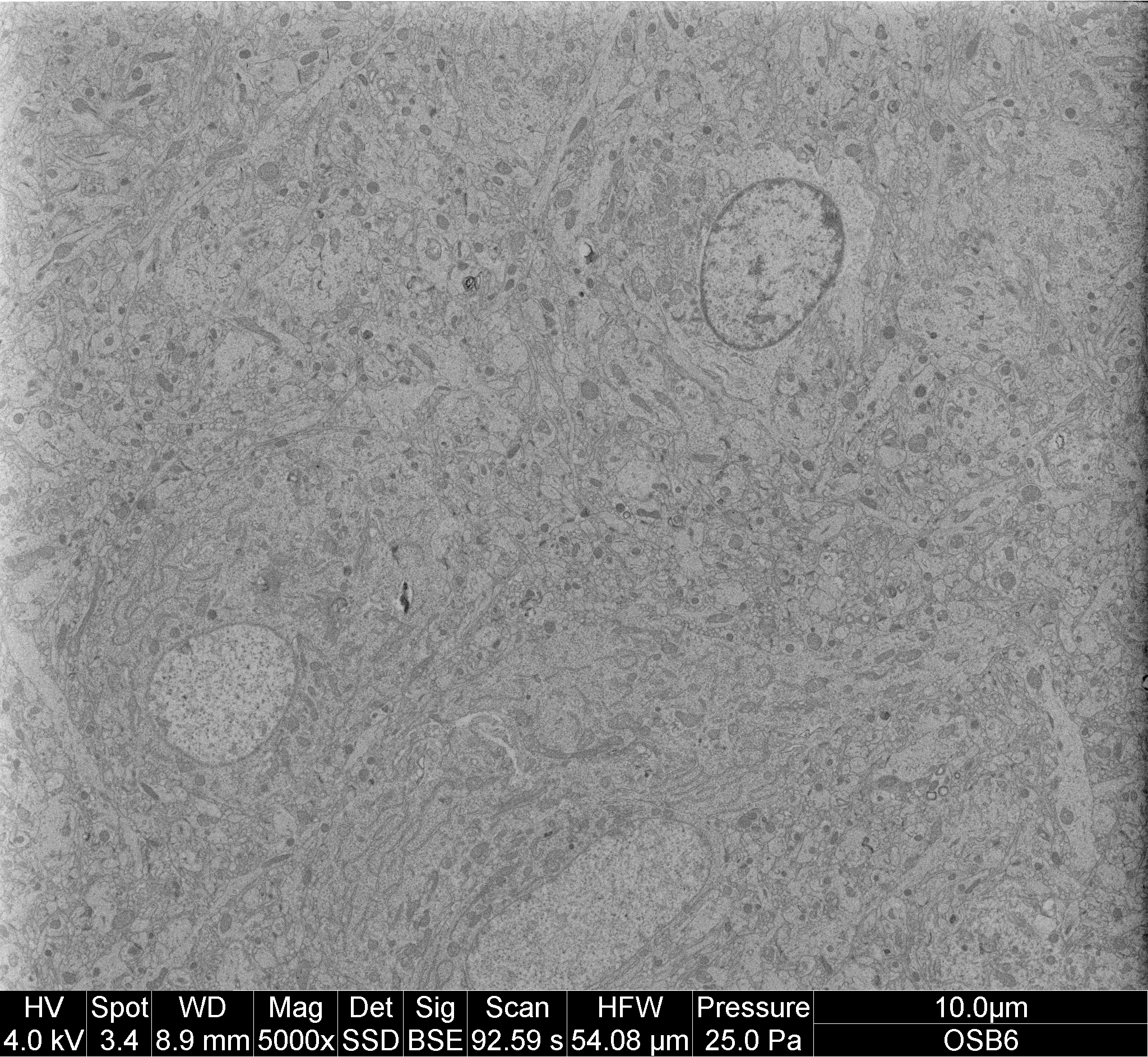

Supplement: Dataset S14 — (251.8 MB ZIP). [file pbio.0020329.sd014.zip › 040604_OS5_st1_1390.tif]

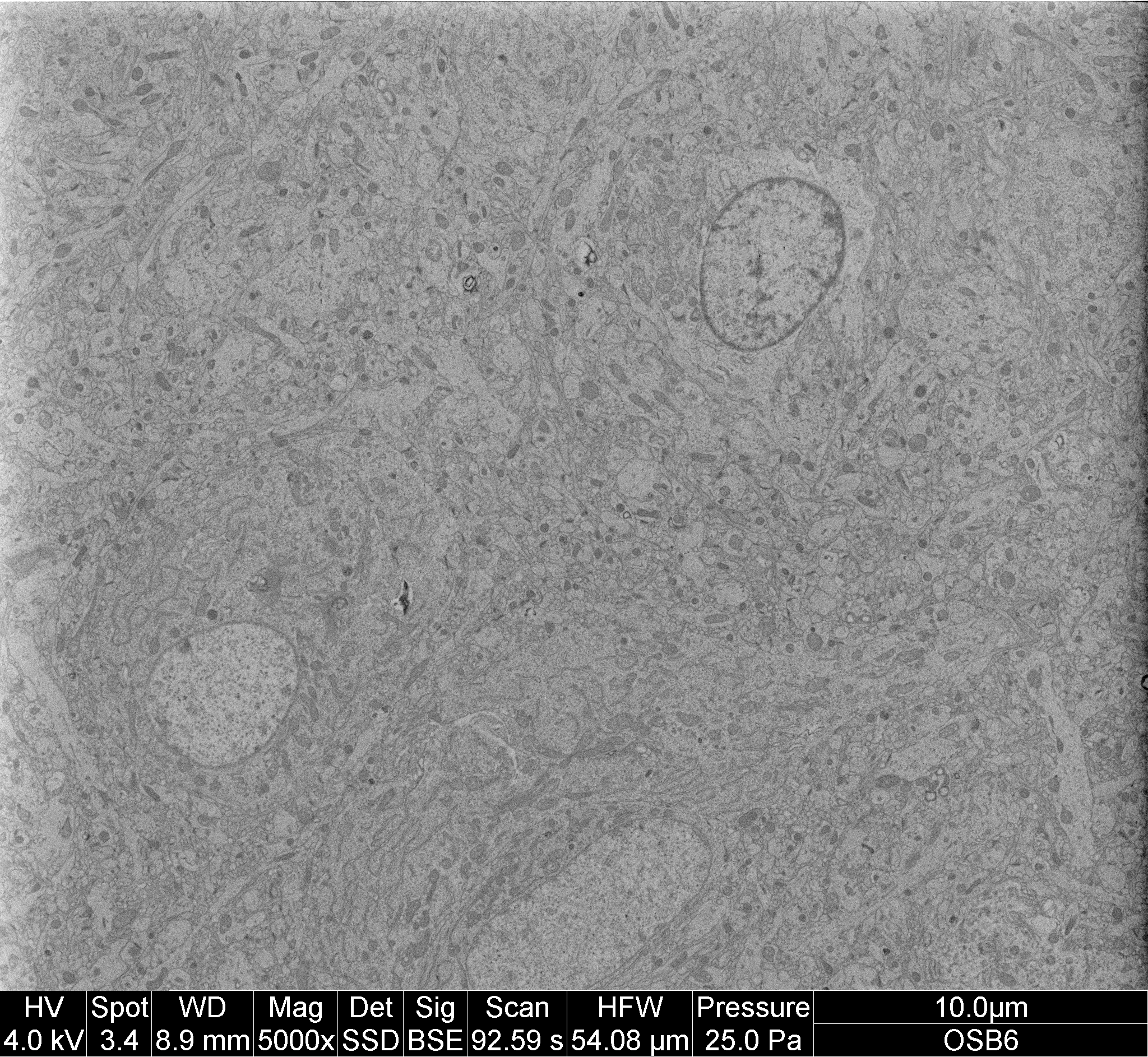

Supplement: Dataset S14 — (251.8 MB ZIP). [file pbio.0020329.sd014.zip › 040604_OS5_st1_1391.tif]

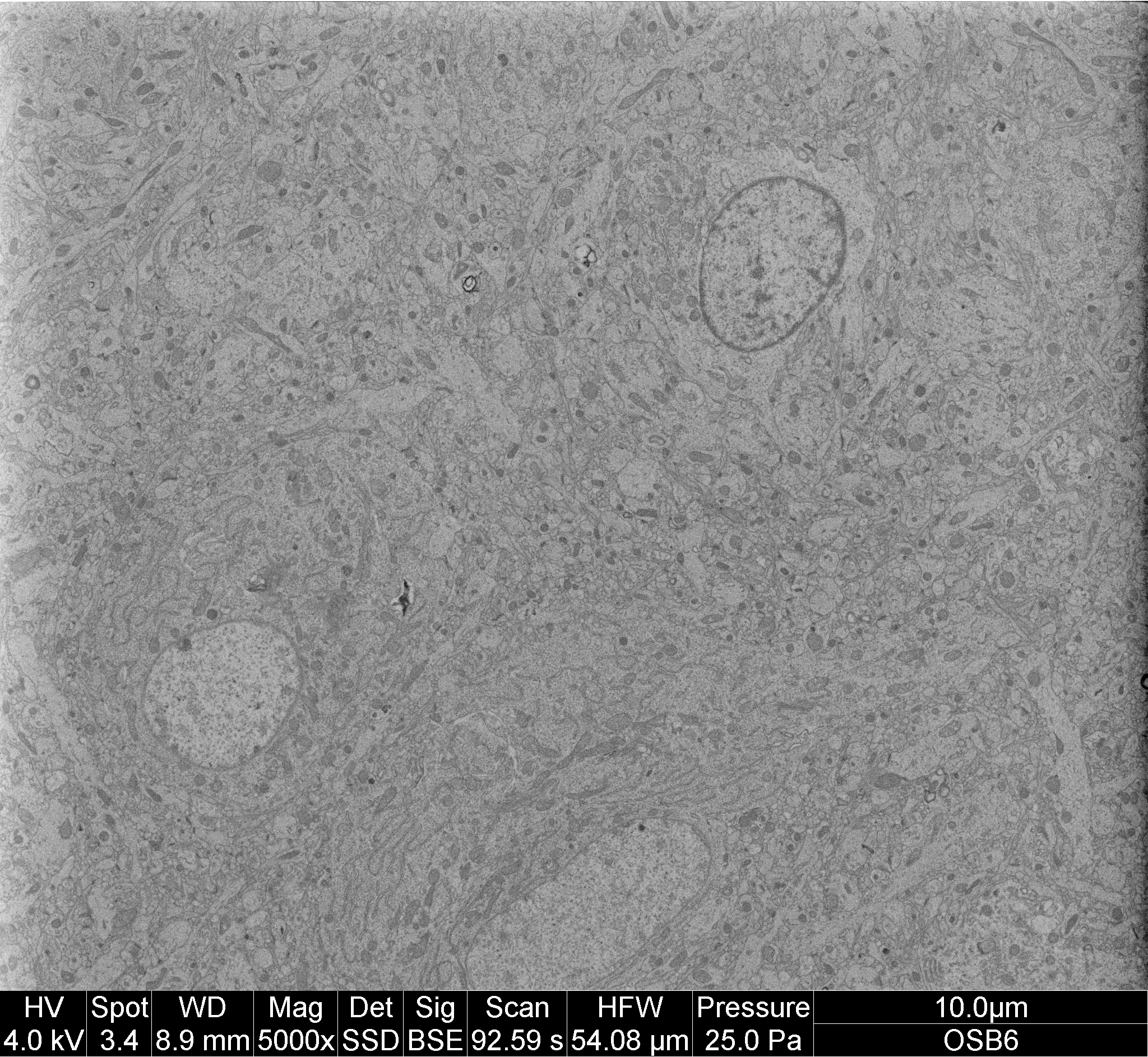

Supplement: Dataset S14 — (251.8 MB ZIP). [file pbio.0020329.sd014.zip › 040604_OS5_st1_1392.tif]

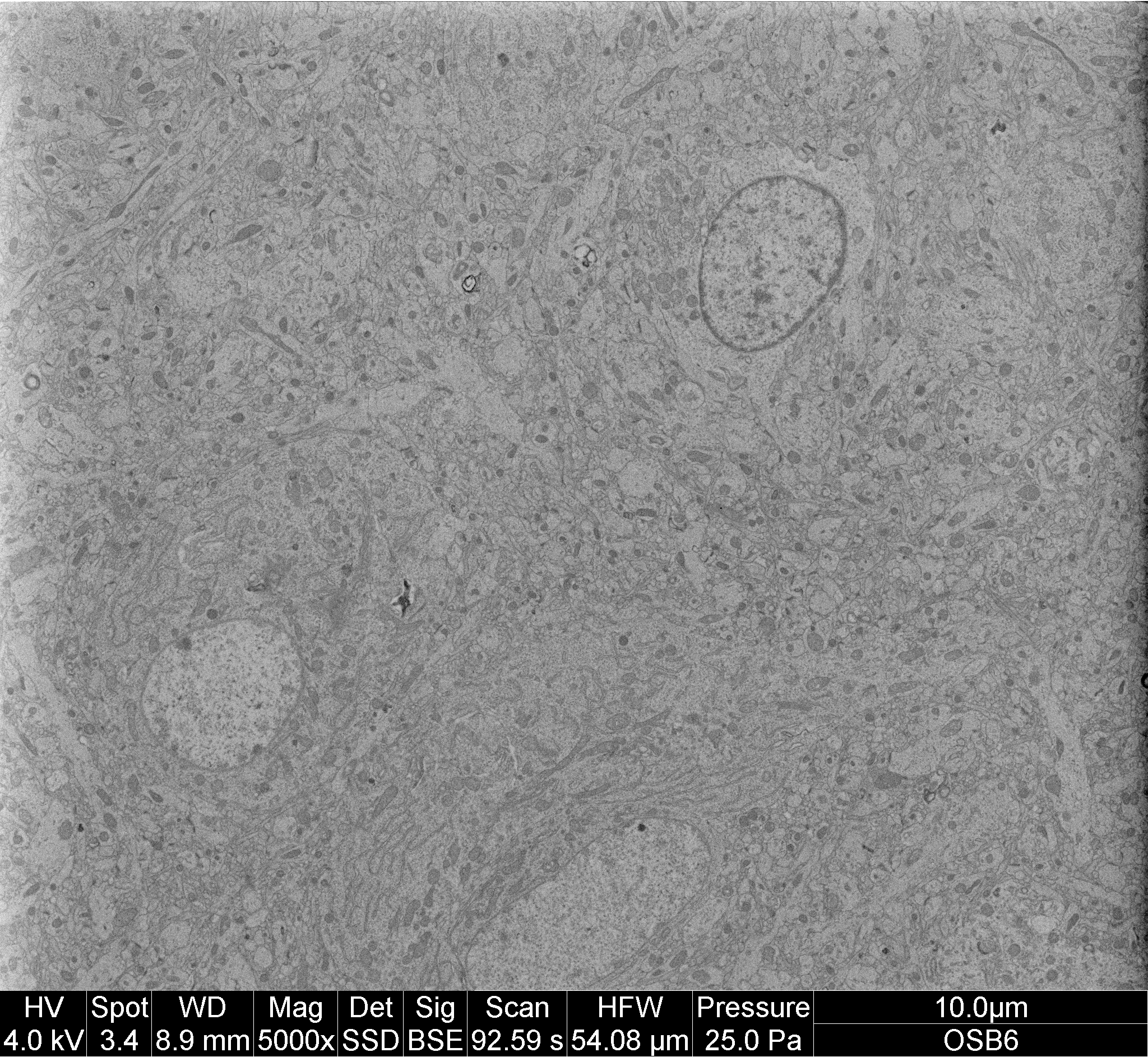

Supplement: Dataset S14 — (251.8 MB ZIP). [file pbio.0020329.sd014.zip › 040604_OS5_st1_1393.tif]

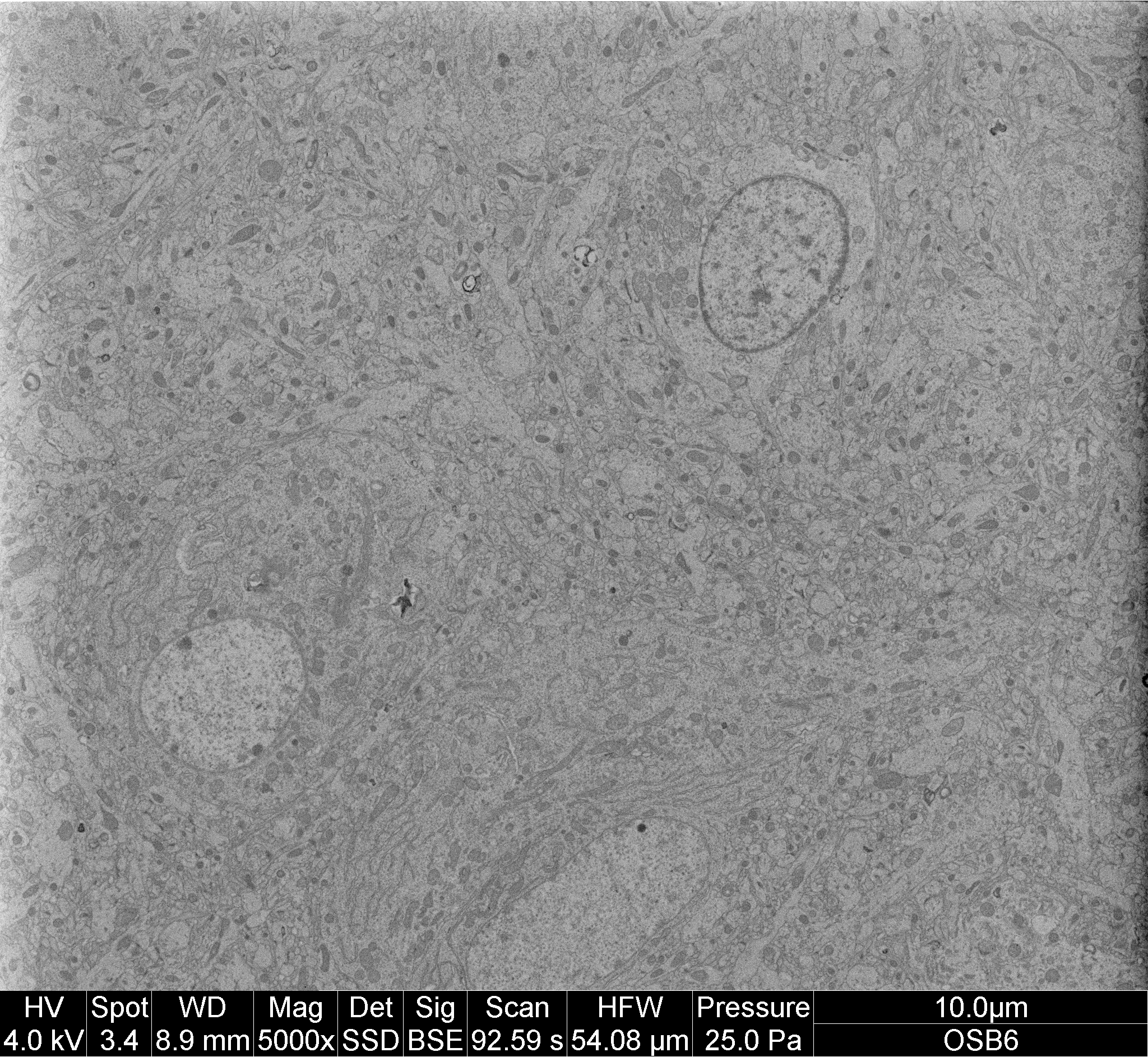

Supplement: Dataset S14 — (251.8 MB ZIP). [file pbio.0020329.sd014.zip › 040604_OS5_st1_1394.tif]

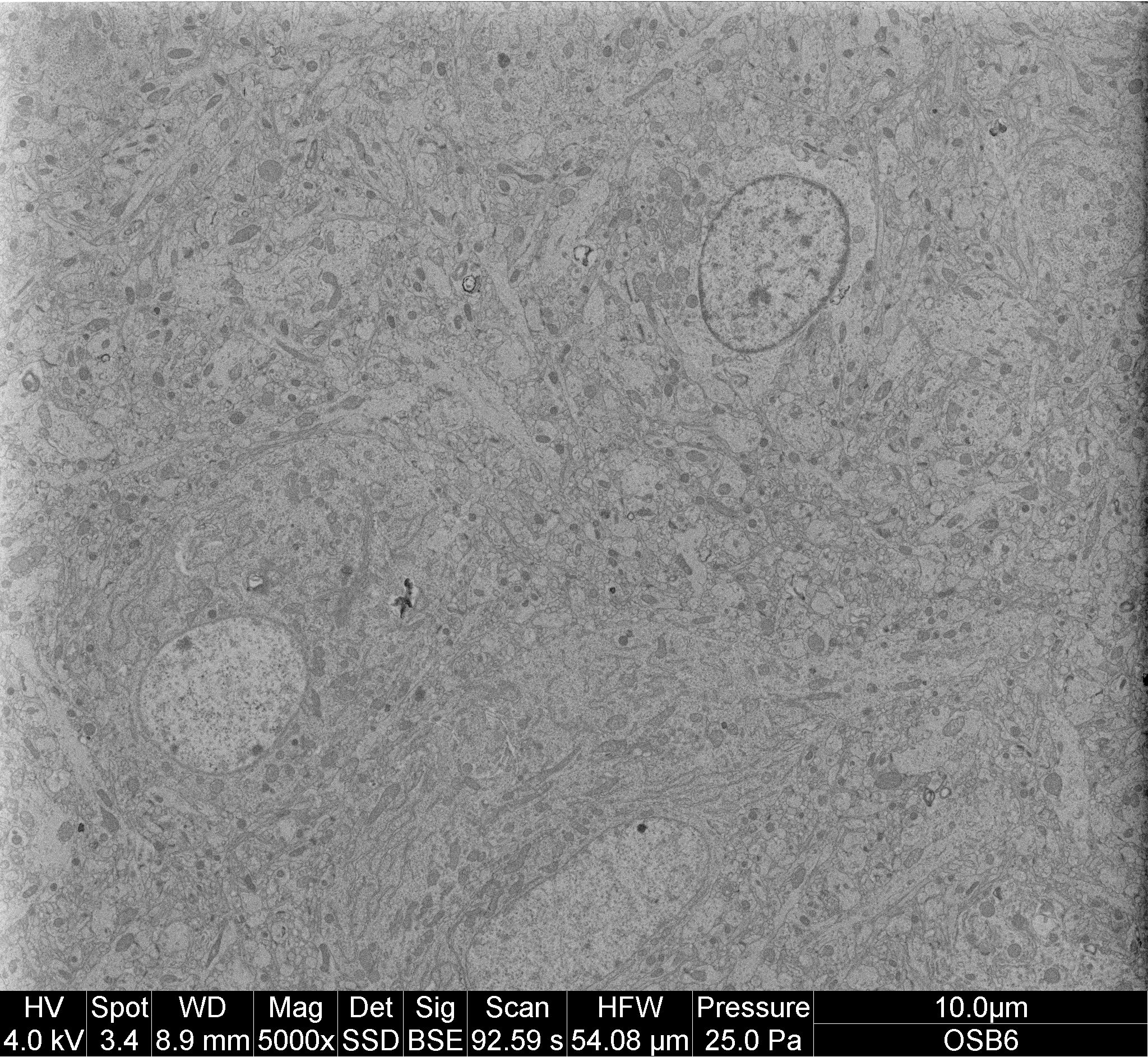

Supplement: Dataset S14 — (251.8 MB ZIP). [file pbio.0020329.sd014.zip › 040604_OS5_st1_1395.tif]

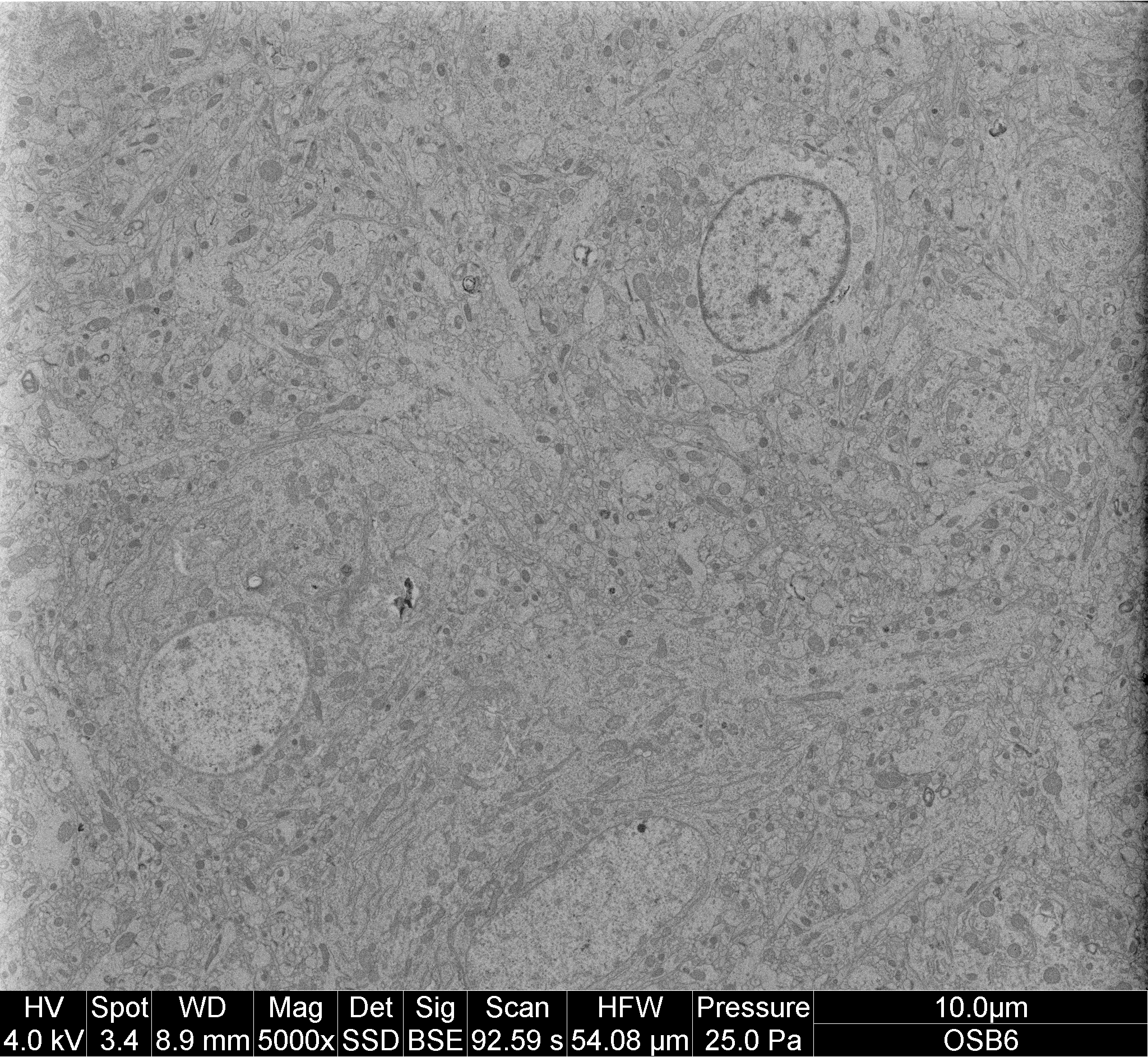

Supplement: Dataset S14 — (251.8 MB ZIP). [file pbio.0020329.sd014.zip › 040604_OS5_st1_1396.tif]

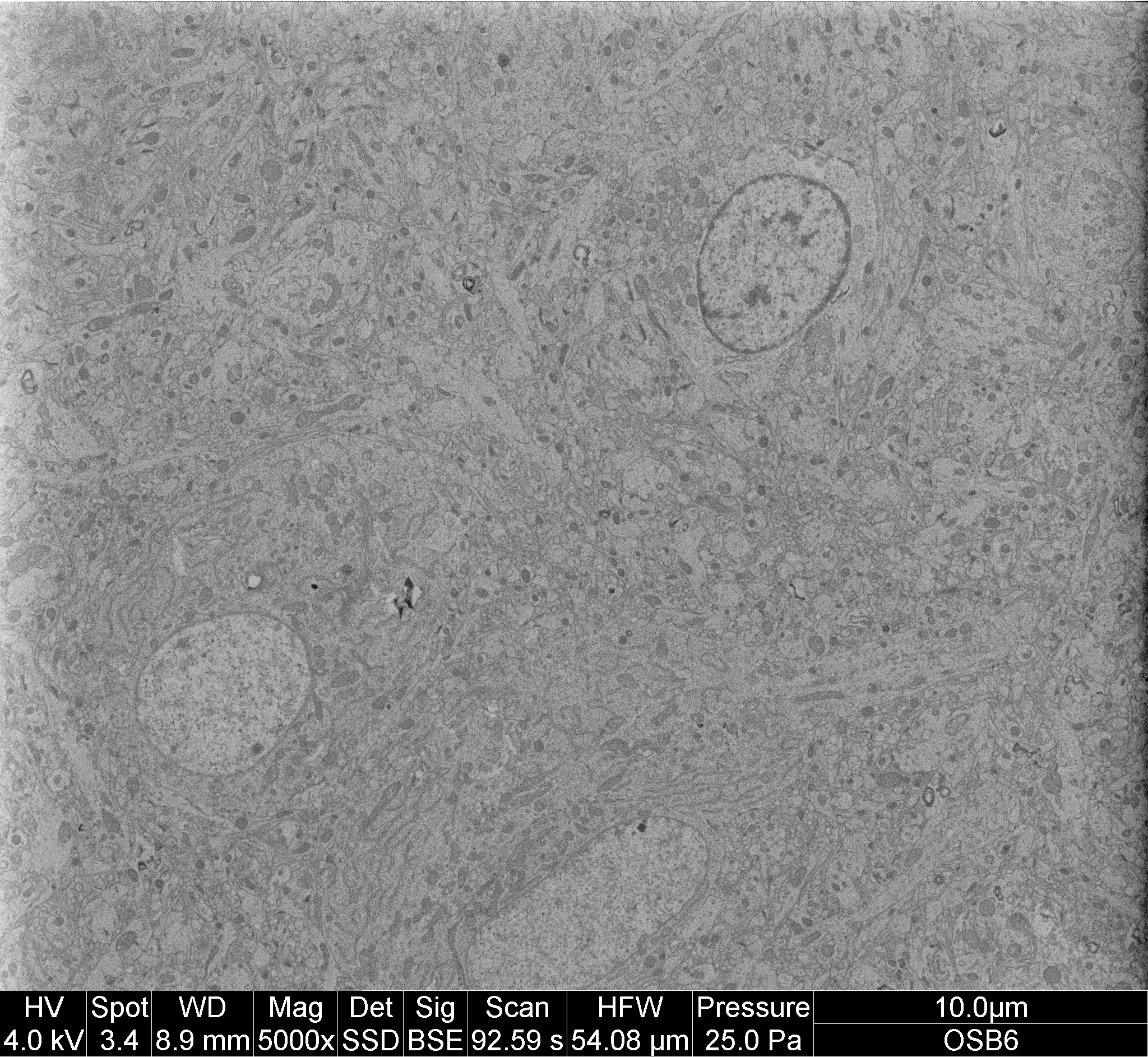

Supplement: Dataset S14 — (251.8 MB ZIP). [file pbio.0020329.sd014.zip › 040604_OS5_st1_1397.tif]

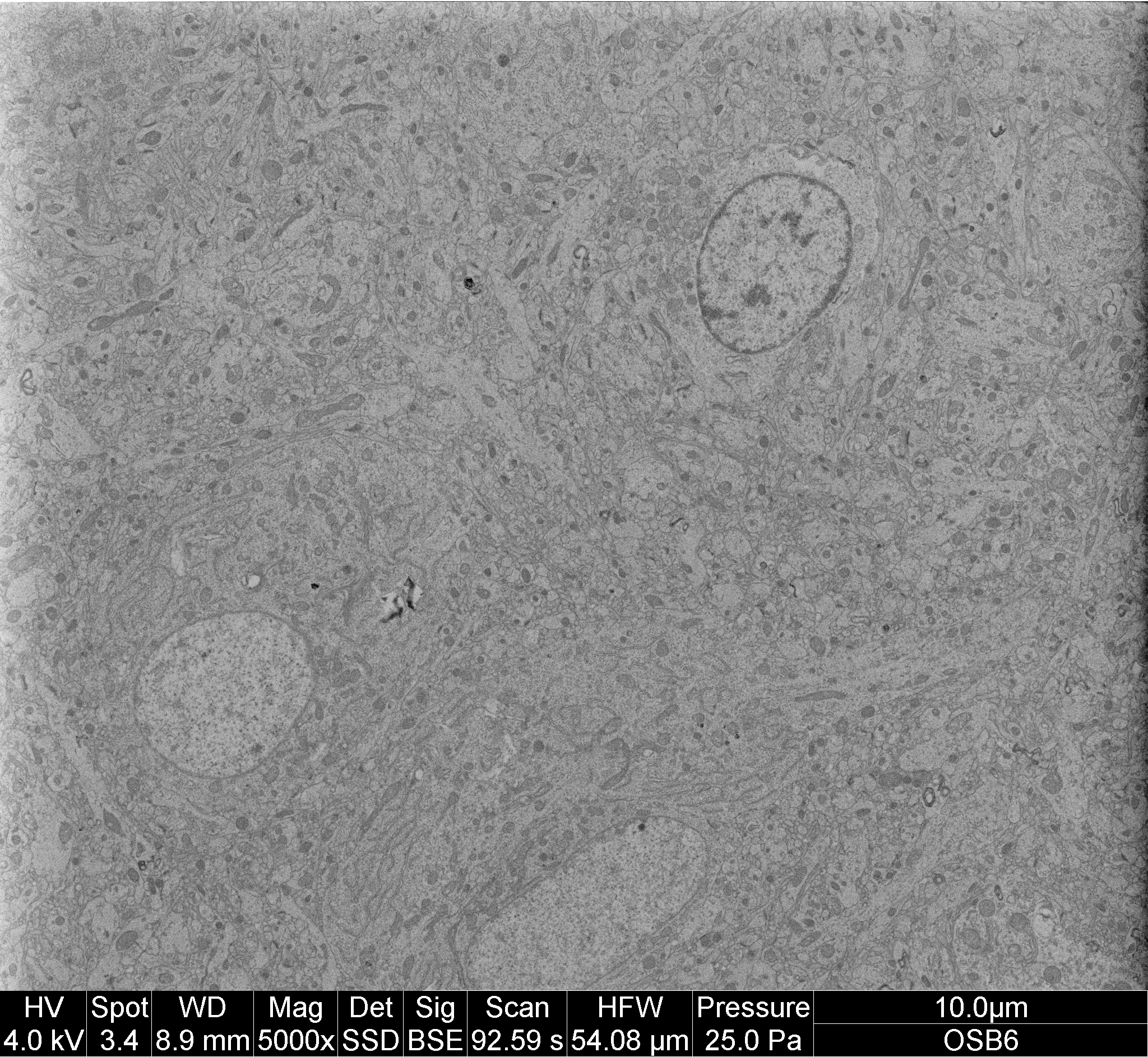

Supplement: Dataset S14 — (251.8 MB ZIP). [file pbio.0020329.sd014.zip › 040604_OS5_st1_1398.tif]

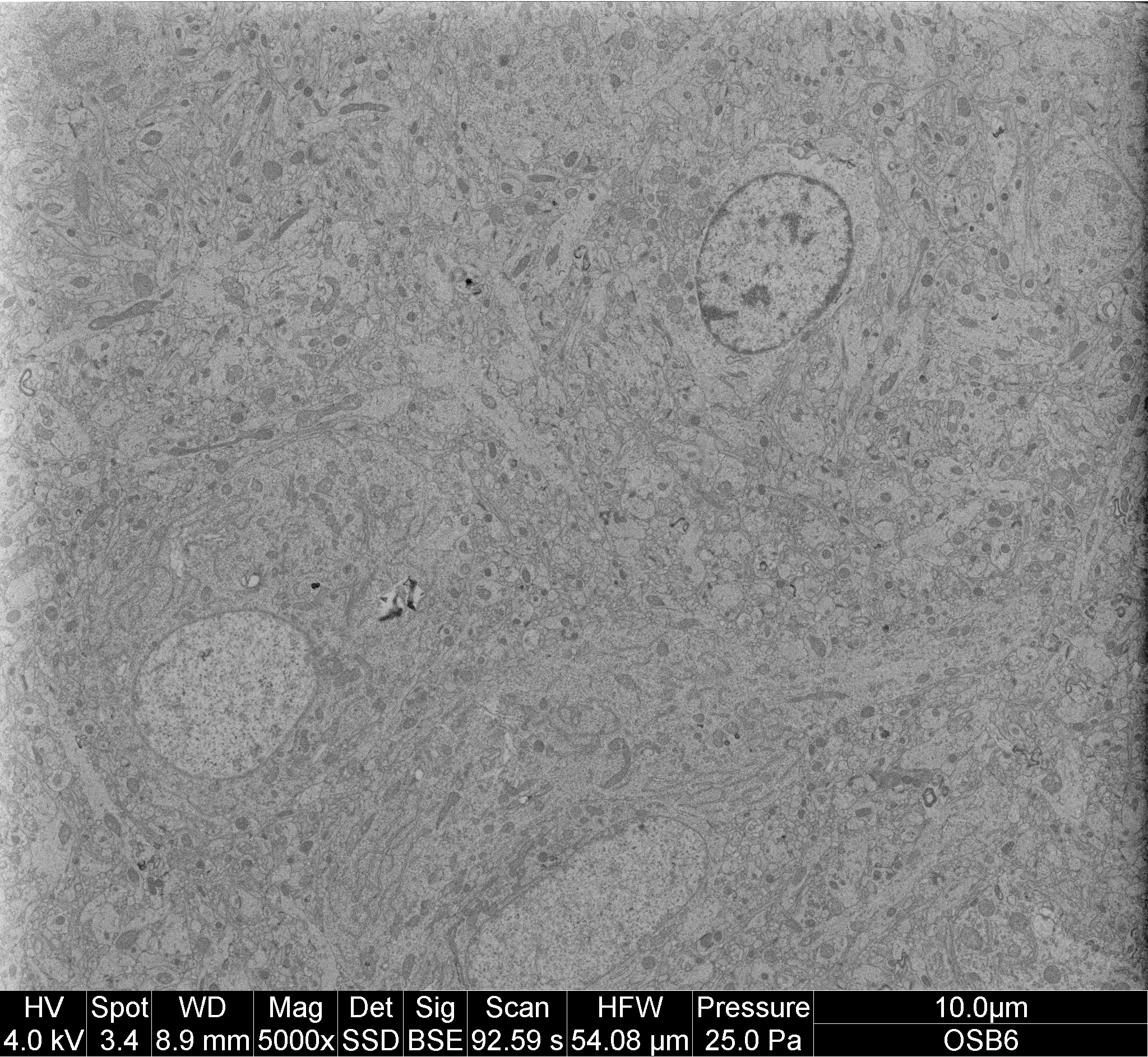

Supplement: Dataset S14 — (251.8 MB ZIP). [file pbio.0020329.sd014.zip › 040604_OS5_st1_1399.tif]

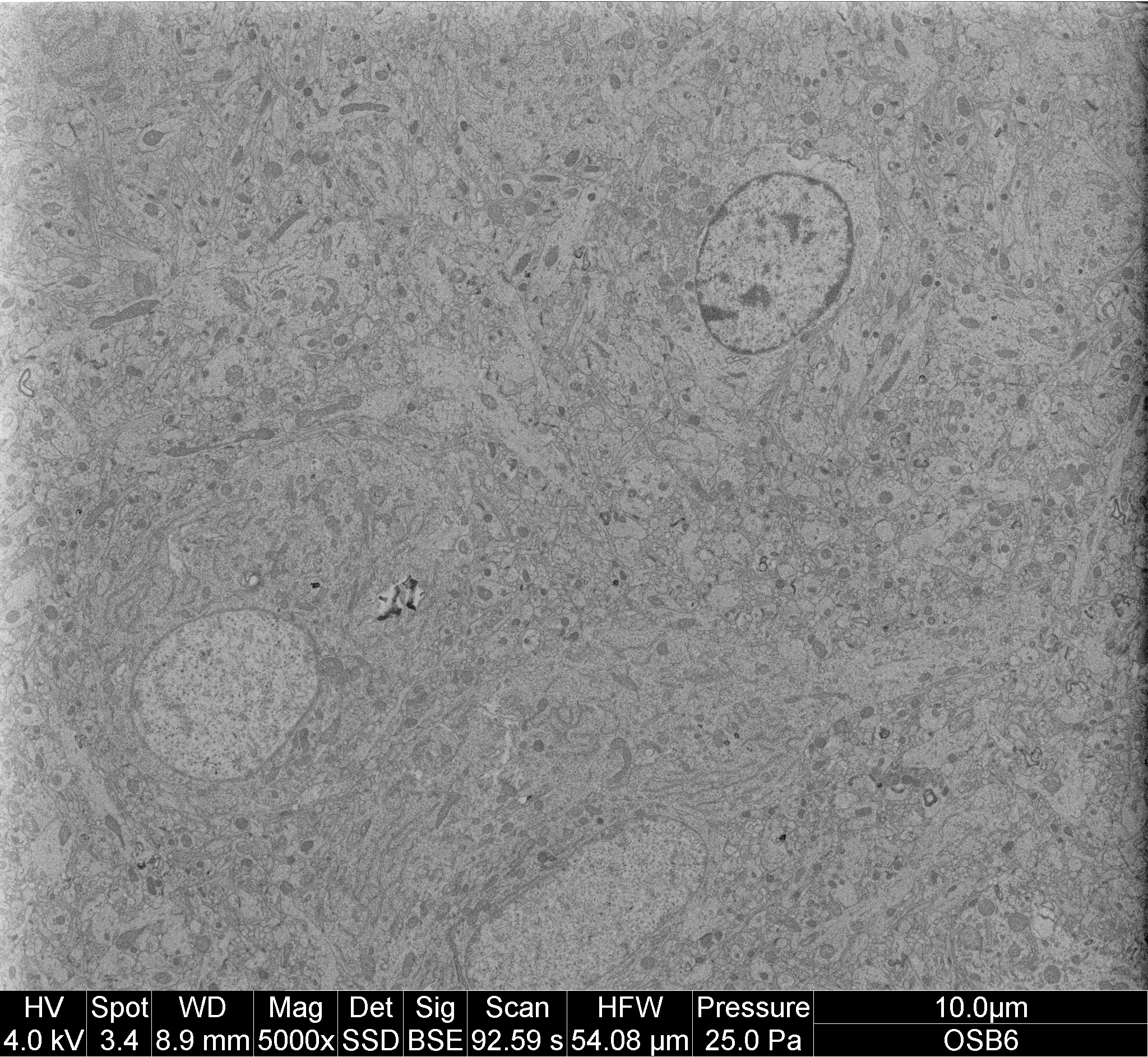

Supplement: Dataset S15 — (250.7 MB ZIP). [file pbio.0020329.sd015.zip › 040604_OS5_st1_1400.tif]
