# Supplementary figures and images for: Serial Block-Face Scanning Electron Microscopy to Reconstruct Three-Dimensional Tissue Nanostructure (part 15 of 21)
Source: PLoS Biol. 2004 Oct 19;2(11):e329. doi: 10.1371/journal.pbio.0020329 (PMC524270; doi:10.1371/journal.pbio.0020329)

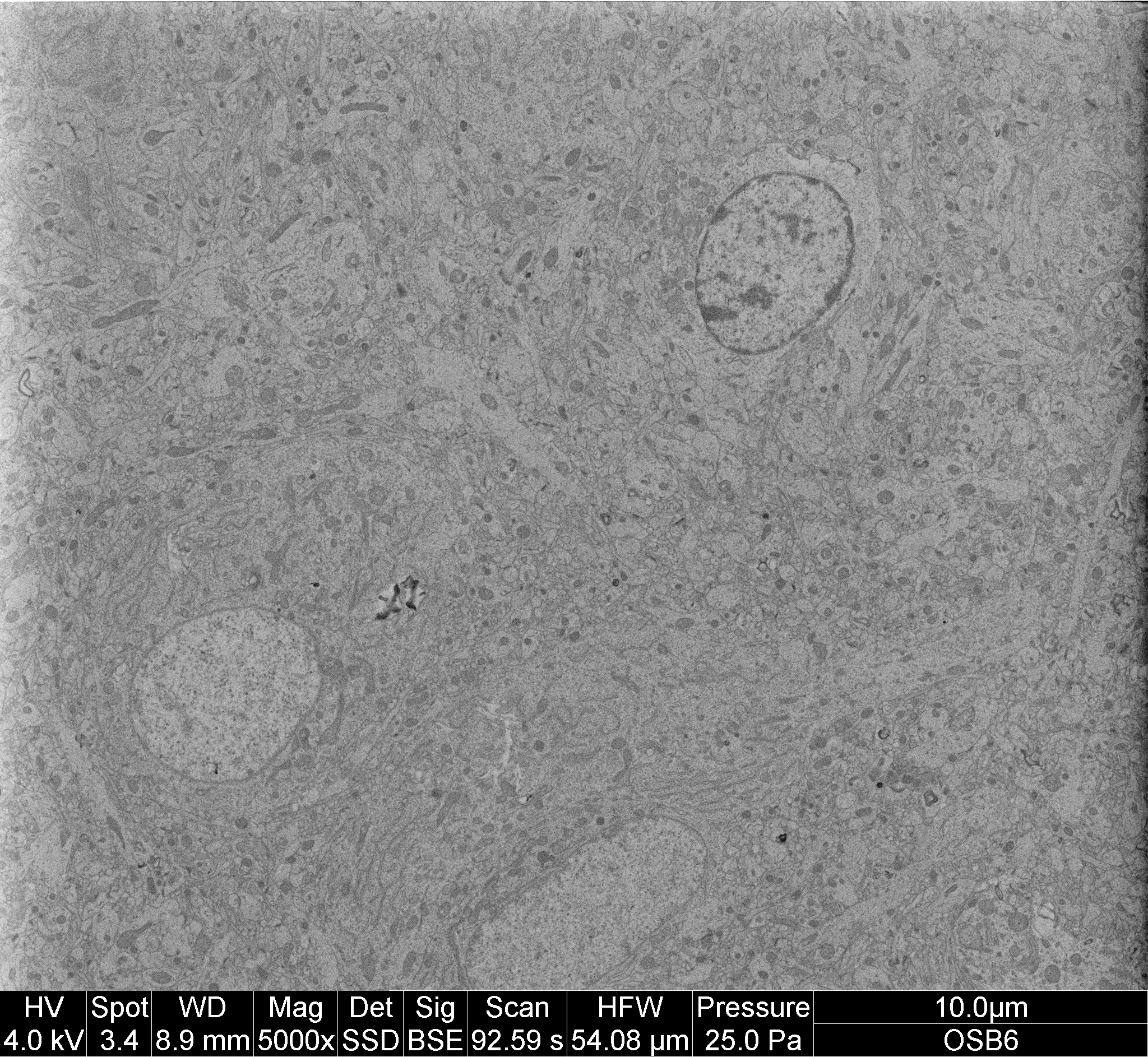

Supplement: Dataset S15 — (250.7 MB ZIP). [file pbio.0020329.sd015.zip › 040604_OS5_st1_1401.tif]

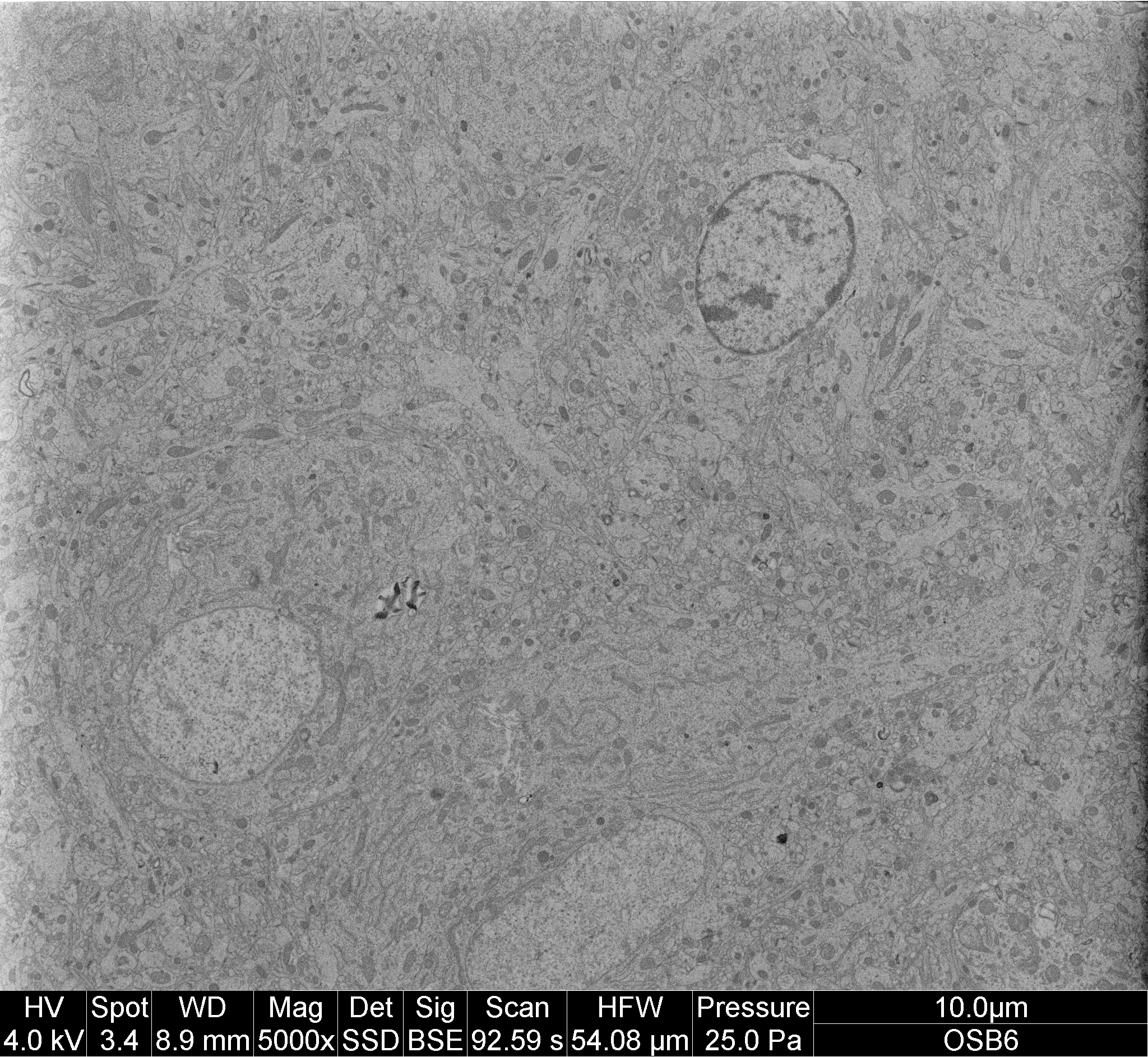

Supplement: Dataset S15 — (250.7 MB ZIP). [file pbio.0020329.sd015.zip › 040604_OS5_st1_1402.tif]

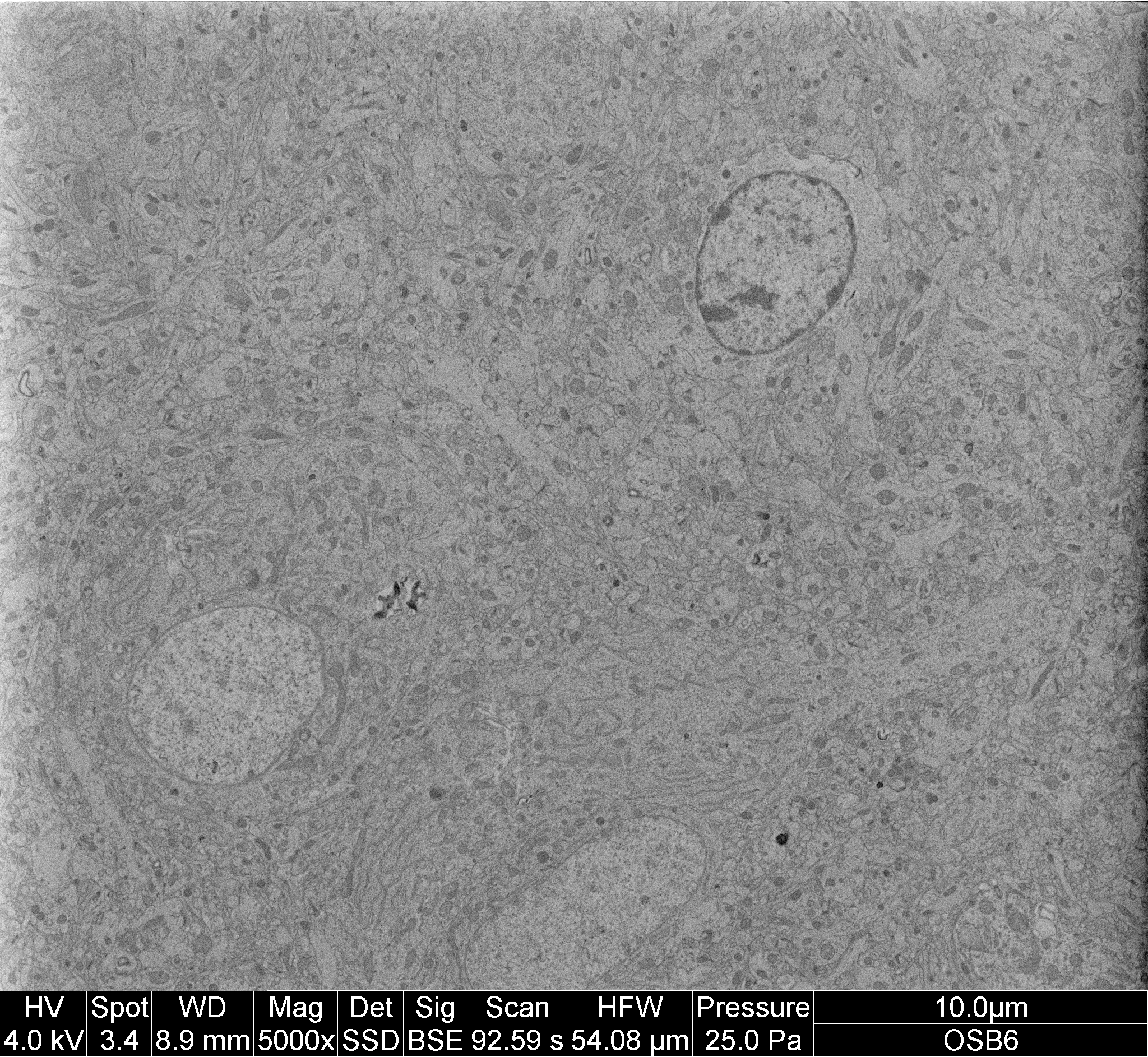

Supplement: Dataset S15 — (250.7 MB ZIP). [file pbio.0020329.sd015.zip › 040604_OS5_st1_1403.tif]

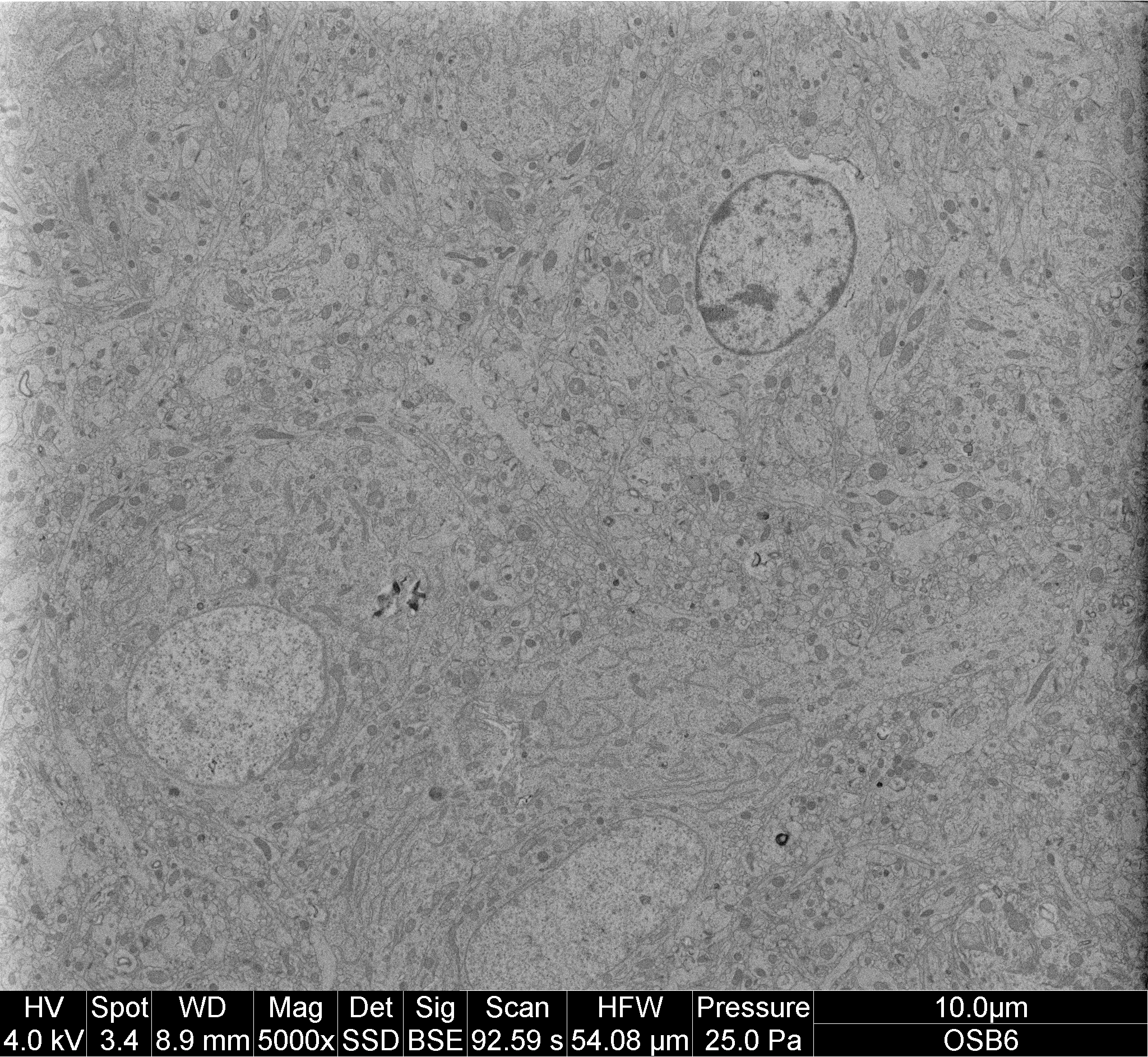

Supplement: Dataset S15 — (250.7 MB ZIP). [file pbio.0020329.sd015.zip › 040604_OS5_st1_1404.tif]

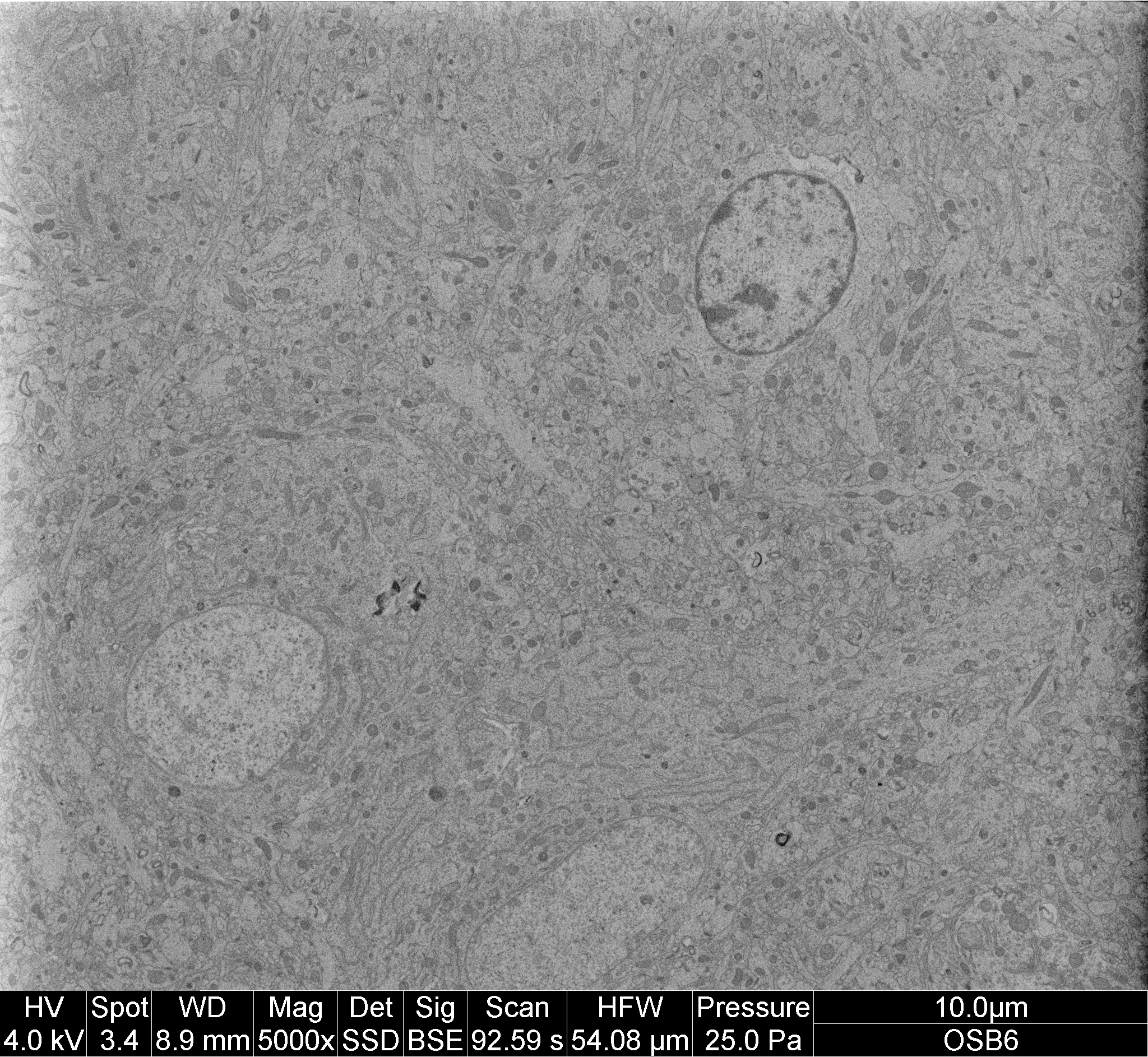

Supplement: Dataset S15 — (250.7 MB ZIP). [file pbio.0020329.sd015.zip › 040604_OS5_st1_1405.tif]

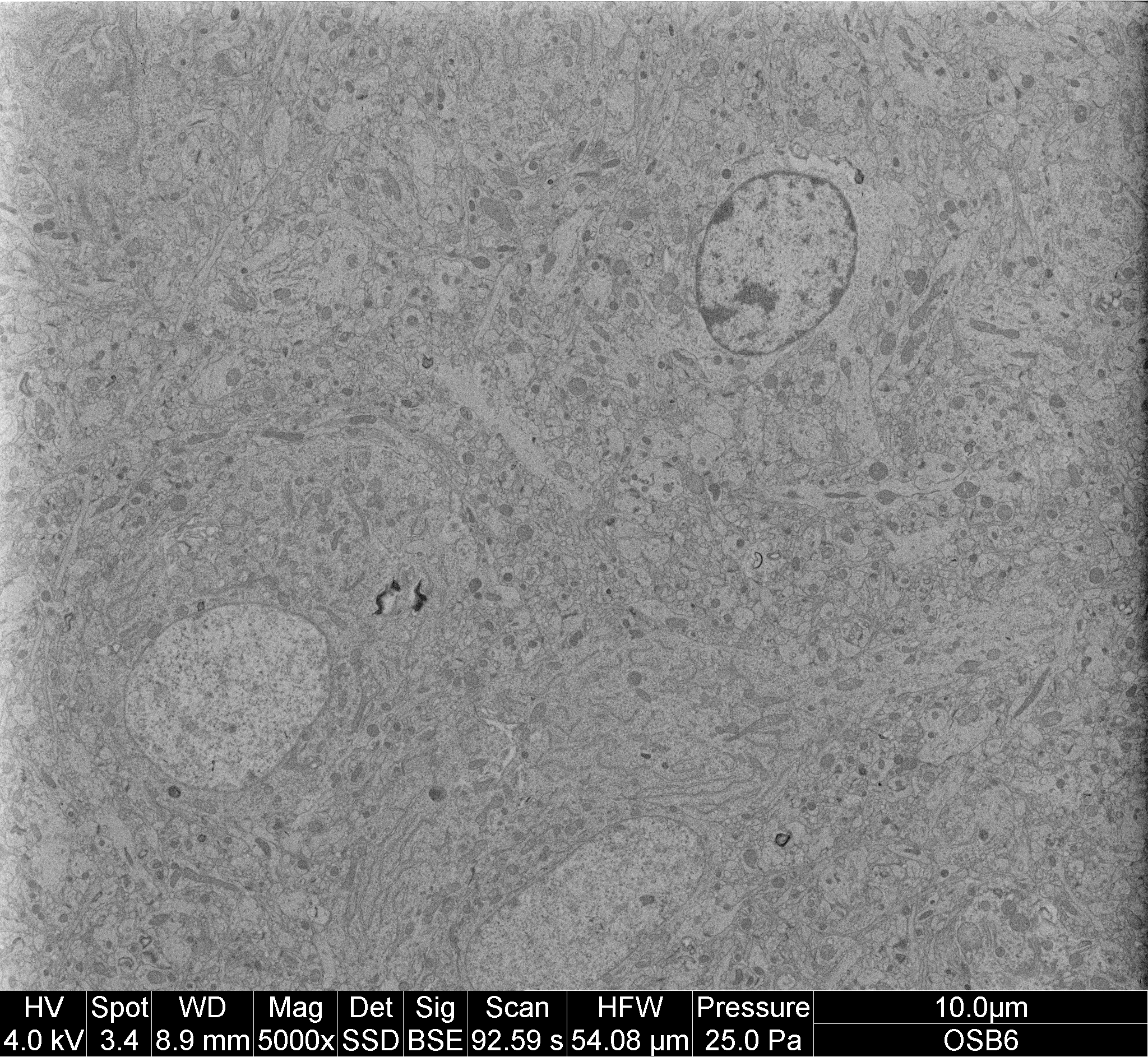

Supplement: Dataset S15 — (250.7 MB ZIP). [file pbio.0020329.sd015.zip › 040604_OS5_st1_1406.tif]

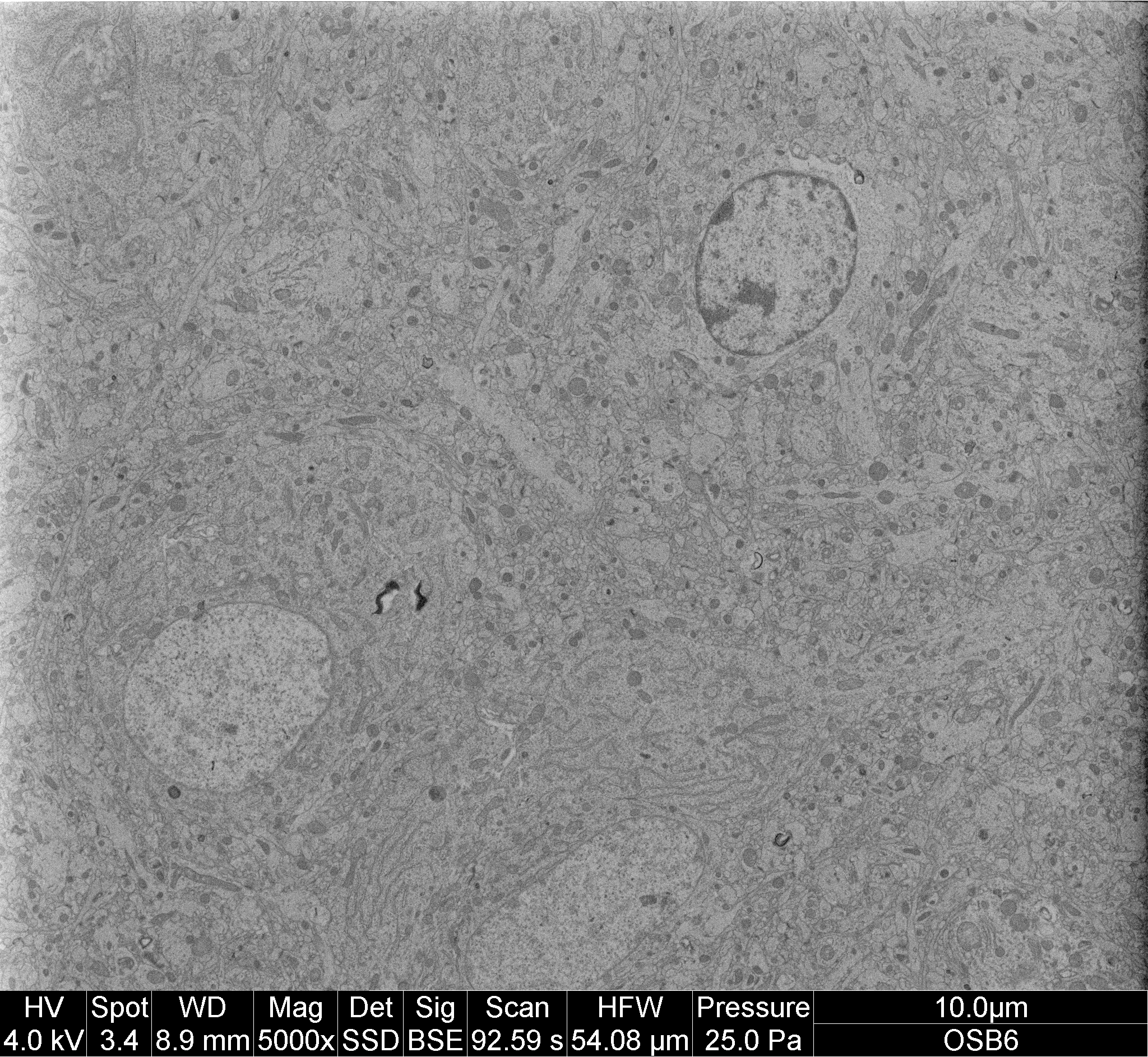

Supplement: Dataset S15 — (250.7 MB ZIP). [file pbio.0020329.sd015.zip › 040604_OS5_st1_1407.tif]

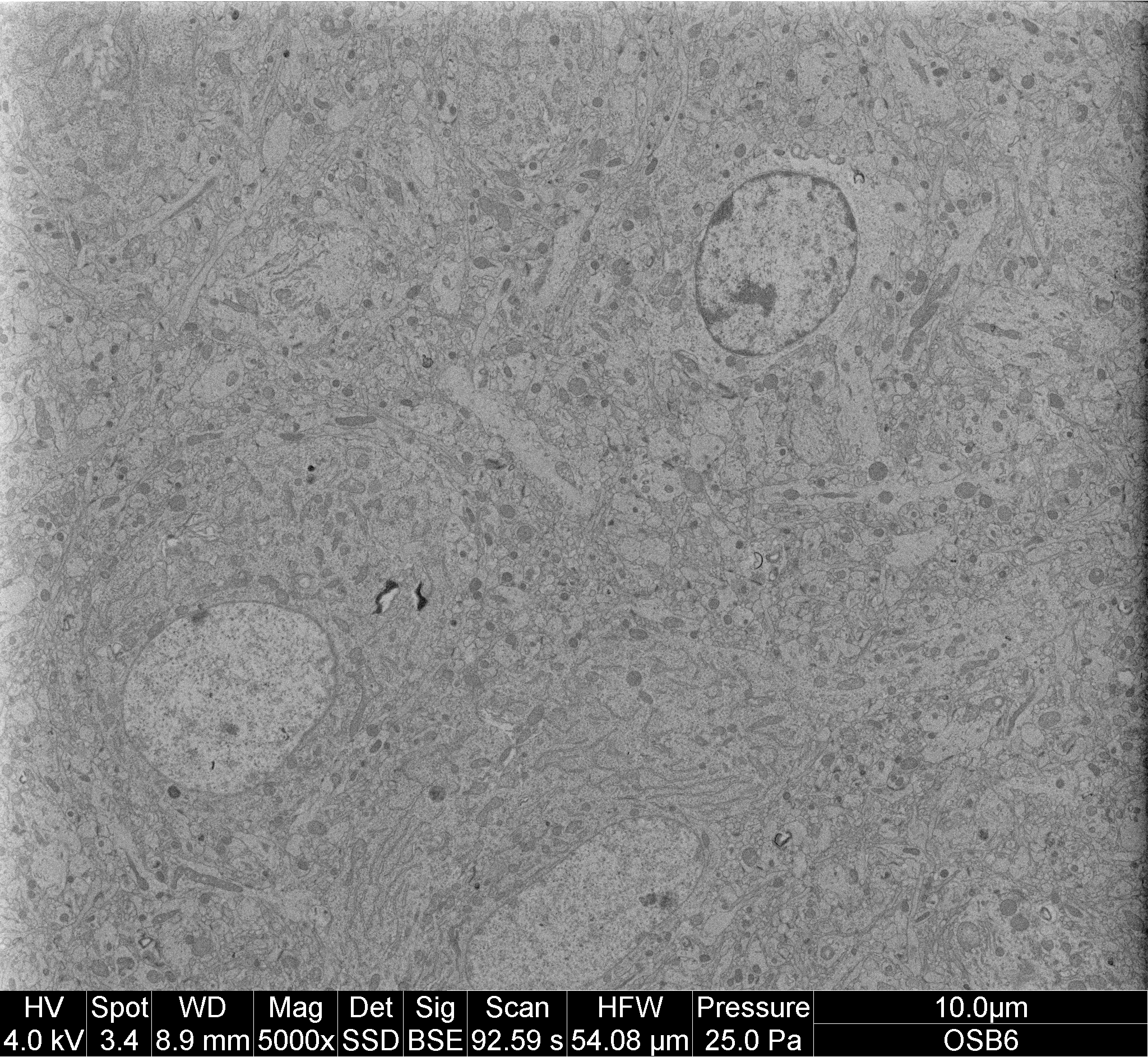

Supplement: Dataset S15 — (250.7 MB ZIP). [file pbio.0020329.sd015.zip › 040604_OS5_st1_1408.tif]

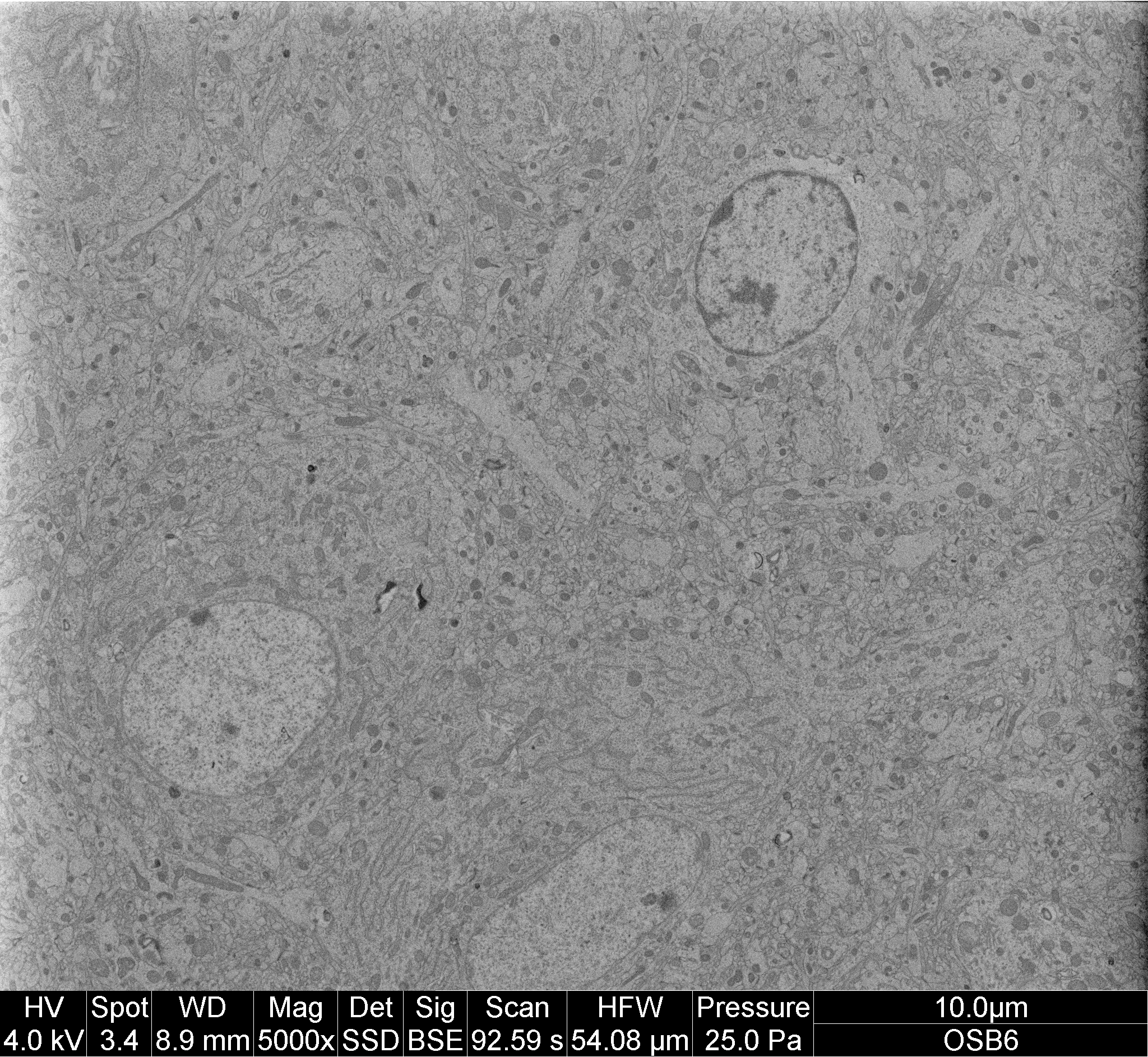

Supplement: Dataset S15 — (250.7 MB ZIP). [file pbio.0020329.sd015.zip › 040604_OS5_st1_1409.tif]

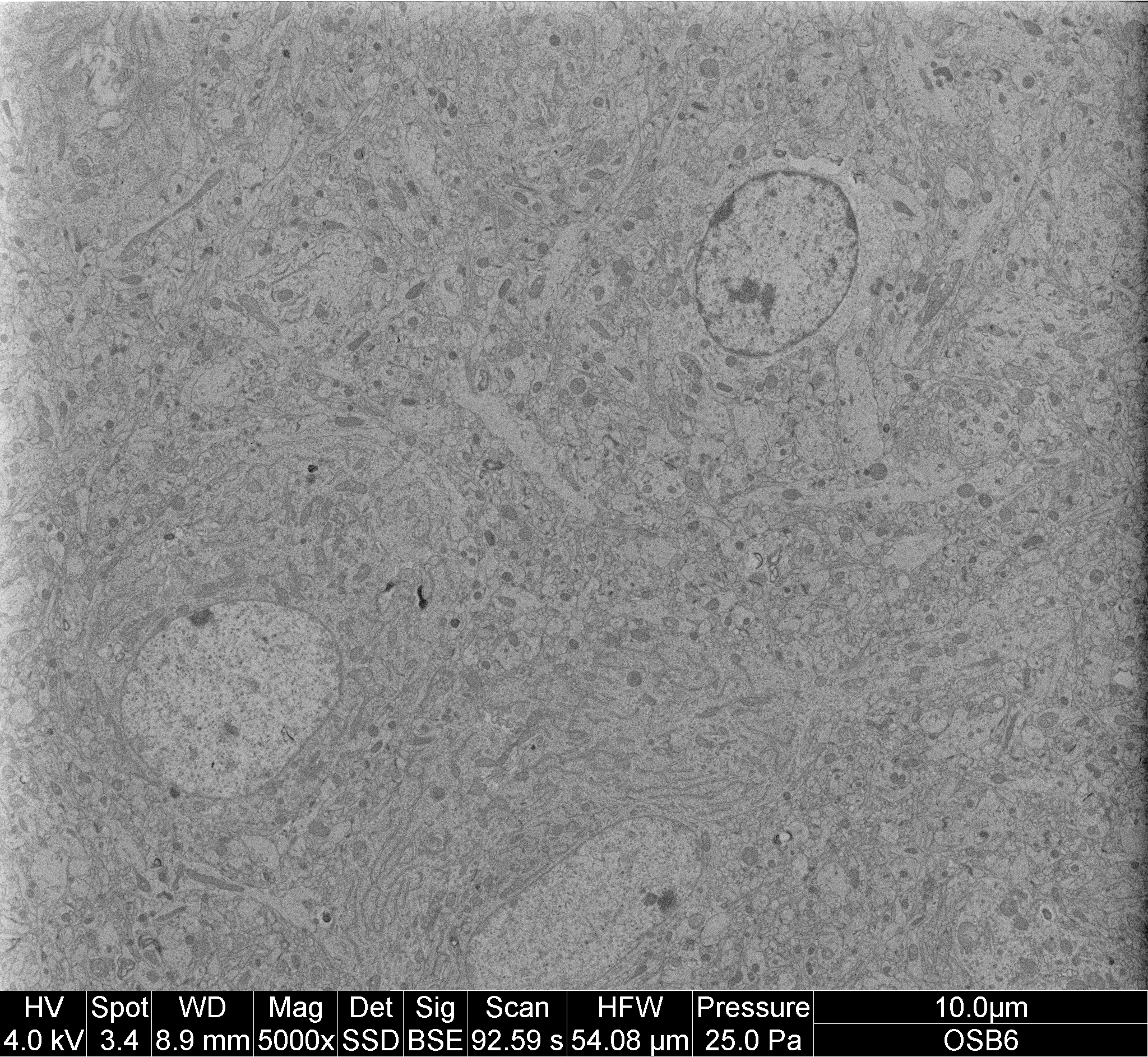

Supplement: Dataset S15 — (250.7 MB ZIP). [file pbio.0020329.sd015.zip › 040604_OS5_st1_1410.tif]

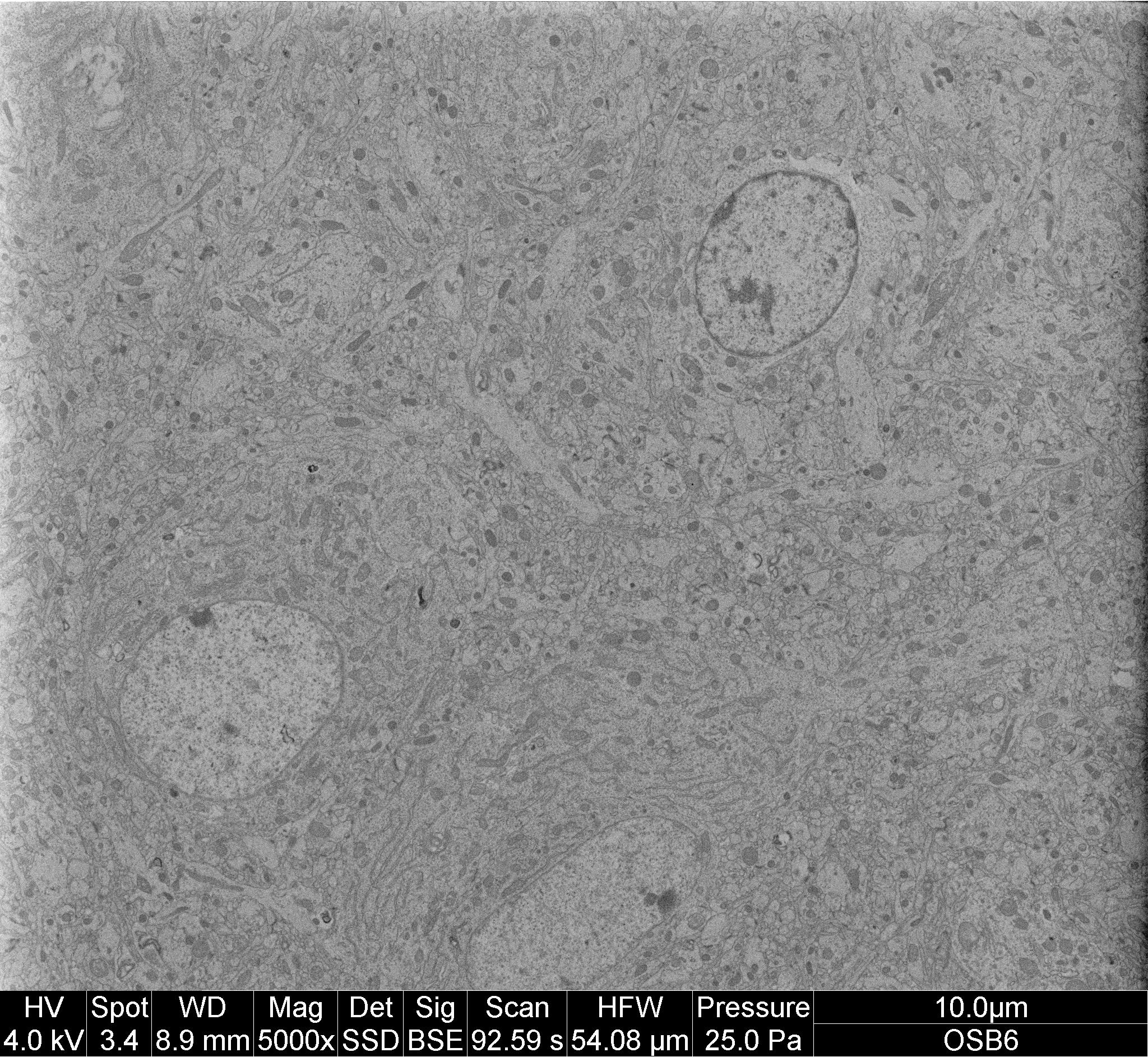

Supplement: Dataset S15 — (250.7 MB ZIP). [file pbio.0020329.sd015.zip › 040604_OS5_st1_1411.tif]

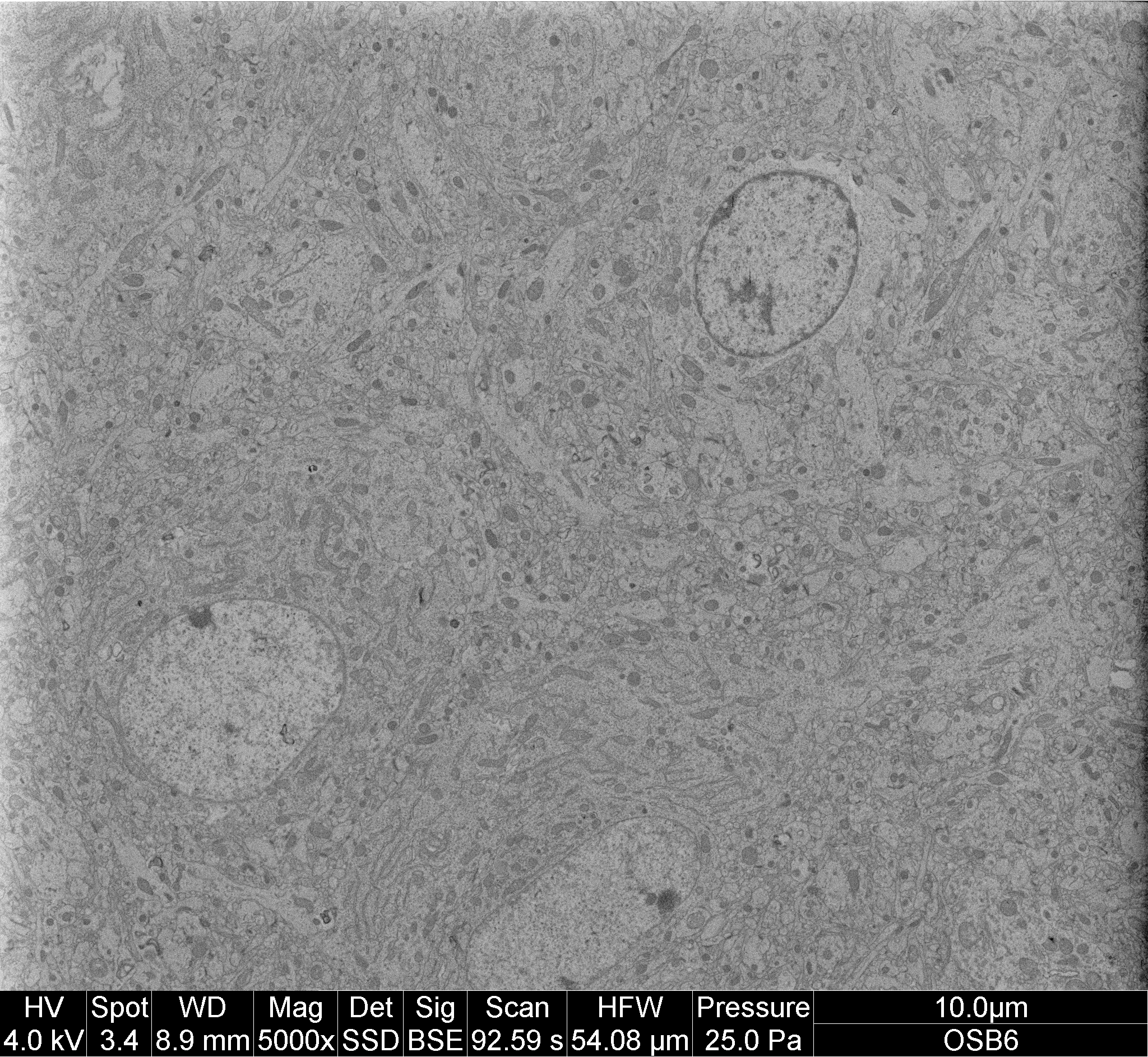

Supplement: Dataset S15 — (250.7 MB ZIP). [file pbio.0020329.sd015.zip › 040604_OS5_st1_1412.tif]

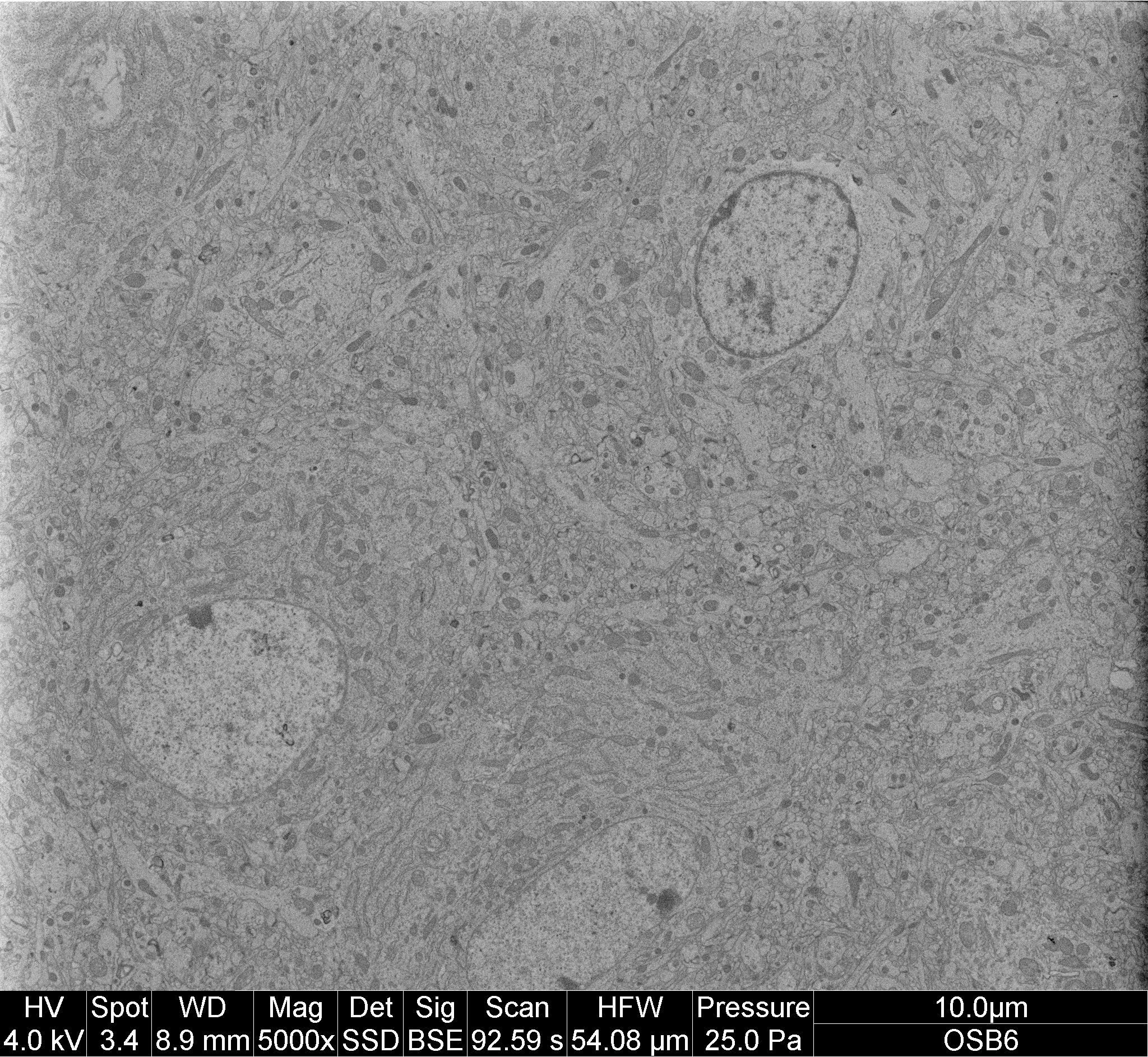

Supplement: Dataset S15 — (250.7 MB ZIP). [file pbio.0020329.sd015.zip › 040604_OS5_st1_1413.tif]

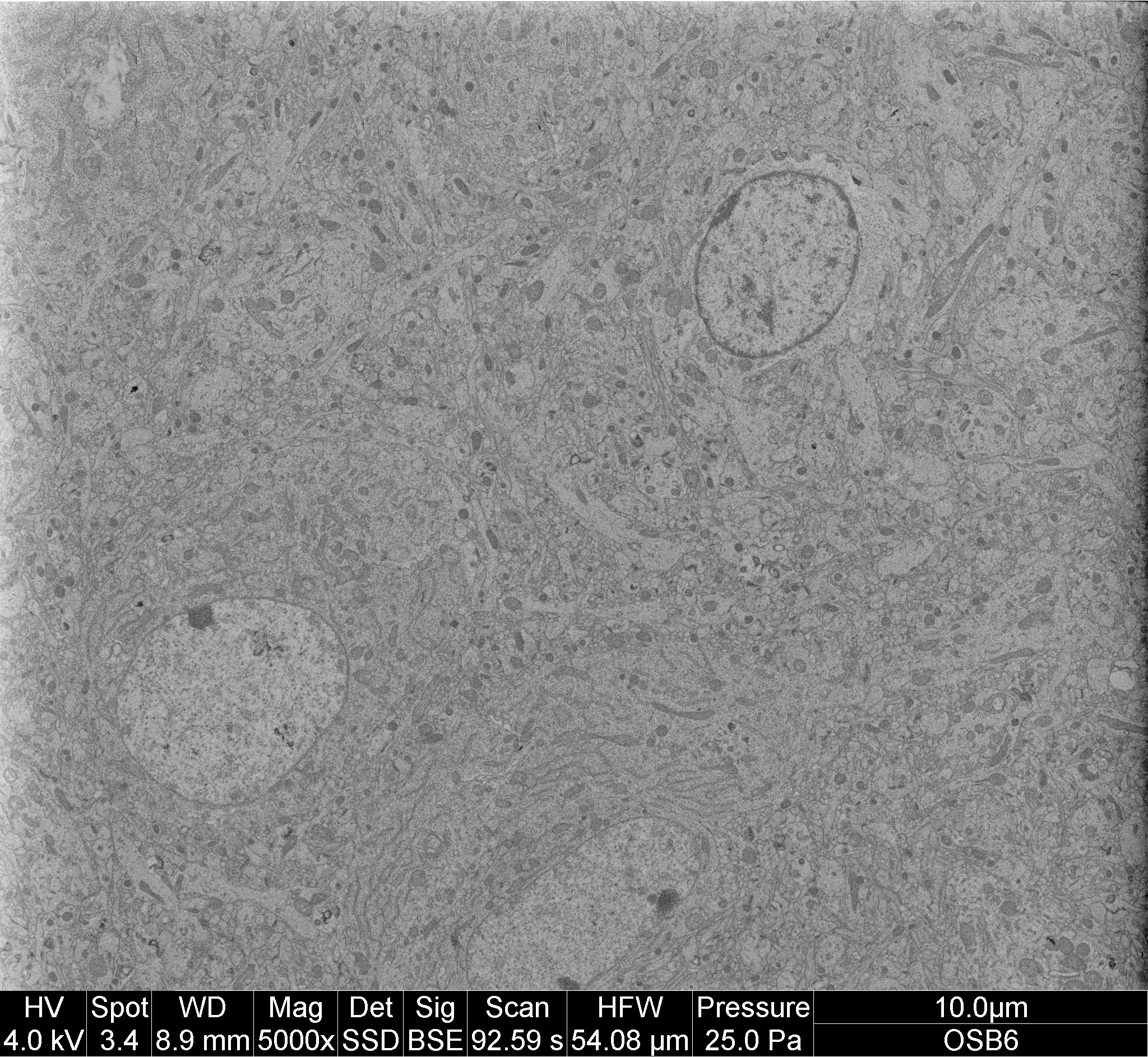

Supplement: Dataset S15 — (250.7 MB ZIP). [file pbio.0020329.sd015.zip › 040604_OS5_st1_1414.tif]

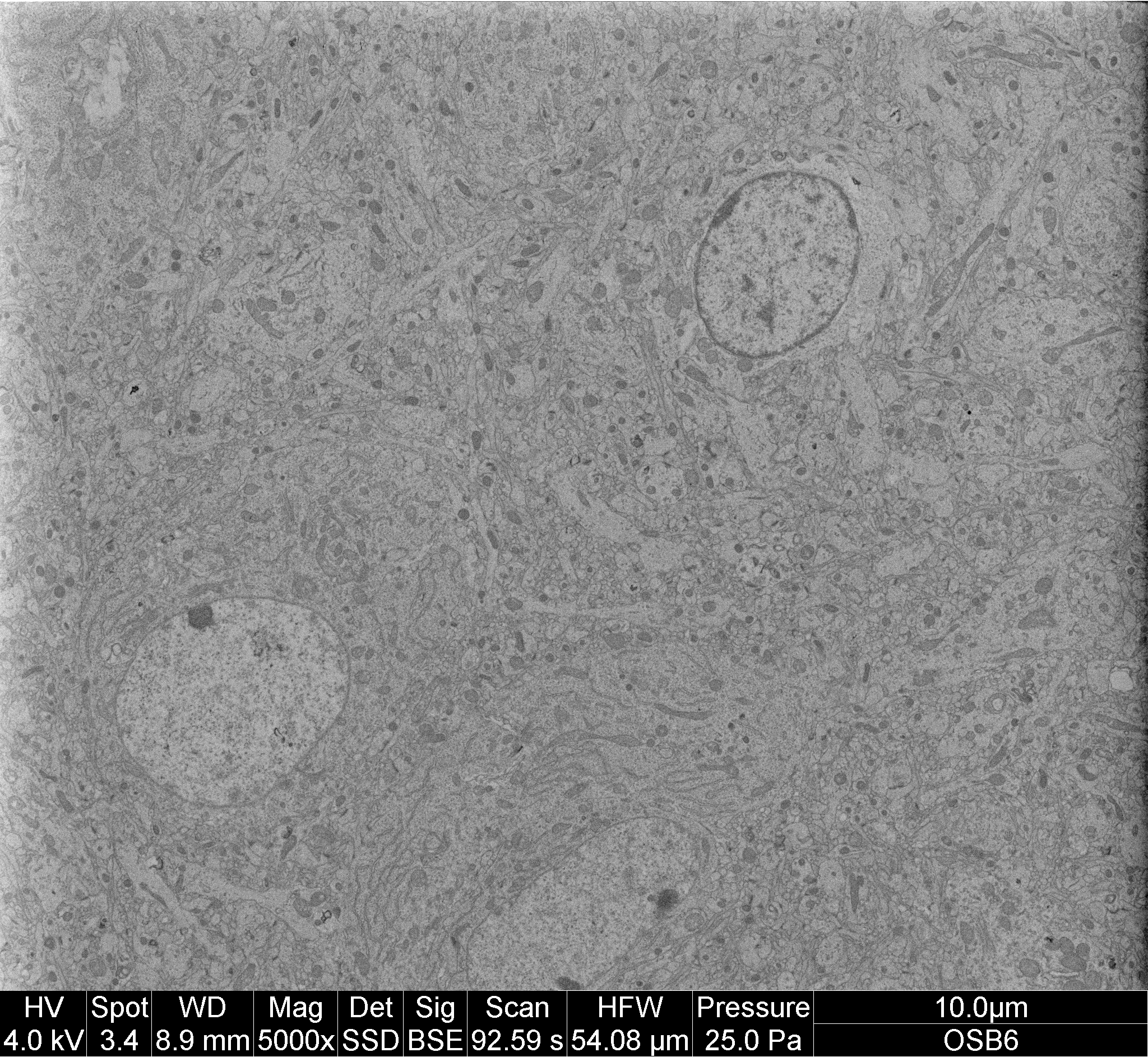

Supplement: Dataset S15 — (250.7 MB ZIP). [file pbio.0020329.sd015.zip › 040604_OS5_st1_1415.tif]

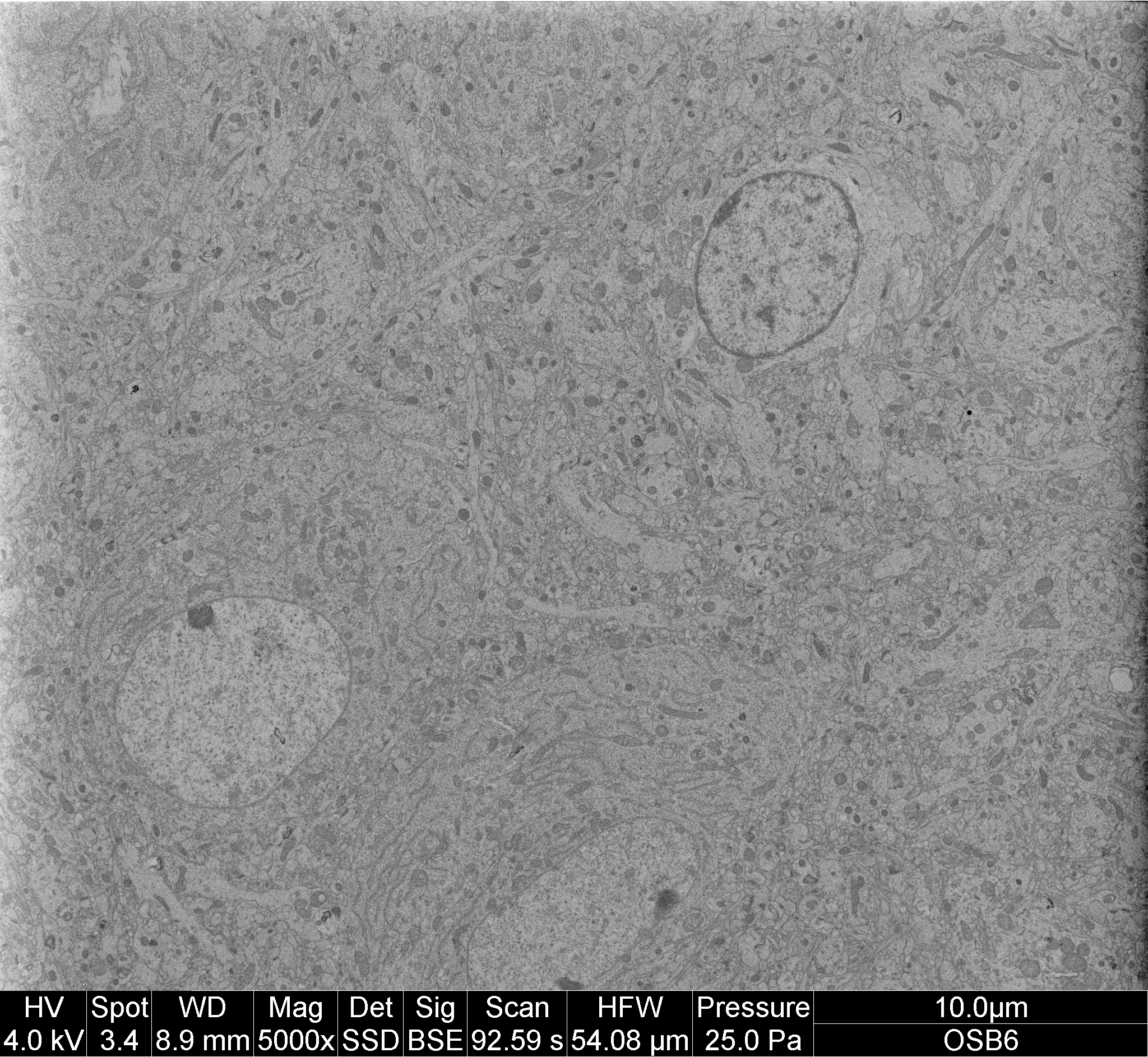

Supplement: Dataset S15 — (250.7 MB ZIP). [file pbio.0020329.sd015.zip › 040604_OS5_st1_1416.tif]

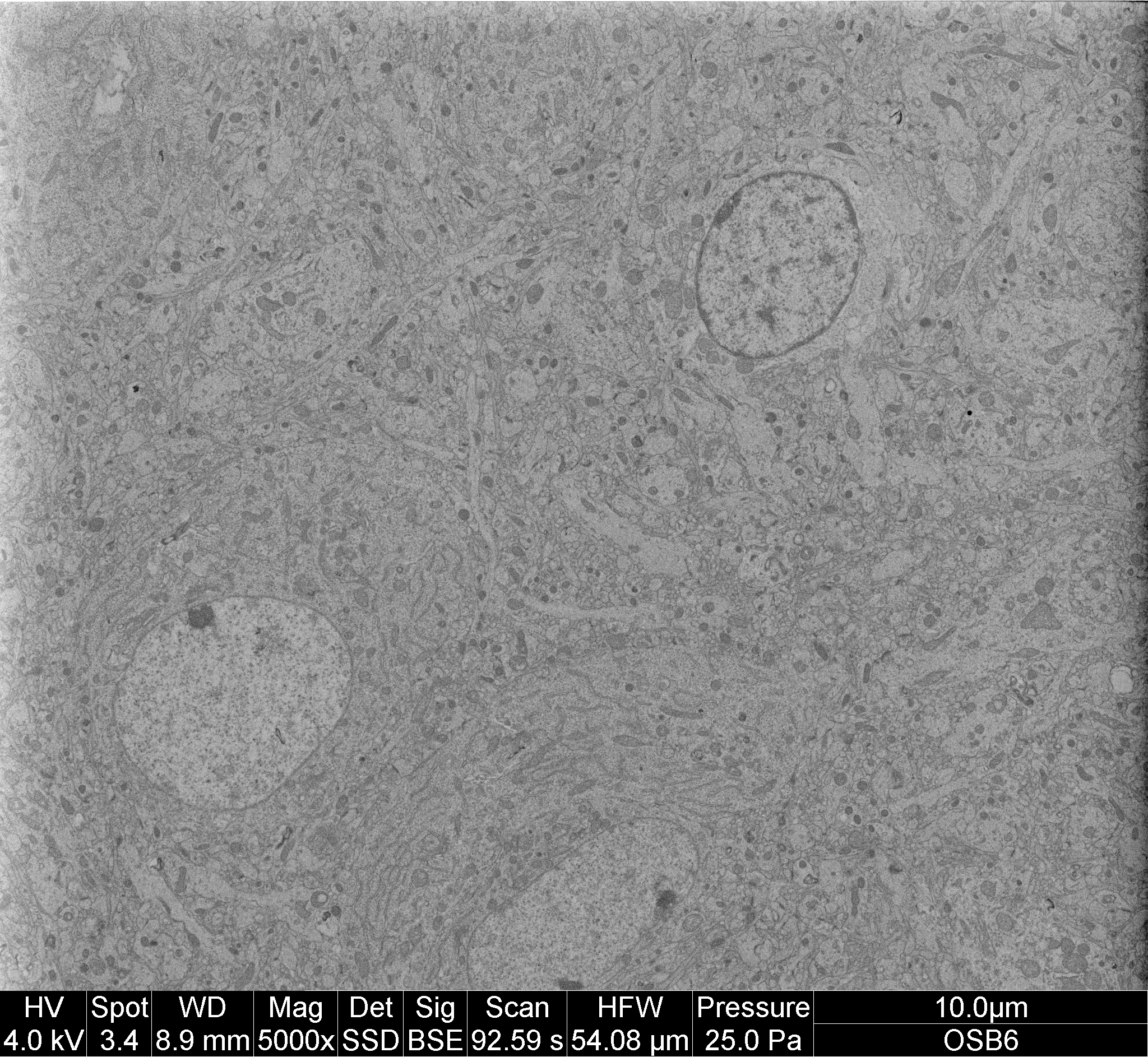

Supplement: Dataset S15 — (250.7 MB ZIP). [file pbio.0020329.sd015.zip › 040604_OS5_st1_1417.tif]

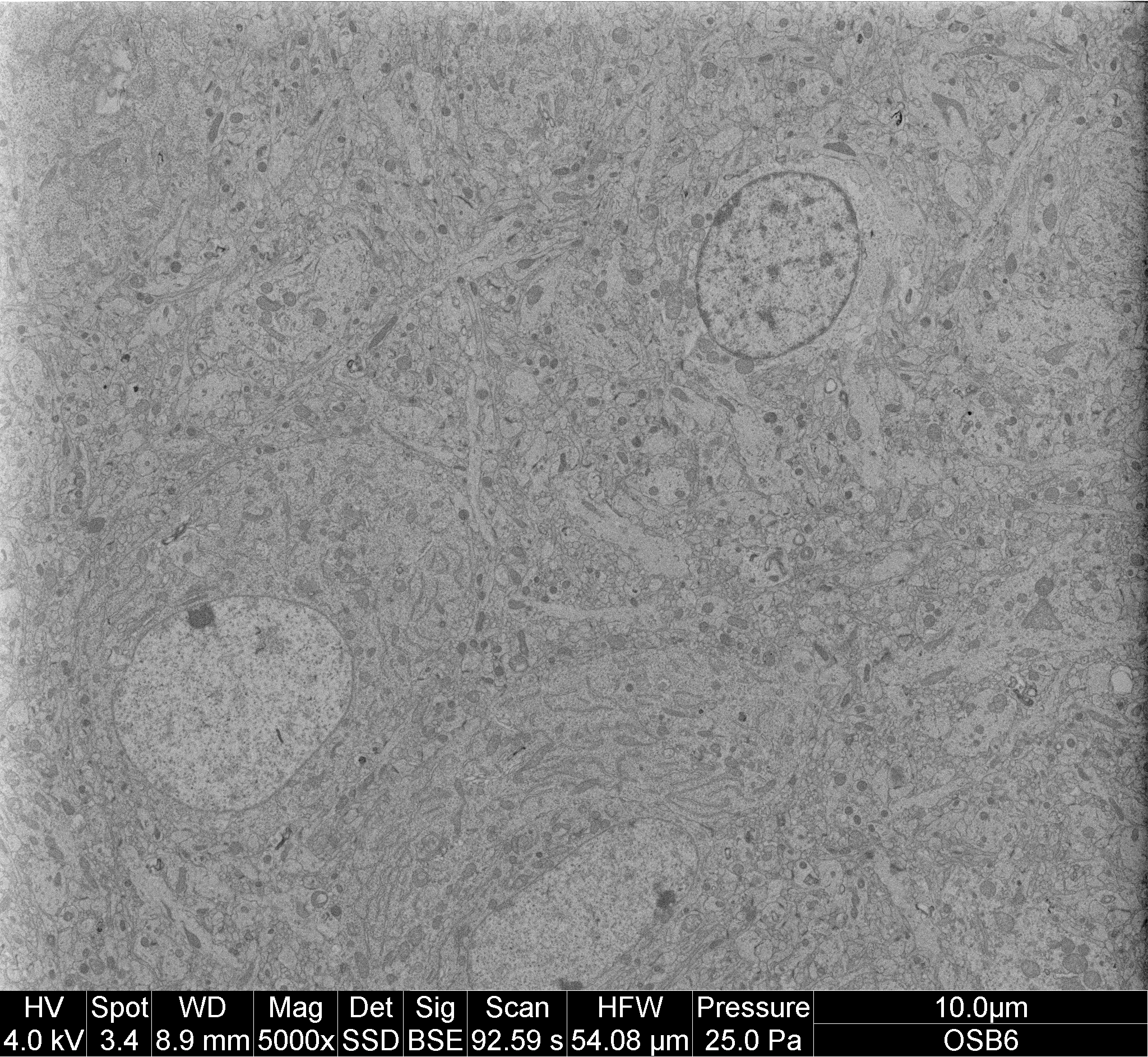

Supplement: Dataset S15 — (250.7 MB ZIP). [file pbio.0020329.sd015.zip › 040604_OS5_st1_1418.tif]

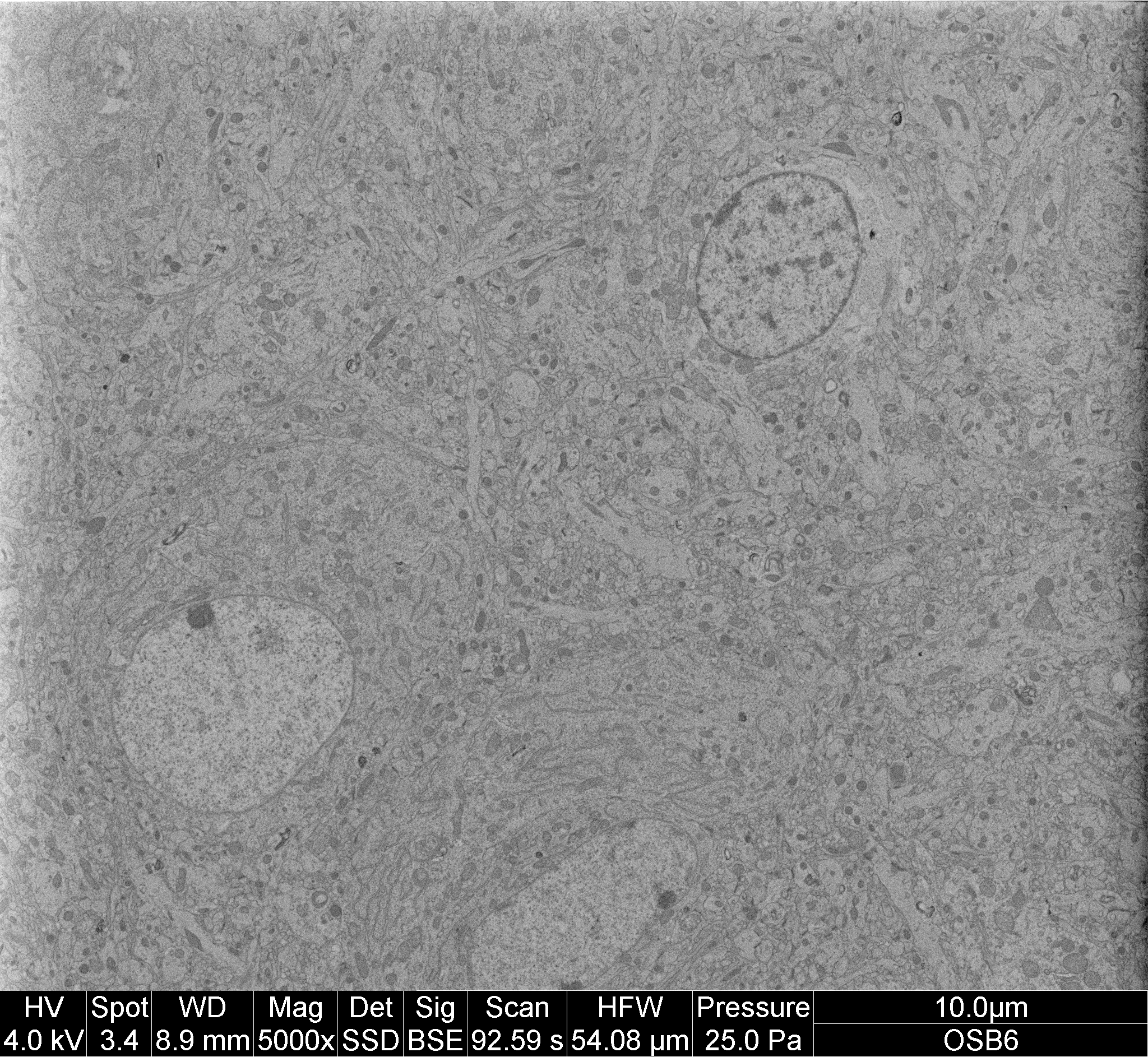

Supplement: Dataset S15 — (250.7 MB ZIP). [file pbio.0020329.sd015.zip › 040604_OS5_st1_1419.tif]

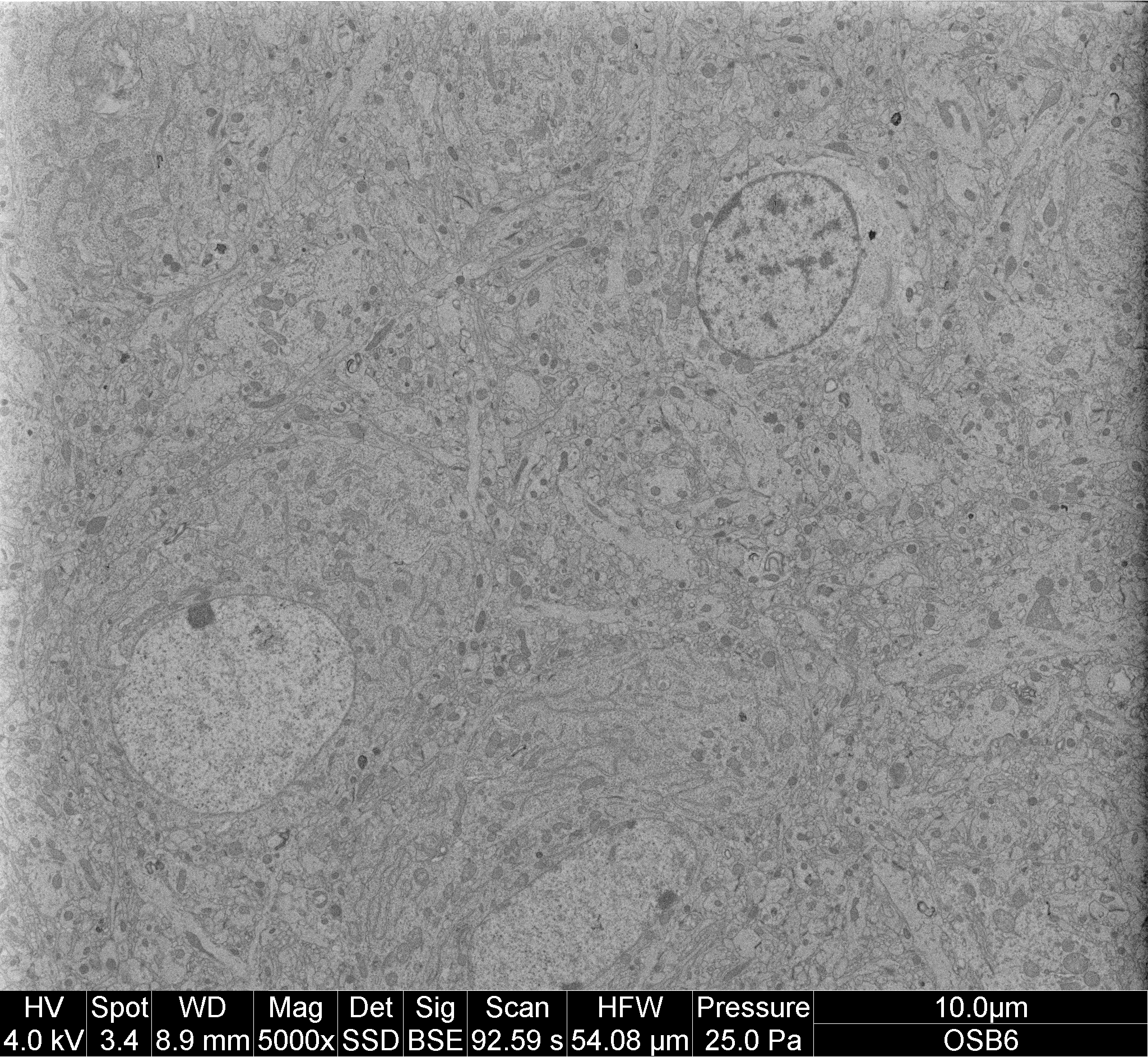

Supplement: Dataset S15 — (250.7 MB ZIP). [file pbio.0020329.sd015.zip › 040604_OS5_st1_1420.tif]

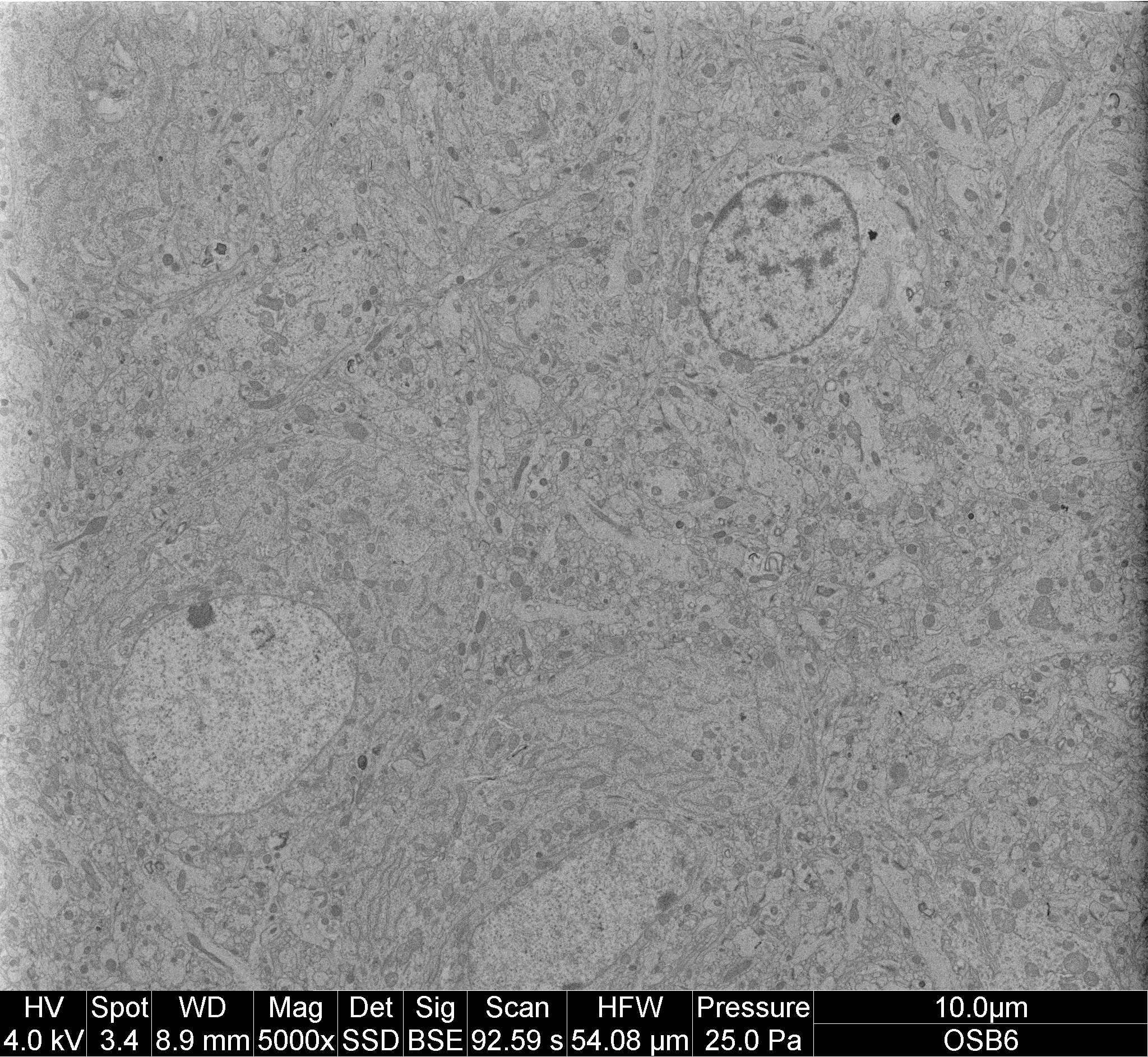

Supplement: Dataset S15 — (250.7 MB ZIP). [file pbio.0020329.sd015.zip › 040604_OS5_st1_1421.tif]

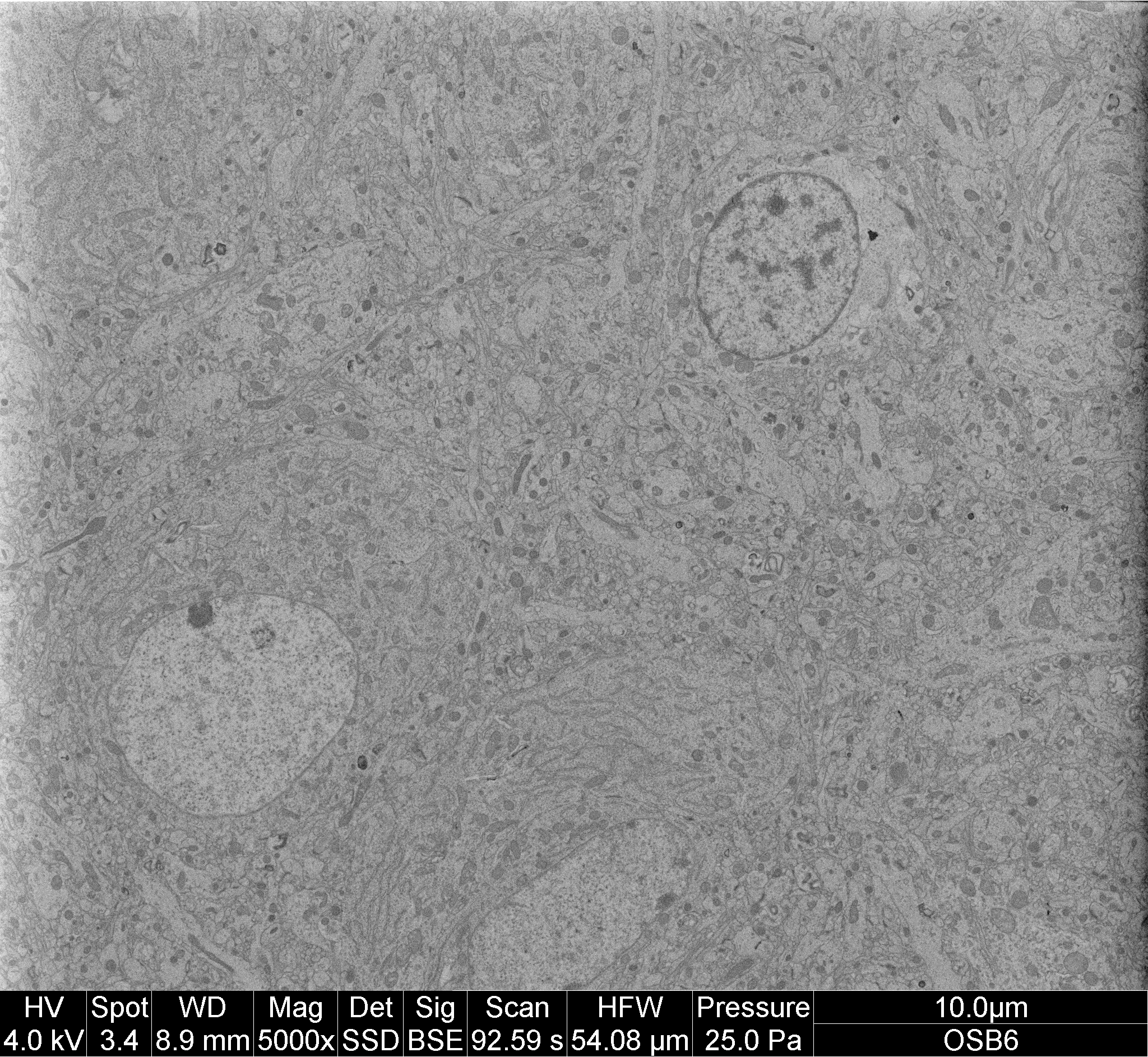

Supplement: Dataset S15 — (250.7 MB ZIP). [file pbio.0020329.sd015.zip › 040604_OS5_st1_1422.tif]

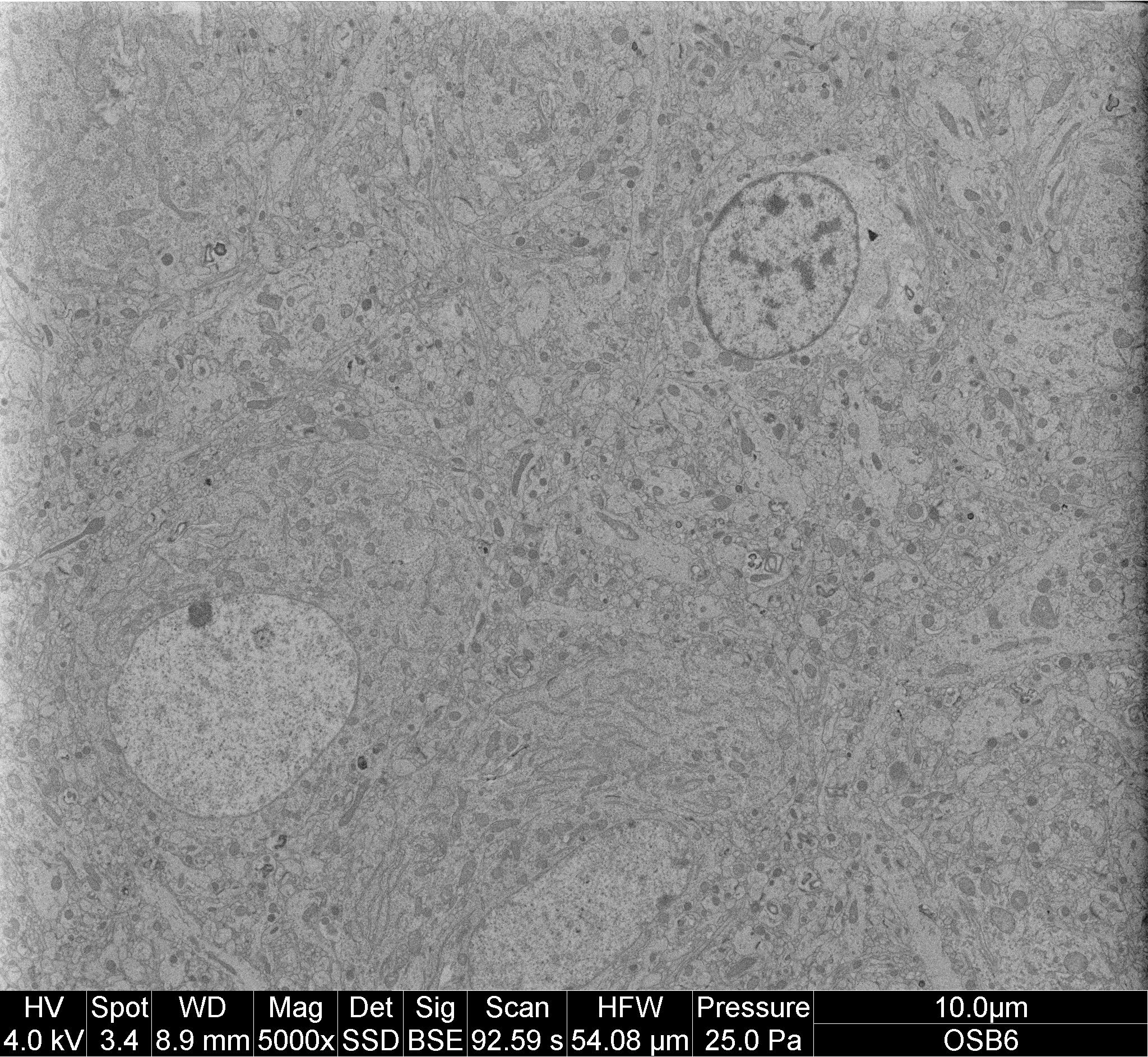

Supplement: Dataset S15 — (250.7 MB ZIP). [file pbio.0020329.sd015.zip › 040604_OS5_st1_1423.tif]

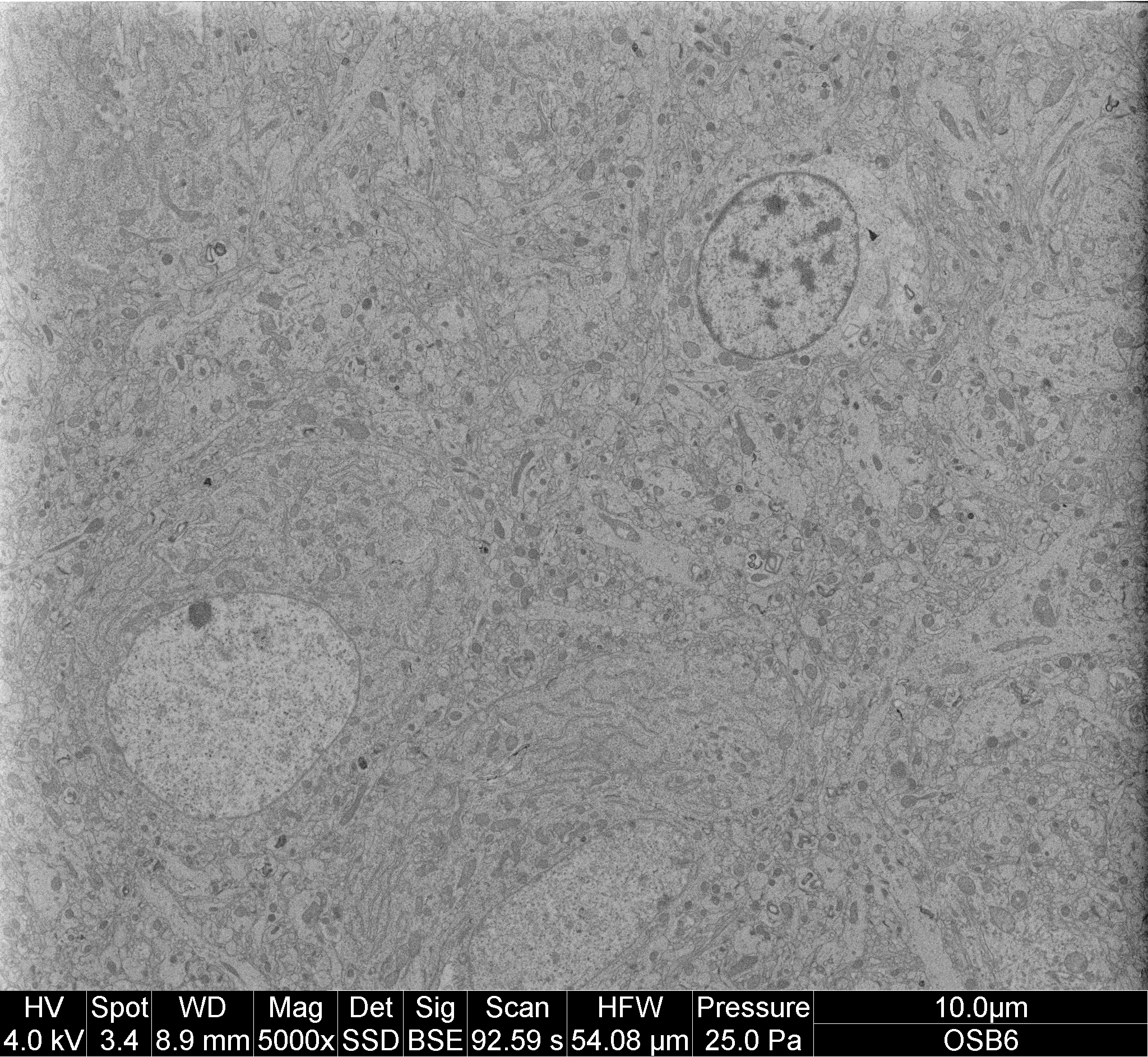

Supplement: Dataset S15 — (250.7 MB ZIP). [file pbio.0020329.sd015.zip › 040604_OS5_st1_1424.tif]

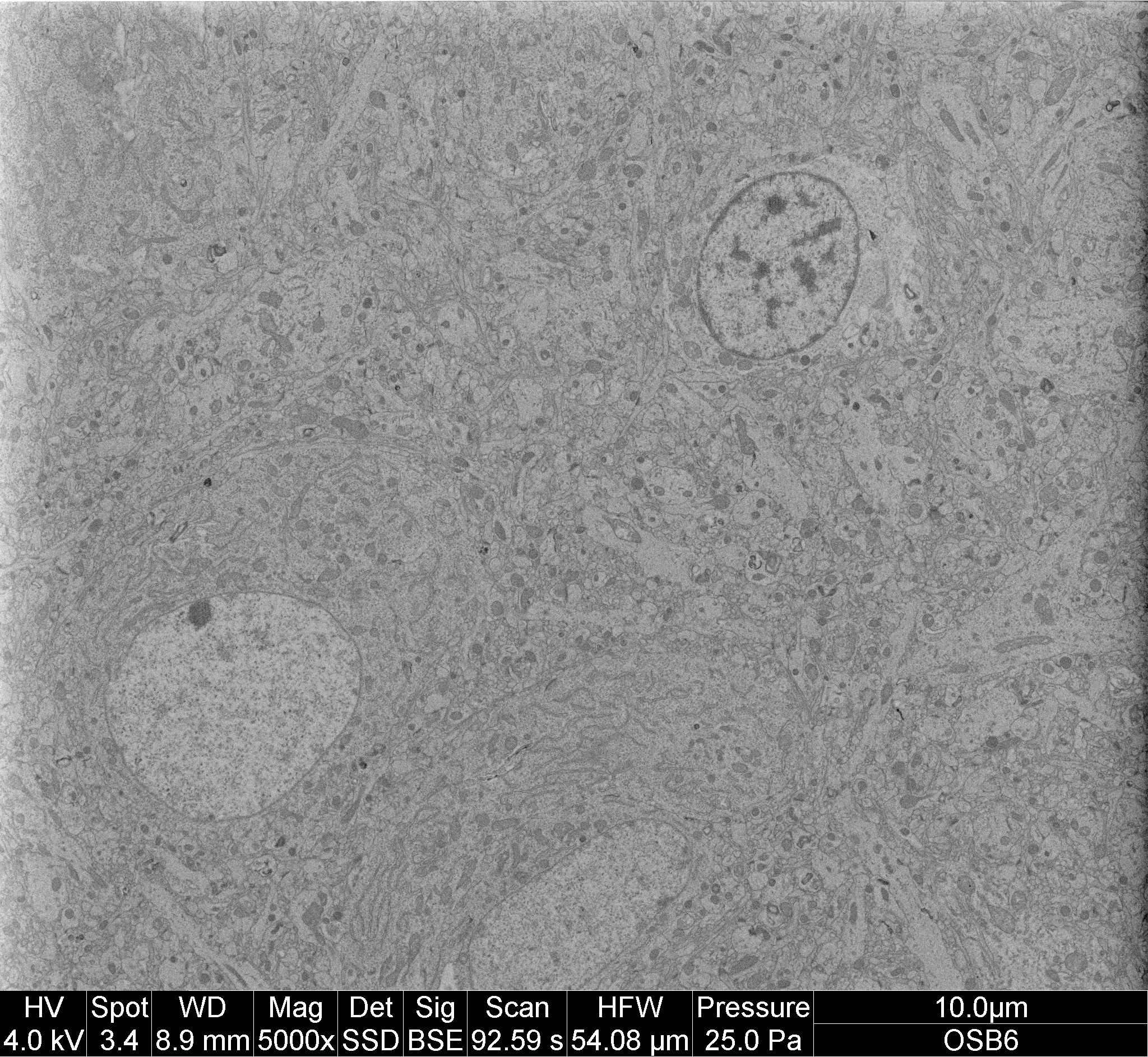

Supplement: Dataset S15 — (250.7 MB ZIP). [file pbio.0020329.sd015.zip › 040604_OS5_st1_1425.tif]

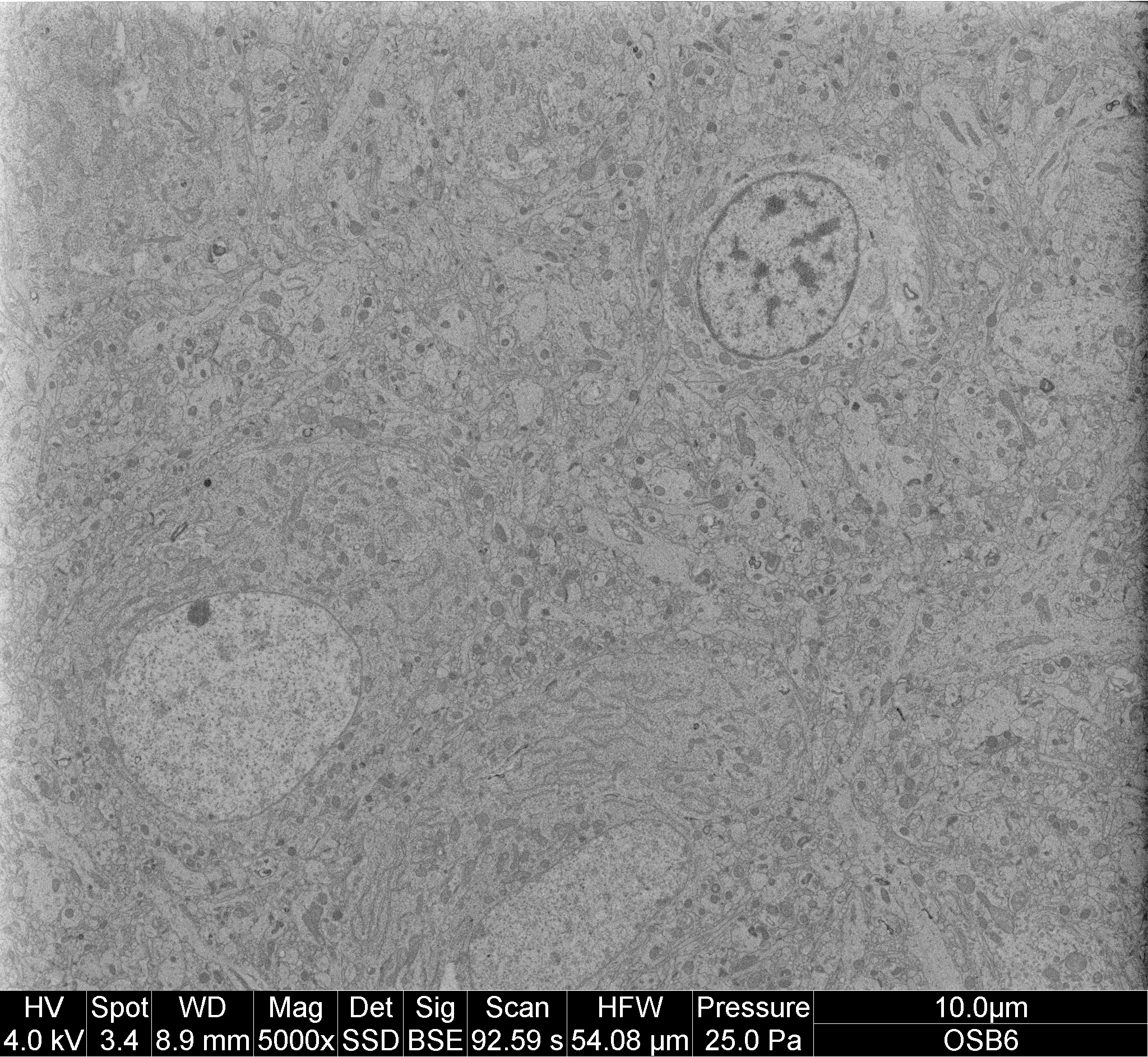

Supplement: Dataset S15 — (250.7 MB ZIP). [file pbio.0020329.sd015.zip › 040604_OS5_st1_1426.tif]

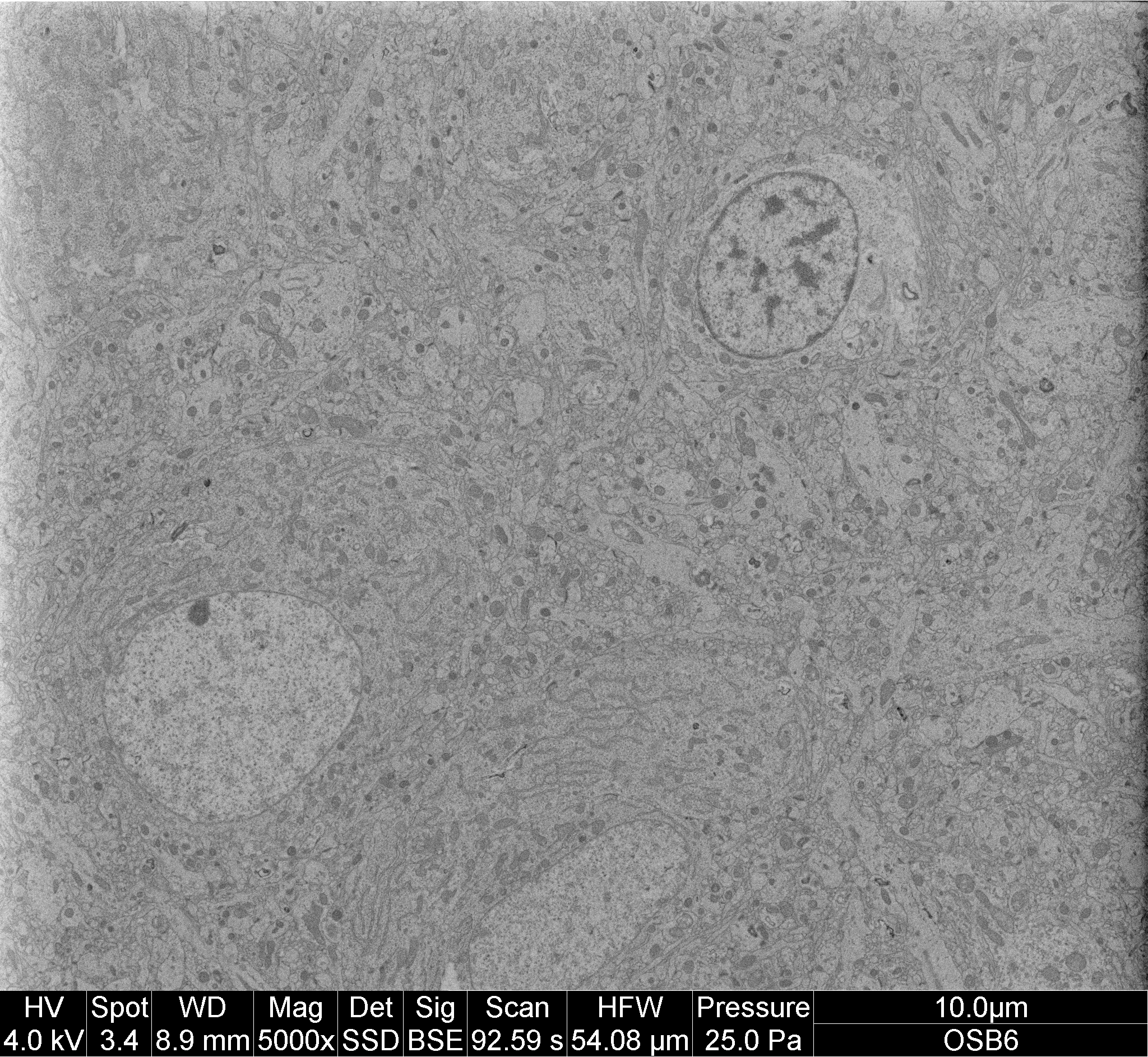

Supplement: Dataset S15 — (250.7 MB ZIP). [file pbio.0020329.sd015.zip › 040604_OS5_st1_1427.tif]

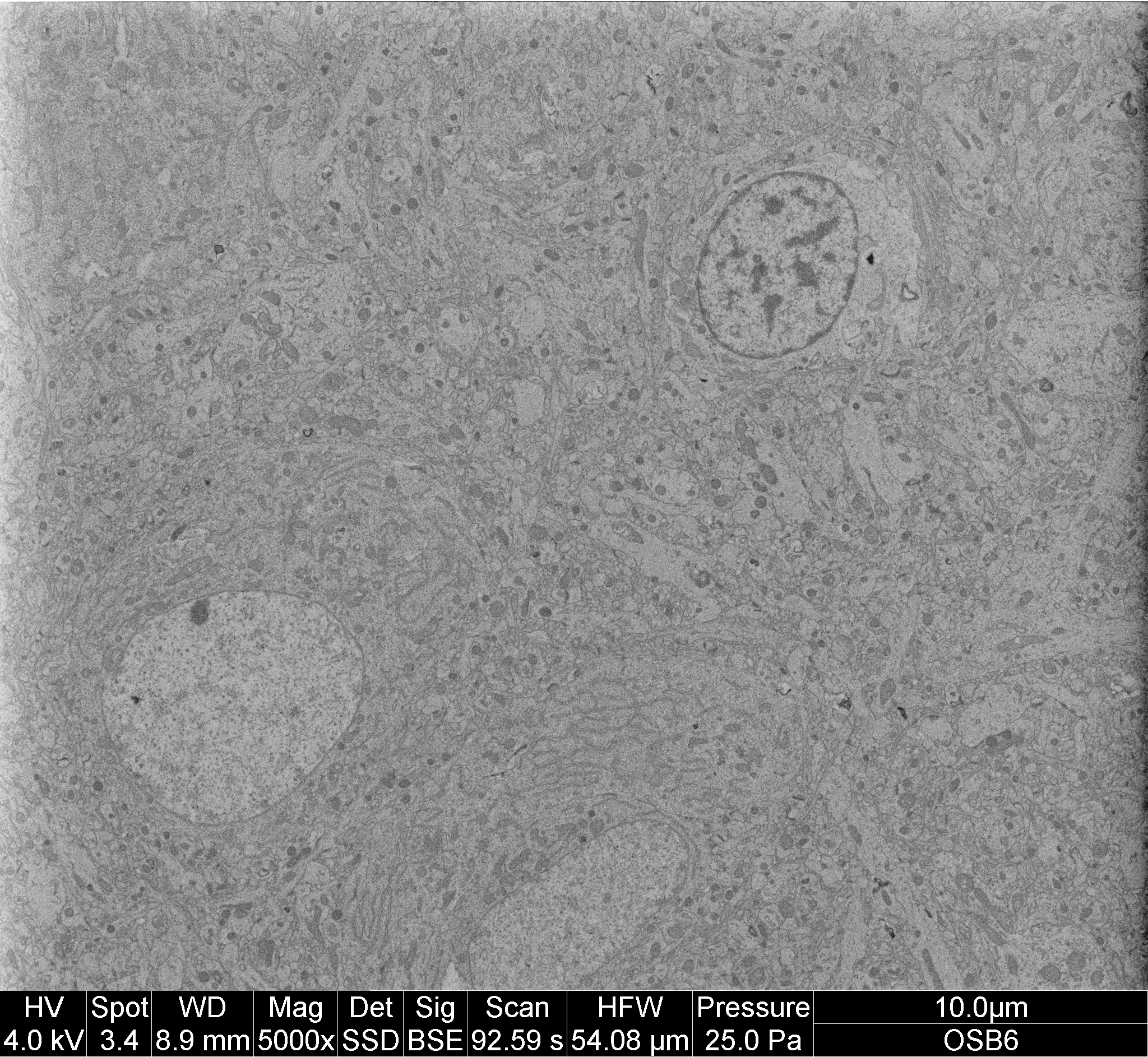

Supplement: Dataset S15 — (250.7 MB ZIP). [file pbio.0020329.sd015.zip › 040604_OS5_st1_1428.tif]

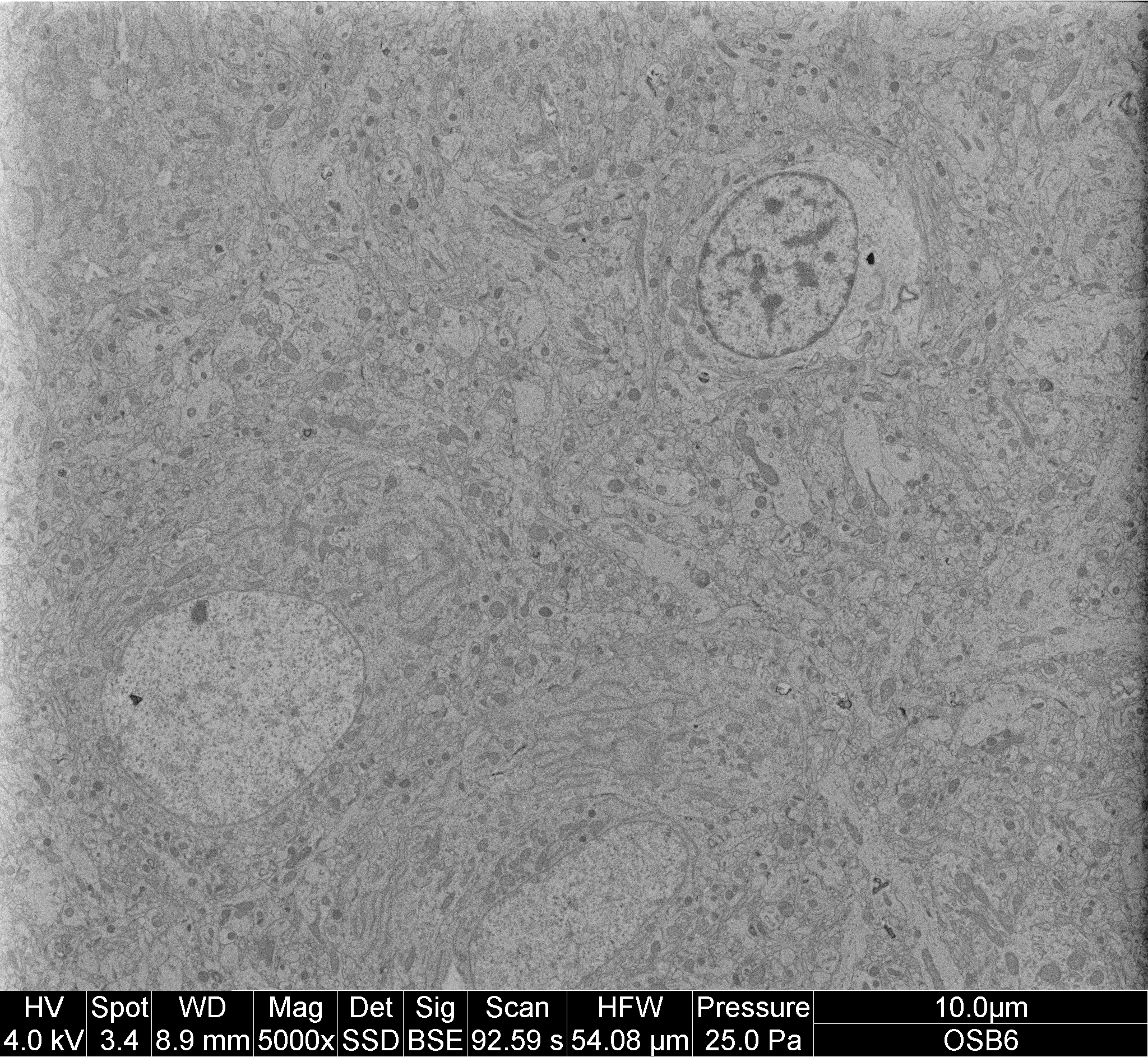

Supplement: Dataset S15 — (250.7 MB ZIP). [file pbio.0020329.sd015.zip › 040604_OS5_st1_1429.tif]

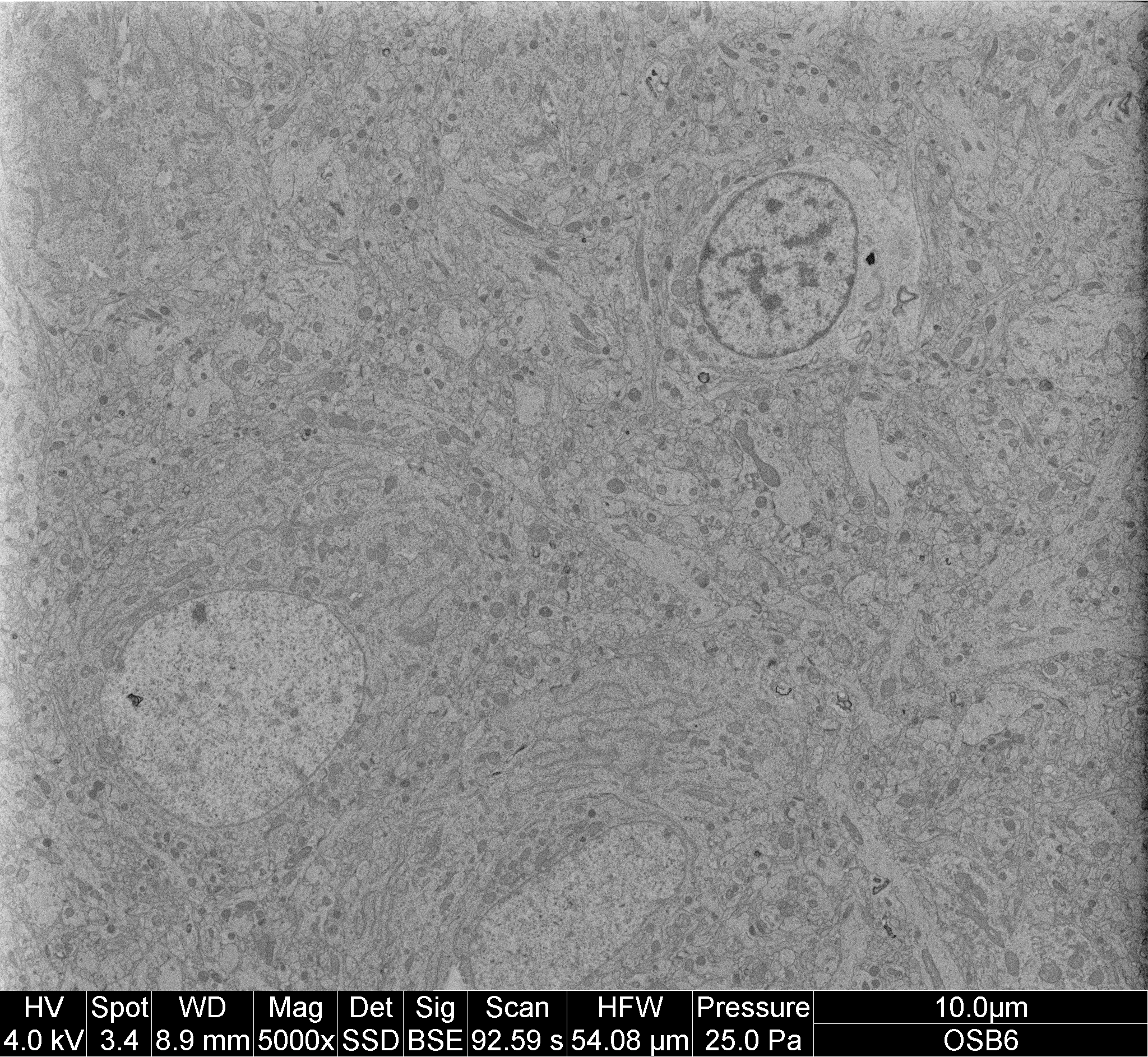

Supplement: Dataset S15 — (250.7 MB ZIP). [file pbio.0020329.sd015.zip › 040604_OS5_st1_1430.tif]

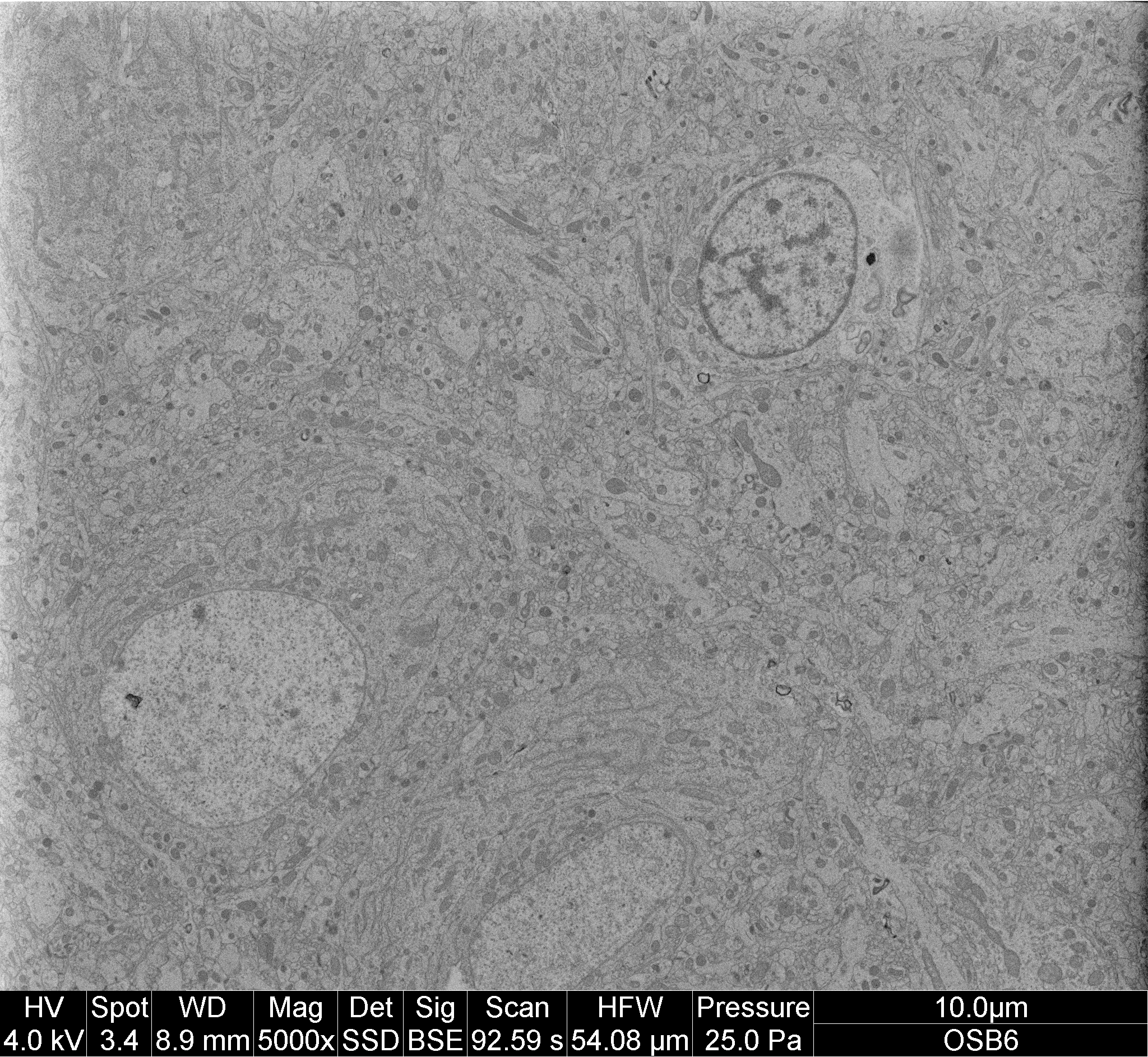

Supplement: Dataset S15 — (250.7 MB ZIP). [file pbio.0020329.sd015.zip › 040604_OS5_st1_1431.tif]

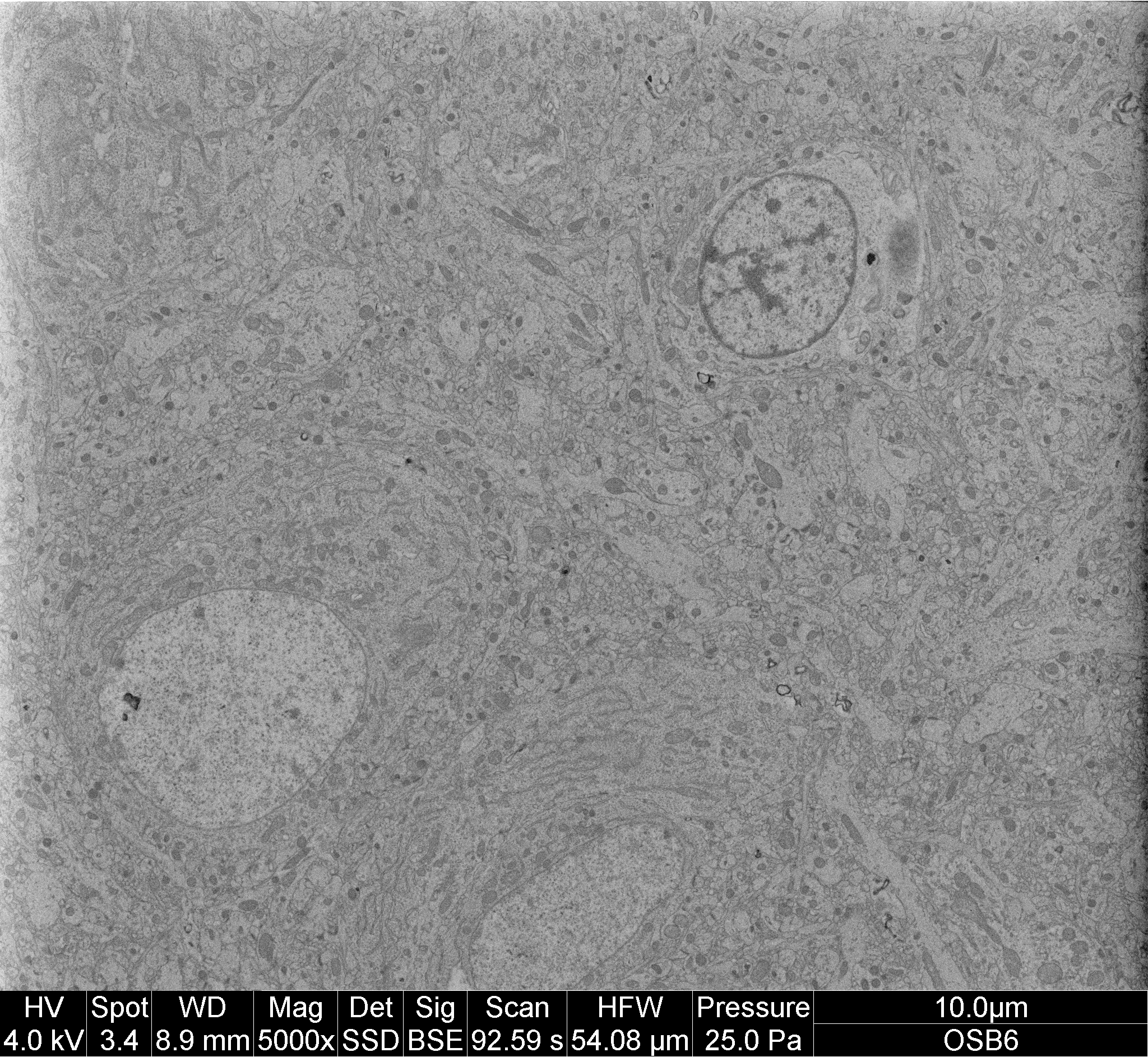

Supplement: Dataset S15 — (250.7 MB ZIP). [file pbio.0020329.sd015.zip › 040604_OS5_st1_1432.tif]

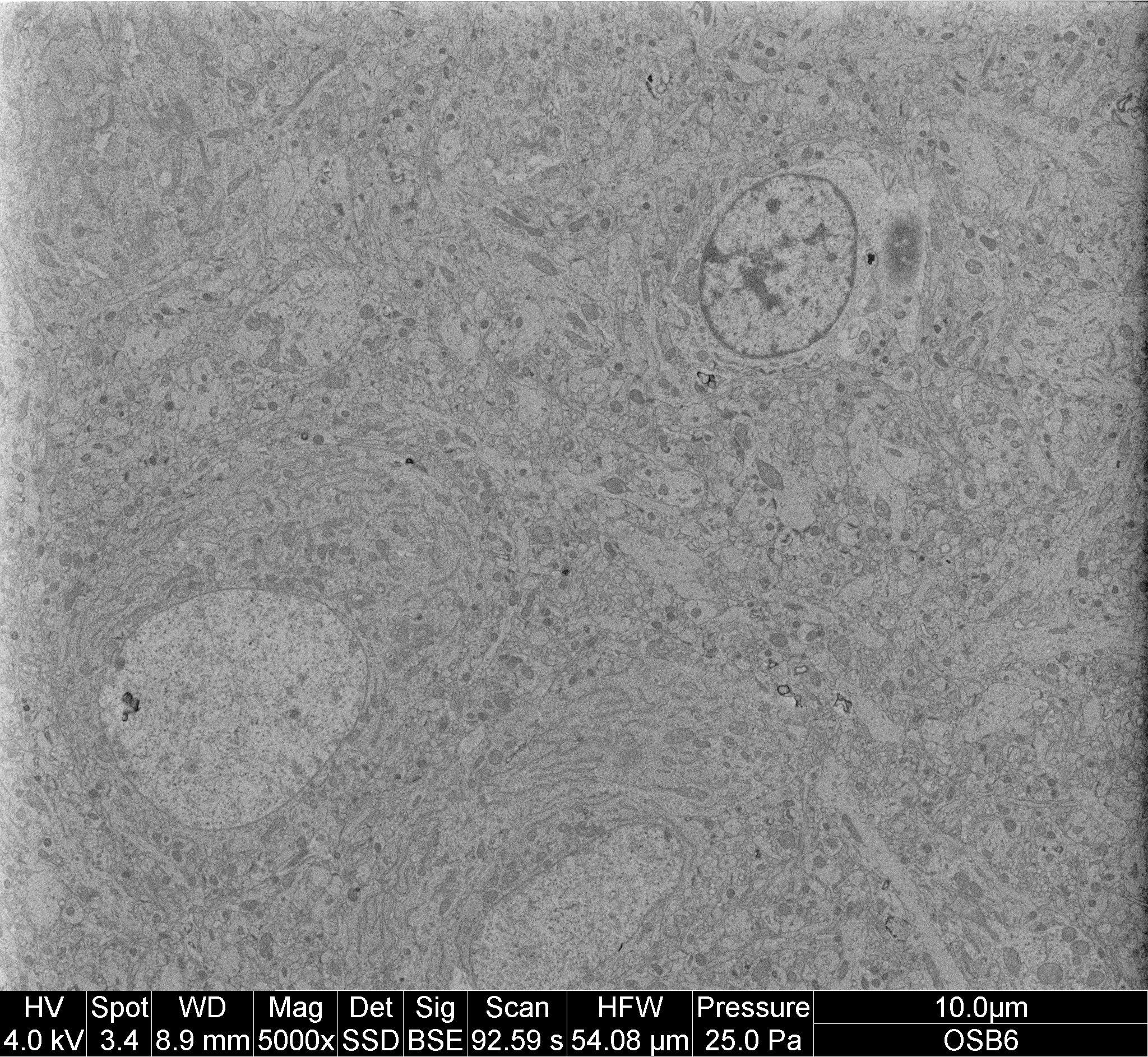

Supplement: Dataset S15 — (250.7 MB ZIP). [file pbio.0020329.sd015.zip › 040604_OS5_st1_1433.tif]

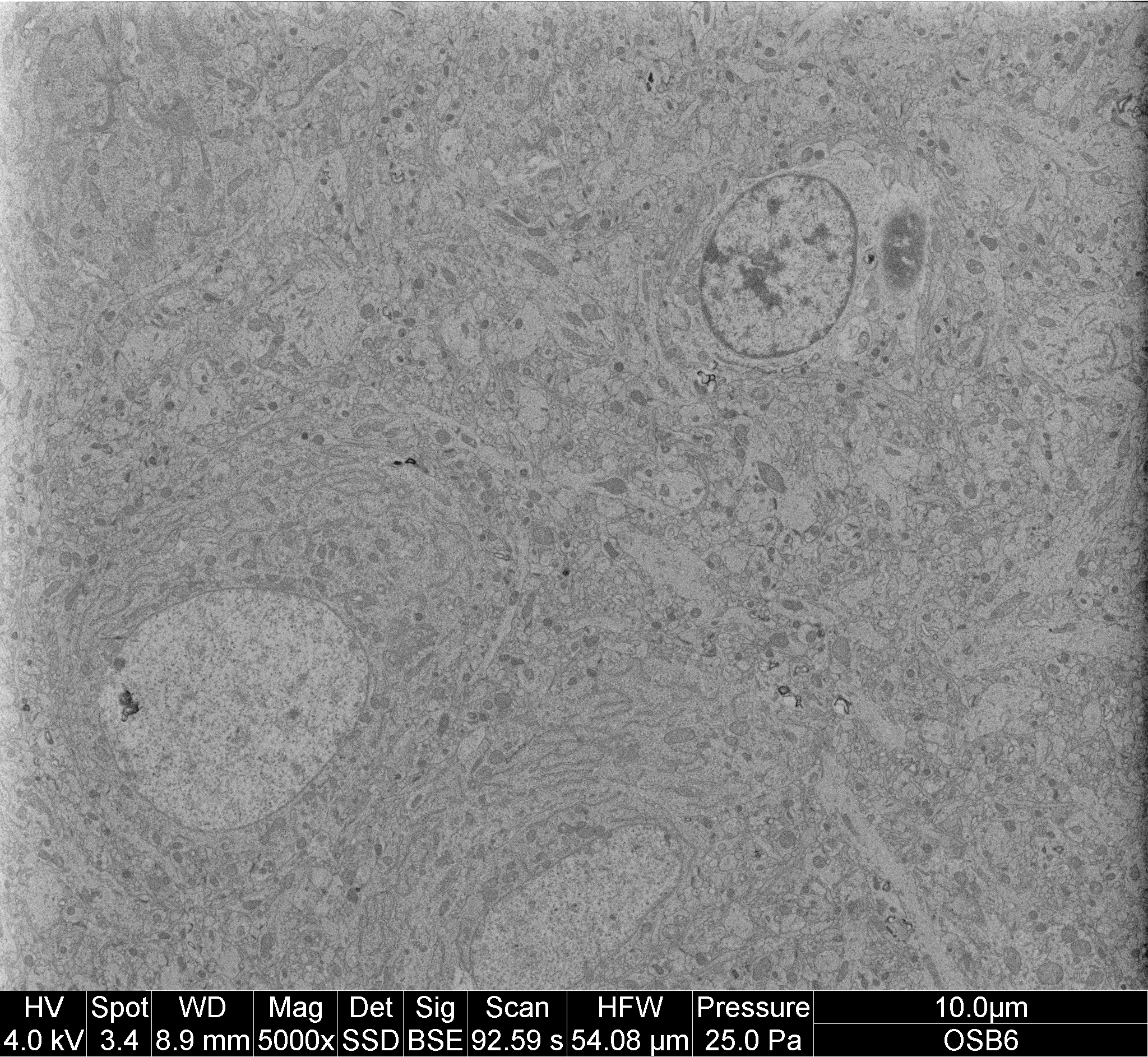

Supplement: Dataset S15 — (250.7 MB ZIP). [file pbio.0020329.sd015.zip › 040604_OS5_st1_1434.tif]

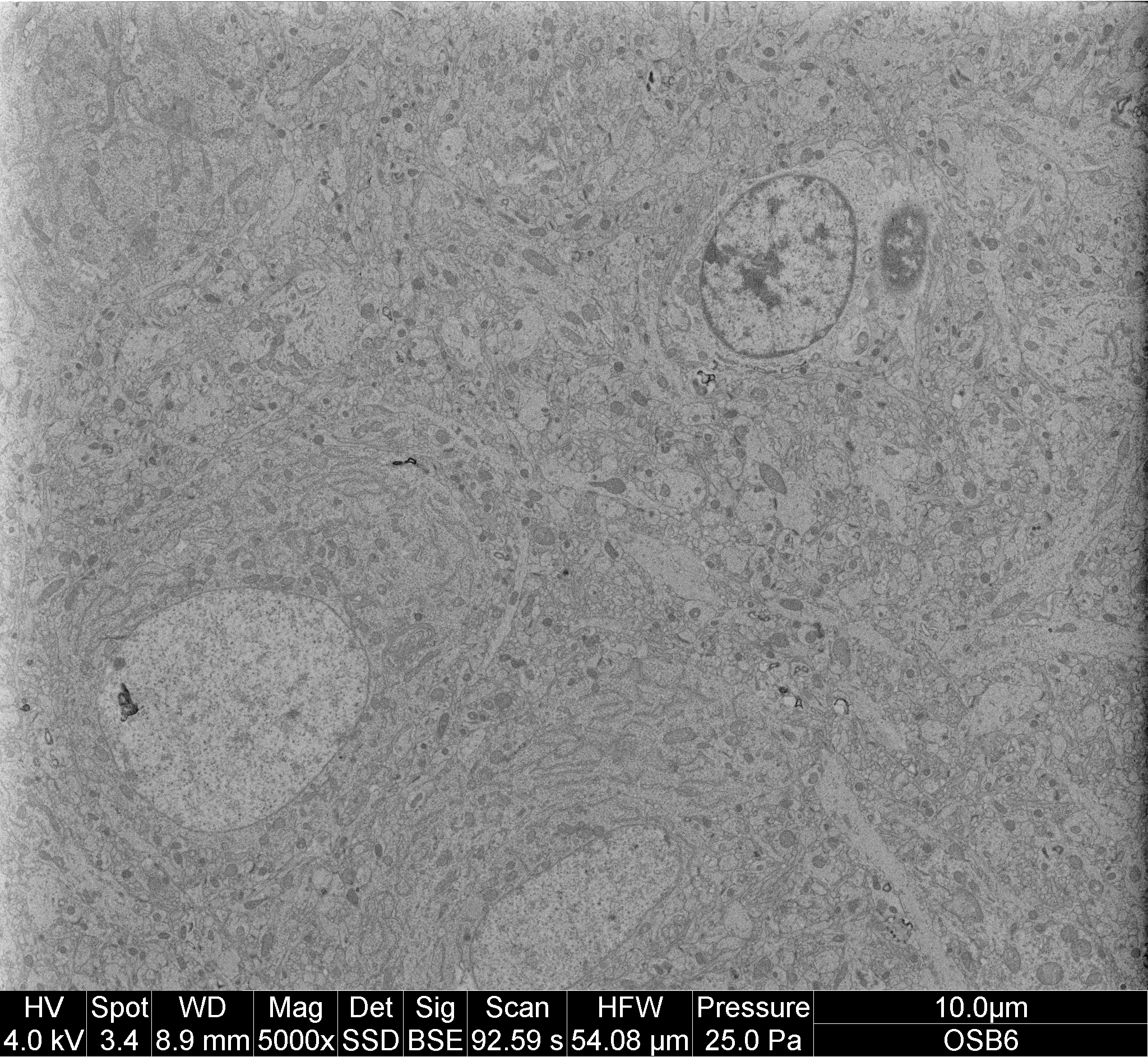

Supplement: Dataset S15 — (250.7 MB ZIP). [file pbio.0020329.sd015.zip › 040604_OS5_st1_1435.tif]

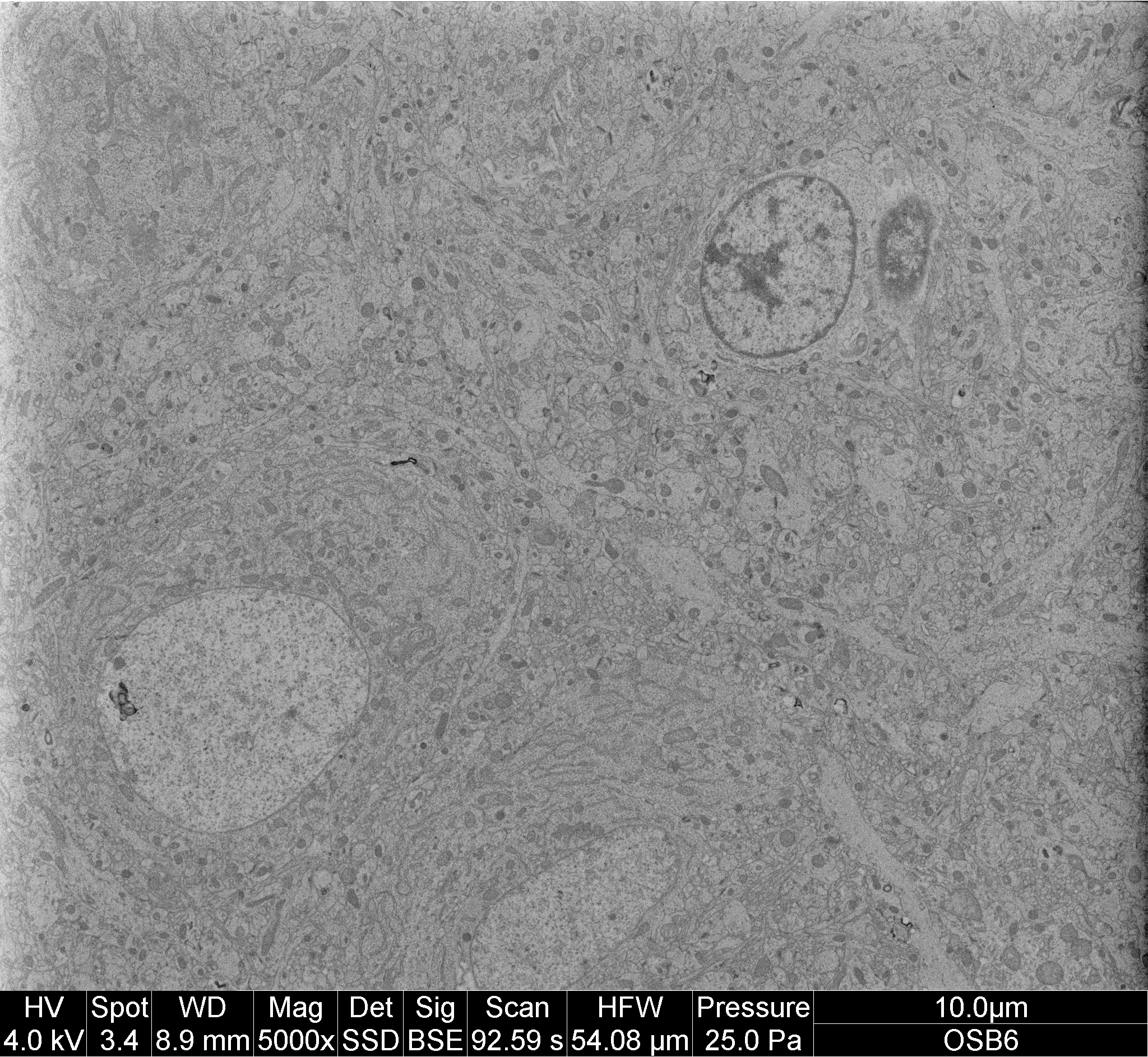

Supplement: Dataset S15 — (250.7 MB ZIP). [file pbio.0020329.sd015.zip › 040604_OS5_st1_1436.tif]

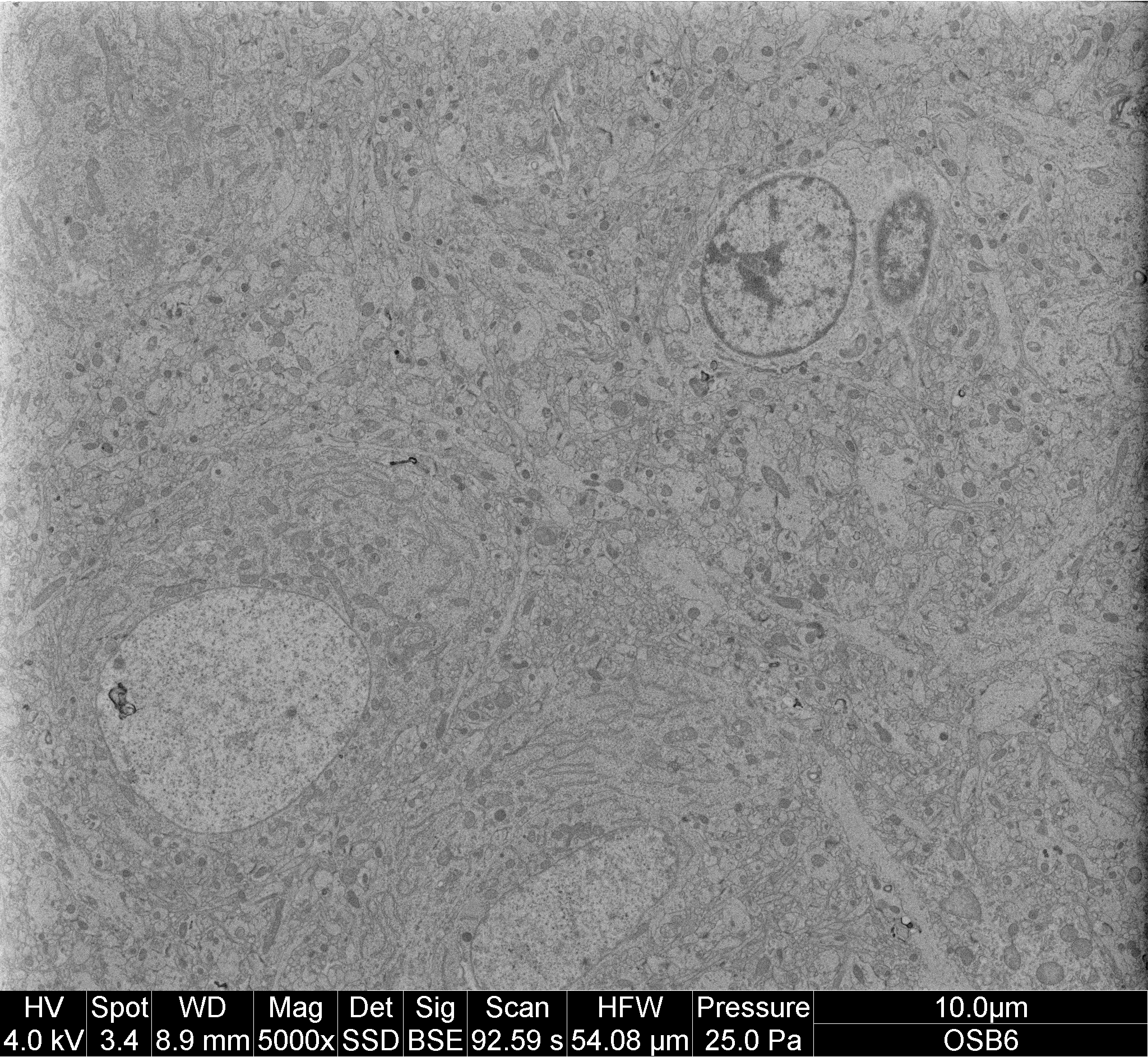

Supplement: Dataset S15 — (250.7 MB ZIP). [file pbio.0020329.sd015.zip › 040604_OS5_st1_1437.tif]

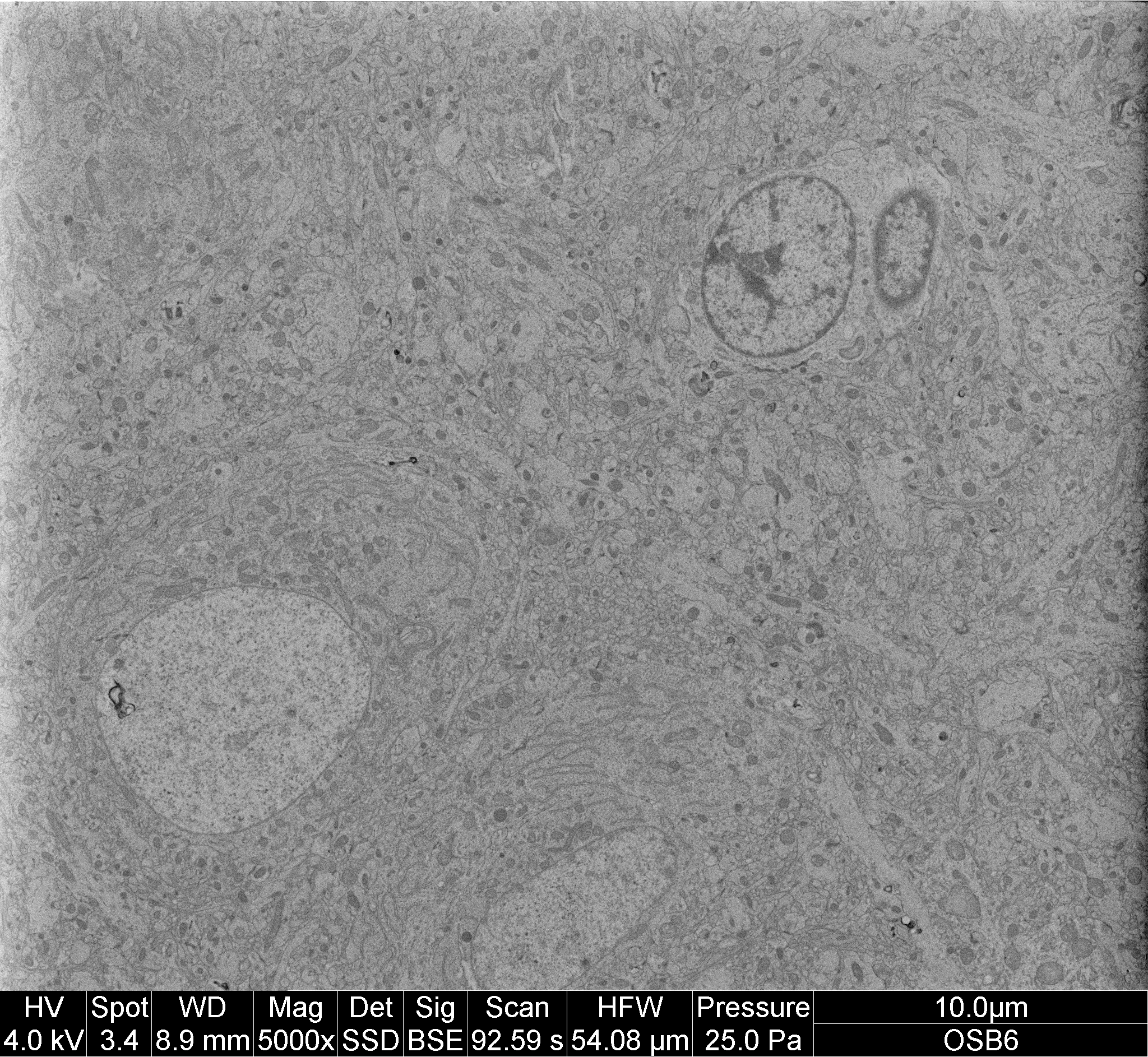

Supplement: Dataset S15 — (250.7 MB ZIP). [file pbio.0020329.sd015.zip › 040604_OS5_st1_1438.tif]

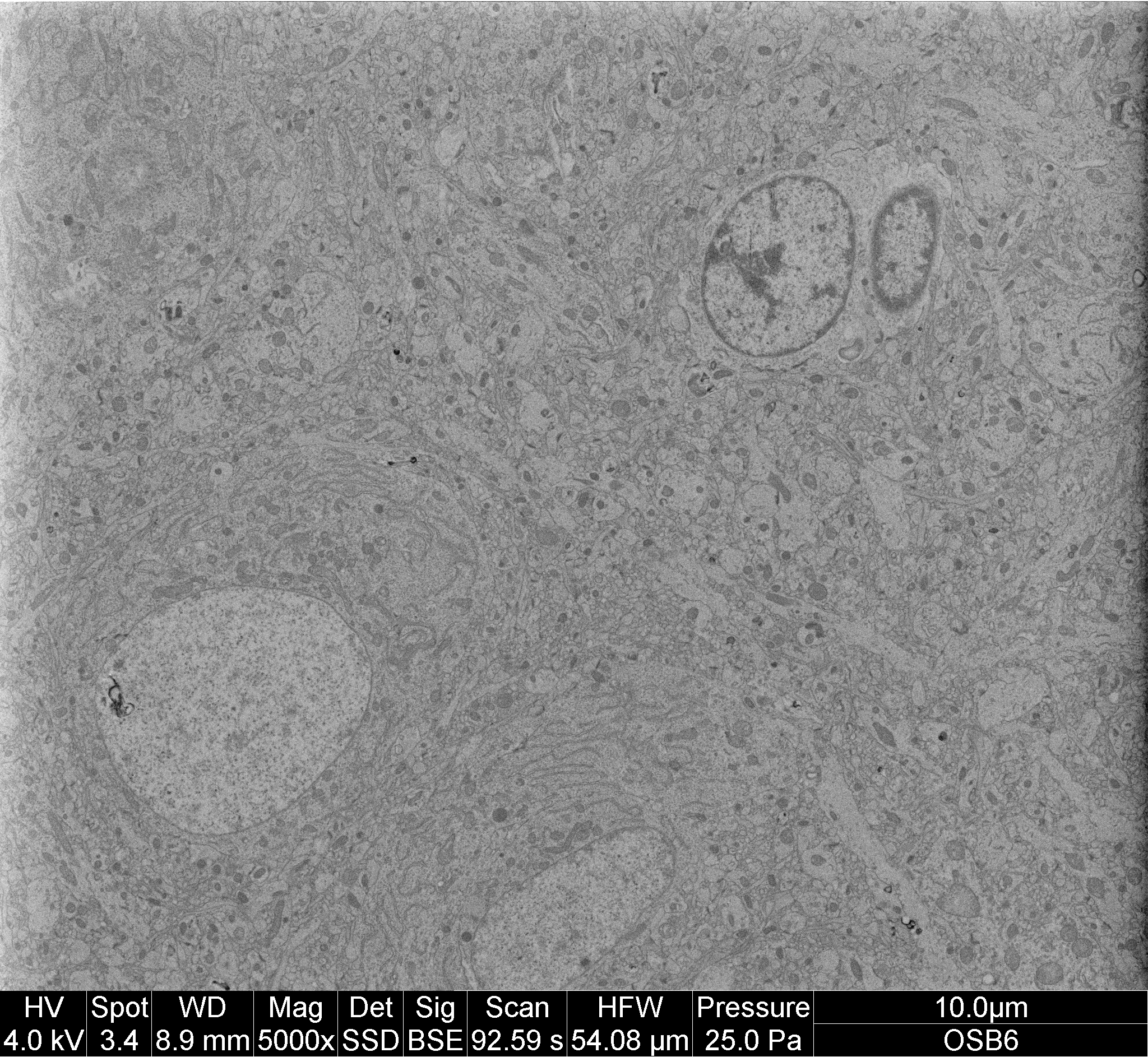

Supplement: Dataset S15 — (250.7 MB ZIP). [file pbio.0020329.sd015.zip › 040604_OS5_st1_1439.tif]

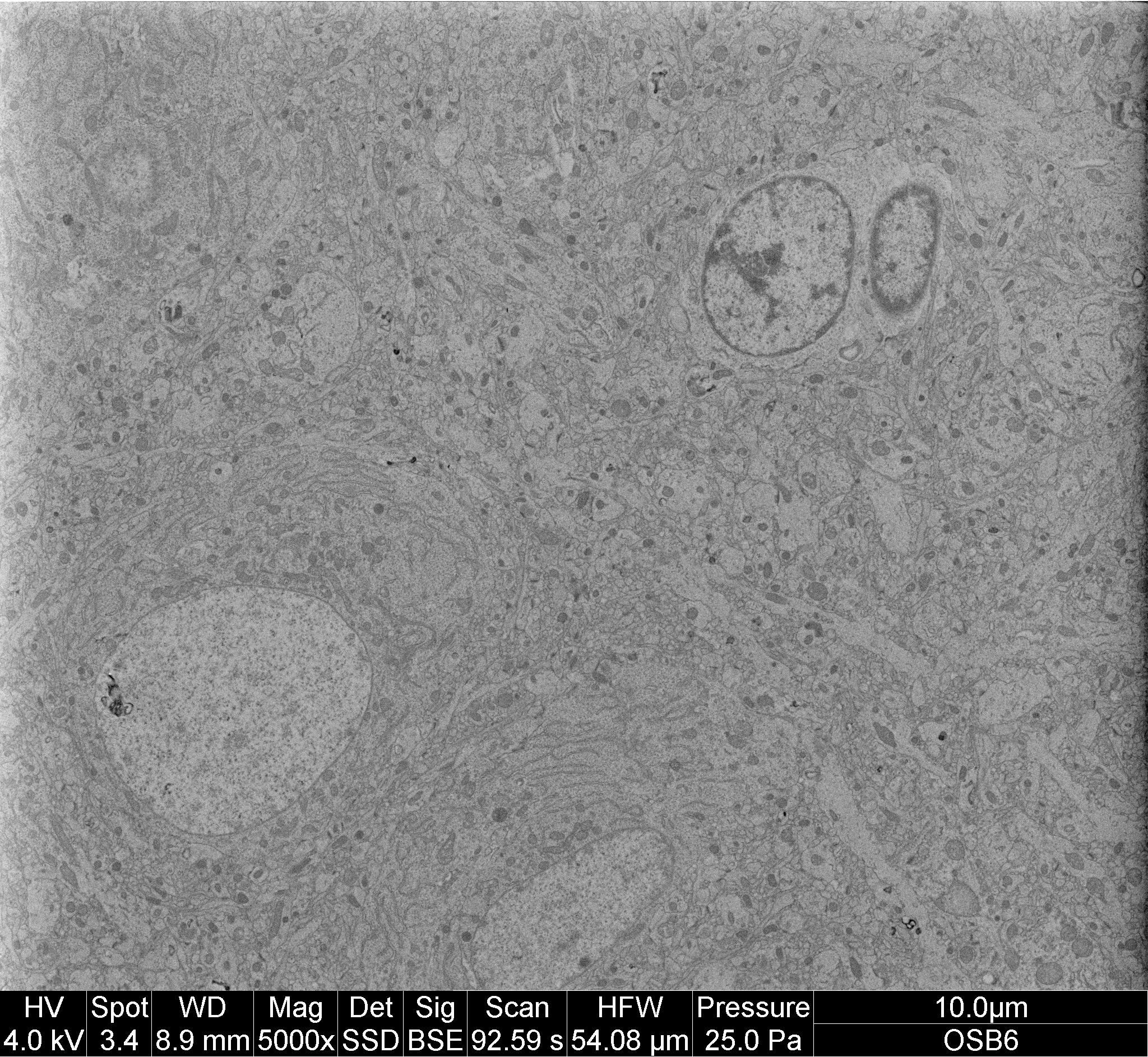

Supplement: Dataset S15 — (250.7 MB ZIP). [file pbio.0020329.sd015.zip › 040604_OS5_st1_1440.tif]

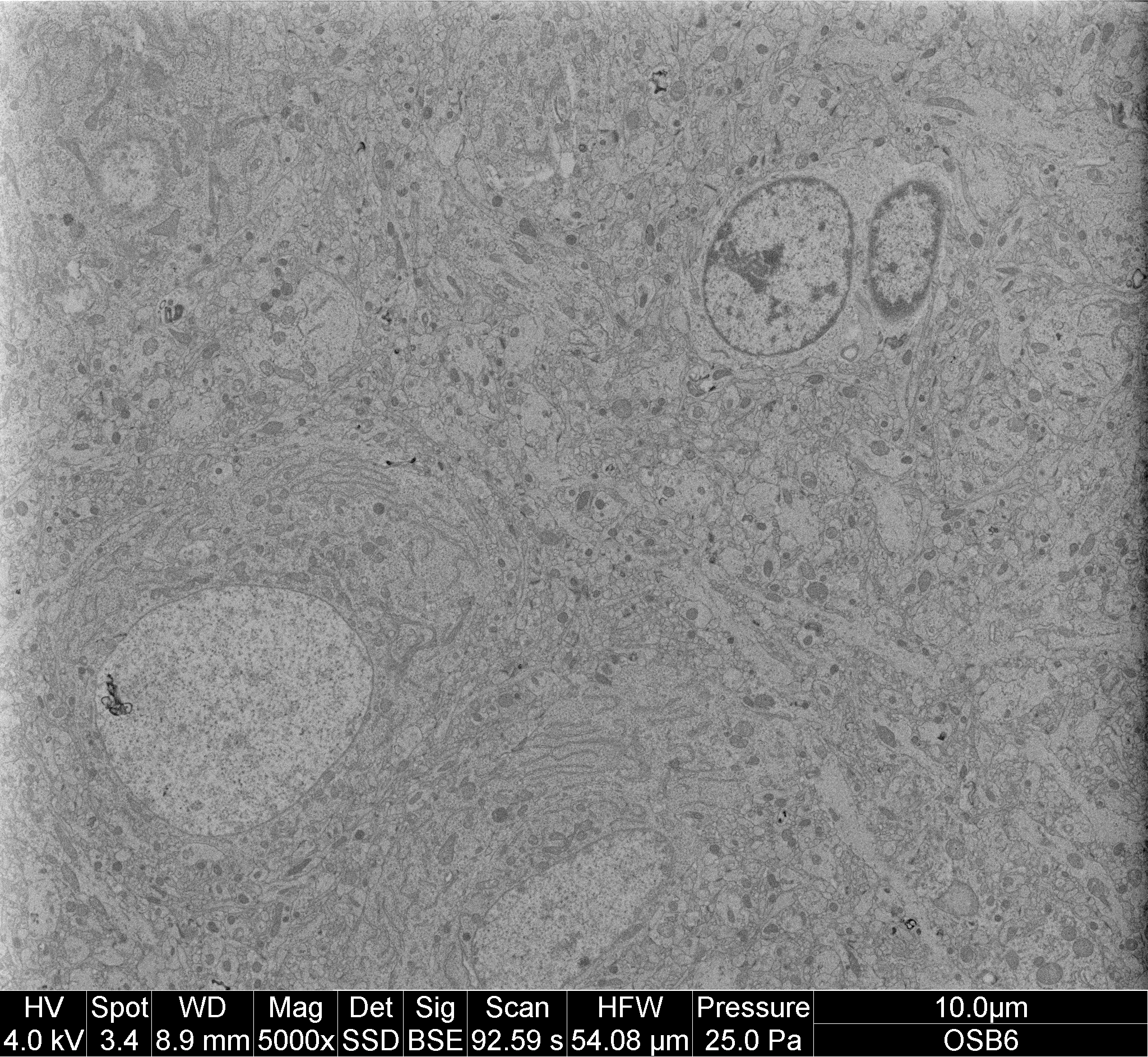

Supplement: Dataset S15 — (250.7 MB ZIP). [file pbio.0020329.sd015.zip › 040604_OS5_st1_1441.tif]

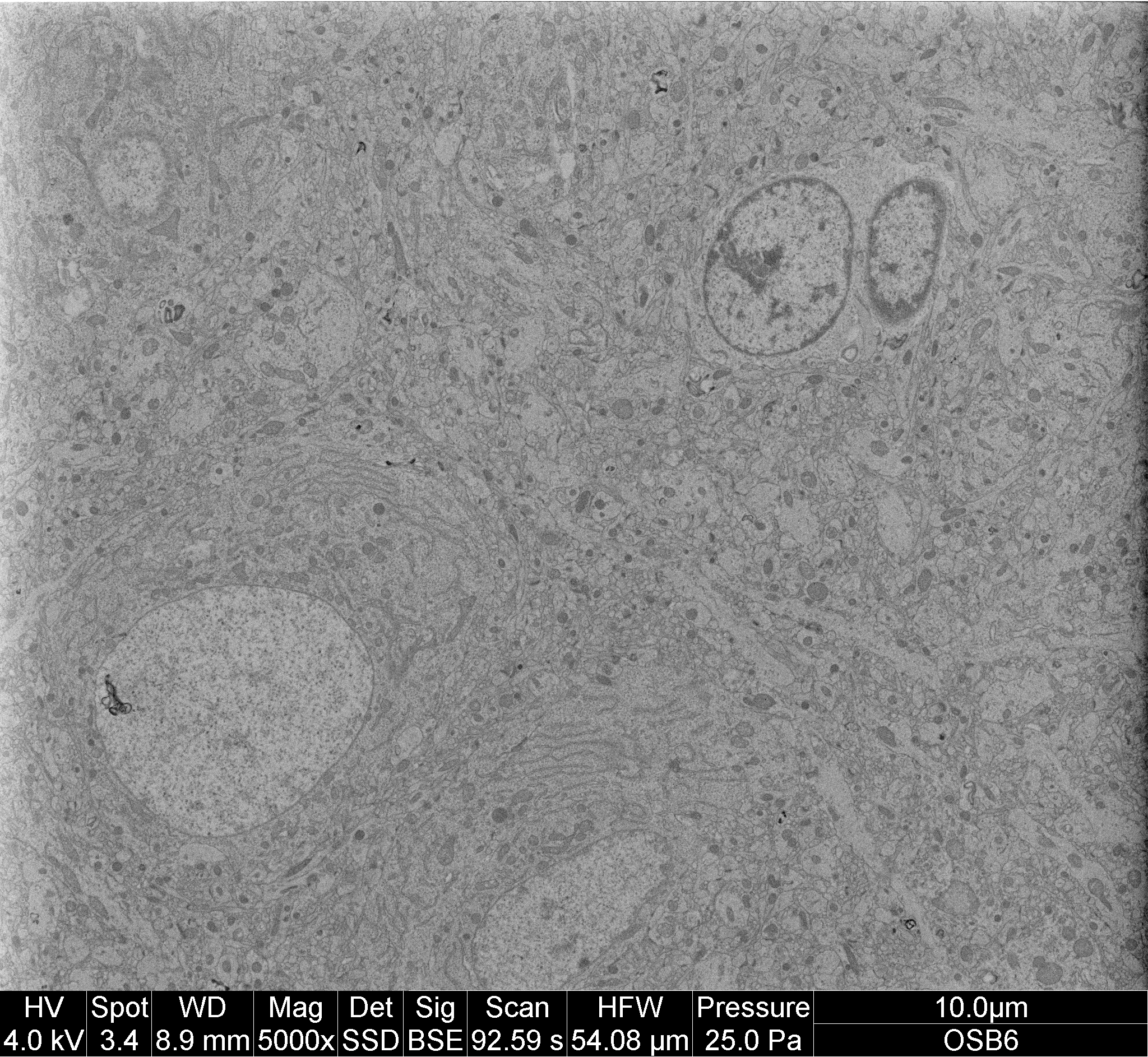

Supplement: Dataset S15 — (250.7 MB ZIP). [file pbio.0020329.sd015.zip › 040604_OS5_st1_1442.tif]

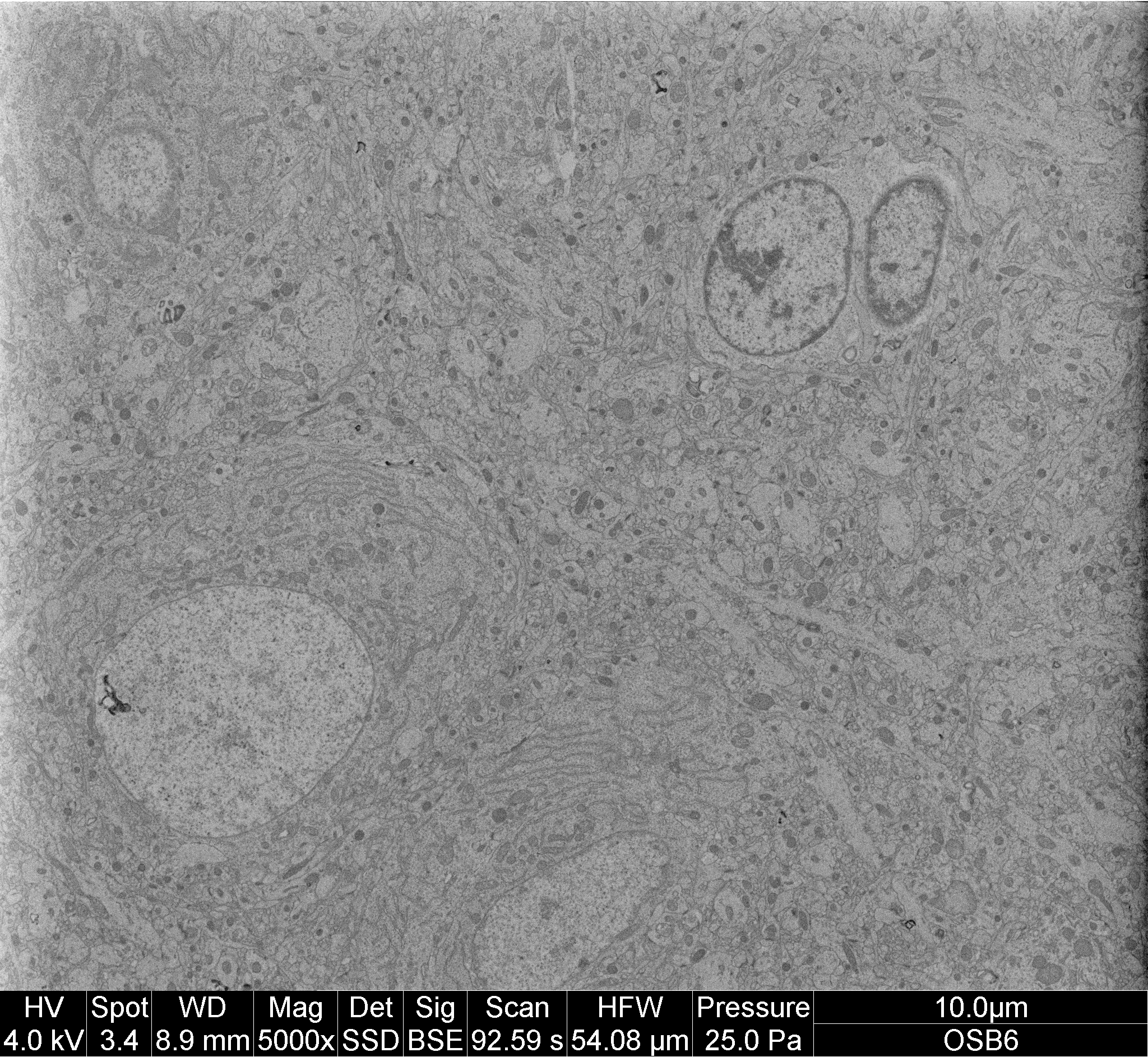

Supplement: Dataset S15 — (250.7 MB ZIP). [file pbio.0020329.sd015.zip › 040604_OS5_st1_1443.tif]

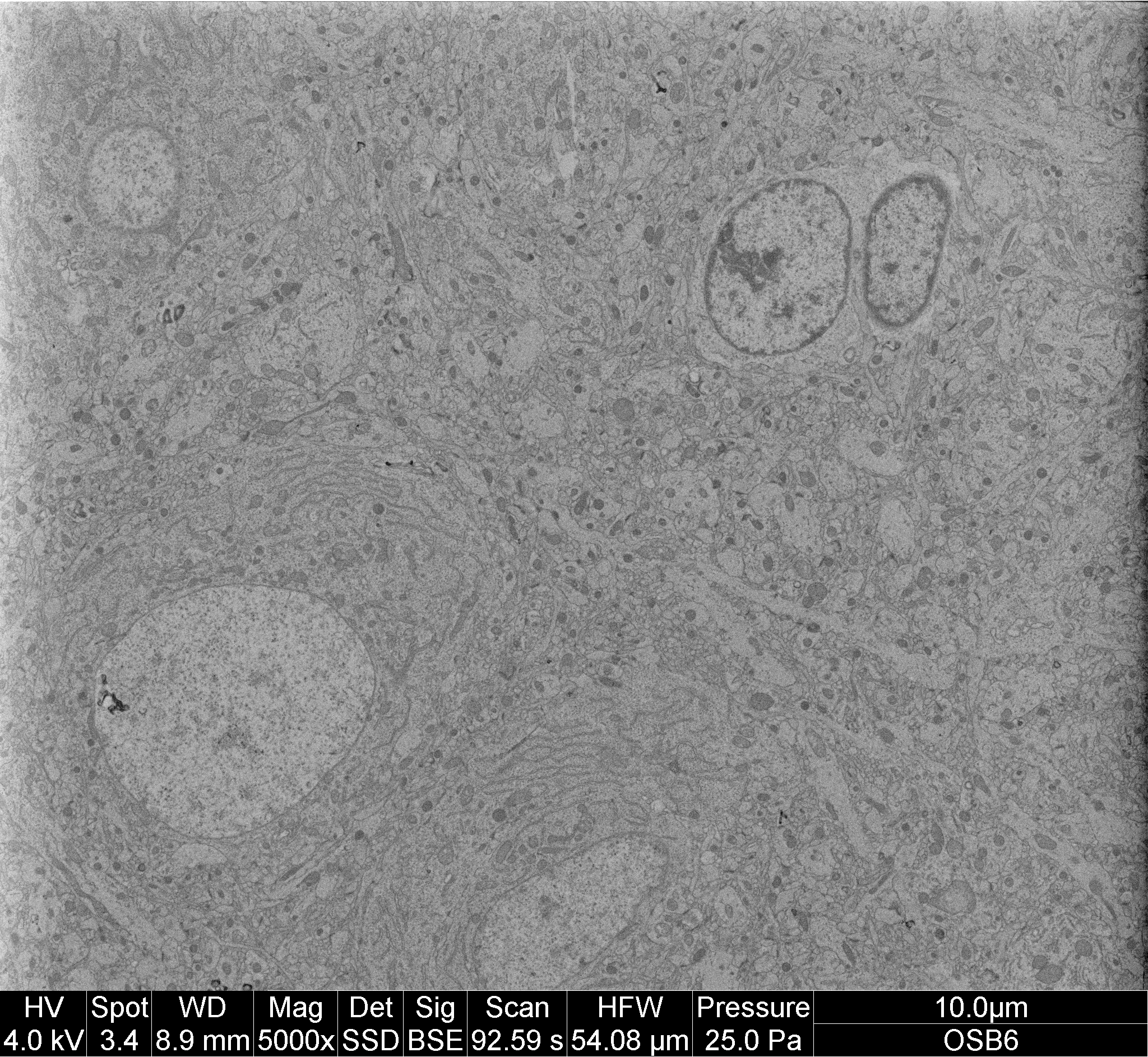

Supplement: Dataset S15 — (250.7 MB ZIP). [file pbio.0020329.sd015.zip › 040604_OS5_st1_1444.tif]

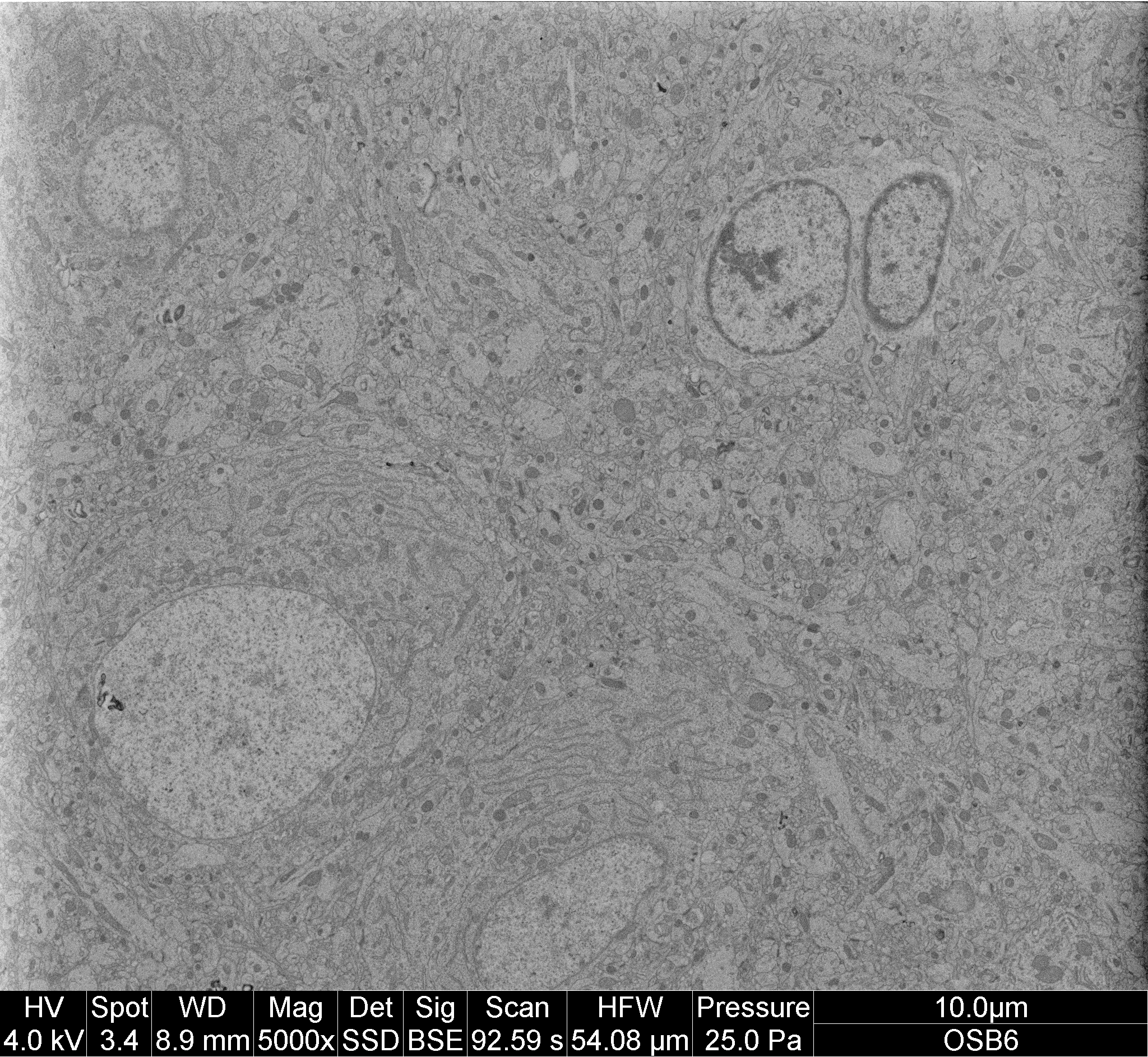

Supplement: Dataset S15 — (250.7 MB ZIP). [file pbio.0020329.sd015.zip › 040604_OS5_st1_1445.tif]

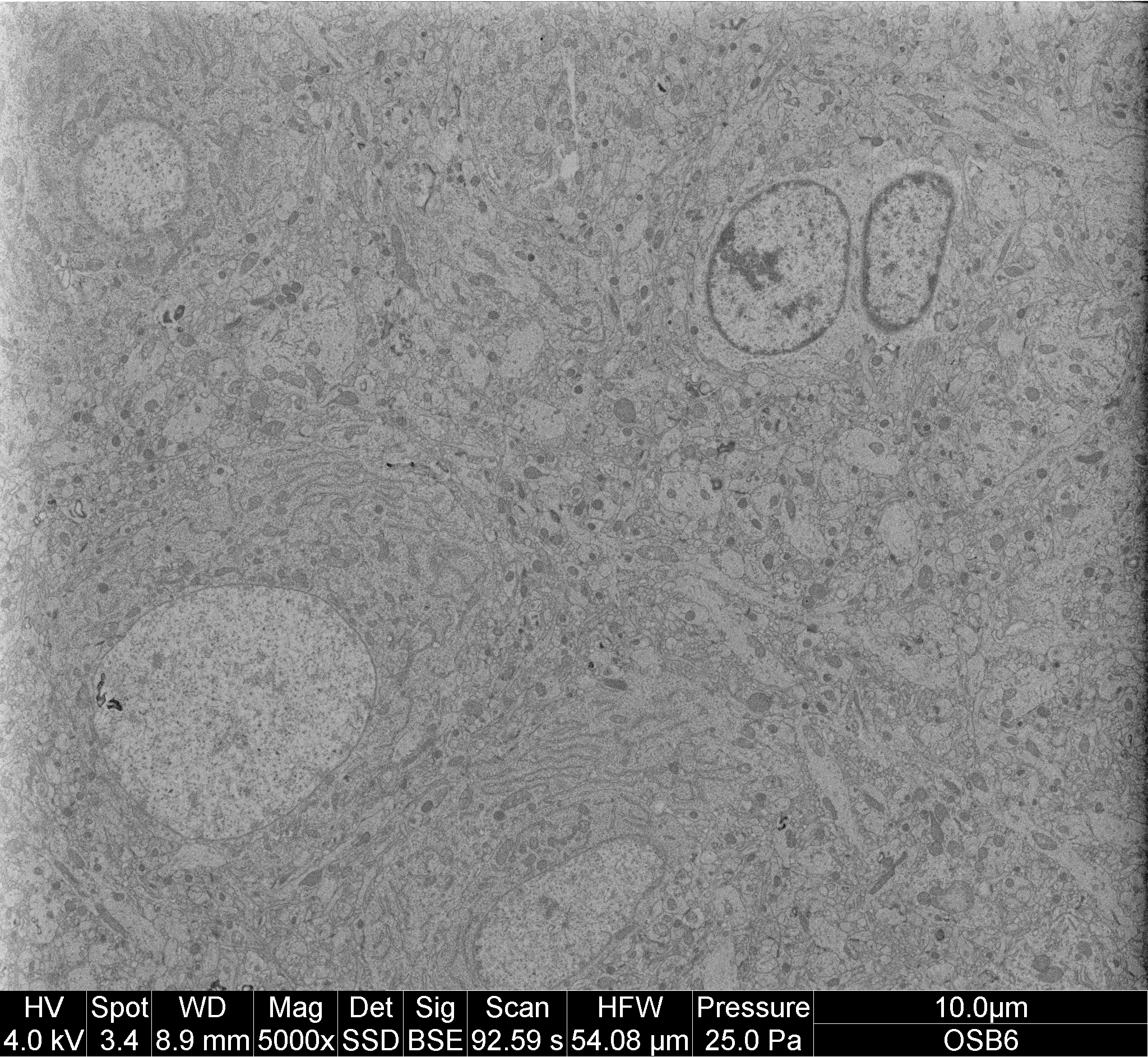

Supplement: Dataset S15 — (250.7 MB ZIP). [file pbio.0020329.sd015.zip › 040604_OS5_st1_1446.tif]

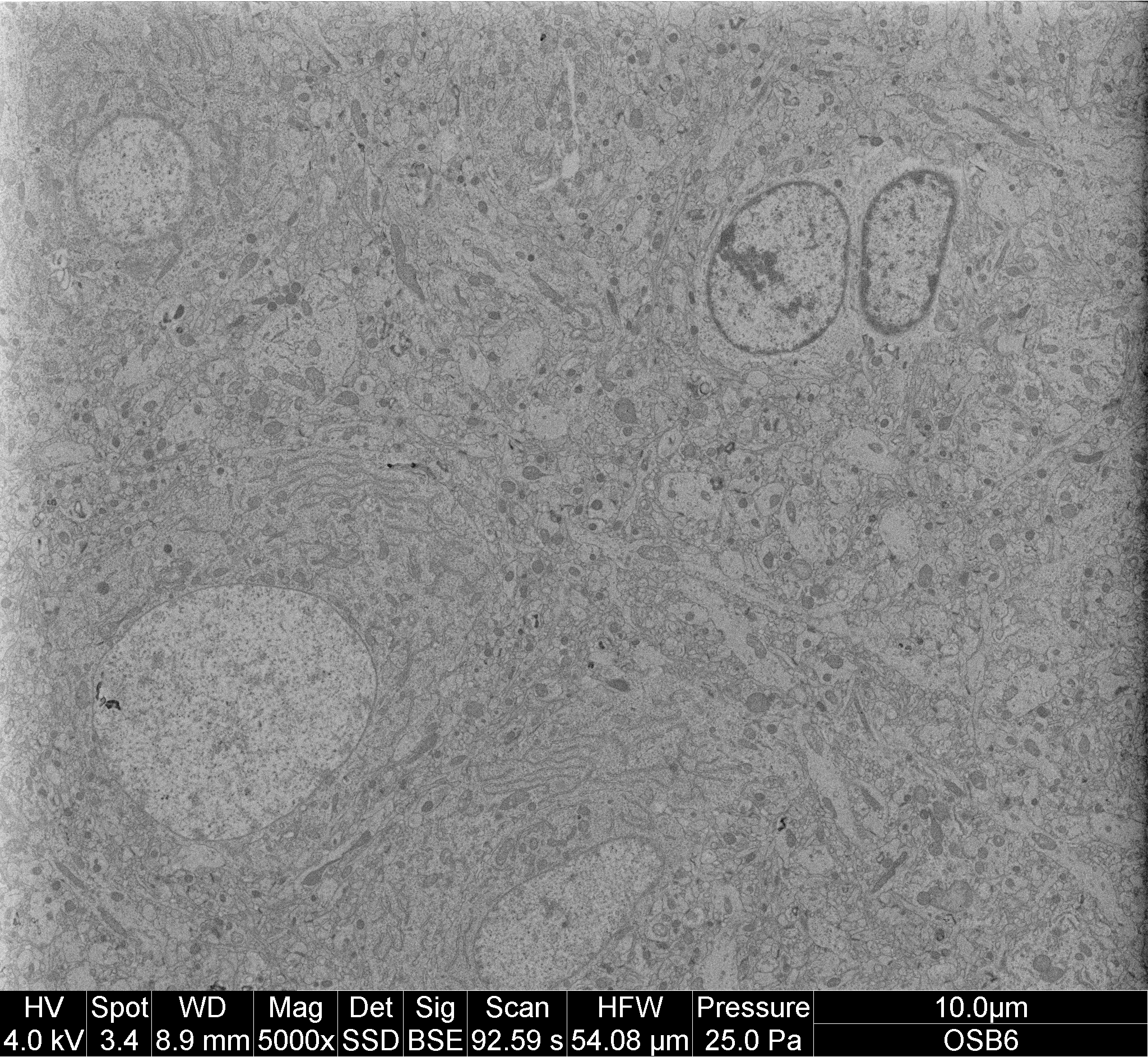

Supplement: Dataset S15 — (250.7 MB ZIP). [file pbio.0020329.sd015.zip › 040604_OS5_st1_1447.tif]

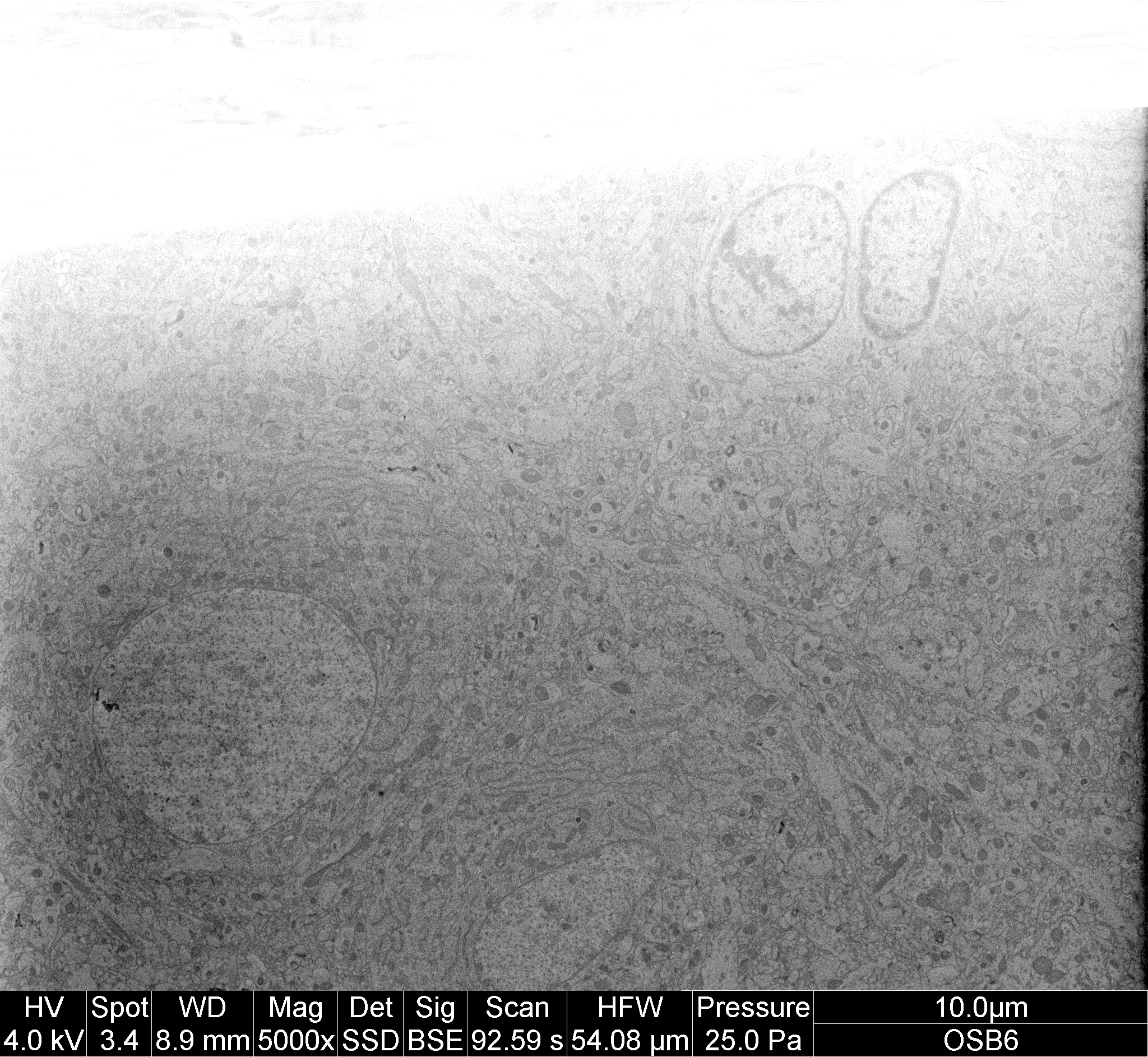

Supplement: Dataset S15 — (250.7 MB ZIP). [file pbio.0020329.sd015.zip › 040604_OS5_st1_1448.tif]

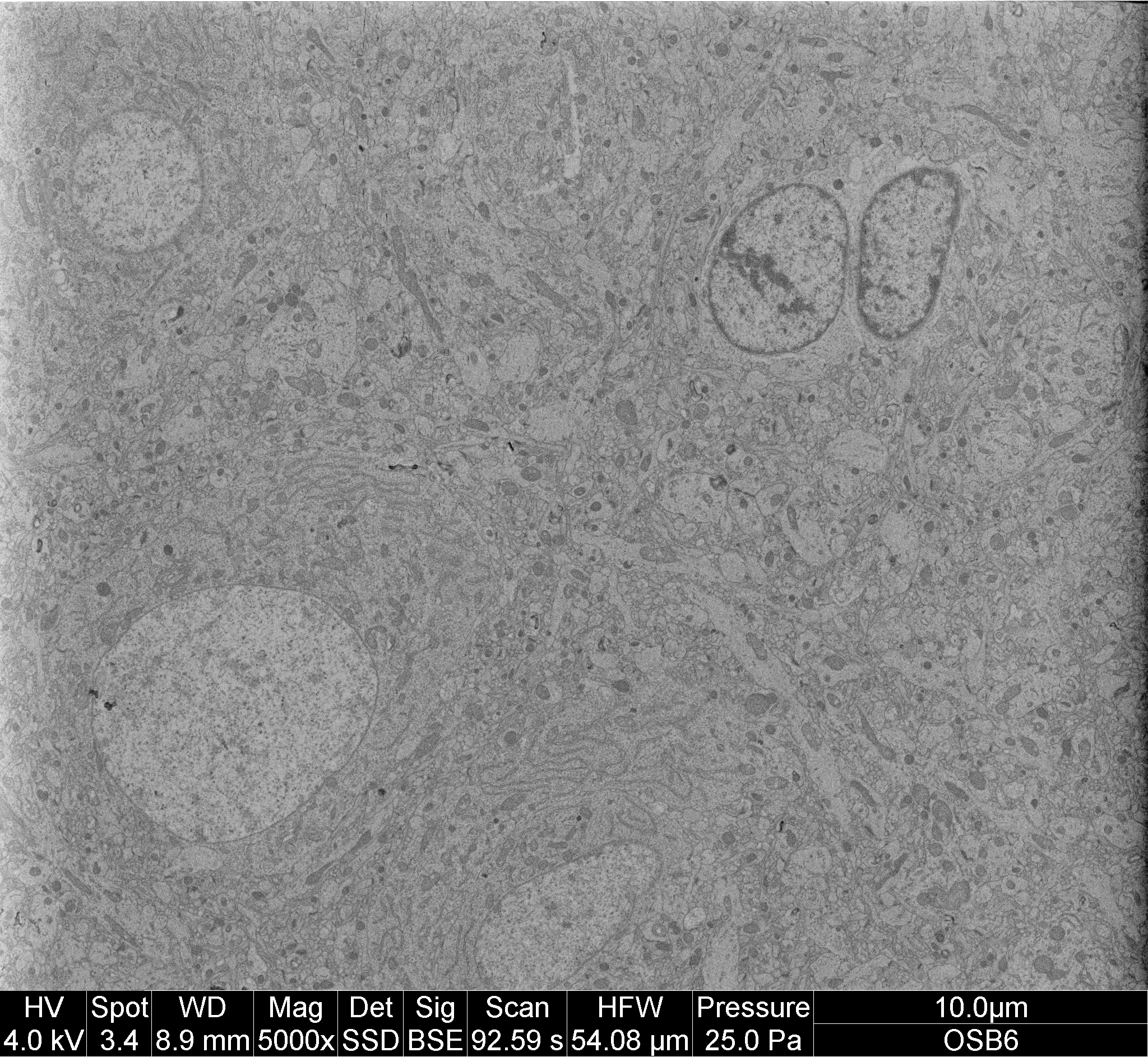

Supplement: Dataset S15 — (250.7 MB ZIP). [file pbio.0020329.sd015.zip › 040604_OS5_st1_1449.tif]

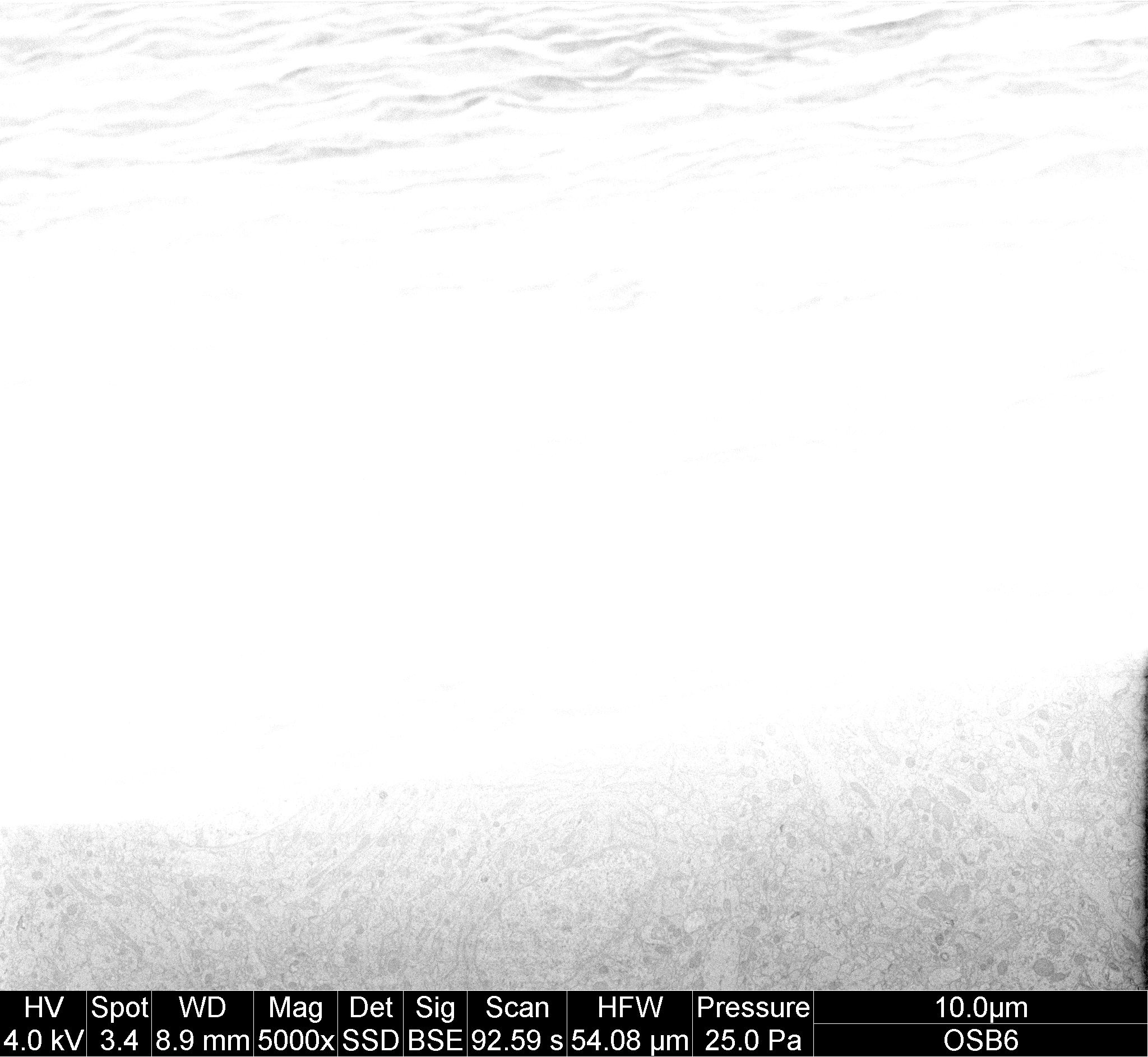

Supplement: Dataset S15 — (250.7 MB ZIP). [file pbio.0020329.sd015.zip › 040604_OS5_st1_1450.tif]

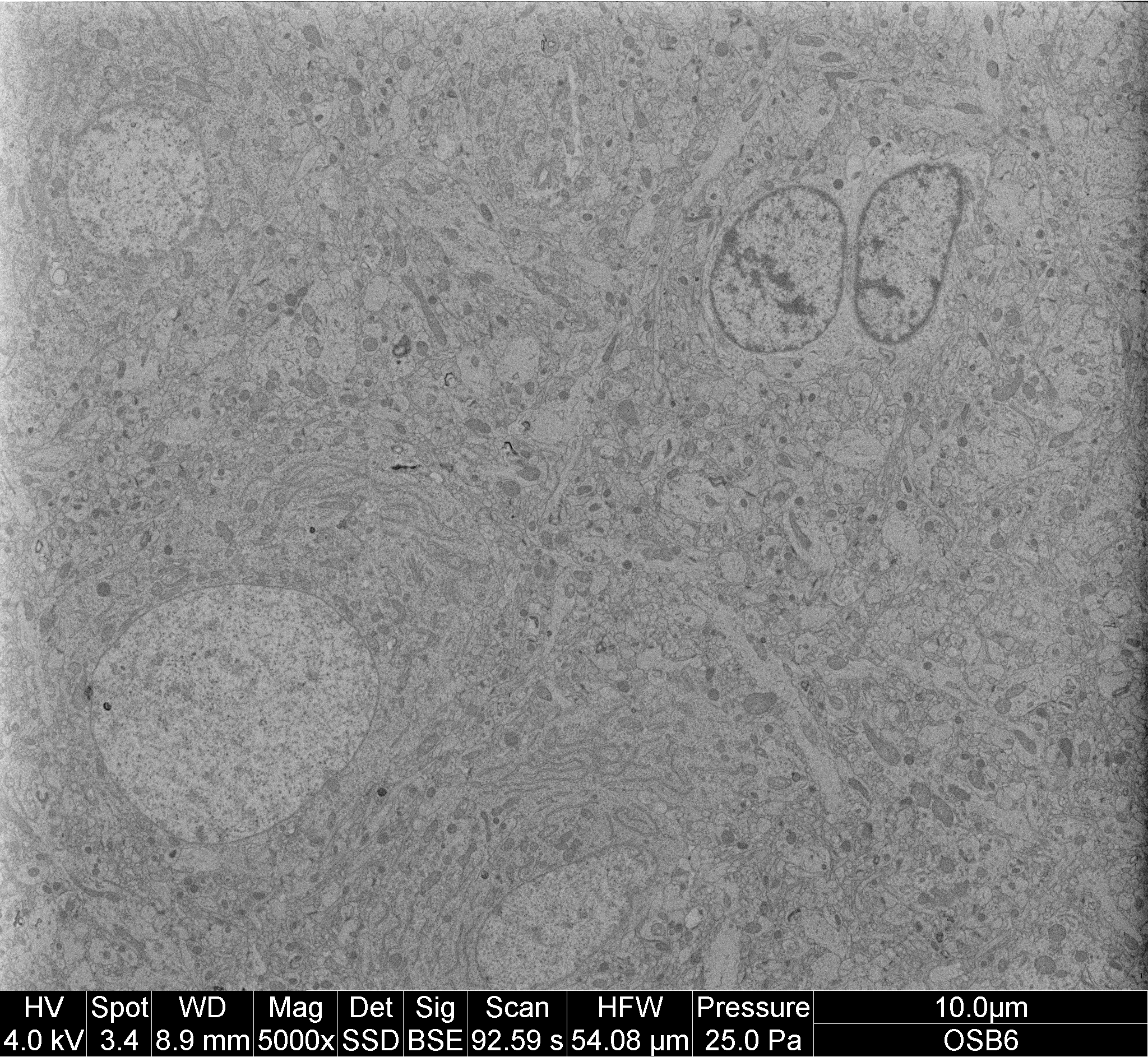

Supplement: Dataset S15 — (250.7 MB ZIP). [file pbio.0020329.sd015.zip › 040604_OS5_st1_1451.tif]

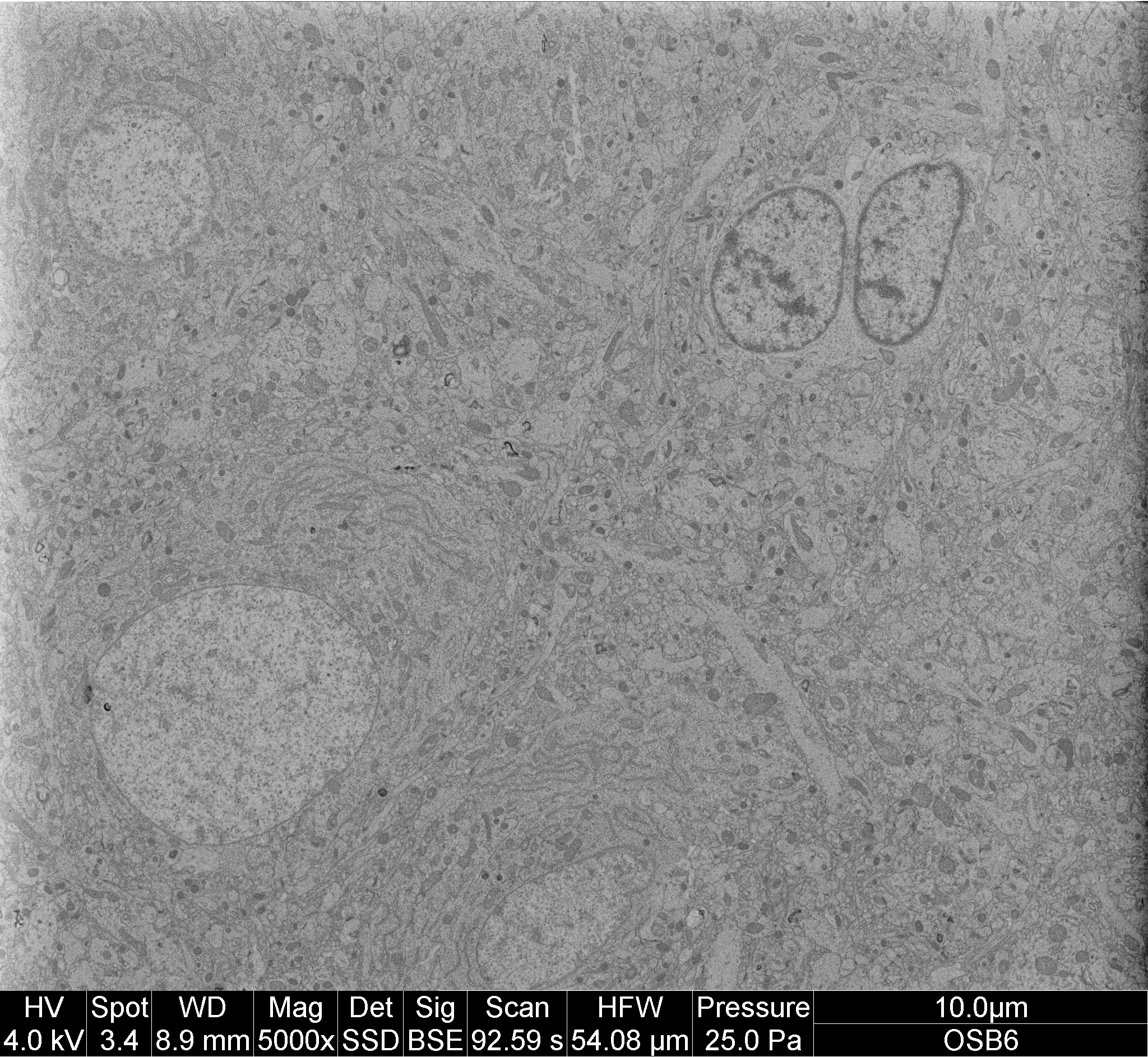

Supplement: Dataset S15 — (250.7 MB ZIP). [file pbio.0020329.sd015.zip › 040604_OS5_st1_1452.tif]

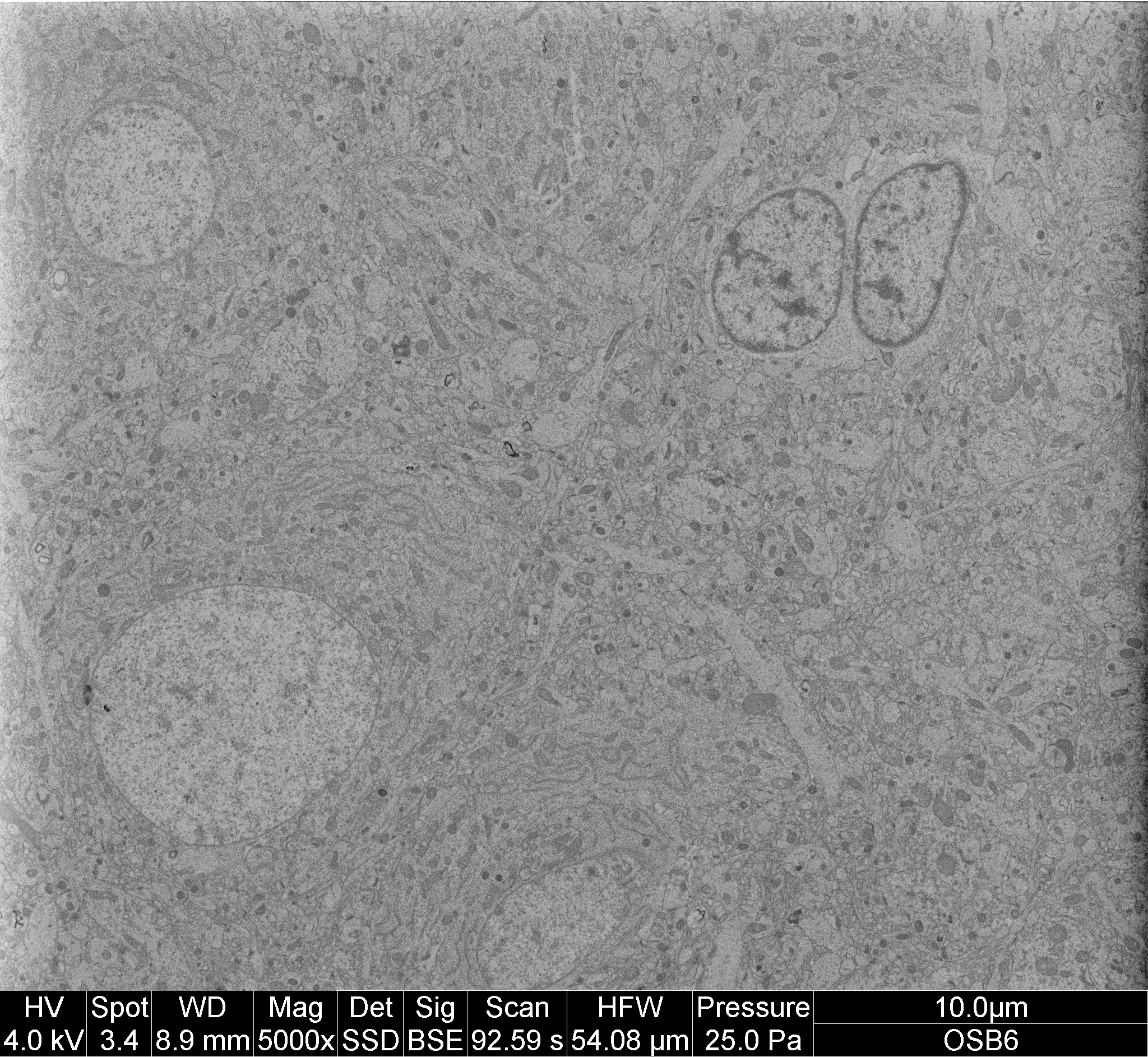

Supplement: Dataset S15 — (250.7 MB ZIP). [file pbio.0020329.sd015.zip › 040604_OS5_st1_1453.tif]

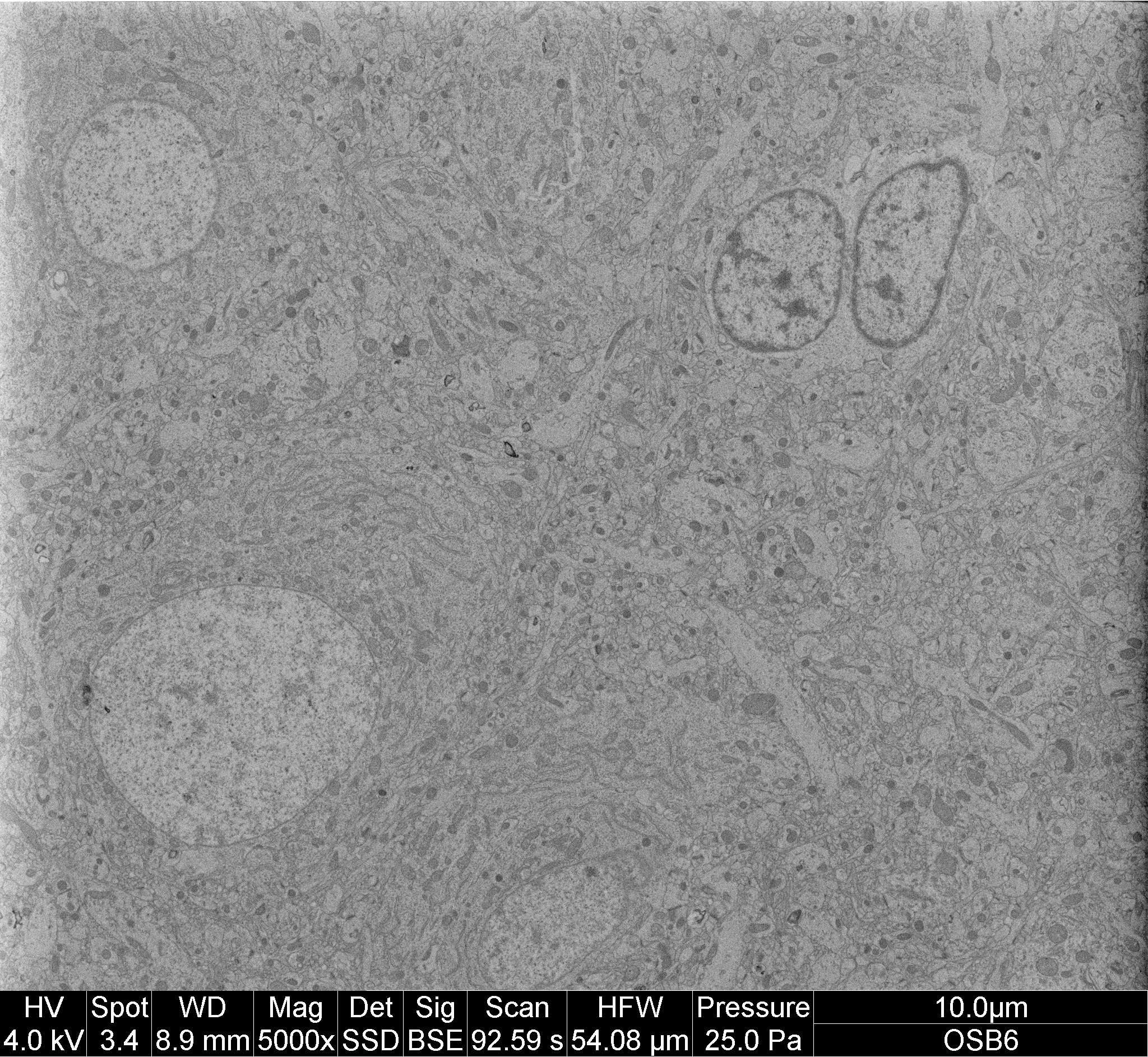

Supplement: Dataset S15 — (250.7 MB ZIP). [file pbio.0020329.sd015.zip › 040604_OS5_st1_1454.tif]

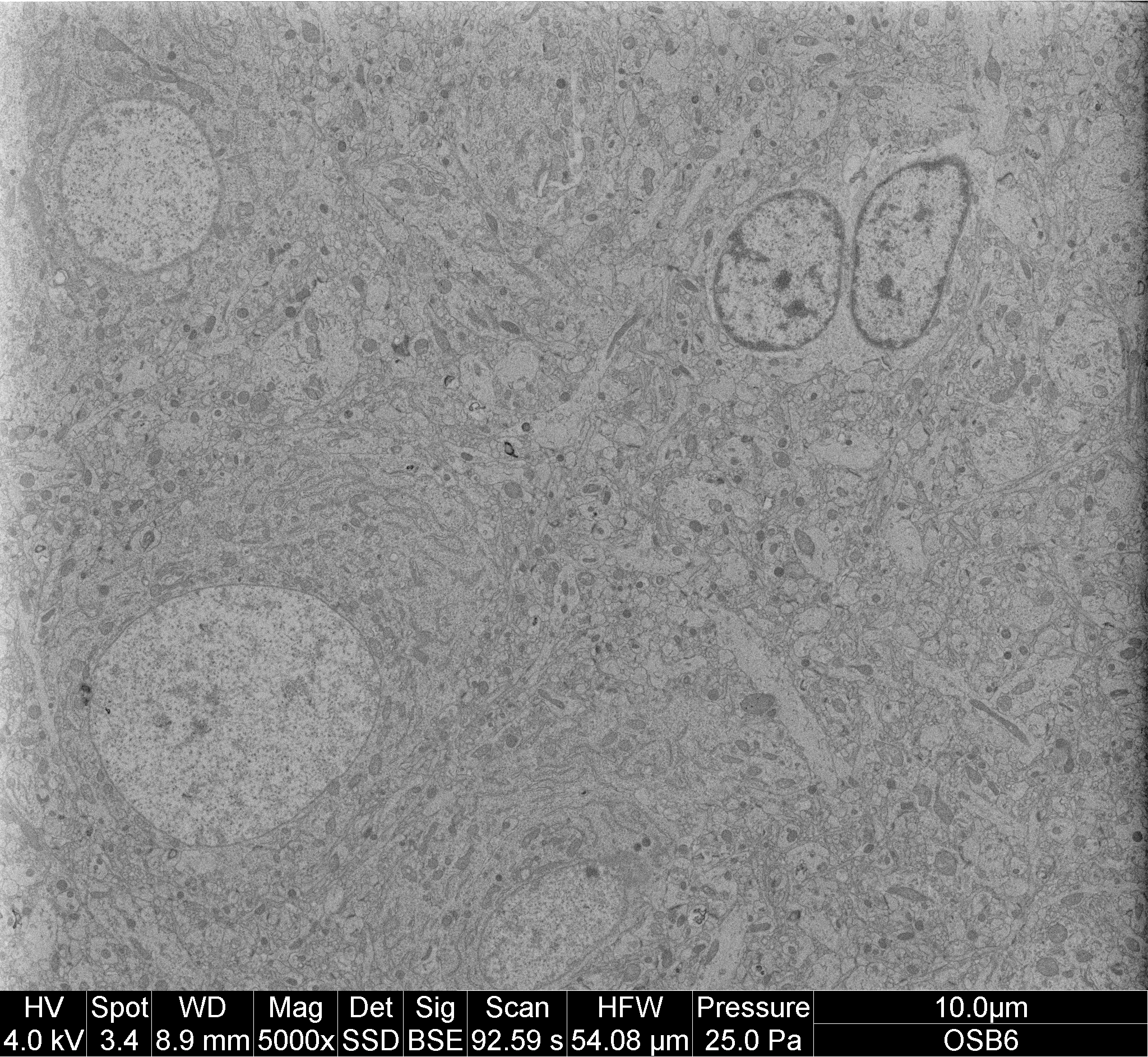

Supplement: Dataset S15 — (250.7 MB ZIP). [file pbio.0020329.sd015.zip › 040604_OS5_st1_1455.tif]

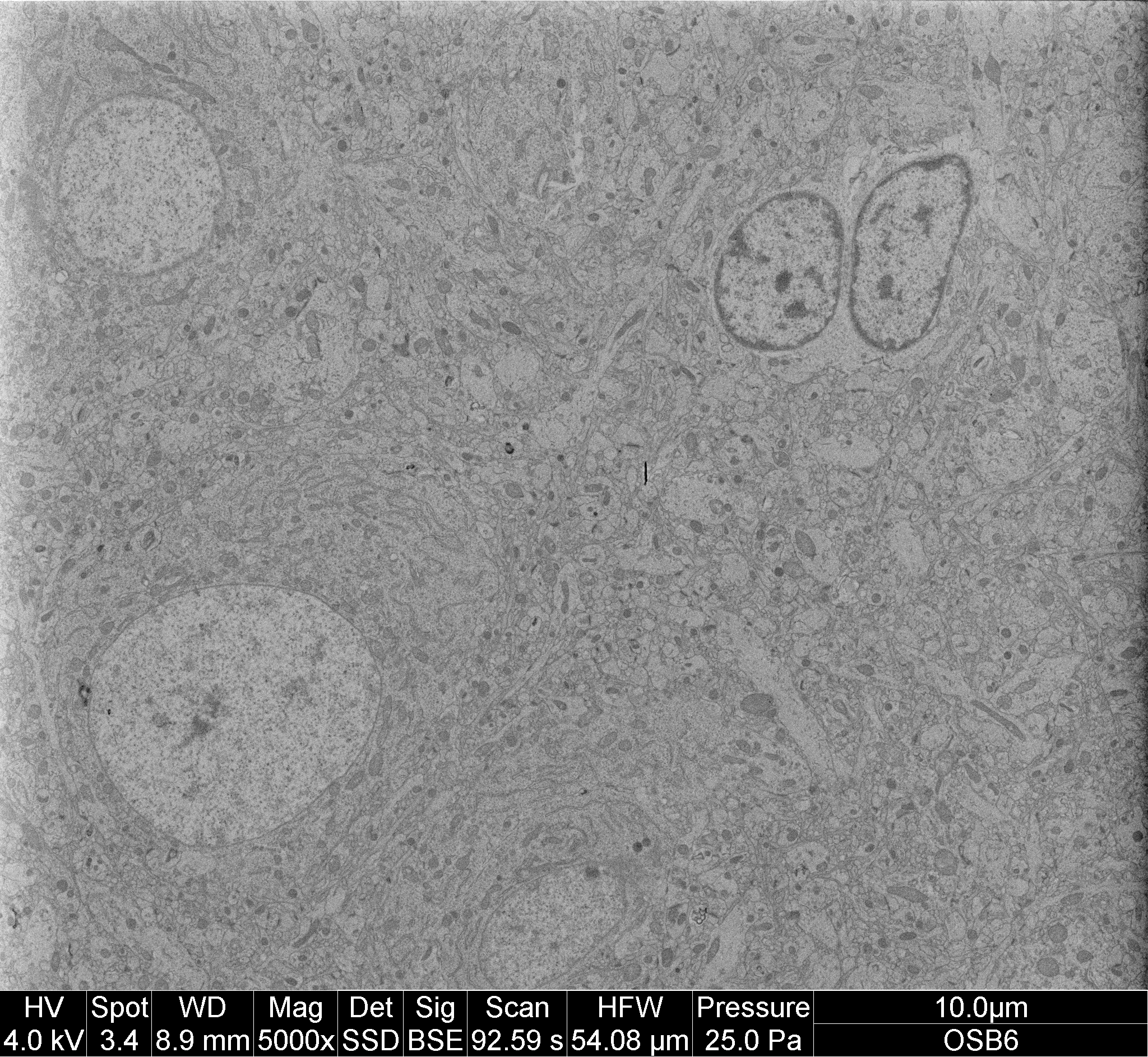

Supplement: Dataset S15 — (250.7 MB ZIP). [file pbio.0020329.sd015.zip › 040604_OS5_st1_1456.tif]

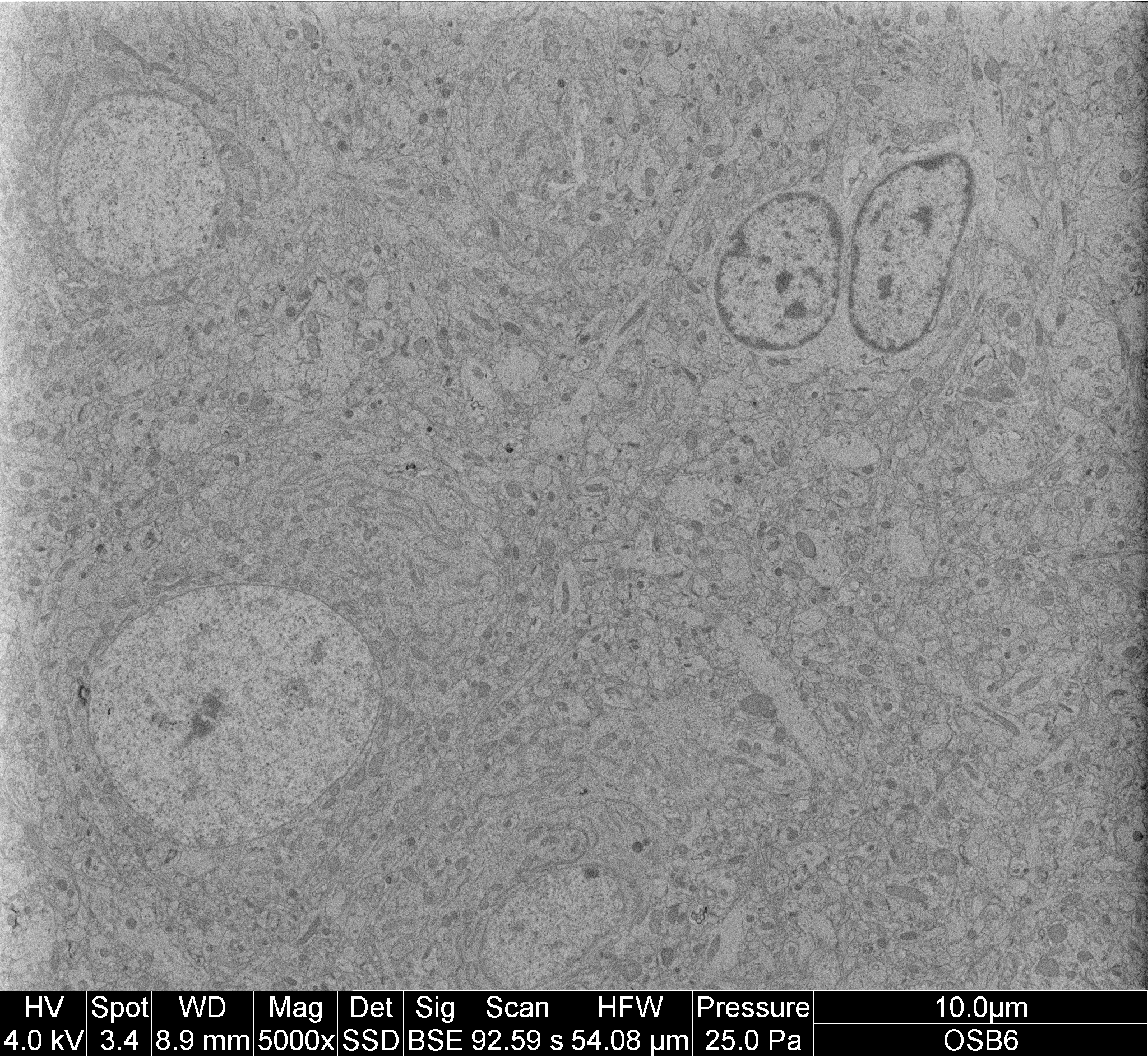

Supplement: Dataset S15 — (250.7 MB ZIP). [file pbio.0020329.sd015.zip › 040604_OS5_st1_1457.tif]

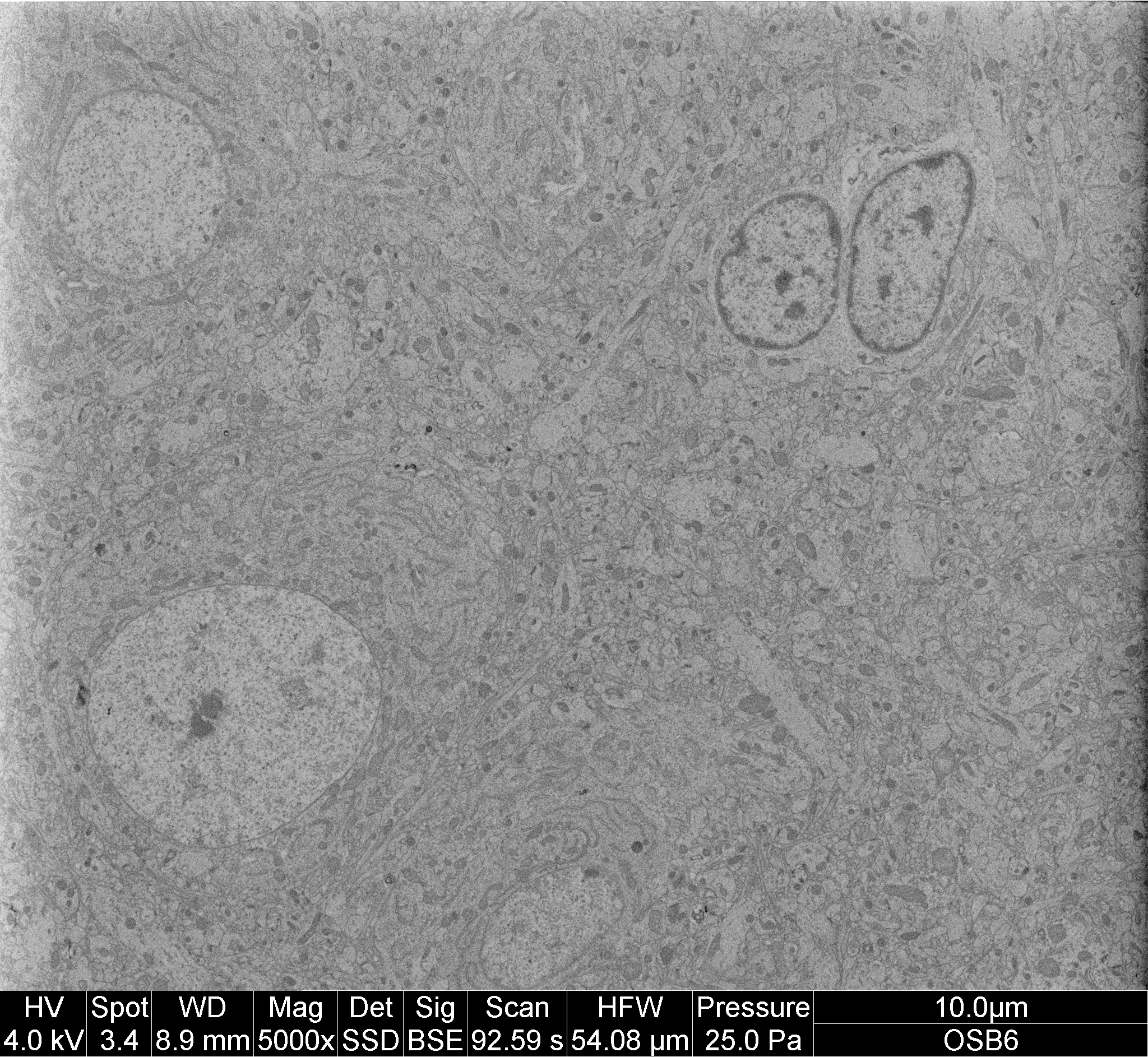

Supplement: Dataset S15 — (250.7 MB ZIP). [file pbio.0020329.sd015.zip › 040604_OS5_st1_1458.tif]

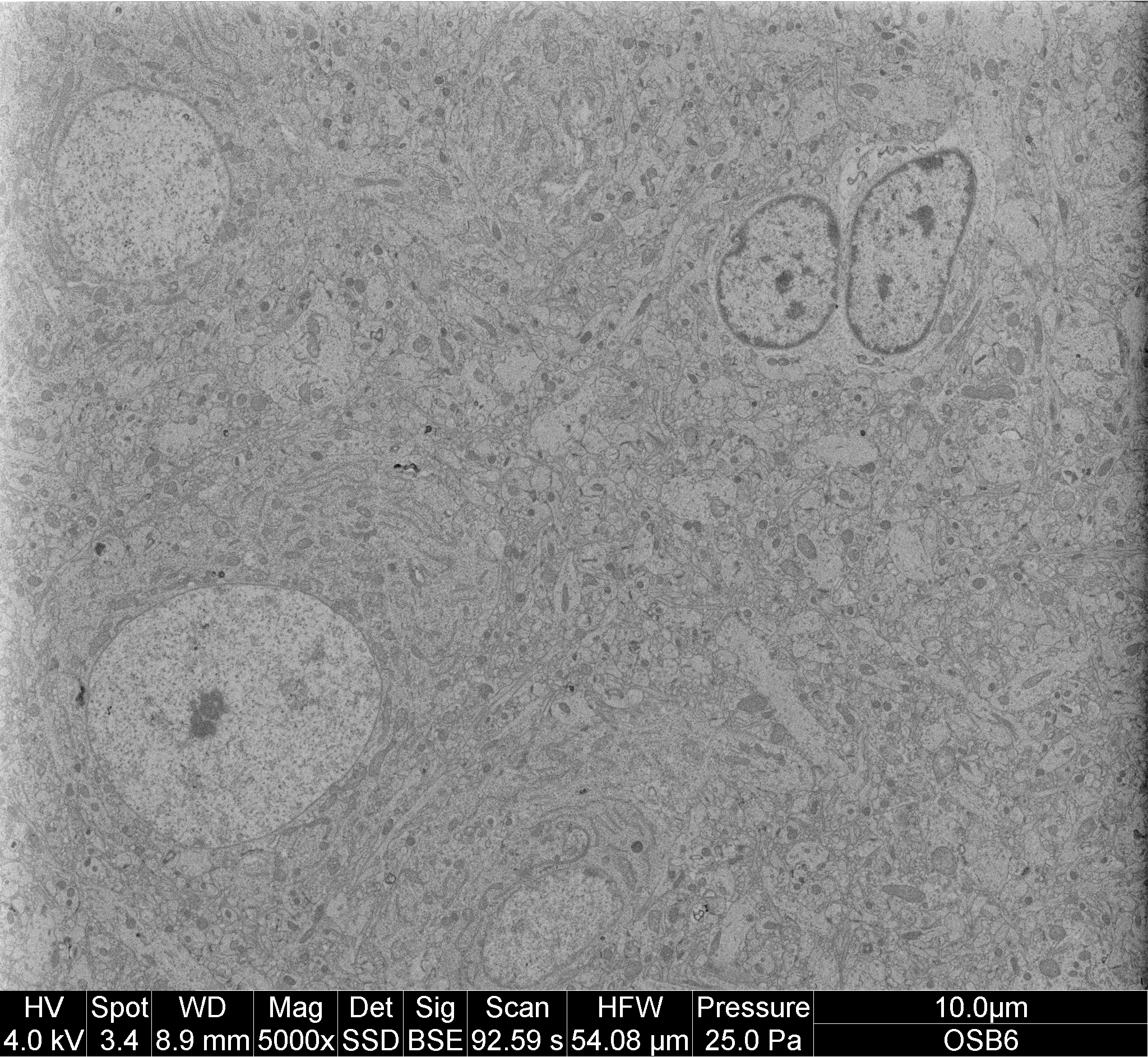

Supplement: Dataset S15 — (250.7 MB ZIP). [file pbio.0020329.sd015.zip › 040604_OS5_st1_1459.tif]

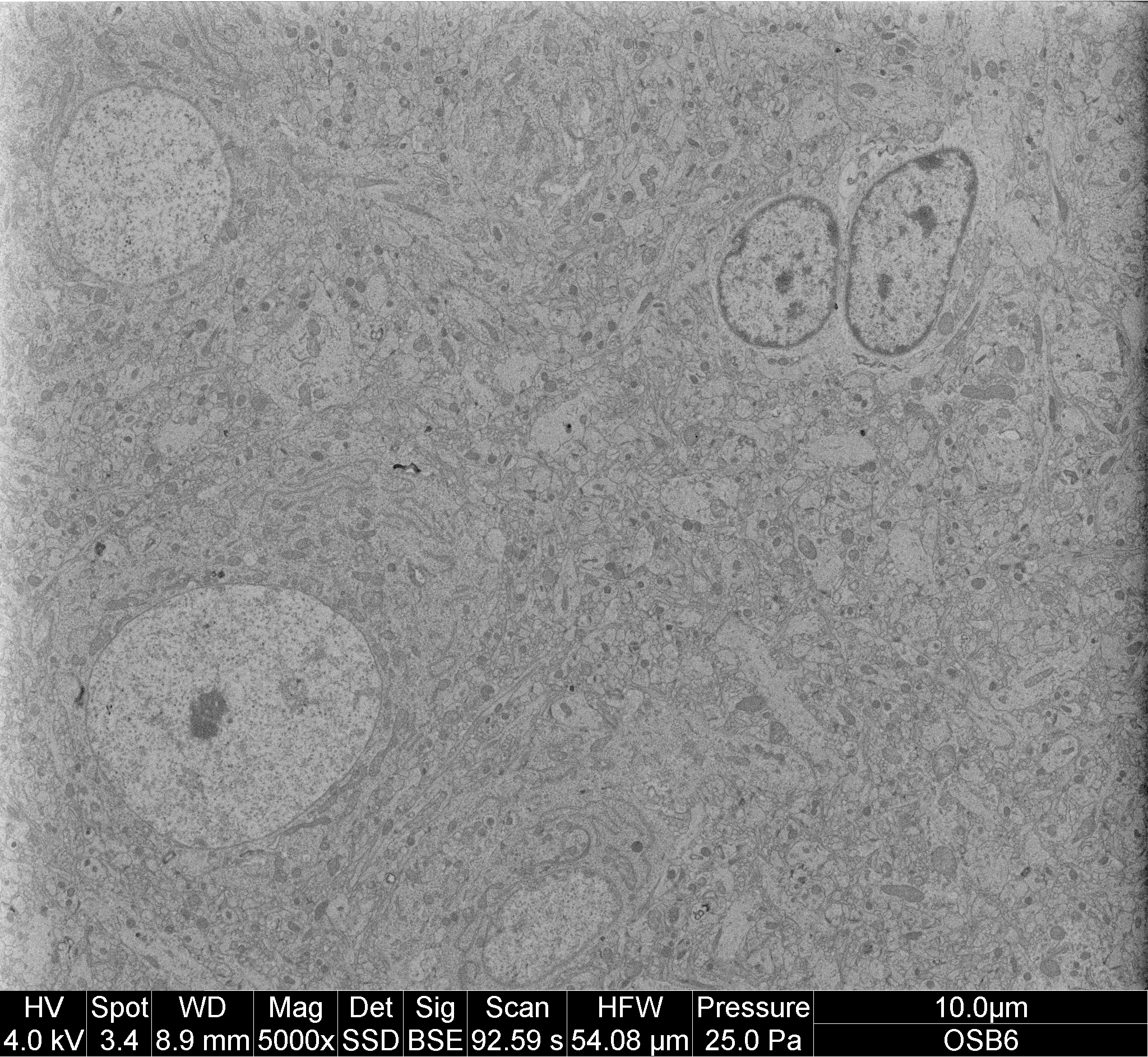

Supplement: Dataset S15 — (250.7 MB ZIP). [file pbio.0020329.sd015.zip › 040604_OS5_st1_1460.tif]

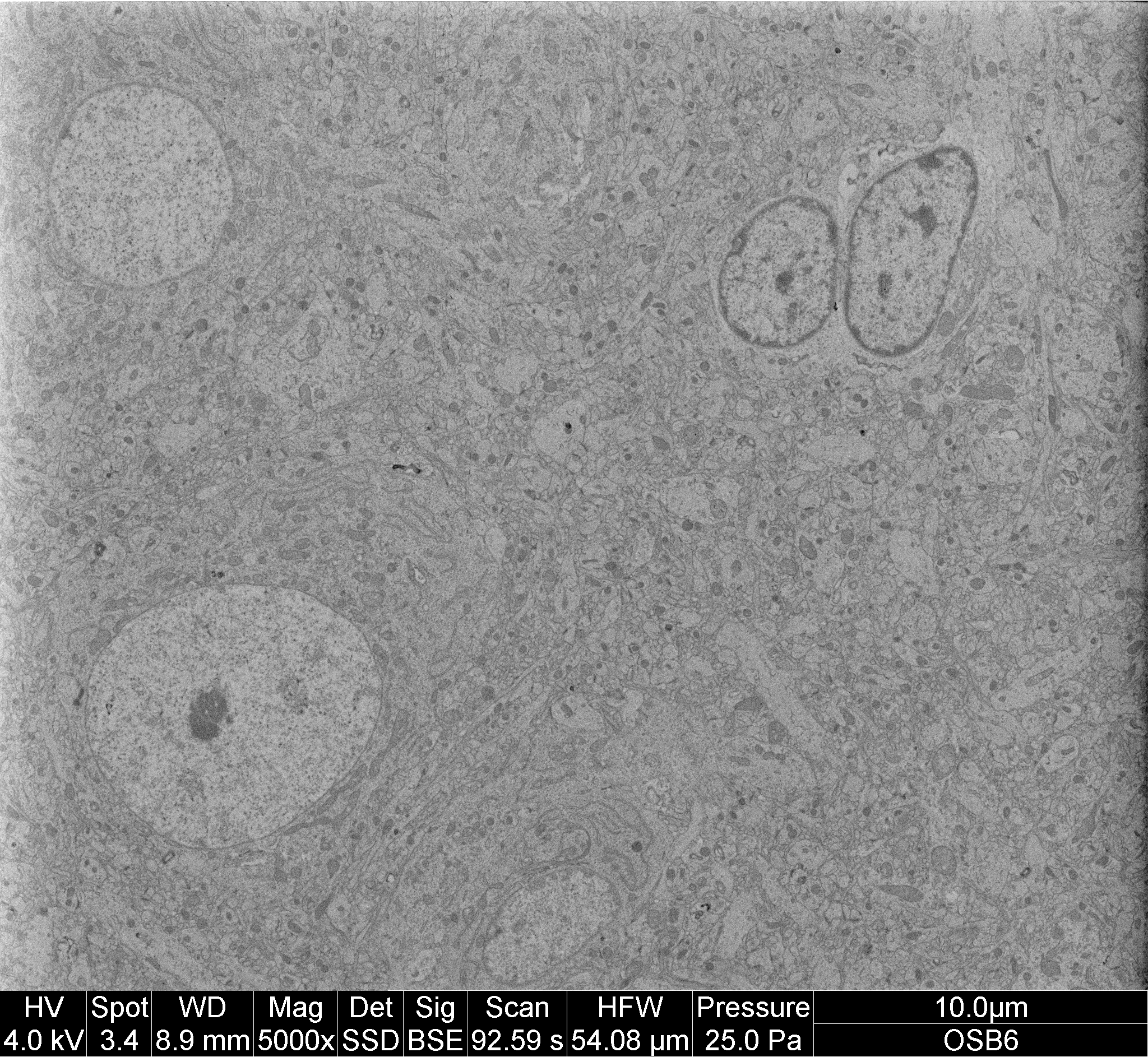

Supplement: Dataset S15 — (250.7 MB ZIP). [file pbio.0020329.sd015.zip › 040604_OS5_st1_1461.tif]

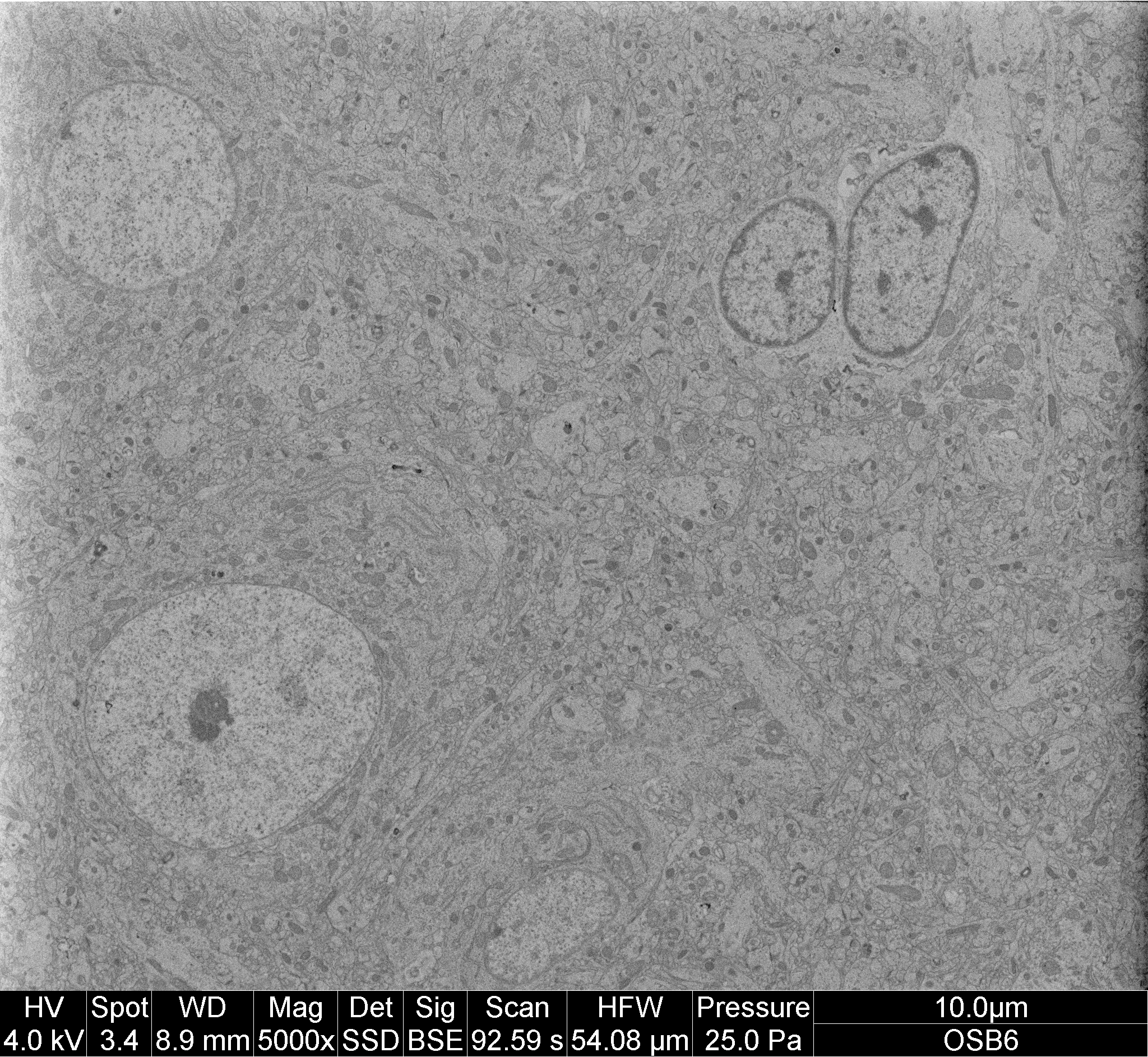

Supplement: Dataset S15 — (250.7 MB ZIP). [file pbio.0020329.sd015.zip › 040604_OS5_st1_1462.tif]

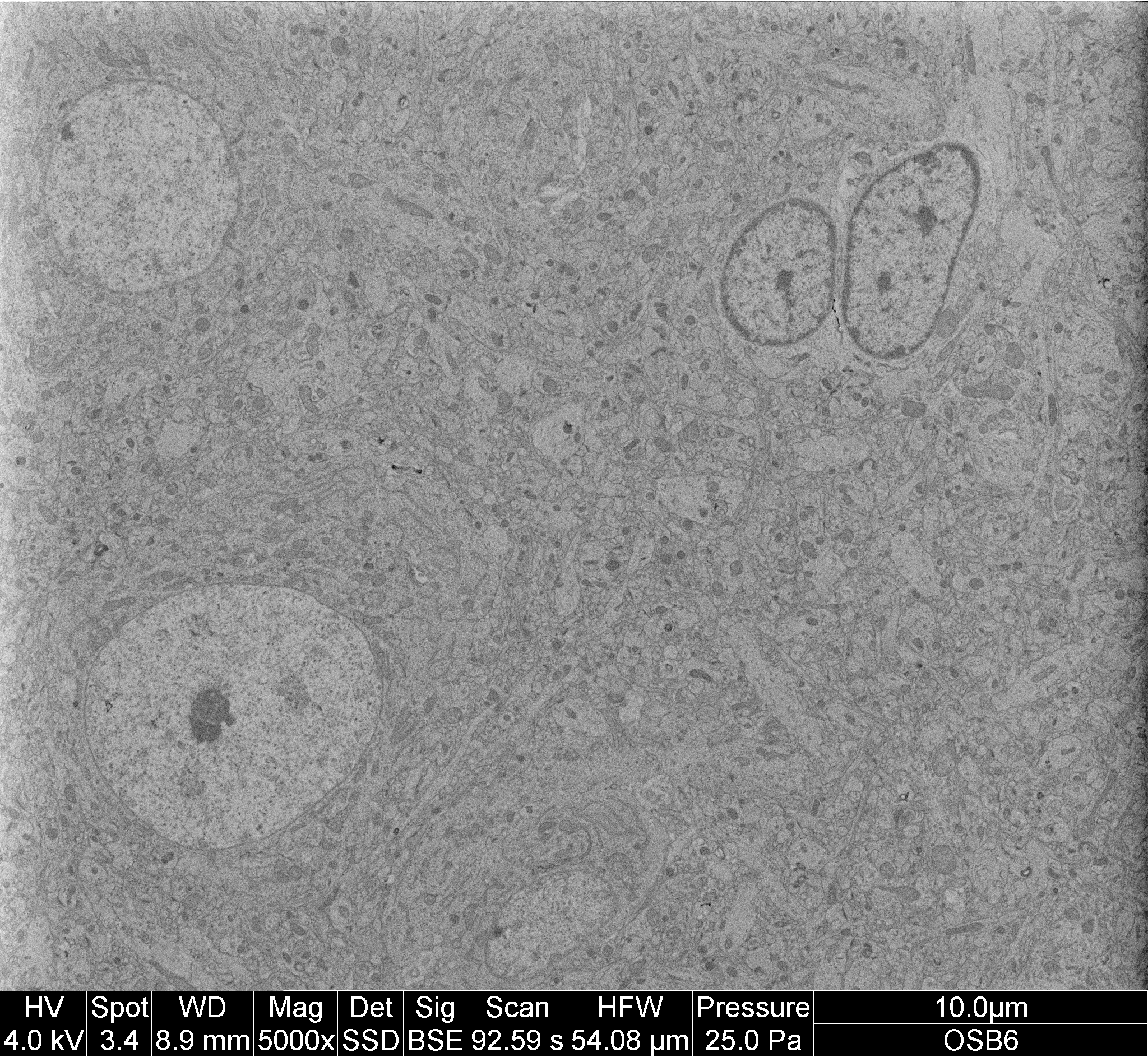

Supplement: Dataset S15 — (250.7 MB ZIP). [file pbio.0020329.sd015.zip › 040604_OS5_st1_1463.tif]

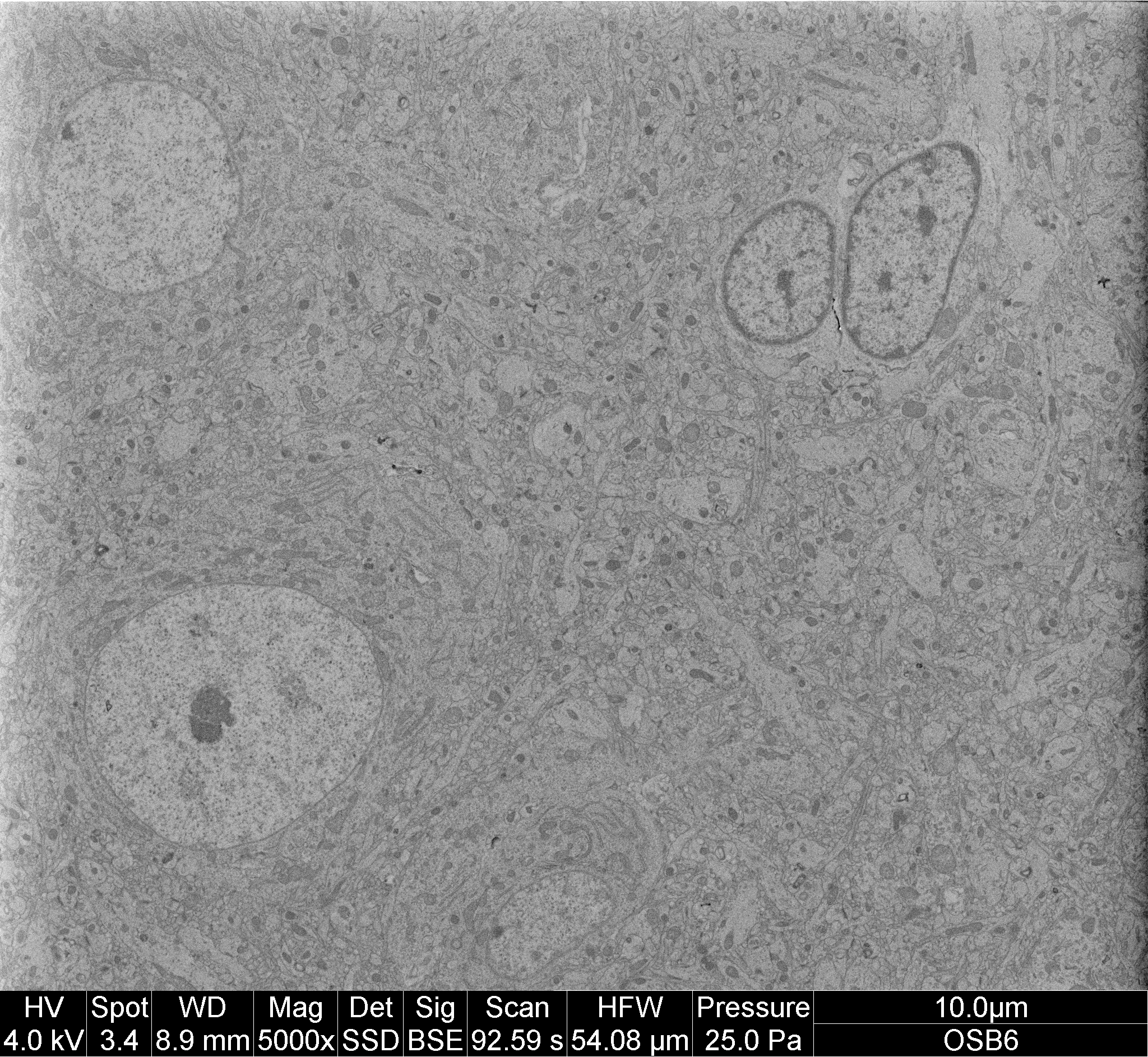

Supplement: Dataset S15 — (250.7 MB ZIP). [file pbio.0020329.sd015.zip › 040604_OS5_st1_1464.tif]

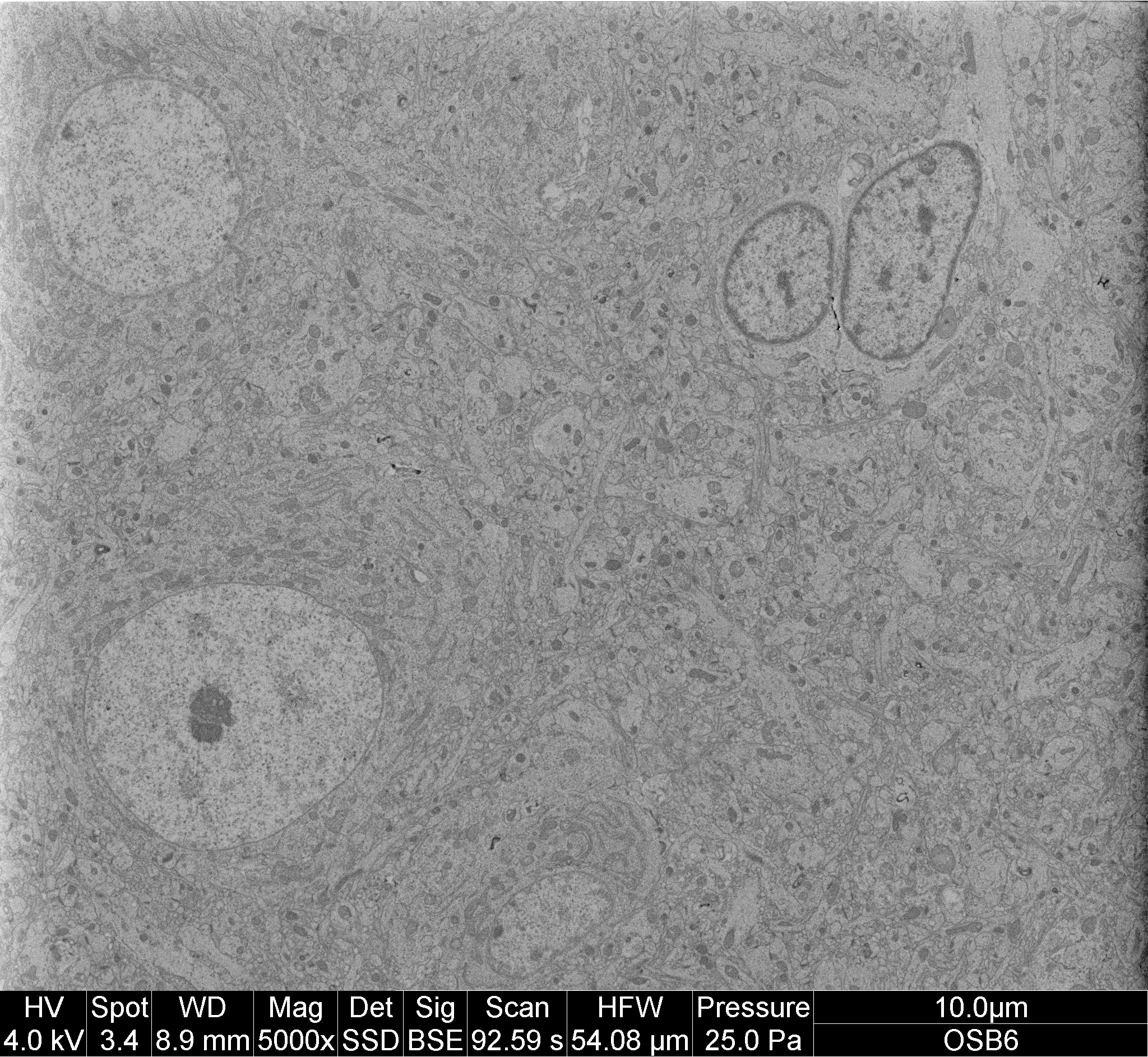

Supplement: Dataset S15 — (250.7 MB ZIP). [file pbio.0020329.sd015.zip › 040604_OS5_st1_1465.tif]

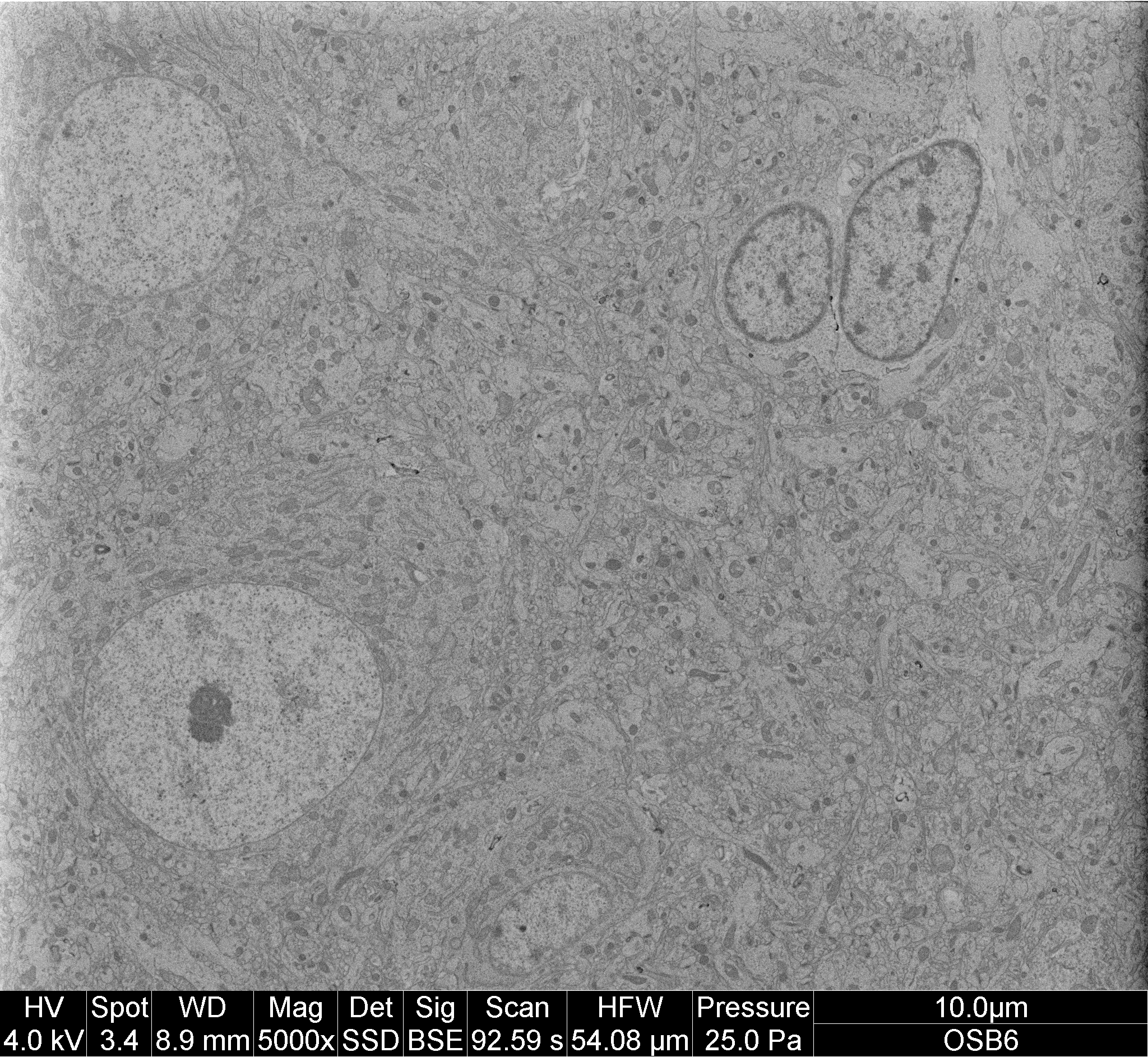

Supplement: Dataset S15 — (250.7 MB ZIP). [file pbio.0020329.sd015.zip › 040604_OS5_st1_1466.tif]

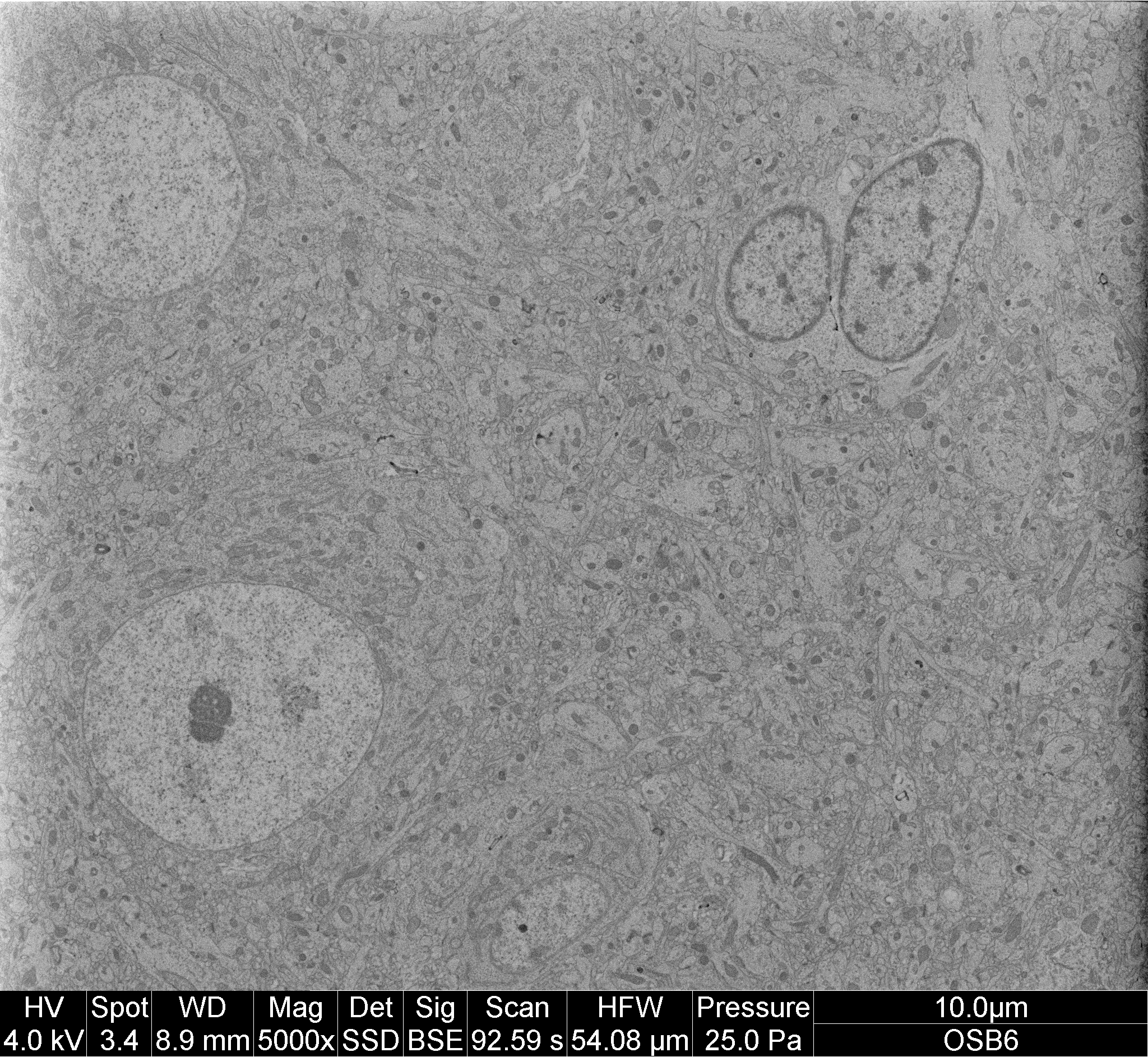

Supplement: Dataset S15 — (250.7 MB ZIP). [file pbio.0020329.sd015.zip › 040604_OS5_st1_1467.tif]

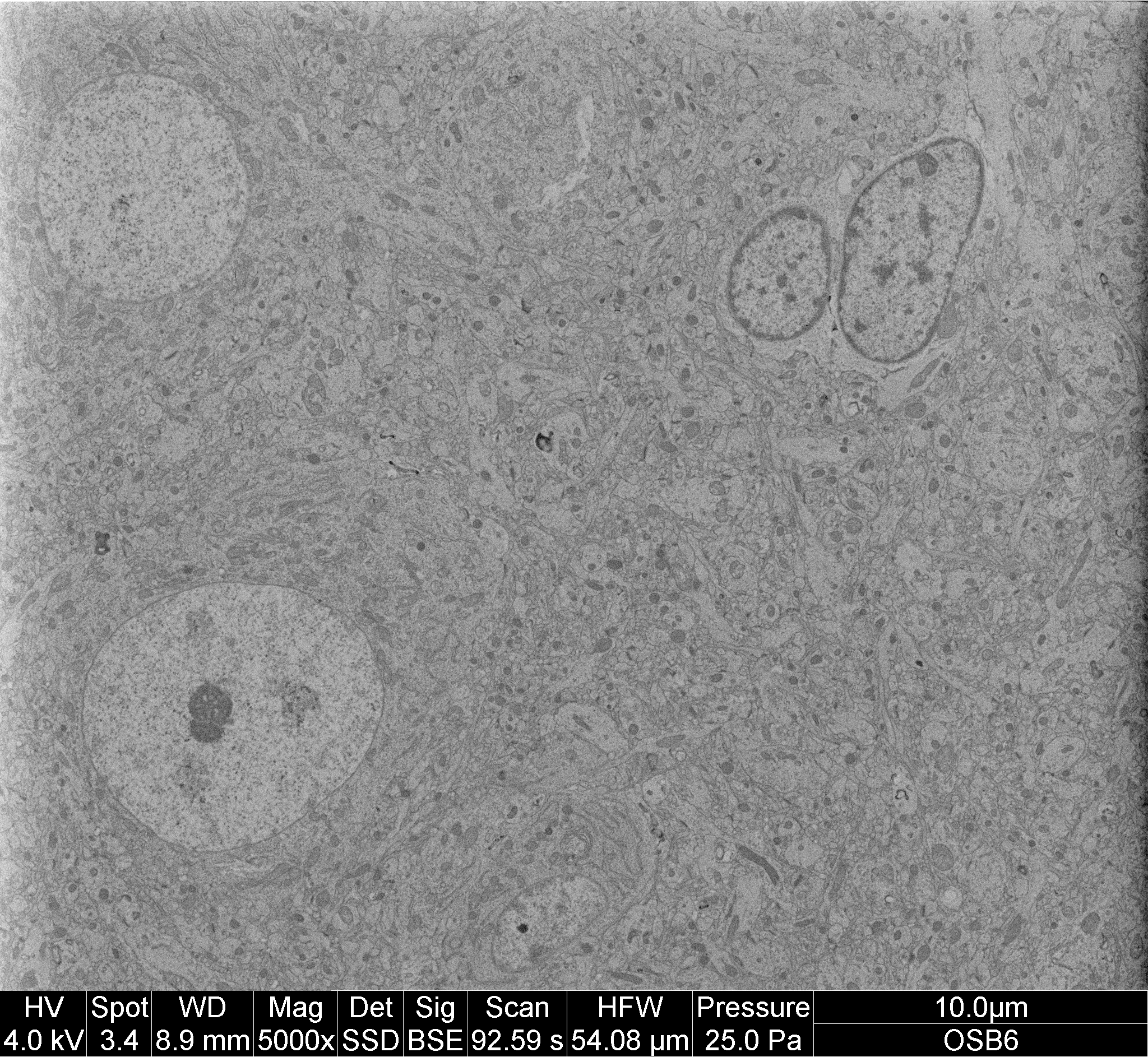

Supplement: Dataset S15 — (250.7 MB ZIP). [file pbio.0020329.sd015.zip › 040604_OS5_st1_1468.tif]

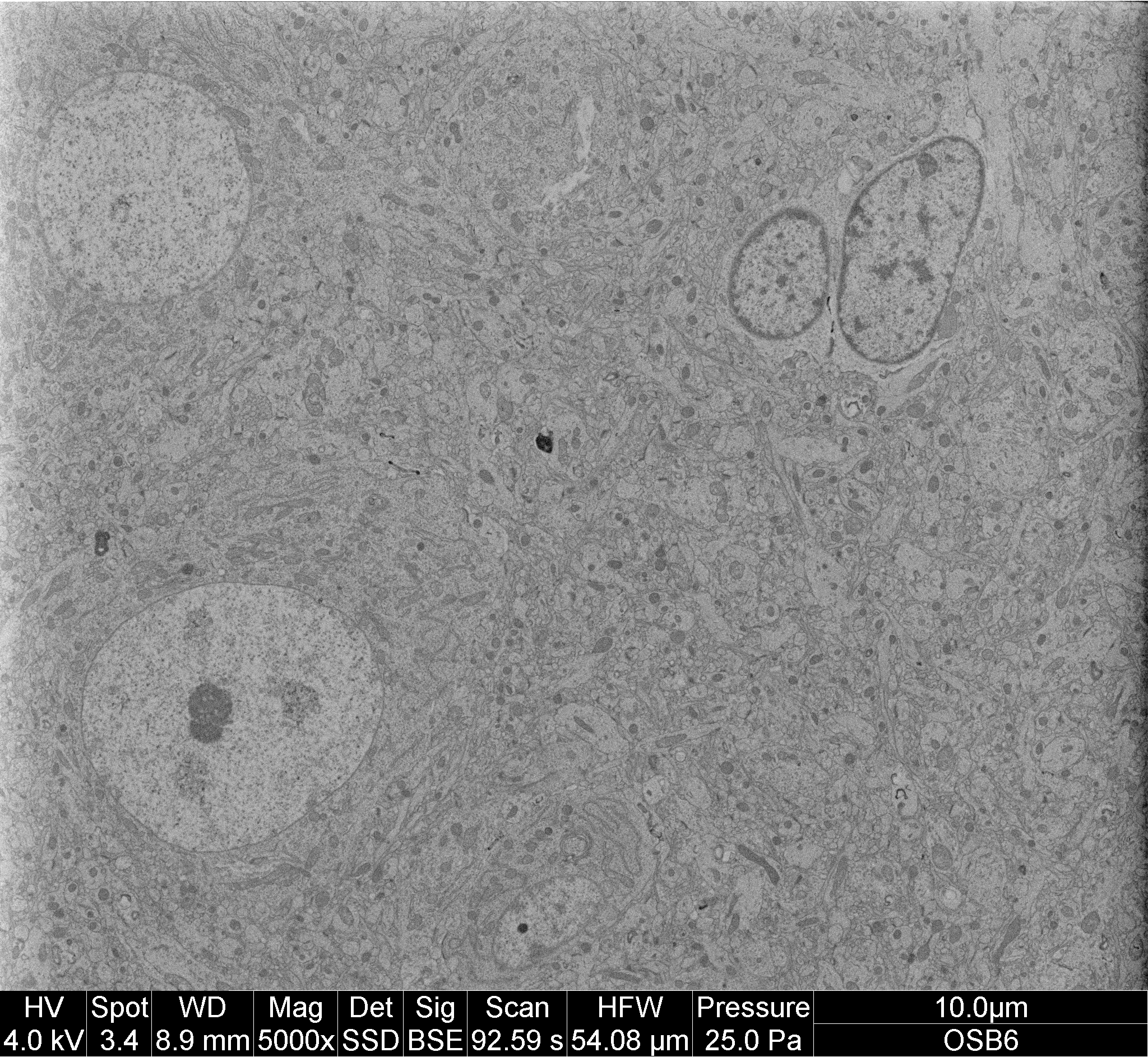

Supplement: Dataset S15 — (250.7 MB ZIP). [file pbio.0020329.sd015.zip › 040604_OS5_st1_1469.tif]

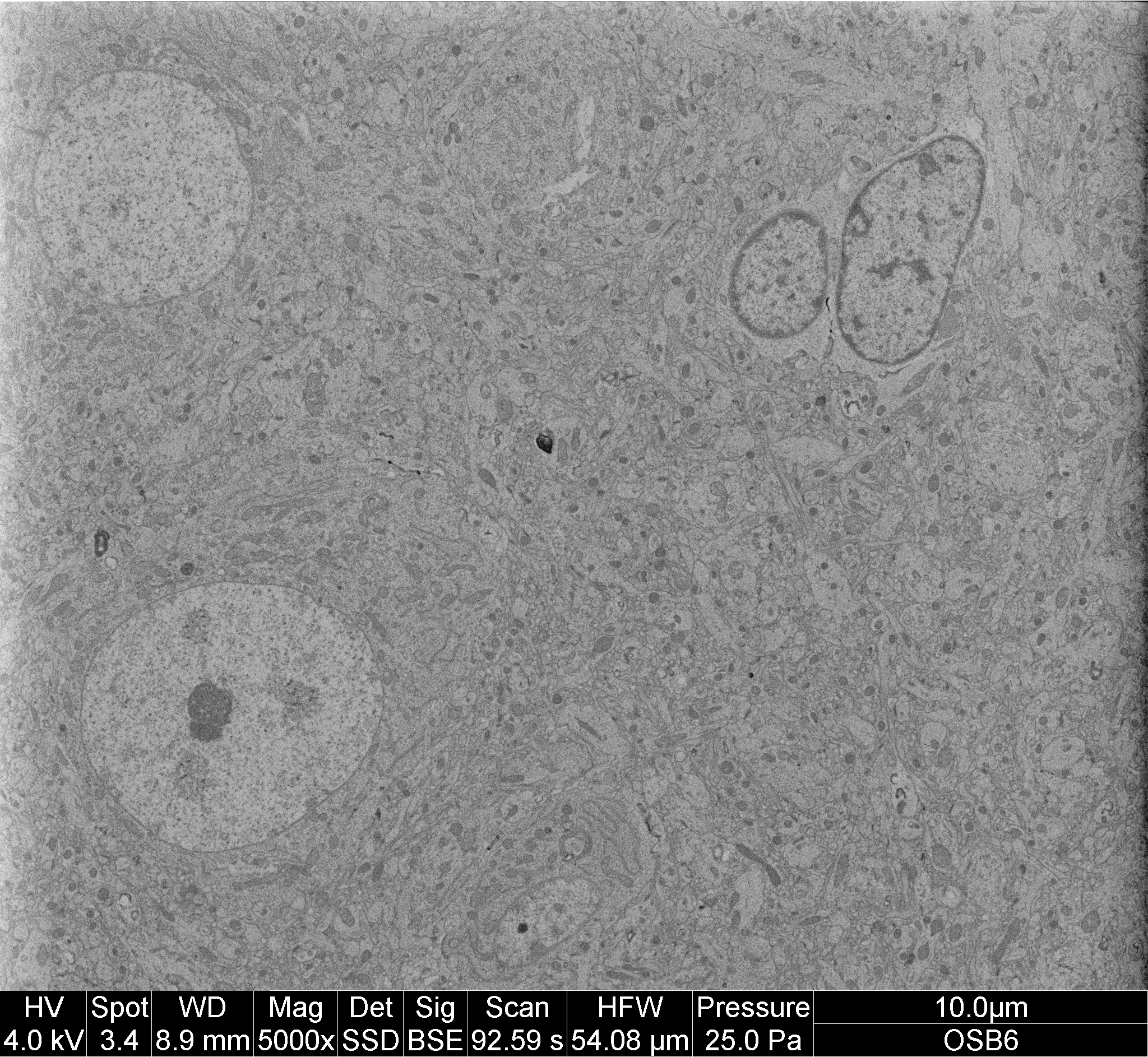

Supplement: Dataset S15 — (250.7 MB ZIP). [file pbio.0020329.sd015.zip › 040604_OS5_st1_1470.tif]

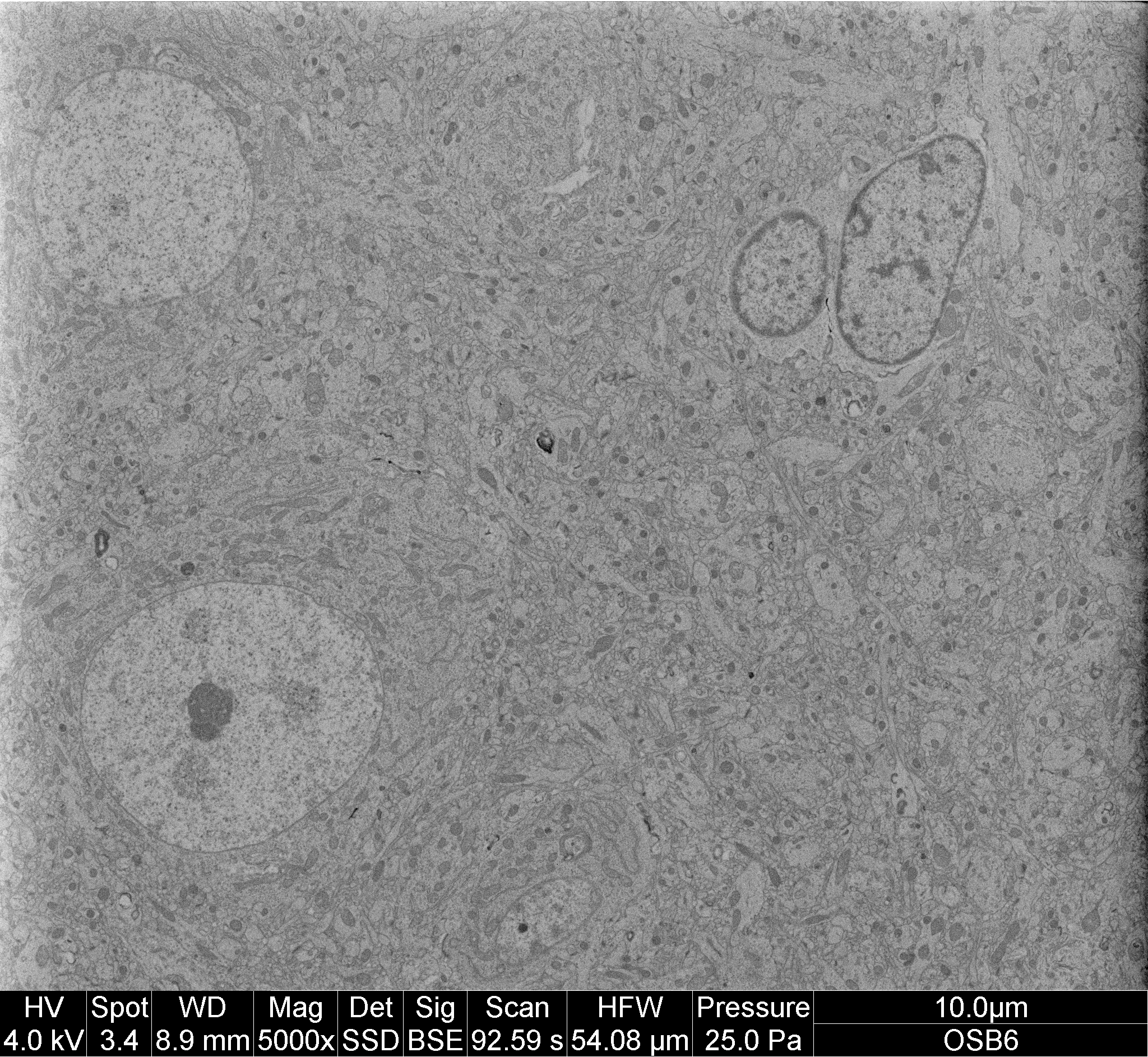

Supplement: Dataset S15 — (250.7 MB ZIP). [file pbio.0020329.sd015.zip › 040604_OS5_st1_1471.tif]

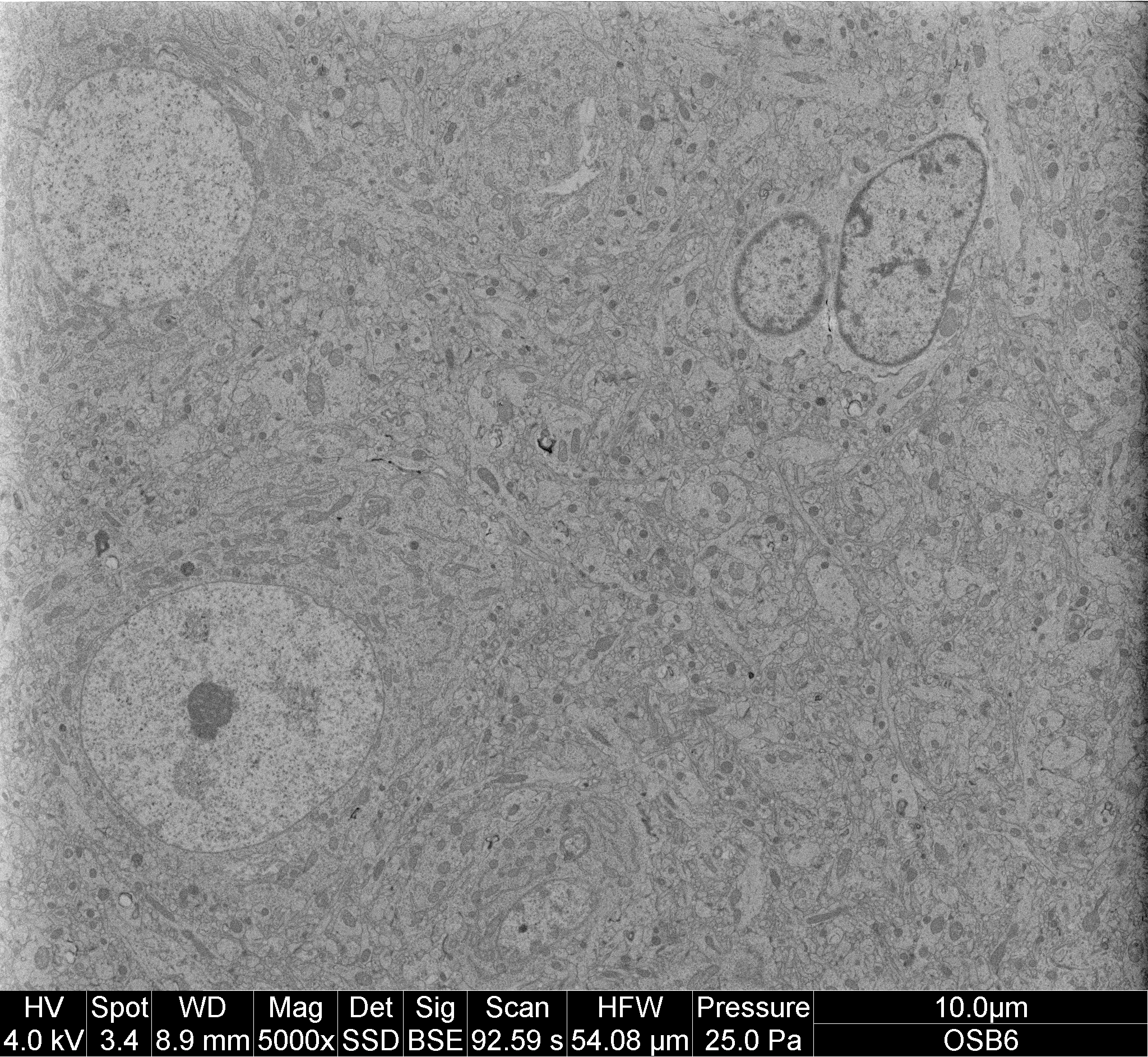

Supplement: Dataset S15 — (250.7 MB ZIP). [file pbio.0020329.sd015.zip › 040604_OS5_st1_1472.tif]

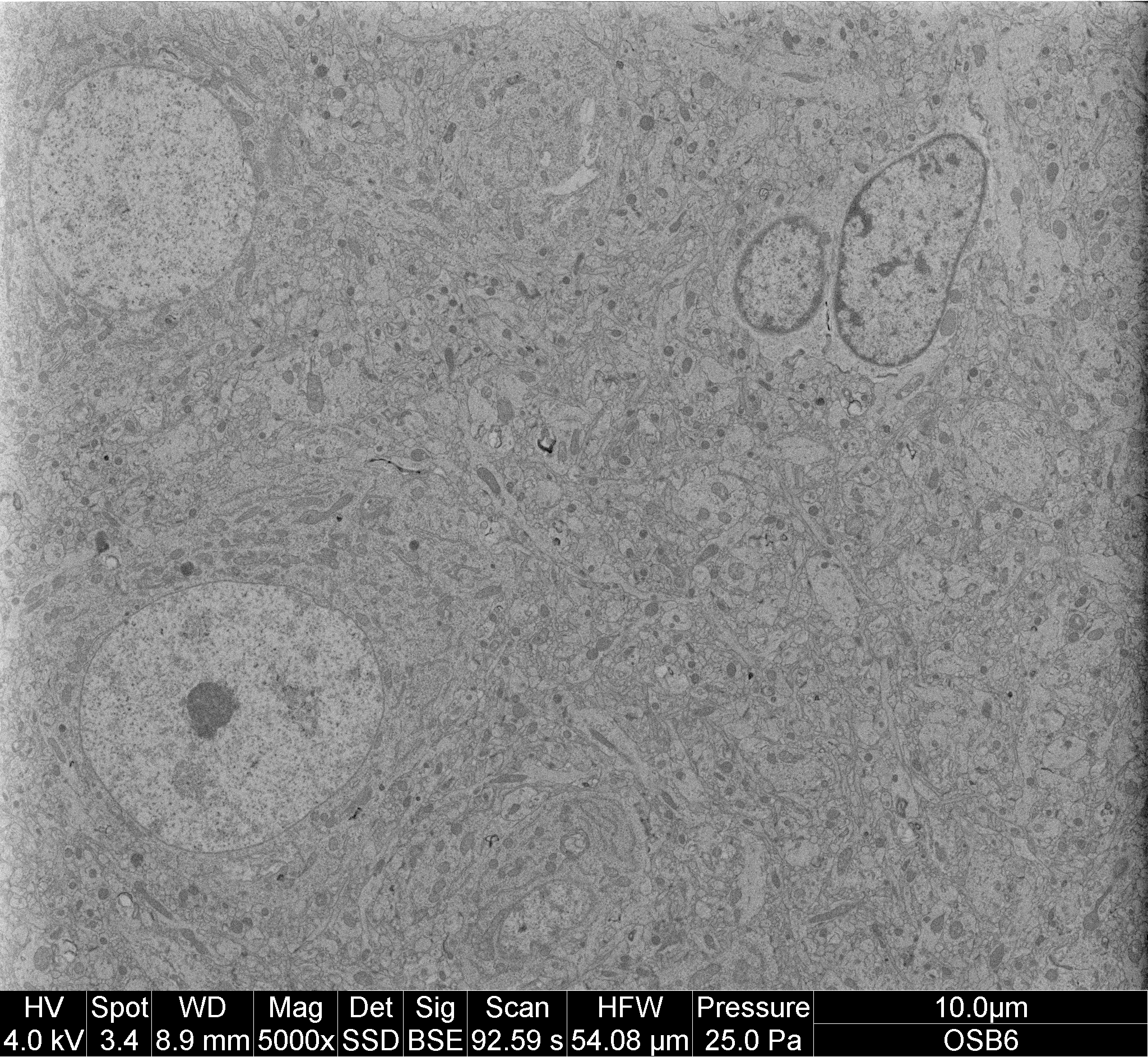

Supplement: Dataset S15 — (250.7 MB ZIP). [file pbio.0020329.sd015.zip › 040604_OS5_st1_1473.tif]

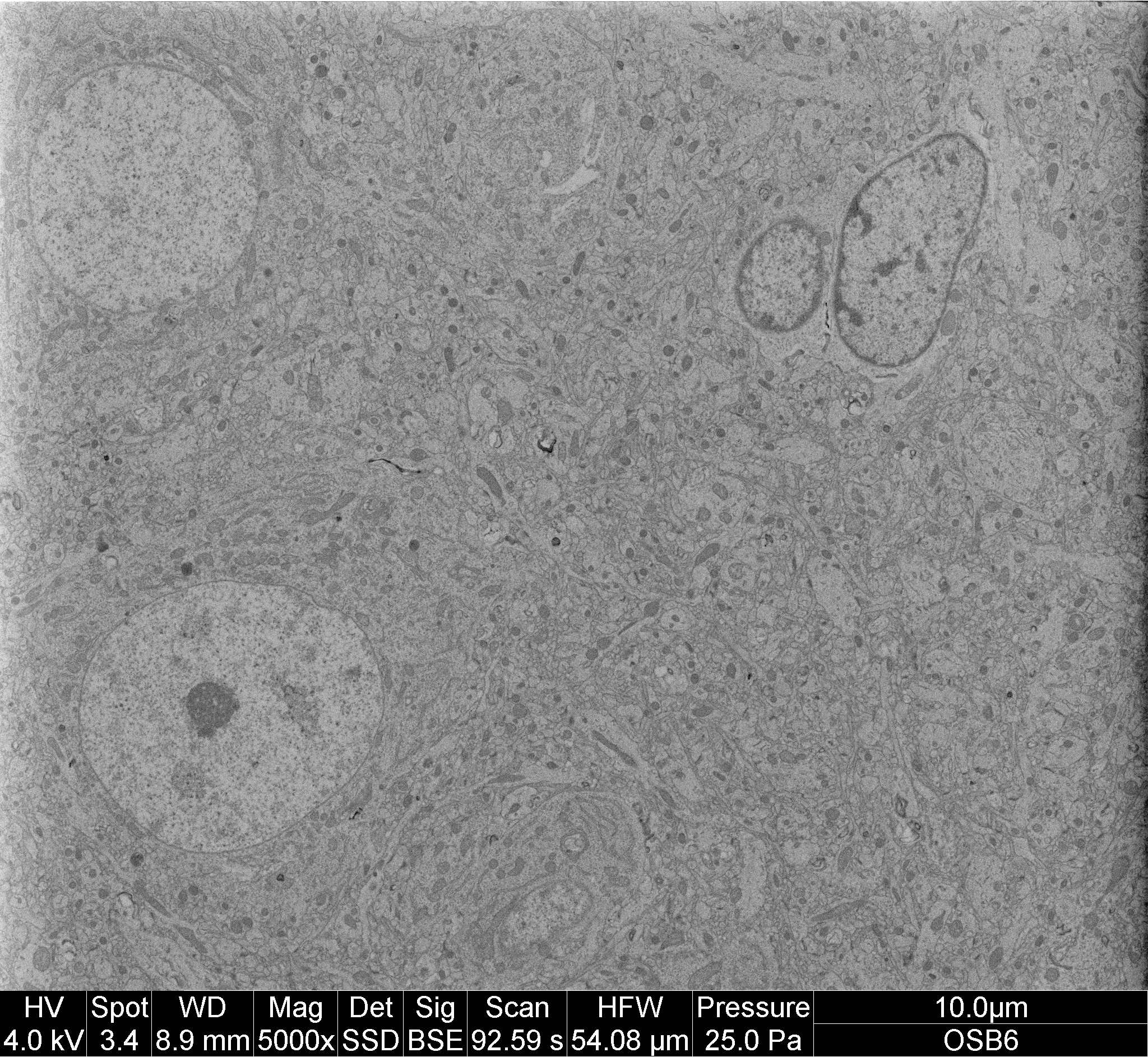

Supplement: Dataset S15 — (250.7 MB ZIP). [file pbio.0020329.sd015.zip › 040604_OS5_st1_1474.tif]

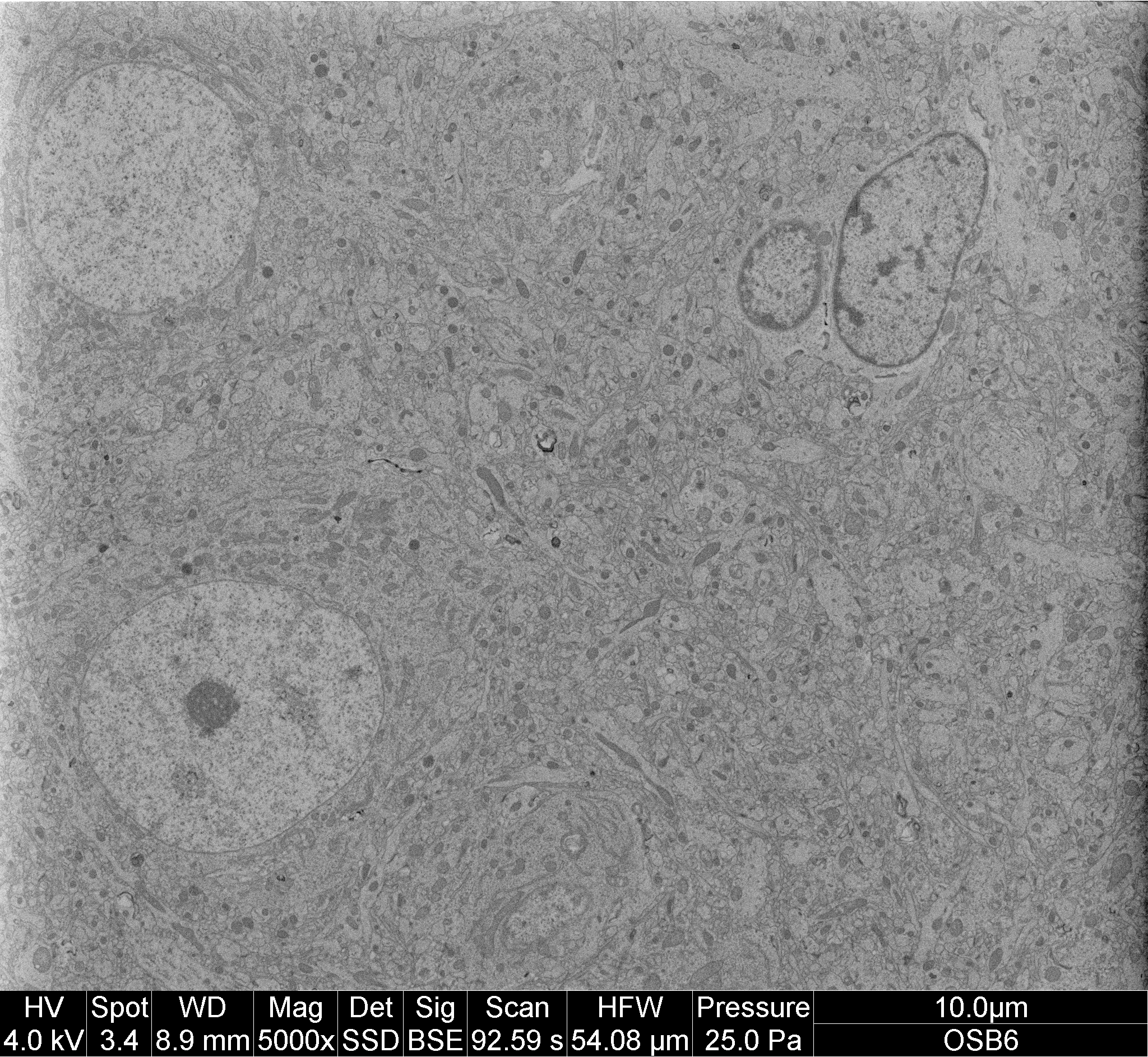

Supplement: Dataset S15 — (250.7 MB ZIP). [file pbio.0020329.sd015.zip › 040604_OS5_st1_1475.tif]

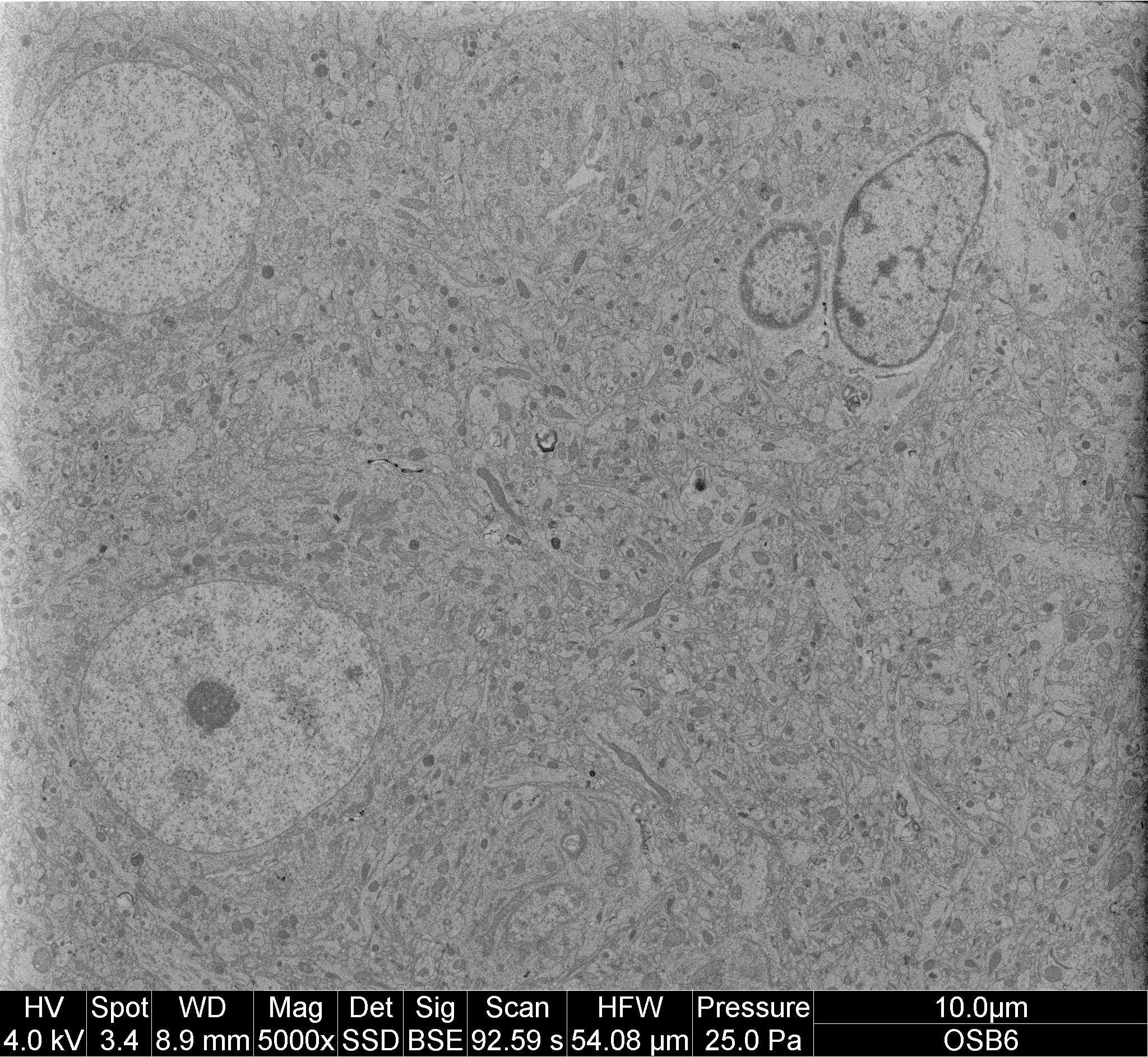

Supplement: Dataset S15 — (250.7 MB ZIP). [file pbio.0020329.sd015.zip › 040604_OS5_st1_1476.tif]

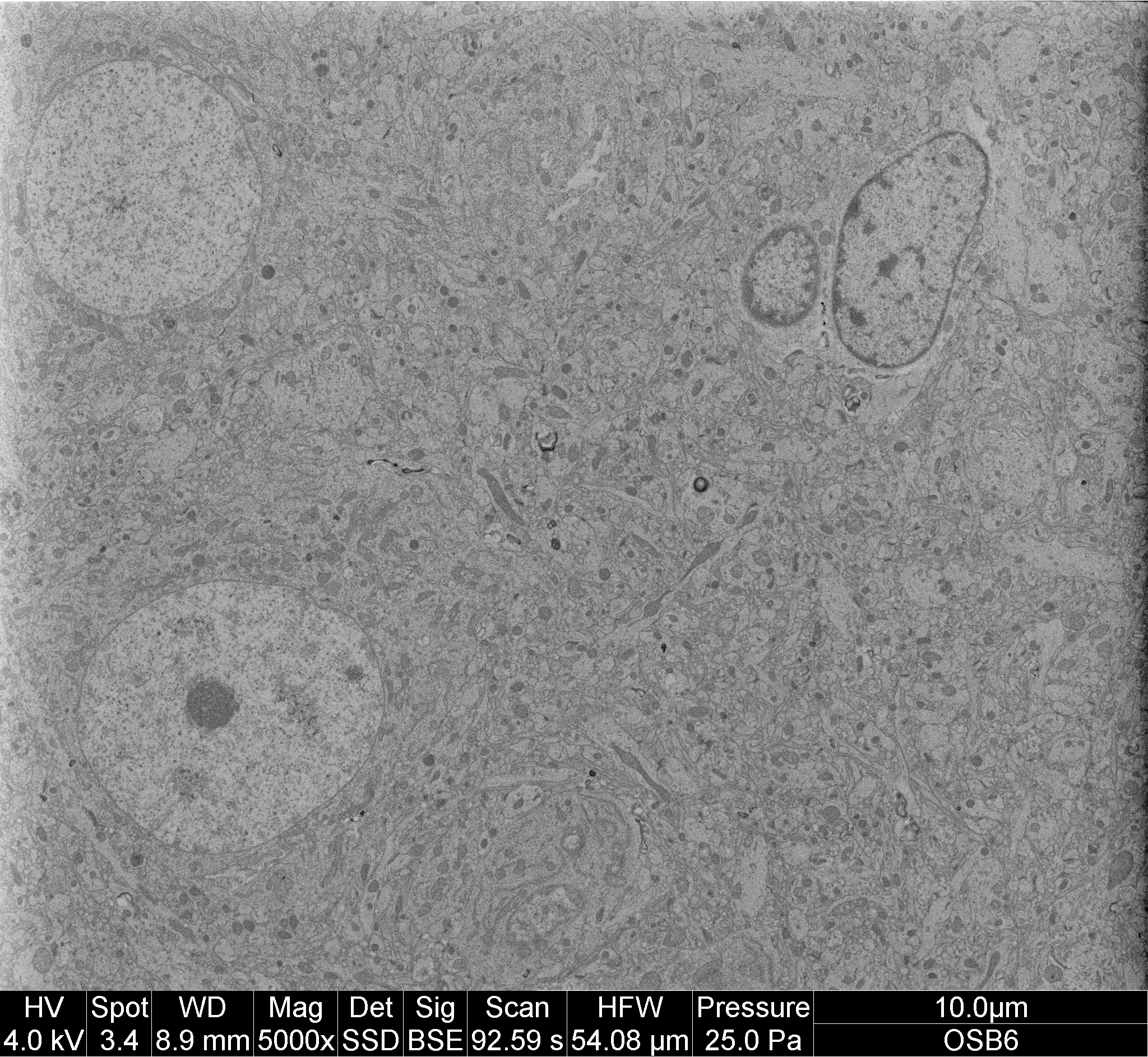

Supplement: Dataset S15 — (250.7 MB ZIP). [file pbio.0020329.sd015.zip › 040604_OS5_st1_1477.tif]

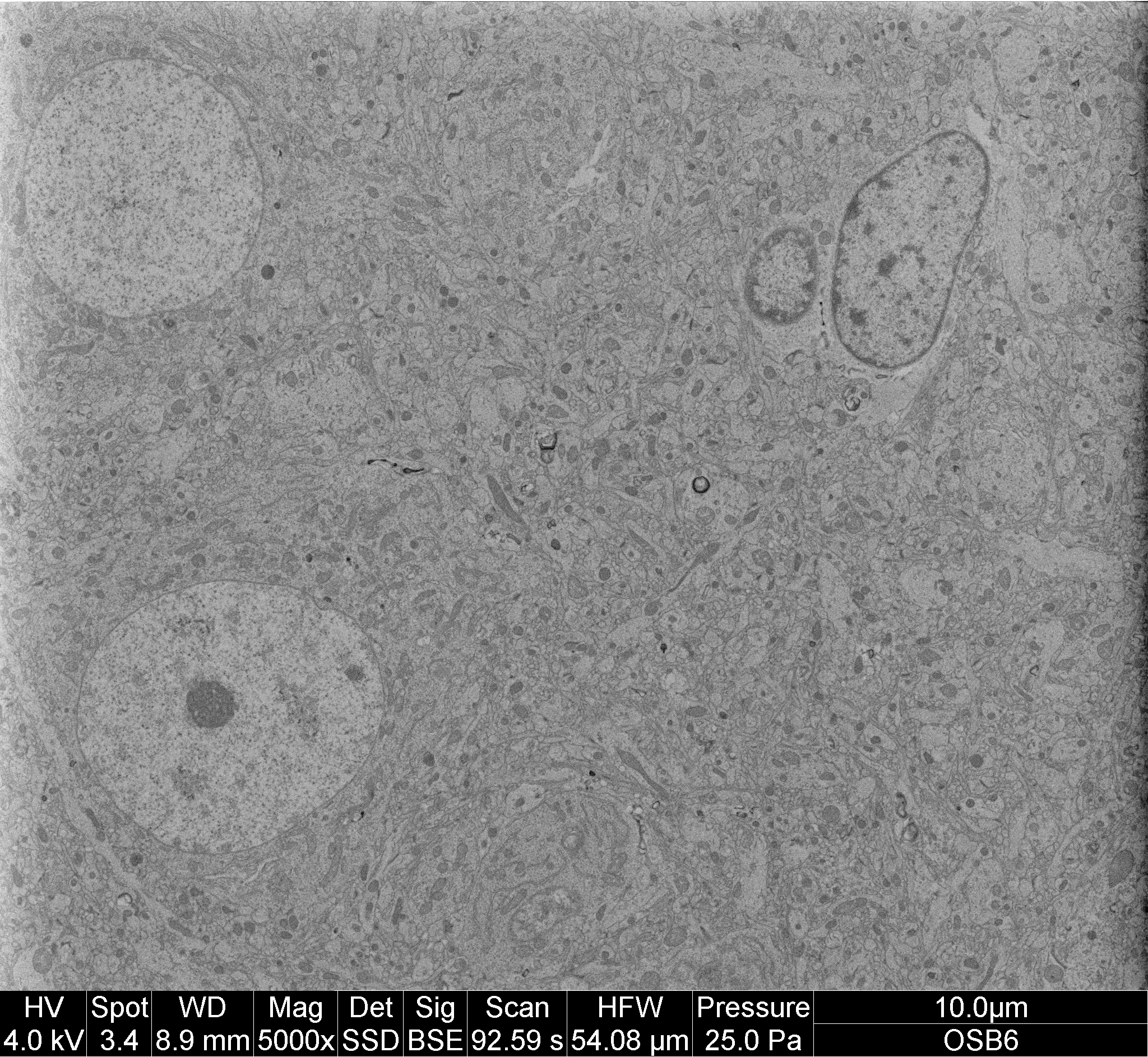

Supplement: Dataset S15 — (250.7 MB ZIP). [file pbio.0020329.sd015.zip › 040604_OS5_st1_1478.tif]

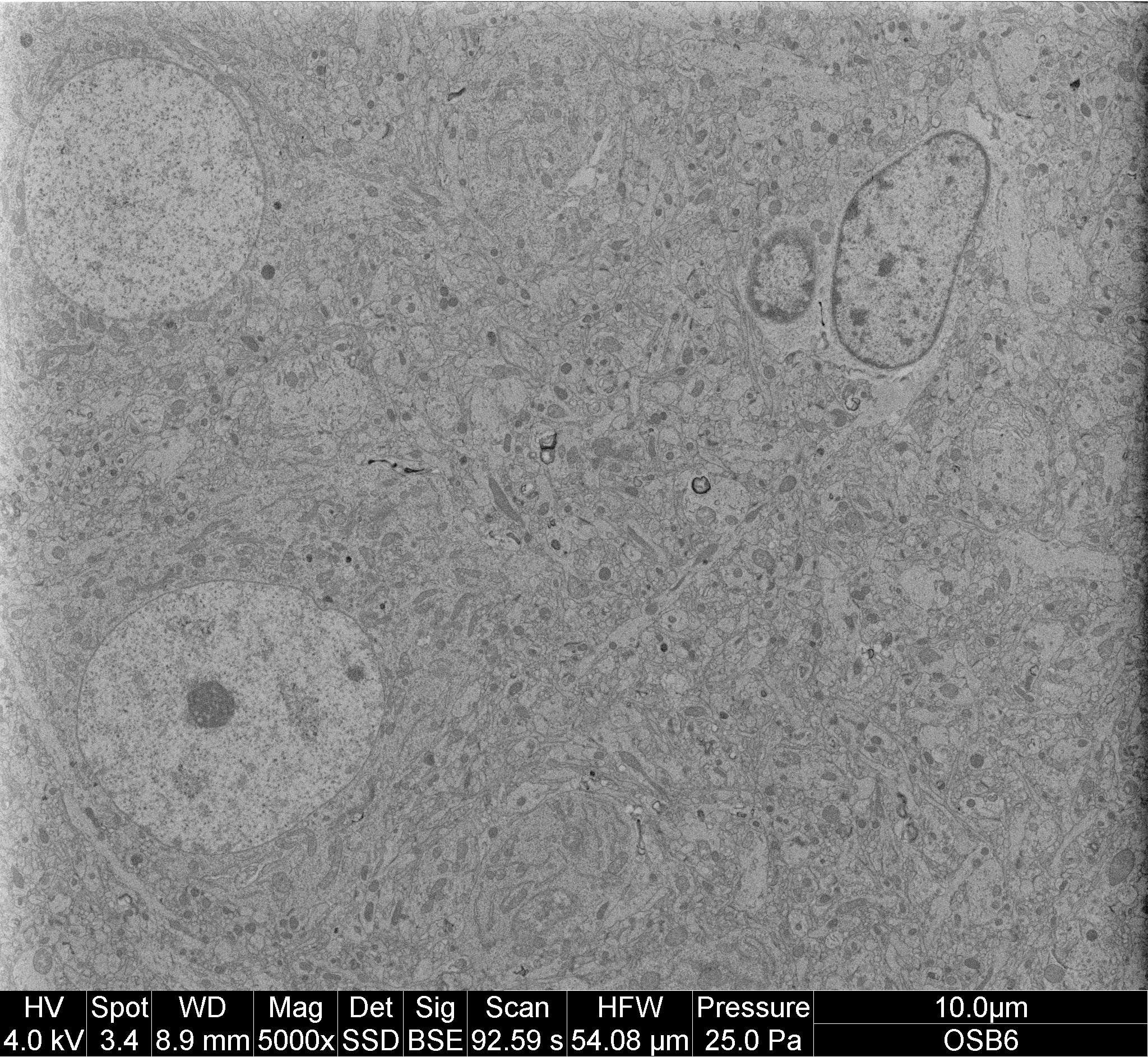

Supplement: Dataset S15 — (250.7 MB ZIP). [file pbio.0020329.sd015.zip › 040604_OS5_st1_1479.tif]

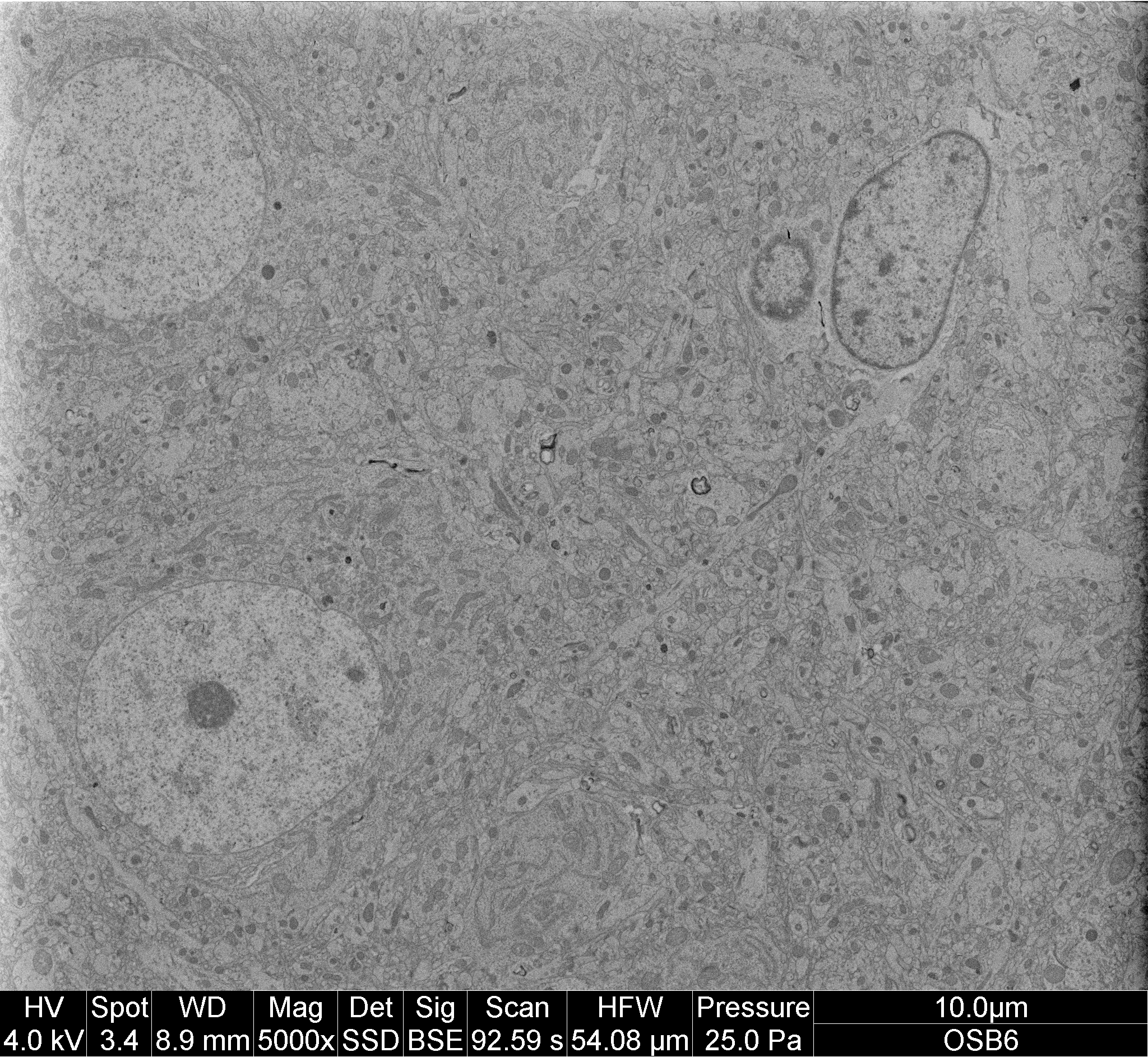

Supplement: Dataset S15 — (250.7 MB ZIP). [file pbio.0020329.sd015.zip › 040604_OS5_st1_1480.tif]

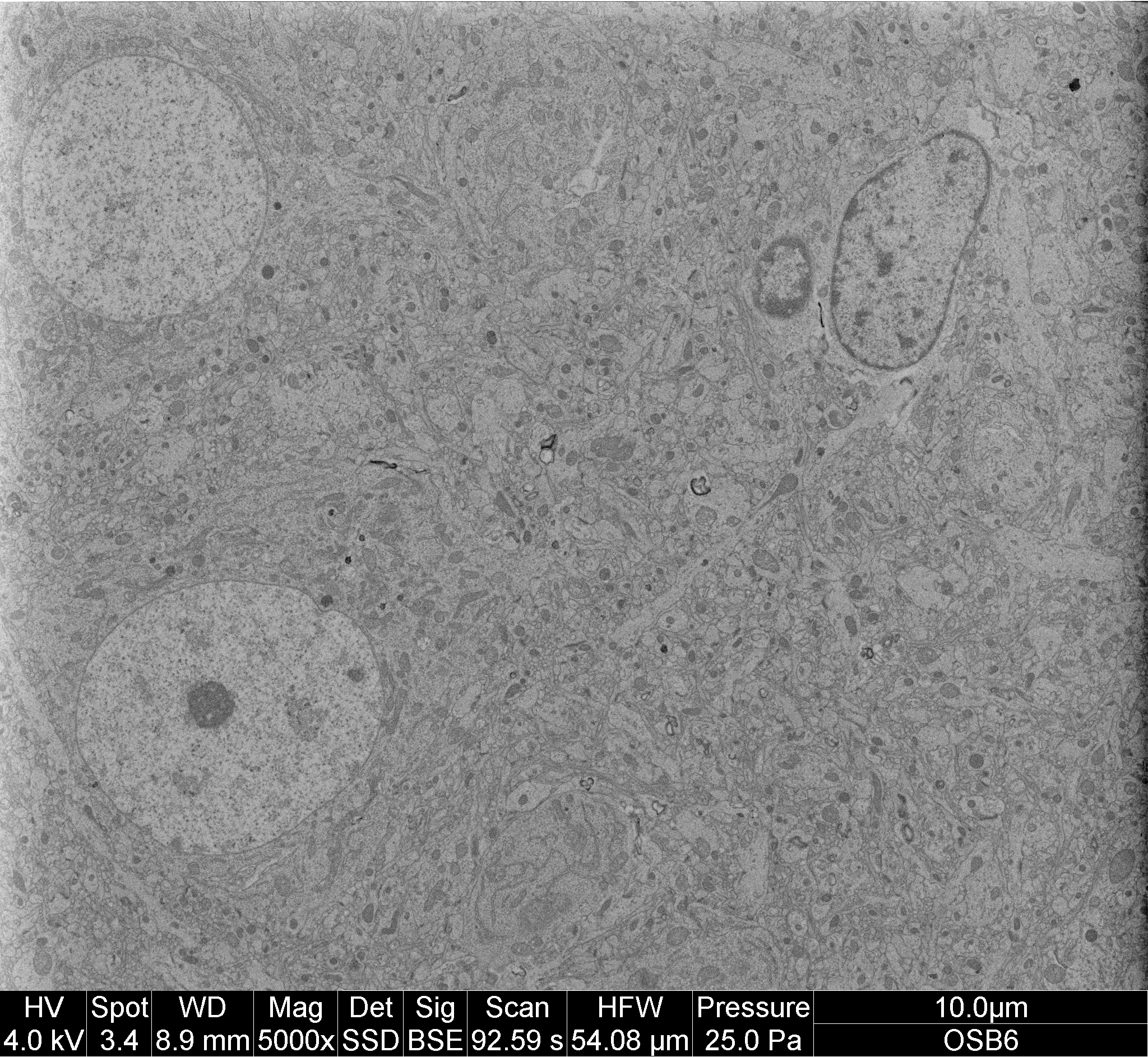

Supplement: Dataset S15 — (250.7 MB ZIP). [file pbio.0020329.sd015.zip › 040604_OS5_st1_1481.tif]

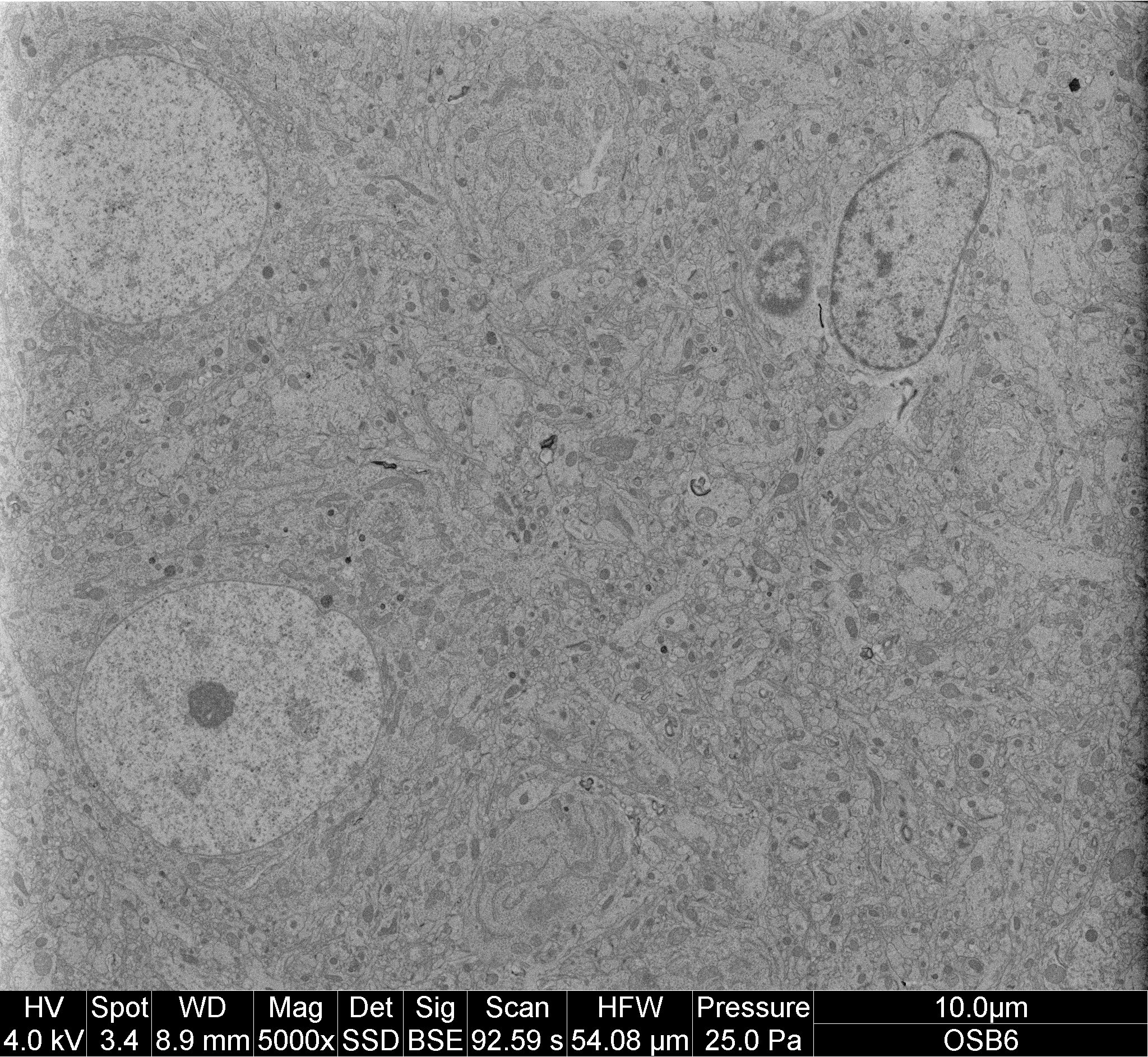

Supplement: Dataset S15 — (250.7 MB ZIP). [file pbio.0020329.sd015.zip › 040604_OS5_st1_1482.tif]

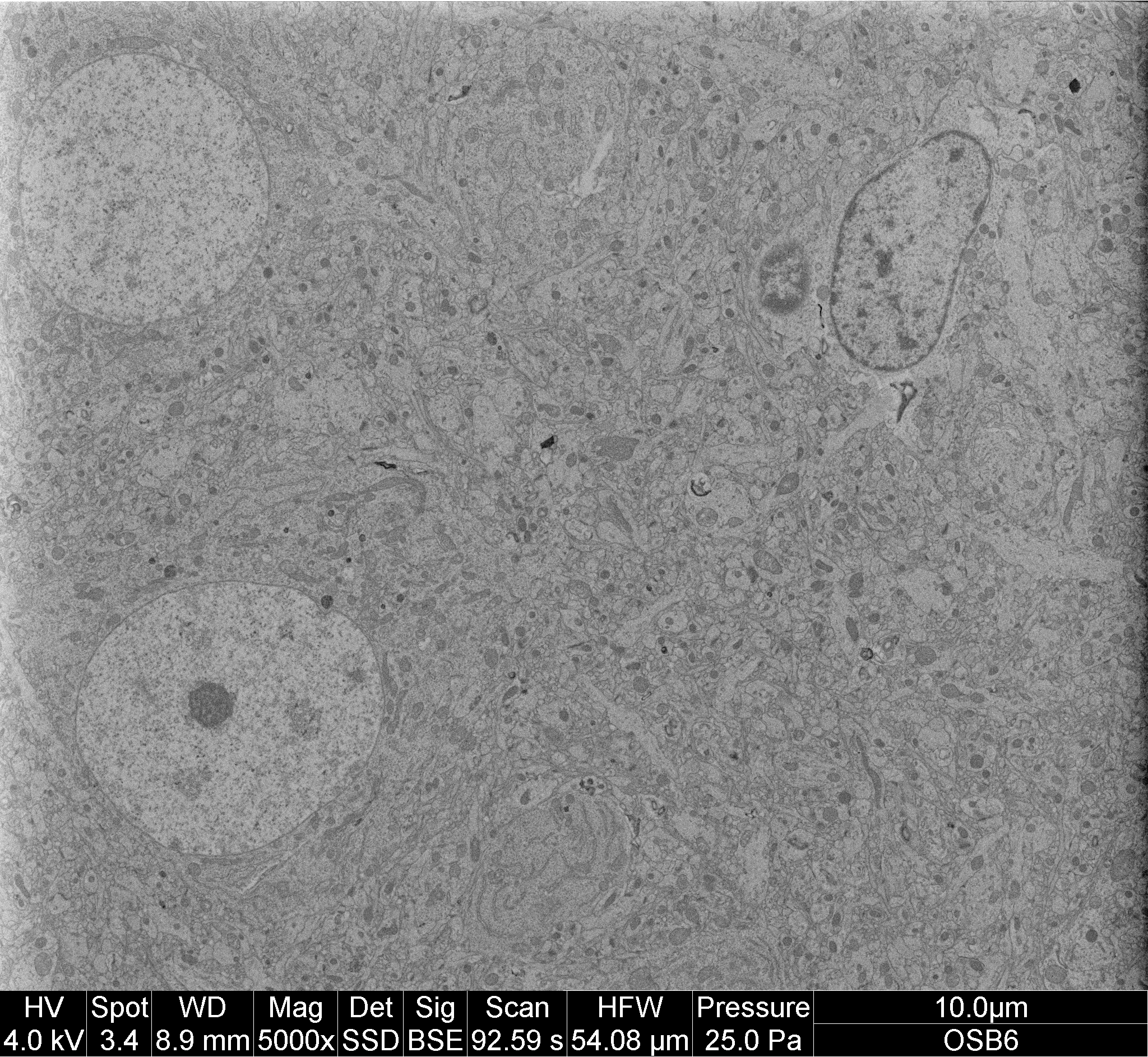

Supplement: Dataset S15 — (250.7 MB ZIP). [file pbio.0020329.sd015.zip › 040604_OS5_st1_1483.tif]

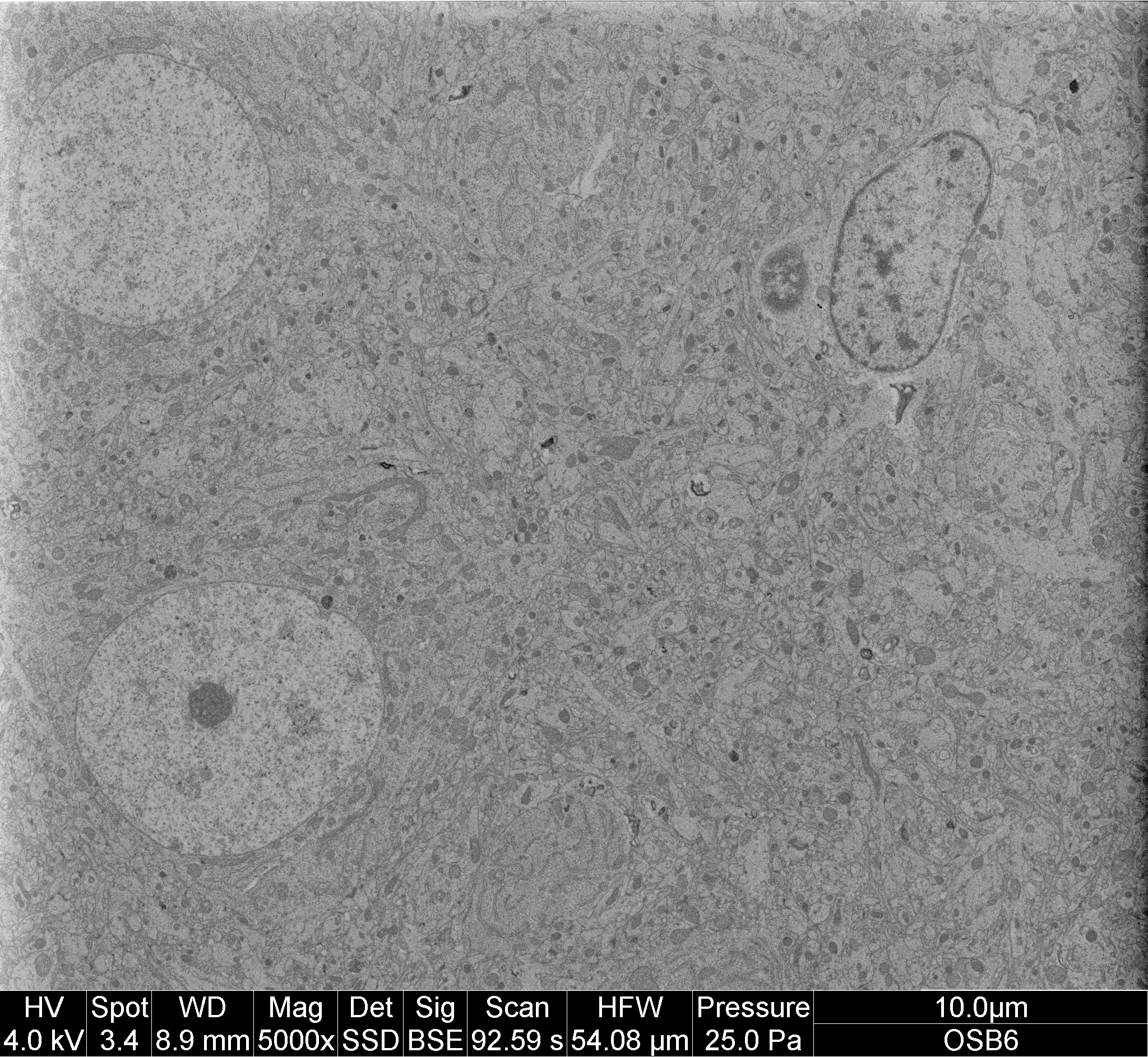

Supplement: Dataset S15 — (250.7 MB ZIP). [file pbio.0020329.sd015.zip › 040604_OS5_st1_1484.tif]

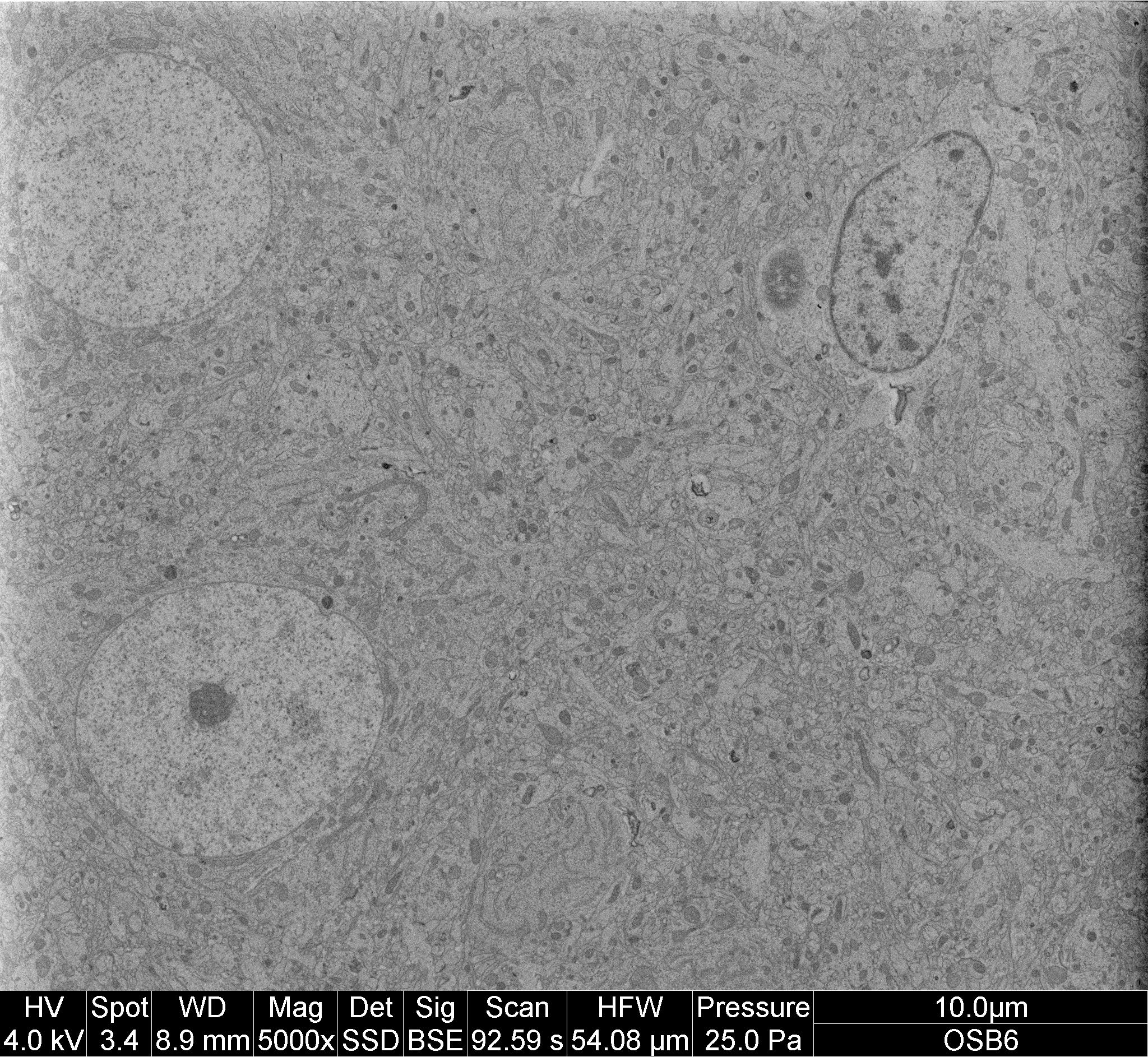

Supplement: Dataset S15 — (250.7 MB ZIP). [file pbio.0020329.sd015.zip › 040604_OS5_st1_1485.tif]

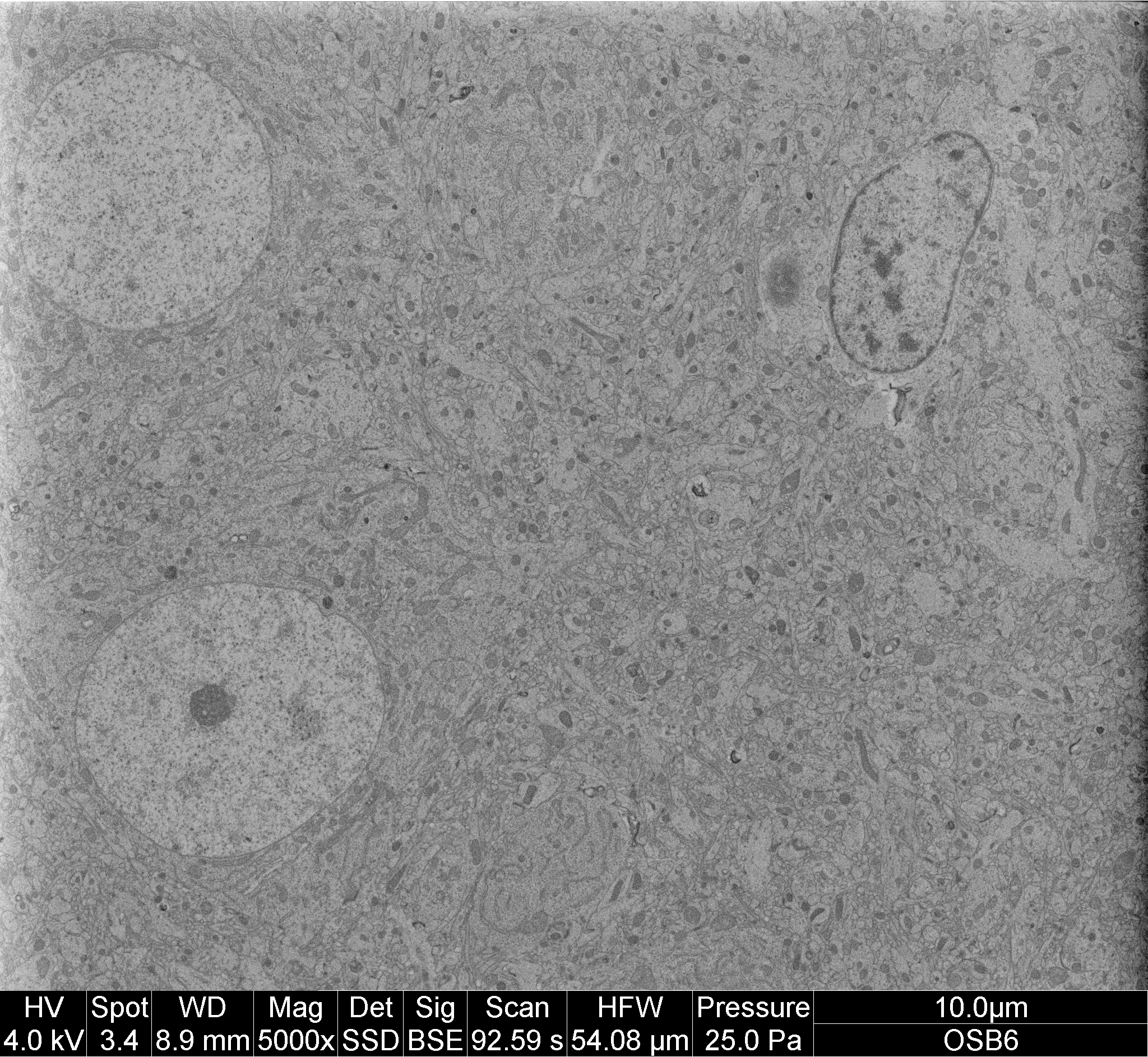

Supplement: Dataset S15 — (250.7 MB ZIP). [file pbio.0020329.sd015.zip › 040604_OS5_st1_1486.tif]

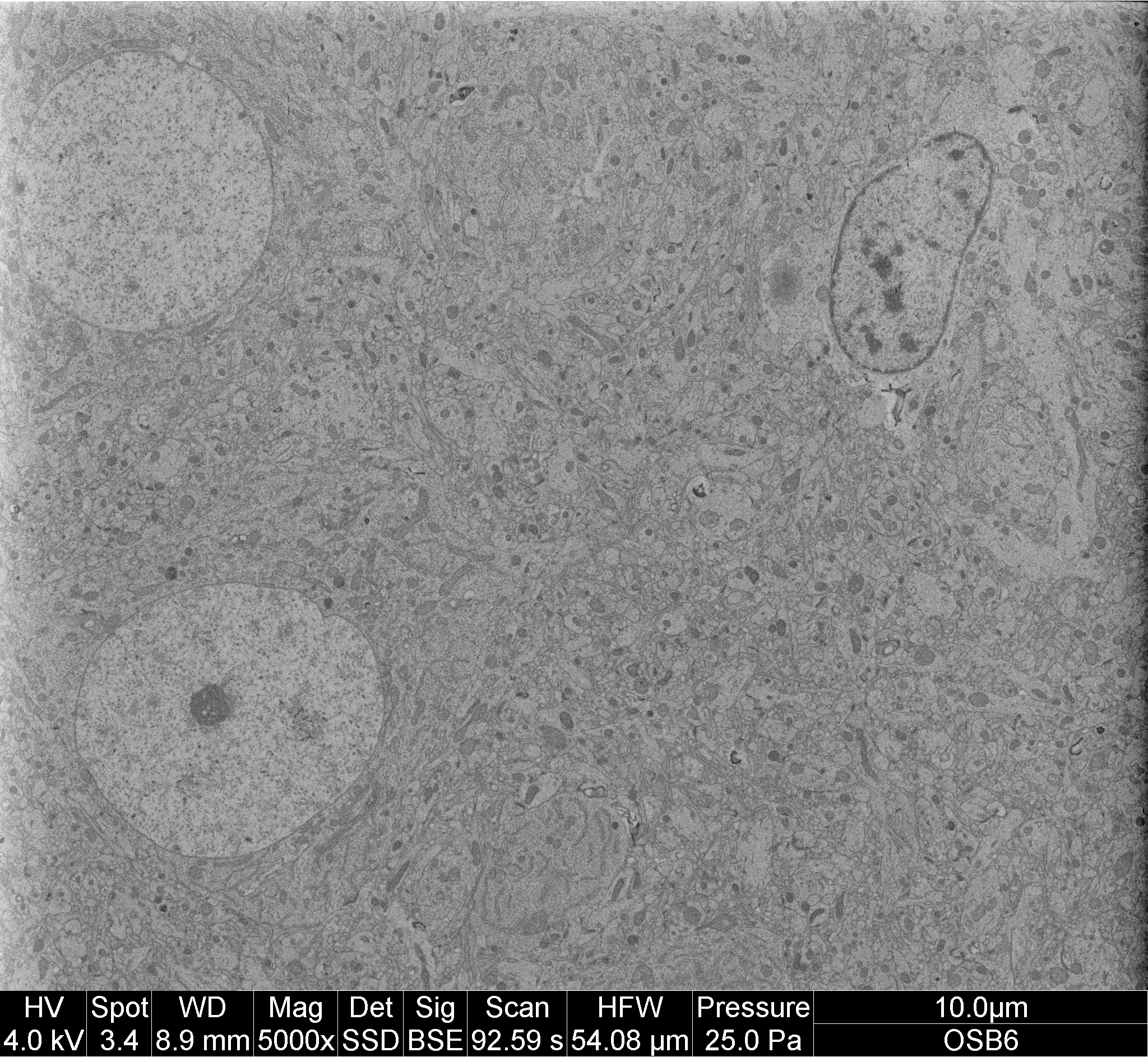

Supplement: Dataset S15 — (250.7 MB ZIP). [file pbio.0020329.sd015.zip › 040604_OS5_st1_1487.tif]

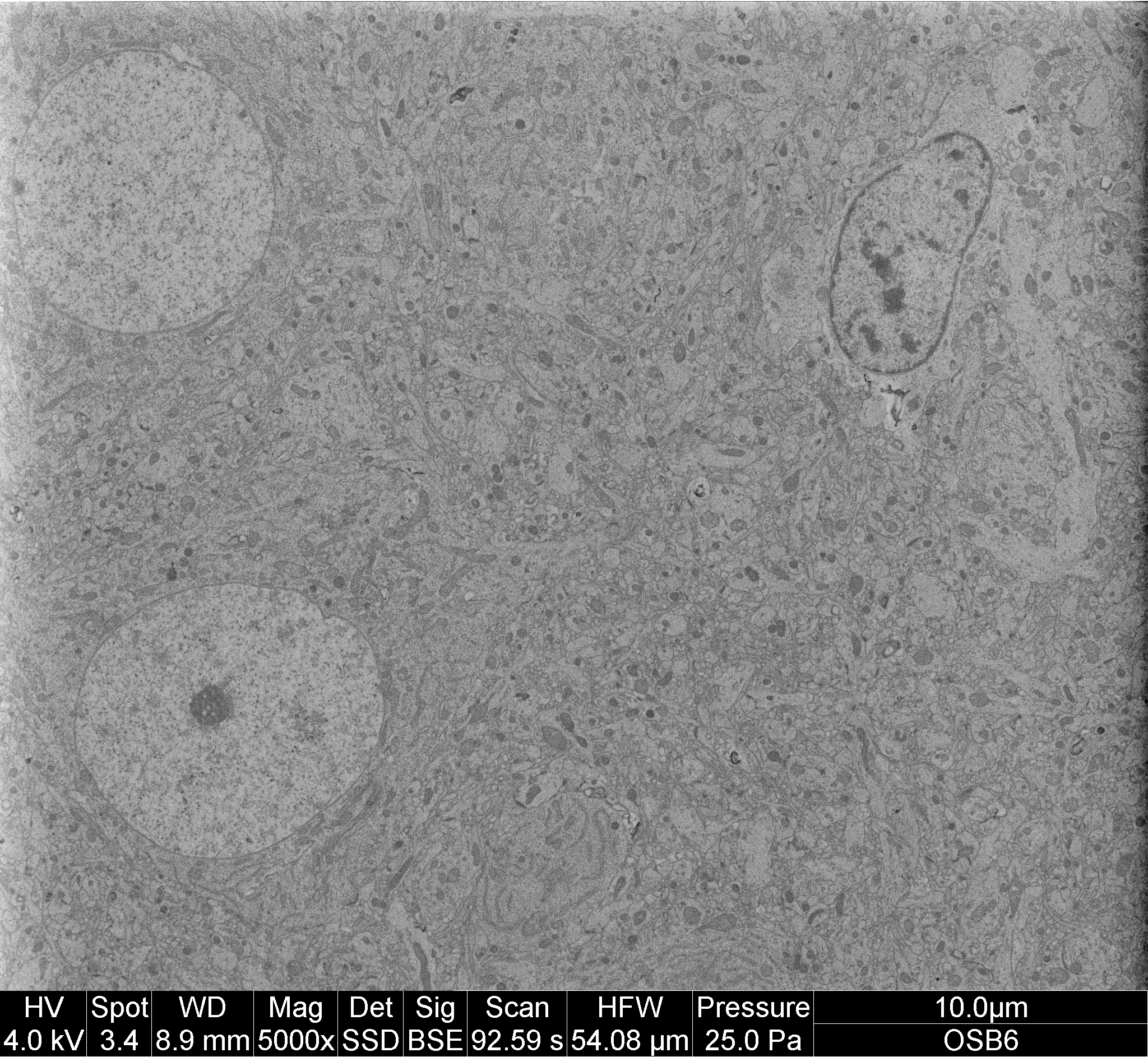

Supplement: Dataset S15 — (250.7 MB ZIP). [file pbio.0020329.sd015.zip › 040604_OS5_st1_1488.tif]

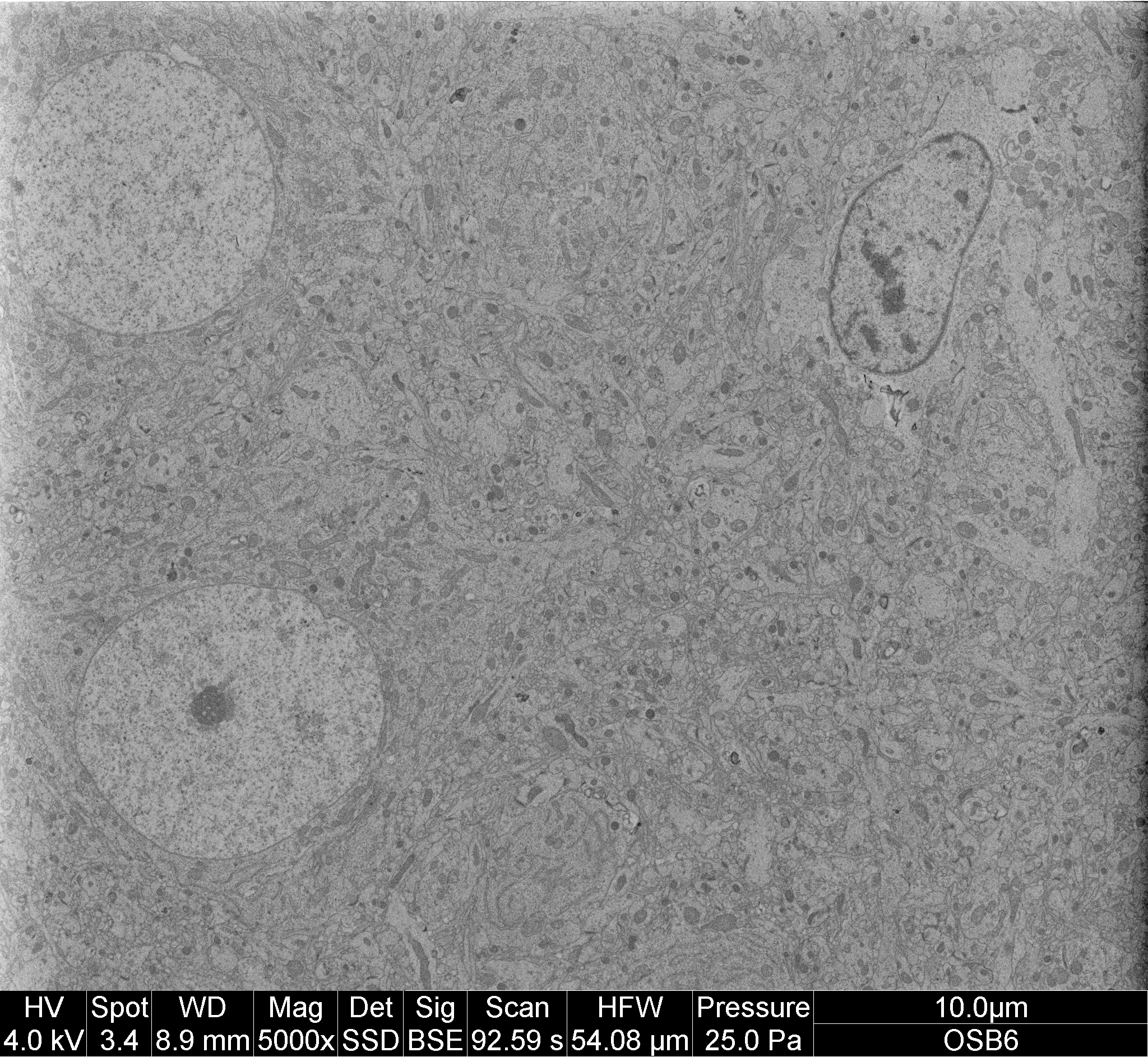

Supplement: Dataset S15 — (250.7 MB ZIP). [file pbio.0020329.sd015.zip › 040604_OS5_st1_1489.tif]

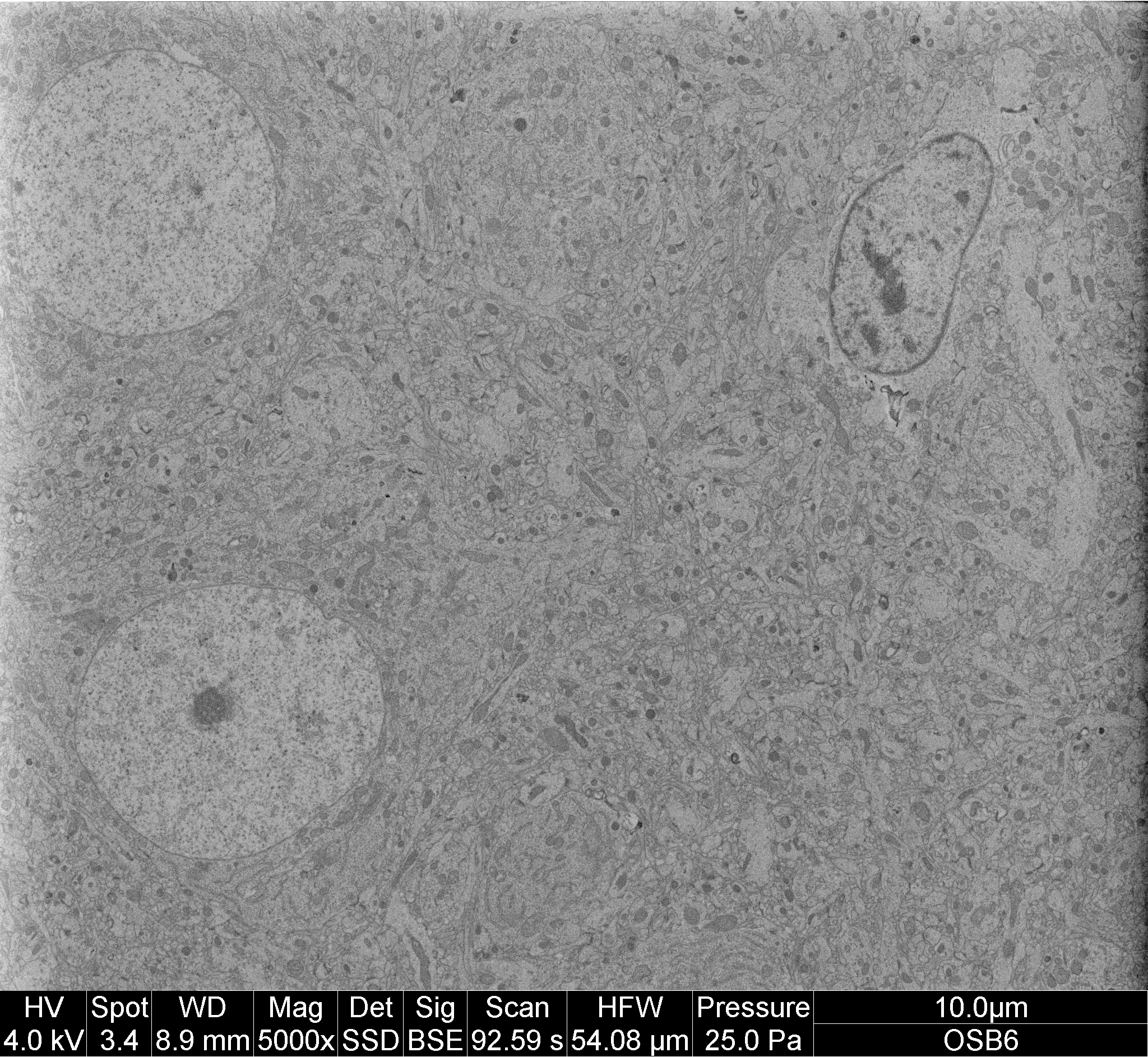

Supplement: Dataset S15 — (250.7 MB ZIP). [file pbio.0020329.sd015.zip › 040604_OS5_st1_1490.tif]

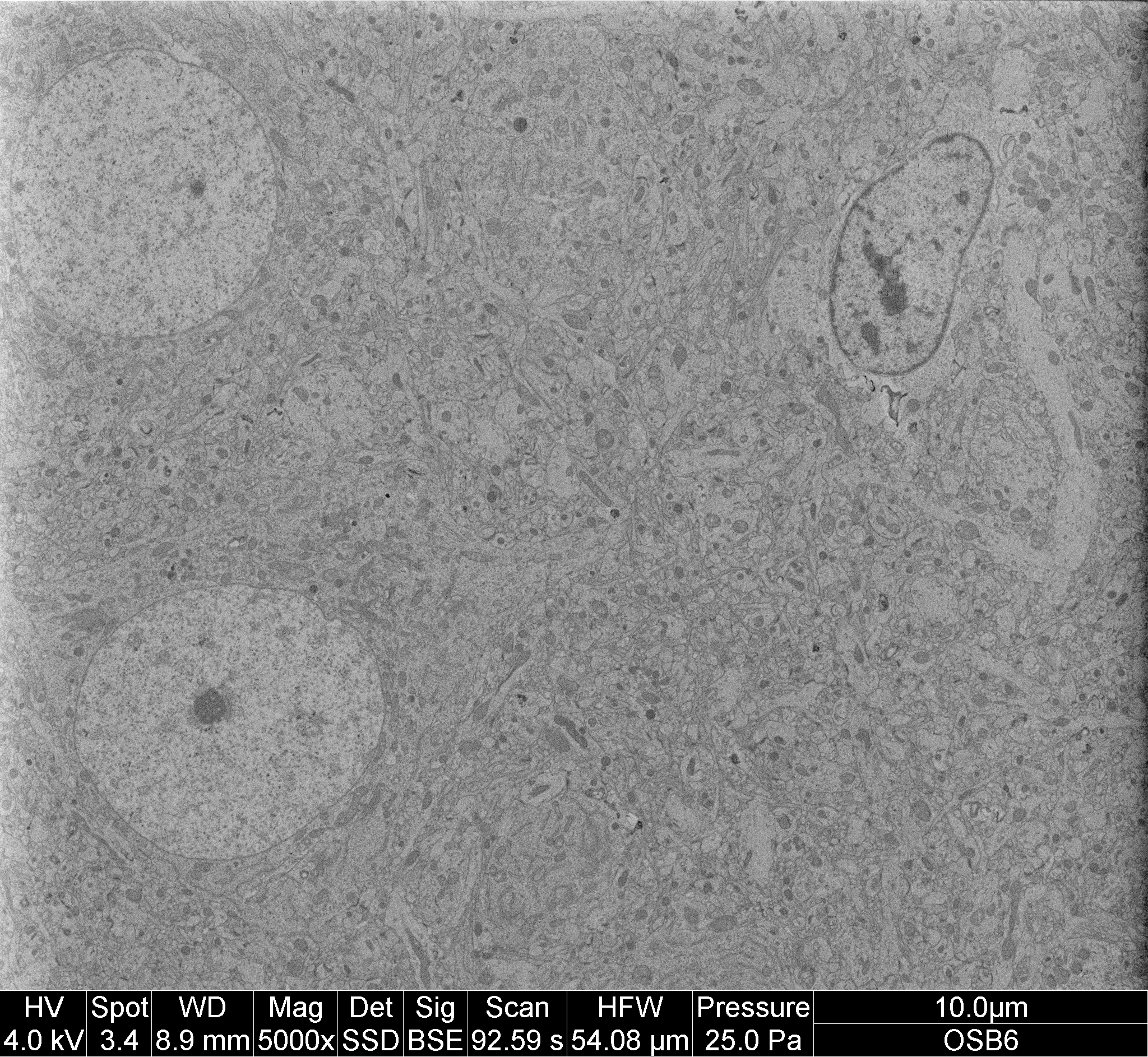

Supplement: Dataset S15 — (250.7 MB ZIP). [file pbio.0020329.sd015.zip › 040604_OS5_st1_1491.tif]

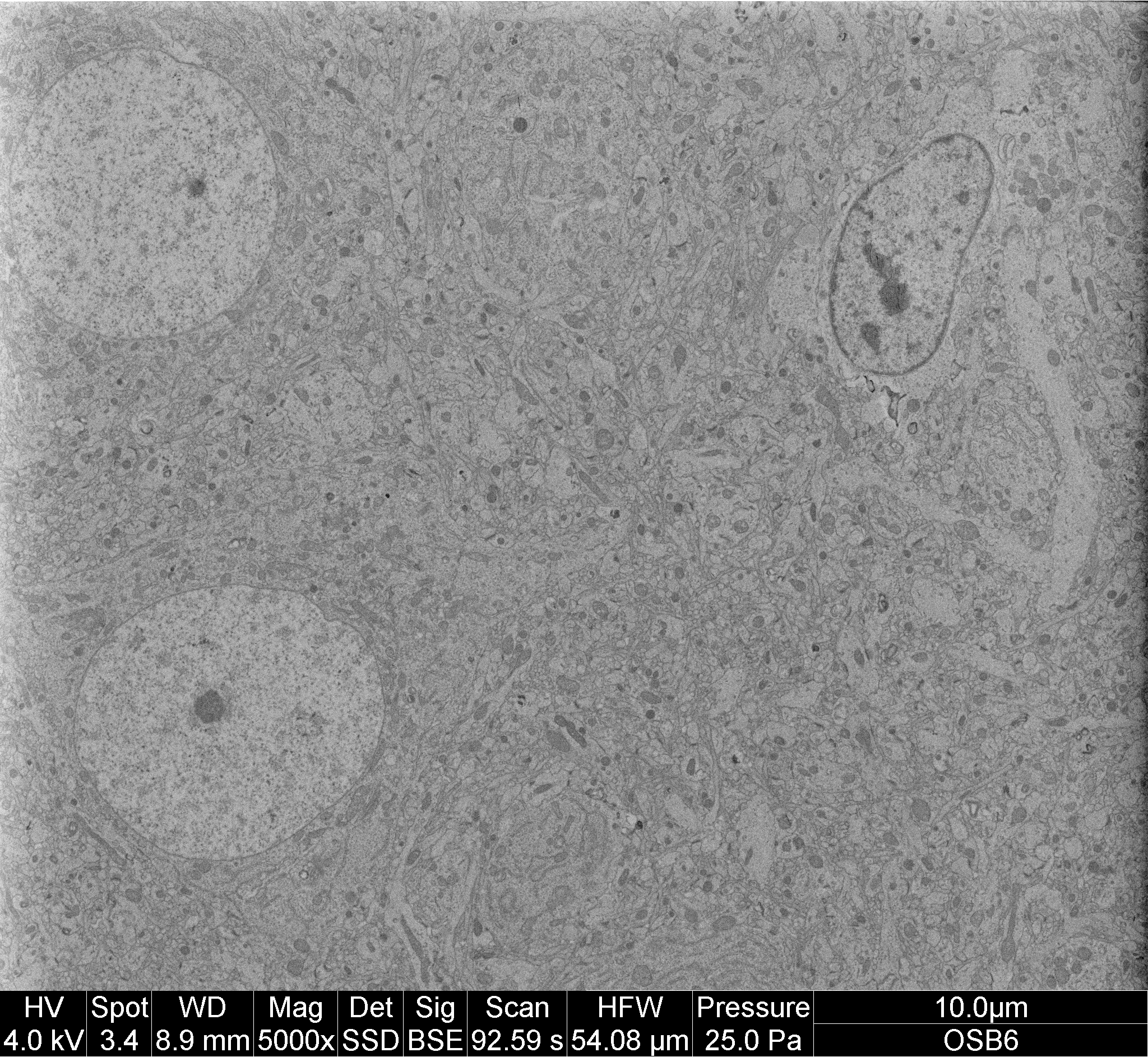

Supplement: Dataset S15 — (250.7 MB ZIP). [file pbio.0020329.sd015.zip › 040604_OS5_st1_1492.tif]

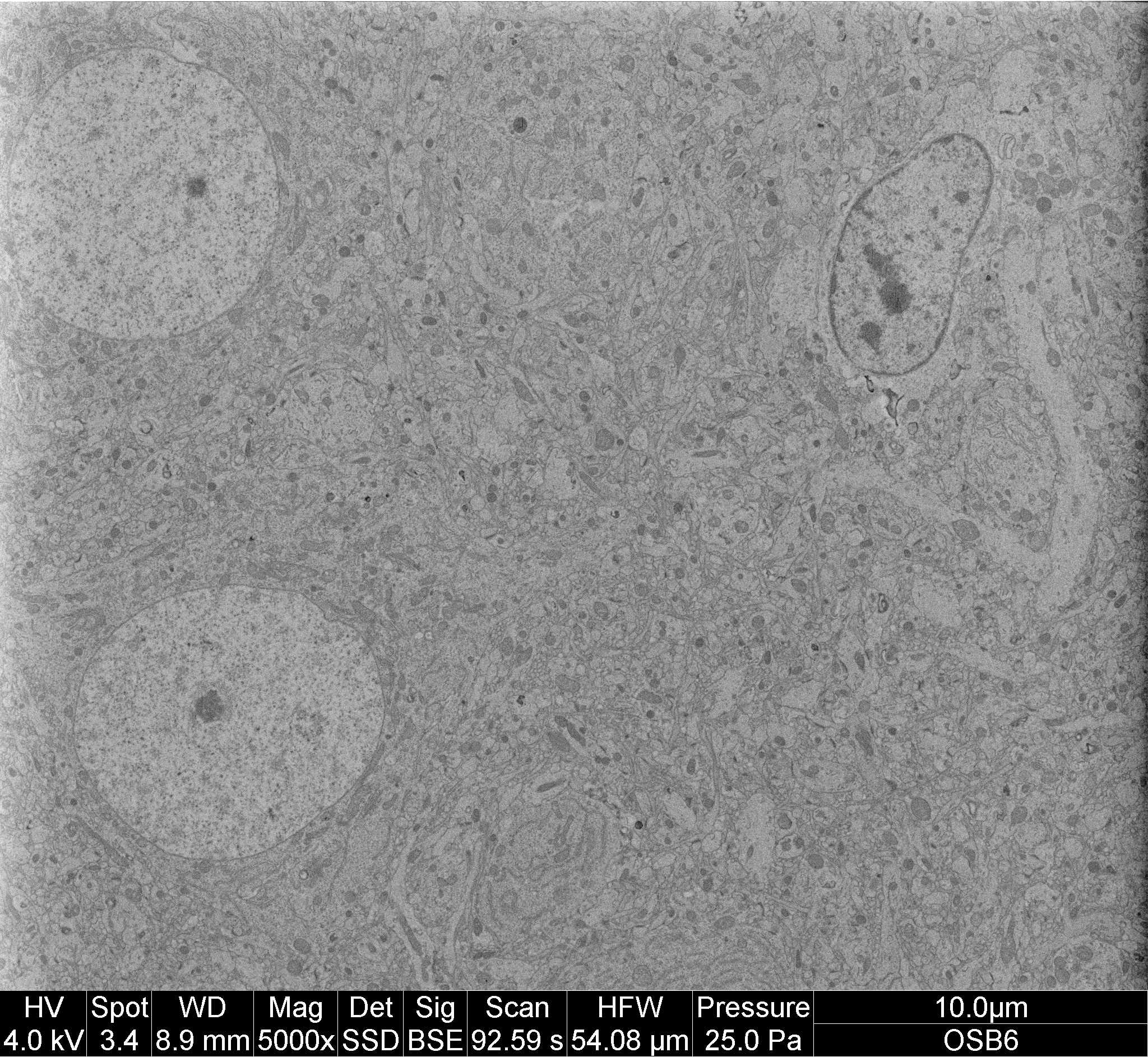

Supplement: Dataset S15 — (250.7 MB ZIP). [file pbio.0020329.sd015.zip › 040604_OS5_st1_1493.tif]

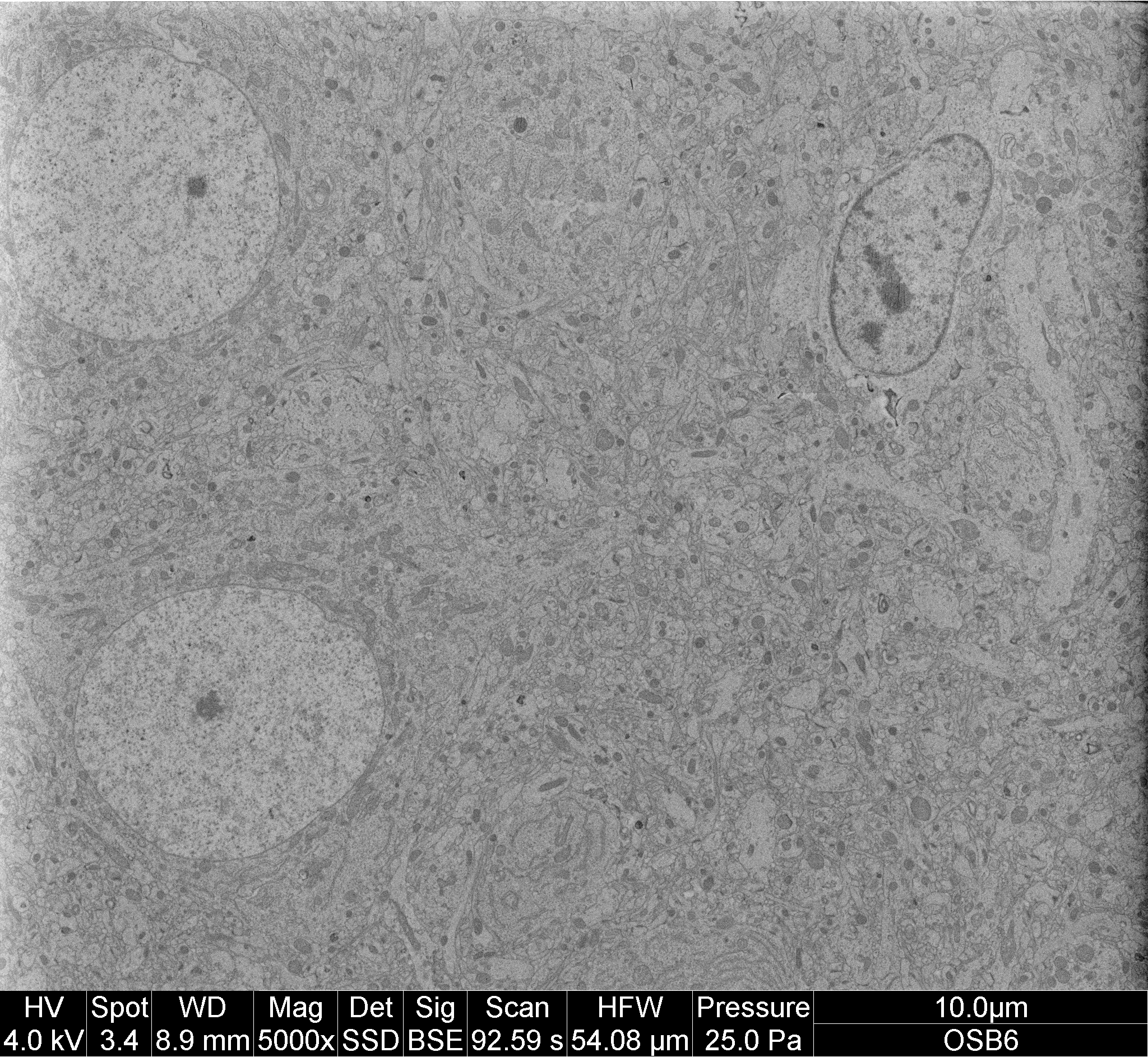

Supplement: Dataset S15 — (250.7 MB ZIP). [file pbio.0020329.sd015.zip › 040604_OS5_st1_1494.tif]

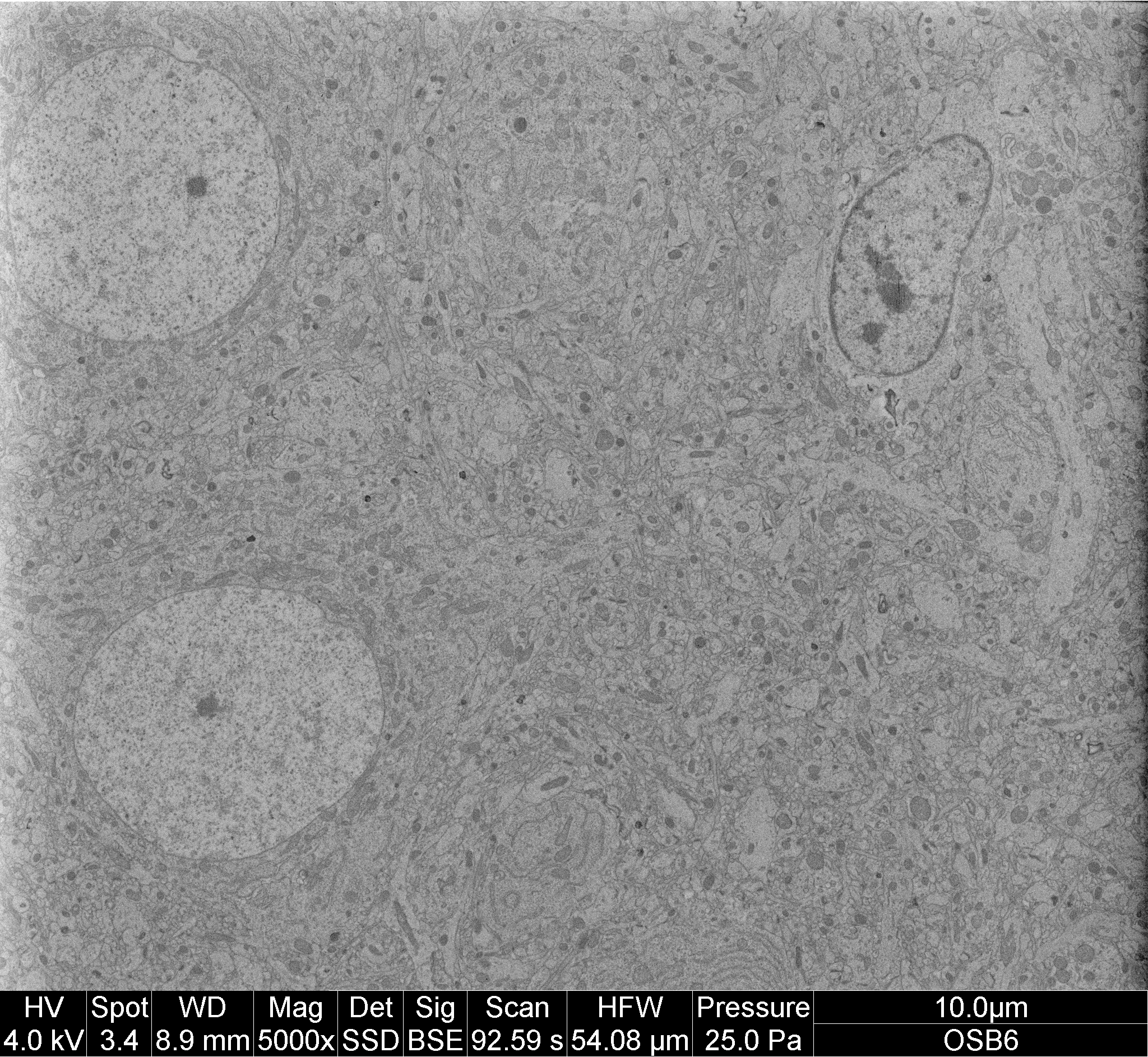

Supplement: Dataset S15 — (250.7 MB ZIP). [file pbio.0020329.sd015.zip › 040604_OS5_st1_1495.tif]

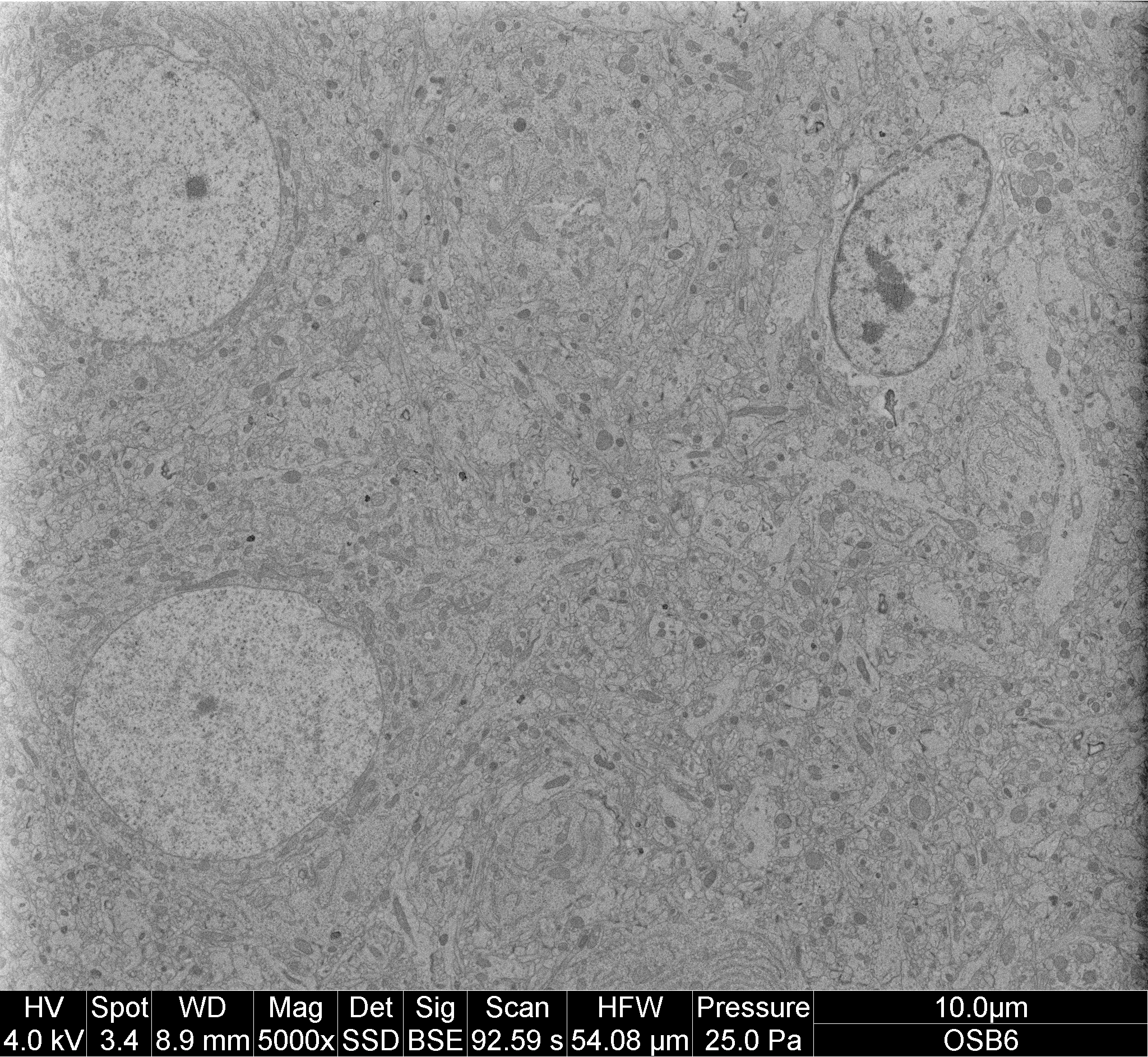

Supplement: Dataset S15 — (250.7 MB ZIP). [file pbio.0020329.sd015.zip › 040604_OS5_st1_1496.tif]

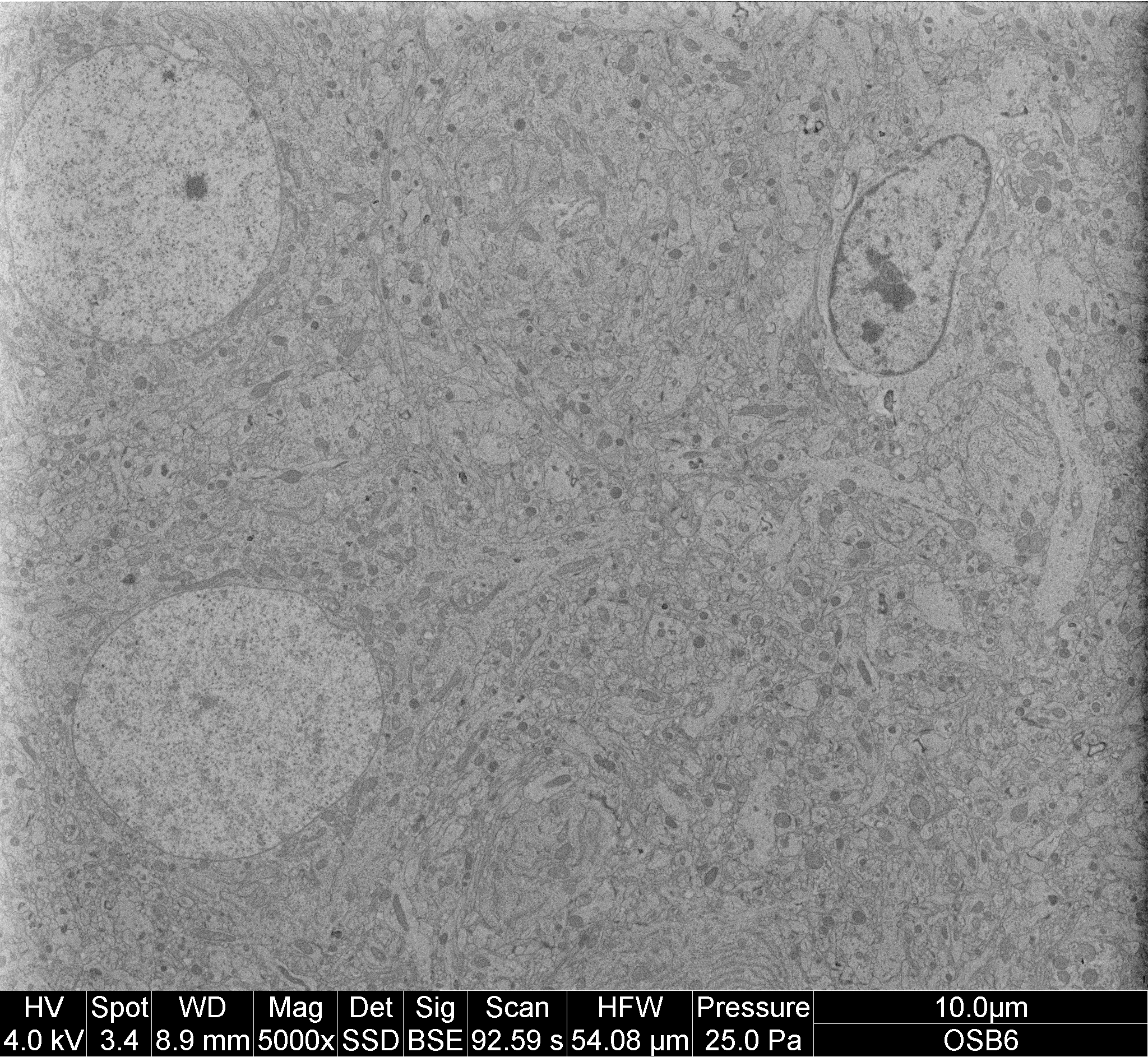

Supplement: Dataset S15 — (250.7 MB ZIP). [file pbio.0020329.sd015.zip › 040604_OS5_st1_1497.tif]

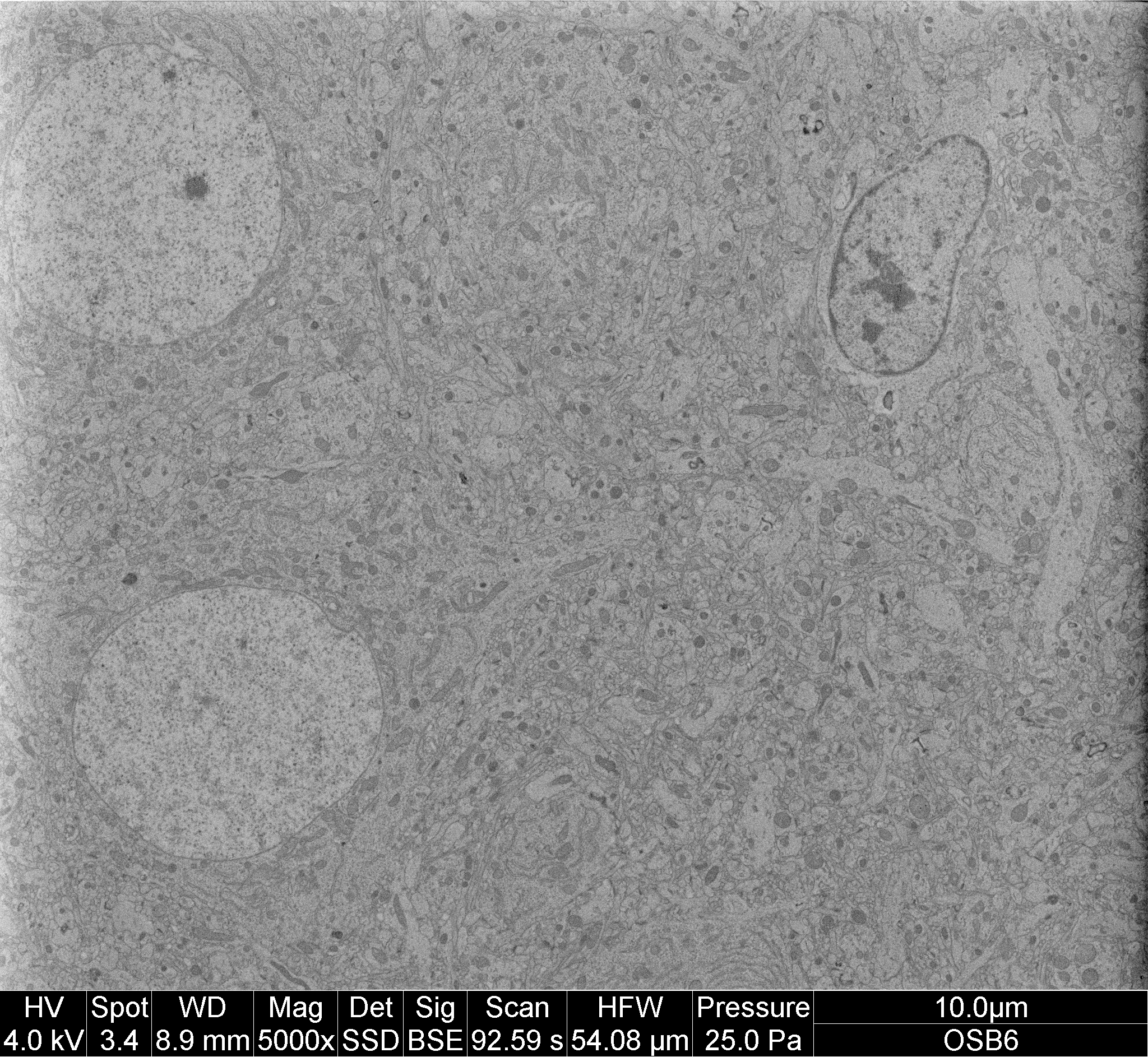

Supplement: Dataset S15 — (250.7 MB ZIP). [file pbio.0020329.sd015.zip › 040604_OS5_st1_1498.tif]

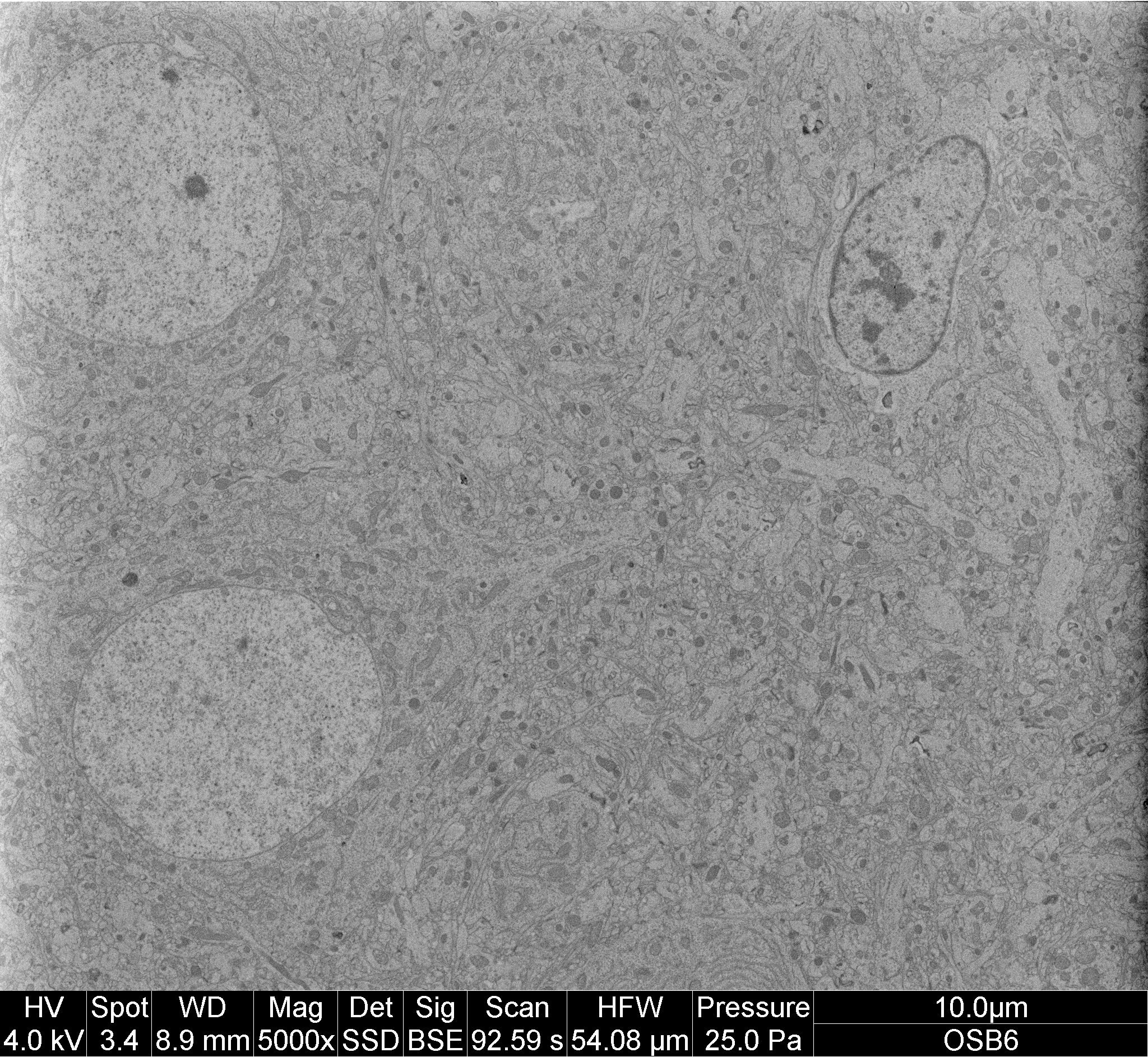

Supplement: Dataset S15 — (250.7 MB ZIP). [file pbio.0020329.sd015.zip › 040604_OS5_st1_1499.tif]

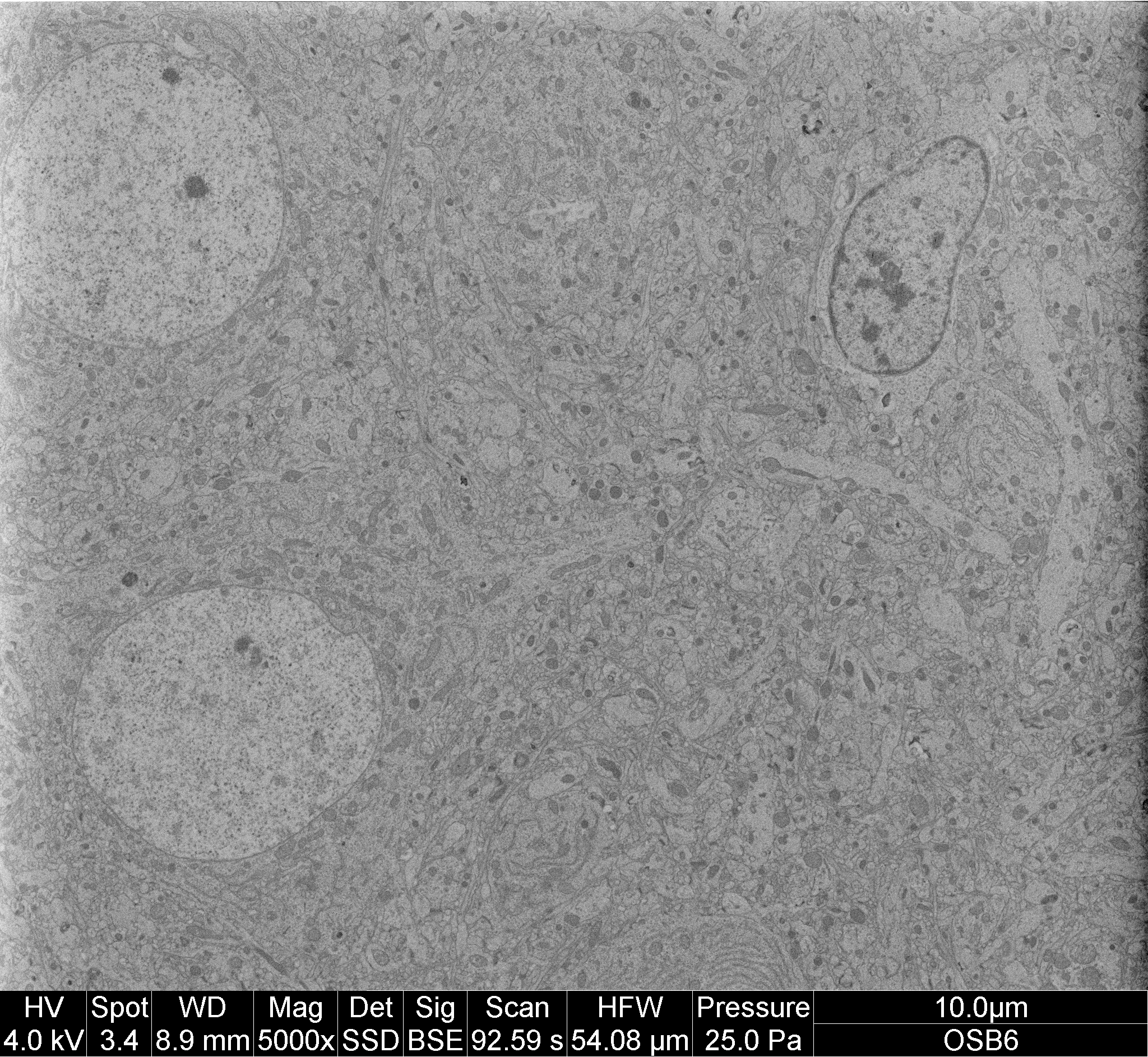

Supplement: Dataset S16 — (251.4 MB ZIP). [file pbio.0020329.sd016.zip › 040604_OS5_st1_1500.tif]
