# Supplementary figures and images for: Serial Block-Face Scanning Electron Microscopy to Reconstruct Three-Dimensional Tissue Nanostructure (part 16 of 21)
Source: PLoS Biol. 2004 Oct 19;2(11):e329. doi: 10.1371/journal.pbio.0020329 (PMC524270; doi:10.1371/journal.pbio.0020329)

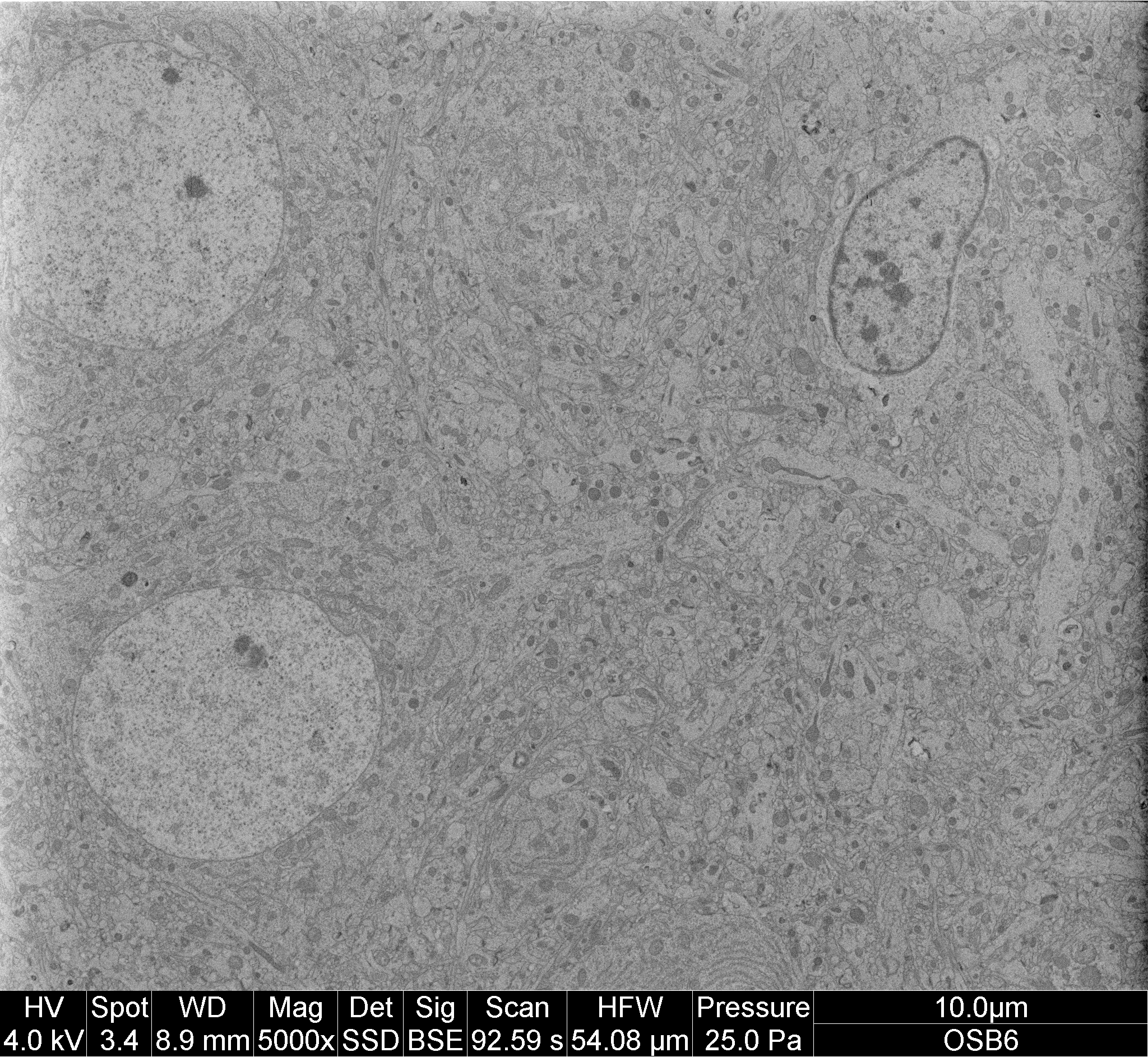

Supplement: Dataset S16 — (251.4 MB ZIP). [file pbio.0020329.sd016.zip › 040604_OS5_st1_1501.tif]

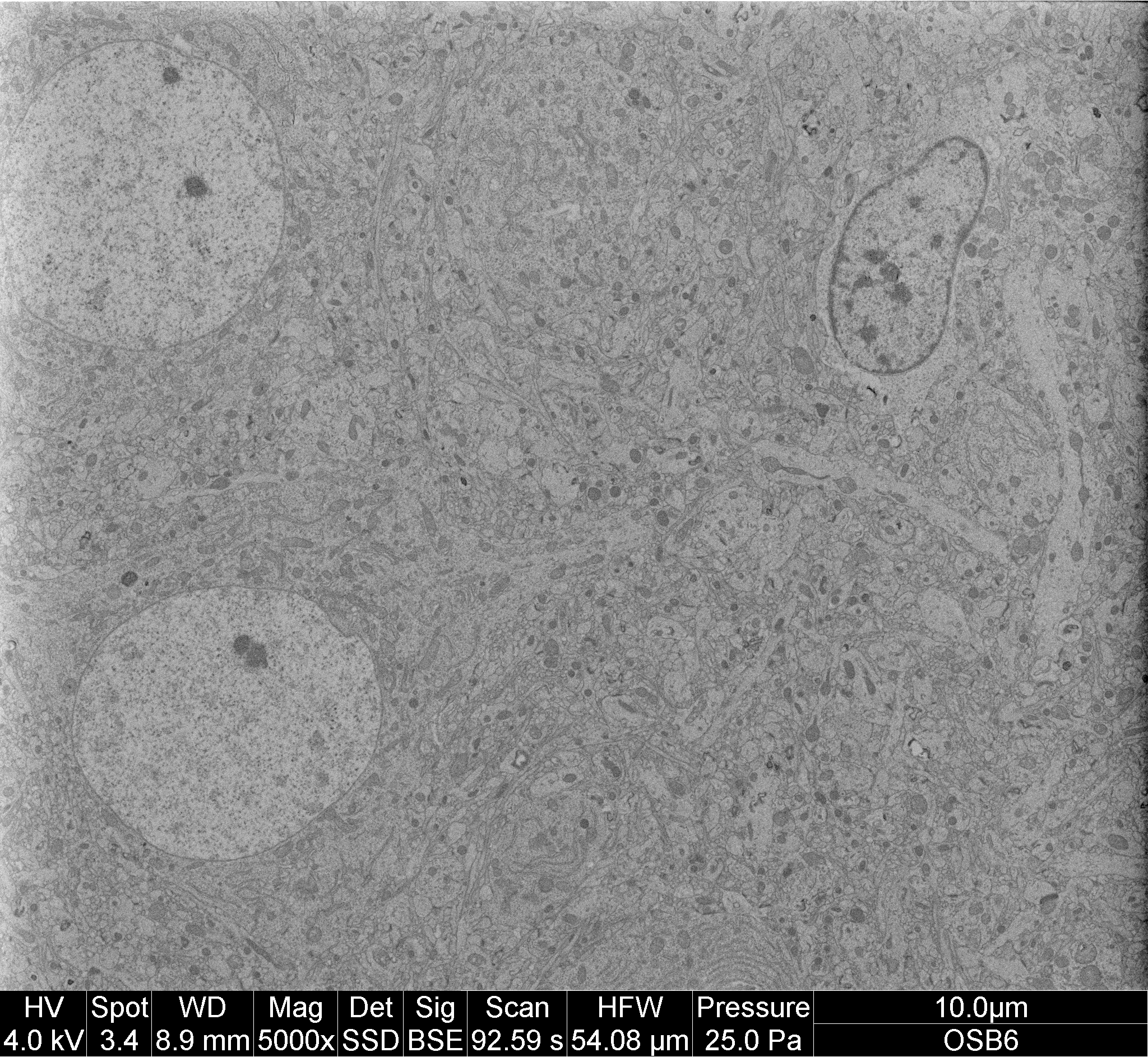

Supplement: Dataset S16 — (251.4 MB ZIP). [file pbio.0020329.sd016.zip › 040604_OS5_st1_1502.tif]

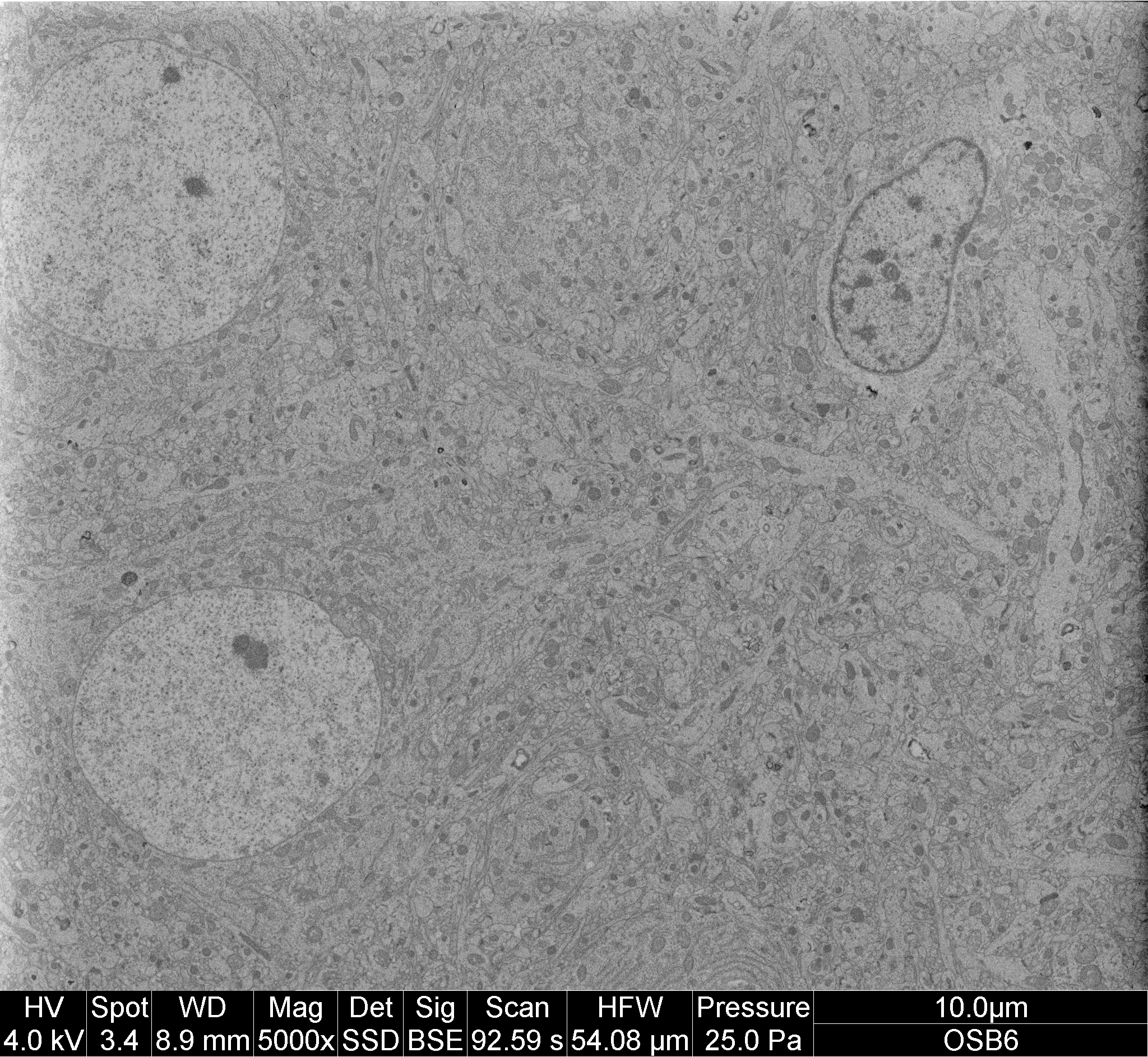

Supplement: Dataset S16 — (251.4 MB ZIP). [file pbio.0020329.sd016.zip › 040604_OS5_st1_1503.tif]

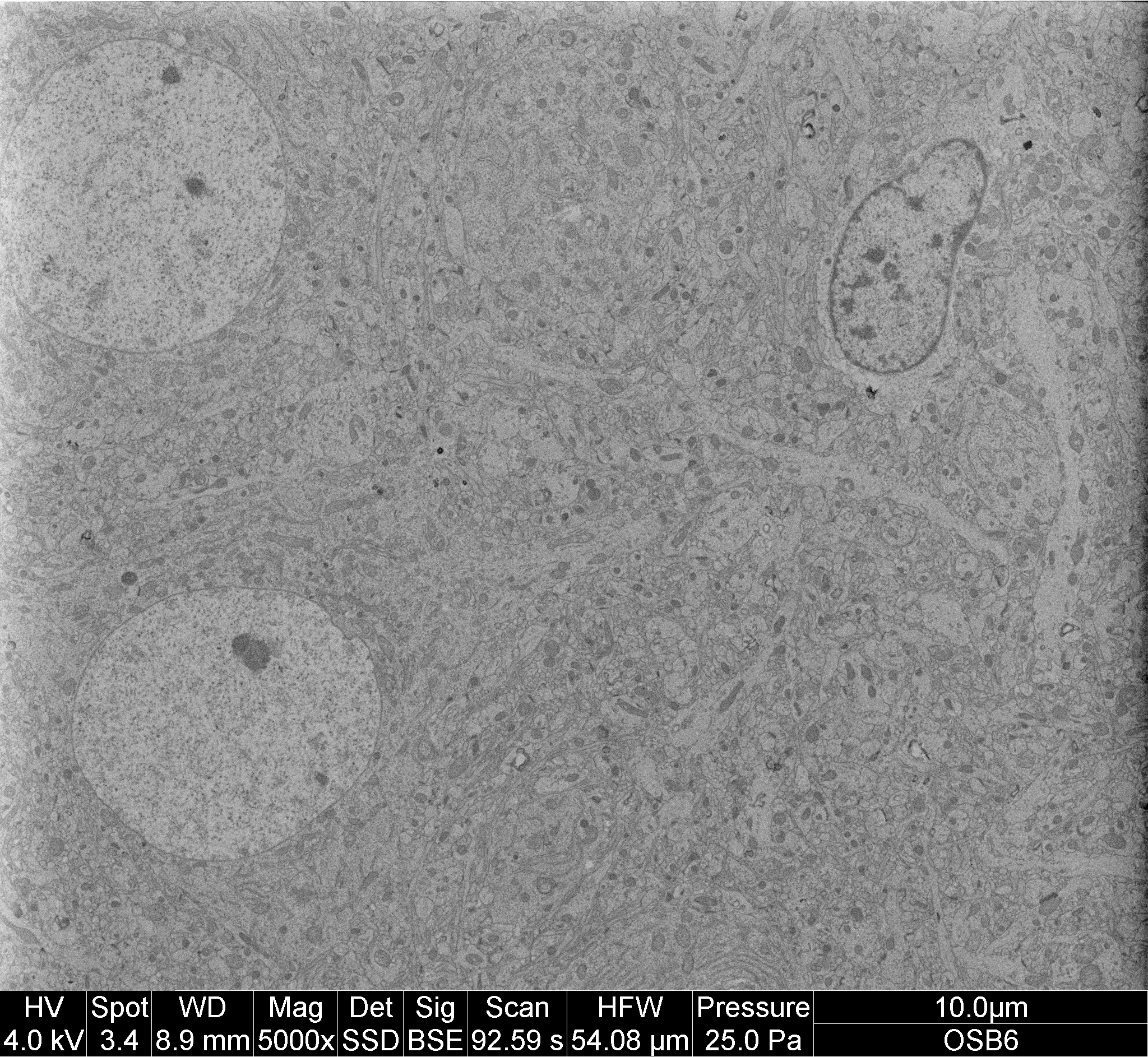

Supplement: Dataset S16 — (251.4 MB ZIP). [file pbio.0020329.sd016.zip › 040604_OS5_st1_1504.tif]

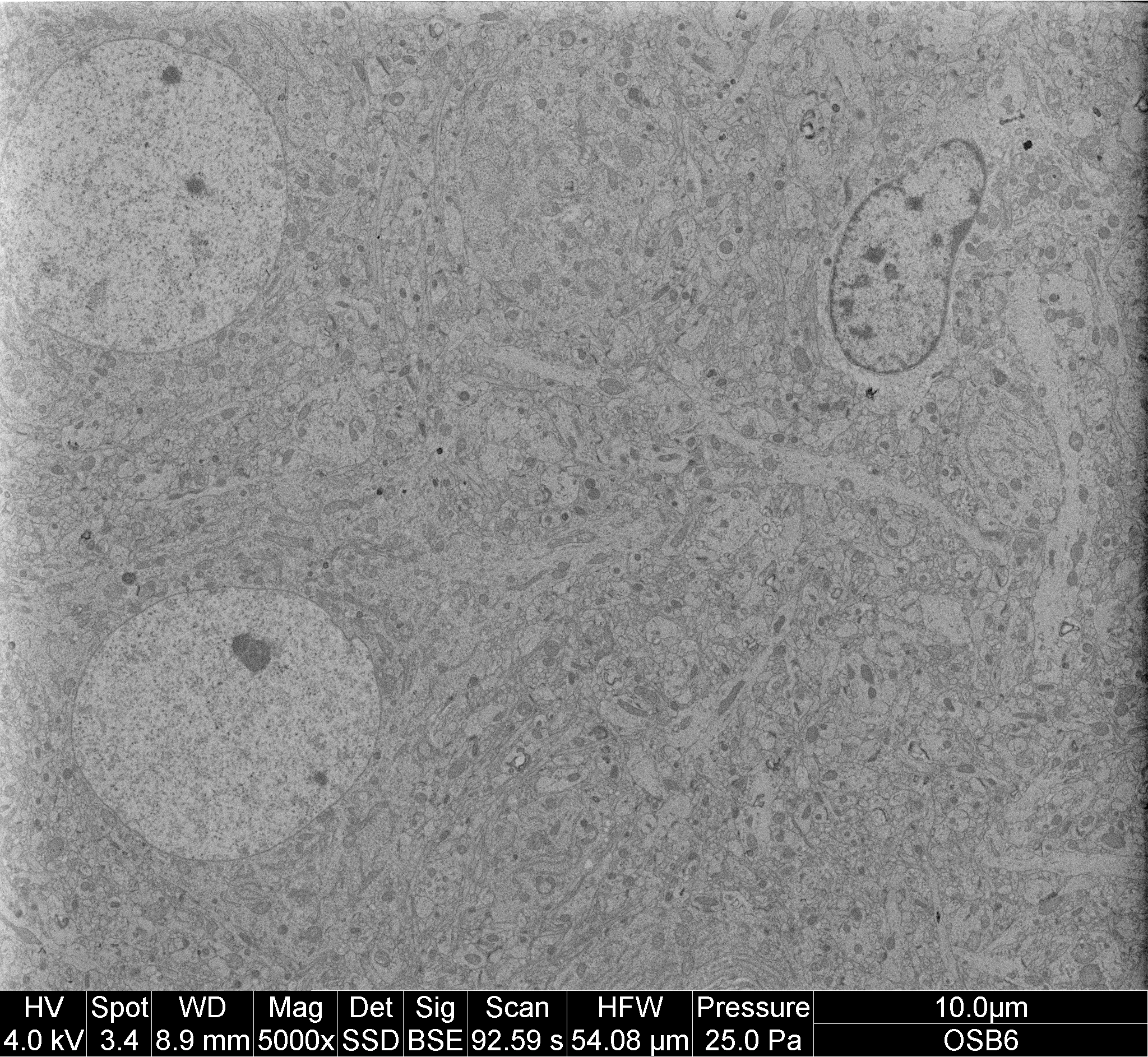

Supplement: Dataset S16 — (251.4 MB ZIP). [file pbio.0020329.sd016.zip › 040604_OS5_st1_1505.tif]

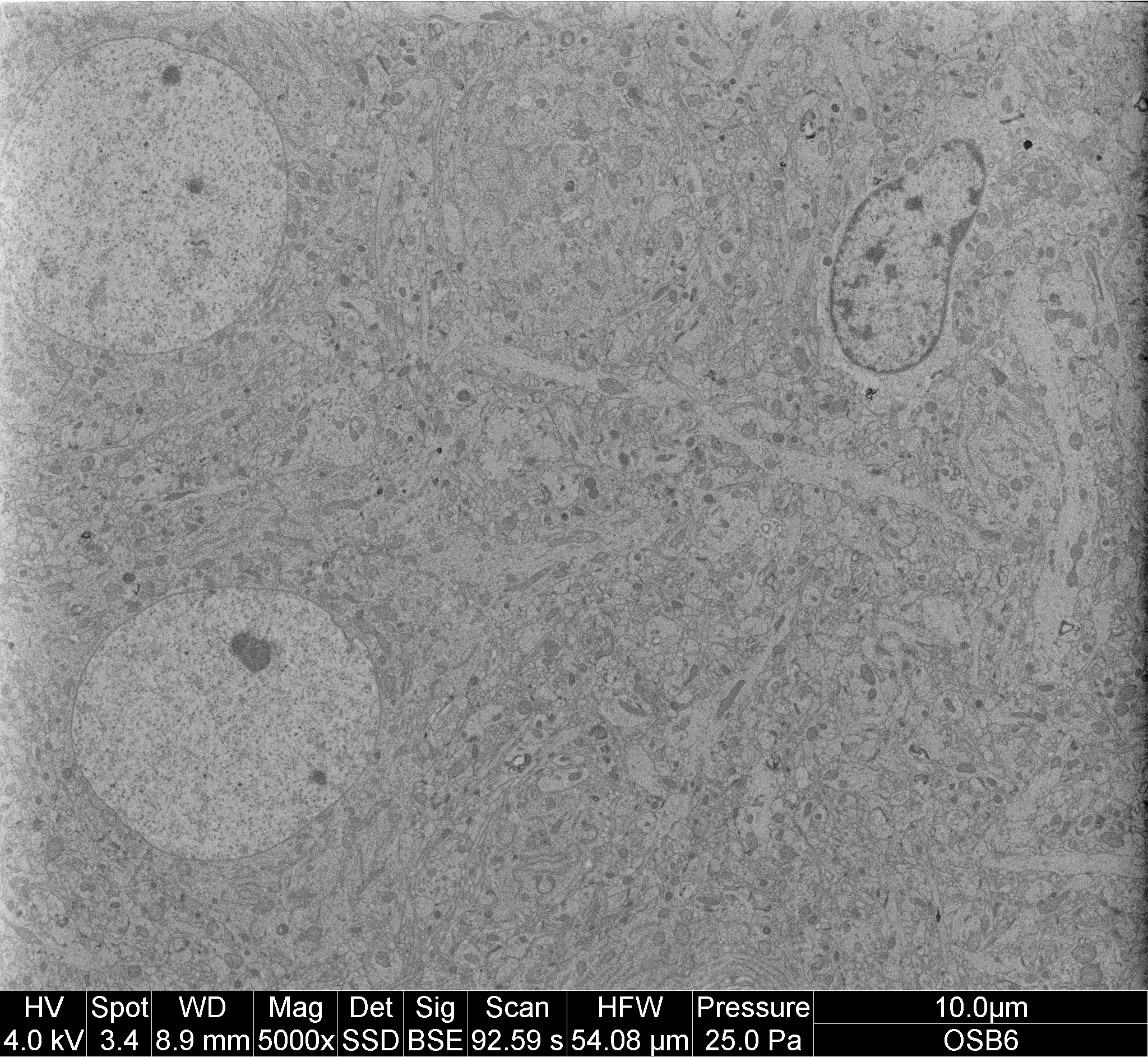

Supplement: Dataset S16 — (251.4 MB ZIP). [file pbio.0020329.sd016.zip › 040604_OS5_st1_1506.tif]

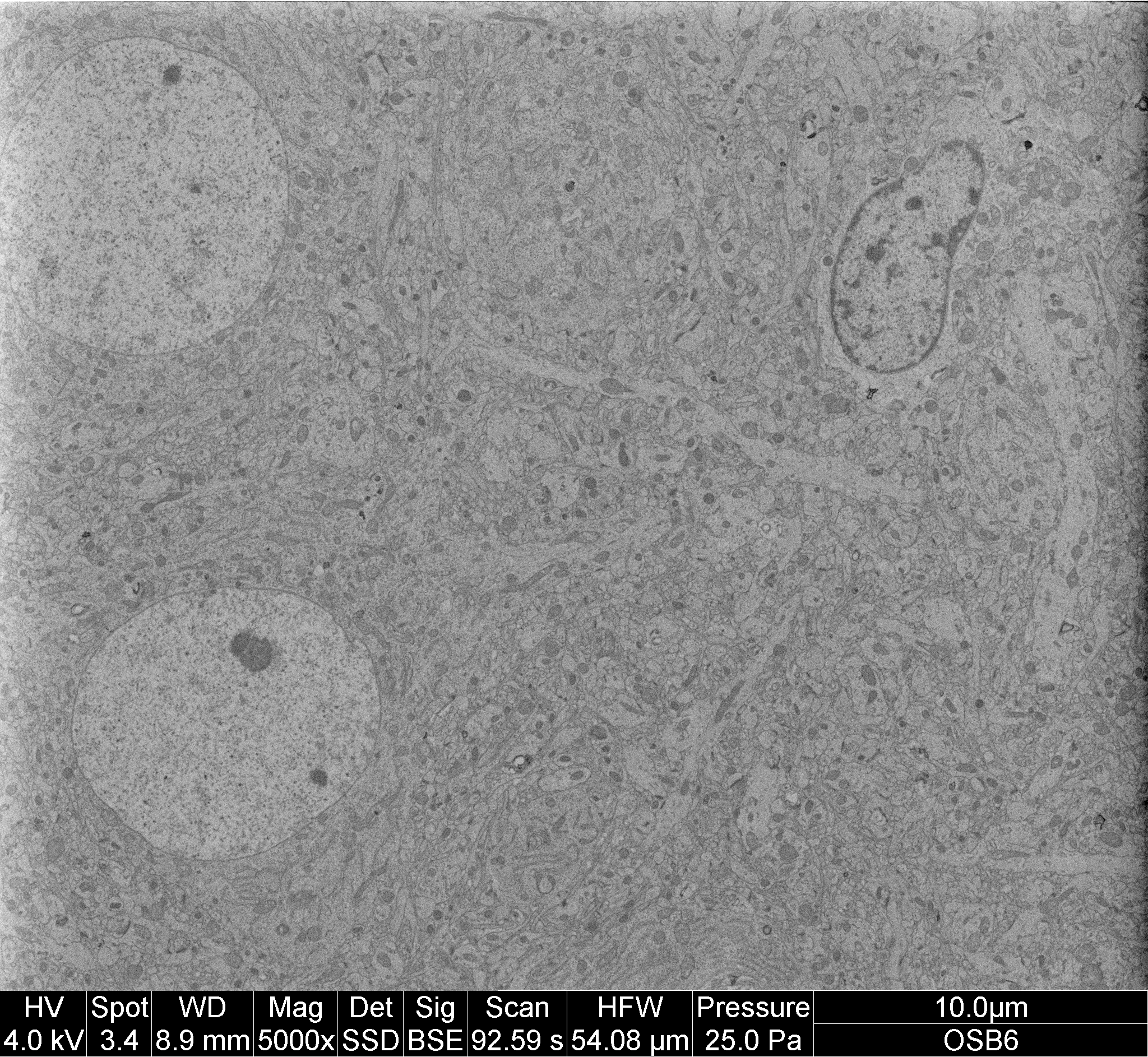

Supplement: Dataset S16 — (251.4 MB ZIP). [file pbio.0020329.sd016.zip › 040604_OS5_st1_1507.tif]

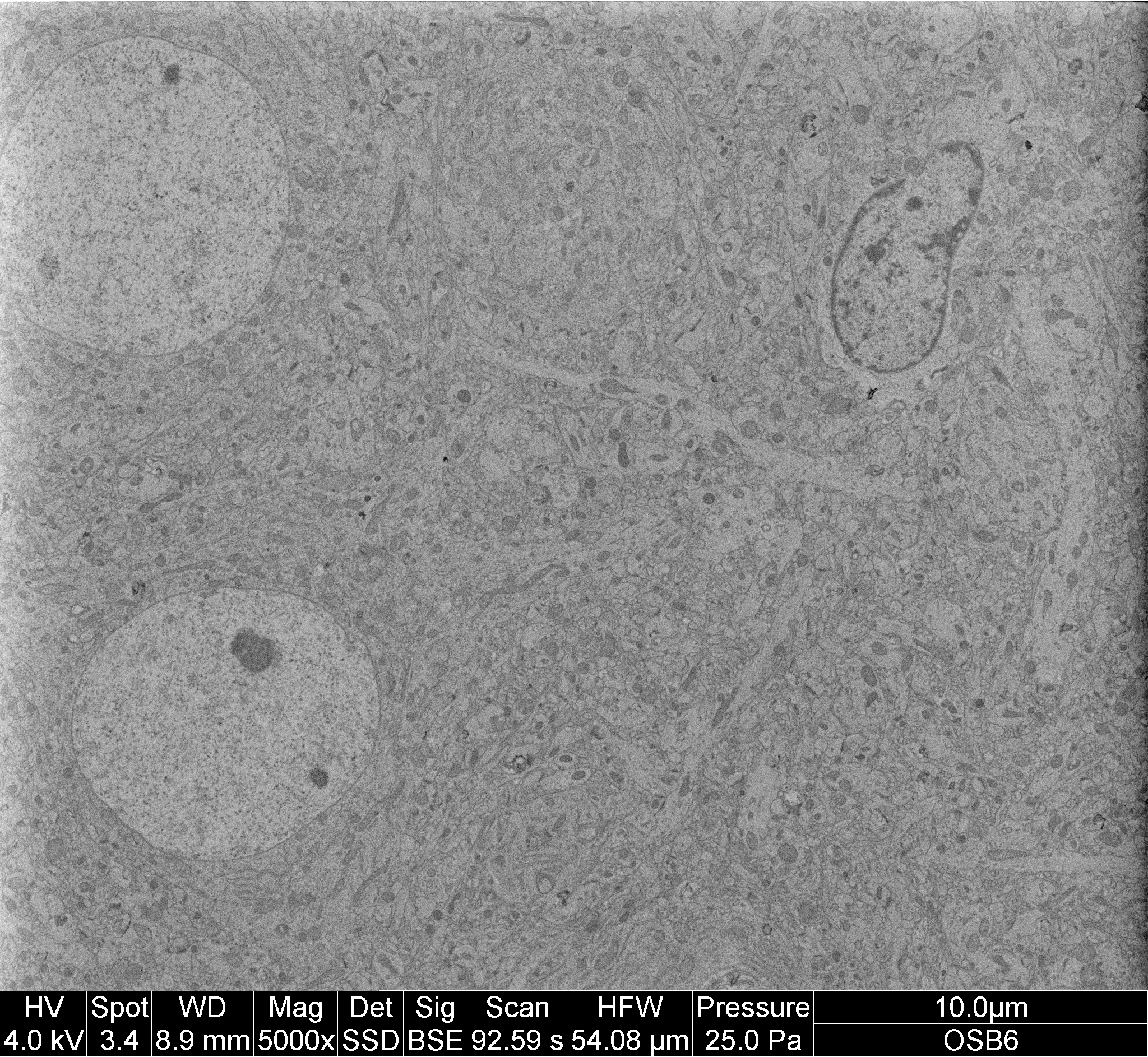

Supplement: Dataset S16 — (251.4 MB ZIP). [file pbio.0020329.sd016.zip › 040604_OS5_st1_1508.tif]

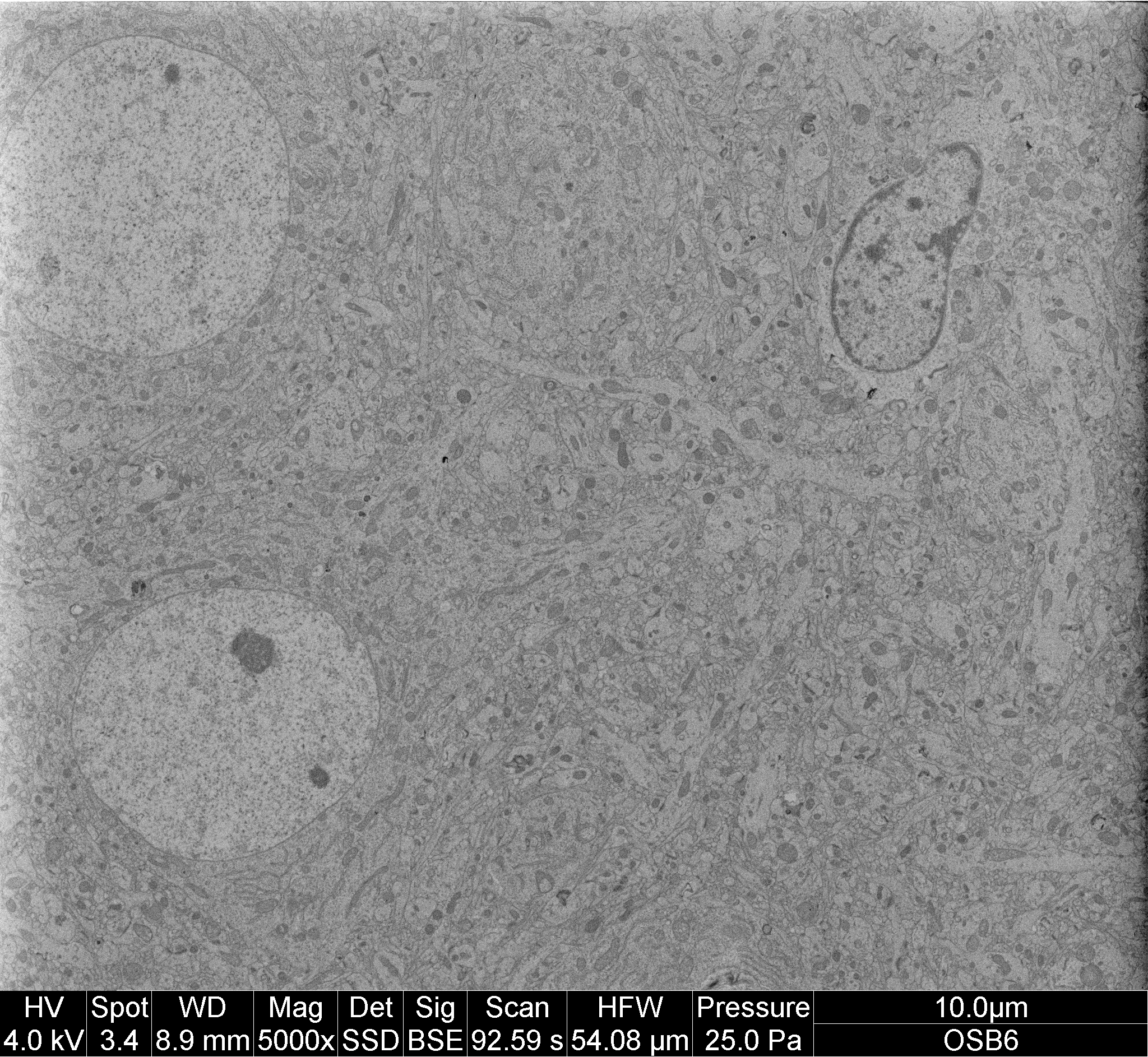

Supplement: Dataset S16 — (251.4 MB ZIP). [file pbio.0020329.sd016.zip › 040604_OS5_st1_1509.tif]

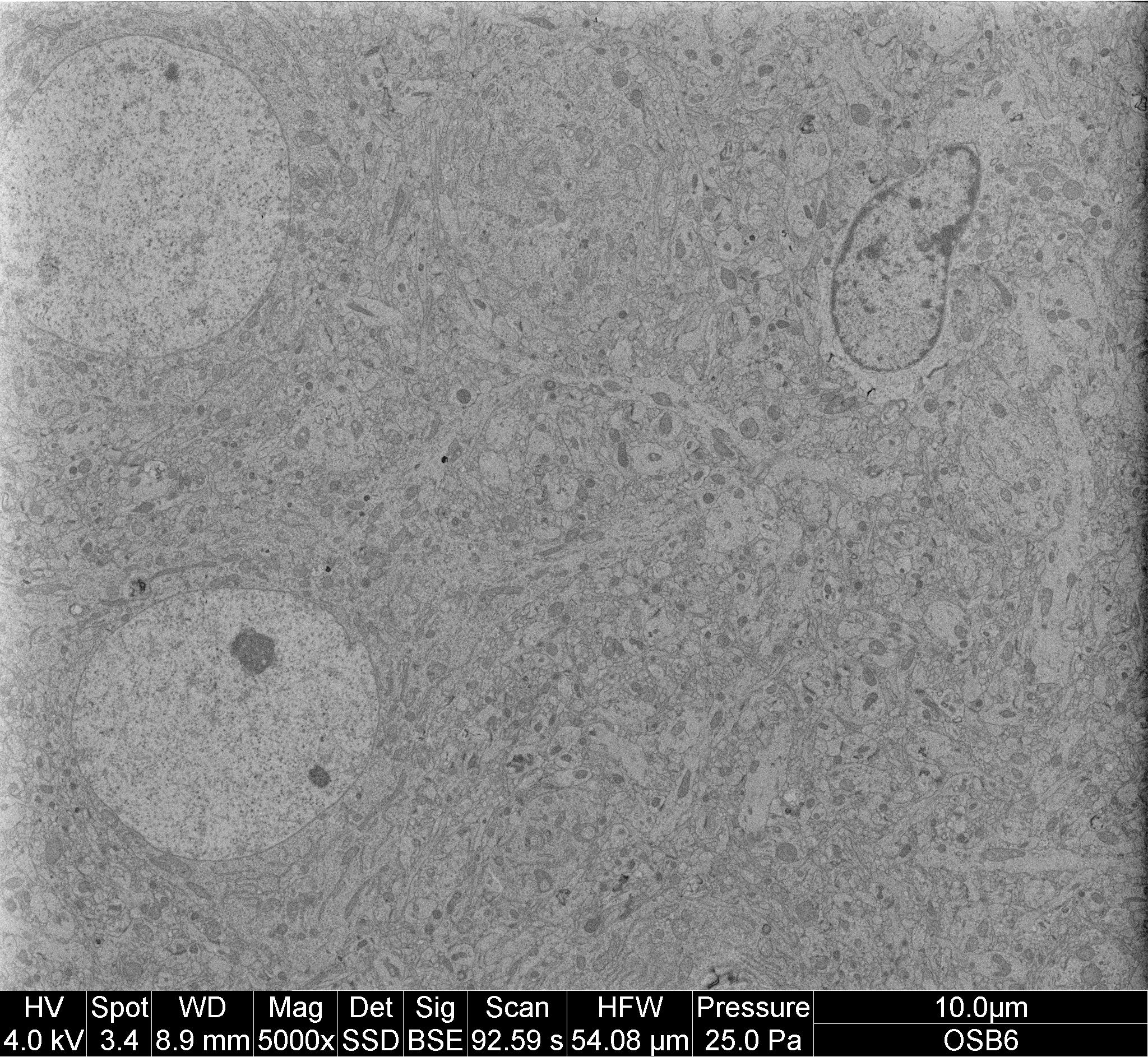

Supplement: Dataset S16 — (251.4 MB ZIP). [file pbio.0020329.sd016.zip › 040604_OS5_st1_1510.tif]

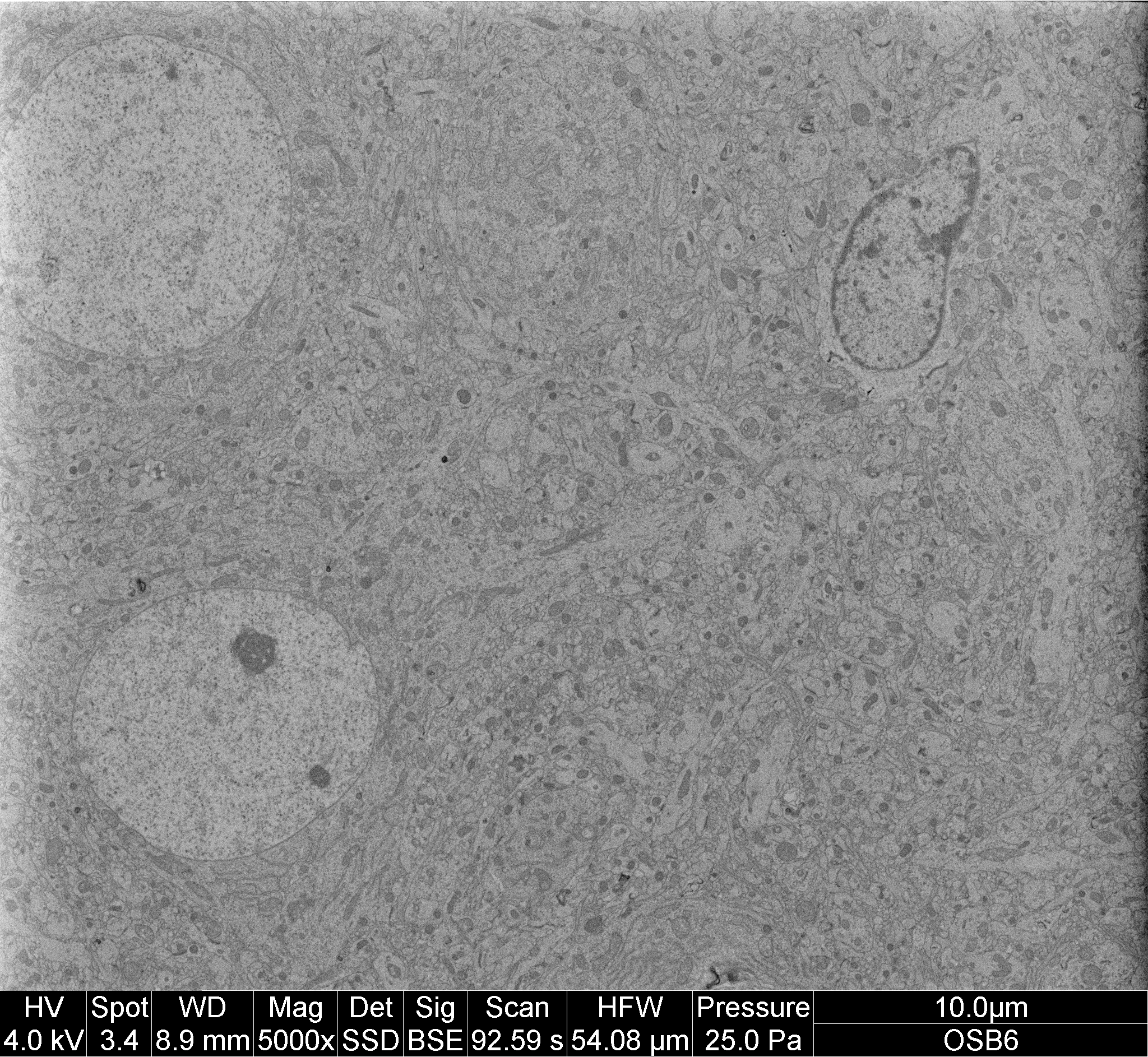

Supplement: Dataset S16 — (251.4 MB ZIP). [file pbio.0020329.sd016.zip › 040604_OS5_st1_1511.tif]

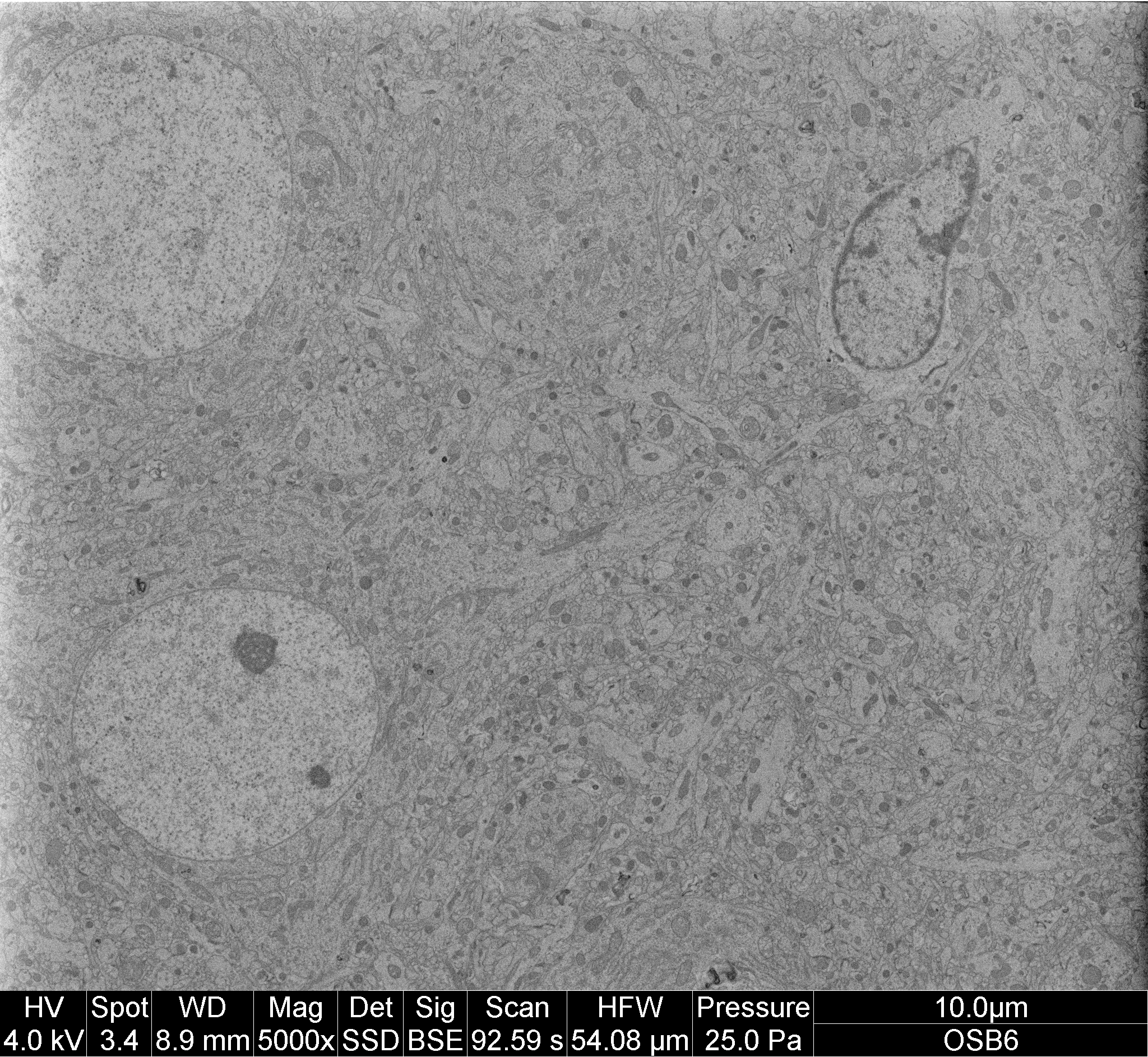

Supplement: Dataset S16 — (251.4 MB ZIP). [file pbio.0020329.sd016.zip › 040604_OS5_st1_1512.tif]

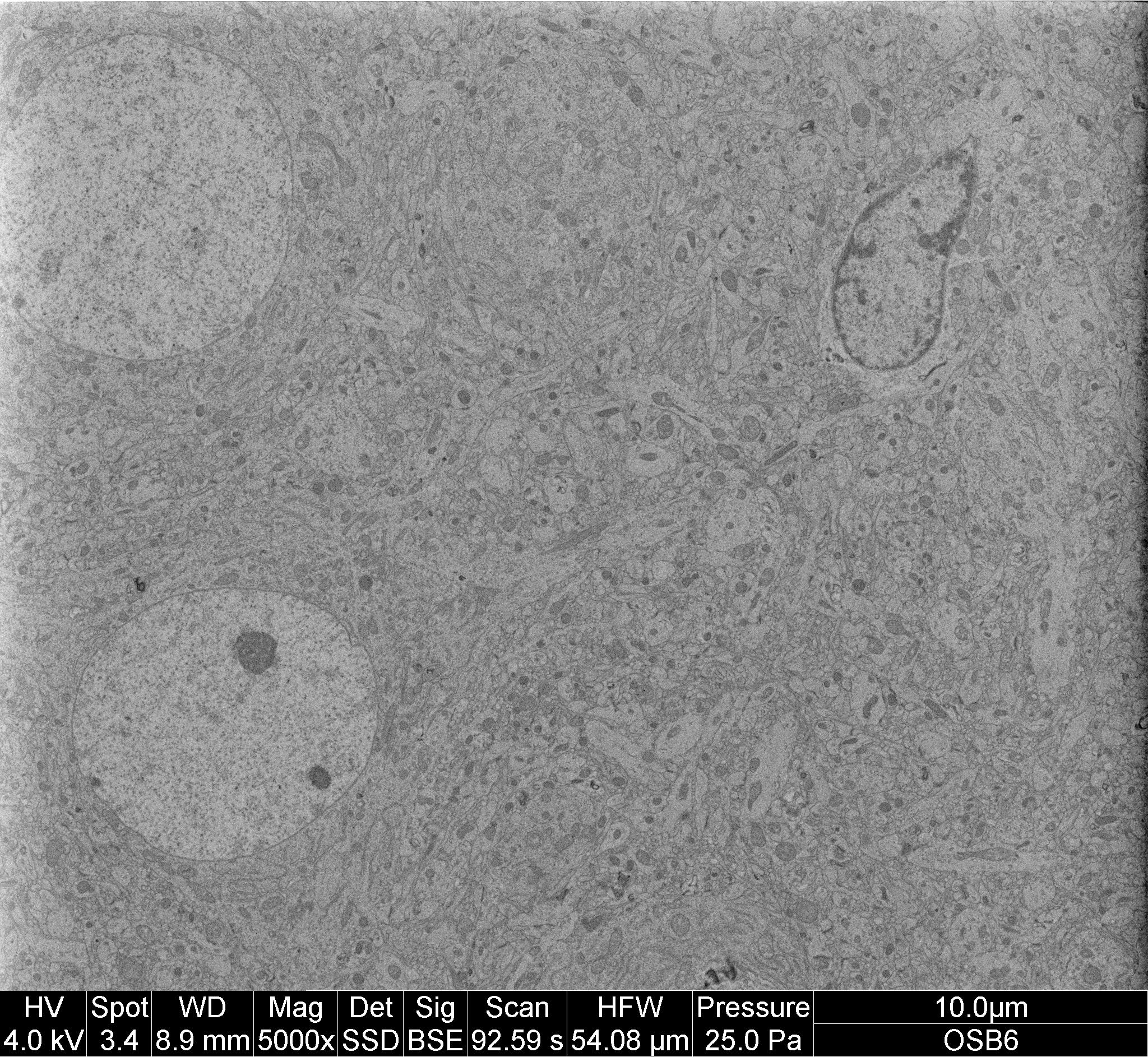

Supplement: Dataset S16 — (251.4 MB ZIP). [file pbio.0020329.sd016.zip › 040604_OS5_st1_1513.tif]

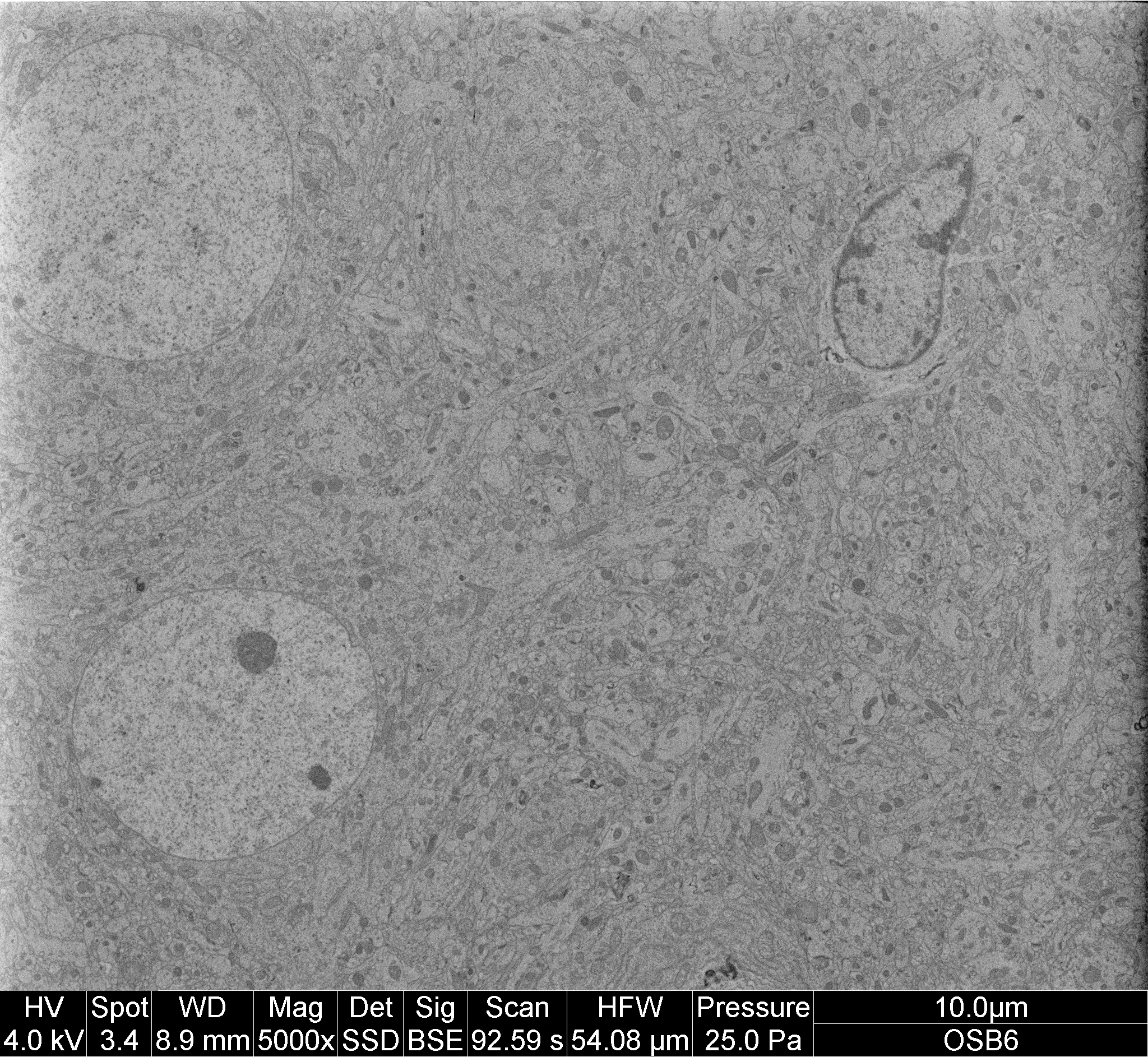

Supplement: Dataset S16 — (251.4 MB ZIP). [file pbio.0020329.sd016.zip › 040604_OS5_st1_1514.tif]

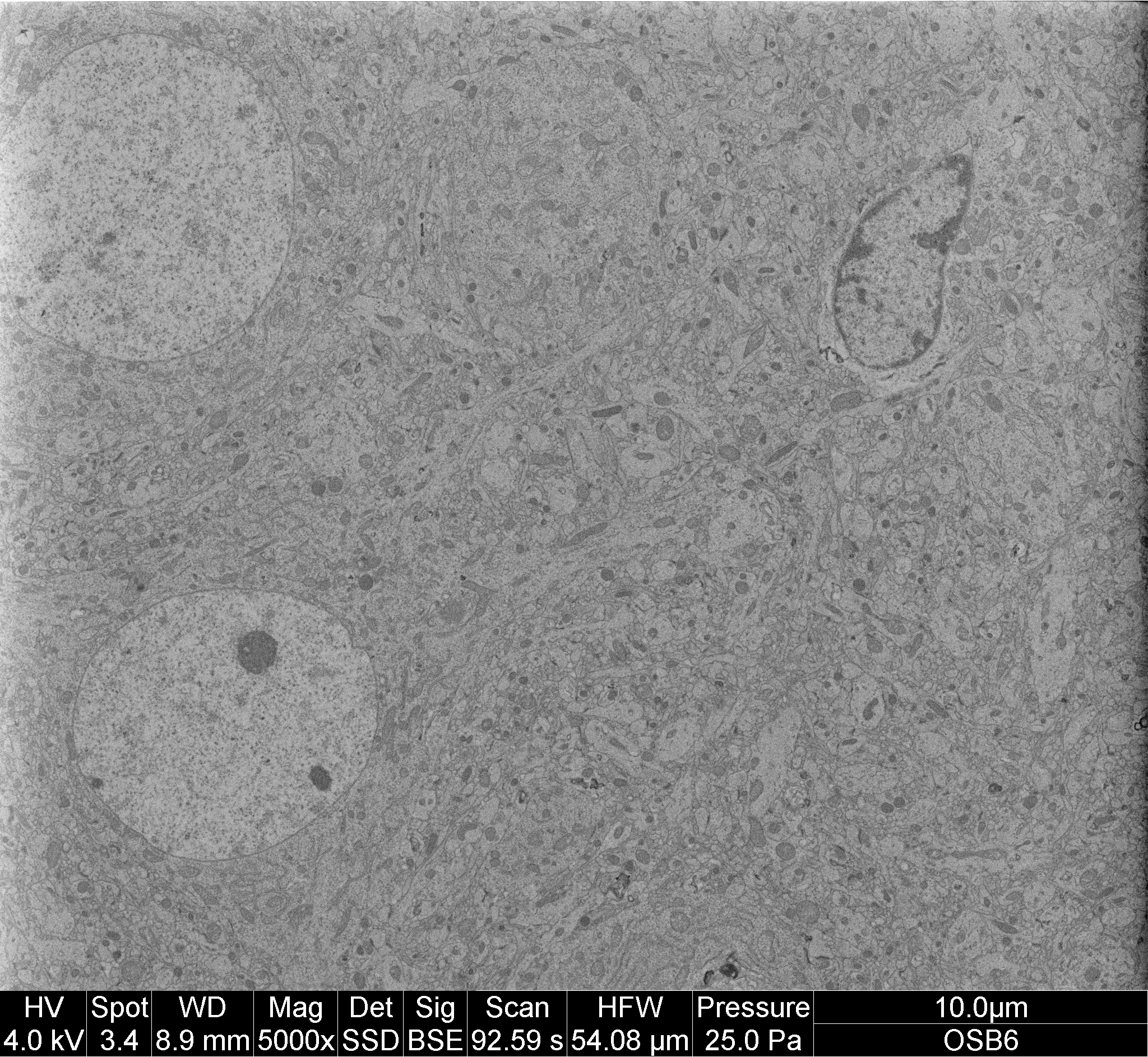

Supplement: Dataset S16 — (251.4 MB ZIP). [file pbio.0020329.sd016.zip › 040604_OS5_st1_1515.tif]

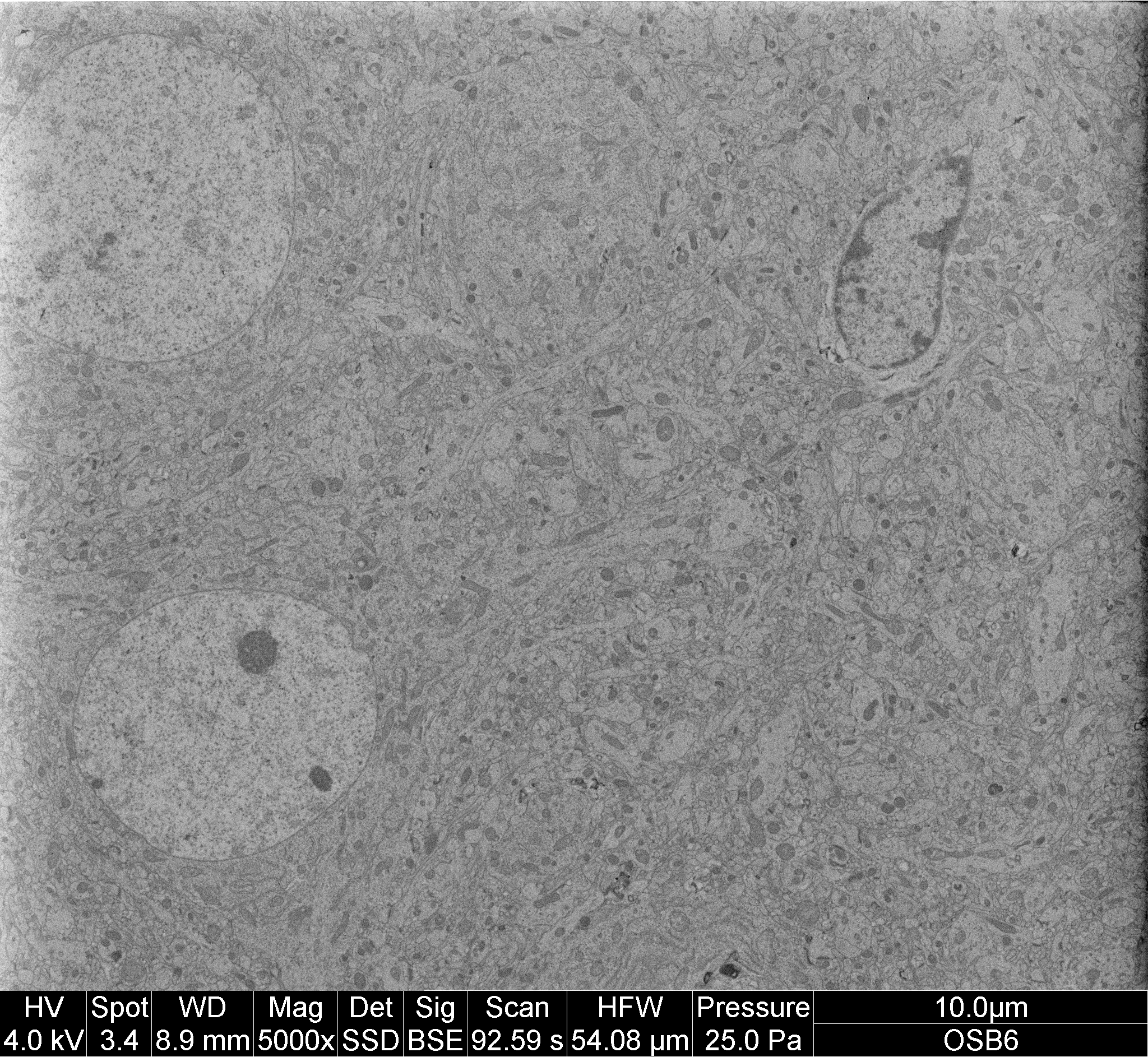

Supplement: Dataset S16 — (251.4 MB ZIP). [file pbio.0020329.sd016.zip › 040604_OS5_st1_1516.tif]

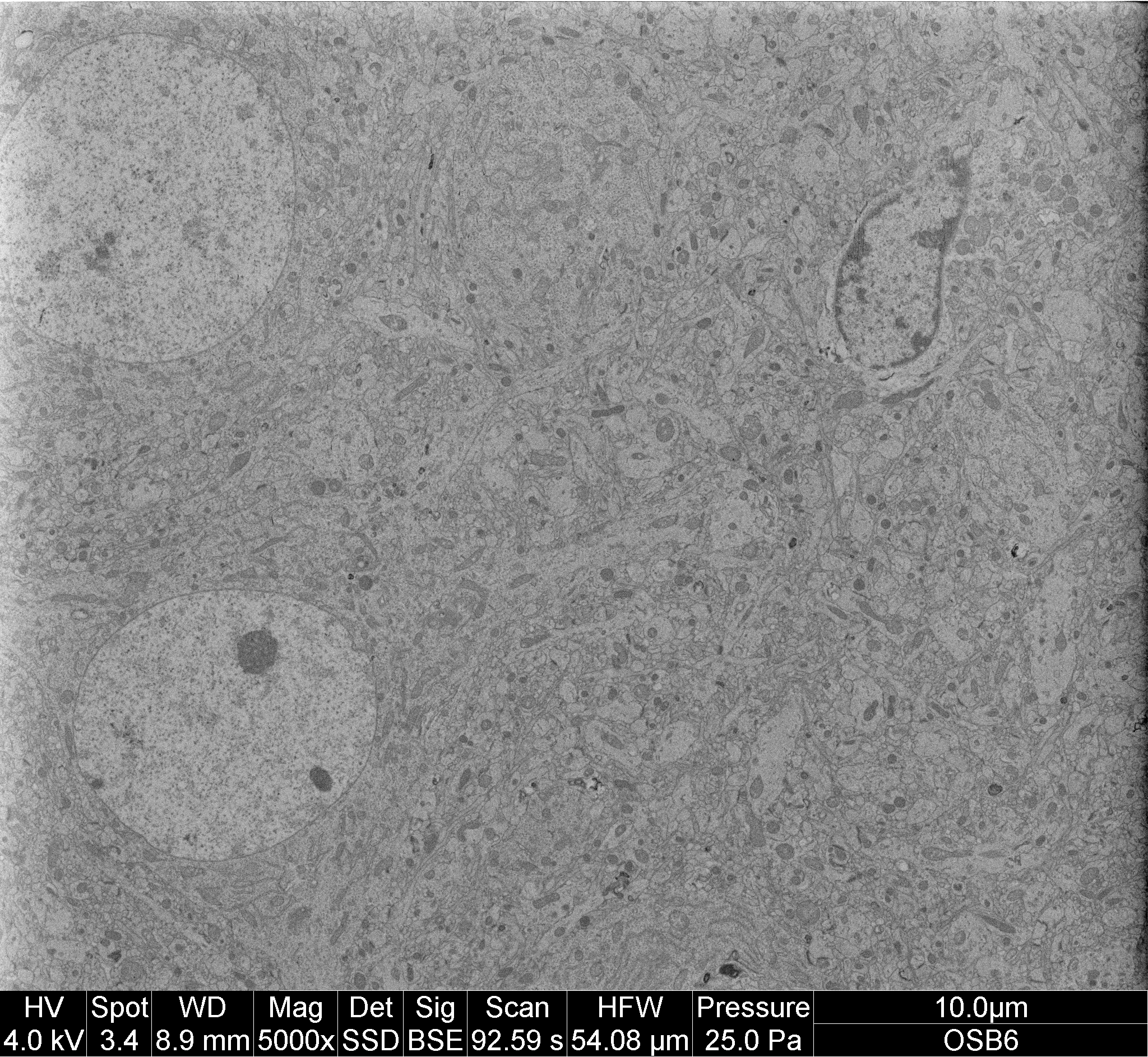

Supplement: Dataset S16 — (251.4 MB ZIP). [file pbio.0020329.sd016.zip › 040604_OS5_st1_1517.tif]

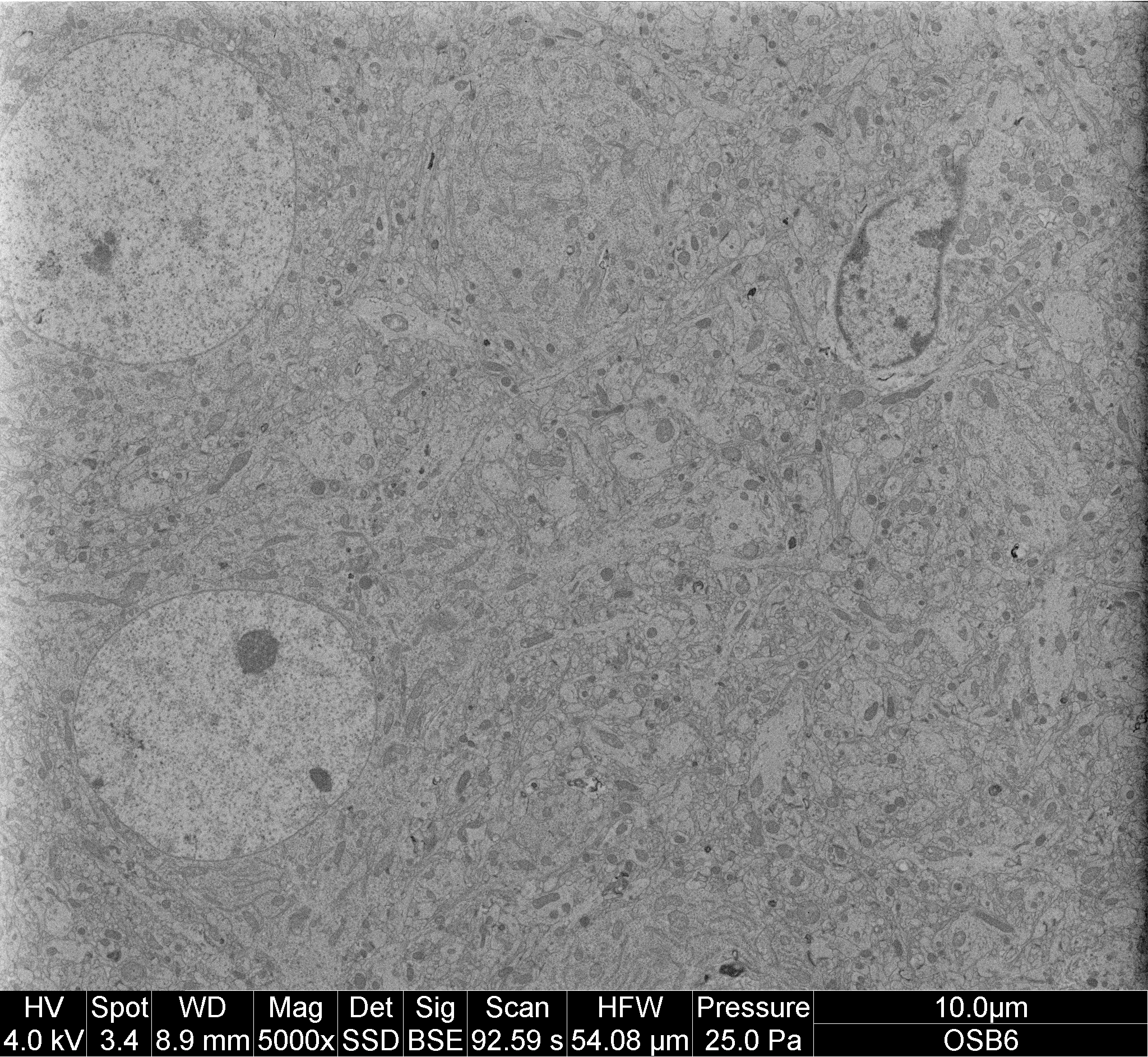

Supplement: Dataset S16 — (251.4 MB ZIP). [file pbio.0020329.sd016.zip › 040604_OS5_st1_1518.tif]

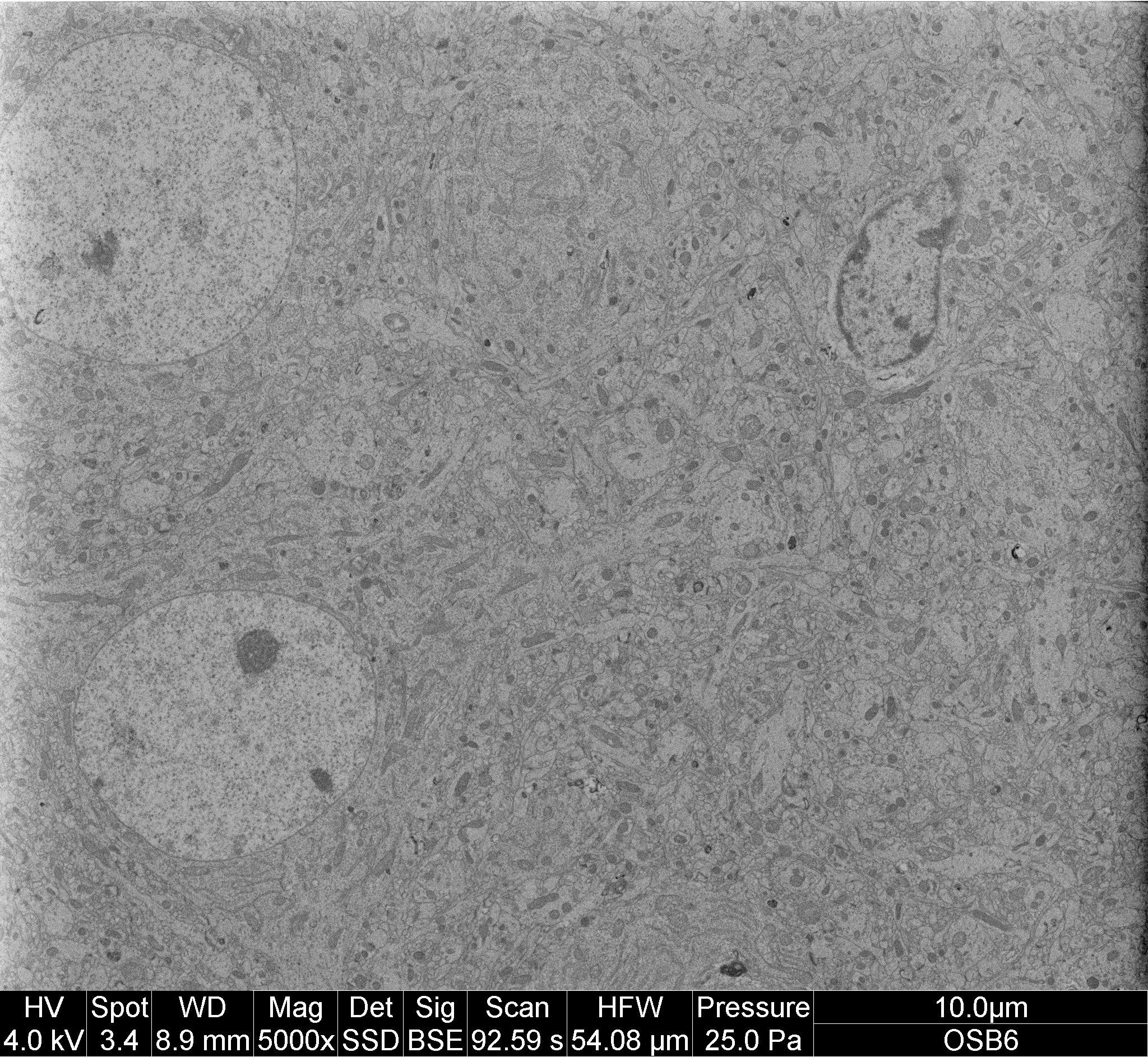

Supplement: Dataset S16 — (251.4 MB ZIP). [file pbio.0020329.sd016.zip › 040604_OS5_st1_1519.tif]

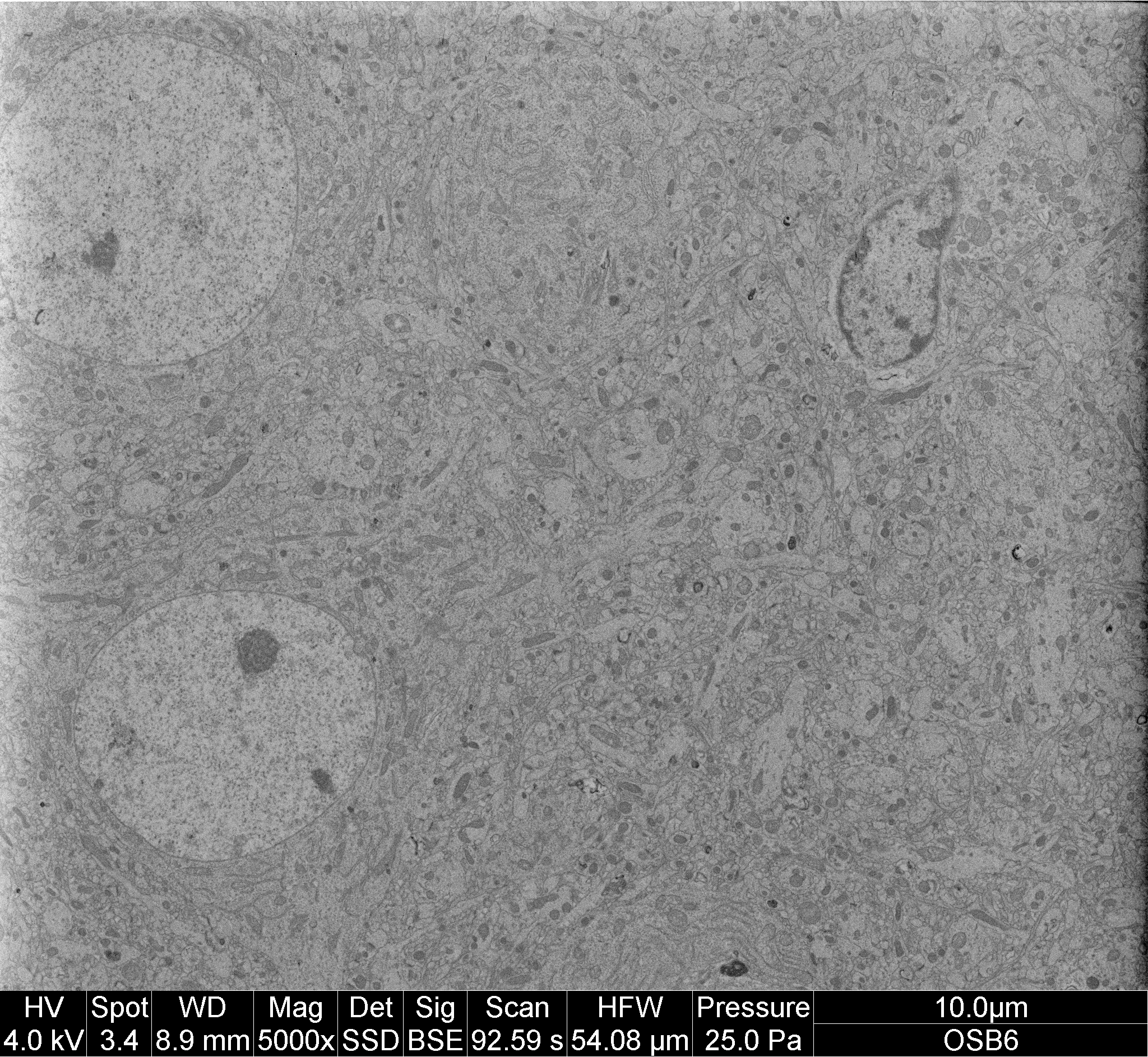

Supplement: Dataset S16 — (251.4 MB ZIP). [file pbio.0020329.sd016.zip › 040604_OS5_st1_1520.tif]

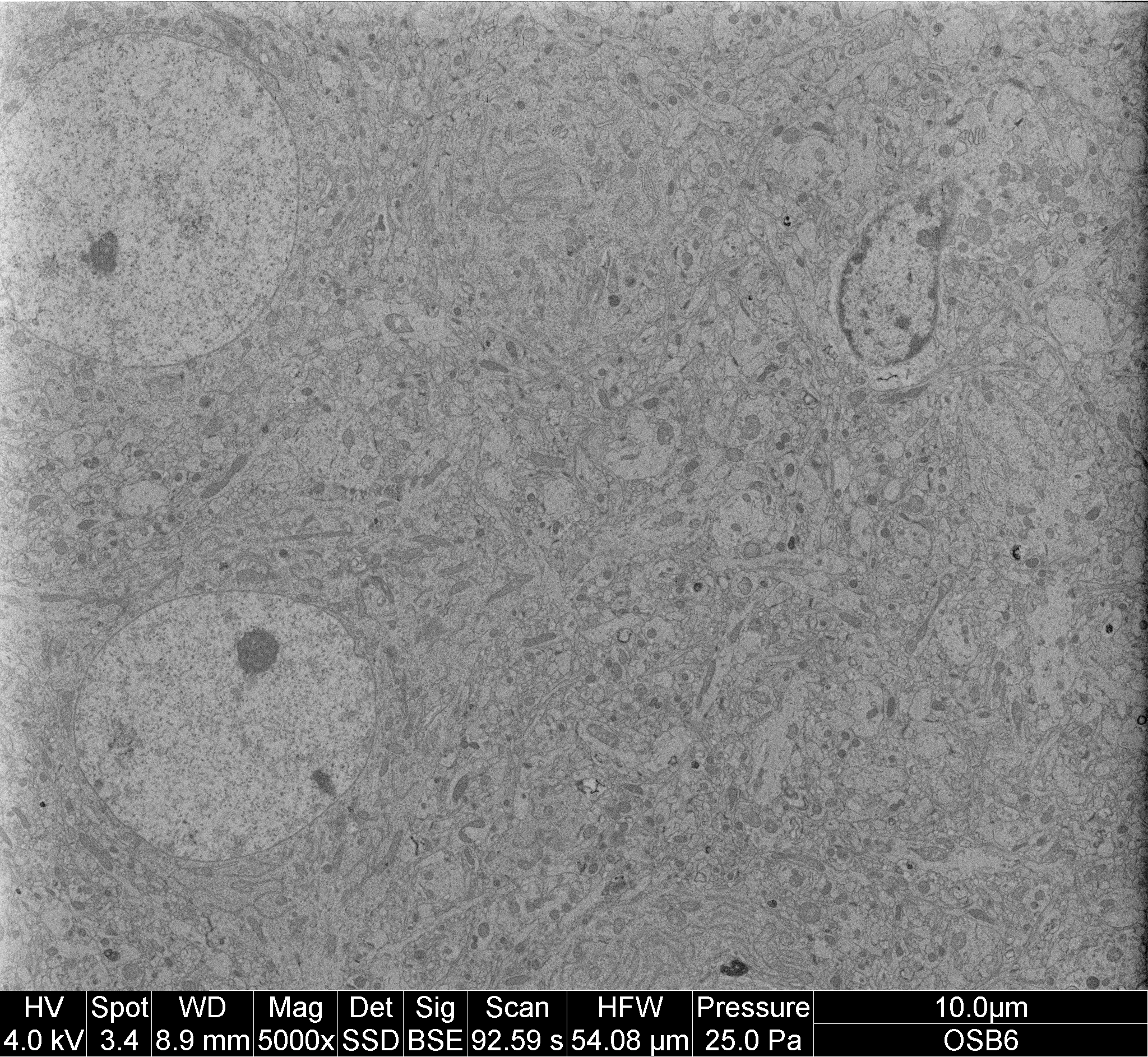

Supplement: Dataset S16 — (251.4 MB ZIP). [file pbio.0020329.sd016.zip › 040604_OS5_st1_1521.tif]

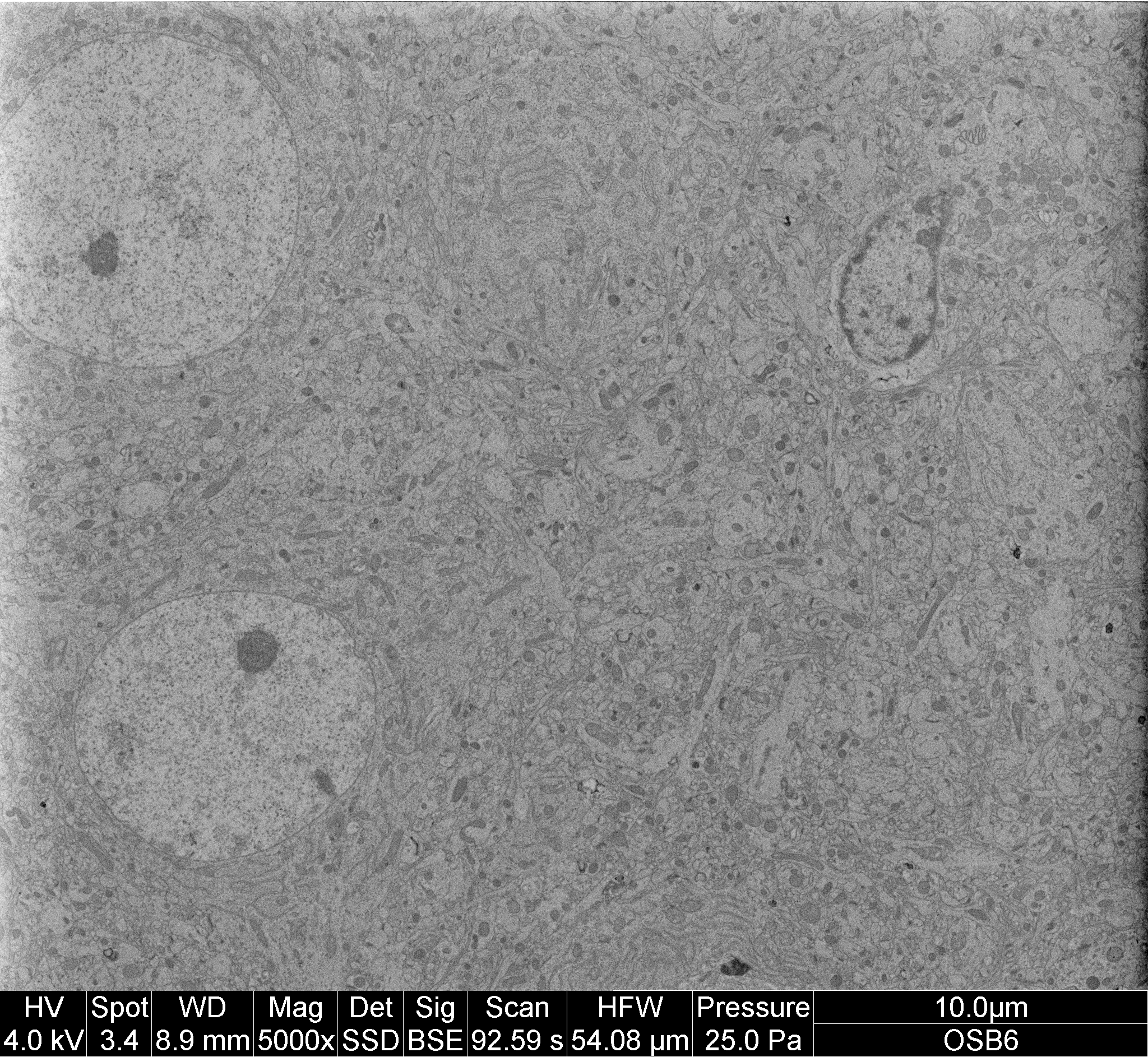

Supplement: Dataset S16 — (251.4 MB ZIP). [file pbio.0020329.sd016.zip › 040604_OS5_st1_1522.tif]

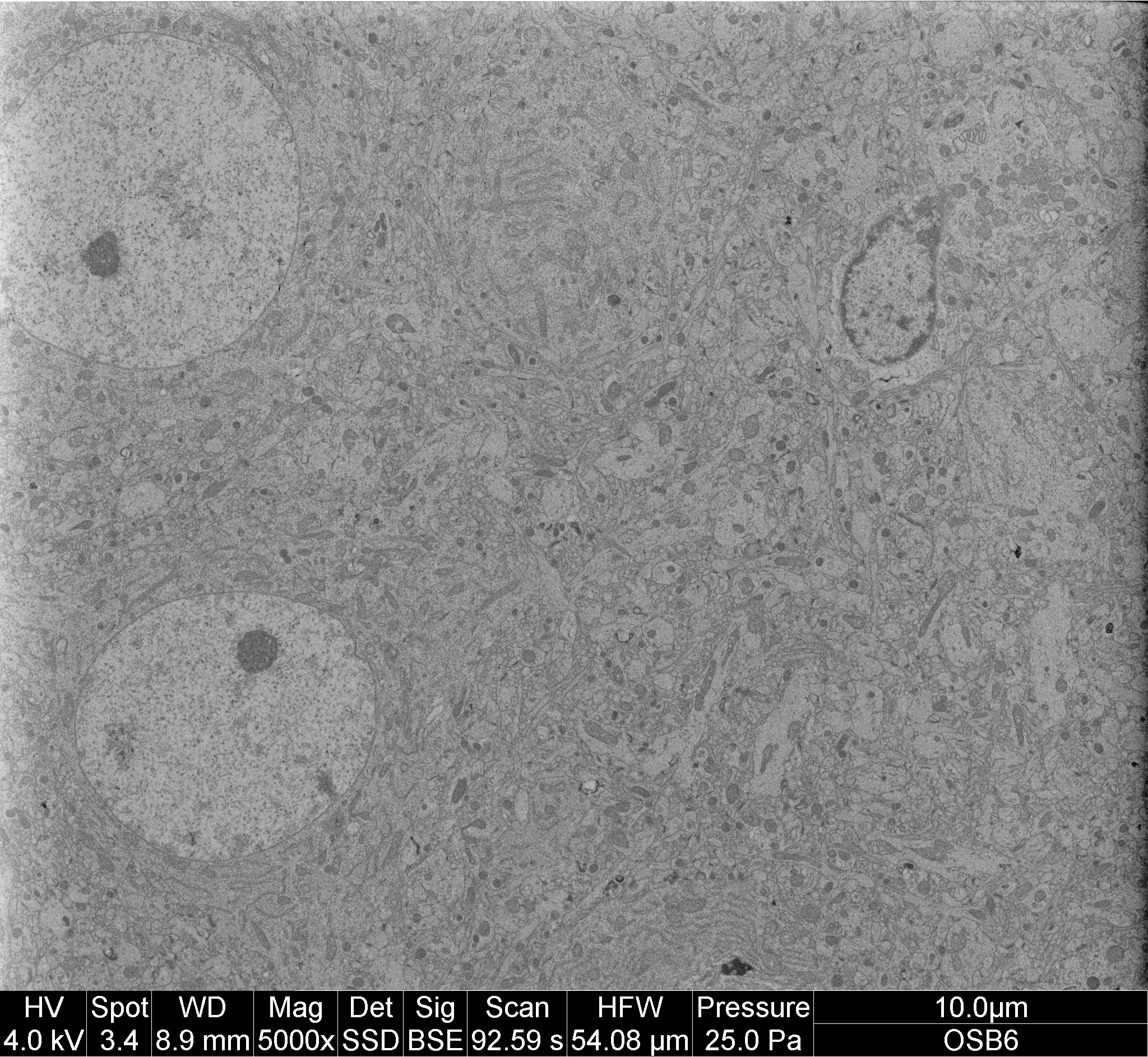

Supplement: Dataset S16 — (251.4 MB ZIP). [file pbio.0020329.sd016.zip › 040604_OS5_st1_1523.tif]

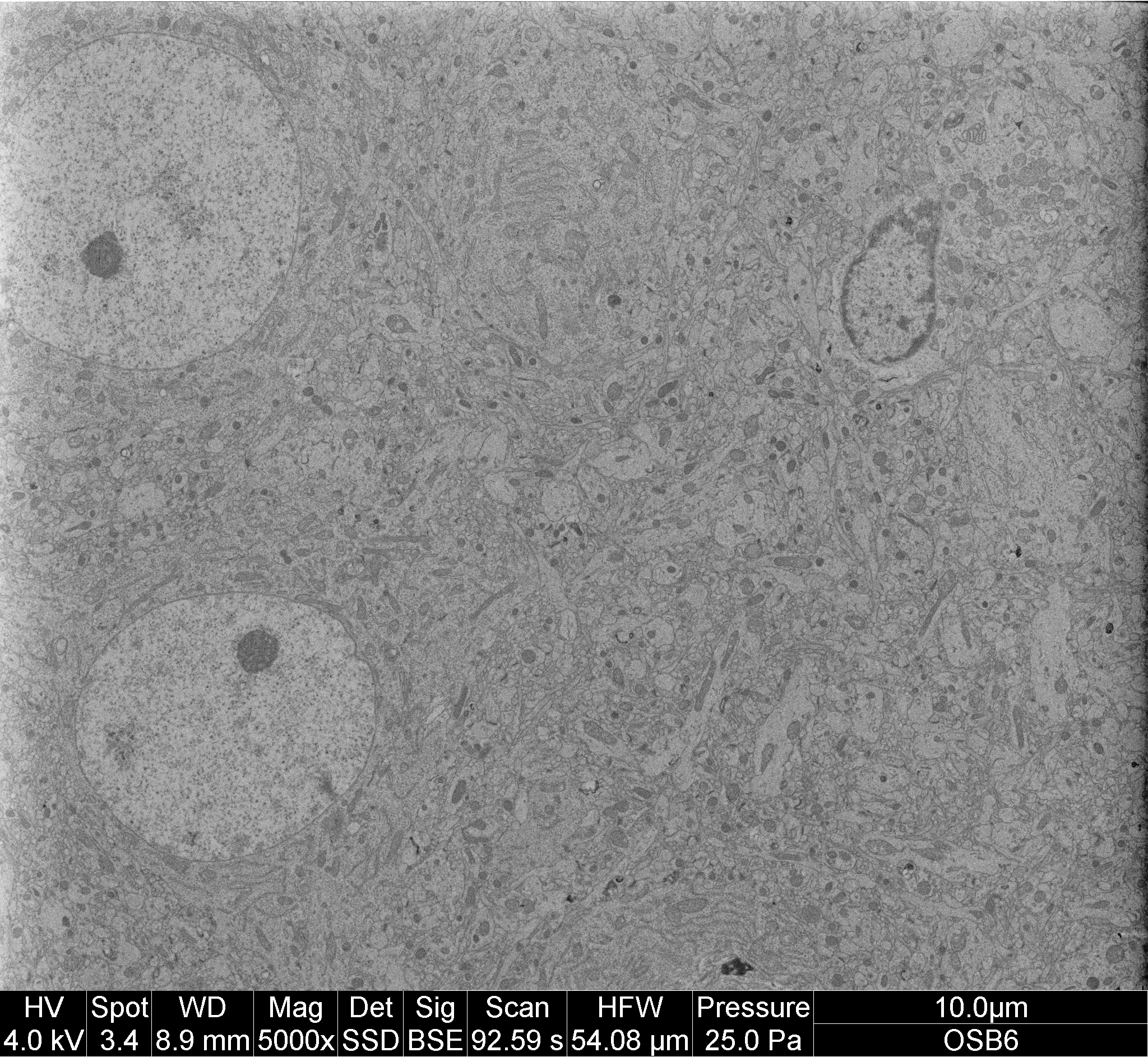

Supplement: Dataset S16 — (251.4 MB ZIP). [file pbio.0020329.sd016.zip › 040604_OS5_st1_1524.tif]

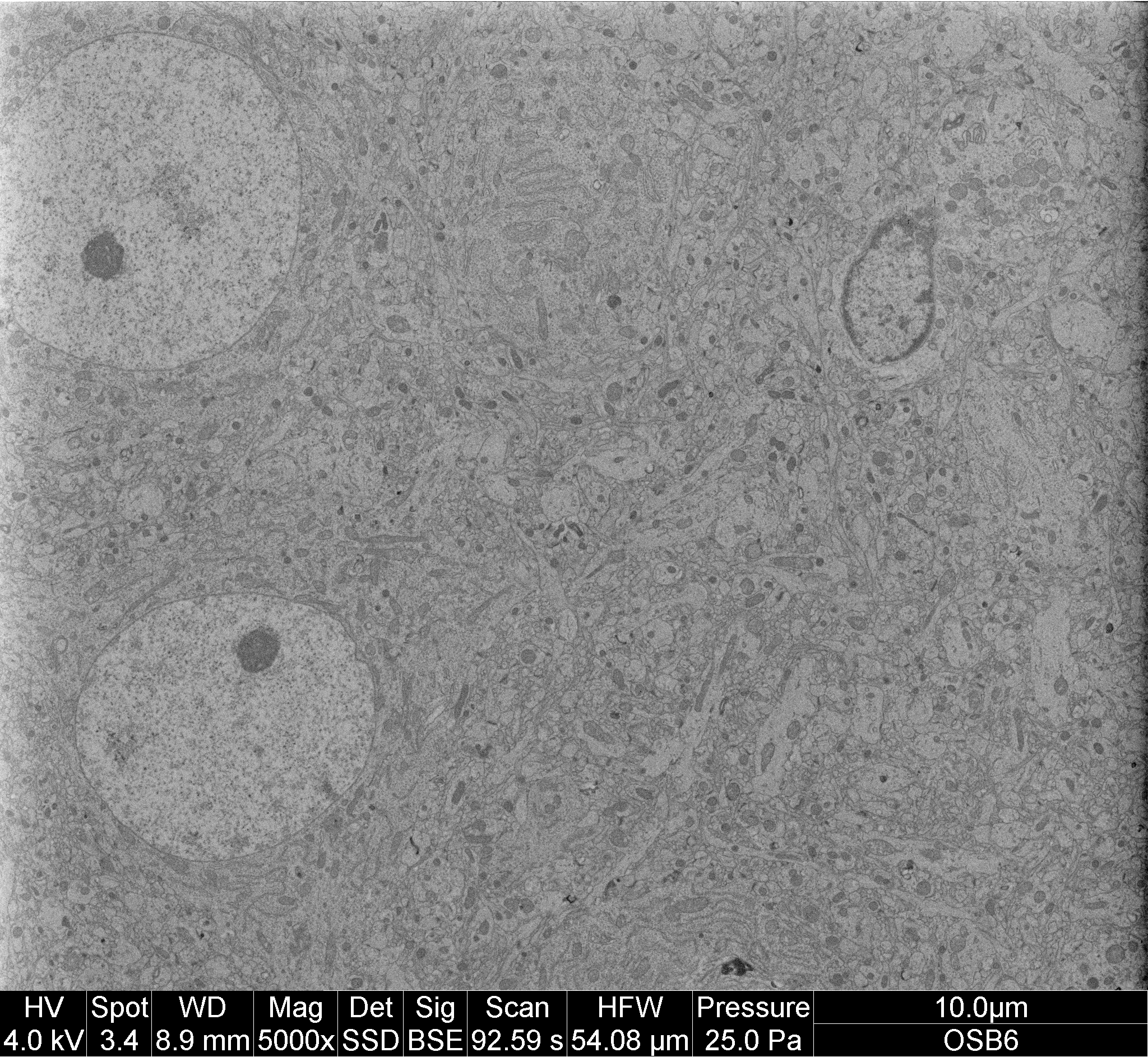

Supplement: Dataset S16 — (251.4 MB ZIP). [file pbio.0020329.sd016.zip › 040604_OS5_st1_1525.tif]

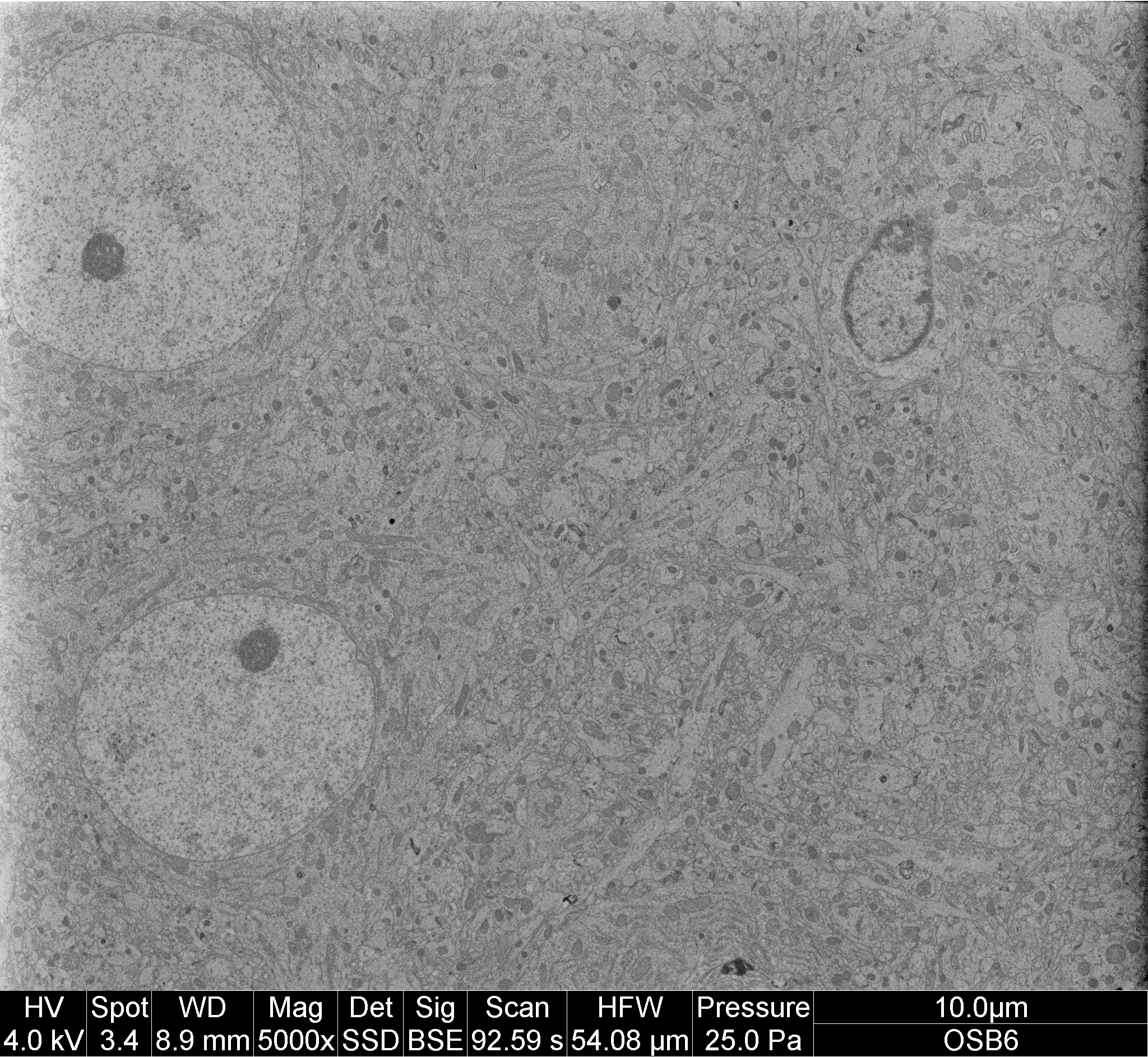

Supplement: Dataset S16 — (251.4 MB ZIP). [file pbio.0020329.sd016.zip › 040604_OS5_st1_1526.tif]

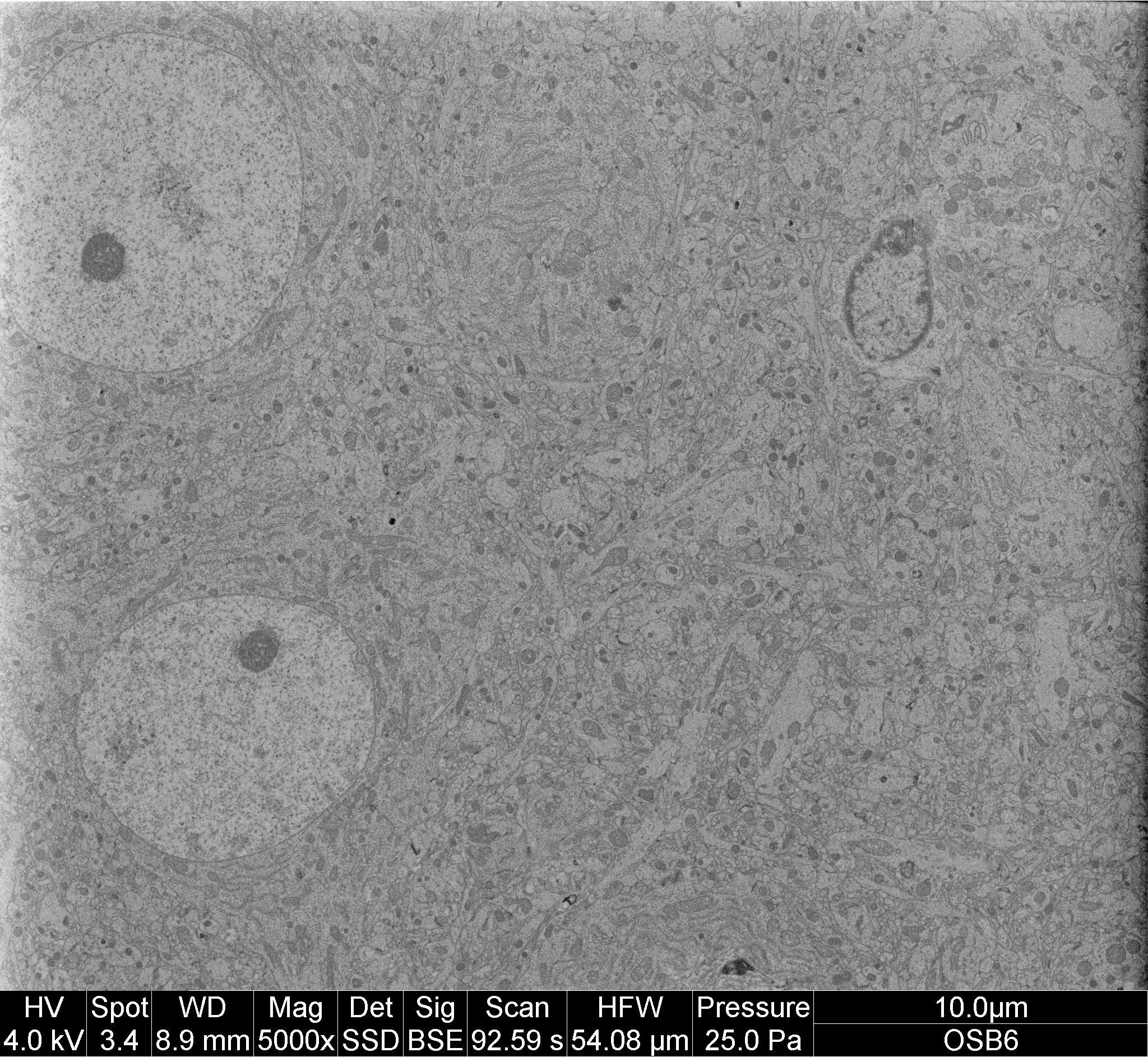

Supplement: Dataset S16 — (251.4 MB ZIP). [file pbio.0020329.sd016.zip › 040604_OS5_st1_1527.tif]

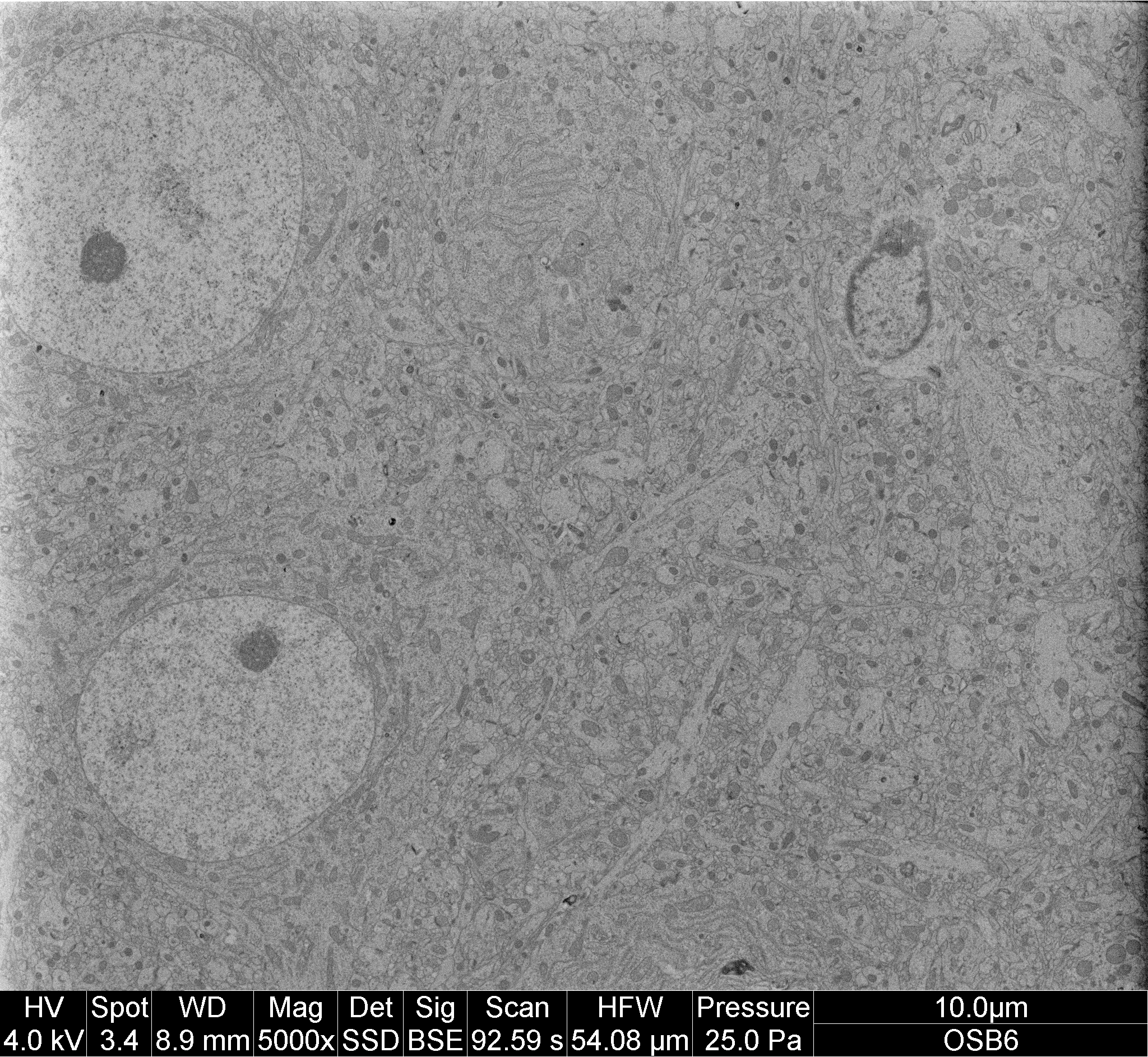

Supplement: Dataset S16 — (251.4 MB ZIP). [file pbio.0020329.sd016.zip › 040604_OS5_st1_1528.tif]

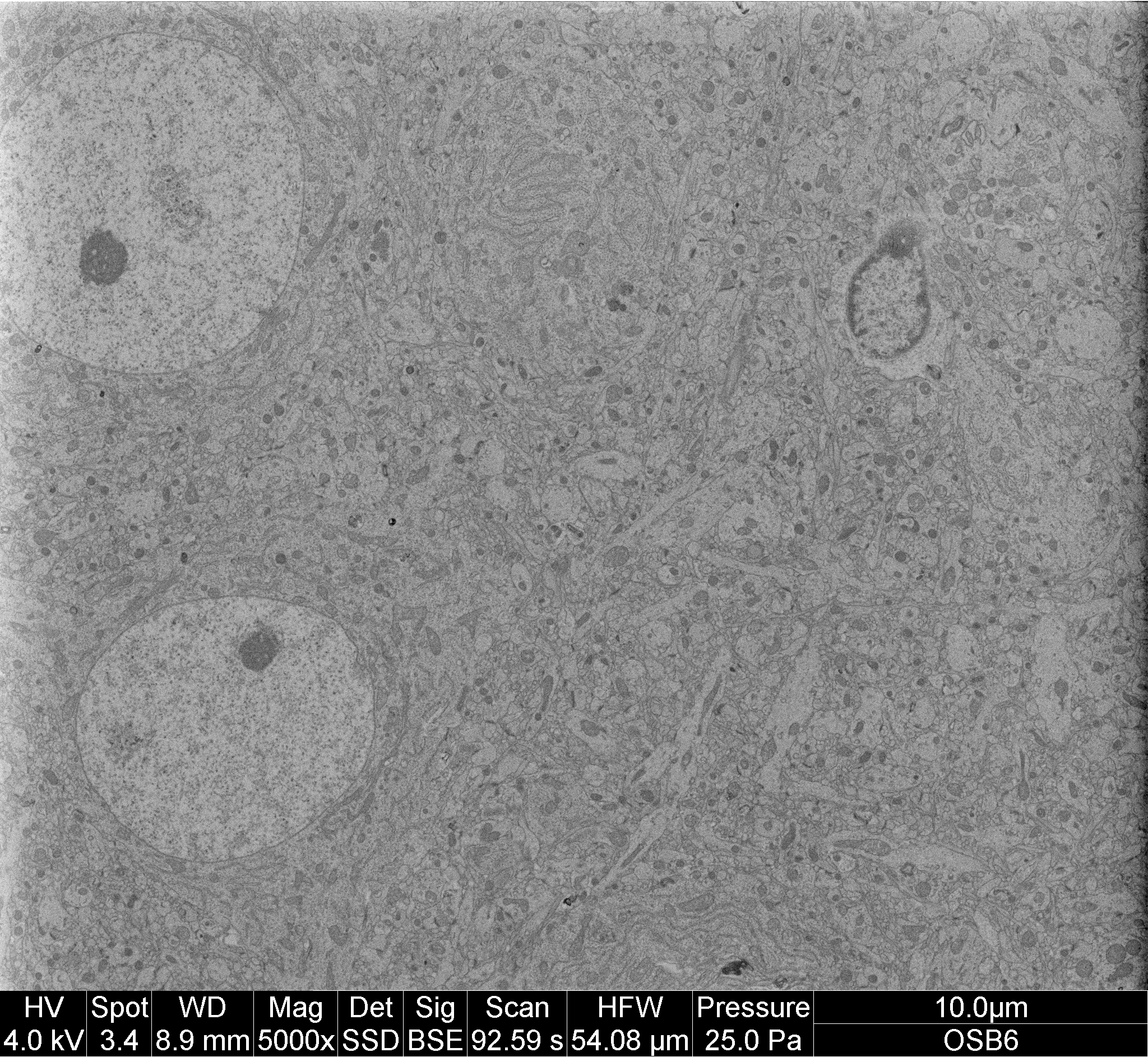

Supplement: Dataset S16 — (251.4 MB ZIP). [file pbio.0020329.sd016.zip › 040604_OS5_st1_1529.tif]

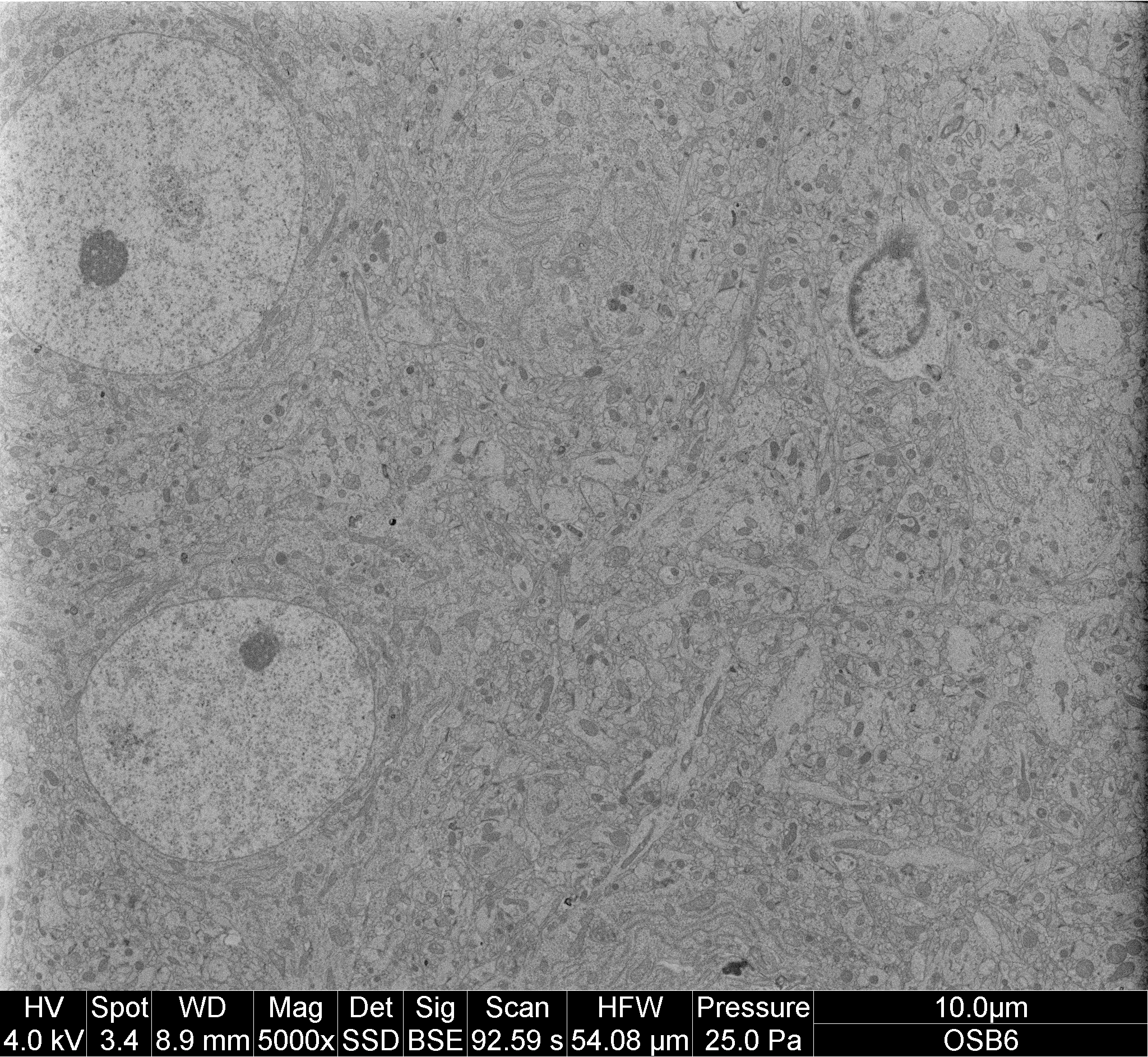

Supplement: Dataset S16 — (251.4 MB ZIP). [file pbio.0020329.sd016.zip › 040604_OS5_st1_1530.tif]

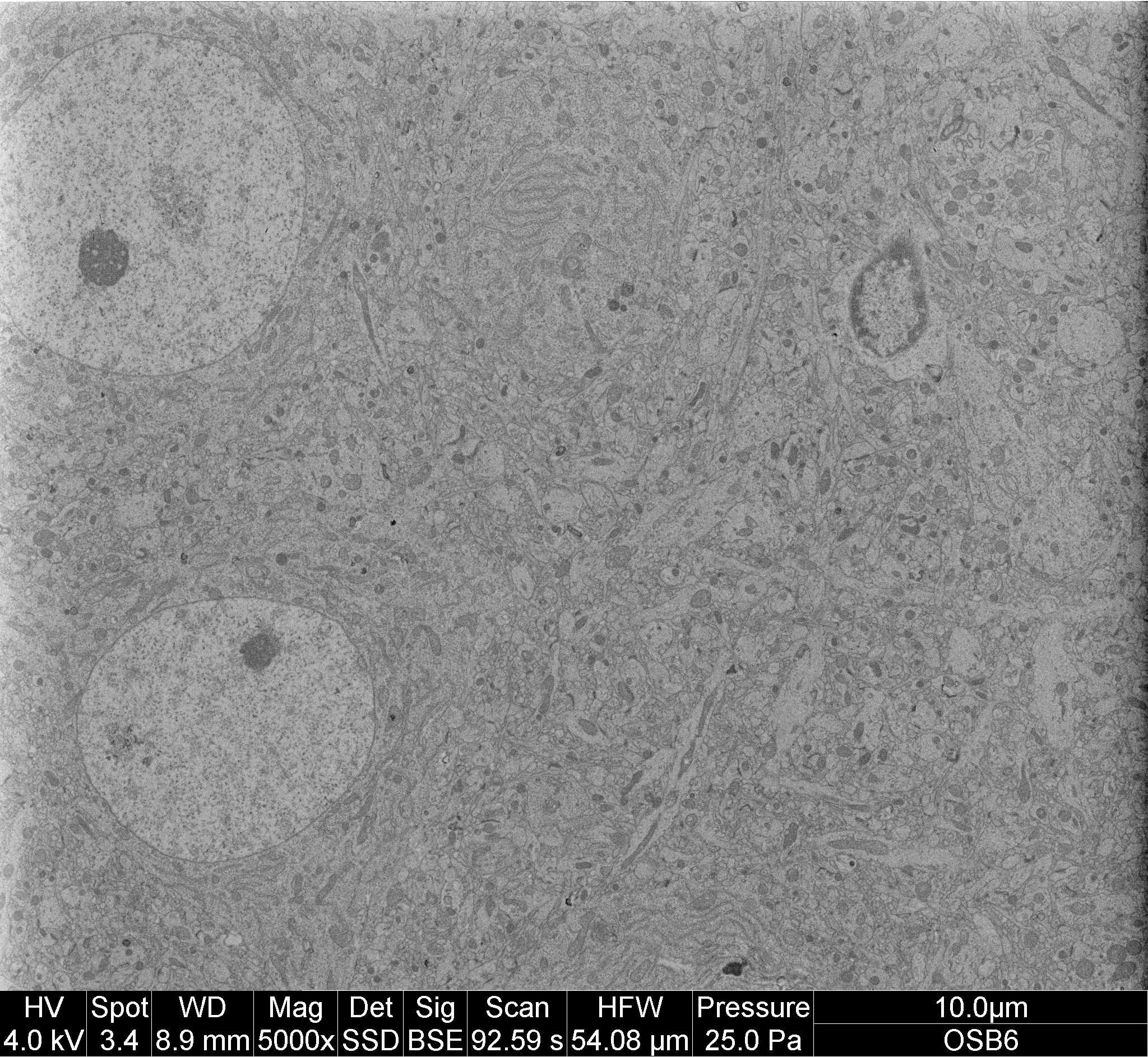

Supplement: Dataset S16 — (251.4 MB ZIP). [file pbio.0020329.sd016.zip › 040604_OS5_st1_1531.tif]

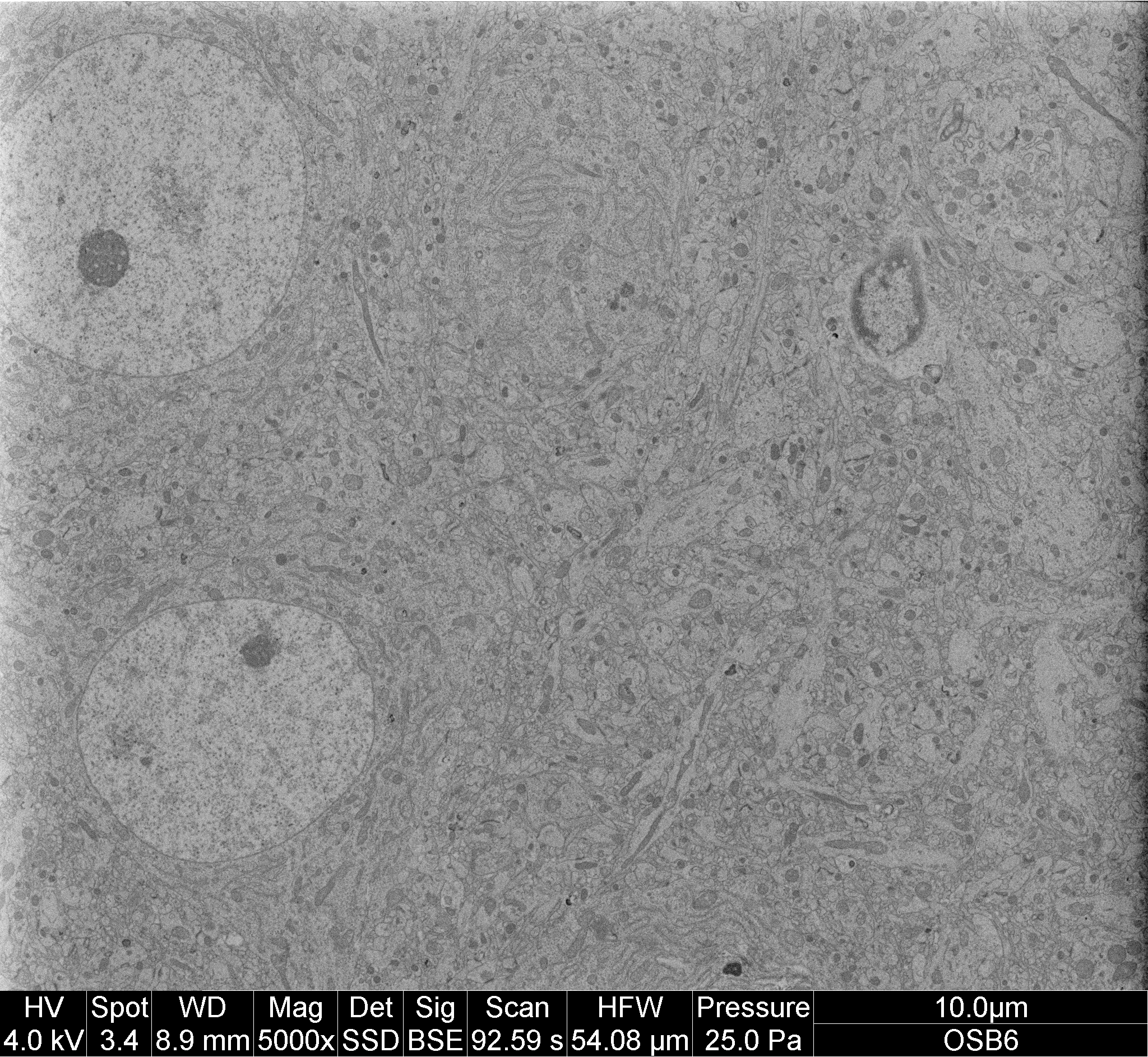

Supplement: Dataset S16 — (251.4 MB ZIP). [file pbio.0020329.sd016.zip › 040604_OS5_st1_1532.tif]

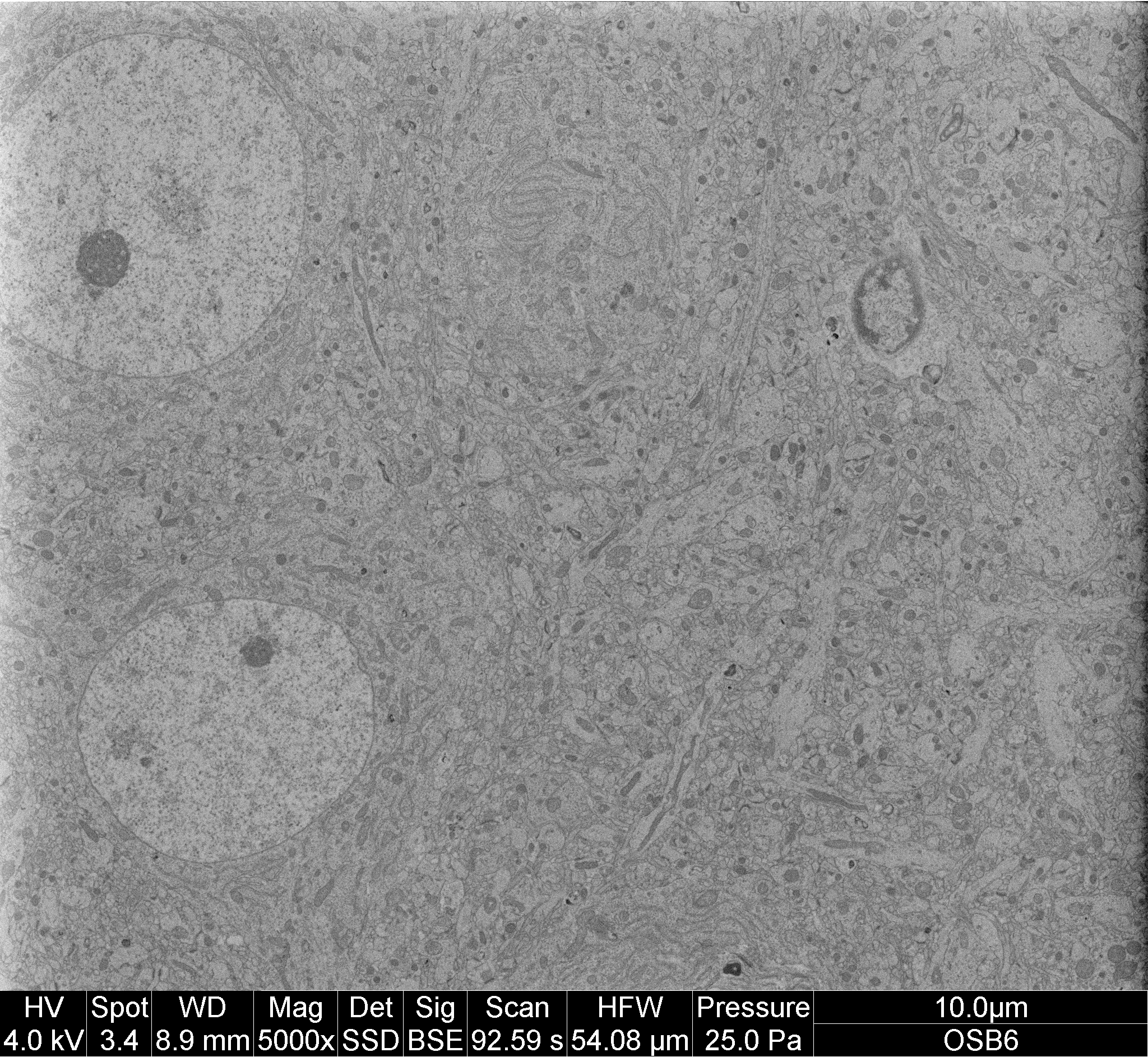

Supplement: Dataset S16 — (251.4 MB ZIP). [file pbio.0020329.sd016.zip › 040604_OS5_st1_1533.tif]

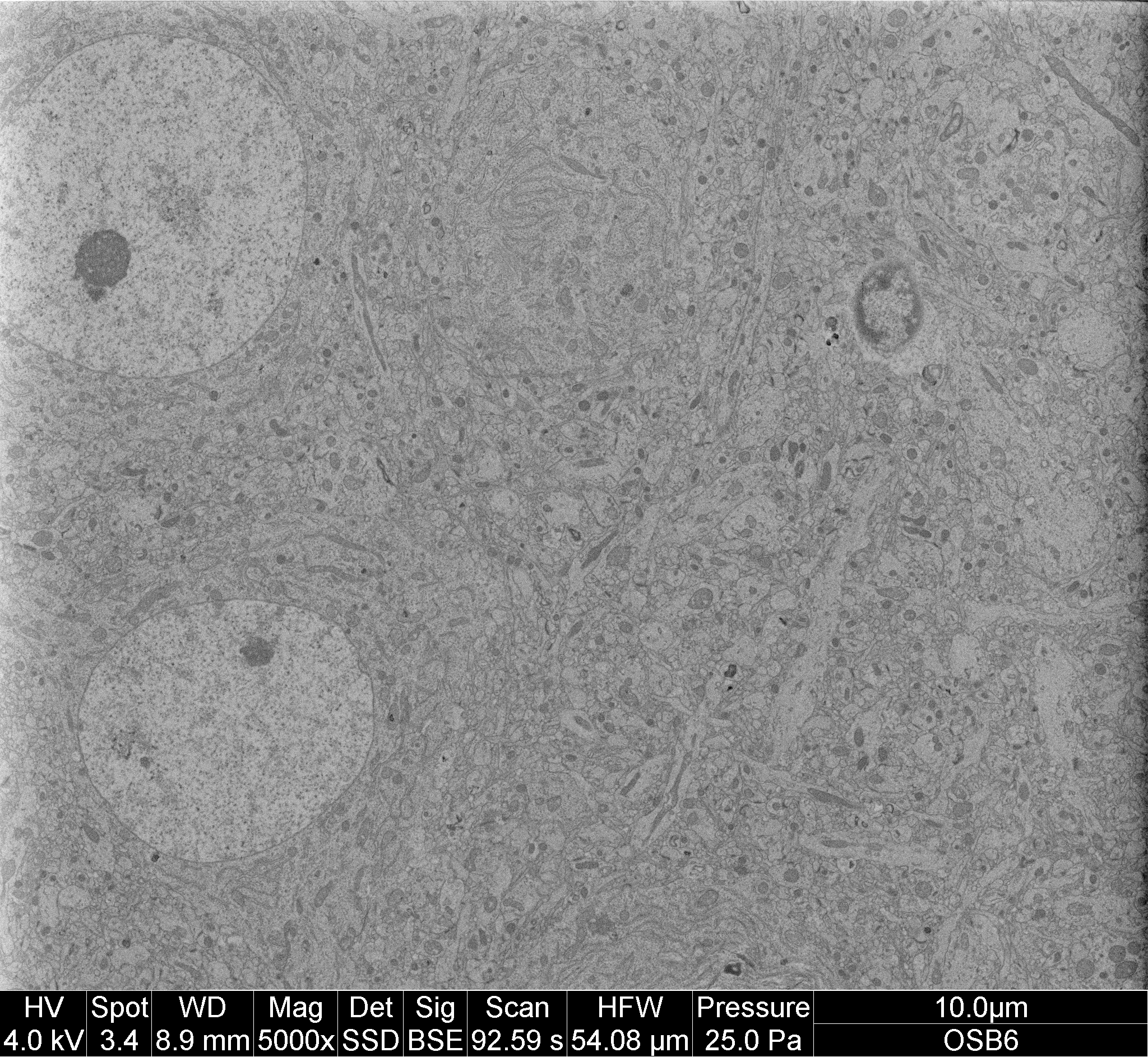

Supplement: Dataset S16 — (251.4 MB ZIP). [file pbio.0020329.sd016.zip › 040604_OS5_st1_1534.tif]

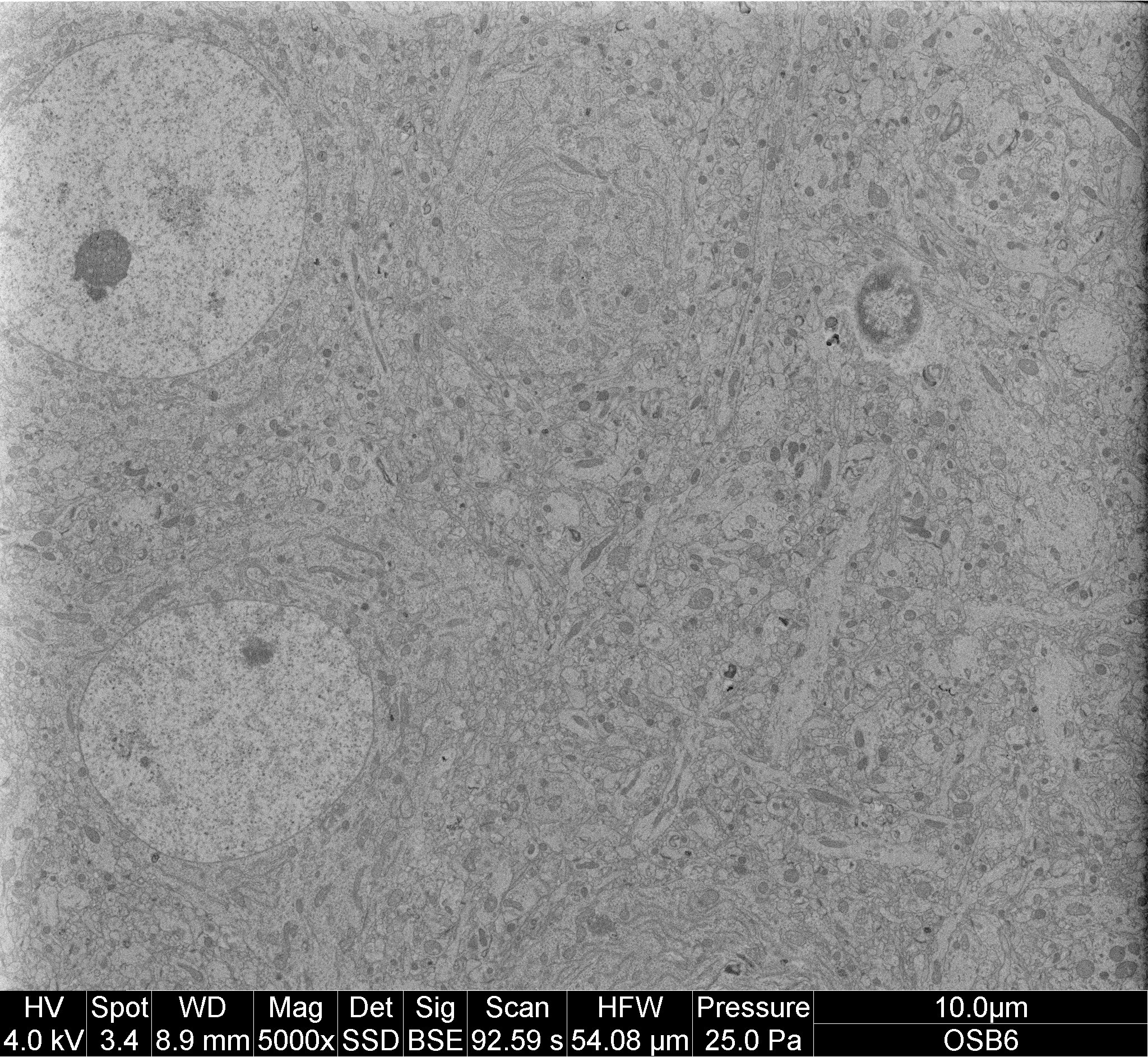

Supplement: Dataset S16 — (251.4 MB ZIP). [file pbio.0020329.sd016.zip › 040604_OS5_st1_1535.tif]

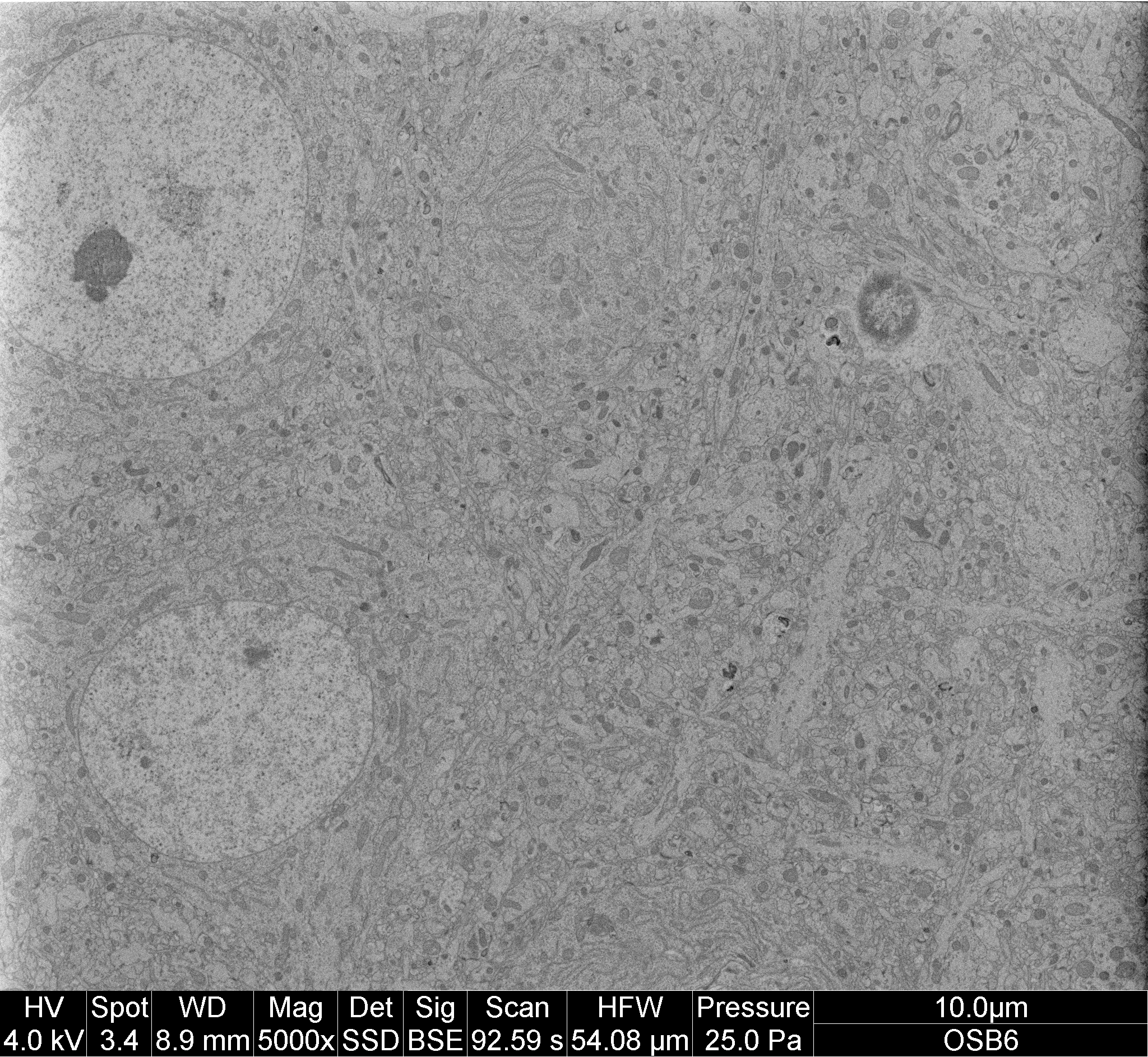

Supplement: Dataset S16 — (251.4 MB ZIP). [file pbio.0020329.sd016.zip › 040604_OS5_st1_1536.tif]

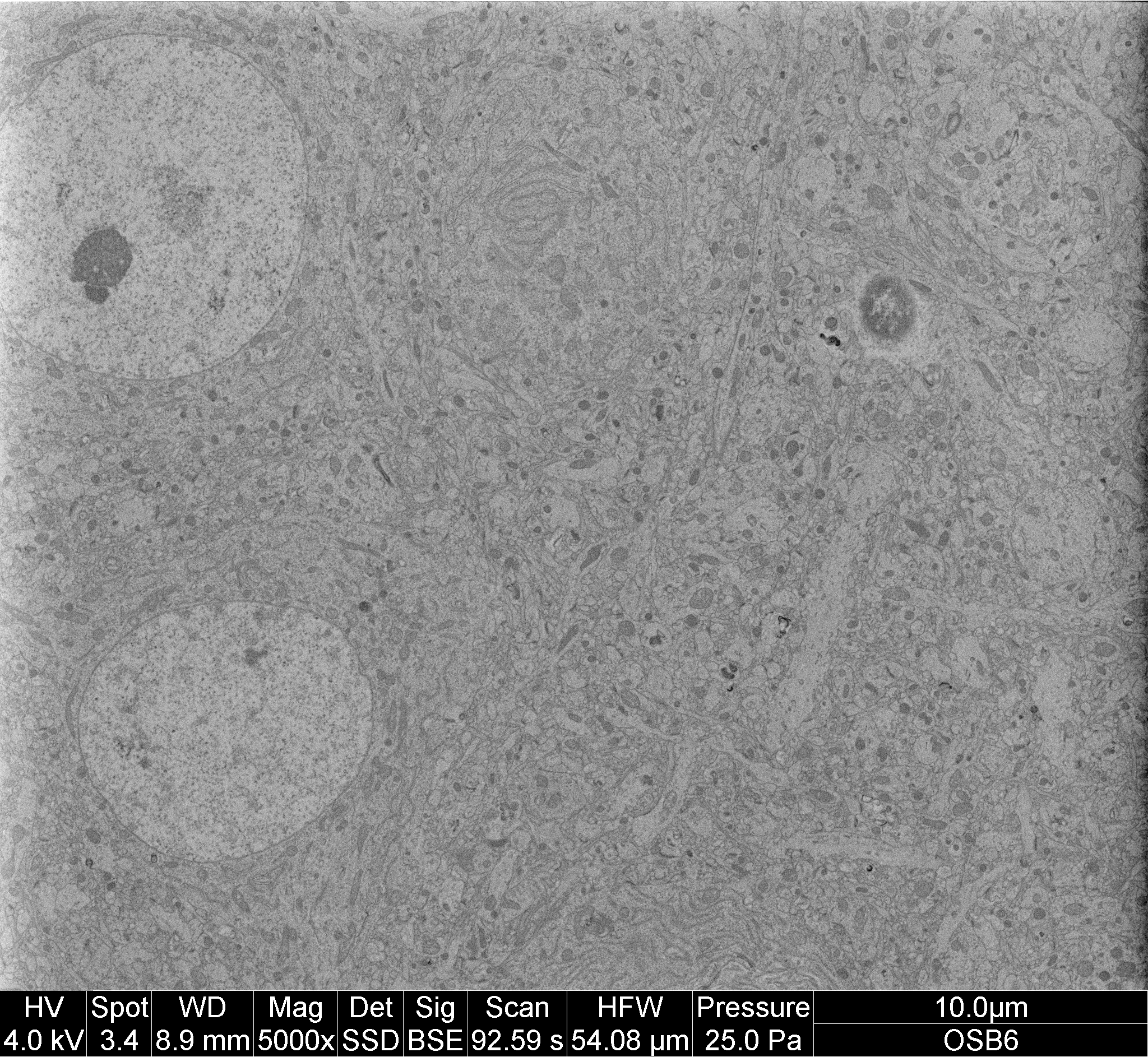

Supplement: Dataset S16 — (251.4 MB ZIP). [file pbio.0020329.sd016.zip › 040604_OS5_st1_1537.tif]

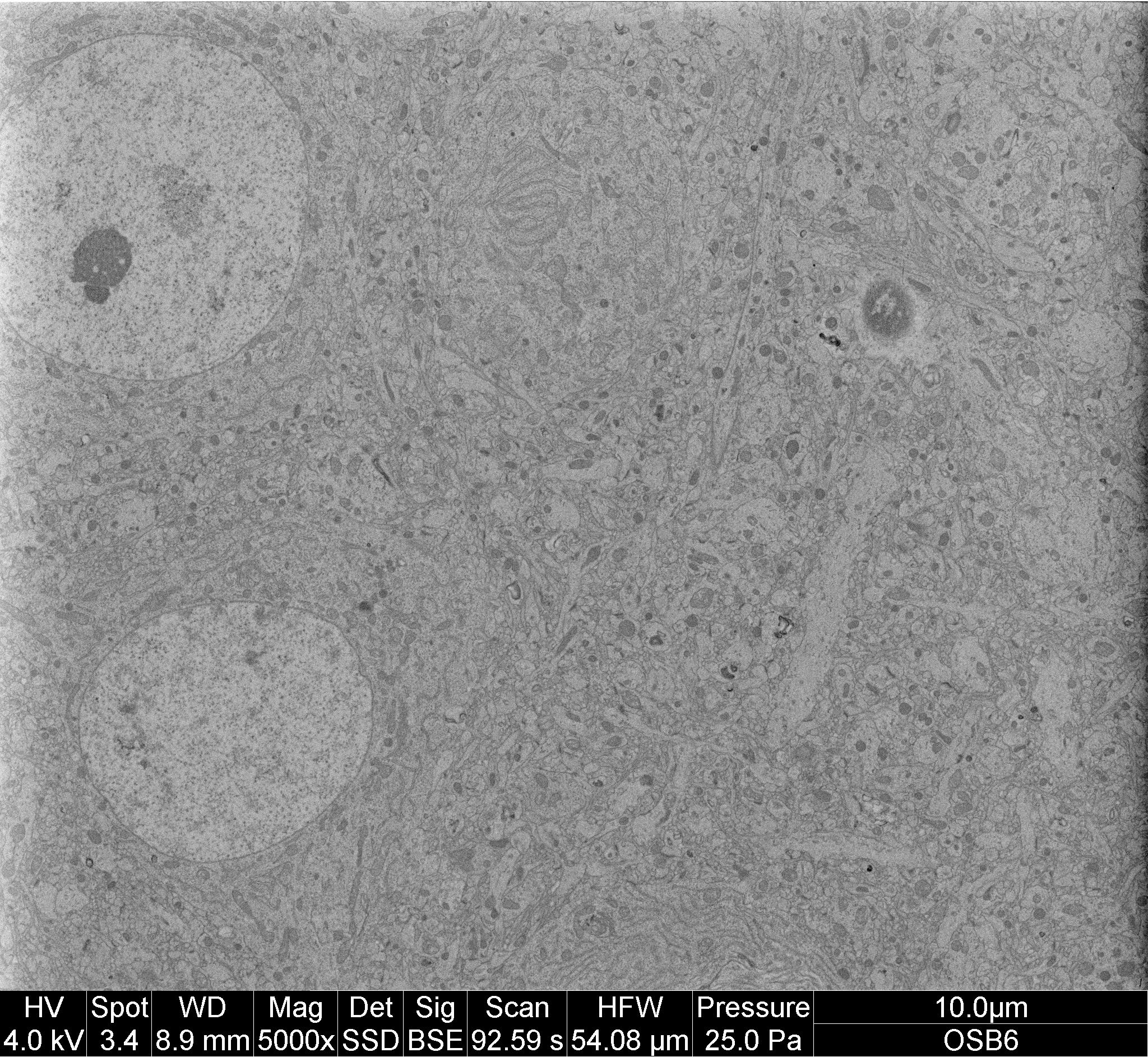

Supplement: Dataset S16 — (251.4 MB ZIP). [file pbio.0020329.sd016.zip › 040604_OS5_st1_1538.tif]

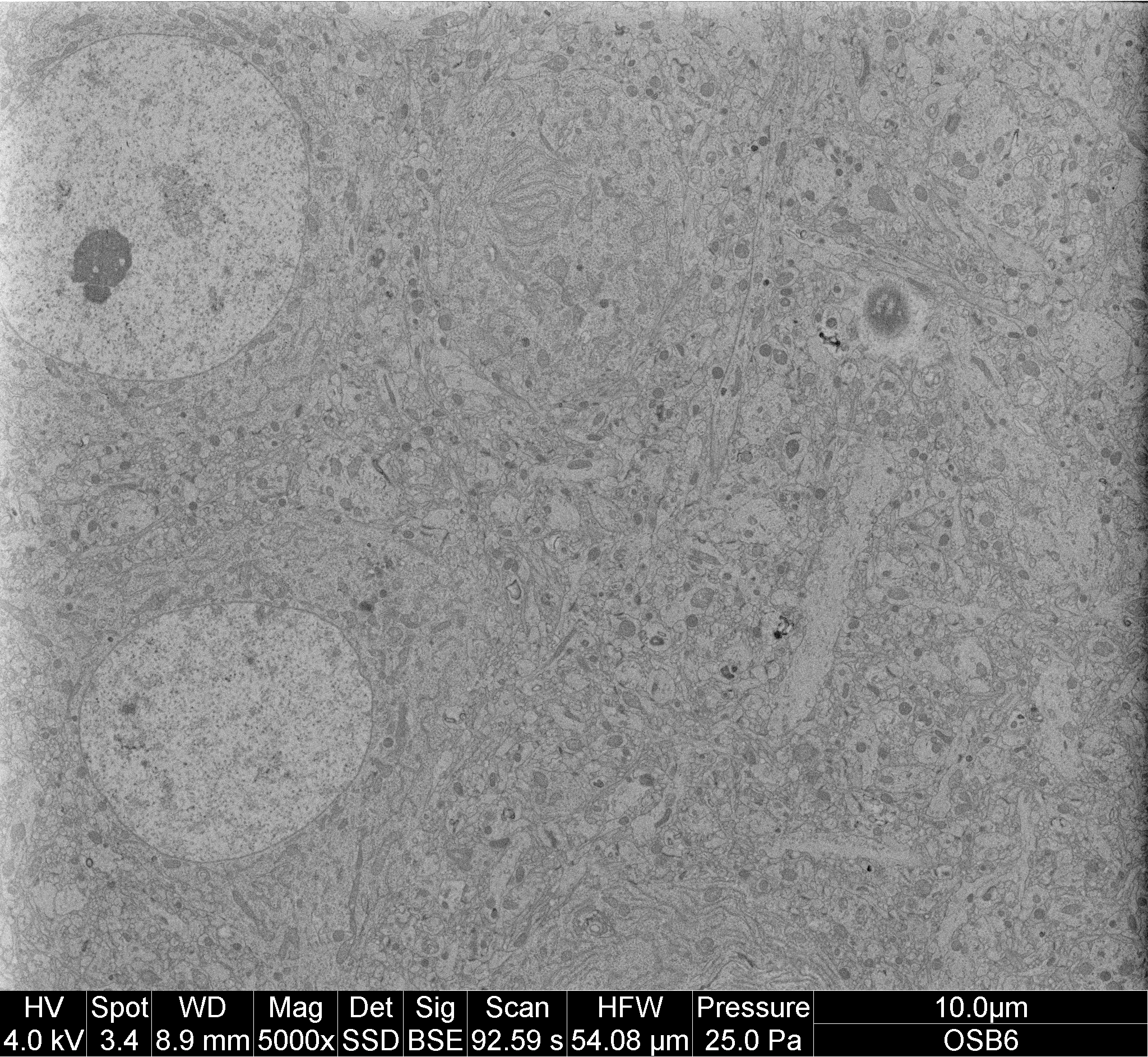

Supplement: Dataset S16 — (251.4 MB ZIP). [file pbio.0020329.sd016.zip › 040604_OS5_st1_1539.tif]

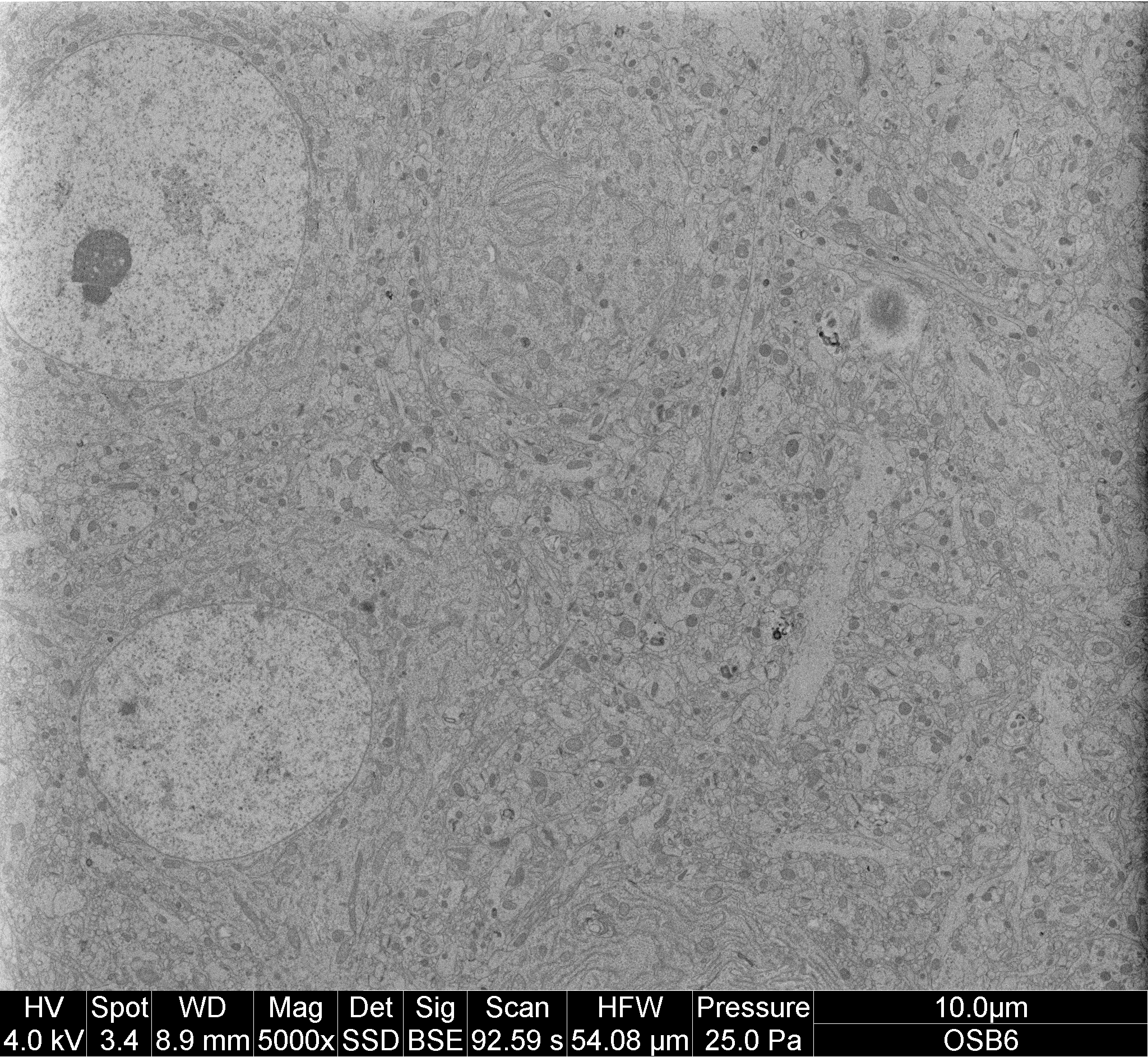

Supplement: Dataset S16 — (251.4 MB ZIP). [file pbio.0020329.sd016.zip › 040604_OS5_st1_1540.tif]

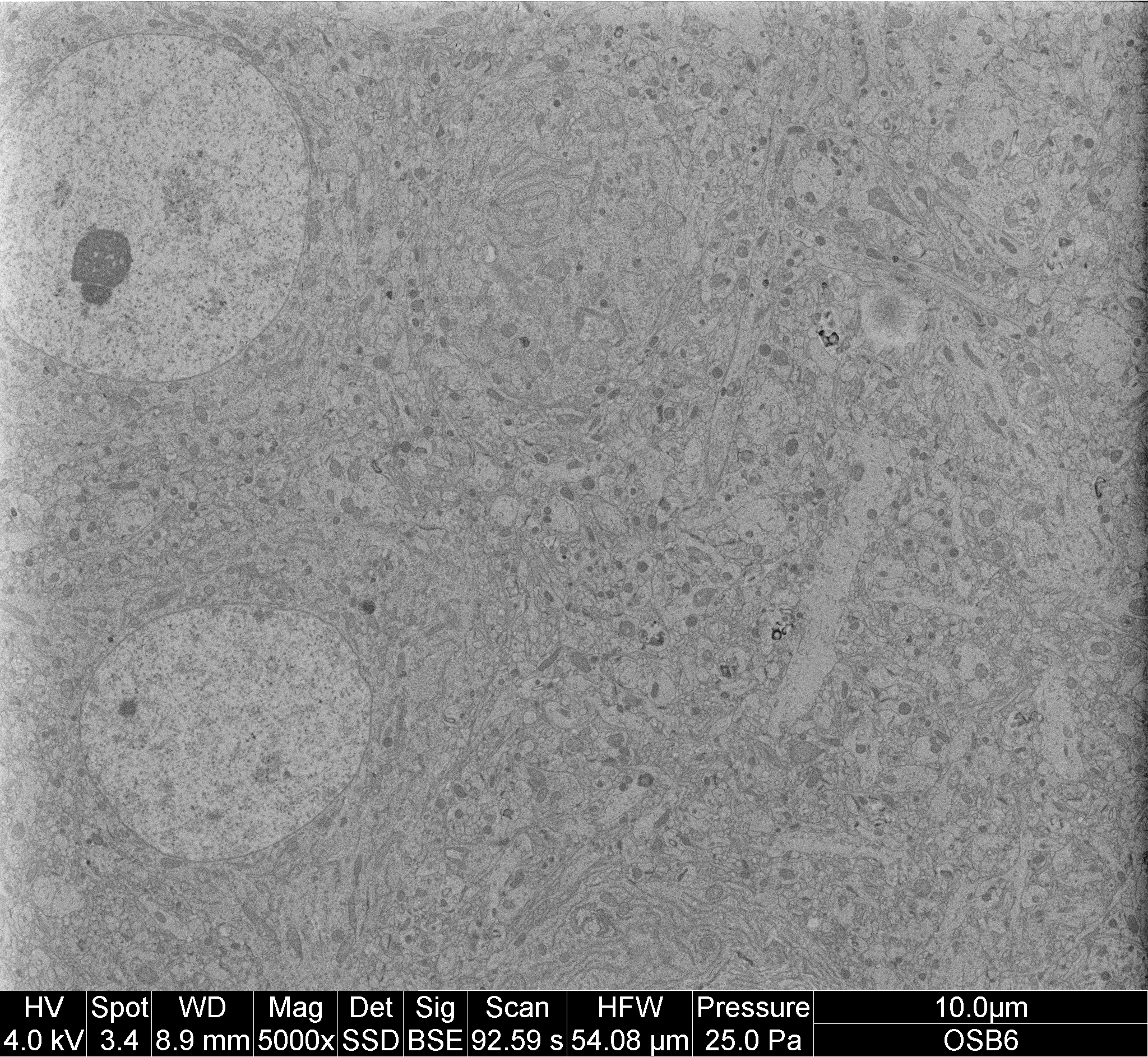

Supplement: Dataset S16 — (251.4 MB ZIP). [file pbio.0020329.sd016.zip › 040604_OS5_st1_1541.tif]

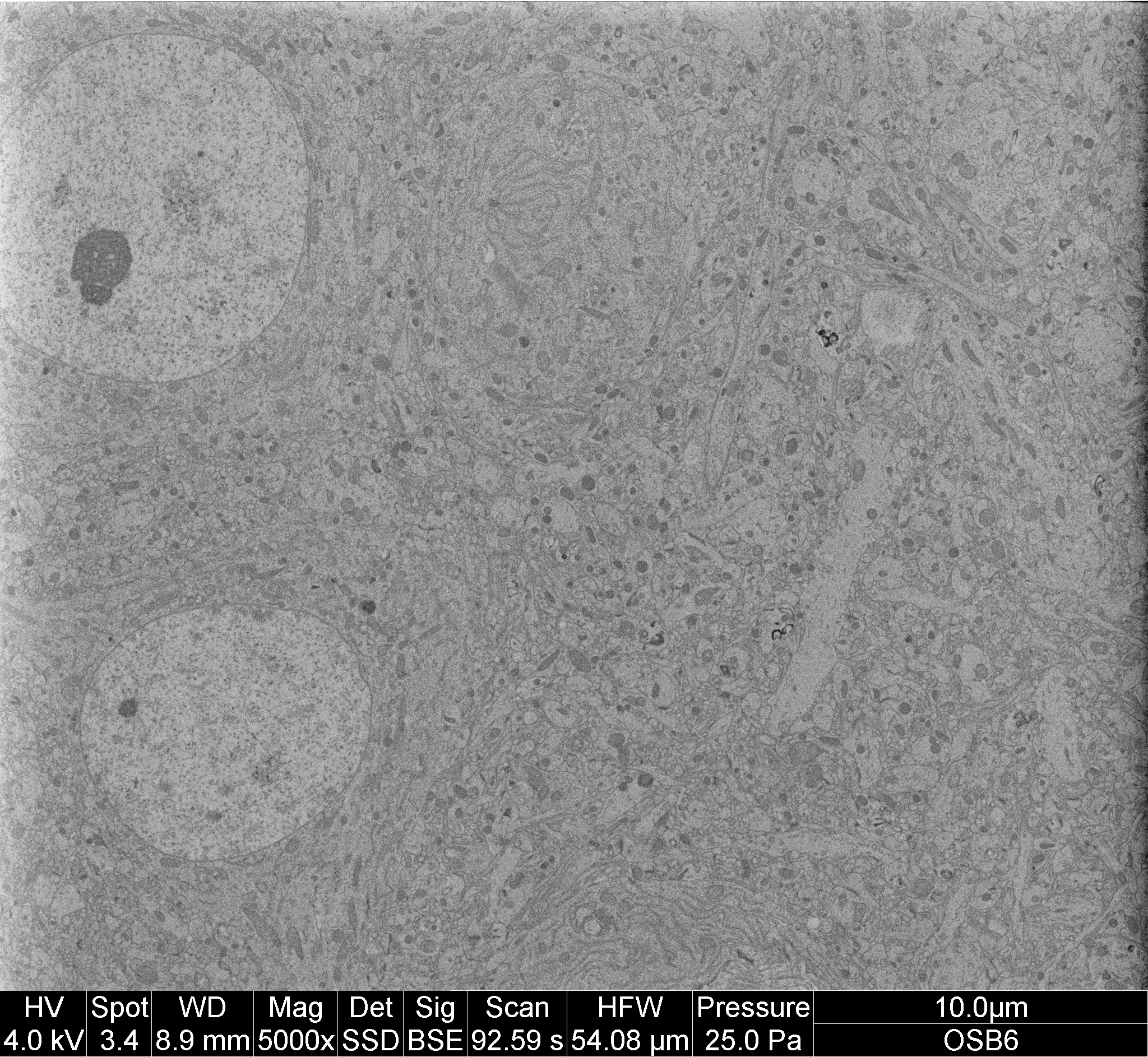

Supplement: Dataset S16 — (251.4 MB ZIP). [file pbio.0020329.sd016.zip › 040604_OS5_st1_1542.tif]

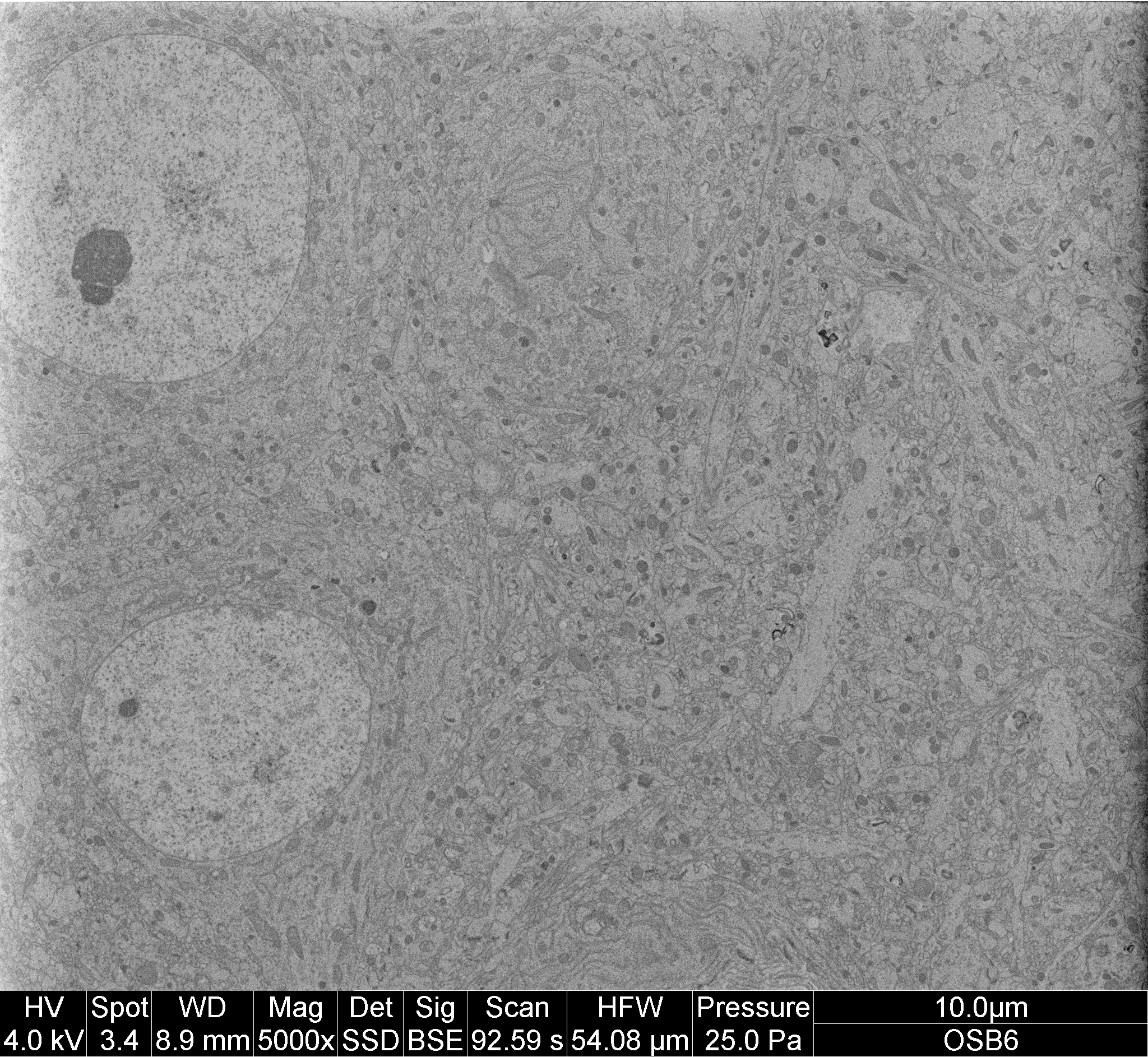

Supplement: Dataset S16 — (251.4 MB ZIP). [file pbio.0020329.sd016.zip › 040604_OS5_st1_1543.tif]

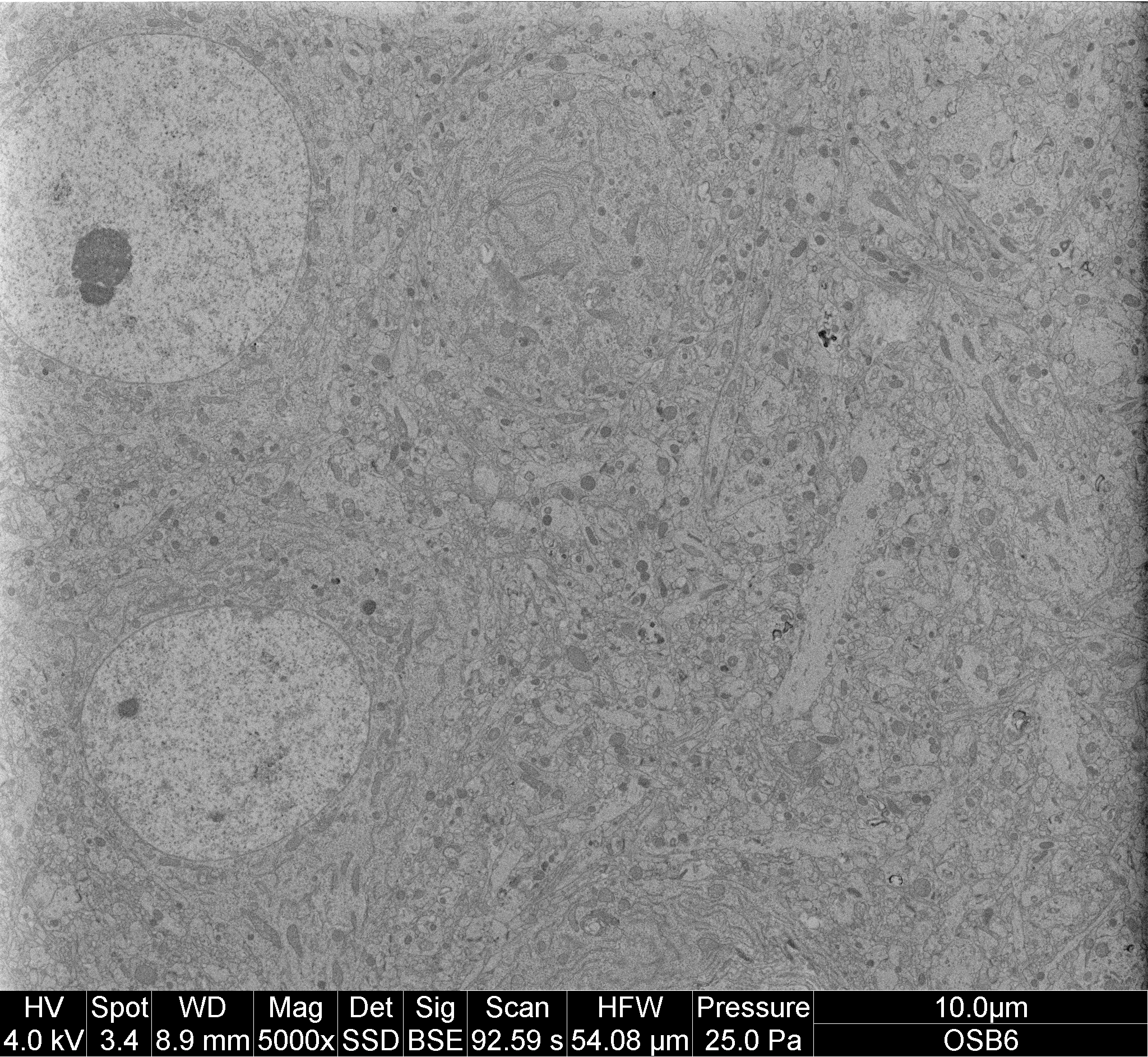

Supplement: Dataset S16 — (251.4 MB ZIP). [file pbio.0020329.sd016.zip › 040604_OS5_st1_1544.tif]

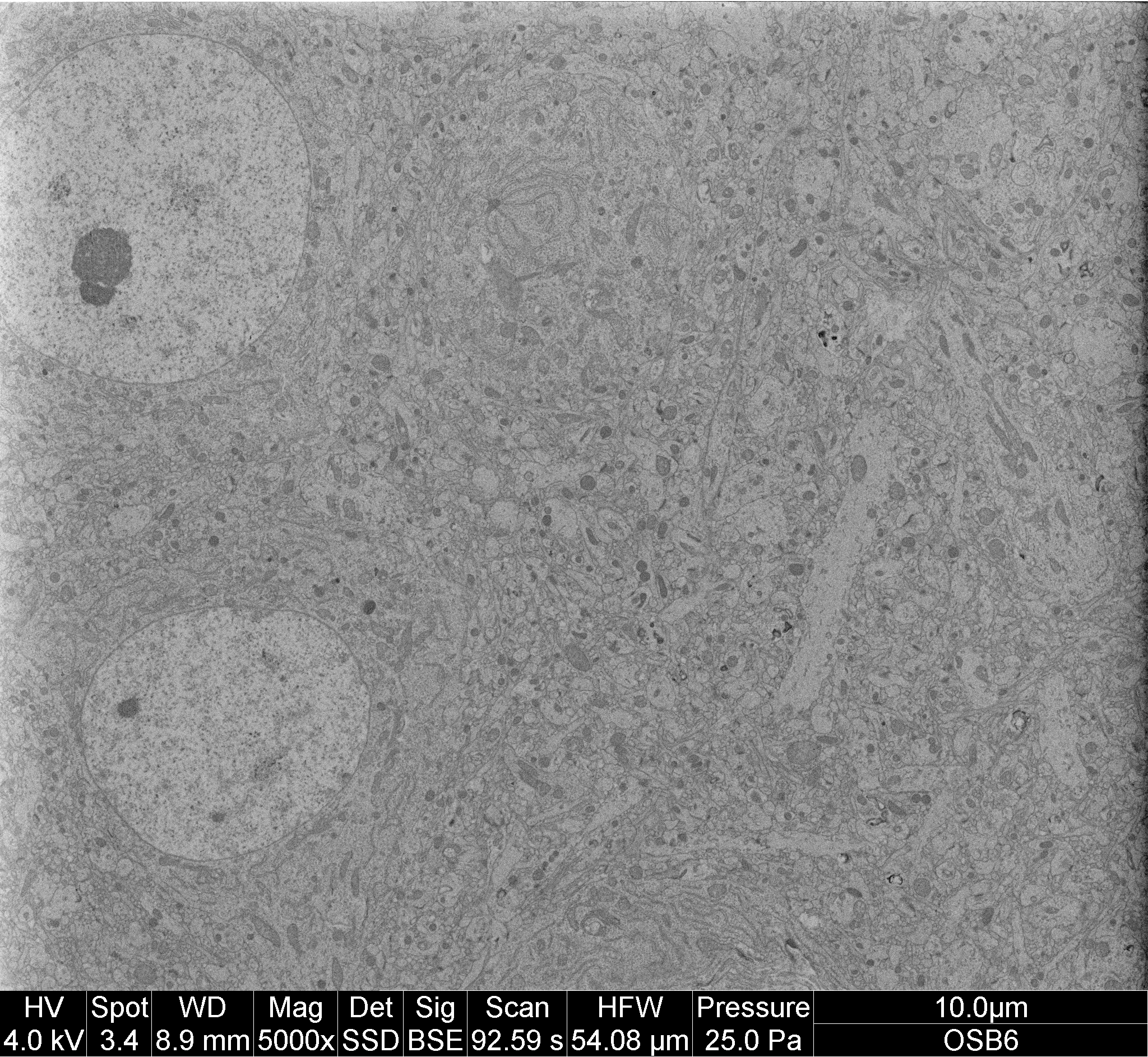

Supplement: Dataset S16 — (251.4 MB ZIP). [file pbio.0020329.sd016.zip › 040604_OS5_st1_1545.tif]

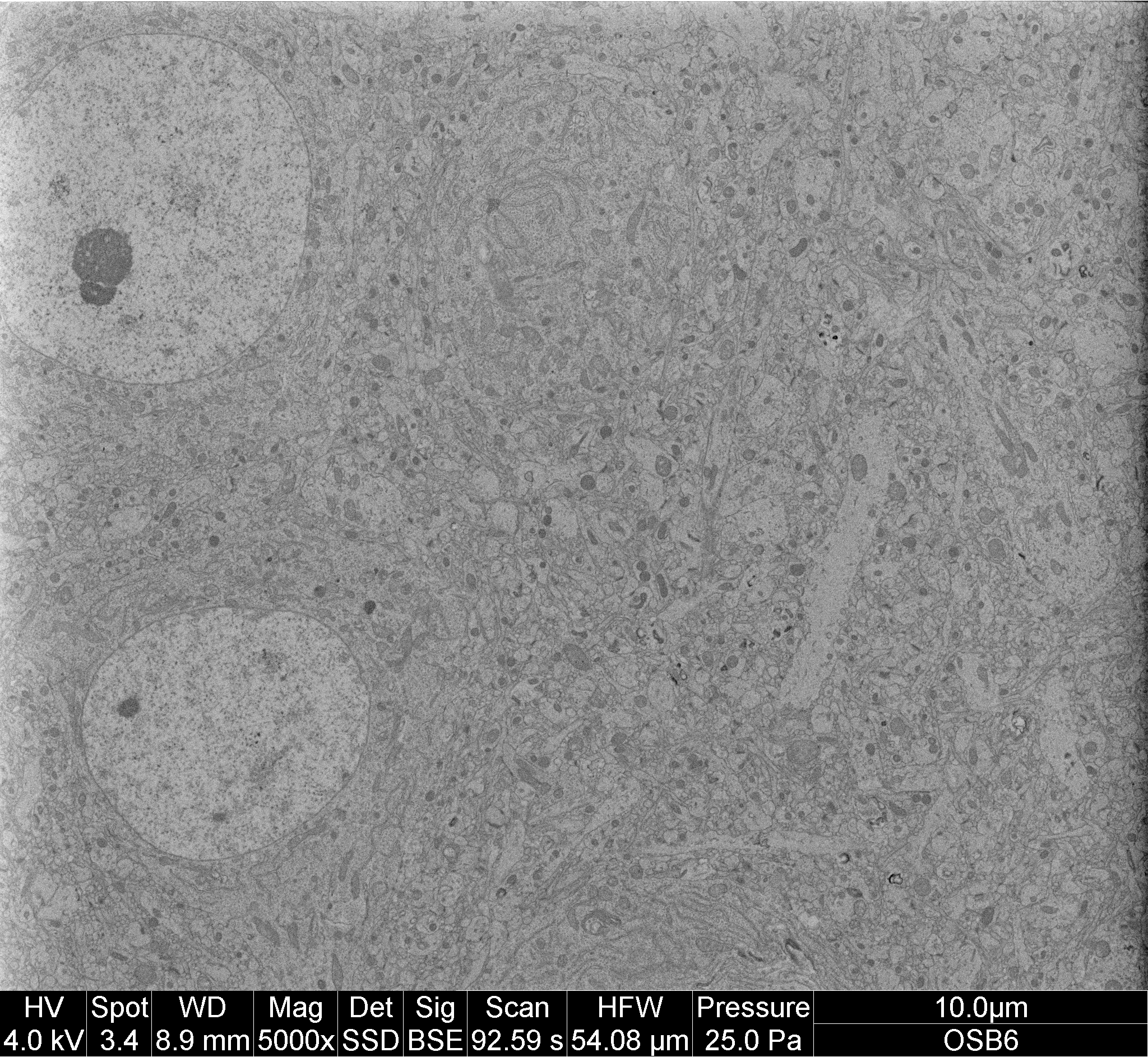

Supplement: Dataset S16 — (251.4 MB ZIP). [file pbio.0020329.sd016.zip › 040604_OS5_st1_1546.tif]

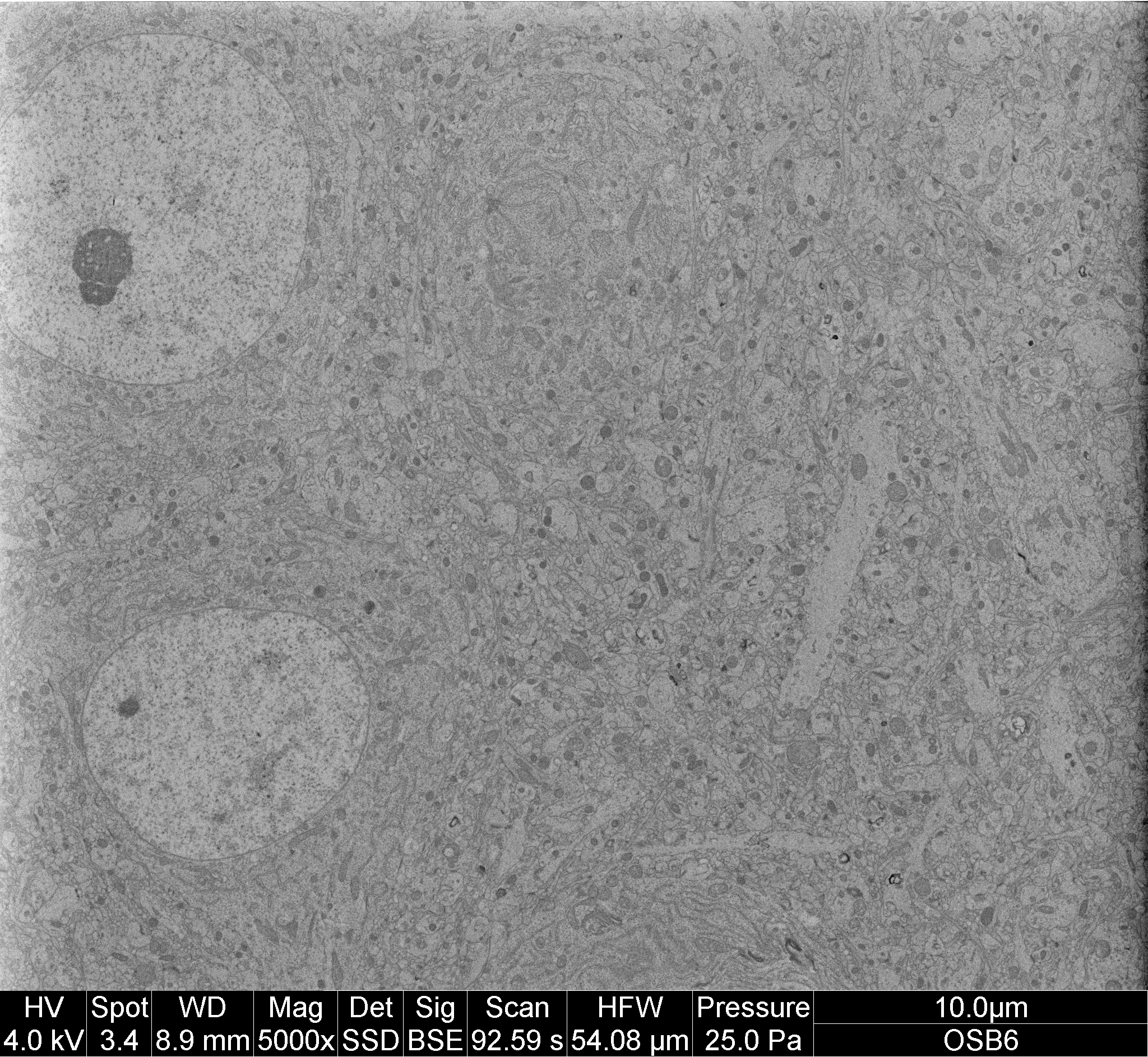

Supplement: Dataset S16 — (251.4 MB ZIP). [file pbio.0020329.sd016.zip › 040604_OS5_st1_1547.tif]

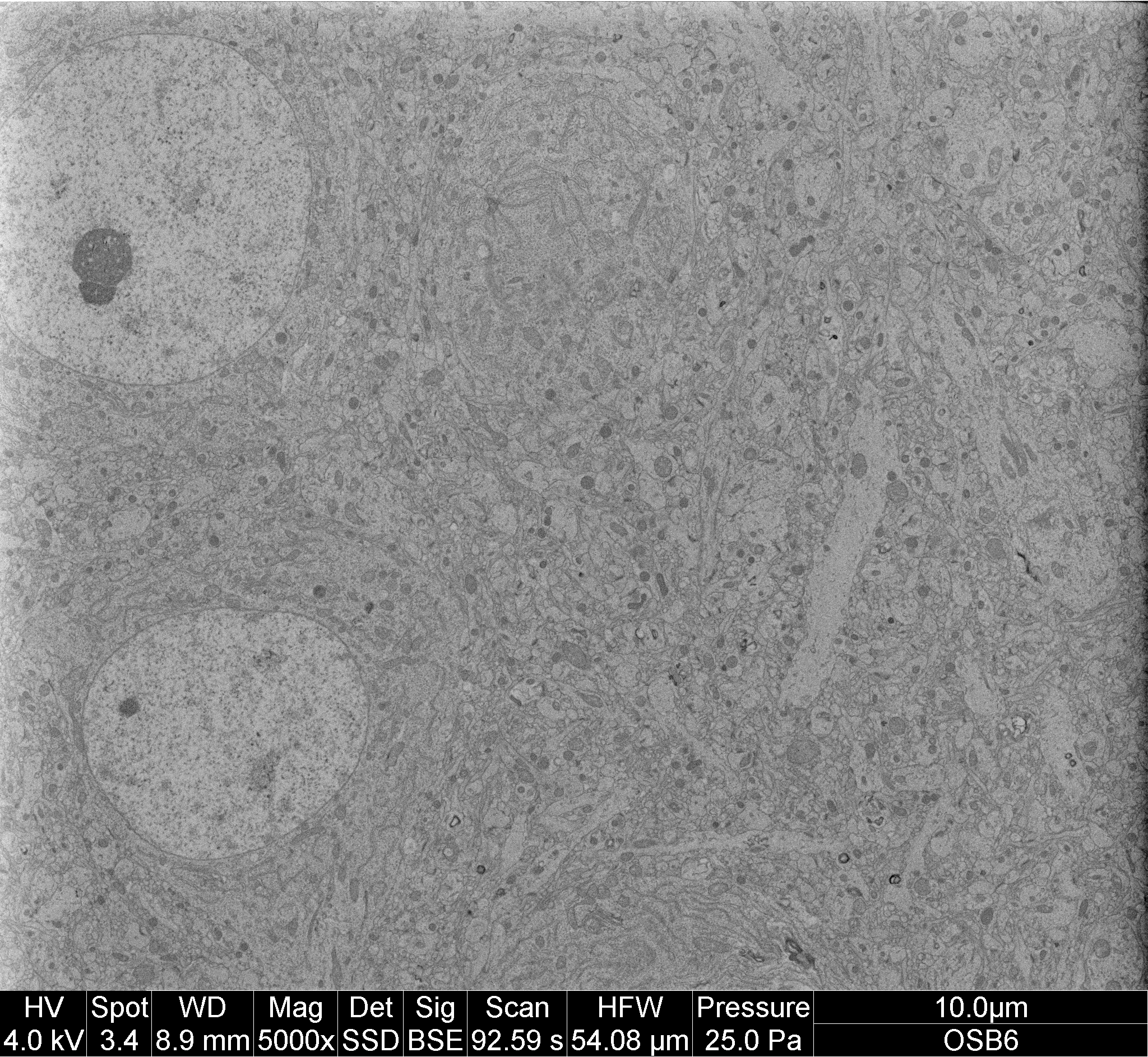

Supplement: Dataset S16 — (251.4 MB ZIP). [file pbio.0020329.sd016.zip › 040604_OS5_st1_1548.tif]

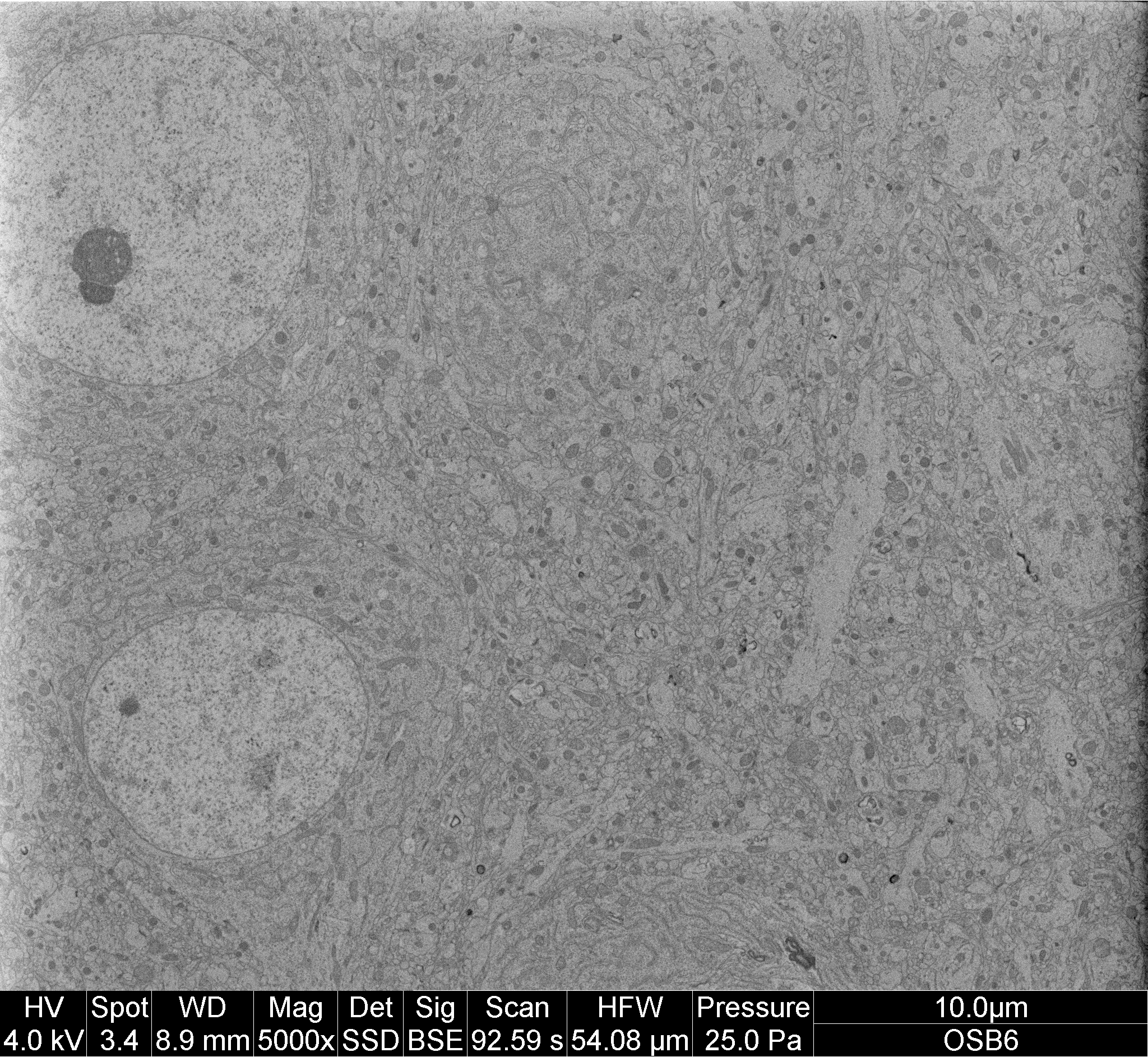

Supplement: Dataset S16 — (251.4 MB ZIP). [file pbio.0020329.sd016.zip › 040604_OS5_st1_1549.tif]

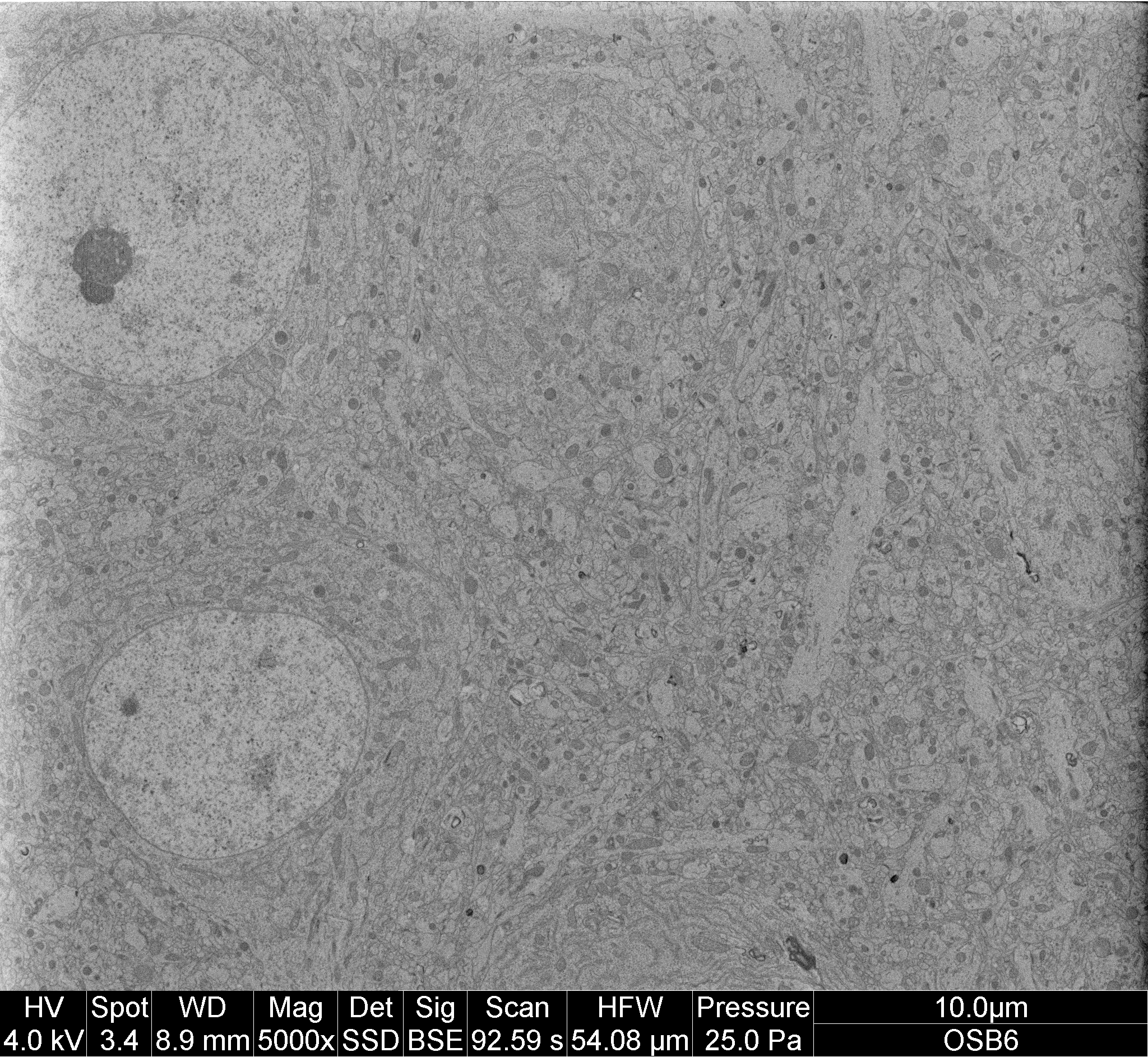

Supplement: Dataset S16 — (251.4 MB ZIP). [file pbio.0020329.sd016.zip › 040604_OS5_st1_1550.tif]

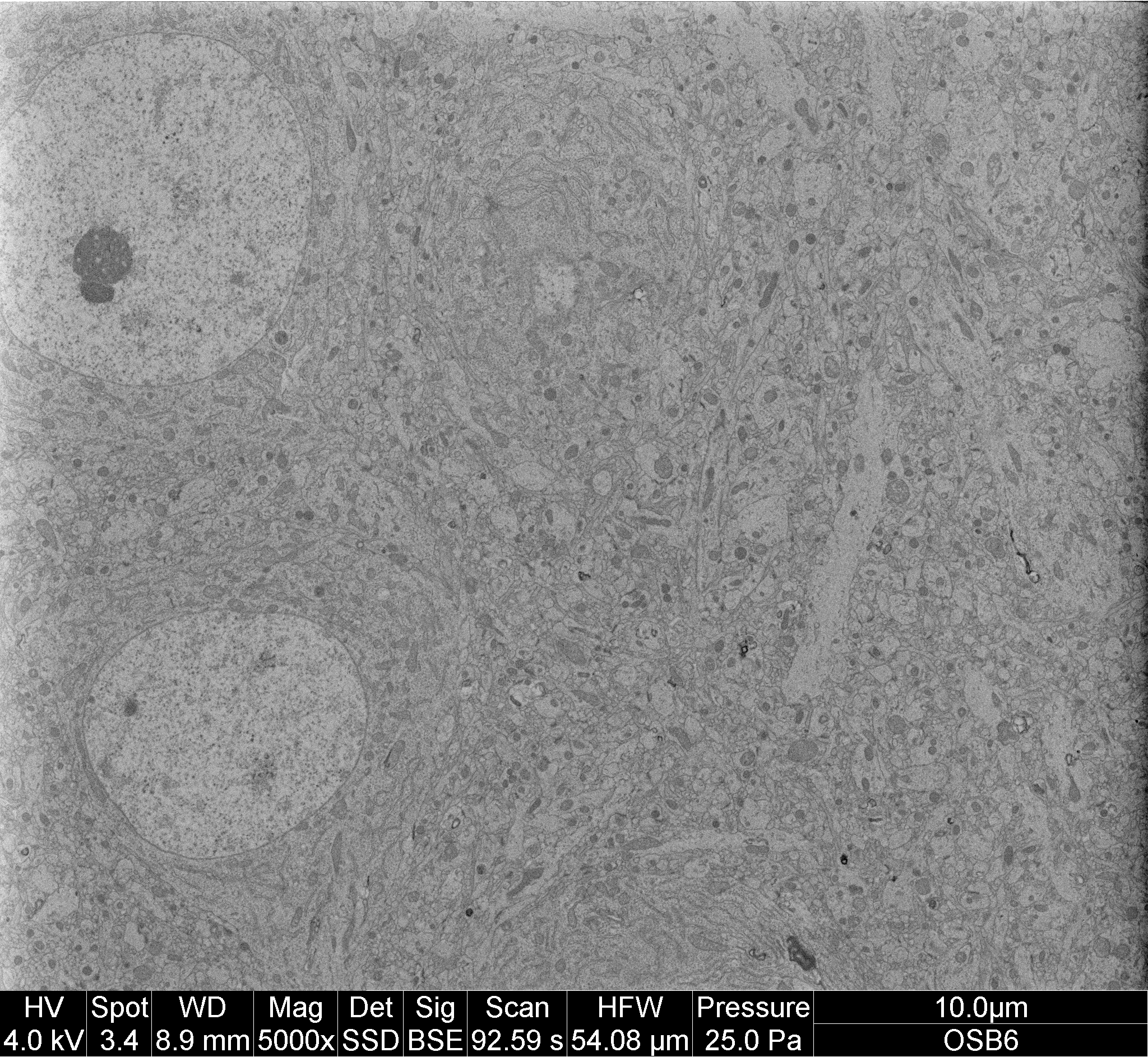

Supplement: Dataset S16 — (251.4 MB ZIP). [file pbio.0020329.sd016.zip › 040604_OS5_st1_1551.tif]

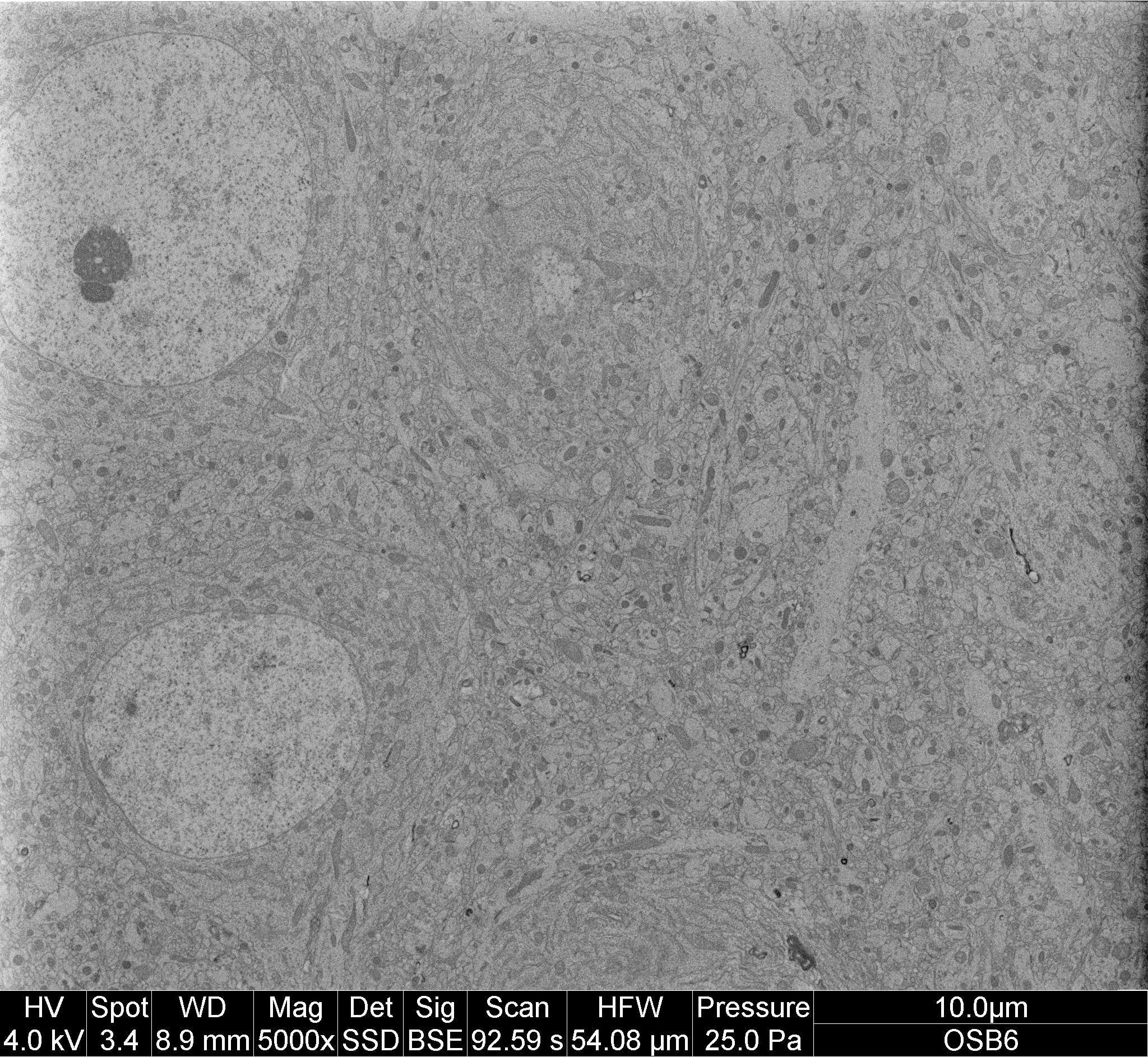

Supplement: Dataset S16 — (251.4 MB ZIP). [file pbio.0020329.sd016.zip › 040604_OS5_st1_1552.tif]

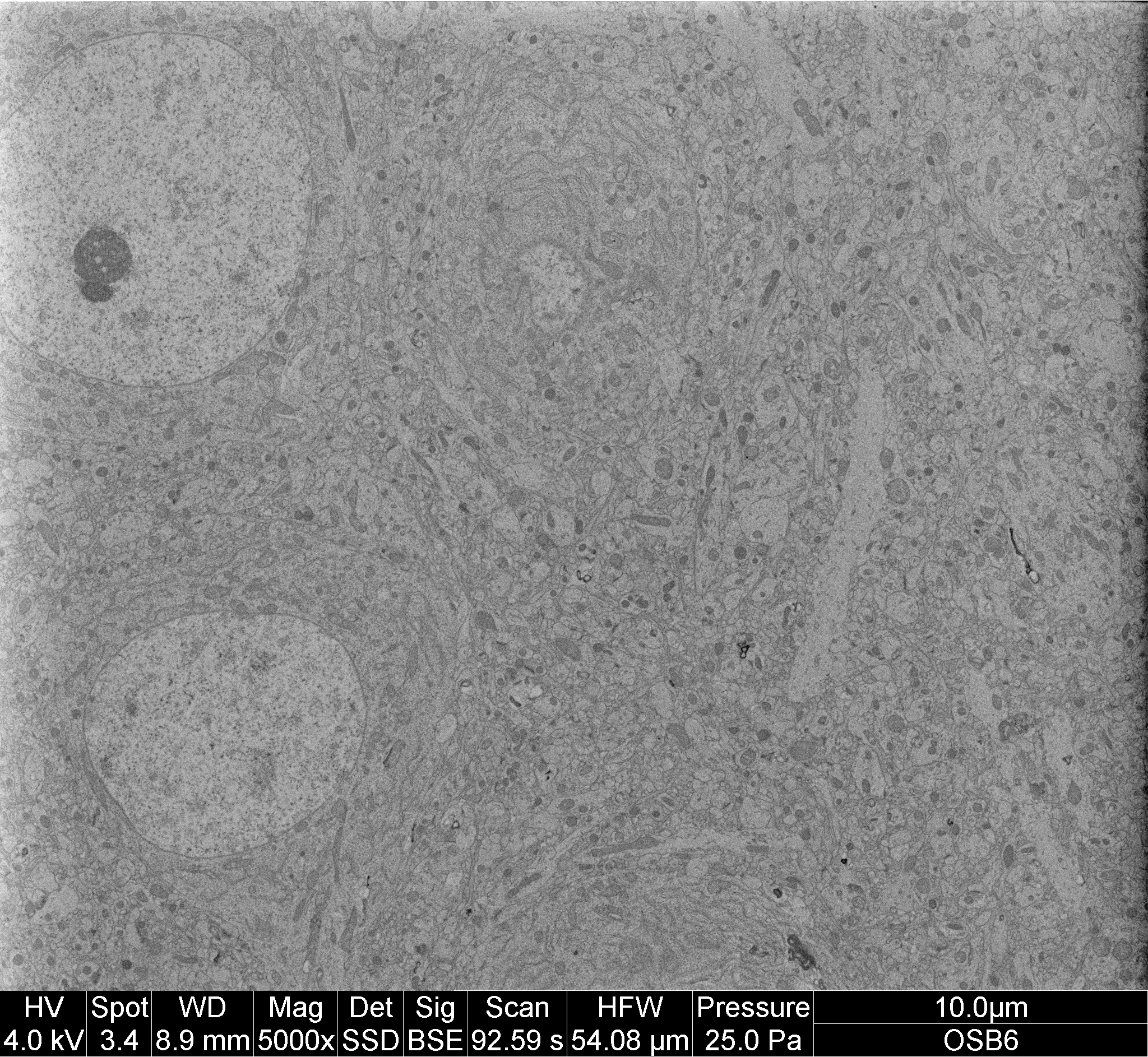

Supplement: Dataset S16 — (251.4 MB ZIP). [file pbio.0020329.sd016.zip › 040604_OS5_st1_1553.tif]

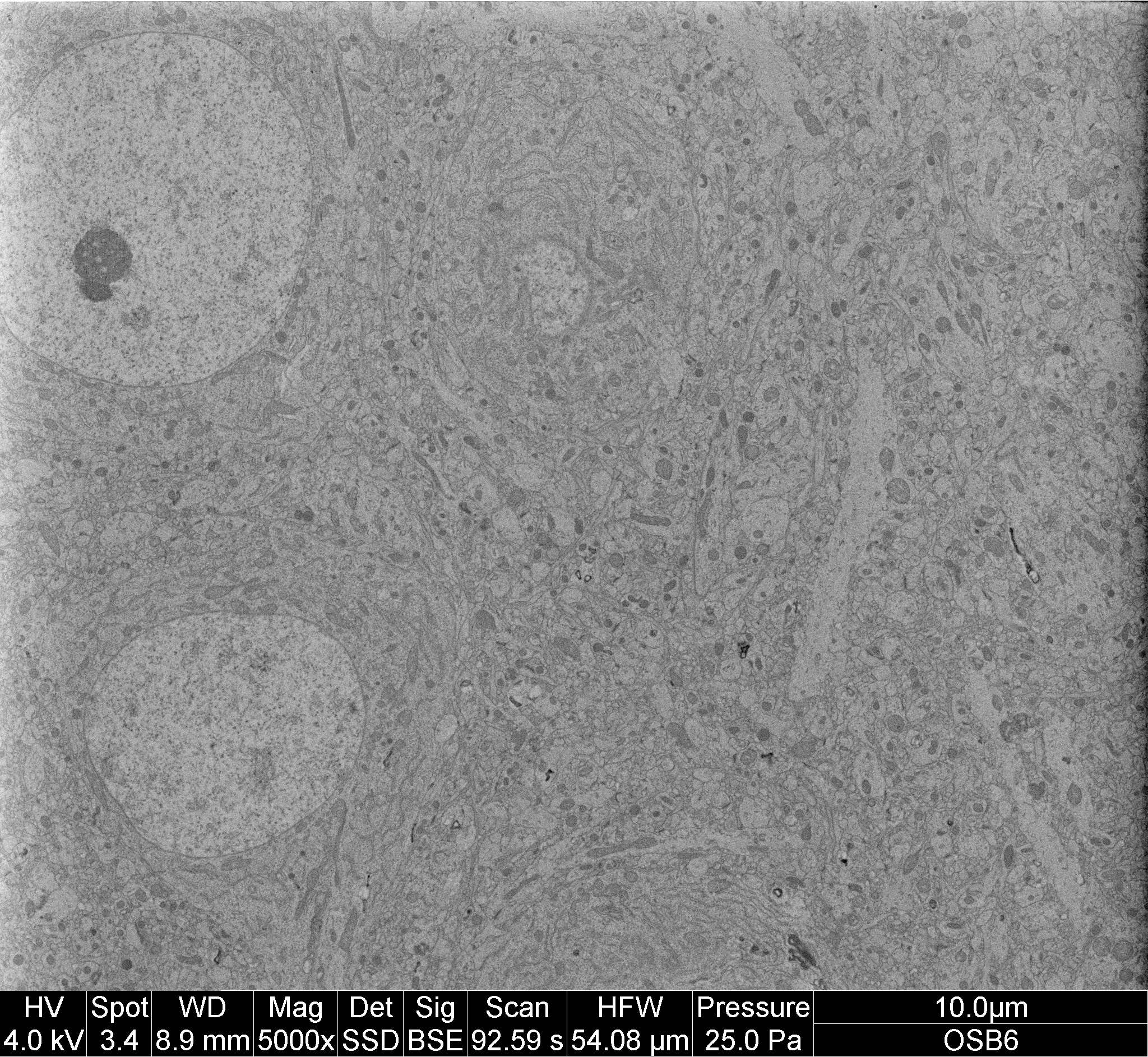

Supplement: Dataset S16 — (251.4 MB ZIP). [file pbio.0020329.sd016.zip › 040604_OS5_st1_1554.tif]

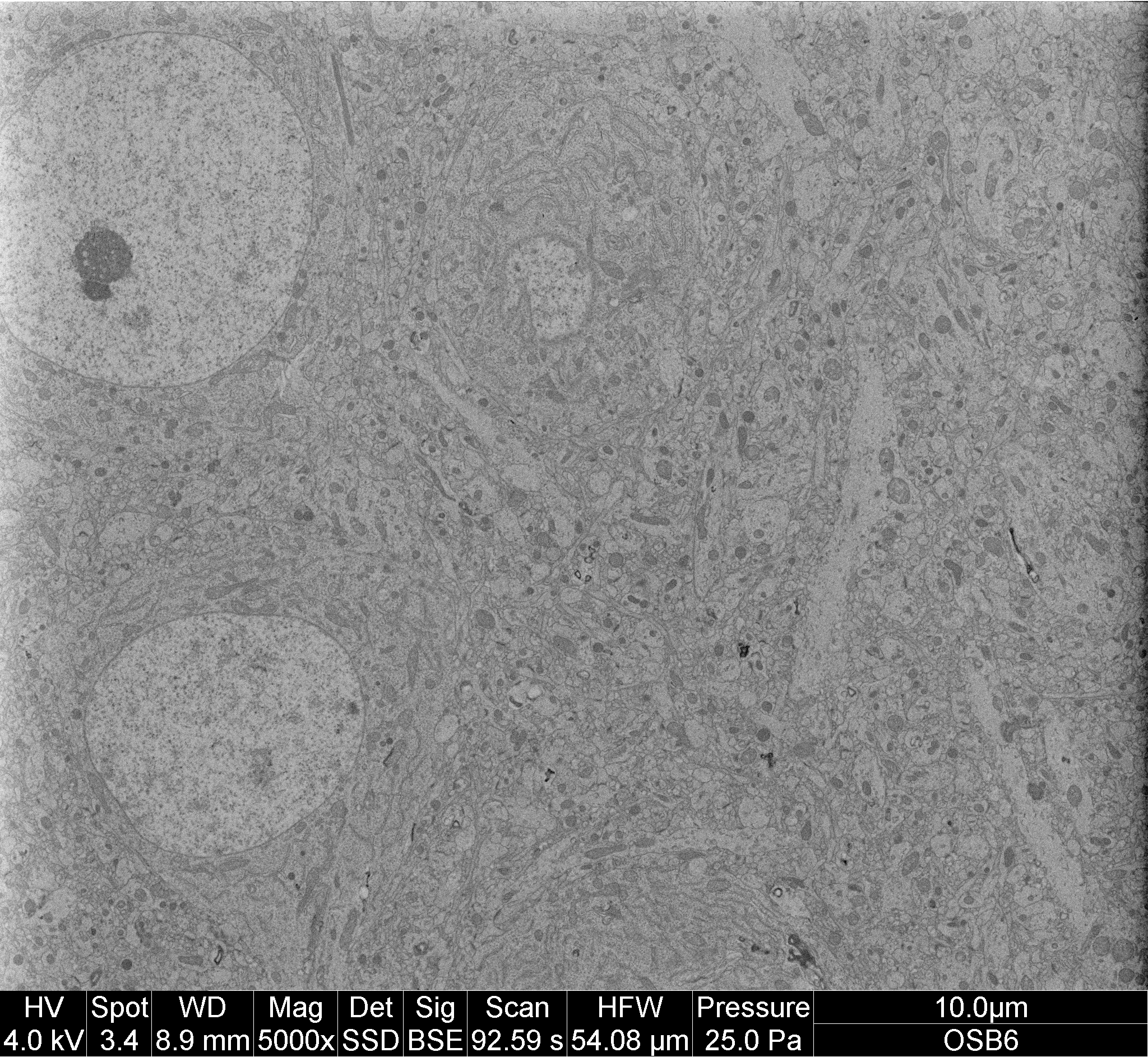

Supplement: Dataset S16 — (251.4 MB ZIP). [file pbio.0020329.sd016.zip › 040604_OS5_st1_1555.tif]

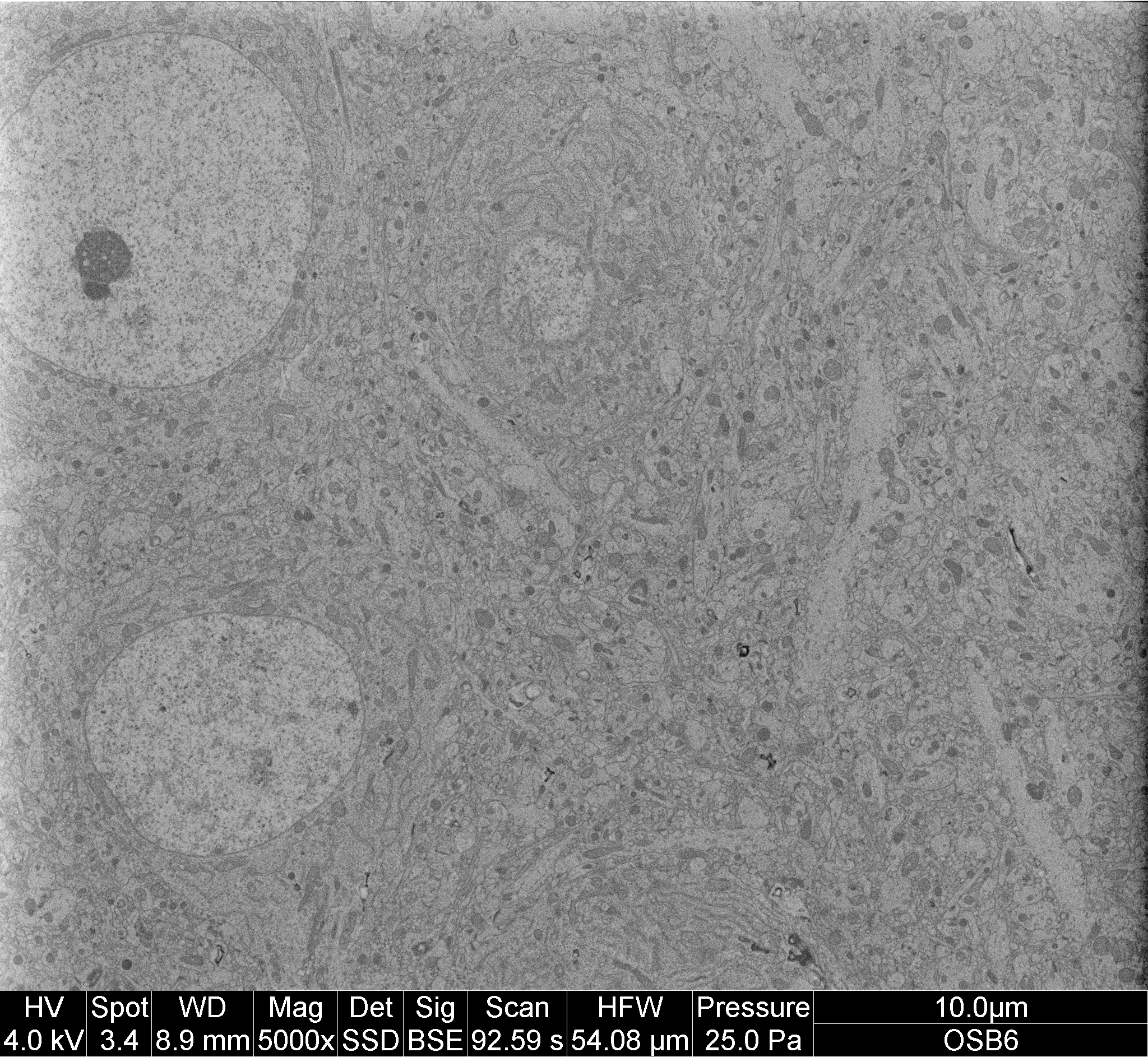

Supplement: Dataset S16 — (251.4 MB ZIP). [file pbio.0020329.sd016.zip › 040604_OS5_st1_1556.tif]

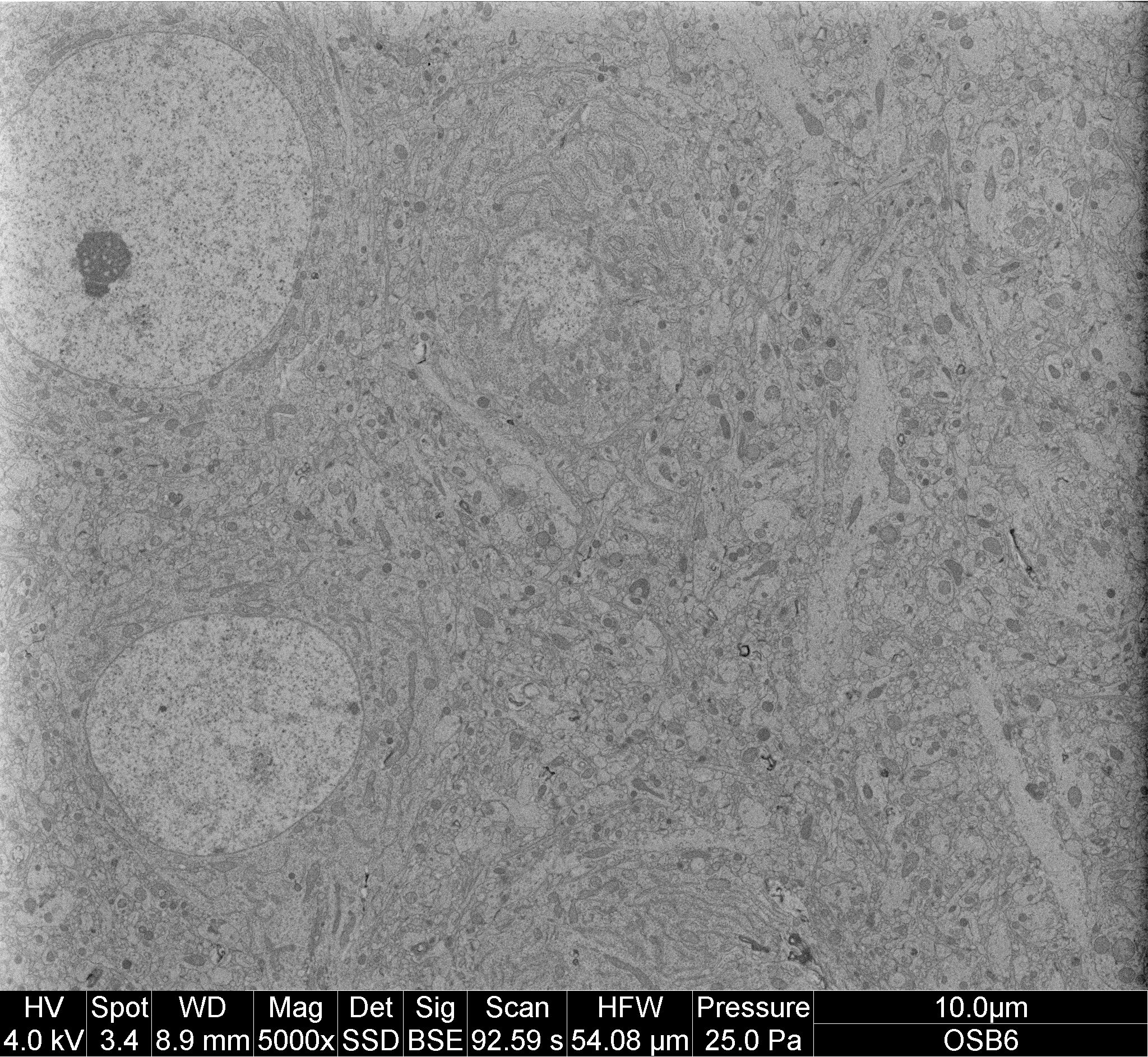

Supplement: Dataset S16 — (251.4 MB ZIP). [file pbio.0020329.sd016.zip › 040604_OS5_st1_1557.tif]

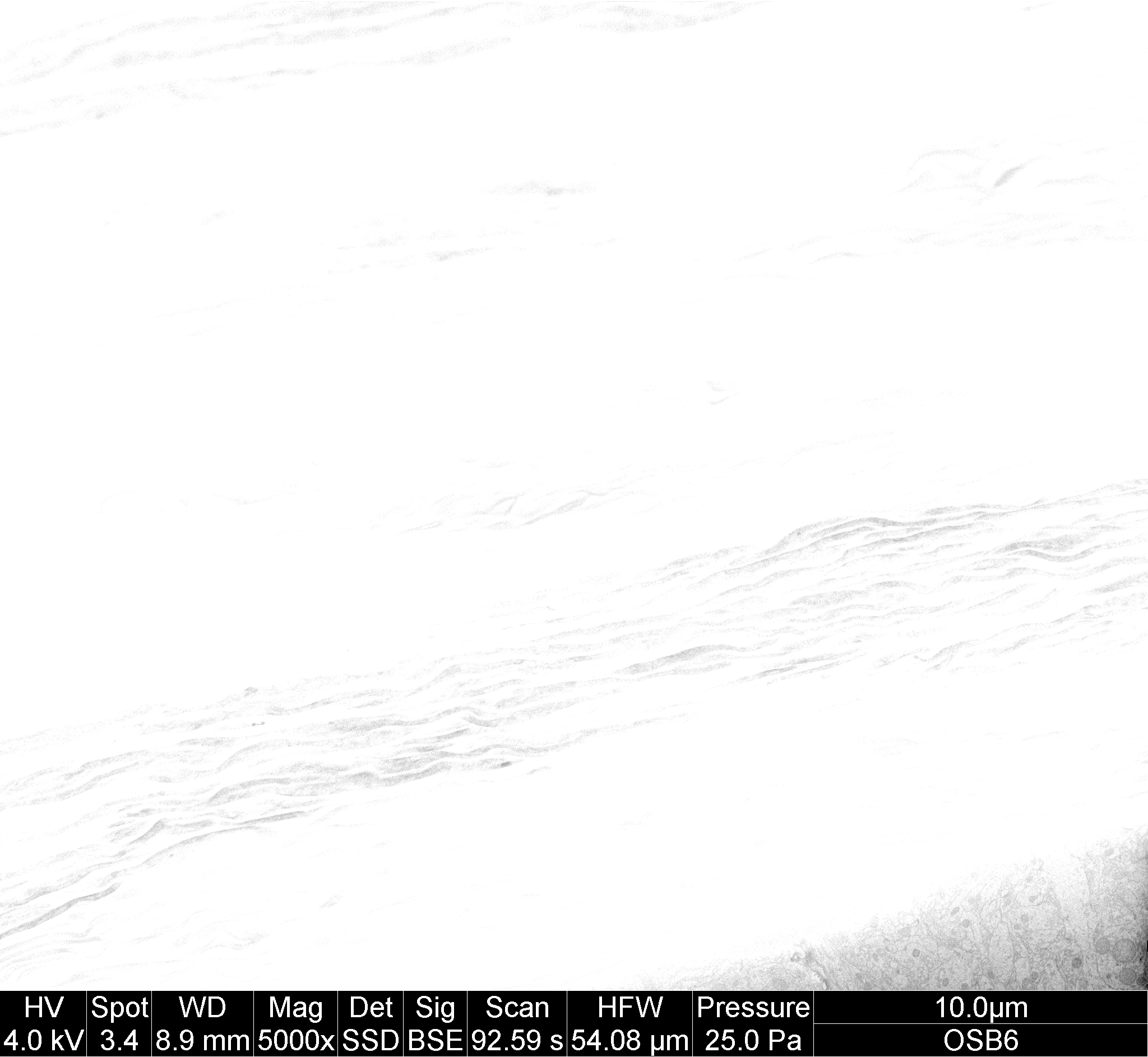

Supplement: Dataset S16 — (251.4 MB ZIP). [file pbio.0020329.sd016.zip › 040604_OS5_st1_1558.tif]

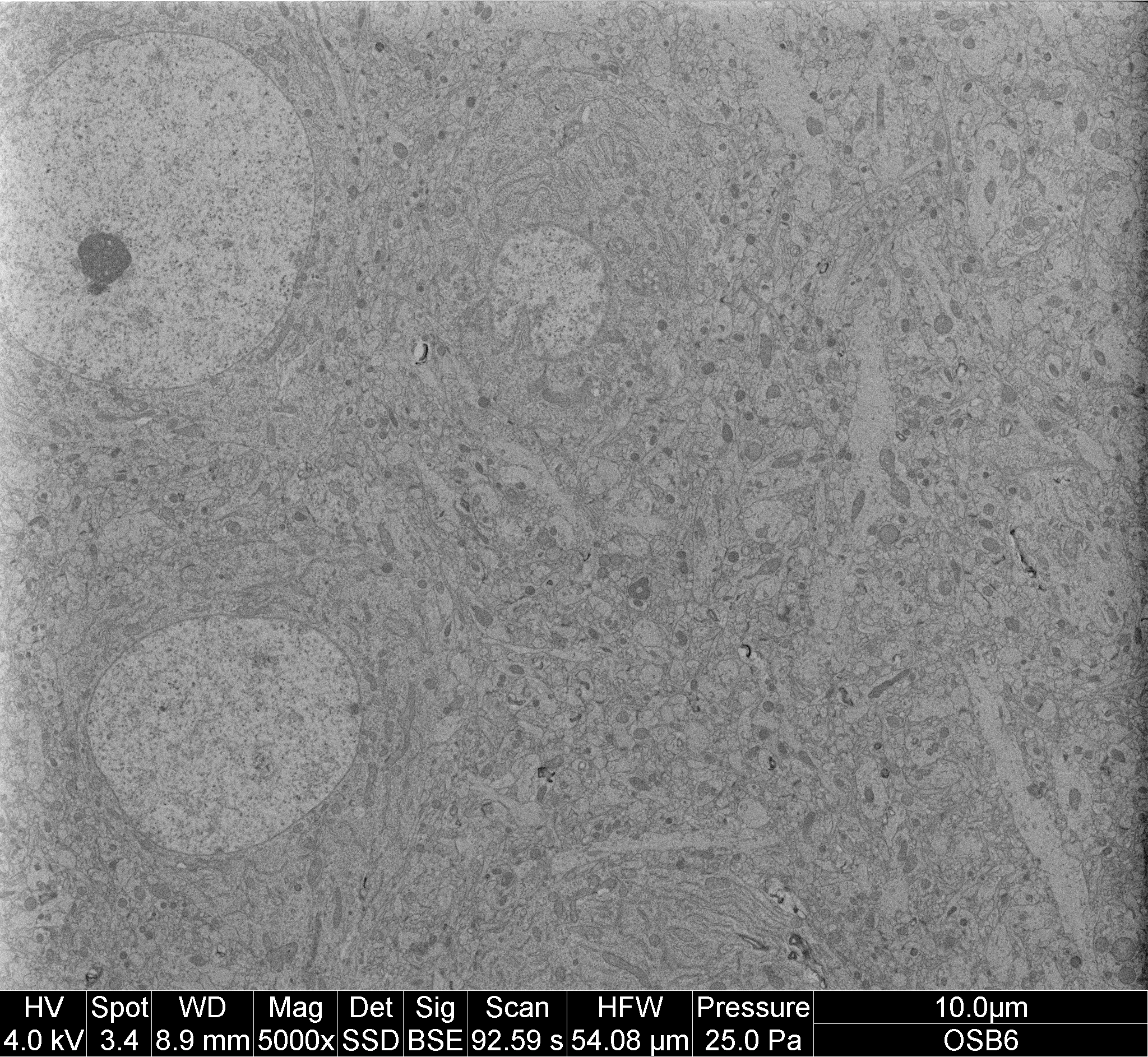

Supplement: Dataset S16 — (251.4 MB ZIP). [file pbio.0020329.sd016.zip › 040604_OS5_st1_1559.tif]

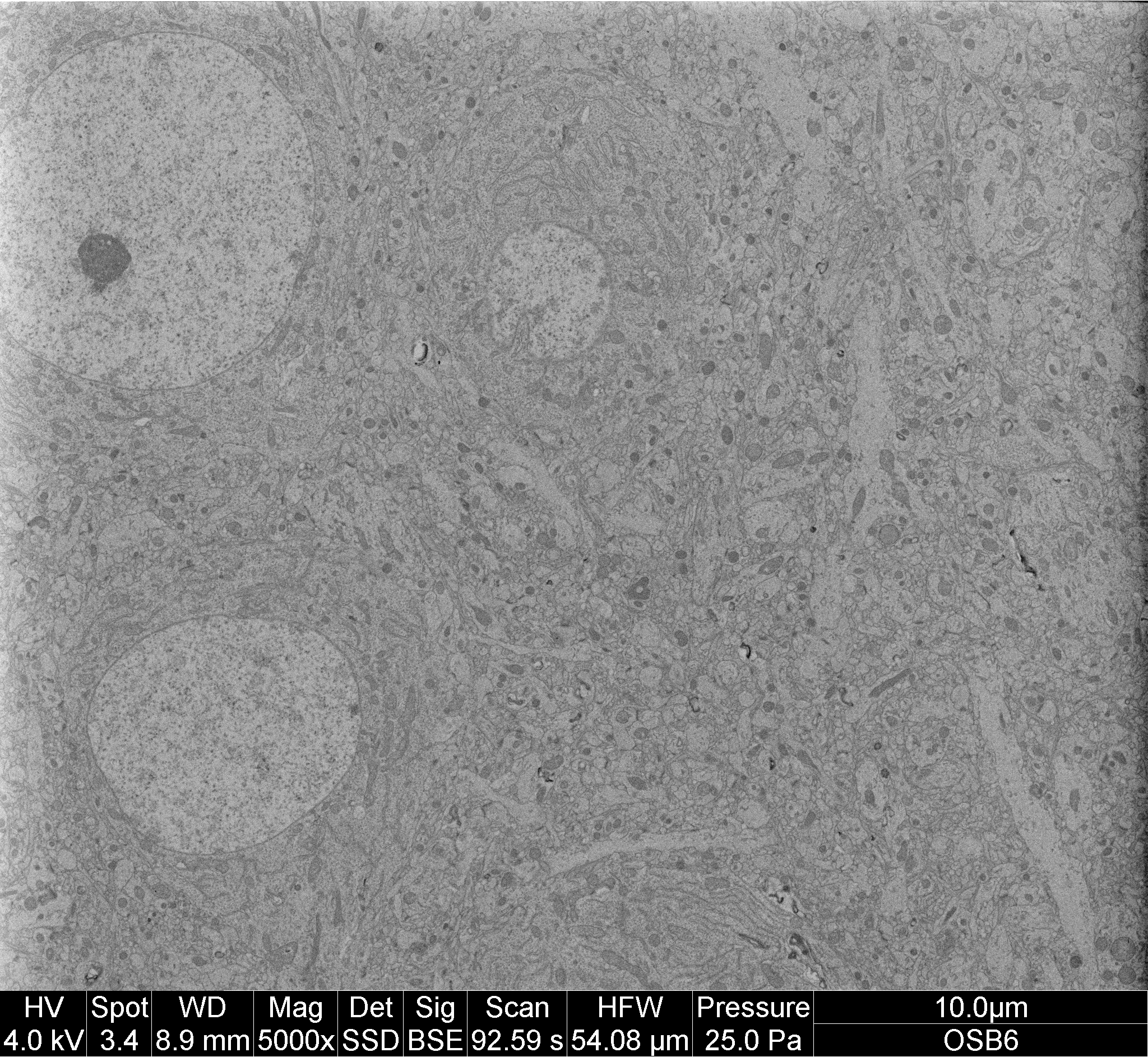

Supplement: Dataset S16 — (251.4 MB ZIP). [file pbio.0020329.sd016.zip › 040604_OS5_st1_1560.tif]

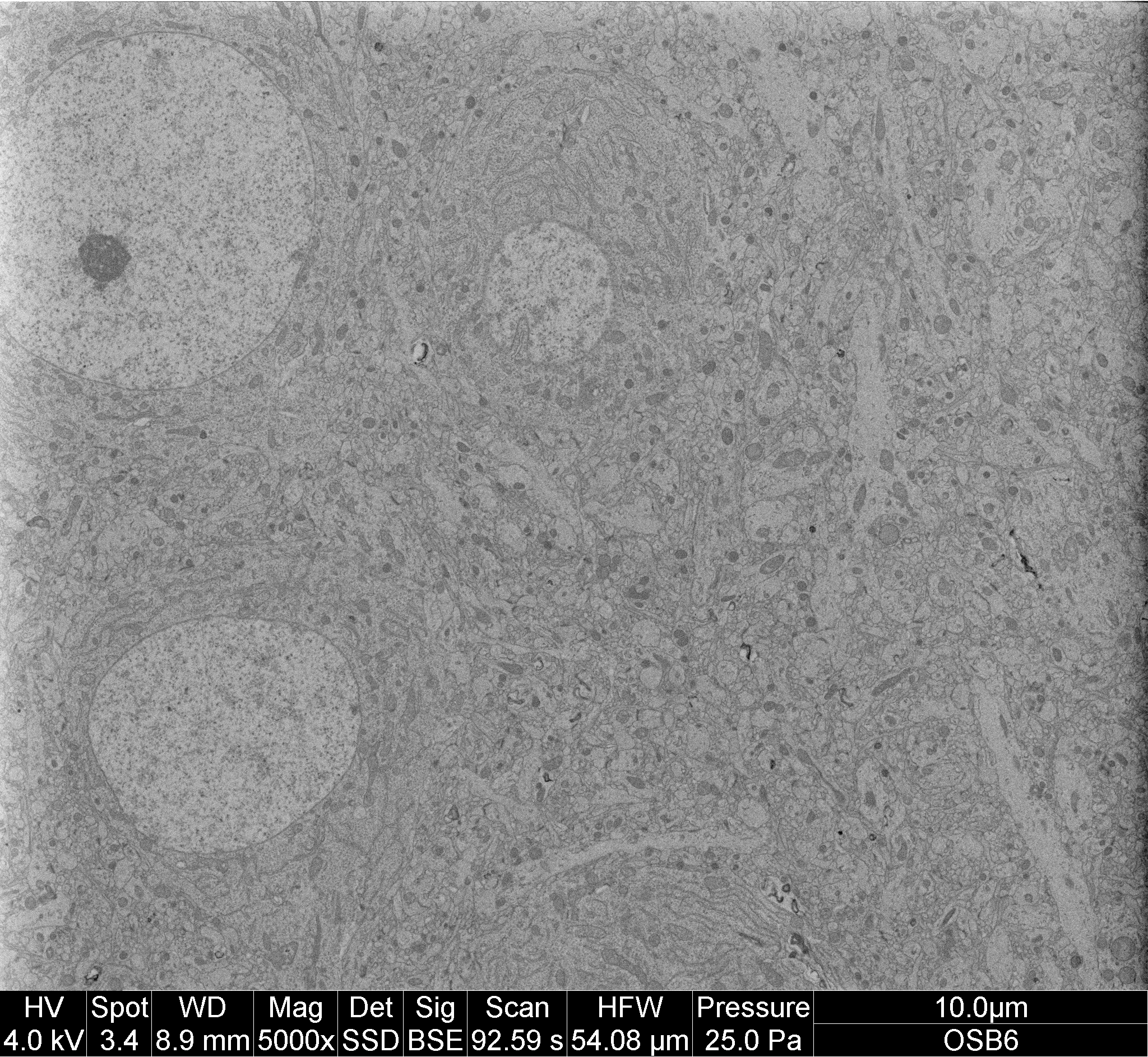

Supplement: Dataset S16 — (251.4 MB ZIP). [file pbio.0020329.sd016.zip › 040604_OS5_st1_1561.tif]

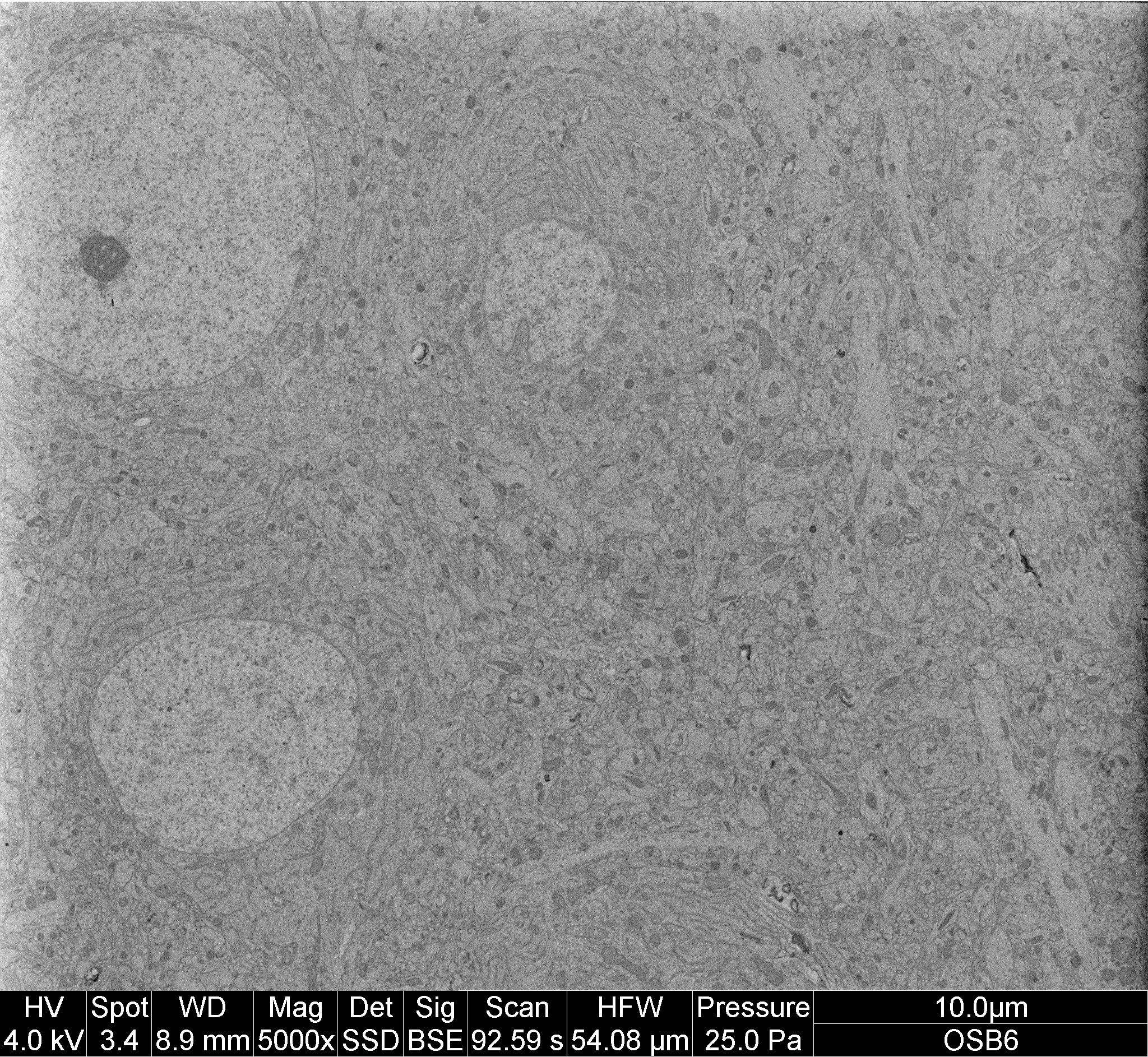

Supplement: Dataset S16 — (251.4 MB ZIP). [file pbio.0020329.sd016.zip › 040604_OS5_st1_1562.tif]

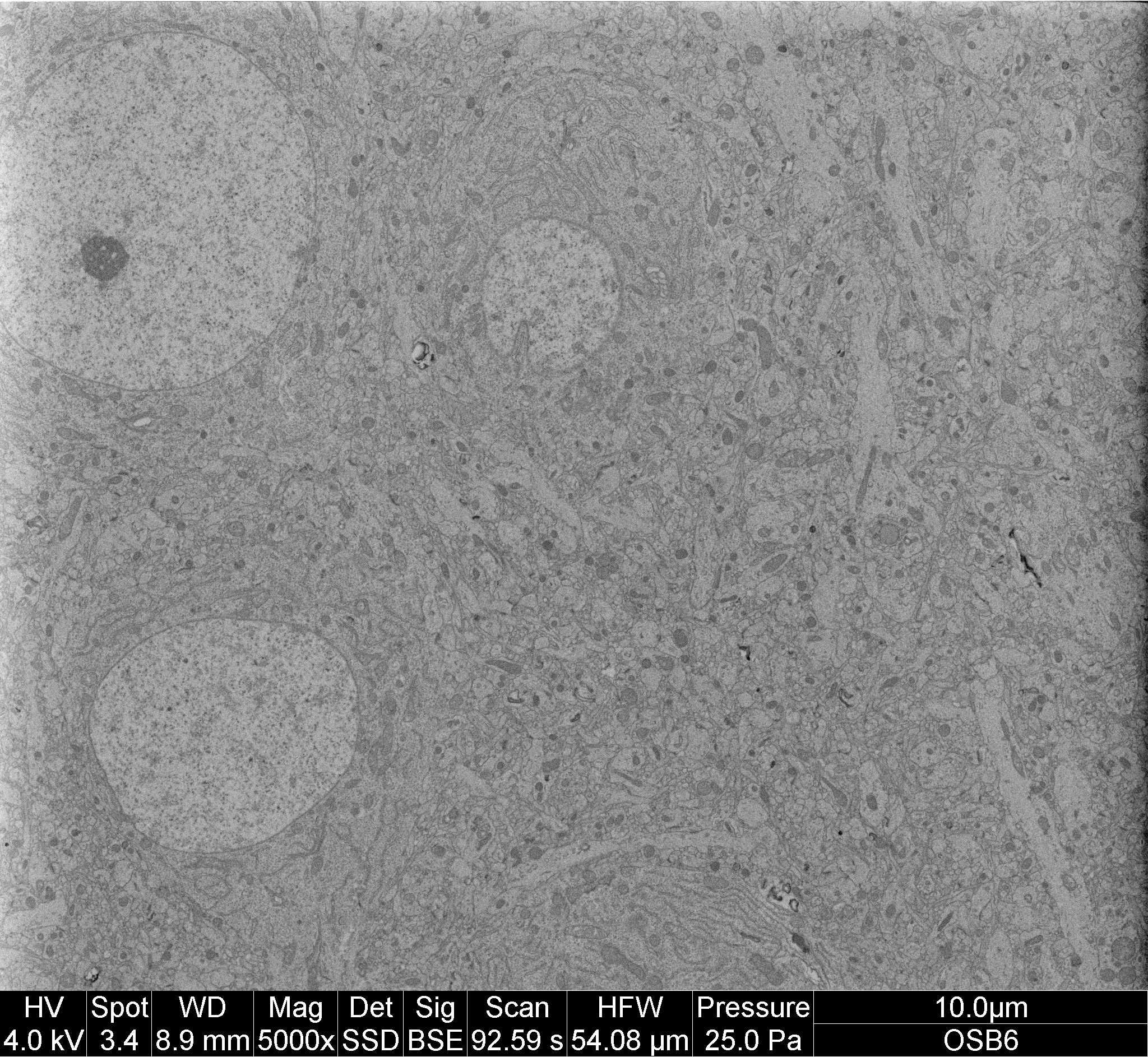

Supplement: Dataset S16 — (251.4 MB ZIP). [file pbio.0020329.sd016.zip › 040604_OS5_st1_1563.tif]

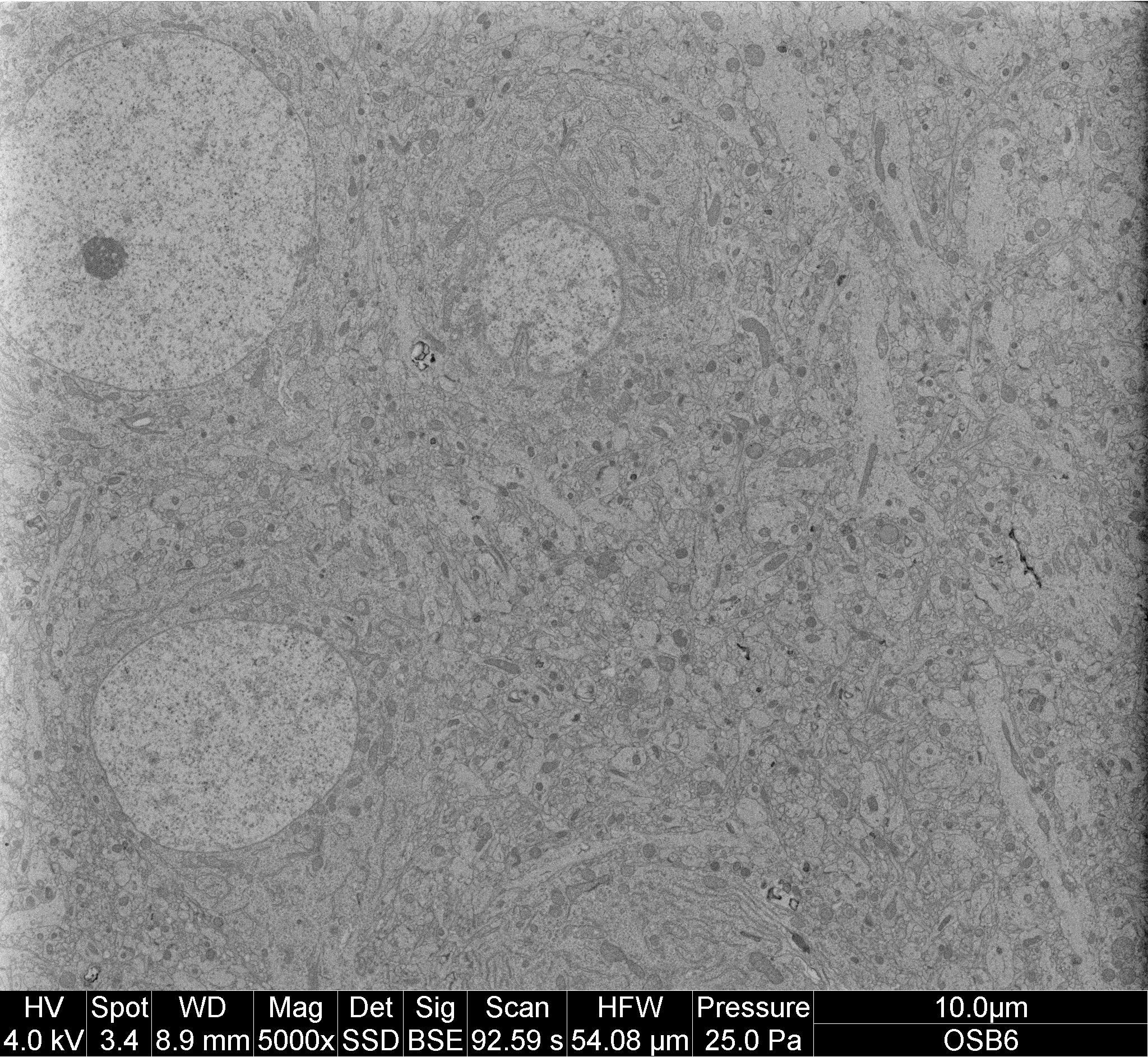

Supplement: Dataset S16 — (251.4 MB ZIP). [file pbio.0020329.sd016.zip › 040604_OS5_st1_1564.tif]

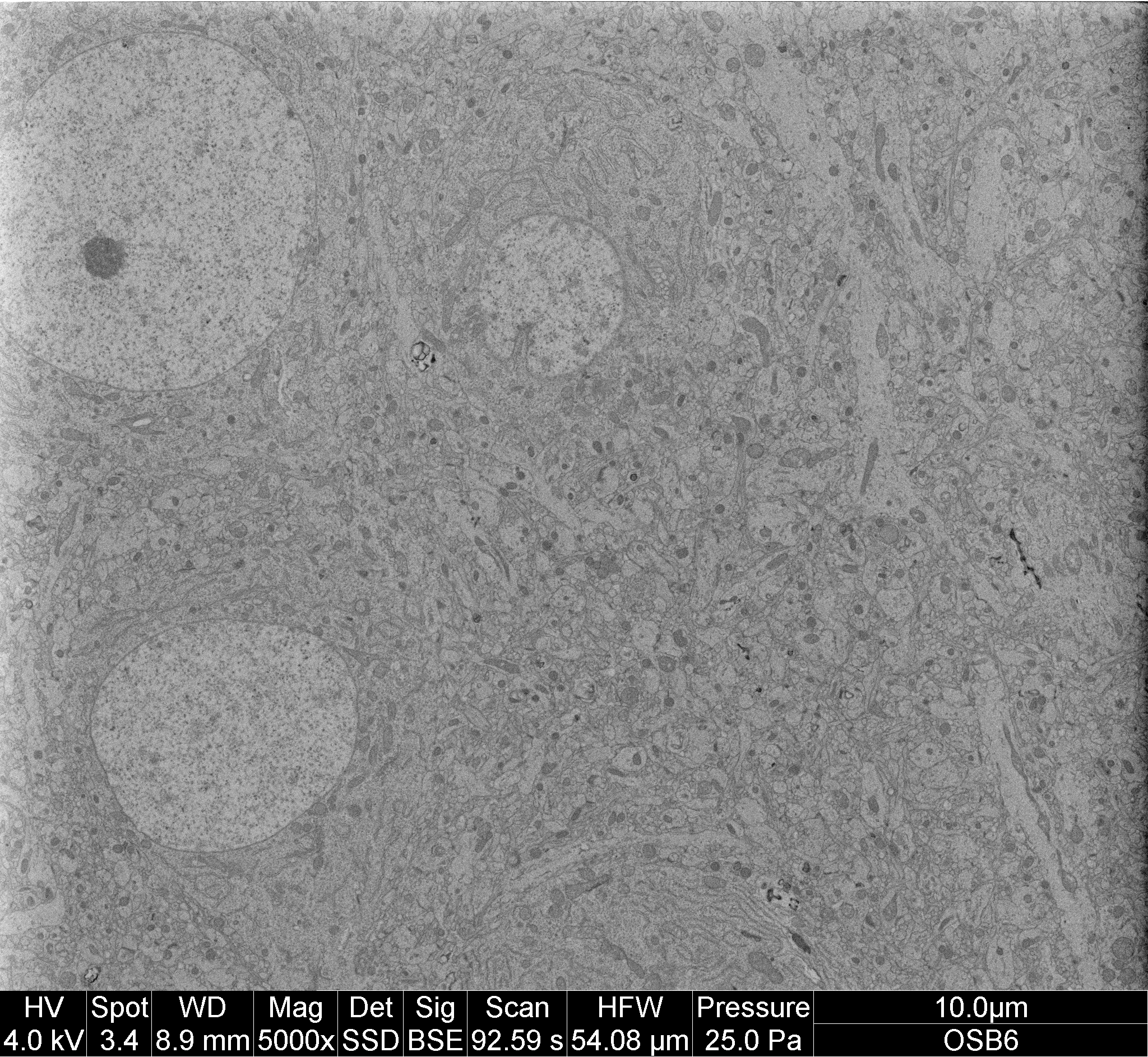

Supplement: Dataset S16 — (251.4 MB ZIP). [file pbio.0020329.sd016.zip › 040604_OS5_st1_1565.tif]

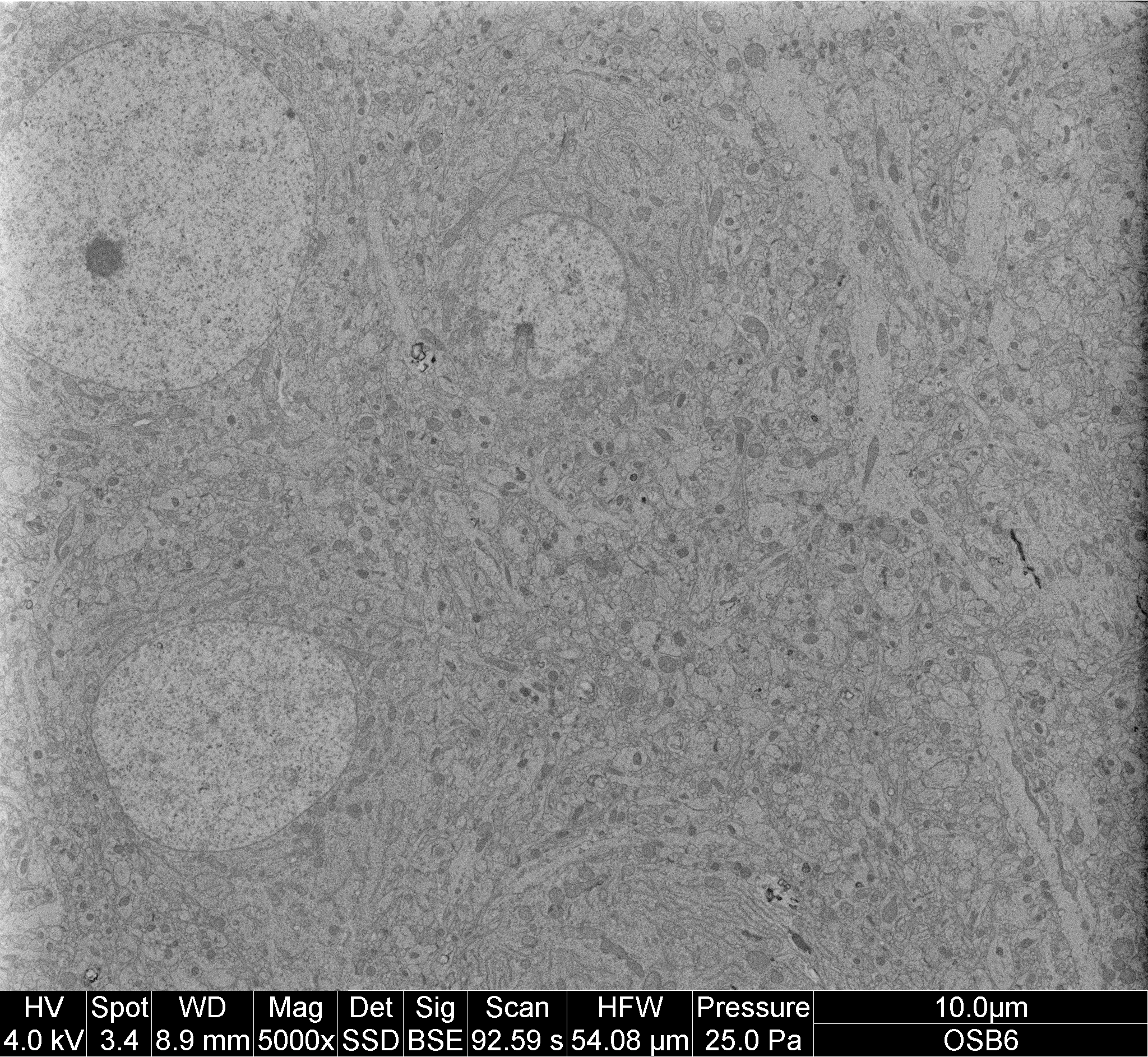

Supplement: Dataset S16 — (251.4 MB ZIP). [file pbio.0020329.sd016.zip › 040604_OS5_st1_1566.tif]

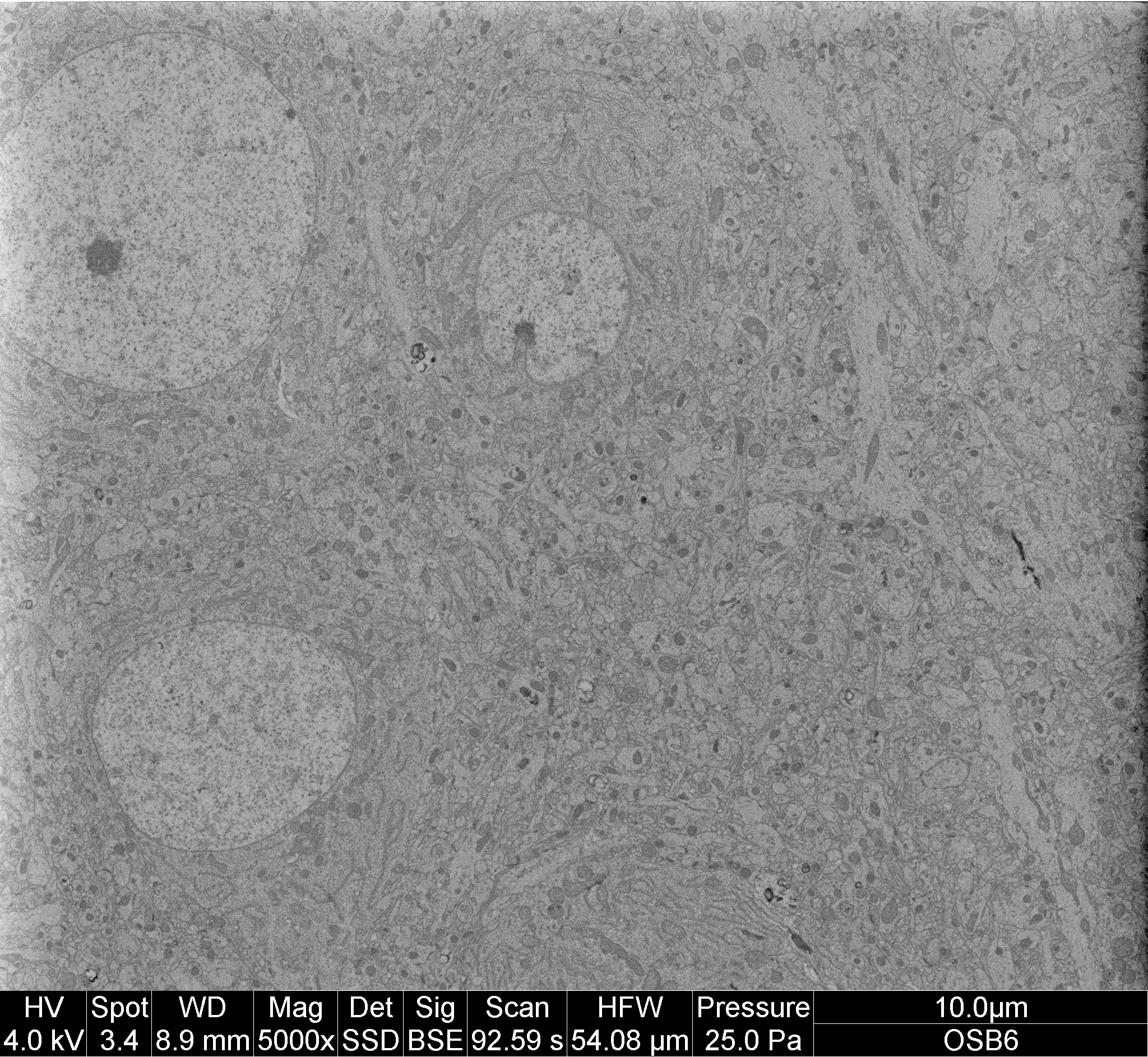

Supplement: Dataset S16 — (251.4 MB ZIP). [file pbio.0020329.sd016.zip › 040604_OS5_st1_1567.tif]

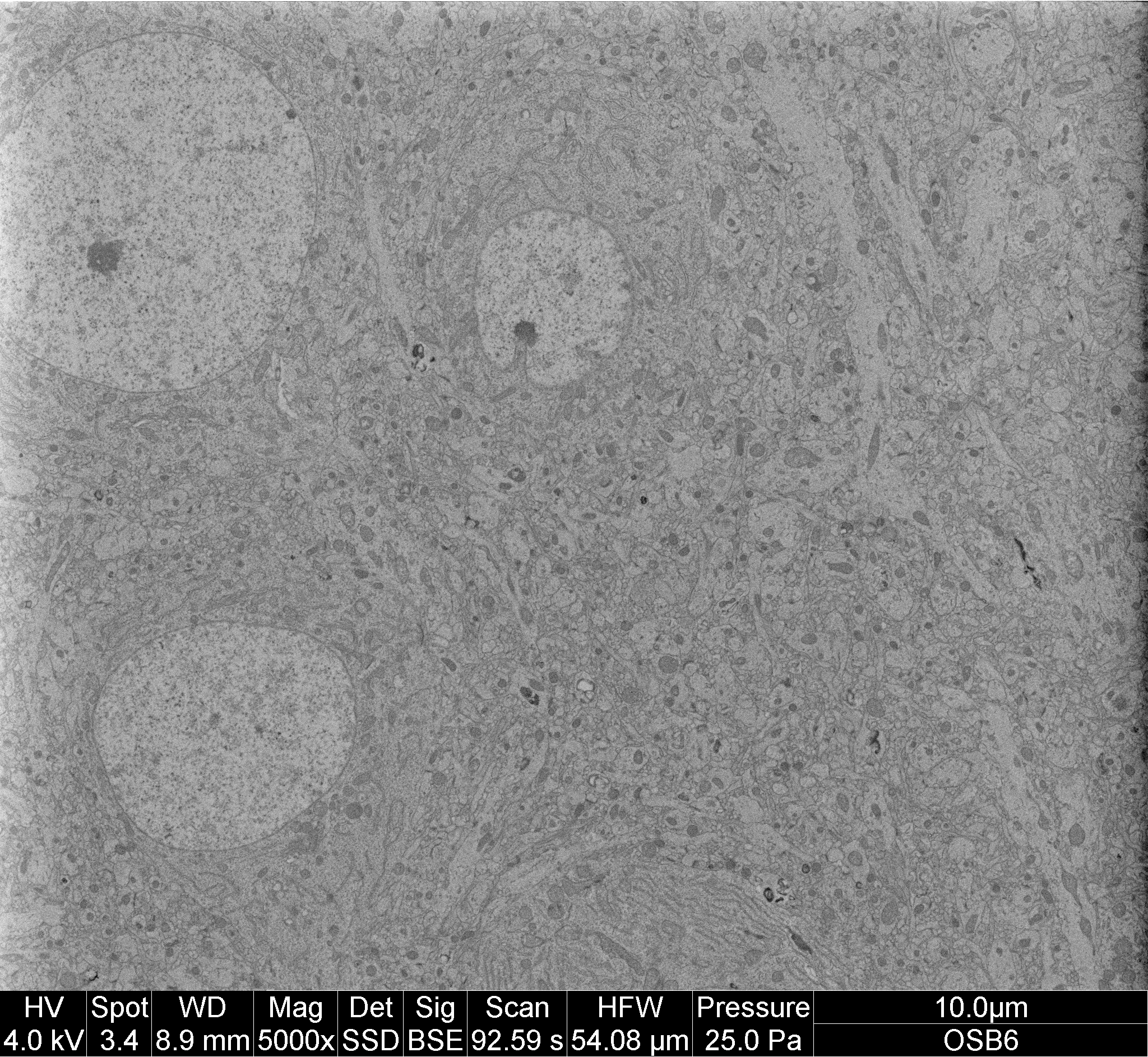

Supplement: Dataset S16 — (251.4 MB ZIP). [file pbio.0020329.sd016.zip › 040604_OS5_st1_1568.tif]

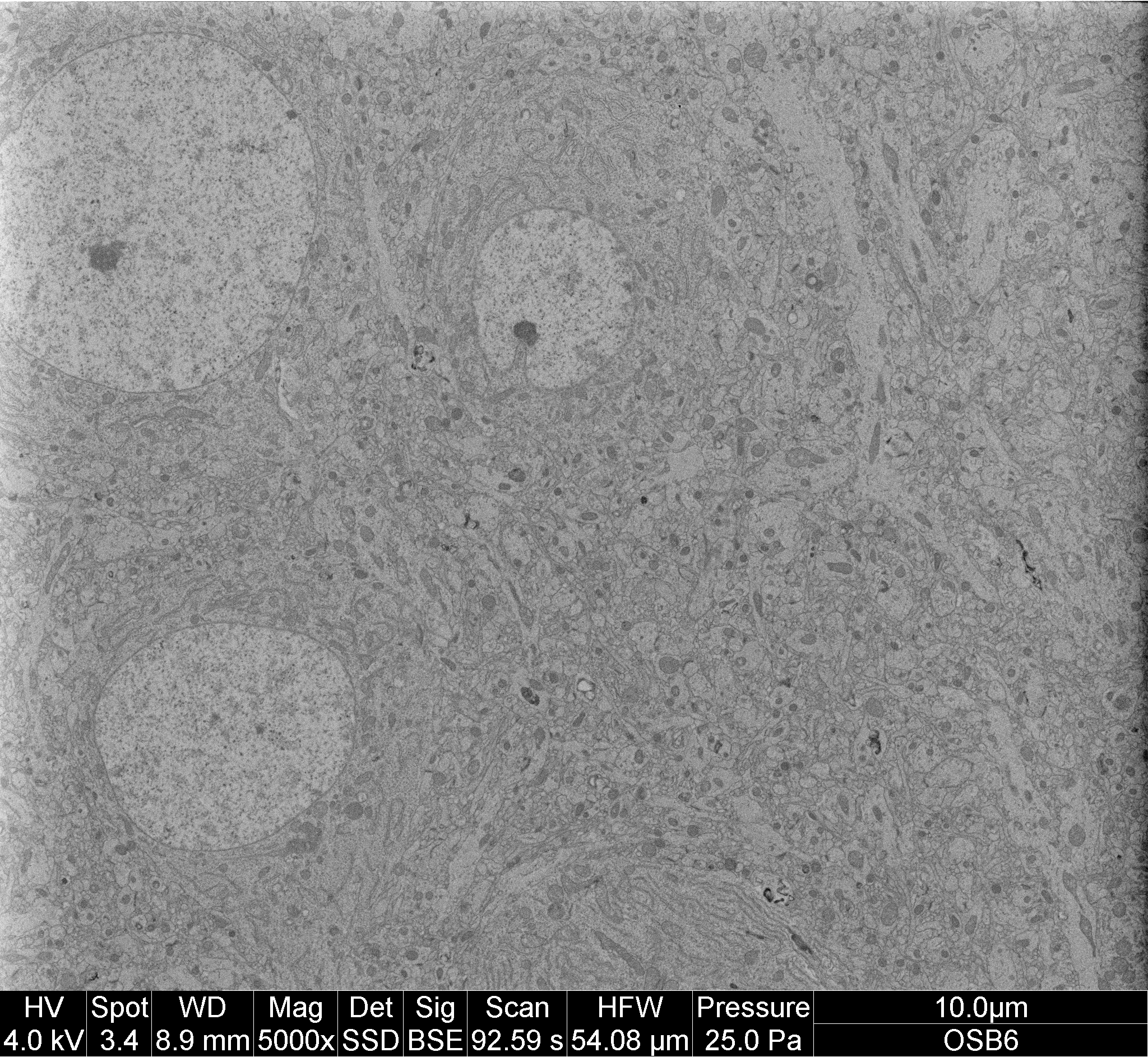

Supplement: Dataset S16 — (251.4 MB ZIP). [file pbio.0020329.sd016.zip › 040604_OS5_st1_1569.tif]

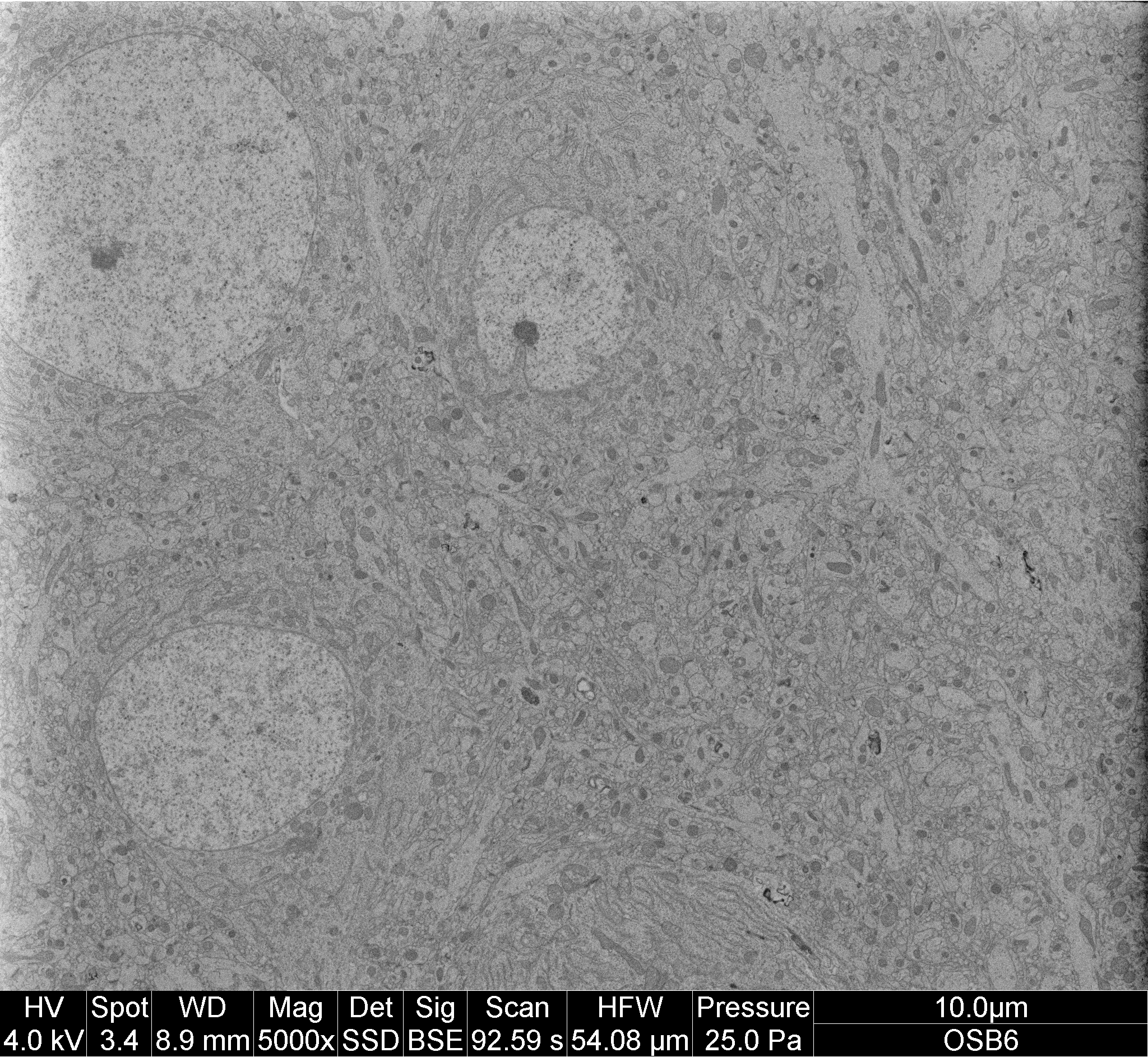

Supplement: Dataset S16 — (251.4 MB ZIP). [file pbio.0020329.sd016.zip › 040604_OS5_st1_1570.tif]

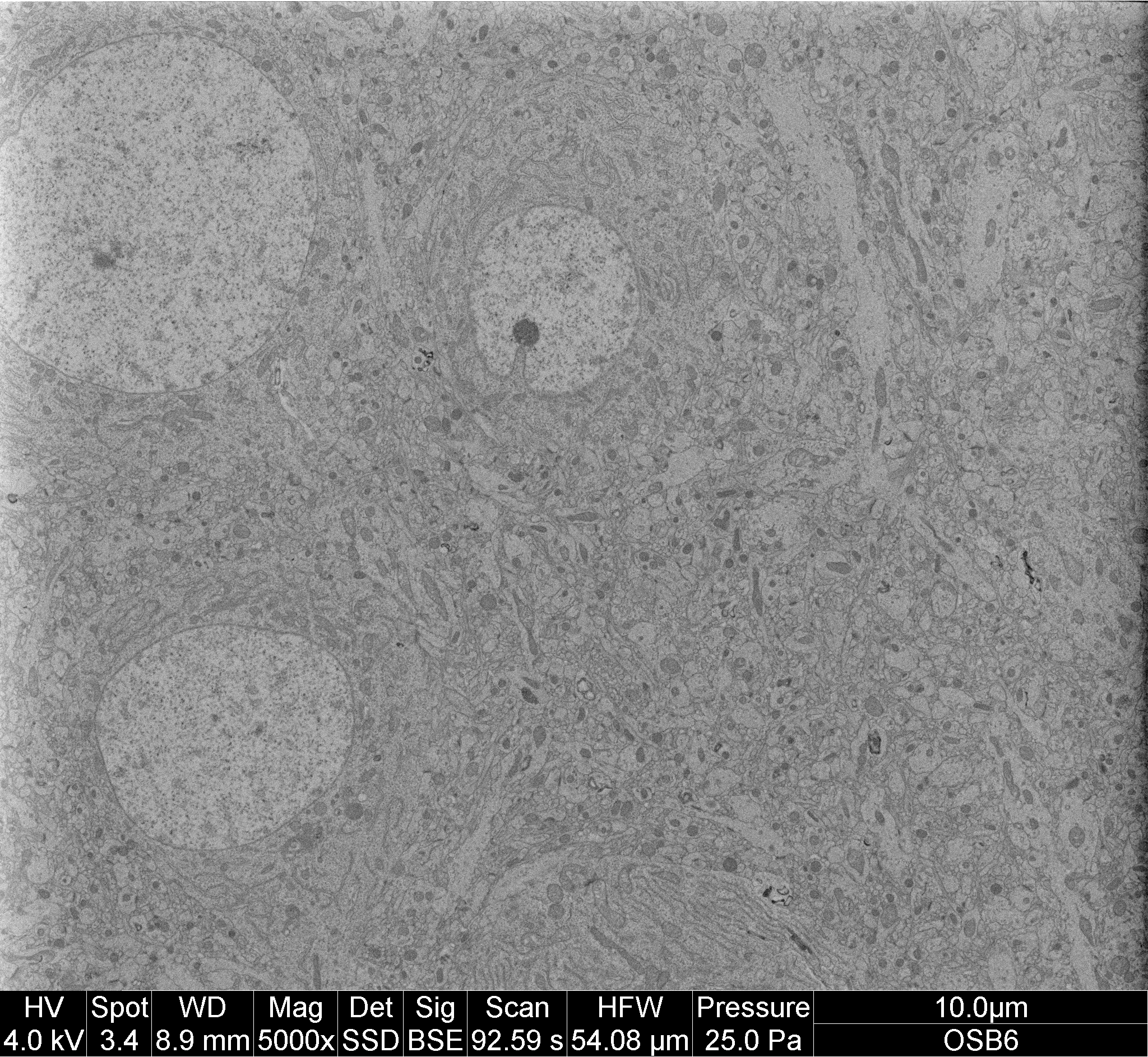

Supplement: Dataset S16 — (251.4 MB ZIP). [file pbio.0020329.sd016.zip › 040604_OS5_st1_1571.tif]

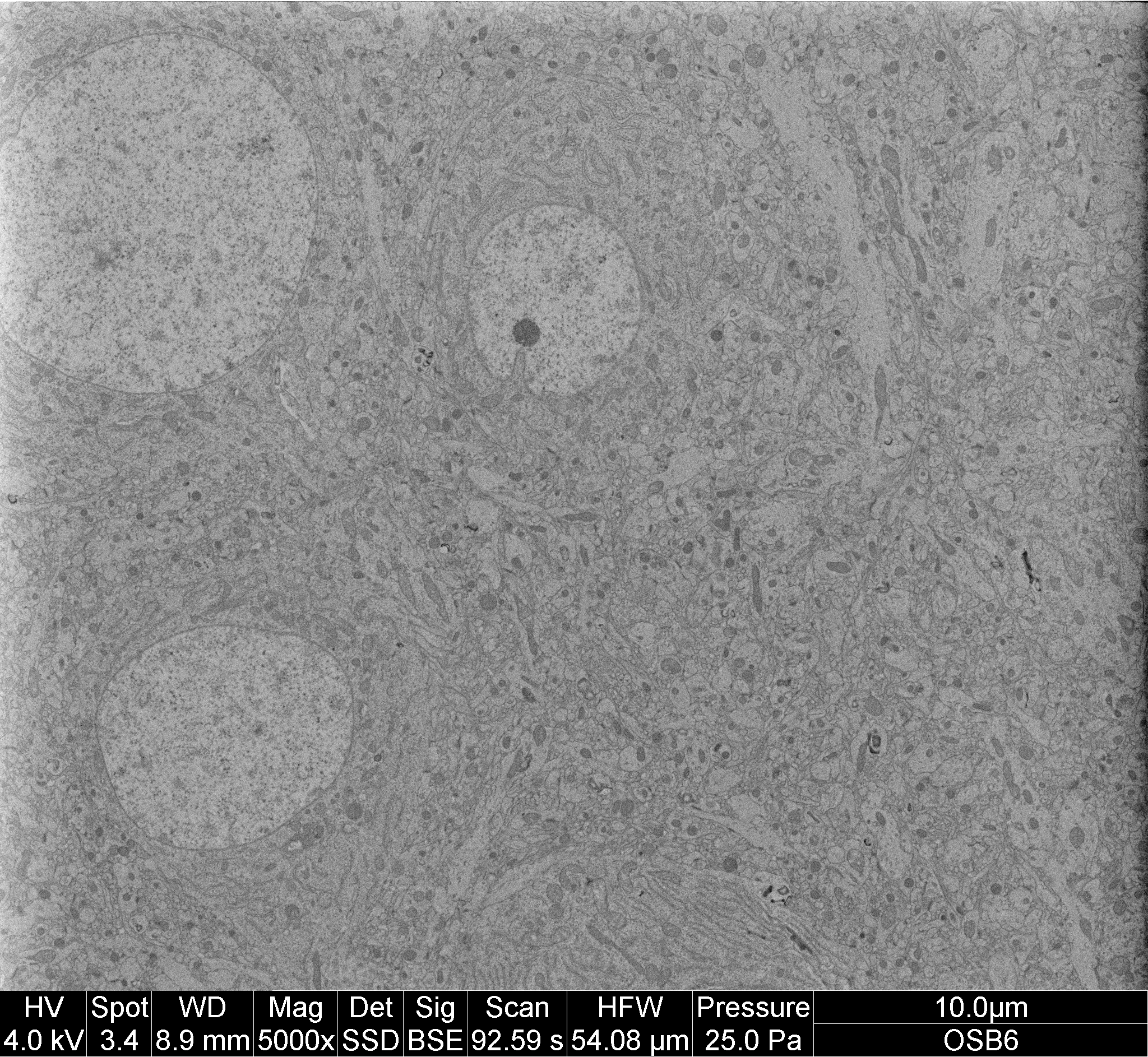

Supplement: Dataset S16 — (251.4 MB ZIP). [file pbio.0020329.sd016.zip › 040604_OS5_st1_1572.tif]

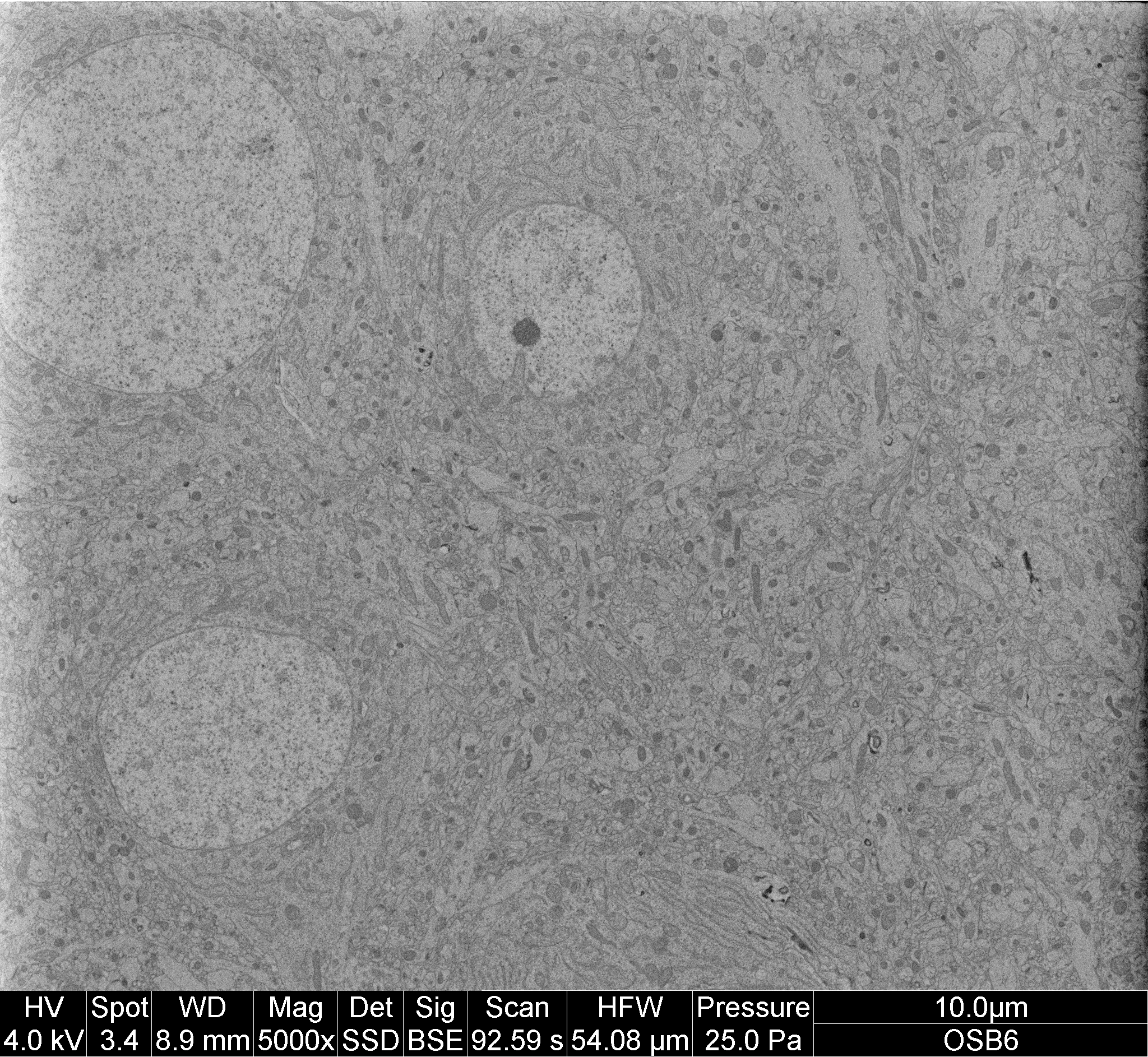

Supplement: Dataset S16 — (251.4 MB ZIP). [file pbio.0020329.sd016.zip › 040604_OS5_st1_1573.tif]

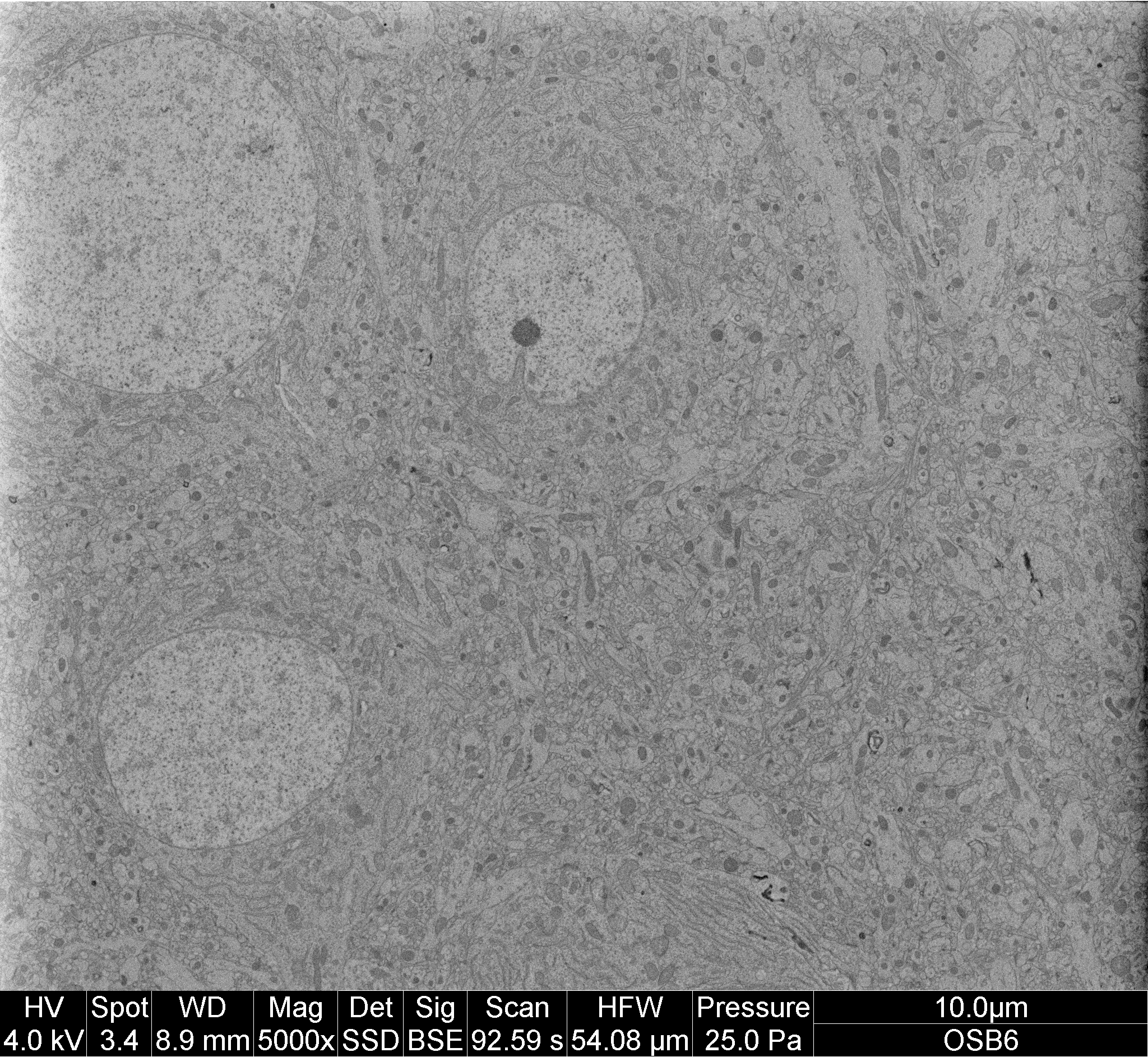

Supplement: Dataset S16 — (251.4 MB ZIP). [file pbio.0020329.sd016.zip › 040604_OS5_st1_1574.tif]

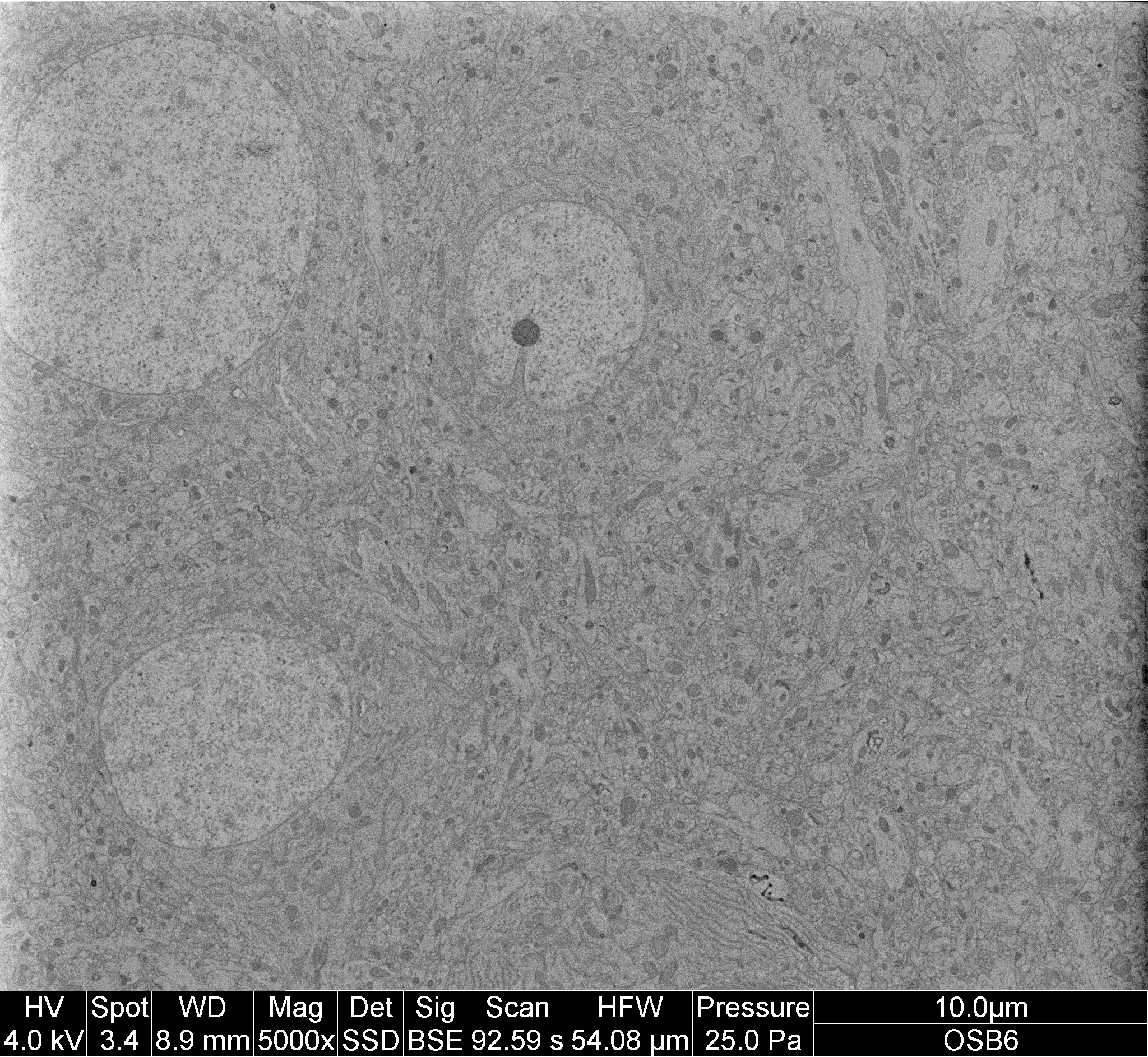

Supplement: Dataset S16 — (251.4 MB ZIP). [file pbio.0020329.sd016.zip › 040604_OS5_st1_1575.tif]

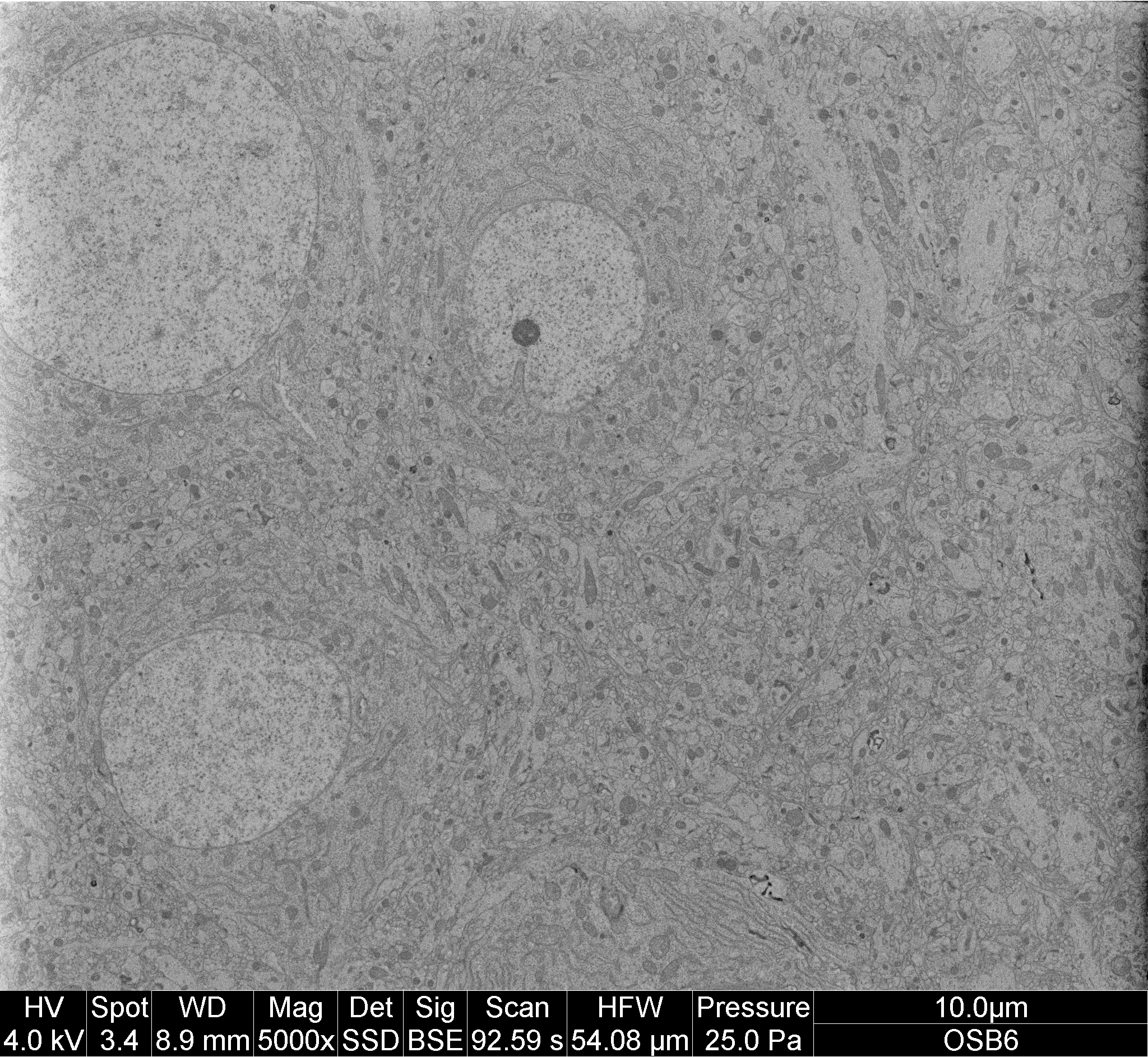

Supplement: Dataset S16 — (251.4 MB ZIP). [file pbio.0020329.sd016.zip › 040604_OS5_st1_1576.tif]

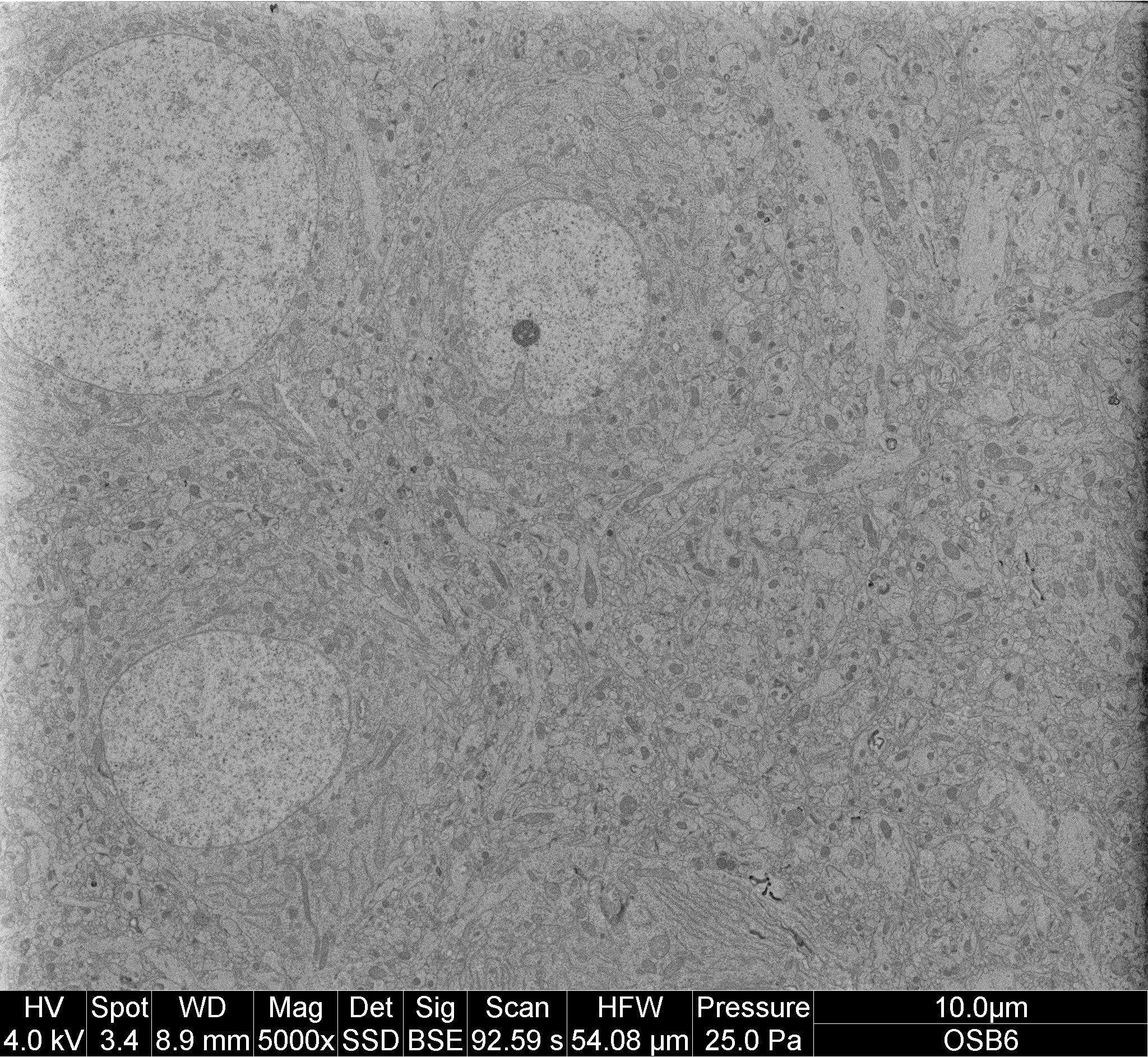

Supplement: Dataset S16 — (251.4 MB ZIP). [file pbio.0020329.sd016.zip › 040604_OS5_st1_1577.tif]

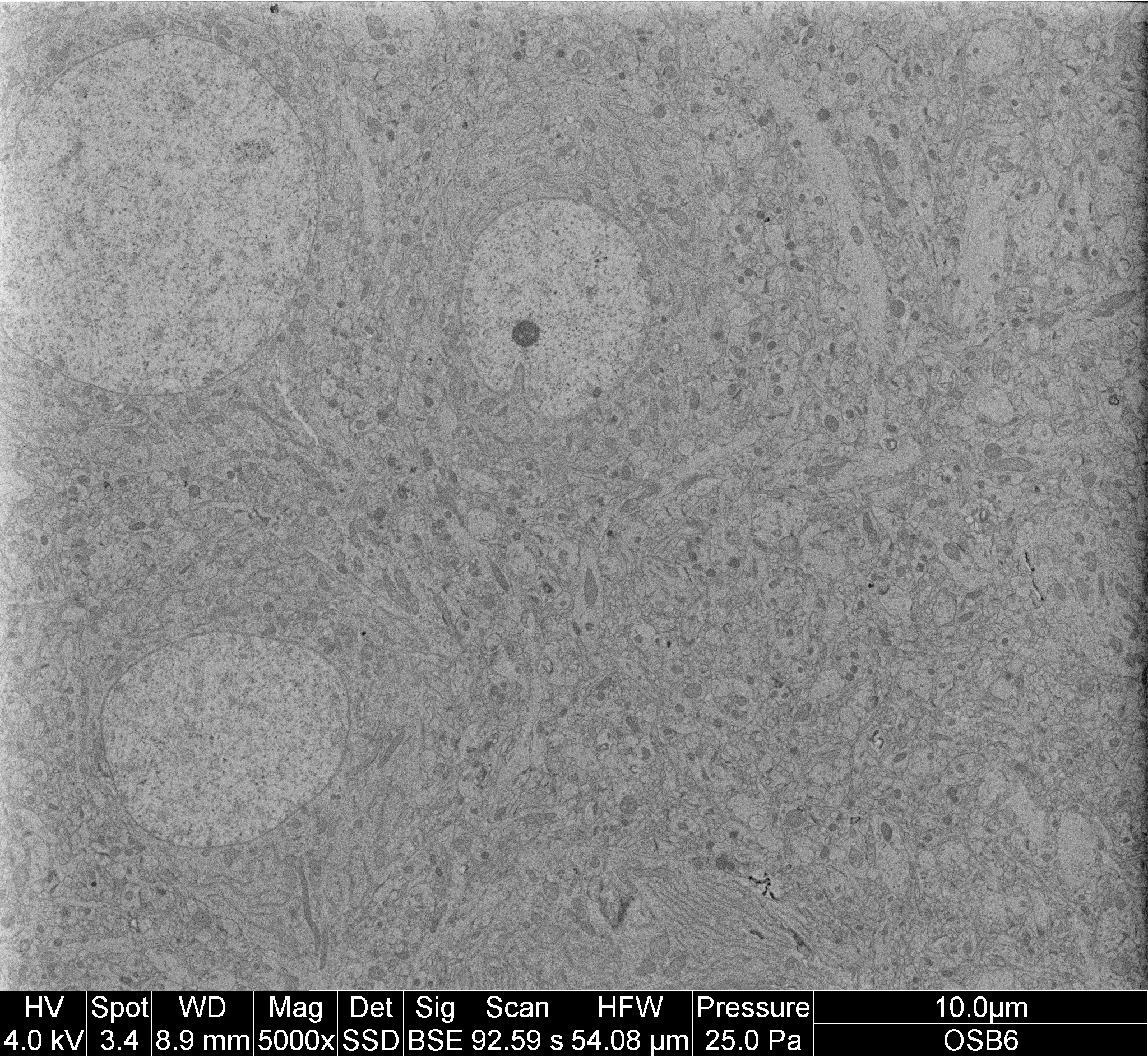

Supplement: Dataset S16 — (251.4 MB ZIP). [file pbio.0020329.sd016.zip › 040604_OS5_st1_1578.tif]

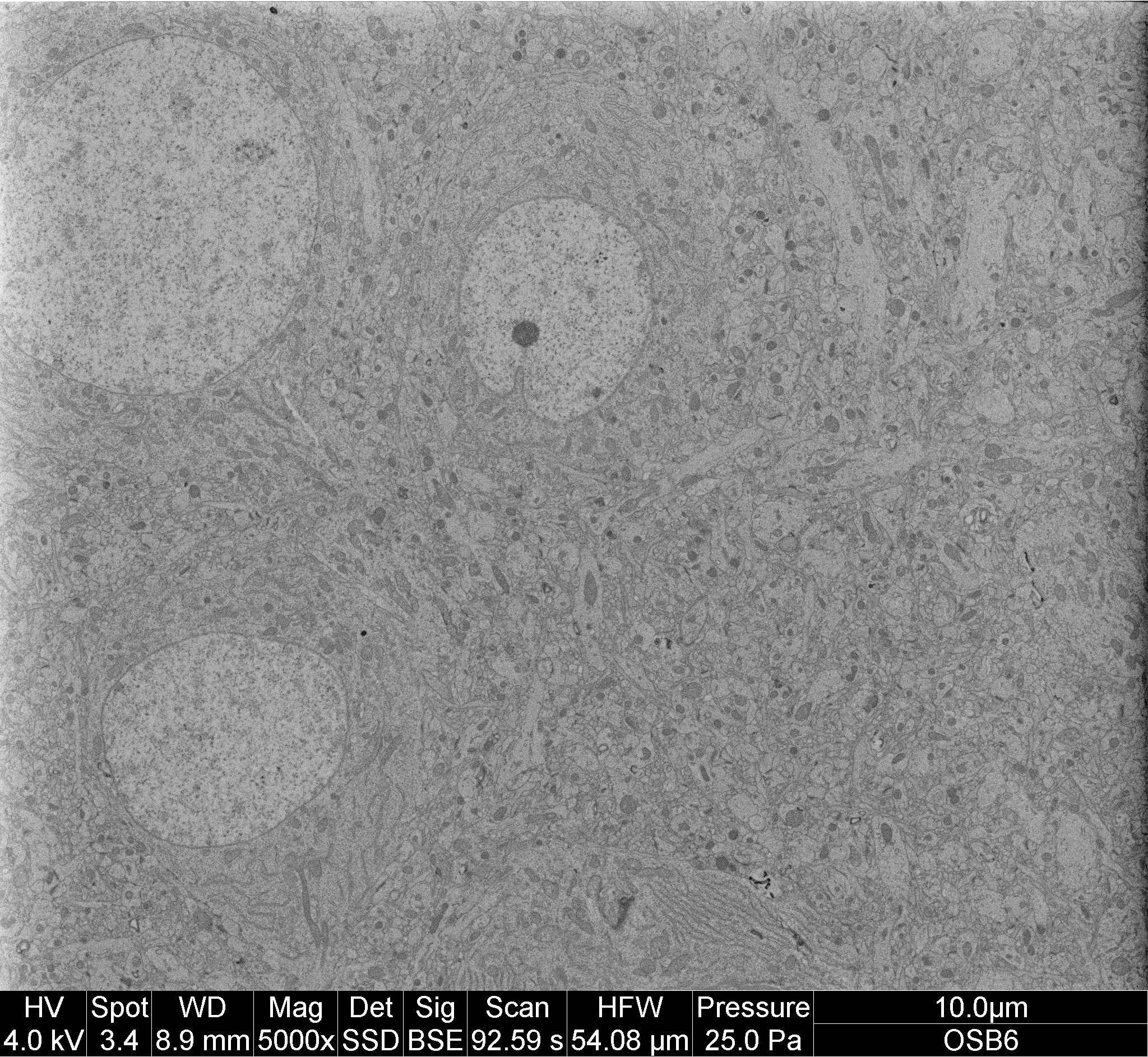

Supplement: Dataset S16 — (251.4 MB ZIP). [file pbio.0020329.sd016.zip › 040604_OS5_st1_1579.tif]

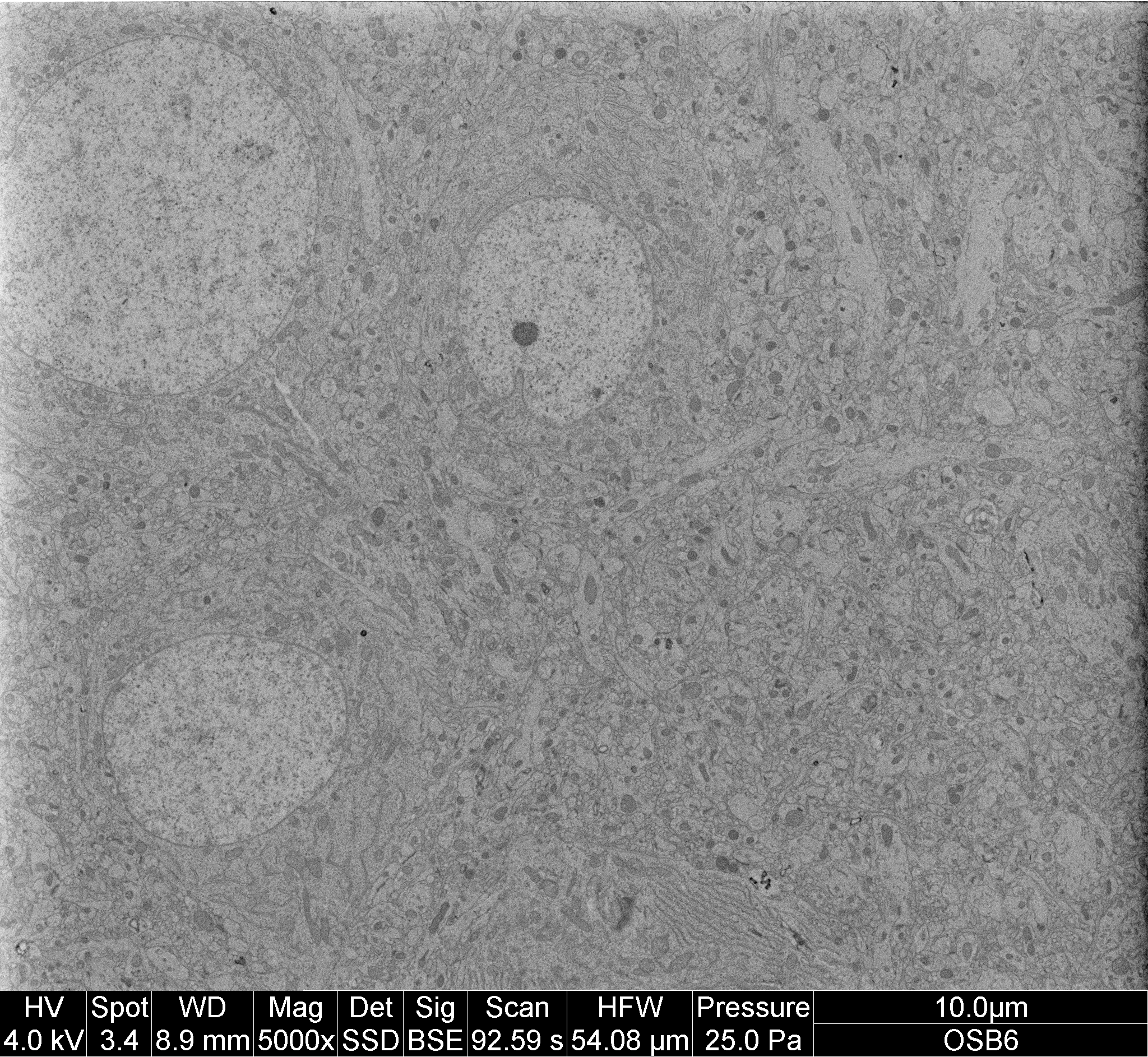

Supplement: Dataset S16 — (251.4 MB ZIP). [file pbio.0020329.sd016.zip › 040604_OS5_st1_1580.tif]

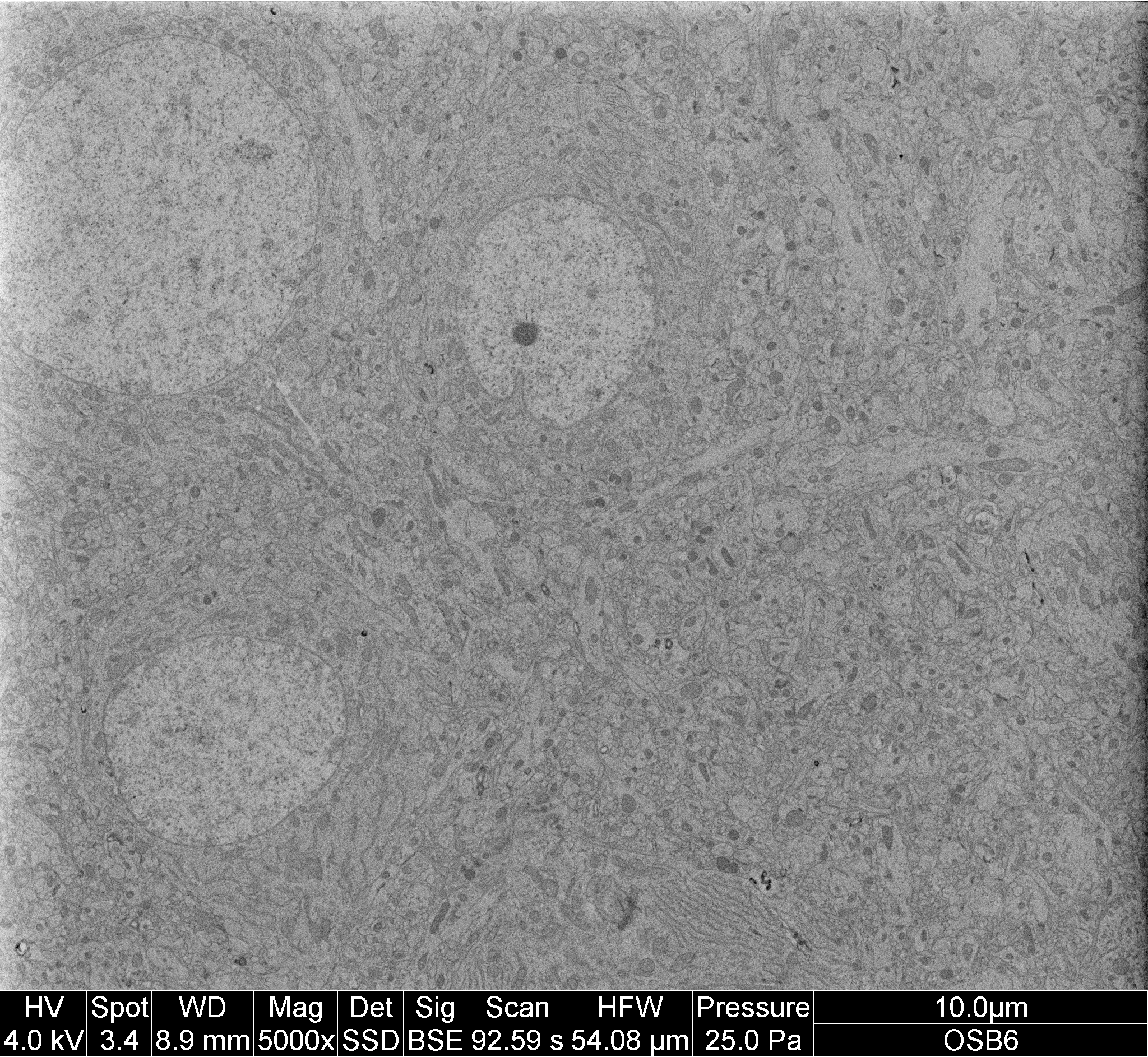

Supplement: Dataset S16 — (251.4 MB ZIP). [file pbio.0020329.sd016.zip › 040604_OS5_st1_1581.tif]

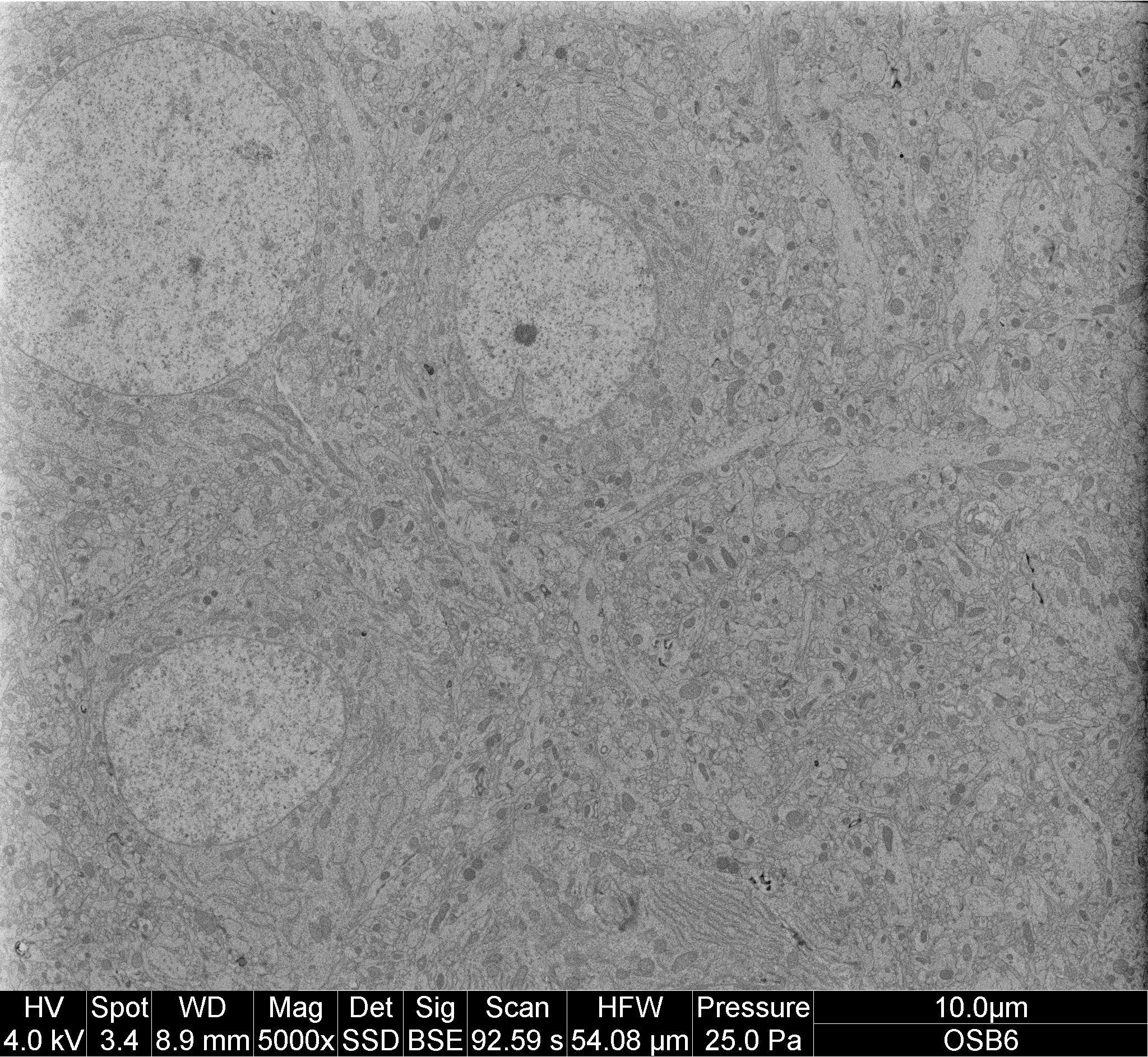

Supplement: Dataset S16 — (251.4 MB ZIP). [file pbio.0020329.sd016.zip › 040604_OS5_st1_1582.tif]

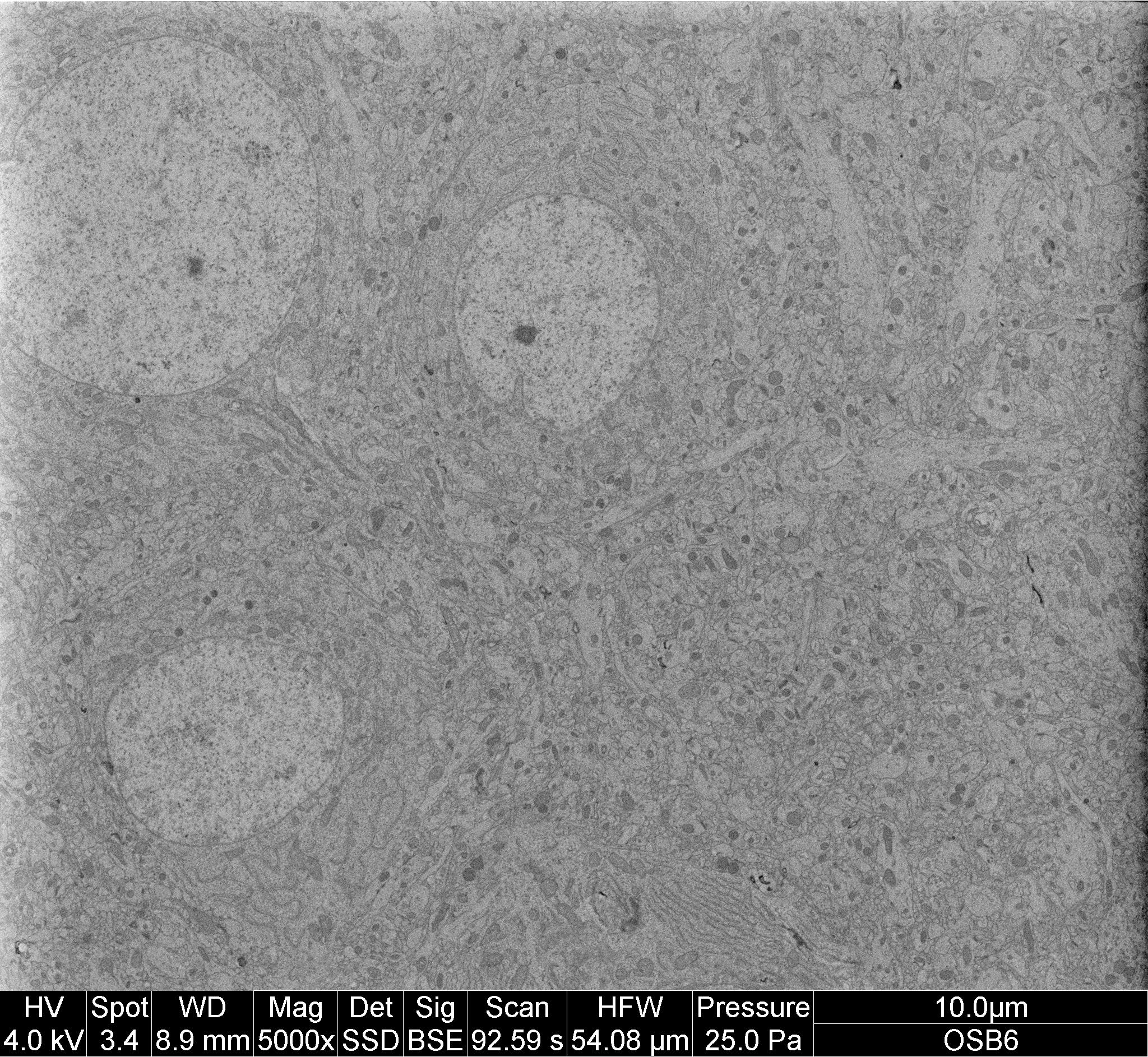

Supplement: Dataset S16 — (251.4 MB ZIP). [file pbio.0020329.sd016.zip › 040604_OS5_st1_1583.tif]

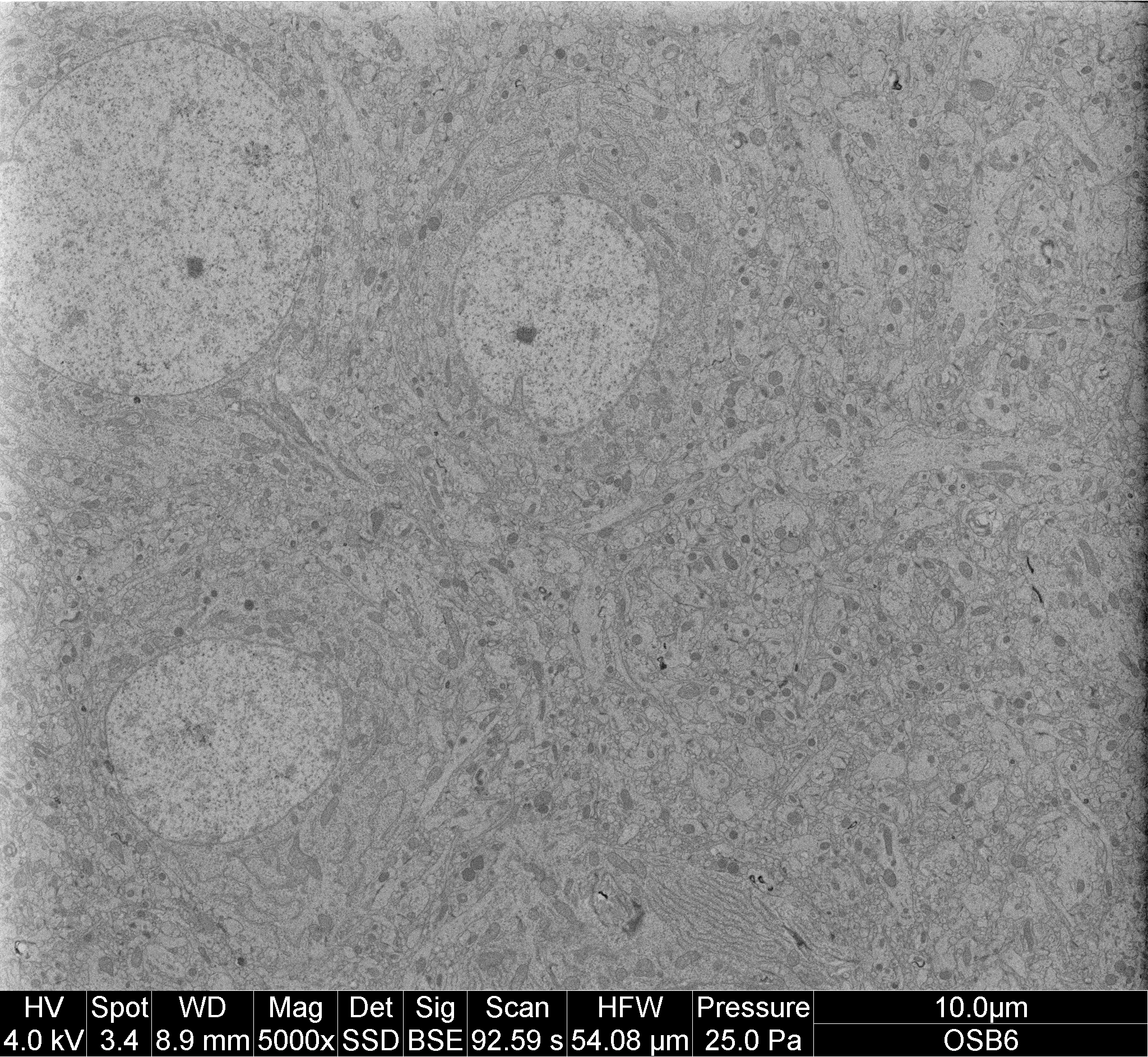

Supplement: Dataset S16 — (251.4 MB ZIP). [file pbio.0020329.sd016.zip › 040604_OS5_st1_1584.tif]

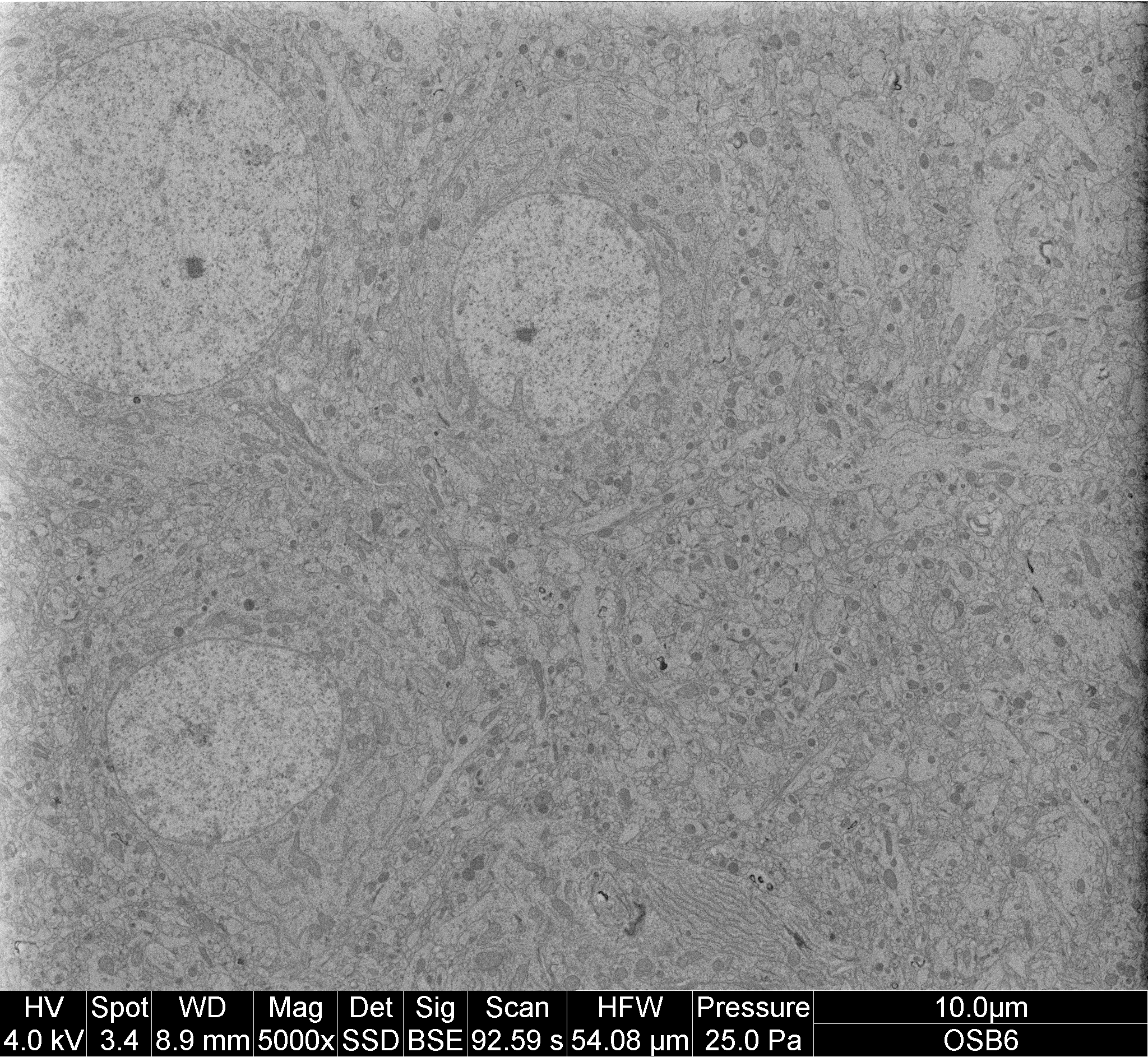

Supplement: Dataset S16 — (251.4 MB ZIP). [file pbio.0020329.sd016.zip › 040604_OS5_st1_1585.tif]

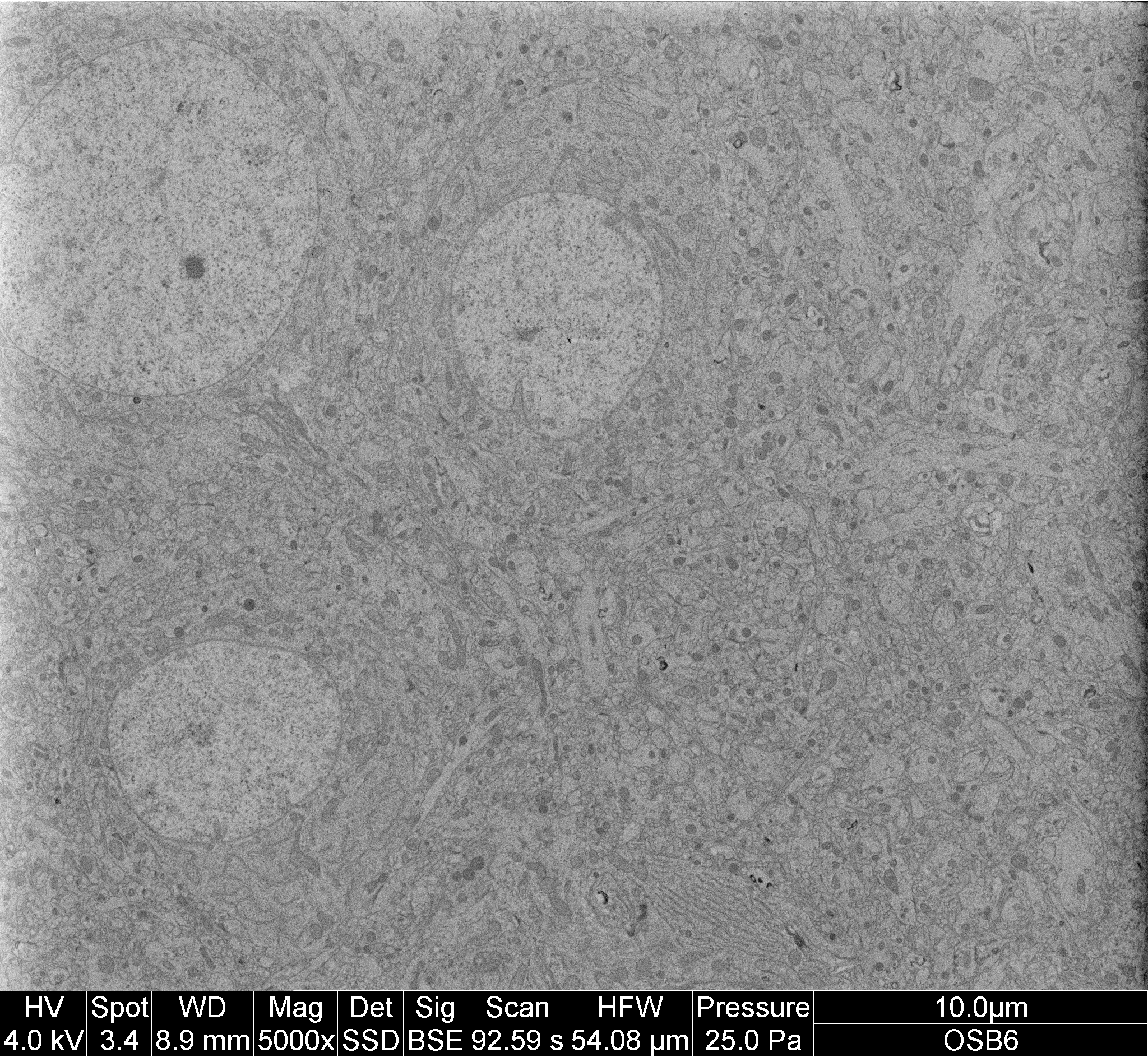

Supplement: Dataset S16 — (251.4 MB ZIP). [file pbio.0020329.sd016.zip › 040604_OS5_st1_1586.tif]

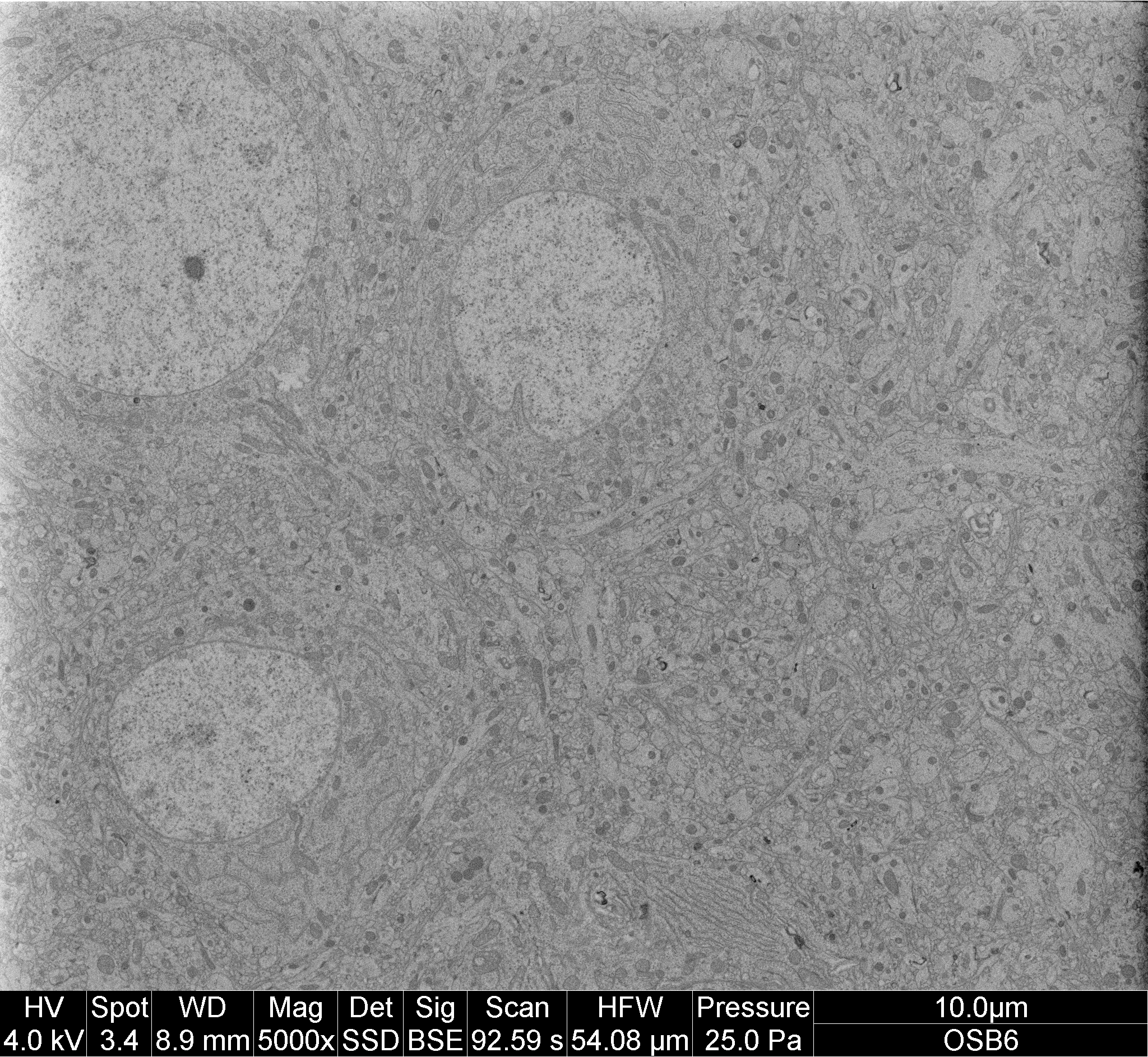

Supplement: Dataset S16 — (251.4 MB ZIP). [file pbio.0020329.sd016.zip › 040604_OS5_st1_1587.tif]

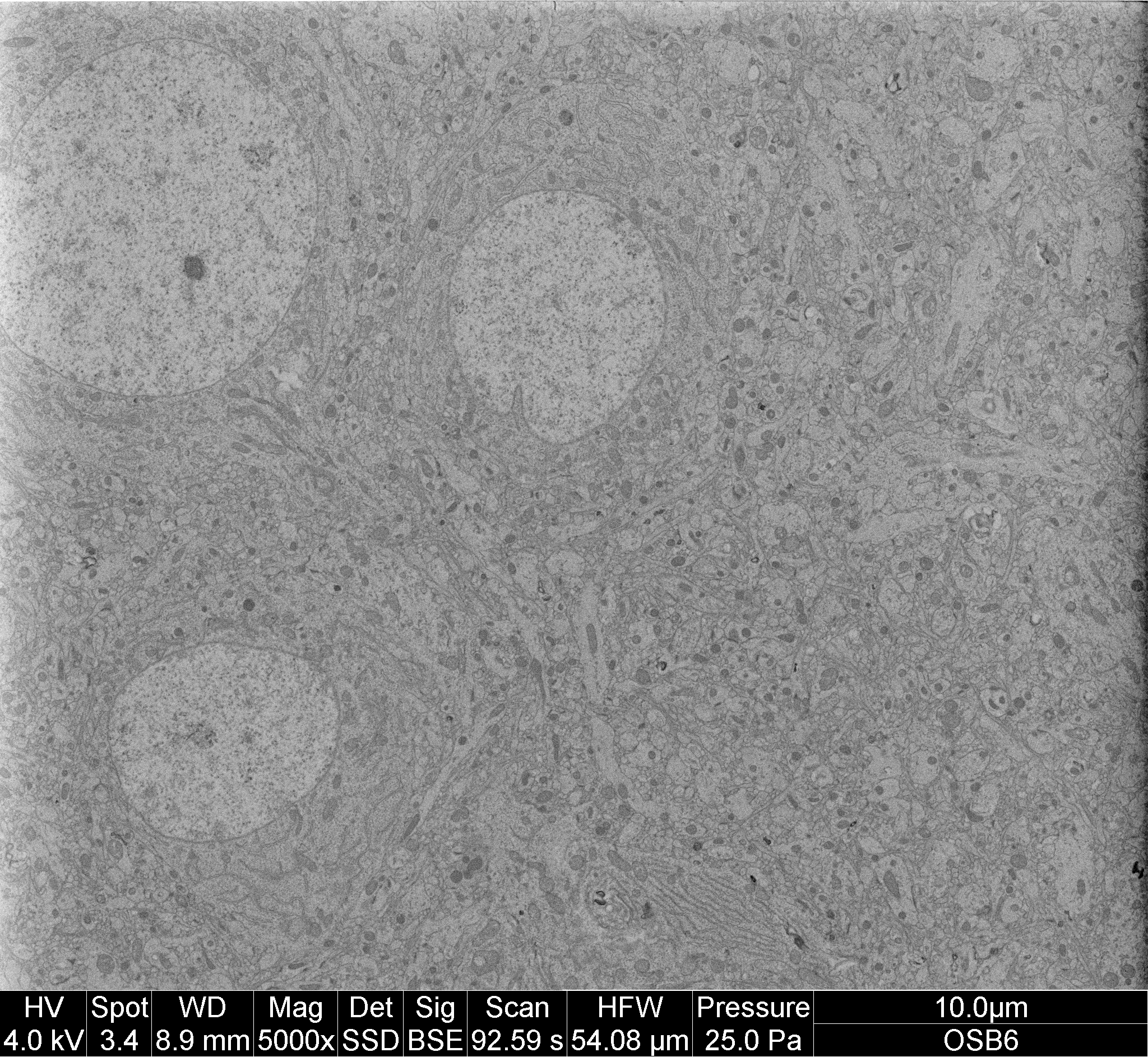

Supplement: Dataset S16 — (251.4 MB ZIP). [file pbio.0020329.sd016.zip › 040604_OS5_st1_1588.tif]

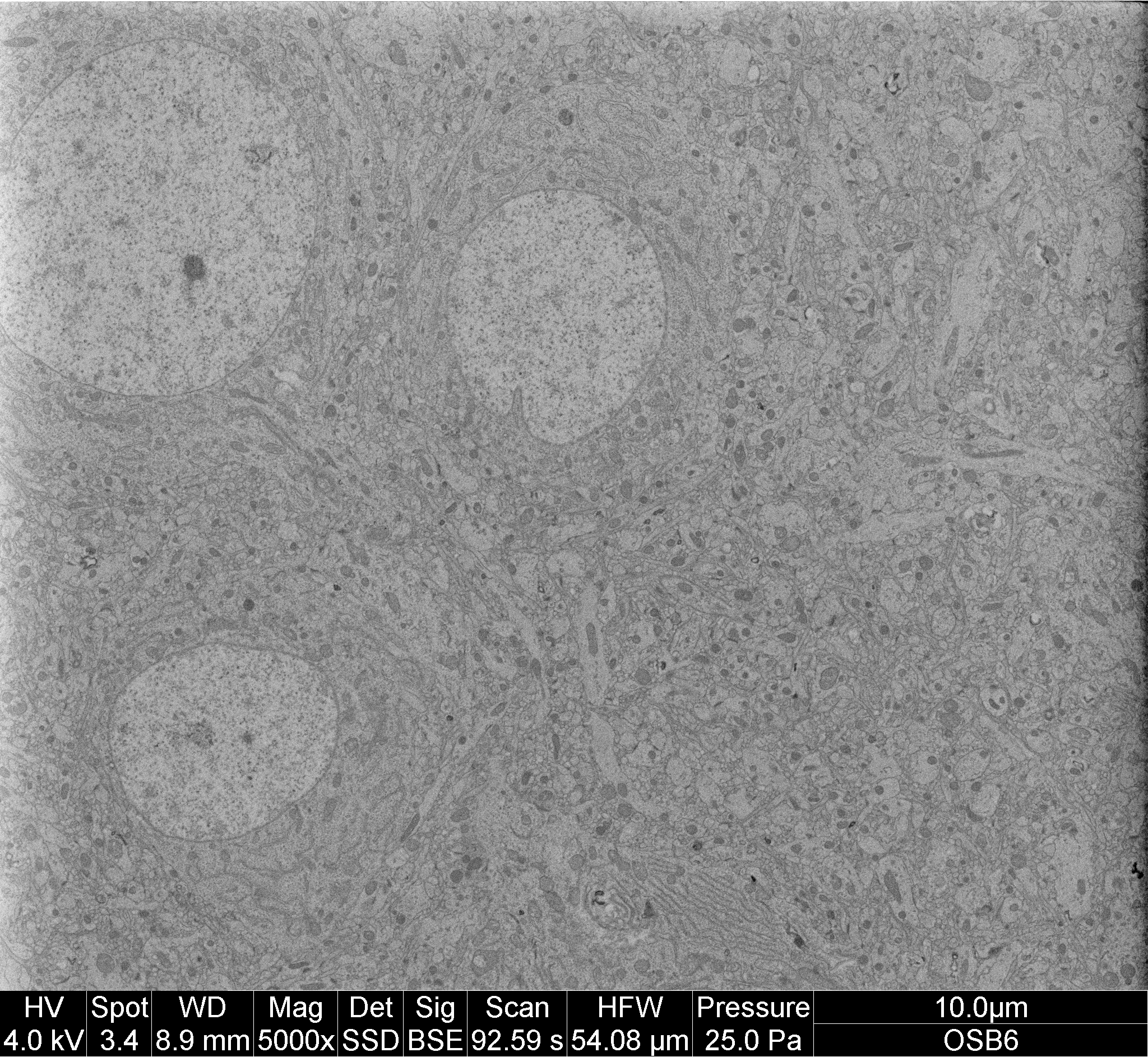

Supplement: Dataset S16 — (251.4 MB ZIP). [file pbio.0020329.sd016.zip › 040604_OS5_st1_1589.tif]

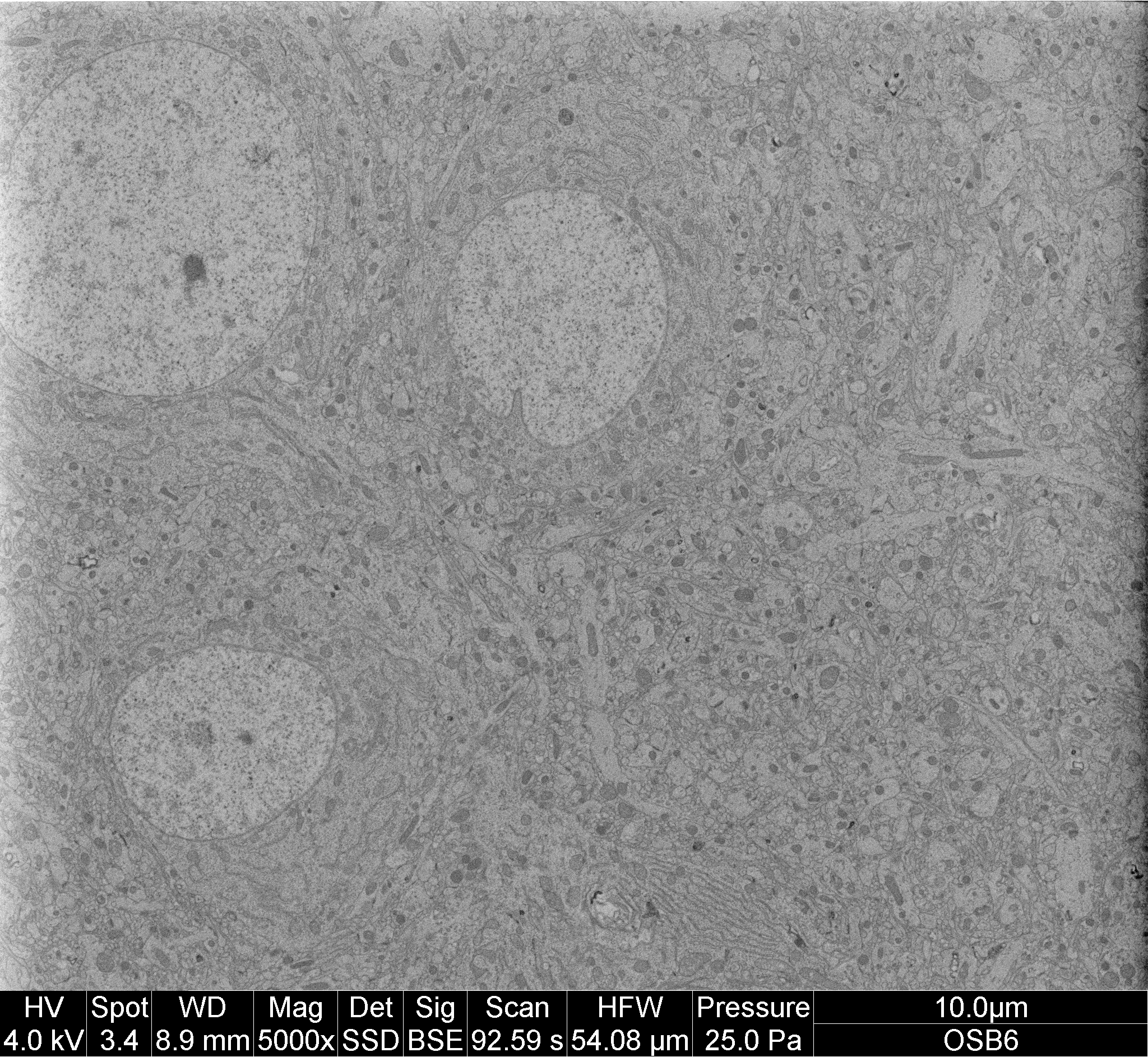

Supplement: Dataset S16 — (251.4 MB ZIP). [file pbio.0020329.sd016.zip › 040604_OS5_st1_1590.tif]

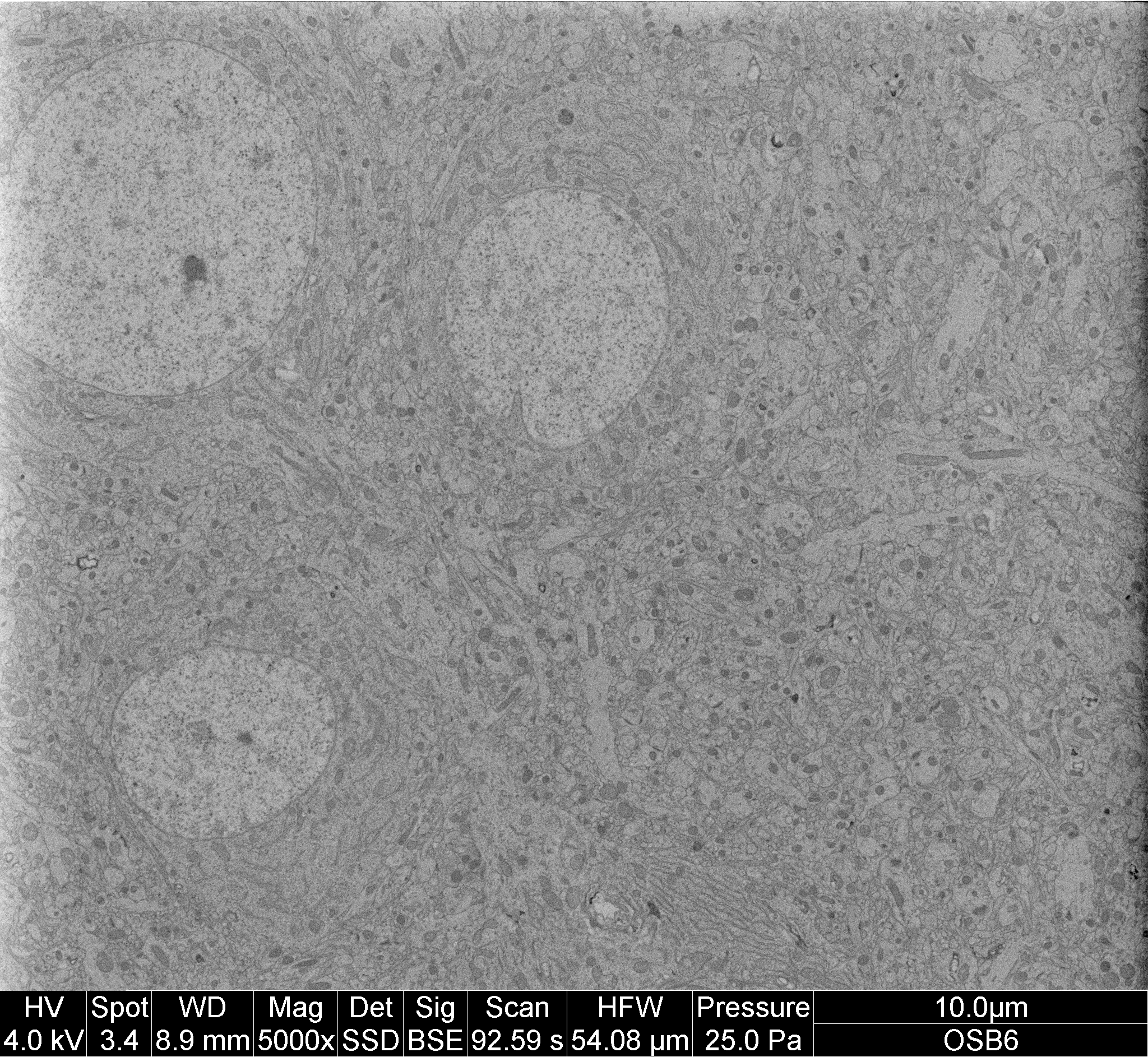

Supplement: Dataset S16 — (251.4 MB ZIP). [file pbio.0020329.sd016.zip › 040604_OS5_st1_1591.tif]

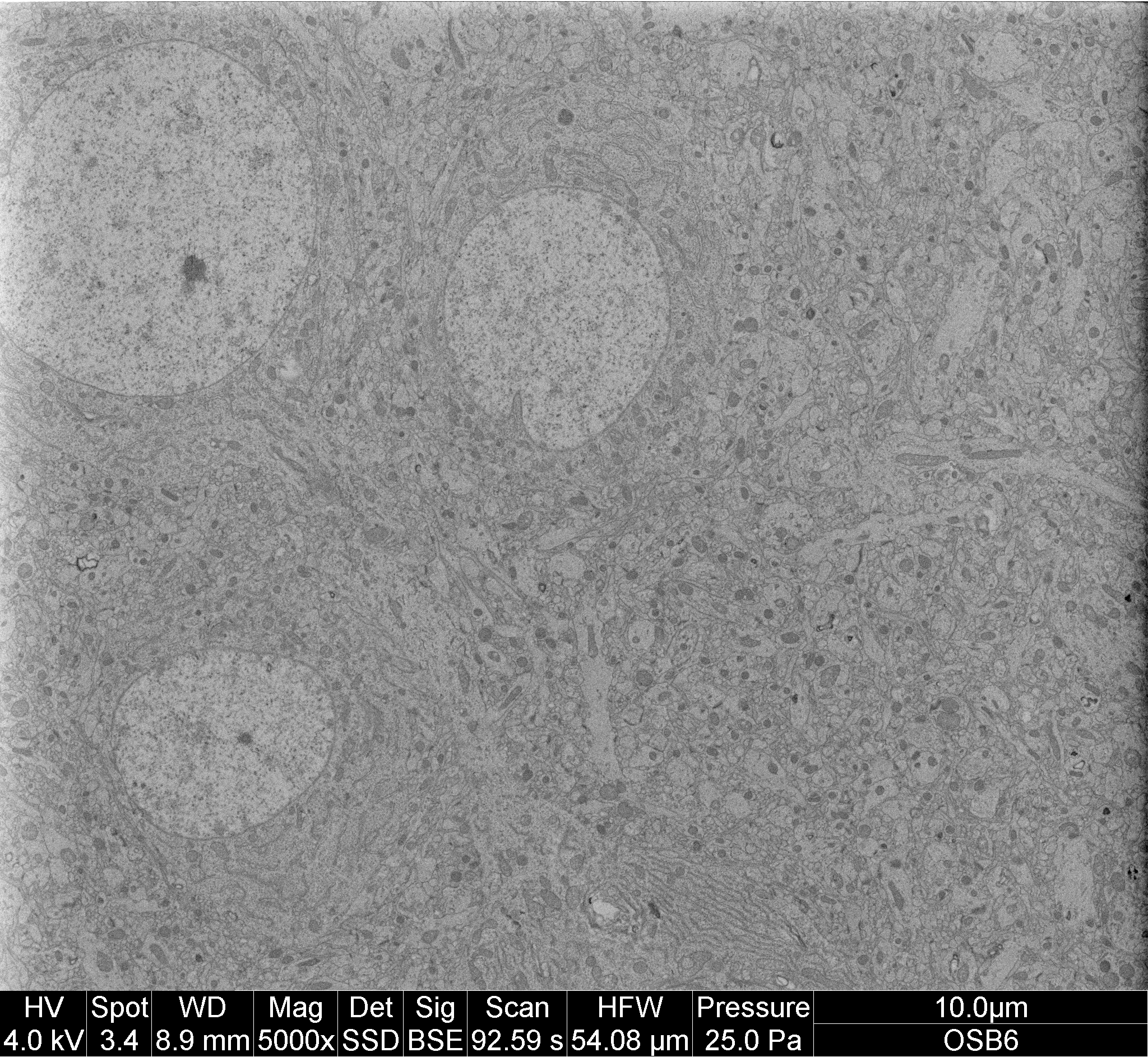

Supplement: Dataset S16 — (251.4 MB ZIP). [file pbio.0020329.sd016.zip › 040604_OS5_st1_1592.tif]

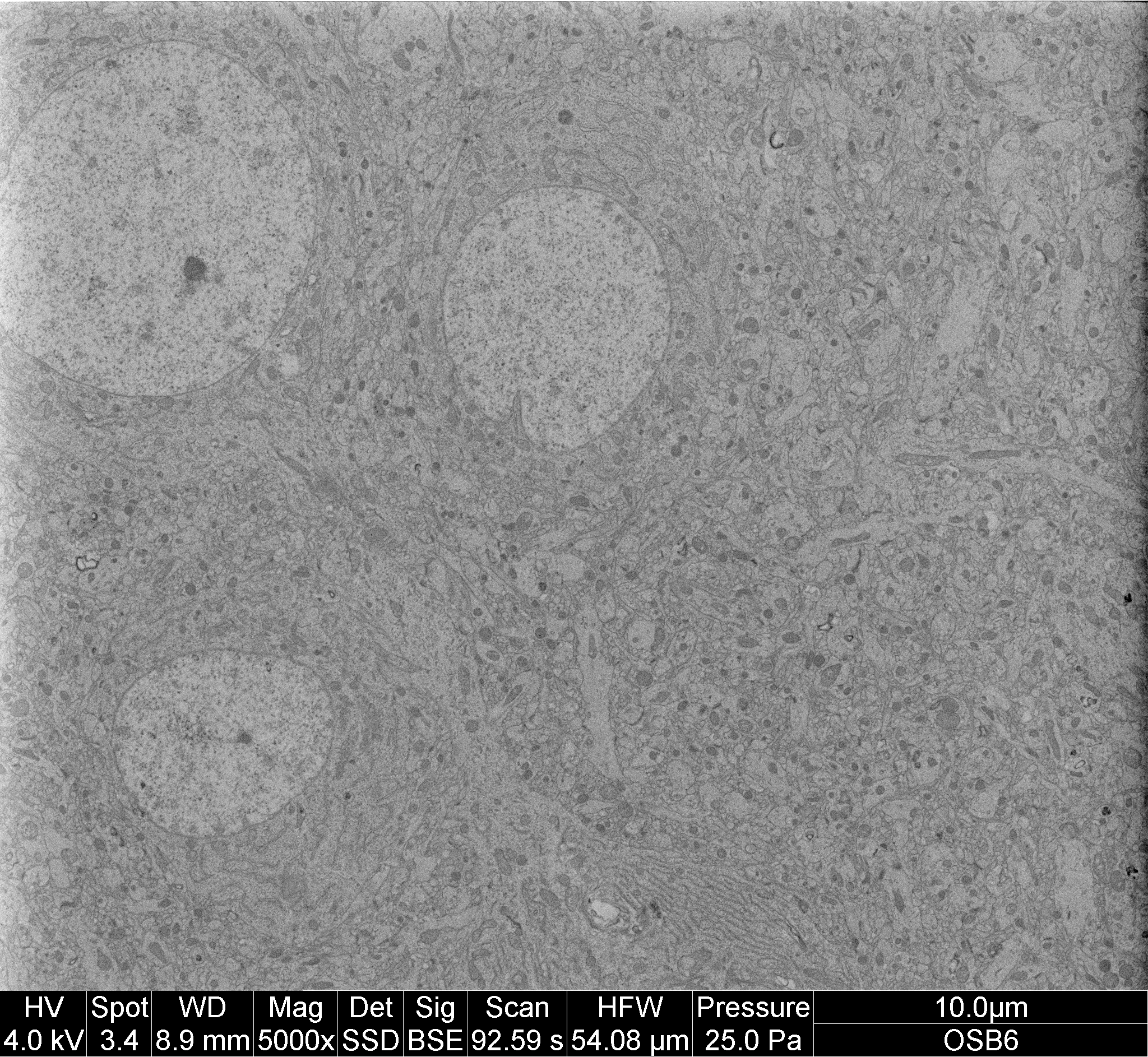

Supplement: Dataset S16 — (251.4 MB ZIP). [file pbio.0020329.sd016.zip › 040604_OS5_st1_1593.tif]

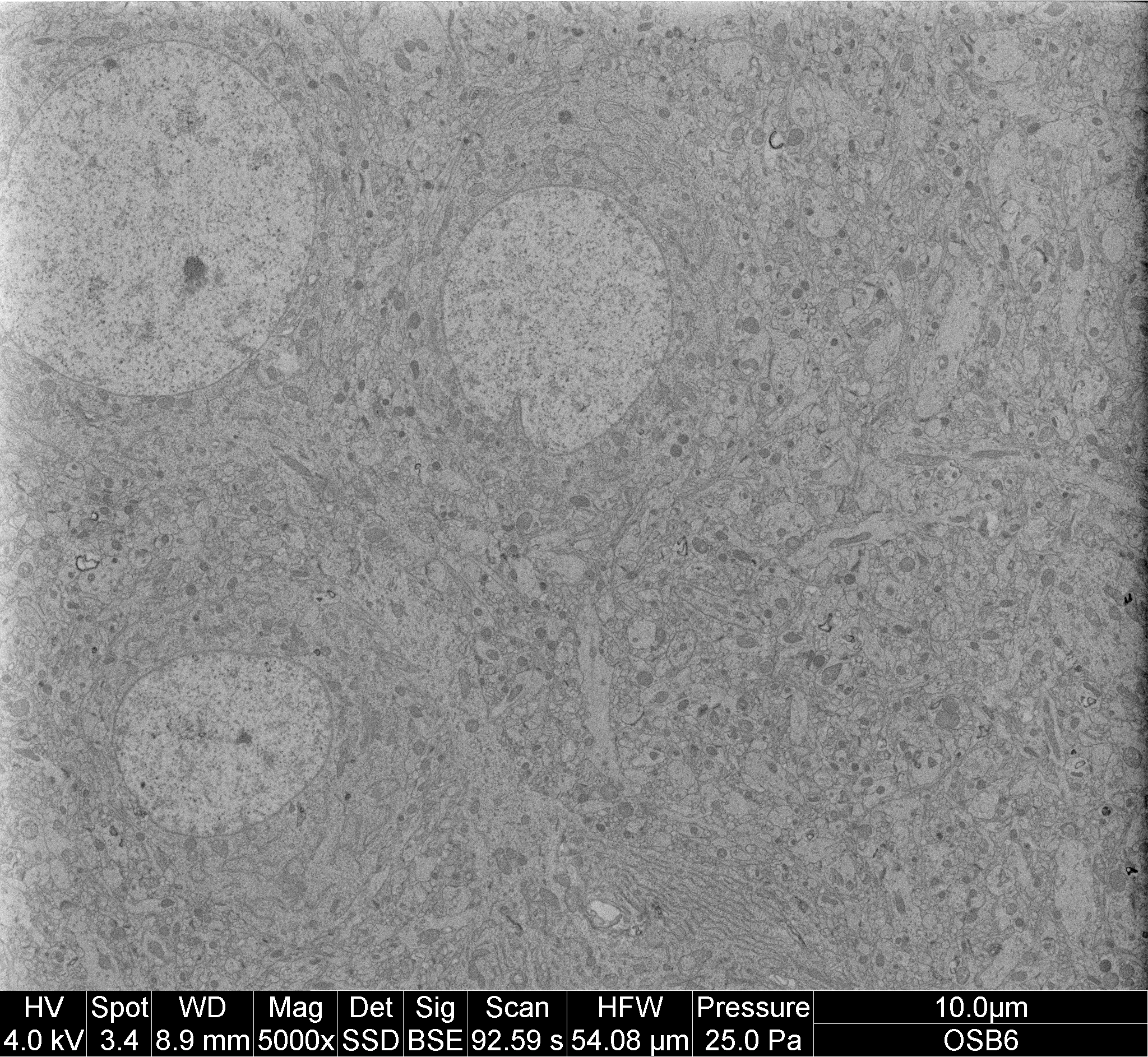

Supplement: Dataset S16 — (251.4 MB ZIP). [file pbio.0020329.sd016.zip › 040604_OS5_st1_1594.tif]

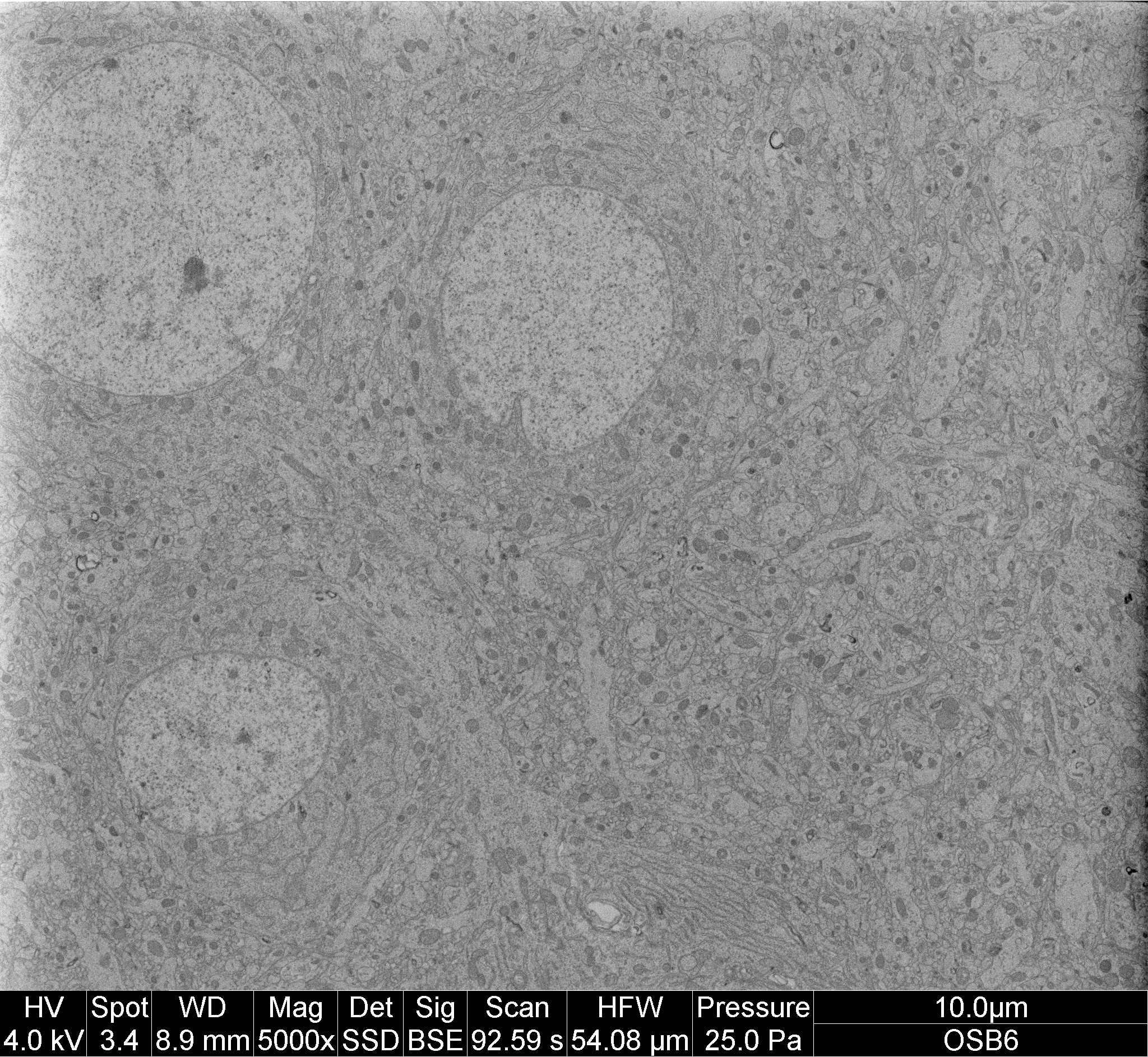

Supplement: Dataset S16 — (251.4 MB ZIP). [file pbio.0020329.sd016.zip › 040604_OS5_st1_1595.tif]

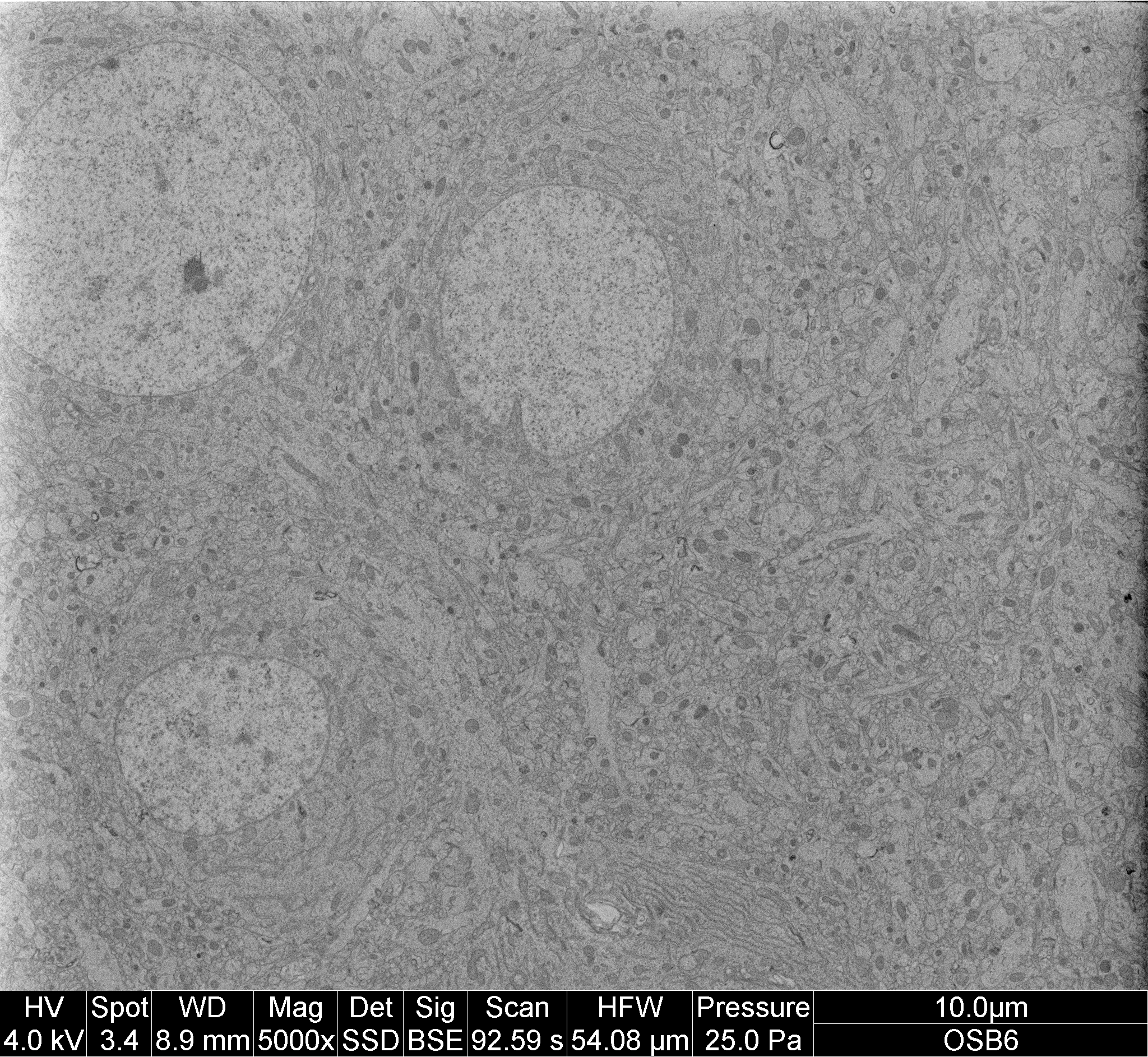

Supplement: Dataset S16 — (251.4 MB ZIP). [file pbio.0020329.sd016.zip › 040604_OS5_st1_1596.tif]

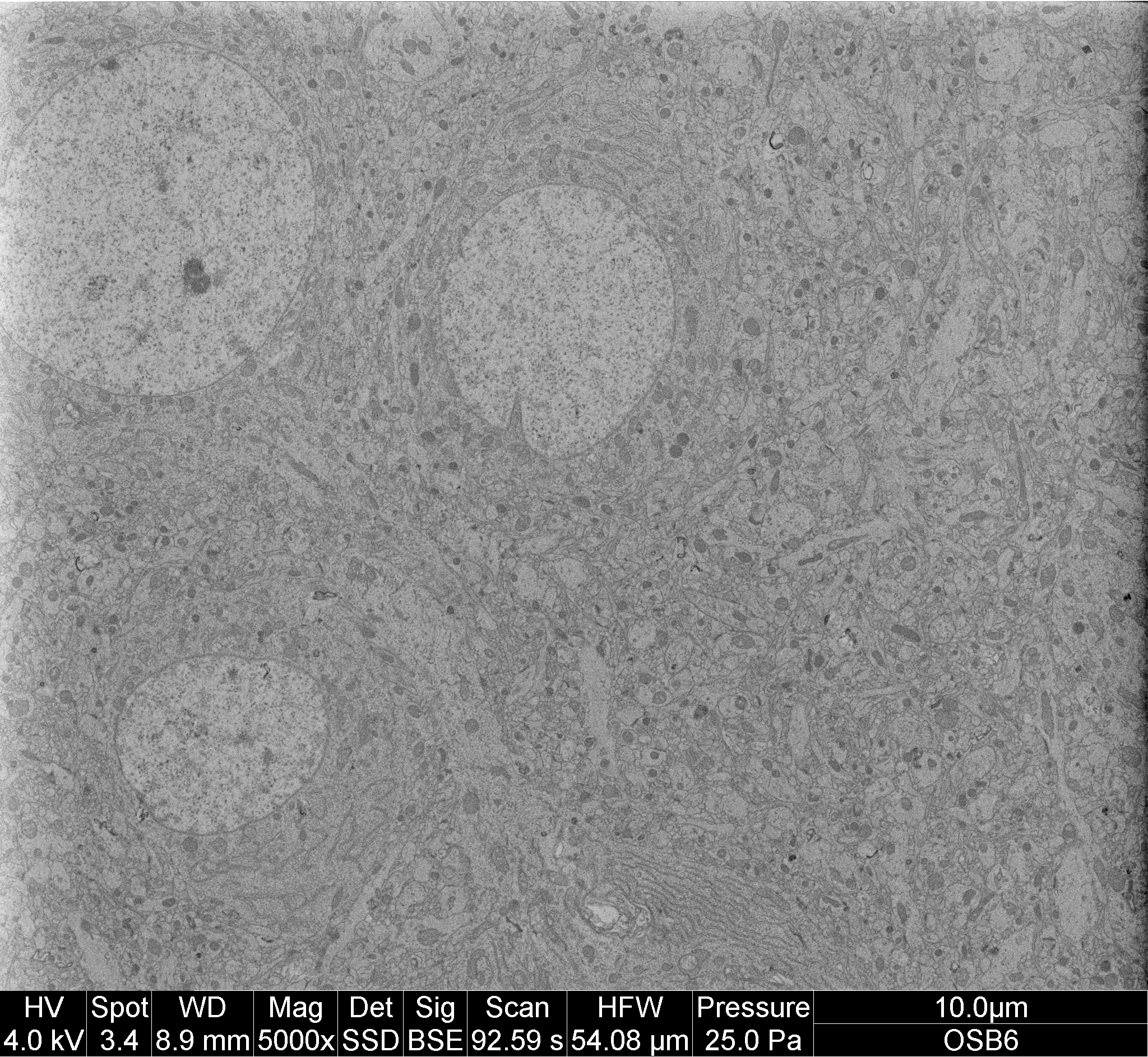

Supplement: Dataset S16 — (251.4 MB ZIP). [file pbio.0020329.sd016.zip › 040604_OS5_st1_1597.tif]

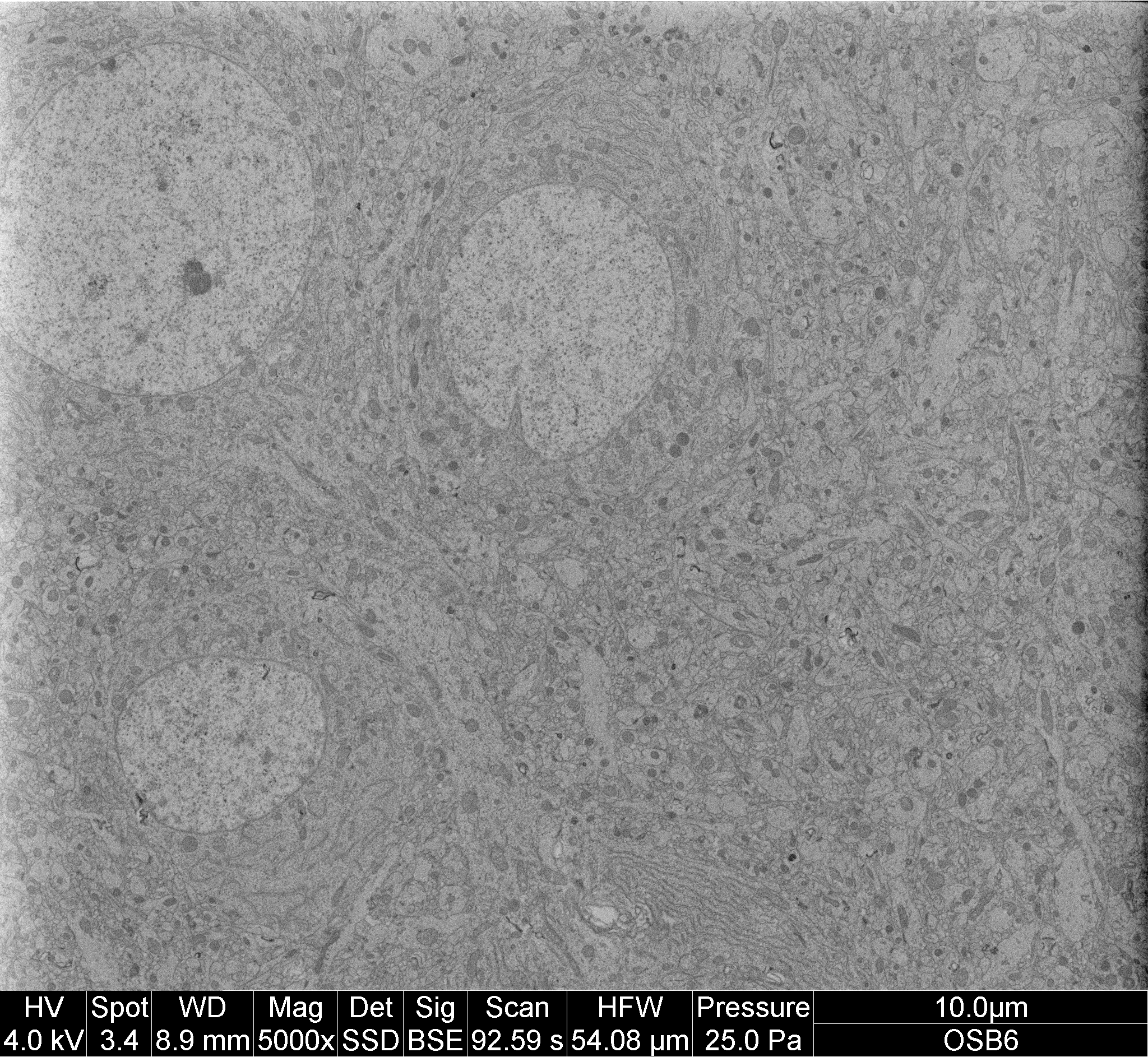

Supplement: Dataset S16 — (251.4 MB ZIP). [file pbio.0020329.sd016.zip › 040604_OS5_st1_1598.tif]

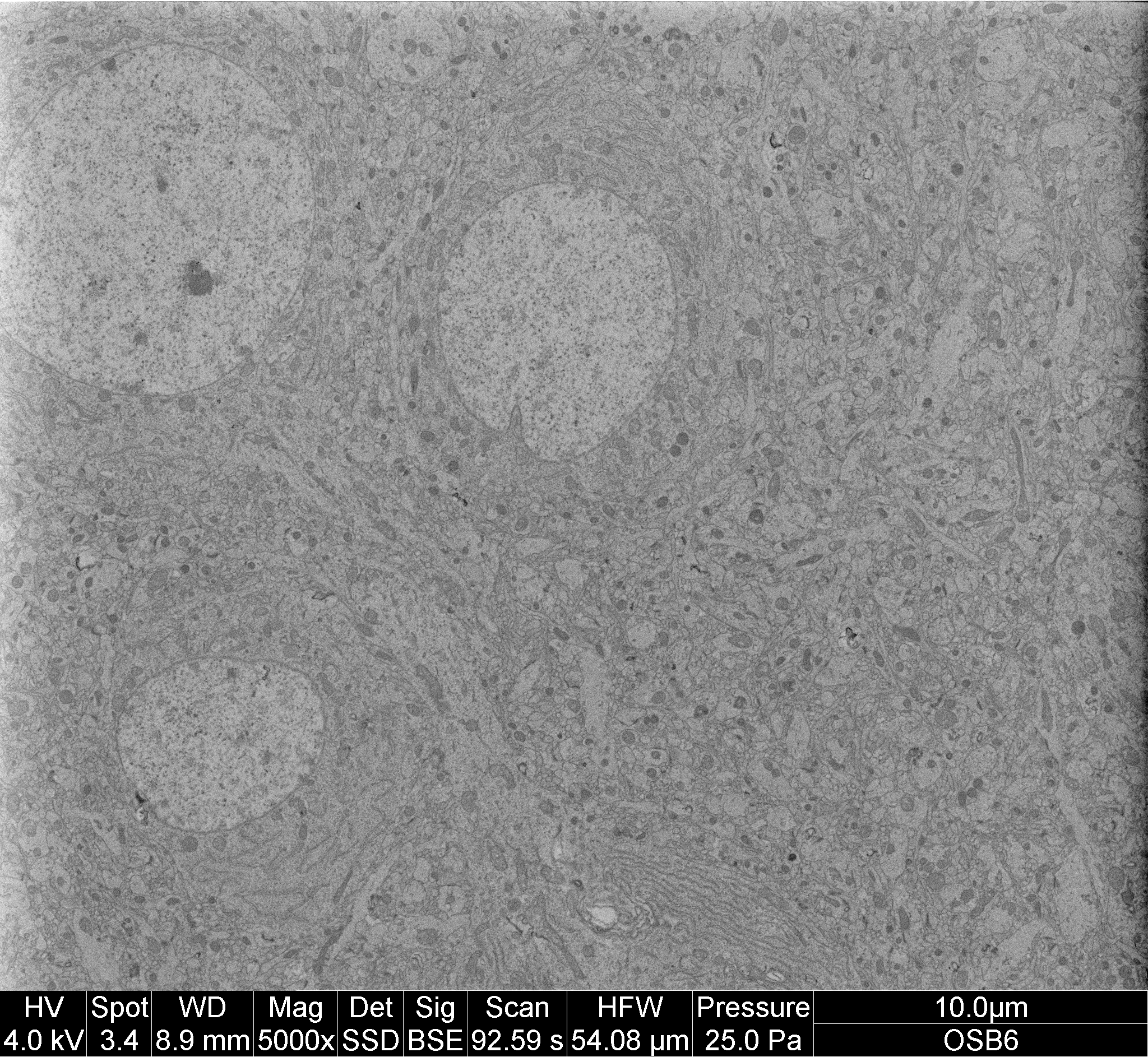

Supplement: Dataset S16 — (251.4 MB ZIP). [file pbio.0020329.sd016.zip › 040604_OS5_st1_1599.tif]

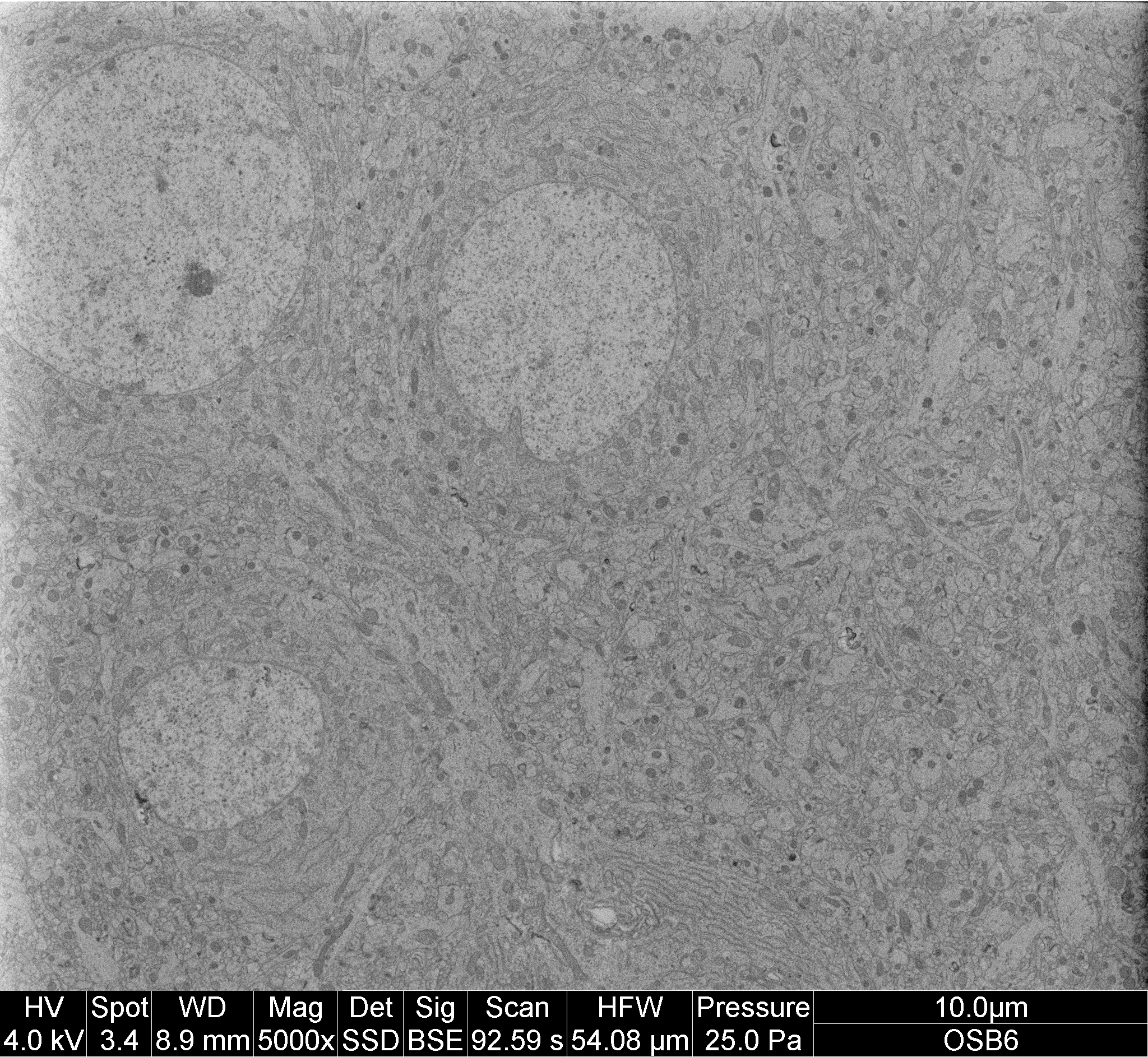

Supplement: Dataset S17 — (252.7 MB ZIP). [file pbio.0020329.sd017.zip › 040604_OS5_st1_1600.tif]
