# Supplementary figures and images for: Serial Block-Face Scanning Electron Microscopy to Reconstruct Three-Dimensional Tissue Nanostructure (part 17 of 21)
Source: PLoS Biol. 2004 Oct 19;2(11):e329. doi: 10.1371/journal.pbio.0020329 (PMC524270; doi:10.1371/journal.pbio.0020329)

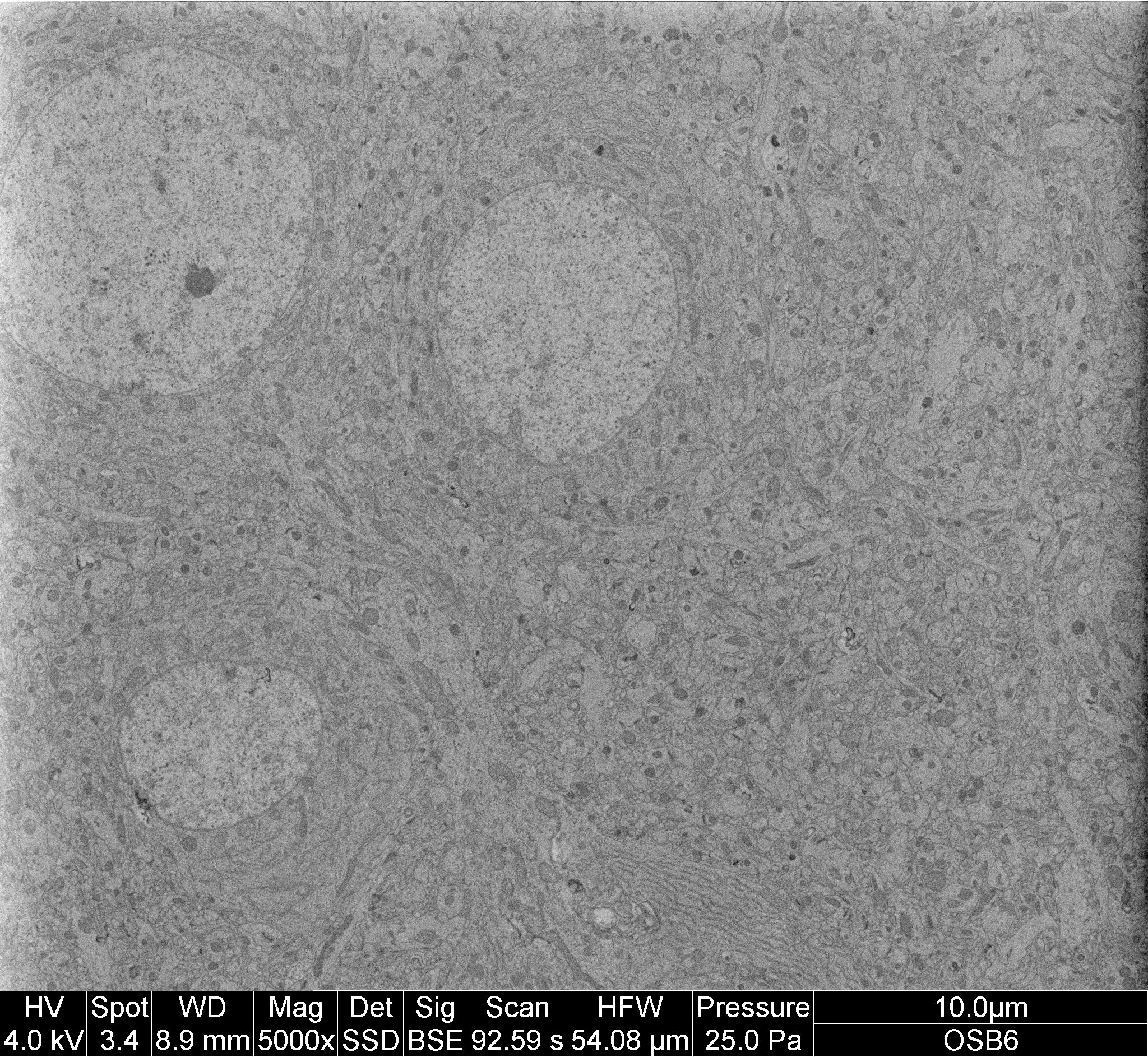

Supplement: Dataset S17 — (252.7 MB ZIP). [file pbio.0020329.sd017.zip › 040604_OS5_st1_1601.tif]

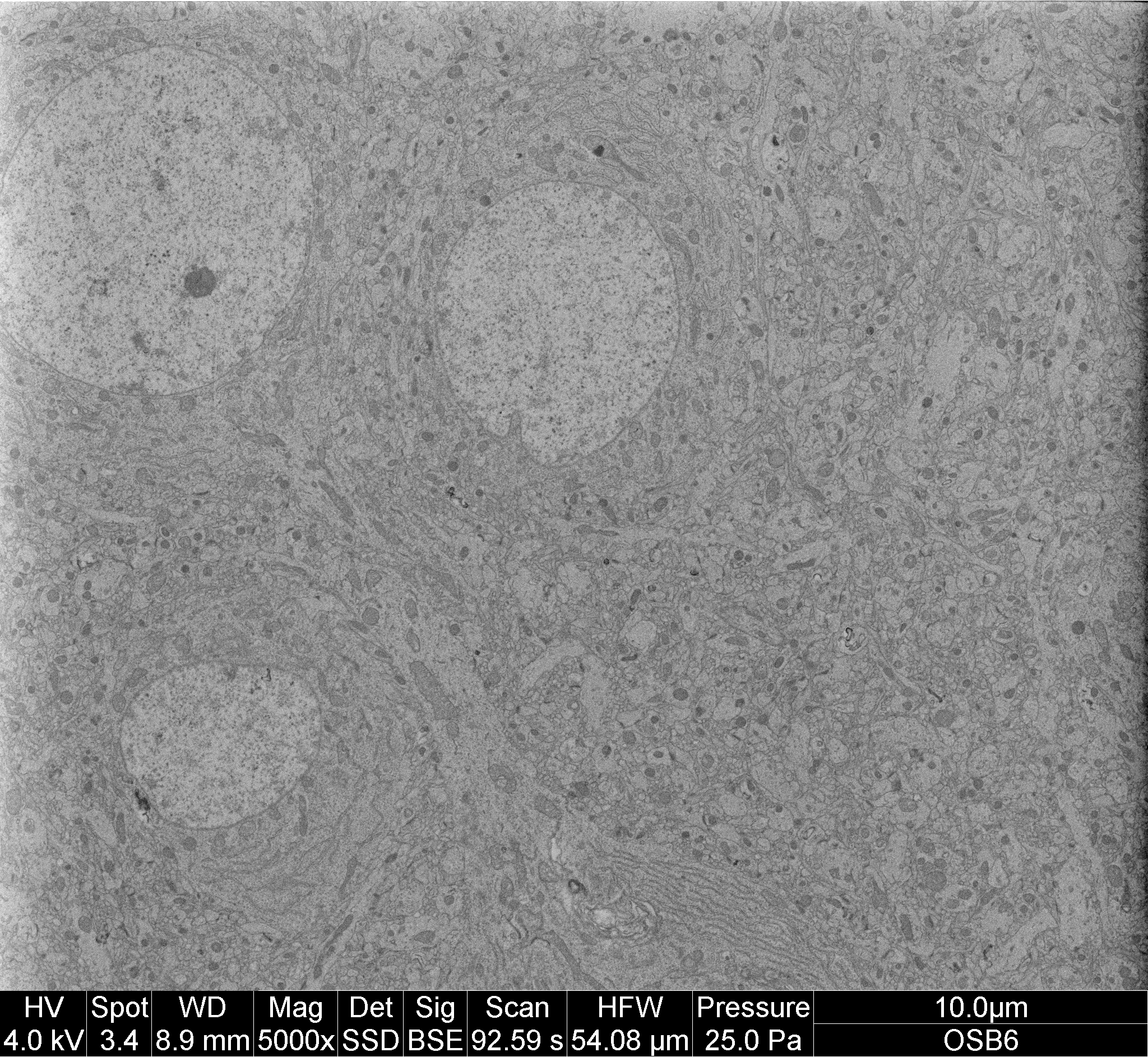

Supplement: Dataset S17 — (252.7 MB ZIP). [file pbio.0020329.sd017.zip › 040604_OS5_st1_1602.tif]

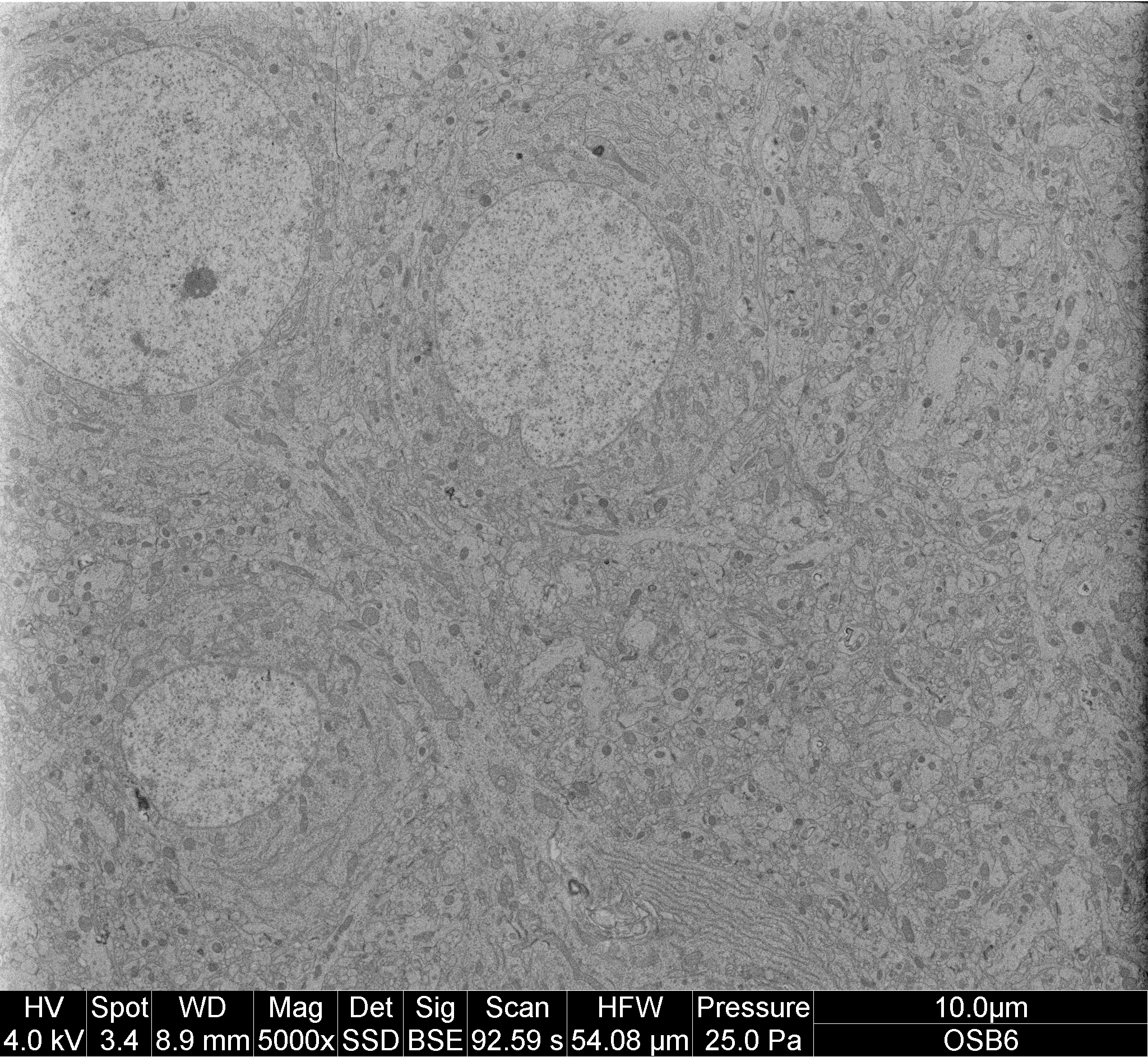

Supplement: Dataset S17 — (252.7 MB ZIP). [file pbio.0020329.sd017.zip › 040604_OS5_st1_1603.tif]

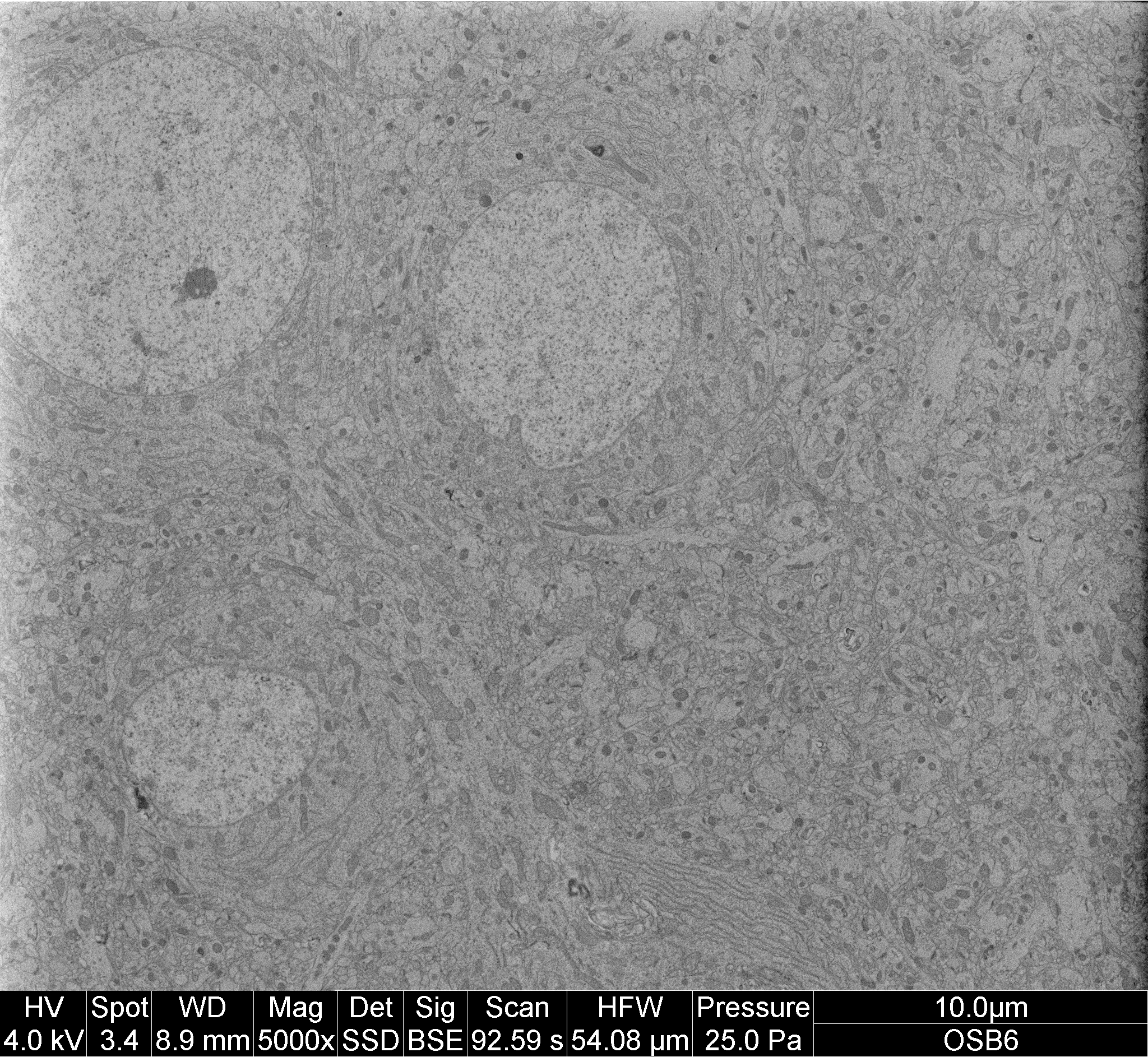

Supplement: Dataset S17 — (252.7 MB ZIP). [file pbio.0020329.sd017.zip › 040604_OS5_st1_1604.tif]

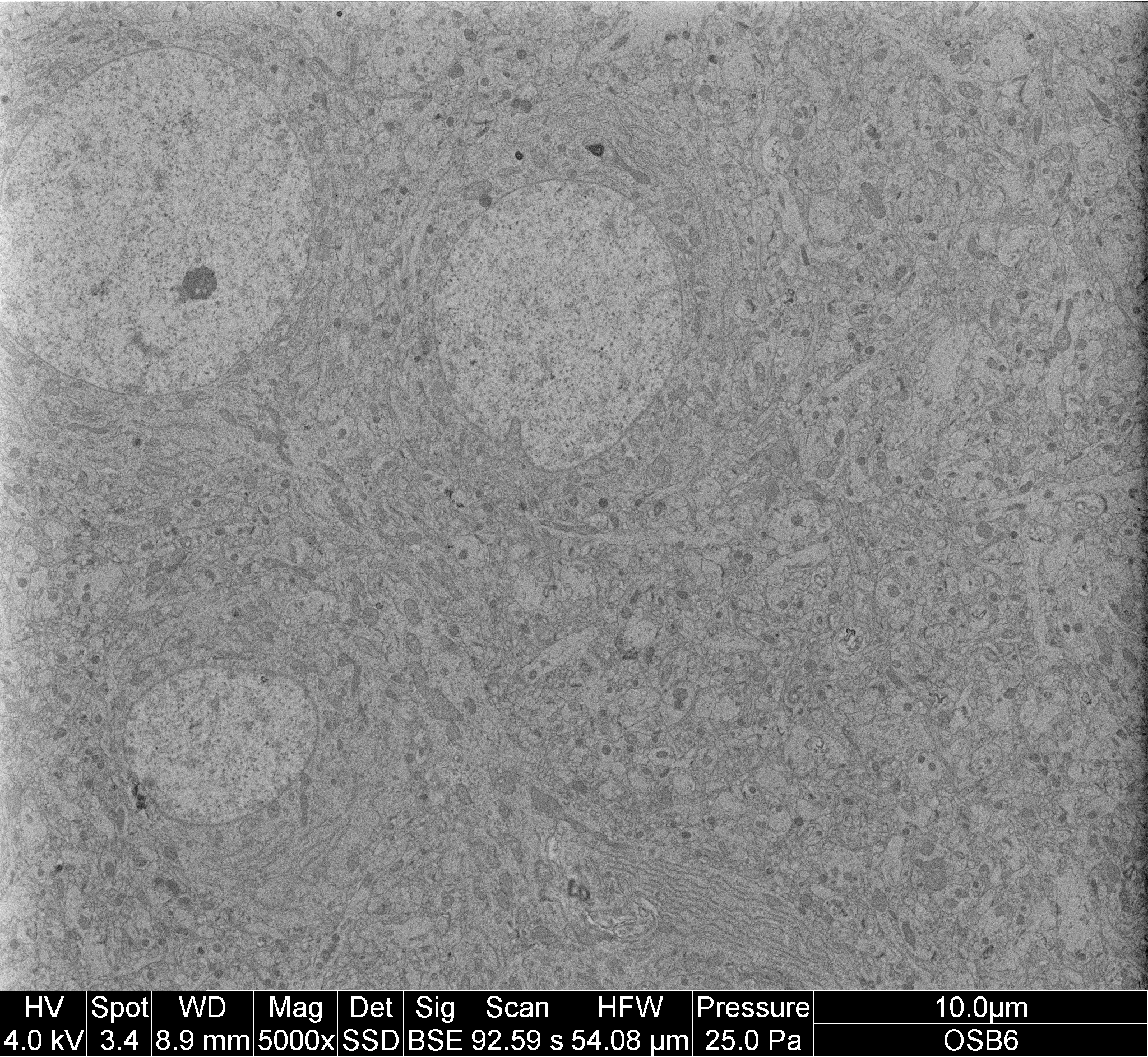

Supplement: Dataset S17 — (252.7 MB ZIP). [file pbio.0020329.sd017.zip › 040604_OS5_st1_1605.tif]

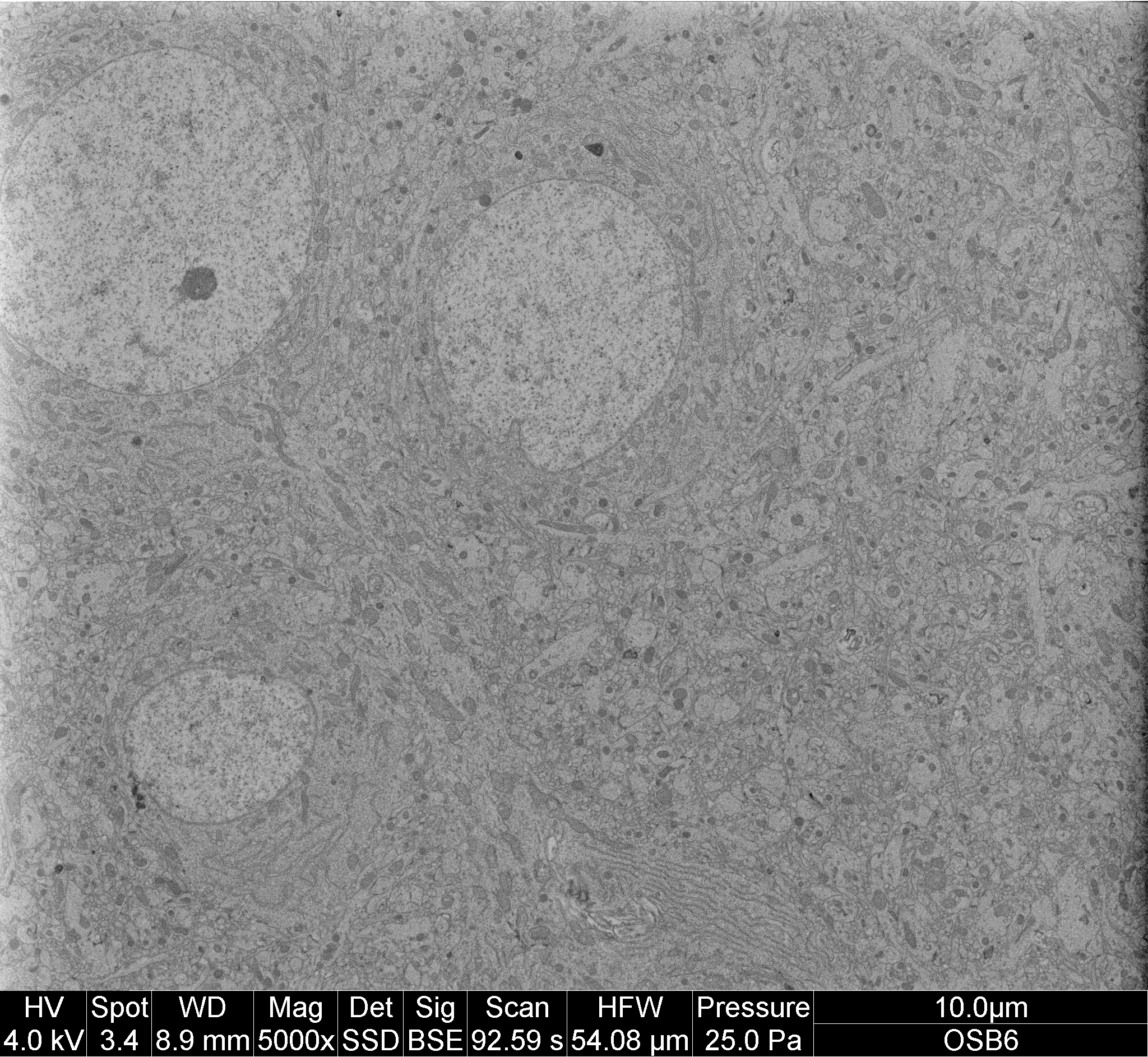

Supplement: Dataset S17 — (252.7 MB ZIP). [file pbio.0020329.sd017.zip › 040604_OS5_st1_1606.tif]

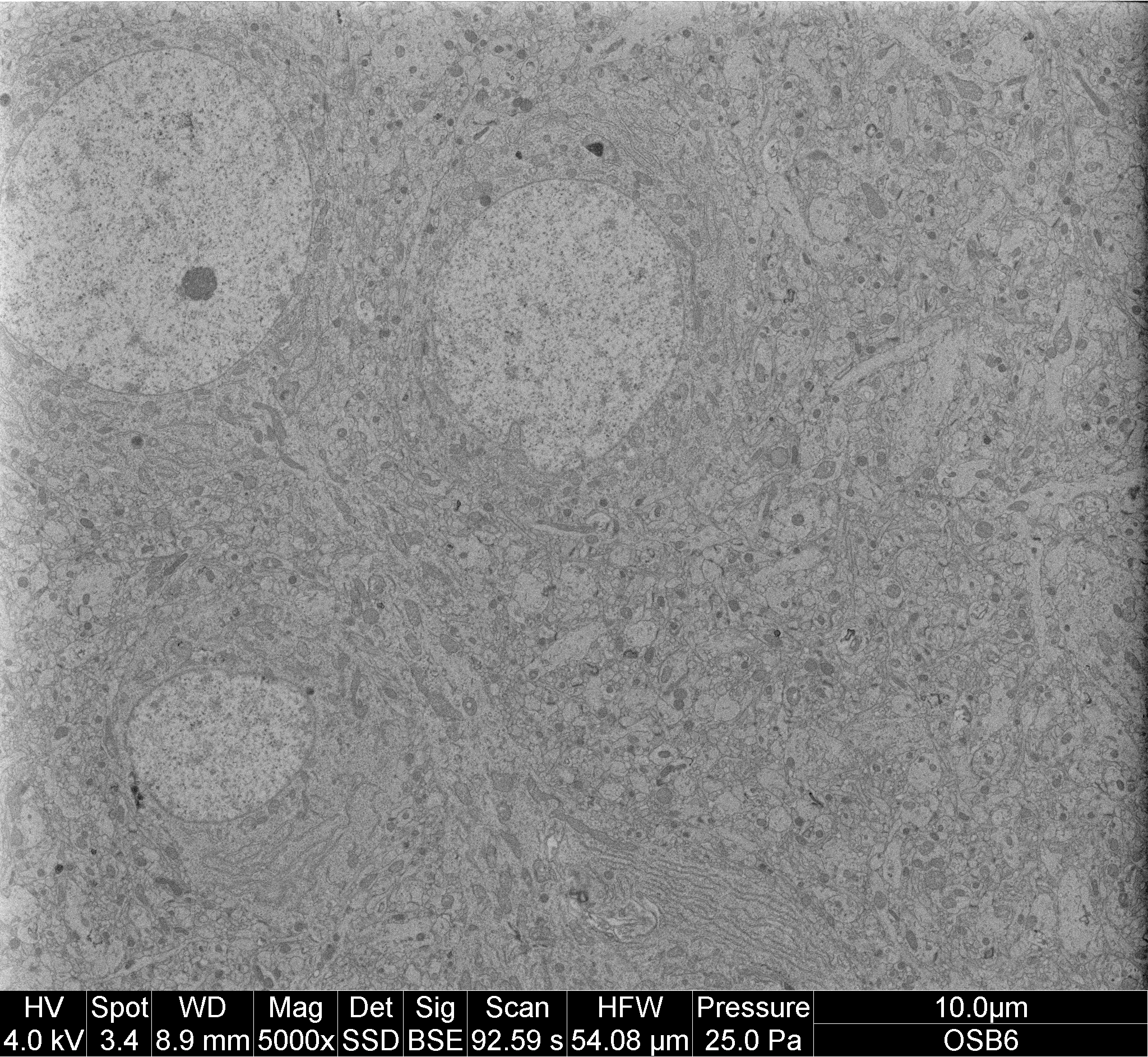

Supplement: Dataset S17 — (252.7 MB ZIP). [file pbio.0020329.sd017.zip › 040604_OS5_st1_1607.tif]

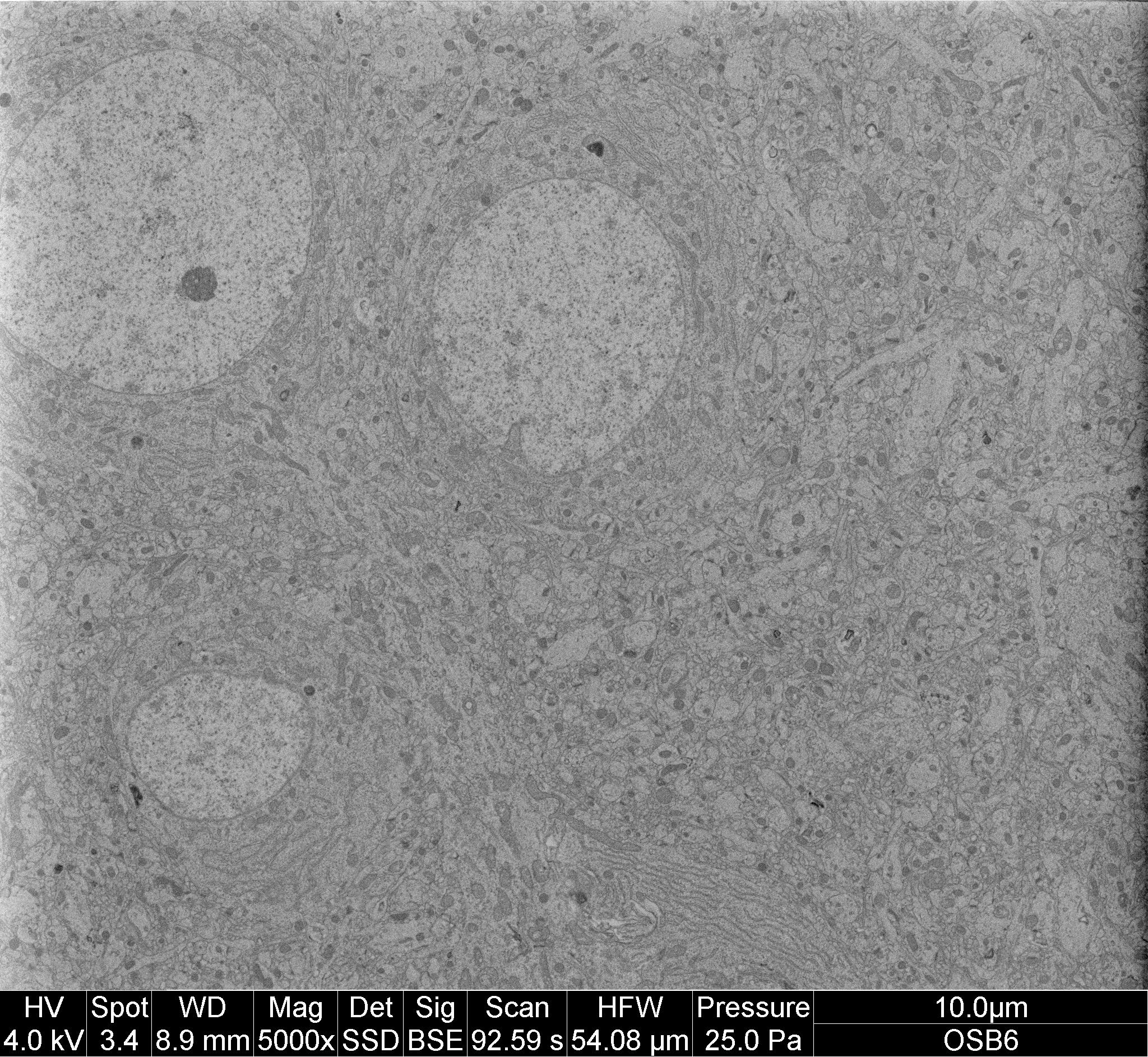

Supplement: Dataset S17 — (252.7 MB ZIP). [file pbio.0020329.sd017.zip › 040604_OS5_st1_1608.tif]

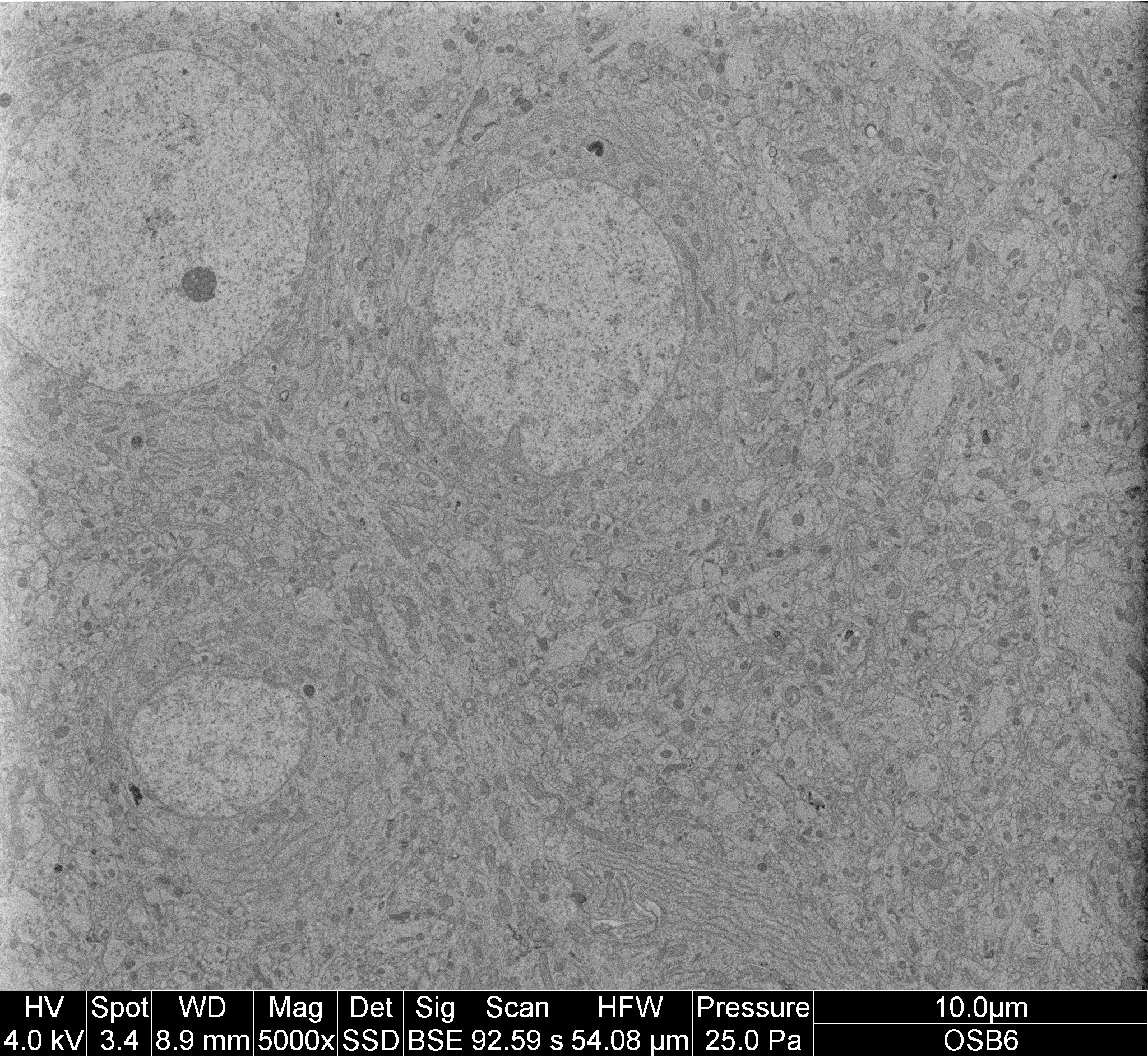

Supplement: Dataset S17 — (252.7 MB ZIP). [file pbio.0020329.sd017.zip › 040604_OS5_st1_1609.tif]

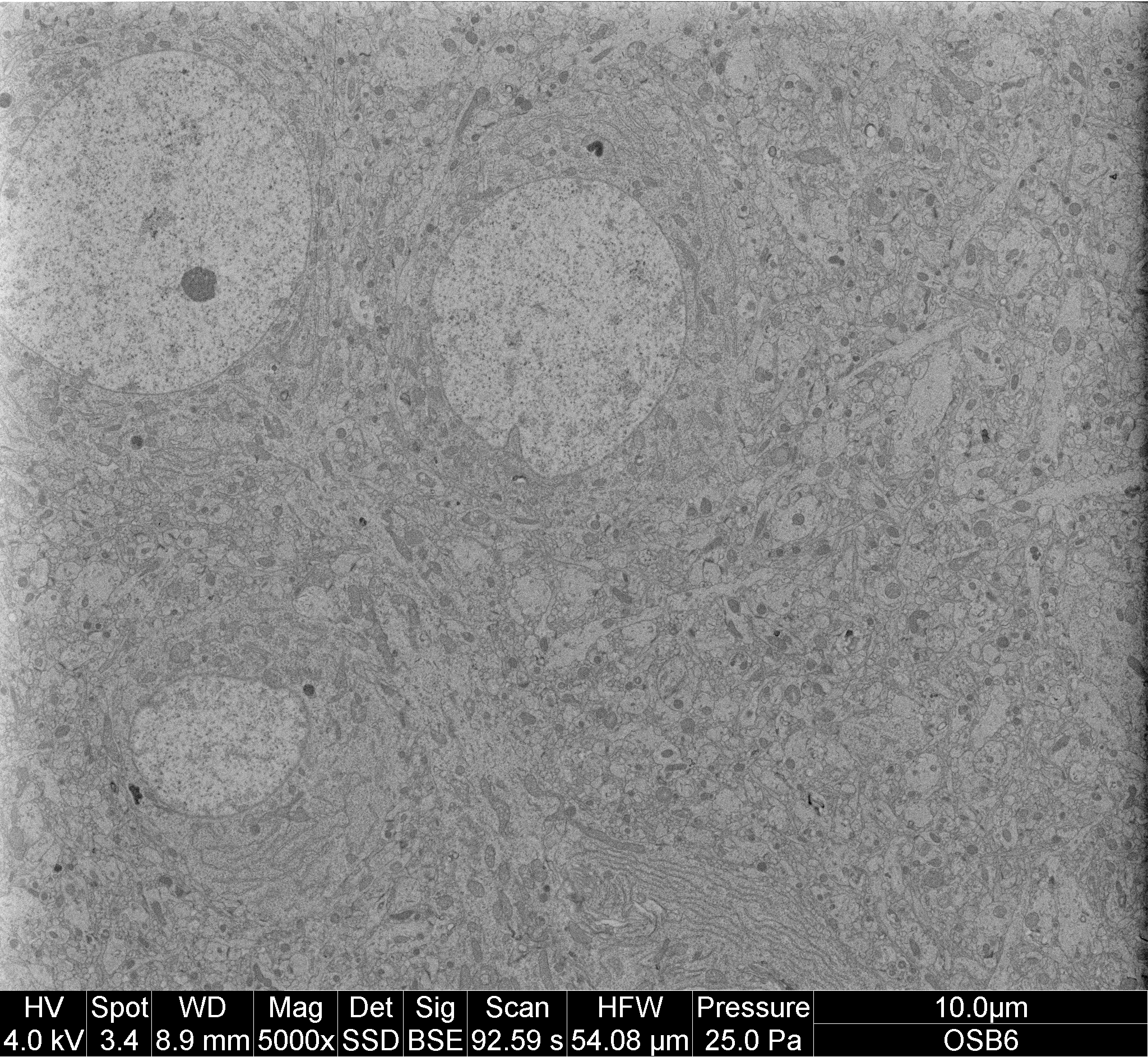

Supplement: Dataset S17 — (252.7 MB ZIP). [file pbio.0020329.sd017.zip › 040604_OS5_st1_1610.tif]

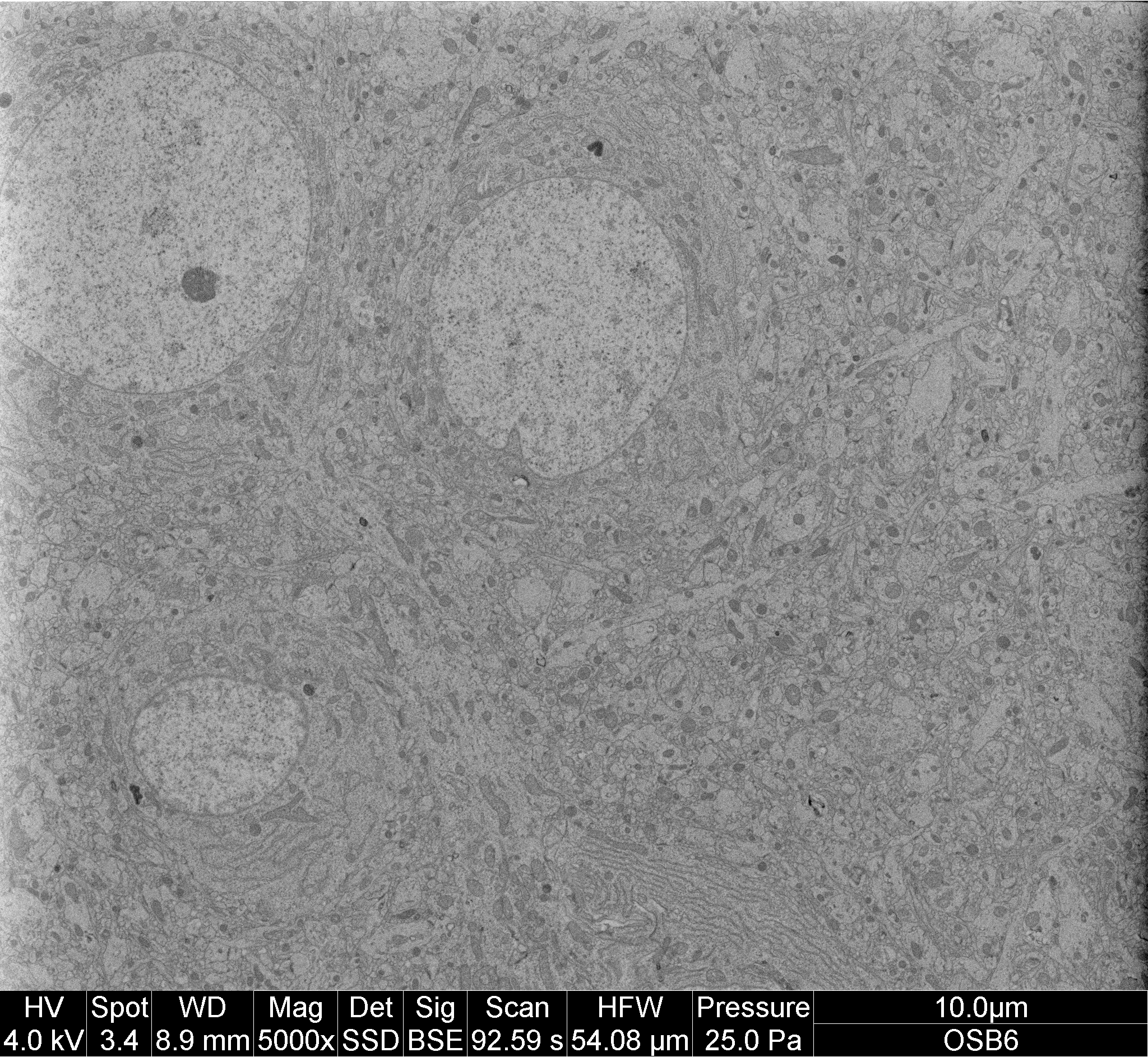

Supplement: Dataset S17 — (252.7 MB ZIP). [file pbio.0020329.sd017.zip › 040604_OS5_st1_1611.tif]

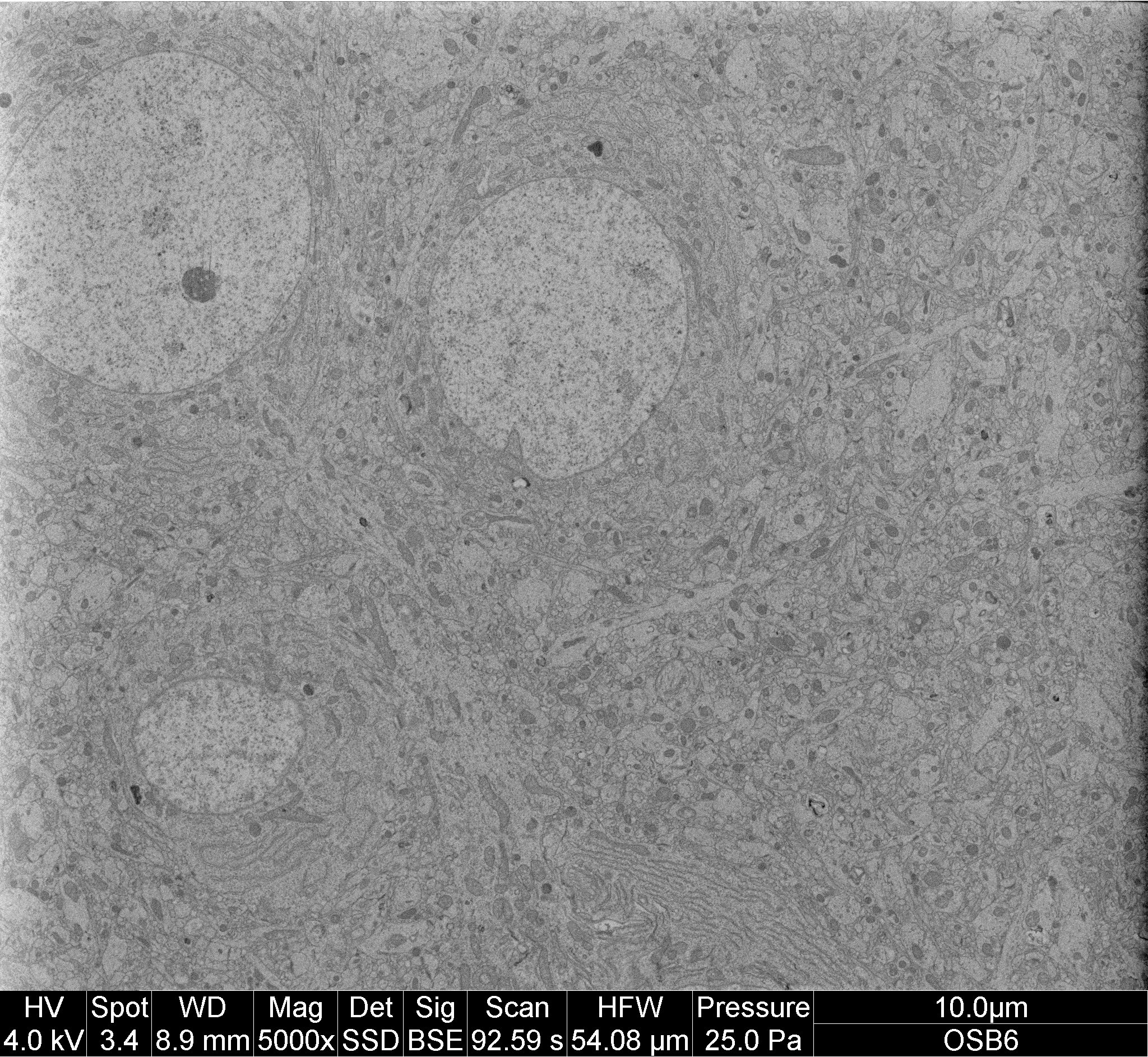

Supplement: Dataset S17 — (252.7 MB ZIP). [file pbio.0020329.sd017.zip › 040604_OS5_st1_1612.tif]

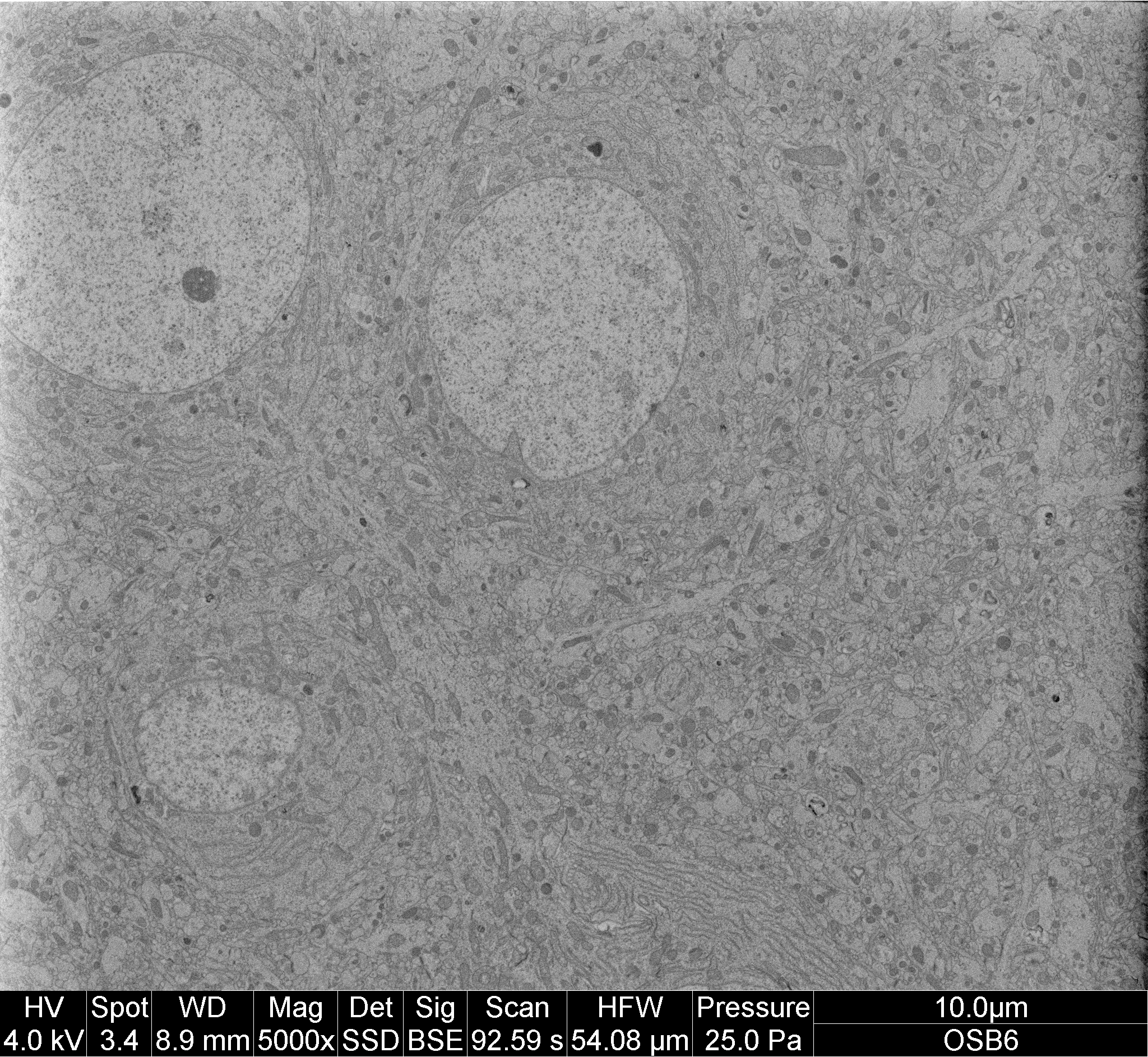

Supplement: Dataset S17 — (252.7 MB ZIP). [file pbio.0020329.sd017.zip › 040604_OS5_st1_1613.tif]

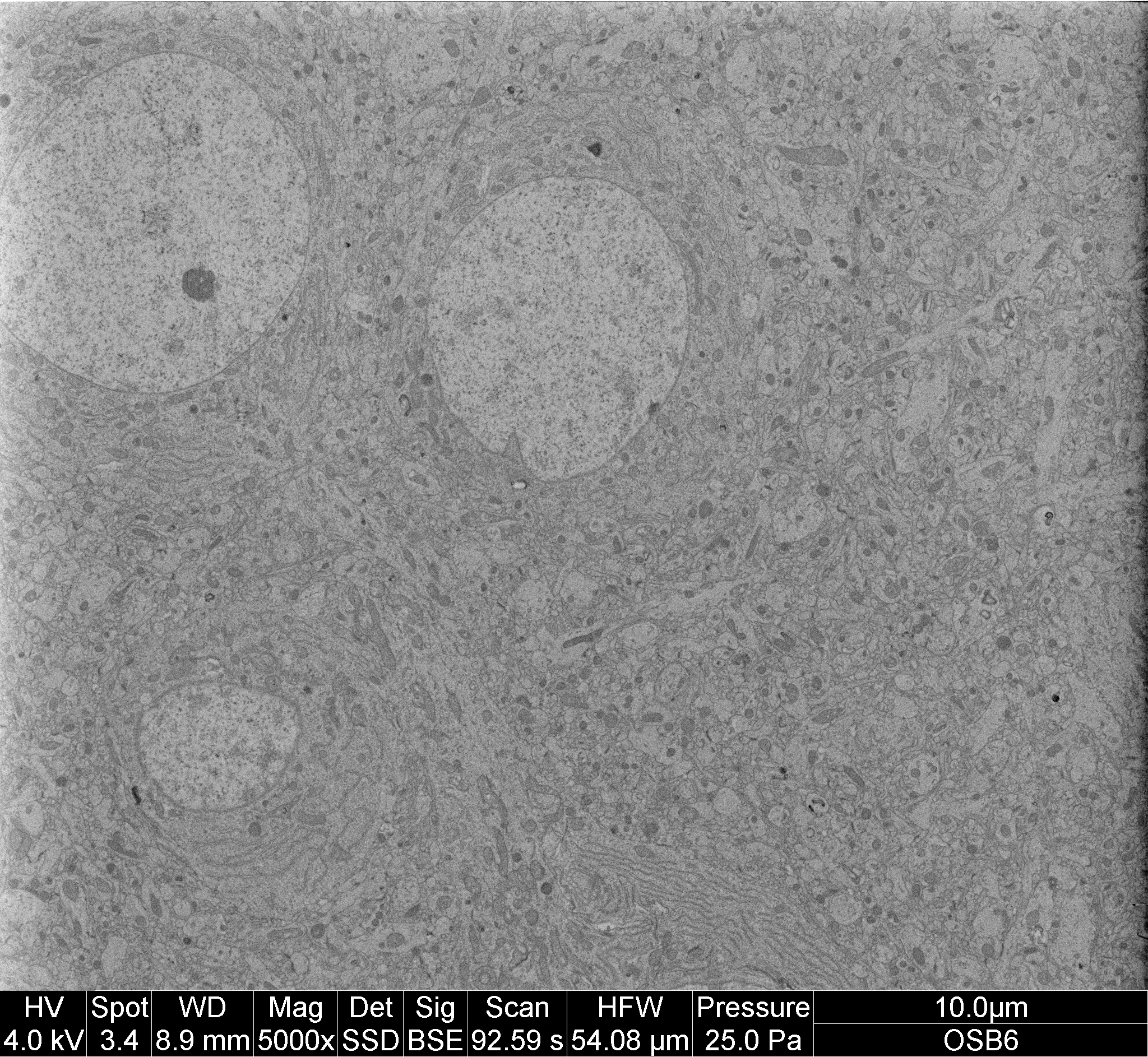

Supplement: Dataset S17 — (252.7 MB ZIP). [file pbio.0020329.sd017.zip › 040604_OS5_st1_1614.tif]

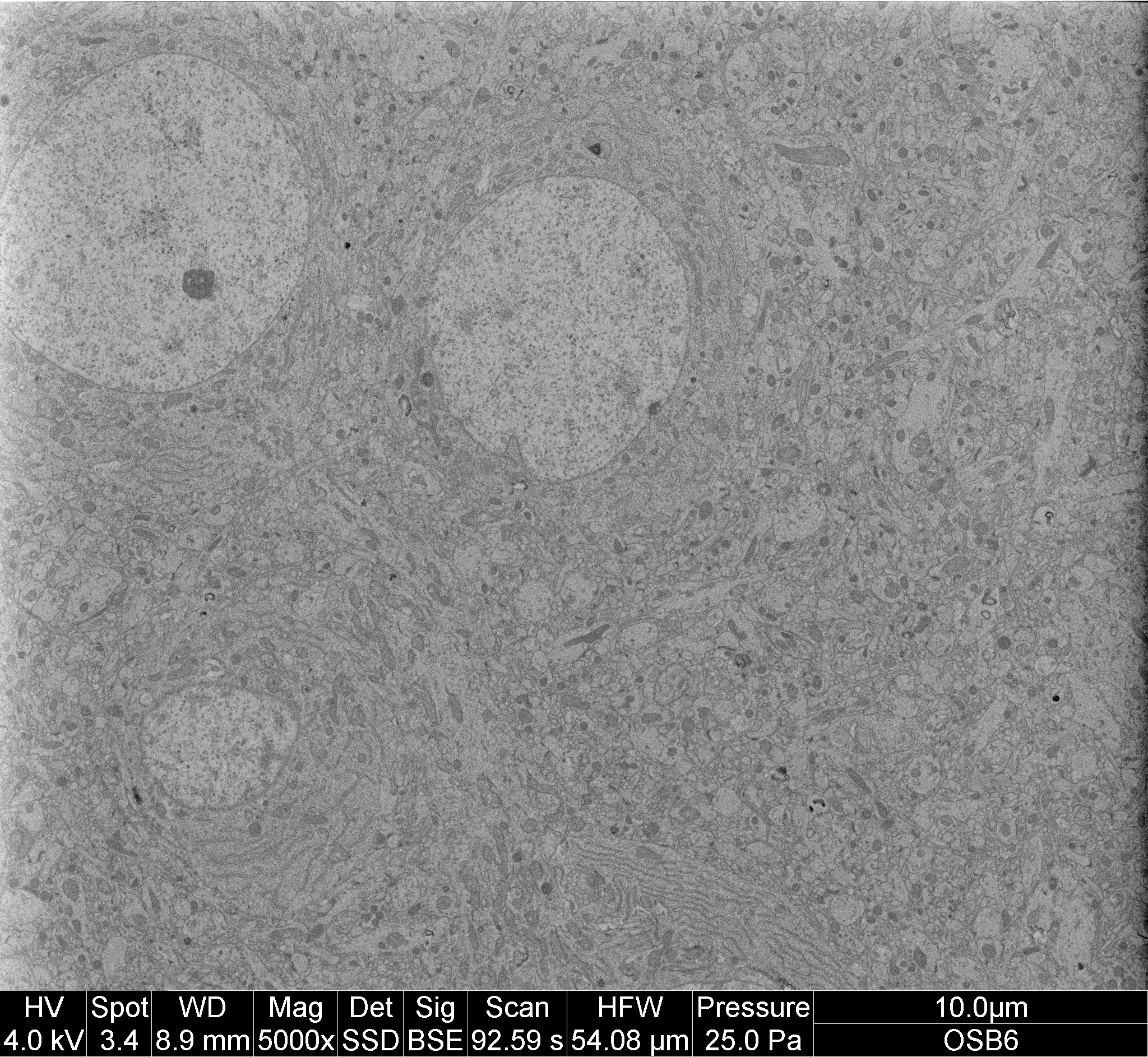

Supplement: Dataset S17 — (252.7 MB ZIP). [file pbio.0020329.sd017.zip › 040604_OS5_st1_1615.tif]

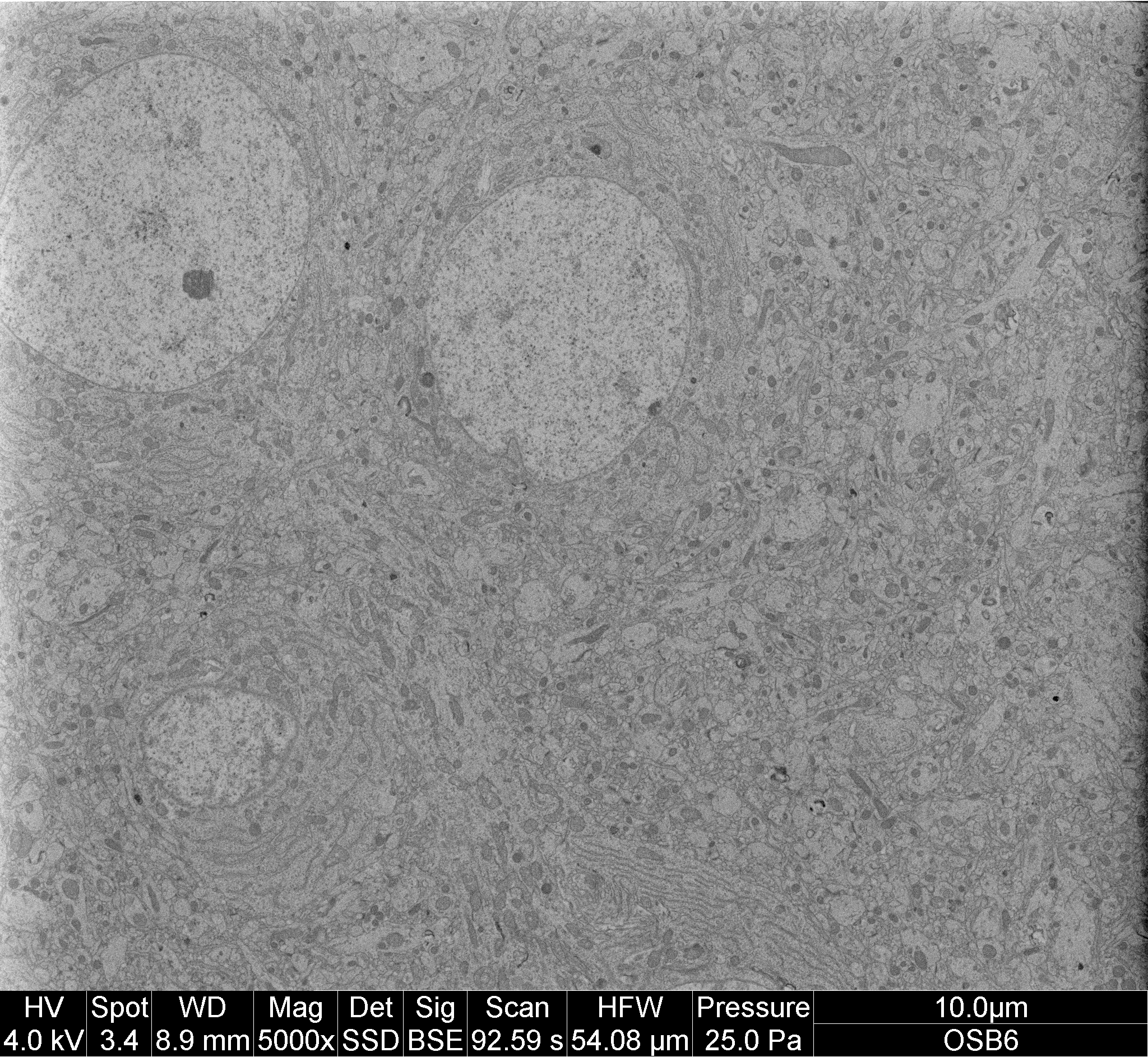

Supplement: Dataset S17 — (252.7 MB ZIP). [file pbio.0020329.sd017.zip › 040604_OS5_st1_1616.tif]

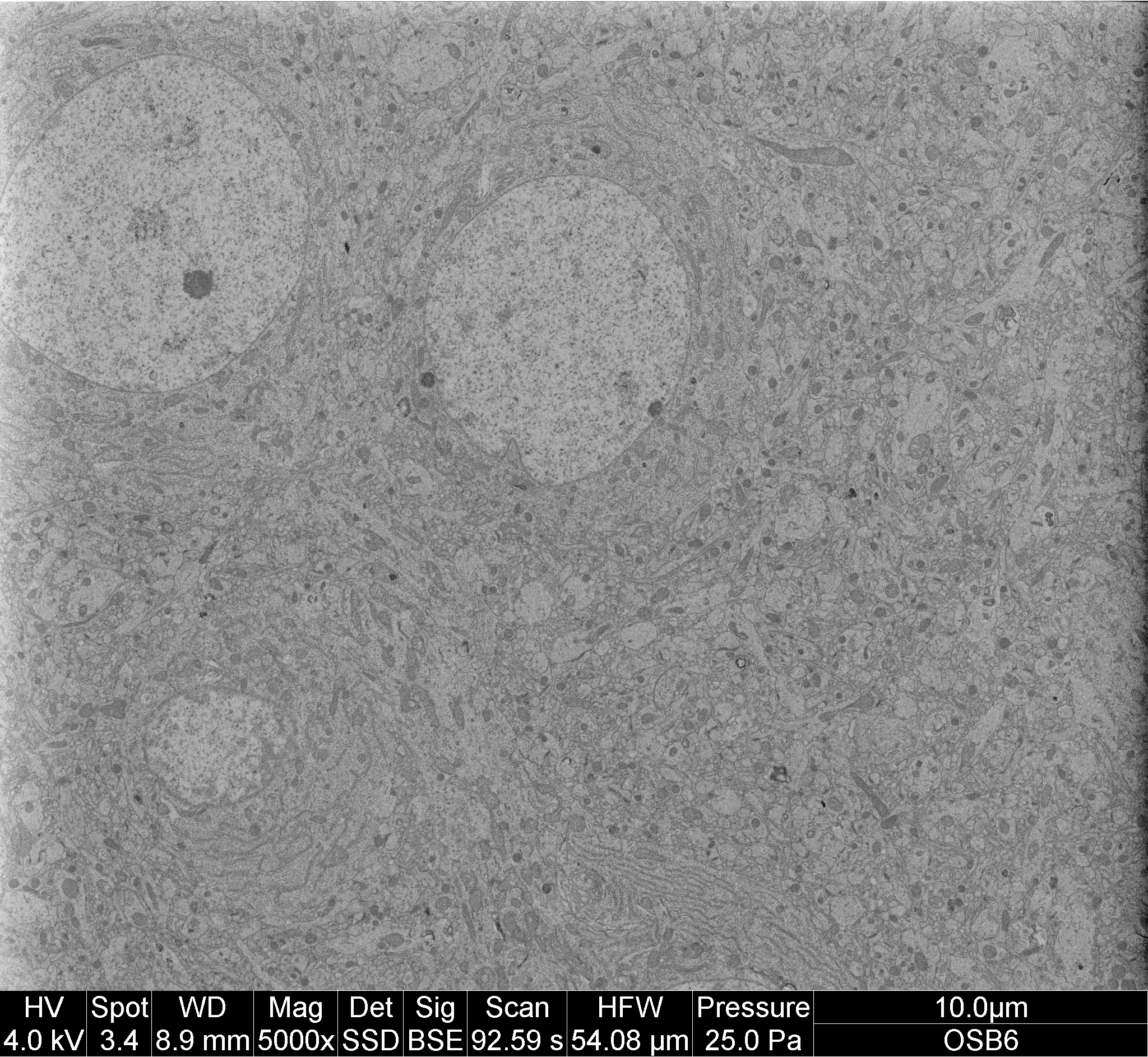

Supplement: Dataset S17 — (252.7 MB ZIP). [file pbio.0020329.sd017.zip › 040604_OS5_st1_1617.tif]

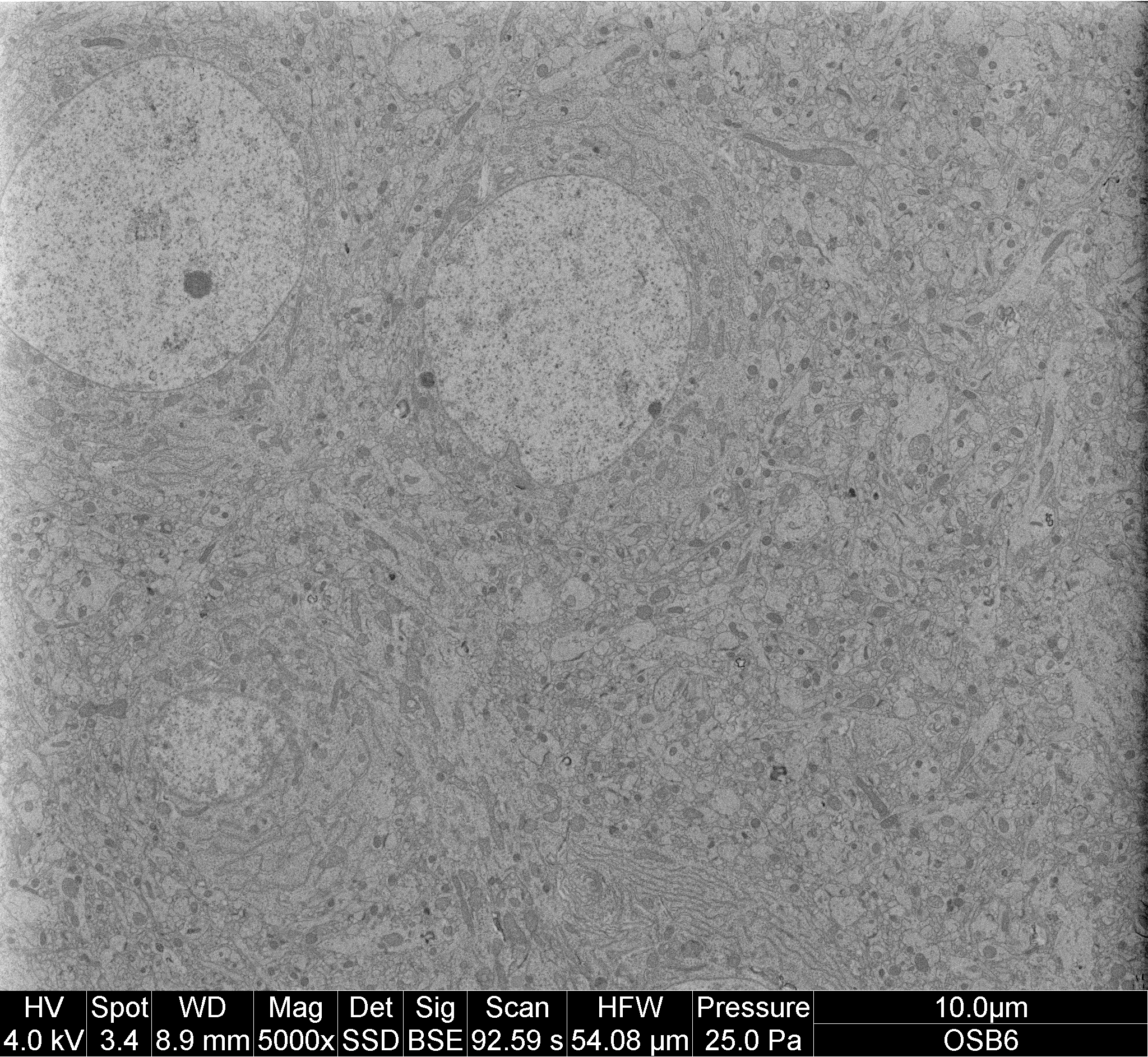

Supplement: Dataset S17 — (252.7 MB ZIP). [file pbio.0020329.sd017.zip › 040604_OS5_st1_1618.tif]

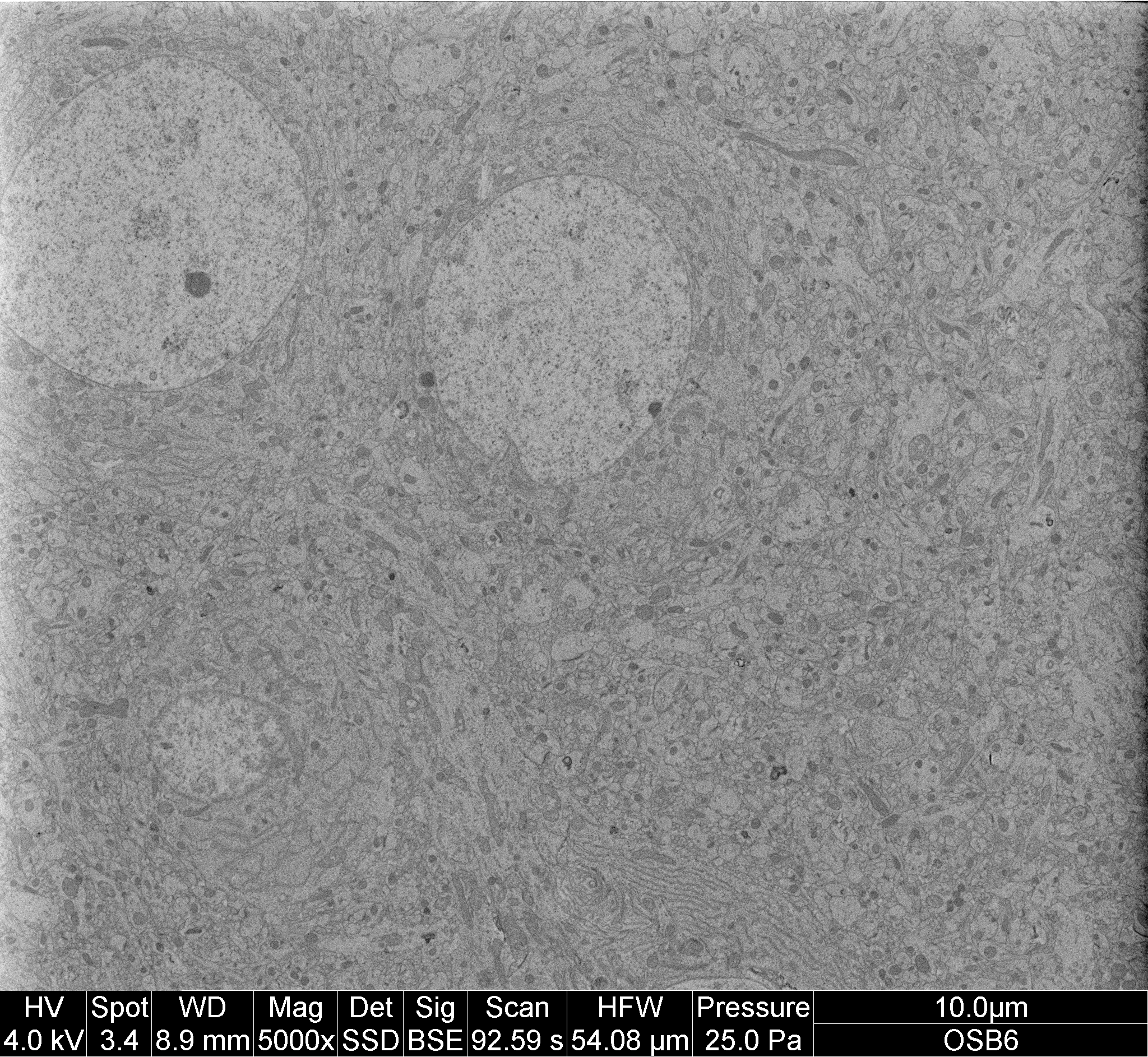

Supplement: Dataset S17 — (252.7 MB ZIP). [file pbio.0020329.sd017.zip › 040604_OS5_st1_1619.tif]

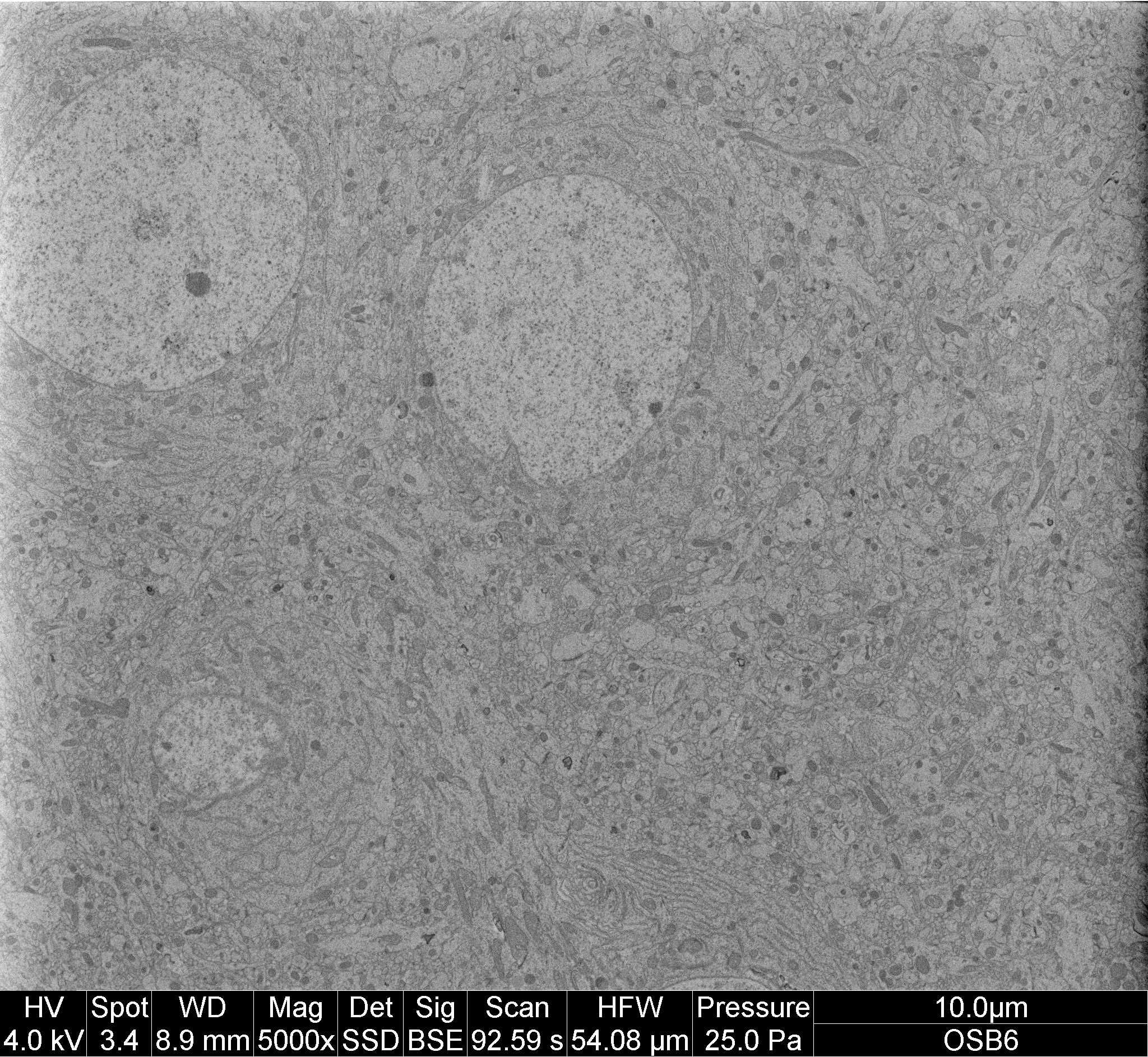

Supplement: Dataset S17 — (252.7 MB ZIP). [file pbio.0020329.sd017.zip › 040604_OS5_st1_1620.tif]

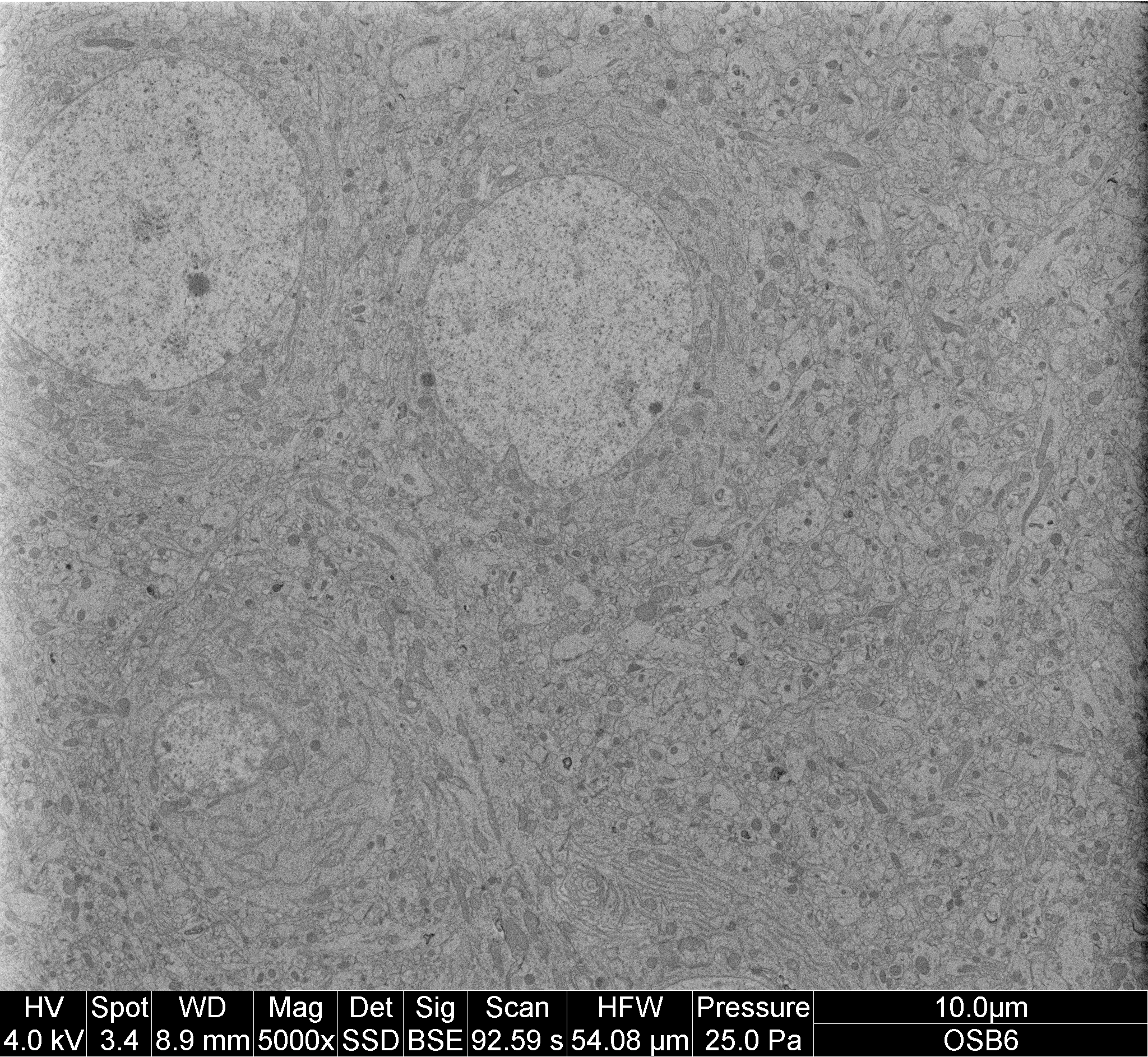

Supplement: Dataset S17 — (252.7 MB ZIP). [file pbio.0020329.sd017.zip › 040604_OS5_st1_1621.tif]

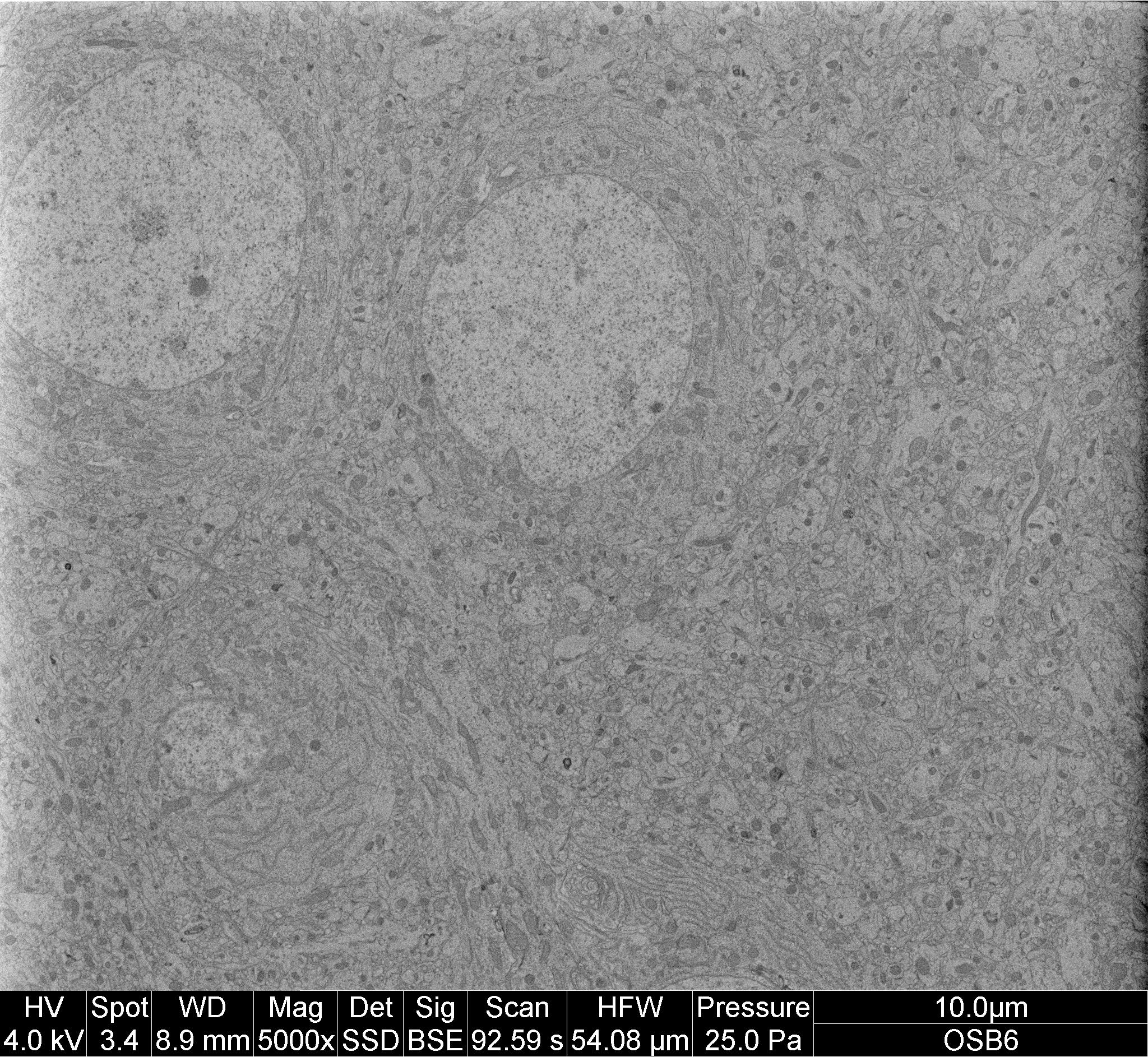

Supplement: Dataset S17 — (252.7 MB ZIP). [file pbio.0020329.sd017.zip › 040604_OS5_st1_1622.tif]

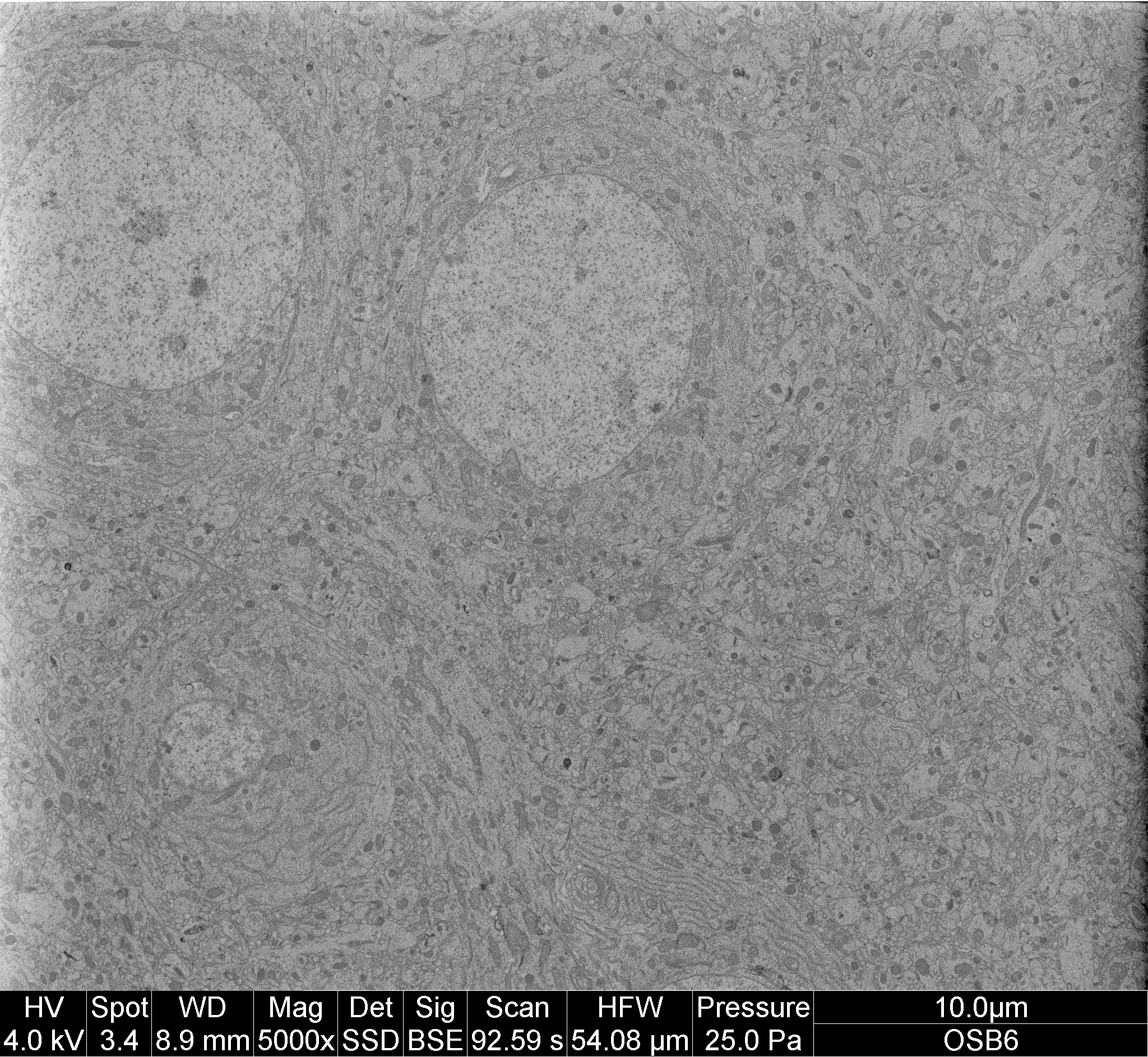

Supplement: Dataset S17 — (252.7 MB ZIP). [file pbio.0020329.sd017.zip › 040604_OS5_st1_1623.tif]

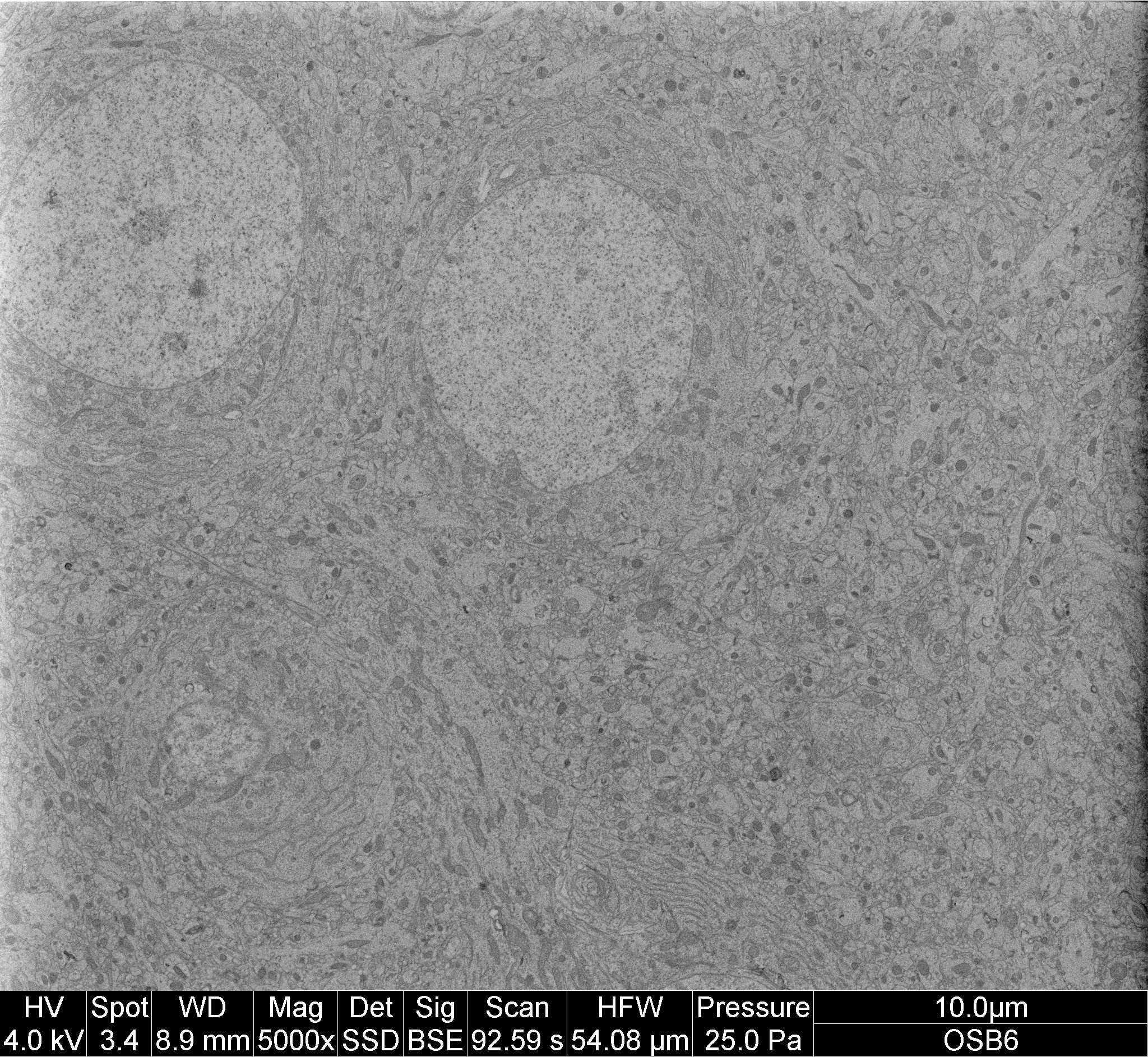

Supplement: Dataset S17 — (252.7 MB ZIP). [file pbio.0020329.sd017.zip › 040604_OS5_st1_1624.tif]

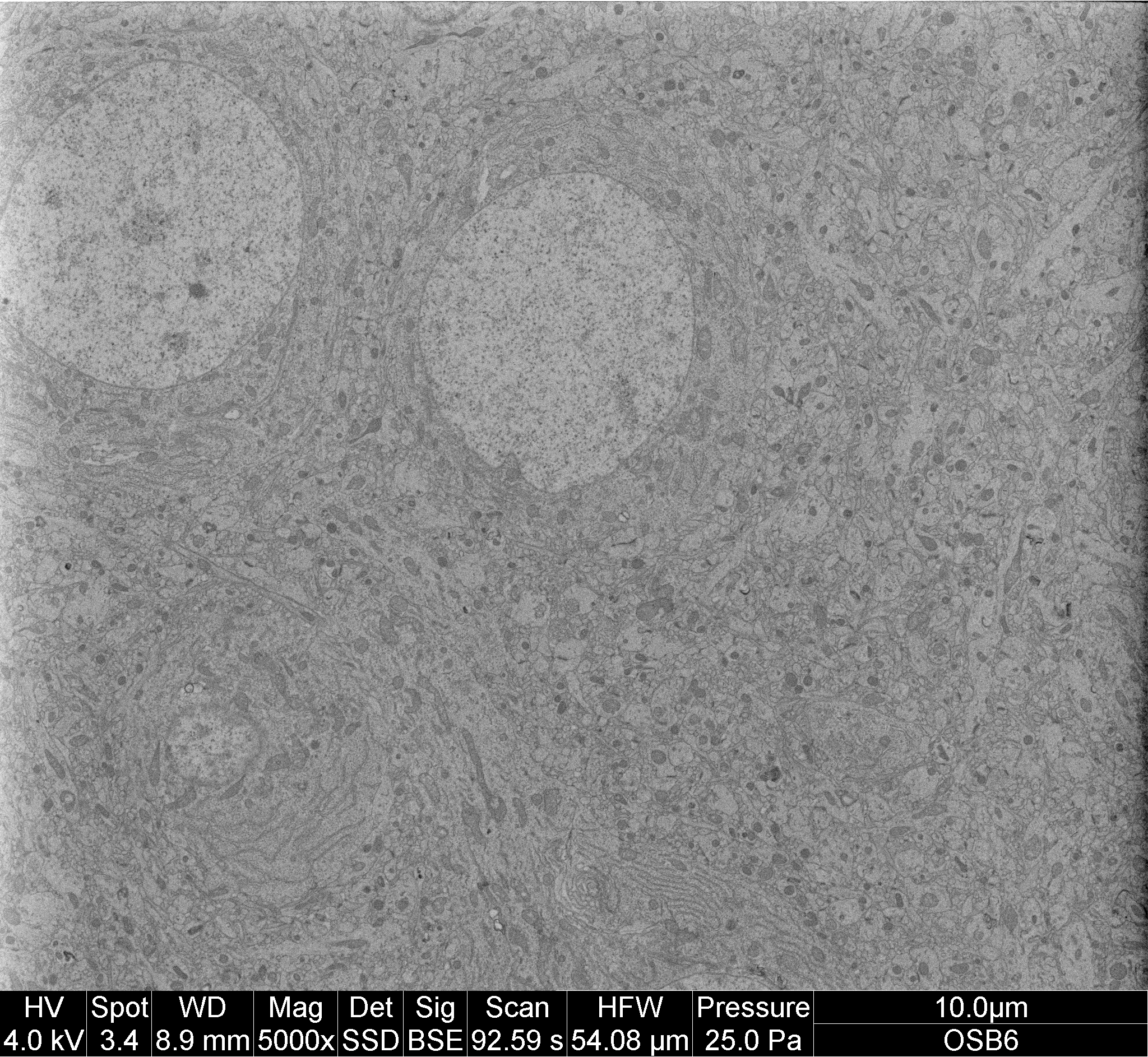

Supplement: Dataset S17 — (252.7 MB ZIP). [file pbio.0020329.sd017.zip › 040604_OS5_st1_1625.tif]

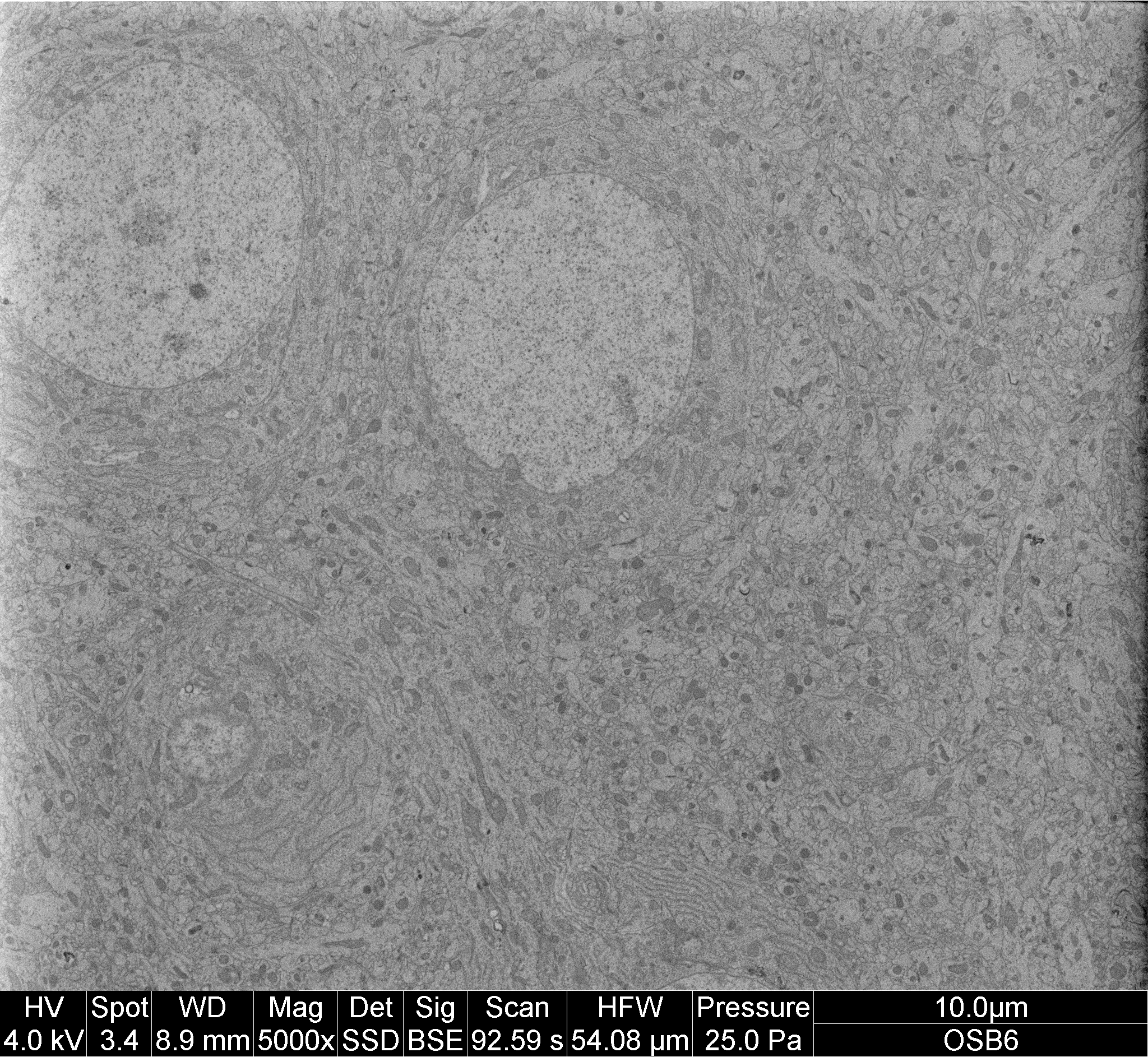

Supplement: Dataset S17 — (252.7 MB ZIP). [file pbio.0020329.sd017.zip › 040604_OS5_st1_1626.tif]

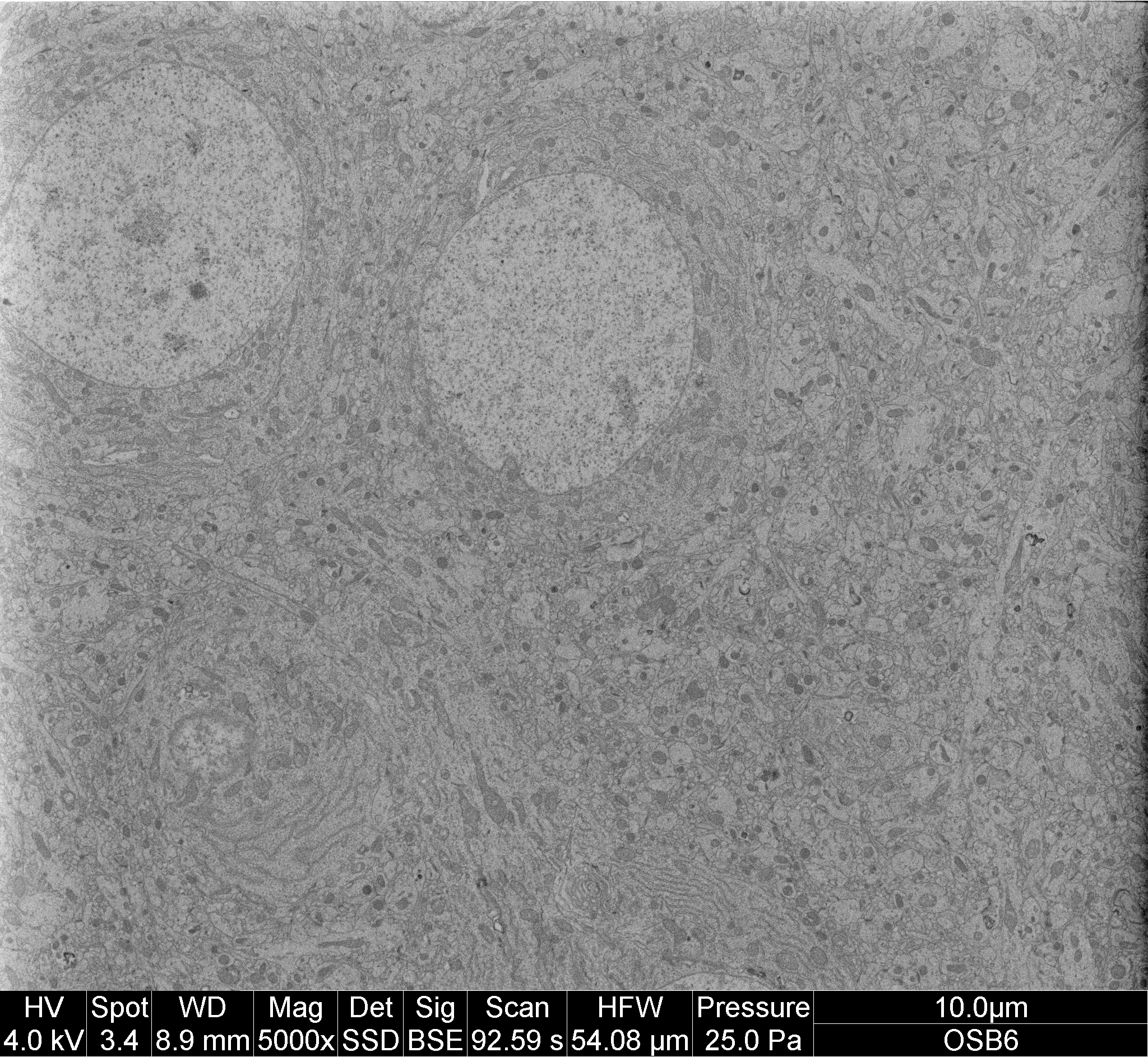

Supplement: Dataset S17 — (252.7 MB ZIP). [file pbio.0020329.sd017.zip › 040604_OS5_st1_1627.tif]

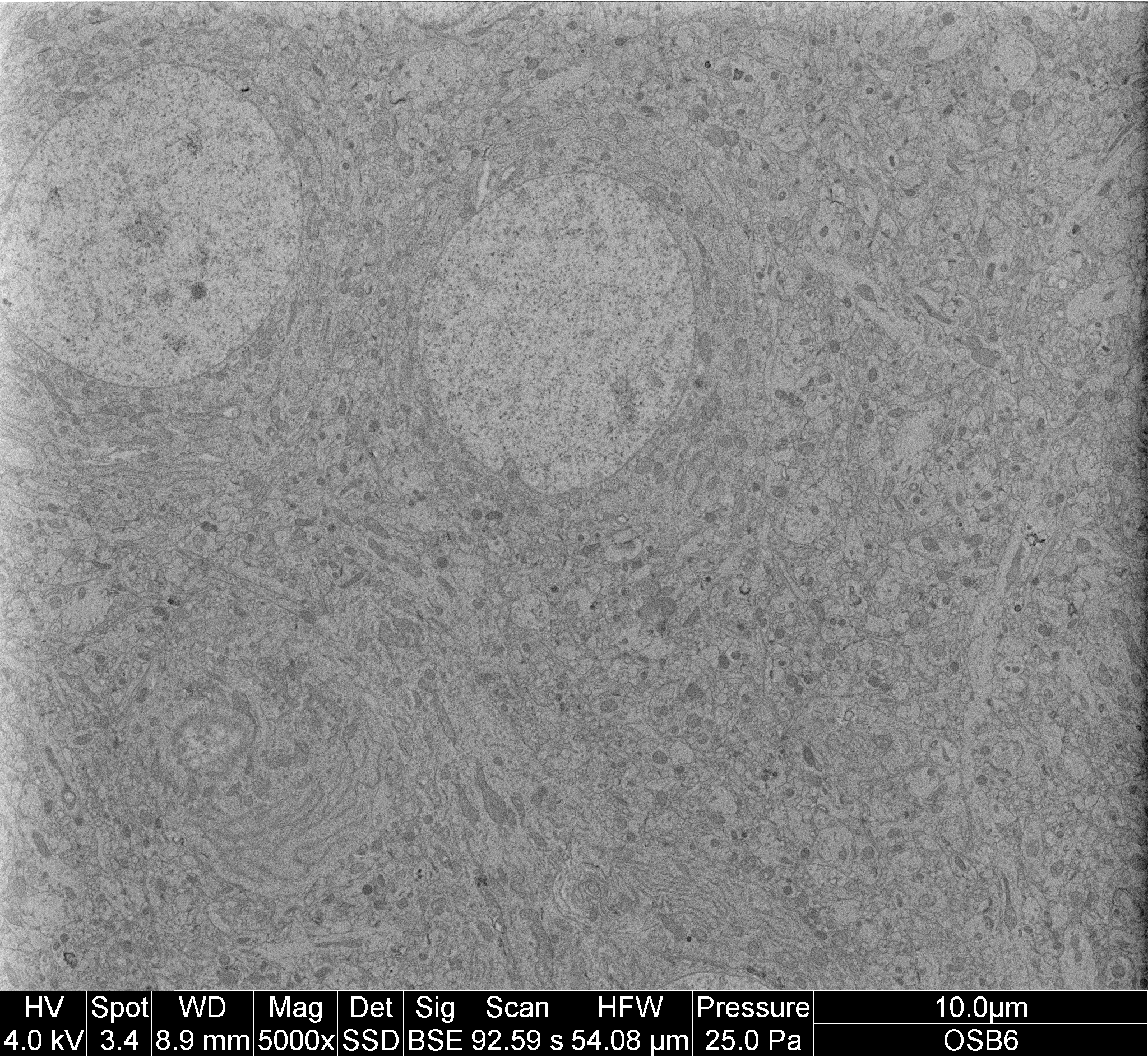

Supplement: Dataset S17 — (252.7 MB ZIP). [file pbio.0020329.sd017.zip › 040604_OS5_st1_1628.tif]

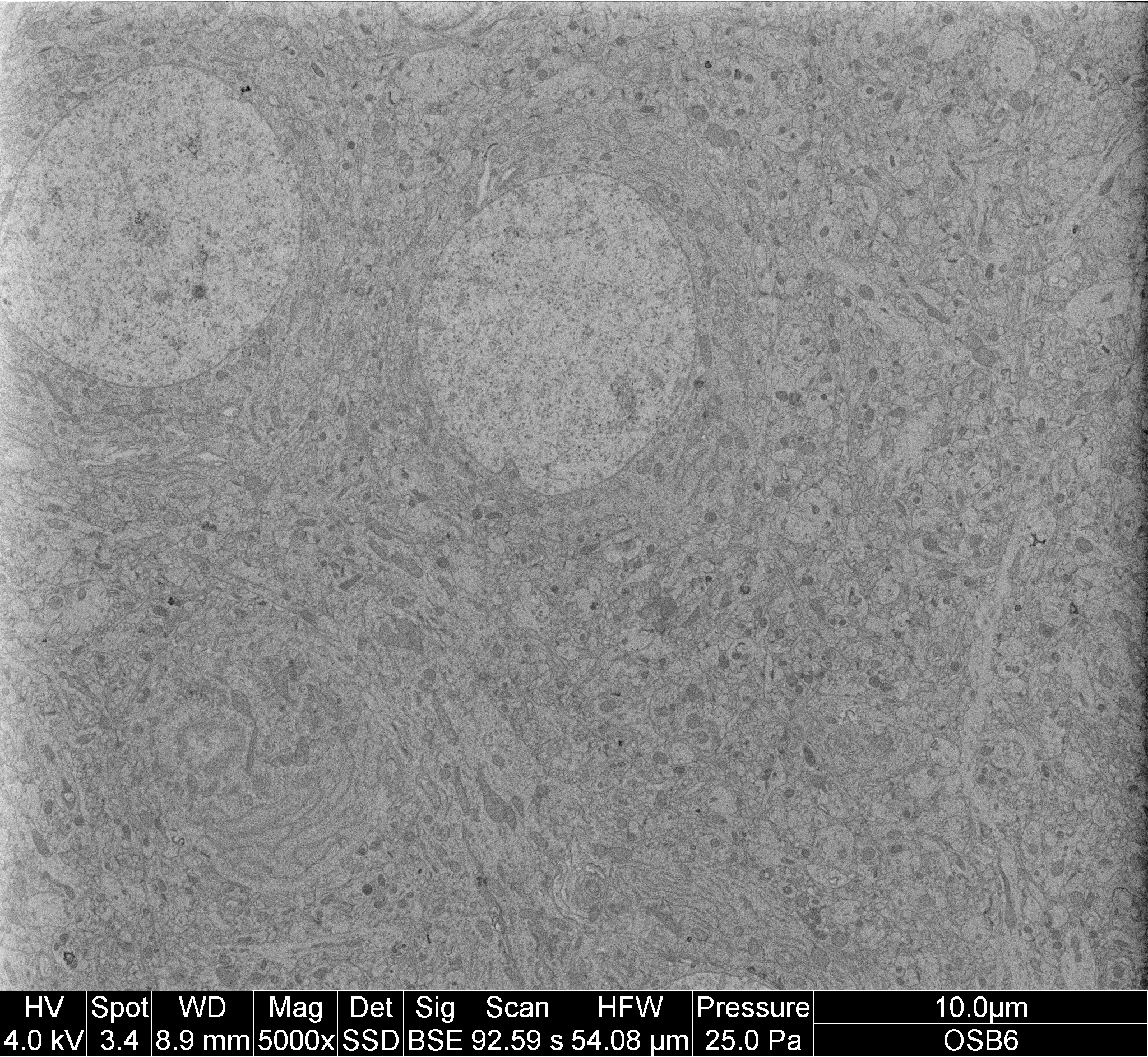

Supplement: Dataset S17 — (252.7 MB ZIP). [file pbio.0020329.sd017.zip › 040604_OS5_st1_1629.tif]

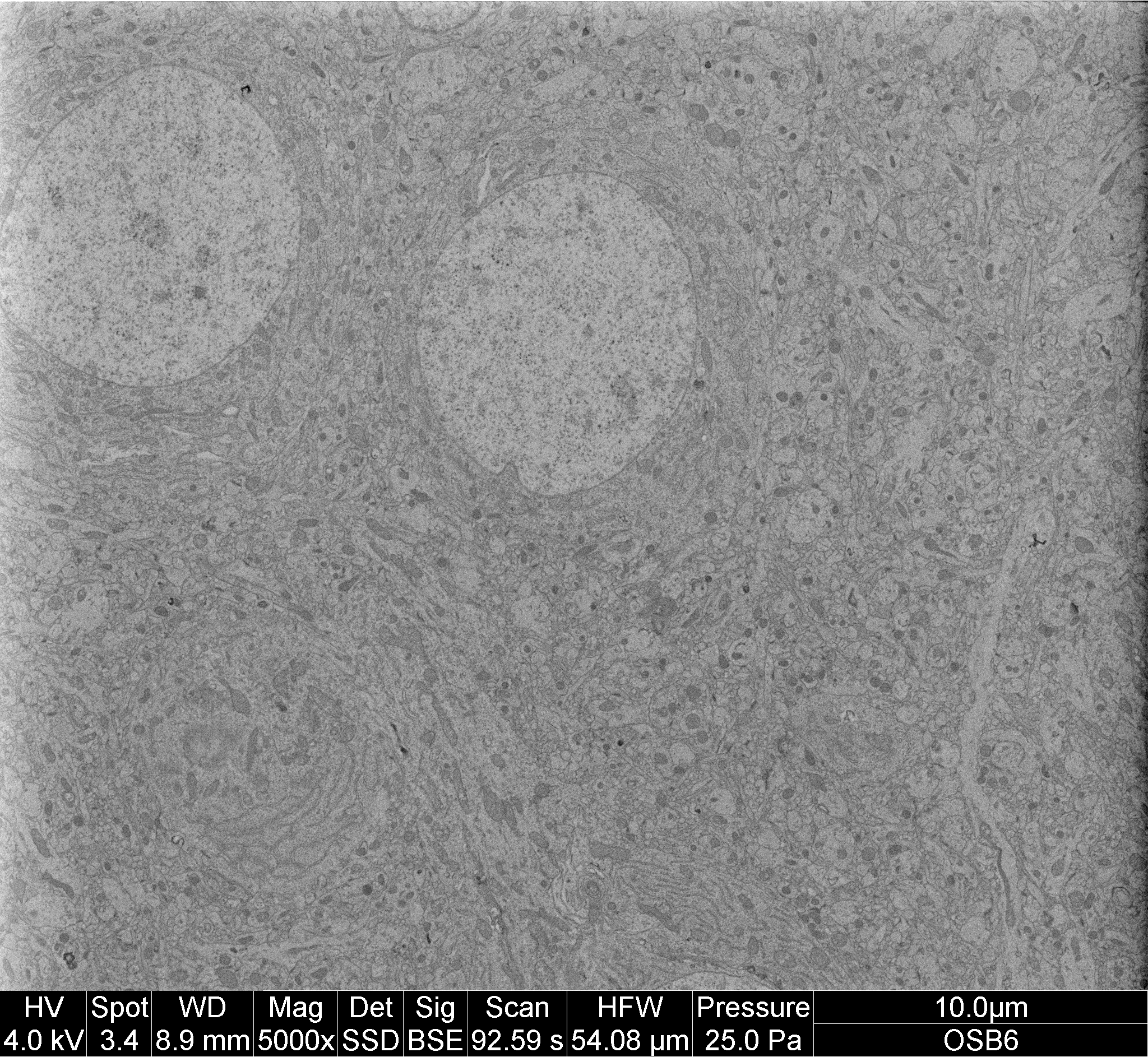

Supplement: Dataset S17 — (252.7 MB ZIP). [file pbio.0020329.sd017.zip › 040604_OS5_st1_1630.tif]

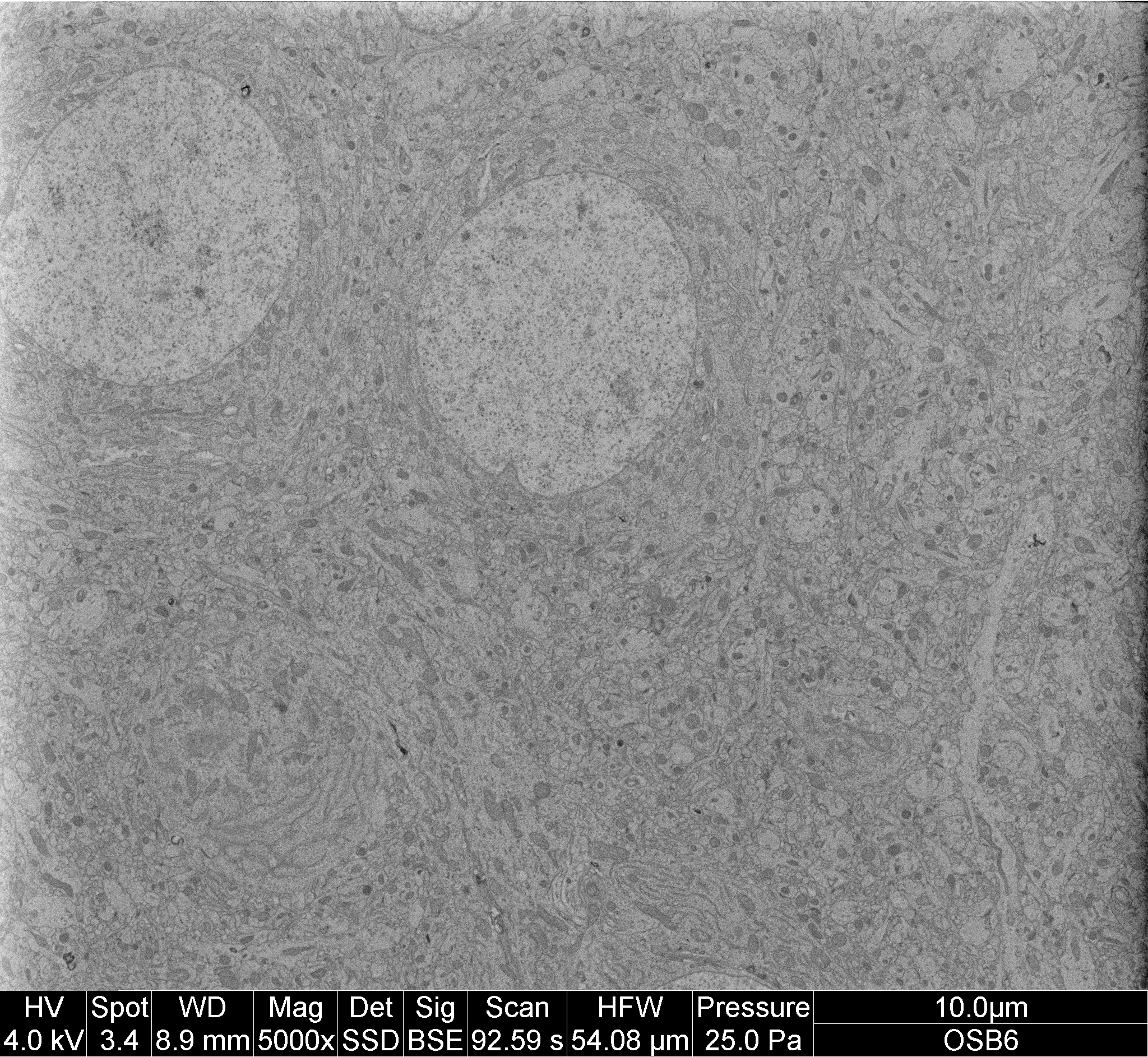

Supplement: Dataset S17 — (252.7 MB ZIP). [file pbio.0020329.sd017.zip › 040604_OS5_st1_1631.tif]

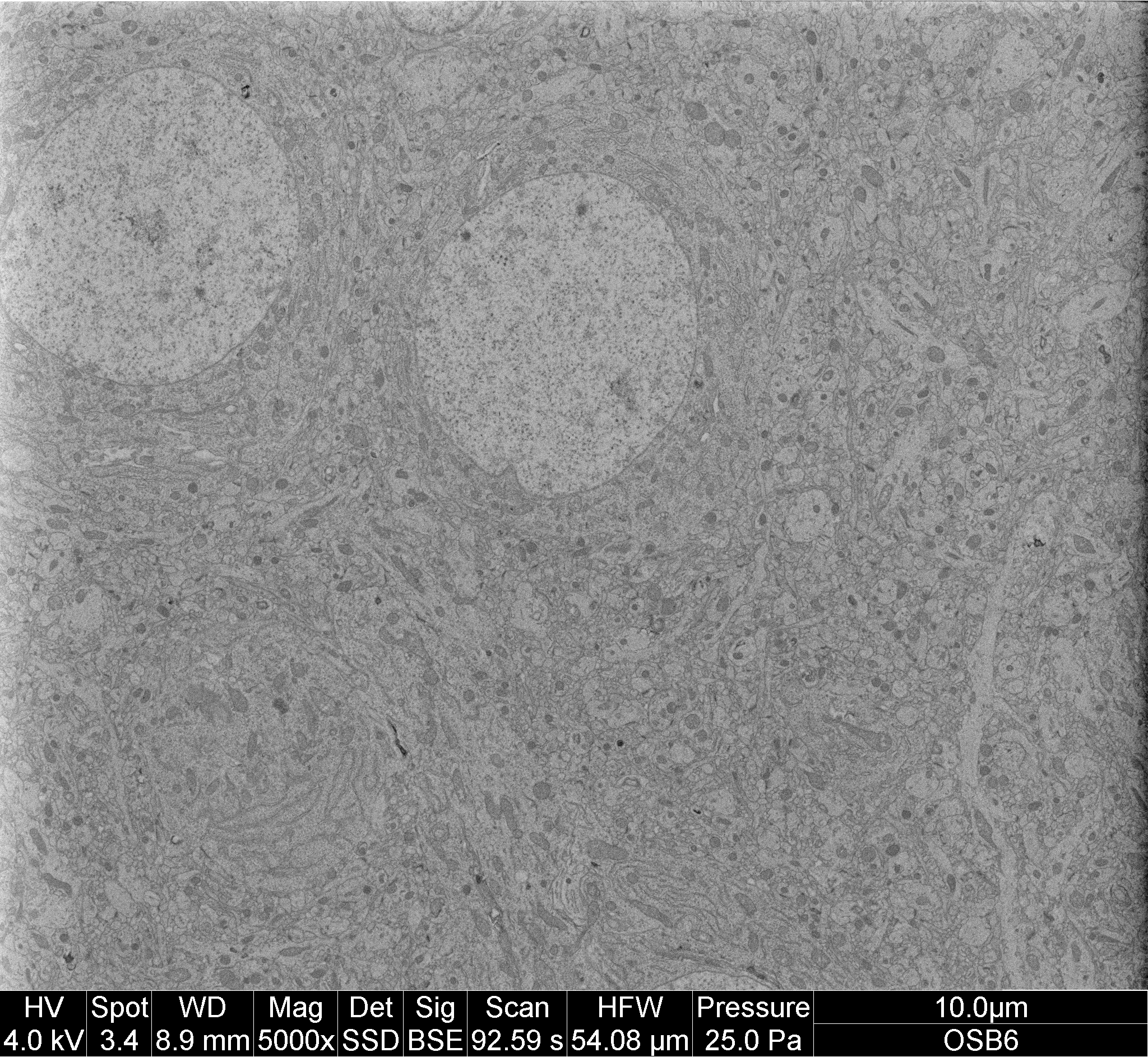

Supplement: Dataset S17 — (252.7 MB ZIP). [file pbio.0020329.sd017.zip › 040604_OS5_st1_1632.tif]

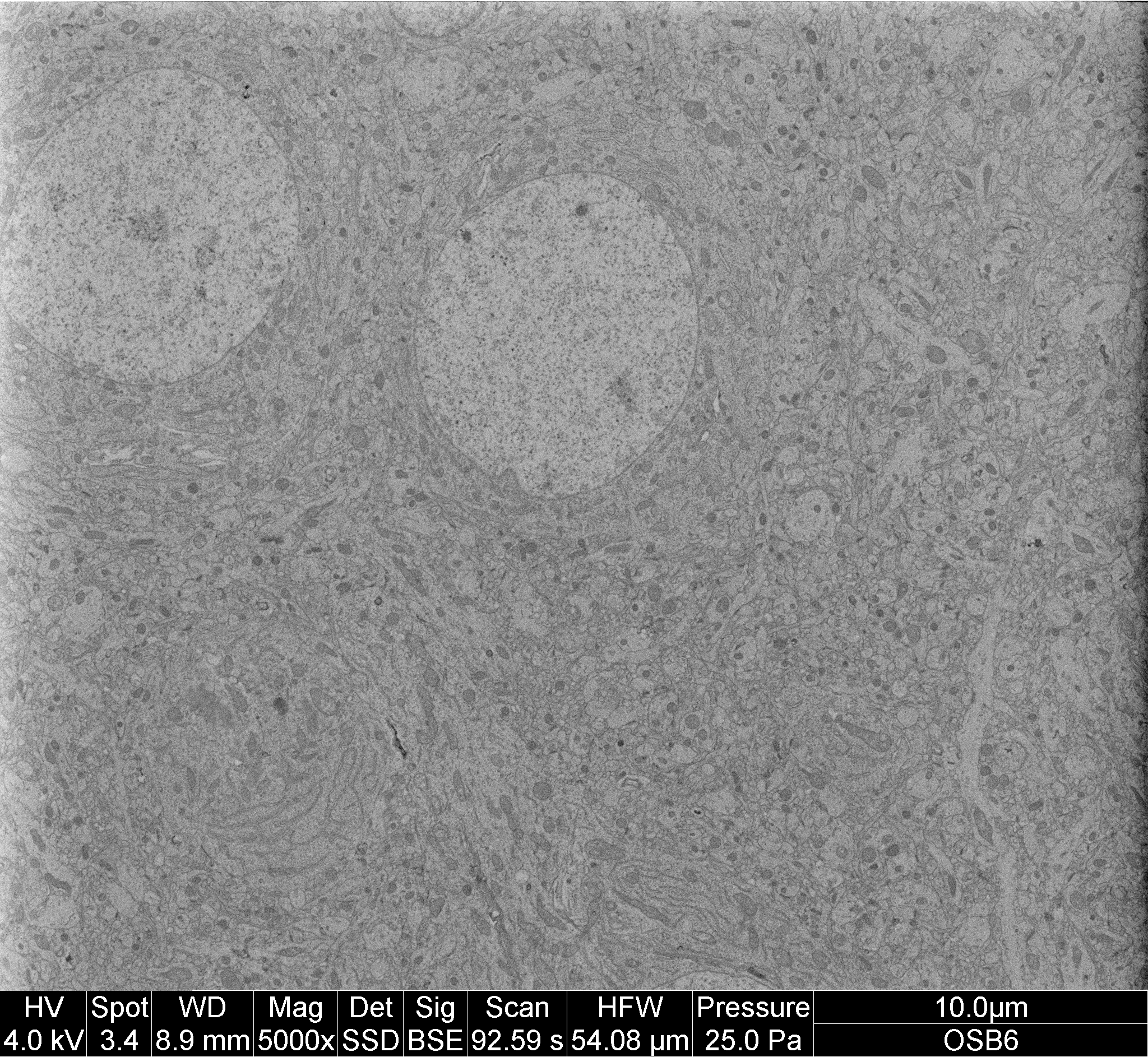

Supplement: Dataset S17 — (252.7 MB ZIP). [file pbio.0020329.sd017.zip › 040604_OS5_st1_1633.tif]

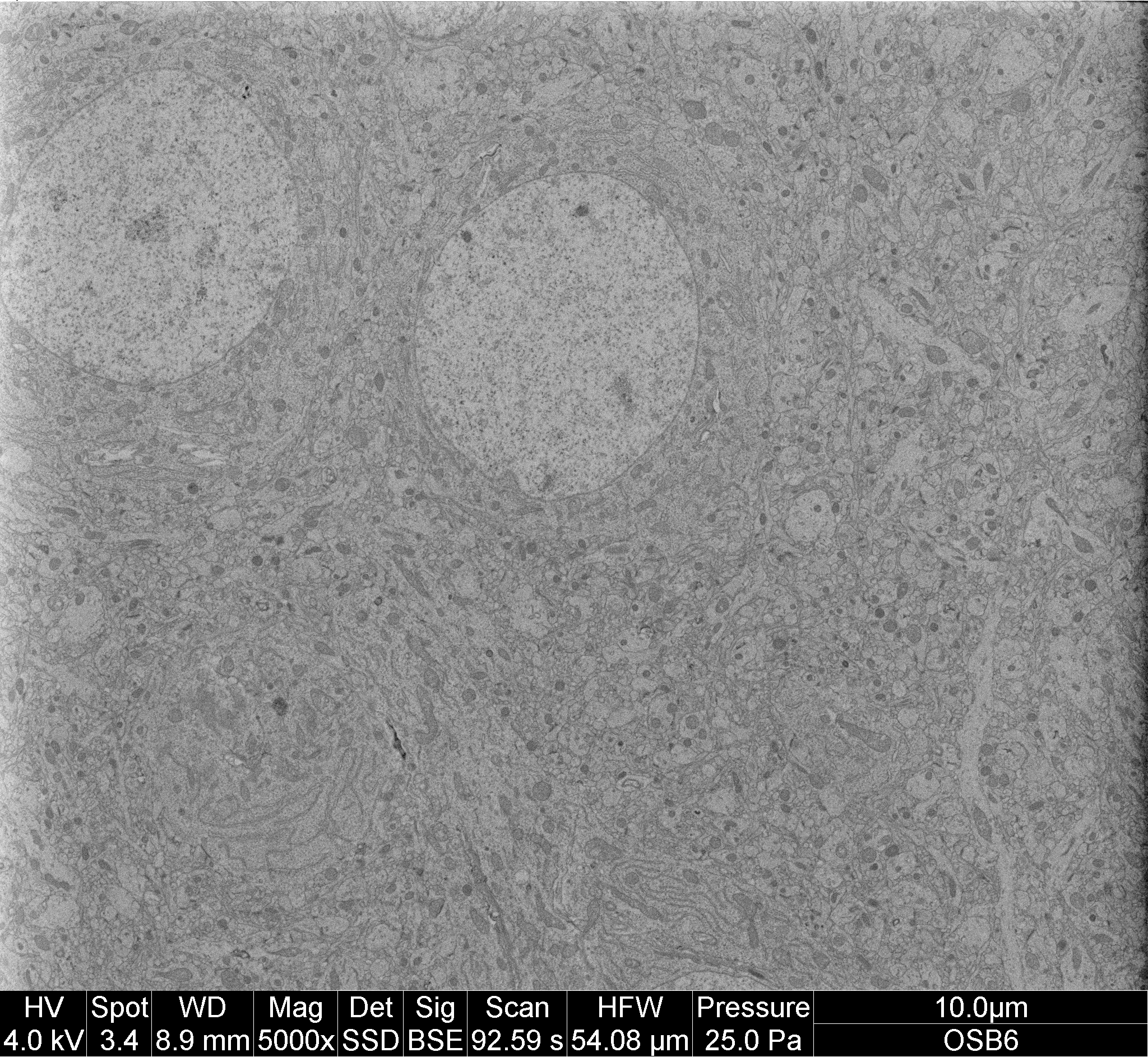

Supplement: Dataset S17 — (252.7 MB ZIP). [file pbio.0020329.sd017.zip › 040604_OS5_st1_1634.tif]

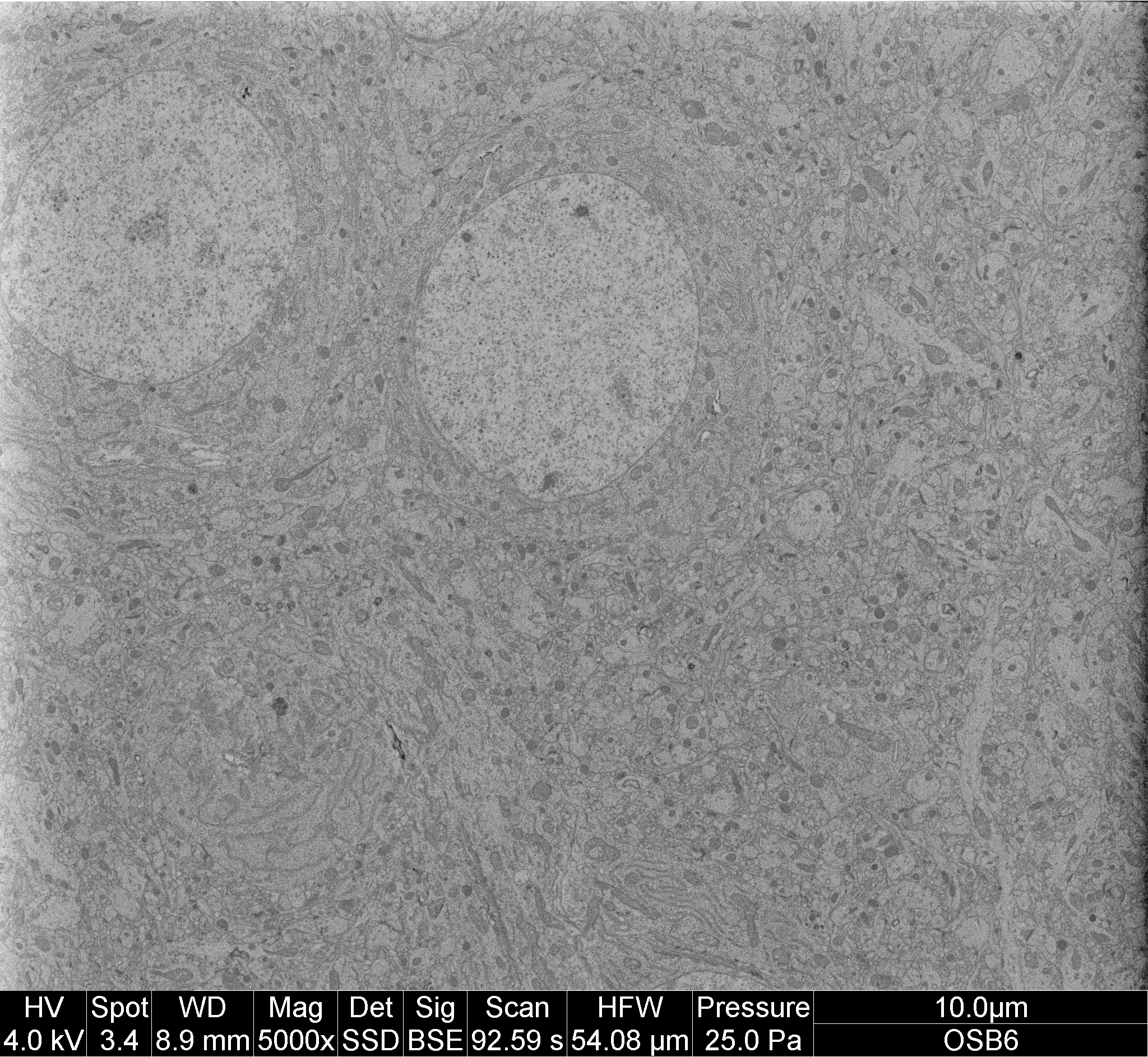

Supplement: Dataset S17 — (252.7 MB ZIP). [file pbio.0020329.sd017.zip › 040604_OS5_st1_1635.tif]

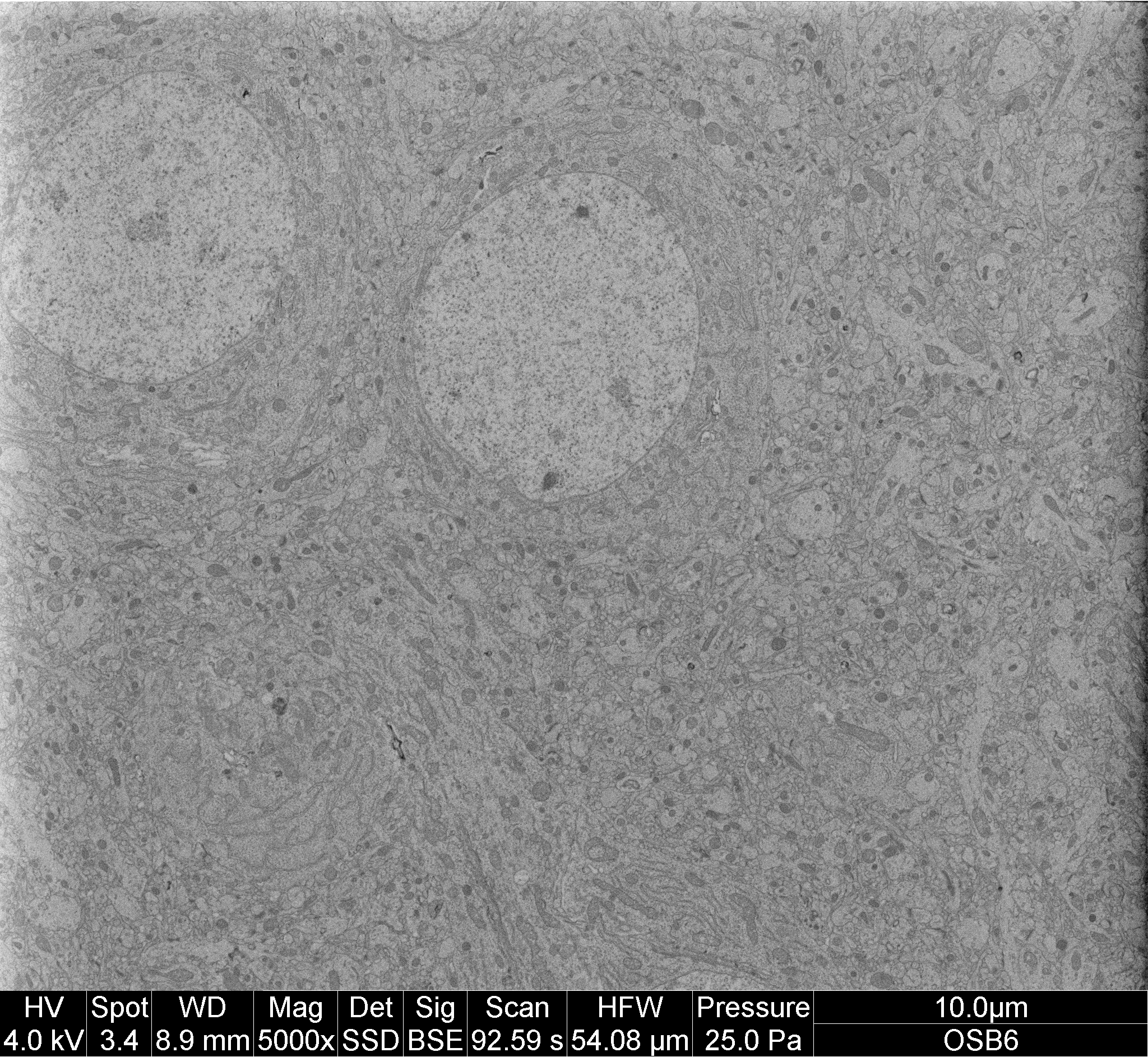

Supplement: Dataset S17 — (252.7 MB ZIP). [file pbio.0020329.sd017.zip › 040604_OS5_st1_1636.tif]

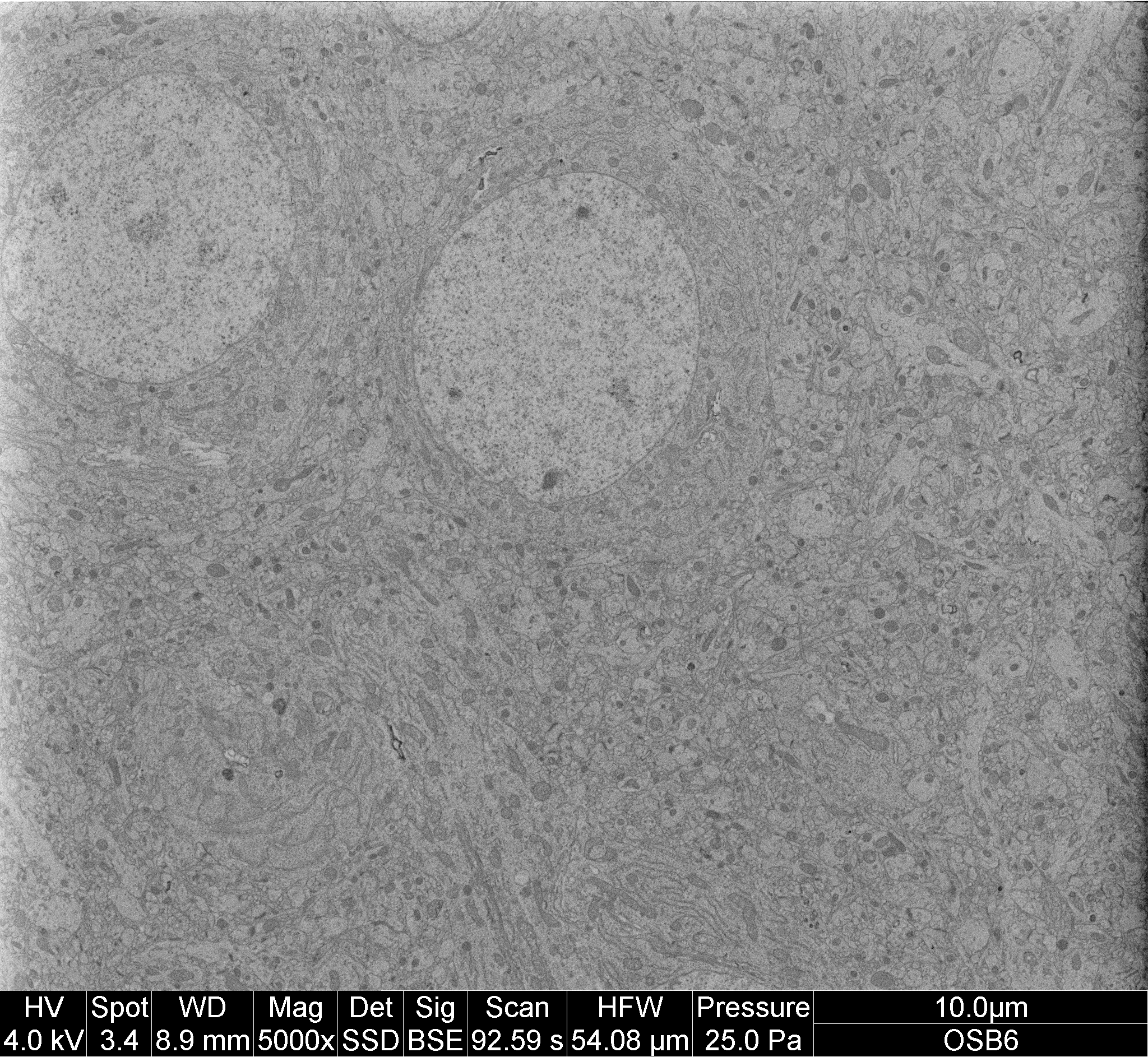

Supplement: Dataset S17 — (252.7 MB ZIP). [file pbio.0020329.sd017.zip › 040604_OS5_st1_1637.tif]

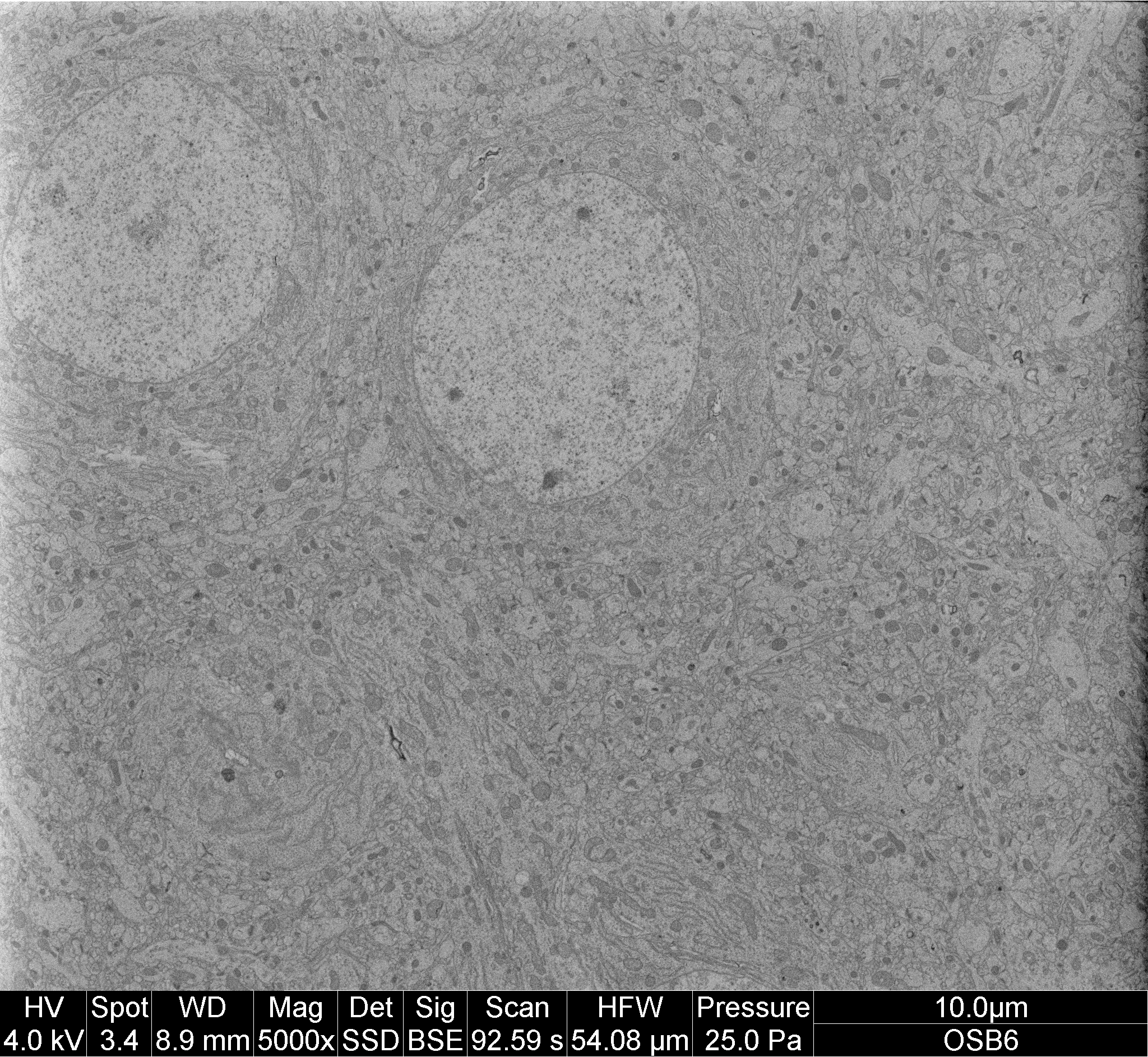

Supplement: Dataset S17 — (252.7 MB ZIP). [file pbio.0020329.sd017.zip › 040604_OS5_st1_1638.tif]

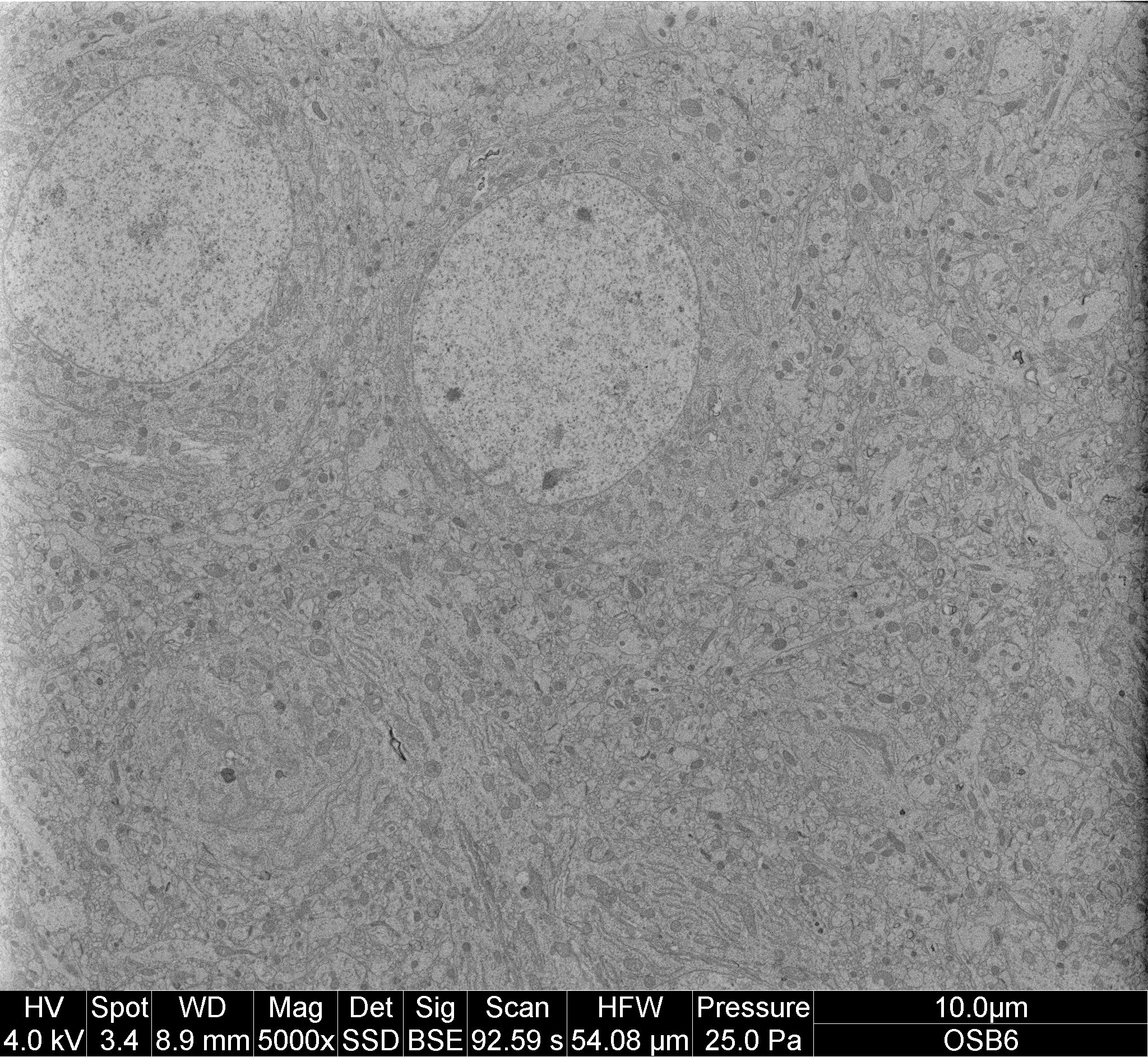

Supplement: Dataset S17 — (252.7 MB ZIP). [file pbio.0020329.sd017.zip › 040604_OS5_st1_1639.tif]

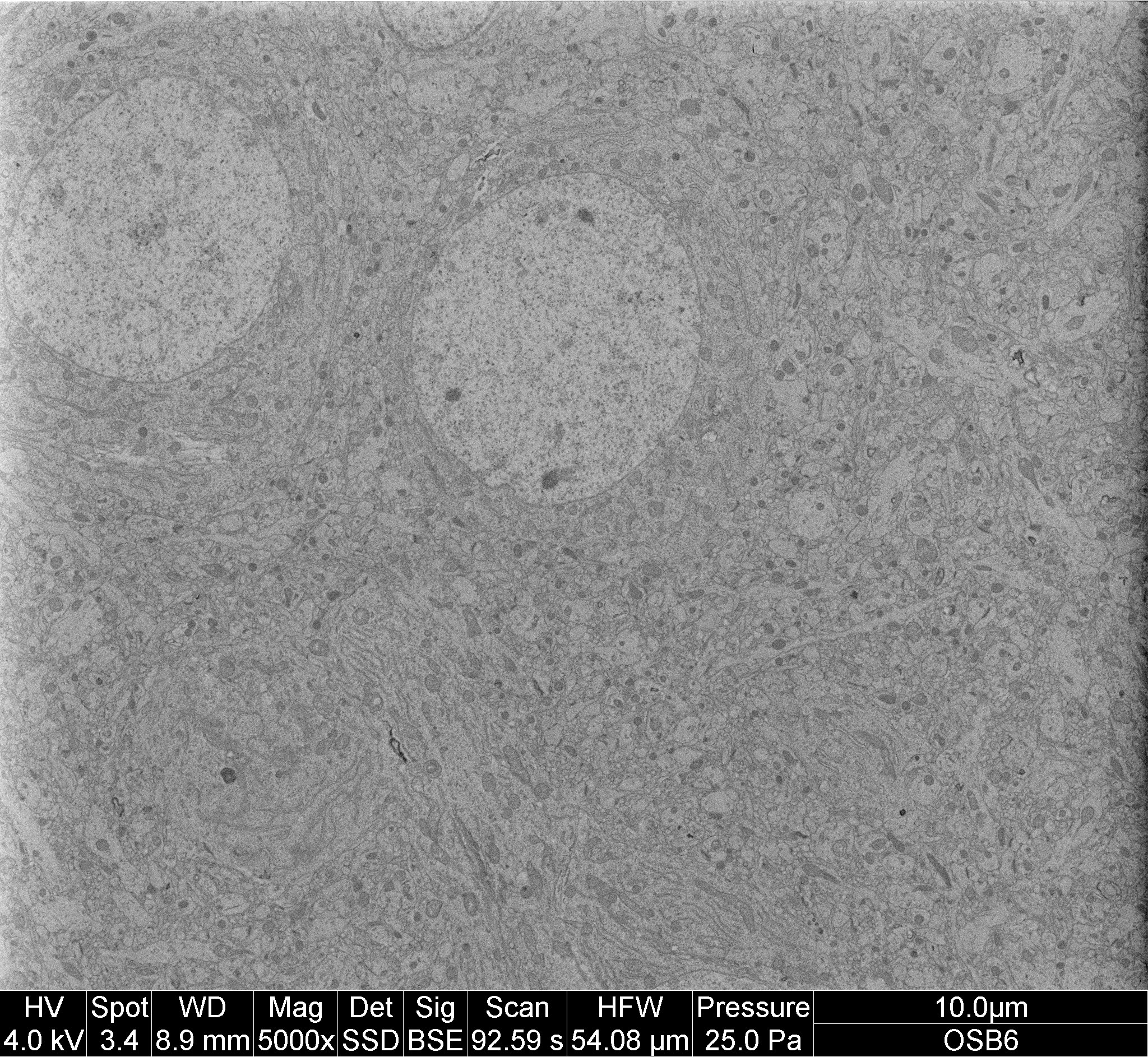

Supplement: Dataset S17 — (252.7 MB ZIP). [file pbio.0020329.sd017.zip › 040604_OS5_st1_1640.tif]

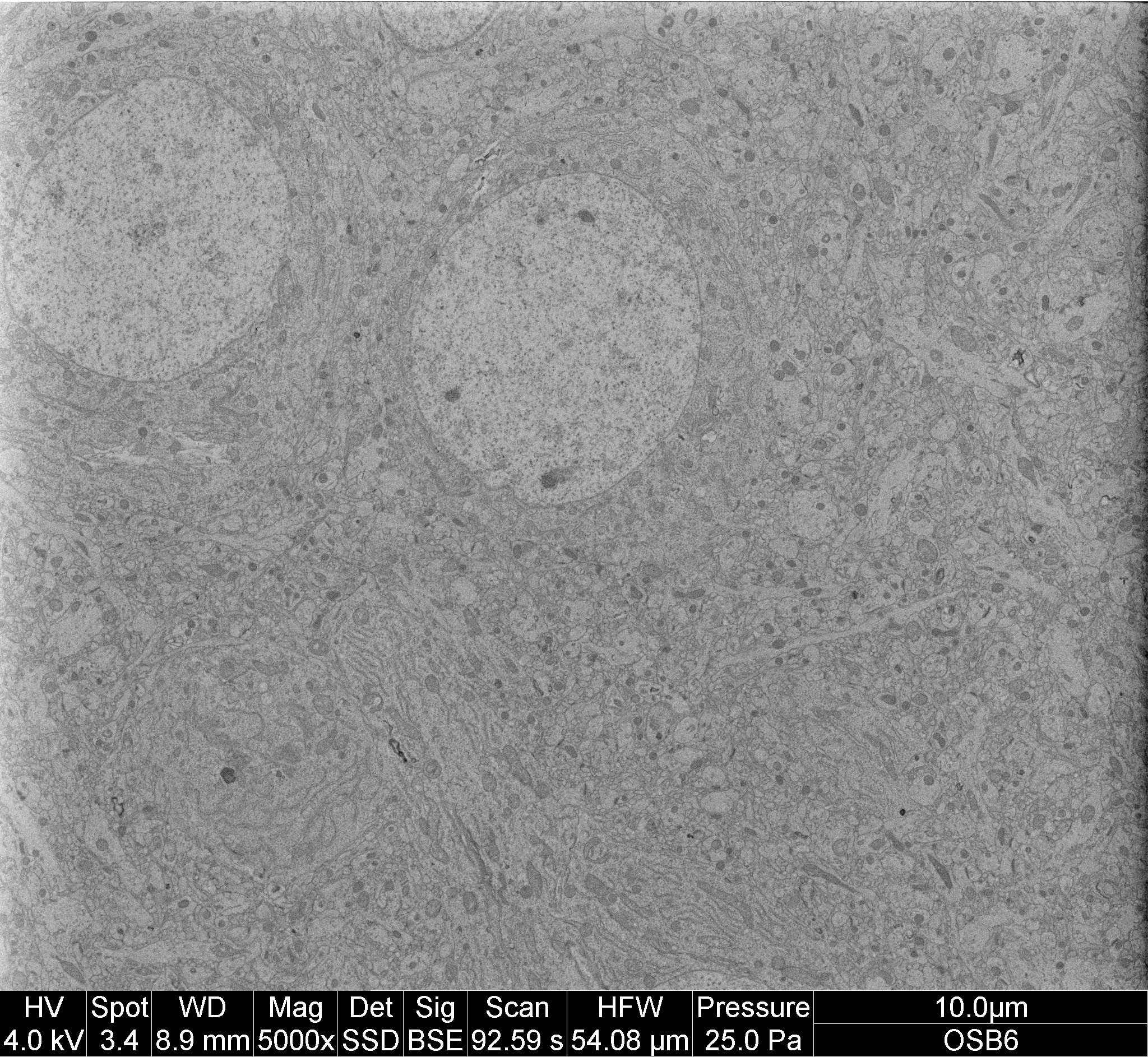

Supplement: Dataset S17 — (252.7 MB ZIP). [file pbio.0020329.sd017.zip › 040604_OS5_st1_1641.tif]

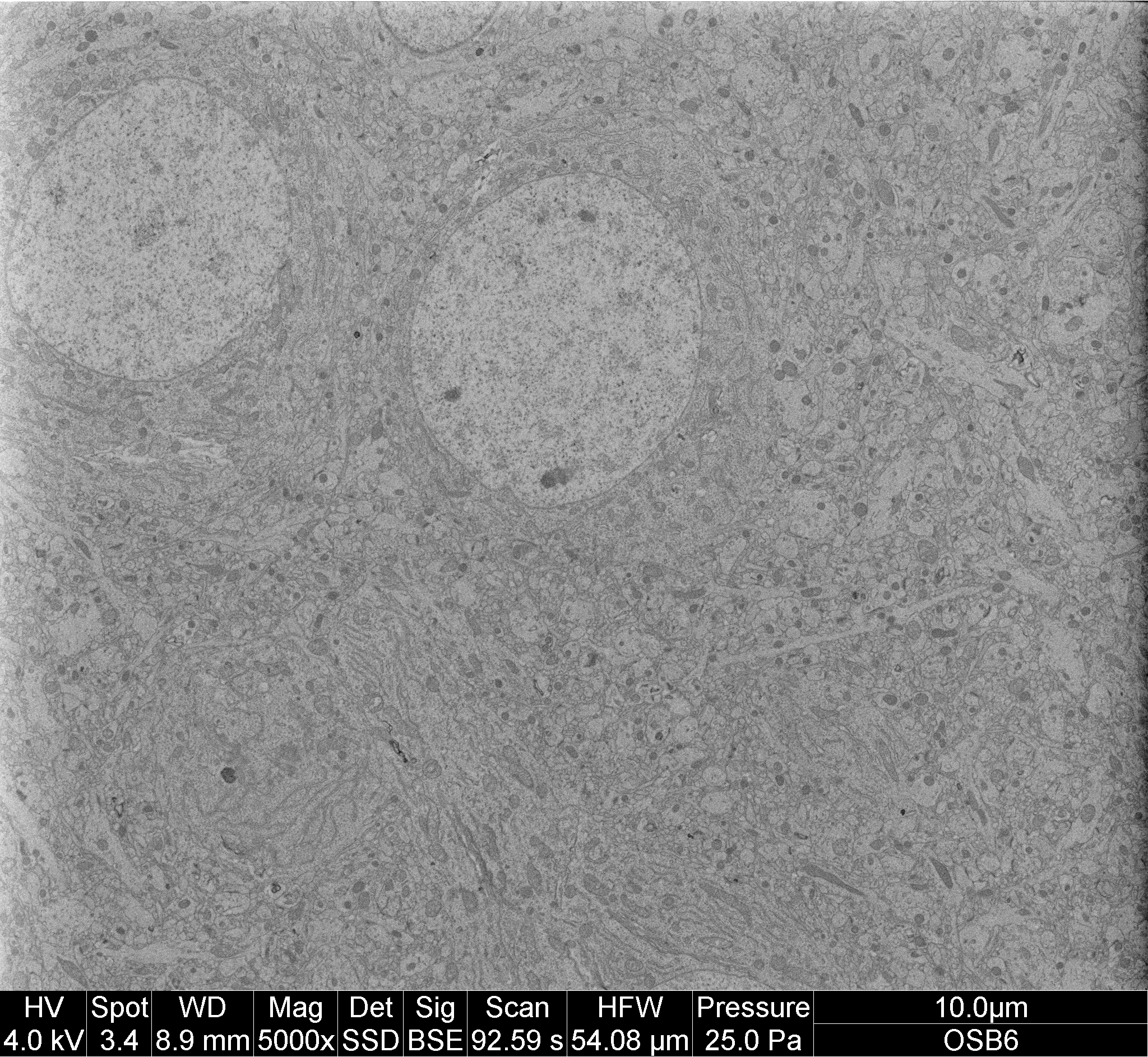

Supplement: Dataset S17 — (252.7 MB ZIP). [file pbio.0020329.sd017.zip › 040604_OS5_st1_1642.tif]

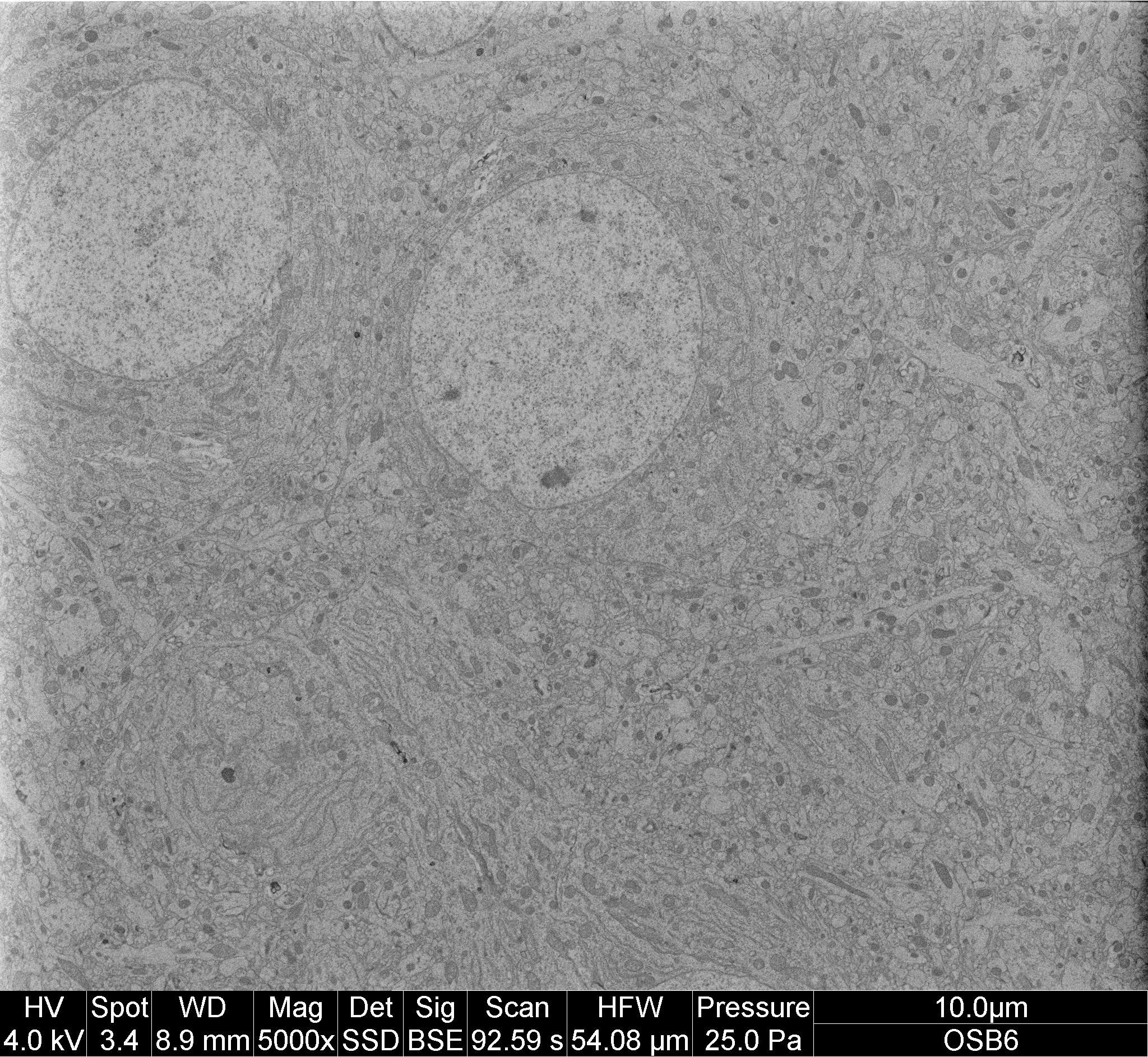

Supplement: Dataset S17 — (252.7 MB ZIP). [file pbio.0020329.sd017.zip › 040604_OS5_st1_1643.tif]

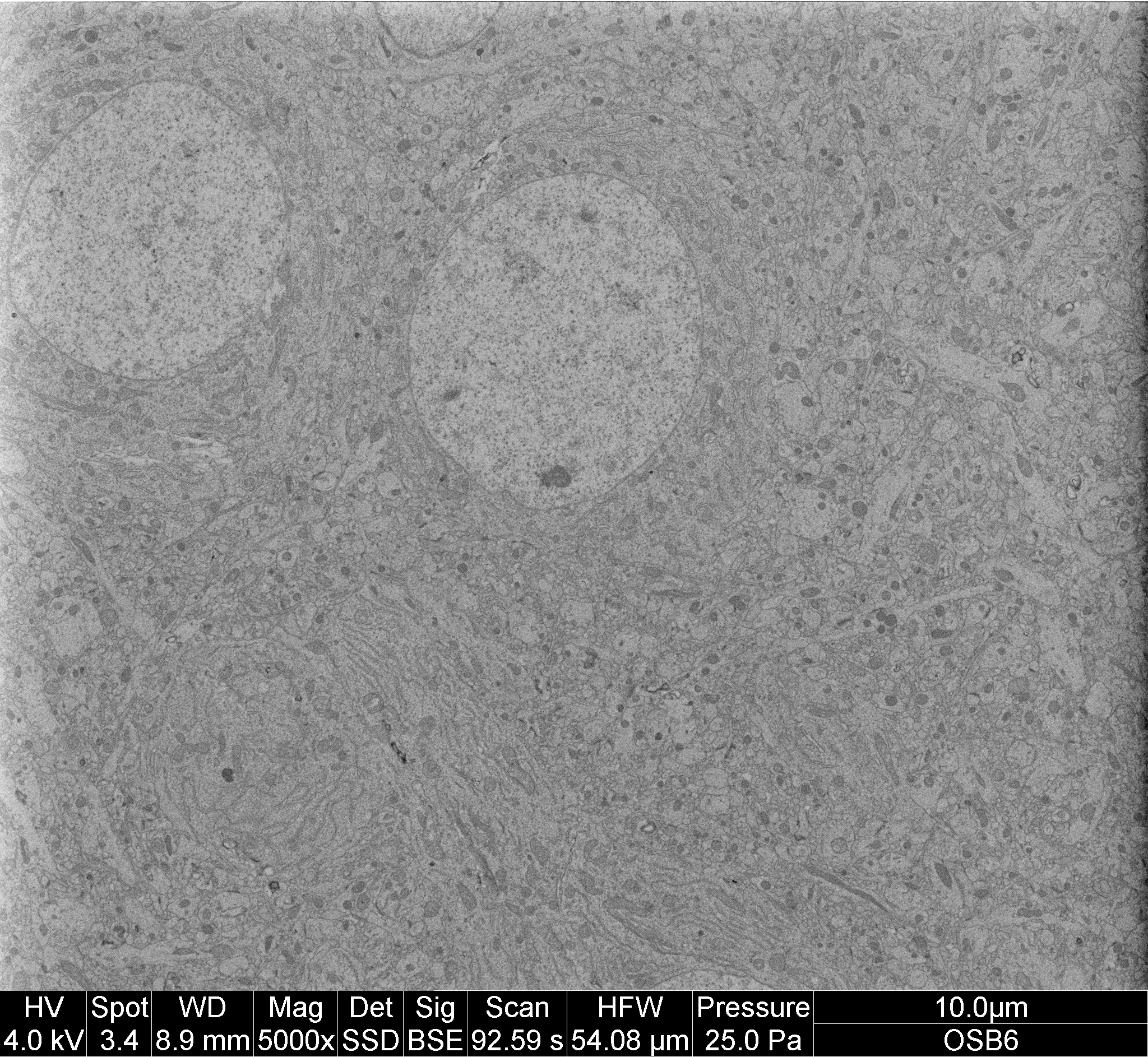

Supplement: Dataset S17 — (252.7 MB ZIP). [file pbio.0020329.sd017.zip › 040604_OS5_st1_1644.tif]

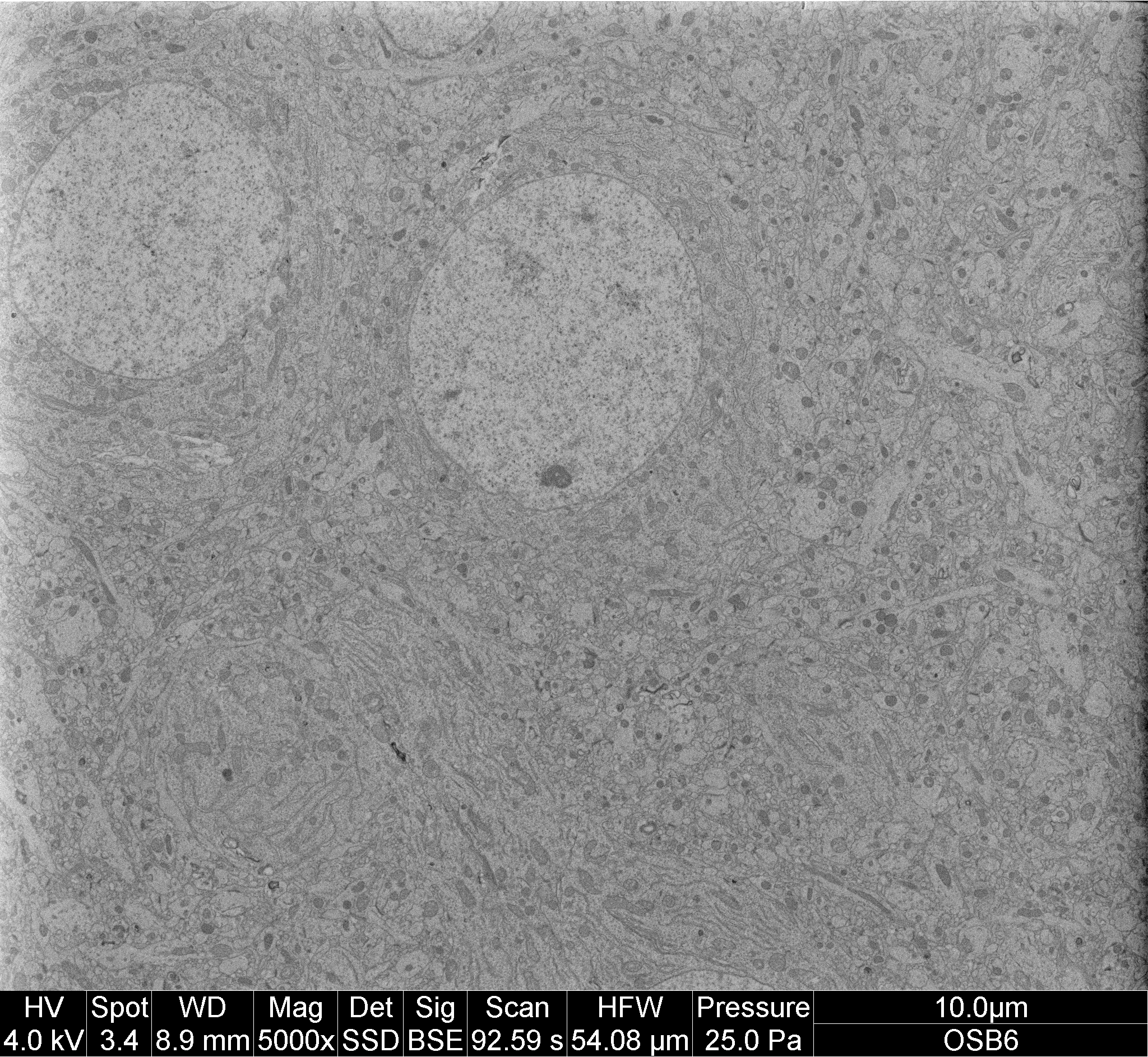

Supplement: Dataset S17 — (252.7 MB ZIP). [file pbio.0020329.sd017.zip › 040604_OS5_st1_1645.tif]

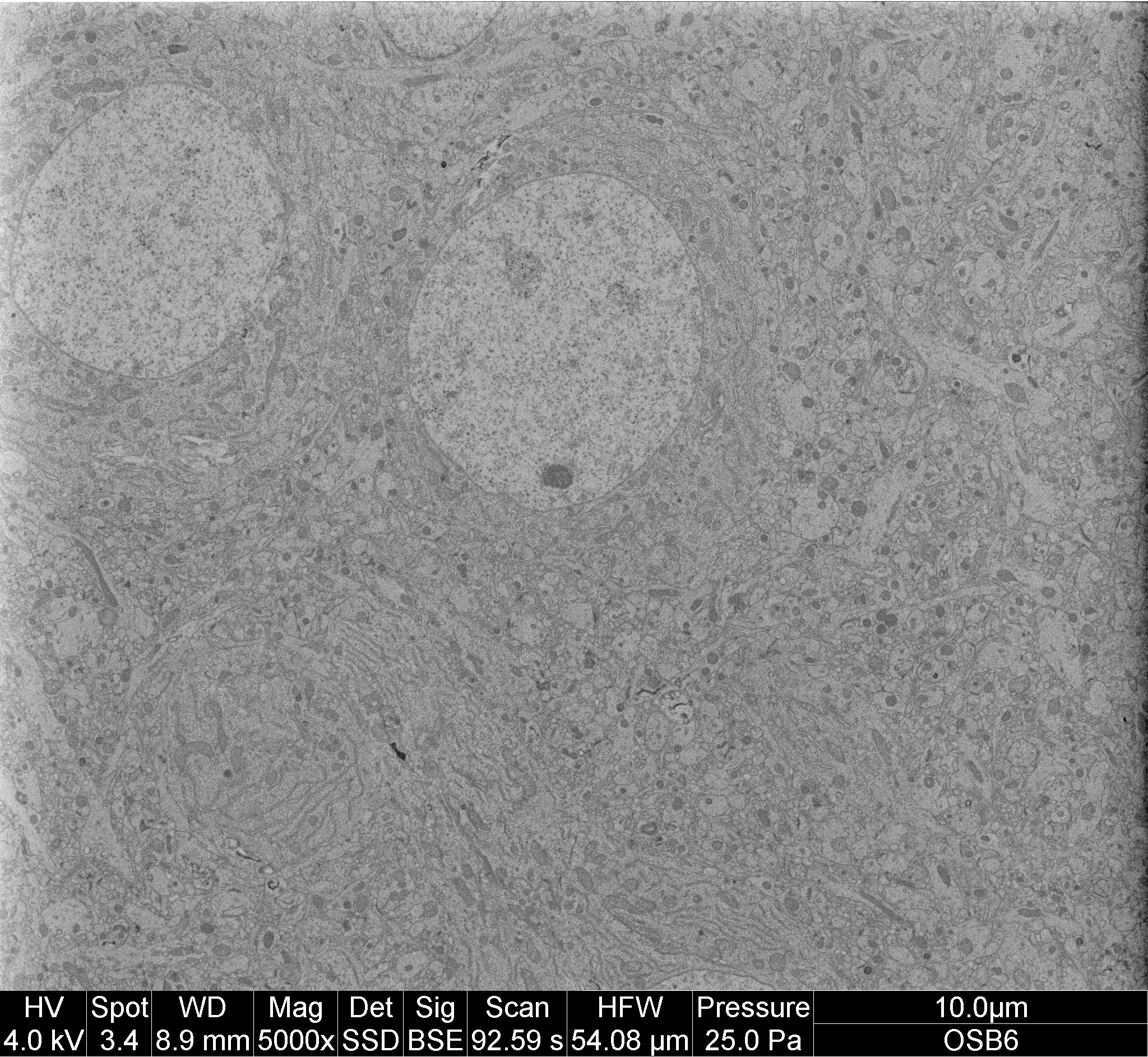

Supplement: Dataset S17 — (252.7 MB ZIP). [file pbio.0020329.sd017.zip › 040604_OS5_st1_1646.tif]

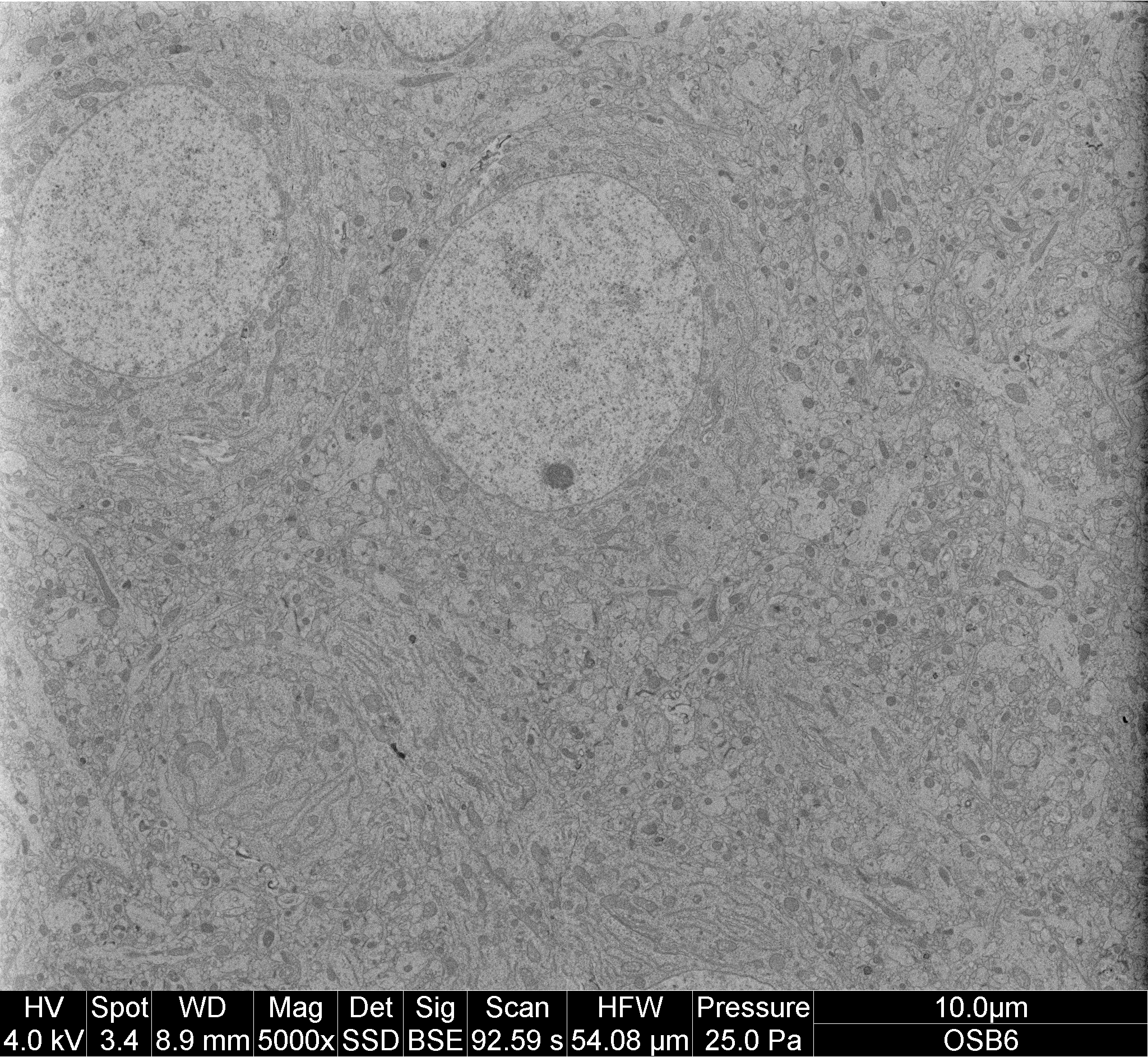

Supplement: Dataset S17 — (252.7 MB ZIP). [file pbio.0020329.sd017.zip › 040604_OS5_st1_1647.tif]

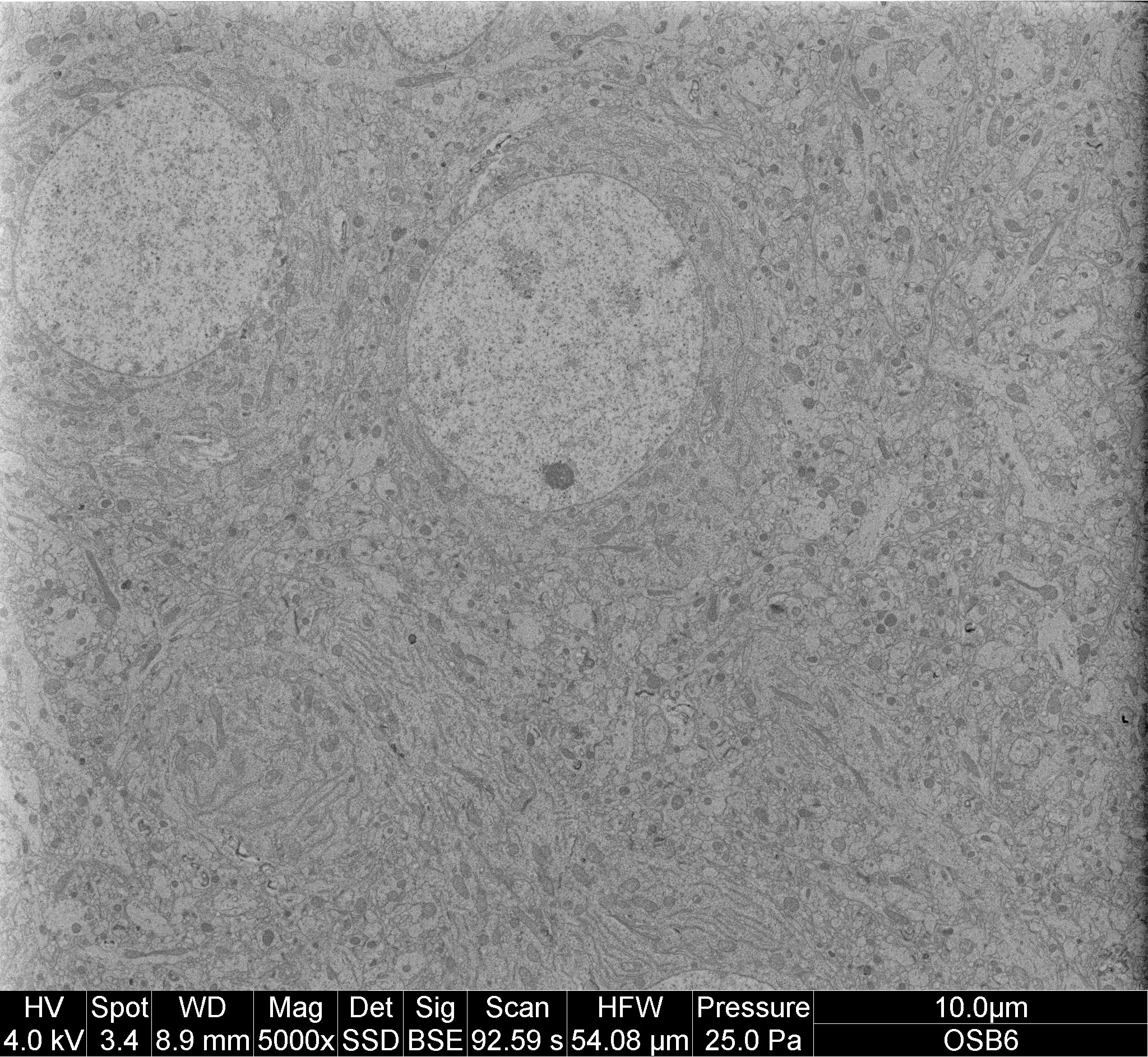

Supplement: Dataset S17 — (252.7 MB ZIP). [file pbio.0020329.sd017.zip › 040604_OS5_st1_1648.tif]

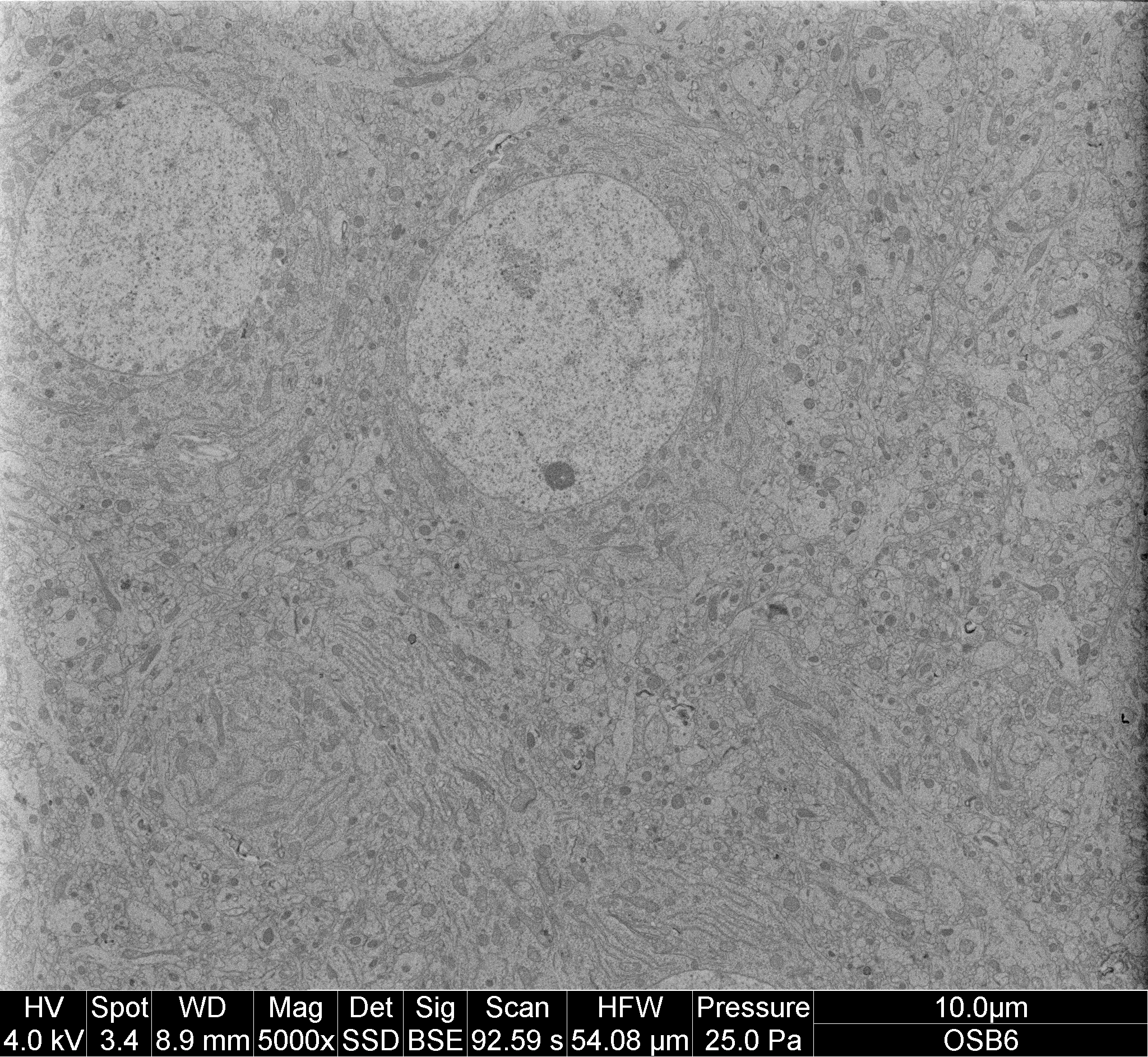

Supplement: Dataset S17 — (252.7 MB ZIP). [file pbio.0020329.sd017.zip › 040604_OS5_st1_1649.tif]

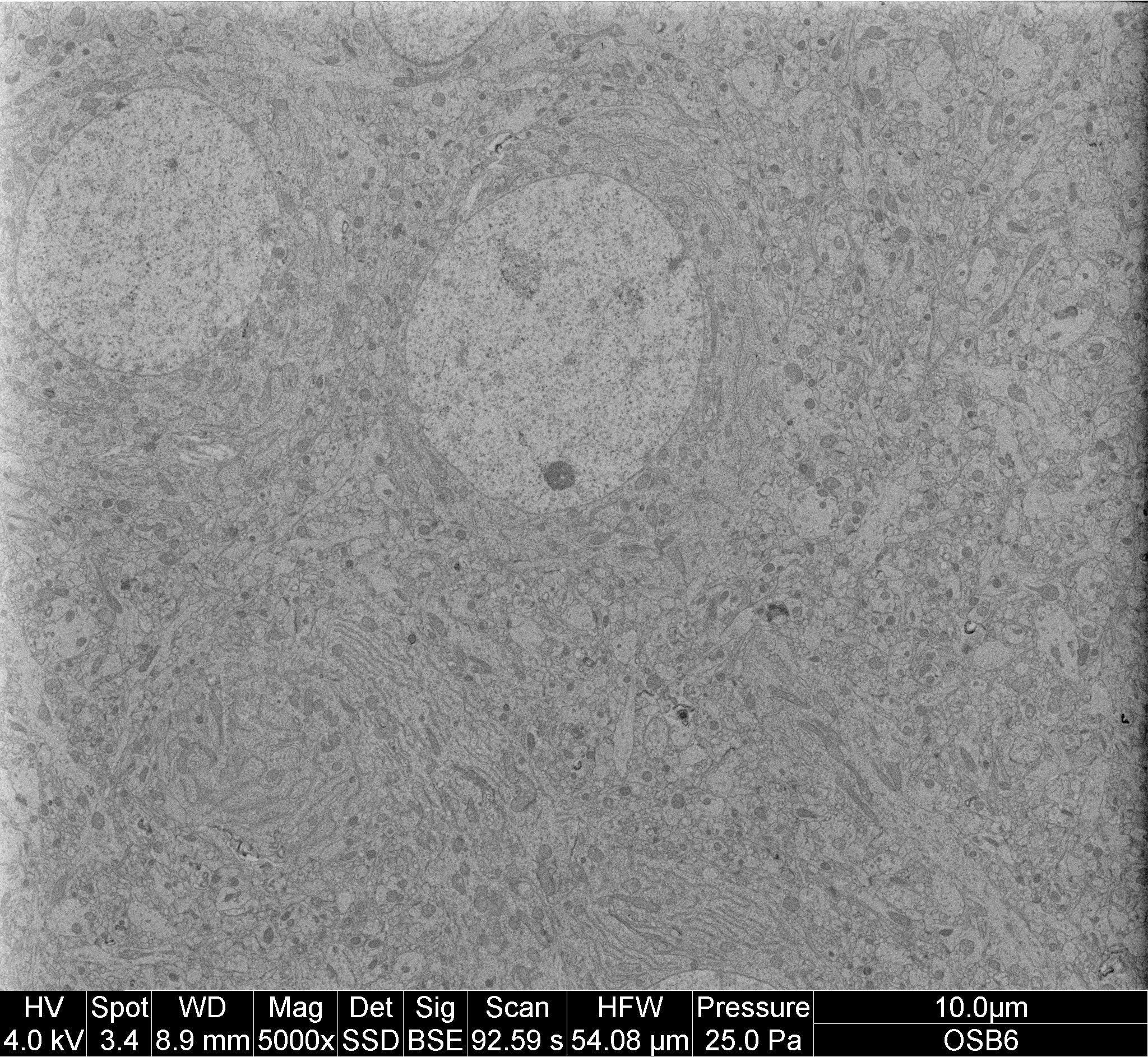

Supplement: Dataset S17 — (252.7 MB ZIP). [file pbio.0020329.sd017.zip › 040604_OS5_st1_1650.tif]

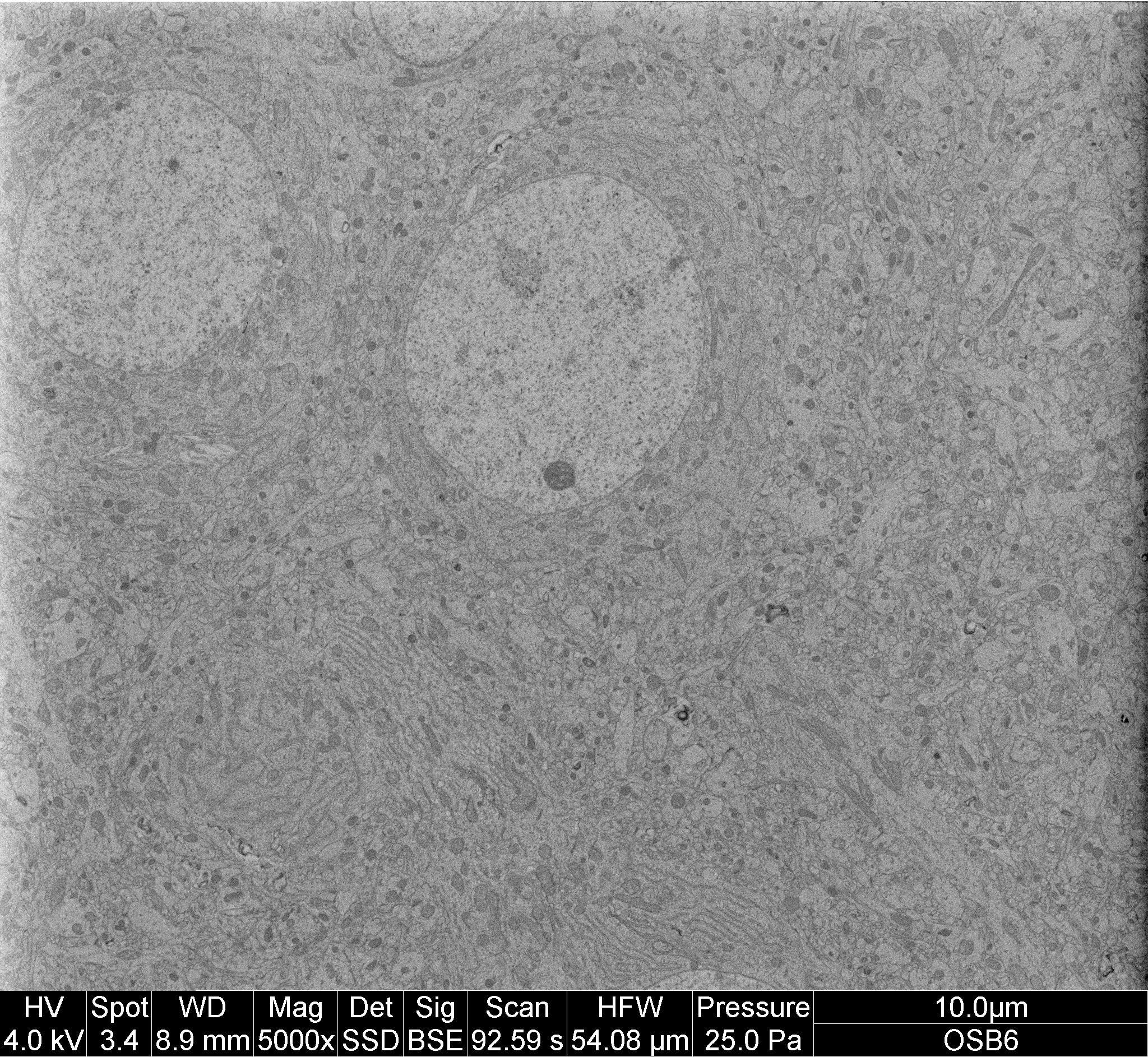

Supplement: Dataset S17 — (252.7 MB ZIP). [file pbio.0020329.sd017.zip › 040604_OS5_st1_1651.tif]

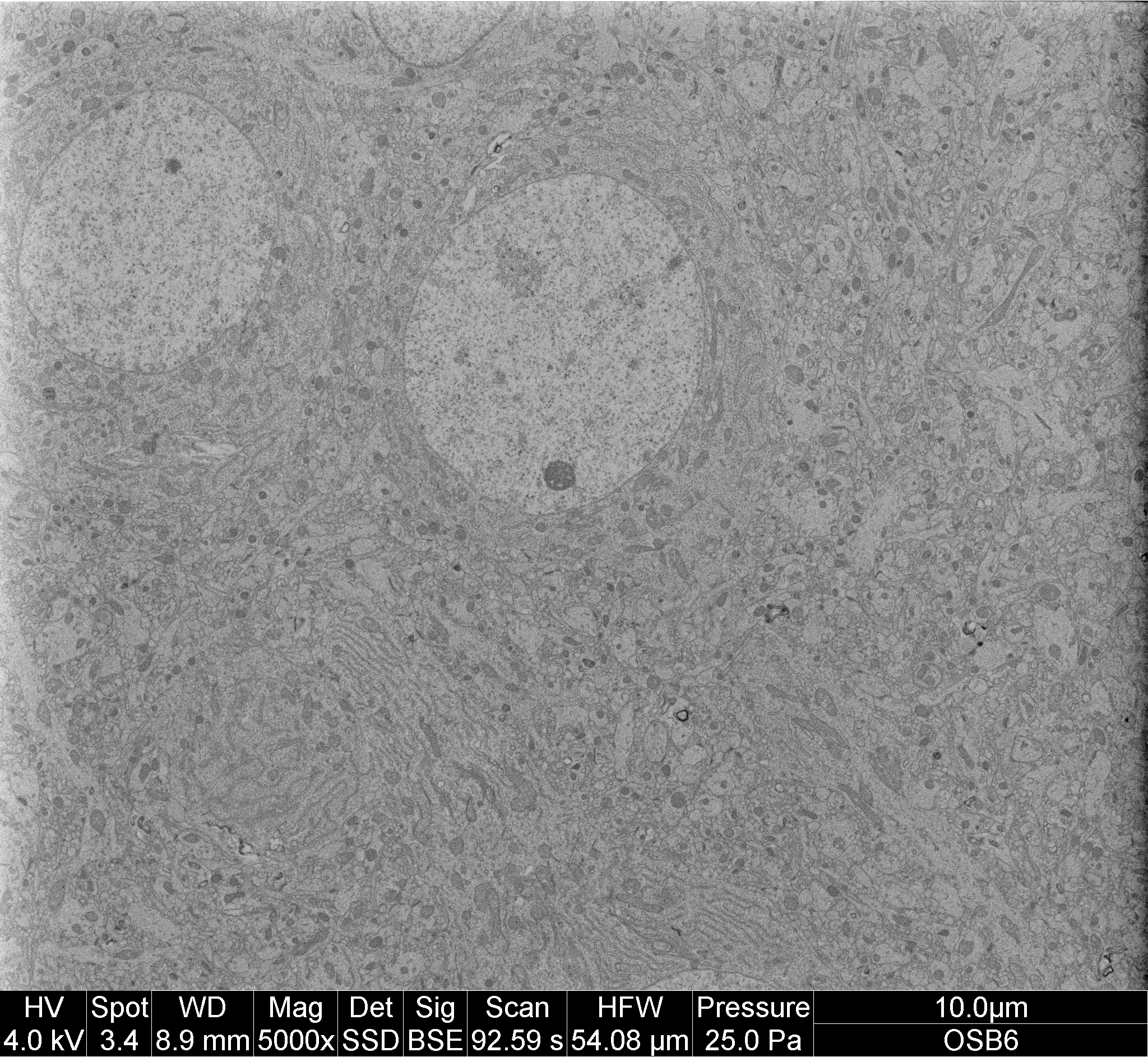

Supplement: Dataset S17 — (252.7 MB ZIP). [file pbio.0020329.sd017.zip › 040604_OS5_st1_1652.tif]

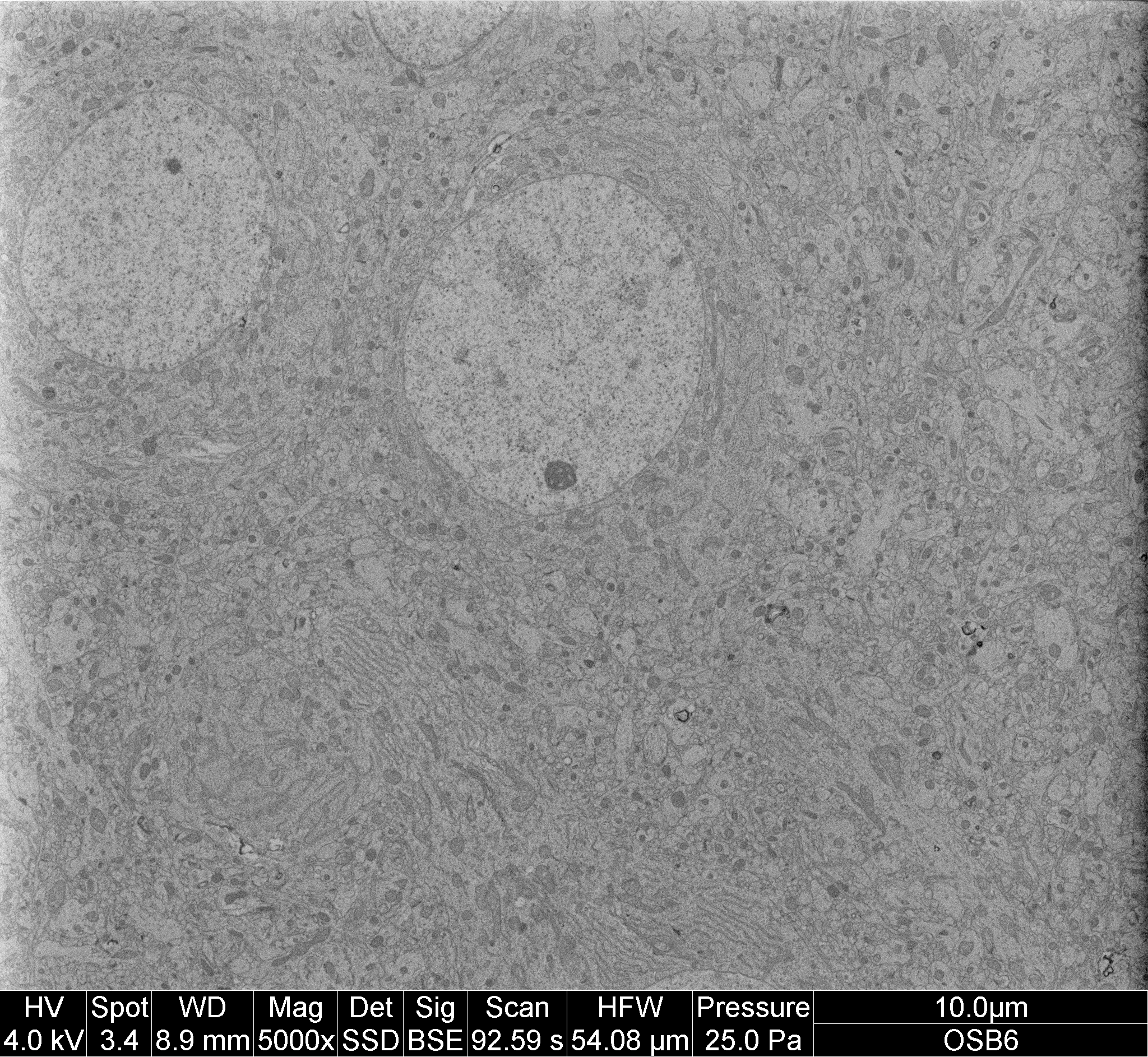

Supplement: Dataset S17 — (252.7 MB ZIP). [file pbio.0020329.sd017.zip › 040604_OS5_st1_1653.tif]

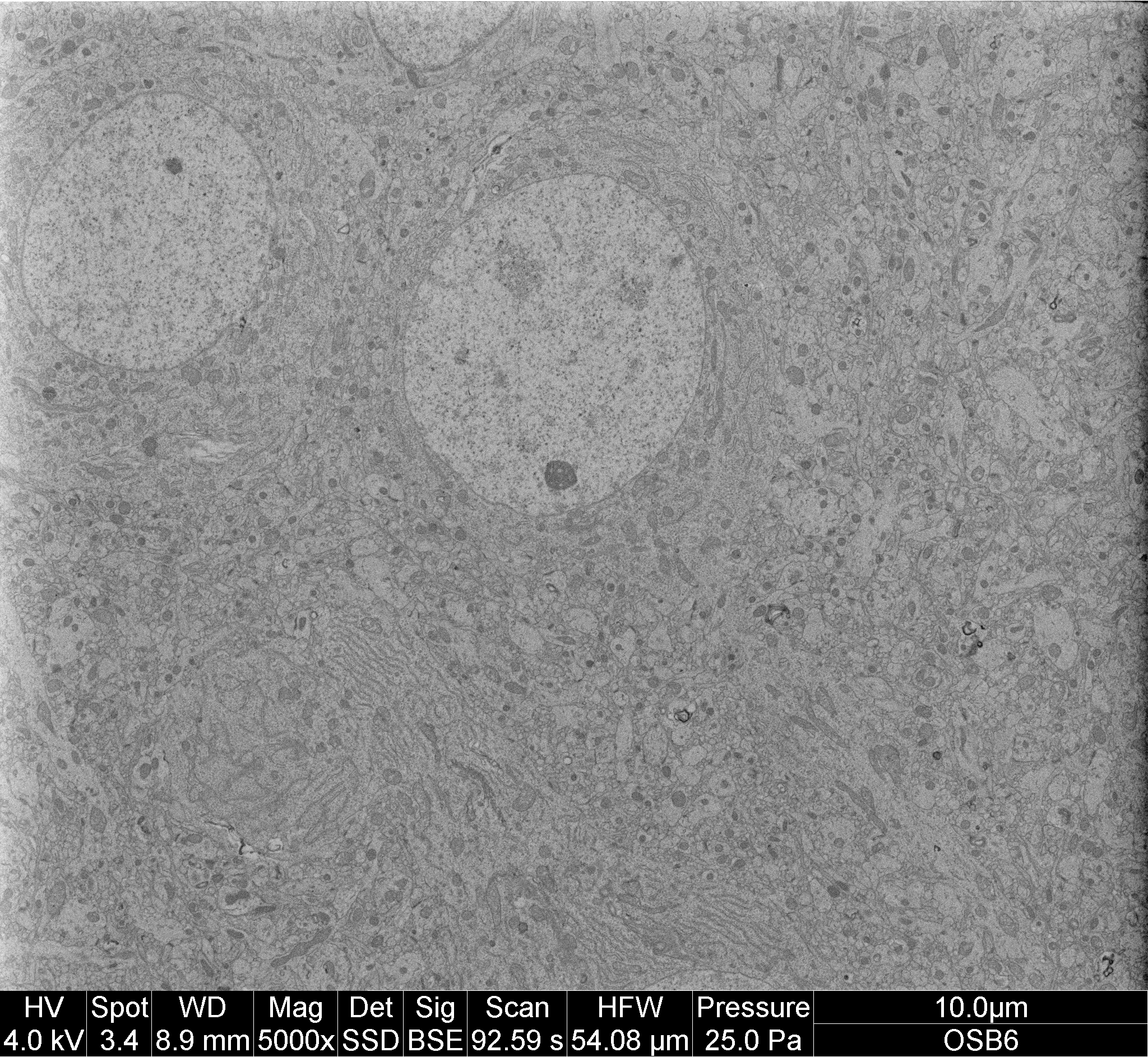

Supplement: Dataset S17 — (252.7 MB ZIP). [file pbio.0020329.sd017.zip › 040604_OS5_st1_1654.tif]

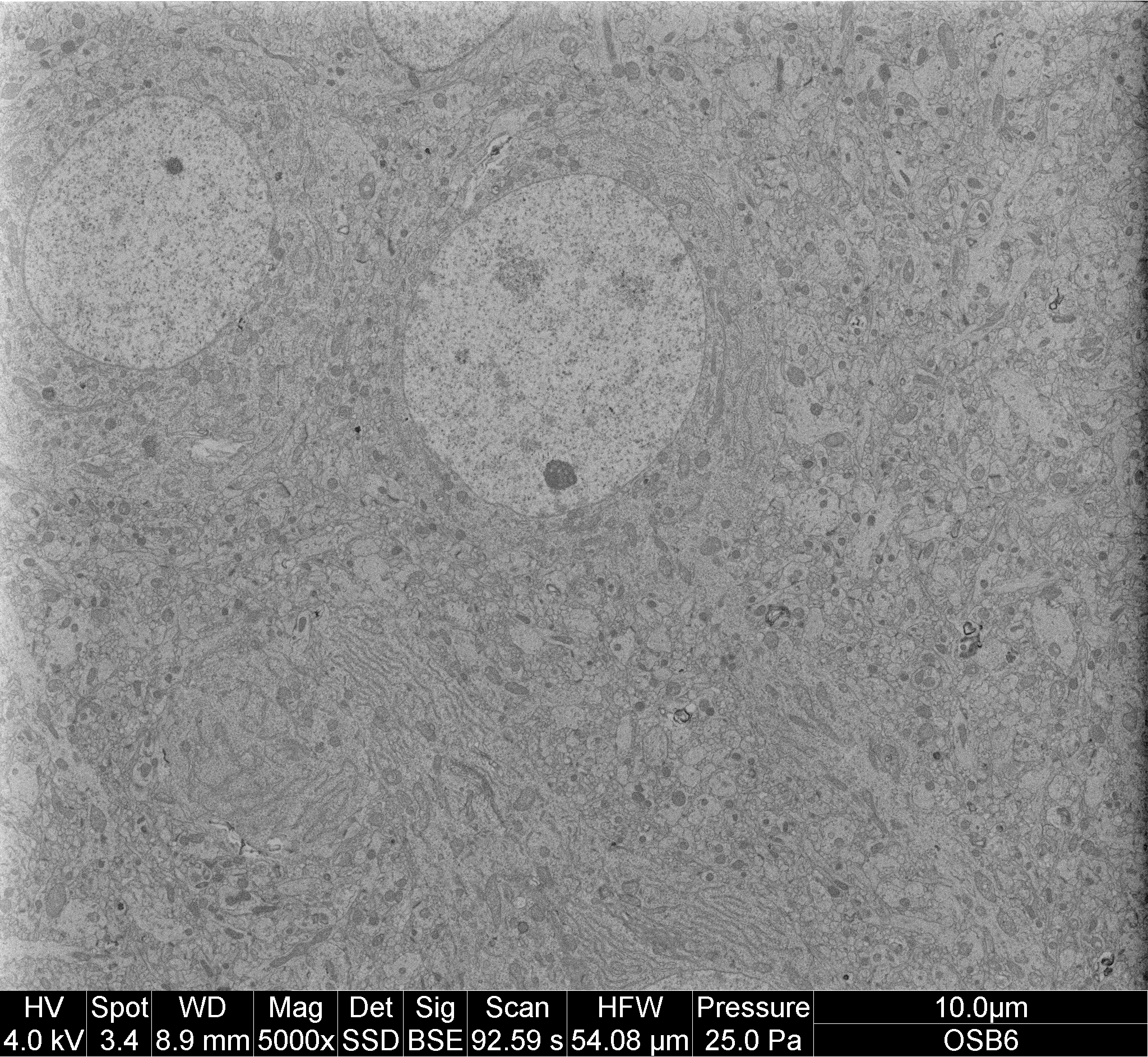

Supplement: Dataset S17 — (252.7 MB ZIP). [file pbio.0020329.sd017.zip › 040604_OS5_st1_1655.tif]

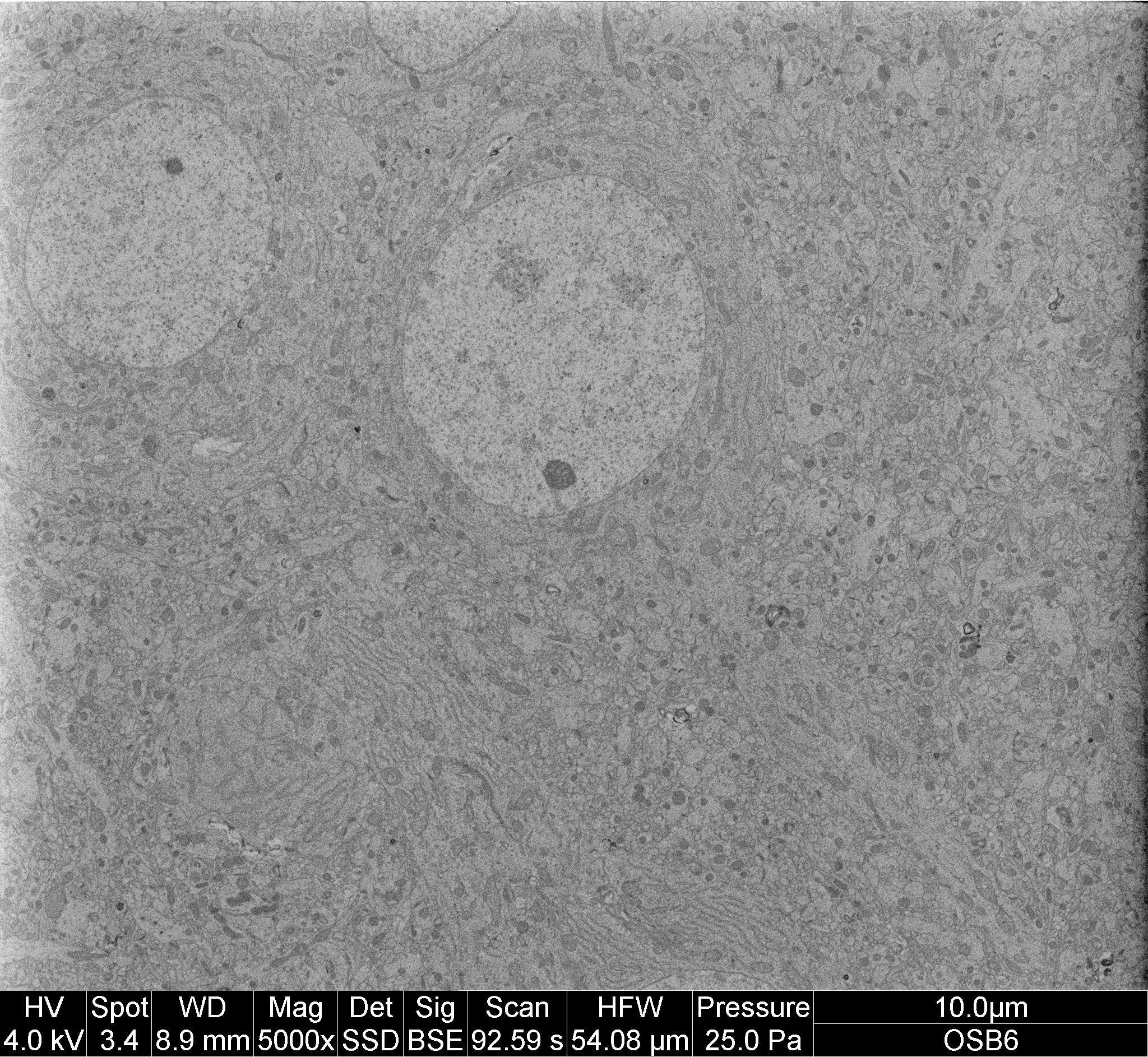

Supplement: Dataset S17 — (252.7 MB ZIP). [file pbio.0020329.sd017.zip › 040604_OS5_st1_1656.tif]

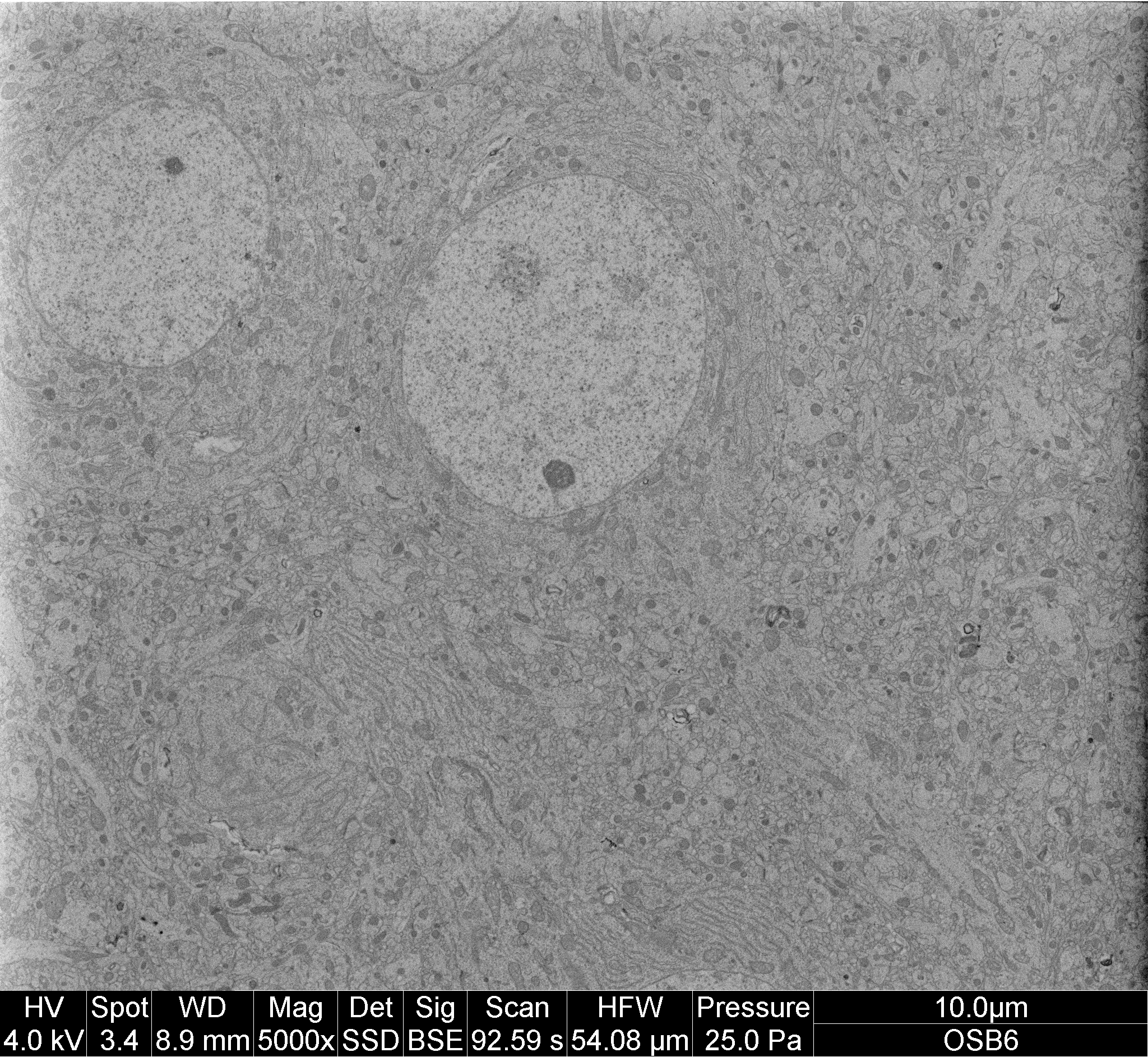

Supplement: Dataset S17 — (252.7 MB ZIP). [file pbio.0020329.sd017.zip › 040604_OS5_st1_1657.tif]

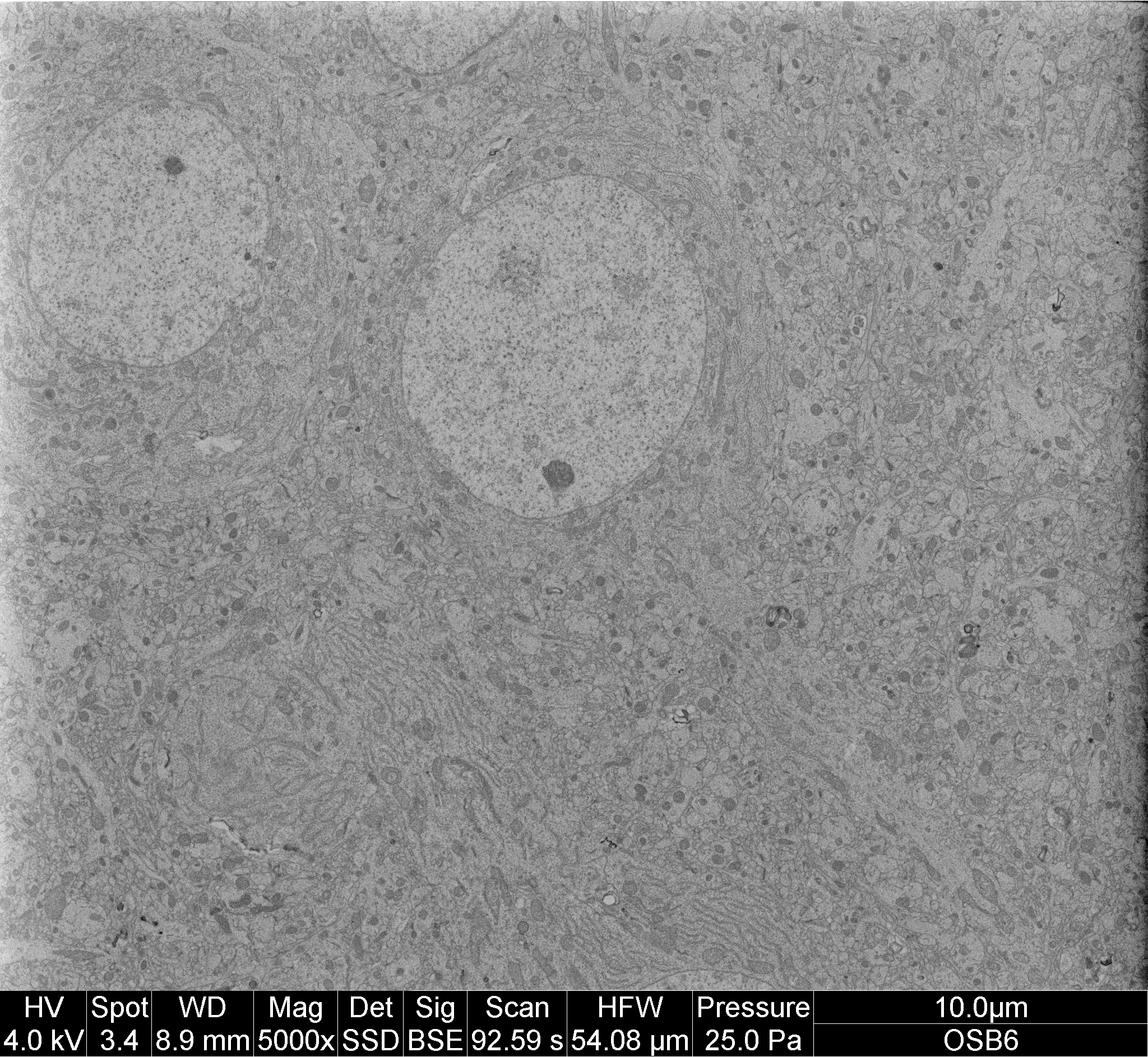

Supplement: Dataset S17 — (252.7 MB ZIP). [file pbio.0020329.sd017.zip › 040604_OS5_st1_1658.tif]

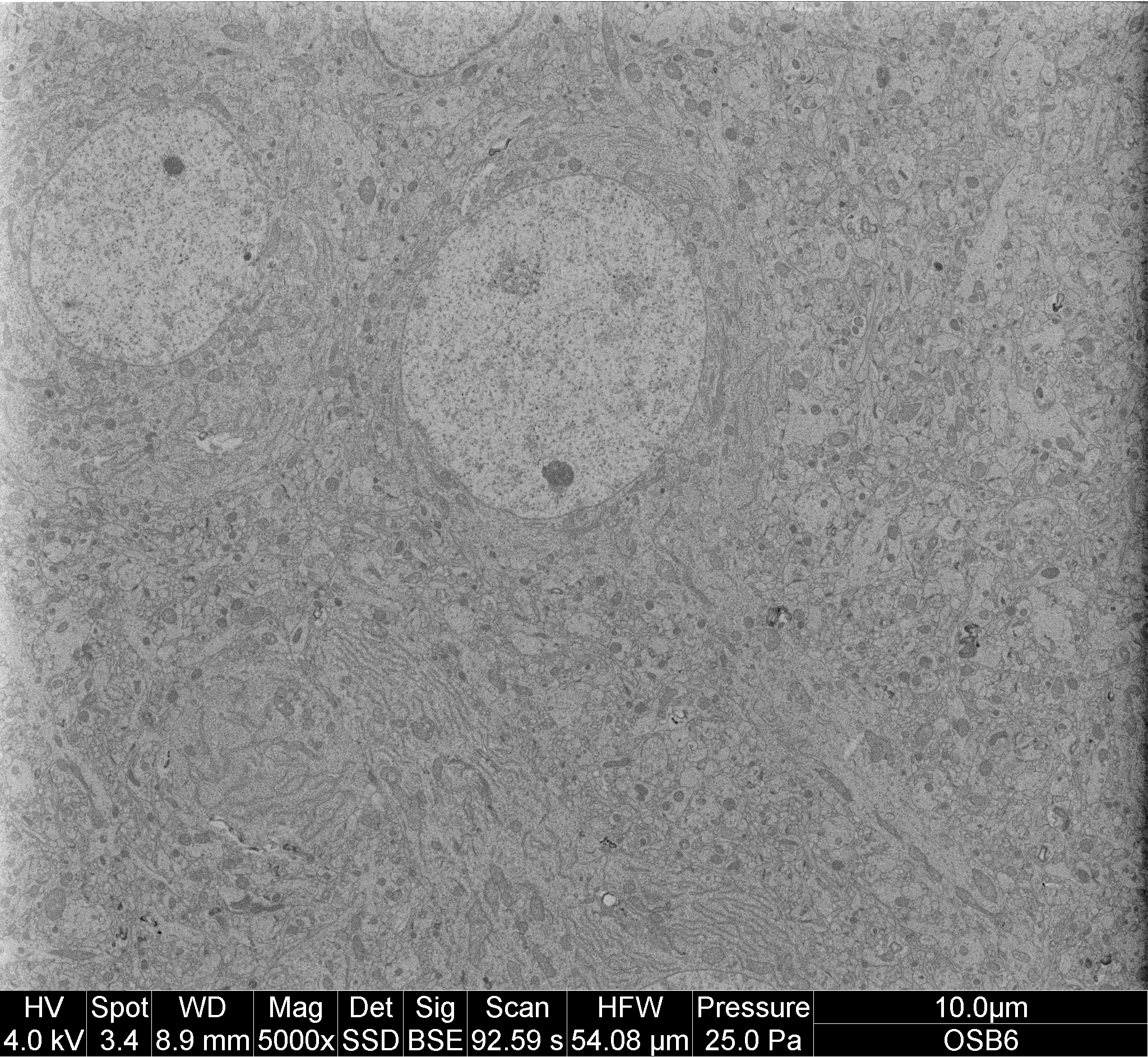

Supplement: Dataset S17 — (252.7 MB ZIP). [file pbio.0020329.sd017.zip › 040604_OS5_st1_1659.tif]

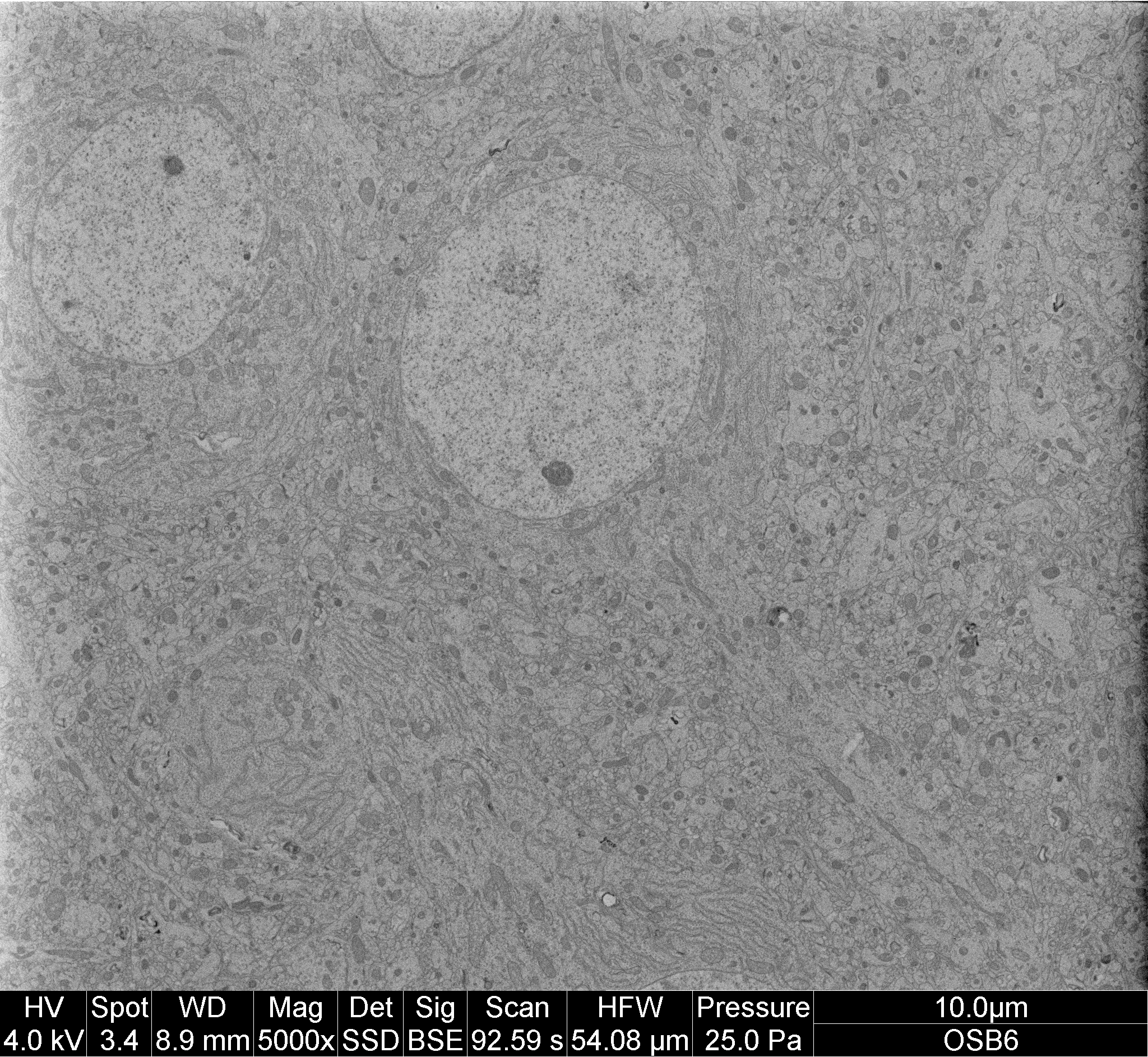

Supplement: Dataset S17 — (252.7 MB ZIP). [file pbio.0020329.sd017.zip › 040604_OS5_st1_1660.tif]

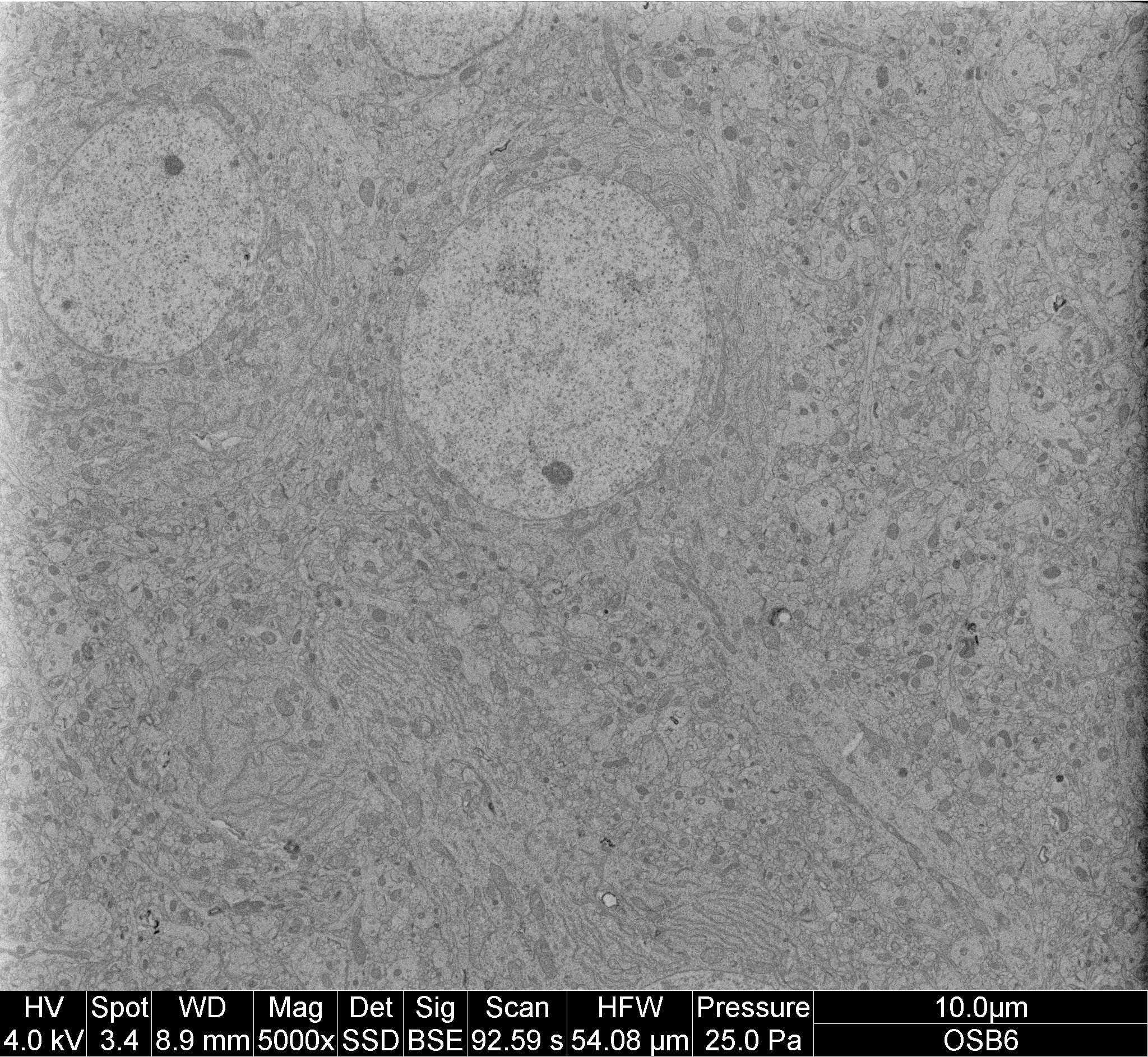

Supplement: Dataset S17 — (252.7 MB ZIP). [file pbio.0020329.sd017.zip › 040604_OS5_st1_1661.tif]

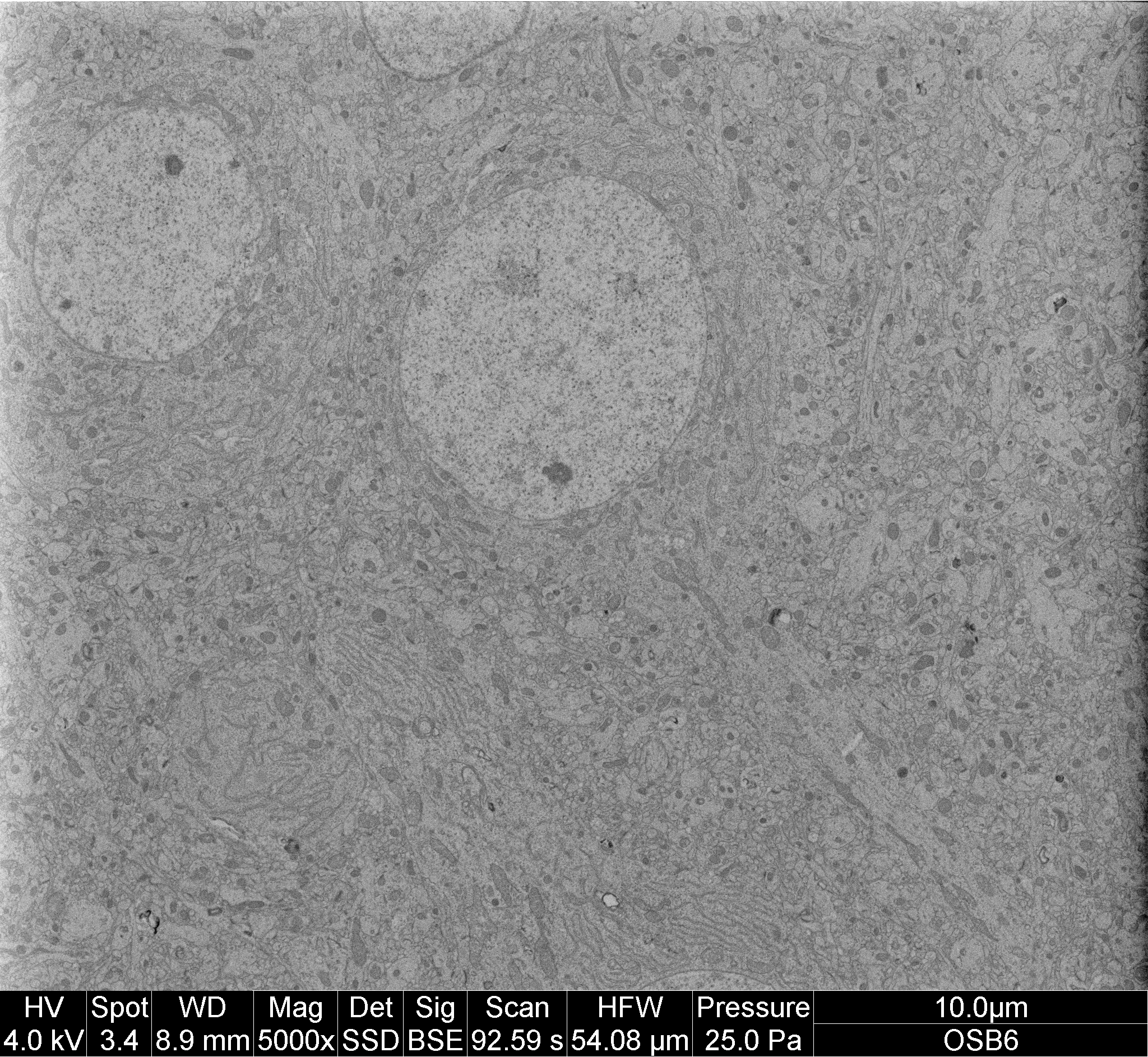

Supplement: Dataset S17 — (252.7 MB ZIP). [file pbio.0020329.sd017.zip › 040604_OS5_st1_1662.tif]

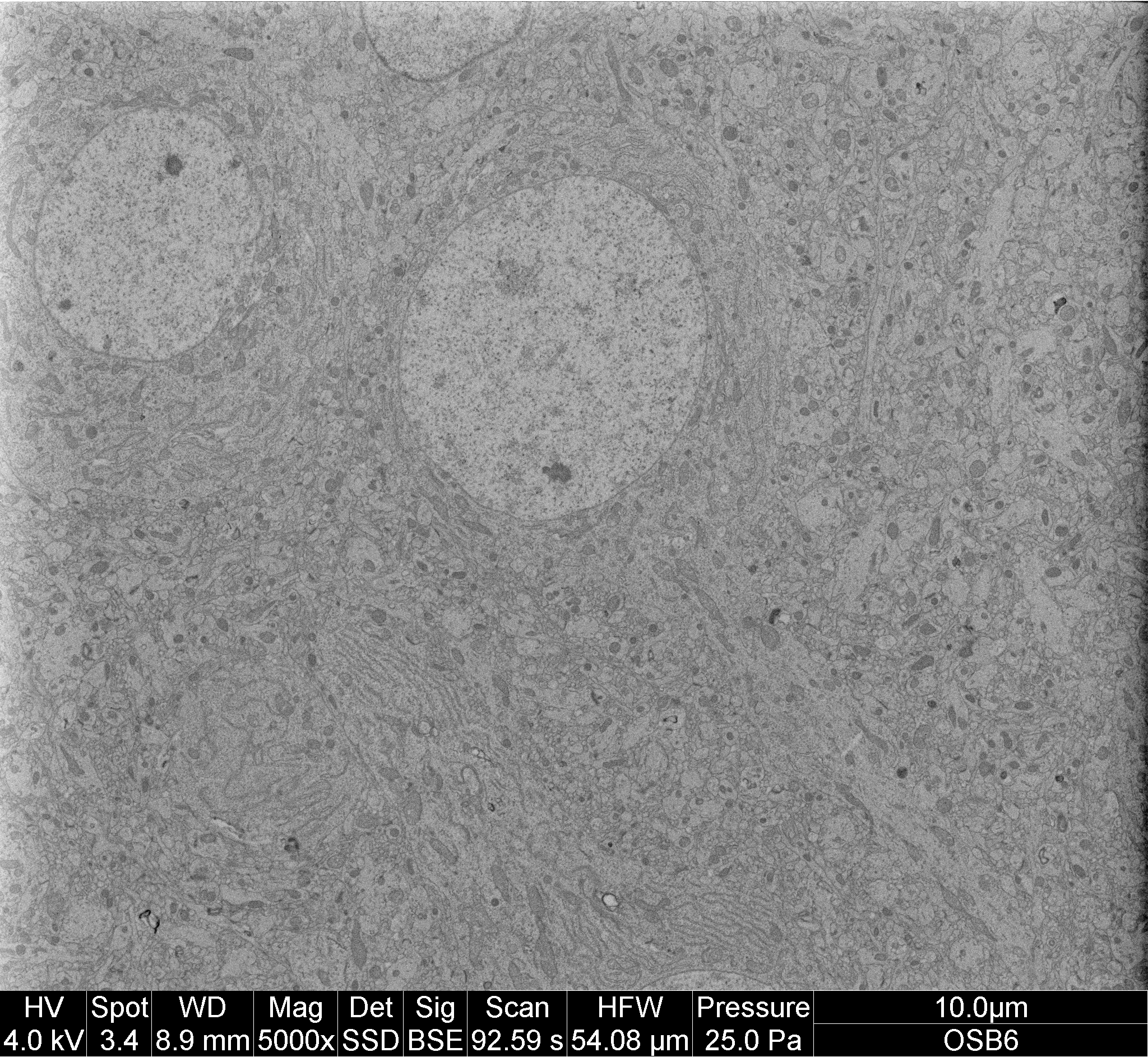

Supplement: Dataset S17 — (252.7 MB ZIP). [file pbio.0020329.sd017.zip › 040604_OS5_st1_1663.tif]

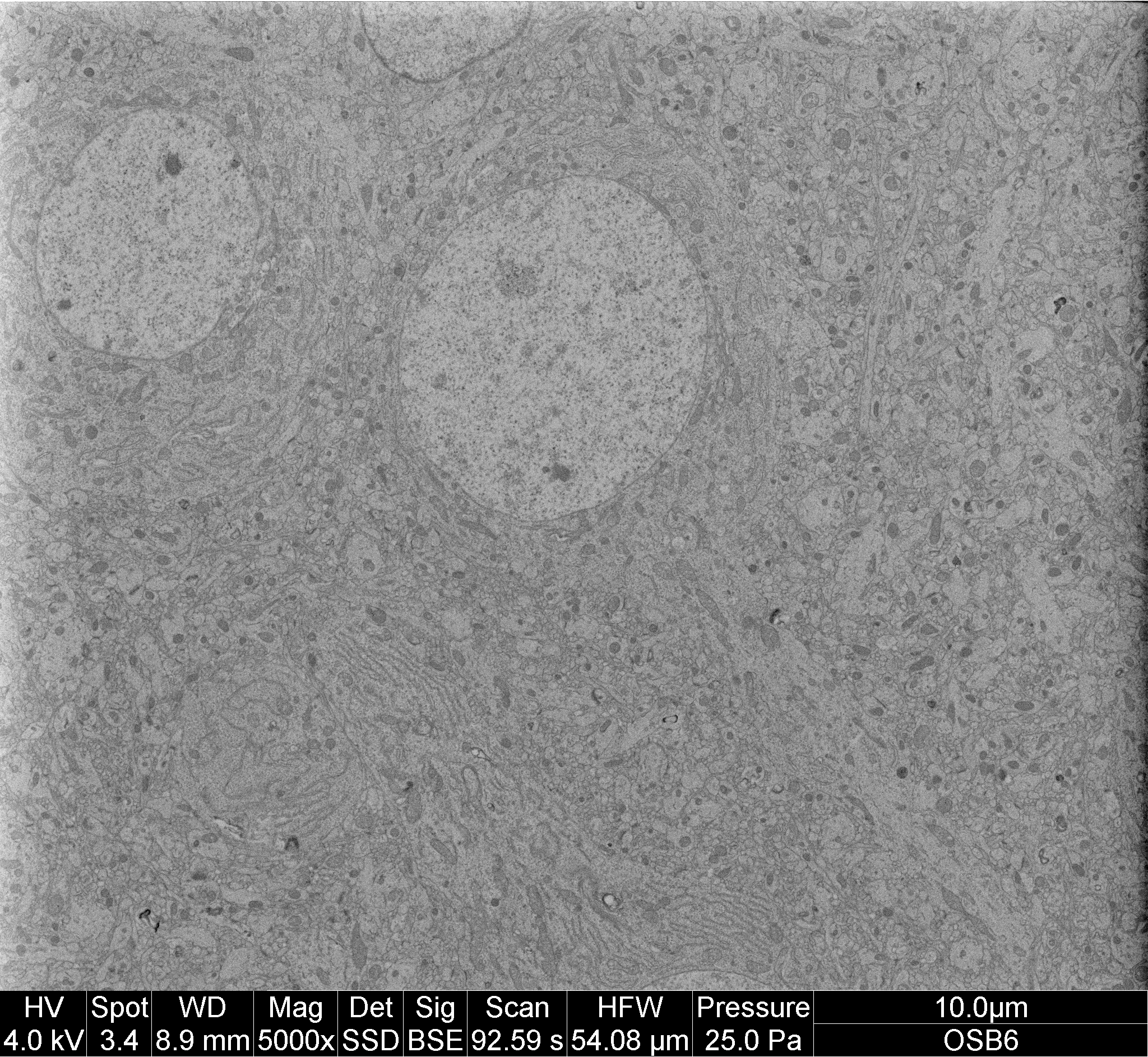

Supplement: Dataset S17 — (252.7 MB ZIP). [file pbio.0020329.sd017.zip › 040604_OS5_st1_1664.tif]

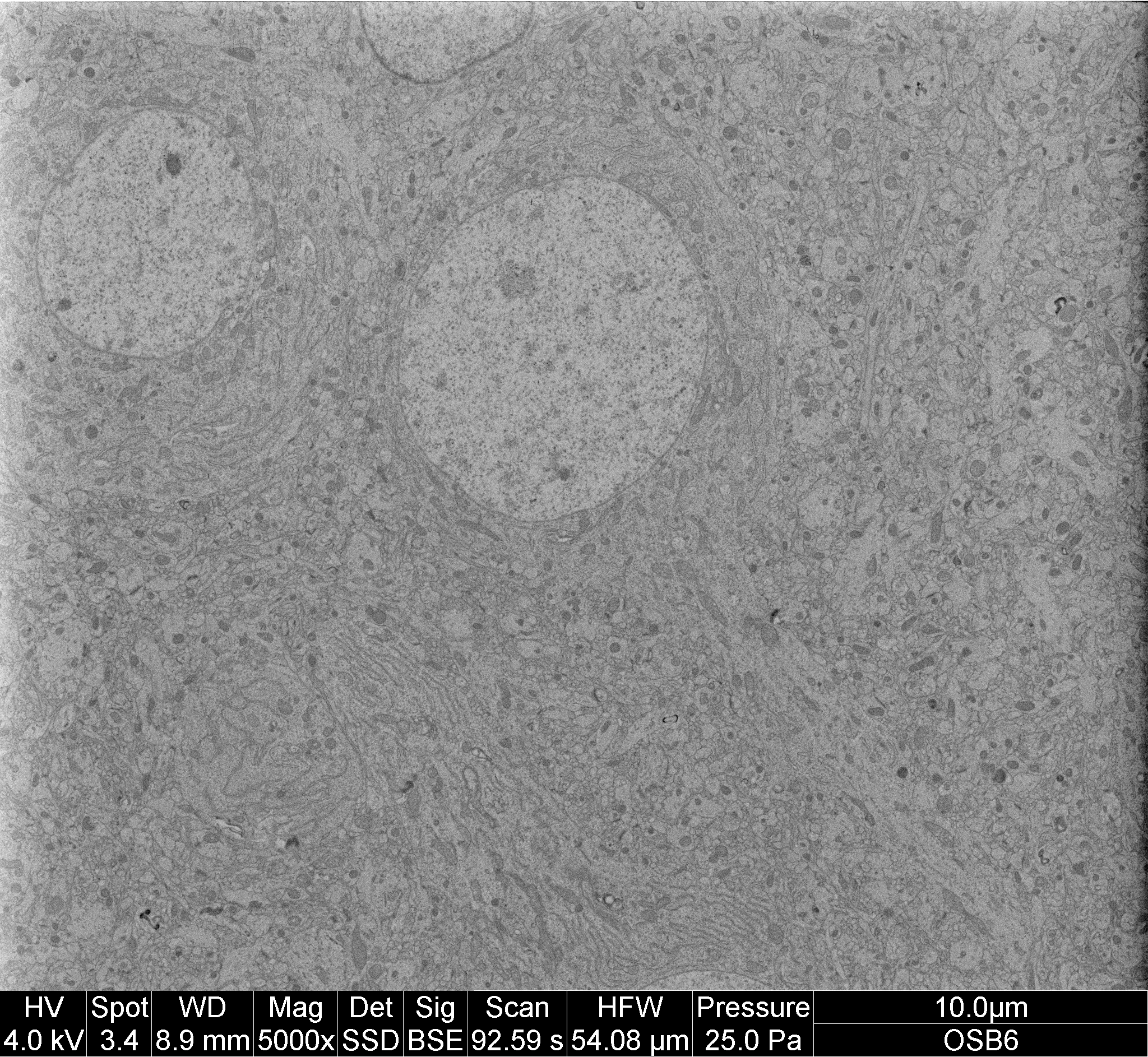

Supplement: Dataset S17 — (252.7 MB ZIP). [file pbio.0020329.sd017.zip › 040604_OS5_st1_1665.tif]

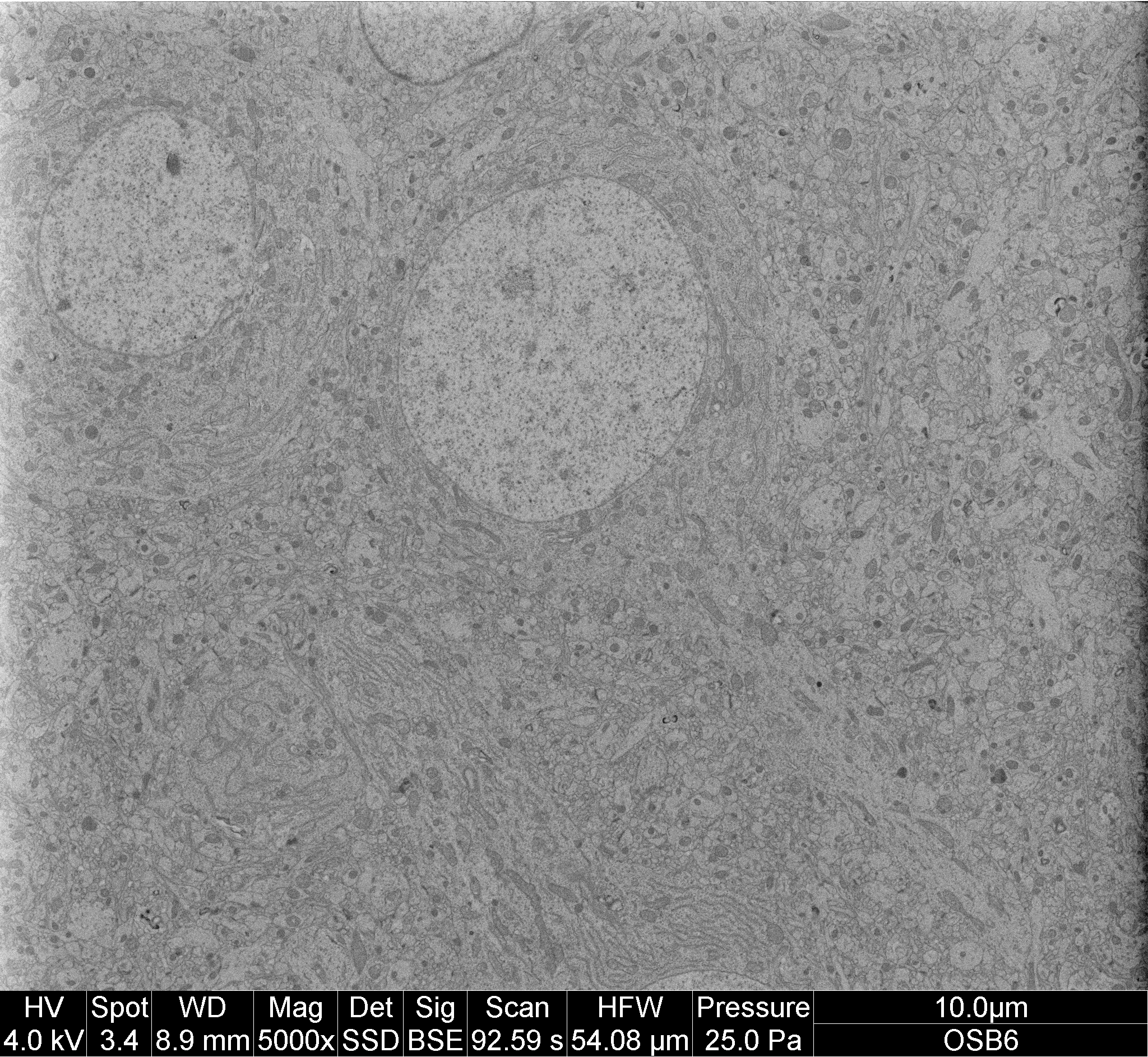

Supplement: Dataset S17 — (252.7 MB ZIP). [file pbio.0020329.sd017.zip › 040604_OS5_st1_1666.tif]

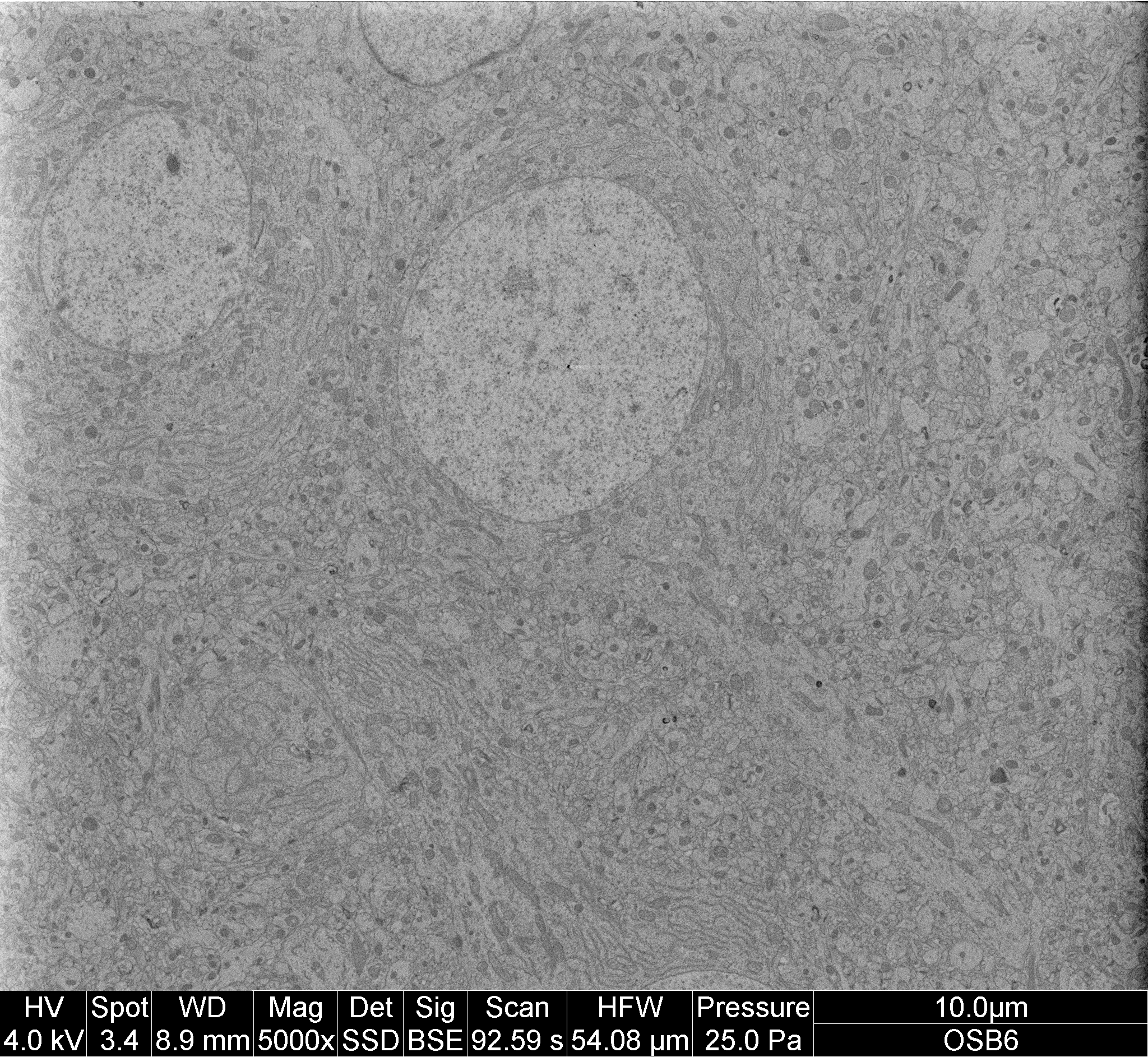

Supplement: Dataset S17 — (252.7 MB ZIP). [file pbio.0020329.sd017.zip › 040604_OS5_st1_1667.tif]

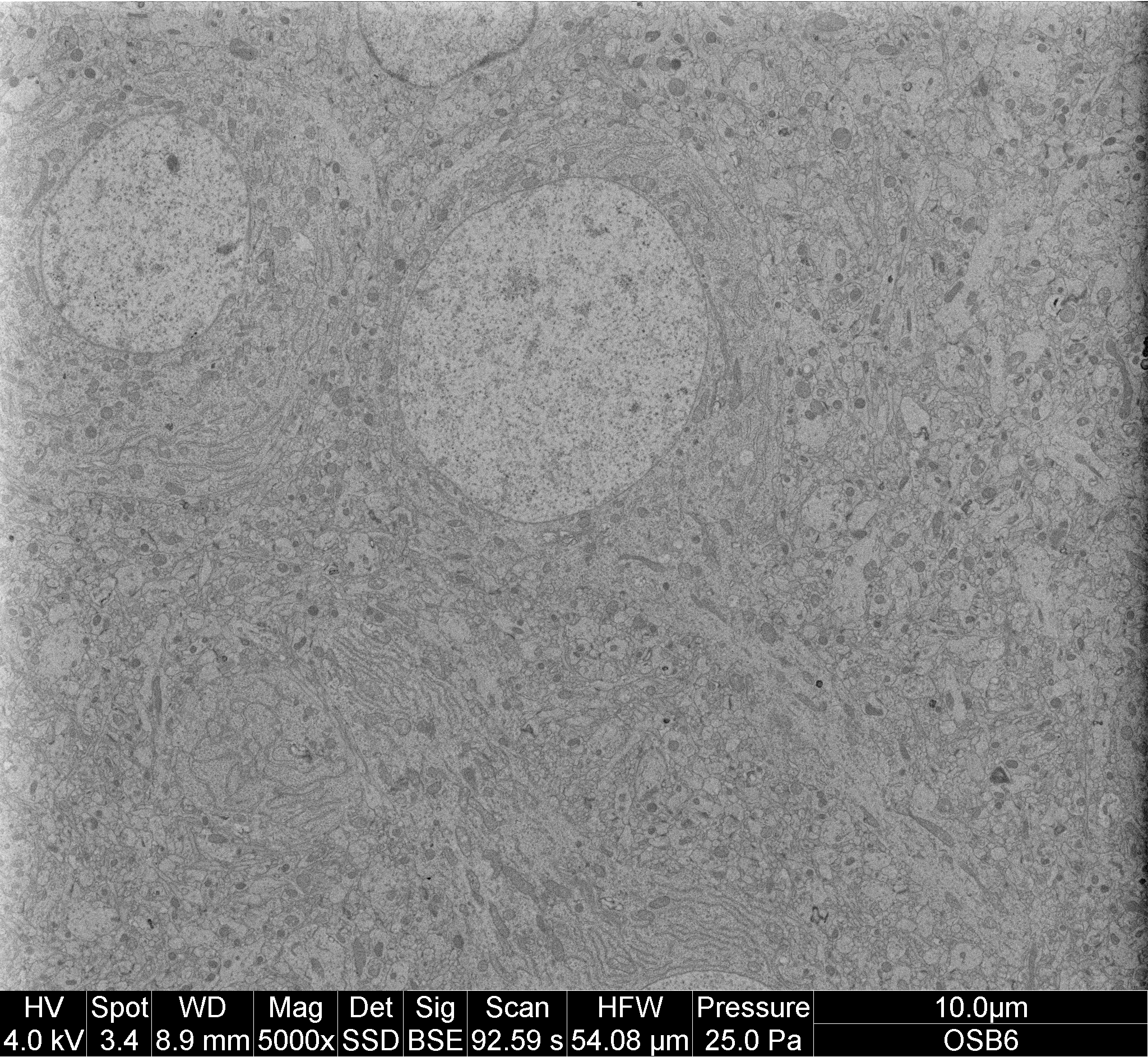

Supplement: Dataset S17 — (252.7 MB ZIP). [file pbio.0020329.sd017.zip › 040604_OS5_st1_1668.tif]

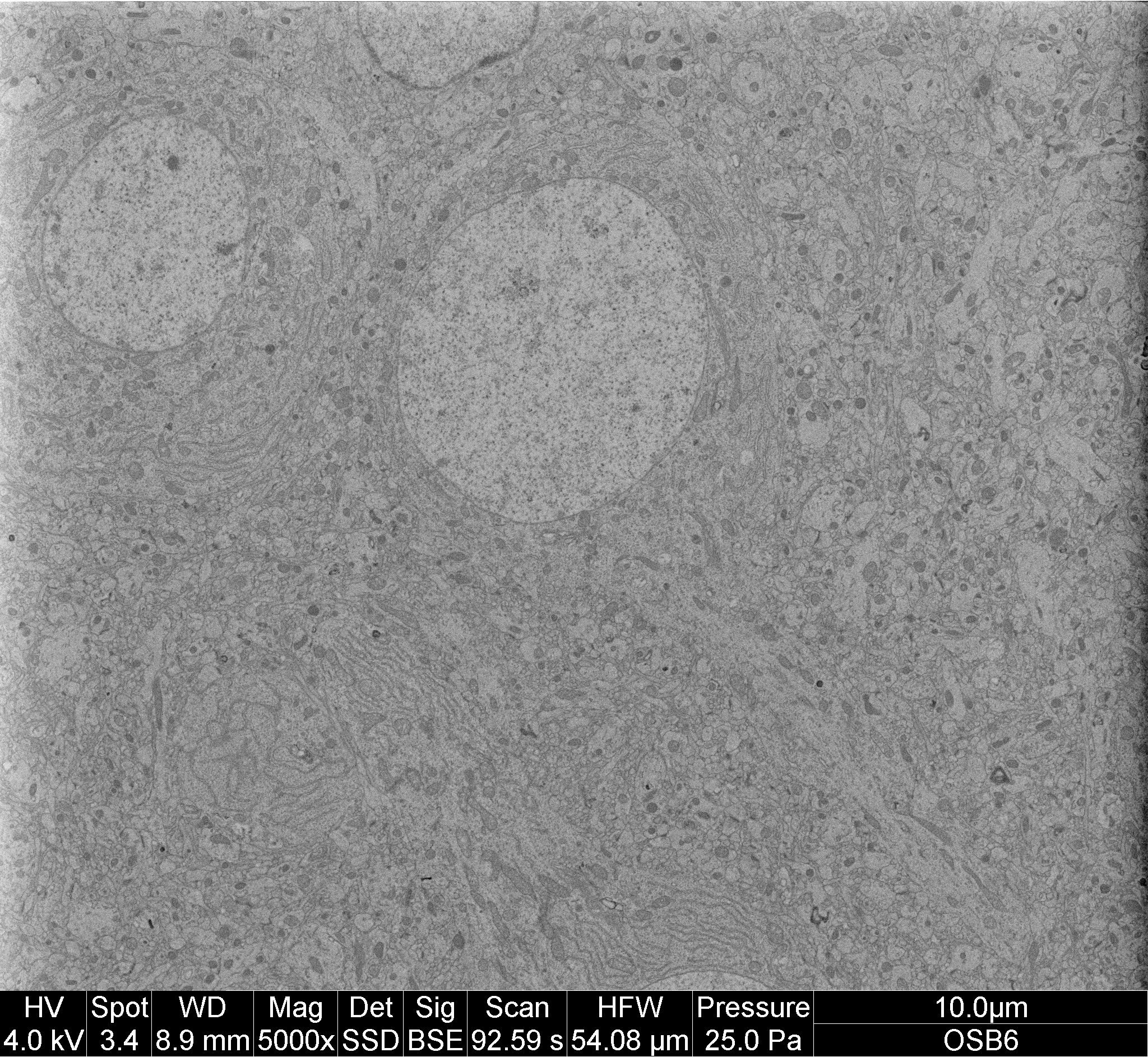

Supplement: Dataset S17 — (252.7 MB ZIP). [file pbio.0020329.sd017.zip › 040604_OS5_st1_1669.tif]

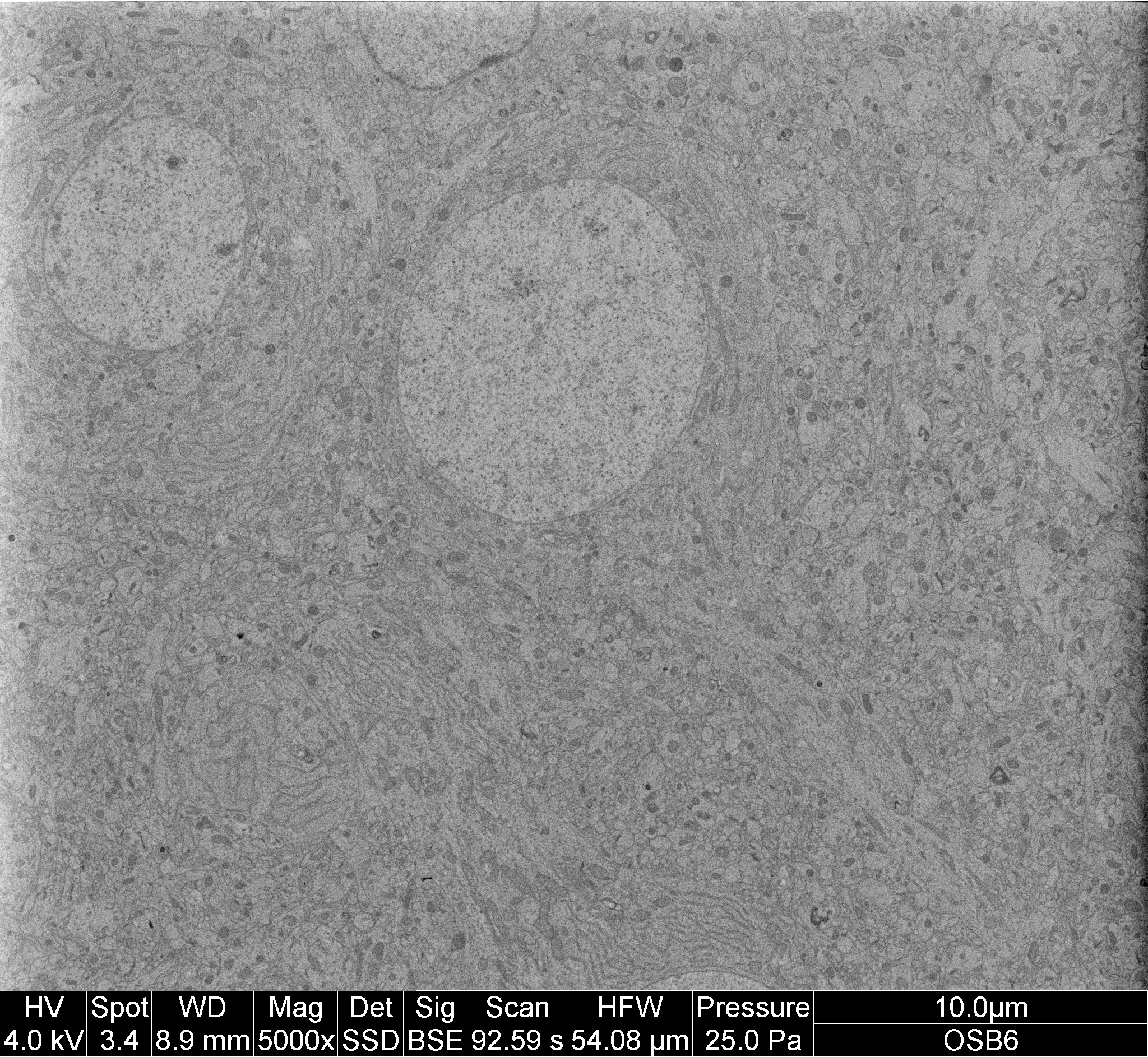

Supplement: Dataset S17 — (252.7 MB ZIP). [file pbio.0020329.sd017.zip › 040604_OS5_st1_1670.tif]

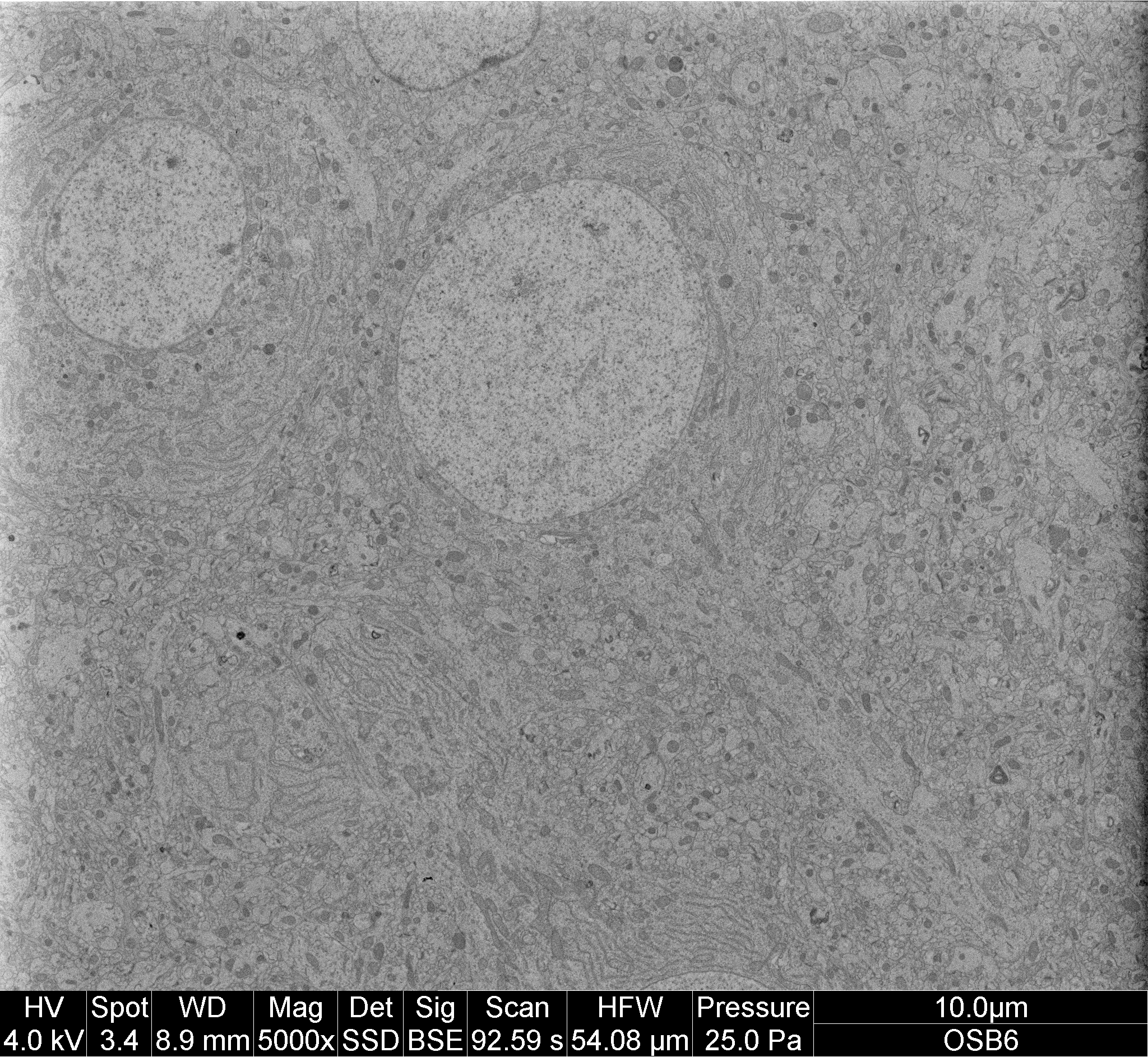

Supplement: Dataset S17 — (252.7 MB ZIP). [file pbio.0020329.sd017.zip › 040604_OS5_st1_1671.tif]

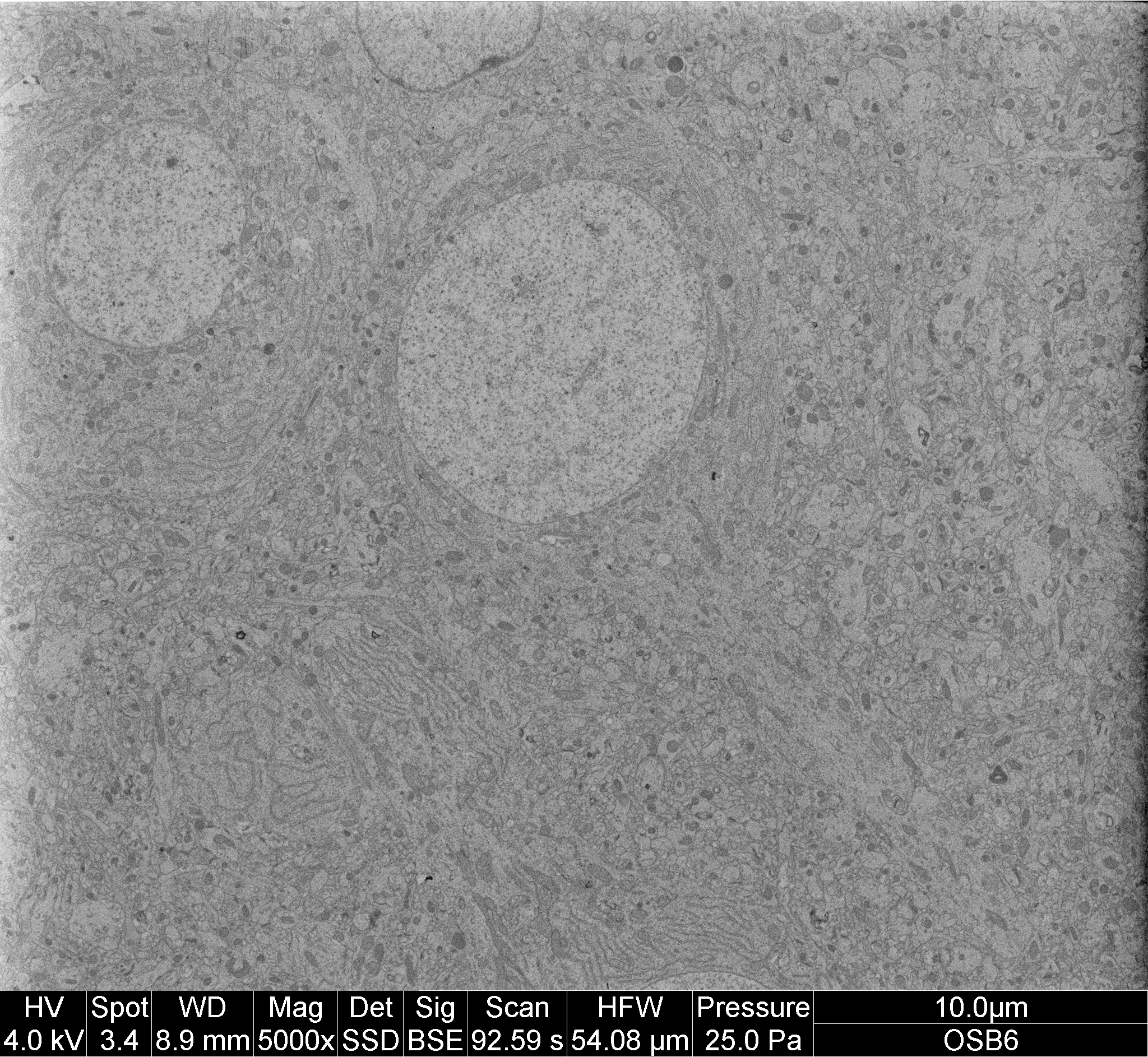

Supplement: Dataset S17 — (252.7 MB ZIP). [file pbio.0020329.sd017.zip › 040604_OS5_st1_1672.tif]

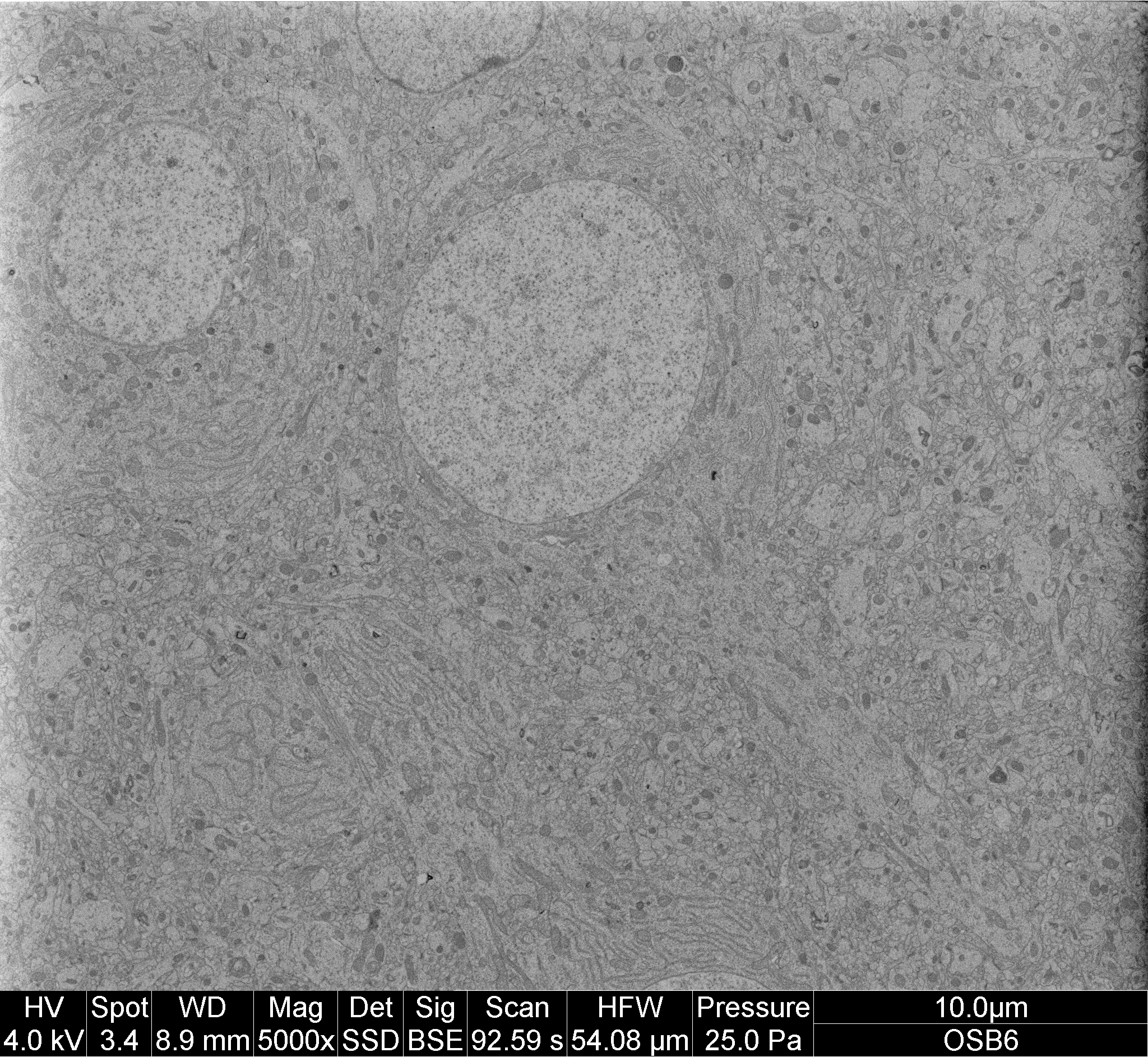

Supplement: Dataset S17 — (252.7 MB ZIP). [file pbio.0020329.sd017.zip › 040604_OS5_st1_1673.tif]

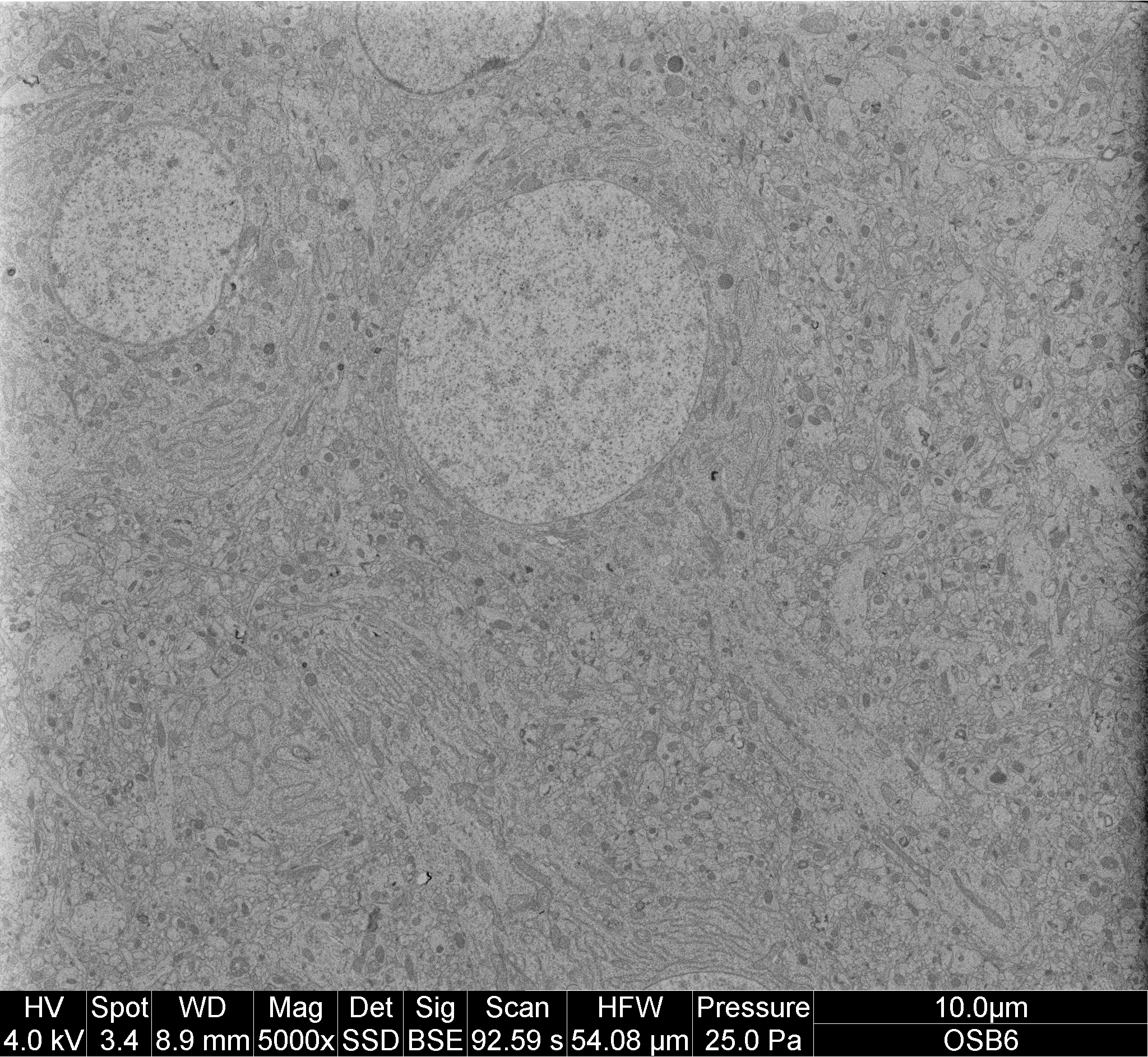

Supplement: Dataset S17 — (252.7 MB ZIP). [file pbio.0020329.sd017.zip › 040604_OS5_st1_1674.tif]

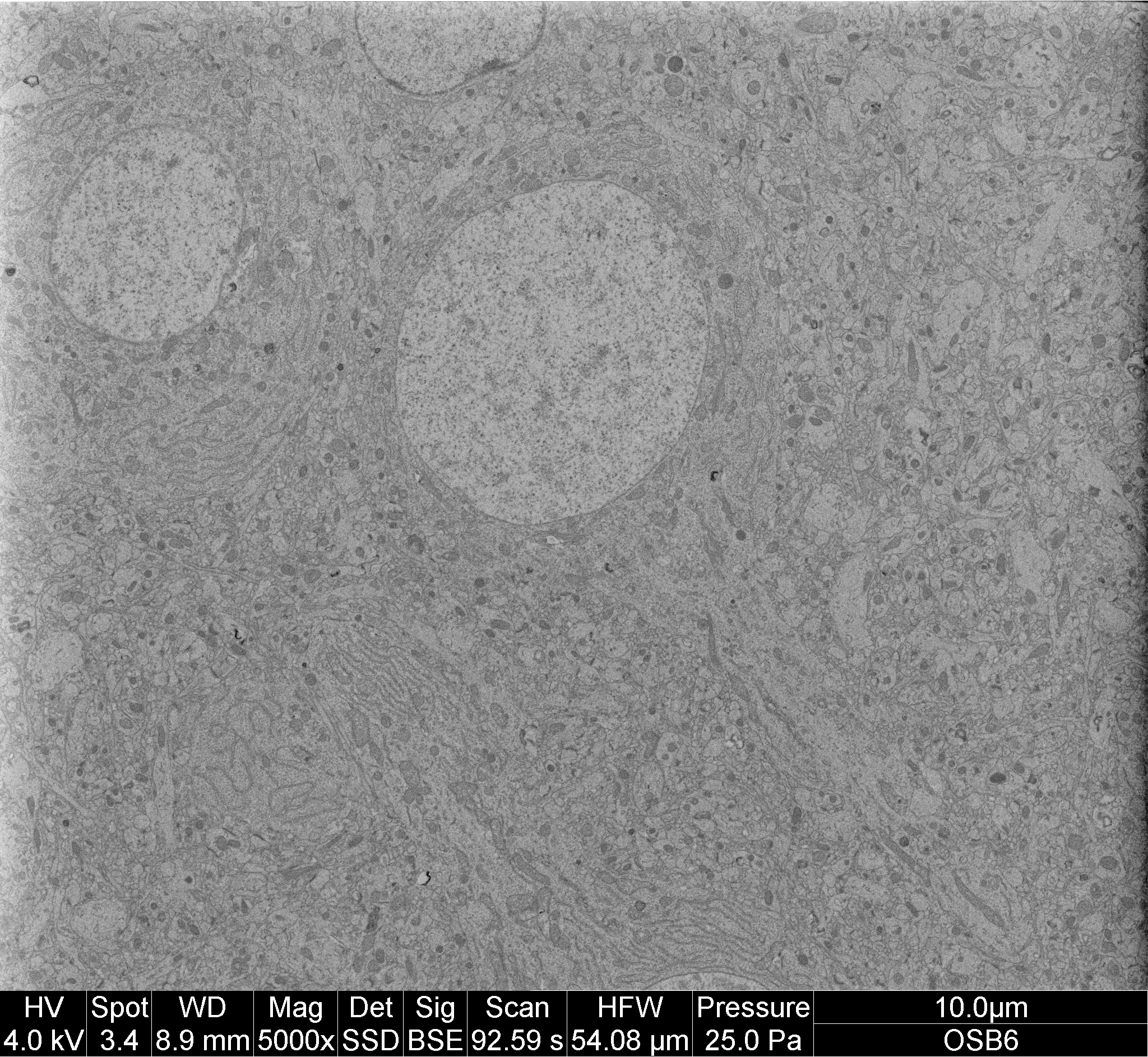

Supplement: Dataset S17 — (252.7 MB ZIP). [file pbio.0020329.sd017.zip › 040604_OS5_st1_1675.tif]

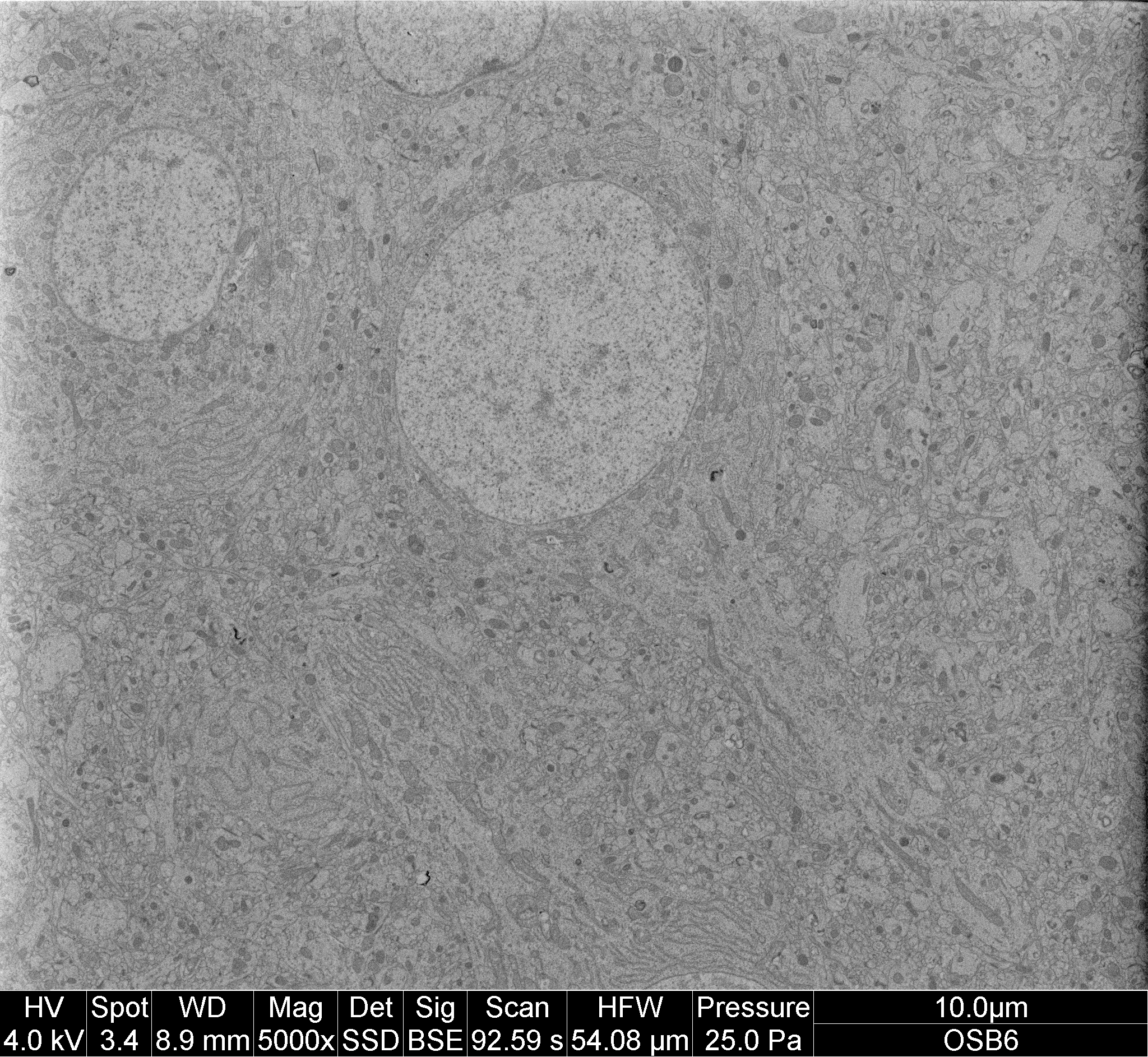

Supplement: Dataset S17 — (252.7 MB ZIP). [file pbio.0020329.sd017.zip › 040604_OS5_st1_1676.tif]

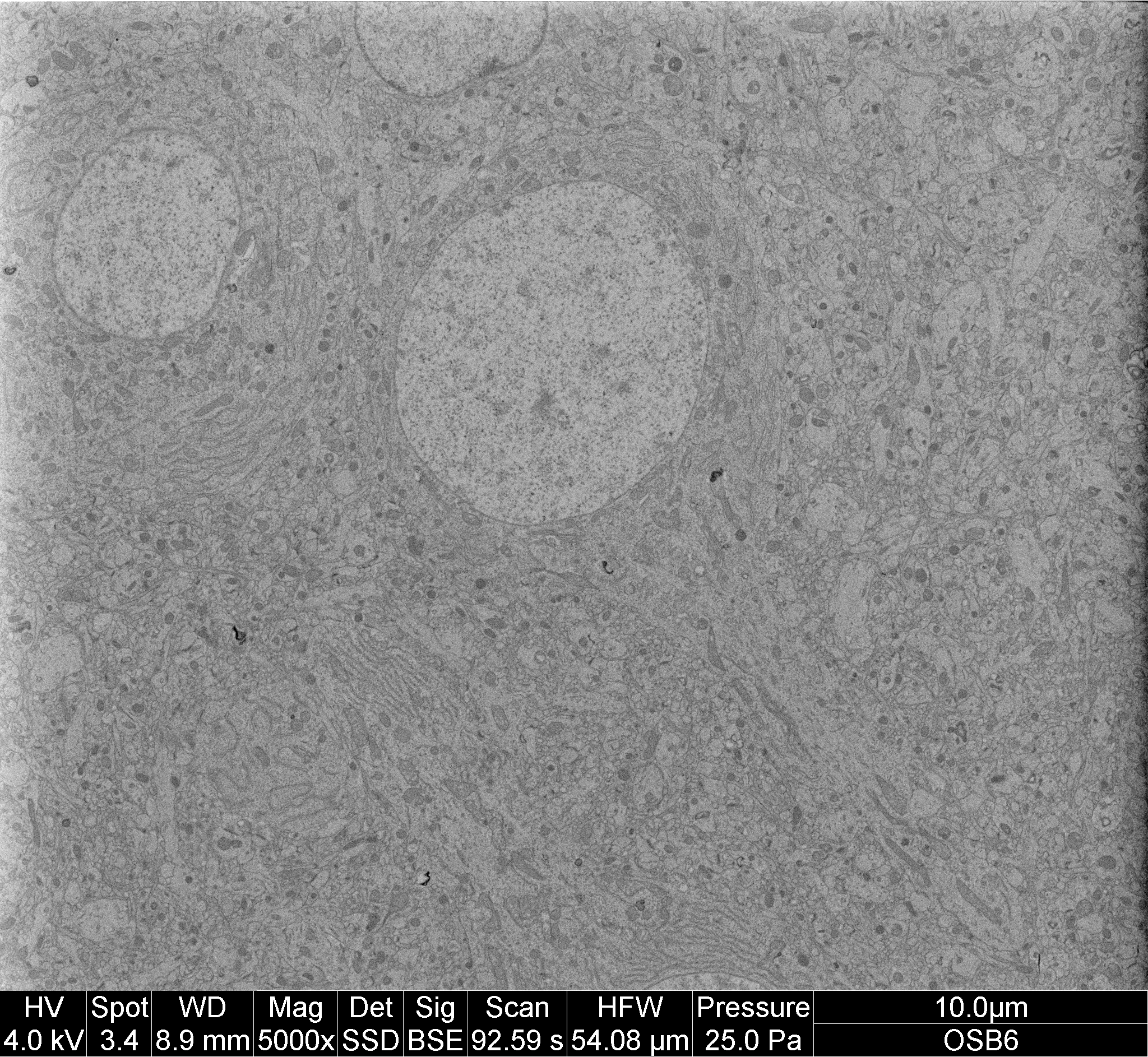

Supplement: Dataset S17 — (252.7 MB ZIP). [file pbio.0020329.sd017.zip › 040604_OS5_st1_1677.tif]

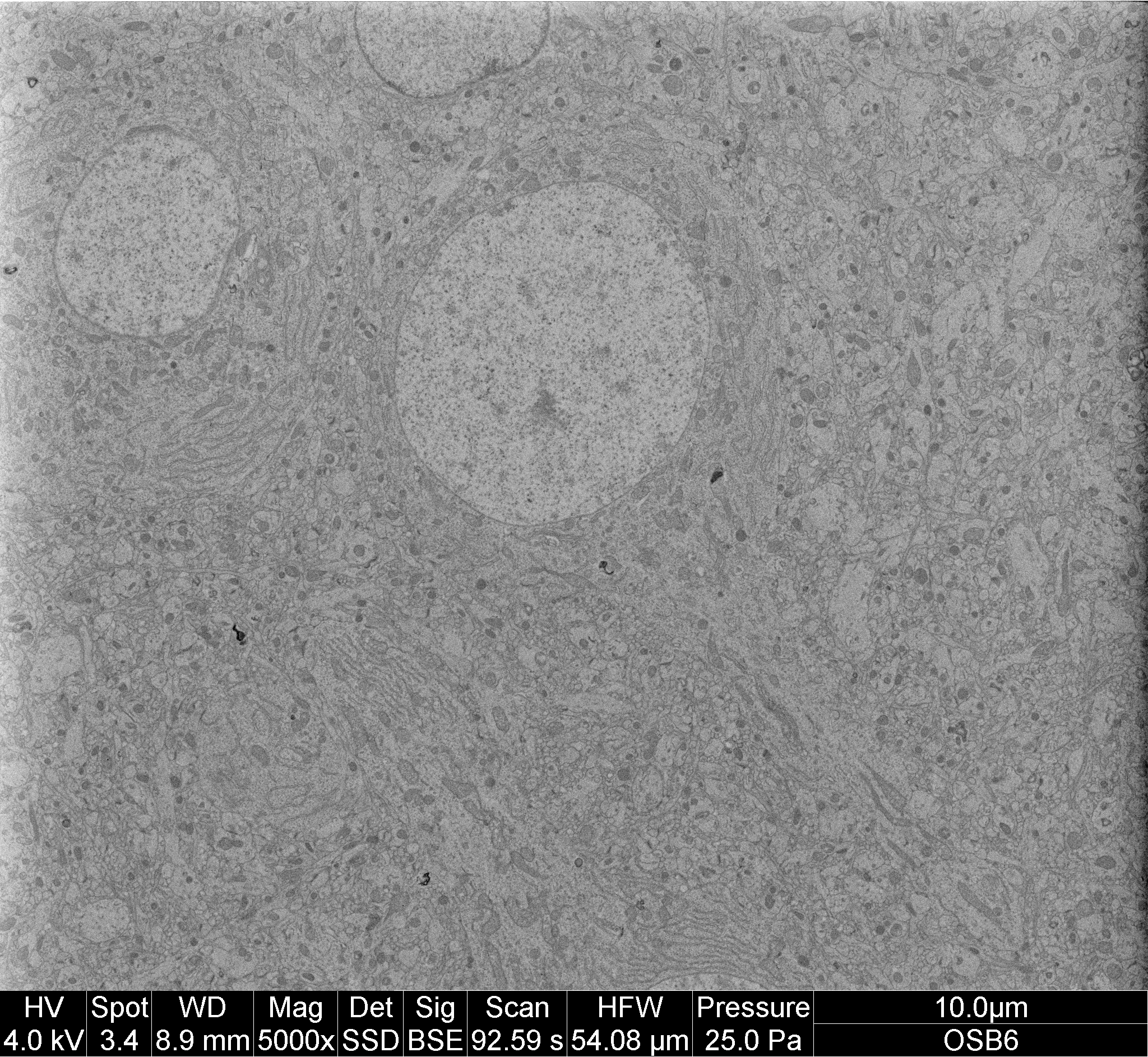

Supplement: Dataset S17 — (252.7 MB ZIP). [file pbio.0020329.sd017.zip › 040604_OS5_st1_1678.tif]

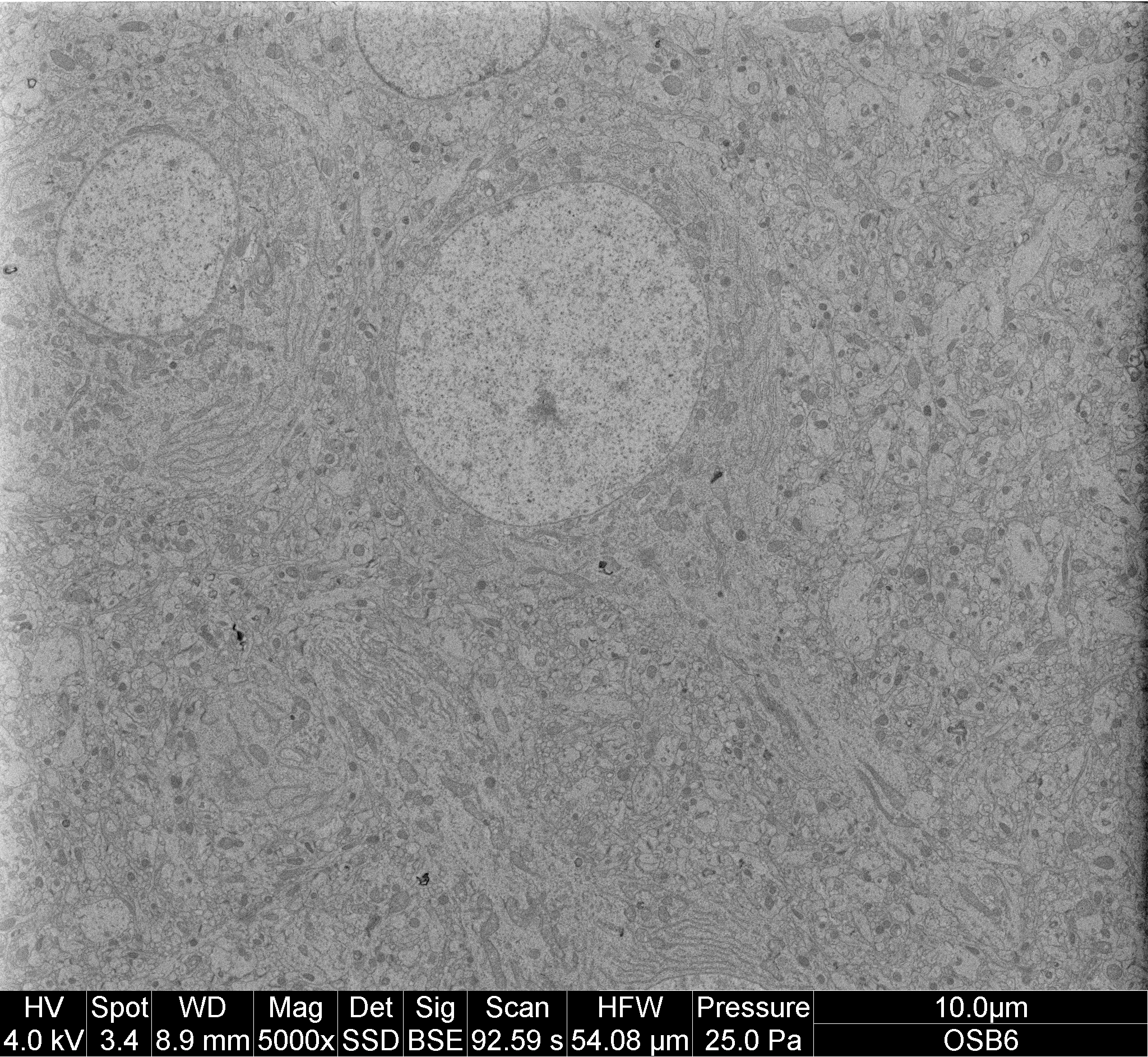

Supplement: Dataset S17 — (252.7 MB ZIP). [file pbio.0020329.sd017.zip › 040604_OS5_st1_1679.tif]

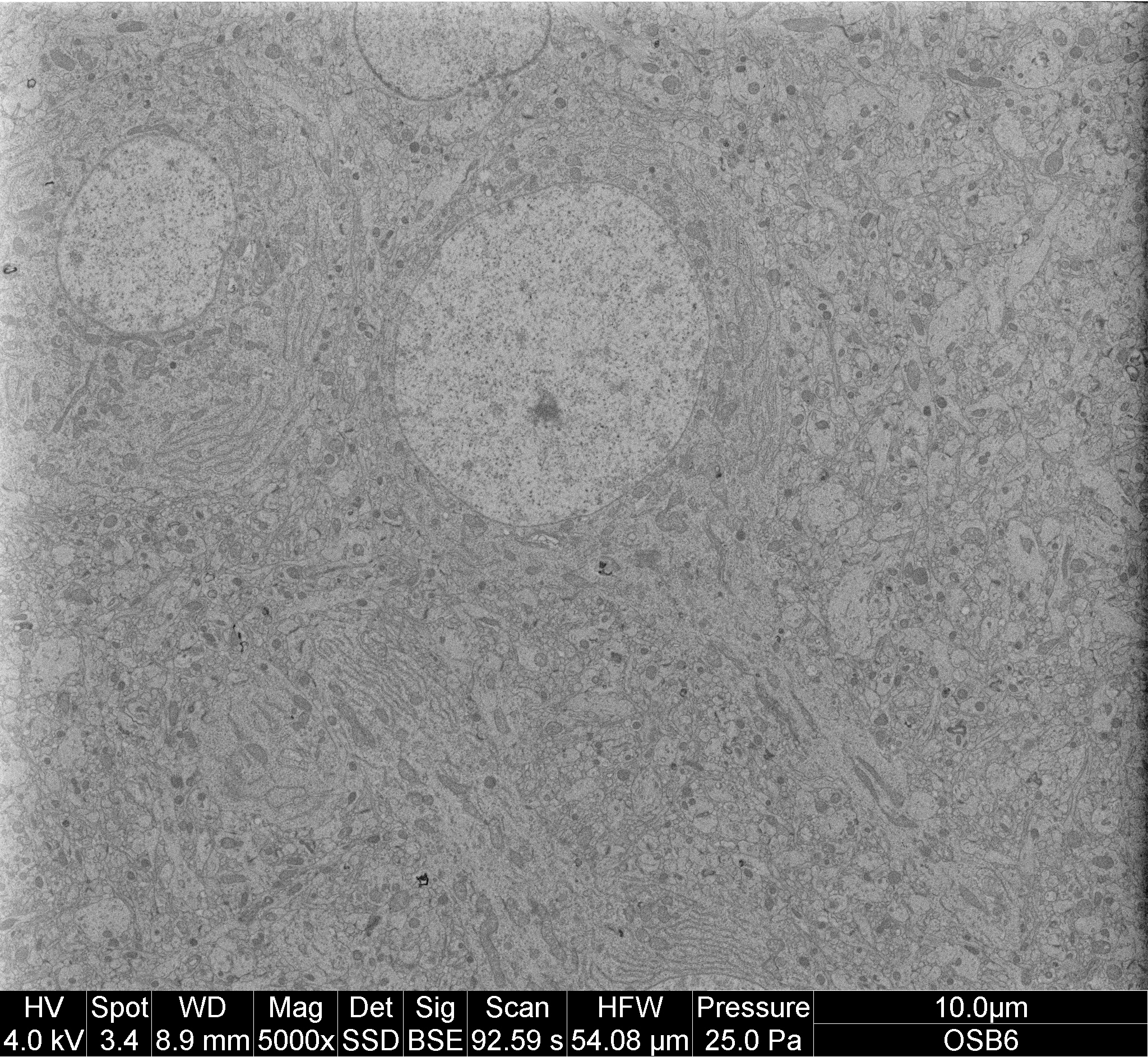

Supplement: Dataset S17 — (252.7 MB ZIP). [file pbio.0020329.sd017.zip › 040604_OS5_st1_1680.tif]

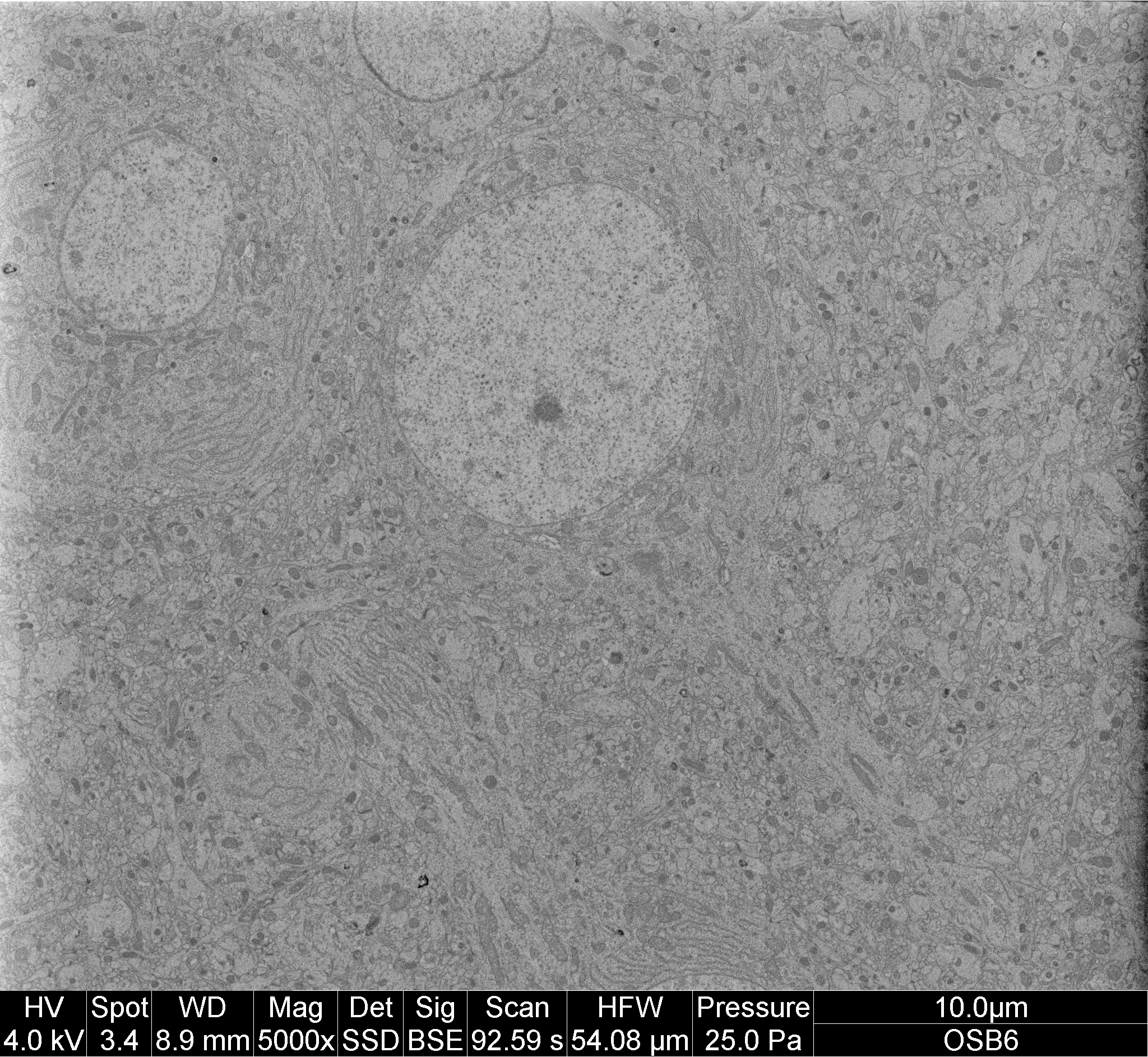

Supplement: Dataset S17 — (252.7 MB ZIP). [file pbio.0020329.sd017.zip › 040604_OS5_st1_1681.tif]

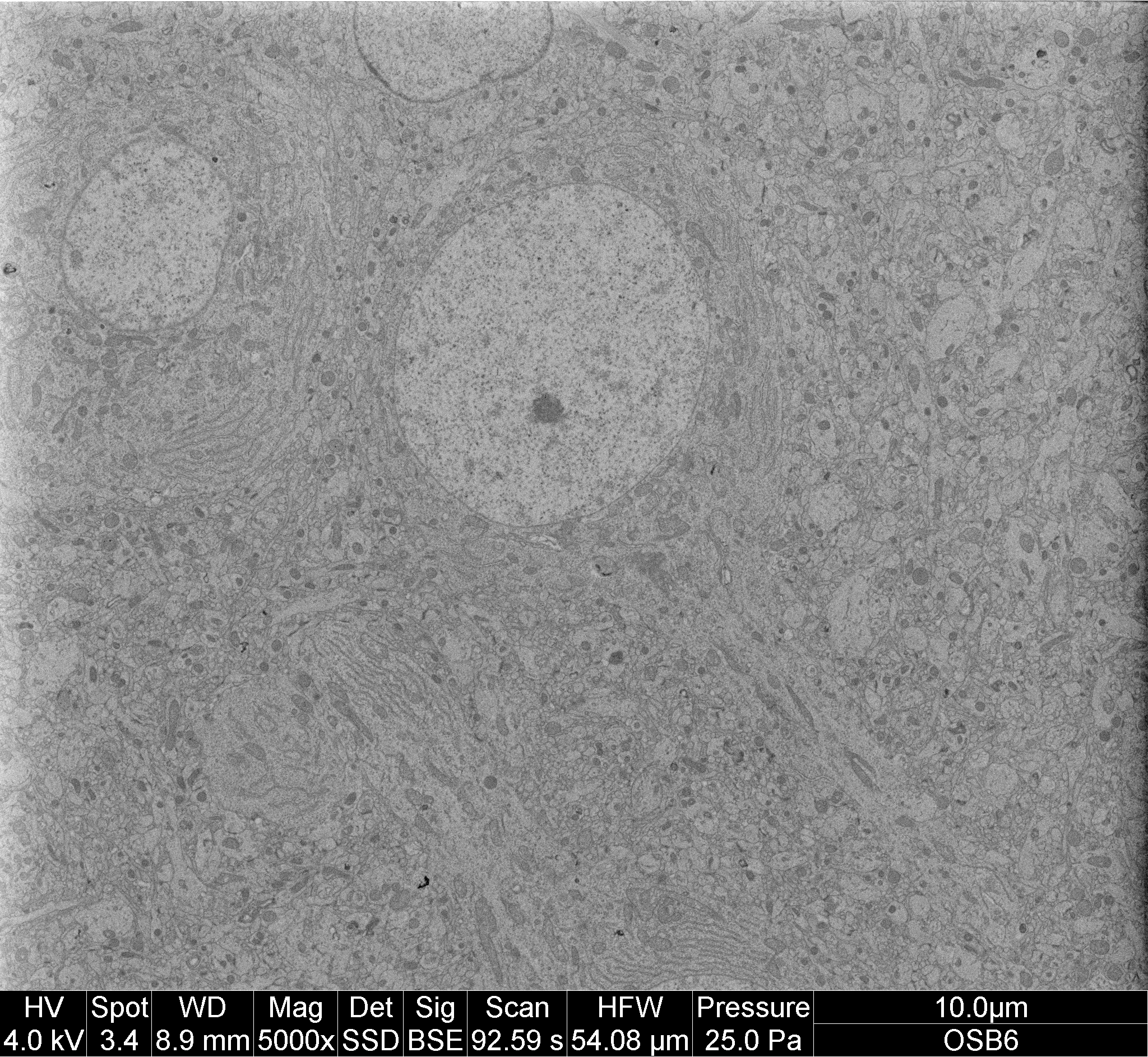

Supplement: Dataset S17 — (252.7 MB ZIP). [file pbio.0020329.sd017.zip › 040604_OS5_st1_1682.tif]

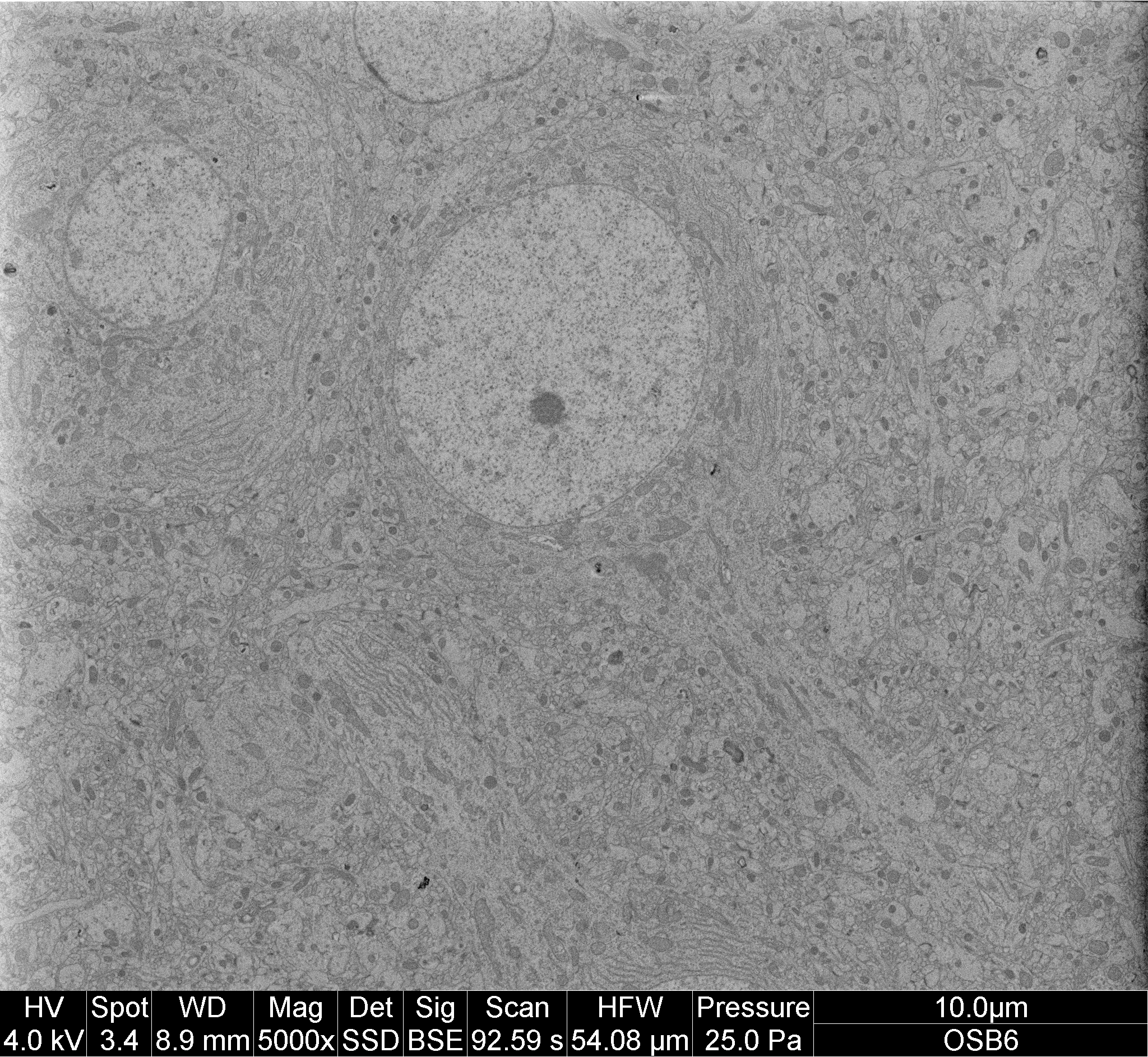

Supplement: Dataset S17 — (252.7 MB ZIP). [file pbio.0020329.sd017.zip › 040604_OS5_st1_1683.tif]

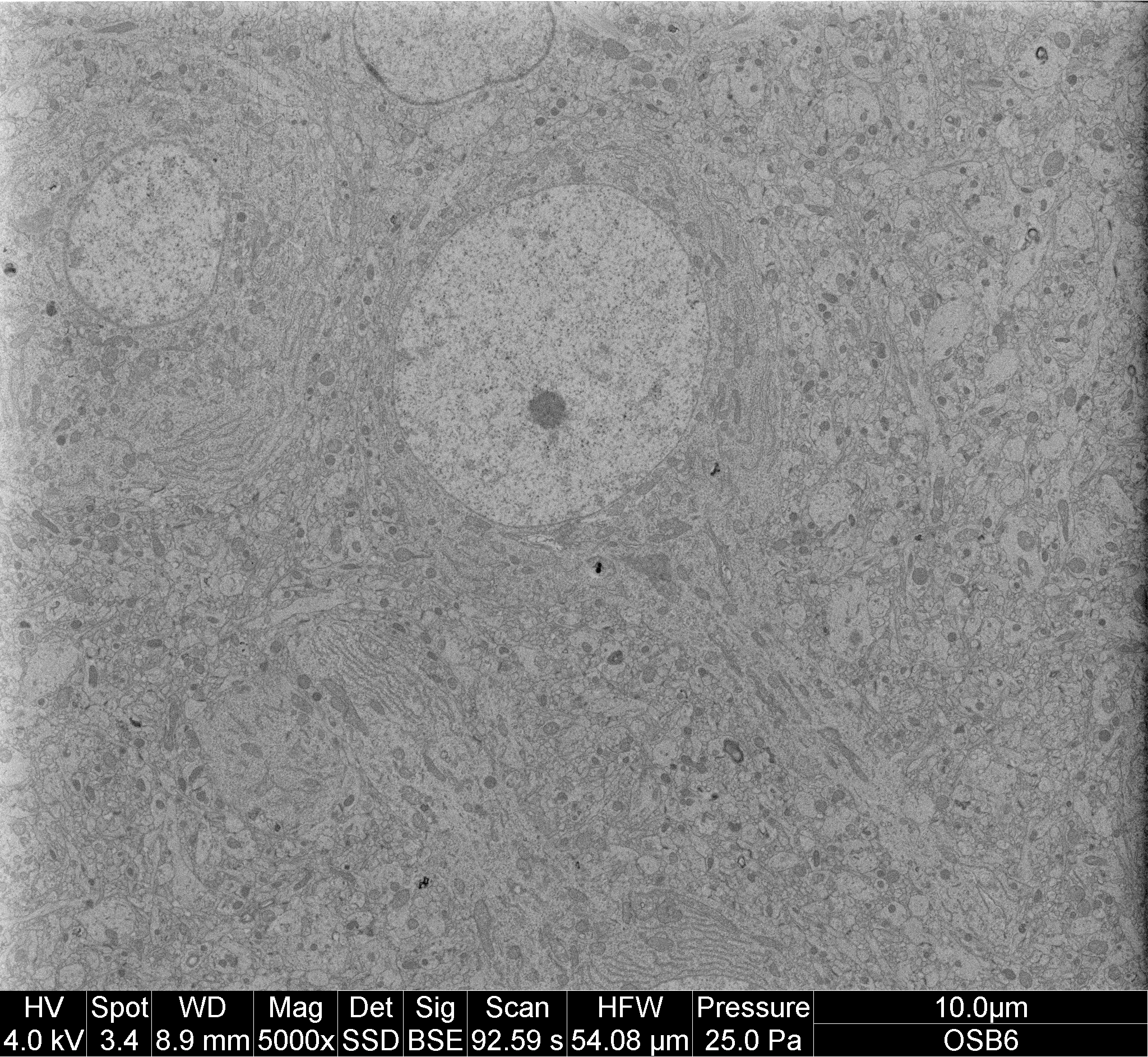

Supplement: Dataset S17 — (252.7 MB ZIP). [file pbio.0020329.sd017.zip › 040604_OS5_st1_1684.tif]

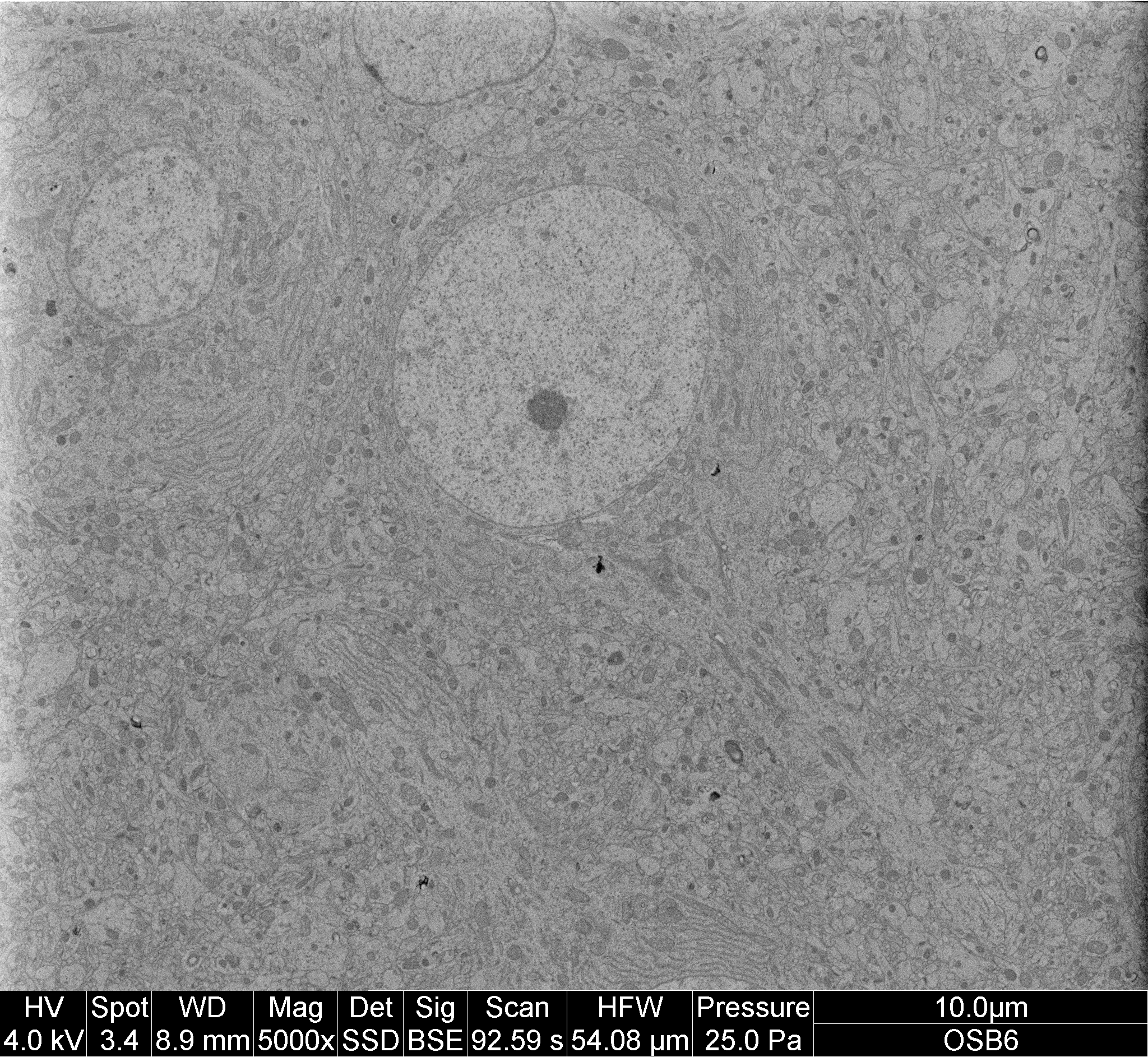

Supplement: Dataset S17 — (252.7 MB ZIP). [file pbio.0020329.sd017.zip › 040604_OS5_st1_1685.tif]

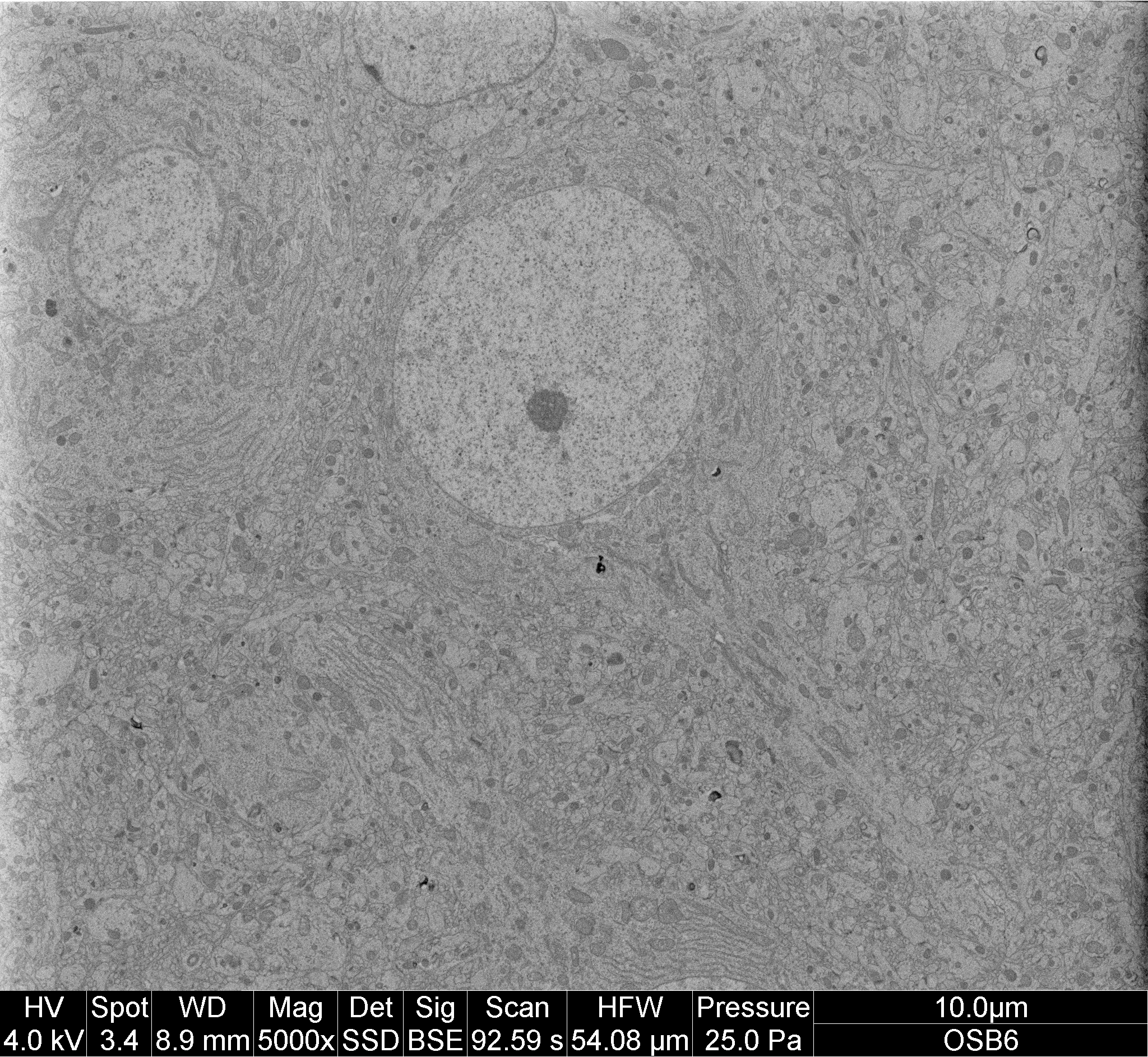

Supplement: Dataset S17 — (252.7 MB ZIP). [file pbio.0020329.sd017.zip › 040604_OS5_st1_1686.tif]

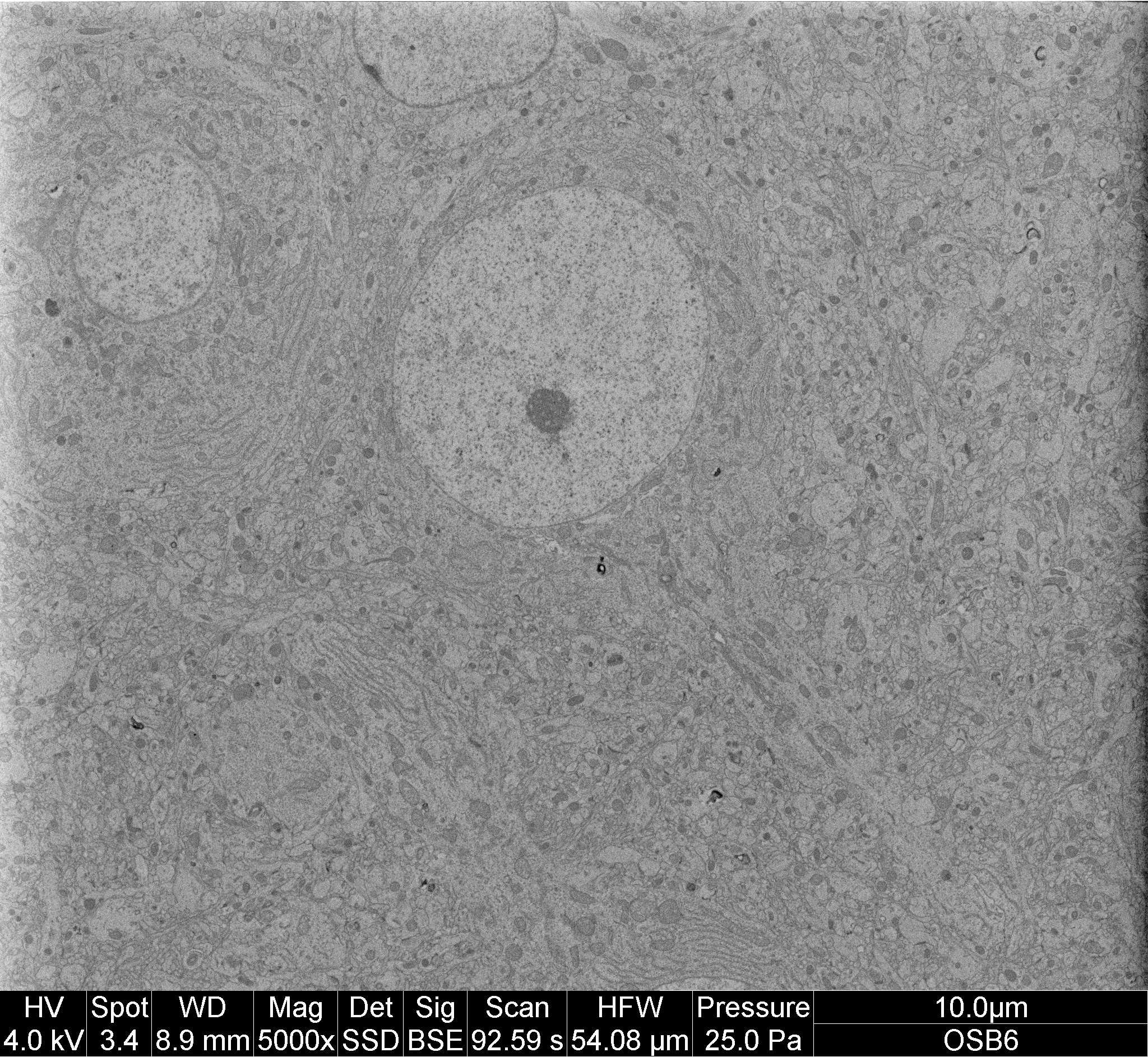

Supplement: Dataset S17 — (252.7 MB ZIP). [file pbio.0020329.sd017.zip › 040604_OS5_st1_1687.tif]

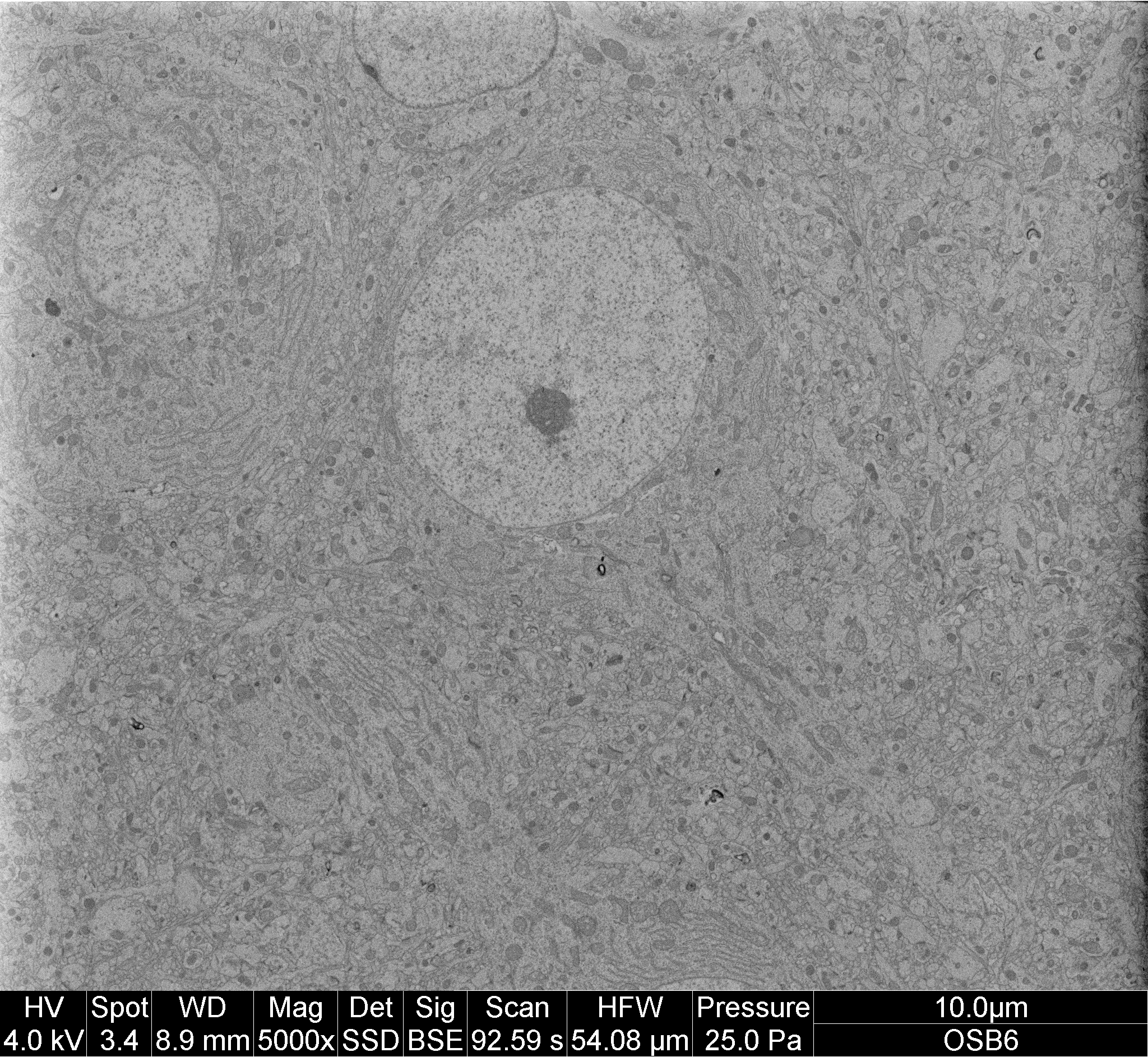

Supplement: Dataset S17 — (252.7 MB ZIP). [file pbio.0020329.sd017.zip › 040604_OS5_st1_1688.tif]

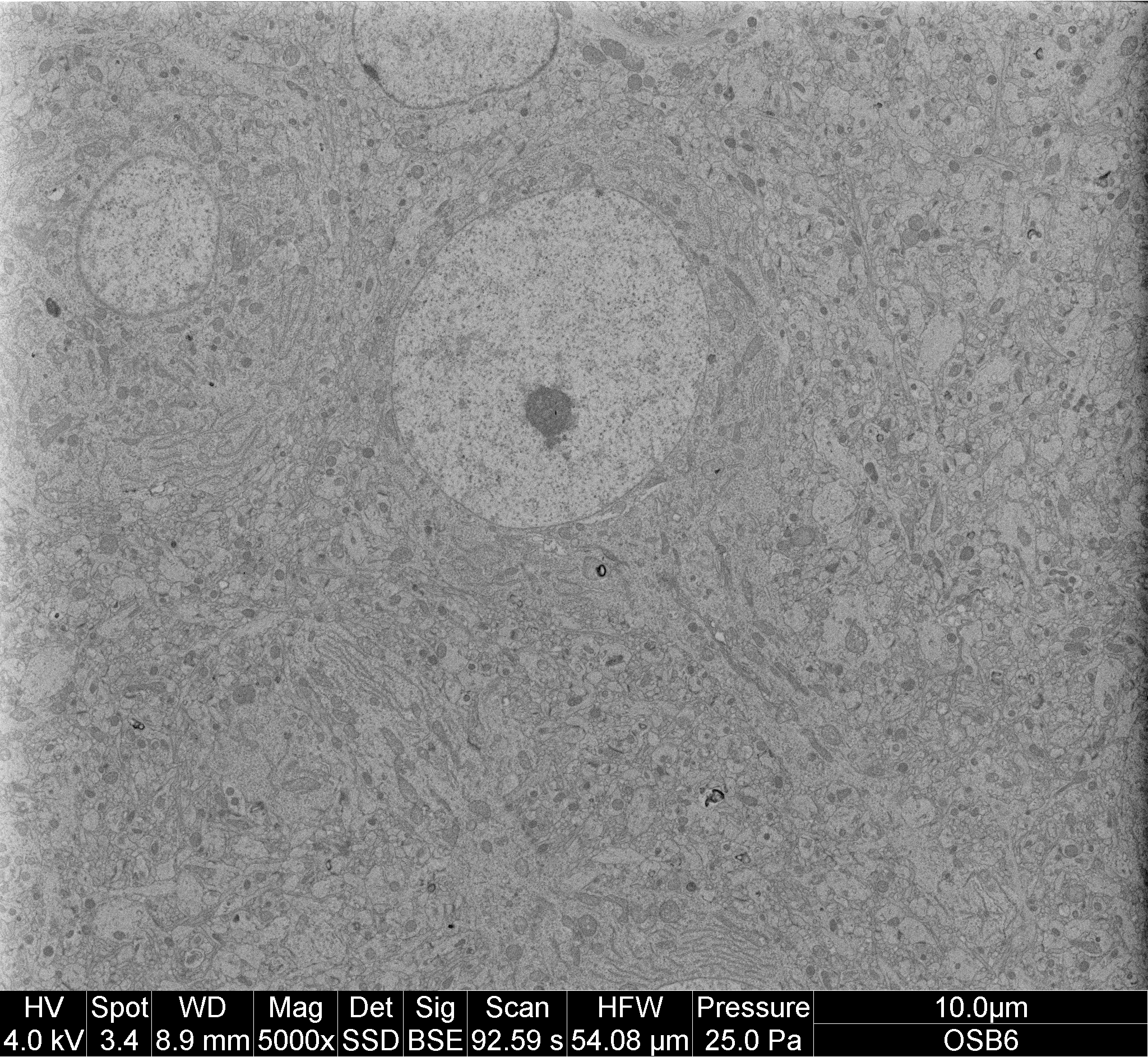

Supplement: Dataset S17 — (252.7 MB ZIP). [file pbio.0020329.sd017.zip › 040604_OS5_st1_1689.tif]

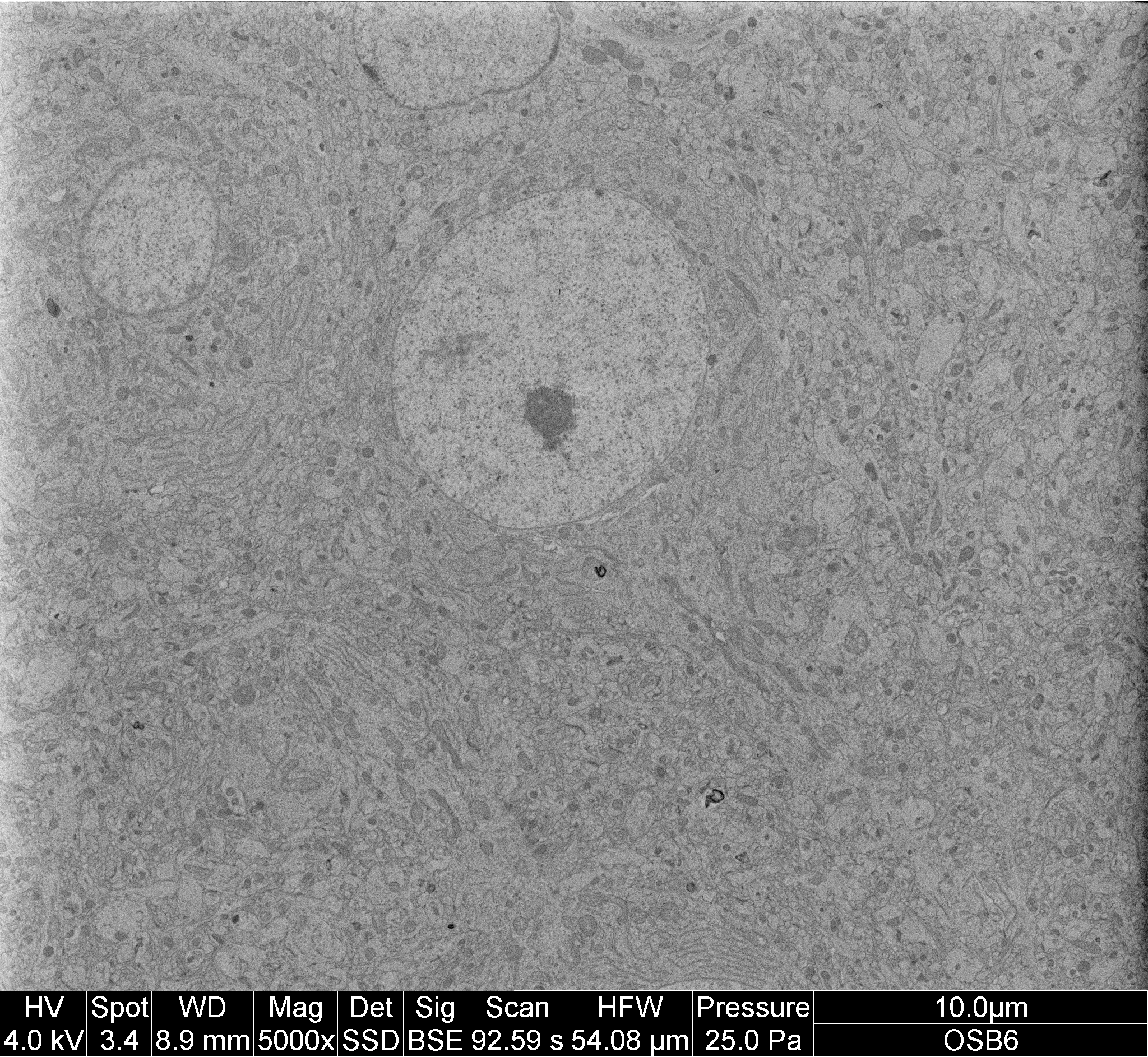

Supplement: Dataset S17 — (252.7 MB ZIP). [file pbio.0020329.sd017.zip › 040604_OS5_st1_1690.tif]

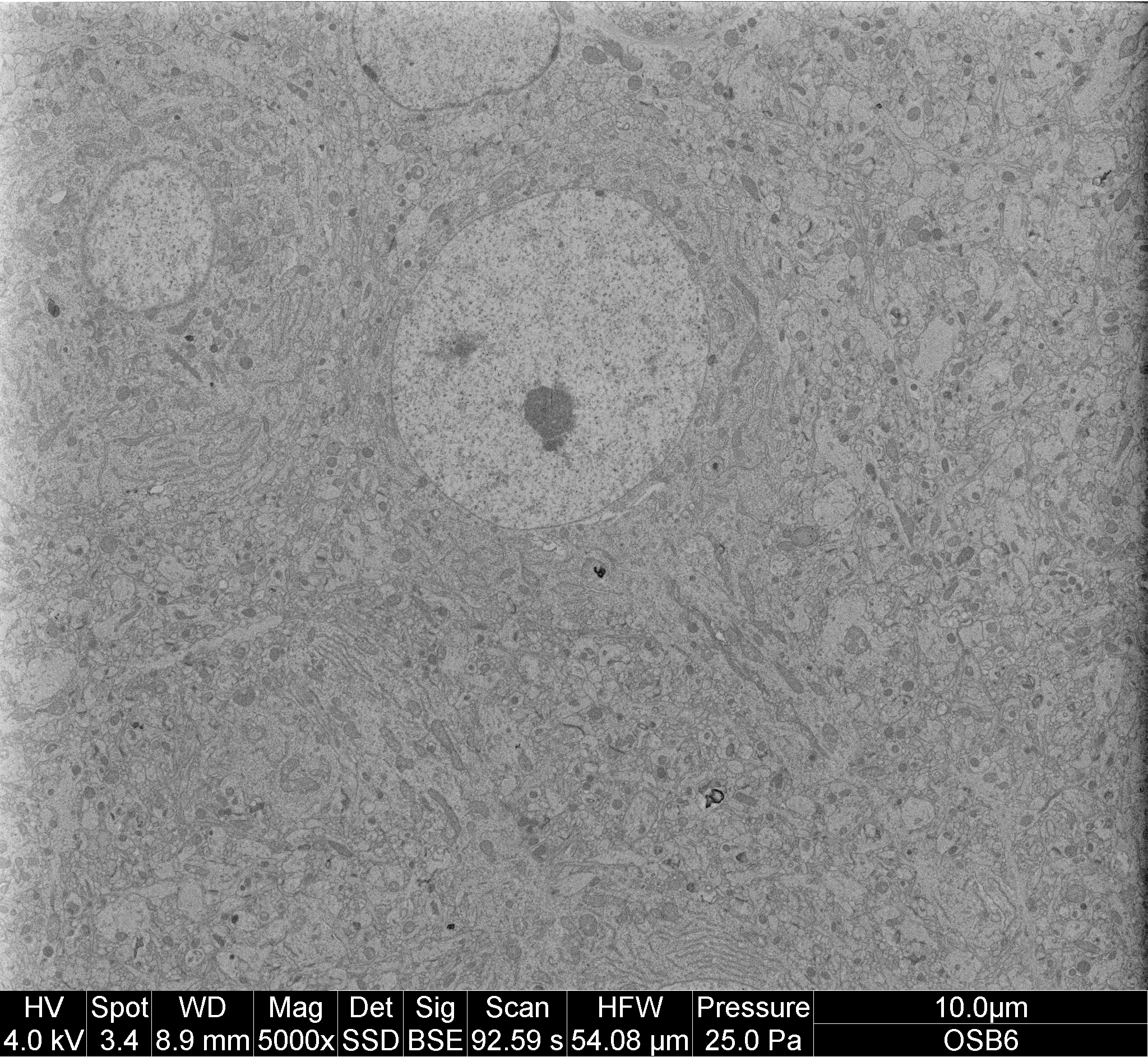

Supplement: Dataset S17 — (252.7 MB ZIP). [file pbio.0020329.sd017.zip › 040604_OS5_st1_1691.tif]

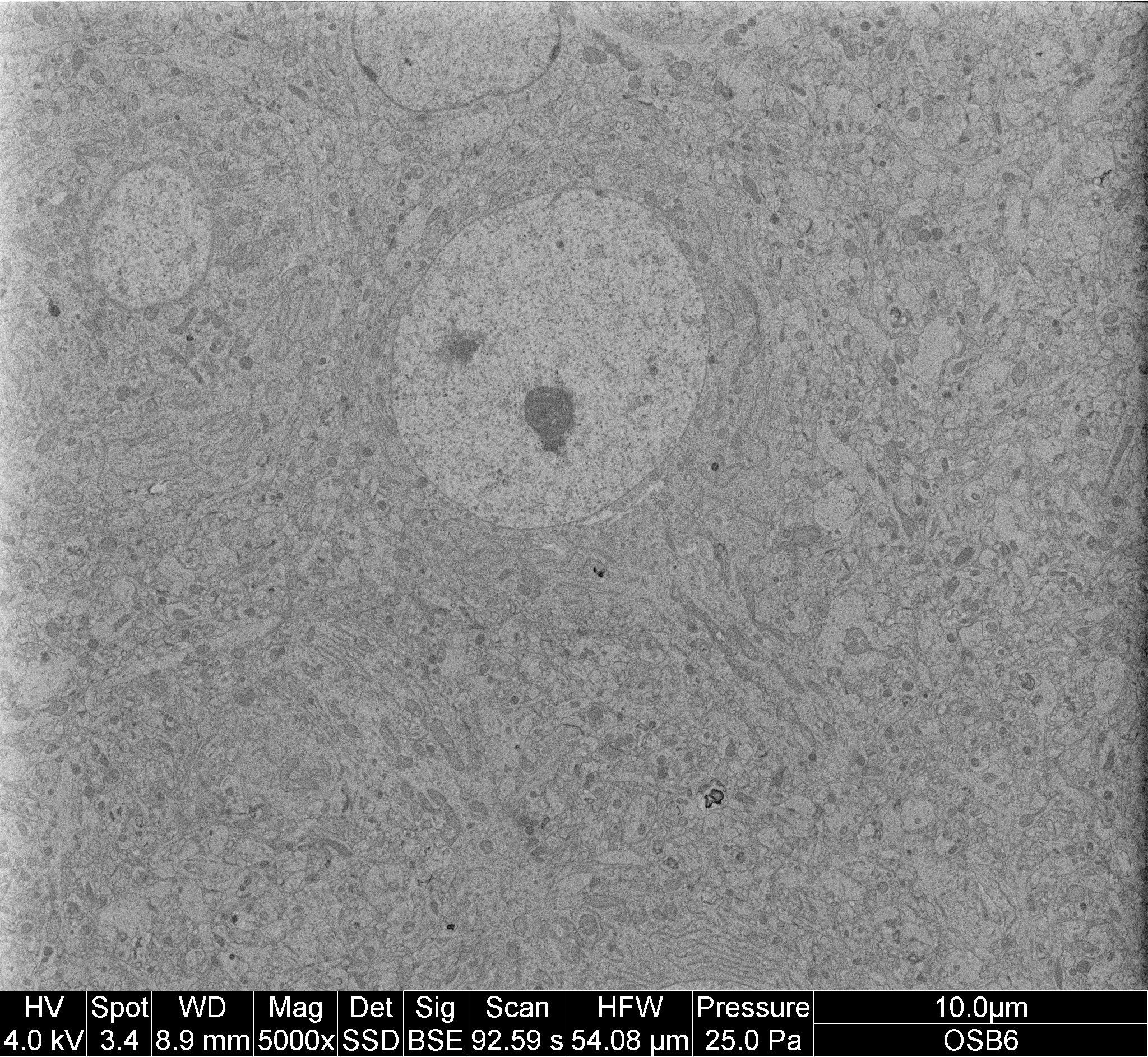

Supplement: Dataset S17 — (252.7 MB ZIP). [file pbio.0020329.sd017.zip › 040604_OS5_st1_1692.tif]

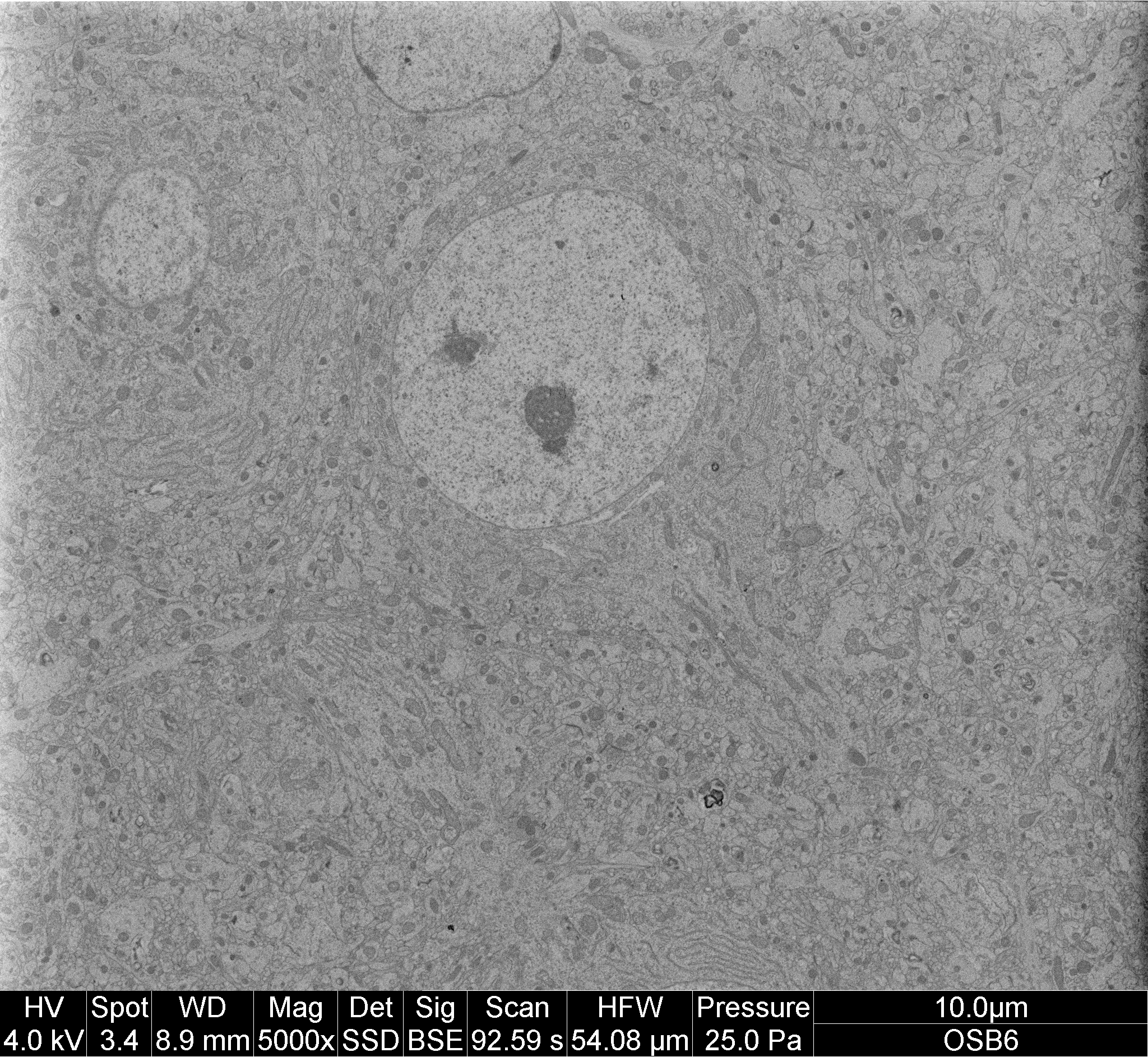

Supplement: Dataset S17 — (252.7 MB ZIP). [file pbio.0020329.sd017.zip › 040604_OS5_st1_1693.tif]

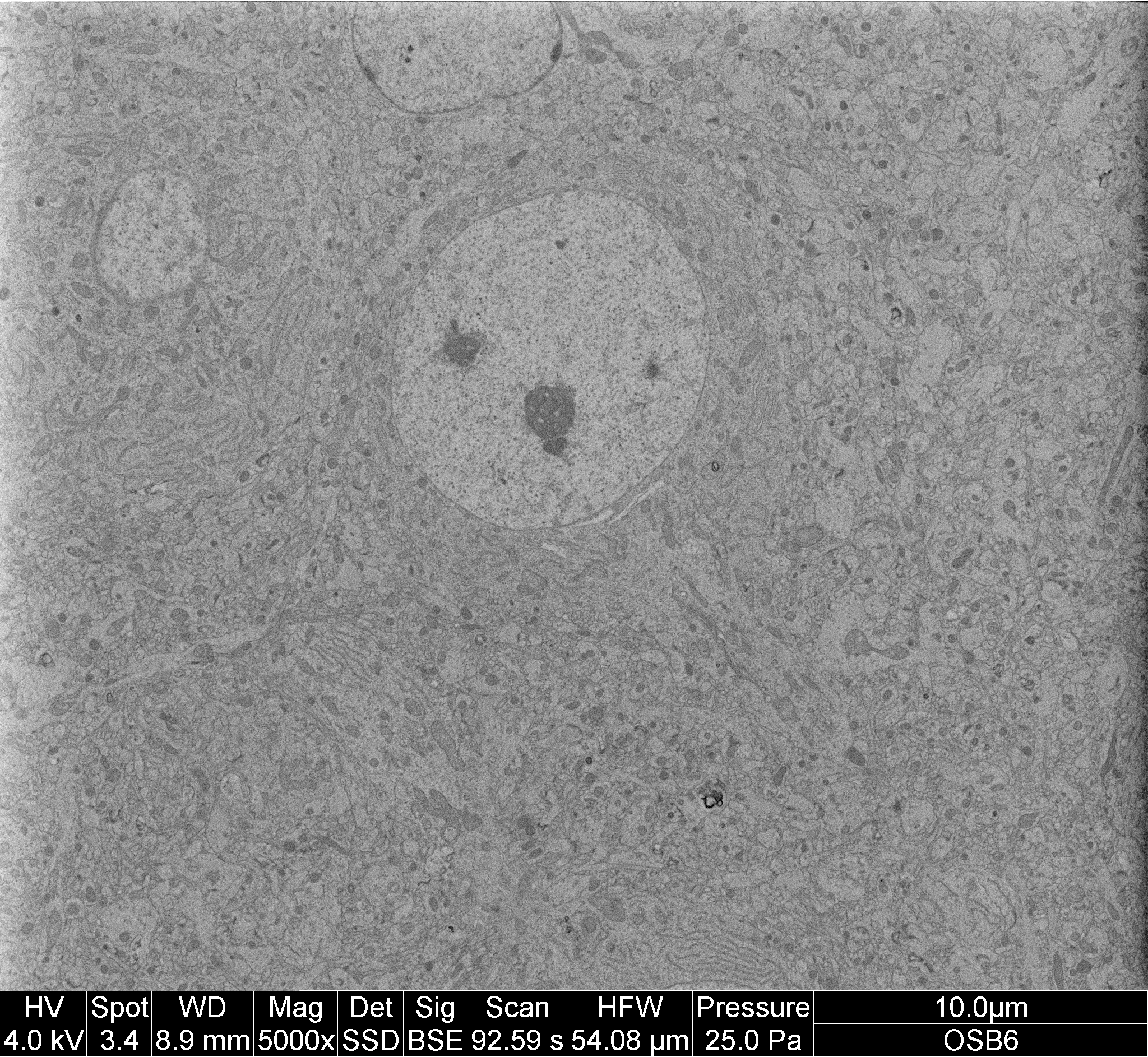

Supplement: Dataset S17 — (252.7 MB ZIP). [file pbio.0020329.sd017.zip › 040604_OS5_st1_1694.tif]

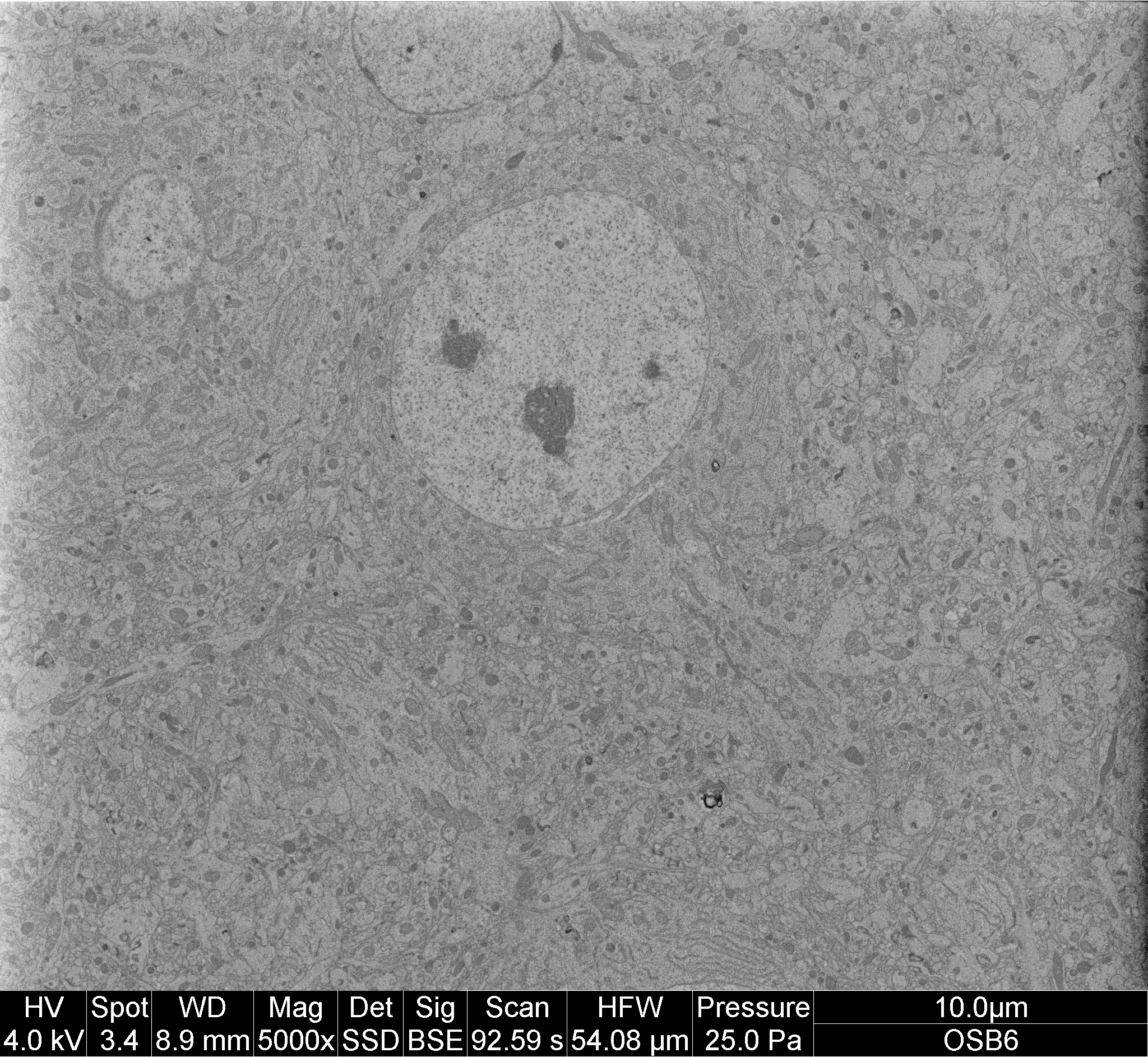

Supplement: Dataset S17 — (252.7 MB ZIP). [file pbio.0020329.sd017.zip › 040604_OS5_st1_1695.tif]

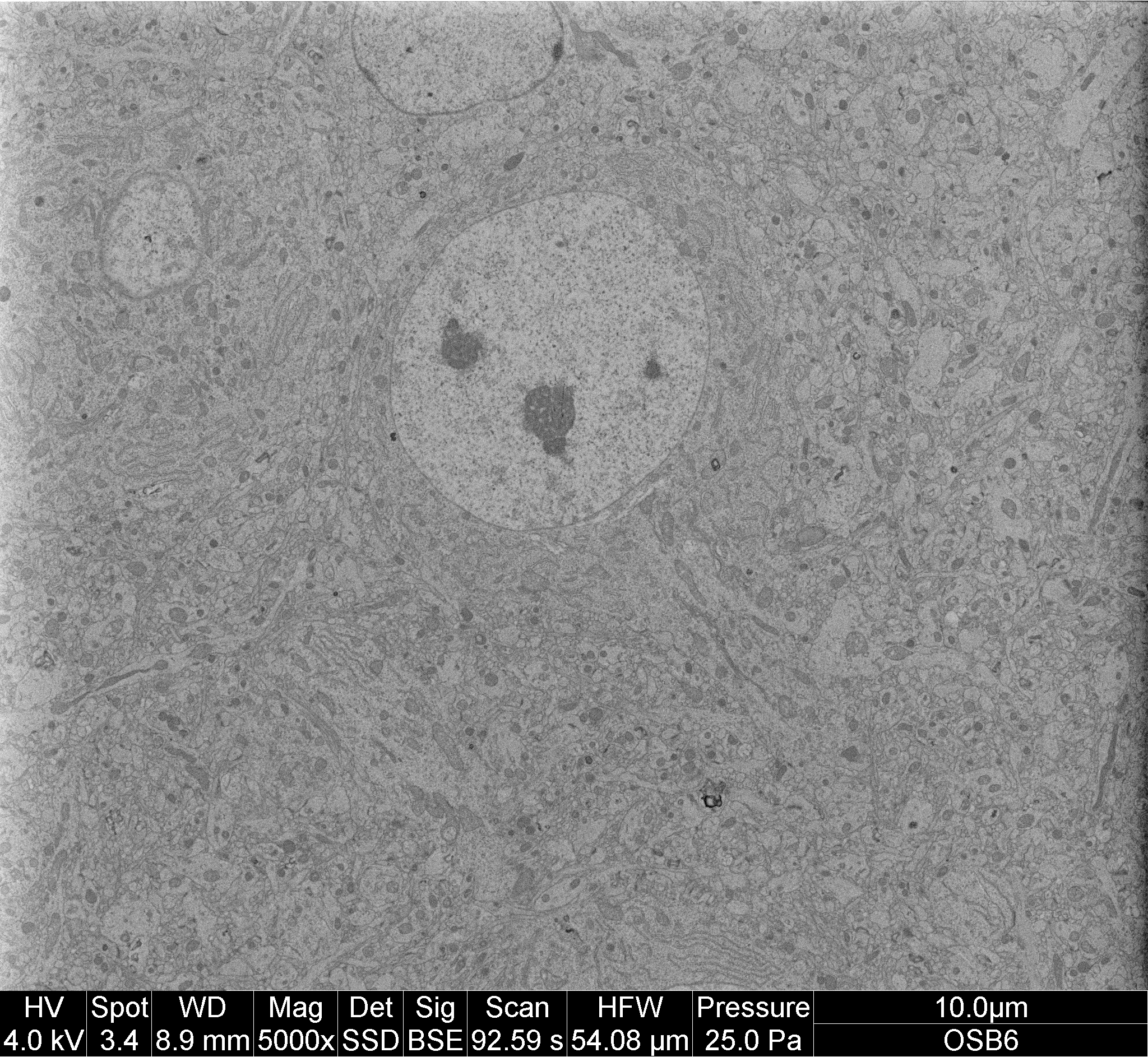

Supplement: Dataset S17 — (252.7 MB ZIP). [file pbio.0020329.sd017.zip › 040604_OS5_st1_1696.tif]

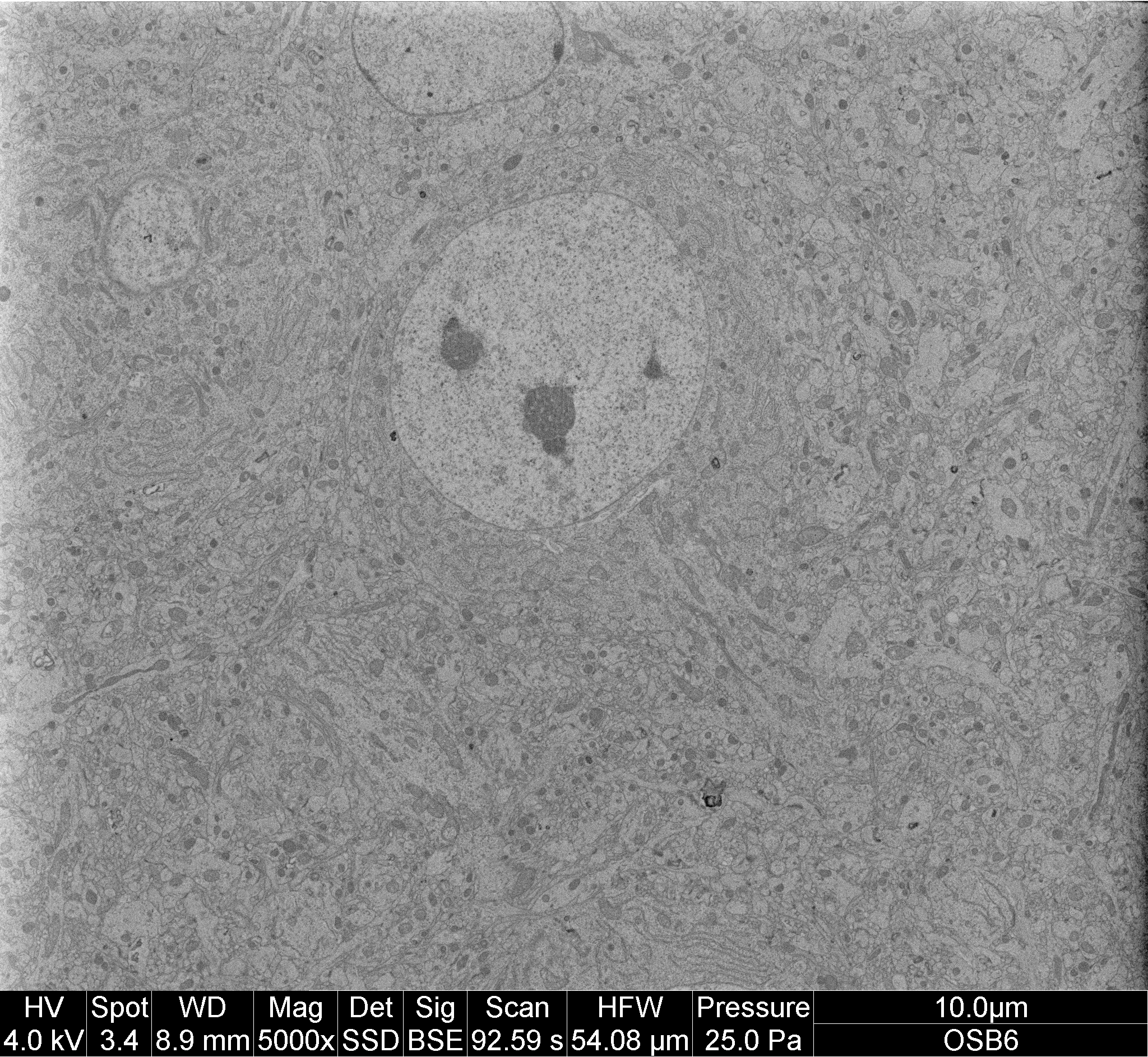

Supplement: Dataset S17 — (252.7 MB ZIP). [file pbio.0020329.sd017.zip › 040604_OS5_st1_1697.tif]

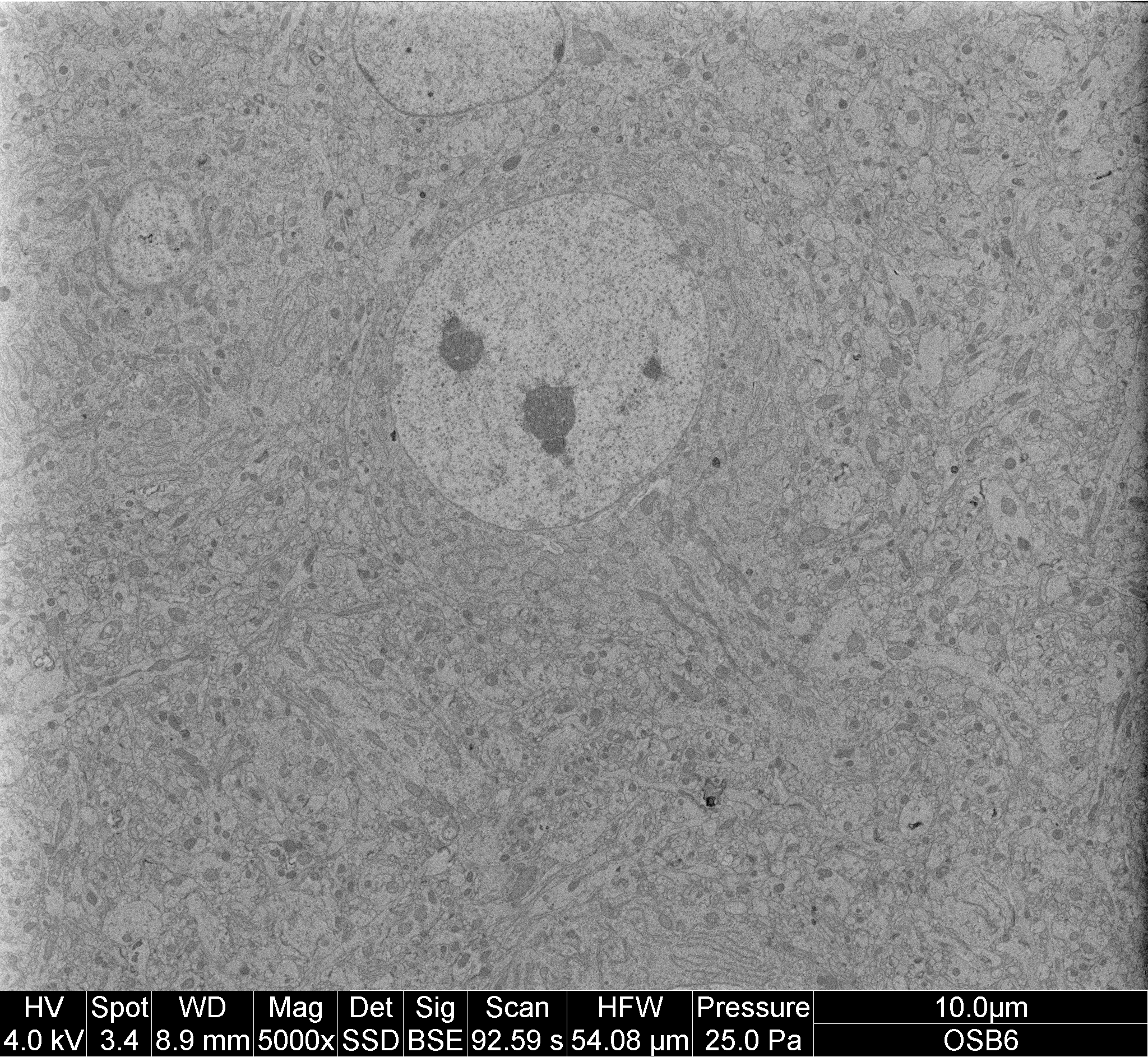

Supplement: Dataset S17 — (252.7 MB ZIP). [file pbio.0020329.sd017.zip › 040604_OS5_st1_1698.tif]

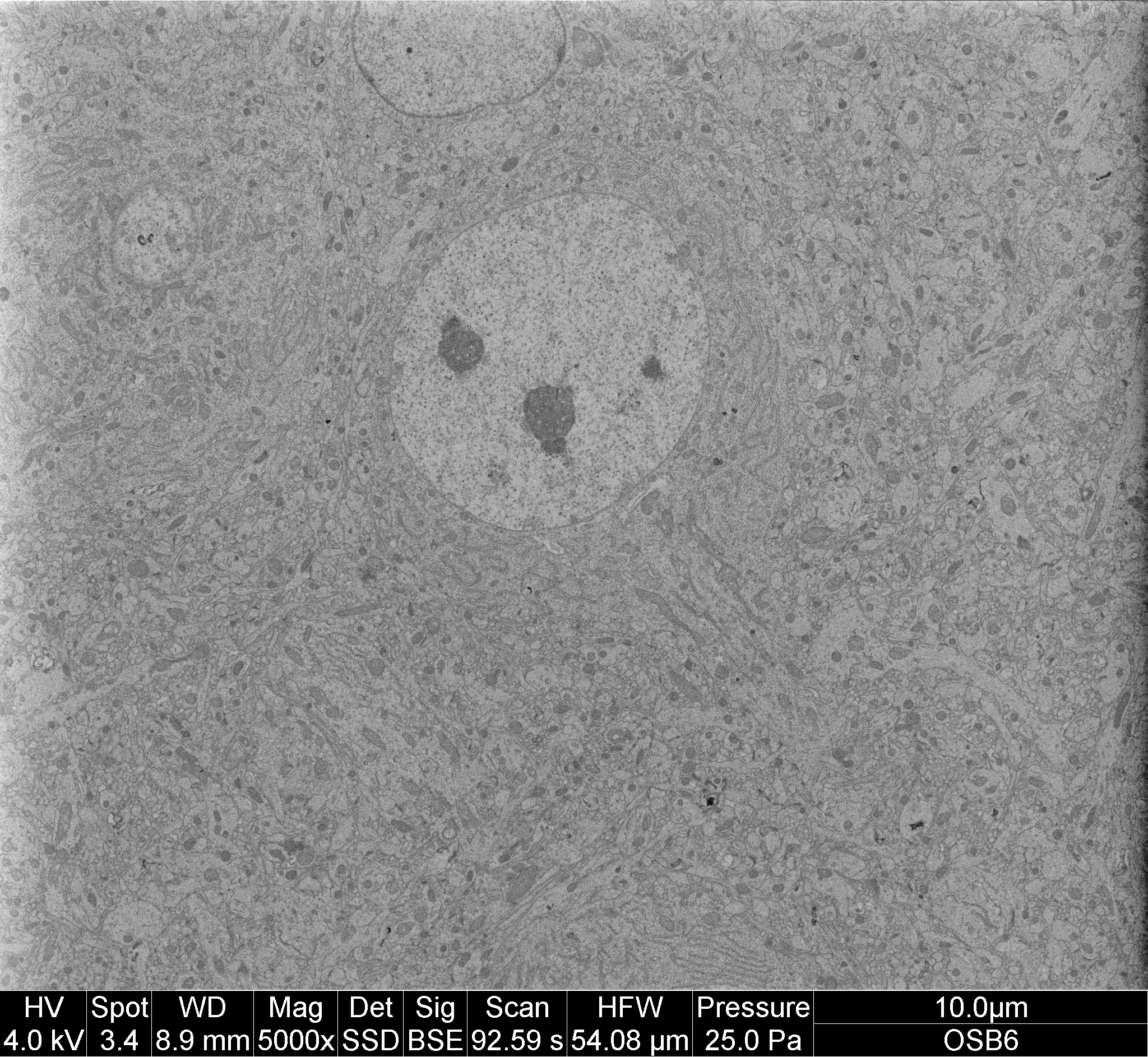

Supplement: Dataset S17 — (252.7 MB ZIP). [file pbio.0020329.sd017.zip › 040604_OS5_st1_1699.tif]

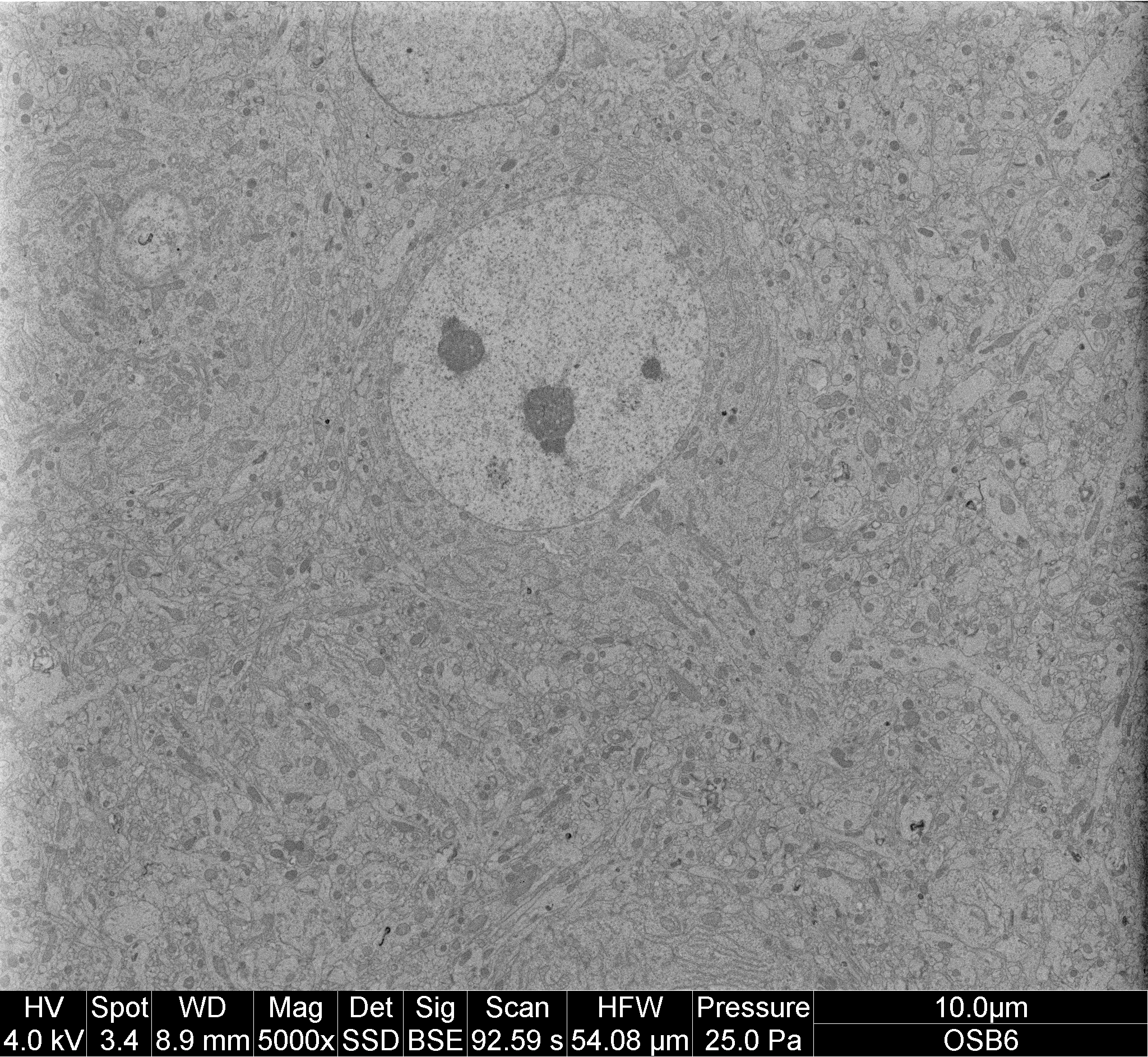

Supplement: Dataset S18 — (250.5 MB ZIP). [file pbio.0020329.sd018.zip › 040604_OS5_st1_1700.tif]
