# Supplementary figures and images for: Serial Block-Face Scanning Electron Microscopy to Reconstruct Three-Dimensional Tissue Nanostructure (part 18 of 21)
Source: PLoS Biol. 2004 Oct 19;2(11):e329. doi: 10.1371/journal.pbio.0020329 (PMC524270; doi:10.1371/journal.pbio.0020329)

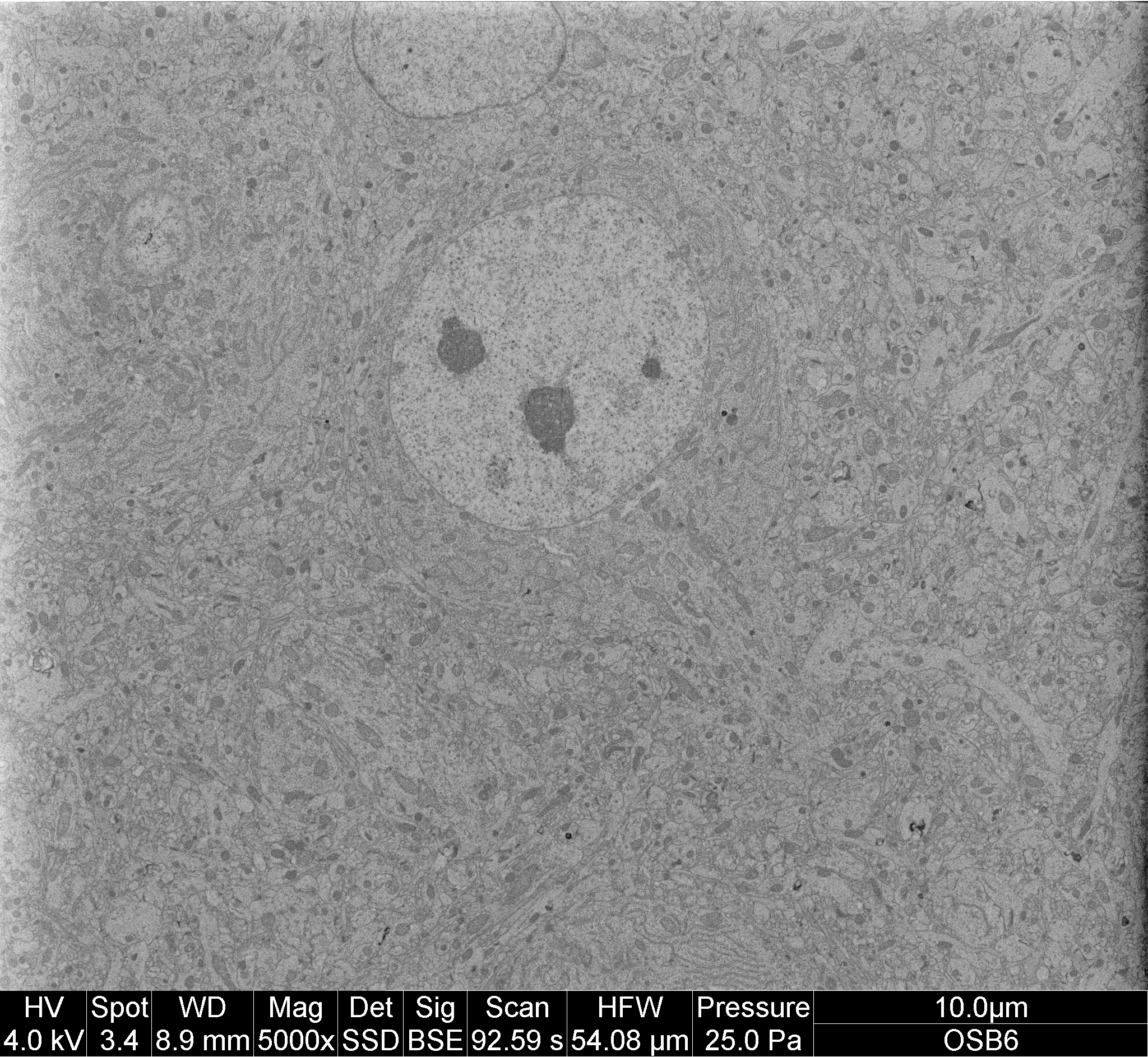

Supplement: Dataset S18 — (250.5 MB ZIP). [file pbio.0020329.sd018.zip › 040604_OS5_st1_1701.tif]

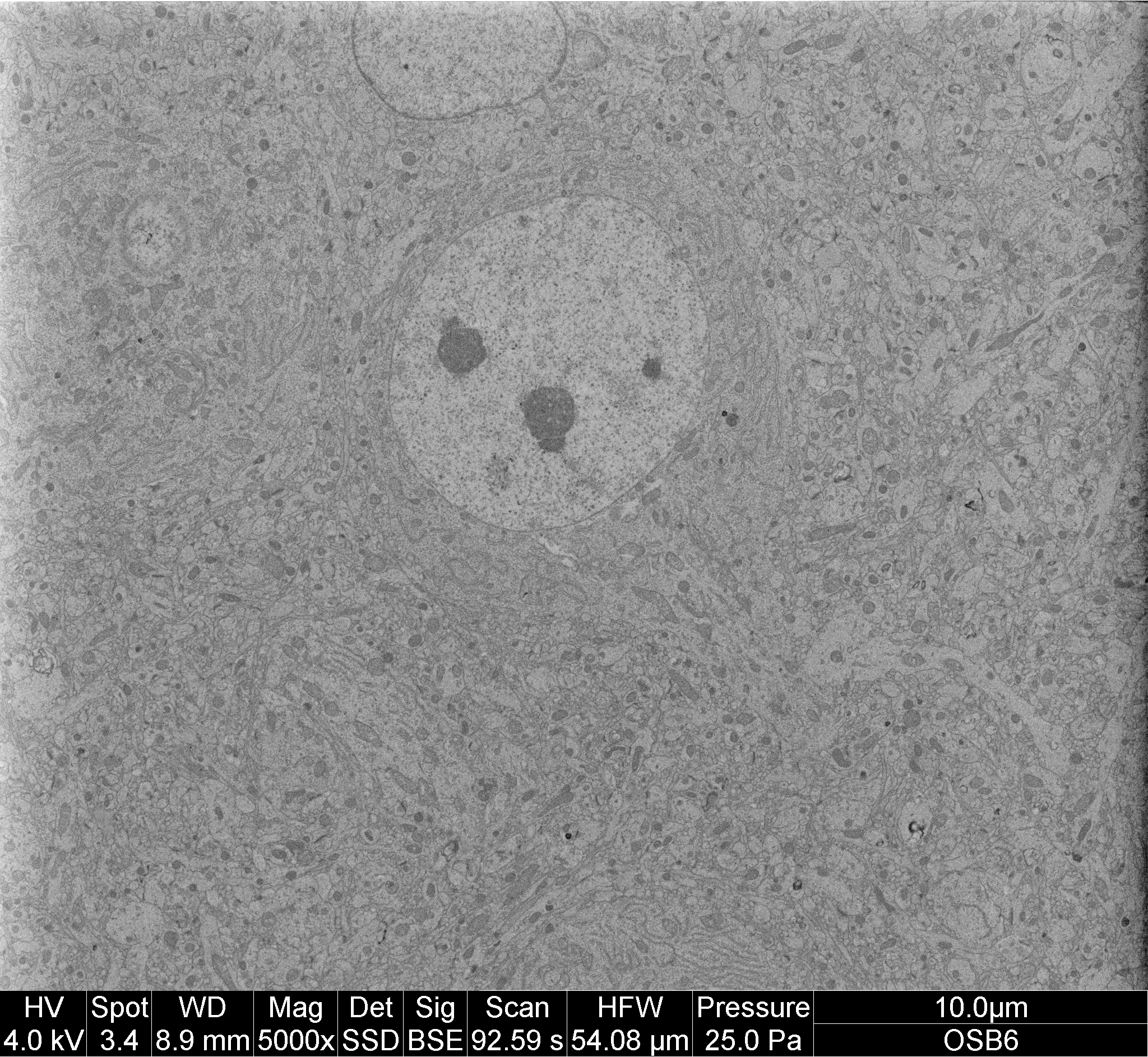

Supplement: Dataset S18 — (250.5 MB ZIP). [file pbio.0020329.sd018.zip › 040604_OS5_st1_1702.tif]

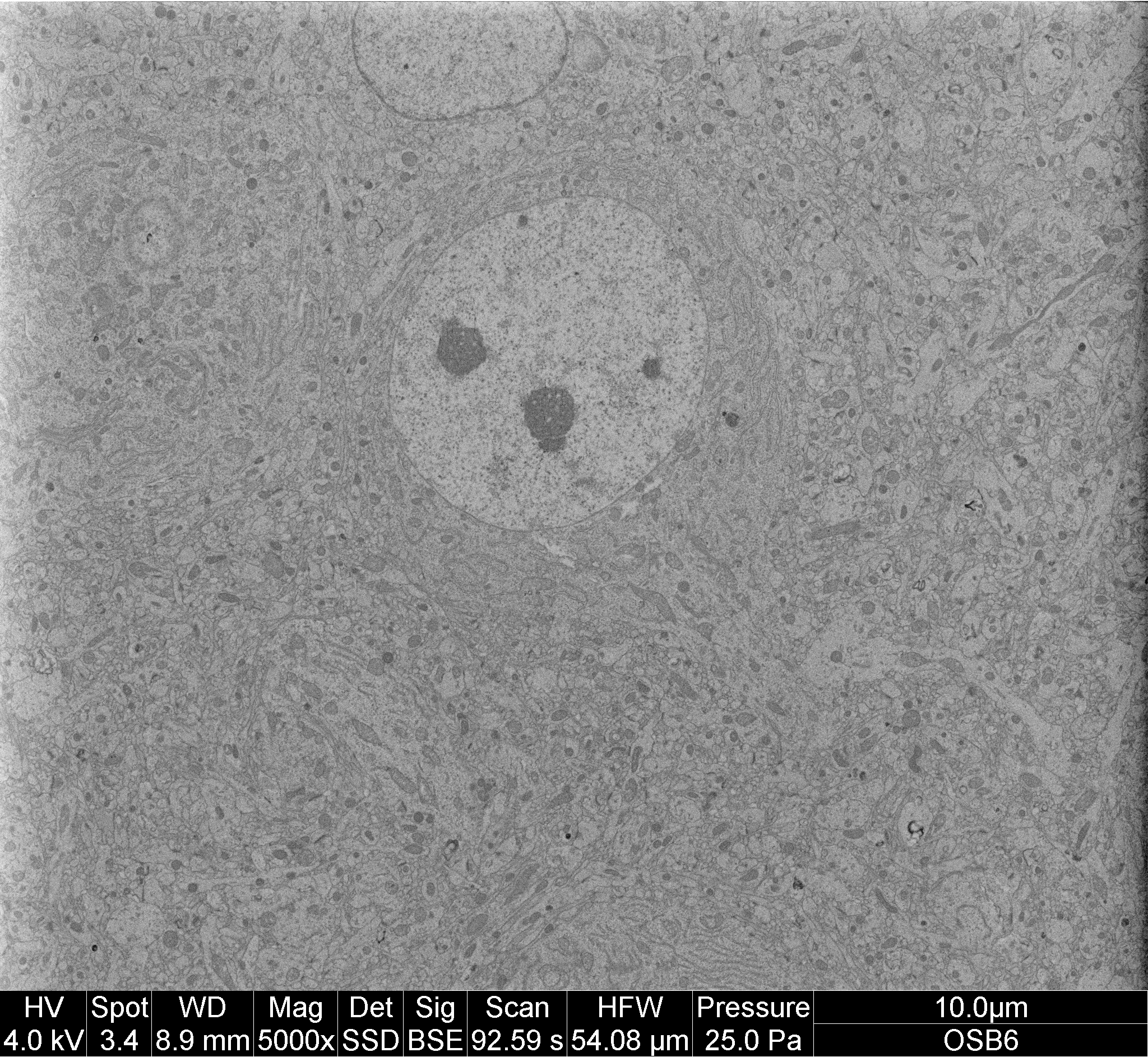

Supplement: Dataset S18 — (250.5 MB ZIP). [file pbio.0020329.sd018.zip › 040604_OS5_st1_1703.tif]

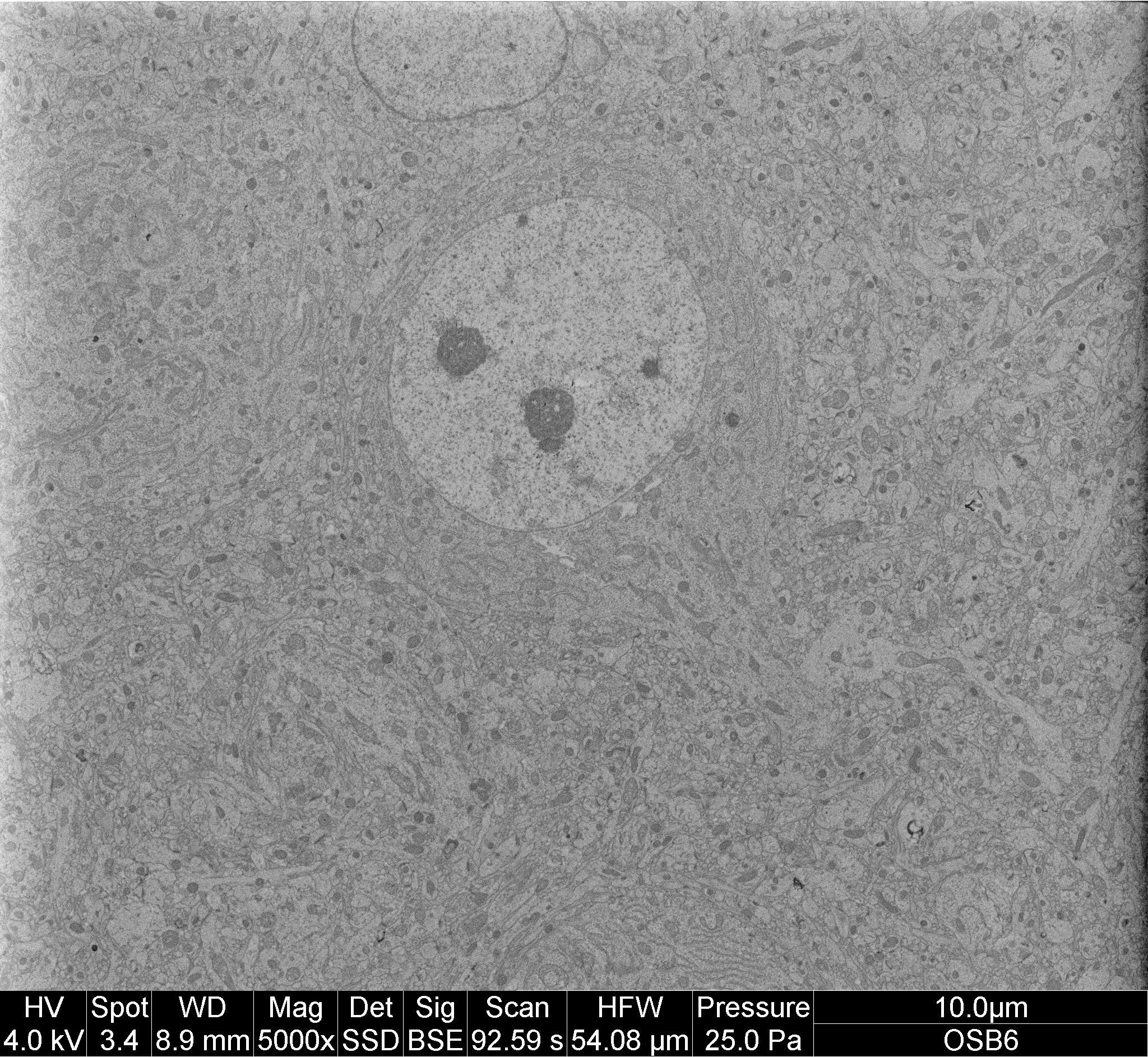

Supplement: Dataset S18 — (250.5 MB ZIP). [file pbio.0020329.sd018.zip › 040604_OS5_st1_1704.tif]

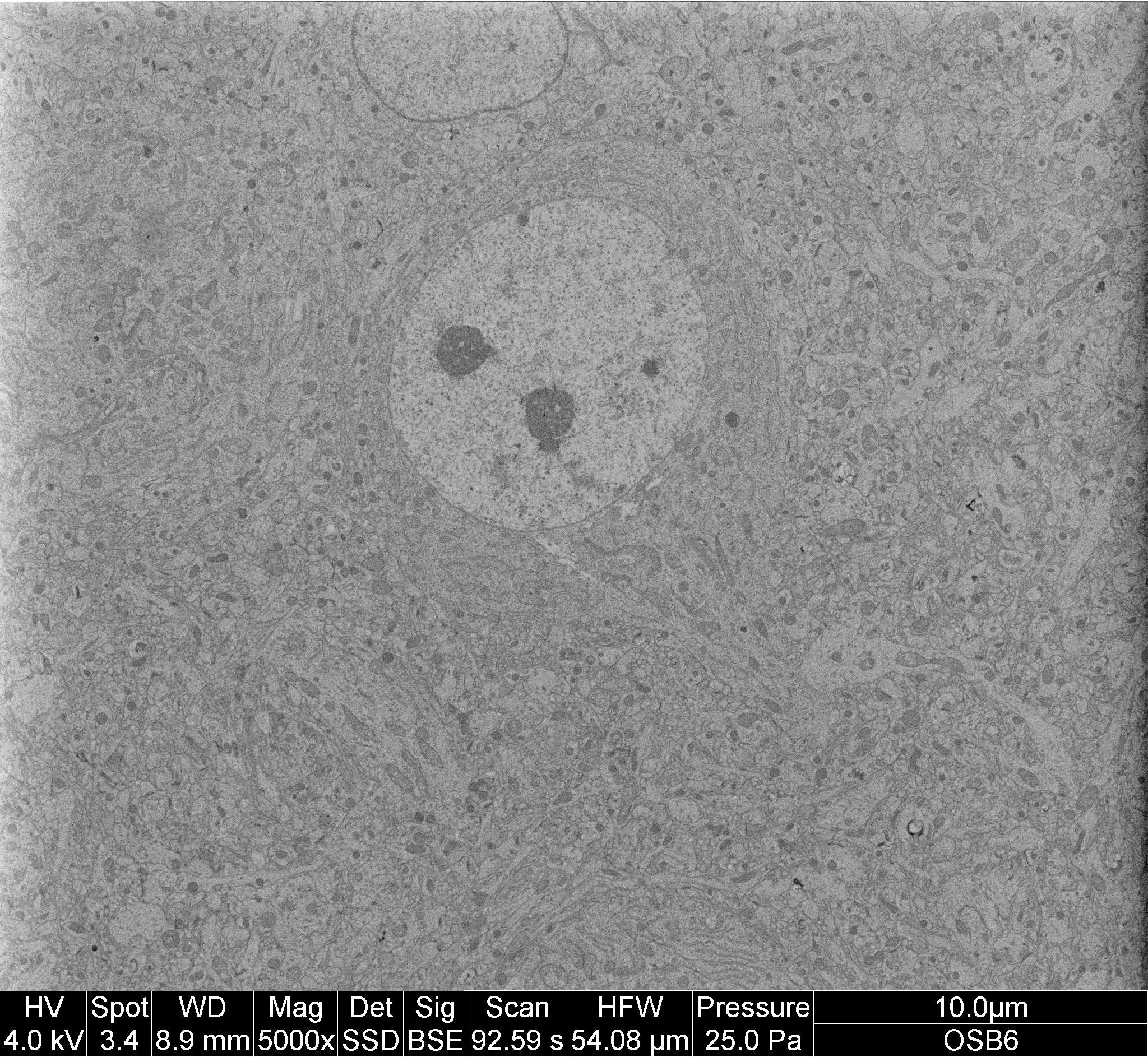

Supplement: Dataset S18 — (250.5 MB ZIP). [file pbio.0020329.sd018.zip › 040604_OS5_st1_1705.tif]

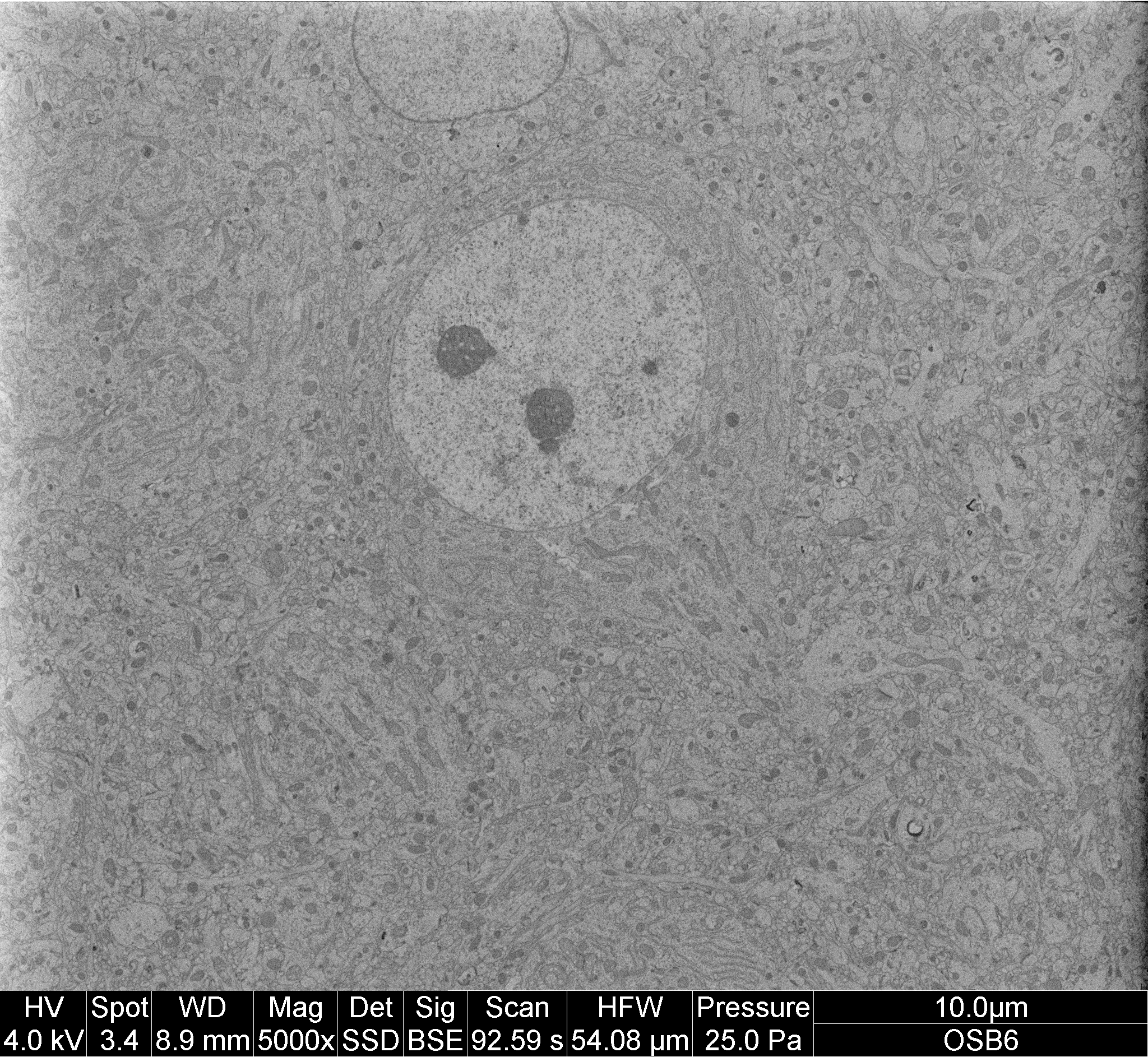

Supplement: Dataset S18 — (250.5 MB ZIP). [file pbio.0020329.sd018.zip › 040604_OS5_st1_1706.tif]

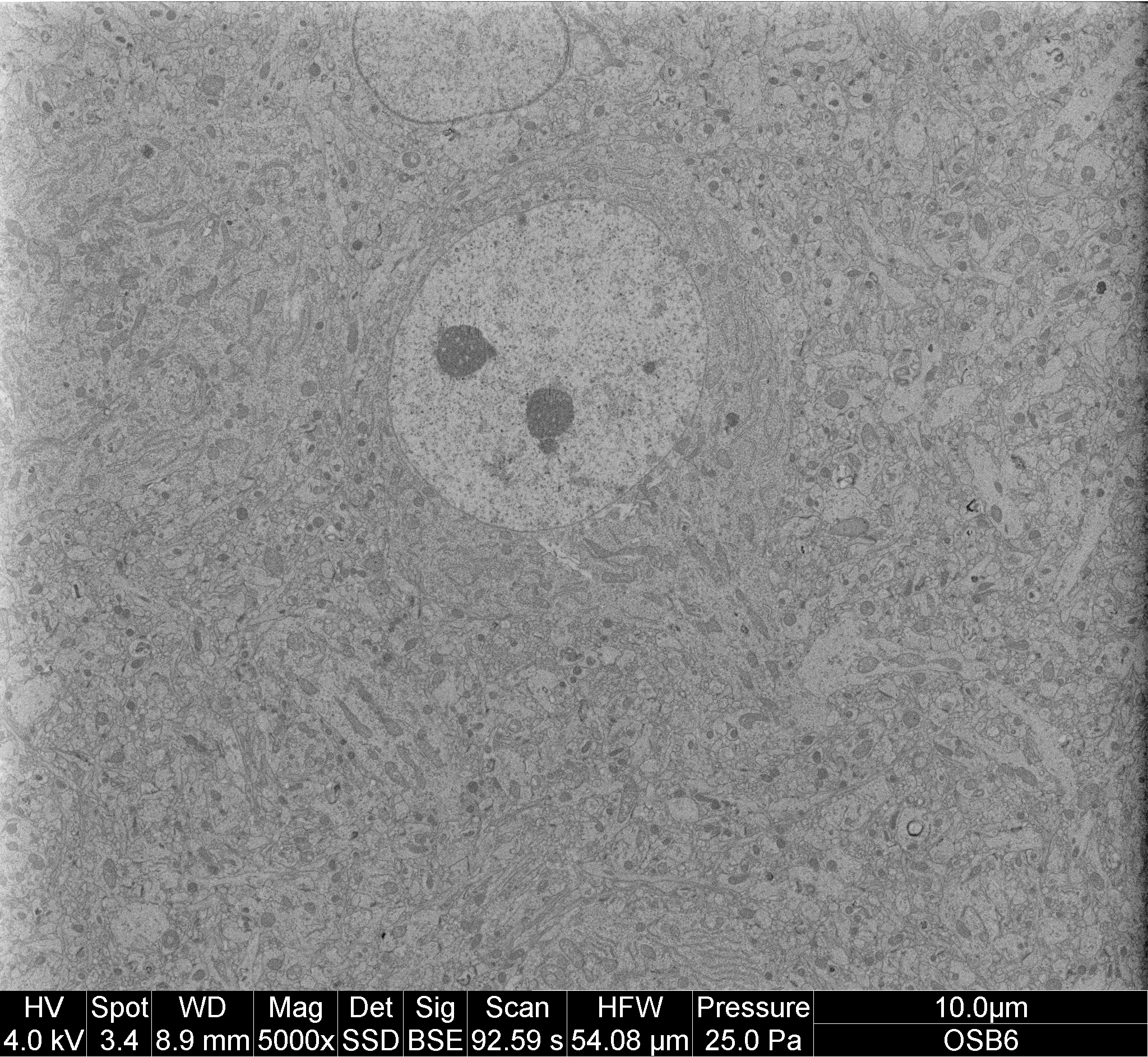

Supplement: Dataset S18 — (250.5 MB ZIP). [file pbio.0020329.sd018.zip › 040604_OS5_st1_1707.tif]

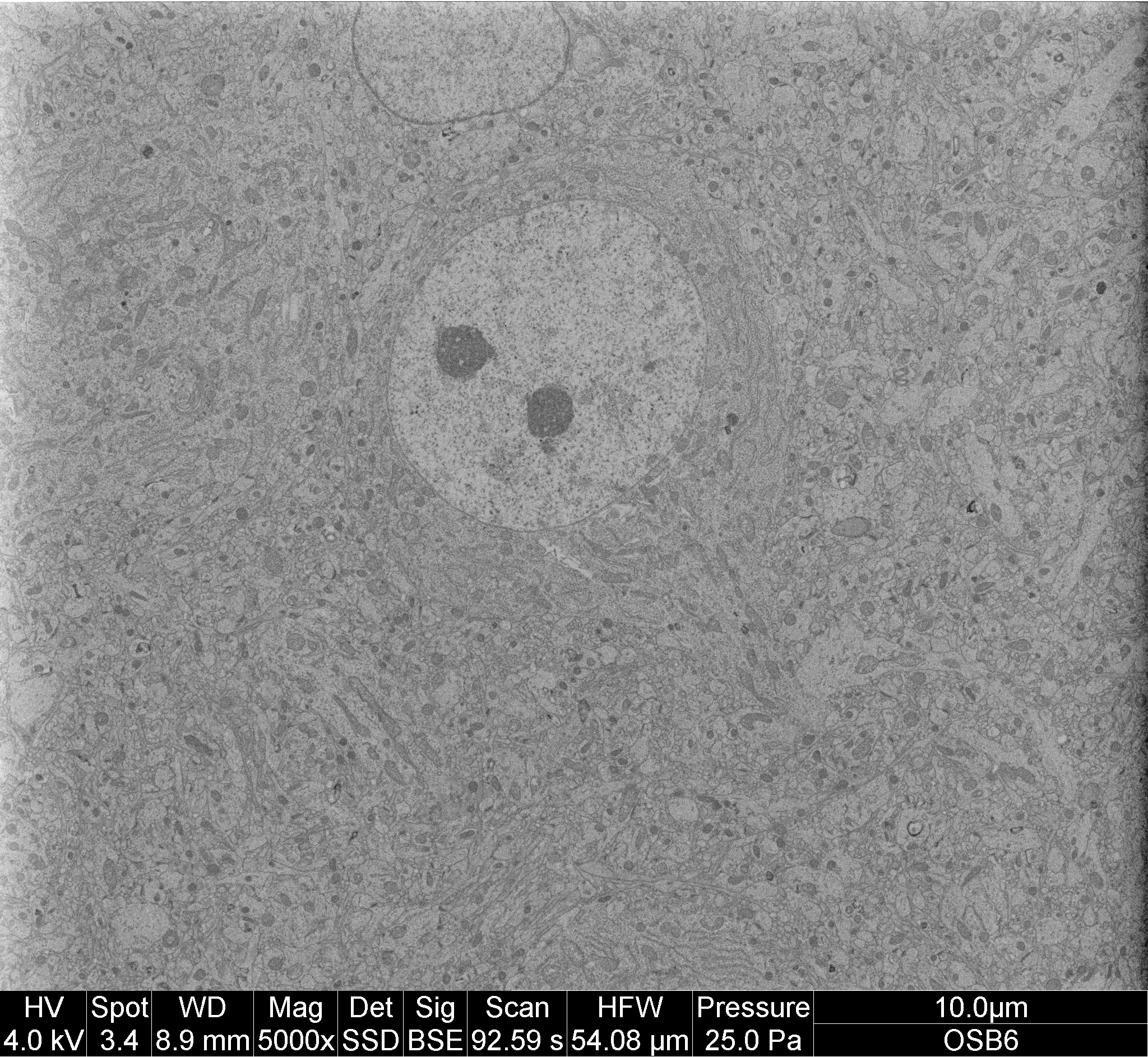

Supplement: Dataset S18 — (250.5 MB ZIP). [file pbio.0020329.sd018.zip › 040604_OS5_st1_1708.tif]

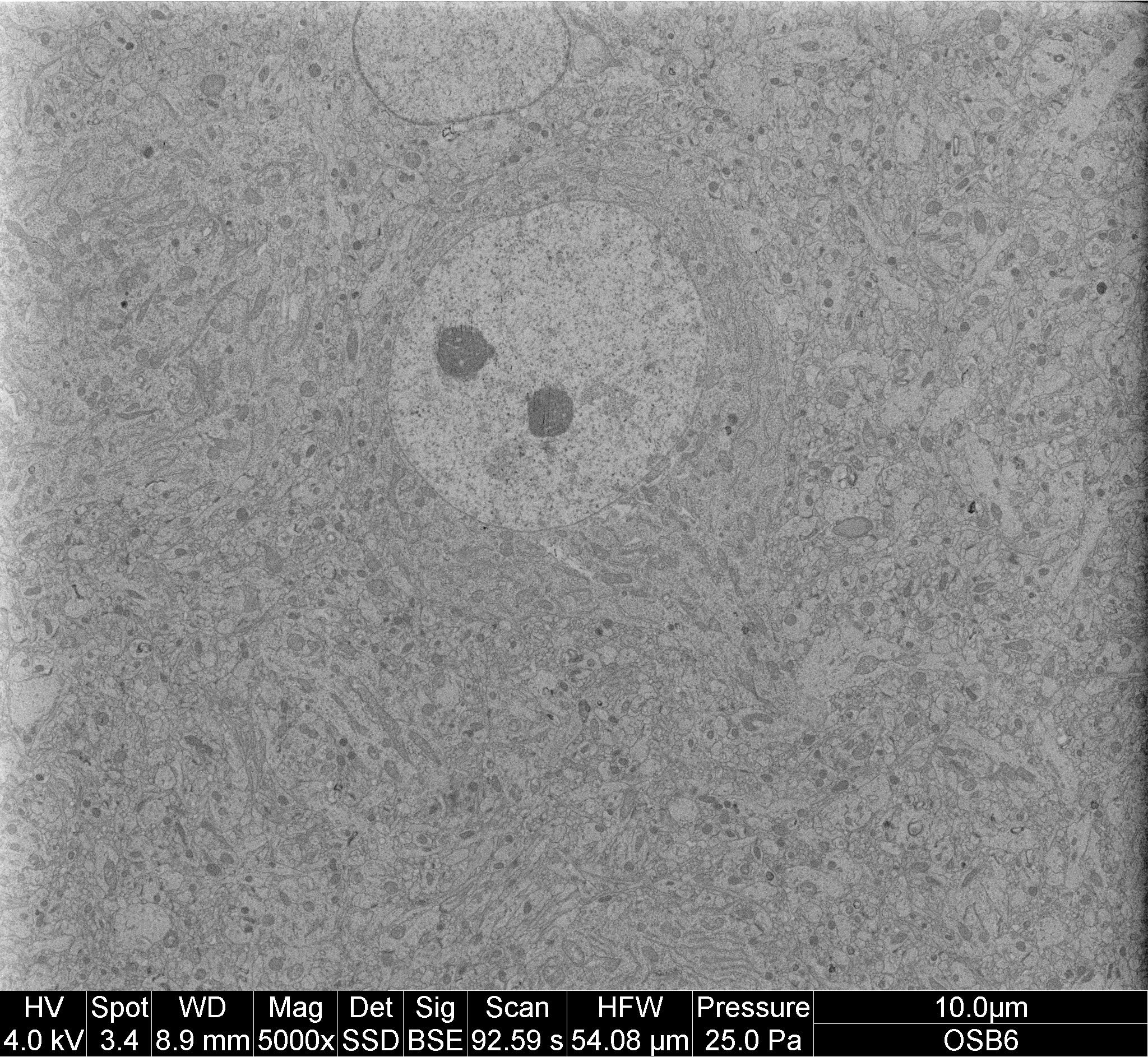

Supplement: Dataset S18 — (250.5 MB ZIP). [file pbio.0020329.sd018.zip › 040604_OS5_st1_1709.tif]

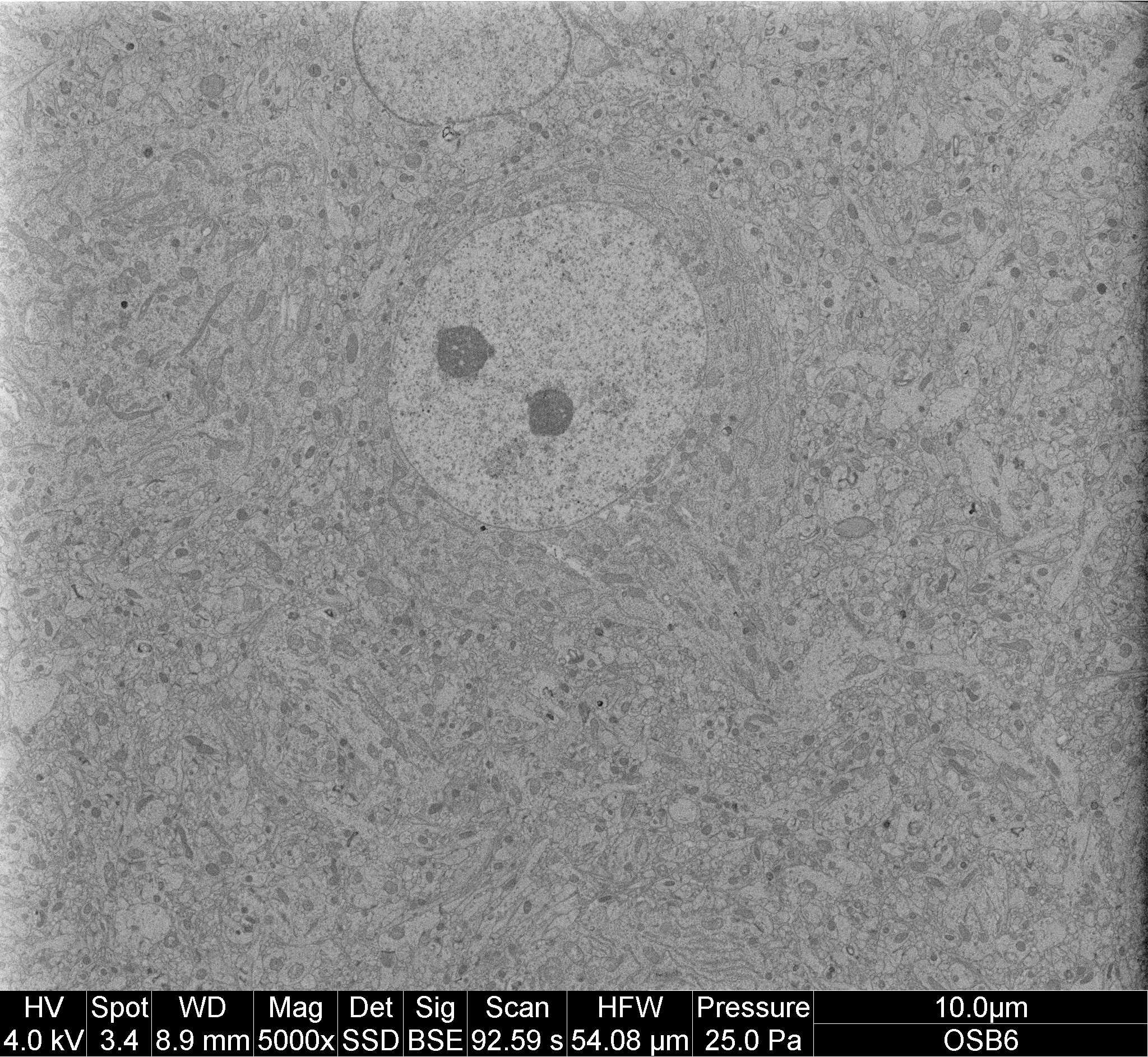

Supplement: Dataset S18 — (250.5 MB ZIP). [file pbio.0020329.sd018.zip › 040604_OS5_st1_1710.tif]

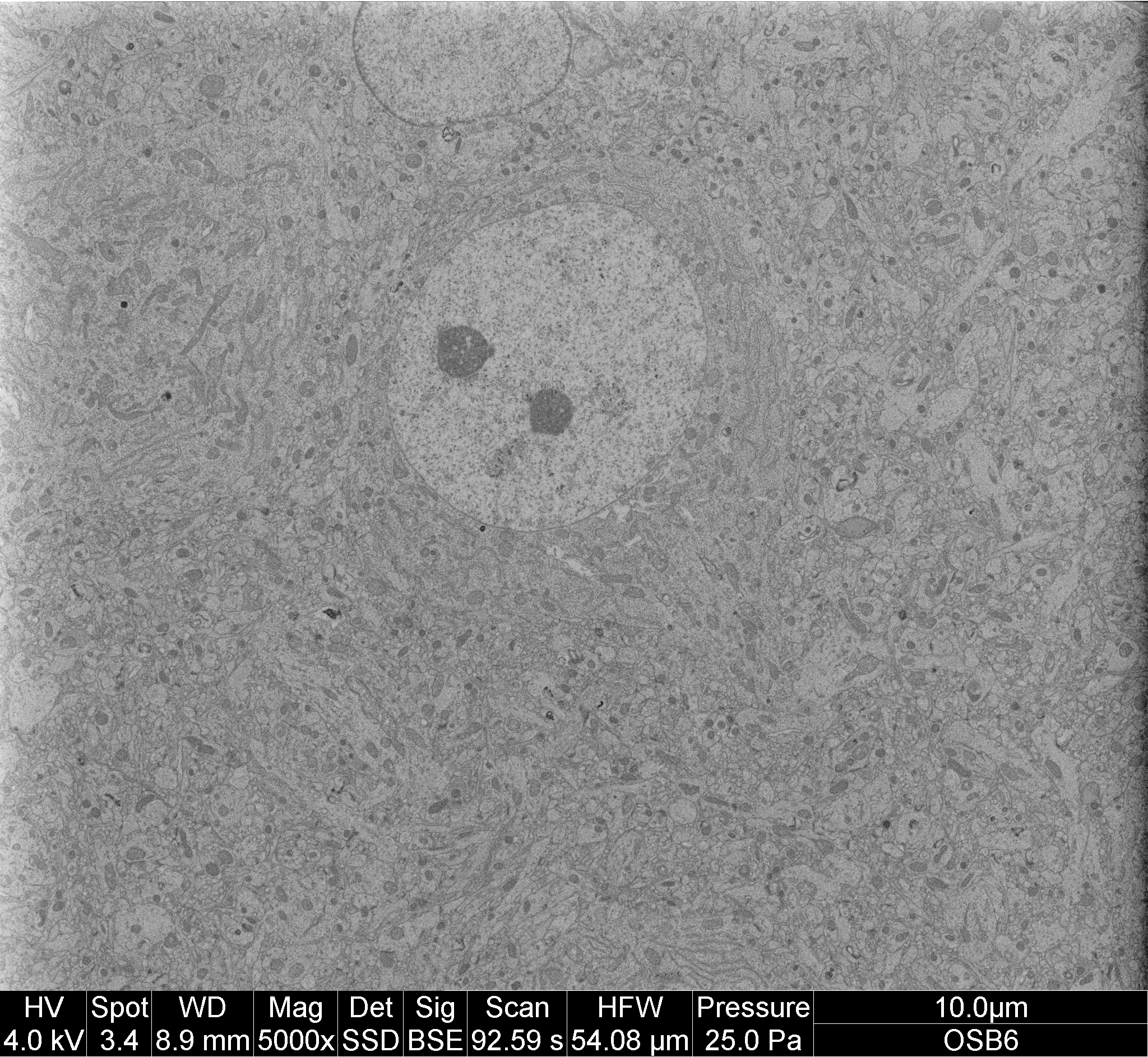

Supplement: Dataset S18 — (250.5 MB ZIP). [file pbio.0020329.sd018.zip › 040604_OS5_st1_1711.tif]

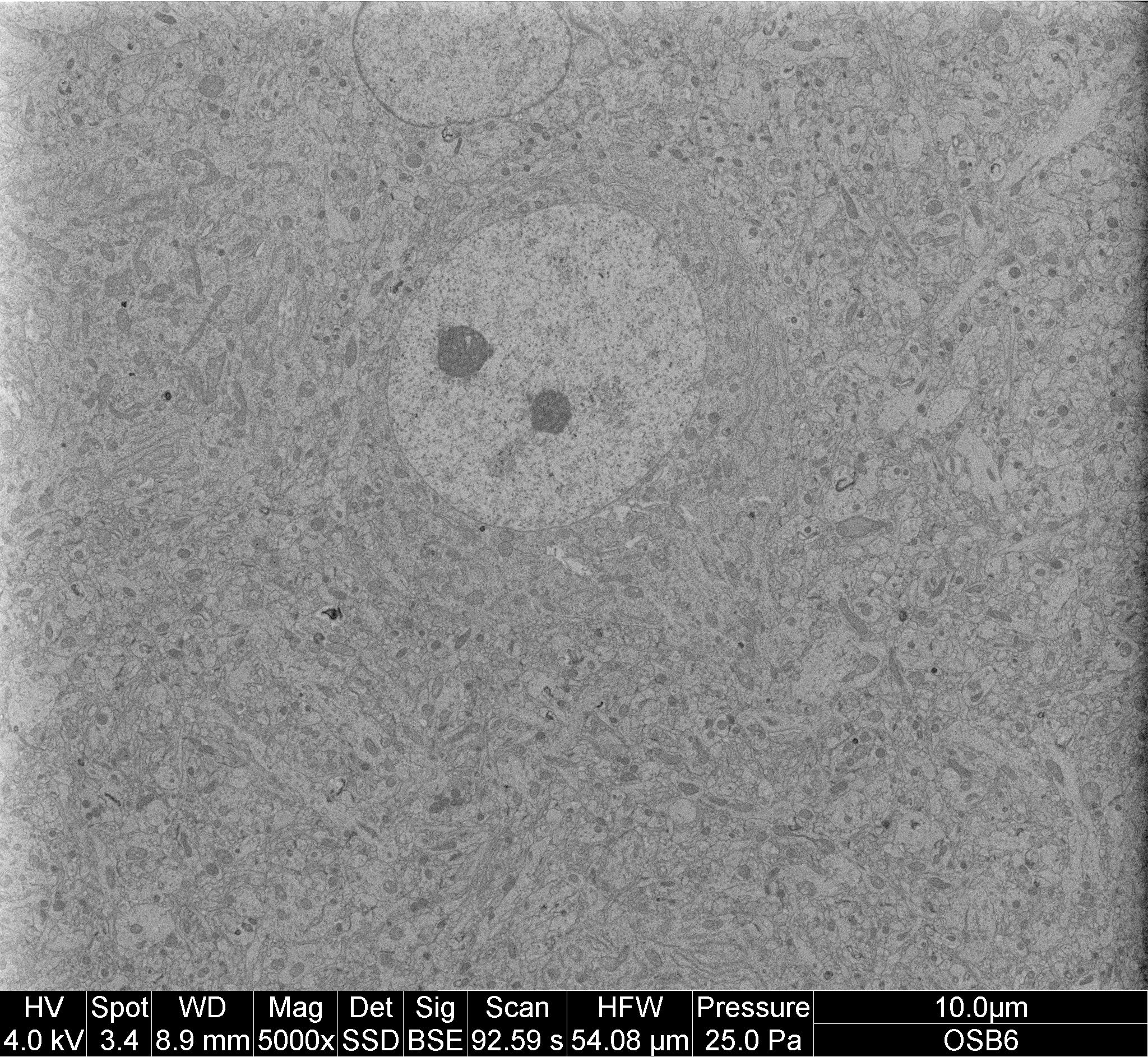

Supplement: Dataset S18 — (250.5 MB ZIP). [file pbio.0020329.sd018.zip › 040604_OS5_st1_1712.tif]

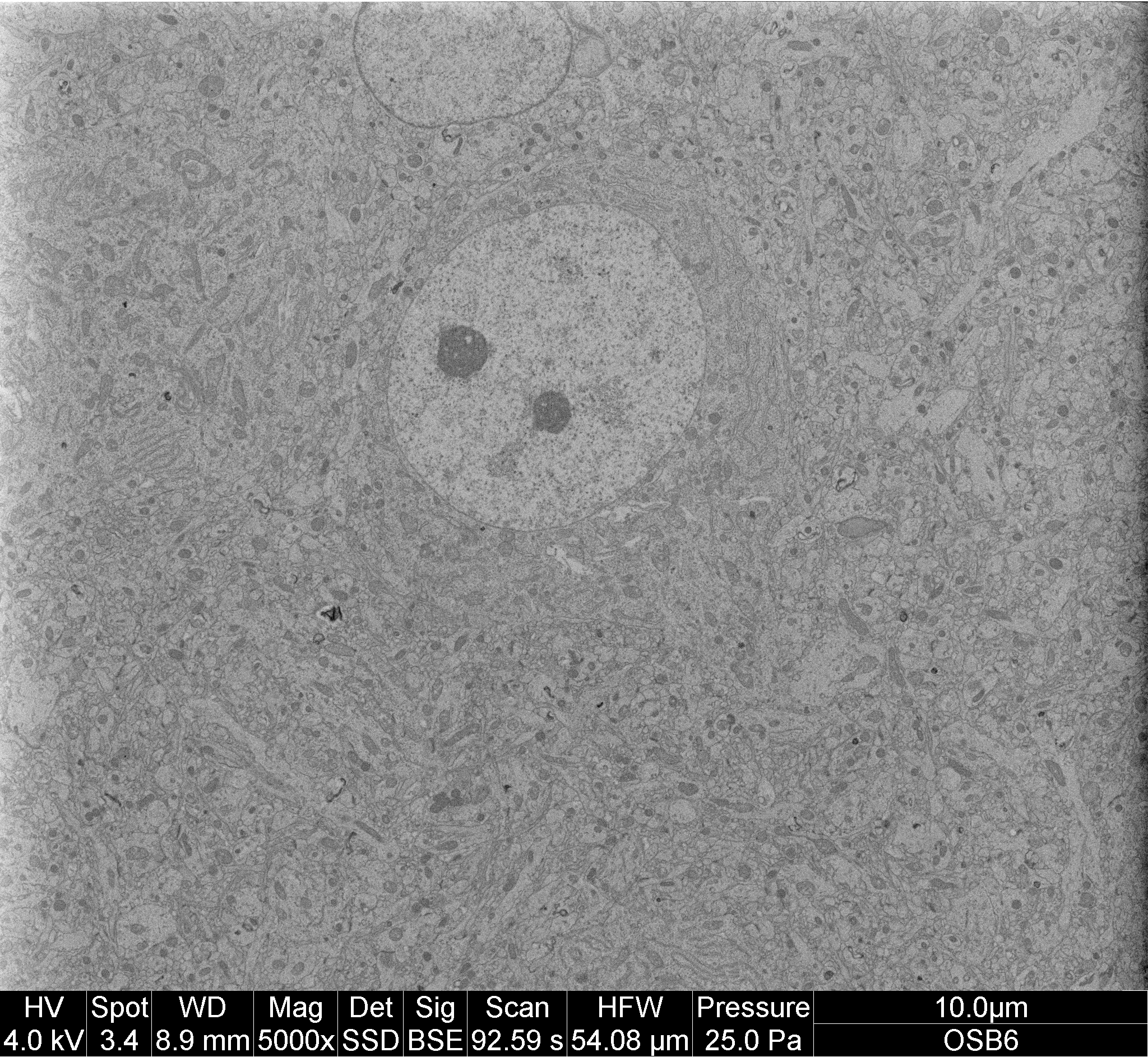

Supplement: Dataset S18 — (250.5 MB ZIP). [file pbio.0020329.sd018.zip › 040604_OS5_st1_1713.tif]

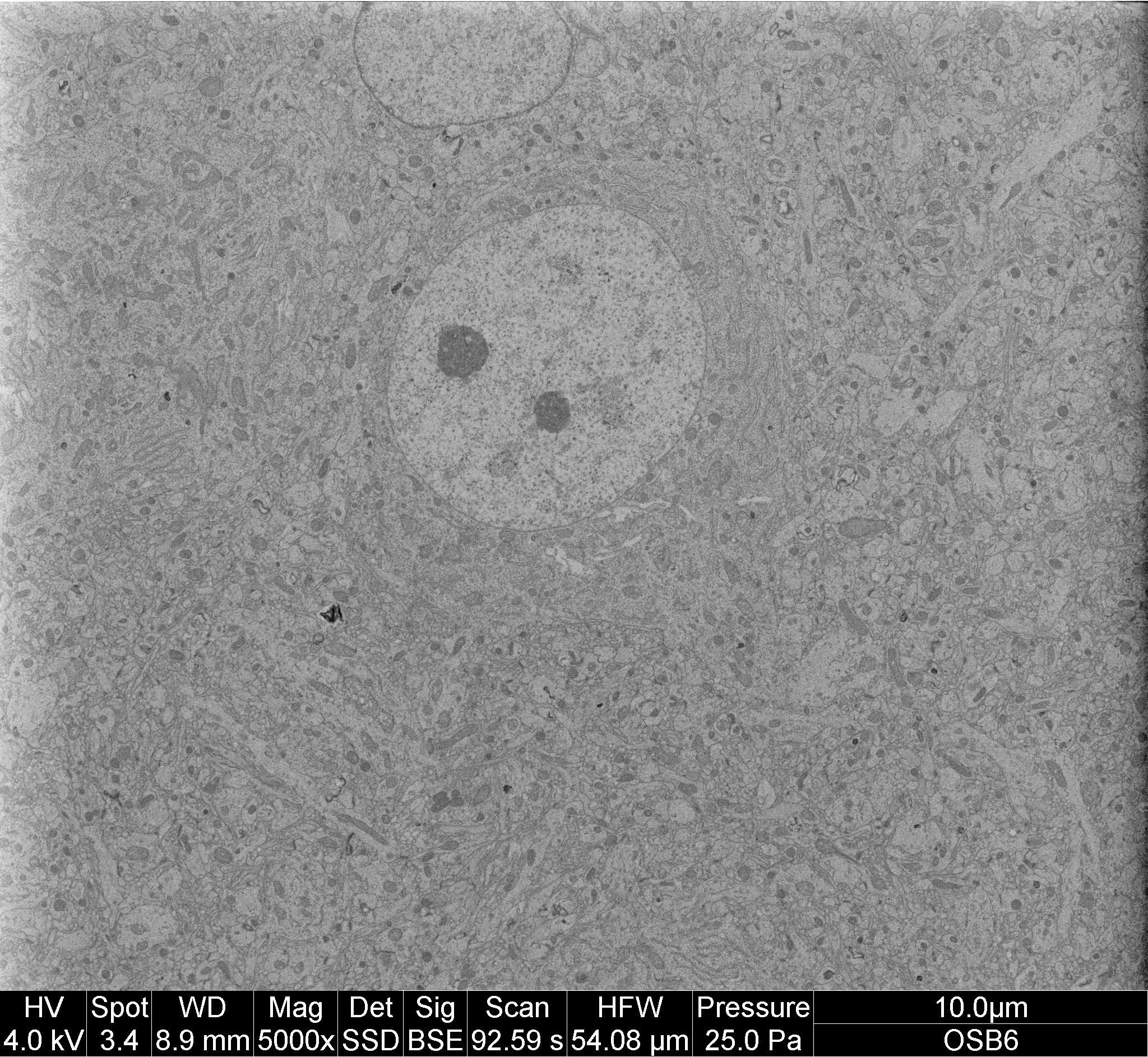

Supplement: Dataset S18 — (250.5 MB ZIP). [file pbio.0020329.sd018.zip › 040604_OS5_st1_1714.tif]

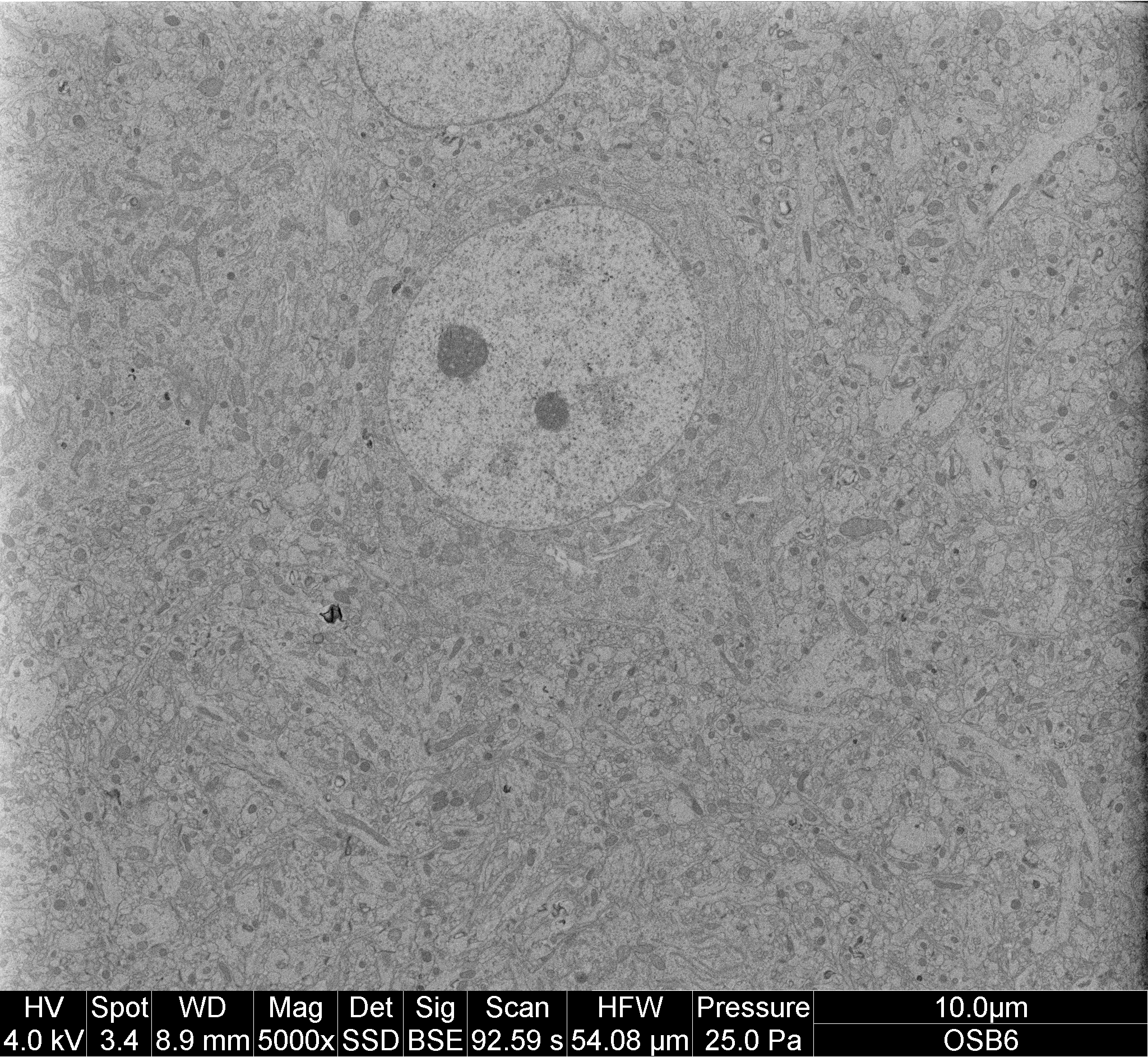

Supplement: Dataset S18 — (250.5 MB ZIP). [file pbio.0020329.sd018.zip › 040604_OS5_st1_1715.tif]

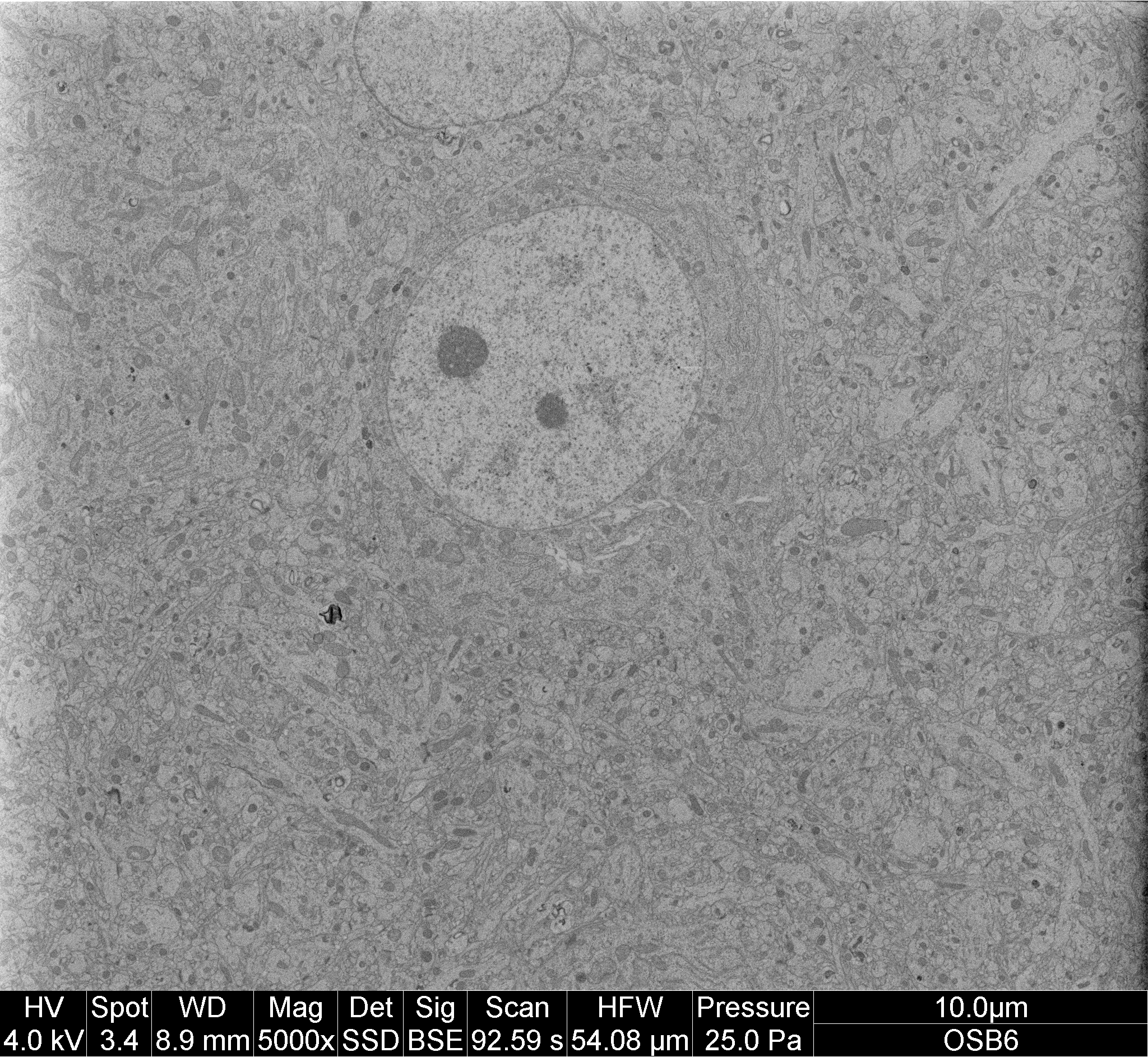

Supplement: Dataset S18 — (250.5 MB ZIP). [file pbio.0020329.sd018.zip › 040604_OS5_st1_1716.tif]

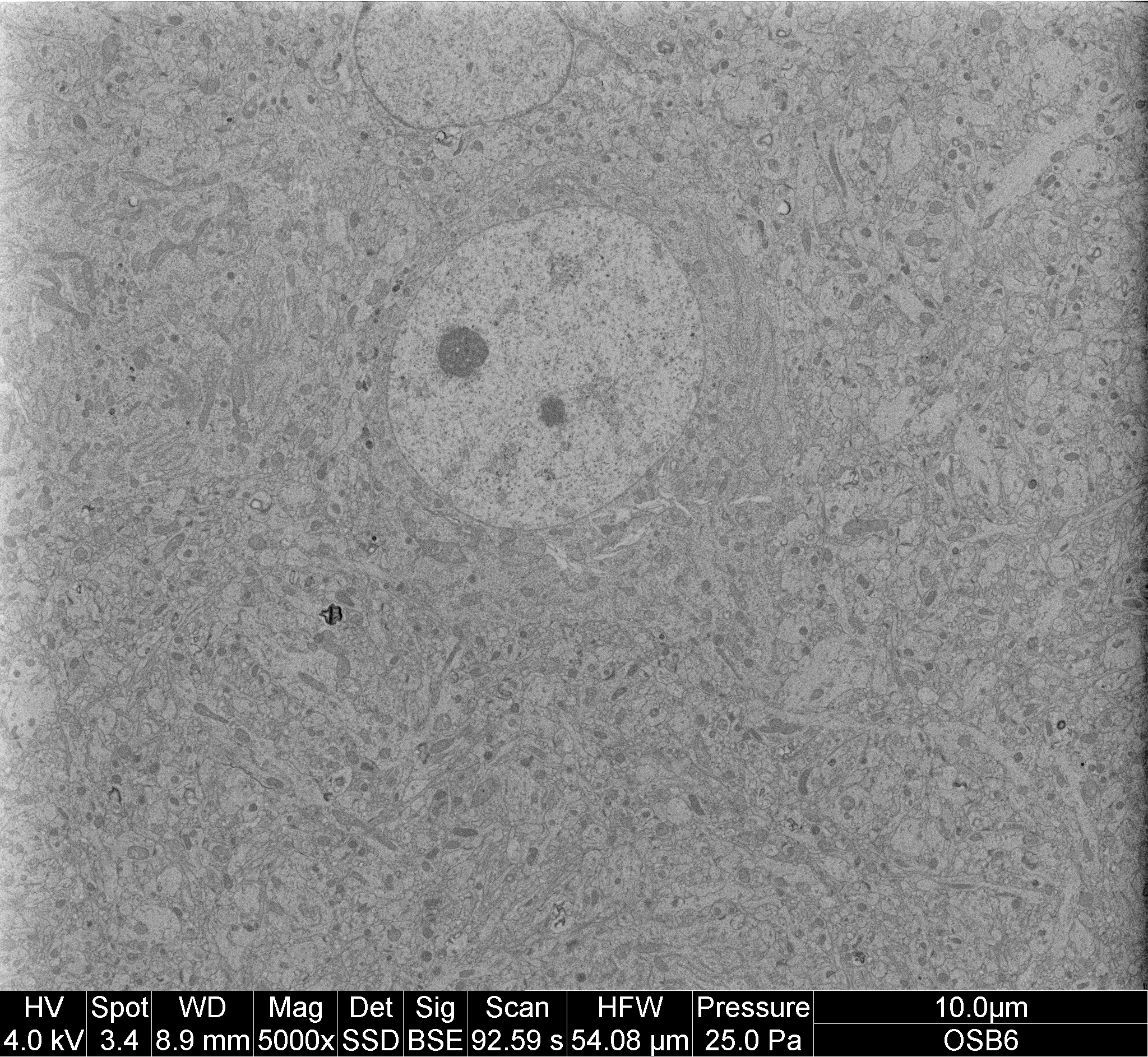

Supplement: Dataset S18 — (250.5 MB ZIP). [file pbio.0020329.sd018.zip › 040604_OS5_st1_1717.tif]

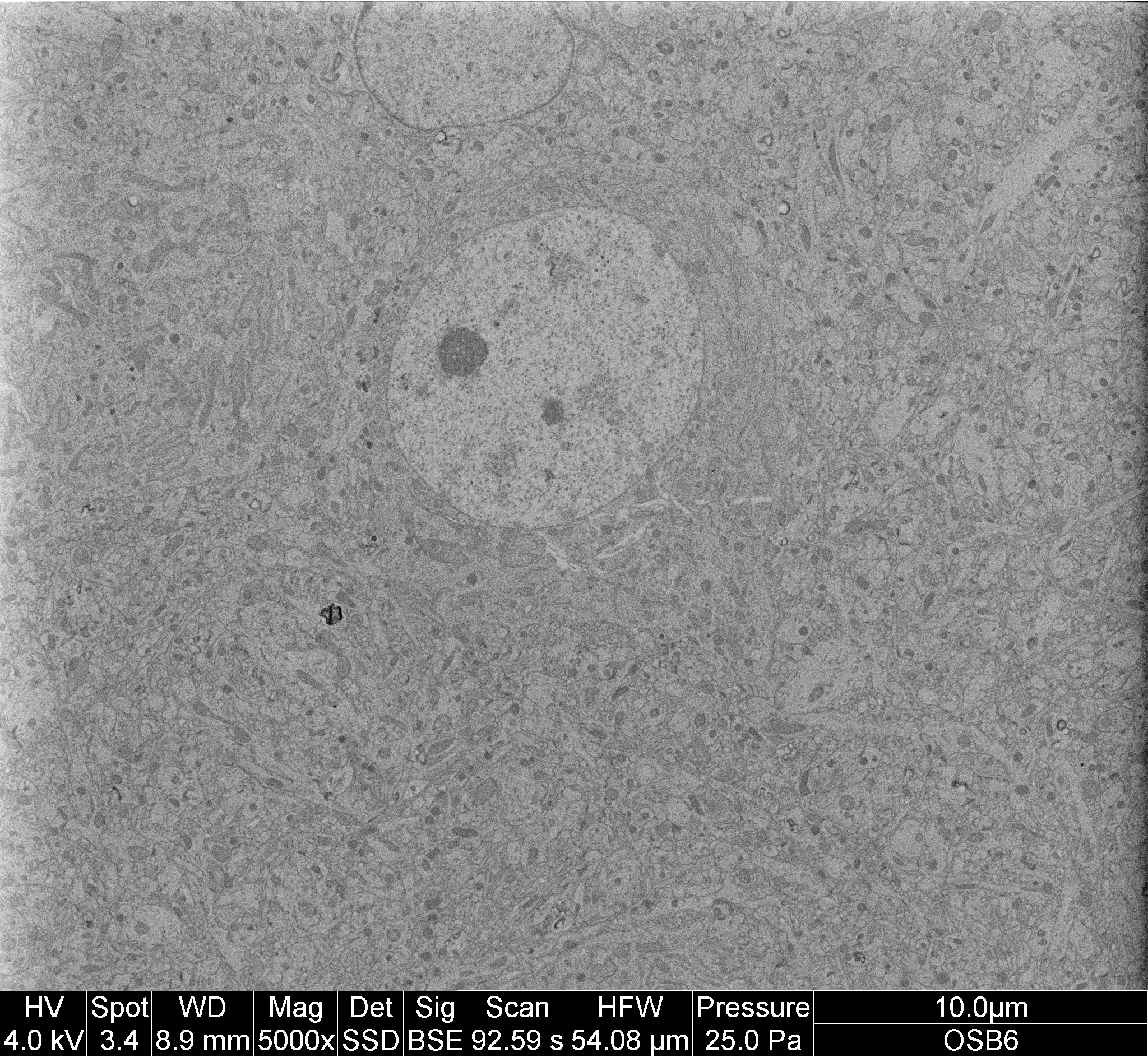

Supplement: Dataset S18 — (250.5 MB ZIP). [file pbio.0020329.sd018.zip › 040604_OS5_st1_1718.tif]

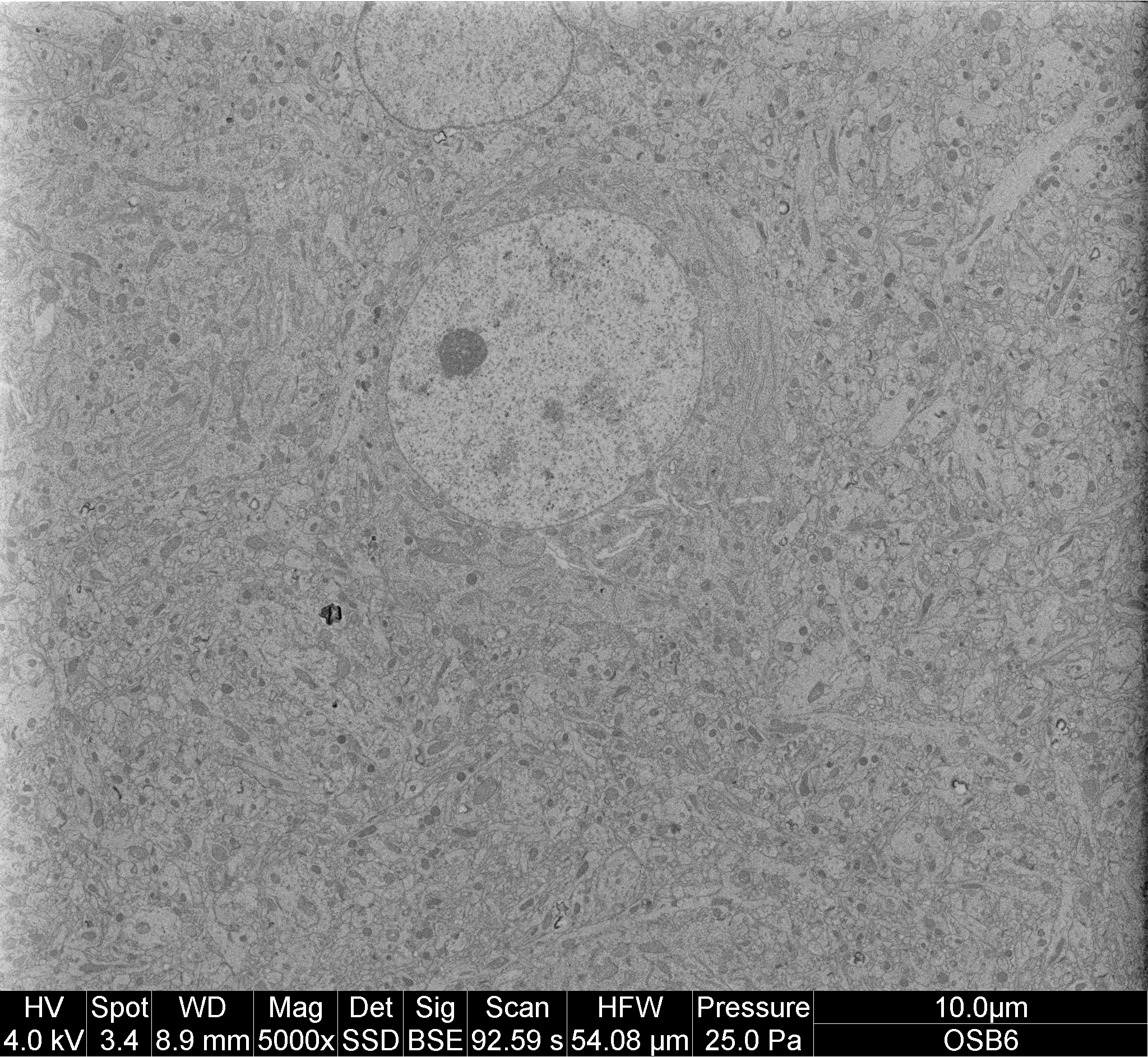

Supplement: Dataset S18 — (250.5 MB ZIP). [file pbio.0020329.sd018.zip › 040604_OS5_st1_1719.tif]

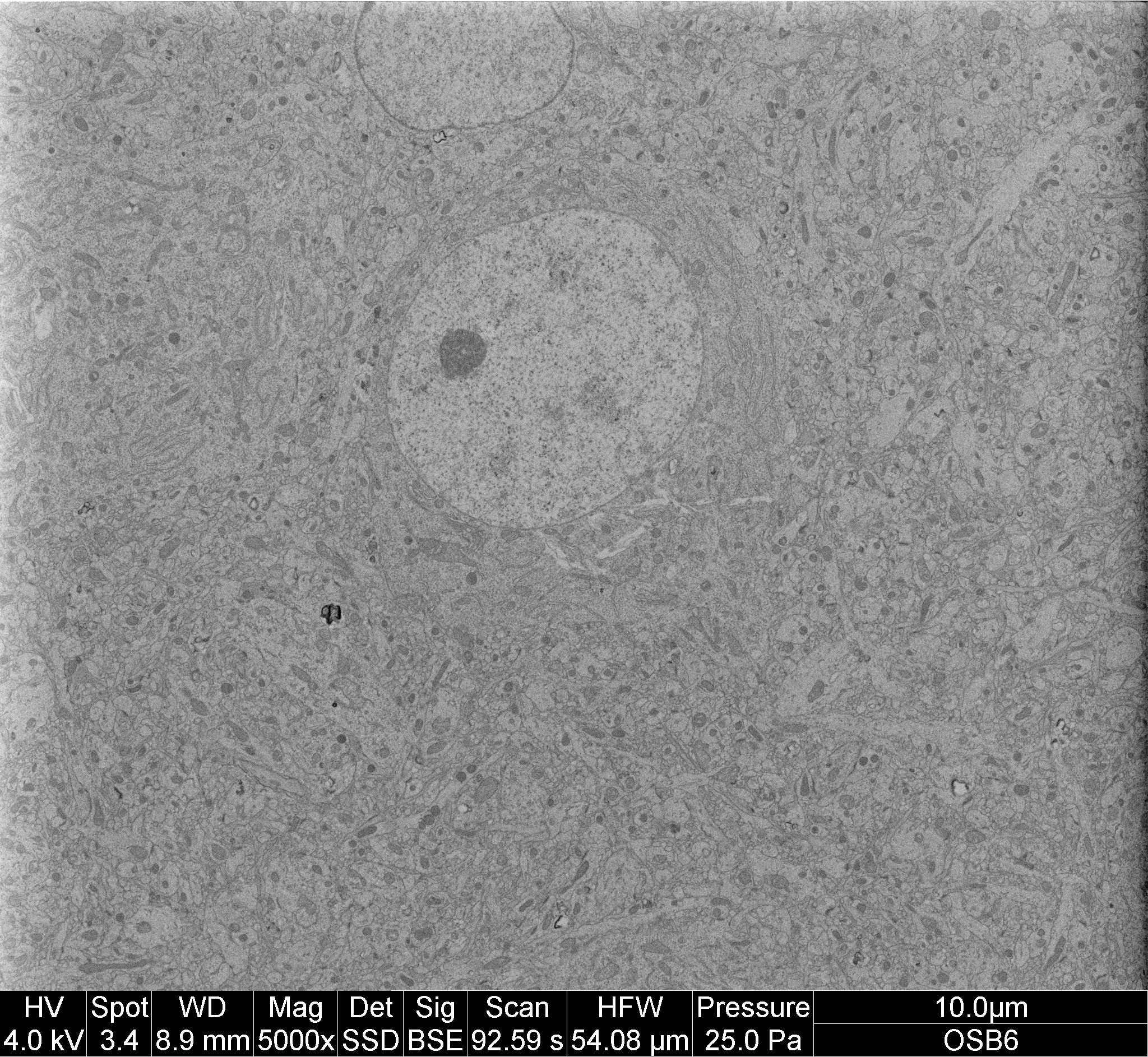

Supplement: Dataset S18 — (250.5 MB ZIP). [file pbio.0020329.sd018.zip › 040604_OS5_st1_1720.tif]

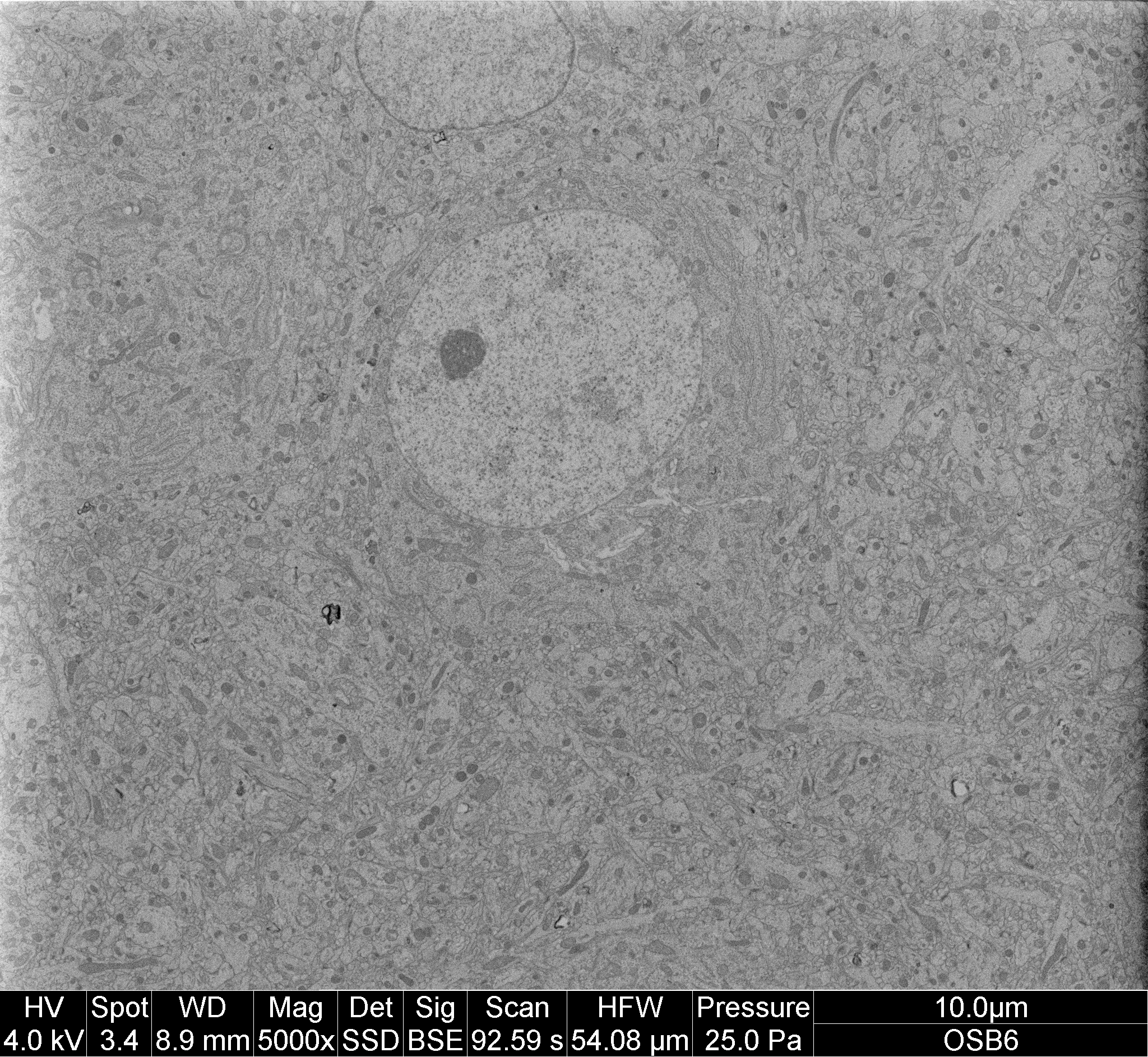

Supplement: Dataset S18 — (250.5 MB ZIP). [file pbio.0020329.sd018.zip › 040604_OS5_st1_1721.tif]

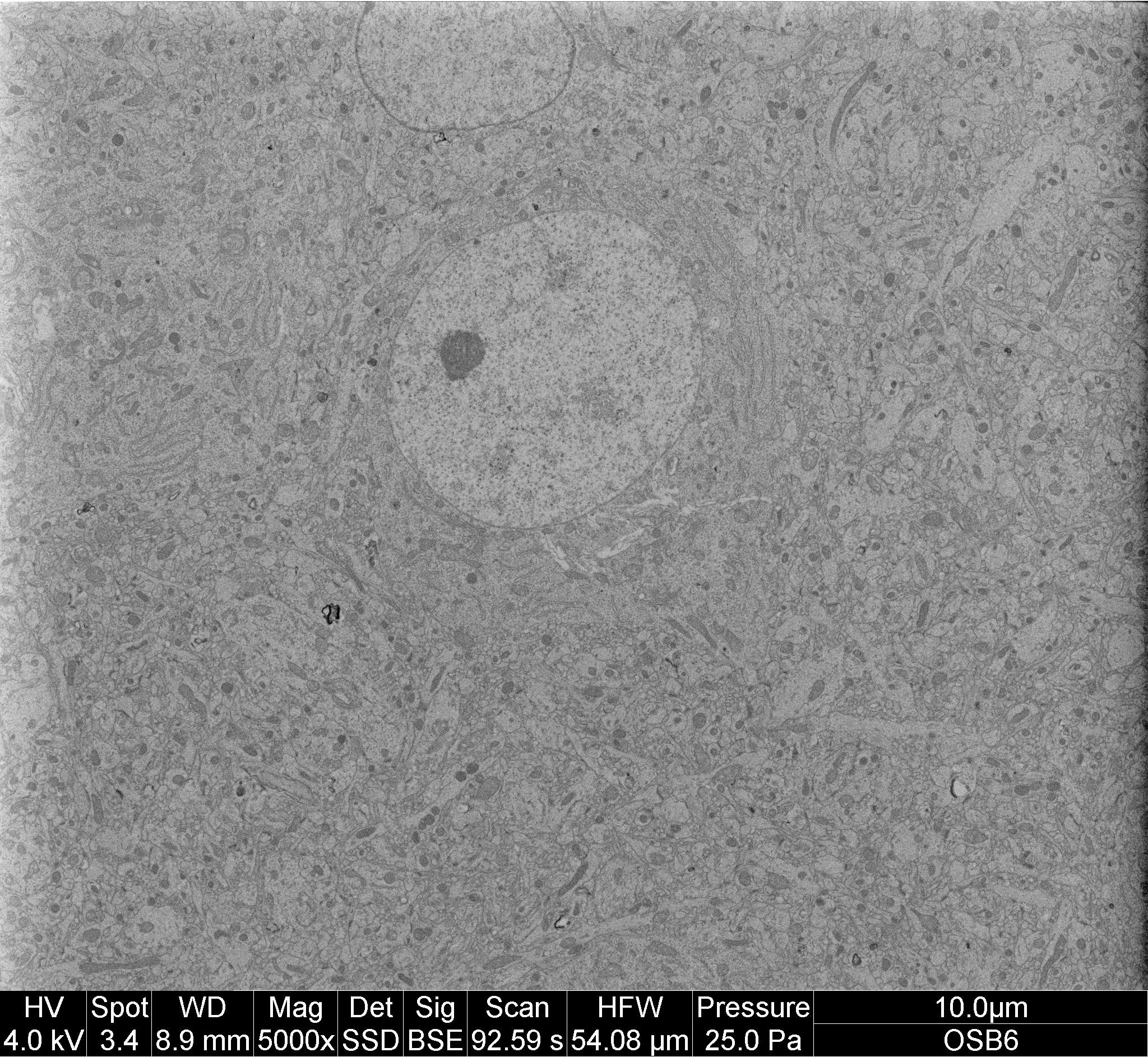

Supplement: Dataset S18 — (250.5 MB ZIP). [file pbio.0020329.sd018.zip › 040604_OS5_st1_1722.tif]

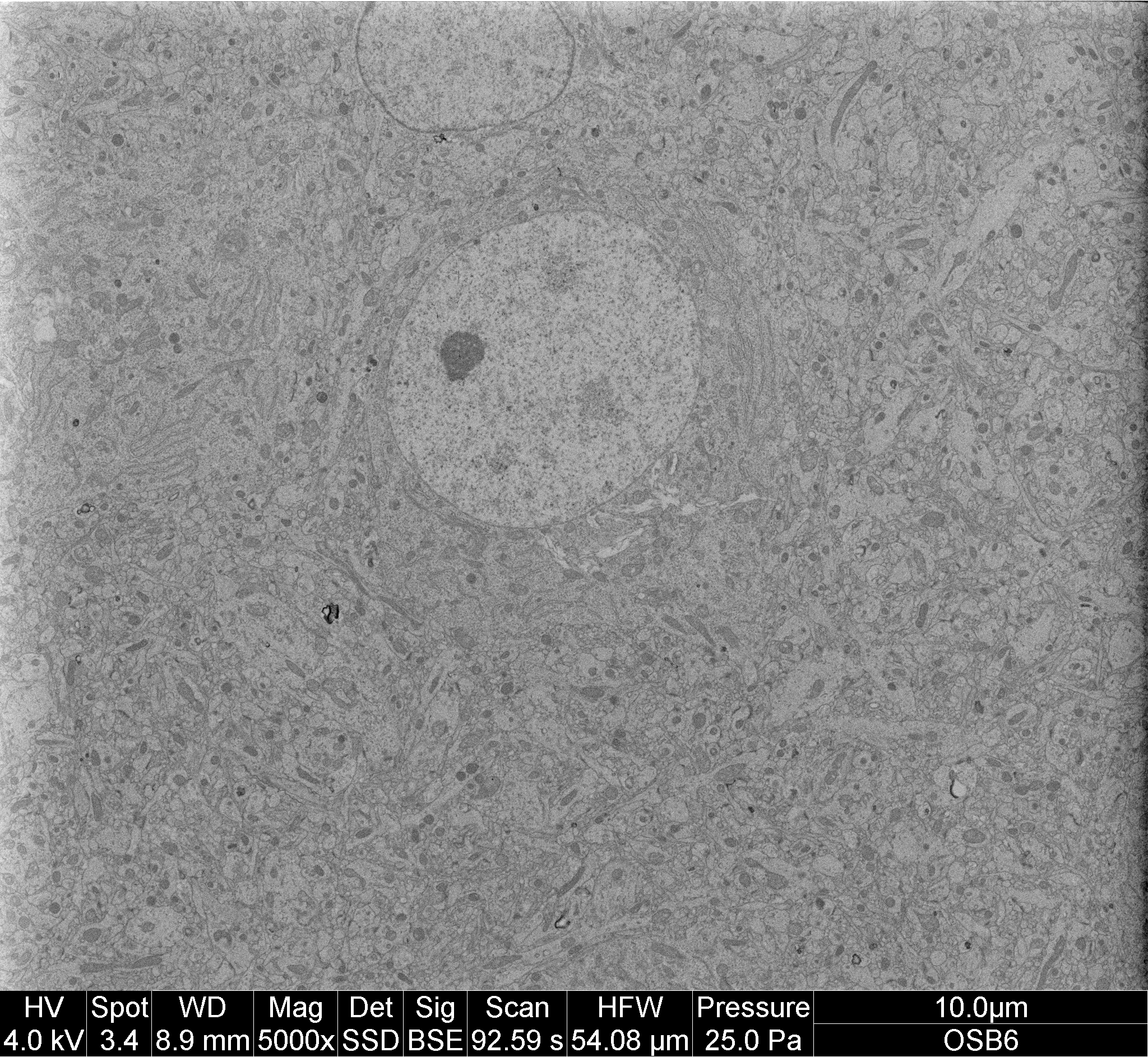

Supplement: Dataset S18 — (250.5 MB ZIP). [file pbio.0020329.sd018.zip › 040604_OS5_st1_1723.tif]

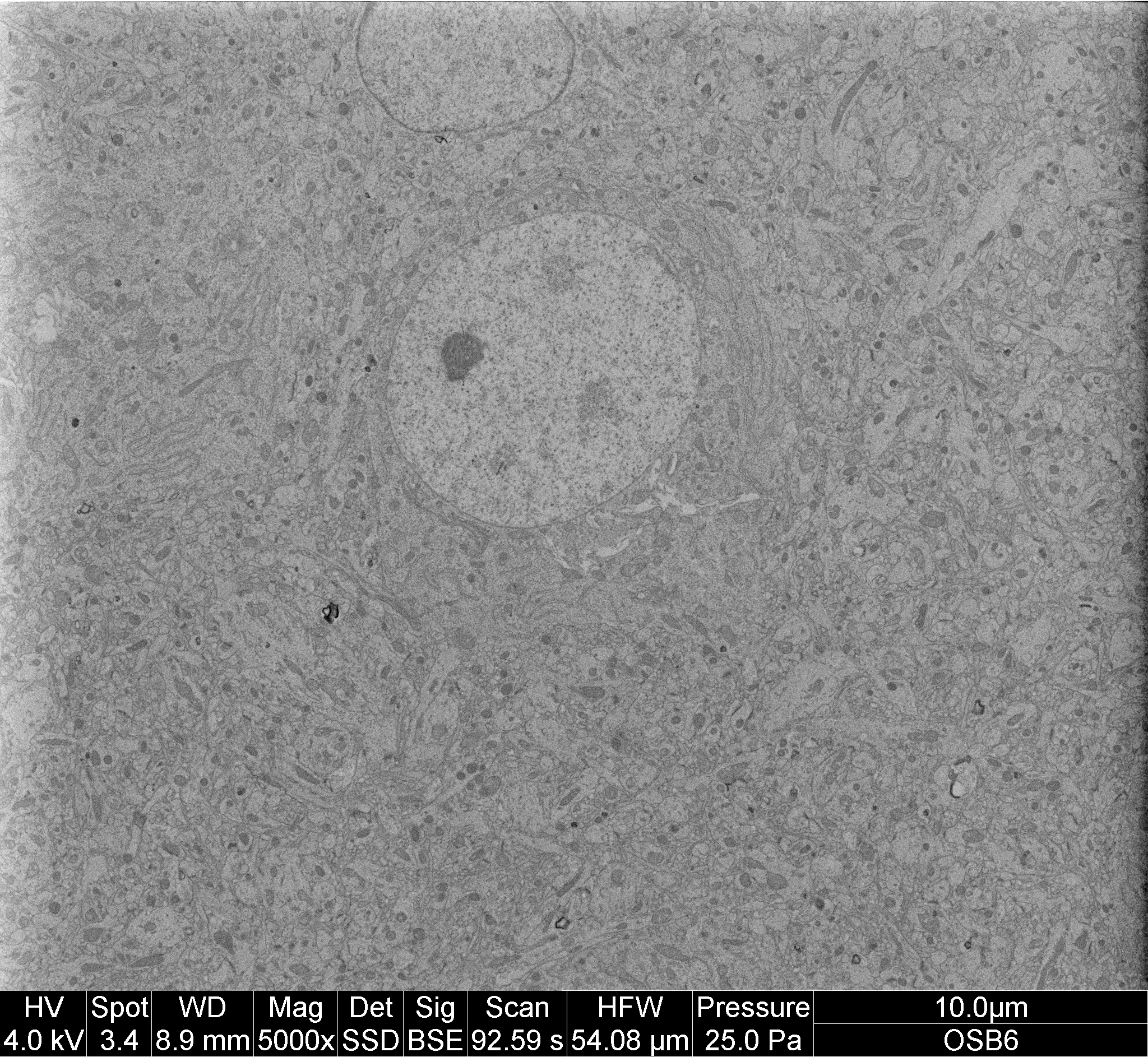

Supplement: Dataset S18 — (250.5 MB ZIP). [file pbio.0020329.sd018.zip › 040604_OS5_st1_1724.tif]

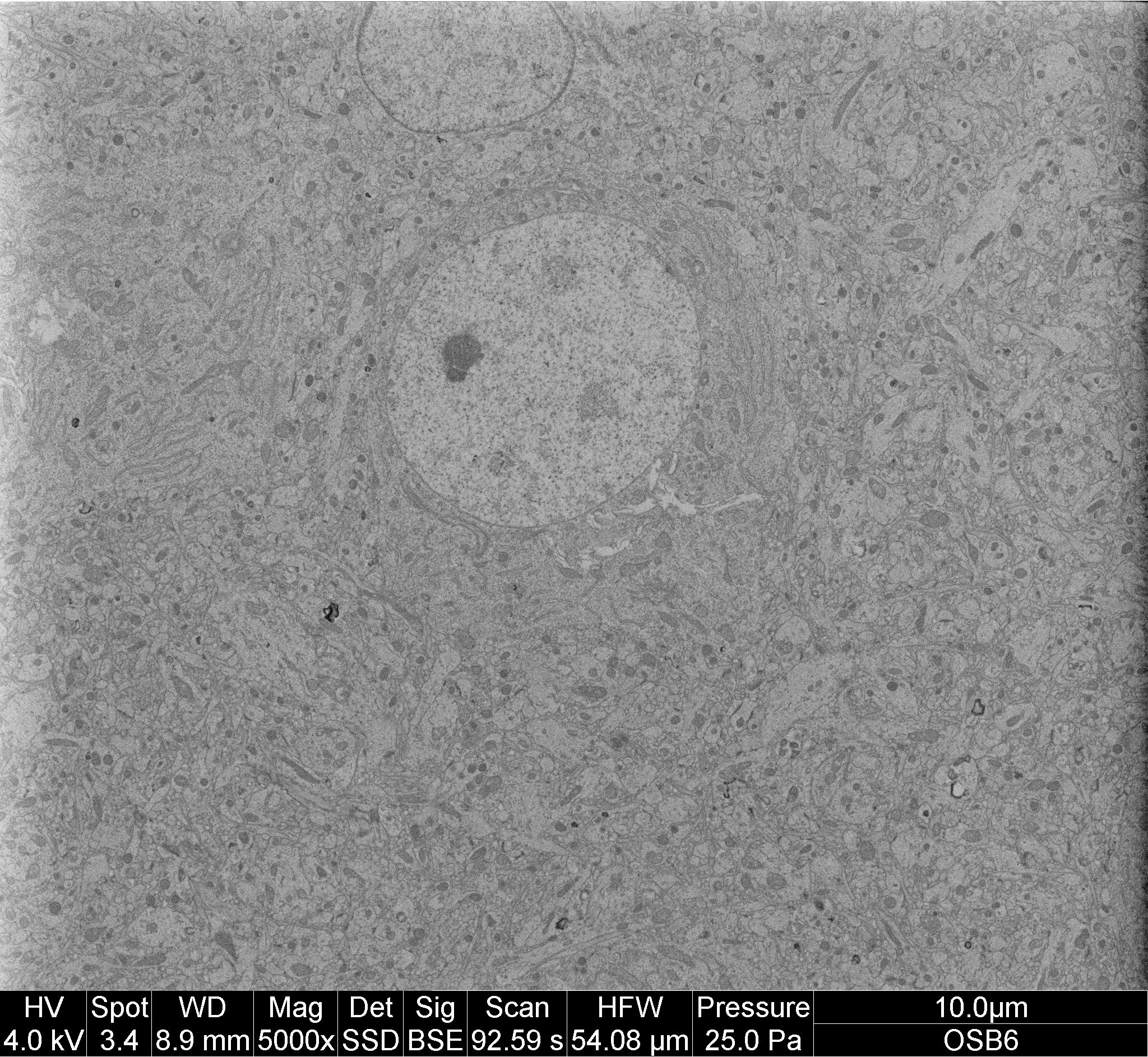

Supplement: Dataset S18 — (250.5 MB ZIP). [file pbio.0020329.sd018.zip › 040604_OS5_st1_1725.tif]

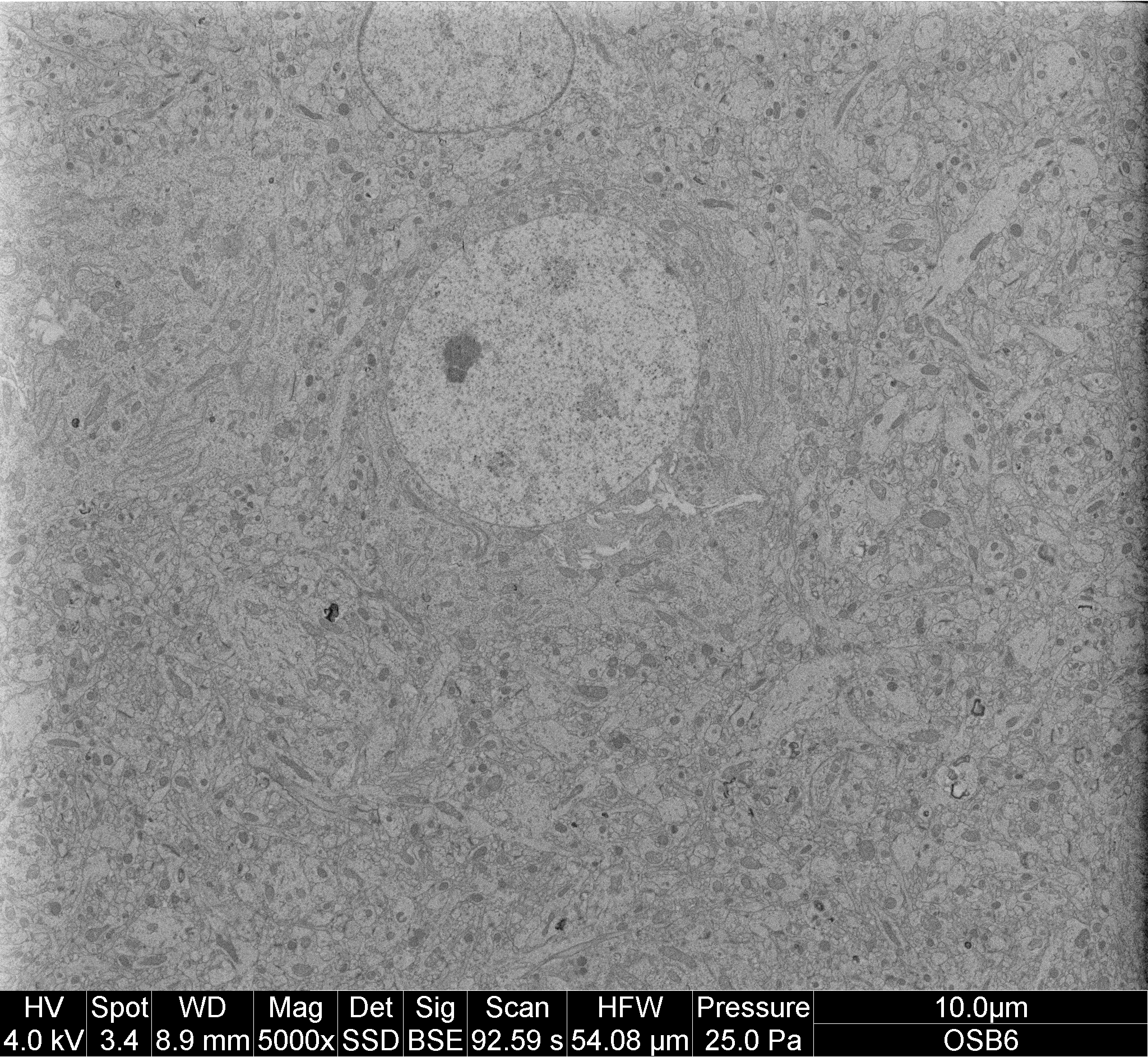

Supplement: Dataset S18 — (250.5 MB ZIP). [file pbio.0020329.sd018.zip › 040604_OS5_st1_1726.tif]

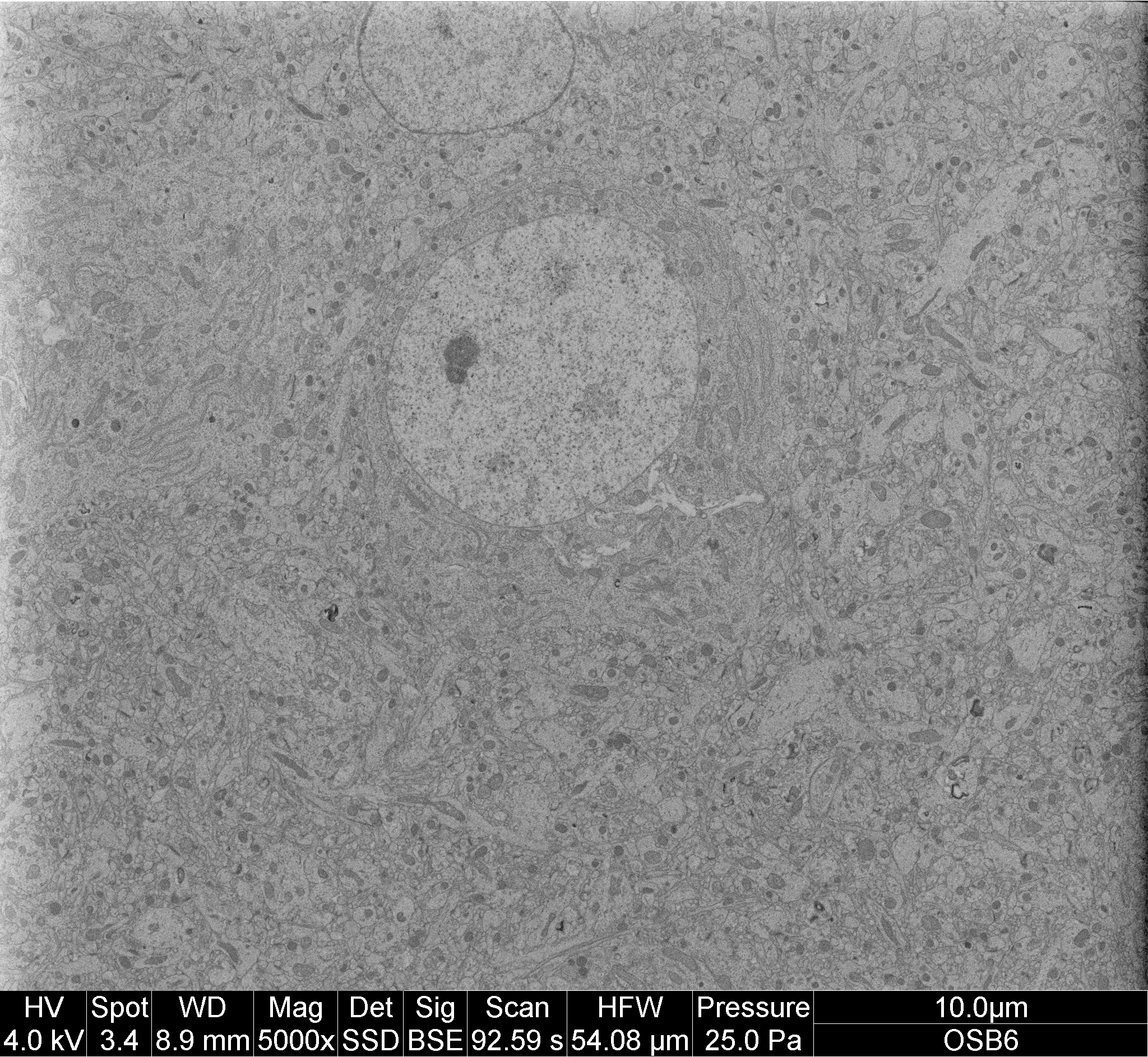

Supplement: Dataset S18 — (250.5 MB ZIP). [file pbio.0020329.sd018.zip › 040604_OS5_st1_1727.tif]

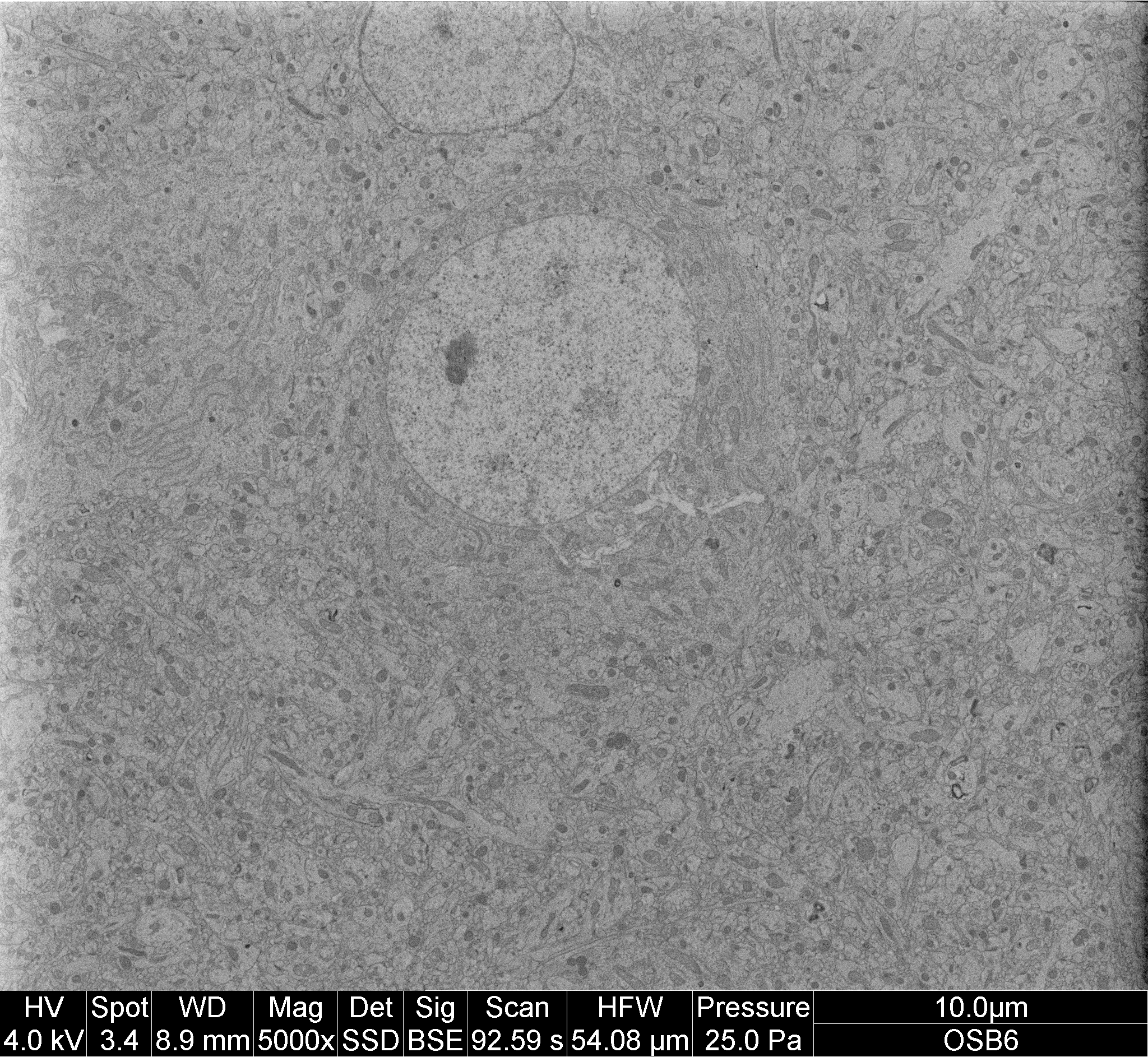

Supplement: Dataset S18 — (250.5 MB ZIP). [file pbio.0020329.sd018.zip › 040604_OS5_st1_1728.tif]

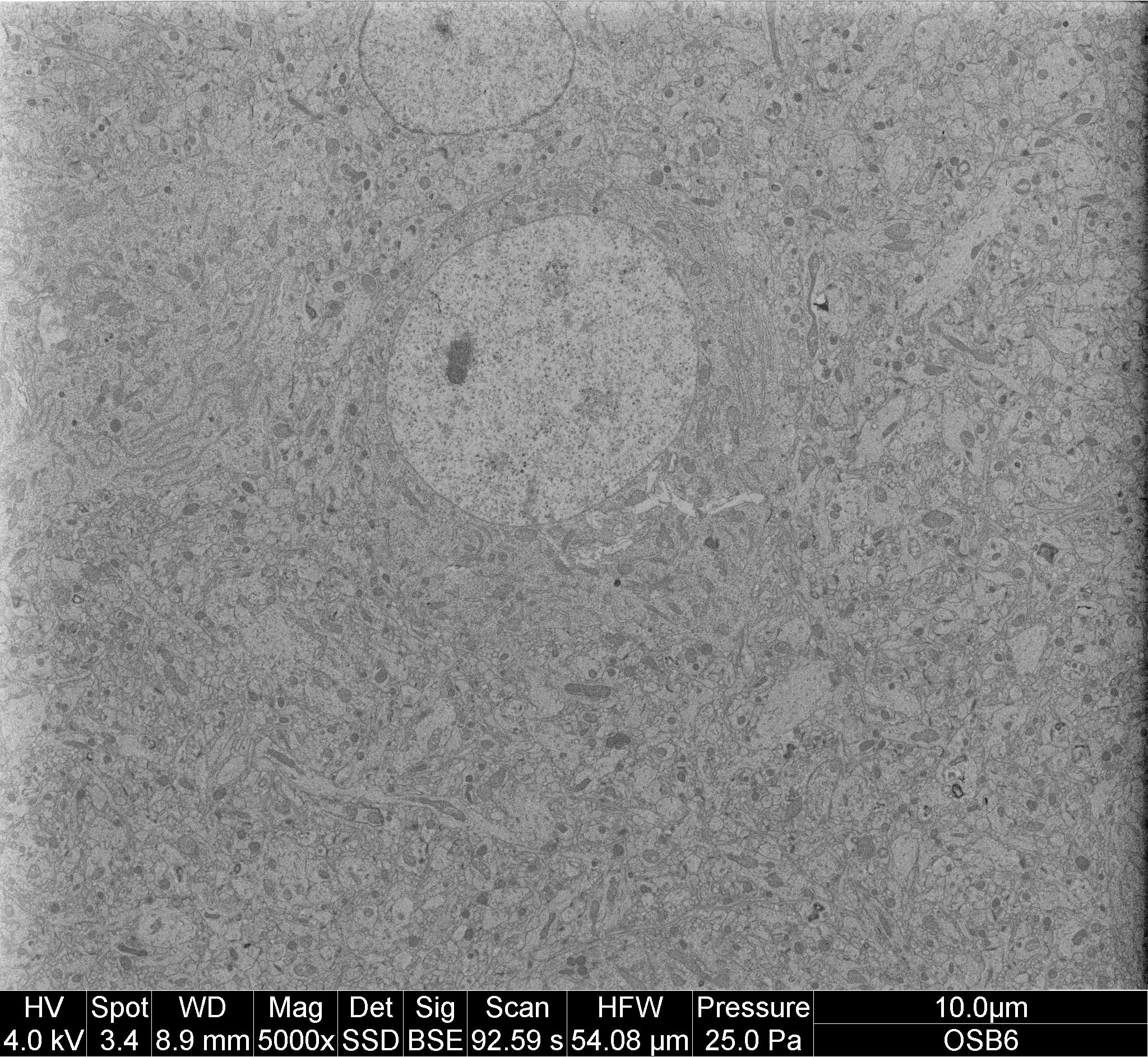

Supplement: Dataset S18 — (250.5 MB ZIP). [file pbio.0020329.sd018.zip › 040604_OS5_st1_1729.tif]

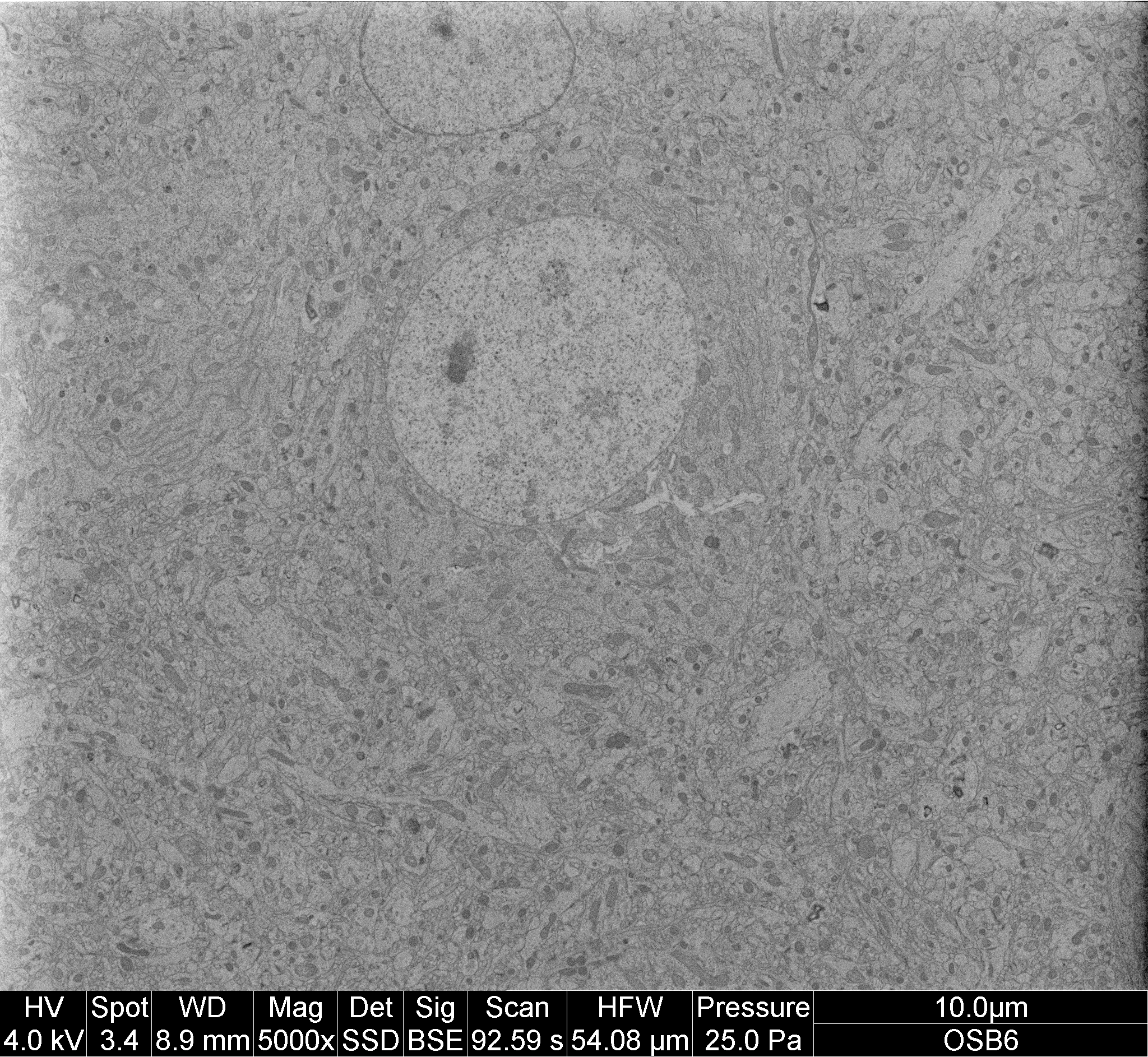

Supplement: Dataset S18 — (250.5 MB ZIP). [file pbio.0020329.sd018.zip › 040604_OS5_st1_1730.tif]

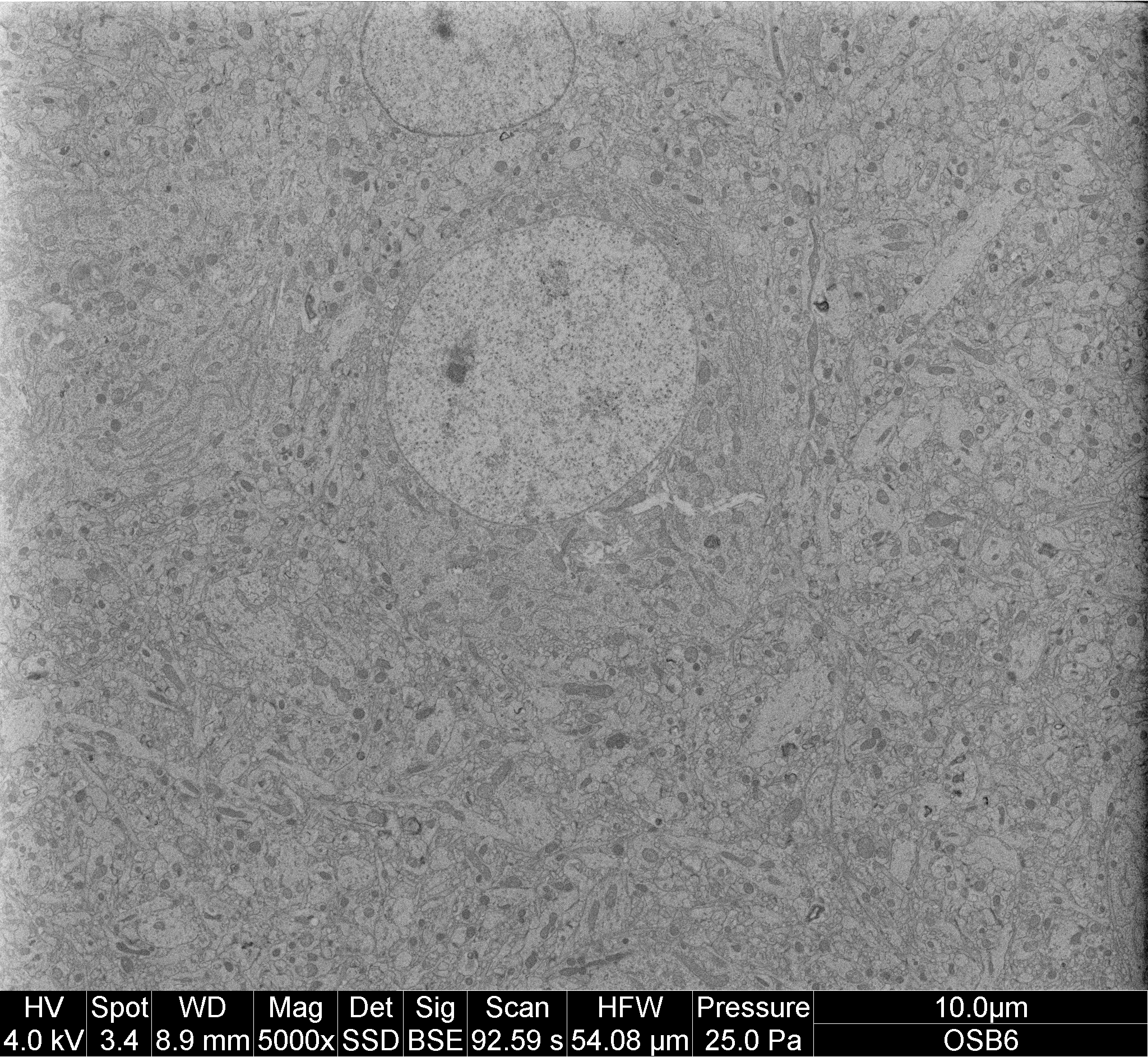

Supplement: Dataset S18 — (250.5 MB ZIP). [file pbio.0020329.sd018.zip › 040604_OS5_st1_1731.tif]

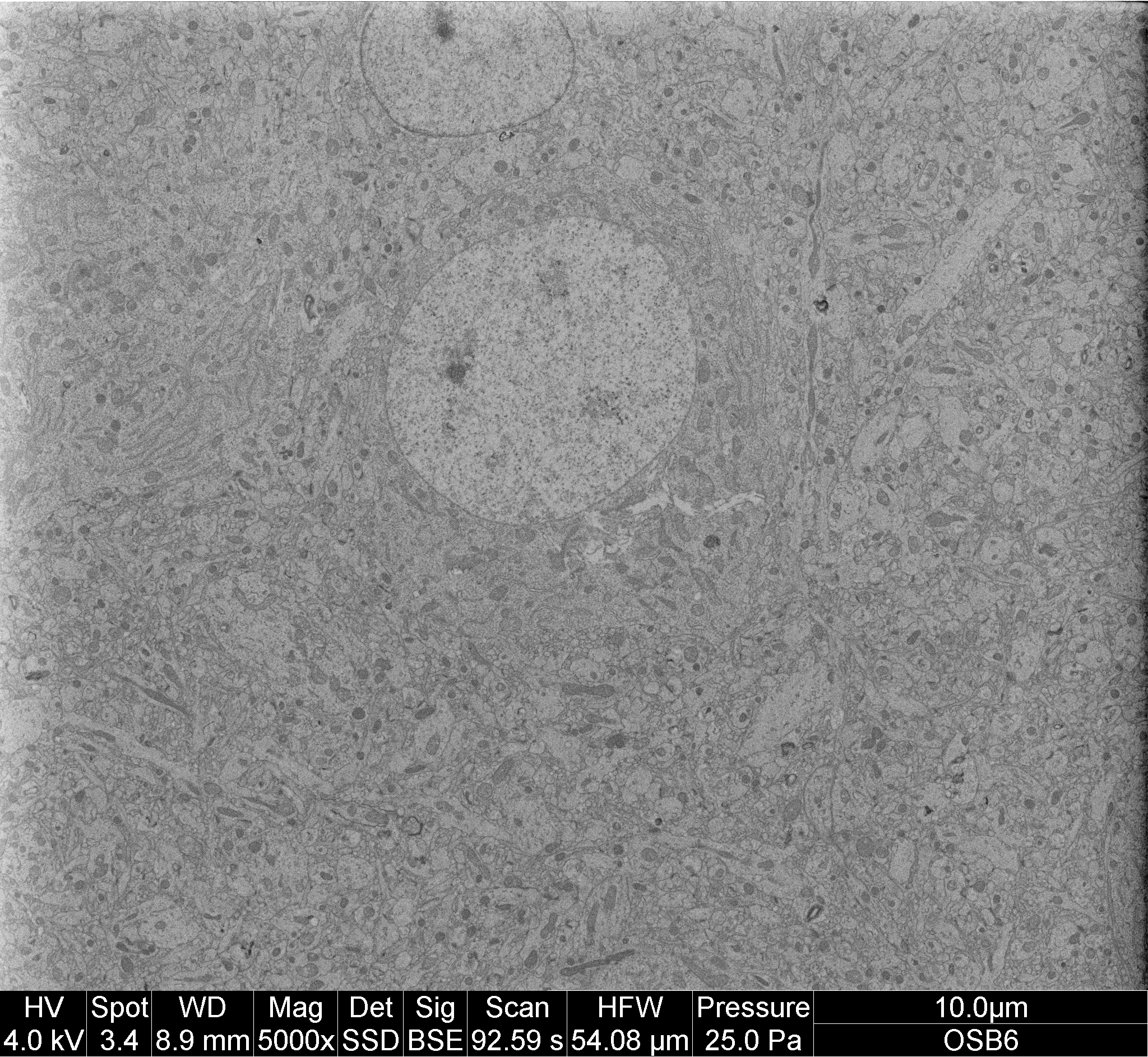

Supplement: Dataset S18 — (250.5 MB ZIP). [file pbio.0020329.sd018.zip › 040604_OS5_st1_1732.tif]

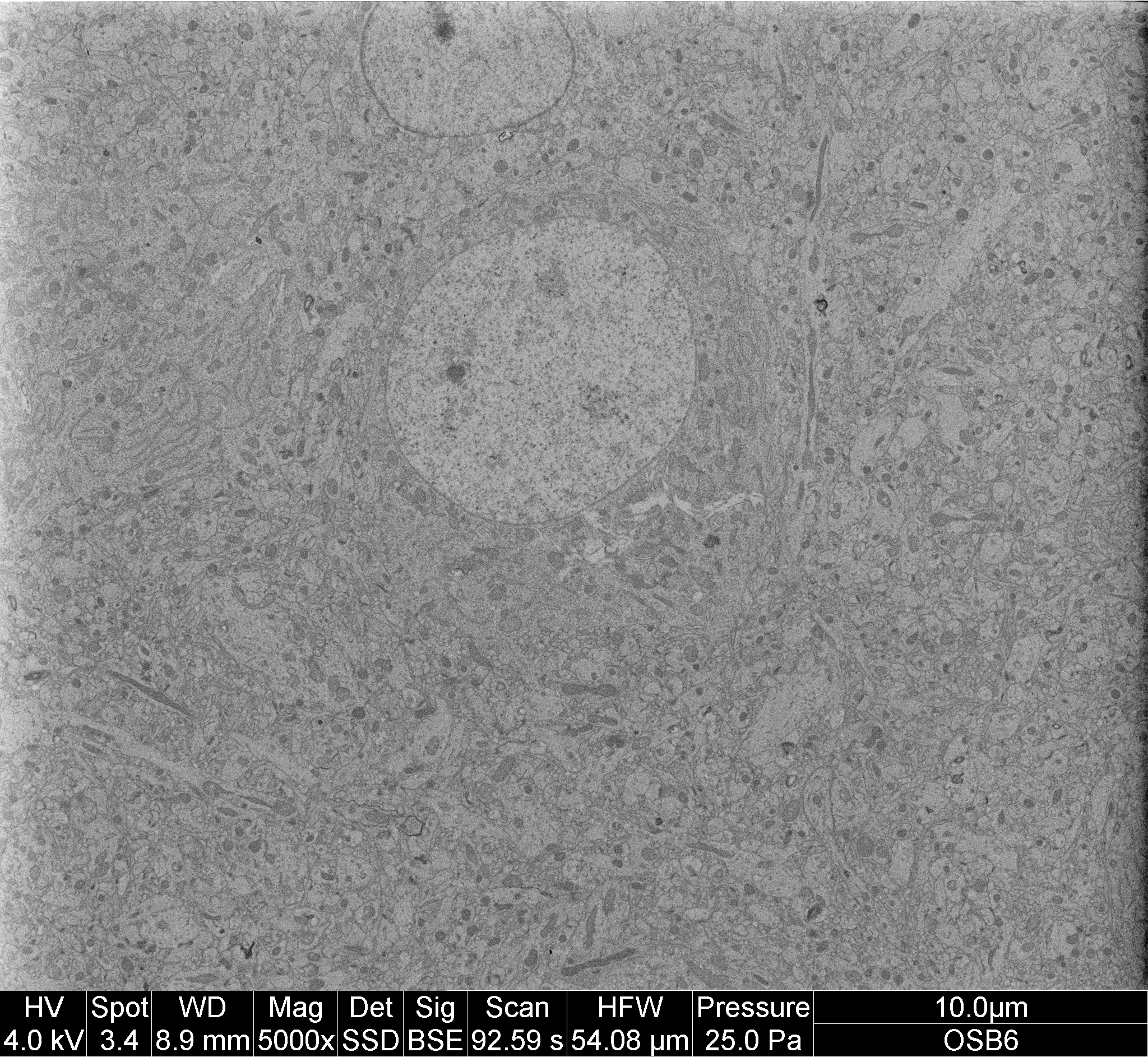

Supplement: Dataset S18 — (250.5 MB ZIP). [file pbio.0020329.sd018.zip › 040604_OS5_st1_1733.tif]

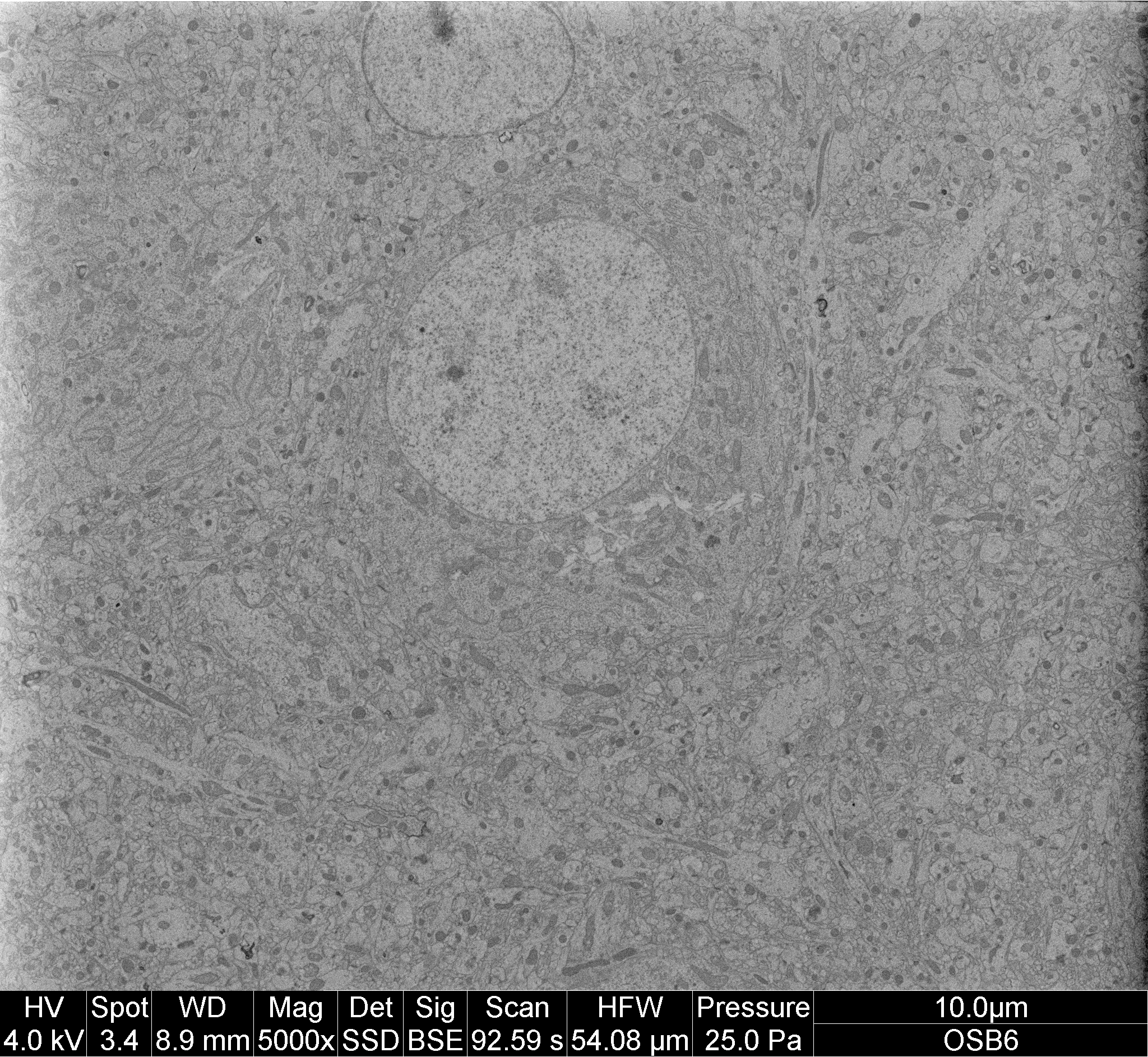

Supplement: Dataset S18 — (250.5 MB ZIP). [file pbio.0020329.sd018.zip › 040604_OS5_st1_1734.tif]

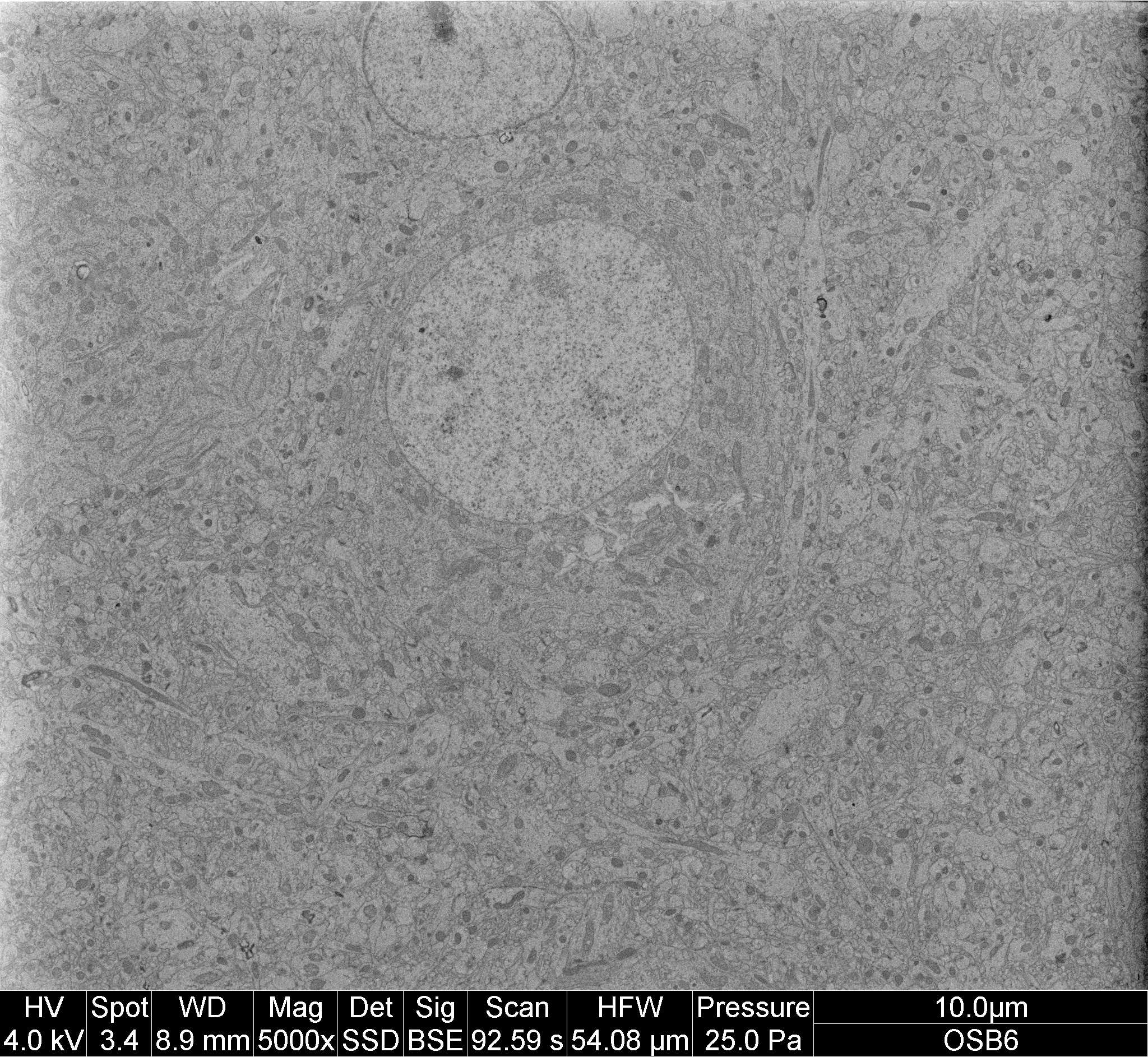

Supplement: Dataset S18 — (250.5 MB ZIP). [file pbio.0020329.sd018.zip › 040604_OS5_st1_1735.tif]

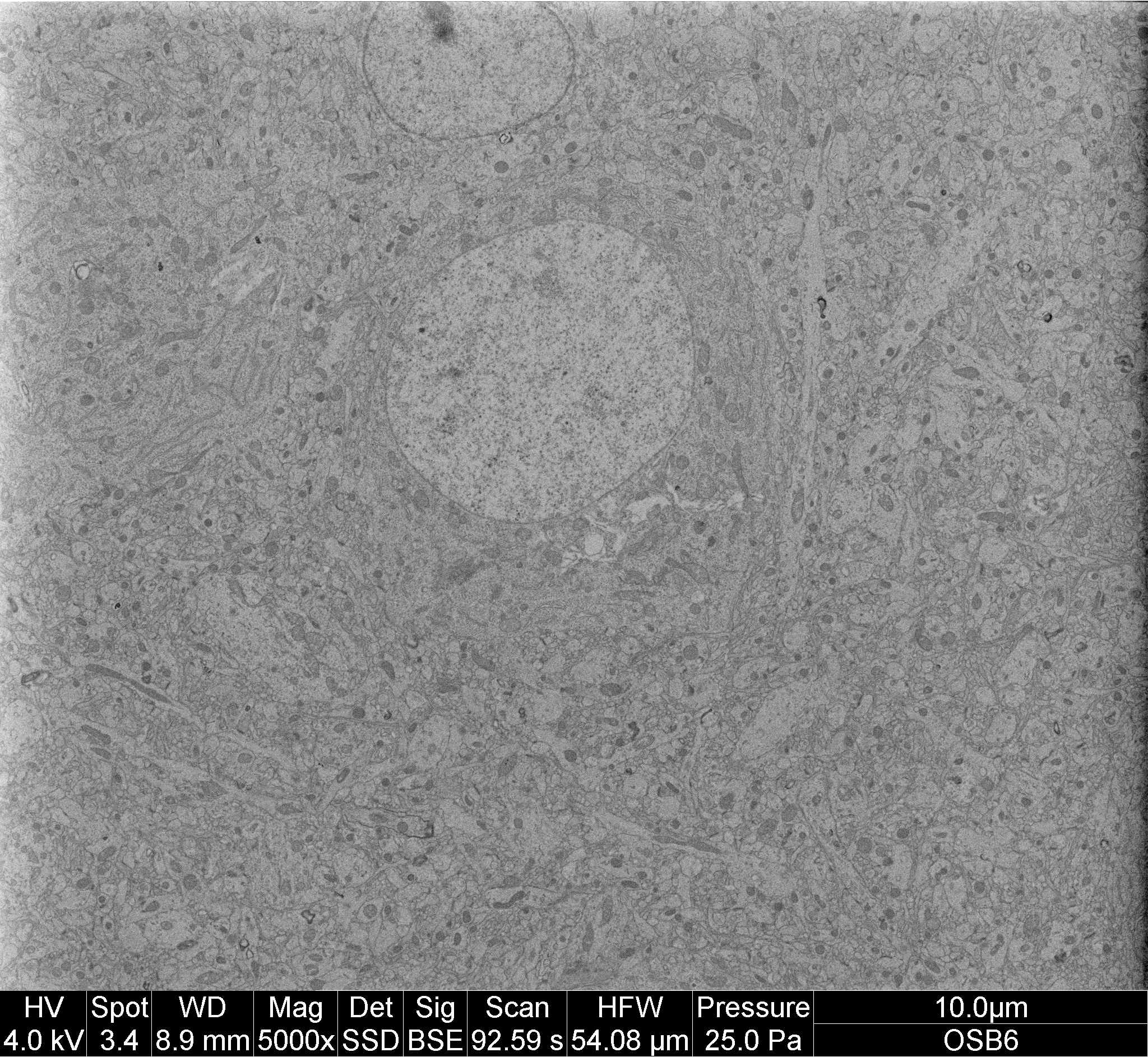

Supplement: Dataset S18 — (250.5 MB ZIP). [file pbio.0020329.sd018.zip › 040604_OS5_st1_1736.tif]

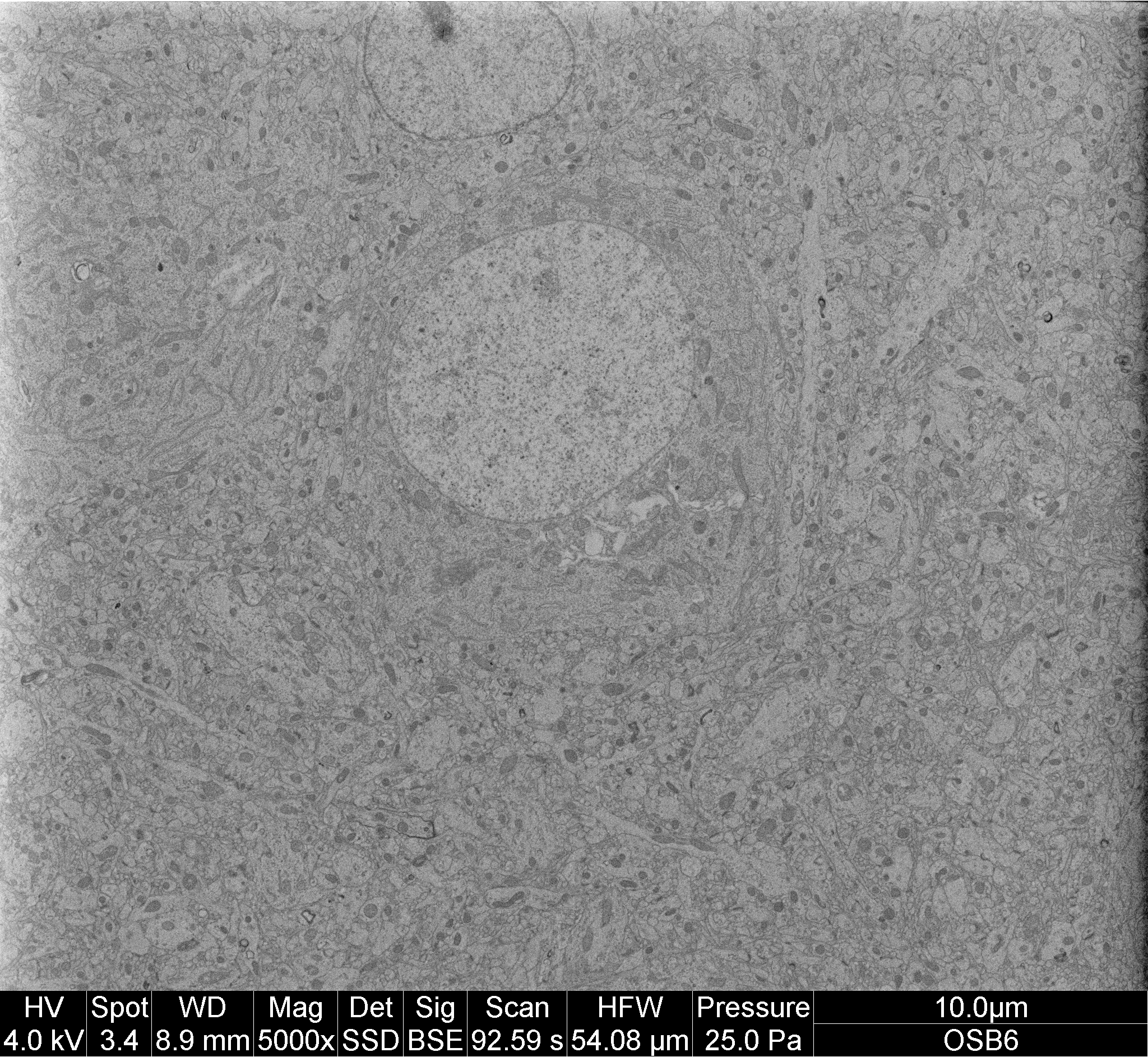

Supplement: Dataset S18 — (250.5 MB ZIP). [file pbio.0020329.sd018.zip › 040604_OS5_st1_1737.tif]

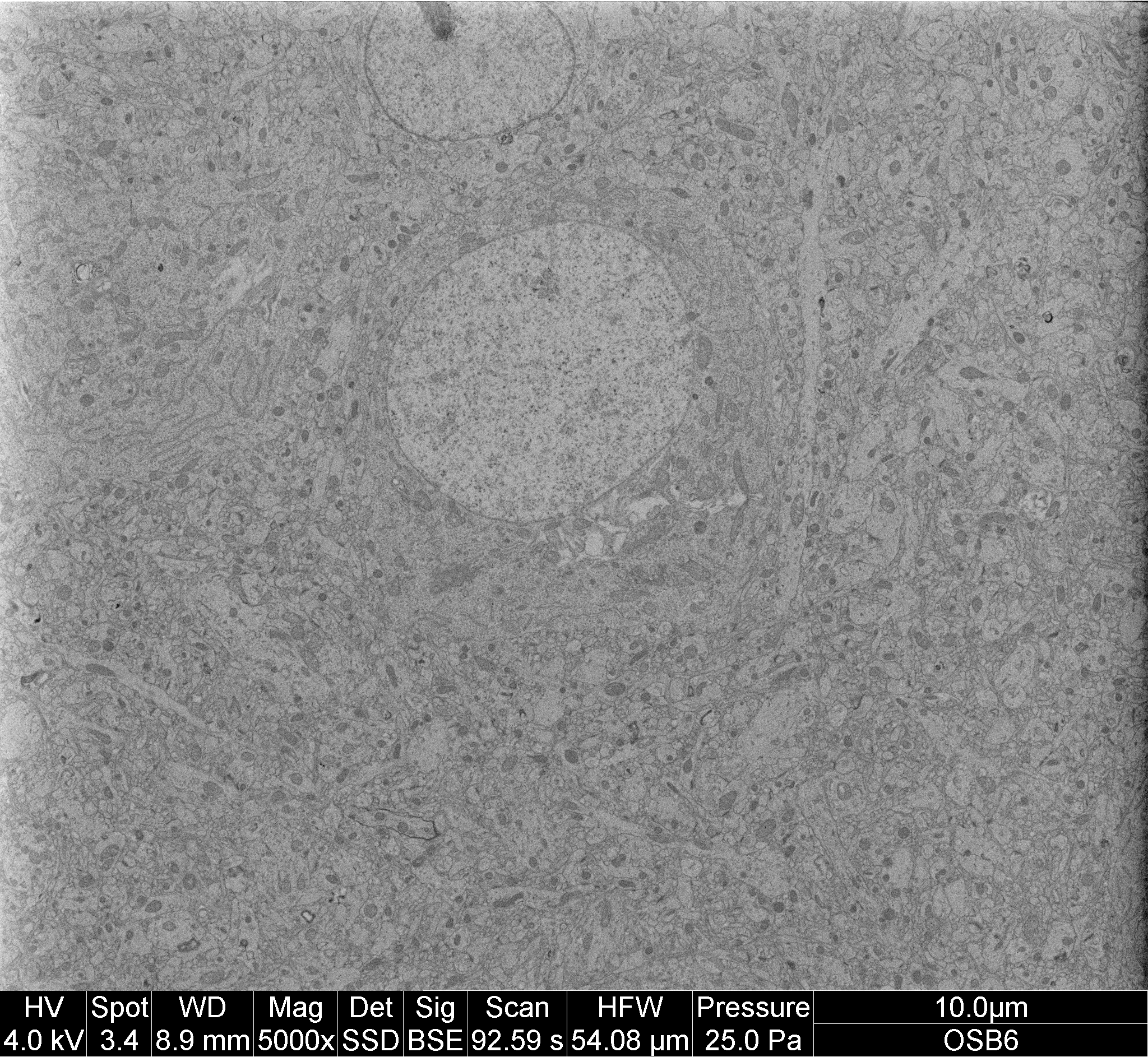

Supplement: Dataset S18 — (250.5 MB ZIP). [file pbio.0020329.sd018.zip › 040604_OS5_st1_1738.tif]

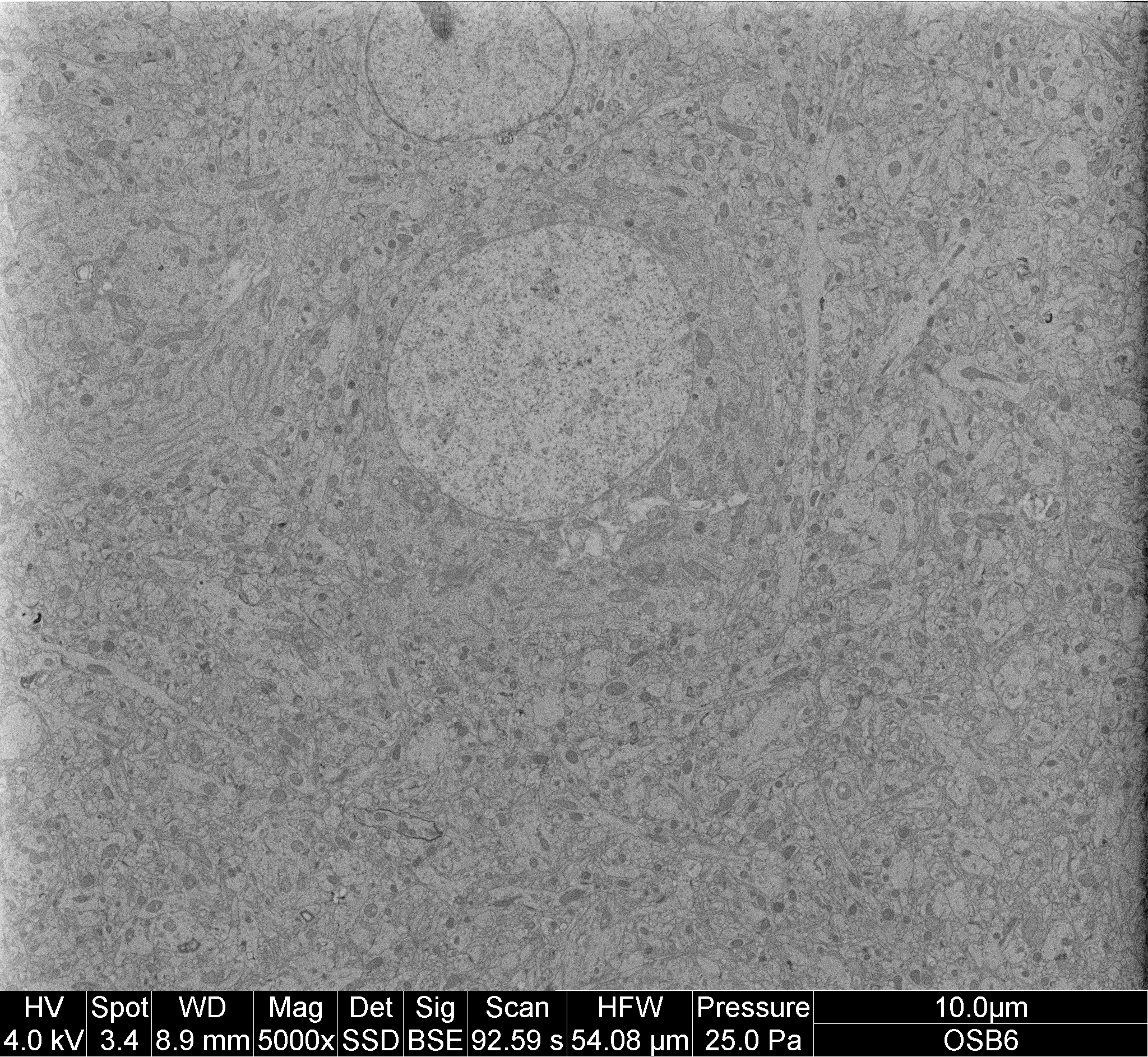

Supplement: Dataset S18 — (250.5 MB ZIP). [file pbio.0020329.sd018.zip › 040604_OS5_st1_1739.tif]

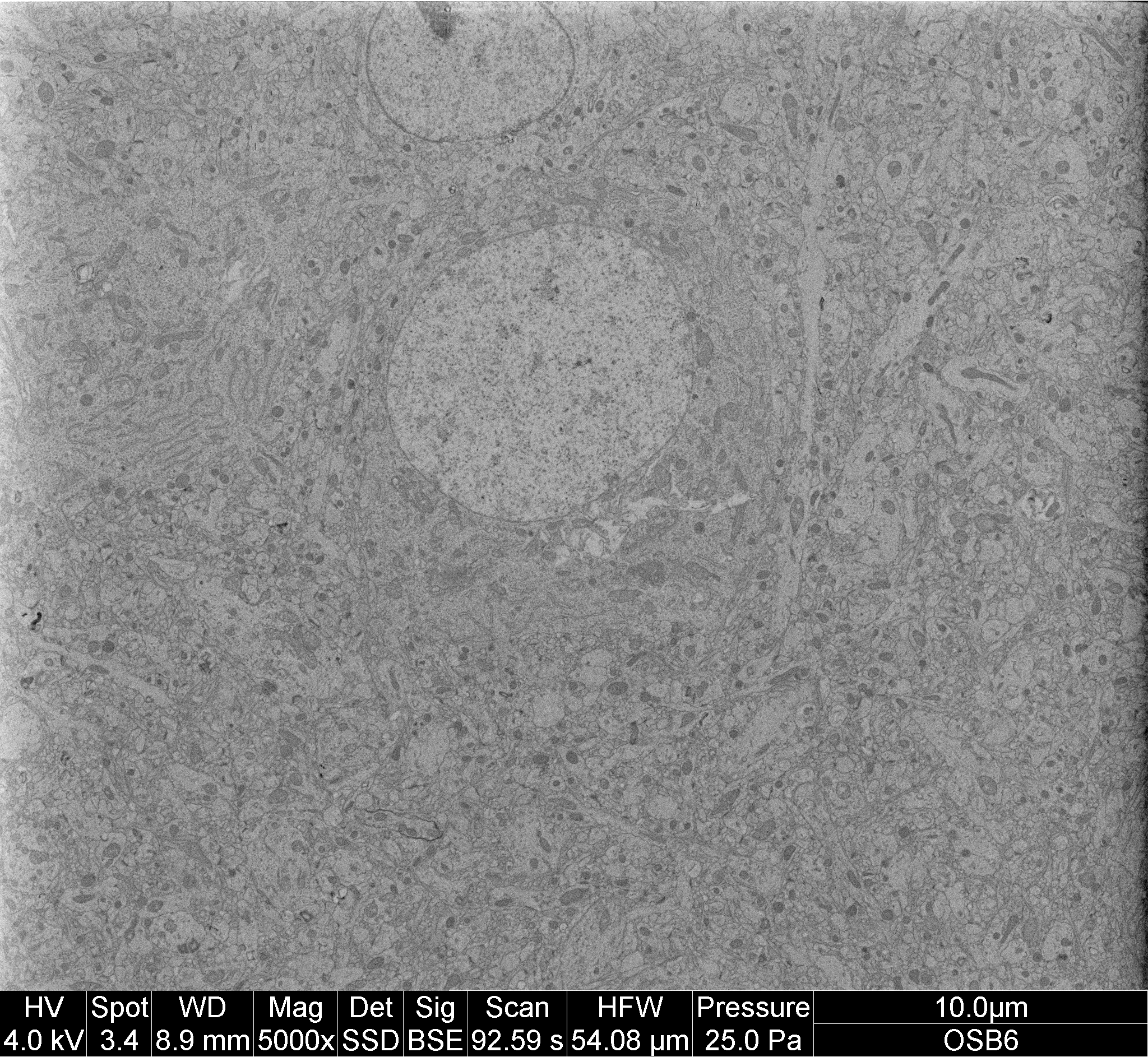

Supplement: Dataset S18 — (250.5 MB ZIP). [file pbio.0020329.sd018.zip › 040604_OS5_st1_1740.tif]

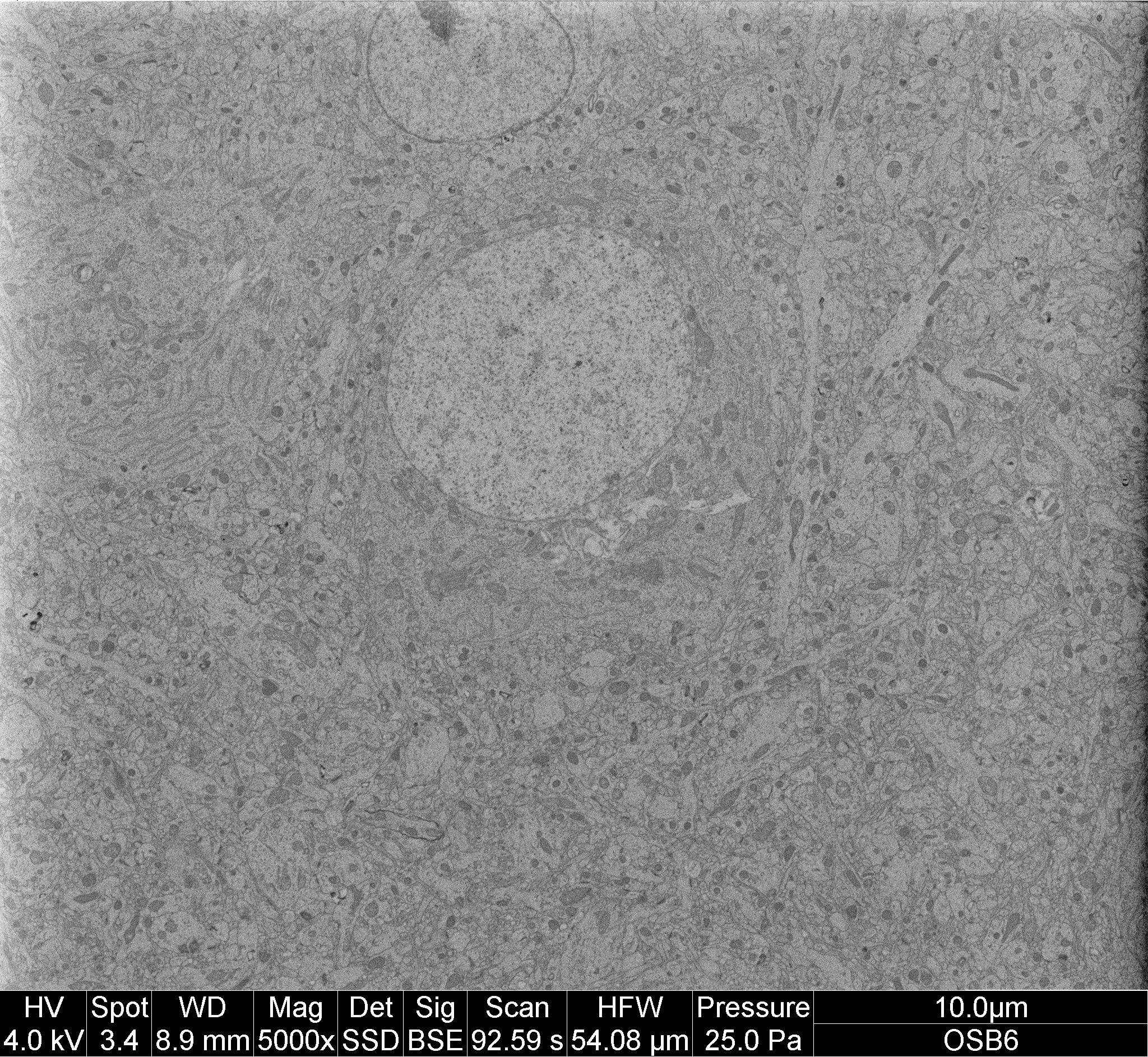

Supplement: Dataset S18 — (250.5 MB ZIP). [file pbio.0020329.sd018.zip › 040604_OS5_st1_1741.tif]

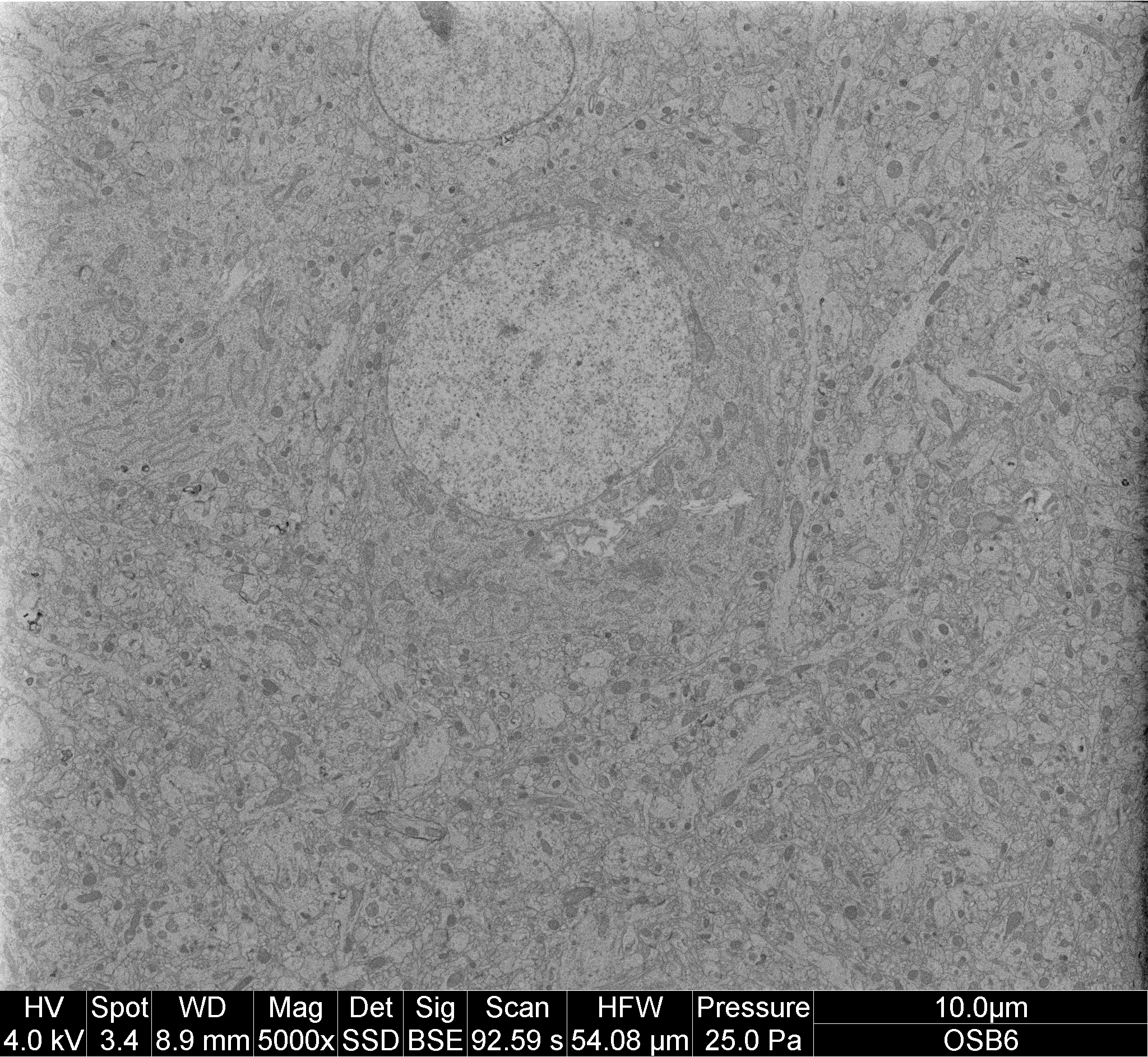

Supplement: Dataset S18 — (250.5 MB ZIP). [file pbio.0020329.sd018.zip › 040604_OS5_st1_1742.tif]

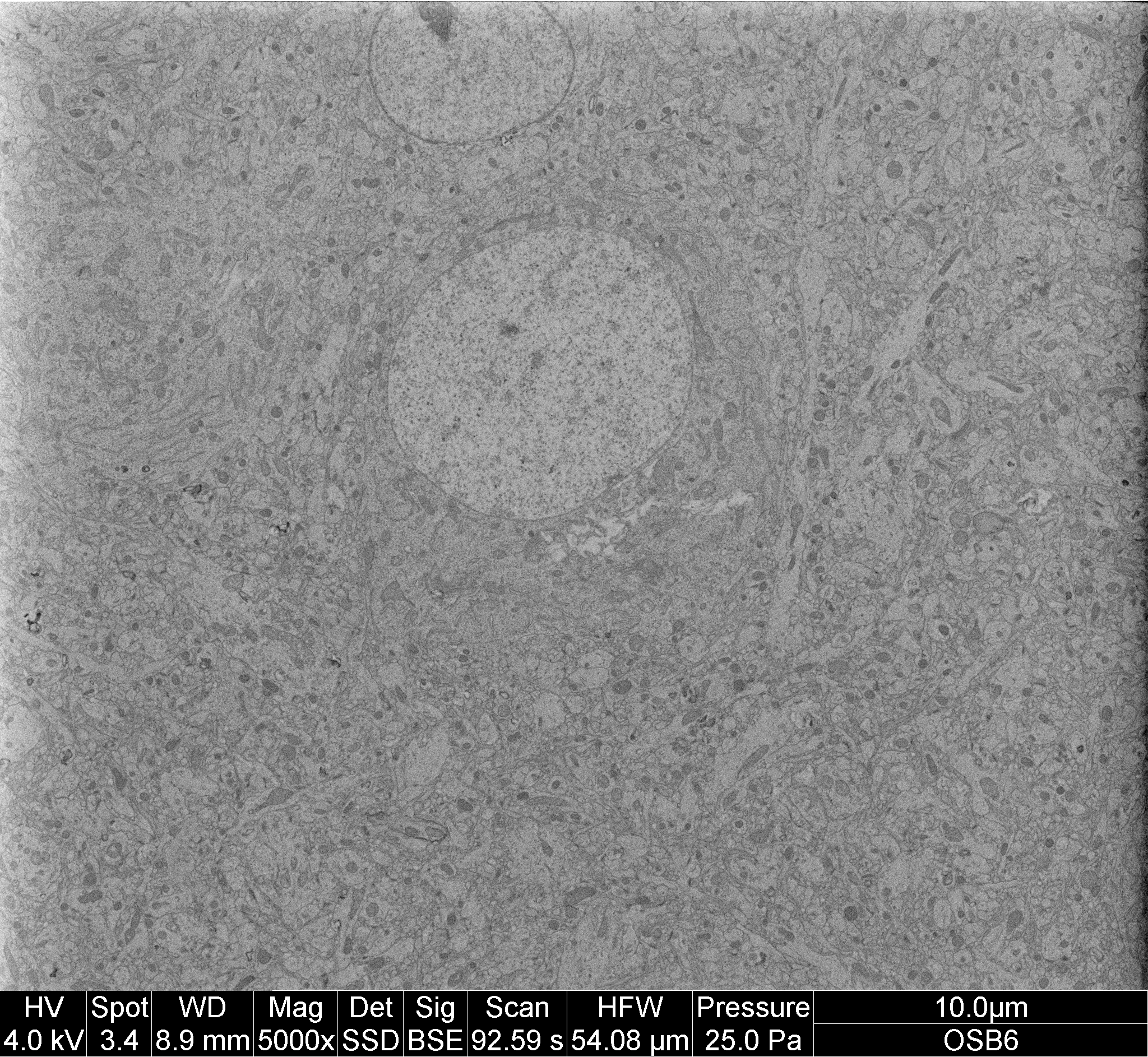

Supplement: Dataset S18 — (250.5 MB ZIP). [file pbio.0020329.sd018.zip › 040604_OS5_st1_1743.tif]

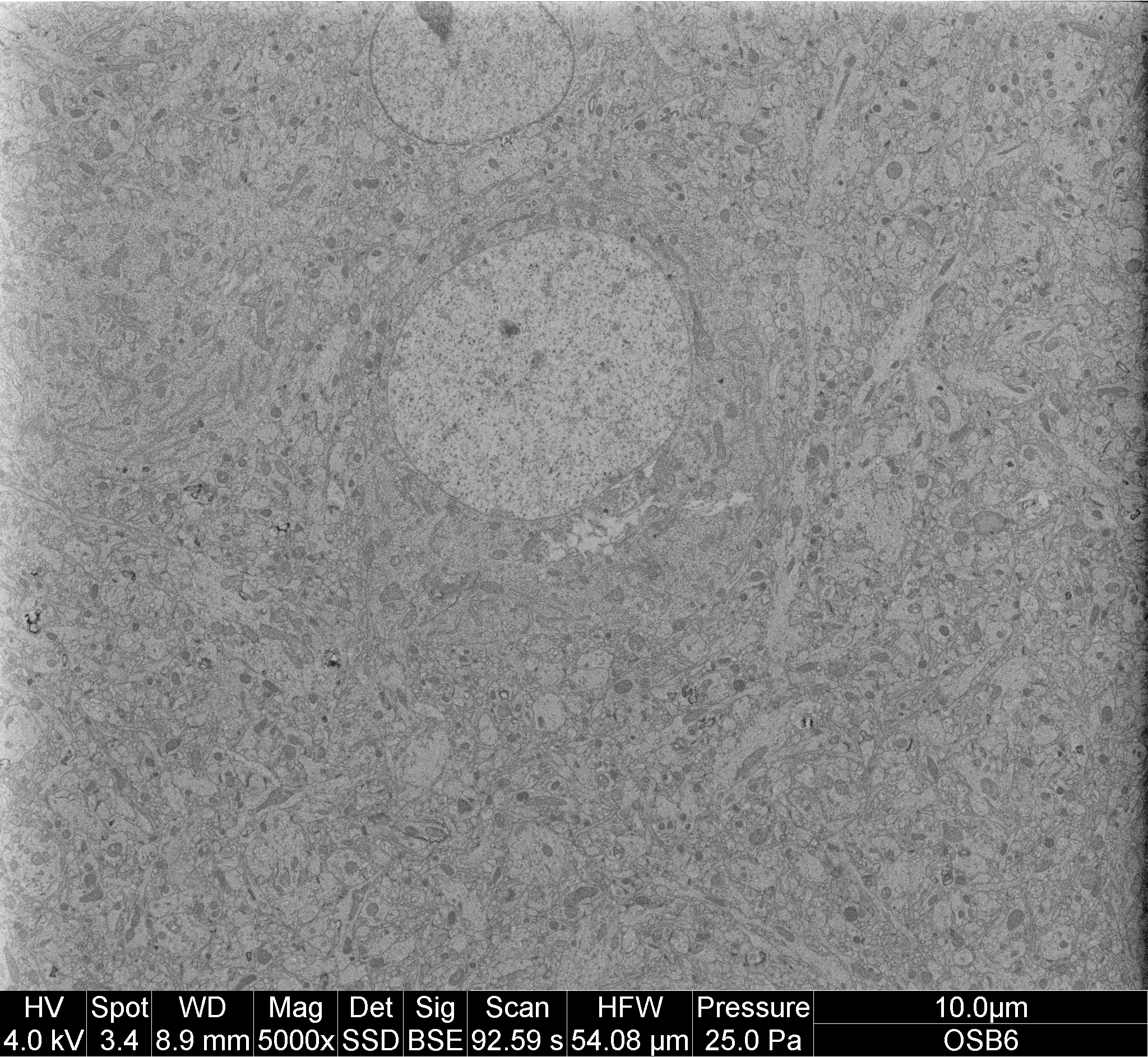

Supplement: Dataset S18 — (250.5 MB ZIP). [file pbio.0020329.sd018.zip › 040604_OS5_st1_1744.tif]

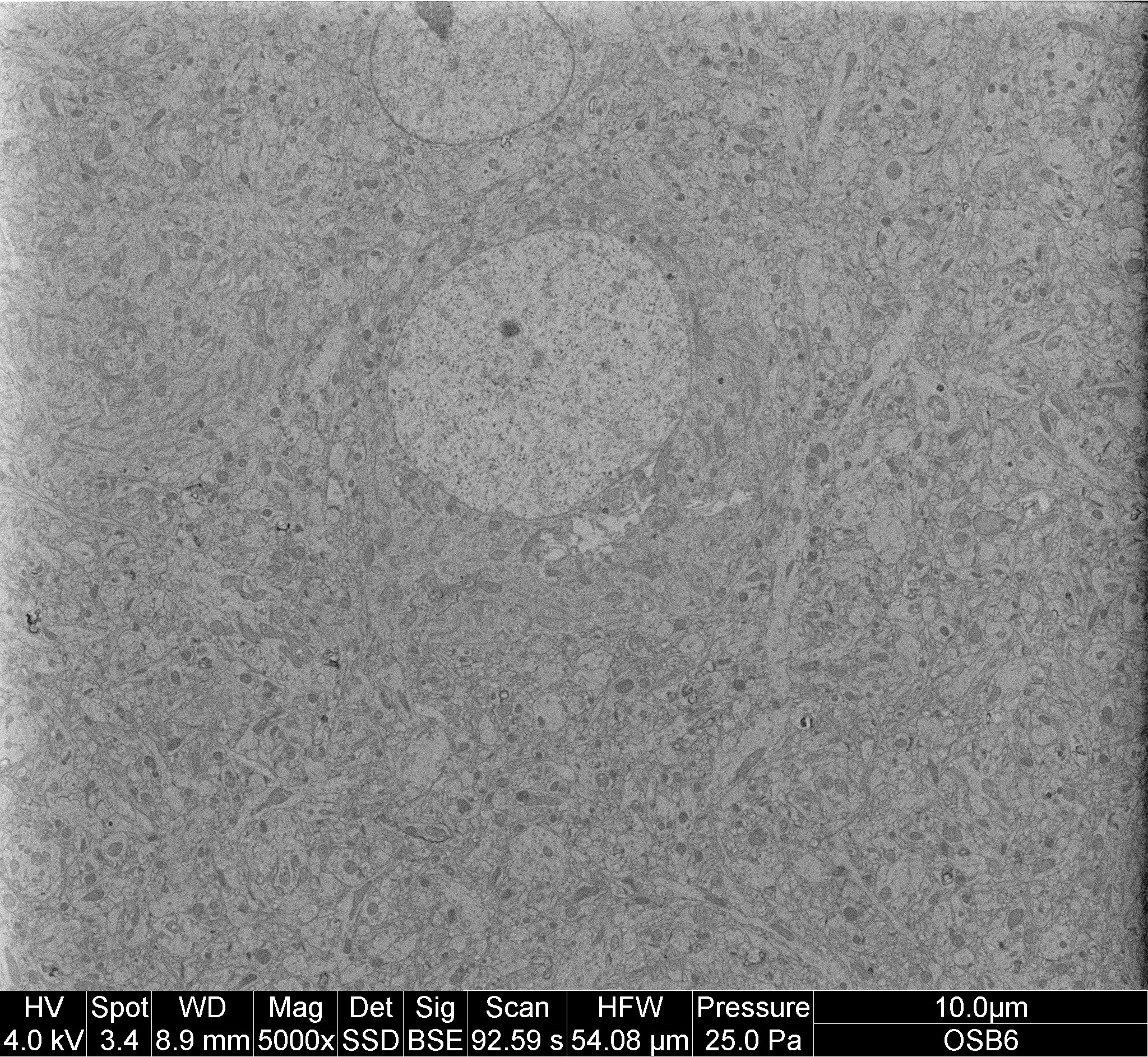

Supplement: Dataset S18 — (250.5 MB ZIP). [file pbio.0020329.sd018.zip › 040604_OS5_st1_1745.tif]

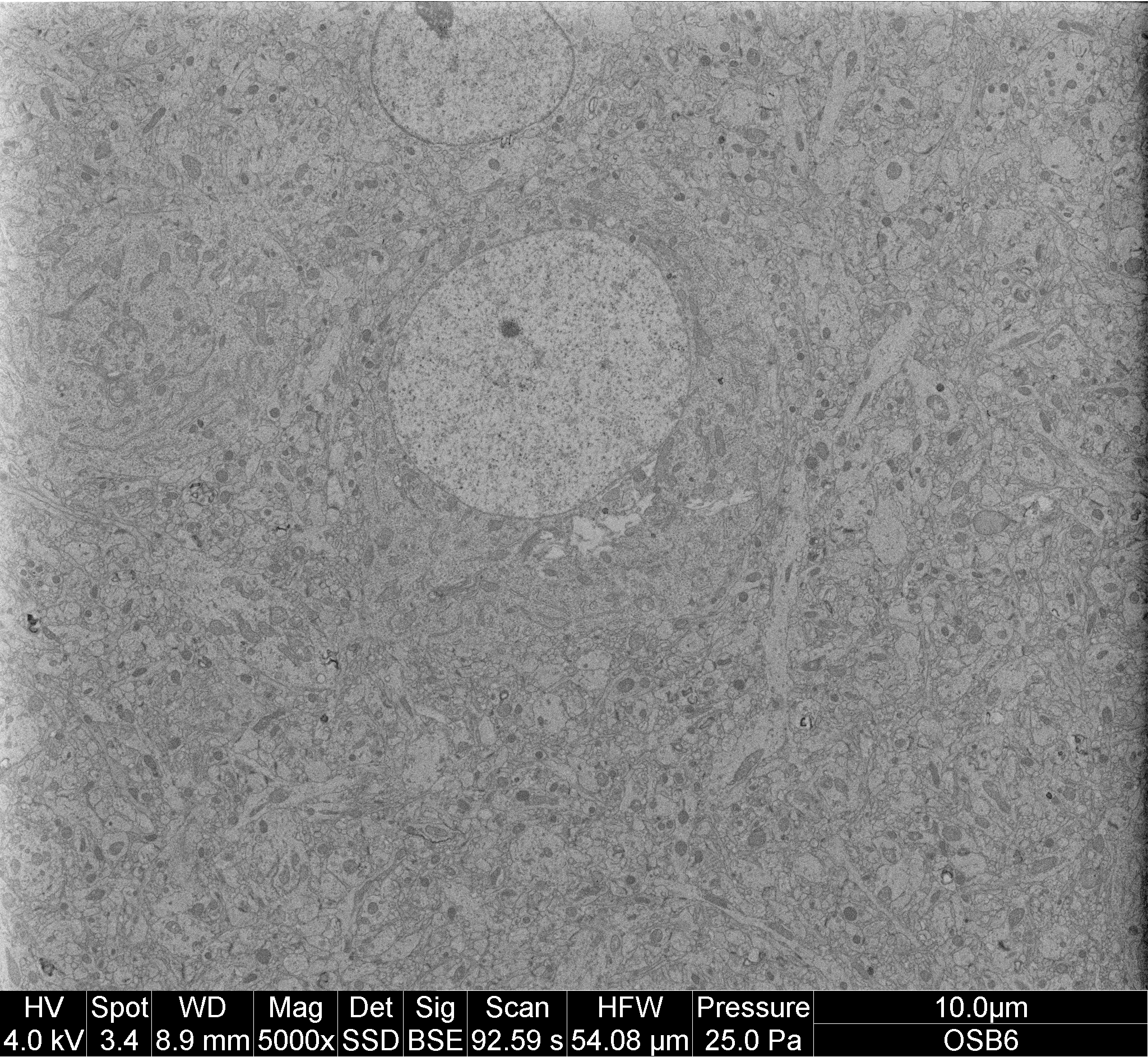

Supplement: Dataset S18 — (250.5 MB ZIP). [file pbio.0020329.sd018.zip › 040604_OS5_st1_1746.tif]

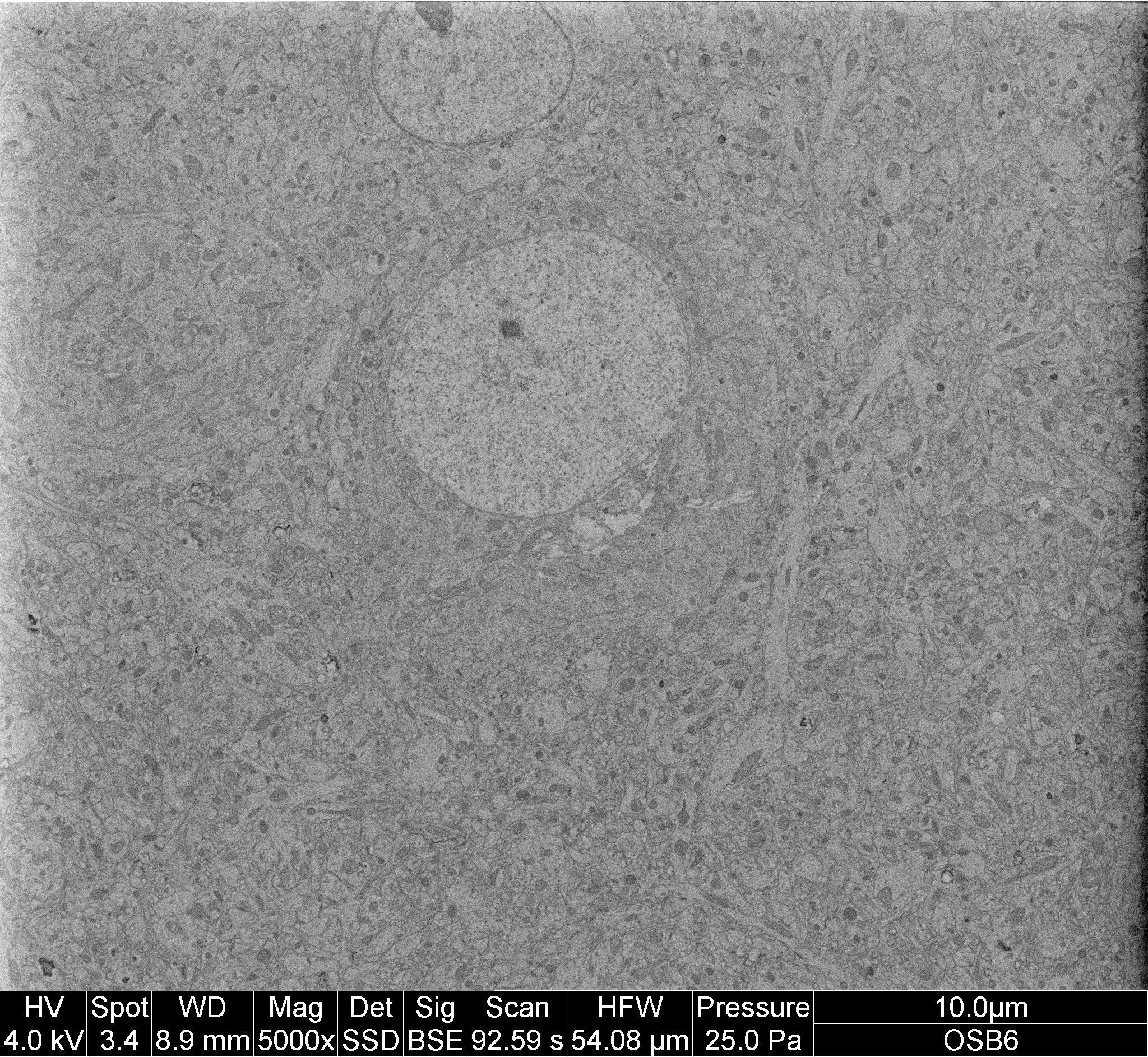

Supplement: Dataset S18 — (250.5 MB ZIP). [file pbio.0020329.sd018.zip › 040604_OS5_st1_1747.tif]

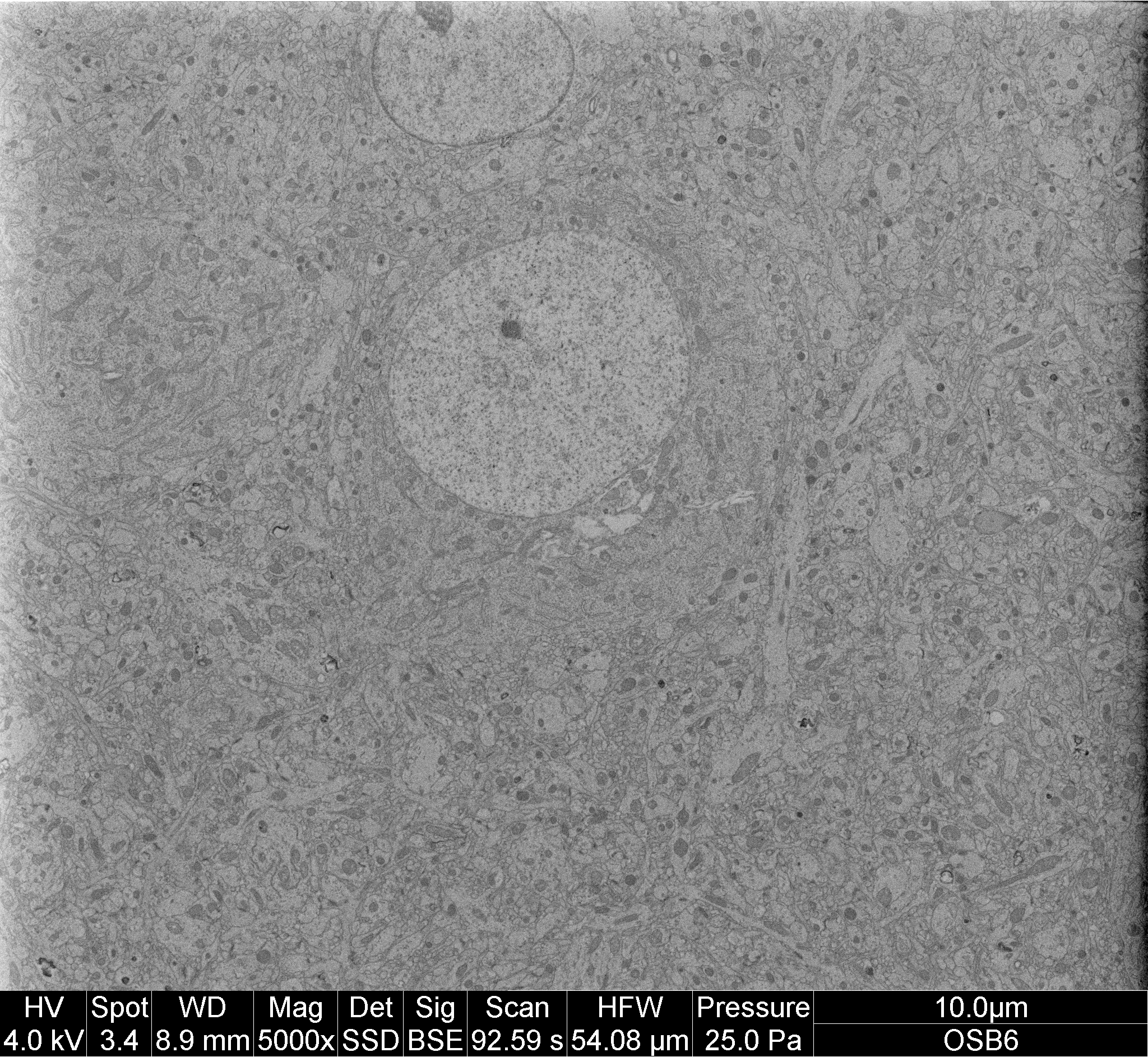

Supplement: Dataset S18 — (250.5 MB ZIP). [file pbio.0020329.sd018.zip › 040604_OS5_st1_1748.tif]

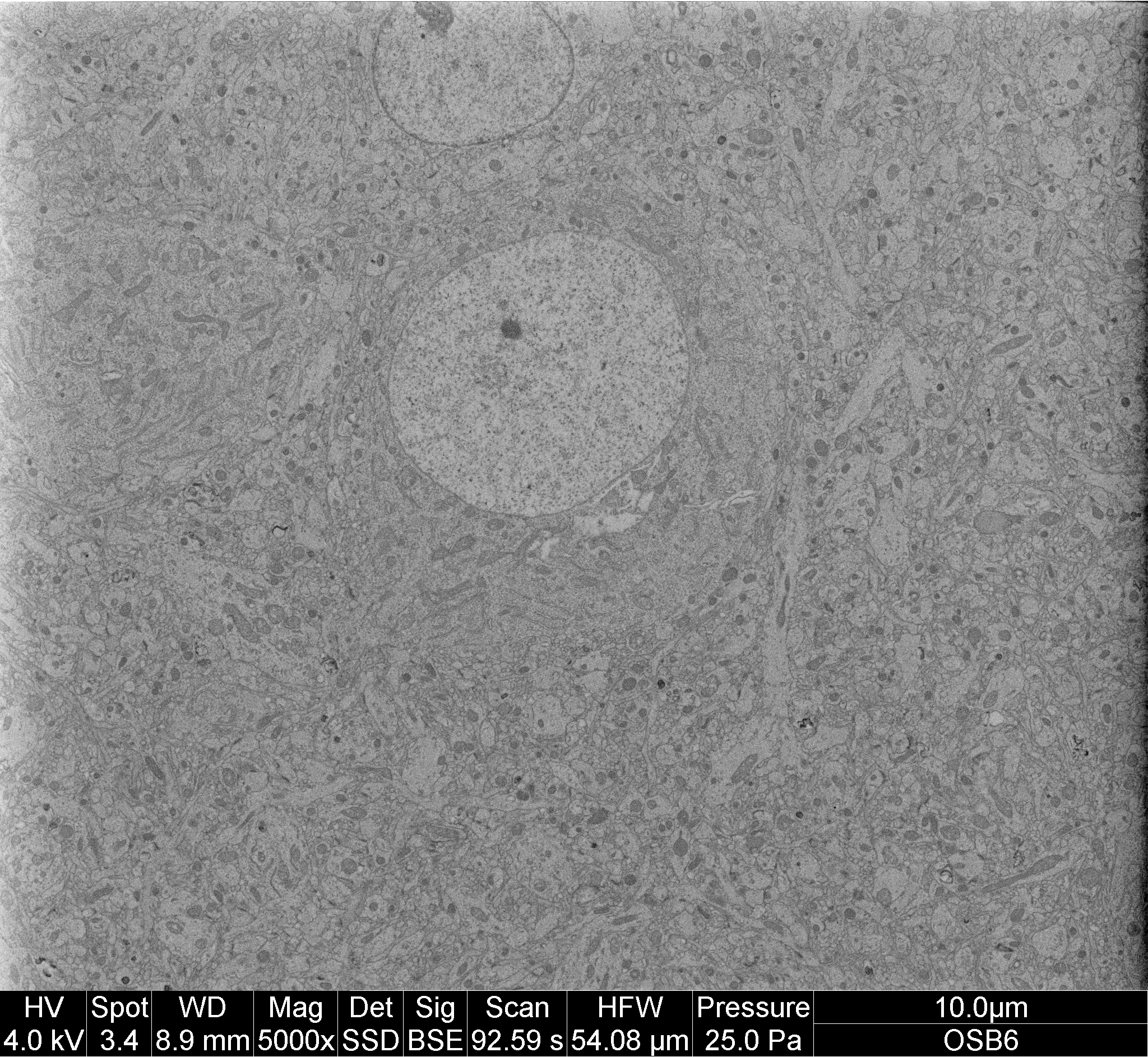

Supplement: Dataset S18 — (250.5 MB ZIP). [file pbio.0020329.sd018.zip › 040604_OS5_st1_1749.tif]

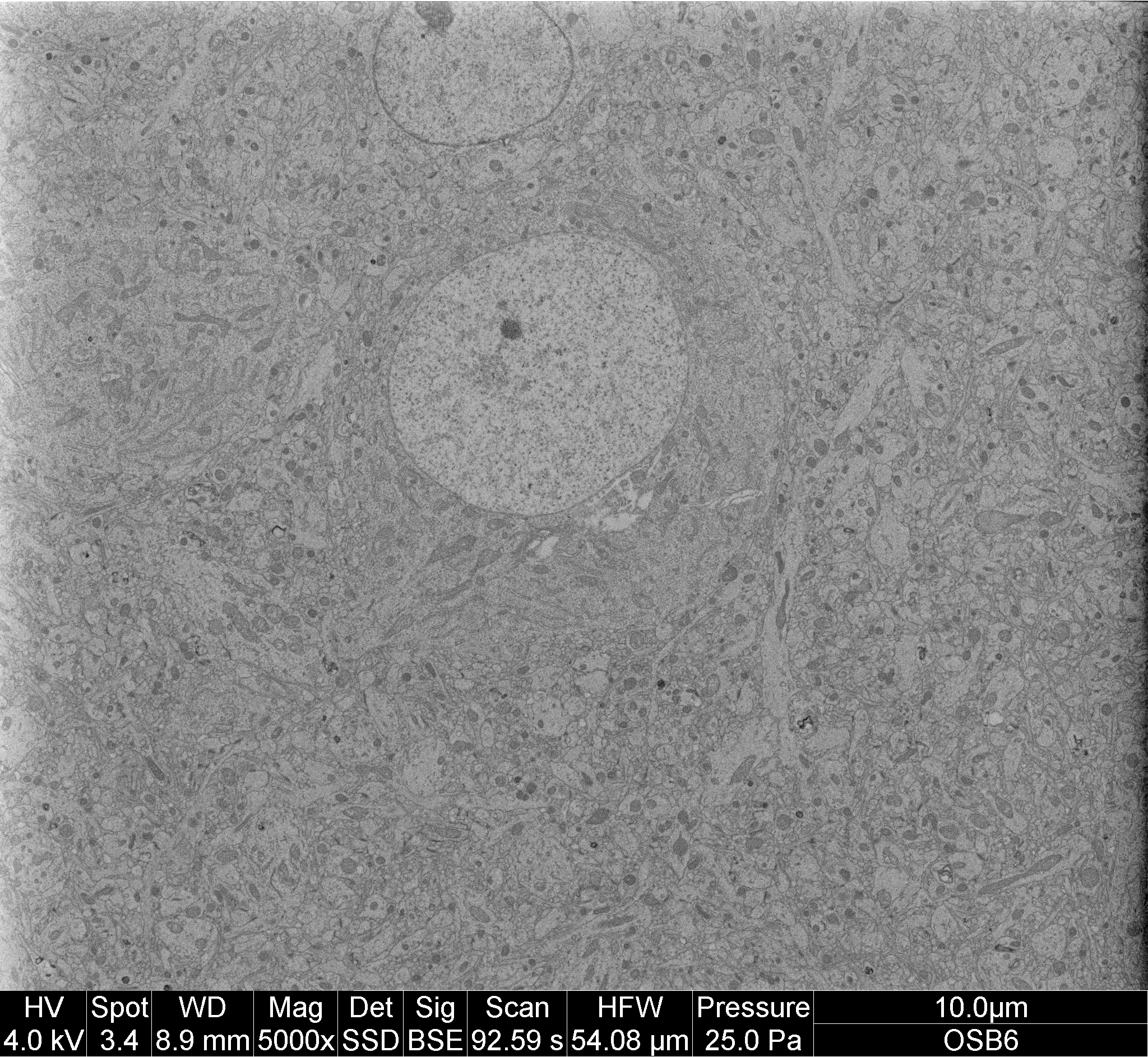

Supplement: Dataset S18 — (250.5 MB ZIP). [file pbio.0020329.sd018.zip › 040604_OS5_st1_1750.tif]

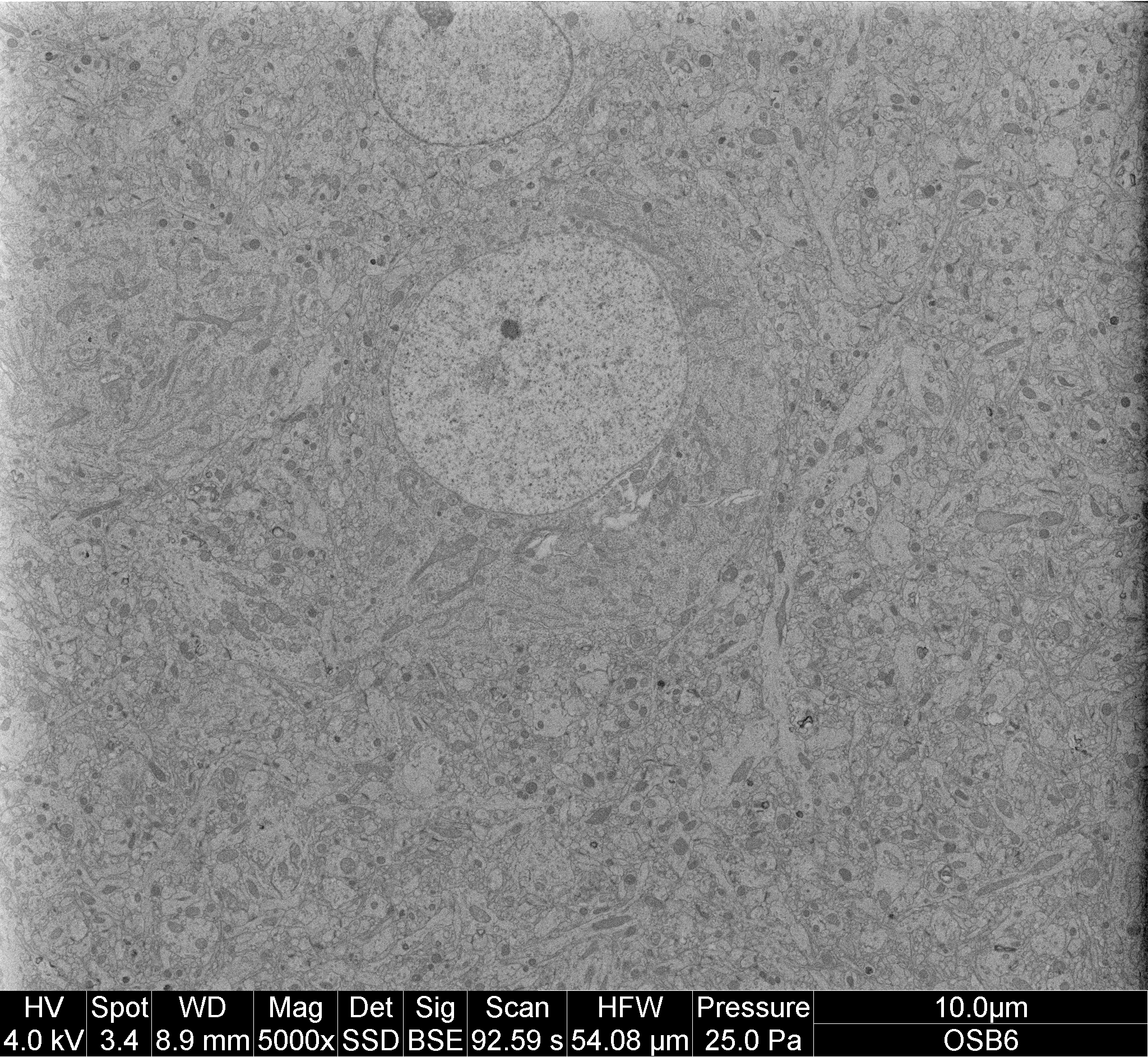

Supplement: Dataset S18 — (250.5 MB ZIP). [file pbio.0020329.sd018.zip › 040604_OS5_st1_1751.tif]

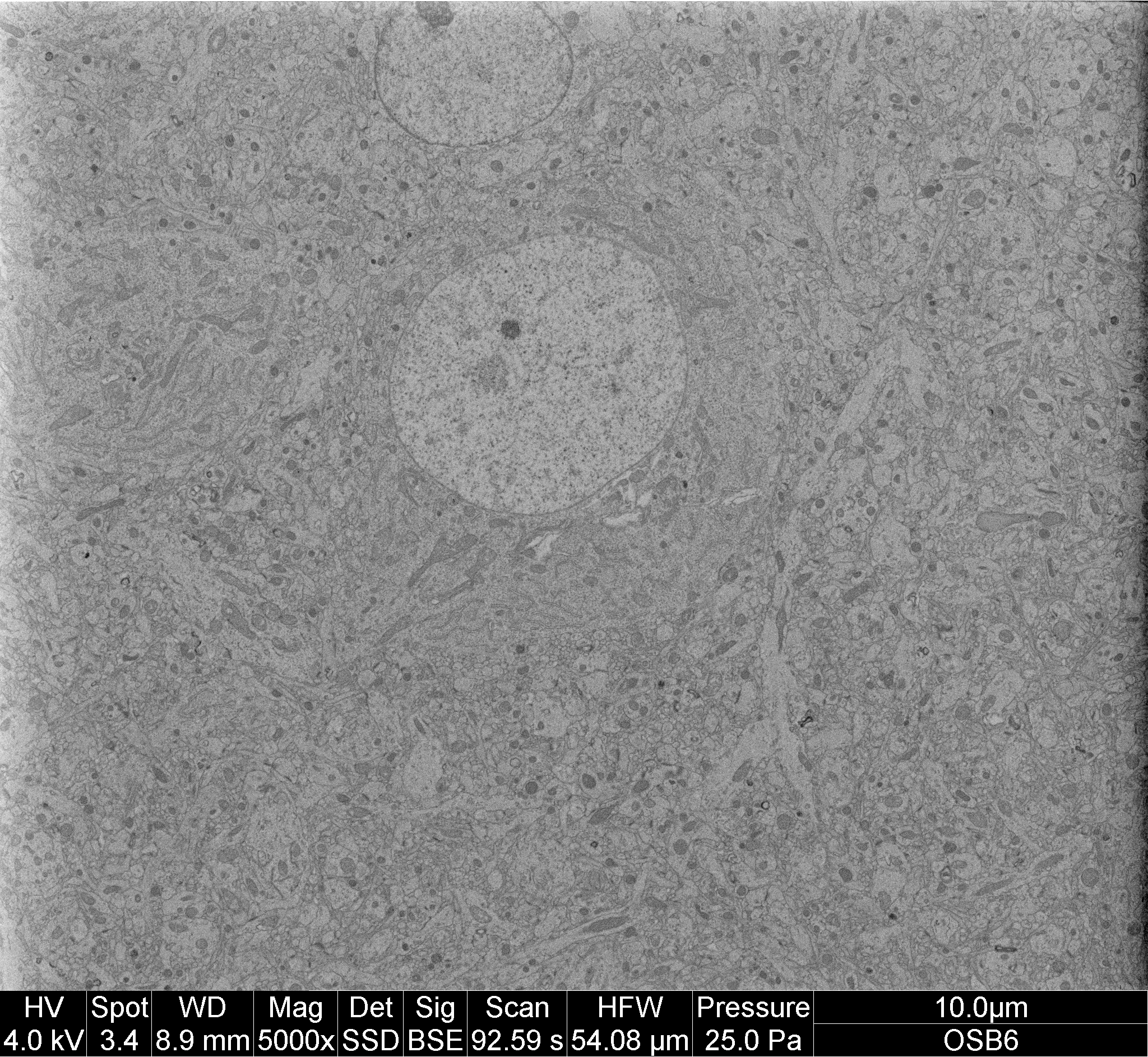

Supplement: Dataset S18 — (250.5 MB ZIP). [file pbio.0020329.sd018.zip › 040604_OS5_st1_1752.tif]

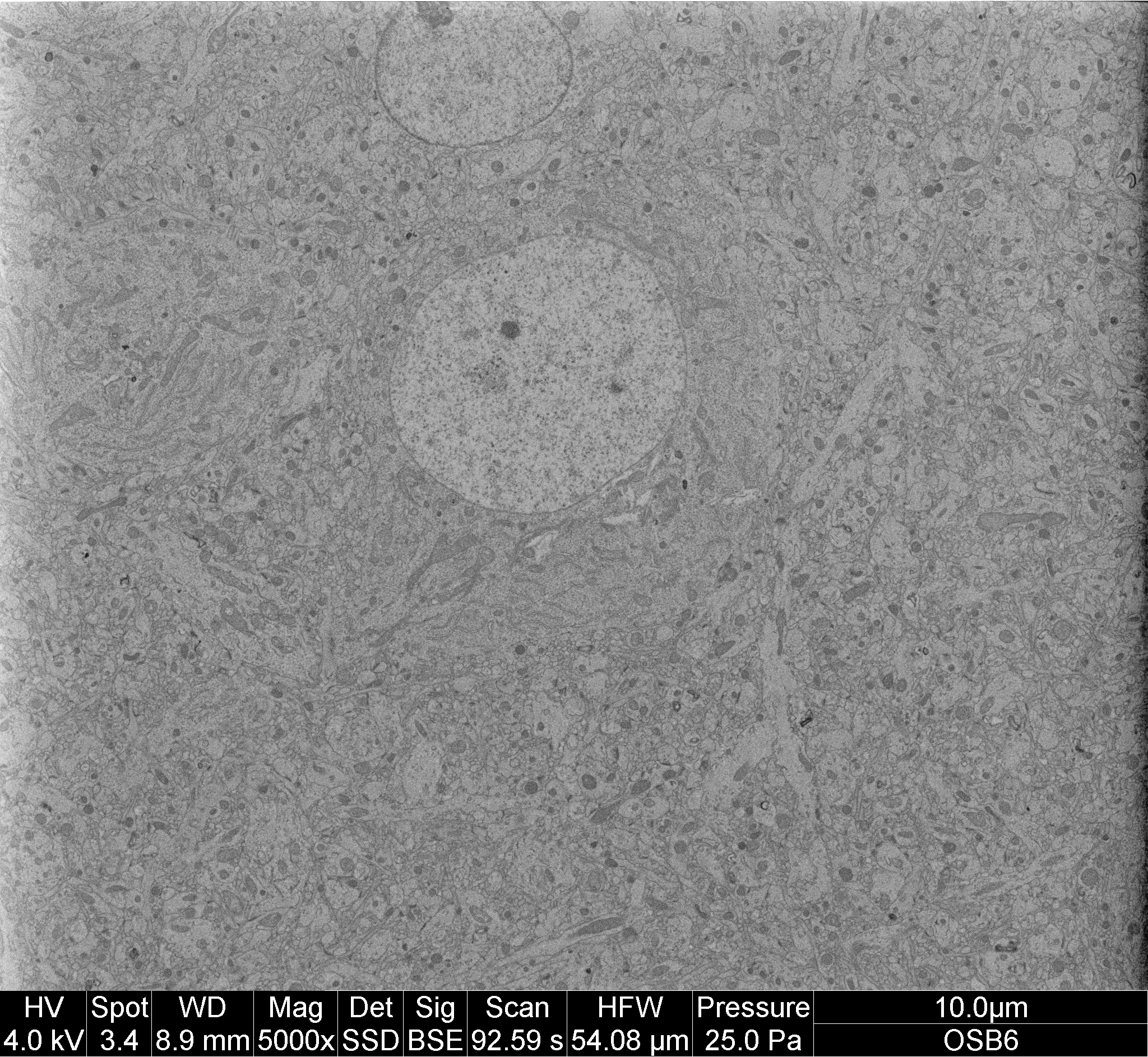

Supplement: Dataset S18 — (250.5 MB ZIP). [file pbio.0020329.sd018.zip › 040604_OS5_st1_1753.tif]

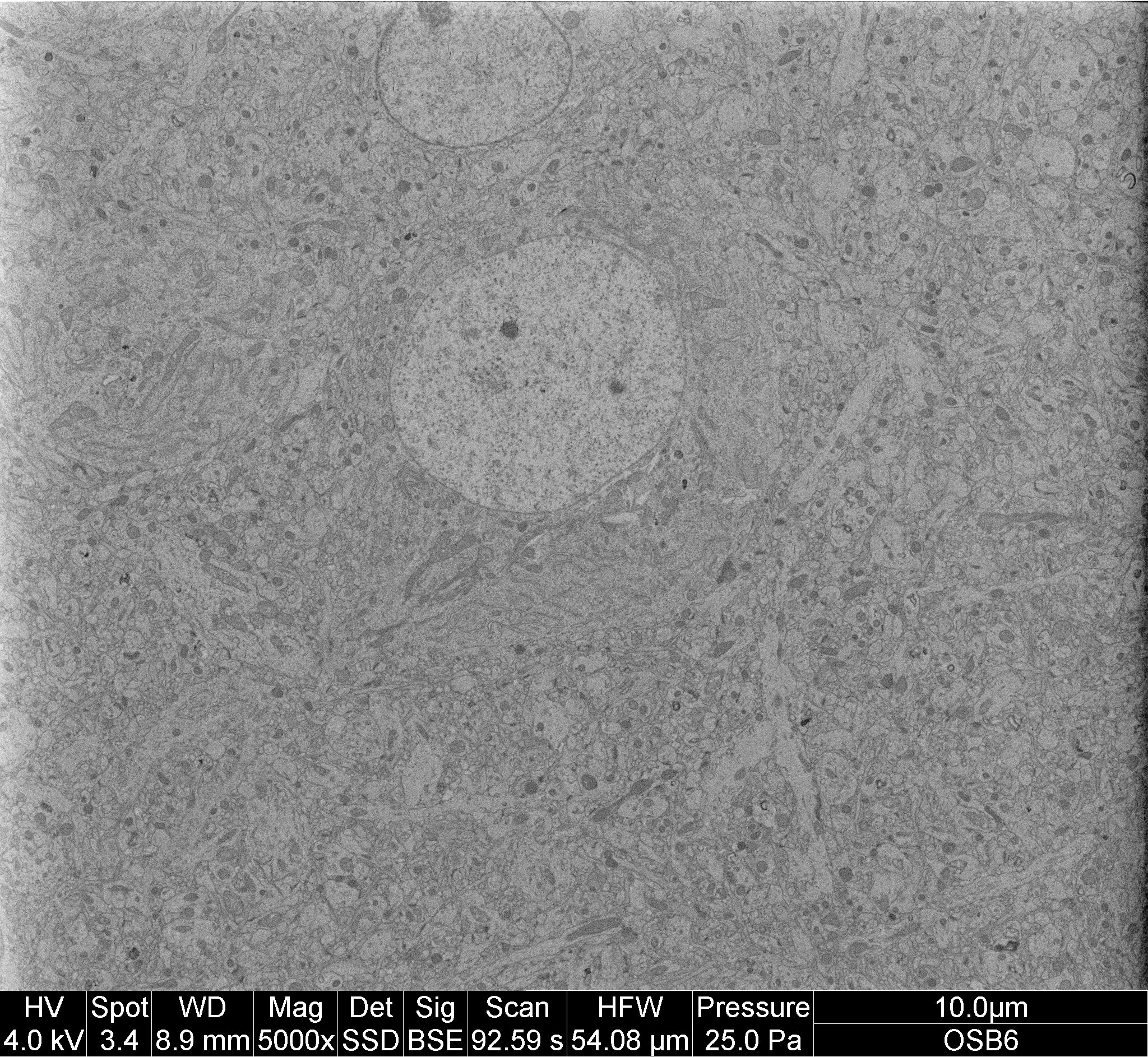

Supplement: Dataset S18 — (250.5 MB ZIP). [file pbio.0020329.sd018.zip › 040604_OS5_st1_1754.tif]

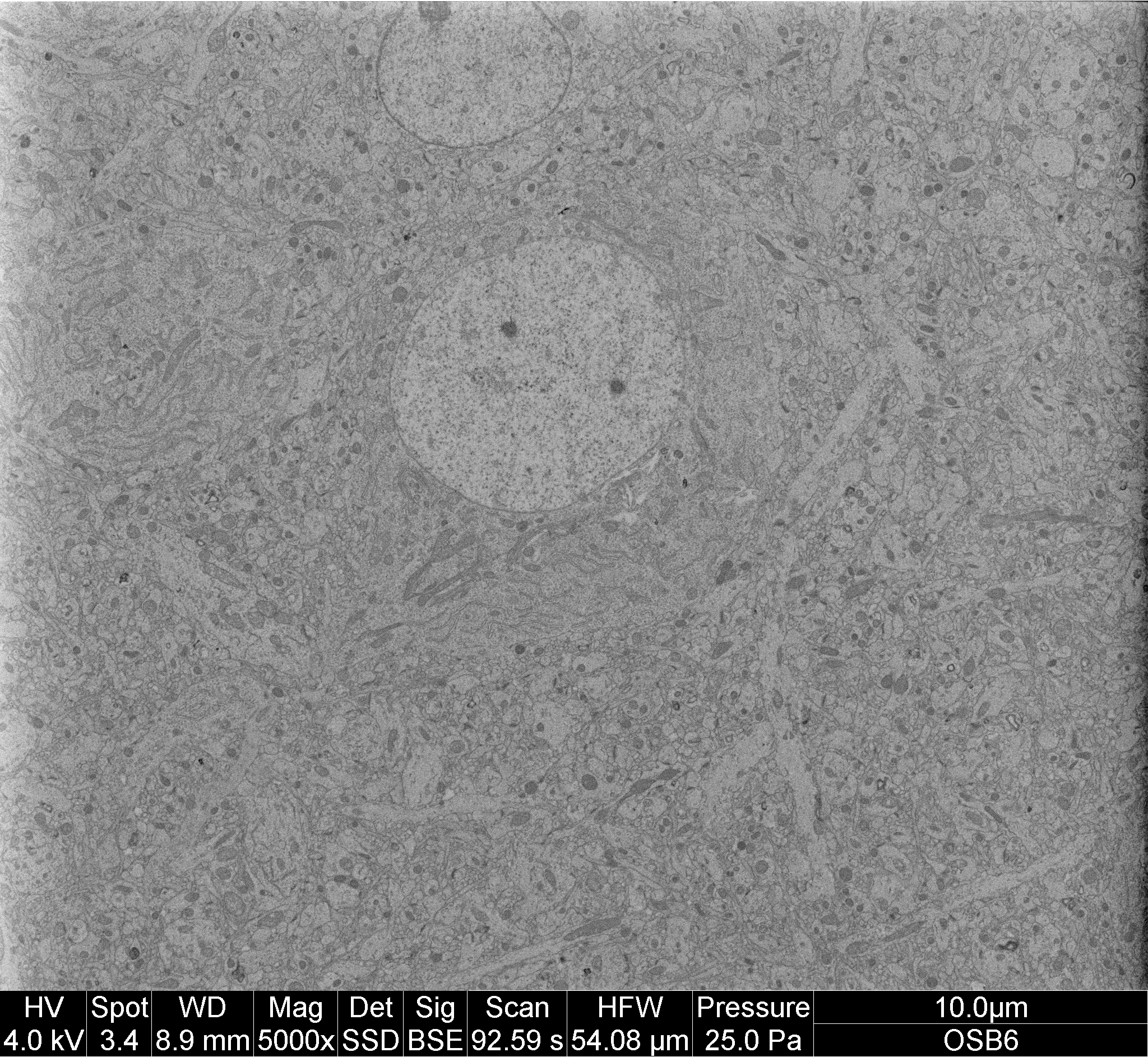

Supplement: Dataset S18 — (250.5 MB ZIP). [file pbio.0020329.sd018.zip › 040604_OS5_st1_1755.tif]

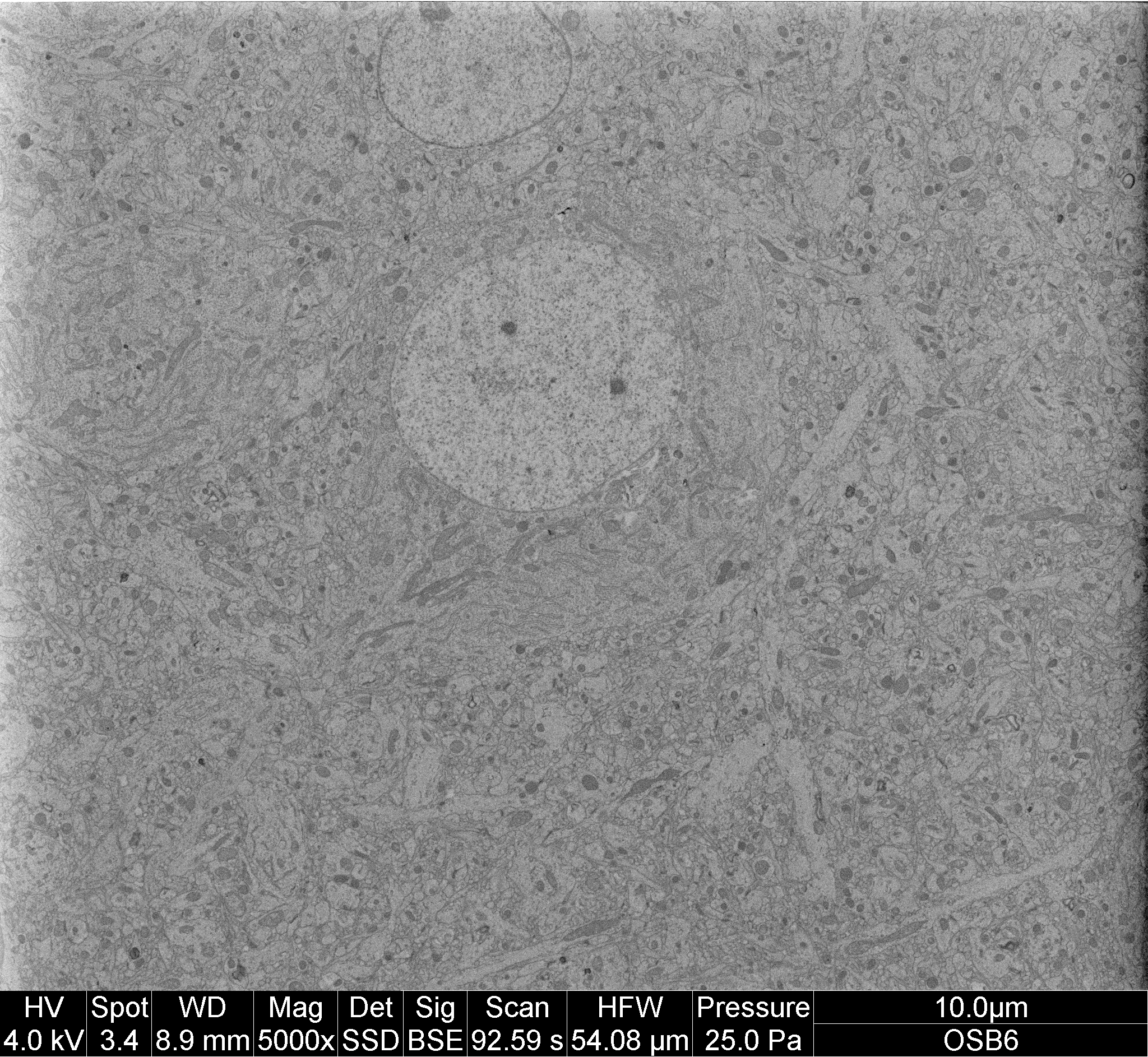

Supplement: Dataset S18 — (250.5 MB ZIP). [file pbio.0020329.sd018.zip › 040604_OS5_st1_1756.tif]

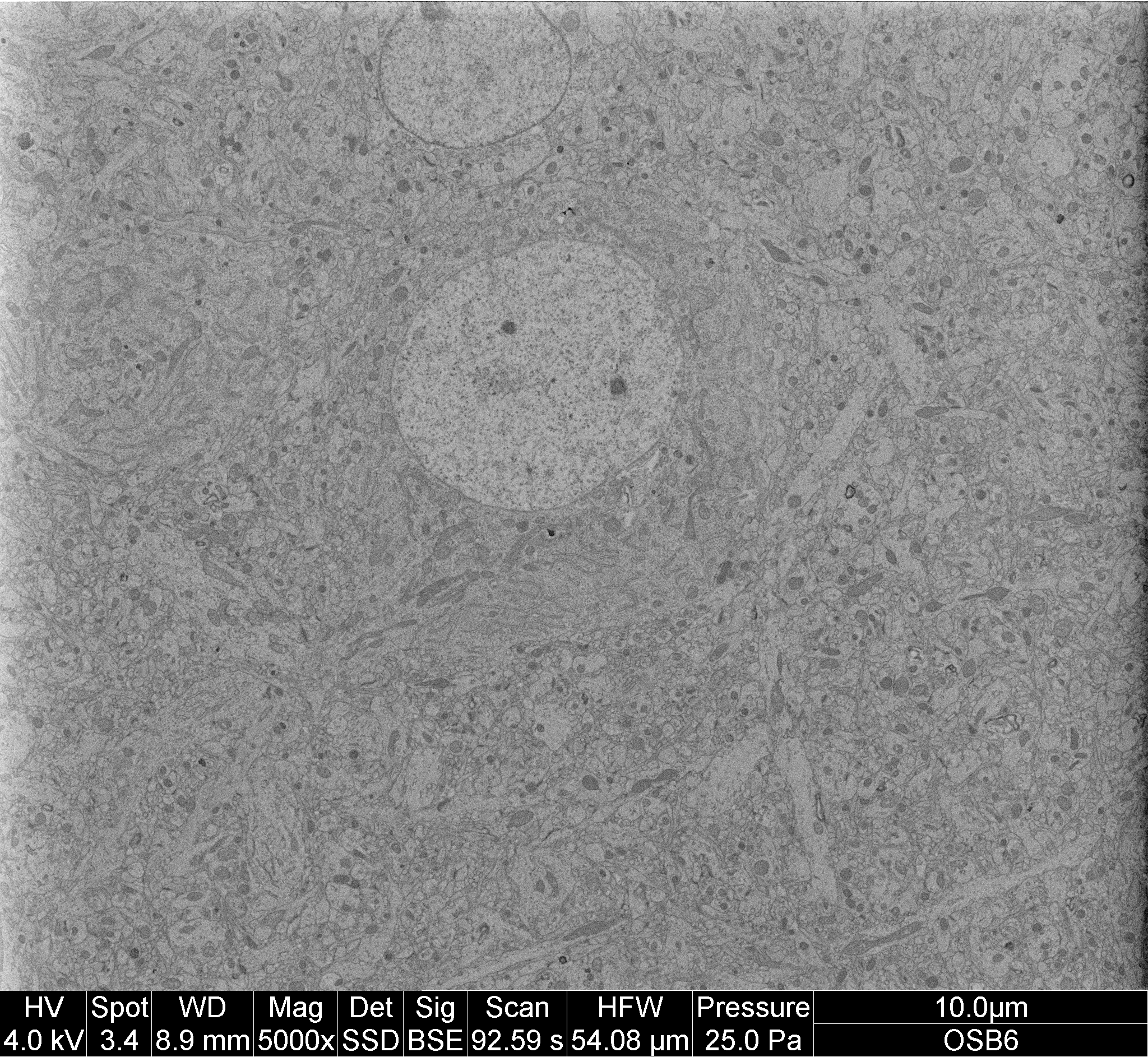

Supplement: Dataset S18 — (250.5 MB ZIP). [file pbio.0020329.sd018.zip › 040604_OS5_st1_1757.tif]

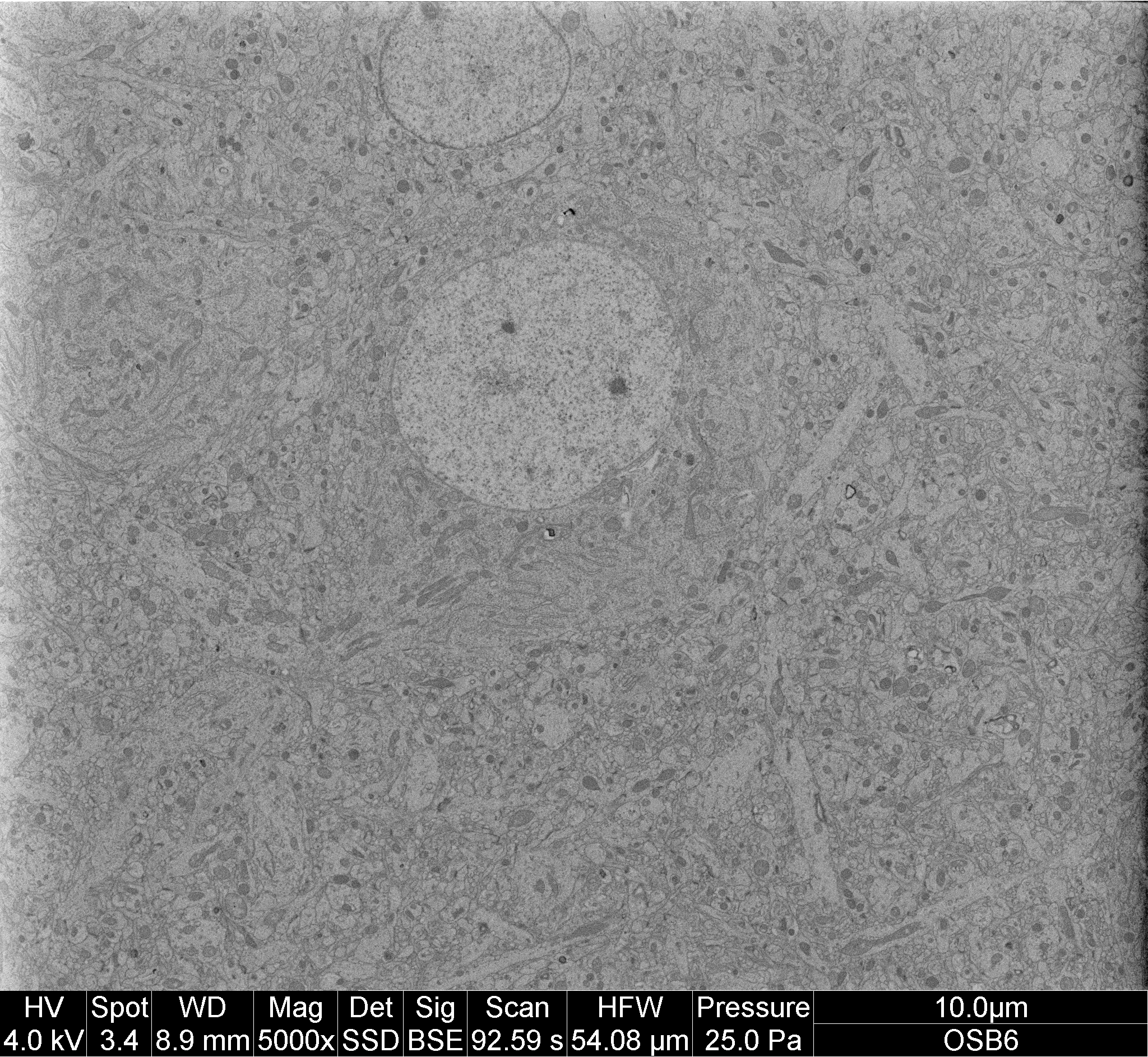

Supplement: Dataset S18 — (250.5 MB ZIP). [file pbio.0020329.sd018.zip › 040604_OS5_st1_1758.tif]

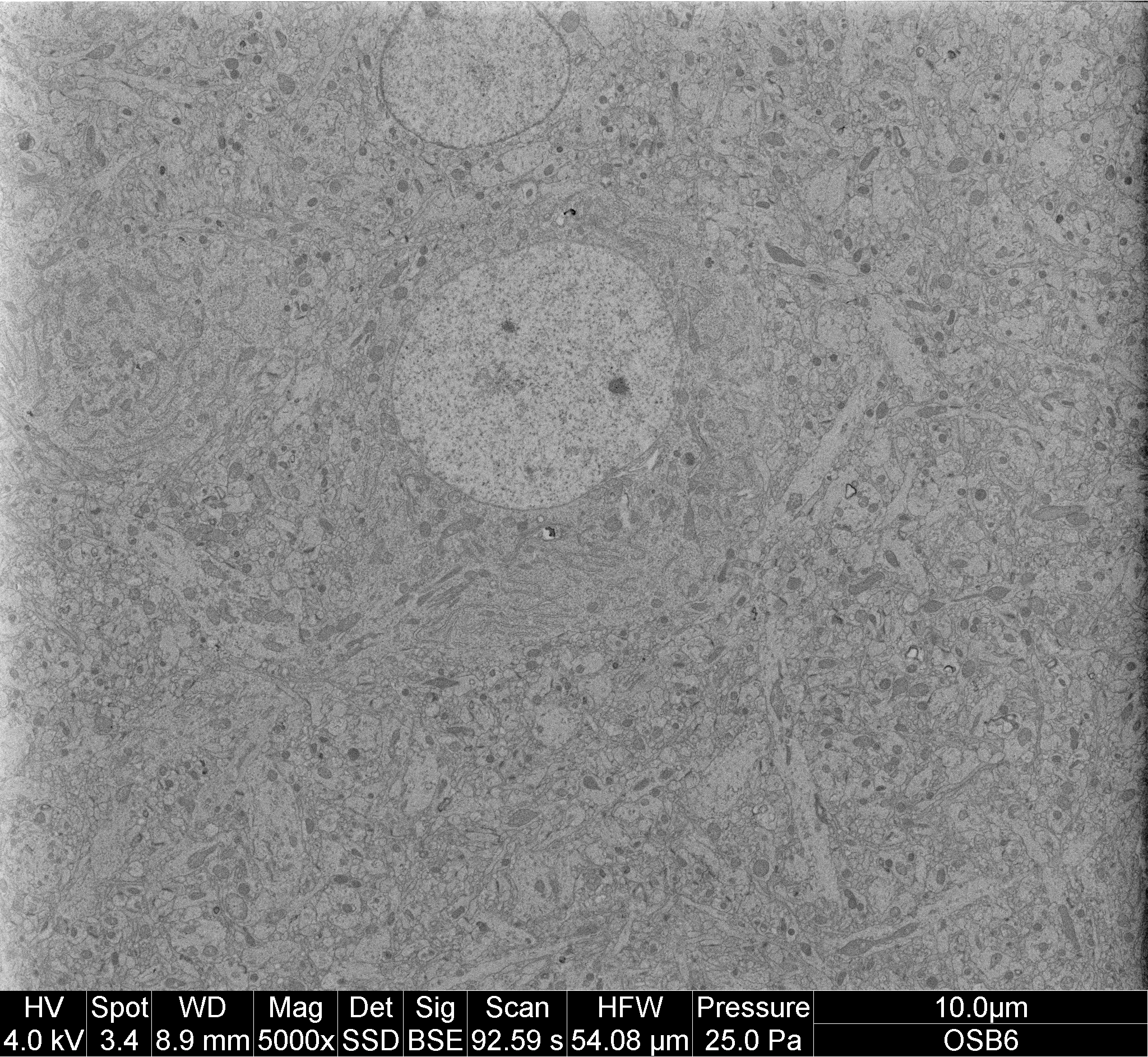

Supplement: Dataset S18 — (250.5 MB ZIP). [file pbio.0020329.sd018.zip › 040604_OS5_st1_1759.tif]

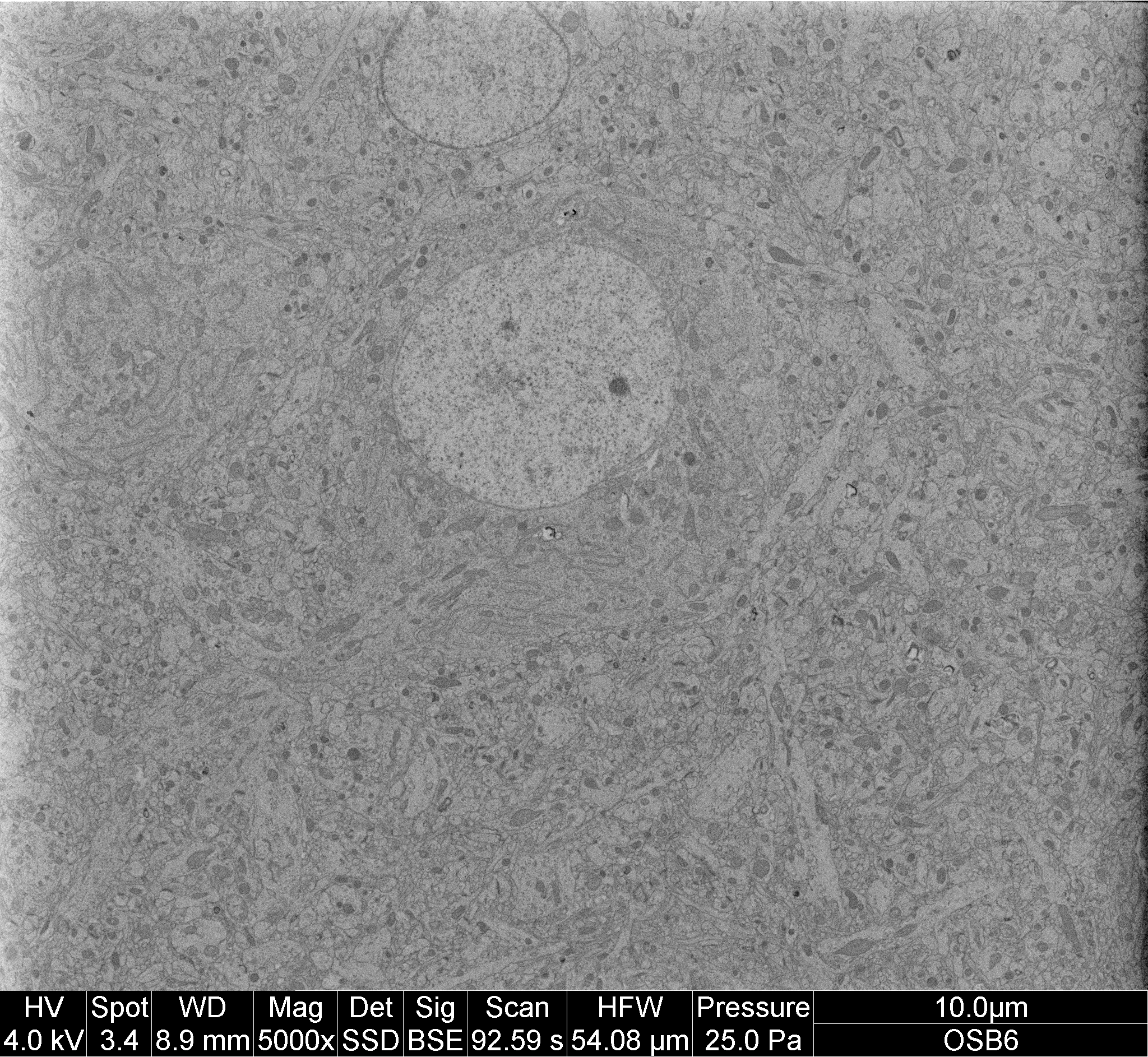

Supplement: Dataset S18 — (250.5 MB ZIP). [file pbio.0020329.sd018.zip › 040604_OS5_st1_1760.tif]

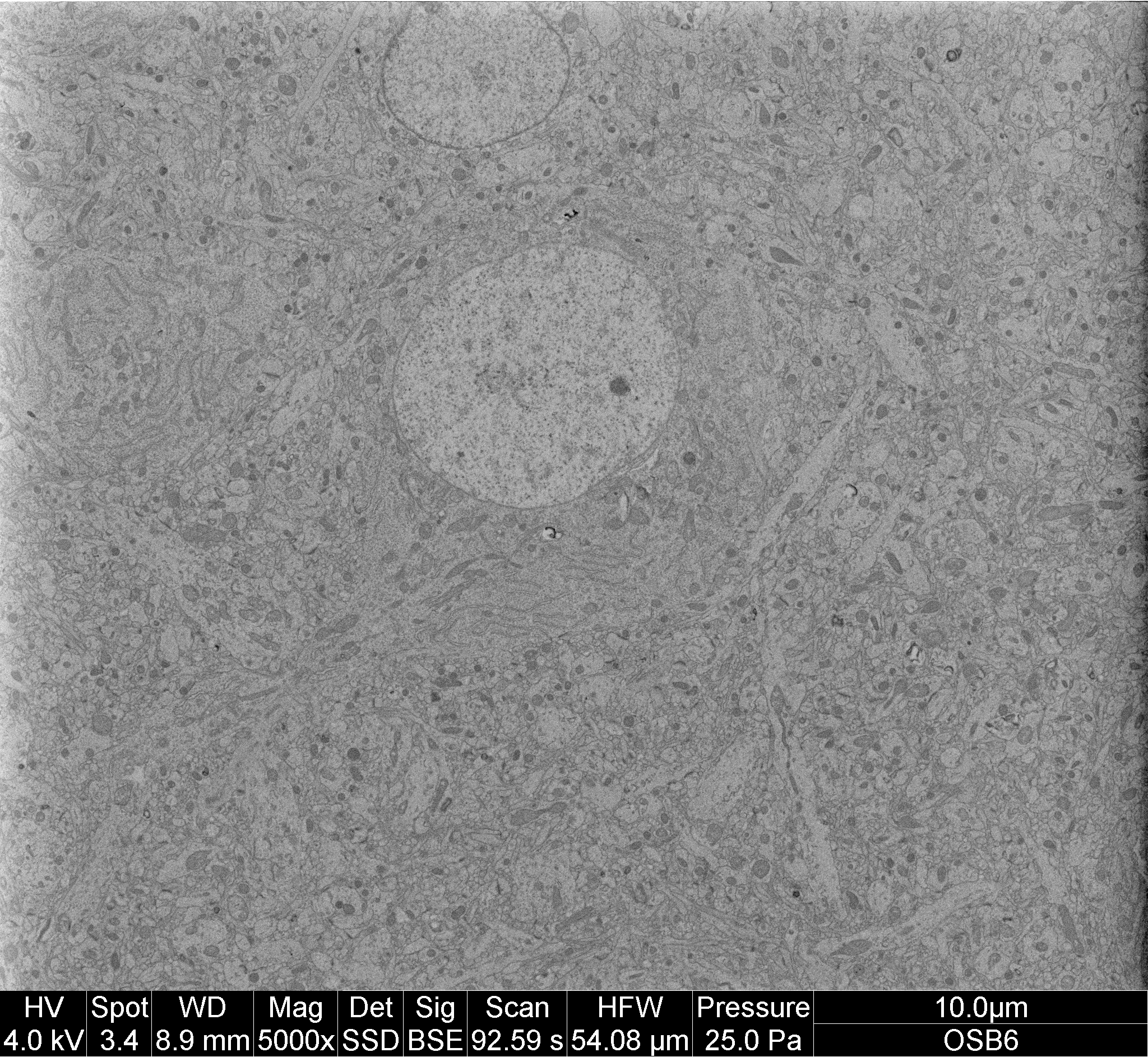

Supplement: Dataset S18 — (250.5 MB ZIP). [file pbio.0020329.sd018.zip › 040604_OS5_st1_1761.tif]

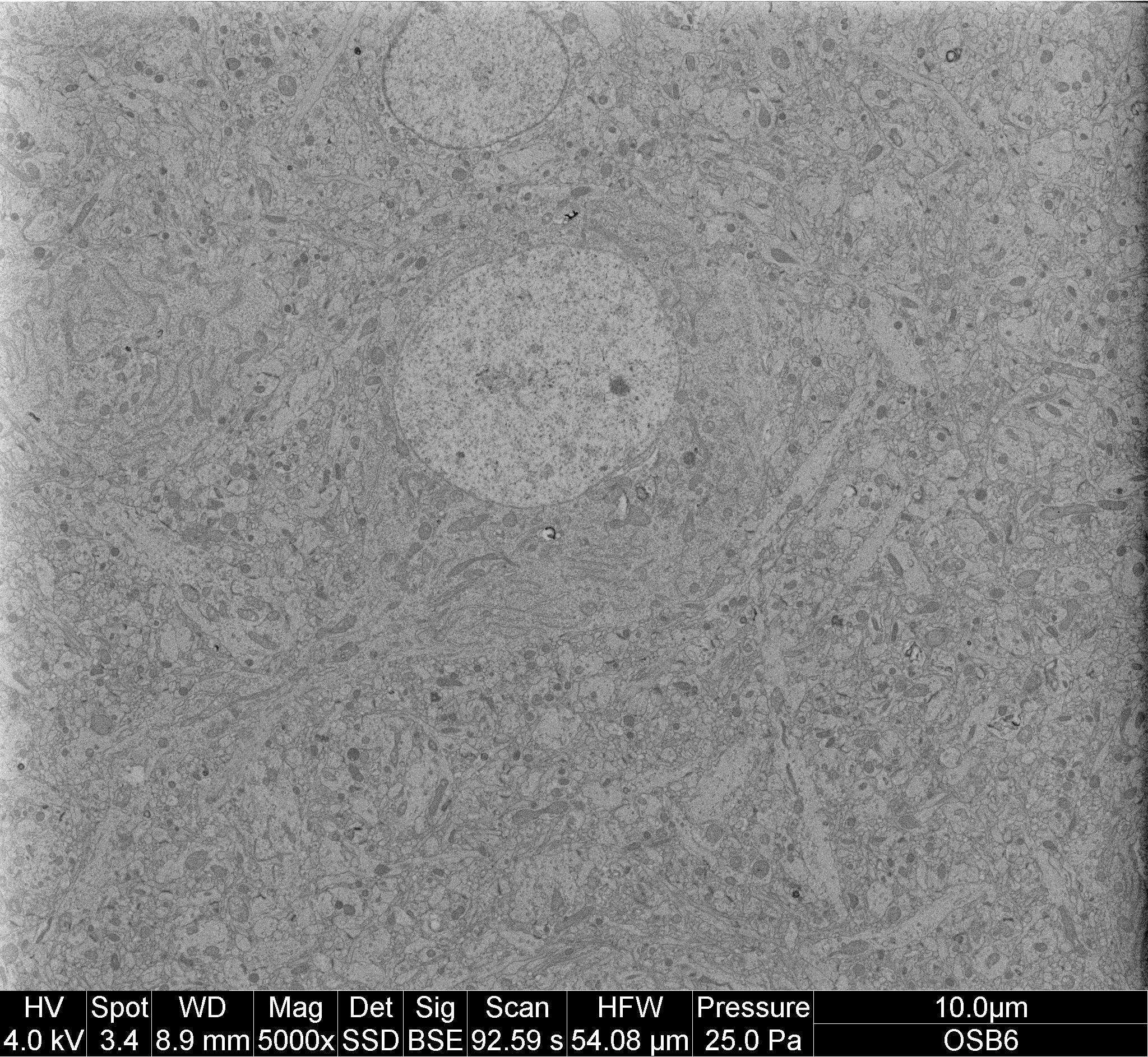

Supplement: Dataset S18 — (250.5 MB ZIP). [file pbio.0020329.sd018.zip › 040604_OS5_st1_1762.tif]

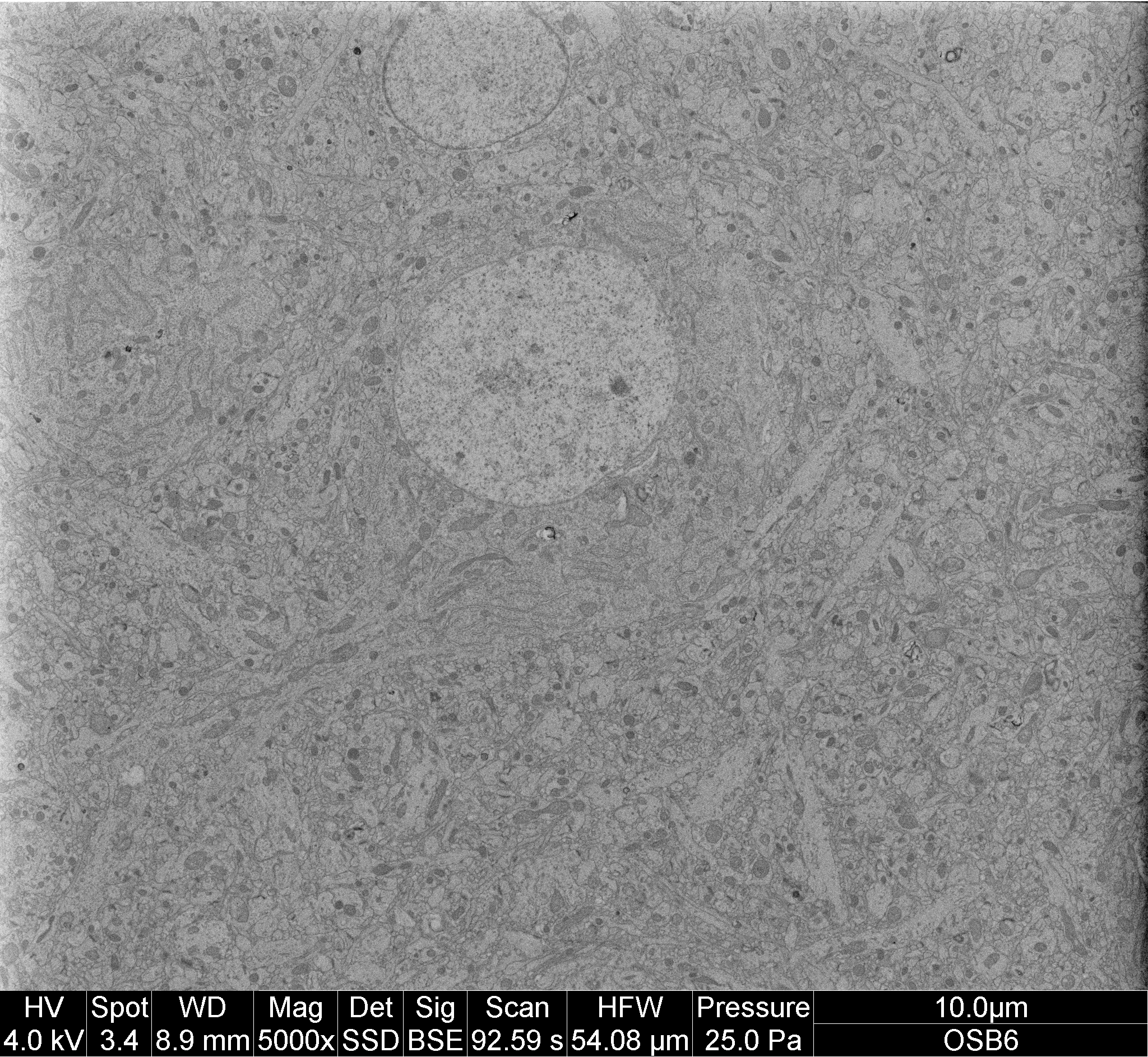

Supplement: Dataset S18 — (250.5 MB ZIP). [file pbio.0020329.sd018.zip › 040604_OS5_st1_1763.tif]

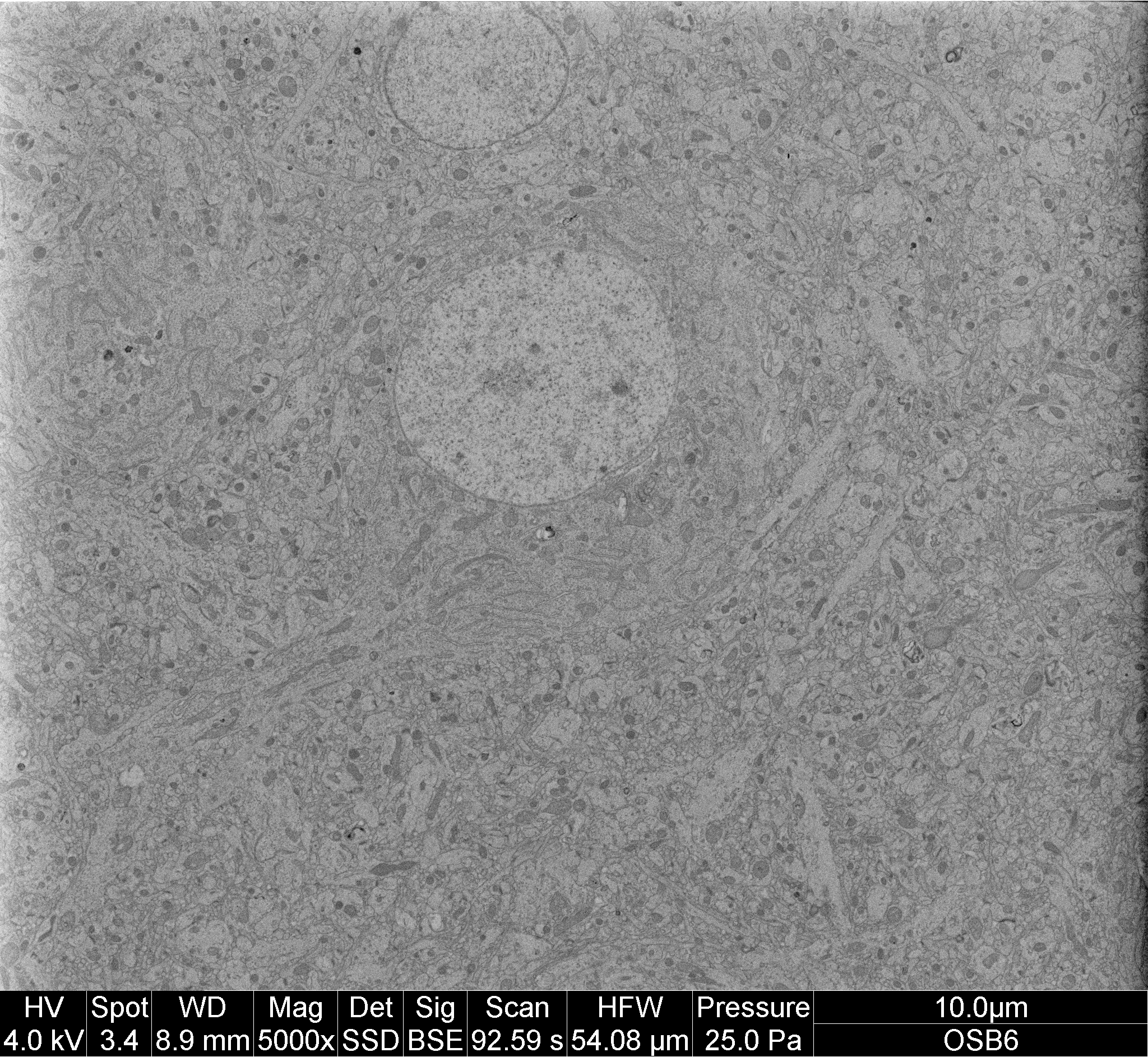

Supplement: Dataset S18 — (250.5 MB ZIP). [file pbio.0020329.sd018.zip › 040604_OS5_st1_1764.tif]

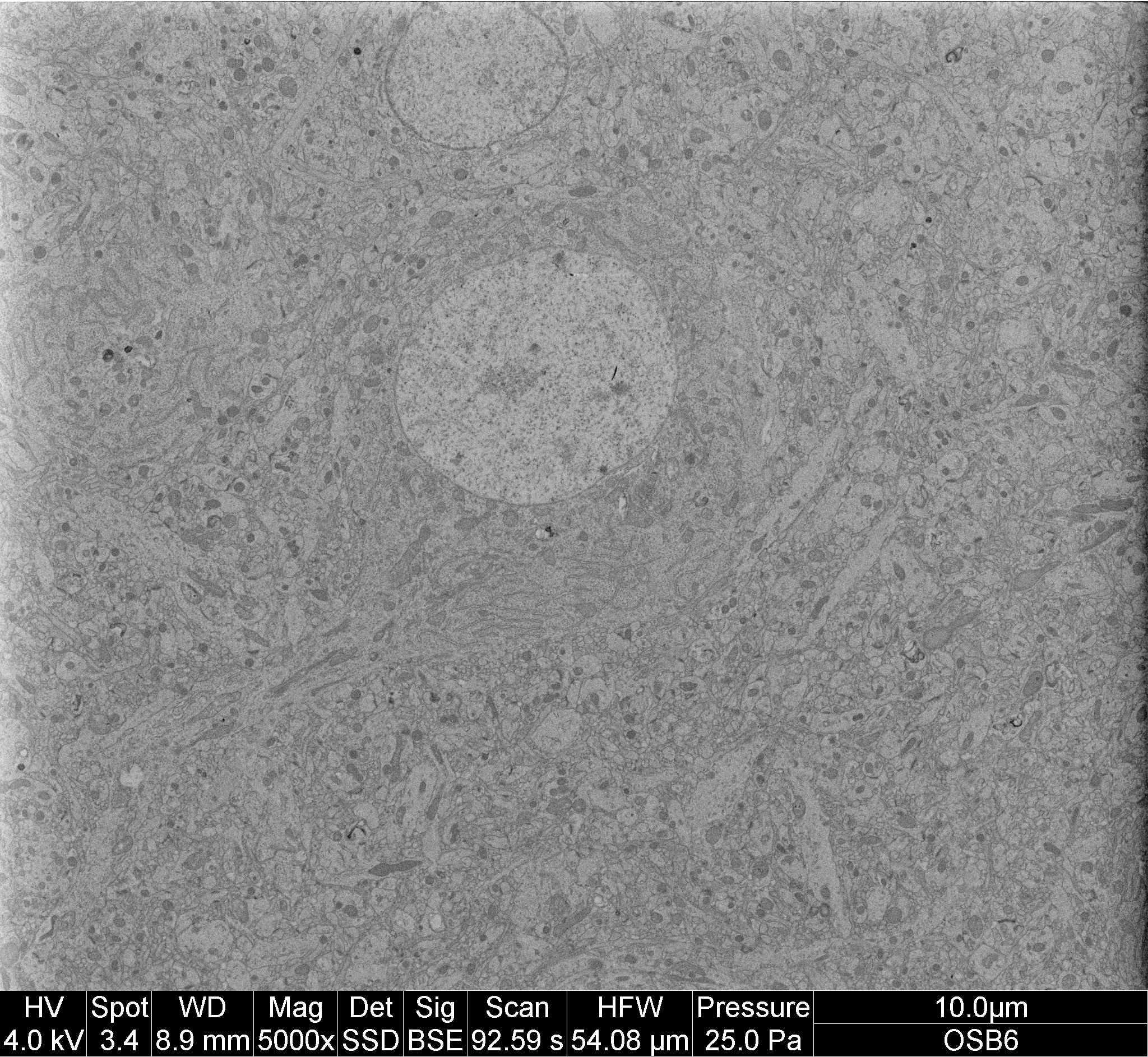

Supplement: Dataset S18 — (250.5 MB ZIP). [file pbio.0020329.sd018.zip › 040604_OS5_st1_1765.tif]

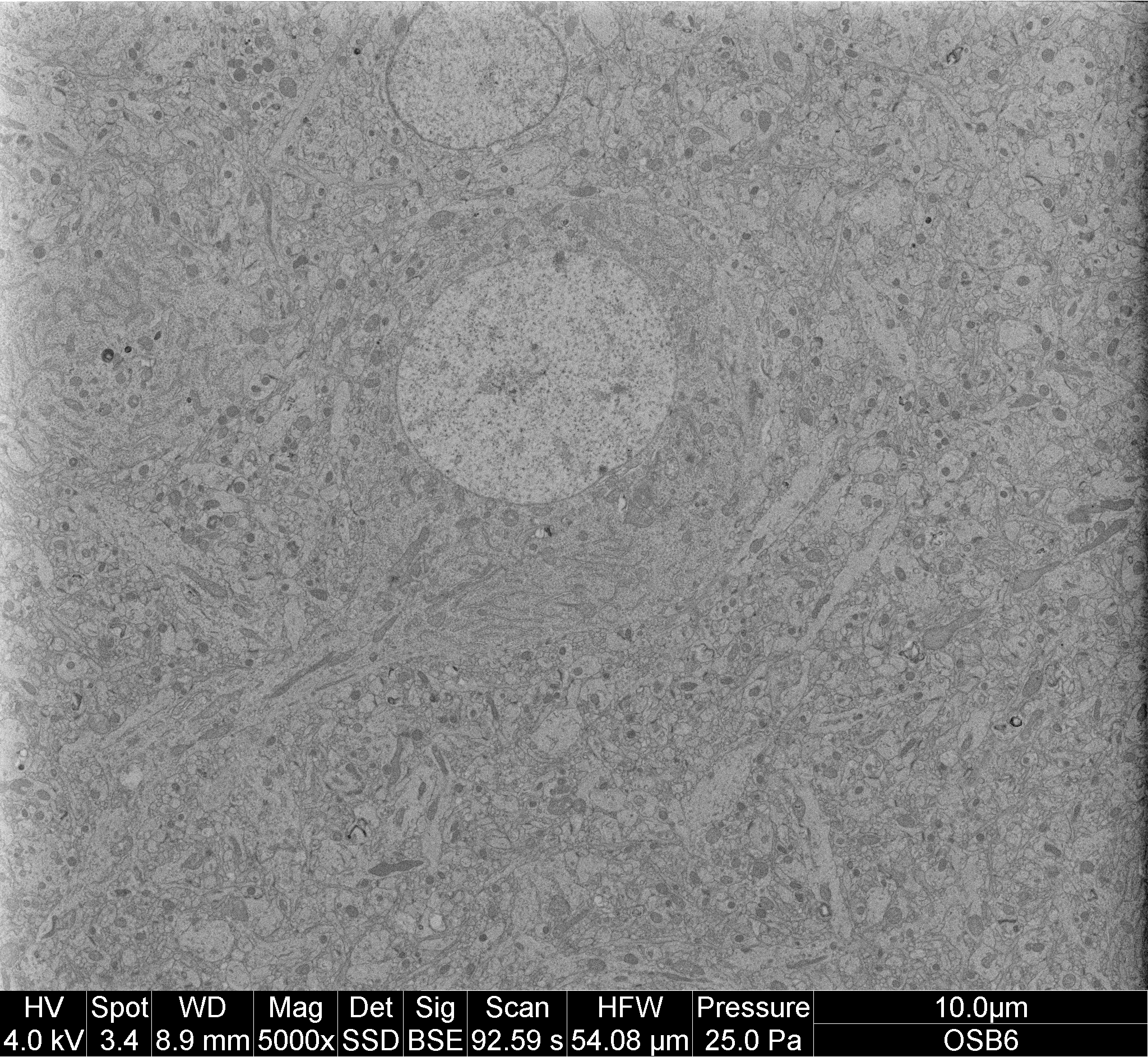

Supplement: Dataset S18 — (250.5 MB ZIP). [file pbio.0020329.sd018.zip › 040604_OS5_st1_1766.tif]

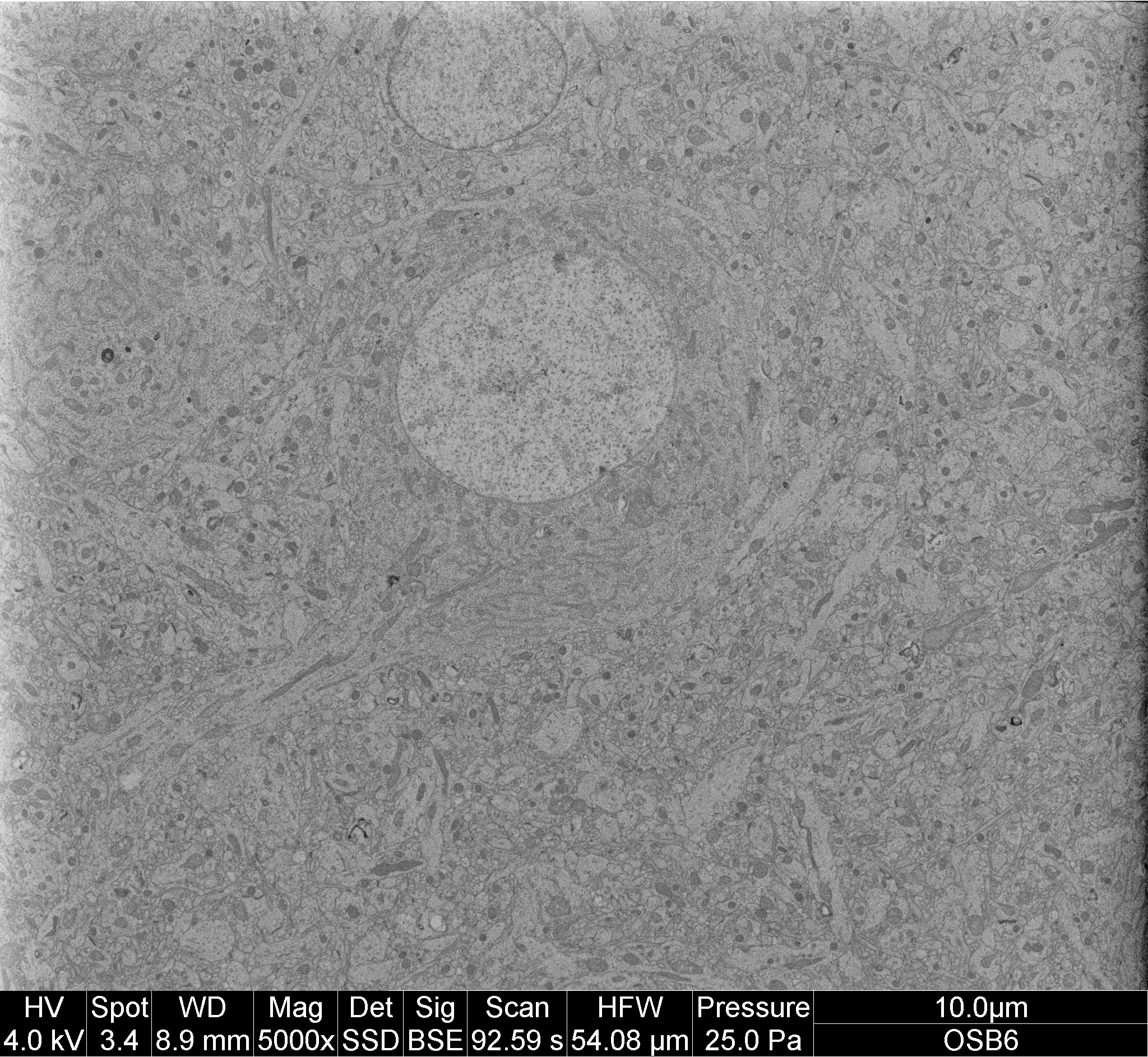

Supplement: Dataset S18 — (250.5 MB ZIP). [file pbio.0020329.sd018.zip › 040604_OS5_st1_1767.tif]

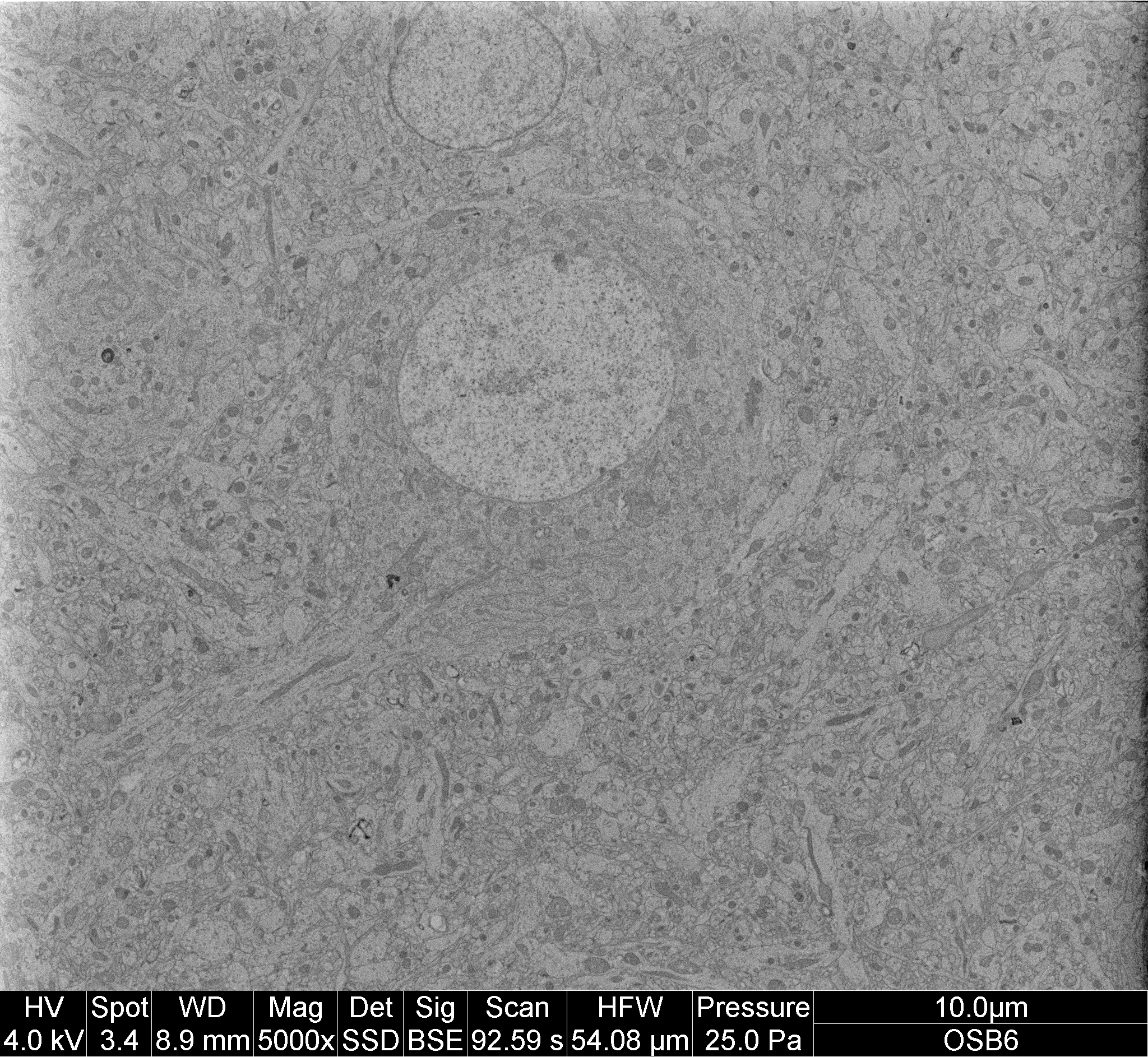

Supplement: Dataset S18 — (250.5 MB ZIP). [file pbio.0020329.sd018.zip › 040604_OS5_st1_1768.tif]

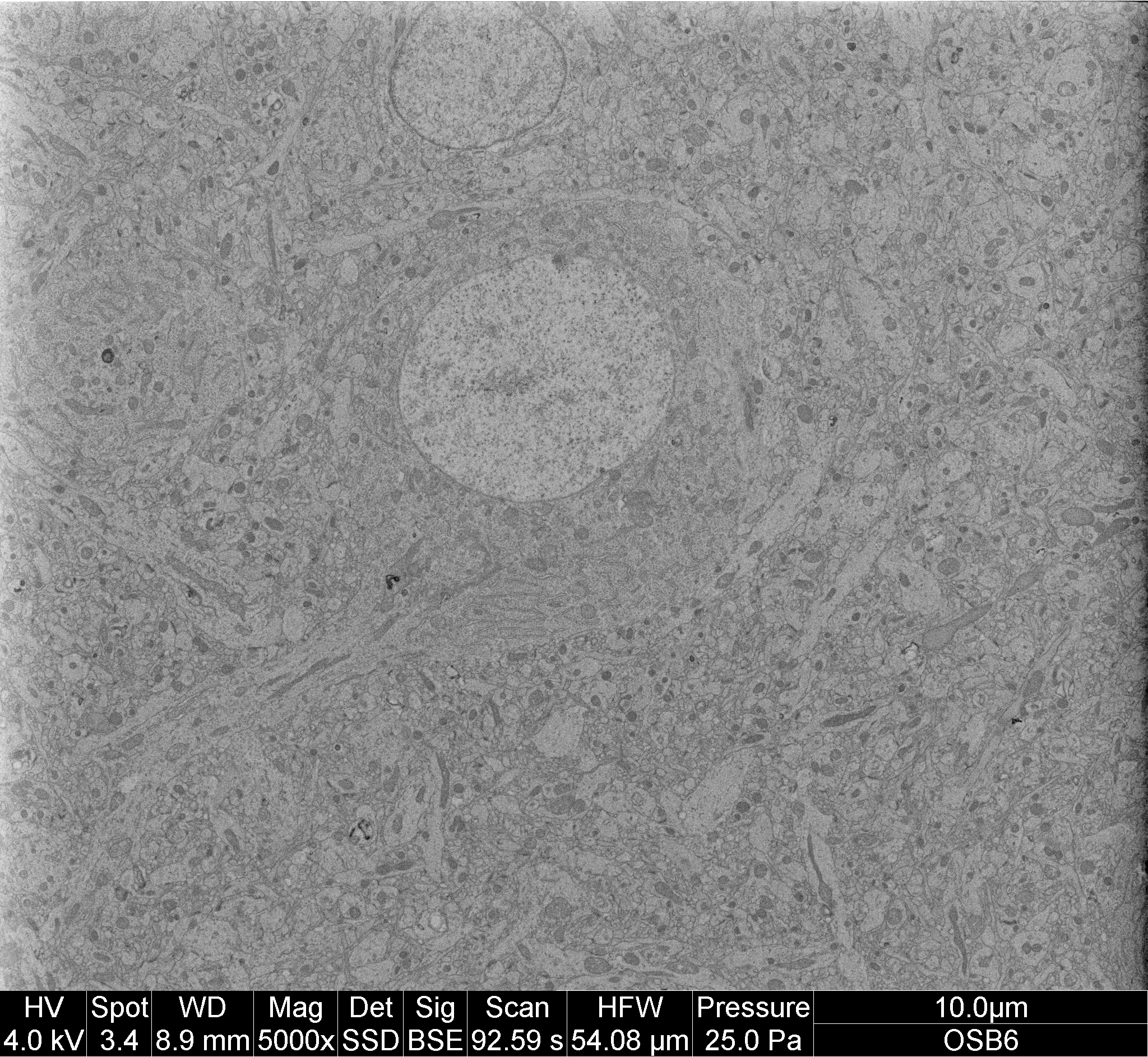

Supplement: Dataset S18 — (250.5 MB ZIP). [file pbio.0020329.sd018.zip › 040604_OS5_st1_1769.tif]

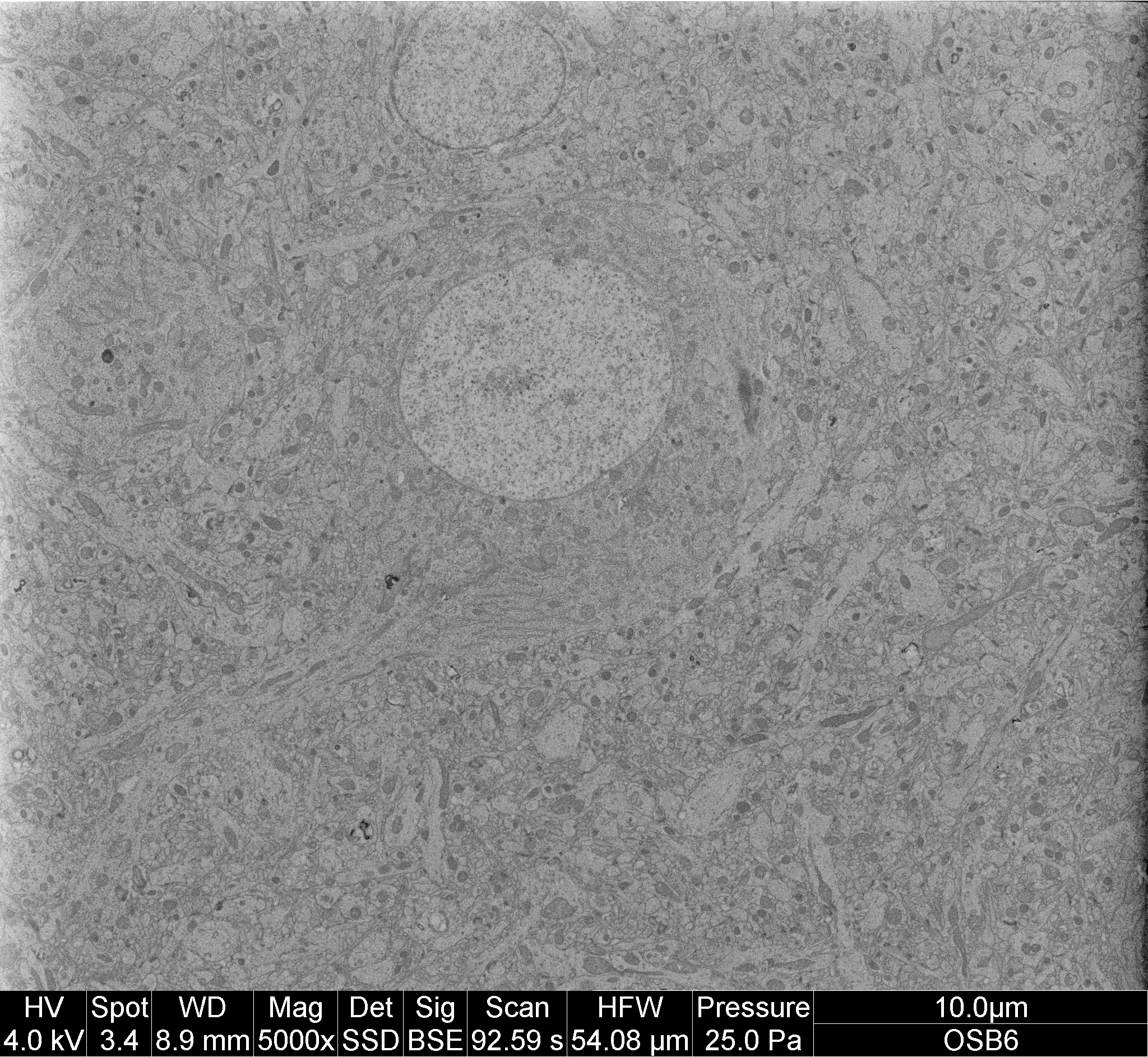

Supplement: Dataset S18 — (250.5 MB ZIP). [file pbio.0020329.sd018.zip › 040604_OS5_st1_1770.tif]

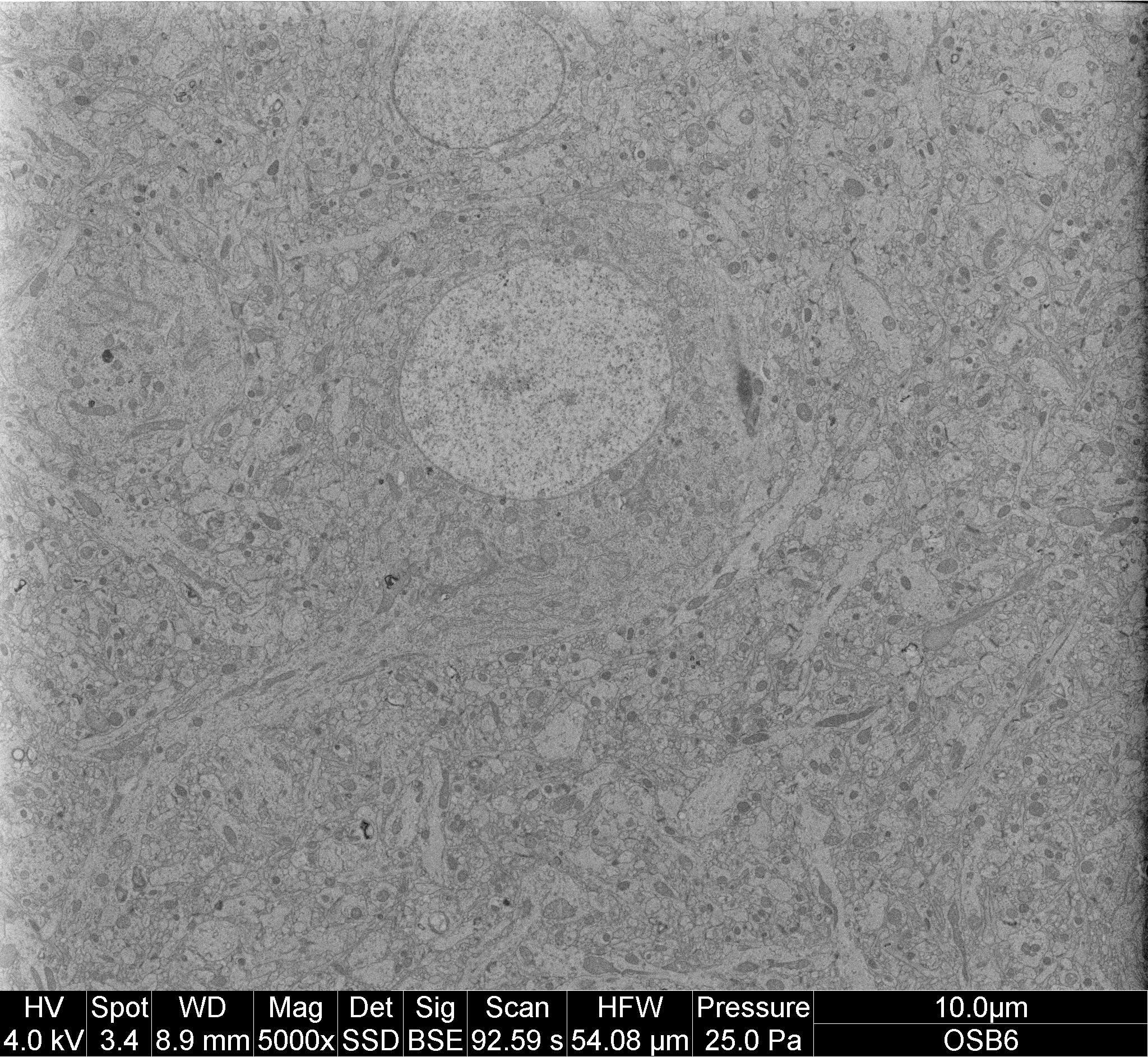

Supplement: Dataset S18 — (250.5 MB ZIP). [file pbio.0020329.sd018.zip › 040604_OS5_st1_1771.tif]

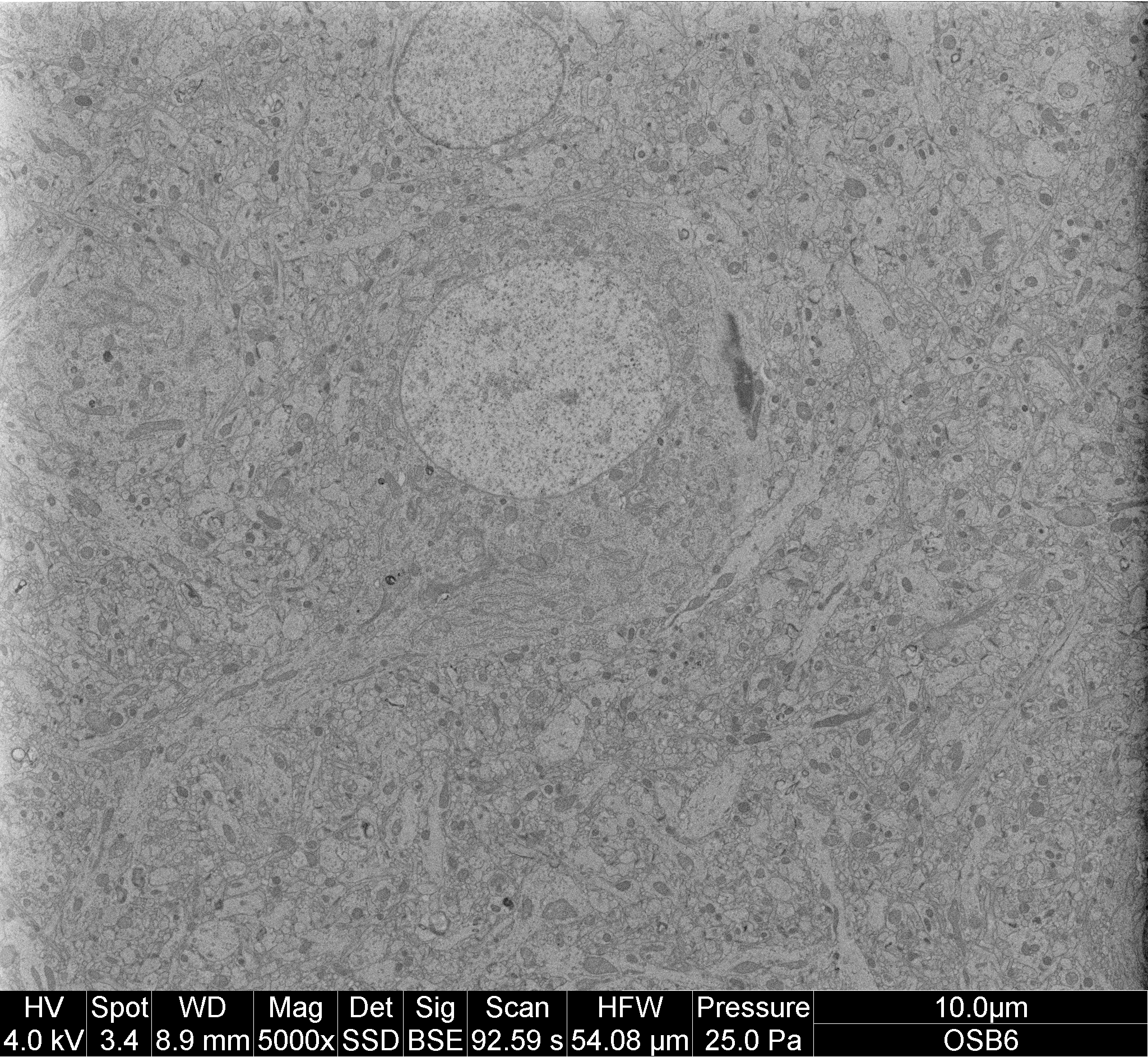

Supplement: Dataset S18 — (250.5 MB ZIP). [file pbio.0020329.sd018.zip › 040604_OS5_st1_1772.tif]

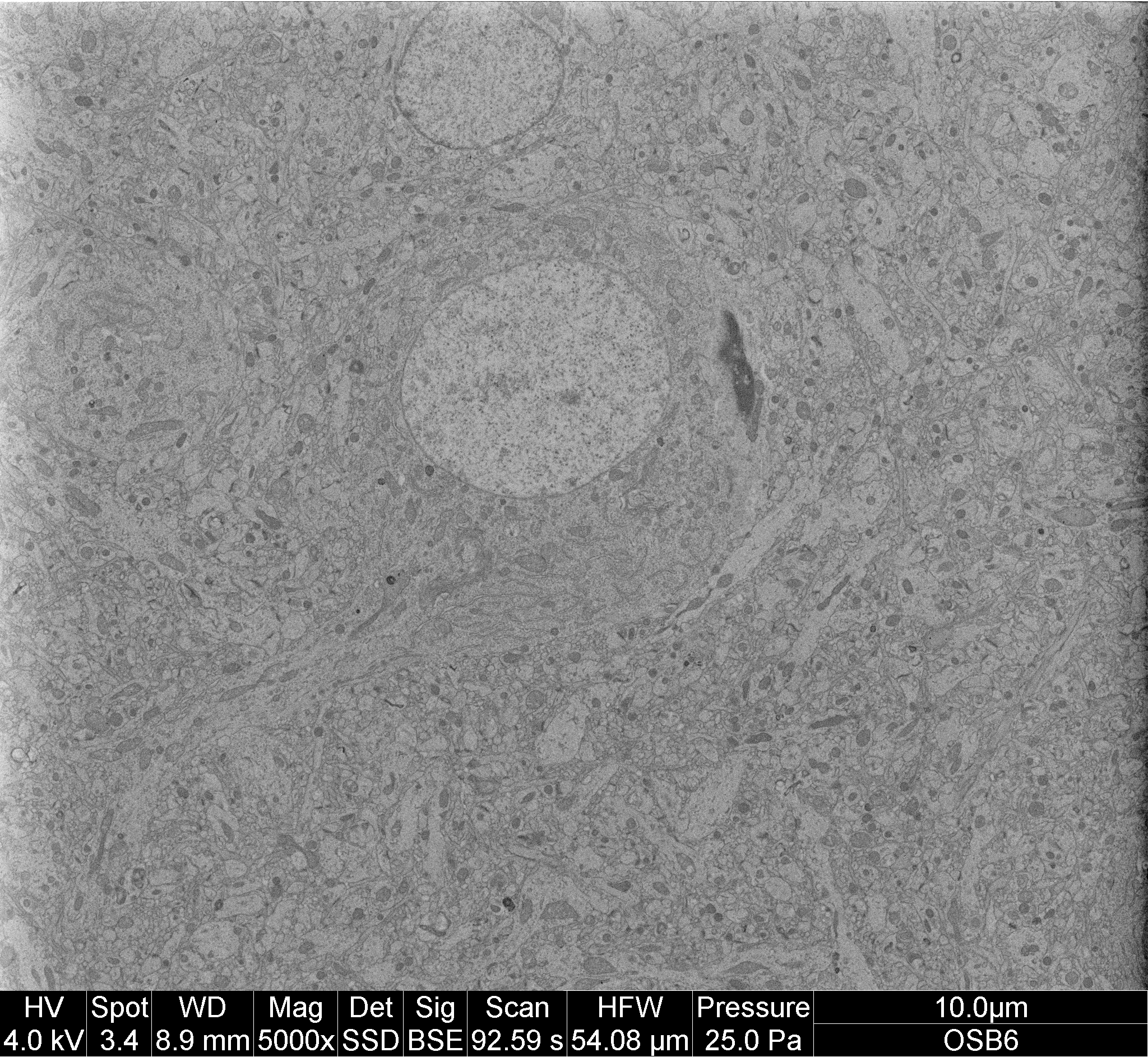

Supplement: Dataset S18 — (250.5 MB ZIP). [file pbio.0020329.sd018.zip › 040604_OS5_st1_1773.tif]

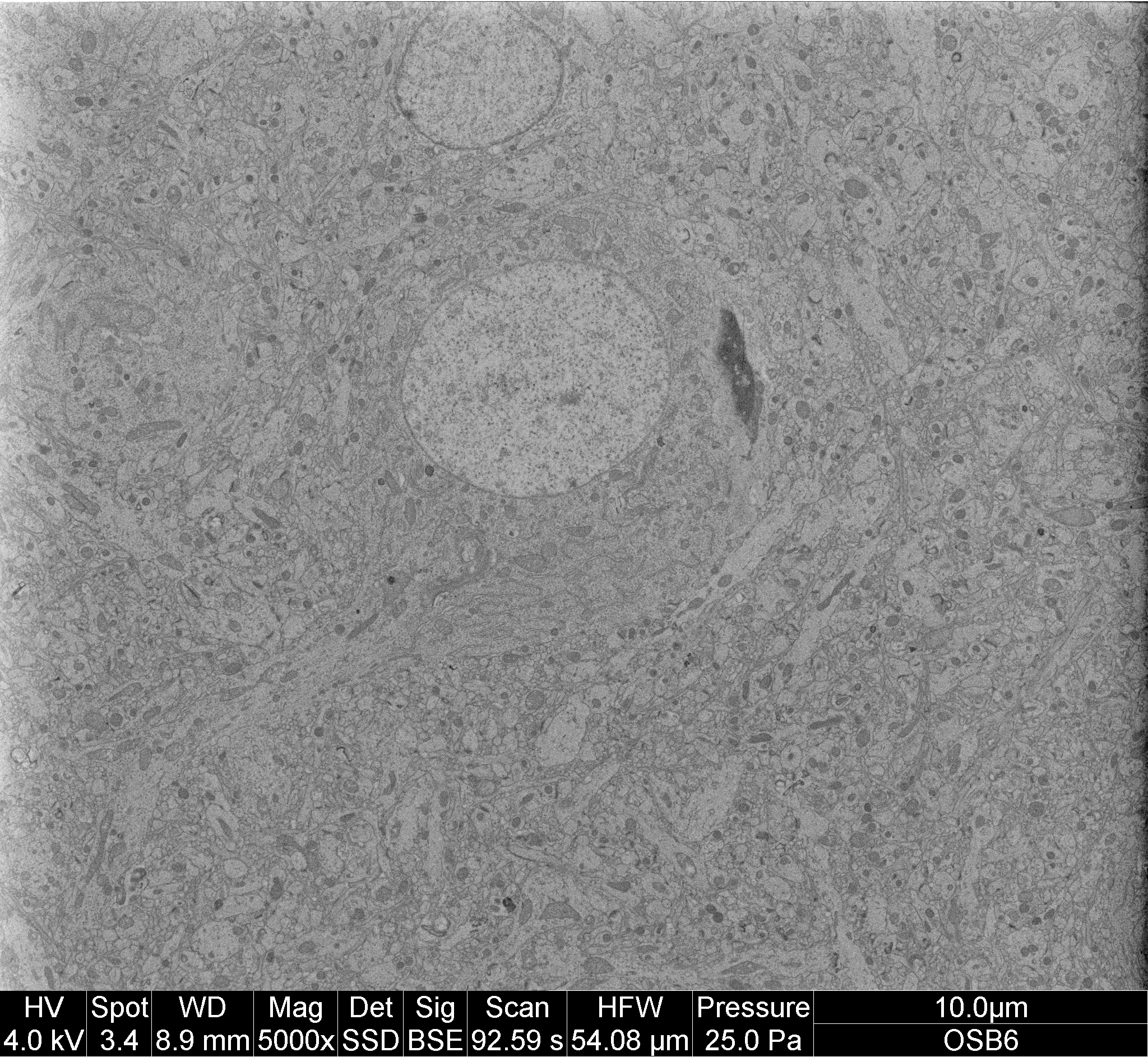

Supplement: Dataset S18 — (250.5 MB ZIP). [file pbio.0020329.sd018.zip › 040604_OS5_st1_1774.tif]

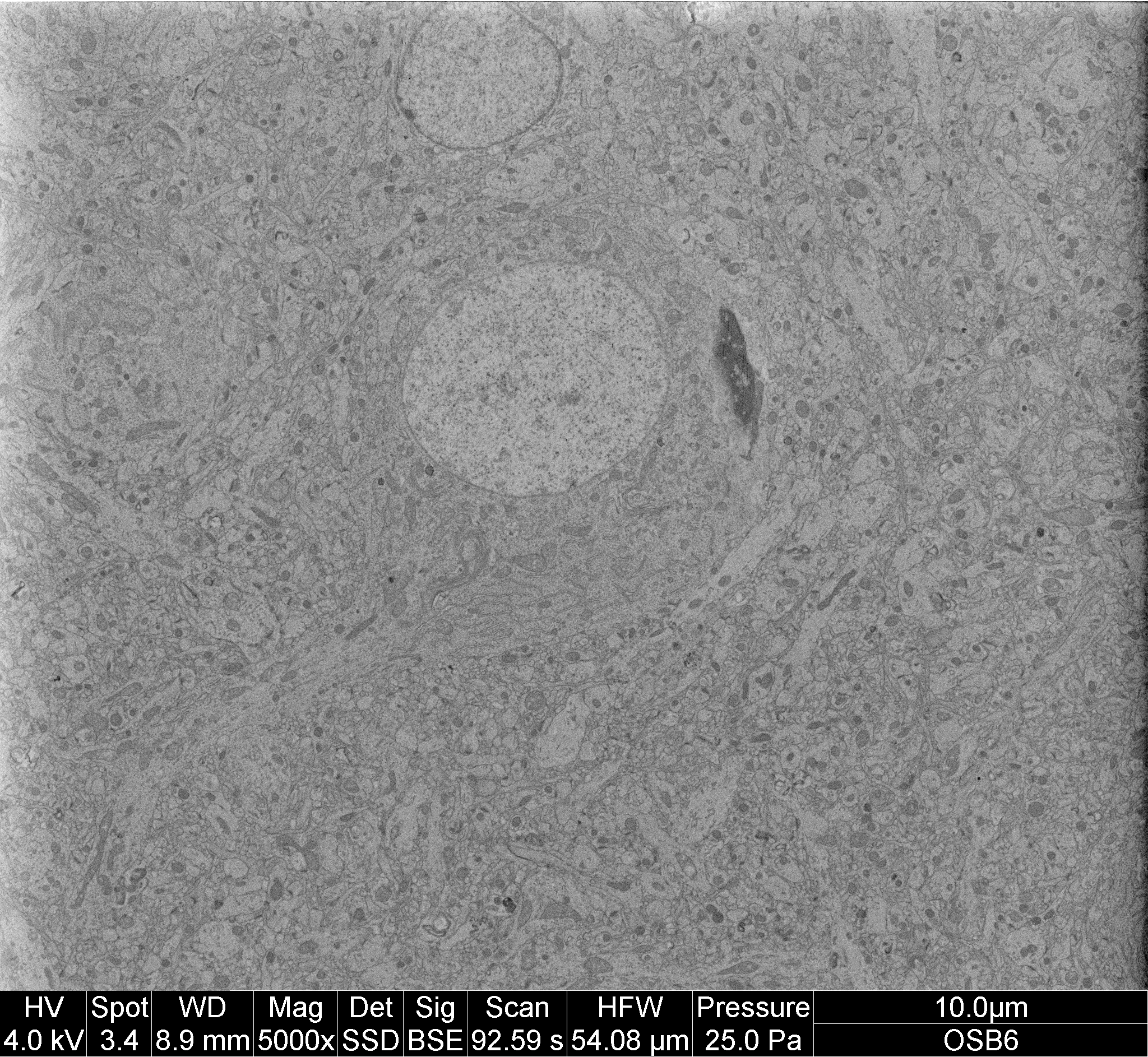

Supplement: Dataset S18 — (250.5 MB ZIP). [file pbio.0020329.sd018.zip › 040604_OS5_st1_1775.tif]

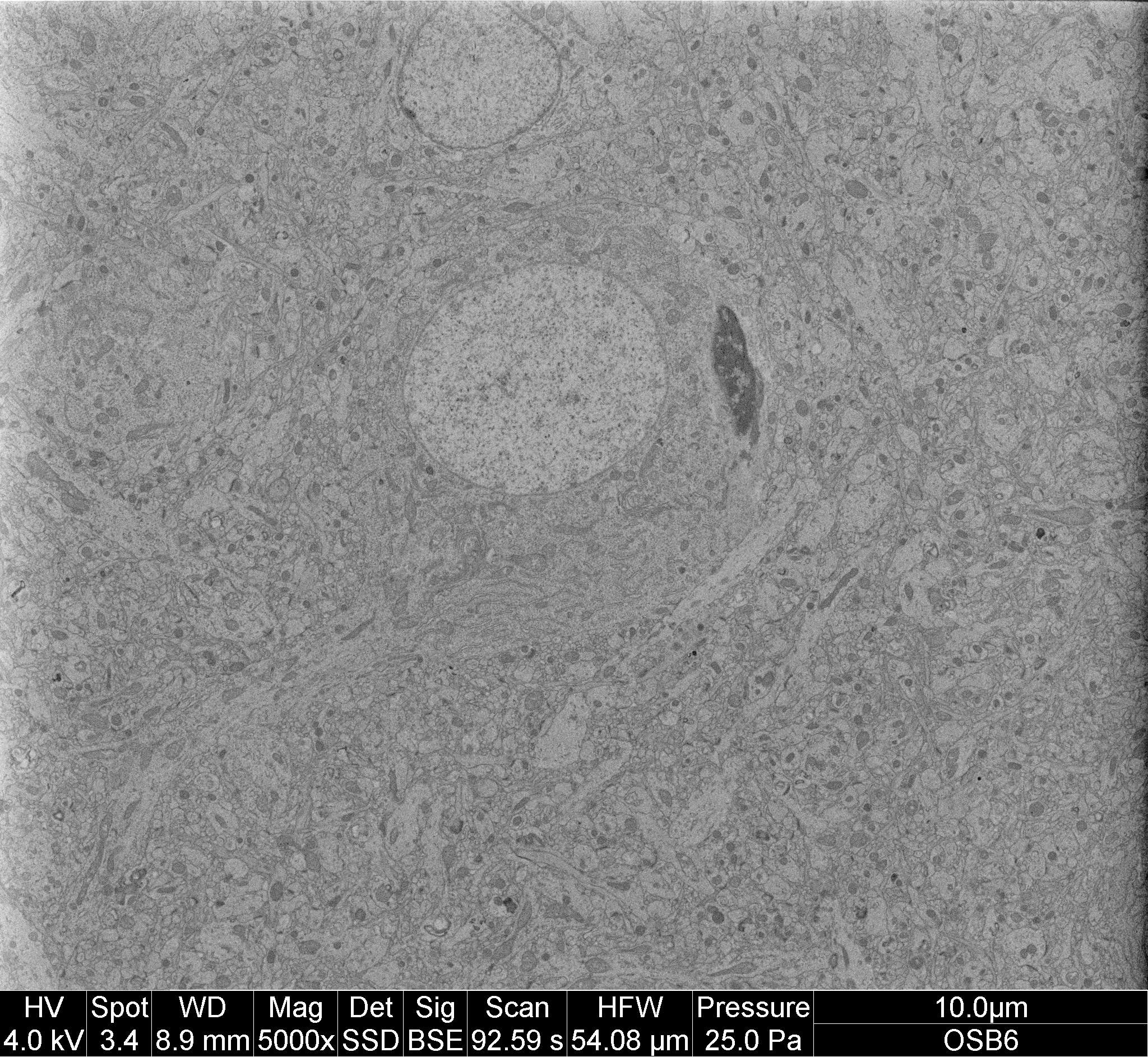

Supplement: Dataset S18 — (250.5 MB ZIP). [file pbio.0020329.sd018.zip › 040604_OS5_st1_1776.tif]

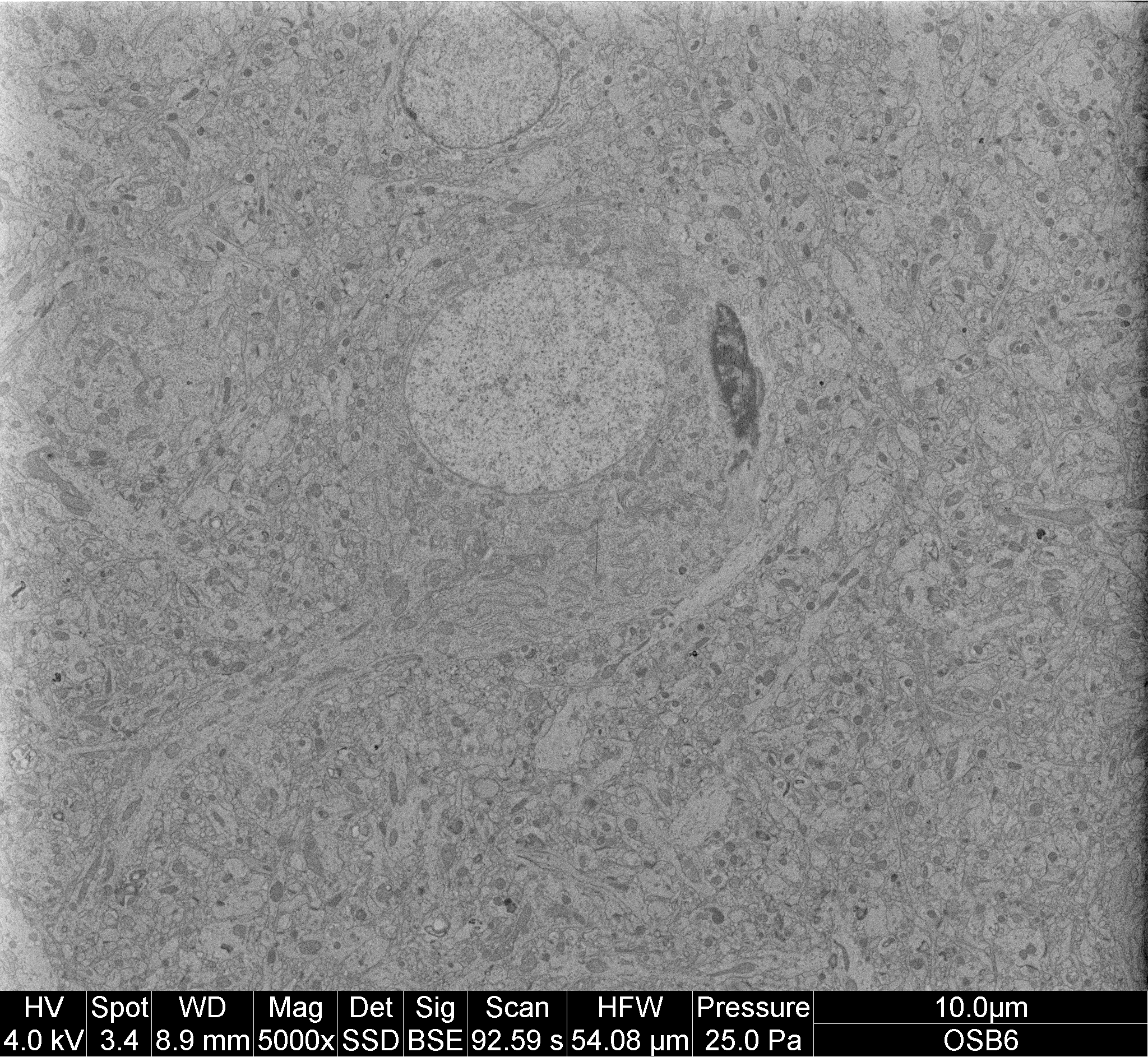

Supplement: Dataset S18 — (250.5 MB ZIP). [file pbio.0020329.sd018.zip › 040604_OS5_st1_1777.tif]

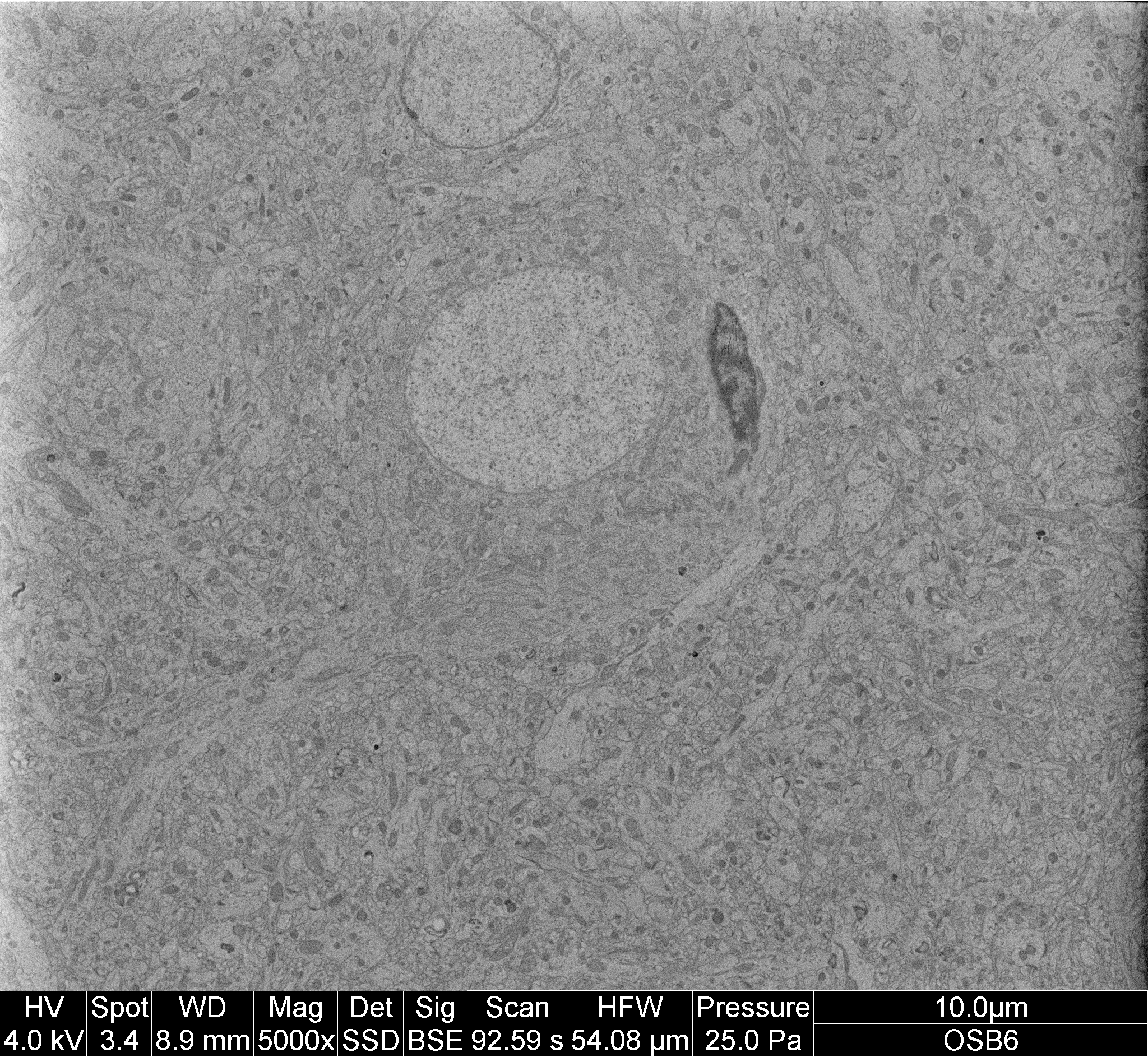

Supplement: Dataset S18 — (250.5 MB ZIP). [file pbio.0020329.sd018.zip › 040604_OS5_st1_1778.tif]

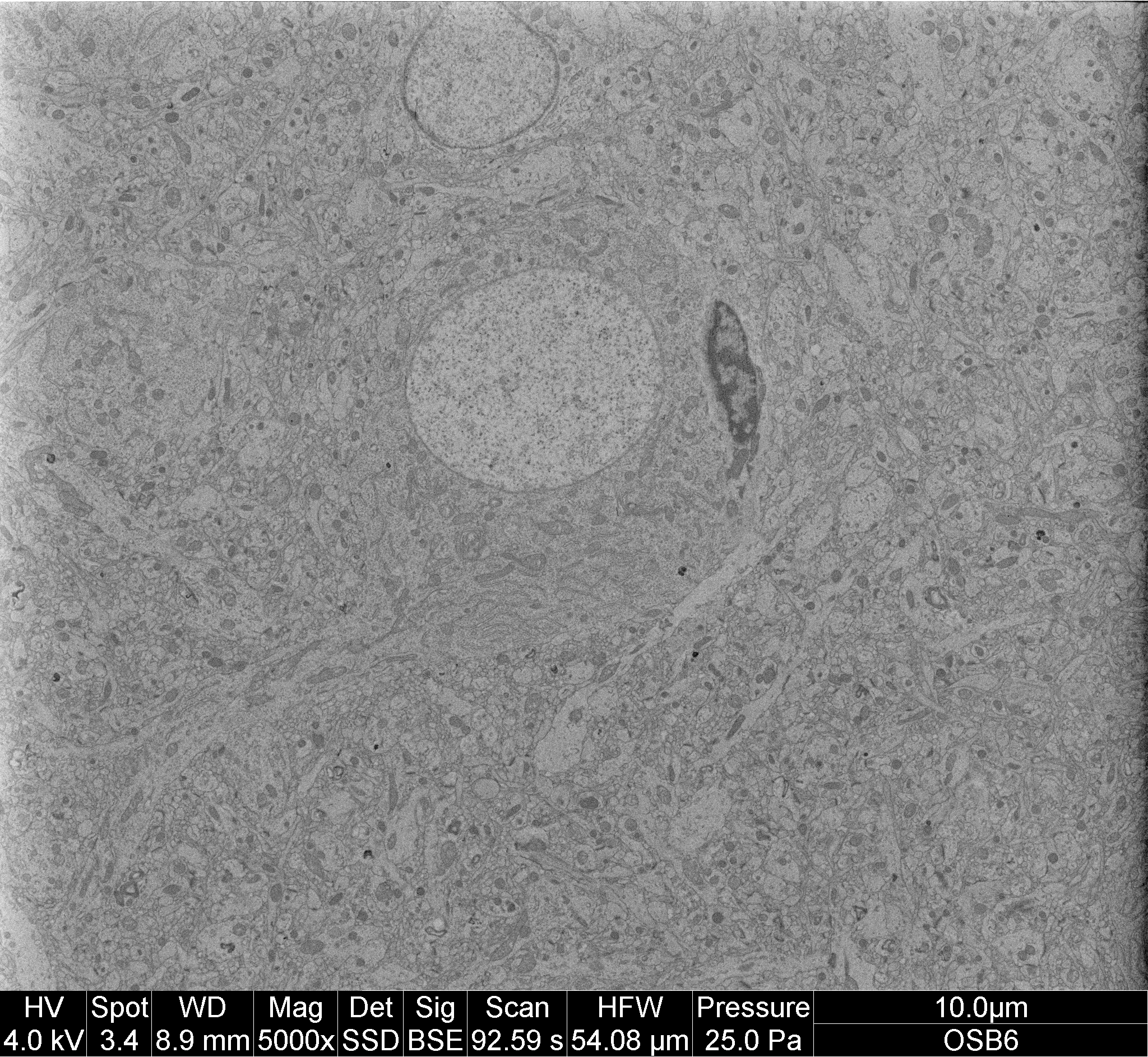

Supplement: Dataset S18 — (250.5 MB ZIP). [file pbio.0020329.sd018.zip › 040604_OS5_st1_1779.tif]

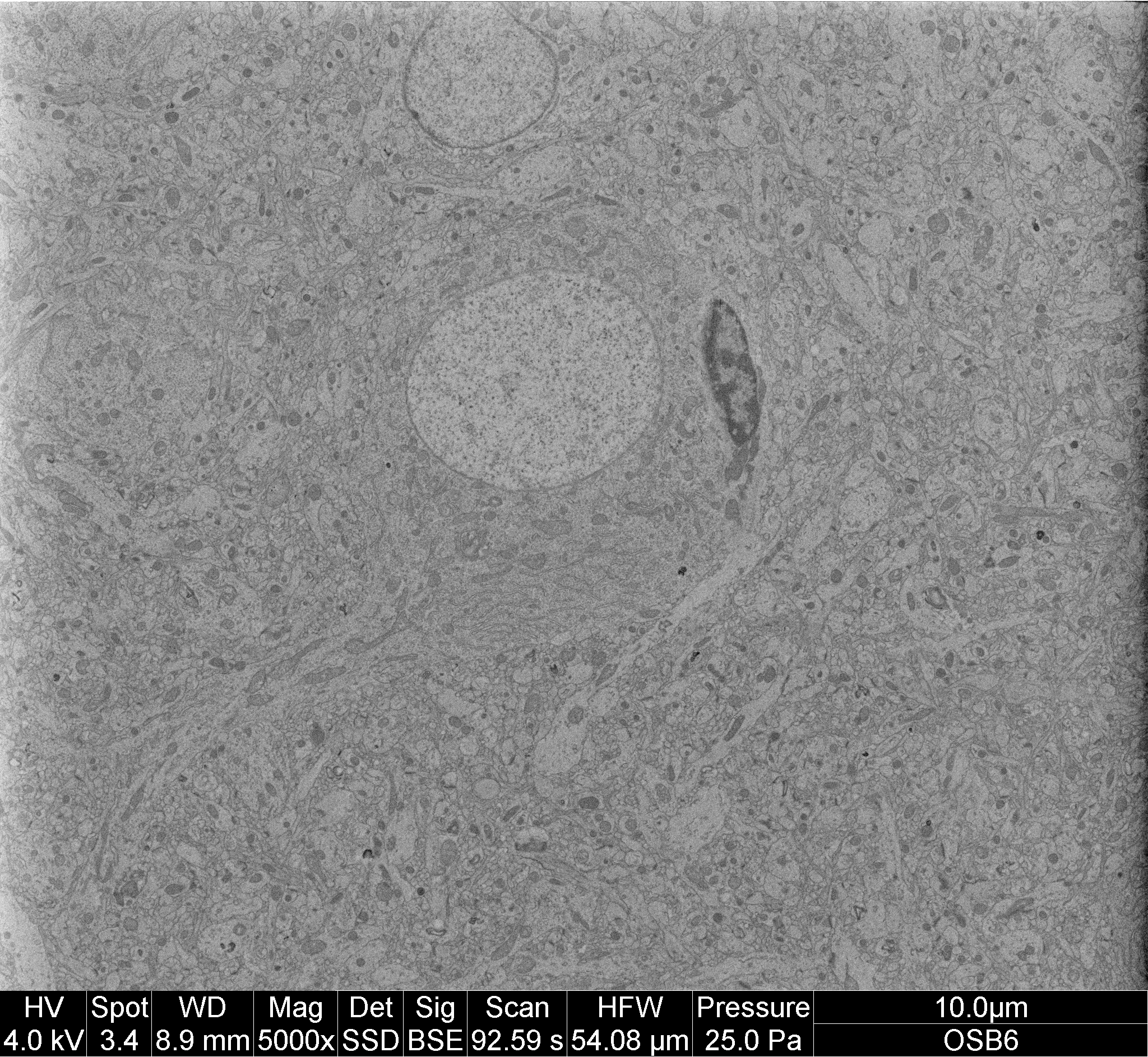

Supplement: Dataset S18 — (250.5 MB ZIP). [file pbio.0020329.sd018.zip › 040604_OS5_st1_1780.tif]

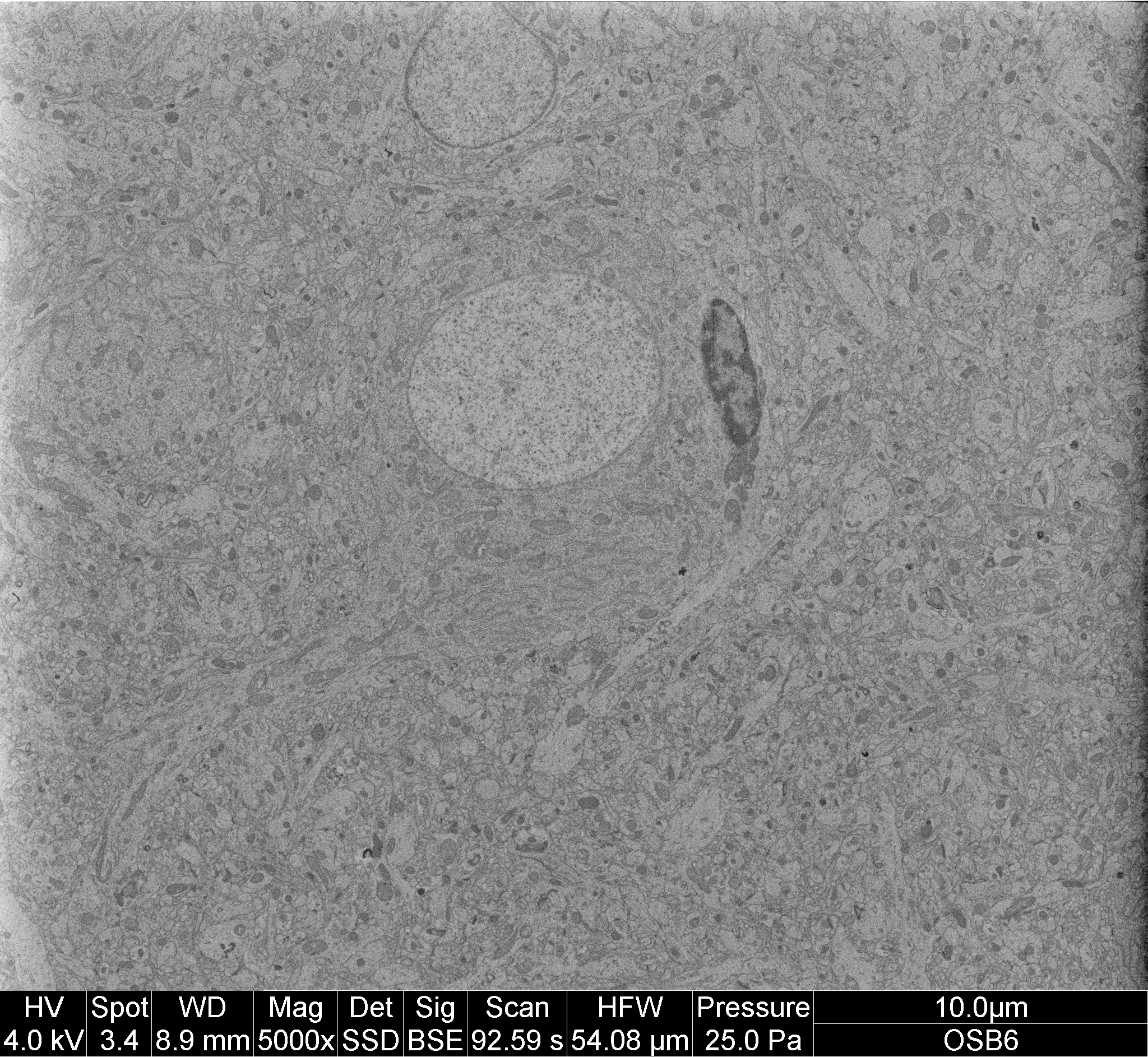

Supplement: Dataset S18 — (250.5 MB ZIP). [file pbio.0020329.sd018.zip › 040604_OS5_st1_1781.tif]

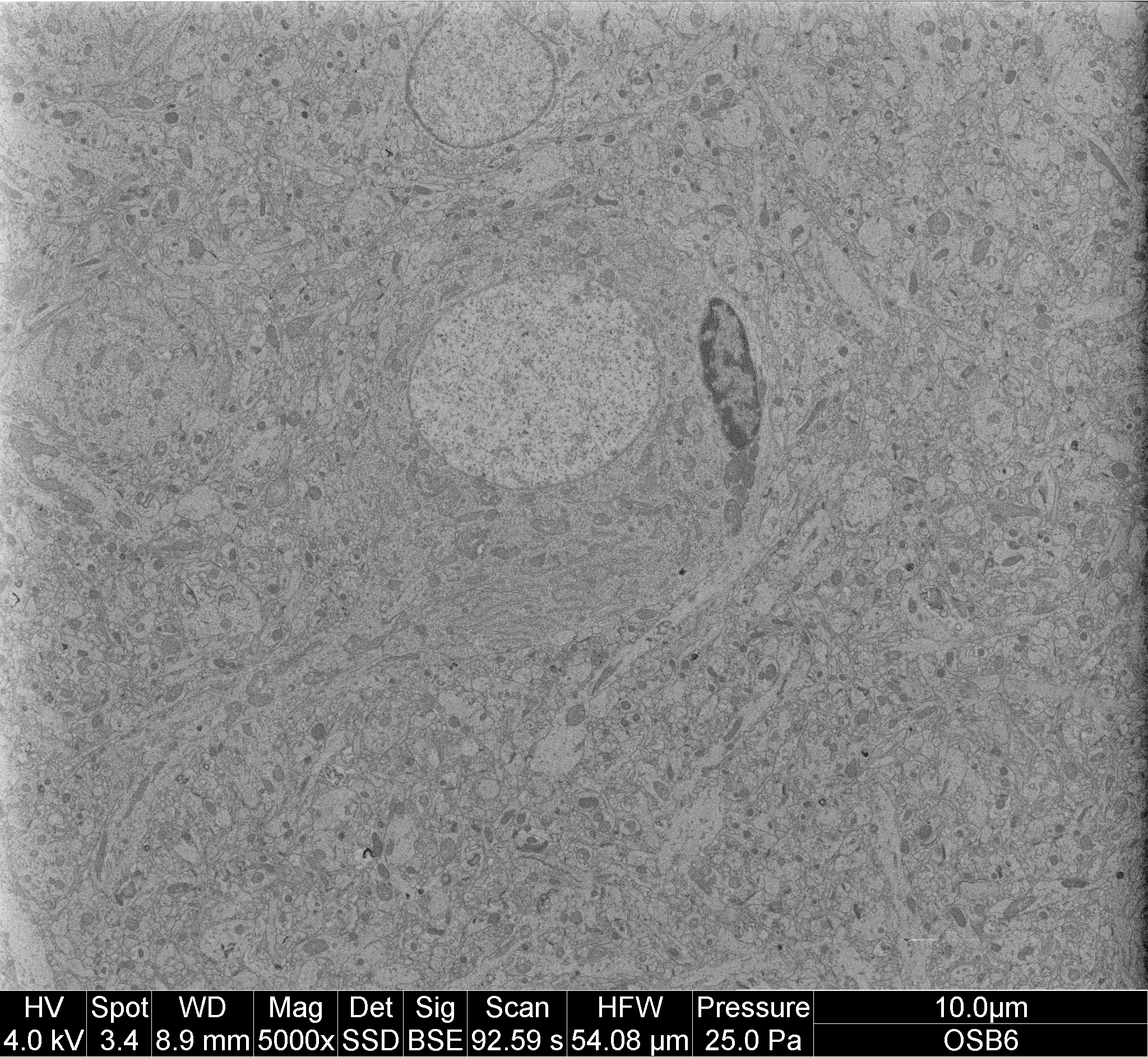

Supplement: Dataset S18 — (250.5 MB ZIP). [file pbio.0020329.sd018.zip › 040604_OS5_st1_1782.tif]

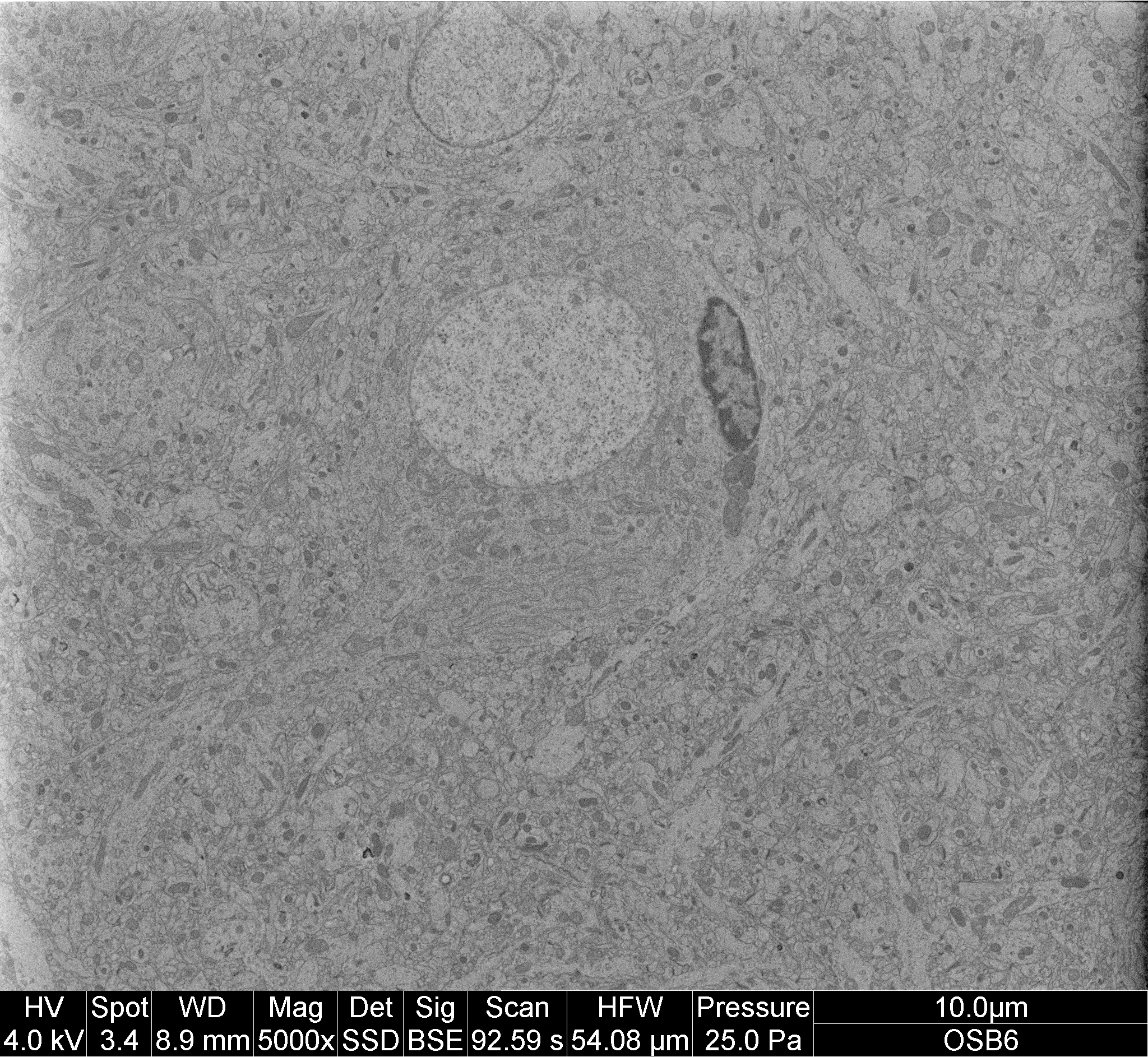

Supplement: Dataset S18 — (250.5 MB ZIP). [file pbio.0020329.sd018.zip › 040604_OS5_st1_1783.tif]

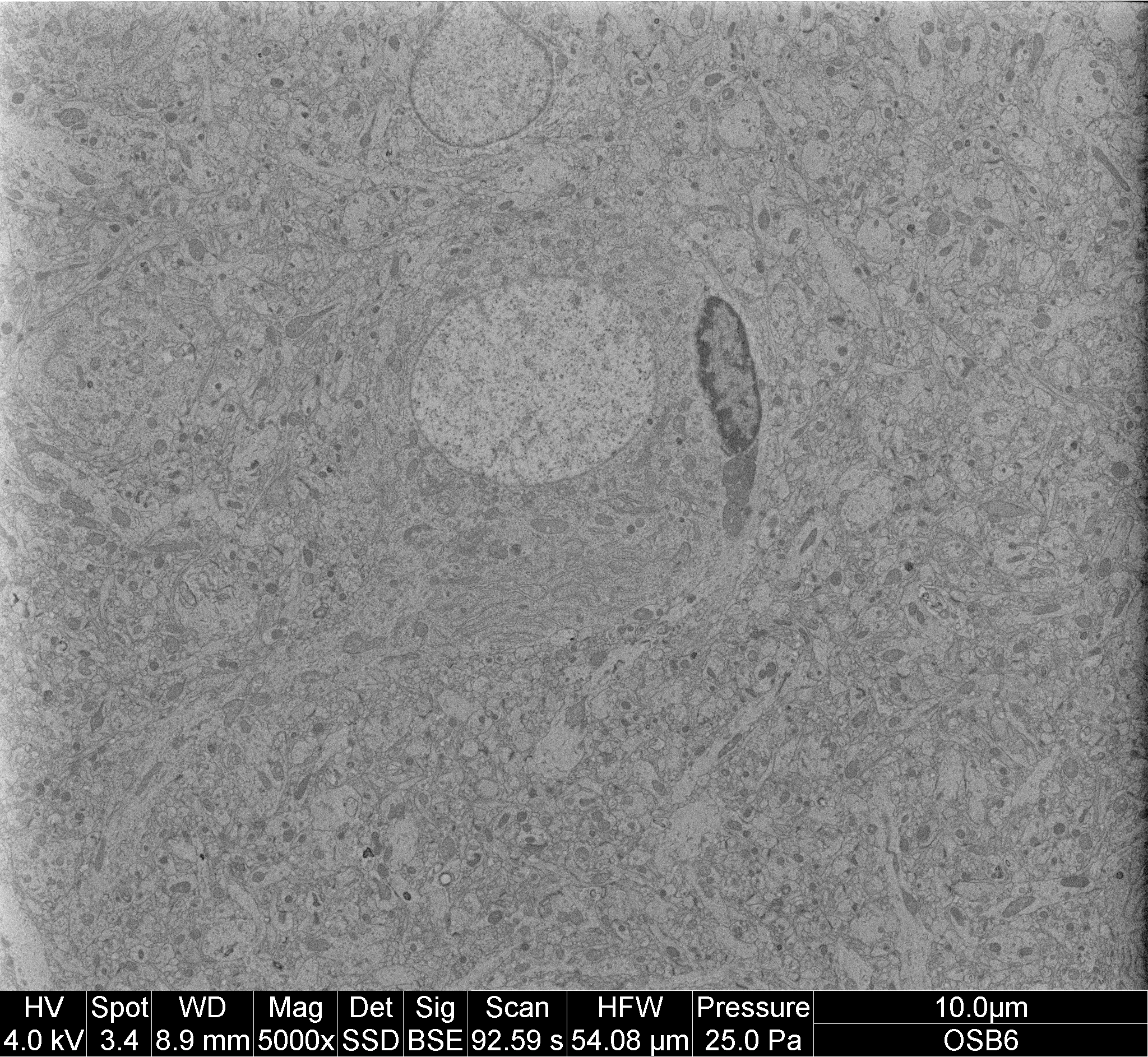

Supplement: Dataset S18 — (250.5 MB ZIP). [file pbio.0020329.sd018.zip › 040604_OS5_st1_1784.tif]

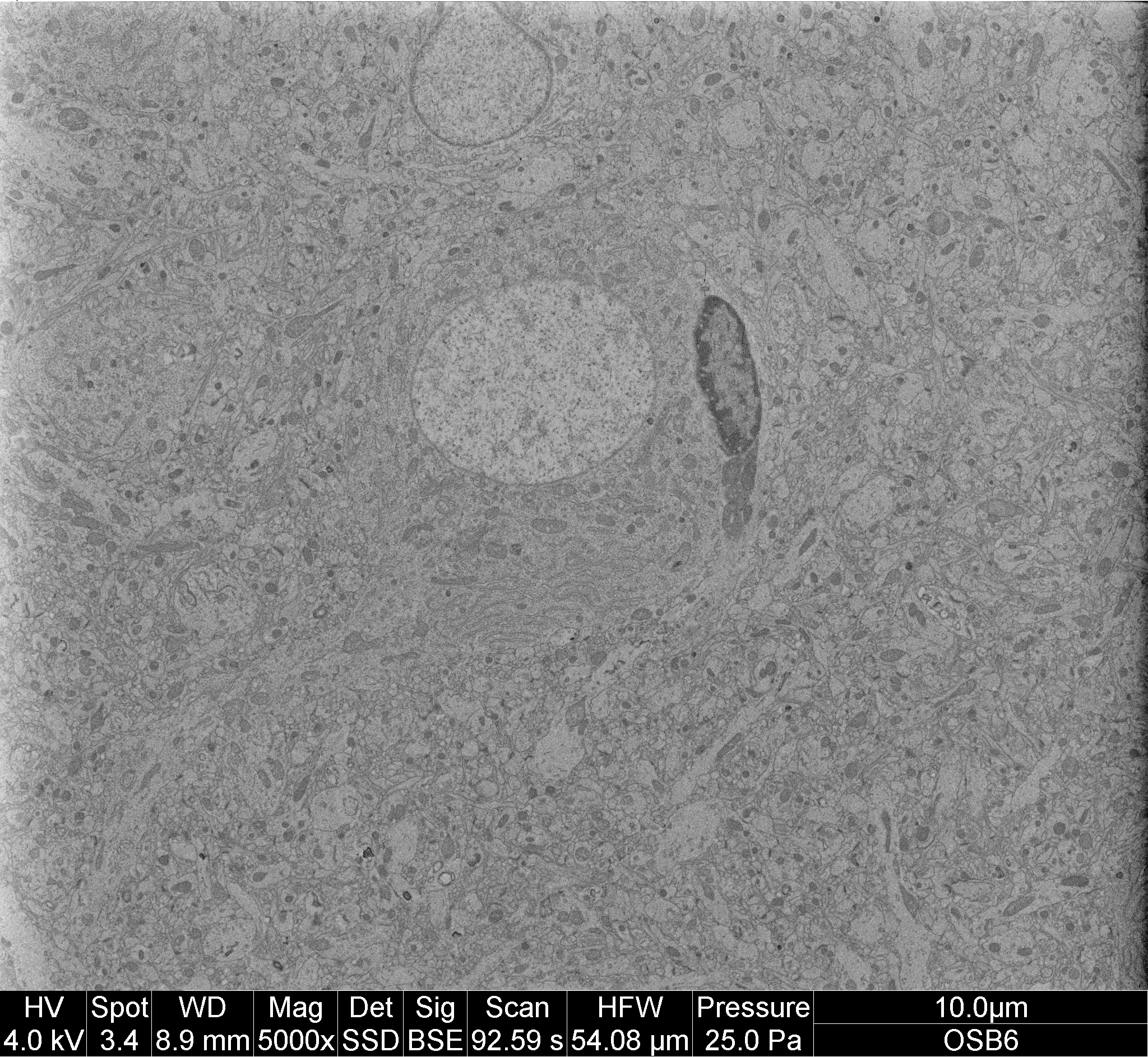

Supplement: Dataset S18 — (250.5 MB ZIP). [file pbio.0020329.sd018.zip › 040604_OS5_st1_1785.tif]

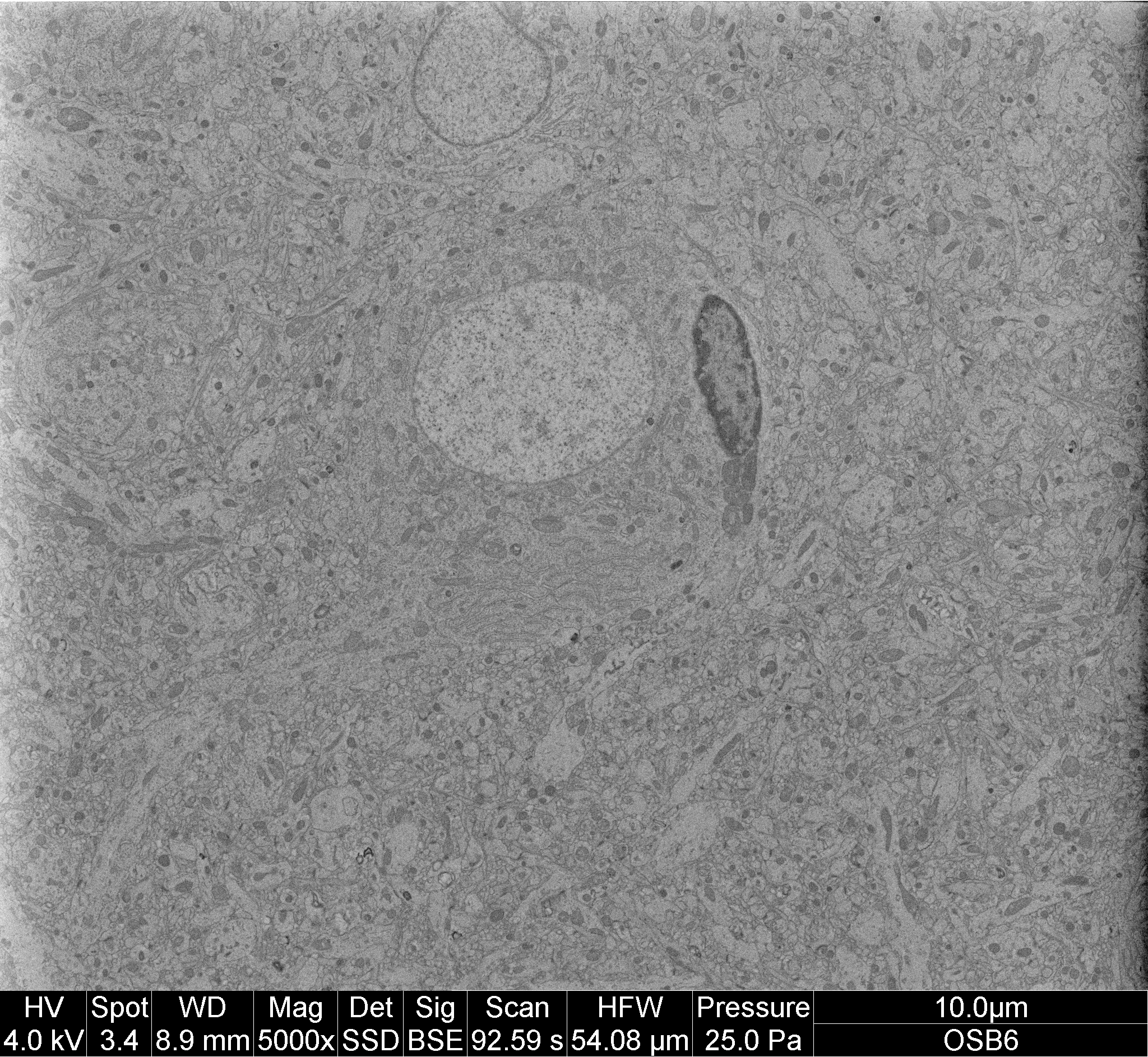

Supplement: Dataset S18 — (250.5 MB ZIP). [file pbio.0020329.sd018.zip › 040604_OS5_st1_1786.tif]

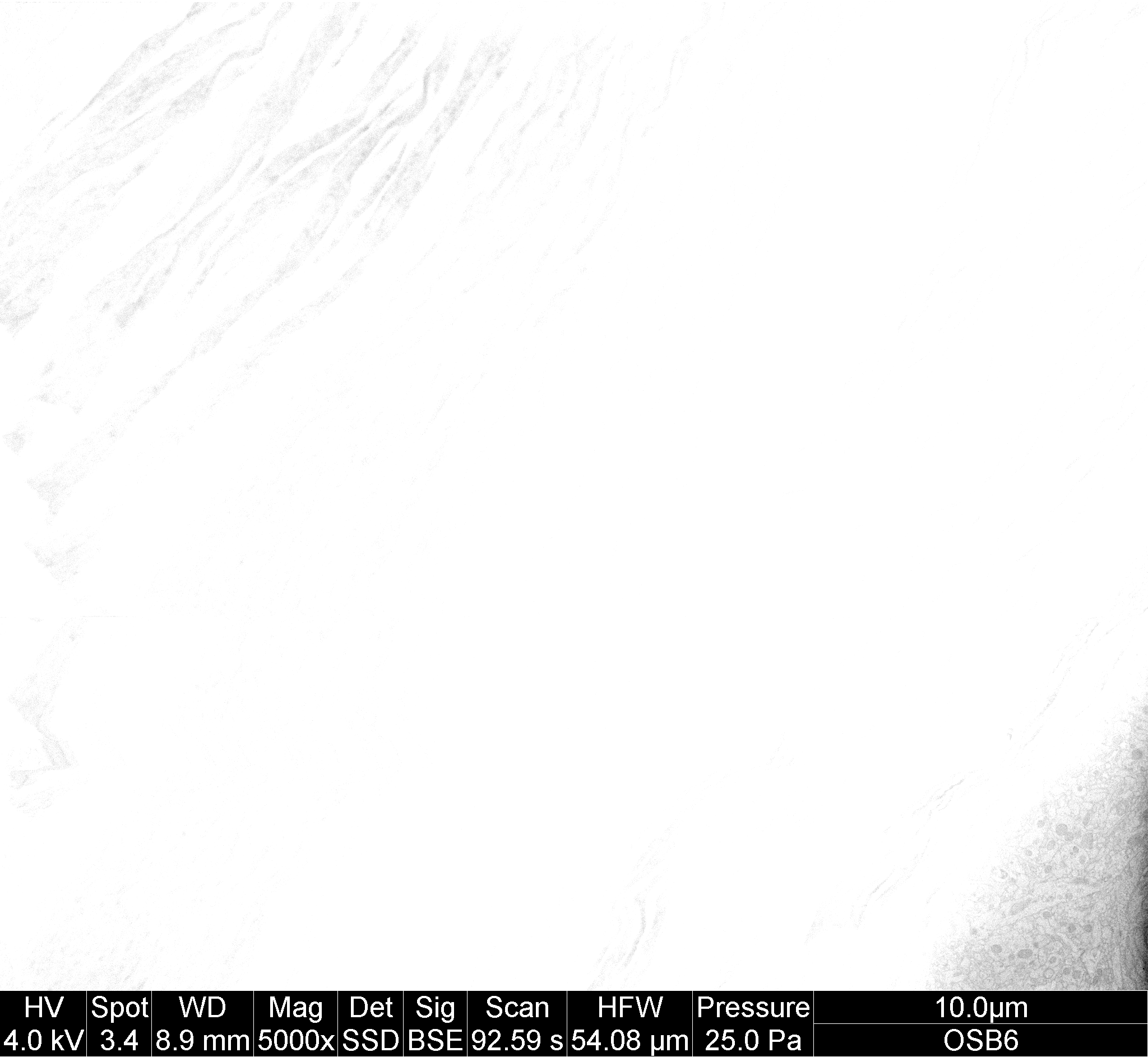

Supplement: Dataset S18 — (250.5 MB ZIP). [file pbio.0020329.sd018.zip › 040604_OS5_st1_1787.tif]

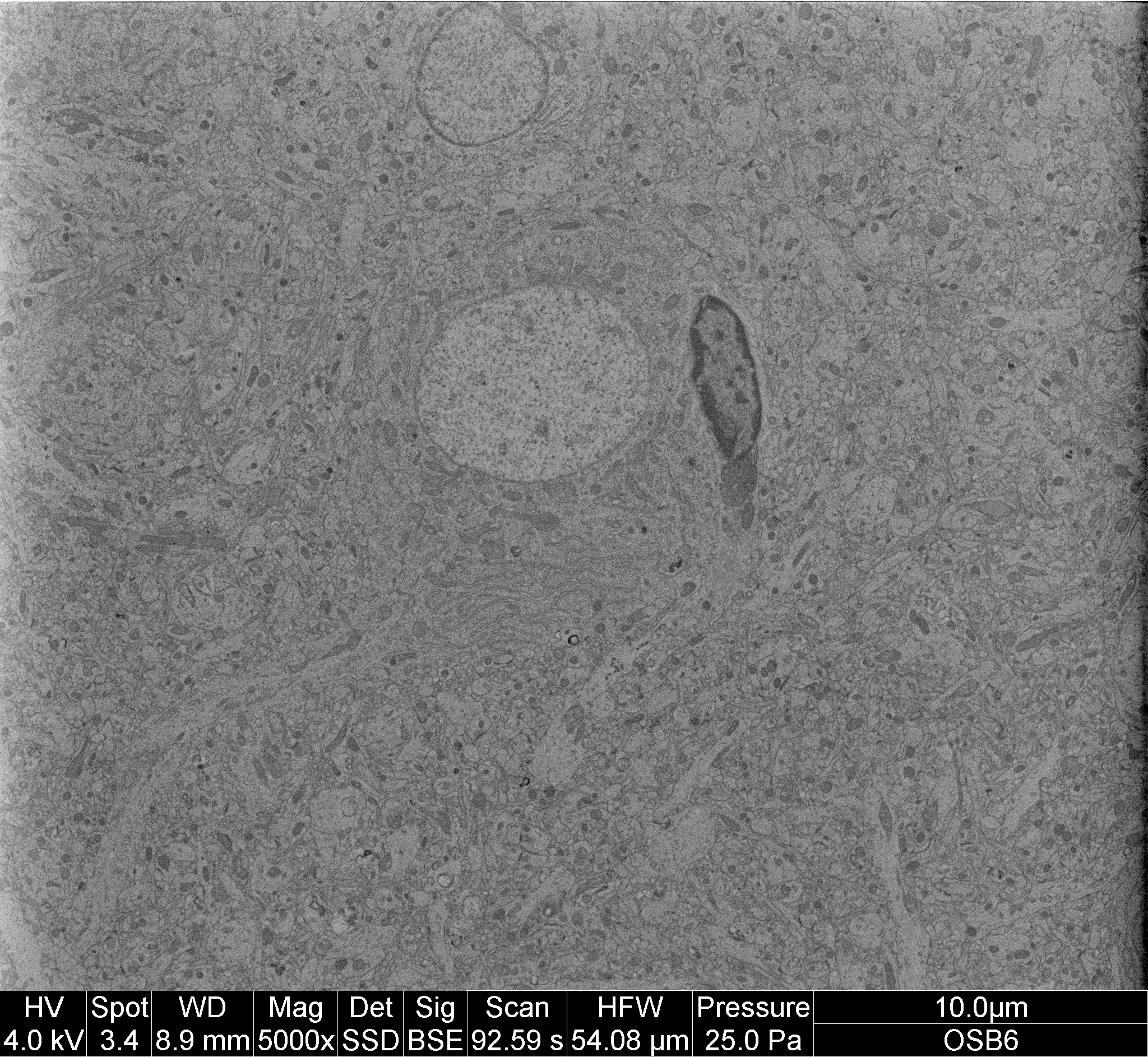

Supplement: Dataset S18 — (250.5 MB ZIP). [file pbio.0020329.sd018.zip › 040604_OS5_st1_1788.tif]

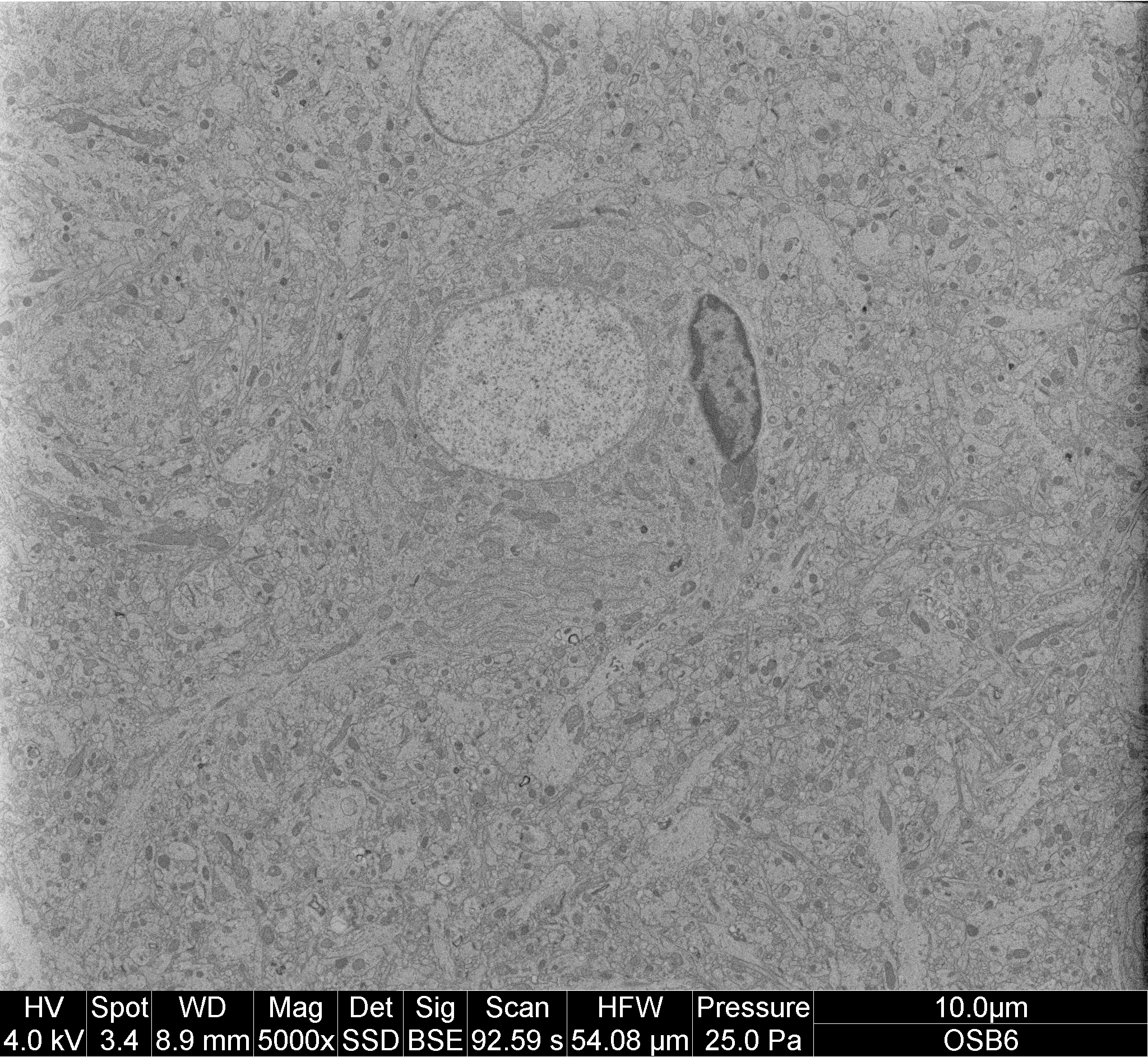

Supplement: Dataset S18 — (250.5 MB ZIP). [file pbio.0020329.sd018.zip › 040604_OS5_st1_1789.tif]

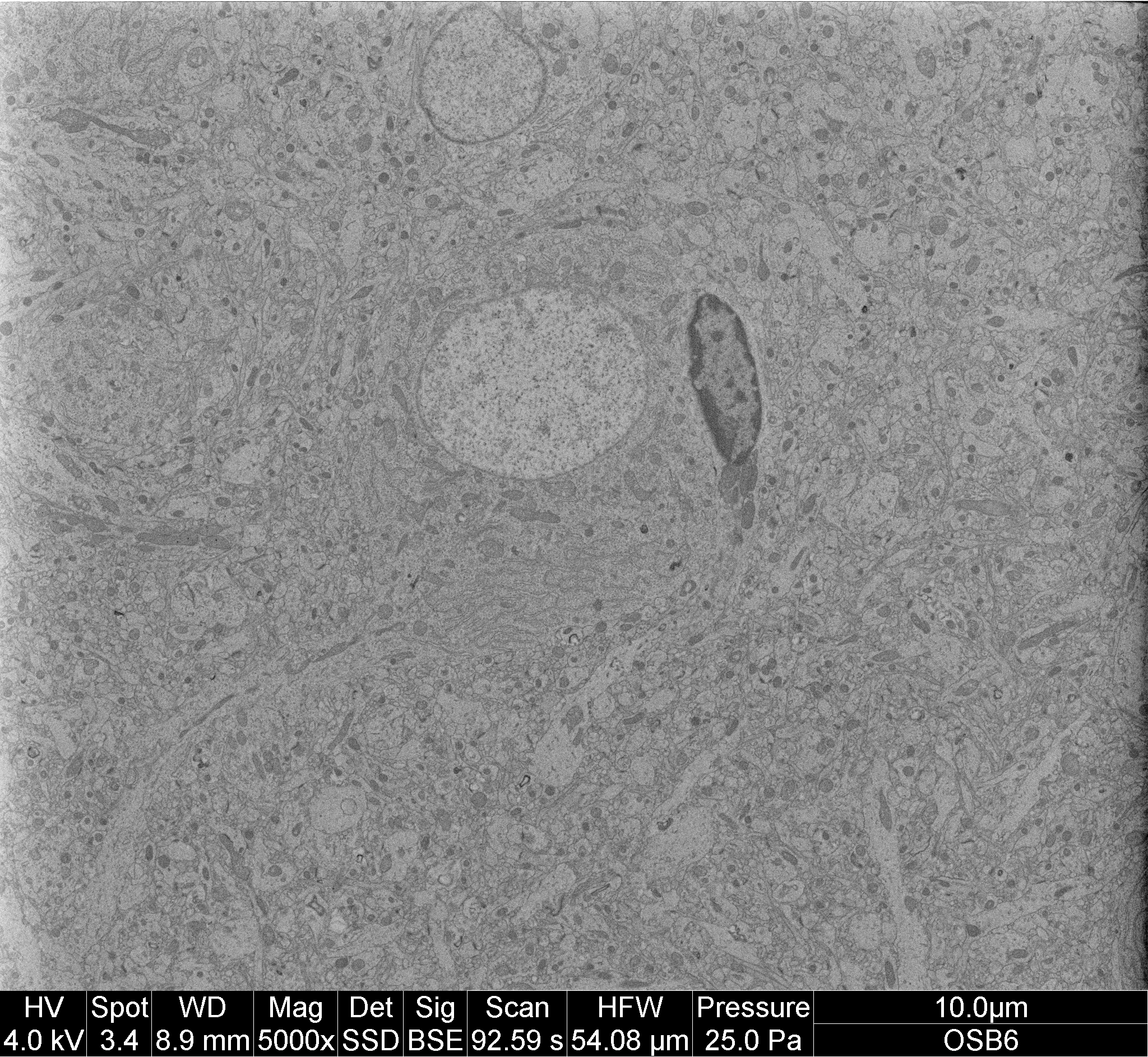

Supplement: Dataset S18 — (250.5 MB ZIP). [file pbio.0020329.sd018.zip › 040604_OS5_st1_1790.tif]

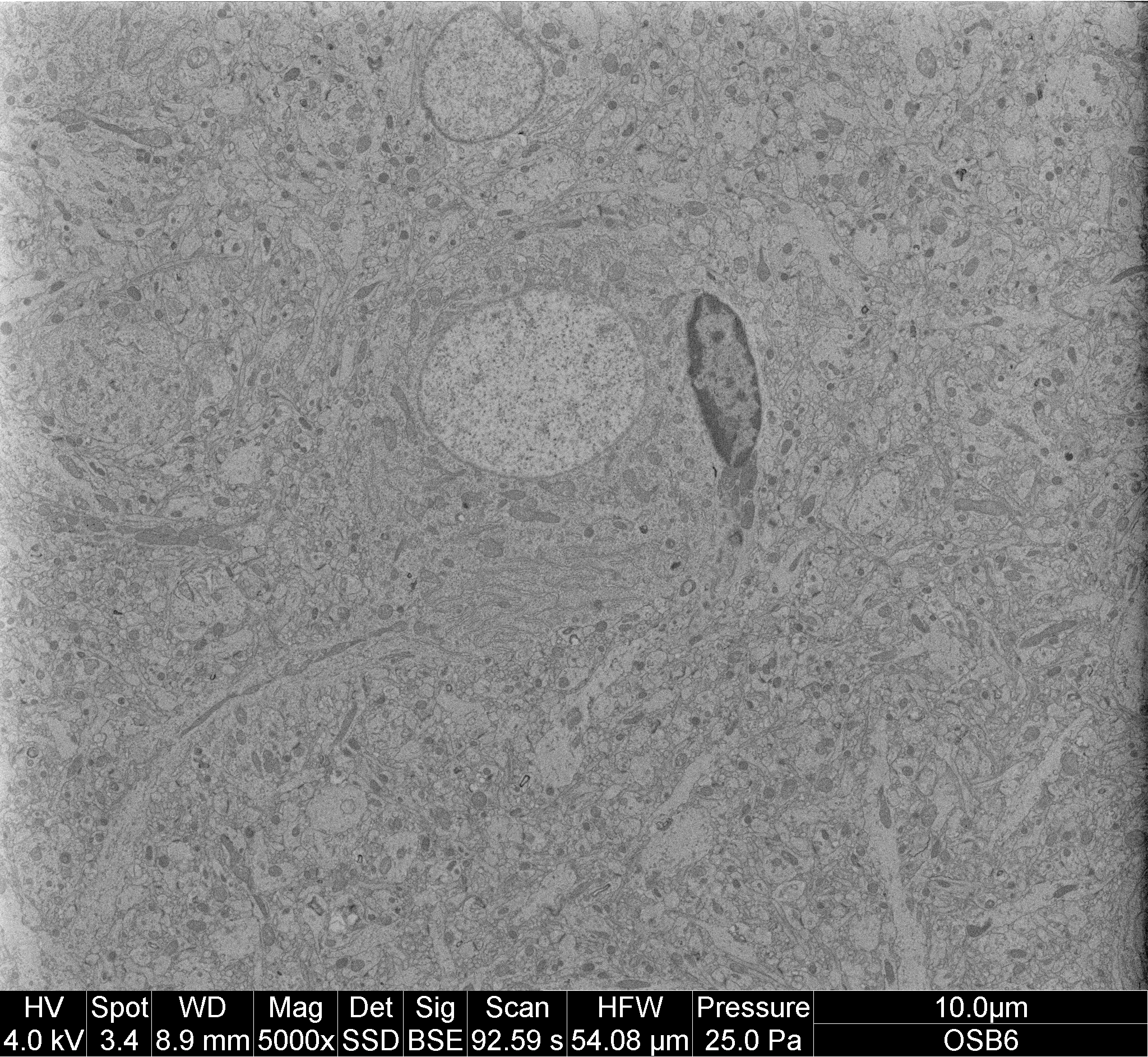

Supplement: Dataset S18 — (250.5 MB ZIP). [file pbio.0020329.sd018.zip › 040604_OS5_st1_1791.tif]

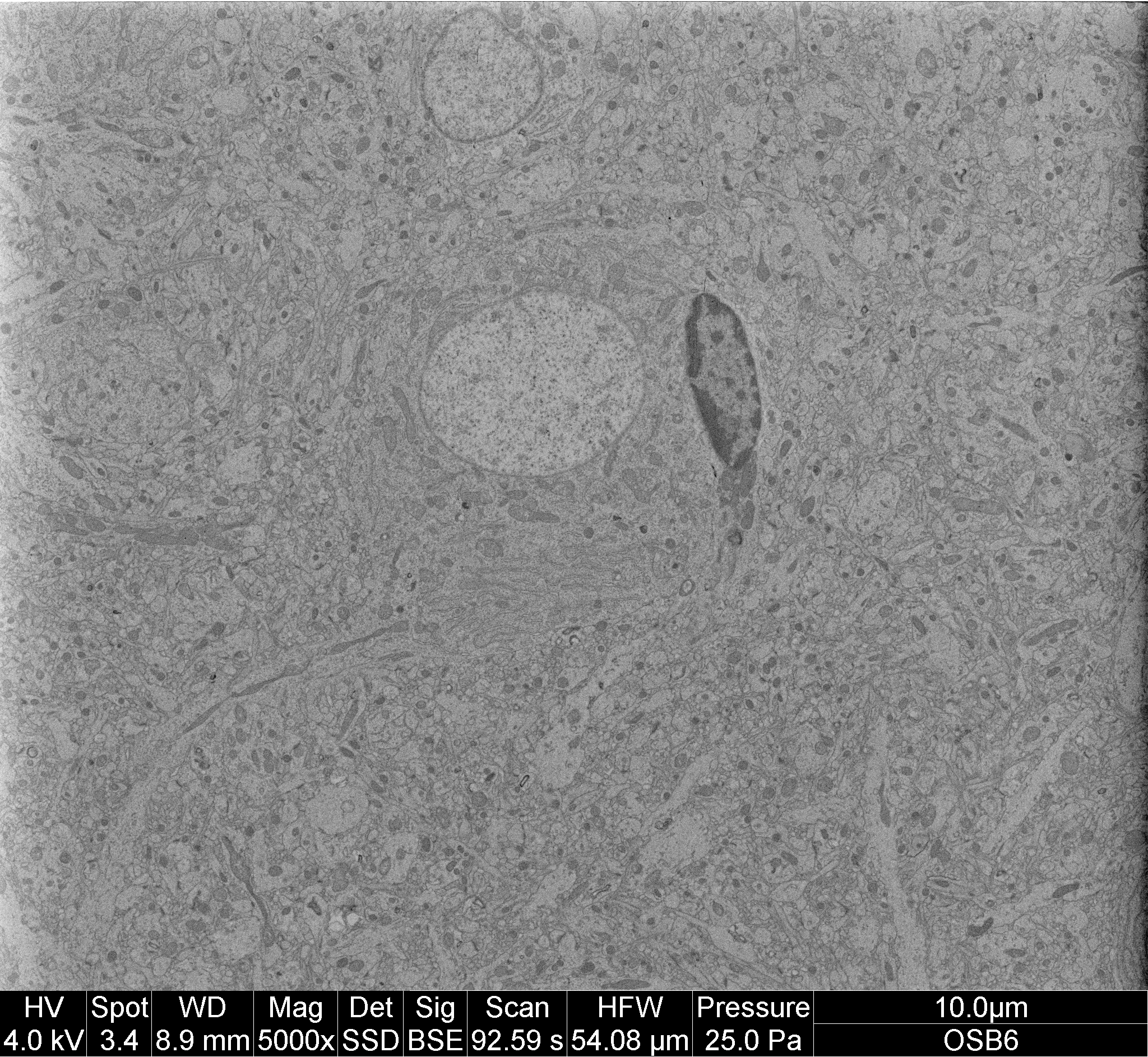

Supplement: Dataset S18 — (250.5 MB ZIP). [file pbio.0020329.sd018.zip › 040604_OS5_st1_1792.tif]

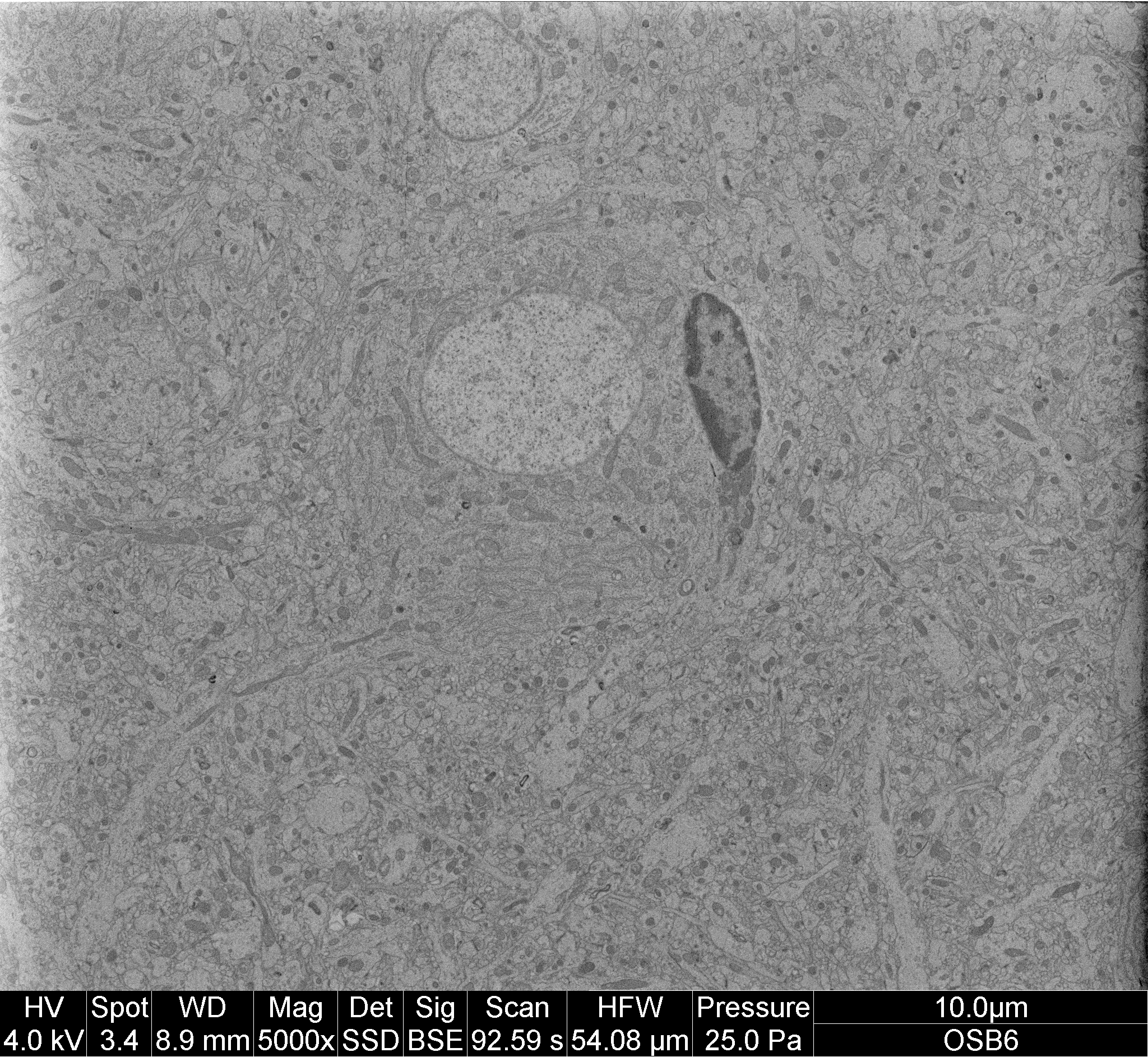

Supplement: Dataset S18 — (250.5 MB ZIP). [file pbio.0020329.sd018.zip › 040604_OS5_st1_1793.tif]

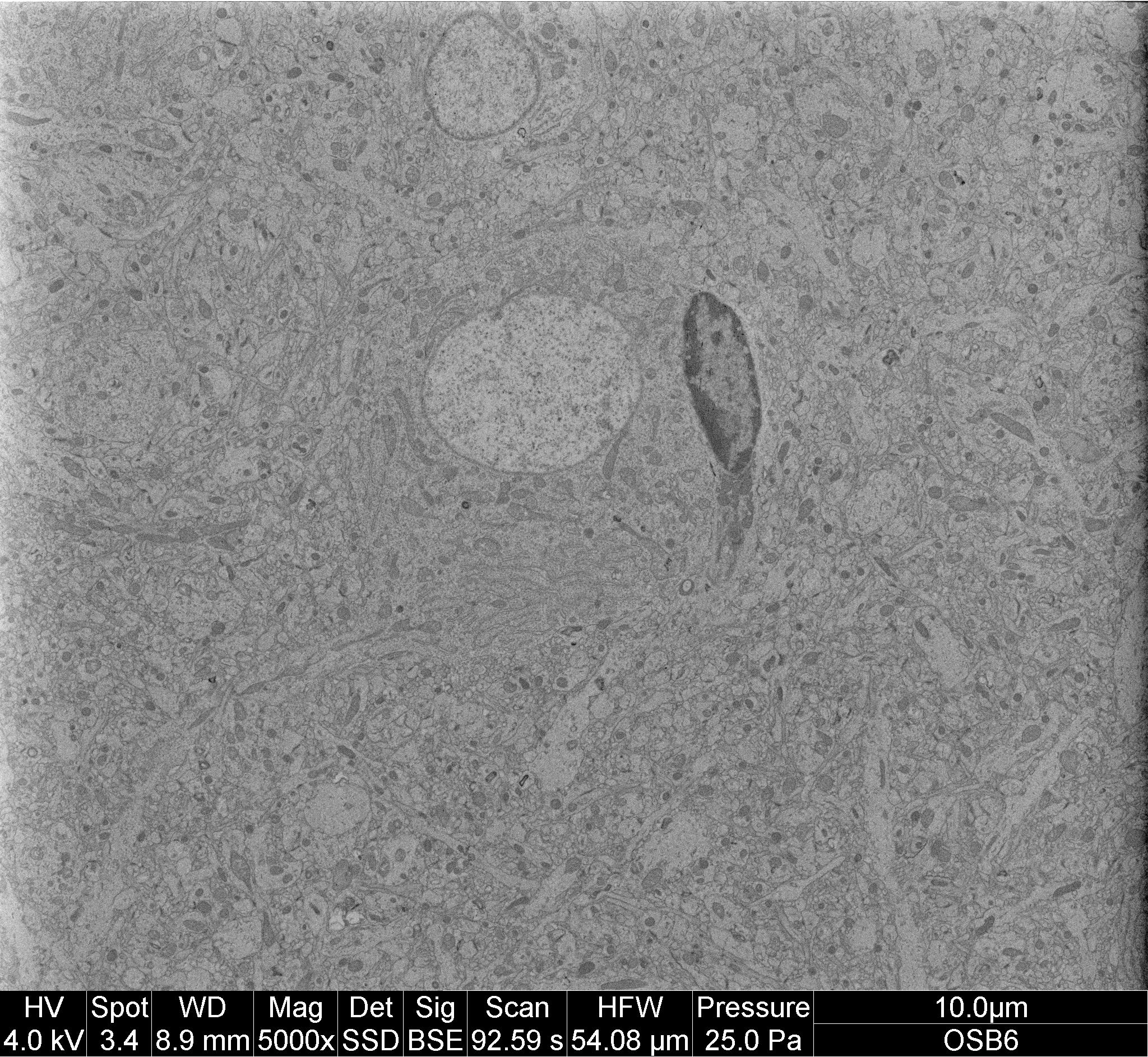

Supplement: Dataset S18 — (250.5 MB ZIP). [file pbio.0020329.sd018.zip › 040604_OS5_st1_1794.tif]

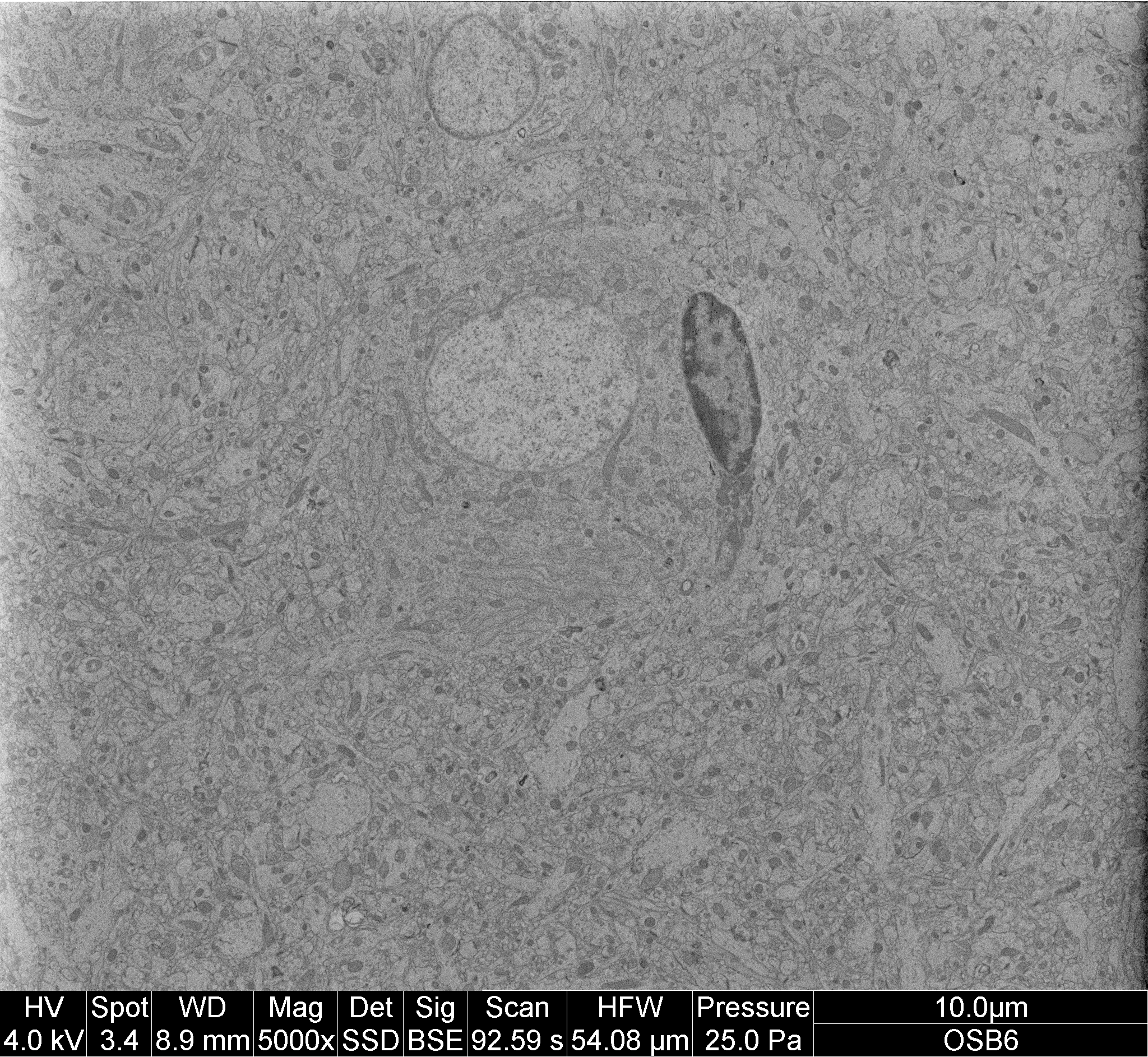

Supplement: Dataset S18 — (250.5 MB ZIP). [file pbio.0020329.sd018.zip › 040604_OS5_st1_1795.tif]

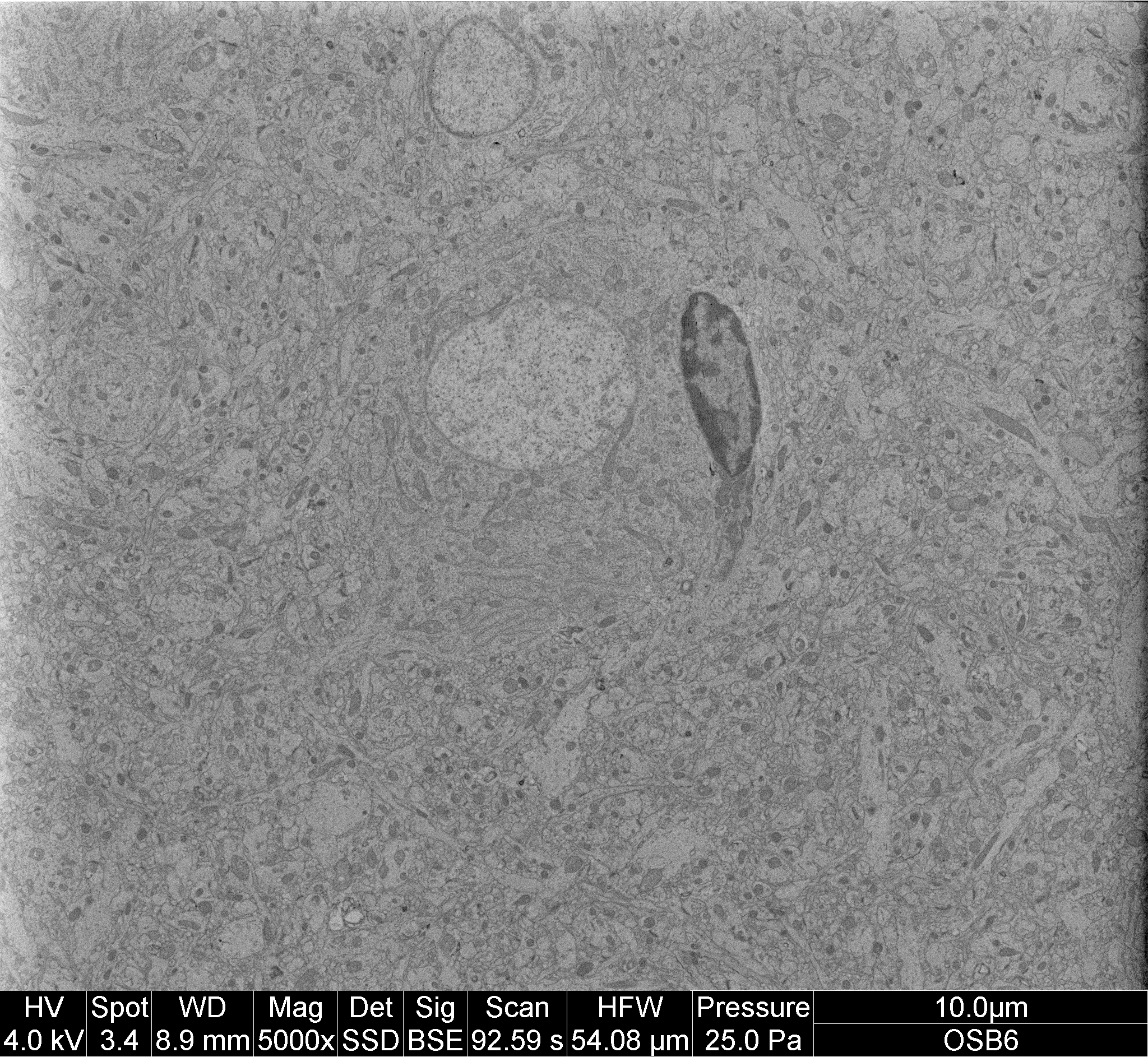

Supplement: Dataset S18 — (250.5 MB ZIP). [file pbio.0020329.sd018.zip › 040604_OS5_st1_1796.tif]

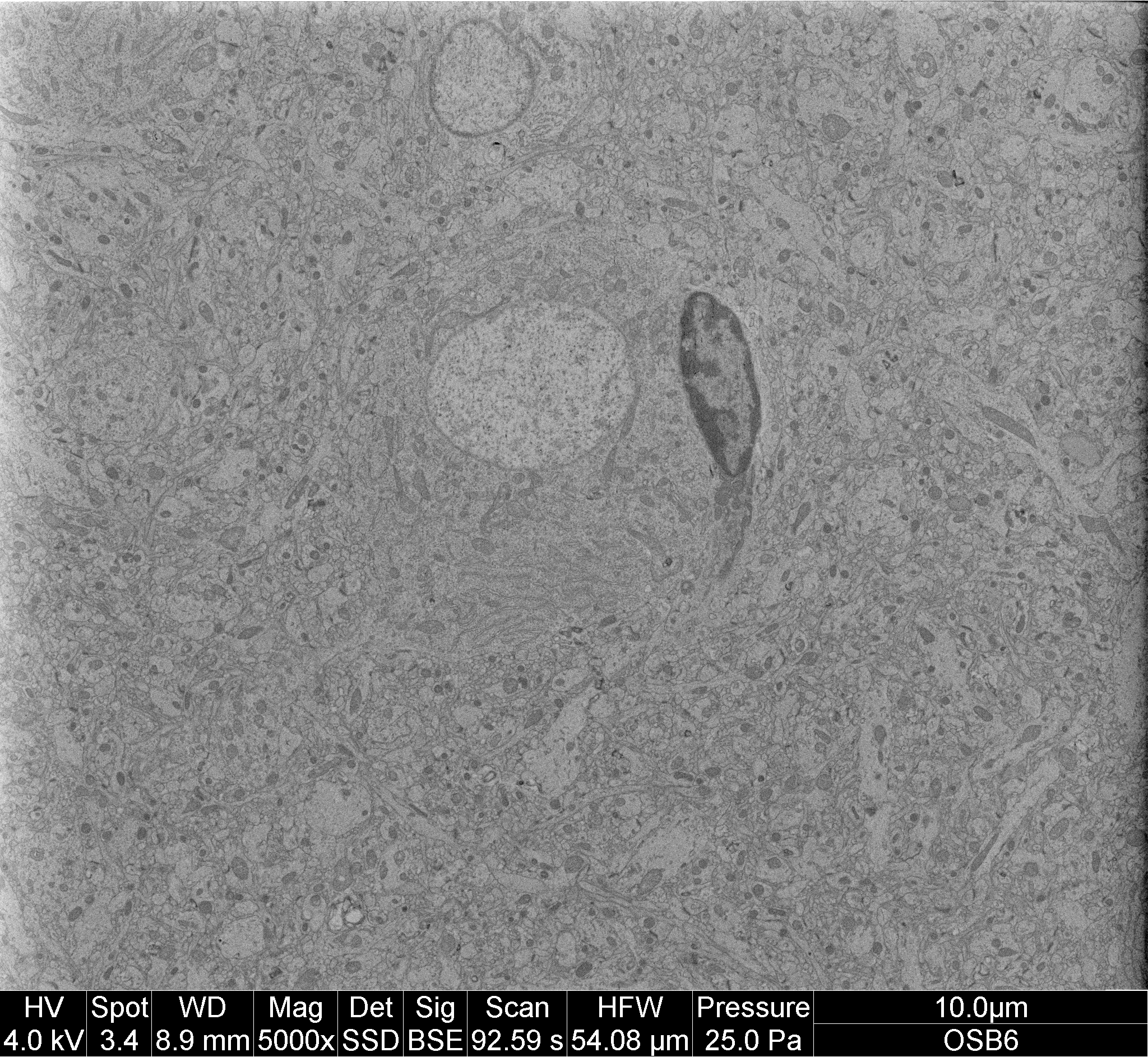

Supplement: Dataset S18 — (250.5 MB ZIP). [file pbio.0020329.sd018.zip › 040604_OS5_st1_1797.tif]

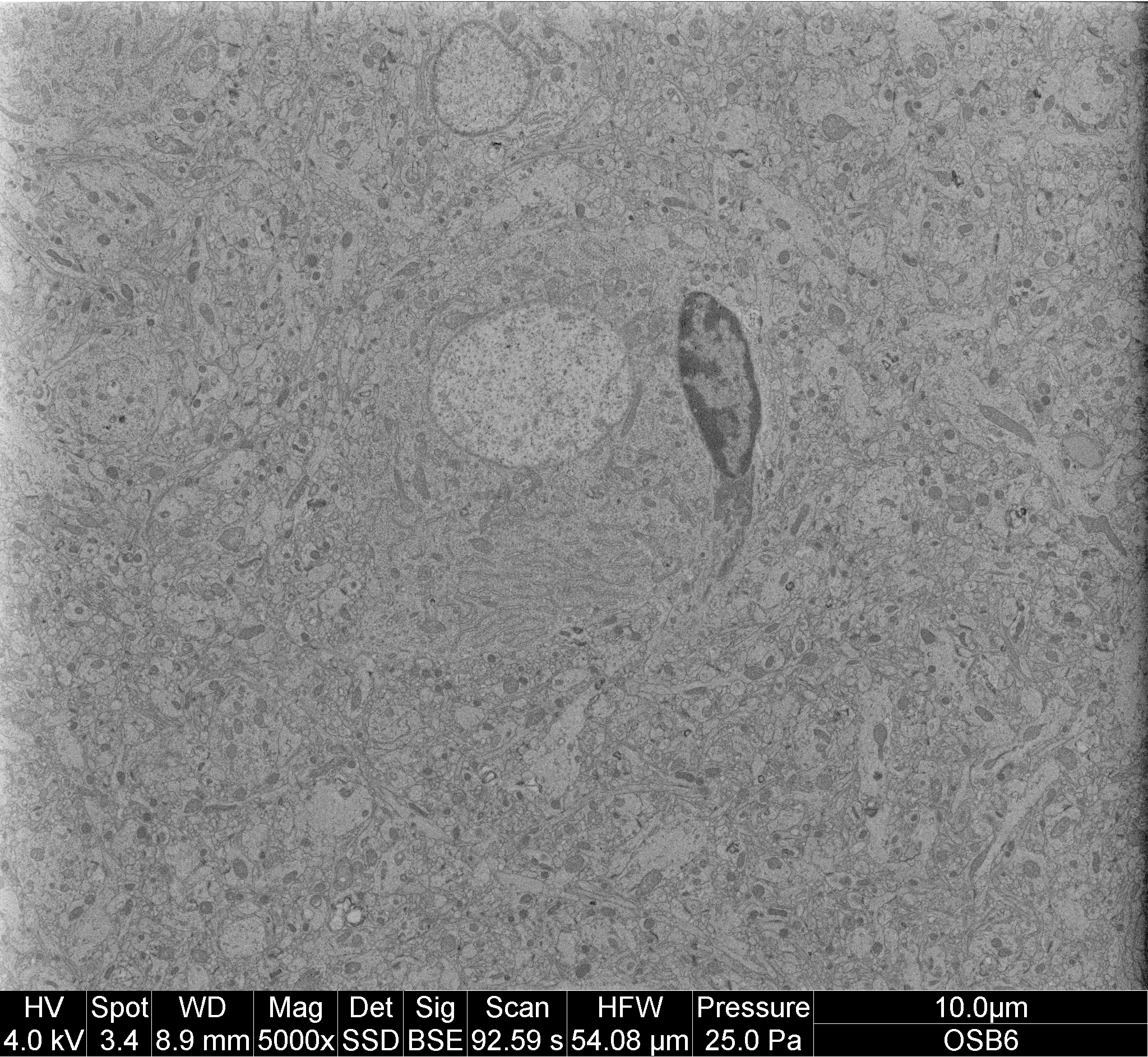

Supplement: Dataset S18 — (250.5 MB ZIP). [file pbio.0020329.sd018.zip › 040604_OS5_st1_1798.tif]

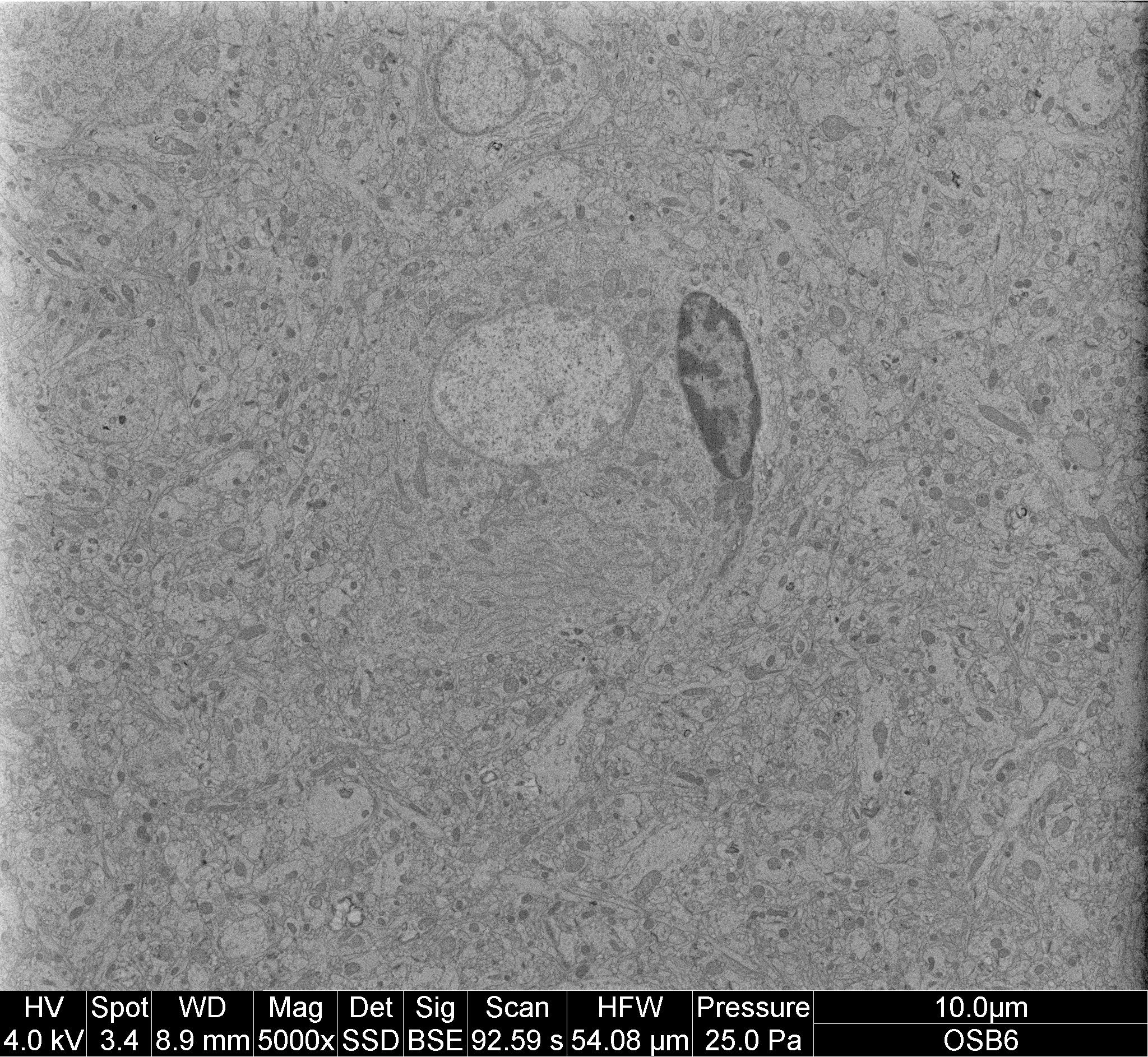

Supplement: Dataset S18 — (250.5 MB ZIP). [file pbio.0020329.sd018.zip › 040604_OS5_st1_1799.tif]

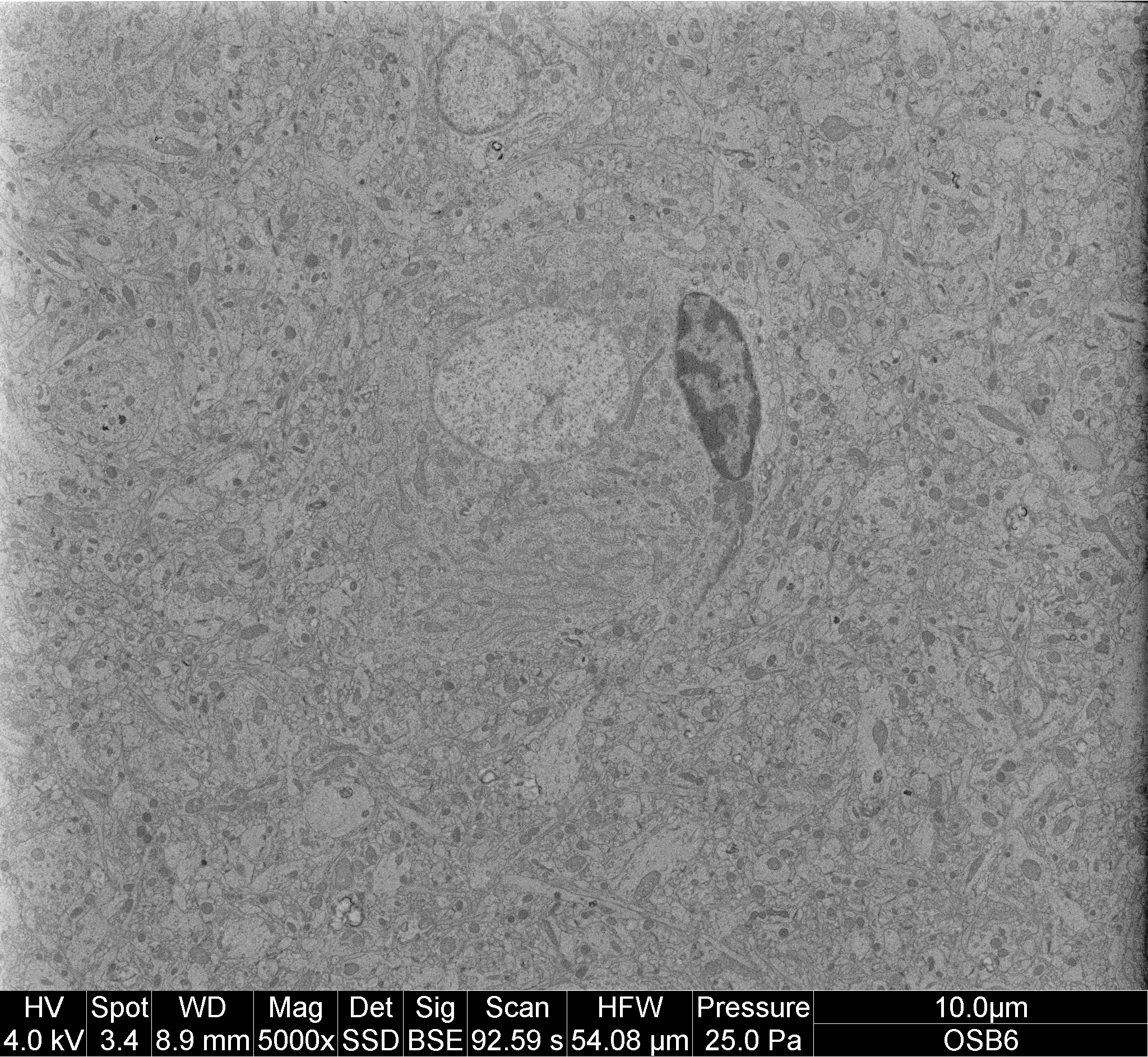

Supplement: Dataset S19 — (253.4 MB ZIP). [file pbio.0020329.sd019.zip › 040604_OS5_st1_1800.tif]
