# Supplementary figures and images for: Serial Block-Face Scanning Electron Microscopy to Reconstruct Three-Dimensional Tissue Nanostructure (part 19 of 21)
Source: PLoS Biol. 2004 Oct 19;2(11):e329. doi: 10.1371/journal.pbio.0020329 (PMC524270; doi:10.1371/journal.pbio.0020329)

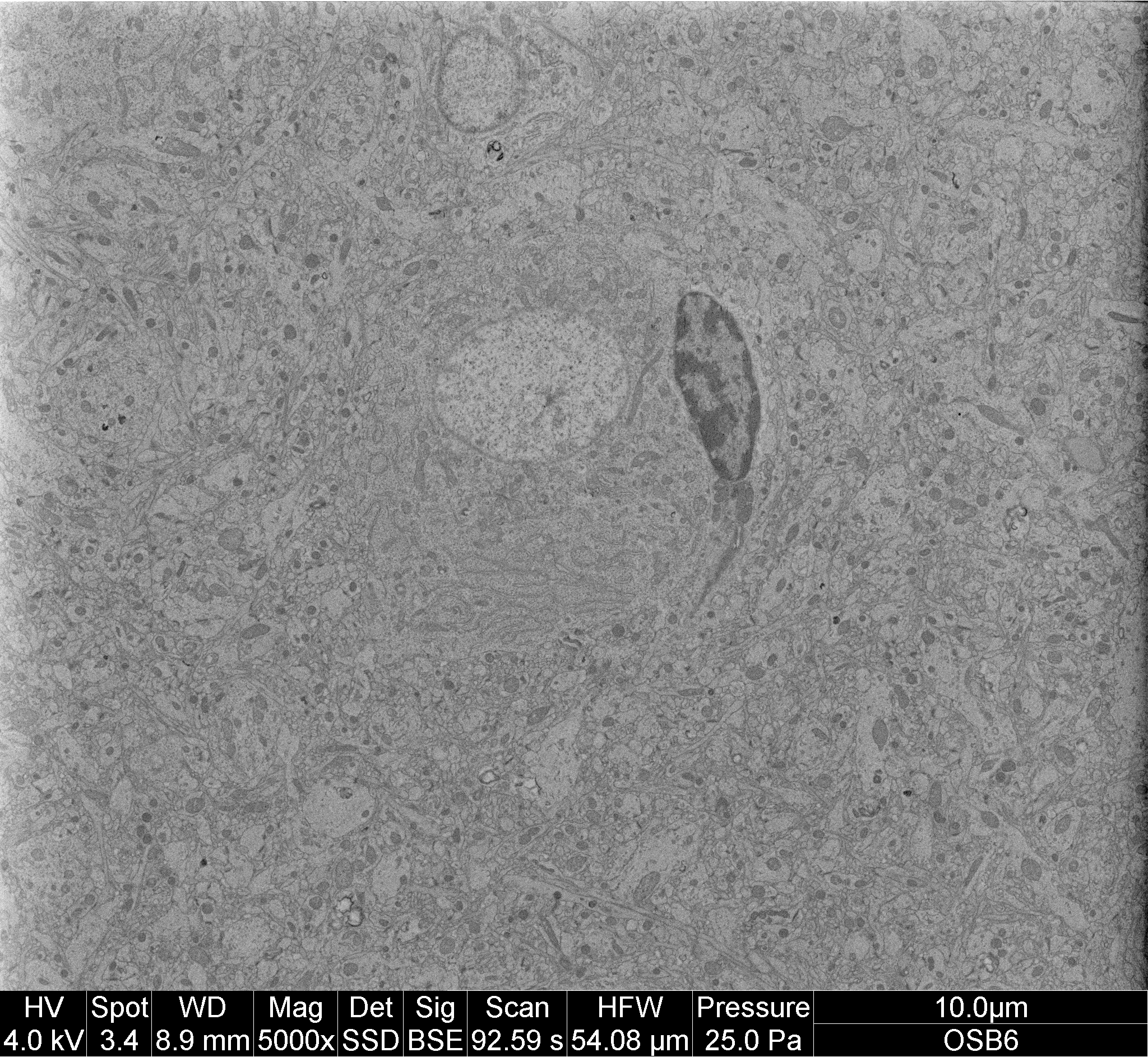

Supplement: Dataset S19 — (253.4 MB ZIP). [file pbio.0020329.sd019.zip › 040604_OS5_st1_1801.tif]

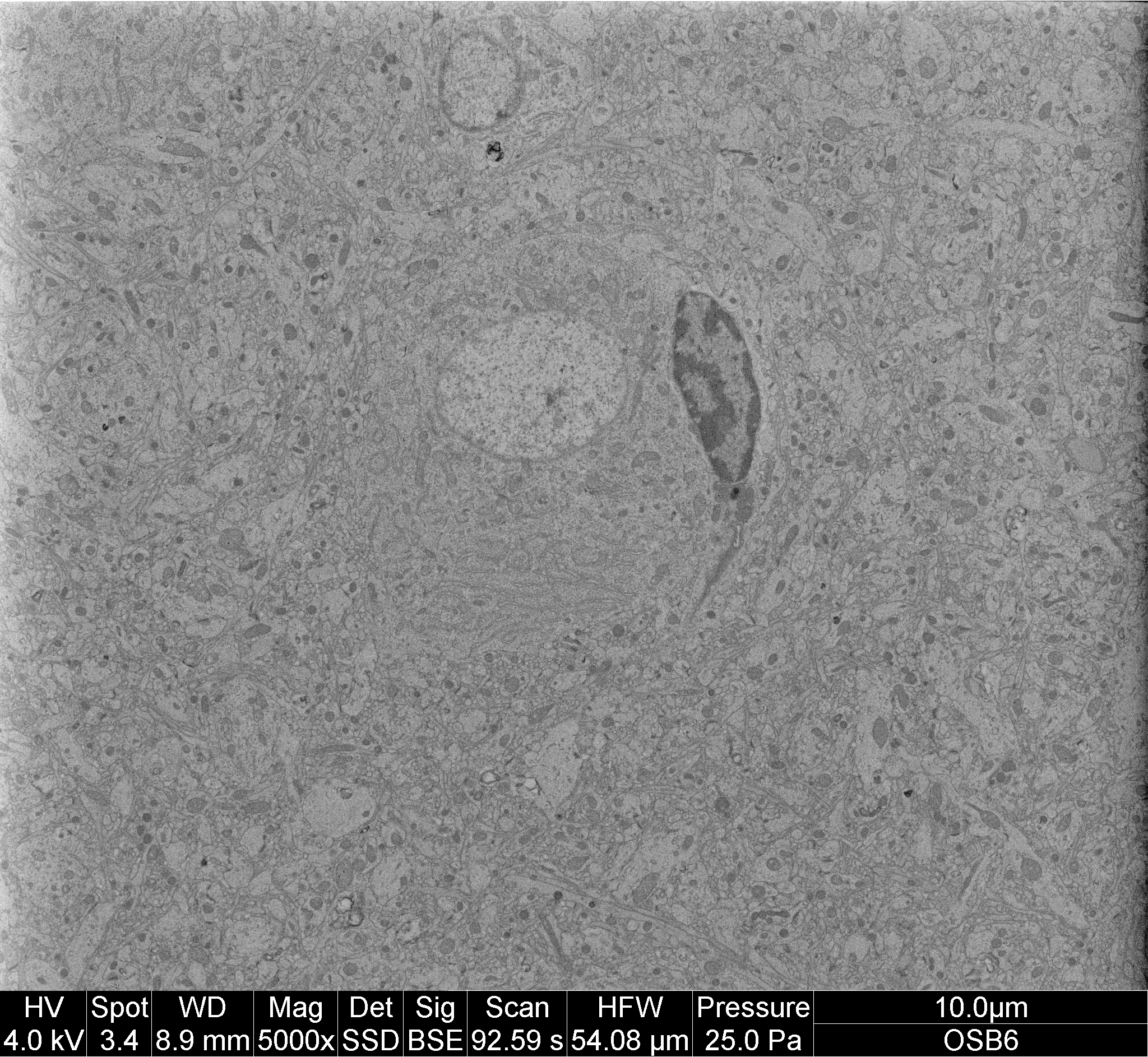

Supplement: Dataset S19 — (253.4 MB ZIP). [file pbio.0020329.sd019.zip › 040604_OS5_st1_1802.tif]

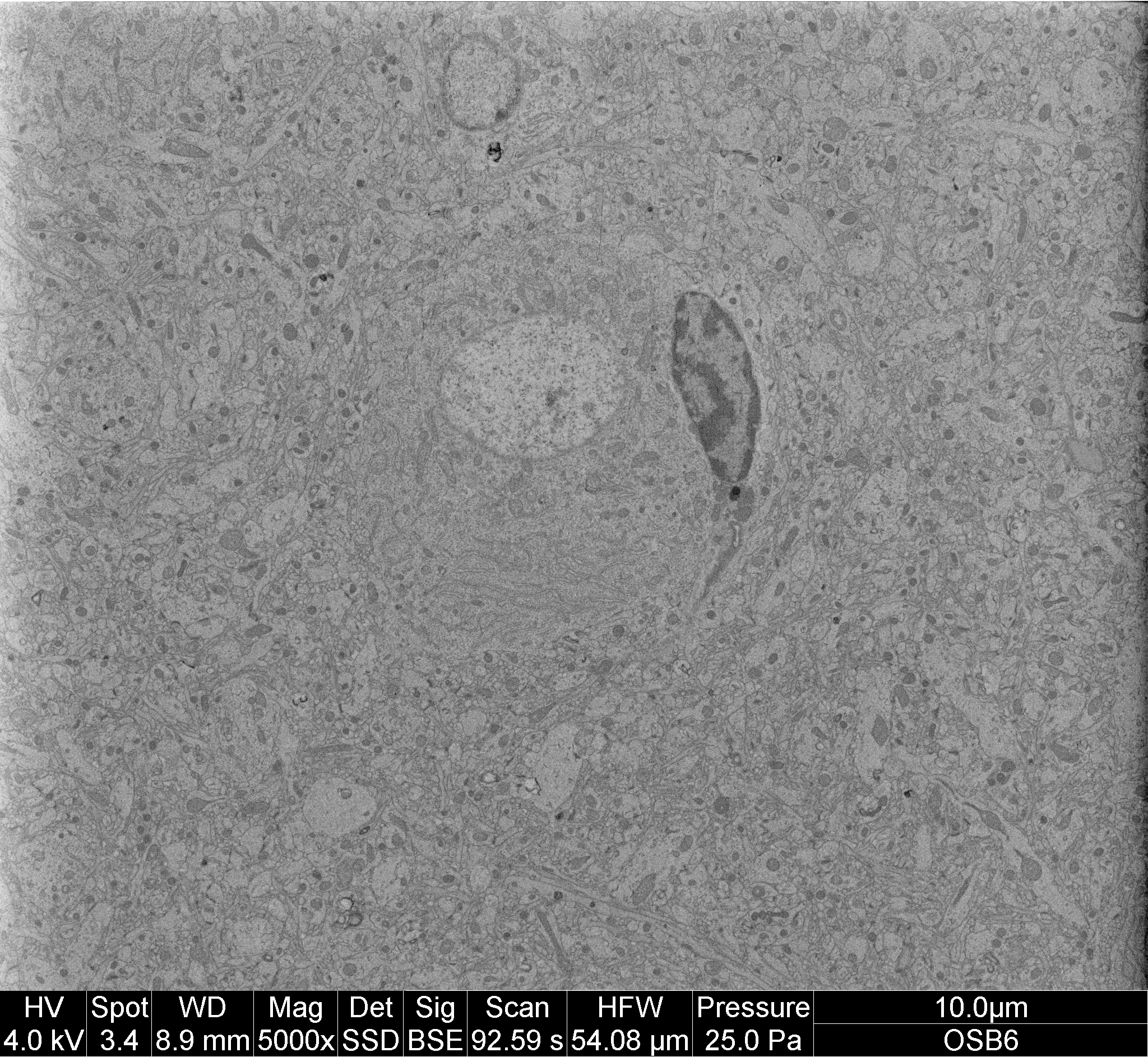

Supplement: Dataset S19 — (253.4 MB ZIP). [file pbio.0020329.sd019.zip › 040604_OS5_st1_1803.tif]

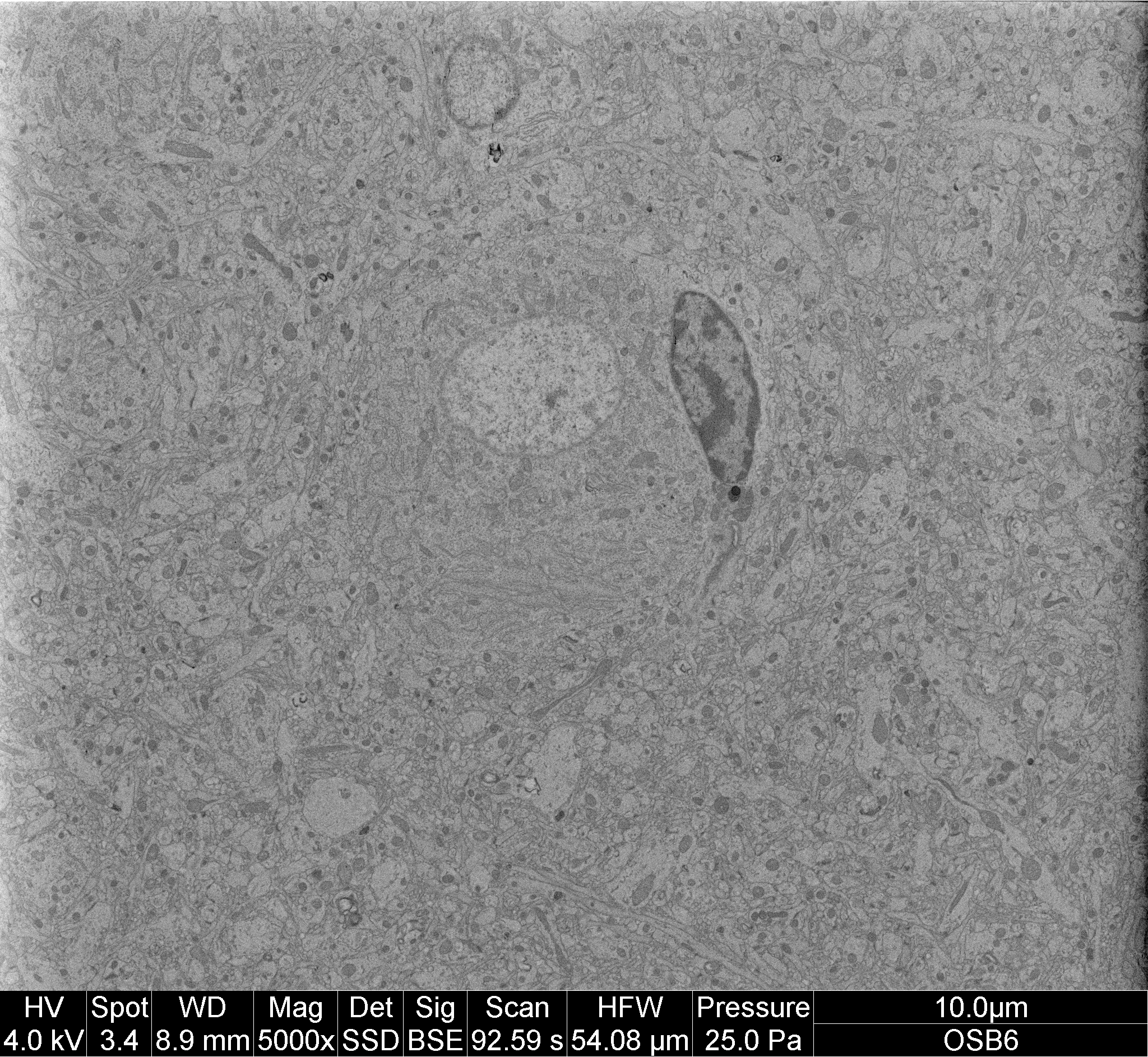

Supplement: Dataset S19 — (253.4 MB ZIP). [file pbio.0020329.sd019.zip › 040604_OS5_st1_1804.tif]

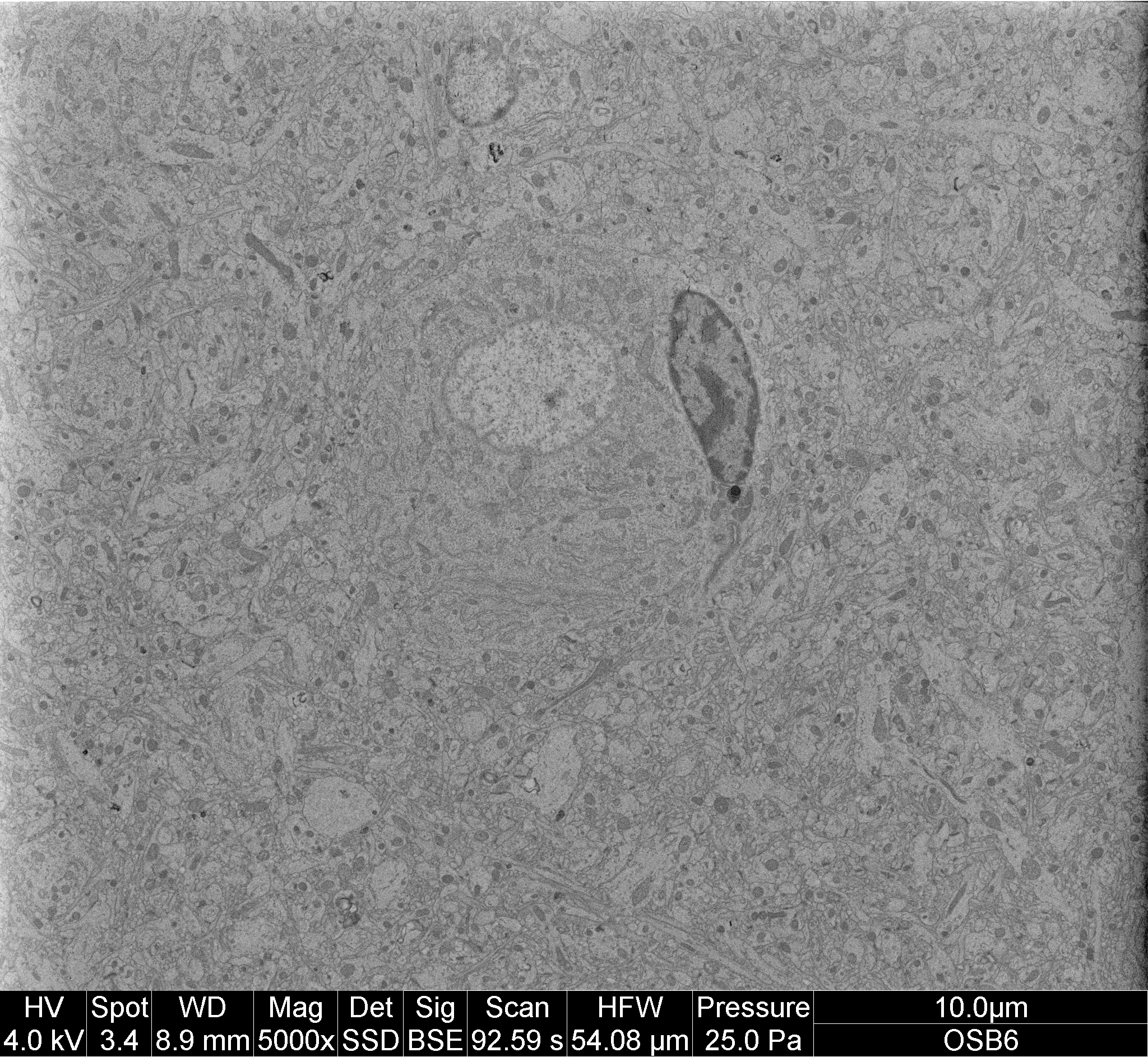

Supplement: Dataset S19 — (253.4 MB ZIP). [file pbio.0020329.sd019.zip › 040604_OS5_st1_1805.tif]

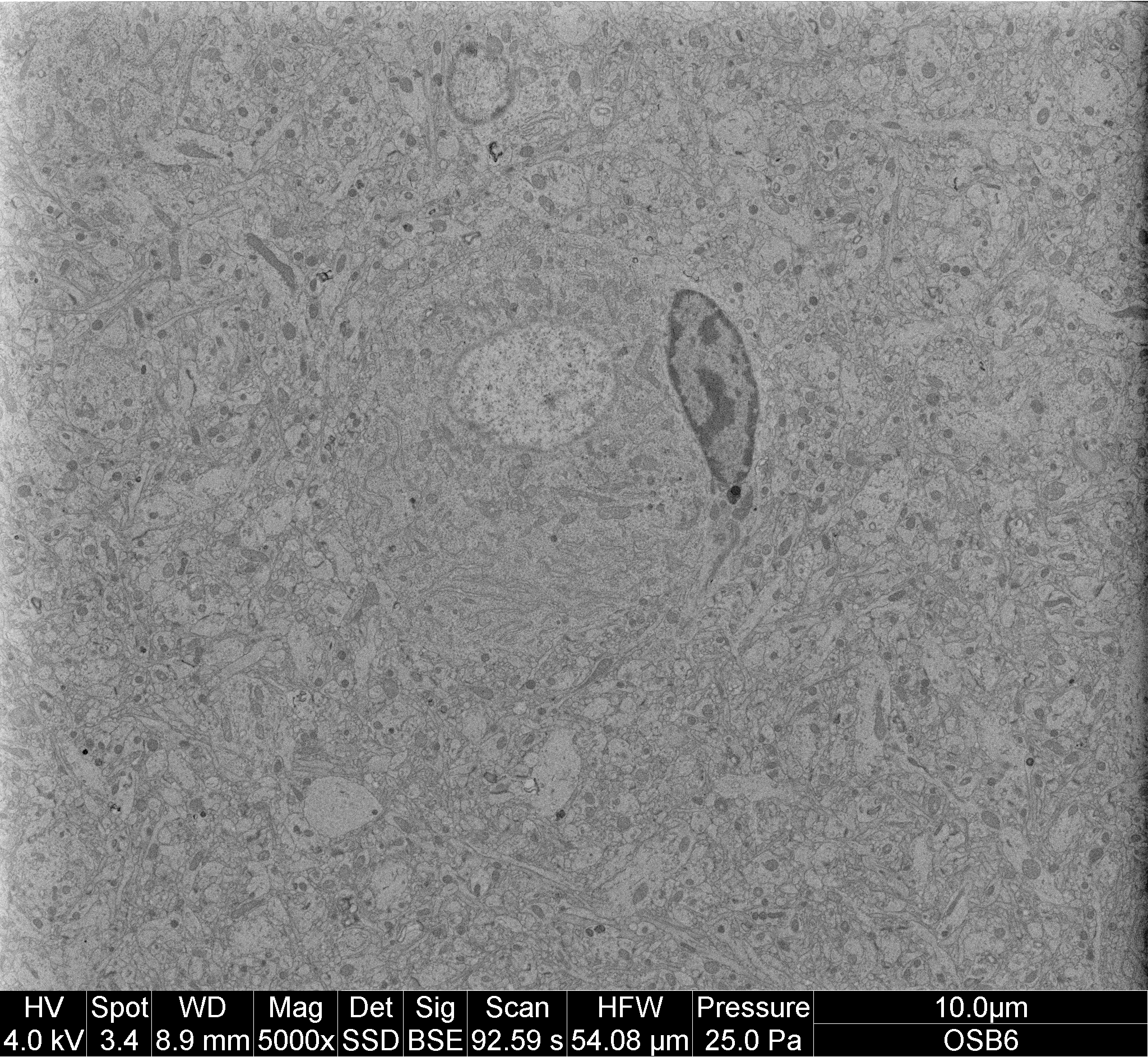

Supplement: Dataset S19 — (253.4 MB ZIP). [file pbio.0020329.sd019.zip › 040604_OS5_st1_1806.tif]

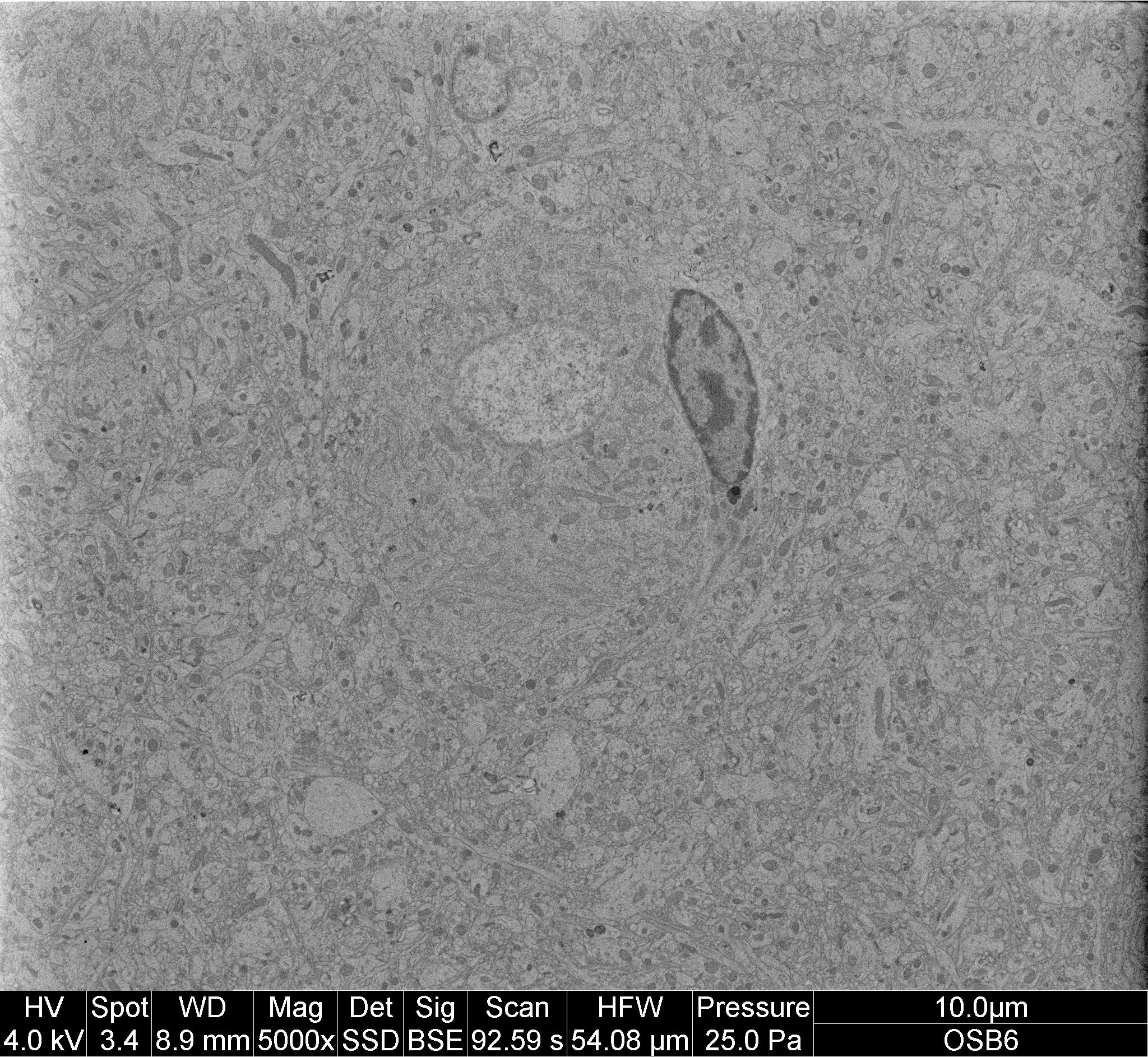

Supplement: Dataset S19 — (253.4 MB ZIP). [file pbio.0020329.sd019.zip › 040604_OS5_st1_1807.tif]

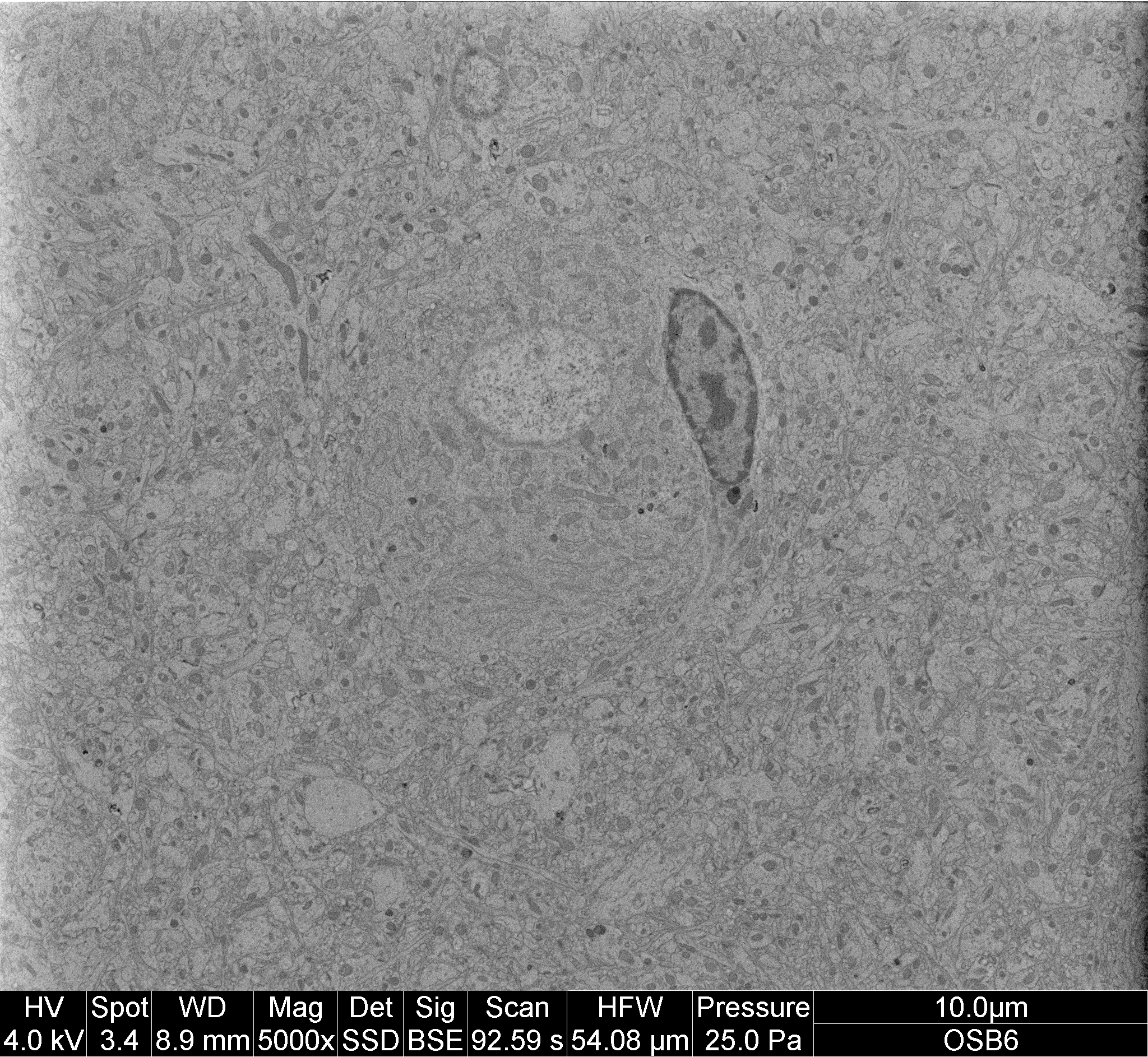

Supplement: Dataset S19 — (253.4 MB ZIP). [file pbio.0020329.sd019.zip › 040604_OS5_st1_1808.tif]

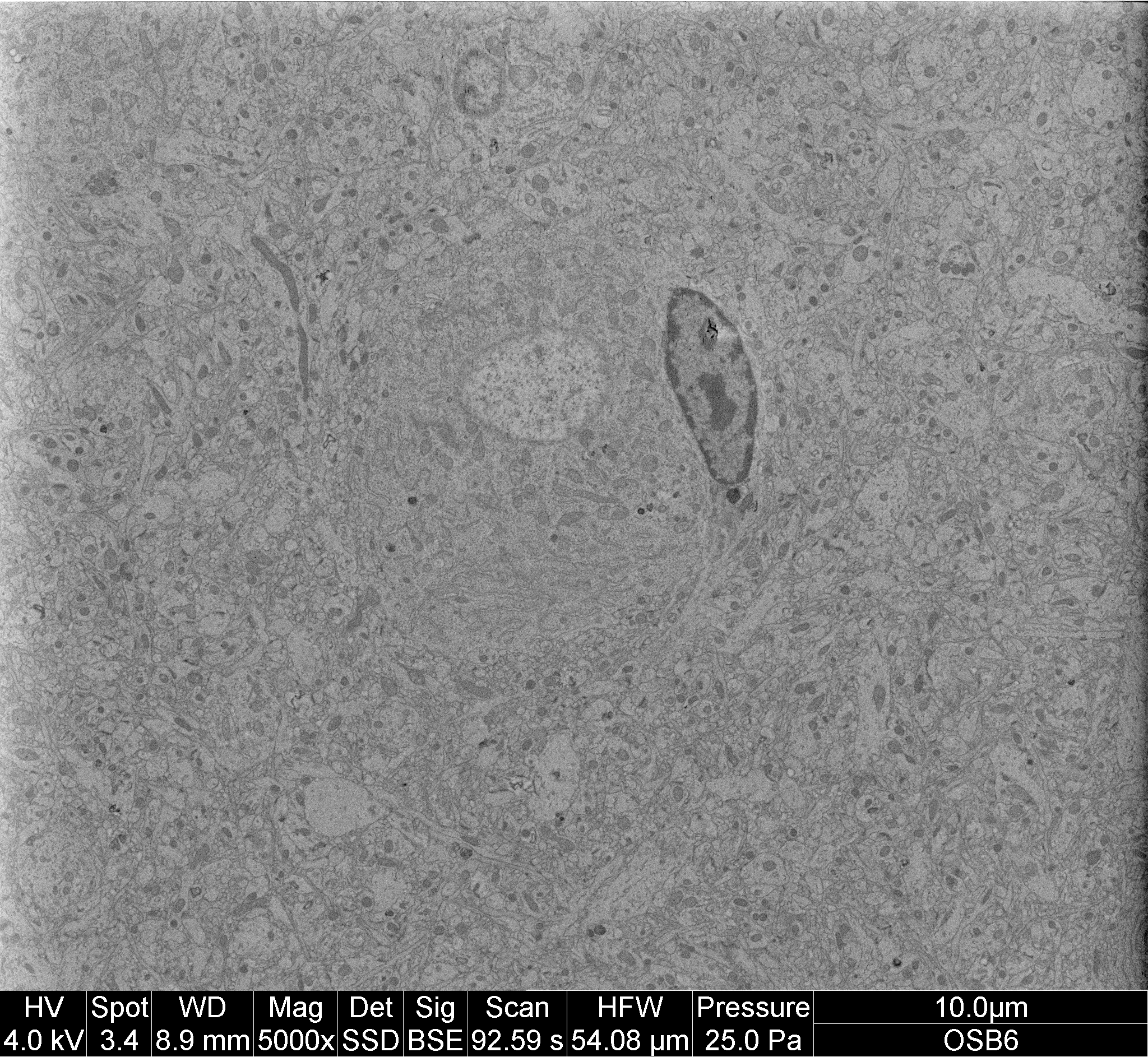

Supplement: Dataset S19 — (253.4 MB ZIP). [file pbio.0020329.sd019.zip › 040604_OS5_st1_1809.tif]

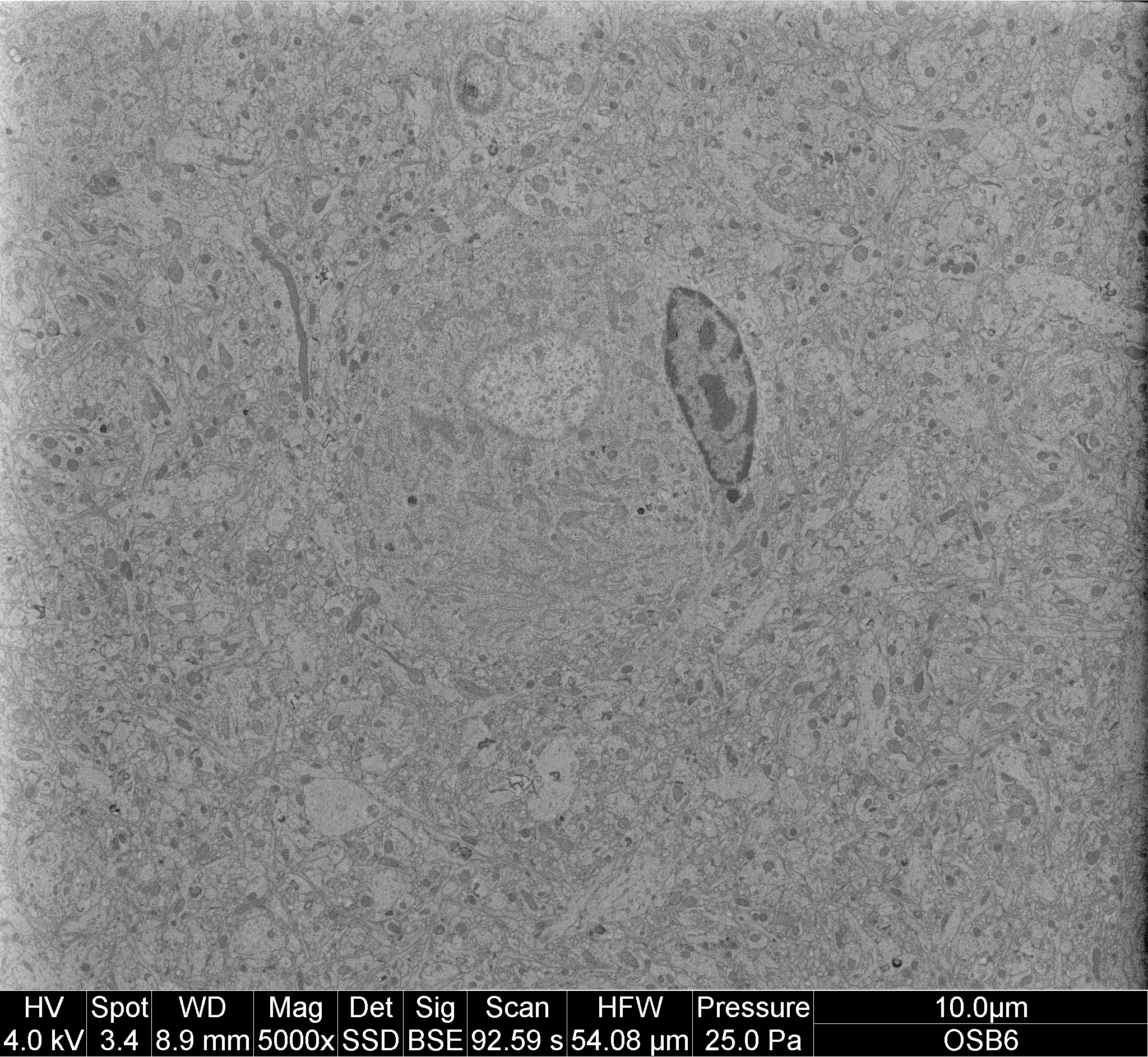

Supplement: Dataset S19 — (253.4 MB ZIP). [file pbio.0020329.sd019.zip › 040604_OS5_st1_1810.tif]

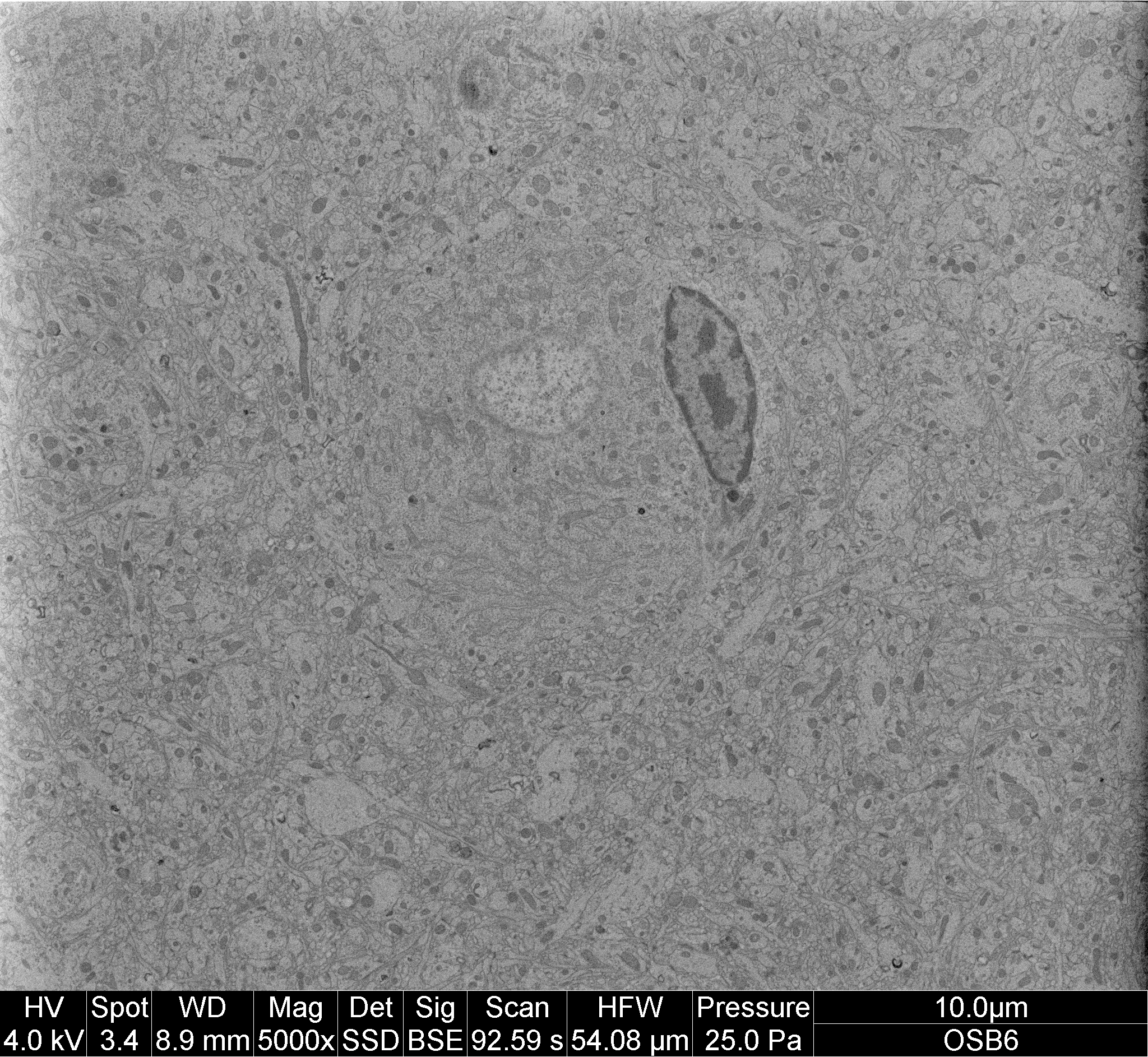

Supplement: Dataset S19 — (253.4 MB ZIP). [file pbio.0020329.sd019.zip › 040604_OS5_st1_1811.tif]

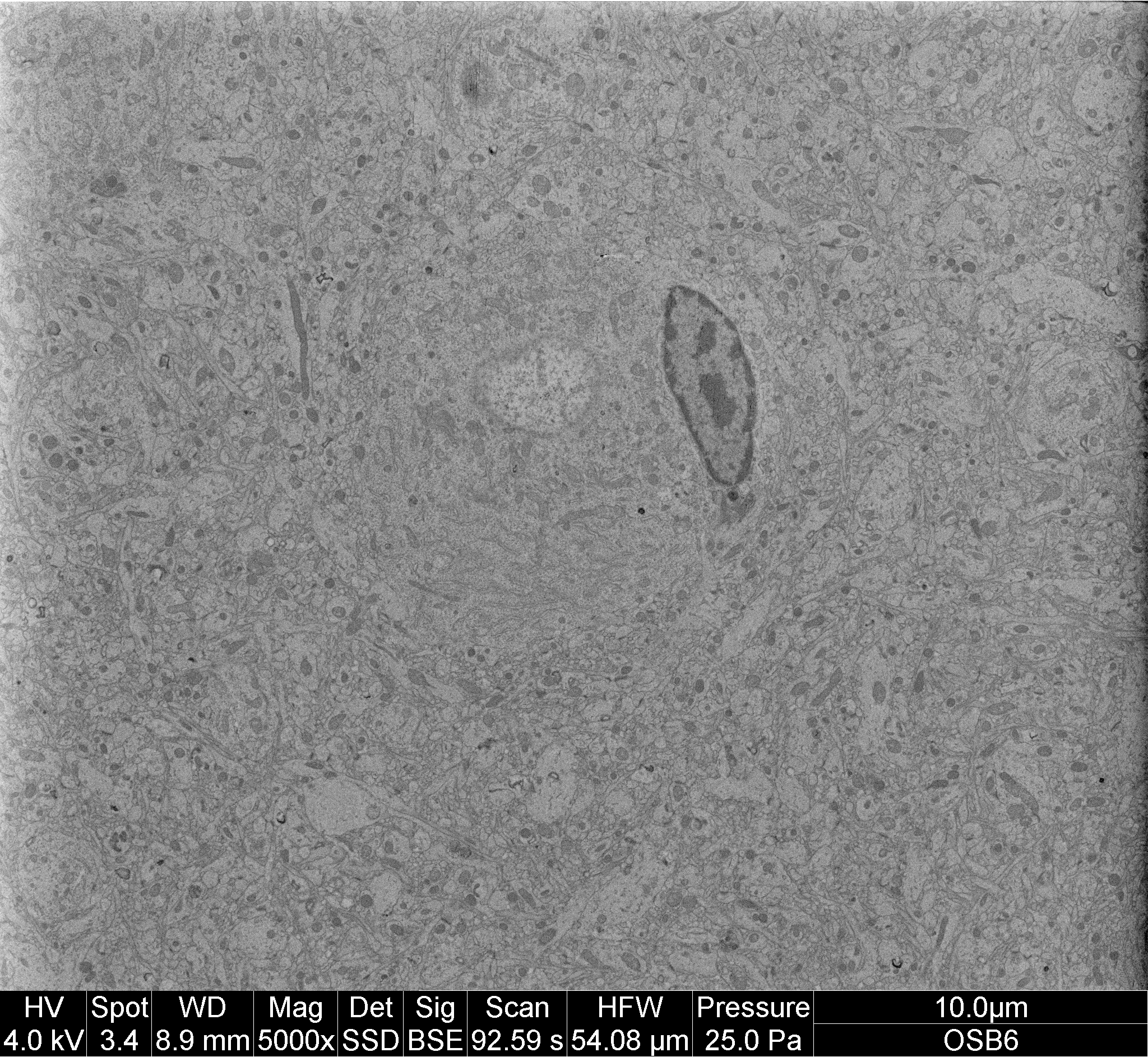

Supplement: Dataset S19 — (253.4 MB ZIP). [file pbio.0020329.sd019.zip › 040604_OS5_st1_1812.tif]

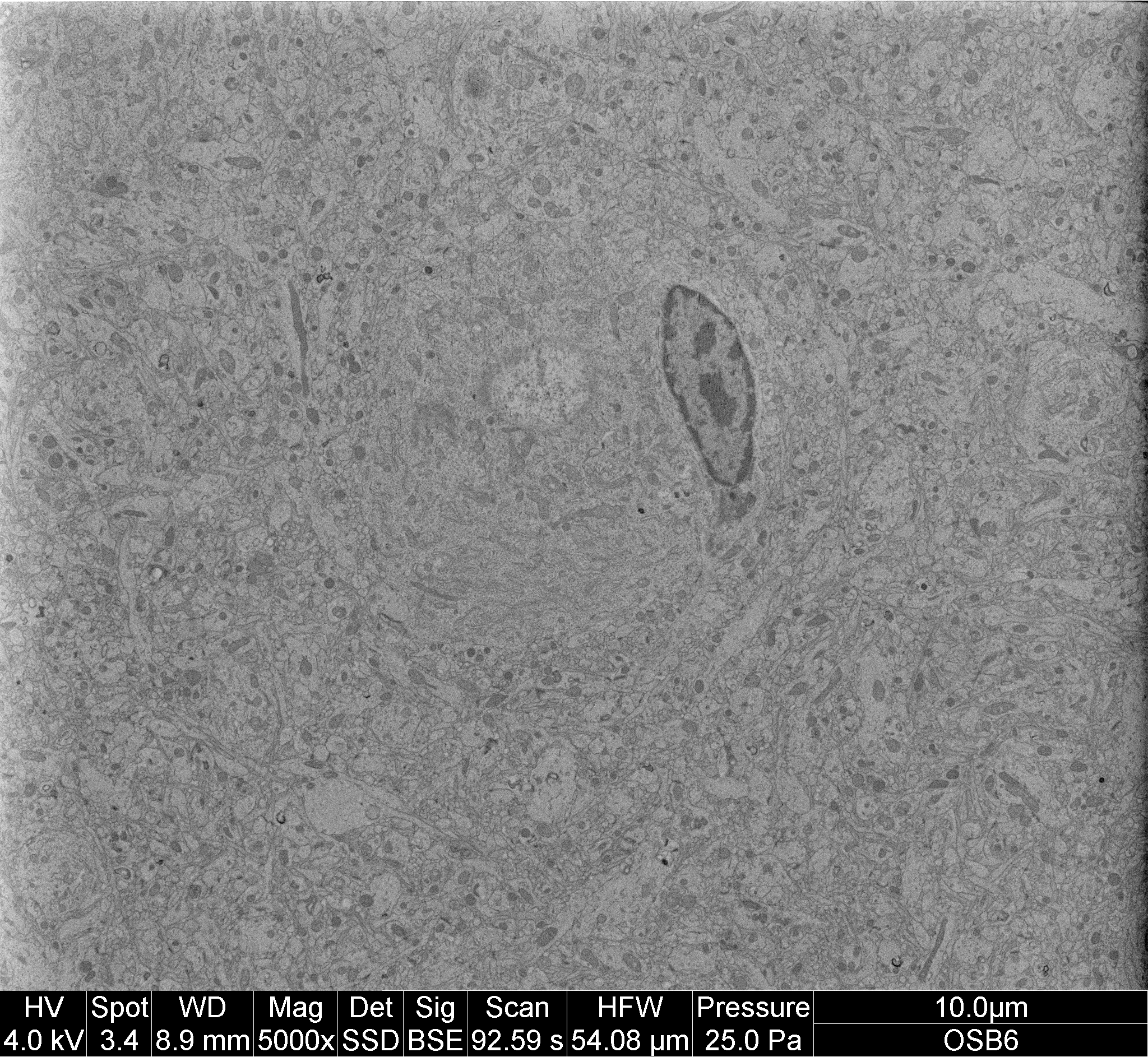

Supplement: Dataset S19 — (253.4 MB ZIP). [file pbio.0020329.sd019.zip › 040604_OS5_st1_1813.tif]

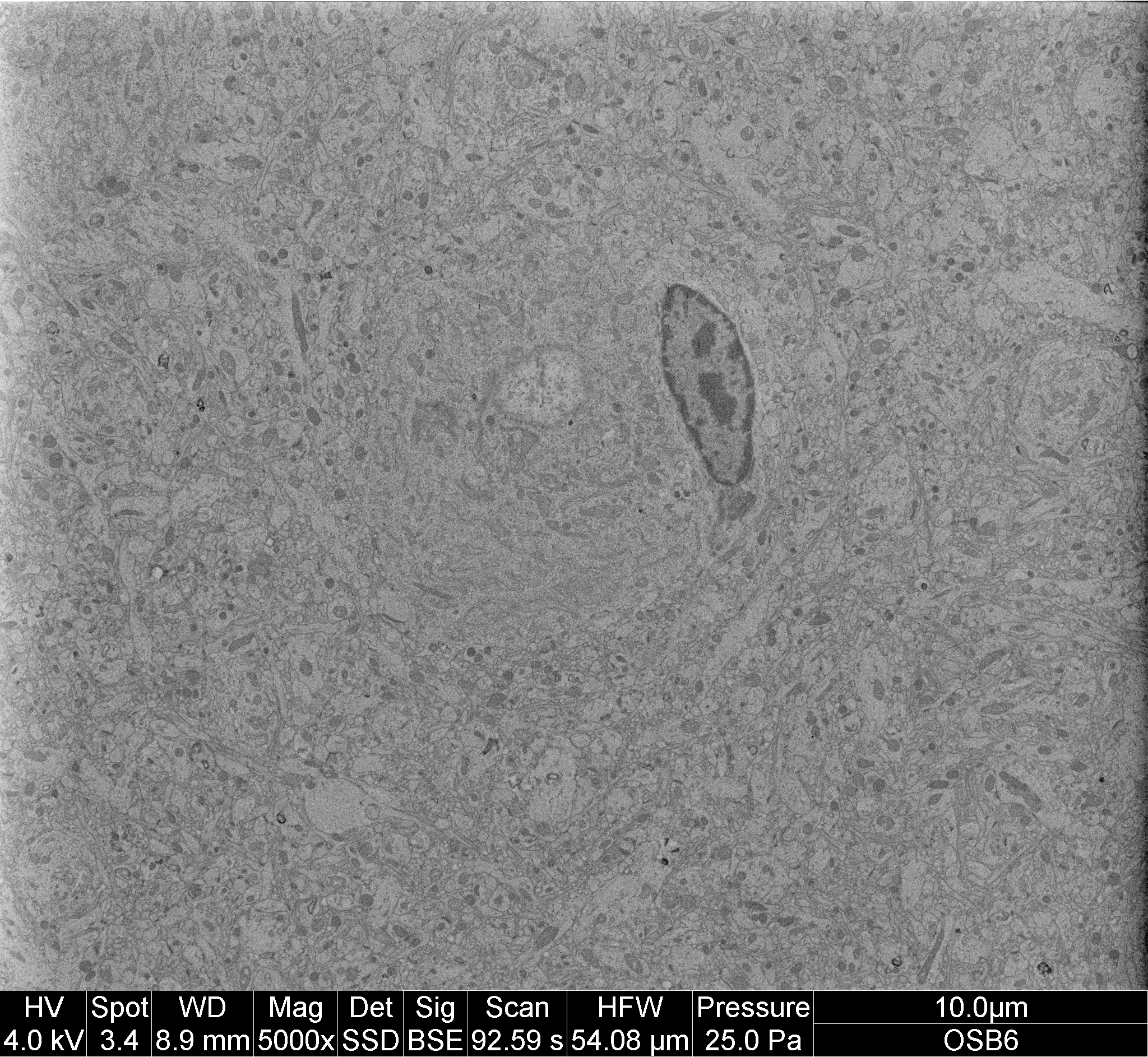

Supplement: Dataset S19 — (253.4 MB ZIP). [file pbio.0020329.sd019.zip › 040604_OS5_st1_1814.tif]

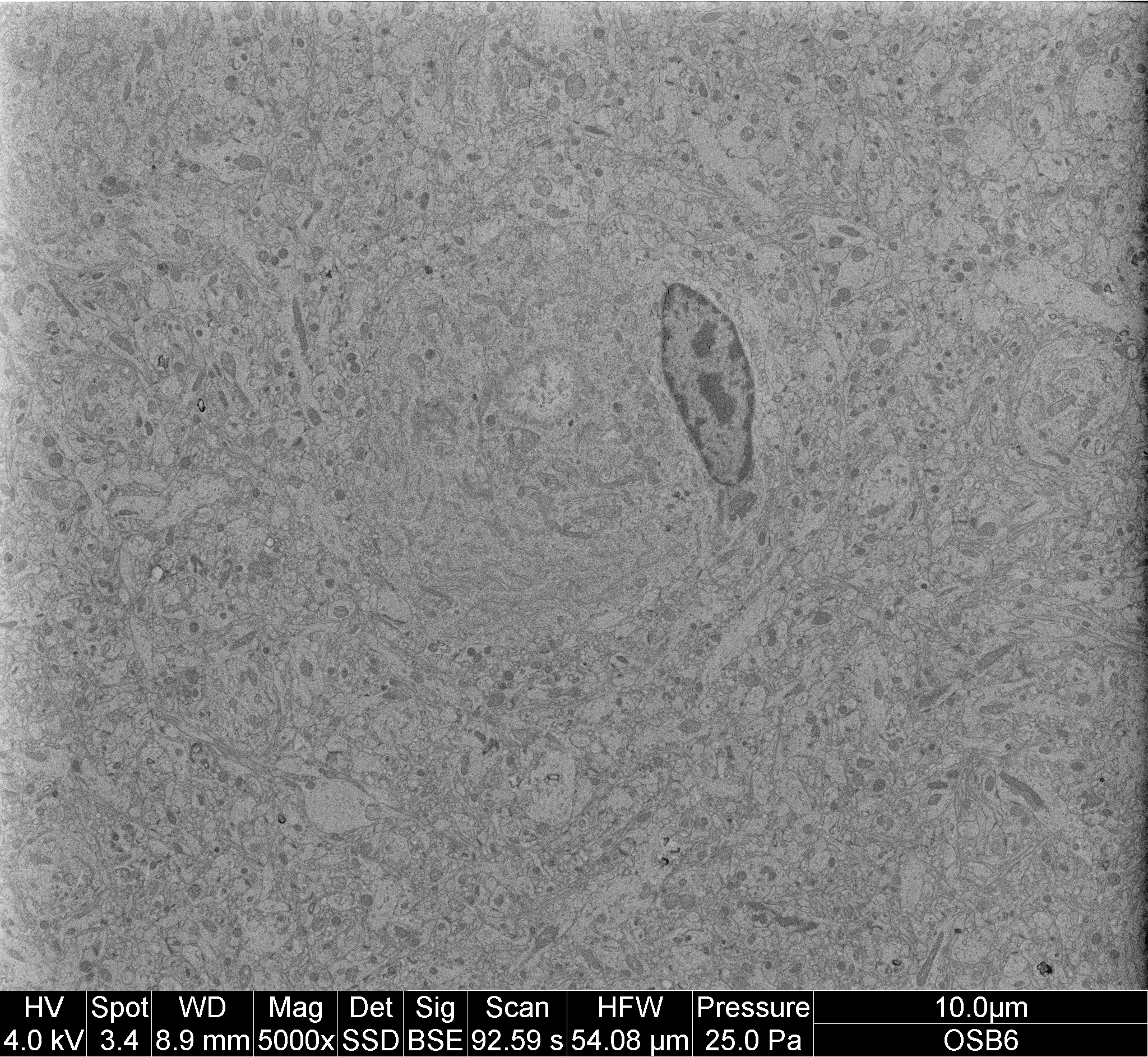

Supplement: Dataset S19 — (253.4 MB ZIP). [file pbio.0020329.sd019.zip › 040604_OS5_st1_1815.tif]

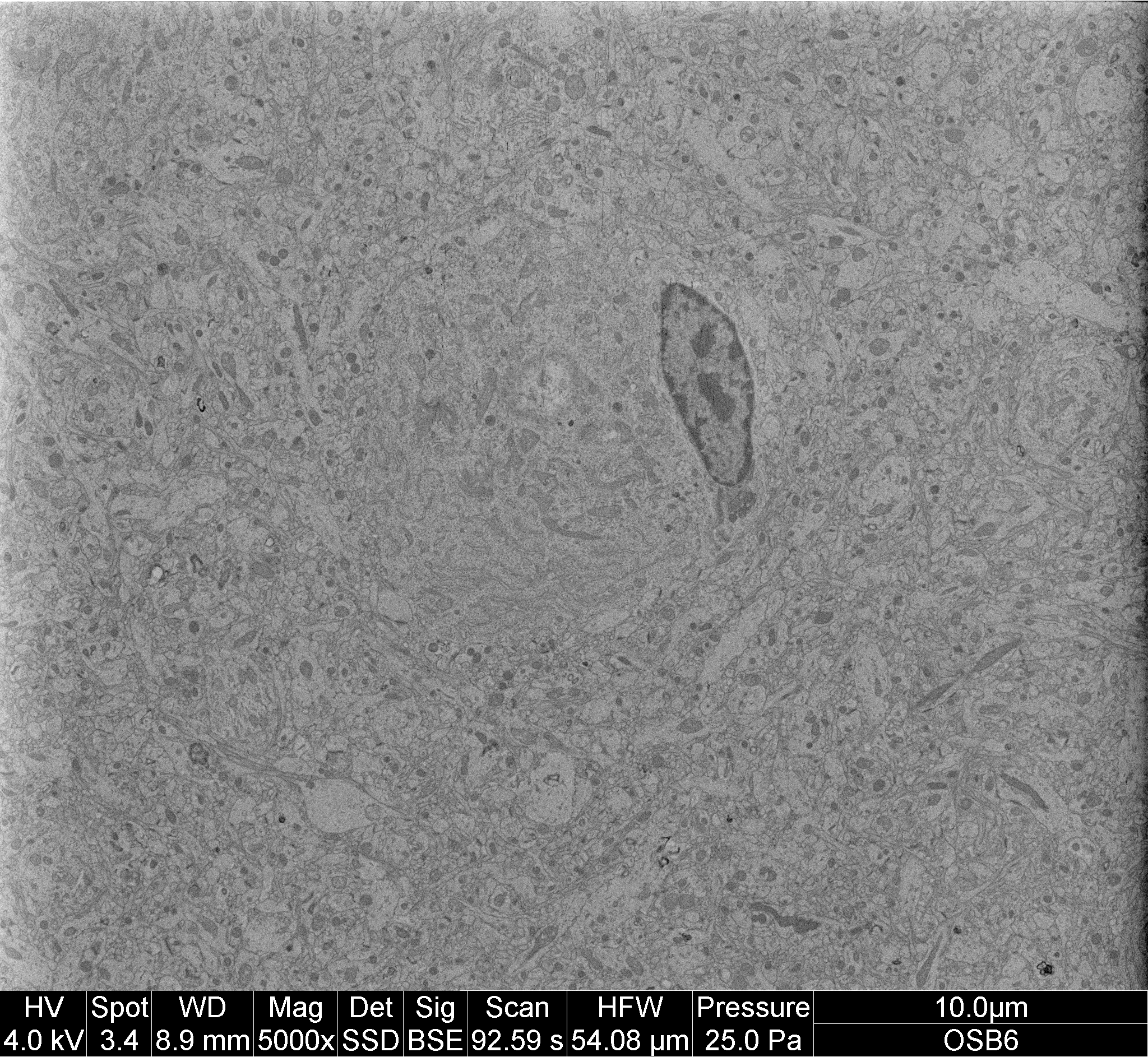

Supplement: Dataset S19 — (253.4 MB ZIP). [file pbio.0020329.sd019.zip › 040604_OS5_st1_1816.tif]

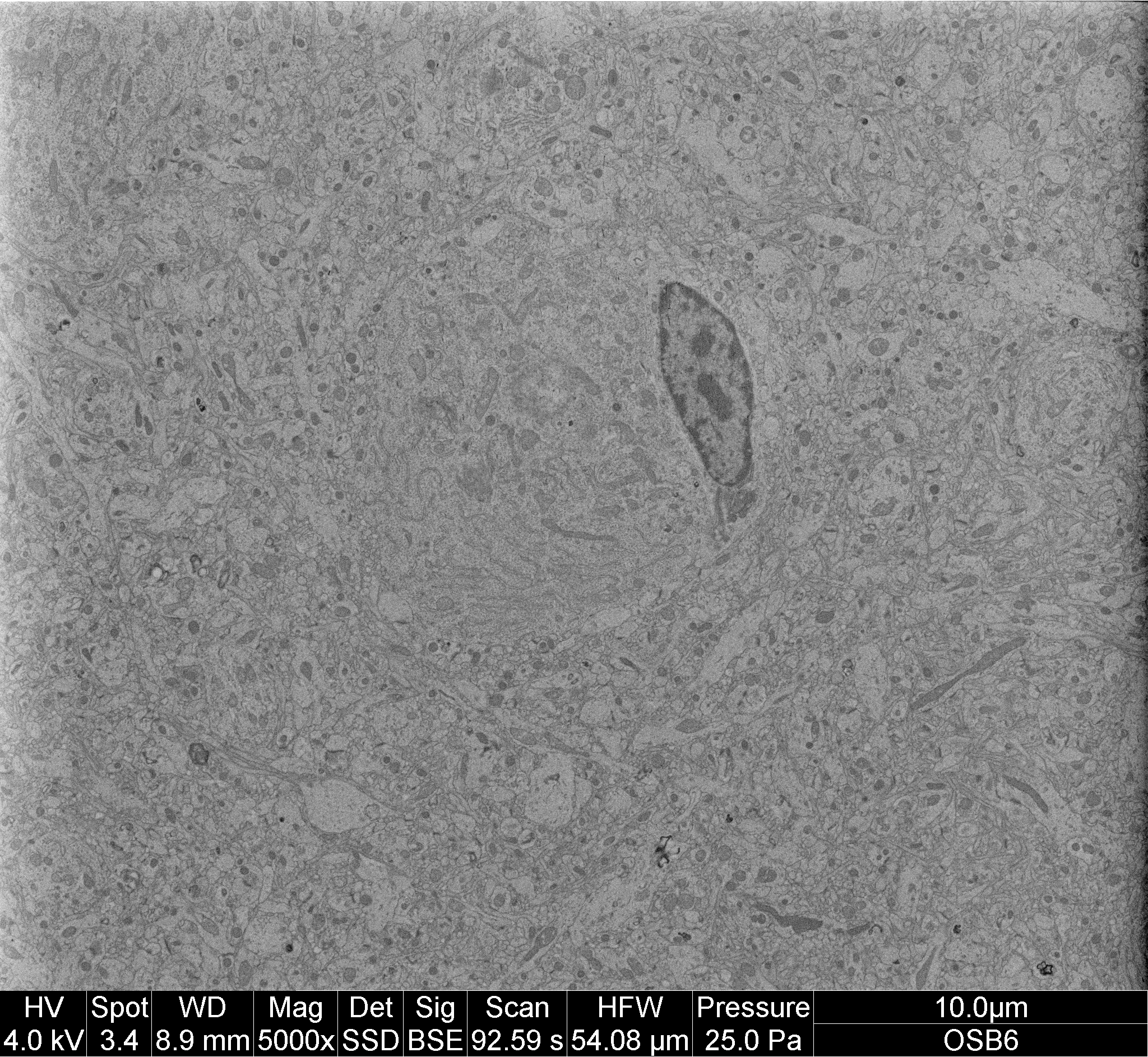

Supplement: Dataset S19 — (253.4 MB ZIP). [file pbio.0020329.sd019.zip › 040604_OS5_st1_1817.tif]

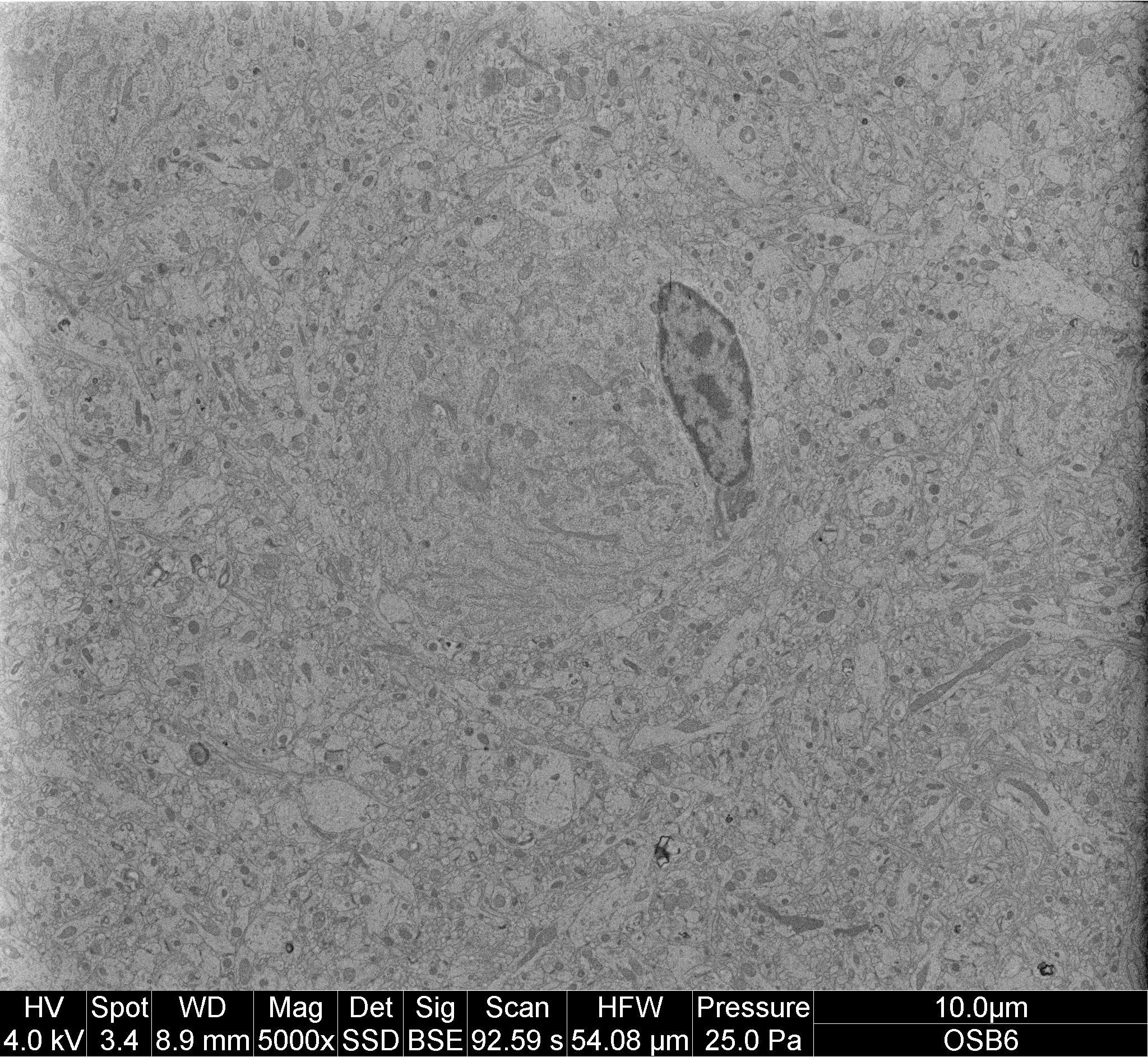

Supplement: Dataset S19 — (253.4 MB ZIP). [file pbio.0020329.sd019.zip › 040604_OS5_st1_1818.tif]

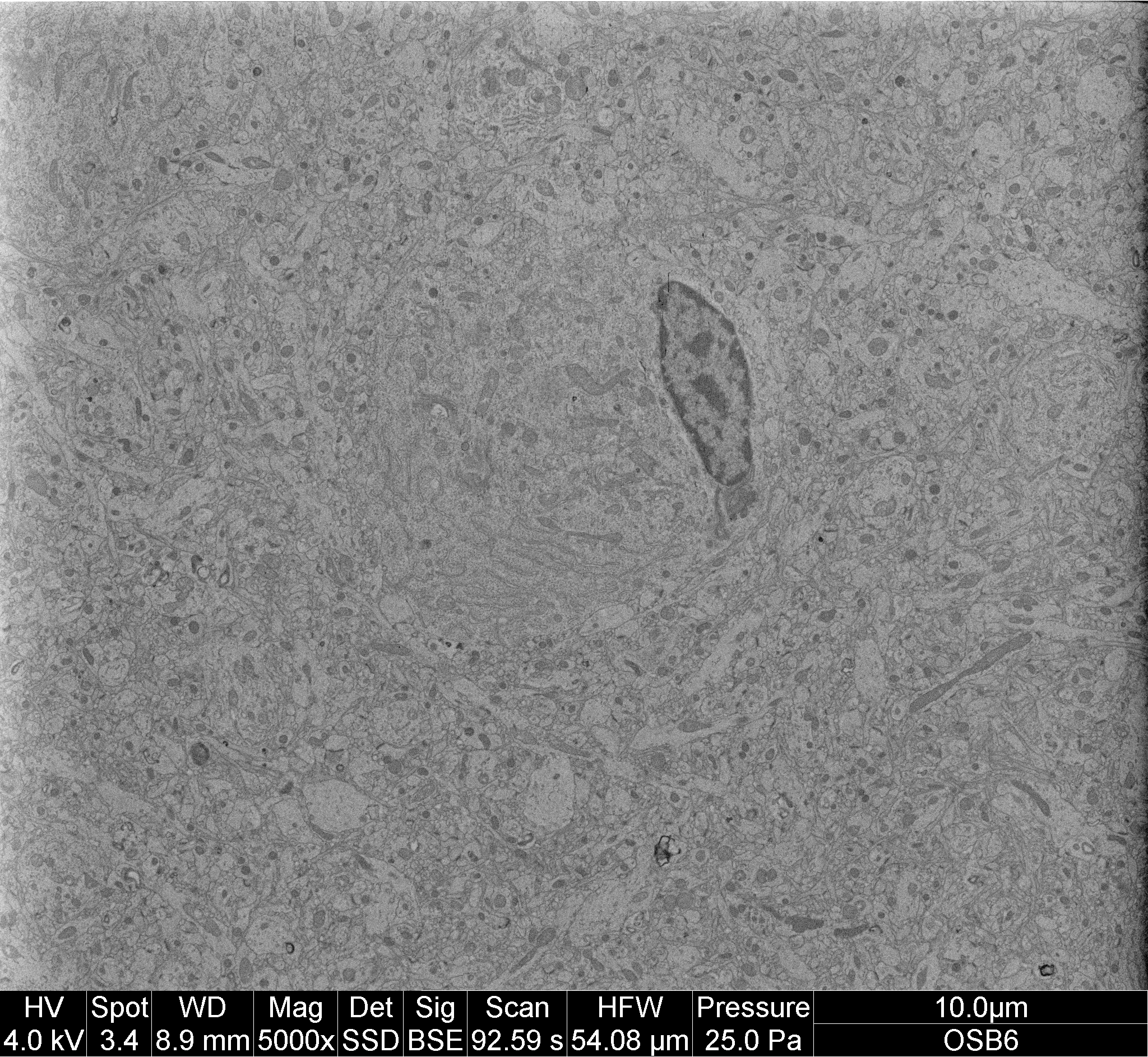

Supplement: Dataset S19 — (253.4 MB ZIP). [file pbio.0020329.sd019.zip › 040604_OS5_st1_1819.tif]

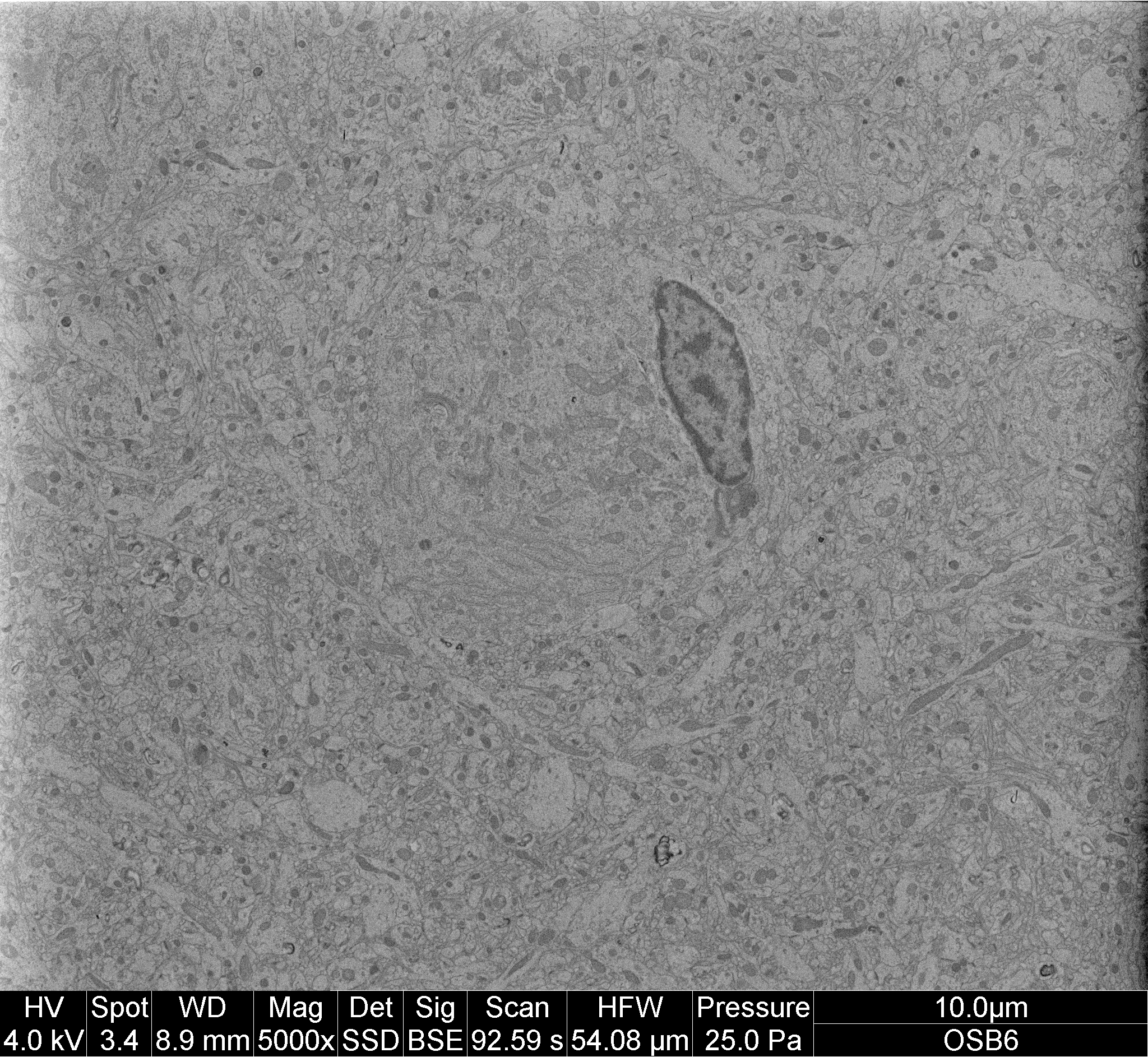

Supplement: Dataset S19 — (253.4 MB ZIP). [file pbio.0020329.sd019.zip › 040604_OS5_st1_1820.tif]

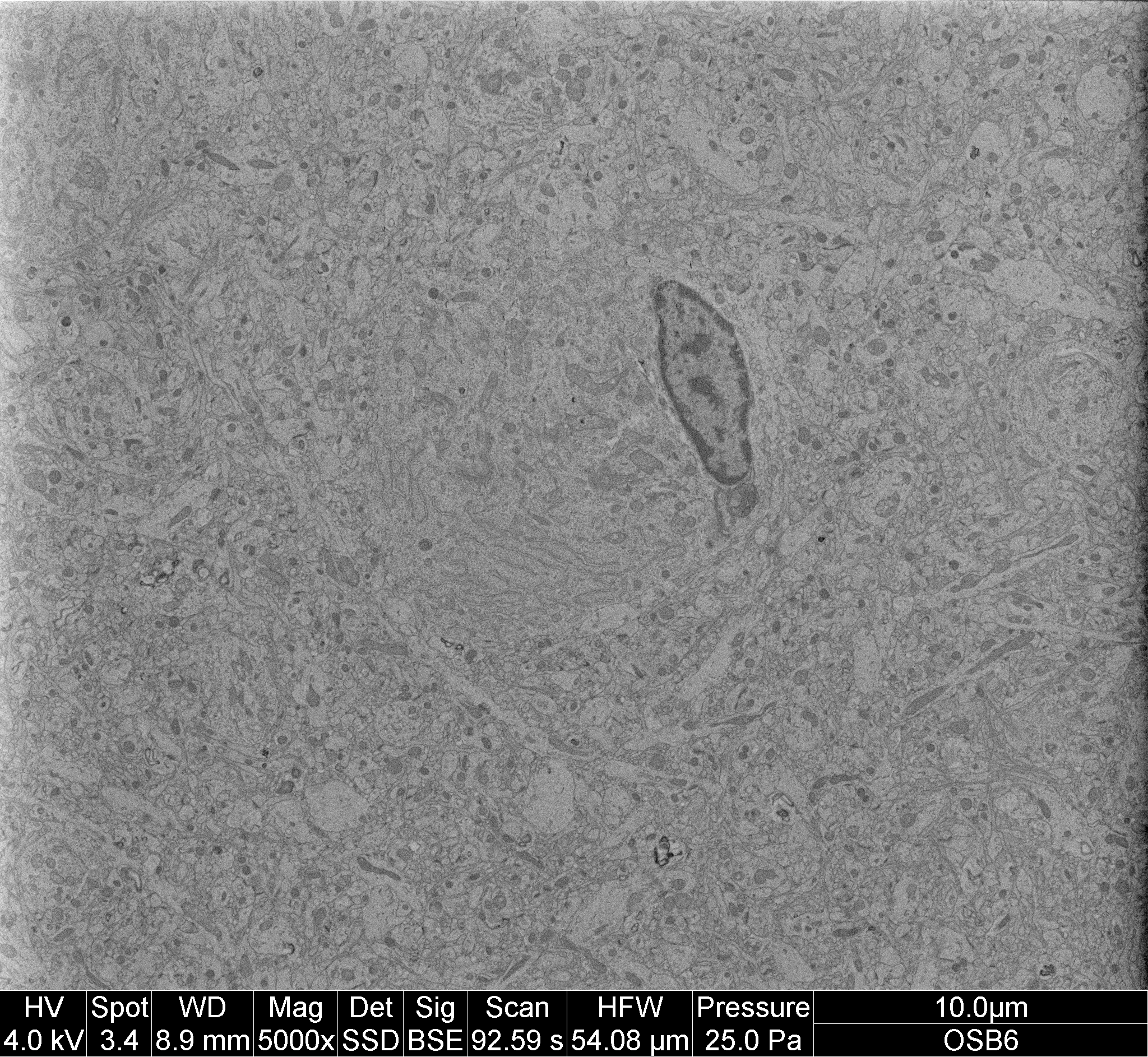

Supplement: Dataset S19 — (253.4 MB ZIP). [file pbio.0020329.sd019.zip › 040604_OS5_st1_1821.tif]

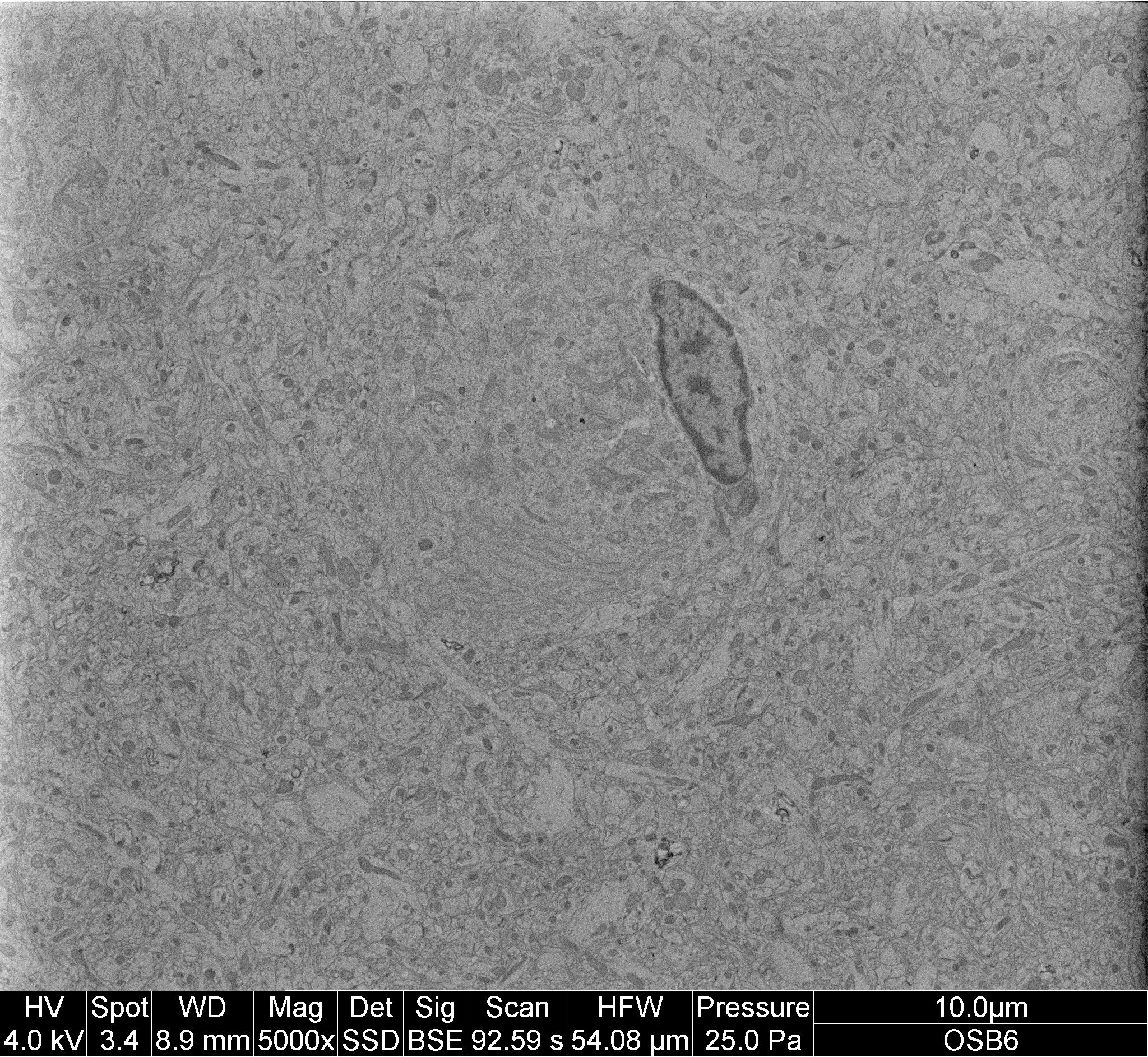

Supplement: Dataset S19 — (253.4 MB ZIP). [file pbio.0020329.sd019.zip › 040604_OS5_st1_1822.tif]

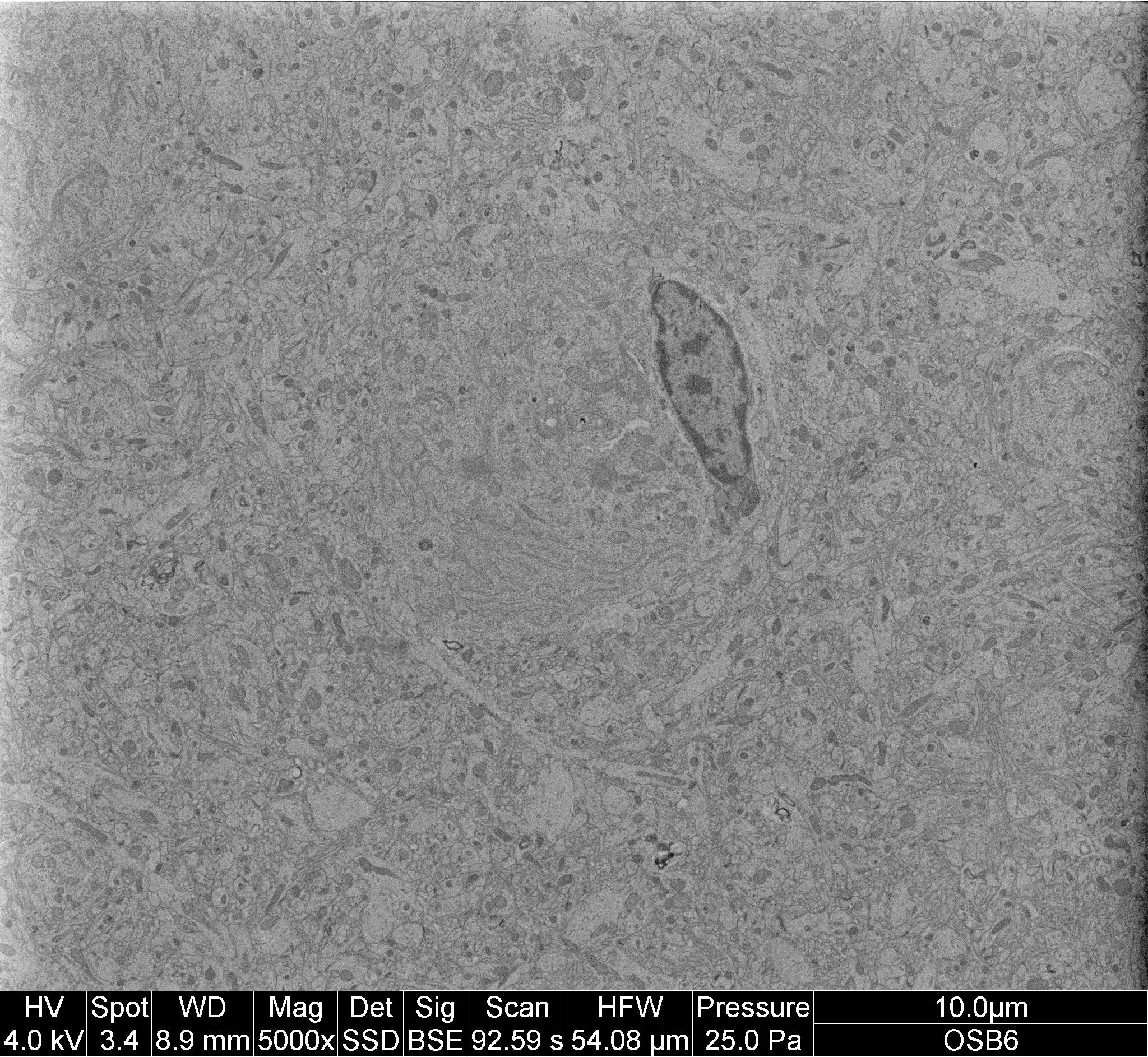

Supplement: Dataset S19 — (253.4 MB ZIP). [file pbio.0020329.sd019.zip › 040604_OS5_st1_1823.tif]

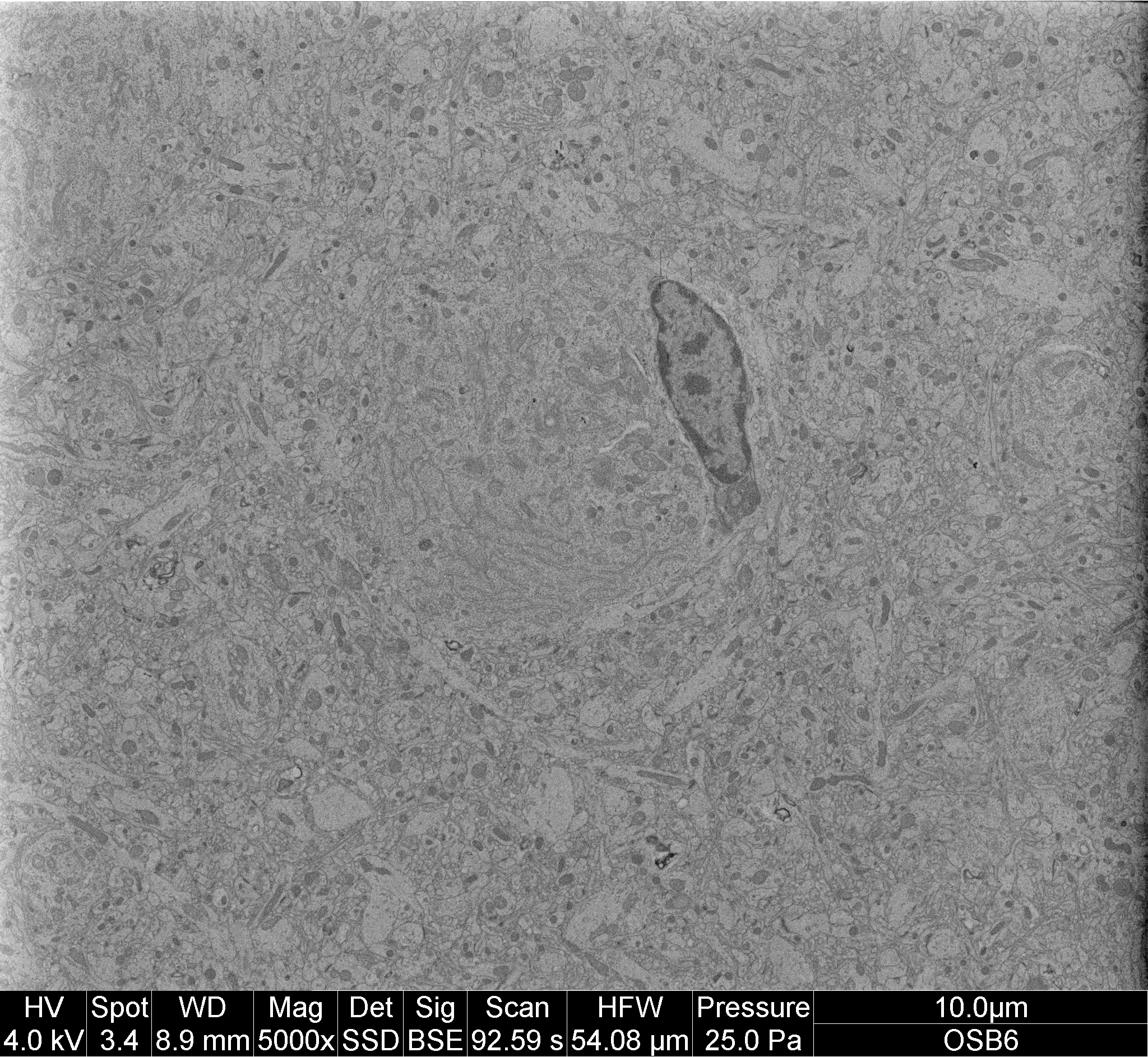

Supplement: Dataset S19 — (253.4 MB ZIP). [file pbio.0020329.sd019.zip › 040604_OS5_st1_1824.tif]

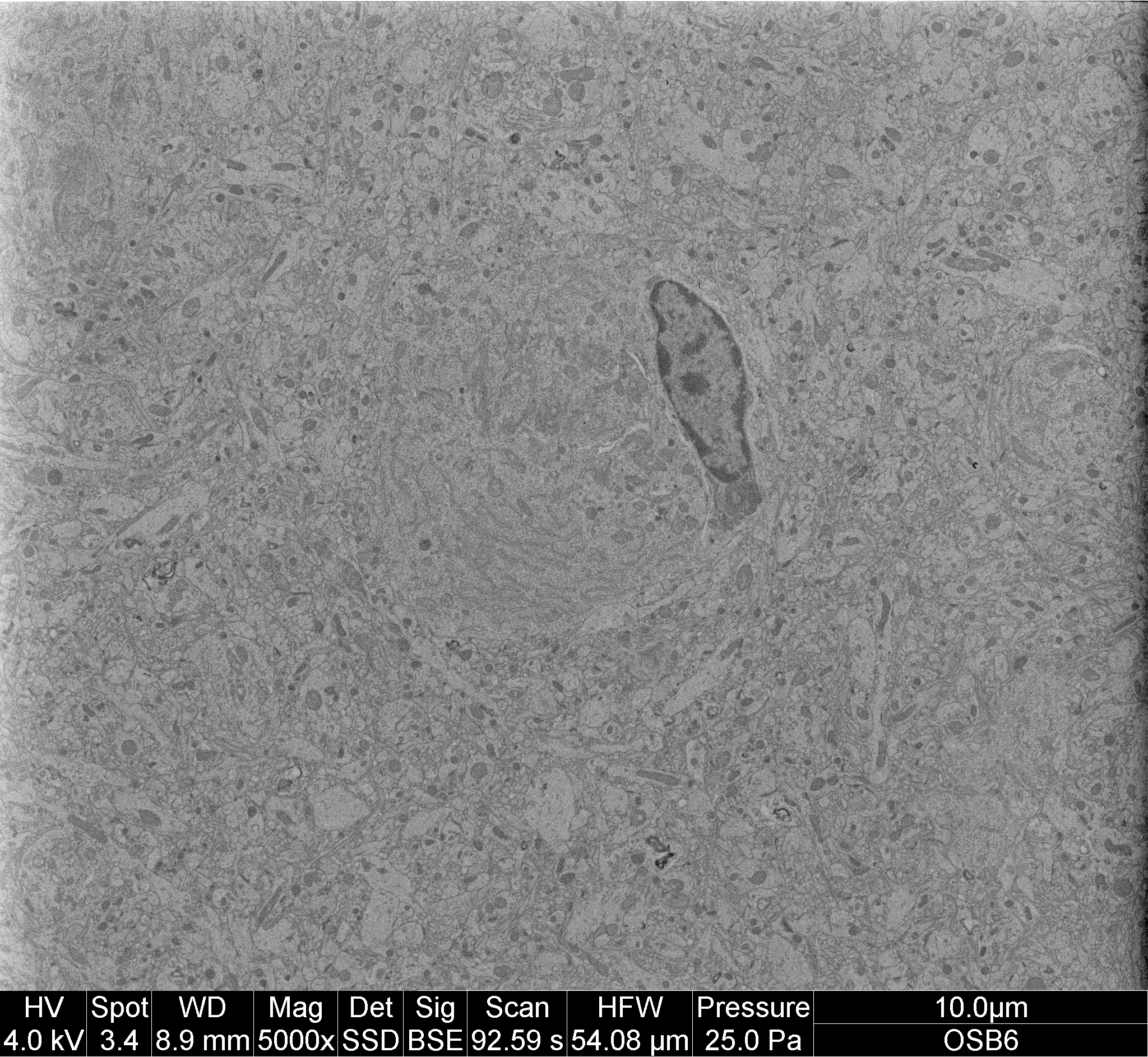

Supplement: Dataset S19 — (253.4 MB ZIP). [file pbio.0020329.sd019.zip › 040604_OS5_st1_1825.tif]

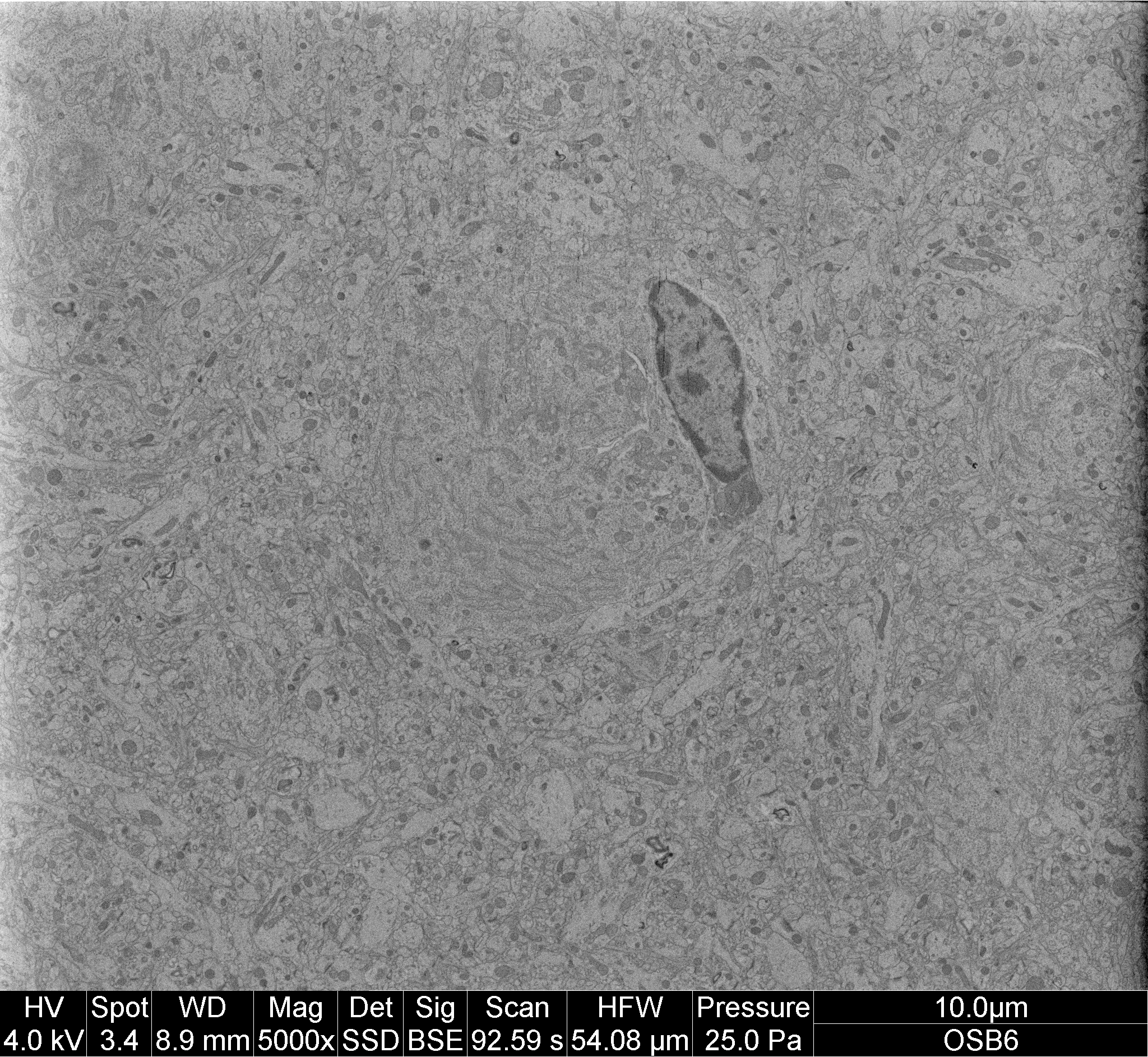

Supplement: Dataset S19 — (253.4 MB ZIP). [file pbio.0020329.sd019.zip › 040604_OS5_st1_1826.tif]

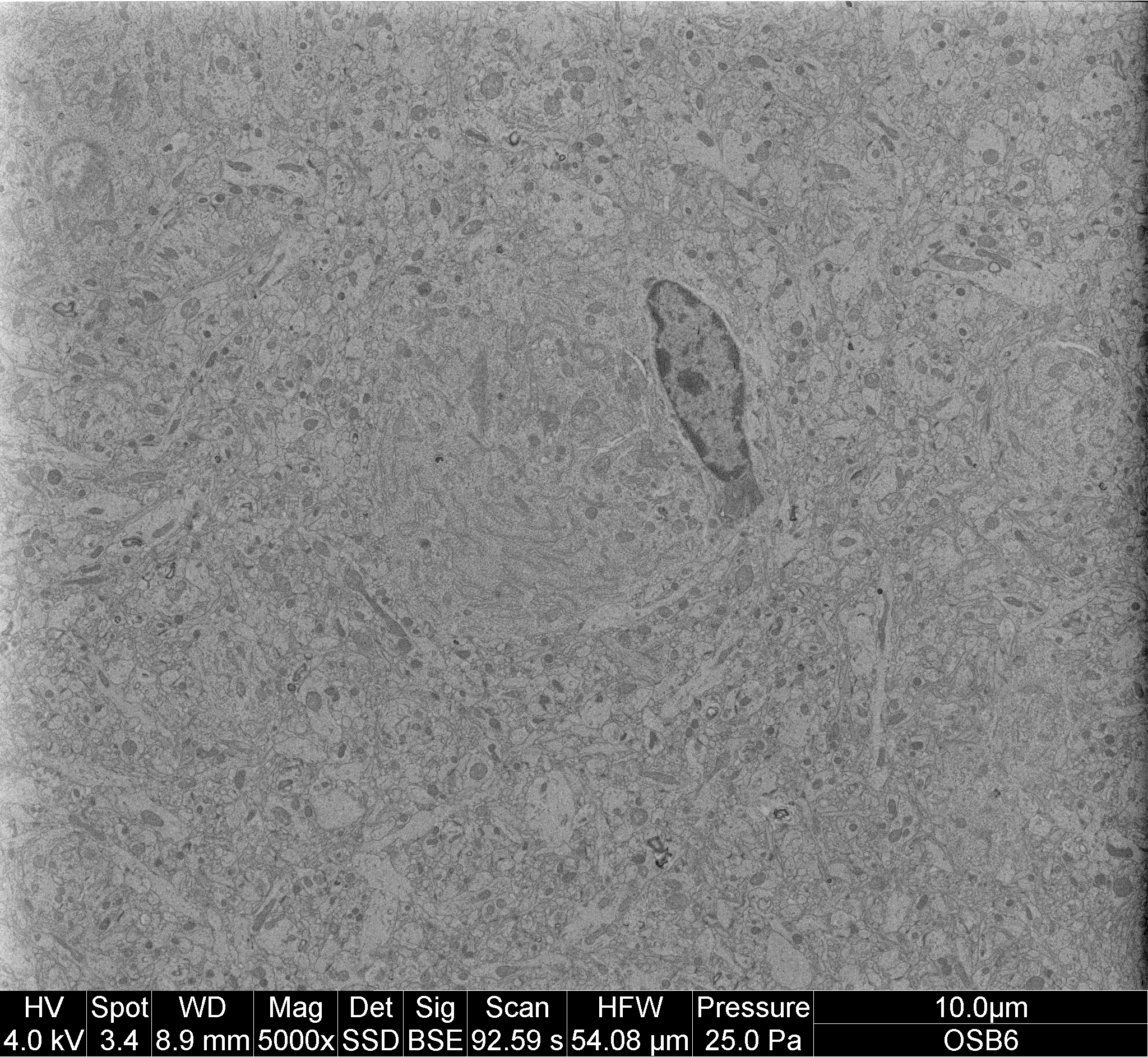

Supplement: Dataset S19 — (253.4 MB ZIP). [file pbio.0020329.sd019.zip › 040604_OS5_st1_1827.tif]

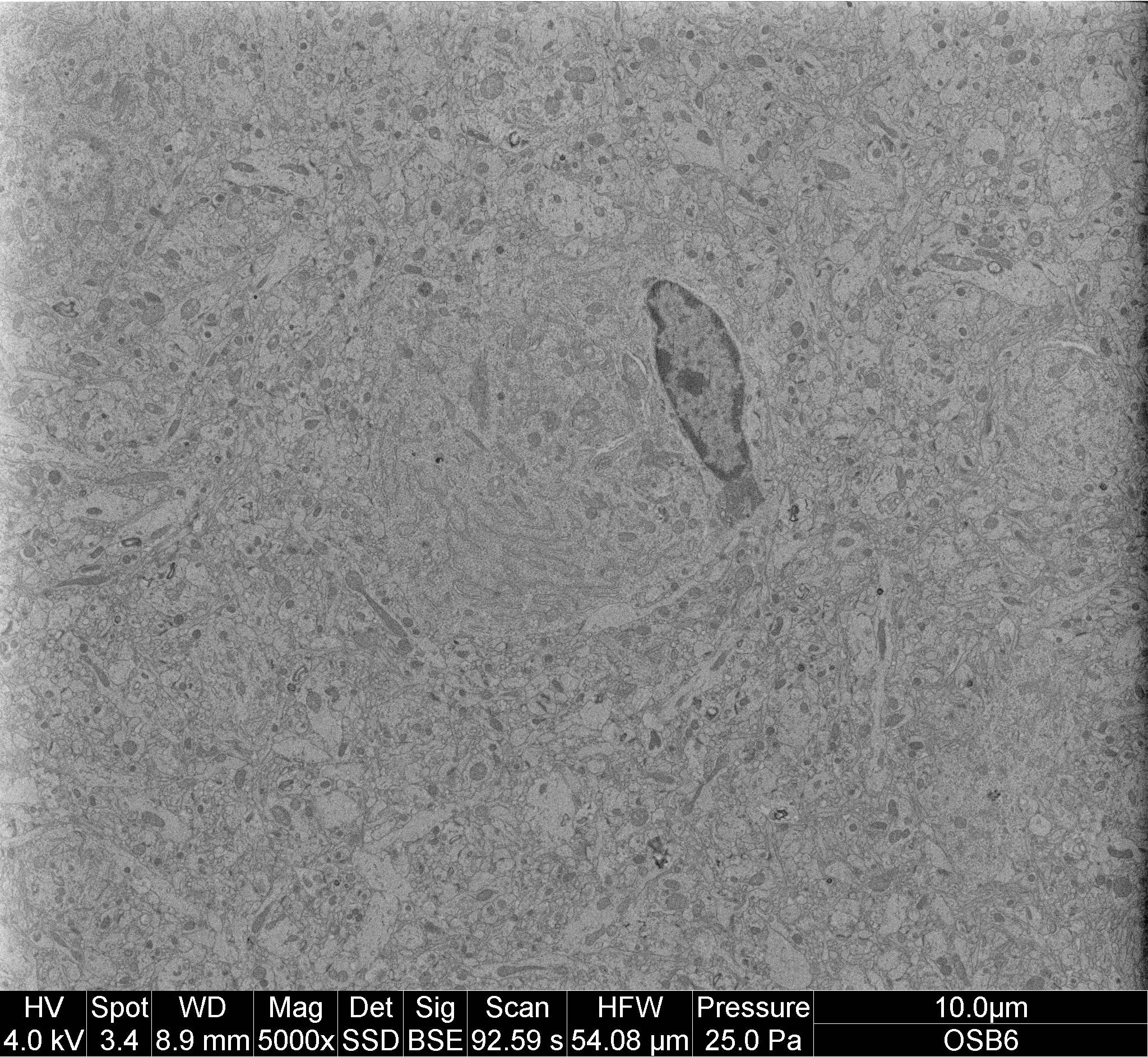

Supplement: Dataset S19 — (253.4 MB ZIP). [file pbio.0020329.sd019.zip › 040604_OS5_st1_1828.tif]

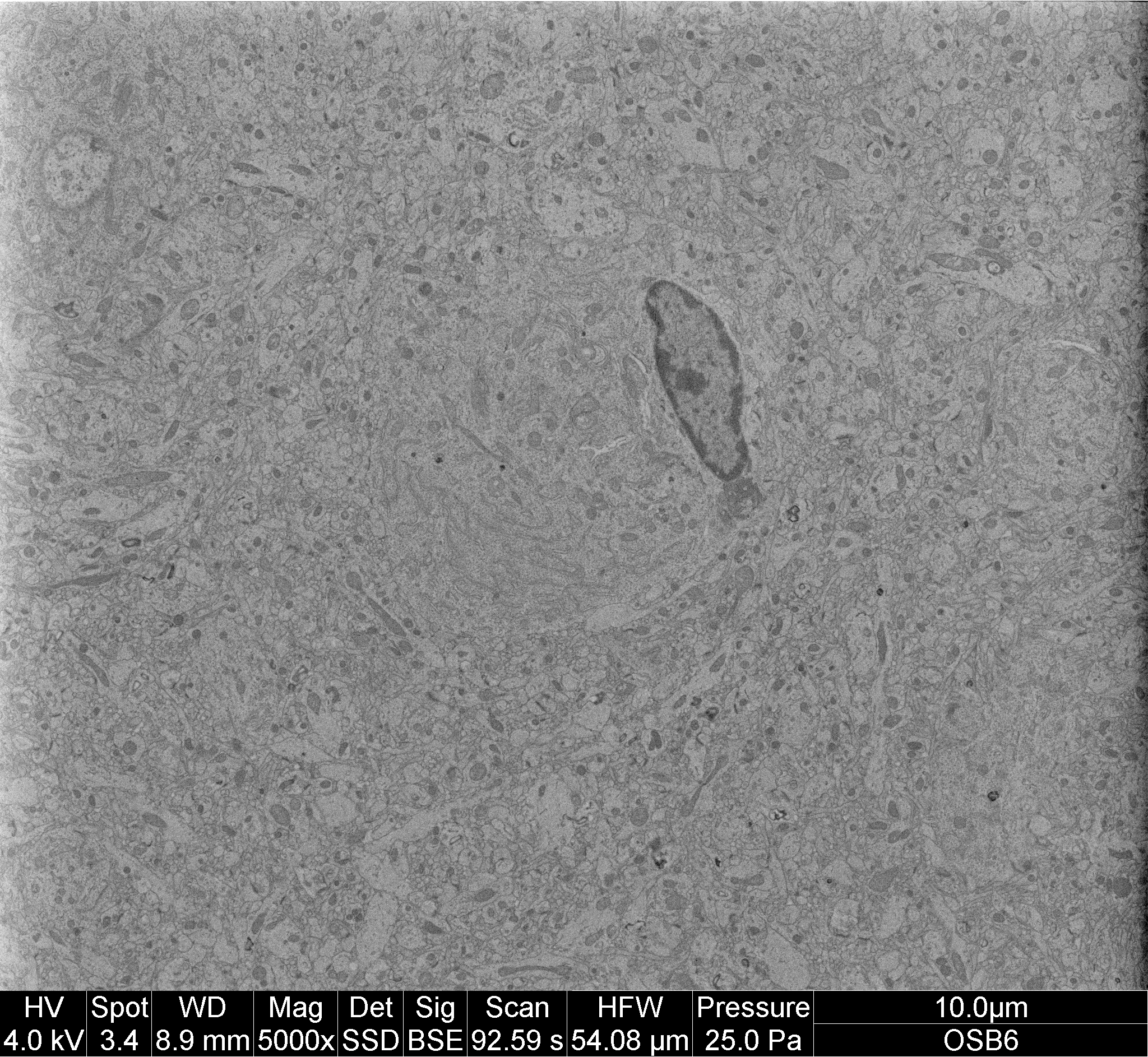

Supplement: Dataset S19 — (253.4 MB ZIP). [file pbio.0020329.sd019.zip › 040604_OS5_st1_1829.tif]

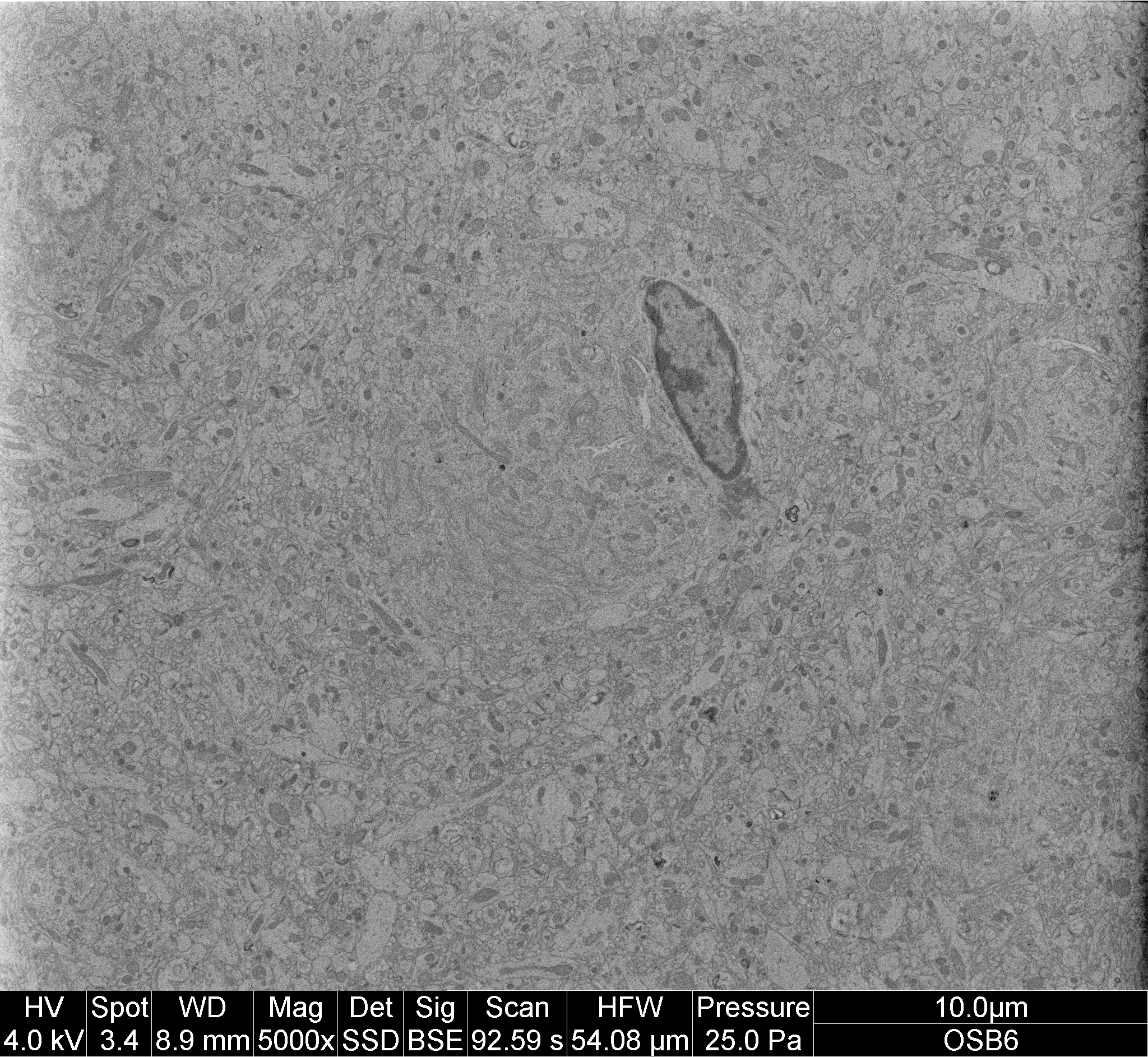

Supplement: Dataset S19 — (253.4 MB ZIP). [file pbio.0020329.sd019.zip › 040604_OS5_st1_1830.tif]

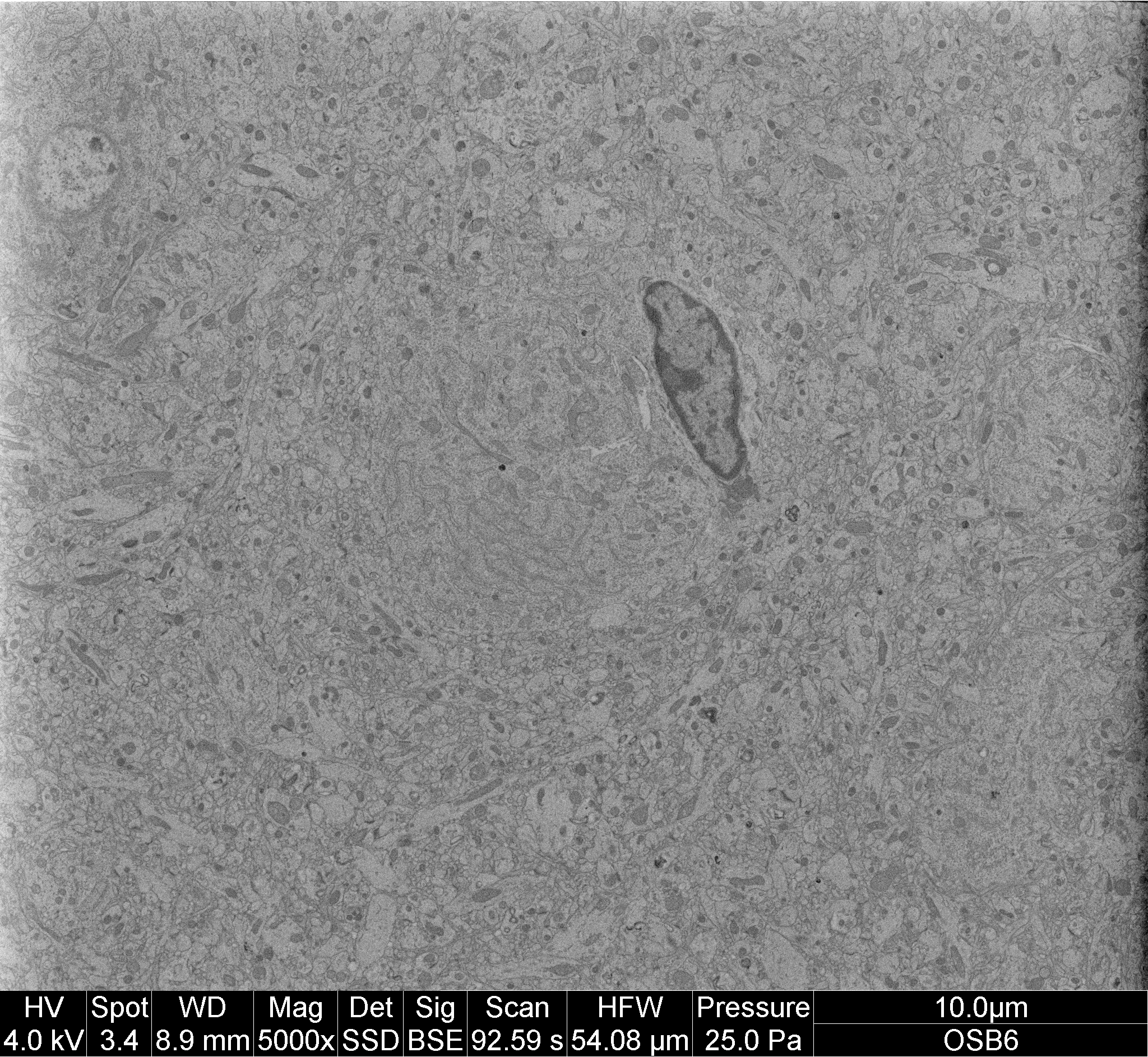

Supplement: Dataset S19 — (253.4 MB ZIP). [file pbio.0020329.sd019.zip › 040604_OS5_st1_1831.tif]

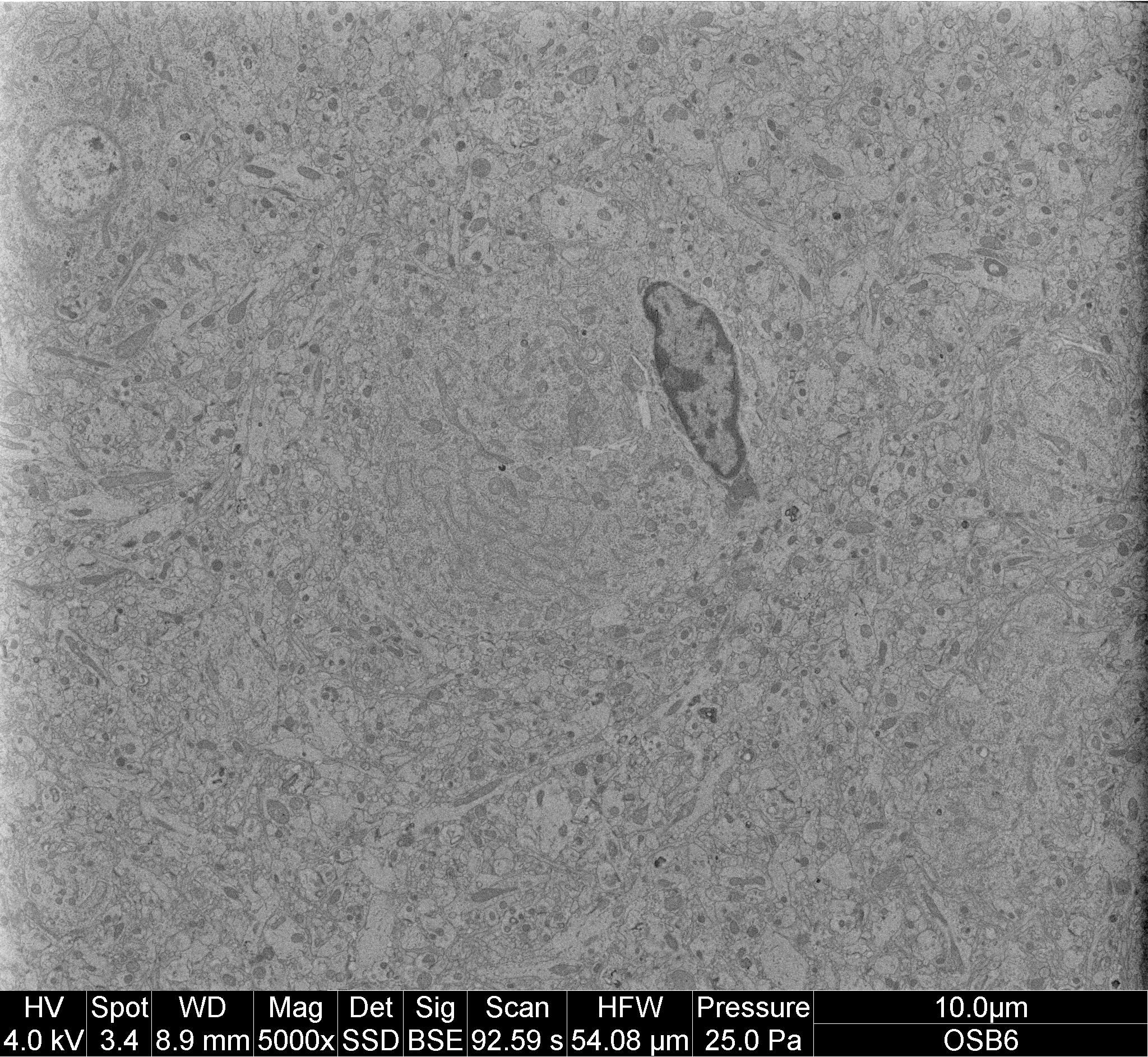

Supplement: Dataset S19 — (253.4 MB ZIP). [file pbio.0020329.sd019.zip › 040604_OS5_st1_1832.tif]

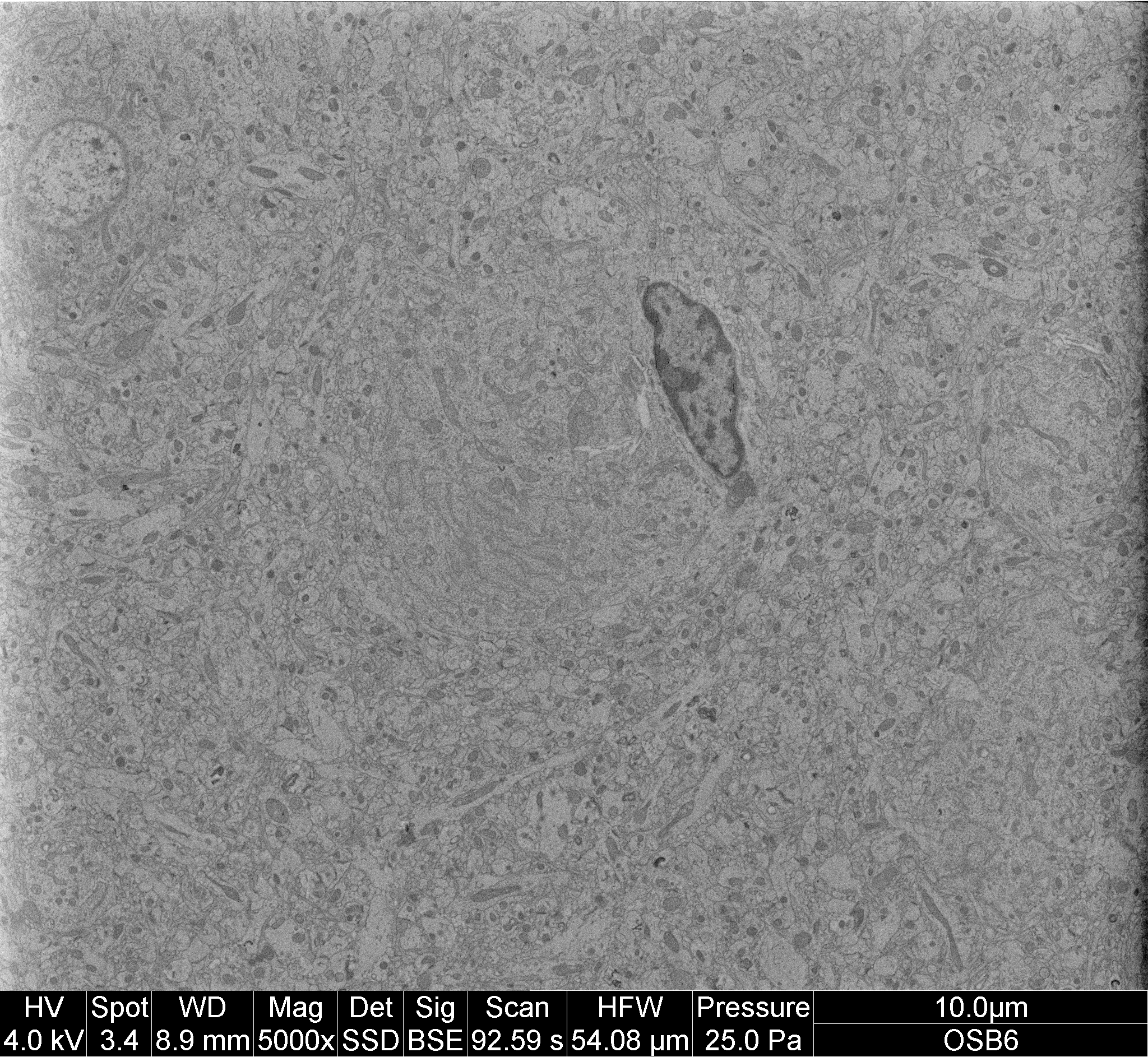

Supplement: Dataset S19 — (253.4 MB ZIP). [file pbio.0020329.sd019.zip › 040604_OS5_st1_1833.tif]

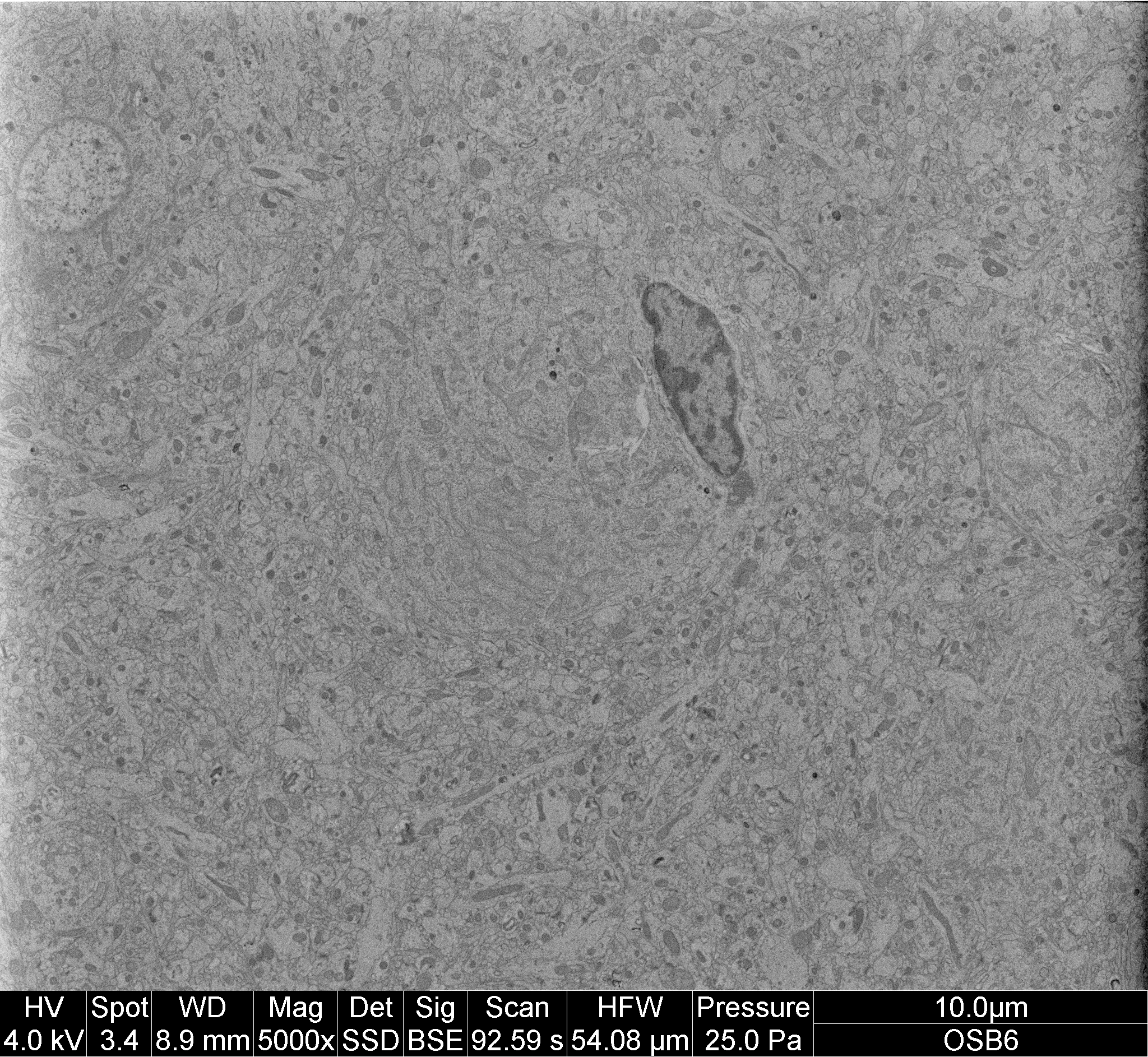

Supplement: Dataset S19 — (253.4 MB ZIP). [file pbio.0020329.sd019.zip › 040604_OS5_st1_1834.tif]

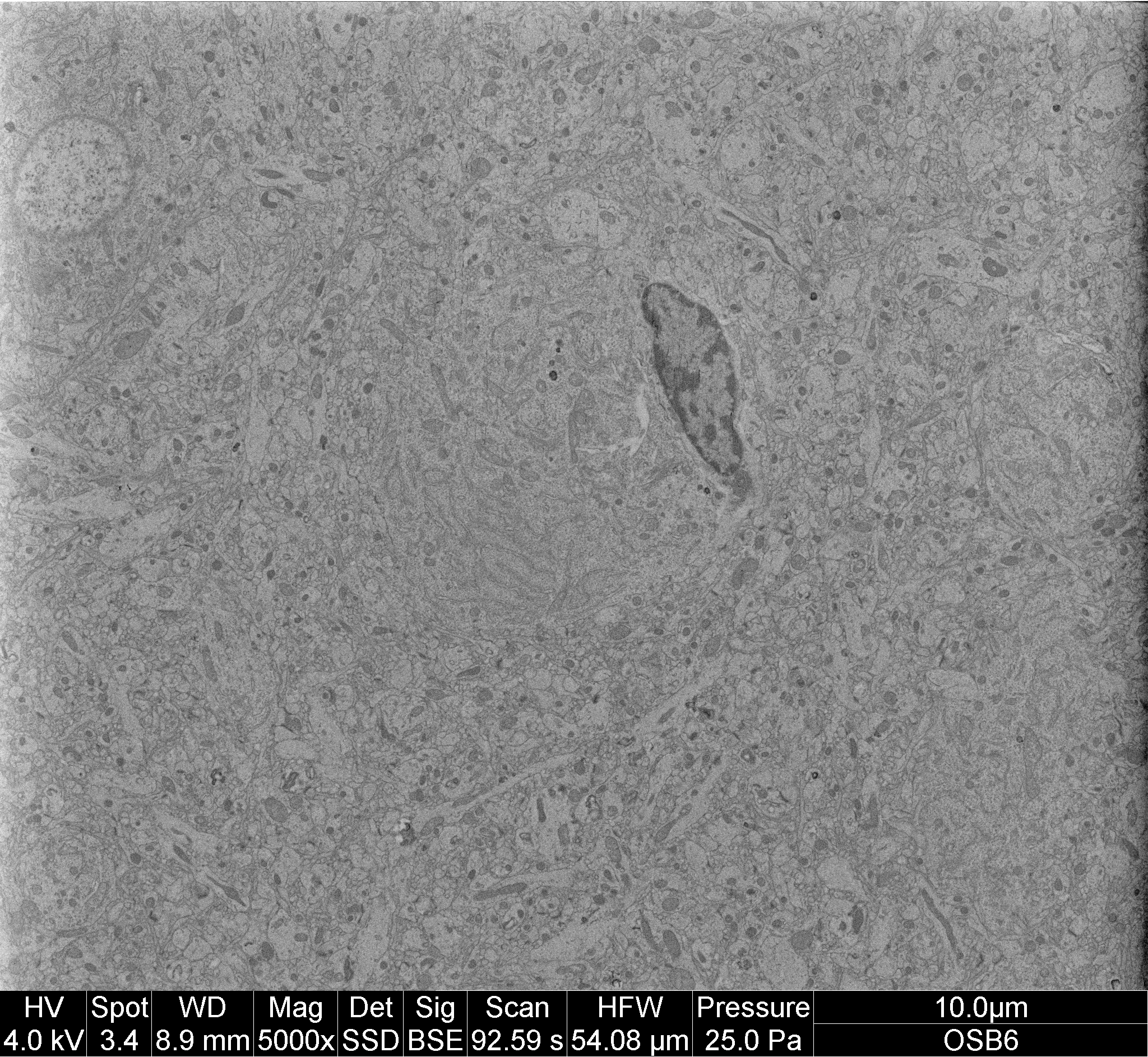

Supplement: Dataset S19 — (253.4 MB ZIP). [file pbio.0020329.sd019.zip › 040604_OS5_st1_1835.tif]

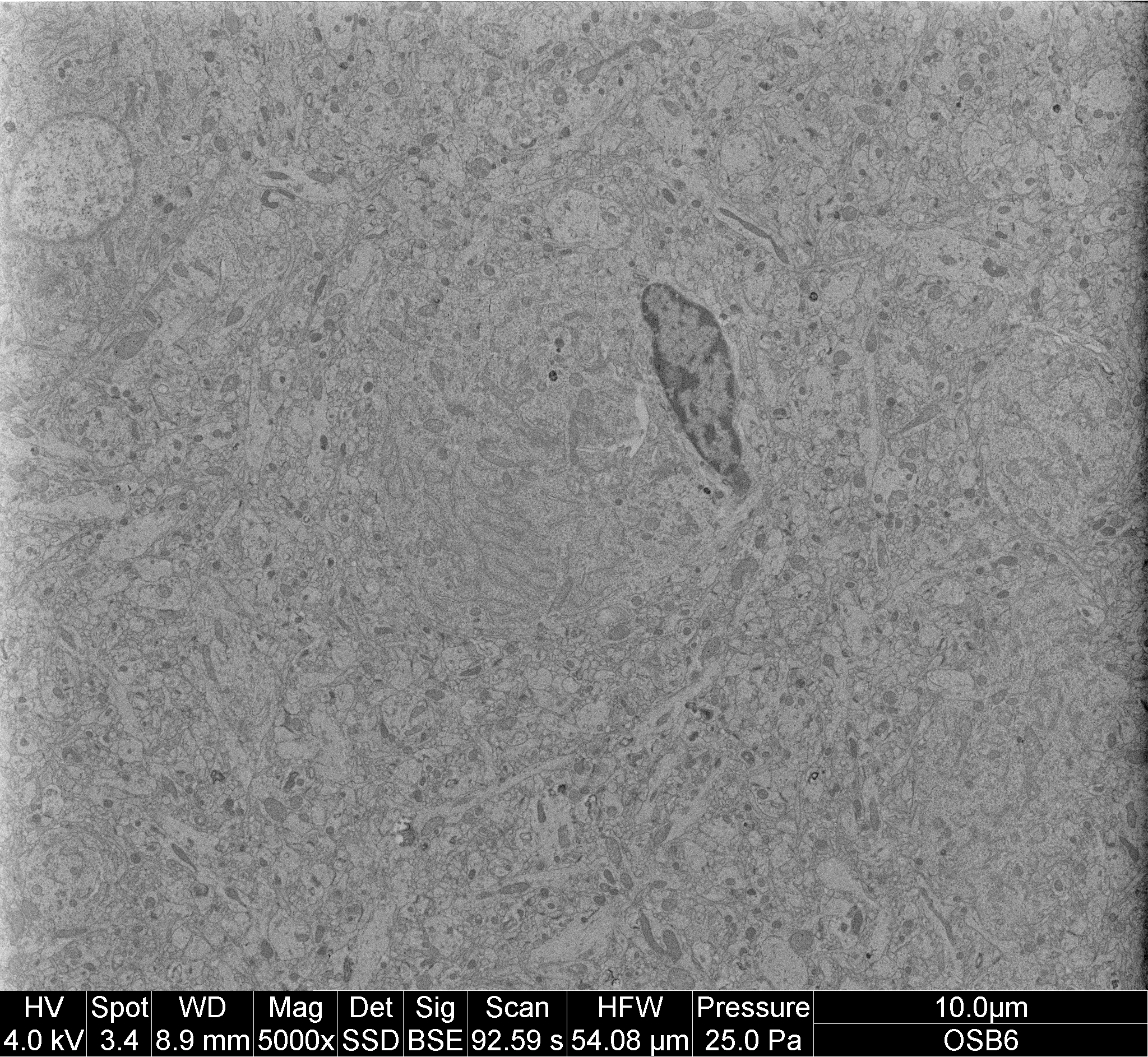

Supplement: Dataset S19 — (253.4 MB ZIP). [file pbio.0020329.sd019.zip › 040604_OS5_st1_1836.tif]

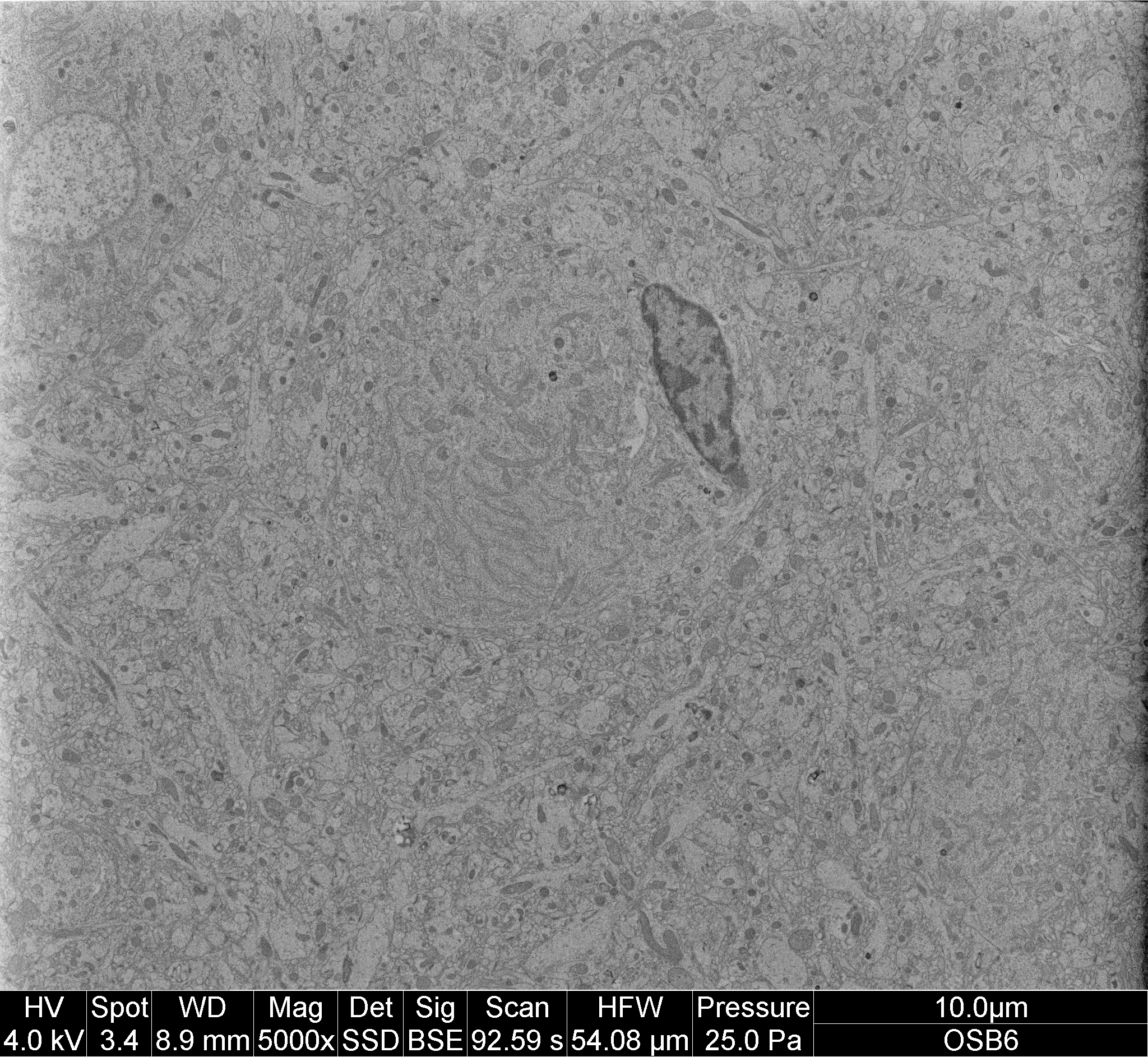

Supplement: Dataset S19 — (253.4 MB ZIP). [file pbio.0020329.sd019.zip › 040604_OS5_st1_1837.tif]

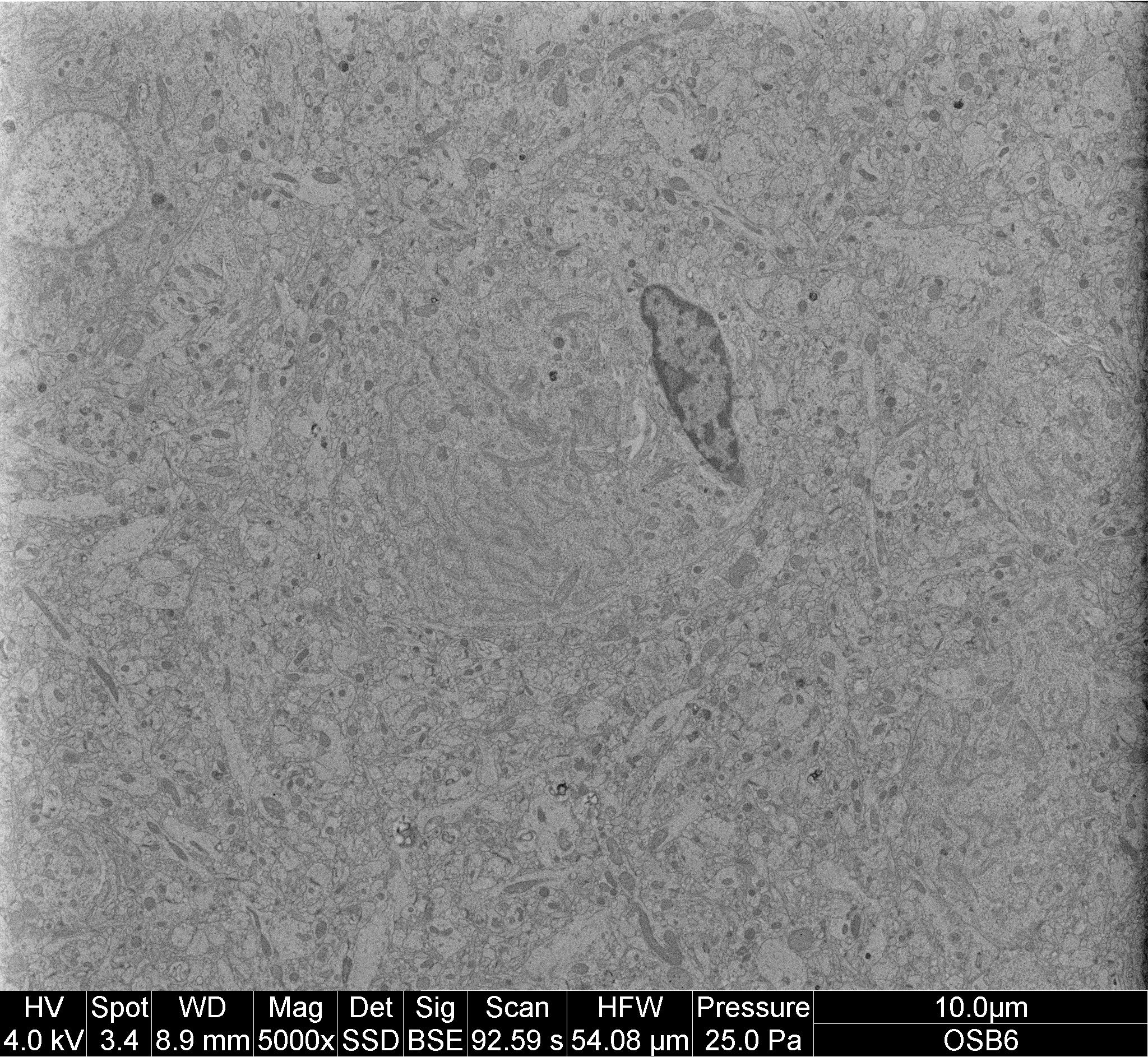

Supplement: Dataset S19 — (253.4 MB ZIP). [file pbio.0020329.sd019.zip › 040604_OS5_st1_1838.tif]

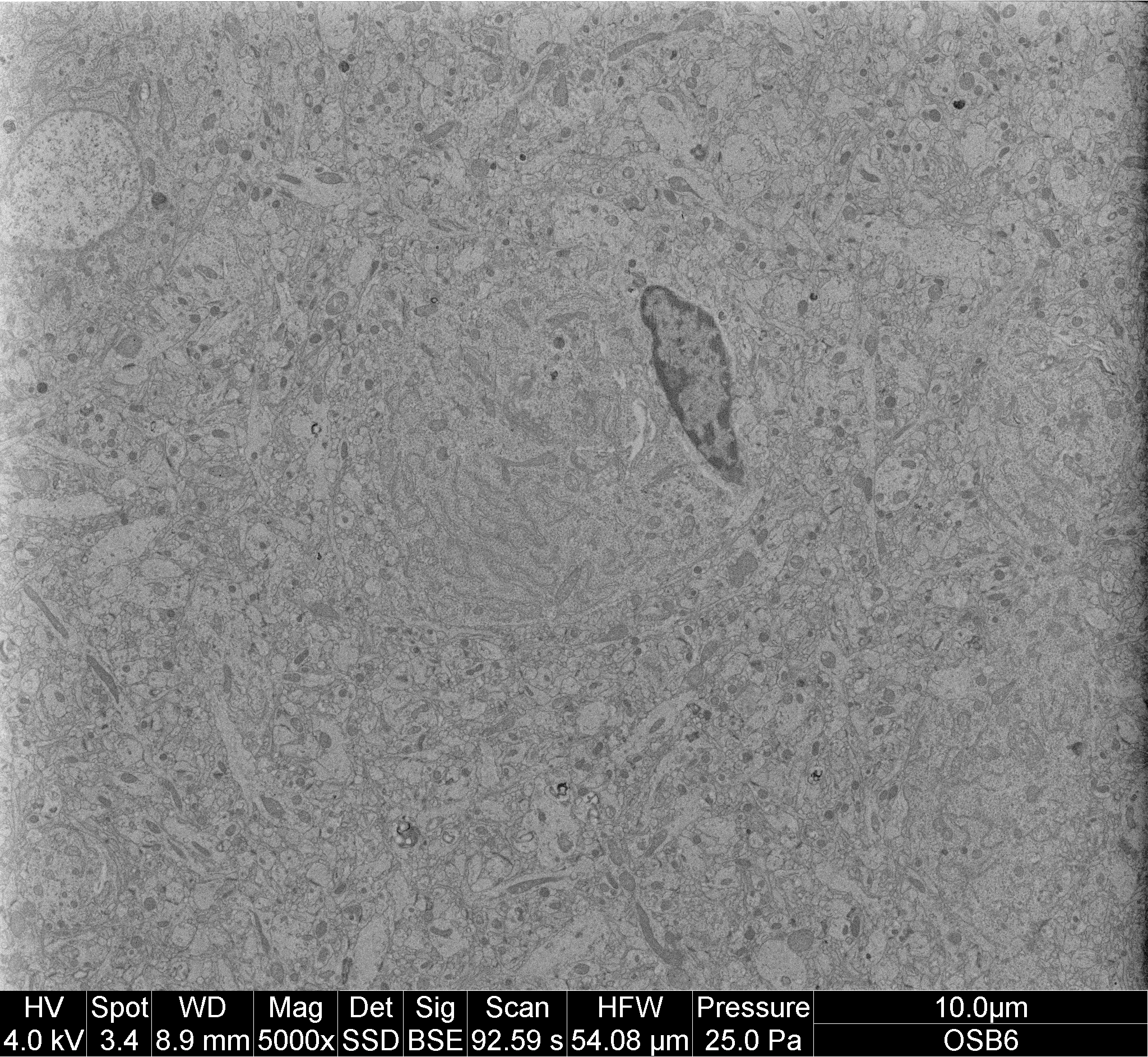

Supplement: Dataset S19 — (253.4 MB ZIP). [file pbio.0020329.sd019.zip › 040604_OS5_st1_1839.tif]

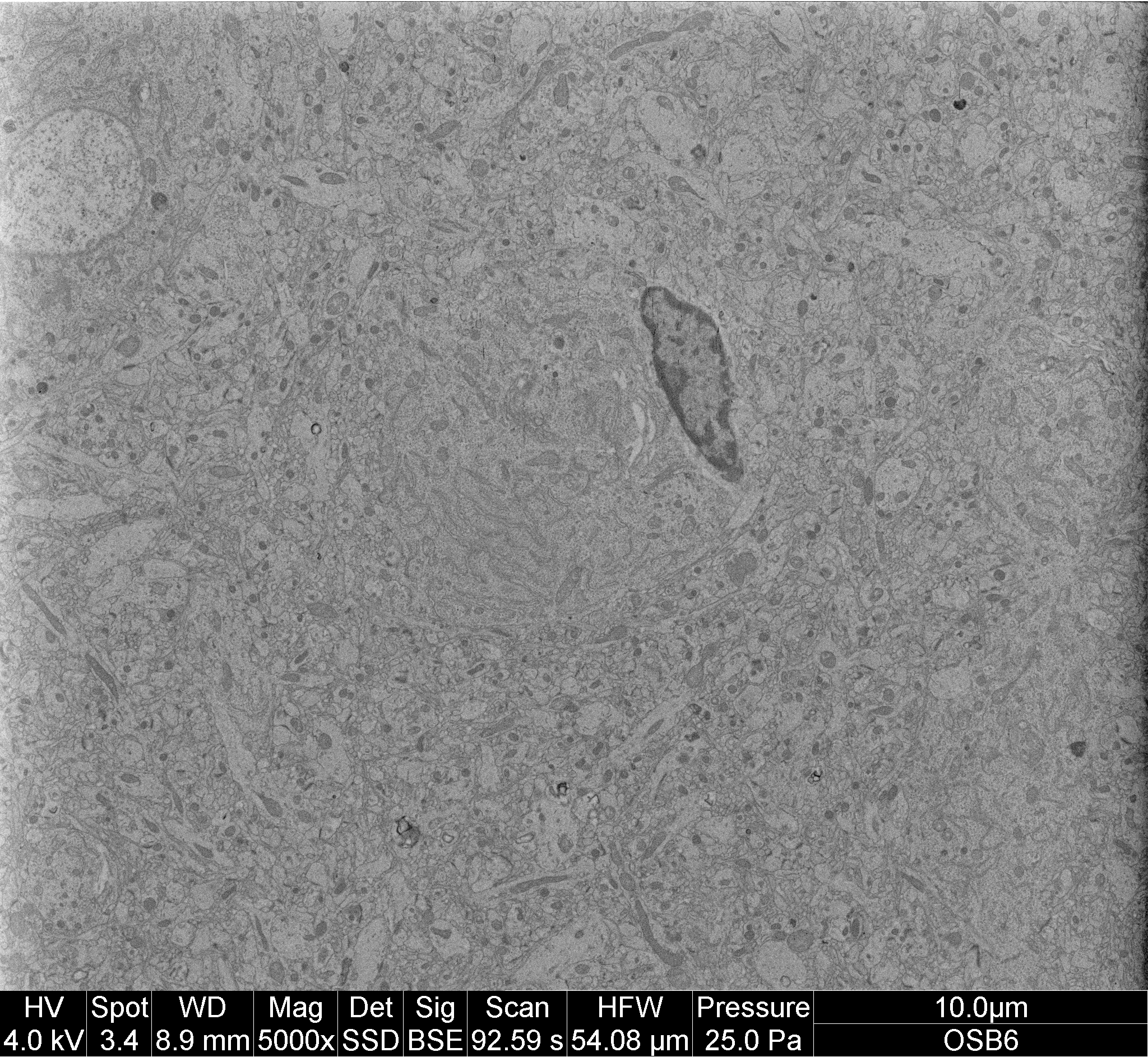

Supplement: Dataset S19 — (253.4 MB ZIP). [file pbio.0020329.sd019.zip › 040604_OS5_st1_1840.tif]

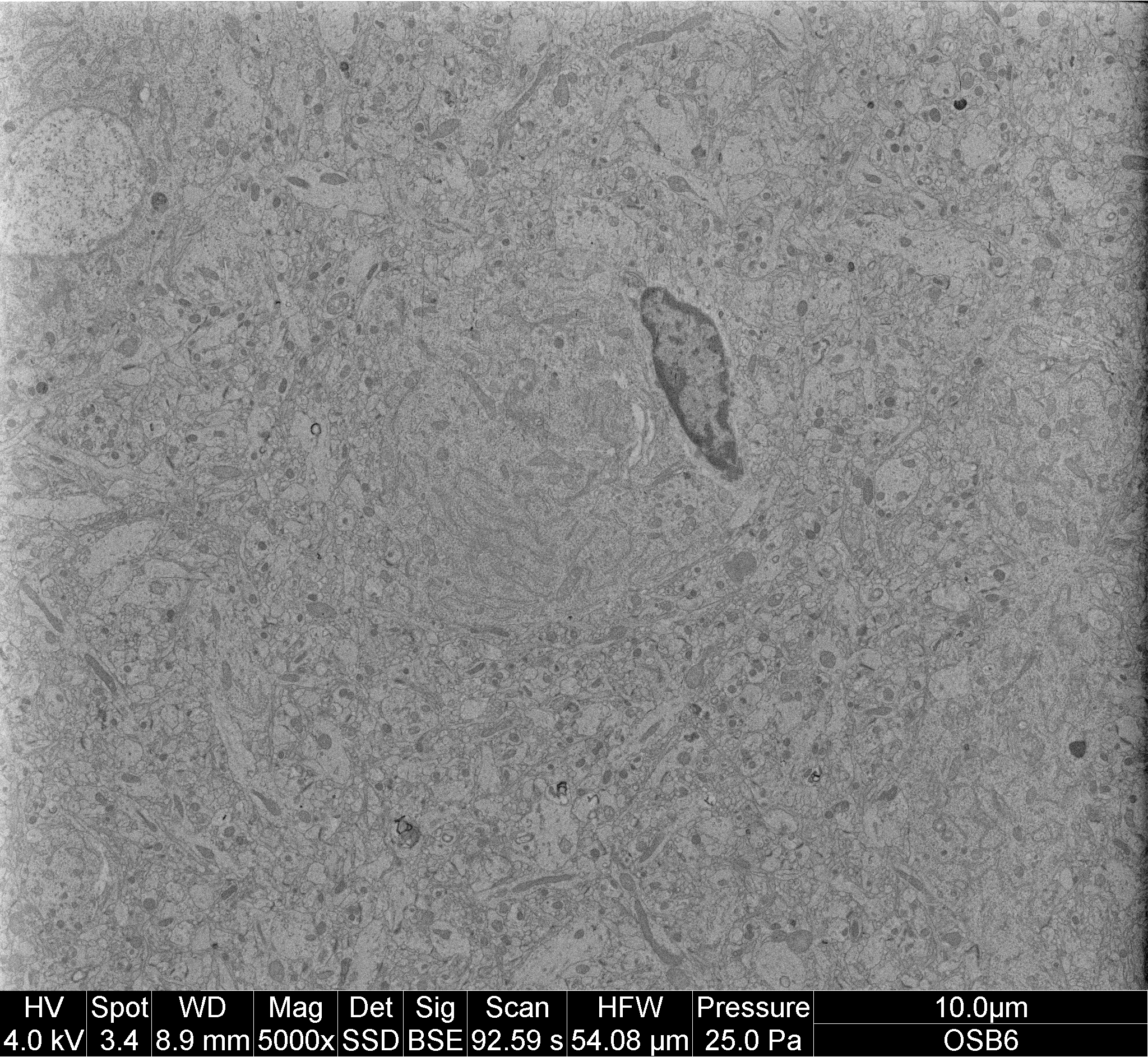

Supplement: Dataset S19 — (253.4 MB ZIP). [file pbio.0020329.sd019.zip › 040604_OS5_st1_1841.tif]

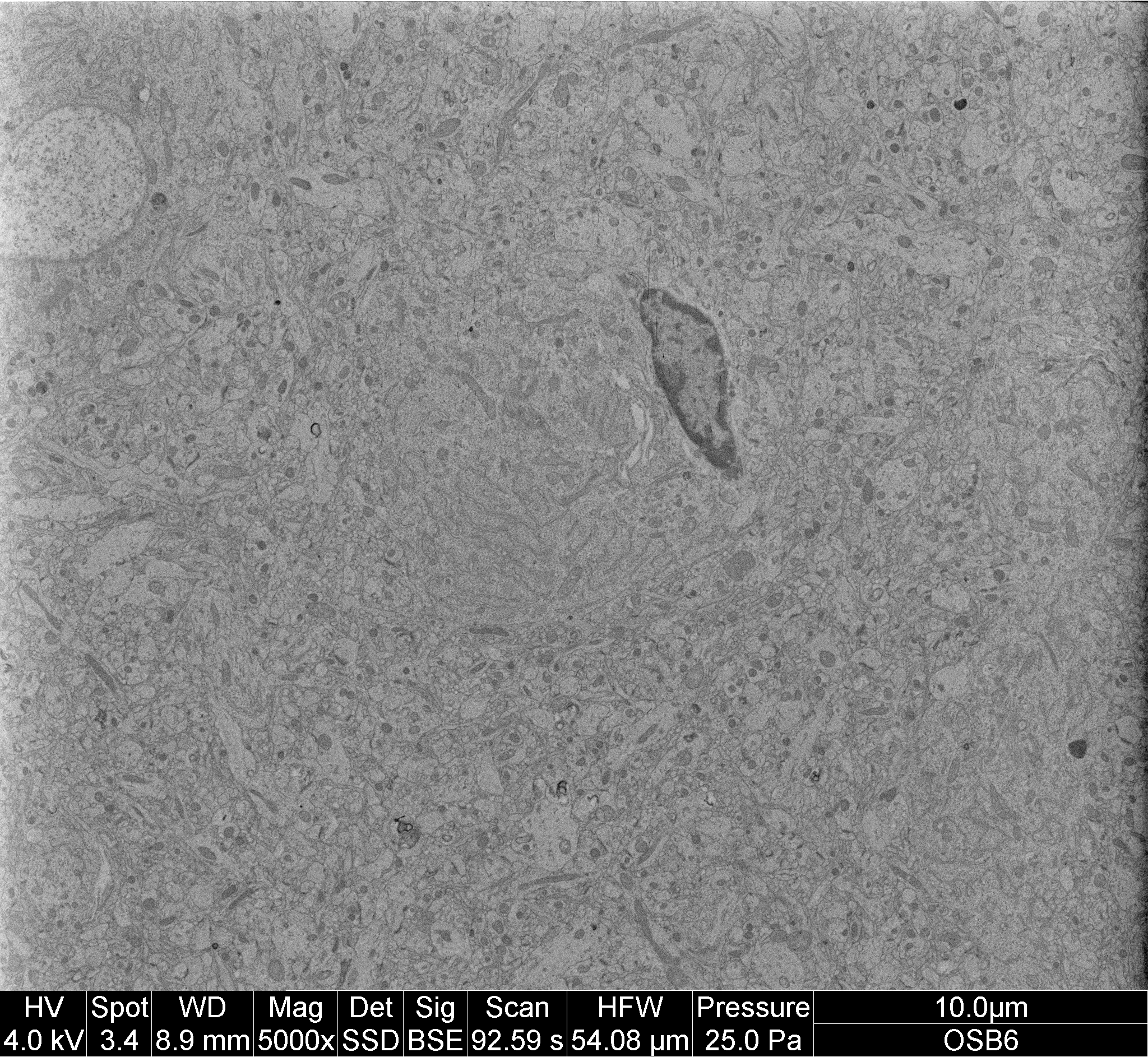

Supplement: Dataset S19 — (253.4 MB ZIP). [file pbio.0020329.sd019.zip › 040604_OS5_st1_1842.tif]

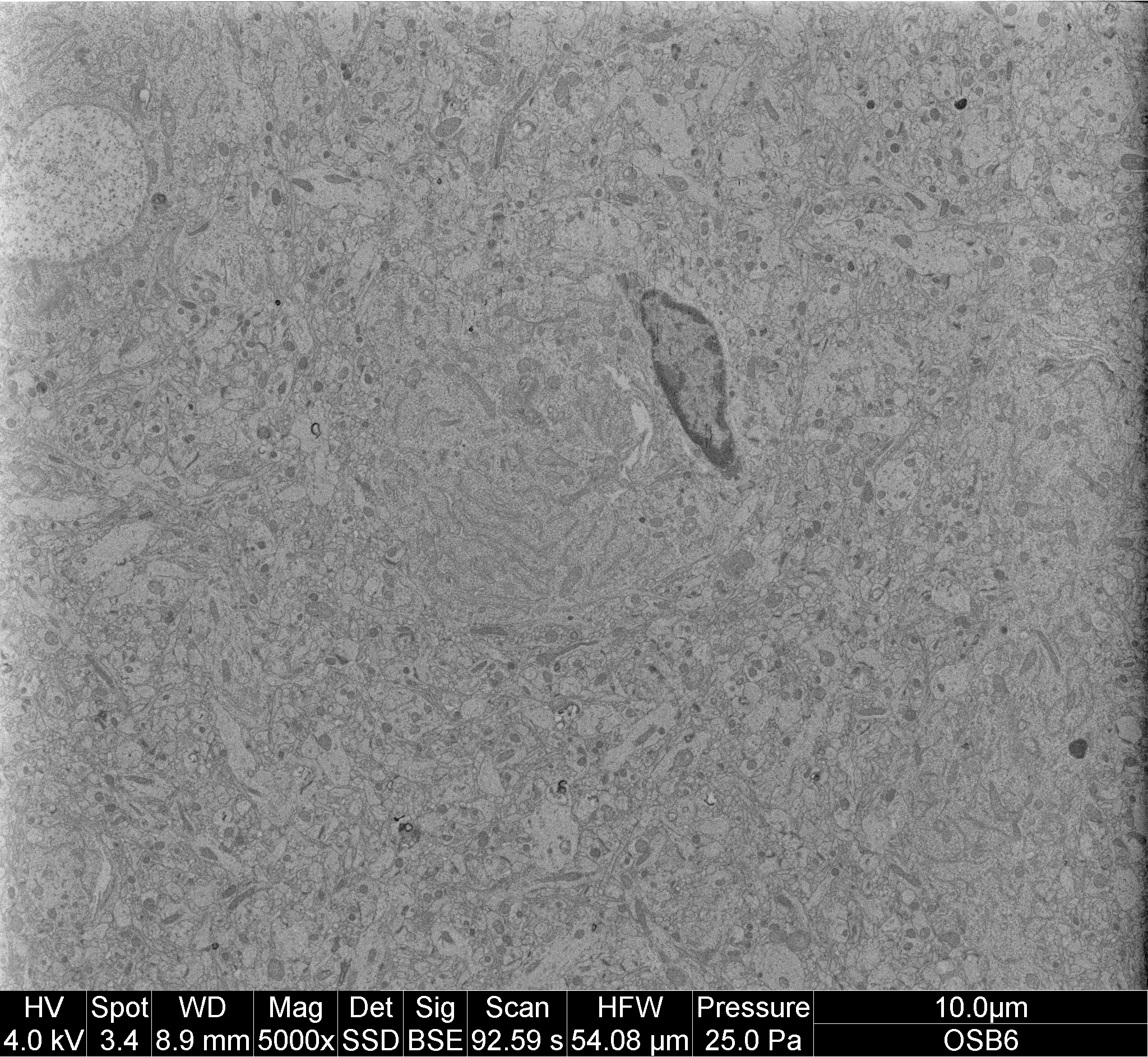

Supplement: Dataset S19 — (253.4 MB ZIP). [file pbio.0020329.sd019.zip › 040604_OS5_st1_1843.tif]

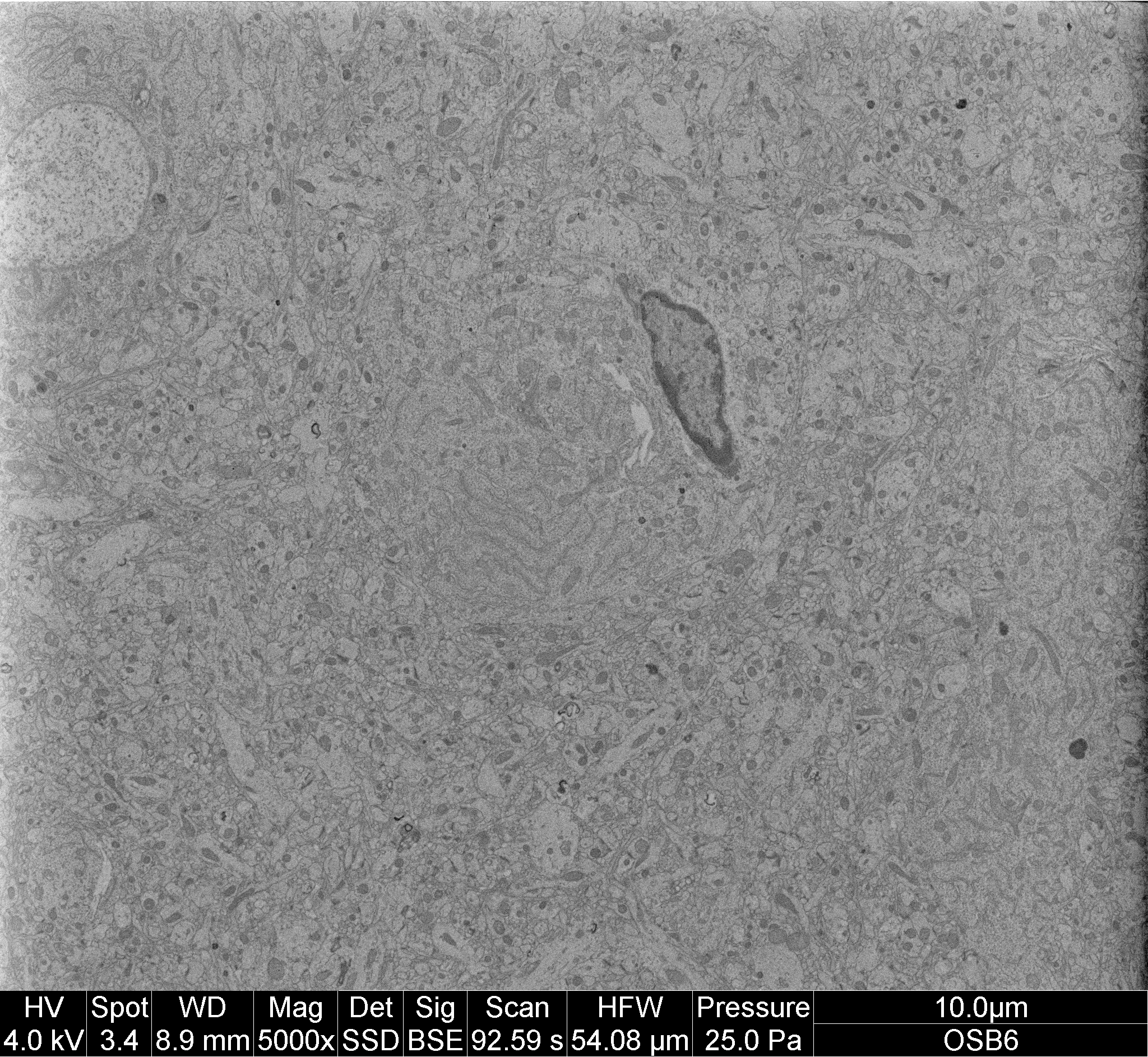

Supplement: Dataset S19 — (253.4 MB ZIP). [file pbio.0020329.sd019.zip › 040604_OS5_st1_1844.tif]

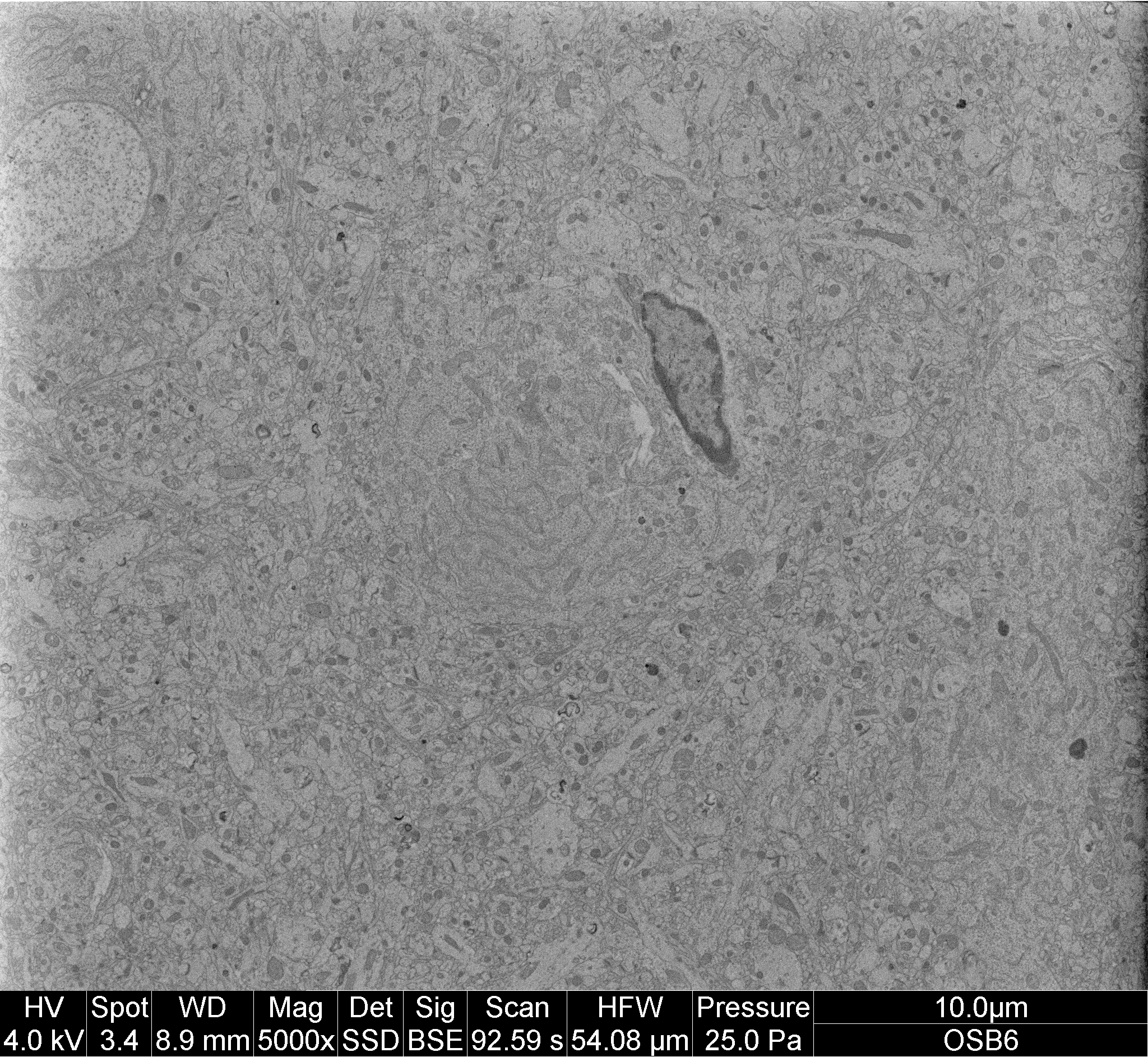

Supplement: Dataset S19 — (253.4 MB ZIP). [file pbio.0020329.sd019.zip › 040604_OS5_st1_1845.tif]

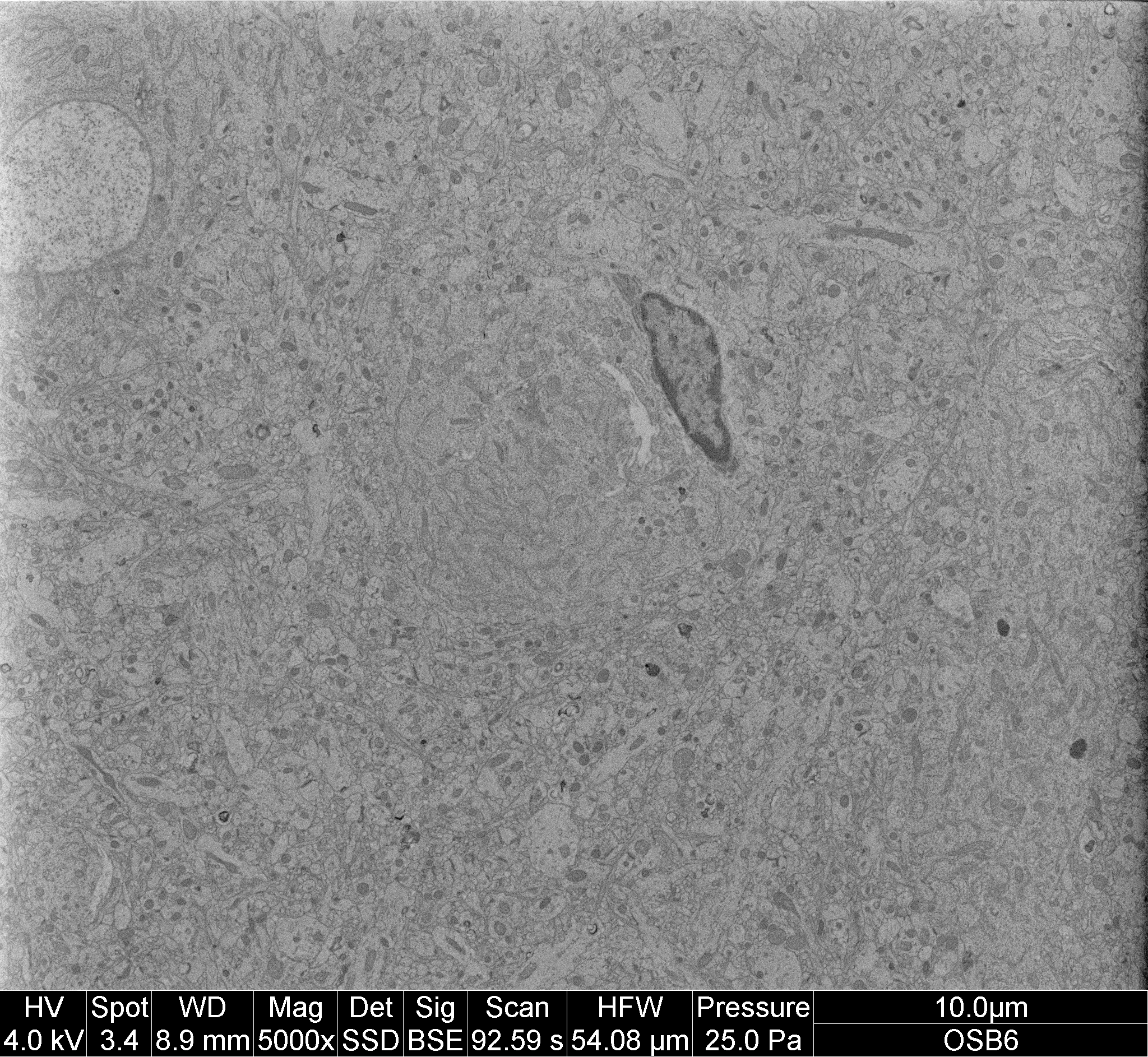

Supplement: Dataset S19 — (253.4 MB ZIP). [file pbio.0020329.sd019.zip › 040604_OS5_st1_1846.tif]

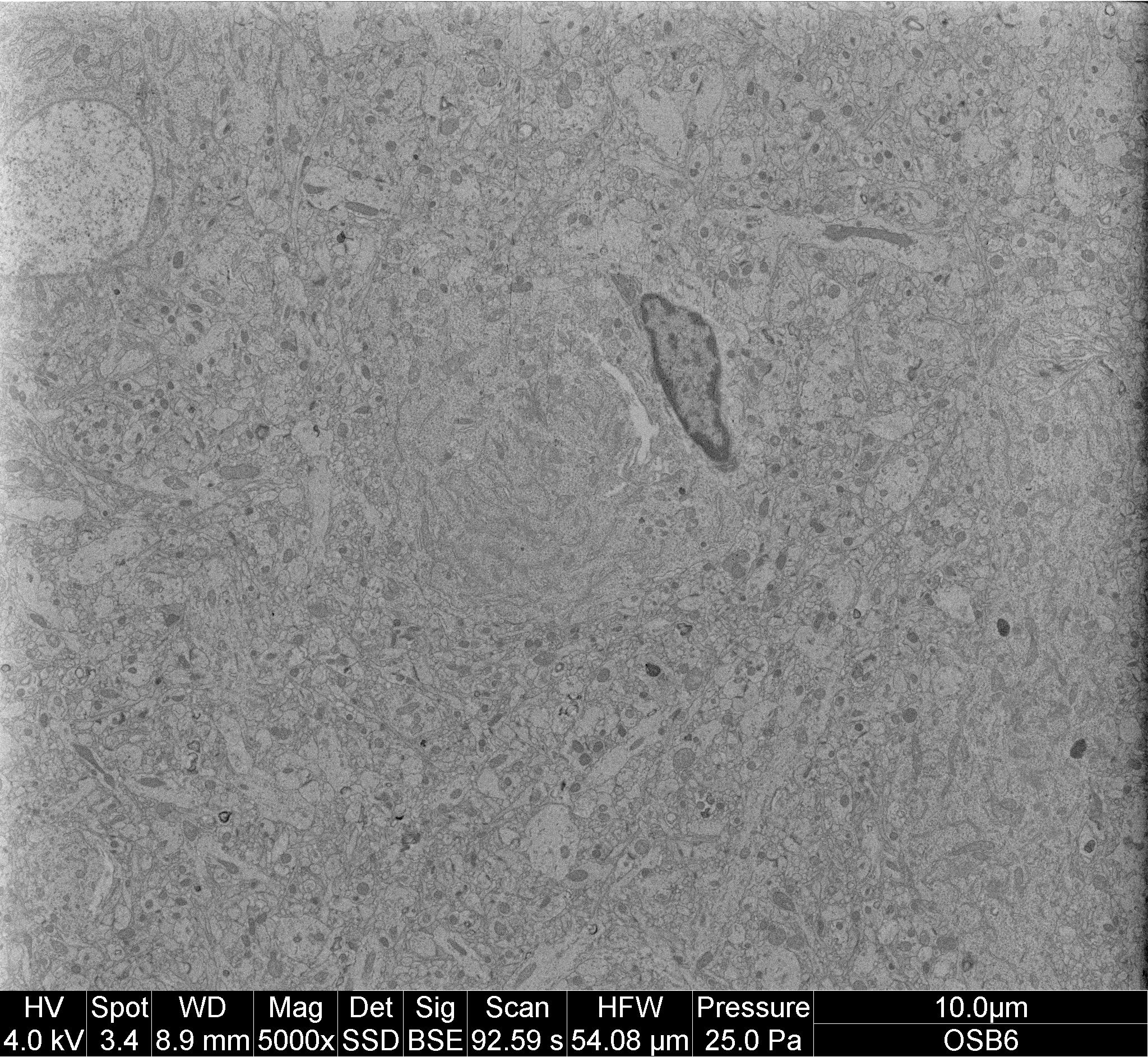

Supplement: Dataset S19 — (253.4 MB ZIP). [file pbio.0020329.sd019.zip › 040604_OS5_st1_1847.tif]

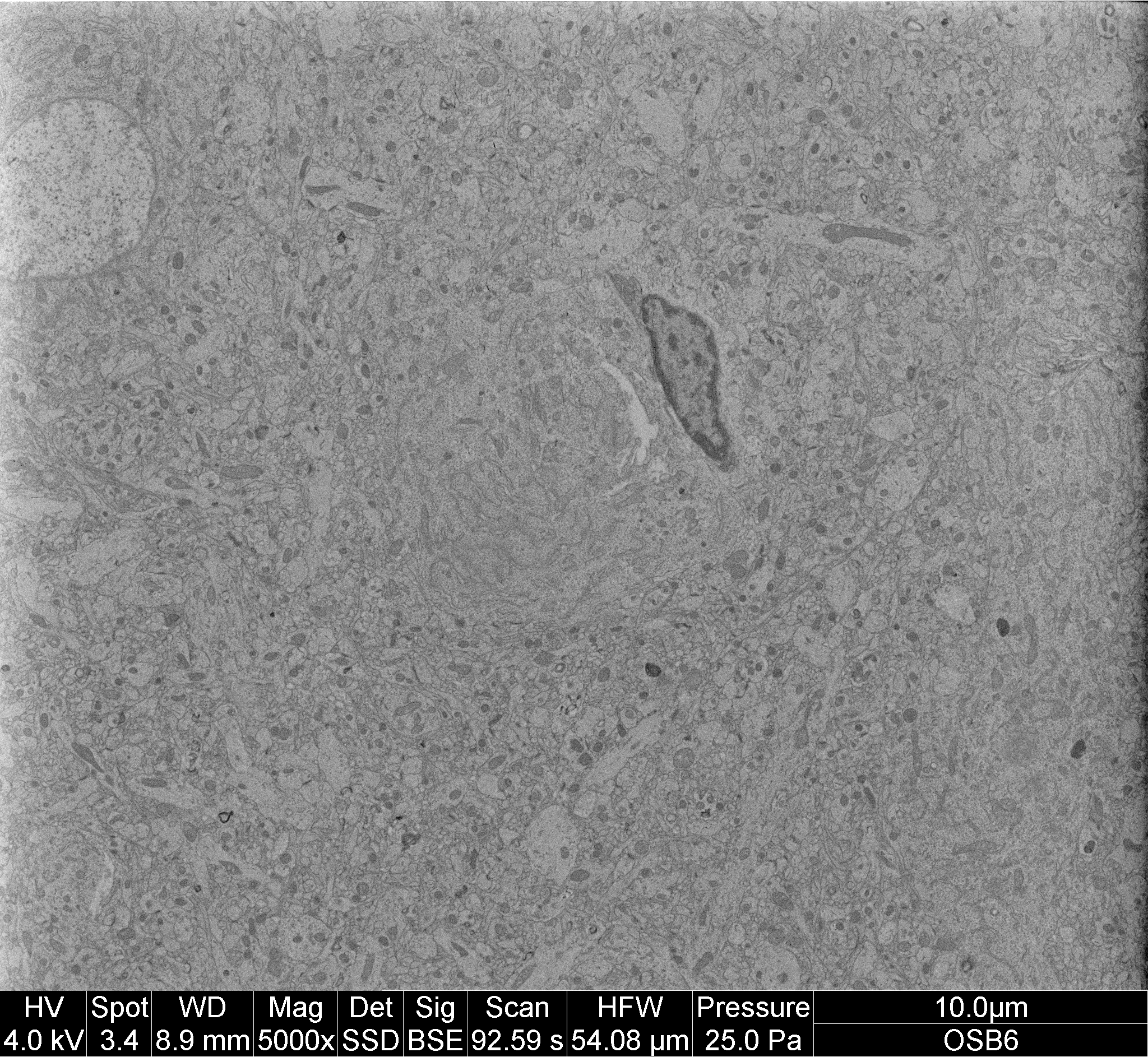

Supplement: Dataset S19 — (253.4 MB ZIP). [file pbio.0020329.sd019.zip › 040604_OS5_st1_1848.tif]

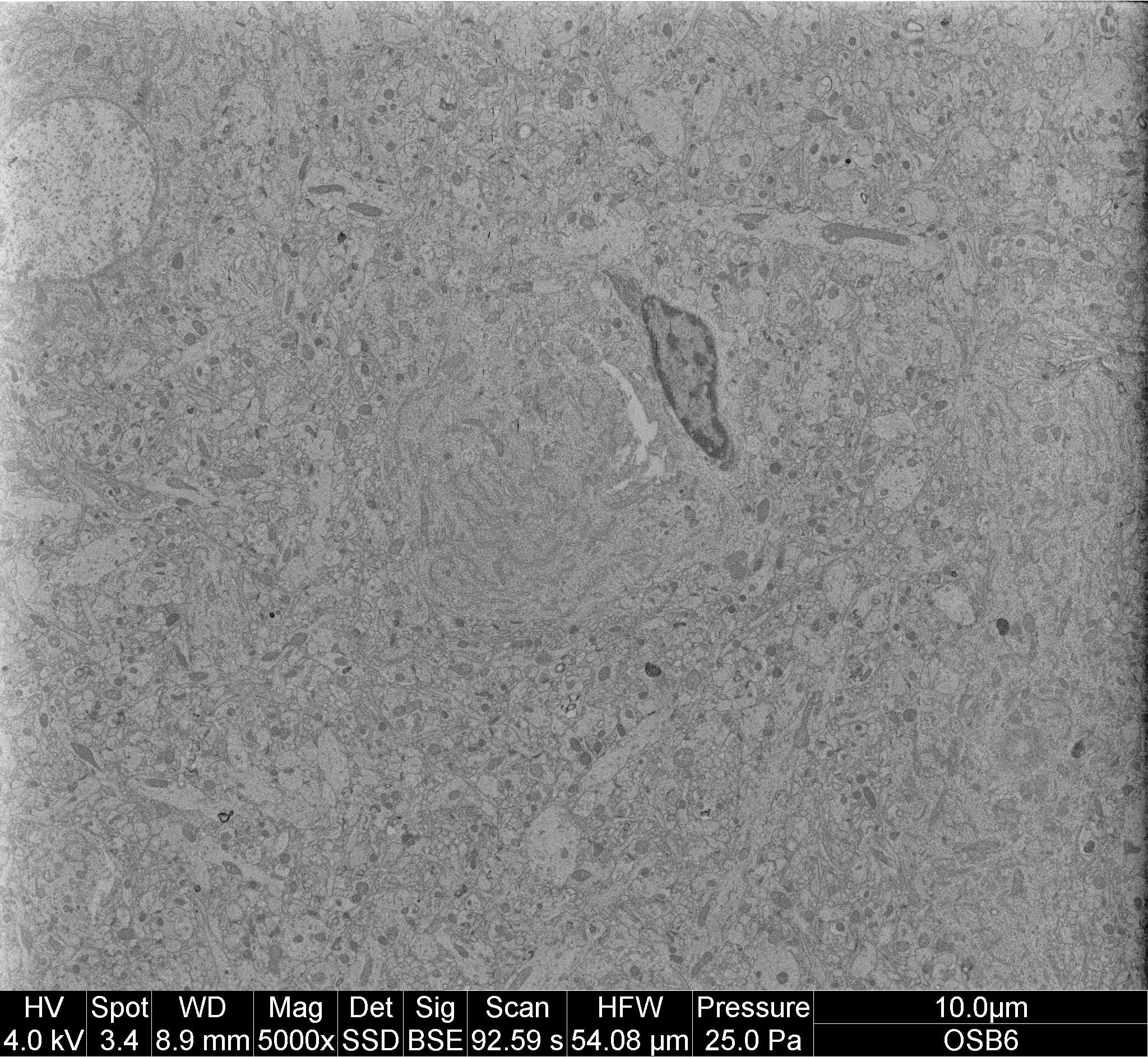

Supplement: Dataset S19 — (253.4 MB ZIP). [file pbio.0020329.sd019.zip › 040604_OS5_st1_1849.tif]

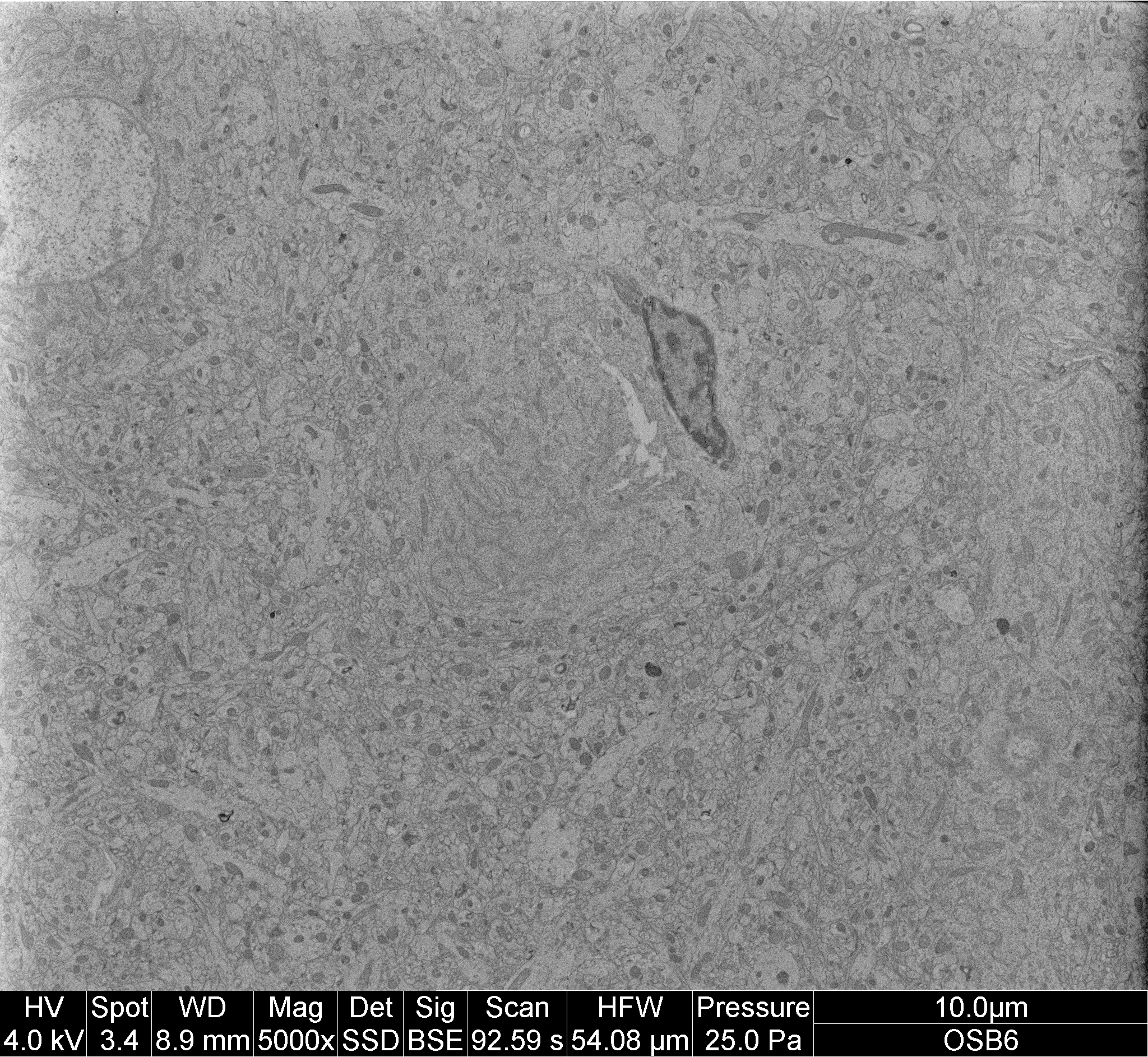

Supplement: Dataset S19 — (253.4 MB ZIP). [file pbio.0020329.sd019.zip › 040604_OS5_st1_1850.tif]

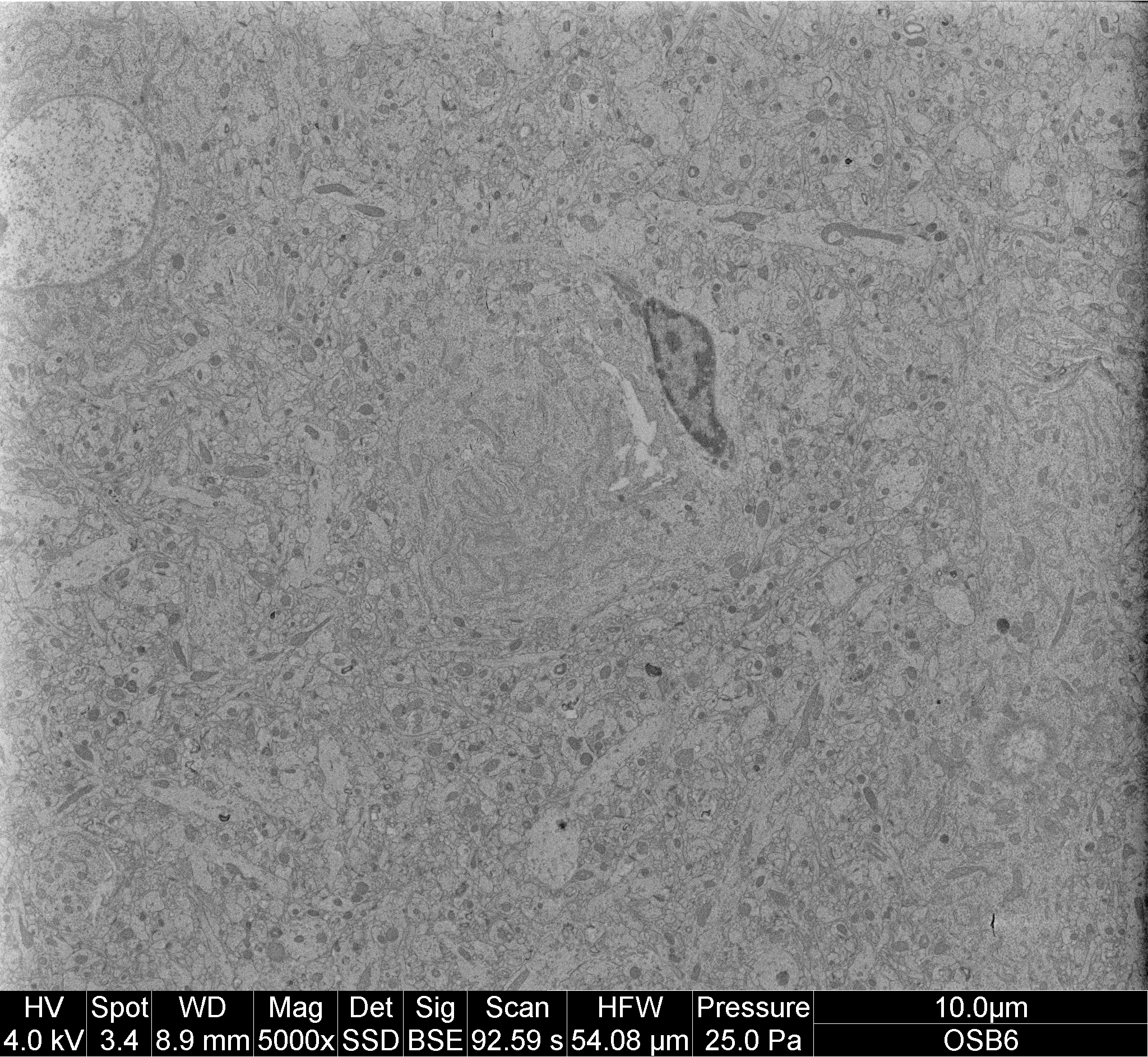

Supplement: Dataset S19 — (253.4 MB ZIP). [file pbio.0020329.sd019.zip › 040604_OS5_st1_1851.tif]

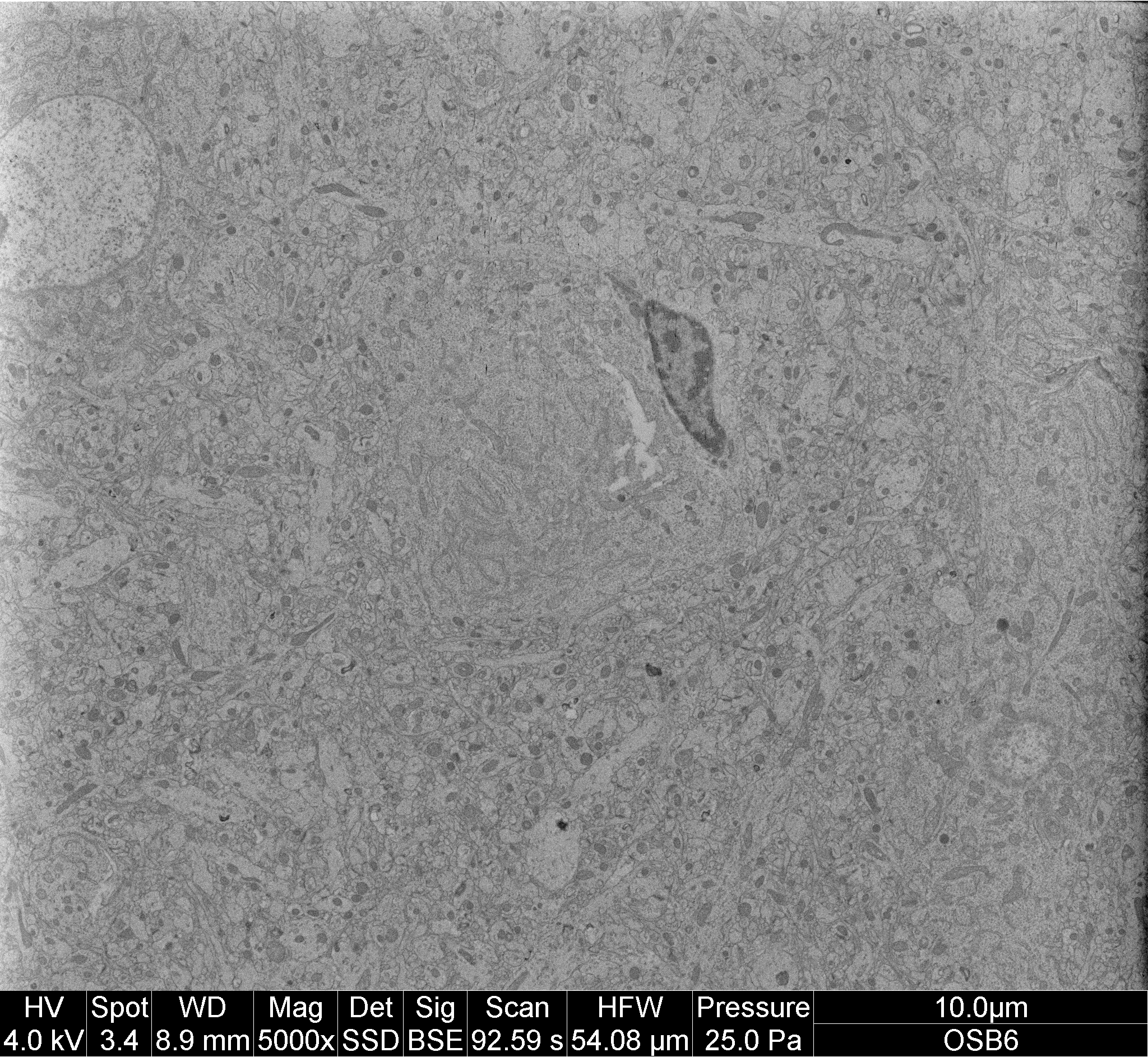

Supplement: Dataset S19 — (253.4 MB ZIP). [file pbio.0020329.sd019.zip › 040604_OS5_st1_1852.tif]

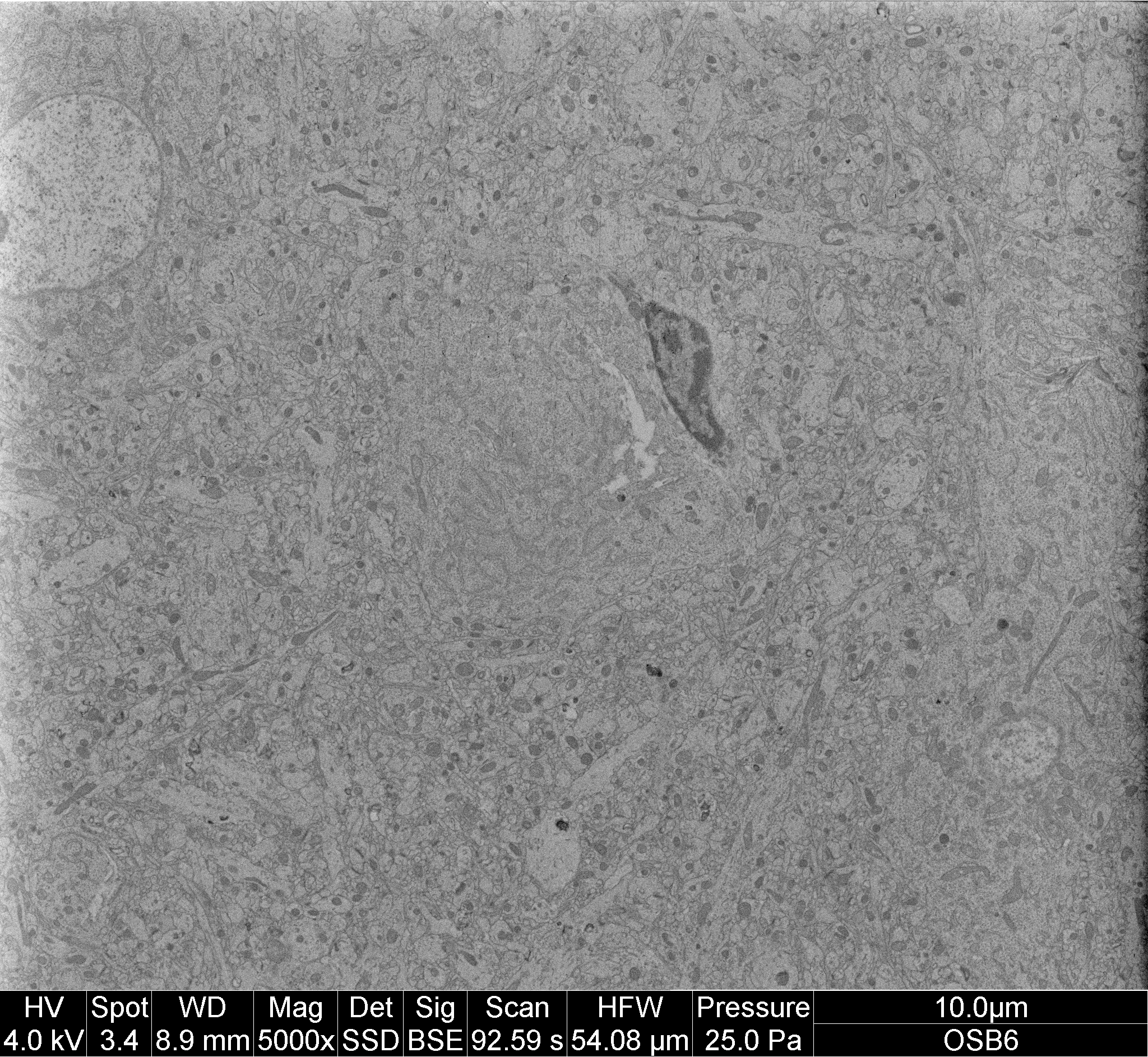

Supplement: Dataset S19 — (253.4 MB ZIP). [file pbio.0020329.sd019.zip › 040604_OS5_st1_1853.tif]

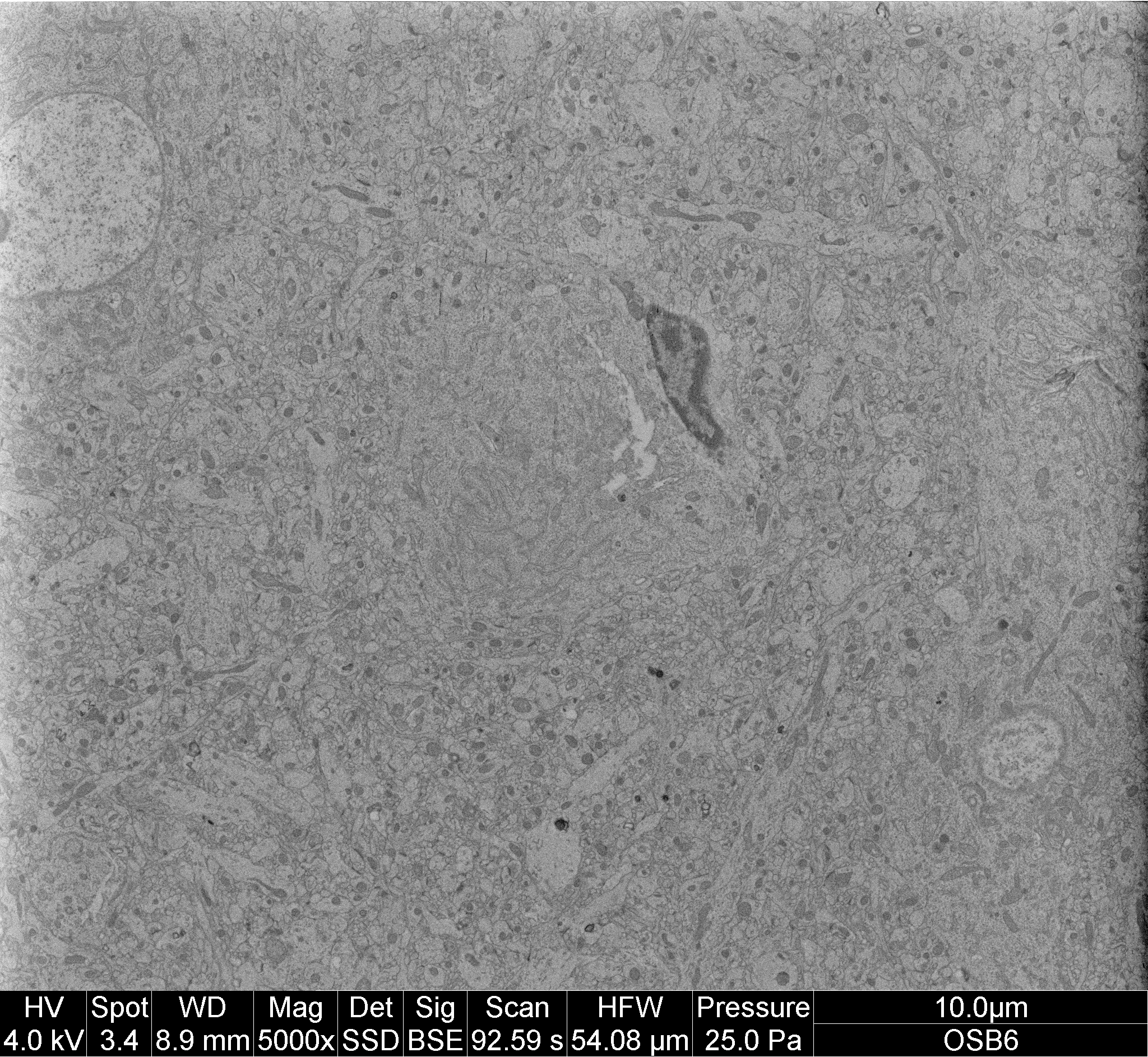

Supplement: Dataset S19 — (253.4 MB ZIP). [file pbio.0020329.sd019.zip › 040604_OS5_st1_1854.tif]

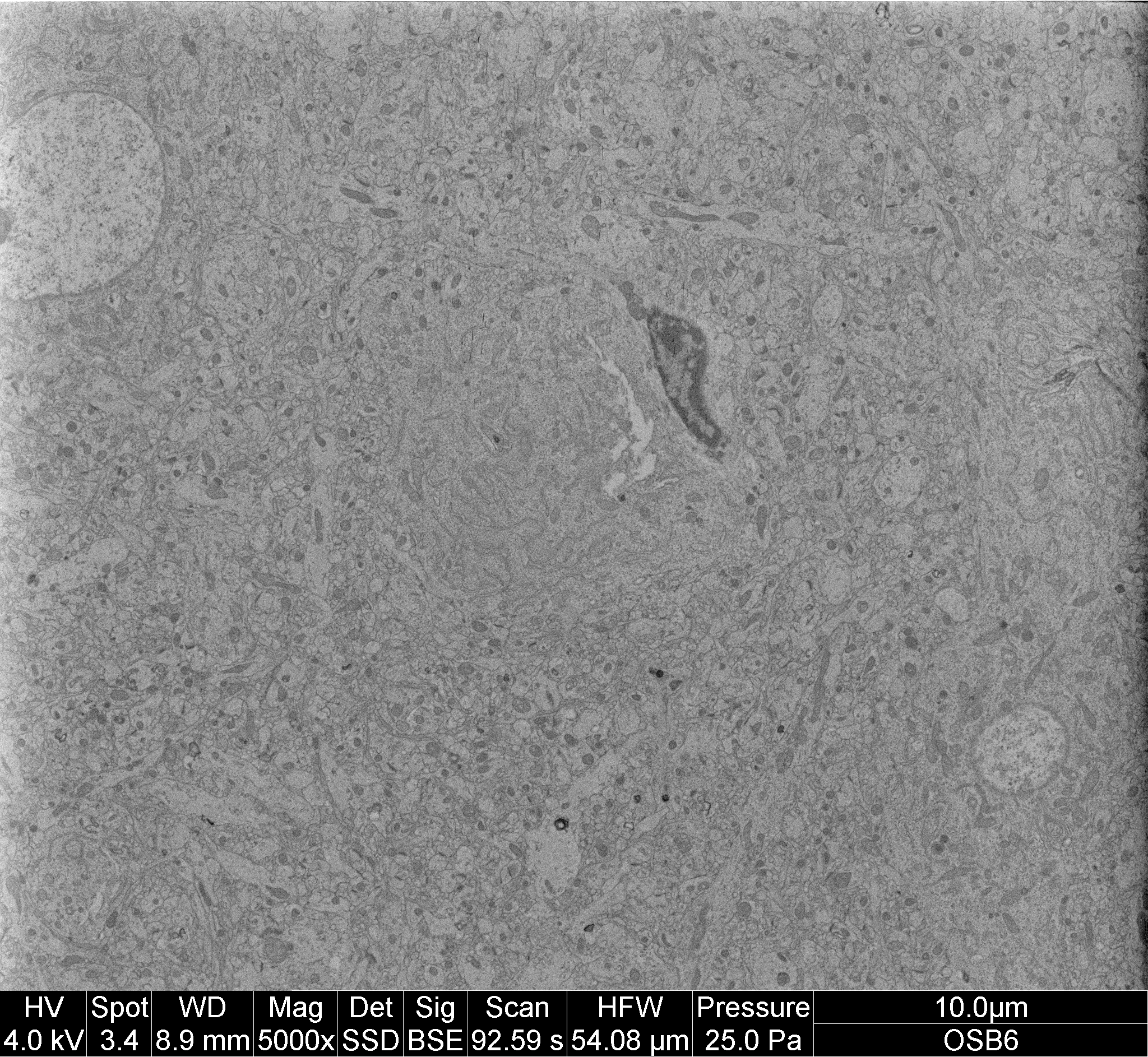

Supplement: Dataset S19 — (253.4 MB ZIP). [file pbio.0020329.sd019.zip › 040604_OS5_st1_1855.tif]

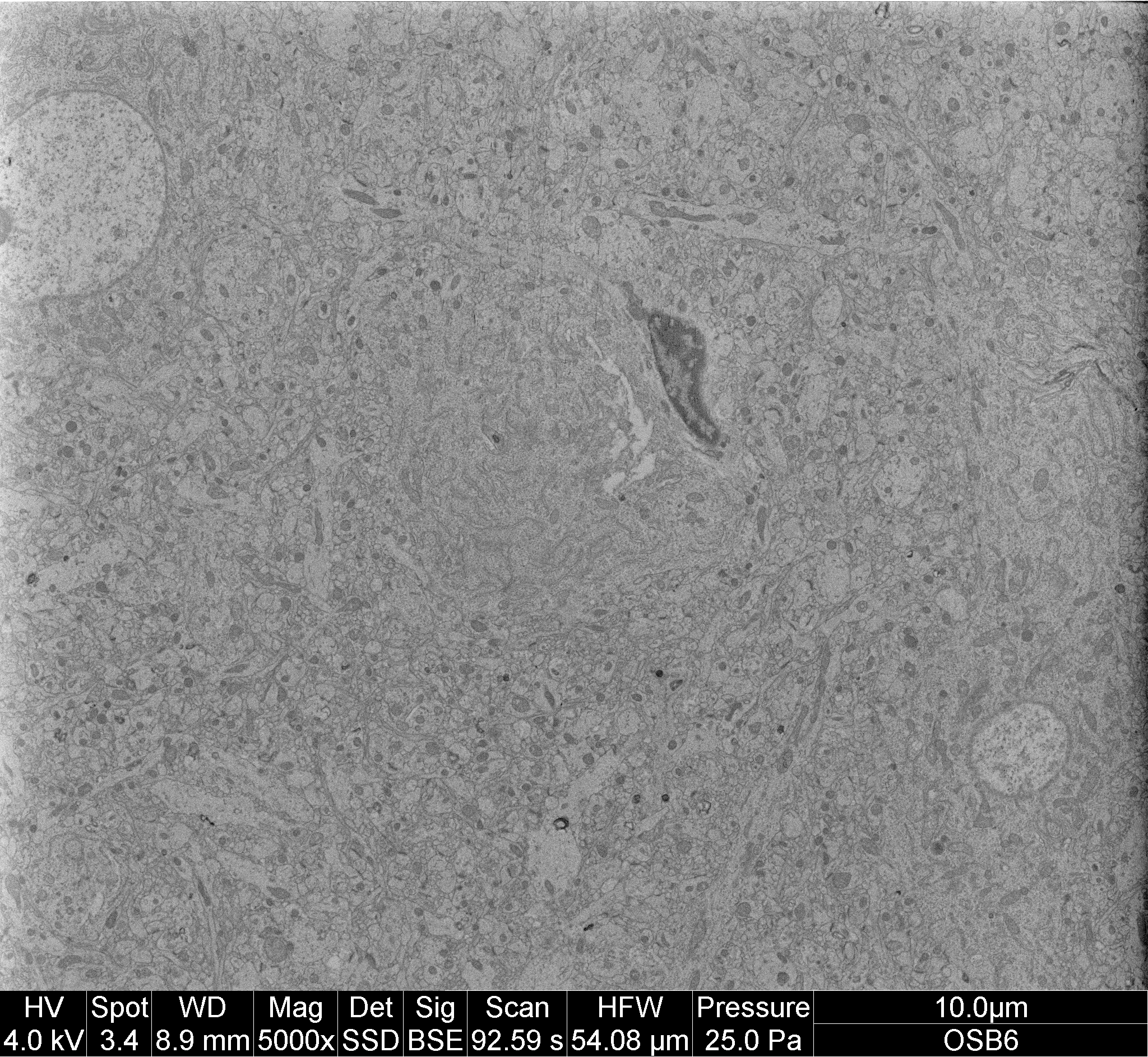

Supplement: Dataset S19 — (253.4 MB ZIP). [file pbio.0020329.sd019.zip › 040604_OS5_st1_1856.tif]

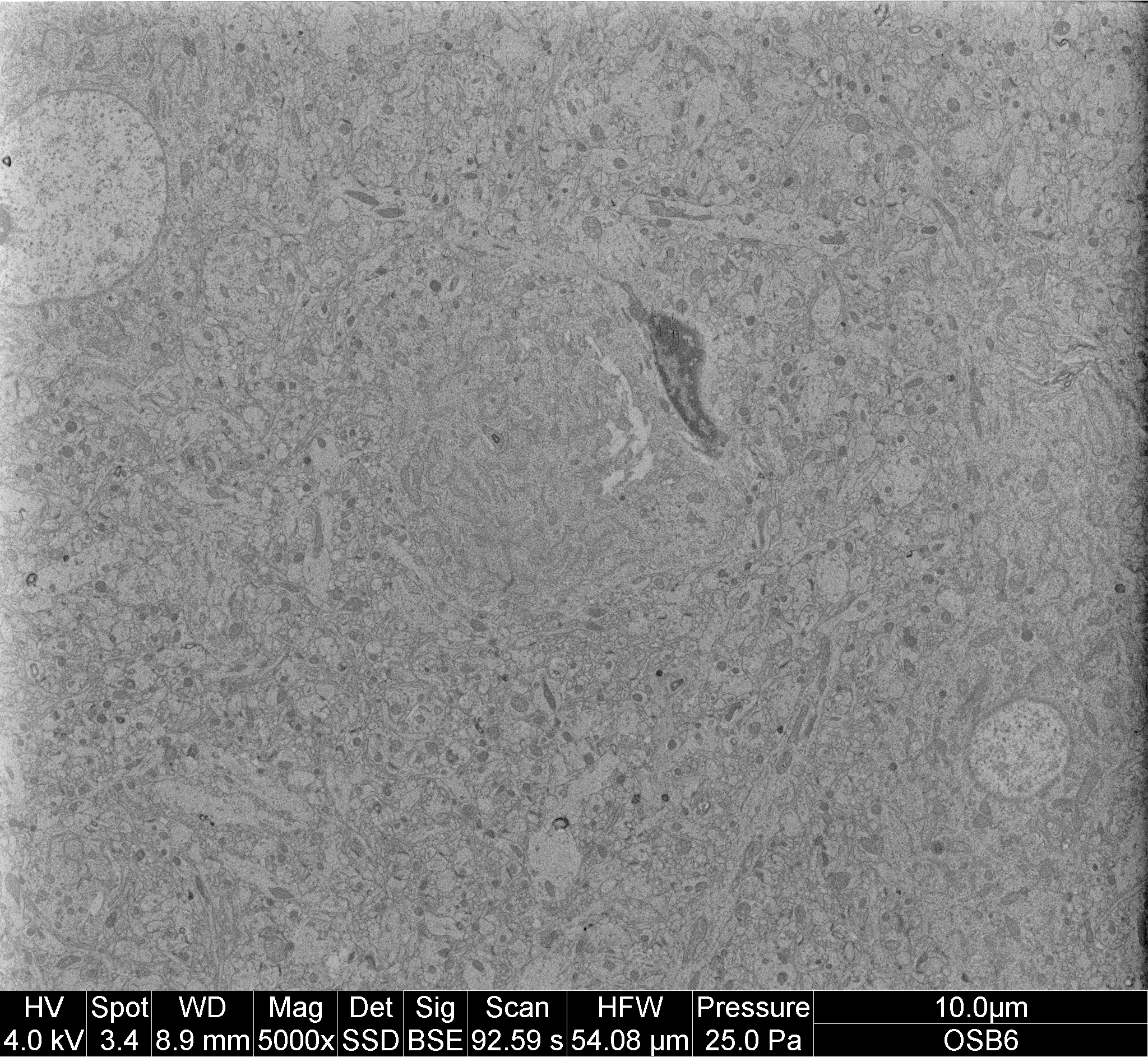

Supplement: Dataset S19 — (253.4 MB ZIP). [file pbio.0020329.sd019.zip › 040604_OS5_st1_1857.tif]

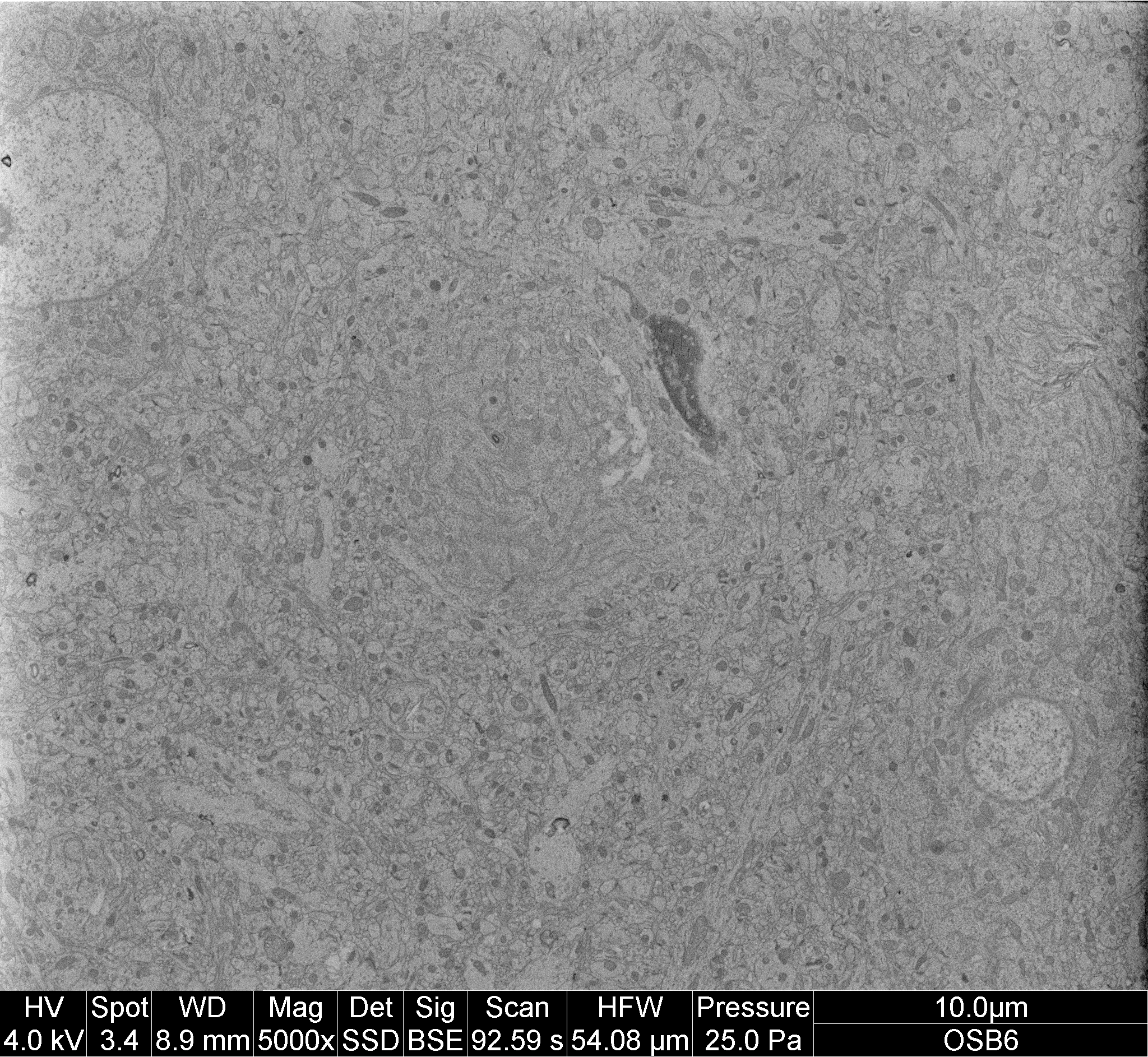

Supplement: Dataset S19 — (253.4 MB ZIP). [file pbio.0020329.sd019.zip › 040604_OS5_st1_1858.tif]

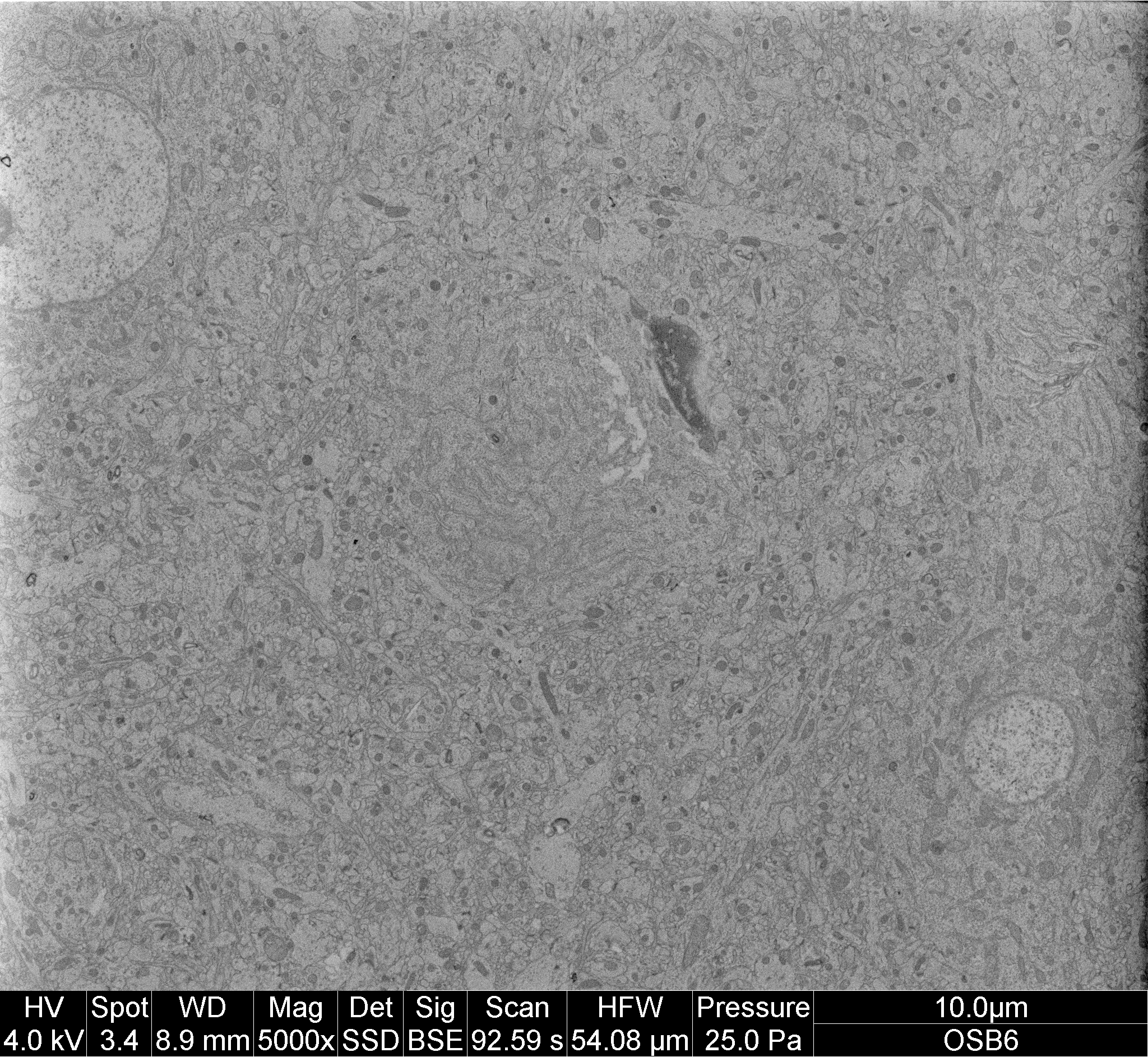

Supplement: Dataset S19 — (253.4 MB ZIP). [file pbio.0020329.sd019.zip › 040604_OS5_st1_1859.tif]

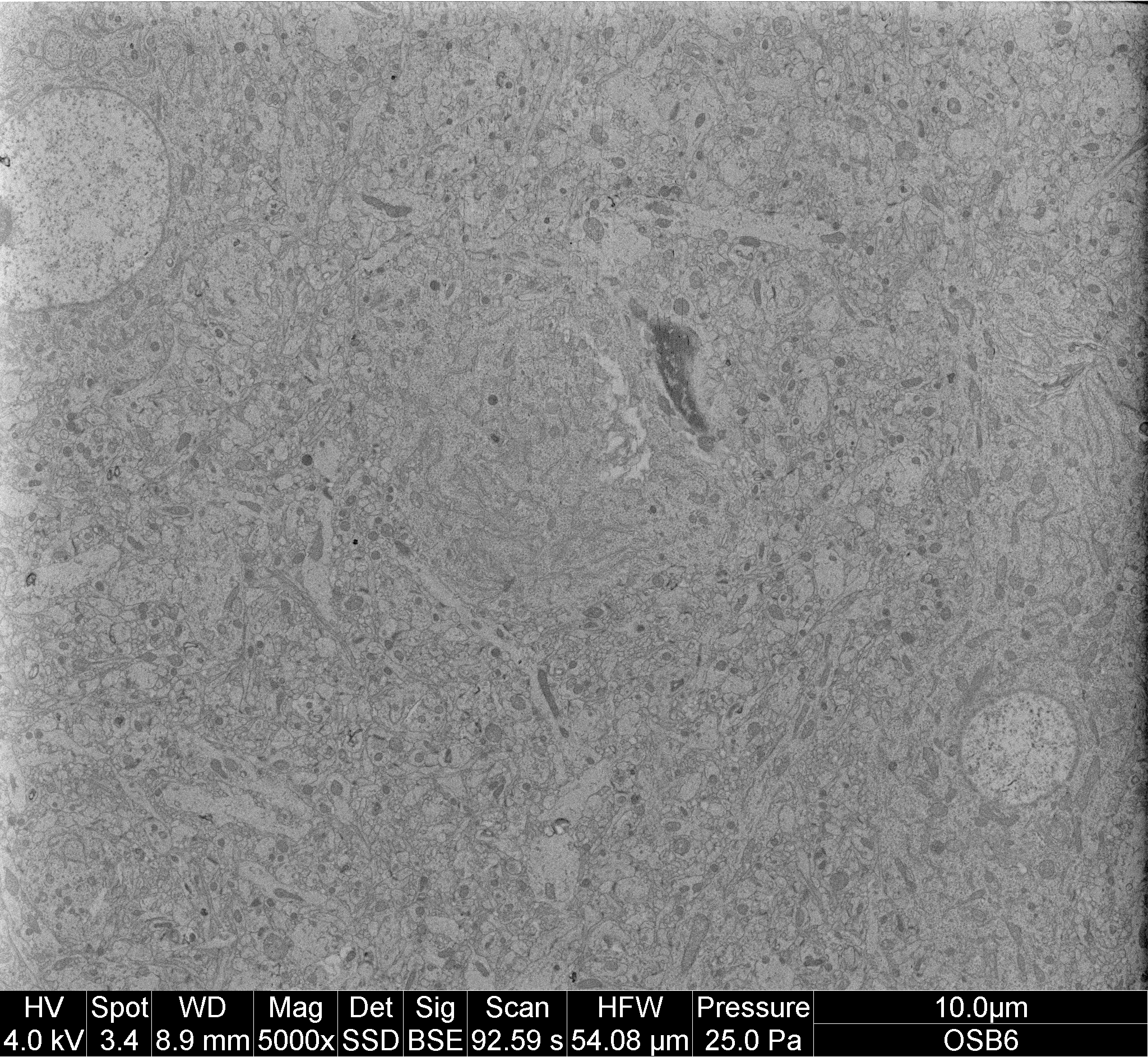

Supplement: Dataset S19 — (253.4 MB ZIP). [file pbio.0020329.sd019.zip › 040604_OS5_st1_1860.tif]

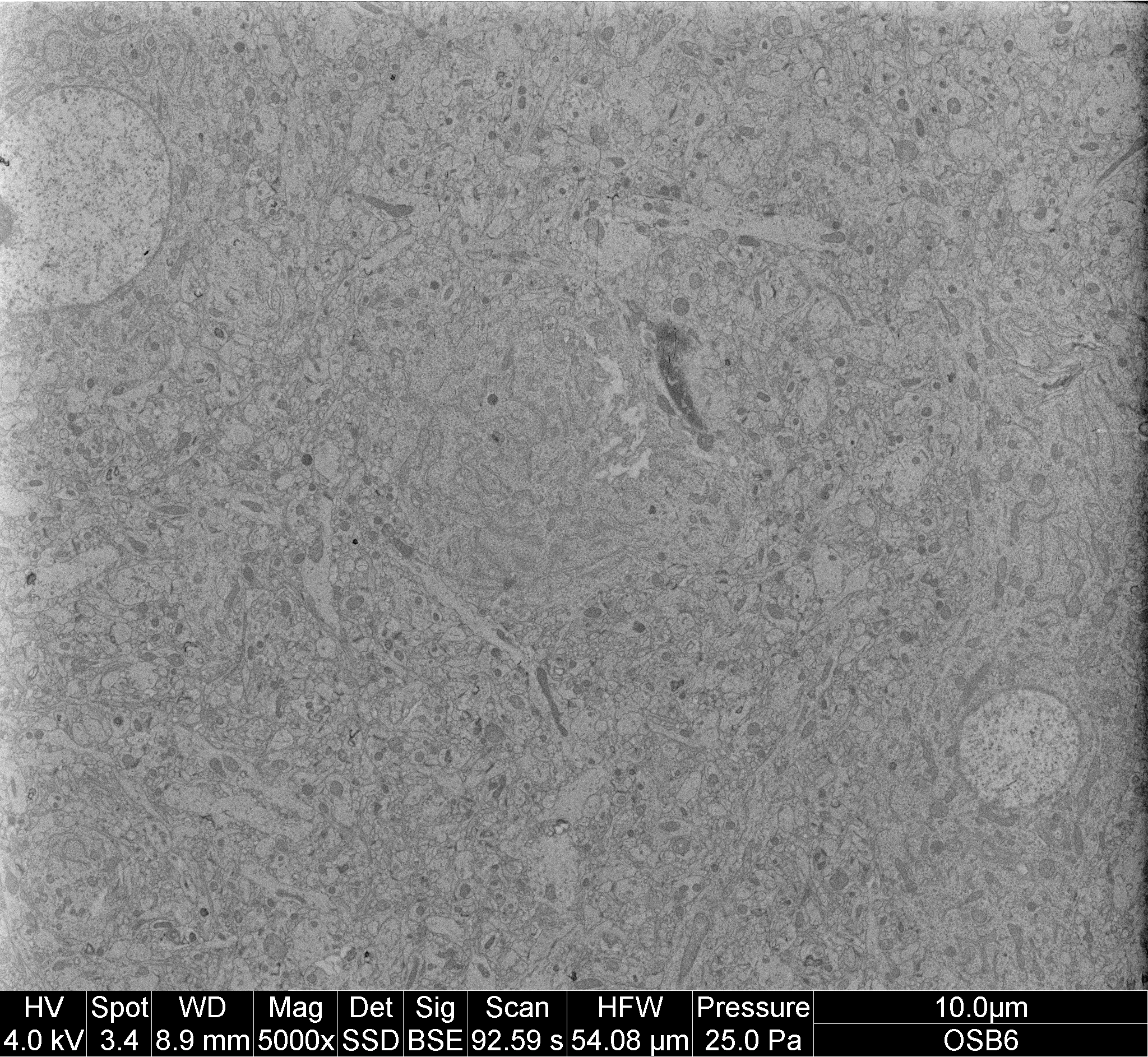

Supplement: Dataset S19 — (253.4 MB ZIP). [file pbio.0020329.sd019.zip › 040604_OS5_st1_1861.tif]

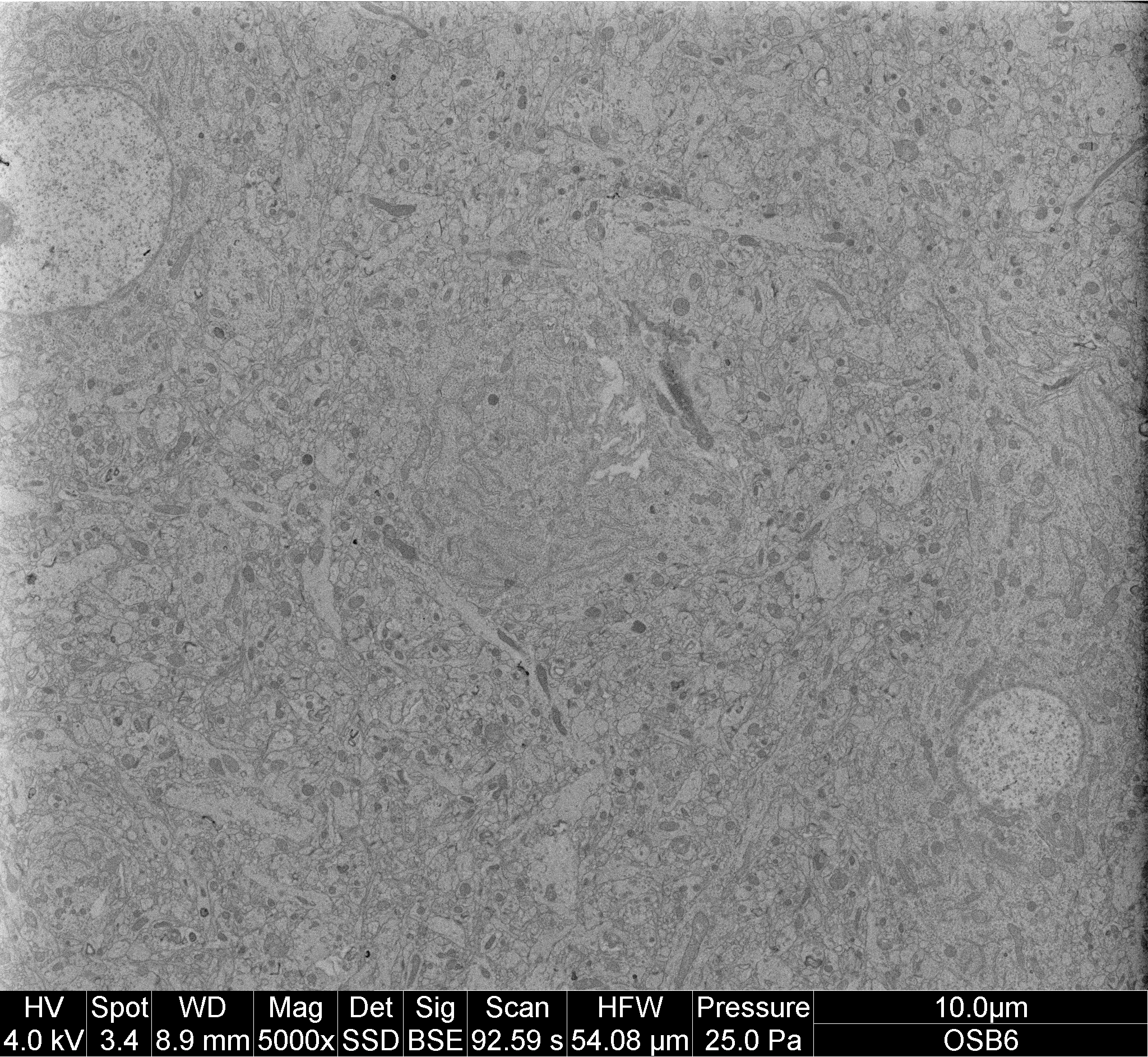

Supplement: Dataset S19 — (253.4 MB ZIP). [file pbio.0020329.sd019.zip › 040604_OS5_st1_1862.tif]

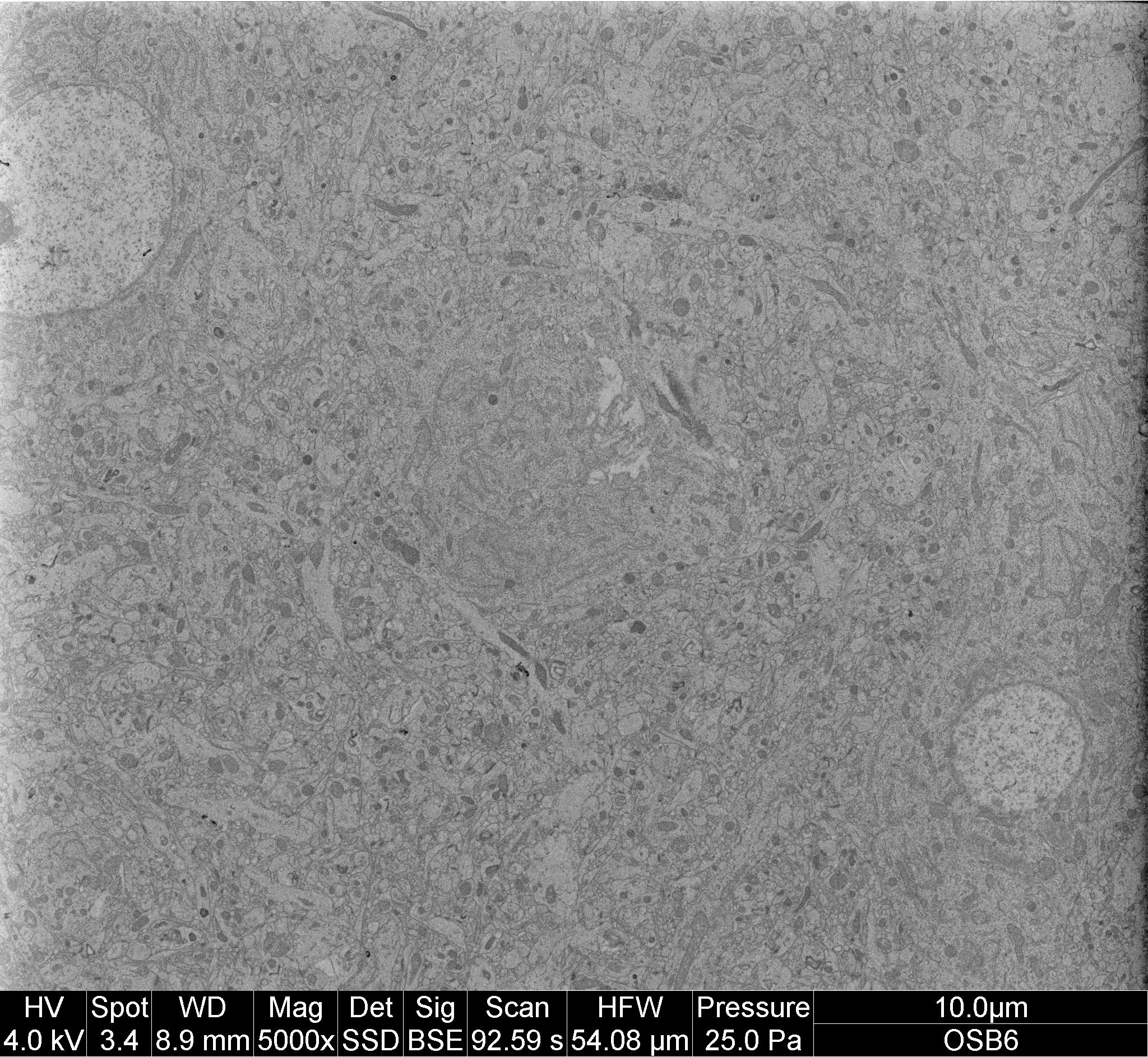

Supplement: Dataset S19 — (253.4 MB ZIP). [file pbio.0020329.sd019.zip › 040604_OS5_st1_1863.tif]

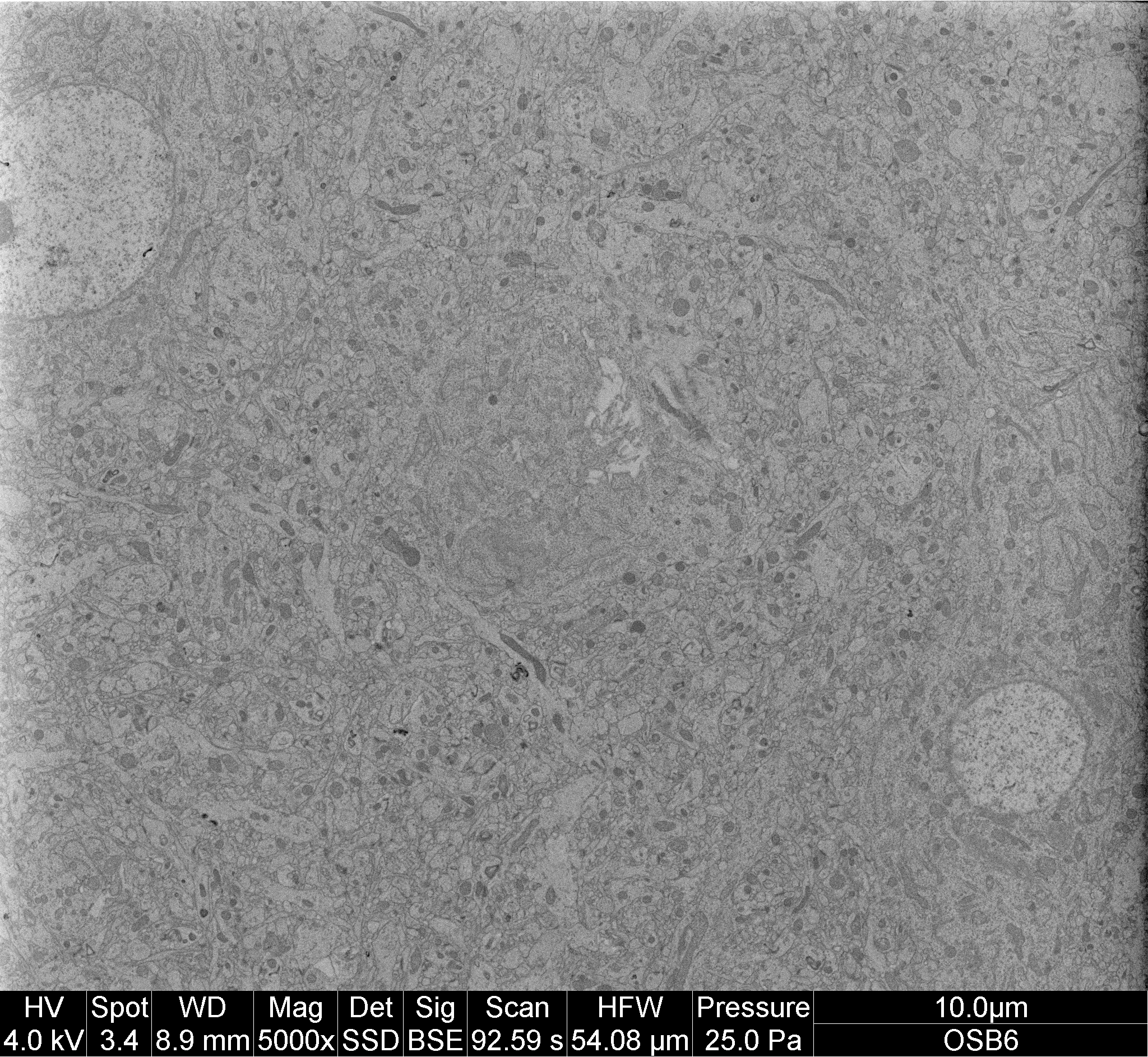

Supplement: Dataset S19 — (253.4 MB ZIP). [file pbio.0020329.sd019.zip › 040604_OS5_st1_1864.tif]

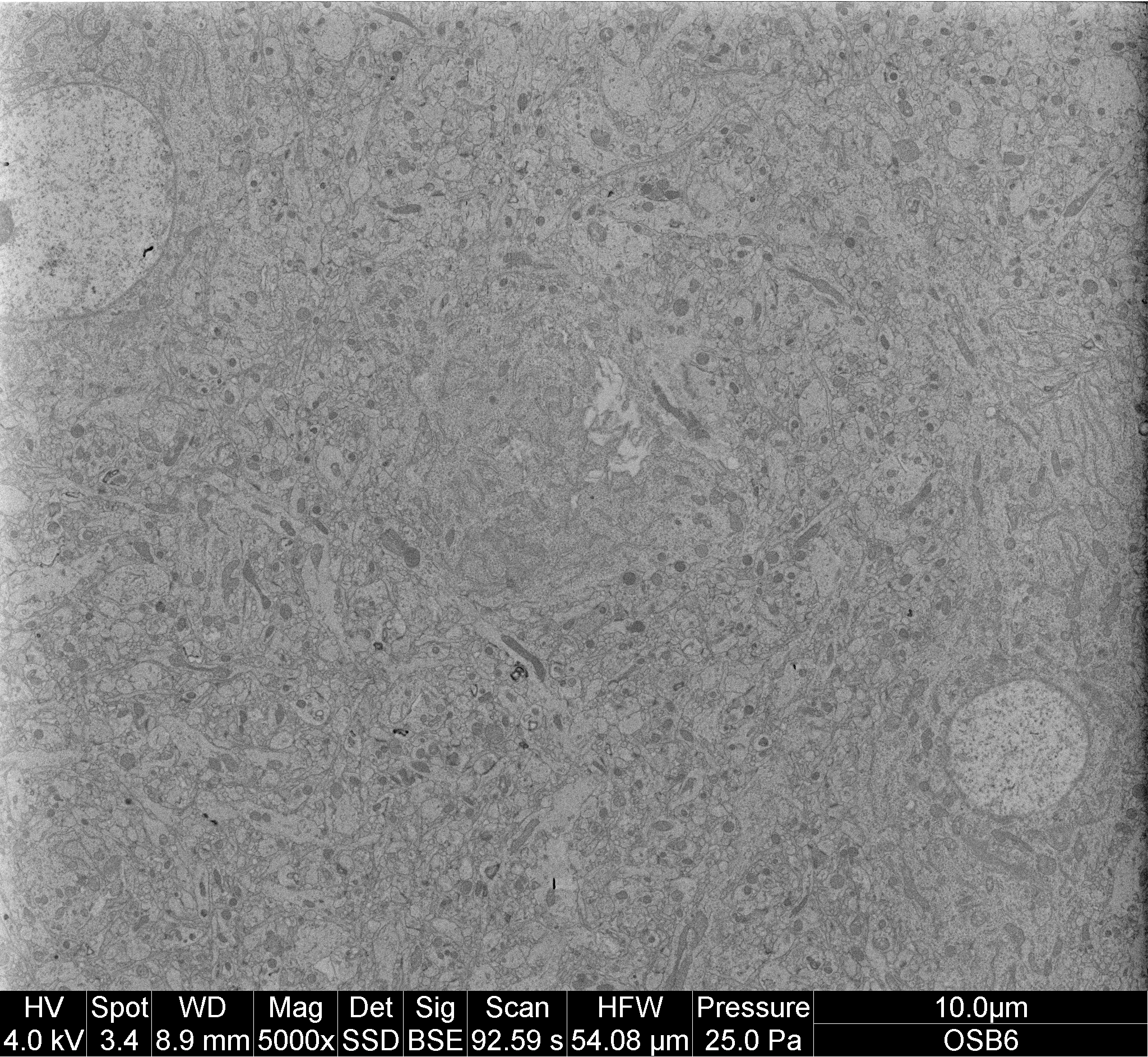

Supplement: Dataset S19 — (253.4 MB ZIP). [file pbio.0020329.sd019.zip › 040604_OS5_st1_1865.tif]

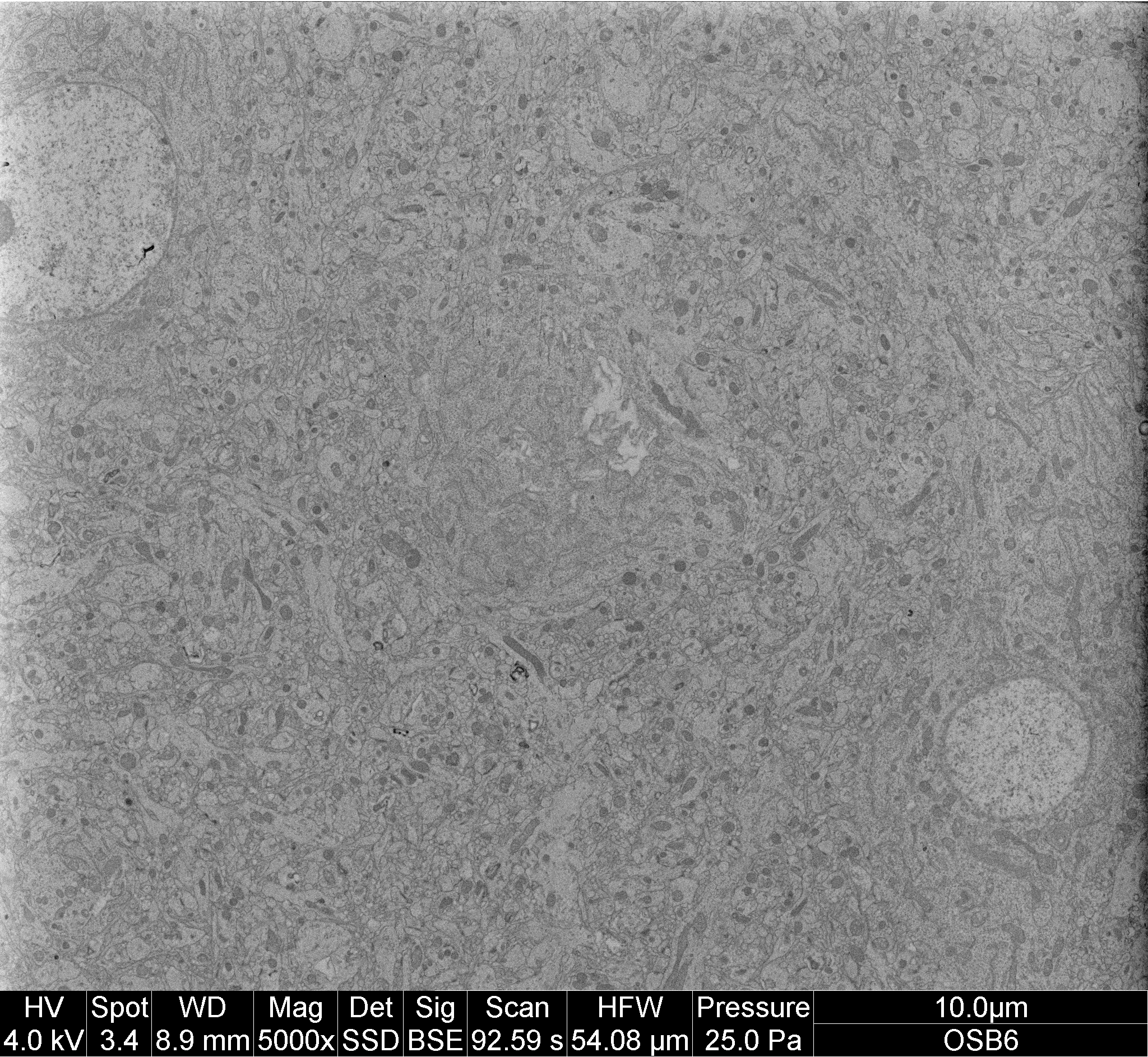

Supplement: Dataset S19 — (253.4 MB ZIP). [file pbio.0020329.sd019.zip › 040604_OS5_st1_1866.tif]

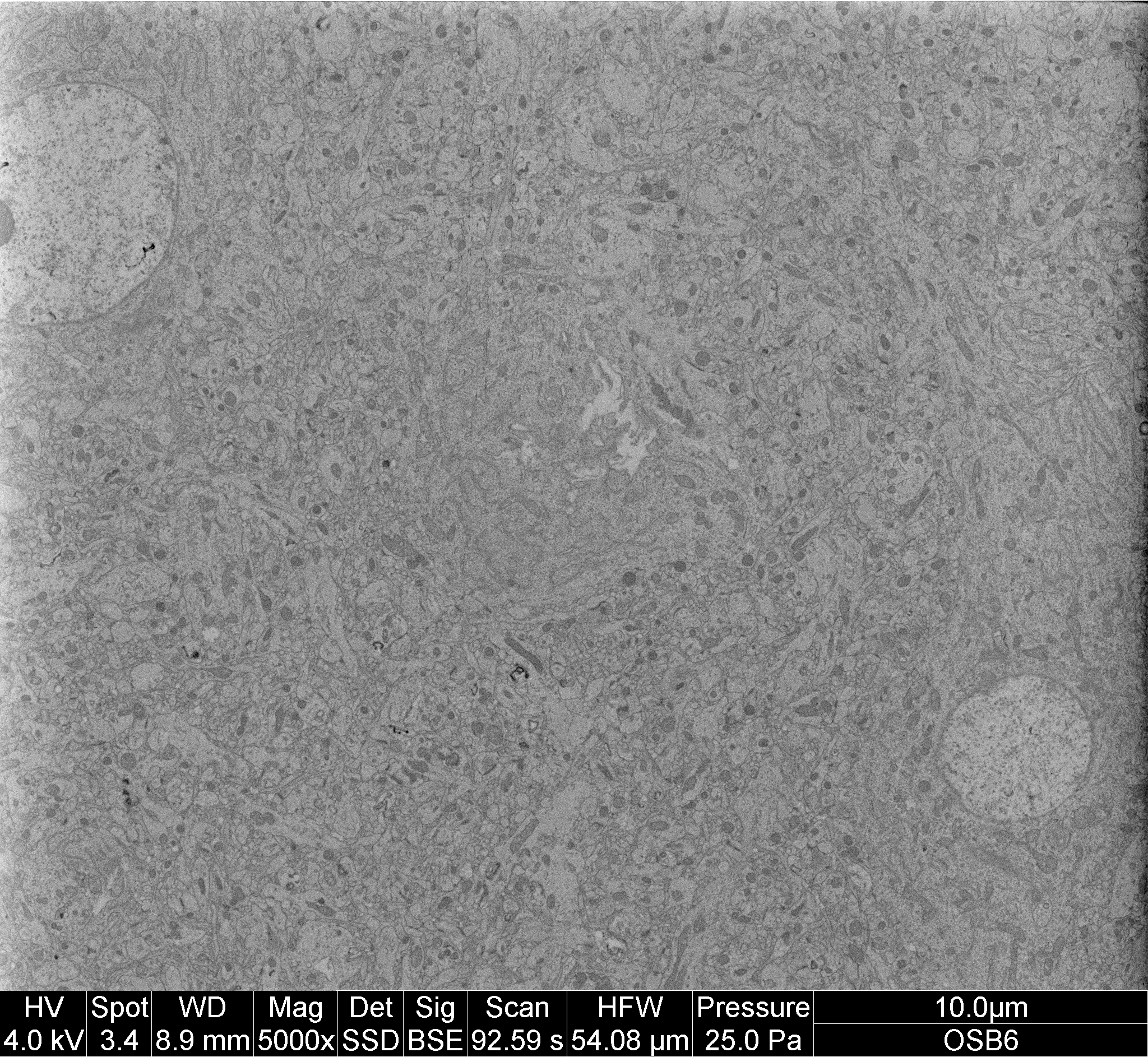

Supplement: Dataset S19 — (253.4 MB ZIP). [file pbio.0020329.sd019.zip › 040604_OS5_st1_1867.tif]

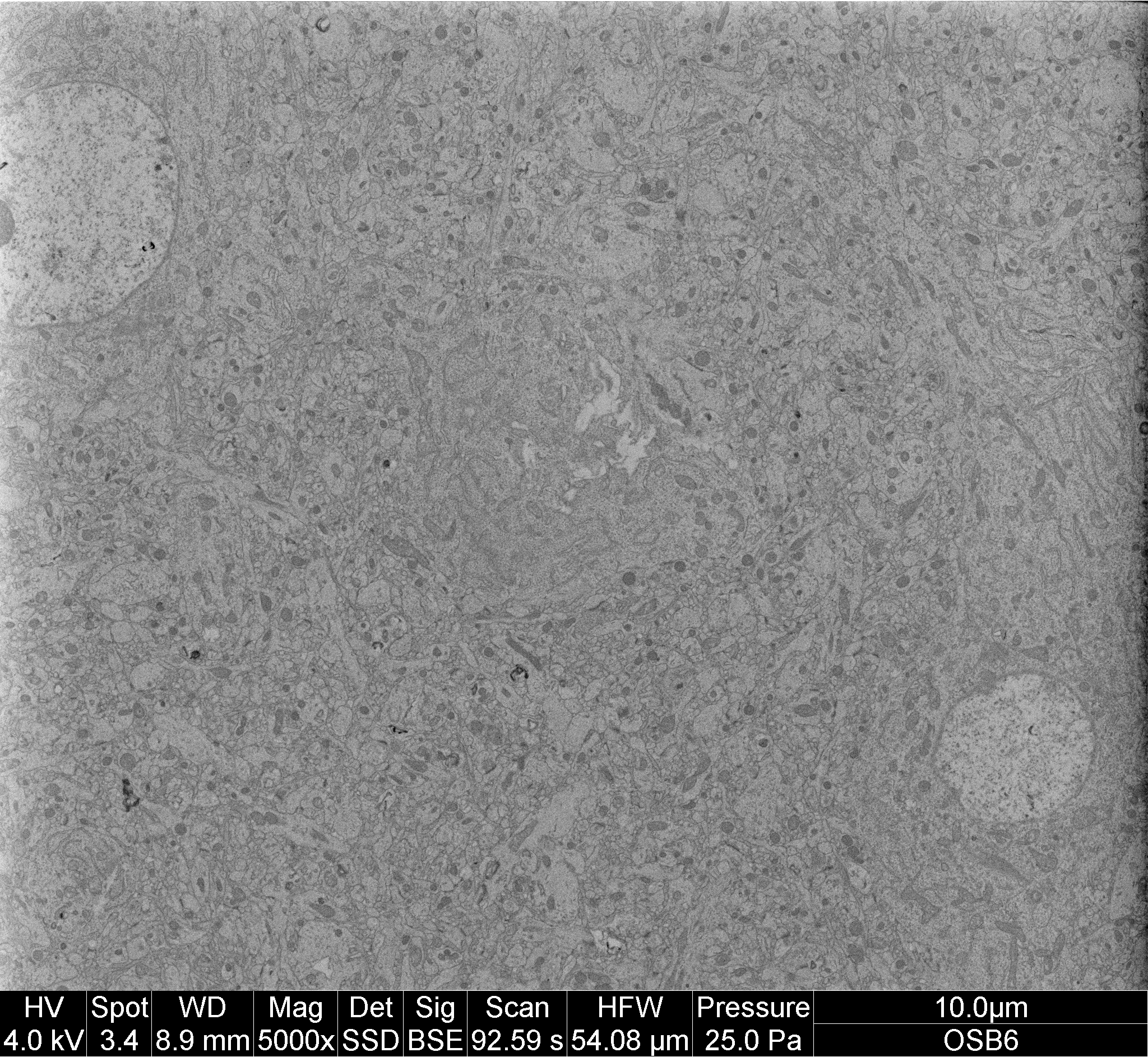

Supplement: Dataset S19 — (253.4 MB ZIP). [file pbio.0020329.sd019.zip › 040604_OS5_st1_1868.tif]

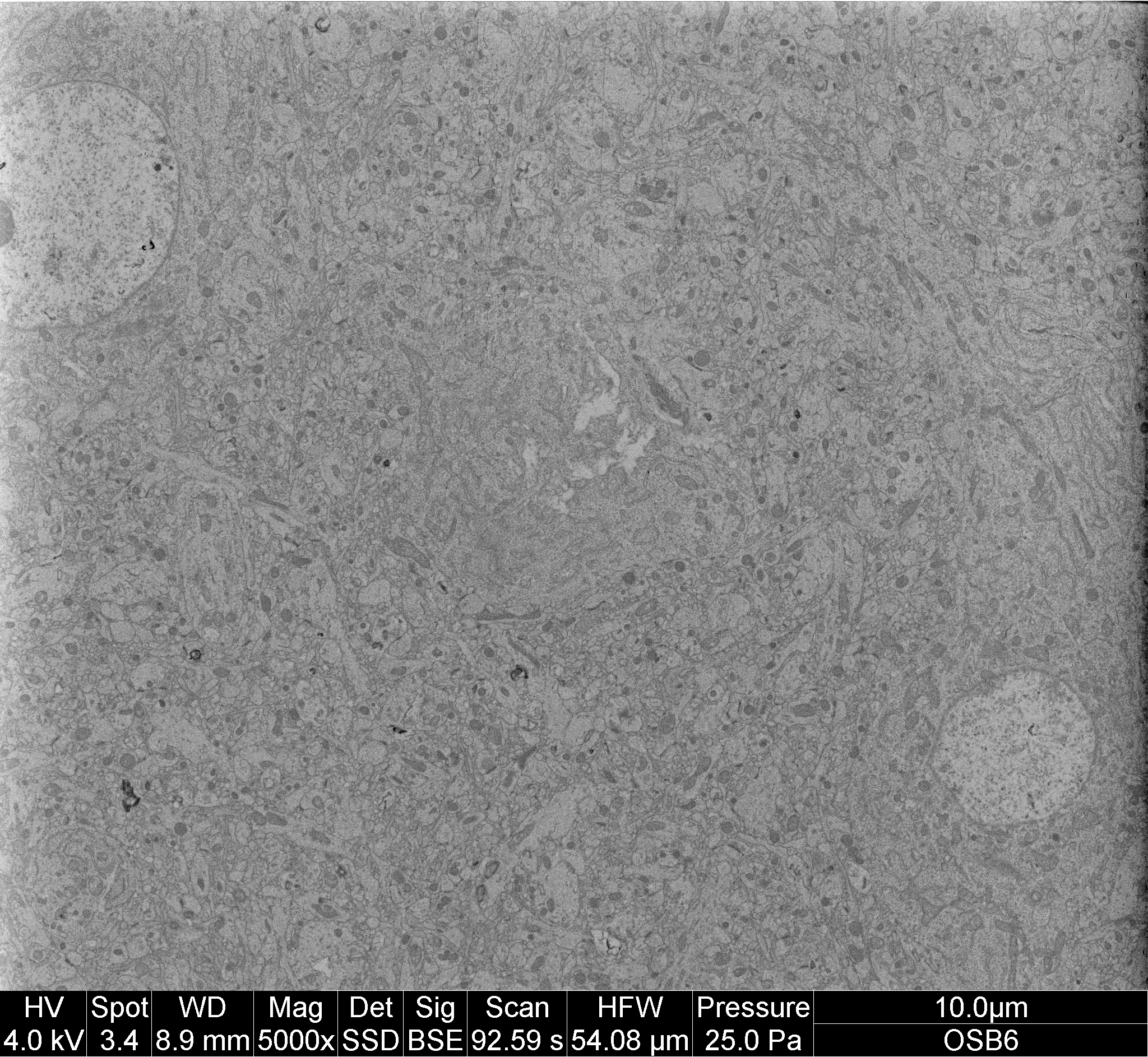

Supplement: Dataset S19 — (253.4 MB ZIP). [file pbio.0020329.sd019.zip › 040604_OS5_st1_1869.tif]

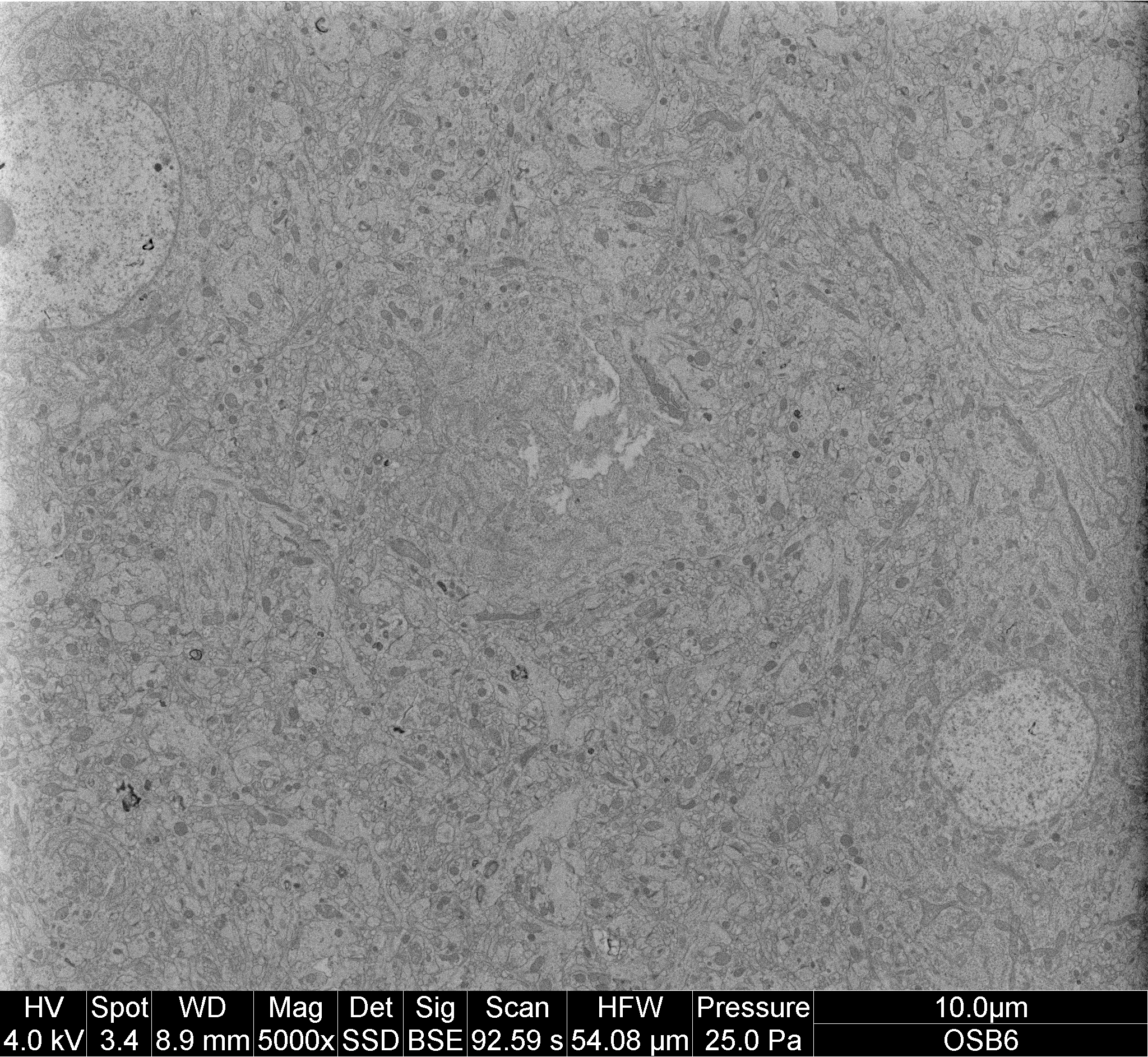

Supplement: Dataset S19 — (253.4 MB ZIP). [file pbio.0020329.sd019.zip › 040604_OS5_st1_1870.tif]

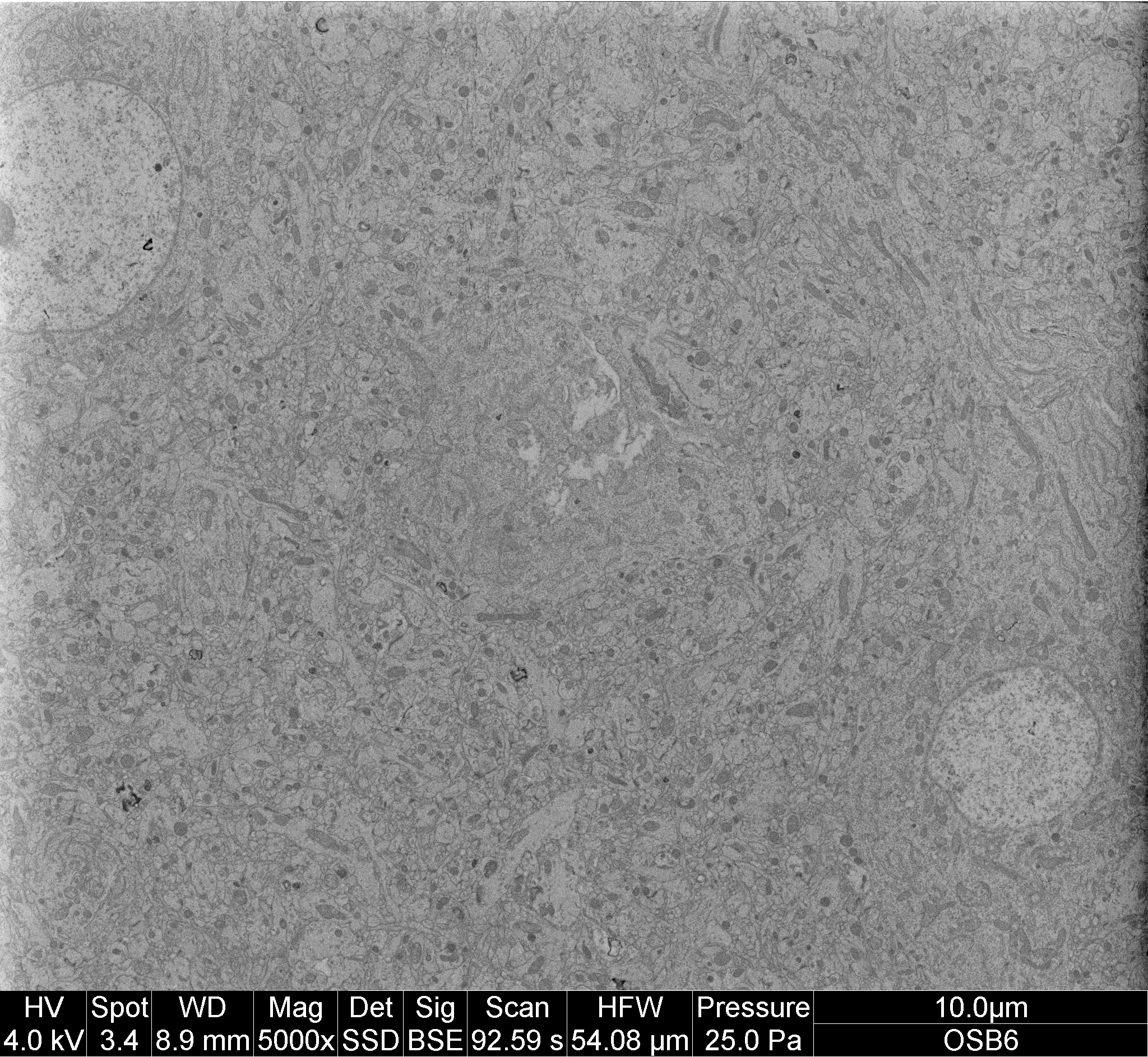

Supplement: Dataset S19 — (253.4 MB ZIP). [file pbio.0020329.sd019.zip › 040604_OS5_st1_1871.tif]

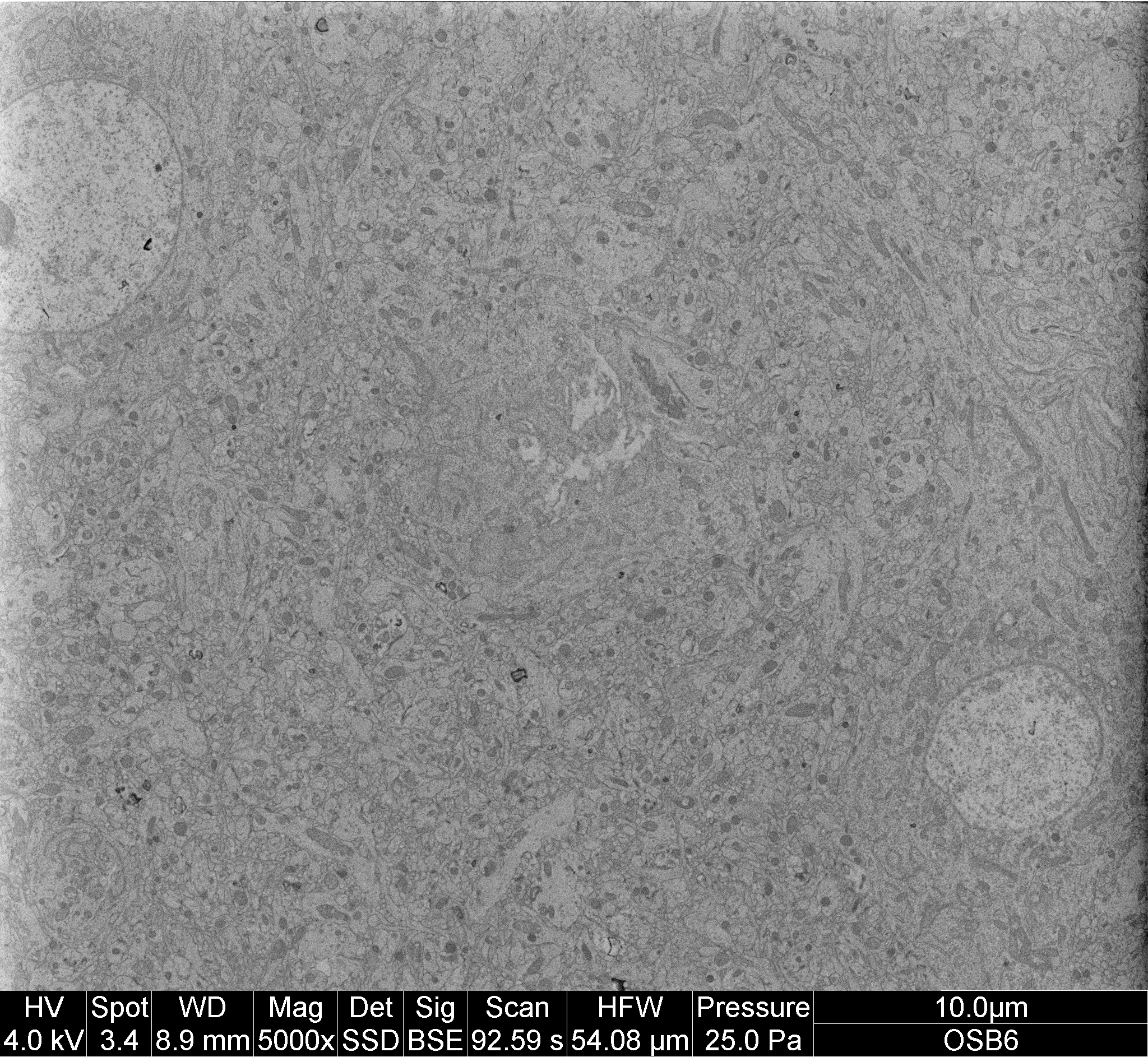

Supplement: Dataset S19 — (253.4 MB ZIP). [file pbio.0020329.sd019.zip › 040604_OS5_st1_1872.tif]

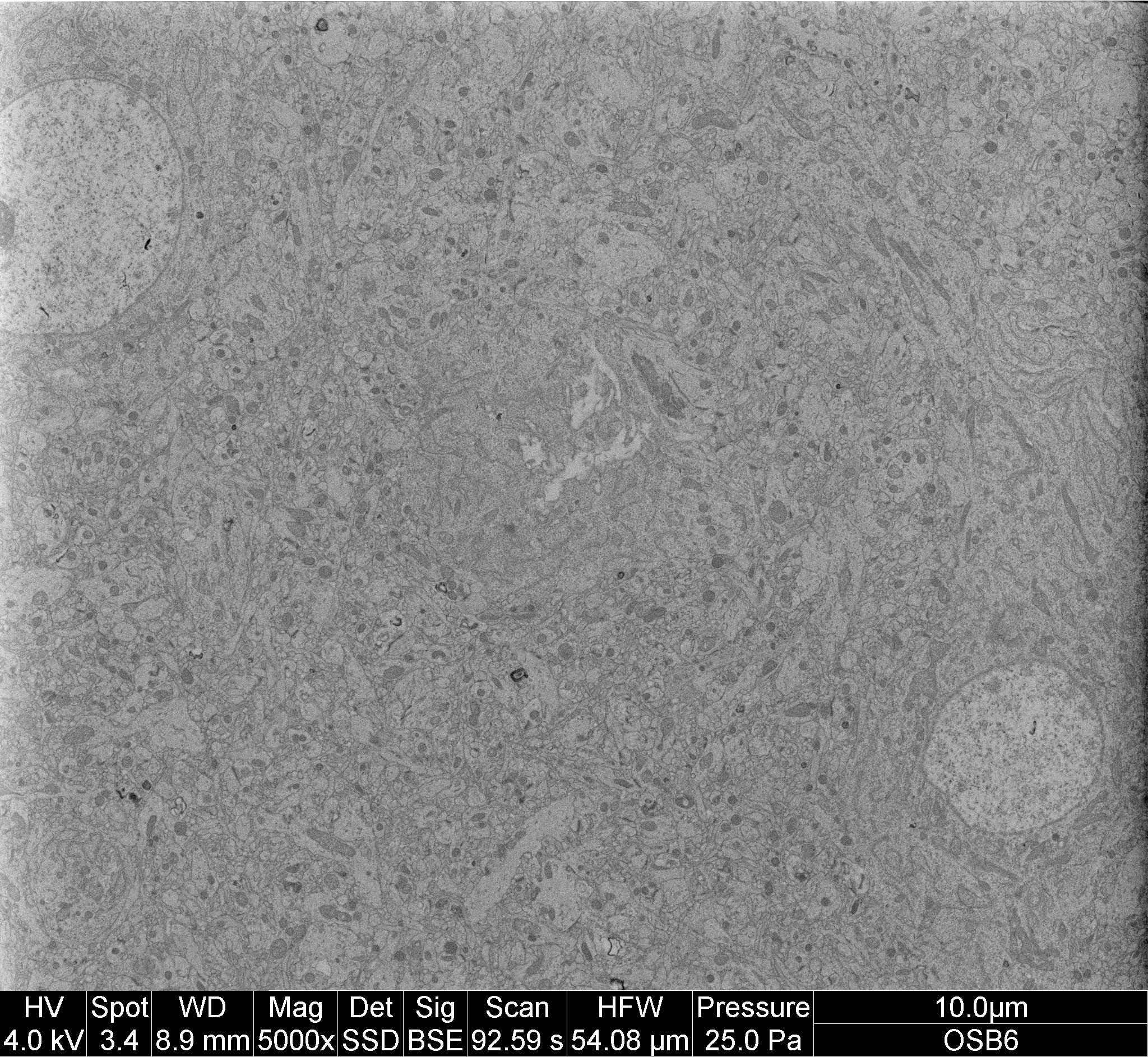

Supplement: Dataset S19 — (253.4 MB ZIP). [file pbio.0020329.sd019.zip › 040604_OS5_st1_1873.tif]

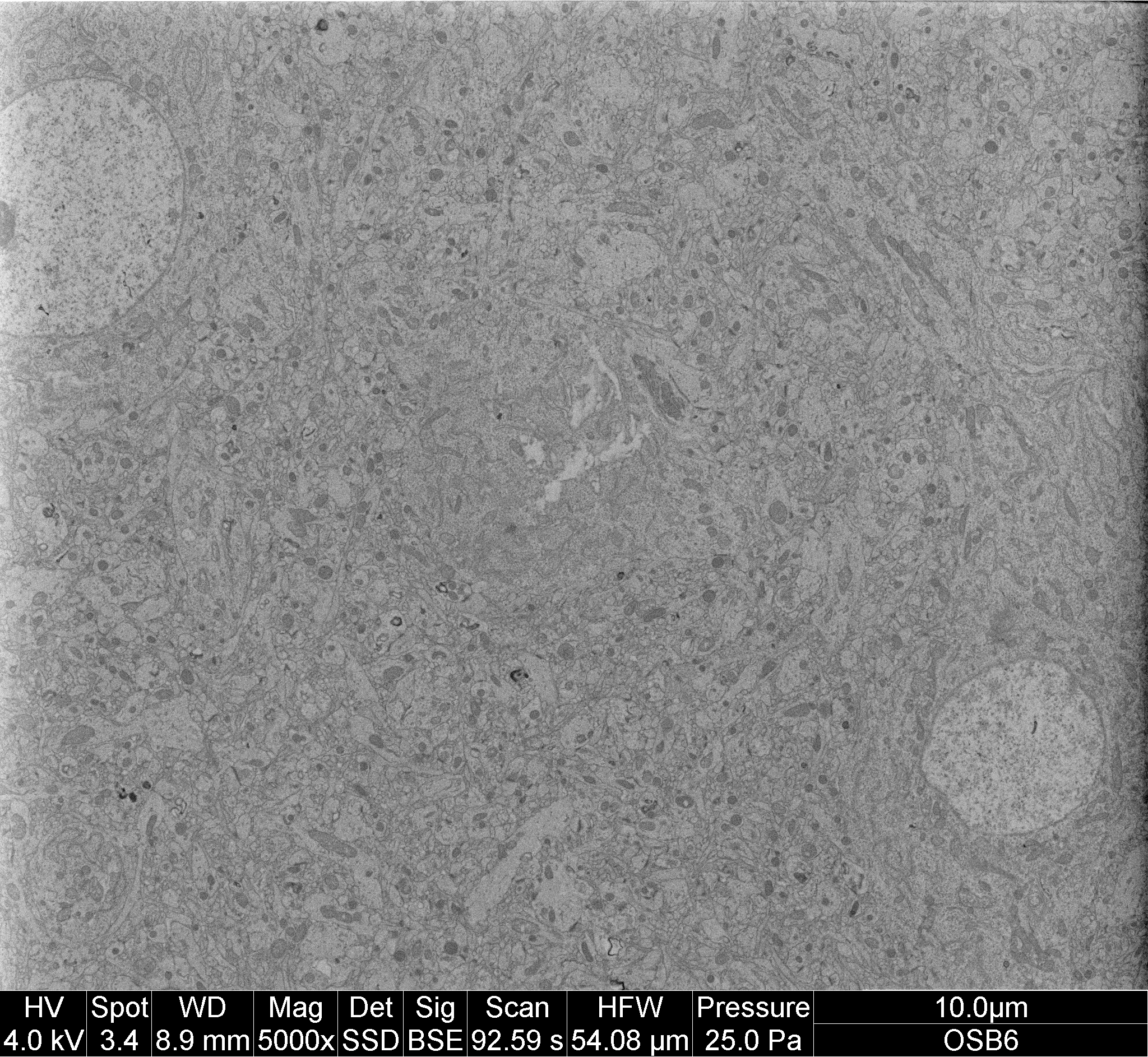

Supplement: Dataset S19 — (253.4 MB ZIP). [file pbio.0020329.sd019.zip › 040604_OS5_st1_1874.tif]

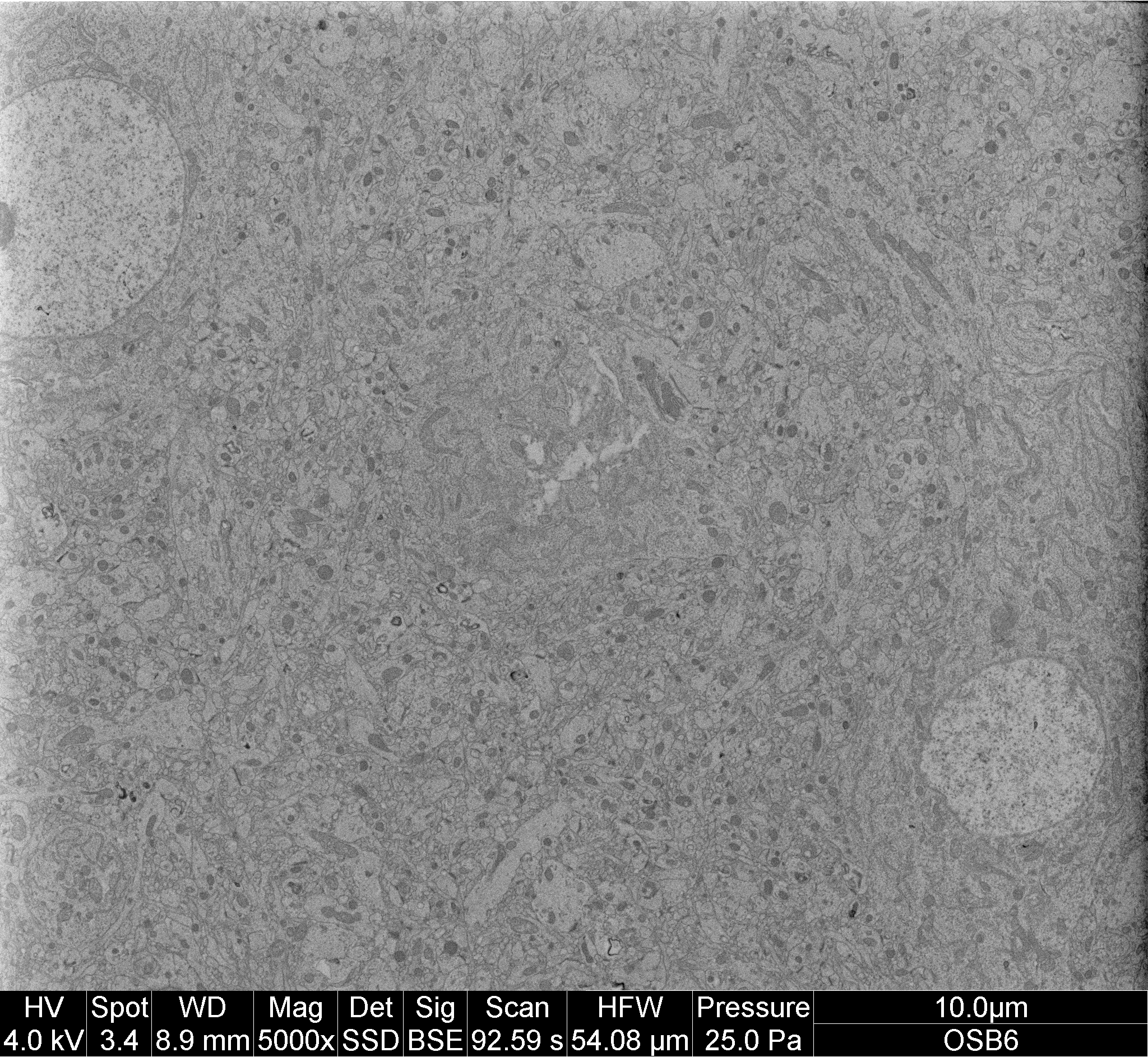

Supplement: Dataset S19 — (253.4 MB ZIP). [file pbio.0020329.sd019.zip › 040604_OS5_st1_1875.tif]

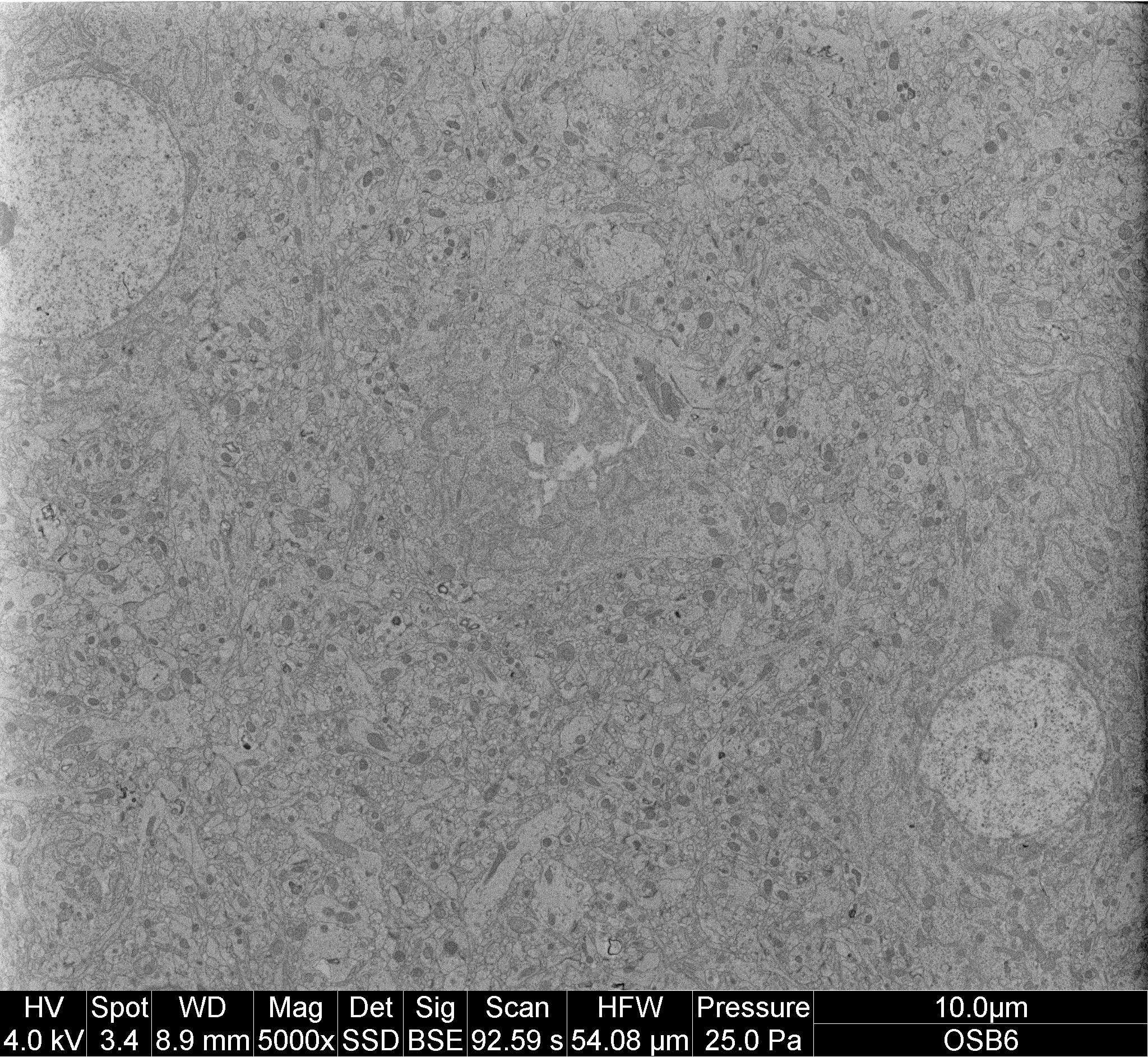

Supplement: Dataset S19 — (253.4 MB ZIP). [file pbio.0020329.sd019.zip › 040604_OS5_st1_1876.tif]

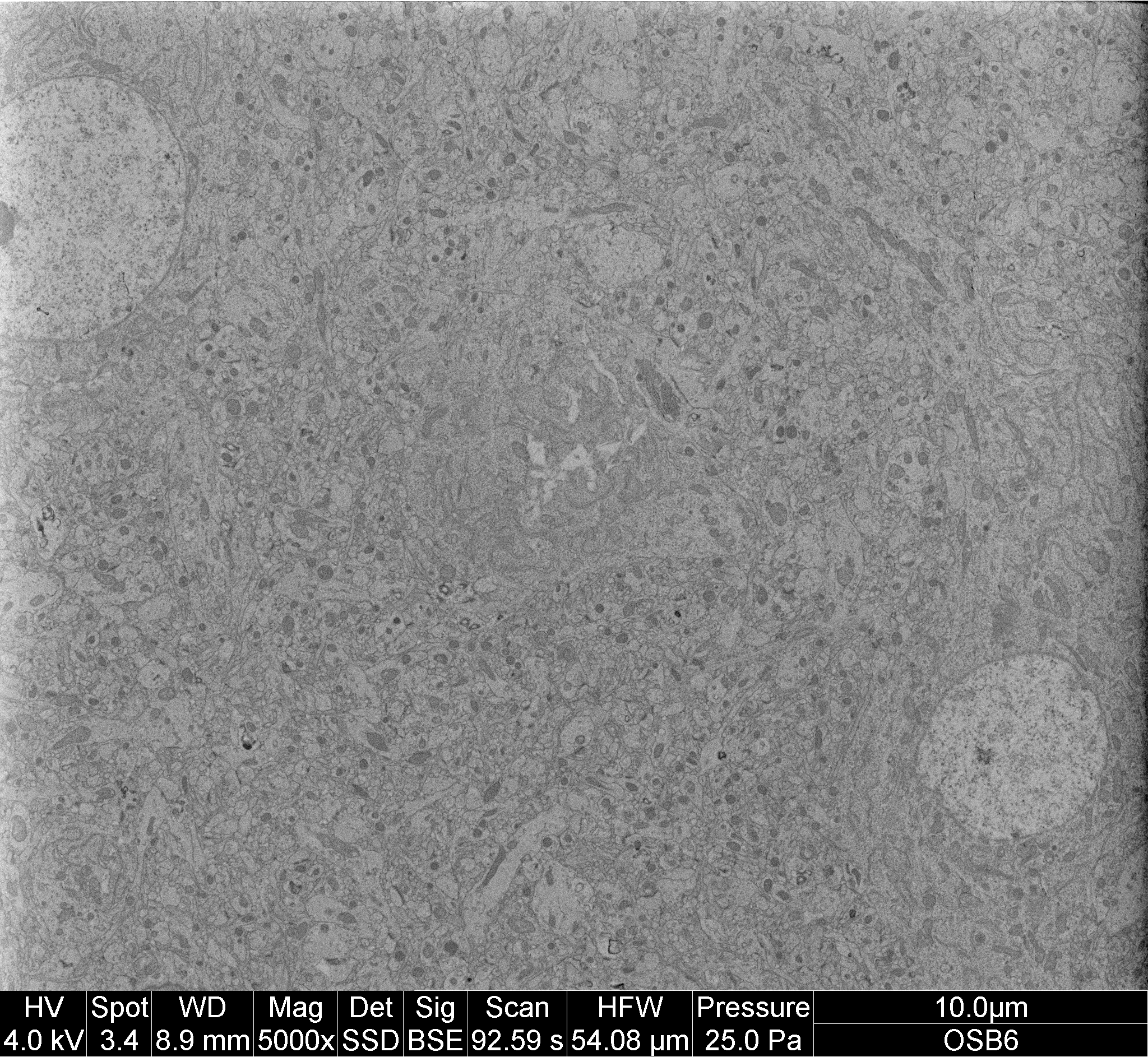

Supplement: Dataset S19 — (253.4 MB ZIP). [file pbio.0020329.sd019.zip › 040604_OS5_st1_1877.tif]

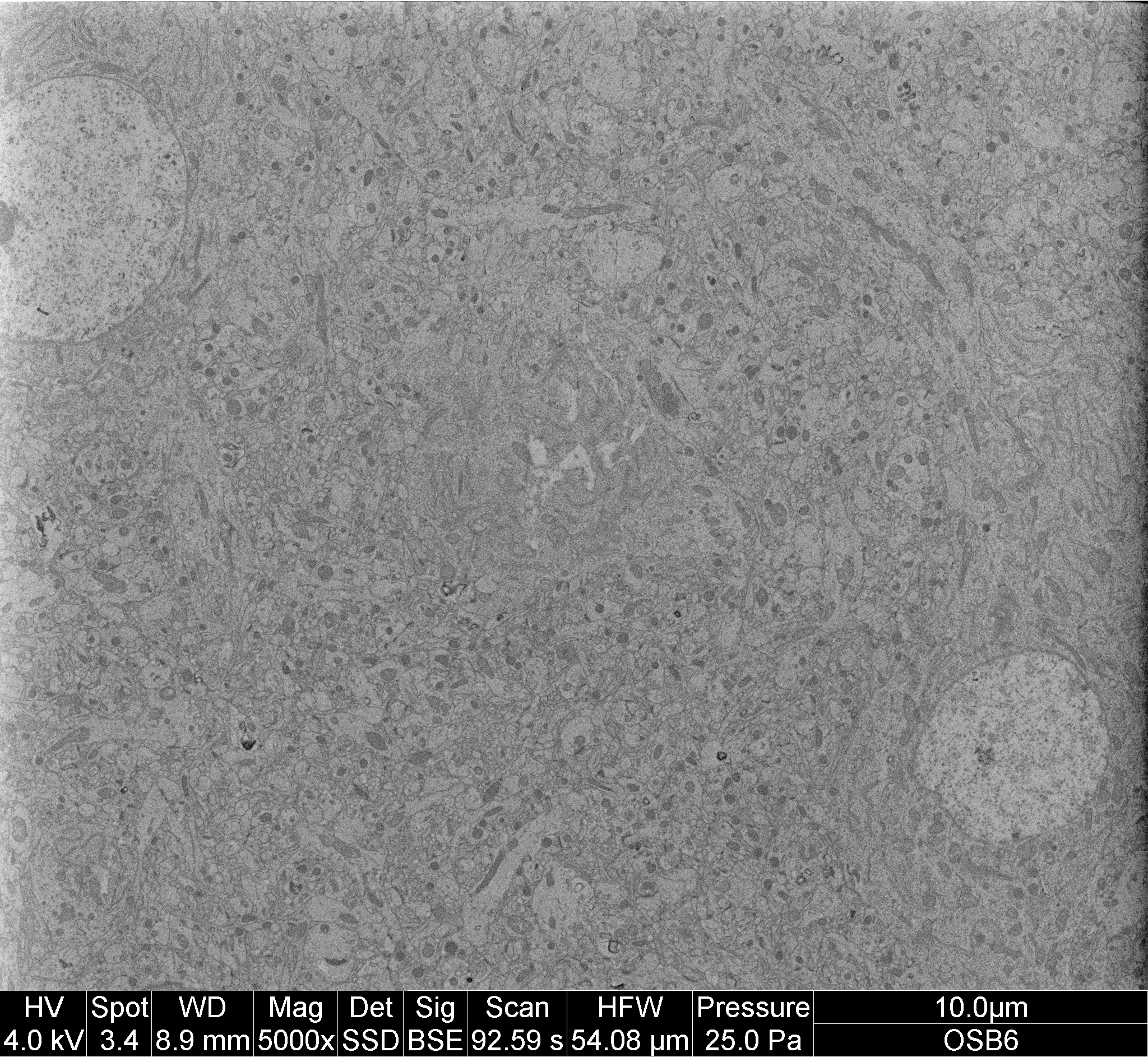

Supplement: Dataset S19 — (253.4 MB ZIP). [file pbio.0020329.sd019.zip › 040604_OS5_st1_1878.tif]

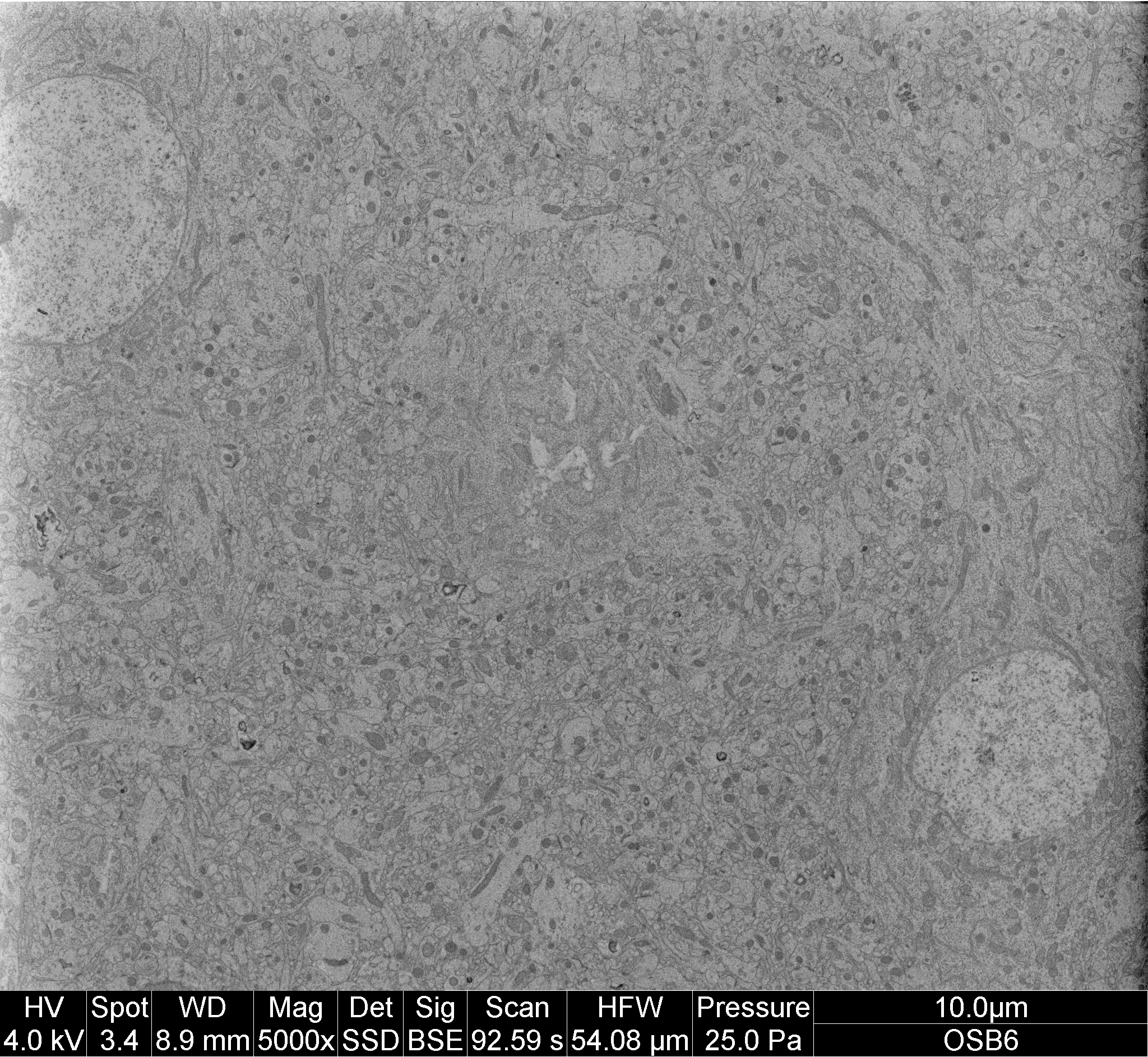

Supplement: Dataset S19 — (253.4 MB ZIP). [file pbio.0020329.sd019.zip › 040604_OS5_st1_1879.tif]

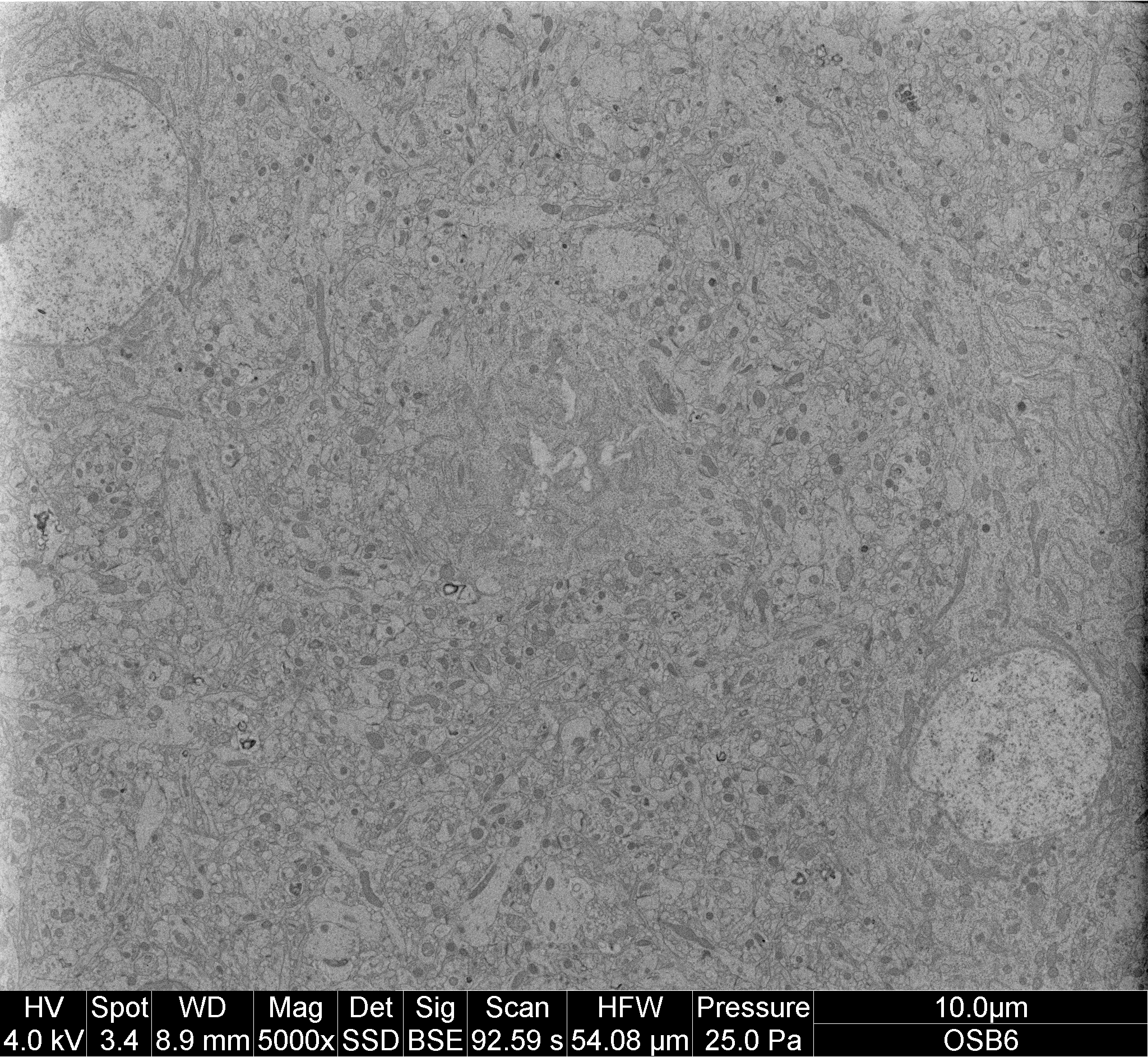

Supplement: Dataset S19 — (253.4 MB ZIP). [file pbio.0020329.sd019.zip › 040604_OS5_st1_1880.tif]

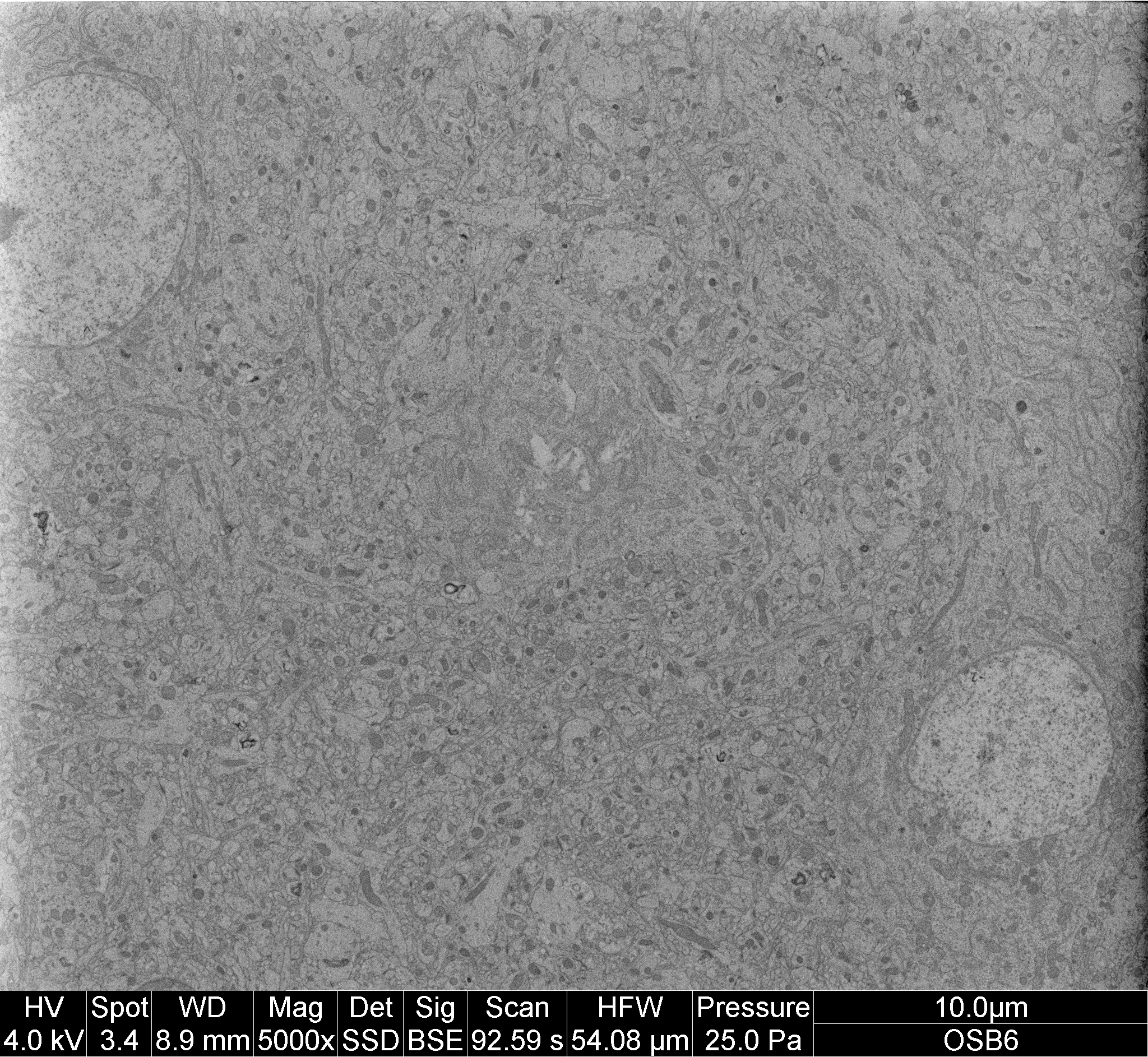

Supplement: Dataset S19 — (253.4 MB ZIP). [file pbio.0020329.sd019.zip › 040604_OS5_st1_1881.tif]

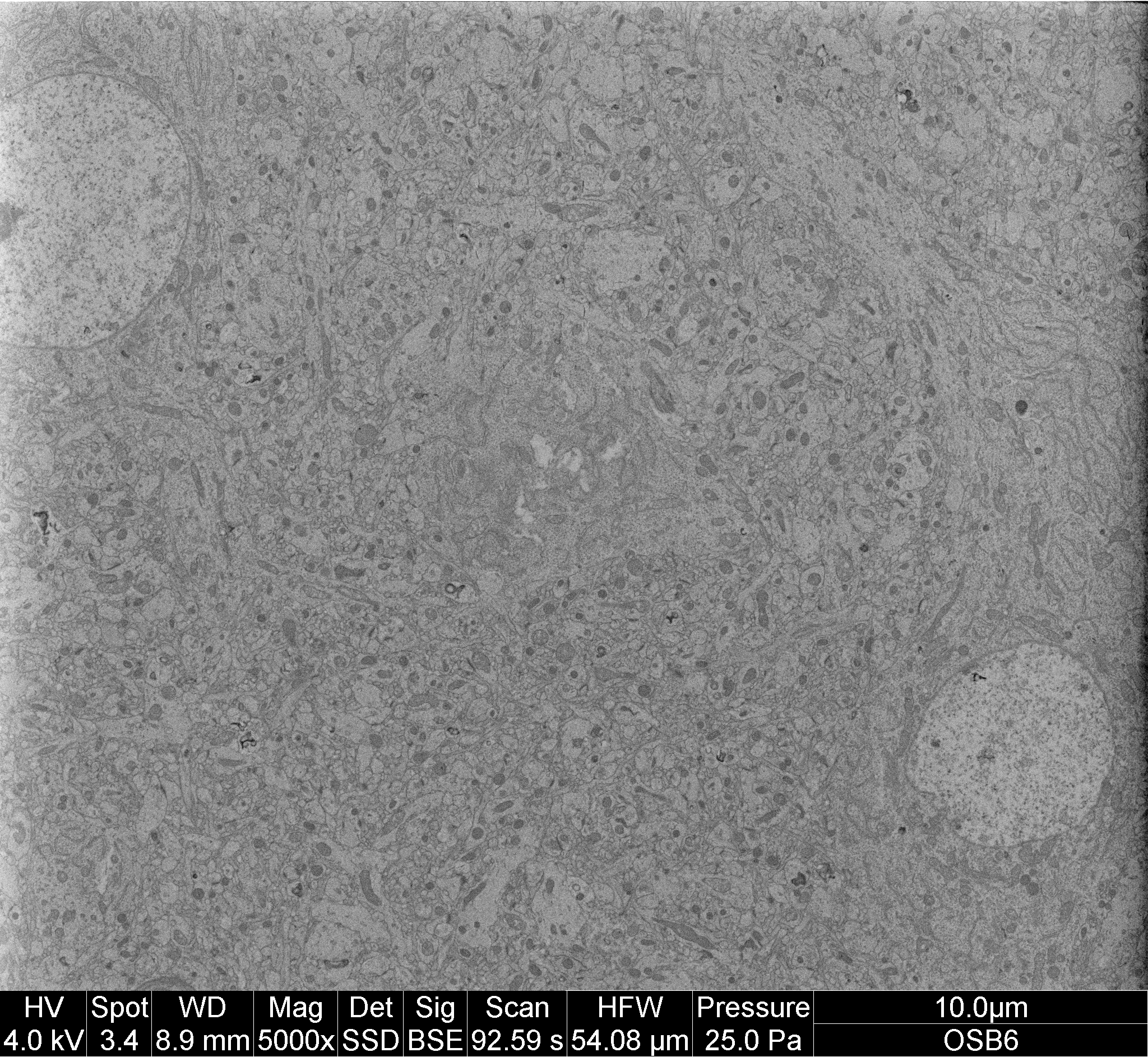

Supplement: Dataset S19 — (253.4 MB ZIP). [file pbio.0020329.sd019.zip › 040604_OS5_st1_1882.tif]

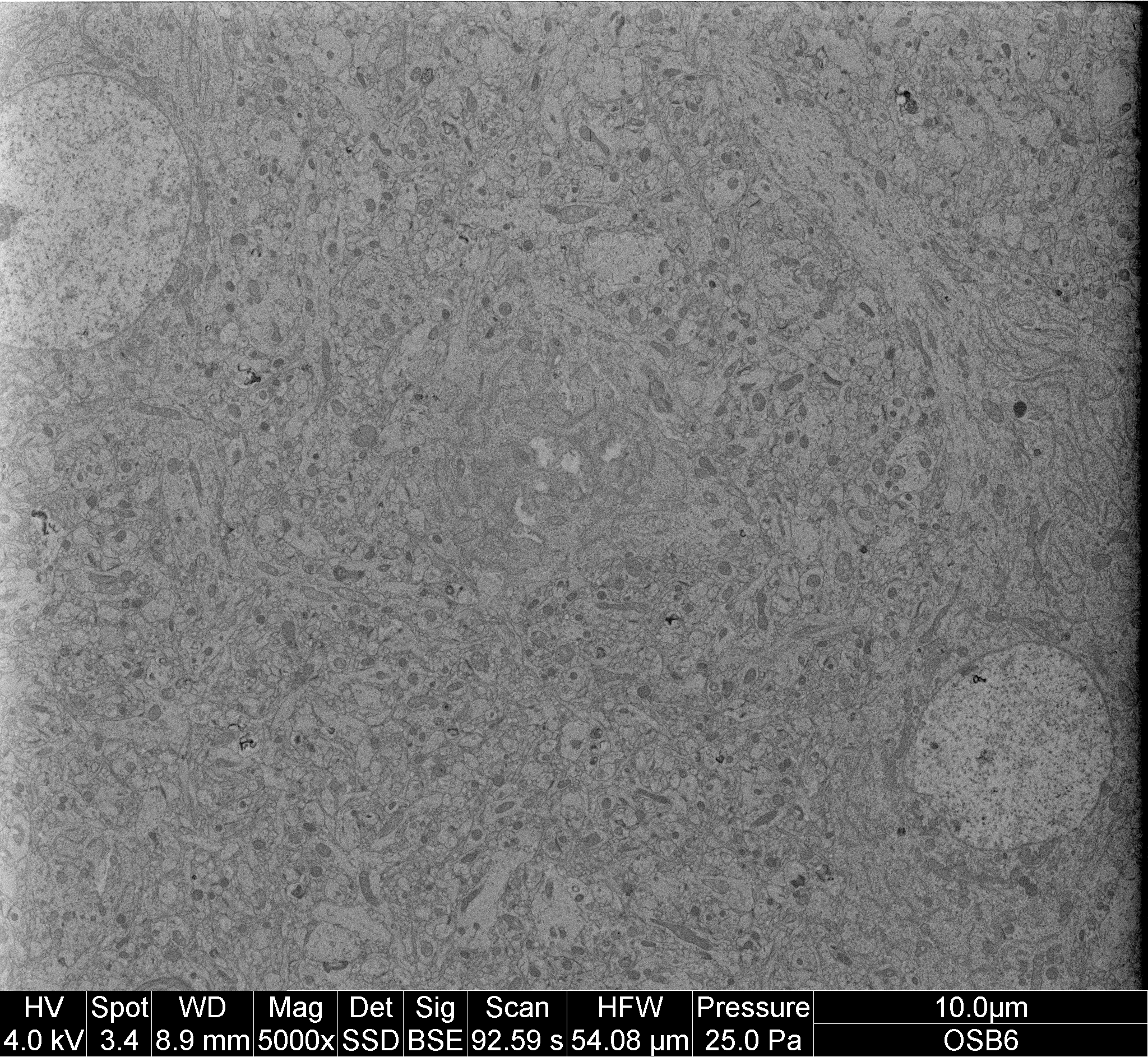

Supplement: Dataset S19 — (253.4 MB ZIP). [file pbio.0020329.sd019.zip › 040604_OS5_st1_1883.tif]

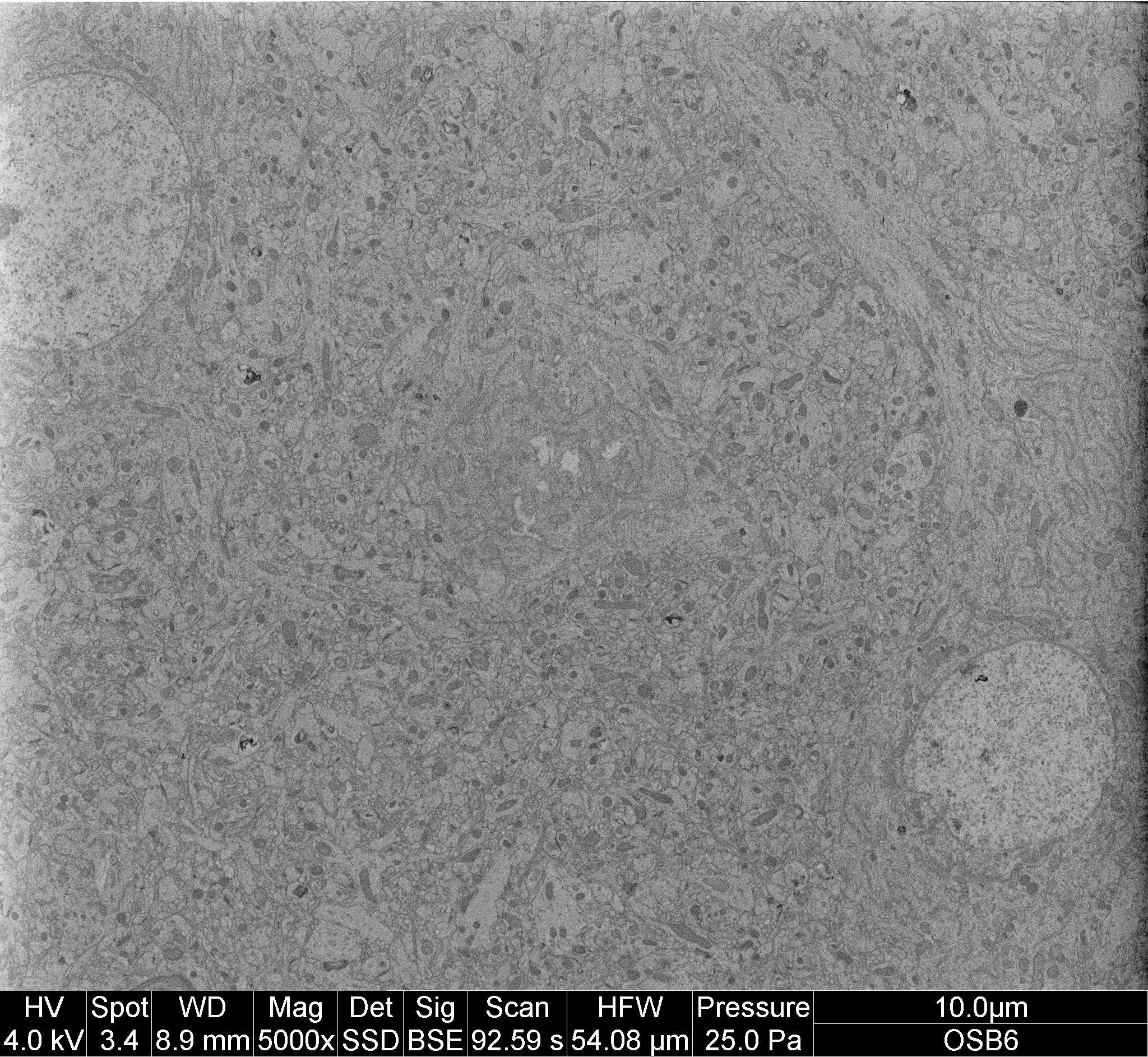

Supplement: Dataset S19 — (253.4 MB ZIP). [file pbio.0020329.sd019.zip › 040604_OS5_st1_1884.tif]

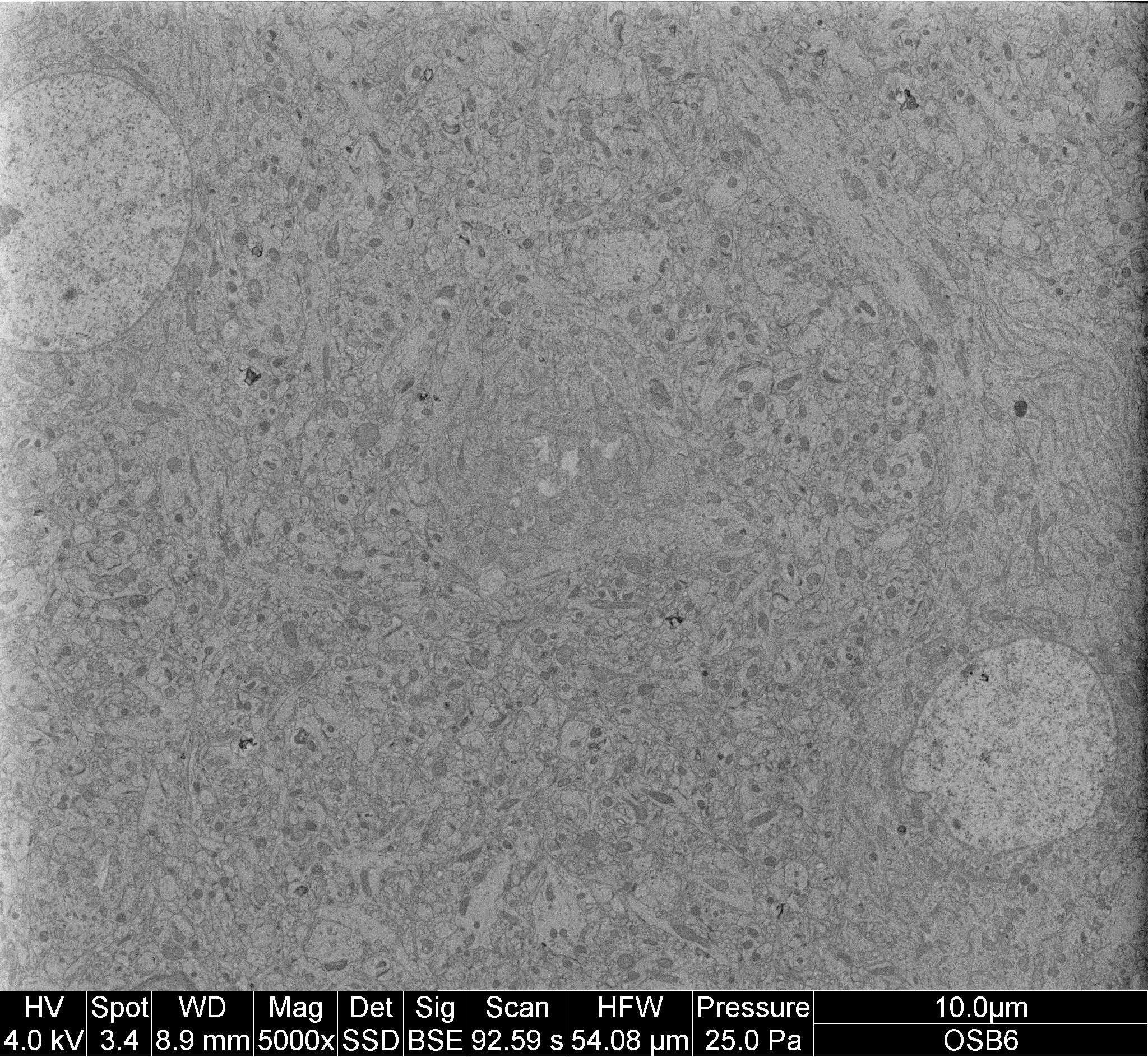

Supplement: Dataset S19 — (253.4 MB ZIP). [file pbio.0020329.sd019.zip › 040604_OS5_st1_1885.tif]

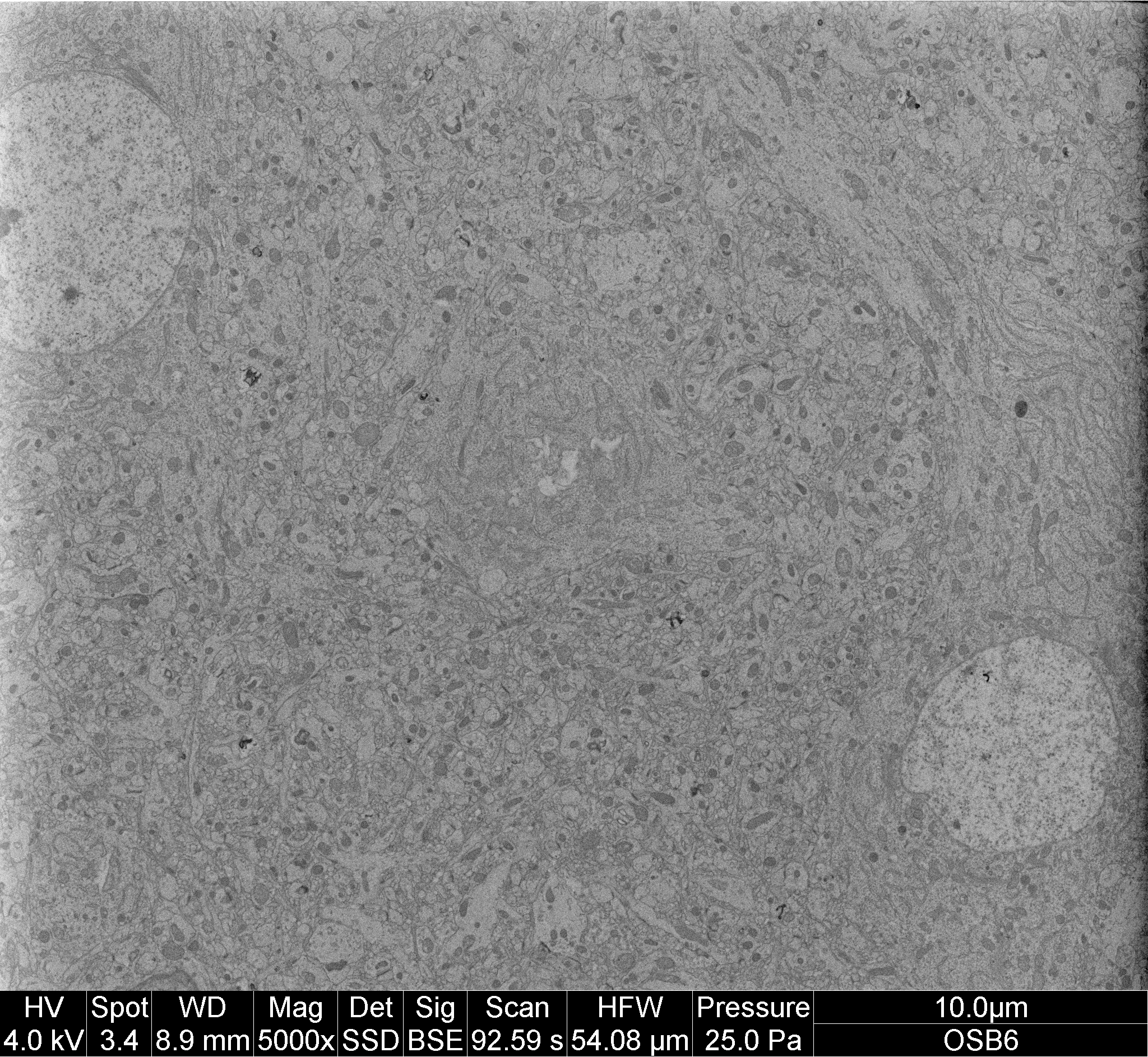

Supplement: Dataset S19 — (253.4 MB ZIP). [file pbio.0020329.sd019.zip › 040604_OS5_st1_1886.tif]

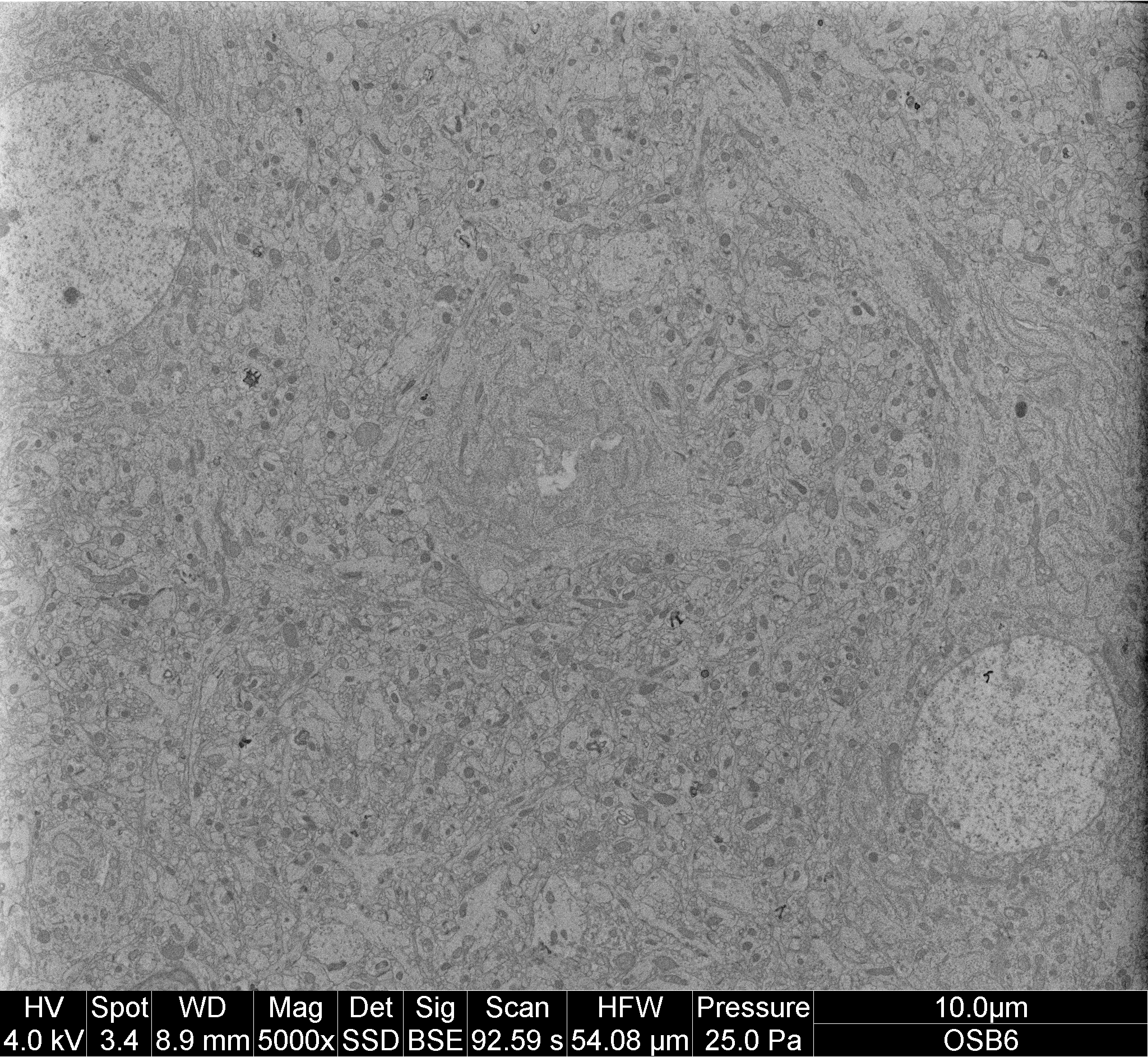

Supplement: Dataset S19 — (253.4 MB ZIP). [file pbio.0020329.sd019.zip › 040604_OS5_st1_1887.tif]

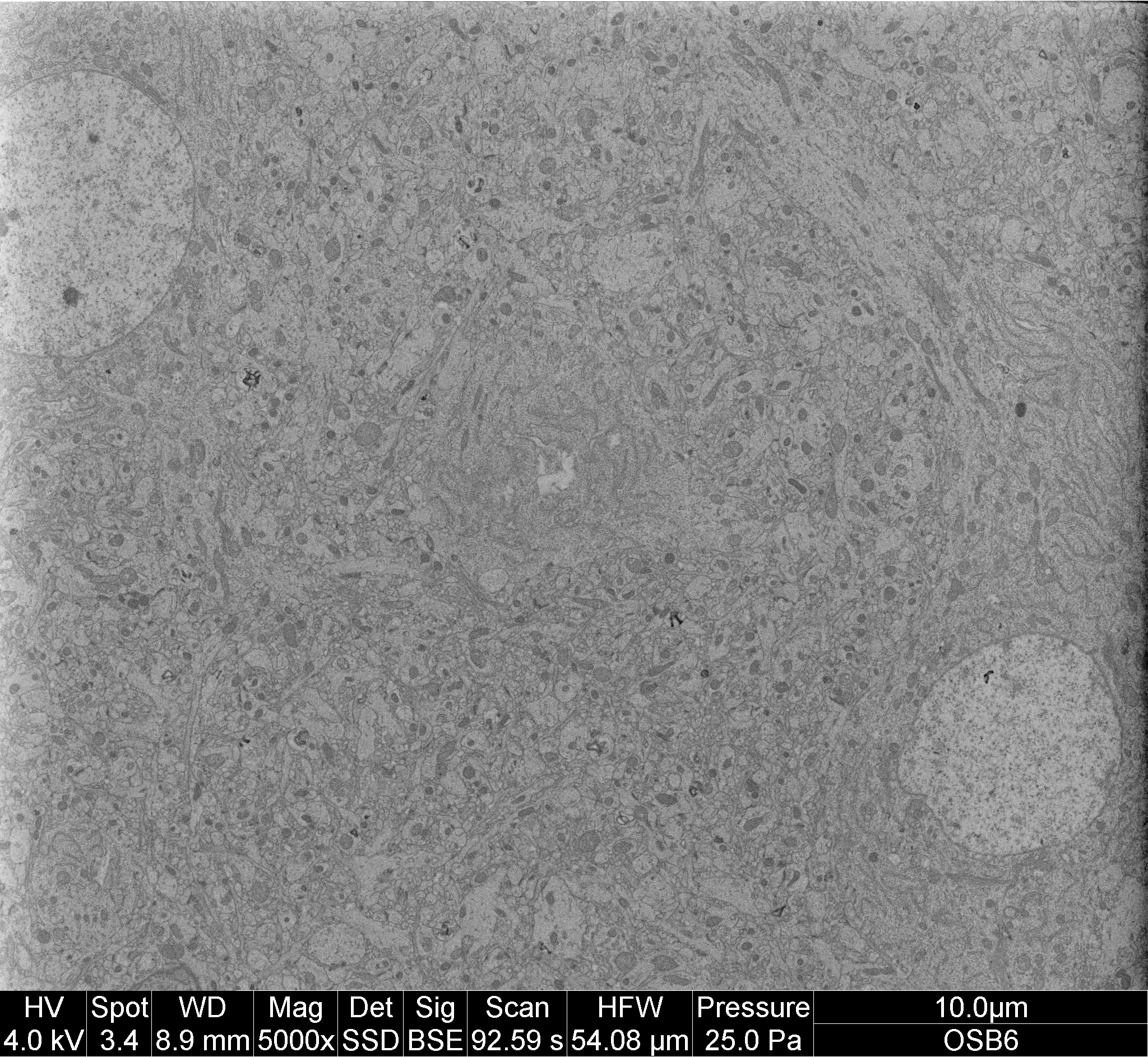

Supplement: Dataset S19 — (253.4 MB ZIP). [file pbio.0020329.sd019.zip › 040604_OS5_st1_1888.tif]

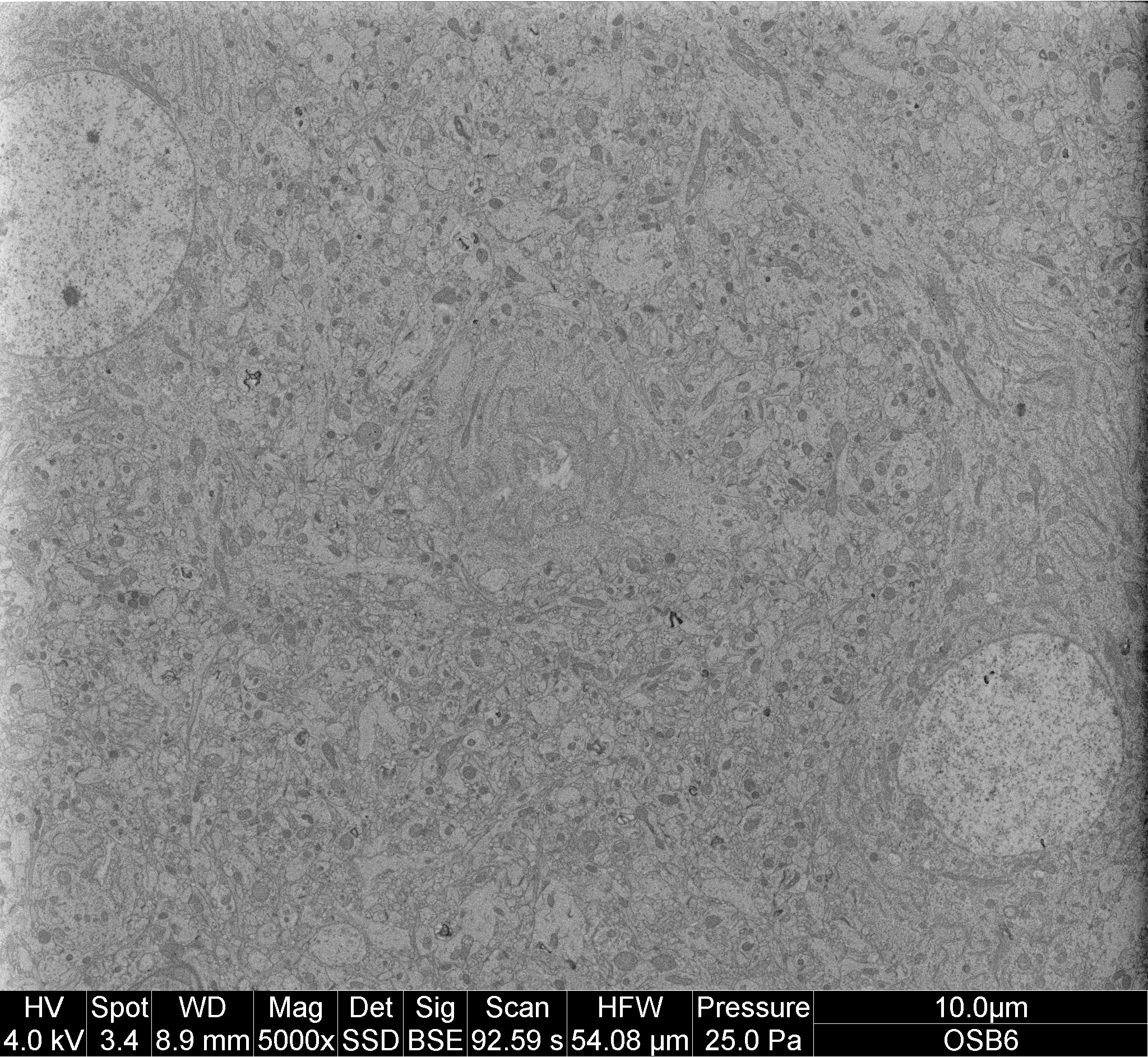

Supplement: Dataset S19 — (253.4 MB ZIP). [file pbio.0020329.sd019.zip › 040604_OS5_st1_1889.tif]

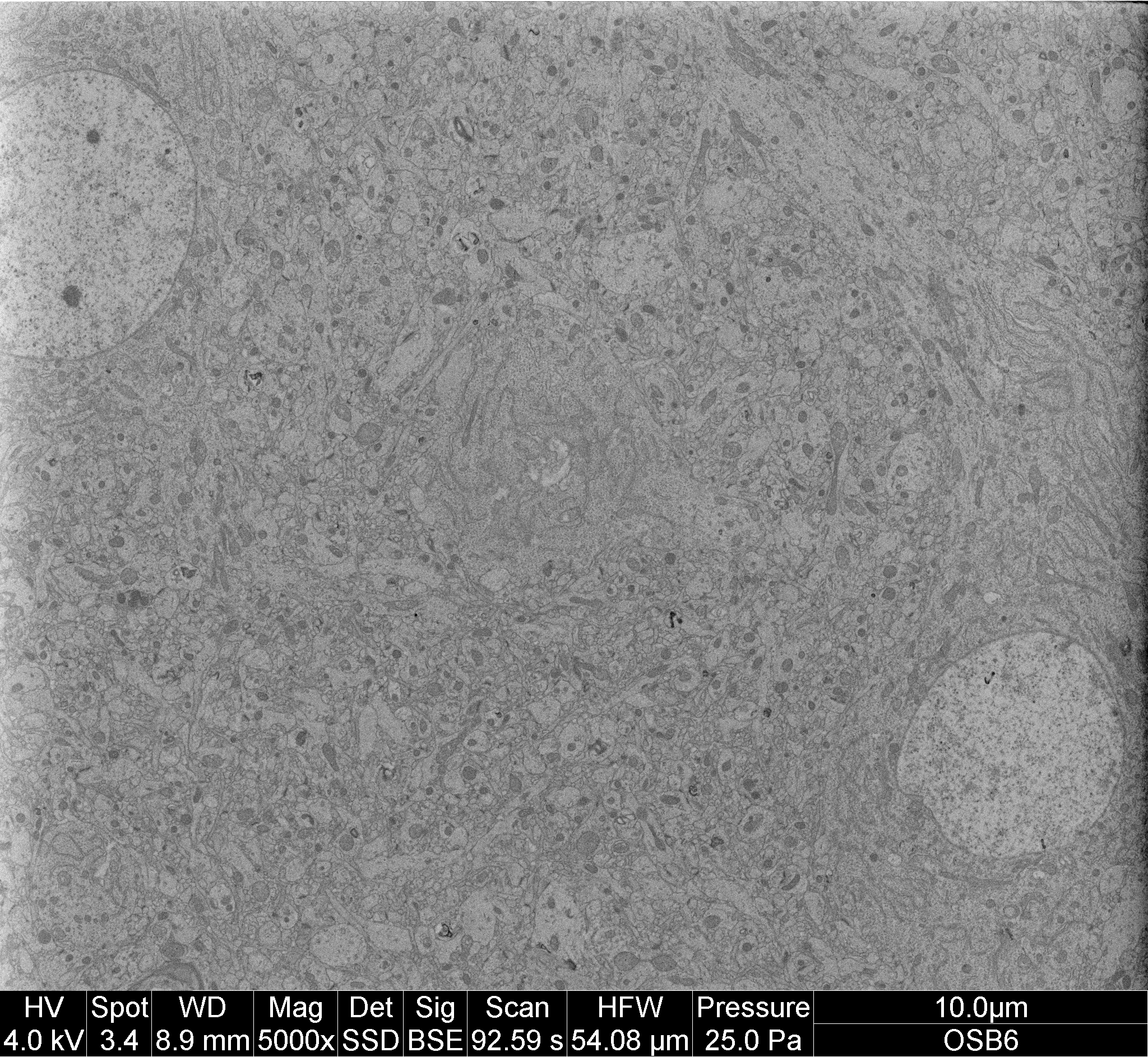

Supplement: Dataset S19 — (253.4 MB ZIP). [file pbio.0020329.sd019.zip › 040604_OS5_st1_1890.tif]

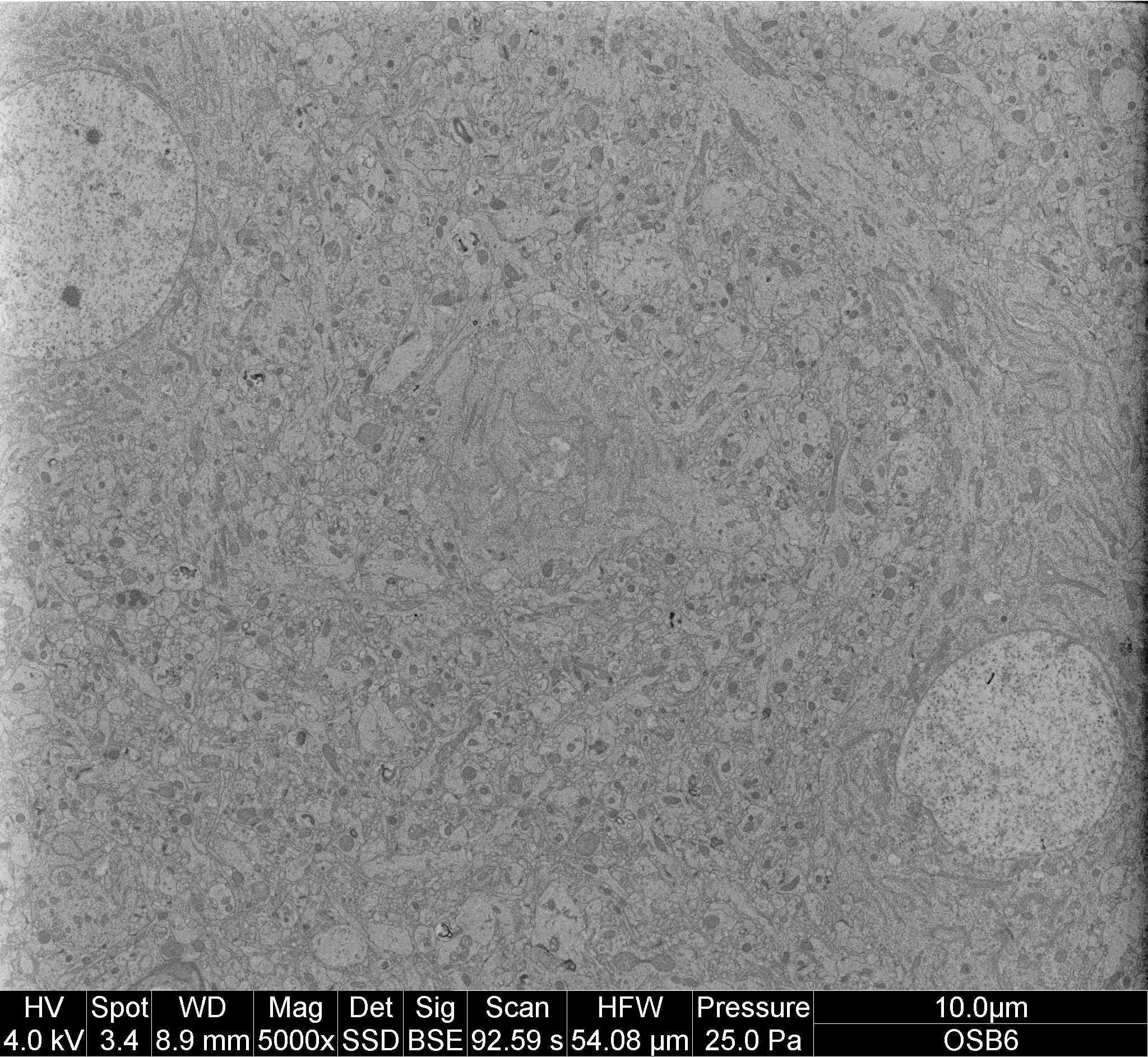

Supplement: Dataset S19 — (253.4 MB ZIP). [file pbio.0020329.sd019.zip › 040604_OS5_st1_1891.tif]

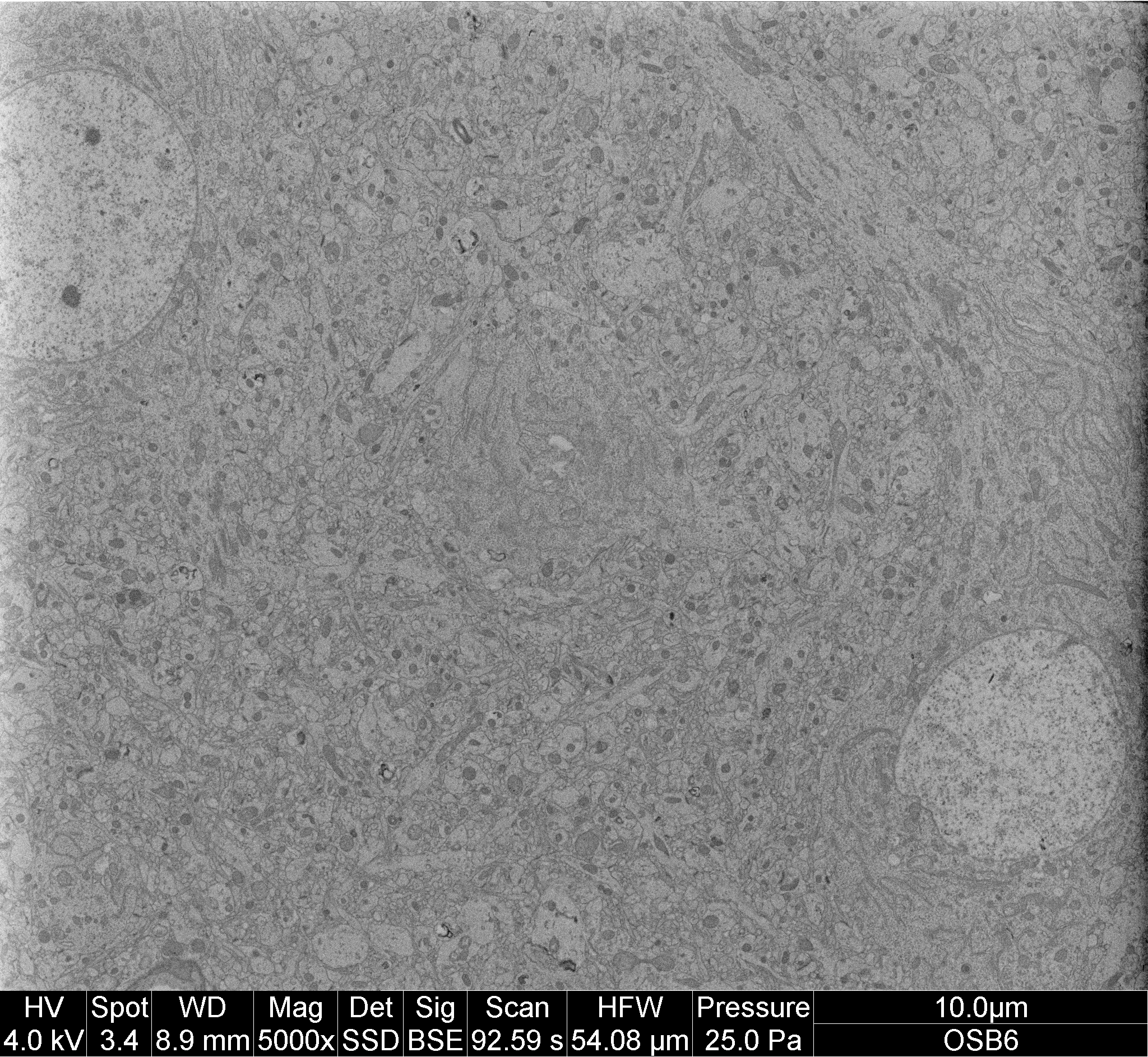

Supplement: Dataset S19 — (253.4 MB ZIP). [file pbio.0020329.sd019.zip › 040604_OS5_st1_1892.tif]

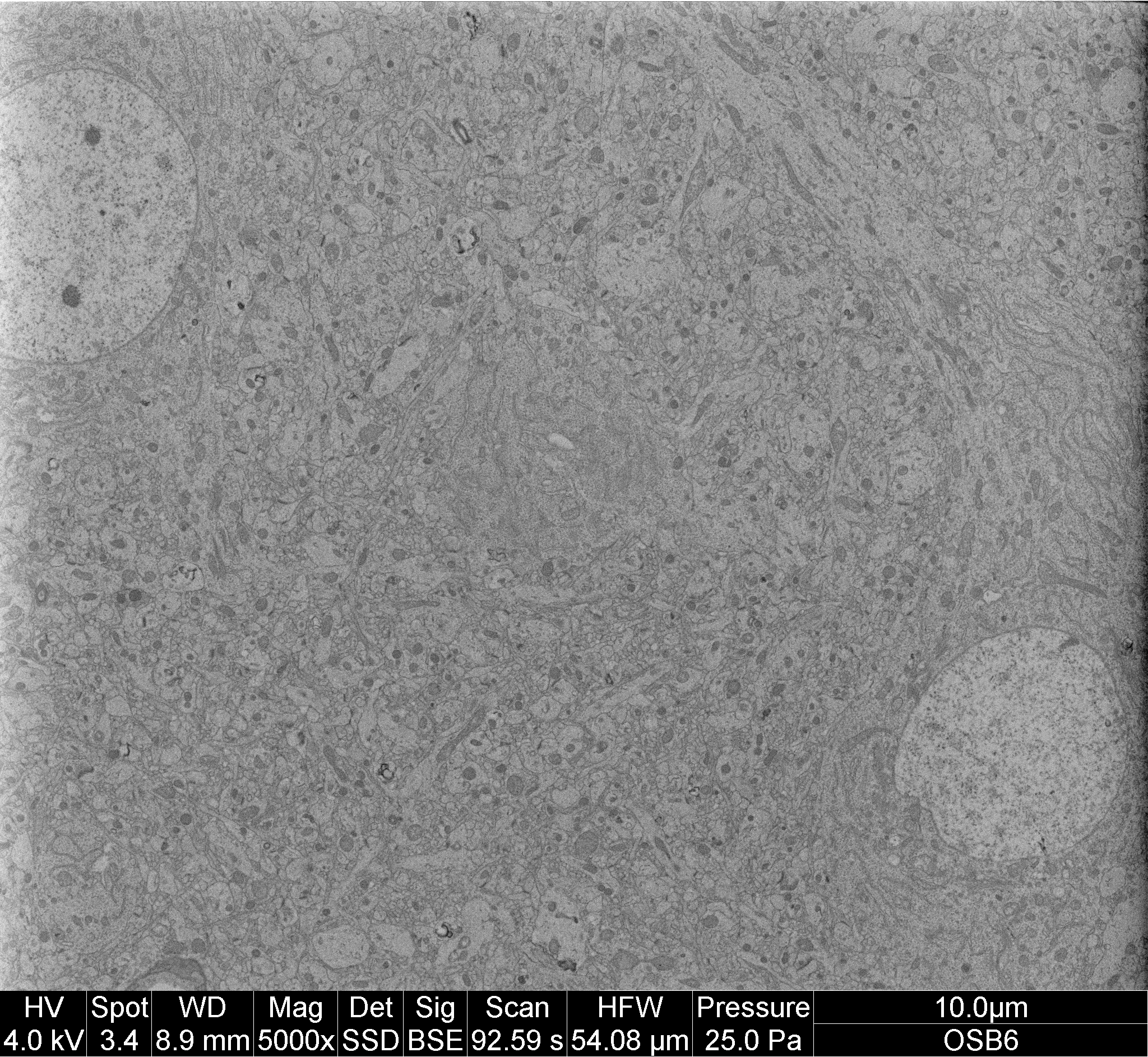

Supplement: Dataset S19 — (253.4 MB ZIP). [file pbio.0020329.sd019.zip › 040604_OS5_st1_1893.tif]

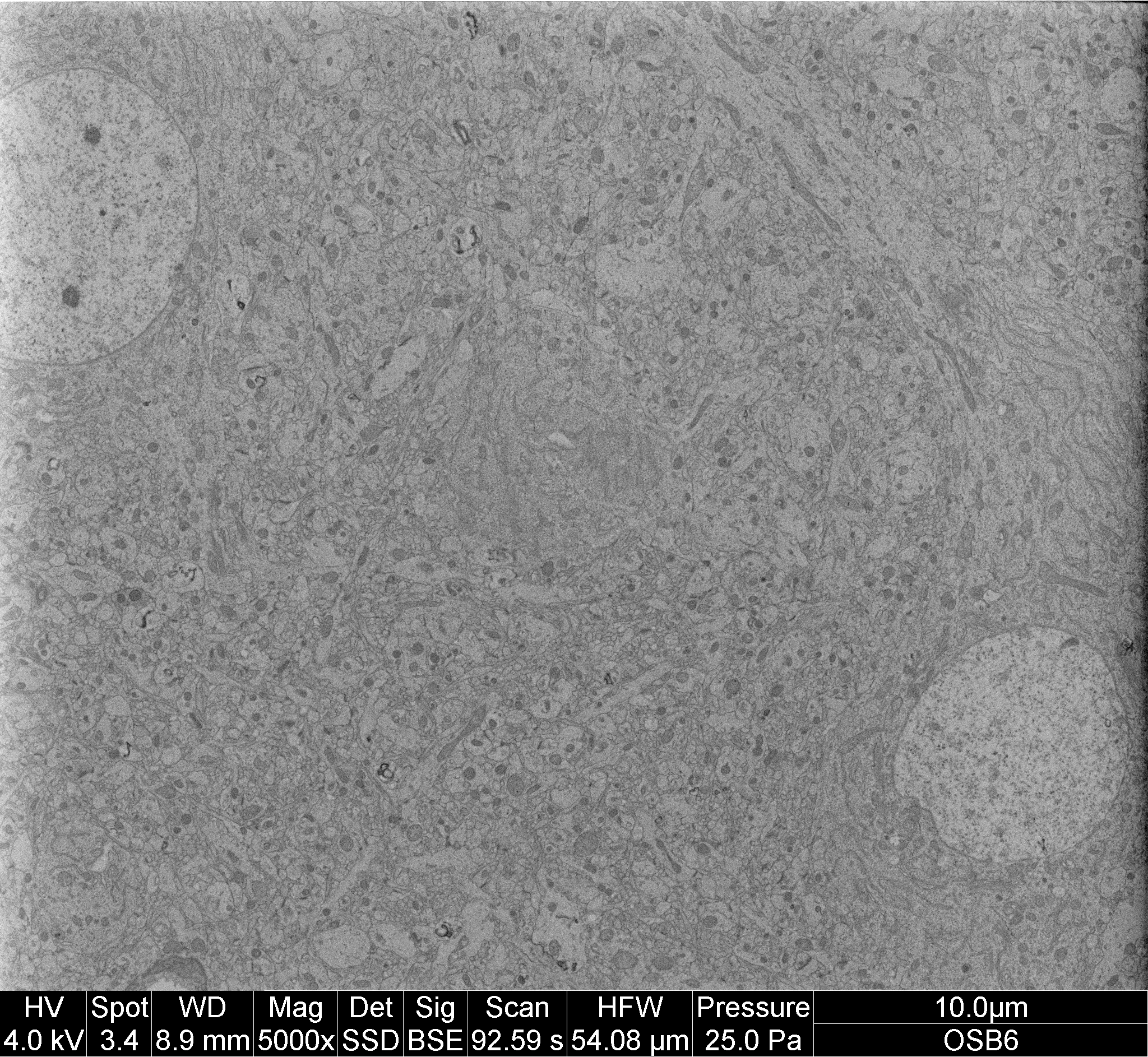

Supplement: Dataset S19 — (253.4 MB ZIP). [file pbio.0020329.sd019.zip › 040604_OS5_st1_1894.tif]

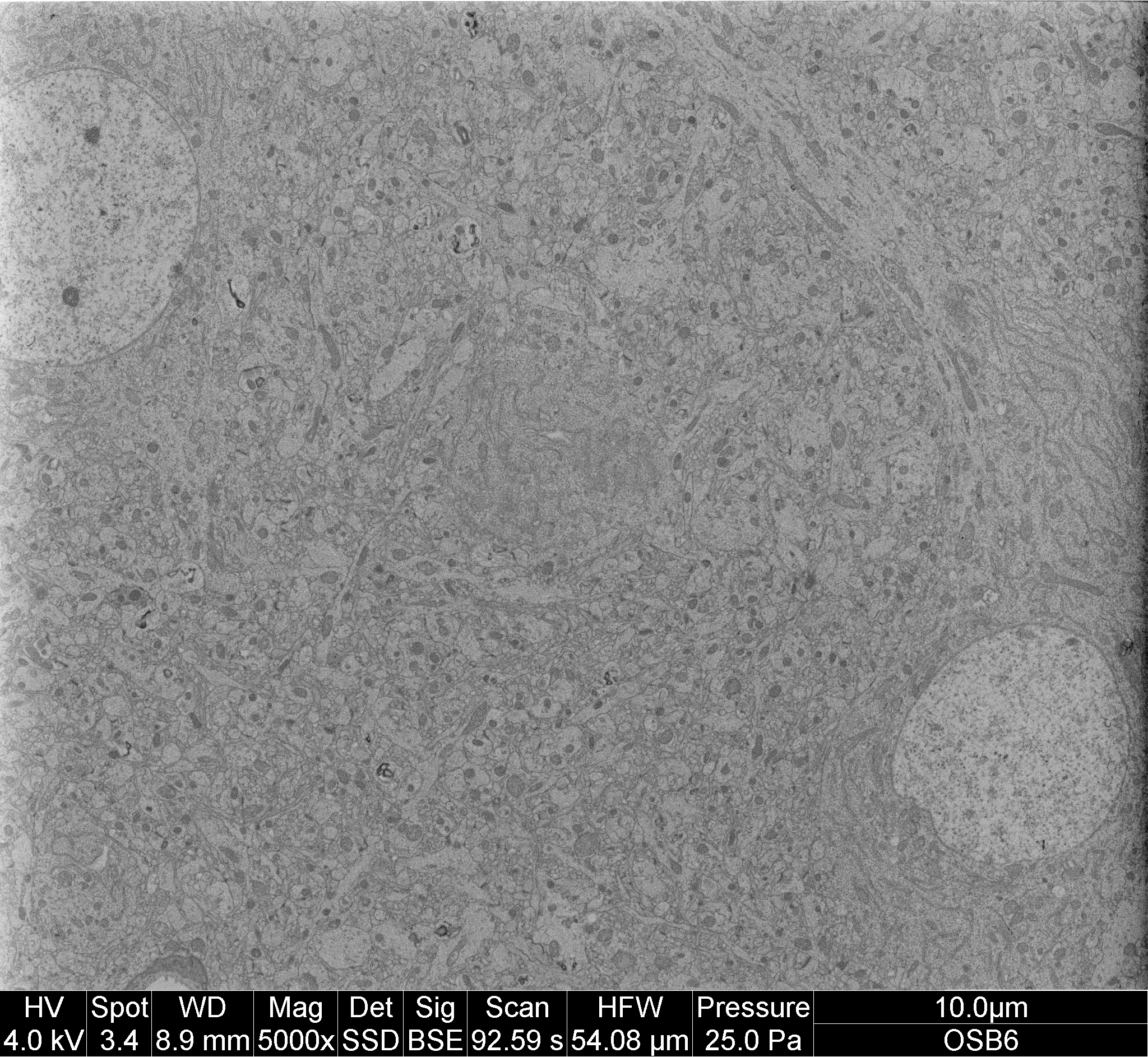

Supplement: Dataset S19 — (253.4 MB ZIP). [file pbio.0020329.sd019.zip › 040604_OS5_st1_1895.tif]

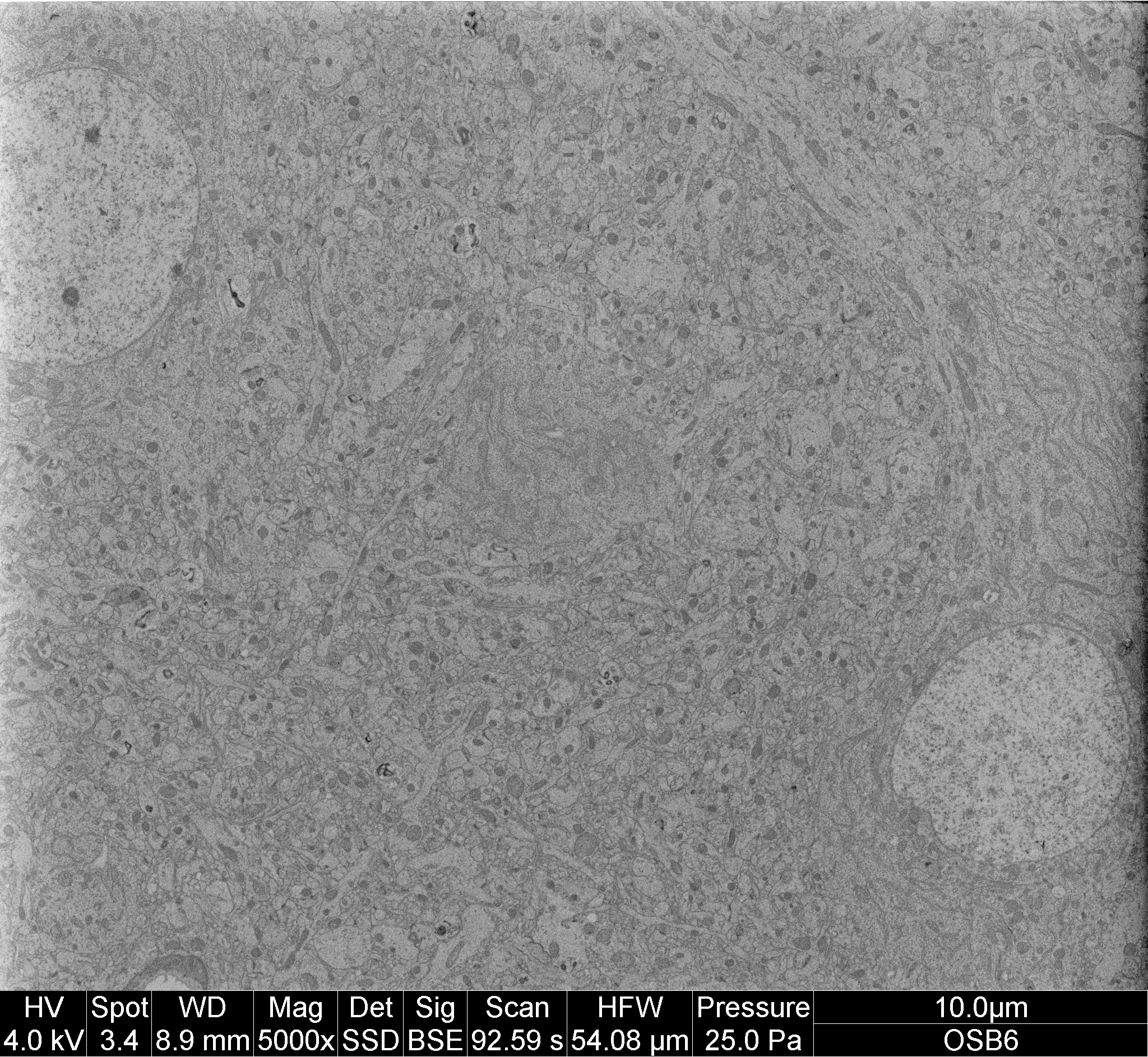

Supplement: Dataset S19 — (253.4 MB ZIP). [file pbio.0020329.sd019.zip › 040604_OS5_st1_1896.tif]

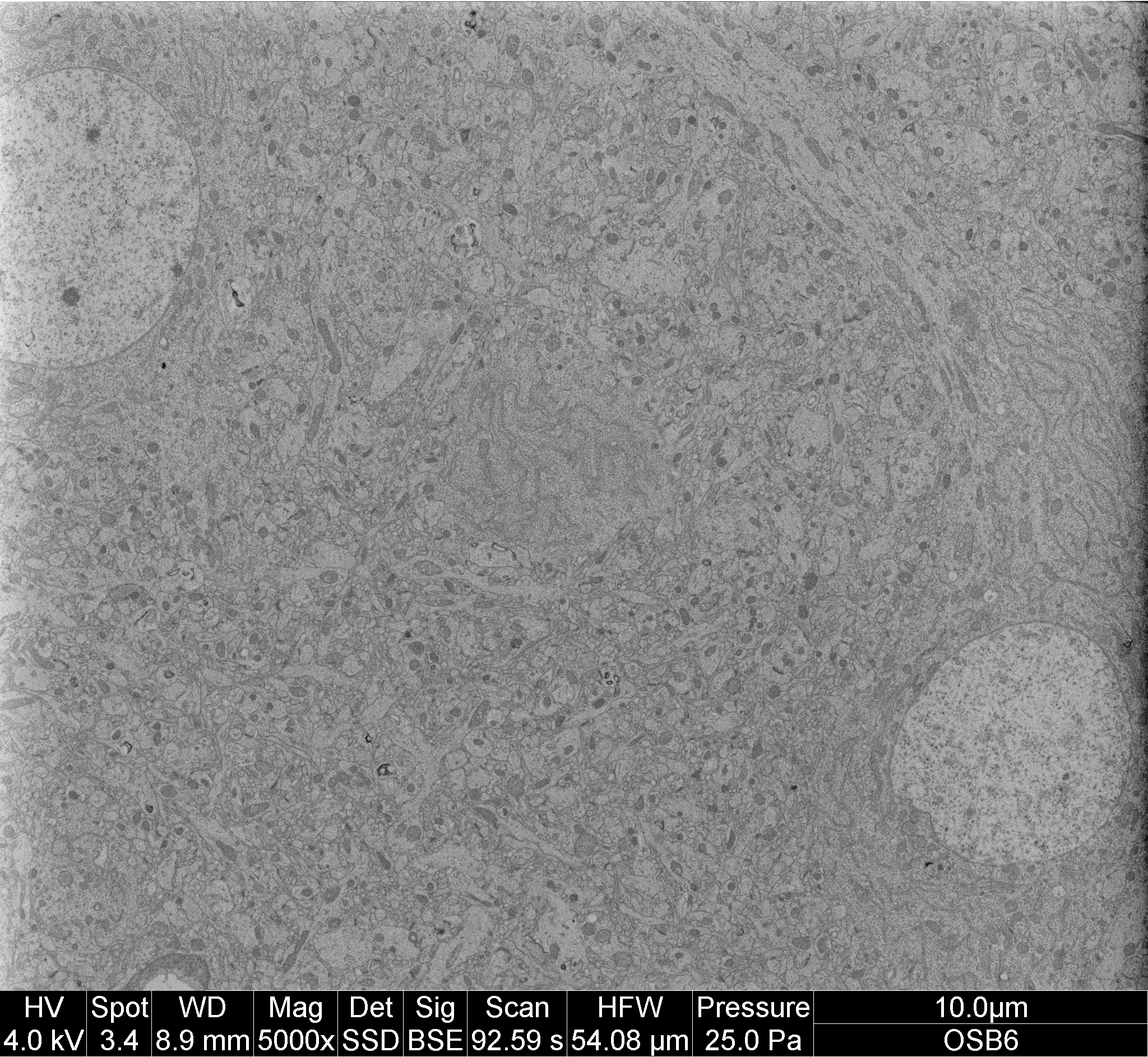

Supplement: Dataset S19 — (253.4 MB ZIP). [file pbio.0020329.sd019.zip › 040604_OS5_st1_1897.tif]

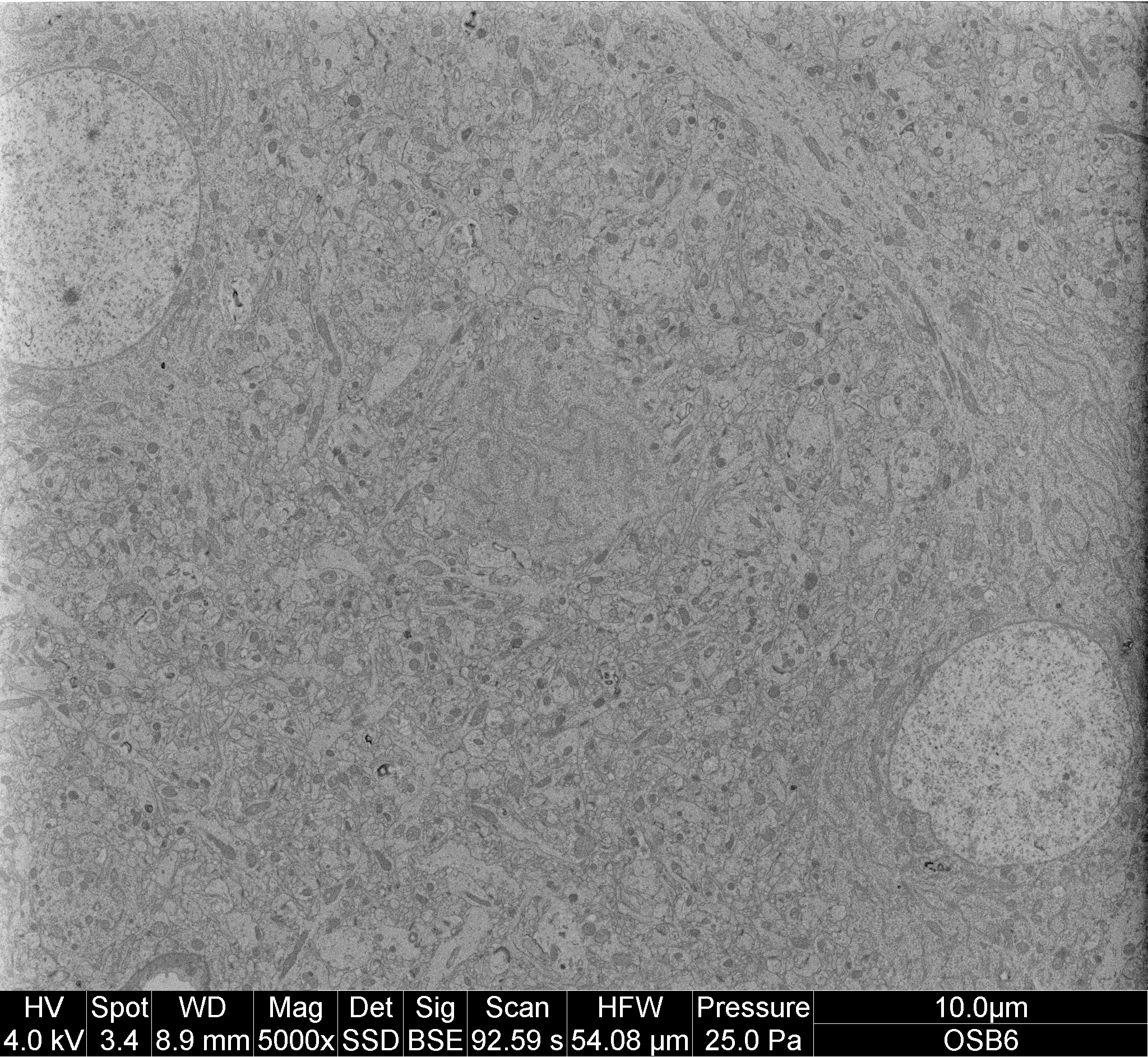

Supplement: Dataset S19 — (253.4 MB ZIP). [file pbio.0020329.sd019.zip › 040604_OS5_st1_1898.tif]

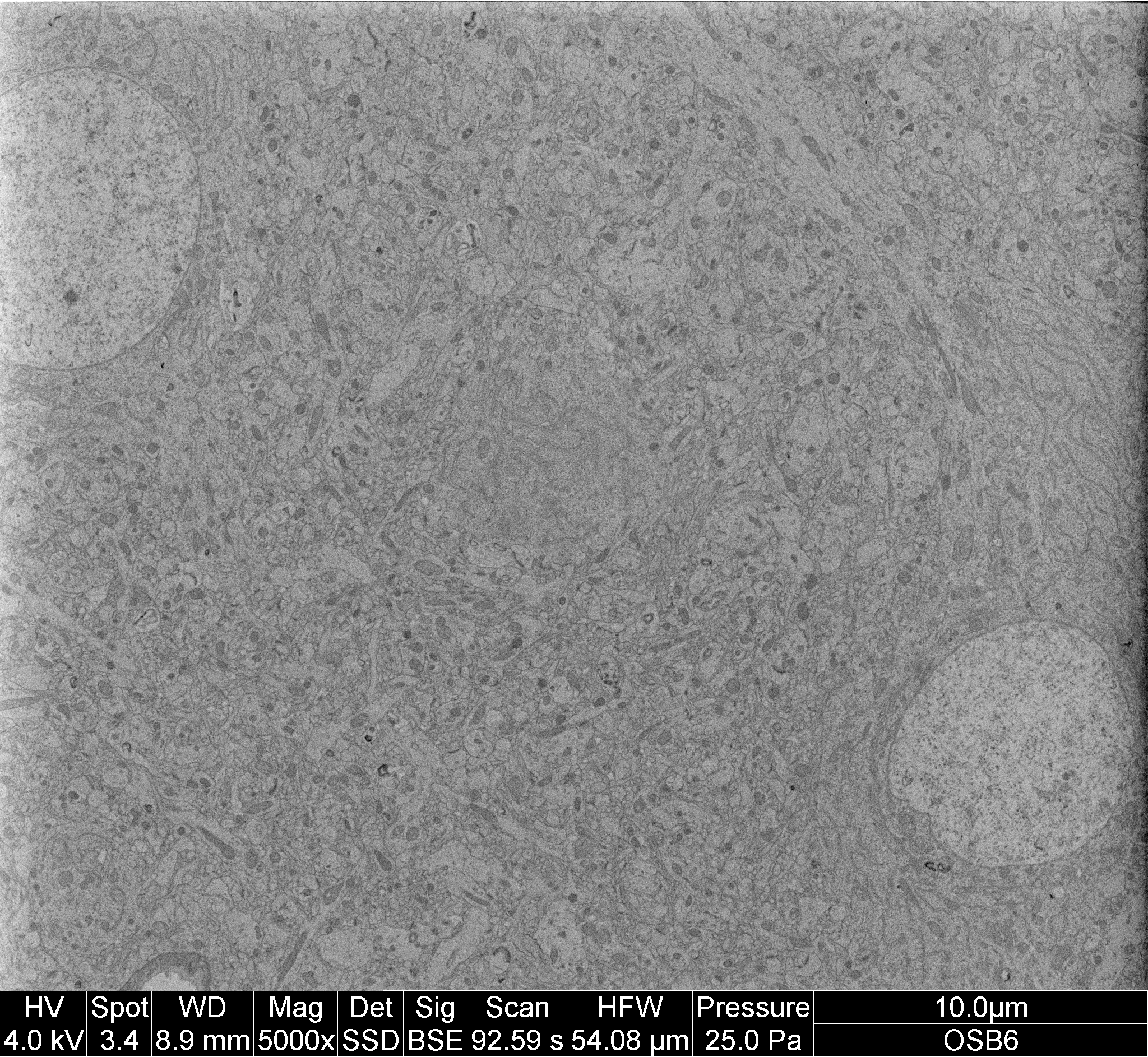

Supplement: Dataset S19 — (253.4 MB ZIP). [file pbio.0020329.sd019.zip › 040604_OS5_st1_1899.tif]

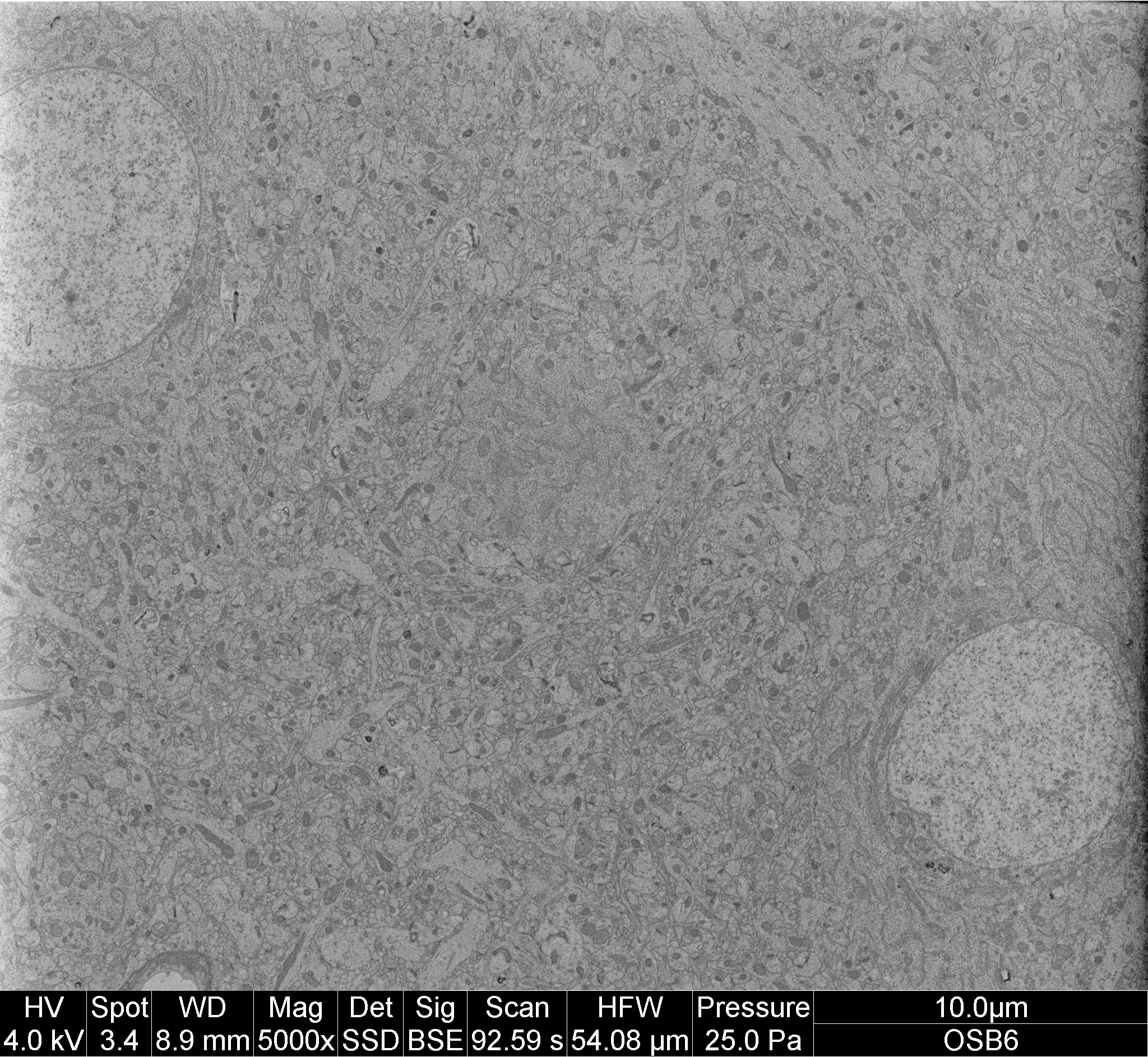

Supplement: Dataset S20 — (254.9 MB ZIP). [file pbio.0020329.sd020.zip › 040604_OS5_st1_1900.tif]
