# Supplementary figures and images for: Serial Block-Face Scanning Electron Microscopy to Reconstruct Three-Dimensional Tissue Nanostructure (part 20 of 21)
Source: PLoS Biol. 2004 Oct 19;2(11):e329. doi: 10.1371/journal.pbio.0020329 (PMC524270; doi:10.1371/journal.pbio.0020329)

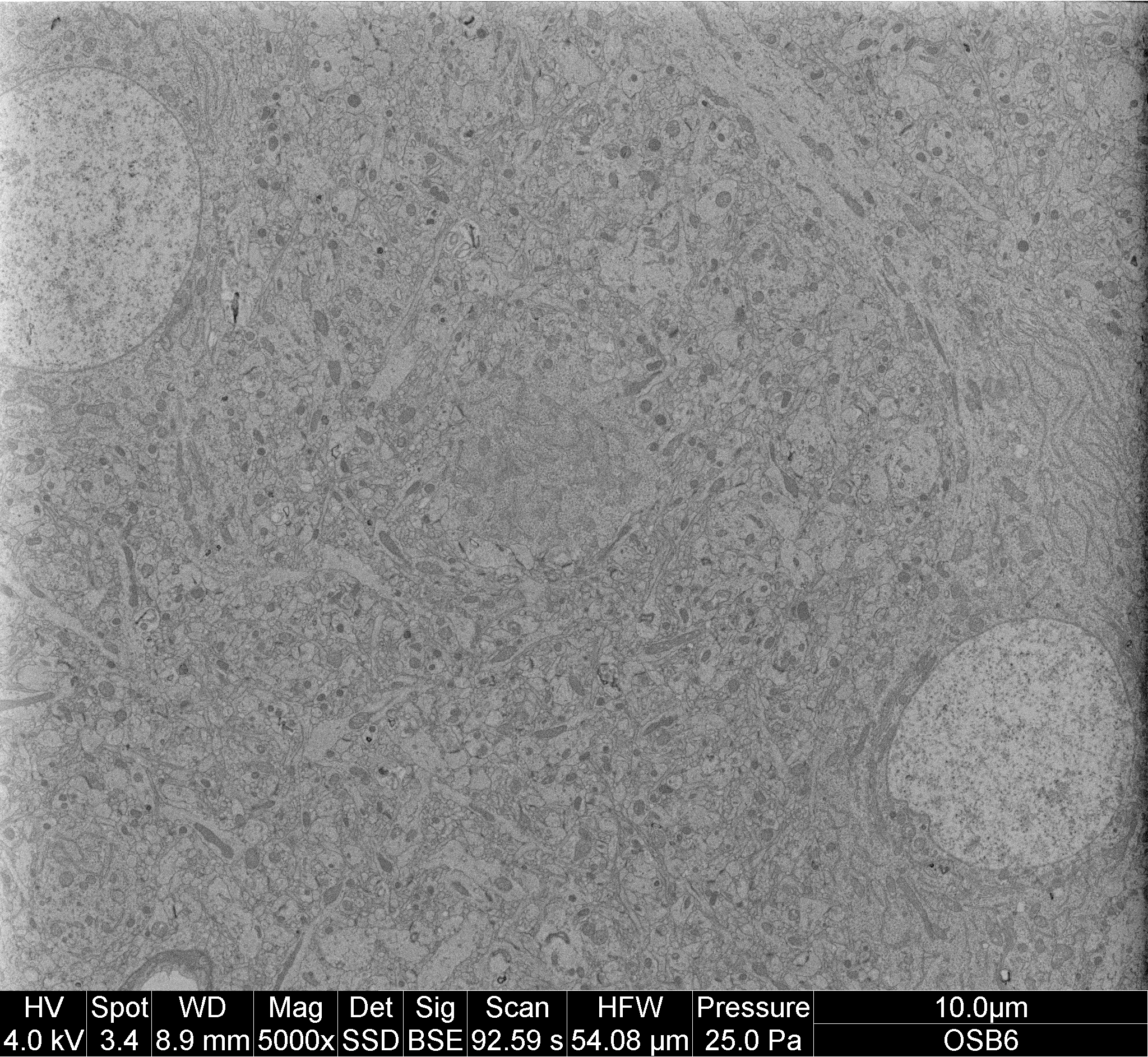

Supplement: Dataset S20 — (254.9 MB ZIP). [file pbio.0020329.sd020.zip › 040604_OS5_st1_1901.tif]

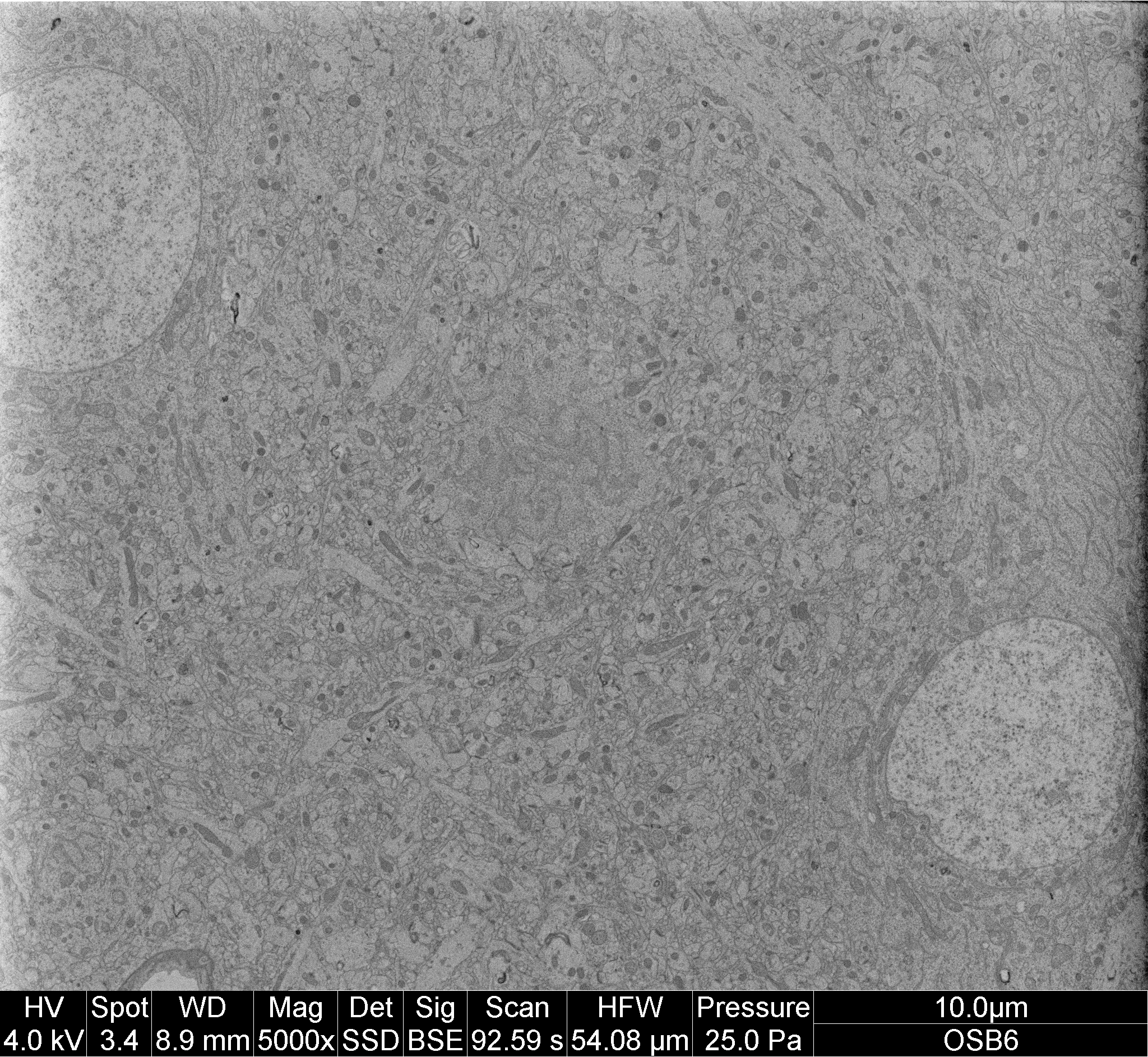

Supplement: Dataset S20 — (254.9 MB ZIP). [file pbio.0020329.sd020.zip › 040604_OS5_st1_1902.tif]

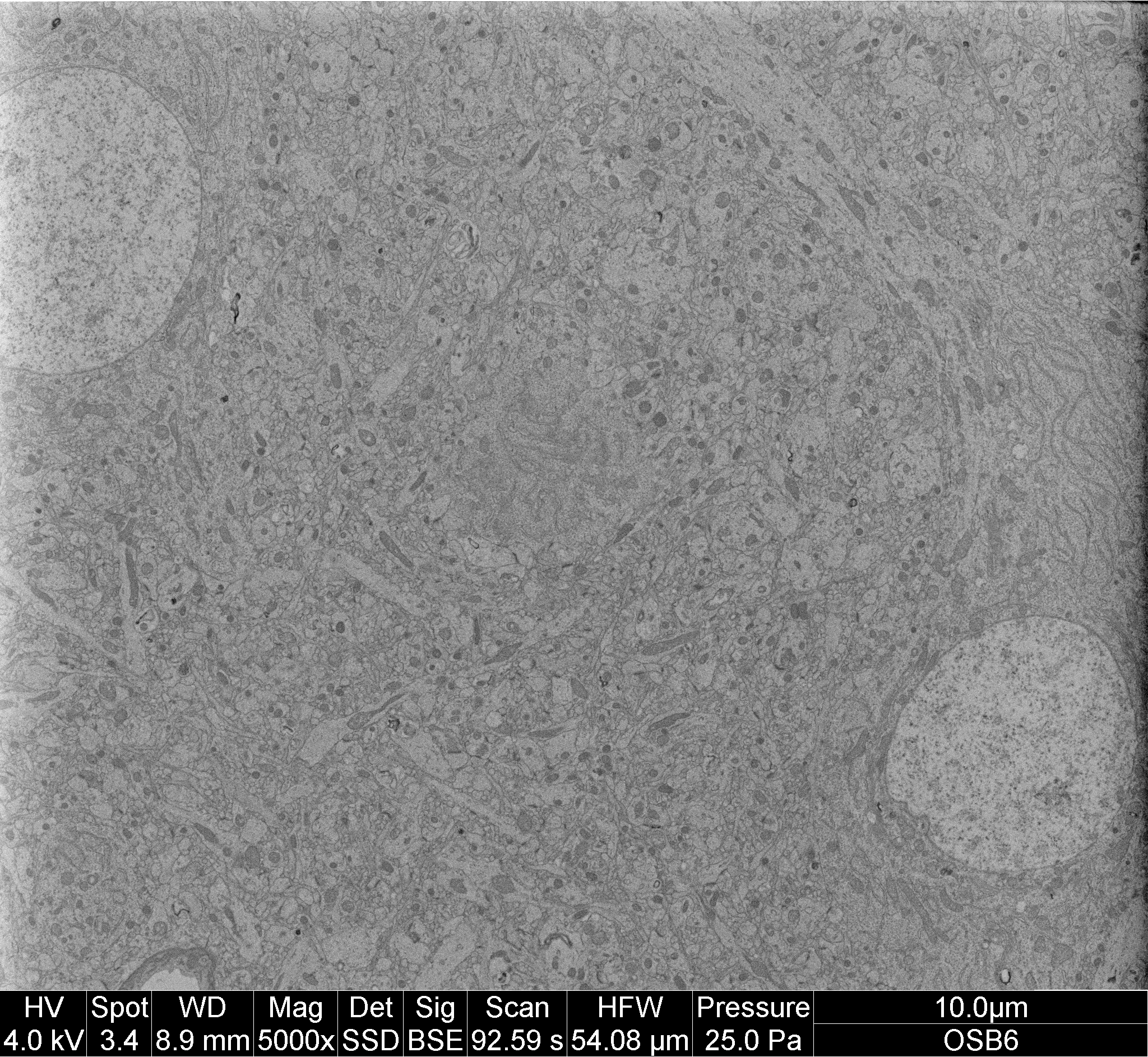

Supplement: Dataset S20 — (254.9 MB ZIP). [file pbio.0020329.sd020.zip › 040604_OS5_st1_1903.tif]

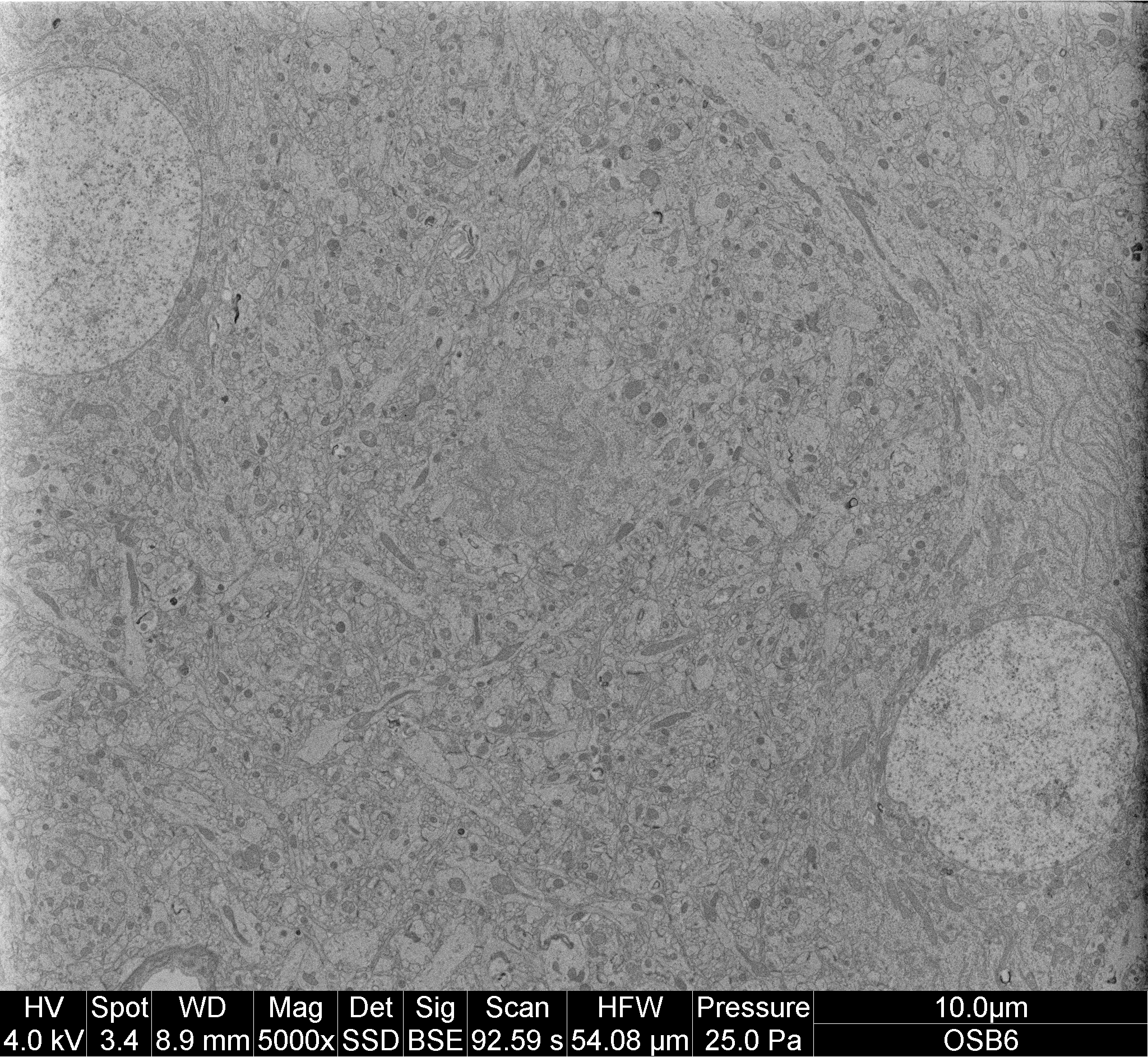

Supplement: Dataset S20 — (254.9 MB ZIP). [file pbio.0020329.sd020.zip › 040604_OS5_st1_1904.tif]

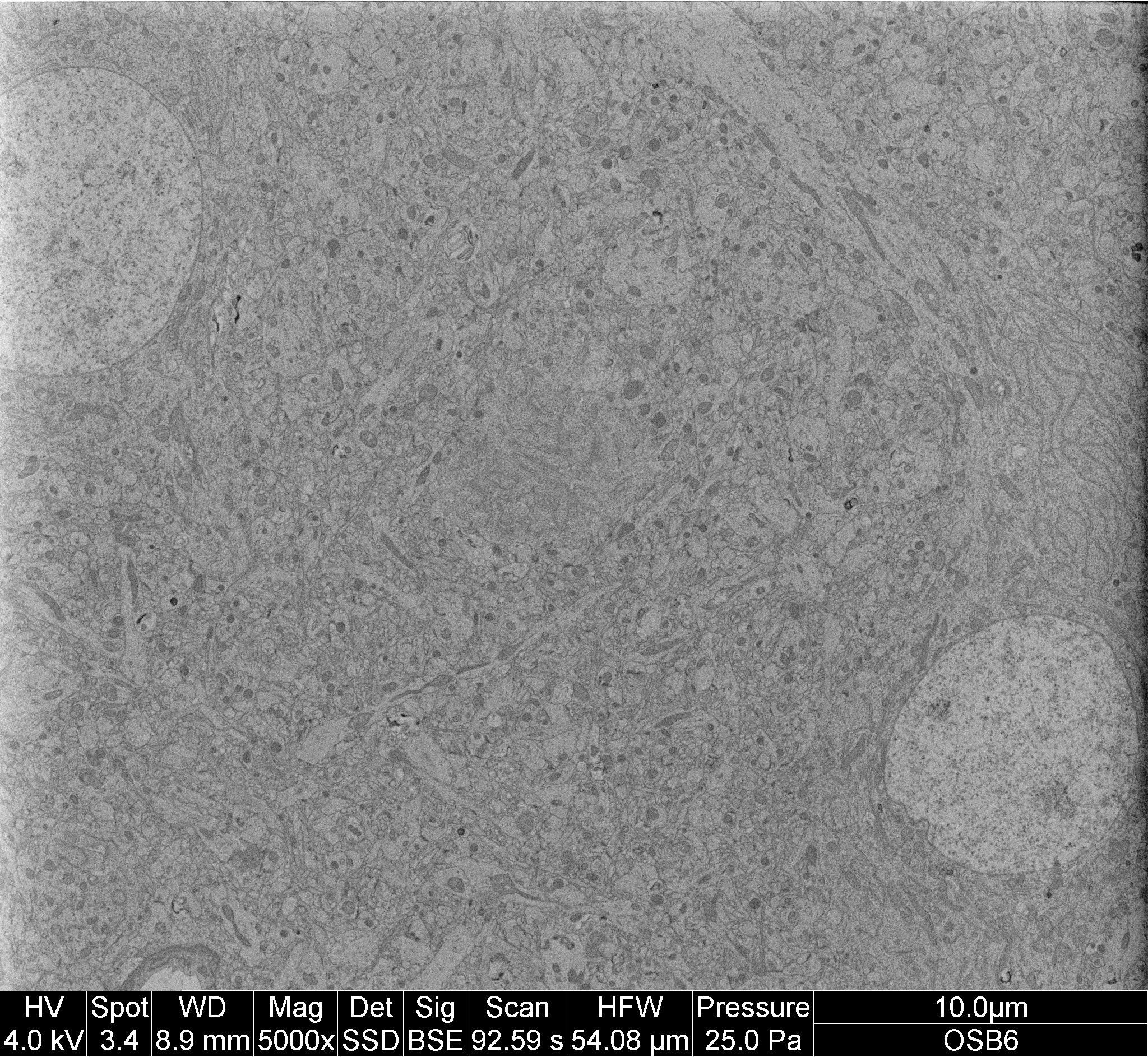

Supplement: Dataset S20 — (254.9 MB ZIP). [file pbio.0020329.sd020.zip › 040604_OS5_st1_1905.tif]

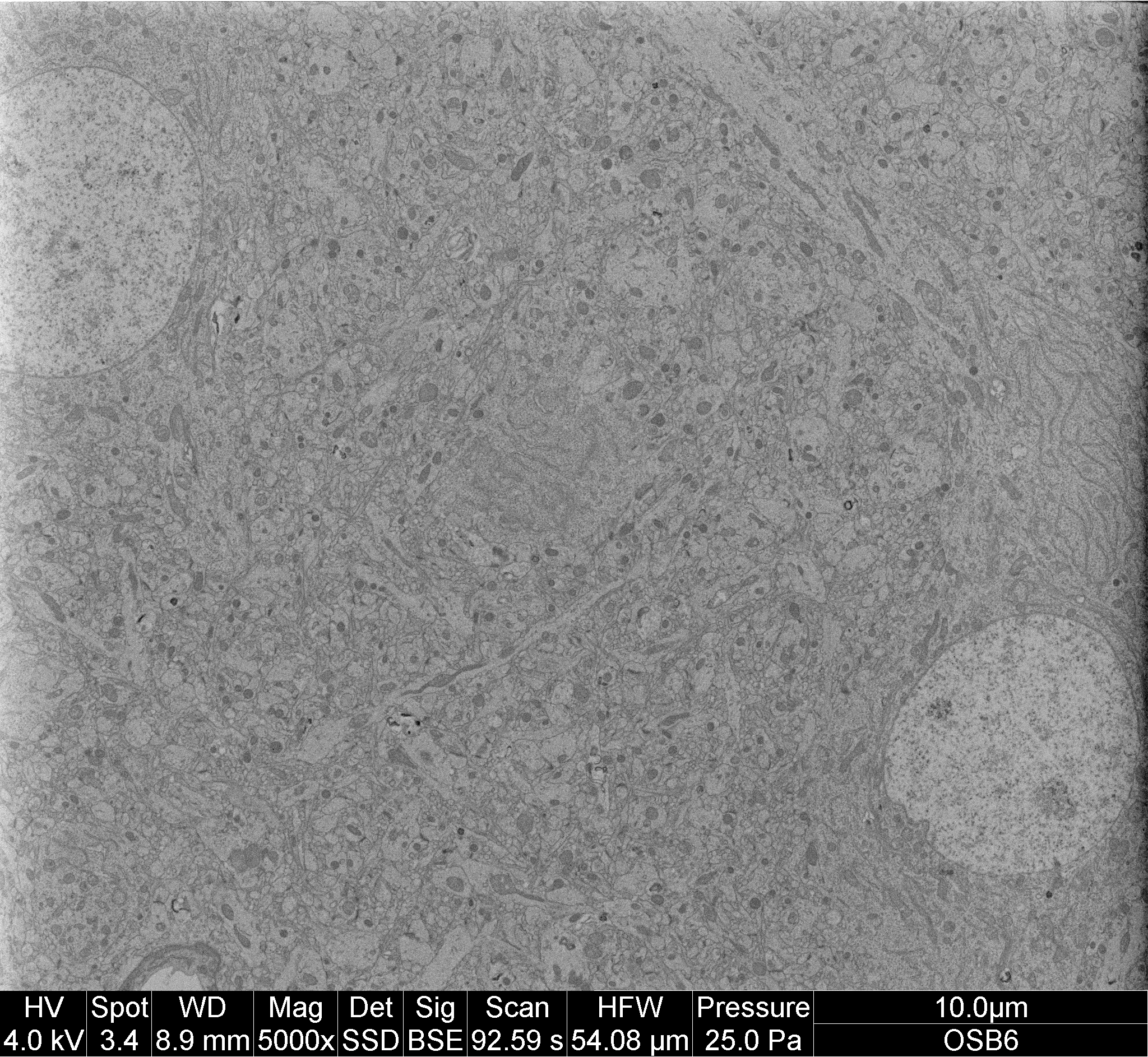

Supplement: Dataset S20 — (254.9 MB ZIP). [file pbio.0020329.sd020.zip › 040604_OS5_st1_1906.tif]

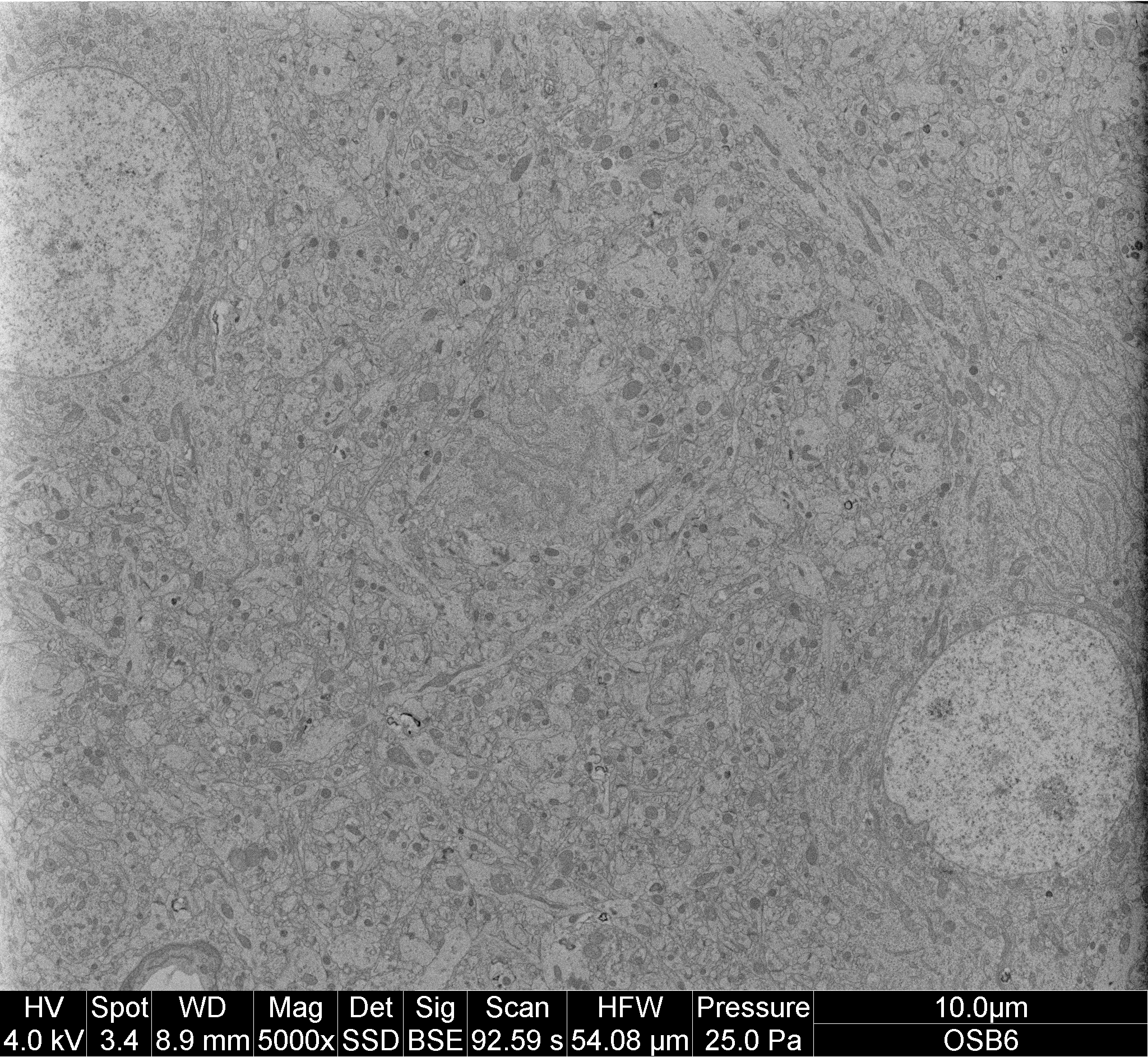

Supplement: Dataset S20 — (254.9 MB ZIP). [file pbio.0020329.sd020.zip › 040604_OS5_st1_1907.tif]

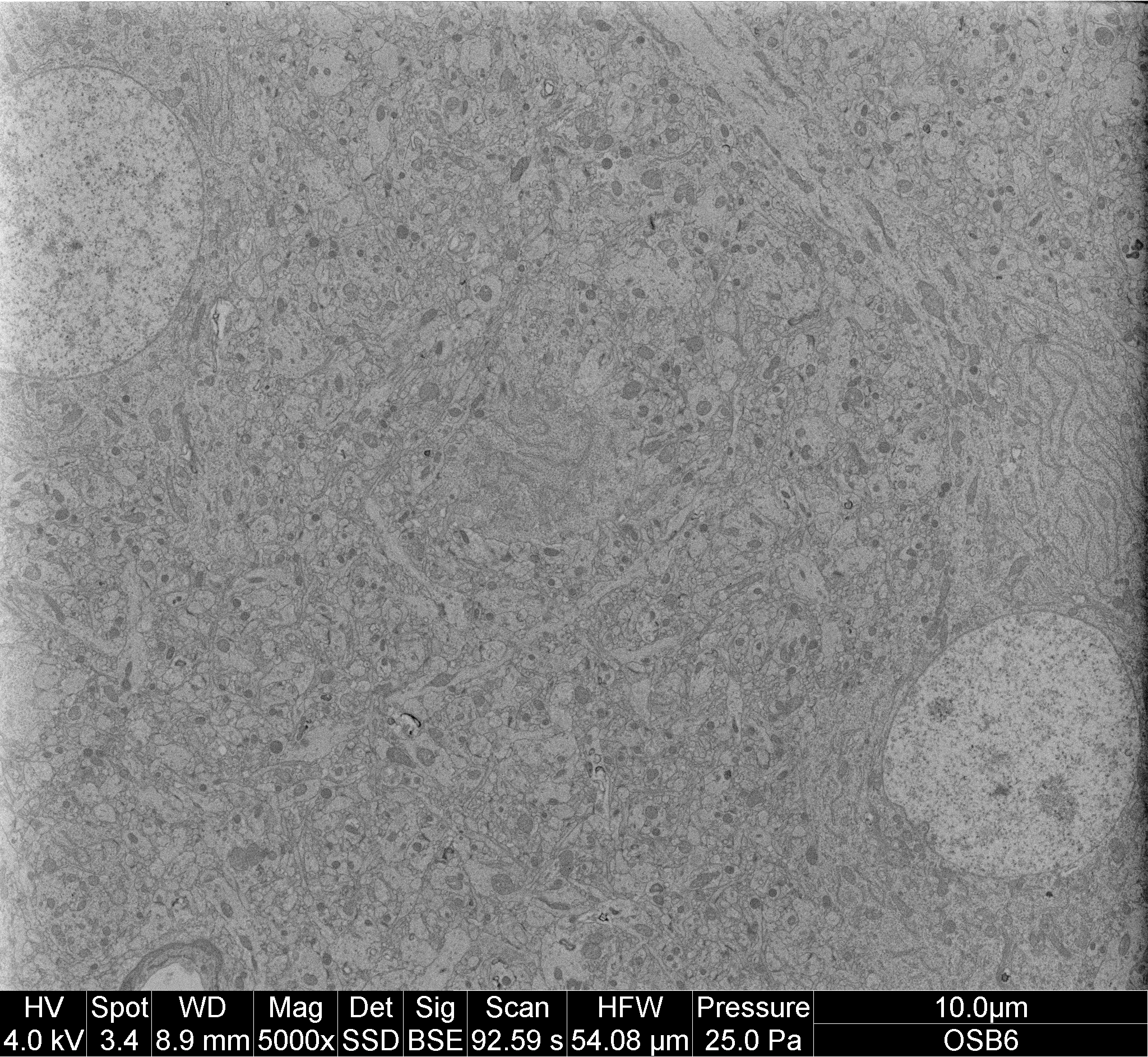

Supplement: Dataset S20 — (254.9 MB ZIP). [file pbio.0020329.sd020.zip › 040604_OS5_st1_1908.tif]

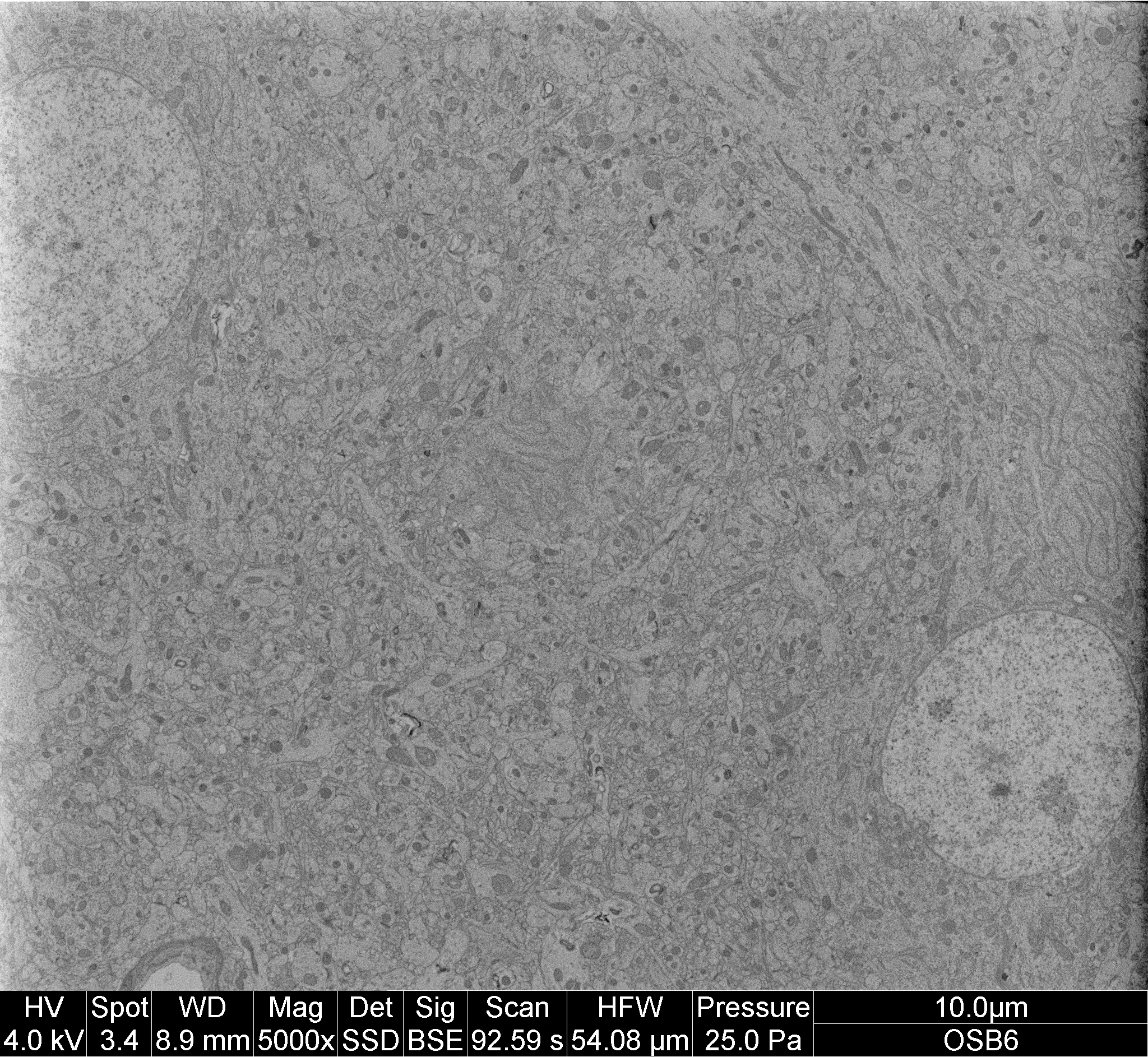

Supplement: Dataset S20 — (254.9 MB ZIP). [file pbio.0020329.sd020.zip › 040604_OS5_st1_1909.tif]

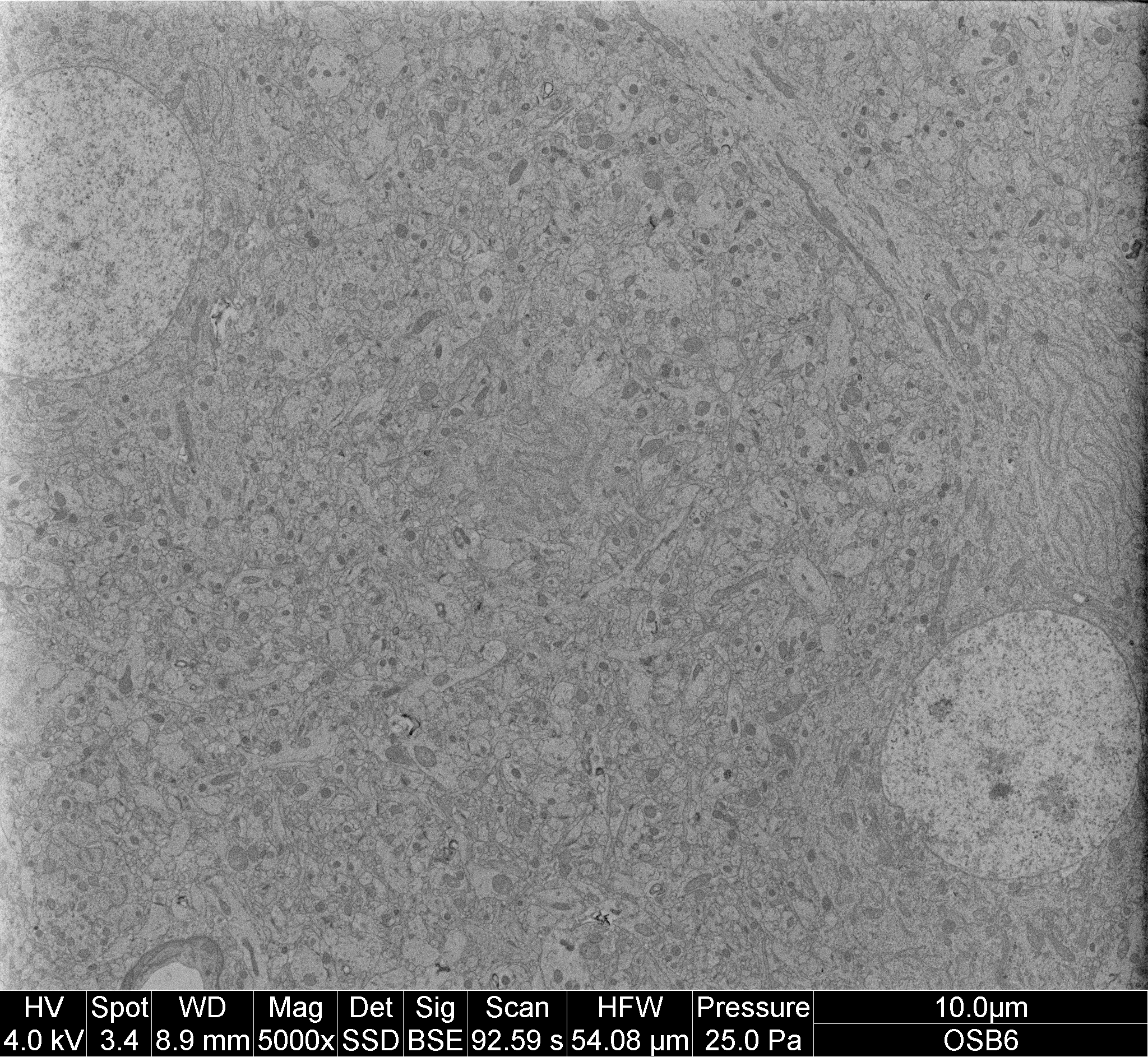

Supplement: Dataset S20 — (254.9 MB ZIP). [file pbio.0020329.sd020.zip › 040604_OS5_st1_1910.tif]

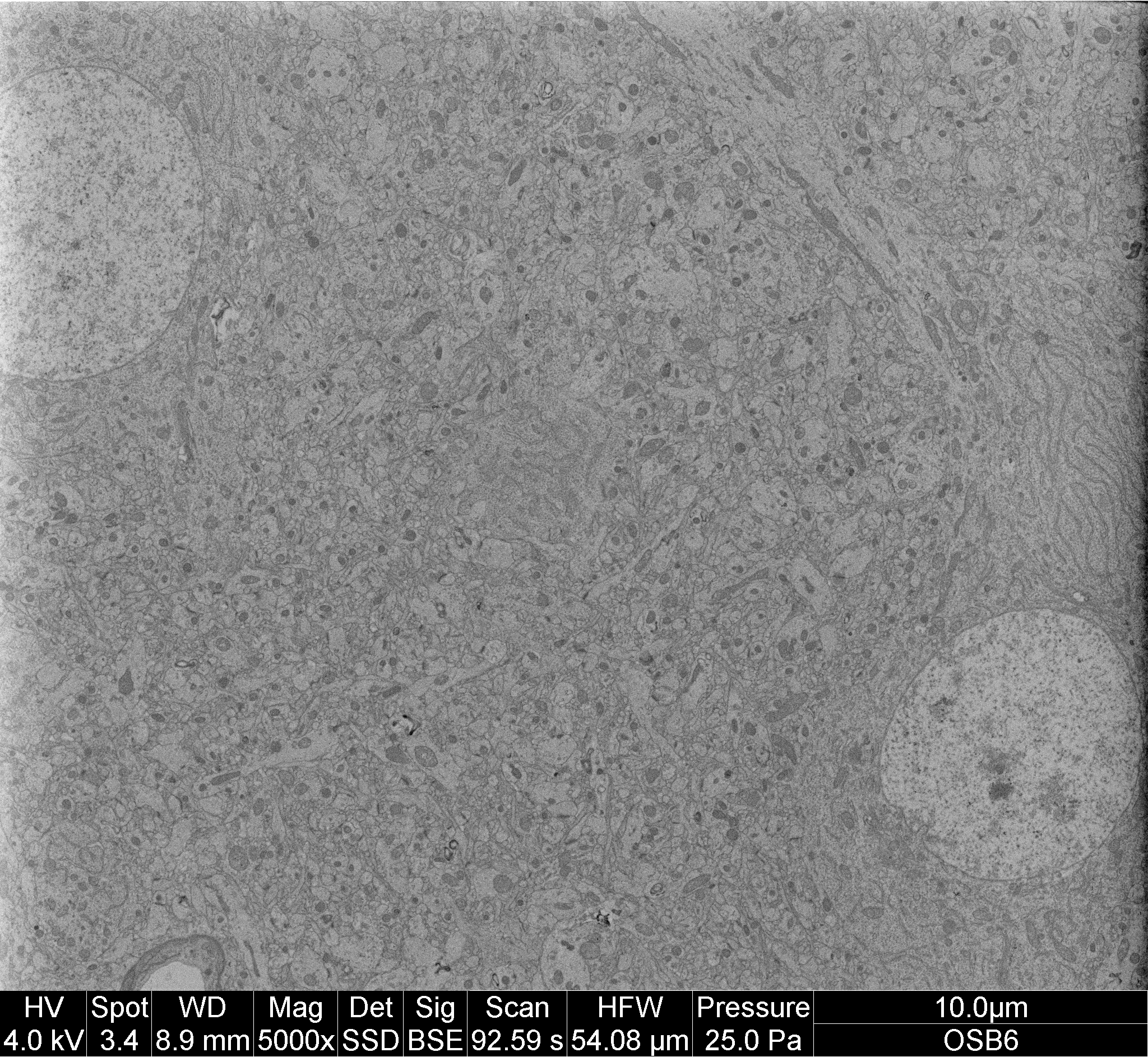

Supplement: Dataset S20 — (254.9 MB ZIP). [file pbio.0020329.sd020.zip › 040604_OS5_st1_1911.tif]

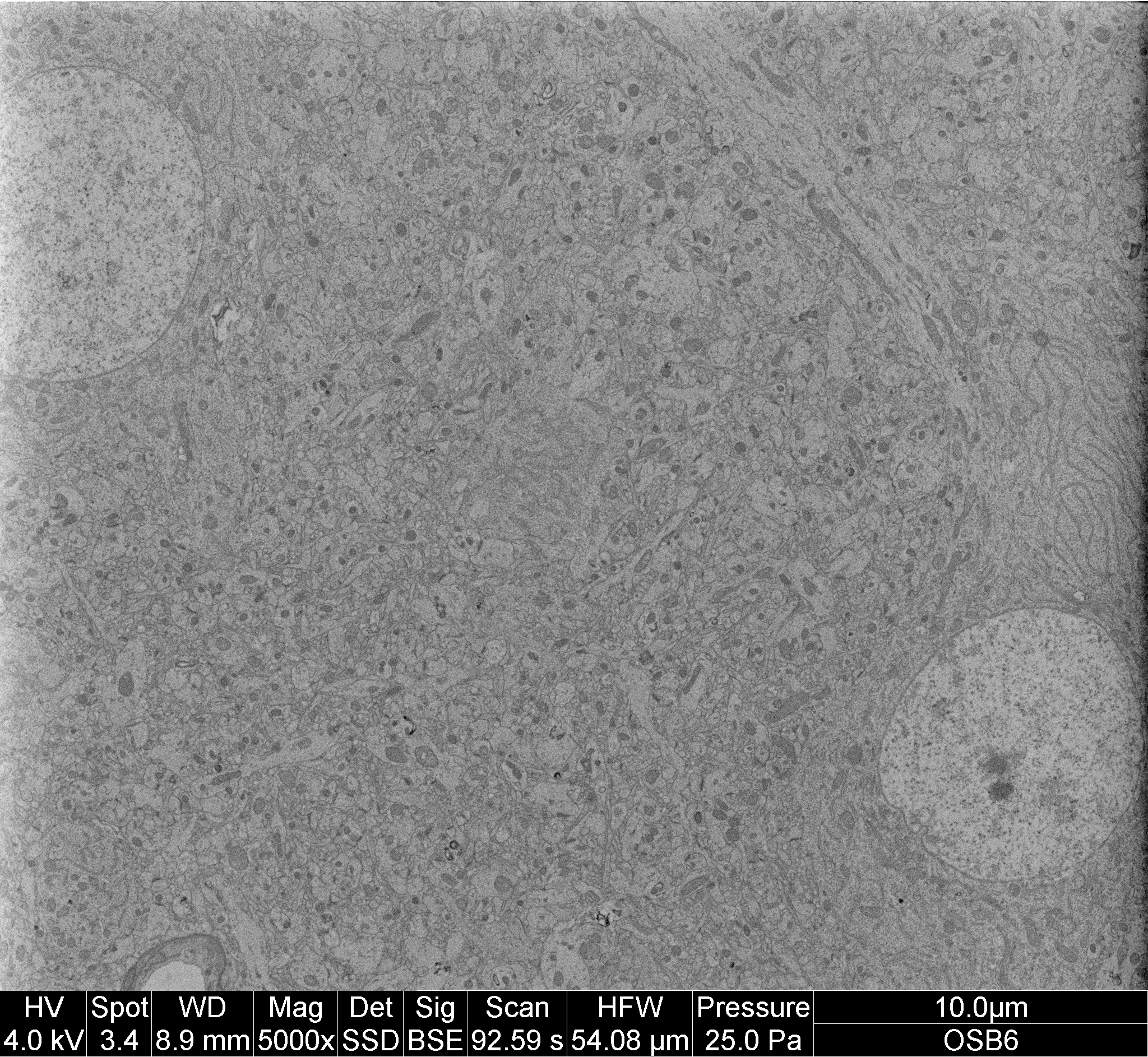

Supplement: Dataset S20 — (254.9 MB ZIP). [file pbio.0020329.sd020.zip › 040604_OS5_st1_1912.tif]

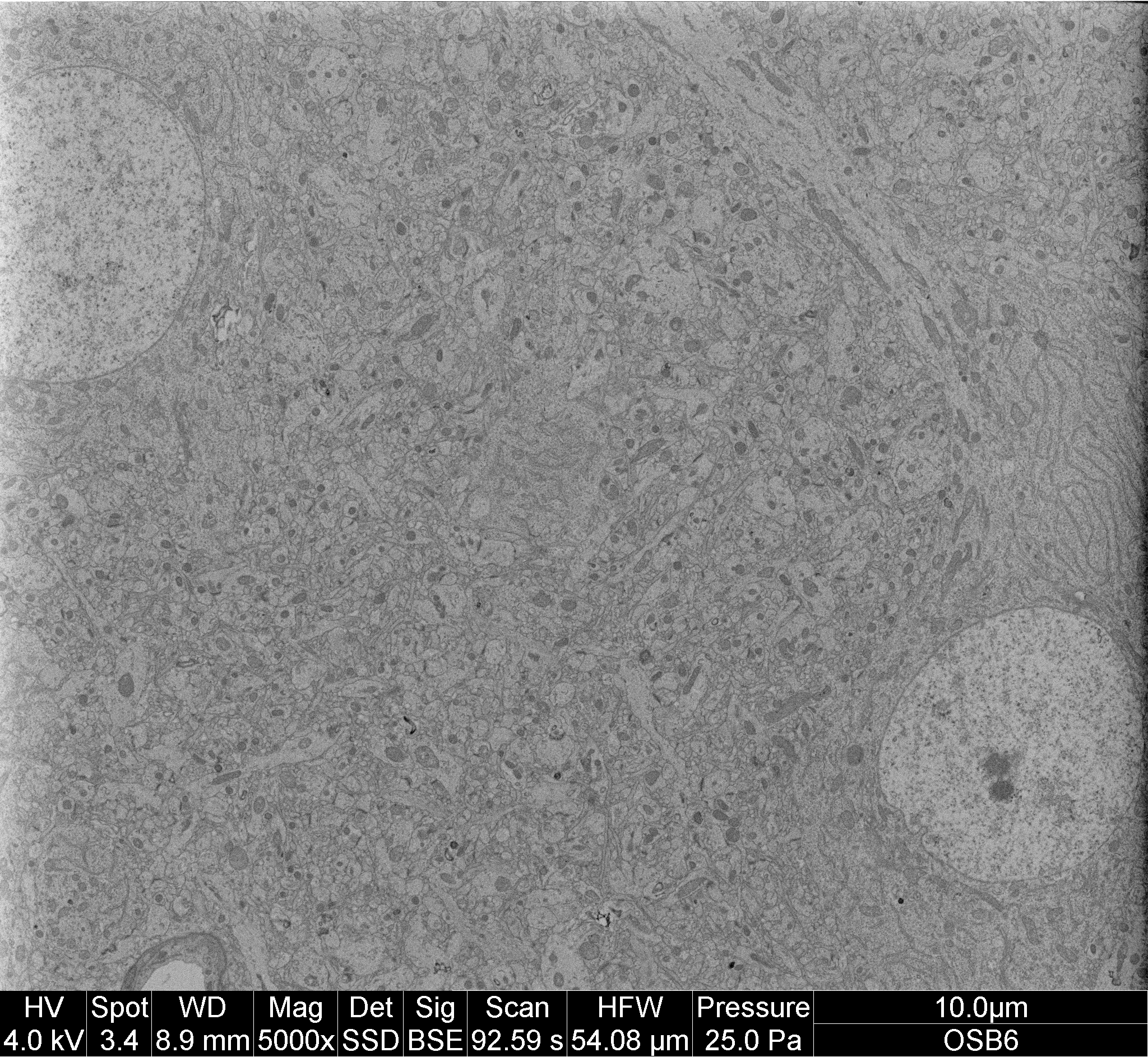

Supplement: Dataset S20 — (254.9 MB ZIP). [file pbio.0020329.sd020.zip › 040604_OS5_st1_1913.tif]

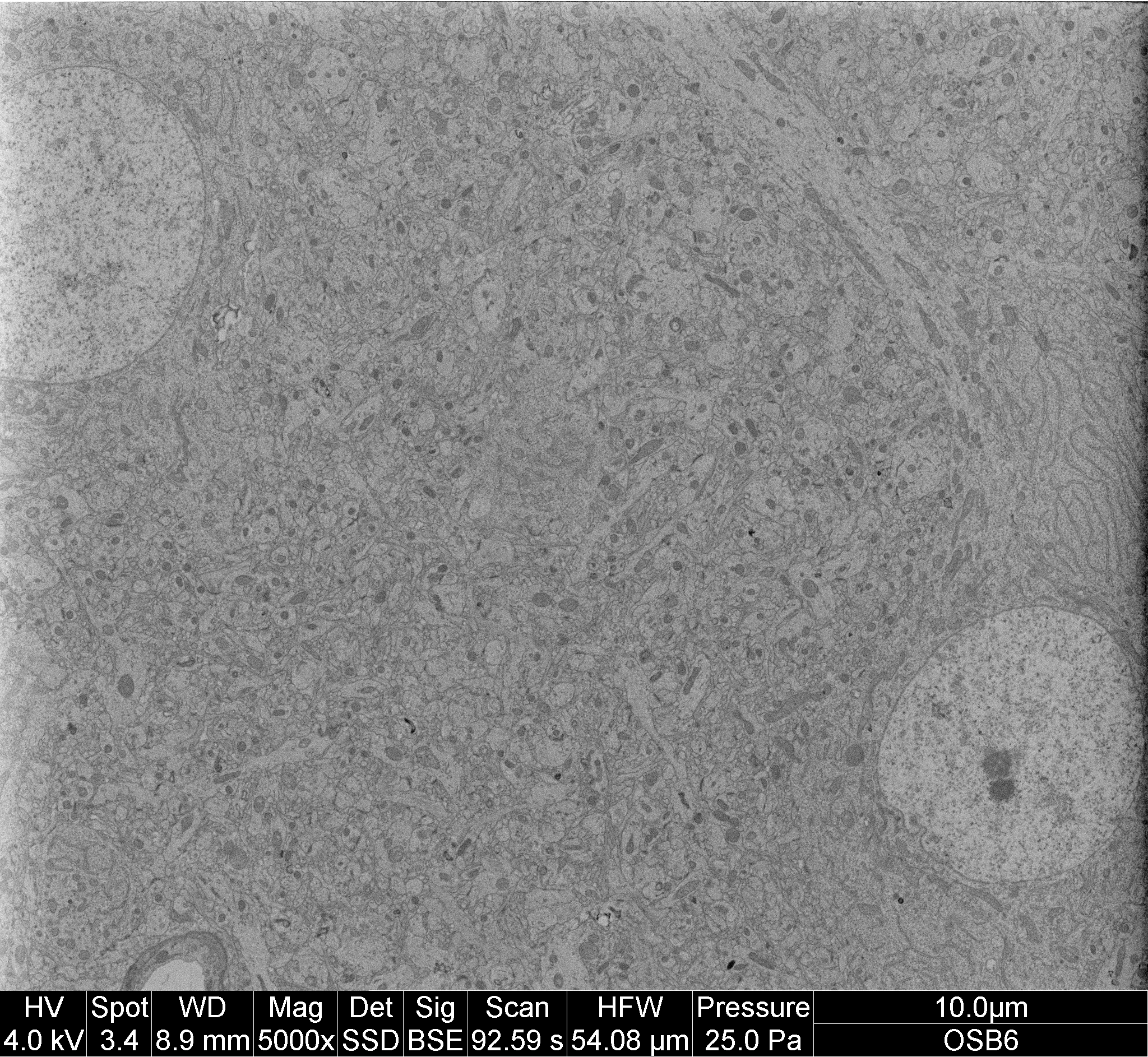

Supplement: Dataset S20 — (254.9 MB ZIP). [file pbio.0020329.sd020.zip › 040604_OS5_st1_1914.tif]

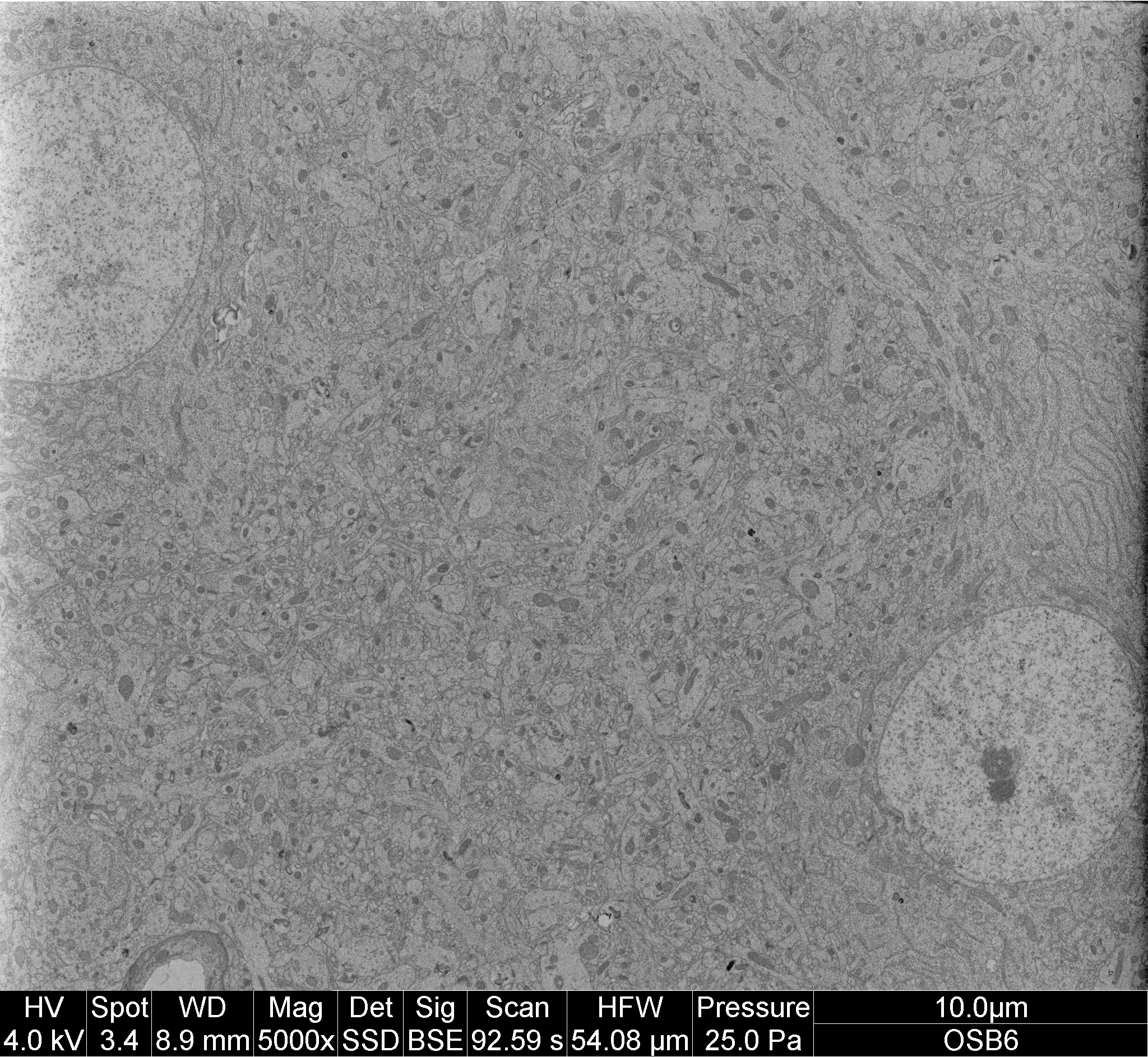

Supplement: Dataset S20 — (254.9 MB ZIP). [file pbio.0020329.sd020.zip › 040604_OS5_st1_1915.tif]

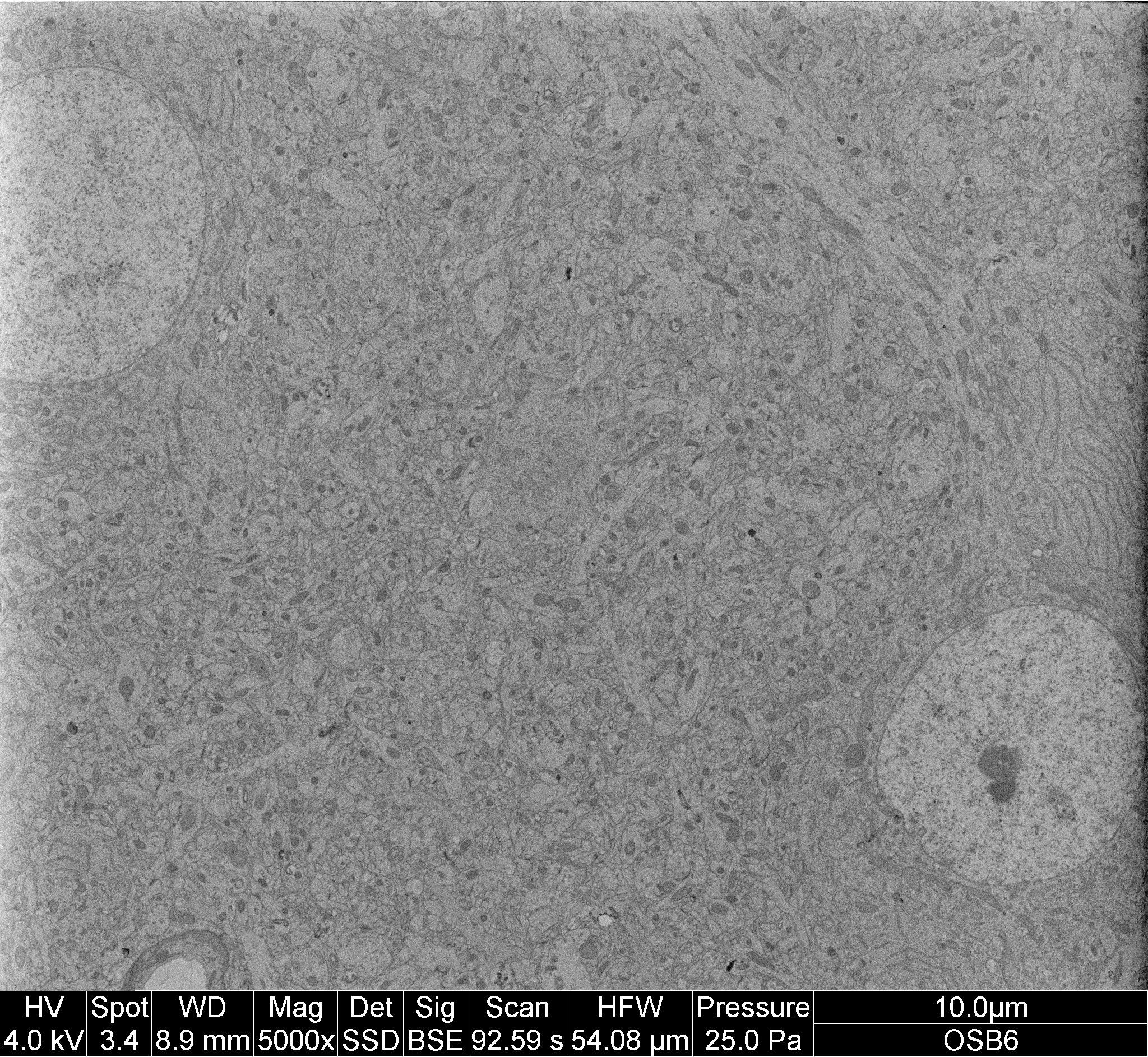

Supplement: Dataset S20 — (254.9 MB ZIP). [file pbio.0020329.sd020.zip › 040604_OS5_st1_1916.tif]

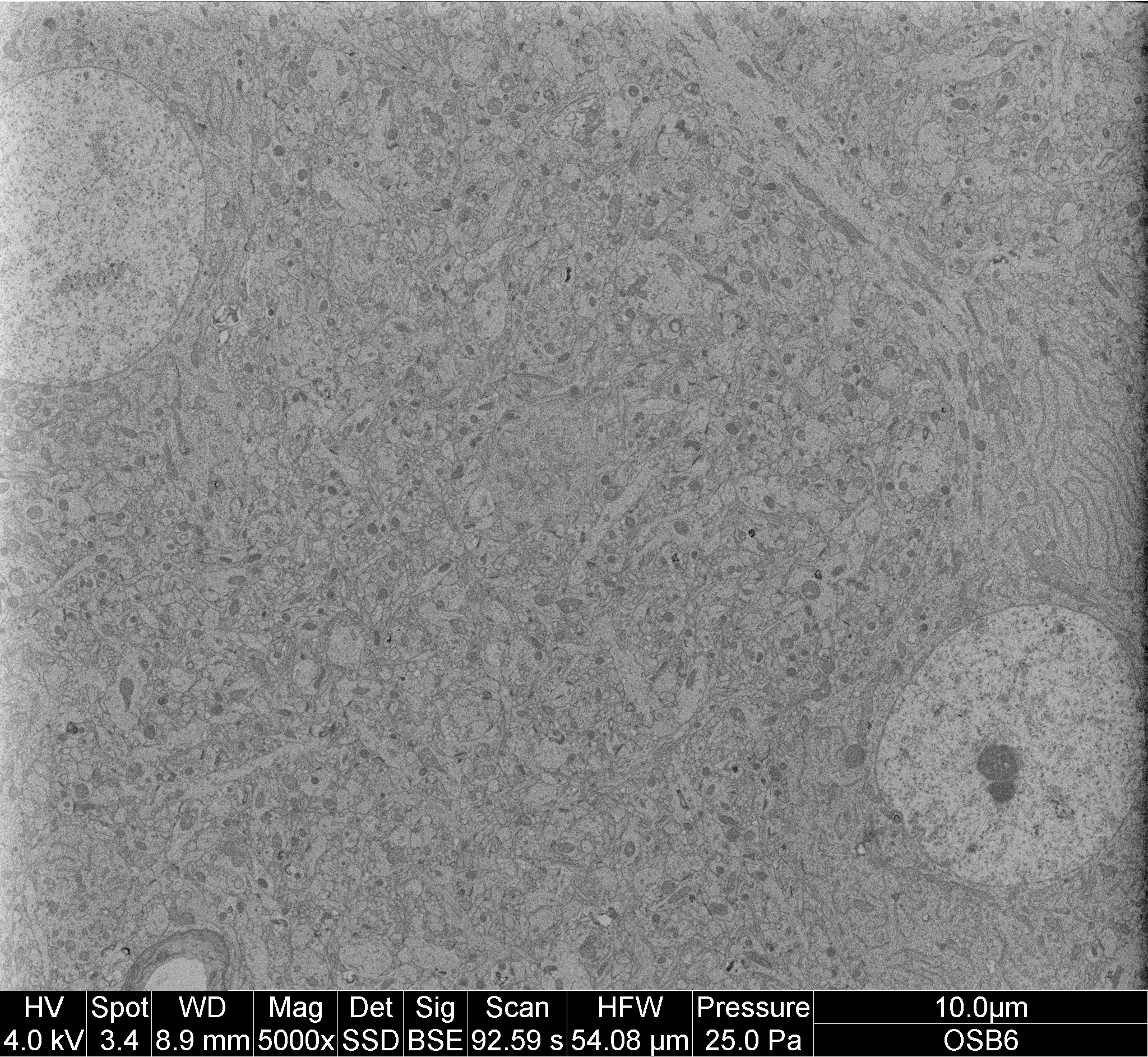

Supplement: Dataset S20 — (254.9 MB ZIP). [file pbio.0020329.sd020.zip › 040604_OS5_st1_1917.tif]

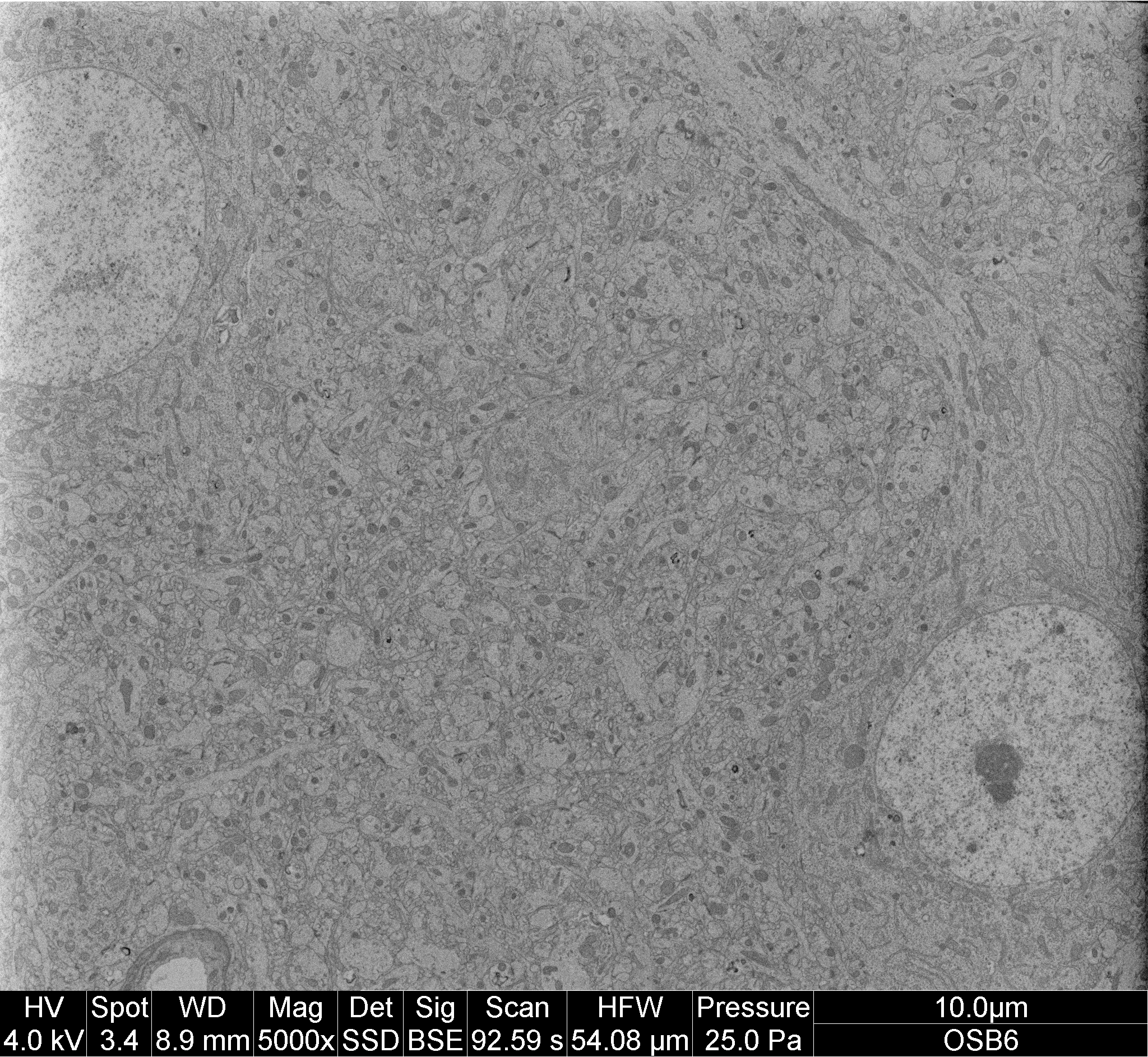

Supplement: Dataset S20 — (254.9 MB ZIP). [file pbio.0020329.sd020.zip › 040604_OS5_st1_1918.tif]

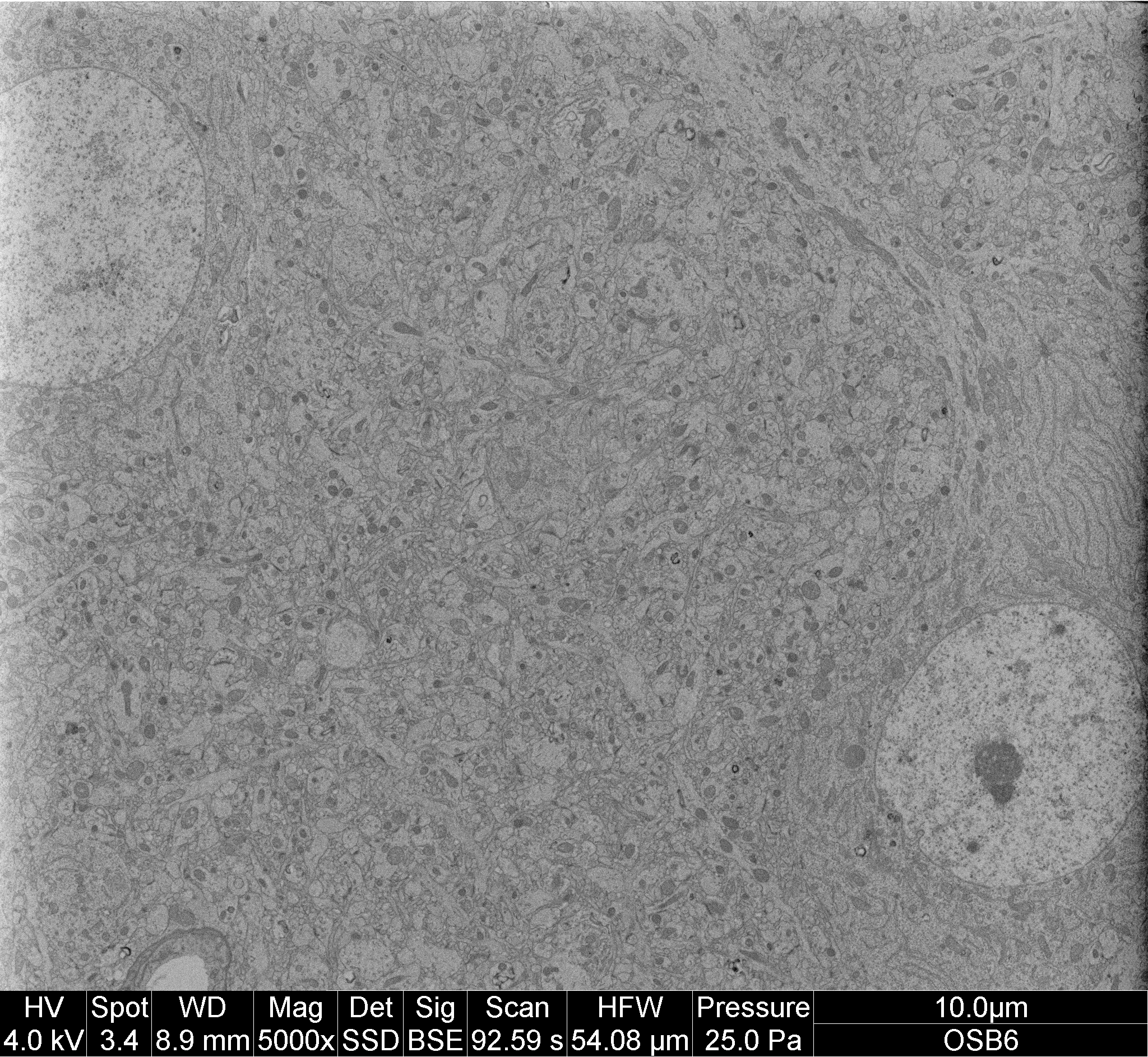

Supplement: Dataset S20 — (254.9 MB ZIP). [file pbio.0020329.sd020.zip › 040604_OS5_st1_1919.tif]

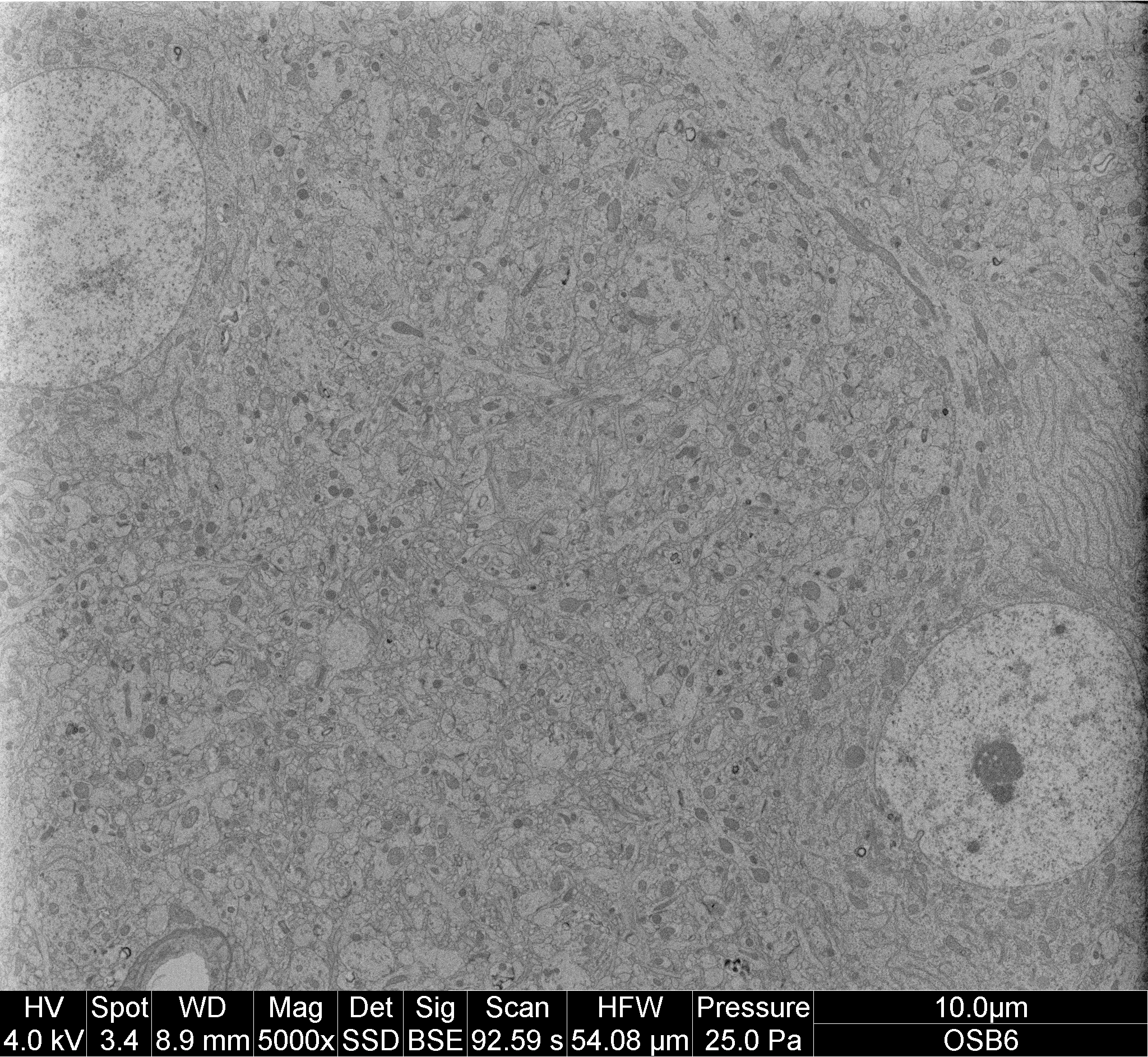

Supplement: Dataset S20 — (254.9 MB ZIP). [file pbio.0020329.sd020.zip › 040604_OS5_st1_1920.tif]

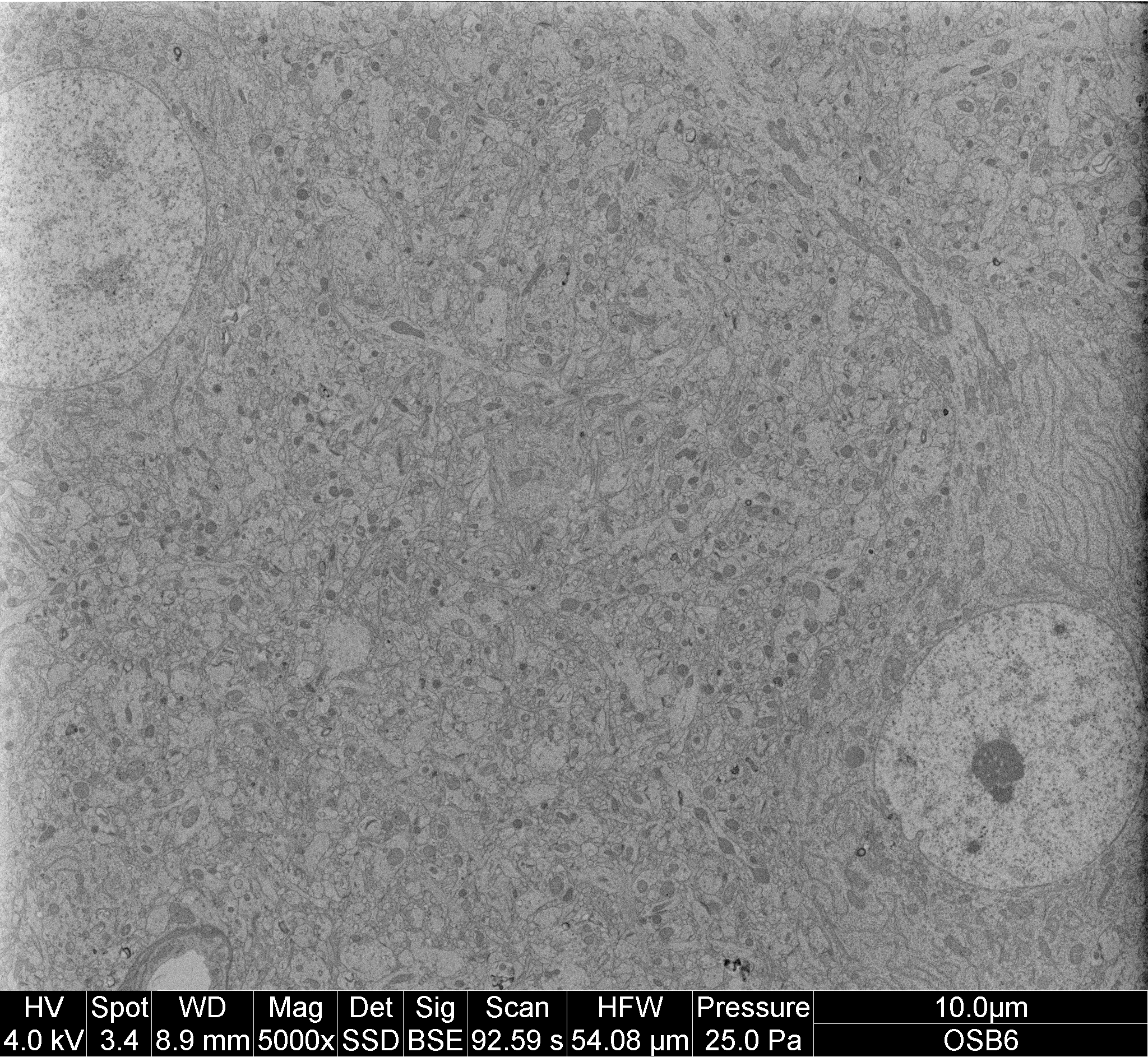

Supplement: Dataset S20 — (254.9 MB ZIP). [file pbio.0020329.sd020.zip › 040604_OS5_st1_1921.tif]

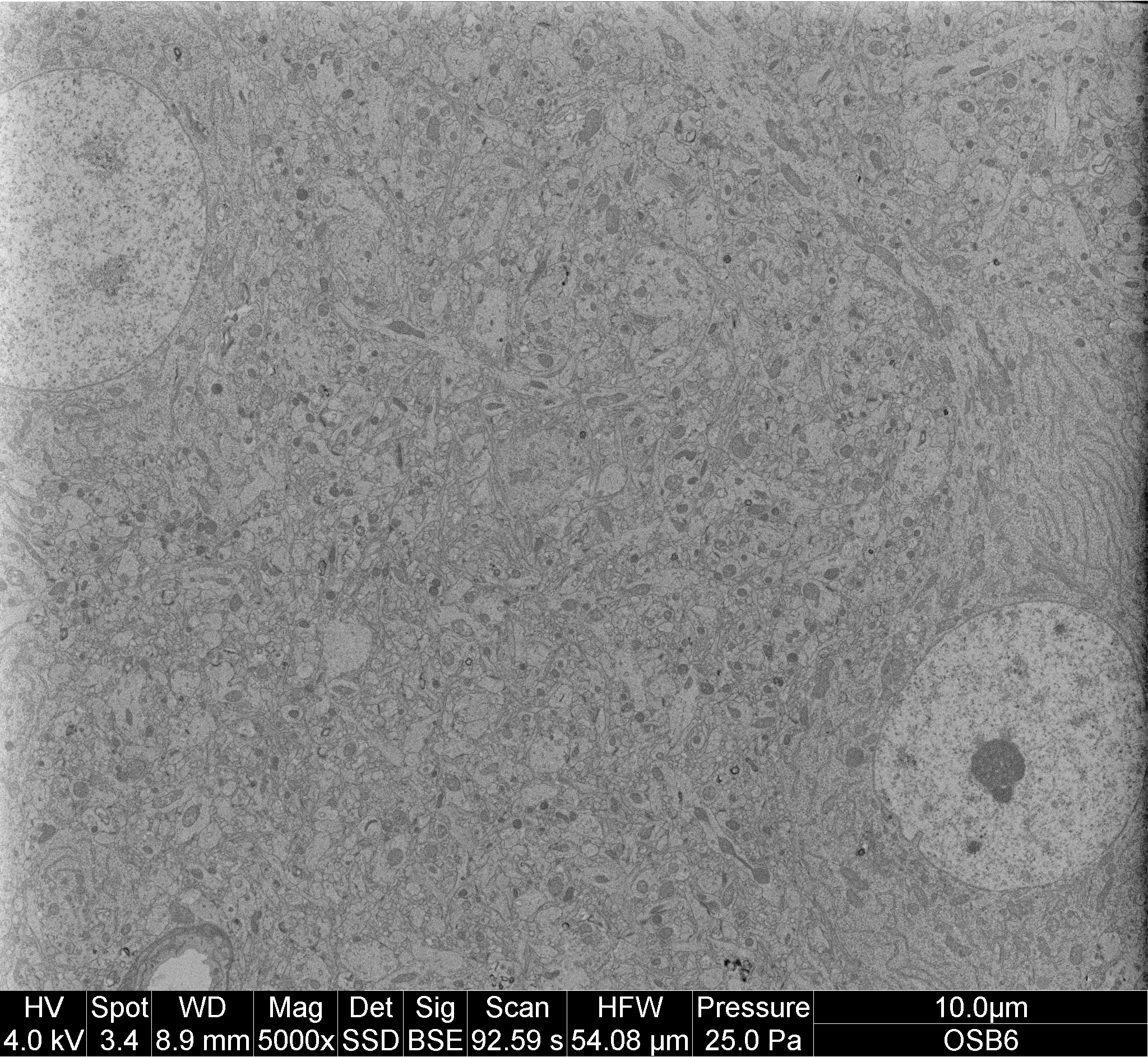

Supplement: Dataset S20 — (254.9 MB ZIP). [file pbio.0020329.sd020.zip › 040604_OS5_st1_1922.tif]

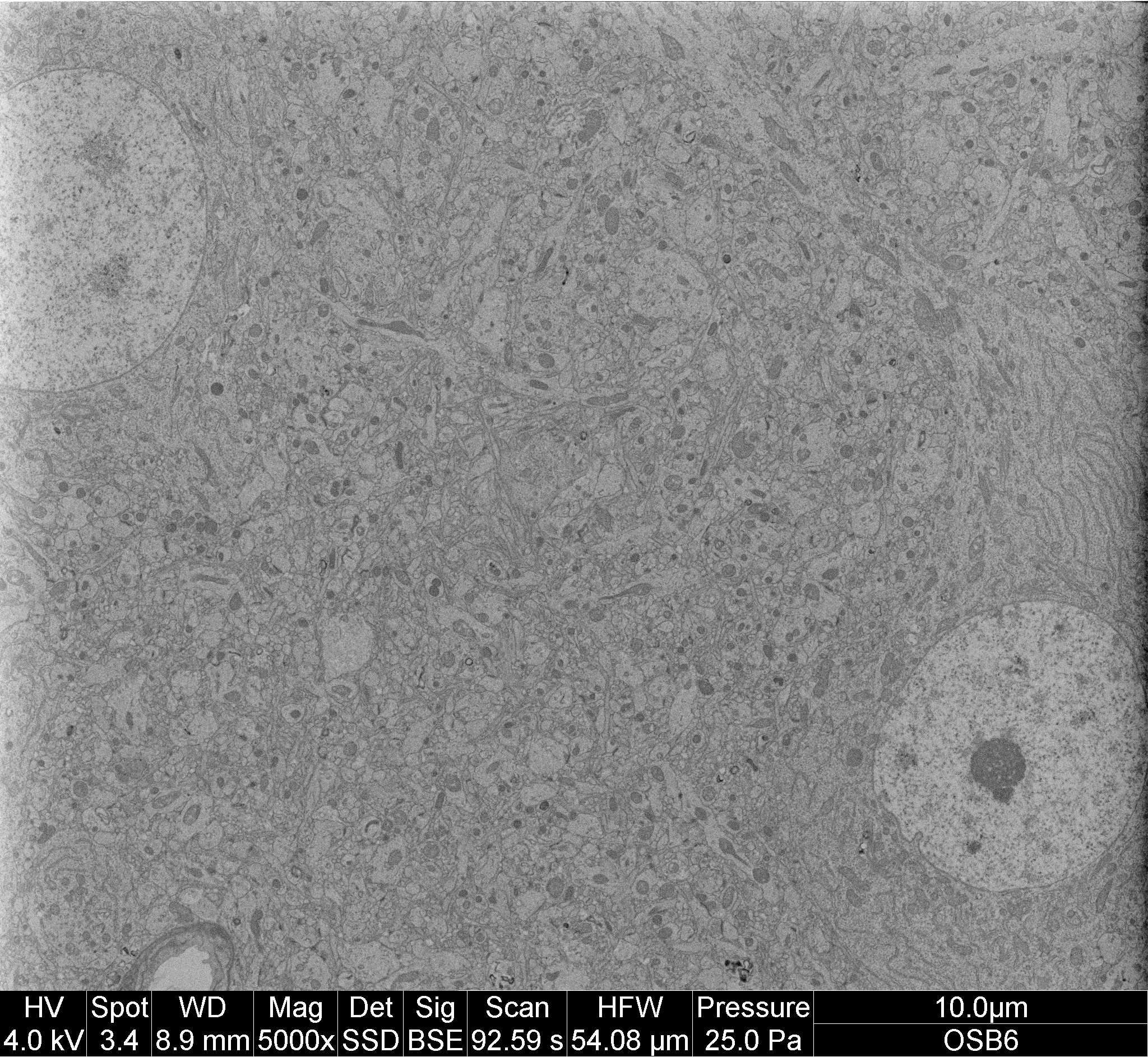

Supplement: Dataset S20 — (254.9 MB ZIP). [file pbio.0020329.sd020.zip › 040604_OS5_st1_1923.tif]

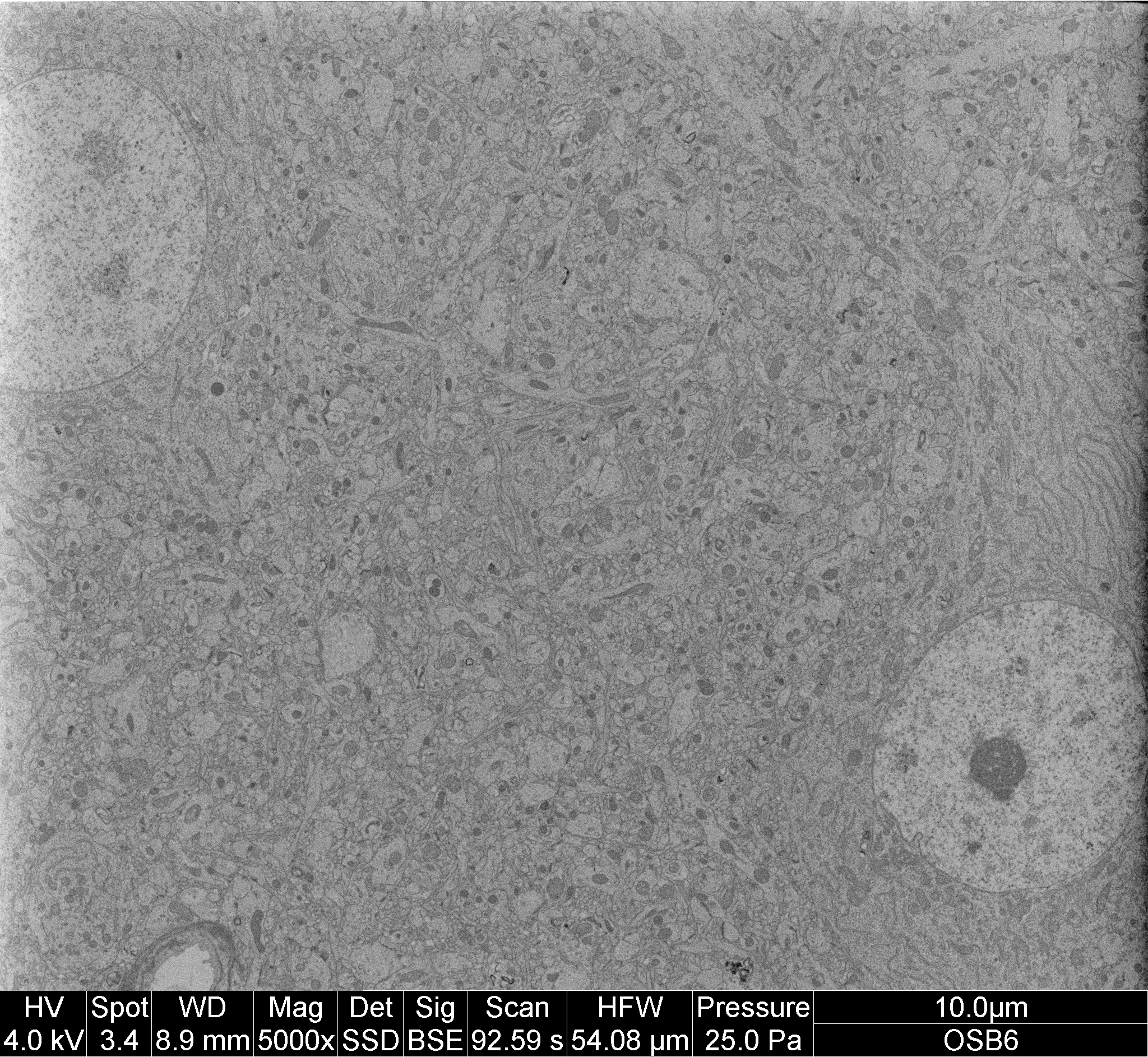

Supplement: Dataset S20 — (254.9 MB ZIP). [file pbio.0020329.sd020.zip › 040604_OS5_st1_1924.tif]

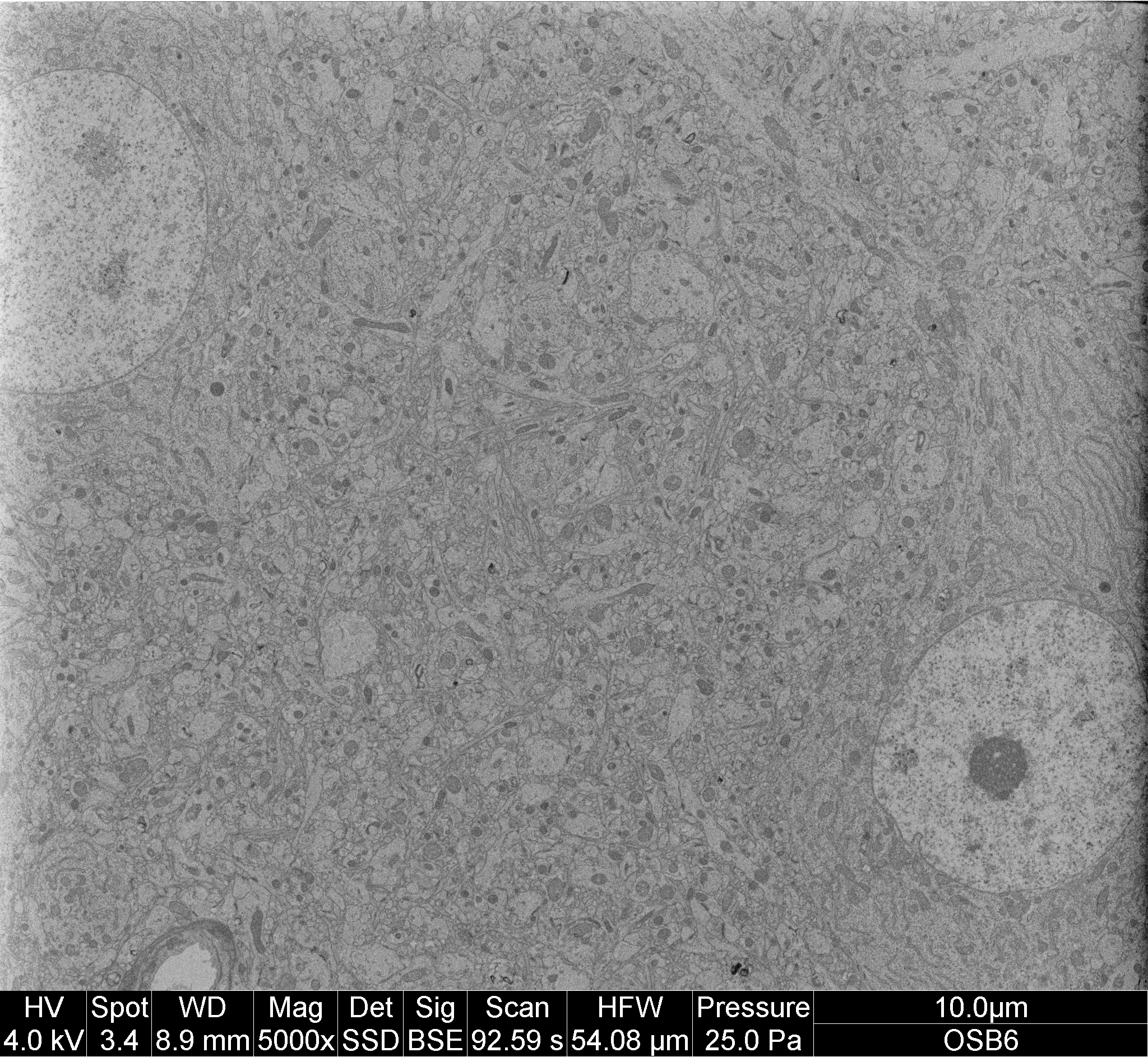

Supplement: Dataset S20 — (254.9 MB ZIP). [file pbio.0020329.sd020.zip › 040604_OS5_st1_1925.tif]

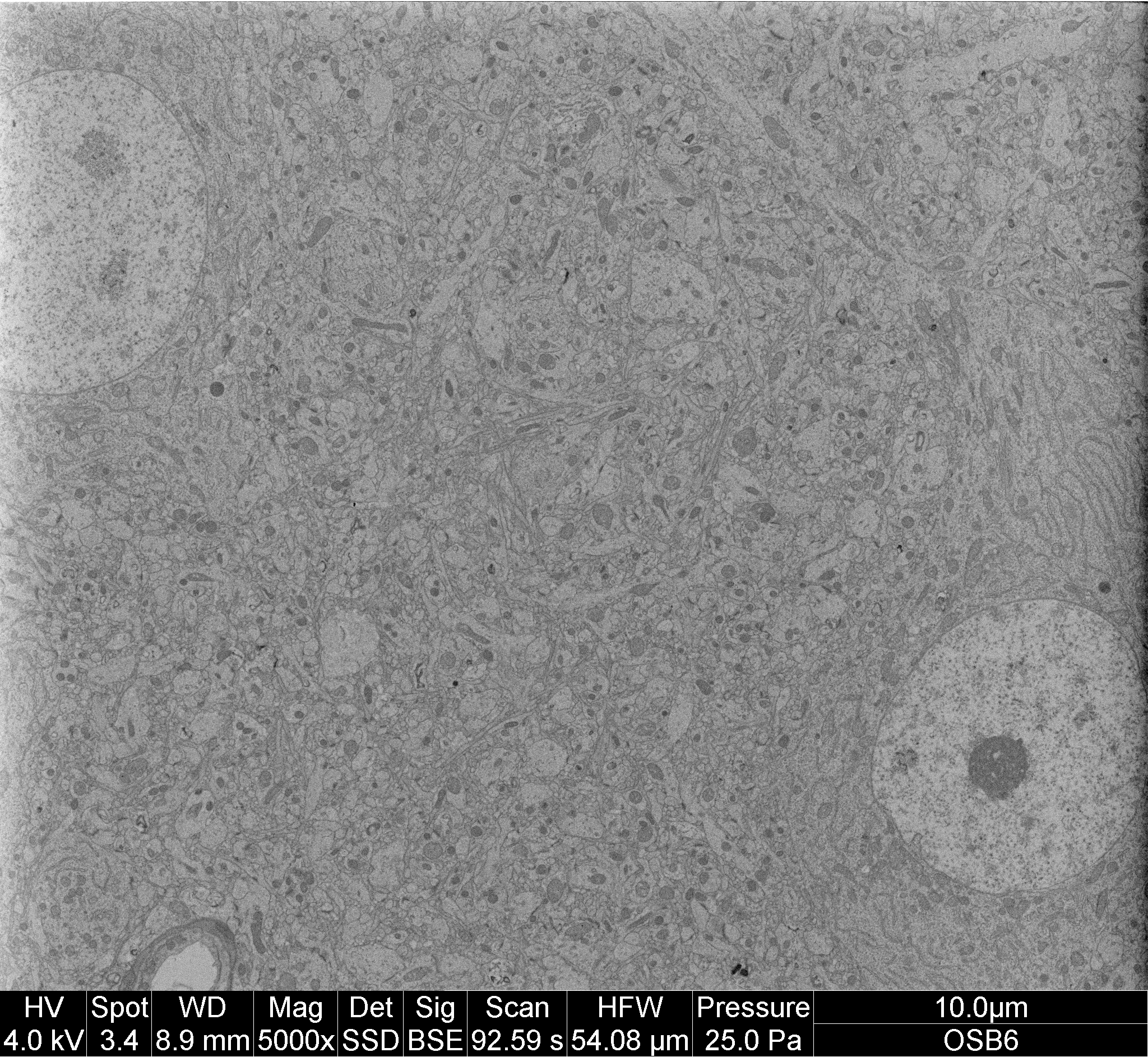

Supplement: Dataset S20 — (254.9 MB ZIP). [file pbio.0020329.sd020.zip › 040604_OS5_st1_1926.tif]

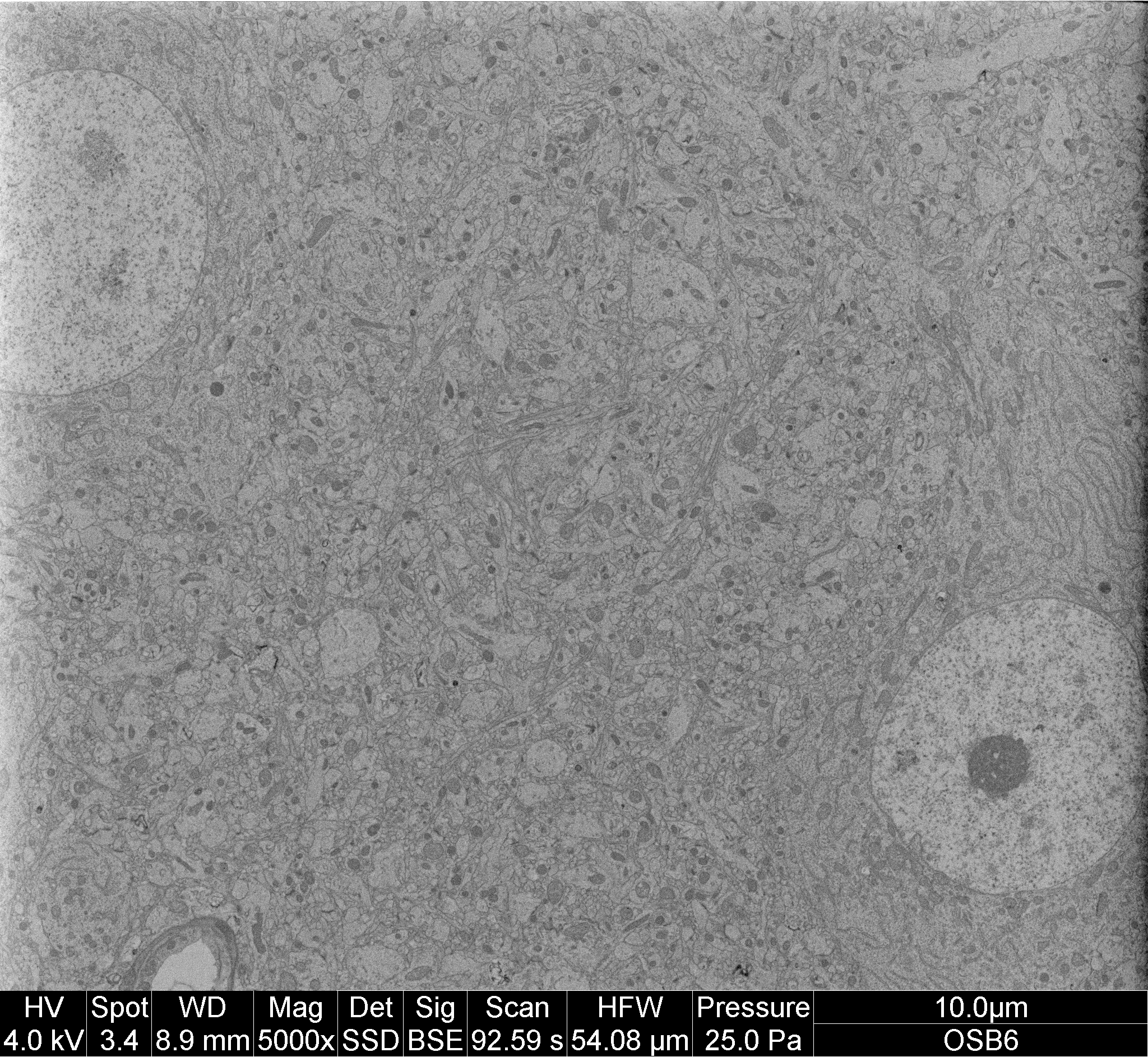

Supplement: Dataset S20 — (254.9 MB ZIP). [file pbio.0020329.sd020.zip › 040604_OS5_st1_1927.tif]

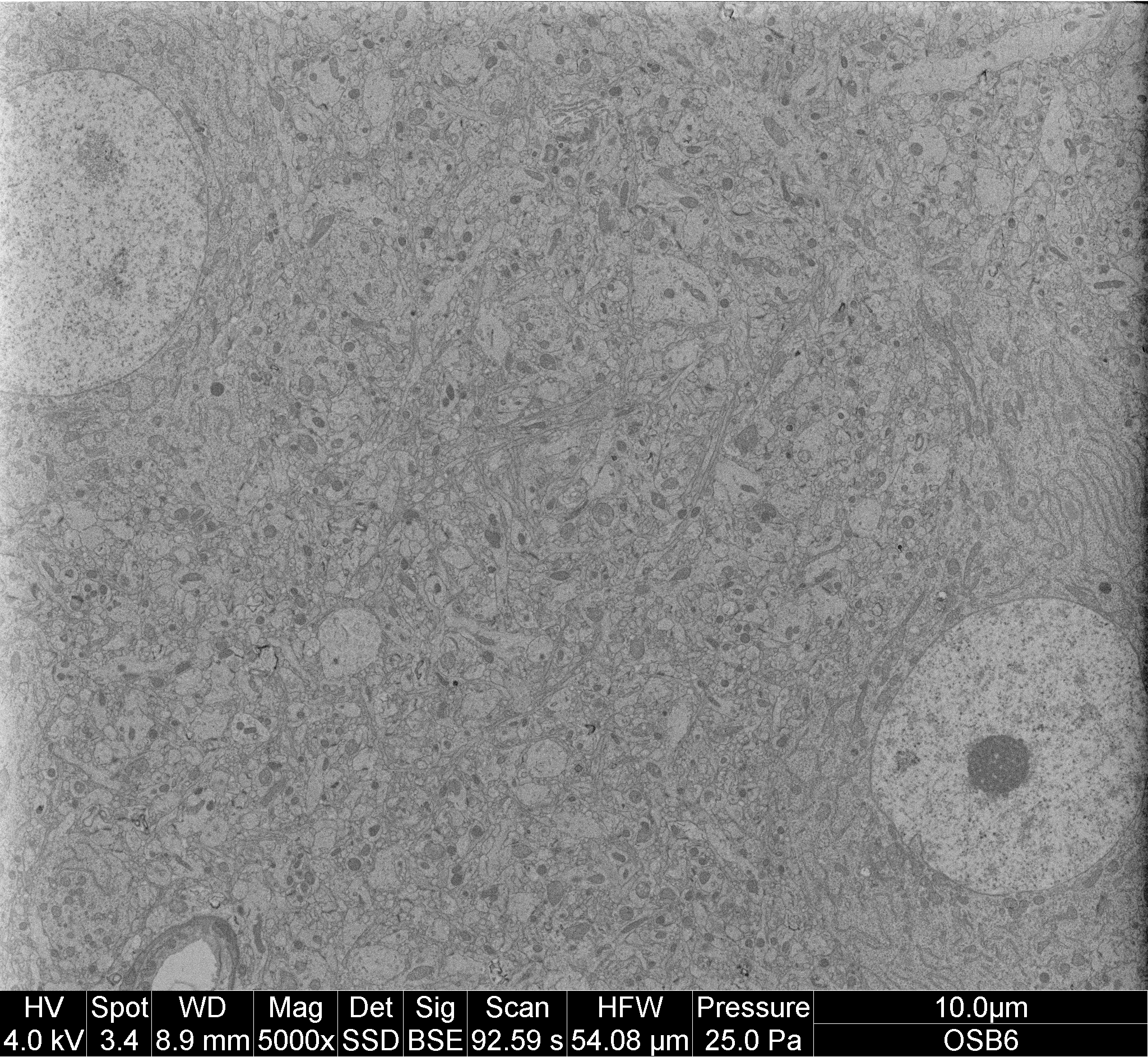

Supplement: Dataset S20 — (254.9 MB ZIP). [file pbio.0020329.sd020.zip › 040604_OS5_st1_1928.tif]

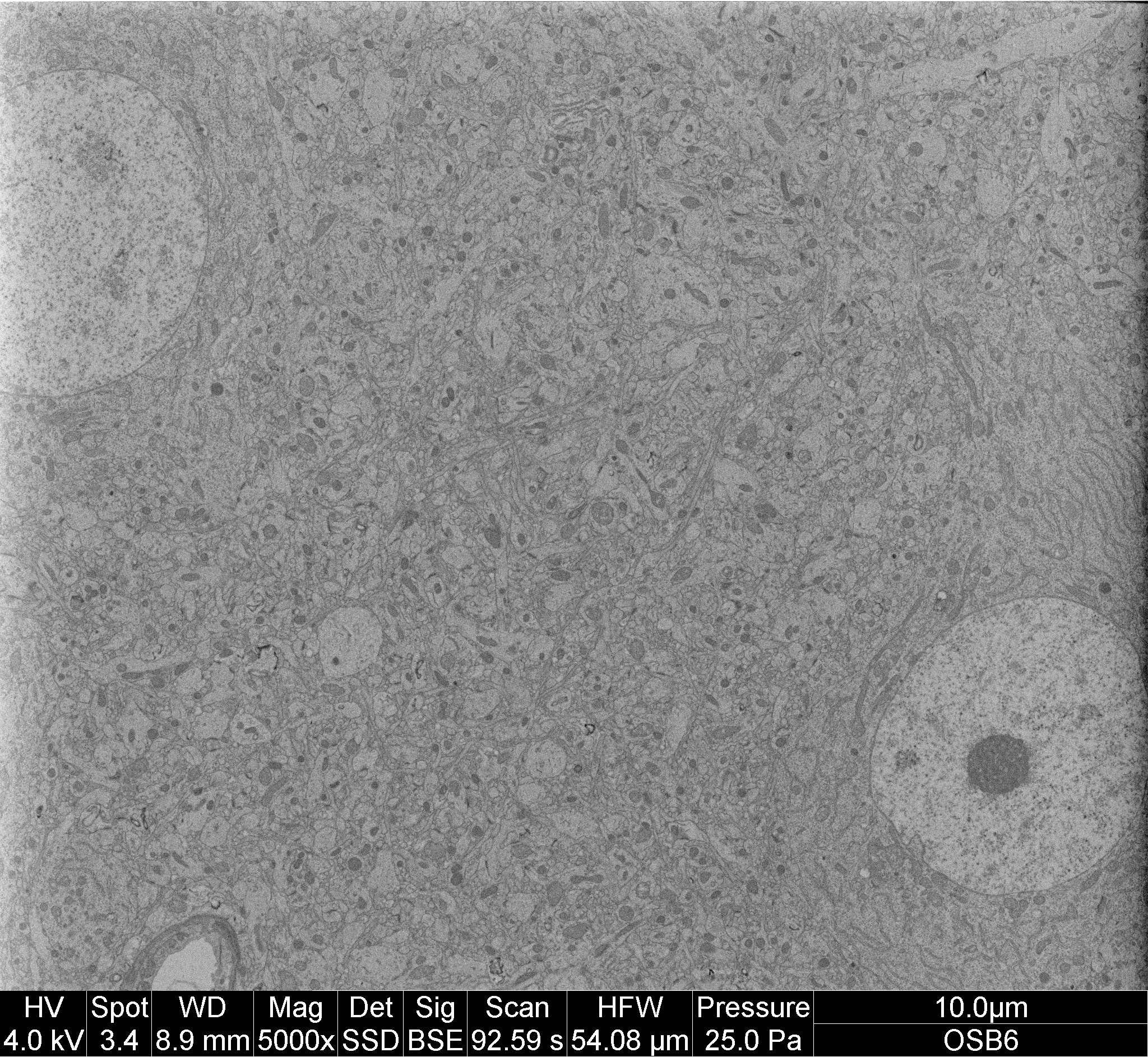

Supplement: Dataset S20 — (254.9 MB ZIP). [file pbio.0020329.sd020.zip › 040604_OS5_st1_1929.tif]

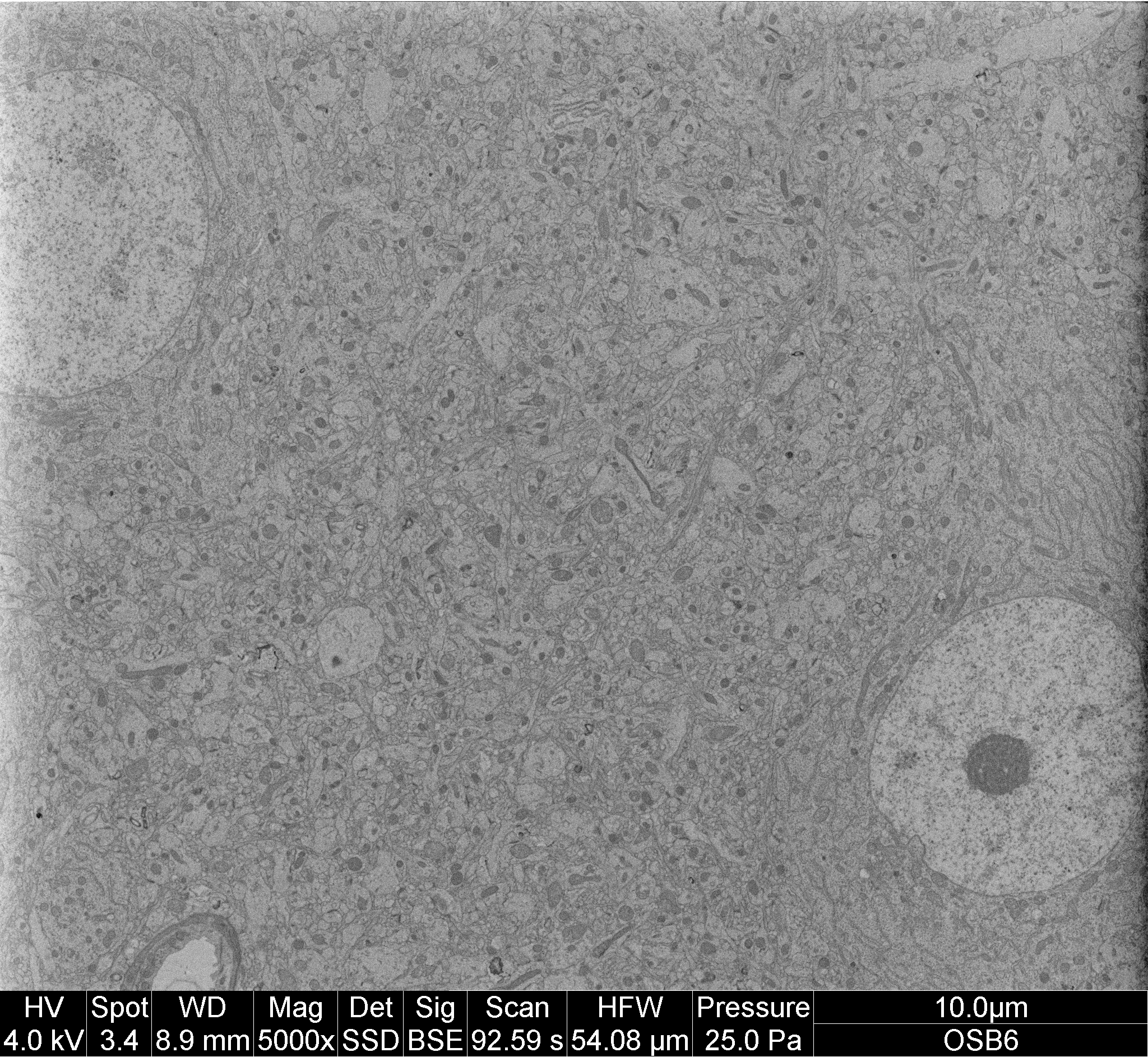

Supplement: Dataset S20 — (254.9 MB ZIP). [file pbio.0020329.sd020.zip › 040604_OS5_st1_1930.tif]

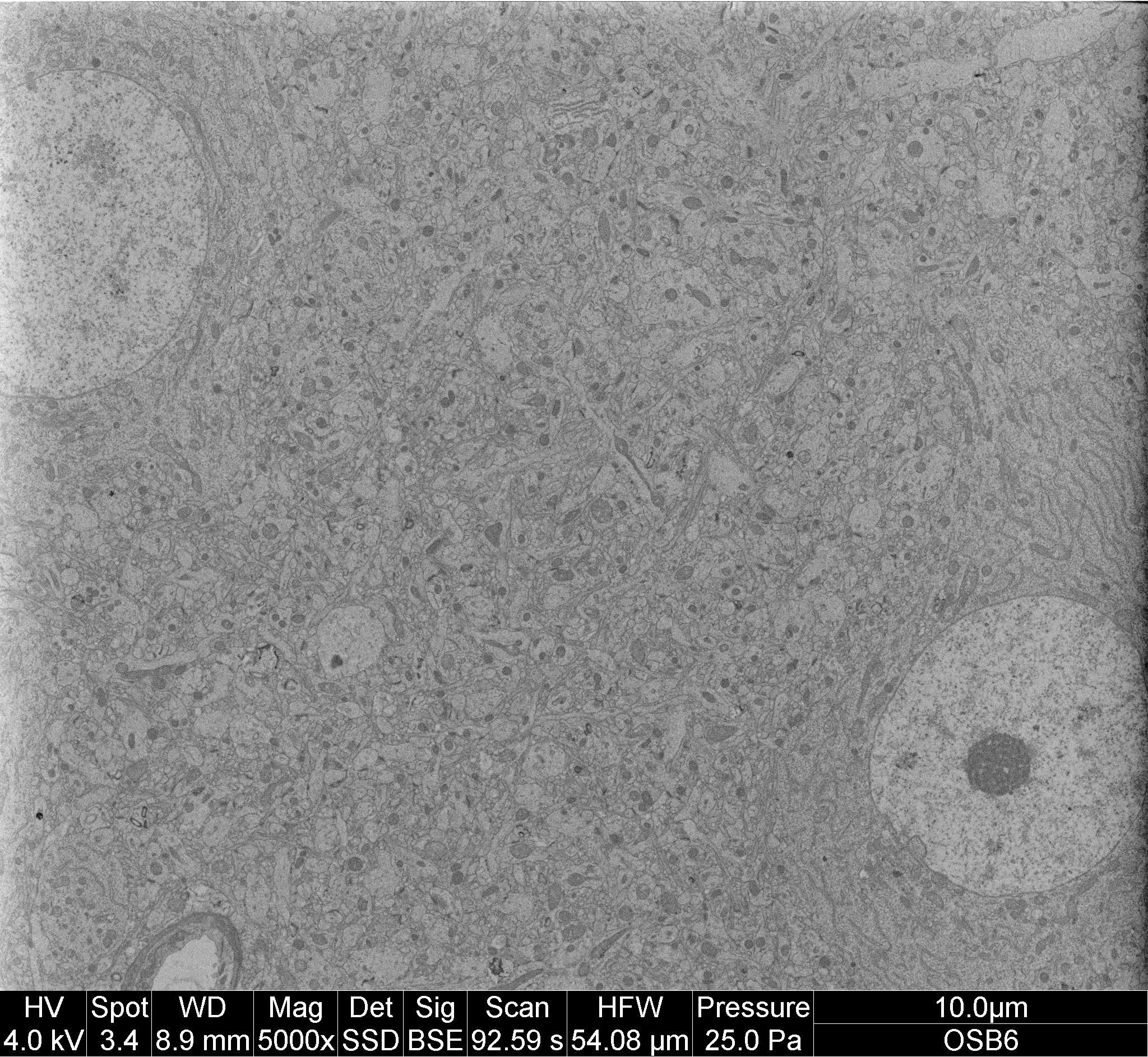

Supplement: Dataset S20 — (254.9 MB ZIP). [file pbio.0020329.sd020.zip › 040604_OS5_st1_1931.tif]

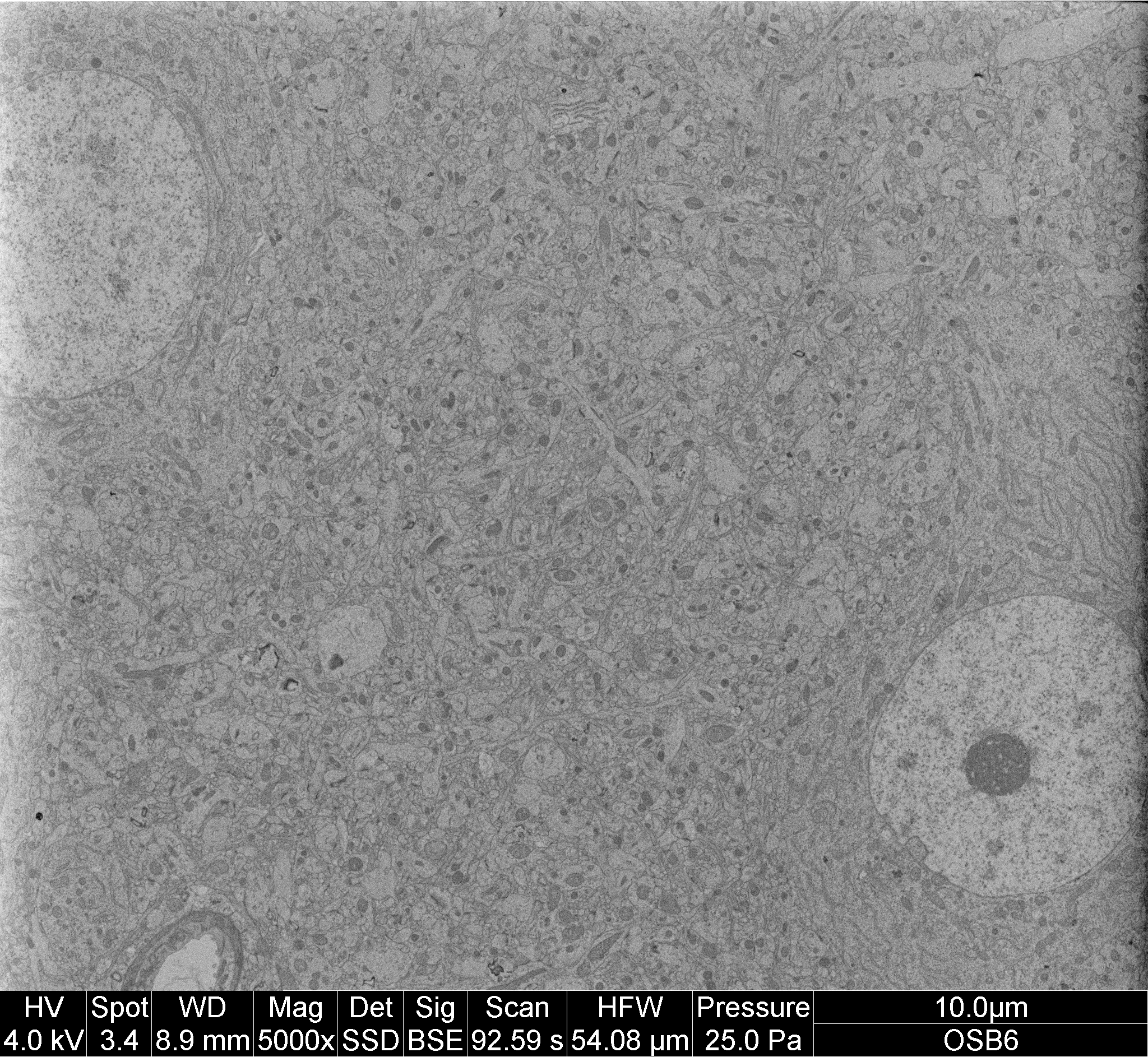

Supplement: Dataset S20 — (254.9 MB ZIP). [file pbio.0020329.sd020.zip › 040604_OS5_st1_1932.tif]

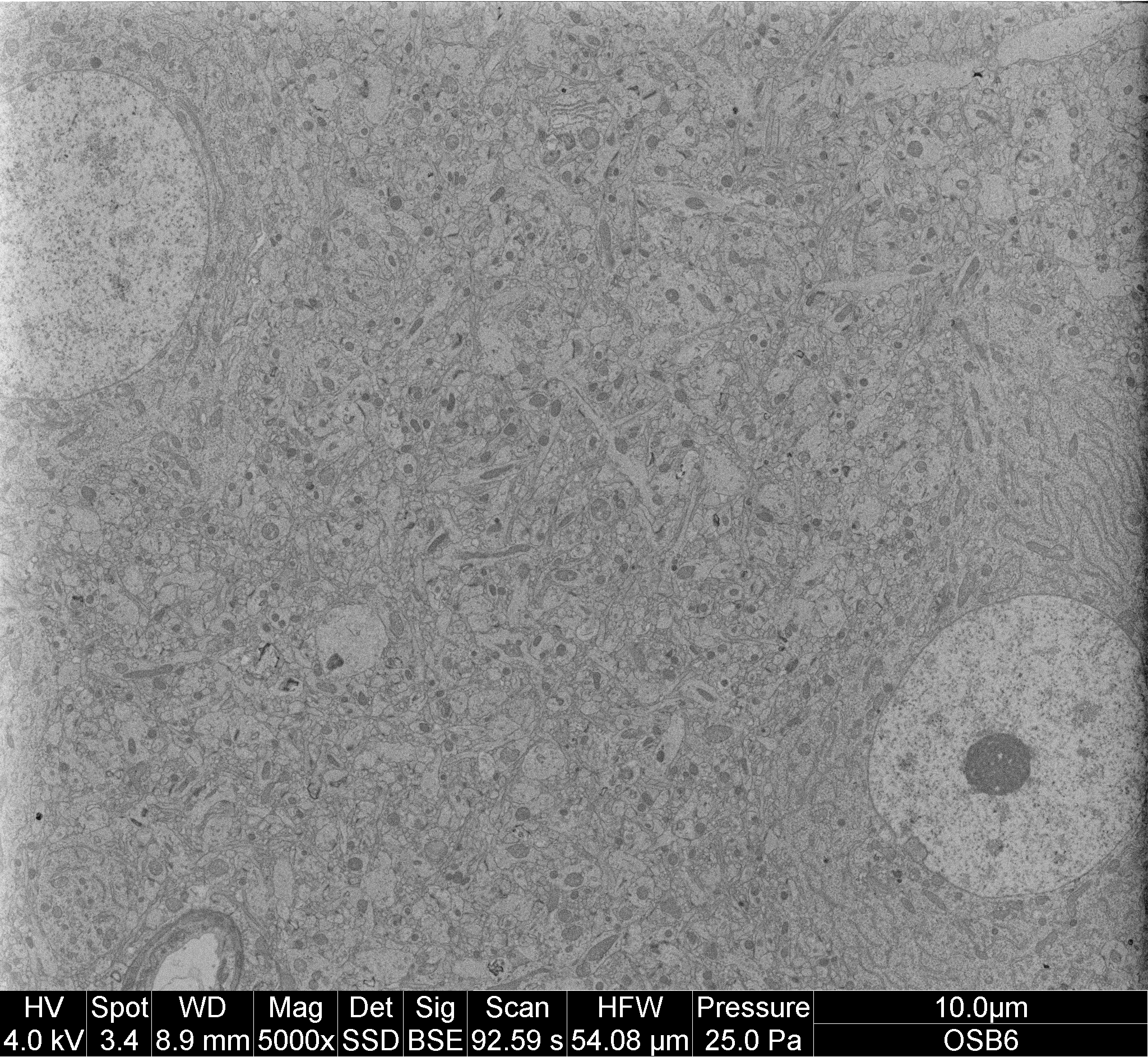

Supplement: Dataset S20 — (254.9 MB ZIP). [file pbio.0020329.sd020.zip › 040604_OS5_st1_1933.tif]

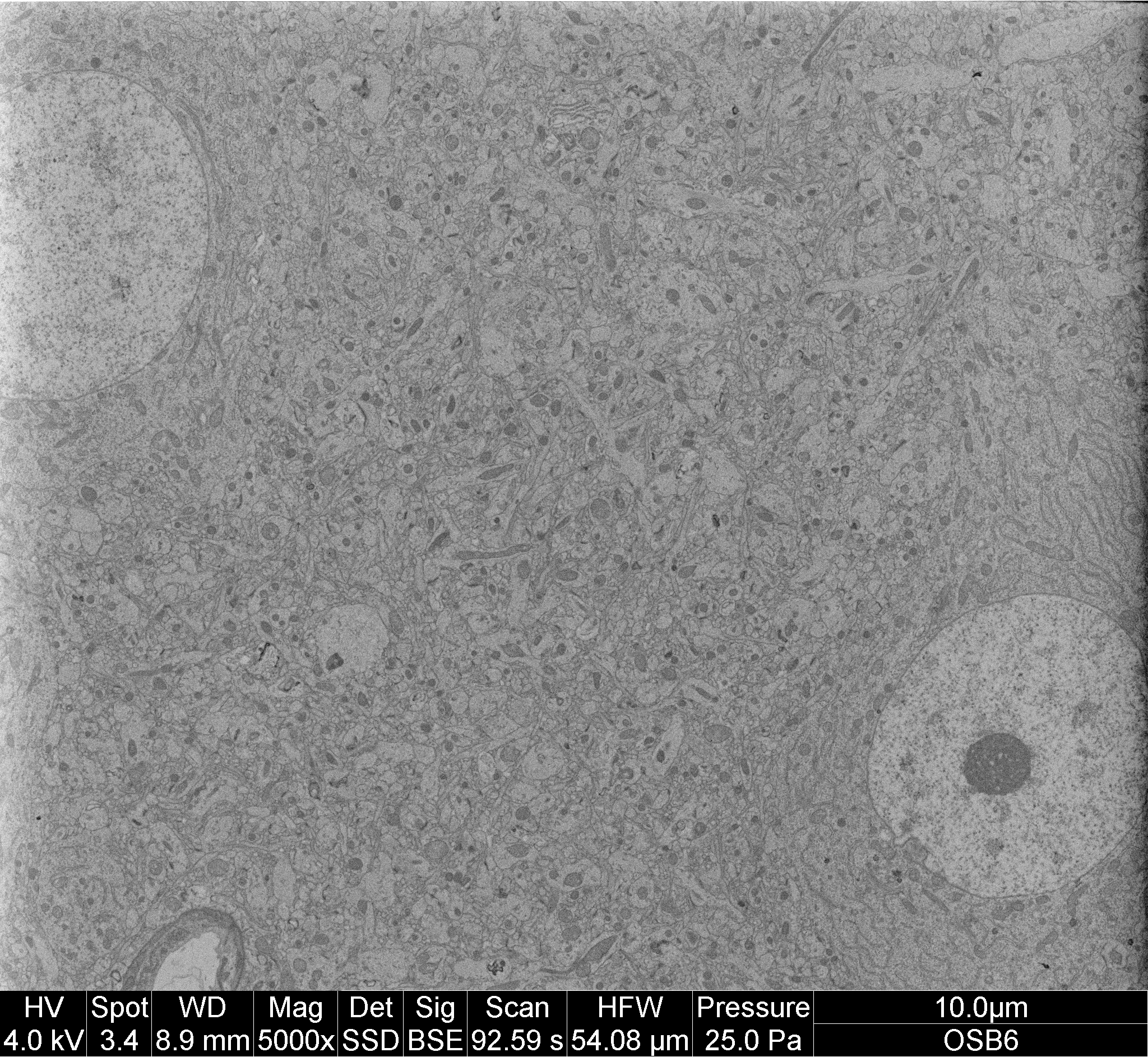

Supplement: Dataset S20 — (254.9 MB ZIP). [file pbio.0020329.sd020.zip › 040604_OS5_st1_1934.tif]

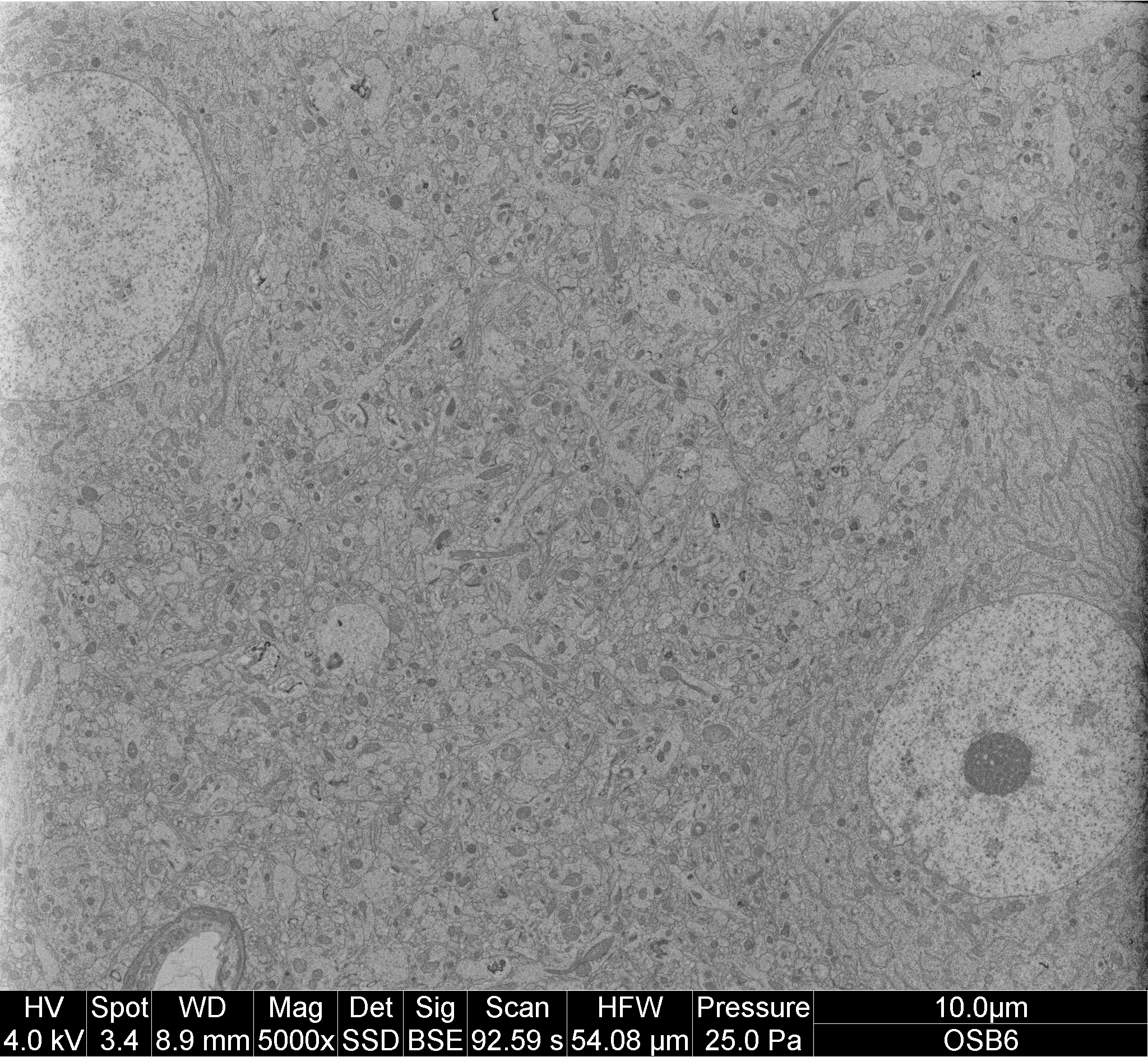

Supplement: Dataset S20 — (254.9 MB ZIP). [file pbio.0020329.sd020.zip › 040604_OS5_st1_1935.tif]

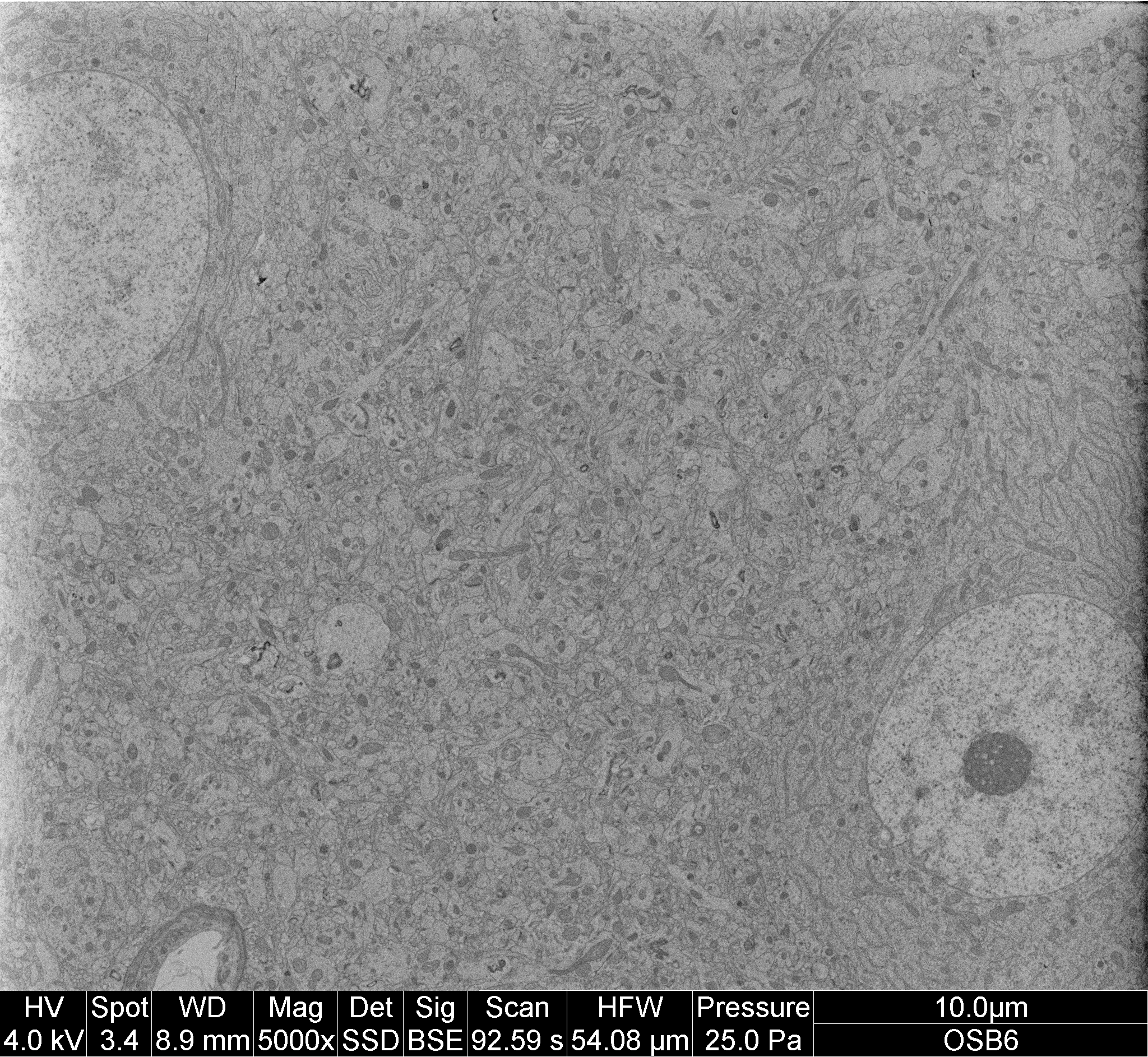

Supplement: Dataset S20 — (254.9 MB ZIP). [file pbio.0020329.sd020.zip › 040604_OS5_st1_1936.tif]

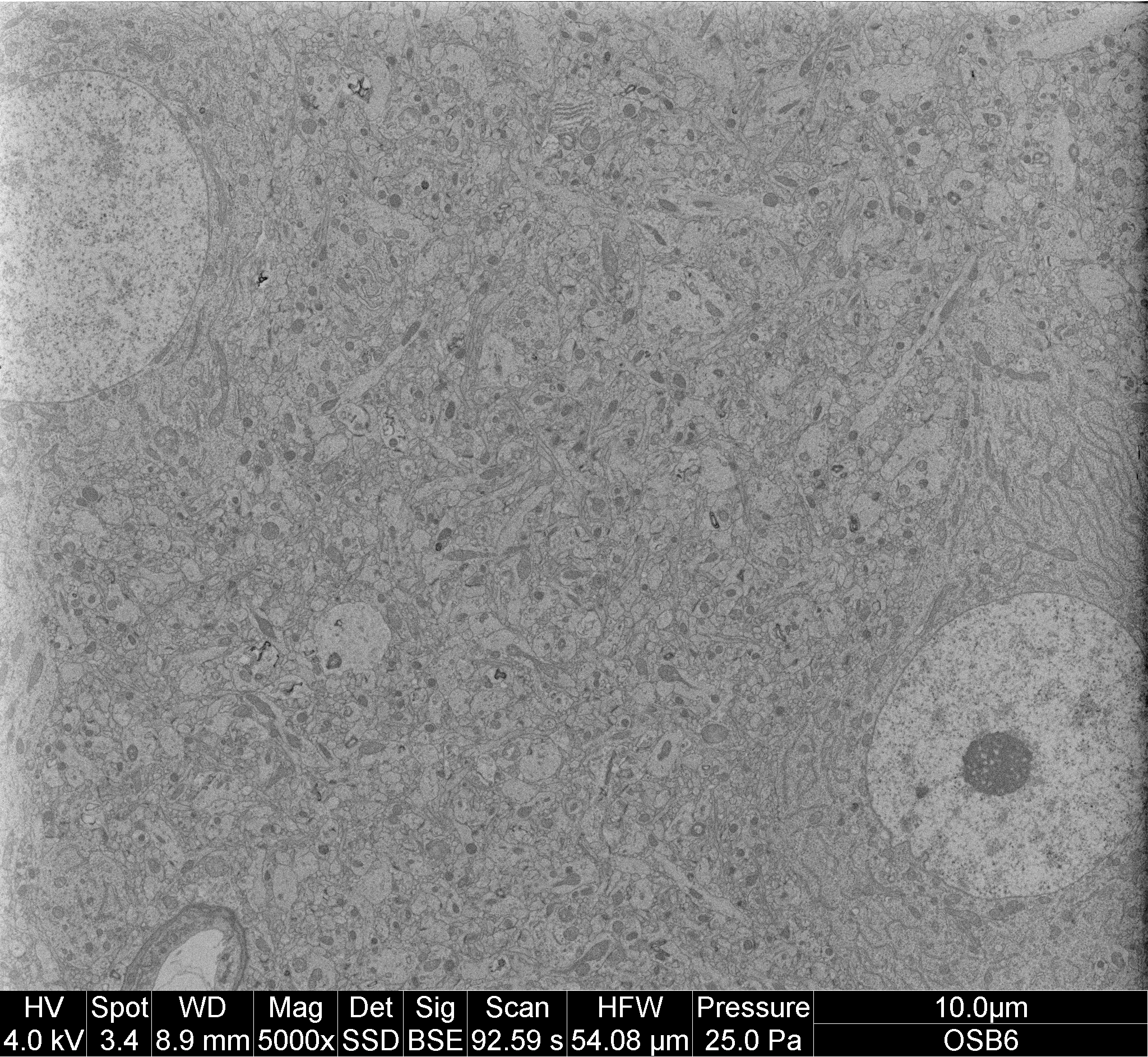

Supplement: Dataset S20 — (254.9 MB ZIP). [file pbio.0020329.sd020.zip › 040604_OS5_st1_1937.tif]

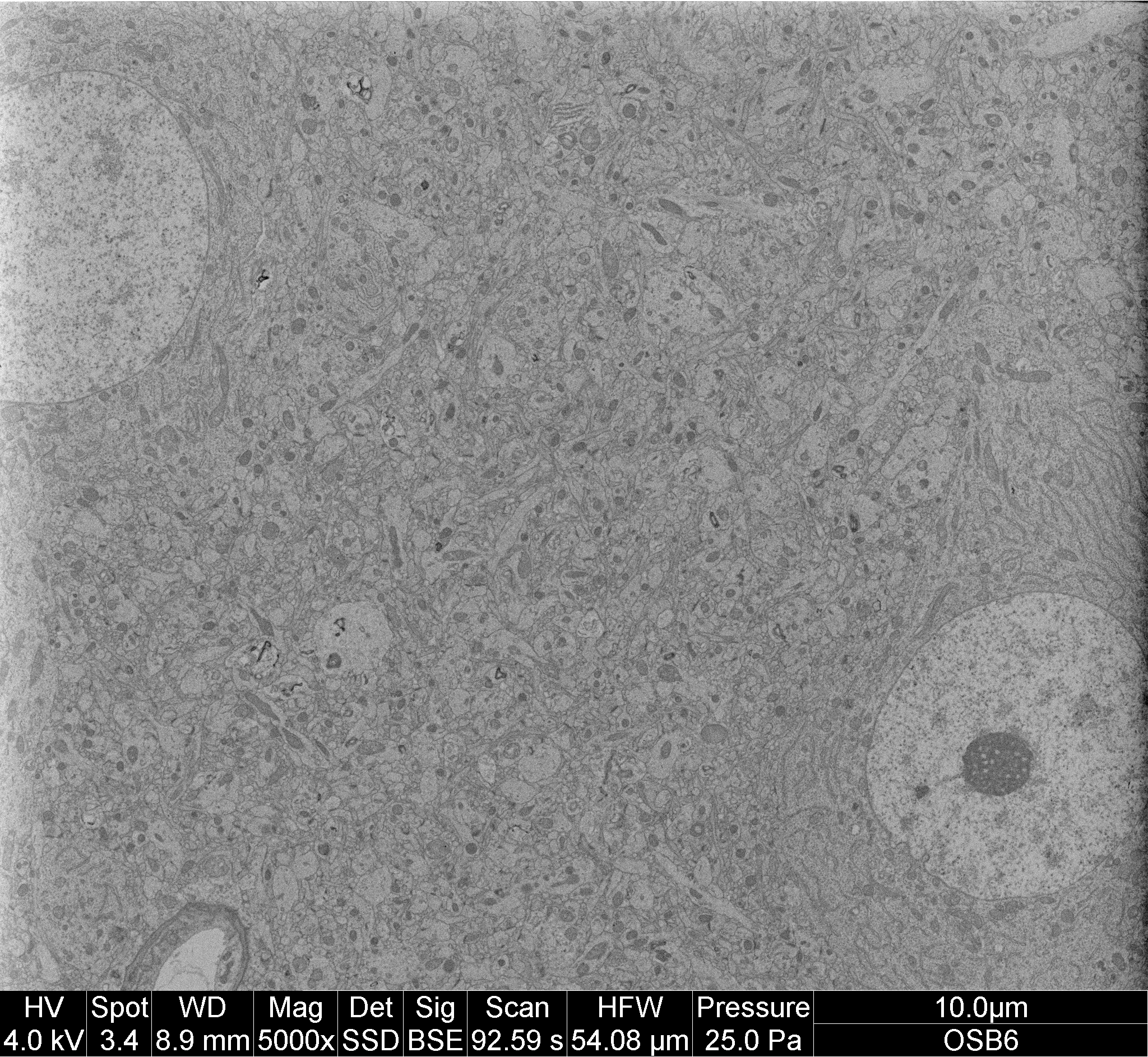

Supplement: Dataset S20 — (254.9 MB ZIP). [file pbio.0020329.sd020.zip › 040604_OS5_st1_1938.tif]

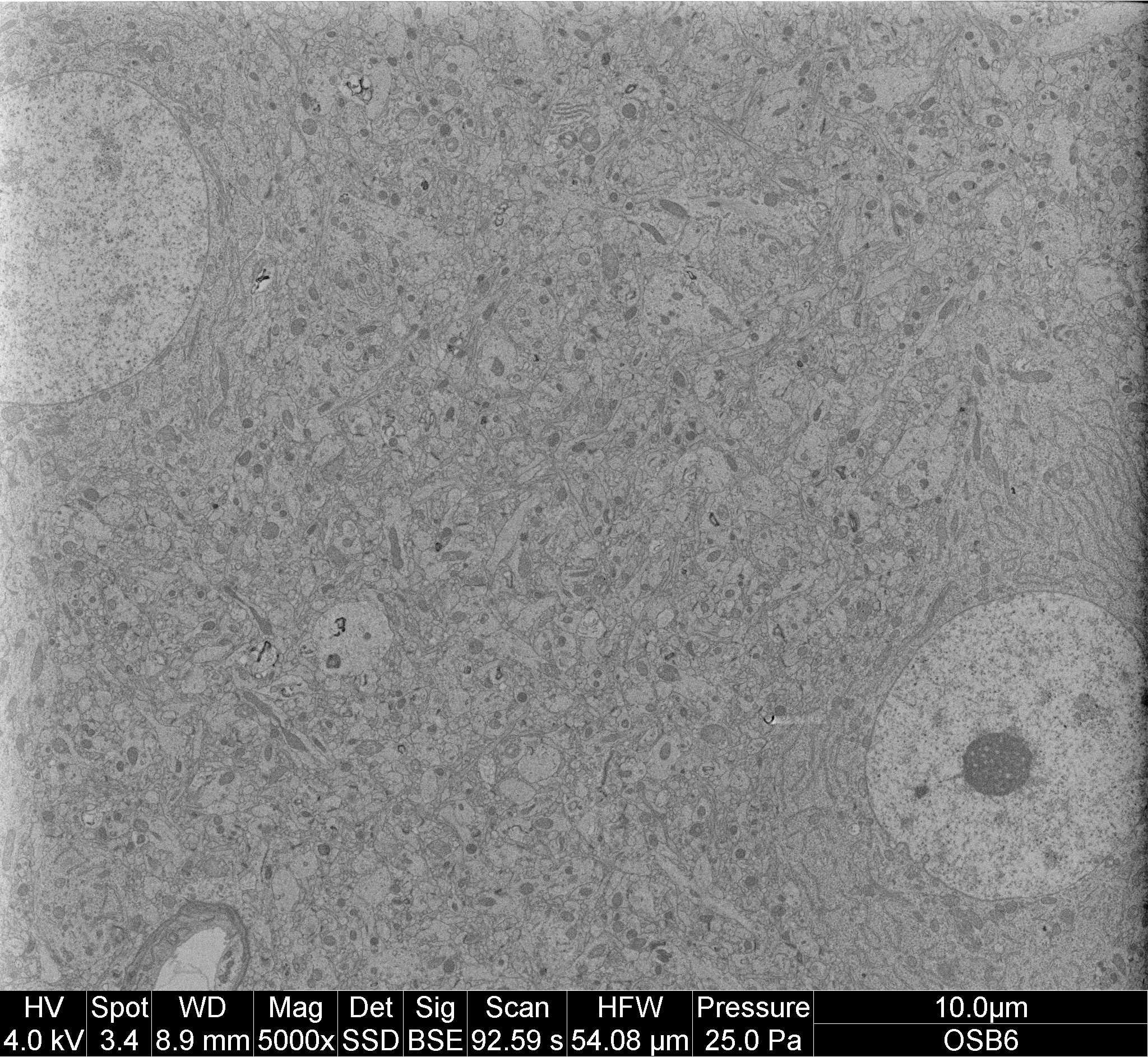

Supplement: Dataset S20 — (254.9 MB ZIP). [file pbio.0020329.sd020.zip › 040604_OS5_st1_1939.tif]

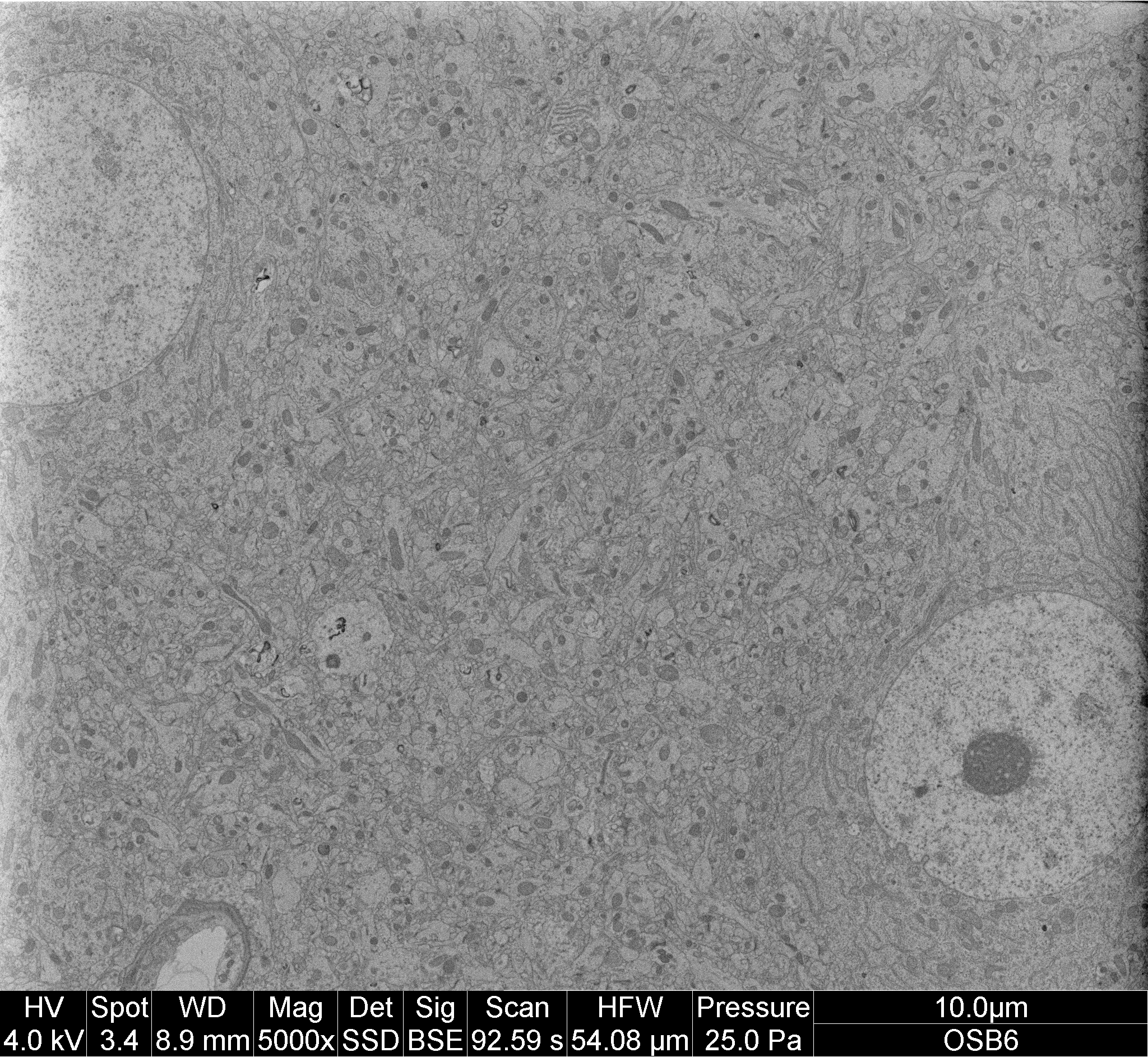

Supplement: Dataset S20 — (254.9 MB ZIP). [file pbio.0020329.sd020.zip › 040604_OS5_st1_1940.tif]

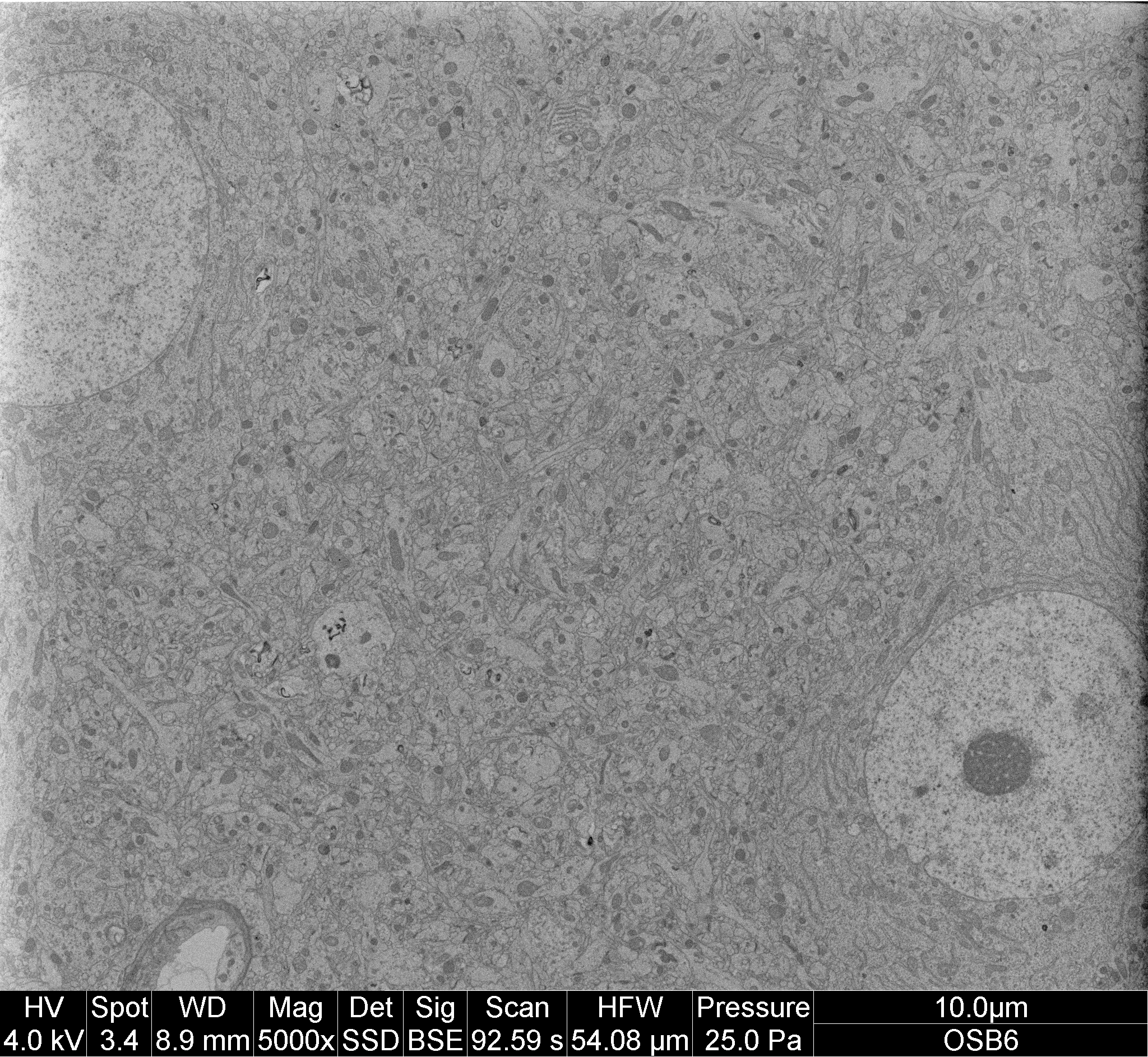

Supplement: Dataset S20 — (254.9 MB ZIP). [file pbio.0020329.sd020.zip › 040604_OS5_st1_1941.tif]

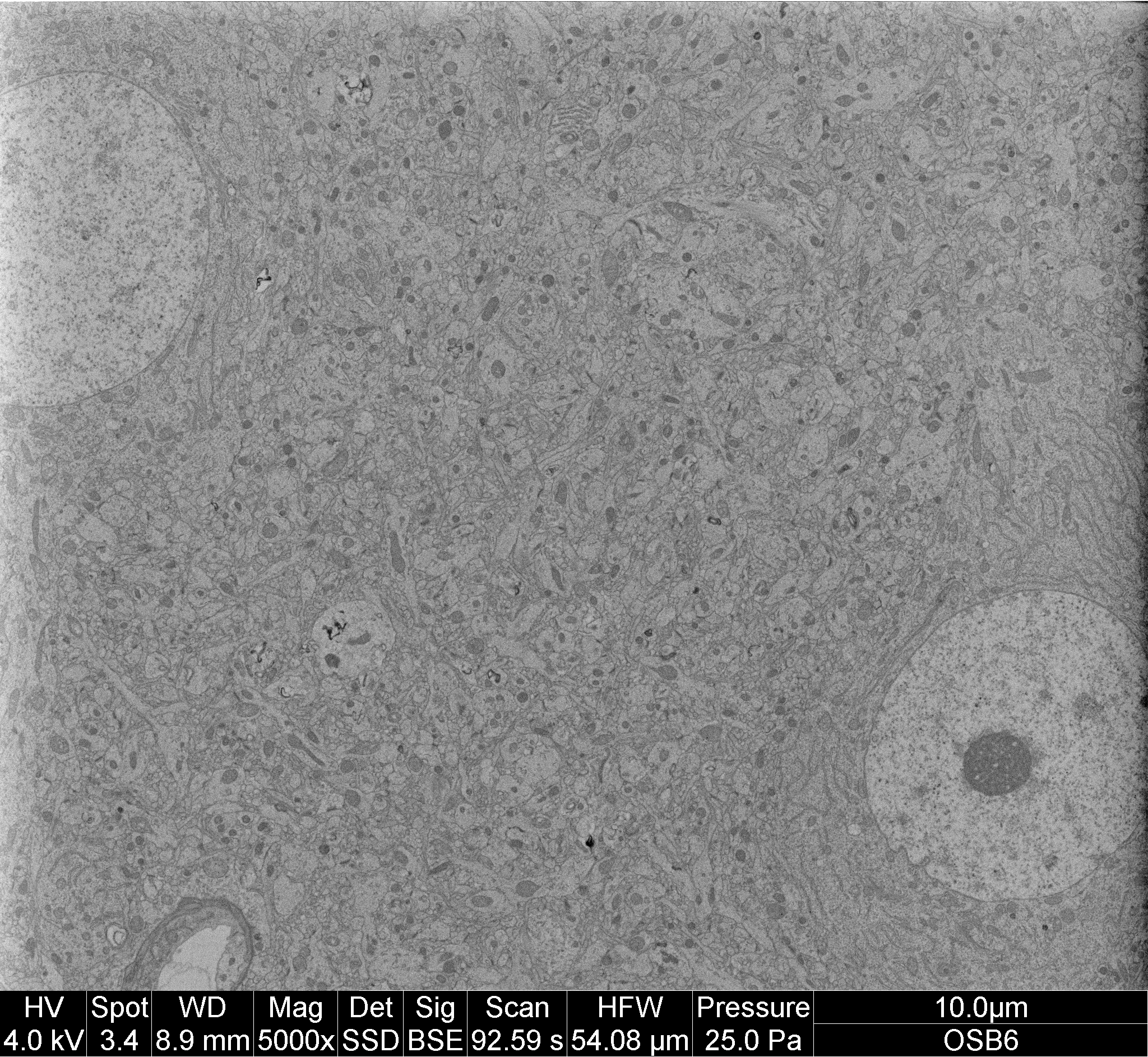

Supplement: Dataset S20 — (254.9 MB ZIP). [file pbio.0020329.sd020.zip › 040604_OS5_st1_1942.tif]

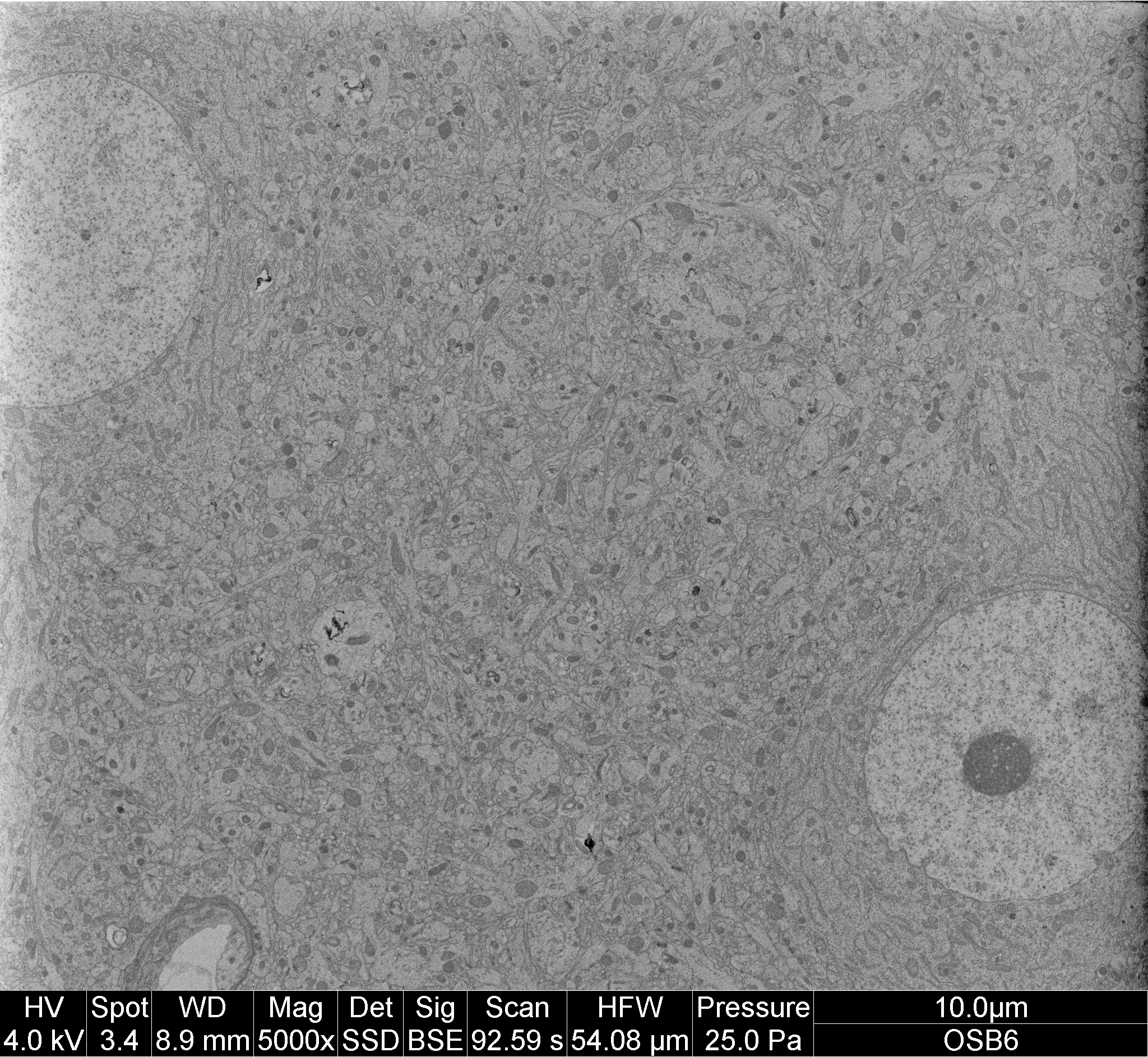

Supplement: Dataset S20 — (254.9 MB ZIP). [file pbio.0020329.sd020.zip › 040604_OS5_st1_1943.tif]

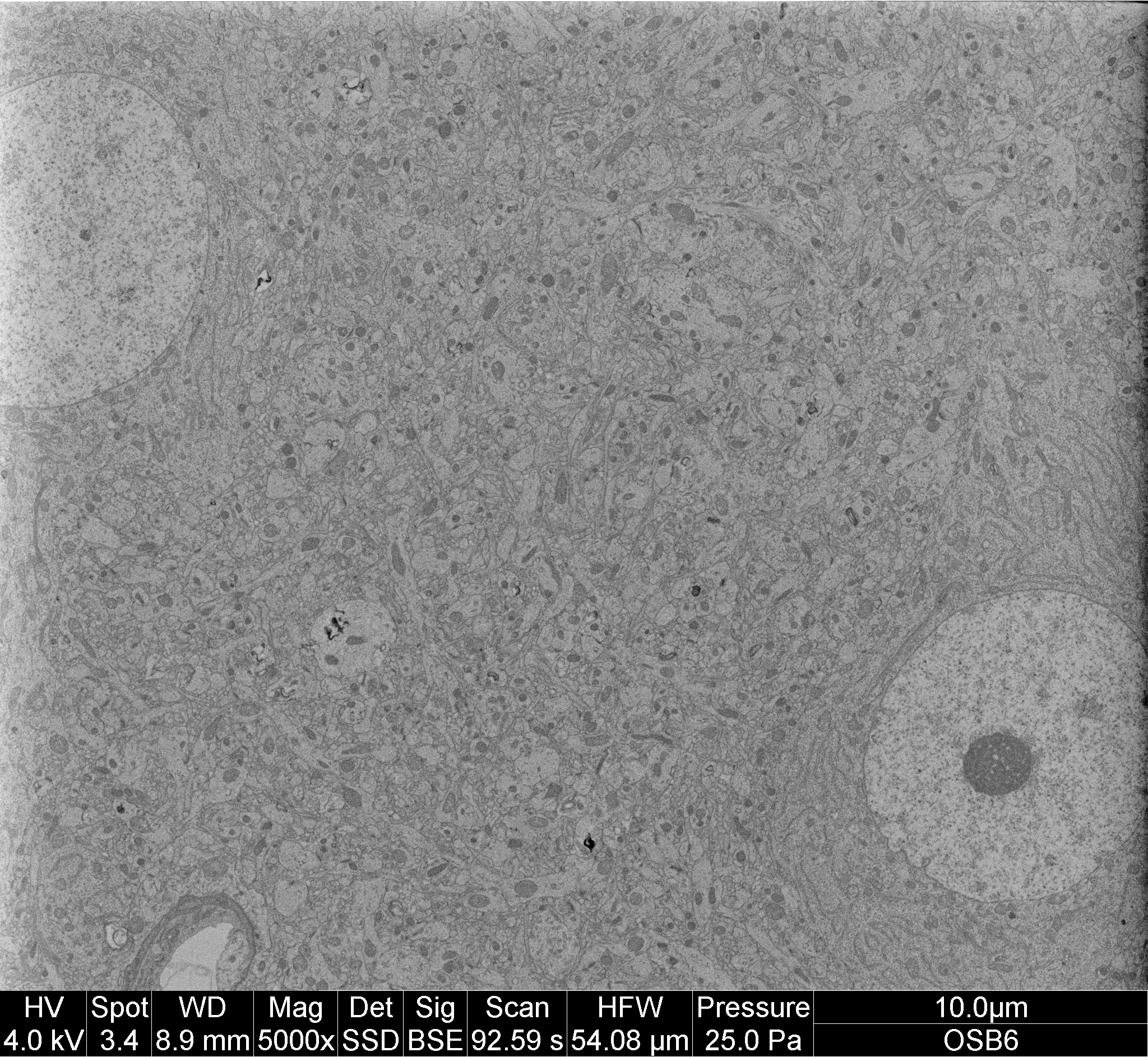

Supplement: Dataset S20 — (254.9 MB ZIP). [file pbio.0020329.sd020.zip › 040604_OS5_st1_1944.tif]

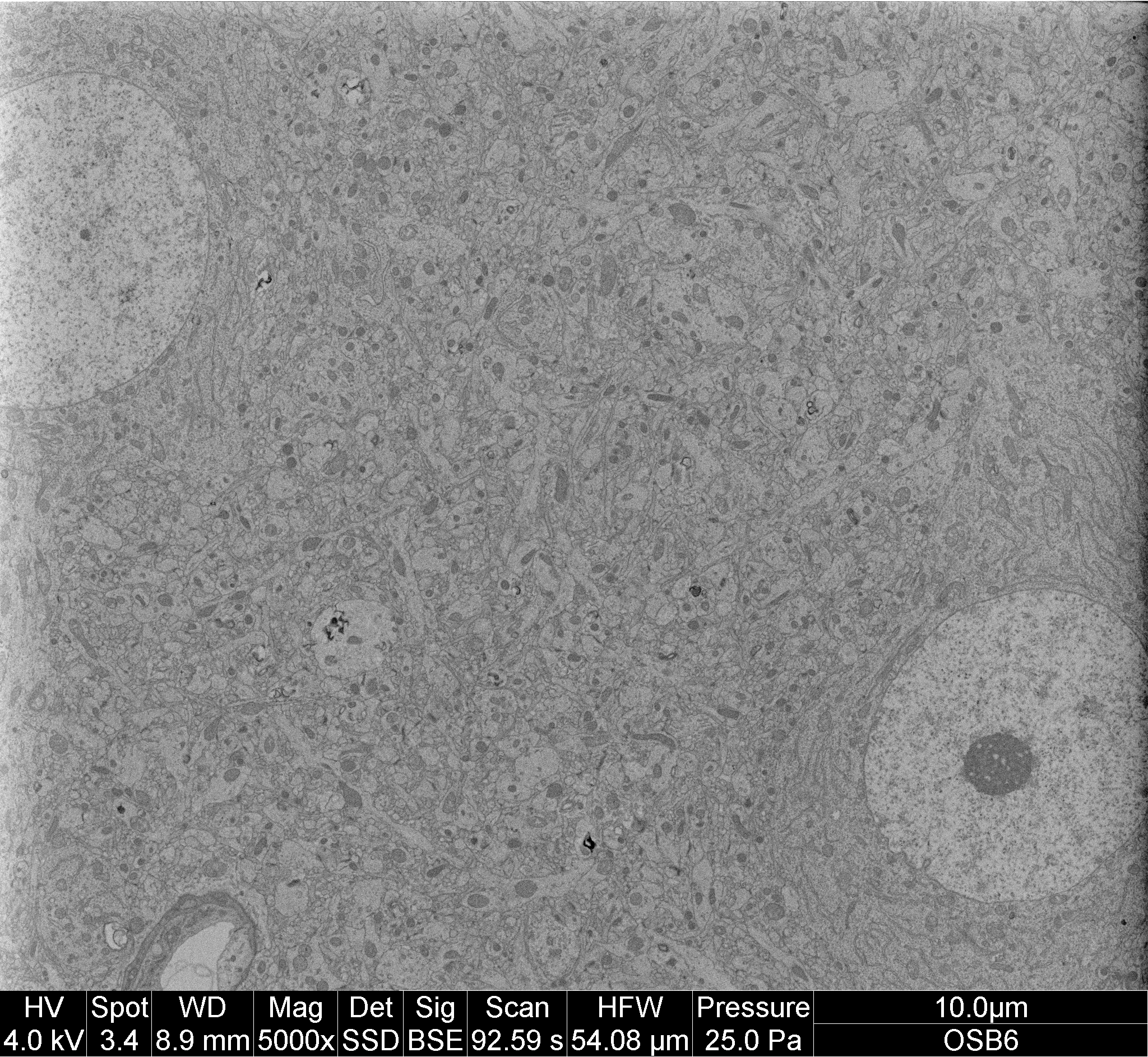

Supplement: Dataset S20 — (254.9 MB ZIP). [file pbio.0020329.sd020.zip › 040604_OS5_st1_1945.tif]

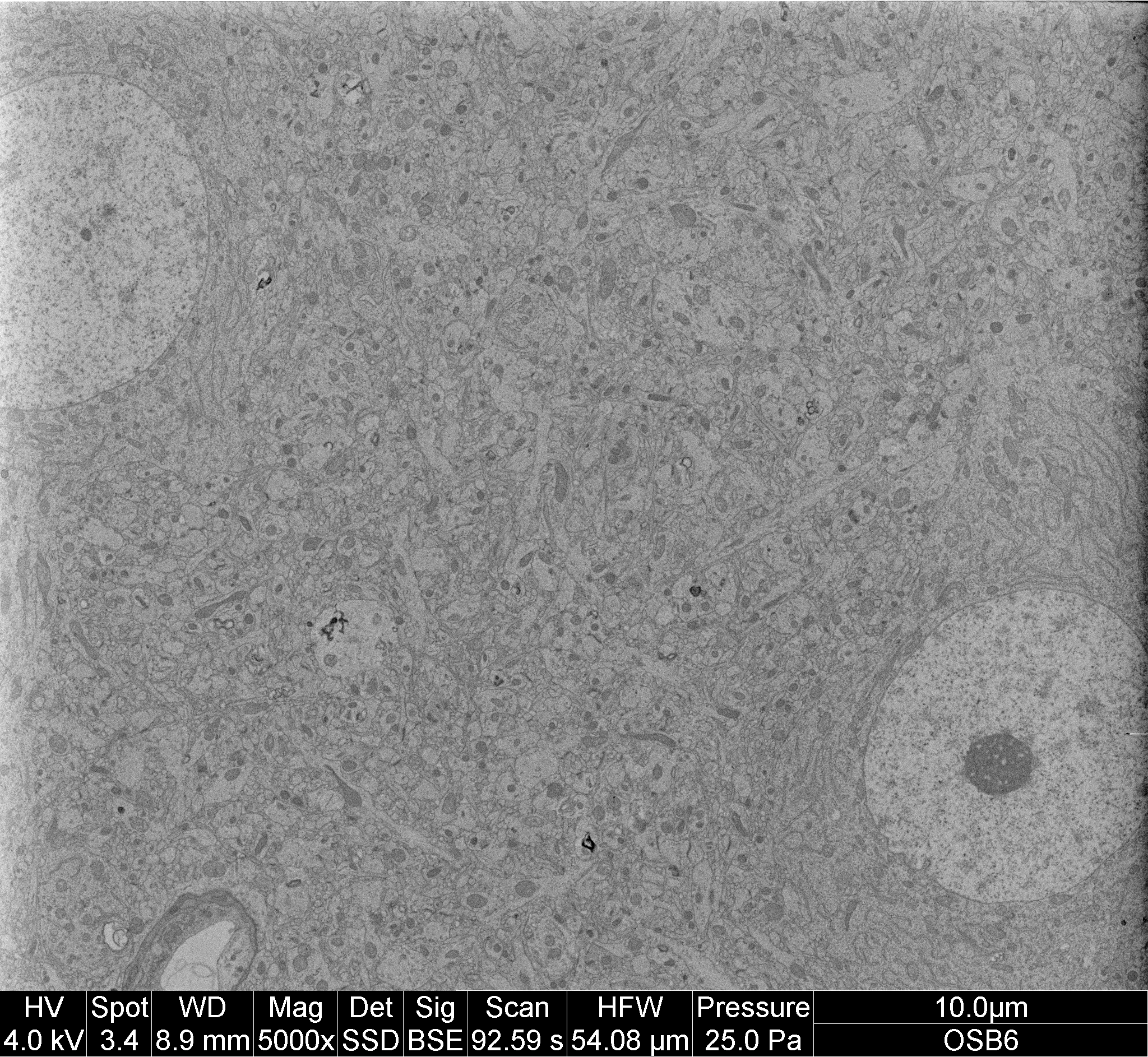

Supplement: Dataset S20 — (254.9 MB ZIP). [file pbio.0020329.sd020.zip › 040604_OS5_st1_1946.tif]

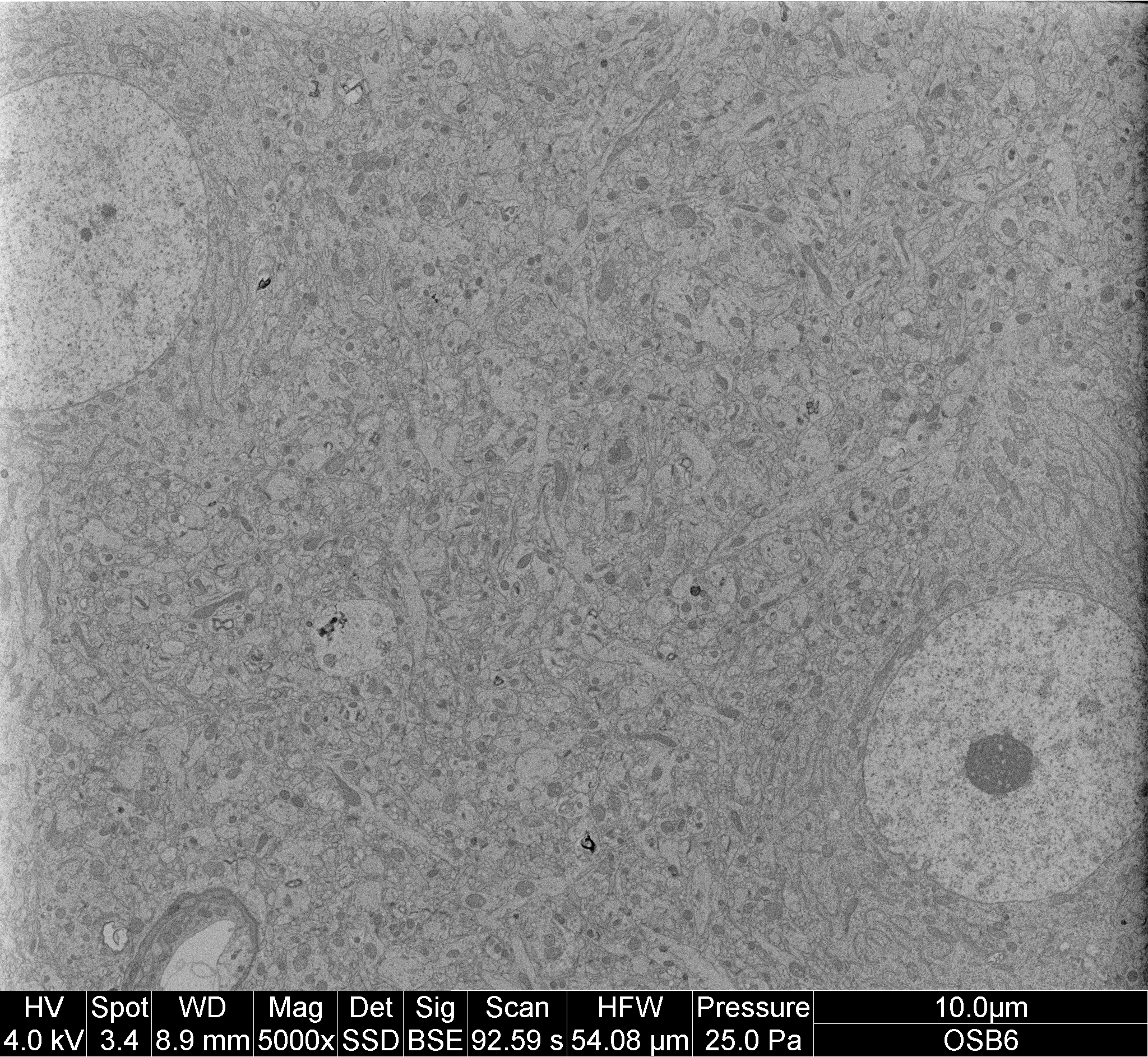

Supplement: Dataset S20 — (254.9 MB ZIP). [file pbio.0020329.sd020.zip › 040604_OS5_st1_1947.tif]

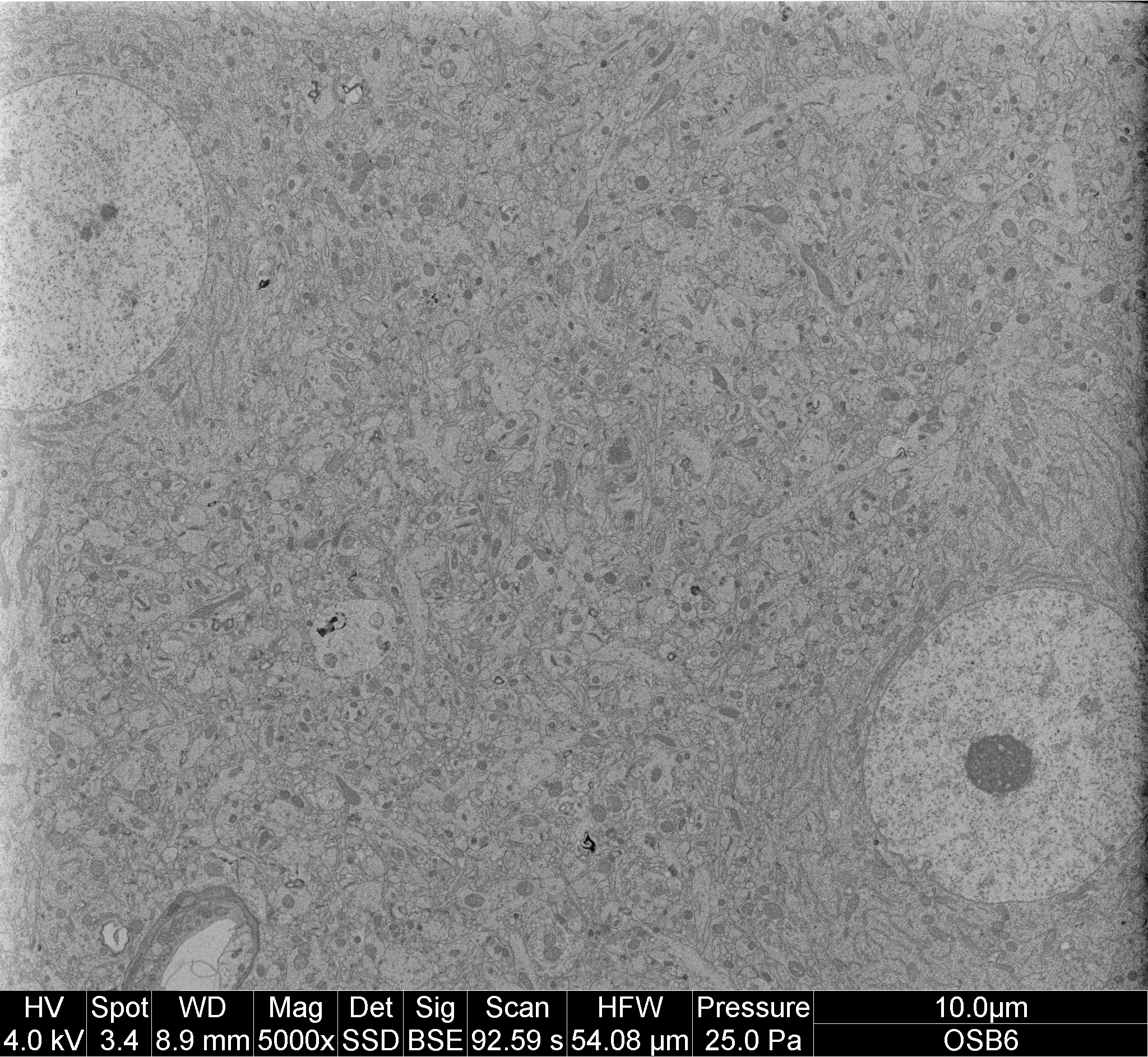

Supplement: Dataset S20 — (254.9 MB ZIP). [file pbio.0020329.sd020.zip › 040604_OS5_st1_1948.tif]

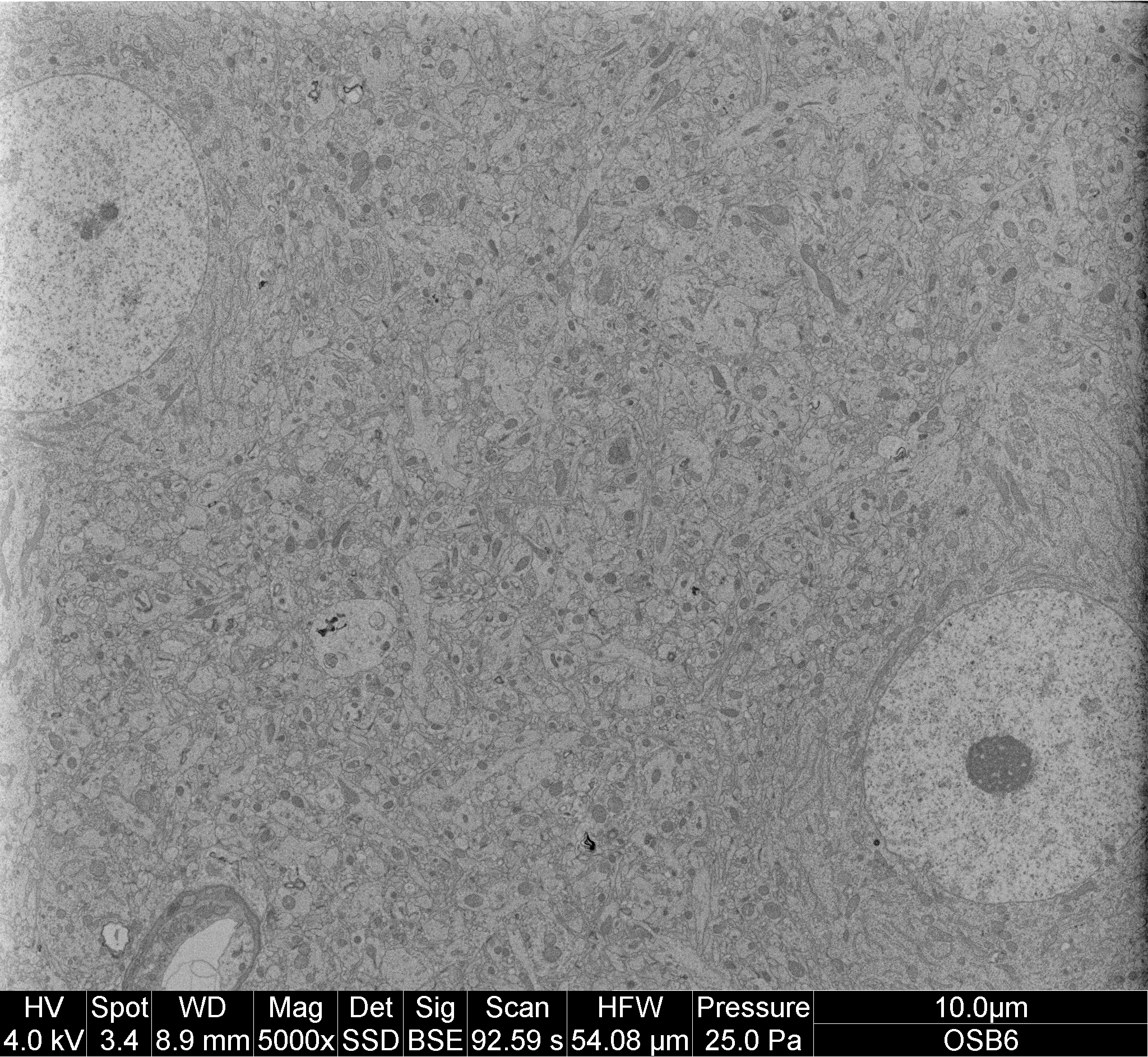

Supplement: Dataset S20 — (254.9 MB ZIP). [file pbio.0020329.sd020.zip › 040604_OS5_st1_1949.tif]

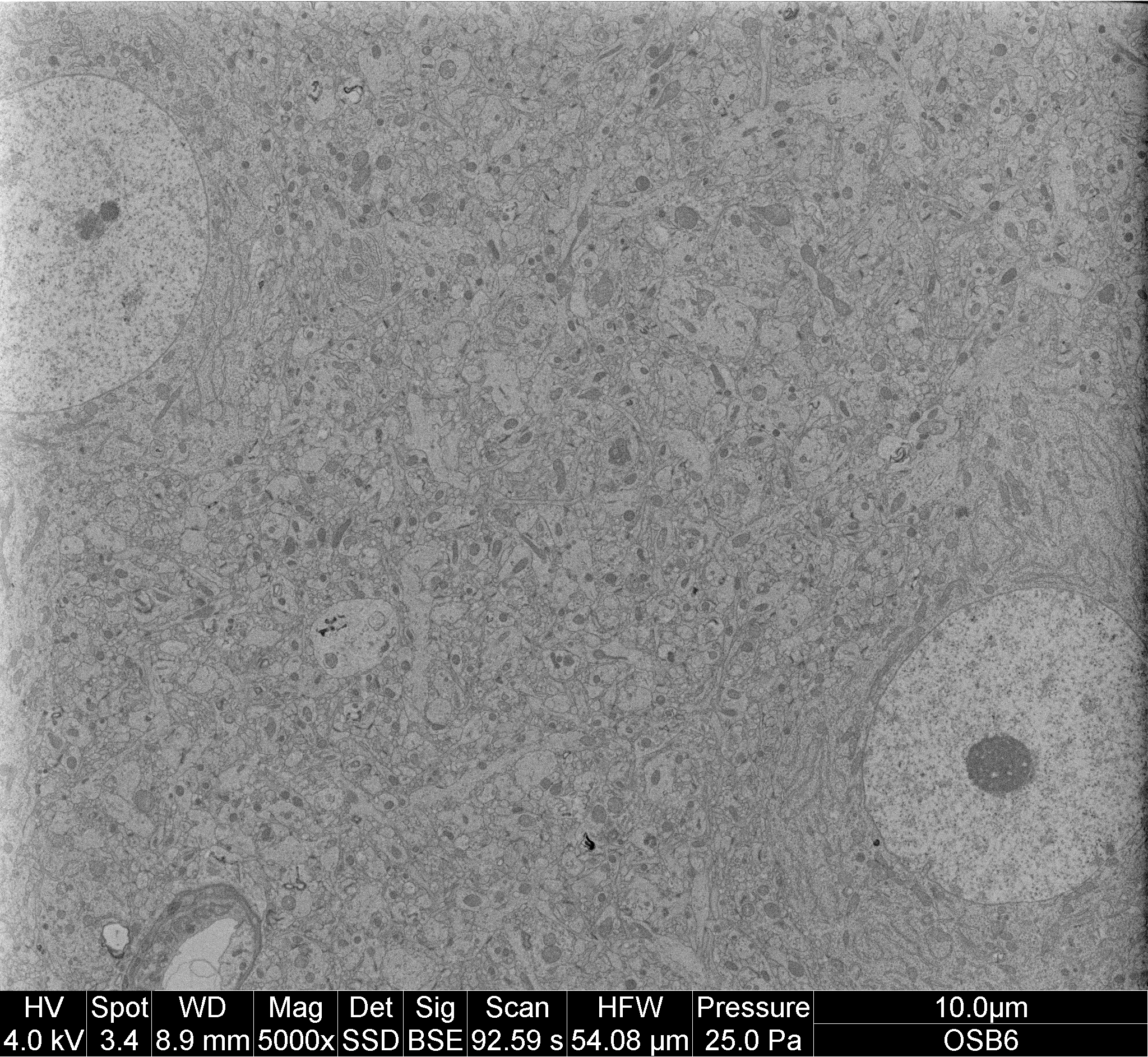

Supplement: Dataset S20 — (254.9 MB ZIP). [file pbio.0020329.sd020.zip › 040604_OS5_st1_1950.tif]

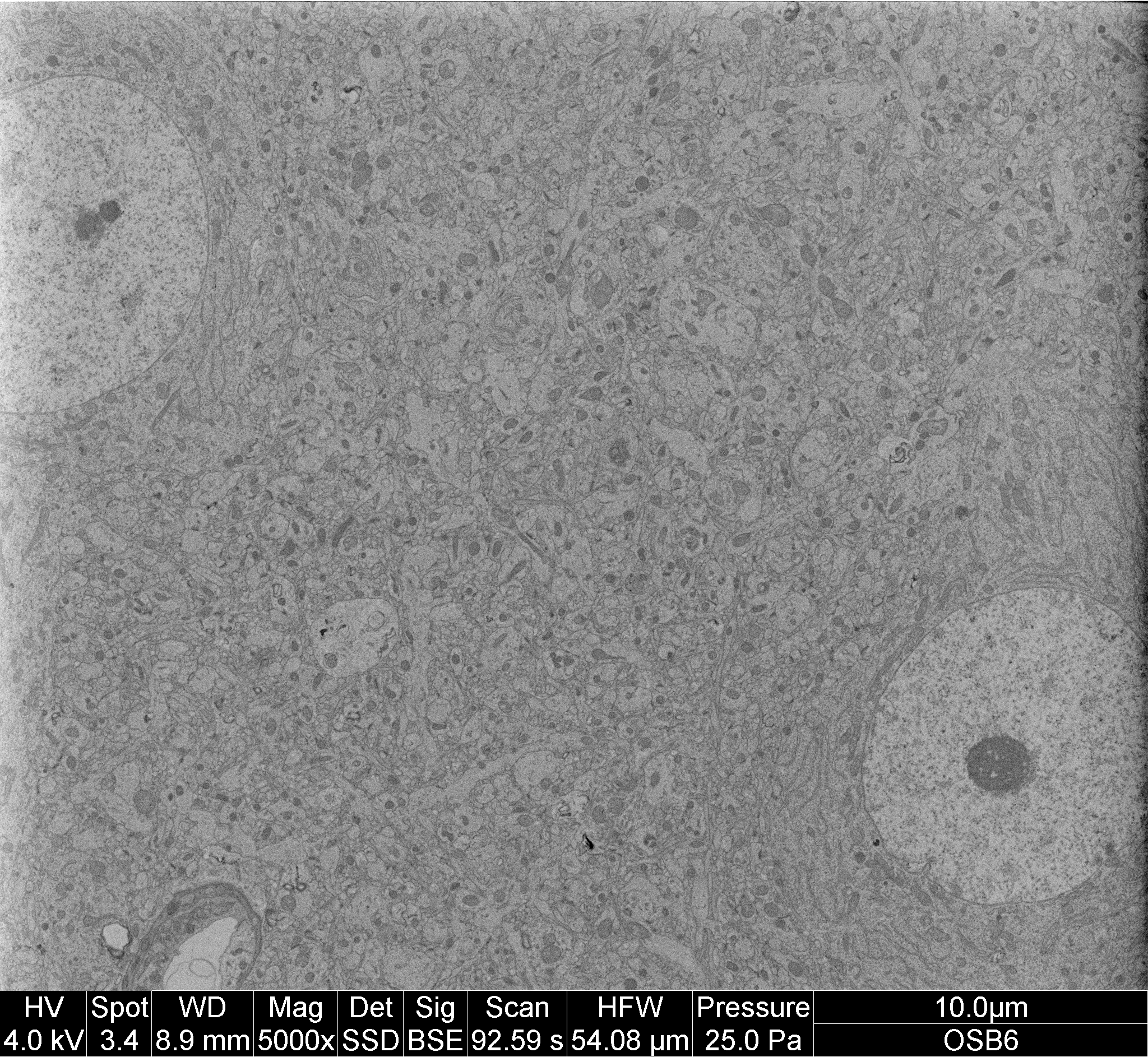

Supplement: Dataset S20 — (254.9 MB ZIP). [file pbio.0020329.sd020.zip › 040604_OS5_st1_1951.tif]

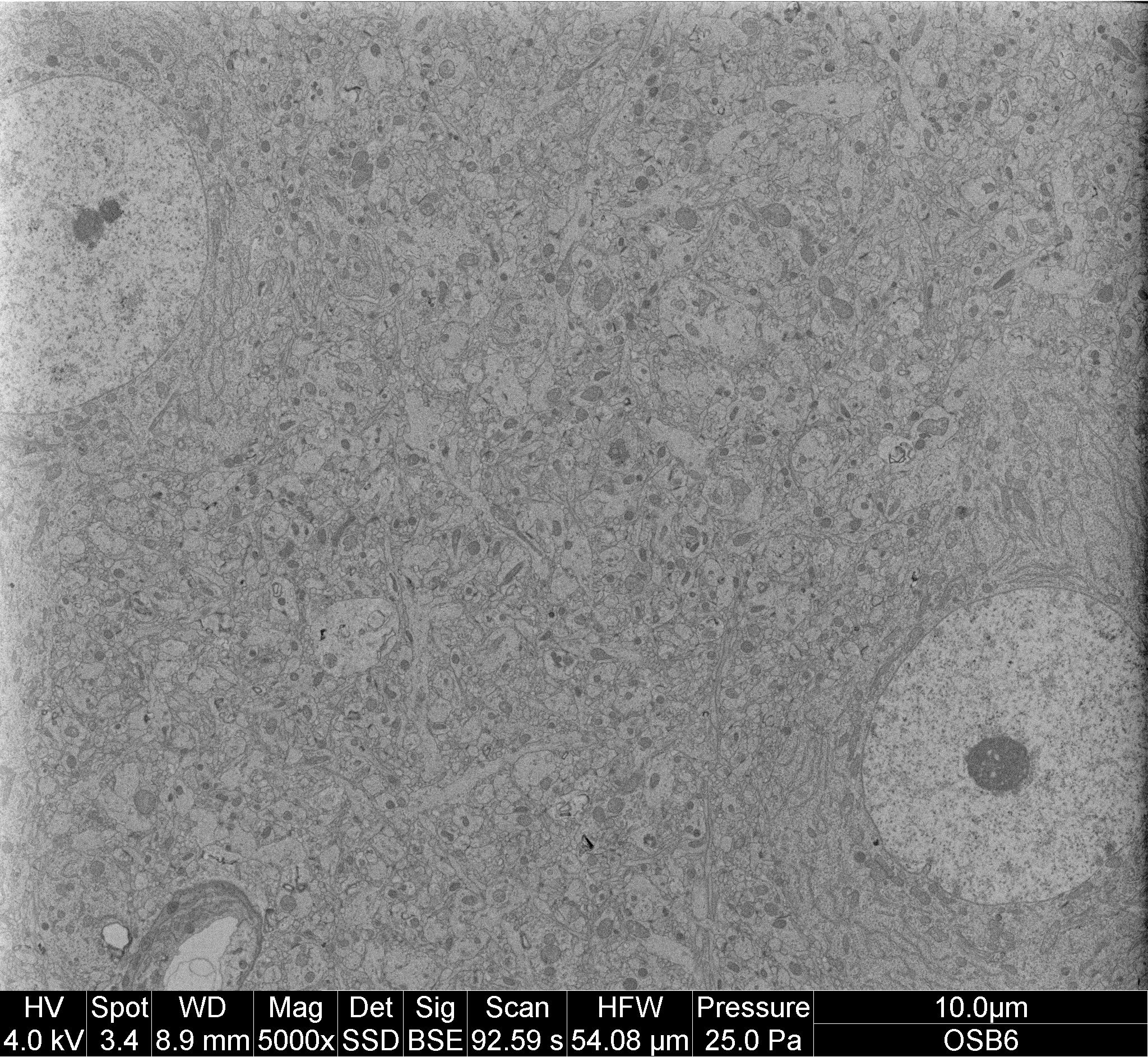

Supplement: Dataset S20 — (254.9 MB ZIP). [file pbio.0020329.sd020.zip › 040604_OS5_st1_1952.tif]

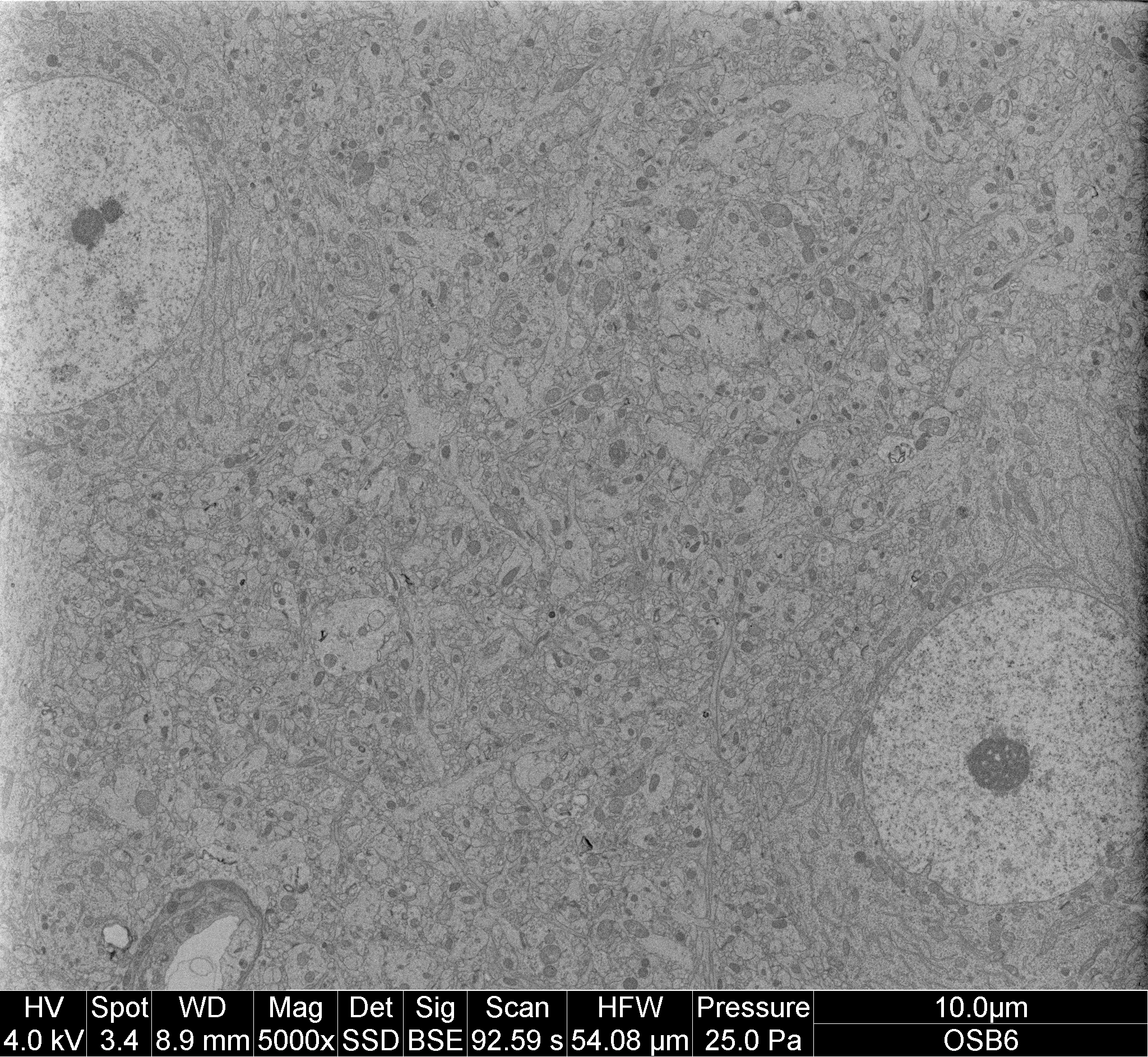

Supplement: Dataset S20 — (254.9 MB ZIP). [file pbio.0020329.sd020.zip › 040604_OS5_st1_1953.tif]

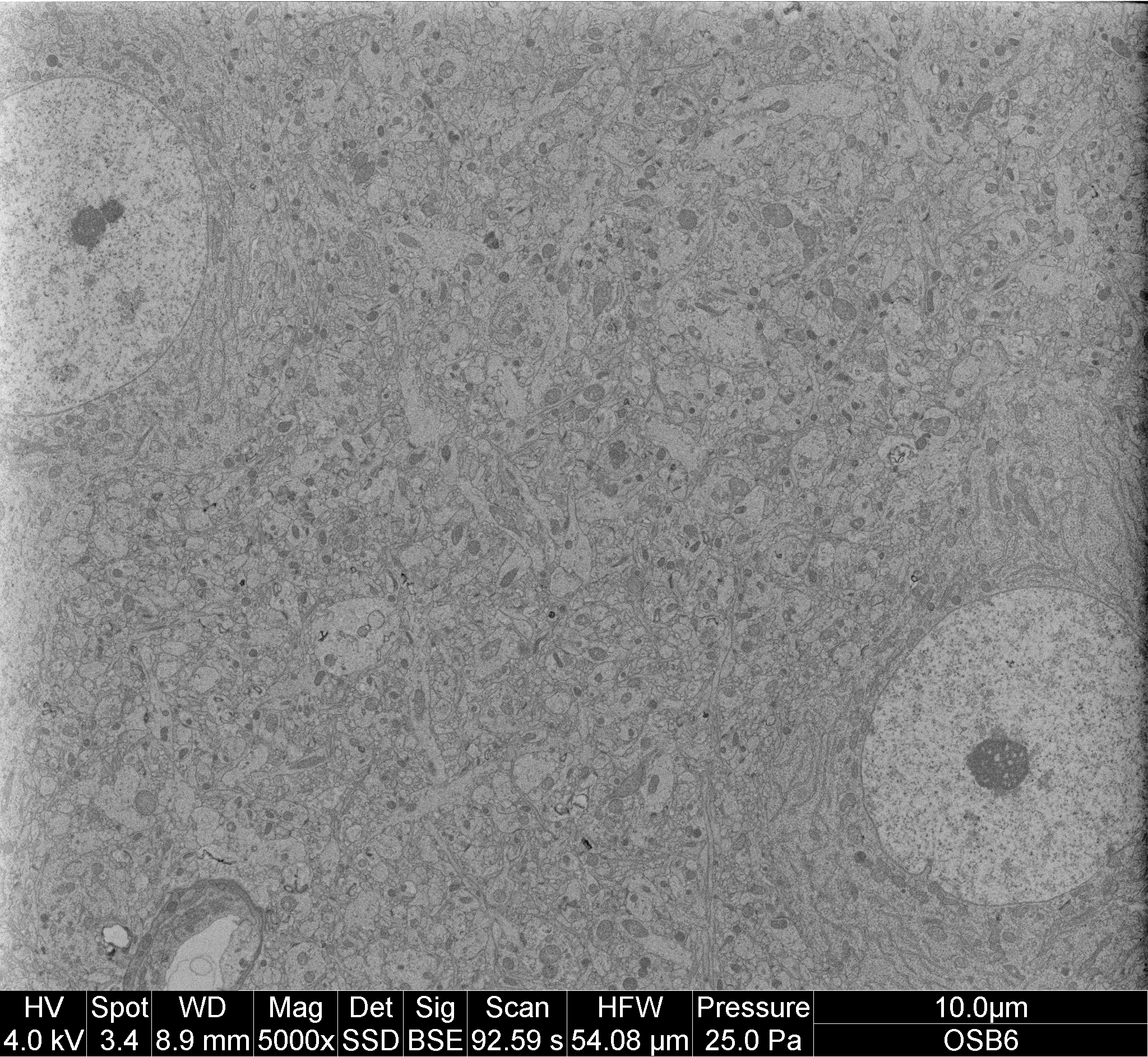

Supplement: Dataset S20 — (254.9 MB ZIP). [file pbio.0020329.sd020.zip › 040604_OS5_st1_1954.tif]

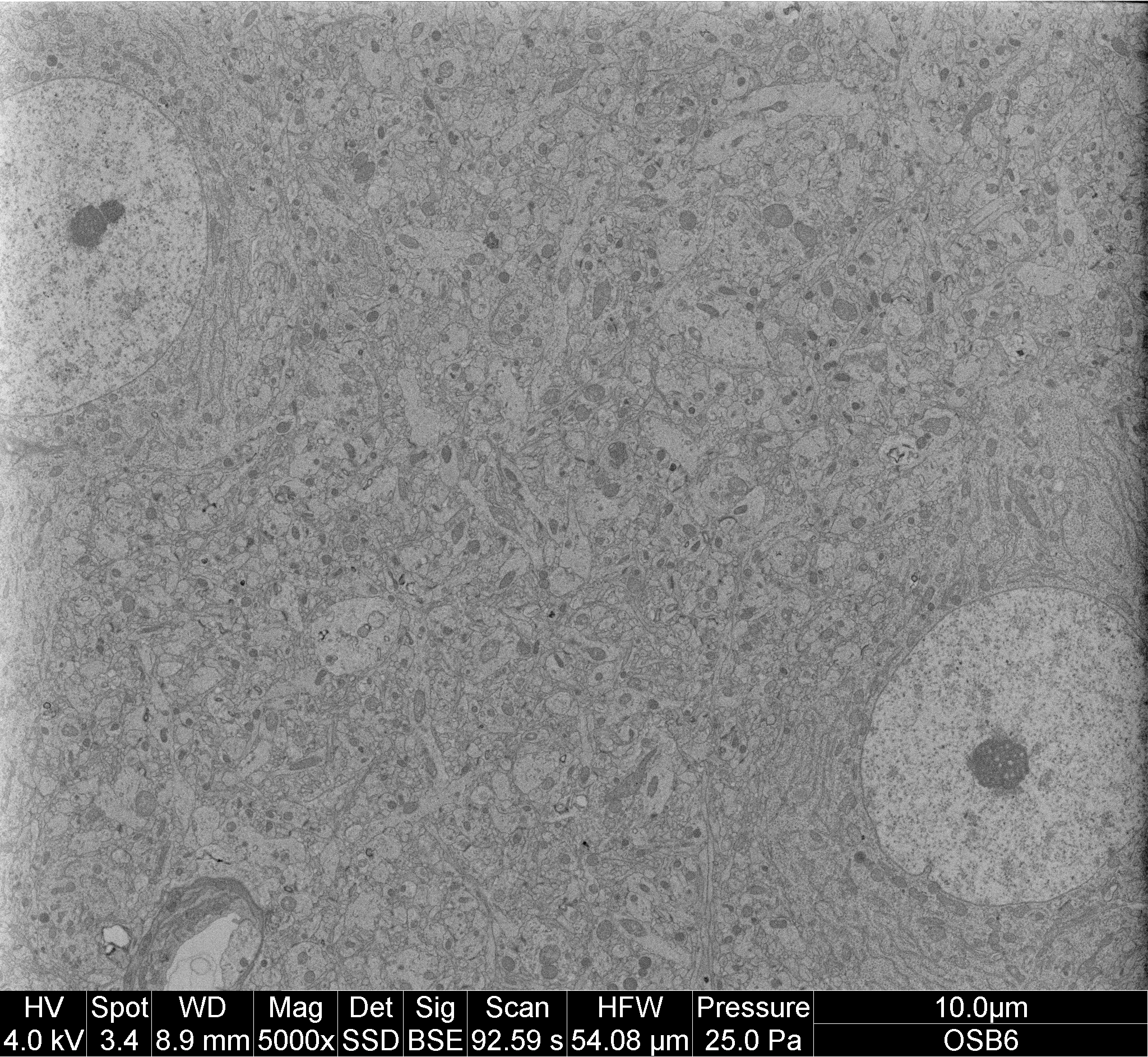

Supplement: Dataset S20 — (254.9 MB ZIP). [file pbio.0020329.sd020.zip › 040604_OS5_st1_1955.tif]

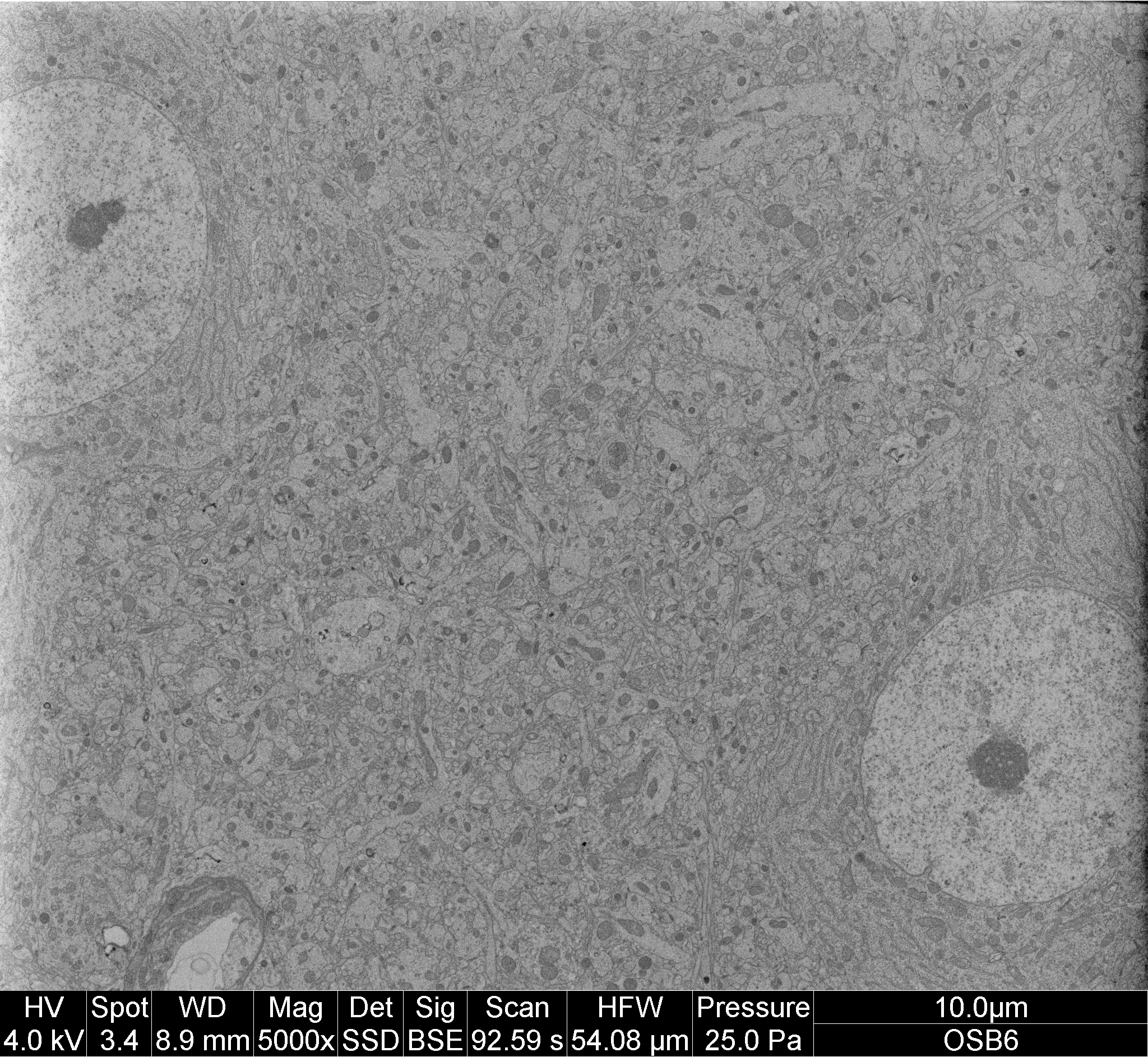

Supplement: Dataset S20 — (254.9 MB ZIP). [file pbio.0020329.sd020.zip › 040604_OS5_st1_1956.tif]

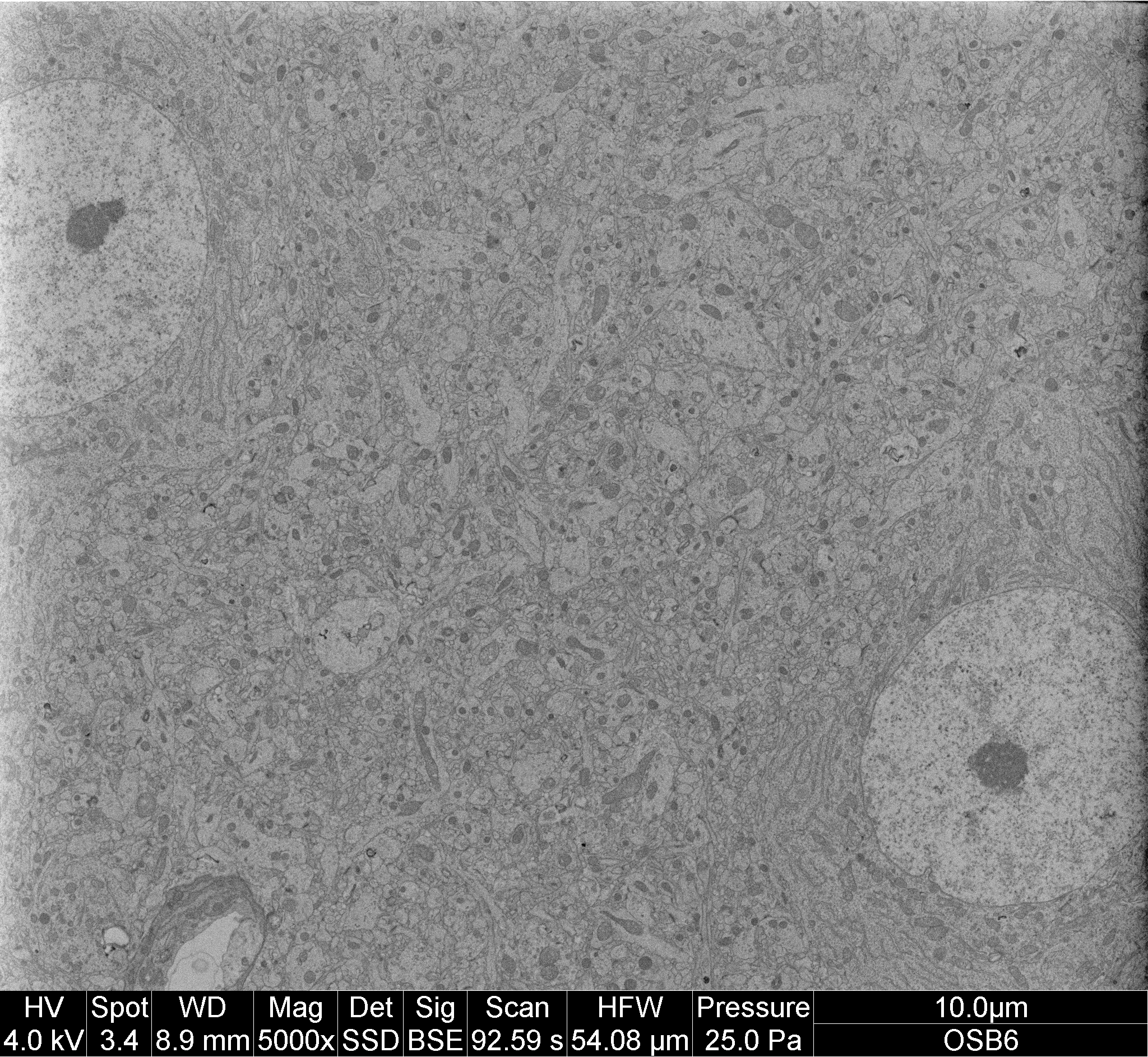

Supplement: Dataset S20 — (254.9 MB ZIP). [file pbio.0020329.sd020.zip › 040604_OS5_st1_1957.tif]

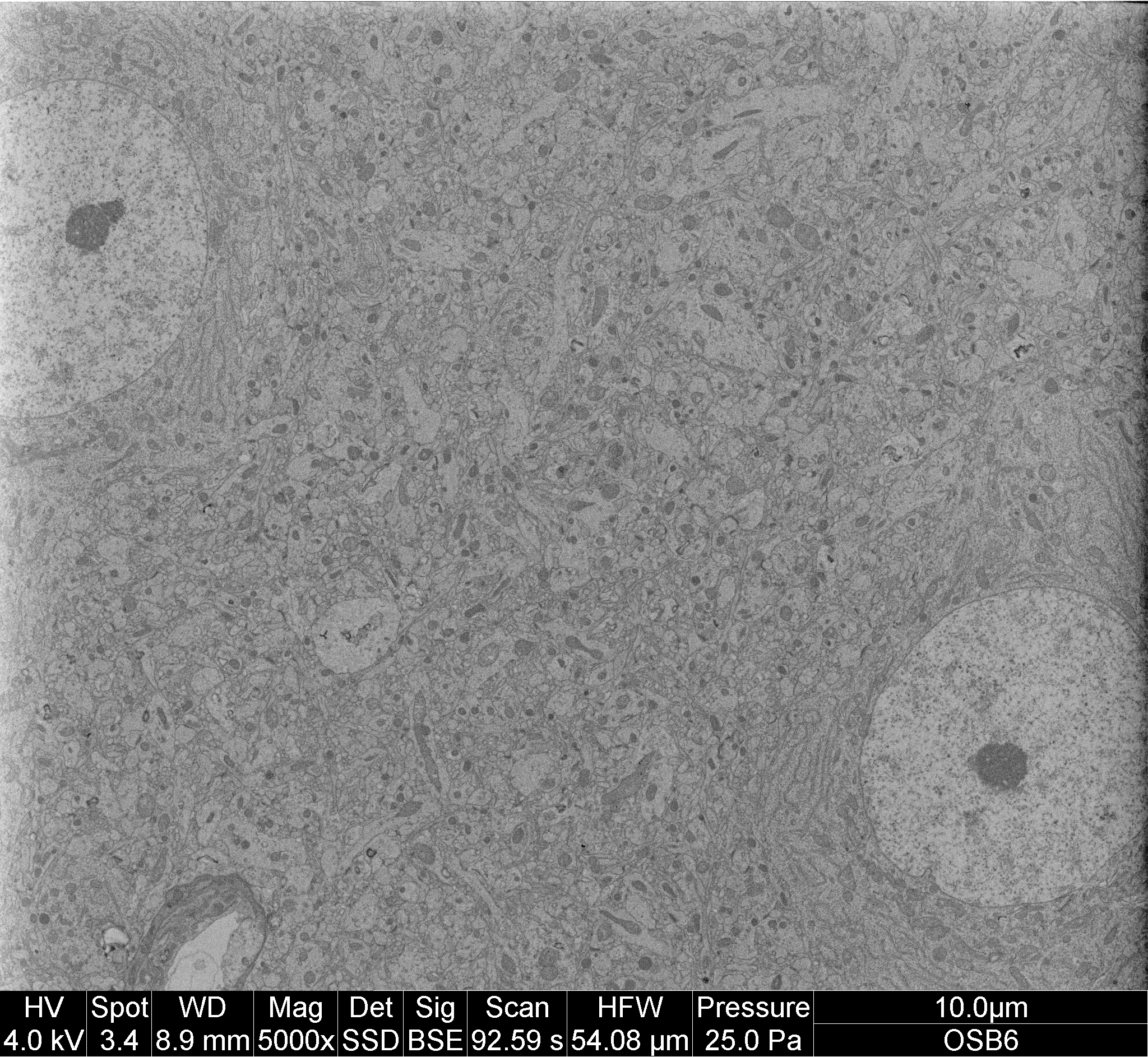

Supplement: Dataset S20 — (254.9 MB ZIP). [file pbio.0020329.sd020.zip › 040604_OS5_st1_1958.tif]

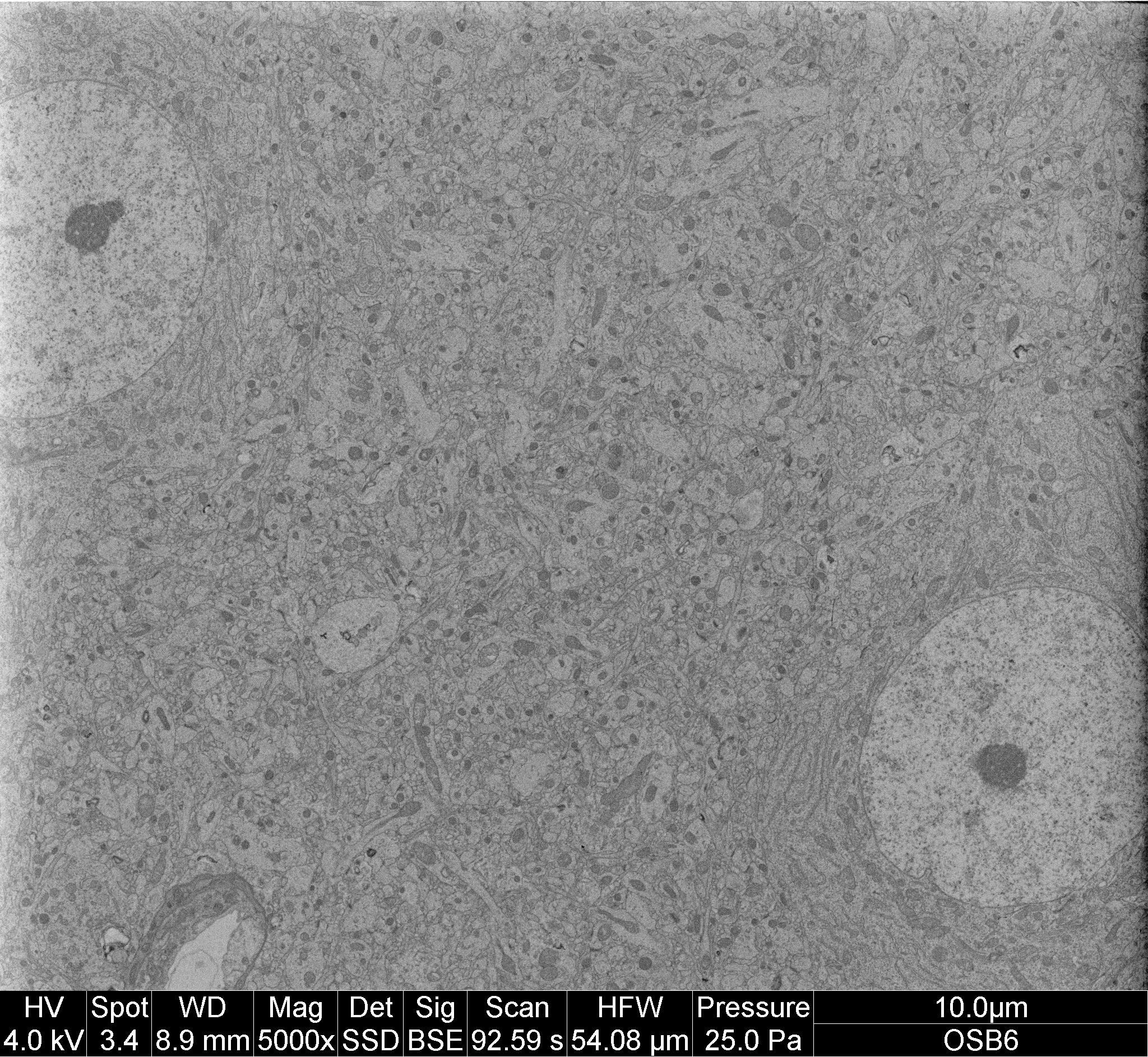

Supplement: Dataset S20 — (254.9 MB ZIP). [file pbio.0020329.sd020.zip › 040604_OS5_st1_1959.tif]

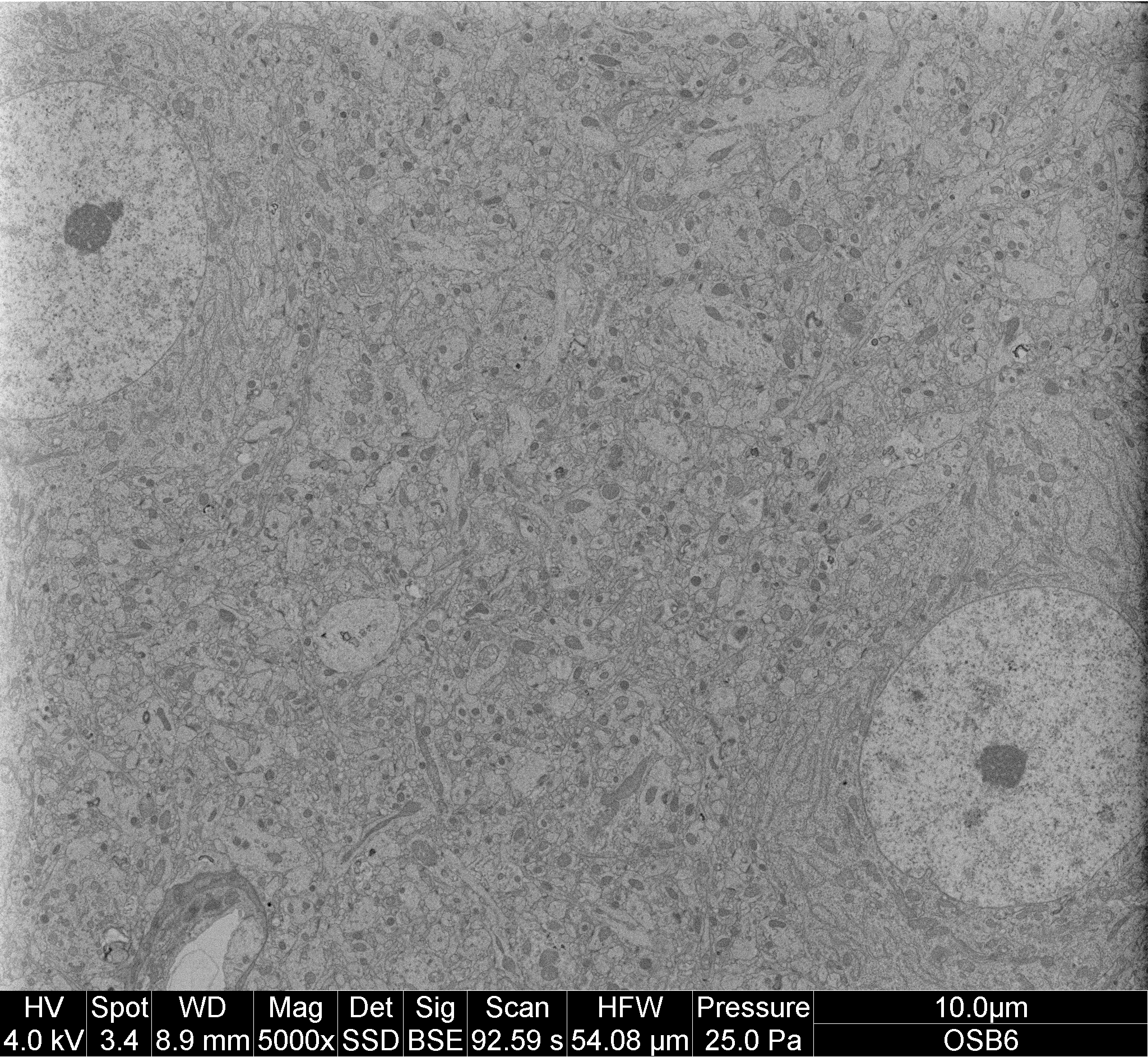

Supplement: Dataset S20 — (254.9 MB ZIP). [file pbio.0020329.sd020.zip › 040604_OS5_st1_1960.tif]

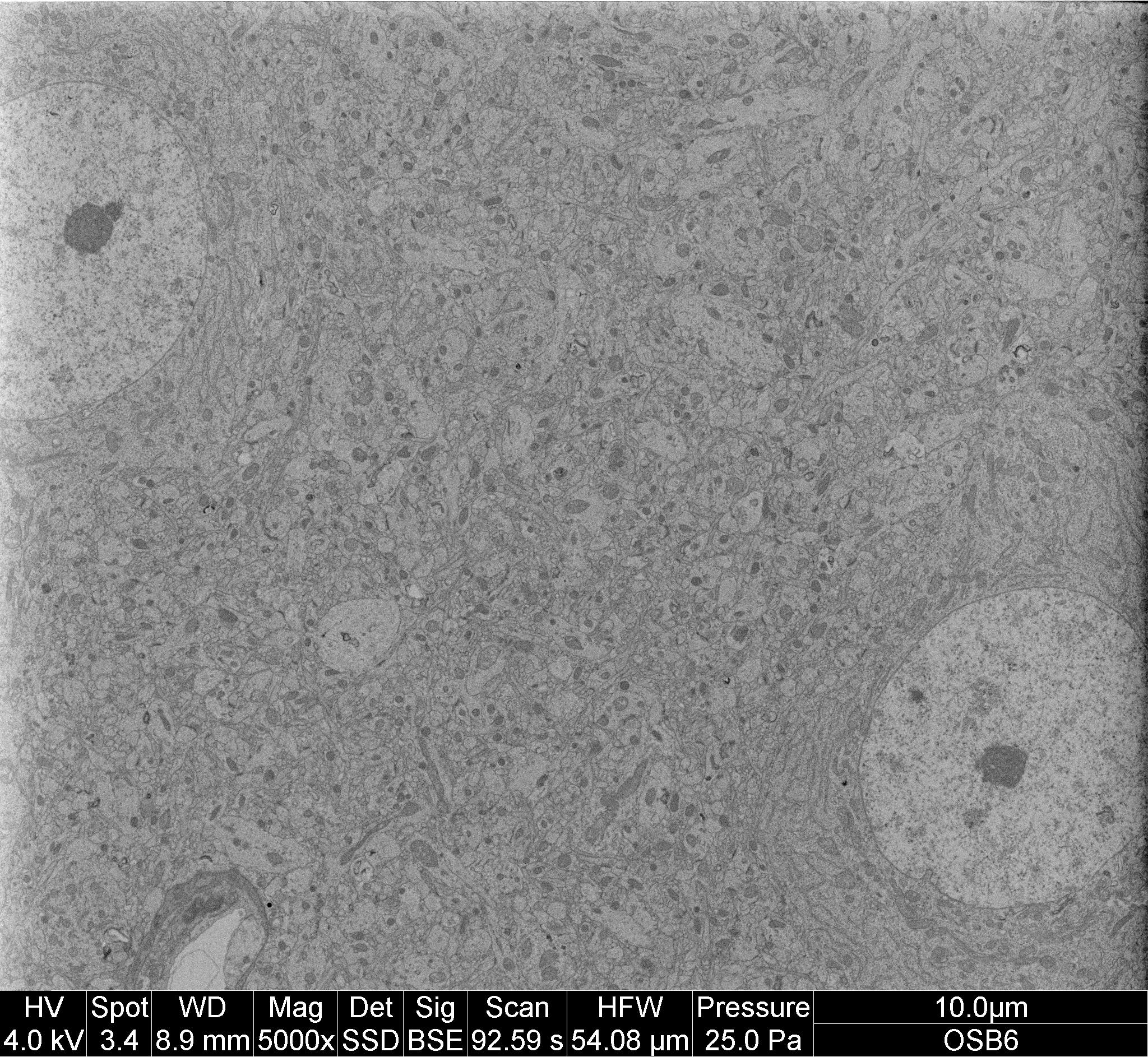

Supplement: Dataset S20 — (254.9 MB ZIP). [file pbio.0020329.sd020.zip › 040604_OS5_st1_1961.tif]

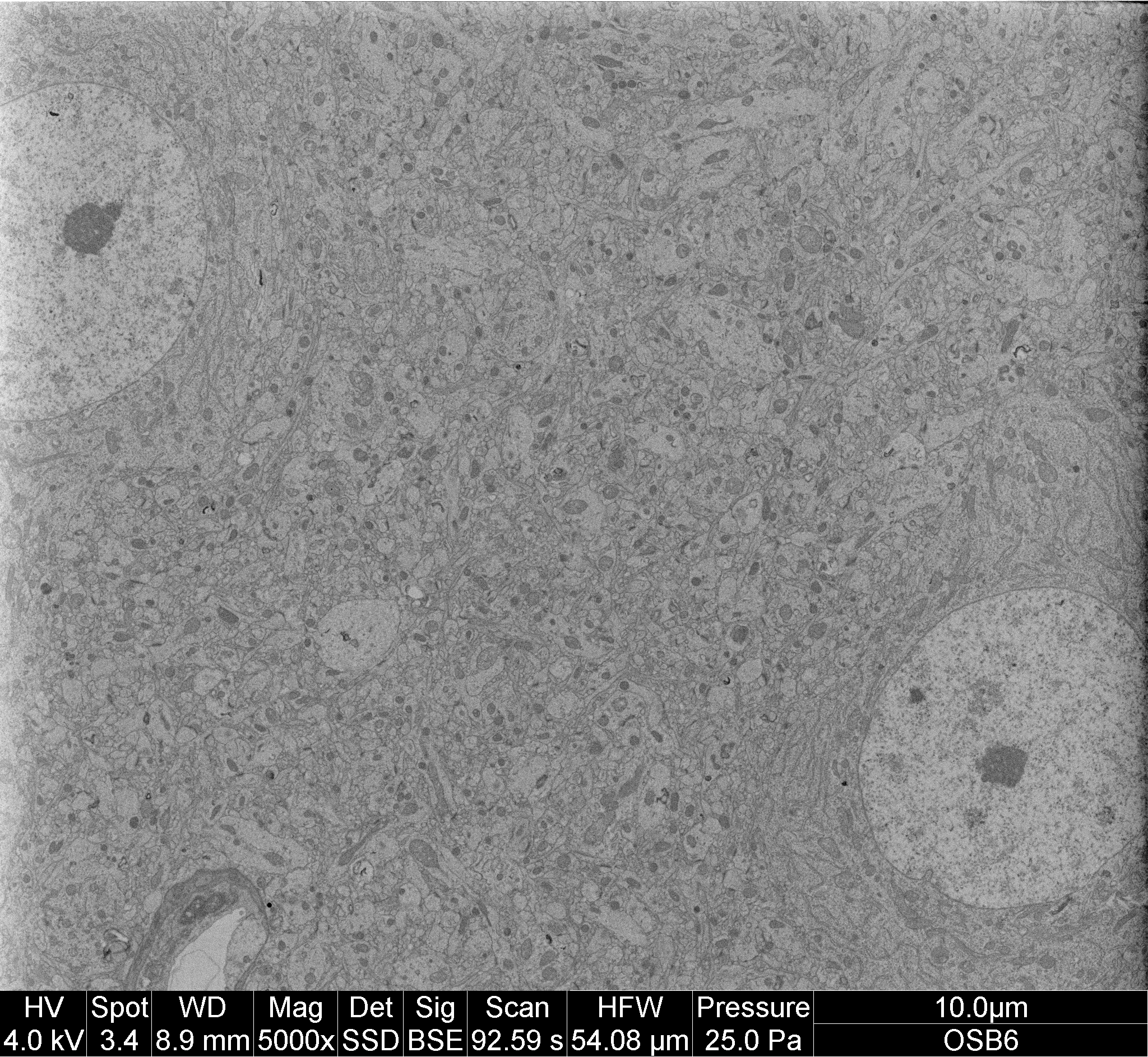

Supplement: Dataset S20 — (254.9 MB ZIP). [file pbio.0020329.sd020.zip › 040604_OS5_st1_1962.tif]

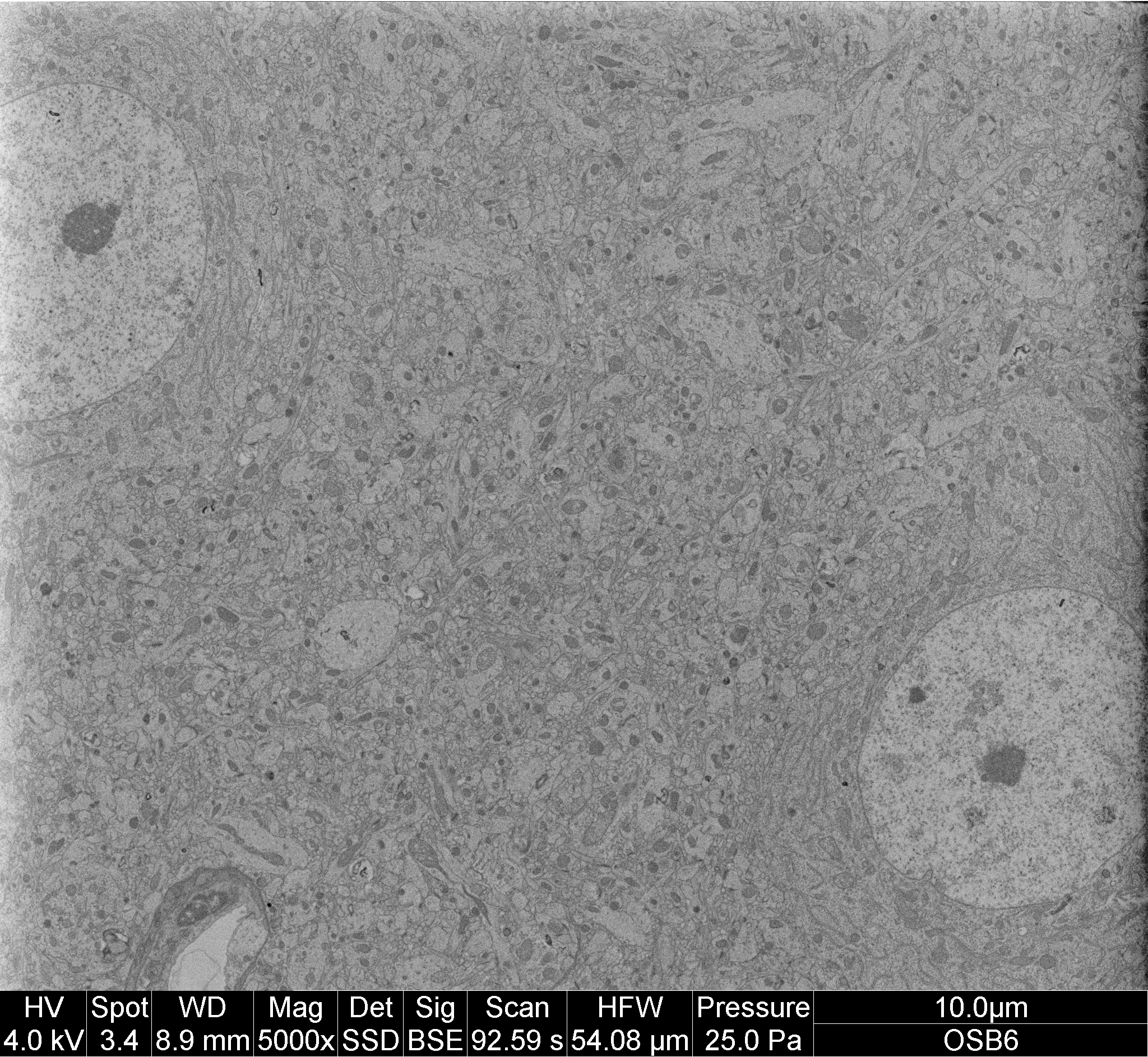

Supplement: Dataset S20 — (254.9 MB ZIP). [file pbio.0020329.sd020.zip › 040604_OS5_st1_1963.tif]

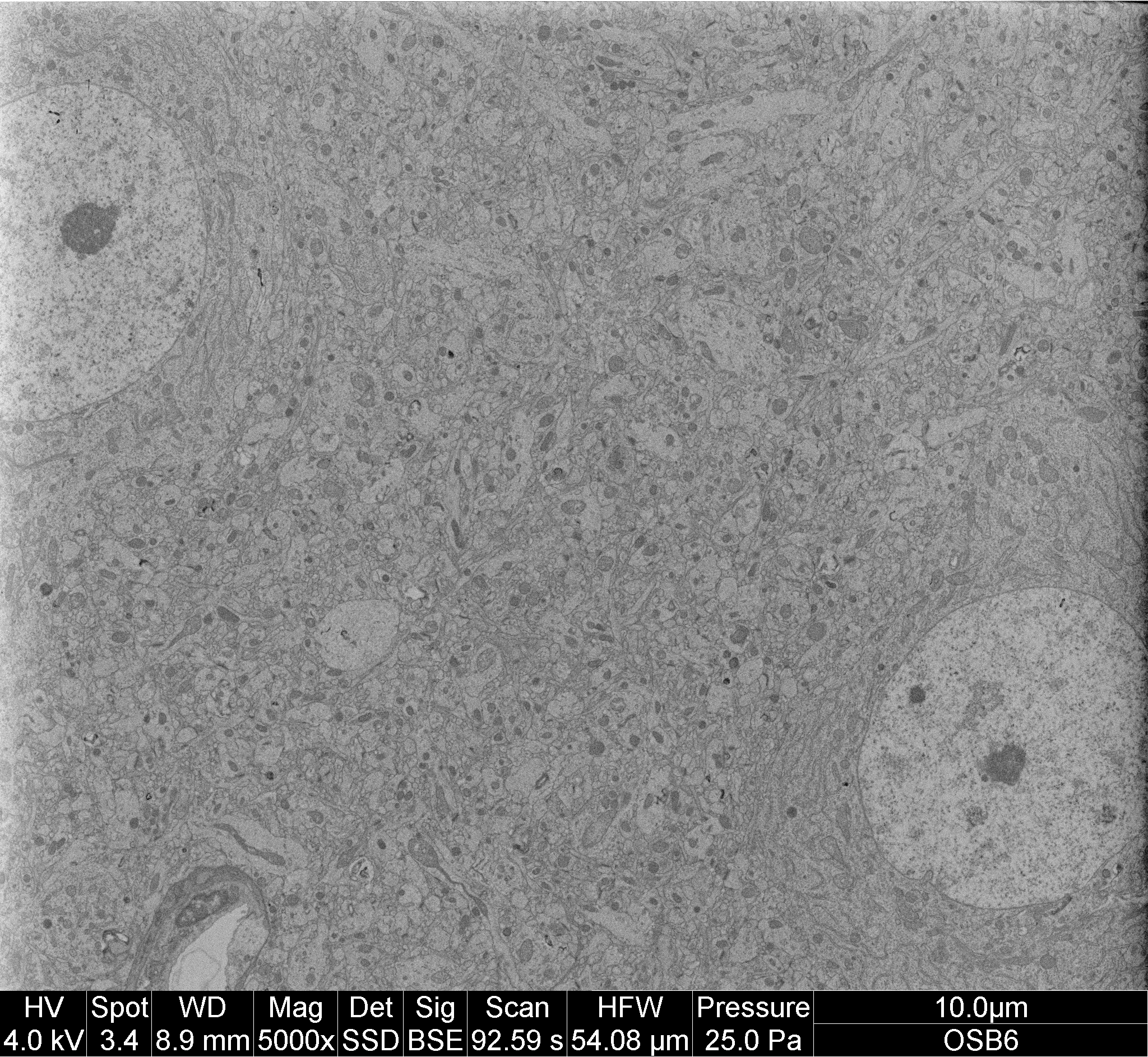

Supplement: Dataset S20 — (254.9 MB ZIP). [file pbio.0020329.sd020.zip › 040604_OS5_st1_1964.tif]

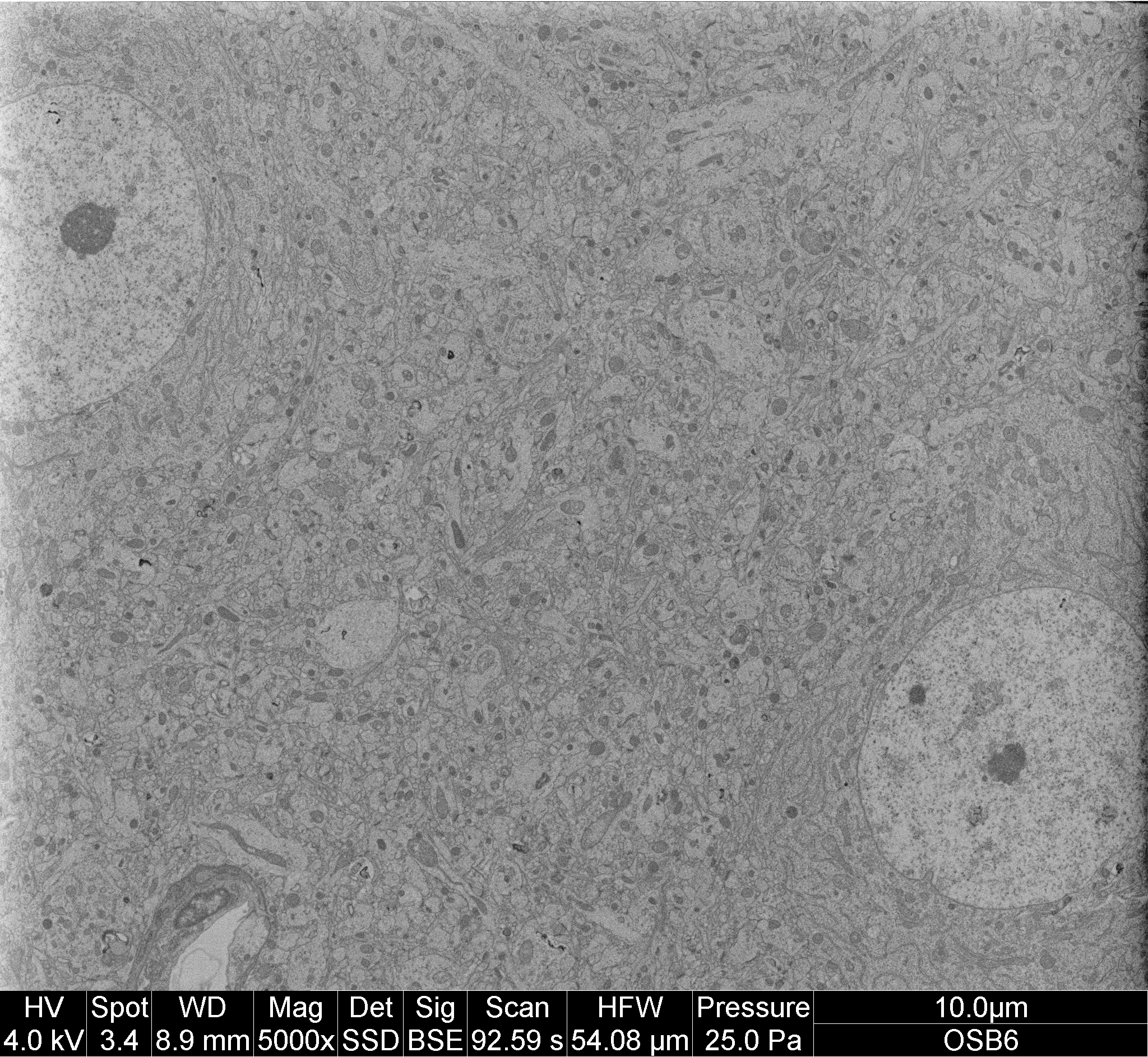

Supplement: Dataset S20 — (254.9 MB ZIP). [file pbio.0020329.sd020.zip › 040604_OS5_st1_1965.tif]

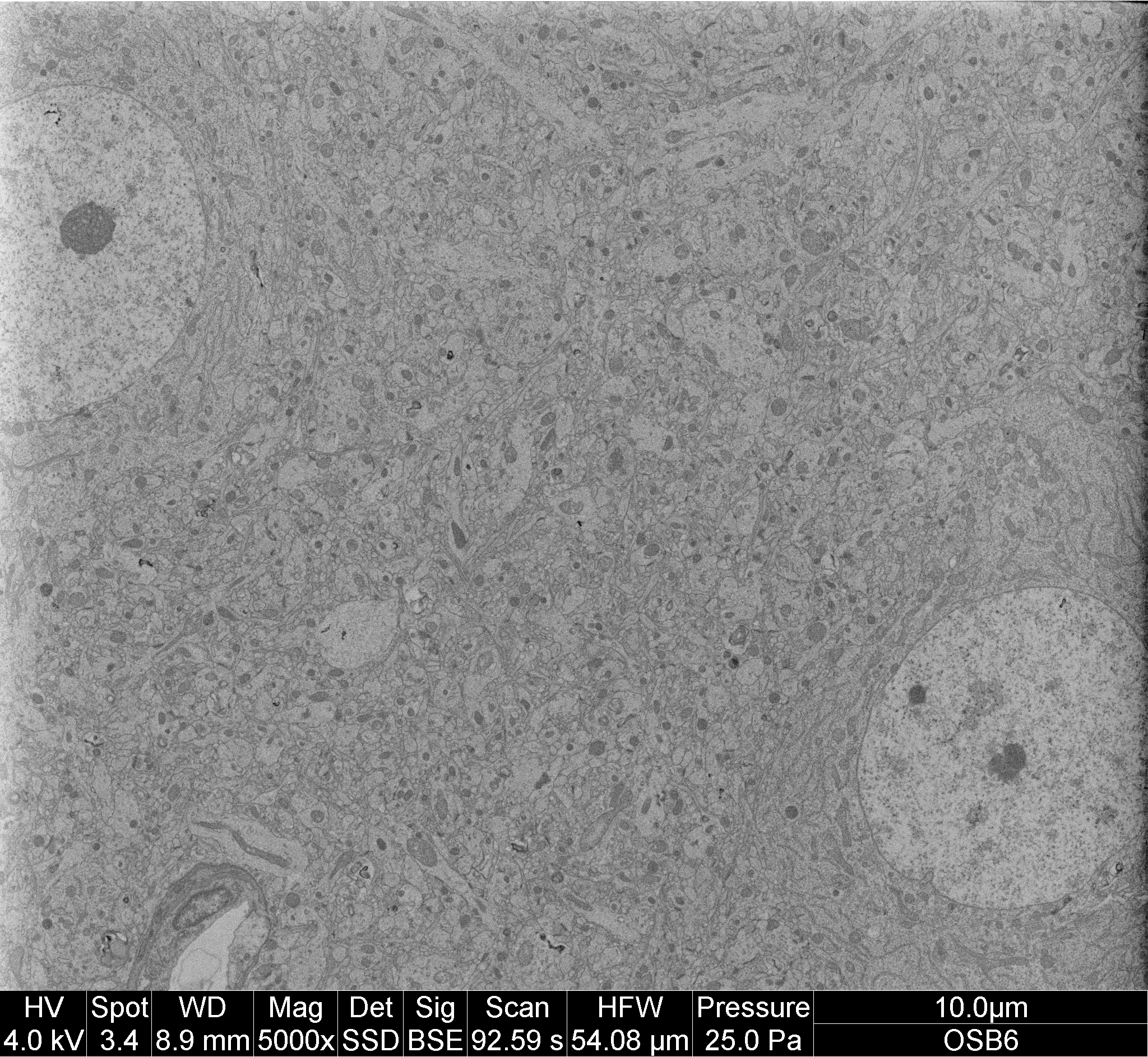

Supplement: Dataset S20 — (254.9 MB ZIP). [file pbio.0020329.sd020.zip › 040604_OS5_st1_1966.tif]

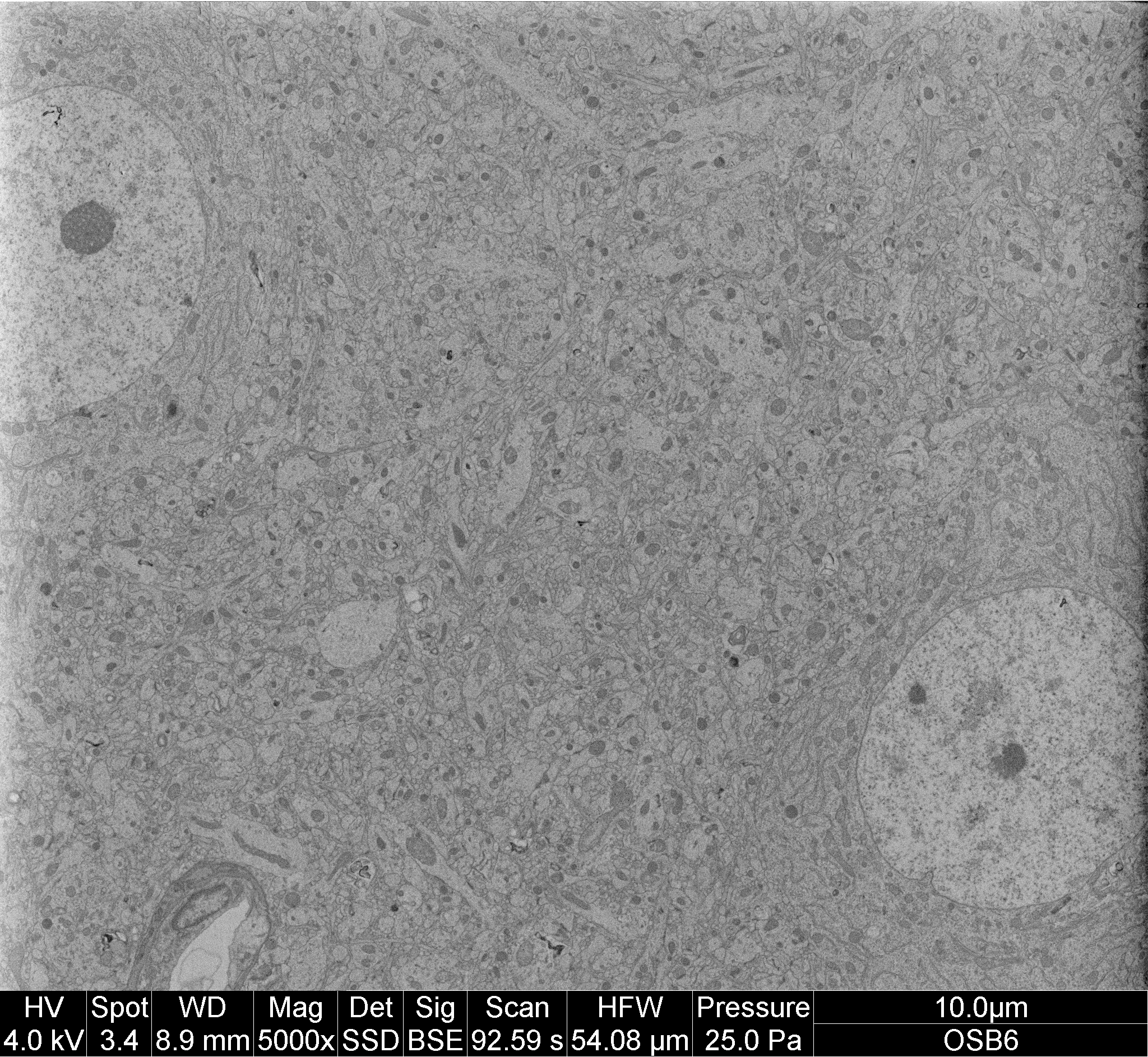

Supplement: Dataset S20 — (254.9 MB ZIP). [file pbio.0020329.sd020.zip › 040604_OS5_st1_1967.tif]

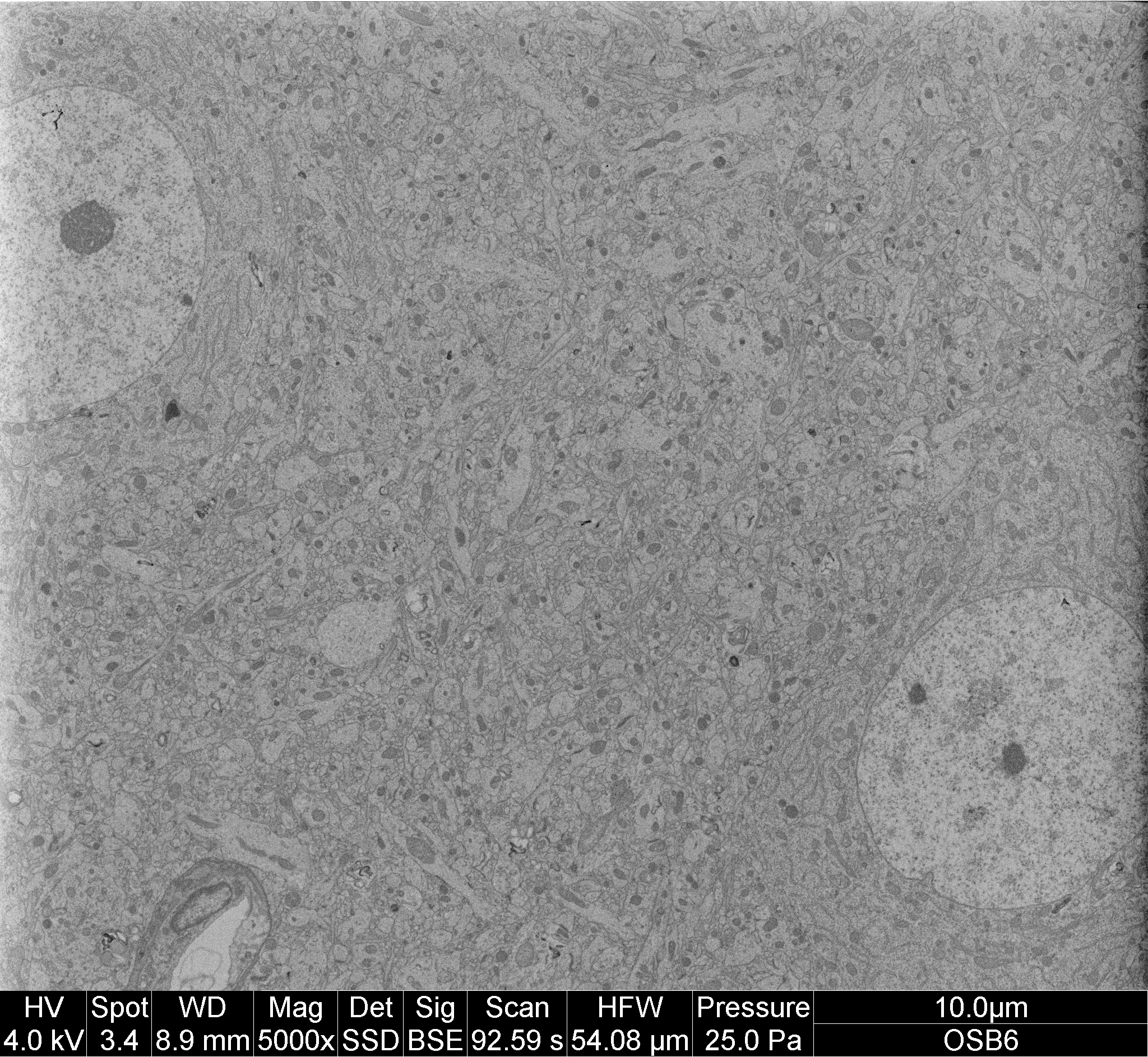

Supplement: Dataset S20 — (254.9 MB ZIP). [file pbio.0020329.sd020.zip › 040604_OS5_st1_1968.tif]

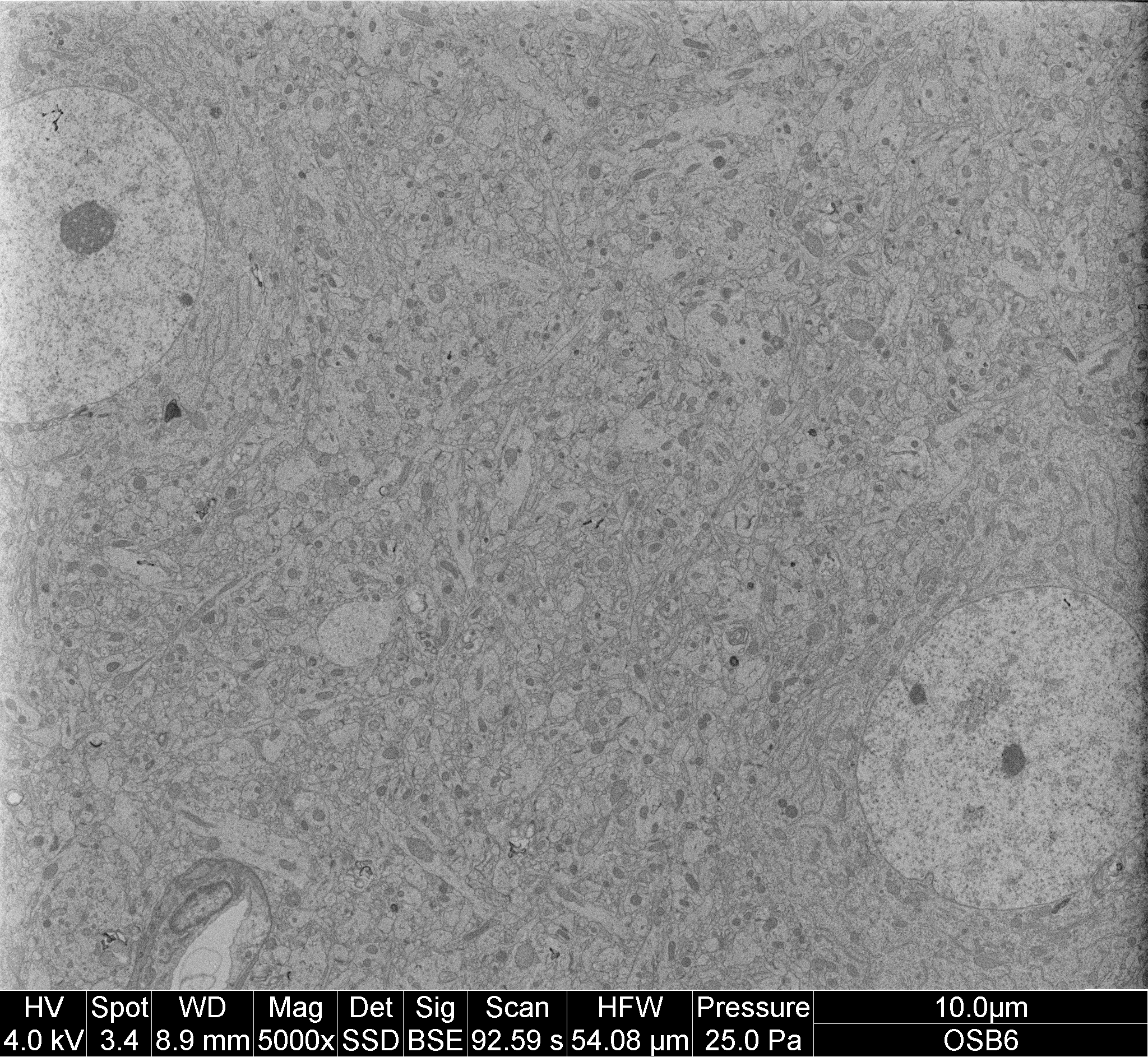

Supplement: Dataset S20 — (254.9 MB ZIP). [file pbio.0020329.sd020.zip › 040604_OS5_st1_1969.tif]

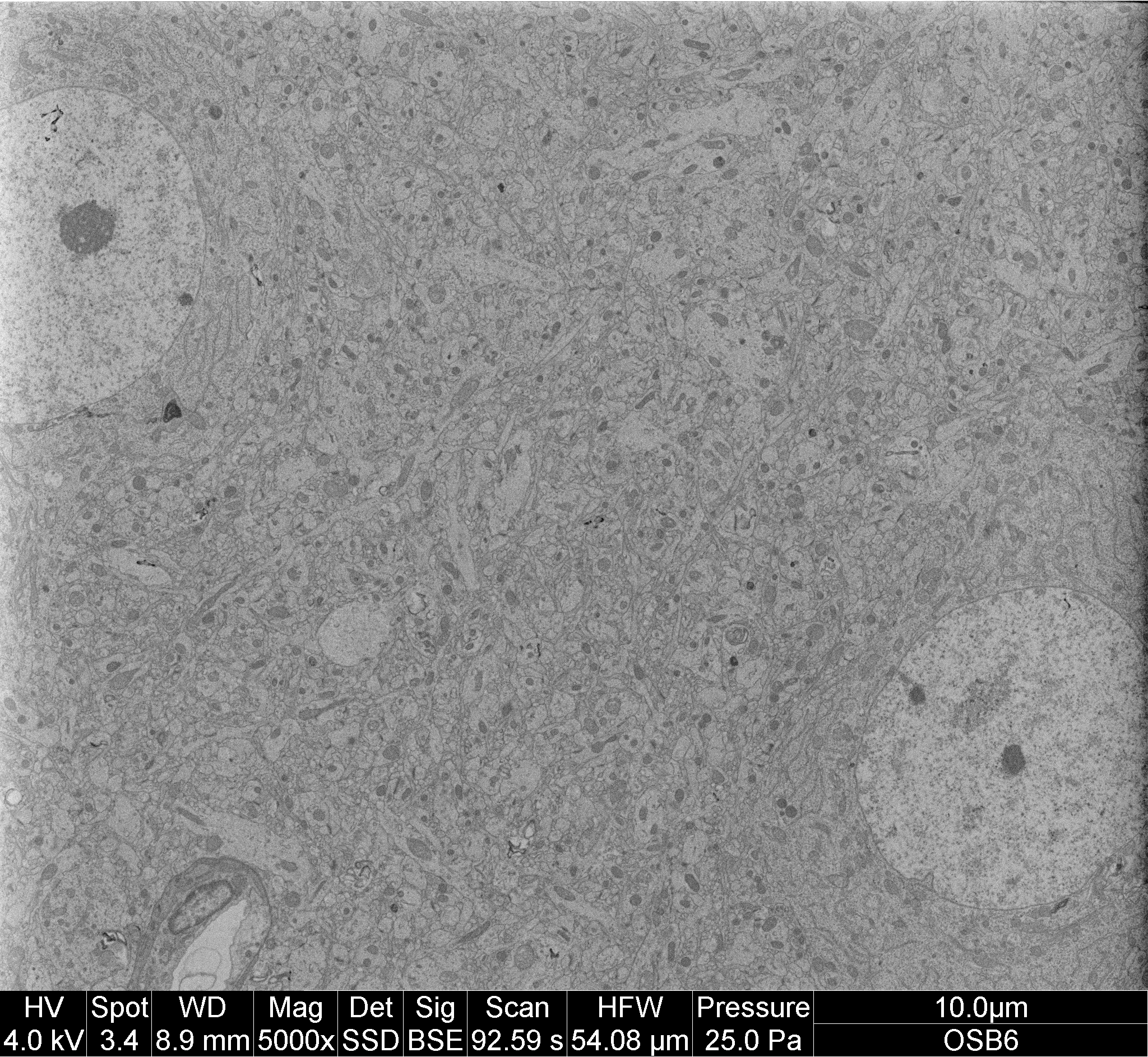

Supplement: Dataset S20 — (254.9 MB ZIP). [file pbio.0020329.sd020.zip › 040604_OS5_st1_1970.tif]

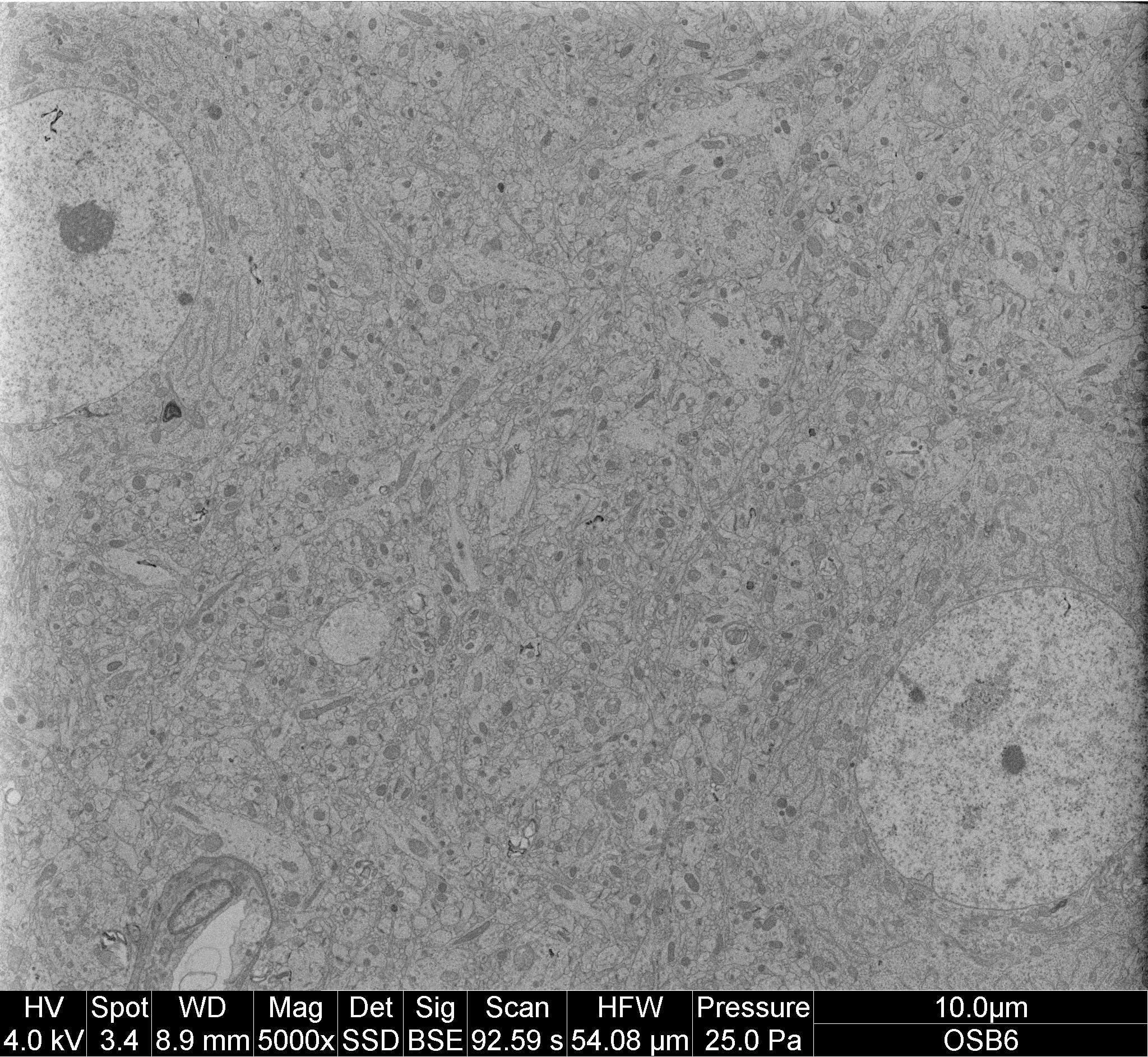

Supplement: Dataset S20 — (254.9 MB ZIP). [file pbio.0020329.sd020.zip › 040604_OS5_st1_1971.tif]

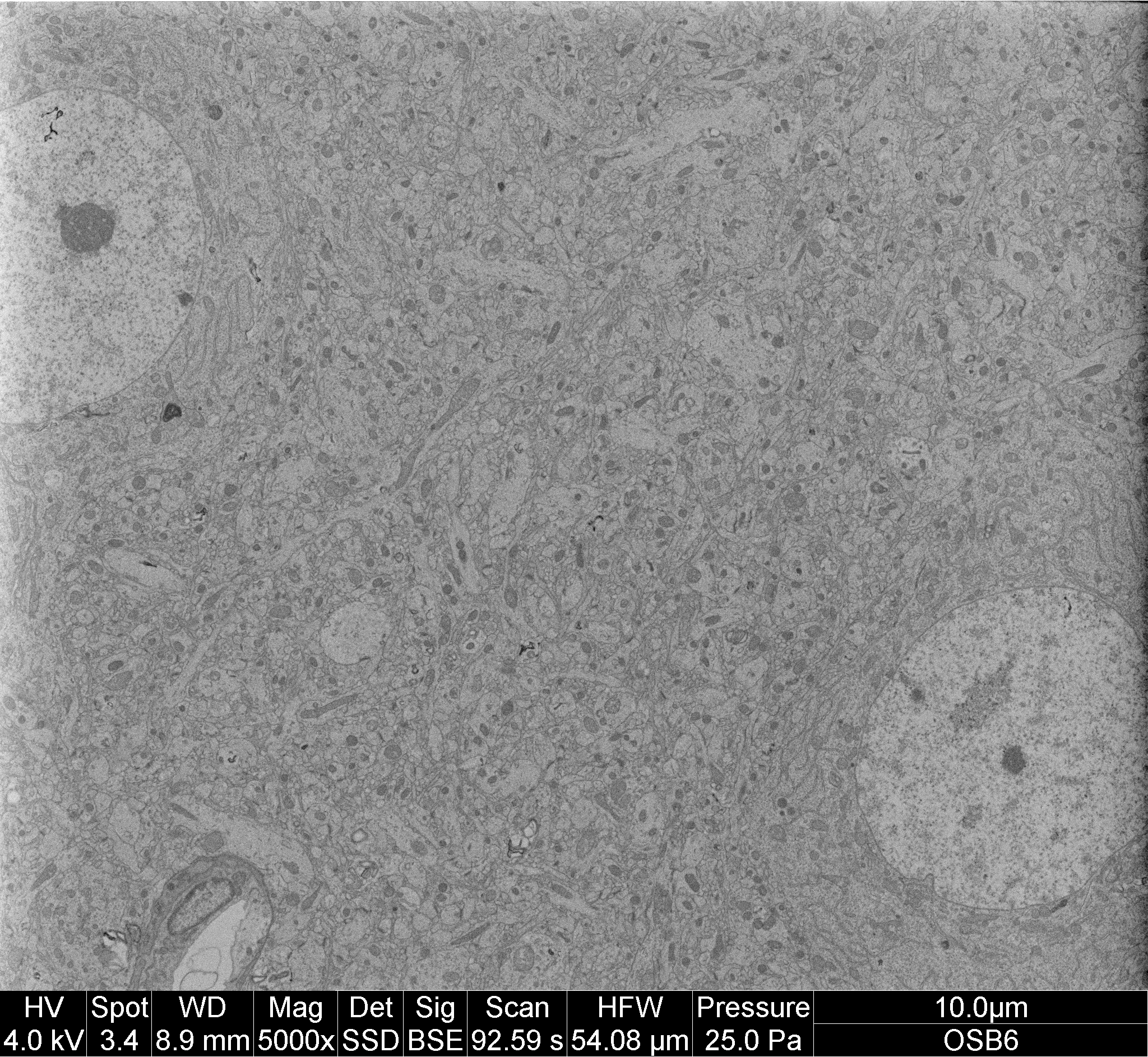

Supplement: Dataset S20 — (254.9 MB ZIP). [file pbio.0020329.sd020.zip › 040604_OS5_st1_1972.tif]

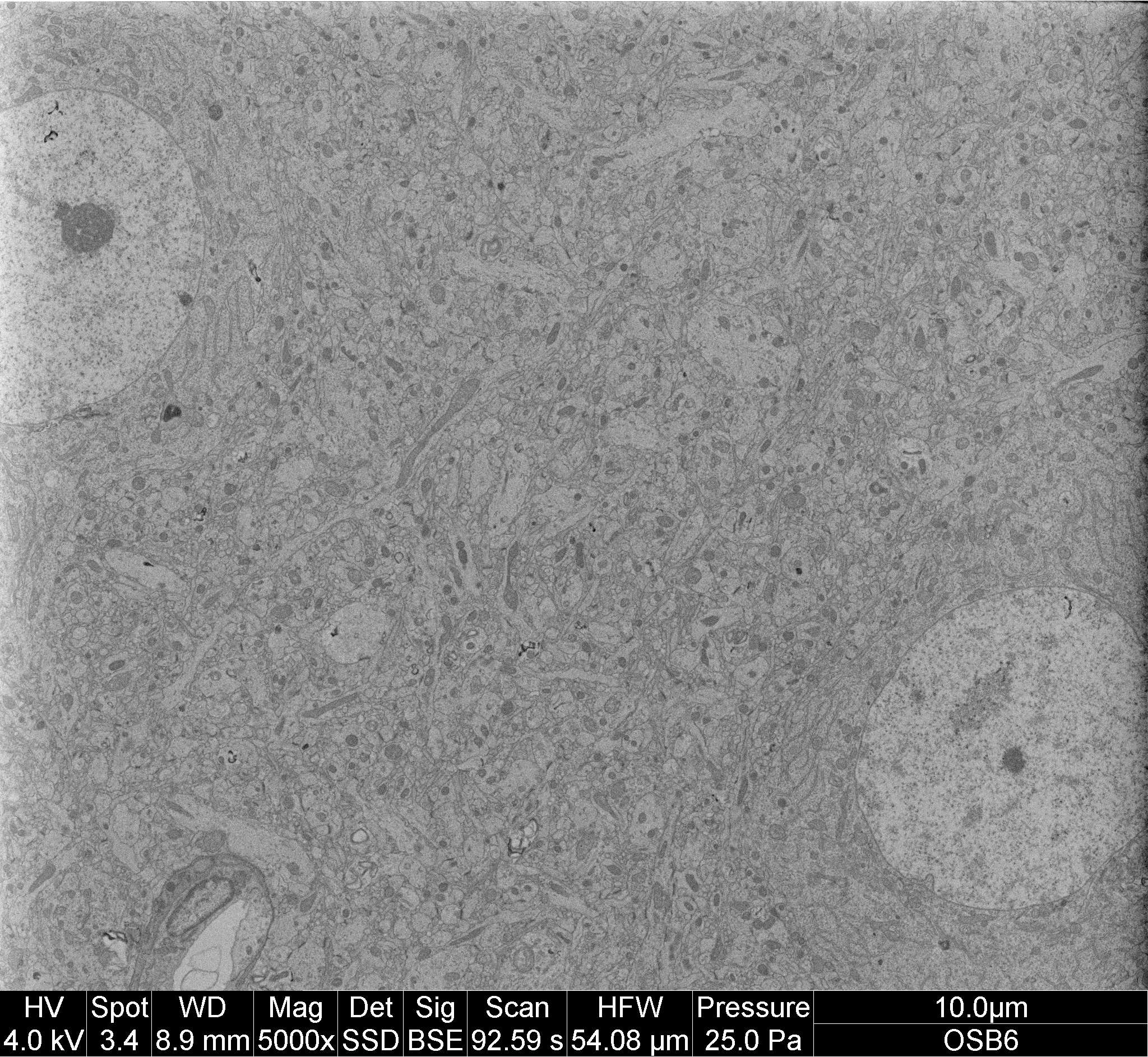

Supplement: Dataset S20 — (254.9 MB ZIP). [file pbio.0020329.sd020.zip › 040604_OS5_st1_1973.tif]

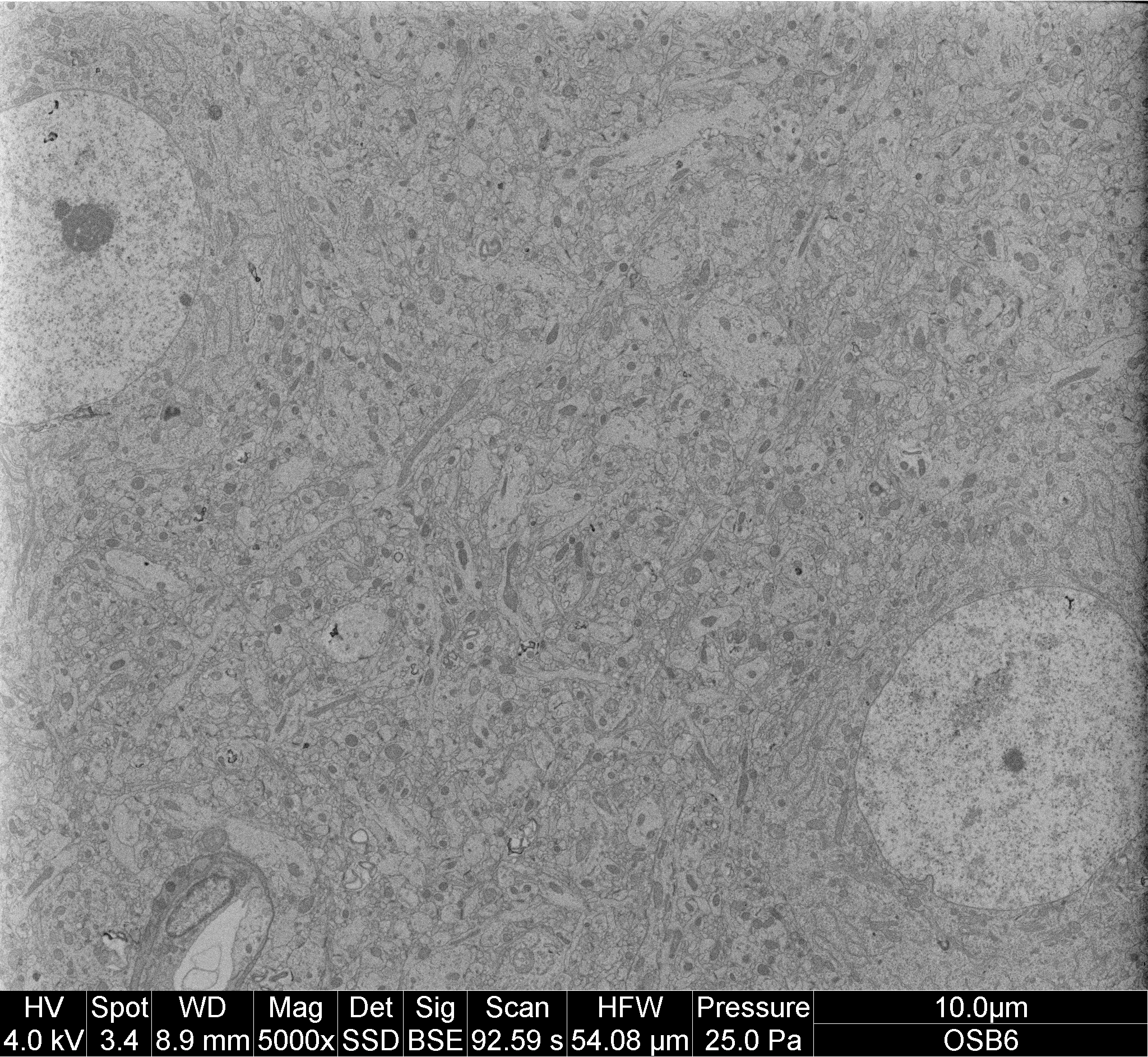

Supplement: Dataset S20 — (254.9 MB ZIP). [file pbio.0020329.sd020.zip › 040604_OS5_st1_1974.tif]

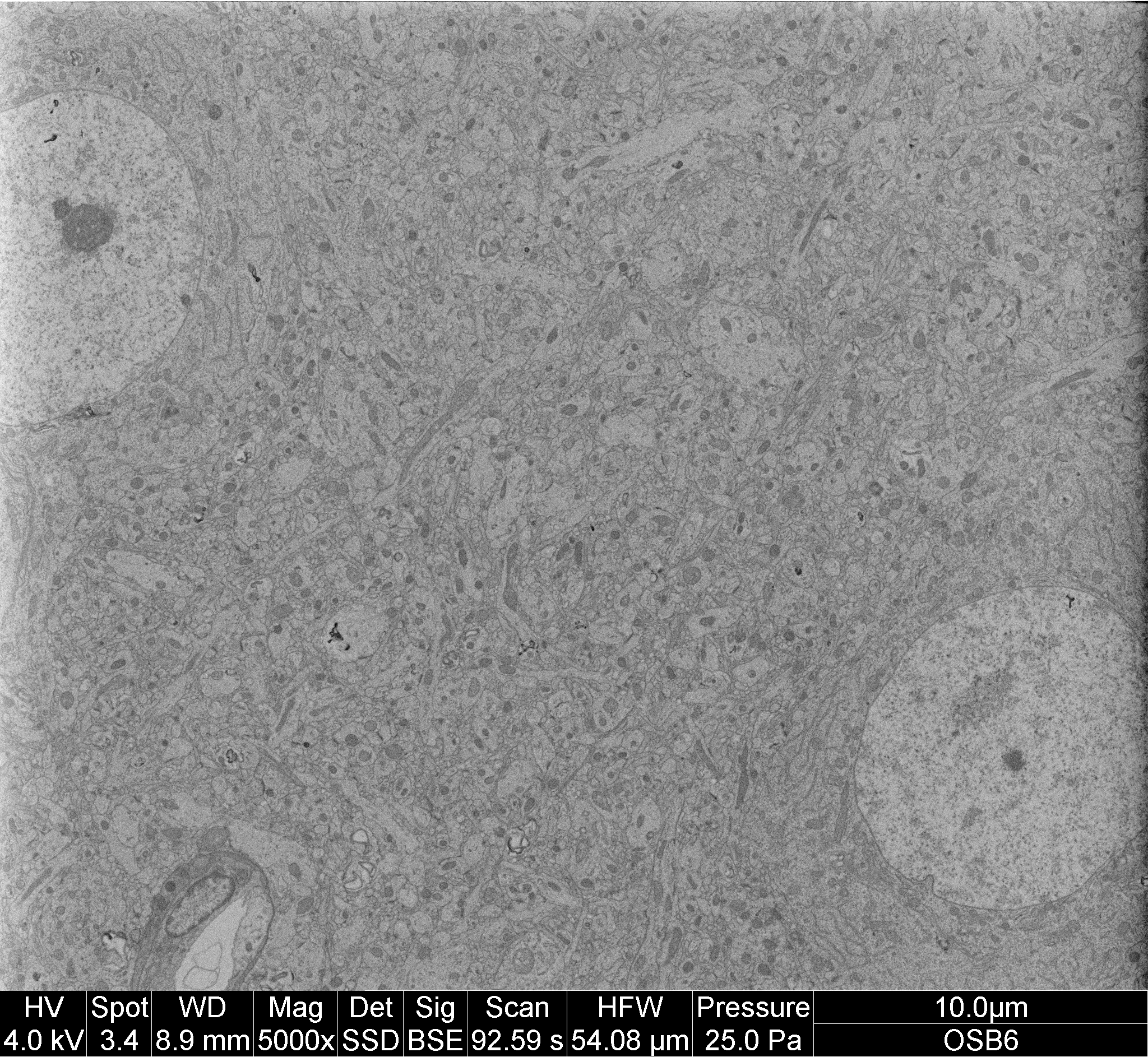

Supplement: Dataset S20 — (254.9 MB ZIP). [file pbio.0020329.sd020.zip › 040604_OS5_st1_1975.tif]

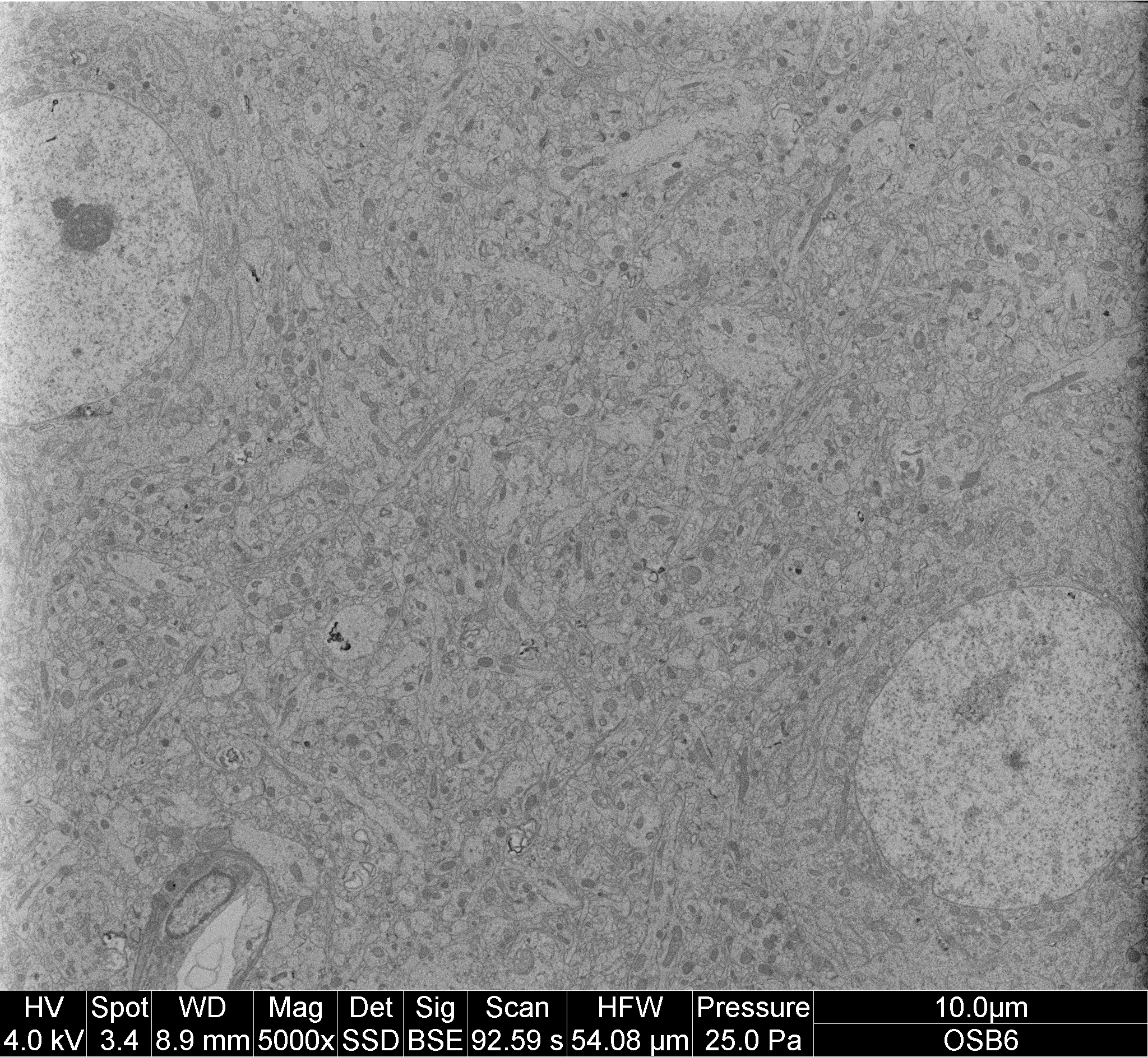

Supplement: Dataset S20 — (254.9 MB ZIP). [file pbio.0020329.sd020.zip › 040604_OS5_st1_1976.tif]

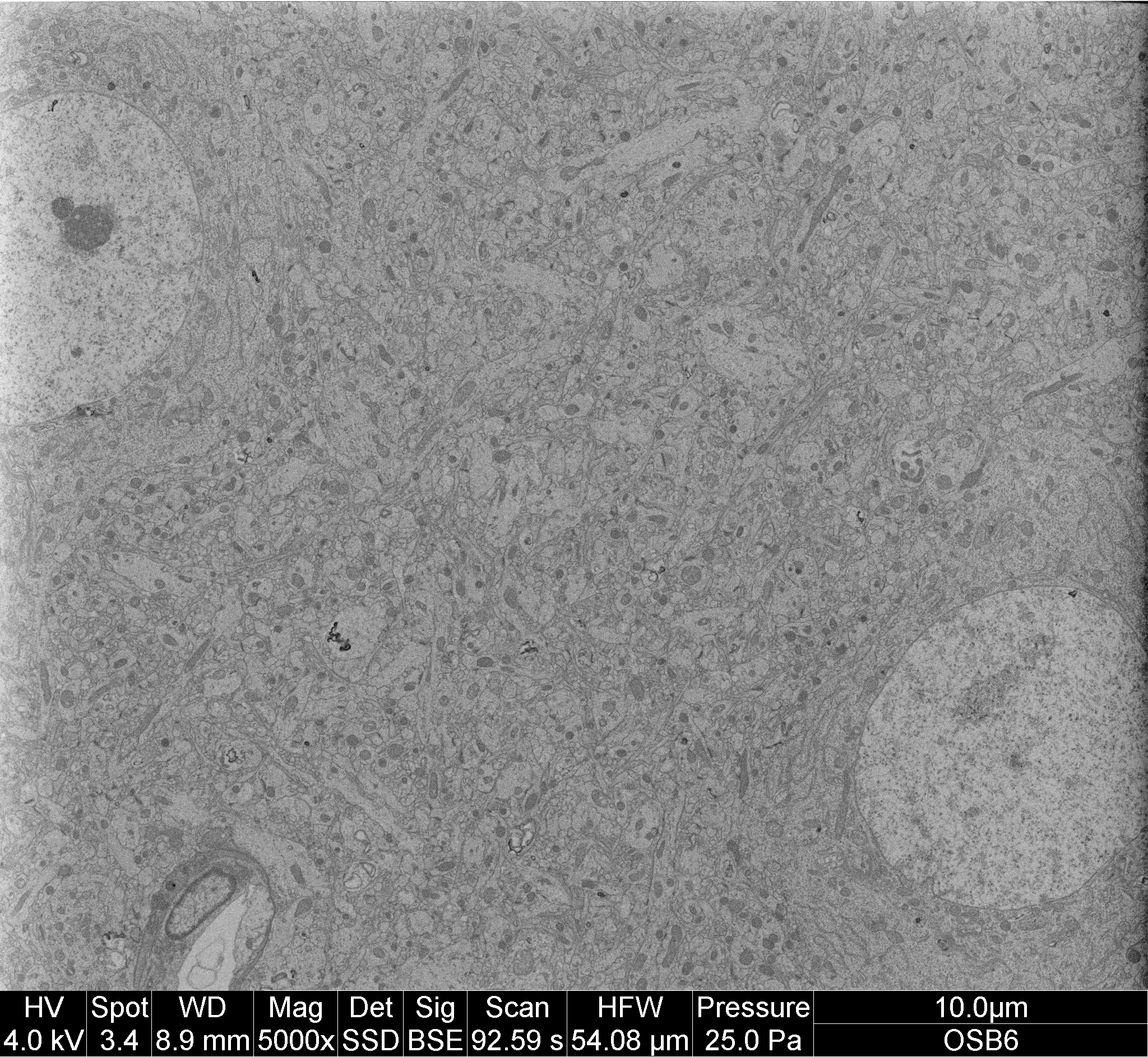

Supplement: Dataset S20 — (254.9 MB ZIP). [file pbio.0020329.sd020.zip › 040604_OS5_st1_1977.tif]

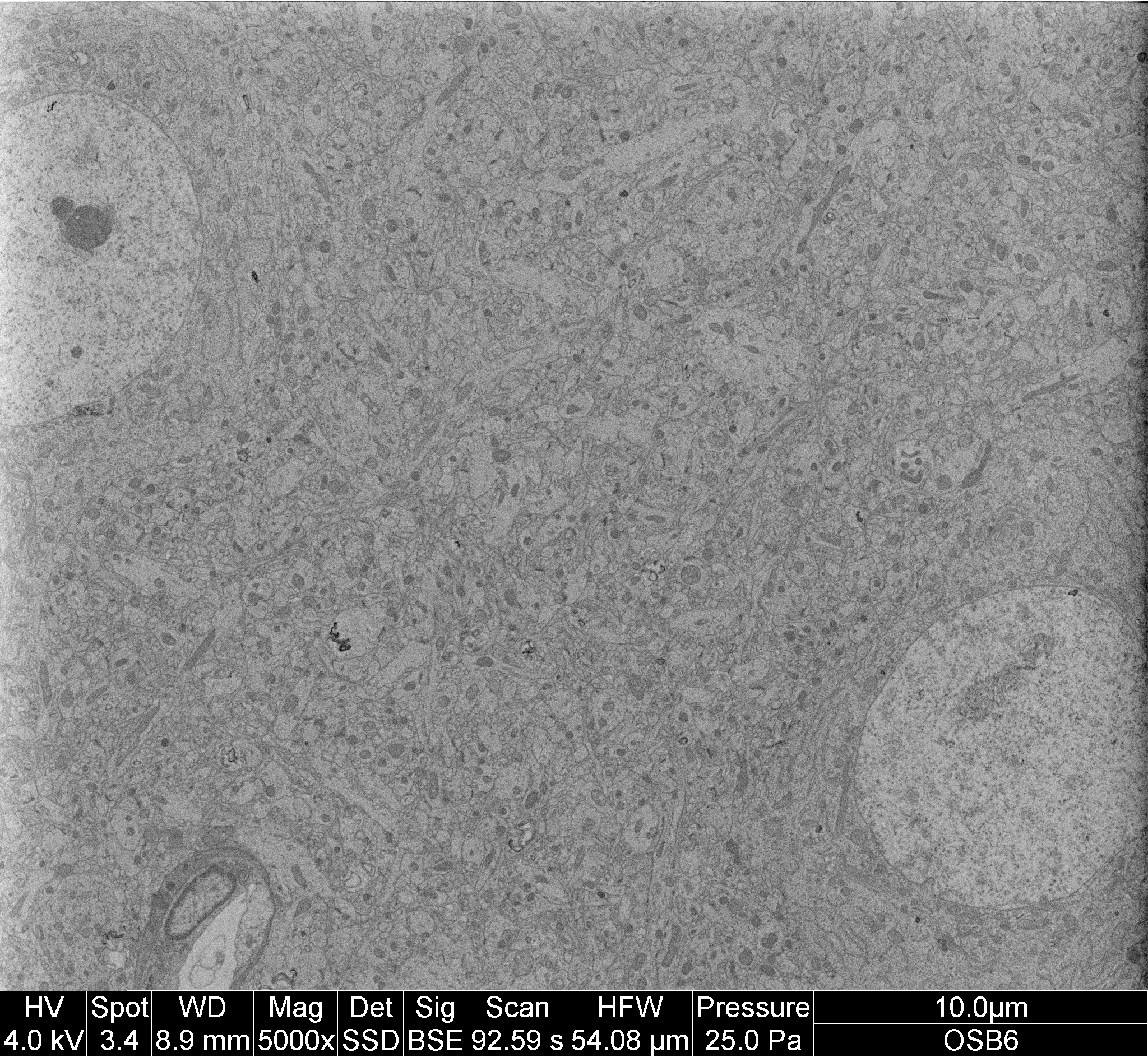

Supplement: Dataset S20 — (254.9 MB ZIP). [file pbio.0020329.sd020.zip › 040604_OS5_st1_1978.tif]

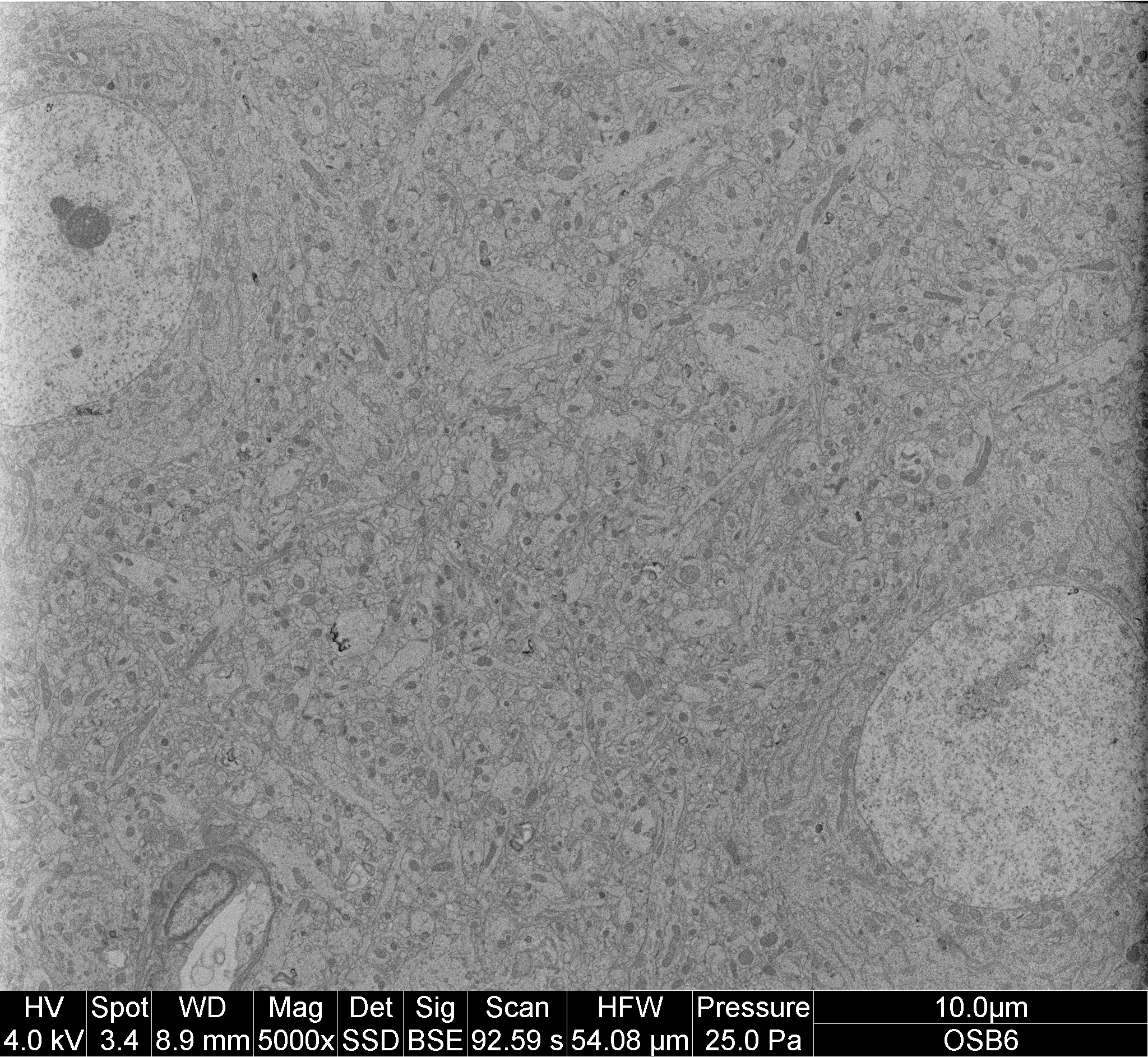

Supplement: Dataset S20 — (254.9 MB ZIP). [file pbio.0020329.sd020.zip › 040604_OS5_st1_1979.tif]

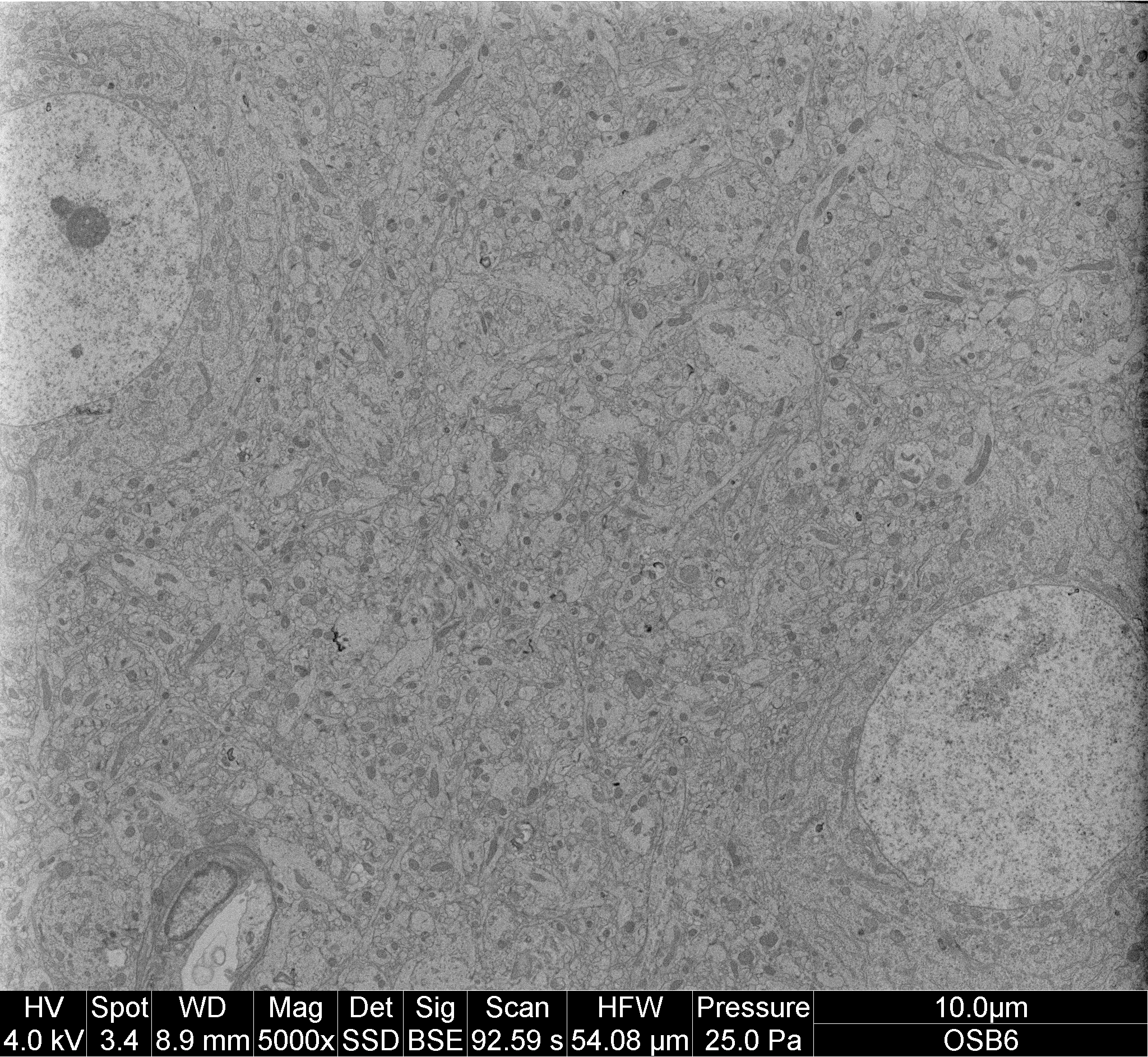

Supplement: Dataset S20 — (254.9 MB ZIP). [file pbio.0020329.sd020.zip › 040604_OS5_st1_1980.tif]

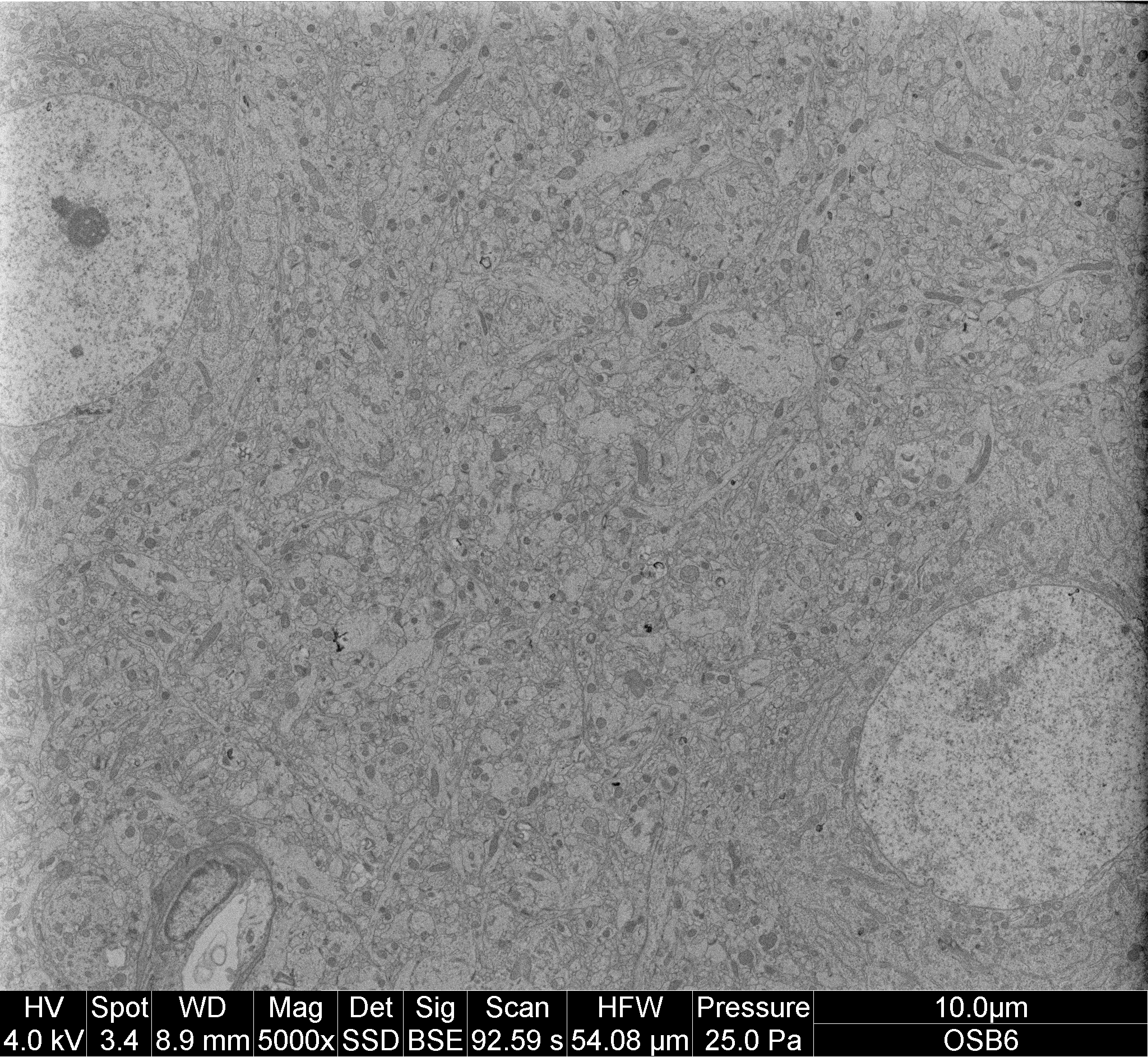

Supplement: Dataset S20 — (254.9 MB ZIP). [file pbio.0020329.sd020.zip › 040604_OS5_st1_1981.tif]

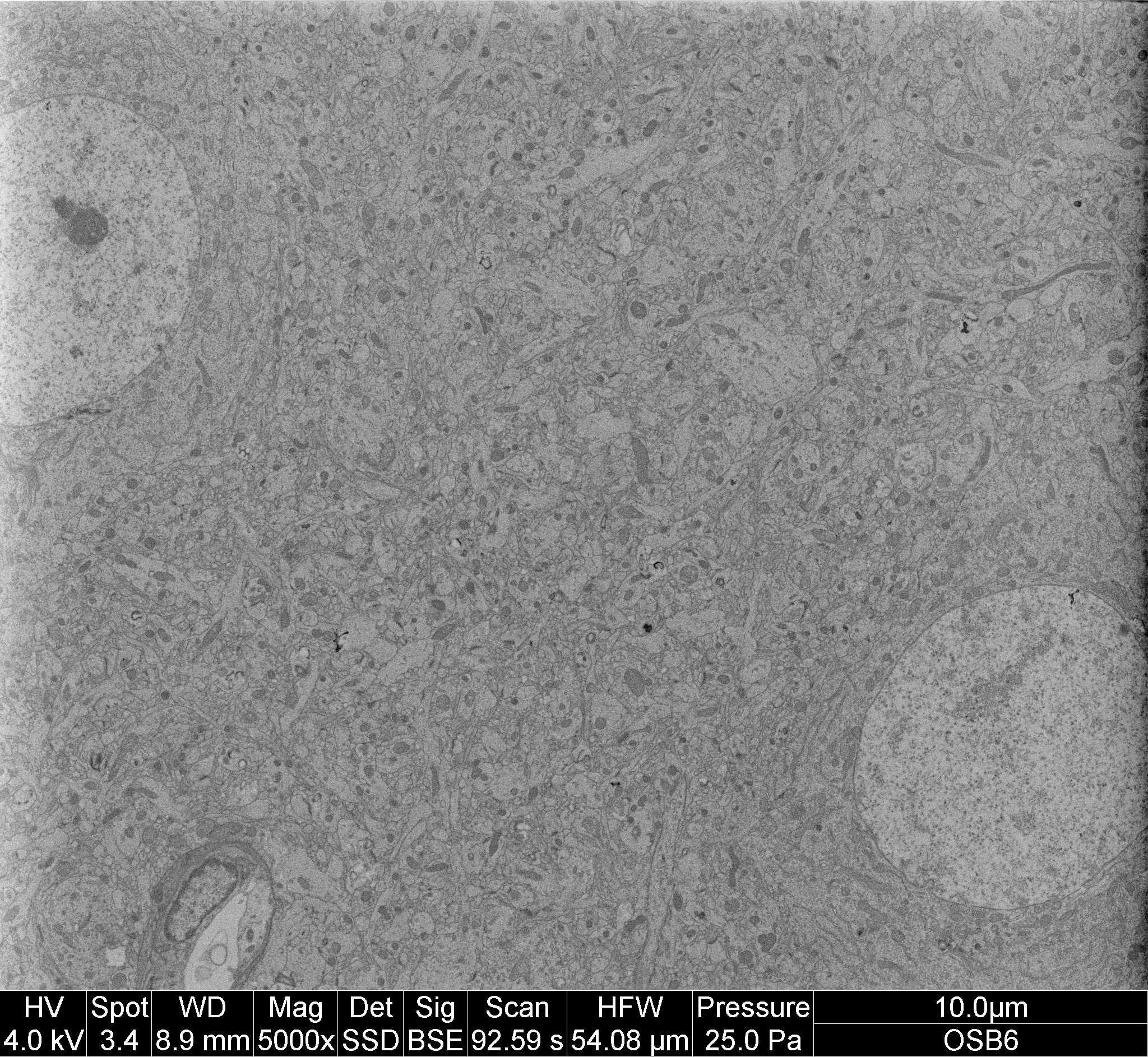

Supplement: Dataset S20 — (254.9 MB ZIP). [file pbio.0020329.sd020.zip › 040604_OS5_st1_1982.tif]

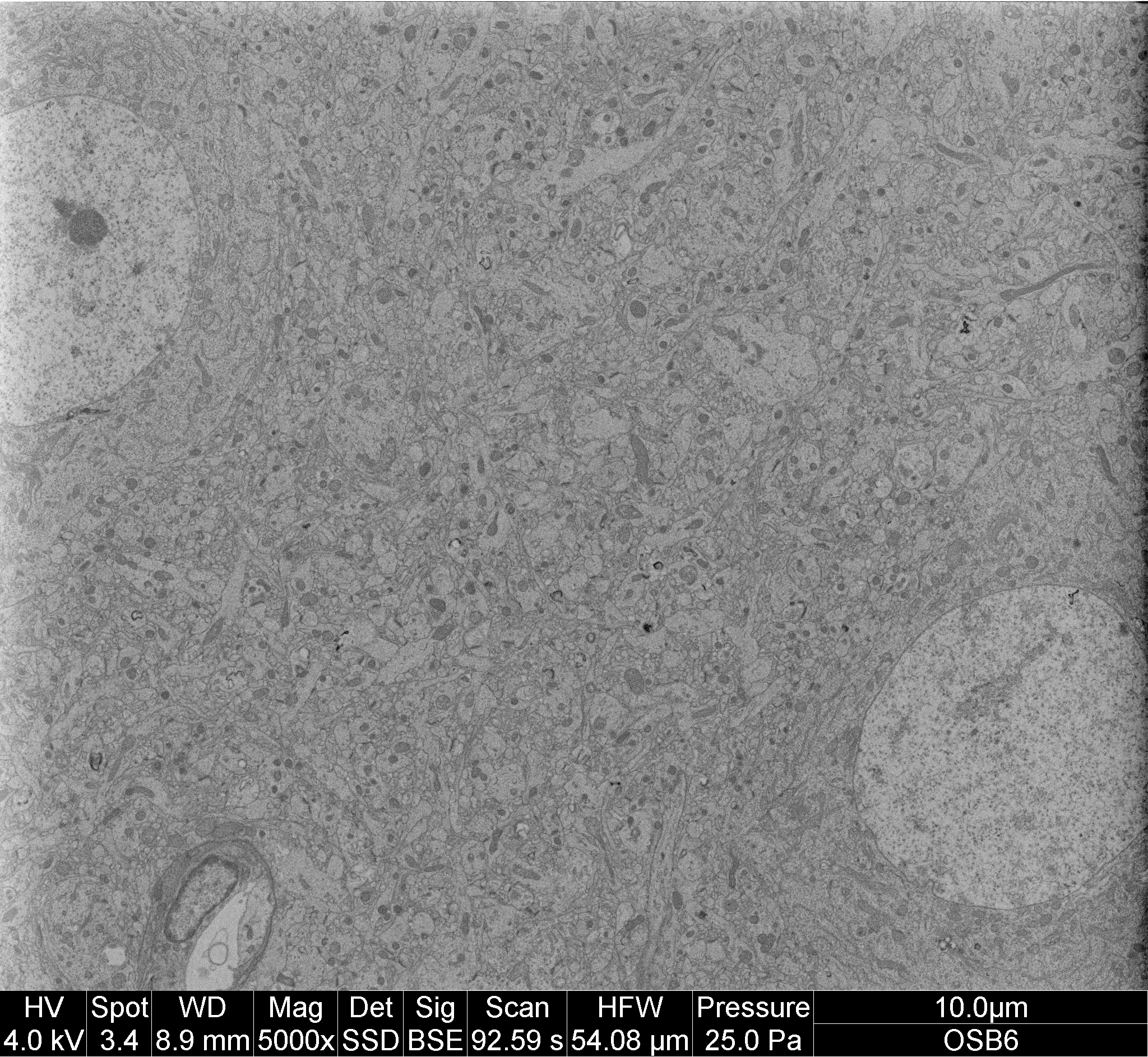

Supplement: Dataset S20 — (254.9 MB ZIP). [file pbio.0020329.sd020.zip › 040604_OS5_st1_1983.tif]

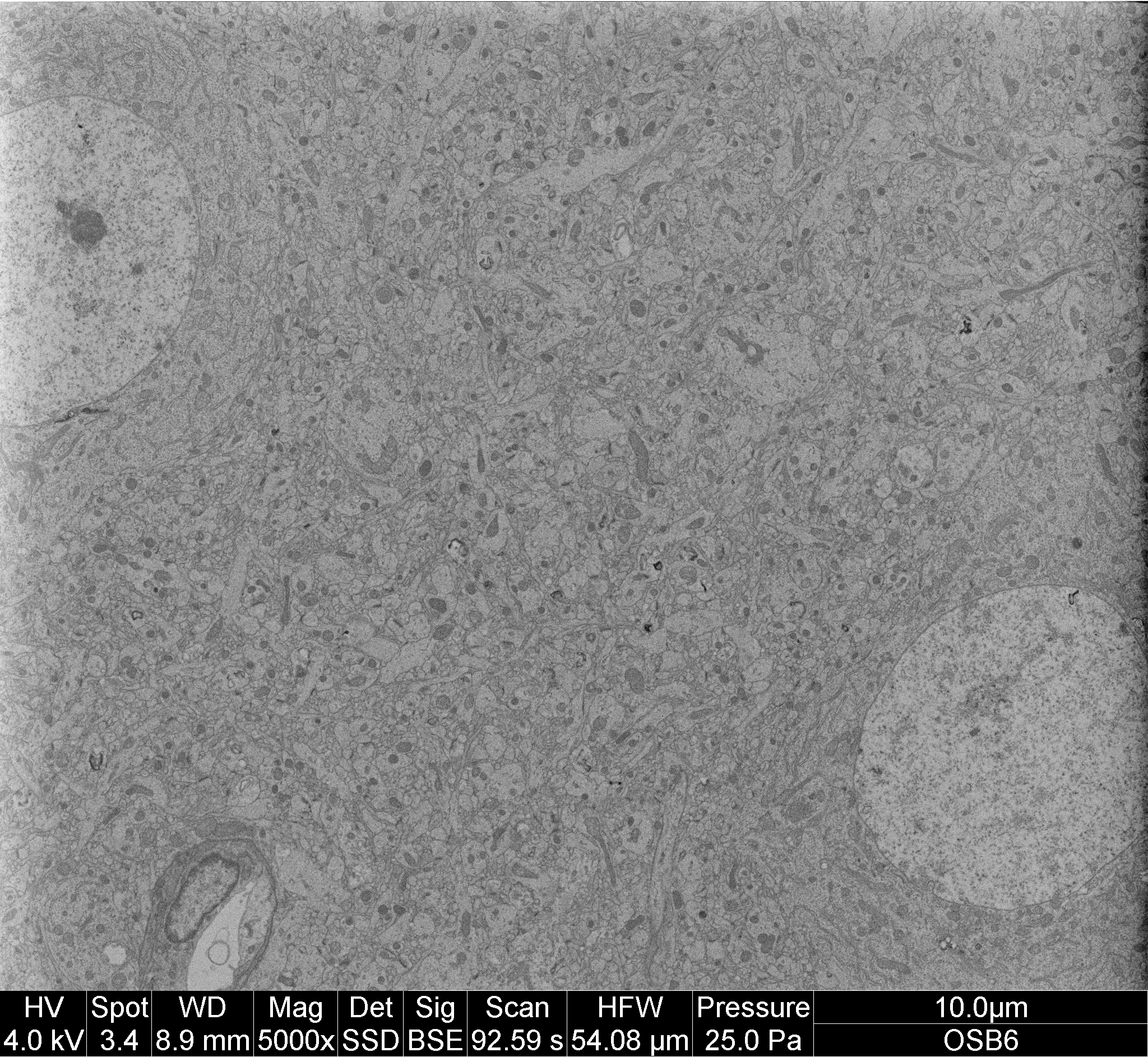

Supplement: Dataset S20 — (254.9 MB ZIP). [file pbio.0020329.sd020.zip › 040604_OS5_st1_1984.tif]

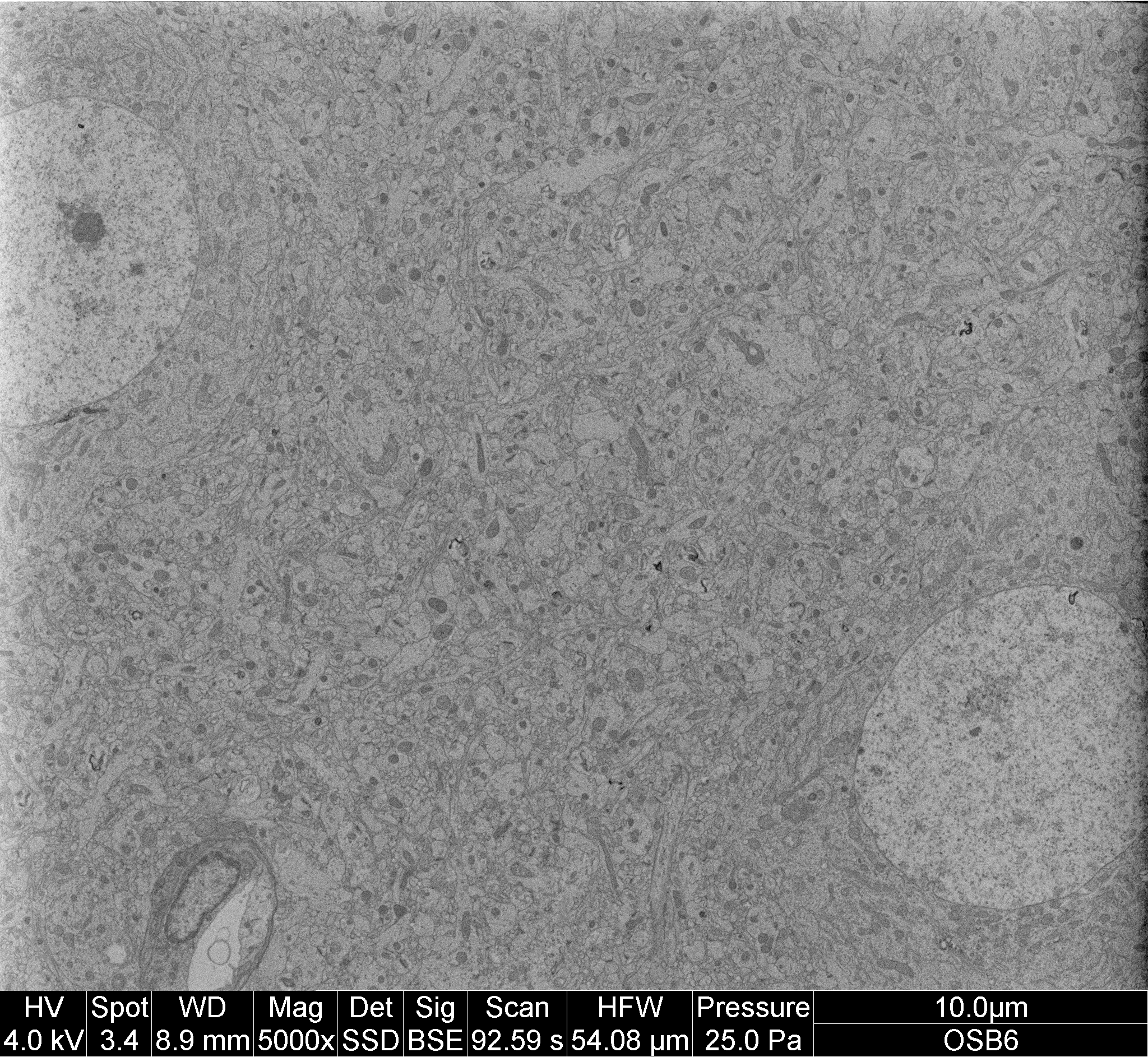

Supplement: Dataset S20 — (254.9 MB ZIP). [file pbio.0020329.sd020.zip › 040604_OS5_st1_1985.tif]

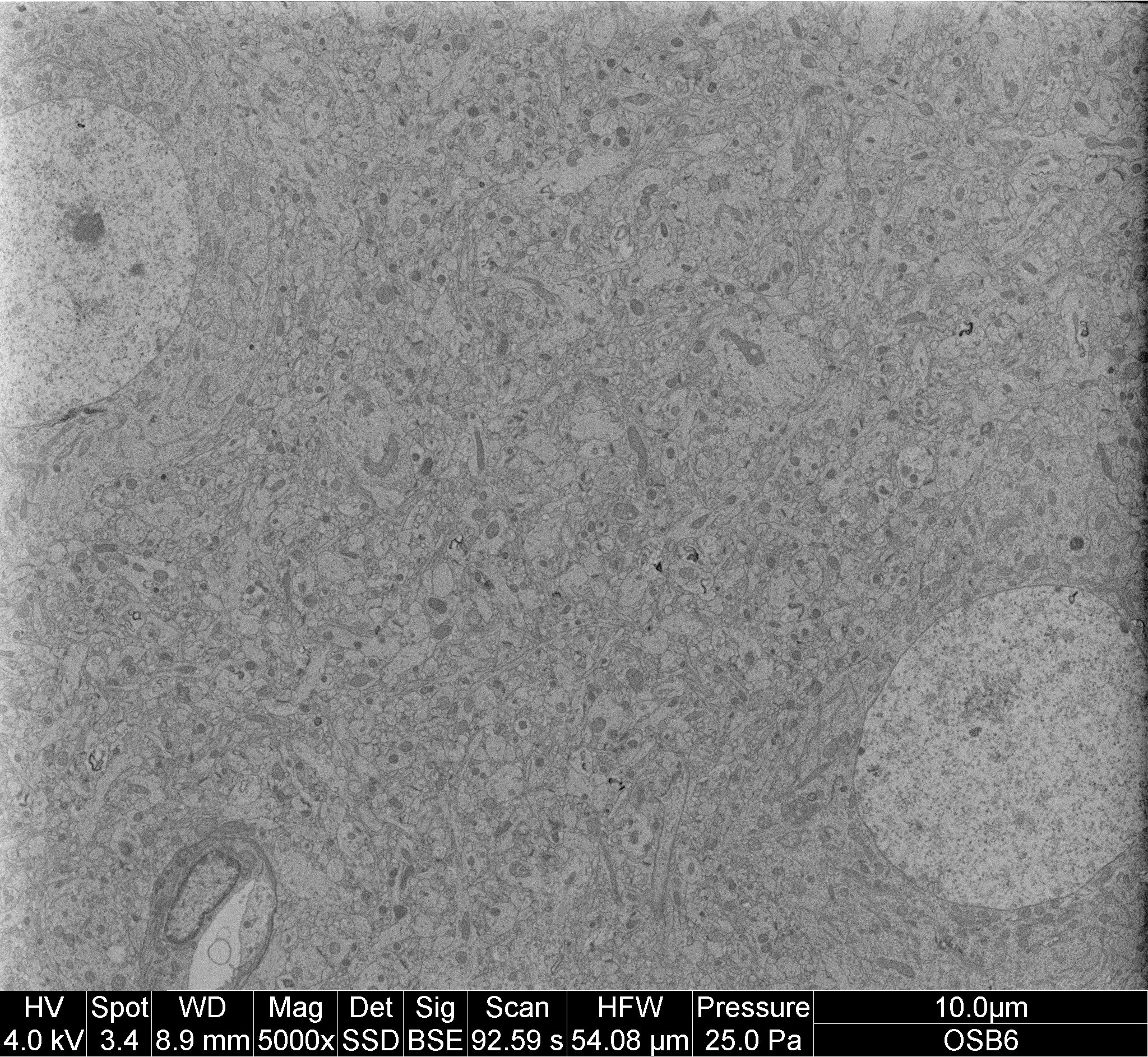

Supplement: Dataset S20 — (254.9 MB ZIP). [file pbio.0020329.sd020.zip › 040604_OS5_st1_1986.tif]

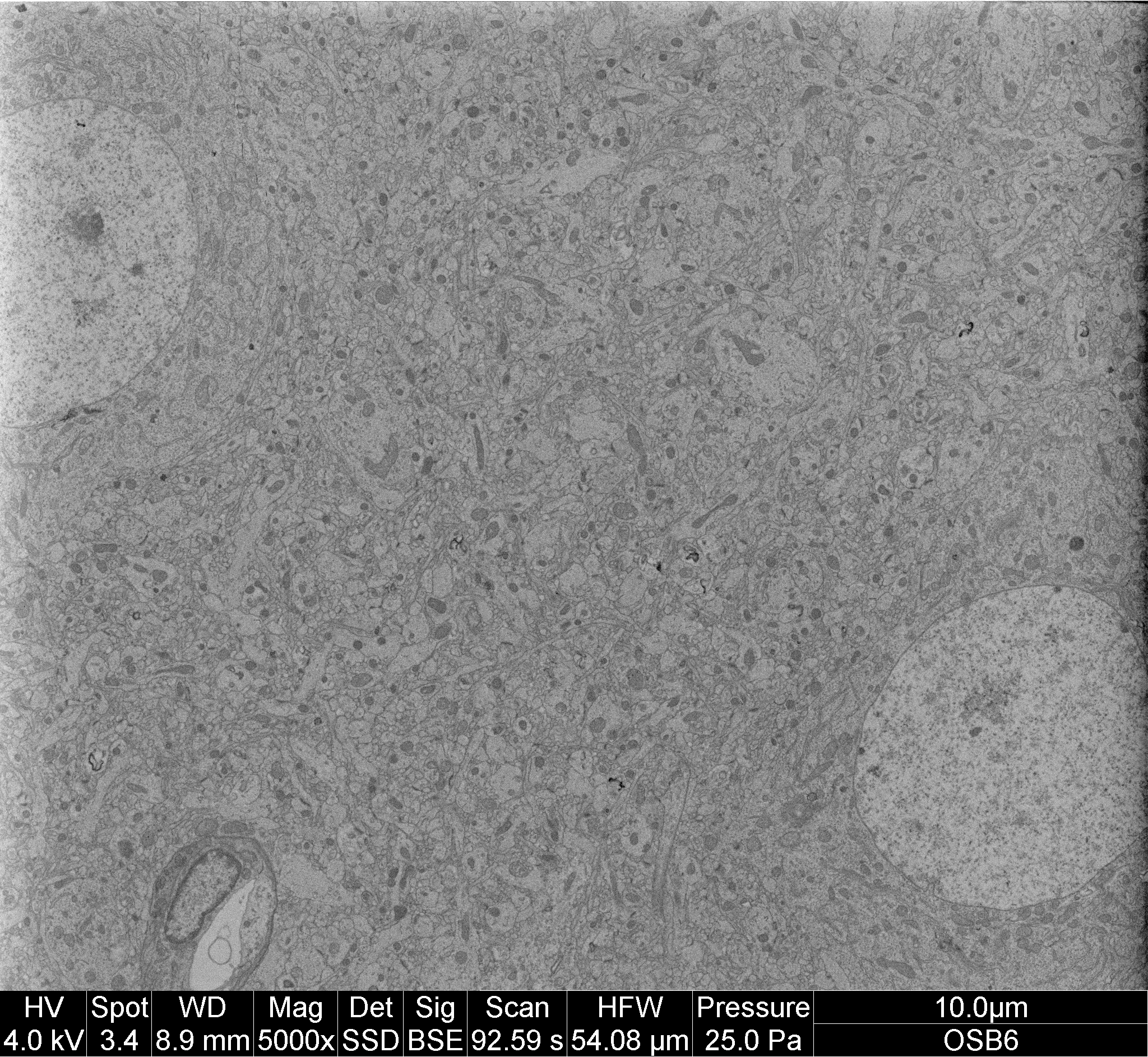

Supplement: Dataset S20 — (254.9 MB ZIP). [file pbio.0020329.sd020.zip › 040604_OS5_st1_1987.tif]

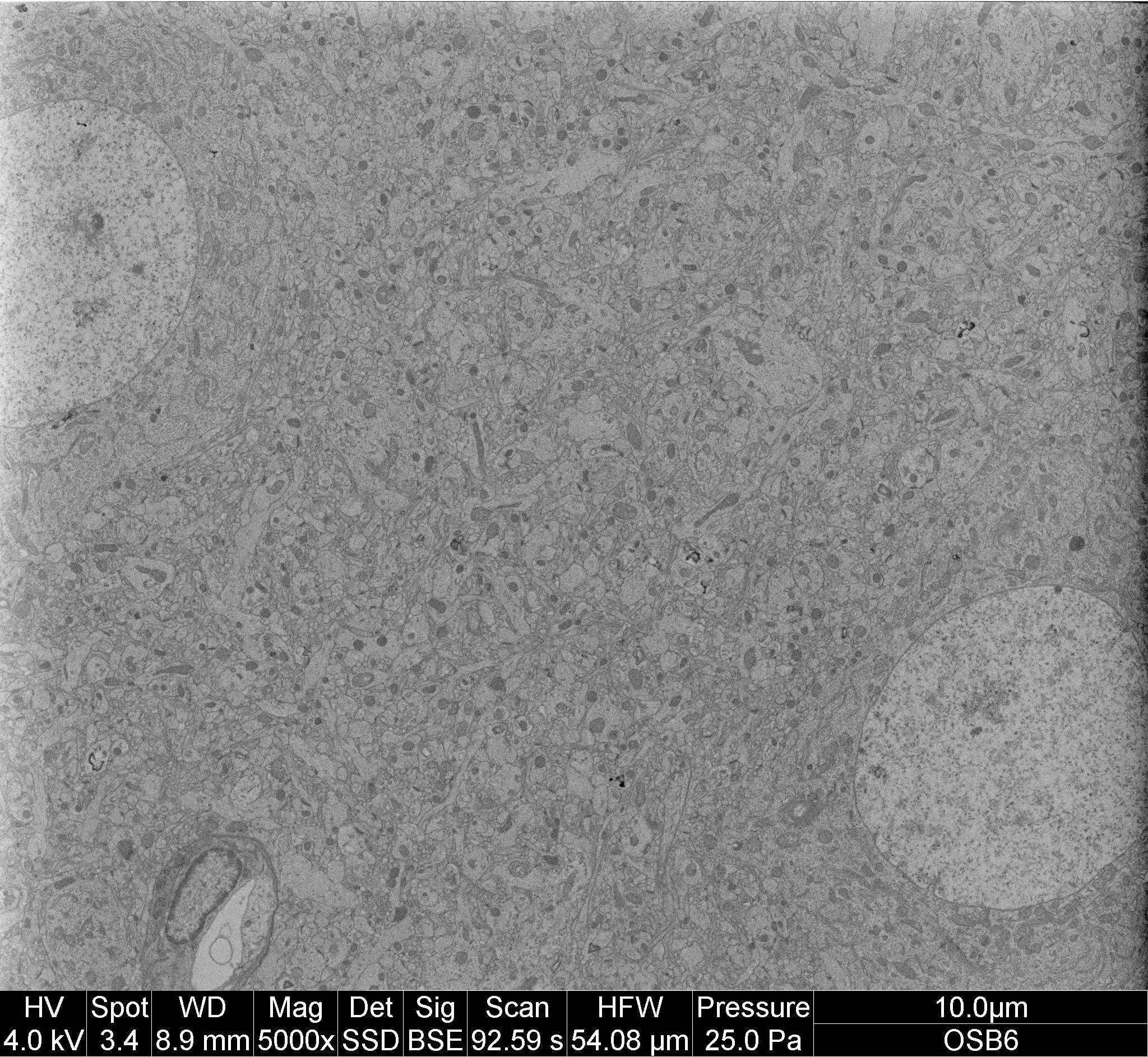

Supplement: Dataset S20 — (254.9 MB ZIP). [file pbio.0020329.sd020.zip › 040604_OS5_st1_1988.tif]

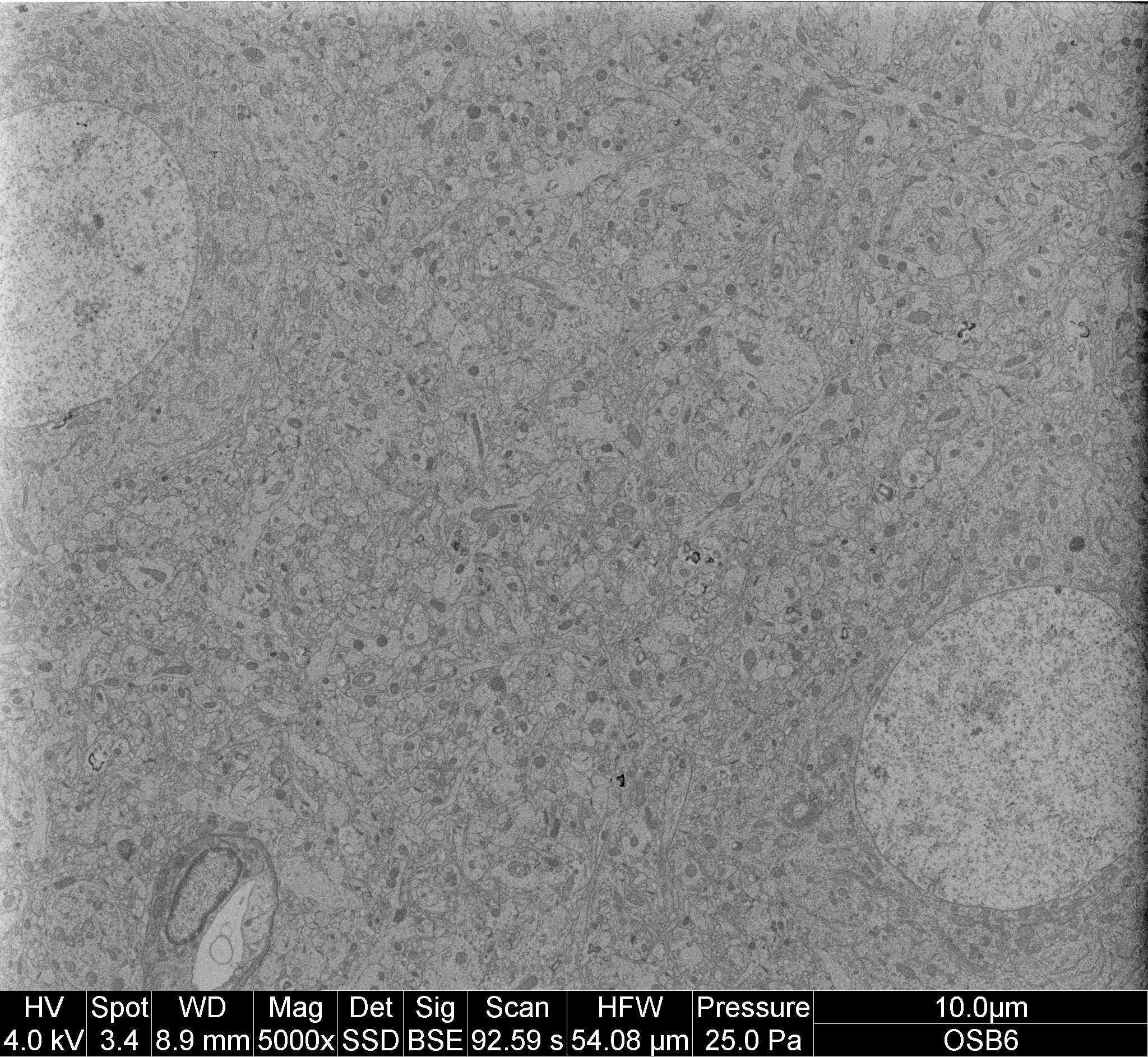

Supplement: Dataset S20 — (254.9 MB ZIP). [file pbio.0020329.sd020.zip › 040604_OS5_st1_1989.tif]

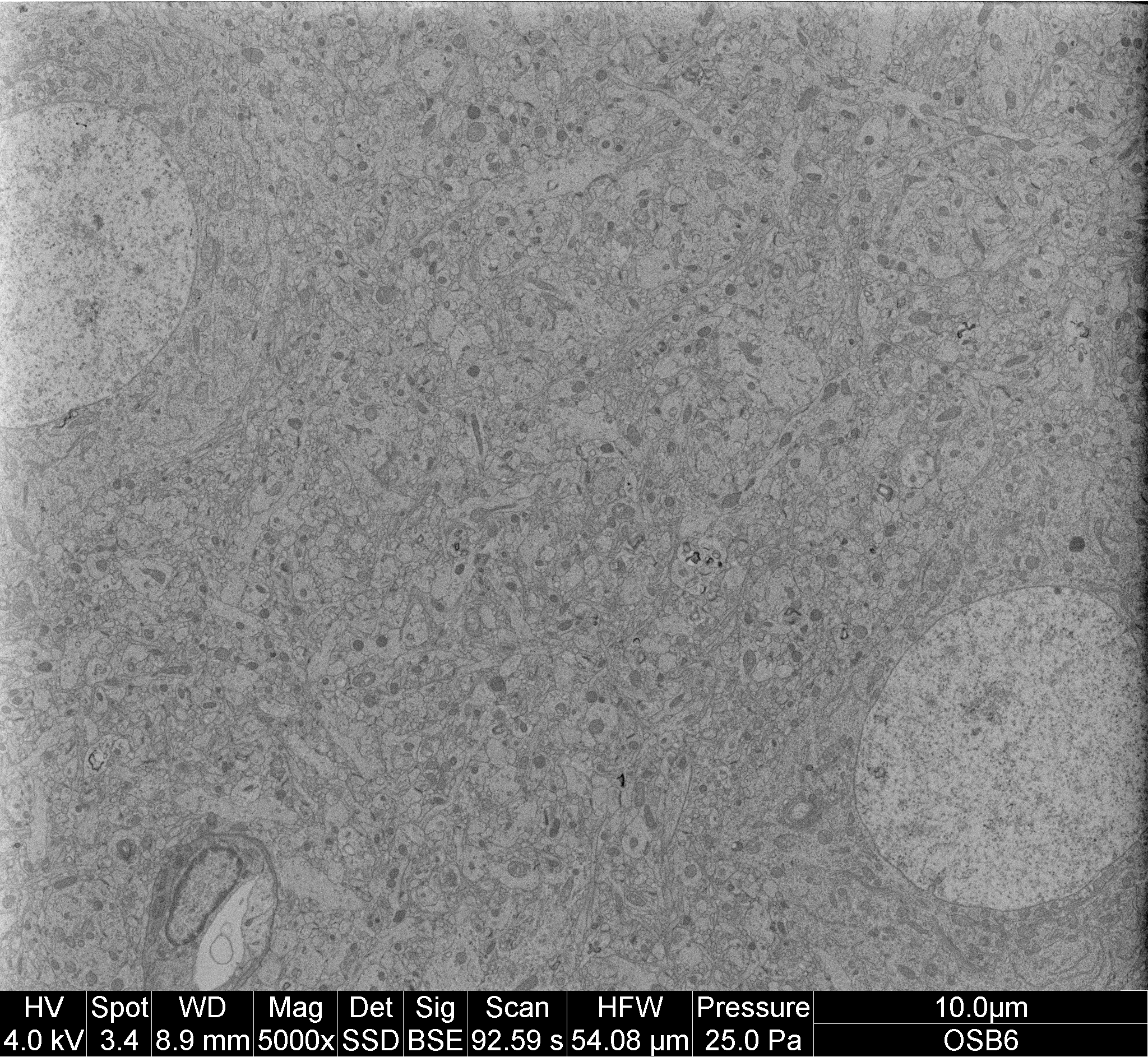

Supplement: Dataset S20 — (254.9 MB ZIP). [file pbio.0020329.sd020.zip › 040604_OS5_st1_1990.tif]

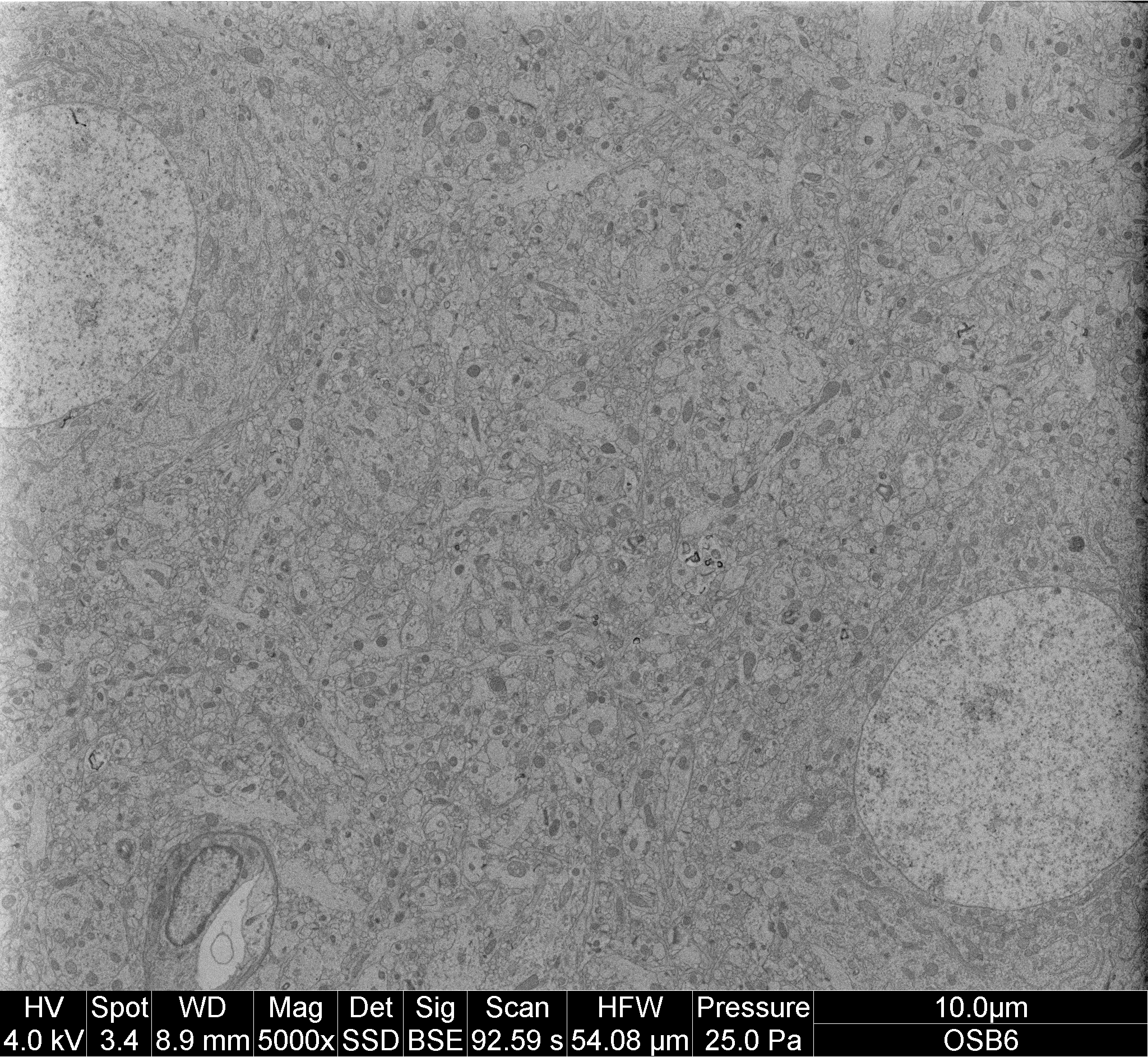

Supplement: Dataset S20 — (254.9 MB ZIP). [file pbio.0020329.sd020.zip › 040604_OS5_st1_1991.tif]

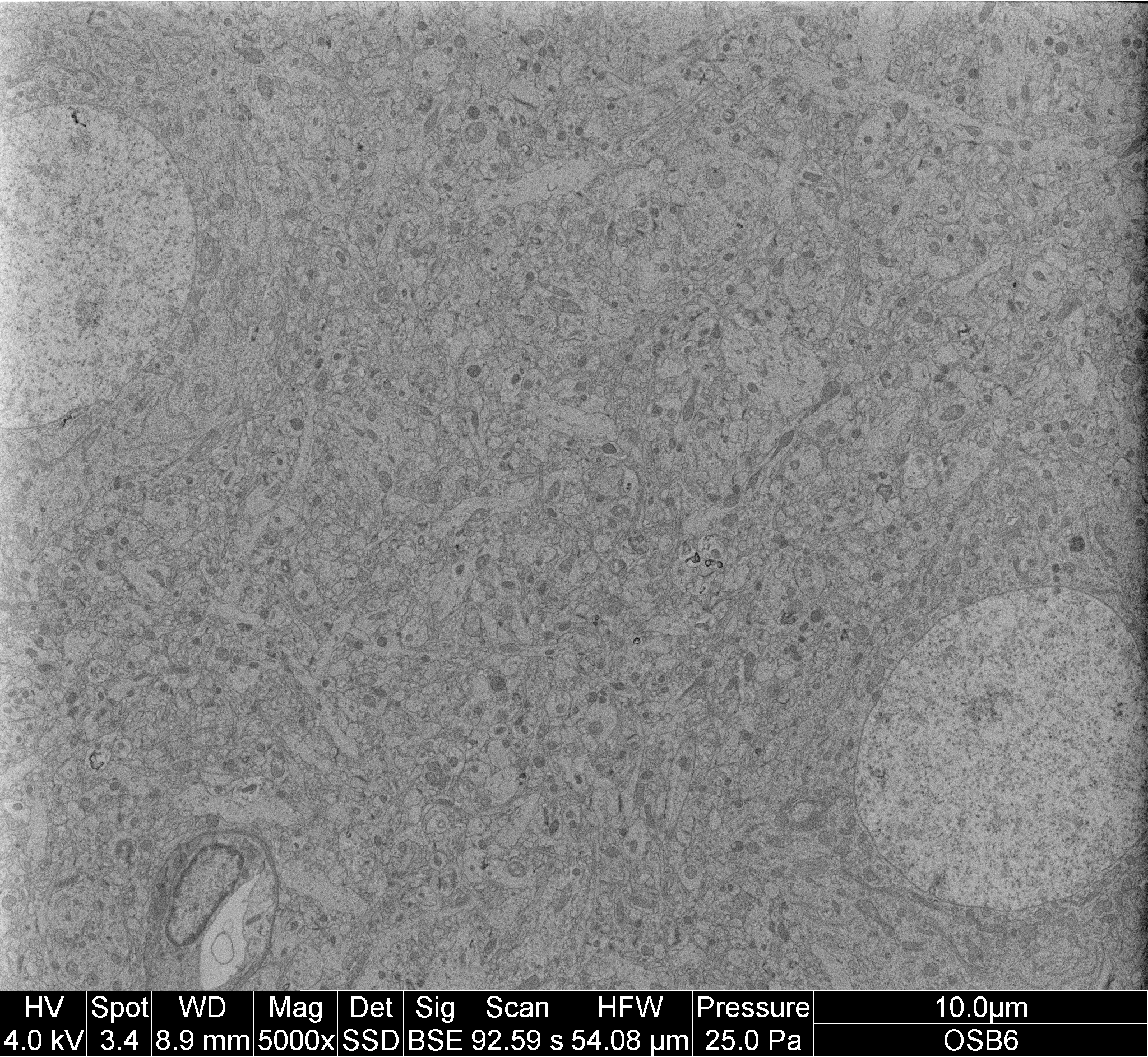

Supplement: Dataset S20 — (254.9 MB ZIP). [file pbio.0020329.sd020.zip › 040604_OS5_st1_1992.tif]

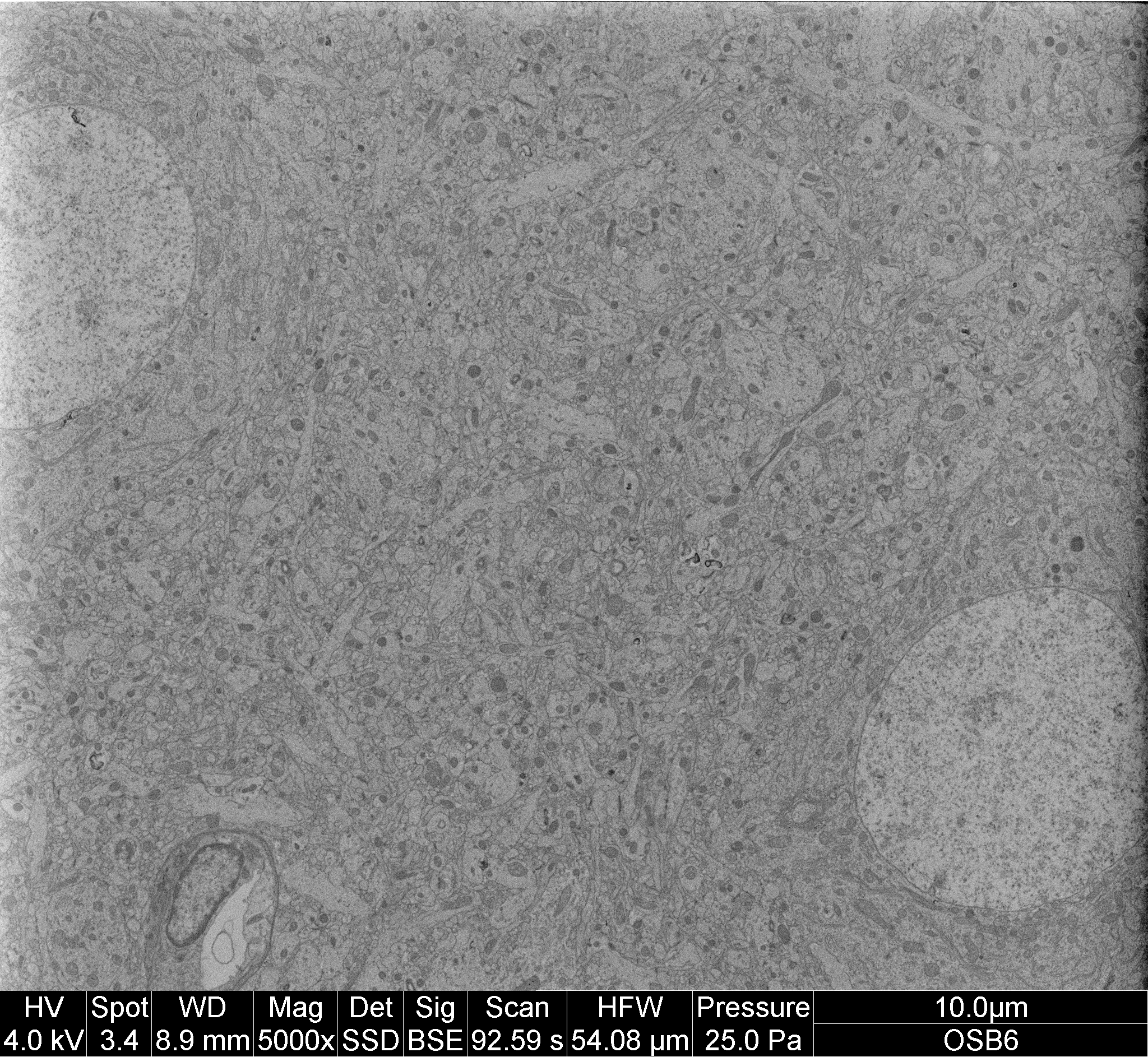

Supplement: Dataset S20 — (254.9 MB ZIP). [file pbio.0020329.sd020.zip › 040604_OS5_st1_1993.tif]

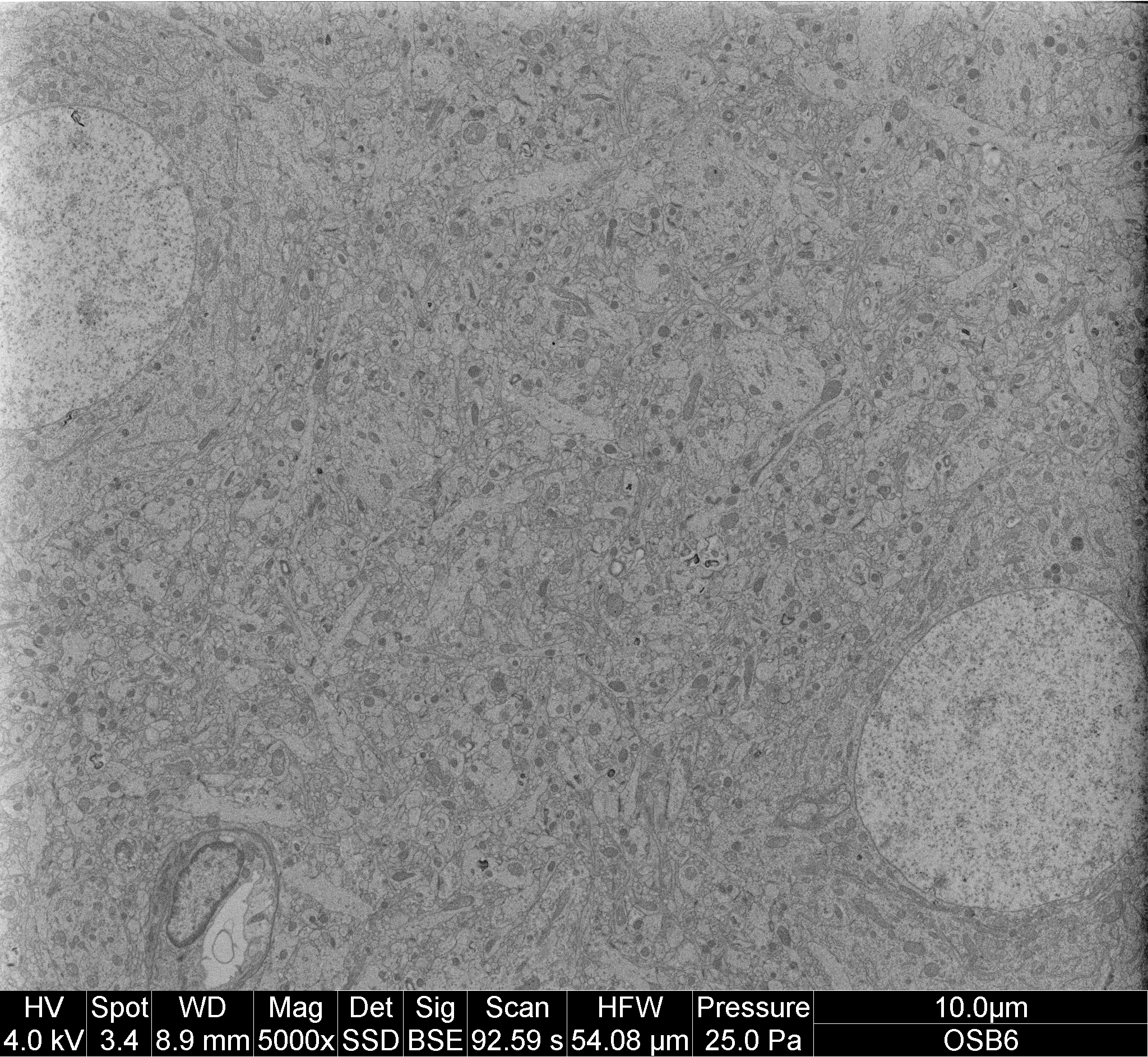

Supplement: Dataset S20 — (254.9 MB ZIP). [file pbio.0020329.sd020.zip › 040604_OS5_st1_1994.tif]

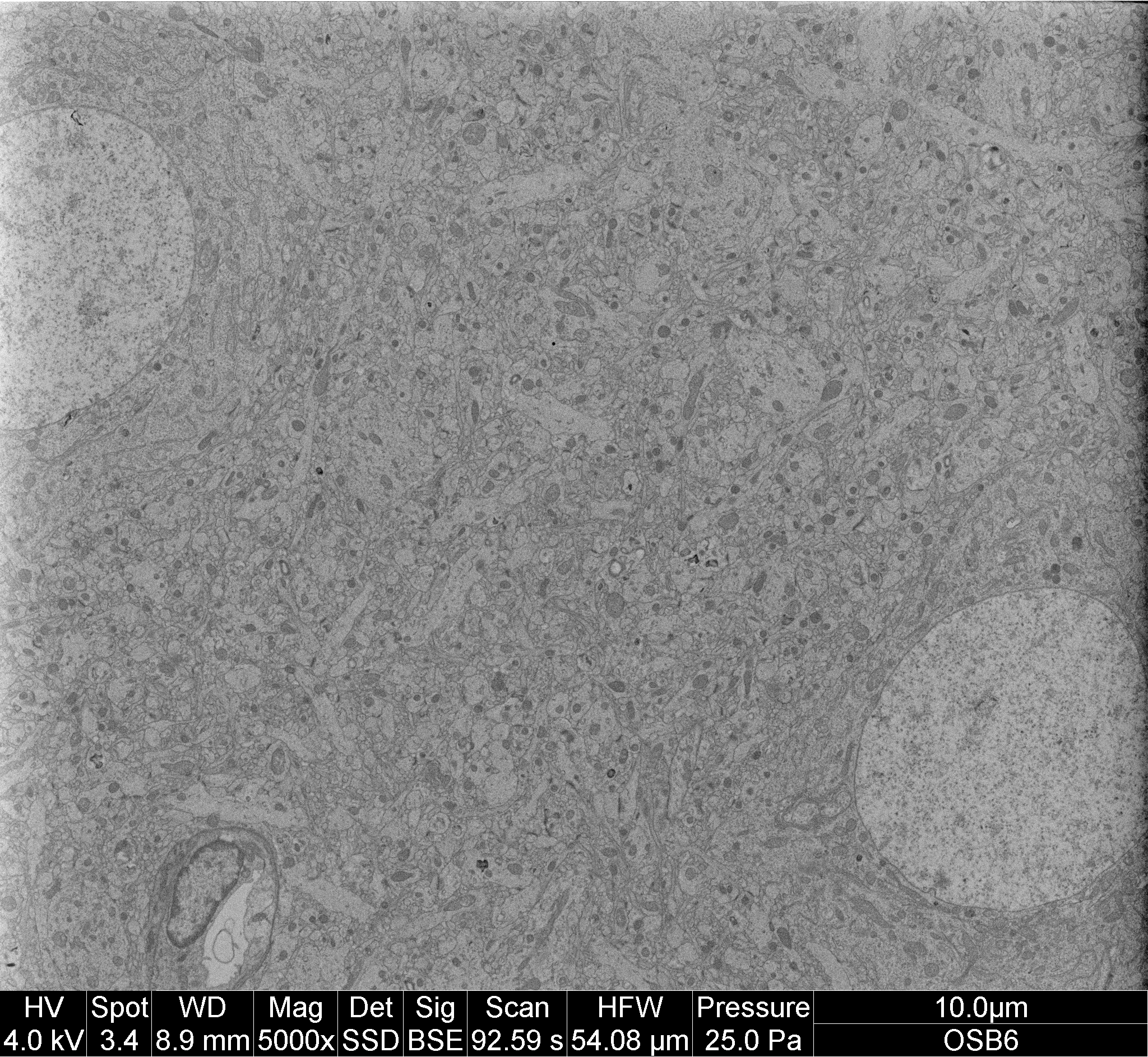

Supplement: Dataset S20 — (254.9 MB ZIP). [file pbio.0020329.sd020.zip › 040604_OS5_st1_1995.tif]

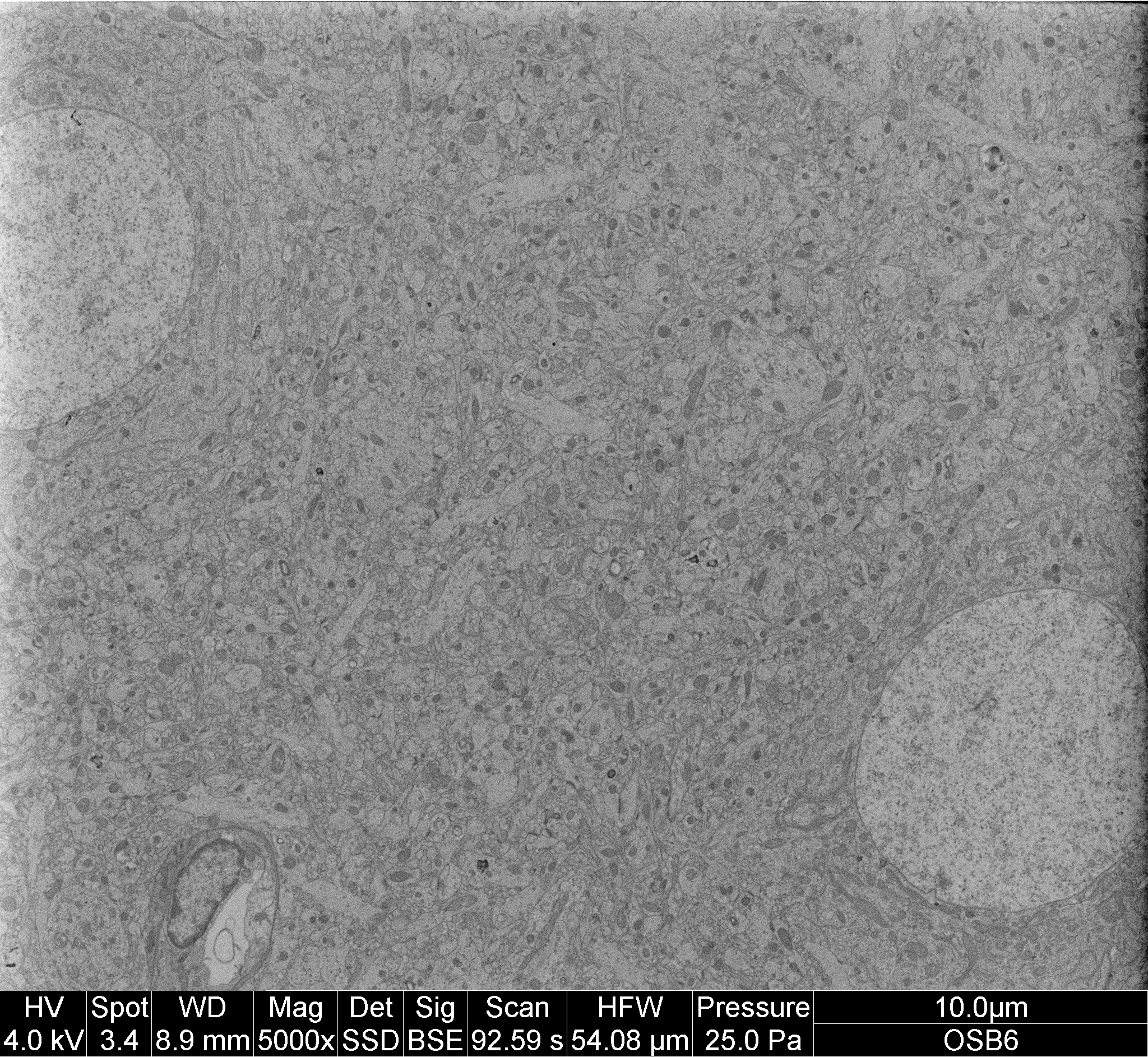

Supplement: Dataset S20 — (254.9 MB ZIP). [file pbio.0020329.sd020.zip › 040604_OS5_st1_1996.tif]

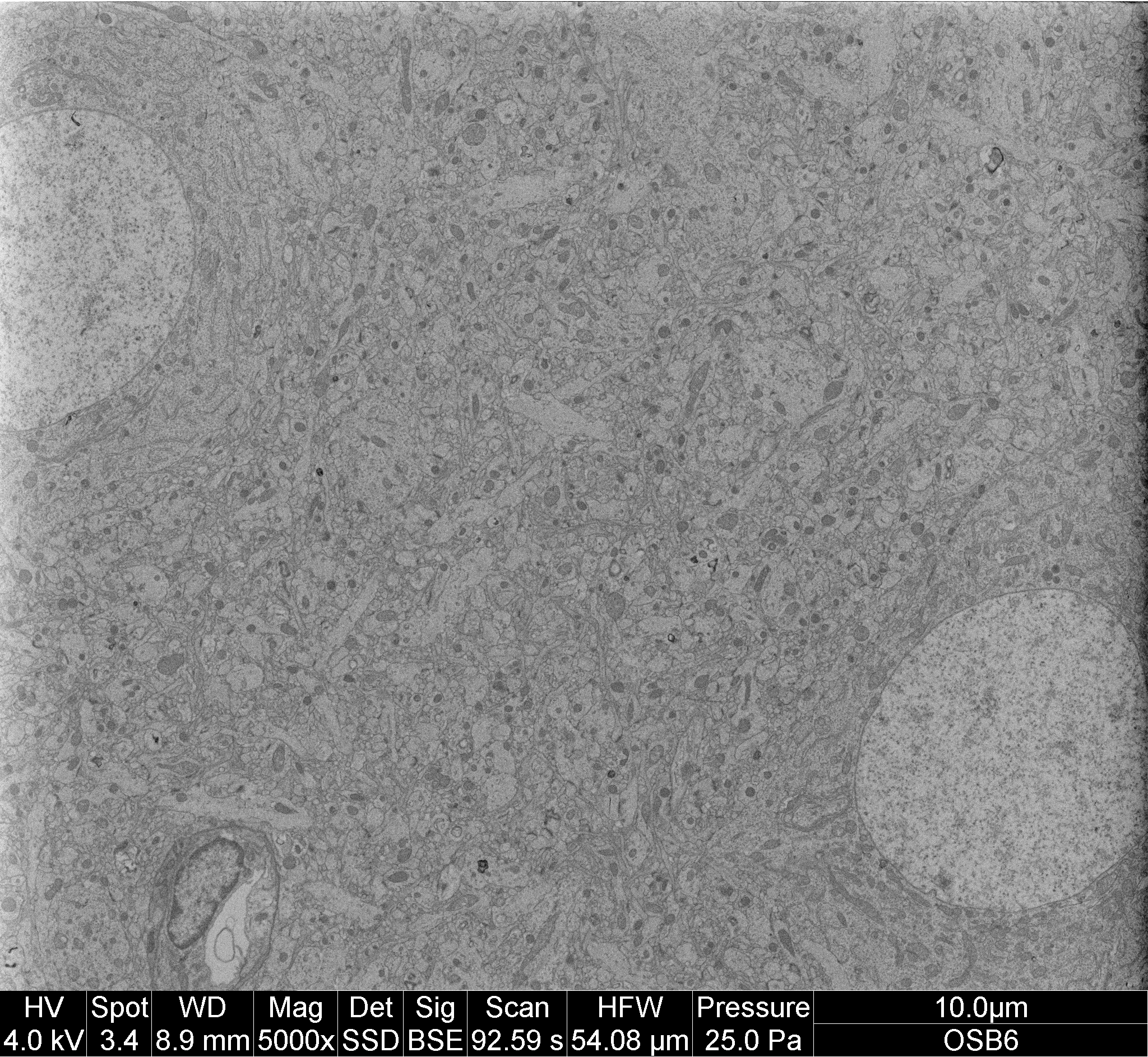

Supplement: Dataset S20 — (254.9 MB ZIP). [file pbio.0020329.sd020.zip › 040604_OS5_st1_1997.tif]

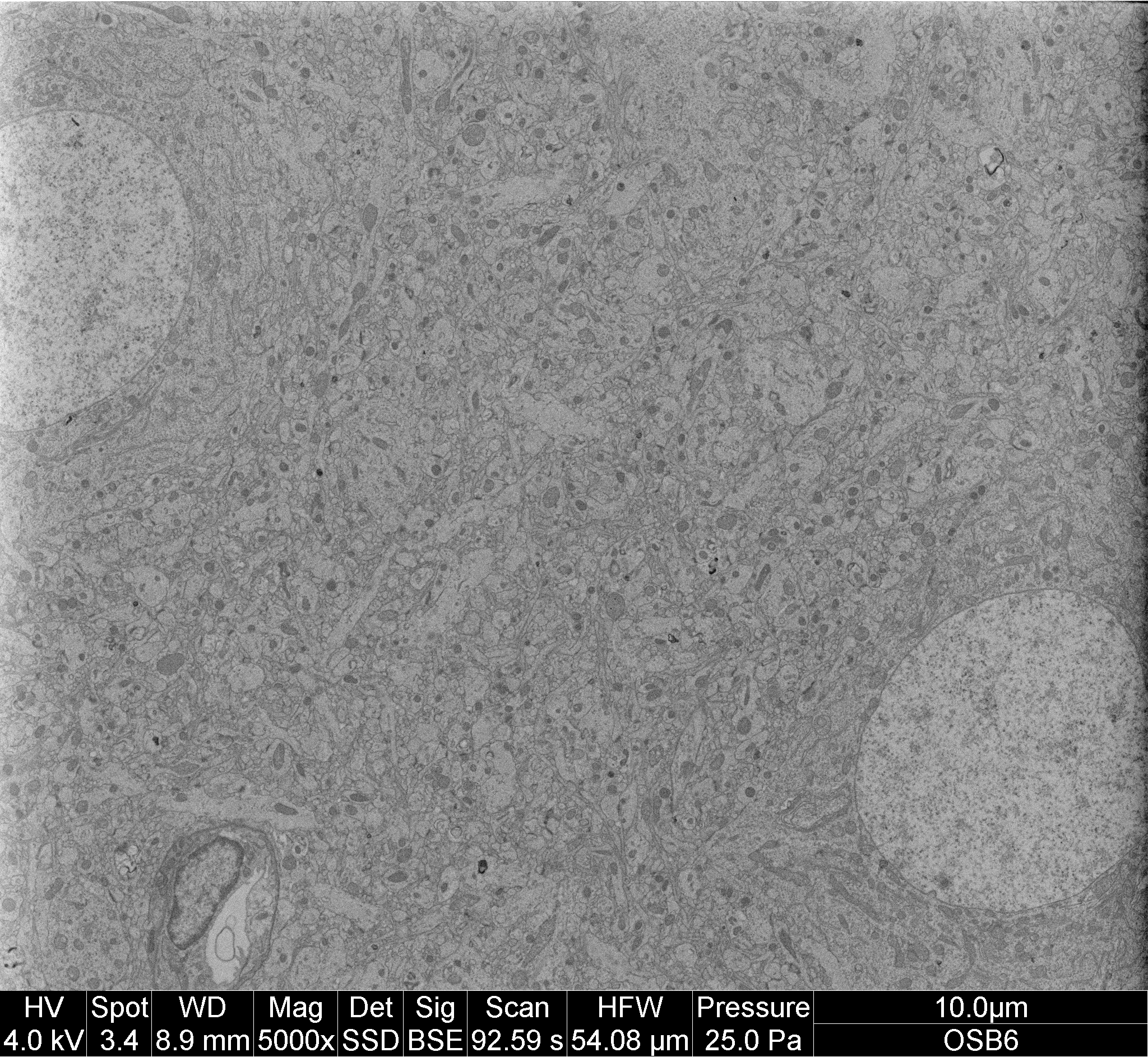

Supplement: Dataset S20 — (254.9 MB ZIP). [file pbio.0020329.sd020.zip › 040604_OS5_st1_1998.tif]

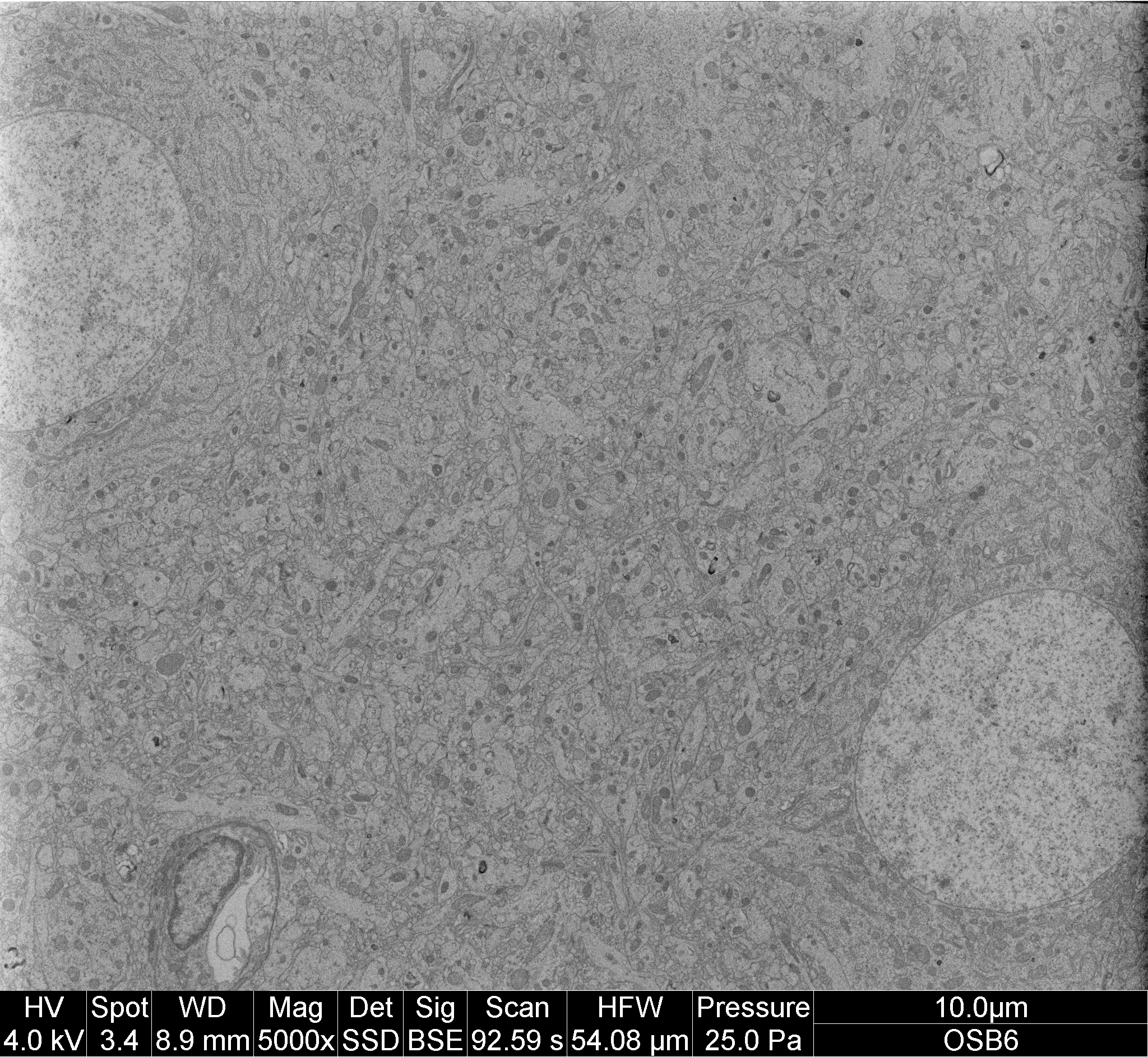

Supplement: Dataset S20 — (254.9 MB ZIP). [file pbio.0020329.sd020.zip › 040604_OS5_st1_1999.tif]

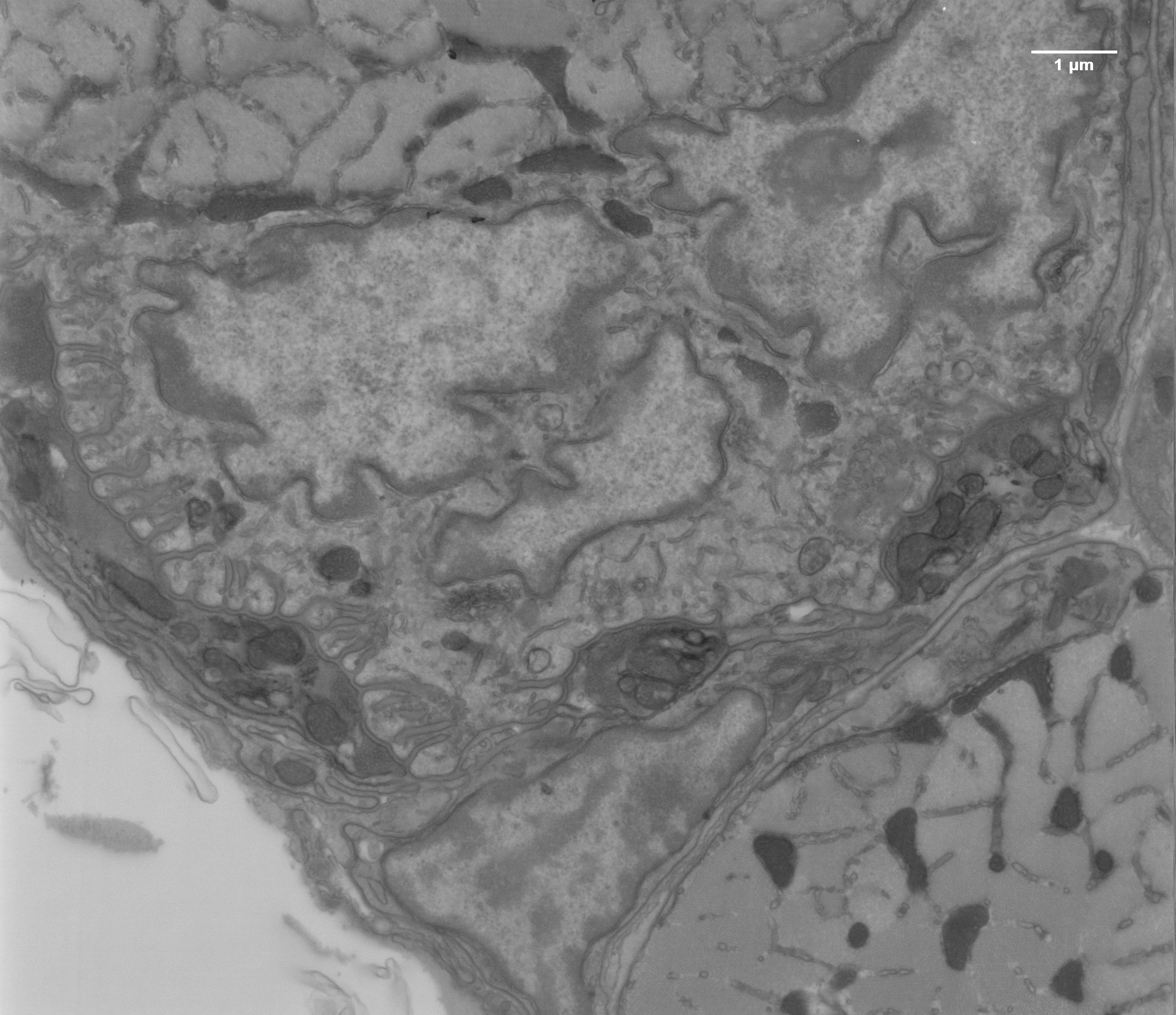

Supplement: Figure S1 — Complete field of view for dataset underlying Figure 1A and 1B. Grayscale is inverted from data taken; no smoothing or contrast enhancement was applied. (10.8 MB TIF). [file pbio.0020329.sg001.tif]
