# Supplementary figures and images for: Serial Block-Face Scanning Electron Microscopy to Reconstruct Three-Dimensional Tissue Nanostructure (part 21 of 21)
Source: PLoS Biol. 2004 Oct 19;2(11):e329. doi: 10.1371/journal.pbio.0020329 (PMC524270; doi:10.1371/journal.pbio.0020329)

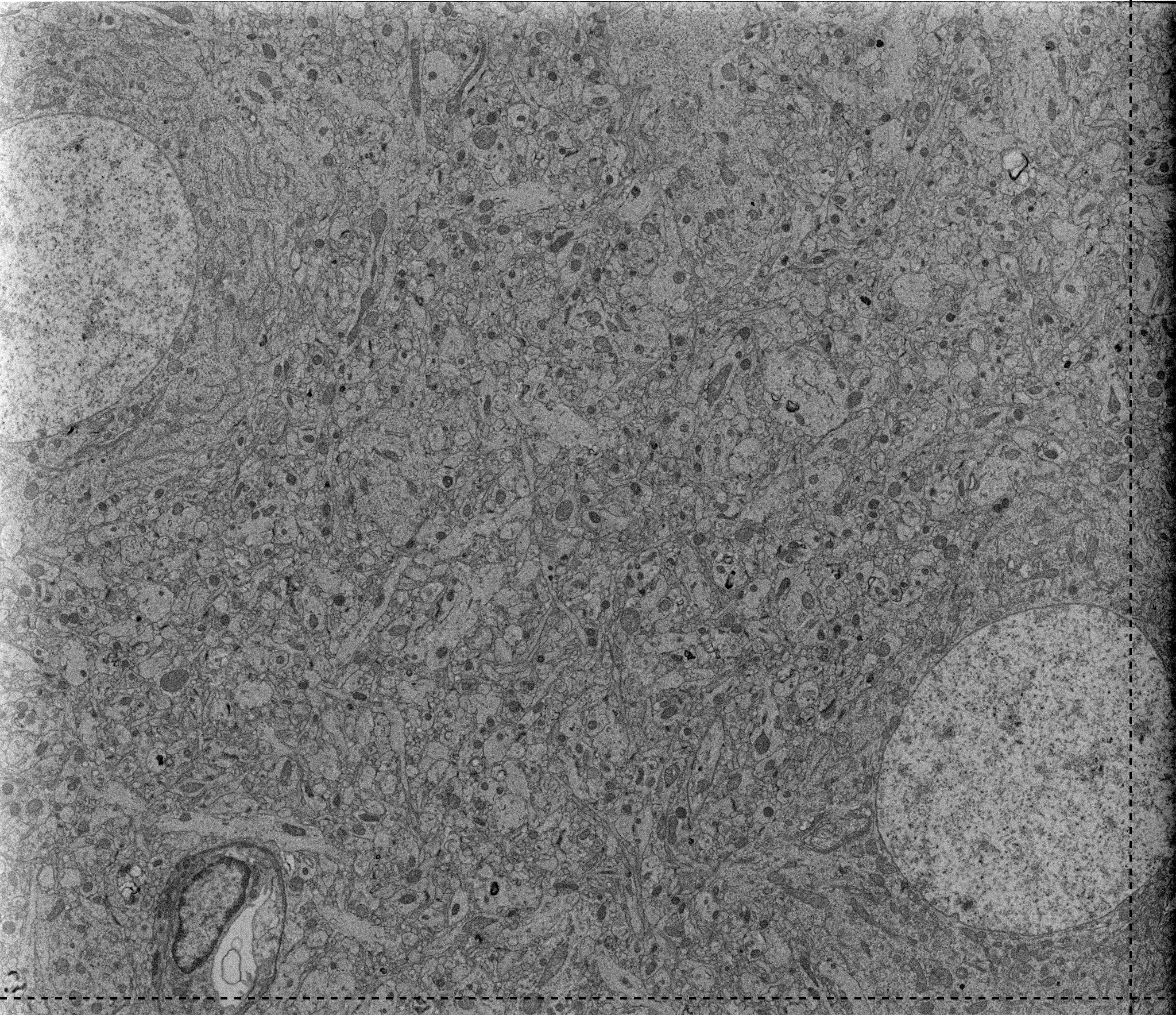

Supplement: Figure S2 — Bottom slice of a stack of 2,000 images taken at 4 keV. Slice thickness was 55 nm. Spotsize was 3.4. Pixel size is 26.7 nm. The pixels correspond to the original data. The area shown corresponds to 54.8 × 47.3 μm. The raw data can be found as numbered TIF images in Datasets S1–S20. (10.6 MB TIF). [file pbio.0020329.sg002.tif]

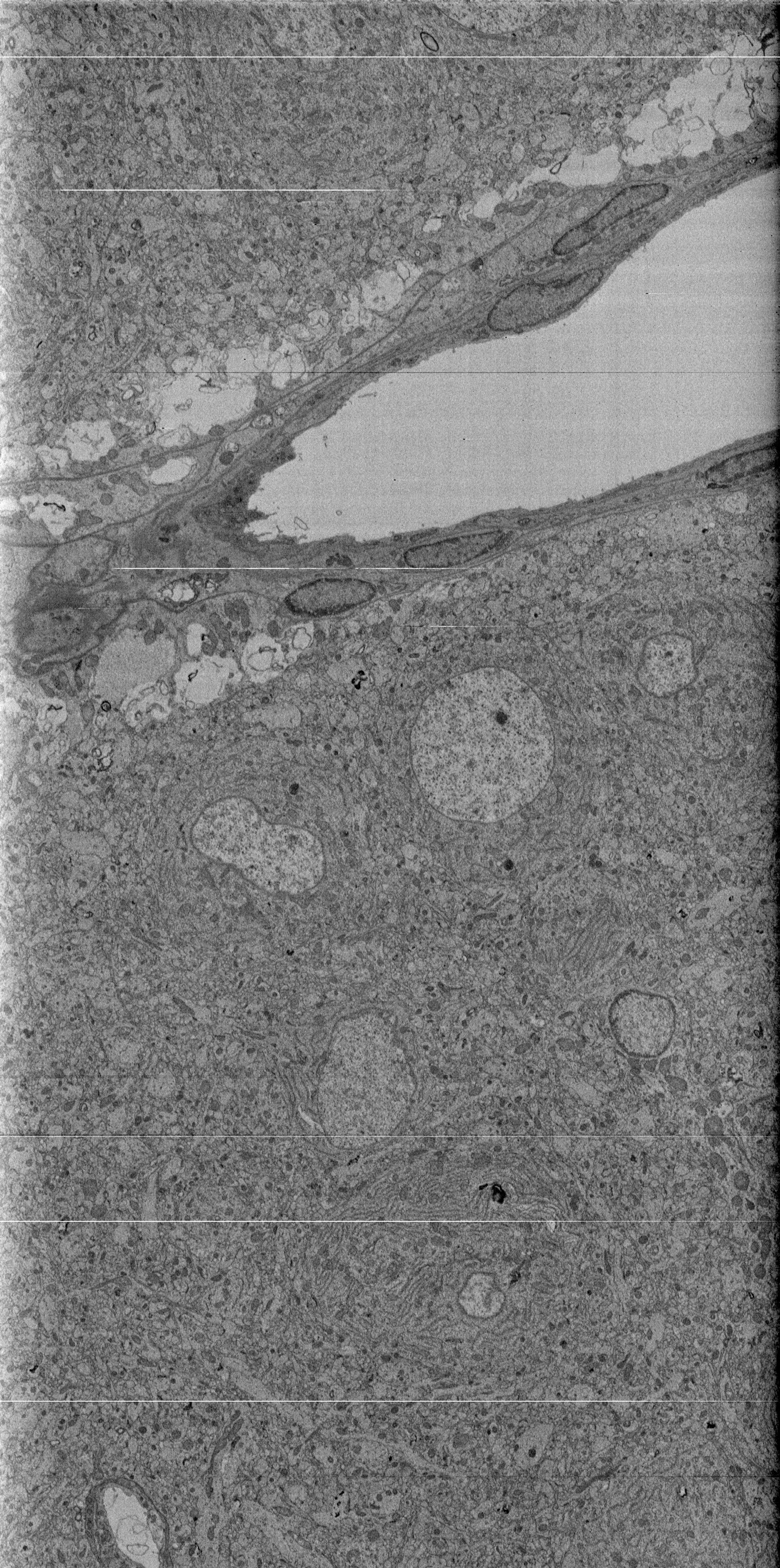

Supplement: Figure S3 — Same volume data as used for Figure S2. The stack was resliced along the horizontal dotted line shown in Figure S2. In the vertical direction the data were interpolated so that each slice now corresponds to slightly more that two pixels. Horizontal white lines are slices with deposited debris. The total stack height was 110 μm. (8.2 MB TIF). [file pbio.0020329.sg003.tif]

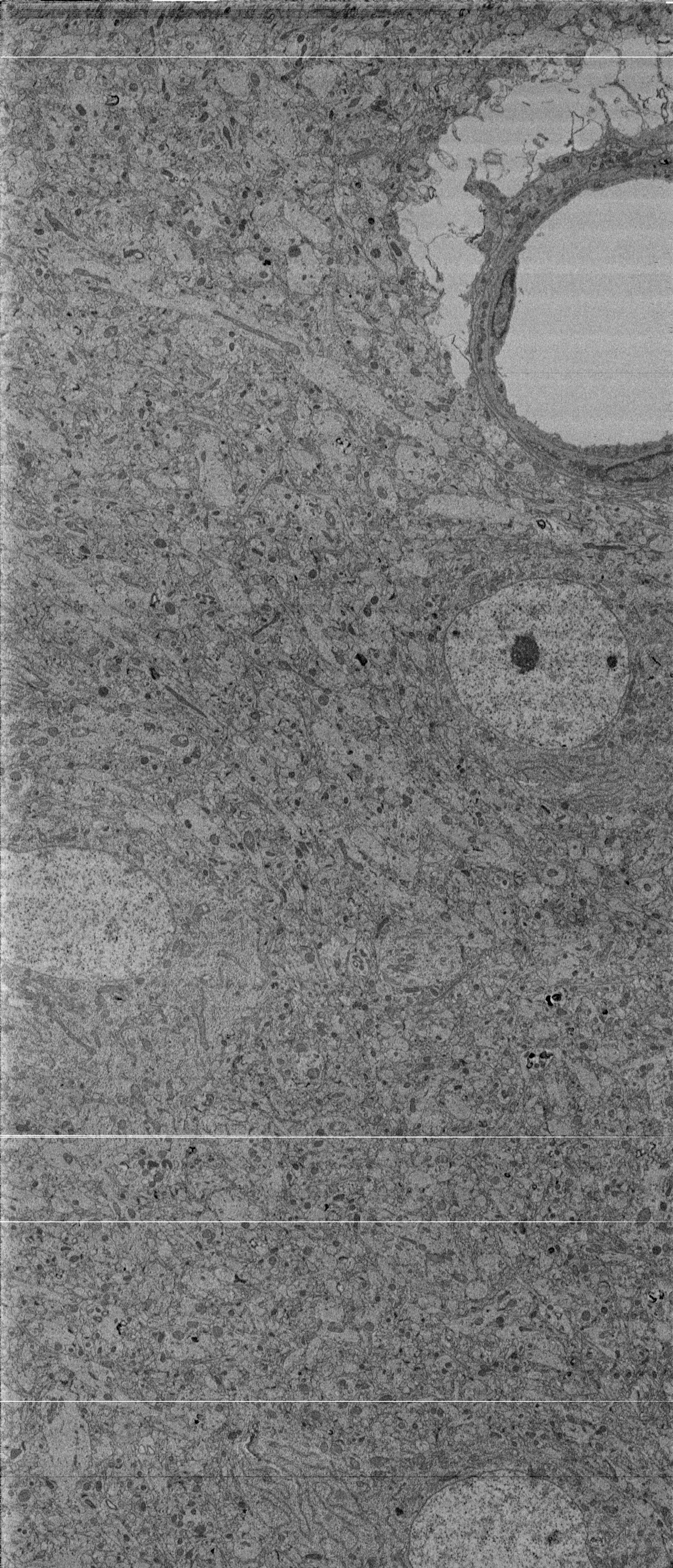

Supplement: Figure S4 — Same as Figure S3 but now resliced along the vertical dotted line in Figure S2. (7.1 MB TIF). [file pbio.0020329.sg004.tif]

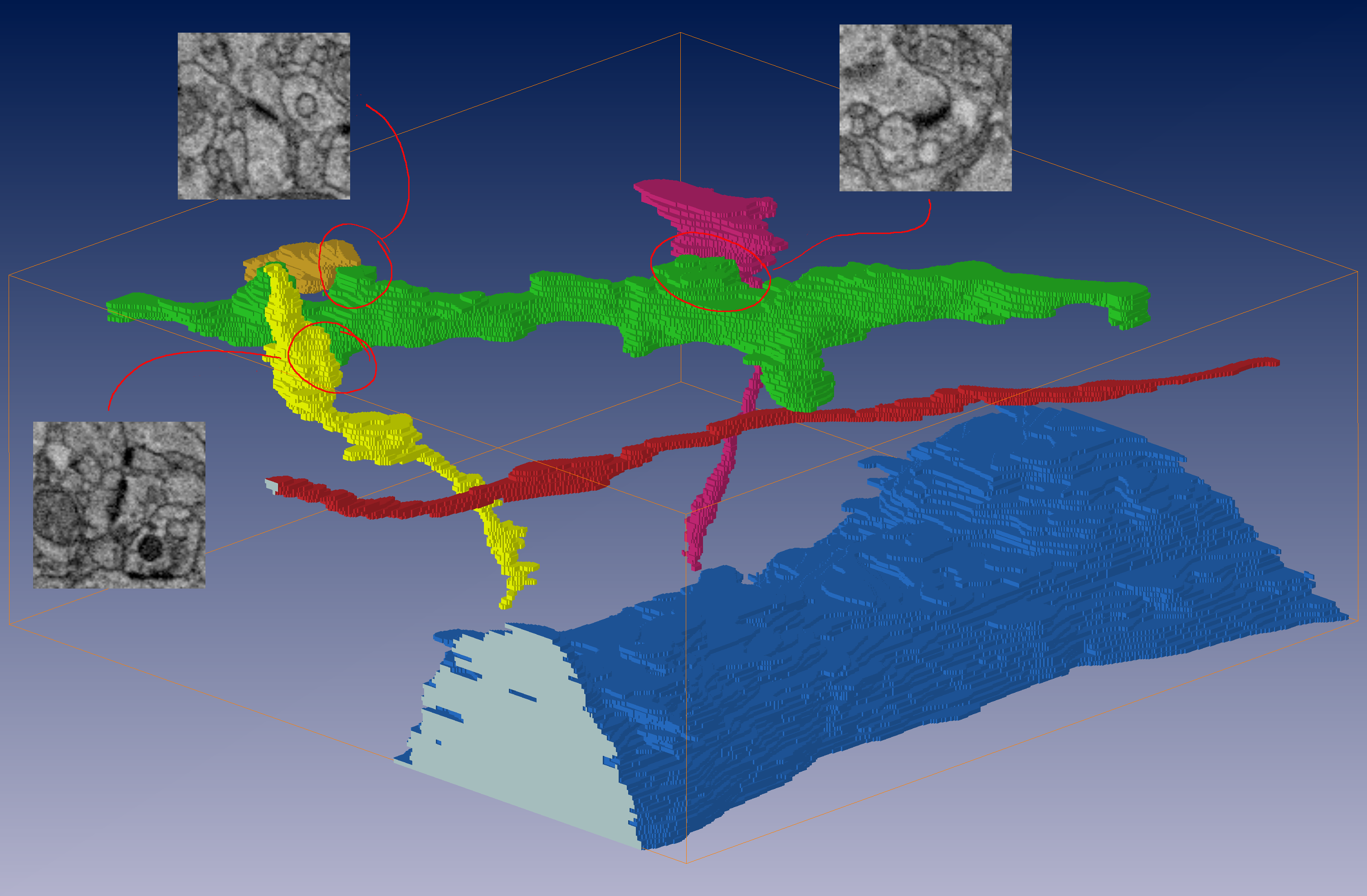

Supplement: Figure S5 — Manual reconstruction of selected processes in cortical tissue (data from Video 2 and Figure 5). Blue, portion of proximal apical dendrite; green, secondary dendrite with three synaptically connected axons (yellow, ocher, and mauve). Insets show the synaptic contacts. Also shown is a passing axon that is not synaptically connected within the volume analyzed. Only the lower part of the stack, which was taken at 4 keV electron energy, was used. (17.5 MB TIF). [file pbio.0020329.sg005.tif]
